# Multi-omic and multi-species meta-analyses of nicotine consumption

**DOI:** 10.1038/s41398-021-01231-y

**Published:** 2021-02-04

**Authors:** Rohan H. C. Palmer, Chelsie E. Benca-Bachman, Spencer B. Huggett, Jason A. Bubier, John E. McGeary, Nikhil Ramgiri, Jenani Srijeyanthan, Jingjing Yang, Peter M. Visscher, Jian Yang, Valerie S. Knopik, Elissa J. Chesler

**Affiliations:** 1 Behavioral Genetics of Addiction Laboratory, Department of Psychology, Emory University, Atlanta, GA USA; 2 The Jackson Laboratory, Bar Harbor, ME USA; 3 Department of Psychiatry and Human Behavior, Brown University, Providence, RI USA; 4 Providence Veterans Affairs Medical Center, Providence, RI USA; 5 Department of Human Genetics, Emory University School of Medicine, Atlanta, GA USA; 6 Institute for Molecular Bioscience, The University of Queensland, Brisbane, QLD Australia; 7 Department of Human Development and Family Studies, Purdue University, West Lafayette, IN USA

**Keywords:** Comparative genomics, Addiction

## Abstract

Cross-species translational approaches to human genomic analyses are lacking. The present study uses an integrative framework to investigate how genes associated with nicotine use in model organisms contribute to the genetic architecture of human tobacco consumption. First, we created a model organism geneset by collecting results from five animal models of nicotine exposure (RNA expression changes in brain) and then tested the relevance of these genes and flanking genetic variation using genetic data from human cigarettes per day (UK BioBank *N* = 123,844; all European Ancestry). We tested three hypotheses: (1) DNA variation in, or around, the ‘model organism geneset’ will contribute to the heritability to human tobacco consumption, (2) that the model organism genes will be enriched for genes associated with human tobacco consumption, and (3) that a polygenic score based off our model organism geneset will predict tobacco consumption in the AddHealth sample (*N* = 1667; all European Ancestry). Our results suggested that: (1) model organism genes accounted for ~5–36% of the observed SNP-heritability in human tobacco consumption (enrichment: 1.60–31.45), (2) model organism genes, but not negative control genes, were enriched for the gene-based associations (MAGMA, H-MAGMA, SMultiXcan) for human cigarettes per day, and (3) polygenic scores based on our model organism geneset predicted cigarettes per day in an independent sample. Altogether, these findings highlight the advantages of using multiple species evidence to isolate genetic factors to better understand the etiological complexity of tobacco and other nicotine consumption.

## Introduction

Contemporary thought on human genetic research is that large genome-wide association studies (GWAS) are required to identify reproducible single nucleotide polymorphism (SNP) associations that can lead to insights into biological systems that underpin a particular phenotype. The agnostic nature of GWAS (i.e., all SNPs being tested without bias) enables the identification of previously unrecognized biological underpinnings for human traits. However, GWASs are not without limitations. One limitation is the stiff penalty for multiple comparisons leading to the need for increasingly large sample sizes. The requirement of sample sizes in the 100’s of thousands to millions (i.e., mega-GWAS) exerts pressure on the depth of phenotyping that may be done (i.e., more intensive and costly phenotypes are untenable for mega-GWAS studies). Additionally, SNPs implicated by GWAS are not always readily associated with gene function. Specifically, the majority of GWAS associations reside in non-coding or intergenic regions^1^. To help make sense of these signals, studies rely upon arbitrarily defined gene regulatory regions (up/downstream of a gene). While GWAS findings have become increasingly reproducible as sample sizes increase, additional sources of data (e.g., gene regulatory and epigenetic data^2^) are needed to understand how SNP effects increase the risk for trait pathology^3^.

Association studies of tobacco consumption assume that variation in the biological sample collected (e.g., DNA extracted from blood and saliva) reflects the genetic influences in the brain that mediate the psychoactive properties of nicotine and other chemicals found in tobacco products. Nicotine causes changes in the neural organization, particularly in the brain’s reward systems, psychomotor, and cognitive processes via its ability to interact with nicotinic acetylcholine receptors (nAChRs)^4,5^. Nicotine takes on the properties of a reinforcer by altering neural circuits, in particular those comprising the dopaminergic systems of the midbrain^6^. Altogether, these properties of tobacco products highlight putative genetic mechanisms that may mediate consumption. The largest tobacco consumption meta-GWAS, to date, has identified 566 genetic variants in 406 loci associated with phenotypes related to tobacco consumption (i.e., initiation, cessation, and heaviness of use)^7^, yet how most of these gene variants contribute to specific tobacco behaviors is unknown.

**Fig. 1:**
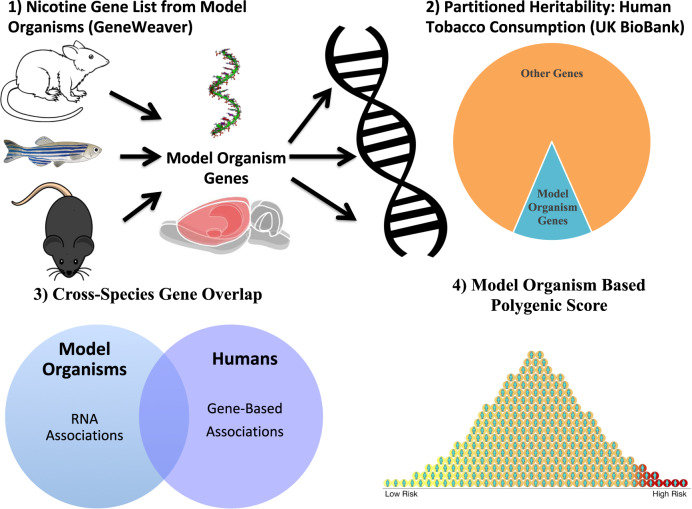
Schematic outlining our study design. Schematic of analytical pipeline used for cross-species analysis. *Panels indicate:* **1)** Derivation of gene list using genes associated with nicotine exposure from various animal paradigms. **2)** Multicomponent SNP-heritability analysis in UKBiobank smokers using mixed linear models. **3)** Examination of gene-list overlap using Jaccard similarity and Fisher exact tests. **4)** Multicomponent polygenic score analysis using GWAS-LOCO summary statistics in an independent sample.

The lack of accessible human brain tissue for tobacco use has precluded understanding of how gene variants contribute to tissue-specific epigenetic and/or expression differences that arise from continued drug exposure. We used a novel and integrative framework that combined human GWAS data with high throughput model organism data to clarify how the genetic liability to human tobacco consumption relates to specific nicotine behaviors in particular brain regions. We hypothesized that genes associated with nicotine exposure paradigms in model organisms will: (1) contribute to the heritability to human tobacco consumption, (2) be enriched for genes associated with human tobacco consumption, and (3) aid in the prediction of a polygenic score of human tobacco consumption in an independent sample (see Fig. 1).

## Materials and methods

### Samples and phenotypes

To find genes associated with nicotine exposure, we used GeneWeaver^8–10^—a genomics and bioinformatics data repository and suite of tools. We created a model organism geneset that included brain-related RNA associations with animal paradigms of nicotine exposure from multiple species: *Mus musculus*, *Rattus norvegicus*, and *Danio rerio* (Table 1; GeneWeaver data gathered in October 2019). Aggregating across all GeneWeaver studies, we identified 786 orthologous genes with humans, which we dubbed “*model organism genes*”.

**Table 1:** Data used to create our (model organism) nicotine gene list.

Author(s)	GeneWeaver ID	Model organism	Nicotine use paradigm	Experimental design	Brain region	Number of genes
Chen et al.^1^	GS87128	*Mus musculus*	Acute nicotine treatment (Adult males; C57BL/6 J mice)	Microarray analysis, WGCNA	VTA	184
Polesskaya et al.^2^	GS14885	*Rattus norvegicus*	Subcutaneous chronic nicotine treatment (Adolescent females; Long evans rats)	Microarray analysis, qRT-PCR, Principle cluster analysis	PFC, NAc & Hippocampus	66
Wang et al.^3^	GS14888, GS14889, GS14890, GS14891, GS14892, GS14893	*Mus musculus*	Nicotine self-administration (Adult males; C3H/HeJ and C56BL/6 J mice)	Microarray analysis, qRT-PCR, WGCNA	Amygdala, Hippocampus, NAc, PFC & VTA	651
Kily et al.^4^	GS14902, GS14903	*Danio rerio*	Nicotine-induced conditioned place preference (Adult males and females; Zebrafish)	Microarray analysis, qRT-PCR	Whole brain	158
Sharp et al.^5^	GS128167	*Rattus norvegicus*	Nicotine self-administration (Adolescent males; Lewis rats)	Microarray analysis, RT-PCR	NAc	188

We used GeneWeaver (https://www.geneweaver.org/) to identify genes associated with nicotine use in model organisms. In total, there were 712 unique genes associated with nicotine use that were also conserved in humans.

We used two independent human datasets from two different countries investigating the same trait—cigarettes per day (CPD; tobacco consumption). All individuals reported using tobacco (e.g., current or former smoker) and were of European ancestry. Our analyses used data from the UK BioBank^11^ (UKB; *N* = 123,844; Age = 58.1, SD = 7.8; Sex = 48.3% Female), which we used for partitioned heritability analyses and gene-based tests, and the National Study of Adolescent Health^12^ (Add Health, Wave IV; *N* = 1667, Age = 28.9, S.D = 1.70; Sex = 49% female) that was used for polygenic score prediction. Ancestry in both samples was determined using principal components analyses and multidimensional scaling^13,14^. This study was approved by the Institutional Review Board at Emory University (IRB00090295).

### Genotype quality control

Human genomic analyses in UKBiobank samples focused on raw and imputed genotypes obtained using the Affymetrix UK BiLEVE Axiom and UK Biobank Axiom® arrays, which genotyped ~850,000 variants (details available here: https://www.ukbiobank.ac.uk/scientists-3/genetic-data/). Analyses in both samples focused on genotyped and imputed SNPs with good quality scores (*r*^2^ > 0.3). PLINK (version 1.9)^15^ was used to filter markers using the following criteria: genotyping rate >99%, minor allele frequency >0.01, Hardy–Weinberg equilibrium *p* value >0.0001, and missing genotype rate <0.10.

### Partitioned heritability of cigarettes per day using nicotine genesets

Our study investigated whether model organism data on nicotine exposure was relevant to the genetics of human tobacco consumption via partitioned heritability analyses. To test this, we used Genome Complex Trait Analysis (GCTA; version 1.92)^16^ with multiple genetic components effects estimated via genome-based restricted maximum likelihood (GREML). To reduce the computational burden and to demonstrate the robustness of our partitioned heritability analyses, we separated the UKB into three separate subsets. Using the GCTA-GREML Power Calculator^17^ we estimated the sample sizes needed to provide at least 70% power to detect SNP-heritability estimates as small as one-third of 1% (i.e., 0.333%)^2^. Power was based on the previously reported SNP-heritability of CPD^7^ and the observed variance of the off-diagonal elements (~6.68 × 10^−4^) of the genomic relatedness matrix for individuals with smoking data in UKBiobank. We used this information to split the UKB sample into three approximately equal subsets (*n*_1_ = 41,263, *n*_2_ = 41,368, *n*_3_ = 41,213), each of which was made constitutionally equivalent by randomly sampling individuals from each quartile of the nicotine consumption distribution; individuals in each subset were no more related than second cousins.

We partitioned the SNP-heritability of tobacco consumption into three regions-of-interest (ROI; and thus set of three genetic relatedness matrices) based on the GeneWeaver model organism genes: (1) protein-coding regions, (2) surrounding regions, and (3) all other variants. Protein coding regions included all SNPs between the start and stop positions for each of the model organism genes that were orthologous in humans. The surrounding regions encompassed genomic loci up and downstream of the 5′ and 3′ ends of each gene, respectively, and captured potential regulatory DNA variants of genomic regions around the model organism genes (e.g., *cis*-expression quantitative trait loci; *cis*-eQTLs). The category “All other variants” reflected aggregate genetic effects from the remainder of the genome, given the corresponding size protein coding and surrounding regions. We investigated five differently sized surrounding regions around the model organism genes as the sizes vary substantially in the literature (5 kilobase pairs (kB), 10 kB, 25 kB, 35 kB, and 50 kB). In order to estimate the contribution of each of these regions to the genetic variance for CPD, we fitted six partitioned heritability models that included variance components including the effects of SNPs within the protein-coding regions identified by the model organism nicotine gene set + buffer around these genes of varying length + effects of all other variants in genome + error (i.e., (1) protein-coding regions + the effect of variants in the remainder of the genome, (2) protein-coding regions + SNP effects within a 5 kB buffer + all other variants in the genome, as well as subsequent models of varying buffer lengths, (3) 10 kB buffer, (4) 25 kB buffer, (5) 35 kB buffer, and (6) 50 kB buffer), which tested the role of the protein-coding regions and surrounding regions of the model organism genes, as well as all other variants and the total heritability of human tobacco consumption, simultaneously. Additional details are provided in the supplementary methods.

The significance of each variance component was assessed using a likelihood ratio test while accounting for covariates (sex, testing site location, age, and age^2^). Population stratification effects were controlled using strict selection for individuals of European Ancestry using genomic principal components and multidimensional scaling^14,18^. Enrichment was calculated to determine whether the observed component-heritability estimates were greater than what would be expected by chance given the observed total genetic variance and ~4.6 million SNPs used in the analysis (i.e., the variance explained that we would expect via a random selection of loci of the same size from the genome). As such, the statistical significance of enrichment was evaluated on whether the expected *h*^2^_SNP_ fell within the 95% confidence interval of the observed *h*^2^_SNP_ (i.e., enrichment >1.96).

$$\begin{array}{l}{\mathrm{Expected}}\;{\mathrm{SNP}} - {\mathrm{heritability}} \\ = \frac{{(\# \;\mathrm{SNP}\;\mathrm{loci}\;\mathrm{of}\;{\it\mathrm{interest}})\;\mathrm{x}\;(\mathrm{Observed}\;\mathrm{SNP} - \mathrm{heritability})}}{{(\mathrm{Total}\;\# \;\mathrm{of}\;\mathrm{SNPs}\;\mathrm{used}\;\mathrm{to}\;\mathrm{estimate}\;\mathrm{Observed}\;\mathrm{SNP} - \mathrm{heritability})}}\end{array}$$

Total SNP-heritability estimates were obtained by pooling results across folds and meta-analyzing using a standard weighted fixed-effect model. Heritability estimates across UKB subsets were combined using fixed-effects inverse-variance meta-analysis implemented in R using the “rmeta” package.

### Gene-based associations

To investigate the overlap of individual genes associated with human tobacco consumption and animal paradigms of nicotine exposure, we compared our model organism genes with human findings from three gene-based tests. First, mixed linear model association analyses (MLMA) were performed in GCTA using the MLMA-leave one chromosome out approach (MLMA-LOCO)^16^. MLMA-LOCO analyses are powerful approaches to assess DNA associations with human traits (fixed effect) and assume a linear model while adjusting for population structure by estimating genomic relatedness matrices (random effect). Second, we used a conventional gene-based association approach: Multi-marker Analysis of GenoMic Annotation (MAGMA) via submitting GWAS summary statistics to Functional Mapping and Annotation (FUMA)^19^ of GWASs (using a 10 kb window to define a gene). Next, we used Hi-C coupled MAGMA (H-MAGMA)^20^, which investigates gene associations by encompassing regulatory SNPs based on chromatin interactions within a cell (*cis*-eQTLs and *trans*-eQTLs [long distance regulatory variants]). We collapsed findings across all cell data for H-MAGMA, including neuronal and astrocyte cell types as well as data from fetal and adult brains. Lastly, we used SMultiXcan^21^, which examines DNA to RNA relationships (*cis-*eQTLs) in specific tissues from GWAS summary statistics by training an elastic neural net algorithm on human donors from the Genotype-Tissue Expression database (GTEx)^22^. We elected to use 13 brain regions from GTEx and utilized multivariate regression from SMultiXcan to predict human *brain-related* gene expression associations with human cigarettes per day. Thus, H-MAGMA characterizes gene-based associations using specific cell-types and developmental stages whereas SMultiXcan reveals the direction of RNA expression in brain tissues for those at high genetic risk for tobacco consumption. All gene-based analyses utilized a Benjamini–Hochberg False Discovery Rate correction for multiple testing (*p*_adj_ < 0.05). To determine whether the overlap of gene-based associations and the model organism genes were more than we would expect by chance, we performed a Fisher’s Exact test—including the 20,809 homologous genes identified by biomaRt^21^.

Using two common negative controls from model organism research, we tested whether gene-based associations of human tobacco consumption were enriched for genes associated with mouse locomotor behavior^23^ and rat sucrose exposure^24^. Similar to our model organism geneset for nicotine exposure, these negative control studies examined RNA associations in similar brain regions and were also identified from GeneWeaver. Collapsing across locomotor behavior and sucrose exposure, our negative control geneset included 845 orthologous genes.

### Polygenic prediction of CPD

To investigate the reproducibility of our model organism geneset in humans, we created polygenic scores from the full UKB sample and tested its predictive utility in AddHealth. We created polygenic scores using the summary-based Best Linear Unbiased Prediction (SBLUP^25^ implemented in GCTA^16^ method, which improves prediction accuracy^26^. These analyses adjusted genetic effect sizes based on linkage disequilibrium patterns using the European reference sample from the 1000 Genomes Project^27^. To adjust for population stratification, our polygenic scores co-varied for the first six principal components extracted from the genetic data of the Add Health sample. Similar to our partitioned heritability approach, we investigated whether polygenic scores were associated with tobacco consumption (1) within-protein coding regions of our nicotine geneset, (2) in the surrounding regions (using a 10 kB surrounding region window to maximize signal to noise ratio as indicated by enrichment analyses in UKB), and (3) all other genomic variants. We also examined whether a PGS using all variants (*n*_SNPs_ = 4656938) was associated with CPD.

## Results

### Model organism nicotine genes

First, using GeneWeaver, we investigated our model organism genes and found that there was a small overlap across studies of the genes associated with nicotine exposure (Supplementary Table 1). For instance, some mouse and rat studies of nicotine use demonstrated significant overlap (Jaccard Similarity 0.01–0.02). Collapsing across the study, we found 21 replicated model organism genes (Supplementary Table 2). Subsequent analyses focused on the SNPs in and around the 786 orthologous model organism genes associated with nicotine exposure.

### Partitioned heritability—human tobacco consumption

Additive genetic factors accounted for 7.6% to 9.5% of the variability in CPD across all subsets of UKB participants (see Table 2). Less than five percent of the SNP-heritability of CPD could be attributed to SNPs within protein-coding regions of the model organism genes, whereas up to 37% of the heritability was observed in the surrounding genomic regions of these genes. The enrichment of heritability started to decline after expanding the region to include a 10kB window of the surrounding genomic regions (directly up/downstream) of the model organism genes. The remaining regions of the genome (i.e., All other variants) were not significantly enriched across models 1 through 6 (see Table 2), indicating that the SNPs in and around genes associated with nicotine exposure in various animal paradigms pointed to important genomic regions underlying human tobacco consumption.

**Table 2:** Partitioned SNP-heritability (*h*^2^_SNP_) of Human Tobacco Consumption with Model Organism Nicotine Genes.

Model components (buffer size)	Subset 1 (*n*_1_ = 41,263)			Subset 2 (*n*_2_ = 41,368)		Subset 3 (*n*_3_ = 41,213)		All (*n* = 123,844)
	# SNPs	*h*^2^_SNP_ (S.E.)	Enrichment	*h*^2^_SNP_ (S.E.)	Enrichment	*h*^2^_SNP_ (S.E.)	Enrichment	% total *h*^*2*^_SNP_
**Model 1 (0 kB)**								
Protein coding regions	81453	0.38% (0.16%)	2.90^c^	0.33% (0.16%)	2.38^c^	0.55% (0.18%)	3.32^c^	4.96%
Surrounding regions	NA	NA	NA	NA	NA	NA	NA	NA
All other variants	4575485	7.14% (0.78%)	0.97^c^	7.59% (0.78%)	0.98	8.85% (0.80%)	0.96^b^	94.92%
Total heritability	4656938	7.53% (0.79%)	NA	7.92% (0.78%)	NA	9.40% (0.81%)	NA	NA
**Model 2 (5 kB)**								
Protein coding regions	81453	0.22% (0.17%)	1.62^c^	0.15% (0.16%)	1.11	0.42% (0.19%)	2.54^c^	3.26%
Surrounding regions	10815	0.45% (0.18%)	25.20^c^	0.58% (0.19%)	31.45^c^	0.34% (0.19%)	15.68^c^	4.95%
All other variants	4564670	6.92% (0.79%)	0.93^c^	7.24% (0.78%)	0.93^c^	8.67% (0.81%)	0.94^c^	91.44%
Total heritability	4656938	7.53% (0.79%)	NA	7.97% (0.78%)	NA	9.42% (0.81%)	NA	NA
**Model 3 (10 kB)**								
Protein coding regions	81453	0.19% (0.17%)	1.27	0.09% (0.16%)	0.63^c^	0.29% (0.18%)	1.77^c^	2.16%
Surrounding regions	21288	0.82% (0.21%)	21.56^c^	0.90% (0.21%)	24.43^c^	0.77% (0.21%)	17.82^c^	9.84%
All other variants	4554197	6.61% (0.78%)	0.90^c^	7.04% (0.77%)	0.90^c^	8.41% (0.80%)	0.91^c^	88.00%
Total heritability	4656938	7.57% (0.78%)	NA	8.03% (0.78%)	NA	9.47% (0.81%)	NA	NA
**Model 4 (25 kB)**								
Protein coding regions	81453	0.12% (0.16%)	0.85	0.11% (0.16%)	0.76	0.28% (0.18%)	1.67^c^	1.92%
Surrounding regions	53341	1.03% (0.23%)	11.56^c^	0.96% (0.23%)	10.50^c^	1.02% (0.24%)	9.36^c^	12.36%
All other variants	4522144	6.36% (0.78%)	0.88^c^	6.93% (0.77%)	0.89^c^	8.18% (0.80%)	0.89^c^	85.71%
Total heritability	4656938	7.59% (0.78%)	NA	8.00% (0.78%)	NA	9.48% (0.81%)	NA	NA
**Model 5 (35 kB)**								
Protein coding regions	81453	0.13% (0.16%)	0.97	0.13% (0.16%)	0.94	0.30% (0.18%)	1.81^c^	2.16%
Surrounding regions	74436	1.04% (0.24%)	8.39^c^	0.95% (0.24%)	7.48^c^	1.05% (0.25%)	6.94^c^	12.50%
All other variants	4501049	6.32% (0.77%)	0.88^c^	6.90% (0.77%)	0.89^c^	8.12% (0.80%)	0.89^c^	85.22%
Total heritability	4656938	7.59% (0.78%)	NA	7.98% (0.78%)	NA	9.45% (0.81%)	NA	NA
**Model 6 (50 kB)**								
Protein coding regions	81453	0.29% (0.16%)	1.97^c^	0.26% (0.16%)	1.84^c^	0.50% (0.18%)	2.98^c^	4.17%
Surrounding regions	841092	3.22% (0.53%)	2.15^c^	3.30% (0.53%)	2.26^c^	2.74% (0.53%)	1.60^c^	36.47%
All other variants	3734393	4.23% (0.74%)	0.66^c^	4.53% (0.73%)	0.70^c^	6.24% (0.77%)	0.82^c^	59.12%
Total heritability	4656938	7.67% (0.79%)	NA	8.09% (0.78%)	NA	9.48% (0.81%)	NA	NA

This table shows the heritability of human tobacco consumption attributed to genes associated with nicotine exposure from various animal paradigms. We report the total heritability, the proportion of heritability within and around model organism genes as well as the degree of enrichment from our partitioned heritability analyses by subset. Note that we performed 6 total models with varying lengths of genomic regions surrounding the model organism genes. NA not applicable, SE standard error. Notations: ^a^*p* < 0.05, ^b^*p* < 0.01, ^c^*p* < 0.001.

### Nicotine/tobacco gene overlap across species

**Fig. 2:**
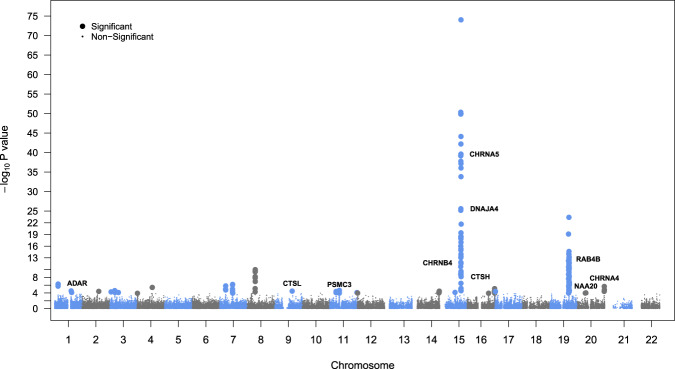
Plot of gene-based test results. Manhattan plot shows results of all three gene-based tests: MAGMA, H-MAGMA (neuron and astrocyte cell types; fetal and adult brain tissues), and S-MultiXcan (13 GTEx brain tissues) for human cigarettes per day. The labeled genes are those identified from our nicotine gene list derived from model organisms.

**Fig. 3:**
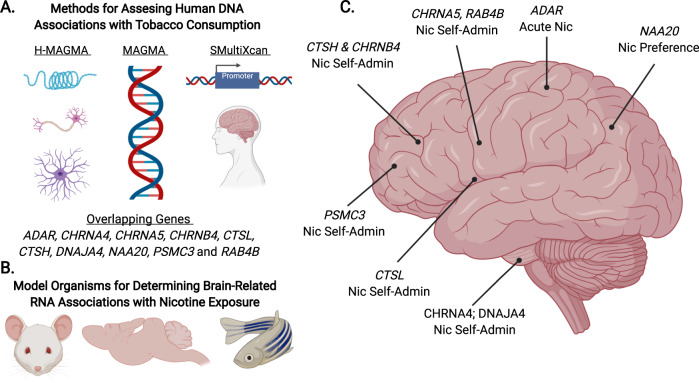
Potential functions of genes associated with nicotine consumption. This figure shows a schematic representation for interpreting our cross-species genetic associations with **A** Human tobacco consumption, **B** Model organism nicotine exposure and inferring their effects in the **C** Human brain. *Note:* Nic means nicotine; Self-Admin refers to self-administration.

We then investigated the overlap of individual genes from model organisms and the gene-based tests associated with human tobacco consumption. Collapsing across MAGMA, H-MAGMA, and SMultiXcan methods, we identified 115 unique genes (with annotated HGNC gene symbols) associated with human cigarettes per day (all *p*_adj_ < 0.05; see Fig. 2; see Supplementary Files 1–3 for gene-based test results). Ten genes were significant across all gene-based tests (*ADAMTS7, C19orf54, CHRNA3, CHRNA5, CYP2A7, GTF2I, HYKK, ITPKC, PSMA4*, and *SNRPA*). Of all human gene-based associations, we found ten genes that were present in our model organism geneset (*ADAR, CHRNA4, CHRNA5, CHRNB4, CTSL, CTSH, DNAJA4, NAA20, PSMC3*, and *RAB4B;* see Fig. 3 for summary). This overlap was more than expected by chance (OR = 2.44, *p* = 0.012, 95% CI [1.13, 4.70]) and no enrichment was observed among our negative control geneset (locomotor behavior and sucrose exposure; OR = 0.458, *p* = 0.450, 95% CI [0.055, 1.70]). Eight out of ten of the overlapping genes from our model organism nicotine exposure genes came from a single study^28^ that investigated chronic nicotine self-administration from five brain regions using two strains of mice. When restricting our gene-based analyses to just the model organism nicotine exposure genes, we found three additional gene-based associations with tobacco consumption (*NUP50, UCHL5, and SDC3*; see Supplementary Fig. 1). Post-hoc examination of the gene-based association signals (–log_10_ *p* values) indicated that model organism genes from *Mus musculus* studies performed better than *Rattus norvegicus*, studies (all *p*_adj_ < 0.001) and better than random genes (via permutations, *p*_adj_ < 0.001; see Supplementary Fig. 2). Collapsing across gene-based tests, the 21 replicated nicotine genes (across studies in model organisms) were not more significant than random (permuted) genes (*p*_adj_ = 0.244).

### Polygenic score analysis

Partitioned polygenic scores for CPD were derived using the GWAS summary statistics from the full UKB sample and predicted CPD in Add Health (see Supplementary Table 3). Restricting polygenic scores to genomic regions of the nicotine genes resulted in significant prediction of cigarettes per day in an independent sample (within the protein coding regions, but not in the 10 kb surrounding regions of these genes). These results further highlight the utility of incorporating model organism data in human genetic studies of substance use.

## Discussion

We found support for all three of our hypotheses. The genes associated with nicotine use in the brains of mice, fish, and rats (1) substantially contributed to the heritability of human tobacco consumption, (2) significantly overlapped with individual genes associated with this genetic predisposition, and (3) aided in polygenic score prediction of tobacco consumption in an independent sample. Our study applies a novel integrative framework for filling the translational space between human and animal genetics research. This line of research may enhance genomic discoveries, help interpret genetic associations with human traits and illuminate what, tissue, cell type and animal paradigm are best suited for genomic follow-up investigation.

Similar to previous research^29^, we found that up to a third of the heritability for the frequency of human tobacco use can be attributed to genomic regions stemming from RNA associations of specific nicotine behaviors in the brain (of model organisms). Our results suggest that the genetic proclivity to human tobacco use is mediated—in part—by RNA associations with voluntary nicotine use, nicotine preference and nicotine’s neuropharmacological properties (mostly) in the brain’s reward circuitry. Similarly, recent genome-wide research identified genome-wide significant loci in neurotransmission and reward learning genes for tobacco use and prioritized non-synonymous protein-coding variants^7^. By using approximately *half of the sample size* from Liu et al., (2019) our findings corroborated the importance of neurotransmission and reward-related genes underlying genetic susceptibility of tobacco consumption. Most cross-species findings appeared to be buried under the genomic significance threshold—demonstrating the strength of our partitioned heritability approach, which captures genes with small effect sizes peppered across the genome. Incorporating model systems allows for studies with small samples to be informative due to larger effect sizes and tighter experimental control and can be used to complement and contextualize human genetic findings.

Our study adds nuance to the genetic mechanisms underlying human tobacco consumption (see Fig. 3). We found cross-species associations with nicotine consumption with established nicotinic acetylcholine receptor genes (*CHRNA4/B4/A5*), as well as unconventional proteasome (*PSMC3*), heat-shock protein (*DNAJA4*), synaptic plasticity, and enzymatic genes (*ADAR, CTSH, CTSL, NAA20, RAB4B*). Most of these genes were contributing to molecular brain mechanisms of nicotine self-administration in C3H/HeJ and C57BL/6 J mice^28^, but *Adar* was associated with acute nicotine use in rats^30^ and *naa20* was linked with nicotine preference in fish^31^. As a whole, genes from animal behavioral paradigms that best aligned with the human trait demonstrated the strongest gene-based effect sizes in humans. Our analyses suggest that humans at high genetic risk for frequent cigarette smoking had increased RNA expression of *CTSH* and *CTSL* in cortical and limbic regions, respectively (see Supplementary File 1). Corroborating this, nicotine-consuming mice had increased RNA expression of *Ctsh* and *Ctsl* in the pre-frontal cortex and limbic reward regions (NAc and hippocampus), respectively. Similarly, elevated pre-frontal cortex *Psmc3* expression was associated with chronic nicotine exposure in mice^28^ and rats^32^ and was associated with human tobacco consumption via regulatory DNA variants in adult brain tissues and neuronal cell-types (see Supplementary File 2). The association of *DNAJA4* with human tobacco consumption was mediated via long-distance gene regulation in neuronal cell-types and demonstrated increased expression in the VTA^28^ and cortical neurons^33^ of nicotine-exposed mice. Therefore, our integrative approach contextualizes otherwise puzzling genetic associations with human traits and characterizes potential mechanisms in relation to specific behaviors, tissues, developmental epochs, and cell-types.

Cross-species polygenic prediction illustrated a novel application for model organism data to be integrated with human GWAS data. In contrast to our partitioned heritability approach, we found significant prediction within protein-coding regions of the model organism genes—instead of the surrounding (potentially regulatory) regions of these genes. This approach furthers the line of research incorporating biologically relevant information for polygenic score approaches^34,35^. While novel, these were *far* from becoming clinically relevant and were limited in their predictive capacity, but this approach offered a way to replicate a priori gene lists in an independent sample.

We urge the reader to interpret the current findings with caution. Human and animal data are very different—ranging from their environments, genetic backgrounds, developmental stages, routes-of-administration, and data types (e.g., DNA versus RNA associations). The animal data was limited to microarray studies and a restricted pallet of behaviors, one tissue type (brain tissue), few samples, and three species. We sought to overcome these limitations by integrating across brain regions, behaviors, and model organisms, but future studies are needed to determine whether these effects are invariant and to determine what paradigms are most relevant to what human traits—especially as the volume of literature increases. The majority of human gene-based associations were not in our model organism nicotine exposure geneset potentially suggesting limited availability of targets for experimental follow-up. But we showed the specificity of this overlap (via our negative control) and also highlighted certain brain regions, behavioral paradigms, and species to follow-up individual genes that were anchored in human biology. Our analyses did not evaluate an exhaustive list of ‘omics data types (methylomics, proteomics, metabolomics, CHiP-seq, ATAC-seq, etc.) and focused on effects in the brain without considering other relevant tissue types or cell types.

Future research is warranted to determine whether our integrative framework generalizes across complex human traits. Traits with different genetic architectures, epigenetic landscapes, and animal models may yield disparate findings. We found that the bulk of our cross-species signal stemmed from mouse models of nicotine use, but it will be important for future research to be conducted across multiple smoking phenotypes and include additional species and studies, as well as incorporate findings from human tissues to benchmark findings with other model organisms. Ideally, integrative genomics comparisons would leverage equitable and minimally error-prone outcomes or endophenotypes across studies. Given the array of animal models for human traits, an inviting avenue of research should clarify the utility of specific tissues, cell types, and animal models in human genetics. With a large enough literature base, we may be able to better refine what tissues and specific mechanisms human genomic signals stem from and ultimately may better characterize the genetic make-up for complex traits. Future studies leveraging these approaches should consider strategies for examining heterogeneity across tissues and cell types, as well as whether the observed effects generalize across human populations (e.g., European, African, Asian, etc). Cross-species genetic research is a fertile territory for methodological innovations. This field is still in its infancy and thus is a ripe area for future research applications.

## Conclusions

In sum, our study identifies biological overlap of nicotine use between human and animal research using integrative genomic models. Our study provides a proof-of-principle that model organism data can be used among standard methods used in human genetics research. Human researchers can take advantage of a rich array of model organism data to aid their interpretations with complex traits—even in small(er) GWASs—and animal researchers can assess the relevance of their findings to corresponding human traits. This study takes a step forward in cross-species research by incorporating a priori information into human genetics analyses and adds to the conversation regarding enhancing the utility of smaller GWASs. Our study suggests that cross-species genetics research is a worthwhile empirical avenue and that the intersection of human and animal biology can help unravel the genetic basis of complex traits.

## Supplementary information

Supplementary Methods

Supplementary Tables and Figures

Supplementary File 1. MAGMA Summary Statistics File

Supplementary File 2. SMULTIxCAN Summary Statistics File

Supplementary File 3. H-MAGMA Summary Statistics File

## References

[1] Maurano MT (2012). Systematic localization of common disease-associated variation in regulatory DNA. Science.

[2] Wu Y (2018). Integrative analysis of omics summary data reveals putative mechanisms underlying complex traits. Nat. Commun..

[3] Vandiedonck C (2018). Genetic association of molecular traits: a help to identify causative variants in complex diseases. Clin. Genet.

[4] Changeux, J.-P., Edelstein, S. & Edelstein, S. J. *Nicotinic Acetylcholine Receptors: From Molecular Biology To Cognition* (Odile Jacob Publishing Corp., 2005).

[5] Besson M (2007). Long-term effects of chronic nicotine exposure on brain nicotinic receptors. Proc. Natl Acad. Sci..

[6] Grenhoff J, Aston-Jones G, Svensson TH (1986). Nicotinic effects on the firing pattern of midbrain dopamine neurons. Acta Physiol. Scand..

[7] Liu M (2019). Association studies of up to 1.2 million individuals yield new insights into the genetic etiology of tobacco and alcohol use. Nat. Genet.

[8] Baker E, Bubier JA, Reynolds T, Langston MA, Chesler EJ (2016). GeneWeaver: data driven alignment of cross-species genomics in biology and disease. Nucleic Acids Res..

[9] Baker EJ, Jay JJ, Bubier JA, Langston MA, Chesler EJ (2012). GeneWeaver: a web-based system for integrative functional genomics. Nucleic Acids Res..

[10] Baker EJ (2009). Ontological discovery environment: A system for integrating gene-phenotype associations. Genomics.

[11] Sudlow C (2015). UK biobank: an open access resource for identifying the causes of a wide range of complex diseases of middle and old age. PLoS Med..

[12] Harris K. M., Udry J. R. *National Longitudinal Study of Adolescent to Adult Health (Add Health), 1994-2008 [Public Use]* (Carolina Population Center, University of North Carolina-Chapel Hill [distributor], Inter-university Consortium for Political and Social Research [distributor], 2018).

[13] 1000 Genomes Project Consortium. (2015). A global reference for human genetic variation. Nature.

[14] Brick LA, Keller MC, Knopik VS, McGeary JE, Palmer RHC (2019). Shared additive genetic variation for alcohol dependence among subjects of African and European ancestry. Addict. Biol..

[15] Chang CC (2015). Second-generation PLINK: rising to the challenge of larger and richer datasets. Gigascience.

[16] Yang J, Lee SH, Goddard ME, Visscher PM (2011). GCTA: a tool for genome-wide complex trait analysis. Am. J. Hum. Genet..

[17] Visscher PM (2014). Statistical power to detect genetic (co)variance of complex traits using SNP data in unrelated samples. PLoS Genet..

[18] Brick LA, Micalizzi L, Knopik VS, Palmer RHC (2019). Characterization of DSM-IV opioid dependence among individuals of European ancestry. J. Stud. Alcohol Drugs.

[19] Watanabe K, Taskesen E, van Bochoven A, Posthuma D (2017). Functional mapping and annotation of genetic associations with FUMA. Nat. Commun..

[20] Sey NYA (2020). A computational tool (H-MAGMA) for improved prediction of brain-disorder risk genes by incorporating brain chromatin interaction profiles. Nat. Neurosci..

[21] Barbeira AN (2018). Exploring the phenotypic consequences of tissue specific gene expression variation inferred from GWAS summary statistics. Nat.Commun..

[22] GTEx Consortium. (2015). Human genomics.The Genotype-Tissue Expression (GTEx) pilot analysis: multitissue gene regulation in humans. Science.

[23] Philip VM (2010). High-throughput behavioral phenotyping in the expanded panel of BXD recombinant inbred strains. Genes Brain Behav..

[24] Rodd ZA (2008). Differential gene expression in the nucleus accumbens with ethanol self-administration in inbred alcohol-preferring rats. Pharmacol. Biochem. Behav..

[25] Robinson MR (2017). Genetic evidence of assortative mating in humans. Nat. Hum. Behav..

[26] Mignogna KM, Bacanu SA, Riley BP, Wolen AR, Miles MF (2019). Cross-species alcohol dependence-associated gene networks: co-analysis of mouse brain gene expression and human genome-wide association data. PLoS ONE.

[27] Kranzler HR (2019). Genome-wide association study of alcohol consumption and use disorder in 274,424 individuals from multiple populations. Nat. Commun..

[28] Wang J (2008). Strain- and region-specific gene expression profiles in mouse brain in response to chronic nicotine treatment. Genes Brain Behav..

[29] Evans LM (2020). The Role of A Priori-Identified Addiction and Smoking Gene Sets in Smoking Behaviors. Nicotine Tob. Res..

[30] Polesskaya OO (2007). Nicotine causes age-dependent changes in gene expression in the adolescent female rat brain. Neurotoxicol. Teratol..

[31] Kily LJ (2008). Gene expression changes in a zebrafish model of drug dependency suggest conservation of neuro-adaptation pathways. J. Exp. Biol..

[32] Kane JK, Konu O, Ma JZ, Li MD (2004). Nicotine coregulates multiple pathways involved in protein modification/degradation in rat brain. Brain Res. Mol. Brain Res..

[33] Wang J (2007). Regulation of platelet-derived growth factor signaling pathway by ethanol, nicotine, or both in mouse cortical neurons. Alcohol. Clin. Exp. Res..

[34] Neuner SM, Heuer SE, Huentelman MJ, O’Connell KMS, Kaczorowski CC (2019). Harnessing genetic complexity to enhance translatability of Alzheimer’s disease mouse models: a path toward precision medicine. Neuron.

[35] Hari Dass SA (2019). A biologically-informed polygenic score identifies endophenotypes and clinical conditions associated with the insulin receptor function on specific brain regions. EBioMedicine.

