# Supplementary material for: Multi-omic and multi-species meta-analyses of nicotine consumption
Source: Transl Psychiatry. 2021 Feb 4;11:98. doi: 10.1038/s41398-021-01231-y (PMC7862377; doi:10.1038/s41398-021-01231-y)
Supplement: Supplementary file 1 — Supplementary Methods [file 41398_2021_1231_MOESM1_ESM.docx]

**Supplementary Material**

**Methods**

*Building an a priori network of genes co-transcriptionally regulated by nicotine*

We first identified studies by literature review or by shared summary statistics archived in the GeneWeaver system. Experimental studies were included if they provided differential expression or whole genome co-expression network analyses along with accessible summary statistics. Literature searches focused on exposure studies utilizing nicotine-specific model organism paradigms, including subcutaneous nicotine treatment, intravenous self-administration (IVSA), nicotine delivered to the animal’s drinking water, and nicotine-induced conditioned place preference (Supplementary Table 1; no studies involving *Drosophila melanogaster* were identified likely because nicotine is a natural insecticide). Priority was given to weighted gene co-expression network analysis (WGCNA) studies to minimize inflation of the Type I error rate typically seen in QTL studies. Next, we merged studies with multiple reported gene sets (i.e., either by region, up/down regulated, or across time) to avoid inflating the replication threshold of individual genes. We then identified orthologous genes using GeneWeaver’s “Combine Gene sets” function which merges multiple gene sets into a single matrix while accounting for orthology across species; none of the identified studies were conducted in human samples.^1^ We verified orthologous genes identified from GeneWeaver by specifying ‘hsapients_homolog_associated_gene_name’ using biomaRt. Lastly, identified gene sets were compared to the current human genome build (hg19) to localize relevant variants that were conserved across species.

*Gene Set Extraction Protocol using GeneWeaver*

Once a list of outputs were generated from a search, the abstract of each gene set would be read in order to determine if the study followed a pre-set inclusion criteria. The inclusion criteria are as followed: (1) the study had to utilize a phenotype of nicotine consumption / exposure as its dependent variable, (2) the gene list extracted from the study had to have been the direct result of experimentation, (3) the study examined RNA associations with nicotine exposure (e.g., microarray, differential expression or WGCNA).

*Data-Screening and Gene list Extraction*

Once data-mining was completed, studies were double-checked to make sure they met inclusion criteria. For every gene set, its PubMed ID (provided in the gene set description) was used to download its publication, which was then read over in full to make sure the study design represented a dependent variable that would be relevant for subsequent analysis. Once passing this screening process, gene sets were then combined if they all belonged to the same study, only differing in neural region or time. This was done utilizing GeneWeaver’s “Boolean gene set logic” tool, which takes several different gene sets and outputs a single gene set containing the union, intersection, or highest-replicated genes through all the studies. In the case of this study, gene sets that belonged to the same publication were submitted through the tool’s “Union” function, a tool which allows a single export of all genes present within all inputted gene sets. “Homology” (a feature provided by GeneWeaver to prevent duplicating genes that differentiate only by species) selected to be “On”^1^. Upon the completion of data-screening, the “Boolean gene set tool” was used to create a final gene list that would be used for analysis.

*Protocol for mapping SNPs in the partitioned heritability analysis*

Sets of SNPs were identified for each model component starting with variants in and around the genes of interest. In addition to investigating gene variants in and around our model organism genes, we examined the effects of all unselected variants (referred to as “other variants”), which belonged to genomic regions that were not within the model organism protein-coding genes nor the surrounding genomic regions.

**REFERENCES**

1. Baker EJ, Jay JJ, Bubier JA, Langston MA, Chesler EJ. GeneWeaver: a web-based system for integrative functional genomics. *Nucleic acids research* 2012; **40**(Database issue)**:** D1067-1076.

2. Visscher PM, Hemani G, Vinkhuyzen AA, Chen GB, Lee SH, Wray NR *et al.* Statistical power to detect genetic (co)variance of complex traits using SNP data in unrelated samples. *PLoS Genet* 2014; **10**(4)**:** e1004269.

3. Liu M, Jiang Y, Wedow R, Li Y, Brazel DM, Chen F *et al.* Association studies of up to 1.2 million individuals yield new insights into the genetic etiology of tobacco and alcohol use. *Nat Genet* 2019; **51**(2)**:** 237-244.
