# Supplementary material for: Multi-omic and multi-species meta-analyses of nicotine consumption
Source: Transl Psychiatry. 2021 Feb 4;11:98. doi: 10.1038/s41398-021-01231-y (PMC7862377; doi:10.1038/s41398-021-01231-y)
Supplement: Supplementary file 2 — Supplementary Tables and Figures [file 41398_2021_1231_MOESM2_ESM.pdf]

Supplementary Table 1. Jaccard Similarity Estimates Across Identified gene sets

Gene set	Jaccard Similarity Estimate				
Merged Kily et al.[30]	1				
Merged Wang et al.[29]	6.00E-03	1			
Chen et al.[27]	0.00E+00	**1.59E-02**	1		
Sharp et al.[31]	3.30E-03	3.50E-03	5.40E-03	1	
Polesskaya et al.[28]	5.50E-03	**8.80E-03**	**8.10E-03**	0.00E+00	1

Note: Gene sets shown do not exactly align with the full complement of GeneWeaver set IDs shown in Table 1 as several sets (within studies) were merged to reduce overrepresentation of genes in a given study. Bolded values indicate $p < 0.05$.

Supplementary Table 2. Replicated Genes Across Gene Weaver Studies

GENE_NAME	STUDY_1	SPECIES_1	BEHAVIOR_1	BRAIN_REGION_1	STUDY_2	SPECIES_2	BEHAVIOR_2	BRAIN_REGION_2
ACTB	Wang	mouse	Nic_Self-Admin	Amyg_Hipp_nAcc_PFC_VTA	Chen	mouse	Acute_Nic	VTA
ALDH7A1	Sharp	rat	Chronic_Nic_Self-Admin	nAcc	Kily	fish	Nic_CPP	Whole_Brain
CCT3	Wang	mouse	Nic_Self-Admin	Amyg_Hipp_nAcc_PFC_VTA	Polesskaya	rat	Chronic_Nic_Treatment	PFC_vStriatum_Hipp
DSTN	Wang	mouse	Nic_Self-Admin	Amyg_Hipp_nAcc_PFC_VTA	Sharp	rat	Chronic_Nic_Self-Admin	nAcc
GABBR1	Wang	mouse	Nic_Self-Admin	Amyg_Hipp_nAcc_PFC_VTA	Chen	mouse	Acute_Nic	VTA
ITPR2	Wang	mouse	Nic_Self-Admin	Amyg_Hipp_nAcc_PFC_VTA	Chen	mouse	Acute_Nic	VTA
KCNK1	Wang	mouse	Nic_Self-Admin	Amyg_Hipp_nAcc_PFC_VTA	Polesskaya	rat	Chronic_Nic_Treatment	PFC_vStriatum_Hipp
KRT5	Kily	fish	Nic_CPP	Whole_Brain	Polesskaya	rat	Chronic_Nic_Treatment	PFC_vStriatum_Hipp
MOBP	Wang	mouse	Nic_Self-Admin	Amyg_Hipp_nAcc_PFC_VTA	Chen	mouse	Acute_Nic	VTA
PPM1B	Kily	fish	Nic_CPP	Whole_Brain	Chen	mouse	Acute_Nic	VTA
PRKACB	Chen	mouse	Acute_Nic	VTA	Polesskaya	rat	Chronic_Nic_Treatment	PFC_vStriatum_Hipp
PSEN1	Wang	mouse	Nic_Self-Admin	Amyg_Hipp_nAcc_PFC_VTA	Polesskaya	rat	Chronic_Nic_Treatment	PFC_vStriatum_Hipp
PSMB5	Wang	mouse	Nic_Self-Admin	Amyg_Hipp_nAcc_PFC_VTA	Chen	mouse	Acute_Nic	VTA
PSMD7	Wang	mouse	Nic_Self-Admin	Amyg_Hipp_nAcc_PFC_VTA	Sharp	rat	Chronic_Nic_Self-Admin	nAcc

RAD21	Sharp	rat	Chronic_Nic_Self-Admin	nAcc	Chen	mouse	Acute_Nic	VTA
RPS28	Wang	mouse	Nic_Self-Admin	Amyg_Hipp_nAcc_PFC_VTA	Sharp	rat	Chronic_Nic_Self-Admin	nAcc
SST	Wang	mouse	Nic_Self-Admin	Amyg_Hipp_nAcc_PFC_VTA	Chen	mouse	Acute_Nic	VTA
SYT4	Wang	mouse	Nic_Self-Admin	Amyg_Hipp_nAcc_PFC_VTA	Chen	mouse	Acute_Nic	VTA
TFDP1	Wang	mouse	Nic_Self-Admin	Amyg_Hipp_nAcc_PFC_VTA	Kily	fish	Nic_CPP	Whole_Brain
UCHL1	Wang	mouse	Nic_Self-Admin	Amyg_Hipp_nAcc_PFC_VTA	Polesskaya	rat	Chronic_Nic_Treatment	PFC_vStriatum_Hipp
YWHAG	Wang	mouse	Nic_Self-Admin	Amyg_Hipp_nAcc_PFC_VTA	Chen	mouse	Acute_Nic	VTA

Table showing the two studies (study 1 and study 2) in GeneWeaver for which a gene was observed to be differentially expressed across two brain tissues (brain region 1/2) for varying smoking/nicotine exposure experimental paradigms.

Supplementary Figure 1 Candidate Gene-Based Tests for Human Tobacco Consumption

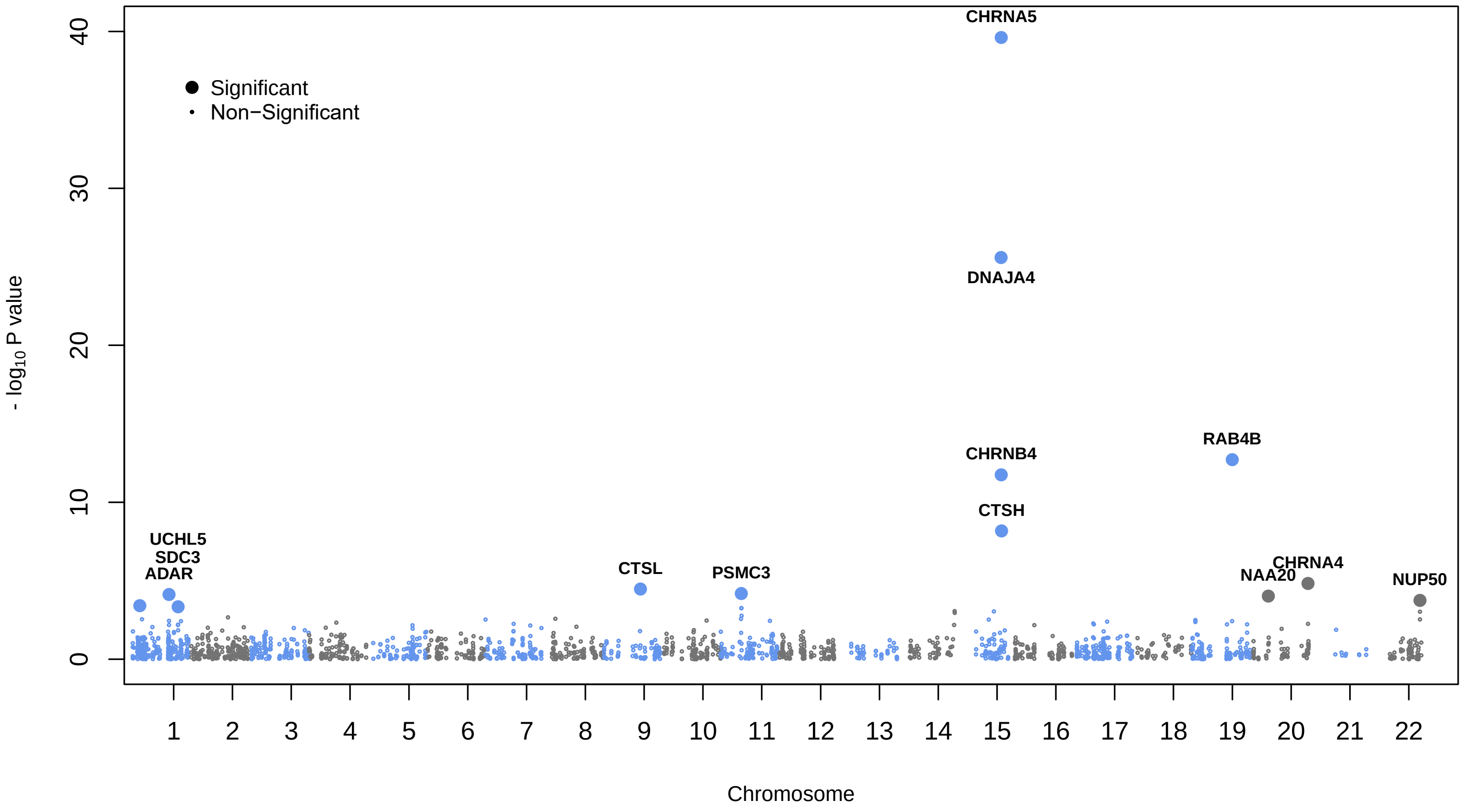

This figure shows the results of the associations of the model organisms nicotine genes via three gene-based tests (MAGMA, H-MAGMA, MultiXcan). Significant genes were required to survive a false discovery rate < 0.05 (padj < 0.05).

Supplementary Figure 2 Genetic Signal of Human Tobacco Consumption from (Model Organisms) Nicotine Genes

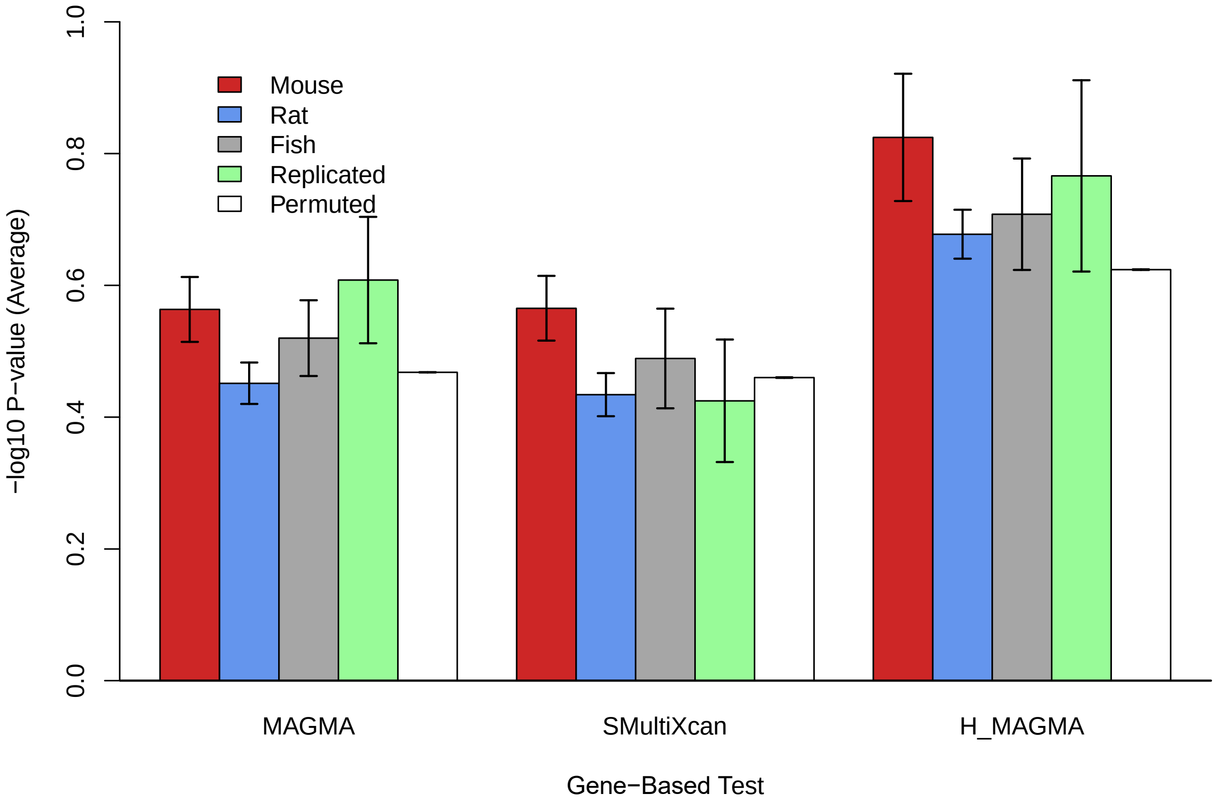

Note: we performed a one-way analysis of variance (ANOVA) to test whether differences existed between species, replicated genes (21 genes associated with nicotine exposure across studies) and a random list of permuted genes (permuted 500 non-model organism genes 10,000 times). Our ANOVA suggested that genes from mouse studies were more associated with human tobacco consumption than rat and fish studies and also than random (permuted) genes ($p < 0.05$).

Supplementary Table 3 Model Organism Based Polygenic Score Predicting Human Tobacco Consumption

Polygenic Score Predicting Cigarettes Per Day in European Ancestry Add Health Participants					
	**β**	**S.E.**	**Z Score**	***P*-value**	**N Variants**
Model Organism Genes (Protein Coding Regions)	0.079	0.027	2.884	0.004*	81453
Model Organism Genes (Surrounding Regions 10 kB)	-0.023	0.028	-0.840	0.401	21288
All Other Variants	0.164	0.025	6.669	< 0.001*	4554197
All Variants	0.174	0.024	7.725	< 0.001*	4656938

Note we fit two polygenic risk score models - one consisting of all variants and one model with multiple components (protein coding genes, surrounding regions, all other variants). Each model controlled for demographics and six principle components.

* $p < .05$
