# Supplementary material for: Multi-omic and multi-species meta-analyses of nicotine consumption
Source: Transl Psychiatry. 2021 Feb 4;11:98. doi: 10.1038/s41398-021-01231-y (PMC7862377; doi:10.1038/s41398-021-01231-y)
Supplement: Supplementary file 3 — Supplementary File 1. MAGMA Summary Statistics File [file 41398_2021_1231_MOESM3_ESM.pdf]

MAGMA Gene Based Test of Human Cigarettes per Day

SYMBOL	GENE	CHR	START	STOP	NSNPS	NPARAM	N	ZSTAT	P	Padj
HYKK	ENSG00000188266	15	78789906	78839714	41	4	123834	7.9347	1.05E-15	2.00E-11
EGLN2	ENSG00000269858	19	41294901	41324338	50	10	123834	7.8387	2.28E-15	2.17E-11
CHRNA3	ENSG00000080644	15	78875394	78923637	71	6	123834	7.6226	1.24E-14	7.86E-11
IREB2	ENSG00000136381	15	78719773	78803798	115	8	123834	7.3726	8.37E-14	3.09E-10
CTC-490E21.12	ENSG00000268797	19	41297202	41414187	249	29	123834	7.3716	8.43E-14	3.09E-10
RAB4B-EGLN2	ENSG00000171570	19	41274147	41324103	94	13	123834	7.3521	9.75E-14	3.09E-10
RAB4B	ENSG00000167578	19	41274121	41312847	83	12	123834	7.2595	1.94E-13	5.27E-10
CHRNB4	ENSG00000117971	15	78906461	79030096	168	16	123834	6.954	1.78E-12	4.23E-09
ADAMTS7	ENSG00000136378	15	79041545	79113773	124	10	123834	6.8971	2.65E-12	5.60E-09
MIA-RAB4B	ENSG00000268975	19	41267553	41302590	77	10	123834	6.4346	6.19E-11	1.18E-07
PSMA4	ENSG00000041357	15	78822747	78851604	42	2	123834	6.1094	5.00E-10	7.31E-07
CHRNA5	ENSG00000169684	15	78847862	78897611	61	6	123834	6.1094	5.00E-10	7.31E-07
CYP2A6	ENSG00000255974	19	41339443	41366352	53	10	123834	6.1094	5.00E-10	7.31E-07
AC027228.1	ENSG00000268838	15	78820023	78841288	28	2	123834	5.9807	1.11E-09	1.51E-06
MORF4L1	ENSG00000185787	15	79092829	79200475	254	11	123834	5.8091	3.14E-09	3.98E-06
CHRNA6	ENSG00000147434	8	42597763	42661535	71	6	123834	5.6153	9.81E-09	1.17E-05
MIA	ENSG00000261857	19	41267553	41293395	51	7	123834	5.2572	7.31E-08	8.17E-05
C19orf54	ENSG00000188493	19	41236761	41267458	34	6	123834	5.1839	1.09E-07	0.000115104
SNRPA	ENSG00000077312	19	41246542	41281294	43	7	123834	5.1451	1.34E-07	0.000134056
BAI2	ENSG00000121753	1	32182718	32240494	25	4	123834	4.8857	5.15E-07	0.000489456
NUMBL	ENSG00000105245	19	41162596	41206877	65	11	123834	4.7245	1.15E-06	0.001040914
CXXC4	ENSG00000168772	4	105379469	105426058	16	3	123834	4.4795	3.74E-06	0.00323136
LTBP4	ENSG00000090006	19	41088789	41145725	76	8	123834	4.4152	5.05E-06	0.00408672
CYP2A7	ENSG00000198077	19	41371344	41398657	91	7	123834	4.4105	5.16E-06	0.00408672
CHRNB3	ENSG00000147432	8	42542519	42602550	145	6	123834	4.326	7.59E-06	0.005770829
CHRNA4	ENSG00000101204	20	61965420	62019753	136	27	123834	4.1757	1.49E-05	0.010893046
ITPKC	ENSG00000086544	19	41213008	41256765	68	9	123834	4.1494	1.67E-05	0.0117568
AL049840.1	ENSG00000224997	14	104167607	104189149	18	5	123834	3.9803	3.44E-05	0.023268414
ADCK4	ENSG00000123815	19	41187434	41234112	71	10	123834	3.9727	3.55E-05	0.023268414
XRCC3	ENSG00000126215	14	104153946	104191841	42	8	123834	3.83	6.41E-05	0.04061376
GTF2I	ENSG00000077809	7	74061994	74185026	82	9	123834	3.8177	6.74E-05	0.041327071
CA14	ENSG00000118298	1	150219554	150247478	2	1	123834	3.714	0.00010198	0.06057612
APH1A	ENSG00000117362	1	150227804	150251980	4	1	123834	3.6752	0.00011881	0.06843456
ZNF280D	ENSG00000137871	15	56912379	57220769	379	16	123834	3.6336	0.00013976	0.077335406
CLU	ENSG00000120885	8	27444434	27482548	39	8	123834	3.6288	0.0001424	0.077335406
MFSD10	ENSG00000109736	4	2922288	2946586	23	7	123834	3.618	0.00014846	0.077406425
MYD88	ENSG00000172936	3	38169969	38194513	21	3	123834	3.6112	0.00015238	0.077406425
CHMP1A	ENSG00000131165	16	89700839	89734253	76	9	123834	3.6063	0.00015529	0.077406425
C1orf54	ENSG00000118292	1	150230600	150263327	7	1	123834	3.6005	0.00015882	0.077406425
BNC2	ENSG00000173068	9	16399501	16880841	773	49	123834	3.5748	0.00017523	0.082647711
ADD1	ENSG00000087274	4	2835584	2941803	63	9	123834	3.5703	0.00017827	0.082647711
ZBTB7A	ENSG00000178951	19	4034362	4076943	45	8	123834	3.5606	0.00018504	0.083652882
RAPSN	ENSG00000165917	11	47449308	47480730	72	7	123834	3.5546	0.00018924	0.083652882
SPATA33	ENSG00000167523	16	89714210	89747680	71	9	123834	3.5228	0.00021352	0.09224064
AC015688.3	ENSG00000266728	17	25940390	25977803	10	4	123834	3.4828	0.00024813	0.104810112
C16orf3	ENSG00000221819	16	90085316	90106309	40	8	123834	3.4569	0.00027318	0.112882727
SHKBP1	ENSG00000160410	19	41072757	41107305	35	9	123834	3.4174	0.00031612	0.127846999
AK5	ENSG00000154027	1	77737736	78035651	748	64	123834	3.3875	0.0003527	0.136834325
ZFYVE21	ENSG00000100711	14	104172067	104210005	21	6	123834	3.3874	0.00035274	0.136834325
DHX40	ENSG00000108406	17	57632886	57695706	32	3	123834	3.3638	0.00038436	0.146118298
KB-1980E6.3	ENSG00000253633	8	103530968	103560896	94	15	123834	3.3125	0.00046227	0.16409088
SPATA2L	ENSG00000158792	16	89752751	89778113	26	6	123834	3.3096	0.0004671	0.16409088
ACAA1	ENSG00000060971	3	38134620	38188733	38	7	123834	3.3088	0.00046848	0.16409088
AC016586.1	ENSG00000205147	19	4031127	4053154	29	4	123834	3.3086	0.00046885	0.16409088
DPEP1	ENSG00000015413	16	89669716	89714839	56	12	123834	3.3051	0.0004748	0.16409088
SLC39A14	ENSG00000104635	8	22214762	22301642	194	13	123834	3.2838	0.00051217	0.173845131
PSMC3	ENSG00000165916	11	47430320	47457993	65	6	123834	3.2578	0.00056141	0.187215461
10-Mar	ENSG00000173838	17	60768675	60895705	154	19	123834	3.2521	0.00057286	0.18774005
SLC22A14	ENSG00000144671	3	38313785	38370066	144	6	123834	3.2386	0.00060065	0.193155004
NPSR1	ENSG00000187258	7	34687851	34927944	559	32	123834	3.2317	0.00061532	0.193155004
TRMT61A	ENSG00000166166	14	103985521	104013410	12	3	123834	3.228	0.00062321	0.193155004
SALL1	ENSG00000103449	16	51159886	51195278	19	4	123834	3.2249	0.00063003	0.193155004
POM121C	ENSG00000135213	7	75036066	75125548	97	15	123834	3.2145	0.00065344	0.197152183
DCDC1	ENSG00000170959	11	30841916	31401357	449	17	123834	3.1554	0.00080138	0.23800986
ZACN	ENSG00000186919	17	74058042	74089747	29	2	123834	3.1489	0.00081939	0.23925888
MAP2K6	ENSG00000108984	17	67400839	67549472	108	18	123834	3.1449	0.00083076	0.23925888
DBNDD1	ENSG00000003249	16	90061273	90096536	70	11	123834	3.1221	0.00089779	0.254704363
WWP2	ENSG00000198373	16	69786209	69985644	346	18	123834	3.1123	0.00092802	0.259408885
NUP50	ENSG00000093000	22	45549722	45593896	97	7	123834	3.108	0.00094181	0.259448181
FNDC5	ENSG00000160097	1	33317869	33348083	24	5	123834	3.0924	0.00099265	0.268356
EPHA3	ENSG00000044524	3	89146674	89541284	480	21	123834	3.0894	0.0010027	0.268356
NOP14	ENSG00000087269	4	2929660	2975112	61	7	123834	3.0854	0.0010165	0.268356
GRK4	ENSG00000125388	4	2955335	3052474	220	15	123834	3.0672	0.0010804	0.2813184
GAS8	ENSG00000141013	16	90076037	90121383	86	12	123834	3.0628	0.0010963	0.281600951
CKB	ENSG00000166165	14	103975996	103999448	24	4	123834	3.0562	0.0011208	0.284055552
USP43	ENSG00000154914	17	9538015	9643008	111	14	123834	3.0386	0.0011883	0.297200084
CYP2B6	ENSG00000197408	19	41487204	41534303	114	11	123834	3.032	0.0012145	0.299808
C1orf51	ENSG00000159208	1	150244953	150269505	18	4	123834	3.0197	0.001265	0.308270769
SETD1A	ENSG00000099381	16	30958615	31006437	31	3	123834	3.0046	0.0013298	0.31983336
LGALS9	ENSG00000168961	17	25946824	25986586	26	4	123834	3.0009	0.0013461	0.31983336
EXOC7	ENSG00000182473	17	74067087	74127657	66	5	123834	2.964	0.0015181	0.351761143
S100PBP	ENSG00000116497	1	33272368	33334476	62	7	123834	2.9629	0.0015238	0.351761143
ING2	ENSG00000168556	4	184416147	184442249	45	9	123834	2.9596	0.0015403	0.351761143
PLCH1	ENSG00000114805	3	155083369	155472856	691	26	123834	2.9568	0.0015545	0.351761143
ZNF521	ENSG00000198795	18	22631890	22942154	389	50	123834	2.9412	0.0016347	0.365557384
HSD3B7	ENSG00000099377	16	30986519	31010473	10	2	123834	2.9275	0.0017087	0.37332149
AC135048.1	ENSG00000268863	16	30986808	31007533	10	2	123834	2.9275	0.0017087	0.37332149
CELF1	ENSG00000149187	11	47477496	47597121	90	12	123834	2.9217	0.0017405	0.375948
SYNDIG1L	ENSG00000183379	14	74862596	74902805	48	9	123834	2.917	0.001767	0.377383551
SPP1	ENSG00000118785	4	88886819	88914562	26	4	123834	2.9091	0.0018124	0.377494748
CHDH	ENSG00000016391	3	53836362	53890417	72	17	123834	2.9073	0.0018231	0.377494748
TCF4	ENSG00000196628	18	52879562	53342018	441	37	123834	2.9066	0.0018271	0.377494748
NT5DC2	ENSG00000168268	3	52548386	52579070	43	6	123834	2.9018	0.0018553	0.379199381
OXSR1	ENSG00000172939	3	38196580	38306979	81	7	123834	2.8963	0.0018879	0.381757481
CCNT2	ENSG00000082258	2	135665805	135726912	89	8	123834	2.8906	0.0019226	0.384681903
DIO1	ENSG00000211452	1	54346912	54386759	113	11	123834	2.885	0.0019568	0.385137353

ORAI3	ENSG00000175938	16	30950387	30977782	13	3	123834	2.8837	0.0019654	0.385137353
ADTRP	ENSG00000111863	6	11702287	11817279	190	27	123834	2.8771	0.002007	0.389276082
CDK10	ENSG00000185324	16	89737145	89772772	45	6	123834	2.8718	0.0020409	0.3918528
TRIB3	ENSG00000101255	20	351261	388203	24	7	123834	2.8654	0.0020824	0.395822592
TAL2	ENSG00000186051	9	108414738	108435367	25	4	123834	2.8602	0.002117	0.396831237
AC005493.1	ENSG00000214841	7	34675709	34709772	111	14	123834	2.8578	0.0021332	0.396831237
NFIB	ENSG00000147862	9	14071842	14408982	168	44	123834	2.8502	0.0021844	0.396831237
FBXL19	ENSG00000099364	16	30924376	30970104	37	4	123834	2.8495	0.0021894	0.396831237
KLC1	ENSG00000126214	14	104018233	104177888	255	13	123834	2.8486	0.0021956	0.396831237
STAB1	ENSG00000010327	3	52519354	52568511	46	7	123834	2.8448	0.0022223	0.396831237
NKX2-1	ENSG00000136352	14	36975602	37000354	19	4	123834	2.8408	0.0022498	0.396831237
RP11-368I7.4	ENSG00000260259	16	89739028	89762977	42	5	123834	2.8399	0.0022565	0.396831237
NALCN	ENSG00000102452	13	101696130	102078843	752	58	123834	2.8372	0.0022756	0.396831237
C4orf6	ENSG00000082929	4	5516296	5539528	39	4	123834	2.831	0.0023203	0.40094784
SOD1	ENSG00000142168	21	33021935	33051244	36	6	123834	2.8232	0.002377	0.407045189
ANP32E	ENSG00000143401	1	150180717	150218504	14	2	123834	2.8134	0.0024507	0.4159188
COA7	ENSG00000162377	1	53142508	53174038	30	4	123834	2.8105	0.0024733	0.416039703
YIPF1	ENSG00000058799	1	54307392	54366407	153	10	123834	2.7979	0.0025721	0.428863832
FAM153A	ENSG00000170074	5	177124982	177220399	16	2	123834	2.7916	0.0026227	0.431678234
DLK2	ENSG00000171462	6	43408090	43434370	20	5	123834	2.7901	0.0026344	0.431678234
USP45	ENSG00000123552	6	99870190	99979604	132	15	123834	2.7872	0.0026587	0.431936492
XPO6	ENSG00000169180	16	28099300	28233241	89	3	123834	2.7728	0.0027789	0.4476384
TOX2	ENSG00000124191	20	42533504	42708256	242	35	123834	2.7653	0.0028437	0.454227308
SYNRG	ENSG00000006114	17	35864900	35979544	86	12	123834	2.7601	0.0028894	0.45768096
NTNG2	ENSG00000196358	9	135027334	135129921	52	10	123834	2.7471	0.0030063	0.467797698
FOXO6	ENSG00000204060	1	41817594	41859262	33	11	123834	2.7469	0.0030081	0.467797698
GSTA4	ENSG00000170899	6	52832751	52870176	103	12	123834	2.7448	0.0030271	0.467797698
YARS	ENSG00000134684	1	33230840	33293754	83	8	123834	2.7396	0.0030757	0.471475045
PTMS	ENSG00000159335	12	6864682	6890116	18	3	123834	2.7335	0.0031329	0.472370902
DGKG	ENSG00000058866	3	185813457	186090026	206	43	123834	2.7321	0.0031463	0.472370902
SARDH	ENSG00000123453	9	136518682	136615077	97	22	123834	2.7296	0.0031708	0.472370902
SIRT6	ENSG00000077463	19	4164106	4192601	22	5	123834	2.7274	0.0031914	0.472370902
ADIPOR2	ENSG00000006831	12	1787740	1907844	172	17	123834	2.726	0.0032058	0.472370902
GNAT3	ENSG00000214415	7	80077987	80151336	96	11	123834	2.7137	0.0033266	0.483949484
RP11-73M18.2	ENSG00000256500	14	104019299	104162261	234	17	123834	2.7129	0.0033353	0.483949484
A3GALT2	ENSG00000184389	1	33762367	33796699	60	9	123834	2.7075	0.0033892	0.4880448
PTPMT1	ENSG00000110536	11	47576982	47605013	20	6	123834	2.6979	0.0034891	0.491579234
STX1B	ENSG00000099365	16	30990577	31031949	18	3	123834	2.6945	0.0035243	0.491579234
NCAM1	ENSG00000149294	11	112821997	113159158	640	28	123834	2.6906	0.0035667	0.491579234
KBTBD4	ENSG00000231880	11	47589277	47609823	20	7	123834	2.6881	0.0035934	0.491579234
TIMP4	ENSG00000157150	3	12184551	12210851	58	10	123834	2.6865	0.0036105	0.491579234
DNAJC24	ENSG00000170946	11	31381387	31463396	83	7	123834	2.6856	0.0036201	0.491579234
TRAF7	ENSG00000131653	16	2195699	2238130	12	2	123834	2.6855	0.0036209	0.491579234
ERAL1	ENSG00000132591	17	27171956	27198085	29	3	123834	2.6852	0.0036241	0.491579234
EBLN2	ENSG00000255423	3	73100810	73122488	35	5	123834	2.6832	0.0036465	0.491579234
BAG5	ENSG00000166170	14	104012881	104039168	21	3	123834	2.6698	0.0037951	0.508008879
TSEN15	ENSG00000198860	1	184010811	184053346	23	4	123834	2.6658	0.0038405	0.510491077
RIF1	ENSG00000080345	2	152256397	152374527	180	16	123834	2.6624	0.0038796	0.5121072
DUSP14	ENSG00000161326	17	35839937	35883603	65	9	123834	2.655	0.003966	0.519901572
AFF1	ENSG00000172493	4	87846154	88072206	336	22	123834	2.6452	0.0040818	0.524518744
SEZ6	ENSG00000063015	17	27271919	27343458	40	5	123834	2.6437	0.0041005	0.524518744
FAM53C	ENSG00000120709	5	137657624	137695416	62	8	123834	2.6428	0.0041108	0.524518744
SCAMP1	ENSG00000085365	5	77646339	77786562	306	11	123834	2.6428	0.0041116	0.524518744
PPP4R2	ENSG00000163605	3	73035936	73128350	207	8	123834	2.6366	0.0041866	0.530525952
PNISR	ENSG00000132424	6	99835927	99883207	86	9	123834	2.6324	0.0042389	0.531056188
FAM222B	ENSG00000173065	17	27072996	27192250	141	5	123834	2.6316	0.004249	0.531056188
SP6	ENSG00000189120	17	45912279	45943240	24	8	123834	2.6296	0.0042746	0.531056188
NDUFAF7	ENSG00000003509	2	37448774	37490546	87	13	123834	2.6081	0.0045528	0.552258199
CCNE1	ENSG00000105173	19	30292805	30325215	35	6	123834	2.6064	0.0045749	0.552258199
MARK3	ENSG00000075413	14	103841729	103980168	267	8	123834	2.605	0.0045932	0.552258199
SCAF4	ENSG00000156304	21	33033346	33114388	112	9	123834	2.6015	0.0046412	0.552258199
CCDC64	ENSG00000135127	12	120417673	120542298	186	12	123834	2.6001	0.0046601	0.552258199
HTR3D	ENSG00000186090	3	183739332	183767157	64	11	123834	2.599	0.0046752	0.552258199
PPP1R13B	ENSG00000088808	14	104190089	104323927	195	7	123834	2.5985	0.0046819	0.552258199
FAM159A	ENSG00000182183	1	53089016	53145355	61	11	123834	2.5958	0.0047183	0.552258199
SORBS1	ENSG00000095637	10	97061528	97331171	507	48	123834	2.5948	0.0047328	0.552258199
TMEM71	ENSG00000165071	8	133687253	133782958	200	15	123834	2.5945	0.0047358	0.552258199
MRPL1	ENSG00000169288	4	78773674	78883944	206	13	123834	2.5899	0.0048007	0.552880887
CHCHD5	ENSG00000125611	2	113331817	113356852	36	8	123834	2.5892	0.0048094	0.552880887
FAM151B	ENSG00000152380	5	79773788	79848382	109	13	123834	2.5879	0.0048284	0.552880887
KBTBD4	ENSG00000123444	11	47583749	47610567	25	7	123834	2.5849	0.0048697	0.554271004
BMP4	ENSG00000125378	14	54406454	54435479	38	12	123834	2.5805	0.0049329	0.5581224
AC069368.3	ENSG00000249240	15	65124082	65233118	211	14	123834	2.5742	0.005023	0.563330033
C6ORF50	ENSG00000145965	6	3972909	3994372	40	5	123834	2.5732	0.0050382	0.563330033
CRYL1	ENSG00000165475	13	20967806	21109996	269	28	123834	2.5628	0.0051916	0.571749778
NCR1	ENSG00000189430	19	55407508	55437508	105	19	123834	2.5623	0.0051992	0.571749778
ZFYVE16	ENSG00000039319	5	79693832	79785169	181	7	123834	2.5614	0.0052131	0.571749778
NUDT18	ENSG00000173566	8	21954383	21976932	29	3	123834	2.5585	0.0052558	0.571749778
TRAF4	ENSG00000076604	17	27061002	27087974	17	3	123834	2.558	0.0052639	0.571749778
PPP3R1	ENSG00000221823	2	68395989	68493369	145	7	123834	2.5481	0.0054154	0.576731803
FAM180B	ENSG00000196666	11	47598198	47620746	20	7	123834	2.5473	0.0054274	0.576731803
COMMD7	ENSG00000149600	20	31280493	31341803	133	9	123834	2.5456	0.0054541	0.576731803
PIAS4	ENSG00000105229	19	3997644	4049384	81	6	123834	2.5446	0.0054697	0.576731803
SLC5A4	ENSG00000100191	22	32604465	32661328	149	18	123834	2.5443	0.0054754	0.576731803
TFIP11	ENSG00000100109	22	26877191	26918471	72	12	123834	2.5397	0.005548	0.576731803
GAPDHS	ENSG00000105679	19	36014314	36046218	55	9	123834	2.5395	0.0055508	0.576731803
SLTM	ENSG00000137776	15	59161244	59235852	52	6	123834	2.5394	0.0055525	0.576731803
CASKIN1	ENSG00000167971	16	2217184	2256526	25	4	123834	2.535	0.0056223	0.580808035
NKX2-8	ENSG00000136327	14	37039784	37061812	48	5	123834	2.5272	0.0057488	0.586757647
C9orf92	ENSG00000205549	9	16193933	16286311	118	18	123834	2.527	0.0057524	0.586757647
C20orf62	ENSG00000168746	20	43070624	43103984	17	3	123834	2.5258	0.0057725	0.586757647
PNO1	ENSG00000115946	2	68374976	68413370	62	6	123834	2.5202	0.0058637	0.592857498
CEP55	ENSG00000138180	10	95246389	95298849	143	16	123834	2.5149	0.0059535	0.5947722
TMEM147	ENSG00000105677	19	36026497	36048428	26	6	123834	2.512	0.0060021	0.5947722
RAPGEF5	ENSG00000136237	7	22147856	22406763	345	33	123834	2.5118	0.0060055	0.5947722
PHF12	ENSG00000109118	17	27222268	27288789	33	4	123834	2.5117	0.0060078	0.5947722
GATA2	ENSG00000179348	3	128188270	128222028	63	13	123834	2.5064	0.0060983	0.600603556
PPP3CC	ENSG00000120910	8	22288332	22408652	127	6	123834	2.5017	0.0061791	0.605424396

TMEM17	ENSG00000186889	2	62717356	62749029	81	15	123834	2.497	0.0062618	0.607363788
PPIF	ENSG00000108179	10	81097225	81125093	36	4	123834	2.497	0.0062628	0.607363788
IQCF3	ENSG00000229972	3	51841620	51874876	41	1	123834	2.4925	0.0063424	0.611961113
IFNAR1	ENSG00000142166	21	34686734	34742168	107	11	123834	2.4858	0.0064626	0.619001729
HOXA7	ENSG00000122592	7	27183335	27207555	37	6	123834	2.4848	0.0064805	0.619001729
CCDC183	ENSG00000213213	9	139680802	139712193	35	4	123834	2.4805	0.0065592	0.619379894
GNLY	ENSG00000115523	2	85902298	85935977	79	7	123834	2.4797	0.0065743	0.619379894
RUSC2	ENSG00000198853	9	35480124	35571895	62	12	123834	2.4781	0.0066048	0.619379894
RGS2	ENSG00000116741	1	192768169	192791403	18	3	123834	2.4775	0.0066148	0.619379894
LPHN3	ENSG00000150471	4	62056976	62954053	1084	72	123834	2.4743	0.0066742	0.6218784
RAB26	ENSG00000167964	16	2180804	2214166	18	3	123834	2.4715	0.0067274	0.623777655
RP11-216L13.17	ENSG00000272896	9	139675831	139705047	51	4	123834	2.4634	0.0068818	0.634996381
CDC25C	ENSG00000158402	5	137610954	137684044	113	10	123834	2.4576	0.0069931	0.639173278
DBH	ENSG00000123454	9	136491482	136534466	64	17	123834	2.4572	0.0070011	0.639173278
NQO2	ENSG00000124588	6	2978221	3029996	184	20	123834	2.4549	0.0070461	0.639173278
DPY19L1	ENSG00000173852	7	34958488	35087883	287	10	123834	2.4539	0.0070663	0.639173278
CREB3L3	ENSG00000060566	19	4143598	4183051	39	6	123834	2.4524	0.0070952	0.639173278
PIPOX	ENSG00000179761	17	27267531	27394234	92	15	123834	2.4488	0.0071664	0.64191744
TTPAL	ENSG00000124120	20	43094526	43133244	38	4	123834	2.4461	0.0072207	0.64191744
ARNTL	ENSG00000133794	11	13288199	13418813	165	14	123834	2.4439	0.0072644	0.64191744
TMEM141	ENSG00000244187	9	139675807	139697709	45	3	123834	2.4422	0.007299	0.64191744
PTCH1	ENSG00000185920	9	98195262	98289339	93	6	123834	2.4405	0.0073332	0.64191744
PAX5	ENSG00000196092	9	36823272	37044103	404	39	123834	2.4387	0.0073701	0.64191744
BCL9	ENSG00000116128	1	147003182	147108017	172	31	123834	2.4374	0.0073969	0.64191744
GMNC	ENSG00000205835	3	190560666	190620218	73	10	123834	2.4362	0.0074207	0.64191744
RP11-474G23.1	ENSG00000273398	2	68348370	68498362	206	10	123834	2.4358	0.0074296	0.64191744
FAM160B2	ENSG00000158863	8	21936670	21972409	48	7	123834	2.4291	0.0075675	0.647868672
COQ3	ENSG00000132423	6	99807276	99852080	93	11	123834	2.4287	0.0075761	0.647868672
BCMO1	ENSG00000135697	16	81262053	81334747	55	11	123834	2.4269	0.007615	0.647868672
FBXO47	ENSG00000204952	17	37082685	37133655	33	4	123834	2.4246	0.0076627	0.647868672
TADA2A	ENSG00000108264	17	35756965	35849835	185	14	123834	2.4243	0.0076689	0.647868672
FAXC	ENSG00000146267	6	99709045	99807938	265	18	123834	2.4209	0.007742	0.649060947
TTC40	ENSG00000171811	10	134611896	134766327	444	31	123834	2.4189	0.0077848	0.649060947
RP11-65D24.2	ENSG00000204398	13	112230548	112334955	226	29	123834	2.4174	0.0078165	0.649060947
FAM63A	ENSG00000143409	1	150959025	150990851	40	7	123834	2.4172	0.0078196	0.649060947
ACPL2	ENSG00000155893	3	140937568	141023748	153	16	123834	2.4146	0.0078753	0.649398857
HIST1H1E	ENSG00000168298	6	26146559	26167343	12	5	123834	2.4139	0.007892	0.649398857
HIST1H4H	ENSG00000158406	6	26271283	26295762	57	7	123834	2.4092	0.007994	0.65495669
PLEKHH2	ENSG00000152527	2	43854412	44005126	297	25	123834	2.4064	0.0080556	0.655266424
PTPRS	ENSG00000105426	19	5148506	5350814	282	51	123834	2.4055	0.0080762	0.655266424
PKD1	ENSG00000008710	16	2128711	2195899	44	4	123834	2.4043	0.0081012	0.655266424
TIMM17A	ENSG00000134375	1	201914619	201949789	39	6	123834	2.3996	0.0082075	0.659762066
RP11-351M8.1	ENSG00000259649	15	81178532	81212118	25	3	123834	2.3987	0.0082262	0.659762066
ZNF655	ENSG00000197343	7	99146029	99184076	34	6	123834	2.3958	0.0082918	0.662229136
ZDHHC16	ENSG00000171307	10	99195927	99227127	38	9	123834	2.3923	0.0083715	0.665796954
CNOT4	ENSG00000080802	7	135036547	135204875	211	21	123834	2.3904	0.0084161	0.66655512
DHX34	ENSG00000134815	19	47842538	47895961	133	10	123834	2.3871	0.0084902	0.6696337
THTPA	ENSG00000259431	14	24015216	24039480	8	2	123834	2.3844	0.0085527	0.669640659
TMEM56-RWDD3	ENSG00000271092	1	95573479	95722781	295	19	123834	2.3822	0.0086057	0.669640659
PGPEP1	ENSG00000130517	19	18441397	18490760	20	5	123834	2.3799	0.008659	0.669640659
NDUFS3	ENSG00000213619	11	47576888	47616114	33	8	123834	2.3785	0.0086925	0.669640659
PTGIS	ENSG00000124212	20	48110411	48194683	55	8	123834	2.3765	0.0087394	0.669640659
HR	ENSG00000168453	8	21961928	22000897	49	7	123834	2.3761	0.0087488	0.669640659
PYGO1	ENSG00000171016	15	55821088	55891145	169	13	123834	2.3754	0.0087657	0.669640659
APOPT1	ENSG00000256053	14	104019299	104083860	101	9	123834	2.3724	0.0088366	0.669640659
CHAMP1	ENSG00000198824	13	115069988	115102796	35	2	123834	2.3703	0.0088859	0.669640659
NFAT5	ENSG00000102908	16	69588997	69748569	275	13	123834	2.37	0.0088943	0.669640659
HOXA6	ENSG00000106006	7	27175015	27200222	39	6	123834	2.3679	0.0089439	0.669640659
MADD	ENSG00000110514	11	47280712	47361582	125	10	123834	2.3674	0.0089573	0.669640659
ISCA2	ENSG00000165898	14	74950423	74973809	27	3	123834	2.3671	0.0089636	0.669640659
KIAA1522	ENSG00000162522	1	33197486	33250571	42	8	123834	2.3663	0.0089835	0.669640659
FOXJ1	ENSG00000129654	17	74122414	74147380	9	2	123834	2.3635	0.0090524	0.671899518
SMAP2	ENSG00000084070	1	40800522	40898998	145	24	123834	2.3622	0.0090845	0.671899518
C9orf172	ENSG00000232434	9	139728867	139751797	4	1	123834	2.3577	0.0091933	0.67345344
MTMR11	ENSG00000014914	1	149890543	149918791	30	3	123834	2.3571	0.0092093	0.67345344
STX4	ENSG00000103496	16	31034210	31064296	33	4	123834	2.357	0.0092118	0.67345344
XRCC5	ENSG00000079246	2	216962187	217081026	168	19	123834	2.3547	0.0092689	0.673844441
PRSS53	ENSG00000151006	16	31084746	31110949	14	2	123834	2.3528	0.0093156	0.673844441
ASPRV1	ENSG00000244617	2	70177226	70199397	30	3	123834	2.3525	0.0093235	0.673844441
GTF2H1	ENSG00000110768	11	18333842	18398591	98	10	123834	2.3498	0.0093906	0.6761232
MLST8	ENSG00000167965	16	2244249	2269417	23	4	123834	2.3422	0.0095863	0.678579527
FBLN7	ENSG00000144152	2	112885962	112955791	102	16	123834	2.342	0.0095906	0.678579527
PRED60	ENSG00000267883	21	47173565	47200005	28	3	123834	2.342	0.0095914	0.678579527
AMIGO1	ENSG00000181754	1	110036797	110062360	25	5	123834	2.3393	0.0096602	0.678579527
JAGN1	ENSG00000171135	3	9922238	9946033	40	6	123834	2.3391	0.009666	0.678579527
ZNF844	ENSG00000223547	19	12165514	12202380	67	5	123834	2.3385	0.0096817	0.678579527
FADS1	ENSG00000149485	11	61557099	61606790	48	5	123834	2.338	0.0096931	0.678579527
DYNC2LI1	ENSG00000138036	2	43991178	44047149	140	10	123834	2.3371	0.009717	0.678579527
RPLP2	ENSG00000177600	11	799647	822880	23	4	123834	2.3334	0.0098145	0.678579527
SERINC3	ENSG00000132824	20	43114862	43160750	48	5	123834	2.3316	0.0098597	0.678579527
ATP5J2-PTCD1	ENSG00000248919	7	99007372	99073820	101	8	123834	2.3303	0.0098955	0.678579527
TP53INP2	ENSG00000078804	20	33282094	33311243	18	4	123834	2.3296	0.0099127	0.678579527
PAQR6	ENSG00000160781	1	156203206	156227881	7	3	123834	2.3235	0.010076	0.678579527
NR1H3	ENSG00000025434	11	47259851	47300396	69	7	123834	2.3226	0.0101	0.678579527
SF3B4	ENSG00000143368	1	149885209	149910236	29	2	123834	2.3208	0.010149	0.678579527
PTCD1	ENSG00000106246	7	99004362	99073787	107	9	123834	2.3202	0.010165	0.678579527
DPH3	ENSG00000154813	3	16289485	16316479	67	7	123834	2.3166	0.010263	0.678579527
RHEB	ENSG00000106615	7	151153098	151227206	68	7	123834	2.3155	0.010292	0.678579527
DHRS13	ENSG00000167536	17	27214799	27240089	9	3	123834	2.3149	0.01031	0.678579527
CALHM3	ENSG00000183128	10	105222561	105248997	45	6	123834	2.3124	0.010378	0.678579527
UBQLN4	ENSG00000160803	1	155995092	156033585	19	5	123834	2.3122	0.010383	0.678579527
CRABP1	ENSG00000166426	15	78622666	78650572	26	7	123834	2.3107	0.010423	0.678579527
MX2	ENSG00000183486	21	42723870	42791317	89	22	123834	2.309	0.01047	0.678579527
ZNF646	ENSG00000167395	16	31075743	31105517	23	3	123834	2.3088	0.010478	0.678579527
NME1-NME2	ENSG00000243678	17	49220920	49259108	100	13	123834	2.3086	0.010484	0.678579527
NME2	ENSG00000011052	17	49220951	49259105	100	13	123834	2.3086	0.010484	0.678579527
SLC22A13	ENSG00000172940	3	38297303	38329806	24	3	123834	2.3078	0.010506	0.678579527

EXOSC1	ENSG00000171311	10	99185899	99215774	38	9	123834	2.3049	0.010587	0.678579527
RP11-1026M7.2	ENSG00000249109	5	177199198	177231397	1	1	123834	2.303	0.01064	0.678579527
TMEM89	ENSG00000183396	3	48648192	48669288	10	2	123834	2.3027	0.010648	0.678579527
VKORC1	ENSG00000167397	16	31092163	31117301	15	2	123834	2.3026	0.010651	0.678579527
C12orf43	ENSG00000157895	12	121430225	121464305	95	14	123834	2.3011	0.010692	0.678579527
B4GALT5	ENSG00000158470	20	48239482	48340415	176	11	123834	2.3003	0.010714	0.678579527
SMARCD1	ENSG00000066117	12	50468755	50504495	22	3	123834	2.2999	0.010727	0.678579527
RP11-196G11.1	ENSG00000255439	16	31084760	31116277	17	3	123834	2.2995	0.010739	0.678579527
C17orf78	ENSG00000167230	17	35722928	35759662	24	6	123834	2.2987	0.010761	0.678579527
BCL6	ENSG00000113916	3	187429165	187473515	76	15	123834	2.2958	0.010843	0.678579527
NPC2	ENSG00000119655	14	74932895	74970880	58	3	123834	2.2958	0.010844	0.678579527
KCTD10	ENSG00000110906	12	109876461	109925349	150	12	123834	2.2946	0.010877	0.678579527
C15orf60	ENSG00000183324	15	73725499	73862355	210	13	123834	2.2938	0.010902	0.678579527
AGTRAP	ENSG00000177674	1	11786141	11824859	20	5	123834	2.2904	0.010998	0.678579527
RWDD3	ENSG00000122481	1	95689711	95722781	78	11	123834	2.2894	0.011028	0.678579527
TTBK1	ENSG00000146216	6	43201418	43265997	74	12	123834	2.2883	0.011059	0.678579527
NRXN1	ENSG00000179915	2	50135643	51269674	1799	93	123834	2.2869	0.011102	0.678579527
SPOCD1	ENSG00000134668	1	32246023	32291652	33	6	123834	2.2858	0.011133	0.678579527
NCOA6	ENSG00000198646	20	33274722	33423452	153	10	123834	2.2857	0.011135	0.678579527
NOS3	ENSG00000164867	7	150678083	150721676	28	7	123834	2.2851	0.011154	0.678579527
DIS3L	ENSG00000166938	15	66575555	66636236	96	16	123834	2.285	0.011157	0.678579527
SLC33A1	ENSG00000169359	3	155528813	155582218	92	9	123834	2.2844	0.011174	0.678579527
SLC26A6	ENSG00000225697	3	48653156	48682926	22	2	123834	2.2789	0.011338	0.68344853
LHX9	ENSG00000143355	1	197871037	197914608	58	9	123834	2.2782	0.011358	0.68344853
BUD31	ENSG00000106245	7	98996264	99027239	44	6	123834	2.2771	0.01139	0.68344853
FSD1	ENSG00000105255	19	4294597	4333840	48	7	123834	2.2769	0.011398	0.68344853
CEP68	ENSG00000011523	2	65273500	65324138	91	10	123834	2.2731	0.011511	0.685590729
KCNQ3	ENSG00000184156	8	133123108	133503200	726	59	123834	2.272	0.011544	0.685590729
AGPAT1	ENSG00000204310	6	32125989	32155873	28	8	123834	2.2715	0.011558	0.685590729
DEF8	ENSG00000140995	16	90004333	90044468	119	12	123834	2.2709	0.011578	0.685590729
HOXA5	ENSG00000106004	7	27170671	27193287	36	7	123834	2.269	0.011635	0.686826335
TLE4	ENSG00000106829	9	82176688	82351658	192	22	123834	2.2666	0.011708	0.687866667
CD200R1	ENSG00000163606	3	112630056	112703969	222	23	123834	2.266	0.011725	0.687866667
SORBS3	ENSG00000120896	8	22392499	22443301	19	3	123834	2.2626	0.01183	0.6918912
CACNA2D2	ENSG00000007402	3	50390233	50551675	73	9	123834	2.2574	0.01199	0.699097914
SBSN	ENSG00000189001	19	36004269	36029253	38	7	123834	2.2489	0.012259	0.70224
ACOX1	ENSG00000161533	17	73927588	73985515	120	14	123834	2.2482	0.012283	0.70224
RBM44	ENSG00000177483	2	238697032	238761451	78	10	123834	2.2461	0.012349	0.70224
CPSF4	ENSG00000160917	7	99026545	99064994	58	6	123834	2.2442	0.012411	0.70224
SERBP1	ENSG00000142864	1	67863493	67906098	19	4	123834	2.2436	0.01243	0.70224
GXYLT2	ENSG00000172986	3	72927224	73057289	216	22	123834	2.2436	0.01243	0.70224
NME4	ENSG00000103202	16	436725	470367	43	7	123834	2.2424	0.012467	0.70224
MBTD1	ENSG00000011258	17	49244786	49347524	194	12	123834	2.2421	0.012476	0.70224
GS1-259H13.10	ENSG00000272647	7	99146291	99215433	73	8	123834	2.2418	0.012488	0.70224
OXNAD1	ENSG00000154814	3	16296706	16401806	221	14	123834	2.238	0.01261	0.70224
C15orf41	ENSG00000186073	15	36861812	37112449	344	28	123834	2.2375	0.012626	0.70224
TNFAIP8L2	ENSG00000163154	1	151119105	151142225	7	3	123834	2.2374	0.012631	0.70224
ADPRHL2	ENSG00000116863	1	36544476	36569533	13	3	123834	2.2368	0.01265	0.70224
NPTN	ENSG00000156642	15	73842355	73936475	134	12	123834	2.2355	0.012694	0.70224
ABCB6	ENSG00000115657	2	220064490	220093712	15	2	123834	2.2341	0.012737	0.70224
PCBP3	ENSG00000183570	21	47053608	47372368	550	15	123834	2.2319	0.012812	0.70224
COL9A2	ENSG00000049089	1	40756159	40793488	79	9	123834	2.2317	0.012817	0.70224
ABLIM2	ENSG00000163995	4	7957039	8170559	215	51	123834	2.2297	0.012882	0.70224
TEN1	ENSG00000257949	17	73965301	74006667	99	13	123834	2.228	0.012941	0.70224
DNAH2	ENSG00000183914	17	7610672	7747062	101	22	123834	2.2272	0.012967	0.70224
NEB	ENSG00000183091	2	152331850	152601001	292	22	123834	2.2272	0.012968	0.70224
SULF2	ENSG00000196562	20	46275092	46425360	202	47	123834	2.2255	0.013023	0.70224
VARS	ENSG00000204394	6	31735295	31773730	51	15	123834	2.2255	0.013025	0.70224
HOOK1	ENSG00000134709	1	60270458	60352050	138	14	123834	2.2249	0.013045	0.70224
GNA12	ENSG00000146535	7	2757746	2893958	288	12	123834	2.2234	0.013094	0.70224
SPA17	ENSG00000064199	11	124533694	124577414	21	2	123834	2.2224	0.013128	0.70224
KCNJ4	ENSG00000168135	22	38812332	38861205	71	13	123834	2.22	0.01321	0.70224
SIAE	ENSG00000110013	11	124493009	124575603	40	3	123834	2.2174	0.013298	0.70224
NCAM2	ENSG00000154654	21	22360633	22925650	1096	72	123834	2.2169	0.013317	0.70224
PAIP1	ENSG00000172239	5	43516369	43567860	80	6	123834	2.2153	0.013368	0.70224
GPR132	ENSG00000183484	14	105505728	105541782	45	4	123834	2.2152	0.013375	0.70224
ABCG2	ENSG00000118777	4	89001416	89162474	227	35	123834	2.2144	0.013399	0.70224
UTP18	ENSG00000011260	17	49327889	49385297	100	9	123834	2.2136	0.013428	0.70224
CSMD2	ENSG00000121904	1	33969609	34641443	779	92	123834	2.213	0.013448	0.70224
IL7	ENSG00000104432	8	79577978	79727758	269	14	123834	2.2115	0.013501	0.70224
SEZ6L	ENSG00000100095	22	26555440	26789562	434	53	123834	2.2106	0.013531	0.70224
PRR24	ENSG00000257704	19	47768142	47788979	19	2	123834	2.2091	0.013584	0.70224
NME1	ENSG00000239672	17	49220897	49249789	78	11	123834	2.2075	0.013638	0.70224
GREB1	ENSG00000196208	2	11664242	11792914	166	26	123834	2.2074	0.013642	0.70224
ATG9A	ENSG00000198925	2	220064494	220104439	29	3	123834	2.2062	0.013684	0.70224
PLEKHA6	ENSG00000143850	1	204177979	204356793	329	27	123834	2.2059	0.013695	0.70224
C5orf28	ENSG00000151881	5	43434354	43493995	106	6	123834	2.2051	0.013722	0.70224
GPR65	ENSG00000140030	14	88461468	88488419	72	9	123834	2.205	0.013726	0.70224
BCL11A	ENSG00000119866	2	60668302	60790702	45	13	123834	2.2046	0.01374	0.70224
RP11-187E13.2	ENSG00000258386	14	32466108	32497891	14	3	123834	2.2035	0.013779	0.70224
KDM6B	ENSG00000132510	17	7733222	7768114	32	8	123834	2.2022	0.013826	0.70224
THRAP3	ENSG00000054118	1	36680017	36780958	64	2	123834	2.2011	0.013866	0.70224
ADAMTS10	ENSG00000142303	19	8635126	8685620	15	5	123834	2.1998	0.013912	0.70224
C1QTNF9B	ENSG00000205863	13	24455238	24486794	47	6	123834	2.1991	0.013935	0.70224
UBE3B	ENSG00000151148	12	109905207	109984507	203	18	123834	2.1987	0.013948	0.70224
LRRC10B	ENSG00000204950	11	61266272	61288482	27	4	123834	2.1986	0.013952	0.70224
TUBB3	ENSG00000258947	16	89977800	90015169	92	13	123834	2.1983	0.013965	0.70224
FAM200A	ENSG00000221909	7	99133931	99166159	23	4	123834	2.1955	0.014066	0.703894677
ARNTL2	ENSG00000029153	12	27475787	27586241	247	35	123834	2.1947	0.014093	0.703894677
SPOCK2	ENSG00000107742	10	73808793	73858790	47	7	123834	2.1943	0.014109	0.703894677
TSPAN5	ENSG00000168785	4	99381518	99589780	214	32	123834	2.1908	0.014233	0.708222157
C11orf94	ENSG00000234776	11	45918085	45938833	8	2	123834	2.1885	0.014317	0.708880825
TNNI2	ENSG00000130598	11	1850219	1872910	2	1	123834	2.1868	0.01438	0.708880825
ACKR3	ENSG00000144476	2	237466430	237501001	37	6	123834	2.1855	0.014428	0.708880825
C5orf20	ENSG00000251380	5	134769905	134793038	60	10	123834	2.1852	0.014438	0.708880825
ZNF668	ENSG00000167394	16	31062164	31095641	32	3	123834	2.1846	0.014459	0.708880825
SNAPC5	ENSG00000174446	15	66772473	66800151	53	7	123834	2.1843	0.01447	0.708880825
DENND1B	ENSG00000213047	1	197463878	197754826	255	8	123834	2.1769	0.014743	0.718794831
ADAM10	ENSG00000137845	15	58877403	59052177	193	13	123834	2.1768	0.014748	0.718794831

MGA	ENSG00000174197	15	41903422	42072141	249	17	123834	2.1751	0.014811	0.719588571
TCF7L2	ENSG00000148737	10	114700009	114937437	176	30	123834	2.1743	0.01484	0.719588571
STK40	ENSG00000196182	1	36795225	36861497	45	5	123834	2.1701	0.014998	0.72086681
MUC20	ENSG00000176945	3	195437753	195477994	2	1	123834	2.1684	0.015062	0.72086681
NAP1L1	ENSG00000187109	12	76428670	76488813	47	4	123834	2.1681	0.015074	0.72086681
MEX3D	ENSG00000181588	19	1544668	1578057	6	3	123834	2.1678	0.015089	0.72086681
VPS45	ENSG00000136631	1	150029369	150127505	94	2	123834	2.1676	0.015094	0.72086681
MET	ENSG00000105976	7	116302444	116448440	121	18	123834	2.1665	0.015137	0.72086681
RTN4RL1	ENSG00000185924	17	1827971	1938639	146	23	123834	2.1658	0.015165	0.72086681
RAB1A	ENSG00000138069	2	65287835	65367240	154	11	123834	2.165	0.015195	0.72086681
YAE1D1	ENSG00000241127	7	39595975	39659919	135	14	123834	2.1639	0.015235	0.72086681
BNIP3L	ENSG00000104765	8	26230414	26373152	268	40	123834	2.1636	0.015246	0.72086681
CCDC9	ENSG00000105321	19	47749237	47785210	38	2	123834	2.1614	0.015332	0.72086681
CDYL	ENSG00000153046	6	4696393	4965785	350	36	123834	2.1579	0.015466	0.72086681
DKFZP434A062	ENSG00000267845	9	139209089	139229640	22	4	123834	2.1579	0.015468	0.72086681
AP1G2	ENSG00000213983	14	24018774	24047279	11	2	123834	2.1576	0.015478	0.72086681
HAO2	ENSG00000116882	1	119901402	119946753	59	7	123834	2.1571	0.015501	0.72086681
C16orf13	ENSG00000130731	16	674429	696358	20	4	123834	2.1569	0.015507	0.72086681
FA2H	ENSG00000103089	16	74736853	74818729	136	22	123834	2.1565	0.015522	0.72086681
DKFZP434O1614	ENSG00000258729	14	96731383	96753060	27	4	123834	2.1558	0.015549	0.72086681
TMEM8A	ENSG00000129925	16	410773	447113	89	13	123834	2.1543	0.015609	0.720880696
CHML	ENSG00000203668	1	241782155	241809232	48	10	123834	2.1524	0.015685	0.720880696
ITGB3	ENSG00000259207	17	45321212	45431658	292	22	123834	2.1519	0.015701	0.720880696
ITGB3	ENSG00000259753	17	45321263	45431658	292	22	123834	2.1519	0.015701	0.720880696
ANKZF1	ENSG00000163516	2	220084479	220111391	37	5	123834	2.1509	0.015743	0.721067335
RGS7BP	ENSG00000186479	5	63792084	63918139	138	18	123834	2.1481	0.015852	0.721837546
CLEC18A	ENSG00000157322	16	69974810	70008141	11	4	123834	2.1477	0.01587	0.721837546
RPS19BP1	ENSG00000187051	22	39915098	39938860	19	3	123834	2.1473	0.015886	0.721837546
PIDD	ENSG00000177595	11	789179	819753	30	5	123834	2.1463	0.015923	0.721837546
NQO1	ENSG00000181019	16	69730899	69770854	65	10	123834	2.1442	0.01601	0.721837546
EFCAB13	ENSG00000178852	17	45390656	45528678	341	13	123834	2.1432	0.01605	0.721837546
OAS2	ENSG00000111335	12	113406200	113459528	82	12	123834	2.1427	0.016068	0.721837546
TENM4	ENSG00000149256	11	78353876	79161992	1071	137	123834	2.1421	0.016091	0.721837546
S100A2	ENSG00000196754	1	153523584	153550366	20	2	123834	2.14	0.016177	0.721837546
HSPB1	ENSG00000106211	7	75921861	75943612	29	5	123834	2.1387	0.01623	0.721837546
ARID2	ENSG00000189079	12	46113448	46311823	81	8	123834	2.1385	0.016239	0.721837546
CALHM1	ENSG00000185933	10	105203144	105228645	27	6	123834	2.1382	0.016252	0.721837546
SETD7	ENSG00000145391	4	140407095	140537853	116	27	123834	2.1362	0.016332	0.721837546
ZC3H8	ENSG00000144161	2	112959102	113022713	55	5	123834	2.1332	0.016454	0.721837546
POU6F1	ENSG00000184271	12	51570719	51621477	45	9	123834	2.1317	0.016516	0.721837546
C6orf48	ENSG00000204387	6	31792385	31817541	43	15	123834	2.1314	0.016528	0.721837546
RAB44	ENSG00000255587	6	36673256	36709870	64	13	123834	2.1312	0.016537	0.721837546
C17orf103	ENSG00000154035	17	21132183	21166722	40	10	123834	2.1251	0.01679	0.721837546
YWHAG	ENSG00000170027	7	75946116	75998348	78	9	123834	2.1249	0.016798	0.721837546
NEU1	ENSG00000204386	6	31815436	31840683	46	12	123834	2.1239	0.016838	0.721837546
GPSM3	ENSG00000213654	6	32148543	32173300	24	8	123834	2.1231	0.016874	0.721837546
C5orf34	ENSG00000172244	5	43476803	43525247	63	4	123834	2.123	0.016878	0.721837546
ZNF788	ENSG00000188474	19	12193078	12235491	51	5	123834	2.1227	0.016888	0.721837546
SAMD15	ENSG00000100583	14	77833032	77867840	55	7	123834	2.1225	0.016896	0.721837546
SV2B	ENSG00000185518	15	91633180	91854539	306	32	123834	2.121	0.016961	0.721837546
SLC39A13	ENSG00000165915	11	47418683	47448047	52	6	123834	2.1187	0.017058	0.721837546
PYDC2	ENSG00000253548	3	191168952	191189245	118	6	123834	2.1185	0.017067	0.721837546
KISS1	ENSG00000170498	1	204149469	204175614	34	6	123834	2.1163	0.017161	0.721837546
OTUD7B	ENSG00000163113	1	149899705	149992625	74	5	123834	2.115	0.017217	0.721837546
TRIM66	ENSG00000166436	11	8623584	8703413	140	14	123834	2.1133	0.017286	0.721837546
ATP5J2	ENSG00000241468	7	99036098	99073954	56	5	123834	2.1128	0.01731	0.721837546
PBX2	ENSG00000204304	6	32142512	32167963	21	7	123834	2.1123	0.017329	0.721837546
PGAM2	ENSG00000164708	7	44092326	44115186	45	4	123834	2.1118	0.017351	0.721837546
PGP	ENSG00000184207	16	2251998	2274808	19	4	123834	2.1103	0.017415	0.721837546
CERS5	ENSG00000139624	12	50513575	50571288	72	5	123834	2.11	0.017427	0.721837546
UBE2Q1	ENSG00000160714	1	154511053	154541504	45	5	123834	2.1094	0.017456	0.721837546
C2orf27A	ENSG00000197927	2	132469948	132534973	132	9	123834	2.1087	0.017487	0.721837546
RNF5	ENSG00000204308	6	32136131	32161930	18	6	123834	2.1083	0.0175	0.721837546
AGER	ENSG00000204305	6	32138745	32162101	18	6	123834	2.1083	0.0175	0.721837546
ARPC1B	ENSG00000130429	7	98961872	99002424	56	6	123834	2.107	0.017559	0.721837546
KDM3B	ENSG00000120733	5	137678285	137782717	182	16	123834	2.1067	0.017573	0.721837546
PSMD9	ENSG00000110801	12	122316637	122366203	104	11	123834	2.1053	0.017632	0.721837546
PLEKHO1	ENSG00000023902	1	150111373	150146916	37	2	123834	2.1046	0.017663	0.721837546
IL12RB2	ENSG00000081985	1	67763047	67872583	192	15	123834	2.1042	0.01768	0.721837546
NEBL	ENSG00000078114	10	21058902	21473116	578	46	123834	2.1042	0.01768	0.721837546
TEKT2	ENSG00000092850	1	36539676	36563876	10	1	123834	2.104	0.017688	0.721837546
PLAC8L1	ENSG00000173261	5	145453949	145493932	83	3	123834	2.1037	0.017703	0.721837546
FOXN3	ENSG00000053254	14	89581215	90095493	604	93	123834	2.1035	0.017711	0.721837546
SLC25A22	ENSG00000177542	11	780475	808316	37	6	123834	2.1018	0.017784	0.721837546
LRRC3DN	ENSG00000269430	21	45869814	45892078	50	5	123834	2.1018	0.017785	0.721837546
TACR1	ENSG00000115353	2	75263590	75436826	197	33	123834	2.1006	0.017837	0.721837546
AUTS2	ENSG00000158321	7	69053905	70268054	1560	51	123834	2.0997	0.017876	0.721837546
SERPINA5	ENSG00000188488	14	95017779	95069457	203	24	123834	2.0955	0.018062	0.721837546
HIST1H2BD	ENSG00000158373	6	26148349	26181577	26	8	123834	2.0947	0.0181	0.721837546
AC003043.1	ENSG00000268413	17	42325297	42365825	37	8	123834	2.0945	0.018107	0.721837546
DLGAP3	ENSG00000116544	1	35321037	35405186	20	4	123834	2.0942	0.018119	0.721837546
DDX19A	ENSG00000168872	16	70370732	70417286	27	6	123834	2.0937	0.018142	0.721837546
ZNF668	ENSG00000232748	16	31062813	31083451	23	3	123834	2.0934	0.018158	0.721837546
SPEF2	ENSG00000152582	5	35607946	35824713	464	27	123834	2.0929	0.018178	0.721837546
MANBAL	ENSG00000101363	20	35908041	35955663	54	7	123834	2.0926	0.018192	0.721837546
REG4	ENSG00000134193	1	120326641	120364283	66	4	123834	2.0913	0.018252	0.721837546
RPL4	ENSG00000174444	15	66780355	66826870	96	11	123834	2.0911	0.018261	0.721837546
MRPS21	ENSG00000187145	1	150256289	150291414	73	6	123834	2.0907	0.018277	0.721837546
NVL	ENSG00000143748	1	224405036	224528089	138	6	123834	2.0894	0.018337	0.721837546
TMEM88	ENSG00000167874	17	7748383	7769417	19	5	123834	2.0893	0.018342	0.721837546
AC073063.1	ENSG00000214314	7	99030552	99050747	32	4	123834	2.0891	0.01835	0.721837546
ADRBK1	ENSG00000173020	11	67023881	67064027	26	5	123834	2.0887	0.018369	0.721837546
BTBD3	ENSG00000132640	20	11861371	11917257	102	6	123834	2.0866	0.018461	0.721837546
NRF1	ENSG00000106459	7	129241555	129406922	312	20	123834	2.0864	0.018469	0.721837546
ZC2HC1A	ENSG00000104427	8	79568282	79642000	127	9	123834	2.0863	0.018473	0.721837546
TMEM56	ENSG00000152078	1	95572894	95673163	174	19	123834	2.0863	0.018476	0.721837546
ZFHX2	ENSG00000136367	14	23980066	24035401	17	5	123834	2.0852	0.018524	0.721837546
KCNK9	ENSG00000169427	8	140603081	140725299	161	24	123834	2.0846	0.018554	0.721837546
ZWILCH	ENSG00000174442	15	66787297	66852115	151	14	123834	2.0842	0.01857	0.721837546

PHC2	ENSG00000134686	1	33779224	33906653	296	16	123834	2.0805	0.018741	0.726548505
YEATS2	ENSG00000163872	3	183405606	183540413	172	9	123834	2.0762	0.018938	0.726548505
UBLCP1	ENSG00000164332	5	158680089	158723044	49	7	123834	2.0747	0.019008	0.726548505
RP11-131H24.4	ENSG00000258987	14	94395901	94420475	48	5	123834	2.0733	0.019073	0.726548505
LAG3	ENSG00000089692	12	6871678	6897621	6	3	123834	2.0726	0.019103	0.726548505
CPNE7	ENSG00000178773	16	89632176	89673654	55	12	123834	2.0716	0.019153	0.726548505
BCDIN3D	ENSG00000186666	12	50221573	50246912	27	5	123834	2.0715	0.019155	0.726548505
IL17RE	ENSG00000163701	3	9934296	9968086	48	3	123834	2.0707	0.019195	0.726548505
TDRD10	ENSG00000163239	1	154464695	154530623	150	7	123834	2.0704	0.019209	0.726548505
HOXA3	ENSG00000105997	7	27135803	27202200	103	12	123834	2.0703	0.019213	0.726548505
SCNM1	ENSG00000163156	1	151119140	151152773	10	4	123834	2.0698	0.019238	0.726548505
HOXB3	ENSG00000120093	17	46616232	46692274	122	20	123834	2.0697	0.019239	0.726548505
FUT4	ENSG00000196371	11	94267017	94293063	33	6	123834	2.0697	0.01924	0.726548505
TRAF2	ENSG00000127191	9	139766364	139831059	85	3	123834	2.0695	0.019249	0.726548505
AASS	ENSG00000008311	7	121705701	121794334	108	12	123834	2.0689	0.019279	0.726548505
HSD17B13	ENSG00000170509	4	88214941	88254058	66	11	123834	2.068	0.019322	0.726548505
VWA7	ENSG00000204396	6	31723367	31755108	28	9	123834	2.0662	0.019407	0.726548505
ZNF789	ENSG00000198556	7	99060464	99111273	46	7	123834	2.0662	0.019407	0.726548505
PRMT7	ENSG00000132600	16	68334877	68402466	130	9	123834	2.0651	0.019458	0.726548505
PLEKHO2	ENSG00000241839	15	65124088	65170206	112	8	123834	2.064	0.019511	0.726548505
TUBB3	ENSG00000198211	16	89975573	90012500	86	12	123834	2.0621	0.019599	0.726548505
C1orf61	ENSG00000125462	1	156364042	156410493	22	6	123834	2.0618	0.019611	0.726548505
CRB1	ENSG00000134376	1	197160592	197457585	251	26	123834	2.0619	0.019611	0.726548505
4-Mar	ENSG00000144583	2	217112588	217246750	151	21	123834	2.0616	0.019625	0.726548505
PDDC1	ENSG00000177225	11	757220	787488	34	7	123834	2.0605	0.019676	0.726548505
PPARGC1A	ENSG00000109819	4	23746664	23915712	273	44	123834	2.0603	0.019685	0.726548505
ARPC1A	ENSG00000241685	7	98913521	98995787	118	10	123834	2.058	0.019795	0.729192558
GIP	ENSG00000159224	17	47025916	47055958	69	7	123834	2.0566	0.01986	0.730171915
HPS5	ENSG00000110756	11	18290223	18353745	112	12	123834	2.0547	0.019953	0.731972809
WFIKKN1	ENSG00000127578	16	669239	694116	35	5	123834	2.054	0.019986	0.731972809
CUEDC1	ENSG00000180891	17	55928604	56042684	105	24	123834	2.0525	0.020061	0.732225958
ANKDD1A	ENSG00000166839	15	65194101	65261042	94	13	123834	2.0515	0.020107	0.732225958
DNAJC10	ENSG00000077232	2	183570999	183669191	121	6	123834	2.0509	0.020136	0.732225958
EPN1	ENSG00000063245	19	56176592	56231224	97	14	123834	2.0507	0.020147	0.732225958
MBP	ENSG00000197971	18	74680783	74855639	305	62	123834	2.0499	0.020187	0.732279573
PPT2-EGFL8	ENSG00000258388	6	32111622	32149755	34	8	123834	2.0474	0.02031	0.733866305
TSSK4	ENSG00000139908	14	24664903	24687568	25	6	123834	2.0471	0.020326	0.733866305
SAMD4A	ENSG00000020577	14	55023815	55270033	286	55	123834	2.0451	0.020424	0.733866305
CLTC	ENSG00000141367	17	57687219	57783671	105	6	123834	2.0447	0.020441	0.733866305
C13orf45	ENSG00000178734	13	76435187	76467947	72	13	123834	2.0437	0.020493	0.733866305
GBP6	ENSG00000183347	1	89819617	89862020	77	9	123834	2.0436	0.020498	0.733866305
CEBPZ	ENSG00000115816	2	37418755	37468856	60	12	123834	2.0435	0.020501	0.733866305
DNAJB2	ENSG00000135924	2	220133989	220161622	20	5	123834	2.0423	0.02056	0.734215205
DBNL	ENSG00000136279	7	44074239	44119055	79	5	123834	2.0417	0.020588	0.734215205
LARS	ENSG00000133706	5	145482601	145572223	266	4	123834	2.0391	0.020718	0.736195828
SRP68	ENSG00000167881	17	74025184	74078734	43	2	123834	2.0391	0.020721	0.736195828
CHMP4A	ENSG00000254505	14	24668789	24693075	25	6	123834	2.0362	0.020867	0.736671549
CCDC37	ENSG00000163885	3	126103782	126165399	104	18	123834	2.036	0.020877	0.736671549
GALR2	ENSG00000182687	17	74060875	74083622	10	1	123834	2.0348	0.020935	0.736671549
MLLT3	ENSG00000171843	9	20331663	20632542	203	31	123834	2.0345	0.02095	0.736671549
2-Sep	ENSG00000168385	2	242244515	242303442	109	12	123834	2.0337	0.020992	0.736671549
VIPAS39	ENSG00000151445	14	77883018	77934295	70	7	123834	2.0325	0.021054	0.736671549
KIF13A	ENSG00000137177	6	17749414	17997854	396	20	123834	2.0281	0.021273	0.736671549
DNPEP	ENSG00000123992	2	220228268	220274744	62	7	123834	2.0279	0.021287	0.736671549
EFCAB10	ENSG00000185055	7	105195567	105251322	48	8	123834	2.0267	0.021346	0.736671549
SH3RF1	ENSG00000154447	4	170005407	170202256	303	22	123834	2.0254	0.021413	0.736671549
SOX6	ENSG00000110693	11	15977995	16771138	941	38	123834	2.025	0.021433	0.736671549
ZNF788	ENSG00000214189	19	12193078	12258050	75	7	123834	2.0239	0.02149	0.736671549
GGT7	ENSG00000131067	20	33422523	33470663	59	6	123834	2.0239	0.02149	0.736671549
TRIM73	ENSG00000178809	7	75014337	75050279	1	1	123834	2.0229	0.021541	0.736671549
MYEOV	ENSG00000172927	11	69051605	69192494	121	19	123834	2.0226	0.021557	0.736671549
MDP1	ENSG00000213920	14	24673143	24695276	25	5	123834	2.0212	0.021628	0.736671549
LRRC3	ENSG00000160233	21	45865369	45888739	63	5	123834	2.0208	0.021652	0.736671549
DECR2	ENSG00000242612	16	441826	472487	25	3	123834	2.0206	0.021662	0.736671549
HOXA9	ENSG00000078399	7	27192054	27220117	22	6	123834	2.0205	0.021668	0.736671549
HOXB2	ENSG00000173917	17	46608256	46633441	43	6	123834	2.0203	0.021677	0.736671549
MAP6D1	ENSG00000180834	3	183523664	183553382	46	4	123834	2.019	0.021742	0.736671549
LHX3	ENSG00000107187	9	139078096	139106955	30	8	123834	2.0183	0.021778	0.736671549
NLRP9	ENSG00000185792	19	56209798	56259768	54	14	123834	2.0177	0.021811	0.736671549
RP11-87C12.2	ENSG00000256950	12	122316715	122369376	116	12	123834	2.0176	0.021818	0.736671549
GYLTL1B	ENSG00000165905	11	45933172	45960647	21	4	123834	2.017	0.021849	0.736671549
COX18	ENSG00000163626	4	73911797	73945472	18	4	123834	2.0169	0.021855	0.736671549
BRICD5	ENSG00000182685	16	2249254	2271951	24	5	123834	2.016	0.021902	0.736671549
C18orf56	ENSG00000176912	18	631320	668340	41	8	123834	2.0144	0.021982	0.736671549
SIGLEC15	ENSG00000197046	18	43395477	43434045	41	6	123834	2.014	0.022007	0.736671549
SH3D21	ENSG00000214193	1	36761988	36800484	27	3	123834	2.0129	0.022061	0.736671549
SFTA3	ENSG00000229415	14	36932493	36993034	26	8	123834	2.0127	0.022075	0.736671549
SYNGR4	ENSG00000105467	19	48857652	48889633	35	8	123834	2.0091	0.022264	0.736671549
NEDD8	ENSG00000129559	14	24676058	24711660	27	5	123834	2.0089	0.022275	0.736671549
CDH19	ENSG00000071991	18	64158320	64281375	154	15	123834	2.0083	0.022306	0.736671549
BPIFC	ENSG00000184459	22	32799834	32870471	153	32	123834	2.0082	0.02231	0.736671549
STK33	ENSG00000130413	11	8403418	8625836	619	20	123834	2.0069	0.022379	0.736671549
SLC45A4	ENSG00000022567	8	142207265	142328404	230	34	123834	2.0066	0.022394	0.736671549
EFR3A	ENSG00000132294	8	132906335	133035889	291	16	123834	2.0058	0.022436	0.736671549
HIST3H2A	ENSG00000181218	1	228635065	228655560	19	3	123834	2.0054	0.022458	0.736671549
HIST3H2BB	ENSG00000196890	1	228635808	228656259	19	3	123834	2.0054	0.022458	0.736671549
BTN2A1	ENSG00000112763	6	26448150	26486849	73	12	123834	2.0052	0.022471	0.736671549
SVOPL	ENSG00000157703	7	138269030	138396097	159	28	123834	2.0033	0.022571	0.736671549
REEP6	ENSG00000115255	19	1481165	1507926	27	4	123834	2.0017	0.022661	0.736671549
GPAM	ENSG00000119927	10	113899624	113985135	103	11	123834	2.0012	0.022684	0.736671549
NOXRED1	ENSG00000165555	14	77850364	77899860	74	7	123834	2.0005	0.022721	0.736671549
IMPDH2	ENSG00000178035	3	49051758	49076841	8	2	123834	2.0002	0.022738	0.736671549
TBCEL	ENSG00000154114	11	120884781	120971484	62	5	123834	2.0002	0.022738	0.736671549
PPP1R32	ENSG00000162148	11	61238592	61268403	46	7	123834	1.9997	0.022768	0.736671549
CADPS	ENSG00000163618	3	62374022	62871054	933	65	123834	1.9973	0.022895	0.736671549
PCDH18	ENSG00000189184	4	138430072	138463648	50	9	123834	1.996	0.022967	0.736671549
NME9	ENSG00000181322	3	137970279	138058728	23	6	123834	1.9948	0.02303	0.736671549
CASQ2	ENSG00000118729	1	116232628	116321402	241	17	123834	1.9941	0.02307	0.736671549
HTR5A-AS1	ENSG00000220575	7	154848777	154873267	34	3	123834	1.9939	0.02308	0.736671549

CTSE	ENSG00000196188	1	206307459	206342104	6	1	123834	1.9938	0.023087	0.736671549
TIFAB	ENSG00000255833	5	134769908	134798089	68	10	123834	1.9937	0.023092	0.736671549
SPTBN4	ENSG00000160460	19	40962148	41092370	105	17	123834	1.9932	0.023118	0.736671549
AARS	ENSG00000090861	16	70276198	70333446	16	6	123834	1.9929	0.023134	0.736671549
GOLT1A	ENSG00000174567	1	204157288	204193220	44	9	123834	1.9911	0.023237	0.736671549
ZMYND11	ENSG00000015171	10	170405	310577	155	13	123834	1.9905	0.023269	0.736671549
ULK2	ENSG00000083290	17	19664142	19781249	116	2	123834	1.9903	0.023278	0.736671549
SESN3	ENSG00000149212	11	94888704	94975705	41	6	123834	1.9901	0.023289	0.736671549
RP1-170O19.20	ENSG00000257184	7	27193154	27229632	23	6	123834	1.9894	0.023326	0.736671549
TUBGCP3	ENSG00000126216	13	113129325	113252481	242	12	123834	1.9893	0.023332	0.736671549
ABCC5	ENSG00000114770	3	183627722	183745803	223	15	123834	1.9891	0.023346	0.736671549
SV2A	ENSG00000159164	1	149864870	149899434	37	2	123834	1.9875	0.023432	0.736671549
IGSF5	ENSG00000183067	21	41107334	41184023	285	14	123834	1.9872	0.023452	0.736671549
MRPL28	ENSG00000086504	16	407384	430527	71	9	123834	1.9863	0.0235	0.736671549
AP006621.5	ENSG00000255284	11	767578	794297	31	6	123834	1.986	0.023514	0.736671549
SLC4A4	ENSG00000080493	4	72043003	72447804	404	27	123834	1.9856	0.023539	0.736671549
SRRD	ENSG00000100104	22	26869843	26900624	54	8	123834	1.9845	0.0236	0.736671549
C9orf153	ENSG00000187753	9	88825180	88884572	76	13	123834	1.9845	0.023602	0.736671549
C7orf69	ENSG00000136275	7	47824889	47869445	79	12	123834	1.9843	0.023614	0.736671549
CCNC	ENSG00000112237	6	99980256	100026849	110	11	123834	1.9833	0.023665	0.736671549
AC023469.1	ENSG00000222031	2	151847734	151915288	136	12	123834	1.9822	0.023729	0.736671549
FKSG61	ENSG00000268156	14	77872741	77893115	31	5	123834	1.9818	0.023748	0.736671549
AC022498.1	ENSG00000213132	3	187886331	187908598	40	11	123834	1.9818	0.023753	0.736671549
AFTPH	ENSG00000119844	2	64741465	64830139	104	4	123834	1.9815	0.02377	0.736671549
MON2	ENSG00000061987	12	62850597	63001363	297	15	123834	1.9799	0.023856	0.736671549
ARL6IP6	ENSG00000177917	2	153564407	153627767	82	10	123834	1.9776	0.023988	0.736671549
AC062017.1	ENSG00000222020	2	240313130	240334058	11	4	123834	1.9775	0.023993	0.736671549
REPIN1	ENSG00000214022	7	150055278	150081133	33	4	123834	1.9774	0.023997	0.736671549
UNC45B	ENSG00000141161	17	33464836	33526364	70	12	123834	1.9736	0.024212	0.736671549
LCE6A	ENSG00000235942	1	152805344	152826459	9	2	123834	1.9729	0.024251	0.736671549
SMIM13	ENSG00000224531	6	11084266	11148964	140	11	123834	1.9728	0.024259	0.736671549
GPD1	ENSG00000167588	12	50487602	50515102	30	3	123834	1.9728	0.02426	0.736671549
NME7	ENSG00000143156	1	169091769	169347205	787	19	123834	1.9727	0.024263	0.736671549
POLM	ENSG00000122678	7	44101846	44132139	47	3	123834	1.9724	0.024283	0.736671549
FEN1	ENSG00000168496	11	61550109	61574716	9	2	123834	1.9723	0.024286	0.736671549
ZNF620	ENSG00000177842	3	40537483	40570227	24	4	123834	1.9719	0.024311	0.736671549
DUXA	ENSG00000258873	19	57655389	57688811	56	7	123834	1.9712	0.02435	0.736671549
MGST2	ENSG00000085871	4	140576922	140671899	147	29	123834	1.9704	0.024394	0.736671549
ZBTB18	ENSG00000179456	1	244204585	244230778	6	3	123834	1.9703	0.024401	0.736671549
SHE	ENSG00000169291	1	154432248	154484589	65	6	123834	1.97	0.024418	0.736671549
MED23	ENSG00000112282	6	131885106	131959369	52	9	123834	1.9692	0.024463	0.736671549
TSPAN19	ENSG00000231738	12	85398094	85440055	10	2	123834	1.9692	0.024467	0.736671549
OR13A1	ENSG00000256574	10	45788102	45821056	116	6	123834	1.9691	0.024473	0.736671549
ACP2	ENSG00000134575	11	47250853	47280457	63	6	123834	1.9682	0.024524	0.736671549
NEDD8-MDP1	ENSG00000255526	14	24673161	24711558	31	6	123834	1.9676	0.024554	0.736671549
IQCF2	ENSG00000184345	3	51885645	51907440	58	5	123834	1.9659	0.024654	0.736671549
CTU1	ENSG00000142544	19	51590863	51621627	17	5	123834	1.9644	0.02474	0.736671549
ST3GAL2	ENSG00000157350	16	70403338	70483140	31	7	123834	1.9644	0.024742	0.736671549
FNBP4	ENSG00000109920	11	47728072	47798995	103	8	123834	1.9643	0.024745	0.736671549
TPD52L2	ENSG00000101150	20	62486596	62532898	65	7	123834	1.964	0.024763	0.736671549
SERPINA4	ENSG00000100665	14	95017428	95046250	86	11	123834	1.964	0.024765	0.736671549
TDRD1	ENSG00000095627	10	115929029	116002063	124	9	123834	1.9603	0.024978	0.7418466
DUSP16	ENSG00000111266	12	12618829	12725317	134	16	123834	1.9579	0.025122	0.744959401
ZNF20	ENSG00000132010	19	12193658	12261222	75	7	123834	1.956	0.025233	0.746248759
GLB1L	ENSG00000163521	2	220091328	220120200	37	4	123834	1.9558	0.025244	0.746248759
LRRIQ1	ENSG00000133640	12	85420092	85667002	196	10	123834	1.953	0.025409	0.748874006
ZNF394	ENSG00000160908	7	99074142	99107947	26	5	123834	1.9524	0.025447	0.748874006
RTCB	ENSG00000100220	22	32773569	32818242	134	20	123834	1.9523	0.025451	0.748874006
TMIGD2	ENSG00000167664	19	4282229	4312428	24	6	123834	1.9508	0.025541	0.749190508
SLC26A1	ENSG00000145217	4	962861	997228	51	10	123834	1.9506	0.025551	0.749190508
B3GALNT2	ENSG00000162885	1	235603238	235677781	140	12	123834	1.9501	0.02558	0.749190508
MAP2K1	ENSG00000169032	15	66669155	66794650	293	16	123834	1.9486	0.025671	0.750235279
FAM114A1	ENSG00000197712	4	38859298	38957360	217	15	123834	1.9466	0.025793	0.750235279
COCH	ENSG00000100473	14	31333720	31374271	32	11	123834	1.9461	0.025822	0.750235279
C2CD5	ENSG00000111731	12	22591517	22707480	141	3	123834	1.9449	0.025895	0.750235279
SPATA4	ENSG00000150628	4	177095789	177126822	55	11	123834	1.9445	0.025918	0.750235279
RAB22A	ENSG00000124209	20	56874752	56952563	155	16	123834	1.9442	0.025933	0.750235279
ADH5	ENSG00000197894	4	99982132	100019952	131	11	123834	1.9429	0.026012	0.750235279
SPEG	ENSG00000072195	2	220289568	220373009	52	7	123834	1.9428	0.02602	0.750235279
EMCN	ENSG00000164035	4	101306498	101811283	1162	42	123834	1.9421	0.026065	0.750235279
ABCC10	ENSG00000124574	6	43385104	43428168	63	10	123834	1.9409	0.026135	0.750235279
ELAVL4	ENSG00000162374	1	50503686	50679458	134	17	123834	1.9405	0.026161	0.750235279
C1QTNF4	ENSG00000172247	11	47601216	47626211	22	6	123834	1.9404	0.026164	0.750235279
MCOLN1	ENSG00000090674	19	7577512	7608895	12	4	123834	1.9403	0.026173	0.750235279
PTK2B	ENSG00000120899	8	27158999	27326903	313	17	123834	1.9397	0.026208	0.750235279
GPR27	ENSG00000170837	3	71793201	71815647	15	5	123834	1.9391	0.026243	0.750235279
LMX1A	ENSG00000162761	1	165161104	165335952	255	32	123834	1.9382	0.026297	0.750235279
ITIH3	ENSG00000162267	3	52818784	52853025	41	5	123834	1.937	0.026374	0.750235279
RNF19B	ENSG00000116514	1	33392046	33440286	58	5	123834	1.9363	0.026414	0.750235279
KCTD13	ENSG00000174943	16	29906333	29948356	30	3	123834	1.936	0.026437	0.750235279
TMEM239	ENSG00000241690	20	2785614	2808712	8	3	123834	1.9352	0.026484	0.750235279
TMEM239	ENSG00000198326	20	2785614	2810930	8	3	123834	1.9352	0.026484	0.750235279
C20orf141	ENSG00000258713	20	2785633	2806479	8	3	123834	1.9352	0.026484	0.750235279
PDAP1	ENSG00000106244	7	98979671	99016452	45	5	123834	1.9334	0.026592	0.751351834
ATP4A	ENSG00000105675	19	36030945	36064560	42	11	123834	1.9332	0.026605	0.751351834
TNFSF14	ENSG00000125735	19	6653148	6680599	17	5	123834	1.9326	0.026642	0.751351834
CENPBD1	ENSG00000177946	16	90026206	90048942	70	11	123834	1.9298	0.026814	0.75508224
PCSK4	ENSG00000115257	19	1471427	1500751	27	5	123834	1.9283	0.026908	0.755085102
DHODH	ENSG00000102967	16	72032487	72068954	63	11	123834	1.9275	0.026958	0.755085102
FAM111B	ENSG00000189057	11	58864658	58904883	30	3	123834	1.9275	0.02696	0.755085102
DES	ENSG00000175084	2	220273099	220301461	16	3	123834	1.9273	0.026973	0.755085102
CLDN8	ENSG00000156284	21	31576324	31598391	35	10	123834	1.9249	0.027123	0.757472987
SLC16A6	ENSG00000108932	17	66253167	66297408	33	7	123834	1.9246	0.027138	0.757472987
ZNF322	ENSG00000181315	6	26626518	26669980	88	9	123834	1.9227	0.027259	0.758857846
CTD-2207O23.10	ENSG00000268614	19	7588481	7614945	22	4	123834	1.9219	0.027312	0.758857846
STAP2	ENSG00000178078	19	4314040	4352783	61	10	123834	1.9206	0.027389	0.758857846
PRUNE	ENSG00000143363	1	150970896	151018189	52	11	123834	1.9206	0.027391	0.758857846
STX19	ENSG00000178750	3	93723213	93757454	20	5	123834	1.9205	0.0274	0.758857846
FAM63B	ENSG00000128923	15	59053391	59164099	147	13	123834	1.9192	0.027481	0.758857846

SEMA6C	ENSG00000143434	1	151094161	151129104	36	6	123834	1.9188	0.027507	0.758857846
CXXC5	ENSG00000171604	5	139016884	139073467	38	5	123834	1.9188	0.027507	0.758857846
PEX16	ENSG00000121680	11	45921220	45950363	15	3	123834	1.9171	0.027613	0.759469292
AHSA1	ENSG00000100591	14	77914213	77945817	40	7	123834	1.9164	0.027656	0.759469292
EGFL8	ENSG00000241404	6	32122360	32146058	23	7	123834	1.9153	0.02773	0.759469292
ZNF124	ENSG00000196418	1	247275277	247345318	147	14	123834	1.915	0.027747	0.759469292
VPS9D1	ENSG00000075399	16	89763542	89797394	41	12	123834	1.9141	0.027806	0.759469292
FAM163B	ENSG00000196990	9	136434079	136461319	32	6	123834	1.9138	0.027824	0.759469292
PPM1A	ENSG00000100614	14	60702470	60775805	102	6	123834	1.9132	0.027863	0.759469292
SETD1B	ENSG00000139718	12	122232086	122280562	31	3	123834	1.9127	0.027893	0.759469292
LCE1A	ENSG00000186844	1	152789949	152810573	8	2	123834	1.9117	0.02796	0.759469292
ST8SIA5	ENSG00000101638	18	44249081	44349220	173	40	123834	1.9116	0.027965	0.759469292
CLSTN1	ENSG00000171603	1	9779084	9894584	89	5	123834	1.9097	0.028087	0.759469292
XPO7	ENSG00000130227	8	21767180	21874096	106	9	123834	1.9096	0.028094	0.759469292
TSR1	ENSG00000167721	17	2215797	2250801	15	7	123834	1.9087	0.028148	0.759469292
ANKRD17	ENSG00000132466	4	73929093	74134515	70	9	123834	1.9083	0.028178	0.759469292
PPDPF	ENSG00000125534	20	62142077	62163559	50	11	123834	1.9067	0.028281	0.759469292
PPT2	ENSG00000221988	6	32111218	32144011	31	9	123834	1.9066	0.028287	0.759469292
HIST1H2BI	ENSG00000168242	6	26263144	26283622	39	6	123834	1.9065	0.028296	0.759469292
NEK8	ENSG00000160602	17	27042915	27080473	25	4	123834	1.906	0.028325	0.759469292
EVA1B	ENSG00000142694	1	36777632	36799755	15	3	123834	1.9055	0.02836	0.759469292
MYO5B	ENSG00000167306	18	47339183	47731463	1029	44	123834	1.9053	0.028368	0.759469292
CPSF4L	ENSG00000187959	17	71234588	71268491	48	3	123834	1.9043	0.028437	0.759469292
DNAJC6	ENSG00000116675	1	65703902	65891552	321	24	123834	1.9041	0.028451	0.759469292
GMPPA	ENSG00000144591	2	220353589	220381710	24	3	123834	1.9041	0.028452	0.759469292
GALNT18	ENSG00000110328	11	11282423	11653552	527	86	123834	1.9026	0.028547	0.759469292
C4orf47	ENSG00000205129	4	186337394	186380980	97	12	123834	1.9023	0.028563	0.759469292
STXBP5	ENSG00000164506	6	147515561	147716866	197	10	123834	1.9023	0.028568	0.759469292
RP11-268J15.5	ENSG00000116883	1	36779335	36804822	14	3	123834	1.9007	0.028673	0.759557577
CCNL2	ENSG00000221978	1	1311091	1344708	60	6	123834	1.9004	0.02869	0.759557577
TNFRSF25	ENSG00000215788	1	6511211	6536255	18	4	123834	1.9	0.028716	0.759557577
EPHB6	ENSG00000106123	7	142542792	142578847	64	6	123834	1.8994	0.028758	0.759557577
ZDBF2	ENSG00000204186	2	207129387	207189148	64	5	123834	1.8989	0.028788	0.759557577
DXO	ENSG00000204348	6	31927587	31950069	31	10	123834	1.8974	0.028891	0.759557577
STK19	ENSG00000204344	6	31928868	31960598	31	10	123834	1.8974	0.028891	0.759557577
DGKZ	ENSG00000149091	11	46344455	46412104	34	5	123834	1.8973	0.028891	0.759557577
SRCRB4D	ENSG00000146700	7	76008651	76049012	112	11	123834	1.8923	0.029223	0.766184727
DGKQ	ENSG00000145214	4	942675	990683	61	10	123834	1.8918	0.029257	0.766184727
COL8A2	ENSG00000171812	1	36550837	36600821	39	5	123834	1.8917	0.029264	0.766184727
C1orf162	ENSG00000143110	1	112006414	112031134	29	9	123834	1.8904	0.029353	0.766630244
CEP57L1	ENSG00000183137	6	109406313	109495135	65	4	123834	1.8895	0.029411	0.766630244
HCN4	ENSG00000138622	15	73602200	73671605	94	11	123834	1.8888	0.029458	0.766630244
SLC22A12	ENSG00000197891	11	64348113	64379820	48	4	123834	1.8882	0.0295	0.766630244
LSM2	ENSG00000204392	6	31755173	31784761	47	16	123834	1.8877	0.029532	0.766630244
APPL1	ENSG00000157500	3	57251765	57317496	73	8	123834	1.8872	0.029565	0.766630244
HTR5A	ENSG00000157219	7	154852034	154887459	66	7	123834	1.886	0.029648	0.766630244
ANXA9	ENSG00000143412	1	150944493	150978110	48	8	123834	1.8851	0.029707	0.766630244
SEC16A	ENSG00000148396	9	139324549	139382141	83	7	123834	1.8851	0.02971	0.766630244
PRKD3	ENSG00000115825	2	37467645	37561951	268	14	123834	1.885	0.029717	0.766630244
ABCG5	ENSG00000138075	2	44029611	44076004	100	13	123834	1.8848	0.02973	0.766630244
ABCE1	ENSG00000164163	4	146009084	146060331	24	5	123834	1.8843	0.029765	0.766630244
CLIC1	ENSG00000213719	6	31688358	31717540	28	11	123834	1.8831	0.029844	0.766844368
SIK1	ENSG00000142178	21	44824395	44857008	43	6	123834	1.8829	0.029854	0.766844368
COX14	ENSG00000178449	12	50495762	50524240	39	3	123834	1.8817	0.029938	0.767028706
OSGIN1	ENSG00000140961	16	83971887	84009937	77	12	123834	1.8802	0.030038	0.767028706
HIST1H3G	ENSG00000256018	6	26261146	26281612	41	7	123834	1.88	0.030056	0.767028706
ZKSCAN5	ENSG00000196652	7	99092274	99142323	42	7	123834	1.8797	0.030071	0.767028706
POC1A	ENSG00000164087	3	52099269	52198706	76	5	123834	1.8789	0.030126	0.767028706
ALPK3	ENSG00000136383	15	85349911	85426713	122	21	123834	1.8782	0.030174	0.767028706
S100A4	ENSG00000196154	1	153506089	153532612	33	1	123834	1.8781	0.030184	0.767028706
S100A3	ENSG00000188015	1	153509805	153531848	33	1	123834	1.8781	0.030184	0.767028706
AL590483.1	ENSG00000268146	1	244217632	244241211	9	3	123834	1.8772	0.030247	0.767243444
TMEM87B	ENSG00000153214	2	112802800	112886895	120	15	123834	1.8752	0.030381	0.767243444
ASCL4	ENSG00000187855	12	108158162	108180421	31	6	123834	1.8751	0.03039	0.767243444
RECQL5	ENSG00000108469	17	73612925	73673269	65	4	123834	1.875	0.030395	0.767243444
FAM26D	ENSG00000164451	6	116840174	116890031	78	9	123834	1.8741	0.030457	0.767243444
BPIFA2	ENSG00000131050	20	31739574	31779218	35	2	123834	1.8739	0.030474	0.767243444
SMPD3	ENSG00000103056	16	68382231	68492591	121	11	123834	1.8739	0.030475	0.767243444
PRPF3	ENSG00000117360	1	150283925	150335671	114	9	123834	1.8729	0.030542	0.767913143
RNF182	ENSG00000180537	6	13914677	13990533	65	4	123834	1.8717	0.030627	0.769033046
MED9	ENSG00000141026	17	17370300	17406540	52	6	123834	1.8699	0.030747	0.771027673
NDUFAF3	ENSG00000178057	3	49047892	49070928	4	2	123834	1.8657	0.031044	0.776694212
TMPO	ENSG00000120802	12	98899290	98954157	113	15	123834	1.865	0.031088	0.776694212
CRADD	ENSG00000169372	12	94061151	94298616	242	35	123834	1.8649	0.031097	0.776694212
FKTN	ENSG00000106692	9	108310411	108413399	141	16	123834	1.8637	0.031181	0.776694212
KDM2A	ENSG00000173120	11	66876740	67035558	90	7	123834	1.8637	0.031181	0.776694212
IL17RC	ENSG00000163702	3	9948758	9985314	34	5	123834	1.8626	0.031259	0.776694212
RHOJ	ENSG00000126785	14	63660832	63769937	182	19	123834	1.8626	0.031259	0.776694212
CCL20	ENSG00000115009	2	228668558	228692272	9	3	123834	1.8611	0.031366	0.776851862
DALRD3	ENSG00000178149	3	49042921	49069726	6	2	123834	1.8595	0.031478	0.776851862
RAP2A	ENSG00000125249	13	98076476	98131382	33	3	123834	1.8594	0.031483	0.776851862
GYS1	ENSG00000104812	19	49461382	49506567	32	5	123834	1.8588	0.031528	0.776851862
SLC25A20	ENSG00000178537	3	48884369	48946426	41	4	123834	1.8588	0.03153	0.776851862
KCNMB1	ENSG00000145936	5	169791675	169826681	34	6	123834	1.8579	0.03159	0.776851862
ECHDC3	ENSG00000134463	10	11774365	11816069	88	7	123834	1.8577	0.031604	0.776851862
ZSCAN10	ENSG00000130182	16	3128891	3159318	30	6	123834	1.8572	0.031639	0.776851862
COL4A3	ENSG00000169031	2	228019281	228189508	573	46	123834	1.8572	0.031642	0.776851862
ICK	ENSG00000112144	6	52856077	52936600	91	12	123834	1.8554	0.031772	0.776851862
NRXN2	ENSG00000110076	11	64363646	64500660	109	6	123834	1.8553	0.031775	0.776851862
TIAF1	ENSG00000221995	17	27390537	27428537	52	4	123834	1.8551	0.031794	0.776851862
OR2W3	ENSG00000238243	1	248021277	248070449	130	19	123834	1.8542	0.031855	0.776851862
PYDC1	ENSG00000169900	16	31217283	31238680	1	1	123834	1.8538	0.031881	0.776851862
ATP6V1G3	ENSG00000151418	1	198482352	198520075	71	9	123834	1.8532	0.031928	0.776851862
ARL13B	ENSG00000169379	3	93688983	93784512	54	8	123834	1.8525	0.031976	0.776851862
NLE1	ENSG00000073536	17	33445772	33479334	15	6	123834	1.8523	0.031992	0.776851862
HOXB4	ENSG00000182742	17	46642875	46667473	38	9	123834	1.8522	0.032001	0.776851862
DMRTC2	ENSG00000142025	19	42338806	42366401	24	2	123834	1.8509	0.032095	0.777977178
PCDH20	ENSG00000197991	13	61973818	62012220	39	4	123834	1.85	0.032154	0.777977178
SLC5A8	ENSG00000256870	12	101539271	101614185	117	12	123834	1.8493	0.032204	0.777977178

PEX11G	ENSG00000104883	19	7531761	7572335	57	11	123834	1.8489	0.032235	0.777977178
ASPHD1	ENSG00000174939	16	29901696	29941185	26	3	123834	1.8487	0.032252	0.777977178
ADAMTS12	ENSG00000151388	5	33513640	33902297	512	48	123834	1.8475	0.032335	0.778100901
SMIM4	ENSG00000168273	3	52558029	52623253	123	8	123834	1.8475	0.032339	0.778100901
SLC47A1	ENSG00000142494	17	19388698	19492347	43	10	123834	1.8448	0.032534	0.778528598
ELFN1	ENSG00000225968	7	1717755	1797590	68	9	123834	1.8441	0.032584	0.778528598
SLC35G3	ENSG00000164729	17	33509539	33531412	32	5	123834	1.8431	0.032661	0.778528598
BTRC	ENSG00000166167	10	103103820	103327078	365	10	123834	1.8424	0.032705	0.778528598
SAP30BP	ENSG00000161526	17	73653196	73714142	50	5	123834	1.8424	0.032706	0.778528598
FOXP1	ENSG00000114861	3	70993844	71643140	528	69	123834	1.8409	0.03282	0.778528598
AC073657.1	ENSG00000268632	15	42063346	42086229	28	7	123834	1.8408	0.032823	0.778528598
HPS4	ENSG00000100099	22	26829389	26889803	112	11	123834	1.8401	0.032877	0.778528598
PTPRZ1	ENSG00000106278	7	121503143	121712090	246	30	123834	1.8389	0.032966	0.778528598
CELSR3	ENSG00000008300	3	48663902	48710348	32	2	123834	1.8381	0.033027	0.778528598
TNN	ENSG00000120332	1	175026994	175127202	258	21	123834	1.8367	0.033129	0.778528598
SPDYE1	ENSG00000136206	7	44030488	44059721	13	2	123834	1.8365	0.033145	0.778528598
SYCP1	ENSG00000198765	1	115387424	115547991	313	15	123834	1.8361	0.033175	0.778528598
PCDHGC5	ENSG00000240764	5	140858808	140902546	61	14	123834	1.835	0.033254	0.778528598
PRKCQ	ENSG00000065675	10	6459105	6632263	266	34	123834	1.8349	0.033261	0.778528598
ACVR2A	ENSG00000121989	2	148592086	148698393	92	8	123834	1.8348	0.033265	0.778528598
ARHGAP26	ENSG00000145819	5	142139949	142618576	665	56	123834	1.8343	0.033302	0.778528598
GPR153	ENSG00000158292	1	6297406	6331035	24	4	123834	1.834	0.033326	0.778528598
CABLES1	ENSG00000134508	18	20704528	20850431	111	13	123834	1.834	0.033326	0.778528598
UPF3A	ENSG00000169062	13	115037059	115081283	63	8	123834	1.8339	0.033336	0.778528598
ADCY6	ENSG00000174233	12	49149975	49192820	39	3	123834	1.833	0.033401	0.778528598
DEGS2	ENSG00000168350	14	100602756	100636500	47	8	123834	1.8319	0.033482	0.778528598
OTOG	ENSG00000188162	11	17558920	17678697	152	17	123834	1.8318	0.033487	0.778528598
ZP3	ENSG00000188372	7	76016835	76081388	144	14	123834	1.8308	0.033566	0.778528598
PSG11	ENSG00000243130	19	43501808	43540664	82	4	123834	1.8304	0.033594	0.778528598
RYR1	ENSG00000196218	19	38914339	39088204	392	32	123834	1.8297	0.033647	0.778528598
ATP5G1	ENSG00000159199	17	46960127	46983233	49	5	123834	1.8291	0.033696	0.778528598
VPS33B	ENSG00000184056	15	91531646	91575833	82	11	123834	1.8284	0.033748	0.778528598
SSR2	ENSG00000163479	1	155968839	156000750	12	3	123834	1.828	0.033777	0.778528598
ZNF362	ENSG00000160094	1	33712146	33776320	50	10	123834	1.8272	0.033833	0.778528598
E2F3	ENSG00000112242	6	20392398	20503941	87	14	123834	1.8272	0.033837	0.778528598
RARB	ENSG00000077092	3	25205823	25649423	867	57	123834	1.8268	0.033862	0.778528598
MTUS1	ENSG00000129422	8	17491304	17668426	530	46	123834	1.8267	0.033876	0.778528598
TRAPPC3L	ENSG00000173626	6	116806152	116876773	104	14	123834	1.8234	0.034123	0.778528598
PIGH	ENSG00000100564	14	68038672	68077004	62	11	123834	1.8224	0.034195	0.778528598
MGST1	ENSG00000008394	12	16490076	16772193	227	25	123834	1.8224	0.0342	0.778528598
C10orf71	ENSG00000177354	10	50497187	50545537	93	15	123834	1.8222	0.03421	0.778528598
HCRTR1	ENSG00000121764	1	32073287	32108119	43	4	123834	1.8212	0.034286	0.778528598
ASB2	ENSG00000100628	14	94390499	94453137	116	19	123834	1.8208	0.034317	0.778528598
PARL	ENSG00000175193	3	183537173	183612721	161	13	123834	1.8208	0.03432	0.778528598
MRPS18C	ENSG00000163319	4	84367085	84400888	49	4	123834	1.8201	0.03437	0.778528598
OR11G2	ENSG00000196832	14	20655495	20676605	54	12	123834	1.8201	0.034371	0.778528598
RRP12	ENSG00000052749	10	99106115	99171127	177	7	123834	1.8201	0.034372	0.778528598
BDKRB1	ENSG00000100739	14	96712161	96745304	54	13	123834	1.8196	0.034407	0.778528598
SOCS7	ENSG00000174111	17	36498111	36566019	81	6	123834	1.8196	0.034413	0.778528598
RSRC2	ENSG00000111011	12	122979190	123021547	45	5	123834	1.8192	0.034441	0.778528598
SERPINE3	ENSG00000253309	13	51899909	51948871	81	5	123834	1.8186	0.034487	0.778528598
CCDC102A	ENSG00000135736	16	57536090	57580511	96	14	123834	1.818	0.034529	0.778528598
OLFM1	ENSG00000130558	9	137957268	138023025	137	21	123834	1.8177	0.034556	0.778528598
SLFN5	ENSG00000166750	17	33560055	33610674	123	14	123834	1.8177	0.034559	0.778528598
SERPINA12	ENSG00000165953	14	94943611	94994181	112	25	123834	1.8172	0.03459	0.778528598
OR2V2	ENSG00000182613	5	180571943	180592890	70	9	123834	1.8151	0.034755	0.778528598
SPRED2	ENSG00000198369	2	65527985	65669771	220	20	123834	1.8149	0.034771	0.778528598
RASD1	ENSG00000108551	17	17387751	17409709	29	4	123834	1.8141	0.034834	0.778528598
OBP2B	ENSG00000171102	9	136070664	136094630	53	5	123834	1.814	0.034842	0.778528598
TPRX1	ENSG00000178928	19	48294500	48332308	53	9	123834	1.8138	0.034853	0.778528598
ZNF536	ENSG00000198597	19	30709197	31214445	494	57	123834	1.8137	0.034863	0.778528598
COL19A1	ENSG00000082293	6	70566463	70929679	513	38	123834	1.8134	0.034881	0.778528598
FLJ27365	ENSG00000197182	22	46439749	46519808	35	10	123834	1.8131	0.034906	0.778528598
PTRH2	ENSG00000141378	17	57741997	57794987	45	6	123834	1.8129	0.034926	0.778528598
SLC6A16	ENSG00000063127	19	49782895	49838482	16	3	123834	1.8125	0.034953	0.778528598
LYSMD1	ENSG00000163155	1	151122224	151148424	8	4	123834	1.8123	0.034973	0.778528598
STRA8	ENSG00000146857	7	134906731	134953244	35	7	123834	1.812	0.034995	0.778528598
RCVRN	ENSG00000109047	17	9789637	9818938	48	8	123834	1.8119	0.034998	0.778528598
FADS2	ENSG00000134824	11	61550452	61644826	128	15	123834	1.8113	0.035045	0.778528598
SMIM5	ENSG00000204323	17	73619514	73647484	15	2	123834	1.8111	0.03506	0.778528598
MEOX2	ENSG00000106511	7	15640837	15736437	194	16	123834	1.8101	0.035138	0.778929231
YWHAH	ENSG00000128245	22	32330447	32363590	68	12	123834	1.8098	0.03516	0.778929231
HSPA12A	ENSG00000165868	10	118420703	118512085	174	16	123834	1.8078	0.035319	0.781042016
ADCY5	ENSG00000173175	3	122991143	123178605	184	14	123834	1.8069	0.035387	0.781042016
CRYZ	ENSG00000116791	1	75161170	75209092	100	15	123834	1.8064	0.035425	0.781042016
C2orf50	ENSG00000150873	2	11263179	11296916	93	10	123834	1.8055	0.035498	0.781042016
CDS2	ENSG00000101290	20	5097432	5188533	164	16	123834	1.8052	0.035519	0.781042016
RP11-529K1.3	ENSG00000260537	16	70323097	70410163	37	8	123834	1.8051	0.03553	0.781042016
OR6F1	ENSG00000169214	1	247865045	247886105	45	7	123834	1.8049	0.035543	0.781042016
FAM181A	ENSG00000140067	14	94375240	94405954	42	9	123834	1.8035	0.035654	0.781202642
AP001094.1	ENSG00000268046	18	8326437	8347038	36	4	123834	1.8031	0.03569	0.781202642
AC074389.6	ENSG00000205971	7	1722446	1743961	27	2	123834	1.8024	0.035742	0.781202642
SLC44A4	ENSG00000204385	6	31820969	31856823	50	13	123834	1.8017	0.035798	0.781202642
POLR2F	ENSG00000100142	22	38338614	38447922	64	4	123834	1.8017	0.035798	0.781202642
AFP	ENSG00000081051	4	74286855	74331891	60	6	123834	1.8016	0.035806	0.781202642
USH2A	ENSG00000042781	1	215786236	216606738	1264	95	123834	1.8012	0.035838	0.781202642
PRRT1	ENSG00000204314	6	32106136	32132150	33	11	123834	1.7994	0.035979	0.782506812
SKIV2L	ENSG00000204351	6	31916857	31947532	37	11	123834	1.7991	0.036001	0.782506812
PRNP	ENSG00000171867	20	4656882	4692236	51	10	123834	1.7979	0.036094	0.782506812
ADIG	ENSG00000182035	20	37199838	37227106	39	8	123834	1.7976	0.036121	0.782506812
ZNF773	ENSG00000152439	19	58001283	58039772	180	5	123834	1.7971	0.036159	0.782506812
VWA2	ENSG00000165816	10	115989018	116061272	127	13	123834	1.7969	0.036174	0.782506812
S100A5	ENSG00000196420	1	153499623	153524241	30	2	123834	1.7968	0.036186	0.782506812
GADD45G	ENSG00000130222	9	92209928	92231470	11	4	123834	1.796	0.036245	0.782715351
C1orf131	ENSG00000143633	1	231349509	231386933	48	8	123834	1.7956	0.036278	0.782715351
PRKAR1B	ENSG00000188191	7	578834	777287	246	32	123834	1.7939	0.036412	0.783940551
AMZ1	ENSG00000174945	7	2709156	2825134	207	21	123834	1.7934	0.036456	0.783940551
NOTCH4	ENSG00000204301	6	32152620	32201844	151	26	123834	1.793	0.036489	0.783940551
SHISA3	ENSG00000178343	4	42389856	42414504	21	6	123834	1.7926	0.036516	0.783940551

GPATCH2	ENSG00000092978	1	217590334	217814424	268	27	123834	1.7923	0.036541	0.783940551
C15orf39	ENSG00000167173	15	75477984	75514510	14	3	123834	1.7918	0.036584	0.78397821
MAPK8IP1	ENSG00000121653	11	45897202	45938016	20	7	123834	1.7896	0.036759	0.785358565
RASAL3	ENSG00000105122	19	15552435	15585382	20	4	123834	1.7894	0.036778	0.785358565
C8orf17	ENSG00000250733	8	140933416	140956190	28	4	123834	1.7889	0.036812	0.785358565
RCCD1	ENSG00000166965	15	91488100	91516349	48	9	123834	1.7888	0.036821	0.785358565
IDUA	ENSG00000127415	4	970785	1008316	42	11	123834	1.7884	0.036855	0.785358565
DIDO1	ENSG00000101191	20	61499090	61579304	159	20	123834	1.7878	0.036904	0.785522096
LCTL	ENSG00000188501	15	66829517	66868317	78	12	123834	1.7867	0.03699	0.785536856
CAPN15	ENSG00000103326	16	567717	614636	44	7	123834	1.7864	0.037017	0.785536856
ANAPC10	ENSG00000164162	4	145878264	146029693	97	10	123834	1.786	0.037046	0.785536856
SMIM20	ENSG00000250317	4	25853452	25941435	103	13	123834	1.7857	0.03707	0.785536856
RPL11	ENSG00000142676	1	24008269	24032915	2	1	123834	1.7838	0.037228	0.787109659
TCEB3C	ENSG00000183791	18	44544573	44566449	15	3	123834	1.7817	0.037396	0.787109659
PCDHGC4	ENSG00000242419	5	140854741	140902546	66	15	123834	1.7816	0.037408	0.787109659
ABCC2	ENSG00000023839	10	101532489	101621949	161	13	123834	1.7814	0.037421	0.787109659
SFXN5	ENSG00000144040	2	73159165	73312747	150	8	123834	1.7813	0.037428	0.787109659
KRTAP13-1	ENSG00000198390	21	31758392	31779136	14	3	123834	1.781	0.037459	0.787109659
MC1R	ENSG00000258839	16	89968527	89997385	68	9	123834	1.7801	0.037528	0.787109659
HYAL3	ENSG00000186792	3	50320262	50346899	12	3	123834	1.7799	0.037545	0.787109659
PGAM1	ENSG00000171314	10	99175917	99203198	39	8	123834	1.779	0.037618	0.787109659
ITIH1	ENSG00000055957	3	52801603	52836078	43	4	123834	1.7788	0.037637	0.787109659
SEC11A	ENSG00000140612	15	85202775	85269947	94	13	123834	1.7778	0.037714	0.787109659
GNG12	ENSG00000172380	1	68157149	68309150	199	24	123834	1.7775	0.037742	0.787109659
AKNA	ENSG00000106948	9	117086436	117166685	72	9	123834	1.7775	0.037745	0.787109659
OR5AC2	ENSG00000196578	3	97796017	97816946	53	4	123834	1.777	0.037787	0.787109659
GALK1	ENSG00000108479	17	73737675	73771792	16	6	123834	1.7769	0.037791	0.787109659
UBXN7	ENSG00000163960	3	196064533	196169345	90	10	123834	1.7756	0.037897	0.787109659
PKIG	ENSG00000168734	20	43150426	43262888	143	12	123834	1.7756	0.037897	0.787109659
PEF1	ENSG00000162517	1	32085463	32120497	36	4	123834	1.7753	0.037927	0.787109659
PCBP1	ENSG00000169564	2	70304585	70326332	30	2	123834	1.7752	0.037931	0.787109659
TMEM254	ENSG00000133678	10	81828402	81862313	117	15	123834	1.7747	0.037977	0.787204816
AL135998.1	ENSG00000215306	14	23276463	23301750	40	8	123834	1.7736	0.038068	0.787684074
NPIPB8	ENSG00000255524	16	28638975	28680003	7	1	123834	1.7734	0.038083	0.787684074
NTM	ENSG00000182667	11	131230373	132216716	1169	134	123834	1.7722	0.038184	0.788127622
TCEB3B	ENSG00000206181	18	44548943	44571988	22	4	123834	1.772	0.038194	0.788127622
FTL	ENSG00000087086	19	49458558	49480135	19	4	123834	1.7713	0.038255	0.788127622
CCDC154	ENSG00000197599	16	1474384	1504557	78	10	123834	1.7708	0.038297	0.788127622
GK2	ENSG00000196475	4	80317508	80339372	10	3	123834	1.77	0.038363	0.788127622
CHIC2	ENSG00000109220	4	54865956	54940857	48	2	123834	1.7694	0.038411	0.788127622
EXOSC6	ENSG00000223496	16	70274134	70295833	6	4	123834	1.769	0.038446	0.788127622
FIGN	ENSG00000182263	2	164439906	164602522	146	20	123834	1.7684	0.038494	0.788127622
HES3	ENSG00000173673	1	6294252	6315638	11	3	123834	1.7679	0.038535	0.788127622
SCAF11	ENSG00000139218	12	46302914	46395903	48	5	123834	1.7678	0.03855	0.788127622
WDR66	ENSG00000158023	12	122345768	122451833	225	19	123834	1.7673	0.03859	0.788127622
LAMB3	ENSG00000196878	1	209778215	209835811	102	14	123834	1.7671	0.038602	0.788127622
AC011239.1	ENSG00000224361	2	23719913	23757214	61	10	123834	1.7666	0.038645	0.78815897
CREB3L1	ENSG00000157613	11	46289212	46352972	14	4	123834	1.7657	0.03872	0.788386183
ADAMTS6	ENSG00000049192	5	64434563	64787747	514	29	123834	1.7648	0.0388	0.788386183
KCNH2	ENSG00000055118	7	150632049	150685403	42	8	123834	1.7644	0.038829	0.788386183
LGALS16	ENSG00000249861	19	40136534	40161287	24	3	123834	1.764	0.038865	0.788386183
PRRG2	ENSG00000126460	19	50073903	50104272	46	4	123834	1.7636	0.038897	0.788386183
HSPA1L	ENSG00000204390	6	31767396	31793437	36	13	123834	1.7635	0.038905	0.788386183
CDKL4	ENSG00000205111	2	39392787	39466729	120	7	123834	1.763	0.038947	0.78839678
MARCKS	ENSG00000155130	6	114168541	114194648	19	6	123834	1.7616	0.03907	0.790045277
AL355531.2	ENSG00000268421	10	131298884	131319525	124	8	123834	1.7601	0.039194	0.790454634
USHBP1	ENSG00000130307	19	17349985	17403595	120	15	123834	1.7599	0.039212	0.790454634
ECI2	ENSG00000198721	6	4105923	4145831	87	8	123834	1.7599	0.039215	0.790454634
ZNHIT1	ENSG00000106400	7	100850949	100877471	37	6	123834	1.759	0.03929	0.791114971
VPS72	ENSG00000163159	1	151132463	151177797	20	5	123834	1.7585	0.039331	0.791114971
TUBA4A	ENSG00000127824	2	220104433	220152892	48	6	123834	1.7575	0.039417	0.791672161
MMS19	ENSG00000155229	10	99208081	99268551	52	7	123834	1.7572	0.039442	0.791672161
SMCP	ENSG00000163206	1	152840793	152867523	14	4	123834	1.7566	0.039494	0.791879696
CD84	ENSG00000066294	1	160500885	160559306	181	16	123834	1.7557	0.039574	0.792479697
SLC22A8	ENSG00000149452	11	62746626	62793311	42	11	123834	1.7551	0.039618	0.792479697
ARHGDIB	ENSG00000111348	12	15084951	15124662	23	5	123834	1.7548	0.039649	0.792479697
PIP5K1A	ENSG00000143398	1	151160425	151232012	53	5	123834	1.7535	0.039761	0.793402176
SNF8	ENSG00000159210	17	46996678	47032479	102	7	123834	1.7524	0.039854	0.793402176
SMG5	ENSG00000198952	1	156209015	156262620	55	4	123834	1.7515	0.039927	0.793402176
LTBP2	ENSG00000119681	14	74954873	75089306	148	21	123834	1.7514	0.039943	0.793402176
CACNA1C	ENSG00000151067	12	2069952	2812108	1066	100	123834	1.7512	0.039953	0.793402176
TMEM115	ENSG00000126062	3	50382180	50407041	5	1	123834	1.751	0.039976	0.793402176
C17orf80	ENSG00000141219	17	71218372	71255091	92	3	123834	1.7505	0.040019	0.793402176
HESX1	ENSG00000163666	3	57221944	57270549	42	5	123834	1.7495	0.040105	0.793402176
FAM153C	ENSG00000204677	5	177423406	177492560	33	6	123834	1.7486	0.040176	0.793402176
UNC119	ENSG00000109103	17	26863725	26889686	3	2	123834	1.7486	0.040176	0.793402176
ACTRT3	ENSG00000184378	3	169474709	169497683	17	2	123834	1.7486	0.040184	0.793402176
RAB35	ENSG00000111737	12	120522899	120565306	44	7	123834	1.7478	0.040248	0.793402176
M6PR	ENSG00000003056	12	9082959	9112551	56	5	123834	1.7478	0.040253	0.793402176
TMEM81	ENSG00000174529	1	205042258	205063645	52	8	123834	1.7473	0.040292	0.793402176
SOCS6	ENSG00000170677	18	67946137	68007436	128	17	123834	1.7468	0.040338	0.793402176
ASPHD2	ENSG00000128203	22	26815239	26850981	55	9	123834	1.7465	0.040363	0.793402176
ITGA9	ENSG00000144668	3	37483606	37875005	552	51	123834	1.745	0.040493	0.794503271
MBLAC2	ENSG00000176055	5	89744020	89780585	32	4	123834	1.7442	0.040566	0.794503271
EPG5	ENSG00000152223	18	43417574	43557240	233	18	123834	1.7432	0.040649	0.794503271
ABCD4	ENSG00000119688	14	74742126	74779759	40	4	123834	1.743	0.040668	0.794503271
GRB2	ENSG00000177885	17	73304157	73411790	217	10	123834	1.7427	0.040694	0.794503271
UBXN6	ENSG00000167671	19	4434996	4467819	54	6	123834	1.7424	0.040719	0.794503271
CLUL1	ENSG00000079101	18	586988	660334	41	11	123834	1.7422	0.040736	0.794503271
MOCS1	ENSG00000124615	6	39857354	39912290	85	11	123834	1.7417	0.040778	0.794503271
REPS1	ENSG00000135597	6	139214630	139319398	278	11	123834	1.7415	0.040796	0.794503271
NAT6	ENSG00000243477	3	50323833	50346852	11	3	123834	1.7411	0.040837	0.794503271
MAN2A2	ENSG00000196547	15	91435448	91475814	53	8	123834	1.7405	0.040888	0.79468211
WNT4	ENSG00000162552	1	22433798	22480462	85	13	123834	1.7397	0.040958	0.794844735
NOB1	ENSG00000141101	16	69765770	69798843	41	7	123834	1.7394	0.04098	0.794844735
SHD	ENSG00000105251	19	4268598	4300721	21	5	123834	1.7388	0.041039	0.795177688
EXOSC2	ENSG00000130713	9	133559108	133590248	54	3	123834	1.7382	0.041091	0.795374468
TRPV6	ENSG00000165125	7	142558956	142593507	72	4	123834	1.7366	0.041227	0.796095297
STAT5B	ENSG00000173757	17	40341186	40438725	50	3	123834	1.7365	0.041241	0.796095297

AGPAT5	ENSG00000155189	8	6555878	6627184	101	15	123834	1.7357	0.041305	0.796095297
CTB-133G6.1	ENSG00000263264	19	7403848	7458271	115	7	123834	1.7343	0.041436	0.796095297
FAM120AOS	ENSG00000188938	9	96198776	96225874	66	10	123834	1.7339	0.041471	0.796095297
UNC45A	ENSG00000140553	15	91463410	91507323	69	6	123834	1.7332	0.041526	0.796095297
ZNF383	ENSG00000188283	19	37698828	37744828	49	7	123834	1.7326	0.041587	0.796095297
IDH2	ENSG00000182054	15	90616277	90655736	39	4	123834	1.7324	0.041597	0.796095297
TMEM222	ENSG00000186501	1	27638651	27672891	45	7	123834	1.7324	0.041601	0.796095297
GAK	ENSG00000178950	4	833064	936161	226	26	123834	1.7322	0.041622	0.796095297
POLR1B	ENSG00000125630	2	113289492	113344635	110	11	123834	1.7314	0.041686	0.796095297
VMO1	ENSG00000182853	17	4678580	4699728	25	6	123834	1.7311	0.041716	0.796095297
RPGRIP1	ENSG00000092200	14	21746098	21829460	125	17	123834	1.7307	0.041755	0.796095297
MC2R	ENSG00000185231	18	13872043	13925706	81	6	123834	1.7298	0.04183	0.796095297
RFTN1	ENSG00000131378	3	16345081	16565533	316	42	123834	1.7293	0.041873	0.796095297
ACE	ENSG00000159640	17	61544422	61609205	38	4	123834	1.7293	0.041876	0.796095297
POLE4	ENSG00000115350	2	75175619	75207255	54	8	123834	1.7293	0.041877	0.796095297
LRRN1	ENSG00000175928	3	3831121	3899387	59	12	123834	1.7291	0.041897	0.796095297
UBE2D3	ENSG00000109332	4	103705540	103800053	149	5	123834	1.7288	0.041924	0.796095297
PRRT4	ENSG00000224940	7	127980379	128011739	23	5	123834	1.7275	0.042041	0.797520287
NAA20	ENSG00000173418	20	19987760	20024299	50	7	123834	1.7269	0.04209	0.797653759
SP5	ENSG00000204335	2	171561861	171584588	31	4	123834	1.7257	0.042197	0.797914415
CNOT6L	ENSG00000138767	4	78624541	78750769	163	11	123834	1.7246	0.042299	0.797914415
AGBL2	ENSG00000165923	11	47671143	47746941	91	8	123834	1.7242	0.04234	0.797914415
OTUD4	ENSG00000164164	4	146021990	146111313	41	8	123834	1.724	0.042358	0.797914415
PTK6	ENSG00000101213	20	62149778	62178723	53	7	123834	1.7219	0.042542	0.797914415
SLC12A9	ENSG00000146828	7	100414442	100474631	84	13	123834	1.7217	0.042559	0.797914415
SNRPB2	ENSG00000125870	20	16700606	16732421	46	10	123834	1.7217	0.042562	0.797914415
HIF1A	ENSG00000100644	14	62152231	62224976	92	9	123834	1.7213	0.042597	0.797914415
CTF1	ENSG00000150281	16	30897928	30924881	10	3	123834	1.7208	0.04264	0.797914415
RP11-181C3.1	ENSG00000245017	12	98869322	98907633	95	14	123834	1.7208	0.042643	0.797914415
TRAPPC11	ENSG00000168538	4	184570420	184644745	135	13	123834	1.7206	0.042666	0.797914415
CCDC85C	ENSG00000205476	14	99967603	100080363	220	17	123834	1.7205	0.042666	0.797914415
ALPPL2	ENSG00000163286	2	233261553	233285424	28	4	123834	1.7199	0.042726	0.797914415
RPRD2	ENSG00000163125	1	150325567	150459042	332	11	123834	1.7194	0.042771	0.797914415
CYB5D1	ENSG00000182224	17	7751064	7775600	25	6	123834	1.7181	0.042887	0.797914415
ALB	ENSG00000163631	4	74252831	74297129	62	5	123834	1.7179	0.04291	0.797914415
NDUFAF2	ENSG00000164182	5	60230956	60458853	435	16	123834	1.7178	0.042919	0.797914415
AL645608.1	ENSG00000268179	1	851264	876445	52	7	123834	1.7171	0.042984	0.797914415
RABL6	ENSG00000196642	9	139692374	139745639	18	2	123834	1.717	0.042988	0.797914415
RNF157	ENSG00000141576	17	74128534	74246454	131	15	123834	1.716	0.04308	0.797914415
NPEPPS	ENSG00000141279	17	45590308	45710642	135	10	123834	1.7155	0.043123	0.797914415
SESN1	ENSG00000080546	6	109297640	109426022	186	5	123834	1.7153	0.043144	0.797914415
TLE3	ENSG00000140332	15	70330129	70400515	50	8	123834	1.7147	0.043199	0.797914415
MAP4K1	ENSG00000104814	19	39068281	39119522	62	7	123834	1.7143	0.043237	0.797914415
ARHGAP28	ENSG00000088756	18	6719717	6925715	232	31	123834	1.7142	0.043246	0.797914415
C2	ENSG00000166278	6	31855562	31923449	93	19	123834	1.7136	0.043305	0.797914415
B3GNTL1	ENSG00000175711	17	80890031	81019686	347	35	123834	1.7132	0.043335	0.797914415
HDAC4	ENSG00000068024	2	239959864	240333348	560	48	123834	1.7128	0.043371	0.797914415
SCRN2	ENSG00000141295	17	45905058	45928699	34	8	123834	1.7127	0.043387	0.797914415
C1orf229	ENSG00000221953	1	247263462	247285719	39	7	123834	1.7125	0.0434	0.797914415
WDR92	ENSG00000243667	2	68340068	68394692	54	7	123834	1.7123	0.043425	0.797914415
RBM27	ENSG00000091009	5	145573113	145728814	198	11	123834	1.7117	0.043478	0.797914415
SH3RF3	ENSG00000172985	2	109735804	110272207	677	53	123834	1.7112	0.043521	0.797914415
IFRD2	ENSG00000214706	3	50315163	50340349	7	2	123834	1.7111	0.043531	0.797914415
DSTYK	ENSG00000133059	1	205101632	205190727	182	12	123834	1.7101	0.043621	0.798793803
KCNA7	ENSG00000104848	19	49560675	49586198	38	5	123834	1.7078	0.043839	0.800757517
TMEM155	ENSG00000164112	4	122670088	122696582	69	9	123834	1.7075	0.043864	0.800757517
TBRG1	ENSG00000154144	11	124482732	124515287	12	4	123834	1.7069	0.043923	0.800757517
CYB561D2	ENSG00000114395	3	50378126	50405891	6	1	123834	1.7064	0.043967	0.800757517
FBXL14	ENSG00000171823	12	1665159	1713331	53	11	123834	1.7063	0.043978	0.800757517
MTCH2	ENSG00000109919	11	47628867	47674175	36	7	123834	1.7063	0.043981	0.800757517
PPIE	ENSG00000084072	1	40147854	40239586	126	25	123834	1.7053	0.04407	0.801610105
CTDSPL	ENSG00000144677	3	37893451	38035960	212	21	123834	1.7035	0.044235	0.803842141
PHF21A	ENSG00000135365	11	45940871	46152985	258	11	123834	1.7022	0.044356	0.803893577
INO80D	ENSG00000114933	2	206848445	206961027	200	17	123834	1.7018	0.044392	0.803893577
PLXNA4	ENSG00000221866	7	131798091	132343447	873	86	123834	1.7018	0.044398	0.803893577
OOSP1	ENSG00000214788	11	59695928	59722807	7	3	123834	1.7017	0.044407	0.803893577
AP3B1	ENSG00000132842	5	77286349	77600579	506	14	123834	1.7011	0.04446	0.804087231
IRF2BP2	ENSG00000168264	1	234730015	234755271	34	7	123834	1.6993	0.044635	0.806484867
IRF3	ENSG00000126456	19	50152826	50179132	16	3	123834	1.6987	0.044686	0.80663959
PAX8	ENSG00000125618	2	113963574	114046527	155	19	123834	1.6975	0.044798	0.80720995
RNF111	ENSG00000157450	15	59147374	59399618	305	20	123834	1.6959	0.044954	0.80720995
POMC	ENSG00000115138	2	25373722	25401772	25	7	123834	1.6944	0.045098	0.80720995
SYTL1	ENSG00000142765	1	27658513	27690421	47	7	123834	1.6943	0.045105	0.80720995
ZMYND10	ENSG00000004838	3	50368541	50394283	9	1	123834	1.6943	0.045108	0.80720995
ACHE	ENSG00000087085	7	100477615	100504594	43	5	123834	1.6935	0.045184	0.80720995
L3MBTL3	ENSG00000198945	6	130324844	130472594	241	23	123834	1.6934	0.045193	0.80720995
STXBP3	ENSG00000116266	1	109279296	109362148	195	8	123834	1.6932	0.045206	0.80720995
ANKRD13D	ENSG00000172932	11	67046018	67079956	27	4	123834	1.6927	0.045252	0.80720995
RP4-758J18.2	ENSG00000224870	1	1324902	1347426	29	4	123834	1.6927	0.045254	0.80720995
CHAF1A	ENSG00000167670	19	4392659	4455015	125	11	123834	1.6925	0.045277	0.80720995
RGPD8	ENSG00000169629	2	113117669	113202065	20	3	123834	1.6924	0.045285	0.80720995
PLA2G16	ENSG00000176485	11	63330667	63394355	23	7	123834	1.6918	0.045342	0.80720995
LASP1	ENSG00000002834	17	37016112	37088023	46	14	123834	1.6918	0.045343	0.80720995
CHRNB2	ENSG00000160716	1	154530257	154562502	30	6	123834	1.6915	0.045374	0.80720995
LRRC31	ENSG00000114248	3	169546967	169597718	73	7	123834	1.6906	0.045454	0.80720995
SP1	ENSG00000185591	12	53763960	53820230	65	4	123834	1.6905	0.045467	0.80720995
MYNN	ENSG00000085274	3	169480619	169517504	39	3	123834	1.6903	0.045482	0.80720995
FAM120A	ENSG00000048828	9	96204004	96338397	292	16	123834	1.6897	0.045544	0.80729088
FAM107B	ENSG00000065809	10	14550556	14826896	558	69	123834	1.689	0.045608	0.80729088
UBE2Z	ENSG00000159202	17	46975731	47016418	98	5	123834	1.6887	0.045635	0.80729088
NELFE	ENSG00000204356	6	31909864	31936887	24	8	123834	1.6876	0.045748	0.80729088
MOCS3	ENSG00000124217	20	49565363	49587820	18	5	123834	1.6852	0.045978	0.80729088
MUC6	ENSG00000184956	11	1002821	1046706	65	9	123834	1.6843	0.046059	0.80729088
LARP7	ENSG00000174720	4	113548120	113588748	59	11	123834	1.6841	0.046081	0.80729088
C10orf118	ENSG00000165813	10	115870621	115943979	94	9	123834	1.6839	0.046097	0.80729088
TXNDC11	ENSG00000153066	16	11762936	11846734	113	12	123834	1.6836	0.046132	0.80729088
KRT16	ENSG00000186832	17	39756030	39782151	34	2	123834	1.6821	0.046273	0.80729088
FIBIN	ENSG00000176971	11	27005628	27028630	75	10	123834	1.6821	0.046277	0.80729088
DNAJB9	ENSG00000128590	7	108200012	108225294	42	6	123834	1.6815	0.046329	0.80729088

DCDC2	ENSG00000146038	6	24161984	24368280	583	38	123834	1.6812	0.046358	0.80729088
CREB5	ENSG00000146592	7	28328940	28875511	648	102	123834	1.681	0.046378	0.80729088
CCDC110	ENSG00000168491	4	186356336	186402913	104	16	123834	1.6809	0.046393	0.80729088
PRKACB	ENSG00000142875	1	84533745	84714181	156	12	123834	1.6806	0.046422	0.80729088
SLC24A3	ENSG00000185052	20	19183290	19713581	918	69	123834	1.6803	0.046449	0.80729088
RDX	ENSG00000137710	11	110035605	110177447	119	6	123834	1.68	0.046474	0.80729088
LSMD1	ENSG00000183011	17	7750003	7798556	57	10	123834	1.679	0.046581	0.80729088
CILP	ENSG00000138615	15	65478337	65513826	11	1	123834	1.6789	0.046584	0.80729088
EBPL	ENSG00000123179	13	50224859	50275611	148	18	123834	1.6772	0.046752	0.80729088
TMX1	ENSG00000139921	14	51696880	51732759	70	9	123834	1.6771	0.046765	0.80729088
OASL	ENSG00000135114	12	121448095	121487045	87	16	123834	1.6764	0.046825	0.80729088
ARIH2	ENSG00000177479	3	48946254	49033815	65	6	123834	1.6764	0.046832	0.80729088
RSPH1	ENSG00000160188	21	43882596	43926464	69	14	123834	1.676	0.046867	0.80729088
PPM1B	ENSG00000138032	2	44385108	44481523	105	8	123834	1.676	0.04687	0.80729088
ISYNA1	ENSG00000105655	19	18535198	18559111	21	2	123834	1.675	0.046971	0.80729088
JAK1	ENSG00000162434	1	65288912	65442187	268	21	123834	1.6749	0.046976	0.80729088
LMBR1L	ENSG00000139636	12	49480919	49514683	25	3	123834	1.6749	0.046976	0.80729088
LHX8	ENSG00000162624	1	75584119	75637218	47	7	123834	1.6746	0.047005	0.80729088
ESYT3	ENSG00000158220	3	138143428	138210528	75	10	123834	1.6745	0.047018	0.80729088
ALDH16A1	ENSG00000161618	19	49946426	49984305	62	6	123834	1.6741	0.047055	0.80729088
NDUFS5	ENSG00000168653	1	39481990	39510308	88	6	123834	1.6741	0.047058	0.80729088
FEM1A	ENSG00000141965	19	4781728	4805571	30	8	123834	1.673	0.047168	0.80729088
SMAD5	ENSG00000113658	5	135458534	135534435	141	3	123834	1.6727	0.04719	0.80729088
ADM5	ENSG00000224420	19	50181921	50203832	14	3	123834	1.6727	0.047192	0.80729088
S100A6	ENSG00000197956	1	153497075	153518720	14	2	123834	1.6712	0.047338	0.80729088
DHRS1	ENSG00000157379	14	24749804	24779039	68	8	123834	1.6712	0.047344	0.80729088
ECHDC2	ENSG00000121310	1	53351656	53402884	37	5	123834	1.6711	0.047349	0.80729088
FASTKD5	ENSG00000215251	20	3117165	3150543	35	7	123834	1.6709	0.047369	0.80729088
CD160	ENSG00000117281	1	145685798	145725614	24	3	123834	1.6708	0.047385	0.80729088
ARMC2	ENSG00000118690	6	109159619	109305186	226	19	123834	1.6705	0.047405	0.80729088
AC003005.4	ENSG00000268107	19	57989086	58026749	154	6	123834	1.6702	0.047435	0.80729088
TMEM184B	ENSG00000198792	22	38605298	38679040	83	10	123834	1.6693	0.047525	0.80729088
RP11-986E7.7	ENSG00000273259	14	95048395	95100983	139	20	123834	1.6689	0.047573	0.80729088
GID8	ENSG00000101193	20	61559471	61586996	49	9	123834	1.6688	0.04758	0.80729088
CACNG4	ENSG00000075461	17	64951026	65039514	114	19	123834	1.6687	0.047589	0.80729088
EPC1	ENSG00000120616	10	32546679	32677726	195	12	123834	1.6679	0.047665	0.80729088
PTPRQ	ENSG00000139304	12	80789774	81082802	607	42	123834	1.6678	0.047674	0.80729088
SPZ1	ENSG00000164299	5	79605444	79627661	85	11	123834	1.6678	0.047682	0.80729088
MRPL20	ENSG00000242485	1	1327288	1352693	30	4	123834	1.6675	0.04771	0.80729088
SSBP4	ENSG00000130511	19	18519674	18555372	29	3	123834	1.6673	0.047731	0.80729088
ATP6V1G1	ENSG00000136888	9	117340026	117370653	66	9	123834	1.6668	0.047777	0.80729088
BCL2L12	ENSG00000126453	19	50158823	50187173	11	2	123834	1.6668	0.04778	0.80729088
NPAS3	ENSG00000151322	14	33394139	34283382	1040	152	123834	1.665	0.047959	0.808996657
ZBTB12	ENSG00000204366	6	31857384	31879769	33	12	123834	1.6644	0.048018	0.808996657
NPHP1	ENSG00000144061	2	110869888	110972643	106	6	123834	1.6635	0.048105	0.808996657
E4F1	ENSG00000167967	16	2263567	2295743	23	4	123834	1.6631	0.048144	0.808996657
TRIM72	ENSG00000177238	16	31215342	31246510	3	1	123834	1.663	0.048161	0.808996657
ERCC8	ENSG00000049167	5	60159658	60250900	180	14	123834	1.6621	0.048247	0.808996657
WBSCR17	ENSG00000185274	7	70587155	71188585	1266	103	123834	1.6621	0.048248	0.808996657
ACMSD	ENSG00000153086	2	135586117	135669604	101	5	123834	1.6618	0.048272	0.808996657
PSMD4	ENSG00000159352	1	151217179	151249955	25	3	123834	1.6611	0.048343	0.808996657
ACOXL	ENSG00000153093	2	111480150	111885799	682	44	123834	1.6604	0.048422	0.808996657
SULT1A1	ENSG00000196502	16	28606903	28644946	16	2	123834	1.6599	0.048465	0.808996657
ZBTB20	ENSG00000181722	3	114046941	114876118	823	55	123834	1.6597	0.04849	0.808996657
MSH5	ENSG00000204410	6	31697725	31742622	47	13	123834	1.6594	0.048515	0.808996657
MSH5-SAPCD1	ENSG00000255152	6	31697797	31742628	47	13	123834	1.6594	0.048515	0.808996657
RBAK	ENSG00000146587	7	5075452	5119119	114	13	123834	1.6591	0.048544	0.808996657
SYNE3	ENSG00000176438	14	95873831	95952173	146	28	123834	1.6581	0.048651	0.808996657
NDUFS7	ENSG00000115286	19	1373526	1405583	30	5	123834	1.6579	0.048669	0.808996657
CISD2	ENSG00000145354	4	103780135	103820399	55	3	123834	1.6562	0.048839	0.808996657
RBBP5	ENSG00000117222	1	205045270	205101143	127	13	123834	1.656	0.048865	0.808996657
SRRT	ENSG00000087087	7	100462733	100496285	40	5	123834	1.6558	0.048886	0.808996657
PNMA2	ENSG00000240694	8	26352202	26381608	88	13	123834	1.6543	0.049036	0.808996657
FCN3	ENSG00000142748	1	27685603	27711315	15	4	123834	1.6539	0.049073	0.808996657
AL645922.1	ENSG00000268923	6	31963945	31984881	2	1	123834	1.6536	0.049105	0.808996657
FLOT2	ENSG00000132589	17	27196353	27234697	22	6	123834	1.653	0.049168	0.808996657
TMEM182	ENSG00000170417	2	103343367	103470352	154	9	123834	1.6525	0.049214	0.808996657
TNFAIP6	ENSG00000123610	2	152204106	152246560	76	8	123834	1.651	0.049369	0.808996657
SLC17A7	ENSG00000104888	19	49922658	49955617	25	3	123834	1.651	0.049374	0.808996657
PLCB2	ENSG00000137841	15	40560377	40610136	26	8	123834	1.6507	0.049403	0.808996657
ACPP	ENSG00000014257	3	132026211	132097142	167	15	123834	1.6506	0.049414	0.808996657
GAMT	ENSG00000130005	19	1387091	1411569	29	5	123834	1.6504	0.049429	0.808996657
FSIP1	ENSG00000150667	15	39882232	40085031	318	17	123834	1.6503	0.049438	0.808996657
KIAA1199	ENSG00000103888	15	81061684	81254117	121	28	123834	1.6502	0.049456	0.808996657
APOA4	ENSG00000110244	11	116681419	116704022	46	10	123834	1.6501	0.049464	0.808996657
C1orf167	ENSG00000215910	1	11811844	11859642	136	10	123834	1.6499	0.049478	0.808996657
SAPCD1	ENSG00000228727	6	31720576	31742628	21	8	123834	1.6493	0.049543	0.808996657
CST8	ENSG00000125815	20	23461738	23486655	47	8	123834	1.6489	0.049585	0.808996657
WT1	ENSG00000184937	11	32399321	32467176	178	18	123834	1.6487	0.04961	0.808996657
PRKAR2A	ENSG00000114302	3	48772030	48895279	59	5	123834	1.6485	0.049624	0.808996657
DCDC2C	ENSG00000214866	2	3741453	3846122	166	22	123834	1.6484	0.04964	0.808996657
RAB40C	ENSG00000197562	16	629357	689272	133	7	123834	1.6483	0.049642	0.808996657
CLEC4A	ENSG00000111729	12	8266228	8301203	26	5	123834	1.6472	0.049756	0.808996657
TTK	ENSG00000112742	6	80703604	80762244	177	8	123834	1.6472	0.049757	0.808996657
NFATC2IP	ENSG00000176953	16	28952128	28988418	19	2	123834	1.6471	0.049765	0.808996657
STK16	ENSG00000115661	2	220100177	220125059	26	4	123834	1.6455	0.049932	0.808996657
DPT	ENSG00000143196	1	168654697	168708502	159	21	123834	1.6454	0.049946	0.808996657
FUS	ENSG00000089280	16	31181431	31213127	4	1	123834	1.6452	0.04996	0.808996657
ALAD	ENSG00000148218	9	116138597	116173613	31	6	123834	1.6451	0.049977	0.808996657
UQCR11	ENSG00000127540	19	1587171	1615480	7	2	123834	1.645	0.049983	0.808996657
SERPINE2	ENSG00000135919	2	224829829	224914036	133	17	123834	1.645	0.049987	0.808996657
C12orf61	ENSG00000221949	12	62985531	63007214	31	7	123834	1.6448	0.050009	0.808996657
AES	ENSG00000104964	19	3042908	3073105	6	3	123834	1.642	0.050298	0.812979918
C20orf112	ENSG00000197183	20	31020862	31182876	148	14	123834	1.6405	0.050445	0.813628873
CSRP1	ENSG00000159176	1	201442658	201488584	59	8	123834	1.6403	0.050466	0.813628873
ROBO2	ENSG00000185008	3	75945846	77709115	2798	154	123834	1.6397	0.050538	0.813628873
LYPD4	ENSG00000183103	19	42331148	42358760	24	2	123834	1.6396	0.050542	0.813628873
RGR	ENSG00000148604	10	85994809	86029716	43	12	123834	1.6393	0.050579	0.813628873
PSMC3IP	ENSG00000131470	17	40714333	40739849	27	4	123834	1.6385	0.050654	0.813628873

SPATA6	ENSG00000132122	1	48751044	48947845	255	20	123834	1.6384	0.050665	0.813628873
OSBPL2	ENSG00000130703	20	60803580	60881268	123	10	123834	1.6378	0.050733	0.813628873
SHANK3	ENSG00000251322	22	51102843	51181726	66	14	123834	1.6377	0.05074	0.813628873
HEY2	ENSG00000135547	6	126058810	126092415	29	3	123834	1.6374	0.050773	0.813628873
PIH1D1	ENSG00000104872	19	49939555	49966754	42	4	123834	1.6371	0.050809	0.813628873
INTS8	ENSG00000164941	8	95815539	95903974	210	16	123834	1.636	0.050925	0.814040343
SPI1	ENSG00000066336	11	47366411	47410127	63	8	123834	1.6355	0.050976	0.814040343
GLTPD2	ENSG00000182327	17	4682254	4703685	22	5	123834	1.6353	0.050989	0.814040343
CAPRIN1	ENSG00000135387	11	34063230	34132703	163	5	123834	1.6352	0.051006	0.814040343
LTB4R	ENSG00000213903	14	24770656	24797242	29	5	123834	1.633	0.051237	0.815643452
NXPH1	ENSG00000122584	7	8463585	8802593	723	42	123834	1.6328	0.05126	0.815643452
CYP2J2	ENSG00000134716	1	60348980	60402462	83	10	123834	1.6324	0.051302	0.815643452
TUBA1A	ENSG00000167552	12	49568579	49593107	21	2	123834	1.6324	0.051303	0.815643452
UQCR11	ENSG00000267059	19	1568338	1615444	12	2	123834	1.6322	0.051321	0.815643452
C3	ENSG00000125730	19	6667715	6740573	116	16	123834	1.6309	0.051456	0.81707427
ATL3	ENSG00000184743	11	63381559	63449393	44	2	123834	1.6305	0.051497	0.81707427
KLRG2	ENSG00000188883	7	139127436	139178458	85	13	123834	1.6296	0.051595	0.817946422
CIDEC	ENSG00000187288	3	9898398	9931938	41	6	123834	1.628	0.051766	0.81997344
NRAS	ENSG00000213281	1	115237090	115269515	26	5	123834	1.6272	0.051845	0.820541016
BRAT1	ENSG00000106009	7	2567511	2605361	61	6	123834	1.6267	0.051898	0.820696493
DEPDC7	ENSG00000121690	11	33027410	33065128	116	6	123834	1.6249	0.05209	0.82244829
LHB	ENSG00000104826	19	49509237	49530338	37	3	123834	1.6243	0.05216	0.82244829
SCGN	ENSG00000079689	6	25642464	25712011	129	16	123834	1.6238	0.052209	0.82244829
ZBTB25	ENSG00000089775	14	64905824	64981931	140	11	123834	1.6238	0.05221	0.82244829
OR2F1	ENSG00000213215	7	143647027	143668108	57	7	123834	1.6235	0.052242	0.82244829
FARP1	ENSG00000152767	13	98784816	99112027	543	74	123834	1.6225	0.052353	0.82244829
UQCR10	ENSG00000184076	22	30153358	30176402	54	8	123834	1.622	0.052402	0.82244829
AMBRA1	ENSG00000110497	11	46407964	46625675	101	5	123834	1.6217	0.052431	0.82244829
OLAH	ENSG00000152463	10	15064226	15125851	150	18	123834	1.6212	0.052492	0.82244829
COL16A1	ENSG00000084636	1	32107848	32179920	42	8	123834	1.6211	0.052499	0.82244829
TMEM143	ENSG00000161558	19	48825613	48877494	86	13	123834	1.6209	0.052516	0.82244829
HCRTR2	ENSG00000137252	6	55029050	55157418	252	19	123834	1.6208	0.052528	0.82244829
MROH2B	ENSG00000171495	5	40988119	41081444	297	23	123834	1.6198	0.052641	0.8235392
PRMT1	ENSG00000126457	19	50169043	50202286	11	3	123834	1.6171	0.052926	0.824987347
SMIM6	ENSG00000259120	17	73632323	73654057	16	2	123834	1.6167	0.052975	0.824987347
P4HTM	ENSG00000178467	3	49017319	49054587	14	3	123834	1.6166	0.052982	0.824987347
MDK	ENSG00000110492	11	46392306	46415375	12	2	123834	1.6159	0.05306	0.824987347
NTF4	ENSG00000225950	19	49549050	49578333	37	6	123834	1.6158	0.05307	0.824987347
CGB	ENSG00000104827	19	49516126	49537590	34	4	123834	1.6154	0.053107	0.824987347
GIPR	ENSG00000010310	19	46161502	46196982	47	7	123834	1.6154	0.053109	0.824987347
LAPTM4B	ENSG00000104341	8	98777285	98875241	240	12	123834	1.6145	0.053206	0.824987347
OR1N2	ENSG00000171501	9	125305391	125326493	126	11	123834	1.6145	0.053213	0.824987347
EPB41L4A	ENSG00000129595	5	111468138	111765013	443	33	123834	1.6136	0.053305	0.824987347
AC011755.1	ENSG00000269686	2	133864577	133886625	51	6	123834	1.6136	0.05331	0.824987347
AC110792.1	ENSG00000269506	4	54917213	54937377	9	2	123834	1.6132	0.053352	0.824987347
TLX3	ENSG00000164438	5	170726288	170749138	27	5	123834	1.6132	0.053355	0.824987347
AL079342.1	ENSG00000203863	6	89664246	89686685	15	6	123834	1.6129	0.053382	0.824987347
YY1	ENSG00000100811	14	100694635	100759129	94	8	123834	1.6126	0.053416	0.824987347
EIF4H	ENSG00000106682	7	73578575	73621431	11	2	123834	1.6125	0.053428	0.824987347
SLC14A1	ENSG00000141469	18	43294092	43342485	116	10	123834	1.6114	0.053541	0.825323047
ZNF652	ENSG00000198740	17	47356568	47449835	173	12	123834	1.6112	0.053565	0.825323047
GOT1L1	ENSG00000169154	8	37781799	37807647	4	1	123834	1.6111	0.05358	0.825323047
C3orf36	ENSG00000221972	3	133636992	133658656	14	3	123834	1.6099	0.053705	0.826510216
CTD-2278I10.6	ENSG00000269307	19	17368227	17403775	89	11	123834	1.6094	0.053761	0.826510216
IQCF5	ENSG00000214681	3	51897737	51919600	39	6	123834	1.609	0.053804	0.826510216
SLC51A	ENSG00000163959	3	195928358	195980049	100	13	123834	1.6088	0.053831	0.826510216
DDAH2	ENSG00000213722	6	31684815	31708394	16	8	123834	1.608	0.053917	0.826510761
HYAL1	ENSG00000114378	3	50327320	50359812	15	4	123834	1.608	0.053918	0.826510761
KLHDC1	ENSG00000197776	14	50149823	50229870	36	4	123834	1.6071	0.054014	0.826817391
TRIM35	ENSG00000104228	8	27132404	27178836	54	6	123834	1.607	0.054025	0.826817391
APOA5	ENSG00000110243	11	116650083	116673136	33	9	123834	1.6061	0.054125	0.826893257
ADAR	ENSG00000160710	1	154544538	154610475	96	7	123834	1.6054	0.054202	0.826893257
FAM104A	ENSG00000133193	17	71193492	71242892	145	4	123834	1.6049	0.054253	0.826893257
HDDC3	ENSG00000184508	15	91464148	91485799	40	4	123834	1.6049	0.054256	0.826893257
HARBI1	ENSG00000180423	11	46614411	46649459	18	2	123834	1.6038	0.054382	0.826893257
PYCARD	ENSG00000103490	16	31202806	31224771	5	1	123834	1.6031	0.054456	0.826893257
C16orf98	ENSG00000261359	16	31203206	31224773	5	1	123834	1.6031	0.054456	0.826893257
ANKLE1	ENSG00000160117	19	17382454	17408455	71	8	123834	1.6026	0.054513	0.826893257
GFRA3	ENSG00000146013	5	137578068	137620360	54	8	123834	1.6023	0.05454	0.826893257
LAMTOR2	ENSG00000116586	1	156014543	156038301	10	5	123834	1.6021	0.054572	0.826893257
RHOF	ENSG00000139725	12	122205664	122251309	65	6	123834	1.6011	0.05468	0.826893257
C1orf106	ENSG00000163362	1	200850176	200894863	45	11	123834	1.6008	0.054706	0.826893257
NGEF	ENSG00000066248	2	233733396	233887982	247	35	123834	1.6008	0.054708	0.826893257
EIF4E3	ENSG00000163412	3	71714567	71813924	139	21	123834	1.6008	0.054712	0.826893257
PPP4R1L	ENSG00000124224	20	56796188	56894495	190	14	123834	1.6001	0.054788	0.826893257
MLXIPL	ENSG00000009950	7	72997524	73048873	102	11	123834	1.6	0.054801	0.826893257
TALDO1	ENSG00000177156	11	737329	775024	44	6	123834	1.6	0.054802	0.826893257
TRIP6	ENSG00000087077	7	100454760	100481076	37	5	123834	1.5999	0.054813	0.826893257
AGBL4	ENSG00000186094	1	48988527	50499585	1456	39	123834	1.5991	0.054902	0.82743541
ENTPD5	ENSG00000187097	14	74414713	74496102	244	19	123834	1.5988	0.054936	0.82743541
GAL3ST4	ENSG00000197093	7	99746867	99776373	17	3	123834	1.5979	0.055036	0.82775385
SMARCC1	ENSG00000173473	3	47616762	47833596	148	4	123834	1.5965	0.055193	0.82775385
LRP5L	ENSG00000100068	22	25737385	25811344	104	16	123834	1.5964	0.055204	0.82775385
SMG6	ENSG00000070366	17	1953133	2217065	485	17	123834	1.5958	0.055263	0.82775385
OR11H6	ENSG00000176219	14	20681791	20702964	113	11	123834	1.5955	0.055295	0.82775385
GADD45B	ENSG00000099860	19	2466120	2488257	13	3	123834	1.5951	0.055348	0.82775385
BOLA1	ENSG00000178096	1	149849440	149882351	31	1	123834	1.5943	0.055429	0.82775385
RTP2	ENSG00000198471	3	187406047	187430345	72	10	123834	1.5936	0.055511	0.82775385
ZNF259	ENSG00000109917	11	116638436	116668766	54	13	123834	1.5935	0.055529	0.82775385
C8orf4	ENSG00000176907	8	40000989	40022821	26	4	123834	1.5932	0.055558	0.82775385
MITF	ENSG00000187098	3	69778586	70027488	274	12	123834	1.5932	0.055563	0.82775385
ARIH2OS	ENSG00000221883	3	48945221	48966818	25	4	123834	1.5929	0.055592	0.82775385
CLEC4C	ENSG00000198178	12	7872011	7914201	111	13	123834	1.5925	0.055631	0.82775385
CACNA1I	ENSG00000100346	22	39956758	40095742	95	15	123834	1.5924	0.055646	0.82775385
MAP2K2	ENSG00000126934	19	4080319	4134126	28	7	123834	1.5924	0.055647	0.82775385
BABAM1	ENSG00000105393	19	17368159	17402058	88	11	123834	1.5921	0.055682	0.82775385
CIDEB	ENSG00000136305	14	24764302	24790636	40	6	123834	1.5916	0.055741	0.82775385
LTB4R2	ENSG00000213906	14	24764940	24791259	40	6	123834	1.5916	0.055741	0.82775385
HOXC8	ENSG00000037965	12	54392832	54417570	8	2	123834	1.5901	0.055911	0.829042371

XXcos-LUCA11.5	ENSG00000272104	3	50378323	50415354	13	2	123834	1.59	0.055915	0.829042371
SRR	ENSG00000167720	17	2196677	2238554	32	5	123834	1.589	0.056029	0.829068018
CD19	ENSG00000177455	16	28933260	28960667	7	2	123834	1.5886	0.056071	0.829068018
ZNF592	ENSG00000166716	15	85281866	85359659	123	13	123834	1.588	0.056138	0.829068018
MCHR2	ENSG00000152034	6	100357786	100452123	110	12	123834	1.5878	0.05616	0.829068018
Z98049.1	ENSG00000269048	6	166935369	166957709	61	4	123834	1.5875	0.056205	0.829068018
C19orf84	ENSG00000262874	19	51881543	51903828	39	5	123834	1.5871	0.056246	0.829068018
TMEM67	ENSG00000164953	8	94757072	94841462	40	4	123834	1.5868	0.056279	0.829068018
FRMD4A	ENSG00000151474	10	13675706	14514141	1610	163	123834	1.5868	0.05628	0.829068018
TIPIN	ENSG00000075131	15	66618544	66689084	157	19	123834	1.586	0.056367	0.829068018
MOXD1	ENSG00000079931	6	132607194	132732684	148	22	123834	1.5857	0.056406	0.829068018
ANAPC1	ENSG00000153107	2	112513848	112652267	193	14	123834	1.5851	0.056468	0.829068018
OR6S1	ENSG00000181803	14	21098855	21119850	82	5	123834	1.5847	0.056519	0.829068018
DNAH17-AS1	ENSG00000268470	17	76484911	76509138	65	14	123834	1.5844	0.056552	0.829068018
TMEM260	ENSG00000070269	14	56945072	57127324	328	34	123834	1.5842	0.056569	0.829068018
HSD17B11	ENSG00000198189	4	88247762	88322538	148	14	123834	1.5842	0.056572	0.829068018
POLR3B	ENSG00000013503	12	106741436	106913976	320	14	123834	1.5819	0.056835	0.829068018
BEND7	ENSG00000165626	10	13470484	13580974	169	14	123834	1.5818	0.05685	0.829068018
LTB4R2	ENSG00000272658	14	24768161	24791252	33	5	123834	1.5815	0.056884	0.829068018
DHFRL1	ENSG00000178700	3	93756680	93792233	17	5	123834	1.5814	0.056888	0.829068018
PRDM12	ENSG00000130711	9	133529981	133568368	70	8	123834	1.5814	0.056895	0.829068018
HMGB4	ENSG00000176256	1	34316076	34340392	16	4	123834	1.5809	0.056953	0.829068018
AASDHPPT	ENSG00000149313	11	105936228	105979437	50	6	123834	1.5805	0.056998	0.829068018
PQLC3	ENSG00000162976	2	11285324	11329000	79	15	123834	1.5804	0.057009	0.829068018
MMAB	ENSG00000139428	12	109981542	110021679	132	15	123834	1.5803	0.057022	0.829068018
CACNA1D	ENSG00000157388	3	53518683	53857760	315	49	123834	1.58	0.05705	0.829068018
DOCK5	ENSG00000147459	8	25032238	25285598	387	38	123834	1.5797	0.057091	0.829068018
ZIC1	ENSG00000152977	3	147101209	147238080	176	16	123834	1.5794	0.057123	0.829068018
SEMA3C	ENSG00000075223	7	80361854	80561675	190	20	123834	1.5793	0.057138	0.829068018
ZCCHC8	ENSG00000033030	12	122947417	122995518	49	3	123834	1.5787	0.057197	0.829291057
LIM2	ENSG00000105370	19	51873163	51901214	41	6	123834	1.5777	0.057312	0.829813479
DPPA3	ENSG00000187569	12	7854050	7880155	47	8	123834	1.5773	0.057358	0.829813479
VAV1	ENSG00000141968	19	6762725	6867377	127	18	123834	1.5773	0.057364	0.829813479
LRRIQ4	ENSG00000188306	3	169529710	169565563	58	6	123834	1.5768	0.057424	0.830049728
CEP70	ENSG00000114107	3	138203186	138323380	179	10	123834	1.5756	0.057554	0.831296681
EIF2A	ENSG00000144895	3	150254465	150312029	110	12	123834	1.5739	0.057751	0.831392791
TSC22D2	ENSG00000196428	3	150116122	150194218	144	16	123834	1.5737	0.05778	0.831392791
CAPN11	ENSG00000137225	6	44116548	44162139	42	11	123834	1.5732	0.057841	0.831392791
LRFN2	ENSG00000156564	6	40349325	40565204	514	40	123834	1.5728	0.057885	0.831392791
ZNF549	ENSG00000121406	19	58028693	58078910	63	6	123834	1.5726	0.057901	0.831392791
C2orf44	ENSG00000163026	2	24242210	24282445	53	10	123834	1.5721	0.057959	0.831392791
UGT2B11	ENSG00000213759	4	70055669	70090449	140	13	123834	1.5717	0.058015	0.831392791
TUBA1B	ENSG00000123416	12	49511565	49535180	27	1	123834	1.5714	0.058041	0.831392791
AC064874.1	ENSG00000222007	2	236672499	236702031	85	2	123834	1.5708	0.058111	0.831392791
GALR1	ENSG00000166573	18	74952505	74990858	137	20	123834	1.5702	0.058184	0.831392791
TAF4B	ENSG00000141384	18	23795900	23981649	305	19	123834	1.5696	0.058249	0.831392791
PPP1R9B	ENSG00000108819	17	48201104	48237991	25	6	123834	1.5696	0.05825	0.831392791
NRN1	ENSG00000124785	6	5988232	6017200	20	6	123834	1.5691	0.058312	0.831392791
MLX	ENSG00000108788	17	40709086	40735257	33	4	123834	1.5691	0.058312	0.831392791
LSMEM2	ENSG00000179564	3	50306458	50335545	8	2	123834	1.5689	0.058331	0.831392791
IGSF21	ENSG00000117154	1	18424240	18714977	431	69	123834	1.5688	0.05835	0.831392791
TOM1	ENSG00000100284	22	35685268	35753985	116	9	123834	1.5683	0.0584	0.831392791
GRPEL1	ENSG00000109519	4	7050633	7079924	52	6	123834	1.5678	0.058467	0.831392791
TSPYL5	ENSG00000180543	8	98275717	98300176	81	9	123834	1.5668	0.058582	0.831392791
ZNF112	ENSG00000062370	19	44820708	44881377	140	14	123834	1.5666	0.058605	0.831392791
GPR55	ENSG00000135898	2	231762033	231835781	113	21	123834	1.5657	0.058714	0.831392791
NOD1	ENSG00000106100	7	30454143	30528400	110	14	123834	1.5654	0.058742	0.831392791
TCEB3CL	ENSG00000234298	18	44538658	44560534	12	3	123834	1.5652	0.058764	0.831392791
AC008498.1	ENSG00000269267	5	60401538	60421762	42	8	123834	1.5649	0.058806	0.831392791
PROS1	ENSG00000184500	3	93581881	93702910	62	11	123834	1.5648	0.058811	0.831392791
PTBP2	ENSG00000117569	1	97177221	97299294	174	11	123834	1.5646	0.058841	0.831392791
NEUROG3	ENSG00000122859	10	71321454	71342994	9	3	123834	1.5641	0.058897	0.831392791
ASIC1	ENSG00000110881	12	50441331	50487394	42	6	123834	1.564	0.058909	0.831392791
FBXO9	ENSG00000112146	6	52906789	52975671	57	6	123834	1.564	0.058913	0.831392791
CCDC144NL	ENSG00000205212	17	20729760	20809453	38	7	123834	1.5633	0.058996	0.831392791
MLTK	ENSG00000091436	2	173930163	174142738	427	35	123834	1.5625	0.05909	0.831392791
SGCG	ENSG00000102683	13	23745091	23909304	602	50	123834	1.5625	0.05909	0.831392791
MAN2B2	ENSG00000013288	4	6566902	6635089	81	15	123834	1.5623	0.059109	0.831392791
AC129492.6	ENSG00000214999	17	7972800	7994883	16	5	123834	1.5623	0.059111	0.831392791
GPR31	ENSG00000120436	6	167559759	167581817	27	2	123834	1.5608	0.059283	0.831392791
PDPR	ENSG00000090857	16	70137529	70205203	25	2	123834	1.5604	0.05933	0.831392791
GALM	ENSG00000143891	2	38883052	38978379	110	11	123834	1.5596	0.059422	0.831392791
ARHGAP22	ENSG00000128805	10	49644077	49874310	385	37	123834	1.5596	0.059423	0.831392791
MICAL3	ENSG00000243156	22	18260415	18517325	526	40	123834	1.5594	0.059454	0.831392791
KDM1B	ENSG00000165097	6	18145560	18234084	196	22	123834	1.559	0.059493	0.831392791
ITGAL	ENSG00000005844	16	30473979	30544506	29	8	123834	1.5589	0.059511	0.831392791
ASB14	ENSG00000239388	3	57292375	57336710	62	8	123834	1.5589	0.059513	0.831392791
DNAJB8	ENSG00000179407	3	128171282	128196091	54	11	123834	1.558	0.059614	0.831392791
FAM231B	ENSG00000268674	1	16855561	16876070	2	1	123834	1.5578	0.059642	0.831392791
ANKMY2	ENSG00000106524	7	16629401	16695442	96	10	123834	1.5577	0.059652	0.831392791
ZBTB1	ENSG00000126804	14	64960430	65010408	109	10	123834	1.5576	0.059659	0.831392791
DDX52	ENSG00000141141	17	35959787	36013493	39	5	123834	1.5573	0.059699	0.831392791
SLC6A11	ENSG00000132164	3	10847885	10992419	214	30	123834	1.5561	0.05984	0.831392791
AC007216.2	ENSG00000269121	16	12052000	12072333	49	6	123834	1.5558	0.059874	0.831392791
ZIC4	ENSG00000174963	3	147093833	147134647	40	7	123834	1.5558	0.059876	0.831392791
RPS19	ENSG00000105372	19	42353988	42386994	24	2	123834	1.5555	0.059915	0.831392791
PGLYRP2	ENSG00000161031	19	15569456	15619767	50	10	123834	1.5552	0.059945	0.831392791
AQP9	ENSG00000103569	15	58420368	58488110	139	19	123834	1.5551	0.059962	0.831392791
KBTBD3	ENSG00000182359	11	105911825	105958492	54	7	123834	1.5548	0.06	0.831392791
SBNO2	ENSG00000064932	19	1097636	1184282	109	16	123834	1.5543	0.060059	0.831392791
THRB	ENSG00000151090	3	24148651	24546773	476	80	123834	1.5539	0.060101	0.831392791
C4A	ENSG00000244731	6	31939801	31980458	23	8	123834	1.5537	0.060128	0.831392791
NDUFAF6	ENSG00000156170	8	95897995	96138683	463	26	123834	1.5534	0.060159	0.831392791
SAMD11	ENSG00000187634	1	850260	889955	71	11	123834	1.5532	0.060185	0.831392791
VRTN	ENSG00000133980	14	74759772	74836711	69	10	123834	1.5532	0.060185	0.831392791
SLC25A2	ENSG00000120329	5	140672196	140693612	69	11	123834	1.5525	0.060268	0.831934745
JDP2	ENSG00000140044	14	75884419	75950814	64	13	123834	1.5515	0.060392	0.832707195
GPC2	ENSG00000213420	7	99757229	99784995	17	4	123834	1.5512	0.060424	0.832707195
PLK2	ENSG00000145632	5	57739809	57766087	103	13	123834	1.5503	0.060532	0.832707195

LCA5L	ENSG00000157578	21	40767770	40827731	107	7	123834	1.5503	0.060539	0.832707195
ZNF487	ENSG00000243660	10	43922282	44001517	235	18	123834	1.5502	0.060543	0.832707195
RANBP9	ENSG00000010017	6	13611730	13721796	115	13	123834	1.5498	0.060593	0.832792295
HMGXB4	ENSG00000100281	22	35643445	35701800	119	4	123834	1.5493	0.060657	0.833033793
AP001885.1	ENSG00000179038	11	66953391	66974638	9	3	123834	1.5484	0.060757	0.833033793
CHRM4	ENSG00000180720	11	46396640	46418107	7	2	123834	1.5483	0.060778	0.833033793
GRIK4	ENSG00000149403	11	120372468	120869613	586	60	123834	1.5479	0.060828	0.833033793
WDFY3	ENSG00000163625	4	85580704	85897544	232	15	123834	1.5475	0.06087	0.833033793
BBS4	ENSG00000140463	15	72968527	73040817	98	11	123834	1.5474	0.060883	0.833033793
HS6ST1	ENSG00000136720	2	128984290	129086151	111	21	123834	1.5469	0.060944	0.833033793
PRCD	ENSG00000214140	17	74513668	74559660	46	6	123834	1.5467	0.06097	0.833033793
WNT11	ENSG00000085741	11	75887369	75931780	26	8	123834	1.5464	0.061005	0.833033793
MAPK15	ENSG00000181085	8	144788429	144814628	30	6	123834	1.5452	0.061147	0.833102421
RFPL3S	ENSG00000205853	22	32745893	32788039	228	19	123834	1.5448	0.061194	0.833102421
NETO1	ENSG00000166342	18	70399549	70545381	240	21	123834	1.5447	0.061215	0.833102421
PLOD3	ENSG00000106397	7	100839258	100871701	37	7	123834	1.5443	0.061263	0.833102421
AC011551.3	ENSG00000268423	19	47040313	47114118	44	10	123834	1.5442	0.061271	0.833102421
AGO3	ENSG00000126070	1	36386319	36548101	77	4	123834	1.5442	0.061273	0.833102421
CCR6	ENSG00000112486	6	167515295	167563184	94	13	123834	1.5434	0.06137	0.833122485
SERTM1	ENSG00000180440	13	37238049	37281976	18	5	123834	1.5428	0.061435	0.833122485
SHF	ENSG00000138606	15	45449412	45503373	70	7	123834	1.5426	0.061467	0.833122485
NPRL2	ENSG00000114388	3	50374761	50398522	7	2	123834	1.5417	0.061578	0.833122485
FGF19	ENSG00000162344	11	69503000	69529410	6	2	123834	1.5411	0.061647	0.833122485
WNT5B	ENSG00000111186	12	1629057	1766409	192	45	123834	1.5409	0.061669	0.833122485
SMOC2	ENSG00000112562	6	168831831	169083984	598	57	123834	1.5407	0.061696	0.833122485
ELL	ENSG00000105656	19	18543473	18642937	149	7	123834	1.54	0.06178	0.833122485
MMD2	ENSG00000136297	7	4935620	5008844	113	16	123834	1.54	0.061783	0.833122485
SGK494	ENSG00000167524	17	26924982	26951218	6	2	123834	1.5393	0.06186	0.833122485
ABT1	ENSG00000146109	6	26587180	26610278	48	9	123834	1.538	0.062028	0.833122485
RNASEL	ENSG00000135828	1	182532769	182568391	47	10	123834	1.5378	0.062044	0.833122485
RGS4	ENSG00000117152	1	163028565	163056592	48	11	123834	1.5371	0.062133	0.833122485
ZNF419	ENSG00000105136	19	57989079	58016048	97	6	123834	1.537	0.062142	0.833122485
CD28	ENSG00000178562	2	204561198	204613635	77	8	123834	1.5357	0.062305	0.833122485
C19orf67	ENSG00000188032	19	14182431	14206687	8	3	123834	1.5357	0.062307	0.833122485
HOXA4	ENSG00000197576	7	27158126	27180418	39	5	123834	1.5355	0.062326	0.833122485
PRKRIR	ENSG00000137492	11	76051000	76102015	58	9	123834	1.5347	0.062425	0.833122485
GLTPD1	ENSG00000224051	1	1250136	1274277	37	4	123834	1.5345	0.062457	0.833122485
DEFB126	ENSG00000125788	20	113010	136392	38	8	123834	1.5345	0.062457	0.833122485
RUVBL2	ENSG00000183207	19	49486705	49529252	60	4	123834	1.5344	0.062464	0.833122485
CLCN7	ENSG00000103249	16	1484935	1535581	181	17	123834	1.5344	0.062471	0.833122485
SOX10	ENSG00000100146	22	38356693	38393429	22	2	123834	1.5338	0.062536	0.833122485
TMEM65	ENSG00000164983	8	125314231	125394933	125	8	123834	1.5337	0.062553	0.833122485
GOT2	ENSG00000125166	16	58731035	58778261	147	15	123834	1.5336	0.06256	0.833122485
KLF9	ENSG00000119138	9	72989503	73039540	47	11	123834	1.5334	0.062585	0.833122485
AL133262.1	ENSG00000267849	6	44390472	44410720	24	4	123834	1.5331	0.06263	0.833122485
HPR	ENSG00000261701	16	72078522	72121145	66	9	123834	1.5326	0.062693	0.833122485
C11orf45	ENSG00000174370	11	128759460	128785930	32	6	123834	1.5323	0.062724	0.833122485
TRAPPC12	ENSG00000171853	2	3373446	3498865	219	24	123834	1.5319	0.062773	0.833122485
DNAH17	ENSG00000187775	17	76409778	76583476	514	52	123834	1.5318	0.062791	0.833122485
RCN2	ENSG00000117906	15	77213960	77252601	35	8	123834	1.5313	0.062847	0.833122485
FCAR	ENSG00000186431	19	55375704	55411838	129	10	123834	1.5313	0.062851	0.833122485
FAM3B	ENSG00000183844	21	42666139	42739358	128	23	123834	1.5311	0.062874	0.833122485
FOXP2	ENSG00000128573	7	113716382	114343827	378	29	123834	1.531	0.062882	0.833122485
CCNK	ENSG00000090061	14	99937506	100011381	59	6	123834	1.5302	0.062982	0.833122485
AL606500.1	ENSG00000268141	1	154575067	154595190	37	4	123834	1.53	0.063009	0.833122485
RUNX1	ENSG00000159216	21	36150098	37386965	1937	124	123834	1.5299	0.06302	0.833122485
MYBPC3	ENSG00000134571	11	47342957	47384253	49	9	123834	1.5292	0.063111	0.833122485
DMRTA1	ENSG00000176399	9	22436840	22465739	17	2	123834	1.5291	0.06312	0.833122485
WWC1	ENSG00000113645	5	167708656	167909308	236	43	123834	1.5288	0.063152	0.833122485
U2AF2	ENSG00000063244	19	56155512	56196081	66	8	123834	1.5288	0.063155	0.833122485
TFAP2A	ENSG00000137203	6	10383419	10429892	33	8	123834	1.5285	0.063194	0.833122485
CCDC176	ENSG00000119636	14	74476056	74559566	250	16	123834	1.5284	0.063203	0.833122485
TLL1	ENSG00000038295	4	166784410	167035047	326	39	123834	1.5279	0.063272	0.833454037
TBL2	ENSG00000106638	7	72973262	73003121	46	7	123834	1.5272	0.063351	0.833670373
CCDC166	ENSG00000255181	8	144778864	144800279	21	5	123834	1.5267	0.063418	0.833670373
IZUMO1	ENSG00000182264	19	49234109	49260166	46	5	123834	1.5267	0.06342	0.833670373
FLJ00388	ENSG00000273395	1	48216979	48237290	12	5	123834	1.5261	0.063487	0.833974358
MAP3K6	ENSG00000142733	1	27671675	27703383	26	5	123834	1.5239	0.063771	0.835539804
TM9SF1	ENSG00000100926	14	24648349	24692679	40	11	123834	1.5238	0.063781	0.835539804
TM9SF1	ENSG00000254692	14	24648363	24692679	40	11	123834	1.5238	0.063781	0.835539804
CTD-2207O23.3	ENSG00000268861	19	7435850	7545131	300	13	123834	1.5238	0.063782	0.835539804
ZNF621	ENSG00000172888	3	40556369	40626176	73	9	123834	1.5233	0.063847	0.835815273
CPNE2	ENSG00000140848	16	57116449	57191878	157	29	123834	1.5222	0.063977	0.83641732
AMHR2	ENSG00000135409	12	53807639	53835318	34	2	123834	1.5221	0.063987	0.83641732
HOXB1	ENSG00000120094	17	46595888	46618359	48	7	123834	1.5218	0.064025	0.83641732
FLJ00418	ENSG00000268927	16	70685570	70709739	18	3	123834	1.5212	0.06411	0.836875742
BRSK2	ENSG00000174672	11	1401129	1493919	261	23	123834	1.5204	0.064201	0.836875742
FUT1	ENSG00000174951	19	49241268	49268647	32	3	123834	1.5189	0.064395	0.836875742
IQCJ-SCHIP1	ENSG00000250588	3	158670024	159625155	1166	72	123834	1.5188	0.064403	0.836875742
ABHD16B	ENSG00000183260	20	62482566	62504341	27	3	123834	1.5186	0.064426	0.836875742
NOSIP	ENSG00000142546	19	50048968	50103519	70	7	123834	1.5182	0.064487	0.836875742
ZNF304	ENSG00000131845	19	57852675	57881266	46	9	123834	1.5179	0.064522	0.836875742
AP5M1	ENSG00000053770	14	57725627	57766797	29	2	123834	1.5179	0.064524	0.836875742
C16orf91	ENSG00000174109	16	1459745	1489345	42	8	123834	1.5179	0.064526	0.836875742
AC027309.1	ENSG00000268553	5	172026245	172046436	14	3	123834	1.5172	0.064614	0.836875742
HSPA1B	ENSG00000204388	6	31785512	31808031	36	14	123834	1.5163	0.064723	0.836875742
RP11-487E13.1	ENSG00000249008	4	178153693	178179927	24	6	123834	1.516	0.064766	0.836875742
CALCR	ENSG00000004948	7	93043799	93214042	325	23	123834	1.5155	0.06482	0.836875742
AFF3	ENSG00000144218	2	100152323	100769201	793	36	123834	1.5153	0.064848	0.836875742
TMC3	ENSG00000188869	15	81613558	81676554	130	12	123834	1.5149	0.064894	0.836875742
COL14A1	ENSG00000187955	8	121062019	121394275	518	40	123834	1.5149	0.064902	0.836875742
SNX27	ENSG00000143376	1	151574541	151681567	91	6	123834	1.5145	0.064948	0.836875742
MPP7	ENSG00000150054	10	28329922	28633415	842	32	123834	1.5139	0.06503	0.836875742
MYH2	ENSG00000125414	17	10414465	10463274	85	6	123834	1.5136	0.065068	0.836875742
RASSF1	ENSG00000068028	3	50357219	50388411	10	2	123834	1.513	0.06514	0.836875742
TMCC1	ENSG00000172765	3	129356635	129622419	204	9	123834	1.5121	0.065249	0.836875742
DLEC1	ENSG00000008226	3	38070696	38175516	178	11	123834	1.5121	0.065251	0.836875742
OPN3	ENSG00000054277	1	241743404	241850678	132	25	123834	1.5117	0.065304	0.836875742
COASY	ENSG00000068120	17	40703485	40728295	32	3	123834	1.5116	0.065312	0.836875742

ZC3H4	ENSG00000130749	19	47557444	47627009	38	3	123834	1.5116	0.065312	0.836875742
TCN1	ENSG00000134827	11	59610273	59644048	11	3	123834	1.5113	0.065354	0.836875742
MRAS	ENSG00000158186	3	138056539	138134375	120	7	123834	1.5109	0.065409	0.836875742
EFNB2	ENSG00000125266	13	107132079	107197462	34	9	123834	1.5108	0.065416	0.836875742
AC004466.1	ENSG00000268069	12	48168706	48189787	26	5	123834	1.5107	0.065437	0.836875742
SLC25A18	ENSG00000182902	22	18033139	18083760	50	12	123834	1.5107	0.065438	0.836875742
SPP2	ENSG00000072080	2	234949323	234995778	120	14	123834	1.5103	0.065483	0.836875742
AC003002.6	ENSG00000269533	19	57891252	57941362	73	9	123834	1.5102	0.065494	0.836875742
FAM111A	ENSG00000166801	11	58900221	58932512	66	2	123834	1.5101	0.065513	0.836875742
GAS1	ENSG00000180447	9	89549279	89572104	40	7	123834	1.5085	0.065716	0.837410075
PRKAG1	ENSG00000181929	12	49386057	49422980	17	2	123834	1.5077	0.06582	0.837410075
ADCY4	ENSG00000129467	14	24777555	24814299	63	9	123834	1.5074	0.065858	0.837410075
NFKB1	ENSG00000109320	4	103412486	103548459	145	13	123834	1.5073	0.06587	0.837410075
TCF12	ENSG00000140262	15	57200821	57601479	772	16	123834	1.506	0.066037	0.837410075
PARP12	ENSG00000059378	7	139713544	139773521	121	18	123834	1.5057	0.066073	0.837410075
C9orf163	ENSG00000196366	9	139367947	139390518	39	5	123834	1.5054	0.066107	0.837410075
ZRANB2	ENSG00000132485	1	71518974	71556980	62	9	123834	1.5042	0.06626	0.837410075
MANSC1	ENSG00000111261	12	12472198	12513475	111	13	123834	1.5037	0.066329	0.837410075
CPSF3L	ENSG00000127054	1	1236965	1270071	46	6	123834	1.5036	0.066342	0.837410075
CTPS1	ENSG00000171793	1	41435007	41488235	88	14	123834	1.5033	0.066375	0.837410075
BAG6	ENSG00000204463	6	31596805	31630482	43	11	123834	1.5033	0.066376	0.837410075
NOP9	ENSG00000196943	14	24759068	24788330	52	7	123834	1.5029	0.066433	0.837410075
AL355490.1	ENSG00000224474	10	99169228	99195831	35	7	123834	1.5028	0.06645	0.837410075
TAL1	ENSG00000162367	1	47671962	47707892	65	9	123834	1.5026	0.06647	0.837410075
C6orf47	ENSG00000204439	6	31616075	31638549	26	11	123834	1.5025	0.066487	0.837410075
CRIPT	ENSG00000119878	2	46833555	46862881	49	6	123834	1.5022	0.066517	0.837410075
ZNF451	ENSG00000112200	6	56941642	57045105	92	5	123834	1.5019	0.066557	0.837410075
ATG2B	ENSG00000066739	14	96737595	96840207	103	7	123834	1.5019	0.066567	0.837410075
MTPAP	ENSG00000107951	10	30588730	30673377	158	19	123834	1.5017	0.066585	0.837410075
ABHD17A	ENSG00000129968	19	1866809	1895546	27	2	123834	1.5016	0.0666	0.837410075
SSTR4	ENSG00000132671	20	23006057	23027314	35	5	123834	1.5012	0.066648	0.837410075
UBOX5	ENSG00000185019	20	3078219	3150842	88	10	123834	1.5008	0.066698	0.837410075
CYGB	ENSG00000161544	17	74513438	74557257	41	6	123834	1.5006	0.066733	0.837410075
GUF1	ENSG00000151806	4	44670444	44712943	67	5	123834	1.5004	0.066756	0.837410075
STOML3	ENSG00000133115	13	39530062	39575203	134	12	123834	1.4988	0.066958	0.837410075
MVK	ENSG00000110921	12	110001060	110045067	109	12	123834	1.4986	0.066985	0.837410075
NPIPA1	ENSG00000183426	16	15006659	15055915	1	1	123834	1.4985	0.066998	0.837410075
ZNF83	ENSG00000167766	19	53087313	53203749	231	33	123834	1.4984	0.067009	0.837410075
GREM1	ENSG00000166923	15	33000175	33036870	20	6	123834	1.4984	0.06701	0.837410075
MYPOP	ENSG00000176182	19	46383278	46415862	82	12	123834	1.4982	0.06704	0.837410075
HP	ENSG00000257017	16	72078491	72104954	35	5	123834	1.4981	0.067058	0.837410075
UFSP1	ENSG00000176125	7	100476346	100497339	23	4	123834	1.4978	0.067093	0.837410075
CDK5R2	ENSG00000171450	2	219814377	219836876	34	3	123834	1.4975	0.06713	0.837410075
DFNB31	ENSG00000095397	9	117154360	117277730	219	22	123834	1.4975	0.067132	0.837410075
MACC1	ENSG00000183742	7	20164278	20267027	165	15	123834	1.4974	0.06715	0.837410075
DEFB1	ENSG00000164825	8	6718097	6745544	104	10	123834	1.4968	0.067224	0.837410075
HIST2H2BF	ENSG00000203814	1	149744245	149793928	12	1	123834	1.4964	0.067273	0.837410075
HIST2H3D	ENSG00000183598	1	149774826	149795236	12	1	123834	1.4964	0.067273	0.837410075
TMEM63A	ENSG00000196187	1	226023237	226080069	143	12	123834	1.496	0.067333	0.837608419
SPDYC	ENSG00000204710	11	64927625	64950715	6	1	123834	1.495	0.067463	0.838000714
HOXB5	ENSG00000120075	17	46658619	46681323	41	10	123834	1.4934	0.067662	0.838000714
C17orf98	ENSG00000214556	17	36981341	37007708	64	11	123834	1.4928	0.06775	0.838000714
ANGEL2	ENSG00000174606	1	213155524	213199168	53	7	123834	1.4926	0.067777	0.838000714
JAM3	ENSG00000166086	11	133928820	134031896	199	9	123834	1.4922	0.067827	0.838000714
FSD1L	ENSG00000106701	9	108200077	108324714	187	18	123834	1.4919	0.067857	0.838000714
TRIM36	ENSG00000152503	5	114450459	114526243	98	12	123834	1.4915	0.067918	0.838000714
CYTH4	ENSG00000100055	22	37668068	37721382	44	10	123834	1.4913	0.067946	0.838000714
EHMT2	ENSG00000204371	6	31837536	31875464	57	15	123834	1.4913	0.067947	0.838000714
IL17C	ENSG00000124391	16	88694999	88716881	13	5	123834	1.4908	0.06801	0.838000714
ZNF548	ENSG00000188785	19	57891218	57922786	48	8	123834	1.4906	0.068036	0.838000714
MKRN3	ENSG00000179455	15	23800454	23883064	87	11	123834	1.4899	0.068124	0.838000714
NECAP1	ENSG00000089818	12	8224807	8260367	33	7	123834	1.4898	0.06814	0.838000714
TJP1	ENSG00000104067	15	29981571	30271068	449	38	123834	1.4897	0.068147	0.838000714
COL24A1	ENSG00000171502	1	86184916	86632626	1149	48	123834	1.4897	0.068148	0.838000714
TMCC2	ENSG00000133069	1	205187304	205252471	134	12	123834	1.4897	0.068157	0.838000714
GFRA1	ENSG00000151892	10	117806444	118042979	344	43	123834	1.4895	0.068184	0.838000714
SPATA22	ENSG00000141255	17	3333313	3427146	225	20	123834	1.4889	0.068254	0.838000714
TM4SF5	ENSG00000142484	17	4665179	4696506	44	10	123834	1.4887	0.068279	0.838000714
PHLDA1	ENSG00000139289	12	76409227	76437712	7	3	123834	1.4879	0.068389	0.838000714
CEBPG	ENSG00000153879	19	33854236	33883592	62	8	123834	1.4877	0.068411	0.838000714
DLEU7	ENSG00000186047	13	51275144	51428075	230	24	123834	1.4873	0.06847	0.838000714
BTN3A3	ENSG00000111801	6	26430700	26463643	73	11	123834	1.4872	0.068478	0.838000714
ASPA	ENSG00000108381	17	3365668	3416713	111	15	123834	1.4872	0.068479	0.838000714
SCPEP1	ENSG00000121064	17	55045466	55094129	46	6	123834	1.4863	0.0686	0.838000714
PMFBP1	ENSG00000118557	16	72136056	72220777	150	16	123834	1.486	0.068645	0.838000714
DSG2	ENSG00000046604	18	29068006	29138971	137	15	123834	1.4858	0.06867	0.838000714
PIAS2	ENSG00000078043	18	44378353	44510123	212	9	123834	1.4857	0.068684	0.838000714
TCL1B	ENSG00000213231	14	96142754	96168980	25	8	123834	1.4855	0.068702	0.838000714
VILL	ENSG00000136059	3	38019550	38058679	81	7	123834	1.485	0.068767	0.838000714
EGLN1	ENSG00000135766	1	231489497	231570790	252	5	123834	1.484	0.068899	0.838000714
ACE	ENSG00000264813	17	61552184	61609209	36	4	123834	1.4835	0.068964	0.838000714
CHD1	ENSG00000153922	5	98180908	98272240	137	12	123834	1.4834	0.068983	0.838000714
HIST2H2AB	ENSG00000184270	1	149849019	149869466	19	1	123834	1.4834	0.068989	0.838000714
CD79A	ENSG00000105369	19	42371190	42395439	8	2	123834	1.4833	0.069004	0.838000714
RALGAPB	ENSG00000170471	20	37091459	37217504	140	10	123834	1.483	0.069038	0.838000714
ZNF17	ENSG00000186272	19	57912531	57943307	44	7	123834	1.4824	0.069123	0.838000714
ILDR2	ENSG00000143195	1	166872443	166954719	78	11	123834	1.4821	0.069155	0.838000714
PCCB	ENSG00000114054	3	135959148	136066738	96	11	123834	1.4815	0.069233	0.838000714
MBD3	ENSG00000071655	19	1566639	1602882	6	1	123834	1.4811	0.069289	0.838000714
LIN28B	ENSG00000187772	6	105394923	105541207	111	13	123834	1.4807	0.069348	0.838000714
TMEM209	ENSG00000146842	7	129794555	129857610	81	10	123834	1.4804	0.069385	0.838000714
STAG1	ENSG00000118007	3	136045077	136481220	487	17	123834	1.4802	0.069406	0.838000714
DPY19L3	ENSG00000178904	19	32886449	32986801	142	14	123834	1.4801	0.069427	0.838000714
MUC3A	ENSG00000228273	7	100537257	100560424	57	10	123834	1.4798	0.069463	0.838000714
MRPL15	ENSG00000137547	8	55037770	55070461	24	3	123834	1.4793	0.069533	0.838000714
UBE2U	ENSG00000177414	1	64659310	64743051	88	9	123834	1.4787	0.069615	0.838000714
PRL	ENSG00000172179	6	22277480	22307730	21	7	123834	1.4785	0.069643	0.838000714
NDFIP2	ENSG00000102471	13	80045287	80140210	116	11	123834	1.4778	0.069732	0.838000714
AC015660.1	ENSG00000269810	15	100028447	100048578	40	6	123834	1.4778	0.069735	0.838000714

HERC3	ENSG00000138641	4	89432199	89639693	317	20	123834	1.4776	0.069752	0.838000714
SLC9B1	ENSG00000164037	4	103796205	103950896	257	5	123834	1.4771	0.069821	0.838000714
DNASE2B	ENSG00000137976	1	84854215	84890701	97	10	123834	1.4771	0.069826	0.838000714
TRAM1	ENSG00000067167	8	71475677	71530622	89	9	123834	1.4769	0.069849	0.838000714
CFB	ENSG00000244255	6	31885475	31929825	54	9	123834	1.4763	0.069927	0.838000714
CFB	ENSG00000243649	6	31885475	31929861	54	9	123834	1.4763	0.069927	0.838000714
ZNF547	ENSG00000152433	19	57864845	57900933	67	10	123834	1.4763	0.069931	0.838000714
OSBPL7	ENSG00000006025	17	45874738	45909200	65	9	123834	1.476	0.069976	0.838000714
TAS1R3	ENSG00000169962	1	1256694	1280686	44	5	123834	1.4752	0.070079	0.838000714
BAG3	ENSG00000151929	10	121400882	121447331	57	9	123834	1.4749	0.070118	0.838000714
ALDH6A1	ENSG00000119711	14	74513553	74561196	141	11	123834	1.4749	0.070125	0.838000714
AP001652.1	ENSG00000224130	11	58662897	58686071	26	3	123834	1.4748	0.070139	0.838000714
AL109927.1	ENSG00000268552	1	28167085	28187255	16	1	123834	1.4747	0.070142	0.838000714
LRRC46	ENSG00000141294	17	45898993	45925079	54	9	123834	1.4732	0.070351	0.838233863
MFAP3	ENSG00000037749	5	153408466	153610038	350	26	123834	1.4731	0.07036	0.838233863
FZD8	ENSG00000177283	10	35917177	35940362	24	3	123834	1.4727	0.070412	0.838233863
MRPS24	ENSG00000062582	7	43896157	43919492	21	5	123834	1.4727	0.070415	0.838233863
COG1	ENSG00000166685	17	71179129	71214646	78	5	123834	1.4716	0.070562	0.838233863
AC003002.4	ENSG00000268133	19	57864934	57919952	92	12	123834	1.4714	0.070595	0.838233863
MYH8	ENSG00000133020	17	10283639	10335267	48	4	123834	1.4713	0.070601	0.838233863
STRN4	ENSG00000090372	19	47212764	47260251	48	8	123834	1.4713	0.070609	0.838233863
CNTN2	ENSG00000184144	1	205002325	205057627	102	17	123834	1.4713	0.07061	0.838233863
KCNMB2	ENSG00000197584	3	177980720	178572217	1053	65	123834	1.4704	0.070733	0.838233863
BARD1	ENSG00000138376	2	215580370	215684428	330	24	123834	1.4702	0.070751	0.838233863
DNAJC5	ENSG00000101152	20	62516518	62577384	123	12	123834	1.4702	0.070754	0.838233863
FKRP	ENSG00000181027	19	47239303	47290245	78	11	123834	1.4701	0.070771	0.838233863
PRDM13	ENSG00000112238	6	100044606	100073454	26	9	123834	1.4699	0.070801	0.838233863
RABIF	ENSG00000183155	1	202838085	202868263	50	7	123834	1.4697	0.070823	0.838233863
GNL3	ENSG00000163938	3	52705172	52738508	42	4	123834	1.4689	0.07093	0.838977872
SLC38A4	ENSG00000139209	12	47148546	47236191	163	20	123834	1.4678	0.071082	0.839910844
CDC25B	ENSG00000101224	20	3757578	3796762	47	7	123834	1.4673	0.071142	0.839910844
RP11-166B2.1	ENSG00000234719	16	12011230	12080500	123	15	123834	1.4669	0.071199	0.839910844
MYH1	ENSG00000109061	17	10385624	10431860	52	5	123834	1.4669	0.071206	0.839910844
TARS2	ENSG00000143374	1	150449887	150490078	93	8	123834	1.4667	0.071234	0.839910844
TMTC2	ENSG00000179104	12	83070659	83538649	759	51	123834	1.4664	0.071274	0.839910844
PCMT1	ENSG00000120265	6	150060579	150142556	152	2	123834	1.4652	0.071431	0.839917461
DTWD1	ENSG00000104047	15	49903177	49947333	103	8	123834	1.4652	0.071438	0.839917461
CDK5RAP1	ENSG00000101391	20	31936645	31999367	25	3	123834	1.4647	0.071502	0.839917461
SEMA6D	ENSG00000137872	15	47466298	48076420	916	51	123834	1.4641	0.071578	0.839917461
ZNF410	ENSG00000119725	14	74343320	74409214	223	15	123834	1.4641	0.071586	0.839917461
FAM175A	ENSG00000163322	4	84372092	84454501	186	7	123834	1.4641	0.071588	0.839917461
RP11-410N8.4	ENSG00000204393	20	31165281	31206695	27	6	123834	1.464	0.071599	0.839917461
HNRNPF	ENSG00000169813	10	43871065	43914614	110	13	123834	1.4635	0.07167	0.839917461
ATP8A1	ENSG00000124406	4	42400390	42669122	373	26	123834	1.4633	0.071691	0.839917461
GJA5	ENSG00000143140	1	147218332	147255484	59	10	123834	1.4623	0.071827	0.839917461
B3GAT2	ENSG00000112309	6	71556382	71676741	185	25	123834	1.4623	0.071827	0.839917461
MGC4294	ENSG00000268941	20	57199828	57220772	28	5	123834	1.4622	0.071843	0.839917461
DHDDS	ENSG00000117682	1	26748773	26807785	69	11	123834	1.4622	0.071849	0.839917461
SOX21	ENSG00000125285	13	95351886	95374389	15	5	123834	1.4618	0.071898	0.839973684
C7orf43	ENSG00000146826	7	99742043	99766338	22	4	123834	1.4611	0.071991	0.840290282
TMEM258	ENSG00000134825	11	61525973	61570274	17	4	123834	1.46	0.07215	0.840290282
FGF21	ENSG00000105550	19	49248816	49271587	16	6	123834	1.4599	0.072156	0.840290282
CA8	ENSG00000178538	8	61089906	61203971	295	22	123834	1.4595	0.072208	0.840290282
RGS5	ENSG00000143248	1	163070911	163301577	637	36	123834	1.4595	0.07221	0.840290282
PDE1B	ENSG00000123360	12	54933134	54983023	35	10	123834	1.4593	0.07224	0.840290282
SNN	ENSG00000184602	16	11752270	11783015	32	4	123834	1.459	0.072283	0.840290282
ZNF439	ENSG00000171291	19	11949541	12004565	103	14	123834	1.4588	0.072317	0.840290282
MAG	ENSG00000105695	19	35773028	35814707	5	3	123834	1.4586	0.072339	0.840290282
GLT8D1	ENSG00000016864	3	52718505	52750048	36	4	123834	1.4577	0.072464	0.840290282
SHISA9	ENSG00000237515	16	12985477	13344272	840	68	123834	1.4576	0.072472	0.840290282
HIST2H2AC	ENSG00000184260	1	149848525	149868961	18	1	123834	1.4574	0.072503	0.840290282
PPFIBP1	ENSG00000110841	12	27666364	27858497	237	27	123834	1.4572	0.072535	0.840290282
PTPRD	ENSG00000153707	9	8304246	10622723	4763	311	123834	1.4571	0.072544	0.840290282
TECPR2	ENSG00000196663	14	102819300	102978818	364	14	123834	1.4563	0.072652	0.841028755
CLIC5	ENSG00000112782	6	45858045	46058132	328	38	123834	1.4557	0.072734	0.841196496
SECISBP2L	ENSG00000138593	15	49270673	49348760	95	10	123834	1.4556	0.072755	0.841196496
SLC19A3	ENSG00000135917	2	228539926	228592728	162	10	123834	1.4545	0.072901	0.842372163
C1QBP	ENSG00000108561	17	5326097	5362150	78	11	123834	1.4539	0.072993	0.842711082
ARG1	ENSG00000118520	6	131884284	131915472	31	8	123834	1.4537	0.073019	0.842711082
NSUN3	ENSG00000178694	3	93771760	93857389	35	8	123834	1.4531	0.073103	0.843168583
ACAP1	ENSG00000072818	17	7229848	7264797	16	3	123834	1.4513	0.073342	0.84511341
ANKRD9	ENSG00000156381	14	102963179	102986136	48	8	123834	1.451	0.073385	0.84511341
FHAD1	ENSG00000142621	1	15563768	15736779	294	28	123834	1.4509	0.073405	0.84511341
AC068039.1	ENSG00000268821	2	172550844	172577314	47	8	123834	1.4506	0.073452	0.845142625
BFAR	ENSG00000103429	16	14716672	14773093	27	5	123834	1.4497	0.073574	0.845614665
SELP	ENSG00000174175	1	169548087	169609431	118	18	123834	1.4496	0.073582	0.845614665
HIST2H2BE	ENSG00000184678	1	149846010	149868232	17	1	123834	1.4477	0.073848	0.846817079
HS3ST2	ENSG00000122254	16	22815498	22937659	172	30	123834	1.4471	0.073938	0.846817079
TCF25	ENSG00000141002	16	89930000	89987792	128	7	123834	1.4462	0.074054	0.846817079
CHMP1B	ENSG00000255112	18	11841395	11864448	23	4	123834	1.4462	0.074055	0.846817079
NUDT17	ENSG00000186364	1	145576115	145599439	4	1	123834	1.4458	0.074122	0.846817079
CDC5L	ENSG00000096401	6	44345262	44428163	85	11	123834	1.4453	0.074186	0.846817079
SH3GL2	ENSG00000107295	9	17569080	17807127	489	37	123834	1.4451	0.074218	0.846817079
CWH43	ENSG00000109182	4	48978264	49074098	61	5	123834	1.4443	0.074331	0.846817079
ZFHX3	ENSG00000140836	16	72806784	73103597	297	47	123834	1.4439	0.07438	0.846817079
SOGA3	ENSG00000255330	6	127749551	127850146	145	14	123834	1.443	0.07451	0.846817079
LRRC32	ENSG00000137507	11	76358568	76391791	12	5	123834	1.4424	0.074589	0.846817079
CNOT6	ENSG00000113300	5	179911412	180015405	331	15	123834	1.4422	0.074622	0.846817079
TMEM233	ENSG00000224982	12	120021264	120089363	114	7	123834	1.4421	0.074632	0.846817079
STARD5	ENSG00000172345	15	81591394	81626524	60	11	123834	1.4418	0.074678	0.846817079
ETNK2	ENSG00000143845	1	204090190	204131307	26	9	123834	1.4418	0.074681	0.846817079
AKAP5	ENSG00000179841	14	64922217	64951221	47	7	123834	1.4417	0.0747	0.846817079
LPGAT1	ENSG00000123684	1	211906799	212014114	265	16	123834	1.4415	0.074725	0.846817079
KCNG2	ENSG00000178342	18	77613668	77670184	114	12	123834	1.4412	0.07477	0.846817079
SIPA1L2	ENSG00000116991	1	232523711	232707304	304	18	123834	1.441	0.074798	0.846817079
KCTD6	ENSG00000168301	3	58467841	58498087	13	4	123834	1.4408	0.074827	0.846817079
ATP2B2	ENSG00000157087	3	10355707	10759716	651	75	123834	1.4407	0.074838	0.846817079
GNAS	ENSG00000087460	20	57404773	57496247	78	15	123834	1.4407	0.074838	0.846817079
APOC3	ENSG00000110245	11	116690422	116713788	48	10	123834	1.4406	0.074847	0.846817079

MUC12	ENSG00000205277	7	100602904	100672230	67	11	123834	1.4401	0.074914	0.846817079
CCIN	ENSG00000185972	9	36159389	36181329	49	5	123834	1.44	0.074934	0.846817079
FLYWCH2	ENSG00000162076	16	2923187	2959383	138	12	123834	1.44	0.074939	0.846817079
ZDHHC11	ENSG00000188818	5	700471	861101	34	4	123834	1.4399	0.074941	0.846817079
KIF21B	ENSG00000116852	1	200928520	201002828	98	10	123834	1.4396	0.074997	0.846817079
PLB1	ENSG00000163803	2	28670012	28876654	420	52	123834	1.4394	0.075024	0.846817079
APOA1	ENSG00000118137	11	116696467	116718666	46	10	123834	1.4394	0.075024	0.846817079
GGA3	ENSG00000125447	17	73222694	73268444	57	5	123834	1.4387	0.075114	0.846817079
LPAR2	ENSG00000064547	19	19724477	19749739	21	5	123834	1.4383	0.07517	0.846817079
SLC25A12	ENSG00000115840	2	172630880	172874766	436	22	123834	1.4383	0.075181	0.846817079
PTGS1	ENSG00000095303	9	125122824	125167982	68	11	123834	1.4377	0.075255	0.846817079
KRTAP23-1	ENSG00000186980	21	31710717	31730924	77	9	123834	1.4374	0.075306	0.846817079
KCNJ11	ENSG00000187486	11	17397406	17420878	60	8	123834	1.4372	0.075328	0.846817079
DVL1	ENSG00000107404	1	1260656	1294730	63	5	123834	1.4372	0.075335	0.846817079
MTRNR2L4	ENSG00000232196	16	3411053	3432283	65	5	123834	1.4365	0.075424	0.846849626
GMPS	ENSG00000163655	3	155578325	155671815	249	10	123834	1.4365	0.075427	0.846849626
SOGA3	ENSG00000214338	6	127749551	127850500	146	14	123834	1.4357	0.075545	0.847673766
NUP85	ENSG00000125450	17	73191754	73241853	53	4	123834	1.4344	0.075725	0.848893245
FAM187B	ENSG00000177558	19	35705703	35729632	76	10	123834	1.4343	0.075743	0.848893245
LAMTOR4	ENSG00000188186	7	99736530	99763567	26	4	123834	1.4336	0.075842	0.849187862
TTC37	ENSG00000198677	5	94789599	94900711	184	8	123834	1.4332	0.075894	0.849187862
C20ORF135	ENSG00000268858	20	62482879	62503217	25	3	123834	1.4324	0.076009	0.849187862
SCN1A	ENSG00000144285	2	166835670	166994523	347	19	123834	1.4323	0.076034	0.849187862
AC002985.3	ENSG00000268193	19	19007822	19055206	38	8	123834	1.4311	0.076203	0.849187862
KPNB1	ENSG00000108424	17	45716842	45772871	69	8	123834	1.431	0.07622	0.849187862
KRTAP5-6	ENSG00000205864	11	1708425	1728985	43	6	123834	1.4309	0.07623	0.849187862
TGOLN2	ENSG00000152291	2	85535147	85565548	58	6	123834	1.4307	0.076259	0.849187862
SYBU	ENSG00000147642	8	110576207	110714020	169	13	123834	1.4304	0.076297	0.849187862
TNFRSF17	ENSG00000048462	16	12048964	12071925	53	6	123834	1.4304	0.076304	0.849187862
TLL2	ENSG00000095587	10	98114363	98283675	249	34	123834	1.4296	0.076416	0.849187862
GTF2IRD1	ENSG00000006704	7	73858120	74026931	107	14	123834	1.4288	0.076529	0.849187862
TMEM57	ENSG00000204178	1	25747388	25836700	85	2	123834	1.4287	0.076541	0.849187862
ULBP1	ENSG00000111981	6	150275143	150304846	84	5	123834	1.4282	0.076624	0.849187862
STARD7	ENSG00000084090	2	96840597	96884563	30	4	123834	1.4279	0.076661	0.849187862
GNPDA2	ENSG00000163281	4	44674217	44738612	117	7	123834	1.4276	0.076709	0.849187862
ESRRB	ENSG00000119715	14	76766957	76978178	362	50	123834	1.4275	0.076718	0.849187862
ALPP	ENSG00000163283	2	233233244	233257599	41	8	123834	1.4274	0.076734	0.849187862
AGO4	ENSG00000134698	1	36263773	36333491	58	7	123834	1.4271	0.076773	0.849187862
DHX30	ENSG00000132153	3	47834399	47901685	40	4	123834	1.4271	0.076774	0.849187862
CEACAM21	ENSG00000007129	19	42045886	42103197	86	10	123834	1.4264	0.076881	0.849187862
PRUNE2	ENSG00000106772	9	79216292	79531003	513	47	123834	1.4263	0.076898	0.849187862
TUSC2	ENSG00000114383	3	50347458	50375682	9	3	123834	1.426	0.076928	0.849187862
THAP5	ENSG00000177683	7	108184987	108220194	40	7	123834	1.4253	0.077037	0.849187862
SPATA16	ENSG00000144962	3	172597148	172869058	367	32	123834	1.4252	0.077051	0.849187862
CYP7B1	ENSG00000172817	8	65490320	65721318	294	15	123834	1.4245	0.077158	0.849187862
MDGA1	ENSG00000112139	6	37590284	37677082	186	28	123834	1.4243	0.077184	0.849187862
ATG13	ENSG00000175224	11	46628826	46706368	45	4	123834	1.424	0.077228	0.849187862
RP11-886H22.1	ENSG00000266997	18	47312163	47373993	122	13	123834	1.4239	0.077231	0.849187862
C14orf180	ENSG00000184601	14	105036021	105066852	50	7	123834	1.4232	0.077343	0.849187862
MASP1	ENSG00000127241	3	186925942	187019810	130	22	123834	1.4231	0.077349	0.849187862
NPNT	ENSG00000168743	4	106805932	106935184	234	19	123834	1.423	0.07737	0.849187862
HMGN1	ENSG00000205581	21	40704241	40731573	65	7	123834	1.4227	0.077417	0.849187862
MBNL2	ENSG00000139793	13	97863688	98056374	163	20	123834	1.4225	0.077442	0.849187862
ANKRD2	ENSG00000165887	10	99322198	99353641	42	7	123834	1.4223	0.077467	0.849187862
CDCP2	ENSG00000157211	1	54588747	54629443	67	13	123834	1.4223	0.07747	0.849187862
MLLT11	ENSG00000213190	1	151020234	151050970	37	6	123834	1.4222	0.077486	0.849187862
FBLN2	ENSG00000163520	3	13563824	13689922	240	36	123834	1.4221	0.077502	0.849187862
PRR13	ENSG00000205352	12	53825389	53850429	17	2	123834	1.4217	0.077563	0.849187862
BUD13	ENSG00000137656	11	116608886	116653704	104	14	123834	1.4213	0.077608	0.849187862
CHRNA2	ENSG00000120903	8	27307279	27347400	77	12	123834	1.4206	0.077722	0.849187862
PSME3	ENSG00000131467	17	40966402	41005774	4	2	123834	1.4206	0.077722	0.849187862
AOC2	ENSG00000131480	17	40986617	41012724	4	2	123834	1.4206	0.077722	0.849187862
ZNF354A	ENSG00000169131	5	178128593	178167703	76	5	123834	1.4205	0.077735	0.849187862
SRMS	ENSG00000125508	20	62162163	62188857	32	5	123834	1.4201	0.077791	0.849311504
CIR1	ENSG00000138433	2	175202750	175270443	45	7	123834	1.4193	0.077903	0.850046053
LRP2	ENSG00000081479	2	169973619	170229195	438	52	123834	1.4184	0.078037	0.850651596
WDR17	ENSG00000150627	4	176976985	177113978	275	13	123834	1.4183	0.078048	0.850651596
AC079341.1	ENSG00000226757	4	122675740	122697962	46	7	123834	1.4175	0.078169	0.850724964
CWC25	ENSG00000108296	17	36946687	36991734	92	11	123834	1.4174	0.078184	0.850724964
IMMP1L	ENSG00000148950	11	31443948	31541192	80	4	123834	1.4174	0.078189	0.850724964
MTSS1L	ENSG00000132613	16	70685107	70729969	41	10	123834	1.4167	0.078285	0.851282197
OAS3	ENSG00000111331	12	113366157	113421054	110	8	123834	1.4162	0.078357	0.851349237
PEX2	ENSG00000164751	8	77882494	77923280	69	8	123834	1.4159	0.0784	0.851349237
C6orf229	ENSG00000260286	6	24787549	24809117	43	10	123834	1.4152	0.078504	0.851349237
RAB32	ENSG00000118508	6	146854829	146886101	26	3	123834	1.4148	0.07856	0.851349237
SPIRE2	ENSG00000204991	16	89874587	89947727	124	11	123834	1.4145	0.078612	0.851349237
ATP1B1	ENSG00000143153	1	169064935	169111960	88	11	123834	1.4133	0.078782	0.851349237
TCEA3	ENSG00000204219	1	23697554	23761233	112	15	123834	1.4133	0.078786	0.851349237
PLCB3	ENSG00000149782	11	64008995	64046622	39	8	123834	1.4132	0.078792	0.851349237
KLHL8	ENSG00000145332	4	88071255	88171466	130	17	123834	1.4131	0.078812	0.851349237
QRICH1	ENSG00000198218	3	49057140	49141796	51	6	123834	1.413	0.078828	0.851349237
RBM26	ENSG00000139746	13	79875962	79990612	236	6	123834	1.4129	0.078849	0.851349237
IRF2BP1	ENSG00000170604	19	46376866	46399376	36	5	123834	1.4124	0.07891	0.851349237
NKTR	ENSG00000114857	3	42632106	42700227	119	9	123834	1.4124	0.078922	0.851349237
LOXL2	ENSG00000134013	8	23144702	23292841	473	34	123834	1.4122	0.078942	0.851349237
C22orf23	ENSG00000128346	22	38329528	38359676	17	3	123834	1.4121	0.078963	0.851349237
ADPGK	ENSG00000159322	15	73033710	73088187	82	10	123834	1.4111	0.079104	0.851467432
CGB7	ENSG00000196337	19	49547531	49572117	20	5	123834	1.4098	0.079302	0.851467432
SPNS1	ENSG00000169682	16	28975542	29005869	16	3	123834	1.4097	0.079315	0.851467432
MYL7	ENSG00000106631	7	44168463	44190931	22	5	123834	1.4094	0.079364	0.851467432
FAM227B	ENSG00000166262	15	49609159	49923128	744	24	123834	1.4084	0.079504	0.851467432
THBS1	ENSG00000137801	15	39863280	39901667	40	4	123834	1.4084	0.079508	0.851467432
DEK	ENSG00000124795	6	18214099	18275054	92	11	123834	1.4083	0.07952	0.851467432
RNFT1	ENSG00000189050	17	58019601	58052122	26	5	123834	1.4082	0.07953	0.851467432
VWA5A	ENSG00000110002	11	123976069	124028428	95	17	123834	1.4079	0.079583	0.851467432
EIF5B	ENSG00000158417	2	99943816	100027789	128	8	123834	1.4077	0.079607	0.851467432
NLRP5	ENSG00000171487	19	56501092	56583179	200	38	123834	1.4076	0.079626	0.851467432
LAT	ENSG00000213658	16	28986147	29012104	10	3	123834	1.4075	0.079641	0.851467432
NKAIN2	ENSG00000188580	6	124115286	125156803	1393	117	123834	1.4068	0.079736	0.851467432

LTC4S	ENSG00000213316	5	179210981	179233648	40	7	123834	1.4066	0.079767	0.851467432
CLDN11	ENSG00000013297	3	170126653	170588169	646	47	123834	1.4066	0.079777	0.851467432
MGC4771	ENSG00000269363	2	232561517	232581621	21	3	123834	1.4065	0.079795	0.851467432
C2orf78	ENSG00000187833	2	74001316	74054274	17	3	123834	1.4059	0.079872	0.851467432
HDAC2	ENSG00000196591	6	114244192	114342472	177	23	123834	1.4056	0.079925	0.851467432
CMAS	ENSG00000111726	12	22189108	22228608	37	5	123834	1.4055	0.07993	0.851467432
PCBP2	ENSG00000197111	12	53825525	53884946	32	5	123834	1.4051	0.079991	0.851467432
C6orf201	ENSG00000185689	6	4069440	4141185	144	12	123834	1.4047	0.080055	0.851467432
PRIM2	ENSG00000146143	6	57169603	57523375	155	13	123834	1.4047	0.080055	0.851467432
C4orf26	ENSG00000174792	4	76471258	76501095	44	9	123834	1.4047	0.080057	0.851467432
TCP11L1	ENSG00000176148	11	33050963	33137489	280	12	123834	1.4039	0.080174	0.851467432
INSC	ENSG00000188487	11	15123970	15278754	258	36	123834	1.4039	0.080181	0.851467432
HSD17B12	ENSG00000149084	11	43567986	43888167	454	23	123834	1.4033	0.080259	0.851467432
EXOC5	ENSG00000070367	14	57660518	57745726	91	3	123834	1.4033	0.080262	0.851467432
BLOC1S2	ENSG00000196072	10	102023713	102056469	57	7	123834	1.4031	0.080297	0.851467432
PPP1R3D	ENSG00000132825	20	58501894	58525352	11	2	123834	1.403	0.080309	0.851467432
IP6K2	ENSG00000068745	3	48715436	48787786	67	6	123834	1.4025	0.080383	0.851467432
SCAF1	ENSG00000126461	19	50135382	50171899	27	6	123834	1.402	0.080456	0.851467432
PCSK5	ENSG00000099139	9	78495560	78987255	868	123	123834	1.402	0.080457	0.851467432
CAPZA3	ENSG00000177938	12	18881045	18902121	28	5	123834	1.4019	0.080472	0.851467432
GZMK	ENSG00000113088	5	54310081	54340398	35	5	123834	1.4017	0.080497	0.851467432
ACTR3C	ENSG00000106526	7	149931005	150030814	179	13	123834	1.4011	0.080593	0.851566829
RFPL4A	ENSG00000223638	19	56260380	56284541	19	4	123834	1.4005	0.080683	0.851566829
DDX49	ENSG00000105671	19	19020484	19049436	16	5	123834	1.4001	0.080736	0.851566829
LYPD6	ENSG00000187123	2	150176499	150340662	164	16	123834	1.3999	0.08077	0.851566829
TMOD1	ENSG00000136842	9	100253462	100374030	137	25	123834	1.3998	0.08078	0.851566829
CAPN8	ENSG00000203697	1	223701349	223863436	220	23	123834	1.3998	0.080782	0.851566829
PKLR	ENSG00000143627	1	155249630	155281225	28	2	123834	1.3996	0.08082	0.851566829
HEATR1	ENSG00000119285	1	236702305	236777804	338	11	123834	1.3986	0.080967	0.851990829
AP003068.23	ENSG00000254614	11	64935077	64959305	11	2	123834	1.3983	0.081011	0.851990829
GNA14	ENSG00000156049	9	80027995	80273223	330	58	123834	1.398	0.081053	0.851990829
REEP2	ENSG00000132563	5	137764706	137792658	44	8	123834	1.3979	0.081073	0.851990829
C9orf91	ENSG00000157693	9	117363486	117418702	180	12	123834	1.3977	0.081103	0.851990829
Z97053.1	ENSG00000269605	20	43224346	43258126	55	8	123834	1.3975	0.081135	0.851990829
LRRC16A	ENSG00000079691	6	25269306	25630758	1100	54	123834	1.3972	0.081174	0.851990829
ARL4C	ENSG00000188042	2	235391685	235415697	25	8	123834	1.3965	0.081277	0.852539729
TYW3	ENSG00000162623	1	75188836	75242362	156	15	123834	1.3963	0.081316	0.852539729
CCNE2	ENSG00000175305	8	95881998	95918906	95	14	123834	1.3957	0.0814	0.852607189
CSPG5	ENSG00000114646	3	47593729	47632282	21	2	123834	1.3956	0.081419	0.852607189
C10orf113	ENSG00000204683	10	21404692	21445488	61	8	123834	1.3953	0.081457	0.852607189
RRAGC	ENSG00000116954	1	39293870	39335495	78	12	123834	1.3944	0.081603	0.853665286
EGR1	ENSG00000120738	5	137791179	137815004	37	6	123834	1.3939	0.081668	0.853875327
GLDC	ENSG00000178445	9	6522464	6655650	161	26	123834	1.3931	0.081787	0.854649421
CXCL1	ENSG00000163739	4	74725110	74746959	32	6	123834	1.3924	0.081907	0.854933205
RALYL	ENSG00000184672	8	85085022	85844079	800	32	123834	1.3923	0.081913	0.854933205
CWF19L1	ENSG00000095485	10	101982055	102037437	107	8	123834	1.3916	0.08202	0.854933205
BNIPL	ENSG00000163141	1	150999046	151030076	34	7	123834	1.3915	0.082036	0.854933205
ZNF878	ENSG00000257446	19	12144620	12177127	50	4	123834	1.3913	0.082065	0.854933205
PRDM1	ENSG00000057657	6	106524195	106567814	32	9	123834	1.391	0.082106	0.854933205
C1orf222	ENSG00000142609	1	1843396	1945276	117	14	123834	1.3909	0.082129	0.854933205
HSPA1A	ENSG00000204389	6	31773291	31795723	22	11	123834	1.3899	0.082277	0.855318611
RSF1	ENSG00000048649	11	77361041	77542063	290	9	123834	1.3898	0.082295	0.855318611
USP38	ENSG00000170185	4	144096070	144154983	95	6	123834	1.3895	0.082347	0.855318611
COMP	ENSG00000105664	19	18883583	18912123	9	2	123834	1.3892	0.08238	0.855318611
CCDC60	ENSG00000183273	12	119762517	119988852	337	41	123834	1.3886	0.082472	0.855318611
RASEF	ENSG00000165105	9	85584500	85688092	77	4	123834	1.3885	0.08249	0.855318611
RP11-276H1.3	ENSG00000175604	16	12170603	12194159	50	7	123834	1.3885	0.082495	0.855318611
MAP3K12	ENSG00000139625	12	53864274	53903847	10	4	123834	1.3878	0.082602	0.855318611
RP11-211G3.3	ENSG00000228804	3	187410101	187461637	111	14	123834	1.3877	0.082611	0.855318611
NUP160	ENSG00000030066	11	47789639	47880107	119	8	123834	1.3874	0.082659	0.855318611
STARD3NL	ENSG00000010270	7	38207824	38280272	167	16	123834	1.3874	0.082661	0.855318611
NT5C1A	ENSG00000116981	1	40114793	40147710	45	10	123834	1.3867	0.082771	0.855990842
ACOT11	ENSG00000162390	1	54997930	55114865	243	26	123834	1.3862	0.082849	0.856279409
TMPRSS13	ENSG00000137747	11	117761358	117810174	60	12	123834	1.3859	0.082889	0.856279409
PBRM1	ENSG00000163939	3	52569368	52729933	283	9	123834	1.3848	0.083061	0.85679534
AC006126.3	ENSG00000266958	19	45680646	45730191	84	8	123834	1.3846	0.083089	0.85679534
PICALM	ENSG00000073921	11	85658727	85790924	304	16	123834	1.3836	0.083235	0.85679534
TRIM21	ENSG00000132109	11	4396127	4424926	29	9	123834	1.3835	0.083249	0.85679534
LAIR1	ENSG00000167613	19	54855362	54892165	34	6	123834	1.3833	0.083282	0.85679534
MPLKIP	ENSG00000168303	7	40155622	40184258	20	6	123834	1.3833	0.083292	0.85679534
NAT10	ENSG00000135372	11	34117149	34179217	81	2	123834	1.3827	0.08338	0.85679534
CCDC33	ENSG00000140481	15	74499613	74638813	156	24	123834	1.3826	0.083396	0.85679534
WDR6	ENSG00000178252	3	49034495	49063386	8	2	123834	1.3823	0.083435	0.85679534
HAS2	ENSG00000170961	8	122614356	122663630	77	11	123834	1.3823	0.083444	0.85679534
DSCR3	ENSG00000157538	21	38585721	38650262	70	12	123834	1.382	0.08348	0.85679534
CDH22	ENSG00000149654	20	44792372	44947137	130	25	123834	1.3816	0.083545	0.85679534
EXOC3L2	ENSG00000130201	19	45705879	45747469	78	5	123834	1.3815	0.08356	0.85679534
FAM83A	ENSG00000147689	8	124181200	124232314	105	7	123834	1.3815	0.08357	0.85679534
TXNDC9	ENSG00000115514	2	99925445	99967165	64	6	123834	1.381	0.083641	0.857060985
RIOK2	ENSG00000058729	5	96486571	96528964	81	13	123834	1.3795	0.083865	0.858893276
KATNAL2	ENSG00000167216	18	44487455	44637658	232	16	123834	1.3791	0.083926	0.858895897
PLA2G10	ENSG00000069764	16	14756405	14798526	14	2	123834	1.3789	0.08397	0.858895897
PARP14	ENSG00000173193	3	122389465	122459687	161	17	123834	1.3786	0.084013	0.858895897
CELF2	ENSG00000048740	10	11037259	11388674	554	56	123834	1.3784	0.084046	0.858895897
VAV3	ENSG00000134215	1	108103782	108517766	833	62	123834	1.3774	0.084188	0.859545435
MDGA2	ENSG00000139915	14	47298826	48154157	1547	57	123834	1.3774	0.0842	0.859545435
JAKMIP1	ENSG00000152969	4	6017926	6212318	217	40	123834	1.3769	0.084273	0.85982887
CTB-60B18.6	ENSG00000267335	19	49516210	49550152	51	5	123834	1.3764	0.084346	0.859882836
MYO15B	ENSG00000266714	17	73574139	73632929	151	17	123834	1.376	0.084403	0.859882836
APOM	ENSG00000204444	6	31610193	31635987	30	11	123834	1.3752	0.084542	0.859882836
LGALS8	ENSG00000116977	1	236671300	236726281	219	7	123834	1.3746	0.084627	0.859882836
POLG	ENSG00000140521	15	89849534	89888092	29	4	123834	1.3745	0.084636	0.859882836
BRWD1	ENSG00000185658	21	40546102	40703485	375	13	123834	1.3739	0.084742	0.859882836
IL10RA	ENSG00000110324	11	117847063	117882196	42	4	123834	1.3738	0.084745	0.859882836
MDGA2	ENSG00000272781	14	47301134	48153999	1543	56	123834	1.3738	0.084748	0.859882836
CTC-512J12.6	ENSG00000267173	19	44823848	44915774	257	21	123834	1.3728	0.084911	0.859882836
EI24	ENSG00000149547	11	125429112	125464575	55	9	123834	1.3725	0.084948	0.859882836
TRDMT1	ENSG00000107614	10	17174253	17254053	220	8	123834	1.3723	0.084992	0.859882836
HIVEP3	ENSG00000127124	1	41962036	42511596	702	53	123834	1.3722	0.085001	0.859882836

TSPAN17	ENSG00000048140	5	176064388	176096058	27	6	123834	1.3719	0.085051	0.859882836
ZC3H6	ENSG00000188177	2	113023171	113107640	126	8	123834	1.3718	0.085055	0.859882836
ADCK3	ENSG00000163050	1	227075237	227185246	132	21	123834	1.3718	0.085057	0.859882836
ZNF136	ENSG00000196646	19	12263879	12310064	42	4	123834	1.3717	0.085079	0.859882836
TRAPPC2P1	ENSG00000256060	19	57864879	57886721	39	7	123834	1.3715	0.085106	0.859882836
OR7E24	ENSG00000237521	19	9351606	9372820	21	4	123834	1.3708	0.08522	0.859882836
MIPEP	ENSG00000027001	13	24294328	24473558	240	17	123834	1.3707	0.085234	0.859882836
UNKL	ENSG00000059145	16	1403206	1474752	78	7	123834	1.3707	0.085241	0.859882836
YRDC	ENSG00000196449	1	38258616	38283857	20	4	123834	1.3704	0.085288	0.859882836
COPE	ENSG00000105669	19	19000323	19040206	42	9	123834	1.3701	0.085326	0.859882836
ALX1	ENSG00000180318	12	85663885	85705562	49	6	123834	1.37	0.08534	0.859882836
RHBDD1	ENSG00000144468	2	227690297	227873931	238	12	123834	1.3699	0.085364	0.859882836
TMEM178A	ENSG00000152154	2	39882122	39955103	31	4	123834	1.3685	0.085582	0.860174972
VPS35	ENSG00000069329	16	46680054	46733430	6	2	123834	1.3683	0.085607	0.860174972
OXSM	ENSG00000151093	3	25814408	25846025	48	5	123834	1.3677	0.085697	0.860174972
WFDC12	ENSG00000168703	20	43742066	43763106	54	6	123834	1.3676	0.085716	0.860174972
APOE	ENSG00000130203	19	45399011	45422650	28	7	123834	1.3669	0.085824	0.860174972
C7orf66	ENSG00000205174	7	108514032	108534644	46	6	123834	1.3668	0.085836	0.860174972
S1PR1	ENSG00000170989	1	101692444	101717074	19	5	123834	1.3668	0.085848	0.860174972
DNM3	ENSG00000197959	1	171800621	172397606	935	50	123834	1.3667	0.085856	0.860174972
MAP1LC3A	ENSG00000101460	20	33124658	33158149	17	3	123834	1.3662	0.085939	0.860174972
FKBP2	ENSG00000173486	11	63998475	64021604	23	4	123834	1.3661	0.085948	0.860174972
HOXA2	ENSG00000105996	7	27129721	27152430	20	4	123834	1.366	0.08597	0.860174972
PPP1R14B	ENSG00000173457	11	64001956	64024413	24	5	123834	1.3654	0.086067	0.860174972
RASSF4	ENSG00000107551	10	45444855	45501339	77	13	123834	1.3653	0.086081	0.860174972
AURKAIP1	ENSG00000175756	1	1299110	1320875	87	8	123834	1.3644	0.086226	0.860174972
FDX1	ENSG00000137714	11	110290607	110345605	107	8	123834	1.3643	0.086233	0.860174972
RP11-41O4.1	ENSG00000266258	18	67992675	68029695	99	10	123834	1.3636	0.086341	0.860174972
CPXM2	ENSG00000121898	10	125455723	125709783	546	63	123834	1.3635	0.086368	0.860174972
WDR64	ENSG00000162843	1	241805580	241975435	318	31	123834	1.3629	0.086462	0.860174972
AC005003.1	ENSG00000213888	22	31732875	31754670	34	9	123834	1.3628	0.086478	0.860174972
ENOSF1	ENSG00000132199	18	660324	722676	183	13	123834	1.3626	0.086501	0.860174972
CLDN15	ENSG00000106404	7	100865373	100892101	37	5	123834	1.3625	0.086514	0.860174972
OR10A6	ENSG00000175393	11	7939180	7960209	75	7	123834	1.3625	0.086527	0.860174972
SLC43A3	ENSG00000134802	11	57164427	57205053	34	7	123834	1.3616	0.086655	0.860174972
SH3BP4	ENSG00000130147	2	235850617	235974358	116	20	123834	1.3611	0.086738	0.860174972
TAB1	ENSG00000100324	22	39785746	39843065	42	3	123834	1.361	0.08675	0.860174972
CTRC	ENSG00000162438	1	15754935	15785737	17	5	123834	1.3606	0.086823	0.860174972
AC027763.2	ENSG00000215067	17	6769954	6925668	141	23	123834	1.3605	0.08684	0.860174972
NUDT7	ENSG00000140876	16	77746411	77786157	184	17	123834	1.3603	0.086868	0.860174972
SLC5A11	ENSG00000158865	16	24847162	24932949	73	14	123834	1.3602	0.086883	0.860174972
NDST1	ENSG00000070614	5	149855381	149947773	135	14	123834	1.3595	0.086999	0.860174972
FGF3	ENSG00000186895	11	69614992	69643792	63	8	123834	1.3594	0.087004	0.860174972
OR52K1	ENSG00000196778	11	4500109	4521138	36	4	123834	1.3593	0.087028	0.860174972
C16orf97	ENSG00000261190	16	52029399	52122386	88	13	123834	1.3588	0.087109	0.860174972
TMEM54	ENSG00000121900	1	33350195	33377039	12	3	123834	1.3586	0.087135	0.860174972
ZNF707	ENSG00000181135	8	144756622	144806068	72	12	123834	1.3585	0.087152	0.860174972
AJUBA	ENSG00000129474	14	23430383	23461851	6	3	123834	1.3584	0.087171	0.860174972
TREM1	ENSG00000124731	6	41225664	41264457	72	14	123834	1.3583	0.087183	0.860174972
GJA9	ENSG00000131233	1	39320175	39357289	72	8	123834	1.3582	0.087198	0.860174972
SPAG7	ENSG00000091640	17	4852521	4881167	31	7	123834	1.3577	0.087272	0.860174972
GABPB2	ENSG00000143458	1	151033054	151108018	119	11	123834	1.3577	0.087284	0.860174972
LY6G6C	ENSG00000204421	6	31676425	31699622	17	9	123834	1.3567	0.087443	0.860174972
RP11-565P22.6	ENSG00000254706	1	162325197	162361905	52	14	123834	1.3558	0.08758	0.860174972
GPR12	ENSG00000132975	13	27319341	27344922	57	5	123834	1.3554	0.087647	0.860174972
RP1-32I10.10	ENSG00000220702	22	44751431	44772164	43	10	123834	1.3549	0.087726	0.860174972
PRDX5	ENSG00000126432	11	64075560	64099283	22	6	123834	1.3547	0.087764	0.860174972
RP5-1021I20.4	ENSG00000258653	14	74308674	74408981	332	14	123834	1.3541	0.087858	0.860174972
CTD-3193O13.9	ENSG00000183248	19	7923605	7949326	27	7	123834	1.3537	0.087914	0.860174972
UBA3	ENSG00000144744	3	69093881	69139559	40	7	123834	1.3534	0.087964	0.860174972
MYCBP	ENSG00000214114	1	39318636	39357289	78	9	123834	1.3532	0.087992	0.860174972
HDLBP	ENSG00000115677	2	242156679	242266476	157	13	123834	1.3531	0.088008	0.860174972
ZNF345	ENSG00000251247	19	37331263	37413931	188	16	123834	1.353	0.088022	0.860174972
CDCA3	ENSG00000111665	12	6943957	6971230	6	3	123834	1.3529	0.088042	0.860174972
TMED8	ENSG00000100580	14	77791364	77853452	107	15	123834	1.3529	0.088049	0.860174972
SUPT20H	ENSG00000102710	13	37573449	37643850	74	4	123834	1.3523	0.088142	0.860174972
ATOH8	ENSG00000168874	2	85968467	86025189	31	8	123834	1.3519	0.088198	0.860174972
ZSWIM2	ENSG00000163012	2	187682562	187723935	104	4	123834	1.3515	0.08826	0.860174972
IPCEF1	ENSG00000074706	6	154465631	154687926	453	50	123834	1.3514	0.088278	0.860174972
GRAMD1B	ENSG00000023171	11	123386344	123508482	170	31	123834	1.3512	0.088313	0.860174972
MNX1	ENSG00000130675	7	156776745	156813345	65	12	123834	1.3512	0.088315	0.860174972
ACP6	ENSG00000162836	1	147091453	147152618	71	7	123834	1.351	0.088343	0.860174972
PSMG1	ENSG00000183527	21	40536695	40565777	90	8	123834	1.3509	0.088365	0.860174972
BCKDK	ENSG00000103507	16	31107428	31134110	34	4	123834	1.3507	0.088397	0.860174972
SLFNL1	ENSG00000171790	1	41471269	41498909	57	9	123834	1.3506	0.088415	0.860174972
MYB	ENSG00000118513	6	135492453	135550311	45	9	123834	1.3504	0.088447	0.860174972
KLHDC3	ENSG00000124702	6	42971951	42999036	35	5	123834	1.3504	0.088448	0.860174972
GMIP	ENSG00000089639	19	19730285	19764476	27	8	123834	1.3503	0.088467	0.860174972
C22orf24	ENSG00000128254	22	32319507	32351504	51	13	123834	1.3499	0.088517	0.860174972
GCNT2	ENSG00000111846	6	10482456	10639601	304	28	123834	1.3498	0.088542	0.860174972
DLGAP2	ENSG00000198010	8	1439532	1666642	375	28	123834	1.3497	0.088553	0.860174972
ACP5	ENSG00000102575	19	11675475	11699823	33	5	123834	1.3491	0.088648	0.860174972
NDC80	ENSG00000080986	18	2561510	2626634	140	15	123834	1.3491	0.088651	0.860174972
DEPDC1	ENSG00000024526	1	68929835	68972904	90	11	123834	1.349	0.088666	0.860174972
KATNA1	ENSG00000186625	6	149906009	149980108	107	2	123834	1.3487	0.08871	0.860174972
PCED1B	ENSG00000179715	12	47463386	47640443	212	27	123834	1.3485	0.088752	0.860174972
RGS12	ENSG00000159788	4	3284755	3451640	215	29	123834	1.3483	0.088787	0.860174972
GPR52	ENSG00000203737	1	174407212	174428683	30	4	123834	1.3477	0.088883	0.860283701
IL12B	ENSG00000113302	5	158731791	158767895	30	5	123834	1.3475	0.088913	0.860283701
NUMB	ENSG00000133961	14	73731815	73940348	319	15	123834	1.3473	0.088934	0.860283701
KRT17	ENSG00000128422	17	39765689	39791094	10	3	123834	1.3459	0.089167	0.860789531
RP11-195F19.5	ENSG00000187186	9	34654160	34676109	39	6	123834	1.3454	0.089249	0.860789531
FDPS	ENSG00000160752	1	155268539	155300457	15	2	123834	1.3453	0.089272	0.860789531
SEMA4A	ENSG00000196189	1	156107157	156157543	42	7	123834	1.3451	0.089298	0.860789531
TSTD2	ENSG00000136925	9	100352362	100405962	34	8	123834	1.3451	0.089302	0.860789531
RP11-422N16.3	ENSG00000248050	8	110646344	110670313	21	4	123834	1.3447	0.089358	0.860789531
POLR3C	ENSG00000186141	1	145582605	145621025	17	2	123834	1.3445	0.089386	0.860789531
C17orf72	ENSG00000224383	17	62065711	62091664	1	1	123834	1.3443	0.089432	0.860789531
MYH4	ENSG00000264424	17	10336607	10382876	54	5	123834	1.344	0.089474	0.860789531

LBH	ENSG00000213626	2	30444397	30556596	190	23	123834	1.3439	0.089496	0.860789531
KMT2D	ENSG00000167548	12	49402758	49463557	28	4	123834	1.3438	0.089499	0.860789531
XKR8	ENSG00000158156	1	28275973	28304607	25	6	123834	1.3436	0.089535	0.860789531
ERVFRD-1	ENSG00000244476	6	11092722	11121965	79	5	123834	1.3434	0.089579	0.860789531
TRH	ENSG00000170893	3	129683148	129706781	29	6	123834	1.3422	0.08976	0.860789531
GINM1	ENSG00000055211	6	149877430	149922884	24	3	123834	1.3419	0.089819	0.860789531
FAM217B	ENSG00000196227	20	58498819	58533735	24	2	123834	1.3417	0.089851	0.860789531
FAM83G	ENSG00000188522	17	18862102	18918117	32	6	123834	1.3417	0.089854	0.860789531
RNMT	ENSG00000101654	18	13716659	13774557	133	15	123834	1.3398	0.090156	0.860789531
ZNF662	ENSG00000182983	3	42937223	42970825	22	4	123834	1.3397	0.090172	0.860789531
CDC42EP4	ENSG00000179604	17	71269763	71318314	26	8	123834	1.3393	0.090237	0.860789531
SETD6	ENSG00000103037	16	58539383	58564431	49	8	123834	1.339	0.090284	0.860789531
RGS16	ENSG00000143333	1	182557758	182583543	20	3	123834	1.3386	0.090347	0.860789531
BAX	ENSG00000087088	19	49448072	49475055	26	5	123834	1.3386	0.090349	0.860789531
ABI2	ENSG00000138443	2	204182942	204322446	118	13	123834	1.3382	0.090413	0.860789531
PDZD7	ENSG00000186862	10	102757440	102800890	22	6	123834	1.3382	0.090421	0.860789531
MPPED2	ENSG00000066382	11	30396040	30618419	337	25	123834	1.3379	0.090459	0.860789531
NEURL4	ENSG00000215041	17	7208947	7242712	37	6	123834	1.3379	0.090459	0.860789531
EDF1	ENSG00000107223	9	139746571	139770738	9	3	123834	1.3377	0.090497	0.860789531
PRKD1	ENSG00000184304	14	30035687	30671104	739	80	123834	1.3377	0.090505	0.860789531
CSTF2T	ENSG00000177613	10	53445247	53469355	57	5	123834	1.3376	0.090513	0.860789531
NCKIPSD	ENSG00000213672	3	48691364	48733797	32	2	123834	1.3371	0.090588	0.860789531
PDXK	ENSG00000160209	21	45128975	45192188	54	13	123834	1.3367	0.090668	0.860789531
HMGN3	ENSG00000118418	6	79900962	79954406	64	6	123834	1.3366	0.090678	0.860789531
SEPHS1	ENSG00000086475	10	13349424	13400297	110	9	123834	1.336	0.090774	0.860789531
CSDE1	ENSG00000009307	1	115249534	115311297	70	7	123834	1.3357	0.090819	0.860789531
TRMT112	ENSG00000173113	11	64073932	64095556	21	5	123834	1.3353	0.090881	0.860789531
TPP1	ENSG00000166340	11	6624000	6650692	37	6	123834	1.3348	0.090978	0.860789531
ZNF138	ENSG00000197008	7	64244766	64304054	150	11	123834	1.3347	0.090991	0.860789531
SH3YL1	ENSG00000035115	2	207730	276398	101	8	123834	1.3345	0.091019	0.860789531
C18orf32	ENSG00000177576	18	46998028	47023622	57	9	123834	1.3345	0.091025	0.860789531
ICAM4	ENSG00000105371	19	10387643	10409198	23	5	123834	1.3344	0.091031	0.860789531
INTS6	ENSG00000102786	13	51918213	52038400	148	11	123834	1.3341	0.091091	0.860789531
OAS1	ENSG00000089127	12	113334582	113379990	98	2	123834	1.3339	0.091112	0.860789531
CENPN	ENSG00000166451	16	81030103	81076719	126	5	123834	1.3339	0.091112	0.860789531
RP11-544M22.13	ENSG00000270149	1	160957747	161018752	104	9	123834	1.3339	0.091126	0.860789531
MYOG	ENSG00000122180	1	203042260	203065164	11	4	123834	1.3333	0.091224	0.860789531
LIG4	ENSG00000174405	13	108849787	108880716	56	8	123834	1.3327	0.091312	0.860789531
QPCT	ENSG00000115828	2	37561717	37610465	100	18	123834	1.3326	0.091333	0.860789531
FAM222A	ENSG00000139438	12	110142033	110218312	73	13	123834	1.3325	0.091351	0.860789531
HCN3	ENSG00000143630	1	155237374	155269639	30	2	123834	1.3324	0.091357	0.860789531
SERP1	ENSG00000120742	3	150249781	150331015	148	15	123834	1.3324	0.091357	0.860789531
FAM134C	ENSG00000141699	17	40721531	40772641	67	5	123834	1.3319	0.091439	0.860789531
CRNKL1	ENSG00000101343	20	20005012	20046690	106	11	123834	1.3318	0.091465	0.860789531
CD7	ENSG00000173762	17	80262744	80285478	30	2	123834	1.3312	0.091566	0.860789531
SMAP1	ENSG00000112305	6	71367479	71581718	337	13	123834	1.3306	0.091654	0.860789531
TSEN34	ENSG00000170892	19	54683789	54707585	36	4	123834	1.3306	0.091662	0.860789531
EPAS1	ENSG00000116016	2	46510806	46623836	107	24	123834	1.3303	0.091703	0.860789531
SPRTN	ENSG00000010072	1	231462850	231500769	52	3	123834	1.3298	0.091784	0.860789531
RAB3A	ENSG00000105649	19	18297594	18324884	42	6	123834	1.3295	0.091837	0.860789531
RP4-583P15.14	ENSG00000273047	20	62359623	62381751	34	4	123834	1.3295	0.091845	0.860789531
LIME1	ENSG00000203896	20	62356815	62380456	36	4	123834	1.3292	0.091892	0.860789531
DNAJC21	ENSG00000168724	5	34919698	34969069	50	7	123834	1.3292	0.091895	0.860789531
UTP23	ENSG00000147679	8	117768742	117871702	155	21	123834	1.3292	0.091895	0.860789531
FKBP1C	ENSG00000198225	6	63911351	63932929	47	3	123834	1.3288	0.091958	0.860789531
PUSL1	ENSG00000169972	1	1233947	1257057	28	5	123834	1.3287	0.091969	0.860789531
PARP15	ENSG00000173200	3	122286449	122367894	181	19	123834	1.3284	0.092028	0.860789531
RP11-9B6.1	ENSG00000248511	4	93208402	93235329	16	3	123834	1.3279	0.0921	0.860789531
FSTL4	ENSG00000053108	5	132522147	132958255	482	82	123834	1.3278	0.092118	0.860789531
UBTD1	ENSG00000165886	10	99248625	99340966	117	11	123834	1.3271	0.092232	0.860789531
CD36	ENSG00000135218	7	79988891	80318593	515	36	123834	1.327	0.092259	0.860789531
TCEB3CL2	ENSG00000266996	18	44532731	44554607	15	4	123834	1.3269	0.092275	0.860789531
LRFN5	ENSG00000165379	14	42066773	42383752	587	25	123834	1.3267	0.092301	0.860789531
C6orf25	ENSG00000204420	6	31676371	31704491	18	9	123834	1.3263	0.092376	0.860789531
UNK	ENSG00000132478	17	73770681	73831886	87	11	123834	1.3262	0.092393	0.860789531
TBX1	ENSG00000184058	22	19734226	19781116	54	5	123834	1.326	0.092418	0.860789531
POMT2	ENSG00000009830	14	77731299	77797227	90	14	123834	1.3257	0.092465	0.860789531
MEAF6	ENSG00000163875	1	37948176	37990375	67	9	123834	1.3257	0.092476	0.860789531
LIN28A	ENSG00000131914	1	26727269	26766213	44	6	123834	1.3253	0.092542	0.860789531
DUSP11	ENSG00000144048	2	73979311	74017284	21	2	123834	1.3251	0.092569	0.860789531
C6orf195	ENSG00000164385	6	2612147	2644837	53	7	123834	1.3245	0.092667	0.860789531
ZNF710	ENSG00000140548	15	90534624	90635438	50	9	123834	1.3243	0.092703	0.860789531
GBP5	ENSG00000154451	1	89714633	89748544	15	4	123834	1.3242	0.092725	0.860789531
CCNI2	ENSG00000205089	5	132073137	132099856	19	1	123834	1.324	0.092745	0.860789531
NYAP2	ENSG00000144460	2	226255364	226528734	341	26	123834	1.3233	0.092866	0.860997627
DYNLRB1	ENSG00000125971	20	33094214	33138762	20	2	123834	1.3224	0.093025	0.860997627
SC5D	ENSG00000109929	11	121153162	121189403	24	3	123834	1.3223	0.093033	0.860997627
ZCCHC6	ENSG00000083223	9	88892648	88979369	96	6	123834	1.3218	0.093113	0.860997627
PTGR2	ENSG00000140043	14	74308547	74363530	173	10	123834	1.3218	0.093115	0.860997627
TNFRSF11A	ENSG00000141655	18	59982520	60068516	137	32	123834	1.3212	0.093212	0.860997627
PTPRK	ENSG00000152894	6	128279924	128851870	549	35	123834	1.321	0.093246	0.860997627
LPP	ENSG00000145012	3	187861072	188618460	1022	120	123834	1.3209	0.093271	0.860997627
C22orf26	ENSG00000182257	22	46435358	46460024	8	2	123834	1.3207	0.0933	0.860997627
SGK1	ENSG00000118515	6	134480384	134649250	220	43	123834	1.3197	0.093468	0.860997627
ATP5F1	ENSG00000116459	1	111981486	112015395	42	5	123834	1.3196	0.093478	0.860997627
C10orf25	ENSG00000165511	10	45483146	45506336	25	5	123834	1.3191	0.093572	0.860997627
C5AR1	ENSG00000197405	19	47783280	47835323	97	6	123834	1.319	0.093585	0.860997627
C1orf226	ENSG00000239887	1	162326698	162366600	52	14	123834	1.3181	0.093732	0.860997627
11-Mar	ENSG00000183654	5	16057248	16190871	109	14	123834	1.3178	0.093789	0.860997627
DHRS7B	ENSG00000109016	17	21016677	21106578	60	6	123834	1.3177	0.093795	0.860997627
PARP10	ENSG00000178685	8	145041321	145096940	41	4	123834	1.3177	0.093801	0.860997627
BTN1A1	ENSG00000124557	6	26491449	26520650	51	9	123834	1.3175	0.093831	0.860997627
MRPS7	ENSG00000125445	17	73247755	73272454	34	4	123834	1.3175	0.093844	0.860997627
PARD3B	ENSG00000116117	2	205400516	206494886	1464	97	123834	1.3172	0.09388	0.860997627
STAMBP	ENSG00000124356	2	74046086	74110786	127	13	123834	1.3168	0.093956	0.860997627
NRD1	ENSG00000078618	1	52244863	52354477	111	15	123834	1.3167	0.093967	0.860997627
BIRC7	ENSG00000101197	20	61857235	61881859	82	11	123834	1.3166	0.093979	0.860997627
AHNAK	ENSG00000124942	11	62191016	62333707	156	20	123834	1.3159	0.094107	0.860997627
NR2F6	ENSG00000160113	19	17332692	17366749	67	14	123834	1.3154	0.094193	0.860997627

SERPINF1	ENSG00000132386	17	1655253	1690868	64	8	123834	1.3153	0.094202	0.860997627
POC5	ENSG00000152359	5	74959949	75023313	181	11	123834	1.3148	0.094282	0.860997627
LATS1	ENSG00000131023	6	149969289	150049392	80	2	123834	1.3147	0.094302	0.860997627
AC004076.7	ENSG00000268533	19	57895551	57959825	84	12	123834	1.3145	0.094343	0.860997627
AC011475.1	ENSG00000226104	19	10741715	10762093	29	4	123834	1.314	0.094426	0.860997627
IMPDH1	ENSG00000106348	7	128022331	128060306	73	14	123834	1.3137	0.094467	0.860997627
C12orf57	ENSG00000111678	12	7042141	7065166	2	1	123834	1.3133	0.094536	0.860997627
OLIG1	ENSG00000184221	21	34432450	34454726	4	2	123834	1.3131	0.094571	0.860997627
KCTD17	ENSG00000100379	22	37437779	37469430	11	4	123834	1.3129	0.094614	0.860997627
RPAIN	ENSG00000129197	17	5312961	5346196	98	6	123834	1.3127	0.094634	0.860997627
CAMSAP3	ENSG00000076826	19	7650788	7693190	43	5	123834	1.3126	0.09466	0.860997627
HNF1A	ENSG00000135100	12	121406346	121450315	105	15	123834	1.3124	0.094692	0.860997627
SQSTM1	ENSG00000161011	5	179223388	179275078	91	15	123834	1.3118	0.09479	0.860997627
MRGPRX1	ENSG00000170255	11	18945360	18971054	7	2	123834	1.3117	0.094806	0.860997627
DCAF4	ENSG00000119599	14	73383040	73436411	133	17	123834	1.3111	0.094915	0.860997627
VSX2	ENSG00000119614	14	74696175	74739441	98	10	123834	1.311	0.094929	0.860997627
PDS5B	ENSG00000083642	13	33150564	33362157	284	5	123834	1.3103	0.095039	0.860997627
PCDHGC3	ENSG00000240184	5	140845580	140902542	89	16	123834	1.3103	0.095043	0.860997627
MIF4GD	ENSG00000125457	17	73252309	73277308	34	4	123834	1.3102	0.095062	0.860997627
LIN52	ENSG00000205659	14	74541499	74677936	369	14	123834	1.3102	0.095071	0.860997627
SPCS1	ENSG00000114902	3	52728971	52752182	30	4	123834	1.31	0.095106	0.860997627
C10orf10	ENSG00000165507	10	45456429	45484258	43	7	123834	1.3099	0.095116	0.860997627
AGRN	ENSG00000188157	1	945503	1001496	63	6	123834	1.3097	0.095154	0.860997627
NFE2L2	ENSG00000116044	2	178082323	178267425	270	15	123834	1.3091	0.095243	0.860997627
CHUK	ENSG00000213341	10	101938055	101999376	109	9	123834	1.3087	0.095321	0.860997627
PDPN	ENSG00000162493	1	13899960	13954452	45	12	123834	1.3086	0.095335	0.860997627
C1QC	ENSG00000159189	1	22960123	22984603	64	7	123834	1.3086	0.095337	0.860997627
KRTAP5-5	ENSG00000185940	11	1641033	1662160	94	13	123834	1.3081	0.095411	0.860997627
IL22RA1	ENSG00000142677	1	24436261	24479611	49	10	123834	1.3079	0.095454	0.860997627
MED18	ENSG00000130772	1	28645513	28672476	29	5	123834	1.3071	0.095594	0.860997627
NENF	ENSG00000117691	1	212596229	212629714	58	9	123834	1.3069	0.095629	0.860997627
MVB12B	ENSG00000196814	9	129079128	129279320	221	23	123834	1.3065	0.095698	0.860997627
PDE4C	ENSG00000105650	19	18308771	18376229	106	11	123834	1.3058	0.095809	0.860997627
EIF2AK1	ENSG00000086232	7	6051881	6108861	182	19	123834	1.3056	0.095845	0.860997627
CCL27	ENSG00000213927	9	34651877	34674045	34	6	123834	1.3055	0.095855	0.860997627
LZTS2	ENSG00000107816	10	102746375	102777593	30	5	123834	1.3051	0.095935	0.860997627
POLE3	ENSG00000148229	9	116159515	116182952	29	3	123834	1.305	0.095945	0.860997627
TTC21B	ENSG00000123607	2	166703985	166820353	116	12	123834	1.3039	0.096136	0.860997627
CELF3	ENSG00000159409	1	151664880	151699290	29	4	123834	1.3037	0.096163	0.860997627
TRNT1	ENSG00000072756	3	3158600	3202563	119	15	123834	1.3037	0.096165	0.860997627
NDUFB5	ENSG00000136521	3	179312478	179355435	49	4	123834	1.3035	0.096196	0.860997627
RHOQ	ENSG00000119729	2	46758945	46820260	70	13	123834	1.3033	0.096242	0.860997627
ZEB1	ENSG00000148516	10	31597424	31828742	185	4	123834	1.303	0.096285	0.860997627
LECT2	ENSG00000145826	5	135247968	135300723	195	13	123834	1.3026	0.096356	0.860997627
MUC3A	ENSG00000169894	7	100537187	100621118	72	13	123834	1.3025	0.09637	0.860997627
GNB3	ENSG00000111664	12	6939118	6966557	14	5	123834	1.3024	0.096385	0.860997627
PAPD4	ENSG00000164329	5	78897943	78992471	181	7	123834	1.302	0.096462	0.860997627
ZNF233	ENSG00000159915	19	44744318	44789470	67	6	123834	1.3012	0.096591	0.860997627
OR2AK2	ENSG00000187080	1	248118535	248139641	121	7	123834	1.3011	0.096617	0.860997627
ZNF625-ZNF20	ENSG00000213297	19	12232932	12277546	49	4	123834	1.3009	0.096643	0.860997627
NUTM2E	ENSG00000228570	10	81591114	81620632	10	2	123834	1.3008	0.096672	0.860997627
ASXL3	ENSG00000141431	18	31148579	31341156	265	18	123834	1.3006	0.096696	0.860997627
LRP11	ENSG00000120256	6	150129934	150196199	71	3	123834	1.3005	0.096721	0.860997627
OR11H4	ENSG00000176198	14	20700898	20721979	39	10	123834	1.3003	0.096756	0.860997627
NUP43	ENSG00000120253	6	150035451	150080801	60	2	123834	1.2995	0.096885	0.860997627
MRPL38	ENSG00000204316	17	73884724	73915899	41	6	123834	1.2995	0.096886	0.860997627
ALAS1	ENSG00000023330	3	52222102	52258343	16	3	123834	1.2993	0.096912	0.860997627
RP11-178C3.1	ENSG00000267318	17	58008269	58060462	41	6	123834	1.2991	0.096957	0.860997627
BTC	ENSG00000174808	4	75659969	75729896	159	11	123834	1.299	0.096969	0.860997627
ZNRF2	ENSG00000180233	7	30313923	30462118	120	15	123834	1.2988	0.097013	0.860997627
MAPKBP1	ENSG00000137802	15	42056632	42130053	101	11	123834	1.2987	0.09702	0.860997627
FBXW12	ENSG00000164049	3	48403709	48452666	57	3	123834	1.2981	0.09712	0.860997627
ZNF205	ENSG00000122386	16	3152561	3180518	25	3	123834	1.298	0.097149	0.860997627
USP12	ENSG00000152484	13	27630293	27756033	227	22	123834	1.2978	0.097169	0.860997627
FSHR	ENSG00000170820	2	49179296	49391676	563	26	123834	1.2977	0.097198	0.860997627
ZNF577	ENSG00000161551	19	52349055	52404203	215	12	123834	1.2967	0.09737	0.860997627
SLC38A1	ENSG00000111371	12	46566846	46673800	85	17	123834	1.2965	0.097397	0.860997627
PRC1	ENSG00000198901	15	91499270	91548859	113	11	123834	1.2964	0.097424	0.860997627
VEGFA	ENSG00000112715	6	43727921	43764224	31	8	123834	1.2959	0.097507	0.860997627
GPANK1	ENSG00000204438	6	31619006	31644060	30	11	123834	1.2954	0.097585	0.860997627
EN1	ENSG00000163064	2	119589747	119615254	27	5	123834	1.2949	0.097683	0.860997627
GALK2	ENSG00000156958	15	49437853	49670066	459	17	123834	1.2945	0.097741	0.860997627
AC139768.1	ENSG00000269339	12	51609860	51630085	17	4	123834	1.2943	0.097783	0.860997627
PLEKHG5	ENSG00000171680	1	6516152	6590121	93	12	123834	1.2939	0.097841	0.860997627
JARID2	ENSG00000008083	6	15236527	15532252	459	42	123834	1.2932	0.097965	0.860997627
SAA1	ENSG00000173432	11	18277721	18301524	54	10	123834	1.2931	0.097985	0.860997627
ADRB1	ENSG00000043591	10	115793806	115816667	11	3	123834	1.2931	0.09799	0.860997627
GNAT1	ENSG00000114349	3	50219045	50243949	13	4	123834	1.293	0.098	0.860997627
ZNRF3	ENSG00000183579	22	29269580	29463475	212	24	123834	1.2927	0.098055	0.860997627
TGS1	ENSG00000137574	8	56675701	56748007	158	8	123834	1.2926	0.098066	0.860997627
RP11-10A14.4	ENSG00000253426	8	8999252	9035646	6	1	123834	1.2925	0.098085	0.860997627
FARP2	ENSG00000006607	2	242285658	242444256	314	19	123834	1.2925	0.098087	0.860997627
C14orf93	ENSG00000100802	14	23446110	23489375	13	6	123834	1.2925	0.098098	0.860997627
CCDC140	ENSG00000163081	2	223152866	223179936	5	2	123834	1.2922	0.09814	0.860997627
AGO1	ENSG00000092847	1	36325409	36405211	44	4	123834	1.2921	0.09816	0.860997627
BEGAIN	ENSG00000183092	14	100993486	101063750	49	8	123834	1.292	0.098172	0.860997627
NUPL1	ENSG00000139496	13	25865662	25933938	21	5	123834	1.2916	0.098241	0.860997627
FAM114A2	ENSG00000055147	5	153359688	153428496	128	5	123834	1.2915	0.098258	0.860997627
P2RY2	ENSG00000175591	11	72919343	72957397	12	4	123834	1.2914	0.098276	0.860997627
GNA15	ENSG00000060558	19	3126191	3173767	77	17	123834	1.2912	0.09831	0.860997627
F11R	ENSG00000158769	1	160955001	161001138	83	6	123834	1.2902	0.098489	0.860997627
LCE1B	ENSG00000196734	1	152774447	152795585	18	5	123834	1.29	0.09852	0.860997627
PAPPA	ENSG00000182752	9	118906083	119174601	130	28	123834	1.2898	0.098552	0.860997627
C3AR1	ENSG00000171860	12	8200898	8229067	15	6	123834	1.2898	0.098554	0.860997627
SCGB2A1	ENSG00000124939	11	61966140	61991408	14	4	123834	1.2897	0.098575	0.860997627
FZD9	ENSG00000188763	7	72838109	72860450	13	3	123834	1.2897	0.098578	0.860997627
NSUN5	ENSG00000130305	7	72706514	72732864	6	2	123834	1.2895	0.098607	0.860997627
RTN3	ENSG00000133318	11	63438918	63537363	60	3	123834	1.2889	0.098718	0.860997627
RPL19	ENSG00000108298	17	37346536	37370980	13	3	123834	1.2888	0.098729	0.860997627

AL133318.1	ENSG00000269120	13	41101138	41121323	32	7	123834	1.2887	0.098746	0.860997627
TRIM11	ENSG00000154370	1	228571374	228604541	36	4	123834	1.2887	0.098755	0.860997627
ITLN2	ENSG00000158764	1	160904815	160934589	40	9	123834	1.2886	0.098764	0.860997627
CYP7A1	ENSG00000167910	8	59392737	59422795	56	9	123834	1.2876	0.098951	0.860997627
AMZ2	ENSG00000196704	17	66233715	66263297	71	11	123834	1.2875	0.098963	0.860997627
KLHL25	ENSG00000183655	15	86292554	86348261	151	22	123834	1.287	0.099049	0.860997627
FCGBP	ENSG00000090920	19	40343963	40450533	124	23	123834	1.2866	0.099115	0.860997627
RARRES2	ENSG00000106538	7	150025408	150048763	31	3	123834	1.2864	0.09914	0.860997627
ZMAT5	ENSG00000100319	22	30116945	30173000	137	12	123834	1.2864	0.099144	0.860997627
IL5RA	ENSG00000091181	3	3101233	3178297	102	22	123834	1.2857	0.099279	0.860997627
STYK1	ENSG00000060140	12	10761538	10836917	103	6	123834	1.2855	0.099305	0.860997627
CUL7	ENSG00000044090	6	42995355	43031683	51	7	123834	1.2854	0.099319	0.860997627
SARNP	ENSG00000205323	12	56136247	56221540	54	7	123834	1.2854	0.099323	0.860997627
RGS7	ENSG00000182901	1	240921554	241530530	1018	101	123834	1.2851	0.099385	0.860997627
SERPINA6	ENSG00000170099	14	94760585	94799731	64	11	123834	1.2848	0.099429	0.860997627
TDRD6	ENSG00000180113	6	46645612	46682056	14	4	123834	1.2848	0.099434	0.860997627
KLRF1	ENSG00000150045	12	9970077	10007606	32	3	123834	1.2845	0.099479	0.860997627
THBS4	ENSG00000113296	5	79277134	79389110	172	12	123834	1.2842	0.099529	0.860997627
PITPNC1	ENSG00000154217	17	65363575	65703372	322	59	123834	1.2831	0.099732	0.860997627
CDH26	ENSG00000124215	20	58523471	58619066	101	16	123834	1.2829	0.099757	0.860997627
KLHL12	ENSG00000117153	1	202850228	202907764	94	8	123834	1.2829	0.099766	0.860997627
PCYT1A	ENSG00000161217	3	195931093	196024828	177	16	123834	1.2827	0.099807	0.860997627
ACBD6	ENSG00000135847	1	180234515	180482089	515	16	123834	1.2826	0.099812	0.860997627
ALOX5	ENSG00000012779	10	45859661	45951561	170	15	123834	1.282	0.099915	0.860997627
ZNF747	ENSG00000261459	16	30527244	30556173	7	3	123834	1.2815	0.1	0.860997627
RABEP1	ENSG00000029725	17	5175558	5299129	338	18	123834	1.2813	0.10004	0.860997627
RP11-762I7.5	ENSG00000257390	12	56141059	56231330	56	7	123834	1.2813	0.10005	0.860997627
UBP1	ENSG00000153560	3	33419828	33492863	172	12	123834	1.2811	0.10008	0.860997627
OCLN	ENSG00000197822	5	68778119	68863931	29	4	123834	1.2809	0.10011	0.860997627
NPFFR1	ENSG00000148734	10	71997085	72053432	94	17	123834	1.2808	0.10013	0.860997627
ESM1	ENSG00000164283	5	54263692	54328499	112	12	123834	1.2804	0.10021	0.860997627
GPR89B	ENSG00000188092	1	147390506	147475753	28	2	123834	1.2798	0.1003	0.860997627
PRKD2	ENSG00000105287	19	47167532	47230384	87	11	123834	1.279	0.10045	0.860997627
ARHGEF18	ENSG00000104880	19	7449999	7547363	263	12	123834	1.2787	0.1005	0.860997627
PIF1	ENSG00000140451	15	65097831	65127867	66	7	123834	1.2785	0.10053	0.860997627
FAIM	ENSG00000158234	3	138317448	138362218	44	4	123834	1.2779	0.10064	0.860997627
TADA2B	ENSG00000173011	4	7033626	7069679	51	6	123834	1.2779	0.10065	0.860997627
PIK3R4	ENSG00000196455	3	130387779	130475673	111	2	123834	1.2777	0.10068	0.860997627
SLC44A2	ENSG00000129353	19	10703133	10765235	92	8	123834	1.2775	0.10071	0.860997627
R3HCC1	ENSG00000104679	8	23117633	23163792	73	12	123834	1.2773	0.10074	0.860997627
CYP21A2	ENSG00000231852	6	31996042	32019447	22	9	123834	1.2771	0.10079	0.860997627
GADD45A	ENSG00000116717	1	68140744	68164021	42	7	123834	1.2763	0.10092	0.860997627
GALNT10	ENSG00000164574	5	153560290	153810544	486	35	123834	1.2754	0.10109	0.860997627
MRPL10	ENSG00000159111	17	45890638	45918900	64	8	123834	1.2746	0.10123	0.860997627
LAPTM5	ENSG00000162511	1	31195316	31240667	25	8	123834	1.2744	0.10126	0.860997627
PPP1R1A	ENSG00000135447	12	54959171	54992443	14	5	123834	1.2739	0.10134	0.860997627
SLC25A19	ENSG00000125454	17	73259073	73295591	30	5	123834	1.2733	0.10145	0.860997627
PTMA	ENSG00000187514	2	232561605	232588251	25	4	123834	1.2732	0.10147	0.860997627
PTPN6	ENSG00000111679	12	7045631	7080479	15	4	123834	1.2728	0.10154	0.860997627
CAMTA2	ENSG00000108509	17	4861287	4900960	76	11	123834	1.2724	0.10162	0.860997627
SLC8A3	ENSG00000100678	14	70500934	70665787	434	33	123834	1.272	0.10168	0.860997627
RET	ENSG00000165731	10	43562475	43635799	182	21	123834	1.272	0.10169	0.860997627
ICAM1	ENSG00000090339	19	10371511	10407291	25	7	123834	1.2718	0.10172	0.860997627
IRX1	ENSG00000170549	5	3586168	3611517	24	3	123834	1.2717	0.10174	0.860997627
SLC7A6OS	ENSG00000103061	16	68308406	68354849	46	7	123834	1.2716	0.10176	0.860997627
RFWD2	ENSG00000143207	1	175903967	176186629	538	18	123834	1.2715	0.10178	0.860997627
PHB2	ENSG00000215021	12	7064490	7089988	21	4	123834	1.2714	0.1018	0.860997627
RCAN1	ENSG00000159200	21	35875440	35997441	342	17	123834	1.2713	0.10182	0.860997627
CTDSP2	ENSG00000175215	12	58203710	58250522	14	4	123834	1.2712	0.10183	0.860997627
FLJ00325	ENSG00000269185	7	108530389	108550655	64	5	123834	1.2704	0.10197	0.860997627
ZIM3	ENSG00000141946	19	57635464	57666570	85	10	123834	1.2704	0.10198	0.860997627
JMJD7	ENSG00000243789	15	42110283	42139779	64	8	123834	1.2702	0.10201	0.860997627
AC104472.1	ENSG00000214919	3	155449933	155471515	43	7	123834	1.2701	0.10202	0.860997627
BRCA2	ENSG00000139618	13	32879611	32983805	160	17	123834	1.27	0.10204	0.860997627
MAP4K3	ENSG00000011566	2	39466407	39674453	329	13	123834	1.2699	0.10205	0.860997627
ZNF57	ENSG00000171970	19	2890896	2928474	173	10	123834	1.2695	0.10214	0.860997627
ESR2	ENSG00000140009	14	64540950	64814830	418	34	123834	1.2688	0.10226	0.860997627
PPP1R8	ENSG00000117751	1	28147289	28188187	29	2	123834	1.2687	0.10227	0.860997627
AKR7A3	ENSG00000162482	1	19599052	19625744	89	6	123834	1.2683	0.10234	0.860997627
NCKAP5L	ENSG00000167566	12	50174929	50232533	105	13	123834	1.2683	0.10234	0.860997627
TEKT4	ENSG00000163060	2	95527178	95552574	14	3	123834	1.2682	0.10236	0.860997627
DRG1	ENSG00000185721	22	31785509	31934726	203	20	123834	1.2681	0.10237	0.860997627
KAAG1	ENSG00000146049	6	24347131	24368512	44	7	123834	1.2679	0.10242	0.860997627
GRINA	ENSG00000178719	8	145054226	145077583	30	2	123834	1.2676	0.10247	0.860997627
OR1J4	ENSG00000239590	9	125271420	125292361	51	8	123834	1.2675	0.10249	0.860997627
TUBG1	ENSG00000131462	17	40751694	40777252	35	4	123834	1.2675	0.10249	0.860997627
SNTB1	ENSG00000172164	8	121537985	121835513	466	43	123834	1.2665	0.10267	0.860997627
KNTC1	ENSG00000184445	12	123001793	123120943	144	11	123834	1.2662	0.10273	0.860997627
PNRC2	ENSG00000189266	1	24275599	24299952	1	1	123834	1.2659	0.10278	0.860997627
SRSF10	ENSG00000188529	1	24281294	24317417	1	1	123834	1.2659	0.10278	0.860997627
GPT	ENSG00000167701	8	145718356	145742557	11	2	123834	1.2656	0.10282	0.860997627
CCDC71	ENSG00000177352	3	49189968	49213754	18	3	123834	1.265	0.10294	0.860997627
C7orf55	ENSG00000164898	7	139014203	139041065	67	4	123834	1.265	0.10294	0.860997627
ATP2C2	ENSG00000064270	16	84392133	84507793	280	42	123834	1.2647	0.10298	0.860997627
METTL1	ENSG00000037897	12	58152254	58176576	18	2	123834	1.2647	0.10299	0.860997627
C1QA	ENSG00000173372	1	22952999	22976101	39	6	123834	1.2646	0.103	0.860997627
ZNF408	ENSG00000175213	11	46712368	46737462	26	5	123834	1.2646	0.103	0.860997627
FAHD2B	ENSG00000144199	2	97739320	97770619	34	2	123834	1.2645	0.10302	0.860997627
WDR61	ENSG00000140395	15	78560177	78602136	67	11	123834	1.2645	0.10303	0.860997627
UBE2E2	ENSG00000182247	3	23234511	23643284	1149	21	123834	1.2644	0.10304	0.860997627
ENAM	ENSG00000132464	4	71484461	71562533	81	6	123834	1.2638	0.10315	0.860997627
METTL21B	ENSG00000123427	12	58155275	58186324	26	2	123834	1.2632	0.10325	0.860997627
SHANK2	ENSG00000162105	11	70303961	70973623	967	90	123834	1.2631	0.10327	0.860997627
RIPK2	ENSG00000104312	8	90759975	90813291	96	8	123834	1.263	0.1033	0.860997627
PALM2	ENSG00000243444	9	112393068	112723755	753	53	123834	1.2615	0.10357	0.860997627
MYO18A	ENSG00000196535	17	27390528	27517430	178	16	123834	1.261	0.10366	0.860997627
VAMP4	ENSG00000117533	1	171659300	171721387	162	9	123834	1.2609	0.10367	0.860997627
HIVEP2	ENSG00000010818	6	143062604	143276338	237	31	123834	1.2606	0.10372	0.860997627
TMOD4	ENSG00000163157	1	151132462	151158760	7	4	123834	1.2604	0.10376	0.860997627

DPM1	ENSG00000000419	20	49541404	49585092	35	6	123834	1.2601	0.10382	0.860997627
RHCE	ENSG00000188672	1	25678740	25766683	68	2	123834	1.2597	0.10389	0.860997627
EVA1A	ENSG00000115363	2	75686428	75806848	146	25	123834	1.2596	0.1039	0.860997627
LAMTOR3	ENSG00000109270	4	100789493	100825647	44	9	123834	1.2595	0.10392	0.860997627
STAT5A	ENSG00000126561	17	40429565	40473961	28	4	123834	1.2594	0.10394	0.860997627
PPP1R16B	ENSG00000101445	20	37424348	37561667	184	27	123834	1.2594	0.10394	0.860997627
BRD9	ENSG00000028310	5	840406	902939	94	3	123834	1.259	0.10402	0.860997627
EXOSC8	ENSG00000120699	13	37562953	37593750	25	3	123834	1.2586	0.10409	0.860997627
AKR1D1	ENSG00000122787	7	137677070	137812732	172	21	123834	1.2583	0.10413	0.860997627
PEX11B	ENSG00000131779	1	145506252	145533730	8	3	123834	1.2582	0.10416	0.860997627
OTOR	ENSG00000125879	20	16719003	16760707	82	11	123834	1.2582	0.10416	0.860997627
HELZ	ENSG00000198265	17	65056554	65252105	175	12	123834	1.2581	0.10418	0.860997627
PDK4	ENSG00000004799	7	95202811	95235803	56	7	123834	1.258	0.1042	0.860997627
BTN3A1	ENSG00000026950	6	26392465	26425444	104	8	123834	1.2576	0.10426	0.860997627
SYCP2	ENSG00000196074	20	58428618	58518710	111	2	123834	1.2574	0.10431	0.860997627
RFPL3	ENSG00000128276	22	32740872	32767148	160	13	123834	1.2571	0.10435	0.860997627
SOX1	ENSG00000182968	13	112711913	112736020	11	3	123834	1.2567	0.10442	0.860997627
GALC	ENSG00000054983	14	88294164	88470009	537	20	123834	1.2567	0.10443	0.860997627
C1QB	ENSG00000173369	1	22969255	22998031	53	7	123834	1.2564	0.10449	0.860997627
HIGD2B	ENSG00000175202	15	72958124	72988490	32	8	123834	1.2561	0.10453	0.860997627
AIG1	ENSG00000146416	6	143371633	143671441	298	36	123834	1.256	0.10456	0.860997627
ILK	ENSG00000166333	11	6614961	6642102	37	6	123834	1.2557	0.10462	0.860997627
TAF12	ENSG00000120656	1	28905835	28979597	46	7	123834	1.2556	0.10463	0.860997627
PHLDB2	ENSG00000144824	3	111441344	111705364	674	45	123834	1.2556	0.10463	0.860997627
ACYP2	ENSG00000170634	2	54187975	54542437	643	33	123834	1.2543	0.10487	0.860997627
KRTAP13-2	ENSG00000182816	21	31733709	31754557	46	8	123834	1.2542	0.10488	0.860997627
CACNA2D4	ENSG00000151062	12	1891123	2038002	264	24	123834	1.2534	0.10502	0.860997627
PIGF	ENSG00000151665	2	46798076	46854258	71	10	123834	1.2533	0.10504	0.860997627
CYP27B1	ENSG00000111012	12	58146117	58172769	16	2	123834	1.2527	0.10515	0.860997627
CINP	ENSG00000100865	14	102798956	102839253	49	10	123834	1.2527	0.10516	0.860997627
ZNF691	ENSG00000164011	1	43302280	43328148	14	3	123834	1.2526	0.10517	0.860997627
ZFP69B	ENSG00000187801	1	40905774	40939390	70	12	123834	1.2525	0.1052	0.860997627
ABHD13	ENSG00000139826	13	108860727	108896603	54	6	123834	1.2522	0.10524	0.860997627
SLC39A8	ENSG00000138821	4	103162198	103362415	422	27	123834	1.2519	0.1053	0.860997627
TAF10	ENSG00000166337	11	6617526	6643898	36	6	123834	1.2516	0.10536	0.860997627
RAB25	ENSG00000132698	1	156020951	156050295	11	6	123834	1.2514	0.1054	0.860997627
ANKRD34B	ENSG00000189127	5	79842574	79876307	43	10	123834	1.2514	0.1054	0.860997627
UNC13D	ENSG00000092929	17	73813306	73850798	57	10	123834	1.2509	0.10549	0.860997627
SMARCD3	ENSG00000082014	7	150925850	150984982	58	10	123834	1.2505	0.10555	0.860997627
EMG1	ENSG00000126749	12	7062408	7115520	42	4	123834	1.2505	0.10555	0.860997627
DAPP1	ENSG00000070190	4	100727990	100801311	155	18	123834	1.2504	0.10558	0.860997627
GTDC1	ENSG00000121964	2	144685635	145100135	350	19	123834	1.2501	0.10563	0.860997627
ESRRA	ENSG00000173153	11	64063044	64094215	25	6	123834	1.2498	0.10568	0.860997627
COL4A4	ENSG00000081052	2	227857427	228038829	294	22	123834	1.2497	0.10571	0.860997627
ROPN1	ENSG00000065371	3	123677368	123721025	33	7	123834	1.2494	0.10576	0.860997627
DPYD	ENSG00000188641	1	97533299	98396605	1050	72	123834	1.2492	0.1058	0.860997627
ERF	ENSG00000105722	19	42741724	42769309	28	1	123834	1.2488	0.10586	0.860997627
MAMDC4	ENSG00000177943	9	139735395	139765249	6	2	123834	1.2488	0.10588	0.860997627
FAM192A	ENSG00000172775	16	57176378	57230028	69	9	123834	1.2487	0.10589	0.860997627
SLC2A4RG	ENSG00000125520	20	62361214	62384858	36	4	123834	1.2482	0.10598	0.860997627
NPTXR	ENSG00000221890	22	39204457	39249987	95	13	123834	1.2479	0.10603	0.860997627
ART5	ENSG00000167311	11	3649733	3673546	26	5	123834	1.2477	0.10607	0.860997627
ENO3	ENSG00000108515	17	4841387	4870426	27	8	123834	1.2476	0.10609	0.860997627
MGAT4B	ENSG00000161013	5	179214597	179243952	45	8	123834	1.2474	0.10613	0.860997627
TCP11L2	ENSG00000166046	12	106685707	106750793	61	12	123834	1.2474	0.10613	0.860997627
LINC00935	ENSG00000257987	12	49111218	49169569	53	9	123834	1.2473	0.10614	0.860997627
RP11-505K9.4	ENSG00000260300	16	83931738	83995058	167	16	123834	1.2472	0.10616	0.860997627
FAM135A	ENSG00000082269	6	71112644	71280877	258	3	123834	1.2471	0.10619	0.860997627
SLC1A5	ENSG00000105281	19	47268140	47301851	79	11	123834	1.2471	0.10619	0.860997627
OR1N1	ENSG00000171505	9	125278637	125299572	83	11	123834	1.2465	0.10629	0.860997627
FANCI	ENSG00000140525	15	89777180	89870492	113	4	123834	1.2464	0.10631	0.860997627
POM121	ENSG00000196313	7	72339936	72431979	5	3	123834	1.2463	0.10633	0.860997627
AIFM2	ENSG00000042286	10	71847979	71902690	84	22	123834	1.2463	0.10633	0.860997627
TXNL1	ENSG00000091164	18	54254439	54328831	116	9	123834	1.2461	0.10637	0.860997627
ADARB1	ENSG00000197381	21	46483768	46656478	290	19	123834	1.246	0.10639	0.860997627
LIMA1	ENSG00000050405	12	50559571	50687329	115	5	123834	1.2456	0.10646	0.860997627
GTF2B	ENSG00000137947	1	89308615	89367627	111	7	123834	1.2452	0.10653	0.860997627
DDX19B	ENSG00000157349	16	70313566	70379186	22	6	123834	1.2451	0.10654	0.860997627
HOXC9	ENSG00000180806	12	54378679	54407121	18	4	123834	1.2448	0.1066	0.860997627
SLC4A1	ENSG00000004939	17	42315753	42355509	29	8	123834	1.2445	0.10667	0.860997627
AC010646.3	ENSG00000269095	19	17336749	17376257	75	16	123834	1.2444	0.10668	0.860997627
SPATC1	ENSG00000186583	8	145076582	145111933	10	2	123834	1.2439	0.10678	0.860997627
DNAJB14	ENSG00000164031	4	100807405	100877883	98	13	123834	1.2437	0.1068	0.860997627
RP11-571M6.15	ENSG00000257921	12	58156811	58190829	32	2	123834	1.2437	0.1068	0.860997627
KCTD16	ENSG00000183775	5	143540396	143875249	388	17	123834	1.2434	0.10686	0.860997627
THBD	ENSG00000178726	20	23016270	23040378	18	3	123834	1.2434	0.10686	0.860997627
LRRC34	ENSG00000171757	3	169501216	169540774	60	6	123834	1.2433	0.10687	0.860997627
MTERFD3	ENSG00000120832	12	107361069	107390944	48	12	123834	1.2433	0.10687	0.860997627
TNS1	ENSG00000079308	2	218654512	218877718	193	44	123834	1.2433	0.10688	0.860997627
EVI5L	ENSG00000142459	19	7885119	7939862	98	12	123834	1.2433	0.10688	0.860997627
MAML1	ENSG00000161021	5	179149851	179233512	145	11	123834	1.2432	0.10689	0.860997627
PLCD1	ENSG00000187091	3	38038987	38081253	79	12	123834	1.2432	0.1069	0.860997627
PCDH9	ENSG00000184226	13	66866967	67814468	1153	87	123834	1.2431	0.10691	0.860997627
TTC23L	ENSG00000205838	5	34828938	34909561	138	20	123834	1.2429	0.10695	0.860997627
ZNF829	ENSG00000185869	19	37369026	37417193	129	11	123834	1.2428	0.10698	0.860997627
ICAM5	ENSG00000105376	19	10390657	10417454	25	6	123834	1.2427	0.107	0.860997627
PLCZ1	ENSG00000139151	12	18826103	18900991	126	20	123834	1.242	0.10712	0.860997627
TSFM	ENSG00000123297	12	58166372	58211854	37	3	123834	1.242	0.10712	0.860997627
ASUN	ENSG00000064102	12	27048114	27101259	82	12	123834	1.2417	0.10717	0.860997627
RHPN1	ENSG00000158106	8	144441057	144476390	63	7	123834	1.2415	0.10721	0.860997627
KLRG1	ENSG00000139187	12	9092640	9173356	146	10	123834	1.241	0.10731	0.860997627
TP53RK	ENSG00000172315	20	45303004	45328418	41	6	123834	1.2405	0.1074	0.860997627
MKX	ENSG00000150051	10	27951804	28044989	118	9	123834	1.2402	0.10745	0.860997627
AL160274.1	ENSG00000268482	9	35351056	35371262	19	3	123834	1.2401	0.10748	0.860997627
PCGF6	ENSG00000156374	10	105052553	105120891	66	7	123834	1.2399	0.1075	0.860997627
CDC42SE1	ENSG00000197622	1	151013447	151052801	53	8	123834	1.2398	0.10752	0.860997627
PDE7B	ENSG00000171408	6	136162834	136526712	278	30	123834	1.2396	0.10756	0.860997627
NFKBIE	ENSG00000146232	6	44215903	44243500	22	6	123834	1.2395	0.10758	0.860997627
HORMAD2	ENSG00000176635	22	30466163	30583064	197	18	123834	1.2394	0.10759	0.860997627

GRIK1-AS2	ENSG00000183653	21	30958360	31013067	114	15	123834	1.2394	0.1076	0.860997627
FAM171B	ENSG00000144369	2	187548698	187640685	111	11	123834	1.2394	0.10761	0.860997627
STX3	ENSG00000166900	11	59470929	59583354	259	6	123834	1.2393	0.10763	0.860997627
OR2C1	ENSG00000168158	16	3395889	3416924	72	7	123834	1.2392	0.10763	0.860997627
FGFR4	ENSG00000160867	5	176503887	176535145	56	10	123834	1.2391	0.10765	0.860997627
RP11-15E18.4	ENSG00000267772	17	58631908	58673984	8	1	123834	1.2391	0.10765	0.860997627
FIP1L1	ENSG00000145216	4	54233810	55171439	1530	95	123834	1.239	0.10767	0.860997627
CNIH2	ENSG00000174871	11	66035661	66062772	16	4	123834	1.2385	0.10777	0.861434886
SPATA31E1	ENSG00000177992	9	90487741	90513814	36	10	123834	1.238	0.10785	0.86154698
VASH2	ENSG00000143494	1	213113862	213175379	96	14	123834	1.2377	0.10791	0.86154698
PLA2G12B	ENSG00000138308	10	74684928	74724536	10	2	123834	1.2377	0.10792	0.86154698
ZNF235	ENSG00000159917	19	44722882	44819199	126	10	123834	1.2371	0.10802	0.861983275
RCN1	ENSG00000049449	11	31823939	32137301	375	37	123834	1.2369	0.10807	0.862020378
ATF7IP2	ENSG00000166669	16	10410291	10587495	377	24	123834	1.2364	0.10816	0.862211165
SGSM1	ENSG00000167037	22	25192236	25333545	273	33	123834	1.2362	0.1082	0.862211165
ZFAND2B	ENSG00000158552	2	220049959	220084373	14	4	123834	1.236	0.10823	0.862211165
MPV17L2	ENSG00000254858	19	18293992	18317758	44	6	123834	1.235	0.10842	0.86283595
MUC4	ENSG00000145113	3	195463636	195549148	98	13	123834	1.2348	0.10845	0.86283595
BECN1	ENSG00000126581	17	40952152	40995367	2	1	123834	1.2347	0.10846	0.86283595
5-Mar	ENSG00000198060	10	94040920	94123721	82	6	123834	1.2346	0.10849	0.86283595
NEDD4	ENSG00000069869	15	56109120	56295944	553	20	123834	1.2343	0.10855	0.86295207
SMAD1	ENSG00000170365	4	146392346	146489231	103	10	123834	1.2337	0.10865	0.863061654
AC006486.9	ENSG00000268643	19	42728735	42769284	34	2	123834	1.2336	0.10869	0.863061654
DHX37	ENSG00000150990	12	125421371	125483668	196	20	123834	1.2335	0.1087	0.863061654
TRIM50	ENSG00000146755	7	72716535	72752085	19	3	123834	1.233	0.10879	0.863372554
UCHL1	ENSG00000154277	4	41248430	41280472	77	11	123834	1.2328	0.10883	0.863372554
FXYD4	ENSG00000150201	10	43857090	43881783	50	9	123834	1.2323	0.10891	0.86360367
SFXN1	ENSG00000164466	5	174894065	174966745	191	15	123834	1.2321	0.10895	0.86360367
DDB2	ENSG00000134574	11	47226493	47270767	69	7	123834	1.2315	0.10907	0.863863371
MCEE	ENSG00000124370	2	71326814	71367369	105	12	123834	1.2314	0.10909	0.863863371
ZNF442	ENSG00000198342	19	12450185	12486719	2	1	123834	1.2309	0.10917	0.863863371
GBX1	ENSG00000164900	7	150835676	150881832	40	8	123834	1.2308	0.1092	0.863863371
DIAPH1	ENSG00000131504	5	140884583	141008622	158	19	123834	1.2308	0.10921	0.863863371
DENND3	ENSG00000105339	8	142117377	142215907	71	13	123834	1.2304	0.10927	0.863978436
PPP1R42	ENSG00000178125	8	67866334	67978839	88	4	123834	1.2301	0.10933	0.864093405
GPR61	ENSG00000156097	1	110072494	110101028	14	5	123834	1.2295	0.10945	0.864682294
TTLL9	ENSG00000131044	20	30448505	30542764	95	13	123834	1.2287	0.10959	0.865262466
COPS2	ENSG00000166200	15	49388268	49457858	102	10	123834	1.2283	0.10966	0.865262466
PRR15L	ENSG00000167183	17	46019333	46045244	52	10	123834	1.2283	0.10966	0.865262466
PLCXD2	ENSG00000240891	3	111383523	111575294	433	34	123834	1.2279	0.10974	0.865534407
SDHB	ENSG00000117118	1	17335217	17390665	131	11	123834	1.2272	0.10987	0.865639917
NUBP1	ENSG00000103274	16	10827643	10873208	37	5	123834	1.2272	0.10987	0.865639917
ASCC2	ENSG00000100325	22	30174597	30244271	121	13	123834	1.2271	0.10989	0.865639917
NPBWR2	ENSG00000125522	20	62727173	62748524	41	6	123834	1.2266	0.10998	0.865989992
FMN1	ENSG00000248905	15	33047747	33496897	1138	96	123834	1.2256	0.11018	0.867205565
RPL15	ENSG00000174748	3	23948036	23975183	44	6	123834	1.2249	0.11031	0.867313485
CYB561	ENSG00000008283	17	61499665	61533739	1	1	123834	1.2246	0.11036	0.867313485
SLAMF6	ENSG00000162739	1	160444820	160503052	92	12	123834	1.2245	0.11039	0.867313485
MEA1	ENSG00000124733	6	42969832	42991706	33	5	123834	1.2243	0.11042	0.867313485
TSC2	ENSG00000103197	16	2087466	2148716	38	8	123834	1.2241	0.11046	0.867313485
AKR1C1	ENSG00000187134	10	4924796	5035475	330	25	123834	1.2238	0.11051	0.867313485
GNAO1	ENSG00000087258	16	56215302	56401356	299	32	123834	1.2232	0.11062	0.867313485
VEGFB	ENSG00000173511	11	63992010	64016259	23	5	123834	1.2232	0.11063	0.867313485
LXN	ENSG00000079257	3	158353611	158400482	122	6	123834	1.2231	0.11064	0.867313485
LINC01118	ENSG00000222005	2	47033807	47059796	28	8	123834	1.2231	0.11065	0.867313485
KRTAP24-1	ENSG00000188694	21	31643629	31665276	62	9	123834	1.2226	0.11074	0.867342456
SSMEM1	ENSG00000165120	7	129837700	129866683	23	5	123834	1.2219	0.11088	0.867342456
ZNF518B	ENSG00000178163	4	10431498	10469034	54	10	123834	1.2217	0.1109	0.867342456
GBP7	ENSG00000213512	1	89587434	89651723	98	8	123834	1.2216	0.11093	0.867342456
PWWP2A	ENSG00000170234	5	159478808	159556430	104	8	123834	1.2212	0.11101	0.867342456
OR6J1	ENSG00000255804	14	23092673	23113716	70	4	123834	1.2209	0.11105	0.867342456
ZNF610	ENSG00000167554	19	52829498	52881031	85	15	123834	1.221	0.11105	0.867342456
AHCYL2	ENSG00000158467	7	128854864	129080052	277	13	123834	1.2209	0.11106	0.867342456
SALL2	ENSG00000165821	14	21979232	22015350	21	2	123834	1.2208	0.11108	0.867342456
BMS1	ENSG00000165733	10	43268249	43340385	199	16	123834	1.2206	0.11111	0.867342456
GSTZ1	ENSG00000100577	14	77777227	77807940	60	12	123834	1.2204	0.11116	0.867376552
DEFB124	ENSG00000180383	20	30043309	30074560	26	5	123834	1.2198	0.11127	0.867878605
OR8D1	ENSG00000196341	11	124169708	124190733	47	6	123834	1.2193	0.11136	0.868224315
ZDHHC11B	ENSG00000206077	5	700475	777067	6	2	123834	1.2189	0.11144	0.868491808
VCL	ENSG00000035403	10	75747872	75889918	136	17	123834	1.2182	0.11158	0.869226492
STK4	ENSG00000101109	20	43585115	43718600	171	9	123834	1.2179	0.11163	0.869259746
AC012493.2	ENSG00000269383	2	100976705	100997007	74	4	123834	1.2173	0.11175	0.869279609
ACAA2	ENSG00000167315	18	47299869	47350330	98	11	123834	1.2171	0.11178	0.869279609
OR8G5	ENSG00000255298	11	124124723	124145763	124	5	123834	1.217	0.11181	0.869279609
RTFDC1	ENSG00000022277	20	55033647	55103943	134	6	123834	1.2161	0.11197	0.869279609
PATZ1	ENSG00000100105	22	31711790	31752218	53	10	123834	1.2159	0.11201	0.869279609
CLDN6	ENSG00000184697	16	3054713	3080072	6	3	123834	1.2158	0.11203	0.869279609
LRTM2	ENSG00000166159	12	1919433	1955918	36	9	123834	1.2156	0.11206	0.869279609
TCP10L2	ENSG00000166984	6	167549902	167620401	150	17	123834	1.2155	0.11209	0.869279609
LIPJ	ENSG00000204022	10	90336510	90376733	35	6	123834	1.2154	0.11212	0.869279609
SS18L2	ENSG00000008324	3	42613332	42646606	66	8	123834	1.2152	0.11215	0.869279609
OR1J2	ENSG00000197233	9	125263081	125284022	23	6	123834	1.215	0.11218	0.869279609
LRRC20	ENSG00000172731	10	72048729	72152382	199	23	123834	1.2148	0.11221	0.869279609
C16orf52	ENSG00000185716	16	22008959	22108855	41	5	123834	1.2143	0.11231	0.869279609
PTPRM	ENSG00000173482	18	7556780	8416859	1429	106	123834	1.2143	0.11232	0.869279609
CASS4	ENSG00000087589	20	54977168	55044396	94	10	123834	1.2142	0.11233	0.869279609
CCDC109B	ENSG00000005059	4	110471361	110619874	217	17	123834	1.214	0.11237	0.869279609
ANKEF1	ENSG00000132623	20	9956774	10047410	197	22	123834	1.2138	0.11241	0.869279609
RAB18	ENSG00000099246	10	27783197	27841143	94	7	123834	1.2131	0.11254	0.869565816
CHD2	ENSG00000173575	15	93416526	93581237	202	22	123834	1.2131	0.11254	0.869565816
RPH3A	ENSG00000089169	12	112998184	113346686	303	39	123834	1.2127	0.11262	0.869565816
COL17A1	ENSG00000065618	10	105781044	105855760	64	11	123834	1.2127	0.11263	0.869565816
CTD-3214H19.4	ENSG00000268400	19	7684682	7717934	66	5	123834	1.2123	0.1127	0.869575832
GDF10	ENSG00000107623	10	48415815	48448976	52	12	123834	1.2119	0.11278	0.869575832
RABGGTB	ENSG00000137955	1	76241879	76270764	63	10	123834	1.2115	0.11285	0.869575832
ANKRD61	ENSG00000157999	7	6061007	6086017	72	9	123834	1.2113	0.1129	0.869575832
RASIP1	ENSG00000105538	19	49213844	49253978	76	7	123834	1.2108	0.11299	0.869575832
ANKRD10	ENSG00000088448	13	111520887	111577416	110	10	123834	1.2101	0.11312	0.869575832
PPP2R5D	ENSG00000112640	6	42942237	42990080	104	12	123834	1.2099	0.11316	0.869575832

NDC1	ENSG00000058804	1	54221133	54314533	84	5	123834	1.2093	0.11327	0.869575832
BRD2	ENSG00000204256	6	32926437	32959282	144	22	123834	1.2092	0.11329	0.869575832
AATF	ENSG00000108270	17	35296175	35424171	87	6	123834	1.2086	0.1134	0.869575832
HDHD3	ENSG00000119431	9	116125699	116149279	38	6	123834	1.2085	0.11342	0.869575832
ASS1	ENSG00000130707	9	133310316	133386661	60	11	123834	1.2082	0.11348	0.869575832
FAM161B	ENSG00000156050	14	74388204	74427117	116	10	123834	1.208	0.11353	0.869575832
TBCB	ENSG00000105254	19	36595191	36626849	38	9	123834	1.208	0.11353	0.869575832
PPIG	ENSG00000138398	2	170430850	170507916	238	15	123834	1.2078	0.11357	0.869575832
RP11-552F3.12	ENSG00000267426	17	73884726	73936210	59	8	123834	1.2076	0.11359	0.869575832
EEF1E1	ENSG00000124802	6	8063593	8112811	93	11	123834	1.2075	0.11361	0.869575832
WDR77	ENSG00000116455	1	111972512	112001998	44	4	123834	1.2074	0.11364	0.869575832
NEK4	ENSG00000114904	3	52734800	52814965	121	8	123834	1.2074	0.11364	0.869575832
IL34	ENSG00000157368	16	70603798	70704585	107	13	123834	1.2068	0.11375	0.869575832
PALM3	ENSG00000187867	19	14154177	14179971	14	2	123834	1.2066	0.1138	0.869575832
CDCA8	ENSG00000134690	1	38148090	38185391	77	6	123834	1.2065	0.11381	0.869575832
STXBP2	ENSG00000076944	19	7691767	7722759	59	5	123834	1.2065	0.11382	0.869575832
LRRC66	ENSG00000188993	4	52849868	52893786	49	5	123834	1.2063	0.11385	0.869575832
PTPRN	ENSG00000054356	2	220144345	220184370	56	8	123834	1.206	0.11392	0.869575832
PHGR1	ENSG00000233041	15	40633234	40658635	20	5	123834	1.2059	0.11393	0.869575832
TMEM175	ENSG00000127419	4	916175	962444	60	12	123834	1.2058	0.11395	0.869575832
TUBGCP5	ENSG00000153575	15	22823395	22883892	86	4	123834	1.2052	0.11406	0.869575832
OR7C2	ENSG00000127529	19	15042301	15063260	52	9	123834	1.2052	0.11406	0.869575832
AK4	ENSG00000162433	1	65603232	65707828	101	10	123834	1.2048	0.11415	0.869575832
ACSM2B	ENSG00000066813	16	20537548	20597749	110	10	123834	1.2045	0.11421	0.869575832
AL645730.2	ENSG00000227839	1	50449990	50471874	16	4	123834	1.2044	0.11422	0.869575832
BAG2	ENSG00000112208	6	57027124	57064631	22	3	123834	1.2043	0.11423	0.869575832
SPATA19	ENSG00000166118	11	133700526	133725433	45	3	123834	1.2042	0.11425	0.869575832
ZDHHC21	ENSG00000175893	9	14601069	14703469	217	29	123834	1.2039	0.11432	0.869575832
SLC31A2	ENSG00000136867	9	115903222	115936417	103	17	123834	1.2036	0.11436	0.869575832
RP11-861L17.3	ENSG00000264472	18	66557018	66579492	59	5	123834	1.2036	0.11437	0.869575832
ARSG	ENSG00000141337	17	66245323	66428872	224	32	123834	1.2036	0.11438	0.869575832
CDK3	ENSG00000250506	17	73986987	74012080	44	8	123834	1.2032	0.11444	0.869575832
MFSD3	ENSG00000167700	8	145724457	145746596	10	2	123834	1.2028	0.11452	0.869575832
DHRS11	ENSG00000108272	17	34938228	34967235	29	5	123834	1.2027	0.11455	0.869575832
C19orf83	ENSG00000267757	19	46134752	46156098	16	5	123834	1.202	0.11469	0.869575832
HGFAC	ENSG00000109758	4	3433614	3461211	10	5	123834	1.2017	0.11474	0.869575832
MTFMT	ENSG00000103707	15	65284845	65331977	57	7	123834	1.2013	0.11481	0.869575832
ZNF787	ENSG00000142409	19	56588732	56642649	44	5	123834	1.2006	0.11496	0.869575832
FBXO48	ENSG00000204923	2	68676551	68704390	42	3	123834	1.2003	0.11502	0.869575832
ENOX1	ENSG00000120658	13	43777654	44371044	914	54	123834	1.2001	0.11506	0.869575832
TPRKB	ENSG00000144034	2	73946231	73974527	35	2	123834	1.2	0.11507	0.869575832
TACC2	ENSG00000138162	10	123738689	124024060	522	64	123834	1.1995	0.11517	0.869575832
TOMM40	ENSG00000130204	19	45383826	45416946	47	10	123834	1.1994	0.11518	0.869575832
LENEP	ENSG00000163352	1	154956062	154976791	18	2	123834	1.1993	0.11521	0.869575832
ANKRD27	ENSG00000105186	19	33077913	33177503	349	15	123834	1.1993	0.11521	0.869575832
TMEM161A	ENSG00000064545	19	19219978	19259322	44	8	123834	1.199	0.11527	0.869575832
SLC1A4	ENSG00000115902	2	65205611	65260999	89	15	123834	1.1984	0.11538	0.869575832
PICK1	ENSG00000100151	22	38442318	38481708	49	8	123834	1.1983	0.1154	0.869575832
NOG	ENSG00000183691	17	54661060	54682951	9	2	123834	1.1981	0.11543	0.869575832
PELO	ENSG00000152684	5	52073774	52109880	103	9	123834	1.1978	0.11549	0.869575832
CLEC4M	ENSG00000104938	19	7818035	7844491	50	8	123834	1.1976	0.11553	0.869575832
GPR179	ENSG00000188888	17	36471413	36509730	18	4	123834	1.1972	0.1156	0.869575832
FAM134B	ENSG00000154153	5	16463147	16627167	137	20	123834	1.1969	0.11568	0.869575832
OR4K1	ENSG00000155249	14	20393767	20414842	18	3	123834	1.1964	0.11576	0.869575832
ACP1	ENSG00000143727	2	254140	288283	47	5	123834	1.1963	0.11579	0.869575832
MVD	ENSG00000167508	16	88708343	88739569	27	7	123834	1.1963	0.1158	0.869575832
SYNDIG1	ENSG00000101463	20	24439835	24657252	256	25	123834	1.1962	0.11581	0.869575832
FUT8	ENSG00000033170	14	65867310	66220839	654	24	123834	1.196	0.11585	0.869575832
GOLGA6L4	ENSG00000184206	15	84894525	84924120	37	10	123834	1.196	0.11586	0.869575832
IL1R1	ENSG00000115594	2	102671004	102806334	191	24	123834	1.1959	0.11587	0.869575832
TMEM50A	ENSG00000183726	1	25654408	25698852	11	1	123834	1.1958	0.1159	0.869575832
PATL1	ENSG00000166889	11	59394189	59446453	56	5	123834	1.1954	0.11596	0.869575832
PRIMA1	ENSG00000175785	14	94174644	94264827	82	21	123834	1.1949	0.11606	0.869575832
PROP1	ENSG00000175325	5	177409236	177433243	36	10	123834	1.948	0.11608	0.869575832
IFIT3	ENSG00000119917	10	91077651	91110728	108	8	123834	1.1945	0.11614	0.869575832
ZSCAN5C	ENSG00000204532	19	56703670	56730821	83	12	123834	1.1937	0.11629	0.869575832
ST18	ENSG00000147488	8	53013399	53383519	476	41	123834	1.1936	0.11631	0.869575832
FKBP6	ENSG00000077800	7	72732167	72782634	61	3	123834	1.1933	0.11638	0.869575832
RICTOR	ENSG00000164327	5	38928021	39084510	247	11	123834	1.1931	0.11642	0.869575832
PPARGC1B	ENSG00000155846	5	149099861	149244585	243	32	123834	1.1931	0.11642	0.869575832
FAM129A	ENSG00000135842	1	184749858	184953682	240	24	123834	1.1927	0.11649	0.869575832
EEF1A2	ENSG00000101210	20	62109366	62140505	84	12	123834	1.1923	0.11658	0.869575832
AC091801.1	ENSG00000217455	7	3169868	3224287	85	14	123834	1.1915	0.11673	0.869575832
TBCA	ENSG00000171530	5	76976991	77174604	511	25	123834	1.1915	0.11674	0.869575832
MUCL1	ENSG00000172551	12	55214303	55262177	162	15	123834	1.1914	0.11674	0.869575832
C4orf46	ENSG00000205208	4	159577831	159603407	10	2	123834	1.1912	0.11679	0.869575832
HSD3B2	ENSG00000203859	1	119947554	119975658	44	5	123834	1.1909	0.11685	0.869575832
VGLL3	ENSG00000206538	3	86977119	87050269	30	6	123834	1.1905	0.11693	0.869575832
ABCG1	ENSG00000160179	21	43609799	43727354	158	29	123834	1.19	0.11702	0.869575832
C11orf58	ENSG00000110696	11	16624679	16788428	181	14	123834	1.1893	0.11716	0.869575832
LIMK2	ENSG00000182541	22	31598225	31686066	114	12	123834	1.1889	0.11723	0.869575832
RNF115	ENSG00000121848	1	145601036	145706218	101	6	123834	1.1886	0.11731	0.869575832
IL17RB	ENSG00000056736	3	53870607	53909827	66	13	123834	1.1885	0.11731	0.869575832
DNMT3B	ENSG00000088305	20	31340191	31407162	138	11	123834	1.1883	0.11735	0.869575832
RXFP2	ENSG00000133105	13	32303674	32387009	128	11	123834	1.1881	0.11739	0.869575832
KIAA1324	ENSG00000116299	1	109646301	109759401	223	29	123834	1.1878	0.11746	0.869575832
FOPNL	ENSG00000133393	16	15949577	15992482	211	5	123834	1.1877	0.11747	0.869575832
ARHGAP1	ENSG00000175220	11	46688630	46732165	39	6	123834	1.1872	0.11757	0.869575832
BHLHE23	ENSG00000125533	20	61627331	61648387	22	8	123834	1.1867	0.11768	0.869575832
PBX4	ENSG00000105717	19	19662522	19739725	87	10	123834	1.1862	0.11778	0.869575832
TOMM5	ENSG00000175768	9	37572643	37602639	50	6	123834	1.186	0.11781	0.869575832
PROSER1	ENSG00000120685	13	39574003	39622252	71	6	123834	1.1852	0.11796	0.869575832
SMIM15	ENSG00000188725	5	60443536	60468301	39	7	123834	1.1851	0.11799	0.869575832
HACE1	ENSG00000085382	6	105165968	105317794	290	11	123834	1.1846	0.11808	0.869575832
TTLL12	ENSG00000100304	22	43552628	43593139	91	14	123834	1.1839	0.11822	0.869575832
INPP5E	ENSG00000148384	9	139313071	139344274	71	9	123834	1.1838	0.11824	0.869575832
AL031663.2	ENSG00000269549	20	44064071	44087220	29	5	123834	1.1838	0.11825	0.869575832
CCDC171	ENSG00000164989	9	15542895	16071661	1065	14	123834	1.1838	0.11826	0.869575832
POLD2	ENSG00000106628	7	44144286	44173957	22	4	123834	1.1833	0.11834	0.869575832

CWC22	ENSG00000163510	2	180799603	180881840	163	11	123834	1.1832	0.11836	0.869575832
CYP17A1	ENSG00000148795	10	104580288	104607290	43	6	123834	1.1832	0.11836	0.869575832
SLC28A2	ENSG00000137860	15	45534428	45578149	126	6	123834	1.1832	0.11836	0.869575832
PROCA1	ENSG00000167525	17	27020215	27048872	16	4	123834	1.183	0.1184	0.869575832
SULT2A1	ENSG00000105398	19	48363723	48399654	61	5	123834	1.1829	0.11843	0.869575832
KAT8	ENSG00000103510	16	31117075	31152714	38	4	123834	1.1828	0.11845	0.869575832
CDH2	ENSG00000170558	18	25520930	25767410	244	31	123834	1.1827	0.11847	0.869575832
LEMD2	ENSG00000161904	6	33728979	33766913	131	11	123834	1.1822	0.11857	0.869575832
COPS5	ENSG00000121022	8	67945314	68006018	63	4	123834	1.1819	0.11863	0.869575832
VHLL	ENSG00000189030	1	156258415	156279428	31	4	123834	1.1815	0.11871	0.869575832
HEMGN	ENSG00000136929	9	100679073	100717138	48	4	123834	1.1814	0.11873	0.869575832
KCTD8	ENSG00000183783	4	44165926	44460824	435	37	123834	1.1813	0.11874	0.869575832
FAM126A	ENSG00000122591	7	22970878	23063749	226	17	123834	1.1808	0.11884	0.869575832
SERINC2	ENSG00000168528	1	31872412	31917525	128	10	123834	1.1802	0.11896	0.869575832
PIK3CG	ENSG00000105851	7	106495723	106557590	51	11	123834	1.1802	0.11896	0.869575832
LPA	ENSG00000198670	6	160942515	161097407	132	11	123834	1.1799	0.11902	0.869575832
GPR37L1	ENSG00000170075	1	202081986	202112720	51	10	123834	1.1799	0.11903	0.869575832
ARSK	ENSG00000164291	5	94880778	94950768	76	7	123834	1.1798	0.11904	0.869575832
PLA2G4B	ENSG00000243708	15	42110283	42150345	77	9	123834	1.1798	0.11904	0.869575832
JMJD7-PLA2G4B	ENSG00000168970	15	42110293	42150353	77	9	123834	1.1798	0.11904	0.869575832
SNAPC1	ENSG00000023608	14	62219075	62273146	111	7	123834	1.1797	0.11906	0.869575832
RNF185	ENSG00000138942	22	31546168	31613005	111	12	123834	1.1797	0.11906	0.869575832
PLXNB1	ENSG00000164050	3	48435261	48481594	28	1	123834	1.1789	0.11922	0.869575832
CYB561D1	ENSG00000174151	1	110026674	110055554	13	5	123834	1.1787	0.11926	0.869575832
10-Sep	ENSG00000186522	2	110290559	110381783	180	16	123834	1.1783	0.11933	0.869575832
RRP36	ENSG00000124541	6	42979383	43011894	45	6	123834	1.1783	0.11933	0.869575832
LEFTY1	ENSG00000243709	1	226063982	226109082	98	9	123834	1.1781	0.11937	0.869575832
RPL17-C18orf32	ENSG00000215472	18	46998051	47027956	72	10	123834	1.177	0.11959	0.869575832
NME6	ENSG00000172113	3	48324754	48353175	54	2	123834	1.1769	0.11961	0.869575832
STK10	ENSG00000072786	5	171459077	171625390	276	28	123834	1.1769	0.11962	0.869575832
IL1R2	ENSG00000115590	2	102598306	102655006	97	6	123834	1.1768	0.11964	0.869575832
VPS25	ENSG00000131475	17	40915454	40941617	3	1	123834	1.1765	0.1197	0.869575832
C1orf111	ENSG00000171722	1	162332993	162356660	35	11	123834	1.1759	0.11983	0.869575832
RHNO1	ENSG00000171792	12	2975424	3008626	69	4	123834	1.1756	0.11988	0.869575832
ITGB4	ENSG00000132470	17	73707408	73763899	20	6	123834	1.1755	0.1199	0.869575832
SPACA7	ENSG00000153498	13	113020633	113099003	266	18	123834	1.1751	0.11997	0.869575832
KIRREL	ENSG00000183853	1	157953063	158080052	212	35	123834	1.1746	0.12008	0.869575832
LHX2	ENSG00000106689	9	126753949	126805580	36	9	123834	1.1746	0.12008	0.869575832
AQP11	ENSG00000178301	11	77290436	77331400	63	4	123834	1.1746	0.12008	0.869575832
AC006486.1	ENSG00000204957	19	42736927	42759125	17	1	123834	1.1743	0.12014	0.869575832
C1orf109	ENSG00000116922	1	38137242	38167914	45	5	123834	1.1742	0.12015	0.869575832
ANKRD65	ENSG00000235098	1	1343800	1367149	20	4	123834	1.1736	0.12028	0.869575832
RBM12B	ENSG00000183808	8	94731584	94763245	39	3	123834	1.1735	0.1203	0.869575832
CENPW	ENSG00000203760	6	126651320	126680021	11	2	123834	1.1734	0.12032	0.869575832
SPINK8	ENSG00000229453	3	48338336	48379831	84	2	123834	1.1733	0.12033	0.869575832
CRBN	ENSG00000113851	3	3180676	3231394	111	13	123834	1.1733	0.12035	0.869575832
DST	ENSG00000151914	6	56312785	56829426	667	18	123834	1.1731	0.12037	0.869575832
S100A12	ENSG00000163221	1	153336184	153358125	24	1	123834	1.1731	0.12038	0.869575832
FAM83H	ENSG00000180921	8	144796103	144825971	42	5	123834	1.173	0.12039	0.869575832
CHMP3	ENSG00000115561	2	86720554	86958245	327	23	123834	1.1726	0.12047	0.869575832
CCDC88B	ENSG00000168071	11	64097695	64135006	46	6	123834	1.172	0.1206	0.869575832
RALA	ENSG00000006451	7	39653082	39757723	148	17	123834	1.1719	0.12063	0.869575832
NXPH2	ENSG00000144227	2	139418342	139547918	256	26	123834	1.1718	0.12064	0.869575832
MYRF	ENSG00000124920	11	61510114	61565990	20	5	123834	1.1716	0.12067	0.869575832
GCN1L1	ENSG00000089154	12	120555007	120642513	64	4	123834	1.1712	0.12076	0.869575832
ORMDL2	ENSG00000123353	12	56201703	56225663	19	4	123834	1.1711	0.12078	0.869575832
TSHZ1	ENSG00000179981	18	72912710	73011905	120	20	123834	1.1711	0.12078	0.869575832
GPSM1	ENSG00000160360	9	139211932	139264057	75	16	123834	1.171	0.1208	0.869575832
STAC2	ENSG00000141750	17	37356789	37392125	16	4	123834	1.1708	0.12085	0.869575832
ORM2	ENSG00000228278	9	117082149	117105532	5	2	123834	1.1699	0.12102	0.869575832
HIST4H4	ENSG00000197837	12	14910933	14934065	41	5	123834	1.1693	0.12113	0.869575832
NUDT15	ENSG00000136159	13	48601703	48631358	74	13	123834	1.1689	0.12122	0.869575832
EMX1	ENSG00000135638	2	73133389	73172020	18	4	123834	1.1688	0.12124	0.869575832
NKAIN4	ENSG00000101198	20	61862136	61914046	159	23	123834	1.1684	0.12132	0.869575832
MICALL1	ENSG00000100139	22	38291664	38348829	46	5	123834	1.1683	0.12133	0.869575832
CDCA7L	ENSG00000164649	7	21930518	21995702	96	13	123834	1.1682	0.12136	0.869575832
AL353354.2	ENSG00000269804	1	28517070	28537419	36	5	123834	1.1681	0.12138	0.869575832
SNAI3	ENSG00000185669	16	88734090	88762901	15	3	123834	1.168	0.1214	0.869575832
PTK7	ENSG00000112655	6	43034006	43139457	162	16	123834	1.1678	0.12145	0.869575832
RBM25	ENSG00000119707	14	73515144	73598122	125	17	123834	1.1676	0.12149	0.869575832
DDIT4	ENSG00000168209	10	74023678	74045794	31	4	123834	1.1671	0.12159	0.869575832
SNTA1	ENSG00000101400	20	31985761	32041698	25	4	123834	1.1666	0.12168	0.869575832
MRGPRE	ENSG00000184350	11	3238928	3263616	51	12	123834	1.1666	0.12169	0.869575832
RP11-1C1.5	ENSG00000249160	5	10495108	10532196	49	11	123834	1.1665	0.12171	0.869575832
CLNS1A	ENSG00000074201	11	77215981	77358850	185	11	123834	1.1664	0.12172	0.869575832
TP53TG3	ENSG00000183632	16	32674852	32698053	1	1	123834	1.1663	0.12174	0.869575832
SYNE2	ENSG00000054654	14	64309683	64703165	676	39	123834	1.1662	0.12177	0.869575832
ZYG11A	ENSG00000203995	1	53298183	53370670	113	7	123834	1.166	0.1218	0.869575832
DTX4	ENSG00000110042	11	58928903	58986060	91	9	123834	1.166	0.12181	0.869575832
PIWIL4	ENSG00000134627	11	94267006	94364587	167	18	123834	1.1659	0.12183	0.869575832
FITM2	ENSG00000197296	20	42921478	42949809	52	6	123834	1.1657	0.12187	0.869575832
SDCBP	ENSG00000137575	8	59455483	59505419	69	3	123834	1.1655	0.12191	0.869575832
SLC8B1	ENSG00000089060	12	113726564	113807298	120	10	123834	1.165	0.12202	0.869575832
ABHD10	ENSG00000144827	3	111687857	111722210	89	6	123834	1.1649	0.12203	0.869575832
C9orf43	ENSG00000157653	9	116162334	116201964	70	6	123834	1.1647	0.12207	0.869575832
ADAM15	ENSG00000143537	1	155013042	155045252	17	7	123834	1.1644	0.12214	0.869575832
UBE2Q2	ENSG00000140367	15	76125622	76203419	75	5	123834	1.1643	0.12214	0.869575832
GAB1	ENSG00000109458	4	144247915	144405721	205	26	123834	1.1643	0.12215	0.869575832
TXLNB	ENSG00000164440	6	139551198	139623276	91	12	123834	1.1642	0.12217	0.869575832
CNIH4	ENSG00000143771	1	224534552	224577161	40	5	123834	1.164	0.12221	0.869575832
SFTPD	ENSG00000133661	10	81687496	81752370	334	16	123834	1.1637	0.12226	0.869575832
CADM1	ENSG00000182985	11	115029938	115385675	304	34	123834	1.1636	0.12229	0.869575832
WBSCR16	ENSG00000174374	7	74431226	74500064	5	1	123834	1.1635	0.12231	0.869575832
DKK2	ENSG00000155011	4	107832959	108214963	533	56	123834	1.1635	0.12232	0.869575832
MEGF11	ENSG00000157890	15	66177417	66556085	573	76	123834	1.1631	0.12239	0.869575832
REV1	ENSG00000135945	2	100006938	100116497	162	9	123834	1.1627	0.12248	0.869575832
TRERF1	ENSG00000124496	6	42182669	42429789	344	27	123834	1.1627	0.12248	0.869575832
BMP6	ENSG00000153162	6	7717030	7891655	292	37	123834	1.1625	0.12251	0.869575832
RUSC1	ENSG00000160753	1	155280687	155310905	9	3	123834	1.1623	0.12255	0.869575832

TMEM104	ENSG00000109066	17	72762622	72845918	111	10	123834	1.1622	0.12257	0.869575832
NUP88	ENSG00000108559	17	5254258	5333480	244	9	123834	1.1622	0.12259	0.869575832
SLC35B4	ENSG00000205060	7	133964084	134011803	79	3	123834	1.1618	0.12265	0.869575832
ANKRA2	ENSG00000164331	5	72838160	72871511	61	7	123834	1.1615	0.12272	0.869575832
HIPK3	ENSG00000110422	11	33268218	33388569	114	10	123834	1.1612	0.12277	0.869575832
HSPBAP1	ENSG00000169087	3	122448846	122522671	200	18	123834	1.1611	0.1228	0.869575832
TYMS	ENSG00000176890	18	647604	683578	81	6	123834	1.1611	0.12281	0.869575832
GMEB2	ENSG00000101216	20	62208955	62268394	168	16	123834	1.1604	0.12294	0.869575832
NYNRIN	ENSG00000205978	14	24857992	24898494	27	12	123834	1.1604	0.12295	0.869575832
SLC35A3	ENSG00000117620	1	100425345	100502535	140	10	123834	1.1601	0.123	0.869575832
C8orf76	ENSG00000189376	8	124222196	124263638	1	1	123834	1.1598	0.12306	0.869575832
CASP6	ENSG00000138794	4	110599785	110634739	48	6	123834	1.1596	0.1231	0.869575832
MGAT3	ENSG00000128268	22	39843349	39898199	21	6	123834	1.1595	0.12312	0.869575832
IRX3	ENSG00000177508	16	54307216	54330675	19	5	123834	1.1595	0.12314	0.869575832
CISH	ENSG00000114737	3	50633921	50659262	19	2	123834	1.1589	0.12325	0.869575832
ZNF230	ENSG00000159882	19	44497100	44528078	24	2	123834	1.1588	0.12328	0.869575832
RGS10	ENSG00000148908	10	121249340	121312220	96	13	123834	1.1587	0.12329	0.869575832
RP4-559A3.7	ENSG00000255835	1	226064396	226121978	111	12	123834	1.1586	0.1233	0.869575832
UTP15	ENSG00000164338	5	72851268	72887794	79	7	123834	1.1584	0.12334	0.869575832
ADCY8	ENSG00000155897	8	131782547	132064672	636	43	123834	1.1573	0.12357	0.869575832
ATP1A2	ENSG00000018625	1	160075549	160123381	90	15	123834	1.157	0.12363	0.869575832
CHPF2	ENSG00000033100	7	150919575	150945908	48	7	123834	1.1569	0.12365	0.869575832
MAN1A2	ENSG00000198162	1	117900071	118081494	427	19	123834	1.1569	0.12366	0.869575832
SART3	ENSG00000075856	12	108906357	108965176	154	11	123834	1.1569	0.12366	0.869575832
C12orf74	ENSG00000214215	12	93086619	93112325	34	4	123834	1.1568	0.12368	0.869575832
FAM25A	ENSG00000188100	10	88770063	88794487	42	10	123834	1.1567	0.12369	0.869575832
C8orf22	ENSG00000168333	8	49956870	49998649	62	7	123834	1.1567	0.1237	0.869575832
LYAR	ENSG00000145220	4	4259428	4301896	90	16	123834	1.1566	0.12372	0.869575832
PTAFR	ENSG00000169403	1	28463677	28530447	41	6	123834	1.1565	0.12374	0.869575832
KHDRBS1	ENSG00000121774	1	32469430	32536451	18	4	123834	1.1562	0.12379	0.869575832
TRRAP	ENSG00000196367	7	98465556	98620866	221	10	123834	1.1563	0.12379	0.869575832
SFMBT1	ENSG00000163935	3	52927588	53090766	229	13	123834	1.156	0.12384	0.869575832
ZNF747	ENSG00000169955	16	30527244	30556668	8	3	123834	1.1559	0.12386	0.869575832
GFM1	ENSG00000168827	3	158352067	158420364	180	8	123834	1.1555	0.12394	0.869575832
GRIK3	ENSG00000163873	1	37251128	37509730	189	25	123834	1.1554	0.12396	0.869575832
NT5C1B-RDH14	ENSG00000250741	2	18726811	18780830	55	6	123834	1.1553	0.12398	0.869575832
NT5C1B	ENSG00000185013	2	18727050	18780838	55	6	123834	1.1553	0.12398	0.869575832
MS4A6A	ENSG00000110077	11	59929081	59962139	38	4	123834	1.1552	0.124	0.869575832
NKX3-2	ENSG00000109705	4	13532454	13556674	13	4	123834	1.1552	0.12401	0.869575832
AL162407.1	ENSG00000234631	10	115664530	115686949	7	3	123834	1.1551	0.12403	0.869575832
IPO5	ENSG00000065150	13	98595912	98686551	31	4	123834	1.1549	0.12406	0.869575832
SCARF2	ENSG00000244486	22	20768874	20802146	53	8	123834	1.1549	0.12406	0.869575832
DOCK1	ENSG00000150760	10	128583978	129260781	906	84	123834	1.1548	0.12408	0.869575832
AL646016.1	ENSG00000268365	1	240619183	240653504	34	5	123834	1.1546	0.12412	0.869575832
CLMP	ENSG00000166250	11	122933035	123075989	367	32	123834	1.1545	0.12416	0.869575832
MIOX	ENSG00000100253	22	50915213	50939077	12	6	123834	1.1543	0.12419	0.869575832
C3orf27	ENSG00000198685	3	128280843	128304929	30	8	123834	1.1542	0.12422	0.869575832
RPL35	ENSG00000136942	9	127610159	127634260	25	2	123834	1.154	0.12424	0.869575832
TLR6	ENSG00000174130	4	38815336	38868438	152	11	123834	1.1537	0.12431	0.869575832
ADRA1B	ENSG00000170214	5	159333790	159409551	117	13	123834	1.1534	0.12436	0.869575832
RABGAP1L	ENSG00000152061	1	174118548	174974445	1425	17	123834	1.1533	0.12439	0.869575832
RIPK3	ENSG00000129465	14	24795227	24819251	63	7	123834	1.1532	0.12442	0.869575832
FGF18	ENSG00000156427	5	170836660	170894627	57	10	123834	1.1531	0.12444	0.869575832
H2AFZ	ENSG00000164032	4	100859243	100881545	36	7	123834	1.1529	0.12448	0.869575832
CLEC11A	ENSG00000105472	19	51216586	51238974	16	5	123834	1.1523	0.12459	0.869652514
TATDN3	ENSG00000203705	1	212955170	212999968	178	11	123834	1.1513	0.12479	0.869652514
OR52R1	ENSG00000176937	11	4814663	4835847	100	8	123834	1.1513	0.1248	0.869652514
CDC25A	ENSG00000164045	3	48188636	48239892	35	4	123834	1.1511	0.12485	0.869652514
MEX3B	ENSG00000183496	15	82324119	82348482	27	7	123834	1.1509	0.12489	0.869652514
LRFN3	ENSG00000126243	19	36416260	36446669	49	4	123834	1.1509	0.12489	0.869652514
CNOT1	ENSG00000125107	16	58543855	58673790	311	12	123834	1.1508	0.12491	0.869652514
TMEM150C	ENSG00000249242	4	83394323	83493510	106	22	123834	1.1507	0.12492	0.869652514
RSPRY1	ENSG00000159579	16	57210049	57284387	100	10	123834	1.1505	0.12496	0.869652514
NAT2	ENSG00000156006	8	18238755	18268728	129	13	123834	1.1504	0.12499	0.869652514
HOXB6	ENSG00000108511	17	46661639	46692354	56	11	123834	1.1502	0.12502	0.869652514
KRTAP27-1	ENSG00000206107	21	31699331	31720012	87	9	123834	1.1501	0.12504	0.869652514
LOXL1	ENSG00000129038	15	74208330	74254478	106	18	123834	1.1494	0.1252	0.869906939
ATAD3C	ENSG00000215915	1	1375069	1415538	5	2	123834	1.1493	0.12521	0.869906939
TACR2	ENSG00000075073	10	71153659	71186623	22	6	123834	1.1487	0.12534	0.869906939
ASCC1	ENSG00000138303	10	73846278	73986892	157	10	123834	1.1486	0.12536	0.869906939
METAP1D	ENSG00000172878	2	172854490	172957158	168	19	123834	1.1484	0.1254	0.869906939
MOCOS	ENSG00000075643	18	33757482	33862120	173	22	123834	1.1484	0.12541	0.869906939
SLC1A1	ENSG00000106688	9	4480444	4597469	117	19	123834	1.1481	0.12546	0.869906939
OSBP	ENSG00000110048	11	59331871	59393617	91	6	123834	1.1481	0.12546	0.869906939
ZNF585A	ENSG00000196967	19	37587636	37673643	153	12	123834	1.1478	0.12553	0.869906939
SP9	ENSG00000217236	2	175189674	175213220	26	8	123834	1.1475	0.12558	0.869906939
AC018470.1	ENSG00000268241	2	175190601	175212151	26	8	123834	1.1475	0.12558	0.869906939
CRYAA	ENSG00000160202	21	44579118	44602915	9	3	123834	1.1469	0.1257	0.870420984
ZNF585B	ENSG00000245680	19	37662481	37719055	66	9	123834	1.1465	0.12579	0.870715808
AC022210.1	ENSG00000269417	2	85112209	85132345	58	2	123834	1.1463	0.12584	0.870715808
AMER3	ENSG00000178171	2	131503008	131535707	21	1	123834	1.1461	0.12588	0.870715808
RP11-934B9.3	ENSG00000258973	14	24791516	24816588	56	7	123834	1.1456	0.12598	0.870833227
HSD17B1	ENSG00000108786	17	40691232	40717231	44	6	123834	1.1456	0.12599	0.870833227
PARVB	ENSG00000188677	22	44385091	44578829	205	45	123834	1.1451	0.12608	0.870833227
PRDM16	ENSG00000142611	1	2975732	3365185	517	75	123834	1.1447	0.12618	0.870833227
C1orf122	ENSG00000197982	1	38262651	38285126	20	4	123834	1.1446	0.12619	0.870833227
AP001579.1	ENSG00000268856	21	46482796	46502927	46	10	123834	1.1442	0.12627	0.870833227
HILPDA	ENSG00000135245	7	128085903	128108472	59	13	123834	1.144	0.12631	0.870833227
OR2V1	ENSG00000185372	5	180541357	180562304	17	3	123834	1.1438	0.12635	0.870833227
SLC7A5	ENSG00000103257	16	87853629	87913094	177	19	123834	1.1438	0.12636	0.870833227
SERPINB7	ENSG00000166396	18	61410169	61482604	205	16	123834	1.1436	0.1264	0.870833227
TRHR	ENSG00000174417	8	110088850	110141813	111	12	123834	1.1434	0.12644	0.870833227
FFAR4	ENSG00000186188	10	95316422	95374237	106	18	123834	1.1433	0.12645	0.870833227
PRRC2A	ENSG00000204469	6	31578497	31615548	58	11	123834	1.1426	0.12661	0.870833227
SLC7A7	ENSG00000155465	14	23232431	23309029	140	19	123834	1.1425	0.12661	0.870833227
TNRC6C	ENSG00000078687	17	75990249	76114916	121	10	123834	1.1426	0.12661	0.870833227
OR7D2	ENSG00000188000	19	9286279	9309493	24	4	123834	1.1425	0.12663	0.870833227
SLC38A10	ENSG00000157637	17	79208800	79279347	140	21	123834	1.1417	0.12678	0.871456311
TENM3	ENSG00000218336	4	183055140	183734177	1172	140	123834	1.1415	0.12684	0.871456311

NUS1	ENSG00000153989	6	117986665	118041803	105	18	123834	1.1414	0.12686	0.871456311
CD93	ENSG00000125810	20	23049986	23076977	14	4	123834	1.1408	0.12697	0.871456311
MTMR3	ENSG00000100330	22	30269144	30436855	302	17	123834	1.1409	0.12697	0.871456311
LIMD1	ENSG00000144791	3	45586886	45737830	330	19	123834	1.1405	0.12704	0.871456311
HIVEP1	ENSG00000095951	6	11998995	12175232	287	26	123834	1.14	0.12715	0.871456311
PSMB10	ENSG00000205220	16	67958405	67980990	6	2	123834	1.1399	0.12716	0.871456311
APCDD1	ENSG00000154856	18	10444625	10499945	71	11	123834	1.1397	0.12721	0.871456311
KRR1	ENSG00000111615	12	75880684	75915416	59	6	123834	1.1395	0.12725	0.871456311
PIGS	ENSG00000087111	17	26870401	26908890	9	3	123834	1.1394	0.12726	0.871456311
SEC24C	ENSG00000176986	10	75494120	75541919	37	3	123834	1.1393	0.12729	0.871456311
CDH18	ENSG00000145526	5	19463060	20585982	1716	75	123834	1.1392	0.12732	0.871456311
TSPAN13	ENSG00000106537	7	16783160	16834161	78	22	123834	1.1387	0.12741	0.871456311
MSANTD4	ENSG00000170903	11	105856350	105903130	30	7	123834	1.1385	0.12745	0.871456311
ASB17	ENSG00000154007	1	76374558	76408116	106	13	123834	1.1384	0.12748	0.871456311
YIF1A	ENSG00000174851	11	66042051	66066641	14	4	123834	1.1383	0.1275	0.871456311
RNF103-CHMP3	ENSG00000249884	2	86722791	86958245	319	21	123834	1.1376	0.12763	0.871746207
GJA8	ENSG00000121634	1	147364946	147391393	46	9	123834	1.1376	0.12764	0.871746207
CSK	ENSG00000103653	15	75064398	75105539	68	9	123834	1.1374	0.12768	0.871746207
RBM24	ENSG00000112183	6	17271577	17304106	46	10	123834	1.1367	0.12783	0.871790875
AVIL	ENSG00000135407	12	58181159	58222487	26	5	123834	1.1367	0.12783	0.871790875
RBM39	ENSG00000131051	20	34281531	34340234	135	6	123834	1.1367	0.12783	0.871790875
FAM69A	ENSG00000154511	1	93297724	93437057	125	4	123834	1.1365	0.12787	0.871790875
PHACTR3	ENSG00000087495	20	58142564	58432766	510	33	123834	1.136	0.12798	0.871790875
ZNF589	ENSG00000164048	3	48272590	48350743	91	4	123834	1.1356	0.12807	0.871960726
TNFRSF12A	ENSG00000006327	16	3058446	3082384	4	2	123834	1.135	0.12818	0.871960726
CDK17	ENSG00000059758	12	96662039	96804338	245	23	123834	1.1349	0.12821	0.871960726
TRIM65	ENSG00000141569	17	73866416	73903084	60	7	123834	1.1343	0.12833	0.871960726
COL15A1	ENSG00000204291	9	101695461	101843069	174	20	123834	1.1343	0.12834	0.871960726
SEC22C	ENSG00000093183	3	42579461	42652572	140	12	123834	1.1341	0.12837	0.871960726
CYP4B1	ENSG00000142973	1	47213510	47295085	107	14	123834	1.134	0.12839	0.871960726
HELQ	ENSG00000163312	4	84318496	84387009	67	7	123834	1.1338	0.12844	0.871960726
REM1	ENSG00000088320	20	30053096	30082708	28	6	123834	1.1338	0.12844	0.871960726
FSCN1	ENSG00000075618	7	5622439	5656286	63	16	123834	1.1336	0.12848	0.871960726
SERPINA3	ENSG00000196136	14	95068714	95100392	39	9	123834	1.1333	0.12855	0.871960726
COL28A1	ENSG00000215018	7	7385834	7585484	433	18	123834	1.133	0.1286	0.871960726
AC092850.1	ENSG00000267819	12	131770067	131792763	7	2	123834	1.133	0.12861	0.871960726
KRTAP25-1	ENSG00000232263	21	31651463	31671832	63	11	123834	1.1325	0.12872	0.871960726
ATP2A2	ENSG00000174437	12	110708561	110798898	63	9	123834	1.1322	0.12878	0.871960726
HPD	ENSG00000158104	12	122267433	122311502	45	8	123834	1.1322	0.12878	0.871960726
HOXC6	ENSG00000197757	12	54374408	54434607	26	7	123834	1.1318	0.12885	0.871960726
ABCB9	ENSG00000150967	12	123395498	123476196	38	7	123834	1.1318	0.12885	0.871960726
ZNF461	ENSG00000197808	19	37118094	37167755	87	9	123834	1.1318	0.12885	0.871960726
PPP4C	ENSG00000149923	16	30077299	30106698	14	2	123834	1.1318	0.12886	0.871960726
MROH9	ENSG00000117501	1	170894612	171043906	287	14	123834	1.1314	0.12893	0.871960726
TRIM24	ENSG00000122779	7	138135079	138284738	127	12	123834	1.1314	0.12895	0.871960726
SCMH1	ENSG00000010803	1	41482872	41717826	490	19	123834	1.1311	0.12901	0.872016495
PDZK1	ENSG00000174827	1	145716918	145774074	2	1	123834	1.1309	0.12905	0.872016495
ZSCAN25	ENSG00000197037	7	99204569	99240030	28	4	123834	1.1304	0.12916	0.872195272
ZNF28	ENSG00000198538	19	53290662	53370853	132	21	123834	1.13	0.12924	0.872195272
TBC1D32	ENSG00000146350	6	121390640	121665891	966	20	123834	1.1299	0.12926	0.872195272
ANAPC7	ENSG00000196510	12	110800705	110851535	80	7	123834	1.1299	0.12926	0.872195272
PLAC8	ENSG00000145287	4	84001201	84068228	28	6	123834	1.1289	0.12948	0.873329606
RNF133	ENSG00000188050	7	122327766	122349210	36	6	123834	1.1287	0.12952	0.873329606
ABCA1	ENSG00000165029	9	107533283	107700518	330	52	123834	1.1274	0.12978	0.873462857
PHC1	ENSG00000111752	12	9056492	9104063	65	9	123834	1.1271	0.12985	0.873462857
DNAJC14	ENSG00000135392	12	56204744	56234608	15	4	123834	1.1265	0.12998	0.873462857
TMCO5A	ENSG00000166069	15	38204140	38269925	70	8	123834	1.1261	0.13006	0.873462857
BPIFB2	ENSG00000078898	20	31585406	31621515	39	11	123834	1.126	0.13009	0.873462857
SPATS2	ENSG00000123352	12	49750367	49931205	285	12	123834	1.1256	0.13016	0.873462857
FIS1	ENSG00000214253	7	100872739	100905597	75	4	123834	1.1254	0.13021	0.873462857
AC012485.1	ENSG00000268396	2	239182329	239202498	31	5	123834	1.1251	0.13027	0.873462857
RECQL4	ENSG00000160957	8	145726667	145753229	11	3	123834	1.1247	0.13035	0.873462857
MCM6	ENSG00000076003	2	136587196	136643996	87	7	123834	1.1247	0.13037	0.873462857
OR7D4	ENSG00000174667	19	9314526	9335542	31	6	123834	1.1245	0.13041	0.873462857
AL353354.1	ENSG00000215902	1	28517013	28537152	35	5	123834	1.1243	0.13044	0.873462857
CEACAM4	ENSG00000105352	19	42115344	42143442	65	12	123834	1.1241	0.13048	0.873462857
UBR4	ENSG00000127481	1	19391000	19546770	215	17	123834	1.1238	0.13054	0.873462857
TRAPPC6A	ENSG00000007255	19	45656186	45691495	34	4	123834	1.1238	0.13055	0.873462857
AC110771.1	ENSG00000269302	4	187101913	187122626	72	6	123834	1.1237	0.13056	0.873462857
TMPRSS12	ENSG00000186452	12	51226703	51291667	141	8	123834	1.1237	0.13057	0.873462857
KLHDC8B	ENSG00000185909	3	49199044	49223917	25	4	123834	1.1235	0.1306	0.873462857
PPP1R16A	ENSG00000160972	8	145693352	145737504	18	3	123834	1.1234	0.13063	0.873462857
EIF5A	ENSG00000132507	17	7200318	7225774	35	6	123834	1.1232	0.13069	0.873462857
NMB	ENSG00000197696	15	85188360	85211794	36	7	123834	1.1231	0.1307	0.873462857
CSPP1	ENSG00000104218	8	67964661	68118498	123	7	123834	1.123	0.13071	0.873462857
RPS6KA1	ENSG00000117676	1	26846252	26911521	78	9	123834	1.1229	0.13075	0.873462857
MKL2	ENSG00000186260	16	14155178	14370630	402	9	123834	1.1228	0.13076	0.873462857
KDM4D	ENSG00000186280	11	94696845	94742682	78	11	123834	1.1227	0.13078	0.873462857
FLAD1	ENSG00000160688	1	154945814	154975587	21	2	123834	1.1225	0.13083	0.873462857
TNNC1	ENSG00000114854	3	52475118	52498086	28	4	123834	1.1223	0.13088	0.873462857
AL354898.1	ENSG00000215428	9	133264029	133292639	36	12	123834	1.1221	0.13091	0.873462857
OR10D3	ENSG00000197309	11	124045923	124066952	61	7	123834	1.1216	0.13103	0.873462857
OR1F1	ENSG00000168124	16	3244247	3265188	33	4	123834	1.1214	0.13107	0.873462857
PSMB6	ENSG00000142507	17	4689439	4711790	12	4	123834	1.1211	0.13112	0.873462857
FAM26F	ENSG00000188820	6	116772533	116794934	52	9	123834	1.1209	0.13116	0.873462857
DLGAP4	ENSG00000080845	20	34884258	35167040	100	14	123834	1.1209	0.13116	0.873462857
RP11-286N22.8	ENSG00000256591	11	61186692	61263294	75	6	123834	1.1207	0.13121	0.873462857
TAS2R1	ENSG00000169777	5	9619109	9722490	117	16	123834	1.1206	0.13122	0.873462857
ITCH	ENSG00000078747	20	32941041	33109198	150	4	123834	1.1206	0.13123	0.873462857
DOCK9	ENSG00000088387	13	99435741	99748879	742	29	123834	1.1205	0.13124	0.873462857
ISCA1	ENSG00000135070	9	88869461	88907676	60	5	123834	1.1201	0.13134	0.873583205
LRRC14	ENSG00000160959	8	145733376	145760557	9	3	123834	1.1201	0.13135	0.873583205
TNK1	ENSG00000174292	17	7273853	7303093	39	7	123834	1.1192	0.13153	0.873663275
SERPINI1	ENSG00000163536	3	167443031	167553356	192	14	123834	1.119	0.13158	0.873663275
DNAJC5B	ENSG00000147570	8	66923795	67022751	207	22	123834	1.119	0.13158	0.873663275
ZNF775	ENSG00000196456	7	150055879	150119558	73	9	123834	1.1188	0.13162	0.873663275
MCHR1	ENSG00000128285	22	41064754	41088818	25	5	123834	1.1184	0.13171	0.873663275
HIC1	ENSG00000177374	17	1947448	1972981	31	8	123834	1.1178	0.13184	0.873663275
DLL1	ENSG00000198719	6	170581294	170609561	26	8	123834	1.1175	0.13189	0.873663275

RBM12B-AS1	ENSG00000212998	8	94742349	94763001	31	2	123834	1.1175	0.13189	0.873663275
AC073342.1	ENSG00000268170	7	142909163	142929360	12	3	123834	1.1174	0.1319	0.873663275
SPOP	ENSG00000121067	17	47666246	47765596	63	14	123834	1.1168	0.13203	0.873663275
ZNF649	ENSG00000198093	19	52382477	52418293	105	6	123834	1.1162	0.13218	0.873663275
QTRT1	ENSG00000213339	19	10802106	10834113	49	6	123834	1.1159	0.13223	0.873663275
RNF217	ENSG00000146373	6	125273691	125423779	128	23	123834	1.1159	0.13224	0.873663275
FUK	ENSG00000157353	16	70478324	70524177	16	6	123834	1.1159	0.13224	0.873663275
SLC23A2	ENSG00000089057	20	4823002	5000939	187	31	123834	1.1155	0.13231	0.873663275
SLC9A1	ENSG00000090020	1	27415306	27503472	62	3	123834	1.1153	0.13235	0.873663275
CERS6	ENSG00000172292	2	169302372	169641644	486	51	123834	1.115	0.13243	0.873663275
DYNC1I2	ENSG00000077380	2	172533919	172614930	134	14	123834	1.1149	0.13244	0.873663275
CEACAM7	ENSG00000007306	19	42167235	42220895	121	12	123834	1.1149	0.13245	0.873663275
GSK3A	ENSG00000105723	19	42724338	42756777	21	1	123834	1.1148	0.13247	0.873663275
RFPL4B	ENSG00000251258	6	112658532	112682498	26	5	123834	1.1147	0.13248	0.873663275
SH3GL1	ENSG00000141985	19	4350367	4410544	85	11	123834	1.1147	0.13249	0.873663275
LARS2	ENSG00000011376	3	45419998	45600913	211	15	123834	1.1146	0.13252	0.873663275
OSTM1	ENSG00000081087	6	108352613	108497058	223	20	123834	1.1145	0.13253	0.873663275
TTYH2	ENSG00000141540	17	72199653	72268155	94	11	123834	1.1144	0.13255	0.873663275
CABP1	ENSG00000157782	12	121068355	121115127	73	13	123834	1.114	0.13264	0.873663275
ZFP62	ENSG00000196670	5	180264611	180298286	64	6	123834	1.1139	0.13267	0.873663275
RIN2	ENSG00000132669	20	19857165	19993101	215	34	123834	1.1138	0.13268	0.873663275
RBAK-RBAKDN	ENSG00000272968	7	5013349	5122852	279	15	123834	1.1136	0.13272	0.873663275
RNF148	ENSG00000235631	7	122331718	122353021	34	6	123834	1.1133	0.13279	0.873663275
NNMT	ENSG00000166741	11	114118509	114194007	67	14	123834	1.1125	0.13296	0.873663275
RIMS4	ENSG00000101098	20	43370449	43448979	69	11	123834	1.1122	0.13302	0.873663275
SERPINF2	ENSG00000167711	17	1636130	1668562	69	8	123834	1.1121	0.13304	0.873663275
MALL	ENSG00000144063	2	110831447	110884143	25	5	123834	1.112	0.13308	0.873663275
UNC5B	ENSG00000107731	10	72962327	73072621	149	25	123834	1.1116	0.13316	0.873663275
AP002884.3	ENSG00000272790	11	112031253	112081394	98	11	123834	1.1116	0.13316	0.873663275
NOL9	ENSG00000162408	1	6571407	6624595	122	2	123834	1.1112	0.13325	0.873663275
TMCO3	ENSG00000150403	13	114135310	114214542	376	11	123834	1.111	0.13329	0.873663275
ZFC3H1	ENSG00000133858	12	71993252	72071505	93	7	123834	1.1109	0.13331	0.873663275
PDLIM7	ENSG00000196923	5	176900395	176934607	5	2	123834	1.1106	0.13337	0.873663275
OPRD1	ENSG00000116329	1	29128654	29200208	109	15	123834	1.1105	0.13339	0.873663275
CBWD2	ENSG00000136682	2	114185268	114263766	8	1	123834	1.1102	0.13345	0.873663275
TAS2R40	ENSG00000221937	7	142909130	142930162	13	2	123834	1.1102	0.13346	0.873663275
KLHDC7A	ENSG00000179023	1	18797424	18822478	68	9	123834	1.1101	0.13347	0.873663275
BICD2	ENSG00000185963	9	95463645	95537094	154	10	123834	1.11	0.13349	0.873663275
ERRFI1	ENSG00000116285	1	8054464	8096368	42	4	123834	1.1099	0.13352	0.873663275
MUC13	ENSG00000173702	3	124614289	124682663	120	19	123834	1.1098	0.13354	0.873663275
COIL	ENSG00000121058	17	55005548	55048415	46	8	123834	1.1097	0.13357	0.873663275
RBM33	ENSG00000184863	7	155427145	155584179	333	9	123834	1.1094	0.13362	0.873663275
UGGT1	ENSG00000136731	2	128838774	128963251	274	12	123834	1.1092	0.13368	0.873663275
FBXL2	ENSG00000153558	3	33308517	33455154	209	14	123834	1.1089	0.13374	0.873663275
DPYSL3	ENSG00000113657	5	146760374	146899619	76	13	123834	1.1086	0.1338	0.873663275
CAPNS2	ENSG00000256812	16	55590584	55611599	16	2	123834	1.1084	0.13385	0.873663275
DCLK1	ENSG00000133083	13	36335478	36715443	729	71	123834	1.1082	0.13388	0.873663275
UNCX	ENSG00000164853	7	1262543	1286954	23	5	123834	1.1082	0.13389	0.873663275
DIRC1	ENSG00000174325	2	189588882	189664831	198	11	123834	1.108	0.13394	0.873689609
RPS6KA4	ENSG00000162302	11	64116620	64149687	59	5	123834	1.1075	0.13405	0.873937778
CTC-479C5.12	ENSG00000261884	16	67953517	67979920	10	2	123834	1.1074	0.13407	0.873937778
CCL16	ENSG00000161573	17	34293529	34318532	64	10	123834	1.1068	0.13418	0.874136509
GRK5	ENSG00000198873	10	120957101	121225131	453	43	123834	1.1067	0.13421	0.874136509
EPYC	ENSG00000083782	12	91347456	91408803	190	10	123834	1.1065	0.13425	0.874136509
BGLAP	ENSG00000242252	1	156201753	156223112	11	3	123834	1.1062	0.13432	0.874136509
NSMAF	ENSG00000035681	8	59486063	59582403	119	12	123834	1.1057	0.13443	0.874136509
METRNL	ENSG00000176845	17	81027567	81062864	41	8	123834	1.1055	0.13448	0.874136509
BLNK	ENSG00000095585	10	97941458	98041344	168	27	123834	1.1051	0.13455	0.874136509
ZNF625	ENSG00000257591	19	12241032	12277546	38	3	123834	1.1051	0.13455	0.874136509
SERGEF	ENSG00000129158	11	17799595	18044709	212	20	123834	1.1047	0.13465	0.874136509
GOLGA6L1	ENSG00000197414	15	22726246	22756002	9	2	123834	1.1044	0.1347	0.874136509
FAM122A	ENSG00000187866	9	71384964	71408609	2	1	123834	1.1039	0.13483	0.874136509
CRYGS	ENSG00000213139	3	186246230	186274491	25	6	123834	1.1035	0.13491	0.874136509
GOLGA1	ENSG00000136935	9	127630646	127720771	82	6	123834	1.1034	0.13492	0.874136509
TCF24	ENSG00000261787	8	67848736	67884825	26	4	123834	1.1027	0.13508	0.874136509
AC114494.1	ENSG00000268950	1	31892192	31912614	58	8	123834	1.1024	0.13515	0.874136509
SLAMF1	ENSG00000117090	1	160567890	160627085	90	17	123834	1.1021	0.1352	0.874136509
C1orf85	ENSG00000198715	1	156249880	156275463	36	3	123834	1.1018	0.13527	0.874136509
SRP54	ENSG00000100883	14	35441163	35508773	241	16	123834	1.1018	0.13527	0.874136509
SHQ1	ENSG00000144736	3	72788428	72921065	58	2	123834	1.1017	0.1353	0.874136509
FAM205A	ENSG00000205108	9	34713052	34739464	41	7	123834	1.1017	0.1353	0.874136509
POLR2I	ENSG00000105258	19	36594612	36616248	31	6	123834	1.1016	0.13533	0.874136509
RP11-298I3.5	ENSG00000259132	14	23405479	23461467	41	5	123834	1.101	0.13545	0.874136509
THSD4	ENSG00000187720	15	71379291	72085722	941	96	123834	1.1007	0.13552	0.874136509
C19orf45	ENSG00000198723	19	7547862	7583336	49	9	123834	1.1005	0.13555	0.874136509
SOCS4	ENSG00000180008	14	55483948	55526206	44	5	123834	1.1004	0.13557	0.874136509
PGK2	ENSG00000170950	6	49743366	49765053	47	8	123834	1.1004	0.13558	0.874136509
RP11-382J12.1	ENSG00000246366	8	71510812	71585514	186	14	123834	1.1004	0.13558	0.874136509
GPS2	ENSG00000132522	17	7204643	7228883	29	6	123834	1.1002	0.13562	0.874136509
TMED3	ENSG00000166557	15	79593404	79714334	210	15	123834	1.1001	0.13564	0.874136509
IDNK	ENSG00000148057	9	86227964	86269045	85	10	123834	1.0998	0.1357	0.874136509
ADAMTS16	ENSG00000145536	5	5130443	5330417	342	50	123834	1.0997	0.13574	0.874136509
CAPZA2	ENSG00000198898	7	116441124	116572103	147	15	123834	1.0996	0.13574	0.874136509
ELF2	ENSG00000109381	4	139939266	140108372	193	16	123834	1.0995	0.13578	0.874136509
THBS2	ENSG00000186340	6	169605875	169664139	142	26	123834	1.0992	0.13585	0.874136509
TRIP13	ENSG00000071539	5	882758	929472	87	2	123834	1.099	0.13589	0.874136509
WDR38	ENSG00000136918	9	127605755	127630160	29	3	123834	1.099	0.13589	0.874136509
CDH6	ENSG00000113361	5	31183857	31339253	122	21	123834	1.0989	0.13591	0.874136509
STMN4	ENSG00000015592	8	27082840	27125937	80	9	123834	1.0989	0.13591	0.874136509
UBL3	ENSG00000122042	13	30328508	30434821	193	11	123834	1.0989	0.13591	0.874136509
SLC17A9	ENSG00000101194	20	61574052	61609949	59	15	123834	1.0987	0.13594	0.874136509
DCLK3	ENSG00000163673	3	36743913	36791352	43	10	123834	1.098	0.13609	0.874457872
TPGS1	ENSG00000141933	19	497299	529654	14	7	123834	1.0976	0.13618	0.874457872
PON2	ENSG00000105854	7	95024175	95074510	105	10	123834	1.0976	0.13619	0.874457872
TNR	ENSG00000116147	1	175274330	175722906	699	76	123834	1.0975	0.13622	0.874457872
SSH3	ENSG00000172830	11	67060919	67090078	30	4	123834	1.0975	0.13622	0.874457872
L3MBTL1	ENSG00000185513	20	42126320	42189590	77	11	123834	1.0967	0.13639	0.875150645
CASC5	ENSG00000137812	15	40876218	40966540	148	9	123834	1.0966	0.13642	0.875150645
SNX6	ENSG00000129515	14	35020300	35109389	158	12	123834	1.096	0.13655	0.875417247

RPEL1	ENSG00000235376	10	104995644	105017773	26	5	123834	1.0956	0.13663	0.875417247
GTF2A1	ENSG00000165417	14	81631796	81697721	83	10	123834	1.0953	0.13669	0.875417247
BBX	ENSG00000114439	3	107231783	107540171	301	20	123834	1.0952	0.13672	0.875417247
INPP5B	ENSG00000204084	1	38316369	38422729	149	12	123834	1.0951	0.13673	0.875417247
PAIP2B	ENSG00000124374	2	71399869	71464213	222	22	123834	1.0948	0.1368	0.875417247
SCN1B	ENSG00000105711	19	35511588	35541352	22	3	123834	1.0947	0.13681	0.875417247
SH2D6	ENSG00000152292	2	85635844	85674152	55	9	123834	1.0947	0.13683	0.875417247
IL6ST	ENSG00000134352	5	55220923	55300821	89	8	123834	1.0939	0.13701	0.87589978
PGM2	ENSG00000169299	4	37818255	37874558	137	12	123834	1.0935	0.13708	0.87589978
C1orf68	ENSG00000198854	1	152681998	152702905	58	2	123834	1.0935	0.1371	0.87589978
C19orf40	ENSG00000131944	19	33453115	33479128	59	12	123834	1.0934	0.13711	0.87589978
COL9A1	ENSG00000112280	6	70914764	71022786	279	25	123834	1.0929	0.13721	0.87589978
FBXO22	ENSG00000167196	15	76186200	76237609	63	4	123834	1.0924	0.13732	0.87589978
NDUFS4	ENSG00000164258	5	52846463	52989168	354	17	123834	1.0922	0.13736	0.87589978
RPL5	ENSG00000122406	1	93287582	93317481	30	3	123834	1.0922	0.13737	0.87589978
CSNK1E	ENSG00000213923	22	38676697	38804527	110	13	123834	1.0921	0.13739	0.87589978
WIBG	ENSG00000170473	12	56285197	56336402	28	2	123834	1.092	0.13741	0.87589978
KIAA1586	ENSG00000168116	6	56901347	56930023	26	3	123834	1.0915	0.13752	0.87589978
NUP93	ENSG00000102900	16	56754017	56888797	242	25	123834	1.0913	0.13756	0.87589978
PDYN	ENSG00000101327	20	1949403	1984732	21	4	123834	1.0913	0.13758	0.87589978
ARPC5L	ENSG00000136950	9	127614409	127650003	36	4	123834	1.0911	0.13761	0.87589978
CNFN	ENSG00000105427	19	42881171	42904439	28	1	123834	1.091	0.13763	0.87589978
RRP8	ENSG00000132275	11	6606305	6634850	38	6	123834	1.0908	0.13768	0.87589978
ZNF768	ENSG00000169957	16	30525325	30548142	3	2	123834	1.0905	0.13775	0.87589978
TULP3	ENSG00000078246	12	2976389	3060306	189	6	123834	1.09	0.13785	0.87589978
RGPD4	ENSG00000196862	2	108433388	108517297	35	3	123834	1.0899	0.13789	0.87589978
ENPP4	ENSG00000001561	6	46087730	46124436	99	7	123834	1.0898	0.1379	0.87589978
SNX29	ENSG00000048471	16	12060594	12678146	1443	100	123834	1.0894	0.13799	0.87589978
NUDT1	ENSG00000106268	7	2271857	2300781	47	9	123834	1.089	0.13809	0.87589978
CTSC	ENSG00000109861	11	88016760	88080955	140	18	123834	1.0886	0.13816	0.87589978
OR5B2	ENSG00000172365	11	58179738	58200786	86	4	123834	1.0885	0.13818	0.87589978
XXbac-BPG181M17.5	ENSG00000248993	6	32895141	32930899	101	13	123834	1.0885	0.13819	0.87589978
DOPEY2	ENSG00000142197	21	37519080	37676572	361	37	123834	1.0882	0.13825	0.87589978
C3orf84	ENSG00000236980	3	49205065	49239291	37	4	123834	1.0882	0.13826	0.87589978
HTR4	ENSG00000164270	5	147820595	148066798	369	27	123834	1.088	0.1383	0.87589978
PKN2	ENSG00000065243	1	89139905	89311938	327	7	123834	1.0878	0.13833	0.87589978
RP4-583P15.15	ENSG00000273154	20	62330216	62380456	85	5	123834	1.0879	0.13833	0.87589978
WRB	ENSG00000182093	21	40742170	40810454	79	5	123834	1.0877	0.13836	0.87589978
STT3B	ENSG00000163527	3	31564130	31689112	176	14	123834	1.0876	0.13838	0.87589978
FKSG48	ENSG00000267885	1	33429268	33449642	21	3	123834	1.0874	0.13843	0.875924581
RPL17	ENSG00000265681	18	47004851	47028906	75	9	123834	1.0869	0.13853	0.876265637
HPGD	ENSG00000164120	4	175401328	175454305	100	17	123834	1.0866	0.13861	0.87648
WNK4	ENSG00000126562	17	40922696	40958954	2	1	123834	1.0861	0.13871	0.876820645
RAB34	ENSG00000109113	17	27031299	27055447	16	2	123834	1.0859	0.13877	0.876908298
MEGF8	ENSG00000105429	19	42819761	42892921	66	2	123834	1.0856	0.13883	0.876995892
UFSP2	ENSG00000109775	4	186310694	186357139	81	11	123834	1.0853	0.13889	0.877083429
CTD-2228K2.5	ENSG00000188242	5	456239	483213	94	10	123834	1.085	0.13896	0.877234035
MGAT5B	ENSG00000167889	17	74854538	74956475	281	33	123834	1.0847	0.13902	0.87726806
FBF1	ENSG00000188878	17	73895655	73947221	63	8	123834	1.0843	0.13911	0.87726806
GLIPR2	ENSG00000122694	9	36126732	36173910	71	11	123834	1.0843	0.13912	0.87726806
BLOC1S3	ENSG00000189114	19	45672003	45695059	22	3	123834	1.0841	0.13915	0.87726806
SNAP25	ENSG00000132639	20	10189478	10298066	105	17	123834	1.0839	0.1392	0.877292308
RPS9	ENSG00000170889	19	54694610	54762862	84	8	123834	1.0836	0.13926	0.877379543
HLA-DMA	ENSG00000204257	6	32906390	32946871	145	16	123834	1.0833	0.13933	0.877529702
ZNF213	ENSG00000085644	16	3169778	3202806	34	2	123834	1.083	0.13941	0.877727905
CBY3	ENSG00000204659	5	179095584	179117975	32	4	123834	1.0826	0.13948	0.877727905
JPH2	ENSG00000149596	20	42730335	42826218	127	30	123834	1.0826	0.1395	0.877727905
CARD14	ENSG00000141527	17	78133791	78193130	78	15	123834	1.0823	0.13956	0.877814851
NAPG	ENSG00000134265	18	10515902	10562758	88	6	123834	1.082	0.13962	0.87790174
NAT14	ENSG00000090971	19	55986371	56008935	19	6	123834	1.0816	0.13971	0.8779108
ASXL1	ENSG00000171456	20	30936155	31037122	95	5	123834	1.0814	0.13975	0.8779108
TPCN1	ENSG00000186815	12	113648855	113746390	129	4	123834	1.0814	0.13976	0.8779108
AL391152.1	ENSG00000212928	14	57661888	57683291	39	2	123834	1.081	0.13985	0.877929276
MC4R	ENSG00000166603	18	58028564	58050001	41	9	123834	1.0807	0.13991	0.877929276
DNM1	ENSG00000106976	9	130955658	131027527	94	14	123834	1.0804	0.13999	0.877929276
TUBG2	ENSG00000037042	17	40801323	40829024	32	3	123834	1.0802	0.14002	0.877929276
HLA-DMB	ENSG00000242574	6	32892406	32918847	81	10	123834	1.0794	0.14021	0.877929276
RAB30	ENSG00000137502	11	82674175	82792965	162	20	123834	1.0793	0.14022	0.877929276
ZXDC	ENSG00000070476	3	126146444	126204762	146	16	123834	1.0791	0.14026	0.877929276
SLC52A3	ENSG00000101276	20	730724	759131	56	14	123834	1.0789	0.14031	0.877929276
KDSR	ENSG00000119537	18	60984959	61044743	47	8	123834	1.0785	0.1404	0.877929276
TMEM106B	ENSG00000106460	7	12240867	12292993	223	11	123834	1.0784	0.14043	0.877929276
SPAG9	ENSG00000008294	17	49029535	49208226	136	9	123834	1.0782	0.14048	0.877929276
UBE2W	ENSG00000104343	8	74682332	74801145	91	7	123834	1.0781	0.1405	0.877929276
SLC6A20	ENSG00000163817	3	45786942	45848027	36	11	123834	1.078	0.14052	0.877929276
PANX3	ENSG00000154143	11	124471386	124500252	14	2	123834	1.078	0.14052	0.877929276
INCA1	ENSG00000196388	17	4881425	4910905	61	9	123834	1.0779	0.14054	0.877929276
ZYG11B	ENSG00000162378	1	53182126	53303014	228	15	123834	1.0778	0.14056	0.877929276
TIGD4	ENSG00000169989	4	153680506	153710916	55	7	123834	1.0777	0.14059	0.877929276
TSTD1	ENSG00000215845	1	160997421	161018780	32	7	123834	1.0773	0.14068	0.877929276
FAM92A1	ENSG00000188343	8	94700789	94753755	47	6	123834	1.0771	0.14072	0.877929276
PDCD5	ENSG00000105185	19	33061974	33088358	32	6	123834	1.077	0.14074	0.877929276
C6orf57	ENSG00000154079	6	71266620	71309272	105	4	123834	1.0764	0.14087	0.877929276
MYLK4	ENSG00000145949	6	2653863	2761200	259	19	123834	1.0762	0.14091	0.877929276
SRC	ENSG00000197122	20	35963088	36044453	105	18	123834	1.0762	0.14092	0.877929276
IGF2BP2	ENSG00000073792	3	185351527	185552844	186	19	123834	1.076	0.14097	0.877929276
SMOX	ENSG00000088826	20	4091627	4178394	149	33	123834	1.0759	0.14098	0.877929276
NCOR2	ENSG00000196498	12	124798961	125062135	357	61	123834	1.0758	0.141	0.877929276
AHI1	ENSG00000135541	6	135594670	135828914	263	18	123834	1.0758	0.14101	0.877929276
MLN	ENSG00000096395	6	33752450	33781788	77	10	123834	1.075	0.1412	0.878373613
CLASP2	ENSG00000163539	3	33527737	33769848	265	16	123834	1.0749	0.14121	0.878373613
PACSIN3	ENSG00000165912	11	47189076	47217994	32	6	123834	1.0748	0.14122	0.878373613
SCGB1D2	ENSG00000124935	11	61999682	62022280	29	5	123834	1.0744	0.14133	0.878770245
FCGR1B	ENSG00000198019	1	120916979	120945937	1	1	123834	1.0738	0.14146	0.878835403
GNAL	ENSG00000141404	18	11678955	11895684	418	25	123834	1.0733	0.14156	0.878835403
TFAP2C	ENSG00000087510	20	55194358	55224339	22	3	123834	1.0733	0.14157	0.878835403
SCGB1C1	ENSG00000188076	11	183080	204573	8	2	123834	1.0732	0.14159	0.878835403
TXNIP	ENSG00000117289	1	145428469	145452635	13	2	123834	1.0721	0.14184	0.878835403
RNF38	ENSG00000137075	9	36326393	36497545	158	16	123834	1.0717	0.14192	0.878835403

CYP4V2	ENSG00000145476	4	187102674	187144610	122	13	123834	1.0717	0.14193	0.878835403
GBP3	ENSG00000117226	1	89462349	89498577	40	5	123834	1.0714	0.142	0.878835403
ZDHHC3	ENSG00000163812	3	44946749	45027677	78	5	123834	1.0711	0.14206	0.878835403
IGF1	ENSG00000017427	12	102779645	102884423	117	14	123834	1.0711	0.14207	0.878835403
PAM	ENSG00000145730	5	102079685	102376809	447	16	123834	1.0709	0.14211	0.878835403
PHOSPHO2	ENSG00000144362	2	170540975	170568218	69	7	123834	1.0706	0.14217	0.878835403
DMRTA2	ENSG00000142700	1	50873222	50899172	24	5	123834	1.0706	0.14218	0.878835403
DCD	ENSG00000161634	12	55028375	55052277	24	6	123834	1.0703	0.14224	0.878835403
GPN3	ENSG00000111231	12	110880289	110917073	47	6	123834	1.0701	0.14229	0.878835403
EXOC8	ENSG00000116903	1	231458480	231483598	19	2	123834	1.07	0.14232	0.878835403
OSTN	ENSG00000188729	3	190907030	190993404	114	13	123834	1.0698	0.14235	0.878835403
DDIT4L	ENSG00000145358	4	101097027	101121939	80	14	123834	1.0697	0.14237	0.878835403
CTD-3148I10.9	ENSG00000269469	19	49956009	49999488	63	7	123834	1.0697	0.14237	0.878835403
MYO3B	ENSG00000071909	2	171024655	171521681	824	94	123834	1.0696	0.1424	0.878835403
FAM151A	ENSG00000162391	1	55064855	55099229	72	11	123834	1.0694	0.14245	0.878835403
LGALS3	ENSG00000131981	14	55580828	55622126	59	5	123834	1.0689	0.14255	0.878835403
ACAP3	ENSG00000131584	1	1217756	1254989	49	8	123834	1.0688	0.14259	0.878835403
HAUS4	ENSG00000092036	14	23405437	23436370	36	4	123834	1.0685	0.14264	0.878835403
PDE10A	ENSG00000112541	6	165730776	166410091	1422	126	123834	1.0684	0.14266	0.878835403
SRRM1	ENSG00000133226	1	24948207	25009758	28	2	123834	1.0681	0.14273	0.878835403
WDR91	ENSG00000105875	7	134858590	134906316	109	3	123834	1.0681	0.14273	0.878835403
RPS6KB1	ENSG00000108443	17	57960447	58037925	77	7	123834	1.068	0.14275	0.878835403
METTL21C	ENSG00000139780	13	103328094	103356854	71	10	123834	1.0679	0.14278	0.878835403
TRPC7	ENSG00000069018	5	135538999	135742730	365	18	123834	1.0678	0.1428	0.878835403
MRPL2	ENSG00000112651	6	43011767	43037544	44	5	123834	1.0678	0.14281	0.878835403
NFKBIA	ENSG00000100906	14	35860717	35883955	29	4	123834	1.0677	0.14282	0.878835403
TMEM256	ENSG00000205544	17	7296294	7317456	15	5	123834	1.0672	0.14294	0.879289165
CEND1	ENSG00000184524	11	777104	800123	23	5	123834	1.0667	0.14305	0.879319664
EIF3K	ENSG00000178982	19	39099735	39137595	35	6	123834	1.0667	0.14306	0.879319664
RWDD1	ENSG00000111832	6	116882530	116928838	96	12	123834	1.0665	0.1431	0.879319664
CEACAM5	ENSG00000105388	19	42202504	42243718	65	6	123834	1.0664	0.14313	0.879319664
MXRA8	ENSG00000162576	1	1278069	1307157	91	8	123834	1.0661	0.14319	0.879404045
ZGPAT	ENSG00000197114	20	62328817	62377494	86	5	123834	1.0655	0.14332	0.87991814
GBP4	ENSG00000162654	1	89636831	89674615	52	6	123834	1.0647	0.1435	0.880138395
SGSH	ENSG00000181523	17	78170515	78204722	56	9	123834	1.0647	0.14351	0.880138395
ZNF865	ENSG00000261221	19	56106771	56138635	19	7	123834	1.0643	0.14359	0.880138395
RNF145	ENSG00000145860	5	158574417	158647061	98	13	123834	1.0643	0.1436	0.880138395
CHST13	ENSG00000180767	3	126233126	126272134	91	12	123834	1.0641	0.14365	0.880138395
TYRO3	ENSG00000092445	15	41839873	41881536	35	7	123834	1.064	0.14366	0.880138395
CHST6	ENSG00000183196	16	75500949	75539282	43	11	123834	1.0639	0.14368	0.880138395
CDKN2AIP	ENSG00000168564	4	184355744	184379351	78	5	123834	1.0628	0.14393	0.880554965
RAB11FIP3	ENSG00000090565	16	465619	583011	157	15	123834	1.0627	0.14395	0.880554965
MFSD4	ENSG00000174514	1	205528013	205582046	87	15	123834	1.0626	0.14399	0.880554965
CD8B	ENSG00000172116	2	87032462	87099047	61	6	123834	1.0626	0.14399	0.880554965
PCP2	ENSG00000174788	19	7686497	7708634	43	3	123834	1.062	0.14411	0.880554965
ZNF777	ENSG00000196453	7	149118454	149168214	24	6	123834	1.0615	0.14423	0.880554965
OR10V1	ENSG00000172289	11	59470296	59491337	50	5	123834	1.0614	0.14425	0.880554965
SOAT1	ENSG00000057252	1	179252925	179337815	285	15	123834	1.0614	0.14426	0.880554965
LY6G6D	ENSG00000244355	6	31673133	31695581	18	9	123834	1.0612	0.14429	0.880554965
SPATC1L	ENSG00000160284	21	47571062	47614390	178	14	123834	1.0612	0.14431	0.880554965
FAM216A	ENSG00000204856	12	110896169	110938190	52	6	123834	1.061	0.14435	0.880554965
TMEFF2	ENSG00000144339	2	192803769	193070435	172	31	123834	1.0609	0.14438	0.880554965
AMT	ENSG00000145020	3	49444211	49470186	30	4	123834	1.0609	0.14438	0.880554965
LPCAT3	ENSG00000111684	12	7075348	7135814	60	4	123834	1.0606	0.14444	0.880554965
CCDC178	ENSG00000166960	18	30507366	31031065	798	18	123834	1.0601	0.14455	0.880554965
IVL	ENSG00000163207	1	152871021	152894362	23	2	123834	1.0599	0.14459	0.880554965
ZBTB47	ENSG00000114853	3	42685176	42719072	31	8	123834	1.0598	0.14462	0.880554965
CLRN2	ENSG00000249581	4	17506788	17538727	63	10	123834	1.0598	0.14462	0.880554965
EDARADD	ENSG00000186197	1	236501562	236658214	355	30	123834	1.0592	0.14476	0.880554965
CRISP1	ENSG00000124812	6	49791970	49854809	82	4	123834	1.0587	0.14487	0.880554965
ZNF507	ENSG00000168813	19	32826500	32888573	115	13	123834	1.0585	0.14491	0.880554965
IGLL5	ENSG00000254709	22	23219960	23248287	6	1	123834	1.0584	0.14493	0.880554965
PRDM4	ENSG00000110851	12	108116643	108165049	97	6	123834	1.0584	0.14494	0.880554965
AGAP1	ENSG00000157985	2	236392733	237050444	635	68	123834	1.0583	0.14497	0.880554965
HNRNPC	ENSG00000092199	14	21667295	21747653	88	7	123834	1.0582	0.14499	0.880554965
WDR19	ENSG00000157796	4	39174024	39297430	116	6	123834	1.058	0.14503	0.880554965
OR5B12	ENSG00000172362	11	58196593	58217646	52	4	123834	1.0579	0.14506	0.880554965
IFFO2	ENSG00000169991	1	19220775	19293180	113	14	123834	1.0573	0.14519	0.880554965
RP11-664I21.6	ENSG00000254943	11	124751200	124772022	46	8	123834	1.0571	0.14523	0.880554965
C2CD4B	ENSG00000205502	15	62445734	62467482	31	7	123834	1.0571	0.14524	0.880554965
SLC35G1	ENSG00000176273	10	95643730	95725819	54	3	123834	1.0564	0.14539	0.880554965
ANKRD36	ENSG00000135976	2	97769233	97940258	111	4	123834	1.0563	0.14542	0.880554965
ROR1	ENSG00000185483	1	64229693	64657181	436	32	123834	1.0562	0.14544	0.880554965
TNIP2	ENSG00000168884	4	2733375	2768103	75	12	123834	1.056	0.14549	0.880554965
CYP39A1	ENSG00000146233	6	46507541	46630523	240	20	123834	1.0557	0.14556	0.880554965
WDR73	ENSG00000177082	15	85175999	85207574	48	7	123834	1.0556	0.14558	0.880554965
AAMDC	ENSG00000087884	11	77522155	77639478	185	12	123834	1.0554	0.14561	0.880554965
NCAPD3	ENSG00000151503	11	134010014	134105348	100	9	123834	1.0555	0.14561	0.880554965
CIC	ENSG00000079432	19	42762689	42809949	34	2	123834	1.0554	0.14562	0.880554965
IFNA1	ENSG00000197919	9	21430440	21451315	38	10	123834	1.0554	0.14563	0.880554965
C11orf71	ENSG00000180425	11	114252165	114281139	25	5	123834	1.0552	0.14567	0.880554965
CCRN4L	ENSG00000151014	4	139926943	139976906	40	8	123834	1.055	0.14571	0.880554965
GSE1	ENSG00000131149	16	85635015	85719810	20	6	123834	1.0549	0.14574	0.880554965
EYA2	ENSG00000064655	20	45513263	45827492	396	55	123834	1.0546	0.1458	0.88063756
ACVR1	ENSG00000115170	2	158582958	158742374	107	18	123834	1.0538	0.14598	0.880676071
ABCA6	ENSG00000154262	17	67064843	67148029	95	7	123834	1.0535	0.14605	0.880676071
TMEM241	ENSG00000134490	18	20767108	21027925	220	28	123834	1.0535	0.14605	0.880676071
KLHL21	ENSG00000162413	1	6640784	6684667	48	7	123834	1.0532	0.14612	0.880676071
TTL	ENSG00000114999	2	113229731	113309316	130	11	123834	1.0532	0.14613	0.880676071
SLC7A6	ENSG00000103064	16	68288433	68345722	57	8	123834	1.0525	0.14629	0.880676071
GPER1	ENSG00000164850	7	1111844	1143451	94	14	123834	1.0524	0.14631	0.880676071
PACS1	ENSG00000175115	11	65827834	66022218	233	13	123834	1.052	0.1464	0.880676071
ACTN1	ENSG00000072110	14	69330860	69456157	260	31	123834	1.0519	0.14642	0.880676071
TMEM18	ENSG00000151353	2	657335	687439	32	6	123834	1.0516	0.1465	0.880676071
ELP4	ENSG00000109911	11	31521297	31815546	260	6	123834	1.0515	0.14651	0.880676071
GDNF	ENSG00000168621	5	37802779	37849788	104	17	123834	1.0513	0.14656	0.880676071
NUDT5	ENSG00000165609	10	12197324	12248143	61	7	123834	1.0509	0.14666	0.880676071
XAB2	ENSG00000076924	19	7674411	7704451	51	4	123834	1.0509	0.14666	0.880676071
HRCT1	ENSG00000196196	9	35896189	35917138	18	5	123834	1.0507	0.14669	0.880676071

KIAA0100	ENSG00000007202	17	26931458	26982472	17	4	123834	1.0507	0.14669	0.880676071
DMRT2	ENSG00000173253	9	1040354	1067552	17	6	123834	1.0504	0.14676	0.880676071
PAPD7	ENSG00000112941	5	6704718	6767161	162	16	123834	1.0502	0.14681	0.880676071
SLC41A2	ENSG00000136052	12	105186331	105362522	280	12	123834	1.0499	0.14687	0.880676071
UBBP4	ENSG00000263563	17	21719601	21741762	24	2	123834	1.0499	0.14688	0.880676071
GABRR1	ENSG00000146276	6	89877220	89950997	132	18	123834	1.0499	0.14689	0.880676071
TPST2	ENSG00000128294	22	26911458	27002681	201	27	123834	1.0498	0.1469	0.880676071
C18orf42	ENSG00000231824	18	5135284	5207502	80	15	123834	1.0497	0.14693	0.880676071
ARL6IP5	ENSG00000144746	3	69124095	69165217	30	4	123834	1.0494	0.147	0.880676071
DPP10	ENSG00000175497	2	115189876	116613328	2283	92	123834	1.0494	0.14701	0.880676071
TAS1R1	ENSG00000173662	1	6605241	6649817	58	3	123834	1.0492	0.14704	0.880676071
AGPS	ENSG00000018510	2	178247372	178418564	255	7	123834	1.049	0.1471	0.880676071
OSR1	ENSG00000143867	2	19541246	19568414	34	6	123834	1.0489	0.14712	0.880676071
TRNAU1AP	ENSG00000180098	1	28869597	28915051	33	6	123834	1.0487	0.14715	0.880676071
PLCXD3	ENSG00000182836	5	41297056	41520730	346	24	123834	1.0482	0.14726	0.881056997
DNAJA2	ENSG00000069345	16	46979299	47017699	7	4	123834	1.0477	0.14738	0.881068275
SUCLA2	ENSG00000136143	13	48500622	48622125	451	17	123834	1.0474	0.14746	0.881068275
MLLT10	ENSG00000078403	10	21813094	22042559	96	11	123834	1.0472	0.14749	0.881068275
ADCY7	ENSG00000121281	16	50270048	50362046	50	10	123834	1.0472	0.14751	0.881068275
CLK2	ENSG00000176444	1	155222659	155258282	38	2	123834	1.047	0.14754	0.881068275
TTLL11	ENSG00000175764	9	124574207	124865885	612	37	123834	1.047	0.14754	0.881068275
WDFY1	ENSG00000085449	2	224710433	224820104	160	5	123834	1.0463	0.14772	0.881220414
ORC4	ENSG00000115947	2	148677968	148789147	135	8	123834	1.0462	0.14773	0.881220414
HS3ST4	ENSG00000182601	16	25693347	26159009	750	92	123834	1.0461	0.14777	0.881220414
PIK3IP1	ENSG00000100100	22	31667579	31698520	48	6	123834	1.0459	0.14781	0.881220414
PINX1	ENSG00000254093	8	10612473	10707394	338	17	123834	1.0456	0.14786	0.881220414
AC104667.3	ENSG00000234949	2	238489663	238514624	77	13	123834	1.0456	0.14788	0.881220414
ZBTB49	ENSG00000168826	4	4281924	4333513	101	8	123834	1.0455	0.14789	0.881220414
GMNN	ENSG00000112312	6	24765159	24796327	48	11	123834	1.0451	0.14799	0.881306958
C12orf23	ENSG00000151135	12	107339497	107382556	81	13	123834	1.045	0.148	0.881306958
TFCP2L1	ENSG00000115112	2	121964163	122052783	95	14	123834	1.0448	0.14805	0.881306958
GNG4	ENSG00000168243	1	235700987	235824054	149	25	123834	1.0446	0.14811	0.881306958
CEACAM18	ENSG00000213822	19	51969838	52004206	133	13	123834	1.044	0.14825	0.881306958
MYL4	ENSG00000198336	17	45267812	45311045	50	11	123834	1.0439	0.14826	0.881306958
LCN15	ENSG00000177984	9	139644086	139670707	53	5	123834	1.0435	0.14835	0.881306958
R3HCC1L	ENSG00000166024	10	99884387	100014654	186	11	123834	1.0433	0.14841	0.881306958
HOXB8	ENSG00000120068	17	46678739	46702478	54	13	123834	1.0432	0.14843	0.881306958
APOBEC3D	ENSG00000243811	22	39400368	39439281	30	4	123834	1.0431	0.14846	0.881306958
ING5	ENSG00000168395	2	242631450	242678893	79	10	123834	1.0428	0.14852	0.881306958
GLUD1	ENSG00000148672	10	88800243	88864623	126	12	123834	1.0428	0.14852	0.881306958
NPM1	ENSG00000181163	5	170804120	170848141	97	6	123834	1.0426	0.14856	0.881306958
NPIPB7	ENSG00000233232	16	28457693	28491868	6	1	123834	1.0425	0.14859	0.881306958
CALM2	ENSG00000143933	2	47377221	47413740	74	10	123834	1.0424	0.1486	0.881306958
ZIC2	ENSG00000043355	13	100624026	100649018	8	3	123834	1.042	0.1487	0.881624953
PRMT6	ENSG00000198890	1	107599267	107611916	2	1	123834	1.0415	0.14882	0.882061291
SLC26A2	ENSG00000155850	5	149330300	149383018	49	7	123834	1.0409	0.14896	0.882231341
PET100	ENSG00000229833	19	7684623	7706842	44	3	123834	1.0409	0.14896	0.882231341
TIMELESS	ENSG00000111602	12	56800903	56853187	58	6	123834	1.0403	0.1491	0.882231341
KIAA1456	ENSG00000250305	8	12793151	12899012	394	27	123834	1.0402	0.14912	0.882231341
ITGAM	ENSG00000169896	16	31261311	31354213	160	9	123834	1.0402	0.14913	0.882231341
KCNK4	ENSG00000182450	11	64048774	64077503	13	4	123834	1.0401	0.14914	0.882231341
ZNF790	ENSG00000197863	19	37298330	37351689	58	8	123834	1.0398	0.14922	0.882231341
DEFB127	ENSG00000088782	20	128111	149804	67	5	123834	1.0398	0.14922	0.882231341
8-Sep	ENSG00000164402	5	132076509	132152933	57	3	123834	1.0395	0.14927	0.882252537
ZBTB7B	ENSG00000160685	1	154965127	155000998	15	4	123834	1.0385	0.14952	0.883374545
TMSB10	ENSG00000034510	2	85122749	85143795	34	3	123834	1.0379	0.14966	0.883374545
WDR43	ENSG00000163811	2	29107509	29181088	201	11	123834	1.0377	0.1497	0.883374545
ROPN1B	ENSG00000114547	3	125677987	125712297	64	10	123834	1.0373	0.1498	0.883374545
CLN5	ENSG00000102805	13	77554795	77586652	64	7	123834	1.0372	0.14982	0.883374545
PLXNA1	ENSG00000114554	3	126697437	126766235	156	16	123834	1.0371	0.14985	0.883374545
FUT10	ENSG00000172728	8	33218342	33340940	231	16	123834	1.0367	0.14994	0.883374545
TSHR	ENSG00000165409	14	81411333	81622646	422	30	123834	1.0366	0.14996	0.883374545
ROBO4	ENSG00000154133	11	124743587	124778396	56	9	123834	1.0365	0.14999	0.883374545
FAM13B	ENSG00000031003	5	137263649	137397650	212	11	123834	1.0363	0.15002	0.883374545
TLCD1	ENSG00000160606	17	27041366	27064953	13	2	123834	1.0361	0.15008	0.883374545
TAF3	ENSG00000165632	10	7850467	8068590	486	29	123834	1.036	0.15009	0.883374545
DUSP22	ENSG00000112679	6	281630	361355	9	4	123834	1.0359	0.15011	0.883374545
CLDN1	ENSG00000163347	3	190013490	190050264	133	18	123834	1.0352	0.15028	0.883374545
ZNF678	ENSG00000181450	1	227741244	227875144	250	14	123834	1.0352	0.15029	0.883374545
TEX40	ENSG00000219435	11	64057863	64082242	13	4	123834	1.0351	0.15031	0.883374545
LRP1B	ENSG00000168702	2	140978992	142899270	5288	208	123834	1.0347	0.15039	0.883374545
LDB3	ENSG00000122367	10	88418206	88505825	192	30	123834	1.0345	0.15046	0.883374545
RNF19A	ENSG00000034677	8	101259288	101358446	139	8	123834	1.0343	0.15049	0.883374545
MMP2	ENSG00000087245	16	55413612	55550603	385	30	123834	1.0341	0.15054	0.883374545
BTNL2	ENSG00000204290	6	32351740	32384905	320	16	123834	1.0334	0.15072	0.883374545
JAZF1	ENSG00000153814	7	27860192	28230362	482	55	123834	1.0334	0.15072	0.883374545
TRIM49	ENSG00000168930	11	89520823	89551743	11	2	123834	1.0334	0.15072	0.883374545
SUPT6H	ENSG00000109111	17	26979109	27039697	32	5	123834	1.0332	0.15074	0.883374545
UQCRC2	ENSG00000140740	16	21953981	22004981	21	3	123834	1.0329	0.15082	0.883374545
CACNB1	ENSG00000067191	17	37319709	37363956	31	7	123834	1.0325	0.15092	0.883374545
C10orf126	ENSG00000204365	10	29125337	29180827	156	13	123834	1.0323	0.15097	0.883374545
TNFRSF10C	ENSG00000173535	8	22931868	22984950	71	10	123834	1.0321	0.15101	0.883374545
CMC2	ENSG00000103121	16	80999698	81063875	267	10	123834	1.0319	0.15105	0.883374545
N4BP1	ENSG00000102921	16	48562637	48664059	143	4	123834	1.0317	0.15111	0.883374545
CTDSP1	ENSG00000144579	2	219252979	219280664	8	3	123834	1.0316	0.15113	0.883374545
INTS7	ENSG00000143493	1	212103741	212218884	341	16	123834	1.031	0.15127	0.883374545
PTPN18	ENSG00000072135	2	131103580	131142982	9	2	123834	1.031	0.15127	0.883374545
SLC5A10	ENSG00000154025	17	18843658	18935908	56	13	123834	1.031	0.15127	0.883374545
NISCH	ENSG00000010322	3	52479134	52537087	68	3	123834	1.031	0.15128	0.883374545
S100A8	ENSG00000143546	1	153352508	153373664	44	1	123834	1.0308	0.15133	0.883374545
CCER1	ENSG00000197651	12	91289399	91358953	118	13	123834	1.0306	0.15137	0.883374545
CYP4A11	ENSG00000187048	1	47384849	47417156	61	5	123834	1.0303	0.15144	0.883374545
GLIS3	ENSG00000107249	9	3814127	4358392	895	117	123834	1.03	0.1515	0.883374545
POLR2E	ENSG00000099817	19	1076594	1105598	46	14	123834	1.03	0.15152	0.883374545
C5orf45	ENSG00000161010	5	179251436	179299173	92	11	123834	1.0298	0.15155	0.883374545
PCDHB17	ENSG00000255622	5	140525577	140548639	58	8	123834	1.0296	0.15159	0.883374545
FBXW11	ENSG00000072803	5	171278553	171443877	101	4	123834	1.0296	0.15159	0.883374545
RPL23A	ENSG00000198242	17	27036411	27061377	16	2	123834	1.0293	0.15168	0.883374545
OVOL3	ENSG00000105261	19	36592104	36614613	33	7	123834	1.029	0.15173	0.883374545

SCP2	ENSG00000116171	1	53382901	53527375	353	13	123834	1.029	0.15174	0.883374545
CHGA	ENSG00000100604	14	93379425	93411638	57	10	123834	1.029	0.15175	0.883374545
ZNF358	ENSG00000198816	19	7570178	7595912	21	4	123834	1.0289	0.15176	0.883374545
RP11-894J14.5	ENSG00000272305	3	52993135	53143469	224	13	123834	1.0287	0.15181	0.883374545
FOXB1	ENSG00000171956	15	60286421	60363929	80	7	123834	1.0287	0.15182	0.883374545
CIRBP	ENSG00000099622	19	1249384	1284879	7	1	123834	1.0286	0.15183	0.883374545
BAIAP3	ENSG00000007516	16	1373602	1409439	51	10	123834	1.0284	0.15188	0.883395055
LMNB1	ENSG00000113368	5	126102315	126182712	143	15	123834	1.0278	0.15203	0.883541128
CCDC106	ENSG00000173581	19	56142428	56174527	49	9	123834	1.0273	0.15214	0.883541128
SLC45A3	ENSG00000158715	1	205616979	205659587	88	18	123834	1.0273	0.15215	0.883541128
DNPH1	ENSG00000112667	6	43183367	43207222	38	6	123834	1.027	0.1522	0.883541128
CYP2F1	ENSG00000197446	19	41610337	41644271	68	7	123834	1.0268	0.15226	0.883541128
GRAP	ENSG00000154016	17	18913986	18960950	10	3	123834	1.0267	0.15227	0.883541128
APOBEC3F	ENSG00000128394	22	39426609	39459915	32	2	123834	1.0267	0.15228	0.883541128
NRG3	ENSG00000185737	10	83625070	84756935	1839	130	123834	1.0265	0.15233	0.883541128
NREP	ENSG00000134986	5	110988318	111343161	468	44	123834	1.0263	0.15238	0.883541128
NCR3LG1	ENSG00000188211	11	17363273	17408888	96	10	123834	1.0263	0.15238	0.883541128
PDZD9	ENSG00000155714	16	21985186	22022433	12	3	123834	1.0257	0.15251	0.883541128
TACSTD2	ENSG00000184292	1	59031099	59053166	96	8	123834	1.0257	0.15252	0.883541128
THRSP	ENSG00000151365	11	77764907	77789397	35	5	123834	1.0256	0.15253	0.883541128
HINT1	ENSG00000169567	5	130484720	130517428	77	4	123834	1.0254	0.15258	0.883541128
ALG6	ENSG00000088035	1	63823261	63914233	109	11	123834	1.0239	0.15293	0.883541128
N6AMT1	ENSG00000156239	21	30234513	30267693	141	10	123834	1.024	0.15293	0.883541128
PEA15	ENSG00000162734	1	160165127	160195166	15	5	123834	1.0235	0.15305	0.883541128
GCNT7	ENSG00000124091	20	55056548	55110981	86	6	123834	1.0234	0.15307	0.883541128
LRRC45	ENSG00000169683	17	79971178	79999027	41	5	123834	1.0231	0.15313	0.883541128
ZNF215	ENSG00000149054	11	6937635	7015863	165	16	123834	1.0229	0.15318	0.883541128
MIDN	ENSG00000167470	19	1238552	1269142	5	2	123834	1.0229	0.15319	0.883541128
QKI	ENSG00000112531	6	163825032	164009628	261	13	123834	1.0227	0.15323	0.883541128
MED4	ENSG00000136146	13	48617459	48679267	158	17	123834	1.0224	0.1533	0.883541128
MAPKAPK3	ENSG00000114738	3	50638951	50696720	41	4	123834	1.0221	0.15336	0.883541128
DAZL	ENSG00000092345	3	16618299	16721813	200	27	123834	1.022	0.15338	0.883541128
SLC7A14	ENSG00000013293	3	170167372	170313863	116	18	123834	1.0219	0.1534	0.883541128
PEX14	ENSG00000142655	1	10522345	10700815	227	17	123834	1.0219	0.15341	0.883541128
RUNDC3A	ENSG00000108309	17	42375781	42406039	37	7	123834	1.0217	0.15346	0.883541128
YIPF7	ENSG00000177752	4	44614086	44690573	224	15	123834	1.0211	0.1536	0.883541128
MEGT1	ENSG00000250641	6	31664681	31695695	32	12	123834	1.0211	0.1536	0.883541128
CCDC173	ENSG00000154479	2	170491935	170560943	207	11	123834	1.0209	0.15365	0.883541128
EDEM3	ENSG00000116406	1	184649365	184734047	82	8	123834	1.0208	0.15367	0.883541128
DDX60	ENSG00000137628	4	169127444	169249958	217	12	123834	1.0208	0.15368	0.883541128
ITGB5	ENSG00000082781	3	124470795	124630265	411	25	123834	1.0207	0.15369	0.883541128
ARFIP1	ENSG00000164144	4	153691089	153849615	263	13	123834	1.0201	0.15383	0.883541128
DCUN1D4	ENSG00000109184	4	52699166	52793003	70	4	123834	1.0201	0.15384	0.883541128
KCNJ5	ENSG00000120457	11	128751251	128800930	71	8	123834	1.0201	0.15385	0.883541128
HRH4	ENSG00000134489	18	22030593	22069921	69	16	123834	1.0198	0.1539	0.883541128
TTC30A	ENSG00000197557	2	178467720	178493694	104	11	123834	1.0197	0.15394	0.883541128
DAAM2	ENSG00000146122	6	39750142	39882648	214	33	123834	1.0196	0.15395	0.883541128
FAM209A	ENSG00000124103	20	55082243	55111198	34	6	123834	1.0196	0.15395	0.883541128
ARRDC5	ENSG00000205784	19	4880449	4912879	46	7	123834	1.0195	0.15398	0.883541128
FAM98B	ENSG00000171262	15	38736328	38789911	40	7	123834	1.0193	0.15404	0.883541128
CPT1C	ENSG00000169169	19	50184155	50226988	53	7	123834	1.0193	0.15404	0.883541128
RBPJL	ENSG00000124232	20	43925491	43955803	52	9	123834	1.0192	0.15405	0.883541128
NR1I3	ENSG00000143257	1	161189456	161218092	47	6	123834	1.0191	0.15407	0.883541128
GATA5	ENSG00000130700	20	61028553	61061026	5	2	123834	1.0186	0.1542	0.883541128
FHL5	ENSG00000112214	6	97000424	97074512	147	11	123834	1.0185	0.15422	0.883541128
QARS	ENSG00000172053	3	49123365	49152553	17	4	123834	1.0176	0.15444	0.883541128
ELK3	ENSG00000111145	12	96578160	96673613	135	25	123834	1.0175	0.15446	0.883541128
ELAC2	ENSG00000006744	17	12885708	12931504	157	8	123834	1.0174	0.15448	0.883541128
PWP2	ENSG00000241945	21	45517171	45561063	62	3	123834	1.0172	0.15452	0.883541128
PRR11	ENSG00000068489	17	57222860	57292066	68	7	123834	1.017	0.15457	0.883541128
FRMPD1	ENSG00000070601	9	37640997	37756901	292	29	123834	1.017	0.15458	0.883541128
TMEM79	ENSG00000163472	1	156242726	156272976	44	3	123834	1.0168	0.15461	0.883541128
NRG4	ENSG00000169752	15	76218310	76362136	197	4	123834	1.0169	0.15461	0.883541128
HOXD1	ENSG00000128645	2	177043307	177065688	15	3	123834	1.0168	0.15462	0.883541128
EPRS	ENSG00000136628	1	220131943	220230000	258	8	123834	1.0165	0.15468	0.883541128
NDRG4	ENSG00000103034	16	58486750	58557532	73	16	123834	1.0162	0.15476	0.883541128
RP11-542C16.2	ENSG00000261915	17	7205980	7232493	31	6	123834	1.0161	0.15479	0.883541128
EPHA8	ENSG00000070886	1	22880057	22940087	93	14	123834	1.016	0.15482	0.883541128
ZC2HC1B	ENSG00000118491	6	144175573	144269483	108	16	123834	1.016	0.15483	0.883541128
VAV2	ENSG00000160293	9	136617016	136867726	602	62	123834	1.0157	0.15488	0.883541128
PNP	ENSG00000198805	14	20927113	20955253	28	6	123834	1.0157	0.15488	0.883541128
ZNF335	ENSG00000198026	20	44567292	44610833	64	11	123834	1.0154	0.15497	0.883581236
RELL1	ENSG00000181826	4	37582422	37697998	235	39	123834	1.0153	0.15498	0.883581236
CXCR5	ENSG00000160683	11	118744475	118778508	20	7	123834	1.0148	0.15509	0.883943244
GLP2R	ENSG00000065325	17	9715523	9805419	172	17	123834	1.0142	0.15525	0.884381804
NCAPG	ENSG00000109805	4	17802525	17856485	77	5	123834	1.0141	0.15526	0.884381804
BCO2	ENSG00000197580	11	112036190	112105422	113	13	123834	1.0138	0.15535	0.884387547
KLF4	ENSG00000136826	9	110237133	110262763	21	3	123834	1.0133	0.15546	0.884387547
ANAPC5	ENSG00000089053	12	121736048	121847699	197	21	123834	1.0132	0.15549	0.884387547
PGM1	ENSG00000079739	1	64048947	64135916	118	19	123834	1.013	0.15553	0.884387547
AHCYL1	ENSG00000168710	1	110517308	110576357	9	2	123834	1.013	0.15554	0.884387547
KCNA1	ENSG00000111262	12	5009071	5050527	46	7	123834	1.0126	0.15563	0.884387547
KCNH8	ENSG00000183960	3	19179946	19587138	191	28	123834	1.0121	0.15575	0.884387547
RP11-47I22.4	ENSG00000258989	14	61985846	62134682	173	37	123834	1.0116	0.15586	0.884387547
MLF2	ENSG00000089693	12	6847170	6886641	73	9	123834	1.0115	0.1559	0.884387547
CHD3	ENSG00000170004	17	7778124	7826078	48	9	123834	1.0114	0.15591	0.884387547
UQCC2	ENSG00000137288	6	33652070	33689504	127	9	123834	1.0113	0.15595	0.884387547
GP6	ENSG00000088053	19	55515073	55559632	156	17	123834	1.0107	0.15607	0.884387547
TM9SF4	ENSG00000101337	20	30687309	30765061	124	8	123834	1.0107	0.15607	0.884387547
GDF3	ENSG00000184344	12	7832378	7858372	75	15	123834	1.0107	0.15608	0.884387547
DISP2	ENSG00000140323	15	40640436	40673257	43	8	123834	1.0106	0.1561	0.884387547
SPRYD3	ENSG00000167778	12	53448388	53483204	25	5	123834	1.0106	0.15611	0.884387547
ARSI	ENSG00000183876	5	149665906	149728870	47	6	123834	1.0098	0.1563	0.884387547
CDK18	ENSG00000117266	1	205463723	205511921	108	20	123834	1.0094	0.15638	0.884387547
VNN3	ENSG00000093134	6	133033926	133065904	75	8	123834	1.0092	0.15643	0.884387547
HEXB	ENSG00000049860	5	73925848	74028472	165	25	123834	1.0091	0.15646	0.884387547
RP11-444E17.6	ENSG00000272442	6	44230931	44356694	194	25	123834	1.009	0.1565	0.884387547
ZNF526	ENSG00000167625	19	42714423	42742353	11	1	123834	1.0088	0.15655	0.884387547
GNPTG	ENSG00000090581	16	1391924	1423352	37	6	123834	1.0086	0.15659	0.884387547

DNAJA4	ENSG00000140403	15	78546428	78584538	56	10	123834	1.0081	0.1567	0.884387547
CNN3	ENSG00000117519	1	95352507	95402834	112	22	123834	1.0076	0.15683	0.884387547
NAMPT	ENSG00000105835	7	105878731	105936772	90	10	123834	1.0074	0.15686	0.884387547
GPR114	ENSG00000159618	16	57566333	57635593	154	17	123834	1.0074	0.15686	0.884387547
DEPDC5	ENSG00000100150	22	32139944	32313012	304	15	123834	1.0075	0.15686	0.884387547
C1orf56	ENSG00000143443	1	151010216	151034462	24	5	123834	1.0073	0.15689	0.884387547
VSTM2L	ENSG00000132821	20	36521499	36583752	76	12	123834	1.0072	0.15691	0.884387547
SURF6	ENSG00000148296	9	136187552	136213235	39	6	123834	1.0072	0.15692	0.884387547
HLX	ENSG00000136630	1	221041699	221068401	37	6	123834	1.0072	0.15693	0.884387547
RAB11FIP5	ENSG00000135631	2	73290510	73393849	208	18	123834	1.0071	0.15694	0.884387547
HIST1H4C	ENSG00000197061	6	26094104	26114518	44	8	123834	1.007	0.15697	0.884387547
SLC36A4	ENSG00000180773	11	92867341	92941130	120	13	123834	1.007	0.15698	0.884387547
ALKBH4	ENSG00000160993	7	102086685	102115323	78	9	123834	1.0066	0.15706	0.884387547
LONRF2	ENSG00000170500	2	100879753	100949195	148	8	123834	1.0061	0.15719	0.884387547
CASP16	ENSG00000228146	16	3184220	3209964	37	2	123834	1.0058	0.15725	0.884387547
PM20D2	ENSG00000146281	6	89845769	89885284	89	8	123834	1.0057	0.15727	0.884387547
TOR3A	ENSG00000186283	1	179040512	179077158	71	12	123834	1.0056	0.15731	0.884387547
TMEM68	ENSG00000167904	8	56598983	56695966	165	13	123834	1.0055	0.15732	0.884387547
TTC28	ENSG00000100154	22	28364004	29085853	1004	30	123834	1.0055	0.15733	0.884387547
UHMK1	ENSG00000152332	1	162457041	162509419	66	3	123834	1.0054	0.15736	0.884387547
SLC18A2	ENSG00000165646	10	118990604	119048941	77	12	123834	1.0053	0.15737	0.884387547
TCTA	ENSG00000145022	3	49439639	49463908	29	4	123834	1.0049	0.15748	0.884387547
SIGLEC10	ENSG00000142512	19	51903275	51931057	47	2	123834	1.0048	0.1575	0.884387547
RPS6KC1	ENSG00000136643	1	213214589	213458116	205	13	123834	1.0047	0.15752	0.884387547
RP11-210M15.2	ENSG00000259495	15	80627195	80705917	240	16	123834	1.0039	0.15772	0.884387547
ARL2BP	ENSG00000102931	16	57269010	57297516	40	5	123834	1.0039	0.15772	0.884387547
TRIM67	ENSG00000119283	1	231287858	231367302	134	19	123834	1.0037	0.15777	0.884387547
KANSL3	ENSG00000114982	2	97248907	97318524	46	8	123834	1.0036	0.15778	0.884387547
PTCD3	ENSG00000132300	2	86323305	86379280	93	7	123834	1.0036	0.15779	0.884387547
CTC-454I21.3	ENSG00000267360	19	37573921	37711414	221	16	123834	1.0035	0.1578	0.884387547
EMILIN2	ENSG00000132205	18	2837028	2925991	42	13	123834	1.0035	0.15782	0.884387547
PLAGL2	ENSG00000126003	20	30770306	30805594	25	4	123834	1.0034	0.15782	0.884387547
MSI1	ENSG00000135097	12	120769133	120816983	44	5	123834	1.0022	0.15813	0.885733608
OR52W1	ENSG00000175485	11	6210376	6231489	31	7	123834	1.0019	0.1582	0.885733608
FAM60A	ENSG00000139146	12	31423518	31489992	19	3	123834	1.0019	0.1582	0.885733608
SLC25A39	ENSG00000013306	17	42386993	42412238	29	7	123834	1.0015	0.15829	0.885883591
LGALS1	ENSG00000100097	22	38061615	38085813	34	7	123834	1.0014	0.15832	0.885883591
IL17RD	ENSG00000144730	3	57114010	57214334	170	15	123834	1.0008	0.15846	0.886275953
RABGGTA	ENSG00000100949	14	24724744	24750945	29	7	123834	1.0007	0.1585	0.886275953
PAQR5	ENSG00000137819	15	69581286	69710119	186	15	123834	1.0005	0.15853	0.886275953
SAP30	ENSG00000164105	4	174281120	174308683	17	2	123834	1.0002	0.15861	0.88640129
TTC30B	ENSG00000196659	2	178403726	178427742	32	3	123834	0.99983	0.1587	0.88640129
TMEM105	ENSG00000185332	17	79275074	79314474	61	10	123834	0.99939	0.1588	0.88640129
VDAC3	ENSG00000078668	8	42239142	42273415	20	3	123834	0.99907	0.15888	0.88640129
KLHL6	ENSG00000172578	3	183195319	183283477	63	9	123834	0.99874	0.15896	0.88640129
L3MBTL4	ENSG00000154655	18	5944705	6425236	660	44	123834	0.9987	0.15897	0.88640129
NFATC4	ENSG00000100968	14	24824879	24858810	81	11	123834	0.99852	0.15901	0.88640129
ZNF567	ENSG00000189042	19	37168514	37228603	115	8	123834	0.99842	0.15904	0.88640129
MRTO4	ENSG00000053372	1	19568033	19596622	97	11	123834	0.99775	0.1592	0.88640129
EFR3B	ENSG00000084710	2	25254999	25388243	161	19	123834	0.99752	0.15926	0.88640129
FIG4	ENSG00000112367	6	110002499	110156631	228	22	123834	0.99743	0.15928	0.88640129
IL2RA	ENSG00000134460	10	6042652	6114288	136	31	123834	0.99738	0.15929	0.88640129
AGL	ENSG00000162688	1	100305640	100399579	198	18	123834	0.99718	0.15934	0.88640129
TUBB8	ENSG00000173876	10	82828	130103	60	10	123834	0.99703	0.15937	0.88640129
PADI2	ENSG00000117115	1	17383256	17455948	196	13	123834	0.99683	0.15942	0.88640129
LMNA	ENSG00000160789	1	156042364	156119880	103	6	123834	0.99685	0.15942	0.88640129
FAM26E	ENSG00000178033	6	116822809	116855955	35	4	123834	0.99671	0.15945	0.88640129
POLR3GL	ENSG00000121851	1	145446236	145480388	9	2	123834	0.99619	0.15958	0.88640129
AMN	ENSG00000166126	14	103378993	103409933	26	8	123834	0.99586	0.15966	0.88640129
RBCK1	ENSG00000125826	20	378142	421610	44	7	123834	0.99584	0.15966	0.88640129
TRAPPC10	ENSG00000160218	21	45422200	45536433	165	6	123834	0.99528	0.1598	0.88640129
C1orf63	ENSG00000117616	1	25558728	25674704	10	3	123834	0.99509	0.15985	0.88640129
TEX12	ENSG00000150783	11	112028095	112053282	43	7	123834	0.99497	0.15987	0.88640129
C2orf48	ENSG00000163009	2	10271509	10361851	150	21	123834	0.99488	0.1599	0.88640129
DGKA	ENSG00000065357	12	56311103	56357811	16	2	123834	0.99489	0.1599	0.88640129
AP000679.2	ENSG00000176984	11	120029685	120052387	36	8	123834	0.99442	0.16001	0.88640129
AGXT2	ENSG00000113492	5	34988206	35058198	127	15	123834	0.99437	0.16002	0.88640129
ORAI2	ENSG00000160991	7	102063553	102107268	78	10	123834	0.99356	0.16022	0.88640129
CHST5	ENSG00000135702	16	75552433	75579145	73	6	123834	0.99339	0.16026	0.88640129
ENDOD1	ENSG00000149218	11	94812974	94875809	135	20	123834	0.99333	0.16027	0.88640129
ZNF587	ENSG00000198466	19	58351225	58386480	69	8	123834	0.99328	0.16029	0.88640129
PPP2R2C	ENSG00000074211	4	6312305	6575327	373	52	123834	0.99316	0.16032	0.88640129
OCM2	ENSG00000135175	7	97603996	97630481	97	8	123834	0.99316	0.16032	0.88640129
TLR9	ENSG00000239732	3	52245096	52283183	31	6	123834	0.99287	0.16039	0.88640129
TWF2	ENSG00000247596	3	52252626	52283276	31	6	123834	0.99287	0.16039	0.88640129
TENM2	ENSG00000145934	5	166701804	167701162	862	112	123834	0.99269	0.16043	0.88640129
KDELR1	ENSG00000105438	19	48875827	48904810	25	7	123834	0.99263	0.16044	0.88640129
GLYATL1	ENSG00000166840	11	58662871	58821000	308	12	123834	0.99247	0.16049	0.88640129
CKS1B	ENSG00000173207	1	154937129	154961725	16	1	123834	0.99236	0.16051	0.88640129
IL16	ENSG00000172349	15	81441916	81615104	298	20	123834	0.99229	0.16053	0.88640129
IGSF9B	ENSG00000080854	11	133768459	133836880	56	13	123834	0.99134	0.16076	0.88640129
KDELR3	ENSG00000100196	22	38854067	38889452	32	11	123834	0.9908	0.16089	0.88640129
DDN	ENSG00000181418	12	49378932	49403092	9	2	123834	0.99063	0.16093	0.88640129
PLEC	ENSG00000178209	8	144979321	145060902	62	4	123834	0.99046	0.16097	0.88640129
TET2	ENSG00000168769	4	106057032	106210973	204	23	123834	0.99014	0.16105	0.88640129
KAT6B	ENSG00000156650	10	76575340	76802380	135	9	123834	0.99017	0.16105	0.88640129
SRSF12	ENSG00000154548	6	89795678	89837800	84	7	123834	0.9898	0.16112	0.88640129
MYH14	ENSG00000105357	19	50681443	50823802	307	36	123834	0.98977	0.16114	0.88640129
KCNT1	ENSG00000107147	9	138584031	138694992	168	24	123834	0.98956	0.16119	0.88640129
FAM168B	ENSG00000152102	2	131795449	131861033	57	4	123834	0.98944	0.16122	0.88640129
RITA1	ENSG00000139405	12	113613331	113640173	39	2	123834	0.98942	0.16123	0.88640129
AC132192.1	ENSG00000268403	11	9471747	9492010	30	4	123834	0.98928	0.16126	0.88640129
LY6G6F	ENSG00000204424	6	31664640	31695581	31	11	123834	0.98926	0.16127	0.88640129
METAP1	ENSG00000164024	4	99906771	99993964	163	13	123834	0.98922	0.16128	0.88640129
C6orf10	ENSG00000204296	6	32246303	32349684	589	24	123834	0.98911	0.16131	0.88640129
C1orf147	ENSG00000162888	1	206654449	206681061	51	8	123834	0.98852	0.16145	0.88640129
KLRK1	ENSG00000213809	12	10514952	10554473	58	5	123834	0.98853	0.16145	0.88640129
ZBTB2	ENSG00000181472	6	151675252	151722683	66	8	123834	0.98841	0.16148	0.88640129
C1orf227	ENSG00000185523	1	212993483	213030991	144	9	123834	0.98805	0.16156	0.88640129

CCDC36	ENSG00000173421	3	49225861	49305537	71	6	123834	0.98789	0.1616	0.88640129
PRKCH	ENSG00000027075	14	61644277	62027694	584	57	123834	0.98773	0.16164	0.88640129
TMEM11	ENSG00000178307	17	21090593	21127937	31	7	123834	0.98767	0.16166	0.88640129
ARFGAP2	ENSG00000149182	11	47175848	47208676	42	7	123834	0.98759	0.16168	0.88640129
MRC1L1	ENSG00000183748	10	17841362	17963178	1	1	123834	0.98747	0.1617	0.88640129
DCN	ENSG00000011465	12	91529025	91586900	31	3	123834	0.98728	0.16175	0.88640129
ZNF804A	ENSG00000170396	2	185453093	185814219	503	15	123834	0.98723	0.16177	0.88640129
ANO6	ENSG00000177119	12	45599770	45844187	438	16	123834	0.9872	0.16177	0.88640129
NKX6-1	ENSG00000163623	4	85403140	85429603	22	5	123834	0.98706	0.16181	0.88640129
RUNX2	ENSG00000124813	6	45285894	45642086	552	43	123834	0.98698	0.16183	0.88640129
ALG5	ENSG00000120697	13	37513912	37584398	87	8	123834	0.98679	0.16187	0.88640129
OPRL1	ENSG00000125510	20	62701526	62741996	45	8	123834	0.98676	0.16188	0.88640129
UGT2B7	ENSG00000171234	4	69907081	69988705	373	10	123834	0.98666	0.16191	0.88640129
GLI2	ENSG00000074047	2	121483199	121760229	281	38	123834	0.98569	0.16214	0.886950685
AGPAT3	ENSG00000160216	21	45275067	45416417	187	21	123834	0.98546	0.1622	0.886950685
ANTXR2	ENSG00000163297	4	80812303	81056608	414	21	123834	0.98473	0.16238	0.886950685
ANKRD34A	ENSG00000181039	1	145460508	145485646	4	2	123834	0.98453	0.16243	0.886950685
HAP1	ENSG00000173805	17	39863994	39900896	78	5	123834	0.9844	0.16246	0.886950685
HMGN2	ENSG00000198830	1	26788941	26812463	37	8	123834	0.98398	0.16256	0.886950685
NAGLU	ENSG00000108784	17	40678190	40706467	50	9	123834	0.98382	0.1626	0.886950685
IGSF8	ENSG00000162729	1	160051130	160078733	25	5	123834	0.98297	0.16281	0.886950685
ITIH2	ENSG00000151655	10	7735232	7801483	161	22	123834	0.98298	0.16281	0.886950685
CAPN9	ENSG00000135773	1	230873130	230947749	162	23	123834	0.98277	0.16286	0.886950685
MMP20	ENSG00000137674	11	102437566	102506063	267	16	123834	0.98277	0.16286	0.886950685
MTF1	ENSG00000188786	1	38265239	38335292	76	9	123834	0.98268	0.16288	0.886950685
C5orf42	ENSG00000197603	5	37096330	37259530	148	10	123834	0.98259	0.1629	0.886950685
NKPD1	ENSG00000179846	19	45643008	45673408	31	6	123834	0.98251	0.16293	0.886950685
UTP14C	ENSG00000253797	13	52588827	52617736	36	6	123834	0.98237	0.16296	0.886950685
GBP2	ENSG00000162645	1	89561815	89626139	143	8	123834	0.98224	0.16299	0.886950685
HS3ST5	ENSG00000249853	6	114366750	114674209	420	42	123834	0.98225	0.16299	0.886950685
KCNC2	ENSG00000166006	12	75423857	75613648	307	23	123834	0.98196	0.16306	0.886950685
ILF3	ENSG00000129351	19	10754937	10813093	58	4	123834	0.98184	0.16309	0.886950685
EVL	ENSG00000196405	14	100427786	100620573	366	24	123834	0.98128	0.16323	0.886950685
ALDOA	ENSG00000149925	16	30054411	30091778	22	3	123834	0.98126	0.16323	0.886950685
RP11-834C11.12	ENSG00000273049	12	54369629	54438672	35	9	123834	0.98105	0.16328	0.886950685
EIF3L	ENSG00000100129	22	38234875	38295414	47	6	123834	0.98038	0.16345	0.886950685
NMUR2	ENSG00000132911	5	151761093	151822929	190	5	123834	0.98026	0.16348	0.886950685
USP4	ENSG00000114316	3	49305264	49388145	63	9	123834	0.97996	0.16355	0.886950685
OCM	ENSG00000122543	7	5909458	5935993	58	14	123834	0.97984	0.16358	0.886950685
ZNF880	ENSG00000221923	19	52863170	52899048	77	9	123834	0.97976	0.1636	0.886950685
SRCIN1	ENSG00000017373	17	36676251	36772183	78	9	123834	0.97949	0.16367	0.886950685
CYP2A13	ENSG00000197838	19	41584377	41612100	80	4	123834	0.97917	0.16375	0.886950685
C3orf22	ENSG00000180697	3	126235842	126287808	109	15	123834	0.97903	0.16378	0.886950685
PPP2R2A	ENSG00000221914	8	26139007	26240196	139	23	123834	0.97875	0.16385	0.886950685
DARC	ENSG00000213088	1	159163097	159186290	20	5	123834	0.97871	0.16386	0.886950685
CNBD2	ENSG00000149646	20	34546512	34628622	85	6	123834	0.97873	0.16386	0.886950685
PGLYRP3	ENSG00000159527	1	153260338	153293194	60	6	123834	0.97861	0.16389	0.886950685
SLC48A1	ENSG00000211584	12	48137699	48186536	57	11	123834	0.97857	0.1639	0.886950685
UGT2B15	ENSG00000196620	4	69502348	69546346	10	2	123834	0.97831	0.16396	0.886950685
XDH	ENSG00000158125	2	31547187	31647581	165	29	123834	0.97803	0.16403	0.886950685
HNRNPR	ENSG00000125944	1	23620264	23680829	10	3	123834	0.97798	0.16404	0.886950685
FAM103A1	ENSG00000169612	15	83644959	83669809	32	8	123834	0.97764	0.16413	0.886950685
NDUFC2-KCTD14	ENSG00000259112	11	77718017	77800911	128	14	123834	0.97756	0.16415	0.886950685
FLT3	ENSG00000122025	13	28567411	28684729	239	16	123834	0.97742	0.16418	0.886950685
EXOSC9	ENSG00000123737	4	122712472	122748176	63	9	123834	0.97711	0.16426	0.886950685
MFSD9	ENSG00000135953	2	103322299	103363347	29	4	123834	0.97688	0.16431	0.886950685
PCIF1	ENSG00000100982	20	44553267	44586662	52	10	123834	0.9768	0.16433	0.886950685
ZNF395	ENSG00000186918	8	28193102	28270218	21	4	123834	0.97677	0.16434	0.886950685
ISCU	ENSG00000136003	12	108946358	108973160	78	9	123834	0.97679	0.16434	0.886950685
CLIP4	ENSG00000115295	2	29310571	29422509	133	16	123834	0.9765	0.16441	0.886950685
TNFSF4	ENSG00000117586	1	173142873	173186452	73	11	123834	0.97611	0.1645	0.886950685
SERPINA9	ENSG00000170054	14	94919054	94956026	113	17	123834	0.97602	0.16453	0.886950685
LACE1	ENSG00000135537	6	108606098	108857999	257	15	123834	0.97596	0.16454	0.886950685
ASB18	ENSG00000182177	2	237092095	237183052	219	15	123834	0.97587	0.16456	0.886950685
EML2	ENSG00000125746	19	46100252	46158887	67	11	123834	0.97561	0.16463	0.886950685
HBB	ENSG00000244734	11	5236694	5260625	15	3	123834	0.97523	0.16472	0.886950685
NDUFC2	ENSG00000151366	11	77769350	77801265	61	7	123834	0.97516	0.16474	0.886950685
PCDHB7	ENSG00000113212	5	140542243	140565957	54	8	123834	0.97506	0.16477	0.886950685
BHLHE22	ENSG00000180828	8	65482814	65506181	21	6	123834	0.97486	0.16481	0.886950685
TNFSF15	ENSG00000181634	9	117536915	117578406	16	6	123834	0.97475	0.16484	0.886950685
GJE1	ENSG00000203733	6	142444227	142466288	28	3	123834	0.97469	0.16486	0.886950685
TRIM29	ENSG00000137699	11	119971983	120066237	133	21	123834	0.97442	0.16492	0.886950685
SFRP2	ENSG00000145423	4	154691744	154720272	48	6	123834	0.97425	0.16497	0.886950685
AASDH	ENSG00000157426	4	57194456	57263666	209	16	123834	0.97411	0.165	0.886950685
PIEZO1	ENSG00000103335	16	88771751	88861619	162	28	123834	0.97401	0.16502	0.886950685
SDS	ENSG00000135094	12	113820250	113874106	85	3	123834	0.97401	0.16503	0.886950685
TRDN	ENSG00000186439	6	123527483	123968238	695	47	123834	0.97376	0.16509	0.886950685
SP8	ENSG00000164651	7	20813906	20836505	2	1	123834	0.97376	0.16509	0.886950685
TSR3	ENSG00000007520	16	1389241	1411912	21	5	123834	0.97313	0.16525	0.886950685
H3F3C	ENSG00000188375	12	31934123	31955175	52	10	123834	0.97295	0.16529	0.886950685
PHLDA3	ENSG00000174307	1	201424620	201448365	19	4	123834	0.97242	0.16542	0.886950685
RP11-690P14.4	ENSG00000269920	10	97723786	97802432	152	21	123834	0.9723	0.16545	0.886950685
CC2D2B	ENSG00000188649	10	97723786	97802441	152	21	123834	0.9723	0.16545	0.886950685
INTS4	ENSG00000149262	11	77579766	77715724	255	12	123834	0.97231	0.16545	0.886950685
PANK1	ENSG00000152782	10	91332745	91415215	232	23	123834	0.97218	0.16548	0.886950685
AC008060.7	ENSG00000218672	7	155164771	155199051	52	10	123834	0.97213	0.16549	0.886950685
SPG21	ENSG00000090487	15	65245362	65292648	105	9	123834	0.97211	0.1655	0.886950685
PCLO	ENSG00000186472	7	82373329	82802246	667	36	123834	0.97208	0.16551	0.886950685
VWA1	ENSG00000179403	1	1360241	1388262	8	2	123834	0.97149	0.16565	0.887396857
ABCA10	ENSG00000154263	17	67133355	67250987	193	11	123834	0.97134	0.16569	0.887396857
HIST1H1T	ENSG00000187475	6	26097640	26118364	42	7	123834	0.97101	0.16577	0.887396857
VTA1	ENSG00000009844	6	142458367	142555826	145	7	123834	0.97096	0.16578	0.887396857
AC114783.1	ENSG00000237524	2	127646472	127669673	57	5	123834	0.97015	0.16599	0.888270811
GSG1L	ENSG00000169181	16	27788850	28084830	409	58	123834	0.96993	0.16604	0.888288297
IQCD	ENSG00000166578	12	113623246	113668899	62	3	123834	0.96921	0.16622	0.888429005
STRA6	ENSG00000137868	15	74461807	74514608	52	5	123834	0.96922	0.16622	0.888429005
OSMR	ENSG00000145623	5	38835960	38955698	218	26	123834	0.96905	0.16626	0.888429005
DYNLL2	ENSG00000264364	17	56150776	56182897	31	6	123834	0.96903	0.16626	0.888429005
DNAH6	ENSG00000115423	2	84733579	85056713	624	13	123834	0.96889	0.1663	0.888429005

NMT2	ENSG00000152465	10	15134583	15220692	183	23	123834	0.96857	0.16638	0.888606642
XRN2	ENSG00000088930	20	21273942	21380463	154	10	123834	0.9678	0.16657	0.888727942
BSPH1	ENSG00000188334	19	48461303	48505427	73	11	123834	0.96771	0.16659	0.888727942
DDX54	ENSG00000123064	12	113584979	113633284	60	3	123834	0.96765	0.16661	0.888727942
CUX2	ENSG00000111249	12	111461828	111798358	243	24	123834	0.9676	0.16662	0.888727942
PDXP	ENSG00000241360	22	38044734	38072941	57	6	123834	0.9673	0.1667	0.888727942
BPI	ENSG00000101425	20	36878551	36975907	149	29	123834	0.9672	0.16672	0.888727942
AC120194.1	ENSG00000213002	8	71475681	71496671	29	5	123834	0.96718	0.16673	0.888727942
TRIM17	ENSG00000162931	1	228585641	228614562	33	4	123834	0.96651	0.16689	0.889295247
MED30	ENSG00000164758	8	118522952	118562501	47	9	123834	0.96635	0.16693	0.889295247
TMEM145	ENSG00000167619	19	42807477	42839214	16	1	123834	0.96591	0.16704	0.889346581
C1QTNF2	ENSG00000145861	5	159764758	159807648	41	8	123834	0.96586	0.16706	0.889346581
WDTC1	ENSG00000142784	1	27551007	27645110	113	9	123834	0.96576	0.16708	0.889346581
HADH	ENSG00000138796	4	108900870	108966331	126	5	123834	0.96493	0.16729	0.890215095
KIF3A	ENSG00000131437	5	132018320	132083330	80	4	123834	0.96419	0.16748	0.890385918
LYSMD2	ENSG00000140280	15	52005208	52053782	55	2	123834	0.96417	0.16748	0.890385918
DCHS1	ENSG00000166341	11	6632556	6687085	62	16	123834	0.96392	0.16754	0.890385918
MAPK1	ENSG00000100030	22	22098789	22231970	209	18	123834	0.96393	0.16754	0.890385918
C9orf84	ENSG00000165181	9	114438453	114567288	385	16	123834	0.96368	0.1676	0.890385918
RAC3	ENSG00000169750	17	79979500	80002080	39	4	123834	0.96353	0.16764	0.890385918
LRAT	ENSG00000121207	4	155538097	155684270	167	10	123834	0.96347	0.16765	0.890385918
AIMP2	ENSG00000106305	7	6038876	6073465	131	16	123834	0.96309	0.16775	0.890430208
ADIRF	ENSG00000148671	10	88717949	88753691	75	13	123834	0.96262	0.16787	0.890430208
SULT1C2	ENSG00000198203	2	108895095	108936371	39	8	123834	0.96234	0.16794	0.890430208
YPEL3	ENSG00000090238	16	30093635	30118236	10	3	123834	0.96235	0.16794	0.890430208
CTTNBP2	ENSG00000077063	7	117340705	117524193	211	27	123834	0.96203	0.16802	0.890430208
CYFIP2	ENSG00000055163	5	156683089	156832606	312	14	123834	0.96197	0.16803	0.890430208
GLP1R	ENSG00000112164	6	39006574	39065519	115	15	123834	0.96169	0.1681	0.890430208
BDNF	ENSG00000176697	11	27666440	27753605	67	7	123834	0.96161	0.16812	0.890430208
PRSS36	ENSG00000178226	16	31140246	31171415	9	3	123834	0.96161	0.16812	0.890430208
SLC13A5	ENSG00000141485	17	6578032	6626886	62	15	123834	0.96149	0.16815	0.890430208
GGNBP2	ENSG00000005955	17	34890737	34956278	92	8	123834	0.96114	0.16824	0.890430208
PIGG	ENSG00000174227	4	482989	543985	168	7	123834	0.96095	0.16829	0.890430208
EPHX3	ENSG00000105131	19	15327730	15354246	3	1	123834	0.96087	0.16831	0.890430208
ECM1	ENSG00000143369	1	150470538	150496265	34	5	123834	0.96084	0.16832	0.890430208
ITGA10	ENSG00000143127	1	145514891	145553868	15	4	123834	0.96038	0.16843	0.890430208
PHF13	ENSG00000116273	1	6663745	6694093	39	6	123834	0.96024	0.16847	0.890430208
PHF7	ENSG00000010318	3	52434673	52467657	16	4	123834	0.95989	0.16855	0.890430208
ASH1L	ENSG00000116539	1	155295059	155542598	101	7	123834	0.95967	0.16861	0.890430208
LPCAT4	ENSG00000176454	15	34641106	34669479	29	6	123834	0.95961	0.16863	0.890430208
ASB10	ENSG00000146926	7	150862785	150894919	34	7	123834	0.95941	0.16868	0.890430208
TBX6	ENSG00000149922	16	30087114	30113208	11	2	123834	0.95899	0.16878	0.890430208
AC008372.1	ENSG00000268208	14	78217711	78238033	68	10	123834	0.9589	0.1688	0.890430208
CABP7	ENSG00000100314	22	30106073	30137828	59	10	123834	0.95882	0.16882	0.890430208
RSPH4A	ENSG00000111834	6	116927642	116964148	94	11	123834	0.95867	0.16886	0.890430208
ATP2B4	ENSG00000058668	1	203585689	203723209	133	15	123834	0.95837	0.16894	0.890430208
EFHD2	ENSG00000142634	1	15726391	15766839	32	9	123834	0.95823	0.16897	0.890430208
ZBP1	ENSG00000124256	20	56168902	56205632	25	9	123834	0.95826	0.16897	0.890430208
MYO18B	ENSG00000133454	22	26128111	26437007	522	55	123834	0.95825	0.16897	0.890430208
hsa-mir-1199	ENSG00000141854	19	14173348	14195874	20	2	123834	0.95792	0.16905	0.890585352
STRADB	ENSG00000082146	2	202242581	202355569	158	12	123834	0.95762	0.16913	0.890585352
CSNK2B	ENSG00000204435	6	31623013	31648120	28	9	123834	0.95758	0.16914	0.890585352
RAB1B	ENSG00000174903	11	66026004	66054963	23	4	123834	0.9568	0.16933	0.890737168
ACSS2	ENSG00000131069	20	33449949	33525769	95	5	123834	0.95661	0.16938	0.890737168
WDR82	ENSG00000164091	3	52278437	52332036	61	6	123834	0.95654	0.1694	0.890737168
CTD-3088G3.8	ENSG00000188897	16	11443702	11627444	494	52	123834	0.95649	0.16941	0.890737168
EIF4ENIF1	ENSG00000184708	22	31822963	31902094	96	14	123834	0.9564	0.16944	0.890737168
SNRNP40	ENSG00000060688	1	31722417	31779662	129	6	123834	0.95633	0.16945	0.890737168
EVPL	ENSG00000167880	17	73990583	74033533	34	7	123834	0.95612	0.16951	0.890806215
ZNF329	ENSG00000181894	19	58627619	58676477	140	14	123834	0.95581	0.16958	0.890927761
ATP6V0A1	ENSG00000033627	17	40600862	40684629	94	10	123834	0.95553	0.16966	0.891101763
NFATC1	ENSG00000131196	18	77145856	77299325	318	24	123834	0.95513	0.16975	0.891259445
SRPK1	ENSG00000096063	6	35790743	35899119	184	6	123834	0.95463	0.16988	0.891259445
GDPD3	ENSG00000102886	16	30106131	30135177	12	3	123834	0.95413	0.17001	0.891259445
AC024940.1	ENSG00000177340	12	31467250	31488879	2	1	123834	0.95409	0.17002	0.891259445
WNT8A	ENSG00000061492	5	137409581	137438054	43	6	123834	0.95349	0.17017	0.891259445
EN2	ENSG00000164778	7	155240824	155267526	29	10	123834	0.95338	0.1702	0.891259445
HYAL2	ENSG00000068001	3	50345221	50370337	6	3	123834	0.95312	0.17026	0.891259445
BZW2	ENSG00000136261	7	16675756	16756148	107	15	123834	0.95314	0.17026	0.891259445
CD48	ENSG00000117091	1	160638536	160691641	87	16	123834	0.95246	0.17043	0.891259445
SEMA3F	ENSG00000001617	3	50182478	50236508	42	7	123834	0.95212	0.17052	0.891259445
SBP1	ENSG00000269490	6	33653196	33673474	70	7	123834	0.95189	0.17058	0.891259445
GNA13	ENSG00000120063	17	62996833	63062957	18	2	123834	0.95162	0.17065	0.891259445
RIN3	ENSG00000100599	14	92970118	93165339	224	47	123834	0.95152	0.17067	0.891259445
MAGEL2	ENSG00000254585	15	23878691	23901175	28	8	123834	0.95128	0.17073	0.891259445
RP11-77K12.7	ENSG00000260092	16	75552430	75589326	95	7	123834	0.95118	0.17076	0.891259445
BUB1	ENSG00000169679	2	111385275	111445691	36	2	123834	0.95072	0.17087	0.891259445
ARHGAP30	ENSG00000186517	1	161006736	161049760	58	9	123834	0.95053	0.17092	0.891259445
ZC3H7A	ENSG00000122299	16	11834442	11901123	75	9	123834	0.94989	0.17108	0.891259445
BRD7	ENSG00000166164	16	50337398	50412845	96	10	123834	0.94988	0.17109	0.891259445
SULT1E1	ENSG00000109193	4	70666498	70735870	157	19	123834	0.94978	0.17111	0.891259445
N4BP2L2	ENSG00000244754	13	32996554	33122970	118	11	123834	0.94979	0.17111	0.891259445
SSBP2	ENSG00000145687	5	80698840	81057616	364	25	123834	0.94974	0.17112	0.891259445
TMEM231	ENSG00000205084	16	75562015	75600184	93	9	123834	0.94936	0.17122	0.891259445
DIP2A	ENSG00000160305	21	47868812	47999926	360	21	123834	0.94935	0.17122	0.891259445
ZNF107	ENSG00000196247	7	64116511	64181404	121	11	123834	0.94933	0.17123	0.891259445
ZMYM1	ENSG00000197056	1	35515387	35591460	73	4	123834	0.94913	0.17128	0.891259445
FAM166B	ENSG00000215187	9	35551828	35573896	21	5	123834	0.94894	0.17132	0.891259445
AAED1	ENSG00000158122	9	99391859	99427585	4	2	123834	0.9485	0.17144	0.891259445
KIAA1239	ENSG00000174145	4	37235842	37461087	300	45	123834	0.94812	0.17153	0.891259445
TMPRSS3	ENSG00000160183	21	43781999	43826955	112	25	123834	0.94781	0.17161	0.891259445
VNN2	ENSG00000112303	6	133055009	133094598	120	15	123834	0.94757	0.17167	0.891259445
LINC00696	ENSG00000256097	3	52087076	52107567	8	2	123834	0.94739	0.17172	0.891259445
GNAI2	ENSG00000114353	3	50253724	50306787	34	5	123834	0.94705	0.17181	0.891259445
MPND	ENSG00000008382	19	4333524	4370083	43	9	123834	0.94683	0.17186	0.891259445
GNGT1	ENSG00000127928	7	93210885	93550577	665	40	123834	0.94631	0.172	0.891259445
TTC29	ENSG00000137473	4	147617790	147877034	476	15	123834	0.94604	0.17206	0.891259445
ZCCHC10	ENSG00000155329	5	132322677	132372296	73	2	123834	0.94595	0.17209	0.891259445
CFD	ENSG00000197766	19	849453	873453	13	3	123834	0.94587	0.17211	0.891259445

SLC2A7	ENSG00000197241	1	9053359	9096404	48	15	123834	0.94572	0.17215	0.891259445
LPIN2	ENSG00000101577	18	2906992	3023313	192	17	123834	0.94557	0.17218	0.891259445
DNAJC8	ENSG00000126698	1	28515967	28569536	115	7	123834	0.94547	0.17221	0.891259445
SLC26A3	ENSG00000091138	7	107395912	107453670	82	12	123834	0.94541	0.17223	0.891259445
GPR88	ENSG00000181656	1	100993693	101017574	38	5	123834	0.94493	0.17235	0.891259445
NDUFA6	ENSG00000184983	22	42471529	42496959	49	4	123834	0.94468	0.17241	0.891259445
OSGIN2	ENSG00000164823	8	90904087	90950116	73	9	123834	0.94436	0.17249	0.891259445
CALD1	ENSG00000122786	7	134419003	134665479	216	37	123834	0.94414	0.17255	0.891259445
HOXA10	ENSG00000253293	7	27200210	27229880	14	5	123834	0.9441	0.17256	0.891259445
DUS1L	ENSG00000169718	17	80005382	80033763	29	6	123834	0.94403	0.17258	0.891259445
AARS2	ENSG00000124608	6	44257391	44291063	74	10	123834	0.94357	0.17269	0.891259445
BRSK1	ENSG00000160469	19	55783440	55833901	67	10	123834	0.9435	0.17271	0.891259445
IL17D	ENSG00000172458	13	21266266	21307237	42	8	123834	0.94347	0.17272	0.891259445
AC009403.2	ENSG00000216895	7	155393925	155447899	76	7	123834	0.94329	0.17277	0.891259445
FERMT2	ENSG00000073712	14	53313986	53429153	140	12	123834	0.94329	0.17277	0.891259445
PFN1	ENSG00000108518	17	4838947	4862356	24	7	123834	0.94299	0.17284	0.891259445
TPBGL	ENSG00000261594	11	74941950	74964742	19	2	123834	0.94295	0.17285	0.891259445
ZNF581	ENSG00000171425	19	56136825	56166988	55	9	123834	0.94293	0.17286	0.891259445
C11orf68	ENSG00000175573	11	65674279	65696588	24	3	123834	0.94262	0.17294	0.891259445
SPATS1	ENSG00000249481	6	44300397	44354904	63	8	123834	0.94256	0.17295	0.891259445
DRAP1	ENSG00000175550	11	65676728	65699032	25	4	123834	0.94258	0.17295	0.891259445
CWC15	ENSG00000150316	11	94685787	94716776	61	10	123834	0.94246	0.17298	0.891259445
CACNA1A	ENSG00000141837	19	13307256	13744804	672	105	123834	0.94231	0.17302	0.891259445
CCDC51	ENSG00000164051	3	48463574	48491866	26	2	123834	0.94216	0.17306	0.891259445
NPY4R	ENSG00000204174	10	47073534	47098320	1	1	123834	0.94213	0.17306	0.891259445
DNAJA3	ENSG00000103423	16	4465806	4516776	61	5	123834	0.9419	0.17312	0.891259445
PRSS8	ENSG00000052344	16	31132756	31157083	16	3	123834	0.94191	0.17312	0.891259445
OR51M1	ENSG00000184698	11	5400607	5421664	146	11	123834	0.94167	0.17318	0.891259445
FGFR1	ENSG00000077782	8	38258656	38336352	73	10	123834	0.94147	0.17323	0.891259445
C2orf27B	ENSG00000186825	2	132542534	132569234	60	6	123834	0.94121	0.1733	0.891259445
MBL2	ENSG00000165471	10	54515140	54541460	51	9	123834	0.94076	0.17342	0.891259445
SECTM1	ENSG00000141574	17	80268900	80301950	52	3	123834	0.94075	0.17342	0.891259445
HIST2H4A	ENSG00000183941	1	149794221	149821339	2	1	123834	0.94069	0.17343	0.891259445
HIST2H3C	ENSG00000203811	1	149801110	149822765	2	1	123834	0.94069	0.17343	0.891259445
HIST2H2AA3	ENSG00000183558	1	149803505	149824478	2	1	123834	0.94069	0.17343	0.891259445
HIST2H2AA4	ENSG00000203812	1	149812643	149833191	2	1	123834	0.94069	0.17343	0.891259445
HIST2H3A	ENSG00000203852	1	149814181	149835836	2	1	123834	0.94069	0.17343	0.891259445
HIST2H4B	ENSG00000182217	1	149815607	149842714	2	1	123834	0.94069	0.17343	0.891259445
DLX3	ENSG00000064195	17	48057369	48082588	45	11	123834	0.9407	0.17343	0.891259445
SLC13A1	ENSG00000081800	7	122743585	122850040	125	15	123834	0.94035	0.17352	0.891259445
MYH7	ENSG00000092054	14	23871947	23914927	67	12	123834	0.93975	0.17367	0.891259445
LYPLAL1	ENSG00000143353	1	219337186	219396207	65	6	123834	0.93968	0.17369	0.891259445
ZBTB32	ENSG00000011590	19	36185429	36217940	47	9	123834	0.93953	0.17373	0.891259445
ALLC	ENSG00000151360	2	3695785	3760261	83	14	123834	0.93951	0.17374	0.891259445
SCRN3	ENSG00000144306	2	175250458	175304303	104	5	123834	0.93941	0.17376	0.891259445
MTL5	ENSG00000132749	11	68464908	68529032	65	4	123834	0.93941	0.17376	0.891259445
TRIM47	ENSG00000132481	17	73860242	73884656	50	5	123834	0.93913	0.17383	0.891259445
C3orf33	ENSG00000174928	3	155470401	155534140	85	12	123834	0.9391	0.17384	0.891259445
PABPN1	ENSG00000100836	14	23780498	23805394	20	6	123834	0.93903	0.17386	0.891259445
AL049829.1	ENSG00000268702	14	23780839	23801484	20	6	123834	0.93903	0.17386	0.891259445
HPGDS	ENSG00000163106	4	95209686	95274027	50	5	123834	0.93894	0.17388	0.891259445
SCNN1D	ENSG00000162572	1	1205816	1237409	68	10	123834	0.93881	0.17391	0.891259445
ECE1	ENSG00000117298	1	21533740	21681997	167	22	123834	0.93831	0.17404	0.891681508
IPMK	ENSG00000151151	10	59941278	60037694	331	8	123834	0.93779	0.17417	0.891681508
C14orf80	ENSG00000185347	14	105946192	105975912	16	3	123834	0.93782	0.17417	0.891681508
HIPK4	ENSG00000160396	19	40875178	40906094	12	5	123834	0.93776	0.17418	0.891681508
BST1	ENSG00000109743	4	15694573	15749936	142	11	123834	0.93736	0.17429	0.891811598
CYP2W1	ENSG00000073067	7	1012835	1039276	60	12	123834	0.937	0.17438	0.891811598
AC055736.1	ENSG00000268716	12	52912915	52932980	35	2	123834	0.93688	0.17441	0.891811598
NICN1	ENSG00000145029	3	49450379	49476759	37	5	123834	0.93681	0.17443	0.891811598
CANX	ENSG00000127022	5	179095629	179167926	108	12	123834	0.93675	0.17444	0.891811598
LCT	ENSG00000115850	2	136535410	136604750	131	10	123834	0.93638	0.17454	0.891854952
LSR	ENSG00000105699	19	35729233	35768867	85	10	123834	0.93632	0.17455	0.891854952
RHBDL2	ENSG00000158315	1	39341479	39417471	173	21	123834	0.93594	0.17465	0.891854952
GAS6	ENSG00000183087	13	114513522	114577046	152	14	123834	0.93577	0.1747	0.891854952
CRY1	ENSG00000008405	12	107375142	107497607	213	13	123834	0.93569	0.17472	0.891854952
TXNL4B	ENSG00000140830	16	72068188	72138330	124	12	123834	0.93564	0.17473	0.891854952
RP11-481A20.11	ENSG00000255098	8	11860545	11883043	4	2	123834	0.93524	0.17483	0.892125809
DTL	ENSG00000143476	1	212198919	212290742	240	23	123834	0.93459	0.175	0.892160343
LTV1	ENSG00000135521	6	144154481	144194949	65	9	123834	0.93451	0.17502	0.892160343
CLN3	ENSG00000261832	16	28467974	28513333	22	2	123834	0.93429	0.17508	0.892160343
CAMK1G	ENSG00000008118	1	209747062	209797283	97	16	123834	0.93412	0.17512	0.892160343
ARHGAP35	ENSG00000160007	19	47411933	47518334	80	9	123834	0.93398	0.17516	0.892160343
ENKUR	ENSG00000151023	10	25260908	25315089	54	5	123834	0.93363	0.17525	0.892160343
PCNA	ENSG00000132646	20	5085599	5117272	44	2	123834	0.93354	0.17527	0.892160343
C10orf107	ENSG00000183346	10	63412719	63536524	308	20	123834	0.93343	0.1753	0.892160343
ST3GAL4	ENSG00000110080	11	126215535	126320239	76	13	123834	0.93327	0.17534	0.892160343
VPS13A	ENSG00000197969	9	79782269	80046457	548	17	123834	0.93323	0.17535	0.892160343
ALG9	ENSG00000086848	11	111642919	111752305	78	7	123834	0.93308	0.17539	0.892160343
TMEM151B	ENSG00000178233	6	44228203	44285243	118	13	123834	0.93304	0.1754	0.892160343
PCDHB8	ENSG00000120322	5	140547371	140570081	47	7	123834	0.93273	0.17548	0.892328475
PTPRC	ENSG00000081237	1	198597801	198736545	169	21	123834	0.93226	0.1756	0.892476664
TMA7	ENSG00000232112	3	48471667	48495616	24	2	123834	0.9322	0.17562	0.892476664
TSN	ENSG00000211460	2	122484679	122535429	52	5	123834	0.93205	0.17565	0.892476664
MRPL44	ENSG00000135900	2	224812121	224842431	36	4	123834	0.93163	0.17576	0.892584845
PDE1C	ENSG00000154678	7	31780793	32348941	1285	96	123834	0.93153	0.17579	0.892584845
CTD-2207O23.12	ENSG00000267952	19	7561938	7594508	34	6	123834	0.93109	0.1759	0.892584845
PDPK1	ENSG00000140992	16	2577965	2663189	1	1	123834	0.93103	0.17592	0.892584845
UGT1A3	ENSG00000243135	2	234627754	234691945	177	18	123834	0.93078	0.17598	0.892584845
KCNH3	ENSG00000135519	12	49922940	49962091	45	5	123834	0.93078	0.17598	0.892584845
HAT1	ENSG00000128708	2	172768958	172858599	141	15	123834	0.93071	0.176	0.892584845
PRSS46	ENSG00000261603	3	46751073	46787919	120	6	123834	0.93051	0.17605	0.892600267
LAMC1	ENSG00000135862	1	182982595	183124727	439	9	123834	0.92995	0.1762	0.893102431
NXPH4	ENSG00000182379	12	57600578	57630232	5	2	123834	0.92967	0.17627	0.893102431
SLC1A6	ENSG00000105143	19	15050846	15143802	255	34	123834	0.92961	0.17629	0.893102431
RALGAPA2	ENSG00000188559	20	20360196	20703131	250	8	123834	0.92924	0.17638	0.893112843
RAB3GAP2	ENSG00000118873	1	220311635	220455796	189	14	123834	0.92908	0.17642	0.893112843
HIAT1	ENSG00000156875	1	100493653	100558933	107	9	123834	0.92898	0.17645	0.893112843
IRAK1BP1	ENSG00000146243	6	79567189	79666157	147	12	123834	0.92885	0.17648	0.893112843

SOX7	ENSG00000258724	8	10572909	10707357	387	20	123834	0.92797	0.17671	0.894038776
ZNF143	ENSG00000166478	11	9471866	9560071	106	9	123834	0.92778	0.17676	0.894053773
SLC25A27	ENSG00000153291	6	46610678	46655930	30	4	123834	0.92739	0.17686	0.894259506
IFNA2	ENSG00000188379	9	21374254	21395396	18	4	123834	0.92697	0.17697	0.894259506
TPD52	ENSG00000076554	8	80860571	81153467	460	23	123834	0.92686	0.177	0.894259506
IRX6	ENSG00000159387	16	55347672	55374672	66	7	123834	0.92649	0.1771	0.894259506
CPXM1	ENSG00000088882	20	2764715	2791283	38	9	123834	0.92648	0.1771	0.894259506
OR1L8	ENSG00000171496	9	125319778	125340838	124	12	123834	0.92644	0.17711	0.894259506
SMIM17	ENSG00000268182	19	57144513	57177134	35	7	123834	0.92634	0.17713	0.894259506
GPR148	ENSG00000173302	2	131476643	131497909	25	1	123834	0.9256	0.17733	0.894680816
PKD2	ENSG00000118762	4	88918820	89008929	135	16	123834	0.92539	0.17738	0.894680816
C12orf79	ENSG00000257242	12	92368756	92546690	302	41	123834	0.92536	0.17739	0.894680816
MAPK1IP1L	ENSG00000168175	14	55508349	55546910	48	5	123834	0.9252	0.17743	0.894680816
INPP5A	ENSG00000068383	10	134341324	134606979	550	23	123834	0.92492	0.1775	0.894680816
BCL2	ENSG00000171791	18	60780579	60997361	304	56	123834	0.92479	0.17754	0.894680816
ZBTB37	ENSG00000185278	1	173827220	173882687	105	10	123834	0.92475	0.17755	0.894680816
CTU2	ENSG00000174177	16	88762871	88791794	75	13	123834	0.92458	0.17759	0.894680816
AL117190.2	ENSG00000267918	14	101285066	101305537	31	6	123834	0.92393	0.17776	0.895278814
RP11-574K11.31	ENSG00000272916	10	75546272	75581409	28	3	123834	0.92361	0.17785	0.895278814
NDST2	ENSG00000166507	10	75551669	75581589	28	3	123834	0.92361	0.17785	0.895278814
F13B	ENSG00000143278	1	196998321	197046397	79	6	123834	0.92292	0.17802	0.895574067
EFNA4	ENSG00000243364	1	155026207	155052029	19	6	123834	0.92291	0.17803	0.895574067
BAZ1B	ENSG00000009954	7	72844728	72946608	101	8	123834	0.92281	0.17805	0.895574067
GDAP1L1	ENSG00000124194	20	42865887	42919013	101	6	123834	0.92215	0.17822	0.896192
ZNF529	ENSG00000186020	19	37015676	37106178	170	15	123834	0.92159	0.17837	0.896471962
CEA	ENSG00000267881	19	42202531	42275190	83	8	123834	0.9216	0.17837	0.896471962
GSC2	ENSG00000063515	22	19126089	19147796	8	2	123834	0.92096	0.17854	0.896929038
PHF2	ENSG00000197724	9	96328689	96451869	267	22	123834	0.92085	0.17857	0.896929038
WDR81	ENSG00000167716	17	1609817	1651893	69	8	123834	0.92032	0.1787	0.896929038
LOH12CR2	ENSG00000205791	12	12498342	12520001	43	8	123834	0.9203	0.17871	0.896929038
IL11RA	ENSG00000137070	9	34640699	34671889	34	7	123834	0.92009	0.17876	0.896929038
APOB	ENSG00000084674	2	21214301	21276945	48	9	123834	0.9196	0.17889	0.896929038
C18orf8	ENSG00000141452	18	21073473	21121746	49	5	123834	0.91953	0.17891	0.896929038
MT1HL1	ENSG00000244020	1	237157403	237177718	14	4	123834	0.91936	0.17895	0.896929038
AP000783.1	ENSG00000254667	11	123291058	123316427	18	4	123834	0.91936	0.17895	0.896929038
9-Mar	ENSG00000139266	12	58138881	58164190	11	2	123834	0.91937	0.17895	0.896929038
CSNK2B-LY6G5B-1181	ENSG00000263020	6	31623879	31651323	29	9	123834	0.91926	0.17898	0.896929038
MAB21L1	ENSG00000180660	13	36037926	36060832	16	4	123834	0.91902	0.17904	0.896993231
GPRC5C	ENSG00000170412	17	72410990	72457792	83	13	123834	0.91858	0.17916	0.897116252
U2AF1	ENSG00000160201	21	44503066	44537697	28	3	123834	0.9183	0.17923	0.897116252
PCDHB6	ENSG00000113211	5	140519683	140542868	56	8	123834	0.91799	0.17931	0.897116252
ARID3C	ENSG00000205143	9	34611376	34638104	32	6	123834	0.91798	0.17931	0.897116252
MFN2	ENSG00000116688	1	12030238	12083571	55	6	123834	0.91791	0.17933	0.897116252
BLID	ENSG00000259571	11	121976062	121996923	33	6	123834	0.91728	0.1795	0.897116252
SOX7	ENSG00000171056	8	10571278	10707357	388	20	123834	0.91695	0.17958	0.897116252
ZNF628	ENSG00000197483	19	55977699	56005854	23	7	123834	0.91694	0.17959	0.897116252
SRGN	ENSG00000122862	10	70837862	70874567	31	6	123834	0.91659	0.17968	0.897116252
NEK6	ENSG00000119408	9	127009885	127125586	166	20	123834	0.91649	0.17971	0.897116252
TMEM229B	ENSG00000198133	14	67903801	68010456	225	35	123834	0.9164	0.17973	0.897116252
ECT2L	ENSG00000203734	6	139107063	139235207	235	34	123834	0.91618	0.17979	0.897116252
LY6G6E	ENSG00000255552	6	31669548	31691842	22	10	123834	0.91608	0.17981	0.897116252
UNG	ENSG00000076248	12	109525379	109558797	31	6	123834	0.91602	0.17983	0.897116252
PTGER3	ENSG00000050628	1	71308036	71523491	412	33	123834	0.91575	0.1799	0.897116252
CRIP3	ENSG00000146215	6	43257448	43286535	34	6	123834	0.9152	0.18004	0.897116252
AC103801.2	ENSG00000229387	11	22636860	22657369	13	3	123834	0.91492	0.18012	0.897116252
SGPP1	ENSG00000126821	14	64140932	64204757	57	2	123834	0.91426	0.18029	0.897116252
PIP4K2B	ENSG00000141720	17	36911942	36966379	94	15	123834	0.91417	0.18031	0.897116252
QDPR	ENSG00000151552	4	17451884	17523857	192	25	123834	0.91409	0.18034	0.897116252
MUM1	ENSG00000160953	19	1275890	1388430	54	13	123834	0.91408	0.18034	0.897116252
APOH	ENSG00000091583	17	64198151	64262643	159	15	123834	0.91356	0.18047	0.897116252
DUSP1	ENSG00000120129	5	172185093	172208198	28	6	123834	0.91347	0.1805	0.897116252
HIATL2	ENSG00000196312	9	99650348	99785862	35	3	123834	0.91341	0.18051	0.897116252
STRA13	ENSG00000169689	17	79966578	79991983	19	4	123834	0.91332	0.18054	0.897116252
THUMPD2	ENSG00000138050	2	39953200	40016407	96	7	123834	0.91312	0.18059	0.897116252
H3F3B	ENSG00000132475	17	73762515	73791974	34	6	123834	0.91313	0.18059	0.897116252
C1D	ENSG00000197223	2	68258262	68348080	52	8	123834	0.91308	0.1806	0.897116252
USF1	ENSG00000158773	1	160999041	161025767	40	7	123834	0.91279	0.18068	0.897116252
NAT8	ENSG00000144035	2	73857959	73879520	75	5	123834	0.91276	0.18068	0.897116252
PEX6	ENSG00000124587	6	42921608	42956958	103	10	123834	0.91259	0.18073	0.897116252
NOM1	ENSG00000146909	7	156732417	156775876	180	12	123834	0.91224	0.18082	0.897116252
11-Sep	ENSG00000138758	4	77860856	77971537	175	14	123834	0.9122	0.18083	0.897116252
DEGS1	ENSG00000143753	1	224353458	224391143	49	6	123834	0.91211	0.18086	0.897116252
SNW1	ENSG00000100603	14	78173942	78237550	219	18	123834	0.91181	0.18093	0.897116252
TUBA1C	ENSG00000167553	12	49572519	49677114	111	17	123834	0.9117	0.18096	0.897116252
RBFOX3	ENSG00000167281	17	77075427	77623550	641	115	123834	0.91148	0.18102	0.897116252
CHST10	ENSG00000115526	2	100998327	101044118	75	9	123834	0.9114	0.18104	0.897116252
MSH4	ENSG00000057468	1	76252567	76388923	407	19	123834	0.91132	0.18106	0.897116252
SYPL2	ENSG00000143028	1	109999180	110034759	57	3	123834	0.91117	0.1811	0.897116252
IARS2	ENSG00000067704	1	220257444	220331380	95	9	123834	0.91102	0.18114	0.897116252
TCTE1	ENSG00000146221	6	44236480	44275458	97	10	123834	0.91095	0.18116	0.897116252
UBE2J2	ENSG00000160087	1	1179289	1219265	94	11	123834	0.91075	0.18121	0.897116252
PIK3CD	ENSG00000171608	1	9701790	9799172	57	11	123834	0.91071	0.18122	0.897116252
TLR9	ENSG00000173366	3	52245097	52275206	20	4	123834	0.91056	0.18126	0.897116252
KLC4	ENSG00000137171	6	42998515	43052837	91	9	123834	0.91045	0.18129	0.897116252
NCSTN	ENSG00000162736	1	160303062	160338742	35	5	123834	0.91039	0.18131	0.897116252
CXCL12	ENSG00000107562	10	44783038	44891941	257	31	123834	0.91032	0.18133	0.897116252
SKIDA1	ENSG00000180592	10	21792407	21824611	9	2	123834	0.90985	0.18145	0.897157826
TRIM22	ENSG00000132274	11	5700919	5768319	285	22	123834	0.90987	0.18145	0.897157826
KCNH6	ENSG00000173826	17	61590695	61636338	12	3	123834	0.90974	0.18148	0.897157826
ATRIP	ENSG00000164053	3	48478114	48517115	29	2	123834	0.9095	0.18154	0.897213157
WRN	ENSG00000165392	8	30881317	31041285	215	20	123834	0.90918	0.18163	0.897213157
ETV3	ENSG00000117036	1	157080983	157118266	27	8	123834	0.90901	0.18167	0.897213157
CHP2	ENSG00000166869	16	23755948	23780272	36	6	123834	0.909	0.18168	0.897213157
ATP8B2	ENSG00000143515	1	154288029	154333783	95	10	123834	0.90838	0.18184	0.897550654
SGCB	ENSG00000163069	4	52876872	52914648	29	4	123834	0.9083	0.18186	0.897550654
F12	ENSG00000131187	5	176819141	176846577	7	2	123834	0.90817	0.18189	0.897550654
CDCP1	ENSG00000163814	3	45113770	45197914	129	20	123834	0.90796	0.18195	0.897613704
RNF34	ENSG00000170633	12	121827844	121878389	125	6	123834	0.90674	0.18227	0.898251987
SLC35B2	ENSG00000157593	6	44211833	44235291	29	7	123834	0.90653	0.18233	0.898251987

FERD3L	ENSG00000146618	7	19174405	19195044	16	5	123834	0.90651	0.18233	0.898251987
C21orf33	ENSG00000160221	21	45543487	45575605	57	2	123834	0.90621	0.18241	0.898251987
LRRC30	ENSG00000206422	18	7221123	7242045	24	3	123834	0.90602	0.18246	0.898251987
ZNF70	ENSG00000187792	22	24073769	24103279	25	5	123834	0.90591	0.18249	0.898251987
TREML4	ENSG00000188056	6	41186062	41216620	140	10	123834	0.90567	0.18255	0.898251987
GPA33	ENSG00000143167	1	167012073	167069868	60	12	123834	0.90556	0.18258	0.898251987
EFTUD1	ENSG00000140598	15	82412571	82565104	294	21	123834	0.90555	0.18259	0.898251987
MPHOSPH10	ENSG00000124383	2	71347444	71387231	94	11	123834	0.90473	0.1828	0.898251987
STX12	ENSG00000117758	1	28089694	28160963	50	2	123834	0.90465	0.18283	0.898251987
GSTM3	ENSG00000134202	1	110266554	110294384	33	7	123834	0.90465	0.18283	0.898251987
AC074091.13	ENSG00000205334	2	27918653	27948599	10	3	123834	0.90458	0.18284	0.898251987
CLSPN	ENSG00000092853	1	36175819	36245568	64	5	123834	0.90448	0.18287	0.898251987
DTX2	ENSG00000091073	7	76080993	76145312	49	5	123834	0.90449	0.18287	0.898251987
PCGF3	ENSG00000185619	4	689537	774428	229	27	123834	0.90434	0.18291	0.898251987
HEMK1	ENSG00000114735	3	50596583	50632366	26	3	123834	0.90426	0.18293	0.898251987
HOXB7	ENSG00000260027	17	46674594	46720934	109	18	123834	0.90425	0.18293	0.898251987
PMS2	ENSG00000122512	7	6002870	6058756	139	17	123834	0.90369	0.18308	0.898506543
DPYSL2	ENSG00000092964	8	26361791	26525694	479	32	123834	0.90329	0.18319	0.898506543
OR2T3	ENSG00000196539	1	248626627	248647634	34	1	123834	0.90306	0.18325	0.898506543
SMURF1	ENSG00000198742	7	98615061	98751723	150	22	123834	0.9029	0.18329	0.898506543
DLL4	ENSG00000128917	15	41211538	41241237	7	3	123834	0.90291	0.18329	0.898506543
ZSCAN18	ENSG00000121413	19	58585205	58639794	152	9	123834	0.90291	0.18329	0.898506543
MAP4	ENSG00000047849	3	47882182	48140769	147	5	123834	0.90279	0.18332	0.898506543
MORC2	ENSG00000133422	22	31311117	31374284	54	5	123834	0.90264	0.18336	0.898506543
C20orf201	ENSG00000171695	20	62704733	62725712	16	6	123834	0.9022	0.18347	0.898679877
H2AFV	ENSG00000105968	7	44856390	44897682	85	9	123834	0.90195	0.18354	0.898679877
AL627171.2	ENSG00000269832	14	50281993	50303258	5	2	123834	0.90173	0.1836	0.898679877
ANKRD16	ENSG00000134461	10	5893580	5941869	160	13	123834	0.90122	0.18373	0.898679877
ZNF326	ENSG00000162664	1	90450671	90511090	56	9	123834	0.90111	0.18377	0.898679877
OXGR1	ENSG00000165621	13	97627973	97656984	23	5	123834	0.9011	0.18377	0.898679877
TTC9	ENSG00000133985	14	71098504	71152077	50	13	123834	0.90109	0.18377	0.898679877
ZBTB43	ENSG00000169155	9	129557285	129610489	19	2	123834	0.90105	0.18378	0.898679877
SCEL	ENSG00000136155	13	78099809	78229398	214	25	123834	0.90045	0.18394	0.898679877
PPM1M	ENSG00000164088	3	52269841	52294613	25	4	123834	0.90037	0.18396	0.898679877
SOX5	ENSG00000134532	12	23672440	24113966	436	76	123834	0.90034	0.18397	0.898679877
RP11-408E5.4	ENSG00000121388	13	19746173	19771496	143	11	123834	0.90026	0.18399	0.898679877
KLRC4-KLRK1	ENSG00000255819	12	10514952	10572745	77	6	123834	0.90018	0.18401	0.898679877
ZNF580	ENSG00000213015	19	56136382	56164835	49	9	123834	0.89969	0.18414	0.898846311
ARHGAP10	ENSG00000071205	4	148643214	149003931	431	26	123834	0.89962	0.18416	0.898846311
SLC47A2	ENSG00000180638	17	19571601	19632292	60	10	123834	0.89938	0.18423	0.898846311
SH2B1	ENSG00000178188	16	28847921	28895526	51	2	123834	0.89901	0.18432	0.898846311
SLIRP	ENSG00000119705	14	78164414	78237447	241	20	123834	0.89866	0.18442	0.898846311
SNAPIN	ENSG00000143553	1	153621130	153644306	21	2	123834	0.89854	0.18445	0.898846311
PAX6	ENSG00000007372	11	31796340	31849509	36	6	123834	0.89838	0.18449	0.898846311
QSOX2	ENSG00000165661	9	139088179	139147687	91	13	123834	0.89793	0.18461	0.898846311
ARFGEF1	ENSG00000066777	8	68075747	68265912	137	12	123834	0.8978	0.18465	0.898846311
CDK1	ENSG00000170312	10	62528089	62564610	75	9	123834	0.89761	0.1847	0.898846311
TLR4	ENSG00000136869	9	120456610	120489149	36	9	123834	0.89729	0.18478	0.898846311
RP11-664D7.4	ENSG00000248801	8	69205703	69253726	37	8	123834	0.89701	0.18486	0.898846311
OR3A2	ENSG00000221882	17	3171193	3192268	63	3	123834	0.89698	0.18486	0.898846311
AC022400.2	ENSG00000267964	10	75521935	75542378	17	2	123834	0.89671	0.18494	0.898846311
FOXC1	ENSG00000054598	6	1600681	1624127	8	4	123834	0.89663	0.18496	0.898846311
EBF2	ENSG00000221818	8	25689246	25912913	319	45	123834	0.89645	0.18501	0.898846311
ROMO1	ENSG00000125995	20	34277194	34298906	37	5	123834	0.89632	0.18504	0.898846311
CLDN22	ENSG00000177300	4	184229220	184251927	39	9	123834	0.89626	0.18506	0.898846311
FAM45A	ENSG00000119979	10	120853598	120907496	85	3	123834	0.89618	0.18508	0.898846311
HIST1H2BC	ENSG00000180596	6	26105101	26134154	64	8	123834	0.8961	0.1851	0.898846311
ALG9	ENSG00000258529	11	111647010	111760149	86	7	123834	0.89606	0.18511	0.898846311
CCZ1	ENSG00000122674	7	5928356	5975605	19	6	123834	0.89601	0.18512	0.898846311
ABHD17C	ENSG00000136379	15	80962025	81057962	189	22	123834	0.89575	0.18519	0.898846311
KLB	ENSG00000134962	4	39398473	39463156	128	20	123834	0.8957	0.18521	0.898846311
TRAK2	ENSG00000115993	2	202231930	202326302	152	12	123834	0.89561	0.18523	0.898846311
SPATA3	ENSG00000173699	2	231850836	231899770	88	12	123834	0.89493	0.18541	0.898846311
ZNF721	ENSG00000182903	4	409604	502945	189	13	123834	0.89488	0.18543	0.898846311
ASAH1	ENSG00000104763	8	17903934	17952494	182	18	123834	0.89486	0.18543	0.898846311
TFRC	ENSG00000072274	3	195744054	195819060	234	26	123834	0.89474	0.18546	0.898846311
DNASE1L2	ENSG00000167968	16	2275817	2298712	13	2	123834	0.89463	0.18549	0.898846311
MSL2	ENSG00000174579	3	135857764	135926083	24	6	123834	0.89457	0.18551	0.898846311
USP42	ENSG00000106346	7	6134515	6211195	149	17	123834	0.8943	0.18558	0.89895633
EDDM3B	ENSG00000181552	14	21226586	21249107	88	10	123834	0.89373	0.18573	0.899189244
AHSG	ENSG00000145192	3	186320712	186349107	51	7	123834	0.89367	0.18575	0.899189244
FHDC1	ENSG00000137460	4	153847504	153910848	68	14	123834	0.89359	0.18577	0.899189244
EHF	ENSG00000135373	11	34632640	34692604	49	11	123834	0.893	0.18593	0.899486577
TSHB	ENSG00000134200	1	115562415	115586941	78	6	123834	0.8925	0.18606	0.899486577
COX7B2	ENSG00000170516	4	46726844	46921252	321	14	123834	0.89253	0.18606	0.899486577
VDAC2	ENSG00000165637	10	76959912	77001206	67	11	123834	0.89235	0.1861	0.899486577
THAP6	ENSG00000174796	4	76429156	76485683	85	10	123834	0.89227	0.18612	0.899486577
AC037199.1	ENSG00000267880	19	51367552	51388040	37	5	123834	0.89201	0.18619	0.899486577
DDX6	ENSG00000110367	11	118610034	118671858	109	9	123834	0.89199	0.1862	0.899486577
TRIML2	ENSG00000179046	4	189002427	189040757	102	15	123834	0.89195	0.18621	0.899486577
CUL9	ENSG00000112659	6	43139913	43202325	82	9	123834	0.8916	0.1863	0.899621899
HUS1	ENSG00000136273	7	47725328	48029178	653	51	123834	0.89143	0.18635	0.899621899
MMP16	ENSG00000156103	8	89034237	89350254	342	31	123834	0.89133	0.18638	0.899621899
TNIP3	ENSG00000050730	4	122042563	122158621	224	12	123834	0.89089	0.1865	0.899972582
INA	ENSG00000148798	10	105026920	105060108	38	5	123834	0.89057	0.18658	0.900130112
FGF7	ENSG00000140285	15	49705293	49790972	191	16	123834	0.88986	0.18677	0.900818107
ST7	ENSG00000004866	7	116583292	116880157	257	23	123834	0.88969	0.18682	0.900830685
IL4	ENSG00000113520	5	131999678	132028368	32	3	123834	0.88912	0.18697	0.901096795
SLC22A6	ENSG00000197901	11	62693857	62762455	82	10	123834	0.88912	0.18697	0.901096795
CX3CR1	ENSG00000168329	3	39294985	39333226	72	16	123834	0.88867	0.18709	0.901182427
OR8D2	ENSG00000197263	11	124179134	124200184	51	7	123834	0.88862	0.1871	0.901182427
TMEM174	ENSG00000164325	5	72459022	72480970	4	2	123834	0.88853	0.18713	0.901182427
LUZP1	ENSG00000169641	1	23400516	23514301	173	13	123834	0.88826	0.1872	0.901291185
WIPI1	ENSG00000070540	17	66407089	66463654	63	13	123834	0.88799	0.18727	0.901399889
COG2	ENSG00000135775	1	230768235	230839728	113	22	123834	0.8875	0.18741	0.90161713
SLC12A7	ENSG00000113504	5	1040499	1122150	129	19	123834	0.88747	0.18741	0.90161713
ADAM21	ENSG00000139985	14	70908874	70936622	80	13	123834	0.8871	0.18751	0.901750288
ZNF44	ENSG00000197857	19	12325501	12415702	165	8	123834	0.88694	0.18756	0.901750288
OSTC	ENSG00000198856	4	109561740	109598976	61	2	123834	0.88686	0.18758	0.901750288

CD86	ENSG00000114013	3	121764213	121849983	94	15	123834	0.88653	0.18766	0.901906771
PDGFRL	ENSG00000104213	8	17423942	17511580	280	38	123834	0.88608	0.18779	0.902303418
TTLL7	ENSG00000137941	1	84320711	84474833	350	12	123834	0.88576	0.18787	0.902375745
ERLIN2	ENSG00000147475	8	37584117	37626619	2	1	123834	0.88565	0.1879	0.902375745
B3GNT6	ENSG00000198488	11	76735385	76763096	26	4	123834	0.88538	0.18798	0.902531912
IQCJ	ENSG00000214216	3	158670717	158994096	349	25	123834	0.88484	0.18812	0.902748033
MAFK	ENSG00000198517	7	1560350	1592679	79	3	123834	0.88483	0.18812	0.902748033
AL138764.1	ENSG00000226209	10	13340145	13360447	44	6	123834	0.88451	0.18821	0.90288
TEKT5	ENSG00000153060	16	10711358	10798802	279	23	123834	0.8842	0.18829	0.90288
C19orf24	ENSG00000228300	19	1265026	1289248	4	1	123834	0.88406	0.18833	0.90288
SEC61A2	ENSG00000065665	10	12161636	12221960	120	7	123834	0.88383	0.18839	0.90288
ZSCAN32	ENSG00000140987	16	3422085	3461065	66	7	123834	0.88356	0.18847	0.90288
DCTN3	ENSG00000137100	9	34603548	34630515	33	6	123834	0.88319	0.18857	0.90288
CAPN5	ENSG00000149260	11	76767979	76847201	103	18	123834	0.88308	0.1886	0.90288
SMC5	ENSG00000198887	9	72863937	72979804	246	15	123834	0.88291	0.18864	0.90288
TBC1D31	ENSG00000156787	8	124044208	124174393	175	21	123834	0.88277	0.18868	0.90288
IFT81	ENSG00000122970	12	110552140	110666602	102	14	123834	0.88269	0.1887	0.90288
DFFA	ENSG00000160049	1	10506579	10542583	22	3	123834	0.8826	0.18873	0.90288
HSPBP1	ENSG00000133265	19	55763599	55801749	36	6	123834	0.88251	0.18875	0.90288
KLF2	ENSG00000127528	19	16425628	16448685	31	6	123834	0.88235	0.18879	0.90288
ZNF510	ENSG00000081386	9	99508147	99550411	53	1	123834	0.88212	0.18886	0.90288
LAMA1	ENSG00000101680	18	6931743	7127813	236	47	123834	0.88211	0.18886	0.90288
BACH1	ENSG00000156273	21	30556392	31013071	557	42	123834	0.88177	0.18895	0.903035177
EOMES	ENSG00000163508	3	27747440	27774206	24	5	123834	0.88153	0.18901	0.903035177
ALOX12B	ENSG00000179477	17	7965954	8001021	38	11	123834	0.88119	0.18911	0.903035177
LAMB2	ENSG00000172037	3	49148547	49180551	11	3	123834	0.88111	0.18913	0.903035177
POLD3	ENSG00000077514	11	74194896	74390162	411	25	123834	0.88112	0.18913	0.903035177
RCC1	ENSG00000180198	1	28822455	28875812	100	7	123834	0.88072	0.18923	0.903285746
HOMER3	ENSG00000051128	19	19030010	19062070	4	2	123834	0.88045	0.18931	0.903357108
USP19	ENSG00000172046	3	49135479	49168371	11	3	123834	0.88035	0.18934	0.903357108
HRH2	ENSG00000113749	5	175075033	175123245	32	6	123834	0.88005	0.18942	0.903475489
ERMN	ENSG00000136541	2	158165137	158194225	10	2	123834	0.87967	0.18952	0.903475489
TMEM61	ENSG00000143001	1	55436465	55467966	57	4	123834	0.87942	0.18959	0.903475489
MMS22L	ENSG00000146263	6	97580037	97741093	211	12	123834	0.87932	0.18961	0.903475489
TRABD2B	ENSG00000269113	1	48216200	48472567	320	22	123834	0.87922	0.18964	0.903475489
RP11-212D19.4	ENSG00000255663	11	114261404	114324654	124	14	123834	0.87917	0.18965	0.903475489
CBX4	ENSG00000141582	17	77796955	77823228	22	5	123834	0.87835	0.18988	0.903911754
SAMD8	ENSG00000156671	10	76849344	76951881	171	14	123834	0.8779	0.19	0.903911754
ETFDH	ENSG00000171503	4	159583277	159640775	28	6	123834	0.87787	0.19001	0.903911754
RWDD2A	ENSG00000013392	6	83893098	83918651	31	7	123834	0.8778	0.19003	0.903911754
BTN2A2	ENSG00000124508	6	26373324	26405102	118	16	123834	0.87772	0.19005	0.903911754
NOP16	ENSG00000048162	5	175800949	175825976	21	3	123834	0.87748	0.19011	0.903911754
TYSND1	ENSG00000156521	10	71887737	71916432	73	13	123834	0.87748	0.19011	0.903911754
ALG11	ENSG00000253710	13	52576534	52613800	56	8	123834	0.87738	0.19014	0.903911754
PRIM1	ENSG00000198056	12	57115380	57156157	30	6	123834	0.87656	0.19036	0.903911754
PROSC	ENSG00000147471	8	37610111	37647283	2	1	123834	0.87654	0.19037	0.903911754
TUBA3C	ENSG00000198033	13	19737910	19765992	154	12	123834	0.87642	0.1904	0.903911754
ATAD3B	ENSG00000160072	1	1397143	1443228	17	3	123834	0.87619	0.19046	0.903911754
VPREB3	ENSG00000128218	22	24084930	24106655	15	3	123834	0.87615	0.19047	0.903911754
SNX8	ENSG00000106266	7	2281405	2403953	128	16	123834	0.87612	0.19048	0.903911754
BIN3	ENSG00000147439	8	22467931	22536661	124	11	123834	0.87594	0.19053	0.903911754
FNIP1	ENSG00000217128	5	130967407	131142710	188	9	123834	0.87588	0.19055	0.903911754
HABP2	ENSG00000148702	10	115300596	115359361	124	23	123834	0.87587	0.19055	0.903911754
NGLY1	ENSG00000151092	3	25750435	25841530	108	4	123834	0.87557	0.19063	0.904065629
CYBA	ENSG00000051523	16	88699691	88727560	17	6	123834	0.87532	0.1907	0.904172013
S100A7A	ENSG00000184330	1	153379000	153405701	21	2	123834	0.87508	0.19077	0.904278344
LMOD3	ENSG00000163380	3	69146023	69182183	35	4	123834	0.87443	0.19094	0.904407555
EXOC3	ENSG00000180104	5	433273	482052	166	13	123834	0.87442	0.19094	0.904407555
ZBTB7C	ENSG00000184828	18	45543044	45947123	652	105	123834	0.87443	0.19094	0.904407555
C22orf42	ENSG00000205856	22	32534993	32565309	79	12	123834	0.87346	0.19121	0.905460807
DNAH12	ENSG00000174844	3	57317727	57540071	656	20	123834	0.87306	0.19132	0.905756055
TMED5	ENSG00000117500	1	93605299	93656285	65	5	123834	0.87262	0.19144	0.905953433
ZBTB22	ENSG00000236104	6	33272183	33295719	21	4	123834	0.87228	0.19153	0.905953433
DRD3	ENSG00000151577	3	113837499	113928254	163	19	123834	0.87216	0.19156	0.905953433
WDR45B	ENSG00000141580	17	80562438	80616429	101	19	123834	0.87204	0.19159	0.905953433
KDM8	ENSG00000155666	16	27204807	27243089	25	6	123834	0.87203	0.1916	0.905953433
AC010368.2	ENSG00000269027	5	35041012	35061198	28	7	123834	0.87134	0.19178	0.90657902
OR1E2	ENSG00000127780	17	3326164	3347135	18	4	123834	0.87096	0.19189	0.906648054
C21orf90	ENSG00000182912	21	45927099	45955835	5	1	123834	0.87095	0.19189	0.906648054
C14orf178	ENSG00000197734	14	78217173	78246085	95	12	123834	0.86995	0.19216	0.906950507
RIC3	ENSG00000166405	11	8117597	8200602	197	17	123834	0.86991	0.19218	0.906950507
ISLR	ENSG00000129009	15	74456012	74479213	16	1	123834	0.86986	0.19219	0.906950507
DUSP7	ENSG00000164086	3	52072935	52100566	13	3	123834	0.86972	0.19223	0.906950507
ATP7B	ENSG00000123191	13	52496809	52595630	165	15	123834	0.86963	0.19225	0.906950507
ADIPOR1	ENSG00000159346	1	202899951	202937700	55	9	123834	0.86951	0.19228	0.906950507
SCARB2	ENSG00000138760	4	77069890	77145046	206	11	123834	0.86875	0.19249	0.906950507
OIT3	ENSG00000138315	10	74643339	74702791	33	4	123834	0.86868	0.19251	0.906950507
ATP5H	ENSG00000167863	17	73024958	73053080	50	8	123834	0.86865	0.19252	0.906950507
ZSCAN5B	ENSG00000197213	19	56690939	56719289	59	8	123834	0.86857	0.19254	0.906950507
LRIG2	ENSG00000198799	1	113605831	113684882	83	8	123834	0.86851	0.19256	0.906950507
FUT11	ENSG00000196968	10	75522049	75550009	20	2	123834	0.86852	0.19256	0.906950507
TRPV5	ENSG00000127412	7	142595267	142640905	85	6	123834	0.86781	0.19275	0.906950507
PCDHB10	ENSG00000120324	5	140561942	140585215	52	6	123834	0.86753	0.19283	0.906950507
SERPINB1	ENSG00000021355	6	2822566	2852240	73	13	123834	0.86749	0.19284	0.906950507
ADCY3	ENSG00000138031	2	25032038	25152708	278	16	123834	0.86726	0.1929	0.906950507
CDK4	ENSG00000135446	12	58131510	58159796	7	2	123834	0.86719	0.19292	0.906950507
TUFM	ENSG00000178952	16	28843732	28867729	27	1	123834	0.86718	0.19292	0.906950507
GJA1	ENSG00000152661	6	121746838	121780873	77	11	123834	0.86699	0.19297	0.906950507
KCTD14	ENSG00000151364	11	77716761	77767237	67	11	123834	0.86691	0.193	0.906950507
HIST1H2AC	ENSG00000180573	6	26114373	26149344	55	9	123834	0.86686	0.19301	0.906950507
ADAM19	ENSG00000135074	5	156812542	157012783	499	28	123834	0.86622	0.19318	0.906950507
BCL2L2-PABPN1	ENSG00000258643	14	23766044	23804578	28	8	123834	0.86614	0.19321	0.906950507
NMI	ENSG00000123609	2	152116979	152156571	121	7	123834	0.86578	0.19331	0.906950507
SH3BP1	ENSG00000100092	22	38020661	38072939	99	9	123834	0.86556	0.19337	0.906950507
AC008387.1	ENSG00000268217	5	72884203	72906845	46	6	123834	0.86498	0.19353	0.906950507
COX4I2	ENSG00000131055	20	30215691	30242809	32	2	123834	0.86493	0.19354	0.906950507
LCN8	ENSG00000204001	9	139638838	139662678	60	5	123834	0.86479	0.19358	0.906950507
RTN4RL2	ENSG00000186907	11	57218022	57255007	11	2	123834	0.86456	0.19364	0.906950507
ARRB1	ENSG00000137486	11	74965226	75072873	89	18	123834	0.86456	0.19364	0.906950507

DEPTOR	ENSG00000155792	8	120875957	121073152	609	24	123834	0.86413	0.19376	0.906950507
HIGD2A	ENSG00000146066	5	175805748	175826772	18	3	123834	0.86397	0.1938	0.906950507
SSTR1	ENSG00000139874	14	38667204	38692272	22	6	123834	0.86394	0.19381	0.906950507
KRTAP11-1	ENSG00000182591	21	32242966	32263874	23	3	123834	0.86393	0.19381	0.906950507
ZNF141	ENSG00000131127	4	321603	388653	199	14	123834	0.8638	0.19385	0.906950507
MLNR	ENSG00000102539	13	49784474	49806513	11	1	123834	0.86362	0.1939	0.906950507
ANXA11	ENSG00000122359	10	81900645	81975328	194	16	123834	0.86352	0.19393	0.906950507
PIGQ	ENSG00000007541	16	606995	644136	59	3	123834	0.86348	0.19394	0.906950507
SLC9A3	ENSG00000066230	5	463425	534447	143	18	123834	0.8633	0.19399	0.906950507
OPALIN	ENSG00000197430	10	98092973	98129092	63	9	123834	0.8632	0.19401	0.906950507
KCTD2	ENSG00000180901	17	73018670	73071984	91	11	123834	0.8631	0.19404	0.906950507
C1QTNF7	ENSG00000163145	4	15331442	15457790	226	17	123834	0.86303	0.19406	0.906950507
CCDC43	ENSG00000180329	17	42740437	42777147	33	9	123834	0.86275	0.19414	0.906950507
FAM186B	ENSG00000135436	12	49966668	50009422	45	5	123834	0.86261	0.19418	0.906950507
RHOA	ENSG00000067560	3	49386578	49460431	100	7	123834	0.86247	0.19421	0.906950507
RFPL2	ENSG00000128253	22	32576422	32610718	109	11	123834	0.86222	0.19428	0.906950507
CST2	ENSG00000170369	20	23794406	23817368	71	10	123834	0.86209	0.19432	0.906950507
VMP1	ENSG00000062716	17	57774553	57929616	154	12	123834	0.86173	0.19442	0.906950507
C3orf18	ENSG00000088543	3	50585462	50618458	31	4	123834	0.86167	0.19443	0.906950507
CTXN1	ENSG00000178531	19	7979372	8001051	35	4	123834	0.8617	0.19443	0.906950507
FBXO18	ENSG00000134452	10	5921535	5989556	170	17	123834	0.86145	0.19449	0.906950507
PCDHB2	ENSG00000112852	5	140464227	140486962	59	3	123834	0.86145	0.1945	0.906950507
TREX1	ENSG00000213689	3	48496445	48519044	7	1	123834	0.86125	0.19455	0.906950507
GCM1	ENSG00000137270	6	52981762	53023627	118	15	123834	0.86051	0.19476	0.906950507
HMGN4	ENSG00000182952	6	26528633	26556482	41	4	123834	0.86046	0.19477	0.906950507
SAMD5	ENSG00000203727	6	147820063	148068683	640	52	123834	0.86028	0.19482	0.906950507
CLVS2	ENSG00000146352	6	123307116	123404072	140	15	123834	0.86004	0.19488	0.906950507
ULBP2	ENSG00000131015	6	150253136	150280371	41	3	123834	0.85993	0.19491	0.906950507
C19orf25	ENSG00000119559	19	1451142	1489555	22	5	123834	0.85985	0.19494	0.906950507
OSM	ENSG00000099985	22	30648818	30672829	10	3	123834	0.85949	0.19504	0.906950507
PI4KB	ENSG00000143393	1	151254273	151310191	30	4	123834	0.85945	0.19505	0.906950507
ELF3	ENSG00000163435	1	201967073	201996316	34	7	123834	0.85942	0.19505	0.906950507
HCN2	ENSG00000099822	19	579893	627159	51	13	123834	0.85944	0.19505	0.906950507
STK24	ENSG00000102572	13	99092455	99240194	235	21	123834	0.85913	0.19513	0.906950507
STPG1	ENSG00000001460	1	24673489	24753424	146	13	123834	0.85871	0.19525	0.906950507
1-Mar	ENSG00000186205	1	220950101	220997735	53	9	123834	0.85871	0.19525	0.906950507
LELP1	ENSG00000203784	1	153165919	153187596	10	2	123834	0.8582	0.19539	0.906950507
BSND	ENSG00000162399	1	55454606	55486556	43	4	123834	0.85809	0.19542	0.906950507
DDX17	ENSG00000100201	22	38869445	38913665	48	7	123834	0.8579	0.19547	0.906950507
C6orf164	ENSG00000203871	6	88096840	88119467	80	4	123834	0.85785	0.19549	0.906950507
MTRF1L	ENSG00000112031	6	153298497	153333820	70	7	123834	0.85767	0.19554	0.906950507
USP6NL	ENSG00000148429	10	11485945	11663753	282	14	123834	0.85739	0.19561	0.906950507
LCAT	ENSG00000213398	16	67963653	67988034	10	2	123834	0.85727	0.19565	0.906950507
GAPT	ENSG00000175857	5	57777262	57802917	71	6	123834	0.85706	0.1957	0.906950507
OR10A3	ENSG00000170683	11	7950081	7971141	70	10	123834	0.85701	0.19572	0.906950507
BCR	ENSG00000186716	22	23511891	23670224	248	25	123834	0.85667	0.19581	0.906950507
RP11-763F8.1	ENSG00000249049	4	92230185	92256457	69	5	123834	0.85662	0.19583	0.906950507
C7orf50	ENSG00000146540	7	1026622	1187896	568	24	123834	0.85655	0.19585	0.906950507
FNDC8	ENSG00000073598	17	33438598	33467751	9	3	123834	0.8565	0.19587	0.906950507
AGAP7	ENSG00000204169	10	51454162	51496327	10	2	123834	0.85636	0.1959	0.906950507
ERGIC1	ENSG00000113719	5	172251278	172389688	195	33	123834	0.85626	0.19593	0.906950507
TBC1D8	ENSG00000204634	2	101614079	101879328	307	23	123834	0.856	0.196	0.906950507
MESP1	ENSG00000166823	15	90281892	90304541	48	3	123834	0.85588	0.19603	0.906950507
GIF	ENSG00000134812	11	59586741	59622974	29	4	123834	0.85584	0.19604	0.906950507
KRT14	ENSG00000186847	17	39728531	39753173	36	3	123834	0.8558	0.19605	0.906950507
MYO1H	ENSG00000174527	12	109775708	109903328	390	23	123834	0.85546	0.19615	0.906950507
KRTAP26-1	ENSG00000197683	21	31681452	31702607	129	11	123834	0.8554	0.19617	0.906950507
NTN4	ENSG00000074527	12	96041583	96194930	310	34	123834	0.85525	0.19621	0.906950507
FAM131C	ENSG00000185519	1	16374264	16410127	87	11	123834	0.85503	0.19627	0.906950507
SCAND1	ENSG00000171222	20	34531539	34557394	50	5	123834	0.85484	0.19632	0.906950507
MANBA	ENSG00000109323	4	103542660	103692151	163	11	123834	0.85467	0.19637	0.906950507
DGKB	ENSG00000136267	7	14174674	15024402	1213	90	123834	0.85467	0.19637	0.906950507
RAD21	ENSG00000164754	8	117848174	117897105	91	7	123834	0.85426	0.19648	0.906950507
CASKIN2	ENSG00000177303	17	73486342	73521664	85	10	123834	0.85417	0.1965	0.906950507
SLC35E2	ENSG00000215790	1	1646277	1687431	4	1	123834	0.8539	0.19658	0.906950507
ATXN2L	ENSG00000168488	16	28824356	28858558	38	2	123834	0.85377	0.19662	0.906950507
ATP6V1C1	ENSG00000155097	8	104023291	104095279	99	10	123834	0.85372	0.19663	0.906950507
ZNF850	ENSG00000267041	19	37195285	37273727	142	13	123834	0.85374	0.19663	0.906950507
TFDP1	ENSG00000198176	13	114229013	114305785	44	3	123834	0.85329	0.19675	0.907202096
ITPRIPL1	ENSG00000198885	2	96981069	97004091	6	2	123834	0.85319	0.19678	0.907202096
SLC10A7	ENSG00000120519	4	147165127	147453123	282	19	123834	0.85295	0.19684	0.907258661
ESPN	ENSG00000187017	1	6474848	6531430	94	9	123834	0.85276	0.1969	0.9073152
KDM3A	ENSG00000115548	2	86657770	86729839	118	11	123834	0.85257	0.19695	0.907325642
ARID5B	ENSG00000150347	10	63651059	63866703	243	39	123834	0.85224	0.19704	0.90752031
SLC35B1	ENSG00000121073	17	47768305	47796376	24	7	123834	0.85165	0.1972	0.907683873
KSR1	ENSG00000141068	17	25773670	25963461	170	18	123834	0.85153	0.19724	0.907683873
RP11-447L10.1	ENSG00000272741	3	195965101	196055093	180	13	123834	0.8508	0.19744	0.907683873
SERINC5	ENSG00000164300	5	79397050	79561898	216	37	123834	0.85058	0.1975	0.907683873
RPLP0	ENSG00000089157	12	120624489	120649038	25	4	123834	0.85056	0.19751	0.907683873
PIGU	ENSG00000101464	20	33138346	33274910	132	8	123834	0.85052	0.19752	0.907683873
KCNQ1	ENSG00000053918	11	2455914	2880339	274	60	123834	0.85047	0.19753	0.907683873
PAGR1	ENSG00000185928	16	29817285	29851948	21	1	123834	0.85041	0.19755	0.907683873
OLFML1	ENSG00000183801	11	7496619	7542608	123	14	123834	0.85029	0.19758	0.907683873
SLC30A8	ENSG00000164756	8	117952512	118198953	269	18	123834	0.85025	0.19759	0.907683873
TEX101	ENSG00000131126	19	43895648	43932763	51	6	123834	0.85003	0.19765	0.907683873
EPHB1	ENSG00000154928	3	134306643	134989309	1188	98	123834	0.84984	0.19771	0.907683873
C2orf49	ENSG00000135974	2	105943816	105975668	84	11	123834	0.84946	0.19781	0.907683873
DGCR14	ENSG00000100056	22	19107792	19142197	36	6	123834	0.84941	0.19783	0.907683873
TMEM64	ENSG00000180694	8	91624223	91813860	246	9	123834	0.84906	0.19792	0.907683873
RESP18	ENSG00000182698	2	220182131	220207899	60	5	123834	0.84841	0.1981	0.907683873
CISD1	ENSG00000122873	10	60018818	60059346	97	7	123834	0.84806	0.1982	0.907683873
TFAP4	ENSG00000090447	16	4297187	4333076	28	4	123834	0.84773	0.19829	0.907683873
ZNF222	ENSG00000159885	19	44519494	44547265	34	3	123834	0.84744	0.19838	0.907683873
HVCN1	ENSG00000122986	12	111055646	111152755	84	5	123834	0.84715	0.19845	0.907683873
HRASLS2	ENSG00000133328	11	63310242	63340855	13	5	123834	0.8471	0.19847	0.907683873
SHH	ENSG00000164690	7	155582680	155614967	11	4	123834	0.8467	0.19858	0.907683873
AIFM3	ENSG00000183773	22	21309396	21345649	26	5	123834	0.84649	0.19864	0.907683873
CTRL	ENSG00000141086	16	67951543	67976317	11	2	123834	0.84613	0.19874	0.907683873
LINC00692	ENSG00000230891	3	25890024	25925191	64	5	123834	0.84609	0.19875	0.907683873

ANKRD49	ENSG00000168876	11	94216795	94242749	32	5	123834	0.84609	0.19875	0.907683873
LCA5	ENSG00000135338	6	80184708	80257175	120	15	123834	0.84582	0.19883	0.907683873
LMBRD1	ENSG00000168216	6	70375694	70517003	400	12	123834	0.84567	0.19887	0.907683873
AC018445.1	ENSG00000269679	18	77265812	77286057	63	7	123834	0.84567	0.19887	0.907683873
MYO7A	ENSG00000137474	11	76829310	76936284	145	20	123834	0.84561	0.19889	0.907683873
TBKBP1	ENSG00000198933	17	45761447	45799416	48	8	123834	0.84556	0.1989	0.907683873
XPR1	ENSG00000143324	1	180591140	180869387	370	21	123834	0.84529	0.19897	0.907683873
CCNI	ENSG00000118816	4	77958311	78007158	43	7	123834	0.84531	0.19897	0.907683873
TAF11	ENSG00000064995	6	34835555	34865866	59	7	123834	0.84515	0.19901	0.907683873
BAD	ENSG00000002330	11	64027302	64062176	25	7	123834	0.84513	0.19902	0.907683873
CHRNB1	ENSG00000170175	17	7338380	7371026	42	6	123834	0.84478	0.19912	0.907683873
OR4X1	ENSG00000176567	11	48275413	48296330	61	5	123834	0.84474	0.19913	0.907683873
DAG1	ENSG00000173402	3	49496146	49583048	101	7	123834	0.84454	0.19918	0.907683873
RUVBL1	ENSG00000175792	3	127773621	127882757	185	8	123834	0.84418	0.19929	0.907683873
TMEM2	ENSG00000135048	9	74288282	74441606	289	12	123834	0.84415	0.19929	0.907683873
XCL2	ENSG00000143185	1	168500003	168523235	52	7	123834	0.84409	0.19931	0.907683873
ZSWIM8	ENSG00000214655	10	75535340	75571551	20	3	123834	0.84391	0.19936	0.907683873
KBTBD6	ENSG00000165572	13	41691705	41716882	12	2	123834	0.84387	0.19937	0.907683873
TMEM184A	ENSG00000164855	7	1571871	1610457	61	9	123834	0.84364	0.19944	0.907683873
CGB2	ENSG00000104818	19	49525169	49546495	26	3	123834	0.84361	0.19945	0.907683873
KLK12	ENSG00000186474	19	51522348	51548486	2	1	123834	0.84359	0.19945	0.907683873
KANSL2	ENSG00000139620	12	49037184	49086021	75	8	123834	0.84347	0.19948	0.907683873
MTHFR	ENSG00000177000	1	11835780	11876977	184	11	123834	0.84319	0.19956	0.907683873
LINC01119	ENSG00000239332	2	47033836	47096146	107	22	123834	0.84315	0.19957	0.907683873
RDH14	ENSG00000240857	2	18725989	18751946	28	8	123834	0.843	0.19961	0.907683873
ROBO3	ENSG00000154134	11	124725282	124761366	54	11	123834	0.84296	0.19962	0.907683873
SOD2	ENSG00000112096	6	160080089	160193561	251	17	123834	0.84273	0.19969	0.907683873
TUBE1	ENSG00000074935	6	112381980	112418732	45	2	123834	0.8427	0.1997	0.907683873
CCDC42	ENSG00000161973	17	8623252	8658537	71	9	123834	0.84236	0.19979	0.907683873
FHL3	ENSG00000183386	1	38452442	38481278	9	4	123834	0.84212	0.19986	0.907683873
EVC2	ENSG00000173040	4	5534499	5721275	359	37	123834	0.84189	0.19993	0.907683873
ATP6V1E2	ENSG00000250565	2	46707889	46779696	102	17	123834	0.84158	0.20001	0.907683873
FRMD6	ENSG00000139926	14	51945818	52207445	436	63	123834	0.84146	0.20005	0.907683873
RAB3B	ENSG00000169213	1	52363628	52466436	158	14	123834	0.84138	0.20007	0.907683873
LRGUK	ENSG00000155530	7	133802052	133959343	288	6	123834	0.8413	0.20009	0.907683873
LCORL	ENSG00000178177	4	17832822	18033499	374	11	123834	0.84113	0.20014	0.907683873
ZNF257	ENSG00000197134	19	22225254	22284282	233	9	123834	0.84098	0.20018	0.907683873
SHANK1	ENSG00000161681	19	51155084	51232707	40	15	123834	0.84077	0.20024	0.907683873
LIPM	ENSG00000173239	10	90552487	90590303	42	11	123834	0.8403	0.20037	0.907683873
TMEM238	ENSG00000233493	19	55880613	55905966	53	12	123834	0.84025	0.20038	0.907683873
C5orf38	ENSG00000186493	5	2742245	2765508	12	3	123834	0.84021	0.20039	0.907683873
MLYCD	ENSG00000103150	16	83922731	83959787	76	5	123834	0.83974	0.20053	0.907683873
IRS1	ENSG00000169047	2	227586033	227674475	123	9	123834	0.83961	0.20056	0.907683873
SLC9B2	ENSG00000164038	4	103931025	104016986	73	10	123834	0.83959	0.20057	0.907683873
RP11-295P9.3	ENSG00000239665	10	13642047	13720299	182	14	123834	0.83949	0.2006	0.907683873
GLYCTK	ENSG00000168237	3	52311105	52339272	30	4	123834	0.83941	0.20062	0.907683873
IAH1	ENSG00000134330	2	9603787	9646672	73	5	123834	0.83932	0.20064	0.907683873
COQ6	ENSG00000119723	14	74406629	74440373	89	9	123834	0.83931	0.20065	0.907683873
TP53AIP1	ENSG00000120471	11	128794626	128823040	39	8	123834	0.83906	0.20072	0.907683873
AC137932.1	ENSG00000268218	16	89324512	89349452	51	6	123834	0.83895	0.20075	0.907683873
ST6GALNAC2	ENSG00000070731	17	74549792	74593038	66	9	123834	0.83887	0.20077	0.907683873
PCF11	ENSG00000165494	11	82858030	82908493	37	2	123834	0.83841	0.2009	0.907683873
LEPROT	ENSG00000213625	1	65876270	65911690	54	12	123834	0.83837	0.20091	0.907683873
HFE2	ENSG00000168509	1	145403095	145427545	7	2	123834	0.83817	0.20097	0.907683873
PCDHB12	ENSG00000120328	5	140578269	140601696	41	7	123834	0.83817	0.20097	0.907683873
C13orf35	ENSG00000197595	13	113291358	113348811	79	13	123834	0.83777	0.20108	0.907683873
UGT1A4	ENSG00000244474	2	234617424	234691945	215	19	123834	0.83765	0.20111	0.907683873
CHCHD3	ENSG00000106554	7	132459629	132776848	338	17	123834	0.8375	0.20115	0.907683873
DBX2	ENSG00000185610	12	45398455	45454882	132	11	123834	0.83749	0.20116	0.907683873
CLDN9	ENSG00000213937	16	3052457	3074506	6	3	123834	0.8374	0.20118	0.907683873
LSM4	ENSG00000130520	19	18407040	18444084	31	6	123834	0.83735	0.2012	0.907683873
PTX3	ENSG00000163661	3	157144578	157171417	29	4	123834	0.83725	0.20123	0.907683873
ZNF350	ENSG00000256683	19	52457596	52500109	123	10	123834	0.8368	0.20135	0.90800968
IGF2	ENSG00000167244	11	2140342	2180833	43	6	123834	0.83647	0.20145	0.908074859
CNTRL	ENSG00000119397	9	123827141	123949888	200	13	123834	0.83641	0.20146	0.908074859
LGR4	ENSG00000205213	11	27377508	27504322	94	15	123834	0.83585	0.20162	0.908199991
HGF	ENSG00000019991	7	81318322	81409754	68	4	123834	0.83581	0.20163	0.908199991
ABCF2	ENSG00000033050	7	150894923	150934316	72	10	123834	0.83554	0.20171	0.908199991
LMO1	ENSG00000166407	11	8235851	8300263	120	29	123834	0.83536	0.20176	0.908199991
HOXC5	ENSG00000172789	12	54416637	54439142	5	2	123834	0.83531	0.20177	0.908199991
CERKL	ENSG00000188452	2	182391403	182555392	278	18	123834	0.83508	0.20183	0.908199991
REG1A	ENSG00000115386	2	79337488	79360545	29	6	123834	0.835	0.20186	0.908199991
ABCC8	ENSG00000006071	11	17404432	17508449	159	26	123834	0.83494	0.20187	0.908199991
KDELC1	ENSG00000134901	13	103426631	103461357	63	6	123834	0.83435	0.20204	0.908749721
ALDH7A1	ENSG00000164904	5	125867533	125941110	102	12	123834	0.8339	0.20217	0.909119319
SLC22A7	ENSG00000137204	6	43253432	43283276	30	5	123834	0.83361	0.20225	0.909207025
SMDT1	ENSG00000183172	22	42465695	42490288	44	4	123834	0.83308	0.2024	0.909207025
SLC20A2	ENSG00000168575	8	42263993	42407069	163	18	123834	0.83299	0.20243	0.909207025
HEG1	ENSG00000173706	3	124674554	124784802	170	16	123834	0.83282	0.20247	0.909207025
LIX1L	ENSG00000152022	1	145467085	145511669	8	3	123834	0.83275	0.20249	0.909207025
C1RL	ENSG00000139178	12	7232183	7271869	43	9	123834	0.83277	0.20249	0.909207025
ZNF491	ENSG00000177599	19	11898482	11930437	52	7	123834	0.83253	0.20255	0.909207025
CTC-241N9.1	ENSG00000245317	5	179276013	179298283	35	7	123834	0.83243	0.20258	0.909207025
STAB2	ENSG00000136011	12	103971051	104170505	376	48	123834	0.83231	0.20262	0.909207025
DZANK1	ENSG00000089091	20	18354011	18457925	223	23	123834	0.83187	0.20274	0.909530781
DKFZP761J1410	ENSG00000105520	19	11456062	11486374	24	5	123834	0.83132	0.2029	0.909863949
ZNF22	ENSG00000165512	10	45485923	45510774	40	5	123834	0.83128	0.20291	0.909863949
MBD3L1	ENSG00000170948	19	8933074	8964016	41	5	123834	0.83087	0.20302	0.910017524
FBXL5	ENSG00000118564	4	15596162	15693302	228	15	123834	0.83079	0.20304	0.910017524
DYNC1H1	ENSG00000197102	14	102420865	102527129	162	13	123834	0.83039	0.20316	0.9102536
NRN1L	ENSG00000188038	16	67908708	67932758	19	4	123834	0.83027	0.20319	0.9102536
AL591806.1	ENSG00000268154	1	161025655	161045943	23	7	123834	0.82989	0.2033	0.9102536
KRTAP21-2	ENSG00000187026	21	32109112	32129551	46	1	123834	0.82986	0.20331	0.9102536
GRIN2C	ENSG00000161509	17	72828162	72867627	51	3	123834	0.82969	0.20336	0.9102536
VPS16	ENSG00000215305	20	2811349	2857378	57	4	123834	0.82963	0.20338	0.9102536
PAPLN	ENSG00000100767	14	73694205	73751348	63	15	123834	0.82922	0.20349	0.91033609
OR2T2	ENSG00000196240	1	248606077	248627130	5	1	123834	0.8291	0.20352	0.91033609
PROCR	ENSG00000101000	20	33749876	33775165	39	4	123834	0.829	0.20355	0.91033609
ZNF285	ENSG00000267508	19	44876459	44915774	140	11	123834	0.82887	0.20359	0.91033609

TSEN54	ENSG00000182173	17	73502141	73530820	83	9	123834	0.8285	0.20369	0.910463098
ZNF30	ENSG00000168661	19	35407807	35446074	84	6	123834	0.82823	0.20377	0.910463098
UGT1A5	ENSG00000240224	2	234611638	234691945	220	20	123834	0.82816	0.20379	0.910463098
ARPC3	ENSG00000111229	12	110862630	110898227	40	5	123834	0.8281	0.20381	0.910463098
UBE2G2	ENSG00000184787	21	46178955	46231934	111	15	123834	0.82747	0.20399	0.911026923
FAM71F1	ENSG00000135248	7	128339115	128381797	91	15	123834	0.82721	0.20406	0.911026923
LYRM5	ENSG00000205707	12	25338150	25372579	80	8	123834	0.82714	0.20408	0.911026923
TGFA	ENSG00000163235	2	70664412	70791325	187	20	123834	0.82652	0.20425	0.911226604
POC1B	ENSG00000139323	12	89803495	89929801	215	13	123834	0.82639	0.20429	0.911226604
TAC1	ENSG00000006128	7	97351220	97379784	57	6	123834	0.8263	0.20432	0.911226604
SLC5A9	ENSG00000117834	1	48678357	48724316	77	12	123834	0.82612	0.20437	0.911226604
TMEM179	ENSG00000258986	14	104931015	105081984	270	22	123834	0.82586	0.20444	0.911226604
MGARP	ENSG00000137463	4	140177317	140211492	50	6	123834	0.82564	0.2045	0.911226604
SYT17	ENSG00000103528	16	19169293	19289652	46	14	123834	0.8252	0.20463	0.911226604
DCUN1D1	ENSG00000043093	3	182645862	182713741	53	6	123834	0.82511	0.20465	0.911226604
TNFAIP2	ENSG00000185215	14	103579779	103613776	40	5	123834	0.82512	0.20465	0.911226604
ZNF267	ENSG00000185947	16	31875079	31938668	49	2	123834	0.82513	0.20465	0.911226604
KLRC1	ENSG00000134545	12	10584863	10617284	56	6	123834	0.82508	0.20466	0.911226604
RAB20	ENSG00000139832	13	111165417	111224080	98	13	123834	0.82494	0.2047	0.911226604
BSG	ENSG00000172270	19	561297	593493	64	8	123834	0.82476	0.20475	0.911235776
ATP1A4	ENSG00000132681	1	160111360	160166767	90	22	123834	0.82457	0.20481	0.911289438
LCE2D	ENSG00000187223	1	152625872	152647135	22	3	123834	0.82416	0.20492	0.911519036
ALDOC	ENSG00000109107	17	26890133	26914282	7	3	123834	0.82394	0.20499	0.911519036
ARID5A	ENSG00000196843	2	97192480	97228375	29	7	123834	0.82334	0.20516	0.911519036
BMPR1B	ENSG00000138696	4	95669119	96089599	831	44	123834	0.82293	0.20527	0.911519036
SMARCA5	ENSG00000153147	4	144424616	144488639	80	5	123834	0.82289	0.20528	0.911519036
SH3PXD2B	ENSG00000174705	5	171742185	171891527	331	21	123834	0.82243	0.20542	0.911519036
JMJD4	ENSG00000081692	1	227908126	227933112	32	7	123834	0.82239	0.20543	0.911519036
RASGRP1	ENSG00000172575	15	38770304	38867776	49	12	123834	0.82236	0.20544	0.911519036
GAST	ENSG00000184502	17	39858578	39882221	78	5	123834	0.82183	0.20559	0.911519036
PCDHB3	ENSG00000113205	5	140470234	140493406	45	3	123834	0.8216	0.20565	0.911519036
DENND5B	ENSG00000170456	12	31525157	31754031	688	24	123834	0.8213	0.20574	0.911519036
A2M	ENSG00000175899	12	9210260	9278825	113	7	123834	0.82116	0.20578	0.911519036
LTBR	ENSG00000111321	12	6474211	6510733	35	7	123834	0.82066	0.20592	0.911519036
CARF	ENSG00000138380	2	203766937	203861786	146	7	123834	0.8206	0.20594	0.911519036
ASH2L	ENSG00000129691	8	37952760	38011594	17	1	123834	0.82048	0.20597	0.911519036
SPRR2E	ENSG00000203785	1	153055611	153088660	41	5	123834	0.82023	0.20604	0.911519036
ITGB3BP	ENSG00000142856	1	63896441	64069392	326	11	123834	0.82007	0.20609	0.911519036
KIAA1671	ENSG00000197077	22	25338697	25603415	393	36	123834	0.81961	0.20622	0.911519036
EPHA7	ENSG00000135333	6	93939738	94139265	295	36	123834	0.81954	0.20624	0.911519036
EDN1	ENSG00000078401	6	12280596	12307427	32	8	123834	0.81853	0.20653	0.911519036
GTF3C3	ENSG00000119041	2	197617756	197674449	33	6	123834	0.81819	0.20662	0.911519036
AGR2	ENSG00000106541	7	16821435	16883057	192	22	123834	0.81781	0.20673	0.911519036
TRNP1	ENSG00000253368	1	27310198	27337389	13	2	123834	0.81769	0.20677	0.911519036
GPR137	ENSG00000173264	11	64027534	64066972	26	7	123834	0.81764	0.20678	0.911519036
KRT27	ENSG00000171446	17	38923060	38948786	29	8	123834	0.81757	0.2068	0.911519036
AP3S2	ENSG00000157823	15	90363831	90447574	94	7	123834	0.81722	0.2069	0.911519036
TMEM205	ENSG00000105518	19	11443452	11467194	28	2	123834	0.81702	0.20696	0.911519036
NKIRAS1	ENSG00000197885	3	23923151	23998082	152	11	123834	0.81662	0.20707	0.911519036
SATB2	ENSG00000119042	2	200124223	200345989	187	13	123834	0.81645	0.20712	0.911519036
PKD1L1	ENSG00000158683	7	47804250	47998088	346	32	123834	0.81639	0.20714	0.911519036
YWHAZ	ENSG00000164924	8	101918753	101975616	85	7	123834	0.81614	0.20721	0.911519036
INIP	ENSG00000148153	9	115436206	115490516	68	11	123834	0.81603	0.20724	0.911519036
CLRN1	ENSG00000163646	3	150633950	150700786	76	11	123834	0.81581	0.2073	0.911519036
FAM210A	ENSG00000177150	18	13653346	13736662	223	18	123834	0.8158	0.20731	0.911519036
GFRA2	ENSG00000168546	8	21537915	21679869	59	17	123834	0.81568	0.20734	0.911519036
MVP	ENSG00000013364	16	29821715	29869355	28	1	123834	0.81558	0.20737	0.911519036
KRTAP21-1	ENSG00000187005	21	32117439	32137746	29	2	123834	0.81546	0.2074	0.911519036
TRPA1	ENSG00000104321	8	72922152	72997852	197	17	123834	0.81537	0.20743	0.911519036
SDSL	ENSG00000139410	12	113850042	113886081	57	2	123834	0.81478	0.2076	0.911519036
SEPW1	ENSG00000178980	19	48271829	48297943	24	6	123834	0.81459	0.20765	0.911519036
PLAG1	ENSG00000181690	8	57063463	57133883	64	5	123834	0.81458	0.20766	0.911519036
ACACA	ENSG00000132142	17	35431923	35776909	300	18	123834	0.81445	0.20769	0.911519036
BTN3A2	ENSG00000186470	6	26355387	26388546	158	14	123834	0.81432	0.20773	0.911519036
LY6G5B	ENSG00000240053	6	31627944	31651553	28	9	123834	0.81422	0.20776	0.911519036
ZNF276	ENSG00000158805	16	89776808	89817311	120	12	123834	0.81416	0.20778	0.911519036
KIFC2	ENSG00000167702	8	145681426	145709585	15	2	123834	0.81393	0.20784	0.911519036
AZGP1	ENSG00000160862	7	99554343	99583780	26	6	123834	0.81361	0.20793	0.911519036
ZNF688	ENSG00000229809	16	30570667	30594055	2	1	123834	0.81327	0.20803	0.911519036
CRMP1	ENSG00000072832	4	5739811	5904785	302	54	123834	0.81305	0.20809	0.911519036
LHFPL5	ENSG00000197753	6	35763070	35811651	79	5	123834	0.81296	0.20812	0.911519036
DUOX1	ENSG00000137857	15	45412131	45467774	82	10	123834	0.81281	0.20816	0.911519036
RCOR3	ENSG00000117625	1	211421719	211499727	160	18	123834	0.81258	0.20823	0.911519036
ZNF471	ENSG00000196263	19	57009212	57051590	115	9	123834	0.81235	0.2083	0.911519036
KCNN3	ENSG00000143603	1	154659931	154852756	198	47	123834	0.81207	0.20837	0.911519036
WFDC5	ENSG00000175121	20	43728093	43753813	59	8	123834	0.81195	0.20841	0.911519036
CCDC149	ENSG00000181982	4	24797739	24991826	396	31	123834	0.81192	0.20842	0.911519036
RGMB	ENSG00000174136	5	98094354	98144347	57	11	123834	0.81191	0.20842	0.911519036
KIAA1958	ENSG00000165185	9	115239127	115441677	306	20	123834	0.81186	0.20844	0.911519036
NIPAL4	ENSG00000172548	5	156877027	156911725	80	11	123834	0.81151	0.20854	0.911519036
SNAPC2	ENSG00000104976	19	7975201	7998135	23	4	123834	0.81117	0.20863	0.911519036
ETV2	ENSG00000105672	19	36122647	36145773	19	5	123834	0.81118	0.20863	0.911519036
TMEM74	ENSG00000164841	8	109609079	109809844	338	24	123834	0.81077	0.20875	0.911519036
HIST1H1A	ENSG00000124610	6	26007260	26028040	29	7	123834	0.81067	0.20878	0.911519036
ABL1	ENSG00000097007	9	133579333	133773062	367	26	123834	0.81027	0.20889	0.911519036
CCDC159	ENSG00000183401	19	11445360	11475620	33	2	123834	0.81014	0.20893	0.911519036
FIZ1	ENSG00000179943	19	56092746	56123336	48	7	123834	0.80998	0.20898	0.911519036
WDR12	ENSG00000138442	2	203729505	203889521	243	10	123834	0.80983	0.20902	0.911519036
ADD2	ENSG00000075340	2	70824750	71005357	313	25	123834	0.80977	0.20903	0.911519036
INSL3	ENSG00000248099	19	17917321	17942383	8	3	123834	0.80979	0.20903	0.911519036
MTAP	ENSG00000099810	9	21792542	21941646	124	15	123834	0.80971	0.20905	0.911519036
C5orf56	ENSG00000197536	5	131736328	131821736	190	19	123834	0.80955	0.2091	0.911519036
ITGAX	ENSG00000140678	16	31356455	31404318	38	8	123834	0.80953	0.20911	0.911519036
KRT74	ENSG00000170484	12	52949566	52977609	84	16	123834	0.80922	0.2092	0.911519036
HIST1H4B	ENSG00000124529	6	26017124	26037480	25	5	123834	0.80881	0.20931	0.911519036
C11orf91	ENSG00000205177	11	33709807	33732347	31	8	123834	0.80855	0.20939	0.911519036
UGT1A6	ENSG00000167165	2	234590253	234691946	269	18	123834	0.80843	0.20942	0.911519036
ABCB11	ENSG00000073734	2	169769448	169897832	225	22	123834	0.80825	0.20947	0.911519036
ZNF687	ENSG00000143373	1	151244094	151274656	17	2	123834	0.80814	0.2095	0.911519036

MAN1C1	ENSG00000117643	1	25933959	26122698	137	11	123834	0.80799	0.20955	0.911519036
AC004817.1	ENSG00000267901	14	71673031	71693462	18	5	123834	0.80797	0.20955	0.911519036
PURA	ENSG00000185129	5	139477362	139506321	8	1	123834	0.80788	0.20958	0.911519036
TAS2R9	ENSG00000121381	12	10951693	10972767	16	2	123834	0.80764	0.20965	0.911519036
MCRS1	ENSG00000187778	12	49940327	49971936	35	3	123834	0.80758	0.20966	0.911519036
BRPF1	ENSG00000156983	3	9763413	9799702	34	10	123834	0.80747	0.2097	0.911519036
KLK6	ENSG00000167755	19	51451887	51482929	16	5	123834	0.80733	0.20974	0.911519036
LRRC24	ENSG00000254402	8	145737761	145762416	8	3	123834	0.8069	0.20986	0.911519036
ELL2	ENSG00000118985	5	95210802	95307775	127	4	123834	0.80682	0.20988	0.911519036
C19orf47	ENSG00000160392	19	40815443	40864434	84	10	123834	0.80673	0.20991	0.911519036
PIGM	ENSG00000143315	1	159987462	160011783	41	3	123834	0.80669	0.20992	0.911519036
ST6GALNAC6	ENSG00000160408	9	130637600	130677687	16	4	123834	0.80661	0.20995	0.911519036
PEX11A	ENSG00000166821	15	90210995	90244014	63	8	123834	0.80659	0.20995	0.911519036
ITPRIPL2	ENSG00000205730	16	19115254	19142946	20	2	123834	0.8064	0.21001	0.911519036
RXRA	ENSG00000186350	9	137198944	137342431	183	18	123834	0.80626	0.21005	0.911519036
KCNK18	ENSG00000186795	10	118947000	118979810	75	5	123834	0.80622	0.21006	0.911519036
SOX13	ENSG00000143842	1	204032243	204106863	39	11	123834	0.80599	0.21012	0.911519036
SSX2IP	ENSG00000117155	1	85099390	85166486	137	14	123834	0.80594	0.21014	0.911519036
IL27RA	ENSG00000104998	19	14132560	14173743	47	6	123834	0.80588	0.21016	0.911519036
ATXN1	ENSG00000124788	6	16289343	16771722	590	86	123834	0.80579	0.21018	0.911519036
CD3EAP	ENSG00000117877	19	45899467	45924024	36	5	123834	0.80575	0.21019	0.911519036
PPP1CC	ENSG00000186298	12	111147485	111190744	60	6	123834	0.80559	0.21024	0.911519036
SETD3	ENSG00000183576	14	99854083	99957216	83	4	123834	0.80533	0.21032	0.911519036
CRISP3	ENSG00000096006	6	49685097	49722150	75	10	123834	0.80509	0.21038	0.911519036
SLC38A11	ENSG00000169507	2	165742696	165822035	144	13	123834	0.80502	0.2104	0.911519036
CTD-2583A14.10	ENSG00000268750	19	58321087	58380309	117	13	123834	0.80491	0.21043	0.911519036
ZNF587B	ENSG00000269343	19	58321099	58380018	117	13	123834	0.80491	0.21043	0.911519036
AC092964.1	ENSG00000203443	3	172024218	172049513	28	3	123834	0.80487	0.21045	0.911519036
ALG14	ENSG00000172339	1	95429963	95548501	263	18	123834	0.8047	0.2105	0.911519036
GPR97	ENSG00000182885	16	57692099	57733975	28	7	123834	0.8045	0.21055	0.911519036
ZNF669	ENSG00000188295	1	247253264	247277674	65	9	123834	0.80443	0.21057	0.911519036
UBE3D	ENSG00000118420	6	83592117	83785560	302	17	123834	0.80442	0.21058	0.911519036
MUC5B	ENSG00000117983	11	1234296	1293406	144	12	123834	0.80428	0.21062	0.911519036
CD37	ENSG00000104894	19	49828428	49856592	24	2	123834	0.80401	0.21069	0.911519036
TMEM52	ENSG00000178821	1	1839029	1860712	38	3	123834	0.80393	0.21072	0.911519036
ANKRD24	ENSG00000089847	19	4173351	4234811	93	16	123834	0.80382	0.21075	0.911519036
TAPBP	ENSG00000231925	6	33257471	33292164	21	4	123834	0.80355	0.21083	0.911519036
C7orf34	ENSG00000165131	7	142626440	142647955	45	4	123834	0.80343	0.21086	0.911519036
OR4K5	ENSG00000176281	14	20378741	20399818	18	3	123834	0.80334	0.21089	0.911519036
FAM83F	ENSG00000133477	22	40380953	40449533	23	4	123834	0.80326	0.21091	0.911519036
DIRC2	ENSG00000138463	3	122503642	122609986	177	20	123834	0.80297	0.211	0.911519036
NRDE2	ENSG00000119720	14	90732580	90808481	108	11	123834	0.80285	0.21103	0.911519036
COX7A2	ENSG00000112695	6	75937391	75970039	41	3	123834	0.80257	0.21111	0.911519036
CANT1	ENSG00000171302	17	76977799	77015949	82	14	123834	0.80242	0.21115	0.911519036
RAB38	ENSG00000123892	11	87836410	87918635	225	19	123834	0.80232	0.21118	0.911519036
APTX	ENSG00000137074	9	32962604	33035166	268	6	123834	0.8023	0.21119	0.911519036
IMPACT	ENSG00000154059	18	21996580	22043499	118	12	123834	0.80215	0.21123	0.911519036
NCAPH	ENSG00000121152	2	96991525	97049583	32	5	123834	0.80195	0.21129	0.911519036
EFCAB7	ENSG00000203965	1	63979043	64048364	136	10	123834	0.8016	0.21139	0.911519036
EPHA4	ENSG00000116106	2	222272747	222448922	129	22	123834	0.80115	0.21152	0.911519036
HSPA8	ENSG00000109971	11	122918197	122943938	84	9	123834	0.80113	0.21153	0.911519036
AP3B2	ENSG00000103723	15	83318033	83388666	101	15	123834	0.801	0.21156	0.911519036
DNAJC4	ENSG00000110011	11	63987750	64011758	22	6	123834	0.80047	0.21172	0.911519036
ALPK2	ENSG00000198796	18	56138479	56306189	310	41	123834	0.80011	0.21182	0.911519036
DCXR	ENSG00000169738	17	79983012	80005608	35	3	123834	0.8001	0.21183	0.911519036
ERVV-2	ENSG00000268964	19	53537991	53564329	11	1	123834	0.80002	0.21185	0.911519036
NUF2	ENSG00000143228	1	163226366	163335554	398	25	123834	0.79976	0.21192	0.911519036
C11orf49	ENSG00000149179	11	46948240	47195936	220	13	123834	0.79978	0.21192	0.911519036
KRT73	ENSG00000186049	12	52991354	53022343	82	12	123834	0.79963	0.21196	0.911519036
ZNF317	ENSG00000130803	19	9241056	9284100	90	7	123834	0.79919	0.21209	0.911519036
DHX58	ENSG00000108771	17	40243422	40274751	40	5	123834	0.79912	0.21211	0.911519036
SH3BP2	ENSG00000087266	4	2784750	2852825	152	21	123834	0.79904	0.21213	0.911519036
RP11-127H5.1	ENSG00000253350	8	105592961	106441488	1197	88	123834	0.799	0.21214	0.911519036
ZNF528	ENSG00000167555	19	52891102	52931665	63	11	123834	0.79842	0.21231	0.911519036
AQP10	ENSG00000143595	1	154283566	154307801	57	7	123834	0.79832	0.21234	0.911519036
C14orf159	ENSG00000133943	14	91516677	91701976	191	29	123834	0.79775	0.21251	0.911519036
TCEA1	ENSG00000187735	8	54869112	54945089	109	5	123834	0.7975	0.21258	0.911519036
C16orf90	ENSG00000215131	16	3533484	3555480	56	7	123834	0.7972	0.21267	0.911519036
VSTM2B	ENSG00000187135	19	30007406	30065386	135	18	123834	0.79714	0.21269	0.911519036
CCL19	ENSG00000172724	9	34679564	34701274	31	6	123834	0.79709	0.2127	0.911519036
CLN3	ENSG00000188603	16	28467983	28516896	27	3	123834	0.79709	0.2127	0.911519036
TTLL6	ENSG00000170703	17	46829597	46904576	147	16	123834	0.79694	0.21274	0.911519036
PAGR1	ENSG00000263136	16	29817832	29841438	11	1	123834	0.79692	0.21275	0.911519036
GATAD2A	ENSG00000167491	19	19486635	19629740	159	8	123834	0.79688	0.21276	0.911519036
DPCD	ENSG00000166171	10	103320317	103379425	73	7	123834	0.79666	0.21282	0.911519036
LUC7L2	ENSG00000269955	7	139016106	139117345	276	5	123834	0.79664	0.21283	0.911519036
PRIMPOL	ENSG00000164306	4	185560767	185626117	170	5	123834	0.79645	0.21289	0.911519036
DLEU2L	ENSG00000116652	1	64004588	64025405	36	6	123834	0.79642	0.2129	0.911519036
GNMT	ENSG00000124713	6	42918496	42941618	71	6	123834	0.79612	0.21298	0.911519036
SRRM2	ENSG00000167978	16	2792330	2832539	14	4	123834	0.79611	0.21298	0.911519036
LHFPL2	ENSG00000145685	5	77771038	78075844	288	26	123834	0.79606	0.213	0.911519036
COPS8	ENSG00000198612	2	237983955	238019109	31	6	123834	0.796	0.21302	0.911519036
ETV1	ENSG00000006468	7	13920853	14041050	235	37	123834	0.79589	0.21305	0.911519036
GTF2H5	ENSG00000272047	6	158579384	158630376	77	8	123834	0.79581	0.21307	0.911519036
AP001468.1	ENSG00000235878	21	47602391	47623673	55	9	123834	0.79582	0.21307	0.911519036
ABCA9	ENSG00000154258	17	66960629	67067205	105	11	123834	0.79579	0.21308	0.911519036
LRRC48	ENSG00000171962	17	17866127	17930203	73	5	123834	0.79553	0.21315	0.911519036
CASP10	ENSG00000003400	2	202037604	202104129	70	5	123834	0.79539	0.21319	0.911519036
MYPN	ENSG00000138347	10	69855912	69981774	318	21	123834	0.79537	0.2132	0.911519036
MARK4	ENSG00000007047	19	45572546	45818541	359	29	123834	0.79522	0.21324	0.911519036
OR52M1	ENSG00000197790	11	4556421	4577374	128	9	123834	0.79518	0.21326	0.911519036
GPR83	ENSG00000123901	11	94100477	94144585	147	15	123834	0.79516	0.21326	0.911519036
CCND1	ENSG00000110092	11	69445855	69479242	37	4	123834	0.795	0.21331	0.911519036
FAM57B	ENSG00000149926	16	30025748	30074299	45	4	123834	0.79489	0.21334	0.911519036
TRIQK	ENSG00000205133	8	93885758	94039901	198	22	123834	0.79485	0.21335	0.911519036
SPAG5	ENSG00000076382	17	26894588	26936297	8	3	123834	0.79482	0.21336	0.911519036
C11orf82	ENSG00000165490	11	82601017	82679319	154	15	123834	0.79479	0.21337	0.911519036
LINC00346	ENSG00000255874	13	111511578	111532162	32	3	123834	0.79426	0.21352	0.911519036
CTSH	ENSG00000103811	15	79203400	79251916	97	9	123834	0.794	0.2136	0.911519036

GPRIN1	ENSG00000169258	5	176012803	176047134	32	6	123834	0.7936	0.21371	0.911519036
LAMA4	ENSG00000112769	6	112419963	112586141	246	24	123834	0.79352	0.21374	0.911519036
CDC27	ENSG00000004897	17	45185069	45276788	151	17	123834	0.79335	0.21379	0.911519036
ABCF3	ENSG00000161204	3	183893811	183921800	42	6	123834	0.79311	0.21386	0.911519036
PPM1J	ENSG00000155367	1	113242616	113268099	12	5	123834	0.79288	0.21392	0.911519036
PSAT1	ENSG00000135069	9	80902059	80955009	94	19	123834	0.79264	0.21399	0.911519036
BAIAP2	ENSG00000175866	17	78998948	79101232	198	32	123834	0.79246	0.21405	0.911519036
FOXH1	ENSG00000160973	8	145688795	145711718	9	2	123834	0.79233	0.21409	0.911519036
ING4	ENSG00000111653	12	6749446	6782314	35	5	123834	0.79226	0.2141	0.911519036
ZBTB48	ENSG00000204859	1	6630061	6659340	20	5	123834	0.79207	0.21416	0.911519036
SPRR2F	ENSG00000244094	1	153074590	153095991	30	5	123834	0.7919	0.21421	0.911519036
FCRL1	ENSG00000163534	1	157754193	157799895	34	4	123834	0.79171	0.21427	0.911519036
FLT1	ENSG00000102755	13	28864489	29079265	285	24	123834	0.79169	0.21427	0.911519036
YTHDF2	ENSG00000198492	1	29053133	29106287	50	5	123834	0.79131	0.21438	0.911519036
KDR	ENSG00000128052	4	55934644	56001756	62	10	123834	0.79108	0.21445	0.911519036
CYP1A2	ENSG00000140505	15	75031185	75058543	44	5	123834	0.79108	0.21445	0.911519036
C7orf55-LUC7L2	ENSG00000146963	7	139015105	139118198	277	4	123834	0.79106	0.21446	0.911519036
LSS	ENSG00000160285	21	47598055	47658738	163	19	123834	0.79099	0.21447	0.911519036
FEV	ENSG00000163497	2	219835809	219860379	3	1	123834	0.79097	0.21448	0.911519036
PRPF18	ENSG00000165630	10	13618927	13682868	107	10	123834	0.79097	0.21448	0.911519036
BRD4	ENSG00000141867	19	15337647	15453356	51	3	123834	0.79083	0.21452	0.911519036
ODF1	ENSG00000155087	8	103553800	103583245	79	12	123834	0.79072	0.21455	0.911519036
CDS1	ENSG00000163624	4	85494132	85582491	83	7	123834	0.79043	0.21464	0.911519036
RHEBL1	ENSG00000167550	12	49448468	49473808	17	3	123834	0.79026	0.21469	0.911519036
NEDD1	ENSG00000139350	12	97291001	97357129	77	6	123834	0.79025	0.21469	0.911519036
FAM109B	ENSG00000177096	22	42460255	42485445	37	3	123834	0.79023	0.2147	0.911519036
HHIPL2	ENSG00000143512	1	222685602	222731445	73	5	123834	0.79007	0.21474	0.911519036
AL356356.1	ENSG00000225996	1	150511897	150534367	9	2	123834	0.7897	0.21485	0.911519036
RP11-944C7.1	ENSG00000258792	14	90085265	90119015	35	7	123834	0.7897	0.21485	0.911519036
WBP1L	ENSG00000166272	10	104493727	104586021	184	19	123834	0.78967	0.21486	0.911519036
BEND6	ENSG00000151917	6	56809773	56902140	124	6	123834	0.78965	0.21487	0.911519036
SLC6A7	ENSG00000011083	5	149559520	149612351	86	12	123834	0.7895	0.21491	0.911519036
EAPP	ENSG00000129518	14	34975135	35018916	73	8	123834	0.78941	0.21494	0.911519036
FAM124B	ENSG00000124019	2	225233415	225276802	83	8	123834	0.78925	0.21498	0.911519036
ZNF524	ENSG00000171443	19	56101388	56124504	18	6	123834	0.78879	0.21512	0.911629782
GBX2	ENSG00000168505	2	237063879	237087012	21	4	123834	0.78868	0.21515	0.911629782
ACER1	ENSG00000167769	19	6296153	6343640	159	11	123834	0.78869	0.21515	0.911629782
SIN3B	ENSG00000127511	19	16930211	17001164	80	10	123834	0.78833	0.21525	0.911772325
GLIS1	ENSG00000174332	1	53961910	54209877	342	23	123834	0.78817	0.2153	0.911772325
ZIC5	ENSG00000139800	13	100605218	100634163	33	6	123834	0.7876	0.21547	0.911772325
NIPAL3	ENSG00000001461	1	24732284	24809466	80	8	123834	0.78755	0.21548	0.911772325
PPID	ENSG00000171497	4	159620286	159654548	23	4	123834	0.7874	0.21552	0.911772325
ENTPD4	ENSG00000197217	8	23233296	23325208	224	21	123834	0.78721	0.21558	0.911772325
C15orf38-AP3S2	ENSG00000250021	15	90367540	90466114	99	8	123834	0.78711	0.21561	0.911772325
KCTD15	ENSG00000153885	19	34276838	34316668	50	12	123834	0.78706	0.21562	0.911772325
FAM150B	ENSG00000189292	2	269558	298851	33	6	123834	0.78677	0.21571	0.911772325
CRY2	ENSG00000121671	11	45858669	45914798	78	12	123834	0.78657	0.21577	0.911772325
CMYA5	ENSG00000164309	5	78975700	79106063	165	24	123834	0.78647	0.2158	0.911772325
PRKAB2	ENSG00000131791	1	146616685	146654129	108	7	123834	0.78561	0.21605	0.911772325
SFI1	ENSG00000198089	22	31874674	32024572	281	15	123834	0.78552	0.21608	0.911772325
PLCL2	ENSG00000154822	3	16834159	17142086	552	28	123834	0.78548	0.21609	0.911772325
TMEM30A	ENSG00000112697	6	75952640	76004684	61	5	123834	0.78538	0.21611	0.911772325
RNF103	ENSG00000239305	2	86820516	86860989	61	7	123834	0.78538	0.21612	0.911772325
FOCAD	ENSG00000188352	9	20648308	21005954	664	29	123834	0.78516	0.21618	0.911772325
SDHD	ENSG00000255292	11	111947627	112074528	130	18	123834	0.78504	0.21622	0.911772325
TESC	ENSG00000088992	12	117466728	117547284	119	14	123834	0.78446	0.21638	0.911772325
ELK4	ENSG00000158711	1	205567071	205611090	71	11	123834	0.78441	0.2164	0.911772325
SKI	ENSG00000157933	1	2150134	2251558	93	14	123834	0.78391	0.21655	0.911772325
BPNT1	ENSG00000162813	1	220220824	220273804	65	11	123834	0.78375	0.21659	0.911772325
ZSWIM4	ENSG00000132003	19	13896274	13953044	27	4	123834	0.78369	0.21661	0.911772325
KLRC4	ENSG00000183542	12	10549983	10572356	30	3	123834	0.7836	0.21664	0.911772325
CREG1	ENSG00000143162	1	167488914	167533004	98	13	123834	0.78341	0.21669	0.911772325
VRK2	ENSG00000028116	2	58124786	58397055	359	20	123834	0.78334	0.21671	0.911772325
USP40	ENSG00000085982	2	234374166	234485428	144	6	123834	0.78327	0.21673	0.911772325
PRR19	ENSG00000188368	19	42796250	42824973	16	1	123834	0.78325	0.21674	0.911772325
ALDH1A1	ENSG00000165092	9	75505578	75705358	174	20	123834	0.78297	0.21682	0.911772325
RGS20	ENSG00000147509	8	54754368	54881863	91	12	123834	0.78287	0.21685	0.911772325
TMEM121	ENSG00000184986	14	105982940	106006539	5	2	123834	0.78273	0.21689	0.911772325
ANKRD62	ENSG00000181626	18	12083852	12139748	161	8	123834	0.78275	0.21689	0.911772325
TREML2	ENSG00000112195	6	41148015	41178932	79	16	123834	0.78268	0.21691	0.911772325
CPEB4	ENSG00000113742	5	173305283	173398979	162	14	123834	0.78265	0.21692	0.911772325
RIC8A	ENSG00000177963	11	197511	225113	57	9	123834	0.78247	0.21697	0.911772325
DAB1	ENSG00000173406	1	57450451	59022406	2812	204	123834	0.78238	0.217	0.911772325
CCNA2	ENSG00000145386	4	122727599	122755087	46	6	123834	0.78235	0.217	0.911772325
LYG1	ENSG00000144214	2	99890701	99931205	68	7	123834	0.78212	0.21707	0.911772325
COMTD1	ENSG00000165644	10	76983727	77005788	32	7	123834	0.78178	0.21717	0.911772325
CLDN14	ENSG00000159261	21	37822919	37958867	351	29	123834	0.78174	0.21718	0.911772325
ABCB5	ENSG00000004846	7	20644830	20826658	294	19	123834	0.78141	0.21728	0.911772325
LPCAT2	ENSG00000087253	16	55532910	55630582	109	7	123834	0.78139	0.21729	0.911772325
ALDH3A2	ENSG00000072210	17	19541449	19590911	51	5	123834	0.7812	0.21734	0.911772325
C6orf120	ENSG00000185127	6	170092233	170116401	49	9	123834	0.78101	0.2174	0.911772325
ZNF616	ENSG00000204611	19	52606344	52653175	85	5	123834	0.78072	0.21748	0.911772325
SGCD	ENSG00000170624	5	155287354	156204799	1349	75	123834	0.7807	0.21749	0.911772325
AKR1CL1	ENSG00000196326	10	5186335	5254449	209	17	123834	0.78065	0.2175	0.911772325
DUSP13	ENSG00000079393	10	76844192	76878979	66	10	123834	0.78065	0.21751	0.911772325
UCN	ENSG00000163794	2	27520268	27541313	18	4	123834	0.78032	0.2176	0.911772325
DCAF16	ENSG00000163257	4	17792278	17822381	46	5	123834	0.78028	0.21761	0.911772325
MXD1	ENSG00000059728	2	70114820	70180077	68	4	123834	0.78022	0.21763	0.911772325
DOCK3	ENSG00000088538	3	50702672	51431629	1029	10	123834	0.78003	0.21769	0.911790653
LGALS7	ENSG00000205076	19	39251611	39274132	64	9	123834	0.77978	0.21776	0.911790653
STXBP6	ENSG00000168952	14	25268862	25529503	517	43	123834	0.77933	0.21789	0.911790653
IKBKE	ENSG00000143466	1	206633791	206680223	79	15	123834	0.7792	0.21793	0.911790653
PDE8B	ENSG00000113231	5	76496274	76735632	297	34	123834	0.77899	0.21799	0.911790653
TBX20	ENSG00000164532	7	35232042	35303758	125	5	123834	0.77897	0.218	0.911790653
SLC2A14	ENSG00000173262	12	7955108	8053744	160	24	123834	0.77893	0.21801	0.911790653
DDI1	ENSG00000170967	11	103897308	103919922	13	4	123834	0.77877	0.21806	0.911790653
MIEF1	ENSG00000100335	22	39885437	39924137	26	5	123834	0.7786	0.21811	0.911790653
TPRXL	ENSG00000180438	3	13968756	14134311	275	9	123834	0.77851	0.21813	0.911790653
ALDH4A1	ENSG00000159423	1	19187926	19239275	107	11	123834	0.77837	0.21818	0.911790653

C1orf213	ENSG00000249087	1	23685490	23708332	37	5	123834	0.77826	0.21821	0.911790653
ASAP3	ENSG00000088280	1	23745056	23821061	150	11	123834	0.77778	0.21835	0.912175121
RETSAT	ENSG00000042445	2	85559211	85591743	30	6	123834	0.77745	0.21845	0.91218374
FKBP1B	ENSG00000119782	2	24262571	24296551	77	9	123834	0.77717	0.21853	0.91218374
LIPA	ENSG00000107798	10	90963326	91184314	365	36	123834	0.77712	0.21854	0.91218374
C15orf54	ENSG00000175746	15	39532885	39557046	29	5	123834	0.77693	0.2186	0.91218374
C1orf216	ENSG00000142686	1	36169476	36195073	26	3	123834	0.77684	0.21863	0.91218374
ZNF543	ENSG00000178229	19	57821877	57852144	83	12	123834	0.7768	0.21864	0.91218374
FAM69C	ENSG00000187773	18	72092963	72135179	45	11	123834	0.77634	0.21877	0.91252582
AL049747.1	ENSG00000268818	22	36013040	36041181	45	5	123834	0.77563	0.21898	0.913126142
ZNF485	ENSG00000198298	10	44091855	44123351	74	11	123834	0.77556	0.21901	0.913126142
AKR1E2	ENSG00000165568	10	4818821	4900254	363	21	123834	0.77504	0.21916	0.913551158
ACADSB	ENSG00000196177	10	124758495	124827827	155	19	123834	0.77466	0.21927	0.913588879
ANKRD33B	ENSG00000164236	5	10554442	10660308	149	14	123834	0.77447	0.21933	0.913588879
DMTN	ENSG00000158856	8	21896506	21950038	89	6	123834	0.77436	0.21936	0.913588879
ABL2	ENSG00000143322	1	179058462	179208819	270	12	123834	0.7743	0.21938	0.913588879
AGR3	ENSG00000173467	7	16889029	16931611	42	9	123834	0.77408	0.21944	0.913588879
FOS	ENSG00000170345	14	75735477	75758933	39	10	123834	0.77366	0.21957	0.913588879
KLLN	ENSG00000227268	10	89608918	89633194	7	4	123834	0.77354	0.2196	0.913588879
PRKAG2	ENSG00000106617	7	151243197	151584210	485	74	123834	0.77342	0.21964	0.913588879
SCN9A	ENSG00000169432	2	167041695	167242503	412	33	123834	0.77303	0.21975	0.913588879
ALOXE3	ENSG00000179148	17	7989218	8032365	41	9	123834	0.77305	0.21975	0.913588879
UBXN10	ENSG00000162543	1	20502578	20532541	26	2	123834	0.77291	0.21979	0.913588879
RPL23	ENSG00000125691	17	36994118	37020096	52	10	123834	0.77286	0.2198	0.913588879
SLC29A1	ENSG00000112759	6	44177242	44211888	22	7	123834	0.77284	0.21981	0.913588879
CASQ1	ENSG00000143318	1	160150285	160181676	17	6	123834	0.77269	0.21985	0.913588879
NOTCH2	ENSG00000134250	1	120444176	120622240	98	6	123834	0.77255	0.21989	0.913588879
PSKH1	ENSG00000159792	16	67917175	67973581	35	5	123834	0.77231	0.21996	0.91368
TSACC	ENSG00000163467	1	156297105	156326786	36	4	123834	0.77195	0.22007	0.913937199
CCL21	ENSG00000137077	9	34699002	34720121	30	5	123834	0.77156	0.22019	0.914077659
MAZ	ENSG00000103495	16	29807427	29833649	5	1	123834	0.77153	0.2202	0.914077659
NEDD9	ENSG00000111859	6	11173531	11392581	343	44	123834	0.7713	0.22027	0.914168594
BMP5	ENSG00000112175	6	55608443	55750362	163	26	123834	0.77105	0.22034	0.914259489
FOXM1	ENSG00000111206	12	2956847	2996206	32	3	123834	0.77056	0.22048	0.914441161
ATPAF2	ENSG00000171953	17	17870723	17952523	95	5	123834	0.77057	0.22048	0.914441161
PAFAH2	ENSG00000158006	1	26276258	26334648	89	5	123834	0.77004	0.22064	0.914513957
ASPM	ENSG00000066279	1	197043258	197125824	112	7	123834	0.76995	0.22066	0.914513957
DAXX	ENSG00000204209	6	33276335	33307046	29	5	123834	0.76994	0.22067	0.914513957
HEXIM2	ENSG00000168517	17	43228067	43257407	15	7	123834	0.76987	0.22069	0.914513957
LEKR1	ENSG00000197980	3	156533270	156773918	480	20	123834	0.76933	0.22085	0.914718606
AC096677.1	ENSG00000269690	1	201582013	201602738	10	4	123834	0.76899	0.22095	0.914718606
MEF2BNB-MEF2B	ENSG00000064489	19	19246376	19312967	138	13	123834	0.76898	0.22095	0.914718606
MEF2B	ENSG00000213999	19	19246377	19313400	138	13	123834	0.76898	0.22095	0.914718606
MRPL54	ENSG00000183617	19	3752662	3778573	27	7	123834	0.76889	0.22098	0.914718606
FBXL3	ENSG00000005812	13	77556740	77611330	112	9	123834	0.76853	0.22109	0.914974683
ZNF716	ENSG00000182111	7	57499883	57543252	146	6	123834	0.7683	0.22115	0.914999818
GPR39	ENSG00000183840	2	133164147	133414132	364	37	123834	0.7677	0.22133	0.914999818
R3HDM1	ENSG00000048991	2	136279025	136492840	223	17	123834	0.7677	0.22133	0.914999818
MGLL	ENSG00000074416	3	127397909	127552051	134	27	123834	0.7677	0.22133	0.914999818
TULP4	ENSG00000130338	6	158723692	158942860	536	18	123834	0.76711	0.22151	0.914999818
PAFAH1B3	ENSG00000079462	19	42791185	42817698	12	1	123834	0.76703	0.22153	0.914999818
GBP1	ENSG00000117228	1	89508002	89541043	75	7	123834	0.76696	0.22155	0.914999818
CDC14B	ENSG00000081377	9	99242523	99392112	82	7	123834	0.7668	0.2216	0.914999818
DUSP6	ENSG00000139318	12	89731009	89757048	52	7	123834	0.76674	0.22162	0.914999818
TAS2R8	ENSG00000121314	12	10948650	10969892	15	2	123834	0.76662	0.22165	0.914999818
PRRT2	ENSG00000167371	16	29813177	29837201	9	1	123834	0.76651	0.22169	0.914999818
RP11-1167A19.2	ENSG00000255038	11	65824748	65847090	18	5	123834	0.76644	0.22171	0.914999818
OVGP1	ENSG00000085465	1	111946936	111980399	77	7	123834	0.7663	0.22175	0.914999818
AC025278.1	ENSG00000204752	19	7001514	7023338	45	6	123834	0.76624	0.22177	0.914999818
TMEM130	ENSG00000166448	7	98434111	98478394	54	6	123834	0.76583	0.22189	0.915221384
PRR12	ENSG00000126464	19	50084900	50139696	117	8	123834	0.76571	0.22192	0.915221384
FNDC7	ENSG00000143107	1	109245279	109295365	48	9	123834	0.76526	0.22206	0.915348626
LRRC61	ENSG00000127399	7	150009728	150045239	47	2	123834	0.76502	0.22213	0.915348626
FAM43A	ENSG00000185112	3	194396622	194419762	48	9	123834	0.765	0.22214	0.915348626
AL160286.1	ENSG00000269064	1	179433716	179467805	246	14	123834	0.76483	0.22219	0.915348626
CHEK2	ENSG00000183765	22	29073731	29148410	127	22	123834	0.76464	0.22224	0.915348626
LL22NC03-75H12.2	ENSG00000218357	22	47847048	47892860	40	5	123834	0.76445	0.2223	0.915348626
CENPV	ENSG00000166582	17	16235848	16266970	32	5	123834	0.76416	0.22238	0.915348626
YME1L1	ENSG00000136758	10	27389383	27454195	203	13	123834	0.76397	0.22244	0.915348626
OTOP2	ENSG00000183034	17	72910370	72940007	12	3	123834	0.76399	0.22244	0.915348626
SENP5	ENSG00000119231	3	196584727	196671585	156	12	123834	0.76384	0.22248	0.915348626
ZNF623	ENSG00000183309	8	144708183	144745900	44	5	123834	0.76369	0.22253	0.915348626
WNT7B	ENSG00000188064	22	46306242	46383009	184	26	123834	0.76325	0.22266	0.915348626
C17orf97	ENSG00000187624	17	250118	283510	42	8	123834	0.76322	0.22267	0.915348626
FUT3	ENSG00000171124	19	5832899	5861485	23	7	123834	0.76269	0.22282	0.915348626
TMEM14E	ENSG00000221962	3	152047484	152068779	29	5	123834	0.76266	0.22283	0.915348626
ZNF382	ENSG00000161298	19	37085719	37129499	58	7	123834	0.76268	0.22283	0.915348626
MAP3K10	ENSG00000130758	19	40687651	40731482	23	4	123834	0.76266	0.22283	0.915348626
IPO7	ENSG00000205339	11	9396169	9479673	48	6	123834	0.76251	0.22288	0.915348626
HTATIP2	ENSG00000109854	11	20375231	20415329	64	7	123834	0.76243	0.2229	0.915348626
ITGAV	ENSG00000138448	2	187444792	187555628	127	13	123834	0.76173	0.22311	0.915348626
SAMD14	ENSG00000167100	17	48177404	48217246	48	11	123834	0.76163	0.22314	0.915348626
HMX3	ENSG00000188620	10	124885478	124907257	9	3	123834	0.76151	0.22318	0.915348626
CCT3	ENSG00000163468	1	156268759	156347664	119	8	123834	0.76121	0.22327	0.915348626
C16orf92	ENSG00000167194	16	30024655	30049057	28	3	123834	0.761	0.22333	0.915348626
UBQLN1	ENSG00000135018	9	86264878	86333118	161	10	123834	0.76091	0.22335	0.915348626
ZNF814	ENSG00000204514	19	58350099	58410442	94	11	123834	0.7609	0.22336	0.915348626
MMP19	ENSG00000123342	12	56219217	56246750	12	4	123834	0.76073	0.22341	0.915348626
KLHL30	ENSG00000168427	2	239037363	239071588	18	7	123834	0.76069	0.22342	0.915348626
THEG	ENSG00000105549	19	351750	386013	29	9	123834	0.7607	0.22342	0.915348626
MRGPRG	ENSG00000182170	11	3229174	3250043	30	7	123834	0.76054	0.22346	0.915348626
LOH12CR1	ENSG00000165714	12	12500013	12629840	232	25	123834	0.76057	0.22346	0.915348626
FYTTD1	ENSG00000122068	3	197454050	197524467	84	8	123834	0.76038	0.22351	0.915348626
PTAR1	ENSG00000188647	9	72314438	72384875	98	10	123834	0.76029	0.22354	0.915348626
CHCHD1	ENSG00000172586	10	75531805	75553410	10	2	123834	0.75998	0.22363	0.915363721
RAVER2	ENSG00000162437	1	65200778	65308915	139	13	123834	0.75995	0.22364	0.915363721
ATP6V1H	ENSG00000047249	8	54618117	54766118	103	2	123834	0.7595	0.22378	0.915549955
OR14A2	ENSG00000241128	1	247876401	247897345	35	3	123834	0.7594	0.22381	0.915549955
TFF3	ENSG00000160180	21	43721777	43745761	30	8	123834	0.75932	0.22383	0.915549955

PNOC	ENSG00000168081	8	28164503	28210872	93	6	123834	0.7589	0.22396	0.915828868
GLIPR1	ENSG00000139278	12	75864460	75907633	92	7	123834	0.75834	0.22412	0.915828868
FGF11	ENSG00000161958	17	7331592	7358256	33	6	123834	0.75815	0.22418	0.915828868
NAGA	ENSG00000198951	22	42444358	42476846	41	4	123834	0.75804	0.22421	0.915828868
CALML6	ENSG00000169885	1	1836266	1858735	35	3	123834	0.75773	0.2243	0.915828868
DSG1	ENSG00000134760	18	28888052	28946992	214	14	123834	0.7577	0.22432	0.915828868
KCNJ3	ENSG00000162989	2	155544811	155724863	324	39	123834	0.75757	0.22435	0.915828868
USPL1	ENSG00000132952	13	31181830	31243686	44	4	123834	0.7576	0.22435	0.915828868
PLEKHG7	ENSG00000187510	12	93105281	93176231	116	11	123834	0.75751	0.22437	0.915828868
NUCB2	ENSG00000070081	11	17219700	17381521	113	21	123834	0.75749	0.22438	0.915828868
CAND2	ENSG00000144712	3	12827971	12923415	185	18	123834	0.75719	0.22447	0.915832623
PTGDR2	ENSG00000183134	11	60608413	60633444	44	8	123834	0.757	0.22453	0.915832623
RFT1	ENSG00000163933	3	53112499	53174478	98	5	123834	0.75674	0.2246	0.915832623
PWWP2B	ENSG00000171813	10	134200672	134241367	30	6	123834	0.75669	0.22462	0.915832623
AMPD1	ENSG00000116748	1	115205719	115248239	74	13	123834	0.75662	0.22464	0.915832623
ADH4	ENSG00000198099	4	100034808	100088949	240	8	123834	0.75626	0.22475	0.915832623
TRAF3	ENSG00000131323	14	103233813	103387837	219	13	123834	0.75618	0.22477	0.915832623
C2orf72	ENSG00000204128	2	231892205	231924434	45	9	123834	0.75585	0.22487	0.915832623
KIF3B	ENSG00000101350	20	30855467	30932814	88	6	123834	0.75565	0.22493	0.915832623
C7orf72	ENSG00000164500	7	50125632	50209426	205	10	123834	0.75557	0.22495	0.915832623
RP11-248J23.6	ENSG00000269948	10	97699725	97754785	118	18	123834	0.75512	0.22509	0.915832623
APOL4	ENSG00000100336	22	36575172	36610886	107	10	123834	0.75481	0.22518	0.915832623
OR7G3	ENSG00000170920	19	9226688	9247626	34	2	123834	0.75456	0.22526	0.915832623
CCDC82	ENSG00000149231	11	96075933	96133087	89	10	123834	0.75444	0.22529	0.915832623
WBP11	ENSG00000084463	12	14929410	14966474	48	10	123834	0.75445	0.22529	0.915832623
ZCCHC24	ENSG00000165424	10	81132081	81215383	158	25	123834	0.7544	0.2253	0.915832623
STMN1	ENSG00000117632	1	26200672	26243482	33	2	123834	0.75437	0.22531	0.915832623
URGCP-MRPS24	ENSG00000270617	7	43896157	43956188	80	10	123834	0.75439	0.22531	0.915832623
FAM229B	ENSG00000203778	6	112398802	112433993	67	1	123834	0.75409	0.2254	0.915832623
POMZP3	ENSG00000146707	7	76229303	76266578	50	5	123834	0.75405	0.22541	0.915832623
CDK2AP2	ENSG00000167797	11	67263968	67286120	15	2	123834	0.75358	0.22555	0.915832623
NLGN2	ENSG00000169992	17	7298193	7333179	24	8	123834	0.75334	0.22562	0.915832623
ATPIF1	ENSG00000130770	1	28552620	28583417	71	6	123834	0.75309	0.2257	0.915832623
MAB21L2	ENSG00000181541	4	151493077	151515843	13	3	123834	0.75305	0.22571	0.915832623
POLR3G	ENSG00000113356	5	89757565	89820370	64	10	123834	0.75303	0.22572	0.915832623
HELB	ENSG00000127311	12	66686325	66747423	90	5	123834	0.753	0.22572	0.915832623
PCDHB16	ENSG00000196963	5	140550980	140575793	52	8	123834	0.75297	0.22573	0.915832623
KIF22	ENSG00000079616	16	29792040	29826706	1	1	123834	0.75297	0.22573	0.915832623
SRSF3	ENSG00000112081	6	36552145	36583377	103	7	123834	0.75274	0.2258	0.915921127
ERLIN1	ENSG00000107566	10	101899851	101958091	90	8	123834	0.75258	0.22585	0.915928483
CENPU	ENSG00000151725	4	185605772	185665287	276	5	123834	0.75223	0.22596	0.916005729
SIM1	ENSG00000112246	6	100822891	100922805	152	17	123834	0.75183	0.22608	0.916005729
ZNF841	ENSG00000197608	19	52557719	52609018	172	12	123834	0.75183	0.22608	0.916005729
FANCF	ENSG00000183161	11	22634079	22657387	15	3	123834	0.75179	0.22609	0.916005729
SLC7A1	ENSG00000139514	13	30073547	30179825	226	21	123834	0.75173	0.22611	0.916005729
AC007377.1	ENSG00000268898	2	40478147	40500117	41	7	123834	0.75124	0.22625	0.916133878
PALB2	ENSG00000083093	16	23604488	23662631	144	13	123834	0.75105	0.22631	0.916133878
USP31	ENSG00000103404	16	23062727	23170591	204	11	123834	0.75089	0.22636	0.916133878
BRINP2	ENSG00000198797	1	177130633	177261558	178	23	123834	0.75078	0.22639	0.916133878
PHF14	ENSG00000106443	7	11003499	11219250	282	14	123834	0.75059	0.22645	0.916133878
NHLRC3	ENSG00000188811	13	39602443	39634246	44	6	123834	0.75031	0.22653	0.916133878
TCFL5	ENSG00000101190	20	61462467	61503115	93	9	123834	0.75028	0.22654	0.916133878
PDE1A	ENSG00000115252	2	182994763	183397919	760	36	123834	0.75001	0.22662	0.916133878
OR2M4	ENSG00000171180	1	248392231	248413166	4	2	123834	0.74993	0.22665	0.916133878
NR4A3	ENSG00000119508	9	102574137	102639173	38	7	123834	0.74982	0.22668	0.916133878
PWP1	ENSG00000136045	12	108069509	108116944	77	8	123834	0.74984	0.22668	0.916133878
CLDN24	ENSG00000185758	4	184232917	184253579	36	8	123834	0.7497	0.22672	0.916133878
C12orf5	ENSG00000078237	12	4420371	4472338	36	7	123834	0.7494	0.22681	0.916302759
RARRES1	ENSG00000118849	3	158404681	158460485	131	11	123834	0.74894	0.22695	0.916586531
PMP2	ENSG00000147588	8	82342561	82369758	50	3	123834	0.74854	0.22707	0.916586531
TMEM33	ENSG00000109133	4	41927137	41972589	118	7	123834	0.74834	0.22713	0.916586531
EIF4G3	ENSG00000075151	1	21122963	21513377	528	14	123834	0.74805	0.22722	0.916586531
NCR2	ENSG00000096264	6	41293393	41328625	108	13	123834	0.74791	0.22726	0.916586531
TGFBRAP1	ENSG00000135966	2	105870871	105956491	193	20	123834	0.7477	0.22732	0.916586531
RNF112	ENSG00000128482	17	19304438	19330589	4	2	123834	0.74755	0.22737	0.916586531
MFSD2B	ENSG00000205639	2	24222951	24296191	130	18	123834	0.74749	0.22738	0.916586531
RP11-867G23.8	ENSG00000255468	11	66105421	66142275	41	3	123834	0.74744	0.2274	0.916586531
TMPRSS6	ENSG00000187045	22	37451476	37515603	90	10	123834	0.74728	0.22745	0.916586531
ZADH2	ENSG00000180011	18	72897063	72931303	70	7	123834	0.7472	0.22747	0.916586531
RP11-497E19.2	ENSG00000258945	14	85984943	86005730	23	3	123834	0.74713	0.22749	0.916586531
ADAMTSL5	ENSG00000185761	19	1495017	1523603	18	5	123834	0.74696	0.22754	0.916586531
CYP3A5	ENSG00000106258	7	99235817	99287621	38	5	123834	0.74681	0.22759	0.916586531
WDR34	ENSG00000119333	9	131385940	131429066	44	4	123834	0.7466	0.22765	0.916586531
GABBR1	ENSG00000204681	6	29513406	29611753	204	26	123834	0.74646	0.2277	0.916586531
MEIOB	ENSG00000162039	16	1873984	1944295	428	5	123834	0.74645	0.2277	0.916586531
RP11-342M21.2	ENSG00000259202	15	67425072	67449277	34	9	123834	0.74614	0.22779	0.916754673
CR1	ENSG00000203710	1	207659492	207823992	217	23	123834	0.74543	0.22801	0.917139002
NUBPL	ENSG00000151413	14	31949162	32340430	840	20	123834	0.74523	0.22807	0.917139002
NSL1	ENSG00000117697	1	212889495	212975124	349	13	123834	0.74495	0.22815	0.917139002
NUTM2G	ENSG00000188152	9	99680592	99712535	7	1	123834	0.74496	0.22815	0.917139002
B4GALT2	ENSG00000117411	1	44434615	44466840	24	6	123834	0.74474	0.22821	0.917139002
OR4S1	ENSG00000176555	11	48317775	48338704	64	5	123834	0.74465	0.22824	0.917139002
CORO7	ENSG00000262246	16	4394543	4485706	109	11	123834	0.74428	0.22835	0.917139002
SP140	ENSG00000079263	2	231057826	231233762	394	25	123834	0.74415	0.22839	0.917139002
KLK3	ENSG00000142515	19	51348171	51374020	65	12	123834	0.744	0.22844	0.917139002
RP11-625H11.1	ENSG00000259458	15	62919371	62947490	34	7	123834	0.74373	0.22852	0.917139002
ORM1	ENSG00000229314	9	117075336	117098755	3	2	123834	0.74369	0.22853	0.917139002
MAP2K3	ENSG00000034152	17	21177984	21228552	17	4	123834	0.74342	0.22861	0.917139002
CCDC70	ENSG00000123171	13	52426117	52450366	41	7	123834	0.74327	0.22866	0.917139002
DNAH5	ENSG00000039139	5	13680440	13954652	670	43	123834	0.74318	0.22869	0.917139002
BMPR1A	ENSG00000107779	10	88506407	88702595	320	21	123834	0.74285	0.22879	0.917139002
POU4F2	ENSG00000151615	4	147550045	147573626	55	5	123834	0.74267	0.22884	0.917139002
OR14K1	ENSG00000153230	1	247891917	247912861	48	4	123834	0.74263	0.22885	0.917139002
ICOS	ENSG00000163600	2	204791471	204836300	91	13	123834	0.74261	0.22886	0.917139002
INSL6	ENSG00000120210	9	5121979	5195668	98	2	123834	0.74257	0.22887	0.917139002
ZNF490	ENSG00000188033	19	12678775	12760912	103	7	123834	0.74196	0.22906	0.917139002
DHH	ENSG00000139549	12	49473204	49498602	17	3	123834	0.74192	0.22907	0.917139002
APITD1-CORT	ENSG00000251503	1	10480159	10522210	61	5	123834	0.74185	0.22909	0.917139002
APITD1	ENSG00000175279	1	10480159	10522210	61	5	123834	0.74185	0.22909	0.917139002

FLG2	ENSG00000143520	1	152311211	152342482	22	2	123834	0.74172	0.22913	0.917139002
CSNK1G1	ENSG00000169118	15	64447716	64658442	250	10	123834	0.74171	0.22913	0.917139002
C15orf38	ENSG00000242498	15	90433159	90466188	26	4	123834	0.74169	0.22914	0.917139002
MYO5A	ENSG00000197535	15	52589480	52831247	514	12	123834	0.74114	0.2293	0.91736
GABRA4	ENSG00000109158	4	46910917	47006424	181	16	123834	0.74109	0.22932	0.91736
ADAMTS9	ENSG00000163638	3	64491333	64683676	187	23	123834	0.74101	0.22934	0.91736
LAX1	ENSG00000122188	1	203724304	203755361	24	2	123834	0.74087	0.22939	0.917366951
TCTN1	ENSG00000204852	12	111041832	111097235	37	6	123834	0.74059	0.22947	0.917474008
ZNF467	ENSG00000181444	7	149451271	149480568	55	6	123834	0.74025	0.22958	0.917474008
SH2D1B	ENSG00000198574	1	162355056	162391928	71	9	123834	0.74007	0.22963	0.917474008
CPLX1	ENSG00000168993	4	768745	829986	142	16	123834	0.73986	0.22969	0.917474008
OR5B21	ENSG00000198283	11	58264648	58285578	16	4	123834	0.73982	0.22971	0.917474008
ZNF286B	ENSG00000249459	17	18551742	18595575	20	3	123834	0.73959	0.22977	0.917474008
PLIN1	ENSG00000166819	15	90197596	90232658	24	6	123834	0.73952	0.2298	0.917474008
WDR33	ENSG00000136709	2	128448596	128578761	255	18	123834	0.739	0.22995	0.917474008
FIGLA	ENSG00000183733	2	70994442	71027775	77	7	123834	0.73888	0.22999	0.917474008
FAM200B	ENSG00000237765	4	15673285	15717188	72	9	123834	0.73875	0.23003	0.917474008
EIF3A	ENSG00000107581	10	120784356	120850316	72	5	123834	0.73865	0.23006	0.917474008
AXL	ENSG00000167601	19	41715108	41777671	60	12	123834	0.73813	0.23022	0.917474008
CCDC18	ENSG00000122483	1	93635476	93754287	193	9	123834	0.73778	0.23032	0.917474008
ENPP5	ENSG00000112796	6	46116924	46148708	57	5	123834	0.73779	0.23032	0.917474008
AC114546.1	ENSG00000212768	15	35260552	35282268	38	7	123834	0.73771	0.23035	0.917474008
ARPP21	ENSG00000172995	3	35670437	35845988	203	24	123834	0.73768	0.23036	0.917474008
HSDL2	ENSG00000119471	9	115132217	115244690	296	13	123834	0.73747	0.23042	0.917474008
NPC1	ENSG00000141458	18	21076148	21176862	106	8	123834	0.73736	0.23045	0.917474008
ZFP90	ENSG00000184939	16	68553993	68619975	184	5	123834	0.73732	0.23047	0.917474008
OR5AU1	ENSG00000169327	14	21613026	21634222	36	8	123834	0.73714	0.23052	0.917474008
CDK13	ENSG00000065883	7	39979636	40146733	206	13	123834	0.73704	0.23055	0.917474008
OR1S2	ENSG00000197887	11	57960674	57981653	48	5	123834	0.73703	0.23055	0.917474008
NEMF	ENSG00000165525	14	50239997	50329921	33	2	123834	0.73696	0.23057	0.917474008
CCNT1	ENSG00000129315	12	49072247	49120681	69	7	123834	0.73692	0.23058	0.917474008
AC003102.1	ENSG00000214921	17	42315758	42337236	14	4	123834	0.73674	0.23064	0.917474008
MT1G	ENSG00000125144	16	56690643	56711977	35	9	123834	0.73656	0.2307	0.917474008
PSG4	ENSG00000243137	19	43686854	43721451	74	11	123834	0.73646	0.23072	0.917474008
ST7-OT4	ENSG00000214188	7	116583953	116748860	105	14	123834	0.73599	0.23087	0.917733105
DOCK10	ENSG00000135905	2	225619807	225917162	555	36	123834	0.73578	0.23093	0.917733105
ZSCAN2	ENSG00000176371	15	85134217	85181027	59	9	123834	0.73578	0.23093	0.917733105
SLC17A8	ENSG00000179520	12	100740857	100825837	116	15	123834	0.73554	0.231	0.917819398
NCF4	ENSG00000100365	22	37247030	37284057	52	10	123834	0.73531	0.23108	0.917945379
CRB2	ENSG00000148204	9	126108449	126152603	52	11	123834	0.73476	0.23124	0.918164211
NUDCD2	ENSG00000170584	5	162863532	162897146	42	6	123834	0.73465	0.23128	0.918164211
CRYM	ENSG00000103316	16	21240195	21324404	58	5	123834	0.73463	0.23128	0.918164211
NACA	ENSG00000196531	12	57096212	57135412	41	6	123834	0.7344	0.23135	0.918217353
MANEAL	ENSG00000185090	1	38249474	38276809	35	6	123834	0.73429	0.23139	0.918217353
AVPR1A	ENSG00000166148	12	63529014	63554722	20	3	123834	0.73408	0.23145	0.918263745
RNF167	ENSG00000108523	17	4833303	4858517	28	7	123834	0.7333	0.23169	0.918590376
MRPS23	ENSG00000181610	17	55906842	55937417	38	8	123834	0.73293	0.2318	0.918590376
TUBD1	ENSG00000108423	17	57926851	57980304	68	7	123834	0.73279	0.23184	0.918590376
SERPINC1	ENSG00000117601	1	173862947	173896516	60	6	123834	0.73276	0.23185	0.918590376
CYP2D6	ENSG00000100197	22	42512501	42536908	42	5	123834	0.73273	0.23186	0.918590376
LACTB2	ENSG00000147592	8	71537553	71591409	145	13	123834	0.73272	0.23187	0.918590376
MRM1	ENSG00000129282	17	34948001	34975407	25	5	123834	0.73184	0.23213	0.918590376
WBP2NL	ENSG00000183066	22	42384729	42464460	112	7	123834	0.73183	0.23214	0.918590376
DLX2	ENSG00000115844	2	172954167	172977628	27	7	123834	0.73156	0.23222	0.918590376
CSTA	ENSG00000121552	3	122034091	122070819	61	9	123834	0.73155	0.23222	0.918590376
UGT1A7	ENSG00000244122	2	234580584	234691945	307	20	123834	0.73138	0.23227	0.918590376
PARK7	ENSG00000116288	1	8004351	8055565	41	5	123834	0.73119	0.23233	0.918590376
BET1L	ENSG00000177951	11	157784	217428	37	6	123834	0.7311	0.23236	0.918590376
ATF4	ENSG00000128272	22	39905700	39928691	16	2	123834	0.73095	0.2324	0.918590376
VASH1	ENSG00000071246	14	77218532	77259354	46	12	123834	0.73039	0.23258	0.918590376
NUP155	ENSG00000113569	5	37278239	37381283	142	10	123834	0.73021	0.23263	0.918590376
TRIM54	ENSG00000138100	2	27495260	27540307	39	5	123834	0.72985	0.23274	0.918590376
UCP3	ENSG00000175564	11	73701326	73730480	25	5	123834	0.72943	0.23287	0.918590376
OR2L13	ENSG00000196071	1	248090493	248274224	436	18	123834	0.72931	0.23291	0.918590376
SEH1L	ENSG00000085415	18	12937132	12997535	107	14	123834	0.72924	0.23293	0.918590376
WDR5B	ENSG00000196981	3	122120715	122144882	39	6	123834	0.72918	0.23295	0.918590376
RAB23	ENSG00000112210	6	57043607	57097078	26	5	123834	0.72911	0.23297	0.918590376
HSPG2	ENSG00000142798	1	22138738	22273790	173	23	123834	0.72886	0.23304	0.918590376
ASPH	ENSG00000198363	8	62403116	62637155	328	26	123834	0.72863	0.23311	0.918590376
NMRAL1	ENSG00000153406	16	4501681	4555764	126	7	123834	0.72862	0.23312	0.918590376
PLEKHG3	ENSG00000126822	14	65160820	65223610	73	7	123834	0.72837	0.23319	0.918590376
RBM7	ENSG00000076053	11	114260752	114294925	57	7	123834	0.72831	0.23321	0.918590376
GID4	ENSG00000141034	17	17932606	17981718	57	4	123834	0.72829	0.23322	0.918590376
DNAH7	ENSG00000118997	2	196592427	196943536	535	18	123834	0.72821	0.23324	0.918590376
RAPGEF3	ENSG00000079337	12	48118455	48174823	94	14	123834	0.72818	0.23325	0.918590376
MRE11A	ENSG00000020922	11	94142895	94237074	139	11	123834	0.72801	0.2333	0.918590376
DOK7	ENSG00000175920	4	3455033	3513200	203	24	123834	0.728	0.23331	0.918590376
LPCAT1	ENSG00000153395	5	1446595	1534092	89	18	123834	0.72774	0.23339	0.918590376
CARS2	ENSG00000134905	13	111283759	111375950	282	14	123834	0.72765	0.23341	0.918590376
AC087239.1	ENSG00000268076	12	25371515	25400840	70	9	123834	0.72753	0.23345	0.918590376
TEAD2	ENSG00000074219	19	49833852	49875714	66	3	123834	0.72746	0.23347	0.918590376
DNAJC1	ENSG00000136770	10	22035466	22302698	260	11	123834	0.72739	0.23349	0.918590376
PHIP	ENSG00000146247	6	79635584	79797953	236	18	123834	0.72678	0.23368	0.918590376
KYNU	ENSG00000115919	2	143625067	143809890	267	19	123834	0.72666	0.23372	0.918590376
SLC19A1	ENSG00000173638	21	46903486	46974325	88	13	123834	0.72643	0.23379	0.918590376
ID3	ENSG00000117318	1	23874409	23896285	19	6	123834	0.72628	0.23383	0.918590376
TCTEX1D1	ENSG00000152760	1	67208142	67254470	86	9	123834	0.72602	0.23391	0.918590376
COPA	ENSG00000122218	1	160249063	160323190	68	8	123834	0.72597	0.23393	0.918590376
SLC30A9	ENSG00000014824	4	41982489	42102474	363	9	123834	0.72595	0.23394	0.918590376
PLEKHB2	ENSG00000115762	2	131852420	132121282	176	5	123834	0.72563	0.23403	0.918590376
PKD2L2	ENSG00000078795	5	137213657	137288436	71	7	123834	0.72556	0.23405	0.918590376
MAPK3	ENSG00000102882	16	30115426	30144827	10	2	123834	0.72537	0.23411	0.918590376
MAU2	ENSG00000129933	19	19421490	19479563	93	7	123834	0.72529	0.23414	0.918590376
GINS1	ENSG00000101003	20	25378363	25443264	152	7	123834	0.72514	0.23418	0.918590376
ARHGEF15	ENSG00000198844	17	8203559	8235829	50	10	123834	0.72505	0.23421	0.918590376
CCDC105	ENSG00000160994	19	15111556	15144081	94	16	123834	0.72491	0.23425	0.918590376
ARHGAP40	ENSG00000124143	20	37220577	37289678	120	19	123834	0.72477	0.2343	0.918590376
OTP	ENSG00000171540	5	76914538	76945513	6	2	123834	0.72472	0.23431	0.918590376
CLEC9A	ENSG00000197992	12	10173276	10228565	28	5	123834	0.72442	0.2344	0.918590376

MTRNR2L1	ENSG00000256618	17	22012437	22033991	57	9	123834	0.72439	0.23441	0.918590376
CACNG2	ENSG00000166862	22	36949968	37109603	214	35	123834	0.72426	0.23445	0.918590376
TMEM219	ENSG00000149932	16	29942206	29994373	40	2	123834	0.72416	0.23448	0.918590376
COMMD8	ENSG00000169019	4	47442885	47475736	37	3	123834	0.72416	0.23449	0.918590376
PRDM7	ENSG00000126856	16	90112974	90168480	103	12	123834	0.72409	0.2345	0.918590376
LMO7	ENSG00000136153	13	76184570	76444004	490	43	123834	0.72367	0.23463	0.918590376
KLHL23	ENSG00000213160	2	170540998	170643499	325	22	123834	0.7236	0.23465	0.918590376
CCDC93	ENSG00000125633	2	118663054	118781709	314	17	123834	0.72357	0.23466	0.918590376
ABCA5	ENSG00000154265	17	67230452	67333385	149	14	123834	0.72356	0.23467	0.918590376
SCAMP3	ENSG00000116521	1	155215770	155242221	31	2	123834	0.72343	0.23471	0.918590376
AQR	ENSG00000021776	15	35137732	35272040	231	16	123834	0.7234	0.23472	0.918590376
RXFP1	ENSG00000171509	4	159226463	159584524	733	15	123834	0.72295	0.23486	0.918590376
PLEKHH3	ENSG00000068137	17	40809932	40839048	18	3	123834	0.72292	0.23486	0.918590376
IMPG1	ENSG00000112706	6	76620832	76792395	165	27	123834	0.72285	0.23489	0.918590376
TNIP1	ENSG00000145901	5	150399506	150483138	174	25	123834	0.7227	0.23493	0.918590376
WDR53	ENSG00000185798	3	196271056	196305545	31	4	123834	0.72222	0.23508	0.918590376
INTS2	ENSG00000108506	17	59932731	60015377	83	7	123834	0.72216	0.2351	0.918590376
CCDC58	ENSG00000160124	3	122068438	122112078	94	12	123834	0.72206	0.23513	0.918590376
EPHA2	ENSG00000142627	1	16440832	16492582	27	6	123834	0.72194	0.23517	0.918590376
C16orf86	ENSG00000159761	16	67690719	67712661	11	2	123834	0.7218	0.23521	0.918590376
ZDHHC14	ENSG00000175048	6	157792165	158109178	462	45	123834	0.72172	0.23523	0.918590376
C15orf62	ENSG00000188277	15	41052159	41074643	17	3	123834	0.72154	0.23529	0.918590376
BMP1	ENSG00000168487	8	22012249	22079839	46	5	123834	0.72115	0.23541	0.918590376
C2orf54	ENSG00000172478	2	241815465	241846306	157	9	123834	0.72072	0.23554	0.918590376
UBE2D4	ENSG00000078967	7	43956037	44005735	72	7	123834	0.72068	0.23555	0.918590376
TMEM133	ENSG00000170647	11	100852811	100874663	44	7	123834	0.72056	0.23559	0.918590376
DUSP2	ENSG00000158050	2	96798905	96821179	15	4	123834	0.72045	0.23562	0.918590376
AL627171.1	ENSG00000197502	14	50290810	50321552	7	1	123834	0.72042	0.23563	0.918590376
ANXA1	ENSG00000135046	9	75756673	75795309	43	10	123834	0.72022	0.23569	0.918590376
C8orf34	ENSG00000165084	8	69232957	69741257	816	63	123834	0.71982	0.23582	0.918590376
CNGA1	ENSG00000198515	4	47927994	48028689	259	12	123834	0.71978	0.23583	0.918590376
USP48	ENSG00000090686	1	21994791	22120099	268	20	123834	0.71967	0.23586	0.918590376
CLTB	ENSG00000175416	5	175809456	175853570	39	6	123834	0.71964	0.23587	0.918590376
RAD51D	ENSG00000185379	17	33416811	33458541	15	6	123834	0.71936	0.23596	0.918590376
FHIT	ENSG00000189283	3	59725036	61247133	3407	244	123834	0.71914	0.23603	0.918590376
NANOS2	ENSG00000188425	19	46406475	46428036	52	9	123834	0.71901	0.23607	0.918590376
SRSF6	ENSG00000124193	20	42076568	42102245	32	9	123834	0.71896	0.23608	0.918590376
ZC3H12C	ENSG00000149289	11	109954087	110052566	155	10	123834	0.71876	0.23614	0.918590376
DHRS3	ENSG00000162496	1	12617939	12687737	130	18	123834	0.71869	0.23617	0.918590376
SDHAF2	ENSG00000167985	11	61187514	61225001	32	2	123834	0.7186	0.23619	0.918590376
BAZ1A	ENSG00000198604	14	35211937	35354853	322	19	123834	0.71862	0.23619	0.918590376
PXN	ENSG00000089159	12	120638250	120713574	49	6	123834	0.71857	0.2362	0.918590376
NOS2	ENSG00000007171	17	26073792	26137525	69	13	123834	0.71852	0.23622	0.918590376
TMPRSS11D	ENSG00000153802	4	68676594	68759750	104	11	123834	0.71801	0.23638	0.919024553
GATA3	ENSG00000107485	10	8085567	8127161	64	9	123834	0.71774	0.23646	0.919043794
C1S	ENSG00000182326	12	7086351	7188336	111	8	123834	0.71765	0.23649	0.919043794
TGM4	ENSG00000163810	3	44906100	44966482	84	8	123834	0.71752	0.23653	0.919043794
CTD-2006C1.10	ENSG00000257355	19	12118703	12173782	80	4	123834	0.71736	0.23658	0.919050202
KRT26	ENSG00000186393	17	38912490	38938414	43	7	123834	0.71664	0.2368	0.919716878
CHST12	ENSG00000136213	7	2433223	2484242	57	7	123834	0.71618	0.23694	0.920072629
CD97	ENSG00000123146	19	14481313	14529537	15	4	123834	0.71568	0.23709	0.920317909
VAC14	ENSG00000103043	16	70711342	70845064	112	13	123834	0.71566	0.2371	0.920317909
TAS2R41	ENSG00000221855	7	143164966	143185889	35	6	123834	0.71456	0.23744	0.921449473
C5	ENSG00000106804	9	123704616	123822554	199	16	123834	0.71411	0.23758	0.921634069
THSD7B	ENSG00000144229	2	137513115	138445287	2247	89	123834	0.71378	0.23768	0.921634069
MEIS3	ENSG00000105419	19	47896381	47932780	59	9	123834	0.71365	0.23772	0.921634069
GMPR	ENSG00000137198	6	16228811	16305780	99	12	123834	0.71364	0.23773	0.921634069
SGTA	ENSG00000104969	19	2744712	2793369	30	2	123834	0.71361	0.23773	0.921634069
AKR1C2	ENSG00000151632	10	5019967	5070223	77	16	123834	0.71344	0.23779	0.921678695
EGFR	ENSG00000146648	7	55076714	55334313	394	59	123834	0.71287	0.23796	0.92177956
HIST1H2BH	ENSG00000197459	6	26241879	26262303	30	7	123834	0.71282	0.23798	0.92177956
FER1L6	ENSG00000214814	8	124854227	125142302	266	17	123834	0.71278	0.23799	0.92177956
CAMKK1	ENSG00000004660	17	3753609	3808185	25	9	123834	0.71272	0.23801	0.92177956
RP13-512J5.1	ENSG00000255767	2	3569574	3605547	54	10	123834	0.71249	0.23808	0.921862832
DNMT3A	ENSG00000119772	2	25445845	25575459	77	17	123834	0.71228	0.23815	0.921946069
EPC2	ENSG00000135999	2	149392009	149555130	296	9	123834	0.71193	0.23825	0.92212459
MBNL1	ENSG00000152601	3	151951617	152193569	225	15	123834	0.71121	0.23848	0.92212459
ADORA3	ENSG00000121933	1	112015970	112116584	142	25	123834	0.71102	0.23854	0.92212459
CTD-2116N17.1	ENSG00000259316	15	64462547	64675980	254	8	123834	0.71095	0.23856	0.92212459
ADCYAP1	ENSG00000141433	18	894944	922173	22	8	123834	0.71094	0.23856	0.92212459
LIN9	ENSG00000183814	1	226408850	226507570	74	10	123834	0.7107	0.23864	0.92212459
IL18	ENSG00000150782	11	112003974	112044840	42	7	123834	0.7106	0.23867	0.92212459
MRPL47	ENSG00000136522	3	179296073	179332442	45	4	123834	0.71047	0.23871	0.92212459
RP11-126K1.2	ENSG00000232671	1	151242500	151264405	13	3	123834	0.70995	0.23887	0.92212459
MYOD1	ENSG00000129152	11	17731115	17753678	10	3	123834	0.70945	0.23902	0.92212459
SKAP2	ENSG00000005020	7	26696681	27044858	651	15	123834	0.70944	0.23903	0.92212459
ZFYVE1	ENSG00000165861	14	73426159	73503920	280	16	123834	0.7093	0.23907	0.92212459
HABP4	ENSG00000130956	9	99202483	99263618	89	4	123834	0.70927	0.23908	0.92212459
FGFRL1	ENSG00000127418	4	993724	1030685	41	9	123834	0.70917	0.23911	0.92212459
TAF6	ENSG00000106290	7	99694693	99727464	27	5	123834	0.70908	0.23914	0.92212459
PGLS	ENSG00000130313	19	17612438	17642097	34	6	123834	0.70886	0.23921	0.92212459
RAX2	ENSG00000173976	19	3759087	3782233	25	8	123834	0.7085	0.23932	0.92212459
AC010336.1	ENSG00000214248	19	7953689	7978427	34	7	123834	0.70844	0.23934	0.92212459
UTP20	ENSG00000120800	12	101663887	101790394	218	19	123834	0.7083	0.23938	0.92212459
RIMS3	ENSG00000117016	1	41076351	41141329	101	14	123834	0.70827	0.23939	0.92212459
FLI1	ENSG00000151702	11	128546430	128693162	211	27	123834	0.70814	0.23943	0.92212459
HHLA1	ENSG00000132297	8	133063733	133133406	181	25	123834	0.70795	0.23949	0.92212459
ZNF791	ENSG00000173875	19	12711732	12752735	65	6	123834	0.70794	0.23949	0.92212459
SP140L	ENSG00000185404	2	231181899	231278447	243	22	123834	0.70786	0.23952	0.92212459
FAM178A	ENSG00000119906	10	102662326	102734893	55	7	123834	0.70751	0.23962	0.92212459
ZNF496	ENSG00000162714	1	247450714	247505148	87	10	123834	0.70746	0.23964	0.92212459
JAKMIP3	ENSG00000188385	10	133908175	134008313	339	32	123834	0.70744	0.23965	0.92212459
RNLS	ENSG00000184719	10	90023621	90354287	440	31	123834	0.70737	0.23967	0.92212459
DNTT	ENSG00000107447	10	98054085	98108321	104	7	123834	0.70735	0.23967	0.92212459
ZMIZ1	ENSG00000108175	10	80818792	81086276	340	58	123834	0.70729	0.23969	0.92212459
UBE3C	ENSG00000009335	7	156921607	157072066	338	13	123834	0.70726	0.2397	0.92212459
FAM120B	ENSG00000112584	6	170589791	170726153	288	14	123834	0.70681	0.23984	0.922416534
MTCH1	ENSG00000137409	6	36925917	36964074	67	10	123834	0.7065	0.23994	0.922416534
DHRS9	ENSG00000073737	2	169911299	169962677	7	3	123834	0.70645	0.23995	0.922416534

CYP20A1	ENSG00000119004	2	204093663	204173009	119	10	123834	0.70641	0.23997	0.922416534
FAT4	ENSG00000196159	4	126227554	126424087	349	29	123834	0.70623	0.24002	0.922422192
MYT1	ENSG00000196132	20	62773144	62883604	137	28	123834	0.70573	0.24018	0.922648013
BAK1	ENSG00000030110	6	33530329	33558019	63	11	123834	0.70555	0.24023	0.922648013
ABR	ENSG00000159842	17	896758	1142315	286	54	123834	0.70549	0.24025	0.922648013
INO80B	ENSG00000115274	2	74672150	74698011	10	2	123834	0.70526	0.24032	0.922648013
C4orf50	ENSG00000181215	4	5948845	6001545	143	20	123834	0.70514	0.24036	0.922648013
HMOX2	ENSG00000103415	16	4514691	4570348	138	7	123834	0.70512	0.24037	0.922648013
IPP	ENSG00000197429	1	46149996	46226322	14	5	123834	0.70488	0.24044	0.922697586
DTD1	ENSG00000125821	20	18558537	18754561	574	19	123834	0.70475	0.24048	0.922697586
ACBD7	ENSG00000176244	10	15109522	15140775	91	16	123834	0.70406	0.2407	0.923355318
RP11-162A12.2	ENSG00000264278	18	74496688	74544251	67	13	123834	0.7038	0.24078	0.923403905
KLHL3	ENSG00000146021	5	136943189	137081779	194	17	123834	0.70366	0.24082	0.923403905
SLC41A3	ENSG00000114544	3	125715198	125830404	343	14	123834	0.70345	0.24089	0.923403905
B3GNT1	ENSG00000174684	11	66102843	66125163	27	4	123834	0.7031	0.241	0.923403905
ATP2A1	ENSG00000196296	16	28879726	28925830	29	2	123834	0.7031	0.241	0.923403905
HIPK2	ENSG00000064393	7	139236316	139487577	229	24	123834	0.70301	0.24102	0.923403905
SLC29A4	ENSG00000164638	7	5304000	5356501	52	12	123834	0.70198	0.24135	0.923403905
ZNF770	ENSG00000198146	15	35260542	35290488	60	10	123834	0.70196	0.24135	0.923403905
TNRC18	ENSG00000182095	7	5336421	5475045	87	26	123834	0.7016	0.24146	0.923403905
KLF15	ENSG00000163884	3	126051478	126086285	25	5	123834	0.70135	0.24154	0.923403905
MORN2	ENSG00000188010	2	39093103	39166213	78	8	123834	0.70118	0.2416	0.923403905
IGIP	ENSG00000182700	5	139495521	139518391	3	1	123834	0.70117	0.2416	0.923403905
C9orf16	ENSG00000171159	9	130912539	130936207	8	2	123834	0.7011	0.24162	0.923403905
KRTAP21-3	ENSG00000231068	21	32080843	32101095	56	1	123834	0.70111	0.24162	0.923403905
C14orf177	ENSG00000176605	14	99167950	99194098	65	8	123834	0.70097	0.24166	0.923403905
RCN3	ENSG00000142552	19	50020875	50060219	81	2	123834	0.70094	0.24167	0.923403905
SEL1L2	ENSG00000101251	20	13819893	13987089	383	34	123834	0.70093	0.24167	0.923403905
APOL1	ENSG00000100342	22	36639056	36673576	46	10	123834	0.70077	0.24172	0.923403905
OR5B3	ENSG00000172769	11	58159937	58180882	89	4	123834	0.70075	0.24173	0.923403905
ACSL3	ENSG00000123983	2	223715652	223819357	174	14	123834	0.70053	0.2418	0.923403905
PSG1	ENSG00000231924	19	43360616	43393974	100	5	123834	0.70054	0.2418	0.923403905
TAAR1	ENSG00000146399	6	132956123	132977142	102	13	123834	0.70045	0.24182	0.923403905
DDX23	ENSG00000174243	12	49213547	49256625	28	4	123834	0.70042	0.24183	0.923403905
IQGAP3	ENSG00000183856	1	156485197	156552396	89	10	123834	0.69998	0.24197	0.923752914
ENTHD2	ENSG00000167302	17	79192077	79222891	35	7	123834	0.6998	0.24203	0.923763614
LRRC8B	ENSG00000197147	1	89980395	90073423	163	11	123834	0.69965	0.24207	0.923763614
RNF138	ENSG00000134758	18	29661818	29721524	30	5	123834	0.69891	0.2423	0.924455721
CRTAM	ENSG00000109943	11	122699208	122753347	70	12	123834	0.69862	0.24239	0.92461351
EIF2B5	ENSG00000145191	3	183842826	184412546	878	102	123834	0.69843	0.24245	0.924656822
CAMP	ENSG00000164047	3	48254837	48276981	16	4	123834	0.69808	0.24256	0.924890768
AMBN	ENSG00000178522	4	71447973	71483005	39	8	123834	0.69758	0.24272	0.925151467
BACE2	ENSG00000182240	21	42529728	42664445	155	34	123834	0.69755	0.24273	0.925151467
VIP	ENSG00000146469	6	153061933	153090900	21	5	123834	0.69717	0.24285	0.925151467
UNC13B	ENSG00000198722	9	35151999	35415335	270	15	123834	0.69712	0.24286	0.925151467
CCDC28B	ENSG00000160050	1	32655987	32680988	19	2	123834	0.69697	0.24291	0.925151467
CCKAR	ENSG00000163394	4	26473022	26502084	59	7	123834	0.6969	0.24293	0.925151467
CDRT15L2	ENSG00000214819	17	20473037	20494224	1	1	123834	0.69636	0.2431	0.925151467
TMEM39B	ENSG00000121775	1	32527632	32578467	41	3	123834	0.6963	0.24312	0.925151467
ZUFSP	ENSG00000153975	6	116946781	116999957	121	11	123834	0.69628	0.24313	0.925151467
CPSF1	ENSG00000071894	8	145608444	145644753	22	3	123834	0.69618	0.24316	0.925151467
ELSPBP1	ENSG00000169393	19	48487908	48538410	155	14	123834	0.69592	0.24324	0.925151467
OR10J3	ENSG00000196266	1	159273460	159294449	39	7	123834	0.69582	0.24327	0.925151467
DLX6	ENSG00000006377	7	96624860	96650351	15	3	123834	0.69561	0.24334	0.925151467
HAUS6	ENSG00000147874	9	19043141	19113117	204	15	123834	0.69548	0.24338	0.925151467
FTCD	ENSG00000160282	21	47546176	47585481	85	15	123834	0.69498	0.24354	0.925151467
INPP5K	ENSG00000132376	17	1387865	1430182	80	7	123834	0.69488	0.24356	0.925151467
PLA2G12A	ENSG00000123739	4	110621145	110661233	37	5	123834	0.69486	0.24357	0.925151467
CORT	ENSG00000241563	1	10499776	10521959	21	2	123834	0.69477	0.2436	0.925151467
TPMT	ENSG00000137364	6	18118542	18165305	99	11	123834	0.69442	0.24371	0.925151467
RAG1	ENSG00000166349	11	36522259	36624706	134	11	123834	0.69435	0.24373	0.925151467
YBX2	ENSG00000006047	17	7181571	7207934	18	6	123834	0.69394	0.24386	0.925151467
HIST1H1C	ENSG00000187837	6	26045968	26066699	31	5	123834	0.69379	0.24391	0.925151467
HOXB9	ENSG00000170689	17	46688518	46713839	60	13	123834	0.69367	0.24394	0.925151467
CCNJL	ENSG00000135083	5	159668659	159782403	159	21	123834	0.69355	0.24398	0.925151467
SPTSSB	ENSG00000196542	3	161052580	161100668	102	11	123834	0.69258	0.24429	0.925151467
CAMK2A	ENSG00000070808	5	149589054	149679854	71	14	123834	0.69243	0.24433	0.925151467
TGM1	ENSG00000092295	14	24708320	24743638	37	9	123834	0.69237	0.24435	0.925151467
RASGRF1	ENSG00000058335	15	79242289	79393115	156	31	123834	0.69233	0.24437	0.925151467
CUX1	ENSG00000257923	7	101448959	101937249	671	41	123834	0.69217	0.24442	0.925151467
METTL2B	ENSG00000165055	7	128106783	128156656	136	18	123834	0.69213	0.24443	0.925151467
DZIP1	ENSG00000134874	13	96220457	96306957	84	14	123834	0.6921	0.24444	0.925151467
G6PC3	ENSG00000141349	17	42138103	42163709	19	4	123834	0.6919	0.2445	0.925151467
CCR7	ENSG00000126353	17	38700021	38731724	11	2	123834	0.69171	0.24456	0.925151467
JUN	ENSG00000177606	1	59236465	59259785	26	4	123834	0.69153	0.24462	0.925151467
LCE2C	ENSG00000187180	1	152637771	152659050	33	3	123834	0.69142	0.24465	0.925151467
RANBP10	ENSG00000141084	16	67747005	67850555	78	6	123834	0.69141	0.24465	0.925151467
SCRG1	ENSG00000164106	4	174295852	174337531	30	5	123834	0.69137	0.24467	0.925151467
NEK5	ENSG00000197168	13	52601093	52713214	207	15	123834	0.6912	0.24472	0.925151467
FOXS1	ENSG00000179772	20	30422103	30443420	20	3	123834	0.69121	0.24472	0.925151467
HMGCLL1	ENSG00000146151	6	55289167	55454012	534	23	123834	0.69117	0.24473	0.925151467
ZNF264	ENSG00000083844	19	57692868	57734724	71	10	123834	0.69118	0.24473	0.925151467
GALNT3	ENSG00000115339	2	166594101	166661192	44	9	123834	0.69114	0.24474	0.925151467
LYG2	ENSG00000185674	2	99848709	99881745	41	8	123834	0.69105	0.24477	0.925151467
OR1A2	ENSG00000172150	17	3090813	3111742	27	7	123834	0.69088	0.24482	0.925151467
TRABD2A	ENSG00000186854	2	85038774	85144132	212	15	123834	0.69068	0.24488	0.925151467
SIRPA	ENSG00000198053	20	1865154	1930543	74	11	123834	0.69062	0.2449	0.925151467
CPPED1	ENSG00000103381	16	12746919	12907874	318	29	123834	0.68989	0.24513	0.925151467
MTRNR2L9	ENSG00000255633	6	62274008	62294534	128	8	123834	0.68983	0.24515	0.925151467
ACTL6A	ENSG00000136518	3	179270668	179316196	55	5	123834	0.68951	0.24525	0.925151467
GPM6A	ENSG00000150625	4	176544085	176933815	562	42	123834	0.68946	0.24527	0.925151467
SLC25A48	ENSG00000145832	5	135160338	135234326	198	20	123834	0.68938	0.24529	0.925151467
COLGALT2	ENSG00000198756	1	183888796	184016863	179	25	123834	0.68928	0.24532	0.925151467
AK2	ENSG00000004455	1	33463585	33556597	81	4	123834	0.68907	0.24539	0.925151467
SIRT3	ENSG00000142082	11	205458	246931	140	13	123834	0.68899	0.24541	0.925151467
RNF175	ENSG00000145428	4	154621277	154691387	124	7	123834	0.6889	0.24544	0.925151467
TRIM16	ENSG00000221926	17	15521274	15597625	98	16	123834	0.68891	0.24544	0.925151467
CTD-2600O9.1	ENSG00000187185	16	57822098	57860850	80	15	123834	0.68887	0.24545	0.925151467
NME8	ENSG00000086288	7	37878199	37950003	281	24	123834	0.68884	0.24546	0.925151467

ARHGAP5	ENSG00000100852	14	32535320	32638934	18	7	123834	0.68873	0.2455	0.925151467
ADRA2C	ENSG00000184160	4	3758075	3780251	28	8	123834	0.68814	0.24568	0.925244012
CSRP2BP	ENSG00000149474	20	18108762	18179031	150	24	123834	0.68777	0.2458	0.925244012
TMEM19	ENSG00000139291	12	72069867	72107836	38	5	123834	0.68773	0.24581	0.925244012
AC003101.1	ENSG00000228768	17	29888161	29912678	27	7	123834	0.68774	0.24581	0.925244012
RP11-520P18.5	ENSG00000261667	8	144151874	144174418	26	4	123834	0.68766	0.24583	0.925244012
B3GNT3	ENSG00000179913	19	17895637	17933891	32	7	123834	0.68751	0.24588	0.925244012
LRRTM1	ENSG00000162951	2	80505483	80541874	23	8	123834	0.68726	0.24596	0.925244012
DHRS4	ENSG00000157326	14	24412795	24448488	106	2	123834	0.68727	0.24596	0.925244012
ITIH4	ENSG00000055955	3	52836991	52875495	52	5	123834	0.68712	0.246	0.925244012
ZCCHC17	ENSG00000121766	1	31759842	31847783	179	8	123834	0.68702	0.24604	0.925244012
SNUPN	ENSG00000169371	15	75880424	75928810	55	4	123834	0.68694	0.24606	0.925244012
SLX4IP	ENSG00000149346	20	10405951	10627477	395	23	123834	0.68626	0.24627	0.925634733
CPSF3	ENSG00000119203	2	9553697	9623230	99	6	123834	0.68613	0.24631	0.925634733
EEFSEC	ENSG00000132394	3	127862297	128137485	381	12	123834	0.68614	0.24631	0.925634733
EMX2	ENSG00000170370	10	119291955	119319056	12	3	123834	0.68582	0.24641	0.925654303
AARD	ENSG00000205002	8	117940438	117966726	20	5	123834	0.68576	0.24643	0.925654303
UGT1A8	ENSG00000241635	2	234516291	234691956	425	28	123834	0.68564	0.24647	0.925654303
PTCHD2	ENSG00000204624	1	11529223	11607641	101	10	123834	0.6855	0.24651	0.925654303
NHLH1	ENSG00000171786	1	160326857	160352638	25	5	123834	0.68486	0.24672	0.926088312
TOP2A	ENSG00000131747	17	38534768	38584202	25	3	123834	0.6848	0.24674	0.926088312
VIPR2	ENSG00000106018	7	158810866	158947649	312	20	123834	0.68435	0.24688	0.926088312
AKT2	ENSG00000105221	19	40726224	40801443	100	11	123834	0.68428	0.2469	0.926088312
ADC	ENSG00000142920	1	33536705	33596131	49	4	123834	0.68402	0.24698	0.926088312
CD200R1L	ENSG00000206531	3	112524556	112575703	188	20	123834	0.68379	0.24706	0.926088312
TMEM216	ENSG00000187049	11	61149159	61176335	8	2	123834	0.68332	0.2472	0.926088312
C19orf80	ENSG00000130173	19	11338178	11362619	22	5	123834	0.6832	0.24724	0.926088312
ULBP3	ENSG00000131019	6	150374286	150400231	53	7	123834	0.68306	0.24728	0.926088312
ZNF638	ENSG00000075292	2	71493691	71672199	445	22	123834	0.68306	0.24729	0.926088312
TNXB	ENSG00000168477	6	31998931	32093111	102	23	123834	0.68304	0.24729	0.926088312
LRIT3	ENSG00000183423	4	110759358	110803471	65	6	123834	0.68283	0.24736	0.926088312
CEACAM8	ENSG00000124469	19	43074393	43109207	13	2	123834	0.68278	0.24737	0.926088312
LCE1C	ENSG00000197084	1	152767311	152789107	30	5	123834	0.68262	0.24742	0.926088312
VPS13B	ENSG00000132549	8	100015494	100899808	1217	15	123834	0.6826	0.24743	0.926088312
RHOU	ENSG00000116574	1	228860824	228892416	76	9	123834	0.68232	0.24752	0.926088312
DSPP	ENSG00000152591	4	88519681	88548062	37	6	123834	0.6821	0.24759	0.926088312
NR3C2	ENSG00000151623	4	148989913	149375850	464	51	123834	0.68209	0.24759	0.926088312
PLEKHH1	ENSG00000054690	14	67990018	68066329	135	23	123834	0.68206	0.2476	0.926088312
TVP23A	ENSG00000166676	16	10844776	10922651	51	8	123834	0.68207	0.2476	0.926088312
NOP2	ENSG00000111641	12	6656029	6687857	17	3	123834	0.68184	0.24767	0.926102655
ANKRD42	ENSG00000137494	11	82894781	82981736	130	12	123834	0.68173	0.24771	0.926102655
KRT19	ENSG00000171345	17	39669869	39694560	33	1	123834	0.68158	0.24775	0.926102655
MTBP	ENSG00000172167	8	121447640	121564373	211	9	123834	0.68123	0.24786	0.926331671
PRMT5	ENSG00000100462	14	23379720	23408794	71	6	123834	0.68084	0.24799	0.926569967
HCN1	ENSG00000164588	5	45249349	45706253	353	19	123834	0.68063	0.24805	0.926569967
CLDN7	ENSG00000181885	17	7153222	7177302	23	4	123834	0.68057	0.24807	0.926569967
CSTF3	ENSG00000176102	11	33088734	33193917	186	11	123834	0.68041	0.24812	0.926574648
MIS12	ENSG00000167842	17	5379605	5404134	77	7	123834	0.68028	0.24817	0.926579328
NFYC	ENSG00000066136	1	41147320	41247275	194	14	123834	0.67986	0.2483	0.926579518
LRP1	ENSG00000123384	12	57512276	57617134	49	5	123834	0.67975	0.24833	0.926579518
INSM2	ENSG00000168348	14	35993248	36016260	4	2	123834	0.6797	0.24835	0.926579518
C1QTNF1	ENSG00000173918	17	77008896	77055870	66	5	123834	0.67937	0.24845	0.926579518
BRD3	ENSG00000169925	9	136885427	136943657	135	11	123834	0.67924	0.24849	0.926579518
PRLH	ENSG00000071677	2	238465217	238485818	64	6	123834	0.67908	0.24854	0.926579518
FAM175B	ENSG00000165660	10	126480354	126535239	72	9	123834	0.67891	0.2486	0.926579518
TCF3	ENSG00000071564	19	1599291	1662604	32	7	123834	0.67888	0.24861	0.926579518
ELAVL1	ENSG00000066044	19	8013463	8080543	107	8	123834	0.6788	0.24863	0.926579518
SVIL	ENSG00000197321	10	29736267	30035710	735	63	123834	0.67845	0.24874	0.926579518
TARSL2	ENSG00000185418	15	102183801	102274807	117	13	123834	0.67843	0.24875	0.926579518
DNASE2	ENSG00000105612	19	12976025	13002282	30	4	123834	0.67818	0.24883	0.926579518
SACS	ENSG00000151835	13	23892965	24017841	180	31	123834	0.67814	0.24884	0.926579518
GABRA2	ENSG00000151834	4	46240444	46487247	367	17	123834	0.6779	0.24892	0.926579518
CYP8B1	ENSG00000180432	3	42887497	42927633	58	7	123834	0.67782	0.24894	0.926579518
MEX3C	ENSG00000176624	18	48690920	48754674	99	6	123834	0.67781	0.24895	0.926579518
NDUFA8	ENSG00000119421	9	124884745	124932098	89	11	123834	0.67757	0.24902	0.926607724
RB1CC1	ENSG00000023287	8	53525016	53668403	92	12	123834	0.67733	0.2491	0.926607724
NFRKB	ENSG00000170322	11	129723531	129775485	56	7	123834	0.67683	0.24926	0.926607724
BCL7B	ENSG00000106635	7	72940686	72982332	59	6	123834	0.67659	0.24933	0.926607724
CYB5R4	ENSG00000065615	6	84559362	84687143	200	7	123834	0.67624	0.24944	0.926607724
AP1B1	ENSG00000100280	22	29713669	29829168	154	18	123834	0.6759	0.24955	0.926607724
MRPL16	ENSG00000166902	11	59563608	59588345	47	6	123834	0.67578	0.24959	0.926607724
TFAP2B	ENSG00000008196	6	50776436	50825326	79	12	123834	0.67555	0.24966	0.926607724
CTD-2510F5.6	ENSG00000265303	17	57264927	57302587	57	5	123834	0.67554	0.24967	0.926607724
SCAI	ENSG00000173611	9	127694887	127915785	262	12	123834	0.67527	0.24975	0.926607724
SCN2A	ENSG00000136531	2	166085912	166258818	321	22	123834	0.67514	0.24979	0.926607724
EML5	ENSG00000165521	14	89068775	89269096	275	12	123834	0.67513	0.2498	0.926607724
MISP	ENSG00000099812	19	741126	774319	17	6	123834	0.67507	0.24982	0.926607724
FPGT	ENSG00000254685	1	74653896	74709770	107	7	123834	0.6749	0.24987	0.926607724
C2orf66	ENSG00000187944	2	197659726	197685000	19	2	123834	0.67472	0.24993	0.926607724
KLF6	ENSG00000067082	10	3808188	3837473	24	4	123834	0.67469	0.24994	0.926607724
CD55	ENSG00000196352	1	207484853	207544311	78	6	123834	0.67449	0.25	0.926607724
GFOD2	ENSG00000141098	16	67698434	67763324	45	5	123834	0.67448	0.25	0.926607724
PUS7	ENSG00000091127	7	105070108	105172714	242	13	123834	0.67435	0.25004	0.926607724
PP13439	ENSG00000183657	3	171499580	171537714	75	9	123834	0.67411	0.25012	0.926607724
RAB17	ENSG00000124839	2	238472965	238520257	136	16	123834	0.67407	0.25013	0.926607724
VASP	ENSG00000125753	19	45999837	46040241	53	9	123834	0.6739	0.25019	0.926607724
DGKI	ENSG00000157680	7	137055783	137541838	337	31	123834	0.67382	0.25021	0.926607724
NANOGNB	ENSG00000205857	12	7907812	7936717	11	2	123834	0.67384	0.25021	0.926607724
DCBLD2	ENSG00000057019	3	98504785	98630533	265	13	123834	0.67342	0.25034	0.926607724
PRLR	ENSG00000113494	5	35038861	35240794	371	26	123834	0.67327	0.25039	0.926607724
UFM1	ENSG00000120686	13	38913986	38947140	41	1	123834	0.67318	0.25042	0.926607724
PCDHB11	ENSG00000197479	5	140569183	140592618	44	7	123834	0.67311	0.25044	0.926607724
LRWD1	ENSG00000161036	7	102095376	102123615	66	8	123834	0.67307	0.25045	0.926607724
PRSS12	ENSG00000164099	4	119191193	119284158	170	21	123834	0.67293	0.25049	0.926607724
RP1-66C13.4	ENSG00000266202	17	26115832	26230391	102	8	123834	0.67288	0.25051	0.926607724
SYF2	ENSG00000117614	1	25539170	25568993	3	2	123834	0.67256	0.25061	0.926607724
DUSP15	ENSG00000149599	20	30425440	30468550	48	11	123834	0.67236	0.25068	0.926607724
FAM133B	ENSG00000234545	7	92180107	92229708	73	10	123834	0.67231	0.25069	0.926607724
PTPN3	ENSG00000070159	9	112127746	112270590	217	19	123834	0.67224	0.25072	0.926607724

SAMD13	ENSG00000203943	1	84754049	84865640	101	15	123834	0.67203	0.25078	0.926607724
RSBN1L	ENSG00000187257	7	77315760	77422339	212	16	123834	0.67198	0.2508	0.926607724
KLK2	ENSG00000167751	19	51354824	51393823	74	13	123834	0.67168	0.25089	0.926607724
LOXL4	ENSG00000138131	10	99997447	100038007	47	5	123834	0.67156	0.25093	0.926607724
CAPS2	ENSG00000180881	12	75659759	75794708	127	13	123834	0.67113	0.25107	0.926607724
TRIM77	ENSG00000214414	11	89433467	89461040	44	2	123834	0.671	0.25111	0.926607724
KCNE3	ENSG00000175538	11	74155886	74188774	41	8	123834	0.67098	0.25112	0.926607724
DIO2	ENSG00000211448	14	80653873	80864100	128	14	123834	0.67089	0.25114	0.926607724
SHC1	ENSG00000160691	1	154924774	154956871	11	1	123834	0.67084	0.25116	0.926607724
HSD17B6	ENSG00000025423	12	57135945	57191574	26	6	123834	0.6708	0.25117	0.926607724
NCOA1	ENSG00000084676	2	24704783	25003571	516	20	123834	0.67073	0.2512	0.926607724
OR11L1	ENSG00000197591	1	247994199	248015254	70	8	123834	0.6705	0.25127	0.926653827
KCNQ5	ENSG00000185760	6	73321520	73918574	950	70	123834	0.67037	0.25131	0.926653827
NFKBIB	ENSG00000104825	19	39380340	39409533	29	6	123834	0.66971	0.25152	0.927108404
NPR1	ENSG00000169418	1	153641113	153676468	26	4	123834	0.66961	0.25155	0.927108404
MYBL2	ENSG00000101057	20	42285754	42355136	120	7	123834	0.66925	0.25167	0.927108404
NBAS	ENSG00000151779	2	15297032	15711454	704	21	123834	0.66896	0.25176	0.927108404
PSEN2	ENSG00000143801	1	227047885	227093806	68	7	123834	0.6688	0.25181	0.927108404
ZC3H12A	ENSG00000163874	1	37930153	37959980	21	4	123834	0.66861	0.25187	0.927108404
PDZD8	ENSG00000165650	10	119030000	119144978	240	11	123834	0.66822	0.252	0.927108404
BCAS4	ENSG00000124243	20	49401431	49503714	197	24	123834	0.6682	0.252	0.927108404
FAM169A	ENSG00000198780	5	74063399	74172776	157	8	123834	0.66817	0.25201	0.927108404
KLK4	ENSG00000167749	19	51399608	51423994	21	6	123834	0.66808	0.25204	0.927108404
KRBOX1	ENSG00000240747	3	42840975	42994284	176	21	123834	0.66796	0.25208	0.927108404
NRG2	ENSG00000158458	5	139216364	139432884	190	21	123834	0.66787	0.25211	0.927108404
EZR	ENSG00000092820	6	159176773	159250444	213	13	123834	0.66764	0.25218	0.927108404
CDIPT	ENSG00000103502	16	29859678	29885057	4	1	123834	0.66736	0.25227	0.927108404
TRIM16L	ENSG00000108448	17	18591311	18649431	48	10	123834	0.66735	0.25227	0.927108404
UGT1A9	ENSG00000241119	2	234570499	234691946	327	21	123834	0.66721	0.25232	0.927108404
MALSU1	ENSG00000156928	7	23328358	23361348	48	4	123834	0.6672	0.25232	0.927108404
CPA2	ENSG00000158516	7	129896667	129939638	48	6	123834	0.66719	0.25233	0.927108404
NT5DC3	ENSG00000111696	12	104154231	104244975	228	20	123834	0.66707	0.25236	0.927108404
AQP3	ENSG00000165272	9	33431152	33457609	44	8	123834	0.6669	0.25242	0.927149635
CGB1	ENSG00000267631	19	49528831	49562006	33	4	123834	0.66663	0.2525	0.927264297
ANG	ENSG00000214274	14	21142336	21177130	54	10	123834	0.66622	0.25263	0.927562496
IGJ	ENSG00000132465	4	71511258	71557534	33	6	123834	0.66586	0.25275	0.927624088
NCOA5	ENSG00000124160	20	44679624	44728591	71	8	123834	0.66574	0.25279	0.927624088
KBTBD7	ENSG00000120696	13	41753969	41778702	13	3	123834	0.66548	0.25287	0.927624088
TMPRSS2	ENSG00000184012	21	42826478	42913043	145	21	123834	0.66528	0.25294	0.927624088
PITPNA	ENSG00000174238	17	1411012	1476110	124	10	123834	0.66522	0.25295	0.927624088
RINT1	ENSG00000135249	7	105162532	105218124	144	6	123834	0.66473	0.25311	0.927624088
PPAPDC3	ENSG00000160539	9	134155081	134194649	57	4	123834	0.66462	0.25315	0.927624088
NADK	ENSG00000008130	1	1672671	1721896	48	7	123834	0.66451	0.25318	0.927624088
C17orf104	ENSG00000180336	17	42723762	42777676	56	9	123834	0.6645	0.25318	0.927624088
VRK1	ENSG00000100749	14	97253641	97408059	281	19	123834	0.66425	0.25326	0.927624088
RWDD2B	ENSG00000156253	21	30366705	30401699	52	7	123834	0.66425	0.25326	0.927624088
ERCC1	ENSG00000012061	19	45900591	45992086	114	19	123834	0.66419	0.25328	0.927624088
SCG5	ENSG00000166922	15	32923877	32999299	94	25	123834	0.66409	0.25332	0.927624088
SIN3A	ENSG00000169375	15	75651720	75758183	64	6	123834	0.66405	0.25333	0.927624088
FOSL1	ENSG00000175592	11	65649520	65678044	26	5	123834	0.66363	0.25346	0.927861159
AL589765.1	ENSG00000240510	1	151672909	151712070	38	6	123834	0.66344	0.25352	0.927861159
C11orf1	ENSG00000137720	11	111739659	111766699	29	3	123834	0.66323	0.25359	0.927861159
TEF	ENSG00000167074	22	41753337	41805330	61	3	123834	0.66324	0.25359	0.927861159
ARHGEF5	ENSG00000050327	7	144042381	144087725	8	1	123834	0.66294	0.25369	0.927864179
CRYGA	ENSG00000168582	2	209015464	209038300	92	13	123834	0.66282	0.25372	0.927864179
WBP1	ENSG00000239779	2	74675456	74698018	9	2	123834	0.66256	0.25381	0.927864179
CTB-58E17.5	ENSG00000227450	17	36895613	36916969	18	8	123834	0.662	0.25398	0.927864179
HES7	ENSG00000179111	17	8013908	8037410	24	5	123834	0.66196	0.254	0.927864179
IFNA6	ENSG00000120235	9	21339834	21361377	52	4	123834	0.66187	0.25403	0.927864179
THNSL1	ENSG00000185875	10	25295587	25325593	59	4	123834	0.66176	0.25406	0.927864179
MT1H	ENSG00000205358	16	56693726	56715041	30	7	123834	0.66152	0.25414	0.927864179
KLHL9	ENSG00000198642	9	21319670	21345379	39	3	123834	0.66146	0.25416	0.927864179
IFNL3	ENSG00000197110	19	39724246	39745646	30	4	123834	0.66138	0.25418	0.927864179
RNF180	ENSG00000164197	5	63451671	63678696	242	13	123834	0.66125	0.25423	0.927864179
ABHD12	ENSG00000100997	20	25265379	25381619	237	7	123834	0.66114	0.25426	0.927864179
TSSK2	ENSG00000206203	22	19108321	19130134	30	5	123834	0.66088	0.25435	0.927864179
DCUN1D2	ENSG00000150401	13	114100134	114155267	130	12	123834	0.66039	0.2545	0.927864179
PITPNB	ENSG00000180957	22	28237657	28326122	116	13	123834	0.66032	0.25453	0.927864179
SPPL3	ENSG00000157837	12	121190313	121352174	423	21	123834	0.66016	0.25457	0.927864179
CTD-2215E18.1	ENSG00000251606	5	96414779	96529354	141	24	123834	0.66006	0.25461	0.927864179
CGB5	ENSG00000189052	19	49537141	49558568	18	2	123834	0.65994	0.25465	0.927864179
HIST1H4A	ENSG00000196176	6	26011907	26032278	28	7	123834	0.65987	0.25467	0.927864179
RGS3	ENSG00000138835	9	116197011	116370018	357	11	123834	0.6597	0.25472	0.927864179
HIST1H2BJ	ENSG00000124635	6	27083676	27110541	28	9	123834	0.65959	0.25476	0.927864179
TEX2	ENSG00000136478	17	62214587	62350661	256	12	123834	0.65941	0.25482	0.927864179
MPRIP	ENSG00000133030	17	16935859	17130993	342	21	123834	0.65938	0.25483	0.927864179
NLRP4	ENSG00000160505	19	56337944	56403220	116	24	123834	0.65934	0.25484	0.927864179
TAS2R7	ENSG00000121377	12	10944131	10965226	15	2	123834	0.6593	0.25485	0.927864179
ATRNL1	ENSG00000107518	10	116843124	117718503	1147	22	123834	0.65927	0.25486	0.927864179
IQSEC3	ENSG00000120645	12	165931	297626	177	33	123834	0.65904	0.25493	0.927926413
NPY1R	ENSG00000164128	4	164235113	164275984	71	12	123834	0.65888	0.25499	0.927926413
ZNF318	ENSG00000171467	6	43264872	43347216	90	9	123834	0.65873	0.25503	0.927926413
SHMT2	ENSG00000182199	12	57613110	57638718	4	2	123834	0.6586	0.25508	0.927926413
GSX1	ENSG00000169840	13	28356780	28378905	29	4	123834	0.6583	0.25517	0.927926413
CLDN10	ENSG00000134873	13	96075858	96242013	408	22	123834	0.65831	0.25517	0.927926413
PPP1R14C	ENSG00000198729	6	150454212	150581493	217	33	123834	0.65803	0.25526	0.928076144
TERT	ENSG00000164362	5	1243262	1305184	25	9	123834	0.65788	0.25531	0.928080413
CLIP2	ENSG00000106665	7	73693805	73830273	168	10	123834	0.65751	0.25543	0.928234296
NKX2-5	ENSG00000183072	5	172649112	172672360	8	4	123834	0.65742	0.25545	0.928234296
SH3BGR	ENSG00000185437	21	40807781	40897433	276	12	123834	0.65707	0.25557	0.928417466
WDR93	ENSG00000140527	15	90224028	90296869	153	8	123834	0.6566	0.25572	0.928417466
MRPL40	ENSG00000185608	22	19409425	19433598	36	7	123834	0.65661	0.25572	0.928417466
CLCF1	ENSG00000175505	11	67121639	67151648	30	3	123834	0.65647	0.25576	0.928417466
C11orf88	ENSG00000183644	11	111375510	111417756	33	3	123834	0.65626	0.25583	0.928417466
CCNG1	ENSG00000113328	5	162854575	162883157	20	5	123834	0.65619	0.25585	0.928417466
AC112205.1	ENSG00000268800	5	162856447	162883821	20	5	123834	0.65619	0.25585	0.928417466
RBFOX1	ENSG00000078328	16	6059095	7773340	4186	328	123834	0.65605	0.2559	0.928417466
NCOA7	ENSG00000111912	6	126092307	126262266	147	17	123834	0.6559	0.25594	0.928417466
MFSD1	ENSG00000118855	3	158439987	158557508	218	20	123834	0.65577	0.25599	0.92842166

CBX8	ENSG00000141570	17	77755931	77785482	14	6	123834	0.65552	0.25607	0.928534636
IFNW1	ENSG00000177047	9	21130631	21152144	19	5	123834	0.65515	0.25619	0.928724211
MPPED1	ENSG00000186732	22	43797202	43913728	237	43	123834	0.65504	0.25622	0.928724211
CHD6	ENSG00000124177	20	40020741	40257133	394	20	123834	0.65471	0.25633	0.928804941
KRAS	ENSG00000133703	12	25347723	25413870	140	14	123834	0.65466	0.25634	0.928804941
AOC3	ENSG00000131471	17	40993201	41020147	3	2	123834	0.65398	0.25656	0.929360472
SLC27A3	ENSG00000143554	1	153736830	153762633	12	2	123834	0.65376	0.25663	0.929360472
RSPH3	ENSG00000130363	6	159383903	159431219	206	7	123834	0.65375	0.25664	0.929360472
PROK2	ENSG00000163421	3	71810807	71844357	33	10	123834	0.65309	0.25685	0.929890892
SIGMAR1	ENSG00000147955	9	34624719	34647806	25	5	123834	0.65294	0.2569	0.929890892
MEGF6	ENSG00000162591	1	3396484	3538059	205	39	123834	0.6528	0.25694	0.929890892
MYF6	ENSG00000111046	12	81091277	81113253	34	6	123834	0.65259	0.25701	0.929890892
RGL2	ENSG00000237441	6	33249431	33277101	25	6	123834	0.65238	0.25708	0.929890892
SLC25A45	ENSG00000162241	11	65132663	65161172	14	4	123834	0.65239	0.25708	0.929890892
PCDHB4	ENSG00000081818	5	140491581	140515201	25	3	123834	0.65138	0.2574	0.930583456
FUBP3	ENSG00000107164	9	133444352	133523739	120	16	123834	0.65085	0.25757	0.930583456
AHR	ENSG00000106546	7	17328246	17395776	19	5	123834	0.65078	0.25759	0.930583456
DRG2	ENSG00000108591	17	17981200	18021285	36	4	123834	0.65077	0.2576	0.930583456
SIRT1	ENSG00000096717	10	69634427	69688147	114	9	123834	0.65072	0.25761	0.930583456
H2AFJ	ENSG00000246705	12	14917270	14940936	36	8	123834	0.65074	0.25761	0.930583456
DHRS4-AS1	ENSG00000197775	14	24397940	24419196	76	1	123834	0.65065	0.25764	0.930583456
CPSF7	ENSG00000149532	11	61160121	61207503	23	1	123834	0.65024	0.25777	0.930583456
INSM1	ENSG00000173404	20	20338765	20361590	6	2	123834	0.65025	0.25777	0.930583456
FSHB	ENSG00000131808	11	30242563	30266808	42	3	123834	0.65014	0.2578	0.930583456
KCNMA1	ENSG00000156113	10	78619359	79408353	1232	110	123834	0.65011	0.25781	0.930583456
ADCK5	ENSG00000173137	8	145586790	145628457	28	3	123834	0.64915	0.25812	0.931431668
PAG1	ENSG00000076641	8	81870045	82034303	188	31	123834	0.64877	0.25824	0.931431668
C8orf82	ENSG00000213563	8	145741117	145764516	9	3	123834	0.6487	0.25827	0.931431668
EIF2B3	ENSG00000070785	1	45306450	45462282	214	7	123834	0.64867	0.25828	0.931431668
EPB41L3	ENSG00000082397	18	5382383	5640699	317	44	123834	0.64863	0.25829	0.931431668
SRF	ENSG00000112658	6	43129037	43159243	52	5	123834	0.64832	0.25839	0.931615539
CORO7-PAM16	ENSG00000103426	16	4380252	4480495	118	12	123834	0.64786	0.25854	0.931888988
NHSL1	ENSG00000135540	6	138733180	139023708	340	32	123834	0.64749	0.25866	0.931888988
F2R	ENSG00000181104	5	76001868	76041606	27	6	123834	0.64744	0.25867	0.931888988
CUL2	ENSG00000108094	10	35287479	35389570	184	6	123834	0.64725	0.25874	0.931888988
C20orf195	ENSG00000125531	20	62174373	62198061	20	8	123834	0.6472	0.25875	0.931888988
GRAMD1C	ENSG00000178075	3	113537029	113676021	86	19	123834	0.64718	0.25876	0.931888988
TMEM30B	ENSG00000182107	14	61734088	61758558	32	8	123834	0.64681	0.25888	0.932016743
SPRY1	ENSG00000164056	4	124307950	124334910	45	10	123834	0.64624	0.25906	0.932016743
PCDHB15	ENSG00000113248	5	140615147	140637799	41	6	123834	0.64618	0.25908	0.932016743
APOLD1	ENSG00000178878	12	12868851	12992909	110	23	123834	0.64581	0.2592	0.932016743
ZNHIT3	ENSG00000108278	17	34832473	34865154	47	8	123834	0.64572	0.25923	0.932016743
RNF7	ENSG00000114125	3	141447046	141476402	62	6	123834	0.64562	0.25926	0.932016743
COLEC11	ENSG00000118004	2	3632426	3702048	105	11	123834	0.64539	0.25934	0.932016743
YY1AP1	ENSG00000163374	1	155619237	155668791	29	4	123834	0.64532	0.25936	0.932016743
FICD	ENSG00000198855	12	108898962	108929614	61	9	123834	0.64524	0.25939	0.932016743
FAM188A	ENSG00000148481	10	15810169	15912519	57	5	123834	0.64511	0.25943	0.932016743
FAM189B	ENSG00000160767	1	155206996	155235274	36	2	123834	0.64498	0.25947	0.932016743
SPATA17	ENSG00000162814	1	217794666	218055038	505	24	123834	0.64489	0.2595	0.932016743
SQLE	ENSG00000104549	8	126000739	126044525	122	12	123834	0.64472	0.25955	0.932016743
NDST3	ENSG00000164100	4	118944773	119189803	647	21	123834	0.64471	0.25956	0.932016743
DBF4B	ENSG00000161692	17	42775976	42839632	83	13	123834	0.64466	0.25957	0.932016743
EREG	ENSG00000124882	4	75220860	75264468	121	9	123834	0.64465	0.25958	0.932016743
LINC01124	ENSG00000222033	2	171558961	171581077	11	5	123834	0.6442	0.25972	0.932343297
ATP6V0B	ENSG00000117410	1	44430159	44453967	21	4	123834	0.6434	0.25998	0.933035374
RPL28	ENSG00000108107	19	55886713	55924612	85	14	123834	0.64312	0.26007	0.933035374
SYT2	ENSG00000143858	1	202549724	202689545	142	22	123834	0.64293	0.26014	0.933035374
IGF2BP3	ENSG00000136231	7	23339828	23520086	228	25	123834	0.64284	0.26017	0.933035374
MRPS34	ENSG00000074071	16	1811891	1833156	26	5	123834	0.64262	0.26024	0.933035374
DERL2	ENSG00000072849	17	5364571	5400131	93	9	123834	0.64255	0.26026	0.933035374
MSTN	ENSG00000138379	2	190910423	190937455	40	4	123834	0.64243	0.2603	0.933035374
RP11-1055B8.6	ENSG00000263053	17	79359275	79385658	18	4	123834	0.64237	0.26032	0.933035374
SDC4	ENSG00000124145	20	43943928	43987064	52	9	123834	0.6422	0.26037	0.933035374
THAP2	ENSG00000173451	12	72046789	72084419	31	4	123834	0.64185	0.26048	0.933035374
LAMB4	ENSG00000091128	7	107653993	107780801	124	9	123834	0.64177	0.26051	0.933035374
CDK14	ENSG00000058091	7	90085738	90849905	1592	54	123834	0.64175	0.26052	0.933035374
RNASE4	ENSG00000258818	14	21142259	21178761	55	10	123834	0.64149	0.2606	0.933035374
AL163636.6	ENSG00000259171	14	21142746	21178735	55	10	123834	0.64149	0.2606	0.933035374
SLC30A4	ENSG00000104154	15	45761809	45825005	32	4	123834	0.64118	0.2607	0.933217627
KRT6B	ENSG00000185479	12	52830435	52855910	77	6	123834	0.64097	0.26077	0.933292442
RGS17	ENSG00000091844	6	153315594	153462384	355	16	123834	0.64017	0.26103	0.934047108
C9orf147	ENSG00000230185	9	115186096	115259484	188	8	123834	0.63984	0.26114	0.934264845
SECISBP2	ENSG00000187742	9	91923421	91984557	79	10	123834	0.63965	0.2612	0.934303651
ZBTB41	ENSG00000177888	1	197112810	197179672	69	7	123834	0.63931	0.26131	0.934503566
DARS2	ENSG00000117593	1	173783641	173837684	52	6	123834	0.63911	0.26137	0.934503566
RP11-293I14.2	ENSG00000258064	12	72057984	72102748	50	6	123834	0.639	0.26141	0.934503566
TAF5	ENSG00000148835	10	105117724	105158822	31	4	123834	0.6387	0.26151	0.934503566
FKBP15	ENSG00000119321	9	115913286	115993641	261	20	123834	0.63853	0.26156	0.934503566
RP11-438J1.1	ENSG00000272410	3	10281056	10337480	78	11	123834	0.63834	0.26163	0.934503566
IGFBP4	ENSG00000141753	17	38589702	38623983	28	5	123834	0.63807	0.26171	0.934503566
PDCD10	ENSG00000114209	3	167391086	167462727	24	3	123834	0.63795	0.26175	0.934503566
INSRR	ENSG00000027644	1	156799855	156838810	38	7	123834	0.63791	0.26177	0.934503566
CALB2	ENSG00000172137	16	71382616	71434341	63	9	123834	0.63789	0.26177	0.934503566
FAHD1	ENSG00000180185	16	1866968	1900208	245	7	123834	0.63773	0.26183	0.934503566
PRF1	ENSG00000180644	10	72347104	72372531	30	9	123834	0.63753	0.26189	0.934503566
PMCH	ENSG00000183395	12	102580237	102601623	31	6	123834	0.63743	0.26192	0.934503566
DR1	ENSG00000117505	1	93801445	93845055	68	5	123834	0.63701	0.26206	0.934503566
NXPE1	ENSG00000095110	11	114382437	114440617	135	5	123834	0.63686	0.26211	0.934503566
RIIAD1	ENSG00000178796	1	151672909	151712281	39	6	123834	0.6367	0.26216	0.934503566
MOS	ENSG00000172680	8	57015501	57036541	36	4	123834	0.63669	0.26216	0.934503566
DCLRE1B	ENSG00000118655	1	114437763	114466708	21	6	123834	0.63661	0.26219	0.934503566
OLFM2	ENSG00000105088	19	9954394	10057228	131	14	123834	0.6366	0.26219	0.934503566
MOGS	ENSG00000115275	2	74678184	74702537	8	2	123834	0.63637	0.26227	0.934613453
PCGF1	ENSG00000115289	2	74722170	74745707	16	2	123834	0.6355	0.26255	0.935435876
TXNDC16	ENSG00000087301	14	52887308	53029240	255	11	123834	0.63488	0.26275	0.935570559
CFHR5	ENSG00000134389	1	196936667	196988804	76	9	123834	0.63484	0.26277	0.935570559
PSG5	ENSG00000204941	19	43660408	43700688	144	14	123834	0.63451	0.26287	0.935570559
ALDH1B1	ENSG00000137124	9	38382661	38408658	36	13	123834	0.6344	0.26291	0.935570559
TNNT3	ENSG00000130595	11	1930792	1969936	40	12	123834	0.63425	0.26296	0.935570559

TPH2	ENSG00000139287	12	72322626	72590398	452	24	123834	0.63409	0.26301	0.935570559
UGT1A1	ENSG00000242366	2	234658894	234691945	74	15	123834	0.63406	0.26302	0.935570559
TINF2	ENSG00000092330	14	24698849	24721880	12	4	123834	0.63397	0.26305	0.935570559
FANCA	ENSG00000187741	16	89793957	89893065	370	14	123834	0.63397	0.26305	0.935570559
EPHB3	ENSG00000182580	3	184269572	184310197	82	14	123834	0.63388	0.26308	0.935570559
TMEM128	ENSG00000132406	4	4227269	4259950	108	6	123834	0.6336	0.26317	0.935715556
SEMA3G	ENSG00000010319	3	52457069	52489101	21	5	123834	0.63329	0.26327	0.935896046
ACTR8	ENSG00000113812	3	53891093	53926229	83	10	123834	0.63291	0.26339	0.936056344
AL139333.1	ENSG00000214479	6	83772692	83792854	29	3	123834	0.63278	0.26344	0.936056344
OR13C3	ENSG00000204246	9	107288030	107309137	42	8	123834	0.63239	0.26356	0.936056344
APLP2	ENSG00000084234	11	129929732	130024699	205	10	123834	0.63202	0.26369	0.936056344
POLE2	ENSG00000100479	14	50100273	50165140	80	4	123834	0.63197	0.2637	0.936056344
CCDC155	ENSG00000161609	19	49881475	49931251	99	5	123834	0.6318	0.26376	0.936056344
F5	ENSG00000198734	1	169473404	169565826	288	20	123834	0.63151	0.26385	0.936056344
DIP2C	ENSG00000151240	10	310130	745683	632	31	123834	0.63145	0.26387	0.936056344
DNAJC15	ENSG00000120675	13	43587339	43693045	143	5	123834	0.63141	0.26389	0.936056344
SLC25A28	ENSG00000155287	10	101360282	101390366	68	7	123834	0.63109	0.26399	0.936056344
ZNF175	ENSG00000105497	19	52064551	52102991	66	14	123834	0.63104	0.26401	0.936056344
BRMS1	ENSG00000174744	11	66094804	66122596	34	4	123834	0.63091	0.26405	0.936056344
CECR1	ENSG00000093072	22	17650194	17712879	105	20	123834	0.63063	0.26414	0.936056344
DNAH11	ENSG00000105877	7	21572833	21951457	903	79	123834	0.63061	0.26415	0.936056344
CFHR2	ENSG00000080910	1	196778898	196938356	144	10	123834	0.6305	0.26418	0.936056344
ANXA5	ENSG00000164111	4	122579110	122628268	147	14	123834	0.63038	0.26422	0.936056344
AL596220.1	ENSG00000268662	1	186354123	186375908	33	8	123834	0.63035	0.26423	0.936056344
MACF1	ENSG00000127603	1	39536988	39962849	588	21	123834	0.63029	0.26425	0.936056344
TCOF1	ENSG00000070814	5	149727202	149789871	62	6	123834	0.63018	0.26429	0.936056344
IQCC	ENSG00000160051	1	32661236	32684288	15	2	123834	0.63015	0.2643	0.936056344
DCST1	ENSG00000163357	1	154996300	155033406	27	6	123834	0.62954	0.2645	0.936385537
TBC1D2B	ENSG00000167202	15	78266378	78380066	300	19	123834	0.62934	0.26456	0.936385537
TOX3	ENSG00000103460	16	52461917	52591714	137	17	123834	0.62936	0.26456	0.936385537
ZNF208	ENSG00000160321	19	22105760	22203751	277	11	123834	0.62925	0.26459	0.936385537
PLA2G3	ENSG00000100078	22	31520795	31546593	33	9	123834	0.6289	0.26471	0.936525547
IL10RB	ENSG00000243646	21	34628663	34679539	74	9	123834	0.62842	0.26486	0.936525547
NGF	ENSG00000134259	1	115818539	115890857	131	29	123834	0.62834	0.26489	0.936525547
RP11-94B19.4	ENSG00000266614	18	73961121	73982069	35	3	123834	0.62835	0.26489	0.936525547
MEF2BNB	ENSG00000254901	19	19277712	19313400	75	8	123834	0.62826	0.26492	0.936525547
CTC-432M15.3	ENSG00000273217	5	130751584	131142676	421	10	123834	0.62811	0.26496	0.936525547
APBA1	ENSG00000107282	9	72032446	72297222	403	25	123834	0.62756	0.26515	0.936525547
FLT3LG	ENSG00000090554	19	49967464	49999488	46	5	123834	0.62746	0.26518	0.936525547
EMC1	ENSG00000127463	1	19532158	19588046	137	15	123834	0.62733	0.26522	0.936525547
ZFYVE27	ENSG00000155256	10	99486878	99530664	97	11	123834	0.62733	0.26522	0.936525547
UBL5	ENSG00000198258	19	9928568	9950791	11	2	123834	0.62698	0.26533	0.936525547
TATDN2	ENSG00000157014	3	10279707	10332902	76	10	123834	0.62694	0.26535	0.936525547
PPARD	ENSG00000112033	6	35300335	35405968	168	17	123834	0.62664	0.26545	0.936525547
PTPRJ	ENSG00000149177	11	47992113	48199670	270	14	123834	0.62601	0.26565	0.936525547
USP32	ENSG00000170832	17	58246455	58509831	155	4	123834	0.62566	0.26577	0.936525547
HIST1H3B	ENSG00000124693	6	26021817	26042288	25	6	123834	0.62551	0.26582	0.936525547
FAM84A	ENSG00000162981	2	14762810	14800933	13	1	123834	0.62524	0.26591	0.936525547
AC011897.1	ENSG00000265629	2	14765215	14800933	13	1	123834	0.62524	0.26591	0.936525547
DEFB133	ENSG00000214643	6	49903814	49927157	34	5	123834	0.62478	0.26606	0.936525547
PDZD3	ENSG00000172367	11	119046166	119070932	14	4	123834	0.62473	0.26607	0.936525547
SETBP1	ENSG00000152217	18	42250138	42658475	344	46	123834	0.62465	0.2661	0.936525547
DNAJC12	ENSG00000108176	10	69546427	69607924	97	12	123834	0.62463	0.26611	0.936525547
SLC2A3	ENSG00000059804	12	8061826	8098871	28	6	123834	0.62456	0.26613	0.936525547
C20orf197	ENSG00000176659	20	58620980	58658008	25	4	123834	0.62436	0.2662	0.936525547
SRFBP1	ENSG00000151304	5	121287656	121421265	151	6	123834	0.62414	0.26627	0.936525547
TEDDM1	ENSG00000203730	1	182357252	182379751	42	5	123834	0.62411	0.26628	0.936525547
HIST3H3	ENSG00000168148	1	228602546	228623026	30	4	123834	0.62405	0.2663	0.936525547
XIRP1	ENSG00000168334	3	39214701	39244087	20	6	123834	0.62406	0.2663	0.936525547
BARX2	ENSG00000043039	11	129235835	129332171	102	27	123834	0.62391	0.26634	0.936525547
SNCG	ENSG00000173267	10	88708375	88733017	37	8	123834	0.62366	0.26643	0.936525547
ADAM18	ENSG00000168619	8	39432008	39597583	196	18	123834	0.62353	0.26647	0.936525547
C4orf32	ENSG00000174749	4	113056553	113126412	90	9	123834	0.62347	0.26649	0.936525547
CADM4	ENSG00000105767	19	44116520	44153991	47	8	123834	0.62331	0.26654	0.936525547
C6ORF165	ENSG00000272514	6	88107701	88184183	211	14	123834	0.62312	0.2666	0.936525547
PDK2	ENSG00000005882	17	48162101	48199516	66	12	123834	0.6231	0.26661	0.936525547
C16orf78	ENSG00000166152	16	49397734	49443342	81	6	123834	0.62297	0.26665	0.936525547
BCL2L2	ENSG00000129473	14	23757999	23790968	16	6	123834	0.62202	0.26696	0.936525547
FDXACB1	ENSG00000255561	11	111734780	111761967	27	3	123834	0.62192	0.267	0.936525547
DKKL1	ENSG00000104901	19	49855040	49888373	92	3	123834	0.62178	0.26704	0.936525547
CREBL2	ENSG00000111269	12	12754761	12808042	66	8	123834	0.62177	0.26705	0.936525547
FAM71C	ENSG00000180219	12	100031531	100053824	19	4	123834	0.62138	0.26717	0.936525547
UGT1A10	ENSG00000242515	2	234535100	234691951	376	26	123834	0.62136	0.26718	0.936525547
SCOC	ENSG00000153130	4	141168440	141316880	222	26	123834	0.62107	0.26728	0.936525547
C11orf63	ENSG00000109944	11	122743391	122840506	183	25	123834	0.62106	0.26728	0.936525547
VPS29	ENSG00000111237	12	110918902	110949922	29	5	123834	0.62091	0.26733	0.936525547
RP5-966M1.6	ENSG00000243696	3	52837298	52879745	57	5	123834	0.62015	0.26758	0.936525547
CTD-2349B8.1	ENSG00000261427	16	19115324	19194900	62	11	123834	0.62009	0.2676	0.936525547
AL161450.1	ENSG00000268155	9	5067163	5094580	58	5	123834	0.61991	0.26766	0.936525547
TP73	ENSG00000078900	1	3559084	3662765	107	22	123834	0.61974	0.26771	0.936525547
MED13	ENSG00000108510	17	60009966	60152643	109	7	123834	0.6194	0.26783	0.936525547
PKP4	ENSG00000144283	2	159303476	159549391	550	18	123834	0.61929	0.26786	0.936525547
ZBTB24	ENSG00000112365	6	109773797	109814440	57	9	123834	0.61903	0.26795	0.936525547
ANAPC16	ENSG00000166295	10	73965787	74005618	30	4	123834	0.61904	0.26795	0.936525547
IYD	ENSG00000009765	6	150680028	150737105	114	9	123834	0.61897	0.26797	0.936525547
SPINK6	ENSG00000178172	5	147572357	147604700	55	2	123834	0.61892	0.26798	0.936525547
EFNA2	ENSG00000099617	19	1276153	1311430	9	3	123834	0.61869	0.26806	0.936525547
SYT12	ENSG00000173227	11	66764249	66828334	18	6	123834	0.61865	0.26807	0.936525547
KCNG4	ENSG00000168418	16	84245823	84283356	74	12	123834	0.61861	0.26809	0.936525547
LYPLA1	ENSG00000120992	8	54948938	55024577	100	4	123834	0.61851	0.26812	0.936525547
SLC4A1AP	ENSG00000163798	2	27876338	27927840	16	4	123834	0.61833	0.26818	0.936525547
LYRM7	ENSG00000186687	5	130496503	130551119	114	7	123834	0.61811	0.26825	0.936525547
ACSL5	ENSG00000197142	10	114123776	114198138	96	10	123834	0.61809	0.26826	0.936525547
FAM162A	ENSG00000114023	3	122093023	122141181	104	12	123834	0.61796	0.2683	0.936525547
MEFV	ENSG00000103313	16	3282028	3316627	55	7	123834	0.61797	0.2683	0.936525547
PTPRG	ENSG00000144724	3	61537243	62293288	1323	116	123834	0.61786	0.26833	0.936525547
OR6K3	ENSG00000203757	1	158676958	158697953	38	4	123834	0.6178	0.26835	0.936525547
C9orf62	ENSG00000178243	9	138225095	138248408	8	3	123834	0.61761	0.26842	0.936525547
AC006435.1	ENSG00000205821	17	2300279	2328731	24	6	123834	0.61761	0.26842	0.936525547

CDH5	ENSG00000179776	16	66390533	66448686	42	9	123834	0.61752	0.26845	0.936525547
AC007375.1	ENSG00000269883	14	77597026	77619339	19	7	123834	0.61707	0.26859	0.936525547
RP11-3B7.1	ENSG00000225399	3	49287518	49308744	14	4	123834	0.61703	0.26861	0.936525547
TPCN2	ENSG00000162341	11	68806365	68868072	88	5	123834	0.61704	0.26861	0.936525547
OXR1	ENSG00000164830	8	107272473	107774922	878	50	123834	0.61685	0.26867	0.936525547
LCE2B	ENSG00000159455	1	152637771	152669877	73	5	123834	0.61672	0.26871	0.936525547
GCM2	ENSG00000124827	6	10863456	10892174	34	8	123834	0.61658	0.26876	0.936525547
HIST1H2AB	ENSG00000137259	6	26023320	26043796	25	6	123834	0.61653	0.26877	0.936525547
KIFC1	ENSG00000237649	6	33349313	33387701	49	10	123834	0.61649	0.26879	0.936525547
STAG3	ENSG00000066923	7	99765186	99829111	84	6	123834	0.6164	0.26882	0.936525547
SPTBN5	ENSG00000137877	15	42130345	42196275	112	17	123834	0.61637	0.26882	0.936525547
RAB33B	ENSG00000172007	4	140364386	140407763	84	8	123834	0.61611	0.26891	0.936525547
KLHL41	ENSG00000239474	2	170356212	170392772	60	11	123834	0.61605	0.26893	0.936525547
EPHB4	ENSG00000196411	7	100390187	100435121	75	9	123834	0.61591	0.26898	0.936525547
SMCR8	ENSG00000176994	17	18208624	18236517	61	5	123834	0.61586	0.26899	0.936525547
SEZ6L2	ENSG00000174938	16	29872480	29920868	8	1	123834	0.61565	0.26906	0.936525547
ZNF254	ENSG00000213096	19	24206276	24322654	310	11	123834	0.61565	0.26906	0.936525547
PDHA2	ENSG00000163114	4	96751239	96772625	90	8	123834	0.6154	0.26915	0.936525547
DOC2A	ENSG00000149927	16	30006830	30044591	46	4	123834	0.61536	0.26916	0.936525547
C15orf52	ENSG00000188549	15	40613653	40643168	32	8	123834	0.61516	0.26922	0.936525547
PLEKHM1	ENSG00000225190	17	43503266	43578115	138	4	123834	0.61509	0.26925	0.936525547
TSNAX	ENSG00000116918	1	231654399	231712270	47	6	123834	0.61477	0.26935	0.936525547
TRIP12	ENSG00000153827	2	230618554	230797955	125	9	123834	0.6146	0.26941	0.936525547
TICRR	ENSG00000140534	15	90108713	90184287	134	8	123834	0.61412	0.26957	0.936525547
CTD-2105E13.6	ENSG00000267706	19	55853669	55880884	24	4	123834	0.61375	0.26969	0.936525547
NNT	ENSG00000112992	5	43592794	43717507	253	8	123834	0.6134	0.26981	0.936525547
USP3	ENSG00000140455	15	63786793	63896839	208	17	123834	0.61314	0.26989	0.936525547
CAMK4	ENSG00000152495	5	110549351	110840584	389	26	123834	0.61286	0.26998	0.936525547
OBFC1	ENSG00000107960	10	105632300	105687963	106	11	123834	0.61282	0.27	0.936525547
GMPR2	ENSG00000100938	14	24691628	24718448	12	4	123834	0.61268	0.27005	0.936525547
PSEN1	ENSG00000080815	14	73593126	73700399	165	15	123834	0.612	0.27027	0.936525547
LIPG	ENSG00000101670	18	47077069	47129272	47	10	123834	0.61182	0.27033	0.936525547
PRPF40B	ENSG00000110844	12	49952001	50048449	82	7	123834	0.61166	0.27038	0.936525547
RPS20	ENSG00000008988	8	56969854	56997069	72	5	123834	0.61161	0.2704	0.936525547
NDUFC1	ENSG00000109390	4	140178034	140233705	86	7	123834	0.61154	0.27042	0.936525547
KRTAP4-1	ENSG00000198443	17	39330354	39351594	68	3	123834	0.61145	0.27045	0.936525547
CCDC96	ENSG00000173013	4	7032579	7054728	25	3	123834	0.61115	0.27055	0.936525547
APOL2	ENSG00000128335	22	36612256	36646000	84	10	123834	0.61111	0.27056	0.936525547
SLC25A35	ENSG00000125434	17	8181081	8208661	60	10	123834	0.6109	0.27063	0.936525547
KRT72	ENSG00000170486	12	52969373	53005322	117	12	123834	0.61074	0.27068	0.936525547
LBX2	ENSG00000179528	2	74714644	74740443	18	2	123834	0.61071	0.27069	0.936525547
PPCDC	ENSG00000138621	15	75305896	75419803	240	16	123834	0.61043	0.27079	0.936525547
SUZ12	ENSG00000178691	17	30254037	30338064	143	3	123834	0.61004	0.27092	0.936525547
ZNF613	ENSG00000176024	19	52420400	52462012	120	11	123834	0.60972	0.27103	0.936525547
GABRR2	ENSG00000111886	6	89956927	90035018	109	23	123834	0.60957	0.27107	0.936525547
TMEM256-PLSCR3	ENSG00000187838	17	7283046	7317416	26	8	123834	0.6095	0.2711	0.936525547
C17orf61-PLSCR3	ENSG00000262481	17	7283053	7317416	26	8	123834	0.6095	0.2711	0.936525547
ARFRP1	ENSG00000101246	20	62319996	62349377	40	4	123834	0.60927	0.27117	0.936525547
PCNXL2	ENSG00000135749	1	233109181	233441459	789	49	123834	0.60914	0.27122	0.936525547
PRPF40A	ENSG00000196504	2	153498107	153584511	138	11	123834	0.6086	0.2714	0.936525547
ZNF554	ENSG00000172006	19	2809872	2845771	64	9	123834	0.60855	0.27141	0.936525547
C1orf21	ENSG00000116667	1	184346192	184608154	300	23	123834	0.60814	0.27155	0.936525547
FKBP4	ENSG00000004478	12	2894119	2924576	30	3	123834	0.60812	0.27155	0.936525547
CPVL	ENSG00000106066	7	29024847	29245067	398	46	123834	0.60764	0.27171	0.936525547
UNC5C	ENSG00000182168	4	96073655	96480357	663	74	123834	0.60762	0.27172	0.936525547
DLGAP5	ENSG00000126787	14	55604830	55668396	83	6	123834	0.60763	0.27172	0.936525547
TFAP2D	ENSG00000008197	6	50671541	50750701	90	8	123834	0.6076	0.27173	0.936525547
FYB	ENSG00000082074	5	39095338	39284630	297	20	123834	0.60745	0.27178	0.936525547
LDLRAD2	ENSG00000187942	1	22128758	22161714	46	14	123834	0.60734	0.27181	0.936525547
TMF1	ENSG00000144747	3	69058978	69111484	35	5	123834	0.60723	0.27185	0.936525547
PCK2	ENSG00000100889	14	24553262	24589807	32	9	123834	0.60719	0.27186	0.936525547
MDFIC	ENSG00000135272	7	114552209	114669256	102	18	123834	0.60709	0.27189	0.936525547
PLEKHA7	ENSG00000166689	11	16789842	17045990	463	34	123834	0.60705	0.27191	0.936525547
LCE4A	ENSG00000187170	1	152670636	152691967	35	3	123834	0.60696	0.27194	0.936525547
FRMD8	ENSG00000126391	11	65144070	65190996	29	6	123834	0.60693	0.27195	0.936525547
SCN3B	ENSG00000166257	11	123489895	123535952	87	6	123834	0.60688	0.27197	0.936525547
PROKR1	ENSG00000169618	2	68860721	68892708	47	12	123834	0.60673	0.27201	0.936525547
NIPAL1	ENSG00000163293	4	47906159	48052188	335	15	123834	0.6067	0.27202	0.936525547
ZC3H18	ENSG00000158545	16	88626789	88708374	136	19	123834	0.60673	0.27202	0.936525547
NSMCE1	ENSG00000169189	16	27226312	27290115	59	6	123834	0.60648	0.2721	0.936525547
BID	ENSG00000015475	22	18206906	18267536	84	19	123834	0.60647	0.2721	0.936525547
RPN1	ENSG00000163902	3	128328817	128409918	186	12	123834	0.60625	0.27217	0.936525547
SRI	ENSG00000075142	7	87824433	87866308	35	3	123834	0.60611	0.27222	0.936525547
BAIAP2L2	ENSG00000128298	22	38470896	38516677	50	9	123834	0.60595	0.27227	0.936525547
KIAA0226	ENSG00000145016	3	197388264	197486598	105	5	123834	0.60588	0.2723	0.936525547
PITX1	ENSG00000069011	5	134353425	134380503	36	2	123834	0.60588	0.2723	0.936525547
H2AFY2	ENSG00000099284	10	71802552	71882036	154	24	123834	0.60587	0.2723	0.936525547
ETF1	ENSG00000120705	5	137831784	137888989	50	8	123834	0.60561	0.27239	0.936525547
ALKBH1	ENSG00000100601	14	78128747	78184363	217	19	123834	0.60546	0.27244	0.936525547
CNPY1	ENSG00000146910	7	155256901	155336557	36	17	123834	0.60503	0.27258	0.936525547
GOLM1	ENSG00000135052	9	88631061	88725088	149	10	123834	0.605	0.27259	0.936525547
PRDM11	ENSG00000019485	11	45105564	45257734	189	23	123834	0.60459	0.27272	0.936525547
DFNA5	ENSG00000105928	7	24727972	24819244	135	9	123834	0.60457	0.27273	0.936525547
KIF7	ENSG00000166813	15	90142020	90208682	84	6	123834	0.60449	0.27276	0.936525547
CHI3L2	ENSG00000064886	1	111733393	111796062	145	15	123834	0.60418	0.27286	0.936525547
ADRA1A	ENSG00000120907	8	26595667	26734790	448	46	123834	0.60404	0.27291	0.936525547
C6orf123	ENSG00000146521	6	168175217	168207539	71	9	123834	0.6039	0.27295	0.936525547
C5orf55	ENSG00000221990	5	431645	453258	70	8	123834	0.60342	0.27311	0.936525547
MTG2	ENSG00000101181	20	60748085	60788624	115	19	123834	0.60344	0.27311	0.936525547
BZW1	ENSG00000082153	2	201665317	201698569	38	6	123834	0.60328	0.27316	0.936525547
RHAG	ENSG00000112077	6	49562871	49614552	152	15	123834	0.60328	0.27316	0.936525547
RTKN	ENSG00000114993	2	74642963	74679549	15	1	123834	0.60326	0.27317	0.936525547
UFL1	ENSG00000014123	6	96959471	97013152	51	7	123834	0.60249	0.27342	0.936525547
PHYH	ENSG00000107537	10	13309796	13354412	73	16	123834	0.60207	0.27356	0.936525547
NANOG	ENSG00000111704	12	7930390	7958655	126	10	123834	0.60194	0.27361	0.936525547
GALNT13	ENSG00000144278	2	154718426	155320361	1211	46	123834	0.6019	0.27362	0.936525547
ZFP91	ENSG00000186660	11	58336584	58398515	73	9	123834	0.60179	0.27366	0.936525547
COL5A2	ENSG00000204262	2	189886622	190054605	376	11	123834	0.60152	0.27375	0.936525547
SPG20OS	ENSG00000120664	13	36910568	36953738	81	9	123834	0.60138	0.27379	0.936525547

HIST1H2BB	ENSG00000196226	6	26033455	26053885	38	7	123834	0.60095	0.27394	0.936525547
CCDC86	ENSG00000110104	11	60599544	60628554	61	13	123834	0.60035	0.27413	0.936525547
SERPINB2	ENSG00000197632	18	61528926	61581124	143	3	123834	0.6003	0.27415	0.936525547
PFDN6	ENSG00000204220	6	33247079	33276178	30	7	123834	0.59907	0.27456	0.936525547
FGF23	ENSG00000118972	12	4467393	4498894	41	8	123834	0.59904	0.27457	0.936525547
SERPINE1	ENSG00000106366	7	100760370	100792547	36	7	123834	0.59884	0.27464	0.936525547
CCKBR	ENSG00000110148	11	6270966	6303357	33	10	123834	0.59855	0.27474	0.936525547
VLDLR	ENSG00000147852	9	2611834	2670053	73	9	123834	0.59848	0.27476	0.936525547
WNT8B	ENSG00000075290	10	102212798	102253501	53	2	123834	0.59842	0.27478	0.936525547
CBX2	ENSG00000173894	17	77741931	77771782	13	5	123834	0.59802	0.27491	0.936525547
DPPA5	ENSG00000203909	6	74052785	74074018	43	8	123834	0.59797	0.27493	0.936525547
MGMT	ENSG00000170430	10	131255448	131576271	847	38	123834	0.59782	0.27498	0.936525547
NSD1	ENSG00000165671	5	176550026	176737216	323	14	123834	0.59741	0.27512	0.936525547
GALNT5	ENSG00000136542	2	158104110	158180723	88	8	123834	0.59715	0.2752	0.936525547
DCHS2	ENSG00000197410	4	155143399	155422930	449	37	123834	0.59713	0.27521	0.936525547
RPL21	ENSG00000122026	13	27815446	27840828	33	5	123834	0.59713	0.27521	0.936525547
C14orf183	ENSG00000168260	14	50540369	50569361	70	10	123834	0.59705	0.27524	0.936525547
SLC29A2	ENSG00000174669	11	66119992	66149685	27	3	123834	0.597	0.27525	0.936525547
RGS13	ENSG00000127074	1	192595275	192639390	62	6	123834	0.59683	0.27531	0.936525547
PDCD11	ENSG00000148843	10	105146405	105216049	62	10	123834	0.59678	0.27533	0.936525547
HIGD1B	ENSG00000131097	17	42913721	42937848	44	8	123834	0.59673	0.27534	0.936525547
PVRIG	ENSG00000213413	7	99805864	99829113	55	5	123834	0.59671	0.27535	0.936525547
C1orf200	ENSG00000179840	1	9702668	9724644	7	2	123834	0.59651	0.27542	0.936525547
LIF	ENSG00000128342	22	30626436	30652840	10	4	123834	0.59652	0.27542	0.936525547
LNPEP	ENSG00000113441	5	96261098	96383219	221	11	123834	0.59648	0.27543	0.936525547
FBXO30	ENSG00000118496	6	146104638	146145889	51	6	123834	0.59631	0.27548	0.936525547
MBD5	ENSG00000204406	2	148768580	149285805	720	32	123834	0.5951	0.27589	0.936525547
KRT7	ENSG00000135480	12	52616304	52655970	57	12	123834	0.59499	0.27592	0.936525547
KIAA0825	ENSG00000185261	5	93478671	93964309	330	34	123834	0.5946	0.27606	0.936525547
MTNR1B	ENSG00000134640	11	92692886	92728232	56	6	123834	0.59434	0.27614	0.936525547
CEACAM19	ENSG00000186567	19	45155545	45197631	33	9	123834	0.59433	0.27615	0.936525547
CIZ1	ENSG00000148337	9	130918343	130976662	54	7	123834	0.59404	0.27624	0.936525547
ELOVL7	ENSG00000164181	5	60037618	60150216	190	17	123834	0.59368	0.27636	0.936525547
VIPR1	ENSG00000114812	3	42520791	42589059	54	15	123834	0.59332	0.27648	0.936525547
POFUT1	ENSG00000101346	20	30785683	30836470	34	4	123834	0.59332	0.27648	0.936525547
PSMG3	ENSG00000157778	7	1596966	1620641	38	5	123834	0.59331	0.27649	0.936525547
CREM	ENSG00000095794	10	35405719	35511886	175	6	123834	0.59292	0.27662	0.936525547
LCMT1	ENSG00000205629	16	25113050	25199552	86	4	123834	0.59291	0.27662	0.936525547
IGDCC3	ENSG00000174498	15	65609465	65680378	67	15	123834	0.59286	0.27664	0.936525547
FCAMR	ENSG00000162897	1	207121310	207153970	44	4	123834	0.59269	0.27669	0.936525547
MESP2	ENSG00000188095	15	90293822	90331982	111	5	123834	0.59248	0.27676	0.936525547
FAM135B	ENSG00000147724	8	139132266	139519065	737	69	123834	0.59247	0.27677	0.936525547
ACTR10	ENSG00000131966	14	58656798	58711750	19	4	123834	0.59237	0.2768	0.936525547
KPNA3	ENSG00000102753	13	50263447	50377057	296	20	123834	0.59235	0.27681	0.936525547
TRIM38	ENSG00000112343	6	25953030	25997384	66	9	123834	0.59214	0.27688	0.936525547
SYNE1	ENSG00000131018	6	152432819	152968936	1010	85	123834	0.59215	0.27688	0.936525547
MICB	ENSG00000204516	6	31452658	31488901	319	22	123834	0.59197	0.27694	0.936525547
TPD52L1	ENSG00000111907	6	125430195	125595553	355	28	123834	0.59185	0.27698	0.936525547
KIF3C	ENSG00000084731	2	26139471	26215618	120	17	123834	0.5918	0.27699	0.936525547
AC005779.2	ENSG00000267545	19	45673080	45715702	43	11	123834	0.59161	0.27706	0.936525547
ADA	ENSG00000196839	20	43238163	43290874	73	15	123834	0.59145	0.27711	0.936525547
STK25	ENSG00000115694	2	242422089	242459145	64	11	123834	0.59133	0.27715	0.936525547
CRISP2	ENSG00000124490	6	49650073	49691274	64	10	123834	0.59129	0.27716	0.936525547
ID2	ENSG00000115738	2	8808975	8834583	24	3	123834	0.59108	0.27723	0.936525547
CAPG	ENSG00000042493	2	85611871	85655555	113	14	123834	0.59105	0.27724	0.936525547
PKD2L1	ENSG00000107593	10	102037903	102100243	81	14	123834	0.59104	0.27725	0.936525547
SIRT4	ENSG00000089163	12	120730119	120761052	36	3	123834	0.5909	0.2773	0.936525547
PCDHB5	ENSG00000113209	5	140504800	140527703	28	7	123834	0.59073	0.27735	0.936525547
PHF10	ENSG00000130024	6	170094001	170134151	76	10	123834	0.59071	0.27736	0.936525547
ZNF835	ENSG00000127903	19	57164020	57193151	57	9	123834	0.59069	0.27736	0.936525547
CCDC80	ENSG00000091986	3	112313407	112378377	83	14	123834	0.5905	0.27743	0.936525547
AMH	ENSG00000104899	19	2239308	2262072	23	7	123834	0.59044	0.27745	0.936525547
OTUD7A	ENSG00000169918	15	31765329	32172992	677	19	123834	0.59037	0.27747	0.936525547
C16orf93	ENSG00000196118	16	30758744	30784031	10	4	123834	0.59033	0.27749	0.936525547
MAP2K7	ENSG00000076984	19	7958728	7989363	31	6	123834	0.59031	0.27749	0.936525547
ILF2	ENSG00000143621	1	153624512	153653524	27	3	123834	0.59029	0.2775	0.936525547
UBE2S	ENSG00000108106	19	55902652	55929145	53	9	123834	0.58957	0.27774	0.936525547
NACAD	ENSG00000136274	7	45110037	45138513	84	10	123834	0.58948	0.27777	0.936525547
RGS9BP	ENSG00000186326	19	33156313	33179206	63	9	123834	0.58942	0.27779	0.936525547
FBLN5	ENSG00000140092	14	92325756	92424331	81	18	123834	0.58933	0.27782	0.936525547
N4BP3	ENSG00000145911	5	177530444	177563088	32	7	123834	0.58915	0.27788	0.936525547
KRTAP9-1	ENSG00000240542	17	39334697	39356891	56	3	123834	0.58904	0.27792	0.936525547
POU2AF1	ENSG00000110777	11	111212977	111336355	275	14	123834	0.58897	0.27794	0.936525547
ARHGEF2	ENSG00000116584	1	155906630	155986861	46	5	123834	0.58887	0.27798	0.936525547
ZAR1L	ENSG00000189167	13	32867837	32899481	69	8	123834	0.58875	0.27801	0.936525547
FGF17	ENSG00000158815	8	21889909	21916320	18	7	123834	0.58869	0.27803	0.936525547
SMARCA4	ENSG00000127616	19	11061598	11186071	247	20	123834	0.58859	0.27807	0.936525547
COBLL1	ENSG00000082438	2	165500134	165710189	384	24	123834	0.58837	0.27814	0.936525547
AL590708.2	ENSG00000232850	9	130880612	130901481	3	1	123834	0.58836	0.27815	0.936525547
SLC25A29	ENSG00000197119	14	100747448	100782884	22	6	123834	0.58826	0.27818	0.936525547
C4orf21	ENSG00000138658	4	113450492	113568151	170	15	123834	0.58811	0.27823	0.936525547
PRPF38B	ENSG00000134186	1	109224945	109254425	3	1	123834	0.58808	0.27824	0.936525547
KHDC3L	ENSG00000203908	6	74062400	74083894	37	8	123834	0.58808	0.27824	0.936525547
CDCA5	ENSG00000146670	11	64823772	64861636	52	9	123834	0.58784	0.27832	0.936525547
ZFP91-CNTF	ENSG00000255073	11	58336645	58402112	78	9	123834	0.58737	0.27848	0.936525547
C12orf76	ENSG00000174456	12	110455872	110521491	100	15	123834	0.58711	0.27857	0.936525547
FAM227A	ENSG00000184949	22	38964125	39062634	183	9	123834	0.587	0.2786	0.936525547
PER2	ENSG00000132326	2	239142679	239208743	87	12	123834	0.58693	0.27863	0.936525547
TMEM220	ENSG00000187824	17	10592332	10643633	102	2	123834	0.58669	0.27871	0.936525547
DHX32	ENSG00000089876	10	127514906	127595005	50	6	123834	0.58653	0.27876	0.936525547
SPTAN1	ENSG00000197694	9	131304866	131405941	78	4	123834	0.58649	0.27877	0.936525547
TUB	ENSG00000166402	11	8030791	8137659	144	28	123834	0.58646	0.27878	0.936525547
GDPD1	ENSG00000153982	17	57287828	57363328	102	6	123834	0.58644	0.27879	0.936525547
ALK	ENSG00000171094	2	29405640	30154432	1178	99	123834	0.5864	0.2788	0.936525547
BCCIP	ENSG00000107949	10	127502115	127552264	44	4	123834	0.58631	0.27883	0.936525547
ACSL6	ENSG00000164398	5	131132683	131357936	238	11	123834	0.58624	0.27886	0.936525547
PVRL4	ENSG00000143217	1	161030785	161069389	33	8	123834	0.58606	0.27892	0.936525547
TEX30	ENSG00000151287	13	103408340	103436161	37	9	123834	0.58603	0.27893	0.936525547
GPX1	ENSG00000233276	3	49384609	49406033	22	3	123834	0.58594	0.27896	0.936525547

SUN3	ENSG00000164744	7	48016745	48078716	64	4	123834	0.58588	0.27898	0.936525547
KRTAP10-8	ENSG00000187766	21	46021996	46042871	50	2	123834	0.58583	0.279	0.936525547
TMEM60	ENSG00000135211	7	77413045	77437897	34	7	123834	0.58577	0.27902	0.936525547
FMO5	ENSG00000131781	1	146636930	146724700	363	15	123834	0.58573	0.27903	0.936525547
RAP1GAP	ENSG00000076864	1	21912708	22005801	168	37	123834	0.58569	0.27904	0.936525547
KRTAP4-3	ENSG00000196156	17	39313484	39334424	42	3	123834	0.58571	0.27904	0.936525547
C2orf91	ENSG00000205086	2	42152508	42191406	52	7	123834	0.58541	0.27914	0.936525547
IST1	ENSG00000182149	16	71869899	71972913	165	12	123834	0.58532	0.27917	0.936525547
KRTAP4-2	ENSG00000244537	17	39323698	39344460	81	2	123834	0.5852	0.27921	0.936525547
DYM	ENSG00000141627	18	46560039	46997717	971	22	123834	0.5849	0.27931	0.936525547
KIF20A	ENSG00000112984	5	137504408	137533404	25	5	123834	0.58488	0.27932	0.936525547
SEMA4D	ENSG00000187764	9	91965702	92123045	289	31	123834	0.58481	0.27934	0.936525547
ICA1L	ENSG00000163596	2	203630690	203746708	134	8	123834	0.58473	0.27937	0.936525547
GALNT4	ENSG00000257594	12	89903185	89930039	64	8	123834	0.5845	0.27944	0.936525547
POC1B-GALNT4	ENSG00000259075	12	89903185	89930039	64	8	123834	0.5845	0.27944	0.936525547
KDM4E	ENSG00000235268	11	94748422	94770760	45	8	123834	0.5844	0.27948	0.936525547
CASR	ENSG00000036828	3	121892530	122015342	397	25	123834	0.58433	0.2795	0.936525547
LATS2	ENSG00000150457	13	21537171	21645686	218	19	123834	0.58419	0.27954	0.936525547
LNX1	ENSG00000072201	4	54315468	54577572	438	38	123834	0.58413	0.27957	0.936525547
KARS	ENSG00000065427	16	75651622	75692541	108	13	123834	0.58398	0.27962	0.936525547
L47234.1	ENSG00000268341	19	45832445	45852639	39	6	123834	0.58367	0.27972	0.936525547
KLHDC2	ENSG00000165516	14	50224326	50259909	16	3	123834	0.58362	0.27974	0.936525547
KIAA0408	ENSG00000189367	6	127751488	127790536	46	2	123834	0.58349	0.27978	0.936525547
C1orf65	ENSG00000178395	1	223556715	223578812	33	4	123834	0.5834	0.27981	0.936525547
ALG2	ENSG00000119523	9	101968708	101994238	28	2	123834	0.58328	0.27985	0.936525547
SH2B2	ENSG00000160999	7	101918405	101972178	31	9	123834	0.58305	0.27993	0.936525547
BANF2	ENSG00000125888	20	17664317	17726516	93	14	123834	0.58302	0.27994	0.936525547
AP4M1	ENSG00000221838	7	99689172	99717968	30	4	123834	0.58275	0.28003	0.936525547
PALM2-AKAP2	ENSG00000157654	9	112532589	112944792	692	80	123834	0.58237	0.28016	0.936525547
LMLN	ENSG00000185621	3	197677071	197780591	186	11	123834	0.58203	0.28027	0.936525547
CTTNBP2NL	ENSG00000143079	1	112928803	113016078	137	4	123834	0.58187	0.28033	0.936525547
PI16	ENSG00000164530	6	36912209	36942613	43	8	123834	0.58157	0.28043	0.936525547
PPAP2C	ENSG00000141934	19	271040	301393	11	5	123834	0.58139	0.28049	0.936525547
PCDHB13	ENSG00000187372	5	140583509	140606993	42	7	123834	0.58126	0.28053	0.936525547
PTPN9	ENSG00000169410	15	75749462	75881630	118	7	123834	0.58118	0.28056	0.936525547
PTGER1	ENSG00000160951	19	14573278	14596174	49	9	123834	0.58094	0.28064	0.936525547
DDX39A	ENSG00000123136	19	14509631	14540192	14	3	123834	0.58077	0.2807	0.936525547
RECK	ENSG00000122707	9	36026430	36134448	167	14	123834	0.58022	0.28088	0.936525547
AIF1	ENSG00000204472	6	31572961	31594798	46	11	123834	0.58009	0.28093	0.936525547
TAAR6	ENSG00000146383	6	132881461	132902498	63	11	123834	0.58008	0.28093	0.936525547
ZPBP	ENSG00000042813	7	49880017	50170925	605	15	123834	0.58005	0.28094	0.936525547
EFCAB12	ENSG00000172771	3	129110164	129157494	79	6	123834	0.58003	0.28095	0.936525547
MYZAP	ENSG00000263155	15	57874139	57987562	291	15	123834	0.57999	0.28096	0.936525547
GYPE	ENSG00000197465	4	144782020	144836716	104	6	123834	0.57981	0.28102	0.936525547
C5orf48	ENSG00000196900	5	125957414	125981979	59	9	123834	0.57966	0.28107	0.936525547
BBS5	ENSG00000163093	2	170325688	170392432	129	18	123834	0.57958	0.2811	0.936525547
RP11-724O16.1	ENSG00000251569	2	170325990	170392432	129	18	123834	0.57958	0.2811	0.936525547
BBC3	ENSG00000105327	19	47714081	47746023	6	2	123834	0.57955	0.28111	0.936525547
APLF	ENSG00000169621	2	68684693	68868004	407	11	123834	0.57947	0.28114	0.936525547
TLX2	ENSG00000115297	2	74730590	74754274	11	2	123834	0.57927	0.2812	0.936525547
OR52B2	ENSG00000255307	11	6180560	6201638	43	8	123834	0.57925	0.28121	0.936525547
DPYSL4	ENSG00000151640	10	133990404	134029280	80	9	123834	0.57916	0.28124	0.936525547
DROSHA	ENSG00000113360	5	31390604	31542303	211	20	123834	0.57908	0.28127	0.936525547
HOXC10	ENSG00000180818	12	54368849	54394063	24	6	123834	0.57906	0.28128	0.936525547
RNF40	ENSG00000103549	16	30763066	30797628	14	4	123834	0.57865	0.28141	0.936525547
ZNF180	ENSG00000167384	19	44969854	45014576	153	15	123834	0.57849	0.28147	0.936525547
NAALADL1	ENSG00000168060	11	64802290	64836021	54	8	123834	0.57827	0.28154	0.936525547
KIAA0930	ENSG00000100364	22	45576219	45646650	150	18	123834	0.57815	0.28158	0.936525547
UAP1	ENSG00000117143	1	162521323	162579627	36	6	123834	0.57794	0.28165	0.936525547
C21orf58	ENSG00000160298	21	47710095	47753789	117	14	123834	0.57796	0.28165	0.936525547
MEIS1	ENSG00000143995	2	66650584	66811001	161	20	123834	0.57791	0.28166	0.936525547
SASH1	ENSG00000111961	6	148583440	148883186	555	73	123834	0.57791	0.28166	0.936525547
POLR2H	ENSG00000163882	3	184069506	184096384	19	5	123834	0.57783	0.28169	0.936525547
FAM206A	ENSG00000119328	9	111686461	111723024	78	10	123834	0.5775	0.2818	0.936525547
SPINK1	ENSG00000164266	5	147194131	147221349	20	5	123834	0.57746	0.28182	0.936525547
ARHGAP12	ENSG00000165322	10	32084365	32227742	197	12	123834	0.57729	0.28187	0.936525547
TMEM207	ENSG00000198398	3	190136444	190177665	82	12	123834	0.57721	0.2819	0.936525547
C9orf156	ENSG00000136932	9	100656771	100694852	72	7	123834	0.57714	0.28192	0.936525547
PLD6	ENSG00000179598	17	17094309	17119629	38	9	123834	0.57713	0.28193	0.936525547
OR13F1	ENSG00000186881	9	107256455	107277547	46	6	123834	0.57709	0.28194	0.936525547
UGT2A3	ENSG00000135220	4	69784181	69827509	128	6	123834	0.57672	0.28206	0.936525547
STX8	ENSG00000170310	17	9143788	9489908	325	42	123834	0.57673	0.28206	0.936525547
UPK1A	ENSG00000105668	19	36147715	36179367	83	7	123834	0.57627	0.28222	0.936525547
BSN	ENSG00000164061	3	49581922	49718978	134	10	123834	0.57618	0.28225	0.936525547
ZNFX1	ENSG00000124201	20	47844483	47904963	189	13	123834	0.57616	0.28225	0.936525547
NABP1	ENSG00000173559	2	192532794	192571385	31	4	123834	0.57615	0.28226	0.936525547
PARP16	ENSG00000138617	15	65516798	65602956	155	12	123834	0.5761	0.28227	0.936525547
AC127496.1	ENSG00000175911	17	78967135	78988932	62	9	123834	0.57606	0.28229	0.936525547
CADM3	ENSG00000162706	1	159131399	159183103	38	9	123834	0.57602	0.2823	0.936525547
RP11-446E24.4	ENSG00000256407	1	54596897	54675746	97	23	123834	0.57572	0.2824	0.936525547
DTX3L	ENSG00000163840	3	122273085	122304050	81	8	123834	0.5756	0.28244	0.936525547
GRM8	ENSG00000179603	7	126068652	126903348	1328	41	123834	0.57556	0.28246	0.936525547
TIMM44	ENSG00000104980	19	7981603	8018805	69	5	123834	0.57492	0.28267	0.936525547
AC026407.1	ENSG00000268432	5	157150003	157171727	42	5	123834	0.57487	0.28269	0.936525547
NAT1	ENSG00000171428	8	18017986	18091198	259	22	123834	0.57459	0.28279	0.936525547
LMTK2	ENSG00000164715	7	97726197	97848945	227	15	123834	0.57435	0.28286	0.936525547
SNX16	ENSG00000104497	8	82701816	82765101	153	7	123834	0.57409	0.28295	0.936525547
RHOD	ENSG00000173156	11	66814289	66849484	24	6	123834	0.57393	0.28301	0.936525547
AL929472.1	ENSG00000230610	1	38282232	38303886	29	5	123834	0.57381	0.28305	0.936525547
C12orf39	ENSG00000134548	12	21669241	21700311	60	12	123834	0.57382	0.28305	0.936525547
ZNF519	ENSG00000175322	18	14047456	14142489	150	15	123834	0.57372	0.28308	0.936525547
NAA15	ENSG00000164134	4	140212609	140351187	221	13	123834	0.57364	0.2831	0.936525547
ICAM2	ENSG00000108622	17	62069954	62107994	4	1	123834	0.57361	0.28311	0.936525547
NCBP2	ENSG00000114503	3	196652273	196679468	41	7	123834	0.57358	0.28312	0.936525547
TUBB6	ENSG00000176014	18	12297668	12354319	145	6	123834	0.57314	0.28327	0.936525547
AL355390.1	ENSG00000177596	13	74977094	75003252	78	15	123834	0.57305	0.28331	0.936525547
SLC25A11	ENSG00000108528	17	4830425	4853546	31	7	123834	0.57236	0.28354	0.936525547
PALM	ENSG00000099864	19	698953	758329	70	17	123834	0.57226	0.28357	0.936525547
HAAO	ENSG00000162882	2	42984229	43029733	125	10	123834	0.5722	0.28359	0.936525547

FAF1	ENSG00000185104	1	50895150	51435935	503	14	123834	0.5721	0.28363	0.936525547
C1orf234	ENSG00000227868	1	23327327	23352343	28	3	123834	0.57203	0.28365	0.936525547
ARGLU1	ENSG00000134884	13	107184021	107230512	14	4	123834	0.57196	0.28368	0.936525547
HSD3B1	ENSG00000203857	1	120039821	120067681	31	1	123834	0.57181	0.28372	0.936525547
ZNF474	ENSG00000164185	5	121455208	121525312	16	3	123834	0.57179	0.28373	0.936525547
WDR89	ENSG00000140006	14	64053757	64118579	120	7	123834	0.57178	0.28374	0.936525547
PCDHB14	ENSG00000120327	5	140592931	140615858	50	6	123834	0.57166	0.28378	0.936525547
GPR183	ENSG00000169508	13	99936784	99969659	60	12	123834	0.57148	0.28384	0.936525547
OGFOD2	ENSG00000111325	12	123449127	123474590	13	3	123834	0.571	0.284	0.936525547
C1GALT1	ENSG00000106392	7	7186565	7298282	422	22	123834	0.57094	0.28402	0.936525547
ZNF622	ENSG00000173545	5	16441628	16475901	41	6	123834	0.57073	0.28409	0.936525547
RP11-385D13.1	ENSG00000251537	17	15464805	15564967	88	14	123834	0.57067	0.28411	0.936525547
GPRIN3	ENSG00000185477	4	90147537	90239161	76	10	123834	0.57044	0.28419	0.936525547
AKAP2	ENSG00000241978	9	112532769	112944792	691	80	123834	0.57043	0.28419	0.936525547
KPNA4	ENSG00000186432	3	160202783	160293376	115	10	123834	0.57039	0.28421	0.936525547
SAC3D1	ENSG00000168061	11	64798373	64822300	46	6	123834	0.57031	0.28423	0.936525547
RRP9	ENSG00000114767	3	51957446	51985957	4	1	123834	0.57025	0.28426	0.936525547
FAM47E-STBD1	ENSG00000118804	4	77162886	77242752	188	16	123834	0.56987	0.28438	0.936525547
MTHFD1	ENSG00000100714	14	64844749	64936722	122	12	123834	0.56957	0.28448	0.936525547
SPATA18	ENSG00000163071	4	52907497	52973458	19	6	123834	0.56923	0.2846	0.936525547
WBP2	ENSG00000132471	17	73831780	73862588	57	9	123834	0.56904	0.28466	0.936525547
IMMP2L	ENSG00000184903	7	110293110	111212573	1102	51	123834	0.56887	0.28472	0.936525547
MRC2	ENSG00000011028	17	60694762	60780958	79	13	123834	0.56877	0.28476	0.936525547
MED27	ENSG00000160563	9	134725494	134965295	208	20	123834	0.56866	0.28479	0.936525547
SRD5A3	ENSG00000128039	4	56202276	56249263	86	10	123834	0.56787	0.28506	0.936525547
IL15RA	ENSG00000134470	10	5980855	6030150	120	20	123834	0.56775	0.2851	0.936525547
HOXC12	ENSG00000123407	12	54338618	54362740	24	5	123834	0.56767	0.28513	0.936525547
FAM81B	ENSG00000153347	5	94717048	94796158	122	13	123834	0.56757	0.28516	0.936525547
OR1S1	ENSG00000172774	11	57972217	57993195	47	6	123834	0.56718	0.2853	0.936525547
SNX5	ENSG00000089006	20	17912241	17959623	127	8	123834	0.56717	0.2853	0.936525547
GABRB3	ENSG00000166206	15	26778693	27194686	670	60	123834	0.56695	0.28537	0.936525547
CCAR2	ENSG00000158941	8	22452145	22489027	83	6	123834	0.56683	0.28541	0.936525547
ATP13A1	ENSG00000105726	19	19746007	19784502	58	9	123834	0.56685	0.28541	0.936525547
KCNA10	ENSG00000143105	1	111049839	111071797	33	9	123834	0.5665	0.28553	0.936525547
RP11-872D17.8	ENSG00000254979	11	57144838	57201532	55	8	123834	0.56649	0.28553	0.936525547
TAGLN3	ENSG00000144834	3	111707511	111742734	99	12	123834	0.56647	0.28554	0.936525547
C21orf37	ENSG00000232560	21	18801208	18831503	30	8	123834	0.56641	0.28556	0.936525547
RP1-4G17.5	ENSG00000262302	17	7140148	7175408	27	4	123834	0.56638	0.28557	0.936525547
FBXO36	ENSG00000153832	2	230777018	230887825	96	11	123834	0.56634	0.28558	0.936525547
ZNF366	ENSG00000178175	5	71728479	71813554	127	9	123834	0.56629	0.2856	0.936525547
AP000758.1	ENSG00000269601	11	120319880	120340047	24	5	123834	0.56627	0.2856	0.936525547
TBC1D9	ENSG00000109436	4	141531919	141687274	167	22	123834	0.56622	0.28562	0.936525547
ADPRM	ENSG00000170222	17	10590911	10624550	72	1	123834	0.56608	0.28567	0.936525547
INTS3	ENSG00000143624	1	153690543	153756555	53	4	123834	0.56604	0.28568	0.936525547
PDZRN3	ENSG00000121440	3	73421584	73684091	285	36	123834	0.56589	0.28574	0.936525547
ABCC12	ENSG00000140798	16	48106884	48199929	33	3	123834	0.56565	0.28582	0.936525547
XXYLT1	ENSG00000173950	3	194779008	195001896	470	32	123834	0.56537	0.28591	0.936525547
HCAR1	ENSG00000196917	12	123094824	123225390	186	18	123834	0.56518	0.28597	0.936525547
FBXO34	ENSG00000178974	14	55728021	55838636	205	8	123834	0.56515	0.28599	0.936525547
DSC1	ENSG00000134765	18	28699199	28752819	121	11	123834	0.5651	0.286	0.936525547
SCYL2	ENSG00000136021	12	100650918	100745502	110	10	123834	0.56507	0.28601	0.936525547
PVRL1	ENSG00000110400	11	119484120	119609794	94	14	123834	0.56503	0.28603	0.936525547
INS-IGF2	ENSG00000129965	11	2143768	2192439	38	7	123834	0.5649	0.28607	0.936525547
NAA60	ENSG00000262621	16	3405099	3546960	356	19	123834	0.56446	0.28622	0.936525547
MAD2L1	ENSG00000164109	4	120966763	120998229	40	8	123834	0.5644	0.28624	0.936525547
ATXN1L	ENSG00000224470	16	71869894	71929171	84	9	123834	0.56427	0.28629	0.936525547
TOMM40L	ENSG00000158882	1	161185793	161210408	27	8	123834	0.56421	0.2863	0.936525547
RNASE8	ENSG00000173431	14	21515981	21536614	14	5	123834	0.56424	0.2863	0.936525547
UBE2E1	ENSG00000170142	3	23837394	23942807	133	17	123834	0.56406	0.28636	0.936525547
ETV5	ENSG00000244405	3	185754097	185838107	86	10	123834	0.56402	0.28637	0.936525547
AC009060.1	ENSG00000269746	16	70229303	70249683	1	1	123834	0.56394	0.2864	0.936525547
GJC1	ENSG00000182963	17	42865816	42918184	71	11	123834	0.56367	0.28649	0.936525547
LRRC8E	ENSG00000171017	19	7943390	7976901	39	8	123834	0.56364	0.2865	0.936525547
HIST1H3A	ENSG00000198366	6	26010718	26031186	27	7	123834	0.56356	0.28653	0.936525547
TMEM163	ENSG00000152128	2	135203330	135486570	441	40	123834	0.56347	0.28656	0.936525547
RAB12	ENSG00000206418	18	8599443	8649379	76	8	123834	0.56329	0.28662	0.936525547
SCNN1A	ENSG00000111319	12	6446009	6496896	28	8	123834	0.56324	0.28664	0.936525547
SLC5A3	ENSG00000198743	21	35435870	35488559	24	2	123834	0.56322	0.28664	0.936525547
SLC5A3	ENSG00000272962	21	35435870	35488561	24	2	123834	0.56322	0.28664	0.936525547
C6orf132	ENSG00000188112	6	42058856	42120357	73	17	123834	0.56316	0.28666	0.936525547
NECAP2	ENSG00000157191	1	16757167	16796573	72	9	123834	0.56301	0.28671	0.936525547
SNAP47	ENSG00000143740	1	227906240	227978927	99	13	123834	0.563	0.28672	0.936525547
PSMD1	ENSG00000173692	2	231911578	232047541	150	14	123834	0.56297	0.28673	0.936525547
CCDC168	ENSG00000175820	13	103371801	103399159	18	2	123834	0.56292	0.28674	0.936525547
POLL	ENSG00000166169	10	103328639	103358027	38	4	123834	0.56216	0.28701	0.936525547
KRT75	ENSG00000170454	12	52807854	52838309	66	7	123834	0.56205	0.28704	0.936525547
DAW1	ENSG00000123977	2	228725770	228799060	254	12	123834	0.56165	0.28718	0.936525547
MSANTD1	ENSG00000188981	4	3236096	3283465	90	9	123834	0.56124	0.28732	0.936525547
COLCA2	ENSG00000214290	11	111159565	111189460	57	8	123834	0.56118	0.28734	0.936525547
SEMA7A	ENSG00000138623	15	74691630	74736808	87	12	123834	0.56115	0.28735	0.936525547
DRD2	ENSG00000149295	11	113270318	113356413	163	20	123834	0.56087	0.28744	0.936525547
MS4A13	ENSG00000204979	11	60272886	60320194	108	6	123834	0.56069	0.2875	0.936525547
RAD9B	ENSG00000151164	12	110929460	110979891	52	5	123834	0.56052	0.28756	0.936525547
SLC9A2	ENSG00000115616	2	103226166	103337777	124	18	123834	0.56	0.28774	0.936525547
DOHH	ENSG00000129932	19	3480819	3510938	27	6	123834	0.56	0.28774	0.936525547
CATSPERB	ENSG00000133962	14	92037040	92257051	360	33	123834	0.55989	0.28778	0.936525547
ZNF782	ENSG00000196597	9	99568754	99647905	51	4	123834	0.55977	0.28782	0.936525547
TTC34	ENSG00000215912	1	2557415	2728286	101	10	123834	0.55967	0.28785	0.936525547
TTC3	ENSG00000182670	21	38435526	38585413	378	8	123834	0.5596	0.28788	0.936525547
FAM47E-STBD1	ENSG00000272414	4	77162874	77242282	187	16	123834	0.55937	0.28796	0.936525547
METTL10	ENSG00000203791	10	126426718	126490439	92	9	123834	0.55927	0.28799	0.936525547
DNAJA1	ENSG00000086061	9	33015209	33049905	114	8	123834	0.5592	0.28801	0.936525547
SMAD3	ENSG00000166949	15	67346101	67497533	173	32	123834	0.55912	0.28804	0.936525547
PTPN21	ENSG00000070778	14	88922122	89031077	156	8	123834	0.55889	0.28812	0.936525547
SYNM	ENSG00000182253	15	99628420	99685798	129	17	123834	0.55883	0.28814	0.936525547
ZNF263	ENSG00000006194	16	3303743	3361401	38	4	123834	0.55868	0.28819	0.936525547
S100A14	ENSG00000189334	1	153576731	153599462	18	3	123834	0.55844	0.28827	0.936525547
ZNF420	ENSG00000197050	19	37488759	37631216	317	10	123834	0.55823	0.28834	0.936525547
NGDN	ENSG00000129460	14	23928897	23989071	74	8	123834	0.55818	0.28836	0.936525547

RP11-147C23.1	ENSG00000237435	1	96393457	96498436	223	19	123834	0.55809	0.28839	0.936525547
CDT1	ENSG00000167513	16	88859621	88885666	52	9	123834	0.55784	0.28848	0.936525547
KTN1	ENSG00000126777	14	56015790	56178244	370	9	123834	0.55773	0.28851	0.936525547
C1orf115	ENSG00000162817	1	220853187	220882499	101	8	123834	0.55758	0.28857	0.936525547
KRBOX1	ENSG00000273291	3	42840938	43107363	334	24	123834	0.55738	0.28863	0.936525547
JADE1	ENSG00000077684	4	129720779	129806379	43	8	123834	0.55734	0.28865	0.936525547
FBXL15	ENSG00000107872	10	104168946	104192893	21	7	123834	0.55732	0.28865	0.936525547
CHAF1B	ENSG00000159259	21	37747676	37801313	115	9	123834	0.55734	0.28865	0.936525547
BTG3	ENSG00000154640	21	18955971	18995265	18	4	123834	0.55721	0.28869	0.936525547
IFNA8	ENSG00000120242	9	21399146	21420184	53	11	123834	0.557	0.28876	0.936525547
WBSCR28	ENSG00000175877	7	73265489	73290223	25	3	123834	0.55684	0.28882	0.936525547
IL6R	ENSG00000160712	1	154367669	154451926	91	9	123834	0.55682	0.28883	0.936525547
CABP2	ENSG00000167791	11	67276383	67300899	20	2	123834	0.55682	0.28883	0.936525547
RP11-463D19.2	ENSG00000258677	8	74590926	74801089	260	13	123834	0.55667	0.28888	0.936525547
KPNA6	ENSG00000025800	1	32563639	32652169	65	2	123834	0.55664	0.28889	0.936525547
RNASEH1	ENSG00000171865	2	3582383	3616206	100	12	123834	0.55663	0.28889	0.936525547
GPR146	ENSG00000164849	7	1074212	1108897	146	13	123834	0.55648	0.28894	0.936525547
CHST9	ENSG00000154080	18	24485595	24775281	566	31	123834	0.55641	0.28896	0.936525547
MPV17	ENSG00000115204	2	27522360	27558547	26	5	123834	0.5564	0.28897	0.936525547
AGTPBP1	ENSG00000135049	9	88151455	88366944	461	11	123834	0.55635	0.28899	0.936525547
RPL10L	ENSG00000165496	14	47110222	47131028	31	6	123834	0.55606	0.28908	0.936525547
GPT2	ENSG00000166123	16	46908290	46975209	9	4	123834	0.55585	0.28916	0.936525547
GRK7	ENSG00000114124	3	141486990	141547332	92	12	123834	0.55577	0.28918	0.936525547
RPS14	ENSG00000164587	5	149812753	149839319	57	4	123834	0.55574	0.2892	0.936525547
NR2E1	ENSG00000112333	6	108477262	108520013	23	6	123834	0.55566	0.28922	0.936525547
PCDHGA12	ENSG00000253159	5	140800185	140902546	190	23	123834	0.55565	0.28923	0.936525547
ZNF277	ENSG00000198839	7	111836643	111993151	197	15	123834	0.55559	0.28924	0.936525547
C1QTNF1-AS1	ENSG00000265096	17	77005291	77033737	26	4	123834	0.55557	0.28925	0.936525547
NAA16	ENSG00000172766	13	41875341	41961166	161	8	123834	0.55551	0.28927	0.936525547
LRRC52	ENSG00000162763	1	165503244	165543198	145	13	123834	0.55547	0.28929	0.936525547
CERCAM	ENSG00000167123	9	131164030	131209626	35	2	123834	0.55542	0.2893	0.936525547
ARL17B	ENSG00000228696	17	44342150	44449130	4	1	123834	0.55543	0.2893	0.936525547
KIAA1009	ENSG00000135315	6	84823960	84947353	167	9	123834	0.55537	0.28932	0.936525547
BBS9	ENSG00000122507	7	33158856	33655680	1052	40	123834	0.55534	0.28933	0.936525547
BMF	ENSG00000104081	15	40370091	40411093	8	3	123834	0.55503	0.28944	0.936525547
C21orf54	ENSG00000229086	21	34527776	34552541	34	9	123834	0.55496	0.28946	0.936525547
KLHL14	ENSG00000197705	18	30242634	30363025	151	9	123834	0.5549	0.28948	0.936525547
PDLIM3	ENSG00000154553	4	186412851	186466766	60	9	123834	0.55461	0.28958	0.936525547
ABHD14A-ACY1	ENSG00000114786	3	51999066	52033199	7	3	123834	0.55443	0.28964	0.936525547
ACY1	ENSG00000243989	3	51999066	52033213	7	3	123834	0.55443	0.28964	0.936525547
NUFIP1	ENSG00000083635	13	45503384	45573618	72	13	123834	0.55428	0.28969	0.936525547
TCEB3	ENSG00000011007	1	24059645	24098549	10	4	123834	0.55405	0.28977	0.936525547
PPP1R13L	ENSG00000104881	19	45872892	45919607	46	8	123834	0.55391	0.28982	0.936525547
DUSP26	ENSG00000133878	8	33438856	33467624	30	2	123834	0.55384	0.28984	0.936525547
THG1L	ENSG00000113272	5	157148205	157178456	52	7	123834	0.55379	0.28986	0.936525547
SH3RF2	ENSG00000156463	5	145306142	145471354	186	6	123834	0.5537	0.28989	0.936525547
PPP4R1	ENSG00000154845	18	9536789	9625238	215	21	123834	0.55365	0.28991	0.936525547
SH2D2A	ENSG00000027869	1	156766035	156796654	38	7	123834	0.55311	0.29009	0.936525547
KIAA1217	ENSG00000120549	10	23973675	24846772	1432	79	123834	0.55295	0.29015	0.936525547
RCHY1	ENSG00000163743	4	76394247	76449974	92	8	123834	0.55283	0.29019	0.936525547
GPX4	ENSG00000167468	19	1093936	1116787	34	9	123834	0.55283	0.29019	0.936525547
ZNF432	ENSG00000256087	19	52524632	52608991	240	14	123834	0.55282	0.29019	0.936525547
PPM1F	ENSG00000100034	22	22263793	22317209	102	10	123834	0.55284	0.29019	0.936525547
TACO1	ENSG00000136463	17	61668231	61695725	3	1	123834	0.55279	0.2902	0.936525547
PHKG2	ENSG00000156873	16	30749591	30782490	11	4	123834	0.55273	0.29022	0.936525547
OLR1	ENSG00000173391	12	10300902	10334737	104	9	123834	0.55265	0.29025	0.936525547
RIC8B	ENSG00000111785	12	107158373	107293090	148	15	123834	0.55243	0.29033	0.936562511
DGCR2	ENSG00000070413	22	19013795	19119967	331	13	123834	0.55232	0.29036	0.936562511
CCDC117	ENSG00000159873	22	29158662	29195283	92	13	123834	0.55189	0.29051	0.936887357
TAAR5	ENSG00000135569	6	132899731	132920877	55	11	123834	0.55176	0.29056	0.936889649
UROS	ENSG00000188690	10	127467146	127521817	73	3	123834	0.55132	0.29071	0.937133363
CKS2	ENSG00000123975	9	91916113	91941618	30	6	123834	0.55111	0.29078	0.937133363
CARTPT	ENSG00000164326	5	71004990	71026875	32	6	123834	0.55095	0.29083	0.937133363
AL139080.1	ENSG00000268735	13	24312243	24333901	35	5	123834	0.55087	0.29086	0.937133363
TECTA	ENSG00000109927	11	120961882	121072202	162	12	123834	0.5507	0.29092	0.937133363
OR10J1	ENSG00000196184	1	159399512	159420600	52	10	123834	0.55048	0.29099	0.937133363
AKT3	ENSG00000117020	1	243641535	244024381	360	14	123834	0.55047	0.291	0.937133363
KLF5	ENSG00000102554	13	73619114	73661676	61	13	123834	0.55037	0.29103	0.937133363
AC140061.12	ENSG00000269529	12	50680489	50701718	23	2	123834	0.55019	0.2911	0.9372
MED22	ENSG00000148297	9	136195160	136224986	47	7	123834	0.55001	0.29115	0.937202235
CLSTN3	ENSG00000139182	12	7272294	7321541	69	8	123834	0.54951	0.29133	0.93737862
EXOC3L4	ENSG00000205436	14	103556481	103586896	26	4	123834	0.54951	0.29133	0.93737862
DDX56	ENSG00000136271	7	44595016	44624650	75	10	123834	0.54937	0.29138	0.93737862
CAPN1	ENSG00000014216	11	64938037	64989477	54	8	123834	0.54926	0.29141	0.93737862
GLRX	ENSG00000173221	5	95077023	95168709	106	18	123834	0.54907	0.29148	0.93737862
GATS	ENSG00000160844	7	99788283	99879855	106	10	123834	0.54892	0.29153	0.93737862
DUPD1	ENSG00000188716	10	76787594	76828272	49	9	123834	0.54886	0.29155	0.93737862
ZNF783	ENSG00000204946	7	148949262	149004393	122	15	123834	0.5486	0.29164	0.937479445
KLC2	ENSG00000174996	11	66014765	66045331	23	5	123834	0.54849	0.29168	0.937479445
CCDC85A	ENSG00000055813	2	56401258	56623308	343	40	123834	0.54815	0.2918	0.937644462
CMTR2	ENSG00000180917	16	71305292	71333618	78	10	123834	0.54806	0.29183	0.937644462
TSPAN2	ENSG00000134198	1	115580632	115642121	126	17	123834	0.54777	0.29192	0.937706631
ZNF174	ENSG00000103343	16	3441235	3469370	45	6	123834	0.54769	0.29195	0.937706631
CAPN7	ENSG00000131375	3	15237659	15304425	50	7	123834	0.54734	0.29207	0.937706631
ARIH1	ENSG00000166233	15	72756667	72889692	122	8	123834	0.54723	0.29211	0.937706631
SAR1A	ENSG00000079332	10	71897045	71940279	121	13	123834	0.54694	0.29221	0.937706631
MMD	ENSG00000108960	17	53459974	53509353	31	6	123834	0.54693	0.29221	0.937706631
CDIP1	ENSG00000089486	16	4550676	4598829	81	6	123834	0.54657	0.29234	0.937706631
BBS7	ENSG00000138686	4	122735595	122801652	85	9	123834	0.54632	0.29242	0.937706631
LRPAP1	ENSG00000163956	4	3498103	3544286	173	17	123834	0.5463	0.29243	0.937706631
RAD52	ENSG00000002016	12	1011243	1109219	147	26	123834	0.54631	0.29243	0.937706631
EDC4	ENSG00000038358	16	67896926	67928406	22	4	123834	0.54617	0.29248	0.937706631
HYI	ENSG00000178922	1	43906824	43929660	20	3	123834	0.54613	0.29249	0.937706631
MBD2	ENSG00000134046	18	51669079	51761158	87	11	123834	0.54602	0.29253	0.937706631
RAB11FIP4	ENSG00000131242	17	29708642	29875236	166	33	123834	0.54598	0.29254	0.937706631
SLC25A30	ENSG00000174032	13	45957451	46002590	57	7	123834	0.54573	0.29263	0.937836965
NKX2-3	ENSG00000119919	10	101282690	101306278	60	6	123834	0.54523	0.2928	0.938223601
ATXN7L2	ENSG00000162650	1	110016101	110045426	16	4	123834	0.54501	0.29287	0.938289729
CBY1	ENSG00000100211	22	39042641	39079859	119	5	123834	0.54477	0.29296	0.93829381

ARV1	ENSG00000173409	1	231104727	231146341	126	11	123834	0.54472	0.29297	0.93829381
SMCO4	ENSG00000166002	11	93201638	93286674	134	14	123834	0.54445	0.29306	0.938363947
HLA-DRB5	ENSG00000198502	6	32475120	32508064	48	3	123834	0.54434	0.2931	0.938363947
AP001350.1	ENSG00000269570	11	58368589	58388759	35	7	123834	0.54422	0.29314	0.938363947
SELK	ENSG00000113811	3	53908437	53936015	64	8	123834	0.54341	0.29342	0.938735928
AC111200.1	ENSG00000229615	2	87079023	87102827	7	2	123834	0.5434	0.29343	0.938735928
SKAP1	ENSG00000141293	17	46200802	46517637	618	25	123834	0.54336	0.29344	0.938735928
ODF3	ENSG00000177947	11	186738	210261	18	4	123834	0.54274	0.29365	0.938735928
3-Sep	ENSG00000100167	22	42362276	42404225	55	6	123834	0.54261	0.2937	0.938735928
CDC42SE2	ENSG00000158985	5	130571186	130744140	164	9	123834	0.54246	0.29375	0.938735928
NKX2-6	ENSG00000180053	8	23549964	23574111	48	8	123834	0.54242	0.29377	0.938735928
CST9L	ENSG00000101435	20	23535369	23559386	44	2	123834	0.54223	0.29383	0.938735928
TMEM167B	ENSG00000215717	1	109622425	109649556	62	10	123834	0.54219	0.29384	0.938735928
MRPL13	ENSG00000172172	8	121383000	121467642	119	9	123834	0.54189	0.29395	0.938735928
JAK2	ENSG00000096968	9	4975033	5138183	226	9	123834	0.54179	0.29398	0.938735928
RUNX3	ENSG00000020633	1	25216002	25301612	79	10	123834	0.54171	0.29401	0.938735928
NME3	ENSG00000103024	16	1810287	1831731	23	5	123834	0.54139	0.29412	0.938735928
MTRNR2L11	ENSG00000270188	1	238097024	238118575	54	5	123834	0.54134	0.29414	0.938735928
CENPE	ENSG00000138778	4	104016963	104129566	159	9	123834	0.54131	0.29415	0.938735928
MCM7	ENSG00000166508	7	99680351	99709563	33	4	123834	0.54131	0.29415	0.938735928
HPCAL4	ENSG00000116983	1	40134320	40167361	45	10	123834	0.54115	0.2942	0.938735928
AP003041.2	ENSG00000267981	11	129926836	129954012	96	5	123834	0.54103	0.29424	0.938735928
MATN4	ENSG00000124159	20	43912085	43947169	81	10	123834	0.54105	0.29424	0.938735928
OPA3	ENSG00000125741	19	46020685	46115470	96	20	123834	0.54098	0.29426	0.938735928
TNFSF13B	ENSG00000102524	13	108893588	108970832	72	14	123834	0.54077	0.29433	0.938735928
L3MBTL2	ENSG00000100395	22	41591209	41637275	89	4	123834	0.54047	0.29444	0.938735928
CEACAM1	ENSG00000079385	19	43001456	43075386	37	2	123834	0.54041	0.29446	0.938735928
TMEM177	ENSG00000144120	2	120426743	120454083	89	5	123834	0.53995	0.29461	0.938735928
RNFT2	ENSG00000135119	12	117166096	117301436	202	20	123834	0.53984	0.29465	0.938735928
KCNC1	ENSG00000129159	11	17746359	17814602	21	7	123834	0.53954	0.29476	0.938735928
ZDHHC5	ENSG00000156599	11	57425219	57478659	47	5	123834	0.53944	0.29479	0.938735928
PDE6C	ENSG00000095464	10	95362345	95435767	120	19	123834	0.5394	0.29481	0.938735928
SLC16A8	ENSG00000100156	22	38464141	38490100	20	7	123834	0.53928	0.29485	0.938735928
SFT2D3	ENSG00000173349	2	128448597	128471385	61	11	123834	0.5391	0.29491	0.938735928
HTR1D	ENSG00000179546	1	23506993	23531222	75	5	123834	0.53908	0.29492	0.938735928
RP11-404P21.8	ENSG00000258691	14	96661181	96740266	142	23	123834	0.53829	0.29519	0.938735928
AC117834.1	ENSG00000212999	8	93885865	93908013	18	6	123834	0.5382	0.29522	0.938735928
RRH	ENSG00000180245	4	110739150	110775760	56	3	123834	0.53818	0.29523	0.938735928
CEP104	ENSG00000116198	1	3718645	3783778	310	16	123834	0.53794	0.29531	0.938735928
GRM4	ENSG00000124493	6	33979628	34133399	298	33	123834	0.53793	0.29531	0.938735928
PTGDR	ENSG00000168229	14	52724431	52753442	65	11	123834	0.53791	0.29532	0.938735928
SCO1	ENSG00000133028	17	10573654	10611692	34	1	123834	0.53773	0.29538	0.938735928
ZNF568	ENSG00000198453	19	37397231	37499602	402	14	123834	0.53745	0.29548	0.938735928
FAM19A1	ENSG00000183662	3	68043359	68604776	1091	53	123834	0.5374	0.29549	0.938735928
NRAP	ENSG00000197893	10	115338475	115433886	244	21	123834	0.53734	0.29552	0.938735928
SEC11C	ENSG00000166562	18	56796709	56836068	94	11	123834	0.53724	0.29555	0.938735928
HLTF	ENSG00000071794	3	148737914	148814341	120	13	123834	0.53704	0.29562	0.938735928
MSMB	ENSG00000138294	10	51539498	51572517	23	6	123834	0.53705	0.29562	0.938735928
AC005358.1	ENSG00000273452	17	12616199	12671542	23	6	123834	0.53674	0.29572	0.938735928
WISP3	ENSG00000112761	6	112365275	112402171	43	6	123834	0.53642	0.29583	0.938735928
ADNP	ENSG00000101126	20	49495585	49557958	89	11	123834	0.53616	0.29592	0.938735928
C3orf62	ENSG00000188315	3	49296035	49325342	21	5	123834	0.53613	0.29593	0.938735928
CAPN2	ENSG00000162909	1	223879295	223973720	175	9	123834	0.53611	0.29594	0.938735928
EPHX1	ENSG00000143819	1	225987794	226043260	107	21	123834	0.53605	0.29596	0.938735928
CD163L1	ENSG00000177675	12	7489281	7642493	232	33	123834	0.53596	0.29599	0.938735928
RBM42	ENSG00000126254	19	36109932	36138588	25	4	123834	0.53596	0.29599	0.938735928
DHX8	ENSG00000067596	17	41551233	41631831	102	4	123834	0.53585	0.29603	0.938735928
DYDC2	ENSG00000133665	10	82094501	82137829	118	7	123834	0.53579	0.29605	0.938735928
CTD-2192J16.20	ENSG00000269693	19	12626534	12701789	39	6	123834	0.53578	0.29605	0.938735928
FAM154B	ENSG00000188659	15	82545151	82587271	60	12	123834	0.53577	0.29606	0.938735928
KITLG	ENSG00000049130	12	88876570	88984628	162	7	123834	0.53567	0.29609	0.938735928
SMPDL3A	ENSG00000172594	6	123100315	123140865	35	6	123834	0.53551	0.29615	0.938735928
SPG20	ENSG00000133104	13	36865775	36954317	156	8	123834	0.53545	0.29617	0.938735928
RAB19	ENSG00000146955	7	140093843	140136050	68	11	123834	0.535	0.29633	0.93877925
AL645608.2	ENSG00000269308	1	808043	829983	2	1	123834	0.53496	0.29634	0.93877925
C4orf19	ENSG00000154274	4	37445563	37635117	435	48	123834	0.53493	0.29635	0.93877925
GBA	ENSG00000177628	1	155194243	155224490	41	3	123834	0.53481	0.29639	0.93877925
TMEM255B	ENSG00000184497	13	114452216	114524926	104	12	123834	0.53463	0.29645	0.93877925
LEPR	ENSG00000116678	1	65876248	66117242	450	25	123834	0.53456	0.29648	0.93877925
SLC15A1	ENSG00000088386	13	99326055	99414908	144	35	123834	0.53436	0.29655	0.938844504
PCDHGA11	ENSG00000253873	5	140790762	140901835	193	23	123834	0.53398	0.29668	0.93906989
BPHL	ENSG00000137274	6	3108608	3163812	55	3	123834	0.53386	0.29672	0.93906989
ADAM11	ENSG00000073670	17	42826399	42869214	66	9	123834	0.53369	0.29678	0.939103419
CRYGB	ENSG00000182187	2	208997297	209020892	64	10	123834	0.53341	0.29688	0.939172313
MAMDC2	ENSG00000165072	9	72648497	72851886	492	15	123834	0.53328	0.29692	0.939172313
C17orf62	ENSG00000178927	17	80390465	80418705	60	7	123834	0.53321	0.29695	0.939172313
ANXA10	ENSG00000109511	4	169003666	169118841	207	17	123834	0.53282	0.29708	0.939334132
CPED1	ENSG00000106034	7	120618731	120947498	544	22	123834	0.53277	0.2971	0.939334132
CCDC89	ENSG00000179071	11	85384893	85407320	25	6	123834	0.53221	0.29729	0.93963424
TTC1	ENSG00000113312	5	159426120	159502550	126	10	123834	0.53186	0.29741	0.93963424
FASTKD1	ENSG00000138399	2	170376259	170440385	147	18	123834	0.53183	0.29742	0.93963424
KRT15	ENSG00000171346	17	39659995	39688781	42	4	123834	0.53175	0.29745	0.93963424
MT1X	ENSG00000187193	16	56706336	56728108	34	9	123834	0.53127	0.29761	0.93963424
C17orf64	ENSG00000141371	17	58459790	58518782	52	3	123834	0.53123	0.29763	0.93963424
ITPK1	ENSG00000100605	14	93393259	93592665	344	17	123834	0.53104	0.2977	0.93963424
C2CD4C	ENSG00000183186	19	395438	419139	20	7	123834	0.53072	0.29781	0.93963424
GMEB1	ENSG00000162419	1	28985244	29055865	67	6	123834	0.53048	0.29789	0.93963424
NME5	ENSG00000112981	5	137440866	137485132	20	4	123834	0.53047	0.29789	0.93963424
KCTD11	ENSG00000213859	17	7245208	7268258	3	1	123834	0.53027	0.29796	0.93963424
ANKRD6	ENSG00000135299	6	90132889	90353553	299	24	123834	0.53013	0.29801	0.93963424
LRP12	ENSG00000147650	8	105491459	105611252	143	9	123834	0.5299	0.29809	0.93963424
PNPO	ENSG00000108439	17	46008872	46035654	53	8	123834	0.52959	0.2982	0.93963424
OTUB2	ENSG00000089723	14	94482675	94525276	68	9	123834	0.52951	0.29823	0.93963424
KCNQ2	ENSG00000075043	20	62027542	62113993	129	21	123834	0.52896	0.29842	0.93963424
ADAM17	ENSG00000151694	2	9618615	9705921	139	10	123834	0.52876	0.29849	0.93963424
ETNPPL	ENSG00000164089	4	109653196	109694210	70	5	123834	0.52862	0.29854	0.93963424
FBXL12	ENSG00000127452	19	9910943	9948492	26	3	123834	0.52851	0.29857	0.93963424
NKAIN1	ENSG00000084628	1	31642592	31722401	190	14	123834	0.52843	0.2986	0.93963424
SLC15A4	ENSG00000139370	12	129267739	129318528	134	11	123834	0.52826	0.29866	0.93963424

LILRA4	ENSG00000239961	19	54834456	54860421	11	5	123834	0.52827	0.29866	0.93963424
RAB31	ENSG00000168461	18	9698162	9872548	265	51	123834	0.52821	0.29868	0.93963424
DEDD2	ENSG00000160570	19	42692750	42734292	20	1	123834	0.52818	0.29869	0.93963424
MIB2	ENSG00000197530	1	1540795	1575990	20	4	123834	0.52815	0.2987	0.93963424
MRVI1	ENSG00000072952	11	10584638	10725535	247	36	123834	0.52804	0.29874	0.93963424
CDC42	ENSG00000070831	1	22369120	22429437	122	14	123834	0.52801	0.29875	0.93963424
SYNGAP1	ENSG00000197283	6	33377847	33431466	46	9	123834	0.52797	0.29876	0.93963424
MRPS28	ENSG00000147586	8	80820952	80952524	99	8	123834	0.52754	0.29891	0.93963424
GPR1	ENSG00000183671	2	207030040	207092771	91	13	123834	0.52743	0.29895	0.93963424
TRIM3	ENSG00000110171	11	6459843	6505689	25	5	123834	0.52735	0.29897	0.93963424
UBE2J1	ENSG00000198833	6	90026344	90072567	53	12	123834	0.52735	0.29898	0.93963424
MT1F	ENSG00000198417	16	56681606	56704610	76	11	123834	0.52726	0.29901	0.93963424
SEC61B	ENSG00000106803	9	101974346	102002897	43	2	123834	0.52717	0.29904	0.93963424
THAP3	ENSG00000041988	1	6674926	6705646	48	7	123834	0.52676	0.29918	0.93963424
SYT14	ENSG00000143469	1	210101538	210347636	262	9	123834	0.52674	0.29919	0.93963424
RBM43	ENSG00000184898	2	152094454	152128393	55	6	123834	0.52667	0.29921	0.93963424
AP000867.1	ENSG00000187811	11	71282956	71328487	99	8	123834	0.52654	0.29926	0.93963424
C9orf135	ENSG00000204711	9	72425709	72531148	150	17	123834	0.52645	0.29929	0.93963424
EMP2	ENSG00000213853	16	10612279	10684555	111	22	123834	0.52642	0.2993	0.93963424
OR51I2	ENSG00000187918	11	5464638	5485707	89	11	123834	0.52634	0.29933	0.93963424
PRSS55	ENSG00000184647	8	10373056	10421676	94	11	123834	0.5263	0.29934	0.93963424
GHRL	ENSG00000157017	3	10317359	10344631	32	7	123834	0.52622	0.29937	0.93963424
HSPA2	ENSG00000126803	14	64992623	65019955	67	10	123834	0.52621	0.29937	0.93963424
FAM110D	ENSG00000197245	1	26475511	26499119	42	7	123834	0.52464	0.29992	0.941084198
ZNF224	ENSG00000267680	19	44588492	44622479	66	6	123834	0.52433	0.30002	0.941084198
TMOD2	ENSG00000128872	15	52033758	52118565	87	6	123834	0.52431	0.30003	0.941084198
ACSBG2	ENSG00000130377	19	6125258	6203112	211	11	123834	0.52432	0.30003	0.941084198
SNX13	ENSG00000071189	7	17820385	17990124	195	10	123834	0.52381	0.30021	0.941273438
MED19	ENSG00000156603	11	57461186	57489693	29	3	123834	0.52374	0.30023	0.941273438
PRCP	ENSG00000137509	11	82524544	82691626	240	21	123834	0.52364	0.30026	0.941273438
TMEM190	ENSG00000160472	19	55878204	55899612	35	9	123834	0.52327	0.30039	0.941273438
PARP3	ENSG00000041880	3	51966361	51992883	2	1	123834	0.52319	0.30042	0.941273438
C14orf166	ENSG00000087302	14	52446193	52481420	53	12	123834	0.5231	0.30045	0.941273438
RP11-723G8.2	ENSG00000265352	18	69389851	69465364	225	16	123834	0.5231	0.30045	0.941273438
KRTAP19-6	ENSG00000186925	21	31903854	31924183	31	3	123834	0.52284	0.30054	0.941273438
NUP214	ENSG00000126883	9	133990948	134120057	164	8	123834	0.52269	0.3006	0.941273438
PLAC9	ENSG00000189129	10	81881438	81915115	72	13	123834	0.52247	0.30067	0.941273438
IRX2	ENSG00000170561	5	2735959	2762969	21	4	123834	0.52231	0.30073	0.941273438
CFHR4	ENSG00000134365	1	196809371	196898102	111	7	123834	0.52226	0.30074	0.941273438
NRXN3	ENSG00000021645	14	78698734	80340762	2221	152	123834	0.52218	0.30077	0.941273438
SAMSN1	ENSG00000155307	21	15847549	15965723	186	30	123834	0.52196	0.30085	0.941273438
UROC1	ENSG00000159650	3	126190124	126246616	80	20	123834	0.52162	0.30097	0.941273438
TMEM74B	ENSG00000125895	20	1151205	1176059	6	3	123834	0.52146	0.30102	0.941273438
SULT1C4	ENSG00000198075	2	108984367	109014513	50	8	123834	0.52136	0.30106	0.941273438
UBXN4	ENSG00000144224	2	136489189	136552625	114	9	123834	0.52123	0.3011	0.941273438
SREBF1	ENSG00000072310	17	17703713	17750325	19	3	123834	0.52108	0.30115	0.941273438
PIGL	ENSG00000108474	17	16110505	16262115	196	10	123834	0.52083	0.30124	0.941273438
PCDHGB7	ENSG00000254122	5	140787427	140902546	198	24	123834	0.5207	0.30129	0.941273438
SOWAHC	ENSG00000198142	2	110361911	110386563	57	9	123834	0.52065	0.3013	0.941273438
ADSS	ENSG00000035687	1	244561796	244625436	115	15	123834	0.52057	0.30133	0.941273438
ZDHHC6	ENSG00000023041	10	114180058	114216672	17	6	123834	0.52059	0.30133	0.941273438
RNF181	ENSG00000168894	2	85812848	85834736	30	6	123834	0.52008	0.3015	0.941273438
C7orf71	ENSG00000222004	7	26667490	26696889	41	3	123834	0.52	0.30153	0.941273438
TTC24	ENSG00000187862	1	156539519	156566562	56	4	123834	0.51996	0.30154	0.941273438
RAD54L2	ENSG00000164080	3	51565160	51712683	74	9	123834	0.51974	0.30162	0.941273438
ZNF91	ENSG00000167232	19	23477793	23588362	278	10	123834	0.51958	0.30168	0.941273438
DTHD1	ENSG00000197057	4	36273244	36357378	187	29	123834	0.5194	0.30174	0.941273438
TUBB2A	ENSG00000137267	6	3143903	3167760	55	3	123834	0.5194	0.30174	0.941273438
TP53INP1	ENSG00000164938	8	95928200	95971639	92	9	123834	0.51938	0.30175	0.941273438
C10orf105	ENSG00000214688	10	73461458	73507581	62	8	123834	0.5192	0.30181	0.941273438
URGCP	ENSG00000106608	7	43905493	43976010	116	11	123834	0.51907	0.30186	0.941273438
KRTAP5-4	ENSG00000241598	11	1632188	1653368	92	9	123834	0.51878	0.30196	0.941273438
ARF6	ENSG00000165527	14	50349810	50371490	6	1	123834	0.51854	0.30204	0.941273438
FBXO7	ENSG00000100225	22	32860663	32904818	79	7	123834	0.51848	0.30206	0.941273438
CREBBP	ENSG00000005339	16	3765055	3940727	39	8	123834	0.51813	0.30218	0.941273438
OCLM	ENSG00000262180	1	186359704	186380587	31	7	123834	0.51795	0.30225	0.941273438
MNT	ENSG00000070444	17	2277354	2314412	50	4	123834	0.51773	0.30232	0.941273438
PYCR2	ENSG00000143811	1	226097578	226121978	27	6	123834	0.51756	0.30238	0.941273438
C17orf100	ENSG00000212734	17	6544971	6566791	76	8	123834	0.51743	0.30243	0.941273438
PTPLAD2	ENSG00000188921	9	20985306	21041635	131	14	123834	0.51703	0.30257	0.941273438
HRK	ENSG00000135116	12	117283949	117329246	40	7	123834	0.51679	0.30265	0.941273438
NXPH3	ENSG00000182575	17	47643220	47671189	16	7	123834	0.51674	0.30267	0.941273438
ZBTB45	ENSG00000119574	19	59014897	59060278	31	6	123834	0.51667	0.30269	0.941273438
ITPR1	ENSG00000150995	3	4525032	4899524	382	74	123834	0.51658	0.30272	0.941273438
WDR62	ENSG00000075702	19	36535783	36606008	127	13	123834	0.5165	0.30275	0.941273438
FBN2	ENSG00000138829	5	127583601	128004878	829	26	123834	0.51648	0.30276	0.941273438
ZSWIM6	ENSG00000130449	5	60618100	60851997	226	15	123834	0.51622	0.30285	0.941273438
OR2AP1	ENSG00000179615	12	55958199	55979128	43	3	123834	0.51588	0.30297	0.941273438
ITPR2	ENSG00000123104	12	26480342	26996131	902	76	123834	0.51571	0.30303	0.941273438
1-Mar	ENSG00000145416	4	164435450	165315202	1984	92	123834	0.51568	0.30304	0.941273438
TRIM42	ENSG00000155890	3	140386881	140429992	91	8	123834	0.51554	0.30309	0.941273438
TAOK1	ENSG00000160551	17	27707482	27888922	250	13	123834	0.5155	0.3031	0.941273438
CFHR3	ENSG00000116785	1	196733925	196773203	2	1	123834	0.51547	0.30311	0.941273438
RANGRF	ENSG00000108961	17	8181815	8203410	43	8	123834	0.51526	0.30318	0.941273438
EBLN1	ENSG00000223601	10	22487743	22508950	67	6	123834	0.51507	0.30325	0.941273438
EDIL3	ENSG00000164176	5	83226373	83690611	492	48	123834	0.51503	0.30326	0.941273438
PERP	ENSG00000112378	6	138399642	138438648	73	15	123834	0.51498	0.30328	0.941273438
GNB1L	ENSG00000185838	22	19760747	19852462	117	17	123834	0.51493	0.3033	0.941273438
NHLRC4	ENSG00000257108	16	606996	629495	19	2	123834	0.51489	0.30331	0.941273438
CBL	ENSG00000110395	11	119066752	119188859	92	8	123834	0.51446	0.30347	0.941273438
LDOC1L	ENSG00000188636	22	44878452	44904178	44	8	123834	0.51436	0.3035	0.941273438
RABEP2	ENSG00000177548	16	28905742	28957847	18	2	123834	0.51433	0.30351	0.941273438
ASB7	ENSG00000183475	15	101132739	101201910	62	14	123834	0.51416	0.30357	0.941273438
DOK5	ENSG00000101134	20	53082136	53277710	237	22	123834	0.51405	0.30361	0.941273438
CH25H	ENSG00000138135	10	90955694	90977071	32	4	123834	0.5138	0.3037	0.941273438
SFTPA1	ENSG00000122852	10	81360695	81385196	62	8	123834	0.51371	0.30373	0.941273438
AC136297.1	ENSG00000267950	11	1285809	1306033	34	7	123834	0.51357	0.30378	0.941273438
GAS7	ENSG00000007237	17	9803926	10111868	436	84	123834	0.51355	0.30378	0.941273438
CSNK1D	ENSG00000141551	17	80186899	80241607	12	4	123834	0.51355	0.30378	0.941273438

TMEM176B	ENSG00000106565	7	150478373	150508448	61	14	123834	0.51342	0.30383	0.941273438
DEFB125	ENSG00000178591	20	57891	87735	11	4	123834	0.51308	0.30395	0.941273438
NCAN	ENSG00000130287	19	19312782	19373042	89	10	123834	0.51291	0.30401	0.941273438
FAM132B	ENSG00000178752	2	239057623	239087541	48	11	123834	0.51265	0.3041	0.941273438
BCL11B	ENSG00000127152	14	99625624	99747861	74	16	123834	0.51256	0.30413	0.941273438
REXO2	ENSG00000076043	11	114300108	114331001	67	11	123834	0.51251	0.30415	0.941273438
TLR1	ENSG00000174125	4	38782298	38868438	273	15	123834	0.51247	0.30416	0.941273438
DHX36	ENSG00000174953	3	153980335	154052286	99	10	123834	0.51246	0.30417	0.941273438
SPNS2	ENSG00000183018	17	4392133	4452330	56	11	123834	0.51244	0.30417	0.941273438
OPN4	ENSG00000122375	10	88404314	88436605	122	16	123834	0.51227	0.30423	0.941273438
RNASE3	ENSG00000169397	14	21349558	21370507	17	3	123834	0.51226	0.30424	0.941273438
ARMC8	ENSG00000114098	3	137896109	138027231	34	9	123834	0.51222	0.30425	0.941273438
N4BP2L1	ENSG00000139597	13	32964861	33012315	53	10	123834	0.51188	0.30437	0.94149145
SPEF1	ENSG00000101222	20	3748151	3772095	17	3	123834	0.5115	0.3045	0.94164896
SLC2A9	ENSG00000109667	4	9762777	10066560	1035	24	123834	0.51144	0.30452	0.94164896
IMP3	ENSG00000177971	15	75921426	75951047	11	3	123834	0.51099	0.30468	0.941990475
CSRNP3	ENSG00000178662	2	166316157	166555917	274	20	123834	0.5106	0.30481	0.942178654
C5orf27	ENSG00000236882	5	95177936	95205837	58	10	123834	0.51054	0.30484	0.942178654
TRMT1L	ENSG00000121486	1	185077220	185136204	66	4	123834	0.50987	0.30507	0.942736232
C10orf120	ENSG00000183559	10	124447225	124469338	46	9	123834	0.5097	0.30513	0.942768375
RPUSD2	ENSG00000166133	15	40851499	40876659	83	7	123834	0.50945	0.30522	0.942772094
PALD1	ENSG00000107719	10	72228577	72338205	143	26	123834	0.5093	0.30527	0.942772094
PPP1R2	ENSG00000184203	3	195231221	195280209	67	9	123834	0.50928	0.30528	0.942772094
PPP1R37	ENSG00000104866	19	45584654	45661335	160	11	123834	0.50867	0.30549	0.942902014
EMID1	ENSG00000186998	22	29591840	29665586	128	19	123834	0.50816	0.30567	0.942902014
LINC00923	ENSG00000251209	15	98275448	98427780	362	28	123834	0.5081	0.30569	0.942902014
TMEM234	ENSG00000160055	1	32670073	32697972	24	2	123834	0.50778	0.3058	0.942902014
RPL29	ENSG00000162244	3	52017616	52039958	7	3	123834	0.50756	0.30588	0.942902014
C1orf110	ENSG00000185860	1	162784248	162848605	170	22	123834	0.50751	0.3059	0.942902014
SLC9A9	ENSG00000181804	3	142974064	143577373	984	104	123834	0.50742	0.30593	0.942902014
SDF2	ENSG00000132581	17	26965374	26999207	15	5	123834	0.50734	0.30596	0.942902014
DCST2	ENSG00000163354	1	154980996	155016257	21	4	123834	0.50699	0.30608	0.942902014
TOM1L2	ENSG00000175662	17	17736828	17885736	157	3	123834	0.50696	0.30609	0.942902014
TMEM150A	ENSG00000168890	2	85815671	85840319	28	5	123834	0.50694	0.3061	0.942902014
TMEM176A	ENSG00000002933	7	150487491	150512208	59	11	123834	0.50687	0.30612	0.942902014
CD209	ENSG00000090659	19	7794879	7822464	27	7	123834	0.50689	0.30612	0.942902014
SIX3	ENSG00000138083	2	45158902	45183216	39	8	123834	0.50664	0.3062	0.942902014
DENND6A	ENSG00000174839	3	57601184	57688816	178	16	123834	0.50653	0.30624	0.942902014
CNTROB	ENSG00000170037	17	7825419	7863236	53	3	123834	0.5065	0.30625	0.942902014
AP000889.3	ENSG00000268467	11	107452471	107473949	36	3	123834	0.50645	0.30627	0.942902014
TTC31	ENSG00000115282	2	74700200	74732013	16	3	123834	0.5062	0.30636	0.942902014
ZNF785	ENSG00000197162	16	30575061	30607092	8	2	123834	0.50568	0.30654	0.942902014
KIRREL2	ENSG00000126259	19	36336763	36368011	35	1	123834	0.50488	0.30682	0.942902014
ZNF195	ENSG00000005801	11	3350491	3410448	113	7	123834	0.50483	0.30684	0.942902014
ACADM	ENSG00000117054	1	76180036	76263260	238	17	123834	0.50446	0.30697	0.942902014
LRIT1	ENSG00000148602	10	85981349	86011217	57	10	123834	0.50441	0.30698	0.942902014
RP11-89K11.1	ENSG00000259658	15	102267302	102295913	27	6	123834	0.50427	0.30704	0.942902014
RP11-463J10.2	ENSG00000258928	14	52372863	52395289	30	6	123834	0.50416	0.30707	0.942902014
EIF4B	ENSG00000063046	12	53389942	53445993	107	6	123834	0.50395	0.30715	0.942902014
DCDC2B	ENSG00000222046	1	32664695	32691797	22	2	123834	0.50388	0.30717	0.942902014
SMARCAD1	ENSG00000163104	4	95118762	95222443	169	8	123834	0.50373	0.30723	0.942902014
RNF216	ENSG00000011275	7	5649678	5831370	398	20	123834	0.50367	0.30725	0.942902014
RBM8A	ENSG00000131795	1	145497598	145523536	5	1	123834	0.50339	0.30734	0.942902014
RP11-429E11.3	ENSG00000179253	20	60282562	60304804	43	6	123834	0.50311	0.30744	0.942902014
C2orf15	ENSG00000241962	2	99747948	99949204	311	15	123834	0.5031	0.30745	0.942902014
NR2C2AP	ENSG00000184162	19	19302225	19324233	15	3	123834	0.5031	0.30745	0.942902014
TMEM127	ENSG00000135956	2	96904254	96941732	23	5	123834	0.503	0.30748	0.942902014
TPT1	ENSG00000133112	13	45901008	45925505	60	8	123834	0.50292	0.30751	0.942902014
ZMAT4	ENSG00000165061	8	40378109	40765352	864	63	123834	0.50272	0.30758	0.942902014
HTR3C	ENSG00000178084	3	183760835	183788459	51	9	123834	0.50242	0.30768	0.942902014
HEATR5A	ENSG00000129493	14	31750994	31899788	142	10	123834	0.50242	0.30769	0.942902014
PLA2G1B	ENSG00000170890	12	120749914	120775592	25	2	123834	0.50233	0.30772	0.942902014
INHBA	ENSG00000122641	7	41714712	41752706	13	4	123834	0.50207	0.30781	0.942902014
LONRF1	ENSG00000154359	8	12569403	12623582	74	9	123834	0.50192	0.30786	0.942902014
PPARG	ENSG00000132170	3	12318867	12485855	219	16	123834	0.50179	0.30791	0.942902014
CBX7	ENSG00000100307	22	39506172	39558679	74	11	123834	0.50164	0.30796	0.942902014
GYS2	ENSG00000111713	12	21679123	21767781	176	9	123834	0.50161	0.30797	0.942902014
OTOP3	ENSG00000182938	17	72921814	72956087	31	7	123834	0.50151	0.30801	0.942902014
RPE65	ENSG00000116745	1	68884505	68925642	58	9	123834	0.50147	0.30802	0.942902014
ZNF266	ENSG00000174652	19	9513272	9556254	90	5	123834	0.50142	0.30804	0.942902014
MRPL34	ENSG00000130312	19	17393418	17427652	93	12	123834	0.50136	0.30806	0.942902014
SOX2	ENSG00000181449	3	181419714	181442221	5	2	123834	0.50085	0.30824	0.942902014
CNTF	ENSG00000242689	11	58380146	58403198	27	4	123834	0.50083	0.30825	0.942902014
SWT1	ENSG00000116668	1	185116212	185270897	77	6	123834	0.50062	0.30832	0.942902014
APBA3	ENSG00000011132	19	3740817	3771697	36	9	123834	0.50054	0.30835	0.942902014
HHIP	ENSG00000164161	4	145557173	145676423	124	8	123834	0.50044	0.30838	0.942902014
OR13C4	ENSG00000148136	9	107278534	107299490	59	8	123834	0.50028	0.30844	0.942902014
RDH13	ENSG00000160439	19	55540476	55592659	159	21	123834	0.50022	0.30846	0.942902014
FSCB	ENSG00000189139	14	44963545	44986482	98	12	123834	0.50012	0.3085	0.942902014
BAALC	ENSG00000164929	8	104142938	104252533	225	32	123834	0.49995	0.30856	0.942902014
KRT6A	ENSG00000205420	12	52870958	52897041	121	7	123834	0.49992	0.30857	0.942902014
LRRC6	ENSG00000129295	8	133574320	133697838	113	11	123834	0.49988	0.30858	0.942902014
ZNF85	ENSG00000105750	19	21096028	21143503	237	8	123834	0.49931	0.30878	0.942902014
DDX1	ENSG00000079785	2	15721302	15781235	141	9	123834	0.49904	0.30887	0.942902014
DLC1	ENSG00000164741	8	12930870	13383167	1464	65	123834	0.49881	0.30896	0.942902014
POSTN	ENSG00000133110	13	38126720	38182981	73	9	123834	0.4985	0.30907	0.942902014
RAB3D	ENSG00000105514	19	11422722	11466946	54	5	123834	0.49835	0.30912	0.942902014
TENC1	ENSG00000111077	12	53430753	53468156	54	10	123834	0.49829	0.30914	0.942902014
TDP1	ENSG00000042088	14	90411283	90521106	226	10	123834	0.49823	0.30916	0.942902014
DDX10	ENSG00000178105	11	108525752	108821657	378	13	123834	0.49814	0.30919	0.942902014
TANC2	ENSG00000170921	17	61076917	61515060	417	17	123834	0.4981	0.30921	0.942902014
AC002310.13	ENSG00000260869	16	30535548	30579600	14	4	123834	0.49802	0.30923	0.942902014
SIGLEC14	ENSG00000254415	19	52135806	52160054	25	4	123834	0.49765	0.30936	0.942902014
GIN1	ENSG00000145723	5	102411704	102465855	122	3	123834	0.4975	0.30942	0.942902014
RPL32	ENSG00000144713	3	12865984	12893087	50	4	123834	0.49746	0.30943	0.942902014
TSEN2	ENSG00000154743	3	12515931	12591122	209	11	123834	0.49744	0.30944	0.942902014
RFPL1	ENSG00000128250	22	29824572	29848444	57	7	123834	0.49745	0.30944	0.942902014
CA5A	ENSG00000174990	16	87911625	87980135	146	18	123834	0.49688	0.30964	0.942902014
NBEAL1	ENSG00000144426	2	203869602	204101101	255	14	123834	0.49665	0.30972	0.942902014

USP36	ENSG00000055483	17	76773463	76847523	72	8	123834	0.49647	0.30978	0.942902014
ARMC1	ENSG00000104442	8	66504694	66556442	154	8	123834	0.49644	0.30979	0.942902014
TRPC4	ENSG00000133107	13	38200773	38454562	537	42	123834	0.49644	0.30979	0.942902014
S1PR2	ENSG00000267534	19	10324520	10351948	11	4	123834	0.49641	0.3098	0.942902014
C1R	ENSG00000159403	12	7177513	7255203	35	9	123834	0.49626	0.30985	0.942902014
GPR137C	ENSG00000180998	14	53009866	53114431	179	17	123834	0.49622	0.30987	0.942902014
FBLN1	ENSG00000077942	22	45888118	46007015	173	24	123834	0.49621	0.30987	0.942902014
NARS2	ENSG00000137513	11	78137007	78295919	258	3	123834	0.4961	0.30991	0.942902014
PLOD2	ENSG00000152952	3	145777227	145891440	240	11	123834	0.49603	0.30994	0.942902014
MUC19	ENSG00000205592	12	40777197	40974632	883	27	123834	0.49593	0.30997	0.942902014
PDLIM2	ENSG00000120913	8	22425792	22465538	27	3	123834	0.49582	0.31001	0.942902014
RHD	ENSG00000187010	1	25588884	25666936	1	1	123834	0.49557	0.3101	0.942902014
DCAF7	ENSG00000136485	17	61617822	61681639	10	3	123834	0.49522	0.31022	0.942902014
TRIM9	ENSG00000100505	14	51431980	51572779	295	34	123834	0.4952	0.31023	0.942902014
ATF6B	ENSG00000213676	6	32055953	32106030	49	16	123834	0.4947	0.31041	0.942902014
SNRNP200	ENSG00000144028	2	96930074	96981297	34	6	123834	0.49465	0.31042	0.942902014
CLEC12A	ENSG00000172322	12	10093915	10158293	211	17	123834	0.49465	0.31042	0.942902014
DCP1B	ENSG00000151065	12	2045220	2123701	191	9	123834	0.49463	0.31043	0.942902014
RAB7L1	ENSG00000117280	1	205727114	205754588	71	11	123834	0.49451	0.31047	0.942902014
SARS2	ENSG00000104835	19	39395906	39450495	71	8	123834	0.49452	0.31047	0.942902014
CTC-360G5.8	ENSG00000269547	19	39396044	39450495	71	8	123834	0.49452	0.31047	0.942902014
APOBEC3C	ENSG00000244509	22	39400088	39426357	15	3	123834	0.49445	0.3105	0.942902014
TBC1D4	ENSG00000136111	13	75848808	76066250	202	37	123834	0.4944	0.31051	0.942902014
FN1	ENSG00000115414	2	216215163	216310895	160	20	123834	0.49437	0.31052	0.942902014
CCER2	ENSG00000262484	19	39389620	39412798	32	6	123834	0.49425	0.31056	0.942902014
TMUB1	ENSG00000164897	7	150768167	150790633	9	2	123834	0.49423	0.31057	0.942902014
FAM89B	ENSG00000176973	11	65329820	65351669	15	3	123834	0.49393	0.31068	0.942902014
LEFTY2	ENSG00000143768	1	226114298	226139189	16	3	123834	0.49389	0.31069	0.942902014
KLC3	ENSG00000104892	19	45826692	45864778	53	8	123834	0.49378	0.31073	0.942902014
SLX4	ENSG00000188827	16	3621182	3671599	103	3	123834	0.49351	0.31083	0.942902014
C8orf48	ENSG00000164743	8	13414352	13435796	87	14	123834	0.49317	0.31095	0.942902014
AZI1	ENSG00000141577	17	79153393	79206799	111	14	123834	0.49304	0.31099	0.942902014
MTA1	ENSG00000182979	14	105876159	105947066	6	1	123834	0.4928	0.31108	0.942902014
METTL7A	ENSG00000185432	12	51307255	51336300	43	7	123834	0.49259	0.31115	0.942902014
AP5S1	ENSG00000125843	20	3791178	3815949	40	6	123834	0.49256	0.31116	0.942902014
PA2G4	ENSG00000170515	12	56488103	56517691	11	3	123834	0.4923	0.31125	0.942902014
B3GALNT1	ENSG00000169255	3	160791671	160833172	68	7	123834	0.49213	0.31131	0.942902014
CEP89	ENSG00000121289	19	33359902	33472897	381	20	123834	0.49215	0.31131	0.942902014
HIST1H2AG	ENSG00000196787	6	27090832	27113070	23	9	123834	0.49201	0.31136	0.942902014
AC112715.2	ENSG00000224132	2	238155734	238176319	12	2	123834	0.4919	0.3114	0.942902014
MYRIP	ENSG00000170011	3	39840405	40311812	1005	45	123834	0.49172	0.31146	0.942902014
FAM71E2	ENSG00000180043	19	55856276	55884628	26	5	123834	0.49164	0.31149	0.942902014
TANC1	ENSG00000115183	2	159815146	160099170	602	41	123834	0.4915	0.31153	0.942902014
PSAPL1	ENSG00000178597	4	7422022	7446700	55	11	123834	0.49128	0.31162	0.942902014
ACD	ENSG00000102977	16	67681415	67704713	14	3	123834	0.4912	0.31164	0.942902014
MC3R	ENSG00000124089	20	54813788	54834871	52	11	123834	0.49103	0.3117	0.942902014
CDK5RAP3	ENSG00000108465	17	46035176	46069140	78	9	123834	0.49101	0.31171	0.942902014
MUSTN1	ENSG00000272573	3	52857130	52879235	37	3	123834	0.49095	0.31173	0.942902014
EPB41L4B	ENSG00000095203	9	111924255	112093244	181	28	123834	0.4909	0.31175	0.942902014
SLC39A11	ENSG00000133195	17	70632088	71098851	741	103	123834	0.49091	0.31175	0.942902014
SMPD2	ENSG00000135587	6	109751966	109775122	36	7	123834	0.4908	0.31178	0.942902014
RELN	ENSG00000189056	7	103102231	103639963	1237	82	123834	0.49071	0.31181	0.942902014
OR2F2	ENSG00000221910	7	143622259	143643372	67	6	123834	0.49074	0.31181	0.942902014
MYLK2	ENSG00000101306	20	30397111	30432492	57	5	123834	0.4907	0.31182	0.942902014
OCEL1	ENSG00000099330	19	17327013	17350028	40	8	123834	0.49058	0.31186	0.942902014
GLIPR1L1	ENSG00000173401	12	75718419	75774340	58	6	123834	0.49057	0.31187	0.942902014
ZBTB9	ENSG00000213588	6	33412356	33435325	27	6	123834	0.49038	0.31193	0.942933435
CDH8	ENSG00000150394	16	61671146	62080939	521	36	123834	0.49004	0.31205	0.942971933
BEND5	ENSG00000162373	1	49183195	49252641	57	10	123834	0.48991	0.3121	0.942971933
SLC25A10	ENSG00000262660	17	79660401	79697569	81	10	123834	0.48962	0.3122	0.942971933
SLC25A10	ENSG00000183048	17	79660404	79698042	81	10	123834	0.48962	0.3122	0.942971933
HK2	ENSG00000159399	2	75051108	75130486	85	18	123834	0.48947	0.31226	0.942971933
MICA	ENSG00000204520	6	31361356	31393092	350	21	123834	0.48938	0.31229	0.942971933
C22orf15	ENSG00000169314	22	24095208	24118048	17	4	123834	0.48936	0.31229	0.942971933
SPINK14	ENSG00000196800	5	147539296	147564961	79	3	123834	0.4891	0.31238	0.943093875
CNDP1	ENSG00000150656	18	72191675	72264448	102	20	123834	0.48898	0.31243	0.943095036
AC004824.2	ENSG00000269468	1	17645507	17686070	147	10	123834	0.48874	0.31251	0.943158857
LRCH3	ENSG00000186001	3	197508097	197625307	146	14	123834	0.48856	0.31258	0.943158857
LSM1	ENSG00000175324	8	38010839	38044248	17	2	123834	0.4885	0.3126	0.943158857
CDC23	ENSG00000094880	5	137513339	137559032	37	5	123834	0.48827	0.31268	0.943250506
MRPS6	ENSG00000243927	21	35435524	35525334	56	3	123834	0.48775	0.31287	0.943673907
ONECUT2	ENSG00000119547	18	55092917	55168529	173	20	123834	0.48753	0.31294	0.943735288
NEO1	ENSG00000067141	15	73334051	73607547	386	17	123834	0.48707	0.3131	0.943771755
ZNF629	ENSG00000102870	16	30779778	30808523	13	4	123834	0.48705	0.31311	0.943771755
KLKB1	ENSG00000164344	4	187120133	187189625	186	16	123834	0.48691	0.31316	0.943771755
CUTC	ENSG00000119929	10	101452315	101525891	69	8	123834	0.48684	0.31318	0.943771755
FGD2	ENSG00000146192	6	36963422	37006846	108	16	123834	0.48671	0.31323	0.943771755
ZNF821	ENSG00000102984	16	71883583	71939239	75	8	123834	0.48666	0.31325	0.943771755
FXYD6	ENSG00000137726	11	117697693	117758201	53	12	123834	0.48634	0.31336	0.943864212
ATP1A3	ENSG00000105409	19	42460734	42511649	5	2	123834	0.48628	0.31338	0.943864212
RBP5	ENSG00000139194	12	7266280	7291538	33	8	123834	0.48615	0.31343	0.943865247
CBLN3	ENSG00000139899	14	24885738	24910160	49	9	123834	0.48573	0.31358	0.943899481
MGAT4C	ENSG00000182050	12	86362516	87242681	1546	34	123834	0.48568	0.3136	0.943899481
MSL1	ENSG00000188895	17	38268551	38303042	32	4	123834	0.48559	0.31363	0.943899481
PTRF	ENSG00000177469	17	40544470	40585535	80	9	123834	0.48555	0.31364	0.943899481
PCDHGA7	ENSG00000253537	5	140752467	140902546	264	20	123834	0.48538	0.3137	0.9439306
GABRP	ENSG00000094755	5	170180354	170251051	130	20	123834	0.48526	0.31375	0.943931624
MREG	ENSG00000118242	2	216799213	216908819	205	36	123834	0.48466	0.31396	0.944176248
GUCY2C	ENSG00000070019	12	14755576	14859519	95	10	123834	0.48461	0.31398	0.944176248
AC073333.1	ENSG00000267906	7	16818866	16839246	46	11	123834	0.48455	0.314	0.944176248
MICAL1	ENSG00000135596	6	109755265	109797171	61	10	123834	0.48446	0.31403	0.944176248
PPTC7	ENSG00000196850	12	110959120	111031125	63	8	123834	0.48391	0.31423	0.944311922
SLC25A40	ENSG00000075303	7	87452883	87515672	89	7	123834	0.48369	0.3143	0.944311922
FAT3	ENSG00000165323	11	92075262	92639618	719	40	123834	0.48367	0.31431	0.944311922
PHF20L1	ENSG00000129292	8	133777618	133871052	136	8	123834	0.4835	0.31437	0.944311922
COQ2	ENSG00000173085	4	84172689	84216067	47	6	123834	0.4831	0.31451	0.944311922
SORL1	ENSG00000137642	11	121312912	121514402	180	33	123834	0.48302	0.31454	0.944311922
GDI2	ENSG00000057608	10	5797186	5894095	163	19	123834	0.48301	0.31455	0.944311922
PRDX1	ENSG00000117450	1	45966708	45998719	60	4	123834	0.48289	0.31459	0.944311922

UBE2I	ENSG00000103275	16	1345548	1387019	117	9	123834	0.48289	0.31459	0.944311922
CHST11	ENSG00000171310	12	104839073	105165792	533	67	123834	0.48269	0.31466	0.944311922
FMNL2	ENSG00000157827	2	153181751	153516348	529	39	123834	0.48261	0.31468	0.944311922
GNPDA1	ENSG00000113552	5	141361314	141402606	99	7	123834	0.48258	0.3147	0.944311922
RUNDC3B	ENSG00000105784	7	87246864	87471611	249	10	123834	0.48243	0.31475	0.944311922
PCDHGA10	ENSG00000253846	5	140782743	140902546	209	25	123834	0.48224	0.31482	0.944311922
ATP6V1G2	ENSG00000213760	6	31502239	31526204	73	16	123834	0.48215	0.31485	0.944311922
FBXO5	ENSG00000112029	6	153281664	153314714	81	8	123834	0.48209	0.31487	0.944311922
AKR1C3	ENSG00000196139	10	5067546	5159878	371	32	123834	0.48181	0.31497	0.944462811
UBB	ENSG00000170315	17	16274112	16296059	31	5	123834	0.48143	0.3151	0.944584589
PTP4A1	ENSG00000112245	6	64221666	64303492	102	11	123834	0.48142	0.31511	0.944584589
LAMP3	ENSG00000078081	3	182830001	182891627	167	16	123834	0.48115	0.31521	0.944735364
HECA	ENSG00000112406	6	139446249	139511939	150	10	123834	0.48076	0.31534	0.944797957
RMND5A	ENSG00000153561	2	86937296	87015164	54	6	123834	0.48071	0.31536	0.944797957
ALG8	ENSG00000159063	11	77801982	77860706	239	6	123834	0.48065	0.31538	0.944797957
COL11A1	ENSG00000060718	1	103332023	103584052	900	29	123834	0.48028	0.31551	0.945038462
PCID2	ENSG00000126226	13	113821891	113873029	52	3	123834	0.47979	0.31569	0.945428631
TGFB2	ENSG00000092969	1	218509577	218627961	95	12	123834	0.47951	0.31579	0.94557913
BTBD18	ENSG00000233436	11	57500986	57529253	23	3	123834	0.47913	0.31592	0.945730318
HSCB	ENSG00000100209	22	29128019	29163503	62	13	123834	0.47909	0.31594	0.945730318
TBC1D10B	ENSG00000169221	16	30358423	30391585	14	3	123834	0.47874	0.31606	0.945763902
HS3ST3B1	ENSG00000125430	17	14194400	14262721	62	19	123834	0.4786	0.31611	0.945763902
STAT6	ENSG00000166888	12	57479191	57535922	31	7	123834	0.47838	0.31619	0.945763902
ZNF705A	ENSG00000196946	12	8280733	8351133	81	5	123834	0.47836	0.3162	0.945763902
SFSWAP	ENSG00000061936	12	132185626	132294282	142	15	123834	0.47835	0.3162	0.945763902
SUSD3	ENSG00000157303	9	95810989	95857420	82	8	123834	0.47792	0.31635	0.945817266
DDI2	ENSG00000197312	1	15933995	16005539	127	5	123834	0.47784	0.31638	0.945817266
ADAMTS13	ENSG00000160323	9	136269478	136334508	166	13	123834	0.47778	0.3164	0.945817266
PCDHGB4	ENSG00000253953	5	140757452	140902546	253	20	123834	0.47763	0.31646	0.945817266
TBC1D9B	ENSG00000197226	5	179279066	179344859	51	8	123834	0.47713	0.31663	0.945817266
TNS4	ENSG00000131746	17	38622080	38667849	52	9	123834	0.47704	0.31667	0.945817266
ANO7	ENSG00000146205	2	242117924	242174792	63	10	123834	0.47679	0.31676	0.945817266
RANBP3L	ENSG00000164188	5	36238536	36312216	87	7	123834	0.47662	0.31682	0.945817266
SEC13	ENSG00000157020	3	10324815	10372862	55	10	123834	0.47659	0.31683	0.945817266
VWCE	ENSG00000167992	11	61015762	61072896	6	2	123834	0.47658	0.31683	0.945817266
ABRACL	ENSG00000146386	6	139339819	139374439	45	8	123834	0.47638	0.3169	0.945817266
KIF5A	ENSG00000155980	12	57933781	57990415	35	7	123834	0.47617	0.31698	0.945817266
ADAT3	ENSG00000213638	19	1895377	1923444	46	5	123834	0.47594	0.31706	0.945817266
LEPREL1	ENSG00000090530	3	189664517	189850226	317	18	123834	0.4758	0.31711	0.945817266
ARL8A	ENSG00000143862	1	202092532	202123869	32	10	123834	0.47567	0.31715	0.945817266
METTL2A	ENSG00000087995	17	60491228	60537454	12	2	123834	0.47565	0.31716	0.945817266
RAI14	ENSG00000039560	5	34646342	34842732	285	42	123834	0.47532	0.31728	0.945817266
ASPG	ENSG00000166183	14	104542016	104589098	52	10	123834	0.4753	0.31729	0.945817266
ARHGAP32	ENSG00000134909	11	128824955	129159219	622	22	123834	0.47523	0.31731	0.945817266
GPN2	ENSG00000142751	1	27192624	27226788	36	4	123834	0.47499	0.3174	0.945817266
INPP5D	ENSG00000168918	2	233914677	234126549	248	38	123834	0.47468	0.31751	0.945817266
ARL5A	ENSG00000162980	2	152635498	152695006	83	9	123834	0.47459	0.31754	0.945817266
PPIP5K2	ENSG00000145725	5	102445853	102558500	125	6	123834	0.47429	0.31765	0.945817266
ARAP2	ENSG00000047365	4	35939843	36256131	541	39	123834	0.47415	0.3177	0.945817266
GRID1	ENSG00000182771	10	87349312	88136250	1539	94	123834	0.47412	0.31771	0.945817266
TEX37	ENSG00000172073	2	88814169	88839102	65	7	123834	0.47405	0.31773	0.945817266
ITGA2	ENSG00000164171	5	52275156	52400609	352	23	123834	0.47375	0.31784	0.945817266
NUP153	ENSG00000124789	6	17605269	17716656	161	17	123834	0.47363	0.31788	0.945817266
MRPS14	ENSG00000120333	1	174969925	175002561	26	7	123834	0.47361	0.31789	0.945817266
TXLNA	ENSG00000084652	1	32635287	32673886	28	2	123834	0.47355	0.31791	0.945817266
DLL3	ENSG00000090932	19	39979535	40009121	41	5	123834	0.47347	0.31794	0.945817266
IKZF1	ENSG00000185811	7	50333720	50482799	187	27	123834	0.47311	0.31807	0.945817266
MCUR1	ENSG00000050393	6	13776789	13824800	35	8	123834	0.47294	0.31813	0.945817266
WDR59	ENSG00000103091	16	74897468	75044071	157	31	123834	0.47295	0.31813	0.945817266
MTERF	ENSG00000127989	7	91311323	91520034	296	12	123834	0.47288	0.31815	0.945817266
PCDHGA8	ENSG00000253767	5	140762381	140902546	243	27	123834	0.47267	0.31822	0.945817266
RXRB	ENSG00000204231	6	33151365	33178630	43	9	123834	0.47267	0.31823	0.945817266
CTNNAL1	ENSG00000119326	9	111694851	111785809	197	18	123834	0.47246	0.3183	0.945817266
CCDC174	ENSG00000154781	3	14683271	14724166	72	6	123834	0.47243	0.31831	0.945817266
MDFI	ENSG00000112559	6	41594620	41631984	45	10	123834	0.4724	0.31832	0.945817266
HMGCS1	ENSG00000112972	5	43279497	43323614	82	3	123834	0.47239	0.31833	0.945817266
MSRB3	ENSG00000174099	12	65662423	65892024	205	14	123834	0.47228	0.31836	0.945817266
UBTF	ENSG00000108312	17	42272401	42308994	31	5	123834	0.47193	0.31849	0.945817266
IPPK	ENSG00000127080	9	95365466	95442547	175	15	123834	0.47178	0.31854	0.945817266
PTH2	ENSG00000142538	19	49915671	49936698	7	2	123834	0.47156	0.31862	0.945817266
NANOS1	ENSG00000188613	10	120779228	120803854	16	3	123834	0.47142	0.31867	0.945817266
PCDHGB3	ENSG00000262209	5	140739831	140902546	272	21	123834	0.47139	0.31868	0.945817266
C20orf166	ENSG00000174407	20	61137660	61177971	32	6	123834	0.47128	0.31872	0.945817266
SLCO2A1	ENSG00000174640	3	133641540	133781028	175	35	123834	0.47113	0.31877	0.945817266
IKZF5	ENSG00000095574	10	124740322	124778333	94	12	123834	0.47093	0.31884	0.945817266
MROH8	ENSG00000101353	20	35719629	35817991	176	12	123834	0.47089	0.31886	0.945817266
ZFPM2	ENSG00000169946	8	106320920	106826760	733	66	123834	0.47076	0.3189	0.945817266
ADAD1	ENSG00000164113	4	123290121	123360957	97	10	123834	0.47075	0.31891	0.945817266
DNAH3	ENSG00000158486	16	20934433	21180762	390	27	123834	0.47063	0.31895	0.945817266
XBP1	ENSG00000100219	22	29180543	29206585	46	9	123834	0.47038	0.31904	0.945817266
BATF2	ENSG00000168062	11	64745415	64774517	43	7	123834	0.46988	0.31922	0.945817266
BTG2	ENSG00000159388	1	203264619	203288730	35	9	123834	0.46977	0.31926	0.945817266
SCARB1	ENSG00000073060	12	125251402	125377214	117	24	123834	0.46963	0.31931	0.945817266
KCNK1	ENSG00000135750	1	233739750	233818258	89	22	123834	0.46955	0.31934	0.945817266
IRS2	ENSG00000185950	13	110396184	110448915	71	10	123834	0.46949	0.31936	0.945817266
TRIM32	ENSG00000119401	9	119439581	119473579	73	11	123834	0.46943	0.31938	0.945817266
PACRGL	ENSG00000163138	4	20687905	20764530	177	15	123834	0.46934	0.31941	0.945817266
DCSTAMP	ENSG00000164935	8	105341315	105378917	55	11	123834	0.46894	0.31955	0.945817266
COMMD1	ENSG00000173163	2	62105859	62384382	378	17	123834	0.46846	0.31973	0.945817266
PCDHGA6	ENSG00000253731	5	140743651	140902546	270	22	123834	0.46835	0.31977	0.945817266
TCP11	ENSG00000124678	6	35075848	35126387	146	14	123834	0.46833	0.31977	0.945817266
GTF2E2	ENSG00000197265	8	30425835	30525768	129	11	123834	0.46833	0.31977	0.945817266
FPGT-TNNI3K	ENSG00000259030	1	74653919	75019666	411	36	123834	0.46833	0.31978	0.945817266
TNNI3K	ENSG00000116783	1	74653947	75020112	411	36	123834	0.46833	0.31978	0.945817266
PRX	ENSG00000105227	19	40889675	40929273	23	7	123834	0.46826	0.3198	0.945817266
IQCG	ENSG00000114473	3	197605946	197697013	179	14	123834	0.46822	0.31981	0.945817266
AC007773.3	ENSG00000269093	19	32885915	32906466	28	5	123834	0.46823	0.31981	0.945817266
SATB1	ENSG00000182568	3	18376879	18497080	142	19	123834	0.4681	0.31986	0.945817266
PCDHGB6	ENSG00000253305	5	140777770	140902546	216	25	123834	0.46798	0.3199	0.945817266

ADRM1	ENSG00000130706	20	60867149	60893918	33	8	123834	0.46751	0.32007	0.946172715
EPB41L5	ENSG00000115109	2	120760581	120946695	259	8	123834	0.46733	0.32013	0.94620293
ACRBP	ENSG00000111644	12	6737241	6766626	43	7	123834	0.46719	0.32018	0.946203582
SLC46A3	ENSG00000139508	13	29264201	29303107	34	4	123834	0.46706	0.32023	0.946204234
WDPCP	ENSG00000143951	2	63338518	64064977	1016	22	123834	0.46692	0.32028	0.946204887
ENO1	ENSG00000074800	1	8911061	8949308	30	3	123834	0.46649	0.32043	0.946237878
CABP5	ENSG00000105507	19	48523210	48557310	83	7	123834	0.46643	0.32045	0.946237878
TUBB4A	ENSG00000104833	19	6484330	6512859	5	2	123834	0.46636	0.32048	0.946237878
AL096677.1	ENSG00000269407	20	23320015	23344066	66	10	123834	0.46618	0.32054	0.946237878
LRIF1	ENSG00000121931	1	111479807	111516701	41	3	123834	0.46604	0.32059	0.946237878
SFN	ENSG00000175793	1	27179633	27200947	21	3	123834	0.46585	0.32066	0.946237878
DKK4	ENSG00000104371	8	42221586	42244750	40	5	123834	0.46585	0.32066	0.946237878
RNF125	ENSG00000101695	18	29588335	29663176	50	11	123834	0.46536	0.32084	0.946237878
EBAG9	ENSG00000147654	8	110541940	110588225	63	9	123834	0.46516	0.32091	0.946237878
CTBP2	ENSG00000175029	10	126666421	126859739	389	45	123834	0.46503	0.32096	0.946237878
PLA2G4C	ENSG00000105499	19	48541100	48624074	179	28	123834	0.46502	0.32096	0.946237878
IL2	ENSG00000109471	4	123362625	123387880	36	5	123834	0.46448	0.32115	0.946237878
FOXF1	ENSG00000103241	16	86534133	86558076	31	9	123834	0.46433	0.32121	0.946237878
RP11-77K12.1	ENSG00000261717	16	75436582	75508604	219	8	123834	0.46423	0.32124	0.946237878
RLN3	ENSG00000171136	19	14128960	14151854	39	5	123834	0.46406	0.3213	0.946237878
CASC3	ENSG00000108349	17	38286571	38338436	36	3	123834	0.46378	0.3214	0.946237878
SLC1A3	ENSG00000079215	5	36596457	36698436	83	17	123834	0.46374	0.32142	0.946237878
ARHGAP21	ENSG00000107863	10	24862538	25022597	115	9	123834	0.46362	0.32146	0.946237878
PHACTR4	ENSG00000204138	1	28686114	28836881	404	8	123834	0.4633	0.32157	0.946237878
OR2M5	ENSG00000162727	1	248298450	248319388	37	4	123834	0.46321	0.32161	0.946237878
RBBP8NL	ENSG00000130701	20	60975293	61012589	72	15	123834	0.46304	0.32167	0.946237878
CRABP2	ENSG00000143320	1	156659398	156685608	6	1	123834	0.46301	0.32168	0.946237878
RGL4	ENSG00000159496	22	24020323	24051363	65	7	123834	0.46293	0.32171	0.946237878
PCDHGB2	ENSG00000253910	5	140729703	140902546	288	22	123834	0.46285	0.32173	0.946237878
ALDH8A1	ENSG00000118514	6	135228528	135281260	42	4	123834	0.46276	0.32177	0.946237878
WDR49	ENSG00000174776	3	167186472	167381771	111	16	123834	0.46272	0.32178	0.946237878
PIK3R2	ENSG00000268173	19	18253968	18298927	40	7	123834	0.46264	0.32181	0.946237878
MATK	ENSG00000007264	19	3767971	3812127	36	11	123834	0.46258	0.32183	0.946237878
SPG7	ENSG00000197912	16	89547325	89634176	164	7	123834	0.46242	0.32189	0.946237878
CLPS	ENSG00000137392	6	35752759	35775088	13	4	123834	0.46213	0.32199	0.946237878
PCDHGA5	ENSG00000253485	5	140733898	140902546	280	21	123834	0.462	0.32204	0.946237878
PCDHGA4	ENSG00000262576	5	140724768	140902546	293	22	123834	0.46184	0.3221	0.946237878
KCNK2	ENSG00000082482	1	215169118	215420436	573	24	123834	0.46144	0.32224	0.946237878
GPATCH3	ENSG00000198746	1	27206979	27236957	33	5	123834	0.4614	0.32226	0.946237878
FBXO45	ENSG00000174013	3	196285482	196325930	26	6	123834	0.46128	0.3223	0.946237878
EFHD1	ENSG00000115468	2	233460767	233557491	109	14	123834	0.46124	0.32231	0.946237878
PCDH1	ENSG00000156453	5	141222938	141268811	27	6	123834	0.46119	0.32233	0.946237878
KPNA5	ENSG00000196911	6	116992350	117073029	98	10	123834	0.46104	0.32238	0.946237878
GCG	ENSG00000115263	2	162989392	163018914	35	3	123834	0.46103	0.32239	0.946237878
RLTPR	ENSG00000159753	16	67668822	67701472	20	3	123834	0.46095	0.32242	0.946237878
AP1G1	ENSG00000166747	16	71752913	71853104	176	14	123834	0.4609	0.32243	0.946237878
WFDC6	ENSG00000243543	20	44152835	44178134	38	5	123834	0.46089	0.32244	0.946237878
SLC2A4	ENSG00000181856	17	7174986	7201576	14	4	123834	0.46071	0.3225	0.946237878
PSMB5	ENSG00000100804	14	23475752	23514439	24	6	123834	0.46056	0.32256	0.946237878
PTPRA	ENSG00000132670	20	2834830	3029722	219	13	123834	0.46056	0.32256	0.946237878
BAMBI	ENSG00000095739	10	28956271	28981868	7	2	123834	0.46038	0.32262	0.946237878
TMEM45B	ENSG00000151715	11	129675714	129739898	112	15	123834	0.4602	0.32268	0.946237878
GAREML	ENSG00000157833	2	26385960	26422532	48	4	123834	0.46005	0.32274	0.946237878
KRT6C	ENSG00000170465	12	52852300	52877569	136	7	123834	0.45996	0.32277	0.946237878
ATP5A1	ENSG00000152234	18	43654110	43694300	125	9	123834	0.45982	0.32282	0.946237878
KHSRP	ENSG00000088247	19	6403359	6434805	19	6	123834	0.45981	0.32283	0.946237878
CPEB1	ENSG00000214575	15	83201951	83327612	177	16	123834	0.45928	0.32302	0.946388144
DISP1	ENSG00000154309	1	222978406	223189337	448	28	123834	0.4589	0.32315	0.946388144
EFCC1	ENSG00000114654	3	128710472	128769585	37	6	123834	0.45886	0.32317	0.946388144
MAP3K4	ENSG00000085511	6	161402759	161548417	225	19	123834	0.45884	0.32317	0.946388144
ZMPSTE24	ENSG00000084073	1	40713779	40769856	47	9	123834	0.45882	0.32318	0.946388144
SNRNP25	ENSG00000161981	16	93010	117669	20	3	123834	0.45881	0.32318	0.946388144
RTN4IP1	ENSG00000130347	6	107008903	107087373	241	13	123834	0.45847	0.32331	0.946622994
DDX18	ENSG00000088205	2	118562226	118599955	89	13	123834	0.45808	0.32345	0.946799778
C16orf72	ENSG00000182831	16	9175505	9225497	61	7	123834	0.45801	0.32347	0.946799778
LGALS3BP	ENSG00000108679	17	76957320	76986191	42	11	123834	0.45781	0.32355	0.946802548
KLHL38	ENSG00000175946	8	124647767	124675190	84	12	123834	0.45759	0.32362	0.946802548
CUEDC2	ENSG00000107874	10	104173002	104202418	35	10	123834	0.45755	0.32364	0.946802548
DUSP27	ENSG00000198842	1	167053282	167108402	105	13	123834	0.4574	0.32369	0.946802548
VIT	ENSG00000205221	2	36913833	37051935	227	38	123834	0.45731	0.32372	0.946802548
HFE	ENSG00000010704	6	26077509	26108571	64	11	123834	0.45679	0.32391	0.946979612
EFNA3	ENSG00000251246	1	155026224	155069283	35	8	123834	0.45673	0.32393	0.946979612
EFNA3	ENSG00000143590	1	155026224	155070014	35	8	123834	0.45673	0.32393	0.946979612
SELM	ENSG00000198832	22	31490758	31526055	75	12	123834	0.45648	0.32402	0.94701041
VCPKMT	ENSG00000100483	14	50565350	50593318	70	10	123834	0.45634	0.32407	0.94701041
DDX21	ENSG00000165732	10	70705884	70754829	95	12	123834	0.45628	0.32409	0.94701041
TM4SF19	ENSG00000273331	3	196032953	196075244	75	10	123834	0.45563	0.32433	0.947363392
OR51V1	ENSG00000176742	11	5210965	5231930	74	6	123834	0.45538	0.32442	0.947363392
MRGPRX2	ENSG00000183695	11	19066002	19092228	78	8	123834	0.45537	0.32442	0.947363392
CBLN4	ENSG00000054803	20	54562496	54590528	14	3	123834	0.45536	0.32443	0.947363392
GNG10	ENSG00000242616	9	114413615	114442526	53	8	123834	0.45526	0.32446	0.947363392
WISP1	ENSG00000104415	8	134193282	134252587	67	15	123834	0.45481	0.32462	0.947656637
FTSJ2	ENSG00000122687	7	2263866	2291840	39	7	123834	0.45466	0.32468	0.947656637
PIK3R2	ENSG00000105647	19	18253928	18291350	32	5	123834	0.45457	0.32471	0.947656637
C11orf30	ENSG00000158636	11	76145967	76274069	188	20	123834	0.45381	0.32498	0.948299024
NAA11	ENSG00000156269	4	80136848	80257204	154	13	123834	0.45347	0.3251	0.948475287
RPS12	ENSG00000112306	6	133125580	133148703	76	8	123834	0.45332	0.32516	0.948475287
OR10K1	ENSG00000173285	1	158425272	158446344	68	8	123834	0.4532	0.3252	0.948475287
COL6A6	ENSG00000206384	3	130269178	130406999	129	18	123834	0.45311	0.32524	0.948475287
AC107021.1	ENSG00000232013	3	145772358	145794156	43	6	123834	0.45238	0.3255	0.948537327
AL358113.1	ENSG00000269295	9	71859331	71879642	37	12	123834	0.45234	0.32551	0.948537327
ASZ1	ENSG00000154438	7	116993276	117078177	79	9	123834	0.45212	0.32559	0.948537327
UBE2H	ENSG00000186591	7	129460572	129602789	361	8	123834	0.45214	0.32559	0.948537327
PROB1	ENSG00000228672	5	138717635	138740885	13	2	123834	0.45194	0.32566	0.948537327
CERS4	ENSG00000090661	19	8261620	8337305	138	25	123834	0.45186	0.32568	0.948537327
DTYMK	ENSG00000168393	2	242605157	242636406	95	8	123834	0.45185	0.32569	0.948537327
ZNF579	ENSG00000218891	19	56078899	56102211	67	7	123834	0.45179	0.32571	0.948537327
RAPGEF6	ENSG00000158987	5	130749614	130980929	247	10	123834	0.45166	0.32576	0.948537327
TMX2	ENSG00000213593	11	57470072	57518445	45	4	123834	0.45157	0.32579	0.948537327

OR5P2	ENSG00000183303	11	7807452	7828520	66	9	123834	0.45149	0.32582	0.948537327
KRT25	ENSG00000204897	17	38894273	38921584	38	7	123834	0.45129	0.32589	0.948537327
ACO2	ENSG00000100412	22	41855129	41934993	57	3	123834	0.45123	0.32591	0.948537327
ZNF560	ENSG00000198028	19	9567183	9619283	140	4	123834	0.45105	0.32598	0.948595811
ADARB2	ENSG00000185736	10	1218073	1789670	1122	128	123834	0.45081	0.32606	0.948683374
ANXA2	ENSG00000182718	15	60629333	60705082	62	7	123834	0.45016	0.3263	0.949066715
GPR133	ENSG00000111452	12	131428452	131636014	382	40	123834	0.45002	0.32635	0.949066715
DHX15	ENSG00000109606	4	24509064	24596173	122	13	123834	0.44955	0.32652	0.949066715
EPS8L1	ENSG00000131037	19	55573388	55609291	46	6	123834	0.44943	0.32656	0.949066715
SASS6	ENSG00000156876	1	100539119	100608511	101	9	123834	0.44924	0.32663	0.949066715
KIAA1432	ENSG00000107036	9	5619025	5786557	281	18	123834	0.4491	0.32668	0.949066715
AP2A2	ENSG00000183020	11	914894	1022239	202	9	123834	0.44909	0.32668	0.949066715
ELP5	ENSG00000170291	17	7144735	7173259	23	3	123834	0.44893	0.32674	0.949066715
SMPDL3B	ENSG00000130768	1	28251504	28295668	93	8	123834	0.44846	0.32691	0.949066715
RHOBTB3	ENSG00000164292	5	95039226	95170087	119	19	123834	0.44833	0.32696	0.949066715
GCSAML	ENSG00000169224	1	247660360	247750992	155	24	123834	0.44826	0.32698	0.949066715
CAMK2D	ENSG00000145349	4	114362188	114693083	500	41	123834	0.44828	0.32698	0.949066715
TPP2	ENSG00000134900	13	103239353	103341521	319	12	123834	0.44782	0.32714	0.949066715
GLRA3	ENSG00000145451	4	175548065	175760465	399	40	123834	0.44781	0.32715	0.949066715
GHRH	ENSG00000118702	20	35869489	35900238	32	5	123834	0.44766	0.3272	0.949066715
EID2	ENSG00000176396	19	40018890	40040870	44	6	123834	0.44764	0.32721	0.949066715
DBI	ENSG00000155368	2	120114497	120140126	93	9	123834	0.44748	0.32726	0.949066715
RAET1E	ENSG00000164520	6	150194511	150229238	83	4	123834	0.44744	0.32728	0.949066715
ACCS	ENSG00000110455	11	44077475	44115772	66	13	123834	0.44738	0.3273	0.949066715
TNFSF10	ENSG00000121858	3	172213298	172251297	106	11	123834	0.44737	0.32731	0.949066715
GLOD4	ENSG00000167699	17	650337	695581	107	12	123834	0.44737	0.32731	0.949066715
MAK16	ENSG00000198042	8	33332268	33368778	89	7	123834	0.44718	0.32737	0.949066715
RPP25L	ENSG00000164967	9	34600483	34622101	23	5	123834	0.44711	0.3274	0.949066715
GPR107	ENSG00000148358	9	132805705	132912448	292	19	123834	0.44704	0.32742	0.949066715
OR4C3	ENSG00000176547	11	48336472	48357507	45	5	123834	0.44698	0.32744	0.949066715
SLC10A2	ENSG00000125255	13	103686350	103729196	157	22	123834	0.44655	0.3276	0.949385699
TCF21	ENSG00000118526	6	134200276	134226691	39	4	123834	0.44624	0.32771	0.94941513
ZBTB46	ENSG00000130584	20	62365019	62472597	318	16	123834	0.44617	0.32774	0.94941513
DDHD2	ENSG00000085788	8	38072736	38143076	37	2	123834	0.44611	0.32776	0.94941513
EPPIN	ENSG00000101448	20	44155625	44186391	88	5	123834	0.44586	0.32785	0.949503123
CSTF1	ENSG00000101138	20	54957427	54989518	23	8	123834	0.44556	0.32796	0.949503123
WWP1	ENSG00000123124	8	87344967	87500649	370	16	123834	0.44536	0.32803	0.949503123
NCOA2	ENSG00000140396	8	71011997	71326040	374	14	123834	0.44527	0.32806	0.949503123
PCDHGA2	ENSG00000081853	5	140708539	140902546	311	23	123834	0.4452	0.32809	0.949503123
ZNF341	ENSG00000131061	20	32309463	32390075	19	3	123834	0.44516	0.3281	0.949503123
ARL6IP4	ENSG00000182196	12	123454607	123477456	12	2	123834	0.44504	0.32815	0.949503123
NOTCH1	ENSG00000148400	9	139378896	139450314	76	20	123834	0.44492	0.32819	0.949503123
C5orf30	ENSG00000181751	5	102584403	102624361	58	5	123834	0.44446	0.32835	0.949821458
LLGL2	ENSG00000073350	17	73511161	73581289	225	24	123834	0.44394	0.32854	0.950139735
SESN2	ENSG00000130766	1	28576038	28619002	63	7	123834	0.44389	0.32856	0.950139735
PHF1	ENSG00000112511	6	33368176	33394230	25	6	123834	0.4437	0.32863	0.950139814
NDUFS6	ENSG00000145494	5	1791514	1826719	121	10	123834	0.44363	0.32866	0.950139814
OR4D11	ENSG00000176200	11	59261049	59281984	26	2	123834	0.44323	0.3288	0.950371108
MYLPF	ENSG00000180209	16	30372255	30399312	19	2	123834	0.44292	0.32891	0.950371108
HIST1H3F	ENSG00000256316	6	26240370	26260835	34	6	123834	0.44289	0.32892	0.950371108
SIKE1	ENSG00000052723	1	115302100	115333306	14	4	123834	0.44285	0.32894	0.950371108
PCDHGB1	ENSG00000254221	5	140719828	140902546	299	22	123834	0.44264	0.32901	0.950428888
C12orf42	ENSG00000179088	12	103621369	103899749	419	21	123834	0.44228	0.32914	0.950515515
PTBP1	ENSG00000011304	19	787075	822327	47	9	123834	0.4423	0.32914	0.950515515
TRPS1	ENSG00000104447	8	116410724	116831899	320	14	123834	0.44202	0.32924	0.950659869
KHDRBS2	ENSG00000112232	6	62379865	63006132	1158	27	123834	0.44148	0.32943	0.950890566
AC011484.1	ENSG00000204850	19	46987765	47009755	50	11	123834	0.4415	0.32943	0.950890566
TMEM261	ENSG00000137038	9	7786490	7898380	171	25	123834	0.44138	0.32947	0.950890566
AP002348.1	ENSG00000204306	11	120372511	120395732	26	5	123834	0.44124	0.32952	0.950890566
OR9I1	ENSG00000172377	11	57875972	57896916	16	3	123834	0.44098	0.32961	0.951005902
POP4	ENSG00000105171	19	30084924	30118144	74	10	123834	0.44079	0.32968	0.951063506
RBM23	ENSG00000100461	14	23359854	23398393	72	7	123834	0.44031	0.32986	0.951236214
PABPC1	ENSG00000070756	8	101688044	101745037	71	5	123834	0.44027	0.32987	0.951236214
MAST1	ENSG00000105613	19	12934765	12995765	93	8	123834	0.44023	0.32989	0.951236214
SLC27A6	ENSG00000113396	5	127863706	128379335	1610	37	123834	0.43979	0.33005	0.951408153
UPF2	ENSG00000151461	10	11952021	12095169	316	12	123834	0.43971	0.33007	0.951408153
EPPIN-WFDC6	ENSG00000249139	20	44155625	44186065	83	5	123834	0.43949	0.33015	0.951408153
MND1	ENSG00000121211	4	154255801	154346270	183	15	123834	0.43946	0.33016	0.951408153
FBXW8	ENSG00000174989	12	117338761	117478953	202	11	123834	0.43931	0.33022	0.951408153
1-Sep	ENSG00000270466	16	30379755	30403863	15	2	123834	0.43918	0.33026	0.951408153
KCNU1	ENSG00000215262	8	36631842	36803646	185	11	123834	0.43908	0.3303	0.951408153
AL137002.1	ENSG00000268130	13	113745563	113766608	32	6	123834	0.4387	0.33044	0.951604672
YTHDF1	ENSG00000149658	20	61816781	61857586	61	6	123834	0.43854	0.3305	0.951604672
CCR10	ENSG00000184451	17	40820907	40845935	17	3	123834	0.43833	0.33057	0.951604672
TBX19	ENSG00000143178	1	168240278	168293664	53	9	123834	0.43798	0.3307	0.951604672
CYP24A1	ENSG00000019186	20	52759988	52800512	99	15	123834	0.43798	0.3307	0.951604672
ZNF334	ENSG00000198185	20	45119701	45152198	35	4	123834	0.43797	0.33071	0.951604672
MYOCD	ENSG00000141052	17	12559207	12682266	160	23	123834	0.43747	0.33089	0.951604672
NTRK1	ENSG00000198400	1	156775432	156861642	91	17	123834	0.43733	0.33094	0.951604672
ADI1	ENSG00000182551	2	3491132	3533507	21	4	123834	0.43727	0.33096	0.951604672
MACROD2	ENSG00000172264	20	13966015	16043842	3710	236	123834	0.43706	0.33103	0.951604672
POU5F1B	ENSG00000212993	8	128416535	128442311	69	8	123834	0.43699	0.33106	0.951604672
CRIP2	ENSG00000182809	14	105929299	105956499	7	1	123834	0.43683	0.33112	0.951604672
PCDHGA3	ENSG00000254245	5	140713601	140902546	306	23	123834	0.43678	0.33114	0.951604672
AC037459.4	ENSG00000248235	8	22436787	22469597	34	3	123834	0.43674	0.33115	0.951604672
CLN6	ENSG00000128973	15	68489330	68559549	69	3	123834	0.43666	0.33118	0.951604672
CDON	ENSG00000064309	11	125815691	125943230	231	30	123834	0.43646	0.33125	0.951604672
TMEM252	ENSG00000181778	9	71141496	71165783	7	3	123834	0.43591	0.33145	0.951604672
TM4SF19	ENSG00000145107	3	196036213	196075374	70	10	123834	0.43578	0.3315	0.951604672
LILRA3	ENSG00000170866	19	54789854	54819952	26	3	123834	0.43576	0.33151	0.951604672
DPH2	ENSG00000132768	1	44425672	44449041	24	4	123834	0.43571	0.33152	0.951604672
CDKN2C	ENSG00000123080	1	51416417	51450305	27	4	123834	0.43548	0.33161	0.951604672
CD34	ENSG00000174059	1	208047594	208094747	72	12	123834	0.43543	0.33162	0.951604672
CCDC142	ENSG00000135637	2	74689113	74720535	13	3	123834	0.43539	0.33164	0.951604672
KRTAP5-2	ENSG00000205867	11	1608409	1629524	36	5	123834	0.43532	0.33166	0.951604672
SSSCA1	ENSG00000173465	11	65327901	65351413	16	3	123834	0.43529	0.33167	0.951604672
KIF23	ENSG00000137807	15	69696585	69750764	30	2	123834	0.43514	0.33173	0.951604672
UFD1L	ENSG00000070010	22	19427433	19476738	94	12	123834	0.4351	0.33174	0.951604672
RNF223	ENSG00000237330	1	996346	1019687	20	4	123834	0.43503	0.33177	0.951604672

XPOT	ENSG00000184575	12	64788130	64854907	76	10	123834	0.4348	0.33185	0.951632796
RNMTL1	ENSG00000171861	17	675513	705749	49	8	123834	0.43474	0.33188	0.951632796
MRPS12	ENSG00000128626	19	39411188	39433802	43	6	123834	0.43459	0.33193	0.951632796
FLYWCH1	ENSG00000059122	16	2951938	3011209	148	20	123834	0.4348	0.33208	0.951772006
EIF3F	ENSG00000175390	11	7981798	8033409	84	18	123834	0.43409	0.33211	0.951772006
PPFIBP2	ENSG00000166387	11	7524529	7688358	410	51	123834	0.43388	0.33219	0.951772006
PAWR	ENSG00000177425	12	79958759	80094877	111	7	123834	0.43356	0.3323	0.951772006
ARHGAP44	ENSG00000006740	17	12682856	12904960	459	36	123834	0.43357	0.3323	0.951772006
PIGC	ENSG00000135845	1	172329329	172423230	133	16	123834	0.43328	0.33241	0.951772006
GADL1	ENSG00000144644	3	30757692	30946257	277	23	123834	0.43296	0.33252	0.951772006
BMPR2	ENSG00000204217	2	203231659	203442474	265	12	123834	0.43271	0.33261	0.951772006
BCAN	ENSG00000132692	1	156601182	156639324	11	5	123834	0.43251	0.33268	0.951772006
SDK1	ENSG00000146555	7	3331080	4318632	2188	77	123834	0.43233	0.33275	0.951772006
EPSTI1	ENSG00000133106	13	43450524	43576407	109	23	123834	0.43221	0.33279	0.951772006
PCDHGA9	ENSG00000261934	5	140772520	140902546	227	26	123834	0.43219	0.3328	0.951772006
HMG20A	ENSG00000140382	15	77702754	77787949	111	9	123834	0.43215	0.33282	0.951772006
GLUL	ENSG00000135821	1	182340839	182371341	36	6	123834	0.43207	0.33284	0.951772006
NPEPL1	ENSG00000215440	20	57254187	57304294	123	14	123834	0.4321	0.33284	0.951772006
TTI2	ENSG00000129696	8	33320904	33381119	142	9	123834	0.43184	0.33293	0.951772006
DHX33	ENSG00000005100	17	5334232	5382380	93	16	123834	0.43182	0.33293	0.951772006
TMPRSS4	ENSG00000137648	11	117937753	118002605	146	17	123834	0.43173	0.33297	0.951772006
OR4N2	ENSG00000176294	14	20177174	20306531	24	6	123834	0.4317	0.33298	0.951772006
INO80E	ENSG00000169592	16	29996615	30027114	31	3	123834	0.43169	0.33298	0.951772006
ARHGAP19	ENSG00000213390	10	98968996	99062413	227	13	123834	0.43148	0.33306	0.951857537
HM13	ENSG00000101294	20	30092231	30167370	107	2	123834	0.43055	0.3334	0.952138623
SUGP1	ENSG00000105705	19	19376827	19441653	98	7	123834	0.43015	0.33354	0.952138623
AL359878.1	ENSG00000205740	10	1007097	1044281	85	8	123834	0.43008	0.33357	0.952138623
BPIFB6	ENSG00000167104	20	31609454	31641853	47	5	123834	0.43006	0.33358	0.952138623
TSNAXIP1	ENSG00000102904	16	67830668	67876051	35	5	123834	0.42977	0.33368	0.952138623
ZNF497	ENSG00000174586	19	58855723	58884214	27	3	123834	0.42968	0.33371	0.952138623
FCHSD1	ENSG00000197948	5	141008869	141040986	49	14	123834	0.42963	0.33373	0.952138623
IQCE	ENSG00000106012	7	2588632	2664368	158	20	123834	0.42953	0.33377	0.952138623
PITPNM1	ENSG00000110697	11	67249239	67283734	16	3	123834	0.42954	0.33377	0.952138623
AKAP13	ENSG00000170776	15	85913802	86302586	1054	32	123834	0.42946	0.33379	0.952138623
LPIN1	ENSG00000134324	2	11807721	11977535	233	36	123834	0.42942	0.33381	0.952138623
LRFN1	ENSG00000128011	19	39787208	39815976	25	4	123834	0.42932	0.33384	0.952138623
FAM217A	ENSG00000145975	6	4039668	4097578	119	11	123834	0.42928	0.33386	0.952138623
SLC22A23	ENSG00000137266	6	3259196	3467256	413	41	123834	0.42915	0.33391	0.952138623
KIAA1279	ENSG00000198954	10	70738487	70786738	132	15	123834	0.42882	0.33403	0.952138623
DACT3	ENSG00000197380	19	47140869	47174395	18	6	123834	0.4288	0.33403	0.952138623
PRLHR	ENSG00000119973	10	120342916	120365160	76	8	123834	0.42872	0.33406	0.952138623
PHF5A	ENSG00000100410	22	41845721	41874729	25	1	123834	0.42872	0.33406	0.952138623
RP11-315D16.2	ENSG00000260007	15	68476139	68532056	63	2	123834	0.42853	0.33413	0.952195358
OR51B5	ENSG00000242180	11	5353744	5374809	78	10	123834	0.42832	0.33421	0.952223309
IFI30	ENSG00000216490	19	18273972	18298927	14	2	123834	0.42824	0.33424	0.952223309
ADM2	ENSG00000128165	22	50909985	50934869	15	6	123834	0.42789	0.33437	0.952250412
C19orf77	ENSG00000095932	19	3464405	3490540	22	5	123834	0.42784	0.33438	0.952250412
AL161645.2	ENSG00000268925	10	135331026	135352661	68	10	123834	0.4277	0.33443	0.952250412
DEFA4	ENSG00000164821	8	6783344	6805860	40	7	123834	0.42751	0.3345	0.952250412
PRDM10	ENSG00000170325	11	129759601	129882730	194	23	123834	0.42753	0.3345	0.952250412
AF165138.7	ENSG00000243440	21	15954251	16041142	158	15	123834	0.42698	0.3347	0.952677089
PIP4K2A	ENSG00000150867	10	22813778	23013484	312	18	123834	0.42654	0.33486	0.952732968
MTA2	ENSG00000149480	11	62350686	62379312	23	2	123834	0.42651	0.33487	0.952732968
EDDM3A	ENSG00000181562	14	21204051	21226539	76	12	123834	0.4265	0.33487	0.952732968
HHIPL1	ENSG00000182218	14	100101447	100156906	43	6	123834	0.42591	0.33509	0.952778478
GPR180	ENSG00000152749	13	95244157	95296899	69	7	123834	0.4256	0.3352	0.952778478
ANKRD34C	ENSG00000235711	15	79565146	79600580	29	7	123834	0.42546	0.33525	0.952778478
SCP2D1	ENSG00000132631	20	18784370	18805033	37	10	123834	0.42545	0.33526	0.952778478
FASTK	ENSG00000164896	7	150763711	150787953	13	3	123834	0.42525	0.33533	0.952778478
APOA1BP	ENSG00000163382	1	156551554	156574091	67	3	123834	0.42473	0.33552	0.952778478
DLG2	ENSG00000150672	11	83156055	85348966	3737	124	123834	0.42464	0.33555	0.952778478
WRAP73	ENSG00000116213	1	3537331	3579325	48	9	123834	0.42459	0.33557	0.952778478
CLP1	ENSG00000172409	11	57406465	57439340	24	4	123834	0.42456	0.33558	0.952778478
HOMER2	ENSG00000103942	15	83499838	83664661	217	28	123834	0.42431	0.33567	0.952778478
PBOV1	ENSG00000254440	6	138527129	138549627	50	4	123834	0.424	0.33578	0.952778478
CLUAP1	ENSG00000103351	16	3540924	3599048	84	9	123834	0.42377	0.33587	0.952778478
PRKRA	ENSG00000180228	2	179286141	179326239	34	5	123834	0.42366	0.33591	0.952778478
CTD-2021H9.3	ENSG00000258053	12	71431182	71522027	184	10	123834	0.42365	0.33591	0.952778478
ETNK1	ENSG00000139163	12	22768009	22853599	70	5	123834	0.42364	0.33592	0.952778478
MESDC1	ENSG00000140406	15	81283295	81306342	39	4	123834	0.42353	0.33595	0.952778478
TRIM60	ENSG00000176979	4	165943105	165972896	42	7	123834	0.4235	0.33596	0.952778478
DQX1	ENSG00000144045	2	74735258	74763463	13	2	123834	0.42347	0.33598	0.952778478
YPEL5	ENSG00000119801	2	30359807	30393399	33	7	123834	0.42341	0.336	0.952778478
MEX3A	ENSG00000254726	1	156031804	156061789	13	4	123834	0.42337	0.33601	0.952778478
IZUMO2	ENSG00000161652	19	50645805	50676452	29	2	123834	0.42308	0.33612	0.952778478
GFY	ENSG00000261949	19	49917006	49942075	8	2	123834	0.42276	0.33623	0.952778478
EPHA5	ENSG00000145242	4	66175281	66546213	754	48	123834	0.42263	0.33628	0.952778478
CCDC69	ENSG00000198624	5	150550613	150613706	122	22	123834	0.42265	0.33628	0.952778478
C1orf194	ENSG00000179902	1	109638573	109666479	70	10	123834	0.42257	0.3363	0.952778478
TBXAS1	ENSG00000059377	7	139466850	139730125	232	41	123834	0.42259	0.3363	0.952778478
RASSF9	ENSG00000198774	12	86188331	86240348	111	9	123834	0.42243	0.33636	0.952778478
RP11-47I22.3	ENSG00000232774	14	62027258	62135414	103	21	123834	0.4224	0.33637	0.952778478
SLC6A12	ENSG00000111181	12	289243	333736	85	15	123834	0.42231	0.3364	0.952778478
RIPPLY2	ENSG00000203877	6	84552985	84577234	47	3	123834	0.42226	0.33642	0.952778478
BCAS3	ENSG00000141376	17	58744814	59480199	592	28	123834	0.4221	0.33648	0.952778478
CDC42BPA	ENSG00000143776	1	227167566	227516175	1517	19	123834	0.42208	0.33649	0.952778478
PFKFB4	ENSG00000114268	3	48545117	48609448	34	3	123834	0.4217	0.33662	0.95294761
AC006372.1	ENSG00000236669	7	157308477	157330019	54	7	123834	0.42164	0.33665	0.95294761
TSHZ3	ENSG00000121297	19	31755851	31850453	78	17	123834	0.42097	0.33689	0.95347719
SPON2	ENSG00000159674	4	1150720	1212750	89	15	123834	0.42032	0.33712	0.95347719
TMX2-CTNND1	ENSG00000254462	11	57470077	57569058	88	6	123834	0.42014	0.33719	0.95347719
C1QTNF8	ENSG00000184471	16	1130005	1156244	46	5	123834	0.42002	0.33724	0.95347719
FARSB	ENSG00000116120	2	223425255	223531056	213	20	123834	0.41978	0.33732	0.95347719
DNAJB11	ENSG00000090520	3	186275192	186325061	98	7	123834	0.41973	0.33734	0.95347719
MGAT5	ENSG00000152127	2	134867554	135222192	461	65	123834	0.41968	0.33736	0.95347719
GALNT11	ENSG00000178234	7	151712759	151829425	87	2	123834	0.41967	0.33736	0.95347719
PDSS2	ENSG00000164494	6	107463761	107790768	411	29	123834	0.41962	0.33738	0.95347719
CTSW	ENSG00000172543	11	65637280	65661212	33	4	123834	0.41962	0.33738	0.95347719
SCRT2	ENSG00000215397	20	632240	666823	21	3	123834	0.41949	0.33743	0.95347719

RNF8	ENSG00000112130	6	37311748	37372514	13	4	123834	0.41916	0.33755	0.95347719
NINL	ENSG00000101004	20	25423341	25576153	285	13	123834	0.419	0.33761	0.95347719
ILKAP	ENSG00000132323	2	239069042	239122370	165	13	123834	0.41881	0.33768	0.95347719
CHTOP	ENSG00000160679	1	153596525	153628782	12	3	123834	0.41876	0.33769	0.95347719
PISD	ENSG00000241878	22	32004477	32068418	155	12	123834	0.41873	0.33771	0.95347719
GDF2	ENSG00000128802	10	48403092	48426853	22	5	123834	0.41866	0.33773	0.95347719
KDM1A	ENSG00000004487	1	23335941	23420182	73	5	123834	0.41865	0.33774	0.95347719
ZCCHC2	ENSG00000141664	18	60180240	60264942	129	17	123834	0.41838	0.33784	0.953586801
LY6E	ENSG00000160932	8	144089399	144115249	21	4	123834	0.41803	0.33796	0.953586801
SEPSECS	ENSG00000109618	4	25111627	25172204	96	15	123834	0.41796	0.33799	0.953586801
SYK	ENSG00000165025	9	93554069	93670831	272	35	123834	0.41783	0.33804	0.953586801
ARFIP2	ENSG00000132254	11	6486910	6512666	23	4	123834	0.41781	0.33804	0.953586801
C15orf40	ENSG00000169609	15	83647193	83690393	61	13	123834	0.41769	0.33809	0.953586801
NOL7	ENSG00000225921	6	13605559	13642971	33	5	123834	0.41757	0.33813	0.953586801
GP1BA	ENSG00000185245	17	4825592	4848325	35	8	123834	0.41732	0.33822	0.953699119
IQCF1	ENSG00000173389	3	51918892	51947351	24	4	123834	0.41711	0.3383	0.95378321
CCDC85B	ENSG00000175602	11	65647875	65669105	20	4	123834	0.41687	0.33839	0.953895465
PSTPIP2	ENSG00000152229	18	43553502	43662238	231	12	123834	0.41623	0.33862	0.954402278
PHF23	ENSG00000040633	17	7128347	7153041	10	3	123834	0.41543	0.33891	0.955017556
PPAPDC1B	ENSG00000147535	8	38110648	38136761	13	2	123834	0.4153	0.33896	0.955017556
HTT	ENSG00000197386	4	3066408	3255676	317	15	123834	0.41503	0.33906	0.955017556
AL137026.1	ENSG00000269515	10	44863687	44888739	76	11	123834	0.41487	0.33912	0.955017556
ERAP2	ENSG00000164308	5	96201643	96265420	223	12	123834	0.4148	0.33914	0.955017556
C6orf100	ENSG00000204709	6	28901654	28922314	45	8	123834	0.41476	0.33916	0.955017556
CASP7	ENSG00000165806	10	115428942	115500662	156	16	123834	0.41468	0.33919	0.955017556
BAAT	ENSG00000136881	9	104112699	104155801	107	4	123834	0.41429	0.33933	0.955088827
F11	ENSG00000088926	4	187177099	187220835	79	15	123834	0.41427	0.33934	0.955088827
HOXC11	ENSG00000123388	12	54356910	54381427	17	3	123834	0.4141	0.3394	0.955088827
KIAA1024	ENSG00000169330	15	79714858	79774632	108	15	123834	0.41392	0.33947	0.955088827
ATP6V1G2-DDX39B	ENSG00000254870	6	31487996	31524385	125	20	123834	0.41384	0.33949	0.955088827
RPN2	ENSG00000118705	20	35796813	35880022	190	11	123834	0.41378	0.33952	0.955088827
GOLGA8A	ENSG00000175265	15	34661269	34890704	104	11	123834	0.4135	0.33962	0.955088827
YDJC	ENSG00000161179	22	21972378	21994353	15	2	123834	0.41304	0.33979	0.955088827
MPV17L	ENSG00000156968	16	15479611	15517125	48	8	123834	0.41295	0.33982	0.955088827
CYP4F31P	ENSG00000178206	2	132034330	132060127	15	1	123834	0.41293	0.33983	0.955088827
GOLGA6D	ENSG00000140478	15	75565176	75596816	2	1	123834	0.41292	0.33983	0.955088827
DNAJC27	ENSG00000115137	2	25156505	25204963	68	8	123834	0.41277	0.33989	0.955088827
C16orf11	ENSG00000161992	16	600422	625528	15	2	123834	0.41264	0.33993	0.955088827
MBLAC1	ENSG00000214309	7	99714317	99736118	18	5	123834	0.41248	0.33999	0.955088827
RP11-62N21.1	ENSG00000248369	4	133346957	133378390	45	7	123834	0.4124	0.34002	0.955088827
KIR3DL1	ENSG00000167633	19	55225969	55388448	121	9	123834	0.41229	0.34006	0.955088827
BIN1	ENSG00000136717	2	127795603	127874931	166	20	123834	0.41226	0.34007	0.955088827
ALMS1	ENSG00000116127	2	73602886	73847920	355	8	123834	0.41209	0.34014	0.955088827
IFNE	ENSG00000184995	9	21470841	21492312	30	4	123834	0.41199	0.34017	0.955088827
FMNL1	ENSG00000184922	17	43288811	43334687	35	6	123834	0.41165	0.3403	0.955114119
CD320	ENSG00000167775	19	8357011	8383240	55	4	123834	0.41148	0.34036	0.955114119
LRRC16B	ENSG00000186648	14	24511206	24548937	7	3	123834	0.41143	0.34038	0.955114119
TRABD	ENSG00000170638	22	50614344	50648027	67	11	123834	0.41144	0.34038	0.955114119
HERC4	ENSG00000148634	10	69671665	69845105	247	18	123834	0.41098	0.34054	0.955151305
LLGL1	ENSG00000131899	17	18118901	18158189	54	6	123834	0.41094	0.34056	0.955151305
SCAND3	ENSG00000232040	6	28529407	28593989	111	16	123834	0.41091	0.34057	0.955151305
AL353583.1	ENSG00000269879	10	24760273	24780632	21	2	123834	0.41058	0.34069	0.955151305
FMN2	ENSG00000155816	1	240167648	240648489	790	59	123834	0.41011	0.34086	0.955151305
PPP3CA	ENSG00000138814	4	101934566	102279435	484	34	123834	0.40992	0.34093	0.955151305
TOP3A	ENSG00000177302	17	18164742	18228321	118	8	123834	0.40994	0.34093	0.955151305
GNG3	ENSG00000162188	11	62465130	62486673	20	2	123834	0.40981	0.34097	0.955151305
WHSC1L1	ENSG00000147548	8	38117215	38249790	104	4	123834	0.40976	0.34099	0.955151305
FMOD	ENSG00000122176	1	203299756	203330617	52	8	123834	0.40957	0.34106	0.955151305
CALCOCO1	ENSG00000012822	12	54094903	54131529	35	7	123834	0.40952	0.34108	0.955151305
PLBD2	ENSG00000151176	12	113786371	113837203	59	4	123834	0.40932	0.34115	0.955151305
CELA3A	ENSG00000142789	1	22318149	22349032	69	9	123834	0.40921	0.34119	0.955151305
GDA	ENSG00000119125	9	74719511	74882109	306	21	123834	0.40917	0.34121	0.955151305
SUMF1	ENSG00000144455	3	3732498	4518965	1878	80	123834	0.40891	0.3413	0.955151305
KIRREL3	ENSG00000149571	11	126283254	126883355	1013	86	123834	0.40893	0.3413	0.955151305
ZNF468	ENSG00000204604	19	53331261	53370902	62	8	123834	0.40891	0.3413	0.955151305
LETM2	ENSG00000165046	8	38233725	38277045	36	3	123834	0.40885	0.34133	0.955151305
S100A16	ENSG00000188643	1	153569362	153595621	22	4	123834	0.40875	0.34136	0.955151305
C17orf89	ENSG00000224877	17	79203039	79225081	21	6	123834	0.4085	0.34145	0.955151305
PSMD7	ENSG00000103035	16	74320673	74350186	30	5	123834	0.40825	0.34155	0.955151305
TMEM170A	ENSG00000166822	16	75466952	75509395	117	7	123834	0.40814	0.34159	0.955151305
GPR157	ENSG00000180758	1	9150364	9199229	56	9	123834	0.40789	0.34168	0.955151305
ATP4B	ENSG00000186009	13	114293119	114322501	9	3	123834	0.40778	0.34172	0.955151305
CEP192	ENSG00000101639	18	12981361	13135051	375	19	123834	0.4074	0.34186	0.955151305
RP11-1055B8.7	ENSG00000171282	17	79363540	79443357	59	19	123834	0.40735	0.34187	0.955151305
CCDC153	ENSG00000248712	11	119050347	119077479	20	5	123834	0.40694	0.34203	0.955151305
SHMT1	ENSG00000176974	17	18221187	18276856	120	10	123834	0.40678	0.34209	0.955151305
SEC31B	ENSG00000075826	10	102236399	102299628	86	2	123834	0.40668	0.34212	0.955151305
TAGAP	ENSG00000164691	6	159445500	159476184	97	6	123834	0.40655	0.34217	0.955151305
IL22RA2	ENSG00000164485	6	137454968	137504785	93	11	123834	0.4061	0.34233	0.955151305
KIR2DL4	ENSG00000189013	19	55226002	55335972	35	10	123834	0.40612	0.34233	0.955151305
HAGH	ENSG00000063854	16	1835621	1887195	359	12	123834	0.40607	0.34234	0.955151305
AEBP1	ENSG00000106624	7	44133960	44164161	28	5	123834	0.40607	0.34235	0.955151305
ZNF225	ENSG00000256294	19	44606334	44647027	79	8	123834	0.406	0.34237	0.955151305
SAMHD1	ENSG00000101347	20	35508632	35590246	92	12	123834	0.4059	0.34241	0.955151305
ZNF177	ENSG00000188629	19	9425021	9503293	152	7	123834	0.40565	0.3425	0.955151305
ST8SIA2	ENSG00000140557	15	92927058	93021958	89	20	123834	0.4054	0.34259	0.955151305
APLP1	ENSG00000105290	19	36348801	36380693	53	1	123834	0.40523	0.34265	0.955151305
SUN5	ENSG00000167098	20	31561579	31602239	69	7	123834	0.40524	0.34265	0.955151305
C2CD4A	ENSG00000198535	15	62349176	62373116	45	5	123834	0.40515	0.34268	0.955151305
TUT1	ENSG00000149016	11	62332517	62369649	21	2	123834	0.40508	0.34271	0.955151305
ZNF462	ENSG00000148143	9	109615378	109785915	137	19	123834	0.40501	0.34273	0.955151305
DUT	ENSG00000128951	15	48613208	48645570	21	1	123834	0.40478	0.34282	0.955151305
BNIP2	ENSG00000140299	15	59941345	59991733	177	7	123834	0.4047	0.34285	0.955151305
AGGF1	ENSG00000164252	5	76315076	76371059	91	12	123834	0.40449	0.34292	0.955151305
RELL2	ENSG00000164620	5	141006517	141030644	40	12	123834	0.4044	0.34296	0.955151305
ITGA11	ENSG00000137809	15	68584050	68734501	287	27	123834	0.40434	0.34298	0.955151305
SCN4B	ENSG00000177098	11	117994092	118033603	35	7	123834	0.404	0.34311	0.955151305
TMEM129	ENSG00000168936	4	1707679	1733085	104	10	123834	0.40393	0.34313	0.955151305
DNAI2	ENSG00000171595	17	72260386	72321023	65	14	123834	0.40387	0.34315	0.955151305

TRPM2	ENSG00000142185	21	45760046	45872964	141	10	123834	0.40383	0.34317	0.955151305
RNF212	ENSG00000178222	4	1040038	1117350	273	9	123834	0.40369	0.34322	0.955151305
BAG4	ENSG00000156735	8	38024051	38080819	44	1	123834	0.40359	0.34326	0.955151305
FLII	ENSG00000177731	17	18138150	18172230	34	6	123834	0.40338	0.34333	0.955151305
SYT7	ENSG00000011347	11	61272785	61358620	57	4	123834	0.40325	0.34338	0.955151305
DGKD	ENSG00000077044	2	234253153	234390750	199	19	123834	0.40323	0.34339	0.955151305
DIMT1	ENSG00000086189	5	61673081	61709766	68	8	123834	0.40312	0.34343	0.955151305
THPO	ENSG00000090534	3	184079723	184105932	23	6	123834	0.40303	0.34346	0.955151305
OR4D9	ENSG00000172742	11	59272386	59293330	19	2	123834	0.40288	0.34352	0.955151305
DUS2	ENSG00000167264	16	68011649	68123223	97	5	123834	0.40265	0.3436	0.955151305
C17orf112	ENSG00000227011	17	51052880	51075012	14	3	123834	0.40262	0.34361	0.955151305
AADAT	ENSG00000109576	4	170971373	171022850	70	8	123834	0.40249	0.34366	0.955151305
RSPH10B	ENSG00000155026	7	5955181	6020314	3	2	123834	0.40247	0.34367	0.955151305
RAG2	ENSG00000175097	11	36587124	36629829	84	6	123834	0.40242	0.34369	0.955151305
PIEZO2	ENSG00000154864	18	10656480	11158587	961	97	123834	0.40224	0.34375	0.955151305
PATE4	ENSG00000237353	11	125693211	125719964	75	7	123834	0.40222	0.34376	0.955151305
RHBDF1	ENSG00000007384	16	98058	136354	39	7	123834	0.4015	0.34403	0.955592836
ZNF559-ZNF177	ENSG00000270011	19	9424850	9503293	153	7	123834	0.40147	0.34404	0.955592836
SHC3	ENSG00000148082	9	91618060	91803682	346	27	123834	0.40139	0.34407	0.955592836
GUCY2D	ENSG00000132518	17	7895912	7933657	37	8	123834	0.40125	0.34412	0.955592836
C14orf79	ENSG00000140104	14	105442112	105486819	107	7	123834	0.40094	0.34423	0.955758668
NKX2-2	ENSG00000125820	20	21481648	21504664	8	3	123834	0.40027	0.34448	0.956197828
AC012360.2	ENSG00000268809	2	105872490	105892585	28	7	123834	0.40024	0.34449	0.956197828
ZNF563	ENSG00000188868	19	12418291	12454534	37	2	123834	0.39993	0.3446	0.956197828
TTC9C	ENSG00000162222	11	62485541	62517765	31	2	123834	0.39992	0.34461	0.956197828
RNF4	ENSG00000063978	4	2453947	2637047	216	23	123834	0.39974	0.34467	0.956197828
LYRM9	ENSG00000232859	17	26195340	26231778	7	4	123834	0.39968	0.34469	0.956197828
RCOR1	ENSG00000089902	14	103048998	103206913	239	16	123834	0.39916	0.34489	0.956583492
CDV3	ENSG00000091527	3	133282574	133319105	63	8	123834	0.39903	0.34494	0.956583492
ZFP2	ENSG00000198939	5	178312895	178370213	179	11	123834	0.39891	0.34498	0.956583492
CCDC103	ENSG00000167131	17	42966510	42992758	59	9	123834	0.39868	0.34506	0.956645723
LIPT2	ENSG00000175536	11	74192757	74214778	51	6	123834	0.39856	0.34511	0.956645723
AL590235.1	ENSG00000268781	4	3501523	3521678	101	12	123834	0.39839	0.34517	0.956645723
LTB	ENSG00000227507	6	31538302	31560299	36	13	123834	0.39762	0.34545	0.956645723
PADI4	ENSG00000159339	1	17624690	17700499	268	19	123834	0.3976	0.34546	0.956645723
DCAF4L1	ENSG00000182308	4	41973713	41998476	66	7	123834	0.39733	0.34556	0.956645723
SERINC1	ENSG00000111897	6	122754499	122802967	89	8	123834	0.39721	0.3456	0.956645723
ANKRD1	ENSG00000148677	10	92661853	92691033	28	7	123834	0.39719	0.34561	0.956645723
DNAJC3	ENSG00000102580	13	96319393	96457243	117	16	123834	0.39721	0.34561	0.956645723
ANKRD60	ENSG00000124227	20	56783551	56813709	20	6	123834	0.39716	0.34562	0.956645723
FUT6	ENSG00000156413	19	5820621	5849742	56	11	123834	0.39702	0.34568	0.956645723
SPTB	ENSG00000070182	14	65203002	65356601	378	32	123834	0.39697	0.34569	0.956645723
DNAH8	ENSG00000124721	6	38673117	39008301	897	40	123834	0.39694	0.3457	0.956645723
GABARAP	ENSG00000170296	17	7133333	7156089	10	2	123834	0.39672	0.34579	0.956645723
ZNF436	ENSG00000125945	1	23675941	23705935	44	5	123834	0.39642	0.3459	0.956645723
CCNJ	ENSG00000107443	10	97793151	97830627	52	10	123834	0.39627	0.34595	0.956645723
PYGO2	ENSG00000163348	1	154919502	154946329	8	2	123834	0.3961	0.34602	0.956645723
MAML3	ENSG00000196782	4	140627907	141085338	489	64	123834	0.3961	0.34602	0.956645723
MAP3K3	ENSG00000198909	17	61689775	61783663	61	3	123834	0.39585	0.34611	0.956645723
CDC42EP1	ENSG00000128283	22	37946454	37975412	38	6	123834	0.39581	0.34612	0.956645723
TEAD1	ENSG00000187079	11	12685969	12976298	179	26	123834	0.39576	0.34614	0.956645723
MRPL36	ENSG00000171421	5	1788500	1811480	70	8	123834	0.39573	0.34615	0.956645723
CGB8	ENSG00000213030	19	49540895	49562400	22	4	123834	0.39572	0.34616	0.956645723
MEGF9	ENSG00000106780	9	123353091	123486748	96	4	123834	0.39542	0.34627	0.956810606
FIBP	ENSG00000172500	11	65641212	65666010	30	4	123834	0.39513	0.34637	0.956947814
RXFP3	ENSG00000182631	5	33926491	33949023	33	6	123834	0.39487	0.34647	0.957000192
FASLG	ENSG00000117560	1	172618154	172646014	21	4	123834	0.39478	0.3465	0.957000192
PRKCD	ENSG00000163932	3	53180025	53236733	31	7	123834	0.39467	0.34654	0.957000192
IRGM	ENSG00000237693	5	150216085	150290298	204	6	123834	0.39442	0.34664	0.957025882
C22orf39	ENSG00000242259	22	19328891	19445755	175	13	123834	0.39438	0.34665	0.957025882
EIF4E1B	ENSG00000175766	5	176047683	176083642	28	7	123834	0.39398	0.3468	0.957073344
HIRA	ENSG00000100084	22	19308221	19445224	208	14	123834	0.39394	0.34681	0.957073344
OR2A12	ENSG00000221858	7	143782141	143803186	34	3	123834	0.39391	0.34682	0.957073344
AKTIP	ENSG00000166971	16	53514952	53548323	30	5	123834	0.39365	0.34692	0.957073344
DMRTB1	ENSG00000143006	1	53915072	53943161	50	7	123834	0.39353	0.34696	0.957073344
PRKAA2	ENSG00000162409	1	57100995	57191008	216	17	123834	0.39344	0.347	0.957073344
CDK6	ENSG00000105810	7	92224235	92475908	168	19	123834	0.39331	0.34704	0.957073344
UQCRC1	ENSG00000010256	3	48626435	48658409	10	2	123834	0.39324	0.34707	0.957073344
OR2L5	ENSG00000197454	1	248175250	248196188	31	3	123834	0.39303	0.34715	0.957155091
PRSS1	ENSG00000204983	7	142447319	142470923	19	1	123834	0.39233	0.34741	0.95756555
C10orf111	ENSG00000176236	10	15127384	15149318	79	12	123834	0.39229	0.34742	0.95756555
AC019206.1	ENSG00000268328	2	70119482	70139841	27	3	123834	0.39222	0.34745	0.95756555
BAHD1	ENSG00000140320	15	40721920	40770441	28	8	123834	0.39194	0.34755	0.957702291
ZNF675	ENSG00000197372	19	23698433	23880022	285	14	123834	0.39177	0.34761	0.957728784
DNAJC5G	ENSG00000163793	2	27488289	27514367	26	4	123834	0.39126	0.3478	0.958025222
CETN1	ENSG00000177143	18	570367	591524	22	7	123834	0.39096	0.34791	0.958025222
PTPRT	ENSG00000196090	20	40691392	41828610	1700	147	123834	0.3909	0.34793	0.958025222
SNRPD1	ENSG00000167088	18	19182228	19220417	34	4	123834	0.39076	0.34799	0.958025222
TMEM95	ENSG00000182896	17	7248442	7270538	6	2	123834	0.39073	0.348	0.958025222
SLC25A41	ENSG00000181240	19	6416048	6443790	51	3	123834	0.39068	0.34802	0.958025222
NLRP7	ENSG00000167634	19	55424877	55487680	124	16	123834	0.39049	0.34809	0.958079166
RNF2	ENSG00000121481	1	185004496	185081740	43	6	123834	0.39029	0.34816	0.958133094
INPP5J	ENSG00000185133	22	31508717	31540682	54	13	123834	0.38996	0.34828	0.958247561
FAM53A	ENSG00000174137	4	1609642	1696029	140	9	123834	0.38953	0.34844	0.958247561
COL7A1	ENSG00000114270	3	48591506	48642700	19	3	123834	0.38943	0.34848	0.958247561
CDCA4	ENSG00000170779	14	105465910	105497485	105	7	123834	0.38941	0.34849	0.958247561
CHIA	ENSG00000134216	1	111823484	111873188	210	19	123834	0.38926	0.34854	0.958247561
TSPAN9	ENSG00000011105	12	3176521	3405730	485	64	123834	0.38924	0.34855	0.958247561
DTNA	ENSG00000134769	18	32063254	32481808	497	19	123834	0.38871	0.34875	0.958247561
RNASE6	ENSG00000169413	14	21239210	21260626	49	8	123834	0.38776	0.3491	0.958247561
OGFRL1	ENSG00000119900	6	71988506	72028653	114	13	123834	0.38763	0.34914	0.958247561
NFS1	ENSG00000244005	20	34245977	34297281	110	8	123834	0.38735	0.34925	0.958247561
COX15	ENSG00000014919	10	101461601	101501857	41	6	123834	0.38731	0.34926	0.958247561
KRTAP4-11	ENSG00000212721	17	39263433	39284606	128	3	123834	0.38719	0.34931	0.958247561
C10orf2	ENSG00000107815	10	102737124	102764158	34	4	123834	0.38703	0.34937	0.958247561
NOC2L	ENSG00000188976	1	869584	904689	42	6	123834	0.38678	0.34946	0.958247561
CCDC163P	ENSG00000236624	1	45949538	45975751	32	4	123834	0.38657	0.34954	0.958247561
BOLL	ENSG00000152430	2	198581603	198661486	75	6	123834	0.3862	0.34968	0.958247561
EP300	ENSG00000100393	22	41477790	41586081	110	8	123834	0.38603	0.34974	0.958247561

OR4M1	ENSG00000176299	14	20238401	20259516	4	2	123834	0.38596	0.34976	0.958247561
RASL11A	ENSG00000122035	13	27834464	27857827	29	7	123834	0.38555	0.34991	0.958247561
HPS3	ENSG00000163755	3	148837371	148901519	132	18	123834	0.38548	0.34994	0.958247561
FAM110A	ENSG00000125898	20	804358	848106	76	20	123834	0.38549	0.34994	0.958247561
CELA2A	ENSG00000142615	1	15773223	15808586	53	9	123834	0.38542	0.34996	0.958247561
IL11	ENSG00000095752	19	55865757	55891831	38	7	123834	0.38537	0.34998	0.958247561
FAM47E	ENSG00000189157	4	77125193	77214933	250	21	123834	0.38515	0.35006	0.958247561
ZFAND2A	ENSG00000178381	7	1181707	1210395	159	9	123834	0.38502	0.35011	0.958247561
OR9Q2	ENSG00000186513	11	57947906	57968990	43	6	123834	0.38491	0.35015	0.958247561
CARD8	ENSG00000105483	19	48674027	48769203	270	21	123834	0.3848	0.35019	0.958247561
SMIM18	ENSG00000253457	8	30486117	30513581	48	7	123834	0.38443	0.35033	0.958247561
MTRNR2L7	ENSG00000256892	10	37880366	37901859	65	7	123834	0.38432	0.35037	0.958247561
CDC42EP3	ENSG00000163171	2	37859032	37975611	214	30	123834	0.38417	0.35043	0.958247561
KRTAP10-7	ENSG00000205441	21	46010497	46032091	62	2	123834	0.38411	0.35045	0.958247561
FADD	ENSG00000168040	11	70039269	70063496	16	6	123834	0.38409	0.35046	0.958247561
MS4A12	ENSG00000071203	11	60250251	60284903	133	8	123834	0.38404	0.35048	0.958247561
DCTPP1	ENSG00000179958	16	30424940	30451396	2	1	123834	0.38369	0.35061	0.958247561
RP11-527L4.2	ENSG00000261514	17	42005731	42026327	29	6	123834	0.3835	0.35067	0.958247561
UGT8	ENSG00000174607	4	115509611	115609380	135	14	123834	0.38307	0.35083	0.958247561
ELANE	ENSG00000197561	19	841014	866242	8	3	123834	0.38307	0.35084	0.958247561
KLK13	ENSG00000167759	19	51549463	51578371	12	3	123834	0.38298	0.35087	0.958247561
SIDT1	ENSG00000072858	3	113241143	113358425	219	20	123834	0.38295	0.35088	0.958247561
GSN	ENSG00000148180	9	123960072	124105121	174	17	123834	0.38284	0.35092	0.958247561
TRHDE	ENSG00000072657	12	72471046	73069422	907	45	123834	0.38275	0.35095	0.958247561
AL365202.1	ENSG00000203286	9	3442305	3479181	29	4	123834	0.38265	0.35099	0.958247561
SV2C	ENSG00000122012	5	75368997	75659764	521	42	123834	0.38259	0.35101	0.958247561
ANKRD26	ENSG00000107890	10	27270843	27399421	360	17	123834	0.38261	0.35101	0.958247561
ZHX2	ENSG00000178764	8	123783633	123996750	134	38	123834	0.38254	0.35103	0.958247561
EME2	ENSG00000197774	16	1813208	1841709	47	7	123834	0.38232	0.35111	0.958247561
CCDC179	ENSG00000255359	11	22858477	22891972	60	5	123834	0.38229	0.35112	0.958247561
GCOM1	ENSG00000137878	15	57874106	58016943	335	23	123834	0.38203	0.35122	0.958247561
SIL1	ENSG00000120725	5	138272409	138639246	251	18	123834	0.38188	0.35127	0.958247561
OR6K2	ENSG00000196171	1	158659369	158680486	26	4	123834	0.38177	0.35132	0.958247561
GZMB	ENSG00000100453	14	25090160	25113473	39	8	123834	0.38115	0.35155	0.958247561
RIBC2	ENSG00000128408	22	45799572	45838376	87	6	123834	0.38104	0.35159	0.958247561
CILP2	ENSG00000160161	19	19639057	19667468	39	4	123834	0.381	0.3516	0.958247561
UNC5CL	ENSG00000124602	6	40984772	41016928	53	4	123834	0.38094	0.35163	0.958247561
NIN	ENSG00000100503	14	51176481	51307839	151	26	123834	0.38049	0.35179	0.958247561
DDB1	ENSG00000167986	11	61056923	61120068	6	2	123834	0.3803	0.35186	0.958247561
STK32A	ENSG00000169302	5	146604526	146777415	358	26	123834	0.38025	0.35188	0.958247561
AC018867.2	ENSG00000268846	2	187357219	187377356	29	5	123834	0.37992	0.352	0.958247561
OR1C1	ENSG00000221888	1	247910676	247931982	41	4	123834	0.37981	0.35204	0.958247561
JSRP1	ENSG00000167476	19	2242251	2279758	57	11	123834	0.37983	0.35204	0.958247561
TMEM88B	ENSG00000205116	1	1351508	1373167	12	4	123834	0.37978	0.35206	0.958247561
SBSPON	ENSG00000164764	8	73966775	74046323	135	12	123834	0.37946	0.35217	0.958247561
FBLIM1	ENSG00000162458	1	16073102	16123089	28	7	123834	0.3793	0.35223	0.958247561
POLR3F	ENSG00000132664	20	18437771	18475287	67	11	123834	0.37922	0.35226	0.958247561
UNC13A	ENSG00000130477	19	17702137	17809401	116	29	123834	0.37891	0.35238	0.958247561
MGRN1	ENSG00000102858	16	4656494	4750975	170	17	123834	0.37888	0.35239	0.958247561
PLEKHF1	ENSG00000166289	19	30145963	30176364	34	5	123834	0.37865	0.35248	0.958247561
CALML4	ENSG00000129007	15	68473043	68508417	27	3	123834	0.37846	0.35255	0.958247561
HMHA1	ENSG00000180448	19	1055922	1096627	78	16	123834	0.37831	0.3526	0.958247561
CERS1	ENSG00000223802	19	18969361	19017536	51	11	123834	0.37822	0.35263	0.958247561
BEND4	ENSG00000188848	4	42102955	42164895	111	14	123834	0.37814	0.35266	0.958247561
NEGR1	ENSG00000172260	1	71851623	72758417	1077	62	123834	0.37789	0.35276	0.958247561
LYPLA2	ENSG00000011009	1	24107460	24132029	9	3	123834	0.37769	0.35283	0.958247561
SFRP4	ENSG00000106483	7	37935543	38075297	446	41	123834	0.37768	0.35283	0.958247561
DDHD1	ENSG00000100523	14	53500686	53630000	171	8	123834	0.37767	0.35284	0.958247561
RARA	ENSG00000131759	17	38455444	38523094	22	5	123834	0.37761	0.35286	0.958247561
CARD11	ENSG00000198286	7	2935775	3093579	206	43	123834	0.37752	0.3529	0.958247561
MZB1	ENSG00000170476	5	138713169	138735770	13	2	123834	0.37638	0.35332	0.958247561
KCNJ9	ENSG00000162728	1	160041360	160070353	17	4	123834	0.37616	0.3534	0.958247561
C8orf31	ENSG00000177335	8	144110626	144151359	91	5	123834	0.37606	0.35343	0.958247561
SLC17A2	ENSG00000112337	6	25902982	25940946	79	8	123834	0.37602	0.35345	0.958247561
CMTM5	ENSG00000166091	14	23836017	23858981	33	8	123834	0.37563	0.3536	0.958247561
FANCL	ENSG00000115392	2	58376378	58478507	156	16	123834	0.37542	0.35367	0.958247561
UBE2QL1	ENSG00000215218	5	6438736	6505022	66	15	123834	0.37537	0.35369	0.958247561
AC106873.4	ENSG00000228368	7	116595900	116617526	19	5	123834	0.37537	0.35369	0.958247561
CD5	ENSG00000110448	11	60859867	60905324	44	7	123834	0.37533	0.35371	0.958247561
TRAPPC9	ENSG00000167632	8	140732586	141478678	1119	119	123834	0.3753	0.35372	0.958247561
FOSB	ENSG00000125740	19	45961253	45988437	49	9	123834	0.37521	0.35375	0.958247561
ZDHHC19	ENSG00000163958	3	195914320	195948308	67	10	123834	0.37505	0.35381	0.958247561
DCAF8	ENSG00000132716	1	160175505	160264920	77	6	123834	0.37444	0.35404	0.958247561
PDE3B	ENSG00000152270	11	14655269	14902350	229	16	123834	0.37426	0.3541	0.958247561
TCTEX1D2	ENSG00000213123	3	196008090	196055170	111	10	123834	0.37391	0.35423	0.958247561
CAMSAP1	ENSG00000130559	9	138690333	138809074	110	8	123834	0.37377	0.35429	0.958247561
MCCD1	ENSG00000204511	6	31486494	31508009	83	12	123834	0.37373	0.3543	0.958247561
MS4A4E	ENSG00000214787	11	59958726	60020561	141	12	123834	0.37365	0.35433	0.958247561
DNAH14	ENSG00000185842	1	225073964	225596996	1274	23	123834	0.37363	0.35434	0.958247561
ADAMTSL4	ENSG00000143382	1	150511884	150543413	19	5	123834	0.37346	0.3544	0.958247561
SNRNP27	ENSG00000124380	2	70110692	70142707	30	3	123834	0.37306	0.35455	0.958247561
FAM187A	ENSG00000214447	17	42967135	42992758	58	9	123834	0.37297	0.35459	0.958247561
PSIP1	ENSG00000164985	9	15454064	15521017	110	9	123834	0.37277	0.35466	0.958247561
TMEM82	ENSG00000162460	1	16058917	16084477	17	4	123834	0.37268	0.35469	0.958247561
ATXN7L3B	ENSG00000253719	12	74921551	74945223	80	9	123834	0.37262	0.35471	0.958247561
HOGA1	ENSG00000241935	10	99334080	99382559	47	12	123834	0.37258	0.35473	0.958247561
GALT	ENSG00000213930	9	34628130	34661032	32	6	123834	0.37228	0.35484	0.958247561
XXbac-BPG32J3.20	ENSG00000204422	6	31644739	31691849	48	11	123834	0.37223	0.35486	0.958247561
PELI1	ENSG00000197329	2	64309786	64381588	116	12	123834	0.37172	0.35505	0.958247561
LPPR3	ENSG00000129951	19	802518	831967	27	6	123834	0.37171	0.35505	0.958247561
TMC6	ENSG00000141524	17	76096539	76138488	50	10	123834	0.3717	0.35506	0.958247561
DCAF8	ENSG00000258465	1	160177254	160264920	76	6	123834	0.37162	0.35509	0.958247561
BBOX1	ENSG00000129151	11	27052272	27159356	158	17	123834	0.37158	0.3551	0.958247561
NUDT22	ENSG00000149761	11	63983413	64008158	29	8	123834	0.37131	0.3552	0.958247561
LANCL2	ENSG00000132434	7	55423141	55511435	71	6	123834	0.3711	0.35528	0.958247561
NSMCE4A	ENSG00000107672	10	123706603	123744732	102	8	123834	0.37085	0.35537	0.958247561
DPF1	ENSG00000011332	19	38691646	38730354	22	5	123834	0.37079	0.3554	0.958247561
PPP2R3C	ENSG00000092020	14	35544673	35601723	154	16	123834	0.37068	0.35544	0.958247561
NEFM	ENSG00000104722	8	24760525	24786607	20	5	123834	0.37066	0.35545	0.958247561

BOC	ENSG00000144857	3	112919850	113016303	178	23	123834	0.37047	0.35552	0.958247561
C2orf68	ENSG00000168887	2	85823777	85849189	24	4	123834	0.37035	0.35556	0.958247561
FOXK2	ENSG00000141568	17	80467589	80612538	319	34	123834	0.36987	0.35574	0.958247561
CLPTM1L	ENSG00000049656	5	1307859	1355214	82	9	123834	0.36972	0.3558	0.958247561
HSPA4	ENSG00000170606	5	132377654	132452141	110	5	123834	0.36969	0.35581	0.958247561
TBC1D16	ENSG00000167291	17	77896142	78019647	134	29	123834	0.36968	0.35581	0.958247561
RSPO2	ENSG00000147655	8	108901544	109105913	382	28	123834	0.36963	0.35583	0.958247561
BCL2L10	ENSG00000137875	15	52391460	52414972	35	6	123834	0.36924	0.35597	0.958247561
CELA3B	ENSG00000219073	1	22293514	22335135	161	16	123834	0.36911	0.35602	0.958247561
PRKCB	ENSG00000166501	16	23837322	24241932	544	55	123834	0.36893	0.35609	0.958247561
TTYH3	ENSG00000136295	7	2661585	2714436	39	8	123834	0.36886	0.35612	0.958247561
AQP7	ENSG00000165269	9	33374765	33412643	33	6	123834	0.36846	0.35626	0.958247561
SIAH2	ENSG00000181788	3	150448914	150491264	59	2	123834	0.36843	0.35628	0.958247561
HNRNPUL2	ENSG00000214753	11	62470102	62504821	34	3	123834	0.36842	0.35628	0.958247561
MAP3K13	ENSG00000073803	3	184990729	185216885	382	21	123834	0.3683	0.35632	0.958247561
ASRGL1	ENSG00000162174	11	62094920	62170882	155	11	123834	0.36832	0.35632	0.958247561
NIFK	ENSG00000155438	2	122474521	122504499	51	3	123834	0.36817	0.35637	0.958247561
ADAMTS4	ENSG00000158859	1	161144098	161178846	32	8	123834	0.36751	0.35662	0.958247561
SPATA7	ENSG00000042317	14	88841268	88946694	81	7	123834	0.36736	0.35667	0.958247561
DACT1	ENSG00000165617	14	59090685	59125039	58	9	123834	0.36731	0.35669	0.958247561
LAT2	ENSG00000086730	7	73603982	73654161	17	3	123834	0.3673	0.3567	0.958247561
TBC1D28	ENSG00000189375	17	18528319	18575263	15	3	123834	0.36716	0.35675	0.958247561
CYP4A22	ENSG00000162365	1	47593107	47625413	71	7	123834	0.36688	0.35686	0.958247561
CD2AP	ENSG00000198087	6	47435525	47604999	294	9	123834	0.36684	0.35687	0.958247561
SLC1A2	ENSG00000110436	11	35262753	35451610	364	24	123834	0.3668	0.35689	0.958247561
BIVM	ENSG00000134897	13	103441399	103503885	108	10	123834	0.36678	0.35689	0.958247561
IZUMO4	ENSG00000099840	19	2086380	2109592	28	6	123834	0.36654	0.35698	0.958247561
SNRPA1	ENSG00000131876	15	101811715	101845487	23	5	123834	0.36649	0.357	0.958247561
PEAR1	ENSG00000187800	1	156853490	156896226	78	7	123834	0.36646	0.35701	0.958247561
DSCAML1	ENSG00000177103	11	117288489	117698240	715	77	123834	0.36641	0.35703	0.958247561
ZBED6CL	ENSG00000188707	7	150016938	150039808	38	1	123834	0.36613	0.35713	0.958247561
TGFBR1	ENSG00000106799	9	101856320	101926474	85	6	123834	0.3659	0.35722	0.958247561
MYO19	ENSG00000141140	17	34841477	34909284	100	9	123834	0.36579	0.35726	0.958247561
SPINT3	ENSG00000101446	20	44131101	44154264	37	6	123834	0.36571	0.35729	0.958247561
ARHGAP39	ENSG00000147799	8	145744563	145921194	146	6	123834	0.36526	0.35746	0.958247561
FGL2	ENSG00000127951	7	76812688	76839143	24	5	123834	0.36512	0.35751	0.958247561
TGDS	ENSG00000088451	13	95216308	95258511	76	5	123834	0.36477	0.35764	0.958247561
PHF21B	ENSG00000056487	22	45267042	45415880	234	32	123834	0.36475	0.35765	0.958247561
MMP3	ENSG00000149968	11	102696532	102724534	65	8	123834	0.36452	0.35774	0.958247561
C10orf11	ENSG00000148655	10	77350998	78329925	972	78	123834	0.36445	0.35776	0.958247561
METTL3	ENSG00000165819	14	21956277	21989517	52	6	123834	0.36443	0.35777	0.958247561
PLEKHA2	ENSG00000169499	8	38748753	38841428	68	18	123834	0.36426	0.35783	0.958247561
CDH15	ENSG00000129910	16	89228175	89271900	107	12	123834	0.36428	0.35783	0.958247561
TARBP2	ENSG00000139546	12	53884705	53910215	6	3	123834	0.36409	0.3579	0.958247561
NPFF	ENSG00000139574	12	53890474	53911422	6	3	123834	0.36409	0.3579	0.958247561
CCDC42B	ENSG00000186710	12	113577663	113607081	52	5	123834	0.36401	0.35793	0.958247561
CHMP6	ENSG00000176108	17	78955398	78993317	93	14	123834	0.36401	0.35793	0.958247561
CHSY3	ENSG00000198108	5	129230165	129532327	378	16	123834	0.36397	0.35794	0.958247561
PPP1R1C	ENSG00000150722	2	182808968	183006125	211	27	123834	0.36375	0.35802	0.958247561
RP1-309K20.6	ENSG00000272897	20	34210262	34272466	125	8	123834	0.36358	0.35809	0.958247561
LCE2A	ENSG00000187173	1	152660840	152681918	53	5	123834	0.36355	0.3581	0.958247561
TMED4	ENSG00000158604	7	44607493	44631886	54	9	123834	0.36321	0.35822	0.958247561
UBAC2	ENSG00000134882	13	99843028	100048688	426	23	123834	0.36321	0.35823	0.958247561
SLC51B	ENSG00000186198	15	65327708	65355734	10	2	123834	0.36312	0.35826	0.958247561
IRF7	ENSG00000185507	11	602553	625999	56	8	123834	0.36286	0.35836	0.958247561
RPL7A	ENSG00000148303	9	136205069	136228281	37	5	123834	0.36267	0.35843	0.958247561
UBASH3B	ENSG00000154127	11	122516383	122695181	252	39	123834	0.36257	0.35846	0.958247561
PROZ	ENSG00000126231	13	113802968	113836694	35	2	123834	0.36252	0.35848	0.958247561
STX1A	ENSG00000106089	7	73103536	73144002	25	5	123834	0.36244	0.35851	0.958247561
KPNA1	ENSG00000114030	3	122130796	122243792	194	15	123834	0.36237	0.35854	0.958247561
HOXD9	ENSG00000128709	2	176977088	176999853	11	4	123834	0.36196	0.35869	0.958247561
KRTAP4-9	ENSG00000212722	17	39251584	39272740	148	5	123834	0.3618	0.35875	0.958247561
CDHR2	ENSG00000074276	5	175959512	176032975	99	14	123834	0.36173	0.35878	0.958247561
RFX3	ENSG00000080298	9	3208297	3536004	471	25	123834	0.36156	0.35884	0.958247561
HERC5	ENSG00000138646	4	89368268	89437314	9	3	123834	0.36149	0.35887	0.958247561
MTIF2	ENSG00000085760	2	55453731	55506483	109	15	123834	0.36101	0.35904	0.958247561
RAB15	ENSG00000139998	14	65402532	65449494	60	12	123834	0.36103	0.35904	0.958247561
ZNF717	ENSG00000227124	3	75748794	75844734	93	6	123834	0.36088	0.3591	0.958247561
RP11-234B24.6	ENSG00000255639	12	4756800	4863747	181	18	123834	0.36069	0.35916	0.958247561
ATP2B1	ENSG00000070961	12	89971828	90113077	167	15	123834	0.36047	0.35925	0.958247561
CNTD2	ENSG00000105219	19	40718115	40742597	21	5	123834	0.36039	0.35928	0.958247561
SUPT7L	ENSG00000119760	2	27863679	27896676	14	3	123834	0.36033	0.3593	0.958247561
ZNF837	ENSG00000152475	19	58868985	58902427	58	6	123834	0.36029	0.35932	0.958247561
WDR54	ENSG00000005448	2	74638805	74662882	10	1	123834	0.36024	0.35933	0.958247561
PCDHGA1	ENSG00000204956	5	140700252	140902546	330	24	123834	0.36017	0.35936	0.958247561
MYH15	ENSG00000144821	3	108089216	108258169	276	24	123834	0.36012	0.35938	0.958247561
HNRNPUL2-BSCL2	ENSG00000234857	11	62447747	62504856	54	3	123834	0.36001	0.35942	0.958247561
ACO1	ENSG00000122729	9	32374618	32464767	105	16	123834	0.35976	0.35951	0.958247561
ARHGEF12	ENSG00000196914	11	120197787	120370645	196	10	123834	0.35955	0.35959	0.958247561
RPS6KA5	ENSG00000100784	14	91326799	91536980	277	17	123834	0.35947	0.35962	0.958247561
5-Sep	ENSG00000184702	22	19691987	19722295	7	2	123834	0.35921	0.35972	0.958247561
OPTC	ENSG00000188770	1	203453271	203487992	78	14	123834	0.35909	0.35976	0.958247561
OR4X2	ENSG00000172208	11	48256608	48277622	58	5	123834	0.35908	0.35977	0.958247561
GALNT7	ENSG00000109586	4	174079904	174255118	193	18	123834	0.3589	0.35983	0.958247561
DDX28	ENSG00000182810	16	68045179	68067770	15	2	123834	0.35849	0.35999	0.958247561
RCL1	ENSG00000120158	9	4782869	4895917	202	24	123834	0.35821	0.36009	0.958247561
ATG14	ENSG00000126775	14	55823110	55888576	120	8	123834	0.35809	0.36014	0.958247561
NPY	ENSG00000122585	7	24313782	24341484	63	10	123834	0.35783	0.36024	0.958247561
RPS29	ENSG00000213741	14	50033390	50075408	65	5	123834	0.35782	0.36024	0.958247561
CD164	ENSG00000135535	6	109677717	109713762	90	8	123834	0.35763	0.36031	0.958247561
OR1I1	ENSG00000094661	19	15187791	15209032	35	6	123834	0.35745	0.36038	0.958247561
SPAG6	ENSG00000077327	10	22624399	22753153	71	5	123834	0.3574	0.3604	0.958247561
TMEM249	ENSG00000214597	8	145565878	145588574	9	2	123834	0.35735	0.36042	0.958247561
GS1-393G12.13	ENSG00000271698	8	145566888	145589269	9	2	123834	0.35735	0.36042	0.958247561
AC073528.1	ENSG00000268519	12	77939297	77960568	135	10	123834	0.35729	0.36044	0.958247561
HS1BP3	ENSG00000118960	2	20750208	20860849	261	26	123834	0.3572	0.36047	0.958247561
SNURF	ENSG00000214265	15	25190181	25255423	114	10	123834	0.35701	0.36054	0.958247561
ZNF800	ENSG00000048405	7	126976844	127081978	233	20	123834	0.35654	0.36072	0.958247561
DYNLT1	ENSG00000146425	6	159047506	159075771	53	6	123834	0.35633	0.3608	0.958247561

PRSS56	ENSG00000237412	2	233375173	233400422	35	10	123834	0.35615	0.36086	0.958247561
LPXN	ENSG00000110031	11	58284344	58355693	89	9	123834	0.35616	0.36086	0.958247561
TLE1	ENSG00000196781	9	84188598	84314220	167	38	123834	0.35606	0.3609	0.958247561
CCNY	ENSG00000108100	10	35525953	35870852	333	14	123834	0.35592	0.36095	0.958247561
C14orf132	ENSG00000227051	14	96495661	96570417	150	16	123834	0.35591	0.36095	0.958247561
LGALS7B	ENSG00000178934	19	39269851	39292389	74	7	123834	0.35567	0.36104	0.958247561
GTSF1L	ENSG00000124196	20	42344804	42365638	20	6	123834	0.35543	0.36114	0.958247561
DCP1A	ENSG00000162290	3	53307447	53391654	92	9	123834	0.35521	0.36122	0.958247561
STX17	ENSG00000136874	9	102658915	102742618	91	6	123834	0.35491	0.36133	0.958247561
TKT	ENSG00000163931	3	53248723	53300068	81	6	123834	0.3547	0.36141	0.958247561
ADAMTS17	ENSG00000140470	15	100501794	100892210	914	80	123834	0.35462	0.36144	0.958247561
AHRR	ENSG00000063438	5	294291	448406	291	24	123834	0.35413	0.36162	0.958247561
PKNOX1	ENSG00000160199	21	44384620	44463691	354	15	123834	0.35397	0.36168	0.958247561
FAM3C	ENSG00000196937	7	120978905	121046418	83	3	123834	0.35391	0.3617	0.958247561
SYT6	ENSG00000134207	1	114621913	114706541	106	29	123834	0.35365	0.3618	0.958247561
CCDC182	ENSG00000166329	17	55811842	55832673	47	6	123834	0.35358	0.36183	0.958247561
ARVCF	ENSG00000099889	22	19947419	20014331	159	19	123834	0.35355	0.36184	0.958247561
PIP4K2C	ENSG00000166908	12	57974957	58007198	18	5	123834	0.35349	0.36186	0.958247561
FKSG63	ENSG00000269866	16	70248261	70268641	5	1	123834	0.35344	0.36188	0.958247561
CLNK	ENSG00000109684	4	10478019	10696489	499	31	123834	0.35335	0.36191	0.958247561
MRPL53	ENSG00000204822	2	74689085	74710449	9	3	123834	0.35322	0.36196	0.958247561
JPH1	ENSG00000104369	8	75136935	75243563	93	14	123834	0.35317	0.36198	0.958247561
RALGAPA1	ENSG00000174373	14	35997558	36288510	248	9	123834	0.35313	0.362	0.958247561
TBC1D5	ENSG00000131374	3	17188654	18496309	1894	33	123834	0.35293	0.36207	0.958247561
REXO4	ENSG00000148300	9	136261186	136293164	111	5	123834	0.35288	0.36209	0.958247561
SDK2	ENSG00000069188	17	71320523	71650228	310	64	123834	0.35263	0.36218	0.958247561
RBFA	ENSG00000101546	18	77784358	77816397	107	9	123834	0.35257	0.36221	0.958247561
KIR3DL2	ENSG00000240403	19	55351898	55388662	76	3	123834	0.35257	0.36221	0.958247561
SPOCK3	ENSG00000196104	4	167644535	168165947	1103	43	123834	0.35251	0.36223	0.958247561
EMILIN3	ENSG00000183798	20	39978606	40005467	49	7	123834	0.35249	0.36223	0.958247561
POGZ	ENSG00000143442	1	151365200	151441941	70	2	123834	0.35244	0.36226	0.958247561
KCNAB1	ENSG00000169282	3	155745490	156266545	780	49	123834	0.35202	0.36241	0.958247561
SPRED3	ENSG00000188766	19	38869061	38896881	70	4	123834	0.35199	0.36242	0.958247561
FEM1C	ENSG00000145780	5	114846608	114890591	47	9	123834	0.35193	0.36245	0.958247561
RP13-279N23.2	ENSG00000255275	1	19165767	19257615	199	28	123834	0.35189	0.36246	0.958247561
AL450307.1	ENSG00000189275	10	133594734	133632535	77	5	123834	0.35179	0.3625	0.958247561
CCDC114	ENSG00000105479	19	48789714	48835151	99	11	123834	0.35177	0.36251	0.958247561
CCL26	ENSG00000006606	7	75388851	75429214	66	8	123834	0.3515	0.36261	0.958247561
SLC25A24	ENSG00000085491	1	108666658	108753471	168	15	123834	0.35139	0.36265	0.958247561
ST6GALNAC4	ENSG00000136840	9	130660165	130689317	13	3	123834	0.35129	0.36269	0.958247561
TTLL3	ENSG00000214021	3	9839770	9906822	110	11	123834	0.35111	0.36275	0.958247561
CLEC2A	ENSG00000188393	12	10041272	10094980	88	17	123834	0.35105	0.36277	0.958247561
BMP2	ENSG00000125845	20	6738311	6770927	34	11	123834	0.35107	0.36277	0.958247561
SLC9A3R2	ENSG00000065054	16	2065357	2099027	26	4	123834	0.35093	0.36282	0.958247561
GLB1L3	ENSG00000166105	11	134134139	134199458	72	12	123834	0.3508	0.36287	0.958247561
PHAX	ENSG00000164902	5	125925960	125972944	110	12	123834	0.35076	0.36288	0.958247561
VWA3A	ENSG00000175267	16	22093859	22178287	49	9	123834	0.3506	0.36294	0.958247561
SARS	ENSG00000031698	1	109746540	109790791	30	7	123834	0.35049	0.36299	0.958247561
GSPT1	ENSG00000103342	16	11951985	12019939	100	8	123834	0.35037	0.36303	0.958247561
KANK3	ENSG00000186994	19	8377468	8418146	113	12	123834	0.35024	0.36308	0.958247561
NOL12	ENSG00000100101	22	38067680	38180137	189	20	123834	0.34995	0.36319	0.958247561
PLEKHB1	ENSG00000021300	11	73347223	73383864	89	14	123834	0.3496	0.36332	0.958247561
AP001055.1	ENSG00000267913	21	45577818	45603580	39	5	123834	0.34931	0.36343	0.958247561
CACNB3	ENSG00000167535	12	49197577	49232724	17	6	123834	0.34925	0.36345	0.958247561
MMRN1	ENSG00000138722	4	90790683	90885780	156	15	123834	0.34916	0.36348	0.958247561
CD79B	ENSG00000007312	17	61996100	62019714	18	3	123834	0.34911	0.3635	0.958247561
SUPV3L1	ENSG00000156502	10	70929988	70978855	115	12	123834	0.34909	0.36351	0.958247561
GIPC1	ENSG00000123159	19	14578572	14616944	58	12	123834	0.34907	0.36352	0.958247561
WNT7A	ENSG00000154764	3	13847755	13931618	114	24	123834	0.3489	0.36358	0.958247561
MLEC	ENSG00000110917	12	121114672	121149667	80	14	123834	0.3489	0.36358	0.958247561
PHKB	ENSG00000102893	16	47485034	47745434	135	7	123834	0.34888	0.36359	0.958247561
LYPD5	ENSG00000159871	19	44290081	44341358	151	15	123834	0.34883	0.36361	0.958247561
RBM28	ENSG00000106344	7	127927738	127993962	58	5	123834	0.34801	0.36392	0.958247561
PGLYRP4	ENSG00000163218	1	153292596	153331316	137	7	123834	0.34797	0.36393	0.958247561
AC017028.1	ENSG00000213828	2	240073493	240094053	37	4	123834	0.34797	0.36393	0.958247561
RRAGD	ENSG00000025039	6	90064355	90131989	81	14	123834	0.34797	0.36393	0.958247561
DPM2	ENSG00000136908	9	130687378	130710763	40	4	123834	0.34794	0.36394	0.958247561
CPEB3	ENSG00000107864	10	93796449	94060844	256	10	123834	0.34766	0.36405	0.958247561
FAM20B	ENSG00000116199	1	178984939	179055697	189	17	123834	0.34758	0.36408	0.958247561
SZT2	ENSG00000198198	1	43845553	43928321	75	4	123834	0.34753	0.3641	0.958247561
AC090574.1	ENSG00000269197	8	70840403	70860635	55	8	123834	0.34729	0.36418	0.958247561
PIRT	ENSG00000233670	17	10715792	10751762	29	6	123834	0.34711	0.36425	0.958247561
SPATA8	ENSG00000185594	15	97316619	97338845	19	6	123834	0.34677	0.36438	0.958247561
CLIP1	ENSG00000130779	12	122745979	122917179	207	13	123834	0.34659	0.36445	0.958247561
TPRX2P	ENSG00000259009	19	48327701	48374769	90	7	123834	0.34647	0.36449	0.958247561
ZNF273	ENSG00000198039	7	64320550	64401344	248	12	123834	0.34631	0.36455	0.958247561
GJD2	ENSG00000159248	15	35033233	35057166	29	6	123834	0.34595	0.36469	0.958247561
DTNBP1	ENSG00000047579	6	15513032	15673289	221	14	123834	0.34592	0.3647	0.958247561
DZIP1L	ENSG00000158163	3	137770832	137844660	101	6	123834	0.34566	0.3648	0.958247561
PRKCA	ENSG00000154229	17	64288754	64816861	1031	81	123834	0.34559	0.36483	0.958247561
PYGB	ENSG00000100994	20	25218705	25288650	194	9	123834	0.34554	0.36484	0.958247561
CFLAR	ENSG00000003402	2	201970827	202051410	121	10	123834	0.34536	0.36491	0.958247561
C17orf96	ENSG00000179294	17	36817961	36841187	26	5	123834	0.34537	0.36491	0.958247561
HMGB2	ENSG00000164104	4	174242846	174266276	27	3	123834	0.34524	0.36496	0.958247561
RP11-701P16.2	ENSG00000251139	4	185724773	185752443	39	5	123834	0.34512	0.365	0.958247561
BSCL2	ENSG00000168000	11	62447747	62487317	34	3	123834	0.34475	0.36514	0.958247561
THSD1	ENSG00000136114	13	52941305	52990629	112	5	123834	0.34472	0.36515	0.958247561
INSIG1	ENSG00000186480	7	155079486	155111945	96	12	123834	0.34453	0.36522	0.958247561
RNF11	ENSG00000123091	1	51691943	51749127	38	3	123834	0.34452	0.36523	0.958247561
RSPO3	ENSG00000146374	6	127429749	127528910	116	8	123834	0.34446	0.36525	0.958247561
AFG3L2	ENSG00000141385	18	12318943	12387313	83	6	123834	0.34442	0.36526	0.958247561
FOXG1	ENSG00000176165	14	29225050	29248870	3	1	123834	0.34432	0.3653	0.958247561
CACYBP	ENSG00000116161	1	174958300	174990851	31	7	123834	0.34415	0.36537	0.958247561
STMN3	ENSG00000197457	20	62261061	62294780	59	12	123834	0.34411	0.36538	0.958247561
DYDC1	ENSG00000170788	10	82085861	82126511	77	7	123834	0.34406	0.3654	0.958247561
MRPL42	ENSG00000198015	12	93851264	93907545	153	12	123834	0.34388	0.36547	0.958247561
RPS28	ENSG00000233927	19	8376042	8398224	71	9	123834	0.34373	0.36552	0.958247561
CCDC34	ENSG00000109881	11	27342374	27395415	77	11	123834	0.34328	0.36569	0.958247561
CD151	ENSG00000177697	11	822843	849831	47	4	123834	0.34324	0.36571	0.958247561

TMEM178B	ENSG00000261115	7	140764032	141190180	511	53	123834	0.34315	0.36574	0.958247561
ADAMTSL2	ENSG00000197859	9	136387286	136450641	74	6	123834	0.34317	0.36574	0.958247561
OR8A1	ENSG00000196119	11	124429893	124451037	55	9	123834	0.34307	0.36577	0.958247561
CFL2	ENSG00000165410	14	35169593	35194029	73	10	123834	0.34301	0.3658	0.958247561
REG1B	ENSG00000172023	2	79302156	79325145	37	7	123834	0.34298	0.36581	0.958247561
ILDR1	ENSG00000145103	3	121696170	121751051	38	10	123834	0.34278	0.36588	0.958247561
AKNAD1	ENSG00000162641	1	109348520	109516106	328	22	123834	0.34268	0.36592	0.958247561
BCKDHB	ENSG00000083123	6	80806364	81065987	614	16	123834	0.34258	0.36596	0.958247561
RP11-190A12.7	ENSG00000256029	1	159817690	159852843	32	7	123834	0.34252	0.36598	0.958247561
TBL1XR1	ENSG00000177565	3	176727143	176925261	308	21	123834	0.34253	0.36598	0.958247561
RFX1	ENSG00000132005	19	14062350	14127851	8	3	123834	0.34252	0.36598	0.958247561
EPHA1	ENSG00000146904	7	143077382	143115985	31	9	123834	0.3422	0.3661	0.958247561
ABHD8	ENSG00000127220	19	17392940	17431045	110	13	123834	0.34213	0.36613	0.958247561
AKAP11	ENSG00000023516	13	42836289	42907396	108	9	123834	0.34193	0.3662	0.958247561
YIPF4	ENSG00000119820	2	32492979	32551663	70	11	123834	0.34185	0.36623	0.958247561
DDX50	ENSG00000107625	10	70651034	70716603	149	14	123834	0.34185	0.36623	0.958247561
TEX29	ENSG00000153495	13	111958531	112006596	119	18	123834	0.34184	0.36624	0.958247561
AKAP1	ENSG00000121057	17	55152453	55208710	92	16	123834	0.34176	0.36626	0.958247561
TYROBP	ENSG00000011600	19	36385303	36409197	42	4	123834	0.34176	0.36627	0.958247561
GDF1	ENSG00000130283	19	18969361	19016905	50	11	123834	0.34168	0.3663	0.958247561
HCFC1R1	ENSG00000103145	16	3062621	3084287	8	3	123834	0.3416	0.36633	0.958247561
C10orf32	ENSG00000166275	10	104603980	104634718	54	7	123834	0.34148	0.36637	0.958247561
NDUFA7	ENSG00000167774	19	8363167	8396263	105	10	123834	0.34148	0.36637	0.958247561
NDUFA7	ENSG00000267855	19	8363490	8396280	105	10	123834	0.34148	0.36637	0.958247561
CHURC1	ENSG00000258289	14	65371079	65421309	138	10	123834	0.34136	0.36641	0.958247561
GNB1	ENSG00000078369	1	1706729	1832495	106	10	123834	0.3411	0.36651	0.958247561
WDR75	ENSG00000115368	2	190296159	190350291	214	7	123834	0.34104	0.36654	0.958247561
PDCD1LG2	ENSG00000197646	9	5500545	5581282	92	14	123834	0.3409	0.36659	0.958247561
DNAJC25	ENSG00000059769	9	114383632	114442508	122	16	123834	0.34077	0.36664	0.958247561
DNAJC25-GNG10	ENSG00000244115	9	114383666	114442509	122	16	123834	0.34077	0.36664	0.958247561
TMEM38A	ENSG00000072954	19	16761938	16810840	106	9	123834	0.34077	0.36664	0.958247561
SLMO1	ENSG00000141391	18	12397895	12442237	64	14	123834	0.34038	0.36678	0.958247561
TMED7-TICAM2	ENSG00000251201	5	114904339	114971858	86	8	123834	0.34035	0.3668	0.958247561
TICAM2	ENSG00000243414	5	114904339	114971876	86	8	123834	0.34035	0.3668	0.958247561
ITIH5	ENSG00000123243	10	7591232	7718961	172	29	123834	0.34028	0.36682	0.958247561
FEZ2	ENSG00000171055	2	36768570	36883230	136	15	123834	0.34025	0.36683	0.958247561
CNPY4	ENSG00000166997	7	99707236	99733134	19	4	123834	0.34012	0.36688	0.958247561
GNRH2	ENSG00000125787	20	3014268	3036393	20	5	123834	0.33989	0.36697	0.958247561
OR5K2	ENSG00000231861	3	98206448	98227496	26	7	123834	0.33986	0.36698	0.958247561
ATP11B	ENSG00000058063	3	182501288	182649423	222	10	123834	0.33921	0.36723	0.958247561
PRSS50	ENSG00000206549	3	46743605	46864064	266	7	123834	0.33914	0.36725	0.958247561
MED31	ENSG00000108590	17	6536635	6564954	99	9	123834	0.339	0.36731	0.958247561
SOCS3	ENSG00000184557	17	76342864	76366158	21	4	123834	0.33894	0.36733	0.958247561
ACKR2	ENSG00000144648	3	42836244	42939466	141	17	123834	0.33885	0.36736	0.958247561
DEFA6	ENSG00000164822	8	6772215	6793598	43	9	123834	0.33855	0.36748	0.958247561
FBXL6	ENSG00000182325	8	145569091	145593036	12	2	123834	0.33853	0.36748	0.958247561
ABHD3	ENSG00000158201	18	19220858	19294766	62	6	123834	0.3384	0.36753	0.958247561
BCL9L	ENSG00000186174	11	118754584	118806317	58	13	123834	0.33832	0.36756	0.958247561
CCDC24	ENSG00000159214	1	44447031	44472200	20	4	123834	0.33817	0.36762	0.958247561
ARRDC2	ENSG00000105643	19	18101941	18134911	56	9	123834	0.33816	0.36762	0.958247561
LY6G5C	ENSG00000204428	6	31634461	31661817	24	7	123834	0.33808	0.36765	0.958247561
TBC1D14	ENSG00000132405	4	6900969	7044845	303	15	123834	0.33785	0.36774	0.958247561
NR0B2	ENSG00000131910	1	27227980	27250457	33	4	123834	0.33757	0.36784	0.958247561
KLRC3	ENSG00000205810	12	10554911	10583194	32	5	123834	0.33757	0.36784	0.958247561
SLIT1	ENSG00000187122	10	98747795	98955677	275	38	123834	0.33744	0.36789	0.958247561
SORT1	ENSG00000134243	1	109842192	109950573	106	4	123834	0.33733	0.36794	0.958247561
GSTO1	ENSG00000148834	10	105985114	106037217	58	12	123834	0.33715	0.368	0.958247561
CXCL13	ENSG00000156234	4	78422907	78542988	55	14	123834	0.33693	0.36809	0.958247561
LAMP1	ENSG00000185896	13	113941556	113987987	4	2	123834	0.33682	0.36812	0.958247561
MIIP	ENSG00000116691	1	12069523	12102102	14	3	123834	0.33662	0.3682	0.958247561
MLLT4	ENSG00000130396	6	168217602	168382703	211	15	123834	0.33663	0.3682	0.958247561
PGAP1	ENSG00000197121	2	197687728	197802520	145	7	123834	0.33649	0.36825	0.958247561
NRIP2	ENSG00000053702	12	2924514	2954710	39	3	123834	0.33632	0.36832	0.958247561
NOL11	ENSG00000130935	17	65703949	65750647	56	4	123834	0.33618	0.36837	0.958247561
ARL5C	ENSG00000141748	17	37303147	37333737	65	9	123834	0.33602	0.36843	0.958247561
KRTAP4-12	ENSG00000213416	17	39269343	39290419	97	3	123834	0.33577	0.36852	0.958247561
REEP4	ENSG00000168476	8	21985533	22009464	19	3	123834	0.33557	0.3686	0.958247561
MT4	ENSG00000102891	16	56588961	56612869	66	14	123834	0.33497	0.36882	0.958247561
LGI4	ENSG00000153902	19	35605417	35643355	28	2	123834	0.33492	0.36884	0.958247561
ARHGAP19-SLIT1	ENSG00000269891	10	98747795	99062413	528	51	123834	0.33491	0.36885	0.958247561
OR13J1	ENSG00000168828	9	35859375	35880461	33	6	123834	0.3346	0.36896	0.958247561
CBR4	ENSG00000145439	4	169774921	169941426	222	11	123834	0.33442	0.36903	0.958247561
EVI2A	ENSG00000126860	17	29634578	29658902	27	1	123834	0.33443	0.36903	0.958247561
PPP6R1	ENSG00000105063	19	55731148	55780363	54	9	123834	0.33434	0.36906	0.958247561
EPHA10	ENSG00000183317	1	38169552	38240805	173	17	123834	0.33429	0.36908	0.958247561
IAPP	ENSG00000121351	12	21497893	21542912	74	13	123834	0.33419	0.36912	0.958247561
EIF4A2	ENSG00000156976	3	186490994	186517689	21	5	123834	0.33412	0.36914	0.958247561
EPDR1	ENSG00000086289	7	37713446	38001543	734	54	123834	0.33414	0.36914	0.958247561
PLXDC1	ENSG00000161381	17	37209556	37320647	243	26	123834	0.334	0.36919	0.958247561
ZNF212	ENSG00000170260	7	148926742	148962700	83	12	123834	0.33398	0.3692	0.958247561
ANKRD13A	ENSG00000076513	12	110426991	110487568	76	9	123834	0.33391	0.36922	0.958247561
SFXN4	ENSG00000183605	10	120890279	120935179	57	7	123834	0.33378	0.36927	0.958247561
SPATA13	ENSG00000182957	13	24543944	24891212	594	91	123834	0.33374	0.36929	0.958247561
NKG2-E	ENSG00000255641	12	10554911	10598600	42	5	123834	0.33368	0.36931	0.958247561
ESD	ENSG00000139684	13	47335391	47381367	35	5	123834	0.33364	0.36932	0.958247561
TMEM63B	ENSG00000137216	6	44084651	44133256	109	15	123834	0.33363	0.36933	0.958247561
PHOX2A	ENSG00000165462	11	71940121	71966708	22	3	123834	0.33362	0.36933	0.958247561
LRRC37A	ENSG00000176681	17	44360099	44425160	1	1	123834	0.33339	0.36942	0.958247561
AC087645.1	ENSG00000268310	17	76200462	76230740	67	8	123834	0.33338	0.36942	0.958247561
LIN7C	ENSG00000148943	11	27506123	27538320	16	3	123834	0.33323	0.36948	0.958247561
CCDC53	ENSG00000120860	12	102396705	102465927	107	13	123834	0.33302	0.36956	0.958247561
ZNF239	ENSG00000196793	10	44041792	44080066	59	6	123834	0.33296	0.36958	0.958247561
EYA1	ENSG00000104313	8	72099668	72284467	174	35	123834	0.3329	0.36961	0.958247561
S100A7L2	ENSG00000197364	1	153399534	153422425	35	5	123834	0.33271	0.36968	0.958247561
TMEM181	ENSG00000146433	6	158947468	159066460	188	15	123834	0.3327	0.36968	0.958247561
RAB4A	ENSG00000168118	1	229396822	229451641	79	6	123834	0.33246	0.36977	0.958247561
ZBTB34	ENSG00000177125	9	129612944	129658157	25	5	123834	0.3324	0.36979	0.958247561
PRRC2C	ENSG00000117523	1	171444651	171572650	238	15	123834	0.33171	0.37005	0.958247561
PSMA6	ENSG00000100902	14	35737839	35796699	138	12	123834	0.33166	0.37007	0.958247561

RP11-171N4.2	ENSG00000251283	4	157545646	157573606	59	7	123834	0.33156	0.37011	0.958247561
ATG4C	ENSG00000125703	1	63239806	63341184	109	7	123834	0.33154	0.37012	0.958247561
CNTNAP1	ENSG00000108797	17	40824631	40861832	17	4	123834	0.33151	0.37013	0.958247561
AQP4	ENSG00000171885	18	24422002	24455782	45	9	123834	0.3313	0.37021	0.958247561
TM9SF2	ENSG00000125304	13	100143671	100226260	121	14	123834	0.3311	0.37028	0.958247561
CPNE1	ENSG00000214078	20	34203953	34262878	107	8	123834	0.33095	0.37034	0.958247561
RNF139	ENSG00000170881	8	125476979	125510155	51	8	123834	0.33086	0.37038	0.958247561
MED16	ENSG00000175221	19	857962	903218	51	6	123834	0.33076	0.37041	0.958247561
NDUFB8	ENSG00000166136	10	102257203	102299757	68	2	123834	0.33064	0.37046	0.958247561
AC131935.1	ENSG00000268242	11	11737060	11757257	24	6	123834	0.33057	0.37049	0.958247561
LPO	ENSG00000167419	17	56285909	56355879	67	12	123834	0.33047	0.37052	0.958247561
PDRG1	ENSG00000088356	20	30522758	30549895	15	3	123834	0.3304	0.37055	0.958247561
SLC4A10	ENSG00000144290	2	162270843	162851792	881	19	123834	0.33026	0.3706	0.958247561
RP11-603J24.9	ENSG00000257411	12	56485115	56513073	13	4	123834	0.3302	0.37062	0.958247561
SWSAP1	ENSG00000173928	19	11475361	11497627	20	4	123834	0.33006	0.37068	0.958247561
AMDHD1	ENSG00000139344	12	96327071	96372370	111	10	123834	0.32995	0.37072	0.958247561
TRPM1	ENSG00000134160	15	31283264	31463476	252	38	123834	0.32982	0.37077	0.958247561
KRT20	ENSG00000171431	17	39022193	39051479	61	10	123834	0.32963	0.37084	0.958247561
BOD1L2	ENSG00000228075	18	54804293	54827531	33	8	123834	0.32922	0.37099	0.958247561
PSMC1	ENSG00000100764	14	90712839	90748968	95	4	123834	0.32908	0.37105	0.958247561
UCN2	ENSG00000145040	3	48589160	48611206	12	2	123834	0.32903	0.37107	0.958247561
ZNF534	ENSG00000198633	19	52922440	52965568	159	5	123834	0.32889	0.37112	0.958247561
SLC25A17	ENSG00000100372	22	41155634	41225403	38	3	123834	0.32881	0.37115	0.958247561
RPRD1B	ENSG00000101413	20	36651948	36730768	94	5	123834	0.32866	0.3712	0.958247561
ATP6V1B2	ENSG00000147416	8	20044878	20094330	35	7	123834	0.3286	0.37123	0.958247561
NTPCR	ENSG00000135778	1	233076351	233129628	101	8	123834	0.32851	0.37126	0.958247561
NDUFB8	ENSG00000255339	10	102255385	102299638	69	2	123834	0.32846	0.37128	0.958247561
NXT1	ENSG00000132661	20	23321373	23345414	69	10	123834	0.32823	0.37137	0.958247561
HAL	ENSG00000084110	12	96356440	96400143	120	17	123834	0.32797	0.37147	0.958247561
RWDD4	ENSG00000182552	4	184550788	184590378	67	12	123834	0.32788	0.3715	0.958247561
PON3	ENSG00000105852	7	94979256	95035680	78	10	123834	0.32776	0.37155	0.958247561
CRTC1	ENSG00000105662	19	18784487	18903004	130	17	123834	0.32775	0.37155	0.958247561
LAMP5	ENSG00000125869	20	9485005	9521171	61	10	123834	0.32772	0.37156	0.958247561
KXD1	ENSG00000105700	19	18658572	18690197	33	3	123834	0.32764	0.37159	0.958247561
ZNF766	ENSG00000196214	19	52762824	52805977	71	7	123834	0.32731	0.37172	0.958247561
CALHM2	ENSG00000138172	10	105196543	105222660	17	5	123834	0.32713	0.37178	0.958247561
TUSC3	ENSG00000104723	8	15264724	15634158	898	48	123834	0.32688	0.37188	0.958247561
PRAME	ENSG00000185686	22	22880123	22911768	48	2	123834	0.32671	0.37194	0.958247561
RP11-521M14.2	ENSG00000253903	8	26933694	26954766	76	13	123834	0.3267	0.37195	0.958247561
USP22	ENSG00000124422	17	20892910	20957073	151	9	123834	0.32665	0.37196	0.958247561
TMEM135	ENSG00000166575	11	86738886	87044800	1084	32	123834	0.32663	0.37197	0.958247561
CSDC2	ENSG00000172346	22	41946767	41983745	39	2	123834	0.32652	0.37202	0.958247561
SPRY2	ENSG00000136158	13	80900111	80925086	11	3	123834	0.32642	0.37205	0.958247561
SLC5A12	ENSG00000148942	11	26678566	26754974	192	30	123834	0.3263	0.3721	0.958247561
RSAD2	ENSG00000134321	2	6995937	7048370	32	7	123834	0.32617	0.37215	0.958247561
GPATCH4	ENSG00000160818	1	156554279	156581288	71	4	123834	0.32615	0.37216	0.958247561
SETX	ENSG00000107290	9	135126743	135240372	323	12	123834	0.32611	0.37217	0.958247561
C8orf87	ENSG00000254318	8	94136324	94189079	92	12	123834	0.32603	0.3722	0.958247561
EID2B	ENSG00000176401	19	40011630	40033494	41	8	123834	0.32553	0.37239	0.958247561
PPIL6	ENSG00000185250	6	109701418	109772374	154	11	123834	0.32541	0.37244	0.958247561
ANGEL1	ENSG00000013523	14	77243588	77302589	108	15	123834	0.32517	0.37253	0.958247561
HCK	ENSG00000101336	20	30629991	30699659	71	10	123834	0.32456	0.37276	0.958247561
RASA4B	ENSG00000170667	7	102112892	102168228	13	4	123834	0.32443	0.37281	0.958247561
TPX2	ENSG00000088325	20	30317074	30399608	112	7	123834	0.3244	0.37282	0.958247561
VAT1L	ENSG00000171724	16	77812427	78024004	411	62	123834	0.32405	0.37295	0.958247561
NRTN	ENSG00000171119	19	5813813	5838335	63	11	123834	0.32404	0.37295	0.958247561
LGMN	ENSG00000100600	14	93160152	93225047	46	11	123834	0.32387	0.37302	0.958247561
NAT9	ENSG00000109065	17	72756686	72782506	43	5	123834	0.32366	0.3731	0.958247561
IL12RB1	ENSG00000096996	19	18159805	18219754	117	15	123834	0.32364	0.37311	0.958247561
DCTN5	ENSG00000166847	16	23642713	23691195	98	6	123834	0.32359	0.37313	0.958247561
ZNF765	ENSG00000196417	19	53883046	53940574	172	24	123834	0.32357	0.37313	0.958247561
FSIP2	ENSG00000188738	2	186593355	186708017	198	8	123834	0.3234	0.3732	0.958247561
EIF3I	ENSG00000084623	1	32677529	32707205	30	2	123834	0.32331	0.37323	0.958247561
RP11-691N7.6	ENSG00000254732	11	57499635	57570715	58	5	123834	0.32322	0.37326	0.958247561
RFC2	ENSG00000049541	7	73635829	73678774	33	3	123834	0.32321	0.37327	0.958247561
RAMP3	ENSG00000122679	7	45187390	45235901	231	12	123834	0.32317	0.37328	0.958247561
ZNF300	ENSG00000145908	5	150263954	150294545	77	6	123834	0.32311	0.37331	0.958247561
MT1B	ENSG00000169688	16	56675811	56697116	79	10	123834	0.32297	0.37336	0.958247561
DDX11	ENSG00000013573	12	31216779	31267725	113	6	123834	0.32279	0.37343	0.958247561
USP10	ENSG00000103194	16	84723584	84823528	207	22	123834	0.32266	0.37347	0.958247561
NDUFA3	ENSG00000170906	19	54596036	54622564	44	9	123834	0.32266	0.37347	0.958247561
BSPRY	ENSG00000119411	9	116101821	116143513	136	15	123834	0.3226	0.3735	0.958247561
FNDC9	ENSG00000172568	5	156758608	156782729	70	4	123834	0.32245	0.37356	0.958247561
NEK3	ENSG00000136098	13	52696775	52743996	98	12	123834	0.32233	0.3736	0.958247561
ABHD12B	ENSG00000131969	14	51328878	51381688	72	13	123834	0.32232	0.37361	0.958247561
HERC2	ENSG00000128731	15	28346186	28577298	136	5	123834	0.32224	0.37363	0.958247561
SPAG11A	ENSG00000178287	8	7695398	7736389	2	1	123834	0.32199	0.37373	0.958247561
NCALD	ENSG00000104490	8	102688771	103147135	650	54	123834	0.32135	0.37397	0.958247561
CCDC62	ENSG00000130783	12	123248874	123322075	183	11	123834	0.3213	0.37399	0.958247561
NUP210	ENSG00000132182	3	13347737	13471809	331	16	123834	0.321	0.37411	0.958247561
PRSS58	ENSG00000258223	7	141941963	141967878	112	8	123834	0.32085	0.37416	0.958247561
SERAC1	ENSG00000122335	6	158520536	158599312	136	11	123834	0.3208	0.37418	0.958247561
DBF4	ENSG00000006634	7	87495531	87548856	74	7	123834	0.32067	0.37423	0.958247561
THAP8	ENSG00000161277	19	36515887	36555664	61	9	123834	0.32067	0.37423	0.958247561
SH2D7	ENSG00000183476	15	78360150	78407251	106	21	123834	0.32054	0.37428	0.958247561
STEAP1	ENSG00000164647	7	89773689	89804143	62	7	123834	0.32034	0.37435	0.958247561
GRID2	ENSG00000152208	4	93215550	94705707	2338	91	123834	0.32034	0.37436	0.958247561
SFTPA2	ENSG00000185303	10	81305608	81330153	31	7	123834	0.32015	0.37443	0.958247561
TMEM102	ENSG00000181284	17	7328762	7350998	15	3	123834	0.32005	0.37447	0.958247561
C8orf74	ENSG00000171060	8	10520147	10568103	31	5	123834	0.31951	0.37467	0.958247561
AC008686.1	ENSG00000214109	19	13880884	13910972	45	2	123834	0.31935	0.37473	0.958247561
CYP3A43	ENSG00000021461	7	99415636	99473718	74	7	123834	0.31924	0.37477	0.958247561
ALDH1L1	ENSG00000144908	3	125812412	125926837	341	29	123834	0.31889	0.3749	0.958247561
PRSS16	ENSG00000112812	6	27205480	27234403	59	10	123834	0.31885	0.37492	0.958247561
NAALADL2	ENSG00000177694	3	174146363	175533428	2413	155	123834	0.31882	0.37493	0.958247561
RNASE2	ENSG00000169385	14	21413611	21434595	13	3	123834	0.31882	0.37493	0.958247561
WFDC8	ENSG00000158901	20	44169792	44217965	208	5	123834	0.31874	0.37496	0.958247561
MYCT1	ENSG00000120279	6	153009030	153055702	145	11	123834	0.31868	0.37498	0.958247561
IP6K3	ENSG00000161896	6	33679444	33724762	139	17	123834	0.31841	0.37509	0.958247561

AC021218.2	ENSG00000204876	7	155745326	155769037	19	7	123834	0.31832	0.37512	0.958247561
RREB1	ENSG00000124782	6	7097830	7262213	146	22	123834	0.31828	0.37513	0.958247561
OOEP	ENSG00000203907	6	74068278	74114856	61	10	123834	0.31829	0.37513	0.958247561
FAM43B	ENSG00000183114	1	20868932	20891512	27	5	123834	0.31793	0.37527	0.958247561
CAMK2B	ENSG00000058404	7	44246749	44384176	171	23	123834	0.31774	0.37534	0.958247561
NKX1-1	ENSG00000235608	4	1386720	1410119	18	4	123834	0.31719	0.37555	0.958247561
HIST1H4G	ENSG00000124578	6	26236886	26257259	37	6	123834	0.3172	0.37555	0.958247561
TMEM235	ENSG00000204278	17	76217391	76247068	60	10	123834	0.31687	0.37567	0.958247561
HES2	ENSG00000069812	1	6462478	6494730	90	9	123834	0.31674	0.37572	0.958247561
PLA2G7	ENSG00000146070	6	46661938	46713430	39	5	123834	0.3167	0.37574	0.958247561
ITLN1	ENSG00000179914	1	160836329	160864960	54	3	123834	0.31658	0.37578	0.958247561
WWC2	ENSG00000151718	4	184010446	184251930	278	23	123834	0.31658	0.37578	0.958247561
TPM4	ENSG00000167460	19	16167831	16223813	62	10	123834	0.31648	0.37582	0.958247561
PLD3	ENSG00000105223	19	40844363	40896346	53	11	123834	0.3157	0.37611	0.958247561
ZBED2	ENSG00000177494	3	111301747	111324290	33	7	123834	0.31562	0.37614	0.958247561
PSMA5	ENSG00000143106	1	109931653	109979062	50	4	123834	0.31554	0.37617	0.958247561
C11orf87	ENSG00000185742	11	109282846	109309840	2	1	123834	0.31543	0.37622	0.958247561
MIXL1	ENSG00000185155	1	226401319	226424755	25	4	123834	0.31538	0.37624	0.958247561
RMDN1	ENSG00000176623	8	87470486	87536586	170	12	123834	0.31512	0.37633	0.958247561
DERA	ENSG00000023697	12	16054106	16200220	95	10	123834	0.31496	0.37639	0.958247561
SP3	ENSG00000172845	2	174761187	174840430	145	11	123834	0.31487	0.37643	0.958247561
HUNK	ENSG00000142149	21	33235628	33426946	325	42	123834	0.31477	0.37647	0.958247561
EFCAB9	ENSG00000214360	5	171611176	171640456	24	7	123834	0.31458	0.37654	0.958247561
LRRC14B	ENSG00000185028	5	181626	205468	58	8	123834	0.31442	0.3766	0.958247561
ARHGEF40	ENSG00000165801	14	21528429	21568399	43	8	123834	0.31442	0.3766	0.958247561
INSL4	ENSG00000120211	9	5221419	5245304	4	2	123834	0.31433	0.37663	0.958247561
MMACHC	ENSG00000132763	1	45955725	45986739	53	5	123834	0.31419	0.37669	0.958247561
MORC3	ENSG00000159256	21	37682487	37768446	213	9	123834	0.31411	0.37672	0.958247561
NR1D2	ENSG00000174738	3	23976751	24032109	93	8	123834	0.31405	0.37674	0.958247561
SIGLEC11	ENSG00000161640	19	50442242	50474429	42	6	123834	0.3138	0.37684	0.958247561
CASP2	ENSG00000106144	7	142975308	143014789	13	3	123834	0.31342	0.37698	0.958247561
DNMT1	ENSG00000130816	19	10234021	10351962	120	12	123834	0.31336	0.377	0.958247561
CBS	ENSG00000160200	21	44463301	44507053	120	17	123834	0.31338	0.377	0.958247561
GK5	ENSG00000175066	3	141872414	141954449	73	12	123834	0.31331	0.37702	0.958247561
CTD-2545G14.7	ENSG00000262526	17	7133746	7157954	13	2	123834	0.3132	0.37706	0.958247561
TP53BP2	ENSG00000143514	1	223957601	224043674	120	8	123834	0.31269	0.37726	0.958247561
DNAH1	ENSG00000114841	3	52340335	52444507	94	5	123834	0.3126	0.37729	0.958247561
TOX	ENSG00000198846	8	59707977	60041767	399	47	123834	0.31254	0.37732	0.958247561
PSMD13	ENSG00000185627	11	226546	262983	116	12	123834	0.31252	0.37732	0.958247561
KRTAP5-11	ENSG00000204571	11	71282901	71324399	86	8	123834	0.31253	0.37732	0.958247561
ATG5	ENSG00000057663	6	106622351	106783666	312	16	123834	0.31237	0.37738	0.958247561
FAM3D	ENSG00000198643	3	58609673	58662575	79	9	123834	0.3118	0.3776	0.958247561
KLRC2	ENSG00000205809	12	10569453	10604899	41	5	123834	0.31172	0.37763	0.958247561
LINC00998	ENSG00000214194	7	112746773	112768668	26	4	123834	0.31146	0.37772	0.958247561
SLC26A11	ENSG00000181045	17	78183498	78237299	75	13	123834	0.31148	0.37772	0.958247561
BIRC5	ENSG00000089685	17	76200267	76231717	68	8	123834	0.31124	0.37781	0.958247561
USF2	ENSG00000105698	19	35749881	35780724	38	4	123834	0.31115	0.37784	0.958247561
RFC3	ENSG00000133119	13	34382186	34550695	295	15	123834	0.31113	0.37785	0.958247561
TRMT12	ENSG00000183665	8	125453048	125484391	54	7	123834	0.31112	0.37786	0.958247561
TMEM139	ENSG00000178826	7	142967050	142995141	5	2	123834	0.31099	0.3779	0.958247561
TBC1D24	ENSG00000162065	16	2515147	2565735	2	1	123834	0.31098	0.37791	0.958247561
ZFPM1	ENSG00000179588	16	88509725	88613424	143	25	123834	0.31094	0.37792	0.958247561
OVOL2	ENSG00000125850	20	17927623	18049832	216	18	123834	0.31081	0.37797	0.958247561
POFUT2	ENSG00000186866	21	46673843	46717813	89	5	123834	0.3108	0.37798	0.958247561
C9orf129	ENSG00000204352	9	96070481	96118696	140	19	123834	0.31043	0.37812	0.958247561
ERC1	ENSG00000082805	12	1089675	1615099	1176	36	123834	0.31038	0.37814	0.958247561
TMEM247	ENSG00000187600	2	46646329	46721564	116	10	123834	0.30992	0.37831	0.958247561
MTMR6	ENSG00000139505	13	25792307	25872147	106	8	123834	0.30993	0.37831	0.958247561
ALKBH2	ENSG00000189046	12	109515996	109541436	20	7	123834	0.30986	0.37834	0.958247561
MERTK	ENSG00000153208	2	112646056	112797138	338	14	123834	0.30966	0.37841	0.958247561
RNF166	ENSG00000158717	16	88752903	88782829	61	11	123834	0.3095	0.37847	0.958247561
SLC35B3	ENSG00000124786	6	8403301	8445794	142	11	123834	0.30935	0.37853	0.958247561
C2orf81	ENSG00000159239	2	74631304	74658718	15	1	123834	0.30908	0.37863	0.958247561
DNAJC11	ENSG00000007923	1	6684228	6771984	113	11	123834	0.30899	0.37867	0.958247561
TAF8	ENSG00000137413	6	42008251	42065199	117	23	123834	0.30873	0.37876	0.958247561
GCNT3	ENSG00000140297	15	59877074	59942438	54	10	123834	0.30858	0.37882	0.958247561
FTO	ENSG00000140718	16	53727875	54165853	696	64	123834	0.30855	0.37883	0.958247561
NFKBIL1	ENSG00000204498	6	31504647	31536606	97	19	123834	0.30854	0.37884	0.958247561
SLC44A3	ENSG00000143036	1	95275898	95370802	159	25	123834	0.30839	0.37889	0.958247561
RHBDD2	ENSG00000005486	7	75461920	75528244	73	12	123834	0.30827	0.37894	0.958247561
TSPAN31	ENSG00000135452	12	58121796	58153994	10	2	123834	0.30825	0.37894	0.958247561
RBM34	ENSG00000188739	1	235284498	235334772	63	4	123834	0.30805	0.37902	0.958247561
NKG7	ENSG00000105374	19	51864860	51885969	29	7	123834	0.308	0.37904	0.958247561
PCGF2	ENSG00000056661	17	36880150	36916070	21	9	123834	0.3077	0.37916	0.958247561
RD3L	ENSG00000227729	14	104396763	104418645	87	6	123834	0.30765	0.37917	0.958247561
POLR2L	ENSG00000177700	11	827356	852545	49	4	123834	0.3076	0.37919	0.958247561
FAM170B	ENSG00000172538	10	50329199	50352053	101	11	123834	0.3074	0.37927	0.958247561
OR2A2	ENSG00000221989	7	143796607	143817657	67	4	123834	0.30726	0.37932	0.958247561
RFX7	ENSG00000181827	15	56369478	56545483	344	10	123834	0.30705	0.3794	0.958247561
CNIH1	ENSG00000100528	14	54883654	54918149	59	5	123834	0.30698	0.37943	0.958247561
KRTAP19-7	ENSG00000244362	21	31923194	31943633	37	2	123834	0.30692	0.37945	0.958247561
MAT2A	ENSG00000168906	2	85756288	85782403	61	8	123834	0.30652	0.3796	0.958247561
PRAC1	ENSG00000159182	17	46789084	46809884	25	7	123834	0.30639	0.37966	0.958247561
C15orf57	ENSG00000128891	15	40810882	40867256	134	9	123834	0.30614	0.37975	0.958247561
VWA5B1	ENSG00000158816	1	20607412	20691387	154	20	123834	0.30603	0.37979	0.958247561
FASTKD3	ENSG00000124279	5	7849272	7879150	34	8	123834	0.30581	0.37987	0.958247561
TMEM132B	ENSG00000139364	12	125661382	126156917	753	90	123834	0.30546	0.38001	0.958247561
RNF187	ENSG00000168159	1	228664762	228693467	41	6	123834	0.30543	0.38002	0.958247561
CRX	ENSG00000105392	19	48312703	48356587	94	10	123834	0.30498	0.38019	0.958247561
C10orf129	ENSG00000173124	10	96943957	96998685	142	9	123834	0.30479	0.38026	0.958247561
SNX18	ENSG00000178996	5	53803589	53852415	122	17	123834	0.30455	0.38035	0.958247561
NOV	ENSG00000136999	8	120418546	120446593	118	5	123834	0.3045	0.38037	0.958247561
KIF2B	ENSG00000141200	17	51890239	51912573	51	7	123834	0.30447	0.38039	0.958247561
HOXC4	ENSG00000198353	12	54400715	54459813	13	6	123834	0.30444	0.3804	0.958247561
SELV	ENSG00000186838	19	39995753	40021326	25	8	123834	0.30436	0.38043	0.958247561
ZNF354B	ENSG00000178338	5	178276954	178325123	241	10	123834	0.30425	0.38047	0.958247561
CCDC77	ENSG00000120647	12	488439	561811	84	11	123834	0.30425	0.38047	0.958247561
PHF20	ENSG00000025293	20	34349896	34548303	421	12	123834	0.30413	0.38052	0.958247561
C6orf99	ENSG00000203711	6	159280971	159341385	112	17	123834	0.30409	0.38053	0.958247561

RGS18	ENSG00000150681	1	192117587	192164945	27	4	123834	0.30407	0.38054	0.958247561
CTDNEP1	ENSG00000175826	17	7136910	7165810	21	3	123834	0.30399	0.38057	0.958247561
RP11-849H4.2	ENSG00000254469	11	71566555	71649700	312	4	123834	0.30391	0.3806	0.958247561
SPTBN1	ENSG00000115306	2	54673422	54906812	409	20	123834	0.30385	0.38062	0.958247561
NRSN2	ENSG00000125841	20	317426	350304	55	8	123834	0.30385	0.38062	0.958247561
GTPBP4	ENSG00000107937	10	1024338	1075876	115	11	123834	0.30366	0.38069	0.958247561
MINOS1	ENSG00000173436	1	19913477	19966314	95	11	123834	0.3035	0.38076	0.958247561
TWIST2	ENSG00000233608	2	239746673	239805893	18	6	123834	0.30333	0.38082	0.958247561
POLR3H	ENSG00000100413	22	41911808	41950610	46	2	123834	0.30317	0.38088	0.958247561
FCRLA	ENSG00000132185	1	161666762	161694142	71	16	123834	0.30278	0.38103	0.958247561
RP11-307N16.6	ENSG00000273167	13	24543839	24905169	611	92	123834	0.30261	0.38109	0.958247561
IFNLR1	ENSG00000185436	1	24470647	24524449	59	7	123834	0.30242	0.38117	0.958247561
GDF11	ENSG00000135414	12	56127064	56160911	15	3	123834	0.30239	0.38118	0.958247561
VAMP2	ENSG00000220205	17	8052467	8076864	32	4	123834	0.30234	0.3812	0.958247561
WDR96	ENSG00000197748	10	105879646	106002120	192	16	123834	0.30221	0.38125	0.958247561
LRRN4CL	ENSG00000177363	11	62443874	62467371	17	2	123834	0.30207	0.3813	0.958247561
LRRC27	ENSG00000148814	10	134135614	134205010	141	12	123834	0.30199	0.38133	0.958247561
POLR1E	ENSG00000137054	9	37475932	37513694	119	5	123834	0.30178	0.38141	0.958247561
PLCL1	ENSG00000115896	2	198659426	199447305	907	44	123834	0.30174	0.38143	0.958247561
C20orf27	ENSG00000101220	20	3724155	3759034	28	7	123834	0.3017	0.38144	0.958247561
SLC35F3	ENSG00000183780	1	234030679	234470262	689	69	123834	0.30159	0.38148	0.958247561
TMEM253	ENSG00000232070	14	21557096	21581883	38	9	123834	0.30157	0.38149	0.958247561
PCDHB1	ENSG00000171815	5	140420979	140443512	14	3	123834	0.30141	0.38155	0.958247561
TSPO	ENSG00000100300	22	43537520	43569248	48	7	123834	0.30131	0.38159	0.958247561
RNF126	ENSG00000070423	19	637526	673277	17	7	123834	0.30126	0.38161	0.958247561
TGFB3	ENSG00000119699	14	76414442	76459334	26	4	123834	0.30117	0.38164	0.958247561
IVD	ENSG00000128928	15	40687686	40738146	84	8	123834	0.30114	0.38165	0.958247561
AL109659.1	ENSG00000269709	1	48559306	48579878	11	4	123834	0.30113	0.38166	0.958247561
PCMTD2	ENSG00000203880	20	62877094	62936855	55	8	123834	0.30106	0.38168	0.958247561
TSSC1	ENSG00000032389	2	3182696	3391653	584	21	123834	0.30104	0.38169	0.958247561
OR5M1	ENSG00000255012	11	56370031	56390978	50	8	123834	0.30092	0.38174	0.958247561
IL25	ENSG00000166090	14	23832018	23855612	35	8	123834	0.30085	0.38176	0.958247561
RORA	ENSG00000069667	15	60770483	61531518	741	124	123834	0.30087	0.38176	0.958247561
MAD1L1	ENSG00000002822	7	1845429	2282878	1206	22	123834	0.30071	0.38182	0.958247561
SELT	ENSG00000198843	3	150310662	150358222	68	10	123834	0.30063	0.38185	0.958247561
CARKD	ENSG00000213995	13	111257881	111302340	89	8	123834	0.30059	0.38186	0.958247561
CTNND1	ENSG00000198561	11	57510715	57597018	64	6	123834	0.30028	0.38198	0.958247561
UBE2L3	ENSG00000185651	22	21893736	21988323	81	2	123834	0.29991	0.38212	0.958247561
ANKS1B	ENSG00000185046	12	99110235	100388432	1569	106	123834	0.29979	0.38217	0.958247561
CENPO	ENSG00000138092	2	25006005	25055245	133	15	123834	0.29965	0.38222	0.958247561
NSF	ENSG00000073969	17	44658035	44844830	50	4	123834	0.29959	0.38225	0.958247561
ADAT1	ENSG00000065457	16	75620879	75667198	115	12	123834	0.29937	0.38233	0.958247561
DDX39B	ENSG00000198563	6	31487996	31520225	102	17	123834	0.29935	0.38234	0.958247561
RSC1A1	ENSG00000215695	1	15976364	15998217	33	2	123834	0.2992	0.38239	0.958247561
ZNF559	ENSG00000188321	19	9424448	9471838	96	6	123834	0.29921	0.38239	0.958247561
STPG2	ENSG00000163116	4	98095244	99074391	2081	18	123834	0.29908	0.38244	0.958247561
HIST1H1D	ENSG00000124575	6	26224440	26245216	45	6	123834	0.29906	0.38245	0.958247561
GP1BB	ENSG00000203618	22	19700468	19722294	6	2	123834	0.29886	0.38252	0.958247561
CCDC127	ENSG00000164366	5	186986	228330	133	7	123834	0.29883	0.38254	0.958247561
BRD8	ENSG00000112983	5	137465455	137524675	42	4	123834	0.29874	0.38257	0.958247561
TOB2	ENSG00000183864	22	41819496	41853027	48	2	123834	0.29824	0.38276	0.958247561
THEMIS2	ENSG00000130775	1	28189055	28223196	44	5	123834	0.29817	0.38279	0.958247561
AC104841.2	ENSG00000268485	2	242157319	242178850	7	3	123834	0.2979	0.38289	0.958247561
CUTA	ENSG00000112514	6	33374219	33396094	16	5	123834	0.29781	0.38293	0.958247561
RAB11FIP1	ENSG00000156675	8	37706136	37766985	6	2	123834	0.2978	0.38293	0.958247561
CXCR4	ENSG00000121966	2	136861919	136885735	16	4	123834	0.29743	0.38307	0.958247561
PDE4D	ENSG00000113448	5	58254865	59827947	2205	120	123834	0.29715	0.38317	0.958247561
GPSM2	ENSG00000121957	1	109407972	109487167	139	13	123834	0.29712	0.38319	0.958247561
DCTN2	ENSG00000175203	12	57913885	57951114	30	2	123834	0.29712	0.38319	0.958247561
DKFZP779J2370	ENSG00000214397	15	100245908	100268029	22	6	123834	0.29704	0.38322	0.958247561
TRIM59	ENSG00000213186	3	160140233	160213561	97	10	123834	0.29695	0.38325	0.958247561
UBL7	ENSG00000138629	15	74728318	74763523	61	4	123834	0.29682	0.3833	0.958247561
DHDH	ENSG00000104808	19	49426939	49458226	36	5	123834	0.29681	0.38331	0.958247561
DENND4C	ENSG00000137145	9	19220433	19384139	306	16	123834	0.29672	0.38334	0.958247561
IFT88	ENSG00000032742	13	21130585	21275503	285	13	123834	0.29654	0.38341	0.958247561
GOLGA8J	ENSG00000179938	15	30365158	30395702	3	1	123834	0.29652	0.38342	0.958247561
FCGRT	ENSG00000104870	19	50000073	50039590	51	4	123834	0.29649	0.38343	0.958247561
PTGES2	ENSG00000148334	9	130872972	130900741	11	2	123834	0.29639	0.38347	0.958247561
MS4A1	ENSG00000156738	11	60213225	60248233	55	7	123834	0.29636	0.38348	0.958247561
CCT6A	ENSG00000146731	7	56109323	56141682	66	11	123834	0.29592	0.38365	0.958247561
EML3	ENSG00000149499	11	62359690	62390237	22	2	123834	0.29587	0.38366	0.958247561
PPP6C	ENSG00000119414	9	127898852	127962218	113	6	123834	0.29585	0.38367	0.958247561
DVL2	ENSG00000004975	17	7118660	7147864	16	3	123834	0.29585	0.38367	0.958247561
SMTN	ENSG00000183963	22	31450091	31510743	119	13	123834	0.29564	0.38375	0.958247561
CFDP1	ENSG00000153774	16	75317596	75477383	439	10	123834	0.29558	0.38377	0.958247561
ACTC1	ENSG00000159251	15	35070297	35098340	24	9	123834	0.29546	0.38382	0.958247561
PRTFDC1	ENSG00000099256	10	25127536	25251533	239	26	123834	0.29515	0.38394	0.958247561
HIP1	ENSG00000127946	7	75152621	75378280	431	39	123834	0.29476	0.38409	0.958247561
ST6GALNAC1	ENSG00000070526	17	74610843	74649920	100	12	123834	0.29469	0.38412	0.958247561
EIF4E2	ENSG00000135930	2	233404762	233458354	67	4	123834	0.29445	0.38421	0.958247561
ENPP2	ENSG00000136960	8	120559326	120695693	205	24	123834	0.29444	0.38421	0.958247561
AC096582.1	ENSG00000267988	7	45892684	45916045	49	9	123834	0.29439	0.38423	0.958247561
DVL3	ENSG00000161202	3	183863176	183901398	33	7	123834	0.29417	0.38431	0.958247561
PET117	ENSG00000232838	20	18108517	18133813	35	10	123834	0.29412	0.38433	0.958247561
RP11-793H13.10	ENSG00000267281	12	53890472	54029763	186	13	123834	0.29402	0.38437	0.958247561
ATF7	ENSG00000170653	12	53891640	54030199	186	13	123834	0.29402	0.38437	0.958247561
LPPR4	ENSG00000117600	1	99719509	99785146	115	14	123834	0.29394	0.3844	0.958247561
C8orf58	ENSG00000241852	8	22447114	22471663	33	3	123834	0.2937	0.38449	0.958247561
ACADS	ENSG00000122971	12	121153538	121187811	74	10	123834	0.29338	0.38462	0.958247561
CD40	ENSG00000101017	20	44736911	44768502	62	11	123834	0.29338	0.38462	0.958247561
IGSF11	ENSG00000144847	3	118609404	118874915	521	30	123834	0.29313	0.38471	0.958247561
HPCA	ENSG00000121905	1	33341595	33374042	13	3	123834	0.29299	0.38476	0.958247561
PQLC1	ENSG00000122490	18	77652420	77721664	78	11	123834	0.29299	0.38477	0.958247561
PRPF31	ENSG00000105618	19	54608837	54645140	49	11	123834	0.29285	0.38482	0.958247561
ZNF324	ENSG00000083812	19	58968423	58994945	19	4	123834	0.29284	0.38482	0.958247561
PNMAL2	ENSG00000204851	19	46980163	47009755	82	13	123834	0.29282	0.38483	0.958247561
SLC35F4	ENSG00000151812	14	58020640	58458912	1016	58	123834	0.29269	0.38488	0.958247561
GNG8	ENSG00000167414	19	47127333	47147942	33	5	123834	0.29255	0.38493	0.958247561
DNAJC19	ENSG00000205981	3	180691497	180717562	16	2	123834	0.2924	0.38499	0.958247561

NEU2	ENSG00000115488	2	233887382	233909767	24	6	123834	0.29238	0.385	0.958247561
GRAMD3	ENSG00000155324	5	125685824	125842186	182	31	123834	0.29212	0.3851	0.958247561
GYG1	ENSG00000163754	3	148699128	148755419	72	11	123834	0.29206	0.38512	0.958247561
RERG	ENSG00000134533	12	15250717	15511609	275	15	123834	0.29164	0.38528	0.958247561
TTC39B	ENSG00000155158	9	15153620	15317358	269	35	123834	0.29159	0.3853	0.958247561
CCNO	ENSG00000152669	5	54516980	54539508	28	3	123834	0.29115	0.38547	0.958247561
C2orf42	ENSG00000115998	2	70367012	70485747	180	11	123834	0.29106	0.3855	0.958247561
CREB3L2	ENSG00000182158	7	137549725	137696813	193	30	123834	0.29099	0.38553	0.958247561
WTAP	ENSG00000146457	6	160136617	160187351	109	10	123834	0.29073	0.38563	0.958247561
CLK3	ENSG00000179335	15	74880841	74942057	58	6	123834	0.29071	0.38564	0.958247561
CRK	ENSG00000167193	17	1313983	1376456	131	15	123834	0.29068	0.38565	0.958247561
RPS7	ENSG00000171863	2	3612795	3638509	52	10	123834	0.2906	0.38568	0.958247561
NPM2	ENSG00000158806	8	21871636	21904408	23	4	123834	0.29055	0.3857	0.958247561
KIAA1211	ENSG00000109265	4	57026361	57204791	252	43	123834	0.29013	0.38586	0.958247561
TC2N	ENSG00000165929	14	92236095	92343880	104	10	123834	0.29004	0.38589	0.958247561
IL13	ENSG00000169194	5	131981955	132006802	14	4	123834	0.28984	0.38597	0.958247561
AC012123.1	ENSG00000228835	18	30339758	30364376	26	4	123834	0.28982	0.38598	0.958247561
HSPA5	ENSG00000044574	9	127987132	128013609	17	2	123834	0.28976	0.386	0.958247561
EFHB	ENSG00000163576	3	19910964	19998517	195	20	123834	0.28974	0.38601	0.958247561
PALMD	ENSG00000099260	1	100101499	100170097	117	13	123834	0.28965	0.38604	0.958247561
AP3D1	ENSG00000065000	19	2090988	2174464	199	20	123834	0.28966	0.38604	0.958247561
RBPMS	ENSG00000157110	8	30231944	30439778	92	12	123834	0.28961	0.38606	0.958247561
KCNA6	ENSG00000151079	12	4908342	4970277	142	21	123834	0.28943	0.38613	0.958247561
PEX7	ENSG00000112357	6	137133717	137245075	150	12	123834	0.28926	0.38619	0.958247561
RABL5	ENSG00000128581	7	100946975	100975104	104	9	123834	0.28927	0.38619	0.958247561
ETV4	ENSG00000175832	17	41595212	41666988	93	6	123834	0.28913	0.38624	0.958247561
ENAH	ENSG00000154380	1	225664537	225850844	311	12	123834	0.28898	0.3863	0.958247561
AL591025.1	ENSG00000214239	6	159037471	159059322	54	6	123834	0.28891	0.38632	0.958247561
ZBTB16	ENSG00000109906	11	113920315	114131398	255	39	123834	0.28891	0.38632	0.958247561
TPSAB1	ENSG00000172236	16	1280697	1302555	29	7	123834	0.28887	0.38634	0.958247561
LTA	ENSG00000226979	6	31529831	31552101	41	11	123834	0.28878	0.38637	0.958247561
WDR11	ENSG00000120008	10	122600687	122679036	220	10	123834	0.28878	0.38637	0.958247561
KIAA1524	ENSG00000163507	3	108258716	108318491	69	8	123834	0.28871	0.3864	0.958247561
MALRD1	ENSG00000204740	10	19482779	20089330	1780	69	123834	0.28869	0.38641	0.958247561
RSPO1	ENSG00000169218	1	38066951	38110595	53	8	123834	0.28847	0.38649	0.958247561
ANKRD11	ENSG00000167522	16	89324038	89566969	220	20	123834	0.28848	0.38649	0.958247561
PRPF19	ENSG00000110107	11	60648202	60684060	41	3	123834	0.28812	0.38663	0.958247561
SRRM3	ENSG00000177679	7	75821216	75926605	75	8	123834	0.28787	0.38672	0.958247561
EDNRA	ENSG00000151617	4	148392069	148476106	65	10	123834	0.28778	0.38676	0.958247561
RP11-794P6.2	ENSG00000255093	11	111312064	111338876	28	5	123834	0.28774	0.38677	0.958247561
C11orf86	ENSG00000173237	11	66732748	66754475	3	1	123834	0.2877	0.38679	0.958247561
CLEC12B	ENSG00000256660	12	10153226	10181218	42	5	123834	0.28771	0.38679	0.958247561
VPS36	ENSG00000136100	13	52976737	53034763	104	7	123834	0.28768	0.3868	0.958247561
RASSF6	ENSG00000169435	4	74427267	74496348	130	10	123834	0.28745	0.38689	0.958247561
SIGLEC7	ENSG00000168995	19	51635556	51666783	16	1	123834	0.28736	0.38692	0.958247561
ZNF555	ENSG00000186300	19	2831433	2870482	74	9	123834	0.28711	0.38701	0.958247561
TIMM10B	ENSG00000132286	11	6492677	6515909	27	4	123834	0.28707	0.38703	0.958247561
MAPK8	ENSG00000107643	10	49504698	49657403	258	14	123834	0.28697	0.38707	0.958247561
DARS	ENSG00000115866	2	136654247	136753670	86	4	123834	0.2867	0.38717	0.958247561
CHURC1-FNTB	ENSG00000125954	14	65371203	65538521	272	27	123834	0.28634	0.38731	0.958247561
TMCC3	ENSG00000057704	12	94950900	95054338	170	30	123834	0.28615	0.38738	0.958247561
USP16	ENSG00000156256	21	30386950	30436809	53	8	123834	0.28604	0.38743	0.958247561
ANO9	ENSG00000185101	11	407933	452011	39	7	123834	0.28544	0.38765	0.958247561
LEF1	ENSG00000138795	4	108958701	109100112	187	13	123834	0.2853	0.38771	0.958247561
DDX27	ENSG00000124228	20	47825884	47870614	119	10	123834	0.2853	0.38771	0.958247561
INCENP	ENSG00000149503	11	61881445	61930635	151	10	123834	0.28527	0.38772	0.958247561
FAM198A	ENSG00000144649	3	43010759	43111703	145	13	123834	0.28525	0.38773	0.958247561
GOSR2	ENSG00000108433	17	44990483	45115003	311	14	123834	0.28513	0.38777	0.958247561
COL6A3	ENSG00000163359	2	238222646	238333018	147	24	123834	0.28492	0.38785	0.958247561
SAV1	ENSG00000151748	14	51088776	51145049	88	9	123834	0.28489	0.38786	0.958247561
TTC6	ENSG00000139865	14	38055052	38520647	554	32	123834	0.28464	0.38796	0.958247561
THOC3	ENSG00000051596	5	175334876	175471683	11	1	123834	0.28449	0.38802	0.958247561
FETUB	ENSG00000090512	3	186343758	186380930	15	2	123834	0.28436	0.38807	0.958247561
ADAP2	ENSG00000184060	17	29223362	29296340	52	3	123834	0.2841	0.38817	0.958247561
VPS13D	ENSG00000048707	1	12280124	12582099	384	13	123834	0.28405	0.38819	0.958247561
SRPRB	ENSG00000144867	3	133492877	133554616	50	11	123834	0.28405	0.38819	0.958247561
CABLES2	ENSG00000149679	20	60953688	60992341	109	13	123834	0.28394	0.38823	0.958247561
PARD6A	ENSG00000102981	16	67684849	67706681	10	2	123834	0.28389	0.38825	0.958247561
ENKD1	ENSG00000124074	16	67686848	67711168	10	2	123834	0.28389	0.38825	0.958247561
CYTH1	ENSG00000108669	17	76660130	76788379	171	10	123834	0.2837	0.38832	0.958247561
RSL1D1	ENSG00000171490	16	11919056	11955442	58	7	123834	0.28353	0.38838	0.958247561
SPAG1	ENSG00000104450	8	101160134	101281506	158	9	123834	0.28327	0.38848	0.958247561
CAST	ENSG00000153113	5	95850971	96125299	602	45	123834	0.28308	0.38856	0.958247561
AP000708.1	ENSG00000255537	11	125355110	125379424	30	5	123834	0.28305	0.38857	0.958247561
FBXO31	ENSG00000103264	16	87350593	87435748	104	20	123834	0.2826	0.38874	0.958247561
SLC28A3	ENSG00000197506	9	86880372	86965672	205	24	123834	0.28254	0.38877	0.958247561
MKS1	ENSG00000011143	17	56272803	56306966	34	7	123834	0.2824	0.38882	0.958247561
TACC3	ENSG00000013810	4	1713227	1756898	183	14	123834	0.28232	0.38885	0.958247561
NLGN1	ENSG00000169760	3	173104074	174014434	1370	91	123834	0.28222	0.38889	0.958247561
PTRHD1	ENSG00000184924	2	25002603	25026251	61	11	123834	0.28217	0.38891	0.958247561
DNAH10OS	ENSG00000250091	12	124400971	124429531	33	2	123834	0.28212	0.38893	0.958247561
TTC23	ENSG00000103852	15	99666528	99801428	207	24	123834	0.28206	0.38895	0.958247561
ZNF100	ENSG00000197020	19	21895568	21960430	195	14	123834	0.28202	0.38896	0.958247561
TMEM107	ENSG00000179029	17	8066555	8089717	28	5	123834	0.28199	0.38897	0.958247561
RP11-738G5.2	ENSG00000253206	8	50438945	50476973	54	2	123834	0.28197	0.38898	0.958247561
COX6A1P2	ENSG00000226976	6	37002607	37023177	61	7	123834	0.28196	0.38899	0.958247561
PSPH	ENSG00000146733	7	56068744	56129297	119	16	123834	0.2819	0.38901	0.958247561
PPP1R3B	ENSG00000173281	8	8983765	9019084	27	5	123834	0.28187	0.38902	0.958247561
FAM71E1	ENSG00000142530	19	50960042	50990010	37	6	123834	0.28161	0.38912	0.958247561
COL20A1	ENSG00000101203	20	61914538	61976203	104	6	123834	0.28158	0.38913	0.958247561
TRPV4	ENSG00000111199	12	110210890	110281212	90	15	123834	0.28139	0.38921	0.958247561
POLR2M	ENSG00000255529	15	57874231	58084960	408	39	123834	0.28132	0.38923	0.958247561
RPLP1	ENSG00000137818	15	69735123	69758255	18	2	123834	0.28131	0.38924	0.958247561
AC009365.3	ENSG00000183470	7	132323553	132423528	81	12	123834	0.28104	0.38934	0.958247561
ZNF619	ENSG00000177873	3	40508604	40541727	32	3	123834	0.28093	0.38938	0.958247561
PXDC1	ENSG00000168994	6	3712848	3762260	153	15	123834	0.28084	0.38942	0.958247561
PRKRIP1	ENSG00000128563	7	101994319	102077123	111	7	123834	0.28039	0.38959	0.958247561
APRT	ENSG00000198931	16	88865747	88888352	79	11	123834	0.28026	0.38964	0.958247561
TFPT	ENSG00000105619	19	54600320	54629055	49	9	123834	0.28019	0.38966	0.958247561

TCF20	ENSG00000100207	22	42546019	42749622	265	22	123834	0.28015	0.38968	0.958247561
ZC3H15	ENSG00000065548	2	187340883	187384090	60	7	123834	0.28014	0.38969	0.958247561
OR6C75	ENSG00000187857	12	55748895	55769833	70	5	123834	0.28014	0.38969	0.958247561
ESCO1	ENSG00000141446	18	19099242	19190845	65	5	123834	0.28004	0.38972	0.958247561
TSPAN15	ENSG00000099282	10	71201229	71277425	159	17	123834	0.28003	0.38973	0.958247561
CGN	ENSG00000143375	1	151472986	151521168	36	4	123834	0.27983	0.3898	0.958247561
GOLGA7B	ENSG00000269783	10	99617889	99641294	14	3	123834	0.27985	0.3898	0.958247561
PRR25	ENSG00000167945	16	845443	873861	51	13	123834	0.27978	0.38982	0.958247561
FCRL6	ENSG00000181036	1	159760301	159796041	59	6	123834	0.2795	0.38993	0.958247561
ATP5C1	ENSG00000165629	10	7820092	7859778	68	8	123834	0.27951	0.38993	0.958247561
RFC1	ENSG00000035928	4	39279076	39377995	30	4	123834	0.27939	0.38997	0.958247561
MESDC2	ENSG00000117899	15	81229667	81292219	94	6	123834	0.27935	0.38999	0.958247561
FNTB	ENSG00000257365	14	65371203	65539368	273	27	123834	0.27924	0.39003	0.958247561
TRA2B	ENSG00000136527	3	185623694	185665924	44	7	123834	0.27918	0.39005	0.958247561
ADAMTS20	ENSG00000173157	12	43737669	43955724	445	26	123834	0.27916	0.39006	0.958247561
SDCCAG8	ENSG00000054282	1	243409320	243673394	272	22	123834	0.27904	0.39011	0.958247561
CC2D2A	ENSG00000048342	4	15461489	15613180	194	24	123834	0.27873	0.39022	0.958247561
KIAA0754	ENSG00000255103	1	39866151	39892154	28	6	123834	0.27866	0.39025	0.958247561
SPRY4	ENSG00000187678	5	141679992	141716020	21	5	123834	0.27867	0.39025	0.958247561
T	ENSG00000164458	6	166561144	166592188	72	8	123834	0.27852	0.39031	0.958247561
C8orf37	ENSG00000156172	8	96247147	96291429	167	17	123834	0.27852	0.39031	0.958247561
BCL7A	ENSG00000110987	12	122447328	122509948	80	10	123834	0.27842	0.39034	0.958247561
ABCC4	ENSG00000125257	13	95662083	95963687	636	58	123834	0.2784	0.39035	0.958247561
CDH4	ENSG00000179242	20	59817482	60525673	1366	158	123834	0.27833	0.39038	0.958247561
EFCAB4A	ENSG00000177685	11	816144	841991	45	4	123834	0.27822	0.39042	0.958247561
MAGEF1	ENSG00000177383	3	184418156	184439836	13	3	123834	0.27779	0.39059	0.958247561
MSLN	ENSG00000102854	16	800762	828865	33	8	123834	0.27772	0.39061	0.958247561
RRAS	ENSG00000126458	19	50128549	50153458	33	5	123834	0.27771	0.39062	0.958247561
PSG6	ENSG00000170848	19	43396231	43433715	81	4	123834	0.27748	0.39071	0.958247561
SFPQ	ENSG00000116560	1	35631979	35668749	46	3	123834	0.27744	0.39072	0.958247561
ZNF223	ENSG00000267022	19	44519506	44601471	114	7	123834	0.27739	0.39074	0.958247561
HLA-DQB2	ENSG00000232629	6	32713875	32741311	252	10	123834	0.27723	0.3908	0.958247561
ARSA	ENSG00000100299	22	51051182	51076607	47	14	123834	0.27695	0.39091	0.958247561
CHMP7	ENSG00000147457	8	23091150	23129512	69	11	123834	0.27688	0.39094	0.958247561
SEC63	ENSG00000025796	6	108178960	108289393	122	6	123834	0.27615	0.39122	0.958247561
S100A13	ENSG00000189171	1	153581263	153616568	23	4	123834	0.27604	0.39126	0.958247561
PKN1	ENSG00000123143	19	14533865	14592679	65	12	123834	0.27589	0.39132	0.958247561
HSPA9	ENSG00000113013	5	137880571	137921133	49	5	123834	0.27569	0.39139	0.958247561
SESTD1	ENSG00000187231	2	179956419	180139517	362	19	123834	0.27565	0.39141	0.958247561
OR4D6	ENSG00000166884	11	59214411	59235464	78	7	123834	0.27551	0.39146	0.958247561
NFX1	ENSG00000086102	9	33280509	33381155	75	8	123834	0.27547	0.39148	0.958247561
INHA	ENSG00000123999	2	220423884	220450435	29	8	123834	0.2752	0.39158	0.958247561
SLFN13	ENSG00000154760	17	33752115	33785856	43	8	123834	0.27516	0.3916	0.958247561
FLNC	ENSG00000128591	7	128460431	128509328	85	9	123834	0.27505	0.39164	0.958247561
CRH	ENSG00000147571	8	67078620	67100960	45	5	123834	0.27505	0.39164	0.958247561
ARL11	ENSG00000152213	13	50192435	50218008	48	13	123834	0.27498	0.39166	0.958247561
EFTUD2	ENSG00000108883	17	42917311	42987030	159	16	123834	0.27493	0.39169	0.958247561
CCDC14	ENSG00000175455	3	123606152	123690564	86	8	123834	0.27463	0.3918	0.958247561
FBXW7	ENSG00000109670	4	153232410	153467253	329	5	123834	0.27448	0.39186	0.958247561
RNF135	ENSG00000181481	17	29285803	29336929	3	2	123834	0.27439	0.39189	0.958247561
SAE1	ENSG00000142230	19	47606531	47723886	42	5	123834	0.2741	0.39201	0.958247561
IQGAP2	ENSG00000145703	5	75689074	76013957	590	48	123834	0.27399	0.39204	0.958247561
RRM1	ENSG00000167325	11	4105937	4170106	176	10	123834	0.27368	0.39217	0.958247561
RPL9	ENSG00000163682	4	39445744	39470568	80	11	123834	0.27362	0.39219	0.958247561
C11orf89	ENSG00000184682	11	1900375	1922084	30	11	123834	0.27346	0.39225	0.958247561
PKIB	ENSG00000135549	6	122783076	123057518	402	30	123834	0.2734	0.39227	0.958247561
CALN1	ENSG00000183166	7	71234476	71922136	1156	48	123834	0.27325	0.39233	0.958247561
VSTM4	ENSG00000165633	10	50212290	50333554	241	18	123834	0.27282	0.3925	0.958247561
CENPA	ENSG00000115163	2	26977157	27033935	69	5	123834	0.27256	0.3926	0.958247561
GJD4	ENSG00000177291	10	35884338	35907863	31	7	123834	0.27254	0.3926	0.958247561
MS4A5	ENSG00000166930	11	60187062	60232687	102	13	123834	0.27247	0.39263	0.958247561
PARPBP	ENSG00000185480	12	102503956	102601298	156	13	123834	0.27242	0.39265	0.958247561
NCOA4	ENSG00000138293	10	51555108	51600734	21	5	123834	0.27216	0.39275	0.958247561
IRF8	ENSG00000140968	16	85922409	85966215	40	8	123834	0.27203	0.3928	0.958247561
RP11-404L6.2	ENSG00000253787	5	67475704	67504235	52	10	123834	0.27184	0.39287	0.958247561
RP11-20I23.1	ENSG00000260272	16	2536033	2580219	1	1	123834	0.27178	0.3929	0.958247561
ATP6V0C	ENSG00000185883	16	2553871	2580219	1	1	123834	0.27178	0.3929	0.958247561
ATP6C	ENSG00000259784	16	2553966	2587956	1	1	123834	0.27178	0.3929	0.958247561
RAC2	ENSG00000128340	22	37611301	37650488	72	8	123834	0.27175	0.39291	0.958247561
PIN1	ENSG00000127445	19	9935933	9970358	19	5	123834	0.2717	0.39293	0.958247561
HAUS8	ENSG00000131351	19	17150539	17196435	117	12	123834	0.27158	0.39297	0.958247561
C5orf24	ENSG00000181904	5	134171370	134205427	16	4	123834	0.27141	0.39304	0.958247561
NCMAP	ENSG00000184454	1	24872602	24945819	133	19	123834	0.27127	0.39309	0.958247561
KLK11	ENSG00000167757	19	51515472	51541295	6	2	123834	0.27115	0.39314	0.958247561
FAM76A	ENSG00000009780	1	28042490	28099633	75	2	123834	0.2711	0.39316	0.958247561
AC018867.1	ENSG00000213953	2	187351840	187375393	35	5	123834	0.27106	0.39317	0.958247561
MYCN	ENSG00000134323	2	16070686	16097129	37	7	123834	0.27062	0.39334	0.958247561
GNB5	ENSG00000069966	15	52403117	52493566	170	15	123834	0.27031	0.39346	0.958247561
IL9	ENSG00000145839	5	135217935	135241516	35	5	123834	0.27029	0.39347	0.958247561
LHX5	ENSG00000089116	12	113889839	113920085	51	3	123834	0.27001	0.39357	0.958247561
PHTF2	ENSG00000006576	7	77418122	77596818	292	15	123834	0.26994	0.3936	0.958247561
TMX4	ENSG00000125827	20	7947995	8010476	108	10	123834	0.26983	0.39364	0.958247561
KCNA5	ENSG00000130037	12	5143085	5165949	9	4	123834	0.26952	0.39377	0.958247561
C4BPA	ENSG00000123838	1	207267607	207328317	71	11	123834	0.26939	0.39381	0.958247561
IL27	ENSG00000197272	16	28500683	28533372	37	5	123834	0.26927	0.39386	0.958247561
PYROXD1	ENSG00000121350	12	21580549	21633300	250	11	123834	0.26919	0.39389	0.958247561
CYB561A3	ENSG00000162144	11	61106217	61139771	2	1	123834	0.26916	0.3939	0.958247561
TMEM138	ENSG00000149483	11	61119473	61146981	2	1	123834	0.26916	0.3939	0.958247561
TESK1	ENSG00000107140	9	35595367	35620038	28	5	123834	0.26913	0.39391	0.958247561
BAI1	ENSG00000181790	8	143520791	143636370	77	18	123834	0.26865	0.3941	0.958247561
CNTN1	ENSG00000018236	12	41076244	41476220	857	43	123834	0.26843	0.39418	0.958247561
RNF113B	ENSG00000139797	13	98818039	98839519	23	4	123834	0.26835	0.39421	0.958247561
ALDH3B2	ENSG00000132746	11	67419633	67458671	57	5	123834	0.26829	0.39424	0.958247561
CAP1	ENSG00000131236	1	40495905	40548321	142	7	123834	0.26822	0.39427	0.958247561
HTR2B	ENSG00000135914	2	231962944	231999832	29	5	123834	0.26813	0.3943	0.958247561
ZNF260	ENSG00000254004	19	36991597	37029562	85	9	123834	0.26814	0.3943	0.958247561
AGAP5	ENSG00000172650	10	75424033	75467639	4	1	123834	0.26794	0.39437	0.958247561
CCDC135	ENSG00000159625	16	57718705	57775717	58	11	123834	0.26795	0.39437	0.958247561
JPH4	ENSG00000092051	14	24027244	24058009	10	2	123834	0.26782	0.39442	0.958247561

LRRC37B	ENSG00000185158	17	30324891	30390523	47	2	123834	0.26775	0.39444	0.958247561
MIR3654	ENSG00000255508	11	62317075	62369003	34	2	123834	0.26754	0.39453	0.958247561
XPNPEP1	ENSG00000108039	10	111614524	111693311	68	9	123834	0.26746	0.39456	0.958247561
FAM204A	ENSG00000165669	10	120055401	120111840	31	6	123834	0.26735	0.3946	0.958247561
PTPN5	ENSG00000110786	11	18739475	18824268	82	15	123834	0.26729	0.39462	0.958247561
HAUS1	ENSG00000152240	18	43674298	43718299	108	10	123834	0.26727	0.39463	0.958247561
ISM1	ENSG00000101230	20	13192418	13291298	193	22	123834	0.26724	0.39464	0.958247561
HCG27	ENSG00000206344	6	31155537	31181745	383	21	123834	0.26722	0.39465	0.958247561
CDC42EP2	ENSG00000149798	11	65072289	65099900	22	5	123834	0.2671	0.39469	0.958247561
AC007382.1	ENSG00000268688	2	37058327	37078530	25	4	123834	0.26705	0.39472	0.958247561
SOS2	ENSG00000100485	14	50573847	50708276	315	14	123834	0.26701	0.39473	0.958247561
OXA1L	ENSG00000155463	14	23225731	23251007	35	7	123834	0.26688	0.39478	0.958247561
KLHL29	ENSG00000119771	2	23598088	23941481	616	71	123834	0.26683	0.3948	0.958247561
KAT2A	ENSG00000108773	17	40255126	40283376	38	3	123834	0.26669	0.39486	0.958247561
RP11-12J10.3	ENSG00000258539	10	126295649	126490296	227	24	123834	0.26664	0.39487	0.958247561
DDX47	ENSG00000213782	12	12956250	12992915	32	6	123834	0.26661	0.39489	0.958247561
KLHL22	ENSG00000099910	22	20773528	20860170	123	10	123834	0.26608	0.39509	0.958247561
SRPR	ENSG00000182934	11	126122814	126149039	40	8	123834	0.26606	0.3951	0.958247561
RNF20	ENSG00000155827	9	104286133	104335622	48	7	123834	0.26588	0.39517	0.958247561
TMEM110	ENSG00000213533	3	52860235	52941612	120	7	123834	0.26486	0.39556	0.958247561
ANGPT4	ENSG00000101280	20	843296	906977	83	17	123834	0.2648	0.39558	0.958247561
LTN1	ENSG00000198862	21	30290466	30375270	135	15	123834	0.26481	0.39558	0.958247561
RCBTB1	ENSG00000136144	13	50096082	50169719	317	14	123834	0.26473	0.39561	0.958247561
RP11-599B13.6	ENSG00000263620	17	8044134	8076293	33	4	123834	0.26434	0.39576	0.958247561
TMEM200B	ENSG00000253304	1	29435940	29460447	44	6	123834	0.26418	0.39582	0.958247561
ATP6V0E1	ENSG00000113732	5	172400760	172472448	93	14	123834	0.26419	0.39582	0.958247561
JAG2	ENSG00000184916	14	105597318	105645161	39	4	123834	0.26399	0.39589	0.958247561
DDOST	ENSG00000244038	1	20968270	20998000	70	9	123834	0.26395	0.39591	0.958247561
OTX1	ENSG00000115507	2	63267192	63294971	5	2	123834	0.2639	0.39593	0.958247561
RP11-156P1.2	ENSG00000262633	17	44990499	45134520	330	16	123834	0.26375	0.39598	0.958247561
SKP2	ENSG00000145604	5	36142091	36194421	94	13	123834	0.26375	0.39599	0.958247561
SIRPD	ENSG00000125900	20	1504897	1549489	100	14	123834	0.26369	0.39601	0.958247561
PRELP	ENSG00000188783	1	203434956	203470480	77	11	123834	0.26359	0.39605	0.958247561
FGFR2	ENSG00000066468	10	123227848	123367972	98	22	123834	0.26356	0.39606	0.958247561
FGD3	ENSG00000127084	9	95699733	95808518	153	17	123834	0.26349	0.39608	0.958247561
FGF4	ENSG00000075388	11	69577797	69600171	42	9	123834	0.26342	0.39611	0.958247561
ZNF600	ENSG00000189190	19	53257448	53300044	60	11	123834	0.26342	0.39611	0.958247561
FKBPL	ENSG00000204315	6	32086484	32108068	21	9	123834	0.26339	0.39612	0.958247561
MARVELD3	ENSG00000140832	16	71650064	71686017	53	7	123834	0.26324	0.39618	0.958247561
SCGB3A1	ENSG00000161055	5	180007103	180028540	27	5	123834	0.26323	0.39619	0.958247561
HMMR	ENSG00000072571	5	162877209	162928947	80	10	123834	0.2631	0.39624	0.958247561
TAF4	ENSG00000130699	20	60518525	60650866	226	13	123834	0.26296	0.39629	0.958247561
SUSD5	ENSG00000173705	3	33181537	33270707	195	21	123834	0.26283	0.39634	0.958247561
HBD	ENSG00000223609	11	5243908	5266600	10	3	123834	0.26276	0.39637	0.958247561
CATSPER2	ENSG00000166762	15	43910701	43970316	67	5	123834	0.2625	0.39647	0.958247561
MRPL12	ENSG00000262814	17	79660387	79684556	70	8	123834	0.26247	0.39648	0.958247561
OR2Y1	ENSG00000174339	5	180156042	180177099	28	4	123834	0.26244	0.39649	0.958247561
ERP27	ENSG00000139055	12	15056969	15102016	33	3	123834	0.26235	0.39653	0.958247561
RBP1	ENSG00000114115	3	139226276	139268671	80	10	123834	0.26224	0.39657	0.958247561
ETAA1	ENSG00000143971	2	67614451	67647677	68	6	123834	0.26219	0.39659	0.958247561
C9orf50	ENSG00000179058	9	132364504	132393055	72	10	123834	0.2621	0.39662	0.958247561
RAET1G	ENSG00000203722	6	150228014	150254257	39	5	123834	0.26203	0.39665	0.958247561
CROT	ENSG00000005469	7	86964997	87039111	135	12	123834	0.26186	0.39672	0.958247561
RNF122	ENSG00000133874	8	33395273	33434643	66	5	123834	0.26181	0.39673	0.958247561
NUDT8	ENSG00000167799	11	67385409	67407401	64	5	123834	0.26165	0.3968	0.958247561
SPACA4	ENSG00000177202	19	49100000	49120970	34	7	123834	0.26157	0.39683	0.958247561
RAD1	ENSG00000113456	5	34895369	34929094	41	7	123834	0.26151	0.39685	0.958247561
POLR2J	ENSG00000005075	7	102103565	102129354	46	6	123834	0.26123	0.39696	0.95834112
CLC	ENSG00000105205	19	40211890	40238668	90	6	123834	0.26104	0.39703	0.95834112
TRIB1	ENSG00000173334	8	126432563	126460647	12	3	123834	0.26102	0.39704	0.95834112
HIRIP3	ENSG00000149929	16	29993645	30017757	27	3	123834	0.26066	0.39718	0.958407431
HDAC9	ENSG00000048052	7	18116572	19052039	1040	87	123834	0.26042	0.39727	0.958407431
ZNF101	ENSG00000181896	19	19769605	19804318	59	10	123834	0.26041	0.39727	0.958407431
ZNF532	ENSG00000074657	18	56519832	56663712	233	23	123834	0.26031	0.39731	0.958407431
THEGL	ENSG00000249693	4	57386775	57479673	146	19	123834	0.26026	0.39733	0.958407431
TMEM154	ENSG00000170006	4	153529784	153611317	112	19	123834	0.26016	0.39737	0.958407431
TIPARP	ENSG00000163659	3	156381024	156434559	83	6	123834	0.25985	0.39749	0.958549041
HIST1H4E	ENSG00000198518	6	26194858	26216266	79	7	123834	0.25962	0.39758	0.958549041
CYYR1	ENSG00000166265	21	27828528	27955603	214	38	123834	0.25962	0.39758	0.958549041
GABARAPL2	ENSG00000034713	16	75590249	75621779	58	8	123834	0.25923	0.39773	0.958690544
SBDS	ENSG00000126524	7	66442664	66470588	24	5	123834	0.25919	0.39774	0.958690544
NLRP1	ENSG00000091592	17	5392747	5532744	473	18	123834	0.25907	0.39779	0.958690544
OR2AT4	ENSG00000171561	11	74789758	74810799	20	5	123834	0.25878	0.3979	0.958834077
SUPT3H	ENSG00000196284	6	44767054	45355690	1506	23	123834	0.25828	0.39809	0.959170328
ZNF729	ENSG00000196350	19	22459210	22509978	58	8	123834	0.25807	0.39818	0.959207353
NFKBID	ENSG00000167604	19	36368555	36403205	76	4	123834	0.25775	0.3983	0.959207353
HIST1H3C	ENSG00000196532	6	26035639	26056097	37	7	123834	0.25754	0.39838	0.959207353
OTUD6B	ENSG00000155100	8	92072424	92109323	37	4	123834	0.25744	0.39842	0.959207353
CCDC27	ENSG00000162592	1	3658962	3698209	87	12	123834	0.2573	0.39848	0.959207353
IDH3B	ENSG00000101365	20	2629041	2654865	49	8	123834	0.25722	0.3985	0.959207353
LMAN1	ENSG00000074695	18	56985055	57037194	128	9	123834	0.25716	0.39853	0.959207353
TMEM110-MUSTN1	ENSG00000248592	3	52857137	52941578	123	7	123834	0.2571	0.39855	0.959207353
LRPPRC	ENSG00000138095	2	44103647	44233144	222	16	123834	0.25706	0.39857	0.959207353
C8orf33	ENSG00000182307	8	146267764	146291416	43	3	123834	0.25694	0.39861	0.959207353
SLC23A1	ENSG00000170482	5	138692885	138730242	18	4	123834	0.25668	0.39871	0.959301354
WBSCR27	ENSG00000165171	7	73238920	73266865	57	1	123834	0.2566	0.39875	0.959301354
TIAL1	ENSG00000151923	10	121324199	121366541	43	7	123834	0.25629	0.39886	0.959444556
OR2H2	ENSG00000204657	6	29545683	29566745	36	8	123834	0.25609	0.39894	0.959490364
DENND5A	ENSG00000184014	11	9150372	9296937	237	15	123834	0.25599	0.39898	0.959490364
YWHAB	ENSG00000166913	20	43504317	43547173	74	5	123834	0.2554	0.39921	0.959618328
LRRC39	ENSG00000122477	1	100604409	100653771	47	6	123834	0.25535	0.39923	0.959618328
LGR6	ENSG00000133067	1	202153029	202298909	184	19	123834	0.25495	0.39938	0.959618328
PTTG1IP	ENSG00000183255	21	46259500	46303752	137	16	123834	0.25484	0.39942	0.959618328
OC90	ENSG00000258417	8	133026467	133107902	162	21	123834	0.25482	0.39943	0.959618328
RASL11B	ENSG00000128045	4	53718457	53743000	53	7	123834	0.25466	0.39949	0.959618328
TLE6	ENSG00000104953	19	2967444	3005177	25	8	123834	0.25466	0.39949	0.959618328
TBCCD1	ENSG00000113838	3	186253862	186298332	80	6	123834	0.25459	0.39952	0.959618328
C12orf49	ENSG00000111412	12	117143593	117185875	41	9	123834	0.25448	0.39956	0.959618328
CLIC6	ENSG00000159212	21	36031688	36100525	81	6	123834	0.25448	0.39956	0.959618328

SSC5D	ENSG00000179954	19	55989771	56040465	59	14	123834	0.25422	0.39966	0.959618328
DAPK3	ENSG00000167657	19	3948451	3981121	24	6	123834	0.25421	0.39967	0.959618328
BTNL3	ENSG00000168903	5	180405845	180443727	33	6	123834	0.25407	0.39972	0.959618328
ZP4	ENSG00000116996	1	238035705	238064094	54	5	123834	0.25402	0.39974	0.959618328
ASPDH	ENSG00000204653	19	51004857	51027947	14	3	123834	0.2537	0.39986	0.959638833
CUL3	ENSG00000036257	2	225324867	225460110	209	8	123834	0.25366	0.39988	0.959638833
FOXI2	ENSG00000186766	10	129525499	129549450	91	7	123834	0.25361	0.3999	0.959638833
ARHGEF35	ENSG00000213214	7	143873176	144001230	3	1	123834	0.25292	0.40016	0.959865658
C19orf53	ENSG00000104979	19	13874982	13899276	43	2	123834	0.25276	0.40023	0.959865658
CPNE3	ENSG00000085719	8	87487059	87583726	193	19	123834	0.25269	0.40025	0.959865658
LAMA5	ENSG00000130702	20	60873011	60952368	170	17	123834	0.2525	0.40033	0.959865658
SPAG4	ENSG00000061656	20	34193814	34218971	41	6	123834	0.25232	0.4004	0.959865658
MMRN2	ENSG00000173269	10	88685297	88739238	64	12	123834	0.25229	0.40041	0.959865658
NTN5	ENSG00000142233	19	49154664	49186338	10	3	123834	0.25222	0.40043	0.959865658
SIRPB1	ENSG00000101307	20	1534167	1610707	78	12	123834	0.25219	0.40045	0.959865658
OR4B1	ENSG00000175619	11	48228344	48249314	61	7	123834	0.25214	0.40047	0.959865658
SLCO1B1	ENSG00000134538	12	21274136	21402180	364	20	123834	0.252	0.40052	0.959865658
UBA7	ENSG00000182179	3	49832640	49861379	21	4	123834	0.25192	0.40055	0.959865658
PEX19	ENSG00000162735	1	160236602	160266138	29	4	123834	0.25151	0.40071	0.959957898
ZMIZ2	ENSG00000122515	7	44778180	44819477	24	4	123834	0.25152	0.40071	0.959957898
DEFB114	ENSG00000177684	6	49917962	49941818	27	3	123834	0.25142	0.40074	0.959957898
ATP8B1	ENSG00000081923	18	55303658	55480333	269	38	123834	0.25091	0.40094	0.960172812
SLC52A2	ENSG00000185803	8	145567795	145594932	13	2	123834	0.2506	0.40106	0.960172812
ASAH2B	ENSG00000204147	10	52489078	52586255	172	10	123834	0.25061	0.40106	0.960172812
HOXC4	ENSG00000273266	12	54437661	54459814	6	3	123834	0.25043	0.40113	0.960172812
ALKBH8	ENSG00000137760	11	107363452	107446472	166	15	123834	0.25034	0.40116	0.960172812
SP2	ENSG00000167182	17	45963516	46016323	88	10	123834	0.25026	0.40119	0.960172812
GRSF1	ENSG00000132463	4	71671499	71715662	106	7	123834	0.25002	0.40128	0.960172812
TMEM109	ENSG00000110108	11	60671346	60700915	46	4	123834	0.24985	0.40135	0.960172812
ROGDI	ENSG00000067836	16	4836969	4862951	25	6	123834	0.24962	0.40144	0.960172812
CAV3	ENSG00000182533	3	8765486	8893492	271	29	123834	0.24958	0.40146	0.960172812
ABCA3	ENSG00000167972	16	2315882	2400747	35	7	123834	0.24941	0.40152	0.960172812
PPIA	ENSG00000196262	7	44826279	44874163	108	10	123834	0.24936	0.40154	0.960172812
ZNF808	ENSG00000198482	19	53020905	53077717	106	9	123834	0.24921	0.4016	0.960172812
PRR3	ENSG00000204576	6	30514663	30541500	22	5	123834	0.24904	0.40167	0.960172812
TJP2	ENSG00000119139	9	71726209	71880124	333	24	123834	0.24903	0.40167	0.960172812
CYP4F12	ENSG00000186204	19	15773567	15817984	254	12	123834	0.24886	0.40173	0.960172812
DDX43	ENSG00000080007	6	74094471	74137292	48	9	123834	0.24886	0.40174	0.960172812
BICC1	ENSG00000122870	10	60262900	60601195	473	28	123834	0.24877	0.40177	0.960172812
SNX15	ENSG00000110025	11	64784880	64818044	62	7	123834	0.24861	0.40183	0.960172812
OSER1	ENSG00000132823	20	42815136	42849431	57	8	123834	0.24859	0.40184	0.960172812
CRHR2	ENSG00000106113	7	30682200	30749745	70	16	123834	0.24814	0.40201	0.960336326
OMP	ENSG00000254550	11	76803886	76824377	25	4	123834	0.24805	0.40205	0.960336326
EPB41	ENSG00000159023	1	29203603	29456553	342	17	123834	0.24802	0.40206	0.960336326
MROH7	ENSG00000184313	1	55097463	55185939	155	26	123834	0.24764	0.4022	0.960429347
OSCAR	ENSG00000170909	19	54587933	54616000	73	9	123834	0.24764	0.4022	0.960429347
CELSR1	ENSG00000075275	22	46746731	46943067	301	40	123834	0.24751	0.40226	0.960451963
RBM12	ENSG00000244462	20	34226847	34262878	72	6	123834	0.2472	0.40238	0.960616531
MATN1	ENSG00000162510	1	31174124	31206434	100	8	123834	0.24707	0.40243	0.960616531
SIX2	ENSG00000170577	2	45222300	45246569	46	6	123834	0.2469	0.40249	0.960639116
HAMP	ENSG00000105697	19	35761619	35786046	22	3	123834	0.24676	0.40255	0.960661695
BRF2	ENSG00000104221	8	37690786	37717422	1	1	123834	0.24656	0.40262	0.960682977
ETFA	ENSG00000140374	15	76497696	76613813	196	12	123834	0.24647	0.40266	0.960682977
KMT2B	ENSG00000272333	19	36198921	36239779	59	10	123834	0.24622	0.40276	0.960800964
NAGS	ENSG00000161653	17	42071914	42096431	34	7	123834	0.24581	0.40292	0.960989149
SLC7A2	ENSG00000003989	8	17344597	17438082	189	41	123834	0.24575	0.40294	0.960989149
SLC26A9	ENSG00000174502	1	205872176	205922588	64	12	123834	0.24544	0.40306	0.961154746
AE000662.92	ENSG00000259003	14	23015534	23037939	83	12	123834	0.24496	0.40325	0.961159245
ALOX12	ENSG00000108839	17	6889384	6924055	48	5	123834	0.24468	0.40335	0.961159245
C1orf35	ENSG00000143793	1	228278427	228303112	18	4	123834	0.24428	0.40351	0.961159245
CPLX3	ENSG00000213578	15	75108888	75134141	27	6	123834	0.24415	0.40356	0.961159245
CTC-534A2.2	ENSG00000253251	5	64910592	64936718	71	7	123834	0.24388	0.40366	0.961159245
CAMK1	ENSG00000134072	3	9789026	9821676	36	9	123834	0.24379	0.4037	0.961159245
ASTL	ENSG00000188886	2	96779589	96814175	18	4	123834	0.24352	0.4038	0.961159245
CDHR5	ENSG00000099834	11	606565	636078	55	8	123834	0.24347	0.40382	0.961159245
STIP1	ENSG00000168439	11	63942744	63982015	61	9	123834	0.24325	0.40391	0.961159245
G2E3	ENSG00000092140	14	31018329	31099269	110	11	123834	0.24317	0.40394	0.961159245
ITFG2	ENSG00000111203	12	2911788	2978957	62	4	123834	0.24303	0.40399	0.961159245
ICOSLG	ENSG00000160223	21	45632874	45670849	62	14	123834	0.24284	0.40406	0.961159245
LCN2	ENSG00000148346	9	130901350	130925734	12	2	123834	0.2428	0.40408	0.961159245
AC093677.1	ENSG00000269559	4	75013327	75034085	6	1	123834	0.24266	0.40413	0.961159245
PSG9	ENSG00000183668	19	43705943	43783682	152	13	123834	0.24254	0.40418	0.961159245
EIF2AK4	ENSG00000128829	15	40216347	40337797	224	27	123834	0.24248	0.4042	0.961159245
SLC22A16	ENSG00000004809	6	110735890	110807844	201	30	123834	0.24233	0.40426	0.961159245
CLUH	ENSG00000132361	17	2582680	2625957	20	5	123834	0.24217	0.40432	0.961159245
MAP1A	ENSG00000166963	15	43793156	43833818	29	4	123834	0.24214	0.40433	0.961159245
C18orf64	ENSG00000265944	18	6501415	6600652	130	24	123834	0.24173	0.4045	0.961159245
PPM1K	ENSG00000163644	4	89168772	89215921	41	6	123834	0.24168	0.40452	0.961159245
ZKSCAN1	ENSG00000106261	7	99603204	99649312	43	7	123834	0.24148	0.40459	0.961159245
METTL21A	ENSG00000144401	2	208435355	208500652	83	6	123834	0.24143	0.40461	0.961159245
C6	ENSG00000039537	5	41132336	41271540	321	21	123834	0.24137	0.40464	0.961159245
AL359736.1	ENSG00000205850	13	24885264	24905566	34	5	123834	0.24116	0.40471	0.961159245
COBL	ENSG00000106078	7	51073909	51394515	358	25	123834	0.24113	0.40473	0.961159245
LRRTM4	ENSG00000176204	2	76964845	77830445	1633	87	123834	0.24095	0.4048	0.961159245
MRRF	ENSG00000148187	9	125016882	125095743	32	5	123834	0.24075	0.40487	0.961159245
NCLN	ENSG00000125912	19	3175561	3219573	75	8	123834	0.24071	0.40489	0.961159245
CHMP2A	ENSG00000130724	19	59052933	59076491	27	6	123834	0.2407	0.40489	0.961159245
TBCC	ENSG00000124659	6	42702219	42724558	17	3	123834	0.24066	0.40491	0.961159245
TRIOBP	ENSG00000100106	22	38083011	38182563	176	17	123834	0.24055	0.40495	0.961159245
LGALSL	ENSG00000119862	2	64671103	64698515	21	6	123834	0.24035	0.40503	0.961159245
HLA-E	ENSG00000204592	6	30447244	30471982	37	7	123834	0.23994	0.40519	0.961159245
LZTR1	ENSG00000099949	22	21323751	21363327	57	13	123834	0.23992	0.4052	0.961159245
PPEF2	ENSG00000156194	4	76771020	76833724	90	12	123834	0.23982	0.40523	0.961159245
CD226	ENSG00000150637	18	67488394	67639039	285	25	123834	0.23976	0.40526	0.961159245
PDP1	ENSG00000164951	8	94860035	94948294	132	8	123834	0.23967	0.40529	0.961159245
NPIPB11	ENSG00000254206	16	29382675	29425350	13	2	123834	0.23966	0.4053	0.961159245
RPP25	ENSG00000178718	15	75236757	75259805	17	4	123834	0.23963	0.40531	0.961159245
SLC26A4	ENSG00000091137	7	107291080	107368254	40	7	123834	0.23941	0.4054	0.961159245
C9orf40	ENSG00000135045	9	77551497	77577802	12	4	123834	0.23924	0.40546	0.961159245

PLEKHN1	ENSG00000187583	1	891877	921245	43	11	123834	0.23904	0.40554	0.961159245
ACTR1B	ENSG00000115073	2	98262431	98290570	22	3	123834	0.23903	0.40554	0.961159245
RP11-295K3.1	ENSG00000250644	11	1758897	1790281	48	5	123834	0.23869	0.40567	0.961159245
RASGRF2	ENSG00000113319	5	80246491	80535975	399	57	123834	0.23868	0.40568	0.961159245
PAPPA2	ENSG00000116183	1	176422307	176824735	564	27	123834	0.23857	0.40572	0.961159245
RAC1	ENSG00000136238	7	6404154	6453608	132	18	123834	0.23831	0.40582	0.961159245
CFL1	ENSG00000172757	11	65580493	65639497	90	9	123834	0.23826	0.40584	0.961159245
EDC3	ENSG00000179151	15	74912899	74998633	53	8	123834	0.23814	0.40589	0.961159245
STAU2	ENSG00000040341	8	74322604	74669943	640	31	123834	0.23801	0.40594	0.961159245
AXDND1	ENSG00000162779	1	179324855	179533870	978	21	123834	0.23779	0.40602	0.961159245
C9orf171	ENSG00000188523	9	135275430	135458704	222	40	123834	0.23769	0.40606	0.961159245
ITPR3	ENSG00000096433	6	33578142	33674351	225	29	123834	0.2376	0.4061	0.961159245
ACADL	ENSG00000115361	2	211042663	211100215	29	4	123834	0.23756	0.40611	0.961159245
DEFA1B	ENSG00000240247	8	6844288	6885778	8	3	123834	0.23755	0.40611	0.961159245
DEFA3	ENSG00000239839	8	6863391	6885823	8	3	123834	0.23755	0.40611	0.961159245
MRPS33	ENSG00000090263	7	140695854	140725028	42	5	123834	0.23747	0.40615	0.961159245
GABBR2	ENSG00000136928	9	101040391	101481479	882	83	123834	0.23744	0.40616	0.961159245
RP11-166N6.3	ENSG00000260234	3	150590784	150669530	68	15	123834	0.23731	0.40621	0.961159245
SLC2A5	ENSG00000142583	1	9085166	9158537	97	12	123834	0.23719	0.40626	0.961159245
SERPINB6	ENSG00000124570	6	2938393	2982090	89	14	123834	0.23702	0.40632	0.961159245
TTC9B	ENSG00000174521	19	40711965	40734306	13	3	123834	0.23695	0.40635	0.961159245
MAST3	ENSG00000099308	19	18198603	18272502	176	18	123834	0.2369	0.40637	0.961159245
AKAP12	ENSG00000131016	6	151551134	151689692	249	41	123834	0.23663	0.40647	0.961159245
POLR3K	ENSG00000161980	16	86407	113628	37	4	123834	0.23656	0.4065	0.961159245
RP11-399J13.3	ENSG00000273003	11	64771654	64818042	82	10	123834	0.2365	0.40652	0.961159245
TPSD1	ENSG00000095917	16	1296060	1318532	19	6	123834	0.23618	0.40665	0.961159245
AAK1	ENSG00000115977	2	69678532	69911481	127	11	123834	0.23599	0.40672	0.961159245
RP1L1	ENSG00000183638	8	10453859	10579697	118	22	123834	0.23592	0.40675	0.961159245
RND1	ENSG00000172602	12	49240928	49269681	16	4	123834	0.23589	0.40676	0.961159245
KIR3DX1	ENSG00000104970	19	55033909	55067053	51	9	123834	0.23577	0.4068	0.961159245
TSLP	ENSG00000145777	5	110395760	110423722	31	8	123834	0.23564	0.40686	0.961159245
GRTP1	ENSG00000139835	13	113968505	114028446	103	14	123834	0.23563	0.40686	0.961159245
MB	ENSG00000198125	22	35992811	36043998	109	10	123834	0.23552	0.4069	0.961159245
ZFP42	ENSG00000179059	4	188906925	188936204	26	4	123834	0.23529	0.40699	0.961159245
TMEM131	ENSG00000075568	2	98362799	98622388	399	15	123834	0.23478	0.40719	0.961159245
DKFZP761K2322	ENSG00000272268	2	6101712	6124374	21	4	123834	0.23476	0.4072	0.961159245
CTD-2547L24.3	ENSG00000258875	14	91699103	91727426	47	5	123834	0.23476	0.4072	0.961159245
CEPT1	ENSG00000134255	1	111672249	111737724	65	9	123834	0.23466	0.40724	0.961159245
C8orf86	ENSG00000196166	8	38358352	38396180	82	8	123834	0.23458	0.40727	0.961159245
HADHA	ENSG00000084754	2	26403504	26477594	112	3	123834	0.23439	0.40734	0.961159245
TMEM262	ENSG00000187066	11	64842451	64866847	28	6	123834	0.23436	0.40735	0.961159245
IL32	ENSG00000008517	16	3105298	3141908	9	3	123834	0.23436	0.40735	0.961159245
REN	ENSG00000143839	1	204113944	204145465	53	7	123834	0.23435	0.40736	0.961159245
LRRC9	ENSG00000131951	14	60376431	60540277	246	8	123834	0.23393	0.40752	0.961176152
KIF1C	ENSG00000129250	17	4891243	4941696	93	13	123834	0.23387	0.40754	0.961176152
POTEC	ENSG00000183206	18	14497338	14553584	83	8	123834	0.2338	0.40757	0.961176152
SELO	ENSG00000073169	22	50629408	50666045	69	11	123834	0.23371	0.40761	0.961176152
KCND2	ENSG00000184408	7	119903722	120400385	604	28	123834	0.23367	0.40762	0.961176152
H1FOO	ENSG00000178804	3	129252057	129280310	44	4	123834	0.23321	0.4078	0.961257024
CA4	ENSG00000167434	17	58217297	58258260	25	5	123834	0.23309	0.40784	0.961257024
CTD-2144E22.5	ENSG00000179755	16	34246507	34267278	3	2	123834	0.23277	0.40797	0.961257024
IFNA13	ENSG00000233816	9	21357371	21378075	62	5	123834	0.23244	0.4081	0.961257024
MUC8	ENSG00000269676	12	133039334	133060726	23	9	123834	0.23238	0.40812	0.961257024
TEX22	ENSG00000226174	14	105854916	105926443	1	1	123834	0.23216	0.40821	0.961257024
GPR68	ENSG00000119714	14	91688876	91730269	50	6	123834	0.23212	0.40822	0.961257024
KCND3	ENSG00000171385	1	112303284	112541777	349	46	123834	0.2321	0.40823	0.961257024
PPP5D1	ENSG00000230510	19	46974053	47114457	186	33	123834	0.2321	0.40823	0.961257024
SLC44A1	ENSG00000070214	9	107996903	108211452	337	18	123834	0.23202	0.40826	0.961257024
FLJ20373	ENSG00000233404	2	102498955	102521150	24	6	123834	0.23187	0.40832	0.961257024
B3GNT5	ENSG00000176597	3	182961032	183026292	17	2	123834	0.23139	0.40851	0.961257024
ADAM28	ENSG00000042980	8	24141553	24226531	192	9	123834	0.23132	0.40853	0.961257024
PSMB4	ENSG00000159377	1	151362010	151384420	14	2	123834	0.23116	0.40859	0.961257024
ZBED4	ENSG00000100426	22	50237490	50293726	165	8	123834	0.23095	0.40868	0.961257024
IFFO1	ENSG00000010295	12	6637541	6675239	37	5	123834	0.23073	0.40876	0.961257024
INMT	ENSG00000241644	7	30727601	30807218	79	21	123834	0.23045	0.40887	0.961257024
PROK1	ENSG00000143125	1	110983822	111009976	27	7	123834	0.23038	0.4089	0.961257024
AC008271.1	ENSG00000231031	2	15820906	15894463	98	18	123834	0.2303	0.40893	0.961257024
PPP2R2D	ENSG00000175470	10	133737955	133783331	90	7	123834	0.23025	0.40895	0.961257024
PRR5-ARHGAP8	ENSG00000248405	22	45088113	45268586	521	69	123834	0.23	0.40905	0.961257024
CTD-3203P2.2	ENSG00000245888	16	27269526	27311813	32	3	123834	0.22996	0.40906	0.961257024
CORO6	ENSG00000167549	17	27931774	27959925	11	2	123834	0.22985	0.4091	0.961257024
NHLRC1	ENSG00000187566	6	18110718	18132851	63	6	123834	0.22965	0.40918	0.961257024
WDR87	ENSG00000171804	19	38365463	38407317	50	8	123834	0.22966	0.40918	0.961257024
AADACL4	ENSG00000204518	1	12694566	12737097	63	4	123834	0.22963	0.40919	0.961257024
VPS26A	ENSG00000122958	10	70873268	70942617	142	10	123834	0.22944	0.40927	0.961257024
ARHGAP8	ENSG00000241484	22	45088355	45268665	520	70	123834	0.22938	0.40928	0.961257024
PSMG4	ENSG00000180822	6	3221637	3313607	239	10	123834	0.22938	0.40929	0.961257024
RNF220	ENSG00000187147	1	44860866	45127396	250	45	123834	0.22933	0.40931	0.961257024
FAM218A	ENSG00000250486	4	165868100	165890273	28	6	123834	0.22925	0.40934	0.961257024
IL2RB	ENSG00000100385	22	37511878	37581094	59	15	123834	0.22906	0.40941	0.961257024
RP11-362K2.2	ENSG00000258231	12	58927907	59216842	523	38	123834	0.22896	0.40945	0.961257024
TEFM	ENSG00000172171	17	29214354	29243838	54	2	123834	0.22893	0.40946	0.961257024
BPIFB3	ENSG00000186190	20	31633230	31671434	61	8	123834	0.22872	0.40954	0.961257024
ADAM20	ENSG00000134007	14	70979075	71011732	72	8	123834	0.22869	0.40955	0.961257024
ACTG2	ENSG00000163017	2	74109441	74156992	76	10	123834	0.22851	0.40963	0.961257024
TMEM206	ENSG00000065600	1	212527273	212598243	83	13	123834	0.22818	0.40975	0.961257024
GALNT2	ENSG00000143641	1	230183536	230427870	470	47	123834	0.22817	0.40976	0.961257024
LINC01100	ENSG00000242107	3	159723811	159759210	106	9	123834	0.22817	0.40976	0.961257024
NOD2	ENSG00000167207	16	50717514	50776988	38	6	123834	0.22796	0.40984	0.961257024
TAX1BP1	ENSG00000106052	7	27768950	27894183	177	17	123834	0.22789	0.40987	0.961257024
SCUBE2	ENSG00000175356	11	9031071	9169661	168	23	123834	0.22785	0.40988	0.961257024
MED6	ENSG00000133997	14	71037974	71077384	123	8	123834	0.22777	0.40991	0.961257024
FAM35A	ENSG00000122376	10	88843918	88961225	237	16	123834	0.22772	0.40993	0.961257024
GGN	ENSG00000179168	19	38864905	38888722	53	5	123834	0.2275	0.41002	0.961276448
ARL10	ENSG00000175414	5	175782471	175838866	53	9	123834	0.22733	0.41008	0.961276448
LBP	ENSG00000129988	20	36964759	37015665	88	13	123834	0.22731	0.41009	0.961276448
SCAMP5	ENSG00000198794	15	75239560	75323837	132	12	123834	0.22713	0.41016	0.961303161
STX16-NPEPL1	ENSG00000254995	20	57216490	57300466	147	18	123834	0.22686	0.41027	0.961303161
WFDC2	ENSG00000101443	20	44088346	44120172	74	12	123834	0.22675	0.41031	0.961303161

KLK10	ENSG00000129451	19	51505995	51533431	8	3	123834	0.22646	0.41042	0.961303161
FRMD4B	ENSG00000114541	3	69209141	69601734	552	53	123834	0.22645	0.41043	0.961303161
PAEP	ENSG00000122133	9	138443602	138468801	33	5	123834	0.22636	0.41046	0.961303161
SIRT5	ENSG00000124523	6	13564816	13624790	37	6	123834	0.22624	0.41051	0.961303161
WDR27	ENSG00000184465	6	169847307	170112159	651	26	123834	0.22615	0.41054	0.961303161
GIT2	ENSG00000139436	12	110357607	110444194	117	9	123834	0.22609	0.41057	0.961303161
SLA2	ENSG00000101082	20	35230721	35284619	16	6	123834	0.22573	0.4107	0.961303161
FAM83E	ENSG00000105523	19	49094067	49128111	61	11	123834	0.22567	0.41073	0.961303161
DSCAM	ENSG00000171587	21	41372926	42229065	1638	150	123834	0.2256	0.41076	0.961303161
USMG5	ENSG00000173915	10	105138798	105166223	22	4	123834	0.22551	0.41079	0.961303161
GSS	ENSG00000100983	20	33506236	33553620	58	5	123834	0.22547	0.41081	0.961303161
HAUS5	ENSG00000249115	19	36093646	36126251	30	7	123834	0.22533	0.41086	0.961303161
STX11	ENSG00000135604	6	144461663	144519507	45	7	123834	0.2248	0.41107	0.96167613
CARD10	ENSG00000100065	22	37876400	37925549	111	14	123834	0.22463	0.41114	0.961721526
RP11-248J23.7	ENSG00000270099	10	97610305	97694601	164	18	123834	0.22407	0.41135	0.961946047
PIK3C2A	ENSG00000011405	11	17089277	17239530	174	13	123834	0.22391	0.41141	0.961946047
IL21	ENSG00000138684	4	123523783	123552224	62	9	123834	0.22369	0.4115	0.961946047
DNAH9	ENSG00000007174	17	11491748	11883065	497	55	123834	0.22363	0.41152	0.961946047
TMA16	ENSG00000198498	4	164405594	164451691	66	6	123834	0.22349	0.41158	0.961946047
NFE2L3	ENSG00000050344	7	26181860	26236745	84	8	123834	0.22336	0.41163	0.961946047
TMEM213	ENSG00000214128	7	138472695	138532846	74	10	123834	0.22336	0.41163	0.961946047
OR4Q3	ENSG00000182652	14	20205587	20226528	5	1	123834	0.22327	0.41166	0.961946047
CUL1	ENSG00000055130	7	148385006	148508128	137	10	123834	0.22293	0.4118	0.961946047
ADK	ENSG00000156110	10	75900960	76479061	674	16	123834	0.22206	0.41213	0.961946047
CCDC92	ENSG00000119242	12	124393207	124467378	87	4	123834	0.22205	0.41214	0.961946047
C6orf165	ENSG00000213204	6	88107730	88231352	338	17	123834	0.22197	0.41217	0.961946047
ARL14EP	ENSG00000152219	11	30334598	30369774	71	7	123834	0.22183	0.41222	0.961946047
TXNL4A	ENSG00000141759	18	77722867	77803949	171	12	123834	0.22171	0.41227	0.961946047
CLEC18B	ENSG00000140839	16	74432529	74465368	1	1	123834	0.22166	0.41229	0.961946047
AC110781.3	ENSG00000176349	7	1868222	1899567	129	11	123834	0.22155	0.41233	0.961946047
LST1	ENSG00000204482	6	31543901	31566686	45	14	123834	0.22122	0.41246	0.961946047
OBP2A	ENSG00000122136	9	138427985	138451815	25	4	123834	0.22108	0.41252	0.961946047
FUT5	ENSG00000130383	19	5855837	5913798	136	15	123834	0.22107	0.41252	0.961946047
AC024592.12	ENSG00000267740	19	5856182	5913798	136	15	123834	0.22107	0.41252	0.961946047
NFXL1	ENSG00000170448	4	47839257	47926653	178	11	123834	0.22099	0.41255	0.961946047
PGBD5	ENSG00000177614	1	230447392	230571475	260	23	123834	0.22088	0.41259	0.961946047
SLC4A2	ENSG00000164889	7	150744297	150783614	20	5	123834	0.22079	0.41263	0.961946047
KRTAP9-2	ENSG00000239886	17	39372900	39393904	30	4	123834	0.22075	0.41264	0.961946047
METTL4	ENSG00000101574	18	2527524	2581508	144	13	123834	0.22054	0.41273	0.961946047
C15orf53	ENSG00000175779	15	38978799	39002239	49	8	123834	0.22051	0.41274	0.961946047
PIGP	ENSG00000185808	21	38421470	38455470	93	9	123834	0.22036	0.41279	0.961946047
FAM109A	ENSG00000198324	12	111788455	111816925	21	3	123834	0.22017	0.41287	0.961946047
GPLD1	ENSG00000112293	6	24414793	24505433	445	30	123834	0.22009	0.4129	0.961946047
FOXRED1	ENSG00000110074	11	126128950	126158026	58	10	123834	0.21986	0.41299	0.961946047
LHFPL3	ENSG00000187416	7	103959104	104559001	929	73	123834	0.21977	0.41302	0.961946047
SLC22A11	ENSG00000168065	11	64313098	64350347	16	3	123834	0.21977	0.41303	0.961946047
ANKRD54	ENSG00000100124	22	38216862	38255334	32	7	123834	0.21966	0.41307	0.961946047
AC011997.1	ENSG00000222017	2	198547830	198649547	104	7	123834	0.21959	0.4131	0.961946047
PEPD	ENSG00000124299	19	33867856	34022700	386	27	123834	0.21945	0.41315	0.961946047
DNAJC18	ENSG00000170464	5	138734279	138790180	35	4	123834	0.21935	0.41319	0.961946047
KIAA1755	ENSG00000149633	20	36828890	36899174	93	12	123834	0.21912	0.41328	0.961946047
FAM193A	ENSG00000125386	4	2616988	2744292	239	18	123834	0.2191	0.41329	0.961946047
LILRA1	ENSG00000104974	19	55095047	55123555	133	11	123834	0.21902	0.41332	0.961946047
ELAVL2	ENSG00000107105	9	23680102	23836335	234	26	123834	0.21893	0.41335	0.961946047
FCRLB	ENSG00000162746	1	161681353	161707933	85	14	123834	0.21883	0.41339	0.961946047
TBK1	ENSG00000183735	12	64835660	64905888	85	8	123834	0.21876	0.41342	0.961946047
COL5A3	ENSG00000080573	19	10060237	10131147	209	31	123834	0.21876	0.41342	0.961946047
ANKRD63	ENSG00000230778	15	40563645	40584787	3	1	123834	0.21851	0.41351	0.961946047
C19orf66	ENSG00000130813	19	10186798	10213928	5	2	123834	0.21848	0.41353	0.961946047
RP11-145E5.5	ENSG00000264545	9	21792635	22042985	229	32	123834	0.21828	0.4136	0.961946047
TBC1D22B	ENSG00000065491	6	37215548	37310746	138	9	123834	0.2179	0.41376	0.961946047
ATF7IP	ENSG00000171681	12	14508610	14661697	271	15	123834	0.21789	0.41376	0.961946047
HIF1AN	ENSG00000166135	10	102278829	102329755	91	2	123834	0.21786	0.41377	0.961946047
CTNNBIP1	ENSG00000178585	1	9898334	9980394	38	4	123834	0.21755	0.41389	0.961946047
SLC2A1	ENSG00000117394	1	43381052	43434530	99	11	123834	0.21747	0.41392	0.961946047
FCER1G	ENSG00000158869	1	161175024	161200489	20	6	123834	0.21738	0.41396	0.961946047
MAPK10	ENSG00000109339	4	86926276	87525284	1181	37	123834	0.21722	0.41402	0.961946047
STRN	ENSG00000115808	2	37060783	37203615	260	14	123834	0.2172	0.41403	0.961946047
SLC4A3	ENSG00000114923	2	220482049	220516702	15	3	123834	0.2172	0.41403	0.961946047
UBA52	ENSG00000221983	19	18672540	18698360	38	4	123834	0.21709	0.41407	0.961946047
DHX9	ENSG00000135829	1	182798504	182866886	65	5	123834	0.21628	0.41438	0.962319131
SLC35F6	ENSG00000213699	2	26977152	27014099	36	3	123834	0.21619	0.41442	0.962319131
IFIT1B	ENSG00000204010	10	91127813	91154962	39	8	123834	0.21599	0.4145	0.962319131
NAT16	ENSG00000167011	7	100803774	100833557	51	8	123834	0.21571	0.41461	0.962319131
VKORC1L1	ENSG00000196715	7	65328254	65434550	183	11	123834	0.21548	0.4147	0.962319131
ADAMTS14	ENSG00000138316	10	72422559	72532197	201	37	123834	0.21542	0.41472	0.962319131
SPHKAP	ENSG00000153820	2	228834666	229056361	534	19	123834	0.21538	0.41473	0.962319131
TMEM202	ENSG00000187806	15	72680614	72710370	42	3	123834	0.21526	0.41478	0.962319131
DNM2	ENSG00000079805	19	10818755	10954164	226	20	123834	0.21517	0.41482	0.962319131
KLHL17	ENSG00000187961	1	885967	911095	34	6	123834	0.21509	0.41485	0.962319131
HSP90AB1	ENSG00000096384	6	44204824	44231620	32	6	123834	0.21505	0.41486	0.962319131
OR1A1	ENSG00000172146	17	3108915	3129844	92	7	123834	0.21498	0.41489	0.962319131
COPS6	ENSG00000168090	7	99676577	99699823	25	4	123834	0.21491	0.41492	0.962319131
OR51F2	ENSG00000176925	11	4832551	4853686	63	5	123834	0.21486	0.41494	0.962319131
CTD-2370N5.3	ENSG00000265118	17	29622231	29655849	35	1	123834	0.21474	0.41499	0.962319131
KEL	ENSG00000197993	7	142628201	142669768	83	7	123834	0.2145	0.41508	0.962362591
EPHX2	ENSG00000120915	8	27338296	27413081	165	12	123834	0.21442	0.41511	0.962362591
GRIN2B	ENSG00000273079	12	13683165	14143053	778	69	123834	0.21405	0.41526	0.962592937
GLIPR1L2	ENSG00000180481	12	75774850	75836468	162	7	123834	0.21373	0.41538	0.962750681
PRED62	ENSG00000268040	21	47337679	47362477	63	7	123834	0.2136	0.41543	0.962750681
GPR18	ENSG00000125245	13	99896968	99923998	33	9	123834	0.21347	0.41548	0.962750681
AC092811.1	ENSG00000203691	1	225590404	225612040	39	5	123834	0.2133	0.41555	0.962792493
ATAD5	ENSG00000176208	17	29148988	29232887	150	3	123834	0.21316	0.4156	0.962792493
FBXL22	ENSG00000197361	15	63879552	63904627	41	8	123834	0.21303	0.41565	0.962792493
LRRC71	ENSG00000160838	1	156880442	156912886	54	4	123834	0.21281	0.41574	0.962858947
VPS37B	ENSG00000139722	12	123339882	123390991	51	5	123834	0.2127	0.41578	0.962858947
KCNS3	ENSG00000170745	2	18049114	18552882	1240	94	123834	0.21239	0.4159	0.962968612
KRTAP3-3	ENSG00000212899	17	39139686	39160385	125	9	123834	0.21222	0.41597	0.962968612
NRL	ENSG00000129535	14	24539316	24594223	43	10	123834	0.21208	0.41602	0.962968612

UBALD2	ENSG00000185262	17	74251283	74277375	47	8	123834	0.21206	0.41603	0.962968612
VIMP	ENSG00000131871	15	101801022	101827705	28	5	123834	0.21174	0.41615	0.963129088
PDE12	ENSG00000174840	3	57532003	57562571	58	9	123834	0.21111	0.4164	0.963590358
TUFT1	ENSG00000143367	1	151502781	151566059	41	8	123834	0.21069	0.41657	0.963672075
GAB2	ENSG00000033327	11	77916343	78139394	274	3	123834	0.21064	0.41658	0.963672075
TSPAN4	ENSG00000214063	11	832808	877116	54	5	123834	0.21054	0.41662	0.963672075
SERPING1	ENSG00000149131	11	57354860	57392326	40	4	123834	0.21047	0.41665	0.963672075
DOPEY1	ENSG00000083097	6	83767385	83891069	137	9	123834	0.21023	0.41675	0.963672075
WDR41	ENSG00000164253	5	76711795	76926436	574	26	123834	0.20984	0.4169	0.963672075
HCST	ENSG00000126264	19	36383382	36405279	39	4	123834	0.20976	0.41693	0.963672075
MIF	ENSG00000240972	22	24226191	24247414	61	9	123834	0.20958	0.417	0.963672075
DOK2	ENSG00000147443	8	21756384	21781371	33	7	123834	0.20926	0.41712	0.963672075
PUS1	ENSG00000177192	12	132403745	132438406	46	6	123834	0.20923	0.41713	0.963672075
KRTAP4-16P	ENSG00000241241	17	39247754	39268461	144	7	123834	0.20915	0.41717	0.963672075
GRIN2D	ENSG00000105464	19	48888132	48958188	87	13	123834	0.20889	0.41727	0.963672075
EXTL2	ENSG00000162694	1	101327943	101371554	31	6	123834	0.20853	0.41741	0.963672075
EEF1B2	ENSG00000114942	2	207014309	207037652	41	4	123834	0.20849	0.41742	0.963672075
SSUH2	ENSG00000125046	3	8651086	8796726	120	31	123834	0.2085	0.41742	0.963672075
RCBTB2	ENSG00000136161	13	49053095	49117369	54	6	123834	0.20849	0.41742	0.963672075
AGXT	ENSG00000172482	2	241797896	241829919	80	7	123834	0.20832	0.41749	0.963672075
RPRML	ENSG00000179673	17	45045523	45066614	72	9	123834	0.20806	0.41759	0.963672075
HTR6	ENSG00000158748	1	19981780	20016055	70	8	123834	0.20803	0.4176	0.963672075
FAM65B	ENSG00000111913	6	24787601	25052238	510	45	123834	0.20791	0.41765	0.963672075
TPR	ENSG00000047410	1	186270784	186354825	145	12	123834	0.2078	0.41769	0.963672075
LHX1	ENSG00000132130	17	35284084	35311917	32	6	123834	0.2078	0.41769	0.963672075
NTSR2	ENSG00000169006	2	11788304	11820290	43	9	123834	0.20777	0.41771	0.963672075
AC018512.1	ENSG00000268488	15	44065041	44085231	25	3	123834	0.20773	0.41772	0.963672075
AL031663.1	ENSG00000268111	20	44085910	44117452	71	11	123834	0.20761	0.41777	0.963672075
TRMT13	ENSG00000122435	1	100588706	100626053	35	5	123834	0.20751	0.41781	0.963672075
MCM3AP	ENSG00000160294	21	47645047	47716211	193	16	123834	0.20735	0.41787	0.963672075
FABP9	ENSG00000205186	8	82360576	82383814	52	4	123834	0.20733	0.41788	0.963672075
NARF	ENSG00000141562	17	80406056	80458413	116	10	123834	0.20706	0.41798	0.963672075
NPPB	ENSG00000120937	1	11907521	11928988	29	6	123834	0.20681	0.41808	0.963672075
COQ10B	ENSG00000115520	2	198308147	198350032	29	3	123834	0.20648	0.41821	0.963672075
UBE2M	ENSG00000130725	19	59057079	59080327	34	6	123834	0.20638	0.41825	0.963672075
RP11-426L16.10	ENSG00000271810	1	113235236	113264055	24	6	123834	0.2063	0.41828	0.963672075
LGALS13	ENSG00000105198	19	40083164	40108116	25	5	123834	0.20627	0.41829	0.963672075
MYO1C	ENSG00000197879	17	1357392	1406106	54	15	123834	0.20589	0.41844	0.963672075
TRIM69	ENSG00000185880	15	45011186	45070027	181	11	123834	0.20582	0.41847	0.963672075
TOLLIP	ENSG00000078902	11	1285601	1340884	124	17	123834	0.20574	0.4185	0.963672075
ZNF608	ENSG00000168916	5	123962608	124094500	196	27	123834	0.20569	0.41852	0.963672075
CACNG6	ENSG00000130433	19	54485542	54525923	69	13	123834	0.2056	0.41855	0.963672075
DIS3L2	ENSG00000144535	2	232815955	233219060	551	23	123834	0.20538	0.41864	0.963672075
ARHGEF10L	ENSG00000074964	1	17856330	18034369	143	20	123834	0.2053	0.41867	0.963672075
AC007557.1	ENSG00000223874	2	217725495	217746362	32	6	123834	0.2053	0.41867	0.963672075
SNCAIP	ENSG00000064692	5	121637049	121809914	113	10	123834	0.20524	0.41869	0.963672075
AMMECR1L	ENSG00000144233	2	128609204	128653496	63	10	123834	0.20512	0.41874	0.963672075
ACBD3	ENSG00000182827	1	226322380	226384431	78	10	123834	0.20501	0.41878	0.963672075
GSTO2	ENSG00000065621	10	106018631	106074703	56	7	123834	0.20471	0.4189	0.963672075
CTC1	ENSG00000178971	17	8120191	8161362	64	10	123834	0.20468	0.41891	0.963672075
ANGPTL7	ENSG00000171819	1	11239398	11266038	39	3	123834	0.20446	0.419	0.963672075
RHOH	ENSG00000168421	4	40182673	40258587	75	17	123834	0.20441	0.41902	0.963672075
CHCHD6	ENSG00000159685	3	126413063	126689249	412	21	123834	0.20415	0.41912	0.963672075
CNR1	ENSG00000118432	6	88839583	88886078	36	4	123834	0.20398	0.41918	0.963672075
ZNF227	ENSG00000131115	19	44701700	44751421	54	6	123834	0.20379	0.41926	0.963672075
REST	ENSG00000084093	4	57764075	57812010	113	12	123834	0.20365	0.41931	0.963672075
NUPR1	ENSG00000176046	16	28538606	28560495	35	5	123834	0.20329	0.41945	0.963672075
TSC22D1	ENSG00000102804	13	44997655	45161283	216	9	123834	0.20315	0.41951	0.963672075
SLC11A1	ENSG00000018280	2	219236752	219271617	13	6	123834	0.20313	0.41952	0.963672075
RAB3C	ENSG00000152932	5	57868048	58165213	517	41	123834	0.20313	0.41952	0.963672075
RAB36	ENSG00000100228	22	23477513	23516537	79	6	123834	0.20312	0.41952	0.963672075
PCDH17	ENSG00000118946	13	58195944	58313445	159	19	123834	0.20306	0.41955	0.963672075
SLC30A10	ENSG00000196660	1	219848769	220141989	710	33	123834	0.2029	0.41961	0.963672075
PMM1	ENSG00000100417	22	41962898	41995894	28	2	123834	0.2029	0.41961	0.963672075
GRWD1	ENSG00000105447	19	48939030	48970279	50	6	123834	0.20281	0.41964	0.963672075
COMMD4	ENSG00000140365	15	75618232	75644268	24	4	123834	0.20273	0.41967	0.963672075
SLC43A1	ENSG00000149150	11	57242007	57293259	33	4	123834	0.20271	0.41968	0.963672075
ARHGEF33	ENSG00000214694	2	39107021	39212590	98	12	123834	0.20225	0.41986	0.963968943
RNF213	ENSG00000173821	17	78224665	78382586	260	30	123834	0.20195	0.41998	0.964128
CD1E	ENSG00000158488	1	158313254	158337343	68	7	123834	0.20166	0.42009	0.964264065
USP39	ENSG00000168883	2	85819979	85886403	68	8	123834	0.20111	0.42031	0.964650837
CRYGC	ENSG00000163254	2	208982861	209004554	67	9	123834	0.20098	0.42036	0.964650837
KDM4C	ENSG00000107077	9	6710863	7185648	1288	82	123834	0.20028	0.42063	0.965033889
GM2A	ENSG00000196743	5	150581711	150660001	107	18	123834	0.20021	0.42066	0.965033889
RP11-167N24.6	ENSG00000258247	12	95760984	95784672	92	11	123834	0.20007	0.42071	0.965033889
KERA	ENSG00000139330	12	91434268	91461760	90	12	123834	0.20002	0.42073	0.965033889
TBX21	ENSG00000073861	17	45800610	45833485	68	10	123834	0.19957	0.42091	0.965076136
MXD3	ENSG00000213347	5	176718462	176749758	34	9	123834	0.19953	0.42093	0.965076136
TPM1	ENSG00000140416	15	63324831	63374114	113	13	123834	0.1995	0.42094	0.965076136
PRICKLE2	ENSG00000163637	3	64069543	64441152	452	64	123834	0.19917	0.42107	0.965076136
PARD3	ENSG00000148498	10	34388488	35114253	1050	42	123834	0.19912	0.42108	0.965076136
CARHSP1	ENSG00000153048	16	8936799	8972866	97	14	123834	0.19887	0.42118	0.965076136
A1CF	ENSG00000148584	10	52549169	52655435	86	11	123834	0.19853	0.42132	0.965076136
DSCR8	ENSG00000198054	21	39483545	39570110	126	11	123834	0.19837	0.42138	0.965076136
SPATA24	ENSG00000170469	5	138722252	138749777	15	3	123834	0.19832	0.4214	0.965076136
MAP3K7CL	ENSG00000156265	21	30439792	30558210	251	17	123834	0.19832	0.4214	0.965076136
LIPC	ENSG00000166035	15	58692768	58871151	266	34	123834	0.19823	0.42143	0.965076136
SMIM7	ENSG00000214046	19	16731562	16781253	70	9	123834	0.19816	0.42146	0.965076136
PABPC3	ENSG00000151846	13	25660300	25683389	23	5	123834	0.1981	0.42148	0.965076136
KREMEN1	ENSG00000183762	22	29459066	29574321	236	19	123834	0.19806	0.4215	0.965076136
SLAMF8	ENSG00000158714	1	159786540	159817039	35	7	123834	0.19804	0.42151	0.965076136
RAB6A	ENSG00000175582	11	73376683	73482182	222	19	123834	0.19773	0.42163	0.965083018
ZNF48	ENSG00000180035	16	30379427	30421429	26	3	123834	0.19737	0.42177	0.965083018
COMT	ENSG00000093010	22	19919130	19967498	112	18	123834	0.1972	0.42183	0.965083018
FAM102A	ENSG00000167106	9	130692858	130752792	128	9	123834	0.19713	0.42186	0.965083018
CTNNBL1	ENSG00000132792	20	36312408	36510531	237	15	123834	0.19709	0.42188	0.965083018
ETS1	ENSG00000134954	11	128318656	128467453	137	28	123834	0.19701	0.42191	0.965083018
KIAA0195	ENSG00000177728	17	73427240	73506171	186	13	123834	0.19687	0.42197	0.965083018
PDLIM4	ENSG00000131435	5	131583364	131619147	68	8	123834	0.19684	0.42198	0.965083018

ST20	ENSG00000180953	15	80181182	80226044	155	13	123834	0.19657	0.42208	0.965083018
SRSF1	ENSG00000136450	17	56070721	56094707	20	3	123834	0.19658	0.42208	0.965083018
MEP1B	ENSG00000141434	18	29755032	29810367	76	8	123834	0.19654	0.42209	0.965083018
HDAC7	ENSG00000061273	12	48166505	48236915	75	15	123834	0.19619	0.42223	0.965083018
CLEC7A	ENSG00000172243	12	10259376	10292857	73	7	123834	0.19595	0.42233	0.965083018
ARPP19	ENSG00000128989	15	52829242	52872080	53	4	123834	0.19583	0.42237	0.965083018
TBC1D21	ENSG00000167139	15	74155949	74191555	45	9	123834	0.19567	0.42243	0.965083018
SPSB3	ENSG00000162032	16	1816713	1853701	141	9	123834	0.19551	0.4225	0.965083018
BIVM-ERCC5	ENSG00000270181	13	103449704	103534748	137	13	123834	0.19541	0.42254	0.965083018
BARHL2	ENSG00000143032	1	91167096	91192794	21	8	123834	0.19535	0.42256	0.965083018
SFRP5	ENSG00000120057	10	99516508	99541709	38	3	123834	0.19532	0.42257	0.965083018
COX6B2	ENSG00000160471	19	55850674	55876182	24	3	123834	0.1952	0.42262	0.965083018
CHN1	ENSG00000128656	2	175654091	175880097	165	9	123834	0.19516	0.42263	0.965083018
TRAF3IP1	ENSG00000204104	2	239219082	239319541	214	16	123834	0.19518	0.42263	0.965083018
YBEY	ENSG00000182362	21	47696251	47727665	112	11	123834	0.19436	0.42295	0.965643769
CTD-2117L12.1	ENSG00000250127	5	57827394	57864070	65	9	123834	0.19428	0.42298	0.965643769
SLC6A17	ENSG00000197106	1	110683108	110754824	43	7	123834	0.19392	0.42312	0.965643769
INPPL1	ENSG00000165458	11	71924745	71960149	40	7	123834	0.19379	0.42317	0.965643769
FRY	ENSG00000073910	13	32595437	32880794	443	43	123834	0.19342	0.42332	0.965643769
PET112	ENSG00000059691	4	152581656	152692175	184	8	123834	0.19336	0.42334	0.965643769
ARHGEF38	ENSG00000236699	4	106463777	106639250	316	23	123834	0.19327	0.42337	0.965643769
OLA1	ENSG00000138430	2	174927175	175123426	369	16	123834	0.19325	0.42338	0.965643769
MVB12A	ENSG00000141971	19	17506531	17554533	79	10	123834	0.19313	0.42343	0.965643769
RFNG	ENSG00000169733	17	79995778	80019707	32	7	123834	0.19305	0.42346	0.965643769
BPTF	ENSG00000171634	17	65811640	65990494	207	5	123834	0.19278	0.42357	0.965643769
EVI2B	ENSG00000185862	17	29620784	29651130	28	1	123834	0.19268	0.4236	0.965643769
TNF	ENSG00000232810	6	31533344	31556113	36	12	123834	0.19254	0.42366	0.965643769
VSIG10L	ENSG00000186806	19	51824790	51855378	40	6	123834	0.19254	0.42366	0.965643769
DHX57	ENSG00000163214	2	39014871	39113075	106	8	123834	0.19244	0.4237	0.965643769
ACTA2	ENSG00000107796	10	90684831	90761147	125	20	123834	0.19239	0.42372	0.965643769
KCNK15	ENSG00000124249	20	43364421	43389675	35	5	123834	0.19229	0.42376	0.965643769
SF3B14	ENSG00000115128	2	24280454	24309313	74	8	123834	0.1922	0.42379	0.965643769
POLB	ENSG00000070501	8	42185972	42239326	81	6	123834	0.19204	0.42385	0.965664725
ERCC6L2	ENSG00000182150	9	98627983	98786842	312	16	123834	0.1914	0.42411	0.965834623
TSSK6	ENSG00000178093	19	19613227	19636838	36	4	123834	0.19125	0.42416	0.965834623
KLHDC10	ENSG00000128607	7	129700350	129785560	120	12	123834	0.19123	0.42417	0.965834623
SERPINA1	ENSG00000197249	14	94833084	94867030	77	18	123834	0.19087	0.42431	0.965834623
TRPV1	ENSG00000196689	17	3458738	3510392	74	14	123834	0.19073	0.42437	0.965834623
TOP3B	ENSG00000100038	22	22301397	22347213	85	9	123834	0.1907	0.42438	0.965834623
SAMD1	ENSG00000141858	19	14188652	14211848	7	3	123834	0.19066	0.42439	0.965834623
SLC28A1	ENSG00000156222	15	85417885	85528876	208	38	123834	0.19066	0.4244	0.965834623
ZNF98	ENSG00000197360	19	22563821	22725287	275	20	123834	0.19037	0.42451	0.965834623
NUBP2	ENSG00000095906	16	1822902	1849192	91	8	123834	0.19014	0.4246	0.965834623
ZNF114	ENSG00000178150	19	48665575	48800865	367	31	123834	0.19015	0.4246	0.965834623
GAR1	ENSG00000109534	4	110726666	110755893	45	4	123834	0.1901	0.42462	0.965834623
ABCC3	ENSG00000108846	17	48702138	48779613	44	16	123834	0.19001	0.42465	0.965834623
C20orf85	ENSG00000124237	20	56715960	56746206	19	3	123834	0.18982	0.42473	0.965834623
UGGT2	ENSG00000102595	13	96443834	96715736	404	17	123834	0.18972	0.42476	0.965834623
AC110615.1	ENSG00000268788	4	76861883	76888526	53	7	123834	0.18955	0.42483	0.965834623
MPG	ENSG00000103152	16	117006	145852	37	6	123834	0.18956	0.42483	0.965834623
KIFAP3	ENSG00000075945	1	169880467	170064349	445	9	123834	0.18943	0.42488	0.965834623
CCL4	ENSG00000129277	17	34420983	34443014	41	4	123834	0.18941	0.42489	0.965834623
PNRC1	ENSG00000146278	6	89780470	89804879	45	4	123834	0.18925	0.42495	0.965855506
SH2D4A	ENSG00000104611	8	19161128	19263729	152	21	123834	0.189	0.42504	0.965897258
ZNF283	ENSG00000167637	19	44321444	44363307	135	9	123834	0.18894	0.42507	0.965897258
GPR111	ENSG00000164393	6	47614172	47675533	202	4	123834	0.18849	0.42525	0.966190772
ZBED3	ENSG00000132846	5	76357897	76393148	50	9	123834	0.18814	0.42538	0.966300574
VENTX	ENSG00000151650	10	135040908	135065433	47	8	123834	0.1881	0.4254	0.966300574
EMC10	ENSG00000161671	19	50969657	50996608	32	6	123834	0.18767	0.42557	0.966425374
EHD3	ENSG00000013016	2	31446880	31502313	47	12	123834	0.18755	0.42561	0.966425374
PIGZ	ENSG00000119227	3	196663214	196705931	37	9	123834	0.18744	0.42566	0.966425374
BANP	ENSG00000172530	16	87972850	88120924	354	17	123834	0.18722	0.42574	0.966425374
NDUFS2	ENSG00000158864	1	161156894	161194185	37	10	123834	0.18721	0.42575	0.966425374
C11orf74	ENSG00000166352	11	36606051	36704823	308	9	123834	0.18719	0.42576	0.966425374
TICAM1	ENSG00000127666	19	4805944	4841716	41	9	123834	0.18651	0.42602	0.966900079
SLC6A6	ENSG00000131389	3	14434076	14540857	109	22	123834	0.1862	0.42614	0.967005628
IFITM10	ENSG00000244242	11	1743640	1781821	54	5	123834	0.18611	0.42618	0.967005628
CCDC94	ENSG00000105248	19	4237076	4279087	60	2	123834	0.18591	0.42626	0.967005628
CAMKK2	ENSG00000110931	12	121665497	121746111	139	20	123834	0.18588	0.42627	0.967005628
IGFBP1	ENSG00000146678	7	45917956	45943267	31	7	123834	0.18568	0.42635	0.967024346
GEMIN7	ENSG00000142252	19	45572453	45604782	75	7	123834	0.1856	0.42638	0.967024346
BAP1	ENSG00000163930	3	52425029	52454366	13	3	123834	0.18545	0.42644	0.967032839
PDE6A	ENSG00000132915	5	149227519	149334356	121	24	123834	0.18521	0.42653	0.967032839
FAM189A1	ENSG00000104059	15	29402457	29872927	691	59	123834	0.18473	0.42672	0.967032839
CDC42EP5	ENSG00000167617	19	54966210	54994411	46	11	123834	0.18473	0.42672	0.967032839
OR2C3	ENSG00000196242	1	247683434	247707141	41	6	123834	0.18463	0.42676	0.967032839
RP11-180C1.1	ENSG00000250038	4	28353901	28414486	166	12	123834	0.18446	0.42682	0.967032839
RYK	ENSG00000163785	3	133774147	133979689	361	18	123834	0.18406	0.42698	0.967032839
DDX46	ENSG00000145833	5	134084469	134200823	60	10	123834	0.18394	0.42703	0.967032839
FST	ENSG00000134363	5	52766239	52792964	54	10	123834	0.18388	0.42705	0.967032839
DPYS	ENSG00000147647	8	105332552	105489281	256	29	123834	0.18388	0.42705	0.967032839
APOA2	ENSG00000158874	1	161182082	161203421	19	6	123834	0.1838	0.42708	0.967032839
CCDC132	ENSG00000004766	7	92851653	92998338	149	19	123834	0.18365	0.42715	0.967032839
FAM162B	ENSG00000183807	6	117063363	117096886	59	11	123834	0.18343	0.42723	0.967032839
CLEC14A	ENSG00000176435	14	38713308	38735574	9	3	123834	0.18339	0.42725	0.967032839
KRTAP5-10	ENSG00000204572	11	71266609	71289980	76	9	123834	0.18321	0.42731	0.967032839
CYP4X1	ENSG00000186377	1	47417036	47526423	135	11	123834	0.18307	0.42737	0.967032839
NLRP3	ENSG00000162711	1	247569458	247622410	137	25	123834	0.18306	0.42737	0.967032839
EMB	ENSG00000170571	5	49682026	49749082	117	7	123834	0.18233	0.42766	0.967032839
BHLHE40	ENSG00000134107	3	5010801	5037008	22	6	123834	0.1823	0.42767	0.967032839
SLC2A10	ENSG00000197496	20	45328126	45374965	89	9	123834	0.18229	0.42768	0.967032839
UBE2E3	ENSG00000170035	2	181821975	181951312	205	15	123834	0.18124	0.42809	0.967032839
VGF	ENSG00000128564	7	100795790	100818874	48	7	123834	0.1811	0.42815	0.967032839
FAM155A	ENSG00000204442	13	107810883	108529083	1212	134	123834	0.18074	0.42829	0.967032839
NTF3	ENSG00000185652	12	5531278	5640702	99	22	123834	0.18053	0.42837	0.967032839
CDR2	ENSG00000140743	16	22347257	22458486	33	7	123834	0.1805	0.42838	0.967032839
MGME1	ENSG00000125871	20	17939556	17981765	105	7	123834	0.18025	0.42848	0.967032839
PCYOX1	ENSG00000116005	2	70474518	70518323	72	4	123834	0.18012	0.42853	0.967032839
SDHA	ENSG00000073578	5	208356	266815	286	5	123834	0.17976	0.42867	0.967032839

CENPK	ENSG00000123219	5	64803593	64868998	174	10	123834	0.17972	0.42869	0.967032839
AK9	ENSG00000155085	6	109804059	110022420	511	10	123834	0.17965	0.42871	0.967032839
FDXR	ENSG00000161513	17	72848619	72879156	33	3	123834	0.17943	0.4288	0.967032839
PRSS57	ENSG00000185198	19	675521	705460	77	10	123834	0.17941	0.42881	0.967032839
OPRK1	ENSG00000082556	8	54128284	54174257	165	13	123834	0.17939	0.42882	0.967032839
NBPF6	ENSG00000186086	1	108908421	109023624	47	3	123834	0.17936	0.42883	0.967032839
PCSK6	ENSG00000140479	15	101830818	102075405	386	72	123834	0.17927	0.42886	0.967032839
ASIC4	ENSG00000072182	2	220368892	220413494	25	5	123834	0.17921	0.42889	0.967032839
VPS37A	ENSG00000155975	8	17094080	17169936	129	16	123834	0.17911	0.42893	0.967032839
PLOD1	ENSG00000083444	1	11984262	12045595	75	8	123834	0.17899	0.42897	0.967032839
C7orf57	ENSG00000164746	7	48065108	48110901	87	8	123834	0.17892	0.429	0.967032839
NLRP8	ENSG00000179709	19	56449198	56509995	172	26	123834	0.17891	0.429	0.967032839
TMEM200C	ENSG00000206432	18	5872071	5905954	37	9	123834	0.1789	0.42901	0.967032839
NSA2	ENSG00000164346	5	74052817	74082737	42	5	123834	0.17888	0.42902	0.967032839
OR2L3	ENSG00000198128	1	248213984	248234922	36	4	123834	0.17872	0.42908	0.967032839
CCDC101	ENSG00000176476	16	28555236	28613111	90	7	123834	0.17862	0.42912	0.967032839
PPIP5K1	ENSG00000168781	15	43815660	43892430	32	3	123834	0.17851	0.42916	0.967032839
C1QTNF9	ENSG00000240654	13	24871304	24906673	61	9	123834	0.17846	0.42918	0.967032839
ENTPD7	ENSG00000198018	10	101409263	101475997	90	6	123834	0.17835	0.42922	0.967032839
TRAPPC13	ENSG00000113597	5	64910543	64972060	140	9	123834	0.1783	0.42924	0.967032839
ERN2	ENSG00000134398	16	23691647	23734821	48	9	123834	0.17832	0.42924	0.967032839
MAN2C1	ENSG00000140400	15	75638133	75670971	18	4	123834	0.17809	0.42932	0.967032839
B4GALNT3	ENSG00000139044	12	559530	682675	208	42	123834	0.17808	0.42933	0.967032839
LGSN	ENSG00000146166	6	63975856	64039882	38	7	123834	0.17782	0.42943	0.967032839
PTF1A	ENSG00000168267	10	23471256	23493181	12	3	123834	0.17756	0.42953	0.967032839
ACTL9	ENSG00000181786	19	8797751	8819172	30	6	123834	0.17751	0.42955	0.967032839
SLC12A4	ENSG00000124067	16	67967377	68013504	30	4	123834	0.17738	0.4296	0.967032839
C2orf43	ENSG00000118961	2	20873788	21032882	197	22	123834	0.17712	0.42971	0.967032839
STAR	ENSG00000147465	8	37991167	38018783	2	1	123834	0.17701	0.42975	0.967032839
PPFIA4	ENSG00000143847	1	202985626	203057868	103	15	123834	0.17676	0.42985	0.967032839
GZMH	ENSG00000100450	14	25065686	25088905	35	6	123834	0.17672	0.42987	0.967032839
KIAA1468	ENSG00000134444	18	59844491	59984355	361	16	123834	0.17665	0.42989	0.967032839
PRELID1	ENSG00000169230	5	176720775	176743960	25	8	123834	0.17661	0.42991	0.967032839
IL22	ENSG00000127318	12	68632022	68657387	83	8	123834	0.17661	0.42991	0.967032839
SLBP	ENSG00000163950	4	1684527	1724282	97	11	123834	0.17634	0.43001	0.967032839
IL17B	ENSG00000127743	5	148740887	148793765	54	7	123834	0.17633	0.43002	0.967032839
PFAS	ENSG00000178921	17	8140936	8183809	80	12	123834	0.17632	0.43002	0.967032839
TAS2R60	ENSG00000185899	7	143130546	143151502	22	5	123834	0.17622	0.43006	0.967032839
HNRNPUL1	ENSG00000105323	19	41758401	41823503	67	5	123834	0.17621	0.43006	0.967032839
USP18	ENSG00000184979	22	18622666	18670164	65	9	123834	0.17614	0.43009	0.967032839
CTD-2210P24.4	ENSG00000205106	11	45782983	45803909	52	10	123834	0.1761	0.43011	0.967032839
CYP3A7	ENSG00000160870	7	99292660	99342819	69	2	123834	0.17603	0.43014	0.967032839
ELMOD1	ENSG00000110675	11	107451817	107547505	167	23	123834	0.17593	0.43018	0.967032839
TWSG1	ENSG00000128791	18	9324765	9412418	209	16	123834	0.17588	0.43019	0.967032839
DAZAP1	ENSG00000071626	19	1397568	1445719	33	5	123834	0.17549	0.43035	0.967032839
AL121761.2	ENSG00000268628	20	19728296	19748679	28	8	123834	0.1754	0.43038	0.967032839
AP001421.1	ENSG00000267937	21	38878740	38898973	24	4	123834	0.17535	0.4304	0.967032839
PIAS1	ENSG00000033800	15	68336517	68493096	103	7	123834	0.17518	0.43047	0.967032839
SEC14L1	ENSG00000129657	17	75072798	75223179	330	30	123834	0.17508	0.43051	0.967032839
C1QL4	ENSG00000186897	12	49716200	49740971	14	3	123834	0.17501	0.43053	0.967032839
GET4	ENSG00000239857	7	906189	946073	77	12	123834	0.1748	0.43062	0.967032839
TIGD3	ENSG00000173825	11	65112238	65135084	13	3	123834	0.17481	0.43062	0.967032839
CNGB1	ENSG00000070729	16	57907503	58015020	90	26	123834	0.17481	0.43062	0.967032839
PIP5KL1	ENSG00000167103	9	130673158	130703076	38	5	123834	0.17422	0.43085	0.967032839
C4orf45	ENSG00000164123	4	159804286	159969912	308	14	123834	0.17417	0.43086	0.967032839
ASCC3	ENSG00000112249	6	100946070	101339248	745	18	123834	0.17401	0.43093	0.967032839
PNPLA2	ENSG00000177666	11	808902	835573	26	6	123834	0.17386	0.43099	0.967032839
ARPC4	ENSG00000241553	3	9824179	9859410	45	7	123834	0.17381	0.43101	0.967032839
PSMD3	ENSG00000108344	17	38127050	38164213	88	8	123834	0.17377	0.43102	0.967032839
PANK4	ENSG00000157881	1	2429972	2468039	38	4	123834	0.17369	0.43105	0.967032839
C11orf31	ENSG00000211450	11	57498825	57520986	15	3	123834	0.17369	0.43105	0.967032839
RERGL	ENSG00000111404	12	18223803	18483041	611	30	123834	0.17368	0.43106	0.967032839
PLLP	ENSG00000102934	16	57280004	57328599	117	16	123834	0.17367	0.43106	0.967032839
TMEM41B	ENSG00000166471	11	9292201	9346327	123	11	123834	0.17363	0.43108	0.967032839
ZBTB4	ENSG00000174282	17	7352685	7397582	115	9	123834	0.17351	0.43113	0.967032839
SUCNR1	ENSG00000198829	3	151581431	151612405	64	12	123834	0.17313	0.43127	0.967032839
RP11-176H8.1	ENSG00000203546	14	31793518	31936647	181	11	123834	0.17285	0.43139	0.967032839
RP11-683L23.1	ENSG00000173213	18	37225	83545	24	4	123834	0.17283	0.43139	0.967032839
QRFP	ENSG00000188710	9	133758815	133779225	40	8	123834	0.17268	0.43145	0.967032839
CLCNKB	ENSG00000184908	1	16360272	16393803	126	17	123834	0.17237	0.43157	0.967032839
GRN	ENSG00000030582	17	42412614	42440470	19	5	123834	0.17233	0.43159	0.967032839
SLC25A3	ENSG00000075415	12	98977369	99005946	47	7	123834	0.17203	0.43171	0.967032839
EFNB3	ENSG00000108947	17	7598520	7624696	22	6	123834	0.172	0.43172	0.967032839
LBR	ENSG00000143815	1	225579204	225626627	81	10	123834	0.17194	0.43174	0.967032839
IL12A	ENSG00000168811	3	159696537	159723806	42	6	123834	0.17194	0.43174	0.967032839
OR51A4	ENSG00000205497	11	4957355	4978356	82	8	123834	0.1719	0.43176	0.967032839
OR8B8	ENSG00000197125	11	124300022	124321054	15	4	123834	0.17173	0.43182	0.967032839
TOMM22	ENSG00000100216	22	39067953	39090818	53	4	123834	0.17172	0.43183	0.967032839
LIMD2	ENSG00000136490	17	61763262	61788532	18	2	123834	0.17168	0.43184	0.967032839
B3GALT2	ENSG00000162630	1	193138175	193165784	50	6	123834	0.17157	0.43189	0.967032839
MYRFL	ENSG00000166268	12	70209084	70362877	409	26	123834	0.17147	0.43193	0.967032839
TTF2	ENSG00000116830	1	117592925	117660075	119	16	123834	0.17133	0.43198	0.967032839
RNF6	ENSG00000127870	13	26696253	26806791	191	26	123834	0.17115	0.43205	0.967075648
XRCC4	ENSG00000152422	5	82363317	82659606	518	17	123834	0.17058	0.43228	0.967130977
OR10A5	ENSG00000166363	11	6856883	6877936	115	5	123834	0.17054	0.43229	0.967130977
SGSM2	ENSG00000141258	17	2230792	2294352	90	15	123834	0.1705	0.43231	0.967130977
PAK4	ENSG00000130669	19	39606410	39683456	92	11	123834	0.17044	0.43233	0.967130977
ARID3B	ENSG00000179361	15	74823518	74900472	96	6	123834	0.17035	0.43237	0.967130977
FLNB	ENSG00000136068	3	57984127	58167982	196	22	123834	0.17033	0.43238	0.967130977
SIK3	ENSG00000160584	11	116704118	116979153	820	22	123834	0.16962	0.43265	0.967621038
SLC37A1	ENSG00000160190	21	43906118	44011550	223	18	123834	0.16923	0.43281	0.967789444
RNASEH2B	ENSG00000136104	13	51473814	51554592	102	15	123834	0.16903	0.43289	0.967789444
HID1	ENSG00000167861	17	72936838	72979261	36	9	123834	0.16882	0.43297	0.967789444
TBC1D1	ENSG00000065882	4	37882708	38150796	500	58	123834	0.16869	0.43302	0.967789444
LRRC3B	ENSG00000179796	3	26654297	26762267	131	18	123834	0.16841	0.43313	0.967789444
ZNF697	ENSG00000143067	1	120152045	120200396	40	6	123834	0.16825	0.43319	0.967789444
ELMSAN1	ENSG00000156030	14	74171825	74266988	136	26	123834	0.16795	0.43331	0.967789444
GPX3	ENSG00000211445	5	150390124	150418554	37	7	123834	0.16778	0.43338	0.967789444
ARL2	ENSG00000213465	11	64771585	64799656	46	8	123834	0.16775	0.43339	0.967789444

NGFR	ENSG00000064300	17	47562655	47602379	25	10	123834	0.16775	0.43339	0.967789444
PCNXL4	ENSG00000126773	14	60548629	60645851	172	5	123834	0.16773	0.4334	0.967789444
VANGL1	ENSG00000173218	1	116174574	116250845	109	18	123834	0.1677	0.43341	0.967789444
AP2M1	ENSG00000161203	3	183882477	183911879	33	6	123834	0.16766	0.43343	0.967789444
ITGA1	ENSG00000213949	5	52073730	52265040	372	42	123834	0.1673	0.43357	0.967789444
ZNF503	ENSG00000165655	10	77147588	77171664	2	1	123834	0.16706	0.43366	0.967789444
SNX20	ENSG00000167208	16	50690211	50725264	24	3	123834	0.167	0.43368	0.967789444
SNX3	ENSG00000112335	6	108522426	108592464	89	8	123834	0.16681	0.43376	0.967789444
APOBR	ENSG00000184730	16	28495970	28520291	20	4	123834	0.16678	0.43377	0.967789444
DMAP1	ENSG00000178028	1	44669127	44696353	23	6	123834	0.1667	0.4338	0.967789444
PYGL	ENSG00000100504	14	51314609	51421454	202	26	123834	0.16661	0.43384	0.967789444
STON2	ENSG00000140022	14	81717000	81912809	329	27	123834	0.16661	0.43384	0.967789444
KIAA1462	ENSG00000165757	10	30291729	30414423	108	17	123834	0.16648	0.43389	0.967789444
NUDC	ENSG00000090273	1	27216729	27283353	65	7	123834	0.16643	0.43391	0.967789444
C11orf65	ENSG00000166323	11	108169246	108348258	262	7	123834	0.16631	0.43396	0.967789444
FANCD2	ENSG00000144554	3	10058098	10153614	200	3	123834	0.16613	0.43403	0.967789444
CTNNA2	ENSG00000066032	2	79402357	80885905	2349	190	123834	0.16607	0.43405	0.967789444
HDAC1	ENSG00000116478	1	32747687	32809236	4	1	123834	0.16595	0.4341	0.967789444
OTOA	ENSG00000155719	16	21679835	21782050	68	11	123834	0.1657	0.4342	0.967898862
RABEPK	ENSG00000136933	9	127952821	128006437	74	7	123834	0.16508	0.43444	0.967936614
CDHR4	ENSG00000187492	3	49818165	49847268	29	5	123834	0.16494	0.43449	0.967936614
LMAN1L	ENSG00000140506	15	75095057	75128099	47	6	123834	0.16488	0.43452	0.967936614
PVR	ENSG00000073008	19	45137098	45176850	47	8	123834	0.16465	0.43461	0.967936614
ENOPH1	ENSG00000145293	4	83341715	83392244	70	11	123834	0.16455	0.43465	0.967936614
DLX5	ENSG00000105880	7	96639704	96664409	23	5	123834	0.16451	0.43466	0.967936614
KRTAP20-2	ENSG00000184032	21	31997555	32017938	25	3	123834	0.16437	0.43472	0.967936614
MITD1	ENSG00000158411	2	99767890	99807521	67	5	123834	0.16433	0.43474	0.967936614
ABHD16A	ENSG00000204427	6	31644726	31681221	40	9	123834	0.16398	0.43487	0.967936614
SEMA3E	ENSG00000170381	7	82983222	83288326	783	32	123834	0.1639	0.43491	0.967936614
RGCC	ENSG00000102760	13	42021695	42055018	19	5	123834	0.16384	0.43493	0.967936614
PHRF1	ENSG00000070047	11	566486	622222	154	14	123834	0.1637	0.43498	0.967936614
FAM149A	ENSG00000109794	4	187015573	187103821	155	24	123834	0.16352	0.43505	0.967936614
BCL7C	ENSG00000099385	16	30834947	30916281	47	4	123834	0.16348	0.43507	0.967936614
NPPA	ENSG00000175206	1	11895766	11918402	64	8	123834	0.16342	0.4351	0.967936614
ACADVL	ENSG00000072778	17	7110444	7138592	19	3	123834	0.16314	0.4352	0.967936614
CHRM3	ENSG00000133019	1	239539865	240088750	689	60	123834	0.16311	0.43522	0.967936614
EEF1E1-BLOC1S5	ENSG00000265818	6	8005959	8112763	312	18	123834	0.16308	0.43523	0.967936614
C16orf96	ENSG00000205832	16	4596491	4660715	87	7	123834	0.1628	0.43534	0.967936614
CEBPA	ENSG00000245848	19	33780840	33803470	33	3	123834	0.16281	0.43534	0.967936614
CHCHD4	ENSG00000163528	3	14143580	14176370	41	7	123834	0.16267	0.43539	0.967936614
CYP1A1	ENSG00000140465	15	75001883	75027951	12	3	123834	0.16255	0.43544	0.967936614
DCAF11	ENSG00000100897	14	24573404	24604451	42	6	123834	0.16249	0.43546	0.967936614
CYP11A1	ENSG00000140459	15	74620100	74670081	77	9	123834	0.16249	0.43546	0.967936614
IKBKAP	ENSG00000070061	9	111619797	111706396	267	20	123834	0.16241	0.43549	0.967936614
CD46	ENSG00000117335	1	207915402	207978858	105	8	123834	0.16199	0.43566	0.967996733
OR4N5	ENSG00000184394	14	20601895	20622821	38	6	123834	0.16186	0.43571	0.967996733
GRIA1	ENSG00000155511	5	152859175	153203429	873	48	123834	0.16183	0.43572	0.967996733
ZWINT	ENSG00000122952	10	58106989	58131036	21	5	123834	0.16175	0.43575	0.967996733
AKR1C4	ENSG00000198610	10	5227425	5270912	167	10	123834	0.16109	0.43601	0.967996733
BEST3	ENSG00000127325	12	70027140	70103256	221	19	123834	0.16087	0.4361	0.967996733
AC099552.4	ENSG00000217825	7	154978273	155000165	62	6	123834	0.16075	0.43615	0.967996733
RP3-468K18.5	ENSG00000257065	6	144118229	144175754	94	11	123834	0.16044	0.43627	0.967996733
VSIG8	ENSG00000243284	1	159814106	159842447	17	7	123834	0.16042	0.43628	0.967996733
BPIFA3	ENSG00000131059	20	31795116	31825564	23	2	123834	0.16035	0.4363	0.967996733
GLG1	ENSG00000090863	16	74475856	74651012	394	17	123834	0.1602	0.43636	0.967996733
ASB13	ENSG00000196372	10	5670830	5718558	95	16	123834	0.16005	0.43642	0.967996733
TNFSF11	ENSG00000120659	13	43126872	43192149	124	11	123834	0.15992	0.43647	0.967996733
PDZD2	ENSG00000133401	5	31629517	32121037	690	117	123834	0.15989	0.43648	0.967996733
GDAP2	ENSG00000196505	1	118396107	118482253	51	7	123834	0.15978	0.43653	0.967996733
ITFG1	ENSG00000129636	16	47178298	47508060	185	7	123834	0.15973	0.43655	0.967996733
CSGALNACT1	ENSG00000147408	8	19251672	19625540	736	62	123834	0.15969	0.43656	0.967996733
SERPINB10	ENSG00000242550	18	61554408	61613345	212	6	123834	0.15966	0.43657	0.967996733
TTC7B	ENSG00000165914	14	90996932	91292823	561	46	123834	0.15957	0.43661	0.967996733
CDX1	ENSG00000113722	5	149536358	149574120	49	9	123834	0.15943	0.43666	0.967996733
KCNN4	ENSG00000104783	19	44260685	44295409	63	10	123834	0.15917	0.43677	0.967996733
ANKRD13B	ENSG00000198720	17	27906787	27951779	14	3	123834	0.15903	0.43682	0.967996733
MUC17	ENSG00000169876	7	100653353	100712020	80	11	123834	0.15867	0.43697	0.967996733
SLC27A5	ENSG00000083807	19	58980879	59033780	40	7	123834	0.15857	0.437	0.967996733
LINC00955	ENSG00000216560	4	3568596	3602438	10	2	123834	0.15852	0.43702	0.967996733
CAPN12	ENSG00000182472	19	39210827	39270544	181	17	123834	0.15852	0.43702	0.967996733
SCAMP4	ENSG00000227500	19	1895213	1936016	60	9	123834	0.15836	0.43709	0.967996733
OPLAH	ENSG00000178814	8	145096167	145128735	18	2	123834	0.15819	0.43715	0.967996733
FAM208B	ENSG00000108021	10	5716801	5815703	121	13	123834	0.15815	0.43717	0.967996733
CENPT	ENSG00000102901	16	67852060	67891714	35	4	123834	0.15804	0.43721	0.967996733
ARF4	ENSG00000168374	3	57547090	57593947	105	11	123834	0.15779	0.43731	0.967996733
C10orf131	ENSG00000173088	10	97657360	97708480	106	16	123834	0.15766	0.43736	0.967996733
NEUROG2	ENSG00000178403	4	113424672	113447328	37	7	123834	0.15754	0.43741	0.967996733
TMEM101	ENSG00000091947	17	42078556	42111314	56	9	123834	0.15742	0.43746	0.967996733
LTK	ENSG00000062524	15	41785836	41816085	31	6	123834	0.15737	0.43748	0.967996733
TACR3	ENSG00000169836	4	104497188	104650973	278	17	123834	0.15724	0.43753	0.967996733
SPATS2L	ENSG00000196141	2	201160604	201356986	277	29	123834	0.15706	0.4376	0.967996733
GGCT	ENSG00000006625	7	30526237	30601095	95	11	123834	0.15702	0.43762	0.967996733
TRIM23	ENSG00000113595	5	64875507	64931802	134	9	123834	0.15697	0.43764	0.967996733
PSMB7	ENSG00000136930	9	127105745	127187723	54	7	123834	0.15658	0.43779	0.967996733
AP1M2	ENSG00000129354	19	10673347	10707991	94	10	123834	0.15649	0.43782	0.967996733
SH3PXD2A	ENSG00000107957	10	105338285	105625301	440	49	123834	0.15631	0.43789	0.967996733
PIFO	ENSG00000173947	1	111878910	111905635	112	16	123834	0.15625	0.43792	0.967996733
ERI3	ENSG00000117419	1	44676742	44830932	166	14	123834	0.15619	0.43794	0.967996733
SLC22A25	ENSG00000196600	11	62921248	63007124	206	9	123834	0.156	0.43802	0.967996733
PAFAH1B1	ENSG00000007168	17	2486504	2598909	94	11	123834	0.15597	0.43803	0.967996733
SLC8A1	ENSG00000183023	2	40314410	40848193	935	65	123834	0.15589	0.43806	0.967996733
ATOH7	ENSG00000179774	10	69980386	70001871	45	8	123834	0.15585	0.43808	0.967996733
OR5D13	ENSG00000198877	11	55530914	55551858	82	7	123834	0.15576	0.43811	0.967996733
TIMM10B	ENSG00000265264	11	6492715	6540208	84	7	123834	0.15571	0.43813	0.967996733
WDR16	ENSG00000166596	17	9469944	9556776	57	12	123834	0.15559	0.43818	0.967996733
PHB	ENSG00000167085	17	47471414	47502246	30	5	123834	0.15557	0.43819	0.967996733
KCNH4	ENSG00000089558	17	40298909	40343296	27	4	123834	0.15546	0.43823	0.967996733
XPO5	ENSG00000124571	6	43480072	43553812	79	7	123834	0.15532	0.43829	0.967996733
AP000350.4	ENSG00000218537	22	24226613	24251117	76	10	123834	0.15467	0.43854	0.967996733

PDHX	ENSG00000110435	11	34927376	35052138	459	19	123834	0.15443	0.43863	0.967996733
SLAMF9	ENSG00000162723	1	159911282	159934044	34	6	123834	0.15439	0.43865	0.967996733
SLC18B1	ENSG00000146409	6	133080509	133129701	143	9	123834	0.15436	0.43866	0.967996733
AREGB	ENSG00000205595	4	75470629	75500486	14	3	123834	0.15423	0.43871	0.967996733
CSF1R	ENSG00000182578	5	149422854	149502935	85	21	123834	0.15418	0.43873	0.967996733
GORAB	ENSG00000120370	1	170491270	170532587	22	3	123834	0.15404	0.43879	0.967996733
DYRK3	ENSG00000143479	1	206798881	206867764	73	13	123834	0.15388	0.43885	0.967996733
TRIM28	ENSG00000130726	19	59045458	59072089	24	6	123834	0.1538	0.43888	0.967996733
SLC6A3	ENSG00000142319	5	1382909	1455545	80	11	123834	0.15368	0.43893	0.967996733
KIFC3	ENSG00000140859	16	57782129	57906957	258	30	123834	0.15366	0.43894	0.967996733
PPOX	ENSG00000143224	1	161126200	161157803	25	5	123834	0.15361	0.43896	0.967996733
TDRD9	ENSG00000156414	14	104384799	104529004	399	14	123834	0.15362	0.43896	0.967996733
SLC25A23	ENSG00000125648	19	6426090	6475214	77	7	123834	0.15356	0.43898	0.967996733
HTR7	ENSG00000148680	10	92490578	92627671	272	13	123834	0.15319	0.43912	0.968043537
HGS	ENSG00000185359	17	79640356	79680168	139	10	123834	0.15301	0.43919	0.968043537
PPM1E	ENSG00000175175	17	56823230	57068983	277	13	123834	0.15282	0.43927	0.968043537
CYHR1	ENSG00000187954	8	145664965	145701060	17	4	123834	0.15244	0.43942	0.968043537
CCDC167	ENSG00000198937	6	37440696	37477698	54	9	123834	0.15216	0.43953	0.968043537
USO1	ENSG00000138768	4	76639777	76745382	179	10	123834	0.15202	0.43958	0.968043537
KIF2A	ENSG00000068796	5	61591989	61843076	410	15	123834	0.15205	0.43958	0.968043537
DLG4	ENSG00000132535	17	7083209	7133021	31	6	123834	0.15196	0.43961	0.968043537
STARD13	ENSG00000133121	13	33667272	33934767	347	33	123834	0.15192	0.43963	0.968043537
SMG8	ENSG00000167447	17	57276761	57302608	42	4	123834	0.15187	0.43965	0.968043537
ENTPD3	ENSG00000168032	3	40418647	40480110	87	2	123834	0.1518	0.43967	0.968043537
LZTS3	ENSG00000088899	20	3133263	3164192	27	6	123834	0.15181	0.43967	0.968043537
CLDN17	ENSG00000156282	21	31528241	31548971	49	4	123834	0.15163	0.43974	0.968043537
LSM12	ENSG00000161654	17	42102003	42154987	65	9	123834	0.15157	0.43976	0.968043537
HOXB13	ENSG00000159184	17	46792125	46816540	25	7	123834	0.15155	0.43977	0.968043537
FAM98C	ENSG00000130244	19	38883775	38909728	70	6	123834	0.15135	0.43985	0.968043537
RASSF7	ENSG00000099849	11	550404	574021	88	13	123834	0.15117	0.43992	0.968043537
C1orf192	ENSG00000188931	1	161324521	161347664	103	3	123834	0.15106	0.43996	0.968043537
DLX4	ENSG00000108813	17	48036334	48062321	17	5	123834	0.15087	0.44004	0.968043537
NCR3	ENSG00000204475	6	31546672	31570762	48	14	123834	0.15075	0.44009	0.968043537
RIN1	ENSG00000174791	11	66087713	66114311	27	3	123834	0.15056	0.44016	0.968043537
TINAGL1	ENSG00000142910	1	32032116	32063288	10	5	123834	0.15042	0.44022	0.968043537
PRSS21	ENSG00000007038	16	2857164	2886305	4	2	123834	0.15023	0.44029	0.968043537
NEFH	ENSG00000100285	22	29866219	29897379	33	6	123834	0.15015	0.44032	0.968043537
TRIM55	ENSG00000147573	8	67029131	67097720	118	8	123834	0.15013	0.44033	0.968043537
UGT3A1	ENSG00000145626	5	35941112	36011130	114	13	123834	0.15009	0.44035	0.968043537
B2M	ENSG00000166710	15	44993675	45021075	44	5	123834	0.15	0.44038	0.968043537
TARS	ENSG00000113407	5	33430802	33479644	102	11	123834	0.14987	0.44043	0.968043537
RFXANK	ENSG00000064490	19	19293008	19322678	28	6	123834	0.14974	0.44048	0.968043537
POLR1C	ENSG00000171453	6	43467440	43507323	47	6	123834	0.14963	0.44053	0.968043537
AP000295.9	ENSG00000249624	21	34609079	34665517	85	10	123834	0.14949	0.44058	0.968043537
HEY1	ENSG00000164683	8	80666245	80690098	13	5	123834	0.149	0.44078	0.968300994
CSNK2A3	ENSG00000254598	11	11363489	11384904	9	3	123834	0.14888	0.44082	0.968300994
TIMM23	ENSG00000138297	10	51582080	51633365	10	3	123834	0.14881	0.44085	0.968300994
AKR1A1	ENSG00000117448	1	46006215	46045721	55	7	123834	0.14853	0.44096	0.968430697
ACTR1A	ENSG00000138107	10	104228986	104272482	105	6	123834	0.14819	0.4411	0.968514358
ZDHHC8	ENSG00000099904	22	20106979	20145530	94	7	123834	0.14818	0.4411	0.968514358
DZIP3	ENSG00000198919	3	108298529	108423693	129	10	123834	0.14792	0.4412	0.968571832
MCPH1	ENSG00000147316	8	6254113	6511144	788	52	123834	0.14771	0.44129	0.968571832
KHDRBS3	ENSG00000131773	8	136459700	136678965	362	19	123834	0.1477	0.44129	0.968571832
SCGB1D4	ENSG00000197745	11	62053754	62076536	16	3	123834	0.1476	0.44133	0.968571832
STAT4	ENSG00000138378	2	191884302	192026322	128	17	123834	0.14722	0.44148	0.968789176
STAMBPL1	ENSG00000138134	10	90629491	90744910	166	19	123834	0.14684	0.44163	0.968921402
AC108868.1	ENSG00000269000	2	107089728	107113848	32	6	123834	0.14638	0.44181	0.968921402
DPP9	ENSG00000142002	19	4665236	4734685	100	14	123834	0.14636	0.44182	0.968921402
NUDCD1	ENSG00000120526	8	110243148	110356614	210	12	123834	0.14631	0.44184	0.968921402
ZNF584	ENSG00000171574	19	58902871	58939694	66	5	123834	0.14597	0.44197	0.968921402
2-Mar	ENSG00000117791	1	220911567	220968150	104	10	123834	0.14591	0.442	0.968921402
PPP1R3G	ENSG00000219607	6	5075720	5097455	18	6	123834	0.14581	0.44204	0.968921402
LYRM2	ENSG00000083099	6	90267863	90358472	112	14	123834	0.1458	0.44204	0.968921402
PNPLA3	ENSG00000100344	22	44309619	44370368	137	12	123834	0.1458	0.44204	0.968921402
NEK7	ENSG00000151414	1	198116093	198301550	490	20	123834	0.14576	0.44205	0.968921402
RNF14	ENSG00000013561	5	141327893	141379856	118	7	123834	0.14555	0.44214	0.968960959
SPPL2C	ENSG00000185294	17	43912256	43934438	100	3	123834	0.14546	0.44217	0.968960959
SFTPC	ENSG00000168484	8	22004426	22031992	10	2	123834	0.14531	0.44223	0.968980731
KRTAP4-7	ENSG00000240871	17	39230459	39251396	118	3	123834	0.14459	0.44252	0.969255062
TMEM151A	ENSG00000179292	11	66049341	66074135	8	4	123834	0.14454	0.44254	0.969255062
STAP1	ENSG00000035720	4	68414446	68483055	131	17	123834	0.14441	0.44259	0.969255062
HIF3A	ENSG00000124440	19	46790303	46856690	151	20	123834	0.14438	0.4426	0.969255062
CBFA2T2	ENSG00000078699	20	32067881	32247842	44	6	123834	0.14427	0.44264	0.969255062
ZNF792	ENSG00000180884	19	35437258	35464953	47	7	123834	0.14339	0.44299	0.969255062
CHCHD10	ENSG00000250479	22	24098021	24120630	16	4	123834	0.14334	0.44301	0.969255062
COMMD3	ENSG00000148444	10	22594903	22619235	2	1	123834	0.14319	0.44307	0.969255062
COMMD3-BMI1	ENSG00000269897	10	22595317	22628471	2	1	123834	0.14319	0.44307	0.969255062
BMI1	ENSG00000168283	10	22600140	22630413	2	1	123834	0.14319	0.44307	0.969255062
CCDC150	ENSG00000144395	2	197494278	197638214	192	10	123834	0.14312	0.4431	0.969255062
TKTL2	ENSG00000151005	4	164382257	164405047	32	6	123834	0.14305	0.44313	0.969255062
SULT1A2	ENSG00000197165	16	28593264	28618430	23	3	123834	0.14289	0.44319	0.969255062
SYT9	ENSG00000170743	11	7250009	7500273	497	61	123834	0.14282	0.44321	0.969255062
OGFOD3	ENSG00000181396	17	80337099	80386513	87	10	123834	0.14284	0.44321	0.969255062
ATP6V1E1	ENSG00000131100	22	18064902	18121584	179	11	123834	0.14276	0.44324	0.969255062
TMEM132A	ENSG00000006118	11	60681935	60714631	60	5	123834	0.14274	0.44325	0.969255062
EIF2S3L	ENSG00000180574	12	10648201	10685734	114	6	123834	0.14246	0.44336	0.969255062
RBM47	ENSG00000163694	4	40415272	40642892	204	49	123834	0.14237	0.44339	0.969255062
DPP4	ENSG00000197635	2	162838751	162941052	121	12	123834	0.14226	0.44344	0.969255062
THEM4	ENSG00000159445	1	151836060	151892284	97	6	123834	0.14214	0.44348	0.969255062
C14orf142	ENSG00000170270	14	93659239	93683439	42	8	123834	0.14212	0.44349	0.969255062
PMF1-BGLAP	ENSG00000260238	1	156172773	156223123	56	5	123834	0.14198	0.44355	0.969255062
CRYAB	ENSG00000109846	11	111769289	111804446	30	4	123834	0.14187	0.44359	0.969255062
C17orf51	ENSG00000212719	17	21418051	21487722	24	1	123834	0.14177	0.44363	0.969255062
PKIA	ENSG00000171033	8	79418374	79527502	174	12	123834	0.14092	0.44397	0.969774967
C19orf35	ENSG00000188305	19	2264631	2292181	48	8	123834	0.14092	0.44397	0.969774967
CNNM2	ENSG00000148842	10	104668050	104859978	386	16	123834	0.14076	0.44403	0.969794581
SNRPG	ENSG00000143977	2	70498494	70530903	43	4	123834	0.14036	0.44419	0.970023555
ATP6V1F	ENSG00000128524	7	128492880	128515898	48	5	123834	0.1402	0.44425	0.970023555
CDC73	ENSG00000134371	1	193081147	193233031	265	10	123834	0.13999	0.44433	0.970023555

SERPINA11	ENSG00000186910	14	94898801	94929127	114	10	123834	0.13989	0.44437	0.970023555
FSTL3	ENSG00000070404	19	666392	693385	72	10	123834	0.13984	0.44439	0.970023555
MYF5	ENSG00000111049	12	81100691	81123452	19	4	123834	0.13959	0.44449	0.970029716
BLOC1S4	ENSG00000186222	4	6707842	6729387	10	4	123834	0.13926	0.44462	0.970029716
KRTAP4-8	ENSG00000204880	17	39243233	39264393	157	7	123834	0.13924	0.44463	0.970029716
SFXN2	ENSG00000156398	10	104464295	104513249	57	8	123834	0.13891	0.44476	0.970029716
PRMT3	ENSG00000185238	11	20399076	20540840	260	12	123834	0.13857	0.44489	0.970029716
CDC16	ENSG00000130177	13	114990362	115048198	101	9	123834	0.13853	0.44491	0.970029716
UNC119B	ENSG00000175970	12	121138238	121171443	87	12	123834	0.13848	0.44493	0.970029716
RYR2	ENSG00000198626	1	237195505	238007288	1208	131	123834	0.13836	0.44498	0.970029716
COX4I1	ENSG00000131143	16	85822239	85850650	31	4	123834	0.13809	0.44508	0.970029716
KRI1	ENSG00000129347	19	10653761	10686713	81	11	123834	0.13785	0.44518	0.970029716
SNX7	ENSG00000162627	1	99117236	99236056	295	15	123834	0.13778	0.44521	0.970029716
MAS1	ENSG00000130368	6	160317974	160339108	26	7	123834	0.13759	0.44528	0.970029716
ZNF572	ENSG00000180938	8	125975540	126001631	60	2	123834	0.13751	0.44531	0.970029716
RLN2	ENSG00000107014	9	5289868	5314969	15	2	123834	0.13734	0.44538	0.970029716
FAM179A	ENSG00000189350	2	29169477	29294239	268	22	123834	0.13693	0.44554	0.970029716
DGCR6	ENSG00000183628	22	18883541	18911751	61	11	123834	0.13673	0.44562	0.970029716
MYOF	ENSG00000138119	10	95056186	95252074	430	36	123834	0.13669	0.44564	0.970029716
FOXE3	ENSG00000186790	1	47871744	47893723	32	4	123834	0.1366	0.44568	0.970029716
BBS1	ENSG00000174483	11	66268077	66311098	67	4	123834	0.13658	0.44568	0.970029716
SPAG16	ENSG00000144451	2	214139113	215285225	2224	58	123834	0.13655	0.44569	0.970029716
ATG9B	ENSG00000181652	7	150699297	150731586	21	8	123834	0.13647	0.44573	0.970029716
PRSS35	ENSG00000146250	6	84212194	84245423	48	7	123834	0.13639	0.44576	0.970029716
PTGFRN	ENSG00000134247	1	117442679	117542980	191	18	123834	0.13627	0.4458	0.970029716
NCBP1	ENSG00000136937	9	100385908	100446030	31	7	123834	0.13614	0.44586	0.970029716
ZNF597	ENSG00000167981	16	3476104	3503542	47	3	123834	0.13613	0.44586	0.970029716
ANTXRL	ENSG00000198250	10	47647581	47711443	74	11	123834	0.13606	0.44588	0.970029716
CRHR1	ENSG00000120088	17	43689267	43923194	844	6	123834	0.13605	0.44589	0.970029716
PAPOLB	ENSG00000218823	7	4887364	4911625	59	8	123834	0.13602	0.4459	0.970029716
GOLGA6L2	ENSG00000174450	15	23674645	23702381	40	4	123834	0.13598	0.44592	0.970029716
KAT6A	ENSG00000083168	8	41776997	41919508	127	9	123834	0.13593	0.44594	0.970029716
OC90	ENSG00000253117	8	133026467	133081627	121	13	123834	0.13568	0.44604	0.970029716
RP4-576H24.4	ENSG00000260861	20	1510790	1610655	129	17	123834	0.13554	0.44609	0.970029716
KLF7	ENSG00000118263	2	207928861	208041991	136	24	123834	0.13551	0.4461	0.970029716
CIB2	ENSG00000136425	15	78386948	78433886	93	13	123834	0.13543	0.44614	0.970029716
MAP4K5	ENSG00000012983	14	50875219	51037844	167	13	123834	0.13532	0.44618	0.970029716
MYO3A	ENSG00000095777	10	26213196	26511456	1040	16	123834	0.13519	0.44623	0.970029716
CYP4Z1	ENSG00000186160	1	47523160	47593991	154	5	123834	0.1347	0.44643	0.970353509
DMC1	ENSG00000100206	22	38904954	38976291	73	11	123834	0.13447	0.44652	0.970438161
LRCH1	ENSG00000136141	13	47117303	47337175	374	23	123834	0.13383	0.44677	0.970526118
RYR3	ENSG00000198838	15	33593163	34168303	1060	102	123834	0.1338	0.44678	0.970526118
AC017081.1	ENSG00000269633	2	207210463	207230843	22	3	123834	0.13375	0.4468	0.970526118
CCDC158	ENSG00000163749	4	77224154	77353021	163	13	123834	0.13341	0.44694	0.970526118
EIF1B	ENSG00000114784	3	40341175	40363915	33	2	123834	0.13338	0.44695	0.970526118
AC008914.1	ENSG00000268280	5	55214468	55234569	10	4	123834	0.13337	0.44695	0.970526118
STX7	ENSG00000079950	6	132757006	132844337	155	20	123834	0.13324	0.447	0.970526118
AC006116.20	ENSG00000267710	19	56774138	56831819	63	14	123834	0.13324	0.447	0.970526118
AMN1	ENSG00000151743	12	31814071	31892108	142	20	123834	0.1332	0.44702	0.970526118
SPHAR	ENSG00000213029	1	229430129	229451248	31	4	123834	0.13278	0.44718	0.97055051
RP11-159D12.5	ENSG00000266086	17	56056399	56092614	40	3	123834	0.13249	0.4473	0.97055051
ATP8B4	ENSG00000104043	15	50140435	50485014	597	44	123834	0.13241	0.44733	0.97055051
CSNK1A1L	ENSG00000180138	13	37667398	37689803	71	4	123834	0.13233	0.44736	0.97055051
B4GALT3	ENSG00000158850	1	161131100	161157287	20	5	123834	0.13219	0.44742	0.97055051
MTFR1	ENSG00000066855	8	66546969	66693496	284	14	123834	0.13218	0.44742	0.97055051
TAS2R10	ENSG00000121318	12	10967916	10988957	23	4	123834	0.13192	0.44752	0.97055051
STK32B	ENSG00000152953	4	5043169	5512725	1293	52	123834	0.13154	0.44767	0.97055051
NR6A1	ENSG00000148200	9	127269554	127543589	65	2	123834	0.13154	0.44767	0.97055051
PSMD6	ENSG00000163636	3	63986225	64019658	32	6	123834	0.13142	0.44772	0.97055051
WDR3	ENSG00000065183	1	118462343	118519165	37	6	123834	0.13135	0.44775	0.97055051
FUT2	ENSG00000176920	19	49189228	49219207	44	5	123834	0.13134	0.44775	0.97055051
GPR139	ENSG00000180269	16	20032807	20095239	138	16	123834	0.13124	0.44779	0.97055051
WSCD1	ENSG00000179314	17	5665554	6037747	515	75	123834	0.13114	0.44783	0.97055051
C5AR2	ENSG00000134830	19	47825404	47856606	77	3	123834	0.13087	0.44794	0.97055051
CBR3	ENSG00000159231	21	37497210	37528864	109	11	123834	0.13085	0.44795	0.97055051
MPPE1	ENSG00000154889	18	11872621	11919222	84	16	123834	0.13079	0.44797	0.97055051
GPR141	ENSG00000187037	7	37713399	37883390	336	26	123834	0.13053	0.44807	0.97055051
NTMT1	ENSG00000148335	9	132361163	132408209	103	13	123834	0.13017	0.44822	0.97055051
PINK1	ENSG00000158828	1	20949948	20988004	103	10	123834	0.13004	0.44827	0.97055051
PPP1R21	ENSG00000162869	2	48657737	48752525	185	15	123834	0.12992	0.44832	0.97055051
MROH7-TTC4	ENSG00000271723	1	55097459	55217981	265	24	123834	0.12967	0.44841	0.97055051
CEACAM3	ENSG00000170956	19	42290369	42325591	49	2	123834	0.12967	0.44841	0.97055051
TFCP2	ENSG00000135457	12	51477446	51576926	206	17	123834	0.12962	0.44843	0.97055051
OR56A4	ENSG00000183389	11	6013216	6034424	64	7	123834	0.12947	0.44849	0.97055051
HMCN2	ENSG00000148357	9	133036882	133319510	217	46	123834	0.12945	0.4485	0.97055051
C14orf144	ENSG00000228032	14	104700541	104722473	59	5	123834	0.12944	0.4485	0.97055051
TRPM7	ENSG00000092439	15	50834670	50989012	374	14	123834	0.1293	0.44856	0.97055051
FBXL4	ENSG00000112234	6	99306420	99405849	129	12	123834	0.12924	0.44858	0.97055051
RBM48	ENSG00000127993	7	92148087	92177319	46	5	123834	0.12879	0.44876	0.97055051
CCDC102B	ENSG00000150636	18	66372446	66732426	773	43	123834	0.12877	0.44877	0.97055051
ZNF846	ENSG00000196605	19	9852669	9913856	91	6	123834	0.12864	0.44882	0.97055051
OR2A4	ENSG00000180658	6	132011520	132032635	28	4	123834	0.12854	0.44886	0.97055051
HSPE1-MOB4	ENSG00000270757	2	198355137	198425450	59	6	123834	0.1283	0.44896	0.97055051
NDUFS1	ENSG00000023228	2	206969541	207034327	139	9	123834	0.12824	0.44898	0.97055051
FBLL1	ENSG00000188573	5	167946121	167967639	33	5	123834	0.12817	0.44901	0.97055051
ECI1	ENSG00000167969	16	2279396	2312301	22	4	123834	0.12808	0.44904	0.97055051
INTS12	ENSG00000138785	4	106593784	106827143	404	10	123834	0.12785	0.44914	0.97055051
ZNF440	ENSG00000171295	19	11915099	11956016	91	3	123834	0.1278	0.44915	0.97055051
KIAA1644	ENSG00000138944	22	44629547	44718731	89	17	123834	0.1278	0.44915	0.97055051
HADHB	ENSG00000138029	2	26456038	26523336	76	4	123834	0.12769	0.4492	0.97055051
ADAMTS19	ENSG00000145808	5	128785958	129084376	608	24	123834	0.1276	0.44923	0.97055051
MRPL14	ENSG00000180992	6	44071194	44105194	100	12	123834	0.12748	0.44928	0.97055051
AC006967.1	ENSG00000269446	7	156892674	156913764	49	5	123834	0.12733	0.44934	0.97055051
GRM7	ENSG00000196277	3	6801688	7793215	1360	126	123834	0.12723	0.44938	0.97055051
OR51B6	ENSG00000176239	11	5362738	5383676	137	10	123834	0.12723	0.44938	0.97055051
RHO	ENSG00000163914	3	129237483	129264012	40	4	123834	0.12702	0.44946	0.970589117
RP11-81K2.1	ENSG00000262039	17	47438102	47564350	124	19	123834	0.12692	0.4495	0.970589117
C11orf35	ENSG00000185522	11	544855	570779	78	14	123834	0.12669	0.44959	0.970673185
1-Sep	ENSG00000180096	16	30379454	30417312	24	2	123834	0.12639	0.44971	0.970821997

RAD51	ENSG00000051180	15	40976972	41034354	85	6	123834	0.12602	0.44986	0.970899035
HEPN1	ENSG00000221932	11	124779146	124800573	27	10	123834	0.12595	0.44988	0.970899035
ACSS3	ENSG00000111058	12	81321594	81660533	775	27	123834	0.12583	0.44993	0.970899035
ACACB	ENSG00000076555	12	109544400	109716031	327	28	123834	0.12579	0.44995	0.970899035
GPX5	ENSG00000224586	6	28483702	28512729	37	8	123834	0.12543	0.45009	0.970969002
CD44	ENSG00000026508	11	35150417	35263949	180	30	123834	0.12533	0.45013	0.970969002
DHX38	ENSG00000140829	16	72117461	72156811	100	9	123834	0.12529	0.45015	0.970969002
FAM177A1	ENSG00000151327	14	35504113	35592336	268	17	123834	0.12508	0.45023	0.970969002
TMEM150B	ENSG00000180061	19	55814169	55855416	81	11	123834	0.12489	0.45031	0.970969002
FAM136A	ENSG00000035141	2	70513107	70539222	28	2	123834	0.1248	0.45034	0.970969002
BTNL9	ENSG00000165810	5	180457225	180498523	78	11	123834	0.12479	0.45034	0.970969002
OR6C3	ENSG00000205329	12	55715485	55736420	65	5	123834	0.12398	0.45066	0.971548744
VWC2	ENSG00000188730	7	49803257	49971546	219	16	123834	0.12363	0.4508	0.971692408
CYB5R2	ENSG00000166394	11	7676331	7708453	98	13	123834	0.12354	0.45084	0.971692408
TRIO	ENSG00000038382	5	14133811	14542235	446	34	123834	0.12344	0.45088	0.971692408
AC026310.1	ENSG00000268865	12	25045243	25067615	36	8	123834	0.12302	0.45104	0.971804726
PSD	ENSG00000059915	10	104152376	104191296	36	11	123834	0.12286	0.45111	0.971804726
C22orf31	ENSG00000100249	22	29444660	29467832	36	6	123834	0.1227	0.45117	0.971804726
SCYL3	ENSG00000000457	1	169808772	169873408	149	11	123834	0.12256	0.45123	0.971804726
NANP	ENSG00000170191	20	25583571	25614811	80	7	123834	0.12253	0.45124	0.971804726
MAP3K7	ENSG00000135341	6	91213292	91306764	214	16	123834	0.1225	0.45125	0.971804726
WDSUB1	ENSG00000196151	2	160082304	160153310	227	13	123834	0.1224	0.45129	0.971804726
FGD5	ENSG00000154783	3	14850469	14985895	345	23	123834	0.12218	0.45138	0.971883561
NCS1	ENSG00000107130	9	132924857	133009583	157	26	123834	0.12195	0.45147	0.971883561
CASC10	ENSG00000204682	10	21771587	21796191	12	4	123834	0.12192	0.45148	0.971883561
CTD-2162K18.4	ENSG00000267260	19	37254055	37277980	43	7	123834	0.12127	0.45174	0.972333136
RTTN	ENSG00000176225	18	67661029	67883181	409	15	123834	0.12089	0.45189	0.97254587
VCAM1	ENSG00000162692	1	101175298	101214601	24	8	123834	0.12064	0.45199	0.972597868
SYNPO2L	ENSG00000166317	10	75394639	75433561	18	2	123834	0.12047	0.45206	0.972597868
ZFAND5	ENSG00000107372	9	74956341	74990163	58	5	123834	0.12024	0.45215	0.972597868
RP11-1099M24.7	ENSG00000262730	17	7819249	7843940	25	5	123834	0.1202	0.45216	0.972597868
C1orf172	ENSG00000175707	1	27266053	27296897	22	3	123834	0.12017	0.45217	0.972597868
CCT8	ENSG00000156261	21	30418126	30456118	43	8	123834	0.11984	0.45231	0.972743378
RPP30	ENSG00000148688	10	92621473	92678312	72	8	123834	0.11976	0.45234	0.972743378
ZNF200	ENSG00000010539	16	3262325	3296221	53	6	123834	0.11922	0.45255	0.973084887
SNRPD2	ENSG00000125743	19	46180712	46205827	23	5	123834	0.11889	0.45268	0.97325432
RHOC	ENSG00000155366	1	113233728	113260056	26	5	123834	0.11851	0.45283	0.973373174
ENDOV	ENSG00000173818	17	78378965	78421886	94	17	123834	0.11811	0.45299	0.973373174
MOB4	ENSG00000115540	2	198370295	198428423	47	5	123834	0.11806	0.45301	0.973373174
CTD-3074O7.11	ENSG00000256349	11	66266550	66311084	69	4	123834	0.11798	0.45304	0.973373174
C9orf96	ENSG00000198870	9	136233117	136281220	134	5	123834	0.11788	0.45308	0.973373174
PATE3	ENSG00000236027	11	125648006	125671495	69	7	123834	0.11768	0.45316	0.973373174
CHRM2	ENSG00000181072	7	136543416	136715002	259	19	123834	0.11755	0.45321	0.973373174
LYPD6B	ENSG00000150556	2	149884621	150081776	270	19	123834	0.11753	0.45322	0.973373174
RP11-1021N1.1	ENSG00000261130	16	15479611	15619205	73	13	123834	0.11704	0.45342	0.973373174
ACOX2	ENSG00000168306	3	58480863	58533046	56	12	123834	0.11688	0.45348	0.973373174
SVIP	ENSG00000198168	11	22825345	22861845	17	3	123834	0.11683	0.4535	0.973373174
SOX11	ENSG00000176887	2	5822799	5851516	27	6	123834	0.11675	0.45353	0.973373174
DCTN1	ENSG00000204843	2	74578281	74629214	43	4	123834	0.11651	0.45362	0.973373174
FAM89A	ENSG00000182118	1	231144704	231185992	74	19	123834	0.11642	0.45366	0.973373174
ITGB1BP1	ENSG00000119185	2	9533604	9573676	71	8	123834	0.11631	0.45371	0.973373174
C10orf115	ENSG00000204566	10	23482745	23538745	38	6	123834	0.11627	0.45372	0.973373174
OR7A17	ENSG00000185385	19	14981138	15002264	76	10	123834	0.1161	0.45379	0.973373174
SOX17	ENSG00000164736	8	55360495	55383448	27	5	123834	0.1159	0.45387	0.973373174
EFCAB8	ENSG00000215529	20	31436729	31559006	176	22	123834	0.11584	0.45389	0.973373174
INTS1	ENSG00000164880	7	1499913	1555489	76	2	123834	0.11571	0.45394	0.973373174
PIGW	ENSG00000184886	17	34880847	34905159	40	5	123834	0.11572	0.45394	0.973373174
AC011530.4	ENSG00000268434	19	46272695	46299231	25	4	123834	0.11529	0.45411	0.973373174
MPDZ	ENSG00000107186	9	13095703	13289589	92	19	123834	0.11509	0.45419	0.973373174
ALKBH3	ENSG00000166199	11	43892361	43951816	149	14	123834	0.11503	0.45421	0.973373174
ZNF33B	ENSG00000196693	10	43074555	43143992	129	10	123834	0.115	0.45422	0.973373174
CATSPER3	ENSG00000152705	5	134293596	134357392	29	6	123834	0.11496	0.45424	0.973373174
ERICH2	ENSG00000204334	2	171630296	171665481	46	4	123834	0.11489	0.45427	0.973373174
EHBP1L1	ENSG00000173442	11	65333509	65370121	27	4	123834	0.11468	0.45435	0.973373174
KIAA1407	ENSG00000163617	3	113672984	113785460	54	4	123834	0.11455	0.4544	0.973373174
HDAC5	ENSG00000108840	17	42144114	42211070	64	6	123834	0.1144	0.45446	0.973373174
EDN2	ENSG00000127129	1	41934446	41960344	17	5	123834	0.11438	0.45447	0.973373174
ANGPT1	ENSG00000154188	8	108251721	108520283	542	38	123834	0.11427	0.45451	0.973373174
PRSS45	ENSG00000188086	3	46773582	46864048	205	7	123834	0.11408	0.45459	0.973373174
CRTC3	ENSG00000140577	15	91063157	91198577	240	15	123834	0.11408	0.45459	0.973373174
FAM132A	ENSG00000184163	1	1167826	1192102	52	7	123834	0.11403	0.45461	0.973373174
IL1RL2	ENSG00000115598	2	102793433	102866462	200	13	123834	0.114	0.45462	0.973373174
C1orf233	ENSG00000228594	1	1523392	1545476	7	3	123834	0.11397	0.45463	0.973373174
POGK	ENSG00000143157	1	166798681	166835581	21	3	123834	0.11383	0.45469	0.973391995
AHCTF1	ENSG00000153207	1	246992400	247105280	123	12	123834	0.1136	0.45478	0.97342703
RPL26L1	ENSG00000037241	5	172375732	172406774	76	9	123834	0.11347	0.45483	0.97342703
AL590714.1	ENSG00000268387	1	161180160	161202296	20	6	123834	0.1134	0.45486	0.97342703
LSP1	ENSG00000269099	13	25581541	25601675	62	5	123834	0.11306	0.45499	0.973595623
RP11-293M10.1	ENSG00000258740	14	75695319	75745986	59	11	123834	0.11289	0.45506	0.973635804
CALM1	ENSG00000198668	14	90852846	90884605	25	4	123834	0.11252	0.45521	0.973836787
NMRK2	ENSG00000077009	19	3923101	3952414	12	4	123834	0.11228	0.4553	0.973836787
SPIC	ENSG00000166211	12	101859199	101890775	60	10	123834	0.11207	0.45538	0.973836787
UPK3B	ENSG00000243566	7	76129745	76658340	610	46	123834	0.11194	0.45544	0.973836787
YPEL4	ENSG00000166793	11	57402560	57427417	11	2	123834	0.1119	0.45545	0.973836787
KRT77	ENSG00000189182	12	53073410	53107247	65	11	123834	0.1117	0.45553	0.973836787
TMEM92	ENSG00000167105	17	48338767	48368844	19	9	123834	0.1117	0.45553	0.973836787
FBXL18	ENSG00000155034	7	5460966	5563429	199	27	123834	0.11128	0.4557	0.973836787
CELF6	ENSG00000140488	15	72567068	72622470	80	7	123834	0.11119	0.45573	0.973836787
LAMC2	ENSG00000058085	1	183145373	183224035	138	11	123834	0.11106	0.45579	0.973836787
PINLYP	ENSG00000234465	19	44070952	44098116	36	9	123834	0.11067	0.45594	0.973836787
USE1	ENSG00000053501	19	17316155	17340638	54	11	123834	0.11031	0.45608	0.973836787
NLRP12	ENSG00000142405	19	54286857	54337648	88	17	123834	0.11028	0.4561	0.973836787
HSPE1	ENSG00000115541	2	198354718	198378181	21	3	123834	0.11019	0.45613	0.973836787
AP1S1	ENSG00000106367	7	100787678	100814877	36	6	123834	0.10984	0.45627	0.973836787
PRMT2	ENSG00000160310	21	48045079	48095036	97	10	123834	0.10973	0.45631	0.973836787
PRB2	ENSG00000121335	12	11534476	11663975	297	16	123834	0.10955	0.45638	0.973836787
NOTCH3	ENSG00000074181	19	15260444	15321792	102	5	123834	0.10944	0.45643	0.973836787
DUS3L	ENSG00000141994	19	5774843	5801249	62	6	123834	0.10915	0.45654	0.973836787
FLT4	ENSG00000037280	5	180018506	180086624	130	20	123834	0.10897	0.45661	0.973836787

OAZ1	ENSG00000104904	19	2259485	2283487	47	8	123834	0.10891	0.45664	0.973836787
MPZL1	ENSG00000197965	1	167680429	167771156	122	15	123834	0.1087	0.45672	0.973836787
CAV2	ENSG00000105971	7	115917434	116158595	459	32	123834	0.10863	0.45675	0.973836787
GCC2	ENSG00000135968	2	109055017	109135871	223	12	123834	0.1084	0.45684	0.973836787
ADAMTSL4-AS1	ENSG00000203804	1	150523480	150543969	19	5	123834	0.10836	0.45685	0.973836787
LMBRD2	ENSG00000164187	5	36088514	36162063	69	10	123834	0.10818	0.45693	0.973836787
BRIX1	ENSG00000113460	5	34905481	34936101	36	6	123834	0.10804	0.45698	0.973836787
TIA1	ENSG00000116001	2	70426576	70485792	89	4	123834	0.10796	0.45701	0.973836787
GABRA1	ENSG00000022355	5	161264197	161336975	148	14	123834	0.10796	0.45701	0.973836787
AC104809.3	ENSG00000226321	2	241848202	241942728	97	11	123834	0.10765	0.45714	0.973836787
RING1	ENSG00000204227	6	33166272	33190499	23	6	123834	0.1075	0.4572	0.973836787
MBD4	ENSG00000129071	3	129139787	129168878	54	3	123834	0.1071	0.45736	0.973836787
ZNF207	ENSG00000010244	17	30667136	30718905	49	7	123834	0.1071	0.45736	0.973836787
KRT3	ENSG00000186442	12	53173469	53199901	50	13	123834	0.10705	0.45738	0.973836787
SLC34A2	ENSG00000157765	4	25646923	25690370	38	11	123834	0.10696	0.45741	0.973836787
SCFD1	ENSG00000092108	14	31081318	31215018	221	15	123834	0.10679	0.45748	0.973836787
ATP5G2	ENSG00000135390	12	54016510	54081192	142	9	123834	0.10673	0.4575	0.973836787
CHL1	ENSG00000134121	3	228279	461090	388	43	123834	0.10658	0.45756	0.973836787
HLA-C	ENSG00000204525	6	31226526	31249907	568	25	123834	0.10648	0.4576	0.973836787
TFAP2E	ENSG00000116819	1	36028971	36070929	21	3	123834	0.10645	0.45761	0.973836787
GFAP	ENSG00000131095	17	42972376	43004305	73	14	123834	0.10602	0.45778	0.973836787
CTD-2267D19.3	ENSG00000266208	17	38506907	38530945	8	2	123834	0.10584	0.45785	0.973836787
GJD3	ENSG00000183153	17	38507235	38530067	8	2	123834	0.10584	0.45785	0.973836787
KRTAP5-3	ENSG00000196224	11	1618795	1639693	70	8	123834	0.10572	0.4579	0.973836787
TMEM14B	ENSG00000137210	6	10737992	10862986	117	19	123834	0.10566	0.45793	0.973836787
AC132872.2	ENSG00000268852	17	80204131	80226080	3	2	123834	0.10538	0.45804	0.973836787
KRTAP4-6	ENSG00000198090	17	39285685	39306739	42	6	123834	0.10534	0.45805	0.973836787
MB21D1	ENSG00000164430	6	74113238	74171999	68	14	123834	0.10532	0.45806	0.973836787
MRPL17	ENSG00000158042	11	6692013	6714632	49	4	123834	0.10513	0.45813	0.973836787
BMP2K	ENSG00000138756	4	79687496	79847526	184	4	123834	0.10483	0.45826	0.973836787
LAYN	ENSG00000204381	11	111401005	111442264	44	6	123834	0.10481	0.45826	0.973836787
MROH1	ENSG00000179832	8	145192919	145326843	50	3	123834	0.10476	0.45828	0.973836787
ROM1	ENSG00000149489	11	62369194	62392592	13	2	123834	0.10467	0.45832	0.973836787
SMG1	ENSG00000157106	16	18806175	18947776	261	10	123834	0.1046	0.45835	0.973836787
NDNF	ENSG00000173376	4	121946768	122004176	108	13	123834	0.10443	0.45841	0.973836787
RP11-111M22.2	ENSG00000179240	11	76082357	76135663	91	14	123834	0.10434	0.45845	0.973836787
VAMP5	ENSG00000168899	2	85801531	85830535	56	9	123834	0.1043	0.45846	0.973836787
RMDN3	ENSG00000137824	15	41018082	41058049	42	4	123834	0.10419	0.45851	0.973836787
YTHDC1	ENSG00000083896	4	69166105	69225807	47	6	123834	0.10409	0.45855	0.973836787
SLC39A3	ENSG00000141873	19	2722202	2750150	31	6	123834	0.10408	0.45855	0.973836787
C2orf71	ENSG00000179270	2	29273842	29307127	52	8	123834	0.10397	0.4586	0.973836787
RFTN2	ENSG00000162944	2	198422948	198550769	138	8	123834	0.10381	0.45866	0.973836787
TMC8	ENSG00000167895	17	76116851	76149049	51	9	123834	0.10353	0.45877	0.973836787
RNF165	ENSG00000141622	18	43896772	44053103	168	24	123834	0.10345	0.4588	0.973836787
F2	ENSG00000180210	11	46730730	46771056	54	6	123834	0.1032	0.4589	0.973836787
NUPR1L	ENSG00000185290	7	56172374	56194093	69	12	123834	0.10308	0.45895	0.973836787
BCL2L1	ENSG00000171552	20	30242255	30321792	59	5	123834	0.10305	0.45896	0.973836787
FAM186A	ENSG00000185958	12	50710013	50800405	115	7	123834	0.10303	0.45897	0.973836787
TMC1	ENSG00000165091	9	75126717	75461267	703	25	123834	0.10288	0.45903	0.973836787
CDH11	ENSG00000140937	16	64967656	65170015	417	32	123834	0.10258	0.45915	0.973836787
CEP72	ENSG00000112877	5	602387	677283	173	15	123834	0.10245	0.4592	0.973836787
ZFYVE20	ENSG00000131381	3	15101580	15150670	81	6	123834	0.10241	0.45922	0.973836787
ZC3H7B	ENSG00000100403	22	41687526	41766151	68	5	123834	0.10238	0.45923	0.973836787
CCDC81	ENSG00000149201	11	86075778	86144151	159	19	123834	0.10192	0.45941	0.973836787
ERMP1	ENSG00000099219	9	5755076	5843117	124	11	123834	0.10188	0.45943	0.973836787
HSPA6	ENSG00000173110	1	161484036	161506681	14	2	123834	0.10151	0.45957	0.973836787
KCNAB3	ENSG00000170049	17	7815177	7843121	33	5	123834	0.10081	0.45985	0.973836787
TERF2IP	ENSG00000166848	16	75671684	75805770	297	14	123834	0.10043	0.46	0.973836787
LRRC4B	ENSG00000131409	19	51010149	51081302	29	8	123834	0.10042	0.46001	0.973836787
MFAP5	ENSG00000197614	12	8779942	8825484	62	2	123834	0.10031	0.46005	0.973836787
KCNS1	ENSG00000124134	20	43710951	43739753	39	8	123834	0.10017	0.4601	0.973836787
C15orf37	ENSG00000259642	15	80205113	80227194	73	7	123834	0.10015	0.46011	0.973836787
CALML5	ENSG00000178372	10	5530658	5551533	18	6	123834	0.10012	0.46012	0.973836787
ZC3H13	ENSG00000123200	13	46518600	46636894	187	11	123834	0.10012	0.46012	0.973836787
USP50	ENSG00000170236	15	50782759	50848905	151	13	123834	0.10008	0.46014	0.973836787
GHRHR	ENSG00000106128	7	30968284	31042869	136	22	123834	0.10002	0.46017	0.973836787
ZNF3	ENSG00000166526	7	99651656	99690171	37	6	123834	0.099832	0.46024	0.973836787
IFNL2	ENSG00000183709	19	39749154	39770732	30	4	123834	0.099817	0.46024	0.973836787
PPP1R15B	ENSG00000158615	1	204362515	204390919	19	5	123834	0.099712	0.46029	0.973836787
PLSCR4	ENSG00000114698	3	145900126	145978966	220	18	123834	0.099651	0.46031	0.973836787
RASSF3	ENSG00000153179	12	64994293	65101347	172	15	123834	0.099661	0.46031	0.973836787
CSF1	ENSG00000184371	1	110442864	110483614	55	11	123834	0.099546	0.46035	0.973836787
GDPD4	ENSG00000178795	11	76917603	77022732	230	18	123834	0.099517	0.46036	0.973836787
LRRC53	ENSG00000162621	1	74925562	74988298	39	5	123834	0.099485	0.46038	0.973836787
C14orf23	ENSG00000186960	14	29231910	29292493	12	3	123834	0.099461	0.46039	0.973836787
TCEB2	ENSG00000103363	16	2811415	2837298	5	2	123834	0.099384	0.46042	0.973836787
PGM3	ENSG00000013375	6	83860869	83913655	49	9	123834	0.099294	0.46045	0.973836787
RPS5	ENSG00000083845	19	58887767	58916173	29	4	123834	0.099113	0.46052	0.973836787
CLCN2	ENSG00000114859	3	184053973	184089439	17	5	123834	0.098597	0.46073	0.973836787
OR3A1	ENSG00000180090	17	3184929	3205876	78	7	123834	0.098531	0.46076	0.973836787
GBAS	ENSG00000146729	7	56009486	56077874	165	20	123834	0.098214	0.46088	0.973836787
RANBP1	ENSG00000099901	22	20093461	20124878	80	7	123834	0.098155	0.4609	0.973836787
LSP1	ENSG00000130592	11	1864200	1923497	69	18	123834	0.097996	0.46097	0.973836787
RAPH1	ENSG00000173166	2	204249068	204410133	135	12	123834	0.097861	0.46102	0.973836787
SLC35A4	ENSG00000176087	5	139934041	139958688	16	4	123834	0.097714	0.46108	0.973836787
NRG1	ENSG00000157168	8	31486902	32632548	2184	104	123834	0.097618	0.46112	0.973836787
WDR7	ENSG00000091157	18	54308574	54708828	726	39	123834	0.097577	0.46113	0.973836787
AC106876.2	ENSG00000222001	2	233867324	233890595	23	7	123834	0.097394	0.46121	0.973836787
FEZ1	ENSG00000149557	11	125305646	125376213	97	13	123834	0.097373	0.46122	0.973836787
SLC35C1	ENSG00000181830	11	45815623	45844566	55	12	123834	0.097274	0.46125	0.973836787
EMP3	ENSG00000142227	19	48814766	48843810	69	10	123834	0.097102	0.46132	0.973836787
DENND2D	ENSG00000162777	1	111719796	111757157	26	9	123834	0.097078	0.46133	0.973836787
RFX4	ENSG00000111783	12	106966685	107166581	286	18	123834	0.096433	0.46159	0.973836787
HES1	ENSG00000114315	3	193843934	193866521	24	5	123834	0.096292	0.46164	0.973836787
KCNJ10	ENSG00000177807	1	159997257	160050038	21	6	123834	0.096272	0.46165	0.973836787
BTG4	ENSG00000137707	11	111328251	111393079	67	7	123834	0.096115	0.46171	0.973836787
USP47	ENSG00000170242	11	11852970	11990870	168	14	123834	0.095747	0.46186	0.973836787
LIAS	ENSG00000121897	4	39450620	39489273	151	16	123834	0.0957	0.46188	0.973836787
ZNF586	ENSG00000083828	19	58271023	58341307	128	13	123834	0.095537	0.46194	0.973836787

STIL	ENSG00000123473	1	47705811	47789819	163	8	123834	0.09549	0.46196	0.973836787
HSPD1	ENSG00000144381	2	198341305	198391461	34	4	123834	0.095505	0.46196	0.973836787
GPR115	ENSG00000153294	6	47643600	47699757	175	5	123834	0.095436	0.46198	0.973836787
C15ORF37	ENSG00000257028	15	80205507	80227194	72	7	123834	0.095448	0.46198	0.973836787
HOPX	ENSG00000171476	4	57504155	57558065	75	13	123834	0.095349	0.46202	0.973836787
BST2	ENSG00000130303	19	17503748	17526457	71	8	123834	0.095277	0.46205	0.973836787
KCNN1	ENSG00000105642	19	18052102	18120889	57	11	123834	0.095259	0.46205	0.973836787
DCBLD1	ENSG00000164465	6	117764980	117901021	299	18	123834	0.095165	0.46209	0.973836787
PNPLA8	ENSG00000135241	7	108100866	108220110	207	17	123834	0.09499	0.46216	0.973836787
CCM2	ENSG00000136280	7	45029074	45126068	266	13	123834	0.09488	0.46221	0.973836787
CBLN2	ENSG00000141668	18	70193915	70315756	183	20	123834	0.09478	0.46224	0.973836787
CXCL17	ENSG00000189377	19	42922440	42957200	20	2	123834	0.094637	0.4623	0.973836787
ICMT	ENSG00000116237	1	6271253	6306032	37	4	123834	0.094527	0.46235	0.973836787
ARHGAP27	ENSG00000159314	17	43461275	43521787	107	5	123834	0.094368	0.46241	0.973836787
CMTM1	ENSG00000089505	16	66590294	66623040	48	3	123834	0.094284	0.46244	0.973836787
FABP7	ENSG00000164434	6	123090620	123115219	22	7	123834	0.094114	0.46251	0.973836787
TPRG1L	ENSG00000158109	1	3531566	3556691	41	8	123834	0.094014	0.46255	0.973836787
DAB2IP	ENSG00000136848	9	124319336	124557809	226	38	123834	0.093872	0.46261	0.973836787
ALDH1A2	ENSG00000128918	15	58235622	58800065	1049	92	123834	0.093831	0.46262	0.973836787
TSC1	ENSG00000165699	9	135756735	135830020	76	7	123834	0.093815	0.46263	0.973836787
DFFB	ENSG00000169598	1	3763845	3811993	166	20	123834	0.093727	0.46266	0.973836787
CADM2	ENSG00000175161	3	84998132	86133579	2096	43	123834	0.093293	0.46284	0.973836787
LILRB1	ENSG00000104972	19	55075346	55158979	334	13	123834	0.093196	0.46287	0.973836787
FAM167B	ENSG00000183615	1	32702834	32724457	27	3	123834	0.093165	0.46289	0.973836787
GPX2	ENSG00000176153	14	65395870	65419531	56	7	123834	0.093038	0.46294	0.973836787
WARS2	ENSG00000116874	1	119563839	119693294	281	16	123834	0.092843	0.46301	0.973836787
NUTF2	ENSG00000102898	16	67870635	67916470	39	4	123834	0.092628	0.4631	0.973836787
C14orf2	ENSG00000156411	14	104368625	104404606	107	8	123834	0.092578	0.46312	0.973836787
CEBPB	ENSG00000172216	20	48797376	48819212	7	2	123834	0.092554	0.46313	0.973836787
CAMSAP2	ENSG00000118200	1	200698686	200839832	235	18	123834	0.092476	0.46316	0.973836787
GAPDH	ENSG00000111640	12	6633093	6657537	35	5	123834	0.092029	0.46334	0.973836787
PAPPA-AS1	ENSG00000256040	9	119150439	119172885	20	5	123834	0.092002	0.46335	0.973836787
CHMP5	ENSG00000086065	9	33254940	33291977	29	4	123834	0.091816	0.46342	0.973836787
NGB	ENSG00000165553	14	77721826	77747655	31	7	123834	0.091737	0.46345	0.973836787
SIGLEC5	ENSG00000268500	19	52105344	52160142	70	17	123834	0.091655	0.46349	0.973836787
ARHGAP20	ENSG00000137727	11	110437766	110593912	255	14	123834	0.091517	0.46354	0.973836787
NR1H4	ENSG00000012504	12	100857486	100968191	94	9	123834	0.091422	0.46358	0.973836787
SDC3	ENSG00000162512	1	31332314	31391608	33	12	123834	0.091318	0.46362	0.973836787
TMTC3	ENSG00000139324	12	88526073	88603664	102	5	123834	0.091252	0.46365	0.973836787
ARL14EPL	ENSG00000268223	5	115377163	115405815	90	6	123834	0.091144	0.46369	0.973836787
ERCC2	ENSG00000104884	19	45843095	45884176	47	7	123834	0.091017	0.46374	0.973836787
MUS81	ENSG00000172732	11	65614597	65645124	37	4	123834	0.090954	0.46376	0.973836787
PREX2	ENSG00000046889	8	68854353	69159265	637	50	123834	0.090818	0.46382	0.973836787
SF3A2	ENSG00000104897	19	2226520	2258678	29	8	123834	0.090782	0.46383	0.973836787
NFATC2	ENSG00000101096	20	49993494	50189370	202	40	123834	0.090728	0.46385	0.973836787
RP11-144F15.1	ENSG00000257545	12	106879736	107178696	416	36	123834	0.090606	0.4639	0.973836787
NDUFA10	ENSG00000130414	2	240821867	240974819	322	25	123834	0.0906	0.46391	0.973836787
RPS21	ENSG00000171858	20	60952172	60973576	66	10	123834	0.090537	0.46393	0.973836787
EEF1D	ENSG00000104529	8	144651867	144691711	99	10	123834	0.090503	0.46394	0.973836787
MGP	ENSG00000111341	12	15024115	15048860	32	1	123834	0.090525	0.46394	0.973836787
IFNGR1	ENSG00000027697	6	137508621	137550586	13	3	123834	0.090199	0.46406	0.973836787
TRIML1	ENSG00000184108	4	189050573	189078897	110	17	123834	0.090147	0.46409	0.973836787
ERCC5	ENSG00000134899	13	103487194	103538345	75	9	123834	0.090116	0.4641	0.973836787
PI4K2B	ENSG00000038210	4	25152263	25290714	308	22	123834	0.089973	0.46415	0.973836787
AC139100.2	ENSG00000267270	18	77895807	77946315	99	11	123834	0.089935	0.46417	0.973836787
AGPAT9	ENSG00000138678	4	84447067	84537028	194	11	123834	0.089478	0.46435	0.973836787
GYPC	ENSG00000136732	2	127403509	127464246	98	18	123834	0.089294	0.46442	0.973836787
RGSL1	ENSG00000121446	1	182409256	182539734	295	13	123834	0.089111	0.4645	0.973836787
CHST14	ENSG00000169105	15	40753160	40775353	18	3	123834	0.089091	0.4645	0.973836787
AGTR1	ENSG00000144891	3	148405571	148470795	100	14	123834	0.088854	0.4646	0.973836787
ZNF446	ENSG00000083838	19	58975384	59002597	27	4	123834	0.088858	0.4646	0.973836787
MAP1LC3C	ENSG00000197769	1	242148792	242172375	28	6	123834	0.088825	0.46461	0.973836787
C4orf36	ENSG00000163633	4	87787358	87867354	100	10	123834	0.08879	0.46462	0.973836787
CNTN6	ENSG00000134115	3	1124260	1455901	645	72	123834	0.088718	0.46465	0.973836787
G3BP2	ENSG00000138757	4	76557966	76659709	195	14	123834	0.08849	0.46474	0.973836787
UGP2	ENSG00000169764	2	64058074	64128696	86	12	123834	0.088362	0.46479	0.973836787
KTI12	ENSG00000198841	1	52487775	52509488	29	3	123834	0.087919	0.46497	0.973836787
PARP1	ENSG00000143799	1	226538392	226605780	119	9	123834	0.087828	0.46501	0.973836787
DNAJC22	ENSG00000178401	12	49730700	49761309	36	3	123834	0.08761	0.46509	0.973836787
SGPL1	ENSG00000166224	10	72565717	72650930	190	18	123834	0.08758	0.46511	0.973836787
LDB2	ENSG00000169744	4	16493164	16910432	599	71	123834	0.087463	0.46515	0.973836787
S1PR5	ENSG00000180739	19	10613623	10638607	32	6	123834	0.087477	0.46515	0.973836787
TNFAIP3	ENSG00000118503	6	138178351	138214449	47	6	123834	0.087423	0.46517	0.973836787
ZNF699	ENSG00000196110	19	9394951	9430514	55	6	123834	0.087374	0.46519	0.973836787
GRAMD1A	ENSG00000089351	19	35475688	35527375	77	8	123834	0.087302	0.46522	0.973836787
CDADC1	ENSG00000102543	13	49812047	49877618	63	2	123834	0.086915	0.46537	0.973836787
TMEM171	ENSG00000157111	5	72406119	72437644	30	5	123834	0.086665	0.46547	0.973836787
TNFSF9	ENSG00000125657	19	6521010	6545931	15	7	123834	0.086621	0.46549	0.973836787
TNNI3	ENSG00000129991	19	55653137	55679141	36	6	123834	0.08633	0.4656	0.973836787
ARL9	ENSG00000196503	4	57361509	57400614	85	11	123834	0.086248	0.46563	0.973836787
POU3F2	ENSG00000184486	6	99272580	99296660	34	4	123834	0.086226	0.46564	0.973836787
PCED1A	ENSG00000132635	20	2805960	2831836	6	2	123834	0.086037	0.46572	0.973836787
UBE2T	ENSG00000077152	1	202290785	202321108	19	3	123834	0.085931	0.46576	0.973836787
COL6A5	ENSG00000172752	3	130054359	130213688	410	25	123834	0.085386	0.46598	0.973836787
OR7G1	ENSG00000161807	19	9215274	9236439	70	7	123834	0.085188	0.46606	0.973836787
PSMA1	ENSG00000129084	11	14505329	14675181	134	14	123834	0.085001	0.46613	0.973836787
TTC38	ENSG00000075234	22	46653858	46699905	117	7	123834	0.084943	0.46615	0.973836787
LRRK1	ENSG00000154237	15	101449420	101620317	331	24	123834	0.08475	0.46623	0.973836787
ISG20	ENSG00000172183	15	89169384	89209714	85	15	123834	0.084651	0.46627	0.973836787
ZNF552	ENSG00000178935	19	58305209	58336281	50	6	123834	0.084515	0.46632	0.973836787
CHRND	ENSG00000135902	2	233380703	233411377	45	11	123834	0.084426	0.46636	0.973836787
PLAUR	ENSG00000011422	19	44140247	44184699	63	12	123834	0.08441	0.46637	0.973836787
OXER1	ENSG00000162881	2	42979642	43001401	26	5	123834	0.084282	0.46642	0.973836787
TCTE3	ENSG00000184786	6	170130210	170161655	64	8	123834	0.084271	0.46642	0.973836787
BAG1	ENSG00000107262	9	33237818	33274761	51	3	123834	0.084218	0.46644	0.973836787
DMRT1	ENSG00000137090	9	831690	979090	291	29	123834	0.083656	0.46667	0.973836787
ARSJ	ENSG00000180801	4	114811440	114910883	114	8	123834	0.083438	0.46675	0.973836787
EFEMP2	ENSG00000172638	11	65623912	65651063	43	5	123834	0.083372	0.46678	0.973836787
C6orf118	ENSG00000112539	6	165683153	165733096	84	12	123834	0.08325	0.46683	0.973836787

P4HA2	ENSG00000072682	5	131517531	131641008	158	16	123834	0.083165	0.46686	0.973836787
OR6C1	ENSG00000205330	12	55704346	55725408	42	5	123834	0.083101	0.46689	0.973836787
AQP12A	ENSG00000184945	2	241621262	241647900	19	2	123834	0.08292	0.46696	0.973836787
LYZL2	ENSG00000151033	10	30890718	30928691	127	14	123834	0.082818	0.467	0.973836787
MIPOL1	ENSG00000151338	14	37657118	38031566	530	12	123834	0.082652	0.46706	0.973836787
CLCC1	ENSG00000121940	1	109462130	109516111	105	15	123834	0.082617	0.46708	0.973836787
PPWD1	ENSG00000113593	5	64849063	64893376	110	8	123834	0.082589	0.46709	0.973836787
TBC1D2	ENSG00000095383	9	100951311	101027915	230	25	123834	0.082498	0.46713	0.973836787
TCHP	ENSG00000139437	12	110328069	110431646	157	10	123834	0.08217	0.46726	0.973836787
CCDC11	ENSG00000172361	18	47743563	47802892	81	7	123834	0.082144	0.46727	0.973836787
DLX1	ENSG00000144355	2	172939468	172964405	24	6	123834	0.082062	0.4673	0.973836787
PIBF1	ENSG00000083535	13	73346197	73600591	423	17	123834	0.08205	0.4673	0.973836787
VNN1	ENSG00000112299	6	132992729	133045188	107	11	123834	0.081891	0.46737	0.973836787
KLF14	ENSG00000174595	7	130407401	130428888	31	7	123834	0.081824	0.46739	0.973836787
SURF1	ENSG00000148290	9	136208610	136233552	40	4	123834	0.081706	0.46744	0.973836787
VPS8	ENSG00000156931	3	184519931	184780402	392	18	123834	0.081513	0.46752	0.973836787
RP11-795F19.5	ENSG00000267127	18	77784346	77864968	259	14	123834	0.081317	0.4676	0.973836787
C17orf74	ENSG00000184560	17	7318934	7340887	17	3	123834	0.081249	0.46762	0.973836787
OR4S2	ENSG00000174982	11	55408380	55429315	2	1	123834	0.080983	0.46773	0.973836787
UNC5D	ENSG00000156687	8	35082975	35664068	697	30	123834	0.080959	0.46774	0.973836787
PIM1	ENSG00000137193	6	37127979	37153202	20	3	123834	0.080914	0.46776	0.973836787
CRIP1	ENSG00000213145	14	105942654	105965284	6	2	123834	0.080743	0.46782	0.973836787
CRIP1	ENSG00000257341	14	105943536	105968604	6	2	123834	0.080743	0.46782	0.973836787
DENR	ENSG00000139726	12	123227321	123265611	47	4	123834	0.080685	0.46785	0.973836787
RPGRIP1L	ENSG00000103494	16	53621595	53747850	223	18	123834	0.080643	0.46786	0.973836787
DRAXIN	ENSG00000162490	1	11741786	11795914	39	11	123834	0.080503	0.46792	0.973836787
AGAP6	ENSG00000204149	10	51738078	51780259	5	2	123834	0.080418	0.46795	0.973836787
RHBG	ENSG00000132677	1	156329003	156365011	36	9	123834	0.080106	0.46808	0.973836787
ANXA13	ENSG00000104537	8	124683034	124759647	68	11	123834	0.080093	0.46808	0.973836787
NPAS2	ENSG00000170485	2	101426614	101623291	305	43	123834	0.080013	0.46811	0.973836787
EIF4EBP2	ENSG00000148730	10	72154135	72198374	54	9	123834	0.079973	0.46813	0.973836787
CRYBA2	ENSG00000163499	2	219844911	219868143	3	1	123834	0.079698	0.46824	0.973836787
GAD1	ENSG00000128683	2	171659723	171727661	93	11	123834	0.079631	0.46827	0.973836787
CPT1A	ENSG00000110090	11	68512088	68621878	154	17	123834	0.079214	0.46843	0.973836787
GRHPR	ENSG00000137106	9	37412663	37446987	51	9	123834	0.079179	0.46845	0.973836787
NDUFAF4	ENSG00000123545	6	97327189	97355757	64	9	123834	0.078954	0.46853	0.973836787
ZFPL1	ENSG00000162300	11	64841695	64865870	31	6	123834	0.078966	0.46853	0.973836787
RPL13	ENSG00000167526	16	89617065	89640950	11	3	123834	0.078965	0.46853	0.973836787
ZNF573	ENSG00000189144	19	38216734	38317940	166	17	123834	0.078811	0.46859	0.973836787
TSPYL6	ENSG00000178021	2	54470315	54493409	43	5	123834	0.07878	0.4686	0.973836787
ARAP3	ENSG00000120318	5	141022968	141071788	64	18	123834	0.078701	0.46864	0.973836787
ANKRD30BL	ENSG00000163046	2	132895164	133025542	75	4	123834	0.078286	0.4688	0.973836787
OR2H1	ENSG00000204688	6	29414958	29442105	38	6	123834	0.077789	0.469	0.973836787
TRPM6	ENSG00000119121	9	77327411	77513010	139	23	123834	0.077729	0.46902	0.973836787
NUAK1	ENSG00000074590	12	106447118	106543811	94	22	123834	0.07752	0.46911	0.973836787
GDF15	ENSG00000130513	19	18475541	18509986	37	5	123834	0.077437	0.46914	0.973836787
LYPD3	ENSG00000124466	19	43954939	43979812	35	8	123834	0.077389	0.46916	0.973836787
F2RL2	ENSG00000164220	5	75901307	75929259	92	14	123834	0.077349	0.46917	0.973836787
MARVELD1	ENSG00000155254	10	99462930	99487907	29	8	123834	0.077318	0.46919	0.973836787
ZNF558	ENSG00000167785	19	8910380	8953004	76	7	123834	0.077202	0.46923	0.973836787
LEPREL4	ENSG00000141696	17	39948199	39978856	24	2	123834	0.07709	0.46928	0.973836787
FCGR2A	ENSG00000143226	1	161465220	161503803	20	5	123834	0.076835	0.46938	0.973836787
DESI1	ENSG00000100418	22	41984032	42027100	27	2	123834	0.076753	0.46941	0.973836787
CYLC2	ENSG00000155833	9	105747593	105790770	120	10	123834	0.07667	0.46944	0.973836787
GFM2	ENSG00000164347	5	74007029	74073196	97	8	123834	0.076465	0.46952	0.973836787
CMTR1	ENSG00000137200	6	37390995	37460603	65	10	123834	0.076342	0.46957	0.973836787
RP11-1102P16.1	ENSG00000253379	8	72305675	72514260	305	26	123834	0.076247	0.46961	0.973836787
PREP	ENSG00000085377	6	105715440	105860959	237	25	123834	0.076218	0.46962	0.973836787
PPM1H	ENSG00000111110	12	63027762	63338817	508	64	123834	0.076235	0.46962	0.973836787
NR1I2	ENSG00000144852	3	119489331	119547332	102	16	123834	0.076062	0.46968	0.973836787
ADAP1	ENSG00000105963	7	927540	1005043	114	27	123834	0.075989	0.46971	0.973836787
OLIG3	ENSG00000177468	6	137803336	137825531	31	7	123834	0.075849	0.46977	0.973836787
IL5	ENSG00000113525	5	131867136	131902530	16	2	123834	0.075804	0.46979	0.973836787
CTC-786C10.1	ENSG00000262601	16	85194882	85228969	46	11	123834	0.075784	0.4698	0.973836787
PXDNL	ENSG00000147485	8	52222138	52732005	1371	46	123834	0.075635	0.46985	0.973836787
SLC13A3	ENSG00000158296	20	45176463	45314714	135	24	123834	0.075648	0.46985	0.973836787
FAM49A	ENSG00000197872	2	16720727	16857599	204	36	123834	0.075574	0.46988	0.973836787
IGFALS	ENSG00000099769	16	1830414	1854972	132	9	123834	0.075297	0.46999	0.973836787
AK7	ENSG00000140057	14	96848448	96965764	138	20	123834	0.075244	0.47001	0.973836787
PRCC	ENSG00000143294	1	156710402	156780607	129	13	123834	0.074982	0.47011	0.973836787
SNURF	ENSG00000273173	15	25190133	25233729	54	8	123834	0.074933	0.47013	0.973836787
EP400	ENSG00000183495	12	132424508	132575005	162	11	123834	0.074727	0.47022	0.973836787
JRKL	ENSG00000183340	11	96113153	96250738	165	17	123834	0.07463	0.47025	0.973836787
C6orf211	ENSG00000146476	6	151763422	151801236	94	10	123834	0.074475	0.47032	0.973836787
CLEC1B	ENSG00000165682	12	10128241	10176023	124	12	123834	0.074403	0.47034	0.973836787
ERVW-1	ENSG00000242950	7	92087694	92117300	54	7	123834	0.074393	0.47035	0.973836787
CPSF2	ENSG00000165934	14	92578281	92640755	164	9	123834	0.074209	0.47042	0.973836787
RGS22	ENSG00000132554	8	100963164	101153496	166	18	123834	0.074058	0.47048	0.973836787
TRIM40	ENSG00000204614	6	30093885	30126512	148	11	123834	0.074029	0.47049	0.973836787
TGIF2	ENSG00000118707	20	35191891	35232353	11	5	123834	0.073688	0.47063	0.973836787
GCNT4	ENSG00000176928	5	74313289	74336724	50	8	123834	0.073491	0.47071	0.973836787
GADD45GIP1	ENSG00000179271	19	13054972	13078050	6	2	123834	0.073456	0.47072	0.973836787
CDC45	ENSG00000093009	22	19456982	19518135	94	16	123834	0.073471	0.47072	0.973836787
PAX9	ENSG00000198807	14	37116773	37158920	80	14	123834	0.073422	0.47074	0.973836787
MAP4K2	ENSG00000168067	11	64546290	64580713	16	2	123834	0.073353	0.47076	0.973836787
MBD6	ENSG00000166987	12	57904493	57933931	23	2	123834	0.07335	0.47076	0.973836787
HIST1H2AD	ENSG00000196866	6	26189079	26209471	76	6	123834	0.07332	0.47078	0.973836787
PRDM9	ENSG00000164256	5	23497264	23538706	40	6	123834	0.07309	0.47087	0.973916971
ITGA4	ENSG00000115232	2	182311929	182410914	123	16	123834	0.072565	0.47108	0.974087656
C1orf141	ENSG00000203963	1	67547848	67707536	366	24	123834	0.07241	0.47114	0.974087656
ABHD17B	ENSG00000107362	9	74467368	74535847	30	2	123834	0.072332	0.47117	0.974087656
TMEM244	ENSG00000203756	6	130142389	130192692	191	18	123834	0.07213	0.47125	0.974087656
GTF2A2	ENSG00000140307	15	59920261	59959740	70	4	123834	0.072124	0.47125	0.974087656
TNFRSF9	ENSG00000049249	1	7969907	8010926	34	2	123834	0.07211	0.47126	0.974087656
PER1	ENSG00000179094	17	8033790	8069824	40	6	123834	0.071951	0.47132	0.974105748
CALCRL	ENSG00000064989	2	188197856	188323187	215	17	123834	0.071726	0.47141	0.974185832
GCHFR	ENSG00000137880	15	41046218	41069906	18	4	123834	0.071416	0.47153	0.97419547
CTSD	ENSG00000117984	11	1763982	1795222	46	5	123834	0.071073	0.47167	0.97419547
ANKRD22	ENSG00000152766	10	90571889	90621575	63	11	123834	0.070803	0.47178	0.97419547

VPS18	ENSG00000104142	15	41176628	41206173	31	2	123834	0.070647	0.47184	0.97419547
CLDND1	ENSG00000080822	3	98206756	98251910	53	9	123834	0.070158	0.47203	0.97419547
TM2D1	ENSG00000162604	1	62136718	62201095	88	7	123834	0.070009	0.47209	0.97419547
N6AMT2	ENSG00000150456	13	21292870	21358097	153	12	123834	0.070012	0.47209	0.97419547
PPP1R12C	ENSG00000125503	19	55592281	55638927	21	5	123834	0.069929	0.47213	0.97419547
PLEKHG1	ENSG00000120278	6	150910999	151174799	592	43	123834	0.069407	0.47233	0.97419547
SIGLEC5	ENSG00000105501	19	52104781	52160151	71	17	123834	0.069422	0.47233	0.97419547
TCL1A	ENSG00000100721	14	96166304	96190533	39	5	123834	0.069174	0.47243	0.97419547
SNTG1	ENSG00000147481	8	50812349	51716678	2069	46	123834	0.069098	0.47246	0.97419547
CPNE9	ENSG00000144550	3	9735491	9781592	35	9	123834	0.068933	0.47252	0.97419547
KANSL1	ENSG00000120071	17	44097282	44312733	472	4	123834	0.068903	0.47253	0.97419547
OR6A2	ENSG00000184933	11	6805756	6827128	56	7	123834	0.068817	0.47257	0.97419547
CSN2	ENSG00000135222	4	70810974	70836725	60	5	123834	0.068762	0.47259	0.97419547
GPRC5D	ENSG00000111291	12	13083709	13115081	47	11	123834	0.068744	0.4726	0.97419547
CHADL	ENSG00000100399	22	41615517	41646938	32	5	123834	0.068689	0.47262	0.97419547
MKL1	ENSG00000196588	22	40796285	41042706	277	8	123834	0.06866	0.47263	0.97419547
TTC4	ENSG00000243725	1	55171495	55218330	147	2	123834	0.068513	0.47269	0.97419547
ARPC4-TTLL3	ENSG00000250151	3	9824766	9887085	115	10	123834	0.06834	0.47276	0.97419547
TSRM	ENSG00000236294	7	113081127	113101457	13	4	123834	0.068285	0.47278	0.97419547
KIAA0101	ENSG00000166803	15	64647193	64689886	41	5	123834	0.068057	0.47287	0.97419547
LL22NC03-63E9.3	ENSG00000220891	22	22891750	22919007	64	3	123834	0.067629	0.47304	0.97419547
SMIM22	ENSG00000267795	16	4828398	4856492	35	5	123834	0.067477	0.4731	0.97419547
SYNGR1	ENSG00000100321	22	39735930	39791593	59	8	123834	0.067327	0.47316	0.97419547
ZKSCAN2	ENSG00000155592	16	25237322	25279252	95	11	123834	0.067205	0.47321	0.97419547
PARP9	ENSG00000138496	3	122236771	122293424	77	9	123834	0.067147	0.47323	0.97419547
OR52A5	ENSG00000171944	11	5142922	5163872	20	6	123834	0.06714	0.47323	0.97419547
FMNL3	ENSG00000161791	12	50021724	50111948	102	7	123834	0.067118	0.47324	0.97419547
RNF183	ENSG00000165188	9	116049373	116075656	49	9	123834	0.06709	0.47325	0.97419547
DPY19L4	ENSG00000156162	8	95721931	95816064	117	14	123834	0.066733	0.4734	0.97419547
FAM194B	ENSG00000165837	13	46098657	46199874	177	22	123834	0.066711	0.47341	0.97419547
FXYD6-FXYD2	ENSG00000255245	11	117680878	117757382	70	17	123834	0.066615	0.47344	0.97419547
ST3GAL6	ENSG00000064225	3	98441080	98550045	202	19	123834	0.066479	0.4735	0.97419547
SYT16	ENSG00000139973	14	62443803	62578431	291	39	123834	0.066415	0.47352	0.97419547
TSNARE1	ENSG00000171045	8	143283441	143494601	562	28	123834	0.066177	0.47362	0.97419547
LUZP2	ENSG00000187398	11	24508516	25114150	1561	58	123834	0.066113	0.47364	0.97419547
C17orf102	ENSG00000197322	17	32891142	32916388	39	8	123834	0.065913	0.47372	0.97419547
HRSP12	ENSG00000132541	8	99104572	99139469	67	10	123834	0.065837	0.47375	0.97419547
MTRNR2L8	ENSG00000255823	11	10519434	10540723	47	9	123834	0.065726	0.4738	0.97419547
OVCA2	ENSG00000262664	17	1935327	1956724	25	5	123834	0.065629	0.47384	0.97419547
HSPB3	ENSG00000169271	5	53741445	53762207	48	6	123834	0.065501	0.47389	0.97419547
PJA2	ENSG00000198961	5	108660410	108755695	267	9	123834	0.065278	0.47398	0.97419547
TRIM63	ENSG00000158022	1	26367795	26404927	57	4	123834	0.06514	0.47403	0.97419547
RGS19	ENSG00000171700	20	62694534	62721323	22	6	123834	0.064938	0.47411	0.97419547
C5orf47	ENSG00000185056	5	173390782	173443143	101	7	123834	0.064905	0.47412	0.97419547
ZNF700	ENSG00000196757	19	12025883	12071588	101	7	123834	0.064694	0.47421	0.97419547
HLA-DQA2	ENSG00000237541	6	32699119	32724992	450	15	123834	0.06459	0.47425	0.97419547
AHCY	ENSG00000101444	20	32858074	32909608	26	3	123834	0.064508	0.47428	0.97419547
CRYGD	ENSG00000118231	2	208976331	208999225	62	10	123834	0.06434	0.47435	0.97419547
PDIA4	ENSG00000155660	7	148690154	148735733	75	8	123834	0.064321	0.47436	0.97419547
LIPH	ENSG00000163898	3	185214050	185280401	134	9	123834	0.064278	0.47437	0.97419547
C10orf32-ASMT	ENSG00000270316	10	104604029	104671656	157	13	123834	0.064144	0.47443	0.97419547
TARM1	ENSG00000248385	19	54563149	54594634	57	5	123834	0.063815	0.47456	0.97419547
PITHD1	ENSG00000057757	1	24094895	24124722	7	3	123834	0.063722	0.4746	0.97419547
CTSV	ENSG00000136943	9	99784940	99811925	25	1	123834	0.063547	0.47467	0.97419547
RP11-434D12.1	ENSG00000268279	3	14166717	14199548	51	8	123834	0.062891	0.47493	0.97419547
MYOT	ENSG00000120729	5	137193480	137233540	23	4	123834	0.062782	0.47497	0.97419547
KNSTRN	ENSG00000128944	15	40664922	40696447	51	7	123834	0.06262	0.47503	0.97419547
NDST4	ENSG00000138653	4	115738919	116045032	440	25	123834	0.062468	0.4751	0.97419547
AP001362.1	ENSG00000269018	11	65348665	65369947	15	2	123834	0.062331	0.47515	0.97419547
LRBA	ENSG00000198589	4	151175594	151946879	1061	26	123834	0.062273	0.47517	0.97419547
BLK	ENSG00000136573	8	11341510	11432113	103	17	123834	0.062272	0.47517	0.97419547
STRADA	ENSG00000266173	17	61770192	61829330	86	5	123834	0.062125	0.47523	0.97419547
DYRK1A	ENSG00000157540	21	38728092	38899753	327	15	123834	0.062076	0.47525	0.97419547
ZDHHC22	ENSG00000177108	14	77587613	77619077	29	10	123834	0.061774	0.47537	0.97419547
UGT3A2	ENSG00000168671	5	36025119	36081460	115	15	123834	0.061735	0.47539	0.97419547
XPO4	ENSG00000132953	13	21341469	21487187	268	13	123834	0.061724	0.47539	0.97419547
FBXO17	ENSG00000269190	19	39422044	39476550	62	10	123834	0.061654	0.47542	0.97419547
STK38L	ENSG00000211455	12	27386901	27488892	198	26	123834	0.061466	0.47549	0.97419547
KIF26A	ENSG00000066735	14	104595060	104657231	89	12	123834	0.061324	0.47555	0.97419547
PRDX6	ENSG00000117592	1	173436405	173467946	42	5	123834	0.061242	0.47558	0.97419547
ADH1B	ENSG00000196616	4	100216121	100252558	69	12	123834	0.06105	0.47566	0.97419547
FAT1	ENSG00000083857	4	187498937	187657876	246	39	123834	0.061035	0.47567	0.97419547
LFNG	ENSG00000106003	7	2542163	2578811	39	10	123834	0.061029	0.47567	0.97419547
TOMM34	ENSG00000025772	20	43560771	43599127	21	4	123834	0.060906	0.47572	0.97419547
TOX4	ENSG00000092203	14	21934756	21977319	89	8	123834	0.06029	0.47596	0.97419547
RGS6	ENSG00000182732	14	72389156	73040654	1405	81	123834	0.060292	0.47596	0.97419547
PXMP4	ENSG00000101417	20	32284512	32318125	42	6	123834	0.060307	0.47596	0.97419547
HSBP1	ENSG00000230989	16	83831448	83863342	34	6	123834	0.060162	0.47601	0.97419547
RTP4	ENSG00000136514	3	187076120	187099864	46	9	123834	0.060132	0.47603	0.97419547
C2CD3	ENSG00000168014	11	73713763	73892255	269	12	123834	0.060015	0.47607	0.97419547
PIP5K1C	ENSG00000186111	19	3620181	3710477	116	18	123834	0.059931	0.47611	0.97419547
LMTK3	ENSG00000142235	19	48978528	49026446	54	7	123834	0.059673	0.47621	0.97419547
GPS1	ENSG00000169727	17	79998567	80025346	33	8	123834	0.059566	0.47625	0.97419547
FAM118A	ENSG00000100376	22	45694849	45747836	113	8	123834	0.059419	0.47631	0.97419547
SNAPC3	ENSG00000164975	9	15412702	15475951	123	10	123834	0.059392	0.47632	0.97419547
ERO1L	ENSG00000197930	14	53096634	53172618	78	9	123834	0.059285	0.47636	0.97419547
CPLX4	ENSG00000166569	18	56932388	56995881	100	11	123834	0.058968	0.47649	0.97419547
CAPS	ENSG00000105519	19	5901718	5925888	20	5	123834	0.058795	0.47656	0.97419547
SLCO5A1	ENSG00000137571	8	70569282	70757299	205	32	123834	0.058552	0.47665	0.97419547
PHYHD1	ENSG00000175287	9	131673174	131714320	38	2	123834	0.058465	0.47669	0.97419547
C10orf128	ENSG00000204161	10	50352773	50406630	134	19	123834	0.058258	0.47677	0.97419547
SLC6A9	ENSG00000196517	1	44447172	44507139	37	9	123834	0.058183	0.4768	0.97419547
CCR2	ENSG00000121807	3	46385225	46412419	41	4	123834	0.058157	0.47681	0.97419547
HSF2	ENSG00000025156	6	122710691	122764264	88	8	123834	0.05798	0.47688	0.97419547
ZNF460	ENSG00000197714	19	57781419	57814937	61	7	123834	0.057744	0.47698	0.97419547
AC006547.14	ENSG00000234409	22	20125950	20148399	41	6	123834	0.057508	0.47707	0.97419547
RCAN2	ENSG00000172348	6	46178475	46469709	292	17	123834	0.057425	0.4771	0.97419547
PPP1R3E	ENSG00000235194	14	23754852	23782057	10	5	123834	0.057425	0.4771	0.97419547
HIST1H3D	ENSG00000197409	6	26187068	26209521	78	6	123834	0.057357	0.47713	0.97419547

HOXA13	ENSG00000106031	7	27223122	27249725	21	4	123834	0.057336	0.47714	0.97419547
ATP6V0E2	ENSG00000171130	7	149560057	149587784	34	5	123834	0.057275	0.47716	0.97419547
SS18	ENSG00000141380	18	23586578	23681181	135	8	123834	0.05729	0.47716	0.97419547
FCER1A	ENSG00000179639	1	159249504	159288014	66	8	123834	0.057261	0.47717	0.97419547
NBPF15	ENSG00000243452	1	148545979	148606267	4	1	123834	0.057234	0.47718	0.97419547
MRPL43	ENSG00000055950	10	102719215	102757272	35	4	123834	0.057181	0.4772	0.97419547
OR10R2	ENSG00000198965	1	158439668	158460675	75	7	123834	0.056967	0.47729	0.97419547
DEFA1	ENSG00000206047	8	6825171	6847602	17	6	123834	0.056428	0.4775	0.97419547
KLRB1	ENSG00000111796	12	9737147	9770482	79	12	123834	0.056115	0.47763	0.97419547
ID1	ENSG00000125968	20	30183086	30204318	51	1	123834	0.055976	0.47768	0.97419547
DEFB129	ENSG00000125903	20	197899	220527	38	4	123834	0.055942	0.47769	0.97419547
ST20-MTHFS	ENSG00000259332	15	80127537	80226096	243	19	123834	0.055625	0.47782	0.97419547
C3orf65	ENSG00000163915	3	185420316	185457575	14	5	123834	0.055492	0.47787	0.97419547
SDAD1	ENSG00000198301	4	76852103	76922115	122	11	123834	0.055437	0.4779	0.97419547
BMPER	ENSG00000164619	7	33934523	34205484	266	30	123834	0.055125	0.47802	0.97419547
GPR63	ENSG00000112218	6	97232002	97295353	140	12	123834	0.054741	0.47817	0.97419547
PCBP4	ENSG00000090097	3	51981470	52012426	3	2	123834	0.054629	0.47822	0.97419547
RP11-155D18.14	ENSG00000272762	3	51984317	52018032	3	2	123834	0.054629	0.47822	0.97419547
NR2F2	ENSG00000185551	15	96859167	96893492	1	1	123834	0.054586	0.47823	0.97419547
AP3M1	ENSG00000185009	10	75871524	75920821	84	7	123834	0.054382	0.47832	0.97419547
INS	ENSG00000254647	11	2171009	2192571	6	2	123834	0.054373	0.47832	0.97419547
C17orf59	ENSG00000196544	17	8081652	8103564	28	6	123834	0.054281	0.47836	0.97419547
HRH3	ENSG00000101180	20	60780026	60805323	22	9	123834	0.054261	0.47836	0.97419547
BPIFB1	ENSG00000125999	20	31851286	31907684	86	8	123834	0.05421	0.47838	0.97419547
ACAD8	ENSG00000151498	11	134113389	134145749	46	6	123834	0.054159	0.4784	0.97419547
NMNAT1	ENSG00000173614	1	9993486	10055559	68	7	123834	0.054134	0.47841	0.97419547
MRPS26	ENSG00000125901	20	3016591	3038900	25	5	123834	0.053994	0.47847	0.97419547
ARID4B	ENSG00000054267	1	235284949	235501534	321	9	123834	0.053961	0.47848	0.97419547
RP11-1012A1.4	ENSG00000258466	14	68116537	68167084	83	9	123834	0.053908	0.4785	0.97419547
DHRS2	ENSG00000100867	14	24089324	24124848	9	3	123834	0.053705	0.47859	0.97419547
PAPOLG	ENSG00000115421	2	60973365	61039220	101	10	123834	0.053573	0.47864	0.97419547
PLCH2	ENSG00000149527	1	2347419	2446969	104	19	123834	0.053483	0.47867	0.97419547
PEX1	ENSG00000127980	7	92106334	92167845	82	8	123834	0.053472	0.47868	0.97419547
KIAA2018	ENSG00000176542	3	113357232	113425493	51	4	123834	0.053221	0.47878	0.97419547
ZNF627	ENSG00000198551	19	11660189	11739950	134	12	123834	0.053164	0.4788	0.97419547
KBTBD12	ENSG00000187715	3	127624075	127716514	142	12	123834	0.053048	0.47885	0.97419547
AK3	ENSG00000147853	9	4701155	4752043	101	12	123834	0.05282	0.47894	0.97419547
LMAN2	ENSG00000169223	5	176748563	176788853	12	4	123834	0.052781	0.47895	0.97419547
ITGA6	ENSG00000091409	2	173282082	173381181	275	20	123834	0.052729	0.47897	0.97419547
FAM53B	ENSG00000189319	10	126297861	126442838	154	20	123834	0.05265	0.47901	0.97419547
GNAI3	ENSG00000065135	1	110081233	110146975	55	7	123834	0.052526	0.47905	0.97419547
C3orf83	ENSG00000225526	3	12546433	12612558	143	12	123834	0.052458	0.47908	0.97419547
PRKAB1	ENSG00000111725	12	120095558	120129435	23	5	123834	0.052358	0.47912	0.97419547
NLRP11	ENSG00000179873	19	56286770	56358166	163	29	123834	0.052353	0.47912	0.97419547
AMOTL1	ENSG00000166025	11	94429597	94619918	245	25	123834	0.05225	0.47916	0.97419547
CD70	ENSG00000125726	19	6573194	6614114	49	8	123834	0.052257	0.47916	0.97419547
DNAJC17	ENSG00000104129	15	41050067	41109675	57	7	123834	0.052206	0.47918	0.97419547
FNDC3B	ENSG00000075420	3	171747418	172129455	407	43	123834	0.051819	0.47934	0.97419547
FGGY	ENSG00000172456	1	59752310	60243347	634	37	123834	0.051657	0.4794	0.97419547
RAPGEF1	ENSG00000107263	9	134442157	134625461	329	22	123834	0.051176	0.47959	0.97419547
PIP	ENSG00000159763	7	142819170	142846839	55	6	123834	0.051011	0.47966	0.97419547
CTA-299D3.8	ENSG00000219016	22	48924712	48953199	44	9	123834	0.050641	0.47981	0.97419547
STEAP4	ENSG00000127954	7	87895744	87946206	60	5	123834	0.050602	0.47982	0.97419547
PLK1	ENSG00000166851	16	23678977	23711688	69	7	123834	0.050612	0.47982	0.97419547
PRSS38	ENSG00000185888	1	227993394	228044171	135	20	123834	0.050416	0.4799	0.97419547
ZNF324B	ENSG00000249471	19	58952971	58979200	19	3	123834	0.050275	0.47995	0.97419547
ABAT	ENSG00000183044	16	8758422	8888432	310	52	123834	0.049919	0.48009	0.97419547
XPC	ENSG00000154767	3	14176647	14230283	74	9	123834	0.049823	0.48013	0.97419547
TCTN3	ENSG00000119977	10	97413158	97463900	102	14	123834	0.049832	0.48013	0.97419547
DEPDC4	ENSG00000166153	12	100587447	100670857	110	15	123834	0.049829	0.48013	0.97419547
DSP	ENSG00000096696	6	7531808	7596950	91	16	123834	0.049711	0.48018	0.97419547
GOLGA8B	ENSG00000215252	15	34807408	34890704	76	6	123834	0.049692	0.48018	0.97419547
KRTAP10-2	ENSG00000205445	21	45960240	45981388	26	3	123834	0.04965	0.4802	0.97419547
MS4A4A	ENSG00000110079	11	60038014	60086445	84	11	123834	0.04957	0.48023	0.97419547
KRTAP4-4	ENSG00000171396	17	39305910	39326983	15	3	123834	0.049473	0.48027	0.97419547
RP11-676J12.7	ENSG00000262003	17	802872	824452	34	6	123834	0.049379	0.48031	0.97419547
AC008964.1	ENSG00000269215	5	39095358	39117350	20	4	123834	0.049304	0.48034	0.97419547
C12orf50	ENSG00000165805	12	88363816	88437814	116	9	123834	0.049214	0.48037	0.97419547
KANK1	ENSG00000107104	9	460291	756105	757	34	123834	0.049191	0.48038	0.97419547
CCL28	ENSG00000151882	5	43366747	43422493	75	8	123834	0.049157	0.4804	0.97419547
LY86	ENSG00000112799	6	6578341	6665216	200	35	123834	0.049052	0.48044	0.97419547
MRPL37	ENSG00000116221	1	54639714	54701137	84	14	123834	0.048964	0.48047	0.97419547
DIS3	ENSG00000083520	13	73319540	73366234	64	9	123834	0.048817	0.48053	0.97419547
APPBP2	ENSG00000062725	17	58510520	58613580	15	3	123834	0.04874	0.48056	0.97419547
TMEM245	ENSG00000106771	9	111767432	111892225	348	21	123834	0.048721	0.48057	0.97419547
PPP2R2B	ENSG00000156475	5	145957936	146474347	541	46	123834	0.048636	0.4806	0.97419547
FTMT	ENSG00000181867	5	121177650	121198519	21	4	123834	0.048608	0.48062	0.97419547
ENY2	ENSG00000120533	8	110336553	110368182	35	10	123834	0.048541	0.48064	0.97419547
PMF1	ENSG00000160783	1	156172784	156222874	55	5	123834	0.048266	0.48075	0.974268909
NRROS	ENSG00000174004	3	196356557	196398875	28	10	123834	0.048068	0.48083	0.974268909
MAPK6	ENSG00000069956	15	52234303	52368462	159	11	123834	0.048077	0.48083	0.974268909
ATM	ENSG00000149311	11	108083211	108249829	166	4	123834	0.047932	0.48089	0.974273419
RTBDN	ENSG00000132026	19	12926292	12956242	48	7	123834	0.047713	0.48097	0.974273419
ERN1	ENSG00000178607	17	62106502	62218179	88	8	123834	0.047643	0.481	0.974273419
AL009178.1	ENSG00000269155	6	168186210	168208927	35	2	123834	0.047522	0.48105	0.974273419
PHLPP2	ENSG00000040199	16	71661738	71768604	174	13	123834	0.047286	0.48114	0.974273419
ALPL	ENSG00000162551	1	21825858	21914905	216	27	123834	0.047059	0.48123	0.974273419
AL441883.1	ENSG00000268059	6	13605728	13631126	24	3	123834	0.046936	0.48128	0.974273419
TMEM140	ENSG00000146859	7	134822824	134860967	87	6	123834	0.046651	0.4814	0.974273419
TGFBR3L	ENSG00000260001	19	7971030	7993982	14	5	123834	0.046462	0.48147	0.974273419
SLC4A9	ENSG00000113073	5	139729787	139764728	11	3	123834	0.046379	0.4815	0.974273419
PCDH15	ENSG00000150275	10	55552531	57397702	4097	152	123834	0.046006	0.48165	0.974273419
SLC2A2	ENSG00000163581	3	170704137	170754539	86	6	123834	0.045722	0.48177	0.974273419
IRF2BPL	ENSG00000119669	14	77480888	77505034	38	8	123834	0.045671	0.48179	0.974273419
DAPL1	ENSG00000163331	2	159641829	159729293	147	16	123834	0.045605	0.48181	0.974273419
OR6C76	ENSG00000185821	12	55810038	55830976	35	5	123834	0.04554	0.48184	0.974273419
AC017104.2	ENSG00000233538	2	232306906	232327864	11	3	123834	0.045316	0.48193	0.974273419
XRCC6BP1	ENSG00000166896	12	58325324	58361052	75	6	123834	0.045158	0.48199	0.974273419
LONP1	ENSG00000196365	19	5681845	5730583	53	3	123834	0.045097	0.48202	0.974273419

EXOSC10	ENSG00000171824	1	11116675	11169938	124	4	123834	0.045027	0.48204	0.974273419
MARS2	ENSG00000247626	2	198560087	198583113	35	3	123834	0.044881	0.4821	0.974273419
KIF18A	ENSG00000121621	11	28032167	28139855	119	10	123834	0.044883	0.4821	0.974273419
HYPK	ENSG00000242028	15	44078340	44105241	21	3	123834	0.044764	0.48215	0.974273419
MLH3	ENSG00000119684	14	75470467	75528235	58	3	123834	0.044607	0.48221	0.974273419
AIM1	ENSG00000112297	6	106949730	107028326	314	21	123834	0.044478	0.48226	0.974273419
FAM221B	ENSG00000204930	9	35806388	35838744	32	6	123834	0.044475	0.48226	0.974273419
RNPEP	ENSG00000176393	1	201941500	201985275	96	10	123834	0.044157	0.48239	0.974273419
FAM8A1	ENSG00000137414	6	17590586	17621950	12	4	123834	0.044049	0.48243	0.974273419
DDX24	ENSG00000089737	14	94507266	94557591	104	7	123834	0.043947	0.48247	0.974273419
ATXN7L1	ENSG00000146776	7	105235514	105527050	360	61	123834	0.043912	0.48249	0.974273419
ATP6V1D	ENSG00000100554	14	67751088	67836982	222	8	123834	0.043789	0.48254	0.974273419
SLC38A8	ENSG00000166558	16	84033272	84086241	189	32	123834	0.043674	0.48258	0.974273419
RBP3	ENSG00000107618	10	48371487	48400991	10	3	123834	0.043406	0.48269	0.974273419
ITGB6	ENSG00000115221	2	160946177	161138399	419	30	123834	0.043268	0.48274	0.974273419
TMEM160	ENSG00000130748	19	47539165	47561888	13	1	123834	0.043221	0.48276	0.974273419
ADCY1	ENSG00000164742	7	45603739	45772715	336	33	123834	0.043174	0.48278	0.974273419
FANCD2OS	ENSG00000163705	3	10113001	10159915	99	2	123834	0.043129	0.4828	0.974273419
PLA2G15	ENSG00000103066	16	68269207	68304961	49	5	123834	0.043104	0.48281	0.974273419
PMEL	ENSG00000185664	12	56337889	56377101	19	4	123834	0.042953	0.48287	0.974273419
TLCD2	ENSG00000185561	17	1601064	1623732	12	3	123834	0.042897	0.48289	0.974273419
KCNK7	ENSG00000173338	11	65350326	65373467	15	2	123834	0.042842	0.48291	0.974273419
DYSF	ENSG00000135636	2	71670852	71923898	427	60	123834	0.042633	0.483	0.974273419
OMD	ENSG00000127083	9	95166527	95196743	65	8	123834	0.042439	0.48307	0.974273419
NPRL3	ENSG00000103148	16	124273	198859	90	12	123834	0.042434	0.48308	0.974273419
LPIN3	ENSG00000132793	20	39959560	39999222	75	11	123834	0.042311	0.48313	0.974273419
C5orf64	ENSG00000178722	5	60923535	61057590	187	25	123834	0.042164	0.48318	0.974273419
NNAT	ENSG00000053438	20	36139617	36162092	20	3	123834	0.042152	0.48319	0.974273419
PAX1	ENSG00000125813	20	21676297	21706620	19	3	123834	0.041815	0.48332	0.974321221
FBXO38	ENSG00000145868	5	147753498	147832399	69	3	123834	0.041771	0.48334	0.974321221
MINOS1-NBL1	ENSG00000270136	1	19913473	19994549	138	20	123834	0.041595	0.48341	0.974321221
YIPF3	ENSG00000137207	6	43469565	43494728	31	5	123834	0.041499	0.48345	0.974321221
USP54	ENSG00000166348	10	75247296	75395711	167	4	123834	0.041456	0.48347	0.974321221
KRTAP8-1	ENSG00000183640	21	32175016	32195570	46	4	123834	0.041274	0.48354	0.974353908
LRRIQ3	ENSG00000162620	1	74481699	74673871	251	13	123834	0.041116	0.4836	0.974353908
IFI6	ENSG00000126709	1	27982572	28008729	20	2	123834	0.041012	0.48364	0.974353908
AL136376.1	ENSG00000269279	1	116905890	116936718	41	4	123834	0.040869	0.4837	0.974371513
CSF2	ENSG00000164400	5	131399483	131421859	14	3	123834	0.04065	0.48379	0.974441898
EEF1A1	ENSG00000156508	6	74215473	74243520	52	5	123834	0.040515	0.48384	0.974441898
USP35	ENSG00000118369	11	77889858	77935757	57	6	123834	0.0404	0.48389	0.974441898
RAB5B	ENSG00000111540	12	56357697	56398490	38	5	123834	0.040264	0.48394	0.974441898
SLN	ENSG00000170290	11	107568104	107600419	46	8	123834	0.03996	0.48406	0.974580286
CNDP2	ENSG00000133313	18	72153051	72198366	79	11	123834	0.039616	0.4842	0.97469594
MZF1	ENSG00000099326	19	59063298	59094942	51	8	123834	0.039553	0.48422	0.97469594
SMCO2	ENSG00000165935	12	27609743	27665118	202	12	123834	0.039415	0.48428	0.974713494
AP001024.1	ENSG00000214305	11	107640219	107662316	50	5	123834	0.03914	0.48439	0.974718171
CCDC146	ENSG00000135205	7	76741751	76968850	447	26	123834	0.038953	0.48446	0.974718171
RBBP9	ENSG00000089050	20	18457184	18487887	72	9	123834	0.038871	0.4845	0.974718171
PPP1R3C	ENSG00000119938	10	93378199	93402811	30	7	123834	0.038701	0.48456	0.974718171
LCP1	ENSG00000136167	13	46690055	46796006	147	21	123834	0.038656	0.48458	0.974718171
CST1	ENSG00000170373	20	23718190	23741905	108	13	123834	0.038638	0.48459	0.974718171
SNRPN	ENSG00000128739	15	25058794	25233870	205	42	123834	0.038391	0.48469	0.974727988
ANO5	ENSG00000171714	11	22204722	22314903	75	5	123834	0.038366	0.4847	0.974727988
FAM117A	ENSG00000121104	17	47777694	47876542	47	11	123834	0.03818	0.48477	0.974727988
BLM	ENSG00000197299	15	91250558	91368859	211	14	123834	0.038103	0.4848	0.974727988
TRPT1	ENSG00000149743	11	63981271	64003726	25	8	123834	0.037571	0.48501	0.974877584
ARHGEF11	ENSG00000132694	1	156894632	157025162	229	14	123834	0.037491	0.48505	0.974877584
C7orf61	ENSG00000185955	7	100044238	100071894	16	2	123834	0.037319	0.48512	0.974877584
GEMIN5	ENSG00000082516	5	154256976	154327769	127	9	123834	0.037094	0.48521	0.974877584
RNF222	ENSG00000189051	17	8284022	8311144	20	5	123834	0.036468	0.48545	0.974877584
IKZF4	ENSG00000123411	12	56391443	56442219	31	4	123834	0.036436	0.48547	0.974877584
CD5L	ENSG00000073754	1	157790704	157878046	160	12	123834	0.036187	0.48557	0.974877584
EMR3	ENSG00000131355	19	14719929	14810839	145	19	123834	0.036094	0.4856	0.974877584
VEZF1	ENSG00000136451	17	56038910	56075620	41	3	123834	0.035974	0.48565	0.974877584
OR10H1	ENSG00000186723	19	15907817	15928936	67	8	123834	0.035848	0.4857	0.974877584
CAPZB	ENSG00000077549	1	19655267	19822066	313	17	123834	0.035706	0.48576	0.974877584
GSTCD	ENSG00000138780	4	106619935	106778885	282	8	123834	0.035438	0.48587	0.974877584
ANKS3	ENSG00000168096	16	4736513	4794379	78	8	123834	0.03522	0.48595	0.974877584
SPEM1	ENSG00000181323	17	7313679	7334951	10	3	123834	0.035171	0.48597	0.974877584
ZNF772	ENSG00000197128	19	57968031	57998938	98	6	123834	0.035136	0.48599	0.974877584
RP11-296A16.1	ENSG00000262560	15	44054815	44101331	44	4	123834	0.035069	0.48601	0.974877584
AL022328.1	ENSG00000268427	22	50704103	50724350	17	5	123834	0.035067	0.48601	0.974877584
XIRP2	ENSG00000163092	2	167734997	168126263	1089	29	123834	0.034804	0.48612	0.974877584
PIAS3	ENSG00000131788	1	145565233	145596546	20	2	123834	0.034664	0.48617	0.974877584
GDF7	ENSG00000143869	2	20856424	20883418	25	8	123834	0.034477	0.48625	0.974877584
CD83	ENSG00000112149	6	14107872	14147149	33	6	123834	0.034336	0.4863	0.974877584
GRM1	ENSG00000152822	6	146338782	146768734	553	27	123834	0.034147	0.48638	0.974877584
RPL34	ENSG00000109475	4	109531722	109561568	49	5	123834	0.034122	0.48639	0.974877584
MBD1	ENSG00000141644	18	47783252	47818144	33	4	123834	0.033855	0.4865	0.974877584
C1orf27	ENSG00000157181	1	186334890	186400510	97	13	123834	0.033444	0.48666	0.974877584
POLR1A	ENSG00000068654	2	86237339	86343278	152	10	123834	0.03338	0.48669	0.974877584
ATP1A1	ENSG00000163399	1	116905290	116962883	70	3	123834	0.033194	0.48676	0.974877584
XRN1	ENSG00000114127	3	142015449	142176904	145	11	123834	0.033149	0.48678	0.974877584
COX19	ENSG00000240230	7	928415	1025235	152	34	123834	0.033052	0.48682	0.974877584
GCNT6	ENSG00000205318	6	10623993	10657501	39	2	123834	0.033016	0.48683	0.974877584
NPHP4	ENSG00000131697	1	5912871	6062533	317	13	123834	0.032875	0.48689	0.974877584
IFT122	ENSG00000163913	3	129148968	129249198	165	5	123834	0.032796	0.48692	0.974877584
NTRK3	ENSG00000140538	15	88408230	88809999	354	41	123834	0.032774	0.48693	0.974877584
POLR2A	ENSG00000181222	17	7377685	7427933	158	9	123834	0.032749	0.48694	0.974877584
RP11-386G21.2	ENSG00000253857	8	56064043	56087287	23	3	123834	0.032726	0.48695	0.974877584
GXYLT1	ENSG00000151233	12	42465647	42548681	146	12	123834	0.032478	0.48705	0.974877584
EVPLL	ENSG00000214860	17	18270976	18302961	29	4	123834	0.031941	0.48726	0.974877584
MTOR	ENSG00000198793	1	11156592	11332564	313	7	123834	0.031745	0.48734	0.974877584
ACTR2	ENSG00000138071	2	65444887	65508387	113	11	123834	0.031565	0.48741	0.974877584
TM4SF18	ENSG00000163762	3	149026285	149062201	33	7	123834	0.031505	0.48743	0.974877584
CCDC47	ENSG00000108588	17	61812610	61863711	86	5	123834	0.031508	0.48743	0.974877584
DAB2	ENSG00000153071	5	39361780	39472402	228	23	123834	0.031263	0.48753	0.974877584
SLC38A7	ENSG00000103042	16	58689013	58729008	88	11	123834	0.031177	0.48756	0.974877584
RRN3	ENSG00000085721	16	15143879	15198174	65	9	123834	0.03116	0.48757	0.974877584

BRINP1	ENSG00000078725	9	121905736	122141745	257	29	123834	0.031149	0.48758	0.974877584
CCDC90B	ENSG00000137500	11	82960139	83007450	99	9	123834	0.031092	0.4876	0.974877584
NFAM1	ENSG00000235568	22	42766416	42838401	110	14	123834	0.031095	0.4876	0.974877584
ELN	ENSG00000049540	7	73432119	73494237	55	6	123834	0.030906	0.48767	0.974877584
GTF3C4	ENSG00000125484	9	135535422	135580342	89	6	123834	0.03066	0.48777	0.974877584
ZSCAN21	ENSG00000166529	7	99637390	99672661	32	4	123834	0.030575	0.4878	0.974877584
TRPC3	ENSG00000138741	4	122790182	122882909	72	11	123834	0.030531	0.48782	0.974877584
CCDC13	ENSG00000244607	3	42724155	42824745	130	11	123834	0.030496	0.48784	0.974877584
TRAPPC1	ENSG00000170043	17	7823663	7845441	23	3	123834	0.030479	0.48784	0.974877584
RP11-51F16.8	ENSG00000125695	17	61770205	61839457	103	5	123834	0.030131	0.48798	0.974877584
SF1	ENSG00000168066	11	64522078	64556258	20	1	123834	0.029536	0.48822	0.974877584
KCNA4	ENSG00000182255	11	30021288	30048570	54	7	123834	0.029513	0.48823	0.974877584
AC025262.1	ENSG00000268028	12	65080189	65100329	10	4	123834	0.029508	0.48823	0.974877584
PCDH7	ENSG00000169851	4	30712037	31158422	857	44	123834	0.029435	0.48826	0.974877584
BCL2L11	ENSG00000153094	2	111866955	111936024	69	12	123834	0.029138	0.48838	0.974877584
ANKRD66	ENSG00000230062	6	46704654	46737243	19	2	123834	0.029062	0.48841	0.974877584
LIG1	ENSG00000105486	19	48608702	48683860	185	15	123834	0.028952	0.48845	0.974877584
C1orf127	ENSG00000175262	1	10996528	11052094	88	9	123834	0.028768	0.48852	0.974877584
RGS14	ENSG00000169220	5	176774838	176809602	27	4	123834	0.028763	0.48853	0.974877584
XYLB	ENSG00000093217	3	38378251	38472839	224	16	123834	0.02863	0.48858	0.974877584
VPS37C	ENSG00000167987	11	60887728	60939089	120	7	123834	0.028584	0.4886	0.974877584
TRIAP1	ENSG00000170855	12	120871764	120894215	31	8	123834	0.028577	0.4886	0.974877584
SERPINB8	ENSG00000166401	18	61627159	61682278	64	13	123834	0.028422	0.48866	0.974877584
C19orf68	ENSG00000185453	19	48663949	48710877	87	11	123834	0.0284	0.48867	0.974877584
MRPL55	ENSG00000162910	1	228284380	228307013	14	3	123834	0.027976	0.48884	0.974877584
RP11-613M10.8	ENSG00000256966	9	37502544	37602466	170	19	123834	0.027923	0.48886	0.974877584
C10orf90	ENSG00000154493	10	128103566	128369079	376	48	123834	0.027873	0.48888	0.974877584
AACS	ENSG00000081760	12	125539925	125637873	101	15	123834	0.027841	0.48889	0.974877584
C22orf46	ENSG00000184208	22	42074943	42104140	35	2	123834	0.027852	0.48889	0.974877584
MMEL1	ENSG00000142606	1	2512078	2574481	124	4	123834	0.027836	0.4889	0.974877584
FAM69B	ENSG00000165716	9	139597022	139628502	69	12	123834	0.027822	0.4889	0.974877584
DPEP2	ENSG00000167261	16	68011297	68044489	26	4	123834	0.027781	0.48892	0.974877584
TRMT10A	ENSG00000145331	4	100457866	100495189	107	8	123834	0.02768	0.48896	0.974877584
TCF15	ENSG00000125878	20	574441	601042	27	7	123834	0.027594	0.48899	0.974877584
HRASLS	ENSG00000127252	3	192948914	193005527	64	7	123834	0.027482	0.48904	0.974877584
KRT80	ENSG00000167767	12	52552780	52595784	74	9	123834	0.027478	0.48904	0.974877584
ENDOU	ENSG00000111405	12	48093517	48129350	81	10	123834	0.027392	0.48907	0.974877584
RPAP3	ENSG00000005175	12	48047070	48109844	92	11	123834	0.027381	0.48908	0.974877584
TRAP1	ENSG00000126602	16	3691640	3777598	57	5	123834	0.026907	0.48927	0.974948199
PODN	ENSG00000174348	1	53517854	53561174	146	14	123834	0.026799	0.48931	0.974948199
TMEM114	ENSG00000232258	16	8609502	8632304	43	7	123834	0.026797	0.48931	0.974948199
MRPL4	ENSG00000105364	19	10352577	10380721	27	7	123834	0.02671	0.48935	0.974948199
ADRB2	ENSG00000169252	5	148196156	148218196	22	4	123834	0.026473	0.48944	0.974948199
CKAP2	ENSG00000136108	13	53019564	53060763	81	6	123834	0.026201	0.48955	0.974948199
LSG1	ENSG00000041802	3	194351517	194403206	163	12	123834	0.02612	0.48958	0.974948199
LDLRAD3	ENSG00000179241	11	35955531	36263686	596	63	123834	0.025864	0.48968	0.974948199
AL031320.1	ENSG00000268402	6	143749575	143774295	54	2	123834	0.025742	0.48973	0.974948199
GSTA3	ENSG00000174156	6	52751437	52784483	83	7	123834	0.02568	0.48976	0.974948199
TIMM50	ENSG00000105197	19	39961052	39994422	61	7	123834	0.025636	0.48977	0.974948199
TRAF3IP2	ENSG00000056972	6	111867657	111937481	58	11	123834	0.025606	0.48979	0.974948199
PSMA3	ENSG00000100567	14	58701549	58748730	37	4	123834	0.025441	0.48985	0.974948199
ASIC2	ENSG00000108684	17	31330105	32511983	2002	186	123834	0.025347	0.48989	0.974948199
MAX	ENSG00000125952	14	65462892	65579413	172	19	123834	0.025025	0.49002	0.974948199
NTRK2	ENSG00000148053	9	87273466	87648505	574	41	123834	0.024974	0.49004	0.974948199
NUAK2	ENSG00000163545	1	205261187	205300883	60	5	123834	0.024781	0.49011	0.974948199
L1TD1	ENSG00000240563	1	62650503	62688000	176	19	123834	0.024417	0.49026	0.974948199
S1PR3	ENSG00000213694	9	91596362	91629925	42	9	123834	0.024024	0.49042	0.974948199
NINJ1	ENSG00000131669	9	95873771	95906570	67	12	123834	0.023988	0.49043	0.974948199
MIOS	ENSG00000164654	7	7596503	7658560	140	11	123834	0.023941	0.49045	0.974948199
DHRS4L2	ENSG00000187630	14	24429148	24485617	90	9	123834	0.023938	0.49045	0.974948199
ZDHHC18	ENSG00000204160	1	27143201	27194093	49	5	123834	0.023756	0.49052	0.974948199
ATP10D	ENSG00000145246	4	47477305	47605503	278	24	123834	0.023569	0.4906	0.974948199
IGLON5	ENSG00000142549	19	51805102	51843608	38	7	123834	0.023414	0.49066	0.974948199
DNASE1	ENSG00000213918	16	3651729	3740144	109	4	123834	0.023172	0.49076	0.974948199
REXO1	ENSG00000079313	19	1805248	1858452	56	8	123834	0.023093	0.49079	0.974948199
ZNF93	ENSG00000184635	19	20001722	20056384	122	13	123834	0.023063	0.4908	0.974948199
ASB15	ENSG00000146809	7	123197064	123287951	126	13	123834	0.02278	0.49091	0.974948199
ESF1	ENSG00000089048	20	13684969	13775532	181	18	123834	0.022747	0.49093	0.974948199
SIGLEC12	ENSG00000254521	19	51984611	52015043	141	20	123834	0.022557	0.491	0.974948199
AC079602.1	ENSG00000272214	12	121397641	121420095	51	9	123834	0.022516	0.49102	0.974948199
IL33	ENSG00000137033	9	6205805	6267983	92	11	123834	0.022477	0.49103	0.974948199
DEFB113	ENSG00000214642	6	49926390	49947338	26	2	123834	0.022378	0.49107	0.974948199
TBX10	ENSG00000167800	11	67388774	67417031	63	8	123834	0.022307	0.4911	0.974948199
ITM2B	ENSG00000136156	13	48797294	48847063	37	4	123834	0.022222	0.49114	0.974948199
ELOVL4	ENSG00000118402	6	80614529	80667297	135	11	123834	0.022134	0.49117	0.974948199
GLB1	ENSG00000170266	3	33028100	33148722	237	23	123834	0.021914	0.49126	0.974948199
MYO10	ENSG00000145555	5	16655395	16946372	418	63	123834	0.021857	0.49128	0.974948199
DAK	ENSG00000149476	11	61090682	61130767	2	1	123834	0.021403	0.49146	0.974948199
AC020922.1	ENSG00000267531	19	55841254	55866561	29	3	123834	0.021261	0.49152	0.974948199
HSPA13	ENSG00000155304	21	15733436	15765805	93	13	123834	0.021235	0.49153	0.974948199
CTLA4	ENSG00000163599	2	204722509	204748683	34	5	123834	0.021183	0.49155	0.974948199
THAP4	ENSG00000176946	2	242513820	242586864	129	9	123834	0.021181	0.49155	0.974948199
SUV420H2	ENSG00000133247	19	55841221	55869488	32	3	123834	0.021139	0.49157	0.974948199
ZNF337	ENSG00000130684	20	25644851	25687477	79	7	123834	0.021086	0.49159	0.974948199
AP001925.1	ENSG00000269042	11	108120255	108146480	19	2	123834	0.020962	0.49164	0.974948199
CTAGE4	ENSG00000225932	7	143870559	143893146	9	2	123834	0.020941	0.49165	0.974948199
OR5D18	ENSG00000186119	11	55577086	55598073	49	6	123834	0.020853	0.49168	0.974948199
JOSD1	ENSG00000100221	22	39071548	39107561	71	6	123834	0.020864	0.49168	0.974948199
NRP2	ENSG00000118257	2	206536714	206672857	103	27	123834	0.020606	0.49178	0.974980165
SEPHS2	ENSG00000179918	16	30444952	30467502	3	2	123834	0.020473	0.49183	0.974980165
AC011298.1	ENSG00000268822	2	241614478	241634705	18	2	123834	0.020437	0.49185	0.974980165
DHRS12	ENSG00000102796	13	52332129	52388293	39	9	123834	0.019675	0.49215	0.975473118
GNE	ENSG00000159921	9	36204438	36287053	162	13	123834	0.019403	0.49226	0.975589415
CMTM2	ENSG00000140932	16	66603351	66632178	43	4	123834	0.019198	0.49234	0.975646239
NXPE4	ENSG00000137634	11	114431313	114476484	75	10	123834	0.018098	0.49278	0.976371327
CYP11B1	ENSG00000160882	8	143943772	143971262	95	8	123834	0.017894	0.49286	0.976371327
PRODH	ENSG00000100033	22	18890294	18934066	79	16	123834	0.017906	0.49286	0.976371327
C10orf85	ENSG00000177234	10	122347721	122369629	33	4	123834	0.017472	0.49303	0.976606319
EMC3	ENSG00000125037	3	9994221	10062800	159	4	123834	0.01715	0.49316	0.976699879

COL3A1	ENSG00000168542	2	189829046	189887472	74	10	123834	0.017097	0.49318	0.976699879
C11orf42	ENSG00000180878	11	6216796	6242362	35	6	123834	0.01666	0.49335	0.976934764
MTRF1	ENSG00000120662	13	41780505	41847742	132	9	123834	0.016393	0.49346	0.9770508
EEF1G	ENSG00000254772	11	62317073	62352401	25	2	123834	0.01609	0.49358	0.977124432
KIN	ENSG00000151657	10	7782925	7839990	85	15	123834	0.016055	0.4936	0.977124432
KDM2B	ENSG00000089094	12	121856900	122028920	181	14	123834	0.015558	0.49379	0.977367872
CLDN4	ENSG00000189143	7	73203872	73257014	63	3	123834	0.015361	0.49387	0.977367872
FAM170A	ENSG00000164334	5	118955254	118981517	77	4	123834	0.0149	0.49406	0.977367872
MRPS25	ENSG00000131368	3	15073967	15116842	61	8	123834	0.01486	0.49407	0.977367872
KIAA0232	ENSG00000170871	4	6773102	6895897	50	8	123834	0.014757	0.49411	0.977367872
KIAA1244	ENSG00000112379	6	138473058	138675800	389	20	123834	0.014735	0.49412	0.977367872
LILRB2	ENSG00000131042	19	54767675	54795039	30	6	123834	0.014674	0.49415	0.977367872
RPL38	ENSG00000172809	17	72189721	72216794	58	11	123834	0.014638	0.49416	0.977367872
POU6F2	ENSG00000106536	7	39007598	39542694	865	56	123834	0.014468	0.49423	0.977367872
ALOX5AP	ENSG00000132965	13	31299645	31348556	100	18	123834	0.014342	0.49428	0.977367872
OR51A2	ENSG00000205496	11	4966002	4986943	46	6	123834	0.014321	0.49429	0.977367872
ARL16	ENSG00000214087	17	79638204	79660954	91	8	123834	0.01418	0.49434	0.977367872
TATDN1	ENSG00000147687	8	125490726	125561699	96	12	123834	0.013514	0.49461	0.97752331
LRRK2	ENSG00000188906	12	40580546	40773087	488	29	123834	0.013376	0.49466	0.97752331
EYA3	ENSG00000158161	1	28286855	28425207	169	17	123834	0.012893	0.49486	0.97752331
OR4P4	ENSG00000181927	11	55395834	55416772	1	1	123834	0.012754	0.49491	0.97752331
OR2S2	ENSG00000122718	9	35947105	35968151	37	5	123834	0.012728	0.49492	0.97752331
OSBPL3	ENSG00000070882	7	24826158	25031253	262	47	123834	0.012664	0.49495	0.97752331
ARNT	ENSG00000143437	1	150772181	150859244	71	6	123834	0.012623	0.49496	0.97752331
SOD3	ENSG00000109610	4	24781534	24812464	62	5	123834	0.012435	0.49504	0.97752331
SCIMP	ENSG00000161929	17	5102256	5148155	100	13	123834	0.012421	0.49504	0.97752331
HOXD4	ENSG00000170166	2	177005950	177027954	15	5	123834	0.012401	0.49505	0.97752331
TRAIP	ENSG00000183763	3	49856034	49904007	40	7	123834	0.01202	0.4952	0.97752331
ZNF771	ENSG00000179965	16	30408618	30450920	9	3	123834	0.01201	0.49521	0.97752331
VSIG2	ENSG00000019102	11	124607368	124632134	11	4	123834	0.011928	0.49524	0.97752331
PHGDH	ENSG00000092621	1	120192421	120296838	88	16	123834	0.011688	0.49534	0.97752331
C10orf99	ENSG00000188373	10	85923494	85955050	44	9	123834	0.011557	0.49539	0.97752331
CLEC2D	ENSG00000069493	12	9807565	9858413	194	16	123834	0.011566	0.49539	0.97752331
TIE1	ENSG00000066056	1	43756664	43798779	20	3	123834	0.01145	0.49543	0.97752331
ERAP1	ENSG00000164307	5	96086521	96153803	265	17	123834	0.011414	0.49545	0.97752331
ZNF92	ENSG00000146757	7	64828712	64876038	128	8	123834	0.011323	0.49548	0.97752331
TM6SF2	ENSG00000213996	19	19365173	19394200	50	5	123834	0.0113	0.49549	0.97752331
SYTL3	ENSG00000164674	6	159061046	159195908	233	37	123834	0.010945	0.49563	0.97752331
SF3A3	ENSG00000183431	1	38412647	38466593	79	5	123834	0.010904	0.49565	0.97752331
RDH12	ENSG00000139988	14	68158603	68211169	121	11	123834	0.010671	0.49574	0.97752331
CRELD1	ENSG00000163703	3	9965506	9997097	26	4	123834	0.010627	0.49576	0.97752331
MSTO1	ENSG00000125459	1	155569979	155728153	102	8	123834	0.010446	0.49583	0.97752331
NPAP1	ENSG00000185823	15	24910541	24938593	72	7	123834	0.010461	0.49583	0.97752331
ADAM32	ENSG00000197140	8	38954509	39152430	303	11	123834	0.010298	0.49589	0.97752331
SLC35G6	ENSG00000259224	17	7374721	7396383	76	6	123834	0.010278	0.4959	0.97752331
MFSD6L	ENSG00000185156	17	8690436	8712667	20	4	123834	0.010252	0.49591	0.97752331
IGSF23	ENSG00000216588	19	45106940	45150081	62	9	123834	0.010104	0.49597	0.977540207
AGFG1	ENSG00000173744	2	228326868	228435930	313	16	123834	0.0097008	0.49613	0.977579608
ANKK1	ENSG00000170209	11	113248513	113281140	44	6	123834	0.0095623	0.49619	0.977579608
VSTM1	ENSG00000189068	19	54534079	54577207	56	10	123834	0.0093874	0.49626	0.977579608
SERINC4	ENSG00000184716	15	44076360	44102419	22	3	123834	0.0093497	0.49627	0.977579608
OR6C65	ENSG00000205328	12	55784214	55805289	54	4	123834	0.009312	0.49629	0.977579608
NELL2	ENSG00000184613	12	44892058	45325631	598	31	123834	0.009239	0.49631	0.977579608
DUSP5	ENSG00000138166	10	112247596	112281302	42	6	123834	0.0091526	0.49635	0.977579608
KCNH5	ENSG00000140015	14	63163287	63578755	662	46	123834	0.0090078	0.49641	0.977596486
AC074212.3	ENSG00000237452	19	46226509	46277792	36	3	123834	0.0087376	0.49651	0.977692125
RNF207	ENSG00000158286	1	6255535	6291359	39	4	123834	0.008248	0.49671	0.977984636
PAIP2	ENSG00000120727	5	138667276	138715406	27	5	123834	0.0080176	0.4968	0.977998608
EXTL3	ENSG00000012232	8	28447986	28623116	172	16	123834	0.0079703	0.49682	0.977998608
CTB-54O9.9	ENSG00000267157	19	5774016	5795630	54	6	123834	0.0073905	0.49705	0.978031066
GRIK2	ENSG00000164418	6	101836664	102527958	1349	96	123834	0.0073376	0.49707	0.978031066
CASP3	ENSG00000164305	4	185538850	185580663	54	8	123834	0.0072829	0.49709	0.978031066
HMGCS2	ENSG00000134240	1	120280619	120321528	38	9	123834	0.0069344	0.49723	0.978031066
NMNAT3	ENSG00000163864	3	139269022	139406859	150	23	123834	0.0069125	0.49724	0.978031066
HSPB2	ENSG00000170276	11	111772966	111799574	23	4	123834	0.0065589	0.49738	0.978031066
RP11-156E8.1	ENSG00000272195	1	245122652	245144390	33	3	123834	0.0065119	0.4974	0.978031066
EBI3	ENSG00000105246	19	4219495	4247528	57	7	123834	0.006422	0.49744	0.978031066
CDC42BPG	ENSG00000171219	11	64580859	64622041	26	4	123834	0.0060698	0.49758	0.978031066
KRTAP3-2	ENSG00000212900	17	39145447	39166138	134	10	123834	0.0059818	0.49761	0.978031066
NDUFA11	ENSG00000174886	19	5881287	5914017	87	11	123834	0.0057359	0.49771	0.978031066
CLIC4	ENSG00000169504	1	25061848	25180815	73	4	123834	0.0055727	0.49778	0.978031066
USP15	ENSG00000135655	12	62644119	62821211	319	15	123834	0.005515	0.4978	0.978031066
NKD1	ENSG00000140807	16	50572241	50680647	94	11	123834	0.0053992	0.49785	0.978031066
SKA2	ENSG00000182628	17	57177312	57242630	39	6	123834	0.0052808	0.49789	0.978031066
ST8SIA3	ENSG00000177511	18	55008044	55048962	63	7	123834	0.0052914	0.49789	0.978031066
SP4	ENSG00000105866	7	21457652	21564440	170	23	123834	0.0051987	0.49793	0.978031066
THAP11	ENSG00000168286	16	67866213	67888097	22	3	123834	0.0049854	0.49801	0.978031066
SLC16A12	ENSG00000152779	10	91180051	91326398	131	23	123834	0.0048225	0.49808	0.978031066
TMEM136	ENSG00000181264	11	120185838	120214391	31	6	123834	0.0046353	0.49815	0.978031066
OR14A16	ENSG00000196772	1	247968102	247989031	114	7	123834	0.004428	0.49823	0.978031066
FHL2	ENSG00000115641	2	105964169	106064970	121	25	123834	0.0043749	0.49825	0.978031066
THOC7	ENSG00000163634	3	63809546	63859579	55	11	123834	0.004103	0.49836	0.978031066
CCR3	ENSG00000183625	3	46195096	46318197	214	7	123834	0.003903	0.49844	0.978031066
PTPRR	ENSG00000153233	12	71021853	71324623	598	66	123834	0.0038829	0.49845	0.978031066
LILRA2	ENSG00000239998	19	55074387	55109027	118	6	123834	0.003781	0.49849	0.978031066
SLC16A1	ENSG00000155380	1	113444469	113509635	57	4	123834	0.003669	0.49854	0.978031066
NR3C1	ENSG00000113580	5	142647496	142825077	149	14	123834	0.0035381	0.49859	0.978031066
ELL3	ENSG00000128886	15	44054798	44079741	27	3	123834	0.0034304	0.49863	0.978031066
RB1	ENSG00000139687	13	48867887	49066122	191	12	123834	0.0033708	0.49866	0.978031066
ZNF430	ENSG00000118620	19	21193426	21252856	123	8	123834	0.0031395	0.49875	0.978031066
PFKP	ENSG00000067057	10	3098525	3189904	307	30	123834	0.0030273	0.49879	0.978031066
CYP4F2	ENSG00000186115	19	15978833	16018930	124	14	123834	0.0022428	0.49911	0.978031066
MATN2	ENSG00000132561	8	98871068	99058944	231	29	123834	0.0018959	0.49924	0.978031066
OTOP1	ENSG00000163982	4	4180530	4238616	149	17	123834	0.0018294	0.49927	0.978031066
GPRC5A	ENSG00000013588	12	13033716	13080871	35	7	123834	0.0013609	0.49946	0.978031066
MEF2A	ENSG00000068305	15	100007370	100266671	257	31	123834	0.0012581	0.4995	0.978031066
PCP4L1	ENSG00000248485	1	161218517	161265240	102	13	123834	0.0011381	0.49955	0.978031066
SF3B1	ENSG00000115524	2	198244508	198309815	41	5	123834	0.0010608	0.49958	0.978031066

RRM2B	ENSG00000048392	8	103206730	103261346	113	11	123834	0.00099512	0.4996	0.978031066
NLRP10	ENSG00000182261	11	7970971	7996973	37	7	123834	0.00089296	0.49964	0.978031066
HMGB1	ENSG00000189403	13	31022884	31201734	116	25	123834	0.00074264	0.4997	0.978031066
PLEKHA1	ENSG00000107679	10	124124212	124201867	151	13	123834	0.0007138	0.49972	0.978031066
AL132989.1	ENSG00000268466	14	58744751	58765865	24	2	123834	0.00040084	0.49984	0.978031066
COX5B	ENSG00000135940	2	98252503	98274846	21	3	123834	0.00015645	0.49994	0.978031066
B4GALT1	ENSG00000086062	9	33094080	33177354	178	17	123834	-2.72E-05	0.50001	0.978031066
OR10C1	ENSG00000206474	6	29397083	29418731	74	5	123834	-0.00020851	0.50008	0.978031066
ITPKA	ENSG00000137825	15	41775591	41805747	25	7	123834	-0.00027822	0.50011	0.978031066
UMPS	ENSG00000114491	3	124439213	124474040	120	12	123834	-0.0005997	0.50024	0.978031066
HTRA3	ENSG00000170801	4	8261492	8318838	80	16	123834	-0.00088026	0.50035	0.978031066
COLEC12	ENSG00000158270	18	309361	510722	329	46	123834	-0.00090317	0.50036	0.978031066
C6orf62	ENSG00000112308	6	24695089	24731064	64	16	123834	-0.0010365	0.50041	0.978031066
SEC24B	ENSG00000138802	4	110344928	110472052	164	6	123834	-0.0011648	0.50046	0.978031066
DKFZP434H0512	ENSG00000268926	9	98524605	98547013	30	7	123834	-0.0014056	0.50056	0.978031066
STOX1	ENSG00000165730	10	70577298	70665188	142	19	123834	-0.0014727	0.50059	0.978031066
DNAL1	ENSG00000119661	14	74101578	74180435	180	13	123834	-0.0014726	0.50059	0.978031066
SLC16A14	ENSG00000163053	2	230889698	230943715	67	8	123834	-0.0015949	0.50064	0.978031066
ADAT2	ENSG00000189007	6	143733969	143781810	91	4	123834	-0.001632	0.50065	0.978031066
RP11-366L20.2	ENSG00000197301	12	66235120	66285947	69	8	123834	-0.0016266	0.50065	0.978031066
CCDC40	ENSG00000141519	17	78000435	78084412	261	19	123834	-0.0016549	0.50066	0.978031066
ARL8B	ENSG00000134108	3	5153905	5232596	113	10	123834	-0.0016935	0.50068	0.978031066
NEIL3	ENSG00000109674	4	178220990	178294097	111	20	123834	-0.0017433	0.5007	0.978031066
PPA2	ENSG00000138777	4	106280234	106405238	335	16	123834	-0.0017788	0.50071	0.978031066
AC138393.1	ENSG00000269358	1	224205760	224226371	1	1	123834	-0.0020329	0.50081	0.978031066
AL139099.1	ENSG00000253459	14	50054193	50075882	46	4	123834	-0.0023126	0.50092	0.978031066
XYLT1	ENSG00000103489	16	17185626	17574738	445	64	123834	-0.0023529	0.50094	0.978031066
CLHC1	ENSG00000162994	2	55391927	55469699	139	16	123834	-0.0025042	0.501	0.978031066
SNX32	ENSG00000172803	11	65591112	65634367	55	7	123834	-0.0025707	0.50103	0.978031066
NEIL1	ENSG00000140398	15	75629296	75657592	19	5	123834	-0.0026518	0.50106	0.978031066
SF3B2	ENSG00000087365	11	65808200	65846779	39	7	123834	-0.0027998	0.50112	0.978031066
IFNB1	ENSG00000171855	9	21067104	21087943	44	8	123834	-0.0028649	0.50114	0.978031066
HSD17B8	ENSG00000204228	6	33162419	33184608	24	6	123834	-0.0030665	0.50122	0.978031066
TXNRD1	ENSG00000198431	12	104599557	104754061	381	25	123834	-0.0033642	0.50134	0.978031066
TBC1D26	ENSG00000214946	17	15625561	15659476	3	1	123834	-0.0034922	0.50139	0.978031066
INMT-FAM188B	ENSG00000254959	7	30781753	30941696	376	28	123834	-0.0035272	0.50141	0.978031066
ARSB	ENSG00000113273	5	78063032	78291910	431	27	123834	-0.0035487	0.50142	0.978031066
SMCO3	ENSG00000179256	12	14947584	14977116	45	9	123834	-0.0036642	0.50146	0.978031066
ANKRD37	ENSG00000186352	4	186307175	186331782	33	6	123834	-0.0036996	0.50148	0.978031066
EEPD1	ENSG00000122547	7	36182758	36351152	296	31	123834	-0.0037992	0.50152	0.978031066
FAM167A	ENSG00000154319	8	11268972	11342224	139	21	123834	-0.0039627	0.50158	0.978031066
ZDHHC24	ENSG00000174165	11	66278108	66323709	57	5	123834	-0.0044658	0.50178	0.978031066
KCTD5	ENSG00000167977	16	2722476	2769031	10	2	123834	-0.0045402	0.50181	0.978031066
ANAPC4	ENSG00000053900	4	25368835	25430120	158	13	123834	-0.0045729	0.50182	0.978031066
NKX2-4	ENSG00000125816	20	21366005	21388666	23	6	123834	-0.0046288	0.50185	0.978031066
CKLF	ENSG00000217555	16	66576466	66610154	56	3	123834	-0.0047777	0.50191	0.978031066
C20orf26	ENSG00000089101	20	20023158	20351346	672	32	123834	-0.004884	0.50195	0.978031066
TMED7	ENSG00000134970	5	114939205	114978689	62	5	123834	-0.0049052	0.50196	0.978031066
CKLF-CMTM1	ENSG00000254788	16	66576490	66623038	76	4	123834	-0.0049566	0.50198	0.978031066
PTGR1	ENSG00000106853	9	114302002	114372135	175	27	123834	-0.0050285	0.50201	0.978031066
ELMO2	ENSG00000062598	20	44984688	45071704	64	4	123834	-0.0050603	0.50202	0.978031066
GABRB1	ENSG00000163288	4	46985740	47438461	665	47	123834	-0.0051124	0.50204	0.978031066
GALNS	ENSG00000141012	16	88870142	88933378	204	19	123834	-0.0054576	0.50218	0.978031066
MMP7	ENSG00000137673	11	102381239	102411484	28	6	123834	-0.0058438	0.50233	0.978031066
HIP1R	ENSG00000130787	12	123309000	123357507	118	8	123834	-0.0059129	0.50236	0.978031066
ARHGAP18	ENSG00000146376	6	129887277	130041370	198	37	123834	-0.0059659	0.50238	0.978031066
ING3	ENSG00000071243	7	120580803	120627270	38	6	123834	-0.005969	0.50238	0.978031066
DOK6	ENSG00000206052	18	67058291	67526323	956	97	123834	-0.0060053	0.5024	0.978031066
TMEM126B	ENSG00000171204	11	85329629	85357580	40	6	123834	-0.0060781	0.50242	0.978031066
DGAT1	ENSG00000185000	8	145529954	145560573	6	1	123834	-0.0062103	0.50248	0.978031066
PNPLA6	ENSG00000032444	19	7588890	7636650	114	11	123834	-0.0063767	0.50254	0.978031066
EFCAB3	ENSG00000172421	17	60437579	60503837	51	7	123834	-0.0065475	0.50261	0.978031066
SMIM19	ENSG00000176209	8	42386298	42419603	73	6	123834	-0.0067296	0.50268	0.978031066
UBE2K	ENSG00000078140	4	39689664	39794412	223	15	123834	-0.0067432	0.50269	0.978031066
ZNF488	ENSG00000165388	10	48345024	48383866	14	5	123834	-0.006745	0.50269	0.978031066
RRAGA	ENSG00000155876	9	19039372	19061019	73	10	123834	-0.0069522	0.50277	0.978031066
ZNF701	ENSG00000167562	19	53049075	53100427	129	18	123834	-0.0070628	0.50282	0.978031066
CD74	ENSG00000019582	5	149771200	149802492	26	7	123834	-0.0071533	0.50285	0.978031066
COL11A2	ENSG00000204248	6	33120458	33170276	130	21	123834	-0.0071481	0.50285	0.978031066
CHRD	ENSG00000090539	3	184087861	184118524	20	6	123834	-0.0071612	0.50286	0.978031066
UBE2D1	ENSG00000072401	10	60084735	60140513	86	11	123834	-0.0071914	0.50287	0.978031066
ODAM	ENSG00000109205	4	71052213	71080293	78	6	123834	-0.0073179	0.50292	0.978031066
NCKAP5	ENSG00000176771	2	133419374	134336034	1161	100	123834	-0.00738	0.50294	0.978031066
PAX2	ENSG00000075891	10	102485360	102599698	127	15	123834	-0.0073614	0.50294	0.978031066
ZNF831	ENSG00000124203	20	57756075	57844168	115	20	123834	-0.0073588	0.50294	0.978031066
C1orf210	ENSG00000253313	1	43737554	43761288	22	7	123834	-0.007393	0.50295	0.978031066
SLC15A3	ENSG00000110446	11	60694556	60730002	74	9	123834	-0.0075287	0.503	0.978031066
C1orf100	ENSG00000173728	1	244505937	244562965	78	10	123834	-0.0077045	0.50307	0.978031066
PSMB8	ENSG00000204264	6	32798494	32822480	109	16	123834	-0.0078522	0.50313	0.978031066
RD3	ENSG00000198570	1	211639864	211676259	44	11	123834	-0.0078932	0.50315	0.978031066
CRB3	ENSG00000130545	19	6453788	6477232	25	6	123834	-0.0079732	0.50318	0.978031066
GTSCR1	ENSG00000263417	18	68287755	68327980	89	9	123834	-0.0085183	0.5034	0.978031066
PUM1	ENSG00000134644	1	31394353	31548838	218	14	123834	-0.0087017	0.50347	0.978031066
SULF1	ENSG00000137573	8	70368859	70583150	211	43	123834	-0.0088217	0.50352	0.978031066
SEMA5B	ENSG00000082684	3	122618041	122757452	195	30	123834	-0.00896	0.50357	0.978031066
ME3	ENSG00000151376	11	86142150	86393678	774	43	123834	-0.0090251	0.5036	0.978031066
RUFY3	ENSG00000018189	4	71559921	71683032	298	9	123834	-0.0091156	0.50364	0.978031066
OR1J1	ENSG00000136834	9	125229220	125250235	44	8	123834	-0.0091856	0.50366	0.978031066
SURF2	ENSG00000148291	9	136213428	136238045	48	5	123834	-0.0091925	0.50367	0.978031066
TAP1	ENSG00000168394	6	32802986	32831755	133	17	123834	-0.0092145	0.50368	0.978031066
CLN8	ENSG00000182372	8	1693944	1744738	70	7	123834	-0.0092209	0.50368	0.978031066
MRI1	ENSG00000037757	19	13865346	13895096	52	3	123834	-0.0092319	0.50368	0.978031066
MAP3K19	ENSG00000176601	2	135712061	135815038	170	13	123834	-0.0093396	0.50373	0.978031066
SERF2	ENSG00000140264	15	44059285	44104787	43	4	123834	-0.0096976	0.50387	0.978031066
MALT1	ENSG00000172175	18	56328618	56427371	200	18	123834	-0.0097205	0.50388	0.978031066
DDIT3	ENSG00000175197	12	57900371	57924300	21	2	123834	-0.0099131	0.50395	0.978031066
SAMM50	ENSG00000100347	22	44341301	44416411	161	17	123834	-0.010012	0.50399	0.978031066
C1orf53	ENSG00000203724	1	197861777	197886497	20	5	123834	-0.010028	0.504	0.978031066

GPR126	ENSG00000112414	6	142612991	142777403	140	11	123834	-0.010027	0.504	0.978031066
TNFRSF6B	ENSG00000243509	20	62318021	62340037	19	3	123834	-0.010068	0.50402	0.978031066
NOC4L	ENSG00000184967	12	132618993	132647013	27	4	123834	-0.01012	0.50404	0.978031066
KDELC2	ENSG00000178202	11	108332832	108379159	42	7	123834	-0.01037	0.50414	0.978042004
UXS1	ENSG00000115652	2	106699759	106820795	181	18	123834	-0.010499	0.50419	0.978042004
CTD-3105H18.14	ENSG00000268744	19	12480003	12522088	16	4	123834	-0.010528	0.5042	0.978042004
PIGN	ENSG00000197563	18	59700800	59864351	530	14	123834	-0.010767	0.5043	0.978052936
NT5E	ENSG00000135318	6	86149809	86215500	79	6	123834	-0.010821	0.50432	0.978052936
CENPH	ENSG00000153044	5	68475375	68516184	82	6	123834	-0.01093	0.50436	0.978052936
SACM1L	ENSG00000211456	3	45720548	45796916	193	9	123834	-0.011255	0.50449	0.978205235
CLDN12	ENSG00000157224	7	90003035	90152716	293	25	123834	-0.011421	0.50456	0.978238336
SGIP1	ENSG00000118473	1	66989066	67223982	559	21	123834	-0.011568	0.50461	0.978238336
TF	ENSG00000091513	3	133454800	133507850	74	10	123834	-0.012228	0.50488	0.978251046
FXYD5	ENSG00000089327	19	35635633	35670786	64	9	123834	-0.012381	0.50494	0.978251046
CD3E	ENSG00000198851	11	118165260	118196890	53	5	123834	-0.01241	0.50495	0.978251046
TRPM3	ENSG00000083067	9	73133979	74071820	1846	78	123834	-0.012465	0.50497	0.978251046
C17orf75	ENSG00000108666	17	30641584	30687042	39	6	123834	-0.012834	0.50512	0.978251046
CTB-186H2.3	ENSG00000266956	17	34300692	34327306	68	10	123834	-0.012845	0.50512	0.978251046
KRTAP6-3	ENSG00000212938	21	31954759	31975394	21	3	123834	-0.013033	0.5052	0.978251046
ZNF253	ENSG00000256771	19	19966695	20015483	102	9	123834	-0.01305	0.50521	0.978251046
OR5P3	ENSG00000182334	11	7836584	7857519	117	9	123834	-0.013084	0.50522	0.978251046
ERG	ENSG00000157554	21	39741949	40043704	439	49	123834	-0.013099	0.50523	0.978251046
MADCAM1	ENSG00000099866	19	479176	515347	70	11	123834	-0.013362	0.50533	0.978251046
RERE	ENSG00000142599	1	8402457	8887702	413	15	123834	-0.013468	0.50537	0.978251046
PSMB2	ENSG00000126067	1	36057185	36117445	20	3	123834	-0.013524	0.50539	0.978251046
AC092384.1	ENSG00000269422	16	88884366	88904545	116	10	123834	-0.013519	0.50539	0.978251046
PVRL3	ENSG00000177707	3	110778918	111004410	346	27	123834	-0.013615	0.50543	0.978251046
ZNF219	ENSG00000165804	14	21548205	21582881	58	12	123834	-0.013648	0.50544	0.978251046
TRIM61	ENSG00000183439	4	165865598	165908820	78	8	123834	-0.014172	0.50565	0.978410975
MEDAG	ENSG00000102802	13	31470328	31509709	48	7	123834	-0.014231	0.50568	0.978410975
ATP6V1A	ENSG00000114573	3	113455866	113540903	74	2	123834	-0.014398	0.50574	0.978410975
SAP30L	ENSG00000164576	5	153815517	153850614	33	10	123834	-0.014416	0.50575	0.978410975
SIDT2	ENSG00000149577	11	117039449	117078160	92	12	123834	-0.014498	0.50578	0.978410975
GPR162	ENSG00000250510	12	6920711	6949136	40	10	123834	-0.01463	0.50584	0.978427467
PRR22	ENSG00000212123	19	5772971	5794776	51	5	123834	-0.015095	0.50602	0.978551407
ARHGAP42	ENSG00000165895	11	100548384	100872668	977	43	123834	-0.015148	0.50604	0.978551407
TMEM180	ENSG00000138111	10	104211149	104246802	74	6	123834	-0.015184	0.50606	0.978551407
SIX5	ENSG00000177045	19	46258043	46282484	20	2	123834	-0.015317	0.50611	0.978551407
LRRC73	ENSG00000204052	6	43464707	43488424	29	4	123834	-0.015806	0.50631	0.978653046
AP002353.1	ENSG00000268602	11	107572998	107605135	50	8	123834	-0.01591	0.50635	0.978653046
MEOX1	ENSG00000005102	17	41707756	41749322	54	8	123834	-0.015912	0.50635	0.978653046
TERF2	ENSG00000132604	16	69379464	69452474	64	8	123834	-0.016051	0.5064	0.978653046
AUP1	ENSG00000115307	2	74743772	74767066	7	2	123834	-0.0161	0.50642	0.978653046
LIPT1	ENSG00000144182	2	99761418	99789620	49	4	123834	-0.016508	0.50659	0.978849315
PPP1R12B	ENSG00000077157	1	202307827	202571834	229	9	123834	-0.017116	0.50683	0.978849315
NODAL	ENSG00000156574	10	72182071	72217707	47	8	123834	-0.017128	0.50683	0.978849315
GALNT8	ENSG00000130035	12	4819507	4970277	292	30	123834	-0.017173	0.50685	0.978849315
NOSTRIN	ENSG00000163072	2	169633049	169732024	103	15	123834	-0.017382	0.50693	0.978849315
C10orf95	ENSG00000120055	10	104199594	104221300	12	5	123834	-0.017363	0.50693	0.978849315
CNTN3	ENSG00000113805	3	74301719	74580291	292	33	123834	-0.017397	0.50694	0.978849315
SLC7A10	ENSG00000130876	19	33689570	33726756	41	9	123834	-0.017412	0.50695	0.978849315
EXD2	ENSG00000081177	14	69648228	69719075	58	9	123834	-0.0177	0.50706	0.978849315
ZNF302	ENSG00000089335	19	35158544	35187302	90	12	123834	-0.017803	0.5071	0.978849315
PCNX	ENSG00000100731	14	71364122	71592099	364	14	123834	-0.017901	0.50714	0.978849315
HIST1H2BF	ENSG00000197846	6	26189748	26210942	84	6	123834	-0.01805	0.5072	0.978849315
MMP13	ENSG00000137745	11	102803724	102836463	49	7	123834	-0.018086	0.50721	0.978849315
P4HA3	ENSG00000149380	11	73936846	74032702	159	12	123834	-0.018256	0.50728	0.978849315
AD000671.6	ENSG00000267120	19	36220153	36246333	45	8	123834	-0.018712	0.50746	0.978849315
NHEJ1	ENSG00000187736	2	219930039	220035587	133	8	123834	-0.018783	0.50749	0.978849315
ZNF658	ENSG00000196409	9	40750700	40846415	15	3	123834	-0.018783	0.50749	0.978849315
MX1	ENSG00000157601	21	42782231	42841141	150	15	123834	-0.018765	0.50749	0.978849315
CD300A	ENSG00000167851	17	72452555	72490933	98	18	123834	-0.018802	0.5075	0.978849315
ZNF593	ENSG00000142684	1	26486362	26508551	54	7	123834	-0.019136	0.50763	0.978959245
TCERG1L	ENSG00000176769	10	132880654	133119984	543	78	123834	-0.01919	0.50766	0.978959245
SLC22A18AS	ENSG00000254827	11	2899010	2934970	30	8	123834	-0.019627	0.50783	0.979066223
KLF1	ENSG00000105610	19	12985237	13007995	29	5	123834	-0.019654	0.50784	0.979066223
MYBPC1	ENSG00000196091	12	101952131	102089796	358	36	123834	-0.019727	0.50787	0.979066223
HIPK1	ENSG00000163349	1	114461814	114530426	79	11	123834	-0.020082	0.50801	0.9792368
MAPT	ENSG00000186868	17	43961748	44115700	607	2	123834	-0.020385	0.50813	0.979368793
ZNF132	ENSG00000131849	19	58934181	58961589	43	4	123834	-0.020635	0.50823	0.97945927
RP11-536G4.1	ENSG00000258292	12	96186875	96227085	92	11	123834	-0.020745	0.50828	0.97945927
MYL9	ENSG00000101335	20	35159887	35188228	7	3	123834	-0.02115	0.50844	0.979668274
VWC2L	ENSG00000174453	2	215265789	215453683	279	20	123834	-0.021311	0.5085	0.979681605
CCSER1	ENSG00000184305	4	91038686	92533064	2745	130	123834	-0.021423	0.50855	0.979681605
SPR	ENSG00000116096	2	73104489	73129247	7	1	123834	-0.022012	0.50878	0.979985931
OR5M11	ENSG00000255223	11	56299746	56320757	92	9	123834	-0.022275	0.50889	0.979985931
GRIP1	ENSG00000155974	12	66731211	67207966	946	72	123834	-0.022458	0.50896	0.979985931
PDIA3	ENSG00000167004	15	44028590	44075477	52	4	123834	-0.022648	0.50903	0.979985931
NDUFAF5	ENSG00000101247	20	13755596	13809067	103	15	123834	-0.022696	0.50905	0.979985931
FLVCR1	ENSG00000162769	1	213021597	213082705	289	12	123834	-0.022758	0.50908	0.979985931
RXFP4	ENSG00000173080	1	155901480	155922625	15	3	123834	-0.022967	0.50916	0.979985931
OR56A1	ENSG00000180934	11	6037901	6058971	37	7	123834	-0.023074	0.5092	0.979985931
COL4A1	ENSG00000187498	13	110791318	110969496	319	49	123834	-0.023281	0.50929	0.979985931
MRPL32	ENSG00000106591	7	42961799	42998557	75	6	123834	-0.023432	0.50935	0.979985931
EVC	ENSG00000072840	4	5702924	5840772	327	51	123834	-0.023578	0.50941	0.979985931
ATF6	ENSG00000118217	1	161726084	161943860	445	14	123834	-0.023726	0.50946	0.979985931
RPL35A	ENSG00000182899	3	197666858	197693481	46	5	123834	-0.023952	0.50955	0.979985931
MAP1LC3B	ENSG00000140941	16	87407601	87448385	55	11	123834	-0.023934	0.50955	0.979985931
COX6B1	ENSG00000126267	19	36129125	36159763	48	7	123834	-0.024164	0.50964	0.979985931
STAM2	ENSG00000115145	2	152963315	153042506	116	9	123834	-0.02421	0.50966	0.979985931
TGFBI	ENSG00000120708	5	135354584	135409507	84	8	123834	-0.024237	0.50967	0.979985931
DRAM2	ENSG00000156171	1	111649955	111692838	59	5	123834	-0.024294	0.50969	0.979985931
PURG	ENSG00000172733	8	30843321	30901231	54	9	123834	-0.02464	0.50983	0.979985931
WBSCR22	ENSG00000071462	7	73087355	73129491	75	6	123834	-0.024685	0.50985	0.979985931
CCL11	ENSG00000172156	17	32602687	32625353	16	5	123834	-0.024763	0.50988	0.979985931
CTXN3	ENSG00000205279	5	126974736	127004322	63	9	123834	-0.024801	0.50989	0.979985931
PIK3C3	ENSG00000078142	18	39525171	39677794	172	10	123834	-0.024972	0.50996	0.979985931
MLK4	ENSG00000143674	1	233453514	233530894	172	16	123834	-0.02504	0.50999	0.979985931
NUCKS1	ENSG00000069275	1	205671947	205729404	94	14	123834	-0.025098	0.51001	0.979985931

TTC12	ENSG00000149292	11	113175251	113264266	68	15	123834	-0.025312	0.5101	0.979985931
GEMIN4	ENSG00000179409	17	637654	667239	69	12	123834	-0.025315	0.5101	0.979985931
CDK8	ENSG00000132964	13	26818276	26989375	275	10	123834	-0.025479	0.51016	0.980002151
FAXDC2	ENSG00000170271	5	154188051	154248812	61	7	123834	-0.02588	0.51032	0.98003726
RNASE1	ENSG00000129538	14	21259387	21281437	11	4	123834	-0.025871	0.51032	0.98003726
SSH2	ENSG00000141298	17	27942956	28267294	270	7	123834	-0.025986	0.51037	0.98003726
CLASRP	ENSG00000104859	19	45532298	45584214	12	3	123834	-0.026055	0.51039	0.98003726
FAM174B	ENSG00000185442	15	93150673	93363114	321	29	123834	-0.026404	0.51053	0.98003726
SCN7A	ENSG00000136546	2	167250083	167360757	225	21	123834	-0.026699	0.51065	0.98003726
MEN1	ENSG00000133895	11	64560982	64588766	8	1	123834	-0.026944	0.51075	0.98003726
PDGFA	ENSG00000197461	7	526895	569933	29	5	123834	-0.027083	0.5108	0.98003726
UBASH3A	ENSG00000160185	21	43814008	43877791	193	21	123834	-0.027518	0.51098	0.98003726
NFATC3	ENSG00000072736	16	68108654	68273162	183	6	123834	-0.027848	0.51111	0.98003726
FIBCD1	ENSG00000130720	9	133767825	133824673	96	16	123834	-0.02812	0.51122	0.98003726
SDHC	ENSG00000143252	1	161274047	161342984	254	6	123834	-0.028345	0.51131	0.98003726
KIAA1257	ENSG00000114656	3	128618709	128731533	183	17	123834	-0.028548	0.51139	0.98003726
ATP8A2	ENSG00000132932	13	25936209	26609989	1132	68	123834	-0.028546	0.51139	0.98003726
TXNDC2	ENSG00000168454	18	9875763	9899272	41	8	123834	-0.028561	0.51139	0.98003726
SPTLC1	ENSG00000090054	9	94784281	94887666	290	16	123834	-0.02858	0.5114	0.98003726
VPS13C	ENSG00000129003	15	62134588	62362672	486	19	123834	-0.028589	0.5114	0.98003726
RILPL1	ENSG00000188026	12	123945925	124028265	123	15	123834	-0.028642	0.51142	0.98003726
HELZ2	ENSG00000130589	20	62179439	62215592	51	12	123834	-0.028702	0.51145	0.98003726
RTP1	ENSG00000175077	3	186905274	186929253	48	9	123834	-0.028882	0.51152	0.98003726
DMP1	ENSG00000152592	4	88561459	88595513	78	10	123834	-0.028936	0.51154	0.98003726
NDUFB4	ENSG00000065518	3	120305156	120331347	29	4	123834	-0.029225	0.51166	0.98003726
SMCO1	ENSG00000214097	3	196223748	196252237	28	6	123834	-0.02925	0.51167	0.98003726
DBT	ENSG00000137992	1	100642475	100725390	120	7	123834	-0.029298	0.51169	0.98003726
MATR3	ENSG00000015479	5	138599441	138677360	37	4	123834	-0.029347	0.51171	0.98003726
FNDC1	ENSG00000164694	6	159580429	159703141	276	11	123834	-0.029594	0.5118	0.98003726
B3GALTL	ENSG00000187676	13	31764073	31916413	203	16	123834	-0.029635	0.51182	0.98003726
GALT	ENSG00000258728	9	34636642	34667110	27	5	123834	-0.029695	0.51184	0.98003726
OR2G6	ENSG00000188558	1	248674916	248695964	10	2	123834	-0.029712	0.51185	0.98003726
MLF1	ENSG00000178053	3	158278952	158335041	148	12	123834	-0.029785	0.51188	0.98003726
PNPLA5	ENSG00000100341	22	44265558	44297893	85	12	123834	-0.030002	0.51197	0.98003726
NT5C2	ENSG00000076685	10	104835940	104963056	237	14	123834	-0.030114	0.51201	0.98003726
DNASE1L3	ENSG00000163687	3	58167984	58210424	57	8	123834	-0.030147	0.51202	0.98003726
UHRF1BP1	ENSG00000065060	6	34749857	34860915	240	13	123834	-0.030581	0.5122	0.98003726
BLOC1S6	ENSG00000104164	15	45869321	45918197	47	3	123834	-0.030589	0.5122	0.98003726
ATG4D	ENSG00000130734	19	10644571	10674094	77	10	123834	-0.030841	0.5123	0.98003726
TLK2	ENSG00000146872	17	60526019	60702842	112	6	123834	-0.030945	0.51234	0.98003726
LCMT2	ENSG00000168806	15	43609974	43632803	21	3	123834	-0.031195	0.51244	0.98003726
ARTN	ENSG00000117407	1	44388992	44412913	48	6	123834	-0.031244	0.51246	0.98003726
KRT10	ENSG00000186395	17	38964369	38988847	45	8	123834	-0.031263	0.51247	0.98003726
KCNG3	ENSG00000171126	2	42659157	42731237	48	9	123834	-0.031296	0.51248	0.98003726
CEACAM16	ENSG00000213892	19	45192421	45223986	37	13	123834	-0.031285	0.51248	0.98003726
CA6	ENSG00000131686	1	8995926	9045151	61	17	123834	-0.03133	0.5125	0.98003726
CREBZF	ENSG00000137504	11	85360752	85403951	59	10	123834	-0.031344	0.5125	0.98003726
LMO3	ENSG00000048540	12	16691307	16773528	16	5	123834	-0.031345	0.5125	0.98003726
CPA4	ENSG00000128510	7	129922974	129974020	69	10	123834	-0.031457	0.51255	0.98003726
GPR155	ENSG00000163328	2	175286966	175361822	90	7	123834	-0.032285	0.51288	0.98012753
ZNF812	ENSG00000224689	19	9790600	9821452	102	5	123834	-0.032417	0.51293	0.98012753
OR8B12	ENSG00000170953	11	124402578	124423575	43	6	123834	-0.032543	0.51298	0.98012753
TMEM8B	ENSG00000137103	9	35804448	35864844	62	8	123834	-0.032786	0.51308	0.98012753
VTI1B	ENSG00000100568	14	68103792	68151548	78	9	123834	-0.033026	0.51317	0.98012753
SLC22A2	ENSG00000112499	6	160582093	160708670	257	22	123834	-0.033111	0.51321	0.98012753
EPB41L4A-AS2	ENSG00000268358	5	111745280	111766310	11	2	123834	-0.033214	0.51325	0.98012753
EHBP1	ENSG00000115504	2	62890986	63283622	193	20	123834	-0.033413	0.51333	0.98012753
SGK223	ENSG00000182319	8	8165258	8254008	157	24	123834	-0.033481	0.51335	0.98012753
MKRN1	ENSG00000133606	7	140142840	140189369	91	5	123834	-0.033721	0.51345	0.98012753
CPB1	ENSG00000153002	3	148498889	148587974	157	24	123834	-0.033849	0.5135	0.98012753
DFNB59	ENSG00000204311	2	179306163	179336117	26	5	123834	-0.034011	0.51357	0.98012753
NR2C2	ENSG00000177463	3	14979091	15105107	132	9	123834	-0.034338	0.5137	0.98012753
EFEMP1	ENSG00000115380	2	56083102	56161274	162	17	123834	-0.034372	0.51371	0.98012753
B3GALT6	ENSG00000176022	1	1157629	1180421	84	4	123834	-0.03449	0.51376	0.98012753
RDH5	ENSG00000135437	12	56104151	56128489	8	2	123834	-0.034636	0.51382	0.98012753
CYP3A4	ENSG00000160868	7	99344604	99391888	24	4	123834	-0.034692	0.51384	0.98012753
C9orf57	ENSG00000204669	9	74656292	74697733	61	9	123834	-0.034713	0.51385	0.98012753
IL1B	ENSG00000125538	2	113577328	113604480	27	6	123834	-0.034826	0.51389	0.98012753
ADAMTS1	ENSG00000154734	21	28198066	28227728	54	12	123834	-0.03486	0.5139	0.98012753
AL158091.1	ENSG00000269223	20	62464779	62485273	49	6	123834	-0.034907	0.51392	0.98012753
RPL27	ENSG00000131469	17	41140290	41164976	26	3	123834	-0.035058	0.51398	0.98012753
NPHS1	ENSG00000161270	19	36306866	36370189	60	6	123834	-0.03526	0.51406	0.98012753
GFPT2	ENSG00000131459	5	179717690	179790387	176	20	123834	-0.035355	0.5141	0.98012753
ST6GAL2	ENSG00000144057	2	107408056	107513564	240	21	123834	-0.035848	0.5143	0.98012753
TMBIM6	ENSG00000139644	12	50091508	50168717	156	8	123834	-0.035928	0.51433	0.98012753
PON1	ENSG00000005421	7	94916988	95035673	152	22	123834	-0.035942	0.51434	0.98012753
STRC	ENSG00000242866	15	43881596	44020458	102	6	123834	-0.036195	0.51444	0.98012753
NET1	ENSG00000173848	10	5444514	5510426	103	5	123834	-0.036355	0.5145	0.98012753
TRPM5	ENSG00000070985	11	2415745	2454275	20	4	123834	-0.036794	0.51468	0.98012753
NUP205	ENSG00000155561	7	135232667	135343505	375	14	123834	-0.036846	0.5147	0.98012753
FBXO46	ENSG00000177051	19	46203887	46244162	19	5	123834	-0.036888	0.51471	0.98012753
STH	ENSG00000256762	17	44066616	44087060	78	2	123834	-0.036906	0.51472	0.98012753
NEUROD2	ENSG00000171532	17	37749789	37776030	21	4	123834	-0.036994	0.51475	0.98012753
MYLK3	ENSG00000140795	16	46730891	46834319	9	4	123834	-0.037368	0.5149	0.98012753
TPD52L3	ENSG00000170777	9	6318349	6341900	23	3	123834	-0.037377	0.51491	0.98012753
SFT2D2	ENSG00000213064	1	168185176	168232263	40	4	123834	-0.037662	0.51502	0.98012753
KPRP	ENSG00000203786	1	152720506	152744529	40	3	123834	-0.03779	0.51507	0.98012753
AOC1	ENSG00000002726	7	150511715	150568592	131	13	123834	-0.037888	0.51511	0.98012753
QPRT	ENSG00000103485	16	29664600	29720020	40	7	123834	-0.037894	0.51511	0.98012753
TMED9	ENSG00000184840	5	177009159	177033125	40	5	123834	-0.038037	0.51517	0.98012753
LSAMP	ENSG00000185565	3	115511235	117726095	3934	212	123834	-0.038264	0.51526	0.98012753
LHFPL4	ENSG00000156959	3	9533481	9605486	147	12	123834	-0.038307	0.51528	0.98012753
CTTN	ENSG00000085733	11	70234510	70292690	38	4	123834	-0.038725	0.51545	0.98012753
C19orf69	ENSG00000204978	19	41939063	41960670	20	3	123834	-0.038855	0.5155	0.98012753
ACSM3	ENSG00000005187	16	20611565	20818903	364	14	123834	-0.038961	0.51554	0.98012753
GNL1	ENSG00000204590	6	30499154	30534951	30	5	123834	-0.039047	0.51557	0.98012753
SIRT2	ENSG00000068903	19	39359197	39400502	20	5	123834	-0.0391	0.51559	0.98012753
POLH	ENSG00000170734	6	43533887	43596701	61	7	123834	-0.039248	0.51565	0.98012753

AC092964.2	ENSG00000267979	3	172009438	172029686	22	4	123834	-0.039396	0.51571	0.98012753
USP17L30	ENSG00000228856	4	9354855	9376447	44	5	123834	-0.039391	0.51571	0.98012753
PLA2R1	ENSG00000153246	2	160778519	160929121	250	33	123834	-0.039539	0.51577	0.98012753
DET1	ENSG00000140543	15	89044790	89099906	113	7	123834	-0.039612	0.5158	0.98012753
RAD50	ENSG00000113522	5	131881711	131990313	79	2	123834	-0.03973	0.51585	0.98012753
CCDC63	ENSG00000173093	12	111274573	111355339	103	12	123834	-0.039738	0.51585	0.98012753
HBG1	ENSG00000213934	11	5259313	5281122	31	3	123834	-0.039851	0.51589	0.98012753
ECM2	ENSG00000106823	9	95246365	95308937	131	12	123834	-0.040095	0.51599	0.98012753
AOX1	ENSG00000138356	2	201440591	201551787	177	27	123834	-0.040327	0.51608	0.98012753
SLC23A3	ENSG00000213901	2	219930051	220045549	145	9	123834	-0.040505	0.51615	0.98012753
LGI1	ENSG00000108231	10	95507566	95567916	87	16	123834	-0.040805	0.51627	0.98012753
ARHGEF3	ENSG00000163947	3	56751446	57123357	559	50	123834	-0.040916	0.51632	0.98012753
STRN3	ENSG00000196792	14	31353005	31505607	271	15	123834	-0.041363	0.5165	0.98012753
MON1B	ENSG00000103111	16	77214732	77246302	74	12	123834	-0.041642	0.51661	0.98012753
NAGK	ENSG00000124357	2	71281474	71316935	94	11	123834	-0.041696	0.51663	0.98012753
STK39	ENSG00000198648	2	168800530	169114651	557	31	123834	-0.041689	0.51663	0.98012753
PIK3CA	ENSG00000121879	3	178855902	178967881	233	19	123834	-0.041733	0.51664	0.98012753
CENPB	ENSG00000125817	20	3754498	3777337	18	3	123834	-0.041723	0.51664	0.98012753
CKMT2	ENSG00000131730	5	80519104	80572216	98	10	123834	-0.041853	0.51669	0.98012753
IER3IP1	ENSG00000134049	18	44671413	44712745	101	8	123834	-0.04209	0.51679	0.98012753
LPPR5	ENSG00000117598	1	99345806	99480588	320	31	123834	-0.042254	0.51685	0.98012753
HDAC3	ENSG00000171720	5	140990443	141026437	52	12	123834	-0.042244	0.51685	0.98012753
MYH6	ENSG00000197616	14	23841199	23887486	52	11	123834	-0.042343	0.51689	0.98012753
CYP2E1	ENSG00000130649	10	135323910	135384724	240	12	123834	-0.042363	0.5169	0.98012753
SYPL1	ENSG00000008282	7	105720949	105763022	54	10	123834	-0.042453	0.51693	0.98012753
MPP5	ENSG00000072415	14	67697826	67812536	287	8	123834	-0.042486	0.51694	0.98012753
DCUN1D5	ENSG00000137692	11	102922805	102972944	42	6	123834	-0.042513	0.51695	0.98012753
QRSL1	ENSG00000130348	6	107067453	107126292	148	18	123834	-0.042596	0.51699	0.98012753
UBR5	ENSG00000104517	8	103255240	103435069	388	23	123834	-0.042828	0.51708	0.98012753
CPZ	ENSG00000109625	4	8584387	8631488	117	20	123834	-0.042926	0.51712	0.98012753
FZD10	ENSG00000111432	12	130637004	130660285	25	8	123834	-0.043057	0.51717	0.98012753
OR3A3	ENSG00000159961	17	3313862	3334827	10	2	123834	-0.043054	0.51717	0.98012753
HIGD1A	ENSG00000181061	3	42788669	42856023	80	10	123834	-0.043275	0.51726	0.98012753
TMEM99	ENSG00000167920	17	38965358	39002522	74	10	123834	-0.043295	0.51727	0.98012753
OR4D1	ENSG00000141194	17	56222494	56243517	22	4	123834	-0.043365	0.51729	0.98012753
PTPRE	ENSG00000132334	10	129695325	129894119	363	55	123834	-0.04337	0.5173	0.98012753
CTSB	ENSG00000164733	8	11690033	11736957	113	12	123834	-0.043469	0.51734	0.98012753
MRFAP1L1	ENSG00000178988	4	6699428	6721607	13	3	123834	-0.043639	0.5174	0.98012753
ZBTB38	ENSG00000177311	3	141033055	141178634	176	22	123834	-0.043769	0.51746	0.98012753
GABRG3	ENSG00000182256	15	27206429	27788373	675	90	123834	-0.043885	0.5175	0.98012753
C2orf73	ENSG00000177994	2	54547171	54620879	119	15	123834	-0.043975	0.51754	0.98012753
C12orf60	ENSG00000182993	12	14946506	15069520	182	9	123834	-0.044088	0.51758	0.98012753
KCNIP1	ENSG00000182132	5	169770491	170173636	530	78	123834	-0.04415	0.51761	0.98012753
BTBD2	ENSG00000133243	19	1975447	2044880	77	15	123834	-0.044302	0.51767	0.98012753
HLA-F	ENSG00000204642	6	29680552	29716305	229	21	123834	-0.044483	0.51774	0.98012753
TRAF6	ENSG00000175104	11	36498577	36541822	40	6	123834	-0.044498	0.51775	0.98012753
SUMF2	ENSG00000129103	7	56121695	56158363	86	13	123834	-0.044635	0.5178	0.98012753
PSMF1	ENSG00000125818	20	1083906	1170596	96	17	123834	-0.044747	0.51785	0.98012753
RNF150	ENSG00000170153	4	141770961	142144031	316	48	123834	-0.044802	0.51787	0.98012753
C1orf173	ENSG00000178965	1	75023795	75149422	108	11	123834	-0.044904	0.51791	0.98012753
AC004381.6	ENSG00000005189	16	20807751	20870987	59	9	123834	-0.044936	0.51792	0.98012753
ENPP6	ENSG00000164303	4	184999859	185152383	223	28	123834	-0.045118	0.51799	0.98012753
KCNAB2	ENSG00000069424	1	6041526	6171253	131	26	123834	-0.045144	0.518	0.98012753
KIAA1109	ENSG00000138688	4	123063488	123293913	255	16	123834	-0.04539	0.5181	0.98012753
TIGD5	ENSG00000179886	8	144670074	144692485	52	7	123834	-0.045678	0.51822	0.98012753
SRPK2	ENSG00000135250	7	104741151	105049755	408	11	123834	-0.045771	0.51825	0.98012753
STK17B	ENSG00000081320	2	196988290	197051227	139	8	123834	-0.046132	0.5184	0.98012753
CDKN3	ENSG00000100526	14	54853567	54896936	67	8	123834	-0.046317	0.51847	0.98012753
AHSA2	ENSG00000173209	2	61394553	61428338	42	6	123834	-0.046399	0.5185	0.98012753
EIF3H	ENSG00000147677	8	117644369	117789164	216	15	123834	-0.04647	0.51853	0.98012753
BTG1	ENSG00000133639	12	92526286	92549673	28	7	123834	-0.046497	0.51854	0.98012753
CLDN5	ENSG00000184113	22	19500547	19525068	26	8	123834	-0.047555	0.51896	0.98012753
SPRED1	ENSG00000166068	15	38534527	38659450	303	8	123834	-0.047565	0.51897	0.98012753
DPH1	ENSG00000108963	17	1923404	1956724	29	6	123834	-0.047716	0.51903	0.98012753
NDUFA4	ENSG00000189043	7	10961578	10989883	21	5	123834	-0.048097	0.51918	0.98012753
ABI3BP	ENSG00000154175	3	100458000	100722359	467	34	123834	-0.04838	0.51929	0.98012753
PHKG1	ENSG00000164776	7	56138440	56170689	88	13	123834	-0.048379	0.51929	0.98012753
PRR5	ENSG00000186654	22	45054593	45143561	238	36	123834	-0.048512	0.51935	0.98012753
STC1	ENSG00000159167	8	23689428	23722320	57	9	123834	-0.048695	0.51942	0.98012753
LCE1D	ENSG00000172155	1	152759227	152780657	27	4	123834	-0.048717	0.51943	0.98012753
ZNF223	ENSG00000178386	19	44545520	44582144	51	4	123834	-0.048739	0.51944	0.98012753
GLTSCR1	ENSG00000063169	19	48101453	48216533	178	17	123834	-0.048931	0.51951	0.98012753
NAAA	ENSG00000138744	4	76821809	76872204	119	10	123834	-0.048961	0.51952	0.98012753
LZIC	ENSG00000162441	1	9972173	10013465	54	6	123834	-0.049139	0.5196	0.98012753
LUC7L3	ENSG00000108848	17	48786905	48843574	75	10	123834	-0.049156	0.5196	0.98012753
KHK	ENSG00000138030	2	27299615	27333640	18	2	123834	-0.049222	0.51963	0.98012753
PSMB9	ENSG00000240065	6	32801913	32837362	159	21	123834	-0.049296	0.51966	0.98012753
SMU1	ENSG00000122692	9	33031762	33086665	139	13	123834	-0.049322	0.51967	0.98012753
FAM210B	ENSG00000124098	20	54923971	54953719	59	5	123834	-0.049745	0.51984	0.98012753
RSU1	ENSG00000148484	10	16622610	16869527	364	45	123834	-0.049778	0.51985	0.98012753
CREB3	ENSG00000107175	9	35722332	35747001	12	5	123834	-0.049845	0.51988	0.98012753
TEX19	ENSG00000182459	17	80307123	80331652	52	4	123834	-0.04995	0.51992	0.98012753
GPR19	ENSG00000183150	12	12803825	12859141	72	14	123834	-0.050078	0.51997	0.98012753
IER3IP1	ENSG00000267228	18	44651027	44712652	149	10	123834	-0.050205	0.52002	0.98012753
FBXO10	ENSG00000147912	9	37500889	37598871	169	18	123834	-0.05031	0.52006	0.98012753
MECOM	ENSG00000085276	3	168791287	169391406	941	74	123834	-0.050656	0.5202	0.98012753
MYOM1	ENSG00000101605	18	3056805	3230106	283	37	123834	-0.050858	0.52028	0.98012753
C8orf56	ENSG00000236939	8	104135191	104163703	67	12	123834	-0.050892	0.52029	0.98012753
MRS2	ENSG00000124532	6	24393153	24435810	198	13	123834	-0.050903	0.5203	0.98012753
SP7	ENSG00000170374	12	53710362	53749099	42	6	123834	-0.05112	0.52039	0.98012753
ATP9A	ENSG00000054793	20	50203053	50395173	516	25	123834	-0.051157	0.5204	0.98012753
PPA1	ENSG00000180817	10	71952586	72003667	147	17	123834	-0.051218	0.52042	0.98012753
HAUS3	ENSG00000214367	4	2219191	2253891	72	5	123834	-0.051233	0.52043	0.98012753
OR8S1	ENSG00000197376	12	48891137	48933680	160	13	123834	-0.051259	0.52044	0.98012753
PI15	ENSG00000137558	8	75726772	75777264	105	10	123834	-0.051428	0.52051	0.98012753
LSM11	ENSG00000155858	5	157160703	157197717	62	5	123834	-0.051604	0.52058	0.98012753
GPR21	ENSG00000188394	9	125786806	125807975	18	3	123834	-0.051613	0.52058	0.98012753
LSM10	ENSG00000181817	1	36846839	36873493	65	5	123834	-0.051676	0.52061	0.98012753

RAB42	ENSG00000188060	1	28908712	28931955	6	3	123834	-0.051775	0.52065	0.98012753
ZNF566	ENSG00000186017	19	36926021	36990804	135	12	123834	-0.051822	0.52066	0.98012753
RAB2A	ENSG00000104388	8	61419416	61546186	208	8	123834	-0.051986	0.52073	0.98012753
TMEM230	ENSG00000089063	20	5070486	5103749	49	4	123834	-0.051984	0.52073	0.98012753
ASB4	ENSG00000005981	7	95097756	95179544	145	23	123834	-0.052084	0.52077	0.98012753
COPS7A	ENSG00000111652	12	6822907	6851041	57	7	123834	-0.052075	0.52077	0.98012753
RTEL1-TNFRSF6B	ENSG00000026036	20	62280653	62340037	126	12	123834	-0.052223	0.52082	0.98012753
ERGIC2	ENSG00000087502	12	29480285	29544122	84	8	123834	-0.052243	0.52083	0.98012753
STX16	ENSG00000124222	20	57216328	57264582	49	8	123834	-0.052231	0.52083	0.98012753
TRIB2	ENSG00000071575	2	12847015	12892860	46	5	123834	-0.052482	0.52093	0.98012753
MYO1B	ENSG00000128641	2	192099911	192300115	206	11	123834	-0.052547	0.52095	0.98012753
RBPJ	ENSG00000168214	4	26155077	26446541	227	22	123834	-0.052603	0.52098	0.98012753
HPDL	ENSG00000186603	1	45782545	45804347	5	2	123834	-0.052708	0.52102	0.98012753
AC104532.2	ENSG00000267314	19	5894852	5924718	31	7	123834	-0.052743	0.52103	0.98012753
EPOR	ENSG00000187266	19	11478236	11505018	22	4	123834	-0.052827	0.52107	0.98012753
E2F8	ENSG00000129173	11	19235610	19273167	20	3	123834	-0.052888	0.52109	0.98012753
PPT1	ENSG00000131238	1	40528379	40573375	121	12	123834	-0.052956	0.52112	0.98012753
CLVS1	ENSG00000177182	8	61959717	62424204	804	50	123834	-0.053048	0.52115	0.98012753
TEX33	ENSG00000185264	22	37377163	37413882	112	10	123834	-0.053221	0.52122	0.98012753
SNRPC	ENSG00000124562	6	34715183	34751571	122	7	123834	-0.053232	0.52123	0.98012753
SUOX	ENSG00000139531	12	56380964	56410425	25	4	123834	-0.053608	0.52138	0.98012753
GRK6	ENSG00000198055	5	176820205	176879902	32	5	123834	-0.053695	0.52141	0.98012753
GCSAM	ENSG00000174500	3	111829688	111862152	42	8	123834	-0.053781	0.52145	0.98012753
CAPRIN2	ENSG00000110888	12	30852486	30917885	102	10	123834	-0.05399	0.52153	0.98012753
HLA-DQA1	ENSG00000196735	6	32585956	32624839	1202	28	123834	-0.054231	0.52162	0.98012753
DYNC1LI1	ENSG00000144635	3	32557463	32622366	101	13	123834	-0.054687	0.52181	0.98012753
MUC2	ENSG00000198788	11	1064875	1114419	132	14	123834	-0.054728	0.52182	0.98012753
VAPA	ENSG00000101558	18	9903999	9970018	108	15	123834	-0.054845	0.52187	0.98012753
MTA3	ENSG00000057935	2	42711709	42994087	353	13	123834	-0.054925	0.5219	0.98012753
CISD3	ENSG00000230055	17	36876488	36901297	11	5	123834	-0.055014	0.52194	0.98012753
SNRNP70	ENSG00000104852	19	49578676	49621862	43	10	123834	-0.055075	0.52196	0.98012753
IFNL1	ENSG00000182393	19	39776965	39799313	67	4	123834	-0.055253	0.52203	0.98012753
TIMP3	ENSG00000100234	22	33187687	33269030	90	15	123834	-0.055482	0.52212	0.98012753
ZNF8	ENSG00000083842	19	58780317	58817254	62	8	123834	-0.055634	0.52218	0.98012753
ZNF8	ENSG00000273439	19	58780318	58817254	62	8	123834	-0.055634	0.52218	0.98012753
FXR1	ENSG00000114416	3	180575929	180710541	166	10	123834	-0.055734	0.52222	0.98012753
FAM101B	ENSG00000183688	17	279769	305730	35	5	123834	-0.055782	0.52224	0.98012753
COX11	ENSG00000166260	17	53019263	53056146	100	7	123834	-0.055974	0.52232	0.98012753
PPY	ENSG00000108849	17	42008172	42029836	23	5	123834	-0.056176	0.5224	0.98012753
GEMIN6	ENSG00000152147	2	38968676	39022142	55	5	123834	-0.056349	0.52247	0.98012753
NADK2	ENSG00000152620	5	36182694	36252381	100	8	123834	-0.056639	0.52258	0.98012753
KCNG1	ENSG00000026559	20	49610193	49649666	40	6	123834	-0.056661	0.52259	0.98012753
ATXN7	ENSG00000163635	3	63840233	63999138	205	20	123834	-0.056719	0.52262	0.98012753
NAA60	ENSG00000122390	16	3483611	3546963	177	14	123834	-0.056849	0.52267	0.98012753
IL20	ENSG00000162891	1	207028699	207052568	14	4	123834	-0.05688	0.52268	0.98012753
MSC	ENSG00000178860	8	72743784	72766703	38	5	123834	-0.056875	0.52268	0.98012753
ROCK2	ENSG00000134318	2	11309887	11498456	381	13	123834	-0.056917	0.52269	0.98012753
NLRP13	ENSG00000173572	19	56393065	56453702	97	12	123834	-0.057057	0.52275	0.98012753
GRIA4	ENSG00000152578	11	105470721	105862819	474	28	123834	-0.057088	0.52276	0.98012753
DDX20	ENSG00000064703	1	112287867	112320638	93	9	123834	-0.057255	0.52283	0.98012753
FOXF2	ENSG00000137273	6	1380069	1405832	16	4	123834	-0.057333	0.52286	0.98012753
EXOC6B	ENSG00000144036	2	72393113	73063170	716	15	123834	-0.05744	0.5229	0.98012753
LRRCC1	ENSG00000133739	8	86009382	86068311	68	8	123834	-0.057466	0.52291	0.98012753
NDUFV1	ENSG00000167792	11	67364323	67390006	48	4	123834	-0.057971	0.52311	0.980324858
NDUFA4L2	ENSG00000185633	12	57618686	57644498	5	2	123834	-0.058056	0.52315	0.980324858
ADAMTS15	ENSG00000166106	11	130308869	130356532	57	12	123834	-0.058106	0.52317	0.980324858
RAD51C	ENSG00000108384	17	56759934	56821703	91	11	123834	-0.058851	0.52346	0.980569348
TRPC4AP	ENSG00000100991	20	33580207	33690674	243	12	123834	-0.058881	0.52348	0.980569348
GINS3	ENSG00000181938	16	58318984	58450048	239	24	123834	-0.058905	0.52349	0.980569348
ZBTB3	ENSG00000185670	11	62505791	62531660	18	3	123834	-0.059112	0.52357	0.980569348
CTD-2587H24.4	ENSG00000267110	19	55656125	55682039	42	8	123834	-0.05914	0.52358	0.980569348
COMMD6	ENSG00000188243	13	76089350	76133575	77	10	123834	-0.059215	0.52361	0.980569348
RP11-432B6.3	ENSG00000248710	3	159935241	160177617	245	18	123834	-0.059899	0.52388	0.980582062
MRPS36	ENSG00000134056	5	68503587	68535956	68	5	123834	-0.059917	0.52389	0.980582062
SLC44A5	ENSG00000137968	1	75657816	76086801	702	28	123834	-0.059962	0.52391	0.980582062
FABP3	ENSG00000121769	1	31828472	31859697	67	6	123834	-0.06002	0.52393	0.980582062
CNPPD1	ENSG00000115649	2	220026619	220052828	33	4	123834	-0.060263	0.52403	0.980582062
MST1	ENSG00000173531	3	49711380	49736934	20	5	123834	-0.060348	0.52406	0.980582062
WDR36	ENSG00000134987	5	110417414	110476200	133	13	123834	-0.060385	0.52408	0.980582062
NFIL3	ENSG00000165030	9	94161327	94196144	36	7	123834	-0.060515	0.52413	0.980582062
PDK1	ENSG00000152256	2	173410101	173499823	223	13	123834	-0.060607	0.52416	0.980582062
HTR1B	ENSG00000135312	6	78161948	78183490	33	7	123834	-0.060706	0.5242	0.980582062
EZH2	ENSG00000106462	7	148494475	148591413	93	11	123834	-0.061613	0.52456	0.980582062
ZNF614	ENSG00000142556	19	52506018	52543493	78	7	123834	-0.061694	0.5246	0.980582062
AS3MT	ENSG00000214435	10	104619273	104671656	133	12	123834	-0.061801	0.52464	0.980582062
KNCN	ENSG00000162456	1	47001316	47027199	26	4	123834	-0.06187	0.52467	0.980582062
SLC30A3	ENSG00000115194	2	27466552	27508685	62	7	123834	-0.061871	0.52467	0.980582062
EXT2	ENSG00000151348	11	44107099	44276979	197	18	123834	-0.061895	0.52468	0.980582062
KIAA1731	ENSG00000166004	11	93384805	93473522	49	6	123834	-0.06203	0.52473	0.980582062
BNIP3	ENSG00000176171	10	133771578	133805435	85	5	123834	-0.062224	0.52481	0.980582062
INTS10	ENSG00000104613	8	19664651	19719594	89	13	123834	-0.06229	0.52483	0.980582062
TFB2M	ENSG00000162851	1	246693862	246739626	97	10	123834	-0.062497	0.52492	0.980582062
ABHD5	ENSG00000011198	3	43721605	43785863	119	7	123834	-0.062557	0.52494	0.980582062
RALB	ENSG00000144118	2	120987640	121062289	155	8	123834	-0.062669	0.52499	0.980582062
DGCR6L	ENSG00000128185	22	20291799	20317603	9	2	123834	-0.062676	0.52499	0.980582062
LMO2	ENSG00000135363	11	33870122	33923836	38	9	123834	-0.062843	0.52505	0.980582062
TMEM25	ENSG00000149582	11	118391756	118427995	21	2	123834	-0.062863	0.52506	0.980582062
MYL1	ENSG00000168530	2	211144874	211189914	41	6	123834	-0.062923	0.52509	0.980582062
CDH10	ENSG00000040731	5	24477209	24655087	404	23	123834	-0.062987	0.52511	0.980582062
DIRAS1	ENSG00000176490	19	2704565	2731416	41	7	123834	-0.062995	0.52511	0.980582062
ZMYM6	ENSG00000163867	1	35439523	35507569	87	2	123834	-0.063177	0.52519	0.980582062
LRRC4	ENSG00000128594	7	127657124	127682160	25	7	123834	-0.063616	0.52536	0.980582062
PLIN2	ENSG00000147872	9	19098373	19159288	105	16	123834	-0.063693	0.52539	0.980582062
GNAQ	ENSG00000156052	9	80321003	80656374	295	17	123834	-0.063748	0.52541	0.980582062
IGFBP3	ENSG00000146674	7	45941949	45971473	13	5	123834	-0.063976	0.52551	0.980582062
AC006538.4	ENSG00000267001	19	2707767	2750046	53	10	123834	-0.064022	0.52552	0.980582062
ZFP28	ENSG00000196867	19	57040317	57078169	100	9	123834	-0.064559	0.52574	0.980582062
SMYD4	ENSG00000186532	17	1672779	1743928	175	16	123834	-0.064611	0.52576	0.980582062

CABYR	ENSG00000154040	18	21708942	21751567	56	8	123834	-0.064646	0.52577	0.980582062
INSL5	ENSG00000172410	1	67253424	67276939	22	3	123834	-0.064987	0.52591	0.980582062
GLRX2	ENSG00000023572	1	193055598	193085244	46	5	123834	-0.065137	0.52597	0.980582062
UCKL1	ENSG00000198276	20	62561186	62597769	32	7	123834	-0.065173	0.52598	0.980582062
ZNF284	ENSG00000186026	19	44566297	44603766	52	5	123834	-0.065461	0.5261	0.980582062
OTX2	ENSG00000165588	14	57257425	57287197	10	4	123834	-0.065554	0.52613	0.980582062
ARHGAP24	ENSG00000138639	4	86386267	86933823	1112	54	123834	-0.065662	0.52618	0.980582062
CYB5R1	ENSG00000159348	1	202920997	202946408	38	7	123834	-0.065792	0.52623	0.980582062
STEAP2	ENSG00000157214	7	89786904	89877451	225	14	123834	-0.06583	0.52624	0.980582062
PRPF4	ENSG00000136875	9	116027623	116065185	77	8	123834	-0.06583	0.52624	0.980582062
HIC2	ENSG00000169635	22	21761693	21815752	12	2	123834	-0.065969	0.5263	0.980582062
AGA	ENSG00000038002	4	178341924	178373657	33	10	123834	-0.066026	0.52632	0.980582062
PROKR2	ENSG00000101292	20	5272317	5307378	145	8	123834	-0.066106	0.52635	0.980582062
PITPNM2	ENSG00000090975	12	123458027	123644562	133	10	123834	-0.066288	0.52643	0.980582062
MRPL48	ENSG00000175581	11	73488361	73586178	282	16	123834	-0.066535	0.52652	0.980582062
CWF19L2	ENSG00000152404	11	107187071	107338572	412	21	123834	-0.066519	0.52652	0.980582062
AKR1B15	ENSG00000227471	7	134223888	134274627	90	6	123834	-0.066562	0.52653	0.980582062
ZFYVE28	ENSG00000159733	4	2261309	2430390	275	23	123834	-0.066609	0.52655	0.980582062
KMT2E	ENSG00000005483	7	104644626	104764808	120	6	123834	-0.066817	0.52664	0.980582062
MUC16	ENSG00000181143	19	8949520	9102018	261	25	123834	-0.067219	0.5268	0.980582062
IL17REL	ENSG00000188263	22	50422942	50461088	28	4	123834	-0.067332	0.52684	0.980582062
OLIG2	ENSG00000205927	21	34388153	34411504	13	5	123834	-0.067345	0.52685	0.980582062
HIST1H2BE	ENSG00000197697	6	26173958	26194454	54	5	123834	-0.067418	0.52688	0.980582062
ARMC5	ENSG00000140691	16	31459401	31488487	10	4	123834	-0.067458	0.52689	0.980582062
AC008394.1	ENSG00000233828	5	86502423	86544822	33	5	123834	-0.067479	0.5269	0.980582062
KIF13B	ENSG00000197892	8	28914796	29130641	244	24	123834	-0.06752	0.52692	0.980582062
FAM173B	ENSG00000150756	5	10216442	10260009	109	14	123834	-0.06757	0.52694	0.980582062
TSPO2	ENSG00000112212	6	41000293	41022076	49	5	123834	-0.067596	0.52695	0.980582062
IGFLR1	ENSG00000126246	19	36220058	36243354	42	8	123834	-0.067655	0.52697	0.980582062
VASN	ENSG00000168140	16	4411849	4443529	38	6	123834	-0.06796	0.52709	0.980697801
BPGM	ENSG00000172331	7	134321560	134374565	132	8	123834	-0.068352	0.52725	0.980697801
ZNF813	ENSG00000198346	19	53960989	54016950	82	18	123834	-0.068368	0.52725	0.980697801
KIAA0391	ENSG00000258790	14	35581755	35796680	536	19	123834	-0.068884	0.52746	0.980697801
MRPL30	ENSG00000273155	2	99761461	99821761	101	7	123834	-0.069285	0.52762	0.980697801
TRAF1	ENSG00000056558	9	123654671	123701451	110	6	123834	-0.069314	0.52763	0.980697801
ATXN2	ENSG00000204842	12	111880018	112047480	89	4	123834	-0.069352	0.52765	0.980697801
C9orf85	ENSG00000155621	9	74516426	74610970	46	7	123834	-0.069675	0.52777	0.980697801
TADA1	ENSG00000152382	1	166815747	166855564	21	5	123834	-0.069685	0.52778	0.980697801
FADS6	ENSG00000172782	17	72863428	72899708	53	5	123834	-0.06976	0.52781	0.980697801
CACFD1	ENSG00000160325	9	136315089	136345970	52	9	123834	-0.069969	0.52789	0.980697801
USP2	ENSG00000036672	11	119215925	119262436	38	4	123834	-0.070031	0.52792	0.980697801
SLC35C2	ENSG00000080189	20	44968167	45003043	25	3	123834	-0.070317	0.52803	0.980697801
ZFP41	ENSG00000181638	8	144318991	144354875	79	5	123834	-0.070456	0.52808	0.980697801
KLF12	ENSG00000118922	13	74250226	74718394	676	70	123834	-0.070438	0.52808	0.980697801
NDUFA13	ENSG00000186010	19	19616545	19654285	63	4	123834	-0.070507	0.5281	0.980697801
ISM2	ENSG00000100593	14	77930740	77975210	79	9	123834	-0.070667	0.52817	0.980697801
RP11-379H8.1	ENSG00000261086	15	83199620	83241124	30	7	123834	-0.071052	0.52832	0.980697801
XKR4	ENSG00000206579	8	56004949	56464613	705	50	123834	-0.071176	0.52837	0.980697801
DMWD	ENSG00000185800	19	46276205	46306060	33	6	123834	-0.071217	0.52839	0.980697801
RP5-850E9.3	ENSG00000270299	20	617270	666444	31	6	123834	-0.071365	0.52845	0.980697801
TRMU	ENSG00000100416	22	46716772	46763237	78	7	123834	-0.071703	0.52858	0.980697801
OR1E1	ENSG00000180016	17	3290398	3311704	22	4	123834	-0.071901	0.52866	0.980697801
CCR1	ENSG00000163823	3	46233200	46259887	43	2	123834	-0.071934	0.52867	0.980697801
CTD-2616J11.11	ENSG00000269403	19	51843627	51881353	65	9	123834	-0.071981	0.52869	0.980697801
GRB7	ENSG00000141738	17	37884180	37913544	20	3	123834	-0.072022	0.52871	0.980697801
PLD2	ENSG00000129219	17	4700391	4736729	20	5	123834	-0.072044	0.52872	0.980697801
IQSEC1	ENSG00000144711	3	12928719	13124617	209	43	123834	-0.072108	0.52874	0.980697801
VMAC	ENSG00000187650	19	5894869	5920864	30	7	123834	-0.072126	0.52875	0.980697801
PMM2	ENSG00000140650	16	8872680	8953188	247	23	123834	-0.072591	0.52893	0.980697801
WNT9A	ENSG00000143816	1	228096357	228145599	64	6	123834	-0.072859	0.52904	0.980697801
EIF4G2	ENSG00000110321	11	10808597	10840657	19	3	123834	-0.07286	0.52904	0.980697801
PDXDC1	ENSG00000179889	16	15058448	15243196	114	13	123834	-0.072931	0.52907	0.980697801
IP6K1	ENSG00000176095	3	49751727	49833975	88	6	123834	-0.072958	0.52908	0.980697801
CLEC4E	ENSG00000166523	12	8675901	8703559	37	6	123834	-0.073087	0.52913	0.980697801
AC068620.1	ENSG00000268171	4	57266682	57287131	45	7	123834	-0.07311	0.52914	0.980697801
DOK3	ENSG00000146094	5	176918908	176948275	10	2	123834	-0.0732	0.52918	0.980697801
ABCC1	ENSG00000103222	16	16033434	16246931	424	40	123834	-0.073217	0.52918	0.980697801
FAM64A	ENSG00000129195	17	6337735	6364789	39	8	123834	-0.07325	0.5292	0.980697801
RP11-244H3.4	ENSG00000271741	1	35437134	35507342	91	2	123834	-0.073321	0.52922	0.980697801
HTR1F	ENSG00000179097	3	88029846	88052919	24	2	123834	-0.073448	0.52928	0.980697801
AKIRIN1	ENSG00000174574	1	39446895	39481731	80	6	123834	-0.073485	0.52929	0.980697801
LGALS4	ENSG00000171747	19	39282311	39314004	75	9	123834	-0.073492	0.52929	0.980697801
CD2	ENSG00000116824	1	117287007	117321850	32	5	123834	-0.073999	0.52949	0.980697801
FZD2	ENSG00000180340	17	42624925	42646907	18	5	123834	-0.074114	0.52954	0.980697801
KBTBD11	ENSG00000176595	8	1912044	1965102	77	13	123834	-0.074498	0.52969	0.980697801
TADA3	ENSG00000171148	3	9811544	9844695	43	6	123834	-0.074529	0.52971	0.980697801
SLC9C1	ENSG00000172139	3	111849734	112023105	572	13	123834	-0.074682	0.52977	0.980697801
FAM134A	ENSG00000144567	2	220030947	220060201	38	4	123834	-0.074814	0.52982	0.980697801
ACTR3	ENSG00000115091	2	114637537	114730173	257	13	123834	-0.075104	0.52993	0.980697801
AC007401.2	ENSG00000217075	2	36748948	36789411	47	8	123834	-0.075245	0.52999	0.980697801
TGIF1	ENSG00000177426	18	3401606	3468409	50	14	123834	-0.075356	0.53003	0.980697801
SGMS2	ENSG00000164023	4	108735719	108846203	91	17	123834	-0.075395	0.53005	0.980697801
PLD5	ENSG00000180287	1	242236288	242697998	935	75	123834	-0.075412	0.53006	0.980697801
HOXD3	ENSG00000128652	2	176991340	177047830	51	9	123834	-0.075549	0.53011	0.980697801
MCMBP	ENSG00000197771	10	121578972	121662068	242	12	123834	-0.075558	0.53011	0.980697801
RAB39A	ENSG00000179331	11	107789229	107844208	105	14	123834	-0.07554	0.53011	0.980697801
C12orf54	ENSG00000177627	12	48866286	48900295	139	11	123834	-0.075947	0.53027	0.980697801
UBE2N	ENSG00000177889	12	93789449	93846038	40	9	123834	-0.07599	0.53029	0.980697801
MYOM3	ENSG00000142661	1	24372525	24448665	108	14	123834	-0.076137	0.53034	0.980697801
NKX1-2	ENSG00000229544	10	126125592	126148753	42	6	123834	-0.076289	0.53041	0.980697801
PDCD1	ENSG00000188389	2	242782033	242811060	61	8	123834	-0.076417	0.53046	0.980697801
FOXA3	ENSG00000170608	19	46357247	46387055	46	8	123834	-0.076607	0.53053	0.980697801
AC004076.9	ENSG00000268163	19	57939338	57998904	139	10	123834	-0.076662	0.53055	0.980697801
KRTAP22-2	ENSG00000206106	21	31952424	31972716	16	3	123834	-0.076658	0.53055	0.980697801
DSEL	ENSG00000171451	18	65163819	65194217	25	5	123834	-0.077121	0.53074	0.980697801
ZBTB8A	ENSG00000160062	1	32995028	33081540	137	6	123834	-0.077174	0.53076	0.980697801
OVCH1	ENSG00000187950	12	29555407	29660619	290	16	123834	-0.077216	0.53077	0.980697801
URAD	ENSG00000183463	13	28541851	28572791	22	5	123834	-0.077229	0.53078	0.980697801
C15orf27	ENSG00000169758	15	76342178	76531462	270	27	123834	-0.077254	0.53079	0.980697801

AC138517.1	ENSG00000269071	5	138842108	138862506	6	2	123834	-0.07731	0.53081	0.980697801
GRIK1	ENSG00000171189	21	30899254	31322351	811	43	123834	-0.077298	0.53081	0.980697801
PPAPDC2	ENSG00000205808	9	4652298	4675256	42	4	123834	-0.077497	0.53089	0.980697801
MRAP2	ENSG00000135324	6	84733475	84810600	111	11	123834	-0.077963	0.53107	0.980697801
SLC25A47	ENSG00000140107	14	100779674	100806715	30	5	123834	-0.078071	0.53111	0.980697801
EXOC1	ENSG00000090989	4	56709782	56781200	134	17	123834	-0.078239	0.53118	0.980697801
NDFIP1	ENSG00000131507	5	141478070	141544005	201	15	123834	-0.07827	0.53119	0.980697801
SMAD7	ENSG00000101665	18	46436223	46487081	43	13	123834	-0.078257	0.53119	0.980697801
BLCAP	ENSG00000166619	20	36110874	36166333	44	3	123834	-0.07851	0.53129	0.980697801
C1orf95	ENSG00000203685	1	226726501	226806915	90	16	123834	-0.07871	0.53137	0.980697801
MAPRE2	ENSG00000166974	18	32546892	32733434	265	25	123834	-0.078723	0.53137	0.980697801
MIEF2	ENSG00000177427	17	18153848	18179866	33	6	123834	-0.078775	0.53139	0.980697801
EDN3	ENSG00000124205	20	57865482	57911047	38	10	123834	-0.078844	0.53142	0.980697801
ATMIN	ENSG00000166454	16	81059452	81090963	54	4	123834	-0.078957	0.53147	0.980697801
CLOCK	ENSG00000134852	4	56284070	56423305	430	13	123834	-0.079239	0.53158	0.980697801
EIF4A3	ENSG00000141543	17	78099013	78130982	68	10	123834	-0.07937	0.53163	0.980697801
RP11-613M10.9	ENSG00000255872	9	37578410	38078684	993	42	123834	-0.079518	0.53169	0.980697801
AXIN1	ENSG00000103126	16	327440	412673	141	16	123834	-0.079577	0.53171	0.980697801
ZNF664	ENSG00000179195	12	124446392	124509986	69	5	123834	-0.079691	0.53176	0.980697801
GGPS1	ENSG00000152904	1	235480665	235517847	68	6	123834	-0.079743	0.53178	0.980697801
ATN1	ENSG00000111676	12	7023626	7061484	6	3	123834	-0.079975	0.53187	0.980697801
LDHA	ENSG00000134333	11	18405935	18439972	94	7	123834	-0.080015	0.53189	0.980697801
CCBL2	ENSG00000137944	1	89391456	89468636	84	5	123834	-0.080064	0.53191	0.980697801
KRTAP20-3	ENSG00000206104	21	32005183	32025455	27	3	123834	-0.08015	0.53194	0.980697801
GJB4	ENSG00000189433	1	35215342	35239325	31	7	123834	-0.080298	0.532	0.980697801
SEMA6A	ENSG00000092421	5	115769312	115920630	208	43	123834	-0.080358	0.53202	0.980697801
ATG10	ENSG00000152348	5	81257844	81582676	361	24	123834	-0.080544	0.5321	0.980697801
CNOT8	ENSG00000155508	5	154227113	154266353	55	4	123834	-0.080583	0.53211	0.980697801
PAN3	ENSG00000152520	13	28702643	28879475	177	11	123834	-0.08066	0.53214	0.980697801
TRIM44	ENSG00000166326	11	35674353	35839775	117	13	123834	-0.080854	0.53222	0.980725839
NOX3	ENSG00000074771	6	155706504	155787037	105	22	123834	-0.081045	0.5323	0.980725839
MNS1	ENSG00000138587	15	56703742	56767335	93	11	123834	-0.081077	0.53231	0.980725839
TRIM58	ENSG00000162722	1	248010501	248051507	102	14	123834	-0.081371	0.53243	0.980726049
FAM19A3	ENSG00000184599	1	113253041	113279857	11	5	123834	-0.081603	0.53252	0.980726049
MTHFS	ENSG00000136371	15	80115927	80199721	193	18	123834	-0.081598	0.53252	0.980726049
EFCAB11	ENSG00000140025	14	90251013	90431121	373	27	123834	-0.081859	0.53262	0.980726049
DPP3	ENSG00000254986	11	66237484	66287130	69	4	123834	-0.081917	0.53264	0.980726049
SERTAD1	ENSG00000197019	19	40917499	40941932	20	5	123834	-0.082699	0.53295	0.980726049
NOL8	ENSG00000198000	9	95049640	95097918	82	8	123834	-0.082754	0.53298	0.980726049
DEAF1	ENSG00000177030	11	634233	716715	169	15	123834	-0.082859	0.53302	0.980726049
TMEM242	ENSG00000215712	6	157700418	157754633	74	8	123834	-0.083071	0.5331	0.980726049
MAL	ENSG00000172005	2	95681422	95729737	21	4	123834	-0.08314	0.53313	0.980726049
RTEL1	ENSG00000258366	20	62279163	62338416	126	12	123834	-0.083187	0.53315	0.980726049
MRPL50	ENSG00000136897	9	104139915	104170896	51	8	123834	-0.083256	0.53318	0.980726049
RHOBTB1	ENSG00000072422	10	62619196	62771198	164	18	123834	-0.083279	0.53319	0.980726049
C11orf54	ENSG00000182919	11	93464757	93507915	66	3	123834	-0.083291	0.53319	0.980726049
CRLS1	ENSG00000088766	20	5976736	6030699	118	12	123834	-0.083311	0.5332	0.980726049
ZNF611	ENSG00000213020	19	53196066	53248326	73	9	123834	-0.083432	0.53325	0.980726049
1-Dec	ENSG00000173077	9	117894097	118174923	618	48	123834	-0.083552	0.53329	0.980726049
COL27A1	ENSG00000196739	9	116907840	117084791	276	51	123834	-0.083675	0.53334	0.980726049
SYTL2	ENSG00000137501	11	85395267	85532184	214	16	123834	-0.083796	0.53339	0.980726049
NAPRT1	ENSG00000147813	8	144646955	144670819	69	11	123834	-0.083916	0.53344	0.980726049
TRPV3	ENSG00000167723	17	3403796	3471289	119	24	123834	-0.084044	0.53349	0.980726049
SPSB2	ENSG00000111671	12	6970099	7008522	19	1	123834	-0.084098	0.53351	0.980726049
ATP1B2	ENSG00000129244	17	7539945	7571086	21	5	123834	-0.084178	0.53354	0.980726049
C17orf85	ENSG00000074356	17	3704460	3759545	74	9	123834	-0.084239	0.53357	0.980726049
CREB1	ENSG00000118260	2	208384461	208478155	99	7	123834	-0.084334	0.5336	0.980726049
RAP2B	ENSG00000181467	3	152870029	152896265	8	3	123834	-0.084479	0.53366	0.980741495
PPP2R5A	ENSG00000066027	1	212448879	212545200	163	16	123834	-0.084897	0.53383	0.980770201
TNFSF8	ENSG00000106952	9	117646003	117702697	61	7	123834	-0.084981	0.53386	0.980770201
OXT	ENSG00000101405	20	3042266	3063163	7	2	123834	-0.085329	0.534	0.980770201
CDKN2D	ENSG00000129355	19	10667138	10689735	61	9	123834	-0.085368	0.53402	0.980770201
AL589739.1	ENSG00000269753	1	2248581	2269042	14	5	123834	-0.085481	0.53406	0.980770201
SUMO4	ENSG00000177688	6	149711495	149732177	57	8	123834	-0.085534	0.53408	0.980770201
S100A1	ENSG00000160678	1	153590402	153614513	14	4	123834	-0.085637	0.53412	0.980770201
MOCS2	ENSG00000164172	5	52381509	52415893	97	8	123834	-0.085643	0.53413	0.980770201
CLEC2L	ENSG00000236279	7	139198602	139239730	41	3	123834	-0.085688	0.53414	0.980770201
PTGES	ENSG00000148344	9	132490610	132525326	79	10	123834	-0.086168	0.53433	0.980984632
RP11-272B17.2	ENSG00000257230	12	59907059	59927965	42	8	123834	-0.086242	0.53436	0.980984632
IFRG15	ENSG00000258664	1	179823916	179844311	24	6	123834	-0.086805	0.53459	0.981077584
IQGAP1	ENSG00000140575	15	90921450	91055475	264	11	123834	-0.087029	0.53468	0.981077584
GCNT1	ENSG00000187210	9	79024752	79132332	246	27	123834	-0.087058	0.53469	0.981077584
SLC9A3R1	ENSG00000109062	17	72734791	72775492	39	7	123834	-0.087056	0.53469	0.981077584
KCNV2	ENSG00000168263	9	2707502	2740037	46	8	123834	-0.087209	0.53475	0.981077584
SLK	ENSG00000065613	10	105716959	105798991	145	9	123834	-0.087504	0.53486	0.981077584
UBE3A	ENSG00000114062	15	25572381	25694128	177	11	123834	-0.087599	0.5349	0.981077584
C20orf78	ENSG00000149443	20	18780372	18820797	59	13	123834	-0.087594	0.5349	0.981077584
GCSH	ENSG00000140905	16	81105566	81140008	115	2	123834	-0.087804	0.53498	0.981077584
YWHAQ	ENSG00000134308	2	9714101	9781143	63	8	123834	-0.087818	0.53499	0.981077584
ATP5I	ENSG00000169020	4	656225	678127	28	4	123834	-0.087931	0.53503	0.981077584
KCNIP4	ENSG00000185774	4	20720239	21960422	3086	134	123834	-0.087915	0.53503	0.981077584
ART1	ENSG00000129744	11	3656358	3695646	78	14	123834	-0.088244	0.53516	0.981221306
PNPT1	ENSG00000138035	2	55851400	55931045	231	22	123834	-0.089062	0.53548	0.981417775
HHLA2	ENSG00000114455	3	108005376	108107132	260	18	123834	-0.089102	0.5355	0.981417775
MAPK13	ENSG00000156711	6	36085586	36117842	25	5	123834	-0.089091	0.5355	0.981417775
MYO1G	ENSG00000136286	7	44992261	45028697	60	11	123834	-0.089287	0.53557	0.981417775
GYPA	ENSG00000170180	4	145020457	145071904	118	12	123834	-0.089322	0.53559	0.981417775
TXNDC17	ENSG00000129235	17	6534078	6557861	80	8	123834	-0.089555	0.53568	0.981417775
MAK	ENSG00000111837	6	10752956	10848764	71	10	123834	-0.089638	0.53571	0.981417775
DTX3	ENSG00000178498	12	57988405	58013587	14	5	123834	-0.089625	0.53571	0.981417775
GALNT14	ENSG00000158089	2	31123333	31388068	525	39	123834	-0.089772	0.53577	0.981417775
CRTAP	ENSG00000170275	3	33145471	33199265	49	7	123834	-0.089842	0.53579	0.981417775
HSD17B4	ENSG00000133835	5	118778138	118982894	380	22	123834	-0.089999	0.53586	0.981417775
RFX2	ENSG00000087903	19	5983175	6209583	388	21	123834	-0.090322	0.53598	0.981417775
PPP1CB	ENSG00000213639	2	28964506	29035806	108	8	123834	-0.09033	0.53599	0.981417775
HOOK2	ENSG00000095066	19	12863817	12993554	188	16	123834	-0.090329	0.53599	0.981417775
CYTIP	ENSG00000115165	2	158261131	158355473	103	12	123834	-0.090598	0.53609	0.98150633
VGLL4	ENSG00000144560	3	11587544	11772220	356	33	123834	-0.090847	0.53619	0.981589428
IFNG	ENSG00000111537	12	68538548	68563527	57	6	123834	-0.091098	0.53629	0.981589428

CEBPZ-AS1	ENSG00000218739	2	37413631	37453336	51	4	123834	-0.09114	0.53631	0.981589428
ZP1	ENSG00000149506	11	60625035	60653166	31	6	123834	-0.09128	0.53636	0.981589428
HSPB2-C11orf52	ENSG00000254445	11	111773460	111807595	22	3	123834	-0.091395	0.53641	0.981589428
LMF2	ENSG00000100258	22	50931376	50956135	50	6	123834	-0.091781	0.53656	0.981589428
RP9	ENSG00000164610	7	33124409	33159013	34	3	123834	-0.092017	0.53666	0.981589428
AC020952.1	ENSG00000268953	19	31630362	31650757	13	3	123834	-0.09205	0.53667	0.981589428
RP11-45M22.4	ENSG00000264187	17	17095963	17150499	103	13	123834	-0.092364	0.5368	0.981589428
SCGB2B2	ENSG00000205209	19	35074234	35176604	330	22	123834	-0.092439	0.53683	0.981589428
RDH11	ENSG00000072042	14	68133518	68172531	71	9	123834	-0.092515	0.53686	0.981589428
CLECL1	ENSG00000184293	12	9858456	9895895	131	11	123834	-0.092579	0.53688	0.981589428
MDM4	ENSG00000198625	1	204475511	204552871	133	7	123834	-0.092661	0.53691	0.981589428
SNED1	ENSG00000162804	2	241928255	242044983	126	18	123834	-0.092646	0.53691	0.981589428
C18orf21	ENSG00000141428	18	33542046	33569241	38	4	123834	-0.092662	0.53691	0.981589428
IMMT	ENSG00000132305	2	86361055	86432893	152	6	123834	-0.092873	0.537	0.981619987
C16orf62	ENSG00000103544	16	19556562	19728115	320	27	123834	-0.09295	0.53703	0.981619987
PEAK1	ENSG00000173517	15	77390471	77722486	374	14	123834	-0.093307	0.53717	0.981781477
CHCHD2	ENSG00000106153	7	56159262	56184269	73	11	123834	-0.093773	0.53736	0.982034312
CHD4	ENSG00000111642	12	6669249	6726642	62	5	123834	-0.094003	0.53745	0.982046502
RP11-637O19.3	ENSG00000272162	6	10738027	10940656	197	30	123834	-0.094052	0.53747	0.982046502
ADPRHL1	ENSG00000153531	13	114066260	114117839	91	17	123834	-0.094275	0.53755	0.982083044
TMPPE	ENSG00000188167	3	33121913	33148293	37	5	123834	-0.094624	0.53769	0.982083044
TMEM186	ENSG00000184857	16	8864241	8901505	113	18	123834	-0.094719	0.53773	0.982083044
PADI3	ENSG00000142619	1	17565593	17620728	145	18	123834	-0.094735	0.53774	0.982083044
ANKRD18A	ENSG00000273170	9	38530566	38587204	14	3	123834	-0.094853	0.53778	0.982083044
ZNF764	ENSG00000169951	16	30555085	30579819	8	2	123834	-0.094887	0.5378	0.982083044
COLCA1	ENSG00000196167	11	111154114	111185770	53	8	123834	-0.095103	0.53788	0.982134778
PCNP	ENSG00000081154	3	101282939	101323281	77	9	123834	-0.095551	0.53806	0.98229274
CCDC124	ENSG00000007080	19	18033825	18064800	22	6	123834	-0.095795	0.53816	0.98229274
PEX10	ENSG00000157911	1	2326236	2355236	32	8	123834	-0.09584	0.53818	0.98229274
ZNF155	ENSG00000204920	19	44462014	44512477	81	9	123834	-0.095995	0.53824	0.98229274
ADAMTS2	ENSG00000087116	5	178527852	178782431	341	48	123834	-0.096181	0.53831	0.98229274
SLIT3	ENSG00000184347	5	168078745	168738133	1162	110	123834	-0.096281	0.53835	0.98229274
POLR2K	ENSG00000147669	8	101152812	101176230	27	3	123834	-0.096389	0.53839	0.98229274
THUMPD3	ENSG00000134077	3	9394526	9438475	62	8	123834	-0.096431	0.53841	0.98229274
C2ORF15	ENSG00000273045	2	99747948	99777950	52	4	123834	-0.096827	0.53857	0.98229274
TRIM10	ENSG00000204613	6	30109722	30138711	128	12	123834	-0.096826	0.53857	0.98229274
PAMR1	ENSG00000149090	11	35443370	35561848	161	21	123834	-0.096909	0.5386	0.98229274
KY	ENSG00000174611	3	134311980	134380478	97	5	123834	-0.096972	0.53863	0.98229274
DPF2	ENSG00000133884	11	65091225	65130720	25	4	123834	-0.097085	0.53867	0.98229274
IFI35	ENSG00000068079	17	41148742	41176473	24	3	123834	-0.097132	0.53869	0.98229274
PDE6B	ENSG00000133256	4	609373	674571	130	8	123834	-0.097452	0.53882	0.982362288
PBLD	ENSG00000108187	10	70032417	70102806	158	9	123834	-0.097663	0.5389	0.982362288
ITGBL1	ENSG00000198542	13	102094966	102385456	497	37	123834	-0.09788	0.53899	0.982362288
PFKFB3	ENSG00000170525	10	6176881	6287495	171	44	123834	-0.098009	0.53904	0.982362288
SART1	ENSG00000175467	11	65719160	65757299	74	7	123834	-0.098236	0.53913	0.982362288
NKD2	ENSG00000145506	5	998944	1049058	117	13	123834	-0.098288	0.53915	0.982362288
ZNF480	ENSG00000198464	19	52790430	52839175	108	14	123834	-0.098371	0.53918	0.982362288
NAGPA	ENSG00000103174	16	5064845	5094142	11	2	123834	-0.098439	0.53921	0.982362288
SERPINB9	ENSG00000170542	6	2877500	2913514	54	8	123834	-0.098484	0.53923	0.982362288
DNAL4	ENSG00000100246	22	39164513	39200203	41	7	123834	-0.098603	0.53927	0.982362288
RAMP1	ENSG00000132329	2	238757536	238830756	81	18	123834	-0.098816	0.53936	0.982362288
CELF4	ENSG00000101489	18	34813010	35156000	334	52	123834	-0.098898	0.53939	0.982362288
HNRNPA1L2	ENSG00000139675	13	53181605	53227919	129	5	123834	-0.098923	0.5394	0.982362288
TBX4	ENSG00000121075	17	59519765	59572471	21	7	123834	-0.099482	0.53962	0.982501591
C16orf80	ENSG00000070761	16	58137496	58173354	86	6	123834	-0.099597	0.53967	0.982501591
RFXAP	ENSG00000133111	13	37383361	37413241	10	2	123834	-0.099839	0.53976	0.982501591
RMND1	ENSG00000155906	6	151715989	151783259	165	14	123834	-0.099873	0.53978	0.982501591
TMEM165	ENSG00000134851	4	56252124	56329564	187	14	123834	-0.099909	0.53979	0.982501591
STYX	ENSG00000198252	14	53186898	53251716	74	6	123834	-0.099954	0.53981	0.982501591
CDC7	ENSG00000097046	1	91956408	92001321	77	9	123834	-0.10006	0.53985	0.982501591
FAM86C1	ENSG00000158483	11	71488556	71522282	122	7	123834	-0.10016	0.53989	0.982501591
AL590822.2	ENSG00000269554	1	2111237	2133179	14	3	123834	-0.10046	0.54001	0.982625893
ERI2	ENSG00000196678	16	20781515	20921671	152	11	123834	-0.10092	0.54019	0.98285934
REEP1	ENSG00000068615	2	86431116	86575206	300	12	123834	-0.10146	0.54041	0.983165513
CECR6	ENSG00000183307	22	17587189	17612257	20	5	123834	-0.10177	0.54053	0.983174295
VPS54	ENSG00000143952	2	64109280	64256206	203	25	123834	-0.10183	0.54055	0.983174295
GALNT1	ENSG00000141429	18	33151081	33301798	285	8	123834	-0.10188	0.54057	0.983174295
XKR9	ENSG00000221947	8	71571600	71712606	407	11	123834	-0.10213	0.54067	0.98326209
IL6	ENSG00000136244	7	22755503	22781621	70	11	123834	-0.10235	0.54076	0.983331683
OGFR	ENSG00000060491	20	61426187	61455352	74	16	123834	-0.10279	0.54093	0.983449487
ZNF860	ENSG00000197385	3	32013263	32043120	90	7	123834	-0.1028	0.54094	0.983449487
KLK14	ENSG00000129437	19	51570752	51597502	22	7	123834	-0.10291	0.54098	0.983449487
CLEC6A	ENSG00000205846	12	8598522	8640926	141	3	123834	-0.10313	0.54107	0.983461343
TXNDC12	ENSG00000117862	1	52475803	52531843	64	4	123834	-0.10317	0.54109	0.983461343
MPC1	ENSG00000060762	6	166768407	166806486	27	1	123834	-0.10362	0.54127	0.983636742
ULK1	ENSG00000177169	12	132369196	132417712	58	7	123834	-0.10367	0.54129	0.983636742
LGALS14	ENSG00000006659	19	40184946	40210084	53	7	123834	-0.10392	0.54139	0.98370262
PGR	ENSG00000082175	11	100890355	101011255	255	15	123834	-0.1042	0.54149	0.98370262
CEP170B	ENSG00000099814	14	105321617	105373107	105	10	123834	-0.10423	0.54151	0.98370262
FAM118B	ENSG00000197798	11	126071309	126142881	123	15	123834	-0.1044	0.54157	0.98370262
BATF3	ENSG00000123685	1	212849760	212883327	60	13	123834	-0.10486	0.54176	0.98370262
IRF2	ENSG00000168310	4	185298867	185405734	107	32	123834	-0.10493	0.54178	0.98370262
SLC6A1	ENSG00000157103	3	11024410	11090933	91	22	123834	-0.10501	0.54182	0.98370262
MLXIP	ENSG00000175727	12	122506628	122641894	159	4	123834	-0.10502	0.54182	0.98370262
LRRC25	ENSG00000175489	19	18491954	18518427	42	7	123834	-0.10528	0.54192	0.98370262
KRTAP6-2	ENSG00000186930	21	31960909	31981219	24	3	123834	-0.10549	0.54201	0.98370262
SOCS5	ENSG00000171150	2	46916091	47000268	215	20	123834	-0.10558	0.54204	0.98370262
GLCCI1	ENSG00000106415	7	7998425	8143902	232	20	123834	-0.10583	0.54214	0.98370262
EIF2B2	ENSG00000119718	14	75459614	75486292	37	3	123834	-0.10605	0.54223	0.98370262
CRIPAK	ENSG00000179979	4	1375340	1399780	13	4	123834	-0.10622	0.5423	0.98370262
TMEM125	ENSG00000179178	1	43725665	43749673	36	8	123834	-0.10628	0.54232	0.98370262
MKI67	ENSG00000148773	10	129884923	129934649	113	16	123834	-0.10639	0.54236	0.98370262
OR10G4	ENSG00000254737	11	123876282	123897217	60	9	123834	-0.10643	0.54238	0.98370262
C14orf64	ENSG00000246223	14	98381947	98454461	141	16	123834	-0.10657	0.54244	0.98370262
PPIB	ENSG00000166794	15	64438011	64465404	31	4	123834	-0.10702	0.54262	0.98370262
C6orf89	ENSG00000198663	6	36829646	36906740	175	12	123834	-0.10708	0.54264	0.98370262
F7	ENSG00000057593	13	113750105	113784995	31	5	123834	-0.10712	0.54265	0.98370262
NTN3	ENSG00000162068	16	2511500	2534146	2	1	123834	-0.1072	0.54269	0.98370262

TUBB1	ENSG00000101162	20	57584309	57611709	19	4	123834	-0.10744	0.54278	0.98370262
ZNF644	ENSG00000122482	1	91370859	91497829	157	9	123834	-0.108	0.543	0.98370262
ZNF596	ENSG00000172748	8	172137	207342	47	7	123834	-0.10824	0.5431	0.98370262
TEKT3	ENSG00000125409	17	15197128	15254958	99	12	123834	-0.10902	0.54341	0.98370262
ZNF683	ENSG00000176083	1	26678125	26711013	83	10	123834	-0.10914	0.54345	0.98370262
CIB4	ENSG00000157884	2	26794070	26874236	90	12	123834	-0.10912	0.54345	0.98370262
DEFA5	ENSG00000164816	8	6902831	6924256	55	7	123834	-0.10926	0.5435	0.98370262
PARP4	ENSG00000102699	13	24985064	25096948	470	14	123834	-0.10942	0.54356	0.98370262
COX7C	ENSG00000127184	5	85903721	85926779	6	2	123834	-0.10951	0.5436	0.98370262
BDKRB2	ENSG00000168398	14	96661016	96720666	113	21	123834	-0.10959	0.54363	0.98370262
PLTP	ENSG00000100979	20	44517399	44550794	44	10	123834	-0.10961	0.54364	0.98370262
CDC6	ENSG00000094804	17	38433885	38469171	13	2	123834	-0.1099	0.54375	0.98370262
RORC	ENSG00000143365	1	151768547	151814348	13	4	123834	-0.10991	0.54376	0.98370262
ADAMDEC1	ENSG00000134028	8	24231798	24273526	24	5	123834	-0.10994	0.54377	0.98370262
ETS2	ENSG00000157557	21	40167231	40206879	65	5	123834	-0.11001	0.5438	0.98370262
IFI44L	ENSG00000137959	1	79075607	79121830	39	6	123834	-0.11007	0.54382	0.98370262
TDRD7	ENSG00000196116	9	100164232	100268407	127	9	123834	-0.11015	0.54385	0.98370262
RP11-96O20.4	ENSG00000260170	15	45869549	45978512	171	14	123834	-0.11013	0.54385	0.98370262
NT5DC4	ENSG00000144130	2	113469063	113510456	41	4	123834	-0.1102	0.54387	0.98370262
MCM5	ENSG00000100297	22	35786056	35831423	72	16	123834	-0.11038	0.54394	0.98370262
SNAI1	ENSG00000124216	20	48589536	48615423	68	7	123834	-0.11042	0.54396	0.98370262
EPS8L2	ENSG00000177106	11	684438	737727	75	14	123834	-0.11047	0.54398	0.98370262
AL358333.1	ENSG00000267802	14	52426014	52446247	49	6	123834	-0.11049	0.54399	0.98370262
YJEFN3	ENSG00000250067	19	19617036	19658390	66	4	123834	-0.11053	0.544	0.98370262
C10orf54	ENSG00000107738	10	73497316	73543255	88	18	123834	-0.11072	0.54408	0.98370262
INHBC	ENSG00000175189	12	57818543	57854611	34	4	123834	-0.11091	0.54416	0.98370262
PPME1	ENSG00000214517	11	73872144	73975748	155	8	123834	-0.11094	0.54417	0.98370262
TRIM2	ENSG00000109654	4	154063494	154270472	260	30	123834	-0.11107	0.54422	0.98370262
METTL20	ENSG00000139160	12	31790094	31836048	106	16	123834	-0.11127	0.5443	0.98370262
ZNF799	ENSG00000196466	19	12490830	12522085	14	3	123834	-0.11129	0.54431	0.98370262
LIPE	ENSG00000079435	19	42895659	42941578	11	1	123834	-0.11138	0.54434	0.98370262
ANKRD29	ENSG00000154065	18	21168890	21252849	59	10	123834	-0.11142	0.54436	0.98370262
FCN2	ENSG00000160339	9	137762654	137789924	66	11	123834	-0.11169	0.54446	0.98370262
RP1-27O5.3	ENSG00000254553	1	32920658	33076393	220	8	123834	-0.11171	0.54447	0.98370262
RUNDC1	ENSG00000198863	17	41122582	41155707	25	4	123834	-0.11182	0.54452	0.98370262
HSD17B2	ENSG00000086696	16	82058609	82142139	53	11	123834	-0.11209	0.54463	0.98370262
MYOZ1	ENSG00000177791	10	75381412	75411515	22	3	123834	-0.11214	0.54464	0.98370262
ST8SIA1	ENSG00000111728	12	22206707	22599975	688	47	123834	-0.11213	0.54464	0.98370262
HORMAD1	ENSG00000143452	1	150660536	150703364	81	3	123834	-0.11229	0.5447	0.98370262
C1orf143	ENSG00000228208	1	218673438	218709320	85	3	123834	-0.11234	0.54472	0.98370262
SENP2	ENSG00000163904	3	185290284	185361339	130	9	123834	-0.1124	0.54475	0.98370262
CCNYL1	ENSG00000163249	2	208566264	208636563	139	5	123834	-0.11251	0.54479	0.98370262
THUMPD1	ENSG00000066654	16	20734986	20763406	57	5	123834	-0.11259	0.54482	0.98370262
KIAA0907	ENSG00000132680	1	155872834	155914191	43	4	123834	-0.11302	0.54499	0.98370262
CIT	ENSG00000122966	12	120113595	120325095	296	24	123834	-0.11347	0.54517	0.98370262
SH3GLB1	ENSG00000097033	1	87160259	87223867	87	11	123834	-0.11359	0.54522	0.98370262
LRRC49	ENSG00000137821	15	71135578	71352414	166	11	123834	-0.11361	0.54523	0.98370262
OR8D4	ENSG00000181518	11	123767109	123788183	49	7	123834	-0.11369	0.54526	0.98370262
PSPC1	ENSG00000121390	13	20238896	20367142	173	11	123834	-0.11369	0.54526	0.98370262
APH1B	ENSG00000138613	15	63558217	63611325	77	13	123834	-0.11375	0.54528	0.98370262
MFNG	ENSG00000100060	22	37855101	37892439	31	4	123834	-0.1138	0.5453	0.98370262
HIST1H2BG	ENSG00000187990	6	26206428	26226872	71	6	123834	-0.11415	0.54544	0.98370262
SERPINB3	ENSG00000057149	18	61312431	61339197	23	3	123834	-0.11424	0.54548	0.98370262
AC022532.1	ENSG00000197604	10	72184585	72206314	35	6	123834	-0.11434	0.54552	0.98370262
MYH9	ENSG00000100345	22	36667327	36794063	210	27	123834	-0.11443	0.54555	0.98370262
CBLL1	ENSG00000105879	7	107374142	107412112	49	4	123834	-0.1146	0.54562	0.98370262
LRRC18	ENSG00000165383	10	50107528	50153242	136	18	123834	-0.1148	0.5457	0.98370262
OR4F6	ENSG00000184140	15	102335902	102356876	29	4	123834	-0.11481	0.5457	0.98370262
RAP1GDS1	ENSG00000138698	4	99172535	99375012	259	5	123834	-0.11483	0.54571	0.98370262
SPN	ENSG00000197471	16	29664300	29692187	24	4	123834	-0.1149	0.54574	0.98370262
MORN3	ENSG00000139714	12	122079024	122120537	88	10	123834	-0.1151	0.54582	0.98370262
TMEM51	ENSG00000171729	1	15469028	15556976	269	21	123834	-0.11522	0.54586	0.98370262
UBIAD1	ENSG00000120942	1	11323263	11366106	57	3	123834	-0.11527	0.54589	0.98370262
KCTD4	ENSG00000180332	13	45756988	45785175	37	3	123834	-0.11554	0.54599	0.98370262
CDK5	ENSG00000164885	7	150740899	150765617	22	6	123834	-0.11558	0.54601	0.98370262
IFT80	ENSG00000068885	3	159964774	160127668	152	14	123834	-0.11592	0.54614	0.98370262
C7orf62	ENSG00000164645	7	88413420	88435035	51	7	123834	-0.11608	0.5462	0.98370262
AC093157.1	ENSG00000269175	1	101445761	101467653	18	3	123834	-0.11614	0.54623	0.98370262
MYL10	ENSG00000106436	7	101246605	101282576	75	12	123834	-0.11626	0.54628	0.98370262
C22orf43	ENSG00000189269	22	23940639	23984508	55	7	123834	-0.11632	0.5463	0.98370262
COG6	ENSG00000133103	13	40219764	40375802	282	13	123834	-0.11656	0.5464	0.98370262
SSTR2	ENSG00000180616	17	71151151	71177185	25	7	123834	-0.11657	0.5464	0.98370262
MTHFSD	ENSG00000103248	16	86553782	86598841	74	9	123834	-0.11689	0.54653	0.98370262
IKBIP	ENSG00000166130	12	98997183	99048891	91	13	123834	-0.11701	0.54657	0.98370262
RNF144B	ENSG00000137393	6	18358779	18479105	379	26	123834	-0.11706	0.54659	0.98370262
AL035406.1	ENSG00000268332	1	6090630	6111399	22	5	123834	-0.11735	0.54671	0.98370262
C6orf223	ENSG00000181577	6	43958317	43983695	92	9	123834	-0.11765	0.54683	0.98370262
UFC1	ENSG00000143222	1	161112566	161138646	28	5	123834	-0.11771	0.54685	0.98370262
C3orf52	ENSG00000114529	3	111795182	111859851	105	7	123834	-0.11799	0.54696	0.98370262
TNFAIP8	ENSG00000145779	5	118594387	118745383	310	22	123834	-0.11809	0.547	0.98370262
TUBAL3	ENSG00000178462	10	5425061	5456793	85	6	123834	-0.11821	0.54705	0.98370262
RP11-650K20.3	ENSG00000257327	12	104225229	104248442	57	5	123834	-0.11826	0.54707	0.98370262
OR4D10	ENSG00000254466	11	59234846	59255869	30	5	123834	-0.11828	0.54708	0.98370262
SPECC1L	ENSG00000100014	22	24656786	24823708	207	15	123834	-0.11834	0.5471	0.98370262
YIPF5	ENSG00000145817	5	143527723	143560278	72	5	123834	-0.1184	0.54713	0.98370262
TMEM173	ENSG00000184584	5	138845119	138872520	11	3	123834	-0.11861	0.54721	0.98370262
ZNF784	ENSG00000179922	19	56122108	56145967	22	5	123834	-0.11868	0.54723	0.98370262
SLC2A6	ENSG00000160326	9	136326217	136354259	35	9	123834	-0.1187	0.54724	0.98370262
CCDC67	ENSG00000165325	11	93053137	93181653	337	17	123834	-0.11887	0.54731	0.98370262
TK1	ENSG00000167900	17	76160160	76193314	77	16	123834	-0.11889	0.54732	0.98370262
NMD3	ENSG00000169251	3	160812484	160981320	238	19	123834	-0.11912	0.54741	0.98370262
IFITM2	ENSG00000185201	11	297631	325272	10	4	123834	-0.11913	0.54741	0.98370262
IFITM1	ENSG00000185885	11	303506	325272	10	4	123834	-0.11913	0.54741	0.98370262
ZNF763	ENSG00000197054	19	12025890	12101196	205	8	123834	-0.1194	0.54752	0.98370262
ETFB	ENSG00000105379	19	51838423	51879672	66	9	123834	-0.11957	0.54759	0.98370262
DNTTIP1	ENSG00000101457	20	44410576	44450066	104	11	123834	-0.11958	0.54759	0.98370262
AQP5	ENSG00000161798	12	50345653	50369464	15	5	123834	-0.11959	0.5476	0.98370262
CYSLTR2	ENSG00000152207	13	49270951	49293498	17	5	123834	-0.11964	0.54762	0.98370262
GPX6	ENSG00000198704	6	28461073	28505992	71	12	123834	-0.11968	0.54763	0.98370262

C17orf99	ENSG00000187997	17	76132434	76172258	79	18	123834	-0.11969	0.54764	0.98370262
TNIK	ENSG00000154310	3	170769128	171188197	545	52	123834	-0.11998	0.54775	0.983807238
GLTP	ENSG00000139433	12	110278748	110328293	65	11	123834	-0.12041	0.54792	0.983879185
RP11-67H2.1	ENSG00000253207	8	114602539	114639187	65	8	123834	-0.12065	0.54802	0.983879185
PCP4	ENSG00000183036	21	41229243	41311322	141	25	123834	-0.12095	0.54813	0.983879185
UBE2D2	ENSG00000131508	5	138896016	139018018	92	8	123834	-0.12098	0.54815	0.983879185
CXCL5	ENSG00000163735	4	74851359	74874496	17	1	123834	-0.1212	0.54823	0.983879185
LCE1E	ENSG00000186226	1	152748690	152770902	37	5	123834	-0.12128	0.54826	0.983879185
SLITRK5	ENSG00000165300	13	88314870	88341871	36	5	123834	-0.12127	0.54826	0.983879185
CADPS2	ENSG00000081803	7	121948481	122536813	850	40	123834	-0.12128	0.54827	0.983879185
SREK1IP1	ENSG00000153006	5	64003971	64074512	60	4	123834	-0.12143	0.54833	0.983879185
MYH13	ENSG00000006788	17	10191401	10286447	140	16	123834	-0.12143	0.54833	0.983879185
NPY5R	ENSG00000164129	4	164255091	164283086	55	7	123834	-0.12172	0.54844	0.983879185
TRMT10C	ENSG00000174173	3	101270706	101295290	42	8	123834	-0.12184	0.54849	0.983879185
OR4C5	ENSG00000176540	11	48377037	48398017	24	4	123834	-0.12199	0.54855	0.983879185
TREH	ENSG00000118094	11	118518026	118560399	98	4	123834	-0.12202	0.54856	0.983879185
SIPA1L3	ENSG00000105738	19	38387868	38709012	501	35	123834	-0.12227	0.54866	0.983879185
UBAP1	ENSG00000165006	9	34169003	34262521	157	11	123834	-0.1223	0.54867	0.983879185
RPL36	ENSG00000130255	19	5664958	5701887	36	3	123834	-0.12231	0.54867	0.983879185
ZNF763	ENSG00000267179	19	12025890	12100105	203	8	123834	-0.12286	0.54889	0.983970942
SNX9	ENSG00000130340	6	158234296	158376109	328	10	123834	-0.12294	0.54892	0.983970942
MAEL	ENSG00000143194	1	166948346	167001451	85	10	123834	-0.12295	0.54893	0.983970942
ZC3H14	ENSG00000100722	14	89019253	89089853	66	9	123834	-0.12303	0.54896	0.983970942
CTIF	ENSG00000134030	18	46055417	46399588	489	75	123834	-0.12309	0.54898	0.983970942
MUSK	ENSG00000030304	9	113421051	113573859	246	39	123834	-0.12337	0.54909	0.984051041
PPAT	ENSG00000128059	4	57249528	57311781	129	12	123834	-0.12348	0.54914	0.984051041
AC009892.10	ENSG00000224730	19	55137173	55160343	89	9	123834	-0.12359	0.54918	0.984051041
KANSL1L	ENSG00000144445	2	210876147	211046107	74	9	123834	-0.12391	0.54931	0.984117547
SURF4	ENSG00000148248	9	136218325	136252970	59	6	123834	-0.12399	0.54934	0.984117547
FABP1	ENSG00000163586	2	88412510	88437635	25	6	123834	-0.12413	0.5494	0.984117547
ADAMTS5	ENSG00000154736	21	28280231	28348832	96	14	123834	-0.12453	0.54955	0.984117547
B3GNT2	ENSG00000170340	2	62413248	62461866	33	2	123834	-0.12496	0.54972	0.984117547
PMPCA	ENSG00000165688	9	139295110	139328213	109	13	123834	-0.12511	0.54978	0.984117547
CCNB1	ENSG00000134057	5	68452837	68484072	74	6	123834	-0.12518	0.54981	0.984117547
EPS8	ENSG00000151491	12	15763092	16045263	99	10	123834	-0.12518	0.54981	0.984117547
UBALD1	ENSG00000153443	16	4648884	4675028	69	5	123834	-0.12529	0.54985	0.984117547
TTI1	ENSG00000101407	20	36601409	36671870	40	5	123834	-0.12542	0.5499	0.984117547
C2orf80	ENSG00000188674	2	209020067	209064797	136	21	123834	-0.12589	0.55009	0.984117547
HIST1H2AE	ENSG00000168274	6	26207165	26227711	71	6	123834	-0.126	0.55014	0.984117547
VPS28	ENSG00000160948	8	145639000	145663931	33	1	123834	-0.12618	0.5502	0.984117547
PTCHD3	ENSG00000182077	10	27677116	27713297	128	10	123834	-0.12624	0.55023	0.984117547
RRAS2	ENSG00000133818	11	14289472	14396052	73	9	123834	-0.12638	0.55028	0.984117547
STOX2	ENSG00000173320	4	184764584	184954679	148	30	123834	-0.12683	0.55046	0.984117547
PRR20A	ENSG00000204919	13	57705052	57728073	9	3	123834	-0.127	0.55053	0.984117547
ASGR2	ENSG00000161944	17	6994641	7029019	70	8	123834	-0.1271	0.55057	0.984117547
IGFBP6	ENSG00000167779	12	53481220	53506129	49	5	123834	-0.12713	0.55058	0.984117547
HES5	ENSG00000197921	1	2450184	2471684	19	4	123834	-0.12722	0.55062	0.984117547
NUDT14	ENSG00000183828	14	105629275	105657660	20	1	123834	-0.12722	0.55062	0.984117547
LRRN4	ENSG00000125872	20	6011424	6044695	48	6	123834	-0.12737	0.55068	0.984117547
GAL	ENSG00000069482	11	68441247	68468643	20	4	123834	-0.12749	0.55072	0.984117547
NEUROD6	ENSG00000164600	7	31367075	31390508	21	5	123834	-0.12754	0.55074	0.984117547
FGF14	ENSG00000102466	13	102362134	103064124	820	72	123834	-0.12759	0.55077	0.984117547
ZNF74	ENSG00000185252	22	20738405	20772745	31	4	123834	-0.12765	0.55079	0.984117547
CD80	ENSG00000121594	3	119233140	119288449	157	18	123834	-0.12771	0.55081	0.984117547
AP001888.1	ENSG00000268351	11	69577254	69597718	31	8	123834	-0.12818	0.551	0.984117547
CCSAP	ENSG00000154429	1	229446758	229489041	60	6	123834	-0.12826	0.55103	0.984117547
SPATA2	ENSG00000158480	20	48509928	48542080	76	7	123834	-0.12837	0.55107	0.984117547
CHCHD7	ENSG00000170791	8	57114245	57141357	7	2	123834	-0.12842	0.55109	0.984117547
PTTG2	ENSG00000250254	4	37952056	37972746	62	7	123834	-0.12848	0.55112	0.984117547
C5orf58	ENSG00000234511	5	169649451	169689579	11	4	123834	-0.12859	0.55116	0.984117547
OR13G1	ENSG00000197437	1	247825320	247846365	93	6	123834	-0.12867	0.55119	0.984117547
CYTH3	ENSG00000008256	7	6191407	6322275	164	17	123834	-0.12866	0.55119	0.984117547
ACR	ENSG00000100312	22	51166624	51193762	12	2	123834	-0.12869	0.5512	0.984117547
OR5L1	ENSG00000186117	11	55568854	55589952	59	8	123834	-0.12896	0.5513	0.984117547
ITGA3	ENSG00000005884	17	48123332	48177845	75	9	123834	-0.129	0.55132	0.984117547
EGR4	ENSG00000135625	2	73508058	73530833	10	2	123834	-0.12912	0.55137	0.984117547
JUP	ENSG00000173801	17	39765692	39953183	286	14	123834	-0.12938	0.55147	0.984117547
AGO2	ENSG00000123908	8	141531264	141655718	148	22	123834	-0.12954	0.55153	0.984117547
TXK	ENSG00000074966	4	48058410	48146273	111	13	123834	-0.12954	0.55154	0.984117547
ANKRD46	ENSG00000186106	8	101511980	101582012	91	10	123834	-0.12973	0.55161	0.984117547
CBR1	ENSG00000159228	21	37432239	37455464	57	11	123834	-0.12973	0.55161	0.984117547
DOLK	ENSG00000175283	9	131697809	131719898	17	1	123834	-0.1298	0.55164	0.984117547
RARS2	ENSG00000146282	6	88214096	88309721	266	11	123834	-0.13017	0.55179	0.984117547
CHN2	ENSG00000106069	7	29151890	29563944	613	84	123834	-0.13017	0.55179	0.984117547
UGT2B28	ENSG00000135226	4	70136217	70170768	4	1	123834	-0.13026	0.55182	0.984117547
TAOK2	ENSG00000149930	16	29974962	30013582	34	3	123834	-0.13035	0.55186	0.984117547
CSRP3	ENSG00000129170	11	19193578	19242120	64	14	123834	-0.13038	0.55187	0.984117547
FAM84B	ENSG00000168672	8	127554687	127580638	20	4	123834	-0.13067	0.55198	0.984117547
RP11-139J15.7	ENSG00000271786	10	18921405	18954123	94	9	123834	-0.13072	0.552	0.984117547
GRAMD4	ENSG00000075240	22	46961909	47085688	320	24	123834	-0.13089	0.55207	0.984117547
TRIM25	ENSG00000121060	17	54955270	55001399	17	5	123834	-0.13092	0.55208	0.984117547
C18orf25	ENSG00000152242	18	43743519	43856949	47	6	123834	-0.1311	0.55215	0.984117547
RP11-680G10.1	ENSG00000261567	16	85381069	85403055	15	4	123834	-0.13114	0.55217	0.984117547
DNAJC13	ENSG00000138246	3	132126370	132267876	222	20	123834	-0.13119	0.55219	0.984117547
AC016251.1	ENSG00000205148	15	96821280	96841660	10	3	123834	-0.13127	0.55222	0.984117547
LCLAT1	ENSG00000172954	2	30660092	30877091	632	17	123834	-0.13148	0.5523	0.984167845
XPA	ENSG00000136936	9	100427191	100469639	25	6	123834	-0.13184	0.55244	0.984188845
SLMO2	ENSG00000101166	20	57598200	57627964	22	4	123834	-0.13221	0.55259	0.984188845
SCN5A	ENSG00000183873	3	38579548	38701164	132	29	123834	-0.13226	0.55261	0.984188845
SLC2A12	ENSG00000146411	6	134299835	134383774	87	15	123834	-0.13235	0.55265	0.984188845
CCR5	ENSG00000160791	3	46401633	46427697	49	7	123834	-0.13252	0.55271	0.984188845
G6PC2	ENSG00000152254	2	169747750	169776505	37	7	123834	-0.13253	0.55272	0.984188845
FAM169B	ENSG00000185087	15	98970391	99067611	128	12	123834	-0.13266	0.55277	0.984188845
WARS	ENSG00000140105	14	100790125	100853142	138	7	123834	-0.13275	0.5528	0.984188845
TOMM20	ENSG00000173726	1	235262651	235302251	50	4	123834	-0.13288	0.55286	0.984188845
CSN1S1	ENSG00000126545	4	70786799	70822289	42	5	123834	-0.13299	0.5529	0.984188845
SMAD9	ENSG00000120693	13	37408968	37504902	106	12	123834	-0.13314	0.55296	0.984188845
ZNF728	ENSG00000269067	19	23148270	23195978	111	4	123834	-0.13337	0.55305	0.984188845
JMJD6	ENSG00000070495	17	74698919	74732866	44	6	123834	-0.13359	0.55314	0.984188845

HIST1H4D	ENSG00000188987	6	26178938	26199304	60	5	123834	-0.13441	0.55346	0.984188845
ADCY2	ENSG00000078295	5	7386321	7840194	615	63	123834	-0.1345	0.5535	0.984188845
ESRRG	ENSG00000196482	1	216666588	217321097	1055	100	123834	-0.13452	0.55351	0.984188845
KIAA1549L	ENSG00000110427	11	33553618	33705648	204	23	123834	-0.13453	0.55351	0.984188845
MAPK11	ENSG00000185386	22	50692142	50719196	31	8	123834	-0.13464	0.55355	0.984188845
GNPAT	ENSG00000116906	1	231366953	231423719	80	7	123834	-0.13483	0.55363	0.984188845
WNT2B	ENSG00000134245	1	112999163	113082787	67	14	123834	-0.13494	0.55367	0.984188845
GGTLC2	ENSG00000100121	22	22978780	23000368	14	2	123834	-0.13509	0.55373	0.984188845
NCDN	ENSG00000020129	1	36013074	36042875	20	3	123834	-0.13551	0.5539	0.984188845
NSUN6	ENSG00000241058	10	18824490	18950551	390	16	123834	-0.1356	0.55393	0.984188845
GPCPD1	ENSG00000125772	20	5515085	5601672	91	10	123834	-0.13574	0.55399	0.984188845
GATC	ENSG00000257218	12	120874241	120909389	48	9	123834	-0.13579	0.55401	0.984188845
ZBTB5	ENSG00000168795	9	37428111	37475396	47	8	123834	-0.13583	0.55402	0.984188845
OR5B17	ENSG00000197786	11	58115597	58136542	44	3	123834	-0.13603	0.5541	0.984188845
CORIN	ENSG00000145244	4	47586015	47850123	467	24	123834	-0.13612	0.55414	0.984188845
FAM212A	ENSG00000185614	3	49830687	49852463	14	3	123834	-0.13633	0.55422	0.984188845
TYRP1	ENSG00000107165	9	12675439	12720290	121	11	123834	-0.13667	0.55435	0.984188845
KRTAP20-4	ENSG00000206105	21	31982946	32003169	28	3	123834	-0.13665	0.55435	0.984188845
SCARF1	ENSG00000074660	17	1527152	1559041	30	6	123834	-0.13696	0.55447	0.984188845
F2RL3	ENSG00000127533	19	16989671	17013417	16	4	123834	-0.13697	0.55447	0.984188845
SUPT16H	ENSG00000092201	14	21809631	21862425	76	7	123834	-0.13708	0.55451	0.984188845
ZSCAN5D	ENSG00000267908	19	56741773	56768863	51	10	123834	-0.13706	0.55451	0.984188845
TNNI1	ENSG00000159173	1	201363625	201408994	26	8	123834	-0.1371	0.55452	0.984188845
APBB1IP	ENSG00000077420	10	26717132	26866732	316	32	123834	-0.13712	0.55453	0.984188845
HNRNPU	ENSG00000153187	1	245004468	245037844	14	2	123834	-0.1372	0.55456	0.984188845
TMEM43	ENSG00000170876	3	14156440	14195179	64	9	123834	-0.1372	0.55457	0.984188845
SCGB2A2	ENSG00000110484	11	62027627	62050628	44	4	123834	-0.13722	0.55457	0.984188845
GLT1D1	ENSG00000151948	12	129327972	129479509	230	38	123834	-0.13721	0.55457	0.984188845
MXRA7	ENSG00000182534	17	74658633	74717098	95	9	123834	-0.13723	0.55458	0.984188845
GALNTL6	ENSG00000174473	4	172723405	173972710	1578	72	123834	-0.13726	0.55459	0.984188845
DPH6	ENSG00000134146	15	35499546	35848394	460	23	123834	-0.13727	0.55459	0.984188845
PIK3R1	ENSG00000145675	5	67501548	67607649	112	21	123834	-0.13752	0.55469	0.984253509
RHBDL3	ENSG00000141314	17	30583195	30661680	115	18	123834	-0.13762	0.55473	0.984253509
AGAP2	ENSG00000135439	12	58108980	58145940	19	3	123834	-0.13777	0.55479	0.98426809
RECQL	ENSG00000004700	12	21611845	21664603	136	16	123834	-0.13875	0.55518	0.98486486
XKR6	ENSG00000171044	8	10743555	11068875	491	38	123834	-0.13888	0.55523	0.98486486
CSHL1	ENSG00000204414	17	61976957	62006198	7	2	123834	-0.13944	0.55545	0.985079272
CXXC1	ENSG00000154832	18	47798713	47824674	16	5	123834	-0.13956	0.5555	0.985079272
ATG16L1	ENSG00000085978	2	234108697	234214320	154	13	123834	-0.13966	0.55553	0.985079272
PGBD2	ENSG00000185220	1	249190395	249224145	17	3	123834	-0.13986	0.55561	0.985079272
PEG10	ENSG00000242265	7	94275637	94309007	25	4	123834	-0.13985	0.55561	0.985079272
ZNF429	ENSG00000197013	19	21669484	21749072	184	7	123834	-0.14027	0.55578	0.985196889
KRTAP13-3	ENSG00000240432	21	31787583	31808286	27	2	123834	-0.14028	0.55578	0.985196889
C1orf170	ENSG00000187642	1	900579	927497	42	7	123834	-0.14058	0.5559	0.985225846
GLYATL2	ENSG00000156689	11	58591542	58681688	317	8	123834	-0.14058	0.5559	0.985225846
RBMXL1	ENSG00000213516	1	89435139	89468643	27	5	123834	-0.14084	0.556	0.985311206
ZNF615	ENSG00000197619	19	52484585	52521483	100	8	123834	-0.14118	0.55614	0.985372311
NIF3L1	ENSG00000196290	2	201744050	201778655	28	4	123834	-0.14131	0.55619	0.985372311
CTC-429P9.4	ENSG00000268790	19	16678453	16780926	142	17	123834	-0.14132	0.55619	0.985372311
LRRC23	ENSG00000010626	12	6972733	7033407	57	2	123834	-0.14168	0.55633	0.985528485
GARS	ENSG00000106105	7	30624297	30683649	87	8	123834	-0.14238	0.55661	0.985731697
SLC27A1	ENSG00000130304	19	17569578	17626977	83	11	123834	-0.1424	0.55662	0.985731697
TLDC2	ENSG00000101342	20	35494524	35532638	37	7	123834	-0.14243	0.55663	0.985731697
ALDH9A1	ENSG00000143149	1	165621453	165678100	159	16	123834	-0.14252	0.55667	0.985731697
TMEM185B	ENSG00000226479	2	120968854	120990984	28	4	123834	-0.14274	0.55675	0.985731697
ABHD14A	ENSG00000248487	3	51995442	52025212	5	3	123834	-0.14281	0.55678	0.985731697
PFDN4	ENSG00000101132	20	52814386	52854591	43	12	123834	-0.14324	0.55695	0.985731697
ARL6IP1	ENSG00000170540	16	18792991	18823000	23	4	123834	-0.1436	0.55709	0.985731697
BUB1B	ENSG00000156970	15	40443224	40523337	85	5	123834	-0.14415	0.55731	0.985731697
CLK1	ENSG00000013441	2	201707732	201739422	27	6	123834	-0.14434	0.55738	0.985731697
DCLRE1C	ENSG00000152457	10	14929358	15006431	121	22	123834	-0.14446	0.55743	0.985731697
MAGOHB	ENSG00000111196	12	10748612	10776222	60	6	123834	-0.14451	0.55745	0.985731697
LRMP	ENSG00000118308	12	25163936	25271268	190	30	123834	-0.14467	0.55752	0.985731697
KDM5A	ENSG00000073614	12	379295	508620	231	10	123834	-0.14488	0.5576	0.985731697
PRPF4B	ENSG00000112739	6	4011501	4075217	122	12	123834	-0.14496	0.55763	0.985731697
NUP37	ENSG00000075188	12	102457967	102523902	114	13	123834	-0.14515	0.5577	0.985731697
UTF1	ENSG00000171794	10	135033778	135055062	49	9	123834	-0.14571	0.55793	0.985731697
LGI3	ENSG00000168481	8	21994338	22024597	16	3	123834	-0.14587	0.55799	0.985731697
ZNF211	ENSG00000121417	19	58131761	58164147	100	5	123834	-0.14594	0.55802	0.985731697
PHOX2B	ENSG00000109132	4	41736099	41760987	28	6	123834	-0.14625	0.55814	0.985731697
AC073569.1	ENSG00000269531	12	80188742	80212321	21	2	123834	-0.1463	0.55816	0.985731697
MUC15	ENSG00000169550	11	26570580	26603780	81	11	123834	-0.14642	0.55821	0.985731697
ACVRL1	ENSG00000139567	12	52290692	52327145	53	12	123834	-0.14657	0.55826	0.985731697
FGF12	ENSG00000114279	3	191847184	192495553	838	76	123834	-0.14663	0.55829	0.985731697
POMGNT2	ENSG00000144647	3	43110724	43157568	61	8	123834	-0.14684	0.55837	0.985731697
CXCR2	ENSG00000180871	2	218980012	219011976	55	5	123834	-0.14691	0.5584	0.985731697
EPCAM	ENSG00000119888	2	47562297	47624740	110	18	123834	-0.14705	0.55845	0.985731697
THAP10	ENSG00000129028	15	71163681	71195124	22	3	123834	-0.1473	0.55855	0.985731697
FXYD1	ENSG00000266964	19	35619712	35644013	2	1	123834	-0.14728	0.55855	0.985731697
SLCO1A2	ENSG00000084453	12	21407534	21582528	323	32	123834	-0.14754	0.55865	0.985731697
CPAMD8	ENSG00000160111	19	16993758	17147625	150	30	123834	-0.14764	0.55869	0.985731697
MIP	ENSG00000135517	12	56833286	56872950	27	6	123834	-0.1478	0.55875	0.985731697
PARN	ENSG00000140694	16	14519558	14736585	203	12	123834	-0.14816	0.55889	0.985731697
THEMIS	ENSG00000172673	6	128019217	128249776	247	22	123834	-0.14825	0.55893	0.985731697
PARD6B	ENSG00000124171	20	49338081	49383332	89	10	123834	-0.14836	0.55897	0.985731697
TBCE	ENSG00000116957	1	235520675	235622283	194	17	123834	-0.1485	0.55902	0.985731697
TMEM134	ENSG00000172663	11	67221824	67246743	15	3	123834	-0.14893	0.5592	0.985731697
FLCN	ENSG00000154803	17	17105526	17150502	94	12	123834	-0.14917	0.55929	0.985731697
COL9A3	ENSG00000092758	20	61437596	61482511	107	24	123834	-0.14919	0.5593	0.985731697
DKK1	ENSG00000107984	10	54064056	54087802	23	3	123834	-0.14931	0.55935	0.985731697
ITPRIP	ENSG00000148841	10	106061894	106108162	63	8	123834	-0.1494	0.55938	0.985731697
HOXD8	ENSG00000175879	2	176984422	177007423	15	5	123834	-0.14942	0.55939	0.985731697
CRTC2	ENSG00000160741	1	153910145	153941101	21	3	123834	-0.14961	0.55946	0.985731697
TAF1D	ENSG00000166012	11	93453114	93527557	111	5	123834	-0.14959	0.55946	0.985731697
ZFP41	ENSG00000264668	8	144319280	144368573	121	7	123834	-0.14964	0.55948	0.985731697
PDIA2	ENSG00000185615	16	323152	347215	28	5	123834	-0.14971	0.5595	0.985731697
GSK3B	ENSG00000082701	3	119530170	119823264	334	15	123834	-0.15014	0.55967	0.985731697
FAM189A2	ENSG00000135063	9	71929488	72017371	185	22	123834	-0.15018	0.55969	0.985731697
LINGO3	ENSG00000220008	19	2279774	2318156	37	12	123834	-0.15017	0.55969	0.985731697

AGAP4	ENSG00000188234	10	46311042	46359323	2	1	123834	-0.15046	0.5598	0.985731697
NMU	ENSG00000109255	4	56451396	56512865	111	12	123834	-0.15078	0.55992	0.985731697
LOXHD1	ENSG00000167210	18	44046935	44246996	316	43	123834	-0.15085	0.55995	0.985731697
ADNP2	ENSG00000101544	18	77856915	77915406	132	11	123834	-0.15133	0.56014	0.985731697
C17orf49	ENSG00000258315	17	6907814	6930844	25	2	123834	-0.1514	0.56017	0.985731697
PLEKHF2	ENSG00000175895	8	96136032	96178912	79	9	123834	-0.15146	0.56019	0.985731697
ZNF121	ENSG00000197961	19	9661029	9705209	107	2	123834	-0.15158	0.56024	0.985731697
ZNF154	ENSG00000179909	19	58198735	58230579	36	7	123834	-0.15191	0.56037	0.985731697
PAPL	ENSG00000183760	19	39564553	39612133	110	9	123834	-0.152	0.56041	0.985731697
TGIF2-C20orf24	ENSG00000259399	20	35192956	35250787	18	6	123834	-0.15222	0.56049	0.985731697
GSKIP	ENSG00000100744	14	96819814	96863625	18	2	123834	-0.15243	0.56058	0.985731697
PTK2	ENSG00000169398	8	141657999	142022315	318	7	123834	-0.15247	0.56059	0.985731697
MGAT1	ENSG00000131446	5	180207541	180252652	105	5	123834	-0.15254	0.56062	0.985731697
CD163	ENSG00000177575	12	7613409	7666489	67	12	123834	-0.15261	0.56065	0.985731697
PARP8	ENSG00000151883	5	49951733	50152356	277	13	123834	-0.15271	0.56069	0.985731697
TES	ENSG00000135269	7	115840547	115908837	209	13	123834	-0.15293	0.56077	0.985731697
ZMYM6NB	ENSG00000243749	1	35437136	35460954	33	2	123834	-0.15295	0.56078	0.985731697
IFNA16	ENSG00000147885	9	21206372	21227310	114	7	123834	-0.15299	0.5608	0.985731697
CLPP	ENSG00000125656	19	6351463	6378919	96	10	123834	-0.15317	0.56087	0.985731697
TLR2	ENSG00000137462	4	154612652	154636851	18	5	123834	-0.15324	0.5609	0.985731697
COL25A1	ENSG00000188517	4	109721877	110233813	1003	59	123834	-0.1533	0.56092	0.985731697
CTC-260F20.3	ENSG00000258674	19	19617036	19656885	63	4	123834	-0.15351	0.561	0.985731697
AQP8	ENSG00000103375	16	25217052	25250261	66	9	123834	-0.15357	0.56103	0.985731697
NBPF3	ENSG00000142794	1	21756621	21821498	164	13	123834	-0.15377	0.5611	0.985731697
CD300LG	ENSG00000161649	17	41914516	41950997	16	2	123834	-0.15385	0.56113	0.985731697
CR1L	ENSG00000197721	1	207808458	207921761	329	15	123834	-0.15397	0.56118	0.985731697
FARS2	ENSG00000145982	6	5251277	5781813	867	98	123834	-0.15413	0.56125	0.985731697
HDHD2	ENSG00000167220	18	44623774	44686891	143	11	123834	-0.15421	0.56128	0.985731697
KIAA1614	ENSG00000135835	1	180872290	180930750	55	16	123834	-0.15429	0.56131	0.985731697
C1orf52	ENSG00000162642	1	85705639	85735355	69	14	123834	-0.15455	0.56141	0.985731697
MCAM	ENSG00000076706	11	119169241	119202231	5	2	123834	-0.15476	0.5615	0.985731697
C14orf105	ENSG00000100557	14	57926019	57970585	12	2	123834	-0.15479	0.56151	0.985731697
KISS1R	ENSG00000116014	19	907287	931015	15	4	123834	-0.15495	0.56157	0.985731697
CCNDBP1	ENSG00000166946	15	43467316	43497396	14	3	123834	-0.155	0.56159	0.985731697
SLC22A18	ENSG00000110628	11	2910951	2956476	32	8	123834	-0.15522	0.56168	0.985731697
TM4SF4	ENSG00000169903	3	149181761	149231068	214	19	123834	-0.15532	0.56172	0.985731697
ALOX15B	ENSG00000179593	17	7932335	7962452	38	6	123834	-0.15536	0.56173	0.985731697
TIMMDC1	ENSG00000113845	3	119207379	119253937	124	14	123834	-0.1555	0.56179	0.985731697
ZNF24	ENSG00000172466	18	32902176	32935446	46	6	123834	-0.1555	0.56179	0.985731697
PNMA1	ENSG00000176903	14	74168494	74191128	51	10	123834	-0.15552	0.5618	0.985731697
AVEN	ENSG00000169857	15	34148428	34341377	472	34	123834	-0.15553	0.5618	0.985731697
PTPLB	ENSG00000206527	3	123199667	123314032	238	16	123834	-0.15557	0.56181	0.985731697
ANP32D	ENSG00000139223	12	48856448	48876843	89	7	123834	-0.15568	0.56186	0.985731697
ERMARD	ENSG00000130023	6	170141718	170191680	112	9	123834	-0.15592	0.56195	0.985731697
ALG12	ENSG00000182858	22	50283877	50322106	152	6	123834	-0.15592	0.56195	0.985731697
WDR46	ENSG00000227057	6	33236885	33267304	39	9	123834	-0.15593	0.56196	0.985731697
MKLN1	ENSG00000128585	7	130784855	131191395	577	63	123834	-0.15596	0.56197	0.985731697
HIST1H4F	ENSG00000198327	6	26230561	26250976	41	6	123834	-0.15626	0.56209	0.985731697
BDP1	ENSG00000145734	5	70741442	70873649	242	9	123834	-0.15649	0.56217	0.985731697
MEF2D	ENSG00000116604	1	156423519	156480620	59	7	123834	-0.1566	0.56222	0.985731697
KIAA0196	ENSG00000164961	8	126026502	126114082	232	9	123834	-0.1568	0.5623	0.985731697
SLC5A7	ENSG00000115665	2	108592979	108640450	65	15	123834	-0.15684	0.56232	0.985731697
WDR24	ENSG00000127580	16	724622	750444	22	4	123834	-0.15688	0.56233	0.985731697
CLCN6	ENSG00000011021	1	11856207	11913201	230	14	123834	-0.15691	0.56234	0.985731697
PARG	ENSG00000227345	10	51016325	51140715	111	8	123834	-0.15717	0.56244	0.985731697
ABI1	ENSG00000136754	10	27025522	27160016	308	14	123834	-0.15717	0.56245	0.985731697
RNF32	ENSG00000105982	7	156422975	156479824	79	11	123834	-0.15722	0.56246	0.985731697
SH2B3	ENSG00000111252	12	111833752	111899427	37	4	123834	-0.15732	0.5625	0.985731697
RNASE9	ENSG00000188655	14	21014252	21039090	20	7	123834	-0.15742	0.56254	0.985731697
CSH1	ENSG00000136488	17	61962275	61984021	15	4	123834	-0.1575	0.56257	0.985731697
PARVG	ENSG00000138964	22	44558836	44625413	125	22	123834	-0.15788	0.56273	0.985731697
TNFRSF13B	ENSG00000240505	17	16822849	16885432	112	15	123834	-0.15828	0.56288	0.985731697
WHAMM	ENSG00000156232	15	83468380	83513611	81	13	123834	-0.1585	0.56297	0.985731697
OR5A2	ENSG00000172324	11	59179416	59200449	45	6	123834	-0.15863	0.56302	0.985731697
APOBEC1	ENSG00000111701	12	7791996	7828499	109	17	123834	-0.15908	0.5632	0.985731697
ASNA1	ENSG00000198356	19	12837953	12869137	8	2	123834	-0.15909	0.5632	0.985731697
HEBP2	ENSG00000051620	6	138714668	138753334	49	5	123834	-0.15933	0.56329	0.985731697
CACNA1E	ENSG00000198216	1	181372238	181787219	629	61	123834	-0.15967	0.56343	0.985731697
B3GAT1	ENSG00000109956	11	134238398	134291812	80	8	123834	-0.1597	0.56344	0.985731697
H1F0	ENSG00000189060	22	38191114	38213442	32	4	123834	-0.15971	0.56345	0.985731697
TMEM80	ENSG00000177042	11	685428	715028	52	9	123834	-0.15984	0.5635	0.985731697
CTSG	ENSG00000100448	14	25032728	25055466	38	6	123834	-0.15994	0.56354	0.985731697
SLC39A6	ENSG00000141424	18	33678495	33719348	66	7	123834	-0.16001	0.56356	0.985731697
ACAT2	ENSG00000120437	6	160171360	160210144	94	9	123834	-0.16003	0.56357	0.985731697
CALCOCO2	ENSG00000136436	17	46898350	46953884	117	7	123834	-0.16004	0.56357	0.985731697
EIF2S1	ENSG00000134001	14	67816714	67863233	94	7	123834	-0.16006	0.56358	0.985731697
ACTB	ENSG00000075624	7	5556782	5613415	57	14	123834	-0.16013	0.56361	0.985731697
FAM160A1	ENSG00000164142	4	152320368	152594784	449	9	123834	-0.16025	0.56366	0.985731697
MPP6	ENSG00000105926	7	24602887	24743812	285	4	123834	-0.16033	0.56369	0.985731697
CMKLR1	ENSG00000174600	12	108671821	108743118	95	18	123834	-0.16032	0.56369	0.985731697
ZNF562	ENSG00000171466	19	9749330	9795776	82	5	123834	-0.16033	0.56369	0.985731697
MASP2	ENSG00000009724	1	11076580	11117290	58	5	123834	-0.16037	0.5637	0.985731697
RMND5B	ENSG00000145916	5	177547997	177587566	52	6	123834	-0.16034	0.5637	0.985731697
FXYD7	ENSG00000221946	19	35624154	35655204	15	4	123834	-0.16039	0.56371	0.985731697
HIST1H4I	ENSG00000198339	6	27097076	27118418	22	8	123834	-0.16044	0.56373	0.985731697
GPR15	ENSG00000154165	3	98240743	98261960	15	5	123834	-0.16045	0.56374	0.985731697
TIRAP	ENSG00000150455	11	126142960	126178740	85	13	123834	-0.16102	0.56396	0.985731697
MTRR	ENSG00000124275	5	7841299	7916138	165	23	123834	-0.16128	0.56406	0.985731697
PPP1R17	ENSG00000106341	7	31716329	31758069	58	12	123834	-0.1613	0.56407	0.985731697
GBGT1	ENSG00000148288	9	136018340	136049332	36	9	123834	-0.16129	0.56407	0.985731697
C5orf22	ENSG00000082213	5	31522373	31565165	86	14	123834	-0.16138	0.5641	0.985731697
PTGES3	ENSG00000110958	12	57047127	57092159	66	7	123834	-0.16147	0.56414	0.985731697
ATP10B	ENSG00000118322	5	159980127	160289221	482	48	123834	-0.16171	0.56423	0.985731697
CDKN2A	ENSG00000147889	9	21957751	22005300	31	7	123834	-0.16169	0.56423	0.985731697
DENND1C	ENSG00000205744	19	6457218	6492568	29	6	123834	-0.1618	0.56427	0.985731697
SLC39A10	ENSG00000196950	2	196430701	196612426	217	26	123834	-0.16184	0.56428	0.985731697
C16orf46	ENSG00000166455	16	81077102	81120872	129	2	123834	-0.16193	0.56432	0.985731697
GPR137B	ENSG00000077585	1	236295832	236395165	109	12	123834	-0.16198	0.56434	0.985731697
WDFY2	ENSG00000139668	13	52148644	52346171	167	17	123834	-0.16208	0.56438	0.985731697

KCTD1	ENSG00000134504	18	24024874	24247365	245	51	123834	-0.1621	0.56438	0.985731697
CXADR	ENSG00000154639	21	18874700	18975897	113	11	123834	-0.16226	0.56445	0.985731697
HSD11B1L	ENSG00000167733	19	5670615	5698533	26	2	123834	-0.16246	0.56453	0.985731697
ZNF696	ENSG00000185730	8	144361846	144390231	84	5	123834	-0.16253	0.56456	0.985731697
PCMTD1	ENSG00000168300	8	52720140	52821735	319	12	123834	-0.16273	0.56464	0.985731697
KRTAP10-3	ENSG00000212935	21	45967673	45988643	35	5	123834	-0.1628	0.56466	0.985731697
HPX	ENSG00000110169	11	6442279	6473847	27	2	123834	-0.16291	0.5647	0.985731697
KRBA2	ENSG00000184619	17	8261955	8290029	40	6	123834	-0.16297	0.56473	0.985731697
RP11-101E3.5	ENSG00000251184	9	131693757	131729300	27	1	123834	-0.16313	0.56479	0.985731697
RP11-152F13.10	ENSG00000260836	15	83198783	83234682	21	5	123834	-0.16326	0.56484	0.985731697
UBE2C	ENSG00000175063	20	44431215	44455596	58	9	123834	-0.16324	0.56484	0.985731697
PDSS1	ENSG00000148459	10	26976588	27045727	151	10	123834	-0.16339	0.5649	0.985731697
DOCK8	ENSG00000107099	9	204854	475259	608	56	123834	-0.1635	0.56494	0.985731697
RBM38	ENSG00000132819	20	55956463	55994389	58	10	123834	-0.16387	0.56508	0.985731697
FOXO3	ENSG00000118689	6	108871038	109015977	132	14	123834	-0.164	0.56514	0.985731697
OCIAD1	ENSG00000109180	4	48797229	48873834	47	7	123834	-0.16414	0.56519	0.985731697
PPP3R2	ENSG00000188386	9	104343897	104367283	46	10	123834	-0.1642	0.56521	0.985731697
IRGQ	ENSG00000167378	19	44078521	44110287	41	11	123834	-0.16441	0.5653	0.985731697
PF4V1	ENSG00000109272	4	74708906	74729872	41	8	123834	-0.1645	0.56533	0.985731697
PRR16	ENSG00000184838	5	119789973	120033027	633	44	123834	-0.1645	0.56533	0.985731697
LTBP3	ENSG00000168056	11	65296276	65336401	31	5	123834	-0.16459	0.56537	0.985731697
RP11-125O5.2	ENSG00000257062	12	21190113	21339829	482	24	123834	-0.16459	0.56537	0.985731697
MAP6	ENSG00000171533	11	75287963	75390165	99	7	123834	-0.16477	0.56544	0.985731697
GIPC3	ENSG00000179855	19	3575551	3603539	27	7	123834	-0.16481	0.56545	0.985731697
CTD-2260A17.2	ENSG00000247121	5	96139731	96281513	422	22	123834	-0.16492	0.5655	0.985731697
HNRNPD	ENSG00000138668	4	83263651	83305656	73	8	123834	-0.16498	0.56552	0.985731697
HCAR2	ENSG00000182782	12	123175840	123197890	48	4	123834	-0.16498	0.56552	0.985731697
PRR7	ENSG00000131188	5	176863446	176893283	36	3	123834	-0.16586	0.56587	0.986151069
API5	ENSG00000166181	11	43323513	43376079	61	13	123834	-0.16596	0.5659	0.986151069
GTSE1	ENSG00000075218	22	46682638	46736707	134	7	123834	-0.16614	0.56598	0.986151069
SEC24A	ENSG00000113615	5	133974479	134073513	50	9	123834	-0.16623	0.56601	0.986151069
RILP	ENSG00000167705	17	1539444	1563371	35	5	123834	-0.16625	0.56602	0.986151069
TAF5L	ENSG00000135801	1	229718858	229771794	77	7	123834	-0.16667	0.56619	0.986283871
DDX42	ENSG00000198231	17	61840963	61906677	93	5	123834	-0.16672	0.5662	0.986283871
CELF5	ENSG00000161082	19	3214701	3307074	121	11	123834	-0.16695	0.56629	0.986350254
TH	ENSG00000180176	11	2175159	2203107	20	2	123834	-0.16741	0.56648	0.986436761
TJAP1	ENSG00000137221	6	43435261	43484294	73	5	123834	-0.16751	0.56652	0.986436761
ANKRD45	ENSG00000183831	1	173568700	173649001	63	10	123834	-0.16785	0.56665	0.986436761
FAM196A	ENSG00000188916	10	128923694	129004422	79	6	123834	-0.16798	0.5667	0.986436761
DNAJC30	ENSG00000176410	7	73085299	73107783	65	5	123834	-0.16816	0.56677	0.986436761
TONSL	ENSG00000160949	8	145644165	145679827	39	1	123834	-0.16816	0.56677	0.986436761
ARHGAP11A	ENSG00000198826	15	32897345	32942150	19	6	123834	-0.1682	0.56679	0.986436761
MICALL2	ENSG00000164877	7	1458101	1509138	83	7	123834	-0.16828	0.56682	0.986436761
RAD51L3-RFFL	ENSG00000267618	17	33328368	33458512	60	8	123834	-0.16855	0.56693	0.986436761
SLC4A8	ENSG00000050438	12	51775101	51912980	190	12	123834	-0.16858	0.56694	0.986436761
CYB5A	ENSG00000166347	18	71910530	71969251	102	7	123834	-0.16865	0.56696	0.986436761
RPS26	ENSG00000197728	12	56425637	56448116	21	4	123834	-0.16872	0.56699	0.986436761
DEFB128	ENSG00000185982	20	158494	180322	78	8	123834	-0.16911	0.56714	0.986436761
ICT1	ENSG00000167862	17	72998765	73027356	28	5	123834	-0.16912	0.56715	0.986436761
ZGLP1	ENSG00000220201	19	10405479	10430556	38	7	123834	-0.16912	0.56715	0.986436761
ATP13A5	ENSG00000187527	3	192982579	193106632	259	19	123834	-0.16918	0.56717	0.986436761
KIAA0391	ENSG00000100890	14	35581052	35753271	440	23	123834	-0.16944	0.56727	0.986503223
TSPAN16	ENSG00000130167	19	11396824	11447672	75	10	123834	-0.16971	0.56738	0.986503223
WNT3	ENSG00000108379	17	44829872	44920520	61	9	123834	-0.1705	0.56769	0.986503223
DNAJB6	ENSG00000105993	7	157118075	157220133	159	17	123834	-0.1706	0.56773	0.986503223
C17orf107	ENSG00000205710	17	4792713	4816227	56	8	123834	-0.17085	0.56783	0.986503223
KAT2B	ENSG00000114166	3	20071515	20205896	281	22	123834	-0.17105	0.56791	0.986503223
RBM17	ENSG00000134453	10	6120950	6169420	103	22	123834	-0.17133	0.56802	0.986503223
RPS17L	ENSG00000182774	15	83195504	83219315	6	3	123834	-0.17134	0.56802	0.986503223
FGFBP1	ENSG00000137440	4	15927192	15950363	41	8	123834	-0.17142	0.56805	0.986503223
SLC15A2	ENSG00000163406	3	121602936	121672949	191	6	123834	-0.17144	0.56806	0.986503223
GFI1	ENSG00000162676	1	92930319	92962433	20	3	123834	-0.17146	0.56807	0.986503223
PDGFC	ENSG00000145431	4	157671606	157902546	152	17	123834	-0.17154	0.5681	0.986503223
CACNG8	ENSG00000142408	19	54456294	54503469	56	7	123834	-0.17161	0.56813	0.986503223
FAM213B	ENSG00000157870	1	2507930	2532908	57	3	123834	-0.17176	0.56819	0.986503223
SNX4	ENSG00000114520	3	125155495	125249041	214	17	123834	-0.17177	0.56819	0.986503223
POU3F3	ENSG00000198914	2	105461969	105486929	6	2	123834	-0.17182	0.56821	0.986503223
PATE2	ENSG00000196844	11	125636008	125658714	69	9	123834	-0.17183	0.56822	0.986503223
ST8SIA4	ENSG00000113532	5	100132639	100248970	182	9	123834	-0.17188	0.56823	0.986503223
FLRT1	ENSG00000126500	11	63860660	63896645	53	10	123834	-0.17193	0.56825	0.986503223
IPO11	ENSG00000086200	5	61689799	61934409	342	12	123834	-0.17199	0.56828	0.986503223
PFKL	ENSG00000141959	21	45709934	45757259	71	8	123834	-0.17211	0.56833	0.986503223
TBATA	ENSG00000166220	10	72520995	72555157	67	7	123834	-0.17217	0.56835	0.986503223
PDE7A	ENSG00000205268	8	66619745	66764557	248	15	123834	-0.17263	0.56853	0.986725551
CCDC108	ENSG00000181378	2	219857568	219916249	64	7	123834	-0.1731	0.56871	0.98680343
CCDC169	ENSG00000242715	13	36791182	36881977	360	6	123834	-0.1733	0.56879	0.98680343
LGI2	ENSG00000153012	4	24990469	25042501	129	21	123834	-0.1736	0.56891	0.98680343
ASIC5	ENSG00000256394	4	156740881	156797425	104	10	123834	-0.17377	0.56898	0.98680343
SCUBE1	ENSG00000159307	22	43583289	43749394	257	39	123834	-0.17384	0.56901	0.98680343
PTPRH	ENSG00000080031	19	55682616	55730874	95	12	123834	-0.17404	0.56908	0.98680343
ARMC4	ENSG00000169126	10	28054115	28297977	306	17	123834	-0.1741	0.56911	0.98680343
NPL	ENSG00000135838	1	182748428	182809519	65	7	123834	-0.17437	0.56921	0.98680343
RPP38	ENSG00000152464	10	15129179	15191817	156	18	123834	-0.17456	0.56929	0.98680343
KCNC3	ENSG00000131398	19	50805194	50846772	16	4	123834	-0.17467	0.56933	0.98680343
OR51S1	ENSG00000176922	11	4859427	4880514	23	4	123834	-0.17478	0.56937	0.98680343
PER3	ENSG00000049246	1	7834380	7915237	210	25	123834	-0.17489	0.56942	0.98680343
LARP4	ENSG00000161813	12	50776166	50883787	139	7	123834	-0.17549	0.56965	0.98680343
KLK9	ENSG00000213022	19	51493806	51522890	50	7	123834	-0.17547	0.56965	0.98680343
KRTAP22-1	ENSG00000186924	21	31963414	31983612	27	3	123834	-0.17569	0.56973	0.98680343
SLC19A2	ENSG00000117479	1	169423147	169465241	25	7	123834	-0.17573	0.56975	0.98680343
SLC35D3	ENSG00000182747	6	137233402	137256777	17	2	123834	-0.1759	0.56981	0.98680343
CLTA	ENSG00000122705	9	36180853	36314778	257	16	123834	-0.17595	0.56983	0.98680343
MSX2	ENSG00000120149	5	174141536	174167896	13	3	123834	-0.17598	0.56985	0.98680343
CCDC148	ENSG00000153237	2	159017593	159323265	1041	41	123834	-0.17622	0.56994	0.98680343
BOD1L1	ENSG00000038219	4	13560362	13639347	96	10	123834	-0.1763	0.56997	0.98680343
AP000769.1	ENSG00000173727	11	65212728	65244028	62	4	123834	-0.1765	0.57005	0.98680343
AL121895.1	ENSG00000269650	20	34746461	34768180	30	5	123834	-0.17674	0.57014	0.98680343
RNGTT	ENSG00000111880	6	89309985	89683348	529	14	123834	-0.17686	0.57019	0.98680343
NAMPTL	ENSG00000229644	10	36800649	36823162	17	2	123834	-0.17691	0.57021	0.98680343

PCDH10	ENSG00000138650	4	134060470	134139356	99	17	123834	-0.17699	0.57024	0.98680343
PLXND1	ENSG00000004399	3	129264018	129335661	133	16	123834	-0.17735	0.57038	0.98680343
ZNF749	ENSG00000186230	19	57936697	57966853	44	11	123834	-0.17736	0.57039	0.98680343
TUSC1	ENSG00000198680	9	25666396	25688856	26	4	123834	-0.17756	0.57046	0.98680343
TNKS	ENSG00000173273	8	9403424	9649856	633	14	123834	-0.17759	0.57048	0.98680343
CAMK2G	ENSG00000148660	10	75562259	75644343	59	5	123834	-0.17764	0.5705	0.98680343
LCK	ENSG00000182866	1	32706840	32761766	23	3	123834	-0.17781	0.57056	0.98680343
ORAOV1	ENSG00000149716	11	69457844	69500184	39	3	123834	-0.17784	0.57058	0.98680343
OR2G3	ENSG00000177476	1	247758856	247779838	32	6	123834	-0.17797	0.57062	0.98680343
FBXO25	ENSG00000147364	8	346428	431225	234	19	123834	-0.17806	0.57066	0.98680343
STON1	ENSG00000243244	2	48746522	48836025	188	21	123834	-0.17807	0.57067	0.98680343
AC117395.1	ENSG00000268175	3	149679066	149700535	34	8	123834	-0.17808	0.57067	0.98680343
KCNH1	ENSG00000143473	1	210846555	211317457	884	66	123834	-0.17837	0.57078	0.98680343
PZP	ENSG00000126838	12	9291436	9370966	98	14	123834	-0.17837	0.57079	0.98680343
CLLU1OS	ENSG00000205057	12	92803870	92831924	42	6	123834	-0.17839	0.57079	0.98680343
HAPLN4	ENSG00000187664	19	19356450	19383605	43	4	123834	-0.1786	0.57087	0.98680343
DDX25	ENSG00000109832	11	125763271	125803158	53	11	123834	-0.17872	0.57092	0.98680343
SOBP	ENSG00000112320	6	107801162	107991357	199	28	123834	-0.17894	0.57101	0.98680343
RIMKLB	ENSG00000166532	12	8824196	8945691	35	5	123834	-0.17896	0.57102	0.98680343
VSIG10	ENSG00000176834	12	118491398	118583831	138	28	123834	-0.1792	0.57111	0.98680343
APOBEC3B	ENSG00000179750	22	39368352	39398809	17	3	123834	-0.1793	0.57115	0.98680343
NAA50	ENSG00000121579	3	113425307	113475147	39	1	123834	-0.17973	0.57132	0.98680343
MRPL30	ENSG00000185414	2	99787542	99824089	56	6	123834	-0.17988	0.57137	0.98680343
BCAR3	ENSG00000137936	1	94017347	94322706	645	21	123834	-0.17997	0.57141	0.98680343
GATAD1	ENSG00000157259	7	92066767	92098150	51	7	123834	-0.18008	0.57145	0.98680343
YOD1	ENSG00000180667	1	207207194	207236325	16	2	123834	-0.18013	0.57148	0.98680343
UBTD2	ENSG00000168246	5	171626644	171721075	157	9	123834	-0.18028	0.57153	0.98680343
ITK	ENSG00000113263	5	156559944	156692201	217	26	123834	-0.18043	0.57159	0.98680343
CXCL14	ENSG00000145824	5	134896373	134924969	19	3	123834	-0.18067	0.57169	0.98680343
PPAPDC1A	ENSG00000203805	10	122206466	122359367	349	31	123834	-0.18069	0.57169	0.98680343
PPBP	ENSG00000163736	4	74842755	74863914	20	1	123834	-0.18088	0.57177	0.98680343
STAU1	ENSG00000124214	20	47719878	47814904	152	12	123834	-0.18098	0.57181	0.98680343
HNF1B	ENSG00000108753	17	36036435	36115237	25	11	123834	-0.181	0.57182	0.98680343
RAD18	ENSG00000070950	3	8807088	9015457	585	26	123834	-0.18104	0.57183	0.98680343
IRAK2	ENSG00000134070	3	10196549	10295427	136	19	123834	-0.18109	0.57185	0.98680343
SH2D3A	ENSG00000125731	19	6742171	6777599	35	4	123834	-0.18123	0.57191	0.98680343
AIPL1	ENSG00000129221	17	6287013	6348519	128	25	123834	-0.18131	0.57194	0.98680343
HECTD2	ENSG00000165338	10	93160096	93284586	122	4	123834	-0.18158	0.57204	0.98680343
SMC4	ENSG00000113810	3	160107062	160162750	70	8	123834	-0.18161	0.57206	0.98680343
RPUSD3	ENSG00000156990	3	9869533	9896286	42	7	123834	-0.18178	0.57212	0.98680343
MOK	ENSG00000080823	14	102680837	102781537	185	14	123834	-0.18183	0.57214	0.98680343
NETO2	ENSG00000171208	16	47101614	47187908	36	3	123834	-0.18208	0.57224	0.98680343
NEK9	ENSG00000119638	14	75538822	75604047	84	3	123834	-0.18231	0.57233	0.98680343
SLC7A9	ENSG00000021488	19	33311415	33370672	192	19	123834	-0.18236	0.57235	0.98680343
FAM98A	ENSG00000119812	2	33798725	33834449	33	7	123834	-0.18262	0.57245	0.98680343
MYBPHL	ENSG00000221986	1	109824987	109859663	43	8	123834	-0.18266	0.57247	0.98680343
ACYP1	ENSG00000119640	14	75509924	75546186	36	2	123834	-0.18271	0.57249	0.98680343
DNLZ	ENSG00000213221	9	139243932	139268241	46	9	123834	-0.18278	0.57251	0.98680343
FMO4	ENSG00000076258	1	171273347	171321223	62	7	123834	-0.18306	0.57262	0.98680343
VN1R1	ENSG00000178201	19	57956542	57977854	67	7	123834	-0.1831	0.57264	0.98680343
COX20	ENSG00000203667	1	244988624	245018359	35	4	123834	-0.18314	0.57266	0.98680343
OR8B4	ENSG00000198657	11	124283807	124304789	71	5	123834	-0.18328	0.57271	0.98680343
BACH2	ENSG00000112182	6	90626248	91016627	359	38	123834	-0.18329	0.57272	0.98680343
ANXA6	ENSG00000197043	5	150470273	150547443	148	24	123834	-0.18333	0.57273	0.98680343
EP400NL	ENSG00000185684	12	132558828	132623029	115	8	123834	-0.18339	0.57275	0.98680343
ZNF66	ENSG00000160229	19	20949110	21001922	366	11	123834	-0.18345	0.57278	0.98680343
ZNF879	ENSG00000234284	5	178440753	178472065	49	3	123834	-0.18397	0.57298	0.986837115
RP11-1035H13.3	ENSG00000260342	16	18789385	18822841	27	4	123834	-0.1842	0.57307	0.986837115
AZI2	ENSG00000163512	3	28346494	28400618	57	6	123834	-0.1843	0.57311	0.986837115
RPSAP58	ENSG00000205246	19	23935807	24020937	127	16	123834	-0.18445	0.57317	0.986837115
MYL5	ENSG00000215375	4	657369	685822	27	4	123834	-0.18463	0.57324	0.986837115
ZNF778	ENSG00000170100	16	89274118	89305363	121	7	123834	-0.18522	0.57347	0.986837115
REM2	ENSG00000139890	14	23342374	23366895	25	4	123834	-0.18529	0.5735	0.986837115
KRBA1	ENSG00000133619	7	149401872	149441664	66	7	123834	-0.18569	0.57366	0.986837115
AKR7A2	ENSG00000053371	1	19620459	19648640	36	9	123834	-0.18577	0.57369	0.986837115
ATP6V0A4	ENSG00000105929	7	138381040	138494305	166	36	123834	-0.186	0.57378	0.986837115
QTRTD1	ENSG00000151576	3	113714680	113817269	59	4	123834	-0.18623	0.57387	0.986837115
HELT	ENSG00000187821	4	185929995	185951958	35	9	123834	-0.18637	0.57392	0.986837115
CAPN13	ENSG00000162949	2	30935637	31053408	193	31	123834	-0.18652	0.57398	0.986837115
OSBPL6	ENSG00000079156	2	179049208	179274160	365	34	123834	-0.18651	0.57398	0.986837115
EMC8	ENSG00000131148	16	85795364	85843214	66	5	123834	-0.18676	0.57407	0.986837115
XCL1	ENSG00000143184	1	168535711	168561315	42	6	123834	-0.18698	0.57416	0.986837115
C3orf17	ENSG00000163608	3	112711287	112748708	39	8	123834	-0.18705	0.57419	0.986837115
OR2T8	ENSG00000177462	1	248074320	248095258	34	4	123834	-0.18728	0.57428	0.986837115
ATP5E	ENSG00000124172	20	57590522	57617437	21	5	123834	-0.1873	0.57429	0.986837115
SLC25A33	ENSG00000171612	1	9589541	9655237	53	3	123834	-0.18732	0.5743	0.986837115
RBL2	ENSG00000103479	16	53457889	53535561	90	4	123834	-0.18744	0.57434	0.986837115
C17orf47	ENSG00000181013	17	56608948	56631729	30	6	123834	-0.18747	0.57435	0.986837115
ARHGEF10	ENSG00000104728	8	1762142	1916807	321	56	123834	-0.18753	0.57438	0.986837115
CRYGN	ENSG00000127377	7	151115921	151147899	19	3	123834	-0.18761	0.57441	0.986837115
UPF1	ENSG00000005007	19	18932747	18989045	59	7	123834	-0.18816	0.57462	0.986837115
ETV3L	ENSG00000253831	1	157051836	157079600	34	7	123834	-0.18821	0.57464	0.986837115
NDRG1	ENSG00000104419	8	134239414	134324265	130	22	123834	-0.18823	0.57465	0.986837115
WBP4	ENSG00000120688	13	41625410	41668137	22	5	123834	-0.18874	0.57485	0.986837115
RP11-297M9.1	ENSG00000260362	16	9750742	9780700	92	11	123834	-0.18876	0.57486	0.986837115
IFNA10	ENSG00000186803	9	21196180	21217142	107	7	123834	-0.18903	0.57497	0.986837115
ZNF578	ENSG00000258405	19	52946829	53025407	309	13	123834	-0.18905	0.57497	0.986837115
SST	ENSG00000157005	3	187376694	187398187	33	3	123834	-0.18936	0.5751	0.986837115
NUDT3	ENSG00000272325	6	34237456	34370451	219	8	123834	-0.1896	0.57519	0.986837115
AC025287.1	ENSG00000214325	16	75718367	75744089	67	7	123834	-0.18961	0.57519	0.986837115
CLCN1	ENSG00000188037	7	143003219	143059176	46	7	123834	-0.18966	0.57521	0.986837115
PAQR3	ENSG00000163291	4	79798281	79870592	133	5	123834	-0.18972	0.57524	0.986837115
TTC17	ENSG00000052841	11	43370482	43526483	262	20	123834	-0.18976	0.57525	0.986837115
CCDC134	ENSG00000100147	22	42186683	42232303	83	10	123834	-0.18975	0.57525	0.986837115
CNPY3	ENSG00000137161	6	42886938	42917025	47	9	123834	-0.18981	0.57527	0.986837115
OR51J1	ENSG00000184321	11	5413827	5434777	180	12	123834	-0.18984	0.57528	0.986837115
FAM131B	ENSG00000159784	7	143040493	143069863	29	8	123834	-0.18987	0.57529	0.986837115
SYT1	ENSG00000067715	12	79247773	79855788	931	42	123834	-0.18987	0.57529	0.986837115
MOGAT3	ENSG00000106384	7	100828288	100854302	27	6	123834	-0.18991	0.57531	0.986837115

SLC30A7	ENSG00000162695	1	101351632	101457309	105	8	123834	-0.19002	0.57535	0.986837115
DPH5	ENSG00000117543	1	101445179	101501644	47	4	123834	-0.19041	0.57551	0.986837115
RPAP1	ENSG00000103932	15	41799374	41846467	71	8	123834	-0.19071	0.57562	0.986837115
GOLGA6C	ENSG00000167195	15	75540940	75572540	2	1	123834	-0.19128	0.57585	0.986837115
CTD-2192J16.22	ENSG00000269242	19	12744645	12769211	15	3	123834	-0.19131	0.57586	0.986837115
METTL15	ENSG00000169519	11	28119795	28365054	228	15	123834	-0.19152	0.57594	0.986837115
ZNF234	ENSG00000263002	19	44635710	44673156	39	5	123834	-0.19156	0.57596	0.986837115
SEPP1	ENSG00000250722	5	42789982	42897494	226	21	123834	-0.19168	0.57601	0.986837115
ATG4B	ENSG00000168397	2	242566628	242623272	127	13	123834	-0.19208	0.57616	0.986837115
ADAM30	ENSG00000134249	1	120426156	120449118	22	4	123834	-0.19212	0.57618	0.986837115
CMA1	ENSG00000092009	14	24964559	24987471	55	9	123834	-0.19216	0.57619	0.986837115
BRMS1L	ENSG00000100916	14	36285524	36411531	132	17	123834	-0.19219	0.5762	0.986837115
BVES	ENSG00000112276	6	105534697	105595049	132	9	123834	-0.19224	0.57622	0.986837115
SPATA31D1	ENSG00000214929	9	84593687	84620171	16	5	123834	-0.19225	0.57623	0.986837115
TMEM97	ENSG00000109084	17	26636121	26665707	18	4	123834	-0.19234	0.57626	0.986837115
OR2AE1	ENSG00000244623	7	99463610	99484680	38	6	123834	-0.1924	0.57629	0.986837115
ST6GAL1	ENSG00000073849	3	186638274	186806341	282	41	123834	-0.19251	0.57633	0.986837115
CLCN3	ENSG00000109572	4	170523784	170654824	217	18	123834	-0.1925	0.57633	0.986837115
ZNF77	ENSG00000175691	19	2923216	2954969	34	5	123834	-0.19252	0.57633	0.986837115
JMJD8	ENSG00000161999	16	721671	744529	21	5	123834	-0.19283	0.57646	0.986837115
EPT1	ENSG00000138018	2	26521415	26628759	137	18	123834	-0.19305	0.57654	0.986837115
NLRC5	ENSG00000140853	16	57013397	57127443	139	33	123834	-0.19365	0.57677	0.986837115
ASNS	ENSG00000070669	7	97471430	97511854	85	11	123834	-0.19367	0.57678	0.986837115
GUSB	ENSG00000169919	7	65415671	65457301	74	8	123834	-0.19385	0.57685	0.986837115
UNC80	ENSG00000144406	2	210626717	210874024	303	15	123834	-0.19402	0.57692	0.986837115
OR2AG1	ENSG00000170803	11	6796248	6817245	43	6	123834	-0.19421	0.57699	0.986837115
SPECC1L-ADORA2A	ENSG00000258555	22	24656866	24848324	242	17	123834	-0.19447	0.57709	0.986837115
RP11-503N18.3	ENSG00000249428	4	2441700	2474668	33	6	123834	-0.19458	0.57714	0.986837115
POLR2B	ENSG00000047315	4	57833888	57907334	181	21	123834	-0.19472	0.57719	0.986837115
AL645728.1	ENSG00000215014	1	1500355	1521373	14	2	123834	-0.19479	0.57722	0.986837115
FRMD3	ENSG00000172159	9	85847905	86163461	773	65	123834	-0.19482	0.57723	0.986837115
FCHO2	ENSG00000157107	5	72241808	72396349	181	10	123834	-0.19497	0.57729	0.986837115
OMA1	ENSG00000162600	1	58871056	59022474	436	34	123834	-0.19506	0.57733	0.986837115
FER	ENSG00000151422	5	108073523	108542542	1128	36	123834	-0.19516	0.57737	0.986837115
DPRX	ENSG00000204595	19	54125310	54150263	8	2	123834	-0.19518	0.57737	0.986837115
SSR3	ENSG00000114850	3	156247929	156282973	55	7	123834	-0.19568	0.57757	0.986837115
LRRC72	ENSG00000205858	7	16556505	16631193	109	16	123834	-0.19567	0.57757	0.986837115
UMOD	ENSG00000169344	16	20334374	20377623	65	10	123834	-0.19585	0.57764	0.986837115
C17orf70	ENSG00000185504	17	79496911	79530987	43	8	123834	-0.19586	0.57764	0.986837115
EFNA5	ENSG00000184349	5	106702590	107016596	248	48	123834	-0.19604	0.57771	0.986837115
C3orf20	ENSG00000131379	3	14706606	14824541	164	21	123834	-0.19662	0.57794	0.986837115
UBR7	ENSG00000012963	14	93663401	93705561	85	14	123834	-0.19662	0.57794	0.986837115
HSDL1	ENSG00000103160	16	84145886	84188797	64	12	123834	-0.19662	0.57794	0.986837115
CDK2	ENSG00000123374	12	56350553	56376568	15	3	123834	-0.19664	0.57795	0.986837115
CCAR1	ENSG00000060339	10	70470769	70562134	134	11	123834	-0.19675	0.57799	0.986837115
SLC12A5	ENSG00000124140	20	44640356	44698784	103	9	123834	-0.19688	0.57804	0.986837115
PP13004	ENSG00000205745	7	36108694	36135346	36	8	123834	-0.19697	0.57807	0.986837115
C12orf56	ENSG00000185306	12	64650217	64794972	248	16	123834	-0.19696	0.57807	0.986837115
PPIL1	ENSG00000137168	6	36812603	36852800	91	11	123834	-0.19698	0.57808	0.986837115
RP11-371E8.4	ENSG00000259066	14	93641318	93694932	87	13	123834	-0.19699	0.57808	0.986837115
OR2B3	ENSG00000204703	6	29043985	29065090	37	8	123834	-0.1972	0.57816	0.986837115
CHRM1	ENSG00000168539	11	62666151	62699279	23	4	123834	-0.19723	0.57818	0.986837115
ZNF229	ENSG00000167383	19	44911685	44962766	158	19	123834	-0.19726	0.57819	0.986837115
MICU3	ENSG00000155970	8	16874747	16990153	150	16	123834	-0.19743	0.57825	0.986837115
GALNT9	ENSG00000182870	12	132670924	132915935	172	29	123834	-0.19766	0.57835	0.986837115
MED15	ENSG00000099917	22	20840200	20951919	182	13	123834	-0.1977	0.57836	0.986837115
LRRC56	ENSG00000161328	11	527527	564916	107	15	123834	-0.19777	0.57839	0.986837115
COG4	ENSG00000103051	16	70504471	70567468	41	3	123834	-0.19784	0.57842	0.986837115
ATP9B	ENSG00000166377	18	76819285	77148283	794	20	123834	-0.19786	0.57842	0.986837115
STUB1	ENSG00000103266	16	720224	742870	24	5	123834	-0.19788	0.57843	0.986837115
KHNYN	ENSG00000100441	14	24888492	24920540	127	8	123834	-0.19796	0.57846	0.986837115
LYST	ENSG00000143669	1	235814341	236056940	210	11	123834	-0.19814	0.57853	0.986837115
LCN9	ENSG00000148386	9	138545168	138568268	54	9	123834	-0.19828	0.57859	0.986837115
BRI3	ENSG00000164713	7	97871691	97947162	122	8	123834	-0.19834	0.57861	0.986837115
ORC6	ENSG00000091651	16	46713555	46742306	1	1	123834	-0.19848	0.57867	0.986837115
TOP1	ENSG00000198900	20	39647458	39763127	127	17	123834	-0.19855	0.57869	0.986837115
IFT46	ENSG00000118096	11	118405243	118453685	55	2	123834	-0.19858	0.5787	0.986837115
C1orf204	ENSG00000188004	1	159794264	159835137	24	9	123834	-0.1986	0.57871	0.986837115
RABL2B	ENSG00000079974	22	51195929	51232091	14	1	123834	-0.19861	0.57872	0.986837115
ZNF384	ENSG00000126746	12	6765643	6808738	17	4	123834	-0.19868	0.57874	0.986837115
LRIT2	ENSG00000204033	10	85970254	85995345	64	7	123834	-0.19872	0.57876	0.986837115
FKBP7	ENSG00000079150	2	179318391	179353327	40	7	123834	-0.19875	0.57877	0.986837115
CDHR1	ENSG00000148600	10	85944410	85989377	91	11	123834	-0.19915	0.57893	0.987021387
HMGCL	ENSG00000117305	1	24118375	24175110	41	2	123834	-0.19948	0.57906	0.987069848
C4orf22	ENSG00000197826	4	81246874	81894910	1105	28	123834	-0.19966	0.57913	0.987069848
CCDC152	ENSG00000198865	5	42746903	42812462	167	12	123834	-0.19973	0.57915	0.987069848
GBE1	ENSG00000114480	3	81528850	81821312	350	20	123834	-0.19976	0.57917	0.987069848
ZNF281	ENSG00000162702	1	200364068	200389184	18	5	123834	-0.19994	0.57923	0.987069848
CORO2A	ENSG00000106789	9	100873257	100964922	123	24	123834	-0.20003	0.57927	0.987069848
CEP170	ENSG00000143702	1	243277730	243428650	149	12	123834	-0.20018	0.57933	0.9870836
FOLR2	ENSG00000165457	11	71917645	71942994	40	7	123834	-0.20035	0.5794	0.987114386
IFNA5	ENSG00000147873	9	21294613	21315312	39	3	123834	-0.2005	0.57946	0.98712813
PAFAH1B2	ENSG00000168092	11	117004983	117057610	126	14	123834	-0.20095	0.57963	0.987329245
ZBED5	ENSG00000236287	11	10823621	10890343	98	10	123834	-0.20128	0.57976	0.987363747
OR5L2	ENSG00000205030	11	55584695	55605630	41	7	123834	-0.20132	0.57977	0.987363747
PABPC4L	ENSG00000254535	4	135107488	135132903	56	5	123834	-0.20163	0.5799	0.987363747
VDR	ENSG00000111424	12	48225320	48346831	106	21	123834	-0.20163	0.5799	0.987363747
GPIHBP1	ENSG00000182851	8	144285068	144309044	62	9	123834	-0.20257	0.58026	0.987363747
WDR1	ENSG00000071127	4	10065963	10128573	171	15	123834	-0.20275	0.58033	0.987363747
AFAP1L1	ENSG00000157510	5	148641434	148731365	78	17	123834	-0.20294	0.58041	0.987363747
PAOX	ENSG00000148832	10	135182695	135215198	37	5	123834	-0.20298	0.58042	0.987363747
ZNF433	ENSG00000197647	19	12115547	12156556	51	3	123834	-0.20307	0.58046	0.987363747
PCSK2	ENSG00000125851	20	17196752	17475223	520	42	123834	-0.20316	0.58049	0.987363747
ELMOD3	ENSG00000115459	2	85571517	85628875	88	10	123834	-0.20319	0.58051	0.987363747
AL050302.1	ENSG00000256715	21	14731931	14755386	79	11	123834	-0.2033	0.58055	0.987363747
ZCCHC9	ENSG00000131732	5	80587409	80619116	62	8	123834	-0.20332	0.58056	0.987363747
DNHD1	ENSG00000179532	11	6508490	6624988	141	16	123834	-0.20357	0.58066	0.987363747
KIAA0087	ENSG00000122548	7	26562740	26588407	87	10	123834	-0.20391	0.58079	0.987363747
DACH1	ENSG00000165659	13	72002098	72451330	330	38	123834	-0.20396	0.58081	0.987363747

RP11-433C9.2	ENSG00000224153	3	184109325	184167436	84	15	123834	-0.20425	0.58092	0.987363747
RPL30	ENSG00000156482	8	99027079	99068697	54	9	123834	-0.20445	0.581	0.987363747
WDR76	ENSG00000092470	15	44109161	44170617	66	4	123834	-0.20457	0.58104	0.987363747
AC136604.1	ENSG00000228259	5	179068298	179089445	11	4	123834	-0.20482	0.58114	0.987363747
FAM19A5	ENSG00000219438	22	48875272	49256724	971	113	123834	-0.20489	0.58117	0.987363747
PEX5	ENSG00000139197	12	7331281	7381170	99	13	123834	-0.20495	0.58119	0.987363747
DEFB110	ENSG00000203970	6	49966851	49999694	26	4	123834	-0.20499	0.58121	0.987363747
PANK3	ENSG00000120137	5	167965500	168016605	27	5	123834	-0.20511	0.58126	0.987363747
ASCL2	ENSG00000183734	11	2279725	2302182	49	6	123834	-0.20516	0.58128	0.987363747
SMC3	ENSG00000108055	10	112317449	112374394	122	7	123834	-0.20519	0.58129	0.987363747
PSMD8	ENSG00000099341	19	38855176	38884464	65	9	123834	-0.20524	0.58131	0.987363747
PTDSS2	ENSG00000174915	11	438268	501393	133	12	123834	-0.20538	0.58136	0.987363747
KRT12	ENSG00000187242	17	39007555	39033462	67	12	123834	-0.20548	0.5814	0.987363747
PPIL3	ENSG00000240344	2	201725630	201764026	33	6	123834	-0.20588	0.58156	0.987363747
CTD-2369P2.10	ENSG00000167807	19	10406103	10436685	45	7	123834	-0.20653	0.58181	0.987363747
FDX1L	ENSG00000267673	19	10406103	10436691	45	7	123834	-0.20653	0.58181	0.987363747
ASTE1	ENSG00000034533	3	130722719	130756493	40	5	123834	-0.20666	0.58186	0.987363747
PAQR7	ENSG00000182749	1	26177701	26207744	28	3	123834	-0.2067	0.58188	0.987363747
FXYD3	ENSG00000089356	19	35596732	35625228	44	4	123834	-0.20677	0.5819	0.987363747
ANKUB1	ENSG00000206199	3	149468892	149696172	385	22	123834	-0.20684	0.58193	0.987363747
ZNF367	ENSG00000165244	9	99138223	99190611	103	4	123834	-0.207	0.58199	0.987363747
NAB2	ENSG00000166886	12	57472677	57499259	24	4	123834	-0.20701	0.582	0.987363747
PTPN4	ENSG00000088179	2	120507207	120751394	300	11	123834	-0.20734	0.58213	0.987363747
MACROD1	ENSG00000133315	11	63756030	63943578	210	31	123834	-0.20742	0.58216	0.987363747
DNAJB4	ENSG00000162616	1	78434859	78493648	113	16	123834	-0.208	0.58238	0.987363747
FAM188B	ENSG00000106125	7	30801033	30942002	346	25	123834	-0.20816	0.58245	0.987363747
ACSM1	ENSG00000166743	16	20624559	20720212	165	12	123834	-0.20827	0.58249	0.987363747
DRD4	ENSG00000069696	11	627293	650706	20	8	123834	-0.20835	0.58252	0.987363747
PANX1	ENSG00000110218	11	93852094	93925138	114	15	123834	-0.20863	0.58263	0.987363747
LINGO1	ENSG00000169783	15	77895369	78123242	334	23	123834	-0.20873	0.58267	0.987363747
EPB41L1	ENSG00000088367	20	34669426	34830721	103	10	123834	-0.20895	0.58276	0.987363747
HTRA2	ENSG00000115317	2	74746504	74770472	5	2	123834	-0.20936	0.58291	0.987363747
KRTAP9-3	ENSG00000204873	17	39378715	39399705	37	3	123834	-0.20939	0.58293	0.987363747
TGM2	ENSG00000198959	20	36746863	36804980	80	5	123834	-0.20942	0.58294	0.987363747
RNASEH2A	ENSG00000104889	19	12907394	12934452	75	5	123834	-0.20945	0.58295	0.987363747
LRR1	ENSG00000165501	14	50055415	50091390	81	5	123834	-0.21015	0.58322	0.987363747
ZNF426	ENSG00000130818	19	9628667	9659303	113	2	123834	-0.21015	0.58323	0.987363747
MCCC2	ENSG00000131844	5	70873115	70964531	114	12	123834	-0.21027	0.58327	0.987363747
SRL	ENSG00000185739	16	4229375	4302081	104	19	123834	-0.21039	0.58332	0.987363747
GOT1	ENSG00000120053	10	101146627	101200381	55	9	123834	-0.21046	0.58335	0.987363747
ARF1	ENSG00000143761	1	228260361	228296912	26	4	123834	-0.21072	0.58345	0.987363747
OR5A1	ENSG00000172320	11	59200617	59221667	72	7	123834	-0.21074	0.58345	0.987363747
OR1B1	ENSG00000171484	9	125380858	125401852	46	9	123834	-0.21091	0.58352	0.987363747
HLA-DRB1	ENSG00000196126	6	32536546	32567625	349	12	123834	-0.21093	0.58353	0.987363747
RPL31	ENSG00000071082	2	101608177	101650494	82	9	123834	-0.21105	0.58358	0.987363747
FAM49B	ENSG00000153310	8	130841839	131039375	183	21	123834	-0.21107	0.58359	0.987363747
DLK1	ENSG00000185559	14	101182042	101211539	39	9	123834	-0.21118	0.58363	0.987363747
ARID4A	ENSG00000032219	14	58755103	58850605	85	6	123834	-0.21139	0.58371	0.987363747
AC138647.1	ENSG00000226490	8	142514738	142538837	46	8	123834	-0.21152	0.58376	0.987363747
GH1	ENSG00000259384	17	61984560	62006179	6	1	123834	-0.21154	0.58377	0.987363747
FGF20	ENSG00000078579	8	16839678	16869690	111	20	123834	-0.21181	0.58387	0.987363747
PDIA5	ENSG00000065485	3	122775909	122954074	331	28	123834	-0.21184	0.58388	0.987363747
PMEPA1	ENSG00000124225	20	56213448	56296592	107	14	123834	-0.21182	0.58388	0.987363747
STAC	ENSG00000144681	3	36411836	36599499	469	32	123834	-0.2119	0.58391	0.987363747
ZC2HC1C	ENSG00000119703	14	75520873	75555126	36	2	123834	-0.21208	0.58398	0.987363747
GTPBP8	ENSG00000163607	3	112699765	112743907	59	10	123834	-0.2122	0.58403	0.987363747
CLCA2	ENSG00000137975	1	86879769	86932241	175	15	123834	-0.21225	0.58404	0.987363747
GC	ENSG00000145321	4	72597410	72679758	97	10	123834	-0.21234	0.58408	0.987363747
GALE	ENSG00000117308	1	24112089	24137271	12	2	123834	-0.21239	0.5841	0.987363747
SLC35D1	ENSG00000116704	1	67455015	67529782	159	14	123834	-0.21241	0.5841	0.987363747
CPM	ENSG00000135678	12	69225977	69375350	183	36	123834	-0.21243	0.58411	0.987363747
HYLS1	ENSG00000198331	11	125743509	125780543	84	9	123834	-0.21256	0.58416	0.987363747
CYP46A1	ENSG00000036530	14	100140641	100203638	56	8	123834	-0.21282	0.58427	0.987363747
MMP23B	ENSG00000189409	1	1557474	1580639	2	1	123834	-0.21288	0.58429	0.987363747
DCC	ENSG00000187323	18	49856542	51067784	3337	88	123834	-0.21312	0.58438	0.987363747
OR4M2	ENSG00000182974	15	22358478	22379561	5	1	123834	-0.21314	0.58439	0.987363747
IGSF6	ENSG00000140749	16	21642609	21673981	20	5	123834	-0.21314	0.58439	0.987363747
CD72	ENSG00000137101	9	35599530	35656807	88	13	123834	-0.2132	0.58442	0.987363747
PKHD1L1	ENSG00000205038	8	110364706	110552559	343	11	123834	-0.21328	0.58444	0.987363747
SLC43A2	ENSG00000167703	17	1462561	1542180	52	19	123834	-0.21326	0.58444	0.987363747
NPLOC4	ENSG00000182446	17	79513913	79625495	396	16	123834	-0.21333	0.58447	0.987363747
RANBP17	ENSG00000204764	5	170278874	170737019	669	11	123834	-0.21389	0.58468	0.987363747
DTD2	ENSG00000129480	14	31905242	31936716	51	3	123834	-0.2141	0.58477	0.987363747
AL590867.1	ENSG00000213121	6	153542455	153678623	306	23	123834	-0.21415	0.58478	0.987363747
ZNF454	ENSG00000178187	5	178358192	178403434	126	13	123834	-0.21416	0.58479	0.987363747
IPO13	ENSG00000117408	1	44402611	44443694	52	6	123834	-0.21426	0.58483	0.987363747
APP	ENSG00000142192	21	27242861	27553446	463	39	123834	-0.21434	0.58486	0.987363747
CPB2	ENSG00000080618	13	46617321	46689211	113	8	123834	-0.21442	0.58489	0.987363747
CDH20	ENSG00000101542	18	58990815	59233006	218	52	123834	-0.21442	0.58489	0.987363747
RP11-598P20.5	ENSG00000254673	8	42863567	42942477	64	8	123834	-0.2145	0.58492	0.987363747
CLDND2	ENSG00000160318	19	51860352	51882257	30	6	123834	-0.21457	0.58495	0.987363747
TRIM27	ENSG00000204713	6	28860779	28901766	95	13	123834	-0.21478	0.58503	0.987363747
MBIP	ENSG00000151332	14	36757770	36799882	35	2	123834	-0.21487	0.58506	0.987363747
ZC3H12D	ENSG00000178199	6	149758794	149816197	155	9	123834	-0.21519	0.58519	0.987363747
DSCR4	ENSG00000184029	21	39313728	39503454	214	28	123834	-0.21523	0.58521	0.987363747
RBP4	ENSG00000138207	10	95341444	95371501	75	11	123834	-0.21528	0.58522	0.987363747
TMX3	ENSG00000166479	18	66330925	66392535	106	6	123834	-0.21535	0.58525	0.987363747
EID3	ENSG00000255150	12	104687517	104708980	50	8	123834	-0.21543	0.58528	0.987363747
ACOT1	ENSG00000184227	14	73993818	74020498	5	2	123834	-0.21552	0.58532	0.987363747
SNAI2	ENSG00000019549	8	49820249	49844299	5	3	123834	-0.21562	0.58536	0.987363747
NID2	ENSG00000087303	14	52461521	52545712	276	20	123834	-0.2157	0.58539	0.987363747
OR51I1	ENSG00000167359	11	5451800	5472744	68	10	123834	-0.2158	0.58543	0.987363747
CACNA2D1	ENSG00000153956	7	81565760	82083114	697	75	123834	-0.21584	0.58544	0.987363747
SHC4	ENSG00000185634	15	49105932	49265641	157	23	123834	-0.21586	0.58545	0.987363747
NFIA	ENSG00000162599	1	61320931	61938465	706	104	123834	-0.21593	0.58548	0.987363747
USP13	ENSG00000058056	3	179360543	179517189	131	23	123834	-0.21595	0.58549	0.987363747
FAM19A2	ENSG00000198673	12	62092040	62682931	968	60	123834	-0.21604	0.58552	0.987363747
TECPR1	ENSG00000205356	7	97833936	97891563	72	12	123834	-0.21646	0.58568	0.98737852
REEP3	ENSG00000165476	10	65271123	65394883	225	19	123834	-0.21677	0.58581	0.98737852

CHRNE	ENSG00000108556	17	4791069	4816369	58	8	123834	-0.21677	0.58581	0.98737852
RP11-116D17.1	ENSG00000257726	12	115790817	115812550	27	5	123834	-0.21685	0.58584	0.98737852
SLC26A10	ENSG00000135502	12	58003310	58029934	22	3	123834	-0.21692	0.58587	0.98737852
IRF4	ENSG00000137265	6	381739	421447	50	8	123834	-0.21721	0.58598	0.98737852
EVI5	ENSG00000067208	1	92964253	93267961	502	9	123834	-0.21732	0.58602	0.98737852
MRO	ENSG00000134042	18	48314574	48361772	88	16	123834	-0.21753	0.5861	0.98737852
CBX6	ENSG00000183741	22	39247455	39278319	37	5	123834	-0.21766	0.58615	0.98737852
TPPP	ENSG00000171368	5	650883	703510	93	12	123834	-0.21787	0.58624	0.98737852
BTBD11	ENSG00000151136	12	107702190	108063419	550	45	123834	-0.21806	0.58631	0.98737852
AC121757.1	ENSG00000267873	12	52699128	52719790	93	7	123834	-0.21833	0.58642	0.98737852
CENPP	ENSG00000188312	9	95077766	95392815	645	18	123834	-0.21867	0.58655	0.98737852
DEFB108B	ENSG00000184276	11	71534246	71558756	56	7	123834	-0.21887	0.58662	0.98737852
AFM	ENSG00000079557	4	74337400	74379691	81	8	123834	-0.21897	0.58666	0.98737852
C7orf13	ENSG00000244291	7	156421057	156443348	38	8	123834	-0.21907	0.5867	0.98737852
C21orf140	ENSG00000222018	21	35762615	35783370	56	8	123834	-0.21917	0.58674	0.98737852
MYBPC2	ENSG00000086967	19	50926160	50979578	60	10	123834	-0.21921	0.58676	0.98737852
ABHD15	ENSG00000168792	17	27877565	27904155	14	5	123834	-0.21969	0.58694	0.98737852
CTD-2287O16.3	ENSG00000268714	5	115348088	115404625	138	14	123834	-0.21995	0.58704	0.98737852
TMED10	ENSG00000170348	14	75588173	75653334	73	3	123834	-0.22015	0.58712	0.98737852
AL583828.1	ENSG00000187461	6	13459513	13496415	41	8	123834	-0.2202	0.58714	0.98737852
XYLT2	ENSG00000015532	17	48413453	48450499	107	6	123834	-0.22022	0.58715	0.98737852
ATXN10	ENSG00000130638	22	46057678	46251187	243	17	123834	-0.22055	0.58728	0.98737852
ALPK1	ENSG00000073331	4	113196665	113373776	278	36	123834	-0.22116	0.58751	0.98737852
COL21A1	ENSG00000124749	6	55911388	56268892	980	39	123834	-0.22124	0.58755	0.98737852
TP63	ENSG00000073282	3	189339205	189625068	566	75	123834	-0.22219	0.58792	0.98737852
ZNF160	ENSG00000170949	19	53559859	53616689	113	16	123834	-0.22261	0.58808	0.98737852
RYBP	ENSG00000163602	3	72410976	72506069	42	7	123834	-0.22275	0.58813	0.98737852
ATP13A2	ENSG00000159363	1	17302453	17348423	67	10	123834	-0.22302	0.58824	0.98737852
TINAG	ENSG00000137251	6	54162657	54264950	227	24	123834	-0.22301	0.58824	0.98737852
CACNA1S	ENSG00000081248	1	200998642	201091694	118	26	123834	-0.22313	0.58828	0.98737852
B4GALT7	ENSG00000027847	5	177017101	177047348	52	8	123834	-0.22318	0.5883	0.98737852
DCUN1D3	ENSG00000188215	16	20856247	20921706	75	8	123834	-0.22355	0.58845	0.98737852
ZNF845	ENSG00000213799	19	53827002	53868122	95	16	123834	-0.22376	0.58853	0.98737852
GTF2A1L	ENSG00000242441	2	48834937	48970287	289	28	123834	-0.22386	0.58857	0.98737852
PRKG1	ENSG00000185532	10	52740945	54068110	2600	154	123834	-0.22393	0.58859	0.98737852
SHC2	ENSG00000129946	19	406583	470996	109	16	123834	-0.22411	0.58867	0.98737852
MILR1	ENSG00000271605	17	62451569	62474760	17	2	123834	-0.22416	0.58868	0.98737852
PAK2	ENSG00000180370	3	196456728	196569518	223	23	123834	-0.22446	0.5888	0.98737852
NDUFB3	ENSG00000119013	2	201926156	201960473	50	7	123834	-0.22456	0.58884	0.98737852
SMKR1	ENSG00000240204	7	129132320	129162773	40	8	123834	-0.22477	0.58892	0.98737852
OGG1	ENSG00000114026	3	9781628	9839903	69	12	123834	-0.22482	0.58894	0.98737852
FGF8	ENSG00000107831	10	103519899	103545854	11	3	123834	-0.22487	0.58896	0.98737852
MAL2	ENSG00000147676	8	120167273	120267913	219	22	123834	-0.22489	0.58897	0.98737852
ARL5B	ENSG00000165997	10	18938334	18980568	80	10	123834	-0.2249	0.58897	0.98737852
PRDM8	ENSG00000152784	4	81095033	81135483	87	10	123834	-0.22531	0.58913	0.98737852
WFDC10A	ENSG00000180305	20	44248165	44269835	75	8	123834	-0.22541	0.58917	0.98737852
SET	ENSG00000119335	9	131435703	131468679	10	3	123834	-0.22543	0.58918	0.98737852
ATE1	ENSG00000107669	10	123489939	123698316	460	27	123834	-0.22552	0.58921	0.98737852
TLR3	ENSG00000164342	4	186980306	187019223	47	8	123834	-0.22558	0.58923	0.98737852
STRIP2	ENSG00000128578	7	129064274	129138240	96	10	123834	-0.22593	0.58937	0.98737852
KCNJ12	ENSG00000184185	17	21269509	21333179	9	3	123834	-0.22597	0.58939	0.98737852
MYBBP1A	ENSG00000132382	17	4432192	4468926	23	6	123834	-0.22605	0.58942	0.98737852
CLEC1A	ENSG00000150048	12	10212153	10274226	121	6	123834	-0.22607	0.58943	0.98737852
CPS1	ENSG00000021826	2	211332406	211553831	353	27	123834	-0.22639	0.58955	0.98737852
ARG2	ENSG00000081181	14	68076515	68128437	70	8	123834	-0.22653	0.5896	0.98737852
GPR17	ENSG00000144230	2	128393439	128420213	30	12	123834	-0.22656	0.58962	0.98737852
SERPINI2	ENSG00000114204	3	167149577	167206792	130	11	123834	-0.22658	0.58962	0.98737852
OR5D16	ENSG00000205029	11	55596228	55617214	55	8	123834	-0.22691	0.58975	0.98737852
SYCE1L	ENSG00000205078	16	77223294	77257112	129	15	123834	-0.22691	0.58975	0.98737852
GPBP1	ENSG00000062194	5	56459775	56570506	135	14	123834	-0.22699	0.58978	0.98737852
SSB	ENSG00000138385	2	170638443	170678574	94	13	123834	-0.22708	0.58982	0.98737852
TAF1C	ENSG00000103168	16	84201458	84230669	81	13	123834	-0.22709	0.58982	0.98737852
MED17	ENSG00000042429	11	93507393	93557861	67	5	123834	-0.22713	0.58984	0.98737852
HIST1H2BK	ENSG00000197903	6	27096073	27124619	30	10	123834	-0.22733	0.58991	0.98737852
ZSCAN20	ENSG00000121903	1	33928246	33972107	47	5	123834	-0.22755	0.59	0.98737852
OR4F15	ENSG00000182854	15	102348366	102369350	25	6	123834	-0.22764	0.59004	0.98737852
METTL25	ENSG00000127720	12	82742276	82883015	261	6	123834	-0.22776	0.59009	0.98737852
C7orf49	ENSG00000122783	7	134767115	134865547	220	10	123834	-0.22795	0.59016	0.98737852
POM121L2	ENSG00000158553	6	27243682	27289949	108	11	123834	-0.22827	0.59028	0.98737852
GSTK1	ENSG00000197448	7	142931186	142977947	13	5	123834	-0.22826	0.59028	0.98737852
GGTLC1	ENSG00000149435	20	23955690	23979416	26	6	123834	-0.22829	0.59029	0.98737852
KCNMB3	ENSG00000171121	3	178947530	178994790	101	15	123834	-0.22847	0.59036	0.98737852
ZSCAN5A	ENSG00000131848	19	56722681	56889752	245	45	123834	-0.2287	0.59045	0.98737852
WDR60	ENSG00000126870	7	158639269	158759438	257	16	123834	-0.22872	0.59046	0.98737852
USP21	ENSG00000143258	1	161119240	161145513	26	5	123834	-0.22875	0.59047	0.98737852
KRTAP9-6	ENSG00000212659	17	39411591	39432893	18	3	123834	-0.22884	0.5905	0.98737852
SQRDL	ENSG00000137767	15	45913346	45993492	175	19	123834	-0.22892	0.59053	0.98737852
PF4	ENSG00000163737	4	74836794	74857841	19	2	123834	-0.22901	0.59057	0.98737852
FAM46B	ENSG00000158246	1	27321511	27349327	1	1	123834	-0.22922	0.59065	0.98737852
SDC1	ENSG00000115884	2	20390558	20435194	69	8	123834	-0.22932	0.59069	0.98737852
AREG	ENSG00000109321	4	75300851	75330726	8	3	123834	-0.22934	0.5907	0.98737852
AP001816.1	ENSG00000254531	4	102258937	102280040	6	2	123834	-0.22949	0.59076	0.98737852
LRRN3	ENSG00000173114	7	110721062	110775510	61	7	123834	-0.22956	0.59078	0.98737852
B4GALNT1	ENSG00000135454	12	58007193	58037138	27	4	123834	-0.22974	0.59085	0.98737852
HEATR4	ENSG00000187105	14	73935189	74035651	59	16	123834	-0.22982	0.59088	0.98737852
SRGAP2	ENSG00000163486	1	206506200	206647783	151	12	123834	-0.22993	0.59093	0.98737852
TP53BP1	ENSG00000067369	15	43689407	43812926	181	9	123834	-0.23003	0.59096	0.98737852
C4orf17	ENSG00000138813	4	100422161	100473460	154	18	123834	-0.23008	0.59098	0.98737852
VCAN	ENSG00000038427	5	82757284	82888122	105	22	123834	-0.23024	0.59105	0.98737852
CTSS	ENSG00000163131	1	150692672	150748433	63	4	123834	-0.2303	0.59107	0.98737852
AL445989.1	ENSG00000226974	13	64310934	64331323	32	3	123834	-0.23044	0.59113	0.98737852
C20orf24	ENSG00000101084	20	35224137	35250960	10	3	123834	-0.23063	0.5912	0.98737852
UBD	ENSG00000213886	6	29513292	29537702	47	9	123834	-0.23066	0.59121	0.98737852
ANKS1A	ENSG00000064999	6	34847042	35069179	410	29	123834	-0.23067	0.59121	0.98737852
KCNK10	ENSG00000100433	14	88639113	88803251	340	45	123834	-0.23104	0.59136	0.98737852
IHH	ENSG00000163501	2	219909142	219935189	61	5	123834	-0.23109	0.59138	0.98737852
AP000350.10	ENSG00000251357	22	24194375	24247410	89	14	123834	-0.23128	0.59145	0.98737852
MROH2A	ENSG00000185038	2	234674370	234752069	140	29	123834	-0.2314	0.5915	0.98737852
ESAM	ENSG00000149564	11	124612026	124642186	14	4	123834	-0.2316	0.59157	0.98737852

NDUFB2	ENSG00000090266	7	140380577	140432590	60	5	123834	-0.23173	0.59163	0.98737852
PEX5L	ENSG00000114757	3	179502746	179764841	417	29	123834	-0.23181	0.59166	0.98737852
ALYREF	ENSG00000183684	17	79835713	79859462	1	1	123834	-0.23181	0.59166	0.98737852
AIRE	ENSG00000160224	21	45695721	45728531	67	10	123834	-0.23198	0.59172	0.98737852
RPL26	ENSG00000161970	17	8270838	8296531	45	8	123834	-0.23208	0.59176	0.98737852
FSTL5	ENSG00000168843	4	162295049	163095187	1752	78	123834	-0.23211	0.59177	0.98737852
EML1	ENSG00000066629	14	100194030	100418397	263	27	123834	-0.23222	0.59181	0.98737852
GLRX3	ENSG00000108010	10	131924663	131992785	192	17	123834	-0.23222	0.59182	0.98737852
GHSR	ENSG00000121853	3	172152923	172176246	42	7	123834	-0.2325	0.59193	0.98737852
CYP51A1	ENSG00000001630	7	91731465	91782266	78	2	123834	-0.23267	0.59199	0.98737852
TAS2R39	ENSG00000236398	7	142870512	142891528	23	3	123834	-0.23266	0.59199	0.98737852
RFX5	ENSG00000143390	1	151303116	151329833	11	3	123834	-0.2328	0.59204	0.98737852
NELFCD	ENSG00000101158	20	57546263	57580188	61	9	123834	-0.23296	0.5921	0.98737852
CNNM1	ENSG00000119946	10	101078856	101164087	102	14	123834	-0.23299	0.59211	0.98737852
SLC14A2	ENSG00000132874	18	42782960	43273072	824	68	123834	-0.233	0.59212	0.98737852
TMCO4	ENSG00000162542	1	19998706	20136438	208	25	123834	-0.2331	0.59216	0.98737852
PTPRN2	ENSG00000155093	7	157321750	158390480	1956	157	123834	-0.23343	0.59229	0.98737852
DHCR7	ENSG00000172893	11	71129239	71173914	104	6	123834	-0.23395	0.59249	0.98737852
RPL14	ENSG00000188846	3	40488783	40513861	41	2	123834	-0.23397	0.5925	0.98737852
MYO7B	ENSG00000169994	2	128283378	128405304	212	25	123834	-0.23414	0.59256	0.98737852
VOPP1	ENSG00000154978	7	55493749	55650681	178	14	123834	-0.23421	0.59259	0.98737852
GALP	ENSG00000197487	19	56677389	56707144	44	8	123834	-0.23423	0.5926	0.98737852
HIST1H3E	ENSG00000196966	6	26215383	26235844	55	6	123834	-0.23438	0.59265	0.98737852
AC012313.1	ENSG00000269855	19	58897457	58918407	21	3	123834	-0.23447	0.59269	0.98737852
KCNA3	ENSG00000177272	1	111204310	111227655	15	3	123834	-0.23458	0.59273	0.98737852
COPS3	ENSG00000141030	17	17140141	17194607	128	10	123834	-0.23477	0.59281	0.98737852
ZNF671	ENSG00000083814	19	58221119	58248992	50	7	123834	-0.23485	0.59284	0.98737852
FAR2	ENSG00000064763	12	29292036	29503913	493	26	123834	-0.23487	0.59285	0.98737852
MOGAT2	ENSG00000166391	11	75418864	75454003	42	7	123834	-0.23506	0.59292	0.98737852
TDRD15	ENSG00000218819	2	21336789	21376144	50	7	123834	-0.23524	0.59299	0.98737852
VGLL2	ENSG00000170162	6	117576721	117604728	28	3	123834	-0.23526	0.593	0.98737852
LRRFIP1	ENSG00000124831	2	238526219	238732325	368	27	123834	-0.23567	0.59315	0.98737852
LEMD1	ENSG00000186007	1	205340506	205435082	86	15	123834	-0.23568	0.59316	0.98737852
MMP26	ENSG00000167346	11	4716157	5023659	870	42	123834	-0.23574	0.59318	0.98737852
C14orf166B	ENSG00000100565	14	77282715	77346645	120	22	123834	-0.23595	0.59326	0.98737852
ATP2C1	ENSG00000017260	3	130559439	130745556	203	18	123834	-0.2361	0.59332	0.98737852
SMC2	ENSG00000136824	9	106846541	106913698	90	6	123834	-0.23618	0.59335	0.98737852
KRT83	ENSG00000170523	12	52698085	52725182	94	7	123834	-0.23646	0.59346	0.98737852
KIAA0319L	ENSG00000142687	1	35889091	36033551	93	5	123834	-0.23669	0.59355	0.98737852
OR2J1	ENSG00000204702	6	29058386	29079658	39	8	123834	-0.23669	0.59355	0.98737852
SYCE1	ENSG00000171772	10	135357404	135392876	142	5	123834	-0.23675	0.59357	0.98737852
MAP4K4	ENSG00000071054	2	102303312	102521149	263	14	123834	-0.23682	0.5936	0.98737852
PPP1R1B	ENSG00000131771	17	37772993	37802879	44	6	123834	-0.23681	0.5936	0.98737852
SMC1B	ENSG00000077935	22	45729944	45819500	197	8	123834	-0.23682	0.5936	0.98737852
TROAP	ENSG00000135451	12	49707019	49735514	42	5	123834	-0.23718	0.59374	0.98737852
TGM3	ENSG00000125780	20	2266647	2331724	88	7	123834	-0.23723	0.59376	0.98737852
DCAF12	ENSG00000198876	9	34076385	34137397	155	14	123834	-0.23727	0.59378	0.98737852
UBN1	ENSG00000118900	16	4886666	4942361	124	14	123834	-0.23756	0.59389	0.98737852
KCNF1	ENSG00000162975	2	11042063	11064350	27	7	123834	-0.23785	0.594	0.98737852
TAGLN	ENSG00000149591	11	117060037	117085498	70	10	123834	-0.23805	0.59408	0.98737852
C17orf58	ENSG00000186665	17	65977217	65999765	13	2	123834	-0.2382	0.59414	0.98737852
PSMB3	ENSG00000108294	17	36898989	36930484	37	9	123834	-0.23823	0.59415	0.98737852
CHID1	ENSG00000177830	11	857357	925058	67	7	123834	-0.23845	0.59423	0.98737852
TAS2R16	ENSG00000128519	7	122624759	122645754	49	7	123834	-0.23849	0.59425	0.98737852
AC093323.1	ENSG00000268791	4	6683782	6704189	20	6	123834	-0.23852	0.59426	0.98737852
ANKRD30B	ENSG00000180777	18	14718271	14862737	218	12	123834	-0.23854	0.59427	0.98737852
CD59	ENSG00000085063	11	33709807	33767991	80	16	123834	-0.23857	0.59428	0.98737852
OXCT2	ENSG00000198754	1	40225195	40247020	37	8	123834	-0.23874	0.59435	0.98737852
HLF	ENSG00000108924	17	53332373	53412426	51	13	123834	-0.23891	0.59441	0.98737852
BUB3	ENSG00000154473	10	124903793	124934886	5	1	123834	-0.23898	0.59444	0.98737852
ST6GALNAC3	ENSG00000184005	1	76530404	77110286	903	89	123834	-0.23903	0.59446	0.98737852
MYO9B	ENSG00000099331	19	17176591	17335346	339	42	123834	-0.23921	0.59453	0.98737852
MRPL18	ENSG00000112110	6	160200844	160229468	95	7	123834	-0.23924	0.59454	0.98737852
LRRTM3	ENSG00000198739	10	68675764	68869588	237	26	123834	-0.23935	0.59458	0.98737852
MRPL52	ENSG00000172590	14	23289088	23314246	28	8	123834	-0.23945	0.59462	0.98737852
NEK11	ENSG00000114670	3	130735694	131079309	613	22	123834	-0.23963	0.59469	0.98737852
CCDC172	ENSG00000182645	10	118073940	118149541	153	15	123834	-0.23965	0.5947	0.98737852
C4orf27	ENSG00000056050	4	170640616	170689104	51	13	123834	-0.23973	0.59473	0.98737852
KRTAP20-1	ENSG00000244624	21	31978750	31999003	33	3	123834	-0.23973	0.59473	0.98737852
ADAMTS3	ENSG00000156140	4	73136686	73444516	304	13	123834	-0.23988	0.59479	0.98737852
B3GALT4	ENSG00000235863	6	33234917	33262609	42	8	123834	-0.24031	0.59495	0.98737852
GPR150	ENSG00000178015	5	94945782	94967846	13	3	123834	-0.24036	0.59497	0.98737852
LINS	ENSG00000140471	15	101089574	101153435	191	16	123834	-0.24057	0.59506	0.98737852
NHLRC2	ENSG00000196865	10	115604420	115686953	31	5	123834	-0.24115	0.59528	0.98737852
SIGLEC1	ENSG00000088827	20	3657617	3697775	77	10	123834	-0.24114	0.59528	0.98737852
AVP	ENSG00000101200	20	3053202	3075370	6	2	123834	-0.24139	0.59537	0.98737852
ABCG8	ENSG00000143921	2	44056103	44115605	239	11	123834	-0.24143	0.59539	0.98737852
OR2A14	ENSG00000221938	7	143816151	143837162	68	3	123834	-0.2416	0.59545	0.98737852
PCOLCE	ENSG00000106333	7	100189800	100215798	3	2	123834	-0.24173	0.59551	0.98737852
GOLT1B	ENSG00000111711	12	21644715	21681342	90	14	123834	-0.24179	0.59553	0.98737852
FBN1	ENSG00000166147	15	48690503	48948046	266	10	123834	-0.24205	0.59563	0.98737852
SGCE	ENSG00000127990	7	94204542	94295521	84	6	123834	-0.24211	0.59565	0.98737852
SLC46A2	ENSG00000119457	9	115631200	115663193	67	9	123834	-0.24241	0.59577	0.98737852
DMPK	ENSG00000104936	19	46262975	46295810	29	4	123834	-0.24248	0.59579	0.98737852
FTSJ3	ENSG00000108592	17	61886793	61917372	39	3	123834	-0.2426	0.59584	0.98737852
RP11-315O6.2	ENSG00000255482	11	102327986	102379532	83	13	123834	-0.24262	0.59585	0.98737852
ARHGAP15	ENSG00000075884	2	143838931	144535921	807	45	123834	-0.24269	0.59588	0.98737852
RNF13	ENSG00000082996	3	149520495	149689926	253	14	123834	-0.24286	0.59594	0.98737852
KCNJ14	ENSG00000182324	19	48948766	48979367	60	7	123834	-0.24313	0.59605	0.98737852
TJP3	ENSG00000105289	19	3698107	3760811	48	11	123834	-0.24316	0.59606	0.98737852
CUBN	ENSG00000107611	10	16855963	17181830	812	63	123834	-0.24334	0.59613	0.98737852
APOBEC3G	ENSG00000239713	22	39426924	39493748	101	12	123834	-0.24366	0.59625	0.98737852
MEMO1	ENSG00000162959	2	32080129	32246299	244	13	123834	-0.24369	0.59626	0.98737852
RPUSD4	ENSG00000165526	11	126061993	126091587	50	9	123834	-0.24418	0.59646	0.98737852
ARFGAP1	ENSG00000101199	20	61894137	61931142	61	5	123834	-0.2443	0.5965	0.98737852
GOLGA6A	ENSG00000159289	15	74352198	74384891	3	1	123834	-0.24449	0.59657	0.98737852
RP11-383H13.1	ENSG00000235531	8	72730402	73040628	730	51	123834	-0.24477	0.59668	0.98737852
LAD1	ENSG00000159166	1	201332372	201378736	93	17	123834	-0.24482	0.5967	0.98737852
DSG3	ENSG00000134757	18	29017758	29068665	124	12	123834	-0.24485	0.59671	0.98737852

CRTAC1	ENSG00000095713	10	99614757	99800585	159	21	123834	-0.24487	0.59672	0.98737852
AZU1	ENSG00000172232	19	815097	842017	13	5	123834	-0.245	0.59677	0.98737852
SCN3A	ENSG00000153253	2	165934032	166070577	185	14	123834	-0.24501	0.59678	0.98737852
TRIM4	ENSG00000146833	7	99464581	99527223	123	9	123834	-0.24503	0.59678	0.98737852
TRAM2	ENSG00000065308	6	52352200	52451713	249	31	123834	-0.24511	0.59681	0.98737852
SRSF7	ENSG00000115875	2	38960741	38988636	14	3	123834	-0.24545	0.59694	0.98737852
GTPBP2	ENSG00000172432	6	43563053	43606899	37	5	123834	-0.24545	0.59694	0.98737852
C17orf50	ENSG00000154768	17	34077916	34102098	49	3	123834	-0.24551	0.59697	0.98737852
GSX2	ENSG00000180613	4	54955690	54978672	25	3	123834	-0.24558	0.597	0.98737852
CIITA	ENSG00000179583	16	10961055	11036079	61	16	123834	-0.24574	0.59706	0.98737852
AC002472.13	ENSG00000187905	22	21390249	21428457	44	14	123834	-0.24591	0.59712	0.98737852
GUCY1A3	ENSG00000164116	4	156577863	156663501	164	22	123834	-0.246	0.59716	0.98737852
CSF3R	ENSG00000119535	1	36921644	36958879	58	11	123834	-0.24614	0.59721	0.98737852
QPCTL	ENSG00000011478	19	46185741	46217247	26	6	123834	-0.24654	0.59737	0.98737852
SNX22	ENSG00000157734	15	64433914	64459680	35	4	123834	-0.24669	0.59742	0.98737852
LMO4	ENSG00000143013	1	87784151	87824606	33	4	123834	-0.24671	0.59743	0.98737852
MCL1	ENSG00000143384	1	150537032	150562066	34	5	123834	-0.2468	0.59747	0.98737852
MTR	ENSG00000116984	1	236948610	237077281	241	13	123834	-0.24731	0.59766	0.98737852
SKIV2L2	ENSG00000039123	5	54593588	54731409	251	6	123834	-0.24731	0.59766	0.98737852
SNX31	ENSG00000174226	8	101575116	101685643	259	19	123834	-0.24798	0.59792	0.98737852
OVCH1-AS1	ENSG00000257599	12	29532227	29650421	303	16	123834	-0.24804	0.59795	0.98737852
BCL2L15	ENSG00000188761	1	114410790	114440169	19	4	123834	-0.2481	0.59797	0.98737852
MT2A	ENSG00000125148	16	56632111	56653409	36	11	123834	-0.24809	0.59797	0.98737852
ZCCHC3	ENSG00000177764	20	267737	290965	13	3	123834	-0.24819	0.598	0.98737852
PLA2G6	ENSG00000184381	22	38497502	38611697	297	12	123834	-0.24819	0.59801	0.98737852
TBX3	ENSG00000135111	12	115098059	115131969	16	6	123834	-0.24822	0.59802	0.98737852
HMGA1	ENSG00000137309	6	34194650	34224008	45	7	123834	-0.24833	0.59806	0.98737852
SUN1	ENSG00000164828	7	845528	946072	233	19	123834	-0.24873	0.59822	0.98737852
MRGBP	ENSG00000101189	20	61417805	61441945	52	12	123834	-0.24875	0.59822	0.98737852
GPR6	ENSG00000146360	6	110289514	110311921	28	7	123834	-0.2488	0.59824	0.98737852
PTGIR	ENSG00000160013	19	47113725	47138375	49	6	123834	-0.24893	0.59829	0.98737852
ATIC	ENSG00000138363	2	216166540	216224487	138	12	123834	-0.24924	0.59841	0.98737852
SUV39H2	ENSG00000152455	10	14910819	14956314	52	10	123834	-0.24928	0.59843	0.98737852
PDCD6	ENSG00000249915	5	261736	363971	238	13	123834	-0.24934	0.59845	0.98737852
ITGB2	ENSG00000160255	21	46295868	46361904	96	23	123834	-0.24941	0.59848	0.98737852
AATK	ENSG00000181409	17	79081095	79149877	94	19	123834	-0.24966	0.59857	0.98737852
CCNB2	ENSG00000157456	15	59387277	59427244	40	5	123834	-0.24967	0.59858	0.98737852
SLC17A5	ENSG00000119899	6	74293102	74373878	306	9	123834	-0.24979	0.59862	0.98737852
SNCA	ENSG00000145335	4	90635250	90769466	374	20	123834	-0.24982	0.59864	0.98737852
AL033381.1	ENSG00000176515	6	1070164	1115181	68	11	123834	-0.24987	0.59866	0.98737852
CHGB	ENSG00000089199	20	5882076	5916007	91	11	123834	-0.25002	0.59871	0.98737852
CECR5	ENSG00000069998	22	17608401	17656177	84	9	123834	-0.25002	0.59871	0.98737852
PIGK	ENSG00000142892	1	77544675	77695115	253	12	123834	-0.25007	0.59873	0.98737852
KCNJ13	ENSG00000115474	2	233621174	233651278	58	7	123834	-0.25052	0.59891	0.98737852
CRYBA1	ENSG00000108255	17	27563881	27591512	45	8	123834	-0.25054	0.59891	0.98737852
ORAI1	ENSG00000182500	12	122054455	122090583	38	7	123834	-0.25072	0.59898	0.98737852
PPP1R12A	ENSG00000058272	12	80157343	80339240	177	6	123834	-0.25077	0.59901	0.98737852
PIK3CB	ENSG00000051382	3	138362860	138563780	184	4	123834	-0.2509	0.59905	0.98737852
LCE1F	ENSG00000240386	1	152738848	152759445	45	4	123834	-0.251	0.59909	0.98737852
OR4A47	ENSG00000237388	11	48500269	48521332	53	7	123834	-0.25117	0.59916	0.98737852
CALM3	ENSG00000160014	19	47094331	47124050	25	3	123834	-0.25128	0.5992	0.98737852
FAM124A	ENSG00000150510	13	51786503	51868377	75	18	123834	-0.2514	0.59925	0.98737852
SNTB2	ENSG00000168807	16	69211032	69352955	205	13	123834	-0.25142	0.59925	0.98737852
ACN9	ENSG00000196636	7	96735902	96821075	98	6	123834	-0.25152	0.59929	0.98737852
STOML1	ENSG00000067221	15	74265547	74296963	56	7	123834	-0.25155	0.59931	0.98737852
NUDT4	ENSG00000173598	12	93761659	93807024	57	8	123834	-0.25179	0.5994	0.98737852
AMOTL2	ENSG00000114019	3	134064716	134104321	23	4	123834	-0.25191	0.59945	0.98737852
OR6C2	ENSG00000179695	12	55835998	55856936	50	6	123834	-0.25206	0.5995	0.98737852
MFSD7	ENSG00000169026	4	665618	693230	13	3	123834	-0.25212	0.59952	0.98737852
TTC14	ENSG00000163728	3	180309918	180345616	27	6	123834	-0.25227	0.59958	0.98737852
CAB39L	ENSG00000102547	13	49872786	50028262	172	20	123834	-0.25242	0.59964	0.98737852
SOGA2	ENSG00000168502	18	8695659	8842776	183	32	123834	-0.25251	0.59968	0.98737852
CSRNP1	ENSG00000144655	3	39173346	39206053	58	12	123834	-0.25253	0.59969	0.98737852
TSNAX-DISC1	ENSG00000270106	1	231654399	231964990	257	32	123834	-0.25258	0.5997	0.98737852
CTD-3105H18.16	ENSG00000268870	19	12492763	12561896	283	7	123834	-0.25275	0.59977	0.98737852
F10	ENSG00000126218	13	113767128	113813843	23	7	123834	-0.25316	0.59993	0.98737852
SUCO	ENSG00000094975	1	172491489	172590971	91	9	123834	-0.25374	0.60015	0.98737852
HS3ST6	ENSG00000162040	16	1951464	1978441	68	5	123834	-0.25391	0.60022	0.98737852
DUSP10	ENSG00000143507	1	221864766	221925518	56	11	123834	-0.25415	0.60031	0.98737852
TBPL1	ENSG00000028839	6	134263308	134321570	77	6	123834	-0.25422	0.60034	0.98737852
IPO8	ENSG00000133704	12	30771922	30858920	207	11	123834	-0.25441	0.60041	0.98737852
SLC31A1	ENSG00000136868	9	115973808	116038674	150	15	123834	-0.2545	0.60044	0.98737852
OR2D3	ENSG00000178358	11	6932205	6953264	87	6	123834	-0.25449	0.60044	0.98737852
CES5A	ENSG00000159398	16	55870066	55999943	197	24	123834	-0.25448	0.60044	0.98737852
MYH7B	ENSG00000078814	20	33553206	33600240	93	8	123834	-0.25448	0.60044	0.98737852
MTO1	ENSG00000135297	6	74161301	74228959	118	11	123834	-0.25463	0.6005	0.98737852
KLF3	ENSG00000109787	4	38655817	38712663	45	11	123834	-0.25466	0.60051	0.98737852
LYRM4	ENSG00000214113	6	5092827	5271172	404	28	123834	-0.25476	0.60055	0.98737852
CKS1B	ENSG00000268942	5	61798070	61818309	24	4	123834	-0.25486	0.60059	0.98737852
AL021546.6	ENSG00000111780	12	120866001	120907809	61	10	123834	-0.25493	0.60061	0.98737852
GOLPH3L	ENSG00000143457	1	150608701	150679630	125	4	123834	-0.25528	0.60075	0.98737852
GLYAT	ENSG00000149124	11	58397899	58509447	303	16	123834	-0.25539	0.60079	0.98737852
SMG9	ENSG00000105771	19	44225301	44269142	46	5	123834	-0.25555	0.60085	0.98737852
RP11-849F2.7	ENSG00000263809	17	8261956	8296505	48	8	123834	-0.25566	0.60089	0.98737852
PHPT1	ENSG00000054148	9	139733176	139755488	1	1	123834	-0.25567	0.6009	0.98737852
TMEM120B	ENSG00000188735	12	122140658	122230907	145	14	123834	-0.25572	0.60092	0.98737852
EIF3E	ENSG00000104408	8	109203445	109457562	615	19	123834	-0.25574	0.60093	0.98737852
MPZL2	ENSG00000149573	11	118114118	118145251	97	9	123834	-0.25588	0.60098	0.98737852
DRAM1	ENSG00000136048	12	102261129	102415908	302	32	123834	-0.25592	0.601	0.98737852
GREB1L	ENSG00000141449	18	18812203	19115378	199	11	123834	-0.25597	0.60101	0.98737852
BRD1	ENSG00000100425	22	50156931	50231160	142	13	123834	-0.25599	0.60102	0.98737852
S100A9	ENSG00000163220	1	153320330	153343503	48	2	123834	-0.25638	0.60117	0.98737852
SLC30A1	ENSG00000170385	1	211734910	211762084	34	5	123834	-0.25648	0.60121	0.98737852
KRTAP9-9	ENSG00000198083	17	39401636	39422616	47	4	123834	-0.25685	0.60135	0.98737852
ASPN	ENSG00000106819	9	95208487	95254788	95	9	123834	-0.25703	0.60142	0.98737852
C1orf86	ENSG00000162585	1	2105903	2154159	27	6	123834	-0.25718	0.60148	0.98737852
ALDH5A1	ENSG00000112294	6	24485080	24547435	204	25	123834	-0.25756	0.60163	0.98737852
ATAD2B	ENSG00000119778	2	23961534	24159984	445	15	123834	-0.25809	0.60183	0.98737852
C9orf152	ENSG00000188959	9	112942328	112980469	76	14	123834	-0.25809	0.60183	0.98737852

ZC3H3	ENSG00000014164	8	144509825	144633623	214	19	123834	-0.2583	0.60191	0.98737852
MMADHC	ENSG00000168288	2	150416148	150454330	59	4	123834	-0.25837	0.60194	0.98737852
PARK2	ENSG00000185345	6	161758452	163158803	2637	156	123834	-0.25842	0.60196	0.98737852
FBXO21	ENSG00000135108	12	117571146	117638336	151	7	123834	-0.25848	0.60198	0.98737852
C1QTNF6	ENSG00000133466	22	37566207	37605425	96	10	123834	-0.25867	0.60205	0.98737852
RAB24	ENSG00000169228	5	176718199	176740745	27	7	123834	-0.25876	0.60209	0.98737852
RABGAP1	ENSG00000011454	9	125693112	125877145	230	12	123834	-0.25908	0.60221	0.98737852
RAB6B	ENSG00000154917	3	133533083	133624680	102	20	123834	-0.2594	0.60234	0.98737852
ADH7	ENSG00000196344	4	100323418	100366894	101	19	123834	-0.25947	0.60236	0.98737852
SCG3	ENSG00000104112	15	51963550	52023223	84	9	123834	-0.25946	0.60236	0.98737852
IGF1R	ENSG00000140443	15	99182200	99517759	446	46	123834	-0.25947	0.60236	0.98737852
ART4	ENSG00000111339	12	14968503	15006429	53	7	123834	-0.25958	0.6024	0.98737852
RP11-1070N10.3	ENSG00000258572	14	95972473	95994248	22	5	123834	-0.25957	0.6024	0.98737852
PML	ENSG00000140464	15	74277014	74350153	104	15	123834	-0.25956	0.6024	0.98737852
FASN	ENSG00000169710	17	80026214	80066208	56	8	123834	-0.25959	0.60241	0.98737852
MKKS	ENSG00000125863	20	10371657	10424870	131	5	123834	-0.25958	0.60241	0.98737852
BCL10	ENSG00000142867	1	85721931	85752773	87	16	123834	-0.25988	0.60252	0.98737852
CTAGE9	ENSG00000236761	6	132019581	132042206	22	3	123834	-0.25997	0.60256	0.98737852
TMBIM1	ENSG00000135926	2	219128915	219167309	62	3	123834	-0.26001	0.60257	0.98737852
C4orf40	ENSG00000187533	4	70989333	71052516	74	13	123834	-0.26056	0.60278	0.98737852
LIPI	ENSG00000188992	21	15471134	15593166	285	17	123834	-0.26062	0.60281	0.98737852
ISX	ENSG00000175329	22	35452129	35493380	144	10	123834	-0.26102	0.60296	0.98737852
ZNF385A	ENSG00000161642	12	54752917	54795082	15	4	123834	-0.26117	0.60302	0.98737852
ALDH3A1	ENSG00000108602	17	19631297	19662256	24	3	123834	-0.26127	0.60306	0.98737852
TRMT2A	ENSG00000099899	22	20089389	20114915	61	9	123834	-0.26142	0.60311	0.98737852
PTGES3L	ENSG00000267060	17	41110105	41142545	22	3	123834	-0.26145	0.60313	0.98737852
MBTPS1	ENSG00000140943	16	84077368	84160511	138	24	123834	-0.26156	0.60317	0.98737852
PLEKHM2	ENSG00000116786	1	16000827	16071264	127	7	123834	-0.26174	0.60324	0.98737852
ASIP	ENSG00000101440	20	32772375	32867150	85	2	123834	-0.2619	0.6033	0.98737852
KLK9	ENSG00000269741	19	51489274	51522837	66	8	123834	-0.262	0.60334	0.98737852
KIF20B	ENSG00000138182	10	91451367	91544700	253	7	123834	-0.26202	0.60335	0.98737852
SRP14	ENSG00000140319	15	40317940	40341389	29	6	123834	-0.26271	0.60361	0.98737852
NUDT9	ENSG00000170502	4	88333734	88390606	108	12	123834	-0.26274	0.60363	0.98737852
METTL18	ENSG00000171806	1	169751670	169774107	58	9	123834	-0.26294	0.6037	0.98737852
PRR21	ENSG00000221961	2	240971230	240992399	52	10	123834	-0.26307	0.60375	0.98737852
MAP2K4	ENSG00000065559	17	11914141	12057147	148	12	123834	-0.26314	0.60378	0.98737852
OTOGL	ENSG00000165899	12	80593233	80782870	232	18	123834	-0.26322	0.60381	0.98737852
TMEM192	ENSG00000170088	4	165985574	166139701	334	18	123834	-0.26329	0.60384	0.98737852
EXOSC7	ENSG00000075914	3	45006733	45087558	122	8	123834	-0.26334	0.60386	0.98737852
ACY3	ENSG00000132744	11	67400026	67428130	46	8	123834	-0.26342	0.60389	0.98737852
UNC93B1	ENSG00000110057	11	67748575	67782452	17	3	123834	-0.26343	0.60389	0.98737852
NLK	ENSG00000087095	17	26358763	26533407	25	11	123834	-0.26349	0.60391	0.98737852
PACS2	ENSG00000179364	14	105756900	105874484	19	3	123834	-0.26354	0.60393	0.98737852
WNT9B	ENSG00000158955	17	44900567	44974096	80	8	123834	-0.26364	0.60397	0.98737852
RNF114	ENSG00000124226	20	48542948	48580429	99	9	123834	-0.26364	0.60397	0.98737852
RP11-80A15.1	ENSG00000258744	14	24960800	24987894	70	10	123834	-0.26379	0.60403	0.98737852
CHODL	ENSG00000154645	21	19263580	19649690	335	50	123834	-0.26381	0.60404	0.98737852
KREMEN2	ENSG00000131650	16	3003945	3028384	32	5	123834	-0.26398	0.6041	0.98737852
AC016757.3	ENSG00000186235	2	239123193	239150318	47	8	123834	-0.26407	0.60414	0.98737852
KRTAP6-1	ENSG00000184724	21	31975750	31996249	30	3	123834	-0.26407	0.60414	0.98737852
GML	ENSG00000104499	8	143905663	144007922	322	13	123834	-0.26411	0.60415	0.98737852
NR2C1	ENSG00000120798	12	95404005	95477479	140	17	123834	-0.26417	0.60418	0.98737852
GPR98	ENSG00000164199	5	89815161	90470038	998	51	123834	-0.26431	0.60423	0.98737852
ANKRD30A	ENSG00000148513	10	37404785	37683039	347	16	123834	-0.26433	0.60424	0.98737852
CCDC71L	ENSG00000253276	7	106287211	106311442	30	9	123834	-0.26435	0.60425	0.98737852
HPS6	ENSG00000166189	10	103815147	103837792	7	2	123834	-0.26443	0.60427	0.98737852
SYNJ2	ENSG00000078269	6	158392888	158530208	165	18	123834	-0.26444	0.60428	0.98737852
CXCL9	ENSG00000138755	4	76912428	76938641	42	8	123834	-0.26469	0.60438	0.98737852
CNBD1	ENSG00000176571	8	87868670	88637447	1319	44	123834	-0.26469	0.60438	0.98737852
ZHX1-C8ORF76	ENSG00000259305	8	124228431	124289627	5	2	123834	-0.2654	0.60465	0.98737852
TNC	ENSG00000041982	9	117772806	117890536	246	29	123834	-0.26549	0.60468	0.98737852
VPS37D	ENSG00000176428	7	73072155	73096442	64	5	123834	-0.26577	0.60479	0.98737852
EPN3	ENSG00000049283	17	48599904	48631111	66	9	123834	-0.26628	0.60499	0.98737852
FAM83D	ENSG00000101447	20	37544955	37591703	70	4	123834	-0.26629	0.60499	0.98737852
OR51F1	ENSG00000188069	11	4780209	4801168	109	7	123834	-0.26633	0.60501	0.98737852
PRDM6	ENSG00000061455	5	122414816	122539960	209	24	123834	-0.26647	0.60506	0.98737852
FAM161A	ENSG00000170264	2	62041989	62091278	99	11	123834	-0.2665	0.60507	0.98737852
PRED57	ENSG00000268805	21	46501596	46525493	58	8	123834	-0.26658	0.6051	0.98737852
KLK8	ENSG00000129455	19	51489263	51515967	60	6	123834	-0.2667	0.60515	0.98737852
ZNF732	ENSG00000186777	4	254464	309110	93	6	123834	-0.26676	0.60517	0.98737852
EXOC4	ENSG00000131558	7	132927829	133761342	873	23	123834	-0.26676	0.60517	0.98737852
PDIK1L	ENSG00000175087	1	26427664	26462034	64	1	123834	-0.2669	0.60523	0.98737852
UNC5A	ENSG00000113763	5	176227478	176317897	89	16	123834	-0.26694	0.60524	0.98737852
FRG2B	ENSG00000225899	10	135427399	135450299	28	4	123834	-0.26704	0.60528	0.98737852
BRF1	ENSG00000185024	14	105665623	105791926	130	6	123834	-0.26722	0.60535	0.98737852
GREM2	ENSG00000180875	1	240642873	240785449	110	29	123834	-0.2673	0.60538	0.98737852
USP34	ENSG00000115464	2	61404598	61707904	465	17	123834	-0.26754	0.60547	0.98737852
CSNK1G3	ENSG00000151292	5	122837793	122962739	127	12	123834	-0.26756	0.60548	0.98737852
HES6	ENSG00000144485	2	239136908	239159303	25	6	123834	-0.26758	0.60549	0.98737852
THOC5	ENSG00000100296	22	29891868	29961205	124	9	123834	-0.26772	0.60554	0.98737852
PAPOLA	ENSG00000090060	14	96957770	97043448	87	7	123834	-0.26776	0.60556	0.98737852
SNX11	ENSG00000002919	17	46170719	46210436	114	11	123834	-0.26778	0.60556	0.98737852
SH3BP5L	ENSG00000175137	1	249094648	249130832	29	3	123834	-0.2678	0.60557	0.98737852
TTC13	ENSG00000143643	1	231031989	231124621	238	18	123834	-0.26783	0.60559	0.98737852
CCDC73	ENSG00000186714	11	32613792	32826204	557	18	123834	-0.26808	0.60568	0.98737852
RCSD1	ENSG00000198771	1	167589330	167685486	163	27	123834	-0.26819	0.60572	0.98737852
LECT1	ENSG00000136110	13	53267399	53323947	107	8	123834	-0.26823	0.60574	0.98737852
PARVA	ENSG00000197702	11	12388732	12562348	265	37	123834	-0.26844	0.60582	0.98737852
NCKAP1L	ENSG00000123338	12	54881495	54947726	96	18	123834	-0.26843	0.60582	0.98737852
TMEM217	ENSG00000172738	6	37169956	37235931	76	10	123834	-0.26861	0.60589	0.98737852
IARS	ENSG00000196305	9	94962489	95066038	217	14	123834	-0.26885	0.60598	0.98737852
FAS	ENSG00000026103	10	90740414	90785542	146	16	123834	-0.26892	0.60601	0.98737852
CDKN2B	ENSG00000147883	9	21992902	22019362	29	5	123834	-0.26916	0.6061	0.98737852
MFAP1	ENSG00000140259	15	44086690	44127000	39	3	123834	-0.26926	0.60613	0.98737852
IER3	ENSG00000137331	6	30700976	30722331	39	5	123834	-0.26939	0.60618	0.98737852
TOMM7	ENSG00000196683	7	22842251	22872470	120	13	123834	-0.26948	0.60622	0.98737852
RDH8	ENSG00000080511	19	10113925	10142955	69	16	123834	-0.26969	0.6063	0.98737852
DCTN6	ENSG00000104671	8	30003813	30051156	57	5	123834	-0.26977	0.60633	0.98737852
CTSZ	ENSG00000101160	20	57560240	57592302	43	7	123834	-0.26993	0.60639	0.98737852

ENTPD1	ENSG00000138185	10	97461536	97647023	491	20	123834	-0.27054	0.60663	0.98737852
GKN2	ENSG00000183607	2	69162364	69190102	32	5	123834	-0.27066	0.60667	0.98737852
BANF1	ENSG00000175334	11	65759550	65781620	54	6	123834	-0.27069	0.60668	0.98737852
RARS	ENSG00000113643	5	167903450	167956304	126	6	123834	-0.27079	0.60672	0.98737852
AC026202.1	ENSG00000268509	3	5219227	5239982	14	4	123834	-0.27088	0.60676	0.98737852
CRELD2	ENSG00000184164	22	50301815	50331188	125	6	123834	-0.27099	0.6068	0.98737852
SPRN	ENSG00000203772	10	135224170	135392916	427	30	123834	-0.27109	0.60684	0.98737852
CSF3	ENSG00000108342	17	38161614	38184066	61	6	123834	-0.27136	0.60694	0.98737852
SH3TC1	ENSG00000125089	4	8173799	8253530	76	14	123834	-0.27147	0.60698	0.98737852
MAPK4	ENSG00000141639	18	48076448	48268194	138	23	123834	-0.27145	0.60698	0.98737852
BCAT2	ENSG00000105552	19	49288319	49324286	16	4	123834	-0.27147	0.60699	0.98737852
IFT20	ENSG00000109083	17	26645352	26672515	16	3	123834	-0.2715	0.607	0.98737852
SDE2	ENSG00000143751	1	226160403	226197032	26	4	123834	-0.27156	0.60702	0.98737852
CCDC38	ENSG00000165972	12	96250826	96346752	247	28	123834	-0.27166	0.60706	0.98737852
CCT8L2	ENSG00000198445	22	17061667	17083700	13	4	123834	-0.27166	0.60706	0.98737852
AC090186.1	ENSG00000232941	8	52720143	52740969	57	6	123834	-0.27177	0.6071	0.98737852
MMP17	ENSG00000198598	12	132302938	132346328	72	15	123834	-0.27182	0.60712	0.98737852
HDDC2	ENSG00000111906	6	125531108	125633282	253	16	123834	-0.27189	0.60715	0.98737852
ZNF443	ENSG00000180855	19	12530521	12561926	210	8	123834	-0.272	0.60719	0.98737852
RGL3	ENSG00000205517	19	11485017	11540018	62	5	123834	-0.27228	0.6073	0.987472917
ADAM2	ENSG00000104755	8	39591254	39705808	106	10	123834	-0.27332	0.6077	0.98784516
ZNF839	ENSG00000022976	14	102773714	102819044	70	13	123834	-0.27335	0.60771	0.98784516
ZC3HAV1L	ENSG00000146858	7	138700452	138730775	56	4	123834	-0.27361	0.60781	0.98784516
RPS15A	ENSG00000134419	16	18782617	18811705	19	3	123834	-0.27364	0.60782	0.98784516
TIGD6	ENSG00000164296	5	149362681	149390730	32	3	123834	-0.27367	0.60783	0.98784516
CLIP3	ENSG00000105270	19	36495562	36534245	45	11	123834	-0.27412	0.608	0.98784516
SPATA9	ENSG00000145757	5	94977885	95044415	96	9	123834	-0.27424	0.60805	0.98784516
GPALPP1	ENSG00000133114	13	45553687	45617746	106	10	123834	-0.27443	0.60812	0.98784516
BHLHA15	ENSG00000180535	7	97830739	97852291	20	6	123834	-0.27445	0.60813	0.98784516
WRAP53	ENSG00000141499	17	7579389	7616820	55	8	123834	-0.27466	0.60821	0.98784516
MCTP1	ENSG00000175471	5	94029446	94630279	769	70	123834	-0.27486	0.60829	0.98784516
HELLS	ENSG00000119969	10	96295547	96383662	140	9	123834	-0.27494	0.60832	0.98784516
CKM	ENSG00000104879	19	45799672	45836235	44	9	123834	-0.27515	0.6084	0.98784516
STK32C	ENSG00000165752	10	134010996	134155351	269	36	123834	-0.27521	0.60842	0.98784516
PPP2CB	ENSG00000104695	8	30621973	30681830	101	10	123834	-0.27526	0.60844	0.98784516
P2RX1	ENSG00000108405	17	3789886	3829794	13	4	123834	-0.27545	0.60851	0.98784516
KIAA0368	ENSG00000136813	9	114112972	114257025	190	12	123834	-0.27563	0.60858	0.98784516
ZNF714	ENSG00000160352	19	21254965	21318073	183	10	123834	-0.27578	0.60864	0.98784516
MICALCL	ENSG00000133808	11	12287627	12390691	261	22	123834	-0.27593	0.6087	0.98784516
EPN2	ENSG00000072134	17	19108928	19250028	60	4	123834	-0.27594	0.6087	0.98784516
CACNB2	ENSG00000165995	10	18419606	18840798	766	81	123834	-0.27609	0.60876	0.98784516
SOX18	ENSG00000203883	20	62669076	62690994	16	5	123834	-0.27626	0.60882	0.98784516
LINC00908	ENSG00000263812	18	74230612	74332925	195	26	123834	-0.2765	0.60892	0.98784516
DCLK2	ENSG00000170390	4	150989426	151188609	352	26	123834	-0.27668	0.60899	0.98784516
NOP10	ENSG00000182117	15	34623917	34645378	52	5	123834	-0.27683	0.60905	0.98784516
DSG4	ENSG00000175065	18	28946740	29004875	64	4	123834	-0.27688	0.60906	0.98784516
PPP1R7	ENSG00000115685	2	242078991	242133067	115	9	123834	-0.2769	0.60907	0.98784516
CLSTN2	ENSG00000158258	3	139644027	140306239	1008	95	123834	-0.27698	0.6091	0.98784516
ABHD11	ENSG00000106077	7	73140424	73163197	21	3	123834	-0.27698	0.6091	0.98784516
LRG1	ENSG00000171236	19	4526421	4550486	20	3	123834	-0.27704	0.60913	0.98784516
CRLF1	ENSG00000006016	19	18673030	18728551	68	10	123834	-0.27708	0.60914	0.98784516
PUS3	ENSG00000110060	11	125753381	125783116	59	8	123834	-0.27733	0.60924	0.987923044
CCDC3	ENSG00000151468	10	12928627	13151652	446	54	123834	-0.27781	0.60942	0.988130629
LCOR	ENSG00000196233	10	98582017	98750800	144	7	123834	-0.27796	0.60948	0.988133968
EDNRB	ENSG00000136160	13	78459616	78503903	96	11	123834	-0.27812	0.60954	0.988133968
SOHLH1	ENSG00000165643	9	138575253	138601374	54	6	123834	-0.27832	0.60962	0.988133968
ACOT7	ENSG00000097021	1	6314329	6464451	349	18	123834	-0.27836	0.60963	0.988133968
TRIM37	ENSG00000108395	17	57049999	57194282	140	12	123834	-0.27851	0.60969	0.988146958
TOP1MT	ENSG00000184428	8	144376554	144452149	186	12	123834	-0.27918	0.60995	0.988448409
SPRR2B	ENSG00000196805	1	153032700	153123927	154	14	123834	-0.27926	0.60998	0.988448409
RSRC1	ENSG00000174891	3	157813644	158273519	1197	16	123834	-0.27999	0.61026	0.988612957
TMPRSS11A	ENSG00000187054	4	68765103	68839858	171	18	123834	-0.28074	0.61054	0.988612957
MYCBPAP	ENSG00000136449	17	48575745	48618862	72	9	123834	-0.2811	0.61068	0.988612957
BMP7	ENSG00000101144	20	55733804	55851685	200	22	123834	-0.28125	0.61074	0.988612957
LRRC8A	ENSG00000136802	9	131634391	131690318	23	5	123834	-0.28144	0.61081	0.988612957
HSBP1L1	ENSG00000226742	18	77714561	77740822	26	4	123834	-0.28165	0.61089	0.988612957
AP000695.1	ENSG00000267976	21	37848165	37870058	56	10	123834	-0.28165	0.6109	0.988612957
OR5R1	ENSG00000174942	11	56174734	56195708	77	8	123834	-0.28186	0.61098	0.988612957
UBL4B	ENSG00000186150	1	110645062	110666569	32	5	123834	-0.28199	0.61102	0.988612957
12-Sep	ENSG00000140623	16	4817670	4848522	47	7	123834	-0.28205	0.61105	0.988612957
ZNF396	ENSG00000186496	18	32936661	32967301	53	8	123834	-0.28219	0.6111	0.988612957
XRCC6	ENSG00000196419	22	42007123	42070044	48	5	123834	-0.28222	0.61111	0.988612957
SLC52A1	ENSG00000132517	17	4925895	4965304	78	13	123834	-0.28225	0.61113	0.988612957
KALRN	ENSG00000160145	3	123788870	124455172	1203	76	123834	-0.28244	0.6112	0.988612957
VPS26B	ENSG00000151502	11	134084539	134127686	32	6	123834	-0.28256	0.61124	0.988612957
ANAPC11	ENSG00000141552	17	79838666	79868867	2	1	123834	-0.28267	0.61129	0.988612957
NPB	ENSG00000183979	17	79848621	79870781	2	1	123834	-0.28267	0.61129	0.988612957
PCYT2	ENSG00000185813	17	79848841	79879340	2	1	123834	-0.28267	0.61129	0.988612957
MMP11	ENSG00000099953	22	24100413	24136503	43	7	123834	-0.28273	0.61131	0.988612957
METTL9	ENSG00000197006	16	21598539	21678794	44	8	123834	-0.28289	0.61137	0.988612957
IFNAR2	ENSG00000159110	21	34592206	34647980	108	10	123834	-0.28296	0.61139	0.988612957
CD2BP2	ENSG00000169217	16	30352087	30376682	8	3	123834	-0.28328	0.61152	0.988612957
RP11-113D6.10	ENSG00000189332	11	18220685	18246109	69	5	123834	-0.2834	0.61157	0.988612957
NIT2	ENSG00000114021	3	100043545	100085710	62	9	123834	-0.28346	0.61159	0.988612957
PXK	ENSG00000168297	3	58308607	58421748	409	15	123834	-0.28376	0.6117	0.988612957
ENPEP	ENSG00000138792	4	111276889	111496441	365	30	123834	-0.28384	0.61173	0.988612957
ZNF805	ENSG00000204524	19	57741973	57776503	83	8	123834	-0.28393	0.61177	0.988612957
TMEM158	ENSG00000249992	3	45255958	45277770	47	7	123834	-0.284	0.6118	0.988612957
DENND6B	ENSG00000205593	22	50737459	50775489	60	10	123834	-0.28405	0.61181	0.988612957
SLC36A2	ENSG00000186335	5	150684539	150737151	100	12	123834	-0.28413	0.61184	0.988612957
FOLR4	ENSG00000183560	11	94028759	94050887	51	8	123834	-0.28415	0.61185	0.988612957
PIANP	ENSG00000139200	12	6792958	6819966	14	3	123834	-0.28416	0.61186	0.988612957
CD101	ENSG00000134256	1	117534382	117589167	79	11	123834	-0.28433	0.61192	0.988612957
HSF1	ENSG00000185122	8	145505280	145548385	16	2	123834	-0.28474	0.61208	0.988612957
BDH2	ENSG00000164039	4	103990592	104031040	43	4	123834	-0.28481	0.6121	0.988612957
ACTR6	ENSG00000075089	12	100582900	100645643	104	14	123834	-0.28486	0.61212	0.988612957
PPM1G	ENSG00000115241	2	27594061	27642554	45	4	123834	-0.28493	0.61215	0.988612957
CEP128	ENSG00000100629	14	80933330	81435861	1002	44	123834	-0.28499	0.61218	0.988612957
FBXO28	ENSG00000143756	1	224291789	224359749	109	6	123834	-0.28518	0.61225	0.988612957

TMEM55B	ENSG00000165782	14	20915878	20939771	30	5	123834	-0.28525	0.61227	0.988612957
MEP1A	ENSG00000112818	6	46751127	46817515	114	9	123834	-0.28534	0.61231	0.988612957
PLK5	ENSG00000185988	19	1514073	1545455	42	8	123834	-0.28537	0.61232	0.988612957
CGA	ENSG00000135346	6	87785216	87814824	29	5	123834	-0.2855	0.61237	0.988612957
CSF2RB	ENSG00000100368	22	37299670	37346491	92	14	123834	-0.2855	0.61237	0.988612957
PSMB11	ENSG00000222028	14	23501376	23523269	8	4	123834	-0.28567	0.61244	0.98863875
NDEL1	ENSG00000166579	17	8306449	8403729	69	8	123834	-0.2858	0.61249	0.98863875
ARHGEF26	ENSG00000114790	3	153828792	153985616	257	12	123834	-0.28601	0.61257	0.988683923
GALNT16	ENSG00000100626	14	69715994	69831183	164	30	123834	-0.28676	0.61285	0.988789316
SMUG1	ENSG00000123415	12	54548529	54592778	34	6	123834	-0.28685	0.61289	0.988789316
DDX51	ENSG00000185163	12	132611139	132638880	45	5	123834	-0.28716	0.613	0.988789316
CDKL1	ENSG00000100490	14	50786310	50893179	126	16	123834	-0.2875	0.61314	0.988789316
UTS2	ENSG00000049247	1	7893143	7923572	77	17	123834	-0.28755	0.61315	0.988789316
AP000349.1	ENSG00000267954	22	24114467	24136145	29	4	123834	-0.28798	0.61332	0.988789316
SLC5A1	ENSG00000100170	22	32429019	32519016	192	9	123834	-0.28814	0.61338	0.988789316
KRTAP10-6	ENSG00000188155	21	46001149	46022386	64	4	123834	-0.28818	0.61339	0.988789316
CELF6	ENSG00000273025	15	72549087	72622287	112	11	123834	-0.28825	0.61342	0.988789316
CNBP	ENSG00000169714	3	128878327	128912765	20	3	123834	-0.28832	0.61345	0.988789316
PLCB1	ENSG00000182621	20	8102824	8959003	1430	107	123834	-0.28866	0.61358	0.988789316
MSANTD2	ENSG00000120458	11	124626394	124680569	60	8	123834	-0.28871	0.6136	0.988789316
MFSD2A	ENSG00000168389	1	40410802	40445638	11	4	123834	-0.28913	0.61376	0.988789316
SGOL2	ENSG00000163535	2	201364731	201458505	116	15	123834	-0.2892	0.61379	0.988789316
ZNF221	ENSG00000159905	19	44445375	44481861	97	10	123834	-0.28955	0.61392	0.988789316
PLXDC2	ENSG00000120594	10	20095168	20588785	1337	70	123834	-0.28972	0.61398	0.988789316
LCP2	ENSG00000043462	5	169663241	169735231	36	13	123834	-0.28978	0.61401	0.988789316
PSD4	ENSG00000125637	2	113904902	113976973	81	19	123834	-0.28996	0.61408	0.988789316
FERMT3	ENSG00000149781	11	63964150	64001354	45	11	123834	-0.29005	0.61411	0.988789316
C11orf21	ENSG00000110665	11	2306875	2334279	83	11	123834	-0.29021	0.61417	0.988789316
DDX41	ENSG00000183258	5	176928578	176954470	11	1	123834	-0.29029	0.6142	0.988789316
TMEM86B	ENSG00000180089	19	55728007	55751647	24	5	123834	-0.29055	0.6143	0.988789316
AC010327.2	ENSG00000269808	19	55728587	55751647	24	5	123834	-0.29055	0.6143	0.988789316
EMC2	ENSG00000104412	8	109445830	109509145	165	9	123834	-0.2909	0.61444	0.988789316
DGCR8	ENSG00000128191	22	20057755	20109400	140	12	123834	-0.29107	0.6145	0.988789316
TSC22D4	ENSG00000166925	7	100050982	100086902	29	3	123834	-0.29127	0.61458	0.988789316
KCNH7	ENSG00000184611	2	163217917	163705240	538	29	123834	-0.29143	0.61464	0.988789316
HRC	ENSG00000130528	19	49644455	49668681	34	7	123834	-0.29156	0.61469	0.988789316
CTB-50L17.14	ENSG00000267385	19	4518439	4550079	21	3	123834	-0.29161	0.61471	0.988789316
GRHL3	ENSG00000158055	1	24635812	24700972	56	14	123834	-0.29194	0.61483	0.988789316
CHRNG	ENSG00000196811	2	233394437	233421113	36	7	123834	-0.29197	0.61485	0.988789316
TEX26	ENSG00000175664	13	31496840	31559639	118	14	123834	-0.29231	0.61498	0.988789316
COX5A	ENSG00000178741	15	75202132	75240509	33	5	123834	-0.29244	0.61502	0.988789316
KCNK13	ENSG00000152315	14	90518109	90662201	288	25	123834	-0.29299	0.61524	0.988789316
ATCAY	ENSG00000167654	19	3869862	3938077	45	10	123834	-0.29305	0.61526	0.988789316
GOLGA8M	ENSG00000188626	15	28937100	28993530	5	1	123834	-0.29337	0.61538	0.988789316
OR6C4	ENSG00000179626	12	55934983	55956028	28	4	123834	-0.29342	0.6154	0.988789316
UBQLNL	ENSG00000175518	11	5525623	5547935	35	6	123834	-0.29347	0.61542	0.988789316
RP11-644F5.10	ENSG00000258311	12	56099820	56128487	10	3	123834	-0.29351	0.61543	0.988789316
ELMO1	ENSG00000155849	7	36883961	37498852	1061	83	123834	-0.29367	0.61549	0.988789316
ZNF720	ENSG00000197302	16	31714550	31816190	175	7	123834	-0.29391	0.61559	0.988789316
CD63	ENSG00000135404	12	56109107	56133491	10	3	123834	-0.29406	0.61565	0.988789316
LPAR6	ENSG00000139679	13	48953707	49028840	79	7	123834	-0.29407	0.61565	0.988789316
CCL1	ENSG00000108702	17	32677347	32700250	56	7	123834	-0.2941	0.61566	0.988789316
KEAP1	ENSG00000079999	19	10586796	10624417	58	4	123834	-0.29414	0.61567	0.988789316
LIMCH1	ENSG00000064042	4	41351624	41712061	546	45	123834	-0.29415	0.61568	0.988789316
MAMSTR	ENSG00000176909	19	49205999	49232978	57	5	123834	-0.29428	0.61573	0.988789316
BATF	ENSG00000156127	14	75978768	76023358	51	9	123834	-0.2943	0.61574	0.988789316
PCBD1	ENSG00000166228	10	72632037	72658541	49	10	123834	-0.2944	0.61578	0.988789316
FAM188B2	ENSG00000214237	3	150578832	150622089	42	10	123834	-0.29449	0.61581	0.988789316
EDEM2	ENSG00000088298	20	33693167	33875928	268	17	123834	-0.29473	0.6159	0.988789316
AMPD2	ENSG00000116337	1	110148726	110184673	44	10	123834	-0.29483	0.61594	0.988789316
UCHL3	ENSG00000118939	13	76113619	76190085	179	14	123834	-0.29489	0.61596	0.988789316
IGLL1	ENSG00000128322	22	23905312	23932495	37	6	123834	-0.29519	0.61608	0.988789316
NPY2R	ENSG00000185149	4	156119781	156148230	52	12	123834	-0.29522	0.61609	0.988789316
MPEG1	ENSG00000197629	11	58965983	58990424	36	5	123834	-0.29522	0.61609	0.988789316
RPS10-NUDT3	ENSG00000270800	6	34246547	34403825	251	9	123834	-0.29565	0.61625	0.988789316
RP11-343C2.9	ENSG00000260371	16	69358994	69400209	43	7	123834	-0.29584	0.61633	0.988789316
ABHD14B	ENSG00000114779	3	51992526	52027425	6	2	123834	-0.29603	0.61639	0.988789316
ADAL	ENSG00000168803	15	43612872	43656096	67	5	123834	-0.29605	0.6164	0.988789316
THNSL2	ENSG00000144115	2	88459835	88496146	58	10	123834	-0.29611	0.61643	0.988789316
DEF6	ENSG00000023892	6	35255595	35299548	77	10	123834	-0.29619	0.61646	0.988789316
GCLC	ENSG00000001084	6	53352139	53491768	193	35	123834	-0.2962	0.61646	0.988789316
SLC39A4	ENSG00000147804	8	145625126	145652279	11	3	123834	-0.29626	0.61649	0.988789316
TRAT1	ENSG00000163519	3	108531545	108583852	70	11	123834	-0.29644	0.61655	0.988789316
SIRPG	ENSG00000089012	20	1599798	1648425	126	17	123834	-0.29648	0.61657	0.988789316
TMEM201	ENSG00000188807	1	9638932	9684935	26	3	123834	-0.29653	0.61659	0.988789316
ARHGEF25	ENSG00000240771	12	57993963	58023162	15	4	123834	-0.29658	0.61661	0.988789316
SNIP1	ENSG00000163877	1	37990050	38029903	58	9	123834	-0.29665	0.61663	0.988789316
UCHL5	ENSG00000116750	1	192971380	193039237	75	9	123834	-0.29691	0.61673	0.988789316
MAPKAP1	ENSG00000119487	9	128189672	128479513	364	16	123834	-0.2969	0.61673	0.988789316
POPDC3	ENSG00000132429	6	105596155	105637870	71	7	123834	-0.29694	0.61675	0.988789316
B3GNT7	ENSG00000156966	2	232250254	232275875	36	5	123834	-0.297	0.61677	0.988789316
DPF3	ENSG00000205683	14	73076004	73370809	437	52	123834	-0.2971	0.6168	0.988789316
SFRP1	ENSG00000104332	8	41109481	41177016	59	9	123834	-0.29718	0.61684	0.988789316
FASTKD2	ENSG00000118246	2	207620081	207667233	96	9	123834	-0.29729	0.61688	0.988789316
PLIN5	ENSG00000214456	19	4512543	4545236	21	3	123834	-0.29746	0.61694	0.988789316
PIGX	ENSG00000163964	3	196356646	196472878	131	19	123834	-0.29751	0.61696	0.988789316
RPL18	ENSG00000063177	19	49108585	49132793	31	5	123834	-0.29775	0.61705	0.988789316
MYO5C	ENSG00000128833	15	52474519	52597995	210	27	123834	-0.29777	0.61706	0.988789316
KRT78	ENSG00000170423	12	53221588	53252876	33	4	123834	-0.29794	0.61713	0.988789316
G0S2	ENSG00000123689	1	209838765	209859733	16	5	123834	-0.29813	0.6172	0.988789316
ITSN1	ENSG00000205726	21	35004706	35282165	263	37	123834	-0.29817	0.61721	0.988789316
CALB1	ENSG00000104327	8	91060836	91117703	79	9	123834	-0.2982	0.61722	0.988789316
HMX2	ENSG00000188816	10	124897638	124920188	1	1	123834	-0.2983	0.61726	0.988789316
ORC3	ENSG00000135336	6	88289839	88387169	199	15	123834	-0.2985	0.61734	0.988789316
LCE3A	ENSG00000185962	1	152585310	152605579	65	3	123834	-0.29856	0.61736	0.988789316
PRG4	ENSG00000116690	1	186255405	186293694	35	7	123834	-0.29886	0.61748	0.988789316
LARP1	ENSG00000155506	5	154082462	154207167	148	16	123834	-0.29886	0.61748	0.988789316
SAYSD1	ENSG00000112167	6	39061840	39092965	32	8	123834	-0.29887	0.61748	0.988789316
C9orf24	ENSG00000164972	9	34369017	34407830	51	10	123834	-0.29904	0.61755	0.988789316

MED26	ENSG00000105085	19	16675718	16749873	88	8	123834	-0.29916	0.61759	0.988789316
PKDREJ	ENSG00000130943	22	46641560	46669219	87	6	123834	-0.29919	0.6176	0.988789316
AC019171.1	ENSG00000269210	2	39176764	39197483	22	2	123834	-0.2993	0.61765	0.988789316
ZNF333	ENSG00000160961	19	14790613	14854558	108	13	123834	-0.29932	0.61765	0.988789316
TMEM156	ENSG00000121895	4	38958365	39044542	170	27	123834	-0.29942	0.61769	0.988789316
RNF121	ENSG00000137522	11	71629747	71718643	193	6	123834	-0.29959	0.61775	0.988789316
INPP4B	ENSG00000109452	4	142934313	143778585	1560	60	123834	-0.29993	0.61789	0.988789316
PSMA2	ENSG00000256646	7	42938872	42981773	85	3	123834	-0.3001	0.61795	0.988789316
FGFBP2	ENSG00000137441	4	15951866	15980932	42	8	123834	-0.30021	0.61799	0.988789316
TBPL2	ENSG00000182521	14	55870259	55933444	145	11	123834	-0.30028	0.61802	0.988789316
FBXO43	ENSG00000156509	8	101135588	101168028	32	3	123834	-0.3003	0.61803	0.988789316
MPST	ENSG00000128309	22	37405676	37435863	26	7	123834	-0.30069	0.61817	0.988789316
PDHB	ENSG00000168291	3	58403357	58429584	103	10	123834	-0.30074	0.61819	0.988789316
TNRC6A	ENSG00000090905	16	24731016	24848953	142	12	123834	-0.30083	0.61823	0.988789316
VEPH1	ENSG00000197415	3	156967531	157261408	465	29	123834	-0.30111	0.61834	0.988789316
ASPSCR1	ENSG00000169696	17	79924683	79985282	60	7	123834	-0.30124	0.61838	0.988789316
RASGRP3	ENSG00000152689	2	33651391	33799817	206	38	123834	-0.30148	0.61847	0.988789316
LOX	ENSG00000113083	5	121388890	121423980	37	6	123834	-0.30173	0.61857	0.988789316
OR10H5	ENSG00000172519	19	15894761	15915892	60	8	123834	-0.30174	0.61858	0.988789316
ERBB2IP	ENSG00000112851	5	65212303	65388377	134	8	123834	-0.3018	0.6186	0.988789316
SMARCD2	ENSG00000108604	17	61899444	61930425	50	3	123834	-0.30181	0.6186	0.988789316
C10orf12	ENSG00000155640	10	98731041	98755582	11	2	123834	-0.30183	0.61861	0.988789316
FADS3	ENSG00000221968	11	61630991	61669523	38	6	123834	-0.30208	0.61871	0.988789316
CRNN	ENSG00000143536	1	152371719	152396739	28	4	123834	-0.30233	0.6188	0.988789316
BOP1	ENSG00000170727	8	145476055	145525082	11	2	123834	-0.30241	0.61883	0.988789316
RNF123	ENSG00000164068	3	49716932	49768962	47	5	123834	-0.3027	0.61894	0.988789316
IMP4	ENSG00000136718	2	131089798	131115383	6	2	123834	-0.30272	0.61895	0.988789316
ANKS6	ENSG00000165138	9	101483611	101569247	118	9	123834	-0.30284	0.61899	0.988789316
AQP1	ENSG00000250424	7	30883010	30973427	151	22	123834	-0.30337	0.61919	0.988789316
AQP1	ENSG00000240583	7	30883010	30975131	151	22	123834	-0.30337	0.61919	0.988789316
GPRC5B	ENSG00000167191	16	19858616	19907489	38	5	123834	-0.30338	0.6192	0.988789316
TWISTNB	ENSG00000105849	7	19725085	19758710	101	9	123834	-0.30346	0.61923	0.988789316
PDCL	ENSG00000136940	9	125550668	125600910	50	7	123834	-0.30345	0.61923	0.988789316
LCN10	ENSG00000187922	9	139622619	139647808	54	7	123834	-0.30388	0.61939	0.988789316
MDM1	ENSG00000111554	12	68656223	68736161	104	14	123834	-0.30393	0.61941	0.988789316
MCOLN2	ENSG00000153898	1	85381268	85472796	200	11	123834	-0.30395	0.61942	0.988789316
PCDH8	ENSG00000136099	13	53408109	53432775	24	3	123834	-0.30396	0.61942	0.988789316
SSH1	ENSG00000084112	12	109166466	109261366	82	13	123834	-0.30399	0.61943	0.988789316
ACSF3	ENSG00000176715	16	89144783	89232254	343	18	123834	-0.3042	0.61951	0.988789316
ZNF134	ENSG00000213762	19	58115601	58144724	56	6	123834	-0.30435	0.61957	0.988789316
CYP19A1	ENSG00000137869	15	51490254	51640807	202	24	123834	-0.30442	0.6196	0.988789316
ATP11A	ENSG00000068650	13	113334643	113551482	338	26	123834	-0.30448	0.61962	0.988789316
APOL6	ENSG00000221963	22	36034442	36074456	87	12	123834	-0.3049	0.61978	0.988789316
TNFSF18	ENSG00000120337	1	172999100	173030103	26	4	123834	-0.30578	0.62011	0.988789316
UBQLN3	ENSG00000175520	11	5518530	5541215	31	6	123834	-0.30584	0.62014	0.988789316
GNPNAT1	ENSG00000100522	14	53231912	53268386	33	4	123834	-0.30591	0.62016	0.988789316
ZNRF1	ENSG00000186187	16	75022928	75154892	146	13	123834	-0.30599	0.6202	0.988789316
PFN2	ENSG00000070087	3	149672691	149778575	214	21	123834	-0.30606	0.62022	0.988789316
AKAP8	ENSG00000105127	19	15454196	15500609	30	2	123834	-0.3065	0.62039	0.988789316
MRPL21	ENSG00000197345	11	68648744	68681303	58	6	123834	-0.30658	0.62042	0.988789316
PRDX2	ENSG00000167815	19	12897634	12922694	49	5	123834	-0.30688	0.62053	0.988789316
ZNF76	ENSG00000065029	6	35216686	35273762	95	9	123834	-0.30698	0.62057	0.988789316
KRTAP10-5	ENSG00000241123	21	45989332	46010481	69	4	123834	-0.30716	0.62064	0.988789316
HSP90AA1	ENSG00000080824	14	102537075	102616036	140	13	123834	-0.30719	0.62065	0.988789316
CA3	ENSG00000164879	8	86275665	86371269	132	13	123834	-0.30727	0.62068	0.988789316
TLN2	ENSG00000171914	15	62672725	63146830	572	58	123834	-0.30726	0.62068	0.988789316
PGM5	ENSG00000154330	9	70933224	71155977	113	12	123834	-0.30744	0.62075	0.988789316
FNDC3A	ENSG00000102531	13	49540048	49793915	187	8	123834	-0.30759	0.6208	0.988789316
TMEM59L	ENSG00000105696	19	18708240	18741849	31	5	123834	-0.3079	0.62092	0.988789316
ATP5SL	ENSG00000105341	19	41927223	41956622	30	2	123834	-0.30792	0.62093	0.988789316
CIAO1	ENSG00000144021	2	96921870	96949087	20	5	123834	-0.30802	0.62097	0.988789316
ANGPTL1	ENSG00000116194	1	178808840	178850187	56	8	123834	-0.30808	0.62099	0.988789316
CUL5	ENSG00000166266	11	107869459	107988503	157	11	123834	-0.30822	0.62104	0.988789316
KIF4B	ENSG00000226650	5	154383260	154407685	68	8	123834	-0.30823	0.62105	0.988789316
LYSMD4	ENSG00000183060	15	100245902	100283766	64	9	123834	-0.30824	0.62105	0.988789316
PTPN12	ENSG00000127947	7	77156592	77279388	248	16	123834	-0.30831	0.62108	0.988789316
VEZT	ENSG00000028203	12	95601522	95706566	279	17	123834	-0.30831	0.62108	0.988789316
UTP3	ENSG00000132467	4	71544196	71566267	22	3	123834	-0.30879	0.62126	0.988789316
MOB2	ENSG00000182208	11	1480687	1532477	116	9	123834	-0.30916	0.6214	0.988789316
C8orf47	ENSG00000177459	8	99066539	99115838	39	8	123834	-0.30921	0.62142	0.988789316
BOLA2B	ENSG00000169627	16	30194255	30215627	4	2	123834	-0.3092	0.62142	0.988789316
PANX2	ENSG00000073150	22	50599160	50628723	36	11	123834	-0.3093	0.62145	0.988789316
GMFB	ENSG00000197045	14	54931202	54965914	28	4	123834	-0.30981	0.62165	0.988789316
HDAC10	ENSG00000100429	22	50673612	50699834	38	7	123834	-0.30986	0.62167	0.988789316
RPS27A	ENSG00000143947	2	55449039	55472989	49	9	123834	-0.31008	0.62175	0.988789316
PDIA6	ENSG00000143870	2	10913517	10988103	354	16	123834	-0.31012	0.62176	0.988789316
AQP12B	ENSG00000185176	2	241605835	241632323	40	5	123834	-0.31011	0.62176	0.988789316
VAPB	ENSG00000124164	20	56954178	57036157	147	14	123834	-0.3103	0.62184	0.988789316
COLQ	ENSG00000206561	3	15481640	15573258	171	15	123834	-0.3107	0.62199	0.988789316
TOPORS	ENSG00000197579	9	32530542	32562551	35	5	123834	-0.31092	0.62207	0.988789316
ZNF236	ENSG00000130856	18	74524563	74692683	282	13	123834	-0.31094	0.62208	0.988789316
RNASEK	ENSG00000219200	17	6905736	6927851	27	3	123834	-0.31138	0.62224	0.988789316
AC040977.1	ENSG00000269871	17	6905928	6927755	27	3	123834	-0.31138	0.62224	0.988789316
AL359195.1	ENSG00000204038	10	81999466	82023395	106	8	123834	-0.31157	0.62232	0.988789316
RNH1	ENSG00000023191	11	484512	517300	112	11	123834	-0.31202	0.62249	0.988789316
ODF3L2	ENSG00000181781	19	453346	484983	76	13	123834	-0.31219	0.62255	0.988789316
GIGYF2	ENSG00000204120	2	233552009	233735285	340	15	123834	-0.31227	0.62258	0.988789316
TNFRSF1A	ENSG00000067182	12	6427923	6461280	8	3	123834	-0.31243	0.62264	0.988789316
USP8	ENSG00000138592	15	50706577	50803280	204	14	123834	-0.31259	0.6227	0.988789316
PPM1L	ENSG00000163590	3	160463390	160806695	582	23	123834	-0.31329	0.62297	0.988789316
SPATA6L	ENSG00000106686	9	4543386	4676674	295	24	123834	-0.31335	0.62299	0.988789316
GSAP	ENSG00000186088	7	76930068	77055717	291	20	123834	-0.31351	0.62305	0.988789316
DNAAF2	ENSG00000165506	14	50081892	50111948	66	5	123834	-0.31394	0.62322	0.988789316
MFHAS1	ENSG00000147324	8	8630864	8761155	330	26	123834	-0.31398	0.62323	0.988789316
ME1	ENSG00000065833	6	83910108	84150797	371	15	123834	-0.3142	0.62332	0.988789316
SEC23IP	ENSG00000107651	10	121642223	121712014	200	11	123834	-0.31423	0.62332	0.988789316
CRLF3	ENSG00000176390	17	29086406	29161794	202	3	123834	-0.3145	0.62343	0.988789316
CGREF1	ENSG00000138028	2	27311757	27351995	18	2	123834	-0.31458	0.62346	0.988789316
FAM107A	ENSG00000168309	3	58539844	58623337	127	20	123834	-0.31461	0.62347	0.988789316

PROSER2	ENSG00000148426	10	11855338	11924276	87	10	123834	-0.31488	0.62357	0.988789316
NECAB3	ENSG00000125967	20	32234893	32272269	9	2	123834	-0.31487	0.62357	0.988789316
FZD5	ENSG00000163251	2	208617310	208644287	25	4	123834	-0.31549	0.6238	0.988789316
RP11-343C2.11	ENSG00000260914	16	69323585	69364614	57	5	123834	-0.31555	0.62383	0.988789316
PSMA2	ENSG00000106588	7	42946460	42981822	70	3	123834	-0.31559	0.62384	0.988789316
EMC7	ENSG00000134153	15	34366218	34404149	55	6	123834	-0.31566	0.62387	0.988789316
OR2J3	ENSG00000204701	6	29069668	29090603	40	8	123834	-0.31568	0.62388	0.988789316
ZNF513	ENSG00000163795	2	27590098	27613657	24	4	123834	-0.31609	0.62403	0.988789316
IFT27	ENSG00000100360	22	37144246	37182300	42	4	123834	-0.31616	0.62406	0.988789316
ZNF556	ENSG00000172000	19	2857333	2888515	35	6	123834	-0.31618	0.62407	0.988789316
MSLNL	ENSG00000162006	16	809428	843370	60	11	123834	-0.31654	0.6242	0.988789316
UBXN2B	ENSG00000215114	8	59313823	59374060	123	14	123834	-0.31675	0.62428	0.988789316
EMP1	ENSG00000134531	12	13339650	13379708	30	8	123834	-0.31675	0.62428	0.988789316
AC022819.2	ENSG00000268730	15	99849300	99869828	45	6	123834	-0.31713	0.62443	0.988789316
NHP2L1	ENSG00000100138	22	42059934	42096508	58	4	123834	-0.31717	0.62444	0.988789316
SHISA5	ENSG00000164054	3	48499197	48552259	42	4	123834	-0.31722	0.62446	0.988789316
WEE1	ENSG00000166483	11	9585228	9625004	30	8	123834	-0.31738	0.62452	0.988789316
AVPR1B	ENSG00000198049	1	206213976	206241639	53	6	123834	-0.31747	0.62456	0.988789316
PEX26	ENSG00000215193	22	18550689	18623905	111	18	123834	-0.3176	0.62461	0.988789316
SHOC2	ENSG00000108061	10	112669301	112783425	177	17	123834	-0.31766	0.62463	0.988789316
GJB5	ENSG00000189280	1	35210648	35234113	33	8	123834	-0.31787	0.62471	0.988789316
SMIM11	ENSG00000205670	21	35737779	35790164	84	7	123834	-0.31801	0.62476	0.988789316
CBX3	ENSG00000122565	7	26230782	26262976	44	5	123834	-0.31804	0.62477	0.988789316
PTPLA	ENSG00000165996	10	17621958	17669376	110	13	123834	-0.31805	0.62478	0.988789316
MAFF	ENSG00000185022	22	38587889	38622518	60	6	123834	-0.31828	0.62486	0.988789316
TBC1D10C	ENSG00000175463	11	67161386	67187560	26	3	123834	-0.31836	0.62489	0.988789316
CCND3	ENSG00000112576	6	41892671	42028095	213	42	123834	-0.31841	0.62491	0.988789316
ZNF274	ENSG00000171606	19	58684396	58734928	67	10	123834	-0.31855	0.62496	0.988789316
RP11-386G21.1	ENSG00000253976	8	56037763	56064918	29	7	123834	-0.31862	0.62499	0.988789316
EIF1AD	ENSG00000175376	11	65754016	65779647	69	7	123834	-0.31863	0.625	0.988789316
ARL4D	ENSG00000175906	17	41466327	41488505	40	6	123834	-0.31877	0.62505	0.988789316
C4BPB	ENSG00000123843	1	207252187	207283338	20	4	123834	-0.31905	0.62516	0.988789316
SUGT1	ENSG00000165416	13	53216844	53285044	170	6	123834	-0.31909	0.62517	0.988789316
LIMS2	ENSG00000072163	2	128385956	128449360	128	24	123834	-0.31913	0.62519	0.988789316
PTGES3L-AARSD1	ENSG00000108825	17	41092543	41142545	35	4	123834	-0.31922	0.62522	0.988789316
CACNB4	ENSG00000182389	2	152679290	152965593	401	24	123834	-0.31931	0.62525	0.988789316
AL357673.1	ENSG00000248835	1	54625333	54647997	21	5	123834	-0.31957	0.62535	0.988789316
RNF144A	ENSG00000151692	2	7047523	7218417	281	26	123834	-0.31956	0.62535	0.988789316
DNAJB7	ENSG00000172404	22	41245553	41268130	14	2	123834	-0.31959	0.62536	0.988789316
PDCD4	ENSG00000150593	10	112621565	112669764	77	13	123834	-0.32033	0.62564	0.988789316
PTH	ENSG00000152266	11	13503602	13527728	48	3	123834	-0.32043	0.62568	0.988789316
RND3	ENSG00000115963	2	151314709	151405525	98	14	123834	-0.32093	0.62587	0.988789316
PEG3	ENSG00000198300	19	57311445	57362096	40	6	123834	-0.32117	0.62596	0.988789316
ZNF676	ENSG00000196109	19	22351893	22389753	99	6	123834	-0.32155	0.6261	0.988789316
OR52J3	ENSG00000205495	11	5057756	5078691	168	6	123834	-0.32156	0.62611	0.988789316
RASGRP4	ENSG00000171777	19	38889695	38926945	85	12	123834	-0.32175	0.62618	0.988789316
C16orf71	ENSG00000166246	16	4774273	4809397	74	4	123834	-0.32181	0.6262	0.988789316
KAZALD1	ENSG00000107821	10	102811598	102837888	4	2	123834	-0.32193	0.62625	0.988789316
SIT1	ENSG00000137078	9	35639292	35660937	60	9	123834	-0.32202	0.62628	0.988789316
RAD9A	ENSG00000172613	11	67149176	67175881	24	3	123834	-0.32207	0.6263	0.988789316
RPS6KA2	ENSG00000071242	6	166812852	167329939	760	86	123834	-0.3222	0.62635	0.988789316
C9orf41	ENSG00000156017	9	77585936	77653339	111	14	123834	-0.32271	0.62654	0.988789316
TFDP2	ENSG00000114126	3	141653277	141878386	548	11	123834	-0.32276	0.62656	0.988789316
ERV3-1	ENSG00000213462	7	64441187	64477121	90	7	123834	-0.32291	0.62662	0.988789316
HLCS	ENSG00000159267	21	38113189	38372536	479	29	123834	-0.32314	0.62671	0.988789316
B3GAT3	ENSG00000149541	11	62372768	62399647	9	2	123834	-0.32353	0.62685	0.988789316
SDF4	ENSG00000078808	1	1142288	1177411	109	6	123834	-0.32358	0.62687	0.988789316
MAP7D1	ENSG00000116871	1	36611180	36656450	25	3	123834	-0.32366	0.6269	0.988789316
MFSD6	ENSG00000151690	2	191263081	191383931	225	17	123834	-0.3238	0.62695	0.988789316
SNX19	ENSG00000120451	11	130735331	130796404	216	13	123834	-0.32389	0.62699	0.988789316
TUBGCP4	ENSG00000137822	15	43651419	43709293	76	7	123834	-0.32392	0.627	0.988789316
RP11-1396O13.13	ENSG00000219492	4	9375743	9400709	129	10	123834	-0.32397	0.62702	0.988789316
DYNC1LI2	ENSG00000135720	16	66744796	66795701	79	4	123834	-0.32396	0.62702	0.988789316
TCP1	ENSG00000120438	6	160189530	160220781	102	9	123834	-0.32409	0.62706	0.988789316
PLEK2	ENSG00000100558	14	67843700	67888917	78	9	123834	-0.32432	0.62715	0.988789316
CD207	ENSG00000116031	2	71047347	71072952	136	11	123834	-0.32437	0.62717	0.988789316
SMYD2	ENSG00000143499	1	214444445	214520474	137	17	123834	-0.32454	0.62724	0.988789316
CXCR1	ENSG00000163464	2	219017568	219041718	28	4	123834	-0.32456	0.62724	0.988789316
ADORA2A	ENSG00000128271	22	24803847	24848328	60	8	123834	-0.32463	0.62727	0.988789316
TAF1B	ENSG00000115750	2	9973483	10084545	401	15	123834	-0.32466	0.62728	0.988789316
ZNF862	ENSG00000106479	7	149525456	149574568	29	6	123834	-0.32465	0.62728	0.988789316
ZNF320	ENSG00000182986	19	53357043	53410946	103	12	123834	-0.32482	0.62734	0.988789316
RANBP3	ENSG00000031823	19	5906150	5988153	64	8	123834	-0.32498	0.6274	0.988789316
HERC1	ENSG00000103657	15	63890817	64136141	503	15	123834	-0.32515	0.62747	0.988789316
RABGEF1	ENSG00000154710	7	66137151	66286451	405	15	123834	-0.32519	0.62748	0.988789316
PLAGL1	ENSG00000118495	6	144251437	144395735	138	22	123834	-0.32566	0.62766	0.988789316
C7orf25	ENSG00000136197	7	42938325	42961904	48	3	123834	-0.32568	0.62767	0.988789316
POU2F3	ENSG00000137709	11	120097349	120200653	129	22	123834	-0.32606	0.62781	0.988789316
PDCL3	ENSG00000115539	2	101169152	101203197	34	6	123834	-0.32616	0.62785	0.988789316
DDX31	ENSG00000125485	9	135458384	135555788	135	15	123834	-0.32635	0.62792	0.988789316
SEMA3A	ENSG00000075213	7	83575093	84132040	1264	51	123834	-0.32672	0.62806	0.988789316
UBR3	ENSG00000144357	2	170673968	170950641	868	22	123834	-0.32684	0.62811	0.988789316
MS4A8	ENSG00000166959	11	60457047	60493284	24	2	123834	-0.32694	0.62814	0.988789316
SHCBP1	ENSG00000171241	16	46604466	46665538	11	5	123834	-0.32695	0.62815	0.988789316
C16orf74	ENSG00000154102	16	85713690	85794735	33	6	123834	-0.32699	0.62816	0.988789316
ZNF217	ENSG00000171940	20	52173604	52236446	61	11	123834	-0.32724	0.62826	0.988789316
CD300E	ENSG00000186407	17	72596026	72629897	76	11	123834	-0.32739	0.62832	0.988789316
KRTAP4-5	ENSG00000198271	17	39295178	39316054	33	6	123834	-0.32751	0.62836	0.988789316
NOP58	ENSG00000055044	2	203120439	203178389	79	6	123834	-0.32753	0.62837	0.988789316
SPOCK1	ENSG00000152377	5	136300987	136944068	916	77	123834	-0.32767	0.62842	0.988789316
RP11-761B3.1	ENSG00000273269	2	47283080	47413650	289	32	123834	-0.32769	0.62843	0.988789316
PMP22	ENSG00000109099	17	15123095	15178643	25	6	123834	-0.32772	0.62844	0.988789316
CES1	ENSG00000198848	16	55826763	55877249	35	7	123834	-0.32777	0.62846	0.988789316
TLE2	ENSG00000065717	19	2987636	3057633	72	20	123834	-0.32789	0.6285	0.988789316
RNF10	ENSG00000022840	12	120961283	121025397	172	15	123834	-0.32792	0.62851	0.988789316
TUBB2B	ENSG00000137285	6	3214495	3241964	8	1	123834	-0.32794	0.62852	0.988789316
ABTB2	ENSG00000166016	11	34162535	34389555	332	48	123834	-0.32843	0.62871	0.988789316
RP11-322L20.1	ENSG00000258804	14	87362122	87399099	78	7	123834	-0.32852	0.62874	0.988789316
ZNF346	ENSG00000113761	5	176439697	176518190	124	6	123834	-0.32859	0.62877	0.988789316

AL035252.1	ENSG00000268302	20	25197122	25217370	30	6	123834	-0.32864	0.62879	0.988789316
FAM212B	ENSG00000197852	1	112213252	112308446	238	28	123834	-0.32868	0.6288	0.988789316
KRTAP10-4	ENSG00000215454	21	45983606	46004987	67	4	123834	-0.32875	0.62883	0.988789316
ISL2	ENSG00000159556	15	76619065	76644817	33	8	123834	-0.32884	0.62886	0.988789316
CACNG5	ENSG00000075429	17	64821235	64891603	138	28	123834	-0.32899	0.62892	0.988789316
CDHR3	ENSG00000128536	7	105507242	105686877	289	44	123834	-0.32918	0.62899	0.988789316
CDRT1	ENSG00000181464	17	15458797	15479590	7	3	123834	-0.32938	0.62906	0.988789316
PI4K2A	ENSG00000249967	10	99334131	99443667	131	12	123834	-0.3295	0.62911	0.988789316
SLC18A3	ENSG00000187714	10	50808347	50830765	24	6	123834	-0.32966	0.62917	0.988789316
CRAMP1L	ENSG00000007545	16	1652326	1737909	113	9	123834	-0.32971	0.62919	0.988789316
TMEM39A	ENSG00000176142	3	119138347	119197677	143	13	123834	-0.32988	0.62926	0.988789316
RBBP8	ENSG00000101773	18	20368224	20616451	446	16	123834	-0.32993	0.62927	0.988789316
TMEM72	ENSG00000187783	10	45396648	45442450	43	6	123834	-0.32999	0.6293	0.988789316
CDKN1A	ENSG00000124762	6	36634305	36665116	63	7	123834	-0.3301	0.62934	0.988789316
LIG3	ENSG00000005156	17	33297513	33342083	25	4	123834	-0.33012	0.62935	0.988789316
MTDH	ENSG00000147649	8	98646407	98750998	143	11	123834	-0.33043	0.62946	0.988789316
SLC16A9	ENSG00000165449	10	61400523	61505760	167	25	123834	-0.33043	0.62946	0.988789316
CA2	ENSG00000104267	8	86366081	86403722	33	6	123834	-0.33057	0.62952	0.988789316
FNTA	ENSG00000168522	8	42879337	42950931	65	7	123834	-0.33092	0.62965	0.988789316
DDX59	ENSG00000118197	1	200583024	200649097	170	15	123834	-0.3312	0.62975	0.988789316
NDRG3	ENSG00000101079	20	35270169	35384481	55	15	123834	-0.33134	0.62981	0.988789316
ENPP3	ENSG00000154269	6	131939582	132078553	199	19	123834	-0.33158	0.6299	0.988789316
SFXN3	ENSG00000107819	10	102780991	102810998	12	4	123834	-0.33161	0.62991	0.988789316
SYT5	ENSG00000129990	19	55672567	55701809	102	12	123834	-0.33167	0.62993	0.988789316
MTMR7	ENSG00000003987	8	17145539	17281037	244	28	123834	-0.3317	0.62994	0.988789316
B9D1	ENSG00000108641	17	19230867	19291495	36	4	123834	-0.3318	0.62998	0.988789316
AQP2	ENSG00000167580	12	50334524	50362664	31	8	123834	-0.33182	0.62999	0.988789316
SLC12A6	ENSG00000140199	15	34515460	34640261	353	16	123834	-0.33185	0.63	0.988789316
GABRG1	ENSG00000163285	4	46027786	46136098	239	16	123834	-0.33199	0.63005	0.988789316
C4B	ENSG00000224389	6	31972539	32013195	8	4	123834	-0.33202	0.63006	0.988789316
GSG1	ENSG00000111305	12	13226494	13266619	30	6	123834	-0.33207	0.63008	0.988789316
EAF2	ENSG00000145088	3	121544030	121615373	159	9	123834	-0.33208	0.63009	0.988789316
NTAN1	ENSG00000157045	16	15121710	15159921	52	6	123834	-0.33228	0.63016	0.988789316
OR1D2	ENSG00000184166	17	2985352	3006290	36	5	123834	-0.33235	0.63019	0.988789316
ZNF843	ENSG00000176723	16	31435569	31464346	19	6	123834	-0.33239	0.6302	0.988789316
SBK1	ENSG00000188322	16	28293840	28345170	36	8	123834	-0.33244	0.63022	0.988789316
TRIP11	ENSG00000100815	14	92422335	92517240	184	13	123834	-0.33245	0.63023	0.988789316
PEMT	ENSG00000133027	17	17398877	17505022	148	14	123834	-0.33249	0.63024	0.988789316
RBM19	ENSG00000122965	12	114244543	114414176	464	30	123834	-0.33261	0.63029	0.988789316
CDH17	ENSG00000079112	8	95129399	95239531	186	27	123834	-0.33264	0.6303	0.988789316
FAM159B	ENSG00000145642	5	63976135	64023917	48	4	123834	-0.33269	0.63032	0.988789316
TMEM179B	ENSG00000185475	11	62544887	62567877	12	2	123834	-0.33274	0.63033	0.988789316
ANKS4B	ENSG00000175311	16	21234986	21273750	11	4	123834	-0.33318	0.6305	0.988789316
METTL7B	ENSG00000170439	12	56065330	56088395	13	2	123834	-0.33328	0.63054	0.988789316
SLC22A10	ENSG00000184999	11	62895339	63147190	522	13	123834	-0.33336	0.63057	0.988789316
CTD-3105H18.18	ENSG00000269755	19	12533917	12605630	268	9	123834	-0.33338	0.63058	0.988789316
PARD6G	ENSG00000178184	18	77905115	78015429	156	14	123834	-0.33343	0.6306	0.988789316
SETD4	ENSG00000185917	21	37396839	37461687	163	18	123834	-0.33357	0.63065	0.988789316
NES	ENSG00000132688	1	156628555	156657189	12	3	123834	-0.33377	0.63072	0.988789316
S1PR4	ENSG00000125910	19	3162344	3190329	28	8	123834	-0.33378	0.63073	0.988789316
LRIG3	ENSG00000139263	12	59255931	59324303	129	15	123834	-0.33389	0.63077	0.988789316
DUSP12	ENSG00000081721	1	161709548	161737028	71	11	123834	-0.33398	0.6308	0.988789316
DMKN	ENSG00000161249	19	35978122	36014560	35	11	123834	-0.33427	0.63091	0.988789316
PDE3A	ENSG00000172572	12	20512179	20847315	516	51	123834	-0.33463	0.63105	0.988789316
SLC25A34	ENSG00000162461	1	16052900	16077891	14	3	123834	-0.33475	0.63109	0.988789316
CDH12	ENSG00000154162	5	21740782	22863731	1535	75	123834	-0.33486	0.63114	0.988789316
NAP1L5	ENSG00000177432	4	89607066	89629386	30	7	123834	-0.33503	0.6312	0.988789316
OR5K1	ENSG00000232382	3	98178324	98199420	18	4	123834	-0.33515	0.63124	0.988789316
EHHADH	ENSG00000113790	3	184898412	185009778	137	12	123834	-0.33529	0.6313	0.988789316
PRM1	ENSG00000175646	16	11364693	11385207	34	5	123834	-0.33538	0.63133	0.988789316
CTSK	ENSG00000143387	1	150758684	150790799	23	4	123834	-0.33546	0.63136	0.988789316
BLMH	ENSG00000108578	17	28565218	28629074	68	7	123834	-0.33545	0.63136	0.988789316
SCUBE3	ENSG00000146197	6	35172190	35230856	72	9	123834	-0.33558	0.63141	0.988789316
DENND2C	ENSG00000175984	1	115115469	115223043	185	17	123834	-0.33585	0.63151	0.988789316
PRM2	ENSG00000122304	16	11359496	11380337	31	5	123834	-0.33589	0.63152	0.988789316
PYGM	ENSG00000068976	11	64503861	64537769	17	2	123834	-0.3359	0.63153	0.988789316
OR6C68	ENSG00000205327	12	55876147	55897100	57	6	123834	-0.33592	0.63153	0.988789316
RGS1	ENSG00000090104	1	192534857	192559161	52	7	123834	-0.33597	0.63155	0.988789316
SRSF9	ENSG00000111786	12	120889471	120917596	42	8	123834	-0.33598	0.63156	0.988789316
OR6V1	ENSG00000225781	7	142739417	142760444	44	5	123834	-0.33601	0.63157	0.988789316
RP1-102H19.8	ENSG00000226524	6	88022323	88119459	262	13	123834	-0.33635	0.6317	0.988789316
CASC1	ENSG00000118307	12	25251354	25358096	297	17	123834	-0.33641	0.63172	0.988789316
OR5M10	ENSG00000254834	11	56334212	56355263	55	6	123834	-0.33663	0.6318	0.988789316
RFC4	ENSG00000163918	3	186497669	186534847	41	7	123834	-0.33667	0.63182	0.988789316
CNTNAP5	ENSG00000155052	2	124772864	125682864	1386	84	123834	-0.33689	0.6319	0.988789316
AC124890.1	ENSG00000251695	12	69176125	69197743	22	7	123834	-0.33703	0.63195	0.988789316
ODF2L	ENSG00000122417	1	86804344	86872023	140	11	123834	-0.33717	0.63201	0.988789316
JAG1	ENSG00000101384	20	10608332	10664694	66	16	123834	-0.33717	0.63201	0.988789316
AC105020.1	ENSG00000203392	15	75960672	75983093	24	4	123834	-0.3372	0.63202	0.988789316
HSF4	ENSG00000102878	16	67187288	67213848	11	3	123834	-0.33723	0.63203	0.988789316
PCNT	ENSG00000160299	21	47734036	47875682	366	18	123834	-0.33733	0.63206	0.988789316
GRIK5	ENSG00000105737	19	42492473	42583650	23	2	123834	-0.33736	0.63208	0.988789316
BTBD16	ENSG00000138152	10	124020821	124107677	192	20	123834	-0.33748	0.63212	0.988789316
IDO2	ENSG00000188676	8	39782133	39883910	345	25	123834	-0.33814	0.63237	0.988789316
ERMAP	ENSG00000164010	1	43272795	43320660	40	7	123834	-0.33827	0.63242	0.988789316
GTF2F1	ENSG00000125651	19	6369580	6403992	94	9	123834	-0.33829	0.63243	0.988789316
ENGASE	ENSG00000167280	17	77061021	77094681	43	7	123834	-0.33869	0.63258	0.988789316
PTHLH	ENSG00000087494	12	28101017	28135638	75	12	123834	-0.33875	0.6326	0.988789316
SRSF4	ENSG00000116350	1	29464255	29518499	85	12	123834	-0.33916	0.63276	0.988789316
BLZF1	ENSG00000117475	1	169327208	169375778	140	14	123834	-0.3392	0.63277	0.988789316
CYB5B	ENSG00000103018	16	69448428	69510169	106	13	123834	-0.33989	0.63303	0.988789316
AC007204.1	ENSG00000268461	19	20037769	20058639	54	10	123834	-0.34	0.63307	0.988789316
PDE4B	ENSG00000184588	1	66248197	66850259	864	67	123834	-0.34003	0.63308	0.988789316
NFKBIZ	ENSG00000144802	3	101536835	101589866	31	4	123834	-0.34016	0.63313	0.988789316
RTN2	ENSG00000125744	19	45978547	46010319	57	10	123834	-0.34028	0.63318	0.988789316
SHISA7	ENSG00000187902	19	55930107	55964230	24	7	123834	-0.34042	0.63323	0.988789316
SLC35E1	ENSG00000127526	19	16650642	16693193	42	8	123834	-0.34059	0.63329	0.988789316
TMCO6	ENSG00000113119	5	140009012	140034993	36	4	123834	-0.34077	0.63336	0.988789316
FAM168A	ENSG00000054965	11	73101532	73319234	209	11	123834	-0.34104	0.63346	0.988789316

PCBD2	ENSG00000132570	5	134230596	134353649	82	10	123834	-0.34122	0.63353	0.988789316
FAM181B	ENSG00000182103	11	82433053	82454906	27	5	123834	-0.34129	0.63356	0.988789316
ACCSL	ENSG00000205126	11	44059531	44091527	63	7	123834	-0.34153	0.63365	0.988789316
SBNO1	ENSG00000139697	12	123763656	123859390	253	9	123834	-0.3416	0.63367	0.988789316
SMCHD1	ENSG00000101596	18	2645737	2815015	486	20	123834	-0.34185	0.63377	0.988789316
RP11-297N6.4	ENSG00000255046	8	11645437	11670077	58	11	123834	-0.34218	0.63389	0.988789316
CACTIN	ENSG00000105298	19	3600639	3636813	82	9	123834	-0.34235	0.63395	0.988789316
RNASEK-C17orf49	ENSG00000161939	17	6905954	6930839	29	3	123834	-0.34236	0.63396	0.988789316
CENPC	ENSG00000145241	4	68327521	68421324	233	14	123834	-0.34245	0.63399	0.988789316
MXI1	ENSG00000119950	10	111957363	112057123	199	19	123834	-0.34243	0.63399	0.988789316
GGACT	ENSG00000134864	13	101173810	101251782	67	3	123834	-0.34253	0.63402	0.988789316
TDO2	ENSG00000151790	4	156765890	156851558	270	13	123834	-0.34254	0.63403	0.988789316
MRPL39	ENSG00000154719	21	26947968	26989829	131	7	123834	-0.34278	0.63412	0.988789316
UCK2	ENSG00000143179	1	165786768	165890855	52	9	123834	-0.34285	0.63415	0.988789316
TNFRSF14	ENSG00000157873	1	2477078	2506821	31	3	123834	-0.34298	0.63419	0.988789316
SOHLH2	ENSG00000120669	13	36732345	36881979	448	10	123834	-0.34304	0.63422	0.988789316
CCDC169-SOHLH2	ENSG00000250709	13	36732931	36881979	448	10	123834	-0.34304	0.63422	0.988789316
SYNPO2	ENSG00000172403	4	119799996	119992402	274	31	123834	-0.34307	0.63423	0.988789316
CLLU1	ENSG00000257127	12	92805307	92834778	46	8	123834	-0.34316	0.63426	0.988789316
MAD2L1BP	ENSG00000124688	6	43587277	43618689	31	4	123834	-0.34328	0.6343	0.988789316
ZNF684	ENSG00000117010	1	40987233	41023841	110	9	123834	-0.34343	0.63436	0.988789316
ASXL2	ENSG00000143970	2	25946622	26111385	301	12	123834	-0.34347	0.63438	0.988789316
SKOR2	ENSG00000215474	18	44736293	44785554	58	8	123834	-0.34349	0.63438	0.988789316
TRAPPC2L	ENSG00000167515	16	88912628	88939094	71	6	123834	-0.34354	0.6344	0.988789316
ANXA2R	ENSG00000177721	5	43029335	43053272	39	7	123834	-0.34378	0.6345	0.988789316
CTC-326K19.6	ENSG00000269179	19	50402758	50471648	105	22	123834	-0.34393	0.63455	0.988789316
OR5K3	ENSG00000206536	3	98099510	98120475	58	6	123834	-0.34404	0.63459	0.988789316
C1orf185	ENSG00000204006	1	51557906	51623752	65	4	123834	-0.34406	0.6346	0.988789316
FRAT2	ENSG00000181274	10	99082255	99104458	44	5	123834	-0.34407	0.6346	0.988789316
GSC	ENSG00000133937	14	95224553	95246562	23	8	123834	-0.34417	0.63464	0.988789316
PAXIP1	ENSG00000157212	7	154725397	154804794	91	6	123834	-0.34421	0.63466	0.988789316
HDGF	ENSG00000143321	1	156701899	156746717	70	11	123834	-0.34435	0.63471	0.988789316
ARID3A	ENSG00000116017	19	915781	985939	95	17	123834	-0.34464	0.63482	0.988789316
LACTBL1	ENSG00000215906	1	23269536	23301831	55	10	123834	-0.34476	0.63486	0.988789316
GUCA1B	ENSG00000112599	6	42142139	42172654	100	6	123834	-0.34494	0.63493	0.988789316
THOC6	ENSG00000131652	16	3064028	3087756	13	3	123834	-0.34517	0.63502	0.988789316
TOR2A	ENSG00000160404	9	130483803	130507604	36	6	123834	-0.34523	0.63504	0.988789316
POLD4	ENSG00000175482	11	67108248	67134443	11	1	123834	-0.34533	0.63508	0.988789316
WDR72	ENSG00000166415	15	53795938	54065075	728	31	123834	-0.34536	0.63509	0.988789316
KRTAP5-9	ENSG00000254997	11	71249466	71270653	73	5	123834	-0.34539	0.6351	0.988789316
TNP1	ENSG00000118245	2	217714181	217734787	22	5	123834	-0.34563	0.63519	0.988789316
JMJD1C	ENSG00000171988	10	64916981	65235722	515	16	123834	-0.34563	0.63519	0.988789316
CDC42BPB	ENSG00000198752	14	103388716	103533799	184	18	123834	-0.34567	0.6352	0.988789316
ZIM2	ENSG00000269699	19	57275920	57362097	62	9	123834	-0.34578	0.63525	0.988789316
ATL1	ENSG00000198513	14	50989227	51109786	167	13	123834	-0.34593	0.6353	0.988789316
RPA2	ENSG00000117748	1	28208035	28251257	90	6	123834	-0.34602	0.63534	0.988789316
SPRR2G	ENSG00000159516	1	153112058	153133345	37	3	123834	-0.34605	0.63535	0.988789316
XKR3	ENSG00000172967	22	17254302	17312589	86	9	123834	-0.34605	0.63535	0.988789316
OR6B3	ENSG00000178586	2	240974494	240995489	55	10	123834	-0.34612	0.63537	0.988789316
ZNF705G	ENSG00000215372	8	7203039	7253080	2	1	123834	-0.34644	0.63549	0.988789316
TIGD1	ENSG00000221944	2	233402779	233425226	35	5	123834	-0.34649	0.63551	0.988789316
E2F2	ENSG00000007968	1	23822922	23867712	54	9	123834	-0.34667	0.63558	0.988789316
OR2M2	ENSG00000198601	1	248333288	248354331	82	5	123834	-0.34676	0.63561	0.988789316
AL354808.2	ENSG00000269591	13	20258547	20279240	29	5	123834	-0.34679	0.63563	0.988789316
PRSS54	ENSG00000103023	16	58303901	58338951	137	7	123834	-0.34694	0.63568	0.988789316
PLA2G5	ENSG00000127472	1	20344672	20427683	197	22	123834	-0.34724	0.63579	0.988820095
FKBP8	ENSG00000105701	19	18632561	18664887	8	3	123834	-0.34731	0.63582	0.988820095
WFIKKN2	ENSG00000173714	17	48902011	48929709	36	5	123834	-0.34744	0.63587	0.988820095
OR1Q1	ENSG00000165202	9	125366948	125388026	69	11	123834	-0.34789	0.63604	0.988820095
TROVE2	ENSG00000116747	1	193018552	193070907	59	6	123834	-0.34793	0.63605	0.988820095
C12orf29	ENSG00000133641	12	88417623	88453937	34	6	123834	-0.34796	0.63607	0.988820095
CXCL16	ENSG00000161921	17	4626821	4653217	29	5	123834	-0.34813	0.63613	0.988820095
C1QL3	ENSG00000165985	10	16545742	16574004	41	6	123834	-0.3483	0.63619	0.988820095
ZNF330	ENSG00000109445	4	142132041	142165851	28	1	123834	-0.34832	0.6362	0.988820095
HMBOX1	ENSG00000147421	8	28737911	28932281	153	12	123834	-0.34837	0.63622	0.988820095
USB1	ENSG00000103005	16	58023450	58065522	35	4	123834	-0.34929	0.63656	0.988868756
UGCG	ENSG00000148154	9	114649046	114707649	194	25	123834	-0.34943	0.63662	0.988868756
KCNE1	ENSG00000180509	21	35808988	35894573	124	14	123834	-0.34949	0.63664	0.988868756
CLEC4D	ENSG00000166527	12	8652071	8684962	42	3	123834	-0.34957	0.63667	0.988868756
FBXO16	ENSG00000214050	8	28195804	28357835	52	14	123834	-0.3496	0.63668	0.988868756
SLC34A1	ENSG00000131183	5	176796236	176835849	25	4	123834	-0.34981	0.63676	0.988868756
TBRG4	ENSG00000136270	7	45129699	45161646	92	12	123834	-0.35005	0.63685	0.988868756
KCNJ1	ENSG00000151704	11	128696210	128747268	71	13	123834	-0.35025	0.63692	0.988868756
PBX3	ENSG00000167081	9	128499624	128739656	216	10	123834	-0.35064	0.63707	0.988868756
RIT2	ENSG00000152214	18	40313192	40705657	595	37	123834	-0.3507	0.63709	0.988868756
TLR10	ENSG00000174123	4	38763860	38794611	151	14	123834	-0.35076	0.63712	0.988868756
PRR5L	ENSG00000135362	11	36307838	36496754	430	50	123834	-0.35077	0.63712	0.988868756
LA16c-431H6.6	ENSG00000261732	16	1672260	1746716	86	5	123834	-0.35108	0.63724	0.988868756
STEAP3	ENSG00000115107	2	119971384	120033228	103	15	123834	-0.35124	0.63729	0.988868756
LRRC58	ENSG00000163428	3	120033356	120078186	79	8	123834	-0.35131	0.63732	0.988868756
CCR8	ENSG00000179934	3	39361197	39385171	45	9	123834	-0.35134	0.63733	0.988868756
NDUFA12	ENSG00000184752	12	95280831	95407546	447	35	123834	-0.35139	0.63735	0.988868756
RBL1	ENSG00000080839	20	35614752	35734398	102	10	123834	-0.35143	0.63737	0.988868756
LUZP6	ENSG00000267697	7	135602022	135622198	29	8	123834	-0.35148	0.63739	0.988868756
HNRNPA2B1	ENSG00000122566	7	26219547	26251149	53	5	123834	-0.3516	0.63743	0.988868756
RP11-242G20.1	ENSG00000235645	10	71434655	71462208	99	10	123834	-0.35162	0.63744	0.988868756
CHSY1	ENSG00000131873	15	101705928	101802137	203	21	123834	-0.35177	0.63749	0.988868756
ABCC11	ENSG00000121270	16	48190821	48291479	208	6	123834	-0.35176	0.63749	0.988868756
DHX29	ENSG00000067248	5	54542073	54613550	85	7	123834	-0.35185	0.63752	0.988868756
LTA4H	ENSG00000111144	12	96384606	96447298	120	13	123834	-0.35204	0.6376	0.988868756
RBMS1	ENSG00000153250	2	161118662	161360305	373	25	123834	-0.35215	0.63764	0.988868756
MAGI2	ENSG00000187391	7	77636393	79092890	3081	177	123834	-0.3523	0.63769	0.988868756
AGAP2-AS1	ENSG00000255737	12	58110054	58132139	15	2	123834	-0.35248	0.63776	0.988868756
TEX14	ENSG00000121101	17	56624039	56779416	262	15	123834	-0.35248	0.63776	0.988868756
YARS2	ENSG00000139131	12	32870424	32918836	97	4	123834	-0.35312	0.638	0.989047546
FTH1	ENSG00000167996	11	61717190	61745132	47	10	123834	-0.35321	0.63803	0.989047546
LINC01098	ENSG00000231171	4	178639911	178921904	483	41	123834	-0.35338	0.6381	0.989047546
CYR61	ENSG00000142871	1	86036444	86059645	13	6	123834	-0.35368	0.63821	0.989047546
PTPRB	ENSG00000127329	12	70900630	71041220	281	37	123834	-0.35429	0.63844	0.989047546

RASSF5	ENSG00000136653	1	206670879	206772616	123	18	123834	-0.35456	0.63854	0.989047546
FAM73A	ENSG00000180488	1	78235309	78354106	156	21	123834	-0.35462	0.63856	0.989047546
SSBP3-AS1	ENSG00000198711	1	54693710	54714736	16	4	123834	-0.35522	0.63879	0.989047546
KCNK16	ENSG00000095981	6	39272474	39300744	65	10	123834	-0.35556	0.63892	0.989047546
IL21R	ENSG00000103522	16	27403483	27472115	90	17	123834	-0.35589	0.63904	0.989047546
C11orf52	ENSG00000149300	11	111778756	111807596	14	2	123834	-0.35609	0.63912	0.989047546
APAF1	ENSG00000120868	12	99028919	99139204	158	14	123834	-0.3561	0.63912	0.989047546
POMP	ENSG00000132963	13	29223241	29263062	47	5	123834	-0.35618	0.63915	0.989047546
C6orf58	ENSG00000184530	6	127830600	127922962	210	17	123834	-0.35633	0.6392	0.989047546
SALL3	ENSG00000256463	18	76730275	76772677	44	10	123834	-0.3564	0.63923	0.989047546
VWDE	ENSG00000146530	7	12360511	12453567	302	17	123834	-0.35653	0.63928	0.989047546
PROM2	ENSG00000155066	2	95930201	95967056	14	3	123834	-0.35682	0.63939	0.989047546
OR2G2	ENSG00000177489	1	247741662	247762615	42	5	123834	-0.357	0.63945	0.989047546
HECW2	ENSG00000138411	2	197049094	197468416	695	53	123834	-0.35704	0.63947	0.989047546
HERPUD2	ENSG00000122557	7	35662269	35745181	105	8	123834	-0.35707	0.63948	0.989047546
PRG3	ENSG00000156575	11	57134242	57158623	23	5	123834	-0.35723	0.63954	0.989047546
TYW1	ENSG00000198874	7	66450160	66714501	1064	23	123834	-0.35745	0.63962	0.989047546
BTAF1	ENSG00000095564	10	93673526	93800082	175	12	123834	-0.35783	0.63977	0.989047546
INHBB	ENSG00000163083	2	121093719	121119384	10	4	123834	-0.35787	0.63978	0.989047546
IGSF3	ENSG00000143061	1	117107031	117220375	28	8	123834	-0.35806	0.63985	0.989047546
CDK5R1	ENSG00000176749	17	30803637	30828274	11	3	123834	-0.35818	0.6399	0.989047546
PRPF8	ENSG00000174231	17	1543923	1598176	95	6	123834	-0.35822	0.63991	0.989047546
UBE2Q2L	ENSG00000259511	15	84831242	84860986	6	2	123834	-0.35825	0.63992	0.989047546
OSTF1	ENSG00000134996	9	77693459	77772181	203	27	123834	-0.3584	0.63998	0.989047546
TMEM98	ENSG00000006042	17	31244928	31282124	58	8	123834	-0.35855	0.64003	0.989047546
ACAN	ENSG00000157766	15	89336674	89428585	121	22	123834	-0.35857	0.64004	0.989047546
TAC4	ENSG00000176358	17	47905671	47935379	14	4	123834	-0.359	0.6402	0.989047546
ESRP1	ENSG00000104413	8	95643302	95729694	203	10	123834	-0.35915	0.64026	0.989047546
BIRC3	ENSG00000023445	11	102178215	102220134	12	4	123834	-0.35922	0.64029	0.989047546
TM2D3	ENSG00000184277	15	102151847	102202594	79	8	123834	-0.35955	0.64041	0.989047546
NIPAL2	ENSG00000104361	8	99192061	99316621	130	18	123834	-0.35958	0.64042	0.989047546
GLYR1	ENSG00000140632	16	4843204	4907343	110	13	123834	-0.35961	0.64043	0.989047546
E2F5	ENSG00000133740	8	86079460	86139387	53	5	123834	-0.35976	0.64049	0.989047546
NUP188	ENSG00000095319	9	131699978	131779375	50	3	123834	-0.35977	0.64049	0.989047546
GZMM	ENSG00000197540	19	534034	559919	64	11	123834	-0.35981	0.64051	0.989047546
SCT	ENSG00000070031	11	616431	637143	32	9	123834	-0.36025	0.64067	0.989047546
STEAP1B	ENSG00000105889	7	22449063	22682544	438	43	123834	-0.36034	0.6407	0.989047546
TMEM78	ENSG00000177800	1	229375383	229397557	15	3	123834	-0.36046	0.64075	0.989047546
AIF1L	ENSG00000126878	9	133961863	134008539	67	11	123834	-0.36054	0.64078	0.989047546
CCL24	ENSG00000106178	7	75430983	75462674	108	12	123834	-0.36059	0.6408	0.989047546
TRAPPC3	ENSG00000054116	1	36592173	36625098	27	2	123834	-0.36093	0.64092	0.989047546
PSMC5	ENSG00000087191	17	61894512	61919379	32	3	123834	-0.36092	0.64092	0.989047546
AOAH	ENSG00000136250	7	36542456	36774154	212	29	123834	-0.36093	0.64093	0.989047546
FABP12	ENSG00000197416	8	82427216	82453613	23	3	123834	-0.36105	0.64097	0.989047546
NUP54	ENSG00000138750	4	77025812	77079668	343	9	123834	-0.36117	0.64101	0.989047546
AKT1S1	ENSG00000204673	19	50362295	50391716	28	4	123834	-0.36123	0.64104	0.989047546
NOTO	ENSG00000214513	2	73419386	73449641	64	9	123834	-0.36161	0.64118	0.989047546
AMIGO3	ENSG00000176020	3	49744267	49771349	29	3	123834	-0.36163	0.64119	0.989047546
GMPPB	ENSG00000173540	3	49744277	49771384	29	3	123834	-0.36163	0.64119	0.989047546
ATP5L2	ENSG00000249222	22	43025809	43046607	39	3	123834	-0.36165	0.64119	0.989047546
NRSN1	ENSG00000152954	6	24116350	24165128	116	23	123834	-0.36187	0.64128	0.989047546
HSPA14	ENSG00000187522	10	14870163	14923740	45	7	123834	-0.362	0.64132	0.989047546
KRT85	ENSG00000135443	12	52743790	52771265	63	9	123834	-0.36202	0.64133	0.989047546
LIN54	ENSG00000189308	4	83821126	83944079	192	7	123834	-0.36204	0.64134	0.989047546
HIATL1	ENSG00000148110	9	97126833	97233324	282	13	123834	-0.36205	0.64134	0.989047546
PLEKHA3	ENSG00000116095	2	179335195	179391189	66	9	123834	-0.36225	0.64142	0.989047546
NCOA3	ENSG00000124151	20	46120601	46295621	288	13	123834	-0.36227	0.64142	0.989047546
RFESD	ENSG00000175449	5	94972458	95030477	78	9	123834	-0.36235	0.64146	0.989047546
TLR5	ENSG00000187554	1	223272748	223326624	101	6	123834	-0.36239	0.64147	0.989047546
CD109	ENSG00000156535	6	74395508	74548040	305	17	123834	-0.36245	0.64149	0.989047546
AL160175.1	ENSG00000268457	20	30299001	30319134	15	2	123834	-0.36244	0.64149	0.989047546
DPY30	ENSG00000162961	2	32082878	32274881	289	12	123834	-0.3625	0.64151	0.989047546
LRRN2	ENSG00000170382	1	204576298	204664861	65	10	123834	-0.36257	0.64154	0.989047546
MIEN1	ENSG00000141741	17	37874749	37897040	18	4	123834	-0.36258	0.64154	0.989047546
NARG2	ENSG00000128915	15	60701808	60781359	107	8	123834	-0.36269	0.64158	0.989047546
STXBP1	ENSG00000136854	9	130364544	130467460	68	8	123834	-0.36282	0.64163	0.989047546
PLCG1	ENSG00000124181	20	39755600	39821629	66	11	123834	-0.36293	0.64167	0.989047546
LSM3	ENSG00000170860	3	14209858	14252619	52	8	123834	-0.36333	0.64182	0.989047546
OR51H1P	ENSG00000176904	11	4870886	4891794	36	6	123834	-0.36351	0.64189	0.989047546
L2HGDH	ENSG00000087299	14	50694281	50789266	172	11	123834	-0.36351	0.64189	0.989047546
NEUROD1	ENSG00000162992	2	182527815	182555603	38	6	123834	-0.36361	0.64193	0.989047546
14-Sep	ENSG00000154997	7	55851237	55940482	160	16	123834	-0.36381	0.642	0.989047546
CLMN	ENSG00000165959	14	95638277	95796243	326	23	123834	-0.36381	0.642	0.989047546
SGTB	ENSG00000197860	5	64951755	65028862	83	12	123834	-0.3639	0.64203	0.989047546
ZNF280B	ENSG00000198477	22	22828767	22873505	119	9	123834	-0.36392	0.64204	0.989047546
LRRC69	ENSG00000214954	8	92104060	92241464	248	13	123834	-0.36406	0.64209	0.989047546
CYBRD1	ENSG00000071967	2	172368757	172424643	130	16	123834	-0.36475	0.64235	0.989364706
MAP2	ENSG00000078018	2	210278782	210608842	601	20	123834	-0.36487	0.6424	0.989364706
PI4K2A	ENSG00000155252	10	99334131	99446191	134	12	123834	-0.36535	0.64257	0.989546347
ZNF827	ENSG00000151612	4	146668779	146869787	175	24	123834	-0.36581	0.64275	0.989616885
FAM21A	ENSG00000099290	10	51817648	51903269	29	2	123834	-0.36588	0.64277	0.989616885
NOL10	ENSG00000115761	2	10700892	10840101	495	16	123834	-0.36589	0.64278	0.989616885
DEDD	ENSG00000158796	1	161080764	161112478	28	4	123834	-0.36609	0.64285	0.989616885
CRISPLD1	ENSG00000121005	8	75886750	75956793	153	16	123834	-0.36618	0.64289	0.989616885
L34079.2	ENSG00000268361	19	44048844	44107188	104	19	123834	-0.36688	0.64315	0.989616885
UBXN1	ENSG00000162191	11	62433970	62456567	9	2	123834	-0.3676	0.64341	0.989616885
MCCC1	ENSG00000078070	3	182723006	182843863	183	12	123834	-0.36802	0.64357	0.989616885
SLC35A1	ENSG00000164414	6	88170341	88232054	156	13	123834	-0.36839	0.64371	0.989616885
THYN1	ENSG00000151500	11	134108173	134133264	38	5	123834	-0.36853	0.64376	0.989616885
NF2	ENSG00000186575	22	29989545	30104587	165	14	123834	-0.36866	0.64381	0.989616885
CPEB2	ENSG00000137449	4	14994298	15081777	145	12	123834	-0.36869	0.64382	0.989616885
GUCA1A	ENSG00000048545	6	42113144	42157794	91	10	123834	-0.36868	0.64382	0.989616885
IQCA1	ENSG00000132321	2	237222794	237426185	237	19	123834	-0.36872	0.64383	0.989616885
C7orf31	ENSG00000153790	7	25164316	25229975	190	14	123834	-0.36884	0.64388	0.989616885
ZNF774	ENSG00000196391	15	90885477	90919324	80	7	123834	-0.36888	0.64389	0.989616885
MANSC4	ENSG00000205693	12	27905671	27934209	47	4	123834	-0.369	0.64394	0.989616885
RFFL	ENSG00000092871	17	33323009	33426338	50	6	123834	-0.36935	0.64407	0.989616885
AKAP7	ENSG00000118507	6	131446806	131614675	192	15	123834	-0.36942	0.64409	0.989616885
CAMKMT	ENSG00000143919	2	44579089	45009731	587	18	123834	-0.36957	0.64415	0.989616885

TEX264	ENSG00000164081	3	51686709	51748339	26	7	123834	-0.36983	0.64424	0.989616885
ADGB	ENSG00000118492	6	146910101	147146598	301	31	123834	-0.36981	0.64424	0.989616885
FAM71F2	ENSG00000205085	7	128302342	128336929	132	10	123834	-0.36987	0.64426	0.989616885
PRSS23	ENSG00000150687	11	86492101	86673952	374	40	123834	-0.37	0.64431	0.989616885
OR10H3	ENSG00000171936	19	15842203	15863153	75	8	123834	-0.37021	0.64439	0.989616885
ALS2CR11	ENSG00000155754	2	202342148	202493901	353	17	123834	-0.37035	0.64444	0.989616885
OR11A1	ENSG00000204694	6	29383281	29434848	106	10	123834	-0.3704	0.64446	0.989616885
KIAA1324L	ENSG00000164659	7	86496222	86699015	332	10	123834	-0.37046	0.64448	0.989616885
C3orf58	ENSG00000181744	3	143680640	143777561	242	10	123834	-0.37089	0.64464	0.989616885
GZF1	ENSG00000125812	20	23332787	23363700	90	10	123834	-0.371	0.64468	0.989616885
ZNF423	ENSG00000102935	16	49511435	49901830	542	75	123834	-0.37111	0.64472	0.989616885
OR8B2	ENSG00000204293	11	124242291	124263258	60	2	123834	-0.37127	0.64478	0.989616885
DNALI1	ENSG00000163879	1	38012520	38042458	53	8	123834	-0.37138	0.64482	0.989616885
KIF5B	ENSG00000170759	10	32287938	32355359	117	9	123834	-0.37151	0.64487	0.989616885
CEP290	ENSG00000198707	12	88432793	88545993	119	9	123834	-0.37162	0.64491	0.989616885
FAM115C	ENSG00000170379	7	143308043	143437502	11	2	123834	-0.37168	0.64493	0.989616885
STOML2	ENSG00000165283	9	35089888	35113154	23	5	123834	-0.37178	0.64497	0.989616885
ATP6V1C2	ENSG00000143882	2	10851775	10935236	141	18	123834	-0.3718	0.64498	0.989616885
ACTN4	ENSG00000130402	19	39128289	39232223	241	15	123834	-0.37193	0.64503	0.989616885
AC069547.2	ENSG00000269700	10	52142766	52164321	52	9	123834	-0.37196	0.64504	0.989616885
HEXA	ENSG00000213614	15	72625775	72678817	58	5	123834	-0.37197	0.64504	0.989616885
RAI1	ENSG00000108557	17	17574787	17724767	99	15	123834	-0.37235	0.64519	0.989616885
AC015987.2	ENSG00000236338	7	135601542	135623214	30	8	123834	-0.37242	0.64521	0.989616885
DDA1	ENSG00000130311	19	17410327	17444110	94	10	123834	-0.37287	0.64538	0.989616885
LUM	ENSG00000139329	12	91486406	91515608	84	10	123834	-0.37306	0.64545	0.989616885
ACER3	ENSG00000078124	11	76561911	76747841	425	7	123834	-0.3732	0.6455	0.989616885
COL4A2	ENSG00000134871	13	110948159	111175374	413	62	123834	-0.37342	0.64558	0.989616885
ENG	ENSG00000106991	9	130567291	130627035	54	12	123834	-0.37345	0.64559	0.989616885
C10orf35	ENSG00000171224	10	71380007	71403352	53	6	123834	-0.3735	0.64561	0.989616885
SYT8	ENSG00000149043	11	1838709	1868751	30	3	123834	-0.37356	0.64563	0.989616885
ITGB8	ENSG00000105855	7	20360325	20465377	138	18	123834	-0.37377	0.64571	0.989616885
FZR1	ENSG00000105325	19	3496271	3548328	44	5	123834	-0.37398	0.64579	0.989616885
BRI3BP	ENSG00000184992	12	125468246	125525777	92	12	123834	-0.37402	0.64581	0.989616885
C14orf39	ENSG00000179008	14	60853187	60992261	249	11	123834	-0.37408	0.64583	0.989616885
EXOSC5	ENSG00000077348	19	41882279	41913384	37	2	123834	-0.37423	0.64588	0.989616885
AL390778.1	ENSG00000269408	9	138140512	138161275	10	3	123834	-0.37452	0.64599	0.989616885
CDKAL1	ENSG00000145996	6	20524688	21242635	1075	50	123834	-0.37474	0.64607	0.989616885
FLJ45079	ENSG00000204283	17	75865109	75888659	57	7	123834	-0.37487	0.64612	0.989616885
PSME4	ENSG00000068878	2	54081204	54207977	277	15	123834	-0.37493	0.64614	0.989616885
LDHB	ENSG00000111716	12	21778276	21920791	78	3	123834	-0.37492	0.64614	0.989616885
ACOT12	ENSG00000172497	5	80615824	80699998	132	24	123834	-0.37498	0.64616	0.989616885
CAB39	ENSG00000135932	2	231567560	231695790	131	9	123834	-0.3751	0.64621	0.989616885
MCF2L	ENSG00000126217	13	113538692	113764053	358	47	123834	-0.37588	0.6465	0.989616885
DNAAF3	ENSG00000167646	19	55660031	55688090	83	10	123834	-0.37608	0.64657	0.989616885
FYN	ENSG00000010810	6	111971535	112204655	417	22	123834	-0.37635	0.64667	0.989616885
SF3B3	ENSG00000189091	16	70547691	70618820	61	4	123834	-0.37634	0.64667	0.989616885
AC004899.1	ENSG00000267970	7	48877362	48901221	59	10	123834	-0.3765	0.64673	0.989616885
H6PD	ENSG00000049239	1	9284834	9341396	38	7	123834	-0.37659	0.64676	0.989616885
NR4A2	ENSG00000153234	2	157170944	157208860	21	4	123834	-0.37665	0.64678	0.989616885
GSDMA	ENSG00000167914	17	38109226	38144019	74	9	123834	-0.37671	0.64681	0.989616885
PROX1	ENSG00000117707	1	214146524	214224595	94	13	123834	-0.37686	0.64686	0.989616885
FAIM2	ENSG00000135472	12	50250679	50308000	49	11	123834	-0.37685	0.64686	0.989616885
GOLGB1	ENSG00000173230	3	121372046	121478602	159	14	123834	-0.37698	0.64691	0.989616885
DAZAP2	ENSG00000183283	12	51622076	51675146	74	7	123834	-0.37735	0.64704	0.989616885
GOLGA8N	ENSG00000232653	15	32875657	32906179	1	1	123834	-0.37752	0.64711	0.989616885
CHRDL2	ENSG00000054938	11	74397474	74452430	27	9	123834	-0.37773	0.64718	0.989616885
BLOC1S1	ENSG00000135441	12	56099820	56123871	8	3	123834	-0.37797	0.64727	0.989616885
TBC1D12	ENSG00000108239	10	96152261	96305687	170	11	123834	-0.37829	0.64739	0.989616885
DBN1	ENSG00000113758	5	176873609	176911402	29	4	123834	-0.37843	0.64745	0.989616885
OR9Q1	ENSG00000186509	11	57781353	57959088	366	22	123834	-0.37861	0.64751	0.989616885
MRPS9	ENSG00000135972	2	105644441	105726418	198	6	123834	-0.37933	0.64778	0.989616885
HECTD1	ENSG00000092148	14	31559318	31687010	82	6	123834	-0.37946	0.64783	0.989616885
FREM1	ENSG00000164946	9	14724664	14920993	284	54	123834	-0.37955	0.64786	0.989616885
RTKN2	ENSG00000182010	10	63932794	64038466	175	9	123834	-0.3796	0.64788	0.989616885
RHOT1	ENSG00000126858	17	30459473	30590393	126	7	123834	-0.37964	0.64789	0.989616885
KIF12	ENSG00000136883	9	116838406	116871507	95	13	123834	-0.3798	0.64795	0.989616885
LYRM1	ENSG00000102897	16	20901190	20946328	38	5	123834	-0.37989	0.64798	0.989616885
GPRC6A	ENSG00000173612	6	117103248	117160200	93	10	123834	-0.37996	0.64801	0.989616885
PAX3	ENSG00000135903	2	223054607	223173715	177	23	123834	-0.38001	0.64803	0.989616885
HIST1H2AH	ENSG00000184825	6	27104861	27125317	22	7	123834	-0.38001	0.64803	0.989616885
TUBA3E	ENSG00000152086	2	130939318	130966034	37	2	123834	-0.38019	0.6481	0.989616885
MAP3K5	ENSG00000197442	6	136868185	137123656	196	19	123834	-0.38029	0.64813	0.989616885
PTH2R	ENSG00000144407	2	209214438	209729227	1044	44	123834	-0.38049	0.64821	0.989616885
TMPRSS7	ENSG00000176040	3	111743690	111810116	131	16	123834	-0.38051	0.64822	0.989616885
AUH	ENSG00000148090	9	93966097	94134195	320	16	123834	-0.38057	0.64824	0.989616885
SYT4	ENSG00000132872	18	40837843	40867615	39	9	123834	-0.38071	0.64829	0.989616885
PACRG	ENSG00000112530	6	163138164	163746524	918	80	123834	-0.38073	0.6483	0.989616885
ADAM7	ENSG00000069206	8	24288443	24394483	225	21	123834	-0.38092	0.64837	0.989616885
ABCA4	ENSG00000198691	1	94448393	94596688	267	44	123834	-0.38129	0.64851	0.989616885
FILIP1L	ENSG00000168386	3	99538985	99843357	293	13	123834	-0.38134	0.64852	0.989616885
ATF5	ENSG00000169136	19	50421959	50447192	31	8	123834	-0.38133	0.64852	0.989616885
GLRX5	ENSG00000182512	14	95989840	96021061	38	8	123834	-0.38139	0.64854	0.989616885
ATAD1	ENSG00000138138	10	89501269	89611100	109	3	123834	-0.3815	0.64858	0.989616885
IQUB	ENSG00000164675	7	123082454	123185131	110	9	123834	-0.38154	0.6486	0.989616885
PAQR4	ENSG00000162073	16	3009246	3033490	28	4	123834	-0.38158	0.64861	0.989616885
PRPH2	ENSG00000112619	6	42654340	42700312	102	11	123834	-0.38158	0.64862	0.989616885
APCDD1L	ENSG00000198768	20	57024157	57100187	70	20	123834	-0.38161	0.64862	0.989616885
OR5D14	ENSG00000186113	11	55553032	55573976	60	10	123834	-0.38171	0.64866	0.989616885
PLG	ENSG00000122194	6	161113270	161184347	118	11	123834	-0.38187	0.64872	0.989616885
C19orf12	ENSG00000131943	19	30181721	30216364	59	11	123834	-0.38239	0.64891	0.989616885
AC018755.1	ENSG00000167765	19	52085036	52107630	31	6	123834	-0.38251	0.64896	0.989616885
STC2	ENSG00000113739	5	172731716	172766506	16	5	123834	-0.38275	0.64905	0.989616885
LIPF	ENSG00000182333	10	90414198	90448571	67	13	123834	-0.38278	0.64906	0.989616885
HNRNPA3	ENSG00000170144	2	178067291	178098686	20	3	123834	-0.38295	0.64912	0.989616885
MIER3	ENSG00000155545	5	56205429	56277502	169	12	123834	-0.38321	0.64922	0.989616885
GP9	ENSG00000169704	3	128769610	128791249	11	2	123834	-0.38323	0.64923	0.989616885
LANCL1	ENSG00000115365	2	211285973	211352376	103	6	123834	-0.38328	0.64924	0.989616885
LCE3B	ENSG00000187238	1	152576287	152596574	34	3	123834	-0.38342	0.6493	0.989616885
CCDC79	ENSG00000249961	16	66778879	66845523	86	5	123834	-0.3836	0.64936	0.989616885

LCN6	ENSG00000204003	9	139622619	139652905	77	8	123834	-0.38365	0.64938	0.989616885
SYCP2L	ENSG00000153157	6	10738027	10989553	232	34	123834	-0.38367	0.64939	0.989616885
XRCC2	ENSG00000196584	7	152331864	152383250	136	17	123834	-0.38389	0.64947	0.989616885
CCDC116	ENSG00000161180	22	21977005	22001616	23	6	123834	-0.38406	0.64953	0.989616885
FAM129B	ENSG00000136830	9	130257618	130351268	123	16	123834	-0.38408	0.64954	0.989616885
SFTA2	ENSG00000196260	6	30889130	30909952	48	9	123834	-0.38416	0.64957	0.989616885
STYXL1	ENSG00000127952	7	75615656	75687322	116	11	123834	-0.38432	0.64963	0.989616885
ZCWPW2	ENSG00000206559	3	28380637	28589613	204	13	123834	-0.38444	0.64967	0.989616885
SLC4A7	ENSG00000033867	3	27404214	27535911	336	17	123834	-0.38458	0.64973	0.989616885
UBXN2A	ENSG00000173960	2	24140155	24237779	256	13	123834	-0.38489	0.64984	0.989616885
INTS9	ENSG00000104299	8	28615178	28757759	149	11	123834	-0.38504	0.6499	0.989616885
SGK2	ENSG00000101049	20	42177608	42226877	59	7	123834	-0.3851	0.64992	0.989616885
RALGPS2	ENSG00000116191	1	178684300	178899238	343	16	123834	-0.38524	0.64997	0.989616885
ESRP2	ENSG00000103067	16	68253014	68282005	29	3	123834	-0.38533	0.65	0.989616885
UQCC1	ENSG00000101019	20	33880369	34009944	164	7	123834	-0.38541	0.65003	0.989616885
BEST1	ENSG00000167995	11	61707293	61742987	51	11	123834	-0.38566	0.65012	0.989616885
CCDC19	ENSG00000213085	1	159832154	159879953	71	10	123834	-0.38597	0.65024	0.989616885
CETP	ENSG00000087237	16	56985762	57027757	69	17	123834	-0.38616	0.65031	0.989616885
CMSS1	ENSG00000184220	3	99526678	99907447	331	14	123834	-0.38661	0.65048	0.989616885
C1orf180	ENSG00000180869	1	85083913	85110703	44	9	123834	-0.38667	0.6505	0.989616885
R3HDM4	ENSG00000198858	19	886503	923240	18	6	123834	-0.38679	0.65054	0.989616885
RPRD1A	ENSG00000141425	18	33554350	33657539	132	8	123834	-0.38681	0.65055	0.989616885
ATP5O	ENSG00000241837	21	35265757	35298284	70	10	123834	-0.38689	0.65058	0.989616885
FAM101A	ENSG00000178882	12	124447788	124810570	560	60	123834	-0.38706	0.65064	0.989616885
SNRNP48	ENSG00000168566	6	7580432	7622200	84	10	123834	-0.38707	0.65065	0.989616885
GGCX	ENSG00000115486	2	85761846	85798670	81	12	123834	-0.38735	0.65075	0.989616885
SCN10A	ENSG00000185313	3	38728293	38845501	224	29	123834	-0.38744	0.65078	0.989616885
FLJ00104	ENSG00000205047	16	87721749	87749290	55	10	123834	-0.38749	0.6508	0.989616885
LCN6	ENSG00000267206	9	139628463	139652980	60	6	123834	-0.38783	0.65093	0.989616885
LEMD3	ENSG00000174106	12	65553351	65652107	69	6	123834	-0.38819	0.65106	0.989616885
AC006455.1	ENSG00000230000	7	62799239	62822151	6	2	123834	-0.3882	0.65107	0.989616885
SIX6	ENSG00000184302	14	60965669	60989568	38	4	123834	-0.38824	0.65108	0.989616885
AF131215.5	ENSG00000215346	8	10973980	10997745	48	9	123834	-0.38835	0.65112	0.989616885
OR51E2	ENSG00000167332	11	4691401	4729084	97	15	123834	-0.38839	0.65114	0.989616885
IFT140	ENSG00000187535	16	1550428	1672111	259	14	123834	-0.38864	0.65123	0.989616885
LRRC28	ENSG00000168904	15	99781567	99940934	344	20	123834	-0.38872	0.65126	0.989616885
CMTM8	ENSG00000170293	3	32270171	32421817	263	39	123834	-0.38896	0.65135	0.989616885
AC126614.1	ENSG00000268367	12	57800198	57820536	15	4	123834	-0.38907	0.65139	0.989616885
ZNF776	ENSG00000152443	19	58248164	58279527	47	7	123834	-0.38907	0.65139	0.989616885
PGD	ENSG00000142657	1	10448649	10490201	70	9	123834	-0.38914	0.65141	0.989616885
FAHD2A	ENSG00000115042	2	96058469	96092364	4	1	123834	-0.38931	0.65147	0.989616885
CYP2C8	ENSG00000138115	10	96786530	96839254	175	17	123834	-0.38945	0.65153	0.989616885
ZNF516	ENSG00000101493	18	74059644	74217146	229	26	123834	-0.38947	0.65153	0.989616885
B9D2	ENSG00000123810	19	41850326	41880078	36	4	123834	-0.38964	0.6516	0.989616885
MATN3	ENSG00000132031	2	20181872	20222455	53	10	123834	-0.38966	0.65161	0.989616885
DECR1	ENSG00000104325	8	91003633	91074320	120	9	123834	-0.38973	0.65163	0.989616885
RNF168	ENSG00000163961	3	196185654	196240639	76	7	123834	-0.38982	0.65166	0.989616885
FLOT1	ENSG00000137312	6	30685486	30720510	74	6	123834	-0.3899	0.65169	0.989616885
LHX4	ENSG00000121454	1	180189421	180259380	112	22	123834	-0.39023	0.65182	0.989616885
PDF	ENSG00000258429	16	69352524	69374498	30	5	123834	-0.39031	0.65185	0.989616885
ELP2	ENSG00000134759	18	33699407	33767909	137	11	123834	-0.39039	0.65187	0.989616885
MYSM1	ENSG00000162601	1	59110411	59175764	196	10	123834	-0.39066	0.65197	0.989616885
CECR2	ENSG00000099954	22	17830837	18047850	422	46	123834	-0.39084	0.65204	0.989616885
TNK2	ENSG00000061938	3	195580235	195648816	73	12	123834	-0.39095	0.65208	0.989616885
MAPK8IP2	ENSG00000008735	22	51029114	51062409	80	12	123834	-0.39095	0.65208	0.989616885
AC002472.1	ENSG00000269103	22	21348030	21370736	61	9	123834	-0.39104	0.65212	0.989616885
SULT6B1	ENSG00000138068	2	37384963	37433741	219	12	123834	-0.39106	0.65213	0.989616885
THAP7	ENSG00000184436	22	21343393	21366485	57	9	123834	-0.39117	0.65217	0.989616885
CDA	ENSG00000158825	1	20905441	20955401	175	17	123834	-0.39122	0.65218	0.989616885
RP11-1407O15.2	ENSG00000174093	17	36327717	36431858	21	2	123834	-0.39137	0.65224	0.989616885
PGBD4	ENSG00000182405	15	34384274	34406591	39	5	123834	-0.3914	0.65225	0.989616885
RP11-723O4.6	ENSG00000187695	3	128618717	128700173	150	15	123834	-0.3915	0.65228	0.989616885
FAM182B	ENSG00000175170	20	25734102	25858861	107	8	123834	-0.3916	0.65232	0.989616885
BMP3	ENSG00000152785	4	81942119	81988685	69	8	123834	-0.39172	0.65237	0.989616885
RP11-585F1.10	ENSG00000250210	8	132091	160563	2	1	123834	-0.39175	0.65238	0.989616885
ATF1	ENSG00000123268	12	51147493	51224905	107	9	123834	-0.39182	0.6524	0.989616885
SHCBP1L	ENSG00000157060	1	182859000	182932660	68	7	123834	-0.39198	0.65246	0.989616885
PIWIL2	ENSG00000197181	8	22122810	22225076	171	9	123834	-0.39211	0.65251	0.989616885
RDH10	ENSG00000121039	8	74196847	74247516	89	11	123834	-0.39242	0.65263	0.989616885
NEIL2	ENSG00000154328	8	11617148	11654855	83	12	123834	-0.39249	0.65265	0.989616885
AL031666.2	ENSG00000267882	20	45937246	45959467	8	2	123834	-0.39255	0.65267	0.989616885
NOMO3	ENSG00000103226	16	16316352	16398668	13	2	123834	-0.39257	0.65268	0.989616885
MDH1	ENSG00000014641	2	63805743	63844331	46	5	123834	-0.39355	0.65304	0.989616885
TOR1AIP2	ENSG00000169905	1	179799102	179856938	86	10	123834	-0.39394	0.65319	0.989616885
ATL2	ENSG00000119787	2	38512022	38614427	134	10	123834	-0.39394	0.65319	0.989616885
MURC	ENSG00000170681	9	103330361	103360188	76	10	123834	-0.39403	0.65322	0.989616885
SPATA32	ENSG00000184361	17	43321760	43349479	24	6	123834	-0.39404	0.65323	0.989616885
LUC7L	ENSG00000007392	16	228968	289462	43	5	123834	-0.39444	0.65337	0.989616885
ZNF142	ENSG00000115568	2	219492639	219534378	53	4	123834	-0.39476	0.65349	0.989616885
ADORA2B	ENSG00000170425	17	15838231	15889060	45	4	123834	-0.39477	0.65349	0.989616885
SLX1A	ENSG00000132207	16	30195208	30218882	2	1	123834	-0.39494	0.65355	0.989616885
SULT1A3	ENSG00000261052	16	30195754	30225631	2	1	123834	-0.39494	0.65355	0.989616885
LENG8	ENSG00000167615	19	54950065	54983217	73	11	123834	-0.39515	0.65364	0.989616885
CELA2B	ENSG00000215704	1	15782404	15827895	100	13	123834	-0.39567	0.65383	0.989616885
LDHD	ENSG00000166816	16	75135758	75160669	7	2	123834	-0.3957	0.65384	0.989616885
PPAP2A	ENSG00000067113	5	54710682	54840878	252	8	123834	-0.39575	0.65385	0.989616885
WDFY4	ENSG00000128815	10	49882921	50201001	660	49	123834	-0.39581	0.65388	0.989616885
SULT2B1	ENSG00000088002	19	49045332	49112682	151	23	123834	-0.39611	0.65399	0.989616885
RHBDD3	ENSG00000100263	22	29645841	29674198	35	10	123834	-0.39621	0.65403	0.989616885
KRTAP9-4	ENSG00000241595	17	39395939	39416904	56	3	123834	-0.39647	0.65412	0.989616885
ZNF117	ENSG00000152926	7	64422154	64477062	144	7	123834	-0.39651	0.65414	0.989616885
SRM	ENSG00000116649	1	11104641	11130081	47	4	123834	-0.39659	0.65417	0.989616885
KRTAP5-1	ENSG00000205869	11	1595572	1616513	30	6	123834	-0.39683	0.65425	0.989616885
PSRC1	ENSG00000134222	1	109812178	109835808	40	9	123834	-0.39694	0.6543	0.989616885
TPGS2	ENSG00000134779	18	34349987	34419158	77	4	123834	-0.39705	0.65434	0.989616885
GIPC2	ENSG00000137960	1	78435226	78614133	317	23	123834	-0.39715	0.65437	0.989616885
POLR3E	ENSG00000058600	16	22298730	22356424	46	8	123834	-0.39735	0.65445	0.989616885
SEMG2	ENSG00000124157	20	43839941	43863099	32	3	123834	-0.39759	0.65453	0.989616885
AC004017.1	ENSG00000268948	19	58193868	58214128	14	4	123834	-0.39761	0.65454	0.989616885

RP11-508N12.4	ENSG00000242631	9	109675630	109696455	10	2	123834	-0.3977	0.65458	0.989616885
AC011308.1	ENSG00000268857	2	157342597	157362655	5	3	123834	-0.3979	0.65465	0.989616885
SLC39A1	ENSG00000143570	1	153921575	153950188	19	3	123834	-0.39817	0.65475	0.989616885
G6PC	ENSG00000131482	17	41042814	41075386	31	4	123834	-0.39818	0.65475	0.989616885
STAC3	ENSG00000185482	12	57627236	57654976	7	3	123834	-0.39821	0.65476	0.989616885
METTL16	ENSG00000127804	17	2298856	2425185	183	21	123834	-0.39857	0.6549	0.989616885
ECE2	ENSG00000145194	3	183957438	184020819	65	9	123834	-0.39863	0.65492	0.989616885
CLEC4G	ENSG00000182566	19	7783843	7808792	23	5	123834	-0.39868	0.65493	0.989616885
KLHL5	ENSG00000109790	4	39036659	39138477	232	8	123834	-0.39869	0.65494	0.989616885
C2orf16	ENSG00000221843	2	27789389	27815588	24	4	123834	-0.39879	0.65498	0.989616885
C8orf88	ENSG00000253250	8	91960865	92007485	23	2	123834	-0.39879	0.65498	0.989616885
NUFIP2	ENSG00000108256	17	27572854	27631136	103	11	123834	-0.39892	0.65502	0.989616885
AFMID	ENSG00000183077	17	76173398	76213782	85	20	123834	-0.3989	0.65502	0.989616885
C8orf12	ENSG00000184608	8	11215911	11306167	136	23	123834	-0.39899	0.65505	0.989616885
GUCA2B	ENSG00000044012	1	42609092	42631495	27	5	123834	-0.39902	0.65506	0.989616885
GATAD2B	ENSG00000143614	1	153767201	153905451	178	10	123834	-0.3991	0.65509	0.989616885
EGLN3	ENSG00000129521	14	34383437	34941980	897	97	123834	-0.39925	0.65515	0.989616885
NCK1	ENSG00000158092	3	136571050	136678665	179	5	123834	-0.39939	0.6552	0.989616885
C9orf47	ENSG00000186354	9	91595778	91621055	31	8	123834	-0.39959	0.65527	0.989616885
AARSD1	ENSG00000266967	17	41092543	41126515	22	3	123834	-0.3998	0.65535	0.989616885
KCNJ2	ENSG00000123700	17	68154814	68186189	18	3	123834	-0.39983	0.65536	0.989616885
ZNF709	ENSG00000242852	19	12561998	12634668	174	9	123834	-0.39986	0.65537	0.989616885
DBP	ENSG00000105516	19	49123287	49150695	31	4	123834	-0.40046	0.65559	0.989616885
ZNF654	ENSG00000175105	3	88178254	88203815	40	2	123834	-0.40064	0.65566	0.989616885
PDGFB	ENSG00000100311	22	39609364	39650756	69	7	123834	-0.40115	0.65584	0.989616885
ZNF311	ENSG00000197935	6	28952562	28983093	44	11	123834	-0.40121	0.65587	0.989616885
AC092291.2	ENSG00000269049	16	14554842	14575054	8	2	123834	-0.40128	0.65589	0.989616885
WSB1	ENSG00000109046	17	25611102	25650657	60	9	123834	-0.40142	0.65594	0.989616885
NPAT	ENSG00000149308	11	108017942	108103369	75	5	123834	-0.40149	0.65597	0.989616885
SLC15A5	ENSG00000188991	12	16331419	16440619	301	14	123834	-0.40163	0.65602	0.989616885
PLCD4	ENSG00000115556	2	219462488	219511907	71	4	123834	-0.4017	0.65605	0.989616885
POLG2	ENSG00000256525	17	62463902	62503154	62	3	123834	-0.40198	0.65615	0.989616885
ZFP82	ENSG00000181007	19	36864022	36919558	72	6	123834	-0.40201	0.65616	0.989616885
S100A7	ENSG00000143556	1	153420220	153443177	34	4	123834	-0.40219	0.65623	0.989616885
IL36A	ENSG00000136694	2	113753038	113775621	30	5	123834	-0.40231	0.65627	0.989616885
MAN2B1	ENSG00000104774	19	12747325	12787556	19	3	123834	-0.40234	0.65628	0.989616885
OR10T2	ENSG00000186306	1	158358312	158379256	54	9	123834	-0.40237	0.6563	0.989616885
C16orf47	ENSG00000197445	16	73150536	73188346	56	8	123834	-0.40246	0.65633	0.989616885
IDE	ENSG00000119912	10	94201441	94343833	277	20	123834	-0.40254	0.65636	0.989616885
CDAN1	ENSG00000140326	15	43005757	43039324	36	2	123834	-0.40276	0.65644	0.989616885
ATRAID	ENSG00000138085	2	27424895	27450046	49	5	123834	-0.4028	0.65645	0.989616885
CDKL2	ENSG00000138769	4	76493215	76565900	132	9	123834	-0.40285	0.65647	0.989616885
RP11-480I12.4	ENSG00000184774	1	202779394	202806353	70	7	123834	-0.40332	0.65664	0.989616885
FAM183B	ENSG00000164556	7	38714946	38736637	49	11	123834	-0.40338	0.65667	0.989616885
OR5AN1	ENSG00000176495	11	59121885	59142921	53	3	123834	-0.40341	0.65668	0.989616885
KRTAP9-8	ENSG00000187272	17	39384297	39405256	40	3	123834	-0.40343	0.65668	0.989616885
ANO4	ENSG00000151572	12	101101304	101532419	713	63	123834	-0.40352	0.65672	0.989616885
PRKCE	ENSG00000171132	2	45868484	46425129	803	115	123834	-0.40357	0.65674	0.989616885
ZNF670	ENSG00000135747	1	247098849	247252113	432	21	123834	-0.4037	0.65678	0.989616885
KNDC1	ENSG00000171798	10	134963951	135049916	229	23	123834	-0.40371	0.65679	0.989616885
C2orf74	ENSG00000237651	2	61362243	61401964	106	6	123834	-0.40374	0.6568	0.989616885
TAOK3	ENSG00000135090	12	118577606	118820750	340	14	123834	-0.40403	0.6569	0.989616885
C1orf94	ENSG00000142698	1	34622484	34694732	121	14	123834	-0.4041	0.65693	0.989616885
TMEM248	ENSG00000106609	7	66376212	66433538	114	10	123834	-0.40427	0.65699	0.989616885
PSAP	ENSG00000197746	10	73566055	73621126	130	18	123834	-0.40428	0.657	0.989616885
CASP14	ENSG00000105141	19	15150195	15179104	101	11	123834	-0.40439	0.65704	0.989616885
SYNGR2	ENSG00000108639	17	76154639	76179608	60	14	123834	-0.40453	0.65709	0.989616885
CD53	ENSG00000143119	1	111405775	111452550	70	9	123834	-0.40471	0.65715	0.989616885
GUCD1	ENSG00000138867	22	24926406	24961903	58	6	123834	-0.4047	0.65715	0.989616885
KLF17	ENSG00000171872	1	44574522	44610812	56	11	123834	-0.40475	0.65717	0.989616885
MEGF10	ENSG00000145794	5	126616523	126811429	491	22	123834	-0.40486	0.65721	0.989616885
FLRT3	ENSG00000125848	20	14293634	14328262	56	10	123834	-0.40514	0.65731	0.989616885
NDUFB1	ENSG00000183648	14	92572466	92598261	95	8	123834	-0.40535	0.65739	0.989616885
CKAP4	ENSG00000136026	12	106621655	106708057	84	21	123834	-0.40544	0.65742	0.989616885
PARM1	ENSG00000169116	4	75848305	75985325	149	15	123834	-0.40551	0.65745	0.989616885
OR52K2	ENSG00000181963	11	4460525	4481591	26	6	123834	-0.40576	0.65754	0.989616885
USP5	ENSG00000111667	12	6951292	6985796	6	1	123834	-0.40583	0.65757	0.989616885
TPI1	ENSG00000111669	12	6966283	6990112	6	1	123834	-0.40583	0.65757	0.989616885
COL12A1	ENSG00000111799	6	75784042	75925767	110	13	123834	-0.40589	0.65759	0.989616885
MGAT2	ENSG00000168282	14	50077489	50100198	53	5	123834	-0.40596	0.65761	0.989616885
ASGR1	ENSG00000141505	17	7066750	7092883	18	5	123834	-0.40642	0.65778	0.989716001
XKR7	ENSG00000260903	20	30545805	30601190	43	10	123834	-0.4064	0.65778	0.989716001
KCNN2	ENSG00000080709	5	113686642	113842337	224	32	123834	-0.40661	0.65785	0.989742979
TBX18	ENSG00000112837	6	85387069	85484237	188	17	123834	-0.40684	0.65794	0.989760608
NF1	ENSG00000196712	17	29411945	29719134	271	4	123834	-0.40707	0.65802	0.989760608
JUNB	ENSG00000171223	19	12892310	12914124	25	4	123834	-0.40711	0.65804	0.989760608
SLAMF7	ENSG00000026751	1	160699037	160734611	29	6	123834	-0.40721	0.65807	0.989760608
DIRAS2	ENSG00000165023	9	93362114	93415386	110	16	123834	-0.40748	0.65817	0.989832689
C5orf52	ENSG00000187658	5	157088561	157117151	25	7	123834	-0.40772	0.65826	0.989889722
LRP2BP	ENSG00000109771	4	186275032	186327053	75	12	123834	-0.40787	0.65832	0.989901634
KRTAP19-5	ENSG00000186977	21	31863975	31884435	22	2	123834	-0.40814	0.65842	0.989973688
SELENBP1	ENSG00000143416	1	151326778	151355209	21	4	123834	-0.40845	0.65853	0.990021294
EIF4EBP3	ENSG00000243056	5	139917251	139939163	16	4	123834	-0.40868	0.65861	0.990021294
AIP	ENSG00000110711	11	67240512	67268574	11	3	123834	-0.40875	0.65864	0.990021294
SPPL2A	ENSG00000138600	15	50989506	51068005	188	14	123834	-0.4088	0.65866	0.990021294
NDUFAB1	ENSG00000004779	16	23582323	23617677	82	9	123834	-0.40939	0.65887	0.990258635
JAKMIP2	ENSG00000176049	5	146957990	147172338	280	15	123834	-0.40979	0.65902	0.990386799
NKX6-2	ENSG00000148826	10	134588297	134609556	21	5	123834	-0.41012	0.65914	0.990386799
EPHB2	ENSG00000133216	1	23027332	23251818	113	24	123834	-0.41021	0.65917	0.990386799
CDK15	ENSG00000138395	2	202645184	202770273	124	22	123834	-0.41023	0.65918	0.990386799
INO80	ENSG00000128908	15	41261078	41418552	94	5	123834	-0.41042	0.65925	0.990386799
EXOC6	ENSG00000138190	10	94580935	94829250	392	19	123834	-0.41059	0.65931	0.990386799
MT1A	ENSG00000205362	16	56662578	56683999	77	11	123834	-0.41061	0.65932	0.990386799
PTGFR	ENSG00000122420	1	78759568	79015434	418	34	123834	-0.41085	0.65941	0.99044372
OSBPL5	ENSG00000021762	11	3098346	3197969	192	33	123834	-0.41104	0.65948	0.990470594
C21orf91	ENSG00000154642	21	19151284	19201703	57	6	123834	-0.41143	0.65962	0.990569379
CD69	ENSG00000110848	12	9895082	9923497	79	8	123834	-0.41152	0.65965	0.990569379
CTD-2583A14.9	ENSG00000269476	19	58378133	58437959	67	7	123834	-0.41213	0.65988	0.990622219
AC092782.1	ENSG00000269795	1	243378058	243405911	39	5	123834	-0.41217	0.65989	0.990622219

GAB4	ENSG00000215568	22	17432826	17499112	171	13	123834	-0.41218	0.6599	0.990622219
KCTD7	ENSG00000243335	7	66083868	66286446	554	15	123834	-0.4123	0.65994	0.990622219
GULP1	ENSG00000144366	2	189146396	189470653	474	22	123834	-0.41246	0.66	0.990622219
CST3	ENSG00000101439	20	23598534	23629110	28	2	123834	-0.41249	0.66001	0.990622219
CHD7	ENSG00000171316	8	61581337	61789465	247	12	123834	-0.41259	0.66005	0.990622219
NMS	ENSG00000204640	2	101076944	101109742	133	10	123834	-0.41288	0.66015	0.990650877
C19orf71	ENSG00000183397	19	3529152	3554028	20	4	123834	-0.41315	0.66025	0.990650877
HSPH1	ENSG00000120694	13	31700762	31746525	71	7	123834	-0.41326	0.66029	0.990650877
SLC5A6	ENSG00000138074	2	27412455	27445826	68	6	123834	-0.41348	0.66037	0.990650877
ATPAF1	ENSG00000123472	1	47088409	47149539	77	3	123834	-0.41365	0.66044	0.990650877
TGM7	ENSG00000159495	15	43558478	43604453	33	4	123834	-0.4137	0.66045	0.990650877
COL6A2	ENSG00000142173	21	47508011	47562763	88	16	123834	-0.41371	0.66046	0.990650877
CNOT11	ENSG00000158435	2	101859264	101896778	32	6	123834	-0.41409	0.6606	0.990650877
SLC6A19	ENSG00000174358	5	1191710	1235232	49	9	123834	-0.41421	0.66064	0.990650877
SFTPB	ENSG00000168878	2	85874437	85905864	20	5	123834	-0.41424	0.66065	0.990650877
SPTLC2	ENSG00000100596	14	77962340	78093116	301	32	123834	-0.41434	0.66069	0.990650877
OR10K2	ENSG00000180708	1	158379718	158400656	52	4	123834	-0.41446	0.66073	0.990650877
FGF10	ENSG00000070193	5	44293646	44399808	158	9	123834	-0.41501	0.66093	0.990650877
GJA3	ENSG00000121743	13	20702394	20745188	64	10	123834	-0.41525	0.66102	0.990650877
ZZEF1	ENSG00000074755	17	3897739	4056314	417	14	123834	-0.41594	0.66127	0.990650877
ZMAT3	ENSG00000172667	3	178725011	178800067	103	12	123834	-0.41596	0.66128	0.990650877
CBX1	ENSG00000108468	17	46137414	46188883	133	13	123834	-0.41683	0.6616	0.990650877
ANO2	ENSG00000047617	12	5631035	6065398	813	74	123834	-0.41705	0.66168	0.990650877
FBXO42	ENSG00000037637	1	16563334	16688949	211	8	123834	-0.41727	0.66176	0.990650877
OR52E2	ENSG00000176787	11	5069880	5090857	154	10	123834	-0.41734	0.66179	0.990650877
LIMK1	ENSG00000106683	7	73487263	73546855	77	9	123834	-0.41766	0.6619	0.990650877
NFKB2	ENSG00000077150	10	104143867	104172281	34	11	123834	-0.41798	0.66202	0.990650877
THSD7A	ENSG00000005108	7	11399984	11881824	872	66	123834	-0.41801	0.66203	0.990650877
RAB40B	ENSG00000141542	17	80602849	80666604	94	16	123834	-0.41813	0.66207	0.990650877
ZNF724P	ENSG00000196081	19	23394401	23443192	93	5	123834	-0.41814	0.66208	0.990650877
POP5	ENSG00000167272	12	121006567	121029201	58	9	123834	-0.41818	0.66209	0.990650877
PRKCDBP	ENSG00000170955	11	6330176	6351877	50	9	123834	-0.41821	0.6621	0.990650877
CLEC3B	ENSG00000163815	3	45033040	45087563	97	7	123834	-0.41824	0.66212	0.990650877
SORCS3	ENSG00000156395	10	106390859	107034993	1178	40	123834	-0.41868	0.66227	0.990650877
PRELID2	ENSG00000186314	5	144841362	145224932	597	19	123834	-0.41894	0.66237	0.990650877
CLEC4F	ENSG00000152672	2	71025775	71057732	121	8	123834	-0.41921	0.66247	0.990650877
KIDINS220	ENSG00000134313	2	8855408	8987760	137	15	123834	-0.41961	0.66261	0.990650877
PADI1	ENSG00000142623	1	17521621	17582500	102	21	123834	-0.41993	0.66273	0.990650877
LITAF	ENSG00000189067	16	11631853	11740237	194	30	123834	-0.41999	0.66275	0.990650877
HOXD11	ENSG00000128713	2	176958944	176984722	18	4	123834	-0.42005	0.66278	0.990650877
TP53	ENSG00000141510	17	7555097	7600856	61	10	123834	-0.42028	0.66286	0.990650877
GPR75	ENSG00000119737	2	54070050	54097126	47	8	123834	-0.42042	0.66291	0.990650877
ARHGAP29	ENSG00000137962	1	94604544	94750624	106	14	123834	-0.4207	0.66301	0.990650877
TTC26	ENSG00000105948	7	138808490	138886732	128	5	123834	-0.42077	0.66304	0.990650877
IFT52	ENSG00000101052	20	42209571	42285936	145	7	123834	-0.42134	0.66325	0.990650877
FANCG	ENSG00000221829	9	35063832	35090013	29	5	123834	-0.42144	0.66328	0.990650877
FRYL	ENSG00000075539	4	48489378	48792339	268	11	123834	-0.42151	0.66331	0.990650877
HLA-DOB	ENSG00000241106	6	32770540	32794825	190	21	123834	-0.4217	0.66338	0.990650877
NCAPH2	ENSG00000025770	22	50936645	50971901	74	10	123834	-0.4218	0.66341	0.990650877
OSGEPL1	ENSG00000128694	2	190601386	190637953	49	6	123834	-0.42192	0.66346	0.990650877
HPCAL1	ENSG00000115756	2	10433015	10577743	234	44	123834	-0.42204	0.6635	0.990650877
RHOBTB2	ENSG00000008853	8	22834930	22887712	50	10	123834	-0.4223	0.6636	0.990650877
PYCRL	ENSG00000104524	8	144676083	144701943	51	9	123834	-0.42257	0.66369	0.990650877
ST8SIA6	ENSG00000148488	10	17350382	17506329	489	32	123834	-0.42266	0.66373	0.990650877
MDH1B	ENSG00000138400	2	207592487	207640271	148	11	123834	-0.42273	0.66376	0.990650877
FOXQ1	ENSG00000164379	6	1302675	1324992	24	6	123834	-0.42276	0.66377	0.990650877
FAM171A1	ENSG00000148468	10	15243642	15423061	337	27	123834	-0.42284	0.66379	0.990650877
LAIR2	ENSG00000167618	19	54999100	55031897	69	14	123834	-0.42291	0.66382	0.990650877
DPYSL5	ENSG00000157851	2	27060615	27183219	136	8	123834	-0.42295	0.66383	0.990650877
CYP2R1	ENSG00000186104	11	14889553	14923798	15	4	123834	-0.42298	0.66385	0.990650877
FAM117B	ENSG00000138439	2	203489901	203644480	88	11	123834	-0.42302	0.66386	0.990650877
RALGDS	ENSG00000160271	9	135963107	136049301	108	19	123834	-0.42323	0.66394	0.990650877
TMTC1	ENSG00000133687	12	29643773	29947692	705	62	123834	-0.42332	0.66397	0.990650877
ZNF605	ENSG00000196458	12	133488019	133542892	18	5	123834	-0.42348	0.66403	0.990650877
SPATA12	ENSG00000186451	3	57084469	57119460	50	9	123834	-0.42388	0.66417	0.990650877
ITGA7	ENSG00000135424	12	56068352	56119827	31	3	123834	-0.42389	0.66418	0.990650877
DSC2	ENSG00000134755	18	28635940	28692378	42	8	123834	-0.42412	0.66426	0.990650877
GSG2	ENSG00000177602	17	3617211	3640067	45	4	123834	-0.42422	0.6643	0.990650877
SLCO4C1	ENSG00000173930	5	101559690	101642253	245	23	123834	-0.42445	0.66438	0.990650877
TNP2	ENSG00000178279	16	11351605	11373390	36	5	123834	-0.4245	0.6644	0.990650877
PGLYRP1	ENSG00000008438	19	46512411	46536323	26	4	123834	-0.42449	0.6644	0.990650877
RGS8	ENSG00000135824	1	182605239	182663711	104	12	123834	-0.42456	0.66442	0.990650877
NXF1	ENSG00000162231	11	62549595	62583774	25	2	123834	-0.42467	0.66446	0.990650877
ZNF709	ENSG00000196826	19	12562373	12672288	179	11	123834	-0.42468	0.66446	0.990650877
IL23R	ENSG00000162594	1	67622083	67735662	180	22	123834	-0.42479	0.6645	0.990650877
TTN	ENSG00000155657	2	179380716	179705529	331	22	123834	-0.42482	0.66452	0.990650877
CCDC50	ENSG00000152492	3	191036866	191126459	262	12	123834	-0.42498	0.66458	0.990650877
KMO	ENSG00000117009	1	241685434	241768944	105	15	123834	-0.42517	0.66464	0.990650877
ODC1	ENSG00000115758	2	10570094	10598630	19	4	123834	-0.42539	0.66473	0.990650877
PGF	ENSG00000119630	14	75398537	75432487	29	7	123834	-0.42555	0.66478	0.990650877
AK1	ENSG00000106992	9	130618759	130650022	30	9	123834	-0.42558	0.66479	0.990650877
BCL2A1	ENSG00000140379	15	80243231	80273788	22	2	123834	-0.42575	0.66485	0.990650877
VCP	ENSG00000165280	9	35046061	35083246	48	6	123834	-0.42619	0.66502	0.990650877
NOTUM	ENSG00000185269	17	79900388	79929716	26	4	123834	-0.42624	0.66503	0.990650877
C21orf128	ENSG00000184385	21	43512244	43538644	89	12	123834	-0.42639	0.66509	0.990650877
FGR	ENSG00000000938	1	27928575	27971788	29	4	123834	-0.42648	0.66512	0.990650877
CTBS	ENSG00000117151	1	85005289	85050163	96	11	123834	-0.42656	0.66515	0.990650877
HS6ST3	ENSG00000185352	13	96733093	97495671	1244	56	123834	-0.42671	0.6652	0.990650877
PPM1D	ENSG00000170836	17	58667544	58751849	3	1	123834	-0.42701	0.66531	0.990650877
TNFRSF18	ENSG00000186891	1	1128888	1152071	55	8	123834	-0.42711	0.66535	0.990650877
TMEM204	ENSG00000131634	16	1568689	1615581	113	11	123834	-0.42742	0.66546	0.990650877
CCDC88A	ENSG00000115355	2	55504978	55657057	351	16	123834	-0.42756	0.66551	0.990650877
ADRBK2	ENSG00000100077	22	25950816	26135261	332	10	123834	-0.42756	0.66551	0.990650877
ZMYM5	ENSG00000132950	13	20387622	20447776	156	6	123834	-0.42766	0.66555	0.990650877
SPDYE4	ENSG00000183318	17	8646424	8671877	21	5	123834	-0.42789	0.66563	0.990650877
AC005841.1	ENSG00000206044	12	2948397	2976213	16	3	123834	-0.42794	0.66565	0.990650877
MAPK7	ENSG00000166484	17	19271034	19296857	11	3	123834	-0.42814	0.66573	0.990650877
NT5C3A	ENSG00000122643	7	33043742	33112409	184	8	123834	-0.42821	0.66575	0.990650877
CSNK1G2	ENSG00000133275	19	1931188	1991337	133	13	123834	-0.42822	0.66575	0.990650877

LRRC17	ENSG00000128606	7	102543438	102595396	58	6	123834	-0.42851	0.66586	0.990650877
OGN	ENSG00000106809	9	95136249	95176978	69	7	123834	-0.42859	0.66589	0.990650877
CA12	ENSG00000074410	15	63603577	63684360	112	19	123834	-0.4286	0.66589	0.990650877
RP11-553A10.1	ENSG00000206532	3	110597231	110622323	38	7	123834	-0.42881	0.66597	0.990650877
LYZL4	ENSG00000157093	3	42428570	42462092	15	4	123834	-0.42893	0.66601	0.990650877
FKBP1A	ENSG00000088832	20	1339622	1383806	64	10	123834	-0.429	0.66604	0.990650877
SDR39U1	ENSG00000100445	14	24898972	24922111	128	6	123834	-0.42903	0.66605	0.990650877
ANKFN1	ENSG00000153930	17	54178319	54599020	628	40	123834	-0.42908	0.66607	0.990650877
PAM16	ENSG00000217930	16	4371550	4415608	57	8	123834	-0.42922	0.66612	0.990650877
FMO3	ENSG00000007933	1	171050018	171096959	141	15	123834	-0.42937	0.66617	0.990650877
GJC3	ENSG00000176402	7	99510892	99537243	46	6	123834	-0.42955	0.66624	0.990650877
IER2	ENSG00000160888	19	13251229	13275722	29	6	123834	-0.42964	0.66627	0.990650877
DPP6	ENSG00000130226	7	153574182	154695995	1677	185	123834	-0.4297	0.66629	0.990650877
APIP	ENSG00000149089	11	34864641	34948046	387	13	123834	-0.42986	0.66635	0.990650877
ANO1	ENSG00000131620	11	69914408	70045634	313	37	123834	-0.43031	0.66651	0.990650877
SCN2B	ENSG00000149575	11	118022666	118057388	54	5	123834	-0.43049	0.66658	0.990650877
NDRG2	ENSG00000165795	14	21474922	21549031	58	19	123834	-0.43054	0.6666	0.990650877
USP33	ENSG00000077254	1	78151672	78235537	100	13	123834	-0.43063	0.66663	0.990650877
GSTT2	ENSG00000099984	22	24312339	24336106	4	2	123834	-0.43068	0.66665	0.990650877
CCDC177	ENSG00000255994	14	70027483	70048032	31	6	123834	-0.43081	0.6667	0.990650877
POTEF	ENSG00000196604	2	130821108	130896795	26	2	123834	-0.43111	0.6668	0.990650877
AP001024.2	ENSG00000214306	11	107633129	107657344	36	8	123834	-0.43127	0.66686	0.990650877
SIX1	ENSG00000126778	14	61100133	61134977	30	5	123834	-0.43157	0.66697	0.990650877
GPR171	ENSG00000174946	3	150905619	150930988	52	7	123834	-0.43221	0.66721	0.990650877
FABP6	ENSG00000170231	5	159604374	159675742	215	21	123834	-0.43226	0.66722	0.990650877
FBXL16	ENSG00000127585	16	732500	765829	27	4	123834	-0.43258	0.66734	0.990650877
TAAR8	ENSG00000146385	6	132863832	132884860	37	6	123834	-0.43268	0.66738	0.990650877
LMBR1	ENSG00000105983	7	156451646	156695924	660	21	123834	-0.4327	0.66738	0.990650877
CYP26C1	ENSG00000187553	10	94811021	94838454	34	7	123834	-0.43285	0.66744	0.990650877
RNF141	ENSG00000110315	11	10523225	10572777	112	15	123834	-0.43306	0.66751	0.990650877
SMEK2	ENSG00000138041	2	55764428	55856015	218	21	123834	-0.43311	0.66753	0.990650877
FAM185A	ENSG00000222011	7	102379418	102459672	97	16	123834	-0.43313	0.66754	0.990650877
LYN	ENSG00000254087	8	56782372	56933940	291	21	123834	-0.43316	0.66755	0.990650877
GABPB1	ENSG00000104064	15	50559389	50657605	177	11	123834	-0.43317	0.66755	0.990650877
NPAS1	ENSG00000130751	19	47513077	47559033	20	4	123834	-0.43316	0.66755	0.990650877
CDC34	ENSG00000099804	19	521714	552097	72	12	123834	-0.43349	0.66767	0.990650877
DDX58	ENSG00000107201	9	32445300	32536322	95	11	123834	-0.43351	0.66768	0.990650877
TCEA2	ENSG00000171703	20	62671189	62713700	32	10	123834	-0.43352	0.66768	0.990650877
GOLGA7	ENSG00000147533	8	41337915	41378499	108	13	123834	-0.43366	0.66773	0.990650877
SPHK2	ENSG00000063176	19	49112548	49143974	40	6	123834	-0.43379	0.66778	0.990650877
PLA1A	ENSG00000144837	3	119306689	119358658	78	10	123834	-0.43407	0.66788	0.990650877
FOXL2	ENSG00000183770	3	138653066	138675982	39	4	123834	-0.43407	0.66788	0.990650877
GPR123	ENSG00000197177	10	134874433	134955179	185	30	123834	-0.43408	0.66788	0.990650877
C6orf106	ENSG00000196821	6	34545065	34674636	206	12	123834	-0.43431	0.66797	0.990650877
NT5C	ENSG00000125458	17	73116320	73137890	27	4	123834	-0.43467	0.6681	0.990650877
NUCB1	ENSG00000104805	19	49393307	49436629	79	9	123834	-0.43469	0.6681	0.990650877
SIMC1	ENSG00000170085	5	175655365	175782994	228	15	123834	-0.43478	0.66814	0.990650877
GJB6	ENSG00000121742	13	20786110	20816534	52	7	123834	-0.43481	0.66815	0.990650877
RPL36AL	ENSG00000165502	14	50075237	50097403	57	5	123834	-0.4352	0.66829	0.990650877
MRPL22	ENSG00000082515	5	154310630	154358971	76	7	123834	-0.43546	0.66839	0.990650877
AC079354.2	ENSG00000267889	2	203094292	203114435	30	6	123834	-0.43578	0.6685	0.990650877
ACTR3B	ENSG00000133627	7	152446834	152562463	194	16	123834	-0.43585	0.66853	0.990650877
SUFU	ENSG00000107882	10	104253744	104403292	257	8	123834	-0.43613	0.66863	0.990650877
KLHL42	ENSG00000087448	12	27922953	27965973	52	5	123834	-0.43648	0.66876	0.990650877
RBM11	ENSG00000185272	21	15578451	15610693	140	10	123834	-0.43651	0.66877	0.990650877
SEC61A1	ENSG00000058262	3	127760484	127800526	89	3	123834	-0.43671	0.66884	0.990650877
SLC35G2	ENSG00000168917	3	136527489	136584734	121	5	123834	-0.4368	0.66887	0.990650877
WFDC9	ENSG00000180205	20	44226578	44269907	147	13	123834	-0.43688	0.6689	0.990650877
MAP3K8	ENSG00000107968	10	30712866	30760762	41	4	123834	-0.43692	0.66892	0.990650877
TOM1L1	ENSG00000141198	17	52966748	53049310	177	13	123834	-0.43692	0.66892	0.990650877
CHERP	ENSG00000085872	19	16618700	16663341	64	11	123834	-0.43697	0.66893	0.990650877
NDUFA2	ENSG00000131495	5	140008325	140037370	41	4	123834	-0.43703	0.66895	0.990650877
MOV10L1	ENSG00000073146	22	50518308	50610119	351	12	123834	-0.43708	0.66897	0.990650877
SLC22A9	ENSG00000149742	11	63127261	63187766	123	12	123834	-0.43711	0.66898	0.990650877
SUN2	ENSG00000100242	22	39120730	39200148	79	13	123834	-0.43736	0.66908	0.990650877
FKBP9	ENSG00000122642	7	32987017	33056543	87	15	123834	-0.4376	0.66916	0.990650877
OR2K2	ENSG00000171133	9	114079763	114100800	15	3	123834	-0.43779	0.66923	0.990650877
TSSK3	ENSG00000162526	1	32807122	32839913	2	1	123834	-0.43783	0.66925	0.990650877
FAM229A	ENSG00000225828	1	32816871	32839879	2	1	123834	-0.43783	0.66925	0.990650877
BSDC1	ENSG00000160058	1	32820704	32870332	2	1	123834	-0.43783	0.66925	0.990650877
ZNF34	ENSG00000196378	8	145988499	146022730	51	4	123834	-0.43788	0.66926	0.990650877
AC010642.1	ENSG00000267216	19	58780332	58836563	85	12	123834	-0.43795	0.66929	0.990650877
SLC30A2	ENSG00000158014	1	26353743	26382624	35	3	123834	-0.43803	0.66932	0.990650877
SIX4	ENSG00000100625	14	61166246	61201066	26	3	123834	-0.43845	0.66947	0.990650877
GIT1	ENSG00000108262	17	27890487	27931072	11	2	123834	-0.43855	0.66951	0.990650877
C6orf1	ENSG00000186577	6	34204157	34227247	43	6	123834	-0.43881	0.6696	0.990650877
SNRPE	ENSG00000182004	1	203820731	203849678	78	5	123834	-0.43885	0.66961	0.990650877
IFNA17	ENSG00000234829	9	21217242	21238221	97	6	123834	-0.43896	0.66965	0.990650877
OR2M3	ENSG00000228198	1	248356332	248377392	42	3	123834	-0.43912	0.66971	0.990650877
ISY1	ENSG00000240682	3	128836258	128890136	31	3	123834	-0.43916	0.66973	0.990650877
ABCB1	ENSG00000085563	7	87123175	87352611	296	25	123834	-0.43924	0.66975	0.990650877
LHCGR	ENSG00000138039	2	48849428	48992880	288	30	123834	-0.43936	0.6698	0.990650877
UPK2	ENSG00000110375	11	118785873	118839269	48	10	123834	-0.43964	0.6699	0.990650877
C10orf40	ENSG00000235931	10	61705188	61730671	42	7	123834	-0.44	0.67003	0.990650877
AC024580.1	ENSG00000204533	19	56652314	56673250	54	6	123834	-0.44011	0.67007	0.990650877
PANK2	ENSG00000125779	20	3859486	3917605	99	10	123834	-0.44038	0.67017	0.990650877
KRTAP10-1	ENSG00000215455	21	45948864	45970078	2	1	123834	-0.4405	0.67021	0.990650877
RP11-343C2.7	ENSG00000259900	16	69359179	69395633	37	6	123834	-0.44075	0.6703	0.990650877
C9orf69	ENSG00000238227	9	138996427	139020731	24	7	123834	-0.44103	0.67041	0.990650877
MRPL11	ENSG00000174547	11	66192546	66244209	38	5	123834	-0.44138	0.67053	0.990650877
SUMO2	ENSG00000188612	17	73153408	73189078	30	5	123834	-0.44161	0.67062	0.990650877
OFCC1	ENSG00000181355	6	9586343	10221841	1126	70	123834	-0.44168	0.67064	0.990650877
SLC39A7	ENSG00000112473	6	33158222	33182216	29	7	123834	-0.44187	0.67071	0.990650877
C19orf57	ENSG00000132016	19	13983161	14027265	47	1	123834	-0.44188	0.67071	0.990650877
WFS1	ENSG00000109501	4	6261576	6314992	142	9	123834	-0.44201	0.67076	0.990650877
HDGFRP2	ENSG00000167674	19	4462284	4512223	107	11	123834	-0.44219	0.67082	0.990650877
KLHL36	ENSG00000135686	16	84672131	84711292	63	10	123834	-0.44224	0.67084	0.990650877
TTLL1	ENSG00000100271	22	43425522	43495434	141	19	123834	-0.44239	0.6709	0.990650877
FKBP11	ENSG00000134285	12	49305301	49330257	53	3	123834	-0.44279	0.67104	0.990650877

ZNF296	ENSG00000170684	19	45564758	45589846	29	3	123834	-0.44285	0.67106	0.990650877
PLEK	ENSG00000115956	2	68582305	68634585	261	13	123834	-0.44287	0.67107	0.990650877
HNRNPDL	ENSG00000152795	4	83333717	83361294	56	7	123834	-0.44292	0.67109	0.990650877
TP53I3	ENSG00000115129	2	24290303	24318731	79	6	123834	-0.44297	0.67111	0.990650877
CC2D1A	ENSG00000132024	19	14007014	14051692	19	1	123834	-0.44298	0.67111	0.990650877
ZNF473	ENSG00000142528	19	50518971	50566671	67	9	123834	-0.443	0.67112	0.990650877
XRCC1	ENSG00000073050	19	44037192	44094625	117	18	123834	-0.4431	0.67115	0.990650877
AL590822.1	ENSG00000203301	1	2133360	2155620	10	4	123834	-0.44334	0.67124	0.990650877
PPFIA2	ENSG00000139220	12	81642045	82163332	730	48	123834	-0.44339	0.67126	0.990650877
C14orf164	ENSG00000215277	14	23644525	23752686	192	24	123834	-0.44352	0.6713	0.990650877
SCLT1	ENSG00000151466	4	129776076	130024764	358	18	123834	-0.44358	0.67133	0.990650877
C19orf82	ENSG00000267106	19	9721967	9766572	56	4	123834	-0.44394	0.67146	0.990650877
BOD1	ENSG00000145919	5	173024517	173053663	70	7	123834	-0.44475	0.67175	0.990650877
ROCK1	ENSG00000067900	18	18516867	18701812	69	2	123834	-0.44474	0.67175	0.990650877
TBC1D20	ENSG00000125875	20	406124	453197	64	8	123834	-0.44479	0.67176	0.990650877
BTNL8	ENSG00000113303	5	180316077	180387906	96	5	123834	-0.44482	0.67178	0.990650877
PRRT3	ENSG00000163704	3	9977226	10004078	31	3	123834	-0.44496	0.67182	0.990650877
C1orf105	ENSG00000180999	1	172379828	172447971	109	12	123834	-0.44512	0.67188	0.990650877
CD38	ENSG00000004468	4	15769898	15864853	228	18	123834	-0.44525	0.67193	0.990650877
ZNF681	ENSG00000196172	19	23911997	23951693	56	8	123834	-0.44541	0.67199	0.990650877
KRT81	ENSG00000205426	12	52669697	52695318	48	6	123834	-0.4455	0.67202	0.990650877
ZNF415	ENSG00000170954	19	53601132	53646330	80	11	123834	-0.44558	0.67205	0.990650877
SWAP70	ENSG00000133789	11	9675624	9784538	206	12	123834	-0.44582	0.67214	0.990650877
CTD-2535L24.2	ENSG00000266076	17	63524220	63647151	129	18	123834	-0.44587	0.67215	0.990650877
TUBGCP6	ENSG00000128159	22	50646118	50693421	66	13	123834	-0.44589	0.67216	0.990650877
AC099344.1	ENSG00000269074	2	11593927	11616274	30	2	123834	-0.44614	0.67225	0.990650877
GAPVD1	ENSG00000165219	9	128014073	128139486	101	5	123834	-0.44624	0.67229	0.990650877
OBSL1	ENSG00000124006	2	220405451	220446581	46	8	123834	-0.44647	0.67237	0.990650877
OR4N4	ENSG00000183706	15	22372382	22393507	2	1	123834	-0.4466	0.67242	0.990650877
ZDHHC2	ENSG00000104219	8	17003538	17092308	102	19	123834	-0.44667	0.67244	0.990650877
RAB43	ENSG00000172780	3	128796412	128851644	35	3	123834	-0.44688	0.67252	0.990650877
SPATA5	ENSG00000145375	4	123834229	124250605	1038	21	123834	-0.44729	0.67267	0.990650877
ADAD2	ENSG00000140955	16	84214744	84240774	91	14	123834	-0.44736	0.67269	0.990650877
ZNF226	ENSG00000167380	19	44659226	44692534	18	5	123834	-0.44755	0.67276	0.990650877
C9orf114	ENSG00000198917	9	131571930	131602100	69	6	123834	-0.44762	0.67279	0.990650877
EPM2A	ENSG00000112425	6	145812719	146067160	551	7	123834	-0.44796	0.67291	0.990650877
HHAT	ENSG00000054392	1	210491596	210859638	655	79	123834	-0.448	0.67292	0.990650877
DESI2	ENSG00000121644	1	244806237	244882335	75	10	123834	-0.44803	0.67293	0.990650877
KCNJ15	ENSG00000157551	21	39519128	39689279	343	17	123834	-0.44855	0.67312	0.990650877
KRTAP9-7	ENSG00000180386	17	39421911	39442949	19	2	123834	-0.44862	0.67315	0.990650877
MORN1	ENSG00000116151	1	2242692	2333146	132	24	123834	-0.44897	0.67327	0.990650877
UST	ENSG00000111962	6	149058464	149408126	457	67	123834	-0.44897	0.67328	0.990650877
ANK2	ENSG00000145362	4	113729265	114314896	931	76	123834	-0.4492	0.67336	0.990650877
HBS1L	ENSG00000112339	6	135271516	135434194	219	7	123834	-0.44927	0.67338	0.990650877
CT62	ENSG00000225362	15	71392583	71417833	27	3	123834	-0.44964	0.67352	0.990650877
OR9K2	ENSG00000170605	12	55513465	55534586	92	2	123834	-0.44974	0.67355	0.990650877
CHMP4B	ENSG00000101421	20	32389110	32452172	30	6	123834	-0.44993	0.67362	0.990650877
CMTM4	ENSG00000183723	16	66638653	66740610	158	8	123834	-0.44999	0.67364	0.990650877
VANGL2	ENSG00000162738	1	160360376	160408468	73	11	123834	-0.4502	0.67372	0.990650877
PHLDB3	ENSG00000176531	19	43969106	44019087	88	11	123834	-0.45033	0.67376	0.990650877
UNC79	ENSG00000133958	14	93789565	94184222	598	19	123834	-0.45042	0.6738	0.990650877
PREPL	ENSG00000138078	2	44533420	44599001	107	10	123834	-0.45046	0.67381	0.990650877
PSMA8	ENSG00000154611	18	23703816	23783319	129	8	123834	-0.45071	0.6739	0.990650877
SSFA2	ENSG00000138434	2	182746560	182805465	50	3	123834	-0.45102	0.67401	0.990650877
RPS16	ENSG00000105193	19	39913847	39936588	48	5	123834	-0.45111	0.67404	0.990650877
SLC32A1	ENSG00000101438	20	37343105	37368015	51	10	123834	-0.4512	0.67408	0.990650877
FAM209B	ENSG00000213714	20	55098302	55121576	22	6	123834	-0.45127	0.6741	0.990650877
ABCB4	ENSG00000005471	7	87021013	87119751	167	10	123834	-0.45131	0.67412	0.990650877
C20orf144	ENSG00000149609	20	32240090	32263425	7	2	123834	-0.4515	0.67418	0.990650877
POLA2	ENSG00000014138	11	65019233	65083060	41	4	123834	-0.45156	0.67421	0.990650877
TBXA2R	ENSG00000006638	19	3584504	3616838	47	7	123834	-0.45163	0.67423	0.990650877
SLC16A11	ENSG00000174326	17	6934949	6957242	10	3	123834	-0.45174	0.67427	0.990650877
KCNK12	ENSG00000184261	2	47733720	47808078	72	15	123834	-0.45187	0.67432	0.990650877
ZFAND3	ENSG00000156639	6	37777275	38132400	769	17	123834	-0.45187	0.67432	0.990650877
RHBDL1	ENSG00000103269	16	715666	738268	32	6	123834	-0.45188	0.67432	0.990650877
WDR5	ENSG00000196363	9	136990487	137035093	137	9	123834	-0.45213	0.67441	0.990650877
CNTNAP4	ENSG00000152910	16	76301176	76603135	625	41	123834	-0.45224	0.67445	0.990650877
C9orf53	ENSG00000224854	9	21957137	21977738	11	4	123834	-0.4525	0.67454	0.990650877
CETN3	ENSG00000153140	5	89678078	89715603	9	2	123834	-0.45254	0.67456	0.990650877
IGFL3	ENSG00000188624	19	46613328	46637931	20	4	123834	-0.45254	0.67456	0.990650877
RER1	ENSG00000157916	1	2313267	2346883	40	10	123834	-0.45267	0.67461	0.990650877
RQCD1	ENSG00000144580	2	219423303	219471803	87	5	123834	-0.45293	0.6747	0.990650877
PHYHIP	ENSG00000168490	8	22067222	22099854	32	5	123834	-0.45298	0.67472	0.990650877
SLC41A1	ENSG00000133065	1	205748221	205792876	94	13	123834	-0.45312	0.67477	0.990650877
POLR3D	ENSG00000168495	8	22092617	22122113	54	5	123834	-0.45316	0.67478	0.990650877
GTF2F2	ENSG00000188342	13	45684650	45868237	241	9	123834	-0.45319	0.6748	0.990650877
MDN1	ENSG00000112159	6	90342218	90539442	336	20	123834	-0.45338	0.67486	0.990650877
VIM	ENSG00000026025	10	17260258	17289592	36	5	123834	-0.45365	0.67496	0.990650877
GABARAPL3	ENSG00000238244	15	90880819	90902669	70	6	123834	-0.45367	0.67497	0.990650877
KLK15	ENSG00000174562	19	51318545	51350469	44	10	123834	-0.4537	0.67498	0.990650877
AP4B1	ENSG00000134262	1	114427370	114457823	24	4	123834	-0.45388	0.67504	0.990650877
SLC12A2	ENSG00000064651	5	127409458	127535380	129	7	123834	-0.45401	0.67509	0.990650877
MS4A3	ENSG00000149516	11	59814060	59848601	64	6	123834	-0.45406	0.67511	0.990650877
FUCA2	ENSG00000001036	6	143805948	143842827	32	4	123834	-0.45412	0.67513	0.990650877
C8orf59	ENSG00000176731	8	86116311	86142650	25	4	123834	-0.45431	0.6752	0.990650877
SLC16A7	ENSG00000118596	12	59979848	60186395	393	15	123834	-0.45433	0.6752	0.990650877
ANGPTL6	ENSG00000130812	19	10193014	10223472	16	4	123834	-0.45437	0.67522	0.990650877
LZTS1	ENSG00000061337	8	20093676	20171474	112	25	123834	-0.45451	0.67527	0.990650877
LY6H	ENSG00000176956	8	144229331	144252128	4	2	123834	-0.45457	0.67529	0.990650877
AC008267.1	ENSG00000267941	7	65929543	65950709	47	6	123834	-0.45506	0.67547	0.990650877
MPHOSPH8	ENSG00000196199	13	20197788	20257599	80	6	123834	-0.45518	0.67551	0.990650877
RACGAP1	ENSG00000161800	12	50360706	50436919	75	2	123834	-0.45532	0.67556	0.990650877
SPOPL	ENSG00000144228	2	139249371	139341117	109	11	123834	-0.45546	0.67561	0.990650877
SNX2	ENSG00000205302	5	122100691	122175803	189	13	123834	-0.45576	0.67572	0.990650877
MAGOH	ENSG00000162385	1	53682564	53714282	34	5	123834	-0.45589	0.67577	0.990650877
TARDBP	ENSG00000120948	1	11062414	11095796	48	4	123834	-0.45597	0.67579	0.990650877
HP1BP3	ENSG00000127483	1	21059154	21123816	92	11	123834	-0.45606	0.67583	0.990650877
OR2T7	ENSG00000227152	1	248594508	248615434	1	1	123834	-0.45609	0.67584	0.990650877
ADO	ENSG00000181915	10	64554516	64578238	27	4	123834	-0.45612	0.67585	0.990650877

SBF2	ENSG00000133812	11	9790214	10325754	1293	27	123834	-0.45613	0.67585	0.990650877
AC003006.7	ENSG00000269026	19	58183413	58274943	114	14	123834	-0.45619	0.67588	0.990650877
DPEP3	ENSG00000141096	16	67999566	68024732	21	3	123834	-0.45639	0.67595	0.990650877
SLC30A6	ENSG00000152683	2	32380933	32459448	129	10	123834	-0.45644	0.67596	0.990650877
SLC25A44	ENSG00000160785	1	156153880	156192587	37	5	123834	-0.45651	0.67599	0.990650877
C5orf15	ENSG00000113583	5	133281201	133314478	20	6	123834	-0.45669	0.67605	0.990650877
TRAF5	ENSG00000082512	1	211489957	211558288	99	14	123834	-0.4569	0.67613	0.990650877
DSN1	ENSG00000149636	20	35370194	35412221	17	7	123834	-0.45718	0.67623	0.990650877
PRM3	ENSG00000178257	16	11357144	11377452	30	5	123834	-0.4575	0.67634	0.990650877
TNPO1	ENSG00000083312	5	72102139	72222560	121	11	123834	-0.45759	0.67638	0.990650877
RPIA	ENSG00000153574	2	88981162	89060452	151	6	123834	-0.45764	0.67639	0.990650877
TTLL10	ENSG00000162571	1	1099264	1143315	112	11	123834	-0.45775	0.67643	0.990650877
GLI4	ENSG00000250571	8	144339603	144369101	78	7	123834	-0.45778	0.67645	0.990650877
CACNA1G	ENSG00000006283	17	48628429	48714835	76	13	123834	-0.45786	0.67647	0.990650877
MTG1	ENSG00000148824	10	135197598	135244811	66	7	123834	-0.45787	0.67648	0.990650877
ZMYM4	ENSG00000146463	1	35724568	35897659	124	4	123834	-0.45833	0.67664	0.990650877
ZKSCAN7	ENSG00000196345	3	44586685	44634975	70	5	123834	-0.45833	0.67664	0.990650877
AC011500.1	ENSG00000213922	19	39920212	39942082	52	5	123834	-0.45832	0.67664	0.990650877
GOPC	ENSG00000047932	6	117629374	117933691	727	39	123834	-0.4586	0.67674	0.990650877
VPS4A	ENSG00000132612	16	69335259	69368949	45	5	123834	-0.45881	0.67681	0.990650877
TMEM196	ENSG00000173452	7	19748933	19823221	182	12	123834	-0.45882	0.67682	0.990650877
ING1	ENSG00000153487	13	111355083	111383421	60	4	123834	-0.45884	0.67683	0.990650877
RPS18	ENSG00000231500	6	33229787	33254287	44	7	123834	-0.45942	0.67704	0.990650877
SLFN12	ENSG00000172123	17	33728079	33770302	66	9	123834	-0.45989	0.6772	0.990650877
GPR25	ENSG00000170128	1	200832083	200853306	42	7	123834	-0.45995	0.67722	0.990650877
AMPH	ENSG00000078053	7	38413305	38681167	724	57	123834	-0.46018	0.67731	0.990650877
TMEM59	ENSG00000116209	1	54487347	54529177	48	4	123834	-0.46026	0.67733	0.990650877
MAPK8IP3	ENSG00000138834	16	1746184	1830318	84	8	123834	-0.46028	0.67734	0.990650877
C1QL2	ENSG00000144119	2	119903819	119926465	27	5	123834	-0.46031	0.67735	0.990650877
ABLIM1	ENSG00000099204	10	116180872	116454762	424	41	123834	-0.4603	0.67735	0.990650877
CMPK1	ENSG00000162368	1	47789469	47854511	118	7	123834	-0.46037	0.67738	0.990650877
AC119673.1	ENSG00000268313	1	205672497	205694153	26	7	123834	-0.46045	0.6774	0.990650877
PUM2	ENSG00000055917	2	20438452	20561995	182	12	123834	-0.46052	0.67743	0.990650877
UBFD1	ENSG00000103353	16	23558392	23595710	83	8	123834	-0.46088	0.67756	0.990650877
TEX9	ENSG00000151575	15	56526207	56748195	476	17	123834	-0.46094	0.67758	0.990650877
CKAP2L	ENSG00000169607	2	113483930	113532254	70	6	123834	-0.46108	0.67763	0.990650877
LRRD1	ENSG00000240720	7	91731473	91818845	111	4	123834	-0.46108	0.67763	0.990650877
RABAC1	ENSG00000105404	19	42450833	42473542	7	1	123834	-0.46139	0.67774	0.990650877
HOXA11	ENSG00000005073	7	27211129	27234842	8	3	123834	-0.46155	0.6778	0.990650877
HN1	ENSG00000189159	17	73121343	73174376	60	6	123834	-0.46182	0.6779	0.990650877
LBX1	ENSG00000138136	10	102976733	102999551	17	4	123834	-0.4619	0.67792	0.990650877
PSMA1	ENSG00000256206	11	14505329	14551890	42	4	123834	-0.46189	0.67792	0.990650877
TBX2	ENSG00000121068	17	59467257	59496827	39	4	123834	-0.46206	0.67798	0.990650877
SUSD4	ENSG00000143502	1	223384161	223547544	179	18	123834	-0.46234	0.67808	0.990650877
BBS2	ENSG00000125124	16	56490748	56564195	112	10	123834	-0.4624	0.6781	0.990650877
MPZ	ENSG00000158887	1	161264525	161289762	35	5	123834	-0.46242	0.67811	0.990650877
BCAR1	ENSG00000050820	16	75252928	75311951	112	8	123834	-0.46253	0.67815	0.990650877
PLEKHA5	ENSG00000052126	12	19272648	19539334	516	24	123834	-0.46267	0.6782	0.990650877
ERBB3	ENSG00000065361	12	56463641	56507289	35	6	123834	-0.46269	0.67821	0.990650877
RP11-468E2.6	ENSG00000259371	14	24580560	24611038	37	5	123834	-0.46277	0.67824	0.990650877
TSPYL1	ENSG00000189241	6	116587741	116611066	63	8	123834	-0.46289	0.67828	0.990650877
PAK1	ENSG00000149269	11	77022752	77195680	245	14	123834	-0.46301	0.67832	0.990650877
AL360181.1	ENSG00000268882	10	135182720	135203102	31	3	123834	-0.4632	0.67839	0.990650877
PTPRF	ENSG00000142949	1	43980858	44099343	199	13	123834	-0.46355	0.67852	0.990650877
KLHL1	ENSG00000150361	13	70264726	70692591	1135	52	123834	-0.46366	0.67855	0.990650877
FZD7	ENSG00000155760	2	202889310	202913160	19	2	123834	-0.46378	0.6786	0.990650877
FANK1	ENSG00000203780	10	127575108	127708161	188	13	123834	-0.46385	0.67862	0.990650877
C11orf83	ENSG00000204922	11	62427745	62451159	11	2	123834	-0.46391	0.67864	0.990650877
VPS4B	ENSG00000119541	18	61046423	61099716	35	5	123834	-0.464	0.67868	0.990650877
AC026461.1	ENSG00000268317	16	56652971	56676150	71	11	123834	-0.46404	0.67869	0.990650877
MRFAP1	ENSG00000179010	4	6631818	6654472	31	7	123834	-0.4641	0.67871	0.990650877
AAGAB	ENSG00000103591	15	67483371	67557533	103	6	123834	-0.46447	0.67884	0.990650877
NECAB2	ENSG00000103154	16	83992237	84046381	226	25	123834	-0.46447	0.67884	0.990650877
GOLIM4	ENSG00000173905	3	167716465	167823763	130	11	123834	-0.46466	0.67891	0.990650877
SUDS3	ENSG00000111707	12	118804185	118865840	104	5	123834	-0.46479	0.67896	0.990650877
NXNL2	ENSG00000130045	9	91140016	91209189	53	8	123834	-0.46481	0.67897	0.990650877
PRKCI	ENSG00000163558	3	169930153	170033769	246	7	123834	-0.46502	0.67904	0.990650877
ALKBH7	ENSG00000125652	19	6362444	6385042	81	8	123834	-0.46507	0.67906	0.990650877
ATP5B	ENSG00000110955	12	57021959	57049798	33	5	123834	-0.46512	0.67908	0.990650877
CAD	ENSG00000084774	2	27430258	27476811	99	8	123834	-0.46558	0.67924	0.990650877
PTCH2	ENSG00000117425	1	45275516	45318735	61	5	123834	-0.46565	0.67927	0.990650877
CSTB	ENSG00000160213	21	45182393	45206326	38	7	123834	-0.46575	0.6793	0.990650877
HECTD3	ENSG00000126107	1	45458212	45487001	27	3	123834	-0.46652	0.67958	0.990650877
SIAH3	ENSG00000215475	13	46344405	46435871	120	19	123834	-0.46653	0.67958	0.990650877
TMED6	ENSG00000157315	16	69367151	69395712	24	6	123834	-0.46653	0.67958	0.990650877
CEACAM6	ENSG00000086548	19	42244885	42286113	18	5	123834	-0.46664	0.67962	0.990650877
BLACE	ENSG00000204960	7	155139680	155170629	116	12	123834	-0.46673	0.67965	0.990650877
GGA1	ENSG00000100083	22	37994481	38039571	77	7	123834	-0.46673	0.67965	0.990650877
TSKU	ENSG00000182704	11	76483295	76519198	40	6	123834	-0.46702	0.67976	0.990650877
CHP1	ENSG00000187446	15	41513037	41584043	106	7	123834	-0.46703	0.67976	0.990650877
ARL14	ENSG00000179674	3	160384948	160406233	22	4	123834	-0.46749	0.67992	0.990650877
GPR78	ENSG00000155269	4	8550452	8631486	147	26	123834	-0.46761	0.67997	0.990650877
GLI3	ENSG00000106571	7	41990548	42287469	463	59	123834	-0.46761	0.67997	0.990650877
AC005544.1	ENSG00000214167	17	65017509	65038198	12	4	123834	-0.46796	0.68009	0.990650877
PRR14L	ENSG00000183530	22	32062242	32156126	147	10	123834	-0.46819	0.68018	0.990650877
SMIM12	ENSG00000163866	1	35168338	35335417	237	35	123834	-0.46842	0.68026	0.990650877
GUK1	ENSG00000143774	1	228317663	228346685	22	5	123834	-0.46851	0.68029	0.990650877
CBX5	ENSG00000094916	12	54614724	54683886	24	3	123834	-0.46858	0.68032	0.990650877
FAM73B	ENSG00000148343	9	131788900	131844361	29	5	123834	-0.46879	0.68039	0.990650877
IGSF9	ENSG00000085552	1	159886829	159925386	58	16	123834	-0.46931	0.68057	0.990650877
PQLC2	ENSG00000040487	1	19628820	19665794	58	9	123834	-0.46933	0.68058	0.990650877
DNAJB5	ENSG00000137094	9	34979638	35008897	18	4	123834	-0.46942	0.68062	0.990650877
TAB2	ENSG00000055208	6	149529777	149742749	439	34	123834	-0.46955	0.68066	0.990650877
TUBA8	ENSG00000183785	22	18583097	18639321	79	17	123834	-0.46955	0.68066	0.990650877
RIT1	ENSG00000143622	1	155857599	155891195	43	4	123834	-0.46965	0.6807	0.990650877
AKAP10	ENSG00000108599	17	19797615	19891656	105	6	123834	-0.46965	0.6807	0.990650877
CCL13	ENSG00000181374	17	32673471	32695629	53	8	123834	-0.46967	0.68071	0.990650877
ELMOD2	ENSG00000179387	4	141435312	141484924	68	7	123834	-0.46984	0.68076	0.990650877
THAP9	ENSG00000168152	4	83811837	83851122	49	4	123834	-0.47014	0.68087	0.990650877

CHMP2B	ENSG00000083937	3	87266421	87314698	63	9	123834	-0.47016	0.68088	0.990650877
TLN1	ENSG00000137076	9	35686945	35742392	51	9	123834	-0.47054	0.68101	0.990650877
ZNF248	ENSG00000198105	10	38081751	38157034	219	10	123834	-0.47061	0.68104	0.990650877
SMLR1	ENSG00000256162	6	131138546	131168275	86	15	123834	-0.47065	0.68105	0.990650877
IBSP	ENSG00000029559	4	88710733	88743074	52	13	123834	-0.47071	0.68107	0.990650877
IRF5	ENSG00000128604	7	128567666	128600089	64	12	123834	-0.47076	0.6811	0.990650877
ZNF444	ENSG00000167685	19	56633968	56682262	93	8	123834	-0.471	0.68118	0.990650877
OR10G9	ENSG00000236981	11	123883720	123904655	40	7	123834	-0.47107	0.6812	0.990650877
PSMC6	ENSG00000100519	14	53163890	53205305	42	5	123834	-0.47121	0.68125	0.990650877
FPR3	ENSG00000187474	19	52288416	52339442	110	17	123834	-0.47138	0.68131	0.990650877
OMG	ENSG00000126861	17	29589031	29634557	40	2	123834	-0.4716	0.68139	0.990650877
AP000688.1	ENSG00000268098	21	37392368	37427818	88	11	123834	-0.47165	0.68141	0.990650877
RETN	ENSG00000104918	19	7723930	7745334	10	3	123834	-0.47177	0.68145	0.990650877
C18orf63	ENSG00000206043	18	71973048	72036422	35	4	123834	-0.4719	0.6815	0.990650877
VHL	ENSG00000134086	3	10172692	10203904	75	9	123834	-0.47194	0.68151	0.990650877
FBXL7	ENSG00000183580	5	15490305	15949900	462	34	123834	-0.47243	0.68169	0.990650877
TNFAIP1	ENSG00000109079	17	26652628	26684035	18	4	123834	-0.47248	0.68171	0.990650877
CSMD3	ENSG00000164796	8	113225157	114459328	2270	51	123834	-0.47253	0.68173	0.990650877
C16orf54	ENSG00000185905	16	29743784	29767327	27	1	123834	-0.47253	0.68173	0.990650877
C19orf43	ENSG00000123144	19	12831454	12855589	8	3	123834	-0.47273	0.6818	0.990650877
HAPLN2	ENSG00000132702	1	156579086	156605517	26	4	123834	-0.4728	0.68182	0.990650877
NDUFA5	ENSG00000128609	7	123167051	123208309	69	8	123834	-0.47312	0.68194	0.990650877
KCNC4	ENSG00000116396	1	110743965	110835722	147	13	123834	-0.47332	0.68201	0.990650877
FDFT1	ENSG00000079459	8	11643082	11706818	158	21	123834	-0.47336	0.68202	0.990650877
IBTK	ENSG00000005700	6	82869700	82967471	179	9	123834	-0.47342	0.68204	0.990650877
PABPN1L	ENSG00000205022	16	88918034	88943068	56	3	123834	-0.47354	0.68209	0.990650877
PRPF6	ENSG00000101161	20	62602488	62674453	99	6	123834	-0.47376	0.68217	0.990650877
SCRT1	ENSG00000170616	8	145544228	145569943	6	1	123834	-0.47393	0.68222	0.990650877
PUF60	ENSG00000179950	8	144888514	144922029	28	3	123834	-0.474	0.68225	0.990650877
EARS2	ENSG00000103356	16	23523335	23579052	140	9	123834	-0.47421	0.68232	0.990650877
ZNF680	ENSG00000173041	7	63970262	64033484	210	12	123834	-0.47429	0.68235	0.990650877
GRXCR2	ENSG00000204928	5	145229296	145262531	43	8	123834	-0.47445	0.68241	0.990650877
ZNF214	ENSG00000149050	11	7010549	7051599	104	8	123834	-0.47478	0.68253	0.990650877
SH3GLB2	ENSG00000148341	9	131759315	131800582	41	6	123834	-0.47498	0.6826	0.990650877
CTB-96E2.2	ENSG00000273171	17	26681310	26705033	31	4	123834	-0.47513	0.68265	0.990650877
OR8I2	ENSG00000172154	11	55850753	55871762	137	5	123834	-0.47527	0.6827	0.990650877
TXNDC8	ENSG00000204193	9	113055801	113110127	97	17	123834	-0.4753	0.68271	0.990650877
NTHL1	ENSG00000065057	16	2079816	2107867	15	5	123834	-0.4755	0.68279	0.990650877
MLPH	ENSG00000115648	2	238384071	238473961	298	18	123834	-0.47576	0.68288	0.990650877
AC091150.1	ENSG00000268960	18	45768672	45789208	75	14	123834	-0.47582	0.6829	0.990650877
SLC17A3	ENSG00000124564	6	25823294	25892514	136	15	123834	-0.4759	0.68293	0.990650877
CCL3	ENSG00000006075	17	34405602	34427515	27	5	123834	-0.47595	0.68294	0.990650877
KIAA0753	ENSG00000198920	17	6471468	6554247	203	14	123834	-0.47605	0.68298	0.990650877
TSGA10	ENSG00000135951	2	99603724	99781427	314	10	123834	-0.47609	0.683	0.990650877
C2orf82	ENSG00000182600	2	233711980	233753418	97	12	123834	-0.4764	0.6831	0.990650877
E2F7	ENSG00000165891	12	77405027	77469360	61	17	123834	-0.47655	0.68316	0.990650877
CCL7	ENSG00000108688	17	32587240	32609261	37	6	123834	-0.47655	0.68316	0.990650877
ZNF71	ENSG00000197951	19	57096632	57147849	75	13	123834	-0.47716	0.68338	0.990650877
LDLRAP1	ENSG00000157978	1	25860071	25905377	43	6	123834	-0.47727	0.68342	0.990650877
RAB5C	ENSG00000108774	17	40266994	40317035	69	5	123834	-0.47728	0.68342	0.990650877
DOCK4	ENSG00000128512	7	111356166	111856466	493	75	123834	-0.4773	0.68343	0.990650877
LENG9	ENSG00000182909	19	54962981	54984894	34	6	123834	-0.47733	0.68344	0.990650877
C15orf26	ENSG00000156206	15	81289374	81451516	169	17	123834	-0.47741	0.68347	0.990650877
RP11-159G9.5	ENSG00000229729	3	88098424	88198123	184	3	123834	-0.4775	0.6835	0.990650877
SNX14	ENSG00000135317	6	86205214	86313874	138	14	123834	-0.47752	0.6835	0.990650877
TGFB1	ENSG00000105329	19	41797492	41869816	70	7	123834	-0.47756	0.68352	0.990650877
IDI1	ENSG00000067064	10	1075848	1105110	65	11	123834	-0.47763	0.68354	0.990650877
TIGD7	ENSG00000140993	16	3338832	3365729	15	1	123834	-0.47793	0.68365	0.990650877
APEH	ENSG00000164062	3	49701435	49731396	27	5	123834	-0.47795	0.68366	0.990650877
IGFL4	ENSG00000204869	19	46533006	46590376	56	10	123834	-0.47795	0.68366	0.990650877
METTL24	ENSG00000053328	6	110557131	110689475	229	13	123834	-0.47876	0.68395	0.990650877
DHRS7C	ENSG00000184544	17	9664751	9704614	87	15	123834	-0.47896	0.68402	0.990650877
EXOSC4	ENSG00000178896	8	145123529	145145550	24	2	123834	-0.47899	0.68403	0.990650877
STX18	ENSG00000168818	4	4407469	4554073	189	13	123834	-0.47912	0.68407	0.990650877
C1orf101	ENSG00000179397	1	244607679	244814479	407	14	123834	-0.47929	0.68413	0.990650877
AC079612.1	ENSG00000196758	2	240489995	240517788	45	8	123834	-0.47928	0.68413	0.990650877
NHLH2	ENSG00000177551	1	116368998	116396538	52	8	123834	-0.47953	0.68422	0.990650877
AL035588.1	ENSG00000268275	6	41643964	41664127	50	8	123834	-0.47953	0.68422	0.990650877
TRIM15	ENSG00000204610	6	30120993	30150473	87	9	123834	-0.47969	0.68428	0.990650877
SLC25A36	ENSG00000114120	3	140650672	140708775	102	5	123834	-0.47983	0.68432	0.990650877
CNOT7	ENSG00000198791	8	17076737	17114387	54	8	123834	-0.47993	0.68436	0.990650877
PRPH	ENSG00000135406	12	49677035	49702465	74	5	123834	-0.48015	0.68444	0.990650877
SCN11A	ENSG00000168356	3	38877260	39002052	261	16	123834	-0.48032	0.6845	0.990650877
AURKB	ENSG00000178999	17	8098056	8123918	28	6	123834	-0.48038	0.68452	0.990650877
CAMLG	ENSG00000164615	5	134064191	134097847	17	6	123834	-0.48059	0.6846	0.990650877
HEATR6	ENSG00000068097	17	58110555	58166292	50	2	123834	-0.48068	0.68463	0.990650877
ANKAR	ENSG00000151687	2	190529016	190635919	144	11	123834	-0.48074	0.68465	0.990650877
AKAP9	ENSG00000127914	7	91560181	91749987	255	4	123834	-0.48093	0.68472	0.990650877
OSGEP	ENSG00000092094	14	20904570	20933264	57	8	123834	-0.48116	0.6848	0.990650877
ODF4	ENSG00000184650	17	8233182	8259364	91	7	123834	-0.48141	0.68489	0.990650877
PVRL2	ENSG00000130202	19	45339432	45402485	103	17	123834	-0.48145	0.6849	0.990650877
TRPM4	ENSG00000130529	19	49650998	49725093	86	13	123834	-0.48151	0.68492	0.990650877
RAB14	ENSG00000119396	9	123930415	123995292	67	12	123834	-0.48162	0.68496	0.990650877
MT1E	ENSG00000169715	16	56649387	56671024	48	11	123834	-0.48163	0.68496	0.990650877
POGLUT1	ENSG00000163389	3	119177785	119223555	110	15	123834	-0.48179	0.68502	0.990650877
PTPN22	ENSG00000134242	1	114346433	114424381	77	9	123834	-0.48192	0.68507	0.990650877
MIB1	ENSG00000101752	18	19274918	19460918	163	9	123834	-0.48199	0.68509	0.990650877
NAIF1	ENSG00000171169	9	130813512	130840485	26	2	123834	-0.48206	0.68512	0.990650877
POU5F2	ENSG00000248483	5	93060508	93087343	20	3	123834	-0.4822	0.68517	0.990650877
CD14	ENSG00000170458	5	140001313	140023286	38	4	123834	-0.4824	0.68524	0.990650877
MGAM	ENSG00000257335	7	141597613	141816547	251	32	123834	-0.48255	0.68529	0.990650877
PKN3	ENSG00000160447	9	131454802	131493197	11	5	123834	-0.48268	0.68534	0.990650877
BAZ2B	ENSG00000123636	2	160165490	160483203	620	39	123834	-0.48279	0.68538	0.990650877
HSF5	ENSG00000176160	17	56487528	56575745	80	9	123834	-0.48316	0.68551	0.990650877
ASB6	ENSG00000148331	9	132389171	132414444	39	8	123834	-0.48336	0.68558	0.990650877
PRSS37	ENSG00000165076	7	141526086	141551287	75	5	123834	-0.48348	0.68562	0.990650877
FOXJ3	ENSG00000198815	1	42632210	42811548	216	10	123834	-0.48359	0.68566	0.990650877
SORBS2	ENSG00000154556	4	186496598	186887806	603	74	123834	-0.48361	0.68567	0.990650877
CD47	ENSG00000196776	3	107752145	107819872	35	4	123834	-0.48415	0.68586	0.990650877

KRT1	ENSG00000167768	12	53058520	53084191	70	10	123834	-0.48424	0.68589	0.990650877
TNFRSF10A	ENSG00000104689	8	23037965	23092639	61	10	123834	-0.48435	0.68593	0.990650877
GPR149	ENSG00000174948	3	154045461	154157504	190	20	123834	-0.48443	0.68596	0.990650877
PTEN	ENSG00000171862	10	89612870	89741687	86	16	123834	-0.48443	0.68596	0.990650877
MIA2	ENSG00000150526	14	39689435	39732859	90	12	123834	-0.48455	0.686	0.990650877
ZNF470	ENSG00000197016	19	57068880	57110279	100	8	123834	-0.48462	0.68603	0.990650877
RP11-321N4.5	ENSG00000271793	6	86257696	86334952	83	11	123834	-0.48482	0.6861	0.990650877
MAPK12	ENSG00000188130	22	50673879	50710254	48	9	123834	-0.48486	0.68611	0.990650877
PSPN	ENSG00000125650	19	6365159	6389069	77	8	123834	-0.48489	0.68612	0.990650877
PRSS33	ENSG00000103355	16	2823954	2847949	3	1	123834	-0.48491	0.68613	0.990650877
NFASC	ENSG00000163531	1	204787779	205001950	291	29	123834	-0.48506	0.68618	0.990650877
GNAI1	ENSG00000127955	7	79753271	79858718	133	20	123834	-0.48542	0.68631	0.990763308
CD244	ENSG00000122223	1	160789950	160842692	86	7	123834	-0.48581	0.68645	0.990890158
TBC1D17	ENSG00000104946	19	50370682	50402005	34	5	123834	-0.48606	0.68654	0.990944819
TPK1	ENSG00000196511	7	144139034	144543488	635	38	123834	-0.48661	0.68673	0.991094321
ODF2	ENSG00000136811	9	131207465	131273571	48	5	123834	-0.48669	0.68676	0.991094321
NEXN	ENSG00000162614	1	78344198	78419580	110	14	123834	-0.48681	0.6868	0.991094321
C10orf82	ENSG00000165863	10	118413207	118439775	20	4	123834	-0.48728	0.68697	0.991264386
TNRC6B	ENSG00000100354	22	40430821	40741812	307	11	123834	-0.48756	0.68707	0.991333426
OR10G8	ENSG00000234560	11	123890297	123911341	33	6	123834	-0.4882	0.6873	0.991554909
FLJ00273	ENSG00000269510	9	35032390	35052824	20	4	123834	-0.48832	0.68734	0.991554909
CAP2	ENSG00000112186	6	17383447	17568023	225	28	123834	-0.48844	0.68738	0.991554909
PLRG1	ENSG00000171566	4	155446158	155481587	75	6	123834	-0.48863	0.68745	0.991580634
C2orf76	ENSG00000186132	2	120049801	120134404	220	23	123834	-0.48925	0.68767	0.991761869
CFTR	ENSG00000001626	7	117095838	117366025	248	18	123834	-0.48928	0.68768	0.991761869
CYB5RL	ENSG00000215883	1	54628009	54675709	50	14	123834	-0.48959	0.68779	0.991786295
ARPC2	ENSG00000163466	2	219071817	219129079	80	6	123834	-0.48964	0.68781	0.991786295
CLCA1	ENSG00000016490	1	86924051	86975972	75	14	123834	-0.48986	0.68788	0.991786295
FAM213A	ENSG00000122378	10	82157585	82202753	124	14	123834	-0.49008	0.68796	0.991786295
CTD-2132N18.3	ENSG00000267261	17	40261692	40316934	74	5	123834	-0.49011	0.68797	0.991786295
AC005549.3	ENSG00000268024	17	32571900	32592306	27	4	123834	-0.49022	0.68801	0.991786295
RPL6	ENSG00000089009	12	112832994	112866642	32	6	123834	-0.49057	0.68814	0.991868871
ENSA	ENSG00000143420	1	150563327	150612088	47	7	123834	-0.49138	0.68842	0.991868871
AL117190.3	ENSG00000269375	14	101349265	101370646	8	2	123834	-0.49169	0.68853	0.991868871
CHD5	ENSG00000116254	1	6151853	6250183	143	18	123834	-0.49189	0.6886	0.991868871
ORC2	ENSG00000115942	2	201763696	201838403	60	6	123834	-0.49191	0.68861	0.991868871
DIO3	ENSG00000197406	14	102017688	102039789	16	5	123834	-0.49223	0.68872	0.991868871
FOXD1	ENSG00000251493	5	72730654	72754352	33	5	123834	-0.49226	0.68873	0.991868871
POU2F2	ENSG00000028277	19	42580263	42710737	86	3	123834	-0.49227	0.68874	0.991868871
STX10	ENSG00000104915	19	13244872	13271197	30	6	123834	-0.4923	0.68875	0.991868871
ISY1-RAB43	ENSG00000261796	3	128796418	128889879	57	3	123834	-0.49273	0.6889	0.991868871
PRKAR2B	ENSG00000005249	7	106675094	106812256	217	18	123834	-0.49298	0.68899	0.991868871
CNTNAP2	ENSG00000174469	7	145803453	148128090	4952	220	123834	-0.49301	0.689	0.991868871
GSDMD	ENSG00000104518	8	144625377	144655232	74	11	123834	-0.49325	0.68908	0.991868871
IFNA14	ENSG00000228083	9	21229201	21249978	63	5	123834	-0.49353	0.68918	0.991868871
GTPBP1	ENSG00000100226	22	39091728	39144304	78	8	123834	-0.49352	0.68918	0.991868871
WDR20	ENSG00000140153	14	102595840	102701184	175	16	123834	-0.49399	0.68934	0.991868871
LCE3D	ENSG00000163202	1	152541857	152562980	9	2	123834	-0.49439	0.68948	0.991868871
SMAD6	ENSG00000137834	15	66984566	67084338	142	19	123834	-0.49464	0.68957	0.991868871
VDAC1	ENSG00000213585	5	133297606	133350824	76	10	123834	-0.49471	0.6896	0.991868871
NEURL1B	ENSG00000214357	5	172058269	172128543	70	12	123834	-0.4949	0.68966	0.991868871
SEC14L3	ENSG00000100012	22	30833946	30878036	102	10	123834	-0.49497	0.68969	0.991868871
RALY	ENSG00000125970	20	32571452	32706114	106	7	123834	-0.4952	0.68977	0.991868871
MT1M	ENSG00000205364	16	56656145	56677898	70	10	123834	-0.49524	0.68979	0.991868871
CUL4A	ENSG00000139842	13	113852552	113929399	96	3	123834	-0.49531	0.68981	0.991868871
CDRT1	ENSG00000241322	17	15458798	15532826	47	10	123834	-0.49532	0.68981	0.991868871
ADAMTSL3	ENSG00000156218	15	84312838	84718594	741	33	123834	-0.49542	0.68985	0.991868871
AL161784.1	ENSG00000267987	9	124093625	124120410	47	10	123834	-0.49557	0.6899	0.991868871
SLC37A3	ENSG00000157800	7	139983493	140114233	238	32	123834	-0.49562	0.68992	0.991868871
SMPD4	ENSG00000136699	2	130898981	130950323	61	2	123834	-0.49567	0.68994	0.991868871
TOMM20L	ENSG00000196860	14	58852634	58885419	12	3	123834	-0.49583	0.68999	0.991868871
KCNB1	ENSG00000158445	20	47970414	48109184	301	27	123834	-0.496	0.69005	0.991868871
RP11-279O9.4	ENSG00000249679	4	186382670	186403417	40	4	123834	-0.49613	0.6901	0.991868871
WASL	ENSG00000106299	7	123311989	123399121	141	8	123834	-0.4962	0.69012	0.991868871
CAMKV	ENSG00000164076	3	49885421	49917655	24	3	123834	-0.49655	0.69025	0.991868871
OR52E4	ENSG00000180974	11	5895501	5916527	83	6	123834	-0.49658	0.69026	0.991868871
BCL2L13	ENSG00000099968	22	18101621	18223388	324	16	123834	-0.49689	0.69037	0.991868871
GPHA2	ENSG00000149735	11	64691943	64713360	25	3	123834	-0.49695	0.69039	0.991868871
FIGNL1	ENSG00000132436	7	50501831	50528088	52	9	123834	-0.49712	0.69045	0.991868871
TOR1B	ENSG00000136816	9	132555432	132583560	69	5	123834	-0.49734	0.69053	0.991868871
ACTA1	ENSG00000143632	1	229556992	229579845	20	3	123834	-0.49768	0.69064	0.991868871
AC021860.1	ENSG00000196355	4	38618029	38676430	82	10	123834	-0.49779	0.69069	0.991868871
TRIM34	ENSG00000258659	11	5630994	5675628	81	13	123834	-0.4978	0.69069	0.991868871
FSD2	ENSG00000186628	15	83414114	83484822	71	10	123834	-0.49791	0.69073	0.991868871
HMGA2	ENSG00000149948	12	66207911	66370075	180	22	123834	-0.49858	0.69096	0.991868871
MTX1	ENSG00000173171	1	155168490	155193615	17	3	123834	-0.49863	0.69098	0.991868871
ZNF438	ENSG00000183621	10	31099136	31330866	532	19	123834	-0.49866	0.69099	0.991868871
GDE1	ENSG00000006007	16	19503015	19543467	129	9	123834	-0.49878	0.69103	0.991868871
TNPO2	ENSG00000105576	19	12800008	12844825	67	2	123834	-0.49877	0.69103	0.991868871
JPH3	ENSG00000154118	16	87625441	87741762	151	25	123834	-0.49888	0.69107	0.991868871
CDK7	ENSG00000134058	5	68520668	68583250	121	9	123834	-0.49903	0.69112	0.991868871
TSPAN3	ENSG00000140391	15	77326359	77386326	14	7	123834	-0.49909	0.69114	0.991868871
TNNT1	ENSG00000105048	19	55634162	55670722	25	7	123834	-0.49915	0.69116	0.991868871
WNT16	ENSG00000002745	7	120955421	120991158	55	7	123834	-0.49924	0.6912	0.991868871
SLC16A5	ENSG00000170190	17	73073822	73112257	66	10	123834	-0.49928	0.69121	0.991868871
AC026740.1	ENSG00000268885	5	657759	678839	40	6	123834	-0.49937	0.69124	0.991868871
FAM219A	ENSG00000164970	9	34388182	34468568	132	15	123834	-0.49946	0.69127	0.991868871
NTN1	ENSG00000065320	17	8914859	9157317	446	48	123834	-0.49995	0.69145	0.991868871
TTC16	ENSG00000167094	9	130468345	130503879	37	6	123834	-0.50014	0.69151	0.991868871
MED11	ENSG00000161920	17	4624723	4646905	29	5	123834	-0.50023	0.69154	0.991868871
OVOL1	ENSG00000172818	11	65544493	65574690	30	6	123834	-0.50025	0.69155	0.991868871
BMP8B	ENSG00000116985	1	40212854	40264533	148	12	123834	-0.50034	0.69158	0.991868871
PEX13	ENSG00000162928	2	61234360	61289125	48	8	123834	-0.50035	0.69159	0.991868871
UPP1	ENSG00000183696	7	48118225	48158330	29	5	123834	-0.50036	0.69159	0.991868871
MED24	ENSG00000008838	17	38165350	38227468	71	8	123834	-0.5004	0.6916	0.991868871
CASP12	ENSG00000204403	11	104746445	104779397	24	2	123834	-0.50045	0.69162	0.991868871
CYP4F3	ENSG00000186529	19	15741707	15783635	165	17	123834	-0.50064	0.69169	0.991868871
FAM83C	ENSG00000125998	20	33863534	33890204	19	2	123834	-0.50066	0.6917	0.991868871
FAF2	ENSG00000113194	5	175864629	175947075	71	9	123834	-0.50071	0.69171	0.991868871

SMO	ENSG00000128602	7	128818713	128863386	61	9	123834	-0.50072	0.69171	0.991868871
ITPKB	ENSG00000143772	1	226809391	226937024	66	9	123834	-0.50073	0.69172	0.991868871
SNX33	ENSG00000173548	15	75930247	75964642	14	3	123834	-0.50095	0.6918	0.991908758
CHD9	ENSG00000177200	16	53078945	53373062	488	15	123834	-0.5017	0.69206	0.992203551
PATL2	ENSG00000229474	15	44947930	45013514	21	6	123834	-0.50184	0.69211	0.992203551
GPR176	ENSG00000166073	15	40081233	40223093	119	22	123834	-0.50226	0.69226	0.992237092
TBC1D13	ENSG00000107021	9	131539483	131582711	93	7	123834	-0.50234	0.69228	0.992237092
PLA2G2E	ENSG00000188784	1	20236502	20260110	50	9	123834	-0.50235	0.69229	0.992237092
PIGO	ENSG00000165282	9	35078685	35106591	35	7	123834	-0.50256	0.69236	0.99224998
FFAR2	ENSG00000126262	19	35924809	35952669	35	9	123834	-0.50283	0.69246	0.99224998
IRX4	ENSG00000113430	5	1867541	1897350	42	12	123834	-0.50298	0.69251	0.99224998
MCFD2	ENSG00000180398	2	47119009	47178994	178	15	123834	-0.50312	0.69256	0.99224998
SLITRK3	ENSG00000121871	3	164894508	164924897	40	5	123834	-0.50312	0.69256	0.99224998
RLBP1	ENSG00000140522	15	89743100	89774982	15	3	123834	-0.50342	0.69266	0.992318456
PPP1R35	ENSG00000160813	7	100022905	100044188	20	2	123834	-0.50366	0.69275	0.992324213
YBX3	ENSG00000060138	12	10841683	10885911	72	9	123834	-0.50377	0.69279	0.992324213
PTPN2	ENSG00000175354	18	12775477	12939642	294	21	123834	-0.50392	0.69284	0.992324213
PLD1	ENSG00000075651	3	171308195	171538740	463	17	123834	-0.50408	0.6929	0.992324213
ZNF595	ENSG00000197701	4	43179	98099	41	5	123834	-0.50473	0.69313	0.992324213
CXCL10	ENSG00000169245	4	76932273	76954650	75	5	123834	-0.50514	0.69327	0.992324213
ERO1LB	ENSG00000086619	1	236368855	236455319	286	10	123834	-0.50542	0.69337	0.992324213
C3orf38	ENSG00000179021	3	88188893	88227879	60	3	123834	-0.50558	0.69342	0.992324213
CNTN5	ENSG00000149972	11	98881683	100239616	3145	160	123834	-0.50562	0.69344	0.992324213
RP11-343C2.12	ENSG00000272617	16	69352547	69378774	35	5	123834	-0.50562	0.69344	0.992324213
ZNF594	ENSG00000180626	17	5072831	5105178	46	6	123834	-0.50575	0.69348	0.992324213
GGNBP1	ENSG00000204188	6	33541515	33566803	57	11	123834	-0.50617	0.69363	0.992324213
FAM83B	ENSG00000168143	6	54701569	54816820	266	19	123834	-0.50619	0.69364	0.992324213
SLC1A7	ENSG00000162383	1	53542855	53618289	226	22	123834	-0.50641	0.69372	0.992324213
OPRM1	ENSG00000112038	6	154321631	154578001	520	40	123834	-0.50642	0.69372	0.992324213
NRIP3	ENSG00000175352	11	8992123	9035596	82	9	123834	-0.50646	0.69373	0.992324213
ABCF1	ENSG00000204574	6	30529153	30574956	62	8	123834	-0.50675	0.69383	0.992324213
7-Sep	ENSG00000122545	7	35830542	35954917	163	7	123834	-0.50696	0.69391	0.992324213
FES	ENSG00000182511	15	91416925	91449006	26	6	123834	-0.50703	0.69393	0.992324213
CUZD1	ENSG00000138161	10	124581665	124649146	74	12	123834	-0.50708	0.69395	0.992324213
PRRC1	ENSG00000164244	5	126843301	126900781	107	5	123834	-0.50723	0.694	0.992324213
GVQW1	ENSG00000241043	9	32556787	32578619	21	4	123834	-0.50814	0.69432	0.992324213
USP37	ENSG00000135913	2	219304974	219443084	177	6	123834	-0.50865	0.6945	0.992324213
4-Sep	ENSG00000108387	17	56587611	56628179	50	8	123834	-0.50885	0.69457	0.992324213
GAS2L2	ENSG00000132139	17	34061530	34089897	49	5	123834	-0.50893	0.6946	0.992324213
KRT5	ENSG00000186081	12	52898359	52924471	65	5	123834	-0.509	0.69462	0.992324213
CYP4F11	ENSG00000171903	19	16013177	16055677	151	13	123834	-0.50918	0.69469	0.992324213
FCGR2B	ENSG00000072694	1	161541101	161658444	46	12	123834	-0.50963	0.69485	0.992324213
ZRSR1	ENSG00000212643	5	112217313	112238791	57	5	123834	-0.50989	0.69494	0.992324213
SKOR1	ENSG00000188779	15	68102042	68136899	85	10	123834	-0.50993	0.69495	0.992324213
SLC25A52	ENSG00000141437	18	29329525	29350843	36	6	123834	-0.50996	0.69496	0.992324213
UBC	ENSG00000150991	12	125386150	125411914	25	5	123834	-0.51004	0.69499	0.992324213
MCIDAS	ENSG00000234602	5	54505442	54533143	44	5	123834	-0.51025	0.69506	0.992324213
TRANK1	ENSG00000168016	3	36858311	36996548	190	15	123834	-0.51031	0.69508	0.992324213
FBXO32	ENSG00000156804	8	124500129	124563446	88	19	123834	-0.51053	0.69516	0.992324213
TMOD3	ENSG00000138594	15	52111825	52249492	257	7	123834	-0.51056	0.69517	0.992324213
TFPI2	ENSG00000105825	7	93504709	93530303	73	6	123834	-0.51067	0.69521	0.992324213
C6orf141	ENSG00000197261	6	49508113	49539620	66	6	123834	-0.51083	0.69527	0.992324213
TTLL5	ENSG00000119685	14	76089968	76431421	365	15	123834	-0.51085	0.69527	0.992324213
SLFN11	ENSG00000172716	17	33667324	33710720	59	13	123834	-0.51096	0.69531	0.992324213
ADPRH	ENSG00000144843	3	119288115	119318792	64	4	123834	-0.5112	0.6954	0.992324213
ANKRD35	ENSG00000198483	1	145539230	145578526	34	3	123834	-0.51148	0.69549	0.992324213
OR10H2	ENSG00000171942	19	15828834	15849862	79	8	123834	-0.51176	0.69559	0.992324213
TEX38	ENSG00000186118	1	47124527	47149266	35	3	123834	-0.51193	0.69565	0.992324213
ETHE1	ENSG00000105755	19	44000871	44041396	81	10	123834	-0.51198	0.69567	0.992324213
TEPP	ENSG00000159648	16	58000339	58032020	35	5	123834	-0.51205	0.69569	0.992324213
OS9	ENSG00000135506	12	58077738	58125340	60	7	123834	-0.51206	0.6957	0.992324213
SMAGP	ENSG00000170545	12	51628342	51674674	68	6	123834	-0.51224	0.69576	0.992324213
GSTM4	ENSG00000168765	1	110188703	110218118	26	7	123834	-0.5123	0.69578	0.992324213
COTL1	ENSG00000103187	16	84589200	84661683	124	19	123834	-0.51277	0.69594	0.992324213
FUBP1	ENSG00000162613	1	78399740	78454794	97	13	123834	-0.51298	0.69602	0.992324213
GALR3	ENSG00000128310	22	38209389	38231502	17	5	123834	-0.5134	0.69616	0.992324213
POR	ENSG00000127948	7	75518518	75626173	95	20	123834	-0.51376	0.69629	0.992324213
LINGO4	ENSG00000213171	1	151762740	151788630	8	1	123834	-0.51405	0.69639	0.992324213
OR10Z1	ENSG00000198967	1	158566229	158587170	41	6	123834	-0.51416	0.69643	0.992324213
RAD23A	ENSG00000179262	19	13046669	13074456	10	3	123834	-0.51418	0.69644	0.992324213
C9orf89	ENSG00000165233	9	95848500	95885565	67	12	123834	-0.51429	0.69648	0.992324213
KIAA1683	ENSG00000130518	19	18357908	18395319	93	10	123834	-0.51449	0.69655	0.992324213
BCS1L	ENSG00000074582	2	219513487	219538166	39	3	123834	-0.51457	0.69657	0.992324213
SEC23B	ENSG00000101310	20	18478137	18552059	198	10	123834	-0.51459	0.69658	0.992324213
OLFM4	ENSG00000102837	13	53592894	53636192	54	10	123834	-0.51463	0.69659	0.992324213
IFNA7	ENSG00000214042	9	21191468	21212204	100	8	123834	-0.51465	0.6966	0.992324213
PCDH12	ENSG00000113555	5	141313150	141359304	53	9	123834	-0.51492	0.69669	0.992324213
ANP32B	ENSG00000136938	9	100735643	100788225	39	5	123834	-0.51526	0.69681	0.992324213
STX5	ENSG00000162236	11	62564369	62609560	54	2	123834	-0.5154	0.69686	0.992324213
TMEM9	ENSG00000116857	1	201093900	201150702	92	14	123834	-0.51562	0.69694	0.992324213
CYP2C9	ENSG00000138109	10	96688415	96759147	207	14	123834	-0.51594	0.69705	0.992324213
CCND2	ENSG00000118971	12	4372938	4424516	15	7	123834	-0.51598	0.69706	0.992324213
HGSNAT	ENSG00000165102	8	42985556	43067998	57	7	123834	-0.51608	0.6971	0.992324213
IK	ENSG00000113141	5	140016643	140052064	41	4	123834	-0.51617	0.69713	0.992324213
HRG	ENSG00000113905	3	186368005	186406029	40	8	123834	-0.5162	0.69714	0.992324213
GUCY1A2	ENSG00000152402	11	106534738	106899250	668	42	123834	-0.51622	0.69715	0.992324213
NPFFR2	ENSG00000056291	4	72887521	73023784	258	6	123834	-0.51655	0.69726	0.992324213
HTR1E	ENSG00000168830	6	87637024	87736349	172	16	123834	-0.51656	0.69727	0.992324213
VAMP3	ENSG00000049245	1	7821329	7851492	56	12	123834	-0.51663	0.69729	0.992324213
CCNL1	ENSG00000163660	3	156854297	156888549	67	5	123834	-0.51691	0.69739	0.992324213
NRP1	ENSG00000099250	10	33456420	33635190	283	30	123834	-0.51729	0.69752	0.992324213
ATG3	ENSG00000144848	3	112241356	112290893	131	7	123834	-0.51756	0.69762	0.992324213
ST3GAL5	ENSG00000115525	2	86056267	86126137	133	14	123834	-0.51759	0.69763	0.992324213
CCT4	ENSG00000115484	2	62085224	62125939	91	11	123834	-0.51764	0.69765	0.992324213
TCP10	ENSG00000203690	6	167760521	167807998	96	16	123834	-0.5177	0.69767	0.992324213
AURKA	ENSG00000087586	20	54934445	54977393	40	9	123834	-0.51779	0.6977	0.992324213
MARCO	ENSG00000019169	2	119689742	119762236	142	16	123834	-0.51803	0.69778	0.992324213
STON1-GTF2A1L	ENSG00000068781	2	48747064	49013654	506	43	123834	-0.51812	0.69781	0.992324213
ANKHD1	ENSG00000131503	5	139771399	139939163	93	10	123834	-0.51811	0.69781	0.992324213

ANKHD1-EIF4EBP3	ENSG00000254996	5	139771493	139939154	93	10	123834	-0.51811	0.69781	0.992324213
RAPGEFL1	ENSG00000108352	17	38323263	38361908	36	5	123834	-0.51811	0.69781	0.992324213
SOCS2	ENSG00000120833	12	93953590	93987263	49	6	123834	-0.51821	0.69785	0.992324213
RP11-595B24.2	ENSG00000263588	18	19658021	19688741	63	10	123834	-0.51835	0.69789	0.992324213
FUT9	ENSG00000172461	6	96453860	96673488	535	30	123834	-0.51869	0.69801	0.992324213
TMEM184C	ENSG00000164168	4	148528534	148603195	59	3	123834	-0.51871	0.69802	0.992324213
ICAM3	ENSG00000076662	19	10434452	10460499	50	8	123834	-0.51876	0.69804	0.992324213
COQ5	ENSG00000110871	12	120931077	120982237	143	14	123834	-0.51916	0.69818	0.992324213
PRB4	ENSG00000230657	12	11450017	11473369	29	4	123834	-0.51921	0.69819	0.992324213
LMF1	ENSG00000103227	16	893634	1041318	530	24	123834	-0.51936	0.69825	0.992324213
RP11-944L7.5	ENSG00000273303	3	44597051	44645665	57	5	123834	-0.51949	0.69829	0.992324213
SLC16A3	ENSG00000141526	17	80176273	80229005	30	6	123834	-0.51968	0.69836	0.992324213
NAIP	ENSG00000249437	5	70254310	70330941	1	1	123834	-0.51995	0.69845	0.992324213
ZFP14	ENSG00000142065	19	36817162	36880101	105	8	123834	-0.51997	0.69846	0.992324213
SPRR1B	ENSG00000169469	1	152993678	153015376	99	2	123834	-0.52015	0.69852	0.992324213
RNF25	ENSG00000163481	2	219518587	219547134	44	4	123834	-0.52014	0.69852	0.992324213
TPM3	ENSG00000143549	1	154117784	154177124	48	7	123834	-0.5204	0.69861	0.992324213
ENDOG	ENSG00000167136	9	131570753	131594956	65	6	123834	-0.52058	0.69867	0.992324213
CLYBL	ENSG00000125246	13	100248919	100559387	440	55	123834	-0.52062	0.69868	0.992324213
PPP1CA	ENSG00000172531	11	67155654	67198654	39	4	123834	-0.5207	0.69871	0.992324213
RP11-796G6.2	ENSG00000258512	14	102186774	102208859	36	1	123834	-0.52096	0.6988	0.992324213
AHSP	ENSG00000169877	16	31529185	31550124	72	8	123834	-0.52101	0.69882	0.992324213
USP24	ENSG00000162402	1	55522032	55690786	209	13	123834	-0.52103	0.69883	0.992324213
B3GALT1	ENSG00000172318	2	168665182	168740551	102	15	123834	-0.52111	0.69886	0.992324213
C4orf51	ENSG00000237136	4	146591356	146702184	146	21	123834	-0.52116	0.69887	0.992324213
VAMP8	ENSG00000118640	2	85778685	85819154	88	10	123834	-0.52122	0.69889	0.992324213
PSMD12	ENSG00000197170	17	65324032	65372743	35	7	123834	-0.52122	0.69889	0.992324213
LCN1	ENSG00000160349	9	138403284	138428378	44	7	123834	-0.52146	0.69898	0.992324213
TANGO6	ENSG00000103047	16	68867507	69129083	351	18	123834	-0.52148	0.69899	0.992324213
CCDC74A	ENSG00000163040	2	132275248	132301239	16	1	123834	-0.52159	0.69902	0.992324213
TTYH1	ENSG00000167614	19	54916393	54958080	54	8	123834	-0.52213	0.69921	0.992324213
TXN2	ENSG00000100348	22	36853083	36888077	48	7	123834	-0.52214	0.69921	0.992324213
ENO2	ENSG00000111674	12	7012909	7042861	23	2	123834	-0.52215	0.69922	0.992324213
SPSB1	ENSG00000171621	1	9342939	9439591	117	28	123834	-0.52226	0.69926	0.992324213
OR5AP2	ENSG00000172464	11	56398942	56419947	47	9	123834	-0.52231	0.69927	0.992324213
ALDH18A1	ENSG00000059573	10	97355696	97426463	208	21	123834	-0.52271	0.69941	0.992324213
C5orf49	ENSG00000215217	5	7820491	7861603	50	8	123834	-0.52314	0.69956	0.992324213
A1BG	ENSG00000121410	19	58846544	58874865	15	1	123834	-0.52324	0.6996	0.992324213
NBN	ENSG00000104320	8	90935564	91025456	148	8	123834	-0.5234	0.69965	0.992324213
ECSCR	ENSG00000249751	5	138774245	138852328	4	2	123834	-0.52357	0.69971	0.992324213
BBIP1	ENSG00000214413	10	112648488	112689032	77	13	123834	-0.52382	0.6998	0.992324213
RGS11	ENSG00000076344	16	308300	335980	12	3	123834	-0.52421	0.69993	0.992324213
MOB3A	ENSG00000172081	19	2061037	2106672	41	13	123834	-0.52426	0.69995	0.992324213
NIT1	ENSG00000158793	1	161077876	161105235	30	4	123834	-0.52438	0.69999	0.992324213
ARMC7	ENSG00000125449	17	73096047	73136360	37	6	123834	-0.52451	0.70004	0.992324213
PAXIP1-AS2	ENSG00000214106	7	154710208	154751618	47	4	123834	-0.52464	0.70008	0.992324213
CYP11B2	ENSG00000179142	8	143981975	144009259	87	7	123834	-0.52462	0.70008	0.992324213
NDUFB6	ENSG00000165264	9	32542997	32583160	37	5	123834	-0.52503	0.70022	0.992324213
CARD9	ENSG00000187796	9	139246355	139278133	69	9	123834	-0.52506	0.70023	0.992324213
SLITRK6	ENSG00000184564	13	86356925	86383623	35	4	123834	-0.52505	0.70023	0.992324213
PLD4	ENSG00000166428	14	105381153	105409574	50	5	123834	-0.52507	0.70023	0.992324213
C9orf131	ENSG00000174038	9	35031092	35055988	27	4	123834	-0.52526	0.7003	0.992324213
CS	ENSG00000062485	12	56655483	56704176	55	3	123834	-0.52532	0.70032	0.992324213
RDH16	ENSG00000139547	12	57335219	57363158	48	4	123834	-0.52547	0.70037	0.992324213
C7orf60	ENSG00000164603	7	112449202	112589971	161	16	123834	-0.52555	0.7004	0.992324213
TRIM14	ENSG00000106785	9	100821557	100891494	57	10	123834	-0.52558	0.70041	0.992324213
EXTL1	ENSG00000158008	1	26336375	26372955	38	4	123834	-0.52561	0.70042	0.992324213
HPSE2	ENSG00000172987	10	100208875	101005619	1324	34	123834	-0.5256	0.70042	0.992324213
NTNG1	ENSG00000162631	1	107672629	108036080	390	41	123834	-0.52568	0.70045	0.992324213
MRPL35	ENSG00000132313	2	86416478	86450917	62	4	123834	-0.52575	0.70047	0.992324213
CDK11A	ENSG00000008128	1	1624169	1665766	4	1	123834	-0.52586	0.70051	0.992324213
PPP6R3	ENSG00000110075	11	68218186	68392802	187	9	123834	-0.52618	0.70062	0.992324213
PSMD11	ENSG00000108671	17	30761279	30820336	30	5	123834	-0.52633	0.70067	0.992324213
C4orf48	ENSG00000243449	4	2033689	2055697	6	1	123834	-0.52641	0.7007	0.992324213
ZNF682	ENSG00000197124	19	20097867	20160315	157	11	123834	-0.52684	0.70085	0.992324213
EIF4G1	ENSG00000114867	3	184022283	184063146	18	5	123834	-0.52687	0.70086	0.992324213
SLC20A1	ENSG00000144136	2	113393434	113431404	59	5	123834	-0.52724	0.70099	0.992324213
NCK2	ENSG00000071051	2	106351354	106520730	307	24	123834	-0.52749	0.70107	0.992324213
HBE1	ENSG00000213931	11	5279582	5536847	1072	38	123834	-0.52756	0.7011	0.992324213
ZNF181	ENSG00000197841	19	35215060	35243777	33	8	123834	-0.52756	0.7011	0.992324213
ADORA1	ENSG00000163485	1	203049782	203146533	98	18	123834	-0.52765	0.70113	0.992324213
CHIT1	ENSG00000133063	1	203171955	203252769	121	18	123834	-0.52803	0.70126	0.992324213
C20orf196	ENSG00000171984	20	5721039	5854558	256	34	123834	-0.52827	0.70134	0.992324213
ROR2	ENSG00000169071	9	94315373	94722444	994	71	123834	-0.52834	0.70137	0.992324213
GMFG	ENSG00000130755	19	39808993	39843012	41	6	123834	-0.52835	0.70137	0.992324213
CMTM6	ENSG00000091317	3	32512804	32554900	60	12	123834	-0.52839	0.70139	0.992324213
SLC6A18	ENSG00000164363	5	1215470	1256304	57	7	123834	-0.52849	0.70142	0.992324213
ZNF483	ENSG00000173258	9	114277439	114350124	161	23	123834	-0.52853	0.70143	0.992324213
NTS	ENSG00000133636	12	86258073	86286770	55	5	123834	-0.52866	0.70148	0.992324213
SCARA3	ENSG00000168077	8	27481385	27544293	111	9	123834	-0.52872	0.7015	0.992324213
VAT1	ENSG00000108828	17	41156622	41187140	17	3	123834	-0.52872	0.7015	0.992324213
ZNF484	ENSG00000127081	9	95597874	95650304	105	14	123834	-0.5291	0.70163	0.992324213
UPB1	ENSG00000100024	22	24853206	24934358	102	11	123834	-0.52913	0.70164	0.992324213
MAGI1	ENSG00000151276	3	65329200	66034509	1023	105	123834	-0.52916	0.70165	0.992324213
RAB11A	ENSG00000103769	15	66008392	66194329	281	15	123834	-0.52924	0.70168	0.992324213
DYNLL1	ENSG00000088986	12	120897653	120946296	118	13	123834	-0.52941	0.70174	0.992324213
PDILT	ENSG00000169340	16	20360492	20426059	121	16	123834	-0.52945	0.70175	0.992324213
STOM	ENSG00000148175	9	124091355	124142531	74	11	123834	-0.52946	0.70176	0.992324213
SRD5A1	ENSG00000145545	5	6623456	6679675	152	16	123834	-0.52952	0.70178	0.992324213
FAM177B	ENSG00000197520	1	222900549	222934147	39	8	123834	-0.52957	0.7018	0.992324213
UGT2A2	ENSG00000271271	4	70444135	70515360	237	18	123834	-0.53025	0.70203	0.992398521
AL078585.1	ENSG00000225683	6	163576446	163622840	34	9	123834	-0.53057	0.70214	0.992398521
FAM220A	ENSG00000178397	7	6359040	6398612	69	10	123834	-0.53077	0.70221	0.992398521
MAST4	ENSG00000069020	5	65882176	66475423	729	64	123834	-0.53103	0.7023	0.992398521
PPFIA1	ENSG00000131626	11	70106806	70240509	210	5	123834	-0.53103	0.7023	0.992398521
MRGPRD	ENSG00000172938	11	68737490	68758455	31	5	123834	-0.53109	0.70232	0.992398521
CES3	ENSG00000172828	16	66985140	67019051	24	5	123834	-0.53113	0.70234	0.992398521
MAP10	ENSG00000212916	1	232930643	232956092	33	5	123834	-0.53156	0.70249	0.992398521
PELI3	ENSG00000174516	11	66224216	66254808	27	4	123834	-0.53156	0.70249	0.992398521

ZNF626	ENSG00000188171	19	20792867	20854402	210	16	123834	-0.53158	0.70249	0.992398521
ITGA8	ENSG00000077943	10	15545948	15772124	374	28	123834	-0.53162	0.70251	0.992398521
PIGT	ENSG00000124155	20	44034717	44064884	14	3	123834	-0.53182	0.70257	0.992398521
ZCCHC14	ENSG00000140948	16	87429852	87535651	268	9	123834	-0.53183	0.70258	0.992398521
METTL14	ENSG00000145388	4	119596523	119646588	85	8	123834	-0.53211	0.70267	0.992398521
CALCB	ENSG00000175868	11	14916543	15113888	216	9	123834	-0.53219	0.7027	0.992398521
UPP2	ENSG00000007001	2	158723214	159002666	413	44	123834	-0.5325	0.70281	0.992398521
MYOC	ENSG00000034971	1	171594557	171631823	91	14	123834	-0.53281	0.70292	0.992398521
ARAP1	ENSG00000186635	11	72386114	72514644	203	18	123834	-0.53282	0.70292	0.992398521
NOL6	ENSG00000165271	9	33451439	33483928	51	7	123834	-0.53283	0.70293	0.992398521
OR5T2	ENSG00000181718	11	55989490	56010737	50	5	123834	-0.5329	0.70295	0.992398521
PAAF1	ENSG00000175575	11	73577744	73648790	173	14	123834	-0.5329	0.70295	0.992398521
TYK2	ENSG00000105397	19	10451209	10501352	82	11	123834	-0.53321	0.70305	0.992398521
TTC36	ENSG00000172425	11	118388187	118411912	5	1	123834	-0.53351	0.70316	0.992398521
PFN3	ENSG00000196570	5	176817108	176837637	7	1	123834	-0.53353	0.70317	0.992398521
VARS2	ENSG00000137411	6	30866019	30904236	91	10	123834	-0.53356	0.70318	0.992398521
RAD23B	ENSG00000119318	9	110035418	110104475	166	15	123834	-0.53367	0.70321	0.992398521
WDR37	ENSG00000047056	10	1085478	1188237	182	20	123834	-0.53392	0.7033	0.992451849
ACTBL2	ENSG00000169067	5	56765846	56788636	46	7	123834	-0.53421	0.7034	0.992506776
OR10W1	ENSG00000172772	11	58024264	58045732	77	7	123834	-0.53438	0.70346	0.992506776
FAM105B	ENSG00000154124	5	14654773	14709820	67	7	123834	-0.53473	0.70358	0.992506776
VTN	ENSG00000109072	17	26681290	26710110	38	3	123834	-0.53474	0.70358	0.992506776
KRT86	ENSG00000170442	12	52633084	52712947	162	16	123834	-0.53479	0.7036	0.992506776
MOV10	ENSG00000155363	1	113205763	113253368	48	6	123834	-0.53501	0.70368	0.992545966
RINL	ENSG00000187994	19	39348470	39378919	7	3	123834	-0.53519	0.70374	0.992556943
GPD2	ENSG00000115159	2	157281802	157480247	196	16	123834	-0.53544	0.70383	0.992578892
NOL3	ENSG00000140939	16	67194057	67219643	13	3	123834	-0.53555	0.70386	0.992578892
RBM22	ENSG00000086589	5	150060356	150090669	14	3	123834	-0.53607	0.70404	0.992647832
DLD	ENSG00000091140	7	107521415	107582175	33	5	123834	-0.53611	0.70406	0.992647832
ZSCAN29	ENSG00000140265	15	43640370	43673223	47	4	123834	-0.53637	0.70415	0.992647832
METTL11B	ENSG00000203740	1	170105142	170146931	118	14	123834	-0.53644	0.70417	0.992647832
DYNC2H1	ENSG00000187240	11	102970160	103360591	1412	25	123834	-0.53642	0.70417	0.992647832
TM9SF3	ENSG00000077147	10	98267866	98357209	132	6	123834	-0.53659	0.70423	0.992658794
CCDC177	ENSG00000267909	14	70028216	70050339	35	6	123834	-0.5373	0.70447	0.992691569
NUDT16	ENSG00000198585	3	131090515	131117674	36	4	123834	-0.53806	0.70473	0.992691569
TSPAN14	ENSG00000108219	10	82203922	82302879	154	16	123834	-0.53813	0.70476	0.992691569
CCDC6	ENSG00000108091	10	61538521	61676414	294	14	123834	-0.53819	0.70478	0.992691569
TAS2R13	ENSG00000212128	12	11050525	11072161	59	3	123834	-0.53824	0.70479	0.992691569
MED10	ENSG00000133398	5	6361994	6388707	68	10	123834	-0.53829	0.70481	0.992691569
EIF4E	ENSG00000151247	4	99782835	99861788	139	12	123834	-0.53849	0.70488	0.992691569
BLOC1S5	ENSG00000188428	6	8003800	8074647	240	15	123834	-0.53851	0.70489	0.992691569
NUP62	ENSG00000213024	19	50400082	50443020	69	14	123834	-0.53882	0.70499	0.992691569
EGR3	ENSG00000179388	8	22535172	22560815	4	2	123834	-0.53921	0.70513	0.992691569
BIRC6	ENSG00000115760	2	32572096	32853966	255	20	123834	-0.53927	0.70515	0.992691569
WFDC3	ENSG00000124116	20	44366583	44430571	250	14	123834	-0.53952	0.70524	0.992691569
HOOK3	ENSG00000168172	8	42742075	42895682	177	9	123834	-0.5396	0.70526	0.992691569
TRMT61B	ENSG00000171103	2	29062687	29103167	51	5	123834	-0.53961	0.70527	0.992691569
TBC1D7	ENSG00000145979	6	13256774	13338815	227	17	123834	-0.53963	0.70527	0.992691569
SCGB1D1	ENSG00000168515	11	61947688	61971011	60	5	123834	-0.53964	0.70528	0.992691569
CCDC65	ENSG00000139537	12	49287893	49335623	94	5	123834	-0.53964	0.70528	0.992691569
HRH1	ENSG00000196639	3	11168779	11315243	212	32	123834	-0.53967	0.70529	0.992691569
COPZ2	ENSG00000005243	17	46093533	46125392	71	11	123834	-0.53983	0.70534	0.992691569
AC013468.1	ENSG00000231145	2	190632909	190653157	40	5	123834	-0.53994	0.70538	0.992691569
KIAA0513	ENSG00000135709	16	85051375	85137836	250	27	123834	-0.54037	0.70553	0.992691569
FGFR1OP2	ENSG00000111790	12	27081316	27129583	65	13	123834	-0.54059	0.7056	0.992691569
RP11-998D10.1	ENSG00000255472	14	21490977	21512054	15	5	123834	-0.54065	0.70563	0.992691569
RNASE13	ENSG00000206150	14	21490979	21512944	15	5	123834	-0.54065	0.70563	0.992691569
ZC3H10	ENSG00000135482	12	56501943	56526278	9	2	123834	-0.54069	0.70564	0.992691569
SZRD1	ENSG00000055070	1	16669070	16734640	116	9	123834	-0.5408	0.70568	0.992691569
UTS2B	ENSG00000188958	3	190974957	191058325	145	16	123834	-0.54097	0.70574	0.992691569
FAM180A	ENSG00000189320	7	135403096	135443594	72	13	123834	-0.54111	0.70578	0.992691569
SNTN	ENSG00000188817	3	63628344	63674696	115	12	123834	-0.54114	0.70579	0.992691569
RP5-1052I5.2	ENSG00000267561	1	87448692	87644884	280	19	123834	-0.54122	0.70582	0.992691569
HSD11B2	ENSG00000176387	16	67454555	67481456	31	5	123834	-0.54147	0.70591	0.992744694
ANPEP	ENSG00000166825	15	90318120	90368633	65	7	123834	-0.54194	0.70607	0.992805598
GGH	ENSG00000137563	8	63917638	63961730	128	12	123834	-0.54203	0.7061	0.992805598
COX6A1	ENSG00000111775	12	120865893	120888545	36	8	123834	-0.54205	0.70611	0.992805598
KIF17	ENSG00000117245	1	20980507	21054510	114	25	123834	-0.54262	0.70631	0.992957735
RPS13	ENSG00000110700	11	17085936	17109334	38	7	123834	-0.54277	0.70636	0.992957735
BOK	ENSG00000176720	2	242488136	242523546	54	12	123834	-0.54288	0.70639	0.992957735
NID1	ENSG00000116962	1	236129130	236238462	137	19	123834	-0.54342	0.70658	0.992957735
NRBF2	ENSG00000148572	10	64883050	64924783	78	8	123834	-0.54349	0.7066	0.992957735
FAM150A	ENSG00000196711	8	53436597	53488067	86	9	123834	-0.54371	0.70668	0.992957735
TMEM215	ENSG00000188133	9	32773497	32797397	57	6	123834	-0.54381	0.70672	0.992957735
C11orf24	ENSG00000171067	11	68018803	68049469	37	5	123834	-0.54384	0.70672	0.992957735
BTBD10	ENSG00000148925	11	13399548	13494844	172	10	123834	-0.544	0.70678	0.992957735
C19orf26	ENSG00000099625	19	1218286	1248026	24	3	123834	-0.54431	0.70689	0.992957735
RHOB	ENSG00000143878	2	20636835	20659200	9	3	123834	-0.54442	0.70692	0.992957735
SIRPB2	ENSG00000196209	20	1441386	1482233	77	10	123834	-0.54442	0.70692	0.992957735
RAP1A	ENSG00000116473	1	112074840	112269313	359	29	123834	-0.54446	0.70694	0.992957735
HSPB7	ENSG00000173641	1	16330523	16356089	114	12	123834	-0.54483	0.70706	0.992957735
KRTAP5-8	ENSG00000241233	11	71239071	71260253	66	4	123834	-0.54488	0.70708	0.992957735
DAP3	ENSG00000132676	1	155647751	155718801	56	6	123834	-0.54491	0.70709	0.992957735
DAPK2	ENSG00000035664	15	64189235	64374232	372	28	123834	-0.54509	0.70715	0.992957735
ARFGEF2	ENSG00000124198	20	47528427	47663230	276	13	123834	-0.54523	0.7072	0.992957735
HNRNPL	ENSG00000104824	19	39317028	39352987	48	6	123834	-0.54597	0.70746	0.992957735
RELT	ENSG00000054967	11	73077309	73118519	40	5	123834	-0.54607	0.70749	0.992957735
S100P	ENSG00000163993	4	6684796	6708897	26	6	123834	-0.54644	0.70762	0.992957735
ARGFX	ENSG00000186103	3	121279551	121315481	28	7	123834	-0.54649	0.70763	0.992957735
HS2ST1	ENSG00000153936	1	87370331	87612334	377	15	123834	-0.54662	0.70768	0.992957735
KLHL20	ENSG00000076321	1	173674080	173765840	71	5	123834	-0.54681	0.70774	0.992957735
SP100	ENSG00000067066	2	231270657	231418805	311	26	123834	-0.5469	0.70778	0.992957735
ZNF397	ENSG00000186812	18	32810994	32857097	60	8	123834	-0.5469	0.70778	0.992957735
OXTR	ENSG00000180914	3	8782094	8821314	61	17	123834	-0.54701	0.70782	0.992957735
SPTA1	ENSG00000163554	1	158570496	158666488	136	12	123834	-0.54705	0.70783	0.992957735
RPL41	ENSG00000229117	12	56500370	56521727	8	2	123834	-0.54715	0.70786	0.992957735
CDCA2	ENSG00000184661	8	25306513	25375436	126	5	123834	-0.5472	0.70788	0.992957735
OAZ2	ENSG00000180304	15	64969772	65005480	34	4	123834	-0.54748	0.70798	0.992957735
TCERG1	ENSG00000113649	5	145816874	145901524	170	12	123834	-0.54758	0.70801	0.992957735

SAG	ENSG00000130561	2	234206462	234265701	116	16	123834	-0.54788	0.70811	0.992957735
PPM1N	ENSG00000213889	19	45982035	46015768	50	9	123834	-0.54806	0.70818	0.992957735
AQP6	ENSG00000086159	12	50350977	50380922	17	4	123834	-0.54809	0.70819	0.992957735
CYP26B1	ENSG00000003137	2	72346367	72385167	27	5	123834	-0.54813	0.7082	0.992957735
ZNF45	ENSG00000124459	19	44406781	44449430	97	9	123834	-0.54813	0.7082	0.992957735
SMIM2	ENSG00000139656	13	44707679	44745393	24	4	123834	-0.54816	0.70821	0.992957735
ADAM33	ENSG00000149451	20	3638612	3672893	47	15	123834	-0.54847	0.70832	0.992957735
OR4K15	ENSG00000169488	14	20433603	20454818	81	6	123834	-0.54854	0.70834	0.992957735
CCDC104	ENSG00000163001	2	55736740	55783015	86	13	123834	-0.5486	0.70836	0.992957735
KRTAP17-1	ENSG00000186860	17	39461173	39481947	60	5	123834	-0.549	0.7085	0.993080746
C15orf43	ENSG00000167014	15	45238900	45281427	103	5	123834	-0.54929	0.7086	0.993147677
C2orf70	ENSG00000173557	2	26775450	26812400	27	5	123834	-0.5499	0.70881	0.993340554
DIP2B	ENSG00000066084	12	50888768	51152450	356	13	123834	-0.55046	0.709	0.993340554
UBAC1	ENSG00000130560	9	138814815	138863226	92	9	123834	-0.55063	0.70906	0.993340554
E2F6	ENSG00000169016	2	11574501	11616297	34	2	123834	-0.55073	0.70909	0.993340554
MAD2L2	ENSG00000116670	1	11724537	11761707	40	11	123834	-0.5509	0.70915	0.993340554
CLDN3	ENSG00000165215	7	73173328	73194600	33	3	123834	-0.55094	0.70916	0.993340554
POM121L12	ENSG00000221900	7	53093349	53114617	42	7	123834	-0.55127	0.70927	0.993340554
PTBP3	ENSG00000119314	9	114970715	115105947	298	8	123834	-0.55153	0.70937	0.993340554
MTTP	ENSG00000138823	4	100474918	100555156	251	21	123834	-0.55161	0.70939	0.993340554
CDH9	ENSG00000113100	5	26870709	27131257	356	25	123834	-0.55176	0.70944	0.993340554
C11orf48	ENSG00000162194	11	62420287	62449727	18	2	123834	-0.55267	0.70975	0.993340554
KRTAP10-12	ENSG00000189169	21	46107087	46127959	58	10	123834	-0.55296	0.70986	0.993340554
MTF2	ENSG00000143033	1	93534792	93614638	68	5	123834	-0.55305	0.70988	0.993340554
TTLL2	ENSG00000120440	6	167728574	167782991	111	17	123834	-0.55333	0.70998	0.993340554
GNL2	ENSG00000134697	1	38022417	38071536	81	10	123834	-0.55365	0.71009	0.993340554
ZNF689	ENSG00000156853	16	30603879	30645333	17	2	123834	-0.55372	0.71012	0.993340554
SEMA4G	ENSG00000095539	10	102719275	102755628	31	4	123834	-0.5538	0.71014	0.993340554
AP003774.4	ENSG00000181908	11	64206546	64229126	1	1	123834	-0.5538	0.71014	0.993340554
PDE6H	ENSG00000139053	12	15115956	15144799	70	7	123834	-0.55386	0.71016	0.993340554
BRIP1	ENSG00000136492	17	59748627	59950882	180	14	123834	-0.55384	0.71016	0.993340554
MCU	ENSG00000156026	10	74441889	74657452	126	5	123834	-0.55398	0.71021	0.993340554
OR8H2	ENSG00000181767	11	55862519	55883457	137	5	123834	-0.55416	0.71027	0.993340554
LDHAL6A	ENSG00000166800	11	18467371	18511147	68	11	123834	-0.55449	0.71038	0.993340554
PKMYT1	ENSG00000127564	16	3008025	3040540	49	6	123834	-0.55449	0.71038	0.993340554
RPS27	ENSG00000177954	1	153953235	153974626	10	4	123834	-0.55454	0.71039	0.993340554
DNMT3L	ENSG00000142182	21	45656222	45692099	60	13	123834	-0.55459	0.71041	0.993340554
ACAT1	ENSG00000075239	11	107982243	108028503	20	4	123834	-0.55463	0.71043	0.993340554
GPC5	ENSG00000179399	13	92040929	93529490	2261	131	123834	-0.55464	0.71043	0.993340554
RP11-287D1.3	ENSG00000264324	2	74428731	74600504	154	16	123834	-0.55497	0.71054	0.993340554
TIMM9	ENSG00000100575	14	58865212	58904332	14	2	123834	-0.55502	0.71056	0.993340554
OXCT1	ENSG00000083720	5	41720167	41880621	151	8	123834	-0.55507	0.71058	0.993340554
PECR	ENSG00000115425	2	216851052	216957678	179	25	123834	-0.55512	0.71059	0.993340554
FKBP5	ENSG00000096060	6	35531362	35706360	220	16	123834	-0.55518	0.71061	0.993340554
ADCK2	ENSG00000133597	7	140362953	140406061	66	6	123834	-0.55527	0.71064	0.993340554
TULP2	ENSG00000104804	19	49374222	49411990	102	8	123834	-0.55537	0.71068	0.993340554
ATP6V0D2	ENSG00000147614	8	86989552	87176457	275	17	123834	-0.55564	0.71077	0.993340554
RIPPLY3	ENSG00000183145	21	38368450	38401959	41	7	123834	-0.55658	0.71109	0.993340554
COG8	ENSG00000213380	16	69344043	69383570	52	7	123834	-0.5566	0.7111	0.993340554
KIF18B	ENSG00000186185	17	42992077	43035082	99	17	123834	-0.55673	0.71115	0.993340554
OR5H2	ENSG00000197938	3	97991732	98012676	98	8	123834	-0.55683	0.71118	0.993340554
RAMP2	ENSG00000131477	17	40900465	40925059	5	3	123834	-0.55712	0.71128	0.993340554
MSI2	ENSG00000153944	17	55323212	55772046	458	82	123834	-0.55716	0.71129	0.993340554
SLC18A1	ENSG00000036565	8	19992366	20050717	112	14	123834	-0.55723	0.71131	0.993340554
ZNF561	ENSG00000171469	19	9705356	9742075	45	4	123834	-0.55729	0.71134	0.993340554
MZT2B	ENSG00000152082	2	130929310	130958302	40	2	123834	-0.55733	0.71135	0.993340554
KLHL2	ENSG00000109466	4	166118770	166254308	167	17	123834	-0.55743	0.71138	0.993340554
ZNF565	ENSG00000196357	19	36663188	36747159	170	11	123834	-0.55755	0.71142	0.993340554
COLGALT1	ENSG00000130309	19	17656403	17703971	75	13	123834	-0.55757	0.71143	0.993340554
LDHC	ENSG00000166796	11	18423854	18483605	193	11	123834	-0.55763	0.71145	0.993340554
PLAT	ENSG00000104368	8	42022236	42075242	34	5	123834	-0.55786	0.71153	0.993340554
URB2	ENSG00000135763	1	229751981	229805946	65	6	123834	-0.55831	0.71168	0.993340554
POLR3A	ENSG00000148606	10	79724907	79799303	68	5	123834	-0.5586	0.71178	0.993340554
NLRP14	ENSG00000158077	11	7031677	7102539	235	14	123834	-0.55871	0.71182	0.993340554
ATP5S	ENSG00000125375	14	50769044	50812276	76	3	123834	-0.55884	0.71186	0.993340554
BTBD19	ENSG00000222009	1	45264154	45291257	31	4	123834	-0.5589	0.71188	0.993340554
MYO1E	ENSG00000157483	15	59417113	59675099	355	40	123834	-0.55896	0.71191	0.993340554
C1orf145	ENSG00000162913	1	228381207	228411365	45	4	123834	-0.55953	0.7121	0.993340554
HEPACAM	ENSG00000165478	11	124779089	124816308	46	13	123834	-0.55958	0.71212	0.993340554
ACTL10	ENSG00000182584	20	32244304	32266331	6	2	123834	-0.55993	0.71224	0.993340554
MFAP3L	ENSG00000198948	4	170897748	170964182	60	7	123834	-0.5601	0.71229	0.993340554
NLRX1	ENSG00000160703	11	119027277	119064725	20	4	123834	-0.56031	0.71236	0.993340554
CYSTM1	ENSG00000120306	5	139544227	139671637	174	8	123834	-0.56047	0.71242	0.993340554
MYO1F	ENSG00000142347	19	8575674	8652461	87	10	123834	-0.56046	0.71242	0.993340554
C16orf95	ENSG00000260456	16	87107168	87361022	458	48	123834	-0.56049	0.71243	0.993340554
NOP56	ENSG00000101361	20	2622791	2649039	89	10	123834	-0.5606	0.71247	0.993340554
SOX30	ENSG00000039600	5	157042687	157108488	89	14	123834	-0.56083	0.71254	0.993340554
SERTAD2	ENSG00000179833	2	64848755	64988139	136	11	123834	-0.56097	0.71259	0.993340554
LAMB1	ENSG00000091136	7	107554244	107653700	77	14	123834	-0.56104	0.71261	0.993340554
ZC3HC1	ENSG00000091732	7	129648126	129701291	38	7	123834	-0.56122	0.71268	0.993340554
FOXL1	ENSG00000176678	16	86599974	86625303	17	4	123834	-0.56126	0.71269	0.993340554
CHST3	ENSG00000122863	10	73714123	73783322	95	11	123834	-0.56141	0.71274	0.993340554
OLFML3	ENSG00000116774	1	114512063	114534876	44	7	123834	-0.56144	0.71275	0.993340554
BORA	ENSG00000136122	13	73292061	73340336	51	8	123834	-0.56198	0.71293	0.993340554
DGKE	ENSG00000153933	17	54901460	54956036	81	4	123834	-0.56195	0.71293	0.993340554
DAND5	ENSG00000179284	19	13065973	13095567	2	1	123834	-0.56216	0.713	0.993340554
AC010536.1	ENSG00000226180	16	87717144	87739753	42	7	123834	-0.56286	0.71324	0.993340554
TXNDC15	ENSG00000113621	5	134199493	134247215	33	6	123834	-0.56301	0.71329	0.993340554
GLE1	ENSG00000119392	9	131256979	131314567	54	5	123834	-0.56317	0.71334	0.993340554
AC073610.5	ENSG00000255863	12	49305767	49343446	79	4	123834	-0.56321	0.71335	0.993340554
KATNBL1	ENSG00000134152	15	34422875	34512297	219	11	123834	-0.56342	0.71343	0.993340554
AP003062.1	ENSG00000268936	11	134845246	134866693	87	7	123834	-0.56363	0.7135	0.993340554
AL590560.1	ENSG00000269620	1	159869884	159890159	17	5	123834	-0.56373	0.71353	0.993340554
C12orf40	ENSG00000180116	12	40009969	40312102	475	19	123834	-0.56406	0.71364	0.993340554
MFN1	ENSG00000171109	3	179055480	179122719	89	10	123834	-0.56407	0.71365	0.993340554
MYLK	ENSG00000065534	3	123318896	123613178	317	25	123834	-0.56411	0.71366	0.993340554
IFI16	ENSG00000163565	1	158959758	159034945	212	14	123834	-0.56414	0.71367	0.993340554
POLN	ENSG00000130997	4	2063645	2253848	354	7	123834	-0.56438	0.71375	0.993340554
MAPK9	ENSG00000050748	5	179650143	179729099	131	18	123834	-0.5646	0.71383	0.993340554

WDR55	ENSG00000120314	5	140034261	140063709	28	3	123834	-0.56481	0.7139	0.993340554
CPA3	ENSG00000163751	3	148573043	148624983	133	9	123834	-0.56505	0.71398	0.993340554
SETD9	ENSG00000155542	5	56195087	56231359	79	6	123834	-0.56506	0.71398	0.993340554
MPI	ENSG00000178802	15	75172346	75201798	29	5	123834	-0.56509	0.71399	0.993340554
ACVR1B	ENSG00000135503	12	52335451	52400862	57	11	123834	-0.56527	0.71405	0.993340554
CGGBP1	ENSG00000163320	3	88091094	88209035	214	4	123834	-0.5653	0.71407	0.993340554
FRG1B	ENSG00000149531	20	29601857	29644010	13	3	123834	-0.56533	0.71407	0.993340554
SS18L1	ENSG00000184402	20	60708822	60767540	153	10	123834	-0.56542	0.71411	0.993340554
C6orf52	ENSG00000137434	6	10661651	10705030	8	2	123834	-0.56561	0.71417	0.993340554
DNM1L	ENSG00000087470	12	32822134	32908486	270	4	123834	-0.56564	0.71418	0.993340554
HERC6	ENSG00000138642	4	89289891	89374263	14	5	123834	-0.56576	0.71422	0.993340554
HEATR2	ENSG00000164818	7	756338	839190	214	15	123834	-0.56585	0.71425	0.993340554
KRT28	ENSG00000173908	17	38938455	38966211	34	8	123834	-0.56596	0.71429	0.993340554
MBOAT7	ENSG00000125505	19	54667107	54703733	16	4	123834	-0.56598	0.7143	0.993340554
KLF13	ENSG00000169926	15	31609058	31737868	141	21	123834	-0.56625	0.71439	0.993340554
SCN4A	ENSG00000007314	17	62005914	62060278	52	8	123834	-0.56624	0.71439	0.993340554
DND1	ENSG00000256453	5	140040379	140063171	24	3	123834	-0.56651	0.71448	0.993340554
MFAP2	ENSG00000117122	1	17290997	17317330	33	7	123834	-0.56659	0.71451	0.993340554
RTF1	ENSG00000137815	15	41690606	41785761	139	11	123834	-0.56662	0.71451	0.993340554
IL20RB	ENSG00000174564	3	136655072	136739927	23	4	123834	-0.56669	0.71454	0.993340554
CNOT3	ENSG00000088038	19	54631444	54669419	32	7	123834	-0.56686	0.7146	0.993340554
CCP110	ENSG00000103540	16	19525133	19574730	133	12	123834	-0.56693	0.71462	0.993340554
RP11-712L6.5	ENSG00000255062	11	126154193	126184213	58	11	123834	-0.56717	0.7147	0.993340554
CCDC181	ENSG00000117477	1	169354108	169439907	193	19	123834	-0.56741	0.71478	0.993340554
PLK3	ENSG00000173846	1	45255897	45281662	27	3	123834	-0.5675	0.71481	0.993340554
PKDCC	ENSG00000162878	2	42265160	42295668	73	6	123834	-0.56748	0.71481	0.993340554
MEI1	ENSG00000167077	22	42085503	42205460	205	10	123834	-0.56765	0.71486	0.993340554
ABHD4	ENSG00000100439	14	23057146	23091265	36	10	123834	-0.56785	0.71493	0.993340554
PPCS	ENSG00000127125	1	42911788	42949056	74	5	123834	-0.56799	0.71498	0.993340554
CLPSL2	ENSG00000196748	6	35734371	35757329	12	3	123834	-0.56809	0.71501	0.993340554
C2orf61	ENSG00000239605	2	47262677	47392517	313	38	123834	-0.5682	0.71505	0.993340554
AGAP3	ENSG00000133612	7	150772918	150851523	54	10	123834	-0.56821	0.71505	0.993340554
ABCA13	ENSG00000179869	7	48201055	48697092	1388	48	123834	-0.56855	0.71517	0.993340554
ZSCAN30	ENSG00000186814	18	32821032	32880196	72	9	123834	-0.56859	0.71518	0.993340554
AGT	ENSG00000135744	1	230828269	230860043	89	11	123834	-0.56878	0.71525	0.993340554
PPP2R5C	ENSG00000078304	14	102218135	102404326	384	34	123834	-0.56886	0.71527	0.993340554
FAM219B	ENSG00000178761	15	75182328	75209462	28	4	123834	-0.56886	0.71527	0.993340554
PPP2R5B	ENSG00000068971	11	64675025	64711945	46	4	123834	-0.5695	0.71549	0.993387025
ULK3	ENSG00000140474	15	75118457	75145687	25	5	123834	-0.56951	0.7155	0.993387025
ASTN2	ENSG00000148219	9	119177504	120187348	1647	102	123834	-0.56965	0.71554	0.993387025
RP11-407N17.3	ENSG00000258941	14	39693106	39830397	252	13	123834	-0.56989	0.71562	0.993387025
ZNF319	ENSG00000166188	16	58018572	58044357	31	5	123834	-0.56998	0.71566	0.993387025
SUMO3	ENSG00000184900	21	46215532	46248694	35	6	123834	-0.5705	0.71583	0.993387025
CD248	ENSG00000174807	11	66071958	66094515	20	3	123834	-0.57056	0.71585	0.993387025
CEP250	ENSG00000126001	20	34032985	34109804	72	5	123834	-0.57071	0.7159	0.993387025
SNAP91	ENSG00000065609	6	84252599	84429410	222	17	123834	-0.57089	0.71596	0.993387025
FAM231A	ENSG00000237847	1	16989498	17010007	2	1	123834	-0.57108	0.71603	0.993387025
ZNF365	ENSG00000138311	10	64123951	64441771	620	51	123834	-0.57125	0.71608	0.993387025
GCK	ENSG00000106633	7	44173872	44247769	115	17	123834	-0.57142	0.71614	0.993387025
TNKS2	ENSG00000107854	10	93548069	93635033	167	12	123834	-0.57141	0.71614	0.993387025
GTF2H3	ENSG00000111358	12	124108375	124157153	94	8	123834	-0.57175	0.71625	0.993387025
C10orf53	ENSG00000178645	10	50877697	50928307	104	11	123834	-0.57184	0.71628	0.993387025
SCML4	ENSG00000146285	6	108015308	108155521	231	24	123834	-0.57193	0.71631	0.993387025
NBEA	ENSG00000172915	13	35506424	36257159	1352	40	123834	-0.57202	0.71635	0.993387025
USP7	ENSG00000187555	16	8975951	9068371	126	9	123834	-0.57209	0.71637	0.993387025
ARHGEF7	ENSG00000102606	13	111756906	111968084	361	33	123834	-0.5722	0.71641	0.993387025
EIF3B	ENSG00000106263	7	2383721	2430380	47	6	123834	-0.57231	0.71645	0.993387025
KLHL26	ENSG00000167487	19	18737775	18791309	79	12	123834	-0.57262	0.71655	0.993387025
COL1A2	ENSG00000164692	7	94013873	94070544	77	15	123834	-0.57265	0.71656	0.993387025
SLC7A13	ENSG00000164893	8	87216281	87343375	286	20	123834	-0.57269	0.71657	0.993387025
ABCB10	ENSG00000135776	1	229642329	229704442	54	3	123834	-0.5727	0.71658	0.993387025
HNRNPH1	ENSG00000169045	5	179031179	179071785	79	12	123834	-0.57281	0.71661	0.993387025
PLEKHD1	ENSG00000175985	14	69941409	70005215	104	17	123834	-0.57334	0.71679	0.993417342
PURB	ENSG00000146676	7	44905896	44934960	39	5	123834	-0.57347	0.71684	0.993417342
ADAM9	ENSG00000168615	8	38844388	38972663	143	12	123834	-0.57425	0.7171	0.993417342
ERGIC3	ENSG00000125991	20	34119770	34155405	58	5	123834	-0.57435	0.71714	0.993417342
GOLGA8H	ENSG00000261794	15	30886329	30916764	1	1	123834	-0.57472	0.71726	0.993417342
LIN7B	ENSG00000104863	19	49607581	49631717	28	5	123834	-0.57482	0.71729	0.993417342
DRC1	ENSG00000157856	2	26614784	26689579	85	12	123834	-0.57497	0.71734	0.993417342
ZNF525	ENSG00000203326	19	53858946	53905470	85	12	123834	-0.57514	0.7174	0.993417342
AP000304.12	ENSG00000249209	21	34946993	35294635	335	43	123834	-0.57538	0.71748	0.993417342
GPR113	ENSG00000173567	2	26521041	26579685	61	14	123834	-0.5754	0.71749	0.993417342
MME	ENSG00000196549	3	154731913	154911497	250	27	123834	-0.57558	0.71755	0.993417342
OR51G1	ENSG00000176879	11	4934531	4955637	56	12	123834	-0.57603	0.7177	0.993417342
ANLN	ENSG00000011426	7	36419415	36503400	199	24	123834	-0.57628	0.71779	0.993417342
IWS1	ENSG00000163166	2	128183783	128294462	182	14	123834	-0.57634	0.71781	0.993417342
KRT84	ENSG00000161849	12	52761596	52789436	115	9	123834	-0.57639	0.71782	0.993417342
RTN1	ENSG00000139970	14	60052694	60347684	305	15	123834	-0.57652	0.71787	0.993417342
ZNF550	ENSG00000251369	19	58036625	58081231	27	5	123834	-0.57653	0.71787	0.993417342
GOLGA7B	ENSG00000155265	10	99599996	99641337	38	4	123834	-0.57665	0.71791	0.993417342
ARHGDIG	ENSG00000242173	16	308726	343003	34	8	123834	-0.5767	0.71793	0.993417342
ZCWPW1	ENSG00000078487	7	99988449	100036615	40	3	123834	-0.57676	0.71795	0.993417342
OR6C70	ENSG00000184954	12	55852984	55873922	70	7	123834	-0.57685	0.71798	0.993417342
KCNS2	ENSG00000156486	8	99429250	99455076	22	6	123834	-0.5769	0.718	0.993417342
ETV6	ENSG00000139083	12	11792788	12058336	256	39	123834	-0.57705	0.71805	0.993417342
R3HDM2	ENSG00000179912	12	57633392	57834788	162	7	123834	-0.57717	0.71809	0.993417342
SMARCA2	ENSG00000080503	9	2005342	2203624	214	41	123834	-0.57795	0.71835	0.993417342
CAMTA1	ENSG00000171735	1	6835384	7839766	1082	118	123834	-0.57799	0.71836	0.993417342
MOGAT1	ENSG00000124003	2	223526505	223584649	99	15	123834	-0.57799	0.71836	0.993417342
TDRD12	ENSG00000173809	19	33200659	33330483	259	13	123834	-0.57844	0.71852	0.993417342
TMEM194B	ENSG00000189362	2	191359068	191409448	85	10	123834	-0.57856	0.71856	0.993417342
DICER1	ENSG00000100697	14	95542565	95634347	96	9	123834	-0.57877	0.71863	0.993417342
P2RX4	ENSG00000135124	12	121637660	121681909	114	11	123834	-0.57891	0.71867	0.993417342
OR2A25	ENSG00000221933	7	143761275	143782288	47	6	123834	-0.57927	0.7188	0.993417342
AHDC1	ENSG00000126705	1	27850546	27940942	33	3	123834	-0.57951	0.71888	0.993417342
MED13L	ENSG00000123066	12	116385711	116725143	392	20	123834	-0.57957	0.7189	0.993417342
GPR84	ENSG00000139572	12	54746229	54768271	10	3	123834	-0.57965	0.71893	0.993417342
WDR86	ENSG00000187260	7	151062995	151117813	40	15	123834	-0.57979	0.71897	0.993417342
RAF1	ENSG00000132155	3	12615100	12715725	227	15	123834	-0.57992	0.71901	0.993417342

ZNF551	ENSG00000204519	19	58183337	58238669	65	11	123834	-0.58004	0.71906	0.993417342
HAO1	ENSG00000101323	20	7853628	7931121	85	14	123834	-0.58007	0.71907	0.993417342
PLA2G4A	ENSG00000116711	1	186788085	186968113	316	25	123834	-0.58013	0.71909	0.993417342
CSH2	ENSG00000213218	17	61939372	61961126	49	3	123834	-0.58016	0.7191	0.993417342
STRBP	ENSG00000165209	9	125861779	126040855	127	13	123834	-0.5802	0.71911	0.993417342
CMTM3	ENSG00000140931	16	66627777	66657795	38	5	123834	-0.58065	0.71926	0.993417342
IFRD1	ENSG00000006652	7	112053023	112131072	152	16	123834	-0.58089	0.71934	0.993417342
FEZF2	ENSG00000153266	3	62345356	62369999	11	3	123834	-0.5815	0.71955	0.993417342
SUMO1	ENSG00000116030	2	203060903	203113331	85	10	123834	-0.58158	0.71958	0.993417342
IL3	ENSG00000164399	5	131386222	131408897	20	4	123834	-0.58194	0.7197	0.993417342
EFCAB6	ENSG00000186976	22	43914624	44218217	690	27	123834	-0.58203	0.71973	0.993417342
WDR25	ENSG00000176473	14	100832755	101006640	128	17	123834	-0.58217	0.71978	0.993417342
MUC1	ENSG00000185499	1	155148300	155172707	15	3	123834	-0.58251	0.71989	0.993417342
ALS2	ENSG00000003393	2	202555277	202655912	219	18	123834	-0.58258	0.71991	0.993417342
TRIM31	ENSG00000204616	6	30060674	30090883	112	19	123834	-0.58301	0.72006	0.993417342
SEPN1	ENSG00000162430	1	26116667	26154713	27	2	123834	-0.58315	0.7201	0.993417342
SLC22A17	ENSG00000092096	14	23805515	23832121	28	6	123834	-0.58343	0.7202	0.993417342
RFK	ENSG00000135002	9	78990433	79019433	86	12	123834	-0.58349	0.72022	0.993417342
RAX	ENSG00000134438	18	56924267	56951318	70	7	123834	-0.58351	0.72023	0.993417342
EIF2AK2	ENSG00000055332	2	37316353	37394208	165	9	123834	-0.584	0.72039	0.993417342
KRT33B	ENSG00000131738	17	39509746	39536052	37	6	123834	-0.58446	0.72054	0.993417342
PRR23A	ENSG00000206260	3	138712804	138735110	18	4	123834	-0.58451	0.72056	0.993417342
AVL9	ENSG00000105778	7	32525038	33088516	1371	28	123834	-0.58499	0.72072	0.993417342
SPC25	ENSG00000152253	2	169680642	169779881	126	19	123834	-0.58522	0.7208	0.993417342
SRBD1	ENSG00000068784	2	45605819	45849304	492	24	123834	-0.58537	0.72085	0.993417342
ZMAT2	ENSG00000146007	5	140068265	140096248	39	3	123834	-0.58563	0.72094	0.993417342
SCRIB	ENSG00000180900	8	144863090	144907549	40	5	123834	-0.58563	0.72094	0.993417342
ARL15	ENSG00000185305	5	53169775	53616412	680	58	123834	-0.58566	0.72095	0.993417342
FOLR3	ENSG00000110203	11	71815915	71860936	122	12	123834	-0.58569	0.72096	0.993417342
CTB-102L5.4	ENSG00000267748	19	38769791	38805550	60	10	123834	-0.58614	0.72111	0.993417342
DAP	ENSG00000112977	5	10669342	10771384	236	15	123834	-0.58636	0.72118	0.993417342
CNGA4	ENSG00000132259	11	6245995	6275659	33	7	123834	-0.58644	0.72121	0.993417342
NELL1	ENSG00000165973	11	20681117	21607227	1461	158	123834	-0.58648	0.72122	0.993417342
C11orf85	ENSG00000168070	11	64694989	64749557	112	8	123834	-0.58647	0.72122	0.993417342
SERPINB4	ENSG00000206073	18	61294493	61321532	14	2	123834	-0.58689	0.72136	0.993417342
JOSD2	ENSG00000161677	19	50999255	51024610	5	3	123834	-0.58691	0.72137	0.993417342
AC006946.15	ENSG00000235478	22	17592476	17622994	49	6	123834	-0.58696	0.72139	0.993417342
KIF5C	ENSG00000168280	2	149622819	149893273	151	4	123834	-0.58699	0.7214	0.993417342
SLAIN2	ENSG00000109171	4	48333339	48438229	208	14	123834	-0.5873	0.7215	0.993417342
C12orf36	ENSG00000180861	12	13514023	13550101	46	5	123834	-0.5873	0.7215	0.993417342
KIAA1191	ENSG00000122203	5	175763064	175798971	67	9	123834	-0.58745	0.72155	0.993417342
ENHO	ENSG00000168913	9	34511038	34533039	9	3	123834	-0.5875	0.72157	0.993417342
SNX1	ENSG00000028528	15	64376322	64448289	92	7	123834	-0.58751	0.72157	0.993417342
TNFRSF4	ENSG00000186827	1	1136706	1159518	57	6	123834	-0.58756	0.72159	0.993417342
KRT79	ENSG00000185640	12	53205194	53238079	46	9	123834	-0.58773	0.72164	0.993417342
RP11-977G19.10	ENSG00000144785	12	56669700	56719843	51	3	123834	-0.58784	0.72168	0.993417342
ERBB2	ENSG00000141736	17	37834167	37896679	57	5	123834	-0.58786	0.72169	0.993417342
OR8U1	ENSG00000172199	11	56133100	56154029	70	9	123834	-0.588	0.72173	0.993417342
MYBL1	ENSG00000185697	8	67464410	67536482	61	3	123834	-0.58812	0.72177	0.993417342
POPDC2	ENSG00000121577	3	119345304	119394171	101	11	123834	-0.58849	0.7219	0.993417342
KLHL31	ENSG00000124743	6	53502699	53540506	149	12	123834	-0.58863	0.72195	0.993417342
TNS3	ENSG00000136205	7	47304752	47632156	393	44	123834	-0.58872	0.72198	0.993417342
LRRC55	ENSG00000183908	11	56939221	56969191	44	7	123834	-0.58882	0.72201	0.993417342
FREM3	ENSG00000183090	4	144488455	144631828	135	11	123834	-0.58908	0.7221	0.993417342
VEGFC	ENSG00000150630	4	177594689	177723881	238	10	123834	-0.58919	0.72213	0.993417342
OR51Q1	ENSG00000167360	11	5433341	5454436	137	14	123834	-0.58935	0.72219	0.993417342
NUTM1	ENSG00000184507	15	34625516	34659938	48	5	123834	-0.58979	0.72234	0.993417342
TMPRSS9	ENSG00000178297	19	2379769	2436237	188	20	123834	-0.58984	0.72235	0.993417342
RAD51AP1	ENSG00000111247	12	4637950	4679214	28	5	123834	-0.59011	0.72244	0.993417342
PTPN11	ENSG00000179295	12	112846155	112957717	83	10	123834	-0.59079	0.72267	0.993417342
PCTP	ENSG00000141179	17	53818340	53930191	221	11	123834	-0.59081	0.72267	0.993417342
STK36	ENSG00000163482	2	219526749	219577439	43	4	123834	-0.59082	0.72268	0.993417342
RNF214	ENSG00000167257	11	117093341	117167161	102	13	123834	-0.59086	0.72269	0.993417342
KPTN	ENSG00000118162	19	47968401	47997525	21	4	123834	-0.59095	0.72272	0.993417342
GAL3ST3	ENSG00000175229	11	65799423	65826651	44	5	123834	-0.5913	0.72284	0.993417342
ST5	ENSG00000166444	11	8704898	8942498	445	25	123834	-0.59147	0.7229	0.993417342
CD247	ENSG00000198821	1	167389877	167497847	127	26	123834	-0.5915	0.72291	0.993417342
CREG2	ENSG00000175874	2	101952013	102014057	106	8	123834	-0.59158	0.72293	0.993417342
IFNA21	ENSG00000137080	9	21155636	21176659	47	5	123834	-0.59165	0.72296	0.993417342
ZNF69	ENSG00000198429	19	11988599	12035144	66	10	123834	-0.59182	0.72302	0.993417342
TEX10	ENSG00000136891	9	103054359	103125221	120	9	123834	-0.59194	0.72306	0.993417342
OR8J1	ENSG00000172487	11	56117691	56138764	72	8	123834	-0.59214	0.72312	0.993417342
RBBP6	ENSG00000122257	16	24539014	24594184	110	10	123834	-0.59261	0.72328	0.993417342
TCTEX1D4	ENSG00000188396	1	45261585	45282957	23	3	123834	-0.59266	0.7233	0.993417342
PLSCR2	ENSG00000163746	3	146099208	146223778	221	10	123834	-0.59275	0.72333	0.993417342
WDR74	ENSG00000133316	11	62589814	62619281	42	2	123834	-0.59377	0.72367	0.993417342
C10orf76	ENSG00000120029	10	103595356	103825950	181	19	123834	-0.59409	0.72378	0.993417342
KRTAP13-4	ENSG00000186971	21	31792572	31813216	29	1	123834	-0.59411	0.72378	0.993417342
CD300LD	ENSG00000204345	17	72565504	72598422	87	10	123834	-0.59431	0.72385	0.993417342
AHNAK2	ENSG00000185567	14	105393581	105454694	86	8	123834	-0.59449	0.72391	0.993417342
MYT1L	ENSG00000186487	2	1782885	2345032	998	65	123834	-0.59455	0.72393	0.993417342
MAP1LC3B2	ENSG00000171471	12	116987186	117024425	56	13	123834	-0.59537	0.7242	0.993417342
SULT4A1	ENSG00000130540	22	44210389	44268398	156	14	123834	-0.59574	0.72432	0.993417342
NPHS2	ENSG00000116218	1	179509674	179555087	69	9	123834	-0.59626	0.7245	0.993417342
MYL2	ENSG00000111245	12	111338623	111368526	39	5	123834	-0.59671	0.72465	0.993417342
C11orf72	ENSG00000184224	11	67360351	67384177	29	3	123834	-0.59687	0.7247	0.993417342
UGT2A1	ENSG00000173610	4	70444135	70528965	250	20	123834	-0.59688	0.72471	0.993417342
UGT2A1	ENSG00000270386	4	70444912	70528967	250	20	123834	-0.59688	0.72471	0.993417342
DKFZP434E1119	ENSG00000268635	11	77174416	77198074	14	2	123834	-0.59701	0.72475	0.993417342
AGPAT6	ENSG00000158669	8	41424706	41492520	128	11	123834	-0.5971	0.72478	0.993417342
CALR3	ENSG00000269058	19	16579868	16617003	103	10	123834	-0.59713	0.72479	0.993417342
DBNDD2	ENSG00000244274	20	44024697	44049250	33	3	123834	-0.59727	0.72484	0.993417342
MORN5	ENSG00000185681	9	124912190	124972367	51	9	123834	-0.59758	0.72494	0.993417342
TOR1AIP1	ENSG00000143337	1	179841177	179904135	120	11	123834	-0.59767	0.72497	0.993417342
CTC-487M23.8	ENSG00000272869	5	112186885	112238776	128	9	123834	-0.59781	0.72502	0.993417342
SLC25A16	ENSG00000122912	10	70227756	70297231	212	13	123834	-0.59795	0.72506	0.993417342
GRIN3B	ENSG00000116032	19	990418	1019731	114	21	123834	-0.59793	0.72506	0.993417342
NDUFV3	ENSG00000160194	21	44289754	44343414	199	14	123834	-0.598	0.72508	0.993417342
ARC	ENSG00000198576	8	143682405	143706833	18	3	123834	-0.59855	0.72526	0.993417342

ANKRD18B	ENSG00000230453	9	33514392	33583007	90	12	123834	-0.59856	0.72527	0.993417342
PRG2	ENSG00000186652	11	57144267	57168130	27	4	123834	-0.59868	0.72531	0.993417342
PTRH1	ENSG00000187024	9	130445257	130497152	35	5	123834	-0.59921	0.72548	0.993417342
ZYX	ENSG00000159840	7	143068173	143098204	15	5	123834	-0.59947	0.72557	0.993417342
LGR5	ENSG00000139292	12	71823550	71990090	436	26	123834	-0.59976	0.72567	0.993417342
AAMP	ENSG00000127837	2	219118850	219144980	37	3	123834	-0.59984	0.72569	0.993417342
POT1	ENSG00000128513	7	124452440	124580037	324	5	123834	-0.59996	0.72573	0.993417342
DERL1	ENSG00000136986	8	124015404	124064663	90	13	123834	-0.59998	0.72574	0.993417342
GDF5OS	ENSG00000204183	20	34010827	34033248	49	4	123834	-0.60004	0.72576	0.993417342
ZBTB14	ENSG00000198081	18	5279018	5307052	29	6	123834	-0.60028	0.72584	0.993417342
OR52D1	ENSG00000181609	11	5499915	5520979	67	10	123834	-0.60036	0.72587	0.993417342
MTRNR2L5	ENSG00000249860	10	57348750	57370488	41	4	123834	-0.60042	0.72589	0.993417342
SLCO3A1	ENSG00000176463	15	92386925	92725665	467	50	123834	-0.60044	0.72589	0.993417342
PFDN1	ENSG00000113068	5	139614624	139692706	72	6	123834	-0.60059	0.72594	0.993417342
KSR2	ENSG00000171435	12	117880817	118416788	665	81	123834	-0.60064	0.72596	0.993417342
C1orf137	ENSG00000203864	1	117226734	117259225	13	2	123834	-0.60071	0.72598	0.993417342
IFITM5	ENSG00000206013	11	288200	309526	14	5	123834	-0.60094	0.72606	0.993417342
AADACL2	ENSG00000197953	3	151441704	151489127	79	10	123834	-0.60169	0.72631	0.993417342
EXO5	ENSG00000164002	1	40964413	40992228	74	9	123834	-0.60186	0.72637	0.993417342
C17orf82	ENSG00000187013	17	59479112	59500641	34	4	123834	-0.60191	0.72638	0.993417342
NRIP1	ENSG00000180530	21	16323556	16447321	109	16	123834	-0.60204	0.72642	0.993417342
C16orf45	ENSG00000166780	16	15518152	15728885	99	20	123834	-0.60222	0.72649	0.993417342
WFDC11	ENSG00000180083	20	44267202	44308909	106	9	123834	-0.6023	0.72651	0.993417342
C16orf59	ENSG00000162062	16	2500081	2524964	7	2	123834	-0.60245	0.72656	0.993417342
COL6A1	ENSG00000142156	21	47391651	47434964	46	9	123834	-0.60266	0.72663	0.993417342
HLA-DOA	ENSG00000204252	6	32961955	32987389	104	19	123834	-0.60284	0.72669	0.993417342
C15orf56	ENSG00000176753	15	40532865	40555170	9	3	123834	-0.60291	0.72672	0.993417342
POLR2D	ENSG00000144231	2	128593840	128625731	66	8	123834	-0.60298	0.72674	0.993417342
SPRR2D	ENSG00000163216	1	153002201	153024407	94	2	123834	-0.60304	0.72676	0.993417342
RRBP1	ENSG00000125844	20	17584323	17672940	110	16	123834	-0.60352	0.72692	0.993417342
TBC1D15	ENSG00000121749	12	72223487	72330629	171	11	123834	-0.60357	0.72694	0.993417342
HOXD10	ENSG00000128710	2	176963518	176994670	16	5	123834	-0.60411	0.72711	0.993417342
STARD10	ENSG00000214530	11	72455774	72514726	87	9	123834	-0.6041	0.72711	0.993417342
TRAPPC8	ENSG00000153339	18	29399136	29543099	148	13	123834	-0.60411	0.72711	0.993417342
BCAS2	ENSG00000116752	1	115100178	115134260	48	10	123834	-0.60425	0.72716	0.993417342
SPINK2	ENSG00000128040	4	57666026	57697908	60	9	123834	-0.60426	0.72716	0.993417342
DLEU1	ENSG00000176124	13	50646307	51307372	1111	77	123834	-0.60433	0.72719	0.993417342
HEXDC	ENSG00000169660	17	80366194	80410521	66	10	123834	-0.60441	0.72721	0.993417342
CREB3L4	ENSG00000143578	1	153930010	153956839	17	3	123834	-0.60455	0.72726	0.993417342
MSH3	ENSG00000113318	5	79940467	80182279	537	17	123834	-0.60466	0.7273	0.993417342
STRAP	ENSG00000023734	12	16025325	16066412	13	2	123834	-0.60467	0.7273	0.993417342
ZNF737	ENSG00000237440	19	20708631	20758615	182	11	123834	-0.60504	0.72742	0.993417342
IKBKB	ENSG00000104365	8	42118820	42199973	98	7	123834	-0.60551	0.72758	0.993417342
A2ML1	ENSG00000166535	12	8965068	9049597	94	13	123834	-0.60561	0.72761	0.993417342
ZDHHC13	ENSG00000177054	11	19128646	19207969	133	13	123834	-0.60572	0.72765	0.993417342
TESK2	ENSG00000070759	1	45799555	45966872	177	10	123834	-0.6058	0.72768	0.993417342
PTPN13	ENSG00000163629	4	87505468	87746324	476	9	123834	-0.60602	0.72775	0.993417342
CPQ	ENSG00000104324	8	97647455	98171882	469	37	123834	-0.60612	0.72778	0.993417342
NLRP2	ENSG00000022556	19	55454498	55522510	130	19	123834	-0.60633	0.72785	0.993417342
CYP2S1	ENSG00000167600	19	41688954	41723444	38	5	123834	-0.60664	0.72796	0.993417342
SLC25A21	ENSG00000183032	14	37137636	37652071	920	63	123834	-0.60679	0.728	0.993417342
KRT4	ENSG00000170477	12	53190333	53218335	49	13	123834	-0.60698	0.72807	0.993417342
KRT24	ENSG00000167916	17	38844243	38870002	38	5	123834	-0.60721	0.72815	0.993417342
ADAMTSL1	ENSG00000178031	9	18463892	18920948	753	102	123834	-0.60754	0.72825	0.993417342
C19orf81	ENSG00000235034	19	51142702	51172567	29	7	123834	-0.60752	0.72825	0.993417342
RFPL4AL1	ENSG00000229292	19	56270507	56294545	3	2	123834	-0.60763	0.72828	0.993417342
C20orf202	ENSG00000215595	20	1174098	1198918	23	4	123834	-0.60764	0.72829	0.993417342
RHPN2	ENSG00000131941	19	33459499	33565794	225	23	123834	-0.60768	0.7283	0.993417342
MYH10	ENSG00000133026	17	8367523	8544079	130	7	123834	-0.60772	0.72831	0.993417342
ANKRD32	ENSG00000133302	5	93944052	94085141	282	14	123834	-0.60778	0.72833	0.993417342
POLR2G	ENSG00000168002	11	62519016	62544182	22	2	123834	-0.60784	0.72835	0.993417342
DCLRE1A	ENSG00000198924	10	115584488	115624142	15	4	123834	-0.60815	0.72846	0.993417342
C12orf45	ENSG00000151131	12	105370088	105453515	232	21	123834	-0.60817	0.72846	0.993417342
LILRB4	ENSG00000186818	19	55145340	55191810	109	10	123834	-0.60825	0.72849	0.993417342
ACAD9	ENSG00000177646	3	128588439	128644910	136	11	123834	-0.60835	0.72852	0.993417342
HARS	ENSG00000170445	5	140042758	140081609	41	3	123834	-0.60839	0.72853	0.993417342
IFT43	ENSG00000119650	14	76358479	76560928	270	18	123834	-0.60847	0.72856	0.993417342
MRGPRF	ENSG00000172935	11	68761863	68790877	22	6	123834	-0.60854	0.72858	0.993417342
HEPACAM2	ENSG00000188175	7	92807899	92865837	70	11	123834	-0.60865	0.72862	0.993417342
ILVBL	ENSG00000105135	19	15215795	15246596	52	8	123834	-0.60878	0.72866	0.993417342
CXCL11	ENSG00000169248	4	76944835	76972568	120	4	123834	-0.60897	0.72873	0.993417342
MMP25	ENSG00000008516	16	3086682	3120727	20	4	123834	-0.60915	0.72879	0.993417342
ACTR5	ENSG00000101442	20	37367085	37410834	162	12	123834	-0.6092	0.7288	0.993417342
ZFYVE19	ENSG00000166140	15	41089284	41116767	45	5	123834	-0.60922	0.72881	0.993417342
LRSAM1	ENSG00000148356	9	130203765	130275780	127	8	123834	-0.6093	0.72884	0.993417342
SUV420H1	ENSG00000110066	11	67912330	67991295	40	4	123834	-0.60947	0.72889	0.993417342
INPP4A	ENSG00000040933	2	99051317	99220853	172	5	123834	-0.60962	0.72894	0.993417342
TCIRG1	ENSG00000110719	11	67796483	67828362	18	5	123834	-0.61003	0.72908	0.993417342
TXNRD3	ENSG00000197763	3	126280622	126383998	191	21	123834	-0.61016	0.72912	0.993417342
PRR23B	ENSG00000184814	3	138727873	138749768	21	5	123834	-0.61029	0.72917	0.993417342
MEF2C	ENSG00000081189	5	88003975	88209922	231	21	123834	-0.6103	0.72917	0.993417342
UBR1	ENSG00000159459	15	43225095	43408311	139	10	123834	-0.61033	0.72918	0.993417342
PIK3C2B	ENSG00000133056	1	204381756	204473852	110	14	123834	-0.61042	0.72921	0.993417342
TMEM8C	ENSG00000187616	9	136369708	136403734	15	4	123834	-0.61115	0.72945	0.993417342
CCL2	ENSG00000108691	17	32572304	32594222	31	4	123834	-0.61125	0.72948	0.993417342
C8orf44	ENSG00000213865	8	67569850	67607799	34	4	123834	-0.61145	0.72955	0.993417342
LRRC47	ENSG00000130764	1	3686784	3723068	128	14	123834	-0.61193	0.72971	0.993417342
NDUFA9	ENSG00000139180	12	4748261	4808454	69	11	123834	-0.61196	0.72972	0.993417342
KLK7	ENSG00000169035	19	51469729	51497355	44	8	123834	-0.61199	0.72973	0.993417342
PIK3R5	ENSG00000141506	17	8772233	8879029	112	20	123834	-0.61205	0.72975	0.993417342
UTS2R	ENSG00000181408	17	80322153	80343462	72	6	123834	-0.61222	0.72981	0.993417342
C3orf80	ENSG00000180044	3	159933423	159955999	13	4	123834	-0.6124	0.72986	0.993417342
PLEKHM3	ENSG00000178385	2	208683027	208900284	189	24	123834	-0.61253	0.72991	0.993417342
GNG7	ENSG00000176533	19	2501217	2712707	313	46	123834	-0.61279	0.72999	0.993417342
CEP41	ENSG00000106477	7	130023612	130092274	62	13	123834	-0.61283	0.73	0.993417342
CATSPERG	ENSG00000099338	19	38816415	38871589	145	15	123834	-0.61291	0.73003	0.993417342
RP11-51L5.7	ENSG00000270033	17	60339848	60370276	20	4	123834	-0.61295	0.73004	0.993417342
LOXL3	ENSG00000115318	2	74749541	74792817	14	3	123834	-0.61297	0.73005	0.993417342
ZFP36	ENSG00000128016	19	39887453	39910052	15	4	123834	-0.61305	0.73008	0.993417342

SLC22A24	ENSG00000197658	11	62837412	62921693	305	9	123834	-0.61337	0.73018	0.993417342
IL1RL1	ENSG00000115602	2	102917962	102978497	212	14	123834	-0.61357	0.73025	0.993417342
AC083862.1	ENSG00000229974	7	134822797	134842916	51	4	123834	-0.61358	0.73025	0.993417342
ISL1	ENSG00000016082	5	50668921	50700564	39	6	123834	-0.61361	0.73026	0.993417342
FGFBP3	ENSG00000174721	10	93656346	93679240	30	7	123834	-0.61381	0.73033	0.993417342
RP3-422G23.4	ENSG00000262543	6	138689042	138714212	78	4	123834	-0.61419	0.73046	0.993417342
SCYL1	ENSG00000142186	11	65282548	65316175	18	5	123834	-0.61478	0.73065	0.993417342
TMEM126A	ENSG00000171202	11	85349011	85377591	39	7	123834	-0.6149	0.73069	0.993417342
TMEM91	ENSG00000142046	19	41846816	41899988	43	5	123834	-0.6153	0.73082	0.993417342
NPS	ENSG00000214285	10	129337613	129360935	118	12	123834	-0.61537	0.73084	0.993417342
ZNF43	ENSG00000198521	19	21977752	22044927	124	12	123834	-0.61544	0.73087	0.993417342
TTPA	ENSG00000137561	8	63951112	64008612	135	14	123834	-0.61599	0.73105	0.993417342
COMMD10	ENSG00000145781	5	115410688	115758459	1346	28	123834	-0.61602	0.73106	0.993417342
SGPP2	ENSG00000163082	2	223279236	223435667	237	31	123834	-0.61608	0.73108	0.993417342
CTD-2203A3.1	ENSG00000248789	5	51293865	51386821	234	12	123834	-0.61612	0.73109	0.993417342
SAR1B	ENSG00000152700	5	133926834	133994961	28	5	123834	-0.61612	0.73109	0.993417342
ANKRD40	ENSG00000154945	17	48760551	48795285	33	8	123834	-0.61647	0.73121	0.993417342
NKX3-1	ENSG00000167034	8	23526206	23550440	47	8	123834	-0.61659	0.73125	0.993417342
RP11-192H23.4	ENSG00000258472	17	26772770	26951215	120	12	123834	-0.61677	0.73131	0.993417342
PHYHIPL	ENSG00000165443	10	60926350	61017534	102	5	123834	-0.61701	0.73139	0.993417342
GSTM5	ENSG00000134201	1	110244864	110328050	114	20	123834	-0.61725	0.73146	0.993417342
TIGIT	ENSG00000181847	3	113985760	114039135	86	13	123834	-0.61726	0.73147	0.993417342
SUPT5H	ENSG00000196235	19	39916796	39977310	136	9	123834	-0.61738	0.73151	0.993417342
CEP112	ENSG00000154240	17	63621656	64198202	1407	26	123834	-0.61741	0.73152	0.993417342
MRPS10	ENSG00000048544	6	42164539	42195603	92	5	123834	-0.61776	0.73163	0.993417342
CDH1	ENSG00000039068	16	68761128	68879451	235	21	123834	-0.61775	0.73163	0.993417342
KLHL33	ENSG00000185271	14	20886970	20913801	50	13	123834	-0.61777	0.73164	0.993417342
GNAZ	ENSG00000128266	22	23402540	23477224	76	5	123834	-0.61783	0.73166	0.993417342
ALS2CR12	ENSG00000155749	2	202142994	202232121	167	16	123834	-0.61824	0.73179	0.993417342
ZFAT	ENSG00000066827	8	135480031	135735292	521	34	123834	-0.61862	0.73192	0.993417342
TTC32	ENSG00000183891	2	20086404	20111747	38	7	123834	-0.61888	0.732	0.993417342
BBS10	ENSG00000179941	12	76728254	76752222	23	5	123834	-0.61914	0.73209	0.993417342
PEBP1	ENSG00000089220	12	118563663	118593389	34	9	123834	-0.6192	0.73211	0.993417342
ATP5D	ENSG00000099624	19	1231749	1254824	8	2	123834	-0.61956	0.73223	0.993417342
FCHO1	ENSG00000130475	19	17848527	17909377	85	12	123834	-0.61971	0.73227	0.993417342
NLN	ENSG00000123213	5	65008023	65177553	310	27	123834	-0.61998	0.73236	0.993417342
OR51E1	ENSG00000180785	11	4654650	4686718	123	14	123834	-0.62001	0.73237	0.993417342
OR9G4	ENSG00000172457	11	56500303	56521287	58	10	123834	-0.62014	0.73242	0.993417342
WNT2	ENSG00000105989	7	116906685	116973343	59	13	123834	-0.62053	0.73255	0.993417342
CCDC68	ENSG00000166510	18	52558740	52636739	112	11	123834	-0.62064	0.73258	0.993417342
ATG16L2	ENSG00000168010	11	72515353	72564719	33	6	123834	-0.62079	0.73263	0.993417342
MRPS35	ENSG00000061794	12	27853706	27919228	103	7	123834	-0.62092	0.73267	0.993417342
KRT40	ENSG00000204889	17	39123968	39153387	181	11	123834	-0.62098	0.73269	0.993417342
CYP4F22	ENSG00000171954	19	15609304	15673128	81	18	123834	-0.62109	0.73273	0.993417342
IL17F	ENSG00000112116	6	52091479	52119335	43	8	123834	-0.62119	0.73276	0.993417342
FRK	ENSG00000111816	6	116242312	116391921	261	17	123834	-0.62125	0.73278	0.993417342
WNK1	ENSG00000060237	12	851759	1030618	256	16	123834	-0.62126	0.73279	0.993417342
KATNAL1	ENSG00000102781	13	30766767	30891621	227	31	123834	-0.6215	0.73287	0.993417342
ECH1	ENSG00000104823	19	39296062	39332645	42	6	123834	-0.62163	0.73291	0.993417342
MASTL	ENSG00000120539	10	27433753	27485853	157	12	123834	-0.62183	0.73297	0.993417342
SPATA21	ENSG00000187144	1	16703612	16773919	102	13	123834	-0.62229	0.73312	0.993417342
SUB1	ENSG00000113387	5	32521739	32614185	107	12	123834	-0.62249	0.73319	0.993417342
RSPH6A	ENSG00000104941	19	46288968	46328577	97	12	123834	-0.62251	0.7332	0.993417342
S100A10	ENSG00000197747	1	151945391	151976866	87	7	123834	-0.62258	0.73322	0.993417342
RIOK3	ENSG00000101782	18	21022787	21076567	102	13	123834	-0.62298	0.73335	0.993417342
DCPS	ENSG00000110063	11	126163647	126225644	55	12	123834	-0.6231	0.73339	0.993417342
POU1F1	ENSG00000064835	3	87298554	87335737	60	9	123834	-0.62321	0.73343	0.993417342
SERPIND1	ENSG00000099937	22	21118167	21152008	73	7	123834	-0.62329	0.73345	0.993417342
MRPS18A	ENSG00000096080	6	43629040	43665528	88	8	123834	-0.62333	0.73347	0.993417342
ELAC1	ENSG00000141642	18	48484361	48524491	24	4	123834	-0.62356	0.73354	0.993417342
CPNE5	ENSG00000124772	6	36698552	36817778	200	38	123834	-0.62361	0.73356	0.993417342
HARS2	ENSG00000112855	5	140061011	140088889	35	3	123834	-0.6245	0.73385	0.993417342
SLC22A31	ENSG00000259803	16	89252406	89278072	59	9	123834	-0.62496	0.734	0.993417342
CNR2	ENSG00000188822	1	24187016	24295549	178	10	123834	-0.625	0.73401	0.993417342
AC104534.3	ENSG00000268083	19	39298111	39340818	54	7	123834	-0.625	0.73401	0.993417342
EPS8L3	ENSG00000198758	1	110282702	110316649	34	6	123834	-0.62514	0.73406	0.993417342
NDN	ENSG00000182636	15	23920565	23942450	21	5	123834	-0.62575	0.73426	0.993417342
STX6	ENSG00000135823	1	180931861	181002047	72	9	123834	-0.62592	0.73432	0.993417342
TAF7	ENSG00000178913	5	140688057	140710330	53	7	123834	-0.62597	0.73433	0.993417342
PPFIA3	ENSG00000177380	19	49612646	49664283	65	11	123834	-0.62618	0.7344	0.993417342
FBXL13	ENSG00000161040	7	102443308	102725286	331	16	123834	-0.62624	0.73442	0.993417342
GLCE	ENSG00000138604	15	69442923	69574556	168	10	123834	-0.62626	0.73443	0.993417342
ERBB4	ENSG00000178568	2	212230446	213413565	2299	140	123834	-0.6263	0.73444	0.993417342
TMIE	ENSG00000181585	3	46732823	46762376	37	7	123834	-0.62642	0.73448	0.993417342
OR10Q1	ENSG00000180475	11	57985354	58006390	53	7	123834	-0.62675	0.73459	0.993417342
TOP2B	ENSG00000077097	3	25629475	25716398	88	5	123834	-0.62714	0.73472	0.993417342
NACC2	ENSG00000148411	9	138888383	138997131	131	23	123834	-0.62727	0.73476	0.993417342
HK1	ENSG00000156515	10	71019740	71171638	282	37	123834	-0.62743	0.73481	0.993417342
C2CD4D	ENSG00000225556	1	151800295	151823033	12	4	123834	-0.62774	0.73491	0.993417342
PSKH2	ENSG00000147613	8	87050602	87110850	63	6	123834	-0.62773	0.73491	0.993417342
SEC23A	ENSG00000100934	14	39491123	39588850	145	16	123834	-0.62776	0.73492	0.993417342
PXT1	ENSG00000179165	6	36348328	36420666	174	11	123834	-0.62792	0.73497	0.993417342
MCOLN3	ENSG00000055732	1	85473765	85524182	68	10	123834	-0.62824	0.73508	0.993417342
C10orf62	ENSG00000203942	10	99339450	99360690	22	4	123834	-0.62824	0.73508	0.993417342
OR1L1	ENSG00000173679	9	125413845	125434927	19	4	123834	-0.62848	0.73515	0.993417342
HBCBP	ENSG00000268162	12	57380745	57401335	16	2	123834	-0.62874	0.73524	0.993417342
C12orf65	ENSG00000130921	12	123707463	123752506	107	6	123834	-0.62887	0.73528	0.993417342
TRIM5	ENSG00000132256	11	5674425	5969849	1143	43	123834	-0.62917	0.73538	0.993417342
FAM126B	ENSG00000155744	2	201828441	201946394	127	12	123834	-0.62933	0.73543	0.993417342
ZNF280A	ENSG00000169548	22	22858060	22884613	56	7	123834	-0.62936	0.73544	0.993417342
CPT2	ENSG00000157184	1	53652101	53689869	46	8	123834	-0.62968	0.73555	0.993417342
C17orf77	ENSG00000182352	17	72570818	72600348	73	7	123834	-0.62971	0.73556	0.993417342
AMY2A	ENSG00000243480	1	104149999	104178402	1	1	123834	-0.62981	0.73559	0.993417342
CA11	ENSG00000063180	19	49131199	49159569	26	4	123834	-0.62999	0.73565	0.993417342
TRPV2	ENSG00000187688	17	16308856	16350317	57	8	123834	-0.63011	0.73569	0.993417342
PHF3	ENSG00000118482	6	64335725	64499229	161	9	123834	-0.63018	0.73571	0.993417342
MSGN1	ENSG00000151379	2	17987763	18008368	22	7	123834	-0.63042	0.73579	0.993417342
TMEM9B	ENSG00000175348	11	8958841	8996558	97	8	123834	-0.63058	0.73584	0.993417342
PRADC1	ENSG00000135617	2	73445134	73470366	38	8	123834	-0.6307	0.73588	0.993417342

D2HGDH	ENSG00000180902	2	242663994	242718231	72	11	123834	-0.6307	0.73588	0.993417342
CTSF	ENSG00000174080	11	66320934	66346312	42	6	123834	-0.63082	0.73592	0.993417342
GHDC	ENSG00000167925	17	40330817	40356531	6	3	123834	-0.63082	0.73592	0.993417342
CSE1L	ENSG00000124207	20	47652849	47723489	152	10	123834	-0.63104	0.73599	0.993417342
RBL1	ENSG00000269846	20	35714188	35735581	12	3	123834	-0.6311	0.73601	0.993417342
UMODL1	ENSG00000177398	21	43473068	43573563	296	38	123834	-0.63116	0.73603	0.993417342
TSPAN8	ENSG00000127324	12	71508865	71845678	685	41	123834	-0.63135	0.7361	0.993417342
ATXN3	ENSG00000066427	14	92514896	92582965	287	11	123834	-0.63137	0.7361	0.993417342
ZNF189	ENSG00000136870	9	104151155	104182942	68	7	123834	-0.63156	0.73616	0.993417342
GRIN2A	ENSG00000183454	16	9842376	10286611	1053	36	123834	-0.63154	0.73616	0.993417342
GGA2	ENSG00000103365	16	23464863	23543316	121	7	123834	-0.63177	0.73623	0.993417342
MTFR2	ENSG00000146410	6	136542162	136581473	2	1	123834	-0.63232	0.73641	0.993417342
RPA3-AS1	ENSG00000219545	7	7670342	8053689	656	88	123834	-0.63233	0.73641	0.993417342
KRTDAP	ENSG00000188508	19	35968226	35996460	40	10	123834	-0.63234	0.73642	0.993417342
RFC5	ENSG00000111445	12	118441393	118480935	49	9	123834	-0.63266	0.73652	0.993417342
ZNF514	ENSG00000144026	2	95803075	95841158	22	4	123834	-0.63268	0.73653	0.993417342
MZT2A	ENSG00000173272	2	132212473	132260316	166	8	123834	-0.63275	0.73655	0.993417342
SIGLEC6	ENSG00000105492	19	52012779	52045110	134	14	123834	-0.63301	0.73664	0.993417342
CYTL1	ENSG00000170891	4	5006313	5031199	52	7	123834	-0.63306	0.73665	0.993417342
PNLDC1	ENSG00000146453	6	160211298	160251736	89	10	123834	-0.63313	0.73668	0.993417342
BCAT1	ENSG00000060982	12	24954295	25112393	356	37	123834	-0.63318	0.73669	0.993417342
BCL6B	ENSG00000161940	17	6916339	6943609	25	3	123834	-0.63316	0.73669	0.993417342
TAS2R30	ENSG00000256188	12	11275557	11297243	104	5	123834	-0.63337	0.73675	0.993417342
PABPC1L	ENSG00000101104	20	43528703	43597676	61	9	123834	-0.63349	0.73679	0.993417342
RP11-302B13.5	ENSG00000272822	12	49287286	49361148	163	7	123834	-0.63357	0.73682	0.993417342
PCSK7	ENSG00000160613	11	117065053	117113241	103	15	123834	-0.63395	0.73694	0.993417342
SAMD7	ENSG00000187033	3	169619360	169666963	91	10	123834	-0.63405	0.73697	0.993417342
APC2	ENSG00000115266	19	1436300	1483243	20	4	123834	-0.63407	0.73698	0.993417342
OR8B3	ENSG00000196661	11	124256278	124277264	32	2	123834	-0.63415	0.73701	0.993417342
CNP	ENSG00000173786	17	40108759	40139749	31	5	123834	-0.6346	0.73715	0.993417342
LRRC3C	ENSG00000204913	17	38087727	38111000	53	7	123834	-0.63463	0.73717	0.993417342
SUPT4H1	ENSG00000213246	17	56412539	56440454	34	9	123834	-0.63501	0.73729	0.993417342
RASGRP2	ENSG00000068831	11	64484383	64522928	10	3	123834	-0.63523	0.73736	0.993417342
PAK7	ENSG00000101349	20	9508036	9829689	492	55	123834	-0.63532	0.73739	0.993417342
EIF2AK3	ENSG00000172071	2	88846259	88937094	80	11	123834	-0.63535	0.7374	0.993417342
PSENEN	ENSG00000205155	19	36226015	36247911	34	6	123834	-0.63536	0.7374	0.993417342
SIRT7	ENSG00000187531	17	79859815	79889199	2	1	123834	-0.63549	0.73744	0.993417342
STAT1	ENSG00000115415	2	191819084	191895686	87	15	123834	-0.63559	0.73748	0.993417342
GLTSCR2	ENSG00000105373	19	48238779	48270315	107	9	123834	-0.63559	0.73748	0.993417342
LINGO2	ENSG00000174482	9	27938076	28680283	1477	139	123834	-0.63562	0.73749	0.993417342
WDR52	ENSG00000206530	3	112995777	113170457	536	18	123834	-0.63575	0.73753	0.993417342
LHPP	ENSG00000107902	10	126140403	126316457	421	44	123834	-0.63578	0.73754	0.993417342
TPSG1	ENSG00000116176	16	1261651	1285257	73	11	123834	-0.63577	0.73754	0.993417342
OR2B6	ENSG00000124657	6	27915019	27935960	15	5	123834	-0.63585	0.73756	0.993417342
P2RY1	ENSG00000169860	3	152542736	152565841	32	3	123834	-0.63619	0.73767	0.993417342
AEN	ENSG00000181026	15	89154527	89185513	50	6	123834	-0.63618	0.73767	0.993417342
OR10G6	ENSG00000198674	11	123854870	123875868	53	9	123834	-0.63642	0.73775	0.993417342
TEX15	ENSG00000133863	8	30679060	30758122	132	9	123834	-0.63653	0.73778	0.993417342
C6orf15	ENSG00000204542	6	31069000	31090336	172	15	123834	-0.63666	0.73783	0.993417342
AP2S1	ENSG00000042753	19	47331393	47364249	13	6	123834	-0.63693	0.73791	0.993417342
SLC12A1	ENSG00000074803	15	48473861	48606275	93	8	123834	-0.63707	0.73796	0.993417342
COA4	ENSG00000181924	11	73573712	73598033	61	8	123834	-0.6371	0.73797	0.993417342
NUDT19	ENSG00000213965	19	33172867	33214702	70	8	123834	-0.63729	0.73803	0.993417342
LY75-CD302	ENSG00000248672	2	160618362	160771221	341	27	123834	-0.63758	0.73813	0.993417342
LY75	ENSG00000054219	2	160618362	160771260	341	27	123834	-0.63758	0.73813	0.993417342
PRAMEF12	ENSG00000116726	1	12824984	12848049	27	6	123834	-0.63765	0.73815	0.993417342
DEFB112	ENSG00000180872	6	50001288	50026364	25	4	123834	-0.63764	0.73815	0.993417342
SLC22A5	ENSG00000197375	5	131695444	131741306	109	9	123834	-0.63793	0.73824	0.993417342
C1orf123	ENSG00000162384	1	53669771	53696289	33	6	123834	-0.63796	0.73825	0.993417342
SPINK4	ENSG00000122711	9	33208363	33258565	83	7	123834	-0.63814	0.73831	0.993417342
PYHIN1	ENSG00000163564	1	158890586	158956844	117	13	123834	-0.63821	0.73833	0.993417342
TCHHL1	ENSG00000182898	1	152046620	152071540	7	3	123834	-0.63825	0.73834	0.993417342
CCL14	ENSG00000213494	17	34300327	34339100	128	11	123834	-0.63823	0.73834	0.993417342
SLC17A6	ENSG00000091664	11	22349643	22411049	68	14	123834	-0.63837	0.73838	0.993417342
RP11-758M4.1	ENSG00000254349	8	75502010	75745548	326	26	123834	-0.63842	0.7384	0.993417342
MPP3	ENSG00000161647	17	41868167	41920538	48	4	123834	-0.63853	0.73843	0.993417342
MARS	ENSG00000166986	12	57859228	57921352	60	5	123834	-0.63855	0.73844	0.993417342
LSM14B	ENSG00000149657	20	60687517	60720434	48	3	123834	-0.63859	0.73845	0.993417342
RP11-477N12.3	ENSG00000214978	17	9684544	9735388	96	13	123834	-0.6386	0.73846	0.993417342
PAPSS2	ENSG00000198682	10	89409370	89517462	143	19	123834	-0.63893	0.73857	0.993417342
UVSSA	ENSG00000163945	4	1331054	1391837	132	11	123834	-0.63908	0.73861	0.993417342
STRIP1	ENSG00000143093	1	110564199	110627263	35	3	123834	-0.63945	0.73874	0.993417342
RHBDF2	ENSG00000129667	17	74456973	74507872	89	16	123834	-0.63952	0.73876	0.993417342
MRPL23	ENSG00000214026	11	1958508	2015752	86	14	123834	-0.63966	0.7388	0.993417342
VTCN1	ENSG00000134258	1	117676209	117763556	133	14	123834	-0.63967	0.73881	0.993417342
ZNF286A	ENSG00000187607	17	15592891	15650874	42	6	123834	-0.64016	0.73897	0.993417342
BRCA1	ENSG00000012048	17	41186312	41287500	147	3	123834	-0.64023	0.73899	0.993417342
LPL	ENSG00000175445	8	19749228	19834769	234	13	123834	-0.6403	0.73901	0.993417342
FAM65C	ENSG00000042062	20	49192645	49318065	199	22	123834	-0.64038	0.73904	0.993417342
CA10	ENSG00000154975	17	49697674	50247377	638	43	123834	-0.64044	0.73906	0.993417342
RP11-597K23.2	ENSG00000259518	15	82370935	82400035	26	6	123834	-0.64047	0.73907	0.993417342
SRP19	ENSG00000153037	5	112186919	112215485	66	7	123834	-0.6405	0.73908	0.993417342
MAP3K11	ENSG00000173327	11	65355226	65392853	25	4	123834	-0.64067	0.73913	0.993417342
RSBN1	ENSG00000081019	1	114294454	114365098	67	8	123834	-0.64078	0.73917	0.993417342
BCAS1	ENSG00000064787	20	52543316	52697304	348	32	123834	-0.64104	0.73925	0.993417342
IGSF10	ENSG00000152580	3	151133172	151186497	73	7	123834	-0.64148	0.73939	0.993417342
SRP9	ENSG00000143742	1	225955531	225988168	69	11	123834	-0.64151	0.7394	0.993417342
CCL15-CCL14	ENSG00000161574	17	34300692	34338660	126	11	123834	-0.64155	0.73942	0.993417342
SLIT2	ENSG00000145147	4	20244883	20632184	575	67	123834	-0.64192	0.73954	0.993417342
OSR2	ENSG00000164920	8	99946631	99974332	16	5	123834	-0.64208	0.73959	0.993417342
SUCLG1	ENSG00000163541	2	84640647	84697169	111	7	123834	-0.64228	0.73965	0.993417342
IL1A	ENSG00000115008	2	113521492	113552167	64	4	123834	-0.64238	0.73969	0.993417342
TSTA3	ENSG00000104522	8	144684788	144710218	27	5	123834	-0.64245	0.73971	0.993417342
KIF24	ENSG00000186638	9	34242379	34339198	173	13	123834	-0.64349	0.74005	0.993417342
MKNK2	ENSG00000099875	19	2027470	2061243	25	6	123834	-0.64382	0.74015	0.993417342
AMIGO2	ENSG00000139211	12	47459490	47483734	11	4	123834	-0.64405	0.74023	0.993417342
RPS8	ENSG00000142937	1	45230923	45254451	22	3	123834	-0.64469	0.74043	0.993417342
MAP1S	ENSG00000130479	19	17820051	17855325	77	7	123834	-0.64472	0.74044	0.993417342
CCDC41	ENSG00000173588	12	94690225	94863764	220	20	123834	-0.64481	0.74047	0.993417342

RP11-108K14.8	ENSG00000254536	10	135194338	135243999	74	9	123834	-0.64487	0.74049	0.993417342
PBXIP1	ENSG00000163346	1	154906552	154938599	27	4	123834	-0.64492	0.74051	0.993417342
CXCL2	ENSG00000081041	4	74952752	74975010	16	2	123834	-0.6451	0.74057	0.993417342
MED8	ENSG00000159479	1	43839588	43865479	33	5	123834	-0.64524	0.74061	0.993417342
URB1	ENSG00000142207	21	33673329	33775335	143	5	123834	-0.64526	0.74062	0.993417342
GFRA4	ENSG00000125861	20	3629939	3654046	58	12	123834	-0.64578	0.74079	0.993417342
C19orf73	ENSG00000221916	19	49611655	49632397	29	6	123834	-0.64583	0.7408	0.993417342
PRED58	ENSG00000267857	21	46510728	46544873	79	10	123834	-0.64588	0.74082	0.993417342
MDH2	ENSG00000146701	7	75667369	75706826	82	7	123834	-0.64599	0.74086	0.993417342
KIAA1429	ENSG00000164944	8	95489921	95575757	291	15	123834	-0.64606	0.74088	0.993417342
RP11-17M16.1	ENSG00000220032	18	74197477	74220045	15	3	123834	-0.64605	0.74088	0.993417342
LSM5	ENSG00000106355	7	32514951	32544895	92	9	123834	-0.64636	0.74098	0.993417342
LRP8	ENSG00000157193	1	53701217	53803742	112	17	123834	-0.64643	0.741	0.993417342
KRTAP12-2	ENSG00000221864	21	46076106	46096844	114	7	123834	-0.64647	0.74101	0.993417342
ZEB2	ENSG00000169554	2	145131648	145292147	88	17	123834	-0.64651	0.74103	0.993417342
SFT2D1	ENSG00000198818	6	166723216	166766079	52	5	123834	-0.64661	0.74106	0.993417342
XPNPEP3	ENSG00000196236	22	41243081	41373838	53	4	123834	-0.64674	0.7411	0.993417342
SCTR	ENSG00000080293	2	120187419	120292070	171	18	123834	-0.64686	0.74114	0.993417342
FAM71A	ENSG00000162771	1	212787789	212810120	30	8	123834	-0.64725	0.74127	0.993417342
ADRA2B	ENSG00000222040	2	96768707	96791984	6	1	123834	-0.64725	0.74127	0.993417342
ADIPOQ	ENSG00000181092	3	186550463	186586252	50	12	123834	-0.6473	0.74128	0.993417342
POU4F1	ENSG00000152192	13	79162497	79187673	23	4	123834	-0.64751	0.74135	0.993417342
NFE2L1	ENSG00000082641	17	46115691	46148849	76	11	123834	-0.6477	0.74141	0.993417342
CD274	ENSG00000120217	9	5440503	5480566	44	7	123834	-0.64778	0.74143	0.993417342
INVS	ENSG00000119509	9	102851538	103073282	372	11	123834	-0.64799	0.7415	0.993417342
CTAGE5	ENSG00000150527	14	39724488	39866156	251	17	123834	-0.64825	0.74159	0.993417342
MGC10955	ENSG00000217702	2	74351599	74372521	30	6	123834	-0.64832	0.74161	0.993417342
CEP78	ENSG00000148019	9	80840978	80904606	88	10	123834	-0.64834	0.74162	0.993417342
SLC7A4	ENSG00000099960	22	21373007	21397129	35	6	123834	-0.64845	0.74165	0.993417342
GAREM	ENSG00000141441	18	29694840	30060447	589	32	123834	-0.64848	0.74166	0.993417342
OR2AG2	ENSG00000188124	11	6779218	6800286	63	8	123834	-0.64863	0.74171	0.993417342
AC018816.3	ENSG00000235978	3	4845978	4938977	109	7	123834	-0.64878	0.74176	0.993417342
LMX1B	ENSG00000136944	9	129366722	129473311	159	25	123834	-0.64913	0.74187	0.993417342
PRKDC	ENSG00000253729	8	48675669	48882743	86	7	123834	-0.64919	0.74189	0.993417342
CORO1A	ENSG00000102879	16	30184148	30210397	13	3	123834	-0.64919	0.74189	0.993417342
PCGF5	ENSG00000180628	10	92969908	93054088	132	5	123834	-0.64929	0.74193	0.993417342
GAS2	ENSG00000148935	11	22637188	22844601	245	23	123834	-0.64949	0.74199	0.993417342
EFCAB1	ENSG00000034239	8	49613348	49657870	48	9	123834	-0.65036	0.74227	0.993417342
RASL12	ENSG00000103710	15	65335679	65379028	19	4	123834	-0.65039	0.74228	0.993417342
NOVA2	ENSG00000104967	19	46426992	46486804	22	6	123834	-0.65044	0.7423	0.993417342
ARCN1	ENSG00000095139	11	118433105	118483748	54	3	123834	-0.65052	0.74232	0.993417342
PLA2G2D	ENSG00000117215	1	20428432	20456050	16	4	123834	-0.65059	0.74235	0.993417342
ELOVL2	ENSG00000197977	6	10970992	11054547	93	7	123834	-0.65067	0.74237	0.993417342
KLF16	ENSG00000129911	19	1842399	1873567	18	4	123834	-0.65081	0.74241	0.993417342
RASSF8	ENSG00000123094	12	26101962	26242825	366	28	123834	-0.65085	0.74243	0.993417342
MDM2	ENSG00000135679	12	69191956	69249214	79	10	123834	-0.65084	0.74243	0.993417342
MIER2	ENSG00000105556	19	295575	354798	138	9	123834	-0.65106	0.74249	0.993417342
ZNF544	ENSG00000198131	19	58729960	58798815	148	15	123834	-0.65106	0.74249	0.993417342
GUCY1B3	ENSG00000061918	4	156670144	156738743	86	17	123834	-0.65122	0.74255	0.993417342
EFCAB14	ENSG00000159658	1	47114366	47194824	120	4	123834	-0.65126	0.74256	0.993417342
ATP6V1B1	ENSG00000116039	2	71153012	71202536	45	9	123834	-0.65131	0.74258	0.993417342
SPIDR	ENSG00000164808	8	48163167	48658868	157	17	123834	-0.65149	0.74263	0.993417342
MROH6	ENSG00000204839	8	144638357	144665141	70	11	123834	-0.65153	0.74265	0.993417342
CSMD1	ENSG00000183117	8	2782875	4862494	6914	445	123834	-0.65165	0.74268	0.993417342
FAM195A	ENSG00000172366	16	681813	708474	21	4	123834	-0.65165	0.74269	0.993417342
GPR42	ENSG00000126251	19	35851831	35873855	21	6	123834	-0.65169	0.7427	0.993417342
CIRH1A	ENSG00000141076	16	69155194	69275033	152	15	123834	-0.65184	0.74275	0.993417342
MBD3L3	ENSG00000182315	19	7046220	7068651	3	2	123834	-0.65189	0.74276	0.993417342
CDR2L	ENSG00000109089	17	72973727	73011895	21	4	123834	-0.65212	0.74284	0.993417342
BIN2	ENSG00000110934	12	51664822	51728452	160	9	123834	-0.65229	0.74289	0.993417342
PSTK	ENSG00000179988	10	124703897	124767029	132	16	123834	-0.6523	0.7429	0.993417342
HYAL4	ENSG00000106302	7	123459037	123527532	70	11	123834	-0.65233	0.74291	0.993417342
TTC21A	ENSG00000168026	3	39139152	39190394	111	12	123834	-0.65239	0.74293	0.993417342
PRMT10	ENSG00000164169	4	148548936	148615381	48	3	123834	-0.65258	0.74299	0.993417342
MS4A2	ENSG00000149534	11	59845734	59873444	51	7	123834	-0.65272	0.74303	0.993417342
DOCK6	ENSG00000130158	19	11299971	11383157	109	14	123834	-0.65307	0.74314	0.993417342
GNAT2	ENSG00000134183	1	110135889	110165679	35	8	123834	-0.6531	0.74315	0.993417342
TCF7L1	ENSG00000152284	2	85350533	85547511	338	29	123834	-0.65337	0.74324	0.993417342
PAN2	ENSG00000135473	12	56700007	56737837	31	2	123834	-0.6534	0.74325	0.993417342
BHMT2	ENSG00000132840	5	78355540	78395289	86	9	123834	-0.65342	0.74326	0.993417342
EFCAB4B	ENSG00000130038	12	3705799	3883985	381	37	123834	-0.65346	0.74327	0.993417342
PIH1	ENSG00000268977	16	79005647	79025739	19	6	123834	-0.65354	0.7433	0.993417342
CNST	ENSG00000162852	1	246719746	246841886	131	14	123834	-0.65384	0.74339	0.993417342
PARP11	ENSG00000111224	12	3890213	3992608	199	17	123834	-0.65382	0.74339	0.993417342
AAR2	ENSG00000131043	20	34814381	34868840	14	4	123834	-0.65422	0.74351	0.993417342
USP6	ENSG00000129204	17	5009733	5088329	121	11	123834	-0.6543	0.74354	0.993417342
C3orf30	ENSG00000163424	3	118854997	118888889	129	16	123834	-0.65484	0.74371	0.993417342
GDF5	ENSG00000125965	20	34011145	34052568	54	5	123834	-0.65488	0.74373	0.993417342
MXD4	ENSG00000123933	4	2239159	2274021	55	6	123834	-0.65529	0.74386	0.993417342
KLHDC9	ENSG00000162755	1	161058151	161080136	22	6	123834	-0.65533	0.74387	0.993417342
KRTAP19-3	ENSG00000244025	21	31853782	31874275	25	2	123834	-0.65559	0.74396	0.993417342
FAM154A	ENSG00000155875	9	18917656	19043251	452	23	123834	-0.65568	0.74398	0.993417342
LOR	ENSG00000203782	1	153222176	153244598	45	5	123834	-0.6558	0.74402	0.993417342
FSCN2	ENSG00000186765	17	79485422	79514156	51	12	123834	-0.65588	0.74405	0.993417342
WISP2	ENSG00000064205	20	43333485	43367150	29	8	123834	-0.65587	0.74405	0.993417342
DCAF10	ENSG00000122741	9	37790499	37877663	197	4	123834	-0.65653	0.74426	0.993417342
PLCG2	ENSG00000197943	16	81762702	82001899	504	65	123834	-0.65708	0.74444	0.993417342
FOLR1	ENSG00000110195	11	71890602	71917345	35	6	123834	-0.65718	0.74447	0.993417342
SNX10	ENSG00000086300	7	26321541	26423949	173	23	123834	-0.6573	0.74451	0.993417342
ZNF730	ENSG00000183850	19	23248012	23340021	151	12	123834	-0.65766	0.74462	0.993417342
ABHD2	ENSG00000140526	15	89620690	89755591	104	18	123834	-0.65769	0.74463	0.993417342
DCAF4L2	ENSG00000176566	8	88872973	88896296	15	5	123834	-0.6578	0.74467	0.993417342
SMOC1	ENSG00000198732	14	70310848	70509083	346	45	123834	-0.65812	0.74477	0.993417342
RSL24D1	ENSG00000137876	15	55463004	55499265	137	8	123834	-0.65836	0.74485	0.993417342
HHATL	ENSG00000010282	3	42724155	42754319	23	5	123834	-0.65882	0.74499	0.993417342
SMIM14	ENSG00000163683	4	39537950	39650710	162	19	123834	-0.6588	0.74499	0.993417342
PDX1	ENSG00000139515	13	28484157	28510368	26	4	123834	-0.65881	0.74499	0.993417342
MAP1B	ENSG00000131711	5	71393061	71515395	129	24	123834	-0.65909	0.74508	0.993417342
LEP	ENSG00000174697	7	127871337	127907681	31	3	123834	-0.65909	0.74508	0.993417342

SLCO1B3	ENSG00000111700	12	20953636	21253040	1133	20	123834	-0.65919	0.74511	0.993417342
CIPC	ENSG00000198894	14	77554440	77593630	32	4	123834	-0.65938	0.74518	0.993417342
CTD-3138B18.4	ENSG00000269545	19	58730831	58808173	156	15	123834	-0.65945	0.7452	0.993417342
FCRL4	ENSG00000163518	1	157533539	157577870	113	9	123834	-0.65957	0.74523	0.993417342
C5orf60	ENSG00000204661	5	179058545	179082047	15	6	123834	-0.65963	0.74525	0.993417342
DEFB131	ENSG00000186146	4	9436260	9462240	46	4	123834	-0.65973	0.74529	0.993417342
MMP28	ENSG00000129270	17	34073268	34132711	97	7	123834	-0.65999	0.74537	0.993417342
STK31	ENSG00000196335	7	23739786	23882132	404	23	123834	-0.66025	0.74545	0.993417342
ZNF648	ENSG00000179930	1	182013705	182040847	54	5	123834	-0.66032	0.74547	0.993417342
C19orf44	ENSG00000105072	19	16597122	16642163	94	12	123834	-0.66061	0.74557	0.993417342
SPRR2A	ENSG00000241794	1	153018589	153040013	52	4	123834	-0.66072	0.74561	0.993417342
SLPI	ENSG00000124107	20	43870880	43893205	57	5	123834	-0.66077	0.74562	0.993417342
DHRS7	ENSG00000100612	14	60600838	60646574	90	3	123834	-0.66084	0.74564	0.993417342
ADRA2A	ENSG00000150594	10	112826790	112850658	17	5	123834	-0.66088	0.74566	0.993417342
RP11-123K3.4	ENSG00000258830	12	57633392	57700267	39	5	123834	-0.6609	0.74566	0.993417342
MAPKAPK5	ENSG00000089022	12	112269782	112344343	121	3	123834	-0.66095	0.74568	0.993417342
SRGAP1	ENSG00000196935	12	64228073	64551613	294	34	123834	-0.66108	0.74572	0.993417342
TBCD	ENSG00000141556	17	80699940	80910724	584	20	123834	-0.66126	0.74578	0.993417342
RNASE7	ENSG00000165799	14	21500385	21522393	15	5	123834	-0.66134	0.7458	0.993417342
TANGO2	ENSG00000183597	22	19994537	20063449	144	20	123834	-0.66159	0.74588	0.993417342
FAM214B	ENSG00000005238	9	35094109	35126338	36	6	123834	-0.66212	0.74605	0.993417342
CYP26A1	ENSG00000095596	10	94823232	94847647	36	10	123834	-0.66233	0.74612	0.993417342
ANKRD50	ENSG00000151458	4	125575207	125643887	82	10	123834	-0.66239	0.74614	0.993417342
OPN1SW	ENSG00000128617	7	128402545	128425844	41	5	123834	-0.66249	0.74617	0.993417342
MTRNR2L12	ENSG00000269028	3	96325981	96347000	45	4	123834	-0.66264	0.74622	0.993417342
DTNB	ENSG00000138101	2	25590067	25906503	149	21	123834	-0.6628	0.74627	0.993417342
ORMDL1	ENSG00000128699	2	190625049	190659097	67	7	123834	-0.66334	0.74644	0.993417342
DTWD2	ENSG00000169570	5	118163017	118334240	236	22	123834	-0.66342	0.74647	0.993417342
FOXD2	ENSG00000186564	1	47891689	47916363	34	5	123834	-0.66346	0.74648	0.993417342
OR10S1	ENSG00000196248	11	123837368	123858488	64	9	123834	-0.66374	0.74657	0.993417342
PGRMC2	ENSG00000164040	4	129180397	129219984	41	6	123834	-0.6639	0.74662	0.993417342
TGM6	ENSG00000166948	20	2351554	2423399	178	22	123834	-0.66403	0.74667	0.993417342
MYC	ENSG00000136997	8	128737680	128763674	23	7	123834	-0.6642	0.74672	0.993417342
RPL27A	ENSG00000166441	11	8693958	8746306	60	12	123834	-0.66431	0.74676	0.993417342
BACE1	ENSG00000186318	11	117146402	117196975	31	10	123834	-0.66445	0.7468	0.993417342
CYP27C1	ENSG00000186684	2	127931696	127987654	73	12	123834	-0.66461	0.74685	0.993417342
C3orf49	ENSG00000163632	3	63795038	63844312	52	8	123834	-0.66473	0.74689	0.993417342
CIDEA	ENSG00000176194	18	12244318	12287594	39	12	123834	-0.66477	0.7469	0.993417342
TSPYL4	ENSG00000187189	6	116561151	116585261	51	7	123834	-0.66524	0.74705	0.993417342
AL139147.1	ENSG00000248458	1	67121547	67152710	36	6	123834	-0.6656	0.74717	0.993417342
TRIM7	ENSG00000146054	5	180610924	180642293	105	13	123834	-0.66561	0.74717	0.993417342
AC005008.2	ENSG00000237896	7	80794824	80838431	117	16	123834	-0.66567	0.74719	0.993417342
CCDC137	ENSG00000185298	17	79623394	79650934	121	9	123834	-0.66581	0.74723	0.993417342
BEST4	ENSG00000142959	1	45239257	45263377	26	4	123834	-0.66583	0.74724	0.993417342
ABLIM3	ENSG00000173210	5	148511046	148650105	160	17	123834	-0.66589	0.74726	0.993417342
NMBR	ENSG00000135577	6	142369467	142419936	66	6	123834	-0.66588	0.74726	0.993417342
MYADML2	ENSG00000185105	17	79887521	79915109	15	4	123834	-0.66597	0.74728	0.993417342
FHOD3	ENSG00000134775	18	33867677	34370018	537	56	123834	-0.66616	0.74734	0.993417342
IL10	ENSG00000136634	1	206930947	206955839	22	5	123834	-0.66621	0.74736	0.993417342
FGFR1OP	ENSG00000213066	6	167402670	167476201	329	9	123834	-0.66639	0.74742	0.993417342
FKSG62	ENSG00000259752	15	47415693	47436320	39	6	123834	-0.66654	0.74747	0.993417342
SSR1	ENSG00000124783	6	7258539	7357679	260	21	123834	-0.66664	0.7475	0.993417342
RPP14	ENSG00000255154	3	58293341	58313847	62	5	123834	-0.66716	0.74766	0.993417342
DDC	ENSG00000132437	7	50516134	50643154	457	26	123834	-0.66765	0.74782	0.993417342
SLC4A5	ENSG00000188687	2	74433369	74580541	134	15	123834	-0.66785	0.74788	0.993417342
ALDH1L2	ENSG00000136010	12	105403568	105488355	251	26	123834	-0.66789	0.7479	0.993417342
CA1	ENSG00000133742	8	86229837	86301243	90	13	123834	-0.66793	0.74791	0.993417342
LIN7A	ENSG00000111052	12	81176299	81341704	267	17	123834	-0.66794	0.74791	0.993417342
EMILIN1	ENSG00000138080	2	27291435	27319271	18	2	123834	-0.66804	0.74795	0.993417342
PPAP2B	ENSG00000162407	1	56950419	57120974	280	31	123834	-0.66819	0.74799	0.993417342
GCAT	ENSG00000100116	22	38193912	38223183	41	6	123834	-0.6682	0.748	0.993417342
KCNB2	ENSG00000182674	8	73439626	73860584	870	50	123834	-0.66847	0.74808	0.993417342
CASP5	ENSG00000137757	11	104854962	104903895	118	10	123834	-0.66847	0.74808	0.993417342
CTD-3222D19.2	ENSG00000141979	19	16579878	16749015	269	17	123834	-0.66845	0.74808	0.993417342
GPR32	ENSG00000142511	19	51263721	51284989	13	5	123834	-0.66846	0.74808	0.993417342
AC068987.1	ENSG00000260415	12	52193789	52216636	30	5	123834	-0.66863	0.74813	0.993417342
GTF3C2	ENSG00000115207	2	27538716	27589868	28	5	123834	-0.66916	0.7483	0.993417342
KDM4A	ENSG00000066135	1	44105829	44181186	85	8	123834	-0.66931	0.74835	0.993417342
UBE2L6	ENSG00000156587	11	57309129	57345757	64	7	123834	-0.66929	0.74835	0.993417342
IER5L	ENSG00000188483	9	131927835	131950540	17	2	123834	-0.66982	0.74851	0.993417342
BPIFA1	ENSG00000198183	20	31813801	31841115	34	1	123834	-0.66981	0.74851	0.993417342
CACNG1	ENSG00000108878	17	65030706	65062909	23	7	123834	-0.66984	0.74852	0.993417342
AC090427.1	ENSG00000268550	19	14824747	14845121	31	5	123834	-0.66987	0.74853	0.993417342
BCLAF1	ENSG00000029363	6	136568001	136620989	13	1	123834	-0.66996	0.74856	0.993417342
ZCCHC7	ENSG00000147905	9	37110536	37368146	186	12	123834	-0.67001	0.74857	0.993417342
PSG8	ENSG00000124467	19	43246838	43369843	340	15	123834	-0.67061	0.74877	0.993417342
PELI2	ENSG00000139946	14	56574532	56778244	332	19	123834	-0.6708	0.74883	0.993417342
CRYBG3	ENSG00000233280	3	97530884	97604132	52	7	123834	-0.67086	0.74884	0.993417342
IL18BP	ENSG00000137496	11	71699587	71726761	48	4	123834	-0.67084	0.74884	0.993417342
COQ10A	ENSG00000135469	12	56650642	56674750	19	2	123834	-0.67105	0.74891	0.993417342
ST13	ENSG00000100380	22	41210539	41263026	36	3	123834	-0.67109	0.74892	0.993417342
C1orf189	ENSG00000163263	1	154161848	154188809	15	3	123834	-0.67146	0.74904	0.993417342
SETDB1	ENSG00000143379	1	150888739	150947213	70	5	123834	-0.67158	0.74907	0.993417342
GUCA2A	ENSG00000197273	1	42618362	42640389	25	7	123834	-0.67202	0.74922	0.993417342
VIL1	ENSG00000127831	2	219273815	219328018	25	4	123834	-0.67239	0.74933	0.993417342
PNN	ENSG00000100941	14	39634387	39662422	52	9	123834	-0.67241	0.74934	0.993417342
RRP15	ENSG00000067533	1	218448629	218521325	67	2	123834	-0.67249	0.74936	0.993417342
MAPKAPK2	ENSG00000162889	1	206848289	206917628	105	11	123834	-0.67255	0.74938	0.993417342
GPN1	ENSG00000198522	2	27841114	27884375	41	4	123834	-0.67262	0.7494	0.993417342
ZNF546	ENSG00000187187	19	40480041	40533514	74	4	123834	-0.67265	0.74942	0.993417342
DAPK1	ENSG00000196730	9	90102143	90333548	490	46	123834	-0.67273	0.74944	0.993417342
VPS53	ENSG00000141252	17	401908	634957	585	24	123834	-0.67318	0.74958	0.993417342
RGP1	ENSG00000107185	9	35739203	35768572	19	4	123834	-0.67321	0.74959	0.993417342
ASNSD1	ENSG00000138381	2	190516111	190545557	48	7	123834	-0.67336	0.74964	0.993417342
OARD1	ENSG00000124596	6	40991366	41075526	133	9	123834	-0.67388	0.74981	0.993417342
RGMA	ENSG00000182175	15	93576636	93642433	68	16	123834	-0.67459	0.75003	0.993417342
CYS1	ENSG00000205795	2	10186907	10231071	91	16	123834	-0.67463	0.75004	0.993417342
AP5B1	ENSG00000254470	11	65533364	65558273	35	6	123834	-0.67473	0.75008	0.993417342
WRNIP1	ENSG00000124535	6	2755648	2797186	90	12	123834	-0.67527	0.75025	0.993417342

NANS	ENSG00000095380	9	100809021	100855357	35	6	123834	-0.67534	0.75027	0.993417342
PFN4	ENSG00000176732	2	24328241	24356347	79	6	123834	-0.67549	0.75032	0.993417342
MYOZ3	ENSG00000164591	5	150030436	150068927	44	2	123834	-0.6757	0.75038	0.993417342
GPR182	ENSG00000166856	12	57378230	57400468	17	2	123834	-0.67601	0.75048	0.993417342
TARBP1	ENSG00000059588	1	234517059	234624849	332	35	123834	-0.67639	0.7506	0.993417342
GTF2H4	ENSG00000213780	6	30865961	30891883	57	7	123834	-0.67639	0.7506	0.993417342
SPDYA	ENSG00000163806	2	28995383	29083477	114	9	123834	-0.67642	0.75061	0.993417342
HOXD12	ENSG00000170178	2	176954458	176976408	17	3	123834	-0.67641	0.75061	0.993417342
CDKN1C	ENSG00000129757	11	2894443	2917111	13	4	123834	-0.67643	0.75062	0.993417342
ZNF738	ENSG00000172687	19	21531732	21572104	157	7	123834	-0.67662	0.75068	0.993417342
RAPGEF4	ENSG00000091428	2	173590002	173927621	568	44	123834	-0.67681	0.75074	0.993417342
TAF6L	ENSG00000162227	11	62528775	62564814	29	3	123834	-0.67692	0.75077	0.993417342
TMEM223	ENSG00000168569	11	62529101	62569493	29	3	123834	-0.67692	0.75077	0.993417342
AIM2	ENSG00000163568	1	159022274	159126886	124	14	123834	-0.67696	0.75079	0.993417342
CD4	ENSG00000010610	12	6886024	6939974	71	10	123834	-0.67711	0.75083	0.993417342
VPS41	ENSG00000006715	7	38752563	38981994	552	23	123834	-0.67719	0.75086	0.993417342
IL36G	ENSG00000136688	2	113720780	113753242	49	5	123834	-0.67767	0.75101	0.993417342
AFF4	ENSG00000072364	5	132201071	132309326	193	10	123834	-0.67787	0.75107	0.993417342
FANCM	ENSG00000187790	14	45595143	45680093	86	7	123834	-0.67795	0.7511	0.993417342
SMYD5	ENSG00000135632	2	73431350	73464365	63	10	123834	-0.67799	0.75111	0.993417342
EXD1	ENSG00000178997	15	41464923	41532941	221	10	123834	-0.67803	0.75112	0.993417342
TMEM199	ENSG00000244045	17	26674604	26718716	56	5	123834	-0.67802	0.75112	0.993417342
RASGEF1C	ENSG00000146090	5	179517795	179646153	184	44	123834	-0.6783	0.75121	0.993417342
LRP3	ENSG00000130881	19	33658509	33708694	78	11	123834	-0.67843	0.75125	0.993417342
ACSM2A	ENSG00000183747	16	20452783	20508991	80	3	123834	-0.67881	0.75137	0.993417342
OGDH	ENSG00000105953	7	44636171	44758665	137	11	123834	-0.67885	0.75138	0.993417342
ZNF512B	ENSG00000196700	20	62578055	62690113	131	10	123834	-0.67903	0.75144	0.993417342
ATP13A3	ENSG00000133657	3	194113401	194229093	137	15	123834	-0.67954	0.7516	0.993417342
CLPSL1	ENSG00000204140	6	35738794	35771452	14	5	123834	-0.67964	0.75163	0.993417342
PRMT8	ENSG00000111218	12	3480515	3713139	229	43	123834	-0.67972	0.75166	0.993417342
KCNK6	ENSG00000099337	19	38800484	38829660	75	10	123834	-0.67975	0.75167	0.993417342
COX6A2	ENSG00000156885	16	31429052	31449967	8	3	123834	-0.68018	0.7518	0.993417342
AL356215.1	ENSG00000269766	11	35140091	35160215	56	7	123834	-0.68025	0.75183	0.993417342
GPX7	ENSG00000116157	1	53058044	53084723	49	7	123834	-0.68036	0.75186	0.993417342
MRPL24	ENSG00000143314	1	156697095	156721382	28	6	123834	-0.68057	0.75193	0.993417342
IFT57	ENSG00000114446	3	107869659	107951417	321	5	123834	-0.6806	0.75194	0.993417342
VPS39	ENSG00000166887	15	42440899	42510514	93	8	123834	-0.68062	0.75194	0.993417342
IMPAD1	ENSG00000104331	8	57860492	57916403	59	5	123834	-0.68072	0.75198	0.993417342
TSPAN32	ENSG00000064201	11	2313227	2349430	112	11	123834	-0.68114	0.75211	0.993417342
SERPINA10	ENSG00000140093	14	94739650	94769608	33	5	123834	-0.68138	0.75218	0.993417342
PGM2L1	ENSG00000165434	11	74031363	74119518	199	15	123834	-0.68148	0.75221	0.993417342
F13A1	ENSG00000124491	6	6134318	6331246	297	45	123834	-0.68148	0.75222	0.993417342
FKBP3	ENSG00000100442	14	45574803	45614522	31	2	123834	-0.68184	0.75233	0.993417342
HAND1	ENSG00000113196	5	153844532	153867824	35	9	123834	-0.68193	0.75236	0.993417342
HBZ	ENSG00000130656	16	192686	214502	23	6	123834	-0.6821	0.75241	0.993417342
RP11-105C20.2	ENSG00000260240	16	59733897	59798752	111	14	123834	-0.68226	0.75246	0.993417342
ZNF667	ENSG00000198046	19	56940694	56999434	77	12	123834	-0.68224	0.75246	0.993417342
RPL22L1	ENSG00000163584	3	170572664	170598272	42	7	123834	-0.6827	0.7526	0.993417342
MARK1	ENSG00000116141	1	220691568	220847803	211	11	123834	-0.6829	0.75266	0.993417342
ZCCHC4	ENSG00000168228	4	25304407	25382005	181	17	123834	-0.68291	0.75267	0.993417342
APBB2	ENSG00000163697	4	40802044	41228731	907	60	123834	-0.68311	0.75273	0.993417342
MTX3	ENSG00000177034	5	79265584	79297082	34	4	123834	-0.68335	0.75281	0.993417342
PFKFB2	ENSG00000123836	1	207212801	207264369	29	4	123834	-0.68341	0.75283	0.993417342
UQCRH	ENSG00000173660	1	46759303	46792448	31	9	123834	-0.68359	0.75288	0.993417342
PSMD5	ENSG00000095261	9	123567774	123615262	56	4	123834	-0.68381	0.75295	0.993417342
NOX4	ENSG00000086991	11	89047524	89332779	312	24	123834	-0.68384	0.75296	0.993417342
TFG	ENSG00000114354	3	100418205	100477810	71	6	123834	-0.68392	0.75299	0.993417342
HCAR3	ENSG00000255398	12	123189303	123211439	49	4	123834	-0.68399	0.75301	0.993417342
IQCF6	ENSG00000214686	3	51802580	51823009	9	1	123834	-0.68413	0.75305	0.993417342
MC5R	ENSG00000176136	18	13814154	13836861	109	10	123834	-0.68413	0.75305	0.993417342
PSCA	ENSG00000167653	8	143741726	143774142	99	9	123834	-0.68443	0.75315	0.993417342
C3orf72	ENSG00000206262	3	138656076	138682293	45	3	123834	-0.68445	0.75316	0.993417342
MAT1A	ENSG00000151224	10	82021576	82059440	104	15	123834	-0.68452	0.75318	0.993417342
KIF2C	ENSG00000142945	1	45195490	45243439	68	6	123834	-0.68461	0.75321	0.993417342
HNF4A	ENSG00000101076	20	42974340	43071485	96	9	123834	-0.68467	0.75322	0.993417342
HPSE	ENSG00000173083	4	84203614	84266306	79	5	123834	-0.68479	0.75326	0.993417342
FNBP1	ENSG00000187239	9	132639466	132815473	404	27	123834	-0.6849	0.7533	0.993417342
CD6	ENSG00000013725	11	60729115	60797849	142	24	123834	-0.6851	0.75336	0.993417342
CD300LF	ENSG00000186074	17	72680452	72719117	33	7	123834	-0.68511	0.75336	0.993417342
LMAN2L	ENSG00000114988	2	97361666	97415801	42	3	123834	-0.68529	0.75342	0.993417342
ITFG3	ENSG00000269881	16	289546	329942	34	4	123834	-0.68528	0.75342	0.993417342
TMED1	ENSG00000099203	19	10933114	10956994	15	4	123834	-0.68541	0.75346	0.993417342
PAH	ENSG00000171759	12	103220663	103362188	182	17	123834	-0.68557	0.75351	0.993417342
THBS3	ENSG00000169231	1	155155379	155188842	24	4	123834	-0.68579	0.75358	0.993417342
PIGV	ENSG00000060642	1	27103963	27134889	20	4	123834	-0.68606	0.75366	0.993417342
VCPIP1	ENSG00000175073	8	67530722	67589452	57	4	123834	-0.68626	0.75372	0.993417342
CCDC97	ENSG00000142039	19	41806094	41840788	40	3	123834	-0.68626	0.75373	0.993417342
SHB	ENSG00000107338	9	37909131	38079210	318	15	123834	-0.68673	0.75387	0.993417342
RIMS2	ENSG00000176406	8	104502976	105278322	1965	23	123834	-0.6869	0.75393	0.993417342
AC109829.1	ENSG00000233438	2	27750253	27800011	54	5	123834	-0.68693	0.75394	0.993417342
CCDC74B	ENSG00000152076	2	130886860	130912707	31	1	123834	-0.68696	0.75395	0.993417342
ZNF500	ENSG00000103199	16	4788240	4827625	78	8	123834	-0.68699	0.75396	0.993417342
RP11-324D17.1	ENSG00000259517	16	54269456	54314658	124	7	123834	-0.68707	0.75398	0.993417342
SLC25A38	ENSG00000144659	3	39414839	39448842	92	7	123834	-0.68714	0.754	0.993417342
NPW	ENSG00000183971	16	2049927	2080756	49	8	123834	-0.68749	0.75411	0.993487435
LDLRAD4	ENSG00000168675	18	13207497	13662754	477	65	123834	-0.68777	0.7542	0.993487435
HLA-B	ENSG00000234745	6	31311649	31334965	615	43	123834	-0.6878	0.75421	0.993487435
CCDC8	ENSG00000169515	19	46903629	46926841	67	12	123834	-0.68842	0.75441	0.993498846
PLEKHS1	ENSG00000148735	10	115501213	115553188	100	8	123834	-0.68845	0.75442	0.993498846
METTL12	ENSG00000214756	11	62422781	62445968	15	2	123834	-0.68846	0.75442	0.993498846
PAX7	ENSG00000009709	1	18947500	19085360	141	28	123834	-0.68866	0.75448	0.993498846
FOXA1	ENSG00000129514	14	38049189	38079245	34	8	123834	-0.68866	0.75448	0.993498846
MMP21	ENSG00000154485	10	127445022	127474390	6	3	123834	-0.6889	0.75456	0.993535362
EOGT	ENSG00000163378	3	69014365	69073112	58	6	123834	-0.68913	0.75463	0.993558706
CCDC64B	ENSG00000162069	16	3067683	3096927	25	4	123834	-0.69032	0.755	0.993977005
LRRC70	ENSG00000186105	5	61864562	61887275	22	4	123834	-0.69062	0.7551	0.994036787
CXXC11	ENSG00000188011	2	242801752	242825975	91	9	123834	-0.69078	0.75515	0.994036787
PLEKHG4B	ENSG00000153404	5	130373	200085	161	9	123834	-0.6912	0.75528	0.994129981
PRKACA	ENSG00000072062	19	14192500	14238896	24	2	123834	-0.69147	0.75537	0.994129981

CCDC136	ENSG00000128596	7	128420811	128472186	76	5	123834	-0.69157	0.7554	0.994129981
GHITM	ENSG00000165678	10	85889196	85923001	48	7	123834	-0.69168	0.75543	0.994129981
TCF7	ENSG00000081059	5	133440402	133497556	73	7	123834	-0.69189	0.7555	0.994153271
HSPB6	ENSG00000004776	19	36235469	36258980	41	8	123834	-0.69208	0.75556	0.9941634
FAM184B	ENSG00000047662	4	17620929	17793135	212	8	123834	-0.69316	0.7559	0.99447309
C17orf53	ENSG00000125319	17	42209274	42249844	44	4	123834	-0.69317	0.7559	0.99447309
GUCA1C	ENSG00000138472	3	108616639	108682742	182	22	123834	-0.69336	0.75596	0.994483195
MSR1	ENSG00000038945	8	15955387	16434999	983	43	123834	-0.69384	0.75611	0.994576207
HSPB9	ENSG00000197723	17	40264756	40285371	21	2	123834	-0.69408	0.75618	0.994576207
DHFR	ENSG00000228716	5	79912047	79960802	203	7	123834	-0.69418	0.75622	0.994576207
C1orf64	ENSG00000183888	1	16320731	16345302	140	15	123834	-0.69427	0.75624	0.994576207
SHISA2	ENSG00000180730	13	26608735	26635169	60	7	123834	-0.69468	0.75637	0.994641626
IL23A	ENSG00000110944	12	56722663	56744193	21	2	123834	-0.69495	0.75646	0.994641626
HTR1A	ENSG00000178394	5	63246183	63268334	34	6	123834	-0.69508	0.7565	0.994641626
CD81	ENSG00000110651	11	2387407	2428649	38	5	123834	-0.69537	0.75659	0.994641626
ZBTB39	ENSG00000166860	12	57382618	57410230	21	2	123834	-0.69551	0.75663	0.994641626
STXBP4	ENSG00000166263	17	53036088	53251646	387	14	123834	-0.69561	0.75666	0.994641626
ZNF135	ENSG00000176293	19	58560607	58607677	44	6	123834	-0.6958	0.75672	0.994641626
ZFR2	ENSG00000105278	19	3794022	3879030	143	14	123834	-0.69582	0.75673	0.994641626
AC109583.1	ENSG00000268537	3	46917699	46940171	18	4	123834	-0.69606	0.7568	0.994641626
SLA	ENSG00000155926	8	134038973	134125298	228	16	123834	-0.69624	0.75686	0.994641626
FILIP1	ENSG00000118407	6	75991575	76213454	203	14	123834	-0.69636	0.7569	0.994641626
ZNF286A	ENSG00000255104	17	15593006	15658092	43	6	123834	-0.69643	0.75692	0.994641626
CENPL	ENSG00000120334	1	173758688	173803858	31	4	123834	-0.6966	0.75697	0.994641626
SCAMP2	ENSG00000140497	15	75126071	75175706	48	7	123834	-0.69694	0.75708	0.994717401
ANKRD44	ENSG00000065413	2	197821741	198185897	570	45	123834	-0.69719	0.75716	0.994753752
MYOZ2	ENSG00000172399	4	120046939	120118944	188	18	123834	-0.6974	0.75722	0.994763823
TYR	ENSG00000077498	11	88900620	89038927	224	12	123834	-0.69799	0.75741	0.994944663
AC002553.1	ENSG00000227782	17	15933505	15953994	30	3	123834	-0.69839	0.75753	0.994963036
SLC2A13	ENSG00000151229	12	40138823	40509891	569	44	123834	-0.6994	0.75785	0.994963036
BRE	ENSG00000158019	2	28102808	28571768	614	35	123834	-0.69963	0.75792	0.994963036
CD27	ENSG00000139193	12	6544033	6570884	47	11	123834	-0.69979	0.75797	0.994963036
FAM171A2	ENSG00000161682	17	42420583	42451243	13	5	123834	-0.69981	0.75798	0.994963036
TSKS	ENSG00000126467	19	50233010	50276587	29	9	123834	-0.69981	0.75798	0.994963036
ASAH2	ENSG00000188611	10	51932538	52018370	66	7	123834	-0.70048	0.75819	0.994963036
PRR23C	ENSG00000233701	3	138750944	138773734	38	3	123834	-0.70151	0.75851	0.994963036
LILRB3	ENSG00000204577	19	54710147	54756602	32	6	123834	-0.70151	0.75851	0.994963036
LILRA6	ENSG00000244482	19	54710737	54756649	32	6	123834	-0.70151	0.75851	0.994963036
SPRR4	ENSG00000184148	1	152933142	152955050	16	2	123834	-0.70157	0.75853	0.994963036
MYO15A	ENSG00000091536	17	18002020	18093116	108	13	123834	-0.70157	0.75853	0.994963036
GCLM	ENSG00000023909	1	94340761	94384966	104	13	123834	-0.70165	0.75855	0.994963036
ASB1	ENSG00000065802	2	239325383	239370891	127	12	123834	-0.70168	0.75856	0.994963036
PPL	ENSG00000118898	16	4922508	5020742	166	27	123834	-0.70183	0.75861	0.994963036
FCN1	ENSG00000085265	9	137791431	137819809	39	5	123834	-0.70193	0.75864	0.994963036
ZMYND12	ENSG00000066185	1	42886000	42931938	113	8	123834	-0.70198	0.75865	0.994963036
MCTP2	ENSG00000140563	15	94764767	95033632	285	38	123834	-0.70218	0.75872	0.994963036
IGHMBP2	ENSG00000132740	11	68661310	68718070	133	10	123834	-0.70222	0.75873	0.994963036
ACSM5	ENSG00000183549	16	20410856	20462658	163	9	123834	-0.7025	0.75882	0.994963036
RGPD3	ENSG00000153165	2	107011446	107094832	8	3	123834	-0.7026	0.75885	0.994963036
AP001631.10	ENSG00000228120	21	44569176	44592387	11	2	123834	-0.70261	0.75885	0.994963036
DDO	ENSG00000203797	6	110702974	110746765	65	12	123834	-0.7027	0.75888	0.994963036
ZNF780B	ENSG00000128000	19	40524167	40572116	46	4	123834	-0.70272	0.75889	0.994963036
KLHDC4	ENSG00000104731	16	87720091	87809598	240	18	123834	-0.70298	0.75897	0.994963036
FAM19A4	ENSG00000163377	3	68770917	68991761	318	15	123834	-0.70311	0.759	0.994963036
LINC00999	ENSG00000225119	10	38707074	38746820	88	8	123834	-0.7034	0.7591	0.994963036
NIPSNAP1	ENSG00000184117	22	29940797	29987326	107	8	123834	-0.70349	0.75912	0.994963036
OR5T1	ENSG00000181698	11	56033029	56054146	54	7	123834	-0.70351	0.75913	0.994963036
KRT18	ENSG00000111057	12	53332655	53356685	33	7	123834	-0.70385	0.75924	0.994963036
GAL3ST2	ENSG00000154252	2	242706240	242753623	47	8	123834	-0.7043	0.75938	0.994963036
ITGAD	ENSG00000156886	16	31394633	31447826	33	7	123834	-0.70442	0.75942	0.994963036
SNX25	ENSG00000109762	4	186115391	186301339	363	25	123834	-0.70451	0.75944	0.994963036
SLCO1B7	ENSG00000205754	12	20958673	21255679	1129	20	123834	-0.70465	0.75949	0.994963036
SOX8	ENSG00000005513	16	1021808	1046979	79	15	123834	-0.70482	0.75954	0.994963036
RPL37A	ENSG00000197756	2	217352912	217453903	127	23	123834	-0.70523	0.75967	0.994963036
AFAP1L2	ENSG00000169129	10	116044583	116174515	176	30	123834	-0.70528	0.75968	0.994963036
AC110619.2	ENSG00000218416	2	241378732	241406131	34	9	123834	-0.70541	0.75972	0.994963036
GSTP1	ENSG00000084207	11	67341066	67364131	61	5	123834	-0.7056	0.75978	0.994963036
RTCA	ENSG00000137996	1	100721763	100768325	76	3	123834	-0.70584	0.75985	0.994963036
MEPCE	ENSG00000146834	7	100016413	100041741	22	2	123834	-0.70589	0.75987	0.994963036
9-Sep	ENSG00000184640	17	75266651	75506678	324	36	123834	-0.70592	0.75988	0.994963036
AC010441.1	ENSG00000269636	5	150147867	150186297	61	4	123834	-0.70623	0.75998	0.994963036
SMIM23	ENSG00000185662	5	171202876	171231602	45	7	123834	-0.70624	0.75998	0.994963036
ZNF618	ENSG00000157657	9	116628562	116828871	240	31	123834	-0.70623	0.75998	0.994963036
WIPF2	ENSG00000171475	17	38365556	38450388	64	2	123834	-0.70666	0.76011	0.994963036
CCM2L	ENSG00000101331	20	30588245	30629984	54	6	123834	-0.70687	0.76018	0.994963036
ADSL	ENSG00000239900	22	40732507	40796467	59	3	123834	-0.70715	0.76026	0.994963036
FRAT1	ENSG00000165879	10	99069022	99091672	71	6	123834	-0.70748	0.76037	0.994963036
FAM57A	ENSG00000167695	17	625652	656206	67	13	123834	-0.70764	0.76041	0.994963036
SLC25A25	ENSG00000148339	9	130820480	130881524	114	2	123834	-0.70781	0.76047	0.994963036
INPP5F	ENSG00000198825	10	121475609	121598652	200	10	123834	-0.70785	0.76048	0.994963036
PPP6R2	ENSG00000100239	22	50771733	50893514	256	18	123834	-0.70795	0.76051	0.994963036
MS4A14	ENSG00000166928	11	60136003	60195161	134	12	123834	-0.70816	0.76058	0.994963036
DAOA	ENSG00000182346	13	106108216	106153383	94	5	123834	-0.70822	0.7606	0.994963036
HBQ1	ENSG00000086506	16	220452	241180	12	2	123834	-0.70827	0.76061	0.994963036
MZT1	ENSG00000204899	13	73272495	73311825	22	5	123834	-0.70892	0.76081	0.994963036
ZDHHC23	ENSG00000184307	3	113656748	113694248	15	5	123834	-0.70899	0.76083	0.994963036
PARS2	ENSG00000162396	1	55212571	55240187	52	8	123834	-0.70905	0.76085	0.994963036
GJA4	ENSG00000187513	1	35248599	35271348	23	5	123834	-0.70911	0.76087	0.994963036
SLC13A4	ENSG00000164707	7	135355985	135424006	101	14	123834	-0.70909	0.76087	0.994963036
FCHSD2	ENSG00000137478	11	72537790	72863306	151	16	123834	-0.70924	0.76091	0.994963036
REEP5	ENSG00000129625	5	112202084	112268236	137	10	123834	-0.70997	0.76114	0.994963036
MRPS31	ENSG00000102738	13	41293432	41355309	44	4	123834	-0.71004	0.76116	0.994963036
SERP2	ENSG00000151778	13	44937801	44981850	34	5	123834	-0.71032	0.76125	0.994963036
NAP1L4	ENSG00000205531	11	2955667	3023607	163	14	123834	-0.71043	0.76128	0.994963036
EFS	ENSG00000100842	14	23815611	23844961	42	6	123834	-0.7105	0.7613	0.994963036
OR10A7	ENSG00000179919	12	55604809	55625759	29	3	123834	-0.71054	0.76132	0.994963036
AC023590.1	ENSG00000225885	8	119284378	119422844	189	18	123834	-0.71066	0.76135	0.994963036
ISG15	ENSG00000187608	1	938803	959920	20	3	123834	-0.71087	0.76142	0.994963036
TAAR2	ENSG00000146378	6	132928161	132955414	76	13	123834	-0.71102	0.76146	0.994963036

SMIM3	ENSG00000256235	5	150147508	150186296	62	4	123834	-0.71126	0.76154	0.994963036
SLC22A3	ENSG00000146477	6	160759300	160886014	154	14	123834	-0.71134	0.76156	0.994963036
OPA1	ENSG00000198836	3	193300933	193425612	258	10	123834	-0.71141	0.76158	0.994963036
FGG	ENSG00000171557	4	155515286	155544119	15	3	123834	-0.71161	0.76165	0.994963036
EIF5A2	ENSG00000163577	3	170596204	170636482	57	9	123834	-0.71194	0.76175	0.994963036
TMTC4	ENSG00000125247	13	101246181	101337347	162	9	123834	-0.71193	0.76175	0.994963036
DPCR1	ENSG00000168631	6	30898749	30931998	52	14	123834	-0.71201	0.76177	0.994963036
FDCSP	ENSG00000181617	4	71081788	71110969	87	5	123834	-0.7123	0.76186	0.994963036
PRPSAP1	ENSG00000161542	17	74295567	74390602	187	22	123834	-0.7123	0.76186	0.994963036
LST3	ENSG00000257046	12	20958608	21253179	1123	20	123834	-0.71263	0.76196	0.994963036
ATP10A	ENSG00000206190	15	25912420	26120317	354	61	123834	-0.71262	0.76196	0.994963036
KBTBD13	ENSG00000234438	15	65359154	65382276	13	3	123834	-0.71265	0.76197	0.994963036
MFAP4	ENSG00000166482	17	19276755	19300553	7	2	123834	-0.7127	0.76198	0.994963036
TWF1	ENSG00000151239	12	44177526	44210178	62	11	123834	-0.71286	0.76203	0.994963036
PREX1	ENSG00000124126	20	47230790	47454420	535	26	123834	-0.71288	0.76204	0.994963036
AC005481.5	ENSG00000222012	7	157396715	157421457	31	6	123834	-0.71317	0.76213	0.994963036
DENND4B	ENSG00000198837	1	153891977	153929172	38	4	123834	-0.71332	0.76218	0.994963036
HRAS	ENSG00000174775	11	522242	547287	79	10	123834	-0.71375	0.76231	0.994963036
RUFY1	ENSG00000176783	5	178967559	179047027	153	17	123834	-0.71421	0.76245	0.994963036
CHST4	ENSG00000140835	16	71549136	71582649	26	6	123834	-0.7143	0.76248	0.994963036
PYCR1	ENSG00000183010	17	79880260	79910288	13	4	123834	-0.71451	0.76254	0.994963036
ECT2	ENSG00000114346	3	172458472	172549264	144	11	123834	-0.71452	0.76255	0.994963036
CEP19	ENSG00000174007	3	196423148	196449164	62	6	123834	-0.71457	0.76256	0.994963036
UBN2	ENSG00000157741	7	138905102	139002982	96	8	123834	-0.71472	0.76261	0.994963036
LAMC3	ENSG00000050555	9	133874469	133979860	148	20	123834	-0.71475	0.76262	0.994963036
RPS25	ENSG00000118181	11	118876422	118899401	58	5	123834	-0.71545	0.76284	0.994963036
PTS	ENSG00000150787	11	112087088	112150678	153	5	123834	-0.71584	0.76296	0.994963036
OR7A10	ENSG00000127515	19	14941760	14962689	45	11	123834	-0.71643	0.76314	0.994963036
ZNF692	ENSG00000171163	1	249134205	249163343	22	2	123834	-0.71665	0.76321	0.994963036
RLF	ENSG00000117000	1	40617045	40716593	77	10	123834	-0.71673	0.76323	0.994963036
BROX	ENSG00000162819	1	222875895	222918538	56	10	123834	-0.71699	0.76331	0.994963036
NBL1	ENSG00000158747	1	19957048	19994945	67	14	123834	-0.71722	0.76338	0.994963036
PSD2	ENSG00000146005	5	139165406	139234051	58	6	123834	-0.71732	0.76341	0.994963036
HMSD	ENSG00000221887	18	61606535	61659008	90	9	123834	-0.71738	0.76343	0.994963036
AUNIP	ENSG00000127423	1	26148414	26195903	76	2	123834	-0.71767	0.76352	0.994963036
CYP2C18	ENSG00000108242	10	96433251	96505947	157	12	123834	-0.71809	0.76365	0.994963036
TNNT2	ENSG00000118194	1	201318136	201356890	94	13	123834	-0.71834	0.76373	0.994963036
TAS2R43	ENSG00000255374	12	11233886	11254912	28	2	123834	-0.71852	0.76378	0.994963036
WDHD1	ENSG00000198554	14	55395668	55503823	99	6	123834	-0.71866	0.76382	0.994963036
KLK1	ENSG00000167748	19	51312404	51337043	43	7	123834	-0.71864	0.76382	0.994963036
INSIG2	ENSG00000125629	2	118836028	118878573	59	14	123834	-0.71872	0.76384	0.994963036
RP1-228P16.5	ENSG00000257955	12	48403554	48429165	56	9	123834	-0.71888	0.76389	0.994963036
OR14I1	ENSG00000189181	1	248834628	248855629	61	3	123834	-0.71964	0.76413	0.994963036
OSBPL1A	ENSG00000141447	18	21732008	21987844	174	38	123834	-0.71982	0.76418	0.994963036
PTH1R	ENSG00000160801	3	46909236	46955287	32	6	123834	-0.71987	0.7642	0.994963036
DEFB134	ENSG00000205882	8	11840692	11863821	21	5	123834	-0.72069	0.76445	0.994963036
SEC22A	ENSG00000121542	3	122910774	123002977	234	5	123834	-0.72082	0.76449	0.994963036
MMP9	ENSG00000100985	20	44627547	44655200	69	9	123834	-0.72094	0.76453	0.994963036
PFDN2	ENSG00000143256	1	161060346	161097901	35	6	123834	-0.72121	0.76461	0.994963036
KCTD3	ENSG00000136636	1	215730735	215805149	12	4	123834	-0.72146	0.76469	0.994963036
ACSM4	ENSG00000215009	12	7446880	7491320	82	10	123834	-0.7216	0.76473	0.994963036
AMICA1	ENSG00000160593	11	118054455	118105809	98	8	123834	-0.72176	0.76478	0.994963036
LY9	ENSG00000122224	1	160755864	160808051	91	7	123834	-0.72246	0.76499	0.994963036
SYNCRIP	ENSG00000135316	6	86308053	86363510	53	7	123834	-0.72271	0.76507	0.994963036
AC135178.1	ENSG00000198150	17	8251731	8273538	47	5	123834	-0.7229	0.76513	0.994963036
AGPAT4	ENSG00000026652	6	161541011	161705093	249	35	123834	-0.72299	0.76516	0.994963036
RHOT2	ENSG00000140983	16	708086	734174	54	5	123834	-0.72304	0.76517	0.994963036
JAM2	ENSG00000154721	21	27001584	27099874	123	16	123834	-0.72306	0.76518	0.994963036
AIM1L	ENSG00000176092	1	26638350	26690621	121	15	123834	-0.72315	0.76521	0.994963036
TOMM70A	ENSG00000154174	3	100072275	100130242	88	11	123834	-0.72325	0.76524	0.994963036
IMPG2	ENSG00000081148	3	100931390	101049404	181	11	123834	-0.72338	0.76528	0.994963036
IDO1	ENSG00000131203	8	39749794	39795963	80	9	123834	-0.72341	0.76529	0.994963036
GPR135	ENSG00000181619	14	59885740	59942060	150	13	123834	-0.72352	0.76532	0.994963036
ANKRD52	ENSG00000139645	12	56621591	56662175	31	2	123834	-0.72361	0.76535	0.994963036
HBA2	ENSG00000188536	16	212846	233709	7	1	123834	-0.72362	0.76535	0.994963036
DMBX1	ENSG00000197587	1	46962668	46989898	41	9	123834	-0.72366	0.76536	0.994963036
PTGER2	ENSG00000125384	14	52771023	52805324	71	12	123834	-0.72384	0.76542	0.994963036
SEC61G	ENSG00000132432	7	54809943	54837667	75	6	123834	-0.72407	0.76549	0.994963036
AC008443.1	ENSG00000268397	5	180672720	180694578	53	8	123834	-0.72415	0.76551	0.994963036
ACOT6	ENSG00000205669	14	74067649	74096592	74	13	123834	-0.725	0.76577	0.994963036
TTBK2	ENSG00000128881	15	43020932	43223007	141	12	123834	-0.72507	0.7658	0.994963036
KRTAP19-2	ENSG00000186965	21	31849362	31869755	22	2	123834	-0.72513	0.76581	0.994963036
ZSWIM7	ENSG00000214941	17	15869874	15913031	84	5	123834	-0.72527	0.76586	0.994963036
CYFIP1	ENSG00000068793	15	22882005	23016016	202	43	123834	-0.72531	0.76587	0.994963036
GAN	ENSG00000261609	16	81338557	81434489	194	23	123834	-0.72546	0.76592	0.994963036
ZBTB8OS	ENSG00000176261	1	33055773	33126504	118	3	123834	-0.72585	0.76603	0.994963036
MNAT1	ENSG00000020426	14	61191460	61446671	161	20	123834	-0.72683	0.76633	0.994963036
CHAC1	ENSG00000128965	15	41235160	41258710	34	6	123834	-0.72686	0.76634	0.994963036
EXOC2	ENSG00000112685	6	475133	703117	466	34	123834	-0.72737	0.7665	0.994963036
SLC25A32	ENSG00000164933	8	104400863	104437417	105	9	123834	-0.7275	0.76654	0.994963036
AGMAT	ENSG00000116771	1	15888848	15921605	108	7	123834	-0.72761	0.76657	0.994963036
ACVR2B	ENSG00000114739	3	38485342	38544633	89	6	123834	-0.72768	0.7666	0.994963036
LMOD2	ENSG00000170807	7	123285861	123314344	37	5	123834	-0.72772	0.76661	0.994963036
ONECUT1	ENSG00000169856	15	53039637	53093273	60	12	123834	-0.72878	0.76693	0.994963036
NANOS3	ENSG00000187556	19	13962877	14001571	25	1	123834	-0.72889	0.76696	0.994963036
AKIRIN2	ENSG00000135334	6	88374790	88421927	84	12	123834	-0.7299	0.76727	0.994963036
MRPL45	ENSG00000174100	17	36442989	36489101	34	3	123834	-0.72992	0.76728	0.994963036
CELSR2	ENSG00000143126	1	109782641	109828372	49	8	123834	-0.72996	0.76729	0.994963036
RASAL2	ENSG00000075391	1	178052864	178458644	487	17	123834	-0.73011	0.76734	0.994963036
FAM50B	ENSG00000145945	6	3839620	3861551	34	5	123834	-0.73039	0.76743	0.994963036
IFI27L2	ENSG00000119632	14	94584116	94606590	34	4	123834	-0.73047	0.76745	0.994963036
IRF9	ENSG00000213928	14	24620262	24645774	24	5	123834	-0.73061	0.76749	0.994963036
POLR2C	ENSG00000102978	16	57486299	57515922	53	11	123834	-0.73069	0.76751	0.994963036
PNKD	ENSG00000127838	2	219125115	219221516	164	11	123834	-0.73071	0.76752	0.994963036
SEMG1	ENSG00000124233	20	43825638	43848413	38	3	123834	-0.73076	0.76754	0.994963036
PPP1R14D	ENSG00000166143	15	41097650	41130907	46	5	123834	-0.73083	0.76756	0.994963036
ZNF18	ENSG00000154957	17	11870756	11910827	50	4	123834	-0.73083	0.76756	0.994963036
KIR2DL3	ENSG00000243772	19	55239980	55305776	8	1	123834	-0.73111	0.76764	0.994963036
RP11-863K10.7	ENSG00000183154	8	37582279	37604944	1	1	123834	-0.73116	0.76766	0.994963036

SOX9	ENSG00000125398	17	70107161	70132561	21	4	123834	-0.73126	0.76769	0.994963036
MIER1	ENSG00000198160	1	67380578	67464302	137	10	123834	-0.73136	0.76772	0.994963036
LURAP1L	ENSG00000153714	9	12765020	12832130	109	21	123834	-0.73153	0.76777	0.994963036
MRPS22	ENSG00000175110	3	138714648	139086065	465	27	123834	-0.73172	0.76783	0.994963036
METTL8	ENSG00000123600	2	172163919	172301312	181	19	123834	-0.73176	0.76784	0.994963036
ST3GAL1	ENSG00000008513	8	134457091	134594183	186	45	123834	-0.73201	0.76792	0.994963036
LEUTX	ENSG00000213921	19	40257235	40286775	37	4	123834	-0.73224	0.76799	0.994963036
TOR1A	ENSG00000136827	9	132565223	132596413	91	5	123834	-0.73234	0.76802	0.994963036
SLCO4A1	ENSG00000101187	20	61263797	61327137	64	16	123834	-0.73251	0.76807	0.994963036
FZD3	ENSG00000104290	8	28341729	28441775	122	3	123834	-0.73277	0.76815	0.994963036
ACAP2	ENSG00000114331	3	194985465	195173807	442	12	123834	-0.73284	0.76817	0.994963036
WSCD2	ENSG00000075035	12	108513248	108654314	246	24	123834	-0.733	0.76822	0.994963036
SSTR5	ENSG00000162009	16	1112756	1141454	45	6	123834	-0.73306	0.76824	0.994963036
EXO1	ENSG00000174371	1	242001269	242068450	183	18	123834	-0.7331	0.76825	0.994963036
PBX1	ENSG00000185630	1	164514821	164878533	361	57	123834	-0.73347	0.76837	0.994963036
CD200	ENSG00000091972	3	112041194	112091659	104	11	123834	-0.73401	0.76853	0.994963036
MPHOSPH9	ENSG00000051825	12	123626867	123738561	232	9	123834	-0.73403	0.76853	0.994963036
GTF3C1	ENSG00000077235	16	27460876	27571234	85	5	123834	-0.73423	0.7686	0.994963036
DDR1	ENSG00000204580	6	30834198	30877933	103	12	123834	-0.73427	0.76861	0.994963036
SH2D5	ENSG00000189410	1	21036225	21069330	61	13	123834	-0.73455	0.76869	0.994963036
WDR70	ENSG00000082068	5	37369314	37763537	663	20	123834	-0.73453	0.76869	0.994963036
MPP2	ENSG00000108852	17	41942725	41997068	81	7	123834	-0.73455	0.76869	0.994963036
HTR3E	ENSG00000186038	3	183804852	183834783	26	4	123834	-0.73477	0.76876	0.994963036
RPL3	ENSG00000100316	22	39698887	39726394	76	11	123834	-0.73513	0.76887	0.994963036
PLET1	ENSG00000188771	11	112108876	112141583	88	3	123834	-0.7353	0.76892	0.994963036
M1AP	ENSG00000159374	2	74775010	74885465	38	5	123834	-0.73566	0.76903	0.994963036
UBE2V2	ENSG00000169139	8	48910960	48987268	28	5	123834	-0.7357	0.76904	0.994963036
CCSER2	ENSG00000107771	10	86078342	86288273	537	17	123834	-0.7357	0.76904	0.994963036
OST4	ENSG00000228474	2	27283340	27304641	13	2	123834	-0.73585	0.76909	0.994963036
PPP1R27	ENSG00000182676	17	79781368	79802926	29	1	123834	-0.73593	0.76911	0.994963036
FUCA1	ENSG00000179163	1	24161567	24204784	92	4	123834	-0.73601	0.76914	0.994963036
LRIG1	ENSG00000144749	3	66419221	66561687	126	18	123834	-0.73603	0.76914	0.994963036
MCF2L2	ENSG00000053524	3	182885831	183156566	358	31	123834	-0.73632	0.76923	0.994963036
GKAP1	ENSG00000165113	9	86344336	86454431	180	7	123834	-0.73642	0.76926	0.994963036
C11orf96	ENSG00000187479	11	43936892	43975888	54	14	123834	-0.73642	0.76926	0.994963036
ITGB1	ENSG00000150093	10	33179247	33304720	291	24	123834	-0.73657	0.76931	0.994963036
GJC2	ENSG00000198835	1	228327553	228357527	27	6	123834	-0.73667	0.76934	0.994963036
APEX1	ENSG00000100823	14	20913350	20935927	37	6	123834	-0.73676	0.76937	0.994963036
MRPL3	ENSG00000114686	3	131171056	131231827	99	11	123834	-0.73704	0.76945	0.994963036
GFER	ENSG00000127554	16	2024208	2047750	68	6	123834	-0.73718	0.76949	0.994963036
ATOH1	ENSG00000172238	4	94740042	94761221	22	3	123834	-0.7375	0.76959	0.994963036
SLC26A8	ENSG00000112053	6	35901291	36002645	189	22	123834	-0.73774	0.76966	0.994963036
LYPD1	ENSG00000150551	2	133392426	133439152	28	2	123834	-0.73784	0.76969	0.994963036
AC020629.1	ENSG00000269367	12	42614050	42638075	41	6	123834	-0.73787	0.7697	0.994963036
SLC12A8	ENSG00000221955	3	124791480	125008021	368	47	123834	-0.73792	0.76972	0.994963036
SMTNL1	ENSG00000214872	11	57298979	57328108	45	5	123834	-0.73799	0.76974	0.994963036
EFHC1	ENSG00000096093	6	52275106	52397892	320	28	123834	-0.73823	0.76981	0.994963036
PNLIPRP1	ENSG00000187021	10	118339897	118378687	79	12	123834	-0.7384	0.76986	0.994963036
MCM4	ENSG00000104738	8	48862745	48900720	9	2	123834	-0.73848	0.76989	0.994963036
DOLPP1	ENSG00000167130	9	131833379	131862717	20	1	123834	-0.73848	0.76989	0.994963036
ASB3	ENSG00000270898	2	53887430	54097297	438	25	123834	-0.73855	0.76991	0.994963036
GALNTL5	ENSG00000106648	7	151643464	151727019	119	17	123834	-0.73864	0.76994	0.994963036
EWSR1	ENSG00000182944	22	29653998	29706515	53	11	123834	-0.73871	0.76996	0.994963036
CHST1	ENSG00000175264	11	45660427	45697172	50	5	123834	-0.7391	0.77008	0.994963036
MRP63	ENSG00000173141	13	21740784	21763223	52	7	123834	-0.73911	0.77008	0.994963036
ANKIB1	ENSG00000001629	7	91865548	92040698	102	7	123834	-0.73916	0.77009	0.994963036
FOXD4L1	ENSG00000184492	2	114246661	114268728	1	1	123834	-0.73926	0.77012	0.994963036
DSE	ENSG00000111817	6	116565336	116772424	325	26	123834	-0.73935	0.77015	0.994963036
GCA	ENSG00000115271	2	163165350	163238105	15	1	123834	-0.73936	0.77016	0.994963036
PRNT	ENSG00000180259	20	4701928	4731314	78	8	123834	-0.73964	0.77024	0.994963036
CRYBG3	ENSG00000080200	3	97585819	97673810	86	6	123834	-0.73976	0.77028	0.994963036
PRKAG3	ENSG00000115592	2	219677106	219706809	4	2	123834	-0.74006	0.77037	0.994963036
CAPN14	ENSG00000214711	2	31385924	31466724	310	22	123834	-0.74013	0.77039	0.994963036
BCKDHA	ENSG00000248098	19	41874215	41940910	88	2	123834	-0.74013	0.77039	0.994963036
HLA-G	ENSG00000204632	6	29784744	29808902	252	17	123834	-0.7402	0.77041	0.994963036
MTMR14	ENSG00000163719	3	9681117	9754077	59	6	123834	-0.74057	0.77052	0.994963036
TRIM26	ENSG00000234127	6	30142232	30191204	126	17	123834	-0.74077	0.77058	0.994963036
SOS1	ENSG00000115904	2	39198537	39361486	185	4	123834	-0.74181	0.7709	0.994963036
UPK3A	ENSG00000100373	22	45670863	45701755	51	7	123834	-0.74188	0.77092	0.994963036
CHRNA1	ENSG00000138435	2	175602320	175639200	37	4	123834	-0.74236	0.77106	0.994963036
KRT71	ENSG00000139648	12	52927693	52956931	57	8	123834	-0.74234	0.77106	0.994963036
A4GALT	ENSG00000128274	22	43078127	43127304	137	18	123834	-0.74243	0.77109	0.994963036
ZDHHC1	ENSG00000159714	16	67418322	67460736	51	5	123834	-0.7426	0.77114	0.994963036
UBE2V1	ENSG00000244687	20	48687661	48742496	62	6	123834	-0.74263	0.77115	0.994963036
COL2A1	ENSG00000139219	12	48356748	48408269	106	14	123834	-0.74303	0.77127	0.994963036
PDS5A	ENSG00000121892	4	39814483	39989576	400	25	123834	-0.74306	0.77128	0.994963036
NDUFAF1	ENSG00000137806	15	41669551	41704717	54	7	123834	-0.74311	0.77129	0.994963036
CST6	ENSG00000175315	11	65769312	65790976	34	7	123834	-0.74318	0.77131	0.994963036
CCRL2	ENSG00000121797	3	46438654	46464488	84	7	123834	-0.7433	0.77135	0.994963036
CITED2	ENSG00000164442	6	139683393	139705757	18	5	123834	-0.74347	0.7714	0.994963036
C11orf84	ENSG00000168005	11	63570860	63605190	24	5	123834	-0.74363	0.77145	0.994963036
BAIAP2L1	ENSG00000006453	7	97910963	98040380	230	7	123834	-0.74375	0.77149	0.994963036
CCNF	ENSG00000162063	16	2469395	2518855	69	2	123834	-0.74386	0.77152	0.994963036
CEP135	ENSG00000174799	4	56805037	56909529	142	16	123834	-0.7439	0.77153	0.994963036
RP1-317E23.6	ENSG00000255054	1	26127961	26160235	39	2	123834	-0.74394	0.77155	0.994963036
FAM21C	ENSG00000172661	10	46212648	46298409	54	1	123834	-0.74398	0.77156	0.994963036
LPAR5	ENSG00000184574	12	6718001	6755613	26	6	123834	-0.744	0.77156	0.994963036
UCP1	ENSG00000109424	4	141470588	141499959	49	6	123834	-0.74409	0.77159	0.994963036
DYNC1I1	ENSG00000158560	7	95391866	95749634	423	48	123834	-0.74466	0.77176	0.994963036
NBR1	ENSG00000188554	17	41312498	41373708	82	2	123834	-0.74468	0.77177	0.994963036
ARF3	ENSG00000134287	12	49319506	49361334	99	7	123834	-0.74501	0.77187	0.994963036
CHD1L	ENSG00000131778	1	146704291	146777443	266	12	123834	-0.74506	0.77188	0.994963036
OR4A15	ENSG00000181958	11	55125360	55146394	79	9	123834	-0.74509	0.77189	0.994963036
FOXK1	ENSG00000164916	7	4673388	4821074	186	26	123834	-0.74518	0.77192	0.994963036
CDC40	ENSG00000168438	6	110491344	110585478	201	15	123834	-0.74542	0.77199	0.994963036
TMEM259	ENSG00000182087	19	999647	1031117	129	22	123834	-0.74551	0.77202	0.994963036
SPINT2	ENSG00000167642	19	38724675	38793254	117	16	123834	-0.74556	0.77203	0.994963036
U2AF1L4	ENSG00000161265	19	36223365	36246346	40	7	123834	-0.74585	0.77212	0.994963036
PDLIM1	ENSG00000107438	10	96987329	97060781	162	21	123834	-0.74605	0.77218	0.994963036

CTD-2192J16.24	ENSG00000269590	19	12765642	12790211	9	2	123834	-0.74607	0.77219	0.994963036
SNAP23	ENSG00000092531	15	42773431	42847547	70	5	123834	-0.74619	0.77222	0.994963036
CCT7	ENSG00000135624	2	73450548	73490149	64	9	123834	-0.74638	0.77228	0.994963036
YAP1	ENSG00000137693	11	101971192	102114154	169	11	123834	-0.74691	0.77244	0.994963036
COX6C	ENSG00000164919	8	100875428	100916290	56	5	123834	-0.74696	0.77245	0.994963036
RBMS2	ENSG00000076067	12	56905713	56994745	115	13	123834	-0.74721	0.77253	0.994963036
RTDR1	ENSG00000100218	22	23391593	23497208	98	7	123834	-0.74783	0.77272	0.994963036
TRIM51	ENSG00000124900	11	55640773	55669286	116	7	123834	-0.7479	0.77274	0.994963036
AMER2	ENSG00000165566	13	25725822	25756426	31	6	123834	-0.74795	0.77275	0.994963036
CA13	ENSG00000185015	8	86122816	86206302	91	7	123834	-0.74862	0.77296	0.994963036
ABCA7	ENSG00000064687	19	1030102	1075571	93	15	123834	-0.74896	0.77306	0.994963036
HSD17B14	ENSG00000087076	19	49306274	49349935	29	5	123834	-0.74902	0.77308	0.994963036
TOPBP1	ENSG00000163781	3	133307019	133390737	174	13	123834	-0.7493	0.77316	0.994963036
SPAST	ENSG00000021574	2	32278680	32392706	185	11	123834	-0.74933	0.77317	0.994963036
APOBEC4	ENSG00000173627	1	183605541	183632451	120	3	123834	-0.7495	0.77322	0.994963036
GFRAL	ENSG00000187871	6	55182267	55277291	349	21	123834	-0.7499	0.77334	0.994963036
AL020996.1	ENSG00000223474	1	26135212	26157288	35	2	123834	-0.74991	0.77335	0.994963036
FAM71D	ENSG00000172717	14	67646110	67705267	107	8	123834	-0.75036	0.77348	0.994963036
OR5AS1	ENSG00000181785	11	55787895	55808869	160	6	123834	-0.75043	0.7735	0.994963036
CST9	ENSG00000173335	20	23573047	23596513	56	3	123834	-0.75082	0.77362	0.994963036
NAT8L	ENSG00000185818	4	2051239	2080816	16	2	123834	-0.75086	0.77363	0.994963036
USP41	ENSG00000161133	22	20694868	20755048	14	4	123834	-0.7511	0.7737	0.994963036
DACT2	ENSG00000164488	6	168683510	168730434	40	14	123834	-0.75126	0.77375	0.994963036
PCCA	ENSG00000175198	13	100731269	101192686	453	32	123834	-0.75138	0.77379	0.994963036
SHPRH	ENSG00000146414	6	146175381	146295559	151	7	123834	-0.75187	0.77393	0.994963036
SPATA20	ENSG00000006282	17	48610419	48643213	47	8	123834	-0.75202	0.77398	0.994963036
CD1D	ENSG00000158473	1	158139737	158164686	44	4	123834	-0.7521	0.774	0.994963036
RAVER1	ENSG00000161847	19	10416888	10454316	78	11	123834	-0.75223	0.77404	0.994963036
WNK2	ENSG00000165238	9	95937198	96092854	315	22	123834	-0.75288	0.77424	0.994963036
OR4K13	ENSG00000176253	14	20492003	20512919	83	8	123834	-0.75288	0.77424	0.994963036
LGALS2	ENSG00000100079	22	37956255	37988623	56	10	123834	-0.75291	0.77425	0.994963036
CCT6B	ENSG00000132141	17	33244878	33318097	56	11	123834	-0.75301	0.77428	0.994963036
ACOT13	ENSG00000112304	6	24657263	24715293	81	17	123834	-0.75315	0.77432	0.994963036
RPS11	ENSG00000142534	19	49989622	50012946	33	2	123834	-0.75318	0.77433	0.994963036
TIMM13	ENSG00000099800	19	2415622	2437892	59	8	123834	-0.75354	0.77444	0.994963036
OR4A16	ENSG00000181961	11	55100627	55121707	44	9	123834	-0.75368	0.77448	0.994963036
YTHDF3	ENSG00000185728	8	64071112	64135346	47	6	123834	-0.75371	0.77449	0.994963036
SYNPO	ENSG00000171992	5	149970642	150048782	85	8	123834	-0.75394	0.77456	0.994963036
DRD5	ENSG00000169676	4	9773258	9795632	37	5	123834	-0.75413	0.77461	0.994963036
GPX8	ENSG00000164294	5	54445946	54472899	44	4	123834	-0.75431	0.77467	0.994963036
HOXA1	ENSG00000105991	7	27122612	27145615	16	6	123834	-0.75445	0.77471	0.994963036
SGCA	ENSG00000108823	17	48231575	48263292	32	8	123834	-0.75443	0.77471	0.994963036
OTUB1	ENSG00000167770	11	63743325	63779283	42	4	123834	-0.75464	0.77477	0.994963036
TULP1	ENSG00000112041	6	35455651	35490715	58	10	123834	-0.75489	0.77484	0.994963036
CAAP1	ENSG00000120159	9	26830683	26902802	134	14	123834	-0.75494	0.77486	0.994963036
GNG2	ENSG00000186469	14	52282913	52456060	372	45	123834	-0.75497	0.77487	0.994963036
ABTB1	ENSG00000114626	3	127381778	127409768	26	4	123834	-0.75506	0.77489	0.994963036
SYCE3	ENSG00000217442	22	50979541	51011334	133	11	123834	-0.75508	0.7749	0.994963036
RORB	ENSG00000198963	9	77102281	77318093	256	19	123834	-0.75538	0.77499	0.994963036
CAPSL	ENSG00000152611	5	35894397	35948881	120	12	123834	-0.75542	0.775	0.994963036
FCF1	ENSG00000119616	14	75169847	75213394	39	5	123834	-0.75553	0.77503	0.994963036
KRTAP15-1	ENSG00000186970	21	31802597	31823070	42	1	123834	-0.75557	0.77505	0.994963036
LIN37	ENSG00000267796	19	36229262	36255420	43	8	123834	-0.7559	0.77514	0.994963036
SLC16A13	ENSG00000174327	17	6929394	6953440	17	4	123834	-0.756	0.77518	0.994963036
DNAAF1	ENSG00000154099	16	84168865	84222373	121	20	123834	-0.75622	0.77524	0.994963036
TRAK1	ENSG00000182606	3	42045294	42277381	404	30	123834	-0.75628	0.77526	0.994963036
ST6GALNAC5	ENSG00000117069	1	77323126	77541396	347	29	123834	-0.75631	0.77527	0.994963036
RNF26	ENSG00000173456	11	119195237	119218023	7	3	123834	-0.75656	0.77534	0.994963036
YES1	ENSG00000176105	18	711588	822547	188	15	123834	-0.75675	0.7754	0.994963036
OR10G3	ENSG00000169208	14	22027934	22048875	37	8	123834	-0.75685	0.77543	0.994963036
C14orf28	ENSG00000179476	14	45356498	45386460	52	4	123834	-0.75706	0.77549	0.994963036
FBXW10	ENSG00000171931	17	18637326	18692662	41	8	123834	-0.75706	0.77549	0.994963036
BFSP1	ENSG00000125864	20	17464550	17559865	146	36	123834	-0.75754	0.77564	0.994963036
FAM46C	ENSG00000183508	1	118138556	118180994	88	7	123834	-0.75767	0.77567	0.994963036
SLC6A5	ENSG00000165970	11	20610946	20690831	150	23	123834	-0.7579	0.77574	0.994963036
FBRSL1	ENSG00000112787	12	133056137	133171774	245	27	123834	-0.75821	0.77584	0.994963036
SLC25A42	ENSG00000181035	19	19164808	19233697	97	8	123834	-0.7586	0.77595	0.994963036
UIMC1	ENSG00000087206	5	176322006	176459634	153	10	123834	-0.75863	0.77596	0.994963036
FBXO40	ENSG00000163833	3	121301966	121359139	73	13	123834	-0.7588	0.77602	0.994963036
CTC-554D6.1	ENSG00000258864	5	112152910	112213279	131	11	123834	-0.75888	0.77604	0.994963036
LMCD1	ENSG00000071282	3	8533393	8619805	165	19	123834	-0.75906	0.77609	0.994963036
CASD1	ENSG00000127995	7	94128531	94196331	103	9	123834	-0.75904	0.77609	0.994963036
TP53TG5	ENSG00000124251	20	43992526	44046529	85	5	123834	-0.75909	0.7761	0.994963036
AC007919.2	ENSG00000269496	3	172351483	172371740	15	3	123834	-0.75946	0.77621	0.994963036
C1orf177	ENSG00000162398	1	55261736	55317925	41	7	123834	-0.75951	0.77623	0.994963036
AC104794.4	ENSG00000233502	2	10213908	10236228	67	11	123834	-0.75952	0.77623	0.994963036
TMEM211	ENSG00000206069	22	25321077	25352662	13	3	123834	-0.75955	0.77624	0.994963036
WDR63	ENSG00000162643	1	85454830	85608821	272	23	123834	-0.7596	0.77625	0.994963036
ROPN1L	ENSG00000145491	5	10431636	10482141	53	4	123834	-0.75974	0.77629	0.994963036
CTD-2054N24.2	ENSG00000259363	15	100337228	100427353	351	15	123834	-0.75975	0.7763	0.994963036
WDR90	ENSG00000161996	16	689311	727833	65	5	123834	-0.75976	0.7763	0.994963036
FAM214A	ENSG00000047346	15	52863514	53012014	180	15	123834	-0.75979	0.77631	0.994963036
SEL1L	ENSG00000071537	14	81927893	82010205	82	12	123834	-0.75988	0.77634	0.994963036
RTN4	ENSG00000115310	2	55189325	55349757	254	22	123834	-0.75997	0.77636	0.994963036
MUL1	ENSG00000090432	1	20815943	20844654	32	4	123834	-0.76028	0.77646	0.994963036
UVRAG	ENSG00000198382	11	75516212	75864239	383	9	123834	-0.76049	0.77652	0.994963036
KRTAP5-7	ENSG00000244411	11	71228313	71249210	56	6	123834	-0.76051	0.77653	0.994963036
JTB	ENSG00000143543	1	153936745	153960164	14	3	123834	-0.76092	0.77665	0.994963036
RBMXL2	ENSG00000170748	11	7100165	7122379	82	10	123834	-0.76121	0.77674	0.994963036
SPO11	ENSG00000054796	20	55894815	55929050	80	3	123834	-0.76157	0.77684	0.994963036
ZNF607	ENSG00000198182	19	38177264	38220691	69	8	123834	-0.76179	0.77691	0.994963036
CNOT2	ENSG00000111596	12	70626774	70758773	324	18	123834	-0.76184	0.77692	0.994963036
ZBED6	ENSG00000257315	1	203755437	203779686	56	4	123834	-0.76186	0.77693	0.994963036
RBX1	ENSG00000100387	22	41337351	41379313	18	3	123834	-0.76187	0.77693	0.994963036
CR2	ENSG00000117322	1	207617575	207673240	90	12	123834	-0.76208	0.777	0.994963036
RPL10A	ENSG00000198755	6	35426185	35448562	58	8	123834	-0.7623	0.77706	0.994963036
WIPF3	ENSG00000122574	7	29836102	29966682	246	30	123834	-0.76232	0.77706	0.994963036
ENO4	ENSG00000188316	10	118599023	118681299	25	8	123834	-0.76259	0.77715	0.994963036
CITED4	ENSG00000179862	1	41316729	41338018	64	11	123834	-0.76292	0.77725	0.994963036

SLC25A4	ENSG00000151729	4	186054395	186081536	25	5	123834	-0.76319	0.77733	0.994963036
RP11-770J1.5	ENSG00000254873	11	118257810	118282610	35	5	123834	-0.76325	0.77734	0.994963036
PPP4R4	ENSG00000119698	14	94602465	94756072	270	13	123834	-0.76327	0.77735	0.994963036
CRP	ENSG00000132693	1	159672079	159694379	30	5	123834	-0.76331	0.77736	0.994963036
KRT9	ENSG00000171403	17	39712096	39738310	22	3	123834	-0.76356	0.77744	0.994963036
KIAA1430	ENSG00000164323	4	186070819	186140658	154	10	123834	-0.76373	0.77749	0.994963036
CKAP5	ENSG00000175216	11	46754598	46877847	128	11	123834	-0.76423	0.77764	0.994963036
ZNF80	ENSG00000174255	3	113943483	113966425	47	12	123834	-0.76441	0.77769	0.994963036
PASK	ENSG00000115687	2	242035514	242099679	160	13	123834	-0.76449	0.77771	0.994963036
OR52B6	ENSG00000187747	11	5592107	5613114	95	8	123834	-0.76452	0.77772	0.994963036
HLA-DRA	ENSG00000204287	6	32397619	32422823	319	17	123834	-0.76483	0.77782	0.994963036
FAM46A	ENSG00000112773	6	82191156	82472491	579	29	123834	-0.76552	0.77802	0.994963036
LGALS12	ENSG00000133317	11	63263556	63294246	6	2	123834	-0.76577	0.77809	0.994963036
NUDT16L1	ENSG00000168101	16	4733695	4755860	13	3	123834	-0.76627	0.77824	0.994963036
IL17A	ENSG00000112115	6	52041185	52065436	91	7	123834	-0.76636	0.77827	0.994963036
GLTSCR1L	ENSG00000112624	6	42739765	42846298	89	8	123834	-0.76648	0.77831	0.994963036
ZMYND15	ENSG00000141497	17	4633319	4659411	27	8	123834	-0.76656	0.77833	0.994963036
OR2L8	ENSG00000196936	1	248102160	248123098	107	4	123834	-0.76684	0.77841	0.994963036
AQPEP	ENSG00000172901	5	115288151	115373316	153	25	123834	-0.76689	0.77843	0.994963036
ARHGAP23	ENSG00000225485	17	36574662	36678628	95	12	123834	-0.76699	0.77846	0.994963036
GMDS	ENSG00000112699	6	1614041	2255926	643	71	123834	-0.7671	0.77849	0.994963036
MOB1B	ENSG00000173542	4	71758043	71898166	157	8	123834	-0.76717	0.77851	0.994963036
MRPS17	ENSG00000239789	7	56009512	56034192	54	10	123834	-0.76728	0.77854	0.994963036
CTB-78H18.1	ENSG00000253110	5	171190656	171211743	49	7	123834	-0.76781	0.7787	0.994963036
AC027307.3	ENSG00000268798	19	1430838	1451937	12	3	123834	-0.76824	0.77883	0.994963036
SLC39A9	ENSG00000029364	14	69854732	69939105	137	12	123834	-0.76836	0.77886	0.994963036
RXRG	ENSG00000143171	1	165360159	165424433	145	21	123834	-0.76862	0.77894	0.994963036
NCCRP1	ENSG00000188505	19	39677601	39702524	22	2	123834	-0.76884	0.77901	0.994963036
MUC21	ENSG00000204544	6	30941495	30967680	82	9	123834	-0.76916	0.7791	0.994963036
GPR4	ENSG00000177464	19	46083022	46115466	14	5	123834	-0.76914	0.7791	0.994963036
HEPHL1	ENSG00000181333	11	93744527	93856917	149	14	123834	-0.76925	0.77913	0.994963036
FAM105A	ENSG00000145569	5	14571884	14625116	32	5	123834	-0.76937	0.77916	0.994963036
NIPA1	ENSG00000170113	15	23033277	23110005	61	9	123834	-0.76954	0.77921	0.994963036
ATP8B3	ENSG00000130270	19	1772074	1822275	39	10	123834	-0.76958	0.77923	0.994963036
FKBP10	ENSG00000141756	17	39958932	39989465	49	4	123834	-0.76987	0.77931	0.994963036
WDR31	ENSG00000148225	9	116065502	116112578	127	11	123834	-0.77019	0.77941	0.994963036
MANEA	ENSG00000172469	6	96015419	96067333	157	9	123834	-0.77024	0.77942	0.994963036
FMO1	ENSG00000010932	1	171207638	171265117	95	13	123834	-0.77041	0.77947	0.994963036
CTD-2561J22.3	ENSG00000269237	19	21577961	21684068	216	10	123834	-0.77047	0.77949	0.994963036
OR8K5	ENSG00000181752	11	55916870	55937793	35	5	123834	-0.77055	0.77951	0.994963036
C1orf112	ENSG00000000460	1	169621245	169833221	710	25	123834	-0.77062	0.77953	0.994963036
TRIM52	ENSG00000183718	5	180671417	180698119	67	8	123834	-0.77061	0.77953	0.994963036
HCRT	ENSG00000161610	17	40326078	40347470	5	3	123834	-0.77111	0.77968	0.994963036
ZNF726	ENSG00000213967	19	24087678	24137961	73	6	123834	-0.77121	0.77971	0.994963036
GYPB	ENSG00000250361	4	144907257	145071844	303	15	123834	-0.77143	0.77977	0.994963036
ZFP37	ENSG00000136866	9	115790660	115829039	48	8	123834	-0.77141	0.77977	0.994963036
LDHAL6B	ENSG00000171989	15	59489042	59510705	57	6	123834	-0.77168	0.77985	0.994963036
ARL3	ENSG00000138175	10	104423488	104484164	29	7	123834	-0.77187	0.7799	0.994963036
TM4SF20	ENSG00000168955	2	228216872	228256711	65	15	123834	-0.77194	0.77992	0.994963036
ANKDD1B	ENSG00000189045	5	74897284	74977671	180	19	123834	-0.77201	0.77994	0.994963036
SOAT2	ENSG00000167780	12	53487302	53528322	98	8	123834	-0.77255	0.78011	0.994963036
HIBCH	ENSG00000198130	2	191044461	191218919	354	9	123834	-0.77289	0.78021	0.994963036
ZNF75A	ENSG00000162086	16	3345406	3378852	17	2	123834	-0.77317	0.78029	0.994963036
FAM228B	ENSG00000219626	2	24289396	24402509	292	17	123834	-0.77356	0.7804	0.994963036
NAF1	ENSG00000145414	4	164021225	164098073	115	13	123834	-0.77361	0.78042	0.994963036
HYDIN	ENSG00000157423	16	70831281	71274625	170	13	123834	-0.77376	0.78046	0.994963036
RUFY4	ENSG00000188282	2	218889683	218965304	117	12	123834	-0.77392	0.78051	0.994963036
B3GNT8	ENSG00000177191	19	41921264	41944635	33	1	123834	-0.77423	0.7806	0.994963036
GPD1L	ENSG00000152642	3	32137181	32220205	214	16	123834	-0.77437	0.78064	0.994963036
OR5J2	ENSG00000174957	11	55934094	55955032	50	7	123834	-0.77434	0.78064	0.994963036
RADIL	ENSG00000157927	7	4826686	4933350	305	18	123834	-0.7744	0.78065	0.994963036
SLC38A2	ENSG00000134294	12	46741972	46776650	27	4	123834	-0.77442	0.78066	0.994963036
SLC10A1	ENSG00000100652	14	70232134	70274006	54	4	123834	-0.77457	0.7807	0.994963036
MED29	ENSG00000063322	19	39871943	39901277	27	6	123834	-0.77469	0.78074	0.994963036
NEURL1	ENSG00000107954	10	105243736	105362309	161	25	123834	-0.77485	0.78078	0.994963036
KATNB1	ENSG00000140854	16	57759642	57801162	63	11	123834	-0.77493	0.78081	0.994963036
MAPRE3	ENSG00000084764	2	27183480	27260064	55	3	123834	-0.77506	0.78085	0.994963036
RTN4R	ENSG00000040608	22	20218938	20280769	18	7	123834	-0.7751	0.78086	0.994963036
DCAF13	ENSG00000164934	8	104416942	104465681	102	7	123834	-0.77518	0.78088	0.994963036
HRNR	ENSG00000197915	1	152174558	152206669	72	4	123834	-0.77552	0.78098	0.994963036
CCDC15	ENSG00000149548	11	124814017	124921385	257	12	123834	-0.77554	0.78099	0.994963036
CDK2AP1	ENSG00000111328	12	123735528	123766881	83	9	123834	-0.77578	0.78106	0.994963036
AC078925.1	ENSG00000268091	12	131504221	131524769	44	8	123834	-0.77582	0.78107	0.994963036
CARNS1	ENSG00000172508	11	67172439	67203078	29	4	123834	-0.77609	0.78115	0.994963036
EIF2S2	ENSG00000125977	20	32666104	32710138	37	4	123834	-0.77694	0.7814	0.994963036
ZBTB10	ENSG00000205189	8	81387854	81448500	80	8	123834	-0.777	0.78142	0.994963036
AGBL1	ENSG00000166748	15	86675227	87582283	2073	103	123834	-0.77721	0.78148	0.994963036
MRPS30	ENSG00000112996	5	44799027	44830530	55	3	123834	-0.77767	0.78162	0.994963036
SETD5	ENSG00000168137	3	9429299	9530924	117	9	123834	-0.77798	0.78171	0.994963036
TNFRSF10B	ENSG00000120889	8	22867646	22936692	76	16	123834	-0.77812	0.78175	0.994963036
HSD11B1	ENSG00000117594	1	209849510	209918295	93	7	123834	-0.77838	0.78183	0.994963036
ACTRT2	ENSG00000169717	1	2928046	2949465	21	1	123834	-0.77851	0.78186	0.994963036
ZNF660	ENSG00000144792	3	44609715	44651186	50	5	123834	-0.77849	0.78186	0.994963036
CDK12	ENSG00000167258	17	37607764	37731160	173	7	123834	-0.77859	0.78189	0.994963036
AGBL3	ENSG00000146856	7	134661259	134842715	320	8	123834	-0.779	0.78201	0.994963036
EAF1	ENSG00000144597	3	15458862	15494120	52	7	123834	-0.77937	0.78212	0.994963036
GDAP1	ENSG00000104381	8	75223365	75411107	189	21	123834	-0.77966	0.7822	0.994963036
SARM1	ENSG00000004139	17	26681378	26738065	57	5	123834	-0.77988	0.78227	0.994963036
TMEM55A	ENSG00000155099	8	91996024	92063292	55	8	123834	-0.77992	0.78228	0.994963036
ATAD2	ENSG00000156802	8	124322090	124438590	73	10	123834	-0.78014	0.78235	0.994963036
CCDC83	ENSG00000150676	11	85556144	85641064	208	14	123834	-0.78032	0.7824	0.994963036
FNIP2	ENSG00000052795	4	159680290	159839201	160	8	123834	-0.78054	0.78246	0.994963036
MAFG	ENSG00000197063	17	79866146	79895590	3	1	123834	-0.78052	0.78246	0.994963036
NFIC	ENSG00000141905	19	3349561	3479215	115	25	123834	-0.78062	0.78249	0.994963036
C16orf89	ENSG00000153446	16	5084123	5126111	27	7	123834	-0.78071	0.78251	0.994963036
CCL15	ENSG00000267596	17	34313476	34339084	106	8	123834	-0.78095	0.78258	0.994963036
KIAA0040	ENSG00000235750	1	175116123	175172135	110	12	123834	-0.78104	0.78261	0.994963036
RP11-507M3.1	ENSG00000266118	2	24337236	24423305	214	20	123834	-0.78105	0.78261	0.994963036
PKP2	ENSG00000057294	12	32933679	33059774	272	21	123834	-0.78133	0.7827	0.994963036

HIST1H2BL	ENSG00000185130	6	27765257	27785709	28	6	123834	-0.78151	0.78275	0.994963036
DOK1	ENSG00000115325	2	74766153	74794681	10	2	123834	-0.78157	0.78277	0.994963036
PCDP1	ENSG00000163075	2	120292008	120429827	138	10	123834	-0.78162	0.78278	0.994963036
GCH1	ENSG00000131979	14	55298726	55379570	179	15	123834	-0.78175	0.78282	0.994963036
ART3	ENSG00000156219	4	76922337	77043955	491	19	123834	-0.7819	0.78286	0.994963036
APCS	ENSG00000132703	1	159547615	159568655	55	9	123834	-0.78203	0.7829	0.994963036
IVNS1ABP	ENSG00000116679	1	185255520	185296461	12	2	123834	-0.7822	0.78295	0.994963036
NEDD4L	ENSG00000049759	18	55701599	56078772	572	58	123834	-0.78242	0.78302	0.994963036
KRTAP2-1	ENSG00000212725	17	39192793	39213568	65	7	123834	-0.78281	0.78313	0.994963036
FOXI1	ENSG00000168269	5	169522901	169546727	79	12	123834	-0.78286	0.78314	0.994963036
TMEM183A	ENSG00000163444	1	202966514	203003976	42	7	123834	-0.78305	0.7832	0.994963036
AMTN	ENSG00000187689	4	71374257	71408459	173	12	123834	-0.78316	0.78323	0.994963036
NCAPD2	ENSG00000010292	12	6592522	6651121	152	11	123834	-0.78323	0.78325	0.994963036
DUSP8	ENSG00000184545	11	1565274	1603150	82	7	123834	-0.7833	0.78328	0.994963036
SBF1	ENSG00000100241	22	50873429	50923454	58	12	123834	-0.78332	0.78328	0.994963036
P2RX3	ENSG00000109991	11	57095848	57148073	78	10	123834	-0.78349	0.78333	0.994963036
LRP6	ENSG00000070018	12	12258959	12429946	222	15	123834	-0.78383	0.78343	0.994963036
PHACTR1	ENSG00000112137	6	12707893	13298645	829	47	123834	-0.78414	0.78352	0.994963036
MTFR1L	ENSG00000117640	1	26135131	26169432	54	2	123834	-0.78438	0.78359	0.994963036
PSMD2	ENSG00000175166	3	184006497	184036842	20	4	123834	-0.78467	0.78368	0.994963036
CD164L2	ENSG00000174950	1	27695666	27719870	15	1	123834	-0.78475	0.7837	0.994963036
8-Mar	ENSG00000165406	10	45940035	46100354	229	12	123834	-0.78474	0.7837	0.994963036
SLCO6A1	ENSG00000205359	5	101697486	101844720	395	11	123834	-0.78494	0.78375	0.994963036
IQCK	ENSG00000174628	16	19717778	19878907	166	7	123834	-0.78514	0.78381	0.994963036
CCT5	ENSG00000150753	5	10240033	10276524	88	4	123834	-0.78522	0.78384	0.994963036
LEAP2	ENSG00000164406	5	132198014	132220738	41	6	123834	-0.78522	0.78384	0.994963036
BTBD6	ENSG00000184887	14	105704827	105727430	38	2	123834	-0.7853	0.78386	0.994963036
NRCAM	ENSG00000091129	7	107778068	108107161	713	42	123834	-0.78534	0.78387	0.994963036
SETMAR	ENSG00000170364	3	4334988	4369251	17	7	123834	-0.78537	0.78388	0.994963036
ZNF609	ENSG00000180357	15	64742941	64988264	309	11	123834	-0.78536	0.78388	0.994963036
EMC4	ENSG00000128463	15	34507200	34532357	36	5	123834	-0.78619	0.78412	0.994963036
RPS3A	ENSG00000145425	4	152010725	152035804	27	4	123834	-0.78635	0.78417	0.994963036
PXMP2	ENSG00000176894	12	133254192	133307276	86	9	123834	-0.78657	0.78423	0.994963036
KIAA0247	ENSG00000100647	14	70068313	70191859	118	13	123834	-0.78691	0.78433	0.994963036
IKZF2	ENSG00000030419	2	213854429	214027151	281	19	123834	-0.7871	0.78439	0.994963036
BYSL	ENSG00000112578	6	41878926	41910784	32	9	123834	-0.78712	0.7844	0.994963036
FNDC4	ENSG00000115226	2	27704750	27728112	22	3	123834	-0.78721	0.78442	0.994963036
ULK4	ENSG00000168038	3	41278090	42013922	1625	26	123834	-0.78732	0.78445	0.994963036
MOBP	ENSG00000168314	3	39498689	39580970	205	7	123834	-0.7874	0.78448	0.994963036
CBWD1	ENSG00000172785	9	111041	198979	9	1	123834	-0.7874	0.78448	0.994963036
HAPLN1	ENSG00000145681	5	82923624	83027432	101	16	123834	-0.78831	0.78474	0.994963036
RIPK1	ENSG00000137275	6	3054225	3125421	47	6	123834	-0.78841	0.78477	0.994963036
C3orf35	ENSG00000198590	3	37417760	37486988	69	9	123834	-0.78847	0.78479	0.994963036
PRICKLE1	ENSG00000139174	12	42842140	42994157	202	28	123834	-0.78876	0.78487	0.994963036
FAM230A	ENSG00000188280	22	20682438	20726938	1	1	123834	-0.78882	0.78489	0.994963036
MSMP	ENSG00000215183	9	35742987	35766610	14	3	123834	-0.78884	0.7849	0.994963036
AP000322.53	ENSG00000243627	21	35781013	35806264	8	3	123834	-0.78887	0.78491	0.994963036
PLXNB2	ENSG00000196576	22	50703408	50756056	67	13	123834	-0.78894	0.78493	0.994963036
NYAP1	ENSG00000166924	7	100071550	100102422	28	3	123834	-0.78915	0.78499	0.994963036
OR5F1	ENSG00000149133	11	55751157	55772101	137	6	123834	-0.7896	0.78512	0.994963036
CFH	ENSG00000000971	1	196611008	196726634	182	11	123834	-0.78995	0.78522	0.994963036
P2RX6	ENSG00000099957	22	21354097	21393119	89	17	123834	-0.78998	0.78523	0.994963036
TAP2	ENSG00000250264	6	32771544	32816599	268	28	123834	-0.79026	0.78531	0.994963036
HNRNPK	ENSG00000165119	9	86572998	86605569	38	5	123834	-0.79034	0.78533	0.994963036
STARD3	ENSG00000131748	17	37783318	37829737	80	9	123834	-0.79056	0.7854	0.994963036
TFF2	ENSG00000160181	21	43756466	43781237	83	12	123834	-0.79056	0.7854	0.994963036
GANC	ENSG00000214013	15	42555431	42655864	228	7	123834	-0.7906	0.78541	0.994963036
C10orf91	ENSG00000180066	10	134247831	134272912	24	2	123834	-0.79063	0.78542	0.994963036
RP1-127H14.3	ENSG00000255692	12	120127573	120148816	32	4	123834	-0.79064	0.78542	0.994963036
GSTT2B	ENSG00000133433	22	24289601	24313373	3	1	123834	-0.79063	0.78542	0.994963036
TM4SF1	ENSG00000169908	3	149076809	149105652	37	9	123834	-0.79065	0.78543	0.994963036
AC007421.1	ENSG00000268503	17	7507264	7527427	22	5	123834	-0.79102	0.78553	0.994963036
FRRS1L	ENSG00000260230	9	111882573	111939571	140	19	123834	-0.79111	0.78556	0.994963036
WSB2	ENSG00000176871	12	118460712	118510235	78	12	123834	-0.79138	0.78564	0.994963036
KIAA0226L	ENSG00000102445	13	46906139	47022325	204	10	123834	-0.79146	0.78566	0.994963036
PPP5C	ENSG00000011485	19	46840251	46906238	72	12	123834	-0.79149	0.78567	0.994963036
KCTD21	ENSG00000188997	11	77872295	77909868	31	4	123834	-0.79266	0.78601	0.994963036
SH3TC2	ENSG00000169247	5	148293202	148452726	245	24	123834	-0.79276	0.78604	0.994963036
LYL1	ENSG00000104903	19	13199847	13223975	11	3	123834	-0.79286	0.78607	0.994963036
MOB3B	ENSG00000120162	9	27315207	27539779	402	41	123834	-0.79311	0.78614	0.994963036
RBP7	ENSG00000162444	1	10047264	10086078	43	6	123834	-0.79322	0.78618	0.994963036
TRMT10B	ENSG00000165275	9	37743804	37788969	98	10	123834	-0.7935	0.78626	0.994963036
CAMK2N2	ENSG00000163888	3	183967001	183989251	24	3	123834	-0.7936	0.78629	0.994963036
WNT10B	ENSG00000169884	12	49349123	49375546	32	5	123834	-0.79361	0.78629	0.994963036
CSGALNACT2	ENSG00000169826	10	43623934	43690756	118	10	123834	-0.79388	0.78637	0.994963036
NUP133	ENSG00000069248	1	229567045	229654103	121	3	123834	-0.79429	0.78649	0.994963036
UGT2B10	ENSG00000109181	4	69671711	69706914	99	6	123834	-0.79433	0.7865	0.994963036
SYMPK	ENSG00000125755	19	46308668	46376548	190	11	123834	-0.79449	0.78654	0.994963036
PLA2G2A	ENSG00000188257	1	20291925	20316932	38	8	123834	-0.79482	0.78664	0.994963036
AC145676.2	ENSG00000249852	7	217554	239557	28	6	123834	-0.795	0.78669	0.994963036
NINJ2	ENSG00000171840	12	663462	782945	252	21	123834	-0.79499	0.78669	0.994963036
INSR	ENSG00000171105	19	7102266	7304045	311	38	123834	-0.79499	0.78669	0.994963036
TMEM30C	ENSG00000235156	3	99894531	99923035	16	4	123834	-0.79522	0.78676	0.994963036
IL8	ENSG00000169429	4	74596223	74619433	31	4	123834	-0.79546	0.78683	0.994963036
PEBP4	ENSG00000134020	8	22560769	22867513	381	63	123834	-0.7956	0.78687	0.994963036
N4BP2	ENSG00000078177	4	40048446	40169872	242	23	123834	-0.79616	0.78703	0.994963036
FAM96A	ENSG00000166797	15	64354758	64396217	63	5	123834	-0.79619	0.78704	0.994963036
DUSP23	ENSG00000158716	1	159740722	159762333	38	7	123834	-0.79624	0.78706	0.994963036
MTMR4	ENSG00000108389	17	56556898	56605266	44	7	123834	-0.79657	0.78715	0.994963036
TDP2	ENSG00000111802	6	24640205	24677261	85	13	123834	-0.79674	0.7872	0.994963036
MECR	ENSG00000116353	1	29509385	29567454	119	13	123834	-0.79685	0.78723	0.994963036
NOLC1	ENSG00000166197	10	103901933	103933627	18	5	123834	-0.79688	0.78724	0.994963036
GPATCH1	ENSG00000076650	19	33561786	33631448	141	7	123834	-0.79691	0.78725	0.994963036
HMG20B	ENSG00000064961	19	3562775	3589086	20	6	123834	-0.79704	0.78729	0.994963036
CD180	ENSG00000134061	5	66468103	66502627	50	9	123834	-0.79736	0.78738	0.994963036
DENND1A	ENSG00000119522	9	126131933	126702431	926	43	123834	-0.7975	0.78742	0.994963036
SVOP	ENSG00000166111	12	109294658	109469045	87	18	123834	-0.79756	0.78744	0.994963036
NFU1	ENSG00000169599	2	69612882	69674760	111	6	123834	-0.79781	0.78751	0.994963036
APOC1	ENSG00000130208	19	45407504	45432606	19	5	123834	-0.79791	0.78754	0.994963036

USH1C	ENSG00000006611	11	17505442	17575963	119	23	123834	-0.79838	0.78768	0.994963036
HBM	ENSG00000206177	16	193891	226767	25	6	123834	-0.79843	0.78769	0.994963036
FAM228A	ENSG00000186453	2	24387938	24433718	123	11	123834	-0.79852	0.78772	0.994963036
CTHRC1	ENSG00000164932	8	104373743	104405225	57	9	123834	-0.79866	0.78776	0.994963036
CLCNKA	ENSG00000186510	1	16335370	16370545	148	15	123834	-0.79875	0.78778	0.994963036
MSH2	ENSG00000095002	2	47620108	47799450	279	21	123834	-0.79878	0.78779	0.994963036
SRA1	ENSG00000213523	5	139906925	139947895	24	4	123834	-0.79923	0.78792	0.994963036
CC2D1B	ENSG00000154222	1	52801395	52841865	16	2	123834	-0.79937	0.78796	0.994963036
CCDC84	ENSG00000186166	11	118858852	118896501	96	7	123834	-0.7994	0.78797	0.994963036
TMCO2	ENSG00000188800	1	40701619	40727363	22	7	123834	-0.79948	0.78799	0.994963036
OR13C8	ENSG00000186943	9	107321449	107342411	35	4	123834	-0.79951	0.788	0.994963036
CTD-2528L19.4	ENSG00000267552	19	38170766	38220089	88	10	123834	-0.79961	0.78803	0.994963036
hsa-mir-150	ENSG00000268000	19	49993781	50014614	27	2	123834	-0.80021	0.78821	0.994963036
SLC25A26	ENSG00000144741	3	66109285	66448540	274	21	123834	-0.80065	0.78833	0.994963036
MAATS1	ENSG00000183833	3	119411869	119495949	150	13	123834	-0.80118	0.78849	0.994963036
C3orf14	ENSG00000114405	3	62294648	62331888	15	7	123834	-0.80199	0.78872	0.994963036
C12orf55	ENSG00000188596	12	96873349	97279333	868	29	123834	-0.80198	0.78872	0.994963036
NACA2	ENSG00000253506	17	59657794	59678563	39	5	123834	-0.80203	0.78873	0.994963036
ARHGDIA	ENSG00000141522	17	79815597	79839282	18	1	123834	-0.80219	0.78878	0.994963036
PHTF1	ENSG00000116793	1	114229453	114312111	95	9	123834	-0.8023	0.78881	0.994963036
RPTN	ENSG00000215853	1	152116071	152141704	19	3	123834	-0.80255	0.78888	0.994963036
GH2	ENSG00000136487	17	61947578	61969295	39	4	123834	-0.8026	0.7889	0.994963036
RP13-672B3.2	ENSG00000256632	12	133254635	133301498	79	9	123834	-0.80268	0.78892	0.994963036
SLC22A15	ENSG00000163393	1	116509119	116622675	198	18	123834	-0.80285	0.78897	0.994963036
RP11-160N1.10	ENSG00000204814	9	44857571	44880528	27	4	123834	-0.80292	0.78899	0.994963036
GBF1	ENSG00000107862	10	103995289	104152656	196	20	123834	-0.80311	0.78905	0.994963036
GEMIN2	ENSG00000092208	14	39573427	39616177	101	13	123834	-0.80313	0.78905	0.994963036
CCDC23	ENSG00000177868	1	43262723	43292954	48	7	123834	-0.80318	0.78906	0.994963036
CCDC170	ENSG00000120262	6	151805165	151952328	397	34	123834	-0.8033	0.7891	0.994963036
DDX60L	ENSG00000181381	4	169267886	169468937	498	39	123834	-0.8035	0.78916	0.994963036
SMIM1	ENSG00000235169	1	3679352	3702546	59	5	123834	-0.80378	0.78924	0.994963036
GDF9	ENSG00000164404	5	132186873	132212576	44	6	123834	-0.80379	0.78924	0.994963036
GFPT1	ENSG00000198380	2	69536905	69624382	120	5	123834	-0.80415	0.78935	0.994963036
HSF2BP	ENSG00000160207	21	44939072	45089374	217	10	123834	-0.8045	0.78945	0.994963036
DHX35	ENSG00000101452	20	37580942	37678366	114	7	123834	-0.80462	0.78948	0.994963036
YIF1B	ENSG00000167645	19	38785753	38817913	71	10	123834	-0.80467	0.78949	0.994963036
MYLIP	ENSG00000007944	6	16119356	16158479	37	9	123834	-0.80508	0.78961	0.994963036
NR1D1	ENSG00000126368	17	38239040	38266978	21	5	123834	-0.80533	0.78968	0.994963036
OXLD1	ENSG00000204237	17	79622066	79643665	101	7	123834	-0.80575	0.78981	0.994963036
TNPO3	ENSG00000064419	7	128584948	128705198	209	19	123834	-0.80623	0.78995	0.994963036
ANKRD28	ENSG00000206560	3	15698743	15911278	336	10	123834	-0.8068	0.79011	0.994963036
DIRAS3	ENSG00000162595	1	68501645	68527314	21	1	123834	-0.80701	0.79017	0.994963036
GGT5	ENSG00000099998	22	24605622	24651110	78	15	123834	-0.80775	0.79038	0.994963036
TK2	ENSG00000166548	16	66531906	66596447	87	4	123834	-0.80814	0.79049	0.994963036
KIAA0430	ENSG00000166783	16	15678243	15747023	66	13	123834	-0.80822	0.79052	0.994963036
VWA5B2	ENSG00000145198	3	183938217	183970117	35	4	123834	-0.80831	0.79054	0.994963036
OR51T1	ENSG00000176900	11	4893049	4914113	26	5	123834	-0.80856	0.79061	0.994963036
CHST8	ENSG00000124302	19	34102861	34274414	217	27	123834	-0.8092	0.7908	0.994963036
SIGLECL1	ENSG00000179213	19	51739603	51782584	77	8	123834	-0.80938	0.79085	0.994963036
PDLIM5	ENSG00000163110	4	95363037	95599377	222	15	123834	-0.80942	0.79086	0.994963036
TBX15	ENSG00000092607	1	119415669	119542179	222	24	123834	-0.80946	0.79087	0.994963036
RP11-468E2.4	ENSG00000259529	14	24610427	24646611	25	6	123834	-0.80988	0.791	0.994963036
SMARCE1	ENSG00000073584	17	38771214	38814760	22	3	123834	-0.81041	0.79115	0.994963036
GPBAR1	ENSG00000179921	2	219114219	219138582	32	3	123834	-0.81053	0.79118	0.994963036
MN1	ENSG00000169184	22	28134265	28207486	27	9	123834	-0.81064	0.79122	0.994963036
KIAA1841	ENSG00000162929	2	61283006	61401960	174	13	123834	-0.81119	0.79137	0.994963036
GRB10	ENSG00000106070	7	50647760	50871159	471	23	123834	-0.81131	0.79141	0.994963036
SPIB	ENSG00000269404	19	50912195	50944570	29	7	123834	-0.8115	0.79146	0.994963036
SLC25A21-AS1	ENSG00000258708	14	37631093	37653016	44	5	123834	-0.81156	0.79148	0.994963036
SNX17	ENSG00000115234	2	27583389	27609995	20	4	123834	-0.81182	0.79155	0.994963036
C10orf67	ENSG00000179133	10	23546124	23643774	170	13	123834	-0.81293	0.79187	0.994963036
ARHGEF1	ENSG00000076928	19	42377228	42444302	12	2	123834	-0.81301	0.79189	0.994963036
RPH3AL	ENSG00000181031	17	52293	246045	220	52	123834	-0.81311	0.79192	0.994963036
AC005606.1	ENSG00000269205	16	2021633	2042918	68	7	123834	-0.81317	0.79194	0.994963036
MTRNR2L3	ENSG00000256222	20	55923496	55944878	67	3	123834	-0.81318	0.79194	0.994963036
DEFB136	ENSG00000205884	8	11821446	11842108	33	6	123834	-0.81347	0.79203	0.994963036
CHAT	ENSG00000070748	10	50807141	50911925	135	22	123834	-0.81347	0.79203	0.994963036
TMEM132E	ENSG00000181291	17	32897768	32976337	62	21	123834	-0.81362	0.79207	0.994963036
PDZK1IP1	ENSG00000162366	1	47639265	47666716	15	4	123834	-0.81371	0.79209	0.994963036
C2orf53	ENSG00000186143	2	27349723	27372278	20	3	123834	-0.81394	0.79216	0.994963036
CES2	ENSG00000172831	16	66958347	66988999	15	3	123834	-0.81416	0.79222	0.994963036
MUC7	ENSG00000171195	4	71286209	71358714	255	16	123834	-0.8142	0.79224	0.994963036
VAMP1	ENSG00000139190	12	6561403	6590153	85	8	123834	-0.8145	0.79232	0.994963036
CTC-435M10.3	ENSG00000255730	19	41846758	41940906	117	5	123834	-0.81471	0.79238	0.994963036
AL133373.1	ENSG00000268657	14	92028788	92051383	89	6	123834	-0.81475	0.79239	0.994963036
BANK1	ENSG00000153064	4	102322443	103005969	985	55	123834	-0.81512	0.7925	0.994963036
APOC4	ENSG00000267467	19	45435495	45462820	56	3	123834	-0.81531	0.79255	0.994963036
APOC4-APOC2	ENSG00000224916	19	45435495	45462822	56	3	123834	-0.81531	0.79255	0.994963036
APOC2	ENSG00000234906	19	45439243	45462822	56	3	123834	-0.81531	0.79255	0.994963036
SLMAP	ENSG00000163681	3	57731177	57924895	212	16	123834	-0.81542	0.79258	0.994963036
DYRK2	ENSG00000127334	12	68032118	68069186	37	5	123834	-0.81634	0.79285	0.994963036
TWIST1	ENSG00000122691	7	19050614	19167295	206	10	123834	-0.81637	0.79286	0.994963036
WIZ	ENSG00000011451	19	15522319	15570762	9	2	123834	-0.81646	0.79288	0.994963036
EXOG	ENSG00000157036	3	38527618	38593652	94	8	123834	-0.81683	0.79299	0.994963036
HNRNPLL	ENSG00000143889	2	38779120	38840728	122	10	123834	-0.81689	0.793	0.994963036
PPHLN1	ENSG00000134283	12	42622249	42863517	481	13	123834	-0.81691	0.79301	0.994963036
NEK2	ENSG00000117650	1	211826114	211858960	76	13	123834	-0.817	0.79304	0.994963036
WDR18	ENSG00000065268	19	973174	1004569	78	10	123834	-0.81728	0.79312	0.994963036
RTL1	ENSG00000254656	14	101336992	101361184	18	6	123834	-0.81738	0.79314	0.994963036
CMTM7	ENSG00000153551	3	32423163	32534559	246	30	123834	-0.81749	0.79318	0.994963036
OR4C46	ENSG00000185926	11	51505282	51526211	73	8	123834	-0.8177	0.79324	0.994963036
STK35	ENSG00000125834	20	2072257	2167684	158	22	123834	-0.81777	0.79326	0.994963036
UGDH	ENSG00000109814	4	39490375	39539931	89	12	123834	-0.81785	0.79328	0.994963036
NPAS4	ENSG00000174576	11	66178475	66204178	18	5	123834	-0.81815	0.79336	0.994963036
SNCB	ENSG00000074317	5	176037085	176067530	18	5	123834	-0.81845	0.79345	0.994963036
TACC1	ENSG00000147526	8	38575704	38720546	129	10	123834	-0.81884	0.79356	0.994963036
P4HB	ENSG00000185624	17	79791035	79828570	50	1	123834	-0.81883	0.79356	0.994963036
CTCFL	ENSG00000124092	20	56061035	56110708	67	13	123834	-0.81885	0.79357	0.994963036
RAD51AP2	ENSG00000214842	2	17681851	17709706	67	5	123834	-0.81924	0.79368	0.994963036

GTF3C6	ENSG00000155115	6	111269763	111299093	141	7	123834	-0.81926	0.79368	0.994963036
PP2D1	ENSG00000183977	3	20011453	20063822	125	14	123834	-0.81932	0.7937	0.994963036
CHRM5	ENSG00000184984	15	34250921	34367291	195	18	123834	-0.81976	0.79382	0.994963036
PODXL	ENSG00000128567	7	131175021	131252976	97	20	123834	-0.81981	0.79384	0.994963036
NMT1	ENSG00000136448	17	43118978	43196384	178	17	123834	-0.82008	0.79391	0.994963036
ELOVL3	ENSG00000119915	10	103976085	103999346	27	5	123834	-0.8201	0.79392	0.994963036
SYS1-DBNDD2	ENSG00000254806	20	43981840	44049250	104	5	123834	-0.82068	0.79408	0.994963036
PTCRA	ENSG00000171611	6	42873727	42903573	40	6	123834	-0.82077	0.79411	0.994963036
GRASP	ENSG00000161835	12	52390724	52419673	25	9	123834	-0.82077	0.79411	0.994963036
CTD-2369P2.12	ENSG00000267303	19	10416147	10441354	48	7	123834	-0.82082	0.79413	0.994963036
ARMS2	ENSG00000254636	10	124204169	124226868	39	6	123834	-0.82092	0.79416	0.994963036
PGAM5	ENSG00000247077	12	133277405	133309228	59	9	123834	-0.82109	0.7942	0.994963036
EIF4EBP1	ENSG00000187840	8	37877859	37927883	8	1	123834	-0.82125	0.79425	0.994963036
OR52L1	ENSG00000183313	11	5997122	6018215	41	11	123834	-0.8213	0.79426	0.994963036
RALBP1	ENSG00000017797	18	9465007	9548114	182	16	123834	-0.82143	0.7943	0.994963036
IL31	ENSG00000204671	12	122646577	122668746	45	6	123834	-0.82151	0.79432	0.994963036
LSM7	ENSG00000130332	19	2311516	2338619	66	14	123834	-0.82149	0.79432	0.994963036
CRYBA4	ENSG00000196431	22	27007928	27036636	60	7	123834	-0.82185	0.79442	0.994963036
WNT5A	ENSG00000114251	3	55489743	55533973	21	6	123834	-0.82223	0.79453	0.994963036
TFEB	ENSG00000112561	6	41641716	41713997	104	18	123834	-0.82223	0.79453	0.994963036
SRRM5	ENSG00000226763	19	44090769	44128649	42	6	123834	-0.82252	0.79461	0.994963036
ASB16	ENSG00000161664	17	42237815	42266451	37	4	123834	-0.8226	0.79463	0.994963036
MCC	ENSG00000171444	5	112347796	112834527	1345	45	123834	-0.8228	0.79469	0.994963036
MMP10	ENSG00000166670	11	102631234	102661359	79	15	123834	-0.82299	0.79474	0.994963036
SFR1	ENSG00000156384	10	105871816	105896143	40	2	123834	-0.82303	0.79476	0.994963036
ZNF184	ENSG00000096654	6	27408522	27450897	99	8	123834	-0.82363	0.79493	0.994963036
CYTH2	ENSG00000105443	19	48962289	48995571	56	7	123834	-0.82365	0.79493	0.994963036
ACSBG1	ENSG00000103740	15	78449810	78548030	173	29	123834	-0.82418	0.79508	0.994963036
C9orf117	ENSG00000160401	9	130459268	130488281	14	3	123834	-0.82447	0.79516	0.994963036
OR10G7	ENSG00000182634	11	123898773	123919717	28	6	123834	-0.82467	0.79522	0.994963036
PDCL2	ENSG00000163440	4	56412692	56468379	121	11	123834	-0.8252	0.79537	0.994963036
TPPP3	ENSG00000159713	16	67413712	67437438	29	5	123834	-0.82529	0.7954	0.994963036
GAP43	ENSG00000172020	3	115332171	115450337	91	25	123834	-0.82532	0.79541	0.994963036
ZNF599	ENSG00000153896	19	35238981	35274134	49	9	123834	-0.82537	0.79542	0.994963036
BCAP29	ENSG00000075790	7	107210422	107279615	60	6	123834	-0.82557	0.79548	0.994963036
ZNF512	ENSG00000243943	2	27795897	27868041	82	7	123834	-0.82566	0.7955	0.994963036
IL24	ENSG00000162892	1	207060788	207087484	41	3	123834	-0.8262	0.79566	0.994963036
ARMC12	ENSG00000157343	6	35694809	35726856	14	4	123834	-0.82646	0.79573	0.994963036
GLS	ENSG00000115419	2	191735553	191840278	90	9	123834	-0.82664	0.79578	0.994963036
PI4KA	ENSG00000241973	22	21051979	21223705	390	12	123834	-0.82665	0.79578	0.994963036
HPS1	ENSG00000107521	10	100165955	100216684	146	13	123834	-0.8268	0.79582	0.994963036
E2F1	ENSG00000101412	20	32253489	32284210	11	3	123834	-0.82681	0.79583	0.994963036
OR2A5	ENSG00000221836	7	143737461	143758473	68	5	123834	-0.827	0.79588	0.994963036
RP11-766F14.2	ENSG00000248713	4	100547686	100585805	87	15	123834	-0.82702	0.79589	0.994963036
MTERFD2	ENSG00000122085	2	242001584	242051747	94	7	123834	-0.82706	0.7959	0.994963036
TRIM6-TRIM34	ENSG00000258588	11	5607955	5675628	126	24	123834	-0.82726	0.79596	0.994963036
SNRK	ENSG00000163788	3	43318004	43476256	105	11	123834	-0.82736	0.79598	0.994963036
CTH	ENSG00000116761	1	70866901	70915534	48	10	123834	-0.82751	0.79602	0.994963036
SOCS1	ENSG00000185338	16	11338262	11360036	29	5	123834	-0.82748	0.79602	0.994963036
ATG2A	ENSG00000110046	11	64652007	64694722	55	4	123834	-0.82756	0.79604	0.994963036
FGF5	ENSG00000138675	4	81177753	81267834	143	15	123834	-0.82761	0.79605	0.994963036
APBB3	ENSG00000113108	5	139927853	139983337	52	5	123834	-0.82778	0.7961	0.994963036
NAPB	ENSG00000125814	20	23345159	23412125	187	13	123834	-0.82779	0.7961	0.994963036
IRG1	ENSG00000102794	13	77512632	77542777	49	13	123834	-0.82834	0.79626	0.994963036
KLHDC7B	ENSG00000130487	22	50976462	50999451	98	7	123834	-0.82853	0.79631	0.994963036
MGEA5	ENSG00000198408	10	103534200	103588696	51	8	123834	-0.82858	0.79633	0.994963036
ACSL1	ENSG00000151726	4	185666749	185757972	164	17	123834	-0.82882	0.7964	0.994963036
ELOVL1	ENSG00000066322	1	43819068	43843696	24	4	123834	-0.82885	0.79641	0.994963036
AP002884.2	ENSG00000268472	11	112120988	112142117	82	2	123834	-0.82894	0.79643	0.994963036
TMEM44	ENSG00000145014	3	194298402	194364418	182	22	123834	-0.82898	0.79644	0.994963036
C8B	ENSG00000021852	1	57384883	57441813	109	20	123834	-0.82914	0.79649	0.994963036
FBXL20	ENSG00000108306	17	37405384	37568776	248	8	123834	-0.82996	0.79672	0.994963036
C9orf3	ENSG00000148120	9	97478983	97859441	599	18	123834	-0.83004	0.79674	0.994963036
C1orf186	ENSG00000196533	1	206228872	206298647	50	3	123834	-0.83009	0.79676	0.994963036
NUGGC	ENSG00000189233	8	27869481	27951388	79	22	123834	-0.83009	0.79676	0.994963036
CLEC2B	ENSG00000110852	12	9995583	10032735	80	12	123834	-0.83023	0.7968	0.994963036
TMEM132D	ENSG00000151952	12	129546270	130398211	1414	175	123834	-0.83038	0.79684	0.994963036
C6orf222	ENSG00000189325	6	36273534	36314662	29	8	123834	-0.83042	0.79685	0.994963036
PITPNM3	ENSG00000091622	17	6344584	6469814	142	24	123834	-0.83116	0.79706	0.994963036
SDCCAG3	ENSG00000165689	9	139286377	139315061	78	11	123834	-0.83129	0.7971	0.994963036
AICDA	ENSG00000111732	12	8744762	8775467	30	7	123834	-0.83131	0.7971	0.994963036
SND1	ENSG00000197157	7	127282234	127742661	499	25	123834	-0.83156	0.79717	0.994963036
MED1	ENSG00000125686	17	37550538	37617539	92	4	123834	-0.83168	0.79721	0.994963036
SPHK1	ENSG00000176170	17	74362665	74393941	49	12	123834	-0.8321	0.79732	0.994963036
HTRA1	ENSG00000166033	10	124211041	124284424	169	20	123834	-0.83242	0.79741	0.994963036
KRT13	ENSG00000171401	17	39647233	39671957	38	9	123834	-0.83248	0.79743	0.994963036
SLC9A8	ENSG00000197818	20	48419250	48518779	235	23	123834	-0.83255	0.79745	0.994963036
ERI1	ENSG00000104626	8	8849657	8984256	180	17	123834	-0.83281	0.79752	0.994963036
POLR1D	ENSG00000186184	13	28184903	28251548	143	24	123834	-0.83292	0.79755	0.994963036
CPNE6	ENSG00000100884	14	24530046	24557309	6	3	123834	-0.83292	0.79756	0.994963036
C1QL1	ENSG00000131094	17	43027061	43055439	55	13	123834	-0.83315	0.79762	0.994963036
FAM160A2	ENSG00000051009	11	6222565	6265941	51	6	123834	-0.83349	0.79772	0.994963036
CALR	ENSG00000179218	19	13039392	13065303	16	5	123834	-0.8338	0.7978	0.994963036
SNRPF	ENSG00000139343	12	96242706	96307606	183	20	123834	-0.83387	0.79782	0.994963036
GDPD5	ENSG00000158555	11	75135685	75246948	146	17	123834	-0.83415	0.7979	0.994963036
DEPDC1B	ENSG00000035499	5	59882739	60006017	143	12	123834	-0.83427	0.79794	0.994963036
HIBADH	ENSG00000106049	7	27555061	27712614	411	14	123834	-0.83444	0.79798	0.994963036
MRPL46	ENSG00000259494	15	88992707	89020650	1	1	123834	-0.83444	0.79798	0.994963036
MRPS11	ENSG00000181991	15	89000684	89032222	1	1	123834	-0.83444	0.79798	0.994963036
BTBD8	ENSG00000189195	1	92535862	92623393	86	8	123834	-0.83461	0.79803	0.994963036
OR2W1	ENSG00000204704	6	29001990	29023017	57	9	123834	-0.8346	0.79803	0.994963036
USP28	ENSG00000048028	11	113658596	113756292	156	13	123834	-0.83466	0.79804	0.994963036
MRPS15	ENSG00000116898	1	36911319	36940038	74	6	123834	-0.83485	0.7981	0.994963036
EGFLAM	ENSG00000164318	5	38248511	38475123	169	32	123834	-0.83485	0.7981	0.994963036
ZNF321P	ENSG00000221874	19	53421728	53476076	43	10	123834	-0.83533	0.79823	0.994963036
KRT2	ENSG00000172867	12	53028342	53055948	51	6	123834	-0.83536	0.79824	0.994963036
HMGXB3	ENSG00000113716	5	149369884	149442386	109	7	123834	-0.83568	0.79833	0.994963036
PSG2	ENSG00000242221	19	43558363	43597197	163	10	123834	-0.83567	0.79833	0.994963036
STARD9	ENSG00000159433	15	42857857	43023179	98	6	123834	-0.83577	0.79836	0.994963036

KLF11	ENSG00000172059	2	10172976	10204963	42	7	123834	-0.83608	0.79844	0.994963036
SAA2	ENSG00000134339	11	18250770	18280190	40	11	123834	-0.83618	0.79847	0.994963036
IL4I1	ENSG00000104951	19	50382911	50442796	88	19	123834	-0.83623	0.79849	0.994963036
LRRC8D	ENSG00000171492	1	90276573	90412170	65	15	123834	-0.83636	0.79852	0.994963036
AL121901.1	ENSG00000269117	20	31813091	31833387	30	1	123834	-0.8364	0.79853	0.994963036
MYO6	ENSG00000196586	6	76448909	76639254	285	15	123834	-0.83641	0.79854	0.994963036
OR4E2	ENSG00000221977	14	22123297	22144238	77	11	123834	-0.83641	0.79854	0.994963036
BRK1	ENSG00000254999	3	10147276	10178874	107	5	123834	-0.83667	0.79861	0.994963036
TRAPPC6B	ENSG00000182400	14	39607015	39649736	100	14	123834	-0.83689	0.79867	0.994963036
PROM1	ENSG00000007062	4	15954699	16096001	245	44	123834	-0.83699	0.7987	0.994963036
ECSIT	ENSG00000130159	19	11606731	11649989	87	7	123834	-0.83736	0.7988	0.994963036
C7	ENSG00000112936	5	40899354	40993041	243	21	123834	-0.83749	0.79884	0.994963036
RAE1	ENSG00000101146	20	55916066	55964267	126	5	123834	-0.83807	0.799	0.994963036
C15ORF31	ENSG00000268327	15	59429899	59450054	31	5	123834	-0.83857	0.79915	0.994963036
TNNC2	ENSG00000101470	20	44441853	44472384	66	10	123834	-0.83867	0.79917	0.994963036
RETNLB	ENSG00000163515	3	108452271	108486231	79	10	123834	-0.83906	0.79928	0.994963036
CDK19	ENSG00000155111	6	110921181	111147161	358	18	123834	-0.83958	0.79943	0.994963036
MOG	ENSG00000204655	6	29614758	29650149	138	20	123834	-0.83988	0.79951	0.994963036
USP25	ENSG00000155313	21	17092344	17262377	230	7	123834	-0.84005	0.79956	0.994963036
ASCL1	ENSG00000139352	12	103341464	103364294	14	4	123834	-0.8401	0.79958	0.994963036
HEYL	ENSG00000163909	1	40079825	40115617	41	11	123834	-0.84016	0.79959	0.994963036
PPP2R3A	ENSG00000073711	3	135674515	135876733	171	9	123834	-0.84045	0.79967	0.994963036
CACNG7	ENSG00000105605	19	54402589	54457195	19	4	123834	-0.84046	0.79968	0.994963036
LACRT	ENSG00000135413	12	55014595	55038679	12	3	123834	-0.84067	0.79973	0.994963036
TMEM37	ENSG00000171227	2	120177477	120206096	43	6	123834	-0.84073	0.79975	0.994963036
AC002398.9	ENSG00000188223	19	36226579	36255420	46	8	123834	-0.84074	0.79975	0.994963036
NDNL2	ENSG00000185115	15	29550353	29572033	19	2	123834	-0.84092	0.7998	0.994963036
TMPRSS5	ENSG00000166682	11	113548272	113587095	97	10	123834	-0.84098	0.79982	0.994963036
MPL	ENSG00000117400	1	43793478	43828443	23	3	123834	-0.84105	0.79984	0.994963036
RP11-215A19.2	ENSG00000272297	4	187337700	187486464	390	42	123834	-0.84103	0.79984	0.994963036
EXT1	ENSG00000182197	8	118796729	119134092	491	40	123834	-0.84124	0.79989	0.994963036
RP11-219B4.5	ENSG00000258256	8	86124675	86167946	45	5	123834	-0.84133	0.79992	0.994963036
EIF5	ENSG00000100664	14	103789881	103821362	60	9	123834	-0.84139	0.79994	0.994963036
CAPN3	ENSG00000092529	15	42630301	42714516	174	6	123834	-0.84148	0.79996	0.994963036
NARS	ENSG00000134440	18	55257888	55299445	94	14	123834	-0.84151	0.79997	0.994963036
UHRF2	ENSG00000147854	9	6403151	6517054	197	11	123834	-0.84192	0.80008	0.994963036
PPIC	ENSG00000168938	5	122348945	122382436	85	13	123834	-0.84199	0.8001	0.994963036
PPP2R5E	ENSG00000154001	14	63828075	64020092	341	16	123834	-0.84212	0.80014	0.994963036
ESPNL	ENSG00000144488	2	238998798	239051928	94	14	123834	-0.8423	0.80019	0.994963036
GSTA1	ENSG00000243955	6	52646462	52678708	68	4	123834	-0.84249	0.80024	0.994963036
ADCK1	ENSG00000063761	14	78256426	78411355	498	36	123834	-0.84259	0.80027	0.994963036
TDRD5	ENSG00000162782	1	179550748	179670407	299	14	123834	-0.84264	0.80028	0.994963036
UQCRQ	ENSG00000164405	5	132192252	132213723	38	6	123834	-0.84292	0.80036	0.994963036
CNGB3	ENSG00000170289	8	87556205	87765903	343	33	123834	-0.84315	0.80043	0.994963036
TAF13	ENSG00000197780	1	109595108	109628624	45	10	123834	-0.84336	0.80049	0.994963036
ZNF428	ENSG00000131116	19	44101371	44134026	40	5	123834	-0.84357	0.80055	0.994963036
CCDC66	ENSG00000180376	3	56581189	56665846	324	19	123834	-0.84367	0.80057	0.994963036
DYX1C1	ENSG00000256061	15	55692723	55810432	278	17	123834	-0.84387	0.80063	0.994963036
SLC35F1	ENSG00000196376	6	118218689	118648839	464	45	123834	-0.8442	0.80072	0.994963036
PRKACG	ENSG00000165059	9	71617469	71639039	28	6	123834	-0.84438	0.80077	0.994963036
UACA	ENSG00000137831	15	70936893	71065932	120	9	123834	-0.84452	0.80081	0.994963036
IL26	ENSG00000111536	12	68585131	68629601	74	11	123834	-0.84475	0.80087	0.994963036
OR10J5	ENSG00000184155	1	159494793	159515842	24	4	123834	-0.84481	0.80089	0.994963036
ZNF331	ENSG00000130844	19	54014235	54093523	217	21	123834	-0.84528	0.80102	0.994963036
PDP2	ENSG00000172840	16	66902492	66939657	57	7	123834	-0.84537	0.80105	0.994963036
ZNF407	ENSG00000215421	18	72255106	72787627	623	26	123834	-0.84544	0.80107	0.994963036
GRAP2	ENSG00000100351	22	40287086	40379725	22	6	123834	-0.84586	0.80118	0.994963036
PDGFRB	ENSG00000113721	5	149483400	149545435	55	11	123834	-0.84666	0.80141	0.994963036
C14orf182	ENSG00000214900	14	50438430	50484238	119	17	123834	-0.84675	0.80143	0.994963036
STK3	ENSG00000104375	8	99403631	99965055	527	25	123834	-0.84689	0.80147	0.994963036
C19orf60	ENSG00000006015	19	18689495	18713146	34	8	123834	-0.84689	0.80147	0.994963036
ASAP2	ENSG00000151693	2	9336894	9555812	198	15	123834	-0.84706	0.80152	0.994963036
CNTLN	ENSG00000044459	9	17124980	17513921	928	23	123834	-0.84739	0.80161	0.994963036
CAT	ENSG00000121691	11	34450472	34503609	121	14	123834	-0.84744	0.80162	0.994963036
CHORDC1	ENSG00000110172	11	89924328	89966532	119	12	123834	-0.84753	0.80165	0.994963036
MANF	ENSG00000145050	3	51412478	51436828	15	3	123834	-0.84765	0.80168	0.994963036
BEST2	ENSG00000039987	19	12852516	12879272	8	3	123834	-0.84788	0.80175	0.994963036
ZZZ3	ENSG00000036549	1	78018101	78159104	193	16	123834	-0.84805	0.80179	0.994963036
KIAA1598	ENSG00000187164	10	118633742	118896097	106	8	123834	-0.84804	0.80179	0.994963036
TSPAN11	ENSG00000110900	12	31069362	31159534	170	32	123834	-0.84835	0.80188	0.994963036
AL359693.1	ENSG00000269494	6	82252307	82272537	35	4	123834	-0.84853	0.80193	0.994963036
COX17	ENSG00000138495	3	119363360	119406301	112	10	123834	-0.84856	0.80194	0.994963036
C21orf119	ENSG00000256073	21	33755439	33776254	27	2	123834	-0.84859	0.80195	0.994963036
KRTAP19-1	ENSG00000184351	21	31842018	31862663	26	2	123834	-0.84899	0.80206	0.994963036
FAP	ENSG00000078098	2	163017194	163111661	86	3	123834	-0.84905	0.80207	0.994963036
DAAM1	ENSG00000100592	14	59645364	59848123	390	22	123834	-0.84916	0.8021	0.994963036
QRFPR	ENSG00000186867	4	122240467	122312214	263	20	123834	-0.84917	0.80211	0.994963036
OR7C1	ENSG00000127530	19	14899958	14921023	50	6	123834	-0.8493	0.80214	0.994963036
LRTM1	ENSG00000144771	3	54942264	55011115	36	3	123834	-0.84937	0.80216	0.994963036
BIRC8	ENSG00000163098	19	53782856	53804875	42	8	123834	-0.84947	0.80219	0.994963036
SEC24D	ENSG00000150961	4	119633978	119769838	376	12	123834	-0.84991	0.80231	0.994963036
TP53I13	ENSG00000167543	17	27883070	27910175	11	2	123834	-0.84991	0.80231	0.994963036
ZNF576	ENSG00000124444	19	44090544	44115309	28	6	123834	-0.85005	0.80235	0.994963036
HS3ST3A1	ENSG00000153976	17	13389006	13515244	246	34	123834	-0.85033	0.80243	0.994963036
NCF2	ENSG00000116701	1	183514698	183570011	90	13	123834	-0.85081	0.80256	0.994963036
PTN	ENSG00000105894	7	136902088	137038611	196	33	123834	-0.85118	0.80266	0.994963036
CEBPE	ENSG00000092067	14	23576513	23598825	9	4	123834	-0.85128	0.80269	0.994963036
KIAA0020	ENSG00000080608	9	2710469	2854241	172	30	123834	-0.85166	0.8028	0.994963036
TGFBR2	ENSG00000163513	3	30637994	30745634	112	18	123834	-0.85175	0.80282	0.994963036
ERLEC1	ENSG00000068912	2	54004181	54055956	84	12	123834	-0.8519	0.80286	0.994963036
C9orf38	ENSG00000215066	9	6457618	6480375	43	5	123834	-0.85204	0.8029	0.994963036
MTMR12	ENSG00000150712	5	32217100	32323115	147	13	123834	-0.85223	0.80296	0.994963036
SPIRE1	ENSG00000134278	18	12436511	12668133	504	21	123834	-0.85243	0.80301	0.994963036
CATSPER1	ENSG00000175294	11	65774223	65803988	46	6	123834	-0.85276	0.8031	0.994963036
HIST1H2AI	ENSG00000196747	6	27765899	27786429	30	6	123834	-0.85286	0.80313	0.994963036
FBXO27	ENSG00000161243	19	39471354	39533425	201	12	123834	-0.85302	0.80318	0.994963036
MRPS5	ENSG00000144029	2	95742952	95825179	32	4	123834	-0.85305	0.80319	0.994963036
ZSCAN22	ENSG00000182318	19	58828385	58863698	52	2	123834	-0.85317	0.80322	0.994963036
NRGN	ENSG00000154146	11	124599742	124627106	11	3	123834	-0.85324	0.80324	0.994963036

TEKT1	ENSG00000167858	17	6682452	6745080	171	18	123834	-0.85332	0.80326	0.994963036
RPP14	ENSG00000163684	3	58281974	58320422	144	8	123834	-0.85353	0.80332	0.994963036
C12orf77	ENSG00000226397	12	25136358	25160453	53	11	123834	-0.85362	0.80334	0.994963036
TP53I11	ENSG00000175274	11	44897454	44982840	76	17	123834	-0.8537	0.80336	0.994963036
NHP2	ENSG00000145912	5	177566461	177590968	49	6	123834	-0.85401	0.80345	0.994963036
ADRB3	ENSG00000188778	8	37810516	37834483	16	1	123834	-0.85438	0.80355	0.994963036
C7orf73	ENSG00000243317	7	135337244	135388166	122	14	123834	-0.85449	0.80358	0.994963036
FAM153B	ENSG00000182230	5	175477692	175569261	36	6	123834	-0.85499	0.80372	0.994963036
RP11-831H9.11	ENSG00000255432	11	62407166	62442650	21	2	123834	-0.85539	0.80383	0.994963036
MAPRE1	ENSG00000101367	20	31397699	31448211	118	15	123834	-0.85563	0.8039	0.994963036
ZNF704	ENSG00000164684	8	81530686	81797016	214	8	123834	-0.85579	0.80394	0.994963036
RPTOR	ENSG00000141564	17	78508619	78950171	1236	47	123834	-0.8558	0.80395	0.994963036
ASIC3	ENSG00000213199	7	150735379	150759843	24	6	123834	-0.85598	0.80399	0.994963036
KRTAP10-9	ENSG00000221837	21	46037040	46058294	48	6	123834	-0.85601	0.804	0.994963036
MTRNR2L2	ENSG00000271043	5	79935819	79956855	100	4	123834	-0.85609	0.80403	0.994963036
GPATCH2L	ENSG00000089916	14	76608259	76730685	334	20	123834	-0.85628	0.80408	0.994963036
OPTN	ENSG00000123240	10	13131449	13190291	50	8	123834	-0.85684	0.80423	0.994963036
OR4K17	ENSG00000176230	14	20575566	20596597	32	5	123834	-0.85682	0.80423	0.994963036
SPSB4	ENSG00000175093	3	140760244	140877453	109	11	123834	-0.85776	0.80449	0.994963036
RBM18	ENSG00000119446	9	124989903	125037118	55	7	123834	-0.85782	0.80451	0.994963036
SYNJ2BP	ENSG00000213463	14	70828148	70893778	201	18	123834	-0.85825	0.80462	0.994963036
GJA10	ENSG00000135355	6	90594188	90615839	33	9	123834	-0.85836	0.80465	0.994963036
ANK3	ENSG00000151150	10	61776056	62503248	1423	63	123834	-0.8584	0.80466	0.994963036
GRHL2	ENSG00000083307	8	102494660	102691954	263	28	123834	-0.85847	0.80468	0.994963036
RP11-830F9.6	ENSG00000205018	16	88996197	89027932	34	7	123834	-0.85847	0.80468	0.994963036
OR8H1	ENSG00000181693	11	56047529	56068566	54	7	123834	-0.85918	0.80488	0.994963036
ALDH1A3	ENSG00000184254	15	101407919	101466831	32	7	123834	-0.85938	0.80493	0.994963036
FANCE	ENSG00000112039	6	35410138	35444880	85	12	123834	-0.85939	0.80494	0.994963036
C14orf37	ENSG00000139971	14	58456453	58774857	413	35	123834	-0.85989	0.80507	0.994963036
ESYT1	ENSG00000139641	12	56502034	56548455	16	3	123834	-0.86032	0.80519	0.994963036
ANAPC13	ENSG00000129055	3	134186548	134215558	37	2	123834	-0.86042	0.80522	0.994963036
POU5F1	ENSG00000204531	6	31122119	31158508	293	23	123834	-0.86076	0.80532	0.994963036
MKRN2	ENSG00000075975	3	12588513	12635212	73	9	123834	-0.86086	0.80534	0.994963036
RAD51B	ENSG00000182185	14	68276496	69206935	1211	64	123834	-0.86112	0.80541	0.994963036
CTCF	ENSG00000102974	16	67586310	67683086	70	8	123834	-0.86126	0.80545	0.994963036
SOST	ENSG00000167941	17	41821099	41846156	27	5	123834	-0.86124	0.80545	0.994963036
CDC37	ENSG00000105401	19	10491810	10540797	70	9	123834	-0.86179	0.8056	0.994963036
RBM15B	ENSG00000179837	3	51418731	51445330	19	2	123834	-0.86219	0.80571	0.994963036
HPN	ENSG00000105707	19	35521410	35567475	84	9	123834	-0.86251	0.8058	0.994963036
OR52N1	ENSG00000181001	11	5799084	5820046	47	3	123834	-0.86309	0.80596	0.994963036
PLEKHG6	ENSG00000008323	12	6409602	6447672	13	5	123834	-0.86312	0.80596	0.994963036
CNNM4	ENSG00000158158	2	97416639	97487628	59	4	123834	-0.86323	0.80599	0.994963036
FGA	ENSG00000171560	4	155494278	155521918	13	3	123834	-0.86321	0.80599	0.994963036
SPINT1	ENSG00000166145	15	41126216	41160405	42	5	123834	-0.86346	0.80606	0.994963036
C20orf173	ENSG00000125975	20	34101014	34127481	37	4	123834	-0.86432	0.80629	0.994963036
RIOK1	ENSG00000124784	6	7379729	7428270	111	8	123834	-0.86435	0.8063	0.994963036
RPSA	ENSG00000168028	3	39438180	39464033	144	5	123834	-0.86457	0.80636	0.994963036
GATA6	ENSG00000141448	18	19739404	19792491	23	6	123834	-0.86461	0.80637	0.994963036
RP11-295D22.1	ENSG00000260970	9	122247653	122296403	133	14	123834	-0.86468	0.80639	0.994963036
H3F3A	ENSG00000163041	1	226239552	226269702	22	3	123834	-0.86483	0.80644	0.994963036
OR4D5	ENSG00000171014	11	123800250	123821344	68	9	123834	-0.86484	0.80644	0.994963036
ATP13A4	ENSG00000127249	3	193109866	193320900	343	30	123834	-0.86509	0.8065	0.994963036
ARRDC3	ENSG00000113369	5	90654541	90689176	33	3	123834	-0.86513	0.80652	0.994963036
RASA2	ENSG00000155903	3	141195889	141344184	100	8	123834	-0.86547	0.80661	0.994963036
OR5I1	ENSG00000167825	11	55692932	55713876	124	5	123834	-0.86564	0.80666	0.994963036
TCTN2	ENSG00000168778	12	124145660	124202948	189	7	123834	-0.86609	0.80678	0.994963036
C9orf66	ENSG00000183784	9	203108	225893	46	6	123834	-0.86616	0.8068	0.994963036
APOL3	ENSG00000128284	22	36526372	36572225	65	11	123834	-0.8664	0.80686	0.994963036
OR4L1	ENSG00000176246	14	20518204	20539142	105	9	123834	-0.86643	0.80687	0.994963036
ZNF780A	ENSG00000197782	19	40565059	40606845	94	5	123834	-0.86653	0.8069	0.994963036
GORASP1	ENSG00000114745	3	39128150	39159854	56	10	123834	-0.86683	0.80698	0.994963036
KRTAP19-4	ENSG00000186967	21	31859142	31879451	21	2	123834	-0.86727	0.8071	0.994963036
FFAR3	ENSG00000185897	19	35839362	35861387	13	4	123834	-0.86732	0.80712	0.994963036
PRPF39	ENSG00000185246	14	45543302	45595485	55	3	123834	-0.86739	0.80714	0.994963036
CCBL1	ENSG00000171097	9	131585221	131654773	47	6	123834	-0.86749	0.80716	0.994963036
CDKN1B	ENSG00000111276	12	12857992	12885305	31	7	123834	-0.86763	0.8072	0.994963036
GNG5	ENSG00000174021	1	84954008	84982248	88	12	123834	-0.86769	0.80722	0.994963036
TIMD4	ENSG00000145850	5	156336293	156400266	84	12	123834	-0.86798	0.8073	0.994963036
IGFBPL1	ENSG00000137142	9	38398991	38434444	70	9	123834	-0.86815	0.80734	0.994963036
OR8K1	ENSG00000150261	11	56103421	56124507	63	7	123834	-0.86832	0.80739	0.994963036
TPPP2	ENSG00000179636	14	21482268	21514435	19	7	123834	-0.86846	0.80743	0.994963036
AC091948.1	ENSG00000268126	5	147688550	147711891	54	2	123834	-0.86873	0.8075	0.994963036
FERMT1	ENSG00000101311	20	6045492	6114191	175	20	123834	-0.86873	0.8075	0.994963036
NSFL1C	ENSG00000088833	20	1412807	1464487	83	11	123834	-0.86878	0.80752	0.994963036
CCDC61	ENSG00000104983	19	46488339	46534576	66	12	123834	-0.86888	0.80754	0.994963036
SPINK9	ENSG00000204909	5	147690766	147729412	70	2	123834	-0.86894	0.80756	0.994963036
VPS51	ENSG00000149823	11	64846796	64889332	45	7	123834	-0.86899	0.80757	0.994963036
MYBPH	ENSG00000133055	1	203126939	203154941	57	12	123834	-0.86911	0.80761	0.994963036
ALG3	ENSG00000214160	3	183950089	183977336	25	3	123834	-0.86921	0.80763	0.994963036
CHST2	ENSG00000175040	3	142828173	142851800	21	4	123834	-0.86954	0.80772	0.994963036
KRT33A	ENSG00000006059	17	39492344	39517064	33	5	123834	-0.86969	0.80777	0.994963036
SPTLC3	ENSG00000172296	20	12979627	13157411	547	36	123834	-0.86982	0.8078	0.994963036
NUTM2F	ENSG00000130950	9	97067605	97100926	55	10	123834	-0.87004	0.80786	0.994963036
OR13C5	ENSG00000255800	9	107350650	107371788	96	5	123834	-0.87023	0.80791	0.994963036
UHRF1BP1L	ENSG00000111647	12	100412233	100546626	245	18	123834	-0.87024	0.80792	0.994963036
NPBWR1	ENSG00000183729	8	53840991	53863677	80	8	123834	-0.8703	0.80793	0.994963036
SPEN	ENSG00000065526	1	16164359	16276955	153	16	123834	-0.87032	0.80794	0.994963036
RNF43	ENSG00000108375	17	56419861	56504956	58	12	123834	-0.87043	0.80797	0.994963036
BFSP2	ENSG00000170819	3	133108839	133204066	142	26	123834	-0.87101	0.80813	0.994963036
RAB27B	ENSG00000041353	18	52375091	52572747	366	18	123834	-0.8712	0.80818	0.994963036
SBK3	ENSG00000231274	19	56042023	56066909	28	9	123834	-0.87136	0.80822	0.994963036
AK8	ENSG00000165695	9	135590965	135764164	238	19	123834	-0.87149	0.80826	0.994963036
GPAA1	ENSG00000197858	8	145127493	145151119	29	2	123834	-0.87161	0.80829	0.994963036
BCL3	ENSG00000069399	19	45240962	45273301	49	5	123834	-0.87195	0.80838	0.994963036
RP11-542P2.1	ENSG00000205301	4	141353416	141429531	173	13	123834	-0.87211	0.80843	0.994963036
VPREB1	ENSG00000169575	22	22589087	22609927	63	7	123834	-0.87237	0.8085	0.994963036
SELE	ENSG00000007908	1	169681781	169743846	285	9	123834	-0.87334	0.80876	0.994963036
SKP1	ENSG00000113558	5	133474633	133522729	55	4	123834	-0.87334	0.80876	0.994963036
PDE6G	ENSG00000185527	17	79607489	79640142	158	12	123834	-0.87332	0.80876	0.994963036

ATP1B3	ENSG00000069849	3	141584966	141655356	169	13	123834	-0.87344	0.80879	0.994963036
PRDM14	ENSG00000147596	8	70953886	70993928	70	12	123834	-0.87343	0.80879	0.994963036
CHD8	ENSG00000100888	14	21843353	21934285	113	8	123834	-0.87348	0.8088	0.994963036
TCAIM	ENSG00000179152	3	44369611	44460943	130	9	123834	-0.87367	0.80885	0.994963036
FBXL17	ENSG00000145743	5	107184736	107727799	627	26	123834	-0.87365	0.80885	0.994963036
NAPA	ENSG00000105402	19	47980894	48028497	45	5	123834	-0.87387	0.80891	0.994963036
SLC12A3	ENSG00000070915	16	56889119	56959762	150	18	123834	-0.87392	0.80892	0.994963036
KBTBD2	ENSG00000170852	7	32897784	32943743	68	6	123834	-0.87398	0.80894	0.994963036
TMEM218	ENSG00000150433	11	124956398	124991659	93	17	123834	-0.87415	0.80898	0.994963036
PITX3	ENSG00000107859	10	103979943	104011231	42	6	123834	-0.87422	0.809	0.994963036
SEMA4B	ENSG00000185033	15	90693836	90782911	103	12	123834	-0.87423	0.809	0.994963036
C21orf62	ENSG00000205929	21	34152985	34196053	70	5	123834	-0.87443	0.80906	0.994963036
TRIM33	ENSG00000197323	1	114925399	115063781	267	13	123834	-0.87496	0.8092	0.994963036
DCK	ENSG00000156136	4	71848255	71906631	57	4	123834	-0.87525	0.80928	0.994963036
TEAD4	ENSG00000197905	12	3058496	3159839	204	28	123834	-0.87527	0.80929	0.994963036
CDH24	ENSG00000139880	14	23506271	23536747	17	4	123834	-0.87533	0.8093	0.994963036
SPCS2	ENSG00000118363	11	74650292	74700076	59	7	123834	-0.87544	0.80933	0.994963036
ANO8	ENSG00000074855	19	17424032	17455638	82	10	123834	-0.87548	0.80934	0.994963036
C5orf54	ENSG00000221886	5	159810155	159837104	42	6	123834	-0.87588	0.80945	0.994963036
AL136115.1	ENSG00000212673	1	32369174	32390745	15	3	123834	-0.87612	0.80952	0.994963036
OR1K1	ENSG00000165204	9	125552370	125573395	28	3	123834	-0.87616	0.80953	0.994963036
KLRD1	ENSG00000134539	12	10368657	10479850	169	10	123834	-0.87626	0.80956	0.994963036
OR2B11	ENSG00000177535	1	247604255	247625308	99	12	123834	-0.8765	0.80962	0.994963036
KIF25	ENSG00000125337	6	168386921	168455769	154	26	123834	-0.87653	0.80963	0.994963036
PDGFRA	ENSG00000134853	4	55085264	55174414	125	7	123834	-0.87661	0.80965	0.994963036
SNAPC4	ENSG00000165684	9	139260029	139303249	117	12	123834	-0.87668	0.80967	0.994963036
POLDIP2	ENSG00000004142	17	26663659	26694545	24	4	123834	-0.87695	0.80974	0.994963036
PRTN3	ENSG00000196415	19	830963	858175	11	3	123834	-0.87707	0.80978	0.994963036
KL	ENSG00000133116	13	33580207	33650282	91	7	123834	-0.87714	0.80979	0.994963036
CACNG3	ENSG00000006116	16	24256874	24384122	165	35	123834	-0.87728	0.80983	0.994963036
MSRB1	ENSG00000198736	16	1978211	2003327	13	2	123834	-0.8774	0.80987	0.994963036
TMEM123	ENSG00000152558	11	102257063	102351115	115	7	123834	-0.87758	0.80991	0.994963036
AP4S1	ENSG00000100478	14	31484312	31572818	122	18	123834	-0.8776	0.80992	0.994963036
LRFN4	ENSG00000173621	11	66614118	66637946	12	4	123834	-0.87766	0.80993	0.994963036
ATP5L	ENSG00000167283	11	118261869	118312211	59	6	123834	-0.87767	0.80994	0.994963036
EPHA6	ENSG00000080224	3	96523425	97481304	812	36	123834	-0.87785	0.80999	0.994963036
COA3	ENSG00000183978	17	40937165	40960722	1	1	123834	-0.87784	0.80999	0.994963036
CNTD1	ENSG00000176563	17	40940810	40973605	1	1	123834	-0.87784	0.80999	0.994963036
MTPN	ENSG00000105887	7	135601509	135672101	150	17	123834	-0.8779	0.81	0.994963036
TIAM1	ENSG00000156299	21	32480734	32942290	607	70	123834	-0.87797	0.81002	0.994963036
MYCBP2	ENSG00000005810	13	77608792	77911185	431	18	123834	-0.87829	0.81011	0.994963036
PPRC1	ENSG00000148840	10	103882787	103920082	36	5	123834	-0.87861	0.81019	0.994963036
SMYD1	ENSG00000115593	2	88357299	88422906	60	10	123834	-0.87875	0.81023	0.994963036
VRK3	ENSG00000105053	19	50469724	50539203	160	9	123834	-0.87911	0.81033	0.994963036
KIAA0895L	ENSG00000196123	16	67199505	67227943	18	4	123834	-0.87988	0.81054	0.994963036
TMEM106A	ENSG00000184988	17	41353854	41382061	30	2	123834	-0.87993	0.81055	0.994963036
GPAT2	ENSG00000186281	2	96677694	96715199	17	3	123834	-0.87995	0.81056	0.994963036
ARL1	ENSG00000120805	12	101776898	101811598	53	10	123834	-0.87998	0.81057	0.994963036
ADD3	ENSG00000148700	10	111746126	111905323	121	10	123834	-0.88008	0.81059	0.994963036
ARHGAP31	ENSG00000031081	3	119003220	119149561	331	41	123834	-0.88013	0.8106	0.994963036
ZNF575	ENSG00000176472	19	44019649	44050282	69	13	123834	-0.88019	0.81062	0.994963036
PDGFD	ENSG00000170962	11	103767914	104045107	544	43	123834	-0.88021	0.81063	0.994963036
DNTTIP2	ENSG00000067334	1	94323373	94355474	106	11	123834	-0.88078	0.81078	0.994963036
DOK4	ENSG00000125170	16	57495863	57531239	55	11	123834	-0.88076	0.81078	0.994963036
HES4	ENSG00000188290	1	924342	945552	30	2	123834	-0.88081	0.81079	0.994963036
WDR35	ENSG00000118965	2	20100021	20199892	187	16	123834	-0.88087	0.81081	0.994963036
ZNF251	ENSG00000198169	8	145936298	145991802	34	2	123834	-0.88095	0.81083	0.994963036
EIF2B1	ENSG00000111361	12	124094953	124128313	85	9	123834	-0.88106	0.81086	0.994963036
LTF	ENSG00000012223	3	46467136	46536724	138	18	123834	-0.8812	0.81089	0.994963036
KRTAP10-10	ENSG00000221859	21	46047273	46068370	51	5	123834	-0.8813	0.81092	0.994963036
GPC6	ENSG00000183098	13	93869095	95069655	1865	123	123834	-0.88256	0.81126	0.995178815
FAM92B	ENSG00000153789	16	85121965	85156114	69	19	123834	-0.88257	0.81127	0.995178815
GPC1	ENSG00000063660	2	241365088	241417493	78	15	123834	-0.88281	0.81133	0.995178815
ZNF16	ENSG00000170631	8	146145744	146186274	14	3	123834	-0.88299	0.81138	0.995178815
SMARCB1	ENSG00000099956	22	24119150	24186703	261	9	123834	-0.88301	0.81138	0.995178815
MGAT4A	ENSG00000071073	2	99225569	99357589	169	19	123834	-0.88311	0.81141	0.995178815
TAS2R31	ENSG00000256436	12	11172986	11194006	76	7	123834	-0.88342	0.81149	0.995212718
GPR125	ENSG00000152990	4	22336694	22527677	388	38	123834	-0.88399	0.81165	0.995260253
PTP4A3	ENSG00000184489	8	142392093	142451620	52	10	123834	-0.88412	0.81169	0.995260253
RTP3	ENSG00000163825	3	46528981	46552439	42	5	123834	-0.88418	0.8117	0.995260253
MAB21L3	ENSG00000173212	1	116644376	116687861	73	9	123834	-0.8844	0.81176	0.995260253
TSSK1B	ENSG00000212122	5	112758251	112780728	80	9	123834	-0.88464	0.81183	0.995260253
IDI2	ENSG00000148377	10	1054847	1081799	65	11	123834	-0.88493	0.8119	0.995260253
CAGE1	ENSG00000164304	6	7316889	7399976	185	17	123834	-0.88515	0.81196	0.995260253
KRT32	ENSG00000108759	17	39606063	39633681	129	6	123834	-0.88526	0.81199	0.995260253
PMVK	ENSG00000163344	1	154887210	154919467	46	5	123834	-0.88528	0.812	0.995260253
AC137056.1	ENSG00000267824	16	20489024	20510981	9	3	123834	-0.8855	0.81206	0.995269616
TELO2	ENSG00000100726	16	1533345	1570458	92	17	123834	-0.88578	0.81213	0.995291234
B3GALT5	ENSG00000183778	21	40918369	41055064	194	22	123834	-0.88621	0.81225	0.995328668
NEU4	ENSG00000204099	2	242739920	242768739	49	8	123834	-0.88636	0.81229	0.995328668
SPINK7	ENSG00000145879	5	147681982	147705485	46	2	123834	-0.88658	0.81235	0.995328668
SDHAF1	ENSG00000205138	19	36476090	36497220	20	6	123834	-0.88666	0.81237	0.995328668
TRIM6	ENSG00000121236	11	5607339	5644188	69	16	123834	-0.88715	0.8125	0.995393814
CBLN1	ENSG00000102924	16	49301828	49325742	39	8	123834	-0.88767	0.81264	0.995393814
SAMD10	ENSG00000130590	20	62595466	62621361	35	4	123834	-0.88858	0.81288	0.995393814
ZMYND8	ENSG00000101040	20	45827859	45995567	115	19	123834	-0.88863	0.8129	0.995393814
COPB1	ENSG00000129083	11	14454986	14531573	59	4	123834	-0.88866	0.81291	0.995393814
AVPI1	ENSG00000119986	10	99427181	99457080	65	8	123834	-0.88893	0.81298	0.995393814
ZNF672	ENSG00000171161	1	249122409	249153716	26	4	123834	-0.88959	0.81316	0.995393814
FBN3	ENSG00000142449	19	8120286	8224730	275	35	123834	-0.88977	0.8132	0.995393814
C17orf105	ENSG00000231256	17	41847803	41874980	54	5	123834	-0.89001	0.81327	0.995393814
PID1	ENSG00000153823	2	229705242	230146001	747	71	123834	-0.89019	0.81332	0.995393814
APOBEC3A	ENSG00000128383	22	39338746	39369188	7	2	123834	-0.89106	0.81355	0.995393814
TIMM10	ENSG00000134809	11	57285936	57308276	8	3	123834	-0.89157	0.81369	0.995393814
DNAJC16	ENSG00000116138	1	15843308	15928874	233	11	123834	-0.89176	0.81374	0.995393814
AKAP6	ENSG00000151320	14	32788479	33310567	741	91	123834	-0.89176	0.81374	0.995393814
DCAKD	ENSG00000172992	17	43090708	43148473	125	14	123834	-0.89187	0.81377	0.995393814
CALU	ENSG00000128595	7	128369346	128421861	90	9	123834	-0.89236	0.8139	0.995393814
OR8K3	ENSG00000181689	11	56075783	56096721	72	7	123834	-0.89268	0.81399	0.995393814

SHBG	ENSG00000129214	17	7507382	7546700	47	10	123834	-0.8928	0.81402	0.995393814
C8orf46	ENSG00000169085	8	67362238	67440759	99	15	123834	-0.89284	0.81403	0.995393814
ZNF653	ENSG00000161914	19	11584242	11626738	96	7	123834	-0.89289	0.81404	0.995393814
TMEM229A	ENSG00000234224	7	123660973	123683523	49	4	123834	-0.89312	0.8141	0.995393814
NUDT2	ENSG00000164978	9	34319504	34353709	58	9	123834	-0.89343	0.81419	0.995393814
ARMC6	ENSG00000105676	19	19134384	19180563	61	5	123834	-0.89407	0.81436	0.995393814
TMEM119	ENSG00000183160	12	108973622	109002096	53	9	123834	-0.89414	0.81438	0.995393814
SNX30	ENSG00000148158	9	115503118	115653951	321	11	123834	-0.89436	0.81443	0.995393814
WIF1	ENSG00000156076	12	65434406	65525346	98	10	123834	-0.89445	0.81446	0.995393814
LMNB2	ENSG00000176619	19	2417636	2466994	112	12	123834	-0.89471	0.81453	0.995393814
ADAM23	ENSG00000114948	2	207298263	207495851	218	29	123834	-0.89537	0.81471	0.995393814
ZHX3	ENSG00000174306	20	39797088	39956312	211	15	123834	-0.89595	0.81486	0.995393814
AC007956.1	ENSG00000214670	14	75148879	75189800	40	3	123834	-0.89599	0.81487	0.995393814
MAP3K1	ENSG00000095015	5	56101401	56201979	192	13	123834	-0.89621	0.81493	0.995393814
EIF3M	ENSG00000149100	11	32595344	32637808	119	14	123834	-0.89625	0.81494	0.995393814
C5orf63	ENSG00000164241	5	126368250	126419184	69	4	123834	-0.8963	0.81495	0.995393814
MMP14	ENSG00000157227	14	23295766	23328236	31	9	123834	-0.89629	0.81495	0.995393814
SCIN	ENSG00000006747	7	12600203	12703228	199	27	123834	-0.89641	0.81498	0.995393814
CD3D	ENSG00000167286	11	118199669	118223459	24	5	123834	-0.89672	0.81507	0.995393814
FGD4	ENSG00000139132	12	32542463	32808984	613	39	123834	-0.8968	0.81509	0.995393814
CEP44	ENSG00000164118	4	175194828	175264531	77	13	123834	-0.89689	0.81511	0.995393814
MAML2	ENSG00000184384	11	95699762	96086344	587	88	123834	-0.89773	0.81533	0.995393814
ZNF292	ENSG00000188994	6	87852551	87983914	284	9	123834	-0.89777	0.81535	0.995393814
AREL1	ENSG00000119682	14	75110140	75189818	64	4	123834	-0.89835	0.8155	0.995393814
WNT3A	ENSG00000154342	1	228184752	228258961	71	5	123834	-0.89868	0.81559	0.995393814
PPP1R26	ENSG00000196422	9	138360925	138390739	37	7	123834	-0.8988	0.81562	0.995393814
SPARC	ENSG00000113140	5	151030657	151076726	45	6	123834	-0.89884	0.81563	0.995393814
KAZN	ENSG00000189337	1	14915200	15454539	733	123	123834	-0.89901	0.81568	0.995393814
PHF19	ENSG00000119403	9	123607977	123649606	53	7	123834	-0.89901	0.81568	0.995393814
ZNF574	ENSG00000105732	19	42562629	42595701	8	2	123834	-0.89949	0.8158	0.995393814
C1orf116	ENSG00000182795	1	207181866	207216101	27	2	123834	-0.89977	0.81588	0.995393814
BMP10	ENSG00000163217	2	69082613	69108649	22	2	123834	-0.90062	0.81611	0.995393814
ZPLD1	ENSG00000170044	3	101808088	102208685	508	31	123834	-0.90068	0.81612	0.995393814
STT3A	ENSG00000134910	11	125451607	125505110	151	9	123834	-0.90068	0.81612	0.995393814
ARHGEF28	ENSG00000214944	5	72911983	73247818	398	47	123834	-0.90078	0.81615	0.995393814
COG3	ENSG00000136152	13	46029060	46120765	182	11	123834	-0.90083	0.81616	0.995393814
FABP4	ENSG00000170323	8	82380654	82405498	47	5	123834	-0.9011	0.81623	0.995393814
RHOV	ENSG00000104140	15	41154412	41176487	31	3	123834	-0.90116	0.81625	0.995393814
SUGCT	ENSG00000175600	7	40164575	40910362	752	53	123834	-0.9012	0.81626	0.995393814
KCNJ8	ENSG00000121361	12	21907889	21938515	1	1	123834	-0.90119	0.81626	0.995393814
HDGFL1	ENSG00000112273	6	22559678	22581892	62	8	123834	-0.90127	0.81628	0.995393814
ZBTB26	ENSG00000171448	9	125667845	125703779	47	6	123834	-0.90139	0.81631	0.995393814
OSCP1	ENSG00000116885	1	36871428	36926086	201	9	123834	-0.90187	0.81644	0.995393814
SDIM1	ENSG00000256391	6	166297330	166319725	131	14	123834	-0.90202	0.81648	0.995393814
15-Sep	ENSG00000183291	1	87318132	87390107	136	8	123834	-0.90213	0.8165	0.995393814
REP15	ENSG00000174236	12	27839428	27860566	18	3	123834	-0.90217	0.81652	0.995393814
C11orf80	ENSG00000173715	11	66501922	66620987	61	9	123834	-0.90245	0.81659	0.995393814
6-Mar	ENSG00000145495	5	10343815	10450500	71	3	123834	-0.90255	0.81662	0.995393814
FZD1	ENSG00000157240	7	90883783	90908123	14	2	123834	-0.90263	0.81664	0.995393814
IL17RA	ENSG00000177663	22	17555844	17606583	36	8	123834	-0.90304	0.81675	0.995393814
SOWAHB	ENSG00000186212	4	77806082	77829002	47	4	123834	-0.90314	0.81678	0.995393814
SAT2	ENSG00000141504	17	7519552	7541194	34	8	123834	-0.90376	0.81694	0.995393814
GBA2	ENSG00000070610	9	35726863	35759983	19	5	123834	-0.90418	0.81705	0.995393814
OR56B4	ENSG00000180919	11	6118914	6140065	72	5	123834	-0.90428	0.81708	0.995393814
FAM20A	ENSG00000108950	17	66521254	66607530	82	15	123834	-0.90444	0.81712	0.995393814
SLC37A2	ENSG00000134955	11	124922963	124969131	125	22	123834	-0.9052	0.81732	0.995393814
ARHGEF17	ENSG00000110237	11	73009334	73090136	136	7	123834	-0.90539	0.81737	0.995393814
OR5T3	ENSG00000172489	11	56009676	56030698	52	5	123834	-0.90572	0.81746	0.995393814
WAPAL	ENSG00000062650	10	88185013	88291572	215	18	123834	-0.90594	0.81752	0.995393814
ABCC9	ENSG00000069431	12	21940335	22104336	153	18	123834	-0.90611	0.81756	0.995393814
MYH11	ENSG00000133392	16	15787029	15960890	381	45	123834	-0.90646	0.81765	0.995393814
DPP8	ENSG00000074603	15	65724801	65820042	174	10	123834	-0.90654	0.81768	0.995393814
SRCAP	ENSG00000080603	16	30699530	30765602	19	2	123834	-0.90672	0.81772	0.995393814
FAM216B	ENSG00000179813	13	43345686	43375683	66	7	123834	-0.90678	0.81774	0.995393814
GIMAP7	ENSG00000179144	7	150201918	150228161	65	8	123834	-0.90682	0.81775	0.995393814
CASP4	ENSG00000196954	11	104803593	104850163	71	7	123834	-0.90691	0.81777	0.995393814
RAB13	ENSG00000143545	1	153944127	153968834	12	4	123834	-0.90781	0.81801	0.995393814
NEK1	ENSG00000137601	4	170304426	170543780	460	12	123834	-0.90871	0.81825	0.995393814
CCBE1	ENSG00000183287	18	57088172	57374612	583	53	123834	-0.90879	0.81827	0.995393814
CCDC30	ENSG00000186409	1	42919001	43130335	485	17	123834	-0.90882	0.81828	0.995393814
EPB41L2	ENSG00000079819	6	131150487	131394462	541	24	123834	-0.90891	0.8183	0.995393814
TPO	ENSG00000115705	2	1367995	1557483	453	36	123834	-0.9096	0.81848	0.995393814
BTD	ENSG00000169814	3	15632848	15697329	90	11	123834	-0.90989	0.81856	0.995393814
ALG1L	ENSG00000189366	3	125638118	125665882	79	5	123834	-0.90996	0.81858	0.995393814
ACRV1	ENSG00000134940	11	125531417	125561018	56	8	123834	-0.9102	0.81864	0.995393814
VTI1A	ENSG00000151532	10	114196756	114588503	377	46	123834	-0.91041	0.8187	0.995393814
DCTN4	ENSG00000132912	5	150078002	150148671	92	2	123834	-0.91052	0.81873	0.995393814
SLCO1C1	ENSG00000139155	12	20838289	20916320	132	15	123834	-0.91059	0.81874	0.995393814
GLDN	ENSG00000186417	15	51623826	51710210	146	17	123834	-0.91074	0.81878	0.995393814
CTD-2014B16.3	ENSG00000258847	14	66414015	66481241	87	16	123834	-0.91078	0.81879	0.995393814
WDR4	ENSG00000160193	21	44253204	44309678	220	18	123834	-0.91091	0.81883	0.995393814
TMEM168	ENSG00000146802	7	112392437	112440647	54	5	123834	-0.91109	0.81888	0.995393814
NR2F1	ENSG00000175745	5	92909043	92940321	13	3	123834	-0.9113	0.81893	0.995393814
ZNF37A	ENSG00000075407	10	38373264	38424472	157	8	123834	-0.91131	0.81893	0.995393814
RCE1	ENSG00000173653	11	66600306	66624017	11	4	123834	-0.91194	0.8191	0.995393814
CMIP	ENSG00000153815	16	81468775	81755367	395	60	123834	-0.91223	0.81918	0.995393814
EPS15L1	ENSG00000127527	19	16456050	16592896	178	18	123834	-0.91248	0.81924	0.995393814
AL138847.1	ENSG00000269624	1	63063437	63085249	9	2	123834	-0.91258	0.81927	0.995393814
C11orf44	ENSG00000175728	11	130532851	130597247	25	3	123834	-0.91267	0.81929	0.995393814
TMEM212	ENSG00000186329	3	171551139	171666505	149	18	123834	-0.91286	0.81934	0.995393814
C1orf50	ENSG00000164008	1	43222940	43273968	67	10	123834	-0.913	0.81938	0.995393814
TBX5	ENSG00000089225	12	114781736	114856247	97	19	123834	-0.91299	0.81938	0.995393814
RASA1	ENSG00000145715	5	86553705	86697748	103	7	123834	-0.91304	0.81939	0.995393814
MIA3	ENSG00000154305	1	222781428	222851354	110	11	123834	-0.91315	0.81942	0.995393814
AC018630.1	ENSG00000268301	12	11177087	11197275	69	7	123834	-0.91329	0.81945	0.995393814
ITFG3	ENSG00000167930	16	274545	329942	45	5	123834	-0.91335	0.81947	0.995393814
TST	ENSG00000128311	22	37396900	37425681	54	7	123834	-0.91344	0.81949	0.995393814
FAM24A	ENSG00000203795	10	124660217	124682627	21	4	123834	-0.91352	0.81952	0.995393814
SKA3	ENSG00000165480	13	21717734	21760741	82	9	123834	-0.91366	0.81955	0.995393814

TMEM69	ENSG00000159596	1	46142886	46170115	12	2	123834	-0.91372	0.81957	0.995393814
ABCA12	ENSG00000144452	2	215786266	216013151	281	31	123834	-0.91383	0.8196	0.995393814
RP11-352D3.2	ENSG00000205300	20	4040799	4065812	116	12	123834	-0.91412	0.81967	0.995393814
RPL22	ENSG00000116251	1	6231329	6279449	60	8	123834	-0.91449	0.81977	0.995393814
C15orf48	ENSG00000166920	15	45712727	45750959	28	4	123834	-0.91457	0.81979	0.995393814
FAM20C	ENSG00000177706	7	182969	310711	102	21	123834	-0.91475	0.81984	0.995393814
POLE	ENSG00000177084	12	133190348	133273951	181	9	123834	-0.91474	0.81984	0.995393814
AGRP	ENSG00000159723	16	67506474	67527716	18	5	123834	-0.91492	0.81988	0.995393814
C2orf83	ENSG00000042304	2	228464806	228508036	248	24	123834	-0.91501	0.81991	0.995393814
CLEC16A	ENSG00000038532	16	11028345	11286046	429	22	123834	-0.91521	0.81996	0.995393814
TMED2	ENSG00000086598	12	124059078	124093116	101	8	123834	-0.91528	0.81998	0.995393814
FBXO8	ENSG00000164117	4	175147809	175215415	101	13	123834	-0.9154	0.82001	0.995393814
TAP2	ENSG00000204267	6	32779610	32816557	178	27	123834	-0.91563	0.82007	0.995393814
AC026703.1	ENSG00000181495	5	32778945	32801819	25	4	123834	-0.91567	0.82008	0.995393814
MINK1	ENSG00000141503	17	4726683	4811356	142	12	123834	-0.9157	0.82009	0.995393814
C6orf203	ENSG00000130349	6	107339407	107382546	107	8	123834	-0.91575	0.8201	0.995393814
RAET1L	ENSG00000155918	6	150329470	150356668	32	2	123834	-0.91595	0.82015	0.995393814
TIPRL	ENSG00000143155	1	168138171	168179950	75	7	123834	-0.91604	0.82018	0.995393814
OR4C11	ENSG00000172188	11	55360830	55381874	24	3	123834	-0.91687	0.8204	0.995393814
DDAH1	ENSG00000153904	1	85774164	86053933	444	37	123834	-0.91699	0.82043	0.995393814
GMCL1	ENSG00000087338	2	70046774	70118528	29	3	123834	-0.91761	0.82059	0.995393814
SCD5	ENSG00000145284	4	83540692	83730010	596	36	123834	-0.91776	0.82063	0.995393814
FAM215A	ENSG00000267496	17	41984576	42005326	32	5	123834	-0.91822	0.82075	0.995393814
MAFA	ENSG00000182759	8	144491352	144522576	48	10	123834	-0.91852	0.82083	0.995393814
AP1AR	ENSG00000138660	4	113142893	113201203	76	6	123834	-0.919	0.82095	0.995393814
TAPBPL	ENSG00000139192	12	6550856	6585683	86	11	123834	-0.91898	0.82095	0.995393814
CERS3	ENSG00000154227	15	100930600	101095200	608	26	123834	-0.91901	0.82096	0.995393814
PRAMEF18	ENSG00000204491	1	13464689	13487522	5	1	123834	-0.91923	0.82101	0.995393814
KIAA0556	ENSG00000047578	16	27551454	27801690	189	6	123834	-0.91969	0.82113	0.995393814
FABP2	ENSG00000145384	4	120228405	120253545	138	7	123834	-0.91997	0.82121	0.995393814
ATP12A	ENSG00000075673	13	25244549	25295921	122	17	123834	-0.9208	0.82142	0.995393814
P4HA1	ENSG00000122884	10	74756975	74866732	146	2	123834	-0.92093	0.82146	0.995393814
KIF14	ENSG00000118193	1	200510628	200599862	255	17	123834	-0.92106	0.82149	0.995393814
THAP1	ENSG00000131931	8	42681817	42708468	17	3	123834	-0.92106	0.82149	0.995393814
ZFP36L2	ENSG00000152518	2	43439541	43463748	18	3	123834	-0.92133	0.82156	0.995393814
ESPL1	ENSG00000135476	12	53652083	53697427	66	7	123834	-0.92147	0.8216	0.995393814
AC012360.1	ENSG00000268556	2	105857759	105879601	22	6	123834	-0.92187	0.8217	0.995393814
INTS5	ENSG00000185085	11	62404320	62430774	16	2	123834	-0.92199	0.82173	0.995393814
OR13C2	ENSG00000257019	9	107356924	107377951	106	5	123834	-0.92221	0.82179	0.995393814
CSN3	ENSG00000171209	4	71098305	71127145	138	8	123834	-0.92282	0.82195	0.995393814
FBP2	ENSG00000130957	9	97311002	97366075	127	18	123834	-0.92295	0.82198	0.995393814
HOXD13	ENSG00000128714	2	176947619	176970666	18	3	123834	-0.92309	0.82202	0.995393814
SYT3	ENSG00000213023	19	51114564	51181651	58	13	123834	-0.92352	0.82213	0.995393814
DIAPH3	ENSG00000139734	13	60229717	60748121	913	28	123834	-0.92366	0.82217	0.995393814
FAM78A	ENSG00000126882	9	134123463	134161934	19	4	123834	-0.92382	0.82221	0.995393814
TSPEAR	ENSG00000175894	21	45907775	46141495	546	30	123834	-0.92388	0.82223	0.995393814
SLC25A31	ENSG00000151475	4	128641533	128705448	43	4	123834	-0.92411	0.82228	0.995393814
CD300C	ENSG00000167850	17	72527247	72552282	83	9	123834	-0.92454	0.8224	0.995393814
IL19	ENSG00000142224	1	206962215	207026324	80	13	123834	-0.92495	0.8225	0.995393814
PLBD1	ENSG00000121316	12	14646595	14731283	93	14	123834	-0.92534	0.8226	0.995393814
EBNA1BP2	ENSG00000117395	1	43619846	43746607	339	23	123834	-0.92545	0.82263	0.995393814
IGFN1	ENSG00000163395	1	201149953	201208080	118	19	123834	-0.92544	0.82263	0.995393814
KIAA2013	ENSG00000116685	1	11969648	11996485	55	8	123834	-0.9255	0.82265	0.995393814
RNF169	ENSG00000166439	11	74449913	74563458	161	11	123834	-0.92557	0.82267	0.995393814
BLOC1S5-TXNDC5	ENSG00000259040	6	7871755	8074597	466	42	123834	-0.92564	0.82268	0.995393814
PKNOX2	ENSG00000165495	11	125024583	125313285	326	44	123834	-0.92605	0.82279	0.995393814
ZFP30	ENSG00000120784	19	38094650	38193238	159	15	123834	-0.92625	0.82284	0.995393814
AKT1	ENSG00000142208	14	105225686	105272088	47	10	123834	-0.92629	0.82285	0.995393814
GLRA1	ENSG00000145888	5	151192074	151314403	168	7	123834	-0.92632	0.82286	0.995393814
GIMAP5	ENSG00000196329	7	150409341	150457121	66	8	123834	-0.9271	0.82306	0.995393814
C19orf33	ENSG00000167644	19	38784801	38805649	45	8	123834	-0.92708	0.82306	0.995393814
CMPK2	ENSG00000134326	2	6970701	7016766	15	2	123834	-0.92747	0.82316	0.995393814
ZFP1	ENSG00000184517	16	75172390	75216134	100	8	123834	-0.92763	0.8232	0.995393814
CTNNA1	ENSG00000044115	5	137936656	138280723	329	10	123834	-0.92765	0.82321	0.995393814
C1orf159	ENSG00000131591	1	1007198	1061741	109	6	123834	-0.92795	0.82328	0.995393814
CYP2C19	ENSG00000165841	10	96437911	96623017	408	13	123834	-0.92813	0.82333	0.995393814
PAQR8	ENSG00000170915	6	52216219	52282575	172	32	123834	-0.92845	0.82341	0.995393814
PLIN4	ENSG00000167676	19	4492204	4527716	32	8	123834	-0.92845	0.82341	0.995393814
GPBP1L1	ENSG00000159592	1	46082976	46163785	105	4	123834	-0.9285	0.82343	0.995393814
PYY	ENSG00000131096	17	42020106	42091837	69	12	123834	-0.92868	0.82347	0.995393814
AC009041.2	ENSG00000269047	16	1021252	1041663	76	13	123834	-0.92883	0.82351	0.995393814
SYCN	ENSG00000179751	19	39683471	39704906	21	2	123834	-0.92883	0.82351	0.995393814
DRGX	ENSG00000165606	10	50562237	50613497	61	5	123834	-0.9289	0.82353	0.995393814
CDH7	ENSG00000081138	18	63407488	63558638	307	15	123834	-0.9296	0.82371	0.995393814
SLC3A2	ENSG00000168003	11	62613518	62666352	27	2	123834	-0.92999	0.82381	0.995393814
DOC2B	ENSG00000272636	17	0	41427	69	8	123834	-0.93004	0.82382	0.995393814
G3BP1	ENSG00000145907	5	151140606	151202346	101	8	123834	-0.93005	0.82383	0.995393814
GAS2L1	ENSG00000185340	22	29692572	29718774	26	6	123834	-0.93021	0.82387	0.995393814
BIRC2	ENSG00000110330	11	102207942	102259401	66	4	123834	-0.93042	0.82392	0.995393814
TAS1R2	ENSG00000179002	1	19156093	19196176	147	22	123834	-0.93059	0.82397	0.995393814
USP30	ENSG00000135093	12	109450894	109535831	68	13	123834	-0.93076	0.82401	0.995393814
RPE	ENSG00000197713	2	210857289	210896300	19	4	123834	-0.93082	0.82403	0.995393814
RP11-770J1.4	ENSG00000255384	11	118293836	118315921	15	3	123834	-0.93082	0.82403	0.995393814
IFI44	ENSG00000137965	1	79105481	79139763	37	4	123834	-0.93091	0.82405	0.995393814
NOXO1	ENSG00000196408	16	2018918	2044193	72	7	123834	-0.93121	0.82413	0.995393814
FAM13A	ENSG00000138640	4	89637106	90042549	913	37	123834	-0.93126	0.82414	0.995393814
MTMR10	ENSG00000166912	15	31221144	31293810	140	12	123834	-0.93132	0.82416	0.995393814
IL1RN	ENSG00000136689	2	113854791	113901593	127	12	123834	-0.93191	0.82431	0.995393814
INADL	ENSG00000132849	1	62198149	62639592	532	64	123834	-0.93196	0.82432	0.995393814
TMEM167A	ENSG00000174695	5	82338665	82383682	80	6	123834	-0.93195	0.82432	0.995393814
CENPF	ENSG00000117724	1	214766538	214847931	210	17	123834	-0.93217	0.82438	0.995393814
NECAB1	ENSG00000123119	8	91793778	91981636	162	12	123834	-0.93253	0.82447	0.995393814
FN3K	ENSG00000167363	17	80683451	80719073	90	10	123834	-0.93276	0.82453	0.995393814
GPR142	ENSG00000257008	17	72353546	72378761	10	3	123834	-0.93281	0.82454	0.995393814
TSGA13	ENSG00000213265	7	130343488	130382268	70	10	123834	-0.93295	0.82458	0.995393814
RPF2	ENSG00000197498	6	111293218	111359466	243	15	123834	-0.9332	0.82464	0.995393814
SH2D3C	ENSG00000095370	9	130490596	130551020	81	10	123834	-0.93321	0.82464	0.995393814
LY6K	ENSG00000160886	8	143771529	143796545	58	5	123834	-0.93399	0.82485	0.995393814
PITRM1	ENSG00000107959	10	3169920	3225003	219	19	123834	-0.93403	0.82486	0.995393814

C14orf119	ENSG00000179933	14	23553974	23579665	46	7	123834	-0.93412	0.82488	0.995393814
FH	ENSG00000091483	1	241650903	241693061	26	2	123834	-0.93442	0.82496	0.995393814
NASP	ENSG00000132780	1	46039518	46094566	61	4	123834	-0.9345	0.82498	0.995393814
ELOVL6	ENSG00000170522	4	110957002	111130355	298	42	123834	-0.93494	0.82509	0.995393814
PRR20D	ENSG00000227151	13	57724766	57747787	2	1	123834	-0.93528	0.82518	0.995393814
IRAK4	ENSG00000198001	12	44142747	44193346	87	9	123834	-0.93549	0.82523	0.995393814
RP11-451M19.3	ENSG00000203876	10	111691029	111723649	46	1	123834	-0.93571	0.82529	0.995393814
DEFB132	ENSG00000186458	20	228377	251737	49	9	123834	-0.9358	0.82531	0.995393814
OR9A2	ENSG00000179468	7	142713245	142734282	31	5	123834	-0.93612	0.82539	0.995393814
SRP72	ENSG00000174780	4	57323081	57379839	148	11	123834	-0.93648	0.82549	0.995393814
OGDHL	ENSG00000197444	10	50932689	50980425	70	9	123834	-0.93648	0.82549	0.995393814
EQTN	ENSG00000120160	9	27274656	27307137	56	8	123834	-0.9366	0.82552	0.995393814
COPB2	ENSG00000184432	3	139064442	139118574	107	8	123834	-0.93673	0.82555	0.995393814
ZFP36L1	ENSG00000185650	14	69244377	69273190	58	9	123834	-0.93706	0.82564	0.995393814
RAB2B	ENSG00000129472	14	21917179	21955132	72	9	123834	-0.9372	0.82567	0.995393814
GANAB	ENSG00000089597	11	62382298	62424104	15	2	123834	-0.93732	0.8257	0.995393814
C21orf49	ENSG00000205930	21	34134411	34276043	208	21	123834	-0.9373	0.8257	0.995393814
BPIFB4	ENSG00000186191	20	31657450	31709557	104	13	123834	-0.93742	0.82573	0.995393814
OR10AG1	ENSG00000174970	11	55724975	55745990	150	4	123834	-0.9376	0.82578	0.995393814
LRP10	ENSG00000197324	14	23330822	23360789	21	5	123834	-0.93764	0.82578	0.995393814
PILRB	ENSG00000121716	7	99923737	99975356	59	4	123834	-0.9379	0.82585	0.995393814
OR8H3	ENSG00000181761	11	55879849	55900787	125	4	123834	-0.93791	0.82585	0.995393814
FAM115A	ENSG00000198420	7	143538468	143609291	16	3	123834	-0.9381	0.8259	0.995393814
OLFM3	ENSG00000118733	1	102258130	102472586	424	42	123834	-0.93813	0.82591	0.995393814
METTL6	ENSG00000206562	3	15412782	15492073	123	11	123834	-0.93842	0.82599	0.995393814
TUBA3D	ENSG00000075886	2	132223666	132250507	94	4	123834	-0.93886	0.8261	0.995393814
AC023632.1	ENSG00000212997	8	95548771	95570630	89	11	123834	-0.93902	0.82614	0.995393814
HSD17B7	ENSG00000132196	1	162750492	162792607	50	9	123834	-0.93921	0.82619	0.995393814
SNRNP35	ENSG00000184209	12	123932188	123967701	21	1	123834	-0.93952	0.82627	0.995393814
CD22	ENSG00000012124	19	35800164	35848258	34	9	123834	-0.94018	0.82644	0.995393814
KIAA1033	ENSG00000136051	12	105491102	105572912	171	14	123834	-0.94055	0.82653	0.995393814
NAA30	ENSG00000139977	14	57847262	57892635	30	4	123834	-0.94094	0.82663	0.995393814
KIAA0586	ENSG00000100578	14	58884103	59025216	60	9	123834	-0.94094	0.82663	0.995393814
PRSS22	ENSG00000005001	16	2892728	2918171	34	5	123834	-0.94097	0.82664	0.995393814
RPL8	ENSG00000161016	8	146005150	146027972	37	6	123834	-0.94122	0.8267	0.995393814
SHFM1	ENSG00000127922	7	96100938	96349203	369	23	123834	-0.94132	0.82673	0.995393814
NAE1	ENSG00000159593	16	66826778	66917159	115	8	123834	-0.94166	0.82682	0.995393814
SYDE2	ENSG00000097096	1	85612556	85676729	120	10	123834	-0.94172	0.82683	0.995393814
IMPA2	ENSG00000141401	18	11971024	12040876	191	25	123834	-0.9419	0.82688	0.995393814
KRTAP1-1	ENSG00000188581	17	39186811	39207713	77	8	123834	-0.94196	0.82689	0.995393814
CCDC115	ENSG00000136710	2	131085814	131109922	10	2	123834	-0.9422	0.82696	0.995393814
AURKC	ENSG00000105146	19	57732377	57756916	60	8	123834	-0.94223	0.82696	0.995393814
KIAA1143	ENSG00000163807	3	44769153	44813154	37	4	123834	-0.94265	0.82707	0.995393814
NR1H2	ENSG00000131408	19	50822910	50896239	62	6	123834	-0.94265	0.82707	0.995393814
RAB6C	ENSG00000222014	2	130727235	130750311	18	1	123834	-0.94346	0.82728	0.995393814
CCDC141	ENSG00000163492	2	179684484	179924813	512	42	123834	-0.94351	0.82729	0.995393814
DHTKD1	ENSG00000181192	10	12100971	12175224	153	16	123834	-0.94352	0.82729	0.995393814
SKIL	ENSG00000136603	3	170065466	170124623	107	9	123834	-0.94376	0.82735	0.995393814
CDC26	ENSG00000176386	9	116008115	116047869	85	13	123834	-0.94374	0.82735	0.995393814
CTR9	ENSG00000198730	11	10762534	10811290	54	11	123834	-0.94378	0.82736	0.995393814
OR5M8	ENSG00000181371	11	56247825	56268871	69	9	123834	-0.94387	0.82738	0.995393814
TRIM13	ENSG00000204977	13	50560024	50604617	61	7	123834	-0.94386	0.82738	0.995393814
DLGAP1	ENSG00000170579	18	3486030	4465335	1101	147	123834	-0.9441	0.82744	0.995393814
LILRA5	ENSG00000187116	19	54808353	54834409	19	3	123834	-0.94428	0.82749	0.995393814
SLC35F5	ENSG00000115084	2	114452588	114524400	197	14	123834	-0.94454	0.82755	0.995393814
MCM8	ENSG00000125885	20	5921298	5985852	148	15	123834	-0.94469	0.82759	0.995393814
TRIM8	ENSG00000171206	10	104394253	104428164	48	7	123834	-0.94508	0.82769	0.995393814
OR7G2	ENSG00000170923	19	9202945	9223982	65	4	123834	-0.94514	0.82771	0.995393814
CDC20	ENSG00000117399	1	43814626	43838874	17	4	123834	-0.9452	0.82772	0.995393814
TRIM64C	ENSG00000214891	11	49065266	49090664	65	8	123834	-0.94518	0.82772	0.995393814
DTX1	ENSG00000135144	12	113484514	113545833	116	13	123834	-0.94548	0.82779	0.995393814
KRT37	ENSG00000108417	17	39567005	39590775	55	8	123834	-0.94554	0.82781	0.995393814
ZNF781	ENSG00000196381	19	38148652	38193223	80	10	123834	-0.94563	0.82783	0.995393814
OR4C6	ENSG00000181903	11	55422614	55443671	4	2	123834	-0.94581	0.82788	0.995393814
PLEKHG2	ENSG00000090924	19	39893225	39929054	34	6	123834	-0.94581	0.82788	0.995393814
TBC1D29	ENSG00000266733	17	28874130	28900511	14	3	123834	-0.94604	0.82794	0.995393814
C7orf33	ENSG00000170279	7	148277657	148322952	48	12	123834	-0.94658	0.82807	0.995393814
CXCL6	ENSG00000124875	4	74692214	74724781	40	8	123834	-0.94735	0.82827	0.995393814
DGKH	ENSG00000102780	13	42604176	42840714	507	26	123834	-0.94738	0.82828	0.995393814
LRRC41	ENSG00000132128	1	46716868	46779280	45	9	123834	-0.94744	0.82829	0.995393814
TRAPPC4	ENSG00000196655	11	118879142	118906164	60	6	123834	-0.94772	0.82836	0.995393814
YEATS4	ENSG00000127337	12	69743483	69794576	166	12	123834	-0.9478	0.82838	0.995393814
ZNF146	ENSG00000167635	19	36695504	36739676	90	6	123834	-0.94824	0.8285	0.995393814
BCHE	ENSG00000114200	3	165480692	165565260	244	21	123834	-0.94868	0.82861	0.995393814
ALOX15	ENSG00000161905	17	4524197	4555589	15	4	123834	-0.94881	0.82864	0.995393814
CSPG4	ENSG00000173546	15	75956663	76015189	36	7	123834	-0.94886	0.82866	0.995393814
EPM2AIP1	ENSG00000178567	3	37017357	37044795	34	4	123834	-0.94915	0.82873	0.995393814
KDM7A	ENSG00000006459	7	139774546	139886835	185	12	123834	-0.94965	0.82886	0.995393814
CHRNA9	ENSG00000174343	4	40327346	40367234	117	11	123834	-0.94997	0.82894	0.995393814
NUMA1	ENSG00000137497	11	71703910	71801739	157	5	123834	-0.95016	0.82898	0.995393814
PAICS	ENSG00000128050	4	57291907	57337534	96	10	123834	-0.9502	0.82899	0.995393814
NRBP2	ENSG00000185189	8	144905764	144934200	21	5	123834	-0.95065	0.82911	0.995393814
COMMD2	ENSG00000114744	3	149446257	149480286	67	6	123834	-0.95083	0.82916	0.995393814
FRG1	ENSG00000109536	4	190851943	190894359	76	1	123834	-0.95099	0.8292	0.995393814
TRIM39	ENSG00000204599	6	30284256	30321506	82	16	123834	-0.95126	0.82926	0.995393814
OR6T1	ENSG00000181499	11	123803492	123824580	68	10	123834	-0.95124	0.82926	0.995393814
DDTL	ENSG00000099974	22	24299089	24324721	4	1	123834	-0.95135	0.82929	0.995393814
DDT	ENSG00000099977	22	24303554	24332660	4	1	123834	-0.95135	0.82929	0.995393814
BARHL1	ENSG00000125492	9	135447572	135475653	14	5	123834	-0.95143	0.82931	0.995393814
AC226150.4	ENSG00000255748	12	133777246	133797772	52	5	123834	-0.95184	0.82941	0.995393814
COPZ1	ENSG00000111481	12	54684986	54755633	23	2	123834	-0.95188	0.82942	0.995393814
KRT38	ENSG00000171360	17	39582698	39607173	103	10	123834	-0.95188	0.82942	0.995393814
DNAJC28	ENSG00000177692	21	34850497	34874027	38	6	123834	-0.9522	0.8295	0.995393814
SLC17A1	ENSG00000124568	6	25773125	25842287	167	15	123834	-0.95222	0.82951	0.995393814
CMC1	ENSG00000187118	3	28273075	28376633	101	10	123834	-0.95265	0.82962	0.995393814
BAI3	ENSG00000135298	6	69335259	70109403	1562	59	123834	-0.95272	0.82963	0.995393814
CRYBB1	ENSG00000100122	22	26985242	27024052	73	10	123834	-0.95269	0.82963	0.995393814
C3orf56	ENSG00000214324	3	126901974	126927025	29	8	123834	-0.95294	0.82969	0.995393814
STATH	ENSG00000126549	4	70851647	70878287	34	6	123834	-0.95303	0.82971	0.995393814

CABIN1	ENSG00000099991	22	24397642	24584596	201	16	123834	-0.95308	0.82973	0.995393814
SDC2	ENSG00000169439	8	97495579	97634000	166	27	123834	-0.95336	0.8298	0.995393814
CKMT1A	ENSG00000223572	15	43975084	44001420	10	2	123834	-0.95346	0.82982	0.995393814
PSMA7	ENSG00000101182	20	60701791	60728496	55	4	123834	-0.95355	0.82984	0.995393814
C9orf9	ENSG00000165698	9	135743414	135775588	37	7	123834	-0.95369	0.82988	0.995393814
AGFG2	ENSG00000106351	7	100126834	100175842	45	5	123834	-0.9538	0.82991	0.995393814
TXNDC5	ENSG00000239264	6	7871483	8036646	395	41	123834	-0.95394	0.82994	0.995393814
DKK3	ENSG00000050165	11	11974653	12041316	96	20	123834	-0.95396	0.82995	0.995393814
SVEP1	ENSG00000165124	9	113117531	113352160	708	53	123834	-0.95407	0.82998	0.995393814
STK17A	ENSG00000164543	7	43612357	43676385	133	10	123834	-0.95452	0.83009	0.995393814
CPOX	ENSG00000080819	3	98229976	98322567	118	20	123834	-0.95471	0.83014	0.995393814
AKR1B10	ENSG00000198074	7	134202344	134236160	92	7	123834	-0.95576	0.8304	0.995393814
LILRB5	ENSG00000105609	19	54744263	54771164	15	4	123834	-0.95588	0.83043	0.995393814
SLC40A1	ENSG00000138449	2	190415305	190458484	17	3	123834	-0.95594	0.83045	0.995393814
DOCK2	ENSG00000134516	5	169054251	169520386	879	76	123834	-0.95608	0.83048	0.995393814
OR52E6	ENSG00000205409	11	5852158	5873182	109	5	123834	-0.95633	0.83055	0.995393814
EZH1	ENSG00000108799	17	40842293	40907071	24	2	123834	-0.95718	0.83076	0.995393814
ZNF148	ENSG00000163848	3	124934405	125104198	227	9	123834	-0.95747	0.83083	0.995393814
GORASP2	ENSG00000115806	2	171774974	171833639	94	8	123834	-0.9577	0.83089	0.995393814
KAT7	ENSG00000136504	17	47855917	47916458	30	7	123834	-0.958	0.83097	0.995393814
ATF2	ENSG00000115966	2	175926978	176043110	220	13	123834	-0.95813	0.831	0.995393814
ZNF564	ENSG00000249709	19	12626184	12672356	14	5	123834	-0.95817	0.83101	0.995393814
MTSS1	ENSG00000170873	8	125553031	125750730	348	42	123834	-0.95828	0.83104	0.995393814
MFSD12	ENSG00000161091	19	3528259	3584288	41	11	123834	-0.95837	0.83106	0.995393814
TCF23	ENSG00000163792	2	27361872	27386378	22	4	123834	-0.95894	0.83121	0.995393814
CTAGE1	ENSG00000212710	18	19983564	20007878	6	2	123834	-0.95907	0.83124	0.995393814
ZBTB8B	ENSG00000273274	1	32920670	32972287	41	3	123834	-0.95924	0.83128	0.995393814
PEX3	ENSG00000034693	6	143761944	143821147	73	6	123834	-0.95978	0.83142	0.995393814
C4orf33	ENSG00000151470	4	130004472	130047795	108	11	123834	-0.96006	0.83149	0.995393814
CD300LB	ENSG00000178789	17	72507313	72537613	81	7	123834	-0.96025	0.83154	0.995393814
MS4A15	ENSG00000166961	11	60514426	60554205	12	6	123834	-0.96036	0.83156	0.995393814
ZRANB1	ENSG00000019995	10	126620692	126686758	76	5	123834	-0.96053	0.83161	0.995393814
CERK	ENSG00000100422	22	47070308	47144158	138	16	123834	-0.9606	0.83162	0.995393814
ARMC3	ENSG00000165309	10	23206953	23336518	205	20	123834	-0.96088	0.83169	0.995393814
PGAP3	ENSG00000161395	17	37817375	37863050	77	5	123834	-0.96096	0.83171	0.995393814
REC8	ENSG00000100918	14	24631062	24659463	24	6	123834	-0.9614	0.83182	0.995393814
CHTF18	ENSG00000127586	16	828046	860737	64	10	123834	-0.96157	0.83187	0.995393814
AC131971.1	ENSG00000183562	11	3001093	3022563	49	8	123834	-0.9618	0.83192	0.995393814
TPBG	ENSG00000146242	6	83062923	83090545	18	3	123834	-0.96182	0.83193	0.995393814
ANKRD18A	ENSG00000180071	9	38530566	38630657	42	5	123834	-0.96181	0.83193	0.995393814
YAF2	ENSG00000015153	12	42540906	42642151	151	12	123834	-0.96187	0.83194	0.995393814
RBM15	ENSG00000162775	1	110871128	110899299	45	6	123834	-0.96192	0.83196	0.995393814
FAM195B	ENSG00000225663	17	79770287	79801178	30	2	123834	-0.96222	0.83203	0.995393814
FECH	ENSG00000066926	18	55205515	55264004	84	18	123834	-0.96244	0.83209	0.995393814
ZBTB44	ENSG00000196323	11	130086572	130194581	206	8	123834	-0.96291	0.8322	0.995393814
B4GALT4	ENSG00000121578	3	118920579	118969950	149	18	123834	-0.96332	0.83231	0.995393814
PRSS3	ENSG00000010438	9	33740515	33809230	141	18	123834	-0.96348	0.83235	0.995393814
HAVCR2	ENSG00000135077	5	156502843	156579880	135	11	123834	-0.96354	0.83236	0.995393814
ZAP70	ENSG00000115085	2	98320023	98366325	25	3	123834	-0.96379	0.83243	0.995393814
IFI27L1	ENSG00000165948	14	94537628	94580192	106	13	123834	-0.96391	0.83246	0.995393814
ZNF79	ENSG00000196152	9	130176661	130217651	66	3	123834	-0.96397	0.83247	0.995393814
CST4	ENSG00000101441	20	23656277	23679677	30	5	123834	-0.96402	0.83248	0.995393814
AC096644.1	ENSG00000224867	1	220593286	220618023	61	6	123834	-0.96417	0.83252	0.995393814
TGFBR3	ENSG00000069702	1	92135902	92381892	416	44	123834	-0.96431	0.83255	0.995393814
PLIN3	ENSG00000105355	19	4828353	4877780	61	14	123834	-0.96448	0.8326	0.995393814
ZNF506	ENSG00000081665	19	19886648	19942560	74	8	123834	-0.96455	0.83262	0.995393814
NPC1L1	ENSG00000015520	7	44542134	44590914	39	6	123834	-0.96479	0.83267	0.995393814
AL138815.2	ENSG00000268344	13	26442422	26462679	16	3	123834	-0.96537	0.83282	0.995393814
OR6N1	ENSG00000197403	1	158725466	158746492	73	11	123834	-0.96541	0.83283	0.995393814
COA1	ENSG00000106603	7	43638055	43779316	350	12	123834	-0.96545	0.83284	0.995393814
SDR42E2	ENSG00000183921	16	22167618	22213075	40	7	123834	-0.96564	0.83289	0.995393814
AL035681.1	ENSG00000269104	22	41675388	41695686	24	5	123834	-0.96563	0.83289	0.995393814
ID4	ENSG00000172201	6	19827617	19850915	21	6	123834	-0.96589	0.83295	0.995393814
CASP1	ENSG00000137752	11	104886170	104982158	195	11	123834	-0.96633	0.83306	0.995393814
RPF1	ENSG00000117133	1	84934942	84973473	136	15	123834	-0.96689	0.8332	0.995393814
TIMM22	ENSG00000177370	17	890357	916911	30	3	123834	-0.96702	0.83323	0.995393814
RPS23	ENSG00000186468	5	81559177	81584396	35	8	123834	-0.96706	0.83324	0.995393814
TMEM14C	ENSG00000111843	6	10713148	10741362	19	2	123834	-0.9671	0.83325	0.995393814
COPG1	ENSG00000181789	3	128958449	129006614	58	2	123834	-0.96733	0.83331	0.995393814
APOBEC2	ENSG00000124701	6	41011043	41042250	67	5	123834	-0.96746	0.83334	0.995393814
UROD	ENSG00000126088	1	45467819	45491247	25	2	123834	-0.96758	0.83337	0.995393814
ST3GAL3	ENSG00000126091	1	44161495	44406831	405	17	123834	-0.96769	0.8334	0.995393814
ARID1A	ENSG00000117713	1	27012524	27118595	103	5	123834	-0.96795	0.83346	0.995393814
CLDN18	ENSG00000066405	3	137707577	137762494	71	7	123834	-0.96797	0.83347	0.995393814
SUGP2	ENSG00000064607	19	19091697	19154832	69	7	123834	-0.96826	0.83354	0.995393814
CHAD	ENSG00000136457	17	48531857	48556327	35	5	123834	-0.96828	0.83355	0.995393814
OSBPL9	ENSG00000117859	1	52032851	52264889	160	13	123834	-0.96831	0.83356	0.995393814
CELA1	ENSG00000139610	12	51712227	51750463	50	5	123834	-0.96904	0.83374	0.995393814
CATSPERD	ENSG00000174898	19	5710688	5788745	95	16	123834	-0.96904	0.83374	0.995393814
RP11-219B4.6	ENSG00000257962	8	86148524	86172077	25	4	123834	-0.96935	0.83381	0.995393814
CDNF	ENSG00000185267	10	14851249	14890574	45	7	123834	-0.96959	0.83387	0.995393814
SOSTDC1	ENSG00000171243	7	16491106	16580205	183	25	123834	-0.96977	0.83392	0.995393814
ZNF816	ENSG00000180257	19	53420388	53476164	45	11	123834	-0.96991	0.83396	0.995393814
ROBO1	ENSG00000169855	3	78636390	79826965	1691	73	123834	-0.96998	0.83397	0.995393814
PLEKHJ1	ENSG00000104886	19	2220002	2247703	28	6	123834	-0.97009	0.834	0.995393814
NPM3	ENSG00000107833	10	103531082	103553170	7	3	123834	-0.97013	0.83401	0.995393814
RP11-661C8.3	ENSG00000250173	5	969480	991415	3	2	123834	-0.97025	0.83404	0.995393814
TET3	ENSG00000187605	2	74219840	74345303	127	13	123834	-0.97031	0.83405	0.995393814
PTX4	ENSG00000251692	16	1525887	1548982	83	16	123834	-0.97049	0.8341	0.995393814
SCD	ENSG00000099194	10	102096881	102134591	34	6	123834	-0.97086	0.83419	0.995393814
C8orf49	ENSG00000255394	8	11608896	11630309	32	9	123834	-0.97088	0.8342	0.995393814
SEMA6B	ENSG00000167680	19	4532600	4569820	38	6	123834	-0.97088	0.8342	0.995393814
SAMD12	ENSG00000177570	8	119191698	119644234	693	57	123834	-0.97163	0.83438	0.995393814
SSBP3	ENSG00000157216	1	54681105	54889152	309	45	123834	-0.97186	0.83444	0.995393814
NUP35	ENSG00000163002	2	183972241	184036408	336	19	123834	-0.97202	0.83448	0.995393814
MAEA	ENSG00000090316	4	1273639	1343935	302	13	123834	-0.97209	0.8345	0.995393814
CCDC28A	ENSG00000024862	6	139084657	139124456	80	9	123834	-0.97223	0.83453	0.995393814
COX8C	ENSG00000187581	14	93803537	93824702	80	6	123834	-0.97258	0.83462	0.995393814
RC3H1	ENSG00000135870	1	173890352	174001435	164	7	123834	-0.9729	0.8347	0.995393814

HLA-DQB1	ENSG00000179344	6	32617244	32646160	475	20	123834	-0.97297	0.83471	0.995393814
MUTYH	ENSG00000132781	1	45784835	45816142	15	2	123834	-0.97299	0.83472	0.995393814
MSRA	ENSG00000175806	8	9901778	10296401	674	64	123834	-0.97311	0.83475	0.995393814
PRKG2	ENSG00000138669	4	81999837	82146218	246	18	123834	-0.97315	0.83476	0.995393814
CTD-2140B24.4	ENSG00000256825	12	133697570	133788955	217	10	123834	-0.97397	0.83496	0.995393814
HCLS1	ENSG00000180353	3	121340246	121389774	94	9	123834	-0.97405	0.83498	0.995393814
GPATCH11	ENSG00000152133	2	37301594	37336387	52	4	123834	-0.97418	0.83502	0.995393814
SSPN	ENSG00000123096	12	26264924	26462223	406	45	123834	-0.97426	0.83504	0.995393814
RASD2	ENSG00000100302	22	35926915	35960048	18	5	123834	-0.97428	0.83504	0.995393814
DLG5	ENSG00000151208	10	79540549	79696378	227	17	123834	-0.97501	0.83522	0.995393814
SLC35F2	ENSG00000110660	11	107651717	107809019	444	25	123834	-0.97507	0.83524	0.995393814
TTLL4	ENSG00000135912	2	219565568	219630139	100	3	123834	-0.97554	0.83535	0.995393814
ZRANB3	ENSG00000121988	2	135884486	136298806	561	10	123834	-0.97586	0.83543	0.995393814
AL118506.1	ENSG00000267848	20	62575007	62595495	14	4	123834	-0.97599	0.83547	0.995393814
TMEM221	ENSG00000188051	19	17536318	17569376	24	6	123834	-0.97694	0.8357	0.995393814
AC008948.1	ENSG00000268424	5	101559696	101581279	57	9	123834	-0.977	0.83572	0.995393814
MPO	ENSG00000005381	17	56337217	56368296	31	5	123834	-0.97719	0.83576	0.995393814
SLC26A7	ENSG00000147606	8	92211722	92420378	232	22	123834	-0.97724	0.83577	0.995393814
GPR3	ENSG00000181773	1	27709148	27732318	16	1	123834	-0.97745	0.83583	0.995393814
KRT8	ENSG00000170421	12	53280977	53353738	144	10	123834	-0.97746	0.83583	0.995393814
SLC7A8	ENSG00000092068	14	23584504	23662883	83	17	123834	-0.97759	0.83586	0.995393814
REG3A	ENSG00000172016	2	79374132	79396879	25	4	123834	-0.97763	0.83587	0.995393814
NOA1	ENSG00000084092	4	57819536	57854989	89	16	123834	-0.97772	0.83589	0.995393814
ORC5	ENSG00000164815	7	103756788	103858495	278	11	123834	-0.97811	0.83599	0.995393814
ADAM29	ENSG00000168594	4	175740819	175909331	405	17	123834	-0.97832	0.83604	0.995393814
KDM4B	ENSG00000127663	19	4959125	5163606	356	28	123834	-0.97832	0.83604	0.995393814
ZNF713	ENSG00000178665	7	55945169	56019918	154	18	123834	-0.97841	0.83606	0.995393814
AC000003.2	ENSG00000264194	17	10038831	10060277	48	15	123834	-0.97874	0.83615	0.995393814
PRSS48	ENSG00000189099	4	152188323	152222605	79	6	123834	-0.9792	0.83626	0.995393814
ZCRB1	ENSG00000139168	12	42695880	42729920	61	6	123834	-0.9793	0.83628	0.995393814
OR6Q1	ENSG00000172381	11	57788402	57809403	76	9	123834	-0.97956	0.83635	0.995393814
RMDN2	ENSG00000115841	2	38140330	38304285	242	19	123834	-0.97983	0.83641	0.995393814
RAB21	ENSG00000080371	12	72138654	72194699	75	4	123834	-0.9801	0.83648	0.995393814
APC	ENSG00000134982	5	112033195	112191936	237	15	123834	-0.98025	0.83652	0.995393814
NPCDR1	ENSG00000269415	3	59947215	59967583	103	16	123834	-0.98065	0.83662	0.995393814
FZD4	ENSG00000174804	11	86646721	86676433	43	5	123834	-0.98068	0.83662	0.995393814
PCK1	ENSG00000124253	20	56126136	56151513	46	10	123834	-0.98073	0.83664	0.995393814
PRRG4	ENSG00000135378	11	32841489	32889669	135	13	123834	-0.9809	0.83668	0.995393814
SPRYD4	ENSG00000176422	12	56852301	56874763	14	3	123834	-0.9811	0.83673	0.995393814
ACTN2	ENSG00000077522	1	236839754	236937931	206	30	123834	-0.98135	0.83679	0.995393814
RGL1	ENSG00000143344	1	183595220	183907665	673	36	123834	-0.98153	0.83683	0.995393814
ZNF169	ENSG00000175787	9	97011593	97073736	176	18	123834	-0.98247	0.83707	0.995393814
CP	ENSG00000047457	3	148870197	148949842	151	21	123834	-0.98272	0.83713	0.995393814
INHBE	ENSG00000139269	12	57836106	57863063	32	7	123834	-0.98309	0.83722	0.995393814
GON4L	ENSG00000116580	1	155709508	155839191	122	6	123834	-0.9832	0.83725	0.995393814
FAM178B	ENSG00000168754	2	97531620	97694175	228	17	123834	-0.98324	0.83726	0.995393814
CACNA2D3	ENSG00000157445	3	54146574	55118584	1444	127	123834	-0.98329	0.83727	0.995393814
PLA2G4D	ENSG00000159337	15	42349207	42396752	138	9	123834	-0.98352	0.83732	0.995393814
LSMEM1	ENSG00000181016	7	112110908	112141117	47	7	123834	-0.98367	0.83736	0.995393814
GAS2L3	ENSG00000139354	12	100957461	101032064	56	8	123834	-0.98378	0.83739	0.995393814
EYA4	ENSG00000112319	6	133551736	133863258	465	43	123834	-0.98386	0.83741	0.995393814
ACLY	ENSG00000131473	17	40013161	40096795	128	8	123834	-0.98403	0.83745	0.995393814
AKAP8L	ENSG00000011243	19	15480859	15539952	49	2	123834	-0.98407	0.83746	0.995393814
TFEC	ENSG00000105967	7	115565202	115809950	447	27	123834	-0.98484	0.83765	0.995393814
CTBP1	ENSG00000159692	4	1195236	1253741	121	13	123834	-0.98523	0.83775	0.995393814
SPRYD7	ENSG00000123178	13	50476842	50520626	66	7	123834	-0.98527	0.83775	0.995393814
ATP6V0D1	ENSG00000159720	16	67461917	67525140	52	7	123834	-0.9855	0.83781	0.995393814
HAUS2	ENSG00000137814	15	42831008	42872192	38	4	123834	-0.98578	0.83788	0.995393814
C12orf75	ENSG00000235162	12	105619068	105799875	292	26	123834	-0.9859	0.83791	0.995393814
FOXD3	ENSG00000187140	1	63778730	63800797	19	7	123834	-0.98609	0.83796	0.995393814
UTP6	ENSG00000108651	17	30177923	30238784	120	10	123834	-0.9864	0.83803	0.995393814
KRT82	ENSG00000161850	12	52777715	52810139	107	13	123834	-0.98648	0.83805	0.995393814
TBC1D3H	ENSG00000242384	17	34571086	34818061	13	3	123834	-0.98653	0.83806	0.995393814
TBC1D3G	ENSG00000161583	17	34735936	34818104	13	3	123834	-0.98653	0.83806	0.995393814
C19orf38	ENSG00000214212	19	10937251	10990466	40	6	123834	-0.98691	0.83816	0.995393814
TFF1	ENSG00000160182	21	43772391	43796703	78	13	123834	-0.98768	0.83834	0.995393814
ORMDL3	ENSG00000172057	17	38067294	38093854	46	6	123834	-0.98772	0.83835	0.995393814
AC016885.1	ENSG00000268080	8	94231867	94252388	24	5	123834	-0.98775	0.83836	0.995393814
AC005609.1	ENSG00000249034	5	140230341	140253224	22	5	123834	-0.98788	0.8384	0.995393814
RFX8	ENSG00000196460	2	102003823	102101165	235	12	123834	-0.98832	0.8385	0.995393814
NUSAP1	ENSG00000137804	15	41614892	41683248	108	8	123834	-0.98836	0.83851	0.995393814
WDR65	ENSG00000243710	1	43627820	43730029	287	20	123834	-0.98844	0.83853	0.995393814
AL138815.1	ENSG00000215549	13	26432061	26465095	26	5	123834	-0.98919	0.83872	0.995393814
2-Mar	ENSG00000099785	19	8468154	8513901	81	14	123834	-0.98936	0.83876	0.995393814
ADAM12	ENSG00000148848	10	127690950	128087024	721	80	123834	-0.99056	0.83905	0.995393814
OR6K6	ENSG00000180433	1	158714510	158735681	53	7	123834	-0.99077	0.8391	0.995393814
RPS10	ENSG00000124614	6	34375231	34403902	39	5	123834	-0.9908	0.83911	0.995393814
ACSS1	ENSG00000154930	20	24976868	25049616	98	15	123834	-0.99094	0.83914	0.995393814
SEC14L5	ENSG00000103184	16	4998318	5079159	106	13	123834	-0.9911	0.83918	0.995393814
MAN1A1	ENSG00000111885	6	119488374	119680926	467	19	123834	-0.99145	0.83927	0.995393814
DEFB123	ENSG00000180424	20	30018322	30048060	49	6	123834	-0.99147	0.83927	0.995393814
AC061975.10	ENSG00000268206	17	26628667	26652323	16	3	123834	-0.99175	0.83934	0.995393814
MRPS17	ENSG00000249773	7	55944970	56032932	178	20	123834	-0.99186	0.83937	0.995393814
PATE1	ENSG00000171053	11	125606188	125629762	105	11	123834	-0.99187	0.83937	0.995393814
EML6	ENSG00000214595	2	54940636	55209157	346	43	123834	-0.99192	0.83938	0.995393814
C9orf64	ENSG00000165118	9	86543226	86581901	48	7	123834	-0.99225	0.83946	0.995393814
MRPL9	ENSG00000143436	1	151722119	151746040	37	5	123834	-0.99243	0.83951	0.995393814
STAM	ENSG00000136738	10	17676124	17767913	285	12	123834	-0.99246	0.83951	0.995393814
UCK1	ENSG00000130717	9	134389188	134416655	42	5	123834	-0.99255	0.83954	0.995393814
ASL	ENSG00000126522	7	65530785	65568545	74	8	123834	-0.99263	0.83955	0.995393814
KCNK3	ENSG00000171303	2	26905619	26966288	44	6	123834	-0.99288	0.83962	0.995393814
PLK4	ENSG00000142731	4	128792016	128830350	28	3	123834	-0.99308	0.83966	0.995393814
EXPH5	ENSG00000110723	11	108366158	108474465	151	19	123834	-0.99339	0.83974	0.995393814
LIX1	ENSG00000145721	5	96417574	96488576	65	10	123834	-0.99395	0.83988	0.995393814
B4GALNT2	ENSG00000167080	17	47199822	47257351	125	13	123834	-0.99432	0.83997	0.995393814
ABHD6	ENSG00000163686	3	58213233	58291420	192	16	123834	-0.99446	0.84	0.995393814
AC011366.3	ENSG00000254106	5	109208883	109230452	23	5	123834	-0.99479	0.84008	0.995393814
PRH2	ENSG00000134551	12	11071835	11094940	53	6	123834	-0.99502	0.84014	0.995393814
ANGPT2	ENSG00000091879	8	6347172	6430930	308	25	123834	-0.99552	0.84026	0.995393814

ZBTB42	ENSG00000179627	14	105256933	105281049	21	4	123834	-0.99562	0.84028	0.995393814
RSPH9	ENSG00000172426	6	43602783	43650336	101	9	123834	-0.99573	0.84031	0.995393814
ADCY9	ENSG00000162104	16	3993388	4176186	275	45	123834	-0.99591	0.84035	0.995393814
DUSP3	ENSG00000108861	17	41833489	41866356	56	5	123834	-0.9959	0.84035	0.995393814
SULT1C3	ENSG00000196228	2	108853651	108891807	94	13	123834	-0.99593	0.84036	0.995393814
RPL12	ENSG00000197958	9	130199953	130223684	49	3	123834	-0.99601	0.84038	0.995393814
PRAMEF2	ENSG00000120952	1	12906941	12931764	57	2	123834	-0.99615	0.84041	0.995393814
ZNF232	ENSG00000167840	17	4998836	5036411	52	9	123834	-0.99628	0.84044	0.995393814
SPNS3	ENSG00000182557	17	4326983	4401503	179	18	123834	-0.99657	0.84051	0.995393814
TDRD3	ENSG00000083544	13	60960591	61158012	339	17	123834	-0.99696	0.84061	0.995393814
RP11-1220K2.2	ENSG00000257743	7	141801549	141932124	191	29	123834	-0.9972	0.84067	0.995393814
PHLDA2	ENSG00000181649	11	2939503	2960685	15	5	123834	-0.9973	0.84069	0.995393814
ZNF268	ENSG00000090612	12	133697570	133793698	230	10	123834	-0.99742	0.84072	0.995393814
PMS1	ENSG00000064933	2	190639107	190752355	190	16	123834	-0.99756	0.84075	0.995393814
FAT2	ENSG00000086570	5	150873654	150958505	162	19	123834	-0.99835	0.84094	0.995393814
KRT222	ENSG00000264058	17	38775049	38831393	34	4	123834	-0.99877	0.84105	0.995393814
APLNR	ENSG00000134817	11	56991051	57014709	26	7	123834	-0.999	0.8411	0.995393814
DPY19L2	ENSG00000177990	12	63942693	64072719	404	23	123834	-0.99923	0.84116	0.995393814
ALG10B	ENSG00000175548	12	38700380	38727784	65	7	123834	-0.99941	0.8412	0.995393814
AC090673.2	ENSG00000267942	12	66281518	66327967	35	9	123834	-0.99946	0.84121	0.995393814
PPAN-P2RY11	ENSG00000243207	19	10206899	10235456	24	5	123834	-0.9995	0.84122	0.995393814
PPAN	ENSG00000130810	19	10206965	10235414	24	5	123834	-0.9995	0.84122	0.995393814
IL18R1	ENSG00000115604	2	102917989	103025218	347	16	123834	-0.99951	0.84123	0.995393814
MCMDC2	ENSG00000178460	8	67772984	67844283	61	4	123834	-0.99989	0.84132	0.995393814
ENTHD1	ENSG00000176177	22	40129049	40299868	118	7	123834	-0.99999	0.84134	0.995393814
IGF2BP1	ENSG00000159217	17	47064774	47143012	79	10	123834	-1.0003	0.84142	0.995393814
ENTPD6	ENSG00000197586	20	25166329	25217365	100	13	123834	-1.0006	0.84149	0.995393814
KRTAP12-1	ENSG00000187175	21	46091491	46112078	131	9	123834	-1.0006	0.84149	0.995393814
SHROOM3	ENSG00000138771	4	77346253	77714406	597	44	123834	-1.0014	0.84169	0.995393814
DUSP28	ENSG00000188542	2	241489471	241513431	18	6	123834	-1.0015	0.84171	0.995393814
TUBB	ENSG00000196230	6	30677978	30703203	53	6	123834	-1.0016	0.84172	0.995393814
HBA1	ENSG00000206172	16	216679	237521	11	2	123834	-1.0017	0.84175	0.995393814
MAPK14	ENSG00000112062	6	35985488	36089013	200	15	123834	-1.002	0.84182	0.995393814
WDR83OS	ENSG00000105583	19	12768885	12792170	10	3	123834	-1.0019	0.84182	0.995393814
RPUSD1	ENSG00000007376	16	824974	848397	52	8	123834	-1.002	0.84183	0.995393814
RBP2	ENSG00000114113	3	139161726	139209589	97	9	123834	-1.0021	0.84186	0.995393814
ARMC10	ENSG00000170632	7	102705328	102750205	31	7	123834	-1.0021	0.84186	0.995393814
KIAA1549	ENSG00000122778	7	138506126	138676064	233	24	123834	-1.0025	0.84195	0.995393814
RIMBP2	ENSG00000060709	12	130870682	131210826	662	84	123834	-1.0027	0.84199	0.995393814
SNAP29	ENSG00000099940	22	21203271	21255506	114	8	123834	-1.0028	0.84201	0.995393814
ANXA3	ENSG00000138772	4	79462673	79541597	78	12	123834	-1.0028	0.84203	0.995393814
AC009065.1	ENSG00000167970	16	2293738	2322343	20	3	123834	-1.0029	0.84205	0.995393814
MTNR1A	ENSG00000168412	4	187444809	187486721	88	16	123834	-1.0031	0.84209	0.995393814
TIFA	ENSG00000145365	4	113185698	113217059	37	2	123834	-1.0032	0.84211	0.995393814
OLFML2A	ENSG00000185585	9	127529437	127587164	20	4	123834	-1.0032	0.84211	0.995393814
ZNF431	ENSG00000196705	19	21314827	21383034	82	9	123834	-1.0033	0.84214	0.995393814
KRTAP16-1	ENSG00000212657	17	39453952	39475505	65	5	123834	-1.0034	0.84218	0.995393814
FAM13C	ENSG00000148541	10	60995890	61132939	180	18	123834	-1.0035	0.8422	0.995393814
DOT1L	ENSG00000104885	19	2154148	2242577	121	16	123834	-1.0037	0.84224	0.995393814
COMMD9	ENSG00000110442	11	36285051	36320999	106	12	123834	-1.0038	0.84226	0.995393814
PRDX3	ENSG00000165672	10	120917215	120948345	16	4	123834	-1.0039	0.84229	0.995393814
PIGR	ENSG00000162896	1	207091863	207129811	41	5	123834	-1.004	0.84231	0.995393814
ZSCAN1	ENSG00000152467	19	58535400	58575999	22	4	123834	-1.0043	0.84238	0.995393814
MINPP1	ENSG00000107789	10	89254632	89323217	84	11	123834	-1.0044	0.8424	0.995393814
NADSYN1	ENSG00000172890	11	71154155	71245153	249	14	123834	-1.0047	0.84247	0.995393814
C7orf76	ENSG00000197851	7	96100938	96142835	67	10	123834	-1.0048	0.84251	0.995393814
RAB7A	ENSG00000075785	3	128434965	128543639	216	11	123834	-1.0049	0.84252	0.995393814
PPP1R10	ENSG00000204569	6	30558177	30596389	62	5	123834	-1.0052	0.84261	0.995393814
NABP2	ENSG00000139579	12	56605799	56633638	14	3	123834	-1.0053	0.84261	0.995393814
YWHAE	ENSG00000108953	17	1237566	1313672	166	20	123834	-1.0053	0.84261	0.995393814
ZNF197	ENSG00000186448	3	44616380	44699963	83	8	123834	-1.0056	0.84269	0.995393814
KCNRG	ENSG00000198553	13	50579390	50605058	34	6	123834	-1.0063	0.84287	0.995393814
ZNF25	ENSG00000175395	10	38228500	38275561	94	9	123834	-1.0064	0.84288	0.995393814
AC110084.1	ENSG00000268656	2	27988851	28019186	16	2	123834	-1.0065	0.84291	0.995393814
SAFB2	ENSG00000130254	19	5577010	5634057	52	6	123834	-1.0068	0.84298	0.995393814
VPS52	ENSG00000223501	6	33208049	33249824	73	8	123834	-1.0068	0.84299	0.995393814
KLHL28	ENSG00000179454	14	45383522	45521525	193	7	123834	-1.0069	0.843	0.995393814
ACIN1	ENSG00000100813	14	23517773	23574823	64	9	123834	-1.007	0.84303	0.995393814
CGRRF1	ENSG00000100532	14	54966530	55015567	36	4	123834	-1.0072	0.84308	0.995393814
C1orf174	ENSG00000198912	1	3795689	3826857	66	8	123834	-1.0074	0.84314	0.995393814
DUSP4	ENSG00000120875	8	29180581	29218185	53	6	123834	-1.0079	0.84326	0.995393814
KIAA2026	ENSG00000183354	9	5871596	6017901	133	12	123834	-1.008	0.84328	0.995393814
FURIN	ENSG00000140564	15	91401822	91436688	30	6	123834	-1.0082	0.84331	0.995393814
DCTD	ENSG00000129187	4	183801213	183849089	119	14	123834	-1.0084	0.84337	0.995393814
ATR	ENSG00000175054	3	142158077	142307668	265	10	123834	-1.0085	0.8434	0.995393814
TRIM68	ENSG00000167333	11	4609902	4639489	69	6	123834	-1.0089	0.84349	0.995393814
ANKRD53	ENSG00000144031	2	71195510	71222626	15	4	123834	-1.009	0.84352	0.995393814
TRIM49B	ENSG00000182053	11	49040504	49069579	65	3	123834	-1.0091	0.84353	0.995393814
HIGD1C	ENSG00000214511	12	51337705	51374289	128	11	123834	-1.0091	0.84354	0.995393814
CDH3	ENSG00000062038	16	68660092	68766519	227	16	123834	-1.0093	0.84359	0.995393814
IFIT1	ENSG00000185745	10	91142303	91173745	56	9	123834	-1.0093	0.8436	0.995393814
PRAMEF11	ENSG00000204513	1	12874468	12901264	18	1	123834	-1.0094	0.84362	0.995393814
SSTR3	ENSG00000183473	22	37590278	37618362	54	10	123834	-1.0096	0.84365	0.995393814
SALL4	ENSG00000101115	20	50390581	50429059	118	21	123834	-1.0097	0.84368	0.995393814
WIPI2	ENSG00000157954	7	5219819	5283457	115	18	123834	-1.0097	0.84369	0.995393814
GSTM2	ENSG00000213366	1	110200644	110262171	57	14	123834	-1.0101	0.84377	0.995393814
GPR158	ENSG00000151025	10	25453991	25901155	1231	42	123834	-1.0102	0.8438	0.995393814
KLHL40	ENSG00000157119	3	42717011	42744036	15	4	123834	-1.011	0.84399	0.995393814
TECRL	ENSG00000205678	4	65130975	65285186	351	12	123834	-1.011	0.84399	0.995393814
UBE4B	ENSG00000130939	1	10082890	10251297	154	19	123834	-1.0115	0.8441	0.995393814
OR5AR1	ENSG00000172459	11	56421138	56442163	53	9	123834	-1.0115	0.84411	0.995393814
PDE4DIP	ENSG00000178104	1	144826157	145086186	5	2	123834	-1.0116	0.84414	0.995393814
SCFD2	ENSG00000184178	4	53729149	54242242	729	44	123834	-1.0117	0.84416	0.995393814
FN3KRP	ENSG00000141560	17	80664559	80698204	118	11	123834	-1.0119	0.8442	0.995393814
SLC35A5	ENSG00000138459	3	112270556	112314424	120	8	123834	-1.0119	0.84421	0.995393814
WDR47	ENSG00000085433	1	109502836	109594850	141	15	123834	-1.0123	0.8443	0.995438184
MLKL	ENSG00000168404	16	74695753	74744858	85	15	123834	-1.0127	0.8444	0.995494337
SDR42E1	ENSG00000184860	16	82021221	82055093	17	4	123834	-1.013	0.84446	0.995503329
SLU7	ENSG00000164609	5	159818648	159858718	49	7	123834	-1.0135	0.84458	0.995556671

REV3L	ENSG00000009413	6	111610234	111814918	359	19	123834	-1.0136	0.84461	0.995556671
CARS	ENSG00000110619	11	3012152	3088843	125	12	123834	-1.0139	0.84469	0.99558923
OR10A2	ENSG00000170790	11	6880924	6901976	100	8	123834	-1.0151	0.84496	0.995845714
ABCD3	ENSG00000117528	1	94873933	94994222	22	7	123834	-1.0154	0.84503	0.995850072
IGDCC4	ENSG00000103742	15	65663802	65725410	57	10	123834	-1.0159	0.84517	0.995850072
ZNF35	ENSG00000169981	3	44680219	44712283	23	6	123834	-1.016	0.84518	0.995850072
LNX2	ENSG00000139517	13	28110050	28204541	233	23	123834	-1.0169	0.8454	0.995850072
CHKB-CPT1B	ENSG00000254413	22	50997298	51031394	124	18	123834	-1.0169	0.8454	0.995850072
DNMBP	ENSG00000107554	10	101625334	101779676	357	18	123834	-1.017	0.84541	0.995850072
KLK5	ENSG00000167754	19	51436559	51466349	48	7	123834	-1.0171	0.84545	0.995850072
SYDE1	ENSG00000105137	19	15208214	15235799	19	6	123834	-1.0172	0.84547	0.995850072
CSTL1	ENSG00000125823	20	23410322	23435567	54	9	123834	-1.0172	0.84548	0.995850072
CCDC54	ENSG00000138483	3	107086188	107107481	28	7	123834	-1.0173	0.84549	0.995850072
KRTAP10-11	ENSG00000243489	21	46056331	46077564	63	6	123834	-1.0175	0.84554	0.995850072
ITSN2	ENSG00000198399	2	24415733	24593583	357	12	123834	-1.0178	0.84562	0.995858483
AC061992.1	ENSG00000268965	17	76412409	76432834	97	2	123834	-1.018	0.84566	0.995858483
RSPO4	ENSG00000101282	20	929095	992907	115	19	123834	-1.0186	0.84579	0.995858483
RRS1	ENSG00000179041	8	67331263	67352966	47	9	123834	-1.0189	0.84588	0.995858483
KIAA1161	ENSG00000164976	9	34356668	34386851	40	8	123834	-1.0191	0.84591	0.995858483
GIMAP2	ENSG00000106560	7	150372785	150400729	51	8	123834	-1.0191	0.84592	0.995858483
SLC5A2	ENSG00000140675	16	31484323	31512181	11	3	123834	-1.0192	0.84595	0.995858483
LPAR3	ENSG00000171517	1	85267285	85368896	153	27	123834	-1.0193	0.84597	0.995858483
GNB2L1	ENSG00000204628	5	180653909	180685096	62	8	123834	-1.02	0.84613	0.995858483
PROC	ENSG00000115718	2	128166003	128196822	32	6	123834	-1.02	0.84614	0.995858483
CSRNP2	ENSG00000110925	12	51444990	51487447	83	10	123834	-1.0206	0.84628	0.995858483
ZNF2	ENSG00000163067	2	95821177	95860065	18	4	123834	-1.0208	0.84633	0.995858483
SHPK	ENSG00000262304	17	3458738	3549543	171	27	123834	-1.021	0.84636	0.995858483
COA6	ENSG00000168275	1	234499202	234529795	50	4	123834	-1.0212	0.84643	0.995858483
LRRC2	ENSG00000163827	3	46546913	46631589	217	17	123834	-1.0219	0.8466	0.995858483
SMG7	ENSG00000116698	1	183431351	183577381	162	20	123834	-1.0223	0.84669	0.995858483
NDUFS8	ENSG00000110717	11	67788084	67814111	27	3	123834	-1.0226	0.84674	0.995858483
NTSR1	ENSG00000101188	20	61330189	61404123	131	22	123834	-1.0227	0.84677	0.995858483
BTBD7	ENSG00000011114	14	93693896	93809438	273	14	123834	-1.0228	0.8468	0.995858483
XPO1	ENSG00000082898	2	61694984	61775761	100	7	123834	-1.0236	0.84699	0.995858483
PHF11	ENSG00000136147	13	50059746	50113123	184	15	123834	-1.0236	0.847	0.995858483
ZNF385B	ENSG00000144331	2	180296709	180736232	774	66	123834	-1.0244	0.84719	0.995858483
FAM183A	ENSG00000186973	1	43600824	43632067	112	14	123834	-1.0246	0.84721	0.995858483
SIGLEC8	ENSG00000105366	19	51944101	51971710	11	3	123834	-1.0248	0.84728	0.995858483
KIAA1024L	ENSG00000186367	5	129073772	129112425	67	9	123834	-1.0249	0.8473	0.995858483
EFCAB5	ENSG00000176927	17	28246218	28445470	197	8	123834	-1.0249	0.8473	0.995858483
SULT1B1	ENSG00000173597	4	70576880	70663679	227	19	123834	-1.025	0.84733	0.995858483
RBBP4	ENSG00000162521	1	33106743	33161812	101	2	123834	-1.0251	0.84734	0.995858483
RANBP6	ENSG00000137040	9	6001043	6025618	17	3	123834	-1.0253	0.84739	0.995858483
ESR1	ENSG00000091831	6	151967826	152460754	992	57	123834	-1.0257	0.84748	0.995858483
HINFP	ENSG00000172273	11	118982297	119016752	61	5	123834	-1.026	0.84754	0.995858483
MORN4	ENSG00000171160	10	99364310	99403344	30	2	123834	-1.0261	0.84758	0.995858483
CCL23	ENSG00000167236	17	34330096	34355005	79	7	123834	-1.0261	0.84758	0.995858483
OIP5	ENSG00000104147	15	41591466	41634819	74	7	123834	-1.0267	0.84771	0.995858483
AL354993.1	ENSG00000227164	20	52095495	52116164	69	5	123834	-1.0267	0.84772	0.995858483
CD33	ENSG00000105383	19	51718320	51757115	50	8	123834	-1.0275	0.84791	0.995858483
ERVV-1	ENSG00000269526	19	53507174	53529375	23	3	123834	-1.0277	0.84795	0.995858483
TRIM62	ENSG00000116525	1	33601003	33657660	42	3	123834	-1.0279	0.84799	0.995858483
TPM2	ENSG00000198467	9	35671989	35701017	38	8	123834	-1.0281	0.84806	0.995858483
AGPAT2	ENSG00000169692	9	139557595	139591875	16	2	123834	-1.0283	0.84809	0.995858483
DERL3	ENSG00000099958	22	24166690	24191315	98	9	123834	-1.0284	0.84811	0.995858483
GSDMB	ENSG00000073605	17	38050848	38086107	54	5	123834	-1.0287	0.8482	0.995858483
RNPS1	ENSG00000205937	16	2293117	2328413	23	4	123834	-1.0289	0.84824	0.995858483
CPT1B	ENSG00000205560	22	50997290	51027899	117	18	123834	-1.0294	0.84834	0.995858483
TRIM49D1	ENSG00000223417	11	89634279	89664935	11	2	123834	-1.0295	0.84837	0.995858483
ELOVL5	ENSG00000012660	6	53122196	53223947	216	10	123834	-1.0296	0.84839	0.995858483
FAIM3	ENSG00000162894	1	207067731	207106592	38	3	123834	-1.0298	0.84845	0.995858483
COPRS	ENSG00000172301	17	30168883	30196356	41	8	123834	-1.03	0.8485	0.995858483
CNN1	ENSG00000130176	19	11639532	11671138	49	7	123834	-1.0304	0.84859	0.995858483
DAO	ENSG00000110887	12	109242708	109304819	73	13	123834	-1.0305	0.84861	0.995858483
PVALB	ENSG00000100362	22	37186728	37225523	58	9	123834	-1.0305	0.84861	0.995858483
LYZL1	ENSG00000120563	10	29567990	29617257	169	21	123834	-1.0306	0.84863	0.995858483
FBXO33	ENSG00000165355	14	39856873	39911704	121	11	123834	-1.0306	0.84863	0.995858483
RP11-321F6.1	ENSG00000259471	15	66864528	66988132	231	36	123834	-1.0307	0.84865	0.995858483
PRKCZ	ENSG00000067606	1	1971909	2126834	184	16	123834	-1.0307	0.84866	0.995858483
SEMA4F	ENSG00000135622	2	74871355	74919186	31	2	123834	-1.0317	0.84889	0.995858483
SRGAP3	ENSG00000196220	3	9012275	9414737	463	88	123834	-1.0317	0.84889	0.995858483
SLC17A4	ENSG00000146039	6	25744927	25791419	141	14	123834	-1.0321	0.849	0.995858483
CASP8	ENSG00000064012	2	202088166	202162434	131	20	123834	-1.0326	0.84912	0.995858483
ACOT4	ENSG00000177465	14	74048410	74073200	75	13	123834	-1.0327	0.84912	0.995858483
KLHL35	ENSG00000149243	11	75123438	75151674	22	4	123834	-1.0332	0.84924	0.995858483
IL31RA	ENSG00000164509	5	55137207	55228678	129	16	123834	-1.0332	0.84925	0.995858483
MAST2	ENSG00000086015	1	46242659	46511796	434	9	123834	-1.0336	0.84934	0.995858483
COQ7	ENSG00000167186	16	19068921	19101417	69	9	123834	-1.0336	0.84935	0.995858483
HBEGF	ENSG00000113070	5	139702428	139736216	14	3	123834	-1.0338	0.8494	0.995858483
LMOD1	ENSG00000163431	1	201855580	201925715	71	13	123834	-1.0339	0.84942	0.995858483
TMEM52B	ENSG00000165685	12	10313141	10354400	92	17	123834	-1.0339	0.84942	0.995858483
CD276	ENSG00000103855	15	73966307	74016859	94	11	123834	-1.0341	0.84946	0.995858483
TOE1	ENSG00000132773	1	45795342	45819647	18	2	123834	-1.0343	0.84951	0.995858483
IL37	ENSG00000125571	2	113660548	113686459	91	3	123834	-1.0347	0.8496	0.995858483
PIM3	ENSG00000198355	22	50344161	50367728	5	2	123834	-1.0348	0.84961	0.995858483
SLC25A51	ENSG00000122696	9	37869400	37914350	44	5	123834	-1.0352	0.8497	0.995858483
LACTB	ENSG00000103642	15	63403999	63444260	105	9	123834	-1.0352	0.84971	0.995858483
RPL7	ENSG00000147604	8	74192506	74218024	53	7	123834	-1.0353	0.84973	0.995858483
OGFOD1	ENSG00000087263	16	56475402	56523012	76	8	123834	-1.0359	0.84987	0.995858483
SERHL2	ENSG00000183569	22	42939623	42980388	44	6	123834	-1.0359	0.84987	0.995858483
KCNIP2	ENSG00000120049	10	103575731	103613677	45	9	123834	-1.0359	0.84988	0.995858483
ARHGAP25	ENSG00000163219	2	68896733	69063965	382	38	123834	-1.036	0.8499	0.995858483
SYS1	ENSG00000204070	20	43980577	44015438	56	3	123834	-1.0365	0.85001	0.995858483
KIF26B	ENSG00000162849	1	245308287	245882733	931	120	123834	-1.0365	0.85002	0.995858483
SMEK1	ENSG00000100796	14	91913955	91986898	138	9	123834	-1.0368	0.85009	0.995858483
ZNF347	ENSG00000197937	19	53617325	53672328	106	16	123834	-1.0369	0.85011	0.995858483
KIF15	ENSG00000163808	3	44793209	44924868	84	8	123834	-1.037	0.85013	0.995858483
MORC1	ENSG00000114487	3	108667086	108846989	469	15	123834	-1.0371	0.85015	0.995858483
FAM211B	ENSG00000178026	22	24971588	24999175	27	3	123834	-1.0375	0.85026	0.995858483

GTPBP3	ENSG00000130299	19	17435729	17463544	53	7	123834	-1.0379	0.85033	0.995858483
PARP6	ENSG00000137817	15	72523522	72575340	76	8	123834	-1.0381	0.85039	0.995858483
RAP1GAP2	ENSG00000132359	17	2670350	2951033	269	60	123834	-1.0393	0.85067	0.995858483
ZNF530	ENSG00000183647	19	58101253	58134090	43	6	123834	-1.0393	0.85068	0.995858483
ATF3	ENSG00000162772	1	212728676	212804119	137	22	123834	-1.0398	0.85078	0.995858483
MAP2K5	ENSG00000137764	15	67825047	68109461	491	20	123834	-1.0398	0.85078	0.995858483
KIAA1467	ENSG00000084444	12	13187218	13305455	147	13	123834	-1.0399	0.85081	0.995858483
CEP120	ENSG00000168944	5	122670579	122769286	191	12	123834	-1.04	0.85082	0.995858483
ADAMTS18	ENSG00000140873	16	77271710	77479011	630	38	123834	-1.04	0.85082	0.995858483
TECTB	ENSG00000119913	10	114033493	114074793	74	11	123834	-1.04	0.85084	0.995858483
PRR14	ENSG00000156858	16	30652038	30677761	6	2	123834	-1.0403	0.8509	0.995858483
RASGEF1A	ENSG00000198915	10	43679983	43772367	161	19	123834	-1.0408	0.85102	0.995858483
OR5W2	ENSG00000187612	11	55671126	55692058	152	6	123834	-1.0408	0.85102	0.995858483
SORD	ENSG00000140263	15	45305302	45379383	100	11	123834	-1.0414	0.85115	0.995858483
OR52I1	ENSG00000232268	11	4605037	4626243	56	7	123834	-1.0415	0.85117	0.995858483
SEL1L3	ENSG00000091490	4	25739055	25875382	114	25	123834	-1.0417	0.85121	0.995858483
TRUB1	ENSG00000165832	10	116687952	116747430	111	11	123834	-1.0417	0.85123	0.995858483
SPAG17	ENSG00000155761	1	118486484	118737846	305	10	123834	-1.0421	0.85132	0.995858483
RAB8A	ENSG00000167461	19	16212439	16255044	44	5	123834	-1.0424	0.85139	0.995858483
KIF16B	ENSG00000089177	20	16242749	16564078	722	46	123834	-1.0428	0.85147	0.995858483
OR7A5	ENSG00000188269	19	14893302	14956188	123	17	123834	-1.043	0.85153	0.995858483
CLEC19A	ENSG00000261210	16	19287061	19343467	92	11	123834	-1.0435	0.85163	0.995858483
ZNRF4	ENSG00000105428	19	5445426	5466867	20	7	123834	-1.0439	0.85173	0.995858483
AC009802.1	ENSG00000268706	18	61592042	61619601	69	4	123834	-1.0444	0.85185	0.995858483
GSTM1	ENSG00000134184	1	110220436	110261661	33	7	123834	-1.0445	0.85187	0.995858483
FAM163A	ENSG00000143340	1	179702298	179795333	152	18	123834	-1.0445	0.85187	0.995858483
KRTAP1-3	ENSG00000221880	17	39180142	39201107	76	9	123834	-1.0445	0.85188	0.995858483
RP11-321M21.3	ENSG00000264116	18	73108967	73140995	58	8	123834	-1.0445	0.85188	0.995858483
SYCP3	ENSG00000139351	12	102112426	102143250	45	9	123834	-1.0446	0.85189	0.995858483
ATRN	ENSG00000088812	20	3441687	3641769	412	18	123834	-1.0451	0.85201	0.995858483
ANKH	ENSG00000154122	5	14694910	14881887	251	35	123834	-1.0451	0.85202	0.995858483
SCG2	ENSG00000171951	2	224451658	224477221	14	3	123834	-1.0456	0.85212	0.995858483
AC002451.1	ENSG00000269449	7	95161457	95183854	73	6	123834	-1.0458	0.85217	0.995858483
FGF1	ENSG00000113578	5	141961743	142087617	187	30	123834	-1.0459	0.85221	0.995858483
CYB5R3	ENSG00000100243	22	43003846	43055574	77	8	123834	-1.046	0.85221	0.995858483
LENG1	ENSG00000105617	19	54648899	54673620	17	5	123834	-1.0461	0.85223	0.995858483
TAF1A	ENSG00000143498	1	222721244	222773275	77	9	123834	-1.0461	0.85224	0.995858483
CBLC	ENSG00000142273	19	45271126	45313891	38	6	123834	-1.0462	0.85226	0.995858483
COL22A1	ENSG00000169436	8	139590478	139936249	614	72	123834	-1.0463	0.85228	0.995858483
TBC1D23	ENSG00000036054	3	99969844	100054095	108	9	123834	-1.0464	0.8523	0.995858483
CCDC151	ENSG00000198003	19	11521272	11556603	23	3	123834	-1.0466	0.85237	0.995858483
USP20	ENSG00000136878	9	132586977	132654107	385	12	123834	-1.0468	0.85241	0.995858483
TMPRSS11E	ENSG00000087128	4	69303167	69373322	116	7	123834	-1.0471	0.85248	0.995858483
SMPD1	ENSG00000166311	11	6401655	6426228	15	2	123834	-1.0471	0.85248	0.995858483
GABRB2	ENSG00000145864	5	160705436	160986050	470	32	123834	-1.0472	0.85249	0.995858483
COX7A1	ENSG00000161281	19	36631824	36653771	23	5	123834	-1.0472	0.8525	0.995858483
SH3D19	ENSG00000109686	4	152013903	152256784	416	17	123834	-1.0473	0.85252	0.995858483
RAD54L	ENSG00000085999	1	46703360	46754145	30	6	123834	-1.0474	0.85255	0.995858483
RP1-139D8.6	ENSG00000214732	6	42113279	42141156	46	9	123834	-1.0475	0.85258	0.995858483
EEF2	ENSG00000167658	19	3966054	3995467	15	3	123834	-1.0478	0.85262	0.995858483
DENND4A	ENSG00000174485	15	65940384	66094631	233	10	123834	-1.048	0.85268	0.99586737
TXNRD2	ENSG00000184470	22	19853040	19939341	204	23	123834	-1.0502	0.8532	0.996118679
CLRN3	ENSG00000180745	10	129666105	129701211	42	15	123834	-1.0508	0.85332	0.996118679
CPA1	ENSG00000091704	7	130010180	130037955	30	7	123834	-1.0508	0.85333	0.996118679
TMEM75	ENSG00000256655	8	128949126	128970591	28	4	123834	-1.0508	0.85333	0.996118679
GLI1	ENSG00000111087	12	57843918	57876045	35	7	123834	-1.0512	0.85341	0.996118679
CTGF	ENSG00000118523	6	132259316	132282513	10	4	123834	-1.052	0.8536	0.996118679
RGS21	ENSG00000253148	1	192276122	192346415	73	8	123834	-1.052	0.85361	0.996118679
ZNF736	ENSG00000234444	7	63757837	63825238	265	17	123834	-1.0524	0.85368	0.996118679
TRIP4	ENSG00000103671	15	64669947	64757502	98	8	123834	-1.0525	0.85371	0.996118679
KIR3DL3	ENSG00000242019	19	55225984	55258003	13	3	123834	-1.053	0.85382	0.996118679
ZNF282	ENSG00000170265	7	148882577	148933339	74	13	123834	-1.0535	0.85395	0.996118679
ZNF385D	ENSG00000151789	3	21449915	22424812	2061	148	123834	-1.0536	0.85396	0.996118679
KRT222	ENSG00000213424	17	38800917	38831433	16	2	123834	-1.0539	0.85404	0.996118679
AP003733.1	ENSG00000269089	11	61725453	61746755	30	7	123834	-1.0544	0.85415	0.996118679
LAPTM4A	ENSG00000068697	2	20222411	20261789	74	11	123834	-1.0551	0.85431	0.996118679
SAA4	ENSG00000148965	11	18242896	18268440	37	4	123834	-1.0552	0.85434	0.996118679
RP11-302M6.4	ENSG00000271949	1	90088699	90408809	294	13	123834	-1.0565	0.85464	0.996118679
HECTD4	ENSG00000173064	12	112587992	112829896	184	12	123834	-1.0565	0.85464	0.996118679
KCNIP3	ENSG00000115041	2	95953052	96061825	75	6	123834	-1.0566	0.85465	0.996118679
GPR26	ENSG00000154478	10	125415871	125464123	92	18	123834	-1.0567	0.85467	0.996118679
TTC22	ENSG00000006555	1	55235385	55276940	43	8	123834	-1.0568	0.85471	0.996118679
MAT2B	ENSG00000038274	5	162920120	162956342	53	9	123834	-1.0569	0.85471	0.996118679
FTCDNL1	ENSG00000226124	2	200615267	200725896	108	19	123834	-1.0574	0.85484	0.996118679
C1orf43	ENSG00000143612	1	154169182	154203104	20	4	123834	-1.0576	0.85488	0.996118679
CYB5D2	ENSG00000167740	17	4036462	4100605	140	11	123834	-1.0576	0.85489	0.996118679
MEPE	ENSG00000152595	4	88732563	88777969	106	17	123834	-1.0577	0.8549	0.996118679
PLEKHA8	ENSG00000106086	7	30057020	30180096	200	9	123834	-1.0579	0.85494	0.996118679
ELOF1	ENSG00000130165	19	11651962	11680051	35	6	123834	-1.0579	0.85494	0.996118679
OPCML	ENSG00000183715	11	132274871	133412414	1643	170	123834	-1.058	0.85498	0.996118679
C1orf158	ENSG00000157330	1	12796163	12833847	89	8	123834	-1.0587	0.85513	0.996118679
AL360004.1	ENSG00000196979	9	132892889	132916498	58	7	123834	-1.0588	0.85516	0.996118679
ZNF540	ENSG00000171817	19	38032308	38114998	130	13	123834	-1.0595	0.85532	0.996118679
TMUB2	ENSG00000168591	17	42254338	42279099	24	4	123834	-1.06	0.85542	0.996118679
CLPB	ENSG00000162129	11	71993469	72155692	184	8	123834	-1.0604	0.85552	0.996118679
ENPP7	ENSG00000182156	17	77694681	77726021	43	8	123834	-1.0609	0.85563	0.996118679
EHD2	ENSG00000024422	19	48206600	48256391	66	10	123834	-1.0612	0.8557	0.996118679
SAMD4B	ENSG00000179134	19	39823036	39886350	82	8	123834	-1.0613	0.85573	0.996118679
WIPF1	ENSG00000115935	2	175414300	175557644	84	14	123834	-1.0615	0.85576	0.996118679
LRRC8C	ENSG00000171488	1	90088631	90245462	170	9	123834	-1.0618	0.85585	0.996118679
JUND	ENSG00000130522	19	18380563	18402432	66	7	123834	-1.0621	0.8559	0.996118679
NUDT13	ENSG00000166321	10	74860217	74901586	79	1	123834	-1.0622	0.85593	0.996118679
PTER	ENSG00000165983	10	16468942	16565736	214	16	123834	-1.0623	0.85596	0.996118679
GRHL1	ENSG00000134317	2	10075341	10152411	222	19	123834	-1.0624	0.85597	0.996118679
SYN3	ENSG00000185666	22	32898539	33464358	945	85	123834	-1.0624	0.85597	0.996118679
FRRS1	ENSG00000156869	1	100164259	100242187	196	7	123834	-1.0625	0.85599	0.996118679
PGA3	ENSG00000229859	11	60960779	60990570	4	1	123834	-1.0625	0.85599	0.996118679
OR56B1	ENSG00000181023	11	5747681	5768768	80	8	123834	-1.0625	0.856	0.996118679
NUB1	ENSG00000013374	7	151028785	151085535	91	18	123834	-1.0629	0.85609	0.996118679

PRRX2	ENSG00000167157	9	132417920	132494875	119	18	123834	-1.0631	0.85613	0.996118679
UCN3	ENSG00000178473	10	5396972	5426169	41	6	123834	-1.0632	0.85615	0.996118679
NAPSA	ENSG00000131400	19	50851734	50879087	33	3	123834	-1.0632	0.85616	0.996118679
ORC1	ENSG00000085840	1	52828501	52880131	26	4	123834	-1.0634	0.85621	0.996118679
S100B	ENSG00000160307	21	48008875	48035121	99	9	123834	-1.0639	0.85632	0.996118679
ZPBP2	ENSG00000186075	17	38014417	38044149	70	5	123834	-1.064	0.85633	0.996118679
HDAC11	ENSG00000163517	3	13511224	13557916	59	13	123834	-1.064	0.85634	0.996118679
TBCK	ENSG00000145348	4	106952756	107252652	511	12	123834	-1.064	0.85634	0.996118679
KB-1507C5.2	ENSG00000253320	8	103866528	104000104	230	25	123834	-1.0642	0.85639	0.996118679
BRDT	ENSG00000137948	1	92404928	92489983	202	11	123834	-1.0644	0.85642	0.996118679
KRT76	ENSG00000185069	12	53151939	53181129	80	14	123834	-1.0645	0.85645	0.996118679
ZG16B	ENSG00000162078	16	2870170	2898967	16	5	123834	-1.0647	0.85648	0.996118679
KBTBD8	ENSG00000163376	3	67038727	67071634	27	4	123834	-1.0648	0.85652	0.996118679
LSM6	ENSG00000164167	4	147086837	147131152	103	6	123834	-1.0648	0.85653	0.996118679
PRTG	ENSG00000166450	15	55893744	56045288	257	16	123834	-1.065	0.85655	0.996118679
GOLGA5	ENSG00000066455	14	93250576	93316308	184	9	123834	-1.0651	0.85659	0.996118679
NUP210L	ENSG00000143552	1	153955161	154137592	161	11	123834	-1.0654	0.85665	0.996118679
LETMD1	ENSG00000050426	12	51431745	51464207	73	10	123834	-1.0655	0.85667	0.996118679
RBPMS2	ENSG00000166831	15	65022091	65077786	97	7	123834	-1.0656	0.8567	0.996118679
OR13D1	ENSG00000179055	9	107446660	107467766	50	6	123834	-1.0657	0.85673	0.996118679
LRRC4C	ENSG00000148948	11	40125753	41491323	2122	124	123834	-1.0657	0.85673	0.996118679
GPHN	ENSG00000171723	14	66964125	67658520	1447	16	123834	-1.0667	0.85694	0.996118679
KRT34	ENSG00000131737	17	39523902	39548655	31	7	123834	-1.0667	0.85695	0.996118679
TBR1	ENSG00000136535	2	162262605	162292381	24	5	123834	-1.0669	0.85699	0.996118679
RFX6	ENSG00000185002	6	117188375	117263326	176	10	123834	-1.0669	0.85699	0.996118679
ANTXR1	ENSG00000169604	2	69230310	69486459	387	45	123834	-1.0669	0.857	0.996118679
GAA	ENSG00000171298	17	78065355	78103678	171	18	123834	-1.0671	0.85704	0.996118679
NDUFB9	ENSG00000147684	8	125541344	125590751	93	13	123834	-1.0672	0.85705	0.996118679
FOXD4	ENSG00000170122	9	106237	128417	5	1	123834	-1.0671	0.85705	0.996118679
ABCG4	ENSG00000172350	11	119009722	119043360	37	4	123834	-1.0672	0.85706	0.996118679
PRR15	ENSG00000176532	7	29593427	29616911	53	12	123834	-1.0673	0.85709	0.996118679
ELTD1	ENSG00000162618	1	79345449	79482403	401	22	123834	-1.0674	0.85711	0.996118679
GNRHR	ENSG00000109163	4	68595046	68630078	80	9	123834	-1.0676	0.85714	0.996118679
SLC16A10	ENSG00000112394	6	111398781	111562397	160	14	123834	-1.0682	0.85728	0.996220471
TTC19	ENSG00000011295	17	15892694	15958329	115	7	123834	-1.0686	0.85737	0.99626415
CCDC17	ENSG00000159588	1	46075716	46099729	39	3	123834	-1.0698	0.85765	0.996479296
SPCS3	ENSG00000129128	4	177231115	177263396	111	14	123834	-1.0698	0.85766	0.996479296
AC112693.2	ENSG00000269360	15	93739295	93761277	48	7	123834	-1.0705	0.85781	0.996543366
DNAJB12	ENSG00000148719	10	74082588	74124988	74	2	123834	-1.0706	0.85782	0.996543366
PRDM2	ENSG00000116731	1	14016693	14161574	282	18	123834	-1.0711	0.85794	0.99658419
MTRNR2L6	ENSG00000270672	7	142364104	142385550	81	6	123834	-1.0712	0.85796	0.99658419
SLC37A4	ENSG00000137700	11	118884824	118911616	47	6	123834	-1.0724	0.85823	0.996741722
POLI	ENSG00000101751	18	51785774	51857636	180	9	123834	-1.0724	0.85823	0.996741722
SCRN1	ENSG00000136193	7	29949719	30039905	165	15	123834	-1.0728	0.85833	0.996741722
SEC31A	ENSG00000138674	4	83729814	83832319	160	7	123834	-1.0732	0.8584	0.996741722
AL162431.1	ENSG00000234237	1	180931695	180959868	32	3	123834	-1.0734	0.85846	0.996741722
CTB-129P6.11	ENSG00000267114	19	45443301	45467264	68	5	123834	-1.0734	0.85846	0.996741722
SLC45A1	ENSG00000162426	1	8367886	8414227	25	6	123834	-1.074	0.85859	0.996741722
OR13C9	ENSG00000136839	9	107369529	107390485	79	4	123834	-1.074	0.85859	0.996741722
CST11	ENSG00000125831	20	23421040	23443494	47	7	123834	-1.074	0.85859	0.996741722
TAT	ENSG00000198650	16	71589563	71621033	33	8	123834	-1.0742	0.85862	0.996741722
SCGB1A1	ENSG00000149021	11	62162575	62200667	54	9	123834	-1.0747	0.85876	0.996756446
KLRF2	ENSG00000256797	12	10024088	10058432	89	9	123834	-1.0748	0.85877	0.996756446
ANGPTL5	ENSG00000187151	11	101751405	101797253	76	10	123834	-1.0749	0.85879	0.996756446
SELPLG	ENSG00000110876	12	109005686	109037735	30	8	123834	-1.0761	0.85906	0.996827686
COPS7B	ENSG00000144524	2	232636381	232683963	49	4	123834	-1.0761	0.85907	0.996827686
LIMS1	ENSG00000169756	2	109140857	109313702	358	19	123834	-1.0765	0.85914	0.996827686
FEZF1	ENSG00000128610	7	121931448	121960745	7	3	123834	-1.077	0.85927	0.996827686
CLEC10A	ENSG00000132514	17	6967856	6993626	56	11	123834	-1.0771	0.85927	0.996827686
CARD17	ENSG00000255221	11	104953196	104982158	66	5	123834	-1.0771	0.85929	0.996827686
ALCAM	ENSG00000170017	3	105075753	105305744	427	25	123834	-1.0773	0.85932	0.996827686
ITPA	ENSG00000125877	20	3179514	3214516	63	10	123834	-1.0774	0.85935	0.996827686
PRB3	ENSG00000197870	12	11408857	11432739	31	3	123834	-1.0778	0.85944	0.996827686
CPA6	ENSG00000165078	8	68324360	68668620	468	47	123834	-1.0781	0.8595	0.996827686
NPTX1	ENSG00000171246	17	78430948	78461643	15	4	123834	-1.0784	0.85956	0.996827686
ZNF391	ENSG00000124613	6	27332394	27381683	196	12	123834	-1.0784	0.85957	0.996827686
TAF1L	ENSG00000122728	9	32619452	32645667	9	2	123834	-1.0785	0.85959	0.996827686
FAM129C	ENSG00000167483	19	17624110	17674648	73	14	123834	-1.0789	0.85969	0.996827686
SYT13	ENSG00000019505	11	45251852	45317870	111	10	123834	-1.079	0.85972	0.996827686
MED28	ENSG00000118579	4	17606254	17645728	100	4	123834	-1.0793	0.85977	0.996827686
AKIP1	ENSG00000166452	11	8922686	8951631	75	8	123834	-1.0796	0.85983	0.996827686
ATP5G3	ENSG00000154518	2	176030986	176059335	69	8	123834	-1.0799	0.8599	0.996827686
WTIP	ENSG00000142279	19	34961874	35007258	51	11	123834	-1.0799	0.8599	0.996827686
BTBD1	ENSG00000064726	15	83675174	83746106	123	14	123834	-1.0803	0.85999	0.996827686
RPL13A	ENSG00000142541	19	49980811	50005565	41	3	123834	-1.0805	0.86004	0.996827686
C2orf40	ENSG00000119147	2	106669702	106704615	43	8	123834	-1.0807	0.86008	0.996827686
ZNF750	ENSG00000141579	17	80777311	80808454	80	8	123834	-1.0807	0.86009	0.996827686
FANCC	ENSG00000158169	9	97851336	98089991	133	14	123834	-1.0808	0.86011	0.996827686
CNRIP1	ENSG00000119865	2	68501303	68557183	43	10	123834	-1.0819	0.86036	0.997056632
TMEM86A	ENSG00000151117	11	18704669	18736332	35	7	123834	-1.0831	0.86061	0.997074286
SMARCC2	ENSG00000139613	12	56546767	56593351	14	2	123834	-1.0831	0.86062	0.997074286
PCYOX1L	ENSG00000145882	5	148727570	148759216	43	6	123834	-1.0834	0.86068	0.997074286
METTL13	ENSG00000010165	1	171740788	171793163	144	6	123834	-1.0836	0.86074	0.997074286
MICU1	ENSG00000107745	10	74117098	74395899	310	4	123834	-1.0838	0.86077	0.997074286
MRPL51	ENSG00000111639	12	6591150	6613007	84	5	123834	-1.0839	0.8608	0.997074286
SETD2	ENSG00000181555	3	47047919	47215457	152	8	123834	-1.084	0.86081	0.997074286
FBXW4	ENSG00000107829	10	103360423	103465052	86	9	123834	-1.0842	0.86086	0.997074286
ANK1	ENSG00000029534	8	41500739	41764280	354	46	123834	-1.0844	0.86091	0.997074286
LEPROTL1	ENSG00000104660	8	29942914	30044724	148	11	123834	-1.0853	0.8611	0.997074286
GFOD1	ENSG00000145990	6	13348062	13497894	200	25	123834	-1.0858	0.86121	0.997074286
RP11-1105G2.3	ENSG00000258365	12	94661534	94686620	55	9	123834	-1.0857	0.86121	0.997074286
KCTD18	ENSG00000155729	2	201343675	201394507	44	13	123834	-1.0861	0.86128	0.997074286
CROCC	ENSG00000058453	1	17056768	17309474	72	7	123834	-1.0863	0.86133	0.997074286
C11orf53	ENSG00000150750	11	111116707	111167126	76	8	123834	-1.0863	0.86133	0.997074286
WWTR1	ENSG00000018408	3	149225022	149464501	437	45	123834	-1.0865	0.86136	0.997074286
KCNT2	ENSG00000162687	1	196184909	196588355	470	11	123834	-1.0875	0.86159	0.997074286
JAK3	ENSG00000105639	19	17925589	17968880	35	7	123834	-1.0877	0.86164	0.997074286
BLVRA	ENSG00000106605	7	43788279	43856939	117	11	123834	-1.0879	0.86167	0.997074286
GIMAP8	ENSG00000171115	7	150137718	150186480	34	7	123834	-1.0882	0.86175	0.997074286

SLC45A2	ENSG00000164175	5	33934721	33994835	34	7	123834	-1.0892	0.86197	0.997074286
UBXN11	ENSG00000158062	1	26597819	26654854	146	11	123834	-1.0892	0.86198	0.997074286
SIGLEC9	ENSG00000129450	19	51618165	51649908	5	3	123834	-1.09	0.86215	0.997074286
ZNF90	ENSG00000213988	19	20178803	20247885	80	7	123834	-1.0902	0.86218	0.997074286
PRB1	ENSG00000251655	12	11494757	11558500	42	7	123834	-1.0902	0.86219	0.997074286
ANKRD12	ENSG00000101745	18	9126226	9295983	297	13	123834	-1.0903	0.8622	0.997074286
ERICH1	ENSG00000104714	8	554746	698106	398	26	123834	-1.0903	0.86221	0.997074286
LRP4	ENSG00000134569	11	46868419	46950193	74	8	123834	-1.0903	0.86221	0.997074286
TFAM	ENSG00000108064	10	60134782	60168981	57	10	123834	-1.0905	0.86226	0.997074286
AC010760.1	ENSG00000269182	15	22446895	22470351	6	1	123834	-1.0907	0.86229	0.997074286
POLDIP3	ENSG00000100227	22	42969727	43020968	61	7	123834	-1.0908	0.86233	0.997074286
LCE3E	ENSG00000185966	1	152528130	152549248	20	2	123834	-1.091	0.86236	0.997074286
RRP1	ENSG00000160214	21	45199394	45235174	135	10	123834	-1.091	0.86237	0.997074286
AL031590.1	ENSG00000214854	22	39646865	39667063	50	12	123834	-1.0912	0.8624	0.997074286
TTC7A	ENSG00000068724	2	47133296	47313276	450	42	123834	-1.0912	0.86241	0.997074286
XRRA1	ENSG00000166435	11	74508784	74670245	195	16	123834	-1.0921	0.8626	0.997074286
MTHFD1L	ENSG00000120254	6	151176685	151433023	589	54	123834	-1.0922	0.86263	0.997074286
DGAT2	ENSG00000062282	11	75460557	75522579	49	3	123834	-1.0936	0.86294	0.997074286
CKMT1B	ENSG00000237289	15	43875252	43907099	13	1	123834	-1.0937	0.86296	0.997074286
STK11IP	ENSG00000144589	2	220452582	220491173	9	1	123834	-1.0938	0.86298	0.997074286
KCNE4	ENSG00000152049	2	223906532	224073117	538	25	123834	-1.0945	0.86314	0.997074286
BTBD9	ENSG00000183826	6	38126227	38617924	705	57	123834	-1.0947	0.86318	0.997074286
ACOT8	ENSG00000101473	20	44460360	44496045	80	12	123834	-1.0948	0.8632	0.997074286
C8A	ENSG00000157131	1	57310479	57393894	168	17	123834	-1.0954	0.86332	0.997074286
ACTL7B	ENSG00000148156	9	111606871	111629239	64	10	123834	-1.0955	0.86336	0.997074286
IFIT2	ENSG00000119922	10	91051712	91079033	22	6	123834	-1.096	0.86347	0.997074286
ARPC5	ENSG00000162704	1	183582401	183614892	159	5	123834	-1.0963	0.86353	0.997074286
HTRA4	ENSG00000169495	8	38821683	38856181	36	4	123834	-1.0965	0.86358	0.997074286
TMCO1	ENSG00000143183	1	165686032	165806992	87	10	123834	-1.0973	0.86375	0.997074286
ZBBX	ENSG00000169064	3	166948075	167109134	208	15	123834	-1.0976	0.8638	0.997074286
ERC2	ENSG00000187672	3	55532336	56512391	1859	82	123834	-1.0976	0.86381	0.997074286
C20orf187	ENSG00000125899	20	10998408	11020014	5	2	123834	-1.0979	0.86387	0.997074286
TCAP	ENSG00000173991	17	37810440	37832808	50	6	123834	-1.0981	0.86392	0.997074286
CCDC107	ENSG00000159884	9	35648301	35671508	50	5	123834	-1.0983	0.86396	0.997074286
RNF17	ENSG00000132972	13	25328290	25464059	313	15	123834	-1.0984	0.86399	0.997074286
OR6C6	ENSG00000188324	12	55678072	55699016	25	4	123834	-1.0985	0.86401	0.997074286
CRYZL1	ENSG00000205758	21	34951647	35026232	56	10	123834	-1.0985	0.86401	0.997074286
HTR3B	ENSG00000149305	11	113765399	113827287	110	12	123834	-1.0985	0.86402	0.997074286
THY1	ENSG00000154096	11	119278090	119305695	28	3	123834	-1.0986	0.86403	0.997074286
SLC39A5	ENSG00000139540	12	56613833	56641630	19	3	123834	-1.0986	0.86403	0.997074286
PHACTR2	ENSG00000112419	6	143847982	144162322	580	41	123834	-1.0989	0.86409	0.997074286
PDE6D	ENSG00000156973	2	232587135	232660982	90	6	123834	-1.099	0.86411	0.997074286
TM2D2	ENSG00000169490	8	38836327	38864343	34	4	123834	-1.0992	0.86417	0.997074286
GNA11	ENSG00000088256	19	3084408	3134002	65	12	123834	-1.0994	0.8642	0.997074286
OTOS	ENSG00000178602	2	241068446	241093979	89	10	123834	-1.0994	0.86421	0.997074286
C19orf55	ENSG00000167595	19	36239044	36271930	73	10	123834	-1.0995	0.86422	0.997074286
TMPRSS11B	ENSG00000185873	4	69082371	69121438	45	2	123834	-1.0997	0.86427	0.997074286
CCNG2	ENSG00000138764	4	78068304	78364542	1233	26	123834	-1.0998	0.86429	0.997074286
TMEM53	ENSG00000126106	1	45090910	45150227	67	3	123834	-1.0999	0.86431	0.997074286
SENP7	ENSG00000138468	3	101033049	101242085	482	9	123834	-1.1	0.86433	0.997074286
DNAI1	ENSG00000122735	9	34447412	34530982	100	11	123834	-1.1001	0.86436	0.997074286
TG	ENSG00000042832	8	133869203	134157147	520	35	123834	-1.1006	0.86446	0.997074286
LY96	ENSG00000154589	8	74893587	74951322	118	12	123834	-1.1006	0.86447	0.997074286
COX16	ENSG00000133983	14	70781798	70836448	145	14	123834	-1.1009	0.86453	0.997074286
RIPK4	ENSG00000183421	21	43149529	43197266	118	15	123834	-1.1009	0.86453	0.997074286
KIF11	ENSG00000138160	10	94343043	94425150	273	17	123834	-1.1009	0.86454	0.997074286
TMEM194A	ENSG00000166881	12	57439426	57491846	60	5	123834	-1.1013	0.86462	0.997074286
PCSK1	ENSG00000175426	5	95716119	95779847	51	6	123834	-1.1014	0.86463	0.997074286
ABCD2	ENSG00000173208	12	39933835	40023553	124	15	123834	-1.1019	0.86475	0.997074286
ADAMTS8	ENSG00000134917	11	130264820	130308888	28	10	123834	-1.102	0.86478	0.997074286
KIAA1045	ENSG00000122733	9	34947484	34992541	19	6	123834	-1.1023	0.86483	0.997074286
TMEM40	ENSG00000088726	3	12765024	12820956	102	14	123834	-1.1024	0.86485	0.997074286
DDX5	ENSG00000108654	17	62485734	62514317	36	3	123834	-1.1024	0.86485	0.997074286
SRSF5	ENSG00000100650	14	70183617	70248722	93	9	123834	-1.1026	0.86489	0.997074286
PHLPP1	ENSG00000081913	18	60372672	60657666	287	16	123834	-1.1031	0.865	0.997074286
FAM208A	ENSG00000163946	3	56644161	56727265	269	14	123834	-1.1031	0.86501	0.997074286
TMEM159	ENSG00000011638	16	21159698	21201937	65	8	123834	-1.1037	0.86513	0.997074286
HOXC13	ENSG00000123364	12	54322535	54350328	37	5	123834	-1.1038	0.86515	0.997074286
CD82	ENSG00000085117	11	44575977	44651913	118	18	123834	-1.1042	0.86526	0.997074286
RP11-484M3.5	ENSG00000251012	3	118856222	118916654	176	23	123834	-1.1045	0.86532	0.997074286
KLHL32	ENSG00000186231	6	97362605	97598630	448	31	123834	-1.105	0.86541	0.997074286
SLC2A11	ENSG00000133460	22	24188890	24238496	56	10	123834	-1.1051	0.86544	0.997074286
TLK1	ENSG00000198586	2	171837333	172097824	426	14	123834	-1.1055	0.86553	0.997074286
LRTOMT	ENSG00000184154	11	71781382	71831828	84	3	123834	-1.106	0.86563	0.997074286
MSH6	ENSG00000116062	2	47912669	48047240	224	21	123834	-1.1062	0.86568	0.997074286
PKP1	ENSG00000081277	1	201242580	201312121	173	17	123834	-1.1063	0.86571	0.997074286
NCL	ENSG00000115053	2	232308242	232358352	69	10	123834	-1.1064	0.86572	0.997074286
CTD-2368P22.1	ENSG00000176593	19	58503429	58532600	79	7	123834	-1.1064	0.86573	0.997074286
CNTNAP3	ENSG00000106714	9	39062764	39298312	61	6	123834	-1.1069	0.86583	0.997074286
POU4F3	ENSG00000091010	5	145708587	145730083	35	5	123834	-1.1078	0.86604	0.997074286
SUSD2	ENSG00000099994	22	24567227	24595078	45	8	123834	-1.1082	0.86611	0.997074286
GINS4	ENSG00000147536	8	41376725	41412565	65	9	123834	-1.1083	0.86613	0.997074286
PLXNA2	ENSG00000076356	1	208185587	208427665	299	41	123834	-1.1086	0.86619	0.997074286
FAM172A	ENSG00000113391	5	92943775	93457404	482	15	123834	-1.109	0.86628	0.997074286
RP11-552I14.1	ENSG00000257860	12	103535620	103572087	71	11	123834	-1.109	0.86628	0.997074286
ZNF830	ENSG00000198783	17	33278549	33300205	20	6	123834	-1.109	0.86628	0.997074286
RCC2	ENSG00000179051	1	17723256	17776220	100	9	123834	-1.1095	0.8664	0.997074286
USP29	ENSG00000131864	19	57620506	57653294	68	8	123834	-1.1096	0.86641	0.997074286
ADRA1D	ENSG00000171873	20	4191329	4239721	44	8	123834	-1.1096	0.86641	0.997074286
ANGPTL3	ENSG00000132855	1	63053158	63081830	14	2	123834	-1.1099	0.86647	0.997074286
GRB14	ENSG00000115290	2	165339322	165488358	92	18	123834	-1.1107	0.86664	0.997074286
APBA2	ENSG00000034053	15	29119629	29420518	395	16	123834	-1.1107	0.86666	0.997074286
TPST1	ENSG00000169902	7	65660186	65895530	378	13	123834	-1.1108	0.86667	0.997074286
C15orf59	ENSG00000205363	15	74022141	74055088	74	12	123834	-1.1111	0.86674	0.997074286
DUSP19	ENSG00000162999	2	183933287	183974733	82	12	123834	-1.1113	0.86678	0.997074286
PRR18	ENSG00000176381	6	166709168	166731936	19	5	123834	-1.112	0.86693	0.997074286
AEBP2	ENSG00000139154	12	19546979	19883735	1037	42	123834	-1.1121	0.86695	0.997074286
TEAD3	ENSG00000007866	6	35431374	35474853	83	15	123834	-1.1123	0.867	0.997074286
C5orf66	ENSG00000224186	5	134358970	134701744	284	40	123834	-1.1132	0.86719	0.997074286

WEE2	ENSG00000214102	7	141398153	141441071	34	2	123834	-1.1132	0.86719	0.997074286
GLO1	ENSG00000124767	6	38633701	38680917	118	13	123834	-1.1139	0.86733	0.997074286
AIMP1	ENSG00000164022	4	107226701	107280383	96	7	123834	-1.114	0.86736	0.997074286
HACL1	ENSG00000131373	3	15592211	15653338	79	13	123834	-1.1143	0.86743	0.997074286
GGT6	ENSG00000167741	17	4450222	4474113	27	4	123834	-1.1147	0.86751	0.997074286
CERS2	ENSG00000143418	1	150923059	150957479	42	4	123834	-1.1149	0.86754	0.997074286
UBE4A	ENSG00000110344	11	118220300	118279926	71	6	123834	-1.1149	0.86755	0.997074286
REL	ENSG00000162924	2	61098656	61168745	66	11	123834	-1.1151	0.86759	0.997074286
SLC7A11	ENSG00000151012	4	139075251	139173503	157	20	123834	-1.1152	0.86763	0.997074286
GLIS2	ENSG00000126603	16	4354762	4399598	33	7	123834	-1.1154	0.86765	0.997074286
ZNF571	ENSG00000180479	19	38035684	38095673	100	11	123834	-1.1154	0.86766	0.997074286
FAM91A1	ENSG00000176853	8	124770696	124837692	101	12	123834	-1.1157	0.86772	0.997074286
SCHIP1	ENSG00000151967	3	159547650	159625149	42	9	123834	-1.1158	0.86774	0.997074286
SCAPER	ENSG00000140386	15	76630526	77207785	900	17	123834	-1.1159	0.86778	0.997074286
NAALAD2	ENSG00000077616	11	89854683	89936062	266	13	123834	-1.1163	0.86785	0.997074286
ATP2A3	ENSG00000074370	17	3817169	3877736	89	12	123834	-1.1163	0.86786	0.997074286
TDRP	ENSG00000180190	8	429803	505781	146	19	123834	-1.1166	0.86793	0.997074286
SLC22A1	ENSG00000175003	6	160532821	160589750	94	20	123834	-1.1167	0.86794	0.997074286
ANKRD55	ENSG00000164512	5	55385507	55539186	285	25	123834	-1.1169	0.86798	0.997074286
AC138655.1	ENSG00000269563	2	47789601	47811998	18	3	123834	-1.117	0.868	0.997074286
GAD2	ENSG00000136750	10	26495236	26603487	225	8	123834	-1.117	0.868	0.997074286
ANXA7	ENSG00000138279	10	75124859	75183834	71	2	123834	-1.1173	0.86808	0.997074286
SCNN1B	ENSG00000168447	16	23279552	23402620	139	21	123834	-1.1184	0.86831	0.997074286
FFAR1	ENSG00000126266	19	35832445	35853367	24	5	123834	-1.1186	0.86833	0.997074286
KIAA0319	ENSG00000137261	6	24534332	24656383	290	33	123834	-1.1188	0.86839	0.997074286
OR51G2	ENSG00000176893	11	4925900	4946922	54	10	123834	-1.1188	0.86839	0.997074286
EDEM1	ENSG00000134109	3	5219331	5271642	58	8	123834	-1.1189	0.86842	0.997074286
RBMS3	ENSG00000144642	3	29312473	30061886	1552	96	123834	-1.1192	0.86847	0.997074286
CRYBB3	ENSG00000100053	22	25585817	25613330	73	12	123834	-1.1192	0.86848	0.997074286
ISOC1	ENSG00000066583	5	128420444	128459721	62	7	123834	-1.1193	0.86849	0.997074286
LAMTOR1	ENSG00000149357	11	71786941	71824433	57	3	123834	-1.1193	0.8685	0.997074286
FAM76B	ENSG00000077458	11	95492106	95533573	101	10	123834	-1.1193	0.8685	0.997074286
ANO3	ENSG00000134343	11	26200829	26694835	1777	59	123834	-1.1204	0.86873	0.997074286
CDRT15	ENSG00000223510	17	14128990	14150179	13	2	123834	-1.1208	0.86881	0.997074286
CPO	ENSG00000144410	2	207794278	207844198	47	9	123834	-1.121	0.86885	0.997074286
TMEM14A	ENSG00000096092	6	52525907	52561386	108	13	123834	-1.1219	0.86905	0.997074286
OR2L2	ENSG00000203663	1	248191474	248212607	19	2	123834	-1.122	0.86908	0.997074286
AC132216.1	ENSG00000184566	11	33892189	33913338	11	3	123834	-1.122	0.86908	0.997074286
TMEM189-UBE2V1	ENSG00000124208	20	48687661	48780174	94	9	123834	-1.1221	0.86909	0.997074286
TMEM189	ENSG00000240849	20	48687663	48780335	94	9	123834	-1.1221	0.86909	0.997074286
GATA4	ENSG00000136574	8	11524468	11627511	97	22	123834	-1.1224	0.86915	0.997074286
CCNH	ENSG00000134480	5	86677311	86718836	23	3	123834	-1.1228	0.86923	0.997074286
TSPAN12	ENSG00000106025	7	120417376	120508456	115	10	123834	-1.1229	0.86927	0.997074286
OBSCN	ENSG00000154358	1	228385831	228576577	368	18	123834	-1.1234	0.86938	0.997074286
PKHD1	ENSG00000170927	6	51470098	51962423	1041	41	123834	-1.1236	0.86941	0.997074286
SNX21	ENSG00000124104	20	44452449	44481914	56	10	123834	-1.1239	0.86947	0.997074286
MRPS18B	ENSG00000204568	6	30575486	30604172	40	6	123834	-1.1241	0.86952	0.997074286
CLPTM1	ENSG00000104853	19	45447842	45506599	149	11	123834	-1.1243	0.86956	0.997074286
C11orf16	ENSG00000176029	11	8931623	8964553	99	7	123834	-1.1244	0.86957	0.997074286
LHX6	ENSG00000106852	9	124954856	125001905	21	5	123834	-1.1244	0.86958	0.997074286
UBA2	ENSG00000126261	19	34909257	34970853	78	8	123834	-1.1245	0.86959	0.997074286
LEO1	ENSG00000166477	15	52220222	52274003	82	3	123834	-1.1246	0.86962	0.997074286
KIF1B	ENSG00000054523	1	10260863	10451661	343	11	123834	-1.1248	0.86966	0.997074286
RRNAD1	ENSG00000143303	1	156688234	156716752	29	7	123834	-1.125	0.8697	0.997074286
NEUROG1	ENSG00000181965	5	134859991	134881639	18	4	123834	-1.125	0.86971	0.997074286
TRAF3IP3	ENSG00000009790	1	209919377	209965668	96	10	123834	-1.1254	0.8698	0.997074286
ZNF493	ENSG00000196268	19	21569921	21620375	100	8	123834	-1.1257	0.86985	0.997074286
GNGT2	ENSG00000167083	17	47270153	47297936	47	8	123834	-1.1262	0.86996	0.997074286
TSG101	ENSG00000074319	11	18479883	18558779	137	12	123834	-1.1266	0.87004	0.997074286
REG3G	ENSG00000143954	2	79242812	79265631	98	7	123834	-1.1267	0.87006	0.997074286
FAM131A	ENSG00000175182	3	184043714	184074063	13	6	123834	-1.1267	0.87007	0.997074286
PUS7L	ENSG00000129317	12	44112410	44162620	104	7	123834	-1.1272	0.87018	0.997074286
PCDHAC2	ENSG00000243232	5	140335820	140401936	59	9	123834	-1.128	0.87033	0.997074286
CRAT	ENSG00000095321	9	131847089	131883468	43	1	123834	-1.1281	0.87035	0.997074286
EPPK1	ENSG00000227184	8	144929497	144962632	12	6	123834	-1.1281	0.87037	0.997074286
BSX	ENSG00000188909	11	122838278	122862428	62	5	123834	-1.1282	0.87038	0.997074286
POP1	ENSG00000104356	8	99119525	99182062	145	14	123834	-1.1283	0.87041	0.997074286
FAU	ENSG00000149806	11	64878100	64899945	22	5	123834	-1.1284	0.87042	0.997074286
AC020907.1	ENSG00000179066	19	35586873	35608141	17	2	123834	-1.1284	0.87042	0.997074286
EXOSC3	ENSG00000107371	9	37756975	37811434	111	8	123834	-1.1285	0.87044	0.997074286
OAZ3	ENSG00000143450	1	151725445	151753808	55	5	123834	-1.1286	0.87046	0.997074286
NACC1	ENSG00000160877	19	13218917	13261955	33	6	123834	-1.1289	0.87054	0.997074286
RAB28	ENSG00000157869	4	13352978	13495989	292	8	123834	-1.129	0.87055	0.997074286
TRIM43	ENSG00000144015	2	96247766	96275526	3	2	123834	-1.1293	0.87061	0.997074286
CPE	ENSG00000109472	4	166272346	166429472	393	15	123834	-1.1297	0.87071	0.997074286
FCRL2	ENSG00000132704	1	157705523	157756922	14	2	123834	-1.1299	0.87074	0.997074286
GPR151	ENSG00000173250	5	145882666	145905753	47	8	123834	-1.13	0.87076	0.997074286
TMEM26	ENSG00000196932	10	63156401	63223208	127	16	123834	-1.1303	0.87083	0.997074286
ATOX1	ENSG00000177556	5	151111877	151162093	67	6	123834	-1.1314	0.87105	0.997074286
PRKAR1A	ENSG00000108946	17	66497921	66557460	95	12	123834	-1.1314	0.87105	0.997074286
PNMT	ENSG00000141744	17	37814234	37836728	57	5	123834	-1.1314	0.87107	0.997074286
MSANTD3-TMEFF1	ENSG00000251349	9	103194553	103349918	287	13	123834	-1.132	0.87119	0.997074286
TMEFF1	ENSG00000241697	9	103194560	103349918	287	13	123834	-1.132	0.87119	0.997074286
ALG1	ENSG00000033011	16	5073703	5147380	43	11	123834	-1.1321	0.8712	0.997074286
EXOC3L1	ENSG00000179044	16	67208269	67234107	25	4	123834	-1.1325	0.87128	0.997074286
RP11-1085N6.3	ENSG00000259133	14	57105683	57207253	177	23	123834	-1.1329	0.87137	0.997074286
SAFB	ENSG00000160633	19	5613046	5678489	65	4	123834	-1.133	0.8714	0.997074286
RUNX1T1	ENSG00000079102	8	92957203	93125514	105	3	123834	-1.1336	0.87152	0.997074286
CCK	ENSG00000187094	3	42289317	42317699	22	6	123834	-1.1338	0.87155	0.997074286
GABPA	ENSG00000154727	21	27096881	27154771	70	6	123834	-1.1337	0.87155	0.997074286
SLCO2B1	ENSG00000137491	11	74801608	74927594	270	14	123834	-1.1339	0.87159	0.997074286
RP11-322E11.6	ENSG00000267140	18	33013835	33087904	38	6	123834	-1.1341	0.87162	0.997074286
LPHN2	ENSG00000117114	1	81761845	82468120	1390	123	123834	-1.1353	0.87188	0.997074286
SLC35D2	ENSG00000130958	9	99072988	99155992	218	14	123834	-1.1353	0.87188	0.997074286
RP11-169F17.1	ENSG00000263711	18	70808476	70941715	202	21	123834	-1.1353	0.87188	0.997074286
AC174470.1	ENSG00000215621	17	79770293	79794533	20	2	123834	-1.1355	0.87191	0.997074286
DDR2	ENSG00000162733	1	162591163	162767190	276	20	123834	-1.1358	0.87198	0.997074286
SLC11A2	ENSG00000110911	12	51363184	51432349	140	10	123834	-1.1359	0.87199	0.997074286
ACOT2	ENSG00000119673	14	74024324	74052357	51	10	123834	-1.1359	0.87201	0.997074286

DNAJB1	ENSG00000132002	19	14615582	14650582	44	6	123834	-1.136	0.87203	0.997074286
PDCD2L	ENSG00000126249	19	34885289	34927073	47	7	123834	-1.1362	0.87206	0.997074286
GCC1	ENSG00000179562	7	127210672	127243665	50	10	123834	-1.1368	0.87219	0.997074286
COQ9	ENSG00000088682	16	57471337	57505187	83	14	123834	-1.1368	0.87219	0.997074286
KMT2A	ENSG00000118058	11	118297205	118407539	51	4	123834	-1.1371	0.87225	0.997074286
CHKB	ENSG00000100288	22	51007378	51049884	139	21	123834	-1.1372	0.87227	0.997074286
NLRC4	ENSG00000091106	2	32439522	32500923	81	7	123834	-1.1372	0.87228	0.997074286
PTGS2	ENSG00000073756	1	186630923	186659559	83	7	123834	-1.1374	0.87231	0.997074286
RHCG	ENSG00000140519	15	89988680	90049844	27	4	123834	-1.1375	0.87234	0.997074286
CEP63	ENSG00000182923	3	134194585	134303859	202	4	123834	-1.1379	0.87241	0.997074286
CA7	ENSG00000168748	16	66868282	66898056	37	5	123834	-1.1379	0.87242	0.997074286
PNKP	ENSG00000039650	19	50354461	50381166	35	6	123834	-1.138	0.87244	0.997074286
CTD-2521M24.10	ENSG00000269035	19	17537420	17581613	36	7	123834	-1.1386	0.87257	0.997077295
SLC6A4	ENSG00000108576	17	28511337	28573020	48	7	123834	-1.1387	0.87258	0.997077295
TMPRSS11F	ENSG00000198092	4	68908916	69005598	223	7	123834	-1.1388	0.8726	0.997077295
FOXC2	ENSG00000176692	16	86590857	86612539	16	2	123834	-1.1397	0.8728	0.997152213
NCOR1	ENSG00000141027	17	15922471	16131499	294	8	123834	-1.1398	0.8728	0.997152213
C4orf29	ENSG00000164074	4	128876461	128970866	104	6	123834	-1.1399	0.87284	0.997152213
GHR	ENSG00000112964	5	42413879	42731979	439	30	123834	-1.1403	0.87292	0.997152213
GPR75-ASB3	ENSG00000115239	2	53749810	54097170	667	51	123834	-1.1407	0.873	0.997152213
CASZ1	ENSG00000130940	1	10686661	10866707	136	25	123834	-1.1408	0.87303	0.997152213
OR2Z1	ENSG00000181733	19	8831316	8852371	14	2	123834	-1.1409	0.87305	0.997152213
OR2T1	ENSG00000175143	1	248559296	248580405	83	3	123834	-1.1413	0.87312	0.997152213
GNG13	ENSG00000127588	16	838041	860733	49	8	123834	-1.1417	0.8732	0.997152213
CPA5	ENSG00000158525	7	129974630	130018571	86	12	123834	-1.1419	0.87324	0.997152213
CPSF6	ENSG00000111605	12	69623317	69678138	84	9	123834	-1.1424	0.87336	0.997152213
LRRC57	ENSG00000180979	15	42824720	42851000	23	3	123834	-1.1425	0.87337	0.997152213
SAP25	ENSG00000205307	7	100159855	100181270	21	3	123834	-1.1426	0.8734	0.997152213
LRCH4	ENSG00000077454	7	100159855	100193776	21	3	123834	-1.1426	0.8734	0.997152213
AC051642.1	ENSG00000268085	8	23402287	23430651	82	11	123834	-1.1431	0.8735	0.997206486
BOLA3	ENSG00000163170	2	74352525	74385121	47	8	123834	-1.1436	0.8736	0.997260753
ARHGEF16	ENSG00000130762	1	3360990	3407677	110	9	123834	-1.144	0.87369	0.997303598
AC079354.1	ENSG00000182329	2	202927978	203072585	155	24	123834	-1.1444	0.87378	0.997346438
TMEM144	ENSG00000164124	4	159112756	159186563	55	7	123834	-1.1449	0.87388	0.997400687
GRPEL2	ENSG00000164284	5	148714993	148744146	33	5	123834	-1.1455	0.874	0.997477754
RP11-463C8.4	ENSG00000259164	14	77554654	77701805	274	28	123834	-1.146	0.87411	0.997543401
ZAR1	ENSG00000182223	4	48482269	48506406	32	5	123834	-1.1469	0.8743	0.997640437
C16orf58	ENSG00000140688	16	31490792	31530630	44	5	123834	-1.1469	0.8743	0.997640437
AL136218.1	ENSG00000215462	13	49997495	50030554	53	8	123834	-1.1475	0.87442	0.99771473
TMEM87A	ENSG00000103978	15	42492730	42575861	74	5	123834	-1.1479	0.8745	0.99771473
ACVR1C	ENSG00000123612	2	158373279	158495517	91	13	123834	-1.1489	0.87469	0.99771473
FPR1	ENSG00000171051	19	52238425	52317363	135	26	123834	-1.149	0.87471	0.99771473
C10orf55	ENSG00000222047	10	75659727	75692535	16	3	123834	-1.1491	0.87474	0.99771473
RRP1B	ENSG00000160208	21	45069429	45125958	81	3	123834	-1.1492	0.87476	0.99771473
FLRT2	ENSG00000185070	14	85986488	86105034	194	26	123834	-1.1493	0.87479	0.99771473
KDM5B	ENSG00000117139	1	202686526	202788598	218	16	123834	-1.1495	0.87482	0.99771473
OR2T4	ENSG00000196944	1	248514883	248535929	102	6	123834	-1.1498	0.87488	0.99771473
CTRB1	ENSG00000168925	16	75242898	75268822	81	8	123834	-1.1498	0.87489	0.99771473
EGFL7	ENSG00000172889	9	139543308	139577130	14	4	123834	-1.1506	0.87506	0.99779743
MEI4	ENSG00000269964	6	78390375	78646691	699	18	123834	-1.1508	0.8751	0.99779743
APPL2	ENSG00000136044	12	105557074	105640016	173	16	123834	-1.1509	0.87512	0.99779743
HTN1	ENSG00000126550	4	70906119	70934562	64	8	123834	-1.1515	0.87523	0.997834502
IBA57	ENSG00000181873	1	228343516	228379958	42	6	123834	-1.1517	0.87528	0.997834502
GTPBP10	ENSG00000105793	7	89954537	90030769	183	14	123834	-1.1518	0.87531	0.997834502
RNF146	ENSG00000118518	6	127577755	127619712	71	8	123834	-1.1528	0.87551	0.997988211
PACSIN1	ENSG00000124507	6	34423916	34513006	86	12	123834	-1.1531	0.87557	0.997988211
C2orf47	ENSG00000162972	2	200810040	200883263	99	13	123834	-1.1534	0.87563	0.997988211
PC	ENSG00000173599	11	66605704	66735847	120	12	123834	-1.1542	0.87578	0.997988211
C1orf168	ENSG00000187889	1	57174477	57295369	152	31	123834	-1.1547	0.87589	0.997988211
MOB1A	ENSG00000114978	2	74369655	74416025	75	5	123834	-1.1552	0.876	0.997988211
LAMTOR5	ENSG00000134248	1	110933871	110960564	57	7	123834	-1.1553	0.87602	0.997988211
ZNF727	ENSG00000257482	7	63495821	63548927	171	6	123834	-1.1554	0.87604	0.997988211
CDX2	ENSG00000165556	13	28526274	28555276	50	8	123834	-1.1558	0.87613	0.997988211
FOXN1	ENSG00000109101	17	26823261	26875914	42	5	123834	-1.1559	0.87614	0.997988211
KIF19	ENSG00000196169	17	72312349	72361959	48	13	123834	-1.156	0.87616	0.997988211
ZNF677	ENSG00000197928	19	53721577	53768151	107	8	123834	-1.1561	0.87617	0.997988211
OR1D5	ENSG00000262628	17	2955963	2976901	1	1	123834	-1.1571	0.87638	0.997988211
POLRMT	ENSG00000099821	19	607223	643597	37	9	123834	-1.1575	0.87646	0.997988211
LUZPP1	ENSG00000268324	3	46588888	46611178	81	8	123834	-1.1583	0.87663	0.997988211
BTLA	ENSG00000186265	3	112172815	112228408	68	8	123834	-1.1591	0.87678	0.997988211
AL162424.1	ENSG00000269738	9	125138344	125158928	29	4	123834	-1.1591	0.8768	0.997988211
PRDM5	ENSG00000138738	4	121596074	121854025	737	36	123834	-1.1592	0.87681	0.997988211
KIAA1715	ENSG00000144320	2	176778620	176877567	187	17	123834	-1.1593	0.87683	0.997988211
AC007040.11	ENSG00000258881	2	71156448	71232466	62	14	123834	-1.1596	0.87689	0.997988211
OR52B1P	ENSG00000180909	11	6163006	6183818	25	5	123834	-1.1596	0.87689	0.997988211
DMGDH	ENSG00000132837	5	78283438	78541861	486	27	123834	-1.16	0.87697	0.997988211
HNRNPAB	ENSG00000197451	5	177621508	177648164	28	6	123834	-1.16	0.87698	0.997988211
GPNMB	ENSG00000136235	7	23265586	23324727	64	7	123834	-1.1603	0.87703	0.997988211
MLLT1	ENSG00000130382	19	6202966	6289959	221	18	123834	-1.1605	0.87708	0.997988211
HNF4G	ENSG00000164749	8	76310149	76489078	554	30	123834	-1.1608	0.87713	0.997988211
GPR128	ENSG00000144820	3	100318433	100424323	161	9	123834	-1.1612	0.87722	0.997988211
FAM86A	ENSG00000118894	16	5124305	5157809	32	6	123834	-1.1612	0.87722	0.997988211
CXCR6	ENSG00000172215	3	45972425	45999845	54	6	123834	-1.1616	0.87729	0.997988211
LNP1	ENSG00000206535	3	100110037	100185163	60	11	123834	-1.1616	0.87729	0.997988211
OR52B4	ENSG00000221996	11	4378493	4399616	19	4	123834	-1.1617	0.87732	0.997988211
PGS1	ENSG00000087157	17	76364721	76431195	171	10	123834	-1.1618	0.87734	0.997988211
GPR37	ENSG00000170775	7	124376051	124415681	55	9	123834	-1.162	0.87737	0.997988211
CHST15	ENSG00000182022	10	125757184	125863206	99	17	123834	-1.1623	0.87744	0.997988211
PSMC4	ENSG00000013275	19	40466912	40497348	47	4	123834	-1.1623	0.87745	0.997988211
RNF149	ENSG00000163162	2	101877681	101935163	45	9	123834	-1.1624	0.87746	0.997988211
TEX36	ENSG00000175018	10	127255091	127381713	118	10	123834	-1.1624	0.87747	0.997988211
PLEKHA4	ENSG00000105559	19	49330354	49381889	96	9	123834	-1.1627	0.87752	0.997988211
CNTN4	ENSG00000144619	3	2130497	3109645	1672	189	123834	-1.1629	0.87756	0.997988211
ZNF33A	ENSG00000189180	10	38289578	38364016	164	12	123834	-1.1635	0.87769	0.997988211
ZNF582	ENSG00000018869	19	56877413	56914914	44	11	123834	-1.1637	0.87773	0.997988211
ZNF606	ENSG00000166704	19	58478421	58524717	111	7	123834	-1.1637	0.87773	0.997988211
C9orf116	ENSG00000160345	9	138377027	138403580	35	6	123834	-1.1639	0.87777	0.997988211
PIK3R3	ENSG00000117461	1	46495812	46652160	168	9	123834	-1.1642	0.87782	0.997988211
C5orf17	ENSG00000248874	5	23941457	24188372	745	21	123834	-1.1642	0.87783	0.997988211

OSBPL11	ENSG00000144909	3	125237702	125323934	241	16	123834	-1.1643	0.87786	0.997988211
SNRPD3	ENSG00000100028	22	24941471	25015947	63	9	123834	-1.1648	0.87794	0.998002387
RANGAP1	ENSG00000100401	22	41631615	41692255	79	8	123834	-1.1651	0.87801	0.998002387
TMEM42	ENSG00000169964	3	44893361	44917162	16	4	123834	-1.1657	0.87813	0.998002387
EFCAB2	ENSG00000203666	1	245123007	245300466	267	15	123834	-1.1658	0.87816	0.998002387
SPERT	ENSG00000174015	13	46266446	46298694	48	8	123834	-1.1661	0.87821	0.998002387
EPHX4	ENSG00000172031	1	92485539	92539093	81	7	123834	-1.1662	0.87823	0.998002387
CARD16	ENSG00000204397	11	104902053	104982158	138	9	123834	-1.1662	0.87824	0.998002387
IDH1	ENSG00000138413	2	209090951	209140798	71	9	123834	-1.1669	0.87837	0.998027935
C9orf170	ENSG00000204446	9	89753559	89784471	81	6	123834	-1.1669	0.87838	0.998027935
IL15	ENSG00000164136	4	142547752	142665140	165	13	123834	-1.1671	0.87842	0.998027935
AL359091.2	ENSG00000268211	9	131052367	131074212	40	5	123834	-1.1679	0.87857	0.998079044
PRR20E	ENSG00000234278	13	57731331	57754352	3	1	123834	-1.1679	0.87857	0.998079044
GRM3	ENSG00000198822	7	86263230	86504200	295	24	123834	-1.1688	0.87876	0.998235229
PTPRO	ENSG00000151490	12	15465331	15760333	337	33	123834	-1.1692	0.87884	0.998266447
FGD6	ENSG00000180263	12	95460525	95621258	388	23	123834	-1.1696	0.87892	0.998297661
PCNXL3	ENSG00000197136	11	65373244	65414910	18	4	123834	-1.17	0.87901	0.998340229
GGT1	ENSG00000100031	22	24969718	25034972	29	3	123834	-1.171	0.87921	0.998479259
GRM2	ENSG00000164082	3	51731086	51762629	17	5	123834	-1.1713	0.87926	0.998479259
ZDHHC17	ENSG00000186908	12	77147368	77257476	133	10	123834	-1.1715	0.87929	0.998479259
TSPAN33	ENSG00000158457	7	128774712	128818671	56	9	123834	-1.172	0.87941	0.998487471
PPIL4	ENSG00000131013	6	149815869	149877174	44	1	123834	-1.1721	0.87943	0.998487471
VSNL1	ENSG00000163032	2	17710393	17848285	362	11	123834	-1.1723	0.87946	0.998487471
CAPZA1	ENSG00000116489	1	113151795	113224241	81	8	123834	-1.1726	0.87952	0.998487471
EMR2	ENSG00000127507	19	14833205	14899353	186	21	123834	-1.173	0.8796	0.998487471
SCNN1G	ENSG00000166828	16	23184036	23238204	144	10	123834	-1.1732	0.87964	0.998487471
GNB4	ENSG00000114450	3	179106990	179179378	59	11	123834	-1.1734	0.87968	0.998487471
KHDC1	ENSG00000135314	6	73941037	74030088	184	11	123834	-1.1738	0.87975	0.998487471
CRISPLD2	ENSG00000103196	16	84843590	84964374	192	42	123834	-1.1738	0.87977	0.998487471
KCTD9	ENSG00000104756	8	25275366	25325992	96	4	123834	-1.1751	0.88001	0.99859744
APOF	ENSG00000175336	12	56744353	56766607	12	2	123834	-1.175	0.88001	0.99859744
TUBGCP2	ENSG00000130640	10	135083135	135135841	84	7	123834	-1.1755	0.8801	0.99859744
ARHGAP9	ENSG00000123329	12	57856038	57892597	39	6	123834	-1.1765	0.8803	0.99859744
ARF5	ENSG00000004059	7	127218399	127241759	41	9	123834	-1.1765	0.88031	0.99859744
GCKR	ENSG00000084734	2	27709709	27756554	40	5	123834	-1.1771	0.88043	0.99859744
YPEL1	ENSG00000100027	22	22041833	22100123	93	17	123834	-1.1772	0.88043	0.99859744
TERF1	ENSG00000147601	8	73911099	73970357	85	6	123834	-1.1773	0.88045	0.99859744
KPNA7	ENSG00000185467	7	98761197	98815129	209	12	123834	-1.1776	0.88053	0.99859744
DDX55	ENSG00000111364	12	124076624	124115488	108	10	123834	-1.1777	0.88055	0.99859744
FAN1	ENSG00000198690	15	31186055	31245311	104	11	123834	-1.1779	0.88058	0.99859744
POLQ	ENSG00000051341	3	121140278	121274853	214	13	123834	-1.178	0.8806	0.99859744
RNASEH2C	ENSG00000172922	11	65472367	65498418	60	9	123834	-1.1781	0.88062	0.99859744
CNPY2	ENSG00000257727	12	56693626	56720120	26	2	123834	-1.1784	0.88069	0.99859744
ATG7	ENSG00000197548	3	11303995	11609139	476	16	123834	-1.1785	0.88071	0.99859744
CCDC112	ENSG00000164221	5	114592885	114642528	61	12	123834	-1.1787	0.88074	0.99859744
BHMT	ENSG00000145692	5	78397602	78438108	79	11	123834	-1.1788	0.88076	0.99859744
CBFA2T3	ENSG00000129993	16	88931266	89053612	247	20	123834	-1.1798	0.88096	0.998631284
TMC2	ENSG00000149488	20	2507253	2632430	288	17	123834	-1.1798	0.88096	0.998631284
CTSL	ENSG00000135047	9	90330434	90356308	56	6	123834	-1.1798	0.88097	0.998631284
CACUL1	ENSG00000151893	10	120423679	120524761	193	14	123834	-1.18	0.881	0.998631284
ATP6V0A2	ENSG00000185344	12	124186865	124256302	155	8	123834	-1.1805	0.8811	0.99863399
GABRD	ENSG00000187730	1	1940780	1972192	22	5	123834	-1.1808	0.88115	0.99863399
AGK	ENSG00000006530	7	141240989	141365044	113	5	123834	-1.1808	0.88116	0.99863399
AC079210.1	ENSG00000269657	17	66529369	66552970	37	5	123834	-1.1813	0.88125	0.998676444
LYZ	ENSG00000090382	12	69732121	69758014	80	8	123834	-1.182	0.88139	0.998775553
GRM6	ENSG00000113262	5	178395328	178433207	83	8	123834	-1.1832	0.88164	0.998837549
HNRNPCL1	ENSG00000179172	1	12897261	12918578	66	1	123834	-1.1833	0.88165	0.998837549
RBM20	ENSG00000203867	10	112394155	112609227	241	36	123834	-1.1835	0.88169	0.998837549
GPR108	ENSG00000125734	19	6719925	6747614	10	4	123834	-1.1835	0.88169	0.998837549
KRTAP12-4	ENSG00000212933	21	46064130	46084576	64	6	123834	-1.1838	0.88175	0.998837549
LYVE1	ENSG00000133800	11	10568513	10643236	115	15	123834	-1.1839	0.88176	0.998837549
NSMCE2	ENSG00000156831	8	126093921	126389362	458	35	123834	-1.1842	0.88184	0.998865756
TMEM38B	ENSG00000095209	9	108446825	108548893	243	6	123834	-1.1845	0.88189	0.998865756
PYROXD2	ENSG00000119943	10	100133322	100184941	154	7	123834	-1.1859	0.88217	0.999038284
PIWIL3	ENSG00000184571	22	25105001	25180687	138	14	123834	-1.1859	0.88218	0.999038284
STK38	ENSG00000112079	6	36451669	36525247	157	9	123834	-1.186	0.8822	0.999038284
GTF2E1	ENSG00000153767	3	120451484	120511916	104	12	123834	-1.187	0.88239	0.999193919
SMTNL2	ENSG00000188176	17	4477294	4521614	43	8	123834	-1.1879	0.88257	0.999236336
SLC2A8	ENSG00000136856	9	130149421	130180703	43	5	123834	-1.1882	0.88261	0.999236336
SCCPDH	ENSG00000143653	1	246877349	246941439	148	11	123834	-1.1882	0.88262	0.999236336
SLC10A5	ENSG00000253598	8	82595842	82618409	64	8	123834	-1.1887	0.88271	0.999236336
SIVA1	ENSG00000184990	14	105209437	105244831	52	7	123834	-1.189	0.88279	0.999236336
AMACR	ENSG00000242110	5	33976283	34018220	50	7	123834	-1.1891	0.8828	0.999236336
SENP8	ENSG00000166192	15	72396599	72443311	57	6	123834	-1.1899	0.88296	0.999236336
HENMT1	ENSG00000162639	1	109180912	109214148	35	3	123834	-1.1901	0.883	0.999236336
TIMM21	ENSG00000075336	18	71805746	71836197	99	5	123834	-1.1916	0.88329	0.999236336
HBG2	ENSG00000196565	11	5264420	5677019	1541	65	123834	-1.1918	0.88334	0.999236336
SPRR3	ENSG00000163209	1	152964223	152986332	112	2	123834	-1.1919	0.88335	0.999236336
BHLHE41	ENSG00000123095	12	26262959	26288060	30	6	123834	-1.1923	0.88343	0.999236336
EBF4	ENSG00000088881	20	2663524	2750753	70	10	123834	-1.1924	0.88344	0.999236336
SLC27A2	ENSG00000140284	15	50464393	50538592	185	15	123834	-1.1925	0.88347	0.999236336
MSANTD3	ENSG00000066697	9	103179438	103223511	100	5	123834	-1.193	0.88356	0.999236336
ZNF10	ENSG00000256223	12	133697161	133746051	92	8	123834	-1.1933	0.88362	0.999236336
KRTAP29-1	ENSG00000212658	17	39448078	39469103	63	5	123834	-1.1933	0.88363	0.999236336
FCER2	ENSG00000104921	19	7743644	7777032	131	8	123834	-1.1936	0.88368	0.999236336
EIF1	ENSG00000173812	17	39835137	39858920	35	2	123834	-1.1942	0.8838	0.999236336
SGCZ	ENSG00000185053	8	13937373	15105848	3356	148	123834	-1.1944	0.88384	0.999236336
FOXO1	ENSG00000150907	13	41119804	41250734	174	10	123834	-1.1947	0.8839	0.999236336
MEIG1	ENSG00000197889	10	14991438	15040049	118	7	123834	-1.1949	0.88394	0.999236336
SPINT4	ENSG00000149651	20	44340990	44364469	104	5	123834	-1.1952	0.88399	0.999236336
ZBTB21	ENSG00000173276	21	43396940	43440496	67	14	123834	-1.1957	0.88409	0.999236336
HSPA4L	ENSG00000164070	4	128692976	128771888	59	6	123834	-1.1962	0.88419	0.999236336
FLVCR2	ENSG00000119686	14	76034960	76139557	119	21	123834	-1.1962	0.8842	0.999236336
LPAR1	ENSG00000198121	9	113625543	113810981	260	23	123834	-1.1965	0.88425	0.999236336
RPS27L	ENSG00000185088	15	63408071	63460220	127	13	123834	-1.1967	0.88429	0.999236336
TMPRSS15	ENSG00000154646	21	19631433	19868197	199	35	123834	-1.1972	0.88439	0.999236336
RNPEPL1	ENSG00000142327	2	241495221	241530789	55	8	123834	-1.1976	0.88447	0.999236336
CDK5RAP2	ENSG00000136861	9	123141147	123352448	189	14	123834	-1.1976	0.88447	0.999236336
FBXO11	ENSG00000138081	2	48006455	48142932	236	13	123834	-1.1978	0.8845	0.999236336

CIAPIN1	ENSG00000005194	16	57452081	57491440	101	15	123834	-1.1987	0.88468	0.999236336
AC097381.1	ENSG00000228919	4	7930728	7952022	65	8	123834	-1.1991	0.88475	0.999236336
PTPLAD1	ENSG00000074696	15	65812756	65880687	111	6	123834	-1.1992	0.88478	0.999236336
PPP1R15A	ENSG00000087074	19	49365649	49389314	63	6	123834	-1.1998	0.88488	0.999236336
TTC39A	ENSG00000085831	1	51742930	51820788	89	13	123834	-1.2013	0.88519	0.999236336
METTL5	ENSG00000138382	2	170656591	170691441	87	14	123834	-1.2014	0.88521	0.999236336
TAS2R5	ENSG00000127366	7	141480017	141501166	35	2	123834	-1.2016	0.88524	0.999236336
CD52	ENSG00000169442	1	26634448	26657014	63	8	123834	-1.202	0.88532	0.999236336
OR5AK2	ENSG00000181273	11	56746347	56767342	43	9	123834	-1.2022	0.88535	0.999236336
MPP4	ENSG00000082126	2	202499593	202573417	61	12	123834	-1.2024	0.88539	0.999236336
MPC2	ENSG00000143158	1	167875967	167916278	44	3	123834	-1.2025	0.88541	0.999236336
SLC25A1	ENSG00000100075	22	19153095	19176343	52	8	123834	-1.2033	0.88557	0.999236336
AC068533.7	ENSG00000249319	7	65542756	65627228	181	12	123834	-1.2035	0.8856	0.999236336
SLC9A4	ENSG00000180251	2	103079762	103160431	238	15	123834	-1.2037	0.88565	0.999236336
RP11-618P17.4	ENSG00000266953	19	34877220	34910269	34	6	123834	-1.2039	0.88568	0.999236336
KCTD20	ENSG00000112078	6	36400544	36468920	111	7	123834	-1.2041	0.88572	0.999236336
NOS1AP	ENSG00000198929	1	162029564	162363321	701	59	123834	-1.2044	0.88579	0.999236336
MTIF3	ENSG00000122033	13	27999776	28034739	93	11	123834	-1.2049	0.88588	0.999236336
NPR3	ENSG00000113389	5	32679176	32801819	207	26	123834	-1.2051	0.88591	0.999236336
SLC25A13	ENSG00000004864	7	95739532	95961459	158	8	123834	-1.2054	0.88598	0.999236336
P2RX5	ENSG00000083454	17	3565493	3609698	42	8	123834	-1.2056	0.88601	0.999236336
YTHDC2	ENSG00000047188	5	112839380	112940982	317	13	123834	-1.2056	0.88602	0.999236336
GPR20	ENSG00000204882	8	142356600	142387367	43	8	123834	-1.2058	0.88605	0.999236336
AC016559.1	ENSG00000268012	5	78513714	78533911	28	4	123834	-1.206	0.88609	0.999236336
AL158147.2	ENSG00000269305	9	4849120	4869260	53	9	123834	-1.2062	0.88612	0.999236336
KIF1A	ENSG00000130294	2	241643181	241769725	170	23	123834	-1.2063	0.88614	0.999236336
SHISA8	ENSG00000234965	22	42297297	42320570	45	4	123834	-1.2068	0.88624	0.999236336
IZUMO3	ENSG00000205442	9	24532950	24555944	45	1	123834	-1.2073	0.88635	0.999236336
CIB1	ENSG00000185043	15	90763207	90787279	28	4	123834	-1.2075	0.88638	0.999236336
OR6Y1	ENSG00000197532	1	158506918	158527895	59	6	123834	-1.2081	0.8865	0.999236336
RC3H2	ENSG00000056586	9	125596835	125677620	104	10	123834	-1.2082	0.88651	0.999236336
NWD1	ENSG00000188039	19	16820787	16938774	149	17	123834	-1.2085	0.88658	0.999236336
WDR48	ENSG00000114742	3	39083489	39148155	129	14	123834	-1.2088	0.88663	0.999236336
CLCA4	ENSG00000016602	1	87002761	87056437	56	12	123834	-1.2089	0.88665	0.999236336
ZNF541	ENSG00000118156	19	48013942	48069113	41	4	123834	-1.2089	0.88665	0.999236336
LRRC63	ENSG00000173988	13	46776083	46861501	162	18	123834	-1.2092	0.8867	0.999236336
CTC-273B12.7	ENSG00000268465	19	48959094	48985688	44	5	123834	-1.2092	0.88671	0.999236336
RASL10B	ENSG00000141150	17	34048668	34080540	44	10	123834	-1.2094	0.88674	0.999236336
OTUD3	ENSG00000169914	1	20199006	20249438	93	14	123834	-1.2097	0.8868	0.999236336
FITM1	ENSG00000139914	14	24590484	24612058	25	3	123834	-1.2098	0.88682	0.999236336
CLDN16	ENSG00000113946	3	190030330	190139932	366	23	123834	-1.2102	0.8869	0.999236336
CEP57	ENSG00000166037	11	95513129	95575857	172	12	123834	-1.2113	0.8871	0.999236336
PLAU	ENSG00000122861	10	75658935	75687255	14	3	123834	-1.2117	0.88718	0.999236336
ISG20L2	ENSG00000143319	1	156681683	156708591	23	6	123834	-1.2117	0.88719	0.999236336
TAF2	ENSG00000064313	8	120733015	120855103	244	16	123834	-1.2118	0.88721	0.999236336
JKAMP	ENSG00000050130	14	59941161	59982128	123	8	123834	-1.212	0.88725	0.999236336
DIRC3	ENSG00000231672	2	218138742	218631316	781	51	123834	-1.2122	0.88727	0.999236336
KANK4	ENSG00000132854	1	62692651	62795085	214	25	123834	-1.2122	0.88728	0.999236336
UBR2	ENSG00000024048	6	42521800	42671242	226	17	123834	-1.2126	0.88736	0.999236336
ELFN2	ENSG00000166897	22	37754000	37833505	111	14	123834	-1.2126	0.88736	0.999236336
UBE2R2	ENSG00000107341	9	33807565	33930402	264	14	123834	-1.2127	0.88738	0.999236336
OR10G2	ENSG00000255582	14	22091992	22113096	45	8	123834	-1.2127	0.88739	0.999236336
ZNF746	ENSG00000181220	7	149159885	149204908	16	4	123834	-1.2134	0.8875	0.999236336
CCDC180	ENSG00000197816	9	99990765	100150806	358	10	123834	-1.2135	0.88752	0.999236336
OR52E8	ENSG00000183269	11	5867904	5888964	103	6	123834	-1.214	0.88763	0.999236336
SSRP1	ENSG00000149136	11	57083459	57113351	47	5	123834	-1.2141	0.88764	0.999236336
ZNF441	ENSG00000197044	19	11867815	11904893	103	8	123834	-1.2142	0.88767	0.999236336
FSTL1	ENSG00000163430	3	120101140	120180100	160	19	123834	-1.2143	0.88768	0.999236336
MYO1D	ENSG00000176658	17	30809540	31214195	400	36	123834	-1.2146	0.88774	0.999236336
ATXN7L3	ENSG00000087152	17	42259173	42287481	23	4	123834	-1.2146	0.88774	0.999236336
SREBF2	ENSG00000198911	22	42219109	42313312	155	17	123834	-1.2147	0.88776	0.999236336
TMEM246	ENSG00000165152	9	104225453	104305819	80	9	123834	-1.2151	0.88783	0.999236336
NOVA1	ENSG00000139910	14	26902299	27076960	198	8	123834	-1.2153	0.88788	0.999236336
DISC1	ENSG00000162946	1	231752561	232187018	522	64	123834	-1.2157	0.88795	0.999236336
PCSK9	ENSG00000169174	1	55495221	55540525	82	17	123834	-1.2159	0.88799	0.999236336
DNAJB13	ENSG00000187726	11	73651364	73691411	84	11	123834	-1.2162	0.88805	0.999236336
COX8A	ENSG00000176340	11	63732079	63754015	31	3	123834	-1.2163	0.88807	0.999236336
TEX261	ENSG00000144043	2	71203068	71232075	19	4	123834	-1.2166	0.88813	0.999236336
IBA57-AS1	ENSG00000203684	1	228341787	228363213	19	4	123834	-1.2168	0.88815	0.999236336
FCGR3B	ENSG00000162747	1	161582986	161611753	8	2	123834	-1.2169	0.88818	0.999236336
ZNF354C	ENSG00000177932	5	178477416	178520538	67	8	123834	-1.2171	0.88821	0.999236336
BARX1	ENSG00000131668	9	96703905	96727654	46	5	123834	-1.2171	0.88821	0.999236336
KRTAP3-1	ENSG00000212901	17	39154773	39185576	127	10	123834	-1.2171	0.88821	0.999236336
NGRN	ENSG00000182768	15	90798891	90826463	15	2	123834	-1.2177	0.88832	0.999236336
CLDN23	ENSG00000253958	8	8549448	8571616	85	3	123834	-1.2178	0.88834	0.999236336
NOL4	ENSG00000101746	18	31421064	31814916	433	30	123834	-1.218	0.88838	0.999236336
ZSCAN23	ENSG00000187987	6	28389707	28421279	76	9	123834	-1.2185	0.88848	0.999236336
RP11-729L2.2	ENSG00000267699	18	48484389	48594514	75	5	123834	-1.2188	0.88854	0.999236336
CCDC57	ENSG00000176155	17	80049336	80180706	457	6	123834	-1.219	0.88858	0.999236336
HNRNPA0	ENSG00000177733	5	137077075	137100039	12	3	123834	-1.2192	0.88861	0.999236336
RAB5A	ENSG00000144566	3	19978571	20036667	164	16	123834	-1.2192	0.88862	0.999236336
WASF1	ENSG00000112290	6	110411022	110511207	215	11	123834	-1.2193	0.88863	0.999236336
CRKL	ENSG00000099942	22	21261714	21318037	100	8	123834	-1.2193	0.88864	0.999236336
IKZF3	ENSG00000161405	17	37911198	38030441	211	4	123834	-1.2194	0.88866	0.999236336
AAAS	ENSG00000094914	12	53691240	53728648	40	3	123834	-1.2196	0.8887	0.999236336
TMEM63C	ENSG00000165548	14	77572911	77735838	311	30	123834	-1.22	0.88876	0.999236336
C19orf70	ENSG00000174917	19	5668432	5690907	19	2	123834	-1.22	0.88876	0.999236336
C19orf52	ENSG00000142444	19	11029409	11054211	29	3	123834	-1.2204	0.88885	0.999236336
OR52N2	ENSG00000180988	11	5831544	5852578	108	11	123834	-1.2208	0.88891	0.999236336
NUDT6	ENSG00000170917	4	123799852	123854123	172	19	123834	-1.2209	0.88894	0.999236336
RP11-247C2.2	ENSG00000248540	15	74408256	74431619	2	1	123834	-1.2215	0.88905	0.999236336
GFI1B	ENSG00000165702	9	135810932	135877083	84	18	123834	-1.2218	0.88911	0.999236336
PGAP2	ENSG00000148985	11	3808954	3857601	82	9	123834	-1.2221	0.88916	0.999236336
FCRL3	ENSG00000160856	1	157634111	157680647	80	8	123834	-1.2229	0.88931	0.999236336
MOSPD3	ENSG00000106330	7	100199725	100223007	10	3	123834	-1.2231	0.88935	0.999236336
GCDH	ENSG00000105607	19	12991840	13035021	52	6	123834	-1.2235	0.88943	0.999236336
H2AFY	ENSG00000113648	5	134659590	134745604	70	5	123834	-1.2238	0.88949	0.999236336
OR6N2	ENSG00000188340	1	158736472	158757425	44	11	123834	-1.2241	0.88955	0.999236336
TNFSF13	ENSG00000161955	17	7451609	7474925	35	9	123834	-1.2242	0.88956	0.999236336

C12orf66	ENSG00000174206	12	64570096	64626076	24	3	123834	-1.225	0.88971	0.999236336
LRRC36	ENSG00000159708	16	67350701	67429106	103	7	123834	-1.2253	0.88977	0.999236336
SLITRK1	ENSG00000178235	13	84441344	84466528	20	4	123834	-1.2257	0.88984	0.999236336
RMI1	ENSG00000178966	9	86585626	86628985	49	5	123834	-1.2257	0.88985	0.999236336
C2orf62	ENSG00000158428	2	219211579	219242822	50	9	123834	-1.2259	0.88987	0.999236336
PACSIN2	ENSG00000100266	22	43221418	43421151	477	13	123834	-1.2259	0.88987	0.999236336
FZD6	ENSG00000164930	8	104300661	104355094	147	14	123834	-1.2259	0.88989	0.999236336
ZCCHC11	ENSG00000134744	1	52863954	53029159	171	10	123834	-1.226	0.8899	0.999236336
ZC3H11A	ENSG00000058673	1	203754782	203833252	152	8	123834	-1.2262	0.88995	0.999236336
CES4A	ENSG00000172824	16	67012492	67053661	23	4	123834	-1.2265	0.88999	0.999236336
RNF44	ENSG00000146083	5	175943698	175975026	26	4	123834	-1.2265	0.89	0.999236336
TGM5	ENSG00000104055	15	43514793	43569055	56	4	123834	-1.2266	0.89001	0.999236336
KLF10	ENSG00000155090	8	103651007	103678130	57	10	123834	-1.2268	0.89004	0.999236336
WASF2	ENSG00000158195	1	27720730	27826669	93	2	123834	-1.2268	0.89005	0.999236336
TMEM108	ENSG00000144868	3	132747235	133126636	742	35	123834	-1.2278	0.89023	0.999246438
ONECUT3	ENSG00000205922	19	1742372	1790987	36	7	123834	-1.2282	0.89031	0.999246438
ERH	ENSG00000100632	14	69836848	69875344	74	11	123834	-1.2283	0.89034	0.999246438
CLEC17A	ENSG00000187912	19	14683896	14731969	95	11	123834	-1.229	0.89046	0.999246438
NAA25	ENSG00000111300	12	112454500	112556826	73	8	123834	-1.2294	0.89054	0.999246438
NOX5	ENSG00000255346	15	69212864	69365083	175	13	123834	-1.2295	0.89055	0.999246438
C4orf3	ENSG00000164096	4	120208207	120235600	68	9	123834	-1.2296	0.89059	0.999246438
BCL2L14	ENSG00000121380	12	12192778	12374018	227	26	123834	-1.2298	0.89062	0.999246438
OCA2	ENSG00000104044	15	27990021	28354504	645	39	123834	-1.2302	0.89068	0.999246438
ABCA8	ENSG00000141338	17	66853433	66961533	153	15	123834	-1.2301	0.89068	0.999246438
KNOP1	ENSG00000103550	16	19704902	19739557	18	3	123834	-1.2306	0.89076	0.999246438
DLAT	ENSG00000150768	11	111885538	111945114	33	2	123834	-1.2308	0.8908	0.999246438
FGF22	ENSG00000070388	19	629879	653703	20	6	123834	-1.2312	0.89087	0.999246438
SLC25A15	ENSG00000102743	13	41353548	41394247	80	8	123834	-1.2322	0.89105	0.999246438
ASCL3	ENSG00000176009	11	8949119	8974580	78	7	123834	-1.2322	0.89106	0.999246438
TRMT5	ENSG00000126814	14	61428169	61458076	31	4	123834	-1.2322	0.89107	0.999246438
ACTG1	ENSG00000184009	17	79466997	79500873	117	12	123834	-1.2325	0.89113	0.999246438
GSTA5	ENSG00000182793	6	52686453	52720893	74	6	123834	-1.2328	0.89117	0.999246438
ZFP57	ENSG00000204644	6	29630169	29658887	197	20	123834	-1.2329	0.89119	0.999246438
WLS	ENSG00000116729	1	68554142	68708803	306	48	123834	-1.2336	0.89132	0.999246438
COL8A1	ENSG00000144810	3	99347319	99528070	320	22	123834	-1.2337	0.89134	0.999246438
SDCBP2	ENSG00000125775	20	1280619	1319883	43	8	123834	-1.2338	0.89135	0.999246438
TRMT44	ENSG00000155275	4	8427867	8505258	183	18	123834	-1.234	0.8914	0.999246438
EBF3	ENSG00000108001	10	131623547	131772105	88	23	123834	-1.2342	0.89143	0.999246438
C20orf96	ENSG00000196476	20	241504	281390	60	11	123834	-1.2342	0.89143	0.999246438
PRPF38A	ENSG00000134748	1	52860236	52896511	19	3	123834	-1.2343	0.89145	0.999246438
IL20RA	ENSG00000016402	6	137311108	137376298	72	10	123834	-1.2347	0.89152	0.999246438
HN1L	ENSG00000206053	16	1718257	1762281	41	2	123834	-1.2351	0.8916	0.999246438
SYNE4	ENSG00000181392	19	36484209	36509695	23	7	123834	-1.2351	0.89161	0.999246438
PDC	ENSG00000116703	1	186402698	186440254	47	5	123834	-1.2365	0.89187	0.999246438
EVX1	ENSG00000106038	7	27272164	27300112	39	5	123834	-1.237	0.89196	0.999246438
ANKRD7	ENSG00000106013	7	117844727	117892785	102	7	123834	-1.237	0.89196	0.999246438
SBK2	ENSG00000187550	19	56031100	56058456	41	11	123834	-1.2375	0.89204	0.999246438
FAM71B	ENSG00000170613	5	156578857	156603275	53	7	123834	-1.2376	0.89206	0.999246438
ECD	ENSG00000122882	10	74879913	74938813	125	1	123834	-1.2382	0.89218	0.999246438
GPR144	ENSG00000180264	9	127203423	127249379	15	3	123834	-1.2383	0.89219	0.999246438
XCR1	ENSG00000173578	3	46048516	46079234	81	5	123834	-1.2387	0.89228	0.999246438
MFF	ENSG00000168958	2	228179867	228232550	119	10	123834	-1.2394	0.89241	0.999246438
SOGA1	ENSG00000149639	20	35395845	35502089	56	12	123834	-1.2399	0.89249	0.999246438
RPA1	ENSG00000132383	17	1722996	1813376	92	13	123834	-1.24	0.89251	0.999246438
CABS1	ENSG00000145309	4	71190683	71212833	119	9	123834	-1.2405	0.89261	0.999246438
RP11-455G16.1	ENSG00000178636	4	120103928	120143799	94	12	123834	-1.2407	0.89264	0.999246438
ECHS1	ENSG00000127884	10	135165984	135197193	75	7	123834	-1.241	0.8927	0.999246438
RP1	ENSG00000104237	8	55518627	55553394	34	5	123834	-1.2414	0.89276	0.999246438
FAM96B	ENSG00000166595	16	66955959	66978326	10	3	123834	-1.2413	0.89276	0.999246438
F2RL1	ENSG00000164251	5	76104758	76141140	30	4	123834	-1.2414	0.89278	0.999246438
CCDC125	ENSG00000183323	5	68566002	68638636	106	11	123834	-1.2415	0.8928	0.999246438
HUS1B	ENSG00000188996	6	645939	666963	31	7	123834	-1.2418	0.89284	0.999246438
KAT5	ENSG00000172977	11	65469467	65497075	63	9	123834	-1.2422	0.89292	0.999246438
ANKLE2	ENSG00000176915	12	133292254	133348474	116	19	123834	-1.2425	0.89298	0.999246438
CNTFR	ENSG00000122756	9	34541430	34600121	83	12	123834	-1.2428	0.89303	0.999246438
FRZB	ENSG00000162998	2	183688002	183741890	72	9	123834	-1.243	0.89307	0.999246438
ALDH3B1	ENSG00000006534	11	67766048	67806744	36	4	123834	-1.2431	0.89308	0.999246438
CD1B	ENSG00000158485	1	158287741	158311321	40	6	123834	-1.2432	0.8931	0.999246438
RNF41	ENSG00000181852	12	56588285	56625717	12	3	123834	-1.2432	0.89311	0.999246438
LARP1B	ENSG00000138709	4	128972423	129154086	293	9	123834	-1.2433	0.89313	0.999246438
LETM1	ENSG00000168924	4	1803206	1867974	12	4	123834	-1.2434	0.89315	0.999246438
CAMK1D	ENSG00000183049	10	12381481	12887545	820	101	123834	-1.2438	0.89321	0.999246438
CCDC39	ENSG00000145075	3	180310646	180598793	430	22	123834	-1.244	0.89325	0.999246438
MAGI3	ENSG00000081026	1	113923371	114238545	426	22	123834	-1.2444	0.89332	0.999246438
DSTN	ENSG00000125868	20	17540508	17600564	105	13	123834	-1.2445	0.89334	0.999246438
FGF6	ENSG00000111241	12	4527321	4564780	28	4	123834	-1.2451	0.89345	0.999246438
KCTD12	ENSG00000178695	13	77444312	77470540	35	10	123834	-1.2453	0.89348	0.999246438
OR9G1	ENSG00000174914	11	56457864	56478781	52	7	123834	-1.246	0.89361	0.999246438
SPINK5	ENSG00000133710	5	147395246	147526852	481	16	123834	-1.246	0.89362	0.999246438
FOSL2	ENSG00000075426	2	28605315	28650179	30	4	123834	-1.2465	0.89371	0.999246438
EML4	ENSG00000143924	2	42386490	42569688	306	13	123834	-1.2465	0.89371	0.999246438
AC024257.1	ENSG00000269282	12	48749919	48771738	37	6	123834	-1.2465	0.89371	0.999246438
RNF170	ENSG00000120925	8	42694780	42762433	57	6	123834	-1.2466	0.89373	0.999246438
MYEOV2	ENSG00000172428	2	241055980	241086224	138	12	123834	-1.2467	0.89374	0.999246438
DAGLB	ENSG00000164535	7	6438757	6533821	247	28	123834	-1.2477	0.89393	0.999246438
PDE11A	ENSG00000128655	2	178477980	178983066	1467	42	123834	-1.2479	0.89397	0.999246438
CLTCL1	ENSG00000070371	22	19156986	19289239	273	12	123834	-1.2482	0.89402	0.999246438
CCDC59	ENSG00000133773	12	82607460	82762584	256	14	123834	-1.2485	0.89408	0.999246438
C14orf1	ENSG00000133935	14	76106134	76137532	31	7	123834	-1.2489	0.89415	0.999246438
PHYKPL	ENSG00000175309	5	177625498	177669792	58	12	123834	-1.2492	0.8942	0.999246438
PAF1	ENSG00000006712	19	39866492	39891835	32	7	123834	-1.2493	0.89422	0.999246438
TNFSF12	ENSG00000239697	17	7442208	7474925	65	11	123834	-1.2493	0.89423	0.999246438
TNFSF12-TNFSF13	ENSG00000248871	17	7442416	7474918	65	11	123834	-1.2493	0.89423	0.999246438
MRPL49	ENSG00000149792	11	64879655	64904843	24	5	123834	-1.2497	0.8943	0.999246438
H1FX	ENSG00000184897	3	129023615	129045120	28	3	123834	-1.2501	0.89436	0.999246438
THRA	ENSG00000126351	17	38204543	38260120	33	9	123834	-1.2505	0.89444	0.999246438
HS3ST1	ENSG00000002587	4	11384774	11441389	81	20	123834	-1.251	0.89454	0.999246438
PPP1R14A	ENSG00000167641	19	38731877	38757231	70	9	123834	-1.2511	0.89454	0.999246438
EPB42	ENSG00000166947	15	43388423	43523481	74	10	123834	-1.2512	0.89456	0.999246438

OAF	ENSG00000184232	11	120071475	120111041	99	13	123834	-1.2513	0.89458	0.999246438
APBB1	ENSG00000166313	11	6406355	6450644	29	6	123834	-1.2517	0.89465	0.999265907
GSTA2	ENSG00000244067	6	52604897	52638367	75	6	123834	-1.2534	0.89497	0.999428793
ESYT2	ENSG00000117868	7	158513686	158632944	362	12	123834	-1.2536	0.895	0.999428793
FXR2	ENSG00000129245	17	7484548	7528189	47	9	123834	-1.2539	0.89506	0.999428793
OR8J3	ENSG00000167822	11	55894247	55915194	64	6	123834	-1.2545	0.89517	0.999428793
UBAP1L	ENSG00000246922	15	65375338	65417535	17	3	123834	-1.2548	0.89522	0.999428793
SETD8	ENSG00000183955	12	123858320	123903905	86	10	123834	-1.2552	0.8953	0.999428793
ECHDC1	ENSG00000093144	6	127599855	127674754	116	10	123834	-1.2553	0.89532	0.999428793
ZNF445	ENSG00000185219	3	44471262	44529162	50	6	123834	-1.2558	0.89541	0.999428793
PLXNC1	ENSG00000136040	12	94532499	94711451	269	41	123834	-1.2558	0.89541	0.999428793
CCDC113	ENSG00000103021	16	58255061	58327740	232	17	123834	-1.2559	0.89543	0.999428793
RNASET2	ENSG00000026297	6	167332992	167380679	105	13	123834	-1.256	0.89544	0.999428793
C1ORF220	ENSG00000184909	1	178501950	178527579	76	5	123834	-1.2562	0.89548	0.999428793
OCIAD2	ENSG00000145247	4	48877036	48918954	51	6	123834	-1.2568	0.89559	0.999428793
SETDB2	ENSG00000136169	13	50008429	50079138	153	13	123834	-1.2569	0.8956	0.999428793
VWF	ENSG00000110799	12	6048040	6243936	318	45	123834	-1.2578	0.89577	0.999428793
PTPRU	ENSG00000060656	1	29553028	29663325	122	20	123834	-1.258	0.8958	0.999428793
LPPR1	ENSG00000148123	9	103780991	104097417	648	45	123834	-1.258	0.89581	0.999428793
OR52N5	ENSG00000181009	11	5788864	5809897	2	1	123834	-1.2583	0.89585	0.999428793
TAS2R19	ENSG00000212124	12	11164218	11185219	100	10	123834	-1.2584	0.89587	0.999428793
LARGE	ENSG00000133424	22	33548212	34328829	1523	134	123834	-1.2584	0.89588	0.999428793
SEMA3D	ENSG00000153993	7	84614869	84826171	151	15	123834	-1.2585	0.8959	0.999428793
PRRC2B	ENSG00000130723	9	134259480	134385584	156	8	123834	-1.259	0.89598	0.999442469
SLC9C2	ENSG00000162753	1	173459603	173582233	216	10	123834	-1.2594	0.89606	0.999442469
HEATR5B	ENSG00000008869	2	37185526	37321485	222	22	123834	-1.2595	0.89607	0.999442469
MFSD8	ENSG00000164073	4	128828960	128897150	66	4	123834	-1.2602	0.8962	0.99946041
SPIN1	ENSG00000106723	9	90993334	91103609	159	2	123834	-1.2603	0.89621	0.99946041
DYTN	ENSG00000232125	2	207506345	207593120	251	27	123834	-1.2613	0.89641	0.99946041
RND2	ENSG00000108830	17	41167258	41194057	1	1	123834	-1.2621	0.89654	0.99946041
MS4A10	ENSG00000172689	11	60542821	60578778	40	6	123834	-1.2621	0.89655	0.99946041
OR5K4	ENSG00000196098	3	98062698	98083663	34	6	123834	-1.2624	0.89659	0.99946041
COX10	ENSG00000006695	17	13962813	14121994	248	31	123834	-1.2624	0.8966	0.99946041
C1orf220	ENSG00000213057	1	178501887	178528024	78	5	123834	-1.2624	0.89661	0.99946041
ZFP69	ENSG00000187815	1	40932887	40972015	76	9	123834	-1.2625	0.89662	0.99946041
TTC8	ENSG00000165533	14	89280497	89354335	82	6	123834	-1.2628	0.89667	0.99946041
AP000721.4	ENSG00000256100	11	63732092	63765818	42	3	123834	-1.2631	0.89672	0.99946041
RNF151	ENSG00000179580	16	2006824	2028976	80	9	123834	-1.2638	0.89684	0.99946041
PPP1R9A	ENSG00000158528	7	94526514	94935727	332	22	123834	-1.2638	0.89685	0.99946041
ABCB8	ENSG00000197150	7	150715510	150754869	40	8	123834	-1.2648	0.89703	0.99946041
MKNK1	ENSG00000079277	1	47013090	47092515	92	10	123834	-1.265	0.89706	0.99946041
ECEL1	ENSG00000171551	2	233334537	233362538	40	10	123834	-1.2651	0.89707	0.99946041
ADAM8	ENSG00000151651	10	135065907	135100372	66	5	123834	-1.2651	0.89709	0.99946041
DDRGK1	ENSG00000198171	20	3160996	3195331	91	13	123834	-1.2658	0.89722	0.99946041
AC092687.4	ENSG00000232056	2	10977703	11005081	64	6	123834	-1.2666	0.89735	0.99946041
ZNF19	ENSG00000157429	16	71488453	71608992	118	15	123834	-1.2666	0.89735	0.99946041
ZNF404	ENSG00000176222	19	44366515	44415537	70	3	123834	-1.2672	0.89745	0.99946041
F3	ENSG00000117525	1	94984781	95017356	8	2	123834	-1.2672	0.89746	0.99946041
MED20	ENSG00000124641	6	41863092	41898877	47	10	123834	-1.2672	0.89746	0.99946041
RP11-58C22.1	ENSG00000261833	16	76577314	76679520	128	17	123834	-1.2677	0.89754	0.99946041
SCAF8	ENSG00000213079	6	155044459	155165192	164	11	123834	-1.2678	0.89757	0.99946041
GDF6	ENSG00000156466	8	97144562	97183020	40	7	123834	-1.268	0.89761	0.99946041
ABCC6	ENSG00000091262	16	16232785	16327379	115	18	123834	-1.2684	0.89766	0.99946041
OR2T27	ENSG00000187701	1	248803232	248824185	9	2	123834	-1.2686	0.89771	0.99946041
CHRNA7	ENSG00000175344	15	32312691	32474722	184	17	123834	-1.2695	0.89787	0.99946041
CAV1	ENSG00000105974	7	116154839	116211233	101	11	123834	-1.2705	0.89805	0.99946041
RASL10A	ENSG00000100276	22	29698922	29725668	33	8	123834	-1.271	0.89814	0.99946041
PXDN	ENSG00000130508	2	1625659	1758624	166	28	123834	-1.2716	0.89824	0.99946041
RABL3	ENSG00000144840	3	120395528	120471840	115	16	123834	-1.2718	0.89827	0.99946041
RASGEF1B	ENSG00000138670	4	82337547	82975397	1308	68	123834	-1.2719	0.8983	0.99946041
PSMB1	ENSG00000008018	6	170834205	170872429	79	6	123834	-1.272	0.89832	0.99946041
PTPN1	ENSG00000196396	20	49116891	49211299	148	15	123834	-1.273	0.89849	0.99946041
IL1F10	ENSG00000136697	2	113815547	113843427	146	14	123834	-1.2731	0.89851	0.99946041
OR5H15	ENSG00000233412	3	97877544	97898485	85	3	123834	-1.2732	0.89853	0.99946041
LRRC43	ENSG00000158113	12	122642285	122698018	61	8	123834	-1.2737	0.89862	0.99946041
HYOU1	ENSG00000149428	11	118904900	118937913	41	5	123834	-1.2742	0.8987	0.99946041
NXN	ENSG00000167693	17	692553	893010	376	67	123834	-1.2749	0.89883	0.99946041
HHEX	ENSG00000152804	10	94437945	94465403	40	8	123834	-1.275	0.89885	0.99946041
SREK1	ENSG00000153914	5	65425799	65489443	66	5	123834	-1.2753	0.89889	0.99946041
FBXO6	ENSG00000116663	1	11714181	11744411	35	8	123834	-1.2759	0.89901	0.99946041
CGNL1	ENSG00000128849	15	57658165	57852925	388	49	123834	-1.2766	0.89912	0.99946041
SLC38A9	ENSG00000177058	5	54911673	55079022	676	23	123834	-1.2767	0.89915	0.99946041
IGF2R	ENSG00000197081	6	160380131	160544539	262	31	123834	-1.2769	0.89918	0.99946041
HNMT	ENSG00000150540	2	138711590	138783930	80	4	123834	-1.277	0.89919	0.99946041
PTTG1	ENSG00000164611	5	159838829	159865748	25	7	123834	-1.2771	0.89922	0.99946041
ZFR	ENSG00000056097	5	32344456	32454867	187	15	123834	-1.2773	0.89925	0.99946041
UTRN	ENSG00000152818	6	144596837	145184170	729	59	123834	-1.2776	0.89931	0.99946041
CRCP	ENSG00000241258	7	65569591	65629555	129	10	123834	-1.2776	0.89931	0.99946041
VPS11	ENSG00000160695	11	118928403	118962688	52	4	123834	-1.2776	0.89931	0.99946041
FRMD5	ENSG00000171877	15	44152962	44497450	323	11	123834	-1.2778	0.89935	0.99946041
ZNF501	ENSG00000186446	3	44761088	44788575	24	3	123834	-1.2784	0.89945	0.99946041
CHI3L1	ENSG00000133048	1	203138059	203165877	42	10	123834	-1.279	0.89955	0.99946041
UBE2G1	ENSG00000132388	17	4162554	4279923	127	4	123834	-1.2793	0.8996	0.99946041
SNX24	ENSG00000064652	5	122169134	122375049	558	17	123834	-1.2798	0.89968	0.99946041
SLC3A1	ENSG00000138079	2	44492599	44558633	77	8	123834	-1.2798	0.8997	0.99946041
XAF1	ENSG00000132530	17	6648766	6688966	65	10	123834	-1.2807	0.89985	0.99946041
FAM24B	ENSG00000213185	10	124598594	124649157	53	9	123834	-1.2809	0.89988	0.99946041
NUP98	ENSG00000110713	11	3682313	3829022	301	17	123834	-1.2809	0.89989	0.99946041
MEIS2	ENSG00000134138	15	37171406	37403504	138	36	123834	-1.2809	0.89989	0.99946041
ANHX	ENSG00000227059	12	133784898	133822681	44	6	123834	-1.2816	0.9	0.99946041
OR4C15	ENSG00000181939	11	55311783	55332895	119	8	123834	-1.283	0.90026	0.99946041
TMC7	ENSG00000170537	16	18985256	19085264	178	13	123834	-1.2831	0.90028	0.99946041
PCDHAC1	ENSG00000248383	5	140296302	140401929	163	10	123834	-1.2833	0.9003	0.99946041
CHEK1	ENSG00000149554	11	125485036	125556150	140	13	123834	-1.2833	0.9003	0.99946041
IDH3A	ENSG00000166411	15	78413840	78474291	110	15	123834	-1.2833	0.9003	0.99946041
LYSMD3	ENSG00000176018	5	89801428	89835401	41	4	123834	-1.2834	0.90032	0.99946041
SPRR1A	ENSG00000169474	1	152946557	152968289	51	2	123834	-1.284	0.90043	0.99946041
CLDN20	ENSG00000171217	6	155575147	155607682	120	11	123834	-1.2843	0.90048	0.99946041
NUDT12	ENSG00000112874	5	102874556	102908494	32	4	123834	-1.2845	0.90052	0.99946041

TRAFD1	ENSG00000135148	12	112553305	112601407	31	4	123834	-1.2846	0.90053	0.99946041
ACTL7A	ENSG00000187003	9	111614603	111636035	62	11	123834	-1.2847	0.90056	0.99946041
VSTM2A	ENSG00000170419	7	54600018	54648773	106	11	123834	-1.2854	0.90067	0.99946041
ZNF250	ENSG00000196150	8	146066632	146137553	88	7	123834	-1.2854	0.90067	0.99946041
TNFRSF19	ENSG00000127863	13	24134509	24260232	193	22	123834	-1.2863	0.90082	0.99946041
ERP44	ENSG00000023318	9	102731461	102871322	164	9	123834	-1.2863	0.90083	0.99946041
LAP3	ENSG00000002549	4	17568815	17619595	110	3	123834	-1.2864	0.90084	0.99946041
EPX	ENSG00000121053	17	56260098	56292535	35	8	123834	-1.287	0.90095	0.99946041
KIAA1147	ENSG00000257093	7	141346528	141411953	55	5	123834	-1.2871	0.90097	0.99946041
OR51L1	ENSG00000176798	11	5010213	5031160	42	8	123834	-1.2873	0.90101	0.99946041
CDYL2	ENSG00000166446	16	80621803	80848226	433	42	123834	-1.2875	0.90104	0.99946041
IRF6	ENSG00000117595	1	209949036	209989465	105	11	123834	-1.2878	0.90109	0.99946041
TAPT1	ENSG00000169762	4	16152128	16239033	221	20	123834	-1.2879	0.90111	0.99946041
KRT35	ENSG00000197079	17	39622941	39647392	100	7	123834	-1.288	0.90113	0.99946041
OR52A1	ENSG00000182070	11	5162239	5217612	151	10	123834	-1.2884	0.90119	0.99946041
MAP3K9	ENSG00000006432	14	71179243	71286251	260	15	123834	-1.2885	0.90122	0.99946041
GART	ENSG00000159131	21	34866238	34925797	63	8	123834	-1.289	0.90129	0.99946041
SGSM3	ENSG00000100359	22	40756595	40816293	49	5	123834	-1.2891	0.90131	0.99946041
ARHGEF19	ENSG00000142632	1	16514349	16549104	88	6	123834	-1.2891	0.90132	0.99946041
LALBA	ENSG00000167531	12	48951467	48973849	25	6	123834	-1.2891	0.90132	0.99946041
HBP1	ENSG00000105856	7	106799406	106852974	73	5	123834	-1.2892	0.90134	0.99946041
FP15737	ENSG00000215298	8	23420157	23442974	90	10	123834	-1.2892	0.90134	0.99946041
CSNK2A2	ENSG00000070770	16	58181811	58241824	137	11	123834	-1.2895	0.90138	0.99946041
MPZL3	ENSG00000160588	11	118087409	118133065	125	8	123834	-1.2895	0.90139	0.99946041
SERTAD4	ENSG00000082497	1	210396144	210429976	31	5	123834	-1.2896	0.90141	0.99946041
HKR1	ENSG00000181666	19	37793739	37870267	107	10	123834	-1.2899	0.90145	0.99946041
EIF6	ENSG00000242372	20	33856714	33882788	24	3	123834	-1.2901	0.90148	0.99946041
STXBP5L	ENSG00000145087	3	120616919	121153608	1469	24	123834	-1.2901	0.9015	0.99946041
CD58	ENSG00000116815	1	117047157	117123661	38	6	123834	-1.2906	0.90157	0.99946041
HECW1	ENSG00000002746	7	43142198	43615600	730	71	123834	-1.2906	0.90158	0.99946041
ARHGAP33	ENSG00000004777	19	36255434	36289724	68	9	123834	-1.2908	0.90162	0.99946041
GLT6D1	ENSG00000204007	9	138505502	138541386	87	17	123834	-1.2914	0.90172	0.99946041
HJURP	ENSG00000123485	2	234732062	234773212	146	9	123834	-1.2917	0.90176	0.99946041
FBXW2	ENSG00000119402	9	123504256	123565690	51	4	123834	-1.2917	0.90177	0.99946041
RP11-6L6.2	ENSG00000250673	4	147135709	147160208	32	7	123834	-1.2919	0.90181	0.99946041
CYC1	ENSG00000179091	8	145139930	145162428	30	1	123834	-1.292	0.90183	0.99946041
AMPD3	ENSG00000133805	11	10319860	10539126	440	54	123834	-1.2923	0.90187	0.99946041
RPS6KB2	ENSG00000175634	11	67185931	67212872	21	3	123834	-1.2929	0.90198	0.999524039
LYPD8	ENSG00000266949	1	248892716	248913150	14	2	123834	-1.2935	0.90208	0.99955652
CHAC2	ENSG00000143942	2	53984929	54012333	62	11	123834	-1.294	0.90217	0.99955652
CTRB2	ENSG00000168928	16	75227994	75251083	84	9	123834	-1.2944	0.90224	0.99955652
SMAD4	ENSG00000141646	18	48484410	48621415	86	5	123834	-1.2946	0.90228	0.99955652
RARRES3	ENSG00000133321	11	63294281	63323934	14	4	123834	-1.2949	0.90233	0.99955652
RAB10	ENSG00000084733	2	26246976	26370323	156	4	123834	-1.2951	0.90236	0.99955652
CATSPER4	ENSG00000188782	1	26507052	26539459	63	13	123834	-1.2952	0.90238	0.99955652
AC187652.1	ENSG00000269396	7	292918	314070	1	1	123834	-1.2955	0.90243	0.99955652
ZFP64	ENSG00000020256	20	50658202	50830847	394	33	123834	-1.2963	0.90257	0.999653336
SLC13A2	ENSG00000007216	17	26790311	26834799	55	5	123834	-1.2971	0.9027	0.999702897
GIMAP1	ENSG00000213203	7	150403645	150431372	24	4	123834	-1.2972	0.90272	0.999702897
COQ4	ENSG00000167113	9	131074815	131106351	39	6	123834	-1.2977	0.90281	0.999702897
TTR	ENSG00000118271	18	29161689	29188974	29	4	123834	-1.3001	0.90322	0.999702897
PROL1	ENSG00000171199	4	71253599	71285911	94	14	123834	-1.3012	0.90341	0.999702897
FBXO41	ENSG00000163013	2	73471810	73521559	41	9	123834	-1.3015	0.90346	0.999702897
JADE2	ENSG00000043143	5	133850003	133928918	116	18	123834	-1.3016	0.90348	0.999702897
GSR	ENSG00000104687	8	30525583	30595443	137	10	123834	-1.3017	0.90349	0.999702897
P2RX2	ENSG00000187848	12	133185366	133208972	66	7	123834	-1.3019	0.90352	0.999702897
CDC20B	ENSG00000164287	5	54398799	54479005	133	10	123834	-1.3021	0.90356	0.999702897
MTHFD2	ENSG00000065911	2	74415689	74454692	40	6	123834	-1.3022	0.90358	0.999702897
FCRL5	ENSG00000143297	1	157473167	157532310	86	7	123834	-1.3024	0.90361	0.999702897
ACOX3	ENSG00000087008	4	8358009	8452450	198	20	123834	-1.3025	0.90362	0.999702897
ENC1	ENSG00000171617	5	73913234	73947249	27	3	123834	-1.3031	0.90373	0.999702897
LRRC7	ENSG00000033122	1	70024081	70627628	1004	56	123834	-1.3034	0.90378	0.999702897
AGBL5	ENSG00000084693	2	27255232	27303490	27	2	123834	-1.3034	0.90378	0.999702897
SFMBT2	ENSG00000198879	10	7190586	7463450	483	48	123834	-1.3036	0.90381	0.999702897
CYP27A1	ENSG00000135929	2	219636472	219690016	50	3	123834	-1.3038	0.90385	0.999702897
FBL	ENSG00000105202	19	40315098	40347054	65	11	123834	-1.3041	0.90389	0.999702897
ANKRD36C	ENSG00000174501	2	96504587	96667541	115	8	123834	-1.3042	0.90391	0.999702897
OR2AJ1	ENSG00000177275	1	248087071	248108057	37	4	123834	-1.3044	0.90395	0.999702897
TRUB2	ENSG00000167112	9	131061382	131095021	47	6	123834	-1.3044	0.90396	0.999702897
CD3G	ENSG00000160654	11	118205059	118235876	37	4	123834	-1.3045	0.90397	0.999702897
FRAS1	ENSG00000138759	4	78968724	79475423	1210	39	123834	-1.3048	0.90402	0.999702897
P2RX7	ENSG00000089041	12	121560622	121633876	262	18	123834	-1.3053	0.9041	0.999702897
B4GALNT4	ENSG00000182272	11	359796	392116	44	7	123834	-1.3056	0.90415	0.999702897
POMT1	ENSG00000130714	9	134368289	134409193	80	4	123834	-1.306	0.90422	0.999702897
IL36B	ENSG00000136696	2	113769668	113820444	56	6	123834	-1.3061	0.90424	0.999702897
PALLD	ENSG00000129116	4	169408217	169859608	670	70	123834	-1.3067	0.90435	0.999702897
TOMM6	ENSG00000214736	6	41745400	41767636	38	8	123834	-1.3069	0.90438	0.999702897
KRTAP2-4	ENSG00000213417	17	39211369	39232131	43	3	123834	-1.3073	0.90445	0.999702897
TMEM62	ENSG00000137842	15	43405477	43487344	47	8	123834	-1.3078	0.90454	0.999702897
ELP6	ENSG00000163832	3	47527130	47565251	28	4	123834	-1.308	0.90456	0.999702897
ZNF140	ENSG00000196387	12	133646424	133694130	20	3	123834	-1.3082	0.9046	0.999702897
GCFC2	ENSG00000005436	2	75869126	75948115	146	12	123834	-1.3084	0.90463	0.999702897
TMEM240	ENSG00000205090	1	1460554	1485833	30	4	123834	-1.3086	0.90467	0.999702897
SENP6	ENSG00000112701	6	76301225	76437997	268	14	123834	-1.3088	0.90471	0.999702897
MBOAT4	ENSG00000177669	8	29979340	30012202	43	5	123834	-1.3095	0.90482	0.999702897
SDHD	ENSG00000204370	11	111947497	112000353	33	4	123834	-1.3096	0.90483	0.999702897
TMEM106C	ENSG00000134291	12	48347352	48372661	31	5	123834	-1.3097	0.90484	0.999702897
TAGLN2	ENSG00000158710	1	159877897	159905522	31	10	123834	-1.3097	0.90485	0.999702897
AC092675.3	ENSG00000222000	2	98937852	98982468	105	9	123834	-1.31	0.90491	0.999702897
IRGC	ENSG00000124449	19	44210162	44234173	29	5	123834	-1.3102	0.90494	0.999702897
UQCRFS1	ENSG00000169021	19	29688173	29714448	51	6	123834	-1.3104	0.90497	0.999702897
EBF1	ENSG00000164330	5	158112928	158536769	487	43	123834	-1.3107	0.90503	0.999702897
SLAIN1	ENSG00000139737	13	78262023	78348377	97	11	123834	-1.311	0.90507	0.999702897
STAT3	ENSG00000168610	17	40455342	40550586	104	9	123834	-1.3112	0.9051	0.999702897
VSTM5	ENSG00000214376	11	93541398	93593697	81	11	123834	-1.3117	0.90518	0.999702897
C9orf78	ENSG00000136819	9	132579569	132608142	130	8	123834	-1.312	0.90524	0.999702897
SPARCL1	ENSG00000152583	4	88384487	88462213	172	11	123834	-1.3128	0.90538	0.999702897
CPLX2	ENSG00000145920	5	175213313	175321023	113	19	123834	-1.3129	0.90538	0.999702897
NIP7	ENSG00000132603	16	69363333	69387014	23	4	123834	-1.3132	0.90544	0.999702897

TRMT6	ENSG00000089195	20	5907881	5941182	98	13	123834	-1.3135	0.9055	0.999702897
OR6C74	ENSG00000197706	12	55630982	55652086	40	3	123834	-1.3136	0.90551	0.999702897
RP11-45H22.3	ENSG00000249016	5	54242063	54263674	51	7	123834	-1.3138	0.90554	0.999702897
CAND1	ENSG00000111530	12	67653061	67723731	178	14	123834	-1.3139	0.90556	0.999702897
ZDHHC20	ENSG00000180776	13	21940263	22043509	174	11	123834	-1.3145	0.90566	0.999752362
IFIH1	ENSG00000115267	2	163113589	163185213	74	2	123834	-1.3148	0.90571	0.999752362
IFT172	ENSG00000138002	2	27657238	27722656	57	4	123834	-1.3158	0.90589	0.999764554
METTL22	ENSG00000067365	16	8705540	8750081	91	7	123834	-1.3167	0.90603	0.999764554
DBX1	ENSG00000109851	11	20167701	20192159	27	9	123834	-1.3168	0.90605	0.999764554
PGC	ENSG00000096088	6	41694449	41731847	54	15	123834	-1.3169	0.90606	0.999764554
ZBTB17	ENSG00000116809	1	16258364	16312627	71	12	123834	-1.3173	0.90612	0.999764554
NOC3L	ENSG00000173145	10	96065004	96132716	123	10	123834	-1.3179	0.90623	0.999764554
SCO2	ENSG00000130489	22	50951997	50974868	39	8	123834	-1.318	0.90625	0.999764554
NKX6-3	ENSG00000165066	8	41492697	41518855	54	10	123834	-1.3181	0.90626	0.999764554
AP005482.1	ENSG00000215527	18	12648042	12673470	63	7	123834	-1.3182	0.90629	0.999764554
ARHGEF39	ENSG00000137135	9	35648872	35685863	62	6	123834	-1.3186	0.90635	0.999764554
OCSTAMP	ENSG00000149635	20	45159585	45189213	34	7	123834	-1.3191	0.90643	0.999764554
ACER2	ENSG00000177076	9	19398925	19462018	44	9	123834	-1.3192	0.90646	0.999764554
MRPS27	ENSG00000113048	5	71505236	71626473	103	9	123834	-1.3194	0.90648	0.999764554
INO80C	ENSG00000153391	18	33022194	33087955	35	5	123834	-1.3194	0.90648	0.999764554
KCNMB4	ENSG00000135643	12	70750056	70838072	233	28	123834	-1.3196	0.90651	0.999764554
TRIM45	ENSG00000134253	1	117643682	117675209	42	6	123834	-1.3207	0.90669	0.999875206
SMIM21	ENSG00000206026	18	73111431	73149658	79	8	123834	-1.3231	0.9071	0.999875206
OR51D1	ENSG00000197428	11	4650945	4672068	63	9	123834	-1.3232	0.90712	0.999875206
ST7L	ENSG00000007341	1	113056140	113173447	173	11	123834	-1.3234	0.90715	0.999875206
TRIM39-RPP21	ENSG00000248167	6	30287359	30324631	72	16	123834	-1.3242	0.90728	0.999875206
TLDC1	ENSG00000140950	16	84501681	84597639	221	39	123834	-1.3247	0.90736	0.999875206
GRXCR1	ENSG00000215203	4	42885284	43042675	329	27	123834	-1.3249	0.90739	0.999875206
STARD6	ENSG00000174448	18	51840728	51894334	131	8	123834	-1.3251	0.90743	0.999875206
MMP1	ENSG00000196611	11	102650651	102678891	54	12	123834	-1.3256	0.90752	0.999875206
GRK1	ENSG00000185974	13	114311470	114450708	26	3	123834	-1.3259	0.90756	0.999875206
STMN2	ENSG00000104435	8	80513049	80588410	109	19	123834	-1.3266	0.90767	0.999875206
SF3B5	ENSG00000169976	6	144406018	144426754	32	7	123834	-1.3267	0.9077	0.999875206
FBXW9	ENSG00000132004	19	12788867	12817457	43	2	123834	-1.3269	0.90773	0.999875206
COPS4	ENSG00000138663	4	83945600	84006971	3	1	123834	-1.327	0.90774	0.999875206
RPP21	ENSG00000241370	6	30302908	30324661	34	12	123834	-1.327	0.90774	0.999875206
FOLH1	ENSG00000086205	11	49158187	49240222	240	17	123834	-1.327	0.90775	0.999875206
MTRNR2L13	ENSG00000270394	4	117210016	117231520	36	4	123834	-1.3272	0.90778	0.999875206
NFYB	ENSG00000120837	12	104500855	104542067	73	12	123834	-1.3272	0.90778	0.999875206
OR1M1	ENSG00000170929	19	9193855	9214889	54	6	123834	-1.3274	0.90781	0.999875206
MARK2	ENSG00000072518	11	63596400	63688491	80	5	123834	-1.3276	0.90784	0.999875206
SPACA1	ENSG00000118434	6	88747507	88786550	18	1	123834	-1.3278	0.90788	0.999875206
CCDC147	ENSG00000120051	10	106103522	106224848	121	14	123834	-1.3301	0.90825	0.999875206
HNRNPA1	ENSG00000135486	12	54663977	54690872	11	2	123834	-1.3303	0.90829	0.999875206
SLC10A4	ENSG00000145248	4	48475360	48501213	46	6	123834	-1.3305	0.90832	0.999875206
GARNL3	ENSG00000136895	9	129976544	130165939	167	15	123834	-1.3306	0.90835	0.999875206
COL10A1	ENSG00000123500	6	116430086	116489910	87	11	123834	-1.3307	0.90836	0.999875206
AXIN2	ENSG00000168646	17	63514681	63567765	39	11	123834	-1.3308	0.90838	0.999875206
OR4A5	ENSG00000221840	11	51401378	51422448	110	10	123834	-1.3311	0.90842	0.999875206
PLS1	ENSG00000120756	3	142305229	142442506	249	17	123834	-1.3312	0.90844	0.999875206
FKBP14	ENSG00000106080	7	30040203	30076300	50	6	123834	-1.3314	0.90846	0.999875206
SH3BGRL2	ENSG00000198478	6	80331000	80423372	120	16	123834	-1.3317	0.90853	0.999875206
CCDC130	ENSG00000104957	19	13832574	13884110	56	6	123834	-1.3318	0.90854	0.999875206
TFPI	ENSG00000003436	2	188318957	188440487	175	22	123834	-1.3322	0.9086	0.999875206
FAM32A	ENSG00000105058	19	16286191	16312857	53	7	123834	-1.3322	0.90861	0.999875206
FMO6P	ENSG00000117507	1	171096879	171140707	115	12	123834	-1.3323	0.90862	0.999875206
UBAP2	ENSG00000137073	9	33911691	34058947	338	14	123834	-1.3335	0.90882	0.999875206
ANAPC15	ENSG00000110200	11	71807424	71833826	47	4	123834	-1.3339	0.90887	0.999875206
WFDC1	ENSG00000103175	16	84318252	84373450	67	19	123834	-1.3339	0.90888	0.999875206
FGF2	ENSG00000138685	4	123737863	123829391	273	25	123834	-1.3344	0.90896	0.999875206
PSTPIP1	ENSG00000140368	15	77275700	77339673	40	8	123834	-1.3344	0.90897	0.999875206
ALS2CL	ENSG00000178038	3	46700487	46745194	45	7	123834	-1.3345	0.90898	0.999875206
AGMO	ENSG00000187546	7	15229943	15611640	715	61	123834	-1.3347	0.90901	0.999875206
WNT6	ENSG00000115596	2	219714544	219748955	12	3	123834	-1.335	0.90907	0.999875206
LDB1	ENSG00000198728	10	103857317	103890210	35	5	123834	-1.3355	0.90914	0.999875206
ITGAE	ENSG00000083457	17	3607922	3714537	187	20	123834	-1.3355	0.90914	0.999875206
ME2	ENSG00000082212	18	48395419	48484691	122	9	123834	-1.3371	0.90941	0.999875206
AC015989.1	ENSG00000215431	18	60481423	60502724	28	4	123834	-1.3372	0.90942	0.999875206
SGMS1	ENSG00000198964	10	52055360	52394923	525	25	123834	-1.3374	0.90946	0.999875206
IFNK	ENSG00000147896	9	27514312	27536496	74	8	123834	-1.3378	0.90953	0.999875206
TMEM66	ENSG00000133872	8	29910528	29950723	65	6	123834	-1.338	0.90955	0.999875206
SLC35E2B	ENSG00000189339	1	1582939	1634167	17	3	123834	-1.3382	0.90958	0.999875206
SLC16A4	ENSG00000168679	1	110895470	110943704	88	12	123834	-1.3382	0.90959	0.999875206
WDR26	ENSG00000162923	1	224562845	224634735	77	3	123834	-1.3398	0.90984	0.999875206
KRTAP1-4	ENSG00000204887	17	39175949	39196356	75	8	123834	-1.3398	0.90985	0.999875206
P2RY6	ENSG00000171631	11	72965550	73019662	50	6	123834	-1.3402	0.90991	0.999875206
PRH1	ENSG00000231887	12	11023610	11046854	65	4	123834	-1.3405	0.90996	0.999875206
FRS3	ENSG00000137218	6	41727914	41764280	59	12	123834	-1.3413	0.91008	0.999875206
TMPRSS11BNL	ENSG00000226894	4	69044242	69093798	90	3	123834	-1.3416	0.91014	0.999875206
WHSC1	ENSG00000109685	4	1863151	1993934	15	7	123834	-1.3417	0.91015	0.999875206
RNPC3	ENSG00000185946	1	104058313	104107861	29	6	123834	-1.3421	0.91022	0.999875206
ZNF12	ENSG00000164631	7	6718064	6756554	102	13	123834	-1.3423	0.91024	0.999875206
PABPC4	ENSG00000090621	1	40016488	40052462	75	12	123834	-1.3425	0.91028	0.999875206
CCR4	ENSG00000183813	3	32983066	33007841	31	5	123834	-1.3436	0.91047	0.999875206
MSRB2	ENSG00000148450	10	23374435	23420942	62	9	123834	-1.3436	0.91047	0.999875206
GRP	ENSG00000134443	18	56877400	56908006	53	4	123834	-1.3438	0.91049	0.999875206
FBXO15	ENSG00000141665	18	71730588	71825100	235	8	123834	-1.3438	0.9105	0.999875206
LIFR	ENSG00000113594	5	38465065	38618456	112	12	123834	-1.344	0.91052	0.999875206
ZNF469	ENSG00000225614	16	88483879	88517165	28	4	123834	-1.3449	0.91066	0.999875206
LIPN	ENSG00000204020	10	90511163	90547999	106	13	123834	-1.3449	0.91067	0.999875206
C1orf74	ENSG00000162757	1	209945661	209967904	35	6	123834	-1.3452	0.91071	0.999875206
EGR2	ENSG00000122877	10	64561756	64689660	304	20	123834	-1.3467	0.91097	0.999875206
LSM14A	ENSG00000257103	19	34653409	34730420	113	9	123834	-1.3479	0.91116	0.999875206
ANKRD33	ENSG00000167612	12	52271744	52295448	29	7	123834	-1.3481	0.91119	0.999875206
DNAH10	ENSG00000197653	12	124237042	124430753	267	22	123834	-1.3485	0.91126	0.999875206
URI1	ENSG00000105176	19	30404551	30516611	137	8	123834	-1.3486	0.91127	0.999875206
EMC6	ENSG00000127774	17	3562109	3582962	41	7	123834	-1.3489	0.91132	0.999875206
WDR83	ENSG00000123154	19	12767614	12796646	14	3	123834	-1.3497	0.91145	0.999875206
DNAJC7	ENSG00000168259	17	40118451	40183394	60	7	123834	-1.35	0.91149	0.999875206

AMD1	ENSG00000123505	6	111185973	111226916	113	9	123834	-1.35	0.9115	0.999875206
ZBTB6	ENSG00000186130	9	125660335	125685609	35	6	123834	-1.3505	0.91157	0.999875206
CACNA1H	ENSG00000196557	16	1193241	1281771	121	20	123834	-1.3511	0.91167	0.999875206
DPPA2	ENSG00000163530	3	109002635	109045364	64	8	123834	-1.3513	0.9117	0.999875206
MTERFD1	ENSG00000156469	8	97241626	97283838	33	4	123834	-1.3516	0.91175	0.999875206
RNF219	ENSG00000152193	13	79178426	79243314	86	5	123834	-1.3516	0.91175	0.999875206
MIS18BP1	ENSG00000129534	14	45662393	45732743	85	6	123834	-1.3516	0.91175	0.999875206
NAA38	ENSG00000128534	7	117814086	117842878	106	6	123834	-1.352	0.91181	0.999875206
MEST	ENSG00000106484	7	130116012	130156133	67	9	123834	-1.3528	0.91194	0.999875206
ZFHX4	ENSG00000091656	8	77583454	77789521	284	22	123834	-1.3528	0.91194	0.999875206
TMEM116	ENSG00000198270	12	112359086	112460970	137	6	123834	-1.353	0.91197	0.999875206
ZFYVE26	ENSG00000072121	14	68184091	68293307	142	14	123834	-1.3533	0.91202	0.999875206
TRPC1	ENSG00000144935	3	142432916	142536730	146	9	123834	-1.3536	0.91206	0.999875206
WFDC10B	ENSG00000182931	20	44303292	44343658	105	11	123834	-1.3536	0.91207	0.999875206
ZNF106	ENSG00000103994	15	42695021	42793321	102	8	123834	-1.3536	0.91208	0.999875206
NFYA	ENSG00000001167	6	41030684	41077715	61	5	123834	-1.3539	0.91211	0.999875206
LGALS9B	ENSG00000170298	17	20342708	20380852	3	2	123834	-1.3543	0.91218	0.999875206
ARL6	ENSG00000113966	3	97473365	97529953	55	4	123834	-1.3544	0.91219	0.999875206
C17orf66	ENSG00000172653	17	34171955	34205895	56	6	123834	-1.3548	0.91225	0.999875206
FAM174A	ENSG00000174132	5	99861009	99932445	117	9	123834	-1.3559	0.91243	0.999875206
ZNF695	ENSG00000197472	1	247098849	247181395	184	18	123834	-1.3564	0.91252	0.999875206
PLCE1	ENSG00000138193	10	95743746	96102580	394	30	123834	-1.3567	0.91257	0.999875206
EIF2B4	ENSG00000115211	2	27577219	27603353	17	3	123834	-1.3577	0.91273	0.999875206
C9	ENSG00000113600	5	39274364	39434970	324	27	123834	-1.3579	0.91274	0.999875206
CCDC121	ENSG00000176714	2	27838506	27861879	36	4	123834	-1.3579	0.91275	0.999875206
BTBD17	ENSG00000204347	17	72342555	72368085	19	5	123834	-1.358	0.91277	0.999875206
ZNF679	ENSG00000197123	7	63678852	63737309	159	15	123834	-1.359	0.91292	0.999875206
ZSCAN9	ENSG00000137185	6	28182664	28211260	42	7	123834	-1.359	0.91293	0.999875206
AC005082.1	ENSG00000214871	7	23200760	23244503	98	4	123834	-1.3591	0.91295	0.999875206
GIMAP4	ENSG00000133574	7	150254365	150281041	115	9	123834	-1.3593	0.91297	0.999875206
ENPP1	ENSG00000197594	6	132119156	132226295	168	23	123834	-1.3598	0.91305	0.999875206
PDE8A	ENSG00000073417	15	85513671	85692376	449	21	123834	-1.3602	0.91312	0.999875206
BTF3L4	ENSG00000134717	1	52511797	52566388	42	4	123834	-1.3604	0.91314	0.999875206
AC004528.1	ENSG00000269169	19	989601	1009952	58	14	123834	-1.3606	0.91317	0.999875206
CHRNA10	ENSG00000129749	11	3676817	3702614	41	8	123834	-1.3608	0.9132	0.999875206
CCDC7	ENSG00000216937	10	32725068	32873492	438	20	123834	-1.3608	0.91321	0.999875206
ZFAND4	ENSG00000172671	10	46101043	46178228	170	5	123834	-1.3611	0.91326	0.999875206
CTC-398G3.6	ENSG00000267477	19	11567055	11626513	126	11	123834	-1.3611	0.91326	0.999875206
COL23A1	ENSG00000050767	5	177654619	178027556	661	85	123834	-1.3614	0.91331	0.999875206
ARFGAP3	ENSG00000242247	22	43182508	43264112	223	11	123834	-1.3614	0.91331	0.999875206
KRTAP12-3	ENSG00000205439	21	46067849	46088258	77	7	123834	-1.3623	0.91344	0.999875206
STIM2	ENSG00000109689	4	26849300	27037003	214	19	123834	-1.3631	0.91357	0.999875206
RNF31	ENSG00000092098	14	24605892	24639870	22	7	123834	-1.3633	0.91361	0.999875206
KIAA1328	ENSG00000150477	18	34399069	34822135	614	10	123834	-1.3637	0.91367	0.999875206
AP2B1	ENSG00000006125	17	33895065	34063436	400	20	123834	-1.3639	0.9137	0.999875206
IQCB1	ENSG00000173226	3	121478610	121563926	243	12	123834	-1.3641	0.91374	0.999875206
NKAPL	ENSG00000189134	6	28217098	28238736	22	7	123834	-1.3647	0.91383	0.999875206
PNLIP	ENSG00000175535	10	118295443	118337367	45	3	123834	-1.3649	0.91385	0.999875206
TRPC6	ENSG00000137672	11	101312295	101753293	1279	40	123834	-1.3649	0.91386	0.999875206
OR2T6	ENSG00000198104	1	248540910	248561836	84	4	123834	-1.3651	0.91389	0.999875206
PSME1	ENSG00000092010	14	24595367	24618176	18	3	123834	-1.3653	0.91391	0.999875206
TDGF1	ENSG00000241186	3	46606045	46678033	222	20	123834	-1.3653	0.91392	0.999875206
ZNF624	ENSG00000197566	17	16514051	16567170	130	6	123834	-1.3657	0.91399	0.999875206
PKM	ENSG00000067225	15	72481370	72534164	51	7	123834	-1.3668	0.91416	0.999875206
ZNF479	ENSG00000185177	7	57177321	57217571	88	6	123834	-1.3669	0.91418	0.999875206
AL121963.1	ENSG00000234188	6	116432320	116453093	32	7	123834	-1.3671	0.9142	0.999875206
RAD17	ENSG00000152942	5	68655120	68720628	74	5	123834	-1.3674	0.91424	0.999875206
HSD17B3	ENSG00000130948	9	98987588	99074434	113	17	123834	-1.3674	0.91425	0.999875206
CTDP1	ENSG00000060069	18	77429801	77524510	279	14	123834	-1.3678	0.91432	0.999875206
SAMD3	ENSG00000164483	6	130455460	130696570	855	22	123834	-1.3684	0.9144	0.999875206
ITGA5	ENSG00000161638	12	54779045	54823244	23	4	123834	-1.3685	0.91442	0.999875206
MYADM	ENSG00000179820	19	54359477	54389691	17	3	123834	-1.3689	0.91449	0.999875206
WASF3	ENSG00000132970	13	27121840	27273085	321	18	123834	-1.3702	0.91469	0.999875206
SAMD9L	ENSG00000177409	7	92749368	92787682	50	10	123834	-1.3703	0.9147	0.999875206
OR10P1	ENSG00000175398	12	56020644	56041638	58	8	123834	-1.3704	0.91472	0.999875206
RAD21L1	ENSG00000244588	20	1196700	1287065	128	15	123834	-1.3711	0.91482	0.999875206
DCT	ENSG00000080166	13	95079558	95141936	127	15	123834	-1.372	0.91496	0.999875206
TOPAZ1	ENSG00000173769	3	44273378	44383590	183	9	123834	-1.3727	0.91508	0.999875206
HAS1	ENSG00000105509	19	52206365	52237247	30	9	123834	-1.3728	0.91509	0.999875206
ZSCAN12	ENSG00000158691	6	28336732	28377511	90	8	123834	-1.3731	0.91514	0.999875206
IL7R	ENSG00000168685	5	35842797	35889705	114	10	123834	-1.3732	0.91515	0.999875206
RHOG	ENSG00000177105	11	3838208	3872213	13	4	123834	-1.3732	0.91515	0.999875206
FPGS	ENSG00000136877	9	130546876	130586606	45	5	123834	-1.3735	0.91521	0.999875206
OR9A4	ENSG00000258083	7	141608617	141629709	45	6	123834	-1.3741	0.91529	0.999875206
TAS2R4	ENSG00000127364	7	141468242	141489235	49	2	123834	-1.3751	0.91545	0.999875206
RSAD1	ENSG00000136444	17	48546161	48573336	38	6	123834	-1.3751	0.91545	0.999875206
YPEL2	ENSG00000175155	17	57399050	57489090	97	11	123834	-1.3755	0.91551	0.999875206
RARG	ENSG00000172819	12	53594354	53636764	33	8	123834	-1.3769	0.91573	0.999875206
SNPH	ENSG00000101298	20	1236960	1299972	77	13	123834	-1.3769	0.91573	0.999875206
ANGPTL4	ENSG00000167772	19	8418173	8449257	43	8	123834	-1.377	0.91574	0.999875206
ATHL1	ENSG00000142102	11	279135	306107	8	2	123834	-1.3771	0.91577	0.999875206
PCDHA11	ENSG00000249158	5	140238689	140401929	234	14	123834	-1.3791	0.91607	0.999875206
OR51B2	ENSG00000184881	11	5334541	5355582	81	8	123834	-1.3791	0.91608	0.999875206
TRIM48	ENSG00000150244	11	55019658	55048595	129	10	123834	-1.3792	0.91609	0.999875206
DEFB135	ENSG00000205883	8	11829830	11852099	33	7	123834	-1.3794	0.91611	0.999875206
VWA3B	ENSG00000168658	2	98693579	98939762	477	27	123834	-1.3798	0.91617	0.999875206
MT3	ENSG00000087250	16	56612986	56635000	58	9	123834	-1.3804	0.91627	0.999875206
C7orf65	ENSG00000221845	7	47684842	47711246	47	6	123834	-1.3806	0.91629	0.999875206
FAH	ENSG00000103876	15	80434832	80489288	73	9	123834	-1.3805	0.91629	0.999875206
GABRA5	ENSG00000186297	15	27101510	27204354	64	14	123834	-1.3828	0.91663	0.999875206
NIPA2	ENSG00000140157	15	22994684	23044427	103	15	123834	-1.383	0.91666	0.999875206
PKP3	ENSG00000184363	11	382614	414908	52	6	123834	-1.3832	0.91669	0.999875206
PIK3R6	ENSG00000174083	17	8696041	8780994	61	12	123834	-1.3835	0.91675	0.999875206
TRIM46	ENSG00000163462	1	155135873	155167447	24	4	123834	-1.3839	0.91681	0.999875206
ZNF665	ENSG00000197497	19	53652466	53706639	83	13	123834	-1.3839	0.91681	0.999875206
SLC27A4	ENSG00000167114	9	131092925	131133749	48	7	123834	-1.3841	0.91683	0.999875206
SYNPR	ENSG00000163630	3	63203991	63612597	863	63	123834	-1.3846	0.91692	0.999875206
CCL22	ENSG00000102962	16	57382684	57410102	74	13	123834	-1.3851	0.91699	0.999875206
ZFYVE9	ENSG00000157077	1	52598046	52822358	160	8	123834	-1.3854	0.91704	0.999875206

HERPUD1	ENSG00000051108	16	56955960	56987798	56	6	123834	-1.3855	0.91705	0.999875206
CARD18	ENSG00000255501	11	104998448	105020453	87	5	123834	-1.3858	0.91709	0.999875206
UTP11L	ENSG00000183520	1	38464930	38500496	15	4	123834	-1.3873	0.91732	0.999875206
GNRH1	ENSG00000147437	8	25266776	25292170	23	3	123834	-1.3876	0.91737	0.999875206
AC013449.1	ENSG00000268412	2	26241074	26261481	28	5	123834	-1.3879	0.91742	0.999875206
MLH1	ENSG00000076242	3	37024823	37117380	126	7	123834	-1.3881	0.91745	0.999875206
C1orf87	ENSG00000162598	1	60442941	60549442	142	27	123834	-1.3887	0.91754	0.999875206
COL13A1	ENSG00000197467	10	71551644	71734031	372	71	123834	-1.3887	0.91754	0.999875206
CHMP4C	ENSG00000164695	8	82634669	82681750	80	8	123834	-1.3889	0.91756	0.999875206
PCDHA12	ENSG00000251664	5	140245058	140401929	223	13	123834	-1.3892	0.91762	0.999875206
IMPA1	ENSG00000133731	8	82560196	82608928	106	14	123834	-1.3894	0.91764	0.999875206
CCL25	ENSG00000131142	19	8107651	8137534	56	11	123834	-1.3895	0.91767	0.999875206
COG7	ENSG00000168434	16	23389814	23474501	90	6	123834	-1.3902	0.91777	0.999875206
CTNND2	ENSG00000169862	5	10961952	11914155	1158	105	123834	-1.3911	0.9179	0.999875206
LURAP1	ENSG00000171357	1	46659006	46696933	27	6	123834	-1.3914	0.91795	0.999875206
TEX35	ENSG00000240021	1	178472212	178527734	114	8	123834	-1.3916	0.91798	0.999875206
HIST1H3H	ENSG00000203813	6	27767842	27788314	26	5	123834	-1.3916	0.91798	0.999875206
CCL5	ENSG00000161570	17	34188495	34217797	56	6	123834	-1.3916	0.91798	0.999875206
PCDHA9	ENSG00000204961	5	140217048	140401929	238	14	123834	-1.392	0.91804	0.999875206
TBL3	ENSG00000183751	16	2012038	2042934	101	12	123834	-1.3927	0.91814	0.999875206
NT5C3B	ENSG00000141698	17	39971335	40002523	65	4	123834	-1.393	0.91819	0.999875206
SI	ENSG00000090402	3	164686686	164806283	184	7	123834	-1.3936	0.91829	0.999875206
OR4C16	ENSG00000181935	11	55329604	55350536	122	9	123834	-1.3937	0.91829	0.999875206
CRHBP	ENSG00000145708	5	76238538	76286983	42	5	123834	-1.3939	0.91833	0.999875206
MRPS2	ENSG00000122140	9	138381830	138406519	45	7	123834	-1.3941	0.91836	0.999875206
PAPSS1	ENSG00000138801	4	108501433	108651608	297	17	123834	-1.3942	0.91837	0.999875206
ESCO2	ENSG00000171320	8	27619466	27680157	199	7	123834	-1.396	0.91864	0.999875206
PLEKHG4	ENSG00000196155	16	67301413	67333402	25	4	123834	-1.396	0.91865	0.999875206
TMEM100	ENSG00000166292	17	53786988	53819482	26	5	123834	-1.3973	0.91883	0.999875206
NDUFB7	ENSG00000099795	19	14666890	14692874	37	7	123834	-1.3975	0.91886	0.999875206
IL36RN	ENSG00000136695	2	113806215	113832959	79	5	123834	-1.3976	0.91889	0.999875206
PCDHA8	ENSG00000204962	5	140210907	140401929	243	15	123834	-1.3977	0.9189	0.999875206
PCDHA13	ENSG00000239389	5	140251793	140401929	217	13	123834	-1.3977	0.9189	0.999875206
HMOX1	ENSG00000100292	22	35766354	35800207	64	8	123834	-1.3977	0.9189	0.999875206
MFRP	ENSG00000259159	11	119199652	119227368	24	2	123834	-1.3987	0.91904	0.999875206
C1QTNF5	ENSG00000223953	11	119199652	119227383	24	2	123834	-1.3987	0.91904	0.999875206
MFRP	ENSG00000235718	11	119199652	119227383	24	2	123834	-1.3987	0.91904	0.999875206
PRICKLE4	ENSG00000124593	6	41738087	41767879	43	9	123834	-1.3989	0.91908	0.999875206
IL4R	ENSG00000077238	16	27314989	27386099	74	10	123834	-1.3989	0.91908	0.999875206
MLC1	ENSG00000100427	22	50487820	50534331	167	11	123834	-1.399	0.91909	0.999875206
ZNF891	ENSG00000214029	12	133684740	133717059	63	7	123834	-1.3991	0.9191	0.999875206
GPR160	ENSG00000173890	3	169745717	169813191	60	5	123834	-1.3994	0.91916	0.999875206
C7orf63	ENSG00000105792	7	89864488	89950377	170	14	123834	-1.3995	0.91916	0.999875206
SLC38A6	ENSG00000139974	14	61437832	61560451	130	10	123834	-1.3997	0.9192	0.999875206
FAM179B	ENSG00000198718	14	45421411	45553634	174	7	123834	-1.4002	0.91928	0.999875206
CLPX	ENSG00000166855	15	65430557	65487680	26	3	123834	-1.4003	0.91929	0.999875206
WWOX	ENSG00000186153	16	78123310	79256564	3156	230	123834	-1.4003	0.91929	0.999875206
FAM78B	ENSG00000188859	1	166016674	166146206	235	16	123834	-1.4005	0.91933	0.999875206
PCDHA10	ENSG00000250120	5	140225595	140401929	237	14	123834	-1.4009	0.91938	0.999875206
TGFB1I1	ENSG00000140682	16	31472906	31499281	13	4	123834	-1.4012	0.91942	0.999875206
RPS6	ENSG00000137154	9	19365713	19390252	19	2	123834	-1.4013	0.91944	0.999875206
ALX3	ENSG00000156150	1	110592616	110623322	22	2	123834	-1.4016	0.91948	0.999875206
PPP1R36	ENSG00000165807	14	65006620	65066098	101	13	123834	-1.4018	0.91951	0.999875206
RP11-178L8.4	ENSG00000131152	16	87326482	87377879	68	15	123834	-1.4021	0.91956	0.999875206
CDO1	ENSG00000129596	5	115130430	115162651	9	2	123834	-1.4041	0.91985	0.999875206
SSU72	ENSG00000160075	1	1467053	1520249	55	6	123834	-1.4042	0.91987	0.999875206
ANKRD31	ENSG00000145700	5	74354100	74542703	414	13	123834	-1.406	0.92014	0.999875206
C12orf68	ENSG00000177875	12	48567366	48589709	73	8	123834	-1.4062	0.92017	0.999875206
GPR110	ENSG00000153292	6	46955440	47020099	78	15	123834	-1.4063	0.92019	0.999875206
CCDC138	ENSG00000163006	2	109393213	109511933	173	9	123834	-1.4067	0.92024	0.999875206
PIWIL1	ENSG00000125207	12	130812432	130867182	93	16	123834	-1.407	0.92029	0.999875206
KIF27	ENSG00000165115	9	86441613	86546342	163	9	123834	-1.4076	0.92037	0.999875206
RAB37	ENSG00000172794	17	72656717	72753474	66	15	123834	-1.4082	0.92046	0.999875206
ACTL6B	ENSG00000077080	7	100230720	100264084	15	5	123834	-1.4085	0.9205	0.999875206
TMEM70	ENSG00000175606	8	74874672	74905018	70	7	123834	-1.4088	0.92055	0.999875206
PTPDC1	ENSG00000158079	9	96783076	96882138	89	10	123834	-1.4088	0.92055	0.999875206
SYVN1	ENSG00000162298	11	64879252	64912004	32	7	123834	-1.4089	0.92056	0.999875206
SHROOM1	ENSG00000164403	5	132147833	132176590	20	4	123834	-1.4089	0.92057	0.999875206
CDH13	ENSG00000140945	16	82650408	83840204	2859	241	123834	-1.4093	0.92062	0.999875206
QSOX1	ENSG00000116260	1	180113969	180183165	163	15	123834	-1.4095	0.92066	0.999875206
FOXA2	ENSG00000125798	20	22551643	22576093	22	1	123834	-1.4097	0.92068	0.999875206
WNT1	ENSG00000125084	12	49362398	49385459	3	1	123834	-1.4097	0.92069	0.999875206
LACC1	ENSG00000179630	13	44443420	44478068	126	13	123834	-1.4097	0.92069	0.999875206
SRRM4	ENSG00000139767	12	119409300	119610856	522	34	123834	-1.411	0.92088	0.999875206
C10ORF68	ENSG00000150076	10	32822297	33181802	1061	21	123834	-1.4112	0.92091	0.999875206
MARVELD2	ENSG00000152939	5	68700939	68750157	58	5	123834	-1.4113	0.92093	0.999875206
SH3BP5	ENSG00000131370	3	15286360	15392875	109	21	123834	-1.412	0.92102	0.999875206
TAF15	ENSG00000172660	17	34126459	34201619	126	9	123834	-1.412	0.92103	0.999875206
TNFRSF13C	ENSG00000159958	22	42311045	42332822	39	4	123834	-1.4122	0.92105	0.999875206
PROX2	ENSG00000119608	14	75309736	75340537	45	7	123834	-1.4136	0.92125	0.999875206
MBOAT2	ENSG00000143797	2	8982820	9153942	190	10	123834	-1.4137	0.92128	0.999875206
ANO10	ENSG00000160746	3	43386351	43743086	477	25	123834	-1.414	0.92132	0.999875206
SPATA5L1	ENSG00000171763	15	45684529	45723617	32	3	123834	-1.4142	0.92134	0.999875206
USP44	ENSG00000136014	12	95900336	95955266	140	26	123834	-1.4147	0.92142	0.999875206
COL5A1	ENSG00000130635	9	137523620	137746686	362	44	123834	-1.4151	0.92148	0.999875206
TDG	ENSG00000139372	12	104349582	104392652	142	19	123834	-1.4153	0.92151	0.999875206
TAS2R46	ENSG00000226761	12	11203964	11224893	47	6	123834	-1.4154	0.92153	0.999875206
HUG1	ENSG00000269259	10	102872536	102893624	21	3	123834	-1.4158	0.92158	0.999875206
CDSN	ENSG00000204539	6	31072867	31098223	156	21	123834	-1.416	0.92161	0.999875206
LDLRAD1	ENSG00000203985	1	54464412	54493856	63	6	123834	-1.416	0.92162	0.999875206
HNRNPM	ENSG00000099783	19	8499651	8563998	126	12	123834	-1.4162	0.92164	0.999875206
AC010547.9	ENSG00000261611	16	71471503	71533214	66	10	123834	-1.4166	0.9217	0.999875206
RBM4B	ENSG00000173914	11	66422469	66455392	19	6	123834	-1.4167	0.92172	0.999875206
CD8A	ENSG00000153563	2	87001729	87045519	37	5	123834	-1.4169	0.92174	0.999875206
CCDC87	ENSG00000182791	11	66347640	66370554	10	3	123834	-1.4173	0.9218	0.999875206
C1orf134	ENSG00000204377	1	16545368	16566038	37	4	123834	-1.4174	0.92182	0.999875206
CCDC175	ENSG00000151838	14	59961257	60053549	236	11	123834	-1.4177	0.92186	0.999875206
FOXE1	ENSG00000178919	9	100605536	100628986	91	6	123834	-1.4188	0.92202	0.999875206
BNIP1	ENSG00000113734	5	172561445	172601390	71	12	123834	-1.4195	0.92212	0.999875206

ZNF492	ENSG00000229676	19	22807126	22860472	280	4	123834	-1.4198	0.92216	0.999875206
DUS4L	ENSG00000105865	7	107193929	107228906	44	5	123834	-1.4207	0.9223	0.999875206
FAM194A	ENSG00000163645	3	150367672	150431758	88	16	123834	-1.4215	0.92241	0.999875206
CENPJ	ENSG00000151849	13	25447171	25507018	139	16	123834	-1.4215	0.92242	0.999875206
RBM6	ENSG00000004534	3	49967440	50147478	245	12	123834	-1.4217	0.92245	0.999875206
TIMM8B	ENSG00000150779	11	111945524	111967522	20	4	123834	-1.4221	0.9225	0.999875206
CAPN10	ENSG00000142330	2	241516133	241567122	130	12	123834	-1.4232	0.92265	0.999875206
KIAA0895	ENSG00000164542	7	36353830	36439734	158	19	123834	-1.4236	0.92272	0.999875206
PTP4A2	ENSG00000184007	1	32362022	32420457	42	7	123834	-1.4237	0.92273	0.999875206
TMEM214	ENSG00000119777	2	27245778	27274563	15	2	123834	-1.4239	0.92276	0.999875206
DLG1	ENSG00000075711	3	196759431	197036171	501	23	123834	-1.4239	0.92277	0.999875206
TIGD2	ENSG00000180346	4	90023968	90046050	85	11	123834	-1.4251	0.92293	0.999875206
INF2	ENSG00000203485	14	105145943	105195942	55	7	123834	-1.4262	0.9231	0.999875206
VPRBP	ENSG00000145041	3	51423298	51544010	115	7	123834	-1.4264	0.92312	0.999875206
ZNF583	ENSG00000198440	19	56899335	56957404	37	8	123834	-1.4264	0.92313	0.999875206
OR4D2	ENSG00000255713	17	56237017	56257940	41	6	123834	-1.4266	0.92315	0.999875206
SPICE1	ENSG00000163611	3	113151565	113244034	313	14	123834	-1.4268	0.92318	0.999875206
TTC5	ENSG00000136319	14	20714717	20784153	114	18	123834	-1.4269	0.92319	0.999875206
TMC4	ENSG00000167608	19	54653846	54686944	18	5	123834	-1.4268	0.92319	0.999875206
HK3	ENSG00000160883	5	176297870	176336333	30	7	123834	-1.427	0.9232	0.999875206
UNC93A	ENSG00000112494	6	167674657	167739507	232	26	123834	-1.427	0.92321	0.999875206
MAF	ENSG00000178573	16	79609740	79644611	29	10	123834	-1.4278	0.92332	0.999875206
PCDHA6	ENSG00000081842	5	140197563	140401929	254	11	123834	-1.4283	0.9234	0.999875206
SLC29A3	ENSG00000198246	10	73069015	73133142	134	20	123834	-1.4296	0.92359	0.999875206
C21orf2	ENSG00000160226	21	45738827	45769285	15	3	123834	-1.43	0.92364	0.999875206
SELL	ENSG00000188404	1	169649808	169690839	118	12	123834	-1.4302	0.92367	0.999875206
MFSD5	ENSG00000182544	12	53635035	53658189	39	7	123834	-1.4306	0.92372	0.999875206
SIAH1	ENSG00000196470	16	48380275	48492313	67	6	123834	-1.4306	0.92373	0.999875206
GINS2	ENSG00000131153	16	85699804	85733679	13	1	123834	-1.4307	0.92375	0.999875206
C18orf54	ENSG00000166845	18	51874287	51921588	149	8	123834	-1.4309	0.92377	0.999875206
GNPTAB	ENSG00000111670	12	102129275	102234716	154	14	123834	-1.4313	0.92383	0.999875206
GLRB	ENSG00000109738	4	157987209	158103242	340	20	123834	-1.4315	0.92386	0.999875206
GRIN3A	ENSG00000198785	9	104321635	104510862	503	20	123834	-1.432	0.92393	0.999875206
STIM1	ENSG00000167323	11	3865757	4124439	392	20	123834	-1.4322	0.92396	0.999875206
ZNF202	ENSG00000166261	11	123584885	123622383	96	9	123834	-1.4323	0.92397	0.999875206
BAZ2A	ENSG00000076108	12	56979380	57040600	37	5	123834	-1.4324	0.92398	0.999875206
SPDL1	ENSG00000040275	5	169000638	169041782	100	7	123834	-1.4324	0.92399	0.999875206
OR2T33	ENSG00000177212	1	248426073	248447138	39	2	123834	-1.4329	0.92406	0.999875206
ZKSCAN3	ENSG00000189298	6	28307691	28346947	74	9	123834	-1.433	0.92408	0.999875206
OR4K2	ENSG00000165762	14	20334391	20355468	23	1	123834	-1.4343	0.92426	0.999875206
PDCD7	ENSG00000090470	15	65399717	65436174	15	3	123834	-1.4345	0.92429	0.999875206
3-Mar	ENSG00000173926	5	126193406	126376500	258	6	123834	-1.4347	0.92432	0.999875206
RASAL1	ENSG00000111344	12	113526624	113584044	154	10	123834	-1.4349	0.92434	0.999875206
GRAMD2	ENSG00000175318	15	72442148	72500126	58	7	123834	-1.4351	0.92437	0.999875206
AL391421.1	ENSG00000204049	10	79616633	79639368	11	3	123834	-1.4354	0.92442	0.999875206
MMP15	ENSG00000102996	16	58049470	58090805	22	5	123834	-1.4361	0.92451	0.999875206
PDE4A	ENSG00000065989	19	10517449	10590305	95	10	123834	-1.4361	0.92451	0.999875206
STARD4	ENSG00000164211	5	110821731	110858288	15	4	123834	-1.4363	0.92454	0.999875206
PRRX1	ENSG00000116132	1	170621869	170718560	68	10	123834	-1.4365	0.92458	0.999875206
BTF3	ENSG00000145741	5	72784233	72811460	67	3	123834	-1.4366	0.92459	0.999875206
FAM110B	ENSG00000169122	8	58897068	59126838	391	34	123834	-1.4367	0.9246	0.999875206
BRAF	ENSG00000157764	7	140409127	140634564	280	8	123834	-1.4376	0.92473	0.999875206
MYL6	ENSG00000092841	12	56541945	56567280	6	2	123834	-1.4378	0.92475	0.999875206
SLC39A2	ENSG00000165794	14	21457414	21480030	17	4	123834	-1.4379	0.92477	0.999875206
TFB1M	ENSG00000029639	6	155568643	155645627	216	17	123834	-1.4381	0.92479	0.999875206
PCDHA7	ENSG00000204963	5	140203969	140401929	248	15	123834	-1.4383	0.92483	0.999875206
OLFML2B	ENSG00000162745	1	161942982	162003644	66	15	123834	-1.4384	0.92484	0.999875206
UBE2F	ENSG00000184182	2	238865469	238961236	205	9	123834	-1.4385	0.92485	0.999875206
KIAA0355	ENSG00000166398	19	34735442	34856491	179	14	123834	-1.4386	0.92487	0.999875206
RSG1	ENSG00000132881	1	16548195	16573657	43	5	123834	-1.4388	0.92489	0.999875206
PODXL2	ENSG00000114631	3	127338024	127401652	33	6	123834	-1.4393	0.92497	0.999875206
SMYD3	ENSG00000185420	1	245902642	246680614	2225	73	123834	-1.4395	0.92499	0.999875206
TAMM41	ENSG00000144559	3	11821916	11898393	101	11	123834	-1.4407	0.92516	0.999875206
KMT2C	ENSG00000055609	7	151822010	152143090	222	4	123834	-1.4408	0.92518	0.999875206
ALKBH6	ENSG00000239382	19	36490022	36515141	14	3	123834	-1.441	0.92521	0.999875206
NPR2	ENSG00000159899	9	35782151	35819729	46	5	123834	-1.4415	0.92527	0.999875206
BEAN1	ENSG00000166546	16	66451200	66537432	87	15	123834	-1.4415	0.92528	0.999875206
TRIP10	ENSG00000125733	19	6727936	6761537	25	6	123834	-1.4421	0.92536	0.999875206
TNKS1BP1	ENSG00000149115	11	57057112	57102426	77	7	123834	-1.4422	0.92538	0.999875206
SRSF11	ENSG00000116754	1	70661365	70728735	58	5	123834	-1.4424	0.9254	0.999875206
PRR20B	ENSG00000204918	13	57711622	57734643	4	1	123834	-1.4432	0.92551	0.999875206
MCM3	ENSG00000112118	6	52118807	52159679	93	15	123834	-1.4432	0.92552	0.999875206
FCGR3A	ENSG00000203747	1	161501549	161610917	31	8	123834	-1.4437	0.92559	0.999875206
OR6B2	ENSG00000182083	2	240958841	240979906	18	8	123834	-1.4445	0.92571	0.999875206
ZNF706	ENSG00000120963	8	102180106	102228421	45	5	123834	-1.4446	0.92571	0.999875206
OR5H14	ENSG00000236032	3	97858170	97879249	74	3	123834	-1.4452	0.92579	0.999875206
PENK	ENSG00000181195	8	57339233	57369293	39	4	123834	-1.4454	0.92583	0.999875206
PRSS42	ENSG00000178055	3	46861032	46885585	39	4	123834	-1.4456	0.92586	0.999875206
INTU	ENSG00000164066	4	128534426	128657892	140	9	123834	-1.4456	0.92586	0.999875206
SLC6A13	ENSG00000010379	12	319789	382039	78	13	123834	-1.4458	0.92588	0.999875206
GALNT15	ENSG00000131386	3	16206156	16283499	118	21	123834	-1.4459	0.9259	0.999875206
NIPSNAP3B	ENSG00000165028	9	107516438	107549738	34	6	123834	-1.4466	0.926	0.999875206
ZNF417	ENSG00000173480	19	58401664	58437978	40	3	123834	-1.4468	0.92602	0.999875206
LEPRE1	ENSG00000117385	1	43202006	43242755	60	10	123834	-1.4484	0.92624	0.999875206
DPPA4	ENSG00000121570	3	109034988	109066419	79	7	123834	-1.4484	0.92625	0.999875206
RAB8B	ENSG00000166128	15	63471668	63569981	117	14	123834	-1.4485	0.92626	0.999875206
C19orf10	ENSG00000074842	19	4631386	4680382	106	10	123834	-1.4485	0.92626	0.999875206
TYMP	ENSG00000025708	22	50954181	50978485	42	8	123834	-1.4497	0.92643	0.999875206
STK11	ENSG00000118046	19	1179406	1238428	47	8	123834	-1.4501	0.92649	0.999875206
CNKSR1	ENSG00000142675	1	26493894	26526377	74	9	123834	-1.4503	0.92651	0.999875206
C3orf70	ENSG00000187068	3	184785838	184880802	166	15	123834	-1.4509	0.92659	0.999875206
SLC30A5	ENSG00000145740	5	68379473	68436896	38	5	123834	-1.451	0.92662	0.999875206
TBP	ENSG00000112592	6	170853390	170891958	77	7	123834	-1.4512	0.92663	0.999875206
CTD-3214H19.16	ENSG00000269711	19	7733387	7757740	93	5	123834	-1.4512	0.92663	0.999875206
TRAPPC5	ENSG00000181029	19	7735729	7757744	93	5	123834	-1.4512	0.92663	0.999875206
AC015989.2	ENSG00000269630	18	60495640	60515948	26	3	123834	-1.4514	0.92667	0.999875206
BASP1	ENSG00000176788	5	17055707	17286943	450	47	123834	-1.4515	0.92668	0.999875206
POMK	ENSG00000185900	8	42938658	42988577	48	5	123834	-1.4517	0.92671	0.999875206
CCS	ENSG00000173992	11	66350292	66383490	19	5	123834	-1.4524	0.9268	0.999875206

DGUOK	ENSG00000114956	2	74143953	74196088	72	6	123834	-1.453	0.92689	0.999875206
RP11-201K10.3	ENSG00000273088	1	155131885	155169748	35	5	123834	-1.4535	0.92696	0.999875206
SORCS2	ENSG00000184985	4	7184265	7754554	1140	146	123834	-1.4536	0.92697	0.999875206
TRIM41	ENSG00000146063	5	180639499	180672809	76	11	123834	-1.4536	0.92698	0.999875206
RP4-539M6.19	ENSG00000249590	22	30795244	30834977	82	13	123834	-1.4538	0.927	0.999875206
RPL18A	ENSG00000105640	19	17960685	17984962	57	7	123834	-1.4541	0.92704	0.999875206
NELFA	ENSG00000185049	4	1974441	2053630	28	4	123834	-1.4542	0.92706	0.999875206
SIPA1	ENSG00000213445	11	65395568	65428401	12	3	123834	-1.4543	0.92707	0.999875206
CDK11B	ENSG00000248333	1	1560603	1600473	16	2	123834	-1.4548	0.92714	0.999875206
ZNF416	ENSG00000083817	19	58072934	58100295	33	4	123834	-1.4556	0.92725	0.999875206
VWA9	ENSG00000138614	15	65861091	65913627	80	5	123834	-1.4557	0.92726	0.999875206
KRTAP1-5	ENSG00000221852	17	39172278	39193454	75	8	123834	-1.4557	0.92726	0.999875206
FOXB2	ENSG00000204612	9	79624571	79645869	35	7	123834	-1.4572	0.92748	0.999875206
HDC	ENSG00000140287	15	50524144	50568223	92	10	123834	-1.4576	0.92753	0.999875206
CHPT1	ENSG00000111666	12	102080725	102147918	146	19	123834	-1.4579	0.92757	0.999875206
ARL4A	ENSG00000122644	7	12716481	12740559	60	10	123834	-1.4591	0.92772	0.999875206
ZNF517	ENSG00000197363	8	146014261	146046554	56	6	123834	-1.4594	0.92777	0.999875206
ISOC2	ENSG00000063241	19	55954352	55983710	36	9	123834	-1.4595	0.92779	0.999875206
WAC	ENSG00000095787	10	28811422	28922041	147	10	123834	-1.46	0.92786	0.999875206
AIDA	ENSG00000186063	1	222831355	222896552	85	11	123834	-1.4603	0.92789	0.999875206
ITGB7	ENSG00000139626	12	53575102	53611091	29	4	123834	-1.4608	0.92796	0.999875206
KCTD19	ENSG00000168676	16	67313331	67370666	51	5	123834	-1.4607	0.92796	0.999875206
LRRC59	ENSG00000108829	17	48442420	48484914	117	11	123834	-1.4608	0.92796	0.999875206
ZKSCAN4	ENSG00000187626	6	28202401	28237011	34	8	123834	-1.4615	0.92806	0.999875206
PAPD5	ENSG00000121274	16	50176829	50279221	110	10	123834	-1.4628	0.92823	0.999875206
KIAA1211L	ENSG00000196872	2	99400309	99562722	290	16	123834	-1.4629	0.92826	0.999875206
BRAP	ENSG00000089234	12	112069950	112133790	27	3	123834	-1.4637	0.92836	0.999875206
ACAD11	ENSG00000240303	3	132266982	132389567	153	14	123834	-1.4641	0.92842	0.999875206
ZIK1	ENSG00000171649	19	58079824	58115145	40	4	123834	-1.4646	0.92849	0.999875206
KRT31	ENSG00000094796	17	39539976	39563844	45	8	123834	-1.4647	0.9285	0.999875206
CHPF	ENSG00000123989	2	220393669	220418509	20	2	123834	-1.4652	0.92857	0.999875206
ABHD1	ENSG00000143994	2	27336682	27363680	12	3	123834	-1.4658	0.92865	0.999875206
AC007461.1	ENSG00000267893	17	70026164	70046872	25	4	123834	-1.4669	0.9288	0.999875206
SAP18	ENSG00000150459	13	21704653	21733221	51	7	123834	-1.4678	0.92892	0.999875206
AC013469.1	ENSG00000267963	2	241043907	241074304	140	12	123834	-1.4685	0.92902	0.999875206
SEC14L2	ENSG00000100003	22	30782846	30831305	104	15	123834	-1.4686	0.92902	0.999875206
CRIM1	ENSG00000150938	2	36573069	36788278	202	35	123834	-1.4686	0.92903	0.999875206
ARRB2	ENSG00000141480	17	4603784	4634794	54	5	123834	-1.4688	0.92906	0.999875206
KIT	ENSG00000157404	4	55514085	55616881	120	16	123834	-1.4693	0.92913	0.999875206
USP46	ENSG00000109189	4	53447138	53535502	77	2	123834	-1.4699	0.92921	0.999875206
PCDHA5	ENSG00000204965	5	140191222	140401929	263	12	123834	-1.4706	0.9293	0.999875206
DNAJC9	ENSG00000213551	10	74933120	75018620	150	2	123834	-1.4707	0.92932	0.999875206
QRICH2	ENSG00000129646	17	74260130	74313761	122	10	123834	-1.471	0.92936	0.999875206
MCM2	ENSG00000073111	3	127307066	127351276	21	5	123834	-1.472	0.9295	0.999875206
TMEM117	ENSG00000139173	12	44219770	44793545	1083	25	123834	-1.4725	0.92955	0.999875206
TSHZ2	ENSG00000182463	20	51578946	52121869	743	94	123834	-1.4727	0.92959	0.999875206
FAM149B1	ENSG00000138286	10	74917924	75014262	170	2	123834	-1.4734	0.92967	0.999875206
CNIH3	ENSG00000143786	1	224612362	224938251	390	27	123834	-1.4739	0.92974	0.999875206
RRM2	ENSG00000171848	2	10252455	10281545	24	5	123834	-1.4744	0.92982	0.999875206
TRIM71	ENSG00000206557	3	32849510	32949318	89	12	123834	-1.4746	0.92984	0.999875206
CBLB	ENSG00000114423	3	105364305	105598396	382	19	123834	-1.4746	0.92984	0.999875206
CCDC129	ENSG00000180347	7	31543704	31708334	306	19	123834	-1.4747	0.92985	0.999875206
CPN2	ENSG00000178772	3	194050494	194082057	42	7	123834	-1.4751	0.92991	0.999875206
CENPQ	ENSG00000031691	6	49421091	49470820	75	7	123834	-1.4758	0.93	0.999875206
TAS2R14	ENSG00000212127	12	11080005	11334172	973	14	123834	-1.4775	0.93023	0.999875206
TRAM1L1	ENSG00000174599	4	117994718	118016736	30	6	123834	-1.4779	0.93028	0.999875206
RFWD3	ENSG00000168411	16	74645292	74710779	191	15	123834	-1.4788	0.9304	0.999875206
QSER1	ENSG00000060749	11	32904724	33024862	156	7	123834	-1.4789	0.93042	0.999875206
TMEM198	ENSG00000188760	2	220398385	220425317	26	3	123834	-1.479	0.93043	0.999875206
NSG1	ENSG00000168824	4	4339867	4430785	123	22	123834	-1.4793	0.93047	0.999875206
RBM45	ENSG00000155636	2	178967151	179013738	66	13	123834	-1.4797	0.93053	0.999875206
JMY	ENSG00000152409	5	78522012	78633038	146	11	123834	-1.4801	0.93058	0.999875206
MTURN	ENSG00000180354	7	30164426	30212378	69	8	123834	-1.4802	0.93059	0.999875206
NIPSNAP3A	ENSG00000136783	9	107499969	107532403	48	7	123834	-1.4803	0.9306	0.999875206
NMRK1	ENSG00000106733	9	77665489	77713133	147	19	123834	-1.4803	0.93061	0.999875206
ADM	ENSG00000148926	11	10316227	10338944	41	5	123834	-1.481	0.9307	0.999875206
FBXL8	ENSG00000135722	16	67183834	67208473	6	3	123834	-1.4813	0.93074	0.999875206
GPI	ENSG00000105220	19	34840385	34903061	81	10	123834	-1.4813	0.93074	0.999875206
SYNC	ENSG00000162520	1	33135507	33179197	62	3	123834	-1.4817	0.93079	0.999875206
P2RY14	ENSG00000174944	3	150919905	151006255	78	14	123834	-1.4842	0.93112	0.999875206
GIGYF1	ENSG00000146830	7	100267130	100297071	29	6	123834	-1.4848	0.9312	0.999875206
AL136531.1	ENSG00000203630	20	1306228	1327555	34	6	123834	-1.4851	0.93124	0.999875206
SYNGR3	ENSG00000127561	16	2029661	2054276	63	7	123834	-1.486	0.93136	0.999875206
RIMS1	ENSG00000079841	6	72586406	73122845	659	30	123834	-1.4865	0.93142	0.999875206
NAV3	ENSG00000067798	12	78214685	78616790	773	49	123834	-1.4867	0.93145	0.999875206
MSMO1	ENSG00000052802	4	166238775	166274312	59	7	123834	-1.4871	0.9315	0.999875206
WFDC13	ENSG00000168634	20	44320655	44347456	78	12	123834	-1.4879	0.93161	0.999875206
OR6B1	ENSG00000221813	7	143691022	143712068	35	2	123834	-1.4886	0.9317	0.999875206
SIGIRR	ENSG00000185187	11	395716	427455	22	6	123834	-1.4892	0.93178	0.999875206
ERP29	ENSG00000089248	12	112441120	112471255	36	3	123834	-1.49	0.93188	0.999875206
UBAP2L	ENSG00000143569	1	154182655	154253986	66	6	123834	-1.4912	0.93205	0.999875206
AMBP	ENSG00000106927	9	116812407	116850752	69	9	123834	-1.4914	0.93207	0.999875206
C15orf65	ENSG00000261652	15	55690746	55720962	86	6	123834	-1.4915	0.93209	0.999875206
TECR	ENSG00000099797	19	14617897	14686792	88	15	123834	-1.492	0.93215	0.999875206
C17orf67	ENSG00000214226	17	54859274	54926134	139	10	123834	-1.4921	0.93216	0.999875206
SH3BGRL3	ENSG00000142669	1	26595667	26618007	58	7	123834	-1.4936	0.93236	0.999875206
FAM193B	ENSG00000146067	5	176936789	176991542	37	1	123834	-1.4939	0.9324	0.999875206
USP53	ENSG00000145390	4	120123742	120226672	244	19	123834	-1.4943	0.93245	0.999875206
IGFBP5	ENSG00000115461	2	217526828	217570248	48	2	123834	-1.4944	0.93246	0.999875206
NUDCD3	ENSG00000015676	7	44408720	44540479	212	5	123834	-1.4944	0.93246	0.999875206
ARHGAP11B	ENSG00000187951	15	30906697	31075196	115	9	123834	-1.4951	0.93255	0.999875206
IPO9	ENSG00000198700	1	201788269	201863422	87	10	123834	-1.4953	0.93259	0.999875206
RGS9	ENSG00000108370	17	63123549	63233821	247	25	123834	-1.4955	0.93261	0.999875206
CDCA7	ENSG00000144354	2	174209548	174243725	64	12	123834	-1.4958	0.93265	0.999875206
OR51A7	ENSG00000176895	11	4918600	4939538	51	9	123834	-1.4958	0.93265	0.999875206
TPRA1	ENSG00000163870	3	127281912	127327094	22	7	123834	-1.4964	0.93272	0.999875206
GPR56	ENSG00000205336	16	57634564	57708944	115	23	123834	-1.4973	0.93284	0.999875206
YIPF2	ENSG00000130733	19	11023444	11049357	32	4	123834	-1.4976	0.93288	0.999875206
NXNL1	ENSG00000171773	19	17556234	17581763	24	3	123834	-1.4978	0.93291	0.999875206

RAN	ENSG00000132341	12	131346424	131372223	57	7	123834	-1.4983	0.93297	0.999875206
SLFN12L	ENSG00000205045	17	33781268	33874880	155	29	123834	-1.4986	0.93302	0.999875206
TSPAN1	ENSG00000117472	1	46630745	46661630	18	4	123834	-1.4996	0.93314	0.999875206
FOXR1	ENSG00000176302	11	118832417	118862001	42	6	123834	-1.4996	0.93314	0.999875206
MB21D2	ENSG00000180611	3	192504604	192645950	197	22	123834	-1.4998	0.93316	0.999875206
PIK3AP1	ENSG00000155629	10	98343069	98490271	195	29	123834	-1.5008	0.93329	0.999875206
TCP10L	ENSG00000242220	21	33938862	33969683	60	9	123834	-1.5007	0.93329	0.999875206
OR52I2	ENSG00000226288	11	4598021	4619135	59	6	123834	-1.5012	0.93334	0.999875206
DIXDC1	ENSG00000150764	11	111787868	111903308	51	2	123834	-1.5021	0.93346	0.999875206
NUPL2	ENSG00000136243	7	23211446	23250630	92	4	123834	-1.5022	0.93347	0.999875206
GJB3	ENSG00000188910	1	35236790	35261970	28	6	123834	-1.5034	0.93363	0.999875206
PTGER4	ENSG00000171522	5	40669600	40703837	65	6	123834	-1.5036	0.93366	0.999875206
TMEM132C	ENSG00000181234	12	128741948	129202460	433	76	123834	-1.5036	0.93366	0.999875206
OR52N4	ENSG00000181074	11	5765923	5786959	92	6	123834	-1.5037	0.93368	0.999875206
C11orf57	ENSG00000150776	11	111934810	111965874	20	4	123834	-1.504	0.93372	0.999875206
PEX12	ENSG00000108733	17	33891814	33915882	59	10	123834	-1.5042	0.93374	0.999875206
MYO9A	ENSG00000066933	15	72104632	72420918	370	12	123834	-1.5044	0.93377	0.999875206
TBC1D30	ENSG00000111490	12	65164589	65284812	96	11	123834	-1.5049	0.93382	0.999875206
BMP8A	ENSG00000183682	1	39947318	40001607	84	12	123834	-1.5065	0.93403	0.999875206
RPL39L	ENSG00000163923	3	186828736	186908696	89	15	123834	-1.5069	0.93409	0.999875206
SMR3B	ENSG00000171201	4	71225810	71265961	147	16	123834	-1.5071	0.93411	0.999875206
KCNE2	ENSG00000159197	21	35726323	35753688	18	4	123834	-1.5081	0.93423	0.999875206
TM7SF3	ENSG00000064115	12	27116128	27177367	69	13	123834	-1.5089	0.93434	0.999875206
GALNT6	ENSG00000139629	12	51735031	51796651	47	10	123834	-1.5113	0.93464	0.999875206
TXNRD3NB	ENSG00000206483	3	126280625	126337398	99	15	123834	-1.5114	0.93466	0.999875206
BZRAP1	ENSG00000005379	17	56368592	56416152	79	12	123834	-1.5132	0.93488	0.999875206
ZFP3	ENSG00000180787	17	4971543	5009669	57	10	123834	-1.5138	0.93496	0.999875206
ZSCAN4	ENSG00000180532	19	58170303	58200520	40	4	123834	-1.5142	0.93501	0.999875206
PHC3	ENSG00000173889	3	169794520	169909537	111	8	123834	-1.5148	0.93508	0.999875206
MYOM2	ENSG00000036448	8	1983155	2123475	611	52	123834	-1.5149	0.9351	0.999875206
EDAR	ENSG00000135960	2	109500927	109615828	275	17	123834	-1.5154	0.93516	0.999875206
CSAD	ENSG00000139631	12	53541447	53585135	51	5	123834	-1.5157	0.9352	0.999875206
EVA1C	ENSG00000166979	21	33774314	33897707	209	19	123834	-1.5157	0.93521	0.999875206
RP11-162P23.2	ENSG00000257767	12	112181694	112239222	30	3	123834	-1.5161	0.93525	0.999875206
ANKFY1	ENSG00000185722	17	4057201	4177274	173	12	123834	-1.5162	0.93527	0.999875206
RPS15	ENSG00000115268	19	1428358	1450583	11	2	123834	-1.5162	0.93527	0.999875206
HTN3	ENSG00000205649	4	70884130	70912255	72	9	123834	-1.5163	0.93528	0.999875206
NAV1	ENSG00000134369	1	201582411	201806102	224	36	123834	-1.5164	0.9353	0.999875206
RP11-219A15.1	ENSG00000266302	17	16583575	16717767	17	2	123834	-1.5166	0.93532	0.999875206
IGFL1	ENSG00000188293	19	46723009	46744500	62	7	123834	-1.5171	0.93537	0.999875206
PTPN14	ENSG00000152104	1	214512039	214735792	460	34	123834	-1.5171	0.93538	0.999875206
CCDC126	ENSG00000169193	7	23626998	23694327	49	6	123834	-1.5173	0.93541	0.999875206
KLHDC8A	ENSG00000162873	1	205295220	205336218	110	12	123834	-1.5174	0.93542	0.999875206
C19orf18	ENSG00000177025	19	58459805	58495902	70	6	123834	-1.5186	0.93557	0.999875206
TAC3	ENSG00000166863	12	57393784	57432667	46	3	123834	-1.5191	0.93563	0.999875206
CTNNA3	ENSG00000183230	10	67662276	69465927	4624	150	123834	-1.5198	0.93572	0.999875206
CORO1C	ENSG00000110880	12	109028885	109135372	149	12	123834	-1.5206	0.93582	0.999875206
OTOF	ENSG00000115155	2	26670071	26791566	109	23	123834	-1.5211	0.93589	0.999875206
C22orf29	ENSG00000215012	22	19823661	19852419	52	6	123834	-1.5213	0.93591	0.999875206
ZNF853	ENSG00000236609	7	6645248	6673921	46	6	123834	-1.5216	0.93594	0.999875206
CDH23	ENSG00000107736	10	73146691	73585702	630	92	123834	-1.5216	0.93595	0.999875206
OR51B4	ENSG00000183251	11	5312244	5333226	50	9	123834	-1.5219	0.93598	0.999875206
NSUN2	ENSG00000037474	5	6589352	6643404	188	17	123834	-1.5228	0.93609	0.999875206
ZNF823	ENSG00000197933	19	11822080	11859824	60	5	123834	-1.523	0.93612	0.999875206
ZNF486	ENSG00000256229	19	20268037	20321299	115	5	123834	-1.5233	0.93615	0.999875206
IRF1	ENSG00000125347	5	131807301	131836490	53	3	123834	-1.5235	0.93618	0.999875206
ANXA4	ENSG00000196975	2	69861557	70063596	144	16	123834	-1.5236	0.93619	0.999875206
CEP76	ENSG00000101624	18	12651832	12712776	145	10	123834	-1.5235	0.93619	0.999875206
PHLDB1	ENSG00000019144	11	118467155	118538741	66	9	123834	-1.5238	0.93622	0.999875206
ALPI	ENSG00000163295	2	233310833	233334742	29	6	123834	-1.5242	0.93627	0.999875206
CREBRF	ENSG00000164463	5	172473355	172576291	207	16	123834	-1.5247	0.93633	0.999875206
MTUS2	ENSG00000132938	13	29588748	30087892	1062	50	123834	-1.5254	0.93642	0.999875206
RPL37	ENSG00000145592	5	40815364	40845437	45	7	123834	-1.5259	0.93649	0.999875206
RASSF2	ENSG00000101265	20	4750669	4814291	116	20	123834	-1.5263	0.93654	0.999875206
SLC22A4	ENSG00000197208	5	131620136	131689899	136	9	123834	-1.5267	0.93658	0.999875206
OR5M3	ENSG00000174937	11	56226964	56248014	59	9	123834	-1.5269	0.93661	0.999875206
FXN	ENSG00000165060	9	71640175	71725094	213	15	123834	-1.527	0.93663	0.999875206
GPR156	ENSG00000175697	3	119873492	120013941	339	20	123834	-1.5272	0.93664	0.999875206
CD302	ENSG00000241399	2	160615364	160664753	115	13	123834	-1.5272	0.93665	0.999875206
CLDN25	ENSG00000228607	11	113640469	113661222	51	7	123834	-1.5272	0.93665	0.999875206
ZNF708	ENSG00000182141	19	21463962	21522227	173	11	123834	-1.5275	0.93669	0.999875206
PREB	ENSG00000138073	2	27343624	27367543	15	3	123834	-1.5278	0.93672	0.999875206
TTC33	ENSG00000113638	5	40704577	40766077	97	10	123834	-1.5278	0.93672	0.999875206
CNGA3	ENSG00000144191	2	98952618	99025064	100	9	123834	-1.5279	0.93674	0.999875206
ASF1A	ENSG00000111875	6	119205384	119240332	59	7	123834	-1.5282	0.93676	0.999875206
INPP1	ENSG00000151689	2	191198196	191246391	85	6	123834	-1.5283	0.93678	0.999875206
ABRA	ENSG00000174429	8	107761711	107792473	41	9	123834	-1.5283	0.93678	0.999875206
DBR1	ENSG00000138231	3	137869854	137903791	54	1	123834	-1.5284	0.93679	0.999875206
MAFB	ENSG00000204103	20	39304488	39327880	10	1	123834	-1.5284	0.93679	0.999875206
BBS12	ENSG00000181004	4	123643857	123676098	75	6	123834	-1.5289	0.93685	0.999875206
PLA2G4E	ENSG00000188089	15	42263780	42353388	246	30	123834	-1.529	0.93687	0.999875206
EMC9	ENSG00000100908	14	24598174	24620797	15	3	123834	-1.5304	0.93704	0.999875206
CCDC144A	ENSG00000170160	17	16582851	16717767	18	2	123834	-1.5316	0.93719	0.999875206
NEK10	ENSG00000163491	3	27141576	27420951	612	17	123834	-1.5323	0.93727	0.999875206
MED7	ENSG00000155868	5	156554423	156596030	67	7	123834	-1.5324	0.93729	0.999875206
ZP2	ENSG00000103310	16	21198773	21235831	32	6	123834	-1.533	0.93737	0.999875206
POLD1	ENSG00000062822	19	50877461	50931273	62	5	123834	-1.5338	0.93746	0.999875206
NUDT21	ENSG00000167005	16	56453045	56496111	74	10	123834	-1.5339	0.93748	0.999875206
PDCD2	ENSG00000071994	6	170874383	170903780	50	5	123834	-1.5342	0.93751	0.999875206
FPR2	ENSG00000171049	19	52245279	52283779	53	9	123834	-1.5347	0.93757	0.999875206
AC011294.3	ENSG00000233539	7	46703383	46809449	265	22	123834	-1.5349	0.93759	0.999875206
TRMT1	ENSG00000104907	19	13205716	13238381	21	7	123834	-1.5353	0.93765	0.999875206
ADCYAP1R1	ENSG00000078549	7	31082076	31161089	41	10	123834	-1.5356	0.93768	0.999875206
ZNF414	ENSG00000133250	19	8565462	8589048	72	3	123834	-1.5367	0.93781	0.999875206
RPS3	ENSG00000149273	11	75100530	75143324	40	8	123834	-1.537	0.93785	0.999875206
KLHL7	ENSG00000122550	7	23135353	23227533	198	9	123834	-1.5398	0.9382	0.999875206
USP49	ENSG00000164663	6	41747634	41873099	183	18	123834	-1.5399	0.93821	0.999875206
ADAM22	ENSG00000008277	7	87553458	87842204	277	16	123834	-1.5412	0.93837	0.999875206
RNF24	ENSG00000101236	20	3897956	4006229	198	14	123834	-1.5412	0.93837	0.999875206

S100A11	ENSG00000163191	1	151994982	152030383	20	2	123834	-1.5415	0.9384	0.999875206
NT5M	ENSG00000205309	17	17196649	17260977	88	15	123834	-1.5418	0.93844	0.999875206
HSPB8	ENSG00000152137	12	119606447	119668936	63	12	123834	-1.5421	0.93847	0.999875206
RP11-89N17.1	ENSG00000173862	7	33755593	33778068	20	5	123834	-1.5433	0.93862	0.999875206
C12orf10	ENSG00000139637	12	53683470	53710961	26	2	123834	-1.5433	0.93862	0.999875206
IGFBP7	ENSG00000163453	4	57886939	57986551	254	31	123834	-1.5435	0.93865	0.999875206
NBPF4	ENSG00000196427	1	108755963	108796689	20	3	123834	-1.5443	0.93874	0.999875206
MYCL	ENSG00000116990	1	40351098	40377928	45	9	123834	-1.5444	0.93876	0.999875206
AC090616.2	ENSG00000214708	17	30458245	30480154	22	2	123834	-1.5451	0.93884	0.999875206
CIB3	ENSG00000141977	19	16262179	16294336	66	7	123834	-1.5456	0.93889	0.999875206
KCNJ6	ENSG00000157542	21	38969678	39298749	431	55	123834	-1.5458	0.93892	0.999875206
YKT6	ENSG00000106636	7	44230567	44263893	59	8	123834	-1.546	0.93895	0.999875206
A4GNT	ENSG00000118017	3	137832560	137861229	25	3	123834	-1.547	0.93906	0.999875206
GPR116	ENSG00000069122	6	46810249	46932680	281	27	123834	-1.5473	0.9391	0.999875206
IGSF22	ENSG00000179057	11	18715852	18757777	44	6	123834	-1.5483	0.93922	0.999875206
DKFZP667F0711	ENSG00000212743	10	6382278	6404723	26	6	123834	-1.5493	0.93935	0.999875206
ERCC3	ENSG00000163161	2	128004866	128061752	104	14	123834	-1.5494	0.93936	0.999875206
U2SURP	ENSG00000163714	3	142673339	142789567	125	10	123834	-1.5494	0.93936	0.999875206
RAP1B	ENSG00000127314	12	68994619	69064372	76	12	123834	-1.5504	0.93948	0.999875206
IFI27	ENSG00000165949	14	94561182	94593033	47	9	123834	-1.551	0.93955	0.999875206
TBC1D22A	ENSG00000054611	22	47148518	47581336	786	62	123834	-1.5513	0.93959	0.999875206
TCEANC2	ENSG00000116205	1	54509260	54588192	99	7	123834	-1.5518	0.93965	0.999875206
HEATR3	ENSG00000155393	16	50089852	50150298	138	9	123834	-1.5521	0.93968	0.999875206
GOLGA4	ENSG00000144674	3	37274668	37418242	191	9	123834	-1.5524	0.93971	0.999875206
TFR2	ENSG00000106327	7	100208039	100250402	26	5	123834	-1.5529	0.93977	0.999875206
ASCL5	ENSG00000232237	1	201073081	201106312	40	7	123834	-1.5533	0.93982	0.999875206
PCDHA3	ENSG00000255408	5	140170783	140401929	291	12	123834	-1.5533	0.93982	0.999875206
CARM1	ENSG00000142453	19	10972189	11043453	69	6	123834	-1.5534	0.93984	0.999875206
MR1	ENSG00000153029	1	180993067	181041074	91	13	123834	-1.5539	0.9399	0.999875206
ZNHIT2	ENSG00000174276	11	64873875	64895170	22	4	123834	-1.554	0.93991	0.999875206
ATP5J	ENSG00000154723	21	27078815	27117984	38	4	123834	-1.5541	0.93992	0.999875206
PLA2G4F	ENSG00000168907	15	42423332	42458839	40	7	123834	-1.5542	0.93993	0.999875206
SMARCAL1	ENSG00000138375	2	217267137	217357776	183	16	123834	-1.5546	0.93998	0.999875206
ZNF418	ENSG00000196724	19	58423252	58456761	24	2	123834	-1.5548	0.94001	0.999875206
AC006449.1	ENSG00000269094	17	36874086	36894451	4	2	123834	-1.5551	0.94004	0.999875206
ZFAND1	ENSG00000104231	8	82603569	82655138	148	11	123834	-1.5552	0.94005	0.999875206
POMGNT1	ENSG00000085998	1	46644354	46695977	36	6	123834	-1.5553	0.94007	0.999875206
HMCES	ENSG00000183624	3	128987671	129035029	62	2	123834	-1.5554	0.94007	0.999875206
PCDHA4	ENSG00000204967	5	140176659	140401929	284	12	123834	-1.5563	0.94018	0.999875206
ZNF569	ENSG00000196437	19	37892057	37970177	93	10	123834	-1.5565	0.9402	0.999875206
FAM102B	ENSG00000162636	1	109092711	109197522	134	4	123834	-1.5565	0.94021	0.999875206
AMFR	ENSG00000159461	16	56385364	56469450	180	12	123834	-1.5584	0.94043	0.999875206
HSP90B1	ENSG00000166598	12	104313885	104357423	143	21	123834	-1.5596	0.94058	0.999875206
FOXJ2	ENSG00000065970	12	8175299	8218099	21	4	123834	-1.56	0.94062	0.999875206
FNBP1L	ENSG00000137942	1	93903688	94030218	60	7	123834	-1.5604	0.94066	0.999875206
FOXP4	ENSG00000137166	6	41504164	41580122	128	16	123834	-1.5626	0.94092	0.999875206
KLHL18	ENSG00000114648	3	47314407	47398306	39	3	123834	-1.5632	0.94099	0.999875206
AMY2B	ENSG00000240038	1	104086437	104132156	22	6	123834	-1.5633	0.94101	0.999875206
ODF3B	ENSG00000177989	22	50958139	50981009	46	9	123834	-1.5636	0.94104	0.999875206
ZNF502	ENSG00000196653	3	44744135	44775323	28	5	123834	-1.5638	0.94107	0.999875206
CYP2U1	ENSG00000155016	4	108842525	108884613	83	7	123834	-1.5641	0.94111	0.999875206
NBEAL2	ENSG00000160796	3	47011173	47061193	35	5	123834	-1.5654	0.94126	0.999875206
TDRKH	ENSG00000182134	1	151732583	151773892	76	6	123834	-1.5655	0.94127	0.999875206
NSRP1	ENSG00000126653	17	28432539	28523493	93	3	123834	-1.566	0.94133	0.999875206
NMUR1	ENSG00000171596	2	232377871	232405206	77	7	123834	-1.567	0.94144	0.999875206
GPR22	ENSG00000172209	7	107100463	107126098	28	4	123834	-1.5678	0.94154	0.999875206
CEP85L	ENSG00000111860	6	118771935	119041238	558	21	123834	-1.5682	0.94159	0.999875206
LRRC19	ENSG00000184434	9	26983134	27015691	64	11	123834	-1.5694	0.94172	0.999875206
PNMAL1	ENSG00000182013	19	46959748	46984820	68	10	123834	-1.5698	0.94177	0.999875206
WDR78	ENSG00000152763	1	67268568	67400570	149	17	123834	-1.5699	0.94178	0.999875206
KRTAP2-2	ENSG00000214518	17	39200750	39221482	79	7	123834	-1.5699	0.94178	0.999875206
CNNM3	ENSG00000168763	2	97471982	97509648	18	1	123834	-1.57	0.94179	0.999875206
SLC35E3	ENSG00000175782	12	69129886	69197508	70	17	123834	-1.5702	0.94181	0.999875206
SLC24A2	ENSG00000155886	9	19497450	19796926	455	45	123834	-1.5704	0.94183	0.999875206
CATX-2	ENSG00000267958	2	74408517	74428627	23	3	123834	-1.5706	0.94186	0.999875206
OR56B3P	ENSG00000180913	11	6139840	6160706	38	5	123834	-1.5706	0.94186	0.999875206
TAS2R20	ENSG00000255837	12	11139094	11160474	96	4	123834	-1.5718	0.942	0.999875206
CDC14A	ENSG00000079335	1	100800584	100995833	169	10	123834	-1.572	0.94203	0.999875206
SPTBN2	ENSG00000173898	11	66442719	66506697	29	9	123834	-1.5724	0.94207	0.999875206
FABP5	ENSG00000164687	8	82182598	82207012	20	4	123834	-1.5725	0.94208	0.999875206
EPGN	ENSG00000182585	4	75164190	75191024	35	3	123834	-1.5727	0.94211	0.999875206
SHISA6	ENSG00000188803	17	11134580	11477380	617	64	123834	-1.5732	0.94216	0.999875206
ZNF836	ENSG00000196267	19	52647117	52684896	54	10	123834	-1.5735	0.94219	0.999875206
MGST3	ENSG00000143198	1	165590098	165641033	174	18	123834	-1.5739	0.94224	0.999875206
CPD	ENSG00000108582	17	28695923	28807007	101	5	123834	-1.5741	0.94226	0.999875206
IFITM3	ENSG00000142089	11	309669	337537	9	3	123834	-1.5752	0.9424	0.999875206
AP000275.65	ENSG00000265590	21	33941132	33994591	100	12	123834	-1.5752	0.9424	0.999875206
CST5	ENSG00000170367	20	23846572	23870387	33	6	123834	-1.5761	0.94249	0.999875206
SLC6A2	ENSG00000103546	16	55679516	55750104	127	23	123834	-1.5761	0.9425	0.999875206
WNT10A	ENSG00000135925	2	219735085	219774303	7	2	123834	-1.5765	0.94254	0.999875206
TTC39C	ENSG00000168234	18	21562737	21725574	167	27	123834	-1.5766	0.94256	0.999875206
CTB-167G5.5	ENSG00000268864	19	54092882	54116751	16	2	123834	-1.5767	0.94256	0.999875206
CHRAC1	ENSG00000104472	8	141511397	141537236	22	4	123834	-1.5769	0.94259	0.999875206
KIAA1658	ENSG00000268538	22	30804212	30824469	38	9	123834	-1.5772	0.94262	0.999875206
OOSP2	ENSG00000149507	11	59797748	59825517	19	5	123834	-1.5774	0.94265	0.999875206
TCHH	ENSG00000159450	1	152068793	152096556	7	3	123834	-1.5784	0.94276	0.999875206
PFDN5	ENSG00000123349	12	53679075	53703234	21	3	123834	-1.5796	0.9429	0.999875206
CRYBB2	ENSG00000244752	22	25605489	25637836	45	8	123834	-1.581	0.94306	0.999875206
ZW10	ENSG00000086827	11	113593909	113654533	148	17	123834	-1.581	0.94307	0.999875206
ZNF133	ENSG00000125846	20	18259121	18307640	74	12	123834	-1.5821	0.94318	0.999875206
PTDSS1	ENSG00000156471	8	97263943	97359223	54	4	123834	-1.5833	0.94332	0.999875206
ETV7	ENSG00000010030	6	36312419	36366164	89	17	123834	-1.5837	0.94337	0.999875206
AC132186.1	ENSG00000247270	16	66506775	66529747	21	3	123834	-1.584	0.9434	0.999875206
C21orf59	ENSG00000159079	21	33954389	33995176	84	10	123834	-1.5843	0.94344	0.999875206
OR5C1	ENSG00000148215	9	125541150	125562237	44	4	123834	-1.5848	0.94349	0.999875206
KRIT1	ENSG00000001631	7	91818283	91885480	44	5	123834	-1.5853	0.94355	0.999875206
MTMR2	ENSG00000087053	11	95556046	95668479	215	17	123834	-1.5853	0.94355	0.999875206
C1QTNF3	ENSG00000082196	5	34009553	34053937	65	8	123834	-1.5853	0.94356	0.999875206
PRDM15	ENSG00000141956	21	43208385	43309591	237	33	123834	-1.5857	0.9436	0.999875206

CORO2B	ENSG00000103647	15	68861308	69030145	233	27	123834	-1.5858	0.94361	0.999875206
HAND2	ENSG00000164107	4	174436120	174461380	20	4	123834	-1.586	0.94363	0.999875206
SCLY	ENSG00000132330	2	238959530	239018054	134	12	123834	-1.5863	0.94367	0.999875206
ROS1	ENSG00000047936	6	117599463	117757018	344	26	123834	-1.5865	0.94369	0.999875206
AC131097.4	ENSG00000216921	2	242826136	242854702	104	11	123834	-1.587	0.94375	0.999875206
ACTL8	ENSG00000117148	1	18071808	18163558	111	12	123834	-1.5874	0.94379	0.999875206
LRRC1	ENSG00000137269	6	53649295	53798919	319	15	123834	-1.5882	0.94388	0.999875206
ALDOB	ENSG00000136872	9	104172860	104208105	68	9	123834	-1.5885	0.94391	0.999875206
LRRC38	ENSG00000162494	1	13791445	13850543	95	21	123834	-1.5892	0.94399	0.999875206
RP11-93B14.6	ENSG00000167046	20	61262071	61284656	13	2	123834	-1.5899	0.94407	0.999875206
CLDN19	ENSG00000164007	1	43188764	43215925	40	7	123834	-1.5905	0.94413	0.999875206
PDE9A	ENSG00000160191	21	44063746	44205619	292	29	123834	-1.5911	0.94421	0.999875206
PITX2	ENSG00000164093	4	111528579	111573279	13	2	123834	-1.5912	0.94422	0.999875206
FMO2	ENSG00000094963	1	171144347	171191822	178	14	123834	-1.5915	0.94426	0.999875206
ZNF99	ENSG00000213973	19	22929007	22976909	196	9	123834	-1.5915	0.94426	0.999875206
C20orf194	ENSG00000088854	20	3219951	3398272	549	14	123834	-1.5919	0.9443	0.999875206
LINC01101	ENSG00000268194	2	121211911	121233697	35	10	123834	-1.5924	0.94435	0.999875206
CSRP2	ENSG00000175183	12	77242495	77282840	56	9	123834	-1.5932	0.94445	0.999875206
VWA8	ENSG00000102763	13	42130973	42545256	803	35	123834	-1.5941	0.94454	0.999875206
YLPM1	ENSG00000119596	14	75220069	75332244	150	10	123834	-1.5944	0.94458	0.999875206
C21orf88	ENSG00000184809	21	40959074	40994748	53	10	123834	-1.5949	0.94463	0.999875206
LAMA3	ENSG00000053747	18	21259407	21545030	338	14	123834	-1.5952	0.94467	0.999875206
ADH1A	ENSG00000187758	4	100187524	100222185	70	9	123834	-1.5953	0.94468	0.999875206
GSDMC	ENSG00000147697	8	130750442	130809134	138	18	123834	-1.5953	0.94468	0.999875206
CPNE4	ENSG00000196353	3	131242399	132014254	1757	76	123834	-1.5956	0.94471	0.999875206
SLC6A15	ENSG00000072041	12	85243492	85317394	65	7	123834	-1.5958	0.94474	0.999875206
VN1R2	ENSG00000196131	19	53751545	53775090	54	6	123834	-1.596	0.94475	0.999875206
MS4A18	ENSG00000214782	11	60486789	60521685	32	3	123834	-1.596	0.94476	0.999875206
EGF	ENSG00000138798	4	110824040	110943422	127	9	123834	-1.5961	0.94477	0.999875206
SYT11	ENSG00000132718	1	155819300	155864990	45	6	123834	-1.5978	0.94495	0.999875206
PFKM	ENSG00000152556	12	48488922	48550187	128	13	123834	-1.5991	0.9451	0.999875206
PRR4	ENSG00000111215	12	10967559	11334212	1198	16	123834	-1.5998	0.94518	0.999875206
MOB3C	ENSG00000142961	1	47063387	47092563	37	4	123834	-1.6005	0.94525	0.999875206
TREM2	ENSG00000095970	6	41116244	41140924	10	4	123834	-1.6015	0.94537	0.999875206
LARP6	ENSG00000166173	15	71113863	71156498	53	6	123834	-1.6017	0.94539	0.999875206
SOX12	ENSG00000177732	20	296207	320865	61	5	123834	-1.6021	0.94543	0.999875206
UBE2F-SCLY	ENSG00000258984	2	238865656	239018053	336	12	123834	-1.6028	0.94551	0.999875206
B3GNT4	ENSG00000176383	12	122678090	122703499	9	2	123834	-1.6032	0.94555	0.999875206
MTX2	ENSG00000128654	2	177124123	177212753	95	8	123834	-1.6038	0.94562	0.999875206
ADH6	ENSG00000172955	4	100113795	100150694	50	9	123834	-1.6045	0.94569	0.999875206
SPDYE3	ENSG00000214300	7	99895325	99929819	20	3	123834	-1.6059	0.94586	0.999875206
MON1A	ENSG00000164077	3	49936302	49977606	39	5	123834	-1.6065	0.94592	0.999875206
RP11-5A19.5	ENSG00000265690	16	67187288	67209718	8	3	123834	-1.6068	0.94595	0.999875206
EPS15	ENSG00000085832	1	51809935	51995000	452	16	123834	-1.6069	0.94596	0.999875206
GATM	ENSG00000171766	15	45643322	45704525	107	3	123834	-1.6072	0.946	0.999875206
COG5	ENSG00000164597	7	106832000	107214959	587	12	123834	-1.6076	0.94604	0.999875206
CD96	ENSG00000153283	3	111001566	111394597	643	45	123834	-1.608	0.94609	0.999875206
AKAP3	ENSG00000111254	12	4714674	4768213	121	12	123834	-1.6092	0.94622	0.999875206
RABL2A	ENSG00000144134	2	114374806	114410973	31	6	123834	-1.6097	0.94627	0.999875206
ZNF557	ENSG00000130544	19	7059455	7097979	43	8	123834	-1.6097	0.94627	0.999875206
DSC3	ENSG00000134762	18	28559974	28632781	83	10	123834	-1.61	0.9463	0.999875206
GPR152	ENSG00000175514	11	67208772	67230200	17	2	123834	-1.6102	0.94632	0.999875206
CABP4	ENSG00000175544	11	67209877	67236699	17	2	123834	-1.6102	0.94632	0.999875206
SOX15	ENSG00000129194	17	7481496	7503488	19	6	123834	-1.6105	0.94635	0.999875206
MRAP	ENSG00000170262	21	33654124	33697095	45	6	123834	-1.6114	0.94645	0.999875206
PILRA	ENSG00000085514	7	99955153	100007719	47	3	123834	-1.6123	0.94655	0.999875206
RP11-468E2.2	ENSG00000259522	14	24639601	24669622	24	7	123834	-1.6123	0.94655	0.999875206
SENP3	ENSG00000161956	17	7455192	7485287	50	11	123834	-1.6123	0.94655	0.999875206
C12orf4	ENSG00000047621	12	4586894	4657674	50	3	123834	-1.6124	0.94656	0.999875206
EME1	ENSG00000154920	17	48440581	48468844	78	8	123834	-1.6132	0.94664	0.999875206
EPO	ENSG00000130427	7	100308423	100331323	36	6	123834	-1.6141	0.94675	0.999875206
ZNF793	ENSG00000188227	19	37987841	38049664	85	13	123834	-1.6147	0.94681	0.999875206
SMNDC1	ENSG00000119953	10	112040488	112074709	62	9	123834	-1.616	0.94695	0.999875206
KRTAP19-8	ENSG00000206102	21	32400478	32420795	16	3	123834	-1.6164	0.947	0.999875206
HCFC2	ENSG00000111727	12	104448235	104508639	94	14	123834	-1.6166	0.94702	0.999875206
CA9	ENSG00000107159	9	35663853	35691156	36	8	123834	-1.6174	0.9471	0.999875206
SLC25A46	ENSG00000164209	5	110063837	110110857	70	10	123834	-1.6174	0.94711	0.999875206
OR2T12	ENSG00000177201	1	248447918	248468880	65	3	123834	-1.6182	0.94719	0.999875206
UBE2O	ENSG00000175931	17	74375532	74459288	171	18	123834	-1.6198	0.94737	0.999875206
DCAF17	ENSG00000115827	2	172280727	172351562	153	9	123834	-1.6211	0.9475	0.999875206
DDX4	ENSG00000152670	5	55023845	55122985	206	20	123834	-1.6212	0.94751	0.999875206
NMNAT2	ENSG00000157064	1	183207372	183397737	245	20	123834	-1.6217	0.94757	0.999875206
PLAA	ENSG00000137055	9	26894081	26957461	169	14	123834	-1.6218	0.94758	0.999875206
RAB3IL1	ENSG00000167994	11	61654773	61697741	40	10	123834	-1.6233	0.94774	0.999875206
AL133481.1	ENSG00000235705	10	81256126	81280678	33	3	123834	-1.6246	0.94787	0.999875206
TIAM2	ENSG00000146426	6	155143831	155588857	614	84	123834	-1.6251	0.94793	0.999875206
KIF9	ENSG00000088727	3	47259516	47334941	47	5	123834	-1.626	0.94803	0.999875206
ATG12	ENSG00000145782	5	115153893	115187555	17	3	123834	-1.6264	0.94807	0.999875206
PTPRCAP	ENSG00000213402	11	67192981	67215538	16	1	123834	-1.627	0.94813	0.999875206
C15orf32	ENSG00000183643	15	93004884	93054347	38	8	123834	-1.6272	0.94815	0.999875206
STMND1	ENSG00000230873	6	17092489	17141603	19	7	123834	-1.6273	0.94816	0.999875206
CCDC122	ENSG00000151773	13	44388045	44463827	230	21	123834	-1.6279	0.94823	0.999875206
HOMEZ	ENSG00000215271	14	23731666	23778656	16	8	123834	-1.6286	0.9483	0.999875206
FBXO39	ENSG00000177294	17	6669542	6710420	94	15	123834	-1.6295	0.94839	0.999875206
RMI2	ENSG00000175643	16	11333476	11455619	338	17	123834	-1.6296	0.94841	0.999875206
TMBIM4	ENSG00000228144	12	66507697	66573765	110	7	123834	-1.6314	0.9486	0.999875206
TMBIM4	ENSG00000155957	12	66507709	66573852	110	7	123834	-1.6314	0.9486	0.999875206
C11orf40	ENSG00000171987	11	4582653	4609050	75	11	123834	-1.6316	0.94862	0.999875206
7-Mar	ENSG00000136536	2	160559000	160637538	249	17	123834	-1.6321	0.94867	0.999875206
DLST	ENSG00000119689	14	75338594	75380448	84	8	123834	-1.633	0.94877	0.999875206
TNFRSF11B	ENSG00000164761	8	119925796	119974439	85	11	123834	-1.6332	0.94878	0.999875206
GJB7	ENSG00000164411	6	87982696	88048996	197	11	123834	-1.6333	0.94879	0.999875206
ZSCAN31	ENSG00000235109	6	28282470	28334048	80	12	123834	-1.6342	0.9489	0.999875206
ALG10	ENSG00000139133	12	34165216	34192629	43	6	123834	-1.6343	0.9489	0.999875206
CTD-2545M3.6	ENSG00000142539	19	50909056	50941666	25	6	123834	-1.6349	0.94896	0.999875206
AC022431.2	ENSG00000225940	5	55797394	55912059	247	37	123834	-1.6359	0.94907	0.999875206
MAF1	ENSG00000179632	8	145149402	145172514	27	1	123834	-1.6366	0.94915	0.999875206
CTC-236F12.4	ENSG00000248727	5	55743622	55787773	142	17	123834	-1.6372	0.9492	0.999875206
MMAA	ENSG00000151611	4	146529415	146591187	79	11	123834	-1.6372	0.94921	0.999875206

THEM5	ENSG00000196407	1	151809739	151836173	29	6	123834	-1.6382	0.94931	0.999875206
FJX1	ENSG00000179431	11	35629735	35652419	11	3	123834	-1.6386	0.94935	0.999875206
SCGB3A2	ENSG00000164265	5	147240245	147271754	80	5	123834	-1.639	0.9494	0.999875206
KIF21A	ENSG00000139116	12	39677030	39847192	195	12	123834	-1.6392	0.94942	0.999875206
PSMG2	ENSG00000128789	18	12648737	12735739	194	13	123834	-1.6396	0.94946	0.999875206
EDRF1	ENSG00000107938	10	127398084	127462712	35	4	123834	-1.6406	0.94956	0.999875206
PCDHA2	ENSG00000204969	5	140164444	140401929	304	12	123834	-1.6408	0.94958	0.999875206
TMEM251	ENSG00000153485	14	93641296	93663434	16	6	123834	-1.6408	0.94958	0.999875206
RBM14	ENSG00000239306	11	66374053	66404818	20	5	123834	-1.6411	0.94962	0.999875206
FAR1	ENSG00000197601	11	13680217	13763893	109	13	123834	-1.6413	0.94963	0.999875206
GP2	ENSG00000169347	16	20310894	20349130	62	10	123834	-1.6415	0.94965	0.999875206
SON	ENSG00000159140	21	34904924	34959812	34	6	123834	-1.6416	0.94967	0.999875206
PLSCR5	ENSG00000231213	3	146284342	146334003	230	15	123834	-1.6422	0.94973	0.999875206
ACBD5	ENSG00000107897	10	27474146	27541059	311	12	123834	-1.6429	0.9498	0.999875206
CALY	ENSG00000130643	10	135128927	135160475	50	9	123834	-1.643	0.94981	0.999875206
MIS18A	ENSG00000159055	21	33630530	33661380	31	6	123834	-1.643	0.94981	0.999875206
SWI5	ENSG00000175854	9	131027658	131061269	51	7	123834	-1.6438	0.9499	0.999875206
NPTX2	ENSG00000106236	7	98236609	98269180	40	8	123834	-1.644	0.94992	0.999875206
LIPK	ENSG00000204021	10	90474301	90522543	159	8	123834	-1.6441	0.94992	0.999875206
FRMD1	ENSG00000153303	6	168446425	168492237	83	12	123834	-1.6454	0.95005	0.999875206
NAA35	ENSG00000135040	9	88546061	88647213	55	8	123834	-1.6457	0.95009	0.999875206
GLS2	ENSG00000135423	12	56854736	56892198	34	5	123834	-1.6459	0.95011	0.999875206
MRPL19	ENSG00000115364	2	75863909	75927977	110	11	123834	-1.6465	0.95017	0.999875206
POU2F1	ENSG00000143190	1	167180066	167406582	201	17	123834	-1.6467	0.95019	0.999875206
LYNX1	ENSG00000180155	8	143835752	143869640	47	8	123834	-1.647	0.95022	0.999875206
CCL8	ENSG00000108700	17	32636055	32658421	9	2	123834	-1.647	0.95022	0.999875206
CBFB	ENSG00000067955	16	67053019	67144961	82	6	123834	-1.6481	0.95033	0.999875206
ASB8	ENSG00000177981	12	48531571	48584996	115	13	123834	-1.6486	0.95039	0.999875206
ATAT1	ENSG00000137343	6	30584619	30624600	40	11	123834	-1.6491	0.95044	0.999875206
CCDC91	ENSG00000123106	12	28276182	28742883	901	14	123834	-1.6494	0.95047	0.999875206
VAX2	ENSG00000116035	2	71117720	71170576	70	14	123834	-1.6498	0.95051	0.999875206
SDPR	ENSG00000168497	2	192689028	192721981	19	6	123834	-1.6499	0.95052	0.999875206
EIF5AL1	ENSG00000253626	10	81262357	81286188	26	3	123834	-1.6503	0.95056	0.999875206
AC103809.2	ENSG00000268626	17	72336906	72358271	24	5	123834	-1.6508	0.95061	0.999875206
MTHFD2L	ENSG00000163738	4	74969891	75178816	149	14	123834	-1.6512	0.95065	0.999875206
FSCN3	ENSG00000106328	7	127221463	127252198	48	7	123834	-1.6518	0.95071	0.999875206
C19orf48	ENSG00000167747	19	51290961	51318186	73	10	123834	-1.6519	0.95072	0.999875206
SPTY2D1	ENSG00000179119	11	18617948	18666338	98	14	123834	-1.6545	0.95098	0.999875206
TANK	ENSG00000136560	2	161983419	162102732	130	7	123834	-1.655	0.95104	0.999875206
ERVMER34-1	ENSG00000226887	4	53578785	53627807	48	1	123834	-1.6551	0.95105	0.999875206
OR10H4	ENSG00000176231	19	16049818	16070768	90	3	123834	-1.6551	0.95105	0.999875206
DMXL1	ENSG00000172869	5	118363467	118594833	159	22	123834	-1.6553	0.95107	0.999875206
LY6D	ENSG00000167656	8	143856296	143878008	79	7	123834	-1.6554	0.95108	0.999875206
ATAD3A	ENSG00000197785	1	1437531	1480067	44	5	123834	-1.6567	0.95121	0.999875206
CALML3	ENSG00000178363	10	5556924	5578225	21	7	123834	-1.6568	0.95122	0.999875206
FOXN2	ENSG00000170802	2	48531776	48616433	176	16	123834	-1.657	0.95124	0.999875206
SERPINB13	ENSG00000197641	18	61244223	61281873	26	3	123834	-1.6586	0.9514	0.999875206
HAX1	ENSG00000143575	1	154234987	154258351	19	4	123834	-1.659	0.95144	0.999875206
RAB11B	ENSG00000185236	19	8444865	8479318	48	8	123834	-1.659	0.95145	0.999875206
PPIL2	ENSG00000100023	22	21996559	22064304	156	19	123834	-1.6593	0.95147	0.999875206
PBK	ENSG00000168078	8	27657137	27705612	180	4	123834	-1.6597	0.95151	0.999875206
SOWAHA	ENSG00000198944	5	132139033	132162488	13	2	123834	-1.6603	0.95157	0.999875206
ARRDC4	ENSG00000140450	15	98452784	98527068	241	29	123834	-1.6604	0.95158	0.999875206
CEL	ENSG00000170835	9	135927365	135957248	15	5	123834	-1.6605	0.9516	0.999875206
COPG2	ENSG00000158623	7	130136089	130363598	220	16	123834	-1.6608	0.95162	0.999875206
EIF3D	ENSG00000100353	22	36896897	36935483	121	14	123834	-1.6615	0.95169	0.999875206
MICU2	ENSG00000165487	13	22056836	22188353	230	16	123834	-1.6626	0.95181	0.999875206
WDR88	ENSG00000166359	19	33612996	33676701	106	13	123834	-1.6631	0.95186	0.999875206
LRRC42	ENSG00000116212	1	54401750	54443841	41	5	123834	-1.6634	0.95188	0.999875206
MYL3	ENSG00000160808	3	46889362	46933659	36	8	123834	-1.6634	0.95188	0.999875206
TNFAIP8L1	ENSG00000185361	19	4629530	4665580	67	9	123834	-1.665	0.95204	0.999875206
RP11-500M8.7	ENSG00000272921	12	4704110	4781809	145	14	123834	-1.6653	0.95208	0.999875206
CD9	ENSG00000010278	12	6298881	6357425	15	3	123834	-1.6657	0.95211	0.999875206
ZNF740	ENSG00000139651	12	53564484	53594596	43	5	123834	-1.6659	0.95213	0.999875206
CDC37L1	ENSG00000106993	9	4669559	4718398	87	7	123834	-1.6668	0.95222	0.999875206
MUC22	ENSG00000261272	6	30968251	31013179	316	26	123834	-1.6672	0.95226	0.999875206
PGBD1	ENSG00000137338	6	28239314	28280326	60	10	123834	-1.6676	0.9523	0.999875206
FAAH	ENSG00000117480	1	46849937	46889520	71	8	123834	-1.6681	0.95235	0.999875206
MRPL33	ENSG00000243147	2	27984584	28220954	255	8	123834	-1.6693	0.95247	0.999875206
AJAP1	ENSG00000196581	1	4704792	4862594	338	51	123834	-1.6694	0.95248	0.999875206
PPP2R1A	ENSG00000105568	19	52683292	52740687	176	11	123834	-1.6721	0.95275	0.999875206
RUFY2	ENSG00000204130	10	70090864	70177051	125	6	123834	-1.6734	0.95287	0.999875206
C19orf59	ENSG00000183019	19	7731514	7754712	46	3	123834	-1.674	0.95293	0.999875206
PLCD3	ENSG00000161714	17	43176335	43220721	49	14	123834	-1.6745	0.95298	0.999875206
P2RY11	ENSG00000244165	19	10212214	10236048	23	5	123834	-1.6751	0.95305	0.999875206
B3GNT9	ENSG00000237172	16	67172008	67195117	7	2	123834	-1.6755	0.95308	0.999875206
VAX1	ENSG00000148704	10	118878032	118907812	12	3	123834	-1.676	0.95313	0.999875206
RAB11FIP2	ENSG00000107560	10	119754427	119816114	43	7	123834	-1.6762	0.95315	0.999875206
GEM	ENSG00000164949	8	95251481	95284578	71	9	123834	-1.6764	0.95317	0.999875206
MYO16	ENSG00000041515	13	109238500	109870355	1548	120	123834	-1.6796	0.95349	0.999875206
HRASLS5	ENSG00000168004	11	63218876	63268666	52	7	123834	-1.6808	0.9536	0.999875206
OSBPL10	ENSG00000144645	3	31689382	32129072	588	62	123834	-1.681	0.95361	0.999875206
FAM196B	ENSG00000204767	5	169281268	169417744	184	35	123834	-1.6809	0.95361	0.999875206
PLVAP	ENSG00000130300	19	17452257	17498159	66	14	123834	-1.6827	0.95378	0.999875206
RBM4	ENSG00000173933	11	66374097	66444153	41	7	123834	-1.6832	0.95383	0.999875206
OSBP2	ENSG00000184792	22	31079769	31313811	409	37	123834	-1.6835	0.95386	0.999875206
HDGFRP3	ENSG00000166503	15	83774320	83886770	137	7	123834	-1.6838	0.95389	0.999875206
HSPB11	ENSG00000081870	1	54372247	54421602	64	3	123834	-1.684	0.95391	0.999875206
P2RY13	ENSG00000181631	3	151034100	151057336	49	9	123834	-1.684	0.95391	0.999875206
FBXO3	ENSG00000110429	11	33752485	33806089	135	15	123834	-1.6849	0.954	0.999875206
OR10AD1	ENSG00000172640	12	48586081	48607170	117	7	123834	-1.6859	0.95409	0.999875206
C2CD2L	ENSG00000172375	11	118962908	118999252	58	6	123834	-1.6865	0.95415	0.999875206
IPO4	ENSG00000196497	14	24639425	24668170	25	7	123834	-1.687	0.9542	0.999875206
PDCD6IP	ENSG00000170248	3	33829844	33921194	234	11	123834	-1.6874	0.95424	0.999875206
C3orf67	ENSG00000163689	3	58693092	59045810	332	12	123834	-1.6875	0.95425	0.999875206
SP110	ENSG00000135899	2	231022009	231100444	229	18	123834	-1.6878	0.95428	0.999875206
PIH1D2	ENSG00000150773	11	111924734	111954998	11	3	123834	-1.6882	0.95431	0.999875206
PMAIP1	ENSG00000141682	18	57557180	57581538	3	2	123834	-1.6885	0.95434	0.999875206
SRXN1	ENSG00000271303	20	617259	644014	10	3	123834	-1.6894	0.95442	0.999875206

ZNF165	ENSG00000197279	6	28038753	28067341	62	7	123834	-1.6904	0.95453	0.999875206
ELF5	ENSG00000135374	11	34490340	34545352	119	13	123834	-1.6905	0.95453	0.999875206
FBXO44	ENSG00000132879	1	11704432	11733384	32	8	123834	-1.6906	0.95454	0.999875206
PHOSPHO1	ENSG00000173868	17	47290724	47318128	46	8	123834	-1.6907	0.95455	0.999875206
CPNE8	ENSG00000139117	12	39030624	39311232	573	21	123834	-1.6909	0.95457	0.999875206
EHD1	ENSG00000110047	11	64609114	64665768	62	5	123834	-1.6912	0.9546	0.999875206
UNC50	ENSG00000115446	2	99215042	99244978	40	3	123834	-1.6914	0.95462	0.999875206
BEND3	ENSG00000178409	6	107376386	107446473	64	10	123834	-1.6914	0.95462	0.999875206
PCDHA1	ENSG00000204970	5	140155876	140401929	316	12	123834	-1.6916	0.95464	0.999875206
ZNF131	ENSG00000172262	5	43055278	43202123	346	18	123834	-1.6917	0.95465	0.999875206
HLA-DPA1	ENSG00000231389	6	33022346	33058552	504	9	123834	-1.6917	0.95465	0.999875206
DONSON	ENSG00000159147	21	34921848	34971014	31	6	123834	-1.6922	0.9547	0.999875206
SKA1	ENSG00000154839	18	47891365	47930543	112	8	123834	-1.6927	0.95474	0.999875206
RP11-1084J3.4	ENSG00000273294	5	33977279	34134633	132	16	123834	-1.6929	0.95476	0.999875206
FYCO1	ENSG00000163820	3	45949396	46047316	217	16	123834	-1.6946	0.95493	0.999875206
RP11-204N11.1	ENSG00000258869	14	98205601	98240326	46	8	123834	-1.6947	0.95493	0.999875206
BDH1	ENSG00000161267	3	197226654	197310194	196	23	123834	-1.6948	0.95494	0.999875206
MTFP1	ENSG00000242114	22	30811518	30835045	39	9	123834	-1.6954	0.955	0.999875206
RBM14-RBM4	ENSG00000248643	11	66374097	66423940	31	6	123834	-1.6958	0.95504	0.999875206
TSSC4	ENSG00000184281	11	2411718	2435106	8	3	123834	-1.6962	0.95508	0.999875206
C1orf198	ENSG00000119280	1	230962865	231015335	89	12	123834	-1.6969	0.95514	0.999875206
FLG	ENSG00000143631	1	152264651	152307679	66	2	123834	-1.697	0.95515	0.999875206
C7orf26	ENSG00000146576	7	6619648	6658357	91	8	123834	-1.6978	0.95523	0.999875206
KRTAP2-3	ENSG00000212724	17	39205495	39226344	60	5	123834	-1.6995	0.95539	0.999875206
ZBTB40	ENSG00000184677	1	22768344	22867650	99	9	123834	-1.6998	0.95542	0.999875206
UCP2	ENSG00000175567	11	73675712	73704352	52	6	123834	-1.7007	0.9555	0.999875206
C9orf72	ENSG00000147894	9	27536544	27583864	117	15	123834	-1.701	0.95553	0.999875206
ZSWIM3	ENSG00000132801	20	44476256	44517761	99	9	123834	-1.7014	0.95557	0.999875206
CNKSR3	ENSG00000153721	6	154698638	154841793	341	47	123834	-1.7018	0.9556	0.999875206
RPL7L1	ENSG00000146223	6	42837356	42867663	64	7	123834	-1.703	0.95572	0.999875206
PTCD2	ENSG00000049883	5	71606194	71666052	26	2	123834	-1.7036	0.95577	0.999875206
MUT	ENSG00000146085	6	49388073	49440904	72	7	123834	-1.7036	0.95577	0.999875206
STAT2	ENSG00000170581	12	56725381	56763939	28	2	123834	-1.7055	0.95595	0.999875206
COX7A2L	ENSG00000115944	2	42550686	42662228	139	13	123834	-1.7056	0.95596	0.999875206
C10orf88	ENSG00000119965	10	124680419	124723919	44	8	123834	-1.7061	0.956	0.999875206
ZNF598	ENSG00000167962	16	2037655	2069824	66	7	123834	-1.7063	0.95602	0.999875206
LRP5	ENSG00000162337	11	68070077	68226743	162	20	123834	-1.7065	0.95604	0.999875206
TMEM237	ENSG00000155755	2	202474907	202518293	56	8	123834	-1.7068	0.95607	0.999875206
UQCRB	ENSG00000156467	8	97228148	97257862	27	4	123834	-1.7075	0.95613	0.999875206
KLHL11	ENSG00000178502	17	39999797	40031684	73	5	123834	-1.7079	0.95617	0.999875206
FGFR3	ENSG00000068078	4	1785034	1820599	20	4	123834	-1.7081	0.95619	0.999875206
TAS2R50	ENSG00000212126	12	11128512	11149511	66	4	123834	-1.7085	0.95623	0.999875206
HAS3	ENSG00000103044	16	69129467	69162622	36	8	123834	-1.7107	0.95643	0.999875206
TAX1BP3	ENSG00000213977	17	3556196	3581976	59	9	123834	-1.7108	0.95645	0.999875206
DYNLRB2	ENSG00000168589	16	80564631	80594657	96	8	123834	-1.7111	0.95646	0.999875206
MED21	ENSG00000152944	12	27165479	27229276	166	16	123834	-1.7114	0.95649	0.999875206
DRD1	ENSG00000184845	5	174857042	174881211	34	8	123834	-1.7117	0.95653	0.999875206
SPTSSA	ENSG00000165389	14	34891995	34941562	73	6	123834	-1.7121	0.95656	0.999875206
FARSA	ENSG00000179115	19	13023293	13054851	34	5	123834	-1.7122	0.95657	0.999875206
CCL17	ENSG00000102970	16	57428679	57459974	75	12	123834	-1.7144	0.95677	0.999875206
SHARPIN	ENSG00000179526	8	145143536	145173027	37	1	123834	-1.7149	0.95682	0.999875206
RILPL2	ENSG00000150977	12	123889936	123931264	40	8	123834	-1.716	0.95692	0.999875206
PAK1IP1	ENSG00000111845	6	10684928	10720015	2	1	123834	-1.7173	0.95703	0.999875206
RP11-318A15.7	ENSG00000267168	17	74719060	74744604	4	1	123834	-1.7175	0.95706	0.999875206
SRSF2	ENSG00000161547	17	74720197	74743456	4	1	123834	-1.7175	0.95706	0.999875206
TPRG1	ENSG00000188001	3	188655003	189053093	429	44	123834	-1.718	0.9571	0.999875206
OPN5	ENSG00000124818	6	47739718	47810516	182	15	123834	-1.7197	0.95725	0.999875206
TREML1	ENSG00000161911	6	41107080	41132075	15	2	123834	-1.7198	0.95727	0.999875206
BRPF3	ENSG00000096070	6	36154521	36210567	52	9	123834	-1.7203	0.95731	0.999875206
APMAP	ENSG00000101474	20	24933561	24983615	146	10	123834	-1.7208	0.95736	0.999875206
CASC4	ENSG00000166734	15	44570927	44717956	152	3	123834	-1.721	0.95738	0.999875206
PIK3C2G	ENSG00000139144	12	18390548	18811348	853	41	123834	-1.7212	0.9574	0.999875206
CTSO	ENSG00000256043	4	156835270	156885069	101	19	123834	-1.7227	0.95753	0.999875206
NKIRAS2	ENSG00000168256	17	40153400	40187659	29	4	123834	-1.723	0.95755	0.999875206
CEP85	ENSG00000130695	1	26550691	26615299	169	12	123834	-1.7231	0.95757	0.999875206
ZNF23	ENSG00000167377	16	71471500	71506998	46	7	123834	-1.7231	0.95757	0.999875206
ZHX1	ENSG00000165156	8	124250696	124297781	6	3	123834	-1.7242	0.95766	0.999875206
TTC27	ENSG00000018699	2	32843099	33056118	375	32	123834	-1.7247	0.95771	0.999875206
KLHL10	ENSG00000161594	17	39981937	40014636	85	5	123834	-1.7251	0.95774	0.999875206
SLC24A4	ENSG00000140090	14	92778925	92972596	393	36	123834	-1.7251	0.95775	0.999875206
DNA2	ENSG00000138346	10	70163821	70241879	228	15	123834	-1.7273	0.95794	0.999875206
MTMR9	ENSG00000104643	8	11131925	11195646	102	7	123834	-1.7275	0.95796	0.999875206
SNTG2	ENSG00000172554	2	936554	1381385	1380	52	123834	-1.7278	0.95799	0.999875206
KIAA1919	ENSG00000173214	6	111570551	111602370	62	12	123834	-1.7278	0.95799	0.999875206
BET1	ENSG00000105829	7	93582074	93643694	53	9	123834	-1.7278	0.95799	0.999875206
LCE5A	ENSG00000186207	1	152473320	152494653	32	3	123834	-1.7283	0.95804	0.999875206
CD68	ENSG00000129226	17	7472785	7495429	29	8	123834	-1.7288	0.95808	0.999875206
RAB3GAP1	ENSG00000115839	2	135799835	135943964	179	11	123834	-1.7302	0.9582	0.999875206
FAM221A	ENSG00000188732	7	23709749	23752868	104	15	123834	-1.7307	0.95824	0.999875206
AP000304.1	ENSG00000268265	21	34937431	34957645	14	4	123834	-1.7309	0.95827	0.999875206
TMEM169	ENSG00000163449	2	216936589	216977506	56	7	123834	-1.7322	0.95838	0.999875206
TRIM56	ENSG00000169871	7	100718720	100745017	35	4	123834	-1.7325	0.95841	0.999875206
DYRK4	ENSG00000010219	12	4661370	4733325	84	13	123834	-1.7339	0.95853	0.999875206
KCMF1	ENSG00000176407	2	85188216	85296595	109	7	123834	-1.7347	0.95861	0.999875206
METTL23	ENSG00000181038	17	74712912	74740018	3	1	123834	-1.736	0.95872	0.999875206
HKDC1	ENSG00000156510	10	70970059	71037315	142	25	123834	-1.737	0.9588	0.999875206
ADSSL1	ENSG00000185100	14	105180523	105223662	64	10	123834	-1.7372	0.95883	0.999875206
KRTCAP2	ENSG00000163463	1	155131884	155155951	25	4	123834	-1.7376	0.95886	0.999875206
DKFZP779L1853	ENSG00000269514	12	48582170	48605814	128	9	123834	-1.7376	0.95886	0.999875206
SPG11	ENSG00000104133	15	44844894	44965876	19	5	123834	-1.7377	0.95887	0.999875206
GDPGP1	ENSG00000183208	15	90767040	90795315	61	5	123834	-1.7384	0.95893	0.999875206
MICAL2	ENSG00000133816	11	12105543	12295334	250	48	123834	-1.7393	0.95901	0.999875206
RP11-514O12.4	ENSG00000249141	6	167261582	167379612	208	27	123834	-1.74	0.95907	0.999875206
FGB	ENSG00000171564	4	155474108	155502238	40	2	123834	-1.7402	0.95909	0.999875206
PAQR9	ENSG00000188582	3	142658006	142692178	57	10	123834	-1.7404	0.9591	0.999875206
AP002956.1	ENSG00000268192	11	119017590	119037930	20	2	123834	-1.742	0.95925	0.999875206
HGD	ENSG00000113924	3	120337020	120411418	97	19	123834	-1.7444	0.95946	0.999875206
PAXBP1	ENSG00000159086	21	34096210	34154169	56	10	123834	-1.7464	0.95963	0.999875206
DIEXF	ENSG00000117597	1	209991352	210040910	109	11	123834	-1.7469	0.95968	0.999875206

CXCL3	ENSG00000163734	4	74892306	74914524	4	1	123834	-1.7477	0.95974	0.999875206
ADHFE1	ENSG00000147576	8	67332420	67393836	141	17	123834	-1.7477	0.95974	0.999875206
ZNF570	ENSG00000171827	19	37948487	37986260	44	6	123834	-1.7476	0.95974	0.999875206
SLC10A6	ENSG00000145283	4	87734621	87780416	41	5	123834	-1.7478	0.95975	0.999875206
THADA	ENSG00000115970	2	43383800	43833185	683	18	123834	-1.7483	0.95979	0.999875206
PUS10	ENSG00000162927	2	61157357	61255394	71	12	123834	-1.7492	0.95987	0.999875206
NXPE2	ENSG00000204361	11	114539108	114589362	91	12	123834	-1.7492	0.95987	0.999875206
EIF2D	ENSG00000143486	1	206734620	206795904	93	13	123834	-1.7502	0.95996	0.999875206
RNF215	ENSG00000099999	22	30763835	30827760	142	17	123834	-1.7509	0.96001	0.999875206
KRT23	ENSG00000108244	17	39068948	39103886	133	18	123834	-1.7519	0.9601	0.999875206
SLC35G5	ENSG00000177710	8	11178397	11199717	52	4	123834	-1.7525	0.96016	0.999875206
NDUFB10	ENSG00000140990	16	1999509	2021976	63	7	123834	-1.7526	0.96016	0.999875206
TOB1	ENSG00000141232	17	48929584	48955339	20	3	123834	-1.7534	0.96023	0.999875206
HMHB1	ENSG00000158497	5	143181726	143210284	14	4	123834	-1.7543	0.96031	0.999875206
TTF1	ENSG00000125482	9	135241008	135292209	54	7	123834	-1.7549	0.96036	0.999875206
CAMK2N1	ENSG00000162545	1	20798884	20822713	19	5	123834	-1.7553	0.9604	0.999875206
MRGPRX4	ENSG00000179817	11	18184384	18205827	71	4	123834	-1.7553	0.9604	0.999875206
ZFAND6	ENSG00000086666	15	80341910	80440735	133	11	123834	-1.7558	0.96044	0.999875206
RLN1	ENSG00000107018	9	5324969	5349873	23	4	123834	-1.756	0.96046	0.999875206
AP1S3	ENSG00000152056	2	224606403	224712744	224	19	123834	-1.7563	0.96048	0.999875206
MCM9	ENSG00000111877	6	119124605	119266327	331	11	123834	-1.7563	0.96048	0.999875206
AC116407.2	ENSG00000269402	17	30496379	30516885	27	3	123834	-1.7564	0.96049	0.999875206
C2CD2	ENSG00000157617	21	43295221	43383999	153	37	123834	-1.757	0.96054	0.999875206
HFM1	ENSG00000162669	1	91716323	91880426	253	14	123834	-1.7572	0.96056	0.999875206
GPATCH8	ENSG00000186566	17	42462652	42590798	127	5	123834	-1.7574	0.96057	0.999875206
CEP350	ENSG00000135837	1	179913873	180094015	355	14	123834	-1.7574	0.96058	0.999875206
MPDU1	ENSG00000129255	17	7476847	7506107	36	10	123834	-1.7581	0.96064	0.999875206
RGPD2	ENSG00000185304	2	88045474	88295309	14	3	123834	-1.7589	0.9607	0.999875206
FRS2	ENSG00000166225	12	69854129	69983562	204	14	123834	-1.7592	0.96073	0.999875206
SHPK	ENSG00000197417	17	3501556	3549616	111	15	123834	-1.7592	0.96073	0.999875206
EIF4A1	ENSG00000161960	17	7466024	7492323	38	9	123834	-1.7592	0.96073	0.999875206
P2RX5-TAX1BP3	ENSG00000257950	17	3556357	3609488	76	13	123834	-1.7593	0.96074	0.999875206
SCN8A	ENSG00000196876	12	51974050	52216648	268	13	123834	-1.7598	0.96078	0.999875206
CSNK2A1	ENSG00000101266	20	449116	534465	66	6	123834	-1.7601	0.96081	0.999875206
HSPA12B	ENSG00000132622	20	3703314	3743758	35	13	123834	-1.7614	0.96091	0.999875206
C12orf73	ENSG00000204954	12	104333980	104369486	136	17	123834	-1.762	0.96096	0.999875206
TTLL13	ENSG00000213471	15	90782762	90818199	68	4	123834	-1.762	0.96096	0.999875206
POU3F1	ENSG00000185668	1	38499523	38522450	36	5	123834	-1.7623	0.96099	0.999875206
PDZRN4	ENSG00000165966	12	41572250	41978392	787	42	123834	-1.7627	0.96102	0.999875206
SH2D4B	ENSG00000178217	10	82287658	82416316	271	24	123834	-1.7629	0.96104	0.999875206
OSBPL8	ENSG00000091039	12	76735577	76963589	312	12	123834	-1.763	0.96105	0.999875206
CALCA	ENSG00000110680	11	14978214	15003900	18	1	123834	-1.7634	0.96108	0.999875206
CNN2	ENSG00000064666	19	1016298	1049068	94	20	123834	-1.7633	0.96108	0.999875206
PTCHD4	ENSG00000244694	6	47835764	48046425	513	29	123834	-1.7642	0.96115	0.999875206
MS4A7	ENSG00000166927	11	60135955	60173424	68	6	123834	-1.7647	0.96119	0.999875206
C2orf57	ENSG00000177673	2	232447575	232468994	12	3	123834	-1.765	0.96121	0.999875206
CWC27	ENSG00000153015	5	64054757	64324590	362	12	123834	-1.7665	0.96134	0.999875206
SAMD9	ENSG00000205413	7	92718829	92757336	89	13	123834	-1.7671	0.96139	0.999875206
ODF3L1	ENSG00000182950	15	76006318	76030029	22	3	123834	-1.7671	0.96139	0.999875206
ZNHIT6	ENSG00000117174	1	86105106	86184116	173	17	123834	-1.7676	0.96144	0.999875206
TMEM200A	ENSG00000164484	6	130676879	130774208	234	16	123834	-1.7692	0.96157	0.999875206
RNF130	ENSG00000113269	5	179328651	179509118	260	16	123834	-1.7703	0.96166	0.999875206
OR6P1	ENSG00000186440	1	158522441	158543394	51	5	123834	-1.7704	0.96167	0.999875206
CCDC12	ENSG00000160799	3	46953216	47033500	56	6	123834	-1.771	0.96172	0.999875206
ZNF527	ENSG00000189164	19	37851963	37893968	48	8	123834	-1.7724	0.96184	0.999875206
TM7SF2	ENSG00000149809	11	64869317	64893856	26	5	123834	-1.7727	0.96186	0.999875206
PRKAA1	ENSG00000132356	5	40749481	40808476	90	11	123834	-1.7732	0.9619	0.999875206
PARP2	ENSG00000129484	14	20801741	20836064	90	9	123834	-1.7734	0.96192	0.999875206
AC112721.1	ENSG00000222022	2	238320063	238343919	34	5	123834	-1.7735	0.96193	0.999875206
GPR87	ENSG00000138271	3	151001891	151044740	52	9	123834	-1.7746	0.96202	0.999875206
LONP2	ENSG00000102910	16	48268207	48407033	150	5	123834	-1.7746	0.96202	0.999875206
C3orf79	ENSG00000237787	3	153192284	153230486	104	11	123834	-1.7747	0.96203	0.999875206
HAVCR1	ENSG00000113249	5	156446424	156496130	123	16	123834	-1.7748	0.96204	0.999875206
C8orf44-SGK3	ENSG00000270024	8	67569831	67782707	244	8	123834	-1.775	0.96205	0.999875206
HIST1H2AA	ENSG00000164508	6	25716291	25736790	60	4	123834	-1.7755	0.96209	0.999875206
HIST1H2BA	ENSG00000146047	6	25717137	25737573	60	4	123834	-1.7755	0.96209	0.999875206
ITM2C	ENSG00000135916	2	231719354	231753963	60	14	123834	-1.7764	0.96217	0.999875206
NKAIN3	ENSG00000185942	8	63151150	63922211	1602	52	123834	-1.7766	0.96218	0.999875206
ZDHHC4	ENSG00000136247	7	6607065	6639005	73	8	123834	-1.7788	0.96236	0.999875206
CCPG1	ENSG00000260916	15	55622230	55710708	254	15	123834	-1.7789	0.96237	0.999875206
MRPL27	ENSG00000108826	17	48435218	48460575	58	6	123834	-1.7788	0.96237	0.999875206
DUOXA2	ENSG00000140274	15	45396519	45420619	28	4	123834	-1.779	0.96238	0.999875206
RP11-133K1.2	ENSG00000259288	15	40499629	40578597	50	12	123834	-1.7798	0.96245	0.999875206
PAK6	ENSG00000137843	15	40499629	40579688	50	12	123834	-1.7798	0.96245	0.999875206
CLEC5A	ENSG00000258227	7	141617157	141656807	63	6	123834	-1.78	0.96246	0.999875206
OR1L3	ENSG00000171481	9	125427315	125448432	35	3	123834	-1.7815	0.96259	0.999875206
CEP152	ENSG00000103995	15	48995125	49113343	100	10	123834	-1.7826	0.96268	0.999875206
FOXN4	ENSG00000139445	12	109705784	109757025	49	6	123834	-1.7833	0.96273	0.999875206
S100Z	ENSG00000171643	5	76135826	76227475	74	11	123834	-1.7836	0.96276	0.999875206
TESPA1	ENSG00000135426	12	55331802	55388530	164	7	123834	-1.7843	0.96281	0.999875206
MYL12B	ENSG00000118680	18	3251907	3288282	99	5	123834	-1.7843	0.96281	0.999875206
ISLR2	ENSG00000167178	15	74382652	74440881	4	2	123834	-1.7859	0.96294	0.999875206
PTPN7	ENSG00000143851	1	202106141	202140716	27	8	123834	-1.7885	0.96315	0.999875206
DMBT1	ENSG00000187908	10	124310181	124413252	189	9	123834	-1.7895	0.96323	0.999875206
NR5A2	ENSG00000116833	1	199986730	200156552	277	34	123834	-1.7903	0.96329	0.999875206
CTNNB1	ENSG00000168036	3	41226328	41311587	71	7	123834	-1.791	0.96335	0.999875206
SMC6	ENSG00000163029	2	17835079	17991509	210	16	123834	-1.7922	0.96345	0.999875206
GLT8D2	ENSG00000120820	12	104372762	104467961	212	32	123834	-1.7926	0.96348	0.999875206
C5orf46	ENSG00000178776	5	147250289	147296101	96	9	123834	-1.7927	0.96349	0.999875206
RP11-817J15.3	ENSG00000255798	11	102542041	102564644	86	14	123834	-1.7934	0.96355	0.999875206
TMEM170B	ENSG00000205269	6	11527938	11593757	163	14	123834	-1.7948	0.96366	0.999875206
FBXO24	ENSG00000106336	7	100171605	100208740	12	2	123834	-1.7949	0.96367	0.999875206
CACHD1	ENSG00000158966	1	64926428	65168741	269	36	123834	-1.7952	0.96369	0.999875206
AC073343.1	ENSG00000198580	7	6703376	6725991	56	9	123834	-1.7957	0.96373	0.999875206
POP7	ENSG00000172336	7	100293676	100315118	45	7	123834	-1.7964	0.96379	0.999875206
GPR85	ENSG00000164604	7	112708386	112737833	31	3	123834	-1.7968	0.96381	0.999875206
AANAT	ENSG00000129673	17	74439433	74476199	62	12	123834	-1.7971	0.96384	0.999875206
IQCH	ENSG00000103599	15	67537138	67804598	332	15	123834	-1.7973	0.96385	0.999875206
SEMA4C	ENSG00000168758	2	97515453	97546494	13	3	123834	-1.7985	0.96395	0.999875206

EEF2K	ENSG00000103319	16	22207603	22308554	126	16	123834	-1.799	0.96399	0.999875206
RELB	ENSG00000104856	19	45494688	45551452	35	11	123834	-1.7994	0.96402	0.999875206
LAMA2	ENSG00000196569	6	129194342	129847714	1052	52	123834	-1.7997	0.96405	0.999875206
SPACA3	ENSG00000141316	17	31287394	31334895	123	18	123834	-1.8001	0.96408	0.999875206
ALX4	ENSG00000052850	11	44271994	44341716	95	16	123834	-1.8006	0.96412	0.999875206
KCNV1	ENSG00000164794	8	110965874	110998076	39	5	123834	-1.8015	0.96419	0.999875206
TCN2	ENSG00000185339	22	30992825	31033265	180	21	123834	-1.8016	0.9642	0.999875206
DCP2	ENSG00000172795	5	112302399	112366667	63	8	123834	-1.8019	0.96422	0.999875206
SCARA5	ENSG00000168079	8	27717399	27860244	308	35	123834	-1.8018	0.96422	0.999875206
OR2M7	ENSG00000177186	1	248476894	248497899	85	2	123834	-1.8041	0.96439	0.999875206
EHD4	ENSG00000103966	15	42180950	42274776	141	18	123834	-1.8047	0.96444	0.999875206
TTC18	ENSG00000156042	10	75003517	75128617	290	2	123834	-1.805	0.96446	0.999875206
OR5H6	ENSG00000230301	3	97973088	97994200	108	6	123834	-1.8053	0.96449	0.999875206
[truncated: 75,386 more chars]
