# Supplementary material for: Multi-omic and multi-species meta-analyses of nicotine consumption
Source: Transl Psychiatry. 2021 Feb 4;11:98. doi: 10.1038/s41398-021-01231-y (PMC7862377; doi:10.1038/s41398-021-01231-y)
Supplement: Supplementary file 4 — Supplementary File 2. SMULTIxCAN Summary Statistics File [file 41398_2021_1231_MOESM4_ESM.pdf]

SMultiXcan Gene Based Associations with Human Cigarettes per Day

SYMBOL	GENE	pvalue	n	n_indep	p_i_best	t_i_best	p_i_worst	t_i_worst	eigen_max	eigen_min	eigen_min_kept	z_min	z_max	z_mean	z_sd	tmi	status	Padj
PSMA4	ENSG00000041357.15	9.11E-75	7	6	1.12E-47	Brain_Cerebellar_Hemisphere	0.208357105	Brain_Hippocampus	3.594363174	0.061213637	0.278209238	1.258095819	14.50553561	8.811804005	4.654057564	6	0	1.27E-70
CHRNA3	ENSG00000080644.15	1.53E-34	4	2	8.29E-28	Brain_Cerebellum	0.814606663	Brain_Caudate_basal_ganglia	2.920292675	0.006243077	0.996774342	-10.92996246	-0.234487337	-5.709531813	4.369693572	2	0	1.07E-30
TMED3	ENSG00000166557.12	2.21E-22	2	2	5.30E-23	Brain_Hippocampus	0.202503262	Brain_Cortex	1.020835119	0.979164881	0.979164881	-1.274452032	9.875898294	4.300723131	7.884488328	2	0	1.03E-18
HYKK	ENSG00000188266.13	3.83E-20	7	7	1.07E-13	Brain_Hippocampus	0.937099391	Brain_Hypothalamus	3.440026804	0.145169841	0.145169841	-2.009102164	7.431531126	2.81366038	3.251979963	7	0	1.34E-16
ADAMTS7	ENSG00000136378.14	3.77E-19	8	6	3.93E-15	Brain_Cortex	0.470423982	Brain_Hippocampus	4.84663216	0.006777619	0.214068576	-6.164938088	7.857184785	2.055342416	3.94664666	6	0	1.06E-15
ITPKC	ENSG00000086544.2	9.35E-14	5	5	5.68E-10	Brain_Caudate_basal_ganglia	0.870271234	Brain_Hippocampus	1.834228408	0.350912012	0.350912012	-6.198999491	3.828273135	-0.401565122	3.679525496	5	0	2.18E-10
CHRNA5	ENSG00000169684.13	1.86E-12	13	1	6.39E-13	Brain_Caudate_basal_ganglia	2.90E-10	Brain_Hippocampus	12.62357666	1.59E-06	12.62357666	-7.191807679	-6.304004005	-6.940295044	0.255852918	1	0	3.71E-09
C19orf54	ENSG00000188493.14	2.33E-12	9	9	6.63E-14	Brain_Anterior_cingulate_cortex_BA24	0.932413278	Brain_Amygdala	2.077687916	0.381808461	0.381808461	-7.494949818	1.618138038	-1.529294482	3.060723419	9	0	4.07E-09
LINGO1	ENSG00000169783.12	2.03E-09	10	8	0.002385685	Brain_Caudate_basal_ganglia	0.992381569	Brain_Cerebellum	5.241925125	0.069455557	0.20358286	-3.037475881	2.709222255	0.322175542	1.598356919	8	0	3.16E-06
CEACAM21	ENSG00000007129.17	6.16E-09	6	5	5.72E-08	Brain_Spinal_cord_cervical_c-1	0.536848875	Brain_Cerebellar_Hemisphere	3.139853869	0.049666597	0.474001977	-5.427362537	0.88558747	-1.572432679	2.222787146	5	0	8.51E-06
CTSH	ENSG00000103811.15	6.69E-09	7	7	2.61E-08	Brain_Cortex	0.845266162	Brain_Caudate_basal_ganglia	2.723100099	0.203952269	0.203952269	-2.616055179	5.565638246	1.883934096	2.801573693	7	0	8.51E-06
CYP2A7	ENSG00000198077.10	2.90E-08	7	3	2.66E-10	Brain_Hippocampus	0.655036491	Brain_Substantia_nigra	5.652039461	0.028602404	0.439631432	0.44677643	6.317168215	3.410717412	2.206875713	3	0	3.39E-05
RPS19	ENSG00000105372.6	2.07E-07	3	3	1.54E-05	Brain_Anterior_cingulate_cortex_BA24	0.049518114	Brain_Cerebellum	1.382301503	0.673482695	0.673482695	-4.322611544	1.964103299	-1.627566969	3.237846368	3	0	0.000222426
CRABP1	ENSG00000166426.7	3.36E-07	6	6	3.34E-09	Brain_Frontal_Cortex_BA9	0.25615261	Brain_Hypothalamus	2.972728001	0.163243275	0.163243275	-5.913818856	-1.135531694	-2.535215959	1.887066478	6	0	0.0003358
RCC1L	ENSG00000274523.4	6.38E-07	2	2	3.30E-06	Brain_Cortex	0.000295165	Brain_Putamen_basal_ganglia	1.228631619	0.771368381								

0.771368381	3.619506502	4.651282184	4.135394343	0.729575581
2	0	0.000595543
SNRPA	ENSG00000077312.8	4.34E-06	2	2	1.57E-06	Brain_Cerebellum
0.622342244	Brain_Cerebellar_Hemisphere	1.35210494	0.64789506	0.64789506
0.492533502	4.801769434	2.647151468	3.047089949	2	0
0.003793986
IDH3A	ENSG00000166411.13	8.14E-06	4	3	8.25E-06	Brain_Cerebellum	0.84397089
Brain_Cerebellar_Hemisphere	2.589062982	0.077903822	0.60061939
-0.438636529	4.458560564	1.195682818	2.214468839	3	0
0.006452496
GATSL2	ENSG00000274070.1	8.30E-06	5	5	0.00021917	Brain_Cortex
0.752416876	Brain_Nucleus_accumbens_basal_ganglia	1.742488794
0.187392322	0.187392322	-3.69582982	1.94830926	-1.333669855
2.331839247	5	0	0.006452496
ACSBG1	ENSG00000103740.9	2.40E-05	1	1	2.40E-05
Brain_Spinal_cord_cervical_c-1	2.40E-05	Brain_Spinal_cord_cervical_c-1	1
1	1	4.22354272	4.22354272	4.22354272	NA	1	0	0.017709054
GTF2I	ENSG00000263001.5	2.56E-05	12	2	2.19E-06	Brain_Putamen_basal_ganglia
0.001254019	Brain_Cerebellar_Hemisphere	11.14287432	6.44E-05
0.390194078	3.226299924	4.735482563	4.346388192	0.465681939
2	0	0.017939002
RASGRF1	ENSG00000058335.15	2.74E-05	3	3	7.20E-05
Brain_Cerebellar_Hemisphere	0.945765619	Brain_Cerebellum	1.760444875
0.518226103	0.518226103	-0.382870327	3.96973357	1.172946033
2.42719947	3	0	0.018249292
CTSL	ENSG00000135047.14	3.37E-05	1	1	3.37E-05
Brain_Anterior_cingulate_cortex_BA24	3.37E-05
Brain_Anterior_cingulate_cortex_BA24	1	1	1	4.146762063
4.146762063	4.146762063	NA	1	0	0.021445034
DNAJC24	ENSG00000170946.14	4.08E-05	1	1	4.08E-05	Brain_Cortex
4.08E-05	Brain_Cortex	1	1	1	4.102783507	4.102783507
4.102783507	NA	1	0	0.024831542
B9D2	ENSG00000123810.7	4.80E-05	2	2	8.22E-06	Brain_Hypothalamus
0.85974164	Brain_Hippocampus	1.044445453	0.955554547	0.955554547
-4.459415926	-0.176703056	-2.318059491	3.028335312	2	0
0.028006714
GTF2IRD2	ENSG00000196275.13	6.71E-05	13	1	2.75E-05	Brain_Hypothalamus
0.001031117	Brain_Substantia_nigra	12.53548127	7.06E-06
12.53548127	3.281895824	4.193034437	3.913937219	0.269329277
1	0	0.037538368
ADAR	ENSG00000160710.15	7.59E-05	4	4	0.001301464	Brain_Cortex
0.366772752	Brain_Putamen_basal_ganglia	1.742273967	0.589760564
0.589760564	-3.215656847	1.14277502	-1.051156896	2.397782959	4
0	0.040864456
CH25H	ENSG00000138135.6	0.000115643	5	5	0.019162215
Brain_Hypothalamus	0.95601526	Brain_Putamen_basal_ganglia	2.688399639
0.166313176	0.166313176	-2.342360125	1.965669599	-0.088099616
1.638289326	5	0	0.059924344
SYNDIG1	ENSG00000101463.5	0.000233803	2	2	0.003756721
Brain_Cerebellum	0.012157837	Brain_Caudate_basal_ganglia	1.12278424	0.87721576
0.87721576	2.507529694	2.897898562	2.702714128	0.276032474	2
0	0.116826412
SRR	ENSG00000167720.12	0.000244933	13	4	7.75E-05
Brain_Cerebellar_Hemisphere	0.140821926	Brain_Substantia_nigra
9.437325899	5.76E-05	0.728785444	-1.472737149	3.952057725
3.034190338	1.490600264	4	0	0.118167683
TMEM91	ENSG00000142046.14	0.000265629	3	3	0.003246816
Brain_Cerebellum	0.013463572	Brain_Frontal_Cortex_BA9	1.181633527
0.807973441	0.807973441	2.471265756	2.943349527	2.676910053
0.241842535	3	0	0.123880421
RP11-431D12.1	ENSG00000253839.1	0.000277351	1	1	0.000277351
Brain_Cortex	0.000277351	Brain_Cortex	1	1	1	-3.635584321

-3.635584321 -3.635584321 NA 1 0 0.125174883
PCOLCE ENSG00000106333.12 0.000304074 2 2 5.72E-05
Brain_Spinal_cord_cervical_c-1 0.714440854 Brain_Frontal_Cortex_BA9
1.101576216 0.898423784 0.898423784 -4.024254524 -0.365898449
-2.195076486 2.586848389 2 0 0.132946685
DCAF7 ENSG00000136485.14 0.000401388 3 3 0.001255442
Brain_Hypothalamus 0.511284912 Brain_Cerebellum 1.597824243
0.283617114 0.283617114 -0.953139201 3.22597517 0.538665899
2.331988829 3 0 0.166269998
C8orf44 ENSG00000213865.7 0.000404058 2 2 0.000160052
Brain_Nucleus_accumbens_basal_ganglia 0.224657188 Brain_Substantia_nigra
1.010730483 0.989269517 0.989269517 -3.774930626 -1.214237123
-2.494583874 1.81068374 2 0 0.166269998
SPATA33 ENSG00000167523.13 0.000438064 7 6 9.67E-06
Brain_Hippocampus 0.177661179 Brain_Caudate_basal_ganglia 4.757660561
0.112663073 0.220440255 -4.424425869 -1.347991304 -2.91367749
1.036109748 6 0 0.173037995
RP11-560J1.2 ENSG00000271888.1 0.000445241 1 1 0.000445241
Brain_Hypothalamus 0.000445241 Brain_Hypothalamus 1 1 1
-3.511705511 -3.511705511 -3.511705511 NA 1 0 0.173037995
PSMC3 ENSG00000165916.8 0.000551113 5 3 6.51E-05 Brain_Cerebellum
0.721444315 Brain_Cortex 3.529693715 0.001853746 0.466381735
-3.993536304 -0.356529164 -3.075129558 1.545379895 3 0
0.208395282
KCNC2 ENSG00000166006.12 0.000607862 1 1 0.000607862
Brain_Hypothalamus 0.000607862 Brain_Hypothalamus 1 1 1
-3.428081701 -3.428081701 -3.428081701 NA 1 0 0.221406245
GOLGA8K ENSG00000249931.4 0.00062192 1 1 0.00062192
Brain_Caudate_basal_ganglia 0.00062192 Brain_Caudate_basal_ganglia 1 1 1
-3.421870232 -3.421870232 -3.421870232 NA 1 0 0.221406245
LINC02166 ENSG00000260259.1 0.000632996 8 7 9.24E-06
Brain_Nucleus_accumbens_basal_ganglia 0.535861738 Brain_Hippocampus
4.11449395 0.016610905 0.168004228 -0.710570216 4.434342133
1.789194651 1.754114568 7 0 0.221406245
TACO1 ENSG00000136463.7 0.000664557 1 1 0.000664557
Brain_Hypothalamus 0.000664557 Brain_Hypothalamus 1 1 1
3.403798673 3.403798673 3.403798673 NA 1 0 0.226776076
NUP93 ENSG00000102900.12 0.000687166 5 4 0.00517363
Brain_Anterior_cingulate_cortex_BA24 0.51957175 Brain_Spinal_cord_cervical_c-1
3.523263818 0.035234664 0.184052696 -0.64400568 2.796019357
1.120994105 1.246096234 4 0 0.228908066
SNAPC4 ENSG00000165684.4 0.000769781 6 4 0.002844685
Brain_Caudate_basal_ganglia 0.925452566 Brain_Frontal_Cortex_BA9 4.1905262
0.053790225 0.26966215 -2.984041196 0.093567703 -1.295820572
1.147456242 4 0 0.249500968
RP11-178C3.2 ENSG00000267302.5 0.00078465 2 1 0.000653505
Brain_Cerebellar_Hemisphere 0.000976486 Brain_Cerebellum 1.993624022
0.006375978 1.993624022 3.297215385 3.40837728 3.352796333
0.07860333 1 0 0.249500968
CKB ENSG00000166165.12 0.000859469 1 1 0.000859469 Brain_Amygdala
0.000859469 Brain_Amygdala 1 1 1 -3.332896651 -3.332896651
-3.332896651 NA 1 0 0.267218343
RPS7 ENSG00000171863.12 0.000892541 1 1 0.000892541 Brain_Cerebellum
0.000892541 Brain_Cerebellum 1 1 1 3.322376728 3.322376728
3.322376728 NA 1 0 0.271468283
APH1A ENSG00000117362.12 0.000986484 2 2 0.000307767
Brain_Putamen_basal_ganglia 0.002336827 Brain_Cortex 1.933459148
0.066540852 0.066540852 3.043706933 3.608673283 3.326190108
0.399491537 2 0 0.293657537
ACP6 ENSG00000162836.11 0.001042403 13 2 0.00010538
Brain_Frontal_Cortex_BA9 0.012563203 Brain_Cerebellum 11.33347419
0.00412239 0.722432278 -3.877859144 -2.495916895 -3.2658279

0.345806222	2	0	0.303838725
NUDT1	ENSG00000106268.15	0.001095913	1	1	0.001095913	Brain_Cerebellum
0.001095913	Brain_Cerebellum	1	1	1	-3.264670859	-3.264670859
-3.264670859	NA	1	0	0.312916835
PHYHIP	ENSG00000168490.13	0.001204694	3	3	0.005350001
Brain_Hypothalamus	0.386390909	Brain_Cerebellum	1.067665989
0.949178493	0.949178493	-2.785167929	0.866181004	-1.51100915
2.060484389	3	0	0.325924914
RNF180	ENSG00000164197.11	0.001205967	10	6	0.097726967
Brain_Anterior_cingulate_cortex_BA24	0.428634776	Brain_Putamen_basal_ganglia
6.919028902	0.030157983	0.250444283	-1.65597458	1.288682493
0.276352658	1.175371108	6	0	0.325924914
FUBP1	ENSG00000162613.16	0.001214182	1	1	0.001214182	Brain_Cerebellum
0.001214182	Brain_Cerebellum	1	1	1	-3.235527591	-3.235527591
-3.235527591	NA	1	0	0.325924914
PRR12	ENSG00000126464.13	0.001247894	7	4	0.026302631
Brain_Frontal_Cortex_BA9	0.43446118	Brain_Hypothalamus	5.703158076
0.015139554	0.23095158	-0.781580449	2.221714124	1.543343986
1.082276294	4	0	0.325924914
RP11-324L17.1	ENSG00000272551.1	0.001257948	3	3	0.00716708
Brain_Cortex	0.495792388	Brain_Frontal_Cortex_BA9	1.154293586
0.840963632	0.840963632	-2.607657802	2.688979603	-0.199934406
2.680904745	3	0	0.325924914
ACACA	ENSG00000278540.4	0.001355985	3	2	0.000321084	Brain_Amygdala
0.006800184	Brain_Cortex	2.566812994	0.058923105	0.374263901
2.706474348	3.597667303	3.09805245	0.455313272	2	0
0.344938046
HIST1H4E	ENSG00000276966.2	0.001388518	1	1	0.001388518
Brain_Spinal_cord_cervical_c-1	0.001388518	Brain_Spinal_cord_cervical_c-1
1	1	1	3.197027313	3.197027313	3.197027313	NA	1	0
0.345229743
SPDYE5	ENSG00000170092.14	0.001408346	7	6	0.000104781
Brain_Caudate_basal_ganglia	0.330017158	Brain_Spinal_cord_cervical_c-1
3.415112476	0.030622218	0.142950662	0.974079317	3.879244328
2.486753314	1.103727673	6	0	0.345229743
TMPRSS6	ENSG00000187045.16	0.001454904	1	1	0.001454904
Brain_Nucleus_accumbens_basal_ganglia	0.001454904
Brain_Nucleus_accumbens_basal_ganglia	1	1	1	-3.183531188
-3.183531188	-3.183531188	NA	1	0	0.345229743
POM121C	ENSG00000272391.5	0.001455833	5	4	6.77E-05
Brain_Spinal_cord_cervical_c-1	0.834266141	Brain_Substantia_nigra
2.798479902	0.079674489	0.145869942	0.20923327	3.984203681
2.381415109	1.578766025	4	0	0.345229743
COL20A1	ENSG00000101203.16	0.001494202	3	3	0.000595849
Brain_Nucleus_accumbens_basal_ganglia	0.859985182	Brain_Cerebellum
1.078913056	0.910782368	0.910782368	0.176393027	3.433496737
1.882992228	1.63415278	3	0	0.348422905
PKD2	ENSG00000118762.7	0.001580156	5	5	0.003528318
Brain_Caudate_basal_ganglia	0.093332527	Brain_Cortex	1.610893608
0.477291444	0.477291444	-1.730215153	2.917515795	0.770010224
2.280610695	5	0	0.362425692
PTPRJ	ENSG00000149177.12	0.001697628	7	7	0.00034602
Brain_Caudate_basal_ganglia	0.947792029	Brain_Spinal_cord_cervical_c-1
3.559356523	0.146328777	0.146328777	-1.187770161	3.578161396
1.115647425	1.897056675	7	0	0.376767286
CTD-2189E23.1	ENSG00000267723.1	0.001724351	7	7	0.053971691
Brain_Frontal_Cortex_BA9	0.864668016	Brain_Caudate_basal_ganglia
1.895151691	0.112009677	0.112009677	-1.927063704	1.891287496
0.297292805	1.354917242	7	0	0.376767286
NUMBL	ENSG00000105245.9	0.00178662	2	2	0.001012255	Brain_Cerebellum
0.001198725	Brain_Cerebellar_Hemisphere	1.683327913	0.316672087
0.316672087	-3.287098176	-3.239183341	-3.263140759	0.033880904

2 0 0.376767286
PAPD4 ENSG00000164329.13 0.001805428 1 1 0.001805428 Brain_Cerebellum
0.001805428 Brain_Cerebellum 1 1 1 -3.120502471 -3.120502471
-3.120502471 NA 1 0 0.376767286
RECK ENSG00000122707.11 0.001854036 1 1 0.001854036
Brain_Nucleus_accumbens_basal_ganglia 0.001854036
Brain_Nucleus_accumbens_basal_ganglia 1 1 1 -3.1126694 -3.1126694
-3.1126694 NA 1 0 0.376767286
GDPD3 ENSG00000102886.14 0.001866245 7 6 0.001050645
Brain_Caudate_basal_ganglia 0.989789699 Brain_Putamen_basal_ganglia
2.296288808 0.058782552 0.422226786 -3.276601566 2.203070283
-1.165636433 1.940156 6 0 0.376767286
MAMSTR ENSG00000176909.11 0.001900827 12 9 0.015393818
Brain_Frontal_Cortex_BA9 0.998731237 Brain_Cerebellar_Hemisphere
5.736474738 0.00071382 0.245012146 -2.422978968 1.903004155
0.398002137 1.154516514 9 0 0.376767286
ACE ENSG00000159640.15 0.001909736 4 3 0.000864873 Brain_Cortex
0.744149819 Brain_Cerebellar_Hemisphere 3.032963825 0.057241166
0.271715713 -3.331152201 -0.32636288 -2.312077125 1.382576542
3 0 0.376767286
JAK1 ENSG00000162434.11 0.001917618 5 2 0.016628775 Brain_Cortex
0.986046597 Brain_Caudate_basal_ganglia 4.481754684 0.00081114
0.349827929 -0.017488888 2.394814513 1.787115672 1.043371796
2 0 0.376767286
DHRS1 ENSG00000157379.13 0.001929672 13 2 0.00111992
Brain_Cerebellar_Hemisphere 0.969447285 Brain_Spinal_cord_cervical_c-1
11.49684592 0.001615773 0.687605125 -3.25852761 0.038301512
-1.367540584 0.767692396 2 0 0.376767286
ATP5SL ENSG00000105341.18 0.001957133 10 4 0.004423574
Brain_Cerebellar_Hemisphere 0.926635823 Brain_Anterior_cingulate_cortex_BA24
7.994072424 0.003784908 0.310438506 -1.884268045 2.846262309
-0.1008078 1.263074845 4 0 0.376767286
GPS1 ENSG00000169727.12 0.001965836 1 1 0.001965836
Brain_Caudate_basal_ganglia 0.001965836 Brain_Caudate_basal_ganglia 1 1
1 -3.095345736 -3.095345736 -3.095345736 NA 1 0
0.376767286
TMED4 ENSG00000158604.14 0.002014414 5 5 0.005249667 Brain_Cerebellum
0.956408391 Brain_Frontal_Cortex_BA9 2.740396971 0.09739272 0.09739272
-2.791300711 0.054661187 -1.229988288 1.075297296 5 0
0.379851559
PCBP4 ENSG00000090097.21 0.002036228 2 2 0.021702246
Brain_Hippocampus 0.027644569 Brain_Hypothalamus 1.183571861
0.816428139 0.816428139 -2.295539137 2.202293701 -0.046622718
3.1804481 2 0 0.379851559
KLC1 ENSG00000126214.21 0.002066009 2 2 0.00767453 Brain_Hippocampus
0.955833678 Brain_Cerebellar_Hemisphere 1.663878351 0.336121649
0.336121649 -2.666063055 -0.055382574 -1.360722815 1.846029872
2 0 0.380336017
SLC39A13 ENSG00000165915.13 0.002096939 5 4 0.001967985
Brain_Caudate_basal_ganglia 0.947755138 Brain_Cerebellum 3.738420061
0.05537963 0.19836213 -0.981201081 3.095021626 0.447322926
1.557032807 4 0 0.381016589
GRK4 ENSG00000125388.19 0.002138899 9 4 0.000120346
Brain_Caudate_basal_ganglia 0.00995033 Brain_Spinal_cord_cervical_c-1
7.062832378 0.016865136 0.258972922 2.577550649 3.845419515
3.171672883 0.405567713 4 0 0.383658234
FXYD4 ENSG00000150201.14 0.002166931 5 5 0.000658982 Brain_Cerebellum
0.738510912 Brain_Hippocampus 3.379380976 0.14963229 0.14963229
0.333825935 3.406099252 1.323741094 1.253072523 5 0
0.383766208
LINC01731 ENSG00000234283.1 0.002235116 3 3 0.000155844
Brain_Nucleus_accumbens_basal_ganglia 0.807616369 Brain_Caudate_basal_ganglia

1.208199916 0.807115351 0.807115351 -0.427957354 3.781567269
1.199037377 2.261594564 3 0 0.390893865
LINC01443 ENSG00000266554.1 0.002488198 1 1 0.002488198
Brain_Substantia_nigra 0.002488198 Brain_Substantia_nigra 1 1
1 -3.024772962 -3.024772962 -3.024772962 NA 1 0
0.429782503
PIH1D1 ENSG00000104872.10 0.002549675 4 4 0.000313605
Brain_Cerebellum 0.461381438 Brain_Spinal_cord_cervical_c-1 1.535430082
0.425796668 0.425796668 -3.603794423 -0.736574021 -2.053355147
1.456263888 4 0 0.430536868
TNK1 ENSG00000174292.12 0.002554111 1 1 0.002554111
Brain_Frontal_Cortex_BA9 0.002554111 Brain_Frontal_Cortex_BA9 1 1
1 -3.016856073 -3.016856073 -3.016856073 NA 1 0
0.430536868
CHRNA2 ENSG00000120903.12 0.002622489 2 2 0.000609601
Brain_Cerebellum 0.001983894 Brain_Cerebellar_Hemisphere 1.843544363
0.156455637 0.156455637 3.092632936 3.427306142 3.259969539
0.236649693 2 0 0.433188002
GET4 ENSG00000239857.6 0.002631762 1 1 0.002631762
Brain_Anterior_cingulate_cortex_BA24 0.002631762
Brain_Anterior_cingulate_cortex_BA24 1 1 1 -3.007765432
-3.007765432 -3.007765432 NA 1 0 0.433188002
LINC01431 ENSG00000232645.5 0.002903932 2 2 0.097525912
Brain_Nucleus_accumbens_basal_ganglia 0.973454561 Brain_Caudate_basal_ganglia
1.869884532 0.130115468 0.130115468 -1.656968164 0.033275914
-0.811846125 1.195183049 2 0 0.470410394
NUP50 ENSG00000093000.18 0.002925145 10 2 0.001945351
Brain_Cerebellar_Hemisphere 0.24149526 Brain_Hypothalamus 8.537136913
0.000588883 0.586830416 1.171257573 3.098451064 2.302034741
0.626283963 2 0 0.470410394
SIRT6 ENSG00000077463.14 0.003324469 12 1 0.001747422
Brain_Caudate_basal_ganglia 0.011474211 Brain_Hippocampus 11.33958396
2.22E-16 11.33958396 2.52791506 3.130107736 2.856402116
0.164468381 1 0 0.517684403
KCNQ3 ENSG00000184156.16 0.00338213 4 4 0.020887079
Brain_Spinal_cord_cervical_c-1 0.120706453 Brain_Cerebellum 1.390609527
0.610291871 0.610291871 -1.974273243 2.310019428 0.071974872
2.174110665 4 0 0.517684403
ZNF615 ENSG00000197619.13 0.003411315 4 4 0.020319088
Brain_Spinal_cord_cervical_c-1 0.645778746 Brain_Hippocampus
2.548699391 0.150085564 0.150085564 -0.459634285 2.32040298
1.152559128 1.179363571 4 0 0.517684403
PCAT19 ENSG00000267107.6 0.003431973 8 7 0.01332687
Brain_Hippocampus 0.552631939 Brain_Amygdala 3.137334418
0.014368096 0.277616355 -2.474912834 1.339026693 -1.084237259
1.181483645 7 0 0.517684403
RRP12 ENSG00000052749.13 0.003438263 8 3 0.009865303
Brain_Substantia_nigra 0.032873325 Brain_Cerebellar_Hemisphere
6.565804363 0.00024905 0.297976425 2.133627007 2.580515146
2.323858157 0.160093947 3 0 0.517684403
MIPEP ENSG00000027001.9 0.003441116 12 1 0.001890441
Brain_Caudate_basal_ganglia 0.047145909 Brain_Anterior_cingulate_cortex_BA24
11.26144081 0.000632666 11.26144081 -3.106925404 -1.984987078
-2.826186141 0.319290779 1 0 0.517684403
SMIM17 ENSG00000268182.5 0.003581724 2 2 0.001090167
Brain_Cortex 0.279282382 Brain_Frontal_Cortex_BA9 1.095975749
0.904024251 0.904024251 -3.266159804 -1.081932817 -2.17404631
1.544481714 2 0 0.529473242
PCDHGA3 ENSG00000254245.2 0.003595165 1 1 0.003595165
Brain_Cortex 0.003595165 Brain_Cortex 1 1 1 2.911657565
2.911657565 2.911657565 NA 1 0 0.529473242
RAB4B ENSG00000167578.17 0.003779345 1 1 0.003779345 Brain_Cortex

0.003779345 Brain_Cortex 1 1 1 2.896014868 2.896014868
2.896014868 NA 1 0 0.547650358
CNTN2 ENSG00000184144.11 0.003796875 2 2 0.003537795 Brain_Cerebellum
0.003883512 Brain_Cerebellar_Hemisphere 1.511184356 0.488815644
0.488815644 2.88747201 2.916679175 2.902075592 0.020652585 2
0 0.547650358
YIPF1 ENSG00000058799.14 0.003846568 2 2 0.000856933 Brain_Cortex
0.876871043 Brain_Caudate_basal_ganglia 1.072331134 0.927668866
0.927668866 -3.333718514 -0.154936925 -1.744327719 2.247738018
2 0 0.549156507
CEP57L1 ENSG00000183137.14 0.004049891 1 1 0.004049891
Brain_Hippocampus 0.004049891 Brain_Hippocampus 1 1 1
2.874249366 2.874249366 2.874249366 NA 1 0 0.563172833
PDPR ENSG00000090857.13 0.004106794 3 3 0.037686116
Brain_Cerebellar_Hemisphere 0.199749163 Brain_Cerebellum 2.01682891
0.246682582 0.246682582 -2.078252427 1.639527554 -0.573663802
1.957564908 3 0 0.563172833
CDK10 ENSG00000185324.21 0.004117023 12 8 3.76E-05
Brain_Nucleus_accumbens_basal_ganglia 0.853298628 Brain_Putamen_basal_ganglia
6.400271287 0.008334583 0.236379574 -1.095599905 4.121955977
1.277312866 1.459319177 8 0 0.563172833
NOP14 ENSG00000087269.15 0.0041433 2 2 0.001205575 Brain_Cerebellum
0.079646525 Brain_Cerebellar_Hemisphere 1.696615643 0.303384357
0.303384357 1.752740705 3.237557829 2.495149267 1.049924257
2 0 0.563172833
RABL6 ENSG00000196642.18 0.004146008 1 1 0.004146008 Brain_Cerebellum
0.004146008 Brain_Cerebellum 1 1 1 -2.866833687 -2.866833687
-2.866833687 NA 1 0 0.563172833
CACNA1B ENSG00000148408.12 0.004207751 1 1 0.004207751
Brain_Anterior_cingulate_cortex_BA24 0.004207751
Brain_Anterior_cingulate_cortex_BA24 1 1 1 2.862151933
2.862151933 2.862151933 NA 1 0 0.563180843
F12 ENSG00000131187.9 0.004226573 2 2 0.003923344
Brain_Hypothalamus 0.138884318 Brain_Anterior_cingulate_cortex_BA24
1.046800872 0.953199128 0.953199128 1.479958516 2.884260205
2.182109361 0.992991247 2 0 0.563180843
COA7 ENSG00000162377.5 0.004354006 13 4 0.000376136
Brain_Caudate_basal_ganglia 0.169410101 Brain_Spinal_cord_cervical_c-1
10.04425949 0.001068059 0.491771073 -3.556284773 -1.374101734
-2.875778692 0.6106601 4 0 0.566087256
RP11-713D19.1 ENSG00000273233.1 0.004360209 2 2 0.007273534
Brain_Spinal_cord_cervical_c-1 0.044937906 Brain_Hippocampus
1.034122074 0.965877926 0.965877926 2.005235224 2.684054089
2.344644656 0.479997423 2 0 0.566087256
FAM166B ENSG00000215187.10 0.004369768 3 3 0.002997216
Brain_Cerebellum 0.642529876 Brain_Cortex 1.420462506 0.62575329
0.62575329 -2.96802328 1.44169379 -0.354054988 2.315926065 3 0
0.566087256
COL16A1 ENSG00000084636.17 0.004450692 12 7 0.002061101
Brain_Putamen_basal_ganglia 0.87769451 Brain_Substantia_nigra 7.12464931
0.024173561 0.253322653 -3.081283773 2.082091545 -0.80268444
1.512896503 7 0 0.568775733
FRY ENSG00000073910.19 0.004471827 2 2 0.001336459 Brain_Cerebellum
0.015893364 Brain_Cerebellar_Hemisphere 1.564641088 0.435358912
0.435358912 -3.208033551 -2.411355137 -2.809694344 0.563336709
2 0 0.568775733
RP11-490G2.2 ENSG00000259946.1 0.004563036 1 1 0.004563036
Brain_Nucleus_accumbens_basal_ganglia 0.004563036
Brain_Nucleus_accumbens_basal_ganglia 1 1 1 -2.836364347
-2.836364347 -2.836364347 NA 1 0 0.572012386
MANBAL ENSG00000101363.12 0.004579043 5 5 0.00214892
Brain_Putamen_basal_ganglia 0.904524312 Brain_Cerebellum 2.417558011

0.096676958 0.096676958 -1.428484703 3.068839509 0.269109547
1.690384436 5 0 0.572012386
HIP1 ENSG00000127946.16 0.004738991 1 1 0.004738991
Brain_Hippocampus 0.004738991 Brain_Hippocampus 1 1 1
2.824260077 2.824260077 2.824260077 NA 1 0 0.584417168
SIGMAR1 ENSG00000147955.16 0.004815005 2 2 0.007231363
Brain_Cortex 0.069154887 Brain_Caudate_basal_ganglia 1.015558989
0.984441011 0.984441011 -2.685997483 1.81740654 -0.434295471
3.184387523 2 0 0.584417168
ZFP90 ENSG00000184939.15 0.004871145 6 4 0.012601727
Brain_Caudate_basal_ganglia 0.784514762 Brain_Cortex 4.194264541
0.018733399 0.151481824 -2.494830603 0.273440321 -0.999597161
0.898864177 4 0 0.584417168
RASSF4 ENSG00000107551.20 0.004897632 4 3 0.000927753
Brain_Anterior_cingulate_cortex_BA24 0.363703391 Brain_Cerebellum
2.814415657 0.080443693 0.156866949 -3.311566208 -0.908330959
-1.944965788 1.014118937 3 0 0.584417168
C2CD5 ENSG00000111731.12 0.004900775 3 3 0.029664727 Brain_Cerebellum
0.751888344 Brain_Caudate_basal_ganglia 2.360464497 0.092256492
0.092256492 -2.174538368 0.316150413 -0.99392536 1.250381184
3 0 0.584417168
AC141928.1 ENSG00000250986.1 0.00492897 5 5 0.004952316 Brain_Cortex
0.521369776 Brain_Frontal_Cortex_BA9 1.248373646 0.709270918
0.709270918 -2.810119152 2.509015799 0.579728061 2.025810666
5 0 0.584417168
WNT5B ENSG00000111186.12 0.005305583 1 1 0.005305583
Brain_Substantia_nigra 0.005305583 Brain_Substantia_nigra 1 1
1 2.787869992 2.787869992 2.787869992 NA 1 0
0.607583557
HCN3 ENSG00000143630.9 0.005347993 1 1 0.005347993 Brain_Cerebellum
0.005347993 Brain_Cerebellum 1 1 1 -2.785289639 -2.785289639
-2.785289639 NA 1 0 0.607583557
RP11-384F7.2 ENSG00000239268.2 0.005365617 2 2 0.005480139
Brain_Putamen_basal_ganglia 0.087599105 Brain_Nucleus_accumbens_basal_ganglia
1.018968775 0.981031225 0.981031225 -2.777366252 1.708200722
-0.534582765 3.171774825 2 0 0.607583557
COMMD7 ENSG00000149600.11 0.005395069 4 4 0.001886073
Brain_Cortex 0.264053908 Brain_Nucleus_accumbens_basal_ganglia
2.661503862 0.111075423 0.111075423 1.11686066 3.107609233
2.235149817 0.826755883 4 0 0.607583557
C14orf166 ENSG00000087302.8 0.005413374 2 2 0.008444
Brain_Cerebellar_Hemisphere 0.069387683 Brain_Cerebellum 1.020804417
0.979195583 0.979195583 -1.815887162 2.633780735 0.408946787
3.146390344 2 0 0.607583557
ZNF852 ENSG00000178917.15 0.00542258 2 2 0.001325395
Brain_Substantia_nigra 0.740119092 Brain_Spinal_cord_cervical_c-1
1.007921704 0.992078296 0.992078296 0.331695642 3.210423506
1.771059574 2.035567994 2 0 0.607583557
RPS3 ENSG00000149273.14 0.005519433 7 3 0.16215207
Brain_Frontal_Cortex_BA9 0.69730747 Brain_Cortex 5.774385788
0.001379627 0.330618026 -1.397870131 1.37015358 -0.400713115
0.847049134 3 0 0.607583557
MFSD10 ENSG00000109736.14 0.005520887 2 2 0.001262581
Brain_Cerebellum 0.975540892 Brain_Frontal_Cortex_BA9 1.004316735
0.995683265 0.995683265 0.030659749 3.224352075 1.627505912
2.2582815 2 0 0.607583557
RP11-434H6.7 ENSG00000273437.1 0.005536914 2 2 0.003982509
Brain_Cortex 0.015301119 Brain_Nucleus_accumbens_basal_ganglia 1.37573313
0.62426687 0.62426687 2.425172389 2.879543836 2.652358112
0.321289131 2 0 0.607583557
WDPCP ENSG00000143951.15 0.00558812 7 6 0.008886498
Brain_Frontal_Cortex_BA9 0.864130617 Brain_Caudate_basal_ganglia

4.136342664 0.058749556 0.159792913 -2.552919149 2.616390047
-0.359963267 1.689288399 6 0 0.607583557
CORO7 ENSG00000262246.5 0.00560205 13 7 0.017467677 Brain_Cerebellum
0.653241099 Brain_Frontal_Cortex_BA9 8.493441239 0.013374286
0.287137135 -1.748763534 2.376712924 -0.731865734 1.168944864
7 0 0.607583557
DPY19L1 ENSG00000173852.14 0.005742586 8 6 0.01137662
Brain_Frontal_Cortex_BA9 0.201901443 Brain_Cortex 4.962145003
0.007050184 0.20693148 -2.530912578 2.066736583 -1.400322921
1.478811534 6 0 0.612592871
DPH3 ENSG00000154813.9 0.00574956 2 2 0.001681356
Brain_Frontal_Cortex_BA9 0.924237537 Brain_Hypothalamus 1.179518953
0.820481047 0.820481047 -3.141411515 0.095097307 -1.523157104
2.288557335 2 0 0.612592871
WDR3 ENSG00000065183.15 0.005796371 2 2 0.003915349
Brain_Anterior_cingulate_cortex_BA24 0.179300965 Brain_Amygdala
1.021942277 0.978057723 0.978057723 -2.884902493 1.342910471
-0.770996011 2.989515217 2 0 0.612592871
TYROBP ENSG00000011600.11 0.005823376 2 2 0.002953241
Brain_Cerebellar_Hemisphere 0.169576004 Brain_Hippocampus 1.05686345
0.94313655 0.94313655 -1.373567462 2.972563478 0.799498008
3.073178659 2 0 0.612592871
ALDH16A1 ENSG00000161618.9 0.005927502 13 4 0.002992889
Brain_Cerebellum 0.934497224 Brain_Hypothalamus 10.16288951
0.000178514 0.563282556 0.082187989 2.968467301 1.75084883
0.775121913 4 0 0.618893116
RNF224 ENSG00000233198.3 0.006097498 2 2 0.020869487
Brain_Cerebellar_Hemisphere 0.033967384 Brain_Frontal_Cortex_BA9
1.035981483 0.964018517 0.964018517 -2.310337305 -2.120458668
-2.215397987 0.134264472 2 0 0.631926639
ATP4A ENSG00000105675.8 0.006290833 2 2 0.002952339
Brain_Cerebellar_Hemisphere 0.181841768 Brain_Cortex 1.066325966
0.933674034 0.933674034 1.335105658 2.97265732 2.153881489
1.157923885 2 0 0.63859576
STX1B ENSG00000099365.10 0.006328997 2 2 0.001490089
Brain_Cerebellar_Hemisphere 0.008107601 Brain_Cerebellum 1.798291343
0.201708657 0.201708657 2.647555667 3.176606852 2.91208126
0.37409568 2 0 0.63859576
GNLY ENSG00000115523.16 0.006337043 13 3 0.004402521
Brain_Hypothalamus 0.312093964 Brain_Anterior_cingulate_cortex_BA24
10.8920935 1.79E-06 0.56344247 1.010838017 2.847781016 2.469165447
0.500557773 3 0 0.63859576
RP11-7K24.3 ENSG00000261068.2 0.006384941 1 1 0.006384941
Brain_Cerebellar_Hemisphere 0.006384941 Brain_Cerebellar_Hemisphere 1 1
1 -2.727328643 -2.727328643 -2.727328643 NA 1 0 0.63859576
GTF2H1 ENSG00000110768.11 0.006416429 13 2 0.007216946
Brain_Spinal_cord_cervical_c-1 0.522799392 Brain_Cerebellar_Hemisphere
11.5014293 0.000269585 0.729023551 -2.686664239 0.639036185
-2.110024546 0.858973343 2 0 0.63859576
CCNT2 ENSG00000082258.12 0.006439994 5 4 0.000400577
Brain_Caudate_basal_ganglia 0.447938644 Brain_Putamen_basal_ganglia
3.134769271 0.060258811 0.116360498 -3.539703488 -0.758856098
-2.267180979 1.180146686 4 0 0.63859576
PSMC4 ENSG00000013275.7 0.006534388 1 1 0.006534388
Brain_Putamen_basal_ganglia 0.006534388 Brain_Putamen_basal_ganglia 1 1
1 -2.719686271 -2.719686271 -2.719686271 NA 1 0 0.63859576
MRPS7 ENSG00000125445.10 0.00653555 6 2 0.001707626 Brain_Cortex
0.126701273 Brain_Frontal_Cortex_BA9 5.117711049 0.005548743
0.691742389 1.527240281 3.136868636 2.07648382 0.561569124 2
0 0.63859576
RP11-11M20.4 ENSG00000283078.1 0.006572639 1 1 0.006572639
Brain_Anterior_cingulate_cortex_BA24 0.006572639

Brain_Anterior_cingulate_cortex_BA24 1 1 1 -2.717755497
-2.717755497 -2.717755497 NA 1 0 0.63859576
EDN3 ENSG00000124205.15 0.006669702 1 1 0.006669702 Brain_Cerebellum
0.006669702 Brain_Cerebellum 1 1 1 2.712901036 2.712901036
2.712901036 NA 1 0 0.641307231
TUB ENSG00000166402.8 0.006721067 2 2 0.005119727
Brain_Cerebellar_Hemisphere 0.783359563 Brain_Cerebellum 1.540707149
0.459292851 0.459292851 -2.799402514 -0.274943608 -1.537173061
1.785062011 2 0 0.641307231
MAPK3 ENSG00000102882.11 0.006745752 7 5 0.000911729 Brain_Cerebellum
0.517518673 Brain_Anterior_cingulate_cortex_BA24 2.964923041
0.029361948 0.804540946 -3.316437638 1.505843078 -1.487786613
1.869158259 5 0 0.641307231
DDB2 ENSG00000134574.11 0.006783895 3 2 0.002228948 Brain_Amygdala
0.072230108 Brain_Hypothalamus 2.637867612 0.047525326
0.314607062 1.797665021 3.057898901 2.612399451 0.706610368
2 0 0.641307231
SLX1A ENSG00000132207.17 0.006857971 2 2 0.014606546 Brain_Cortex
0.075568918 Brain_Cerebellar_Hemisphere 1.088264013 0.911735987
0.911735987 -2.441990131 -1.776995878 -2.109493005 0.470221946
2 0 0.641479123
ARHGAP1 ENSG00000175220.11 0.006907571 3 3 0.002111638
Brain_Amygdala 0.796251319 Brain_Cerebellum 1.715377661 0.477277824
0.477277824 -3.074064446 -0.258201605 -1.420525012 1.470795966
3 0 0.641479123
CLDN15 ENSG00000106404.13 0.006959396 13 2 0.024619146
Brain_Substantia_nigra 0.986128331 Brain_Cerebellum 11.07480564
0.000971885 1.074117908 -2.247326851 1.462195162 0.265498694
1.002682587 2 0 0.641479123
PPP1R13B ENSG00000088808.16 0.006969111 3 3 0.000797905
Brain_Caudate_basal_ganglia 0.781119756 Brain_Nucleus_accumbens_basal_ganglia
1.06547631 0.930708244 0.930708244 0.277860097 3.353520655
1.427529807 1.678370593 3 0 0.641479123
TMTC1 ENSG00000133687.15 0.007027024 3 2 0.001783507 Brain_Cortex
0.691030011 Brain_Cerebellum 1.947004553 0.052640987 1.00035446
-3.124098593 0.457293845 -0.756449033 2.050662922 2 0
0.642582349
DHX40 ENSG00000108406.9 0.007179791 5 4 0.000389456 Brain_Cortex
0.361439758 Brain_Hypothalamus 2.71608375 0.062749085 0.343977772
0.912625083 3.547127599 2.381724392 1.154949695 4 0
0.651430507
SLC12A9 ENSG00000146828.17 0.007216906 6 6 0.006066885
Brain_Cerebellum 0.733185555 Brain_Caudate_basal_ganglia 1.6044652
0.556423216 0.556423216 -2.744144344 1.764895653 -0.74632081
1.720897547 6 0 0.651430507
NQO2 ENSG00000124588.19 0.007296929 13 5 0.007170145
Brain_Hippocampus 0.769159047 Brain_Cerebellar_Hemisphere 8.609755163
0.025687673 0.533457939 -2.688836861 1.28740939 -0.019168276
1.217021456 5 0 0.654431595
WFIKKN1 ENSG00000127578.6 0.007524354 13 1 0.0039376
Brain_Anterior_cingulate_cortex_BA24 0.051329289 Brain_Substantia_nigra
12.69565893 0.00010806 12.69565893 -2.883117945 -1.948716511
-2.63840153 0.245242311 1 0 0.666736822
CEP78 ENSG00000148019.12 0.007529442 1 1 0.007529442
Brain_Frontal_Cortex_BA9 0.007529442 Brain_Frontal_Cortex_BA9 1 1
1 2.672473076 2.672473076 2.672473076 NA 1 0
0.666736822
ZNF695 ENSG00000197472.14 0.007711812 1 1 0.007711812
Brain_Cerebellum 0.007711812 Brain_Cerebellum 1 1 1 2.664433485
2.664433485 2.664433485 NA 1 0 0.677925928
DHX34 ENSG00000134815.18 0.007752709 9 4 0.005505575
Brain_Putamen_basal_ganglia 0.642365443 Brain_Nucleus_accumbens_basal_ganglia

6.994588242 0.002780585 0.284401472 0.464394062 2.77586097
1.678490314 0.881218059 4 0 0.677925928
NRP1 ENSG00000099250.17 0.007873169 1 1 0.007873169
Brain_Anterior_cingulate_cortex_BA24 0.007873169
Brain_Anterior_cingulate_cortex_BA24 1 1 1 -2.657461061
-2.657461061 -2.657461061 NA 1 0 0.681200722
GRK1 ENSG00000185974.6 0.007887536 2 2 0.014608317
Brain_Spinal_cord_cervical_c-1 0.026692436 Brain_Substantia_nigra
1.123485264 0.876514736 0.876514736 -2.441946354 2.215986361
-0.112979996 3.293655809 2 0 0.681200722
ATP5G1 ENSG00000159199.13 0.008015304 8 6 0.001541356
Brain_Hippocampus 0.586070039 Brain_Hypothalamus 3.040211181
0.074850253 0.215131796 -1.979918874 3.166782352 0.487956259
1.83423152 6 0 0.681393817
TSPAN5 ENSG00000168785.7 0.008023397 3 3 0.000635338
Brain_Cerebellar_Hemisphere 0.635750741 Brain_Spinal_cord_cervical_c-1
1.408972843 0.5685158 0.5685158 -0.47364828 3.41606211 1.350020582
1.956149307 3 0 0.681393817
EXOSC6 ENSG00000223496.2 0.008081804 13 1 0.005325409
Brain_Nucleus_accumbens_basal_ganglia 0.019424698 Brain_Cortex 12.8466457
1.30E-05 12.8466457 -2.786661401 -2.337278682 -2.632770497
0.132037263 1 0 0.681393817
C1QTNF9 ENSG00000240654.6 0.008084581 6 2 0.001039685
Brain_Amygdala 0.078117094 Brain_Frontal_Cortex_BA9 5.566426142
0.002430371 0.254876483 -3.279561673 -1.761717179 -2.785179384
0.606023933 2 0 0.681393817
SP6 ENSG00000189120.4 0.008141272 4 4 0.003486431
Brain_Hippocampus 0.480391168 Brain_Cortex 1.802728002 0.42111087
0.42111087 -1.581890764 2.921238238 -0.125450744 2.062388794 4
0 0.682063089
MRM2 ENSG00000122687.17 0.008399207 13 5 0.001075373
Brain_Hypothalamus 0.19379565 Brain_Spinal_cord_cervical_c-1 9.714123298
5.93E-18 0.358666472 1.299432191 3.270026809 2.653073073
0.577781533 5 0 0.699483996
CBLC ENSG00000142273.10 0.008452055 1 1 0.008452055
Brain_Hippocampus 0.008452055 Brain_Hippocampus 1 1 1
-2.633456953 -2.633456953 -2.633456953 NA 1 0 0.699720118
REST ENSG00000084093.16 0.008589143 1 1 0.008589143
Brain_Putamen_basal_ganglia 0.008589143 Brain_Putamen_basal_ganglia 1 1
1 -2.627988443 -2.627988443 -2.627988443 NA 1 0
0.706664942
RNF5 ENSG00000204308.7 0.00863696 6 4 0.011434651
Brain_Frontal_Cortex_BA9 0.462884385 Brain_Hypothalamus 3.634004243
0.048367145 0.487069494 -2.529127397 0.799897998 -1.38891532
1.294379708 4 0 0.706664942
TRDN ENSG00000186439.12 0.008713527 2 2 0.030384381 Brain_Cortex
0.079262064 Brain_Cerebellar_Hemisphere 1.183906982 0.816093018
0.816093018 -1.754983886 2.165043152 0.205029633 2.7718777 2
0 0.706789404
ARID3C ENSG00000205143.2 0.008739516 8 3 0.000826295
Brain_Putamen_basal_ganglia 0.113180525 Brain_Nucleus_accumbens_basal_ganglia
7.094401249 0.000840029 0.250581226 -3.343832995 -1.584057984
-2.440991548 0.487783727 3 0 0.706789404
DNAH2 ENSG00000183914.14 0.00883582 2 2 0.002613207
Brain_Caudate_basal_ganglia 0.080790421 Brain_Cerebellar_Hemisphere
1.386763876 0.613236124 0.613236124 1.746118164 3.009915113
2.378016639 0.893639393 2 0 0.710471056
VPS9D1 ENSG00000075399.13 0.00890385 12 6 0.015729527
Brain_Spinal_cord_cervical_c-1 0.987046715 Brain_Cerebellar_Hemisphere
7.537464698 0.006154259 0.355635004 -1.916291281 2.415131567
-0.353566733 1.313024389 6 0 0.711850118
BTN2A2 ENSG00000124508.16 0.009052551 12 8 0.015143897

Brain_Hippocampus 0.863744831 Brain_Frontal_Cortex_BA9 5.488329761
0.034621903 0.253016509 -2.39660019 2.428919434 0.062253297
1.368877004 8 0 0.711972999
NUP88 ENSG00000108559.11 0.009083442 6 3 0.1546061 Brain_Cortex
0.810741603 Brain_Amygdala 4.661013383 0.025263161 0.248862738
-0.239469424 1.423448781 0.671298162 0.568472612 3 0
0.711972999
RMND5A ENSG00000153561.12 0.009135353 1 1 0.009135353
Brain_Cerebellum 0.009135353 Brain_Cerebellum 1 1 1 2.606946897
2.606946897 2.606946897 NA 1 0 0.711972999
MRPL41 ENSG00000182154.7 0.009181968 2 2 0.004041854
Brain_Cerebellar_Hemisphere 0.261055538 Brain_Cerebellum 1.023559652
0.976440348 0.976440348 -1.123899795 2.874876623 0.875488414
2.827561922 2 0 0.711972999
CTPS1 ENSG00000171793.13 0.009287267 8 8 0.005075671
Brain_Putamen_basal_ganglia 0.741347531 Brain_Cortex 3.39975557
0.177471658 0.177471658 -0.933558941 2.802191637 0.554256885
1.244759695 8 0 0.711972999
TEX9 ENSG00000151575.14 0.009328675 9 3 0.004978619
Brain_Anterior_cingulate_cortex_BA24 0.481235736 Brain_Hippocampus
7.886356173 0.000972502 0.316469032 -2.808413916 -0.704316431
-1.205302841 0.62404743 3 0 0.711972999
SLC22A17 ENSG00000092096.16 0.009423654 1 1 0.009423654
Brain_Spinal_cord_cervical_c-1 0.009423654 Brain_Spinal_cord_cervical_c-1
1 1 1 2.596289818 2.596289818 2.596289818 NA 1 0
0.711972999
BAIAP3 ENSG00000007516.13 0.009478869 8 7 0.051840528
Brain_Caudate_basal_ganglia 0.647117714 Brain_Cerebellum 2.312030116
0.043725332 0.466964687 -1.944455654 1.829258568 0.003423574
1.460182067 7 0 0.711972999
RP11-596C23.6 ENSG00000282885.1 0.009520934 1 1 0.009520934
Brain_Spinal_cord_cervical_c-1 0.009520934 Brain_Spinal_cord_cervical_c-1
1 1 1 -2.592759441 -2.592759441 -2.592759441 NA 1 0
0.711972999
RAPSN ENSG00000165917.9 0.009567171 4 4 0.001057133 Brain_Cortex
0.658823309 Brain_Frontal_Cortex_BA9 2.125831251 0.438466439
0.438466439 -0.441538344 3.274862875 1.187117279 1.546855145
4 0 0.711972999
LINC00928 ENSG00000259218.5 0.00957811 1 1 0.00957811
Brain_Caudate_basal_ganglia 0.00957811 Brain_Caudate_basal_ganglia 1 1 1
2.590699458 2.590699458 2.590699458 NA 1 0 0.711972999
NUP160 ENSG00000030066.13 0.009600195 1 1 0.009600195
Brain_Hippocampus 0.009600195 Brain_Hippocampus 1 1 1
2.589906707 2.589906707 2.589906707 NA 1 0 0.711972999
ANKEF1 ENSG00000132623.15 0.009609445 1 1 0.009609445
Brain_Hypothalamus 0.009609445 Brain_Hypothalamus 1 1 1
2.58957514 2.58957514 2.58957514 NA 1 0 0.711972999
HGFAC ENSG00000109758.8 0.009775378 6 5 0.031565046
Brain_Frontal_Cortex_BA9 0.791467264 Brain_Cerebellum 2.610541862
0.067361596 0.244315881 -1.567120744 2.149875733 0.741499812
1.483480505 5 0 0.711972999
SPEF2 ENSG00000152582.13 0.009781378 8 2 0.004607755
Brain_Anterior_cingulate_cortex_BA24 0.550656017 Brain_Amygdala
6.648289776 0.008379531 0.670421649 -2.833248501 -0.596777387
-2.292451463 0.721562152 2 0 0.711972999
MGMT ENSG00000170430.9 0.009814776 13 3 0.161515191
Brain_Hippocampus 0.933566053 Brain_Substantia_nigra 10.49564394
0.016844828 0.488590056 -0.966143836 1.399993746 -0.207278204
0.791096439 3 0 0.711972999
CALHM2 ENSG00000138172.10 0.009817352 4 4 0.02652566
Brain_Putamen_basal_ganglia 0.197296302 Brain_Hypothalamus 2.730596328
0.093947361 0.093947361 -2.21842806 1.289292833 -1.09919356

1.64852315 4 0 0.711972999
EGLN2 ENSG00000269858.5 0.009891429 2 2 0.013613772
Brain_Frontal_Cortex_BA9 0.0202898 Brain_Putamen_basal_ganglia 1.24361133
0.75638867 0.75638867 -2.467296124 -2.320945224 -2.394120674
0.103485714 2 0 0.711972999
RP11-440L14.3 ENSG00000260262.1 0.009899546 1 1 0.009899546
Brain_Cerebellar_Hemisphere 0.009899546 Brain_Cerebellar_Hemisphere 1 1
1 2.579318523 2.579318523 2.579318523 NA 1 0
0.711972999
LINC01480 ENSG00000270164.1 0.009923146 5 5 0.001243429
Brain_Putamen_basal_ganglia 0.956100849 Brain_Nucleus_accumbens_basal_ganglia
2.486951508 0.183148877 0.183148877 0.055047214 3.228726151
1.413518202 1.345334412 5 0 0.711972999
C12orf60 ENSG00000182993.3 0.010217511 13 3 0.026762703
Brain_Substantia_nigra 0.876726807 Brain_Cortex 11.37929529
0.00016636 0.417511674 -2.214961555 1.145064011 -0.265827552
0.717661813 3 0 0.727710219
EGLN3 ENSG00000129521.13 0.010265574 2 2 0.002631289
Brain_Nucleus_accumbens_basal_ganglia 0.816471768 Brain_Cerebellum
1.033512707 0.966487293 0.966487293 -3.007820026 -0.232085292
-1.619952659 1.962740853 2 0 0.727710219
B3GALNT2 ENSG00000162885.12 0.010385138 3 3 0.008614488
Brain_Nucleus_accumbens_basal_ganglia 0.09000425 Brain_Frontal_Cortex_BA9
1.868031916 0.149644766 0.149644766 1.695375291 2.626985998
2.190181114 0.468505826 3 0 0.727710219
SEMA6C ENSG00000143434.15 0.010397173 4 3 0.000957534
Brain_Cortex 0.30947798 Brain_Caudate_basal_ganglia 2.599861889
0.053539327 0.347959672 1.016317999 3.302715619 2.350664533
0.963053464 3 0 0.727710219
GAS8 ENSG00000141013.16 0.01045903 12 6 0.006160085
Brain_Cerebellar_Hemisphere 0.798509409 Brain_Caudate_basal_ganglia
6.256898096 0.020935694 0.312748074 -2.739136216 -0.255276682
-1.560874807 0.747742909 6 0 0.727710219
GS1-166A23.2 ENSG00000272361.2 0.010570269 10 8 0.00312604
Brain_Nucleus_accumbens_basal_ganglia 0.955669522 Brain_Substantia_nigra
3.829885165 0.010680733 0.203014663 -2.95506423 1.381137847
-1.334795836 1.639534919 8 0 0.727710219
AC144831.1 ENSG00000261888.1 0.010625074 2 2 0.008759458
Brain_Cortex 0.300828197 Brain_Anterior_cingulate_cortex_BA24
1.165577982 0.834422018 0.834422018 -2.621302205 -1.034658986
-1.827980595 1.12192618 2 0 0.727710219
ZNHIT1 ENSG00000106400.11 0.010665695 3 2 0.00195544
Brain_Putamen_basal_ganglia 0.008902685 Brain_Caudate_basal_ganglia
2.800256827 0.034307675 0.165435498 -3.096917986 -2.615768671
-2.882635369 0.24484685 2 0 0.727710219
RP11-395G23.3 ENSG00000254615.2 0.010714546 1 1 0.010714546
Brain_Spinal_cord_cervical_c-1 0.010714546 Brain_Spinal_cord_cervical_c-1
1 1 1 2.551873355 2.551873355 2.551873355 NA 1 0
0.727710219
BSDC1 ENSG00000160058.18 0.0107258 3 3 0.025066926
Brain_Putamen_basal_ganglia 0.109552741 Brain_Cerebellar_Hemisphere
1.503098835 0.504533843 0.504533843 -2.118534365 2.240369782
-0.49279042 2.381131961 3 0 0.727710219
COIL ENSG00000121058.4 0.01072831 1 1 0.01072831
Brain_Spinal_cord_cervical_c-1 0.01072831 Brain_Spinal_cord_cervical_c-1 1
1 1 2.551426027 2.551426027 2.551426027 NA 1 0
0.727710219
U91328.19 ENSG00000272462.2 0.010766637 13 7 0.014352479
Brain_Hippocampus 0.970851829 Brain_Cerebellar_Hemisphere 8.703457522
0.040522496 0.307025745 -2.448318323 1.314182922 -0.59776862
1.033219719 7 0 0.727710219
HS3ST2 ENSG00000122254.6 0.010893428 1 1 0.010893428

Brain_Amygdala 0.010893428 Brain_Amygdala 1 1 1 2.546099027
2.546099027 2.546099027 NA 1 0 0.732740155
NCBP2-AS2 ENSG00000270170.1 0.011021459 2 2 0.030314339
Brain_Putamen_basal_ganglia 0.218728707 Brain_Hippocampus 1.336327505
0.663672495 0.663672495 -2.165958752 1.229915614 -0.468021569
2.401245792 2 0 0.737804957
CNNM4 ENSG00000158158.11 0.011196984 5 3 0.061078976 Brain_Amygdala
0.390310248 Brain_Hypothalamus 3.535169444 0.000730441
0.269758165 -1.281940695 1.872923311 -0.446226731 1.305801442
3 0 0.739059879
WDR49 ENSG00000174776.10 0.011281487 4 4 0.000794657 Brain_Cerebellum
0.791870604 Brain_Hypothalamus 1.712913907 0.337647966
0.337647966 -3.354649305 -0.263882314 -1.296805894 1.447799302
4 0 0.739059879
UPF3A ENSG00000169062.14 0.011311236 7 6 0.00210032
Brain_Putamen_basal_ganglia 0.634706929 Brain_Substantia_nigra
3.303911477 0.092188861 0.129590698 -0.522988912 3.075667425
0.817281082 1.201098042 6 0 0.739059879
DRD1 ENSG00000184845.3 0.011388851 1 1 0.011388851
Brain_Hypothalamus 0.011388851 Brain_Hypothalamus 1 1 1
-2.530535656 -2.530535656 -2.530535656 NA 1 0 0.739059879
SV2A ENSG00000159164.9 0.011412644 1 1 0.011412644
Brain_Spinal_cord_cervical_c-1 0.011412644 Brain_Spinal_cord_cervical_c-1
1 1 1 -2.529803455 -2.529803455 -2.529803455 NA 1 0
0.739059879
COCH ENSG00000100473.15 0.011486772 2 2 0.00351182 Brain_Cortex
0.676517286 Brain_Caudate_basal_ganglia 1.076501839 0.923498161
0.923498161 -2.91897715 0.417220289 -1.25087843 2.359047833
2 0 0.739059879
NRG4 ENSG00000169752.16 0.011566944 12 2 0.070808017
Brain_Hypothalamus 0.931713688 Brain_Cerebellum 10.72180989
0.001071701 0.387948793 0.085688948 1.806706733 1.268923772
0.642092752 2 0 0.739059879
TMEM63A ENSG00000196187.11 0.011570634 6 6 0.010156908
Brain_Frontal_Cortex_BA9 0.383746037 Brain_Nucleus_accumbens_basal_ganglia
3.103650406 0.165202449 0.165202449 -1.950481544 2.570441147
1.063773984 1.63697285 6 0 0.739059879
MRPL10 ENSG00000159111.12 0.011588159 8 8 3.69E-05 Brain_Cerebellum
0.839211581 Brain_Frontal_Cortex_BA9 1.708747978 0.345362821
0.345362821 -4.126127029 1.178766131 -1.073343617 1.631134899
8 0 0.739059879
PXYLP1 ENSG00000155893.12 0.011682195 2 2 0.007611904
Brain_Cortex 0.796400478 Brain_Spinal_cord_cervical_c-1 1.367778853
0.632221147 0.632221147 -2.668816421 -0.25800833 -1.463412375
1.704698749 2 0 0.739059879
RNF208 ENSG00000212864.3 0.011775952 1 1 0.011775952
Brain_Amygdala 0.011775952 Brain_Amygdala 1 1 1 -2.518788056
-2.518788056 -2.518788056 NA 1 0 0.739059879
TSHR ENSG00000165409.16 0.011825252 3 3 0.031054605
Brain_Caudate_basal_ganglia 0.452082017 Brain_Putamen_basal_ganglia
1.860702445 0.142973918 0.142973918 0.751948521 2.156372309
1.338022904 0.730455548 3 0 0.739059879
TXNDC11 ENSG00000153066.12 0.011916993 2 2 0.005124619
Brain_Cerebellar_Hemisphere 0.316688638 Brain_Caudate_basal_ganglia
1.003914522 0.996085478 0.996085478 -1.001285836 2.799094199
0.898904181 2.687274494 2 0 0.739059879
TRH ENSG00000170893.3 0.011919646 2 2 0.006643294
Brain_Cerebellar_Hemisphere 0.133059313 Brain_Cerebellum 1.105914036
0.894085964 0.894085964 1.502146366 2.714215488 2.108180927
0.857062295 2 0 0.739059879
HSD17B13 ENSG00000170509.11 0.011946121 13 3 0.000371842
Brain_Cerebellum 0.02197183 Brain_Nucleus_accumbens_basal_ganglia 10.86295246

0.001235798	0.468116143	-3.559302159	-2.290854497	-2.891746361
0.380170968	3	0	0.739059879
SESN1 ENSG00000080546.13	0.011954692	1	1	0.011954692
Brain_Anterior_cingulate_cortex_BA24	0.011954692
Brain_Anterior_cingulate_cortex_BA24	1	1	1	2.513478936
2.513478936	2.513478936	NA	1	0	0.739059879
EGLN1 ENSG00000135766.8	0.01199029 2	2	0.052833299
Brain_Nucleus_accumbens_basal_ganglia 0.477100586	Brain_Hippocampus
1.581496903	0.418503097	0.418503097	-1.936280967	0.710973615
-0.612653676	1.871891667	2	0	0.739059879
CPSF4 ENSG00000160917.14	0.011991037	3	2	0.003257112
Brain_Anterior_cingulate_cortex_BA24	0.016182987	Brain_Frontal_Cortex_BA9
2.793418433	0.053822709	0.152758858	-2.942369336	-2.40476232
-2.706459464	0.274774998	2	0	0.739059879
TMEM163	ENSG00000152128.13	0.012224863	5	5	0.000729995
Brain_Putamen_basal_ganglia 0.78154018 Brain_Frontal_Cortex_BA9	2.397836256
0.117544874	0.117544874	-0.277312477	3.378058737	1.396714182
1.379847416	5	0	0.750166927
MIER1 ENSG00000198160.14	0.012286747	5	5	0.092580648	Brain_Cerebellum
0.80617454 Brain_Cortex	3.135420981	0.21770353 0.21770353 -1.681939107
1.530789114	-0.293197648	1.159281931	5	0	0.750671928
TPD52L1	ENSG00000111907.20	0.012452374	6	5	0.043785743
Brain_Cortex	0.820758611	Brain_Anterior_cingulate_cortex_BA24
2.839390443	0.049883009	0.139736837	-1.074189121	2.016136127
0.745133124	1.270074915	5	0	0.75748335
ZWILCH	ENSG00000174442.11	0.012577108	1	1	0.012577108
Brain_Frontal_Cortex_BA9	0.012577108	Brain_Frontal_Cortex_BA9	1	1
1	2.495524474	2.495524474	2.495524474	NA	1	0
0.758660175
CDC37L1-AS1	ENSG00000273061.1	0.012625669	2	2	0.003266892
Brain_Cerebellar_Hemisphere 0.766381156	Brain_Caudate_basal_ganglia
1.002093236	0.997906764	0.997906764	-0.2971118 2.94144084 1.32216452
2.290002533	2	0	0.758660175
MTAP ENSG00000099810.18	0.012634395	6	5	0.049573366
Brain_Putamen_basal_ganglia 0.598099508	Brain_Anterior_cingulate_cortex_BA24
3.461791788	0.080976389	0.268285223	-1.963626981	1.815246806
-0.414096141	1.610395174	5	0	0.758660175
NXT1 ENSG00000132661.3	0.012741446	1	1	0.012741446
Brain_Cerebellar_Hemisphere 0.012741446	Brain_Cerebellar_Hemisphere 1	1
1	-2.490915382	-2.490915382	-2.490915382	NA	1	0
0.761818686
DDAH2 ENSG00000213722.8	0.012874597	6	6	0.002285106	Brain_Cortex
0.137850124	Brain_Cerebellum 3.210910695	0.114985278	0.114985278
-3.050434376	1.550009414	-1.671181647	1.668833576	6	0
0.762414582
KANK4 ENSG00000132854.18	0.012909179	4	4	0.004586502
Brain_Caudate_basal_ganglia 0.523566629	Brain_Hypothalamus	1.754881474
0.496685663	0.496685663	-1.073930918	2.834725922	0.356946514
1.828368886	4	0	0.762414582
ZNF580	ENSG00000213015.8	0.012917145	2	2	0.003276666
Brain_Hippocampus	0.939890551	Brain_Cerebellum 1.051628597
0.948371403	0.948371403	-0.075407426	2.940515539	1.432554057
2.13257958 2	0	0.762414582
ZNF547	ENSG00000152433.14	0.012995017	3	3	0.002925453
Brain_Frontal_Cortex_BA9	0.559411219	Brain_Cerebellum 1.357731692
0.691209728	0.691209728	-2.975464384	0.58371627 -1.415997043
1.819973659	3	0	0.762414582
SCAMP1	ENSG00000085365.17	0.013023879	5	4	0.002108017
Brain_Putamen_basal_ganglia 0.942724021	Brain_Caudate_basal_ganglia
3.293279658	0.050260806	0.280009088	0.071846558	3.074576485
1.581504952	1.109700307	4	0	0.762414582
C2orf50	ENSG00000150873.11	0.013204444	3	3	0.001189103

Brain_Frontal_Cortex_BA9 0.64643769 Brain_Nucleus_accumbens_basal_ganglia
1.67183415 0.416437026 0.416437026 -3.24148123 -0.458716602
-1.774876712 1.397469099 3 0 0.762468029
SCRT2 ENSG00000215397.3 0.013219896 1 1 0.013219896
Brain_Nucleus_accumbens_basal_ganglia 0.013219896
Brain_Nucleus_accumbens_basal_ganglia 1 1 1 2.477789907
2.477789907 2.477789907 NA 1 0 0.762468029
LAMP3 ENSG00000078081.7 0.013233651 1 1 0.013233651
Brain_Caudate_basal_ganglia 0.013233651 Brain_Caudate_basal_ganglia 1 1
1 2.477418825 2.477418825 2.477418825 NA 1 0
0.762468029
JARID2 ENSG00000008083.13 0.013243217 3 3 0.026496578
Brain_Amygdala 0.991402329 Brain_Caudate_basal_ganglia 1.801553238
0.235000439 0.235000439 -1.692293074 2.218855192 0.179112637
1.961000551 3 0 0.762468029
HTR1A ENSG00000178394.4 0.013303123 1 1 0.013303123 Brain_Cortex
0.013303123 Brain_Cortex 1 1 1 2.475549735 2.475549735
2.475549735 NA 1 0 0.762468029
TSSK3 ENSG00000162526.6 0.013424894 2 2 0.007273018
Brain_Frontal_Cortex_BA9 0.53045974 Brain_Cerebellum 1.200756261
0.799243739 0.799243739 -0.62730437 2.684077791 1.028386711
2.341500781 2 0 0.762468029
ANKDD1A ENSG00000166839.16 0.013470842 5 3 0.002461821
Brain_Cerebellum 0.111609825 Brain_Nucleus_accumbens_basal_ganglia 3.58902752
0.029049765 0.236706202 1.590998892 3.027995131 2.395873275
0.614672404 3 0 0.762468029
NR1H3 ENSG00000025434.18 0.013500475 6 5 0.001741044 Brain_Amygdala
0.962327624 Brain_Nucleus_accumbens_basal_ganglia 3.01782055 0.074772006
0.174870339 -0.168459321 3.131181634 0.986380162 1.236111665
5 0 0.762468029
SH2D7 ENSG00000183476.12 0.013515265 8 4 0.002516159
Brain_Caudate_basal_ganglia 0.804181358 Brain_Nucleus_accumbens_basal_ganglia
6.071708408 0.046456379 0.217840419 -3.021391377 0.707664853
-0.783591108 1.167547527 4 0 0.762468029
MTPAP ENSG00000107951.14 0.013649378 7 4 0.074572655
Brain_Hypothalamus 0.595885214 Brain_Hippocampus 4.753812163
0.015374837 0.396726147 -1.783083847 1.498451114 -0.245870834
1.161460166 4 0 0.766941559
CHTOP ENSG00000160679.12 0.013747487 1 1 0.013747487 Brain_Cerebellum
0.013747487 Brain_Cerebellum 1 1 1 2.463794584 2.463794584
2.463794584 NA 1 0 0.769364384
MUTYH ENSG00000132781.17 0.013892816 5 4 0.048950611
Brain_Nucleus_accumbens_basal_ganglia 0.718532353
Brain_Spinal_cord_cervical_c-1 3.051742873 0.066263608 0.351643073
-0.729880983 1.969021601 0.509768097 1.267552774 4 0
0.770767298
FAM131A ENSG00000175182.14 0.013909004 2 2 0.008302422
Brain_Amygdala 0.260808707 Brain_Hypothalamus 1.050109337
0.949890663 0.949890663 -2.639517171 1.124481753 -0.757517709
2.661549163 2 0 0.770767298
SNRNP70 ENSG00000104852.14 0.013937826 3 3 0.001362616
Brain_Nucleus_accumbens_basal_ganglia 0.93393351 Brain_Cerebellum 1.682033764
0.443342209 0.443342209 -0.082896911 3.202454915 1.586165539
1.643311566 3 0 0.770767298
NQO1 ENSG00000181019.12 0.014051215 1 1 0.014051215
Brain_Nucleus_accumbens_basal_ganglia 0.014051215
Brain_Nucleus_accumbens_basal_ganglia 1 1 1 -2.455951423
-2.455951423 -2.455951423 NA 1 0 0.771297043
CITF22-92A6.1 ENSG00000273145.1 0.014098681 1 1 0.014098681
Brain_Cerebellum 0.014098681 Brain_Cerebellum 1 1 1 2.454739259
2.454739259 2.454739259 NA 1 0 0.771297043
TNN ENSG00000120332.15 0.01411279 3 2 0.003126917 Brain_Cortex

0.215027607 Brain_Hippocampus 2.285782804 0.026783846 0.68743335
1.23985885 2.954977693 2.331143557 0.948309385 2 0
0.771297043
ZNF850 ENSG00000267041.5 0.01419335 3 3 0.120208558 Brain_Cerebellum
0.348814374 Brain_Frontal_Cortex_BA9 1.652722276 0.420354657
0.420354657 -0.936891496 1.553898801 0.673713637 1.396833588
3 0 0.772182682
ANKRD24 ENSG00000089847.12 0.014267336 5 3 0.002487063
Brain_Cerebellum 0.15774728 Brain_Spinal_cord_cervical_c-1 4.327892991
0.00100621 0.260409678 1.412688687 3.024910953 2.491448907
0.667907203 3 0 0.772182682
MRPS21 ENSG00000266472.5 0.014295969 8 5 0.0526634 Brain_Cortex
0.919344674 Brain_Putamen_basal_ganglia 5.916228837 0.000661204
0.253062503 -1.937670788 0.550474524 -0.719083834 0.996986879
5 0 0.772182682
BAIAP2 ENSG00000175866.15 0.014394606 2 2 0.004389676
Brain_Cerebellum 0.507062608 Brain_Spinal_cord_cervical_c-1 1.020369774
0.979630226 0.979630226 -0.663418426 2.848710804 1.092646189
2.483450395 2 0 0.772182682
GMEB2 ENSG00000101216.10 0.01440721 3 2 0.012256208 Brain_Cortex
0.973280261 Brain_Cerebellum 2.38246978 0.04079876 0.576731459
-2.504680425 0.033494488 -0.907033225 1.390840568 2 0
0.772182682
CEACAM4 ENSG00000105352.10 0.014668799 2 2 0.038005122
Brain_Cortex 0.055959644 Brain_Cerebellar_Hemisphere 1.057729578
0.942270422 0.942270422 1.911349797 2.074799493 1.993074645
0.115576388 2 0 0.772182682
NAF1 ENSG00000145414.8 0.014692264 1 1 0.014692264 Brain_Cortex
0.014692264 Brain_Cortex 1 1 1 -2.439876987 -2.439876987
-2.439876987 NA 1 0 0.772182682
SLC22A5 ENSG00000197375.12 0.014717975 8 6 0.008041619
Brain_Cortex 0.778929618 Brain_Cerebellar_Hemisphere 4.296994051
0.028942889 0.168838213 -0.902743864 2.650317389 0.664930274
1.185585251 6 0 0.772182682
ABI1 ENSG00000136754.16 0.014925398 1 1 0.014925398
Brain_Cerebellar_Hemisphere 0.014925398 Brain_Cerebellar_Hemisphere 1 1
1 2.4341842 2.4341842 2.4341842 NA 1 0 0.772182682
CNTNAP5 ENSG00000155052.14 0.014925932 1 1 0.014925932
Brain_Anterior_cingulate_cortex_BA24 0.014925932
Brain_Anterior_cingulate_cortex_BA24 1 1 1 2.434171239
2.434171239 2.434171239 NA 1 0 0.772182682
C6orf132 ENSG00000188112.8 0.014958189 2 2 0.006847804
Brain_Cerebellum 0.074634246 Brain_Cerebellar_Hemisphere 1.857871337
0.142128663 0.142128663 -2.704156447 -1.78270556 -2.243431004
0.65156417 2 0 0.772182682
BIN1 ENSG00000136717.14 0.015067184 4 4 0.001216433
Brain_Cerebellar_Hemisphere 0.906695895 Brain_Amygdala 1.90297309 0.16058411
0.16058411 -3.234998721 0.749477519 -1.187306509 1.826593924 4
0 0.772182682
DUSP16 ENSG00000111266.8 0.015074412 1 1 0.015074412
Brain_Anterior_cingulate_cortex_BA24 0.015074412
Brain_Anterior_cingulate_cortex_BA24 1 1 1 -2.430586383
-2.430586383 -2.430586383 NA 1 0 0.772182682
RAPGEF5 ENSG00000136237.18 0.015106691 1 1 0.015106691
Brain_Spinal_cord_cervical_c-1 0.015106691 Brain_Spinal_cord_cervical_c-1
1 1 1 2.429811169 2.429811169 2.429811169 NA 1 0
0.772182682
RP11-33M22.2 ENSG00000272880.1 0.015168837 1 1 0.015168837
Brain_Hypothalamus 0.015168837 Brain_Hypothalamus 1 1 1
-2.428322758 -2.428322758 -2.428322758 NA 1 0 0.772182682
PICK1 ENSG00000100151.15 0.015334659 7 3 0.004541585
Brain_Caudate_basal_ganglia 0.71234979 Brain_Amygdala 5.760973007

0.002728024 0.306692126 0.368702091 2.837868741 1.824087748
0.758267121 3 0 0.772182682
TMED6 ENSG00000157315.4 0.015347621 3 3 0.00451697
Brain_Putamen_basal_ganglia 0.417973665 Brain_Anterior_cingulate_cortex_BA24
1.591626485 0.411723529 0.411723529 -0.990754928 2.8396031
0.346302146 2.161153763 3 0 0.772182682
C4B ENSG00000224389.8 0.015511474 10 10 0.071426442
Brain_Hypothalamus 0.993158931 Brain_Cerebellar_Hemisphere 3.734034545
0.142911188 0.142911188 -1.802756646 1.788188847 -0.004987287
1.10297714 10 0 0.772182682
CAPN2 ENSG00000162909.17 0.015607969 1 1 0.015607969 Brain_Cerebellum
0.015607969 Brain_Cerebellum 1 1 1 -2.417955877 -2.417955877
-2.417955877 NA 1 0 0.772182682
SREBF2 ENSG00000198911.11 0.015698742 1 1 0.015698742
Brain_Hypothalamus 0.015698742 Brain_Hypothalamus 1 1 1
-2.415845016 -2.415845016 -2.415845016 NA 1 0 0.772182682
GMDS-AS1 ENSG00000250903.8 0.015711864 1 1 0.015711864
Brain_Nucleus_accumbens_basal_ganglia 0.015711864
Brain_Nucleus_accumbens_basal_ganglia 1 1 1 2.415540748
2.415540748 2.415540748 NA 1 0 0.772182682
TIPIN ENSG00000075131.9 0.015757311 13 1 0.009142197 Brain_Amygdala
0.121357418 Brain_Cerebellar_Hemisphere 12.5931078 8.94E-05 12.5931078
1.549101104 2.606690448 2.372737107 0.316663646 1 0
0.772182682
CLEC18C ENSG00000157335.20 0.015780537 3 3 0.009355337
Brain_Cortex 0.573157179 Brain_Cerebellum 1.570990476 0.434196869
0.434196869 -2.059062329 2.598788586 0.367711376 2.335083859
3 0 0.772182682
VPS45 ENSG00000136631.12 0.015946376 2 2 0.018986593
Brain_Frontal_Cortex_BA9 0.026075905 Brain_Anterior_cingulate_cortex_BA24
1.263608197 0.736391803 0.736391803 2.225079446 2.345794106
2.285436776 0.085358155 2 0 0.772182682
RP11-1072A3.4 ENSG00000275263.1 0.016059995 3 3 0.001501998
Brain_Hypothalamus 0.711629959 Brain_Cerebellar_Hemisphere 1.840269211
0.169750415 0.169750415 -3.174297193 0.369667891 -1.809823315
1.907383564 3 0 0.772182682
TPTE2 ENSG00000132958.17 0.016129025 6 6 0.110327155
Brain_Frontal_Cortex_BA9 0.98606892 Brain_Anterior_cingulate_cortex_BA24
2.803883331 0.126944823 0.126944823 -0.289635387 1.596724397
0.819673266 0.788404501 6 0 0.772182682
CDK4 ENSG00000135446.16 0.016129243 2 2 0.021520748
Brain_Hippocampus 0.308546124 Brain_Amygdala 1.277337531
0.722662469 0.722662469 -2.298721686 1.018277446 -0.64022212
2.345472579 2 0 0.772182682
ATP5J2 ENSG00000241468.7 0.016130844 1 1 0.016130844
Brain_Putamen_basal_ganglia 0.016130844 Brain_Putamen_basal_ganglia 1 1
1 2.405941588 2.405941588 2.405941588 NA 1 0
0.772182682
TIAL1 ENSG00000151923.17 0.016269581 4 4 0.010772779 Brain_Cortex
0.924417707 Brain_Nucleus_accumbens_basal_ganglia 1.318583388
0.699969101 0.699969101 -2.187611154 2.549984237 -0.030762929
1.9532716 4 0 0.772182682
GABRB3 ENSG00000166206.14 0.016316754 1 1 0.016316754
Brain_Cerebellum 0.016316754 Brain_Cerebellum 1 1 1 2.401752282
2.401752282 2.401752282 NA 1 0 0.772182682
ZC3H8 ENSG00000144161.12 0.016363014 6 4 0.005313122
Brain_Cerebellar_Hemisphere 0.675709584 Brain_Putamen_basal_ganglia 4.56758167
0.043606791 0.194188643 0.418324904 2.787409923 1.848905882
0.776063695 4 0 0.772182682
LINC00346 ENSG00000255874.2 0.016399664 8 7 0.016035952
Brain_Substantia_nigra 0.552426081 Brain_Cortex 2.265372485
0.038779133 0.540655576 -2.228678085 2.408096266 0.129262977

1.541869316 7 0 0.772182682
HSD17B8 ENSG00000204228.3 0.016438985 7 7 0.001722092
Brain_Putamen_basal_ganglia 0.968690259 Brain_Nucleus_accumbens_basal_ganglia
2.876909955 0.311496332 0.311496332 -1.542499868 3.134394461
0.46522096 1.512120523 7 0 0.772182682
SMG6 ENSG00000070366.13 0.016518418 3 3 0.002086493 Brain_Cerebellum
0.807320506 Brain_Spinal_cord_cervical_c-1 1.685213885 0.423476965
0.423476965 -3.07763648 -0.2438842 -1.786107218 1.43341324 3 0
0.772182682
STK25 ENSG00000115694.14 0.016535688 2 2 0.045474401
Brain_Cerebellar_Hemisphere 0.131340969 Brain_Nucleus_accumbens_basal_ganglia
1.240570708 0.759429292 0.759429292 -2.000239566 1.50883494
-0.245702313 2.481290379 2 0 0.772182682
TRIP6 ENSG00000087077.13 0.016630738 2 2 0.005324704
Brain_Cerebellar_Hemisphere 0.646427869 Brain_Cerebellum 1.069388031
0.930611969 0.930611969 -2.786704343 0.458730276 -1.163987033
2.294868827 2 0 0.772182682
NRDC ENSG00000078618.21 0.016659587 7 6 0.003896025
Brain_Frontal_Cortex_BA9 0.545410603 Brain_Nucleus_accumbens_basal_ganglia
2.693959449 0.004840421 0.283361812 -2.886459795 0.920932252
-1.007363055 1.382243559 6 0 0.772182682
ZNF788 ENSG00000214189.8 0.016710081 2 2 0.004227848
Brain_Cerebellum 0.044841177 Brain_Cerebellar_Hemisphere 1.704935319
0.295064681 0.295064681 2.006141279 2.860641548 2.433391414
0.604222935 2 0 0.772182682
REPIN1 ENSG00000214022.11 0.016750775 4 3 0.01216752 Brain_Cortex
0.987197437 Brain_Amygdala 2.395307016 0.006194792 0.752279302
-2.507248302 1.095702497 -0.934014179 1.777244469 3 0
0.772182682
TUBG1 ENSG00000131462.7 0.016750886 1 1 0.016750886
Brain_Nucleus_accumbens_basal_ganglia 0.016750886
Brain_Nucleus_accumbens_basal_ganglia 1 1 1 2.392130483
2.392130483 2.392130483 NA 1 0 0.772182682
UCKL1 ENSG00000198276.15 0.016799211 1 1 0.016799211 Brain_Cerebellum
0.016799211 Brain_Cerebellum 1 1 1 -2.391073025 -2.391073025
-2.391073025 NA 1 0 0.772182682
CCSER2 ENSG00000107771.15 0.016813318 1 1 0.016813318
Brain_Hypothalamus 0.016813318 Brain_Hypothalamus 1 1 1
2.390764849 2.390764849 2.390764849 NA 1 0 0.772182682
CNTD2 ENSG00000105219.8 0.016862403 1 1 0.016862403 Brain_Cerebellum
0.016862403 Brain_Cerebellum 1 1 1 -2.38969427 -2.38969427
-2.38969427 NA 1 0 0.772182682
CDIPT ENSG00000103502.13 0.016882474 2 2 0.188101739
Brain_Cerebellar_Hemisphere 0.817802587 Brain_Cerebellum 1.847528469
0.152471531 0.152471531 -1.316215453 0.230372167 -0.542921643
1.093602594 2 0 0.772182682
MRPS18C ENSG00000163319.10 0.016945474 1 1 0.016945474
Brain_Cortex 0.016945474 Brain_Cortex 1 1 1 2.387888664
2.387888664 2.387888664 NA 1 0 0.772182682
ABHD14B ENSG00000114779.19 0.016951527 1 1 0.016951527
Brain_Spinal_cord_cervical_c-1 0.016951527 Brain_Spinal_cord_cervical_c-1
1 1 1 -2.387757411 -2.387757411 -2.387757411 NA 1 0
0.772182682
DLK2 ENSG00000171462.14 0.016978032 2 2 0.004335636
Brain_Anterior_cingulate_cortex_BA24 0.688250248 Brain_Substantia_nigra
1.181526089 0.818473911 0.818473911 0.401230744 2.852649878
1.626940311 1.733415093 2 0 0.772182682
SPTLC3 ENSG00000172296.12 0.017038852 1 1 0.017038852
Brain_Amygdala 0.017038852 Brain_Amygdala 1 1 1 2.385868301
2.385868301 2.385868301 NA 1 0 0.772182682
EEA1 ENSG00000102189.16 0.017063815 2 2 0.005180062
Brain_Frontal_Cortex_BA9 0.5520579 Brain_Amygdala 1.008455425

0.991544575	0.991544575	-2.795617819	-0.594679242	-1.695148531
1.556298592	2	0	0.772182682
TYMS	ENSG00000176890.15	0.01707194 1	1	0.01707194
Brain_Anterior_cingulate_cortex_BA24	0.01707194
Brain_Anterior_cingulate_cortex_BA24	1	1	1	2.385154712
2.385154712	2.385154712	NA	1	0	0.772182682
AQR	ENSG00000021776.10	0.017084523	1	1	0.017084523	Brain_Cortex
0.017084523	Brain_Cortex	1	1	1	-2.384883666	-2.384883666
-2.384883666	NA	1	0	0.772182682
TADA2A	ENSG00000276234.4	0.017139155	10	3	0.002843756
Brain_Cerebellar_Hemisphere 0.474147762	Brain_Spinal_cord_cervical_c-1
7.58666831 0.001788125	0.337896862	-2.984141129	-0.715746711
-2.121485142	0.890564621	3	0	0.772182682
SIGLEC7	ENSG00000168995.13	0.017166062	2	2	0.013560955
Brain_Cortex	0.151664391	Brain_Spinal_cord_cervical_c-1	1.002864079
0.997135921	0.997135921	1.433677237	2.468687606	1.951182421
0.73186285 2	0	0.772182682
OR2W3 ENSG00000238243.3	0.017239961	10	7	0.072149578	Brain_Amygdala
0.759625074	Brain_Hypothalamus	6.396124416	0.037539557
0.258846732	-1.79817312	1.345835105	-0.304931098	0.893520268
7	0	0.772182682
SEC61A1	ENSG00000058262.9	0.017375983	4	4	0.018886117
Brain_Hippocampus	0.747239355	Brain_Amygdala	1.492420113
0.466691028	0.466691028	-1.72409808	2.347771241	-0.022319639
1.794261671	4	0	0.772182682
TRIM73	ENSG00000178809.11	0.017384592	4	4	0.006986474
Brain_Hippocampus	0.236919353	Brain_Anterior_cingulate_cortex_BA24
2.186723088	0.178539376	0.178539376	-1.81748562	2.697488297
-0.448450265	2.113245736	4	0	0.772182682
KLHL12	ENSG00000117153.15	0.017447305	8	5	0.047345957
Brain_Putamen_basal_ganglia 0.718661227	Brain_Caudate_basal_ganglia
5.083530689	0.005866198	0.249645925	-1.983192266	0.719043345
-0.449715885	0.959410371	5	0	0.772182682
ADGRE5	ENSG00000123146.19	0.017455472	8	8	0.007489177
Brain_Spinal_cord_cervical_c-1	0.747377322	Brain_Caudate_basal_ganglia
2.210002167	0.17863933 0.17863933 -2.674271599	1.74449848 -0.278492077
1.415201373	8	0	0.772182682
EPHA3 ENSG00000044524.10	0.017492027	3	3	0.05377633 Brain_Cerebellum
0.287160683	Brain_Caudate_basal_ganglia 1.246417818	0.662053906
0.662053906	-1.064370961	1.92863388 0.781118052	1.613968532	3
0	0.772182682
PNOC	ENSG00000168081.8	0.017514746	3	3	0.092943038
Brain_Cerebellar_Hemisphere 0.953532292	Brain_Cerebellum 1.912400024
0.097302957	0.097302957	-0.351785134	1.680073389	0.462186617
1.074463947	3	0	0.772182682
CTSS	ENSG00000163131.10	0.017541023	5	2	0.00743514
Brain_Caudate_basal_ganglia 0.901786597	Brain_Cortex	3.573492011
0.019729871	1.281437778	-2.676698989	-0.123404749	-1.407911524
1.201245544	2	0	0.772182682
FBXO10	ENSG00000147912.12	0.017550861	2	2	0.008772024
Brain_Spinal_cord_cervical_c-1	0.744547752	Brain_Cerebellar_Hemisphere
1.279738074	0.720261926	0.720261926	-2.620813496	0.32583691
-1.147488293	2.083596484	2	0	0.772182682
SP1	ENSG00000185591.9	0.017773522	5	4	0.01327751
Brain_Cerebellar_Hemisphere 0.969593398	Brain_Cortex	3.762790242
0.085160832	0.198891655	-0.306066986	2.476237826	1.178764608
1.238306185	4	0	0.777630976
CCDC150	ENSG00000144395.17	0.017785856	1	1	0.017785856
Brain_Cerebellar_Hemisphere 0.017785856	Brain_Cerebellar_Hemisphere 1	1
1	-2.370046336	-2.370046336	-2.370046336	NA	1	0
0.777630976
XRCC3 ENSG00000126215.13	0.018037509	6	5	0.000176477	Brain_Cortex

0.850682857 Brain_Hippocampus 3.24079853 0.053289637 0.392198011
0.188247221 3.750507766 1.608779062 1.324413477 5 0
0.782416753
LINC02055 ENSG00000254101.5 0.018061029 1 1 0.018061029
Brain_Frontal_Cortex_BA9 0.018061029 Brain_Frontal_Cortex_BA9 1 1
1 -2.364364579 -2.364364579 -2.364364579 NA 1 0
0.782416753
LRRC47 ENSG00000130764.9 0.018072688 5 5 0.007613078
Brain_Substantia_nigra 0.411263619 Brain_Hypothalamus 1.97434032
0.29906764 0.29906764 -2.66876462 0.821671964 -1.434480364
1.420693769 5 0 0.782416753
RPE65 ENSG00000116745.6 0.018119007 1 1 0.018119007 Brain_Cortex
0.018119007 Brain_Cortex 1 1 1 -2.363177132 -2.363177132
-2.363177132 NA 1 0 0.782416753
NOD1 ENSG00000106100.10 0.01852124 1 1 0.01852124 Brain_Caudate_basal_ganglia
0.01852124 Brain_Caudate_basal_ganglia 1 1 1 -2.35502941
-2.35502941 -2.35502941 NA 1 0 0.795131046
EYA2 ENSG00000064655.18 0.018527105 1 1 0.018527105 Brain_Cortex
0.018527105 Brain_Cortex 1 1 1 -2.35491176 -2.35491176
-2.35491176 NA 1 0 0.795131046
TNR ENSG00000116147.16 0.018602152 2 2 0.023561284
Brain_Hypothalamus 0.040409786 Brain_Hippocampus 1.171644768
0.828355232 0.828355232 2.049535444 2.264208662 2.156872053
0.151796889 2 0 0.795910409
SMIM15 ENSG00000188725.7 0.018711415 1 1 0.018711415
Brain_Putamen_basal_ganglia 0.018711415 Brain_Putamen_basal_ganglia 1 1
1 2.351230975 2.351230975 2.351230975 NA 1 0
0.796586992
SETD1A ENSG00000099381.17 0.018816035 2 1 0.009090547
Brain_Cerebellum 0.039500058 Brain_Cerebellar_Hemisphere 1.973912658
0.026087342 1.973912658 2.058939255 2.608630014 2.333784635
0.388690063 1 0 0.796586992
TARS2 ENSG00000143374.16 0.018893847 5 5 0.000421289
Brain_Frontal_Cortex_BA9 0.928577302 Brain_Anterior_cingulate_cortex_BA24
2.841166785 0.181065846 0.181065846 0.08963496 3.526375869
1.710352588 1.308057248 5 0 0.796586992
RP11-139I14.2 ENSG00000237390.1 0.018976911 2 2 0.014036596
Brain_Cerebellum 0.952214682 Brain_Cerebellar_Hemisphere 1.507392133
0.492607867 0.492607867 -2.456325484 -0.059925862 -1.258125673
1.694510423 2 0 0.796586992
SPEG ENSG00000072195.14 0.019045228 2 2 0.007086591
Brain_Cerebellar_Hemisphere 0.073198966 Brain_Cerebellum 1.384525235
0.615474765 0.615474765 -2.692747528 -1.79158813 -2.242167829
0.637215921 2 0 0.796586992
CNOT6L ENSG00000138767.12 0.019061446 8 1 0.011671796
Brain_Caudate_basal_ganglia 0.032538276 Brain_Anterior_cingulate_cortex_BA24
7.512817956 0.000137865 7.512817956 -2.521914833 -2.137734773
-2.272220103 0.133857218 1 0 0.796586992
MLST8 ENSG00000167965.17 0.01909572 13 4 0.002458884
Brain_Substantia_nigra 0.299862908 Brain_Frontal_Cortex_BA9
9.859747981 1.82E-16 0.506473021 -1.036727422 3.028355754
2.076386288 1.154335244 4 0 0.796586992
SMIM8 ENSG00000111850.10 0.019117794 12 4 0.199947781
Brain_Caudate_basal_ganglia 0.906848748 Brain_Nucleus_accumbens_basal_ganglia
8.498585046 0.026637111 0.304072405 -1.141197623 1.281700353
-0.252581337 0.670419179 4 0 0.796586992
CATIP ENSG00000158428.3 0.01920051 3 3 0.025387789 Brain_Cerebellum
0.201681477 Brain_Hypothalamus 1.121320201 0.908145828
0.908145828 -1.675850091 2.235450485 0.612125336 2.03859953 3
0 0.796586992
ZNF180 ENSG00000167384.10 0.019225326 10 4 0.114451634
Brain_Hippocampus 0.900250129 Brain_Cortex 7.467859045

0.020308481 0.347232638 -1.578496317 0.874173878 0.291505709
0.705172929 4 0 0.796586992
VANGL2 ENSG00000162738.5 0.019302135 2 1 0.016813991
Brain_Cerebellar_Hemisphere 0.02583321 Brain_Cerebellum 1.949184393
0.050815607 1.949184393 -2.390750147 -2.228709949 -2.309730048
0.114579723 1 0 0.796586992
NHLH2 ENSG00000177551.5 0.019406446 5 5 0.0531348
Brain_Anterior_cingulate_cortex_BA24 0.633991155 Brain_Frontal_Cortex_BA9
2.968889452 0.102217965 0.102217965 -1.933823766 -0.476116819
-1.200589547 0.633606931 5 0 0.796586992
ATP6V1E2 ENSG00000250565.6 0.019597318 11 11 0.000577368
Brain_Putamen_basal_ganglia 0.876005836 Brain_Hypothalamus 3.495784836
0.277209049 0.277209049 -1.317078305 3.442029285 0.849788697
1.268111428 11 0 0.796586992
MEIS3 ENSG00000105419.17 0.019606309 3 2 0.031151976
Brain_Caudate_basal_ganglia 0.056979834 Brain_Frontal_Cortex_BA9 1.9537845
0.047319207 0.998896293 -2.155125998 1.903465483 -0.753578996
2.302226452 2 0 0.796586992
HADHB ENSG00000138029.13 0.019699668 9 3 0.023487049 Brain_Cerebellum
0.810017622 Brain_Cortex 7.447358124 0.011269421 0.277827433
-2.265417864 1.185267869 0.396025153 1.09004523 3 0
0.796586992
TUBB2A ENSG00000137267.5 0.019705702 10 8 0.005627769
Brain_Cerebellar_Hemisphere 0.823283893 Brain_Cerebellum 5.268729917
0.020205414 0.25122535 -2.768715874 2.281901603 -0.608386764
1.257744265 8 0 0.796586992
BICDL1 ENSG00000135127.11 0.019803346 3 2 0.005838532
Brain_Hypothalamus 0.035629336 Brain_Cerebellum 2.795736628
0.048011981 0.156251391 -2.756714436 -2.101132513 -2.409905833
0.329441841 2 0 0.796586992
ECE2 ENSG00000145194.17 0.0198477 13 5 0.050815384
Brain_Nucleus_accumbens_basal_ganglia 0.869429657 Brain_Cortex
9.957148283 1.83E-17 0.339930092 -1.867844162 1.953035541
-1.03845399 1.075939323 5 0 0.796586992
LGALS3 ENSG00000131981.15 0.01985139 2 2 0.008811632
Brain_Nucleus_accumbens_basal_ganglia 0.384718813 Brain_Cerebellar_Hemisphere
1.04537969 0.95462031 0.95462031 -2.619277169 -0.869234624 -1.744255896
1.237466951 2 0 0.796586992
SYT15 ENSG00000204176.13 0.019873641 10 10 0.034446795
Brain_Nucleus_accumbens_basal_ganglia 0.974485992
Brain_Anterior_cingulate_cortex_BA24 2.518901919 0.118847944
0.118847944 -1.751088183 2.114802367 0.16350159 1.228438809 10
0 0.796586992
MDK ENSG00000110492.15 0.019917493 2 2 0.008201889
Brain_Caudate_basal_ganglia 0.091531703 Brain_Substantia_nigra
1.831345046 0.168654954 0.168654954 -2.643643907 -1.687372737
-2.165508322 0.676185829 2 0 0.796586992
CHD7 ENSG00000171316.11 0.020068317 2 2 0.04379905
Brain_Caudate_basal_ganglia 0.120325708 Brain_Spinal_cord_cervical_c-1
1.175484942 0.824515058 0.824515058 -1.553407935 2.016008854
0.231300459 2.523958816 2 0 0.796586992
PAPPA2 ENSG00000116183.10 0.020071957 2 2 0.273540954
Brain_Cerebellar_Hemisphere 0.422384926 Brain_Cerebellum 1.76904533 0.23095467
0.23095467 -0.802290518 1.094944496 0.146326989 1.341547744 2
0 0.796586992
HSD3B7 ENSG00000099377.13 0.020092548 2 2 0.031348384
Brain_Anterior_cingulate_cortex_BA24 0.863985919 Brain_Cortex
1.589620836 0.410379164 0.410379164 -0.171302494 2.152622176
0.990659841 1.643262893 2 0 0.796586992
2-Sep ENSG00000168385.17 0.020214532 13 1 0.011509403 Brain_Cerebellum
0.08232559 Brain_Hypothalamus 12.49728606 3.14E-05 12.49728606
1.737348983 2.526839681 2.27531267 0.190977213 1 0

0.796586992
GOPC ENSG00000047932.13 0.020227361 1 1 0.020227361
Brain_Frontal_Cortex_BA9 0.020227361 Brain_Frontal_Cortex_BA9 1 1
1 2.32210354 2.32210354 2.32210354 NA 1 0 0.796586992
XAB2 ENSG00000076924.11 0.020458901 1 1 0.020458901
Brain_Anterior_cingulate_cortex_BA24 0.020458901
Brain_Anterior_cingulate_cortex_BA24 1 1 1 2.317823765
2.317823765 2.317823765 NA 1 0 0.796586992
DCDC2 ENSG00000146038.11 0.020557029 4 4 0.001248451
Brain_Nucleus_accumbens_basal_ganglia 0.583624386 Brain_Cerebellum
2.335258019 0.159347519 0.159347519 -3.227573291 -0.548098332
-1.803756231 1.132756385 4 0 0.796586992
LRP4 ENSG00000134569.9 0.020612317 5 4 0.034965089 Brain_Cortex
0.816618359 Brain_Caudate_basal_ganglia 3.470753754 0.008416123
0.139904642 0.231896556 2.108762479 0.648880131 0.816764312
4 0 0.796586992
GPR146 ENSG00000164849.9 0.020677013 2 2 0.075825271
Brain_Cerebellar_Hemisphere 0.97028801 Brain_Cerebellum 1.761898212
0.238101788 0.238101788 -0.037247068 1.775439957 0.869096444
1.281763287 2 0 0.796586992
NUB1 ENSG00000013374.15 0.020725622 3 3 0.01257965
Brain_Spinal_cord_cervical_c-1 0.348527075
Brain_Nucleus_accumbens_basal_ganglia 1.070143671 0.91105757 0.91105757
-2.495452767 1.822875055 0.088290799 2.280963052 3 0
0.796586992
ALDOC ENSG00000109107.13 0.020730317 3 3 0.010381858 Brain_Amygdala
0.776651997 Brain_Spinal_cord_cervical_c-1 1.776132635 0.216656795
0.216656795 -2.56284435 -0.283684731 -1.235199454 1.185220475
3 0 0.796586992
RP11-557H15.4 ENSG00000232310.6 0.020733503 3 3 0.001860211
Brain_Cortex 0.110091653 Brain_Hippocampus 2.402770455
0.168578753 0.168578753 1.59778132 3.111687885 2.393537208
0.759931049 3 0 0.796586992
ETFA ENSG00000140374.15 0.020748149 4 4 0.010232659 Brain_Cerebellum
0.608926361 Brain_Spinal_cord_cervical_c-1 1.580869644 0.313736693
0.313736693 -2.567866382 0.826305487 -0.876745407 1.418423969
4 0 0.796586992
ADPRM ENSG00000170222.11 0.020782814 9 6 0.090393962
Brain_Caudate_basal_ganglia 0.948248042 Brain_Spinal_cord_cervical_c-1
6.695314675 0.002167606 0.245832111 -1.693323215 1.119456838
-0.753966701 0.840723582 6 0 0.796586992
POLDIP2 ENSG00000004142.11 0.020836973 12 3 0.037821347
Brain_Spinal_cord_cervical_c-1 0.828449949 Brain_Hippocampus
9.701283518 0.000223269 0.708597679 -2.076785658 1.971382049
0.287586184 0.922553621 3 0 0.796586992
FADS1 ENSG00000149485.18 0.020851501 9 2 0.00453046
Brain_Putamen_basal_ganglia 0.096410592 Brain_Anterior_cingulate_cortex_BA24
8.366542694 0.001414017 0.399562137 1.662509838 2.838651576
2.421662174 0.329246228 2 0 0.796586992
DACH1 ENSG00000276644.4 0.020865126 1 1 0.020865126 Brain_Amygdala
0.020865126 Brain_Amygdala 1 1 1 2.310416144 2.310416144
2.310416144 NA 1 0 0.796586992
DLG1 ENSG00000075711.20 0.021032565 2 2 0.010519611
Brain_Cerebellar_Hemisphere 0.100505332 Brain_Cerebellum 1.85915739 0.14084261
0.14084261 1.642408706 2.558264274 2.10033649 0.647607683 2 0
0.796586992
FRAT1 ENSG00000165879.8 0.021113238 1 1 0.021113238
Brain_Hippocampus 0.021113238 Brain_Hippocampus 1 1 1
2.305953374 2.305953374 2.305953374 NA 1 0 0.796586992
AGO1 ENSG00000092847.11 0.021160028 1 1 0.021160028
Brain_Cerebellar_Hemisphere 0.021160028 Brain_Cerebellar_Hemisphere 1 1
1 2.305116892 2.305116892 2.305116892 NA 1 0

0.796586992
LRRC46 ENSG00000141294.9 0.021171423 9 6 0.000784787
Brain_Cerebellum 0.99461926 Brain_Anterior_cingulate_cortex_BA24 4.446489366
0.009072392 0.271095991 -3.358105512 1.476432681 -0.5044567
1.694404999 6 0 0.796586992
GREM1 ENSG00000166923.10 0.02120967 10 10 0.000884486 Brain_Amygdala
0.949510311 Brain_Cerebellum 1.600469183 0.567066736 0.567066736
-3.324905095 2.248645294 -0.364152433 1.525993214 10 0
0.796586992
CTNNA2 ENSG00000066032.18 0.021252692 3 3 0.025301564
Brain_Nucleus_accumbens_basal_ganglia 0.877999666 Brain_Hypothalamus
2.325485426 0.173053737 0.173053737 -2.236767133 0.153505483
-1.146786089 1.208934938 3 0 0.796586992
RP11-196G11.2 ENSG00000260911.2 0.021293183 11 3 0.002621841
Brain_Cerebellum 0.146034897 Brain_Frontal_Cortex_BA9 9.262608946
0.002028025 0.517134744 1.453680535 3.00891314 2.586674925
0.413919717 3 0 0.796586992
CTC-297N7.9 ENSG00000264016.2 0.021295478 1 1 0.021295478
Brain_Caudate_basal_ganglia 0.021295478 Brain_Caudate_basal_ganglia 1 1
1 2.302704447 2.302704447 2.302704447 NA 1 0
0.796586992
RP11-368I23.4 ENSG00000273177.1 0.021367236 1 1 0.021367236
Brain_Nucleus_accumbens_basal_ganglia 0.021367236
Brain_Nucleus_accumbens_basal_ganglia 1 1 1 -2.301431806
-2.301431806 -2.301431806 NA 1 0 0.796586992
EVPL ENSG00000167880.7 0.021378975 4 4 0.038800684
Brain_Frontal_Cortex_BA9 0.938478365 Brain_Amygdala 1.899657116
0.398286531 0.398286531 -0.530067994 2.066294465 0.741375418
1.181257335 4 0 0.796586992
PPP3CC ENSG00000120910.14 0.021407813 9 2 0.004625484
Brain_Hypothalamus 0.102472795 Brain_Hippocampus 8.241712315
0.008590497 0.429127166 1.63298194 2.832020825 2.549070965
0.364499737 2 0 0.796586992
RGR ENSG00000148604.13 0.021604252 12 12 0.021715758
Brain_Hypothalamus 0.954594814 Brain_Hippocampus 1.856166495
0.37737979 0.37737979 -2.295303133 2.192498034 0.244058809
1.338740125 12 0 0.79847168
RP3-449M8.6 ENSG00000259005.1 0.02161626 3 3 0.014575035
Brain_Hypothalamus 0.139136721 Brain_Nucleus_accumbens_basal_ganglia
1.316299274 0.690110153 0.690110153 1.479013447 2.4427697
1.923570417 0.486194544 3 0 0.79847168
GANAB ENSG00000089597.16 0.021629674 2 1 0.014395795
Brain_Hippocampus 0.037559057 Brain_Cerebellum 1.942297604
0.057702396 1.942297604 -2.447232485 -2.079634637 -2.263433561
0.259930931 1 0 0.79847168
REEP6 ENSG00000115255.10 0.021762468 6 3 0.005340501 Brain_Cerebellum
0.186554555 Brain_Cerebellar_Hemisphere 5.175625471 0.010193666
0.32161779 -2.785744147 -1.320840491 -2.392182566 0.544991774 3
0 0.801259702
RFX8 ENSG00000196460.12 0.021998594 1 1 0.021998594
Brain_Substantia_nigra 0.021998594 Brain_Substantia_nigra 1 1
1 -2.29039216 -2.29039216 -2.29039216 NA 1 0
0.807827619
VCAN ENSG00000038427.15 0.02208176 2 2 0.009668204
Brain_Cerebellar_Hemisphere 0.991837125 Brain_Cerebellum 1.352867209
0.647132791 0.647132791 -2.587475599 -0.010230825 -1.298853212
1.822387257 2 0 0.808337838
GTF2F1 ENSG00000125651.13 0.022226333 1 1 0.022226333
Brain_Cortex 0.022226333 Brain_Cortex 1 1 1 -2.286477605
-2.286477605 -2.286477605 NA 1 0 0.808337838
MVK ENSG00000110921.13 0.022256828 2 2 0.011164368
Brain_Hippocampus 0.258927694 Brain_Cortex 1.018471546

0.981528454 0.981528454 -2.537511559 1.128929219 -0.70429117
2.592565137 2 0 0.808337838
EXT1 ENSG00000182197.10 0.022401872 2 2 0.009876997 Brain_Cortex
0.293077475 Brain_Putamen_basal_ganglia 1.031862808 0.968137192
0.968137192 -2.580106071 1.051394425 -0.764355823 2.567858627
2 0 0.808337838
CLIP1 ENSG00000130779.19 0.022412379 1 1 0.022412379
Brain_Cerebellar_Hemisphere 0.022412379 Brain_Cerebellar_Hemisphere 1 1
1 -2.283305512 -2.283305512 -2.283305512 NA 1 0
0.808337838
HIST1H1C ENSG00000187837.3 0.022414962 4 3 0.039744333
Brain_Caudate_basal_ganglia 0.404793969 Brain_Cortex 3.650080125
0.054065391 0.140880678 -2.056396296 -0.833090007 -1.481680058
0.609589121 3 0 0.808337838
MTX3 ENSG00000177034.14 0.022471471 1 1 0.022471471
Brain_Cerebellar_Hemisphere 0.022471471 Brain_Cerebellar_Hemisphere 1 1
1 2.282302777 2.282302777 2.282302777 NA 1 0
0.808337838
AARS ENSG00000090861.15 0.022687922 9 2 0.005965022 Brain_Cortex
0.148884383 Brain_Caudate_basal_ganglia 7.747991389 0.000375741
0.732323584 1.443483267 2.749697934 2.521745722 0.416071454
2 0 0.808337838
TSHZ3 ENSG00000121297.6 0.022691476 1 1 0.022691476 Brain_Cerebellum
0.022691476 Brain_Cerebellum 1 1 1 -2.278589539 -2.278589539
-2.278589539 NA 1 0 0.808337838
MDFIC ENSG00000135272.10 0.022708312 3 3 0.006482909
Brain_Spinal_cord_cervical_c-1 0.489393529 Brain_Hypothalamus
1.469692772 0.560260016 0.560260016 -2.72230088 -0.691273744
-1.636865715 1.022709585 3 0 0.808337838
SH2D2A ENSG00000027869.11 0.022745059 1 1 0.022745059
Brain_Hypothalamus 0.022745059 Brain_Hypothalamus 1 1 1
-2.277689917 -2.277689917 -2.277689917 NA 1 0 0.808337838
POU2F2 ENSG00000028277.21 0.022755123 3 3 0.002253385
Brain_Hypothalamus 0.958263577 Brain_Frontal_Cortex_BA9 1.131216877
0.91140179 0.91140179 0.052332726 3.054629796 1.089111977
1.703045337 3 0 0.808337838
VEGFC ENSG00000150630.3 0.022787207 2 2 0.034666995
Brain_Nucleus_accumbens_basal_ganglia 0.108249011 Brain_Cerebellum
1.071273808 0.928726192 0.928726192 -2.112226863 1.606113333
-0.253056765 2.629263567 2 0 0.808337838
MARK3 ENSG00000075413.17 0.022821346 3 3 0.002008755
Brain_Hippocampus 0.045192761 Brain_Frontal_Cortex_BA9 2.449540719
0.110525153 0.110525153 -3.088934822 -2.002855868 -2.604060887
0.55230568 3 0 0.808337838
DEPDC4 ENSG00000166153.16 0.022985688 1 1 0.022985688
Brain_Amygdala 0.022985688 Brain_Amygdala 1 1 1 2.273672442
2.273672442 2.273672442 NA 1 0 0.812102941
SYNRG ENSG00000275066.4 0.023332412 6 6 0.010438798
Brain_Hypothalamus 0.290658234 Brain_Cerebellar_Hemisphere 2.774588119
0.221711129 0.221711129 -2.397845745 2.560944675 -0.443035008
2.034381584 6 0 0.818790077
OSGIN1 ENSG00000140961.12 0.023368394 1 1 0.023368394
Brain_Hypothalamus 0.023368394 Brain_Hypothalamus 1 1 1
-2.267357524 -2.267357524 -2.267357524 NA 1 0 0.818790077
DHRS13 ENSG00000167536.13 0.023391779 3 3 0.006771754
Brain_Cerebellar_Hemisphere 0.996667982 Brain_Frontal_Cortex_BA9
1.509229609 0.567554658 0.567554658 -0.004176077 2.70786513
1.704727689 1.487404268 3 0 0.818790077
TM4SF1 ENSG00000169908.11 0.023438474 1 1 0.023438474
Brain_Hippocampus 0.023438474 Brain_Hippocampus 1 1 1
2.266210898 2.266210898 2.266210898 NA 1 0 0.818790077
PLEKHG1 ENSG00000120278.15 0.023518567 2 2 0.006519427

Brain_Spinal_cord_cervical_c-1 0.482600668 Brain_Putamen_basal_ganglia 1.143566428 0.856433572 0.856433572 -2.720444226 -0.702125874 -1.71128505 1.427166594 2 0 0.818790077
RP11-13A1.1 ENSG00000246363.2 0.023527019 3 3 0.010105278 Brain_Cortex 0.747349543 Brain_Frontal_Cortex_BA9 1.53205713 0.510564261 0.510564261 -0.349690855 2.572205864 0.848217058 1.530340138 3 0 0.818790077
SVIP ENSG00000198168.8 0.023620332 6 6 0.03419396 Brain_Putamen_basal_ganglia 0.784928457 Brain_Frontal_Cortex_BA9 1.620029873 0.470528856 0.470528856 -2.117776982 1.185990599 -0.760545773 1.267710607 6 0 0.818790077
GAS6 ENSG00000183087.14 0.023643141 1 1 0.023643141 Brain_Cerebellum 0.023643141 Brain_Cerebellum 1 1 1 -2.262879127 -2.262879127 -2.262879127 NA 1 0 0.818790077
NLRC5 ENSG00000140853.15 0.02374205 1 1 0.02374205 Brain_Spinal_cord_cervical_c-1 0.02374205 Brain_Spinal_cord_cervical_c-1 1 1 1 2.261277969 2.261277969 2.261277969 NA 1 0 0.820122065
C4orf36 ENSG00000163633.10 0.023829205 2 2 0.00897034 Brain_Cerebellar_Hemisphere 0.653078375 Brain_Cerebellum 1.132602666 0.867397334 0.867397334 -2.613182441 0.449489785 -1.081846328 2.165636299 2 0 0.820122065
AJ239322.1 ENSG00000235615.2 0.023857457 9 9 0.054060831 Brain_Frontal_Cortex_BA9 0.550312741 Brain_Hippocampus 1.869416417 0.280426744 0.280426744 -1.926348837 1.919705935 -0.541737693 1.18477471 9 0 0.820122065
CELF2 ENSG00000048740.18 0.023974241 1 1 0.023974241 Brain_Cerebellum 0.023974241 Brain_Cerebellum 1 1 1 2.257541794 2.257541794 2.257541794 NA 1 0 0.820276344
RP11-491F9.5 ENSG00000260769.1 0.02400332 4 4 0.008504907 Brain_Cerebellar_Hemisphere 0.316264086 Brain_Frontal_Cortex_BA9 2.476174998 0.138836678 0.138836678 -2.631339323 1.002164632 -1.209424322 1.552484587 4 0 0.820276344
ARHGAP11A ENSG00000198826.10 0.024037832 4 4 0.002092152 Brain_Putamen_basal_ganglia 0.663825657 Brain_Hippocampus 1.32583984 0.623428262 0.623428262 -1.282043504 3.076829087 0.404607899 1.91641181 4 0 0.820276344
LINC01089 ENSG00000212694.8 0.024250127 11 3 0.004556566 Brain_Spinal_cord_cervical_c-1 0.284199809 Brain_Amygdala 8.614743349 7.38E-05 0.725085825 1.070932438 2.836817398 2.353892642 0.466295658 3 0 0.823558191
VARS ENSG00000204394.12 0.024251731 2 2 0.006454915 Brain_Anterior_cingulate_cortex_BA24 0.880058468 Brain_Caudate_basal_ganglia 1.10680059 0.89319941 0.89319941 -2.723730552 -0.150895097 -1.437312824 1.819269397 2 0 0.823558191
CENPT ENSG00000102901.12 0.024372064 1 1 0.024372064 Brain_Cerebellar_Hemisphere 0.024372064 Brain_Cerebellar_Hemisphere 1 1 1 -2.251212782 -2.251212782 -2.251212782 NA 1 0 0.824778382
DBNL ENSG00000136279.20 0.024445187 2 2 0.006726546 Brain_Cerebellum 0.045804701 Brain_Cortex 1.659440216 0.340559784 0.340559784 1.997188582 2.710087513 2.353638047 0.504095668 2 0 0.824778382
INAFM2 ENSG00000259330.2 0.02458954 3 3 0.012073119 Brain_Cerebellar_Hemisphere 0.472432123 Brain_Substantia_nigra 1.330377851 0.811866132 0.811866132 -1.269672131 2.509999903 0.652951744 1.890689108 3 0 0.824778382
LMBR1L ENSG00000139636.15 0.024596073 1 1 0.024596073 Brain_Cerebellum 0.024596073 Brain_Cerebellum 1 1 1 2.24768829 2.24768829 2.24768829 NA 1 0 0.824778382
SYCP2L ENSG00000153157.12 0.024628633 8 7 0.015562097 Brain_Cerebellum 0.896116359 Brain_Cerebellar_Hemisphere 4.33269128

0.014579204 0.27074251 -2.419026724 1.456550054 -0.794418742
1.406784747 7 0 0.824778382
AC009014.3 ENSG00000271824.1 0.024641367 3 2 0.022326274
Brain_Cerebellum 0.126815292 Brain_Spinal_cord_cervical_c-1 1.964725686
0.050167974 0.985106339 1.526781748 2.284770743 1.897702934
0.379252375 2 0 0.824778382
KCNH2 ENSG00000055118.14 0.024863057 2 1 0.021551558 Brain_Cerebellum
0.030105913 Brain_Cerebellar_Hemisphere 1.982056527 0.017943473
1.982056527 -2.298179804 -2.168694131 -2.233436968 0.091560198
1 0 0.825888482
FBXW12 ENSG00000164049.14 0.02489389 1 1 0.02489389
Brain_Anterior_cingulate_cortex_BA24 0.02489389
Brain_Anterior_cingulate_cortex_BA24 1 1 1 -2.243045348
-2.243045348 -2.243045348 NA 1 0 0.825888482
KCNK4 ENSG00000182450.12 0.024894702 3 2 0.027460889
Brain_Nucleus_accumbens_basal_ganglia 0.420346539 Brain_Cortex
2.737015196 0.026540035 0.236444769 -2.204903182 -0.805820182
-1.68982469 0.769050137 2 0 0.825888482
SLC7A8 ENSG00000092068.19 0.024910653 6 6 0.01977773 Brain_Cortex
0.500234428 Brain_Nucleus_accumbens_basal_ganglia 1.18806365 0.828770914
0.828770914 -2.330538078 1.708934404 -0.348220879 1.632551947
6 0 0.825888482
RP11-133F8.2 ENSG00000249776.5 0.02502934 1 1 0.02502934
Brain_Hippocampus 0.02502934 Brain_Hippocampus 1 1 1
-2.240949601 -2.240949601 -2.240949601 NA 1 0 0.827861685
SPOCK1 ENSG00000152377.13 0.025089687 3 3 0.004974298
Brain_Cerebellar_Hemisphere 0.821326097 Brain_Spinal_cord_cervical_c-1
1.20839723 0.798129407 0.798129407 -2.808693515 0.950896777
-0.69454548 1.923120802 3 0 0.827900493
DTD2 ENSG00000129480.12 0.025196375 8 5 0.054654159
Brain_Hippocampus 0.914047329 Brain_Hypothalamus 5.083922183
0.001793038 0.266921477 -1.626623023 1.92161548 -0.378894869
1.233706021 5 0 0.829464666
DIAPH1 ENSG00000131504.15 0.025389095 1 1 0.025389095
Brain_Cerebellum 0.025389095 Brain_Cerebellum 1 1 1 -2.23543057
-2.23543057 -2.23543057 NA 1 0 0.830376705
C1R ENSG00000159403.15 0.02540829 1 1 0.02540829
Brain_Nucleus_accumbens_basal_ganglia 0.02540829
Brain_Nucleus_accumbens_basal_ganglia 1 1 1 -2.235138012
-2.235138012 -2.235138012 NA 1 0 0.830376705
SMPD3 ENSG00000103056.11 0.025424246 5 4 0.001555213 Brain_Cerebellum
0.620616451 Brain_Putamen_basal_ganglia 2.578564251 0.079651924
0.390418784 -1.100054877 3.16417871 1.562866978 1.813652689 4
0 0.830376705
SLC22A1 ENSG00000175003.12 0.025510574 1 1 0.025510574
Brain_Amygdala 0.025510574 Brain_Amygdala 1 1 1 -2.233582226
-2.233582226 -2.233582226 NA 1 0 0.830376705
FZD7 ENSG00000155760.2 0.02556226 1 1 0.02556226 Brain_Cerebellum 0.02556226
Brain_Cerebellum 1 1 1 2.232798127 2.232798127 2.232798127
NA 1 0 0.830376705
YPEL3 ENSG00000090238.11 0.025580184 7 3 0.003268969
Brain_Caudate_basal_ganglia 0.922262088 Brain_Cortex 5.786986664
0.000748799 0.22540173 -2.941244036 0.097584682 -1.281916885
0.891831827 3 0 0.830376705
SUPT20H ENSG00000102710.19 0.025736065 2 2 0.011567417
Brain_Frontal_Cortex_BA9 0.030388547 Brain_Caudate_basal_ganglia
1.531516438 0.468483562 0.468483562 -2.525073259 -2.164988753
-2.345031006 0.254618196 2 0 0.832371261
ZNF20 ENSG00000132010.15 0.025800195 7 6 0.033160775
Brain_Nucleus_accumbens_basal_ganglia 0.861325078 Brain_Putamen_basal_ganglia
4.182201098 0.026662834 0.144583238 -1.478405482 2.130131296
-0.44999278 1.275346539 6 0 0.832371261

CYP11A1 ENSG00000140459.17 0.025845655 5 4 0.006817348
Brain_Cerebellar_Hemisphere 0.151185627 Brain_Nucleus_accumbens_basal_ganglia
3.112796279 0.035081206 0.276234551 1.435356176 2.705637217
1.839610398 0.503281078 4 0 0.832371261
ST8SIA2 ENSG00000140557.11 0.025879601 1 1 0.025879601
Brain_Frontal_Cortex_BA9 0.025879601 Brain_Frontal_Cortex_BA9 1 1
1 -2.228013706 -2.228013706 -2.228013706 NA 1 0
0.832371261
ZNF461 ENSG00000197808.11 0.025958626 3 1 0.008687485
Brain_Cortex 0.049123482 Brain_Nucleus_accumbens_basal_ganglia
2.921225609 0.025006543 2.921225609 -2.624113438 -1.967518358
-2.197696174 0.369677854 1 0 0.832998015
TIMM23 ENSG00000265354.3 0.026206793 1 1 0.026206793
Brain_Amygdala 0.026206793 Brain_Amygdala 1 1 1 2.223133588
2.223133588 2.223133588 NA 1 0 0.837564451
SLC26A9 ENSG00000174502.18 0.026220658 1 1 0.026220658
Brain_Spinal_cord_cervical_c-1 0.026220658 Brain_Spinal_cord_cervical_c-1
1 1 1 -2.222927957 -2.222927957 -2.222927957 NA 1 0
0.837564451
RP11-539L10.2 ENSG00000246526.2 0.026447436 1 1 0.026447436
Brain_Nucleus_accumbens_basal_ganglia 0.026447436
Brain_Nucleus_accumbens_basal_ganglia 1 1 1 2.219577864
2.219577864 2.219577864 NA 1 0 0.840843063
P2RX4 ENSG00000135124.14 0.026451497 6 6 0.024591026 Brain_Cortex
0.616697493 Brain_Caudate_basal_ganglia 2.596180751 0.105850822
0.105850822 -2.2477674 1.984516887 0.406994505 1.411045041 6
0 0.840843063
MRPS2 ENSG00000122140.10 0.026503594 1 1 0.026503594
Brain_Hypothalamus 0.026503594 Brain_Hypothalamus 1 1 1
-2.218752099 -2.218752099 -2.218752099 NA 1 0 0.840843063
VRK2 ENSG00000028116.16 0.026662531 2 2 0.024915964
Brain_Hippocampus 0.947596772 Brain_Substantia_nigra 1.532808724
0.467191276 0.467191276 -2.242703145 0.065724995 -1.088489075
1.632305192 2 0 0.843600462
CRYGN ENSG00000127377.8 0.0267111 1 1 0.0267111
Brain_Anterior_cingulate_cortex_BA24 0.0267111
Brain_Anterior_cingulate_cortex_BA24 1 1 1 -2.215713929
-2.215713929 -2.215713929 NA 1 0 0.843600462
CWF19L1 ENSG00000095485.16 0.02684544 13 2 0.009989182
Brain_Substantia_nigra 0.419958396 Brain_Hippocampus 11.8387498
0.00010096 0.473212585 -2.576203545 -0.806493427 -2.187479961
0.498671384 2 0 0.84436232
C3 ENSG00000125730.16 0.026855924 1 1 0.026855924
Brain_Anterior_cingulate_cortex_BA24 0.026855924
Brain_Anterior_cingulate_cortex_BA24 1 1 1 -2.213605568
-2.213605568 -2.213605568 NA 1 0 0.84436232
NES ENSG00000132688.10 0.027006857 1 1 0.027006857
Brain_Cerebellar_Hemisphere 0.027006857 Brain_Cerebellar_Hemisphere 1 1
1 -2.21141868 -2.21141868 -2.21141868 NA 1 0
0.846540308
SMAD7 ENSG00000101665.8 0.02704621 1 1 0.02704621 Brain_Frontal_Cortex_BA9
0.02704621 Brain_Frontal_Cortex_BA9 1 1 1 2.210850239
2.210850239 2.210850239 NA 1 0 0.846540308
SRGAP2B ENSG00000196369.10 0.027110775 6 5 0.071160662
Brain_Anterior_cingulate_cortex_BA24 0.791825068 Brain_Hypothalamus
2.794468502 9.00E-17 0.372026196 -1.804450824 0.496107787
-0.51283168 0.978570967 5 0 0.846667093
RP11-71E19.1 ENSG00000250934.1 0.027192364 4 3 0.0022058
Brain_Cerebellar_Hemisphere 0.91660857 Brain_Nucleus_accumbens_basal_ganglia
1.946660889 0.052909204 0.948702731 0.104706669 3.061025918
1.607121921 1.573440992 3 0 0.847323746
DYRK1B ENSG00000105204.13 0.027326452 3 3 0.002889985

Brain_Spinal_cord_cervical_c-1 0.861868254 Brain_Cerebellar_Hemisphere 1.93256594 0.160048121 0.160048121 -2.979203831 -0.173996448 -1.140963476 1.592681708 3 0 0.84960975
VPS13A ENSG00000197969.11 0.027623842 6 6 0.117971246 Brain_Caudate_basal_ganglia 0.86782838 Brain_Putamen_basal_ganglia 2.952471693 0.132630559 0.132630559 -1.476990117 1.563345951 -0.069715932 1.262938013 6 0 0.85013142
ZMIZ2 ENSG00000122515.14 0.027721156 8 8 0.000495468 Brain_Frontal_Cortex_BA9 0.706883036 Brain_Cerebellar_Hemisphere 3.918282269 0.151939257 0.151939257 0.376045546 3.48319471 1.81406429 1.020259286 8 0 0.85013142
MRTO4 ENSG00000053372.4 0.027743295 9 3 0.090213085 Brain_Amygdala 0.955005465 Brain_Putamen_basal_ganglia 7.543944397 0.037311835 0.364709301 -0.467545001 1.694274756 0.835016175 0.734994379 3 0 0.85013142
PDLIM2 ENSG00000120913.23 0.027780848 2 2 0.037124885 Brain_Cortex 0.576493269 Brain_Frontal_Cortex_BA9 1.776147469 0.223852531 0.223852531 -2.084388037 -0.558514261 -1.321451149 1.078955694 2 0 0.85013142
CD84 ENSG00000066294.14 0.027811835 3 3 0.011945996 Brain_Nucleus_accumbens_basal_ganglia 0.210782829 Brain_Hippocampus 1.426504205 0.622647835 0.622647835 1.251415764 2.513735581 1.88004688 0.631175106 3 0 0.85013142
NCKIPSD ENSG00000213672.7 0.027819887 13 2 0.011383934 Brain_Hippocampus 0.441740908 Brain_Hypothalamus 12.10131814 0.000729582 0.419152673 -2.530687146 -0.769256748 -2.045458709 0.468221296 2 0 0.85013142
IGSF10 ENSG00000152580.8 0.027891852 1 1 0.027891852 Brain_Anterior_cingulate_cortex_BA24 0.027891852 Brain_Anterior_cingulate_cortex_BA24 1 1 1 -2.19880414 -2.19880414 -2.19880414 NA 1 0 0.85013142
CD46 ENSG00000117335.19 0.02791812 13 3 0.012991432 Brain_Hypothalamus 0.974579983 Brain_Anterior_cingulate_cortex_BA24 10.91813349 0.001402718 0.483255708 -0.031864658 2.484004086 1.501369724 0.665230899 3 0 0.85013142
LINC01106 ENSG00000175772.10 0.02793681 4 4 0.009857816 Brain_Caudate_basal_ganglia 0.346167669 Brain_Hypothalamus 2.764850985 0.176801333 0.176801333 0.942048774 2.580777253 1.789521981 0.694128614 4 0 0.85013142
COLEC12 ENSG00000158270.11 0.028011024 4 4 0.020318518 Brain_Cerebellar_Hemisphere 0.198990015 Brain_Cortex 1.465454085 0.519616221 0.519616221 -2.320413524 1.78133077 -0.374163686 2.211622972 4 0 0.85013142
SLC18A2 ENSG00000165646.11 0.028147574 1 1 0.028147574 Brain_Cerebellum 0.028147574 Brain_Cerebellum 1 1 1 2.195223428 2.195223428 2.195223428 NA 1 0 0.85013142
DNMT3B ENSG00000088305.18 0.028401216 3 2 0.009141053 Brain_Anterior_cingulate_cortex_BA24 0.080007719 Brain_Cerebellar_Hemisphere 2.400534259 0.057021756 0.542443985 1.750641283 2.606733287 2.172850538 0.428165368 2 0 0.85013142
RP11-307E17.8 ENSG00000232063.1 0.028445042 6 5 0.038955784 Brain_Substantia_nigra 0.498483402 Brain_Putamen_basal_ganglia 3.842558809 0.11431138 0.218472072 -0.945356428 2.064653636 1.002400845 1.054289774 5 0 0.85013142
RNF144B ENSG00000137393.9 0.028486412 1 1 0.028486412 Brain_Substantia_nigra 0.028486412 Brain_Substantia_nigra 1 1 1 -2.190521849 -2.190521849 -2.190521849 NA 1 0 0.85013142
DDX25 ENSG00000109832.13 0.02861955 11 4 0.023146189 Brain_Anterior_cingulate_cortex_BA24 0.946596962 Brain_Caudate_basal_ganglia 8.678567601 0.003294141 0.349487527 -2.27101304 0.754848453 -0.537296025 0.754994853 4 0 0.85013142
TBC1D14 ENSG00000132405.18 0.028621991 1 1 0.028621991

Brain_Spinal_cord_cervical_c-1 0.028621991 Brain_Spinal_cord_cervical_c-1
1 1 1 -2.188654089 -2.188654089 -2.188654089 NA 1 0
0.85013142
SMARCD1 ENSG00000066117.14 0.028632782 1 1 0.028632782
Brain_Cerebellar_Hemisphere 0.028632782 Brain_Cerebellar_Hemisphere 1 1
1 -2.18850576 -2.18850576 -2.18850576 NA 1 0 0.85013142
KB-1592A4.15 ENSG00000197210.7 0.028705476 2 2 0.008035859
Brain_Cerebellar_Hemisphere 0.133617617 Brain_Cerebellum 1.474434386
0.525565614 0.525565614 -2.650559417 -1.499987591 -2.075273504
0.81357714 2 0 0.85013142
BCKDHB ENSG00000083123.14 0.028740332 4 3 0.021703913
Brain_Anterior_cingulate_cortex_BA24 0.389573432 Brain_Caudate_basal_ganglia
2.956922339 0.055501759 0.399887013 0.860391205 2.295510024
1.665877102 0.710798229 3 0 0.85013142
RP11-1007O24.2 ENSG00000261187.1 0.028746229 1 1 0.028746229
Brain_Putamen_basal_ganglia 0.028746229 Brain_Putamen_basal_ganglia 1 1
1 2.186949245 2.186949245 2.186949245 NA 1 0 0.85013142
LINC02102 ENSG00000248677.1 0.028784791 3 3 0.002790088
Brain_Cortex 0.815262446 Brain_Cerebellar_Hemisphere 1.314905189
0.767958586 0.767958586 -2.989965707 -0.233642609 -1.153031096
1.590832305 3 0 0.85013142
STAU2 ENSG00000040341.17 0.028801564 2 2 0.140086517 Brain_Cerebellum
0.686439244 Brain_Cerebellar_Hemisphere 1.906892919 0.093107081
0.093107081 0.403691972 1.475468923 0.939580447 0.75786075 2
0 0.85013142
TMOD1 ENSG00000136842.13 0.028817845 2 1 0.026079954 Brain_Cerebellum
0.033788895 Brain_Cerebellar_Hemisphere 1.977794841 0.022205159
1.977794841 -2.225019133 -2.12258199 -2.173800562 0.072433999
1 0 0.85013142
RP11-3B7.1 ENSG00000225399.4 0.028951654 2 2 0.02071604 Brain_Cortex
0.036547589 Brain_Anterior_cingulate_cortex_BA24 1.376538411
0.623461589 0.623461589 -2.313120052 -2.090782234 -2.201951143
0.157216579 2 0 0.85013142
CENPO ENSG00000138092.10 0.02896348 4 4 0.02143218 Brain_Frontal_Cortex_BA9
0.975211201 Brain_Putamen_basal_ganglia 2.33980253 0.147071724
0.147071724 -0.793236658 2.300283217 0.192913877 1.445532596
4 0 0.85013142
UNC45A ENSG00000140553.17 0.029003072 1 1 0.029003072
Brain_Cerebellum 0.029003072 Brain_Cerebellum 1 1 1 -2.183444766
-2.183444766 -2.183444766 NA 1 0 0.85013142
ALOX12 ENSG00000108839.11 0.029086949 1 1 0.029086949
Brain_Spinal_cord_cervical_c-1 0.029086949 Brain_Spinal_cord_cervical_c-1
1 1 1 -2.182306101 -2.182306101 -2.182306101 NA 1 0
0.85013142
AC093323.3 ENSG00000170846.16 0.029108034 1 1 0.029108034
Brain_Putamen_basal_ganglia 0.029108034 Brain_Putamen_basal_ganglia 1 1
1 -2.182020313 -2.182020313 -2.182020313 NA 1 0 0.85013142
ORC4 ENSG00000115947.13 0.029110029 5 3 0.003599091
Brain_Frontal_Cortex_BA9 0.076070882 Brain_Amygdala 3.457019796
0.010930703 0.501963947 1.77395326 2.911316648 2.365422133
0.40294771 3 0 0.85013142
TCP11L1 ENSG00000176148.15 0.029434447 9 5 0.003689821
Brain_Cortex 0.605917605 Brain_Frontal_Cortex_BA9 6.340145328
0.006564875 0.313385006 0.51590952 2.903529595 1.475513049
0.694310279 5 0 0.85013142
CLASRP ENSG00000104859.14 0.029480159 2 2 0.008999311
Brain_Substantia_nigra 0.710197782 Brain_Spinal_cord_cervical_c-1
1.039242184 0.960757816 0.960757816 0.371590475 2.612080326
1.4918354 1.584265567 2 0 0.85013142
KDM8 ENSG00000155666.11 0.029486491 6 6 0.00932503
Brain_Nucleus_accumbens_basal_ganglia 0.940864105 Brain_Putamen_basal_ganglia
1.857965593 0.307961479 0.307961479 -2.23174326 2.599902323

0.184021865 1.781315512 6 0 0.85013142
AC007563.1 ENSG00000236295.1 0.029564031 1 1 0.029564031
Brain_Cerebellum 0.029564031 Brain_Cerebellum 1 1 1 2.175882727
2.175882727 2.175882727 NA 1 0 0.85013142
RHCG ENSG00000140519.12 0.02961758 1 1 0.02961758 Brain_Cerebellum 0.02961758
Brain_Cerebellum 1 1 1 -2.175167322 -2.175167322 -2.175167322
NA 1 0 0.85013142
RP11-575F12.2 ENSG00000256001.1 0.029649179 1 1 0.029649179
Brain_Frontal_Cortex_BA9 0.029649179 Brain_Frontal_Cortex_BA9 1 1
1 2.174745693 2.174745693 2.174745693 NA 1 0 0.85013142
RP11-554F20.1 ENSG00000237461.1 0.029727624 1 1 0.029727624
Brain_Hippocampus 0.029727624 Brain_Hippocampus 1 1 1
-2.173700645 -2.173700645 -2.173700645 NA 1 0 0.85013142
XPNPEP3 ENSG00000196236.12 0.029761548 7 7 0.021600229
Brain_Hippocampus 0.973249446 Brain_Spinal_cord_cervical_c-1
2.665607143 0.099912993 0.099912993 -2.297325137 0.661872592
-0.902017009 1.153882825 7 0 0.85013142
RP11-1070N10.4 ENSG00000258390.1 0.029766926 1 1 0.029766926
Brain_Cerebellum 0.029766926 Brain_Cerebellum 1 1 1 -2.173177958
-2.173177958 -2.173177958 NA 1 0 0.85013142
DDX17 ENSG00000100201.20 0.029802951 3 2 0.030305309
Brain_Cerebellar_Hemisphere 0.369895153 Brain_Putamen_basal_ganglia 2.75599537
0.054806524 0.189198107 0.896669776 2.166076923 1.438188094
0.654903888 2 0 0.85013142
PLEK ENSG00000115956.9 0.029864366 5 4 0.002197473
Brain_Nucleus_accumbens_basal_ganglia 0.583321727 Brain_Caudate_basal_ganglia
2.322543602 0.052748967 0.638641482 -3.062158196 0.950121339
-1.424996493 1.677719136 4 0 0.85013142
ADRA2C ENSG00000184160.7 0.029889448 3 3 0.004297664
Brain_Spinal_cord_cervical_c-1 0.630724612 Brain_Cerebellum 1.362492035
0.741578712 0.741578712 -2.855444468 0.931018004 -0.801711222
1.913532649 3 0 0.85013142
TSPAN3 ENSG00000140391.14 0.029895265 2 2 0.0094737 Brain_Cerebellum
0.141092261 Brain_Cerebellar_Hemisphere 1.375249328 0.624750672
0.624750672 -2.594469565 -1.471735708 -2.033102636 0.793892723
2 0 0.85013142
RP11-486I11.2 ENSG00000277369.1 0.030260086 1 1 0.030260086
Brain_Hypothalamus 0.030260086 Brain_Hypothalamus 1 1 1
-2.166669208 -2.166669208 -2.166669208 NA 1 0 0.857794409
RP11-791G15.2 ENSG00000272275.1 0.030287359 2 2 0.01003569
Brain_Frontal_Cortex_BA9 0.533323183 Brain_Cerebellar_Hemisphere
1.007143373 0.992856627 0.992856627 -2.574597157 0.622941168
-0.975827995 2.261001033 2 0 0.857794409
RP1-56K13.5 ENSG00000266588.1 0.030416323 1 1 0.030416323
Brain_Frontal_Cortex_BA9 0.030416323 Brain_Frontal_Cortex_BA9 1 1
1 -2.164626202 -2.164626202 -2.164626202 NA 1 0
0.859658408
KPNA4 ENSG00000186432.8 0.03056327 1 1 0.03056327
Brain_Anterior_cingulate_cortex_BA24 0.03056327
Brain_Anterior_cingulate_cortex_BA24 1 1 1 2.162712881
2.162712881 2.162712881 NA 1 0 0.859658408
WDR17 ENSG00000150627.15 0.030696692 3 3 0.031682296 Brain_Cerebellum
0.661648575 Brain_Cortex 1.143770292 0.850885591 0.850885591
-2.148396176 1.855519311 0.048253779 2.030160087 3 0
0.859658408
SIPA1L2 ENSG00000116991.10 0.030704204 5 5 0.005934992
Brain_Hippocampus 0.70474618 Brain_Putamen_basal_ganglia 2.29038624
0.189788339 0.189788339 -0.378921471 2.75135152 1.678194391
1.243259009 5 0 0.859658408
CIB2 ENSG00000136425.12 0.030719213 12 5 0.008871133 Brain_Cerebellum
0.768143909 Brain_Substantia_nigra 8.293866285 0.014911646
0.457192719 -2.616980818 0.906359667 -1.185983527 0.975283591

5	0	0.859658408
HOXC4	ENSG00000198353.7	0.030721836	1	1	0.030721836
Brain_Nucleus_accumbens_basal_ganglia	0.030721836
Brain_Nucleus_accumbens_basal_ganglia	1	1	1	-2.160657118
-2.160657118	-2.160657118	NA	1	0	0.859658408
USP2	ENSG00000036672.15	0.031018058	3	3	0.003791154	Brain_Cortex
0.941975969	Brain_Frontal_Cortex_BA9	1.156976292	0.801343768
0.801343768	-0.072786556	2.895035743	1.078285452	1.59196042	3
0	0.860205619
GMPPA	ENSG00000144591.18	0.031025066	2	2	0.027948965	Brain_Cerebellum
0.218725353	Brain_Spinal_cord_cervical_c-1	1.098800461	0.901199539
0.901199539	-2.198001978	-1.229924569	-1.713963274	0.684534101
2	0	0.860205619
GMPS	ENSG00000163655.15	0.031226916	2	2	0.044727237	Brain_Amygdala
0.093096604	Brain_Caudate_basal_ganglia	1.012313159	0.987686841
0.987686841	-2.007210664	1.679284535	-0.163963064	2.606745754
2	0	0.860205619
IFRD2	ENSG00000214706.10	0.03131959	1	1	0.03131959	Brain_Cerebellum	0.03131959
Brain_Cerebellum	1	1	1	-2.152988402	-2.152988402	-2.152988402
NA	1	0	0.860205619
RP11-313F23.4	ENSG00000255652.3	0.031355026	1	1	0.031355026
Brain_Amygdala	0.031355026	Brain_Amygdala	1	1	1	2.152537741
2.152537741	2.152537741	NA	1	0	0.860205619
GDAP1L1	ENSG00000124194.16	0.031384417	1	1	0.031384417
Brain_Cerebellum	0.031384417	Brain_Cerebellum	1	1	1	2.152164297
2.152164297	2.152164297	NA	1	0	0.860205619
RPH3A	ENSG00000089169.14	0.031384851	1	1	0.031384851
Brain_Cerebellar_Hemisphere	0.031384851	Brain_Cerebellar_Hemisphere	1	1
1	2.152158785	2.152158785	2.152158785	NA	1	0
0.860205619
LINC00865	ENSG00000232229.5	0.031455549	13	1	0.012754016
Brain_Amygdala	0.497531358	Brain_Cerebellar_Hemisphere	12.41287843
0.000332457	12.41287843	0.678379098	2.490565015	2.090413621
0.469255646	1	0	0.860205619
CALN1	ENSG00000183166.10	0.031468709	1	1	0.031468709
Brain_Caudate_basal_ganglia	0.031468709	Brain_Caudate_basal_ganglia	1	1
1	2.151094908	2.151094908	2.151094908	NA	1	0
0.860205619
RP11-178F10.3	ENSG00000266489.1	0.031509764	7	7	0.034265244
Brain_Hypothalamus	0.842348029	Brain_Amygdala	3.429748501
0.166473709	0.166473709	-2.116936422	0.976082745	-0.039779053
1.123643687	7	0	0.860205619
RGPD8	ENSG00000169629.11	0.031578601	13	4	0.003843936
Brain_Putamen_basal_ganglia	0.645332371	Brain_Nucleus_accumbens_basal_ganglia
9.943036033	0.003448531	0.485221001	-2.890692895	1.55061555
-1.64002818	1.106782179	4	0	0.860205619
VSTM2A	ENSG00000170419.10	0.031609895	1	1	0.031609895
Brain_Cortex	0.031609895	Brain_Cortex	1	1	1	-2.149309239
-2.149309239	-2.149309239	NA	1	0	0.860205619
CAPN15	ENSG00000103326.11	0.031632663	3	3	0.006809099
Brain_Cerebellum	0.620980433	Brain_Putamen_basal_ganglia	1.129893075
0.827949107	0.827949107	-2.706039301	-0.494461298	-1.36961529
1.175732469	3	0	0.860205619
ARHGEF39	ENSG00000137135.17	0.031702491	12	4	0.019938171
Brain_Nucleus_accumbens_basal_ganglia	0.997788061	Brain_Cerebellar_Hemisphere
8.818393473	0.009453597	0.674926429	-1.263900026	2.327509367
-0.117088944	0.836772869	4	0	0.860205619
WDR86	ENSG00000187260.15	0.03174625	3	2	0.009786245
Brain_Nucleus_accumbens_basal_ganglia	0.516829425	Brain_Cerebellum
1.942925459	0.057099972	0.999974569	-2.583292018	-0.648240479
-1.886285087	1.075010438	2	0	0.860205619
C1orf168	ENSG00000187889.12	0.03175677	2	2	0.01128753	Brain_Cerebellum

0.461091257 Brain_Substantia_nigra 1.017550563 0.982449437
0.982449437 -2.533668987 -0.737051129 -1.635360058 1.27040067 2
0 0.860205619
RAD51 ENSG00000051180.16 0.031818293 2 2 0.010828992
Brain_Substantia_nigra 0.420491558 Brain_Caudate_basal_ganglia
1.067808595 0.932191405 0.932191405 -2.548169236 -0.805568737
-1.676868986 1.232204629 2 0 0.860205619
GALT ENSG00000213930.11 0.031848082 3 3 0.003537195
Brain_Putamen_basal_ganglia 0.699328409 Brain_Cortex 1.511410916
0.48476944 0.48476944 -2.916732073 -0.386227202 -1.264541467
1.431791594 3 0 0.860205619
S100A13 ENSG00000189171.14 0.031974363 12 2 0.105861476
Brain_Substantia_nigra 0.629732384 Brain_Spinal_cord_cervical_c-1
10.41956981 0.009766195 0.529811032 -1.617077856 0.572073444
-1.032547461 0.628257779 2 0 0.8606068
ZKSCAN5 ENSG00000196652.11 0.031985958 3 2 0.009266456
Brain_Cortex 0.013137559 Brain_Frontal_Cortex_BA9 2.821862568
0.032082427 0.146055005 -2.602063985 -2.480018424 -2.540706652
0.061025532 2 0 0.8606068
RP1-151F17.2 ENSG00000272341.1 0.032177093 1 1 0.032177093
Brain_Cortex 0.032177093 Brain_Cortex 1 1 1 2.142203706
2.142203706 2.142203706 NA 1 0 0.862519717
PCDHGB1 ENSG00000254221.2 0.032262487 1 1 0.032262487
Brain_Cerebellar_Hemisphere 0.032262487 Brain_Cerebellar_Hemisphere 1 1
1 2.141143257 2.141143257 2.141143257 NA 1 0
0.862519717
CRYGA ENSG00000168582.4 0.032293156 8 5 0.164048415
Brain_Hypothalamus 0.727661647 Brain_Frontal_Cortex_BA9 5.6889705
0.029982096 0.206619211 -1.391583972 0.452151436 -0.828352582
0.644721129 5 0 0.862519717
MCM6 ENSG00000076003.4 0.032303647 5 4 0.03732561 Brain_Cerebellum
0.989616508 Brain_Caudate_basal_ganglia 2.925049834 0.065837535
0.214512078 -2.082184611 0.761212326 -0.387312129 1.246358911
4 0 0.862519717
STPG3 ENSG00000197768.10 0.032445132 1 1 0.032445132 Brain_Cortex
0.032445132 Brain_Cortex 1 1 1 -2.138883167 -2.138883167
-2.138883167 NA 1 0 0.864647308
ZNF669 ENSG00000188295.14 0.032639196 9 3 0.186997152
Brain_Anterior_cingulate_cortex_BA24 0.740947952 Brain_Caudate_basal_ganglia
7.491137584 0.017025456 0.345506727 -0.763350901 1.319514542
0.628107451 0.630611515 3 0 0.865270611
C20orf203 ENSG00000198547.8 0.032734222 13 4 0.002808184
Brain_Hippocampus 0.545485578 Brain_Hypothalamus 10.25225069
1.30E-16 0.397106397 -2.987990353 -0.604538655 -2.58068792
0.663745306 4 0 0.865270611
FMN2 ENSG00000155816.19 0.03274693 2 2 0.014302055
Brain_Frontal_Cortex_BA9 0.383788527 Brain_Substantia_nigra
1.017971196 0.982028804 0.982028804 -2.449586012 -0.870937048
-1.66026153 1.116273387 2 0 0.865270611
MIEF2 ENSG00000177427.12 0.032803312 1 1 0.032803312
Brain_Putamen_basal_ganglia 0.032803312 Brain_Putamen_basal_ganglia 1 1
1 -2.134482405 -2.134482405 -2.134482405 NA 1 0
0.865270611
LSS ENSG00000160285.14 0.032883481 8 6 0.064618871
Brain_Anterior_cingulate_cortex_BA24 0.814979888 Brain_Hypothalamus
4.592229791 0.108328536 0.182411994 -0.935542731 1.847885757
0.151081276 0.959157576 6 0 0.865270611
WDR92 ENSG00000243667.6 0.032996846 8 3 0.003625872
Brain_Hippocampus 0.730455006 Brain_Spinal_cord_cervical_c-1
6.080904181 3.60E-05 0.523383579 0.344520335 2.908999693
2.107052325 0.8066006 3 0 0.865270611
FLVCR2 ENSG00000119686.9 0.03317226 2 2 0.101006949

Brain_Hippocampus 0.108532547 Brain_Spinal_cord_cervical_c-1
1.227137527 0.772862473 0.772862473 -1.604823979 1.639991431
0.017583726 2.29443098 2 0 0.865270611
RASL11A ENSG00000122035.6 0.033189736 1 1 0.033189736
Brain_Cerebellum 0.033189736 Brain_Cerebellum 1 1 1 2.129780543
2.129780543 2.129780543 NA 1 0 0.865270611
CSNK2A2 ENSG00000070770.8 0.033205862 1 1 0.033205862
Brain_Spinal_cord_cervical_c-1 0.033205862 Brain_Spinal_cord_cervical_c-1
1 1 1 -2.12958534 -2.12958534 -2.12958534 NA 1 0
0.865270611
NOXRED1 ENSG00000165555.9 0.033267113 7 5 0.082901295
Brain_Frontal_Cortex_BA9 0.467288801 Brain_Caudate_basal_ganglia
4.651651095 0.002203394 0.205600952 -1.734094623 1.721329663
0.544796364 1.305463336 5 0 0.865270611
NDUFS4 ENSG00000164258.11 0.033268707 1 1 0.033268707
Brain_Cerebellar_Hemisphere 0.033268707 Brain_Cerebellar_Hemisphere 1 1
1 -2.128825423 -2.128825423 -2.128825423 NA 1 0
0.865270611
MIRLET7BHG ENSG00000197182.14 0.033316297 1 1 0.033316297
Brain_Cerebellum 0.033316297 Brain_Cerebellum 1 1 1 2.128250774
2.128250774 2.128250774 NA 1 0 0.865270611
LMO7 ENSG00000136153.19 0.033345437 1 1 0.033345437
Brain_Frontal_Cortex_BA9 0.033345437 Brain_Frontal_Cortex_BA9 1 1
1 -2.127899254 -2.127899254 -2.127899254 NA 1 0
0.865270611
CCL20 ENSG00000115009.11 0.033435316 2 2 0.056492536
Brain_Spinal_cord_cervical_c-1 0.056632306 Brain_Hippocampus
1.069825257 0.930174743 0.930174743 -1.907216671 -1.906137982
-1.906677326 0.000762748 2 0 0.865270611
GFRA2 ENSG00000168546.10 0.033443232 2 2 0.034155043
Brain_Frontal_Cortex_BA9 0.180378764 Brain_Cerebellar_Hemisphere
1.082490065 0.917509935 0.917509935 -2.118236504 -1.339589731
-1.728913117 0.550586414 2 0 0.865270611
PELO ENSG00000152684.10 0.03349668 2 2 0.041972958
Brain_Nucleus_accumbens_basal_ganglia 0.077724292 Brain_Hippocampus
1.067842628 0.932157372 0.932157372 -2.03378817 1.76404566
-0.134871255 2.685474055 2 0 0.865270611
RAVER2 ENSG00000162437.14 0.033519882 2 2 0.020225068
Brain_Cerebellar_Hemisphere 0.394760873 Brain_Cerebellum 1.719960518
0.280039482 0.280039482 0.851015266 2.322146145 1.586580705
1.040246621 2 0 0.865270611
ATG14 ENSG00000126775.8 0.033659457 12 3 0.015229136
Brain_Cerebellar_Hemisphere 0.65074035 Brain_Hippocampus 10.10763383
0.004556017 0.51323597 0.452733922 2.42688371 1.190989861
0.529637274 3 0 0.865643096
RP11-399K21.14 ENSG00000272692.1 0.033682395 5 5 0.003404808
Brain_Spinal_cord_cervical_c-1 0.974211283 Brain_Cerebellar_Hemisphere
1.51273017 0.536356428 0.536356428 -0.267510579 2.928610461
1.217897347 1.361647908 5 0 0.865643096
TBC1D8-AS1 ENSG00000272902.2 0.033754384 1 1 0.033754384
Brain_Nucleus_accumbens_basal_ganglia 0.033754384
Brain_Nucleus_accumbens_basal_ganglia 1 1 1 2.122993653
2.122993653 2.122993653 NA 1 0 0.865643096
NAMPT ENSG00000105835.11 0.033781798 2 2 0.009735751
Brain_Cerebellar_Hemisphere 0.111956474 Brain_Cerebellum 1.699218911
0.300781089 0.300781089 1.58946046 2.585076098 2.087268279
0.704006569 2 0 0.865643096
QPCT ENSG00000115828.15 0.033902424 2 2 0.017852437
Brain_Nucleus_accumbens_basal_ganglia 0.096577932 Brain_Cortex
1.263246213 0.736753787 0.736753787 1.661675123 2.368664546
2.015169835 0.499917015 2 0 0.866227595
ARV1 ENSG00000173409.13 0.033928434 5 3 0.051819666

Brain_Cerebellar_Hemisphere 0.596867272 Brain_Cerebellum 3.927844772
0.031442667 0.252518774 -1.944628838 0.644443091 -0.772302037
0.945283544 3 0 0.866227595
FBF1 ENSG00000188878.19 0.034104634 13 3 0.009806059
Brain_Nucleus_accumbens_basal_ganglia 0.61329839 Brain_Spinal_cord_cervical_c-1
10.60138035 0.002000073 0.679999731 -0.713273764 2.582594209
1.681224504 0.878351284 3 0 0.869140144
MAP7 ENSG00000135525.18 0.034305586 1 1 0.034305586
Brain_Frontal_Cortex_BA9 0.034305586 Brain_Frontal_Cortex_BA9 1 1
1 2.116461383 2.116461383 2.116461383 NA 1 0
0.870664744
MTRF1L ENSG00000112031.15 0.034367576 13 1 0.022613711
Brain_Spinal_cord_cervical_c-1 0.052755748
Brain_Anterior_cingulate_cortex_BA24 12.57520497 0.000171977
12.57520497 1.93691489 2.279898464 2.080175435 0.086934673 1
0 0.870664744
ESR1 ENSG00000091831.22 0.034551234 2 2 0.017252212
Brain_Spinal_cord_cervical_c-1 0.862619573 Brain_Caudate_basal_ganglia
1.45720379 0.54279621 0.54279621 -2.381288131 -0.173040526 -1.277164328
1.561466856 2 0 0.870664744
LAMTOR3 ENSG00000109270.12 0.034658371 11 1 0.025440474
Brain_Substantia_nigra 0.11952978 Brain_Hypothalamus 9.99273475 0.0034709
9.99273475 1.556750303 2.234647887 2.016316779 0.199820391 1
0 0.870664744
HSPA12A ENSG00000165868.13 0.034679543 2 2 0.060547191
Brain_Cerebellum 0.484153796 Brain_Cerebellar_Hemisphere 1.469098658
0.530901342 0.530901342 -0.699637374 1.876787745 0.588575186
1.821807673 2 0 0.870664744
CCDC18 ENSG00000122483.17 0.034679673 5 2 0.030559309
Brain_Spinal_cord_cervical_c-1 0.115938093 Brain_Cerebellum 3.940080535
0.007621671 0.972843007 -2.162764346 -1.57205372 -1.79703089
0.246106156 2 0 0.870664744
LARS ENSG00000133706.17 0.034684413 12 2 0.010863938
Brain_Anterior_cingulate_cortex_BA24 0.18801746 Brain_Frontal_Cortex_BA9
11.11892716 0.001184122 0.383068751 -2.547045126 -1.316466663
-2.263843677 0.353251043 2 0 0.870664744
GSTM4 ENSG00000168765.16 0.034721205 12 4 0.010256884 Brain_Cerebellum
0.975853939 Brain_Cerebellar_Hemisphere 8.811587113 0.019207575
0.424022553 -0.332998764 2.567046556 0.757628974 0.822134425
4 0 0.870664744
ZNF396 ENSG00000186496.10 0.034724532 3 3 0.008030062
Brain_Nucleus_accumbens_basal_ganglia 0.660043339 Brain_Putamen_basal_ganglia
1.704523769 0.485785344 0.485785344 -2.650803168 0.439853331
-1.07960515 1.545977727 3 0 0.870664744
SALL2 ENSG00000165821.11 0.034787331 1 1 0.034787331 Brain_Cerebellum
0.034787331 Brain_Cerebellum 1 1 1 -2.110825294 -2.110825294
-2.110825294 NA 1 0 0.870678982
CLDN12 ENSG00000157224.15 0.034857494 1 1 0.034857494
Brain_Substantia_nigra 0.034857494 Brain_Substantia_nigra 1 1
1 2.110010005 2.110010005 2.110010005 NA 1 0
0.870877142
KB-68A7.1 ENSG00000274225.1 0.035067403 3 3 0.016501579
Brain_Nucleus_accumbens_basal_ganglia 0.338384856 Brain_Cerebellar_Hemisphere
1.590648283 0.442513065 0.442513065 -1.330179789 2.397628784
0.036695853 2.053107793 3 0 0.871659844
CPPED1 ENSG00000103381.11 0.035072755 1 1 0.035072755
Brain_Cortex 0.035072755 Brain_Cortex 1 1 1 2.107517396
2.107517396 2.107517396 NA 1 0 0.871659844
ITGA3 ENSG00000005884.17 0.035075727 5 4 0.065650435
Brain_Anterior_cingulate_cortex_BA24 0.784082327 Brain_Putamen_basal_ganglia
3.312678245 0.099536622 0.396652413 -1.840802987 0.830159281
-0.528447241 1.074212216 4 0 0.871659844

CMAS ENSG00000111726.12 0.035229309 1 1 0.035229309
Brain_Spinal_cord_cervical_c-1 0.035229309 Brain_Spinal_cord_cervical_c-1
1 1 1 2.105712776 2.105712776 2.105712776 NA 1 0
0.872247017
CYC1 ENSG00000179091.4 0.035249653 2 2 0.18459666
Brain_Cerebellar_Hemisphere 0.2452324 Brain_Frontal_Cortex_BA9 1.536512354
0.463487646 0.463487646 -1.162007545 1.32673409 0.082363273
1.759806087 2 0 0.872247017
DYNLL2 ENSG00000264364.2 0.03541551 8 7 0.03036976
Brain_Cerebellar_Hemisphere 0.90889741 Brain_Nucleus_accumbens_basal_ganglia
4.069280114 0.030145768 0.210676837 -1.51153059 2.165234128
0.026939132 1.110262671 7 0 0.872247017
LINC01123 ENSG00000204588.5 0.03564733 6 4 0.002458902 Brain_Cortex
0.607279111 Brain_Hypothalamus 4.110856137 0.078603177
0.210283474 -3.028353583 0.51396121 -2.13893229 1.316690092 4
0 0.872247017
NUMB ENSG00000133961.19 0.035680396 3 3 0.021110186
Brain_Hypothalamus 0.192923578 Brain_Spinal_cord_cervical_c-1
1.235141256 0.695290219 0.695290219 1.301978962 2.306007994
1.778954194 0.503884384 3 0 0.872247017
DLG5 ENSG00000151208.16 0.035703437 8 4 0.016704256
Brain_Hippocampus 0.523847055 Brain_Putamen_basal_ganglia 6.034365342
0.00803967 0.238727608 -2.393153387 1.687684964 -0.93511195
1.20839403 4 0 0.872247017
UBE2E2 ENSG00000182247.9 0.035746524 2 2 0.009868301
Brain_Anterior_cingulate_cortex_BA24 0.98694506 Brain_Substantia_nigra
1.031105484 0.968894516 0.968894516 -2.580410242 -0.016362671
-1.298386456 1.813055425 2 0 0.872247017
ACAA2 ENSG00000167315.17 0.035749453 4 4 0.022116315
Brain_Anterior_cingulate_cortex_BA24 0.620167459 Brain_Cerebellar_Hemisphere
1.784114628 0.254218293 0.254218293 -2.021041719 2.288364291
-0.259008764 1.82125984 4 0 0.872247017
DYRK1A ENSG00000157540.19 0.035781328 1 1 0.035781328
Brain_Cerebellum 0.035781328 Brain_Cerebellum 1 1 1 -2.099403704
-2.099403704 -2.099403704 NA 1 0 0.872247017
LYG1 ENSG00000144214.9 0.035785698 4 2 0.038325437
Brain_Cerebellar_Hemisphere 0.452339451 Brain_Hippocampus 3.073234382
0.038342621 0.810498819 -0.751520529 2.071357103 1.250037677
1.340995234 2 0 0.872247017
CCDC82 ENSG00000149231.11 0.035855881 13 1 0.029952659
Brain_Anterior_cingulate_cortex_BA24 0.059243116
Brain_Nucleus_accumbens_basal_ganglia 12.82672451 2.86E-06 12.82672451
-2.170715844 -1.886384826 -2.084272778 0.075439856 1 0
0.872247017
ADCYAP1 ENSG00000141433.12 0.035891087 2 2 0.012268472
Brain_Cerebellar_Hemisphere 0.602964073 Brain_Cerebellum 1.430689079
0.569310921 0.569310921 0.520142765 2.50432663 1.512234698
1.403029866 2 0 0.872247017
SLC25A34 ENSG00000162461.7 0.035956834 13 2 0.016108491
Brain_Hippocampus 0.885599659 Brain_Nucleus_accumbens_basal_ganglia
11.75116111 1.11E-16 0.466489699 -2.406448135 -0.143874389
-0.909313316 0.51437312 2 0 0.872247017
EML5 ENSG00000165521.15 0.035972163 1 1 0.035972163 Brain_Cortex
0.035972163 Brain_Cortex 1 1 1 2.09724195 2.09724195 2.09724195
NA 1 0 0.872247017
DMTN ENSG00000158856.17 0.03609814 9 8 0.002109683 Brain_Amygdala
0.822910205 Brain_Cerebellar_Hemisphere 3.220587392 0.0066622
0.442786249 -0.223803502 3.07434071 1.422364724 1.07127223 8 0
0.873227322
LINC00926 ENSG00000247982.6 0.036155713 9 9 0.01054573
Brain_Caudate_basal_ganglia 0.915546505 Brain_Cerebellar_Hemisphere
2.834304655 0.157035314 0.157035314 -1.158993884 2.557401881

0.445951566 1.147377778 9 0 0.873227322
MYPOP ENSG00000176182.5 0.036253608 1 1 0.036253608 Brain_Cortex
0.036253608 Brain_Cortex 1 1 1 -2.094071542 -2.094071542
-2.094071542 NA 1 0 0.873227322
ZNF501 ENSG00000186446.11 0.036350843 11 9 0.04705919
Brain_Caudate_basal_ganglia 0.935616915 Brain_Frontal_Cortex_BA9
4.542354563 0.057480992 0.243410078 -1.321111181 1.985767105
0.248471038 0.899560673 9 0 0.873227322
NPIPB2 ENSG00000234719.8 0.036359322 13 1 0.027491942
Brain_Anterior_cingulate_cortex_BA24 0.062488775 Brain_Caudate_basal_ganglia
12.76723101 4.41E-05 12.76723101 1.862811619 2.204460962
2.073512428 0.107936262 1 0 0.873227322
ACOT1 ENSG00000184227.7 0.036414162 13 9 0.000999587
Brain_Hypothalamus 0.940107466 Brain_Caudate_basal_ganglia 7.055504149
0.009730962 0.284161742 -0.345520357 3.290643075 0.874603606
0.936467197 9 0 0.873227322
NAPG ENSG00000134265.12 0.036459886 10 4 0.024780061
Brain_Hippocampus 0.698803083 Brain_Hypothalamus 7.805567576
0.00149957 0.310060818 -0.506665583 2.244814227 1.389201757
0.892507278 4 0 0.873227322
SELENBP1 ENSG00000143416.20 0.036521801 10 7 0.017818106
Brain_Spinal_cord_cervical_c-1 0.910818289 Brain_Cerebellum 5.731157911
9.62E-18 0.32301278 -0.760420888 2.369376479 1.191835255
1.120085477 7 0 0.873227322
ARL2BP ENSG00000102931.7 0.036751019 1 1 0.036751019
Brain_Cortex 0.036751019 Brain_Cortex 1 1 1 -2.088519273
-2.088519273 -2.088519273 NA 1 0 0.873227322
GOLT1B ENSG00000111711.9 0.036859838 3 3 0.006480823
Brain_Frontal_Cortex_BA9 0.38168676 Brain_Cortex 1.502177896
0.510801325 0.510801325 -2.722407209 -0.874792608 -1.565170939
1.008408241 3 0 0.873227322
CLEC18A ENSG00000157322.17 0.036967866 13 1 0.010245282
Brain_Substantia_nigra 0.298924136 Brain_Anterior_cingulate_cortex_BA24
12.48017608 0.000444569 12.48017608 -2.567438965 -1.038743303
-2.041904227 0.393226244 1 0 0.873227322
RILP ENSG00000167705.11 0.037009929 2 2 0.041286236
Brain_Cerebellar_Hemisphere 0.185636608 Brain_Cerebellum 1.110438175
0.889561825 0.889561825 -1.323597944 2.040643894 0.358522975
2.378878217 2 0 0.873227322
LONRF2 ENSG00000170500.12 0.037030765 3 3 0.070833692
Brain_Cortex 0.177400816 Brain_Putamen_basal_ganglia 1.142832776
0.853347832 0.853347832 -1.517260645 1.80654218 -0.353173235
1.872264043 3 0 0.873227322
CCDC154 ENSG00000197599.12 0.037080592 12 1 0.023211006
Brain_Cerebellar_Hemisphere 0.090402654 Brain_Hypothalamus 10.99447599
0.001925917 10.99447599 -2.269943595 -1.693277523 -1.996519273
0.169833273 1 0 0.873227322
TRMT61A ENSG00000166166.12 0.037342798 5 5 0.006697957
Brain_Putamen_basal_ganglia 0.584969069 Brain_Substantia_nigra
2.179977551 0.231783855 0.231783855 -2.711499879 1.250160767
-1.14410935 1.600602285 5 0 0.873227322
UBXN6 ENSG00000167671.11 0.037367808 12 4 0.023474876
Brain_Caudate_basal_ganglia 0.98211802 Brain_Hypothalamus 8.832949312
0.005225791 0.399065684 -0.373389574 2.265616464 0.656256501
0.904416317 4 0 0.873227322
AC008991.1 ENSG00000267683.1 0.037373778 2 2 0.019020844
Brain_Spinal_cord_cervical_c-1 0.236854903 Brain_Hypothalamus
1.063383178 0.936616822 0.936616822 1.182884555 2.345122203
1.764003379 0.821826122 2 0 0.873227322
RP5-875H18.9 ENSG00000276851.1 0.037418838 2 2 0.017297387
Brain_Cerebellum 0.131887072 Brain_Cortex 1.846059797 0.153940203
0.153940203 -2.380324733 -1.506701948 -1.94351334 0.617744596

2	0	0.873227322
LINC01266	ENSG00000224957.5	0.037426467	2	2	0.123466199	Brain_Cerebellar_Hemisphere	0.559877591	Brain_Cerebellum	1.641675809	0.358324191	0.358324191	-1.540386383	0.583023336	-0.478681523	1.501477412	2	0	0.873227322
NEU4	ENSG00000204099.11	0.03747331	4	4	0.0026001	Brain_Nucleus_accumbens_basal_ganglia	0.334873086	Brain_Cerebellum	1.702924392	0.315947649	0.315947649	0.964344802	3.011442106	1.738256618	0.88643951	4	0	0.873227322
SEL1L3	ENSG00000091490.10	0.037487006	7	6	0.011780974	Brain_Putamen_basal_ganglia	0.853068035	Brain_Cerebellar_Hemisphere	2.432896877	0.043011847	0.4026476	-2.518637896	1.990224379	0.076824755	1.509289133	6	0	0.873227322
FCN3	ENSG00000142748.12	0.037496661	1	1	0.037496661	Brain_Cortex	0.037496661	Brain_Cortex	1	1	1	2.080314875	2.080314875	2.080314875	NA	1	0	0.873227322
SEC16A	ENSG00000148396.18	0.037539891	1	1	0.037539891	Brain_Cerebellum	0.037539891	Brain_Cerebellum	1	1	1	-2.079843487	-2.079843487	-2.079843487	NA	1	0	0.873227322
CCDC9	ENSG00000105321.12	0.037585072	2	2	0.010692757	Brain_Cerebellum	0.452184912	Brain_Cortex	1.211849619	0.788150381	0.788150381	0.751777438	2.552582529	1.652179984	1.273361491	2	0	0.873227322
CLUL1	ENSG00000079101.16	0.037641218	11	11	0.020908389	Brain_Cerebellar_Hemisphere	0.563308949	Brain_Cerebellum	3.094481489	0.269186253	0.269186253	-1.526949804	2.309634657	1.215235531	1.02078862	11	0	0.873227322
CFAP46	ENSG00000171811.13	0.03779472	5	5	0.001733641	Brain_Cerebellar_Hemisphere	0.876038172	Brain_Nucleus_accumbens_basal_ganglia	2.335988202	0.09943406	0.09943406	-0.602440906	3.132432838	1.599462735	1.819834814	5	0	0.873227322
SGCB	ENSG00000163069.12	0.037846913	3	2	0.045523001	Brain_Cortex	0.146163775	Brain_Cerebellum	1.970144187	0.032797131	0.997058682	1.453216059	1.999789476	1.774524211	0.285663792	2	0	0.873227322
RARG	ENSG00000172819.16	0.037880573	1	1	0.037880573	Brain_Amygdala	0.037880573	Brain_Amygdala	1	1	1	-2.076144666	-2.076144666	-2.076144666	NA	1	0	0.873227322
BEND4	ENSG00000188848.15	0.037953661	1	1	0.037953661	Brain_Hippocampus	0.037953661	Brain_Hippocampus	1	1	1	2.075354832	2.075354832	2.075354832	NA	1	0	0.873227322
CHDH	ENSG00000016391.10	0.037972178	6	3	0.009512838	Brain_Nucleus_accumbens_basal_ganglia	0.5898562	Brain_Cerebellum	4.000920313	0.016477735	0.201545254	-2.593052005	0.765261237	-1.118724138	1.504721424	3	0	0.873227322
THRAP3	ENSG00000054118.13	0.037986148	3	3	0.0109665	Brain_Cerebellum	0.856653628	Brain_Hypothalamus	1.133337172	0.871951171	0.871951171	-1.138357479	2.543764495	0.528680865	1.865571548	3	0	0.873227322
EI24	ENSG00000149547.14	0.038229082	2	2	0.03445059	Brain_Cerebellum	0.131974768	Brain_Substantia_nigra	1.034663658	0.965336342	0.965336342	1.506360061	2.114757863	1.810558962	0.430202212	2	0	0.873227322
TACC2	ENSG00000138162.18	0.03823219	3	3	0.016237099	Brain_Anterior_cingulate_cortex_BA24	0.967492916	Brain_Substantia_nigra	1.075123388	0.950686628	0.950686628	-0.040752865	2.403542052	1.305250655	1.240832536	3	0	0.873227322
NELFA	ENSG00000185049.14	0.038303793	2	2	0.014518617	Brain_Spinal_cord_cervical_c-1	0.268812277	Brain_Cerebellum	1.15242317	0.84757683	0.84757683	1.10580189	2.444169179	1.774985535	0.946368586	2	0	0.873227322
ADAMTSL2	ENSG00000197859.9	0.038312171	4	4	0.004581328

Brain_Cerebellar_Hemisphere 0.679852529 Brain_Cortex 1.222391566
0.816603563 0.816603563 -2.835086527 0.412664375 -1.101710842
1.337611181 4 0 0.873227322
SEMA6B ENSG00000167680.15 0.038346574 1 1 0.038346574
Brain_Nucleus_accumbens_basal_ganglia 0.038346574
Brain_Nucleus_accumbens_basal_ganglia 1 1 1 2.071130799
2.071130799 2.071130799 NA 1 0 0.873227322
C5AR1 ENSG00000197405.7 0.038645535 2 2 0.012711347 Brain_Cerebellum
0.033271999 Brain_Cerebellar_Hemisphere 1.697365818 0.302634182
0.302634182 -2.491755614 -2.128785642 -2.310270628 0.256658528
2 0 0.873227322
LCN15 ENSG00000177984.6 0.038745318 9 2 0.021554024 Brain_Cortex
0.978513044 Brain_Cerebellum 7.951529645 0.000463944 0.766054065
-0.026933161 2.298136448 1.838557501 0.737861724 2 0
0.873227322
AMZ1 ENSG00000174945.13 0.03876327 9 6 0.004976468
Brain_Caudate_basal_ganglia 0.708507104 Brain_Putamen_basal_ganglia
5.617476651 0.043286508 0.213188971 -2.808553058 0.7573427
-0.93008161 1.172660444 6 0 0.873227322
F5 ENSG00000198734.10 0.038840582 2 2 0.153300204
Brain_Frontal_Cortex_BA9 0.565699888 Brain_Cortex 1.933274565
0.066725435 0.066725435 0.574395957 1.427971008 1.001183482
0.603568706 2 0 0.873227322
GALR1 ENSG00000166573.5 0.038843988 1 1 0.038843988
Brain_Anterior_cingulate_cortex_BA24 0.038843988
Brain_Anterior_cingulate_cortex_BA24 1 1 1 -2.065835782
-2.065835782 -2.065835782 NA 1 0 0.873227322
CNTN6 ENSG00000134115.12 0.038971659 2 2 0.011199322
Brain_Cerebellar_Hemisphere 0.088526333 Brain_Cerebellum 1.735010635
0.264989365 0.264989365 -2.536417212 -1.70322306 -2.119820136
0.589157235 2 0 0.873227322
IMMT ENSG00000132305.20 0.039011989 10 6 0.139308473
Brain_Spinal_cord_cervical_c-1 0.91591268 Brain_Anterior_cingulate_cortex_BA24
7.255024882 0.004789812 0.306785134 -1.10350595 1.478371112
-0.398998014 0.801215318 6 0 0.873227322
GNAO1 ENSG00000087258.14 0.039047081 1 1 0.039047081
Brain_Putamen_basal_ganglia 0.039047081 Brain_Putamen_basal_ganglia 1 1
1 2.063690388 2.063690388 2.063690388 NA 1 0
0.873227322
CRY2 ENSG00000121671.11 0.039216157 2 2 0.010991851
Brain_Cerebellar_Hemisphere 0.974823829 Brain_Hypothalamus 1.053028021
0.946971979 0.946971979 -2.542957765 0.031558889 -1.255699438
1.820458184 2 0 0.873227322
TTI2 ENSG00000129696.12 0.039229517 1 1 0.039229517
Brain_Cerebellar_Hemisphere 0.039229517 Brain_Cerebellar_Hemisphere 1 1
1 2.061771277 2.061771277 2.061771277 NA 1 0
0.873227322
CTB-129P6.11 ENSG00000267114.1 0.039241215 1 1 0.039241215
Brain_Substantia_nigra 0.039241215 Brain_Substantia_nigra 1 1
1 2.061648479 2.061648479 2.061648479 NA 1 0
0.873227322
SERPINB1 ENSG00000021355.12 0.039244285 2 2 0.08618573
Brain_Cerebellar_Hemisphere 0.200000464 Brain_Cerebellum 1.298440837
0.701559163 0.701559163 -1.715870595 1.281550244 -0.217160176
2.119496601 2 0 0.873227322
HAR1B ENSG00000231133.6 0.039271042 11 11 0.046838628 Brain_Cortex
0.965332711 Brain_Anterior_cingulate_cortex_BA24 2.686570287
0.252381396 0.252381396 -1.98775649 1.609446884 -0.347292532
1.235660954 11 0 0.873227322
C17orf97 ENSG00000187624.8 0.039316698 11 8 0.015657959
Brain_Nucleus_accumbens_basal_ganglia 0.693505721 Brain_Putamen_basal_ganglia
4.617472564 0.000404722 0.276962078 -2.416792053 2.265717587

0.535366686	1.403556297	8	0	0.873227322
ELAVL2	ENSG00000107105.14	0.039435561	2	2	0.023712288	Brain_Putamen_basal_ganglia	0.316461855	Brain_Cortex	1.069672553	0.930327447	0.930327447	-2.261759144	-1.001755168	-1.631757156	0.890957356	2	0	0.873227322
AC007204.2	ENSG00000267268.1	0.039587926	7	4	0.110021648	Brain_Hippocampus	0.970013117	Brain_Caudate_basal_ganglia	5.321061604	0.025010202	0.226090063	-0.70206103	1.598095844	0.080515953	0.739203572	4	0	0.873227322
CISD3	ENSG00000277972.1	0.039635938	5	5	0.027215534	Brain_Cortex	0.600945642	Brain_Frontal_Cortex_BA9	1.641554519	0.662589256	0.662589256	-1.787626335	2.208412462	0.035126623	1.558890626	5	0	0.873227322
RECQL4	ENSG00000160957.12	0.039692998	6	6	0.024231331	Brain_Nucleus_accumbens_basal_ganglia	0.927955773	Brain_Cerebellar_Hemisphere	1.876269891	0.236642978	0.236642978	-1.78551689	2.253441411	0.153776003	1.393096895	6	0	0.873227322
SERPINA1	ENSG00000197249.13	0.039715134	1	1	0.039715134	Brain_Frontal_Cortex_BA9	0.039715134	Brain_Frontal_Cortex_BA9	1	1	1	2.056699561	2.056699561	2.056699561	NA	1	0	0.873227322
RNF125	ENSG00000101695.8	0.039784396	1	1	0.039784396	Brain_Anterior_cingulate_cortex_BA24	0.039784396	Brain_Anterior_cingulate_cortex_BA24	1	1	1	-2.0559805	-2.0559805	-2.0559805	NA	1	0	0.873227322
RAB5A	ENSG00000144566.10	0.040050043	1	1	0.040050043	Brain_Caudate_basal_ganglia	0.040050043	Brain_Caudate_basal_ganglia	1	1	1	-2.053232409	-2.053232409	-2.053232409	NA	1	0	0.873227322
CTD-2541J13.1	ENSG00000260578.1	0.040069207	1	1	0.040069207	Brain_Cerebellar_Hemisphere	0.040069207	Brain_Cerebellar_Hemisphere	1	1	1	2.053034752	2.053034752	2.053034752	NA	1	0	0.873227322
PNPLA6	ENSG00000032444.15	0.040108103	4	4	0.016157133	Brain_Cerebellum	0.913672638	Brain_Caudate_basal_ganglia	1.927034906	0.256869338	0.256869338	-2.405346619	0.508444227	-0.395600653	1.350609078	4	0	0.873227322
SPIRE2	ENSG00000204991.10	0.040189466	12	10	0.003966479	Brain_Hippocampus	0.95280647	Brain_Substantia_nigra	4.380829909	0.020306809	0.227698266	-2.307558298	2.880815378	0.62412618	1.548157058	10	0	0.873227322
ITGAV	ENSG00000138448.11	0.040322056	9	7	0.019007693	Brain_Cerebellum	0.967251983	Brain_Spinal_cord_cervical_c-1	3.838836545	0.04602563	0.155610543	-1.234681959	2.34538007	0.47680248	1.352092391	7	0	0.873227322
CCER2	ENSG00000262484.1	0.040343879	1	1	0.040343879	Brain_Nucleus_accumbens_basal_ganglia	0.040343879	Brain_Nucleus_accumbens_basal_ganglia	1	1	1	-2.050210651	-2.050210651	-2.050210651	NA	1	0	0.873227322
DHRS4L2	ENSG00000187630.15	0.040413375	13	4	0.026487748	Brain_Substantia_nigra	0.958766128	Brain_Cerebellum	10.09232596	0.003399687	0.391176832	-0.355201428	2.218984955	1.117104028	0.688129386	4	0	0.873227322
KIF1BP	ENSG00000198954.7	0.040417602	4	4	0.225274301	Brain_Hypothalamus	0.587238244	Brain_Anterior_cingulate_cortex_BA24	3.325396277	0.169788063	0.169788063	-1.212622191	0.542842507	-0.55085311	0.760754492	4	0	0.873227322
BMP3	ENSG00000152785.6	0.04042792	2	2	0.255906598	Brain_Cerebellum	0.365420516	Brain_Cerebellar_Hemisphere	1.674518097	0.325481903	0.325481903	-1.136119398	0.905084702	-0.115517348	1.443349261	2	0	0.873227322
MITF	ENSG00000187098.14	0.04044626	2	2	0.011431417	Brain_Cortex

0.84655879 Brain_Substantia_nigra 1.02263835 0.97736165 0.97736165
0.193511003 2.529226695 1.361368849 1.651600405 2 0
0.873227322
PDZD2 ENSG00000133401.15 0.040511419 1 1 0.040511419 Brain_Cortex
0.040511419 Brain_Cortex 1 1 1 -2.048496046 -2.048496046
-2.048496046 NA 1 0 0.873227322
SLA2 ENSG00000101082.13 0.040513487 1 1 0.040513487
Brain_Cerebellar_Hemisphere 0.040513487 Brain_Cerebellar_Hemisphere 1 1
1 -2.048474917 -2.048474917 -2.048474917 NA 1 0
0.873227322
TBC1D10B ENSG00000169221.13 0.04052812 1 1 0.04052812 Brain_Cortex
0.04052812 Brain_Cortex 1 1 1 2.048325455 2.048325455
2.048325455 NA 1 0 0.873227322
BHLHE41 ENSG00000123095.5 0.040619155 1 1 0.040619155
Brain_Nucleus_accumbens_basal_ganglia 0.040619155
Brain_Nucleus_accumbens_basal_ganglia 1 1 1 -2.047396646
-2.047396646 -2.047396646 NA 1 0 0.873227322
RSPO4 ENSG00000101282.8 0.040660611 6 6 0.009854383
Brain_Frontal_Cortex_BA9 0.606669194 Brain_Cerebellar_Hemisphere
2.917199226 0.112081175 0.112081175 0.514833757 2.580897511
1.490345757 0.69816305 6 0 0.873227322
IL34 ENSG00000157368.10 0.040687597 3 2 0.055864036
Brain_Putamen_basal_ganglia 0.888741149 Brain_Frontal_Cortex_BA9
2.551723509 0.058462256 0.389814235 0.139897281 1.9120948
1.296663593 1.002473832 2 0 0.873227322
ADH1C ENSG00000248144.5 0.040708032 3 3 0.009355063
Brain_Caudate_basal_ganglia 0.685458801 Brain_Putamen_basal_ganglia
1.380802053 0.702692016 0.702692016 -2.598798643 1.500007712
-0.501272128 2.051097499 3 0 0.873227322
CLCN7 ENSG00000103249.17 0.040724618 6 2 0.016662589
Brain_Hippocampus 0.059711568 Brain_Cerebellar_Hemisphere 4.408355168
0.025518515 1.283048814 1.882917362 2.394069547 2.090716431
0.234571073 2 0 0.873227322
TCF25 ENSG00000141002.19 0.04074736 1 1 0.04074736 Brain_Substantia_nigra
0.04074736 Brain_Substantia_nigra 1 1 1 -2.04609159
-2.04609159 -2.04609159 NA 1 0 0.873227322
DTWD1 ENSG00000104047.14 0.040757675 3 3 0.005529858
Brain_Nucleus_accumbens_basal_ganglia 0.669430258 Brain_Frontal_Cortex_BA9
1.137466383 0.893536242 0.893536242 -2.774429745 -0.426930077
-1.387911012 1.230244076 3 0 0.873227322
C1RL ENSG00000139178.10 0.040878061 4 4 0.060736279
Brain_Caudate_basal_ganglia 0.173510543 Brain_Frontal_Cortex_BA9
1.305273689 0.729857332 0.729857332 -1.875410445 1.72743479
-0.84247915 1.729886611 4 0 0.873227322
ARNT2 ENSG00000172379.20 0.041026753 3 3 0.009981985
Brain_Substantia_nigra 0.689381146 Brain_Cortex 2.034555151
0.209668061 0.209668061 -2.576452733 0.487361997 -0.829595258
1.576499548 3 0 0.873227322
TXLNB ENSG00000164440.14 0.041062186 4 4 0.002997131
Brain_Substantia_nigra 0.360401295 Brain_Anterior_cingulate_cortex_BA24
2.486797642 0.231927241 0.231927241 -0.914600693 2.968032003
1.565930048 1.706805021 4 0 0.873227322
NDUFAF7 ENSG00000003509.15 0.041117376 4 4 0.010429098
Brain_Putamen_basal_ganglia 0.100227907 Brain_Cerebellum 2.998001032
0.134274627 0.134274627 -2.561267629 -1.643749741 -2.156263624
0.472357424 4 0 0.873227322
PHC2 ENSG00000134686.18 0.041128827 1 1 0.041128827
Brain_Spinal_cord_cervical_c-1 0.041128827 Brain_Spinal_cord_cervical_c-1
1 1 1 2.042228937 2.042228937 2.042228937 NA 1 0
0.873227322
AP006621.5 ENSG00000255284.1 0.041130499 13 3 0.004108009
Brain_Cerebellar_Hemisphere 0.226956168 Brain_Substantia_nigra

11.14432246 0.000538683 0.572899251 1.208236859 2.869746611
2.105572368 0.571132514 3 0 0.873227322
LINC00607 ENSG00000235770.5 0.041221748 2 2 0.013038298
Brain_Hypothalamus 0.606878145 Brain_Caudate_basal_ganglia 1.021102037
0.978897963 0.978897963 -2.482721506 -0.514534787 -1.498628147
1.391718176 2 0 0.873838606
RLF ENSG00000117000.8 0.041349835 1 1 0.041349835 Brain_Cortex
0.041349835 Brain_Cortex 1 1 1 -2.040004931 -2.040004931
-2.040004931 NA 1 0 0.874894752
BLOC1S3 ENSG00000189114.6 0.041396635 4 4 0.007257796
Brain_Cortex 0.388479451 Brain_Cerebellum 2.706586441 0.271709276
0.271709276 -2.684778184 -0.862378167 -1.75537715 0.752476548
4 0 0.874894752
ATP5E ENSG00000124172.9 0.04149949 1 1 0.04149949 Brain_Frontal_Cortex_BA9
0.04149949 Brain_Frontal_Cortex_BA9 1 1 1 -2.038504651
-2.038504651 -2.038504651 NA 1 0 0.875166114
TYK2 ENSG00000105397.13 0.041534579 3 3 0.182747853 Brain_Cortex
0.471426358 Brain_Cerebellum 1.916733738 0.335228843 0.335228843
-0.720160212 1.332341934 0.606849719 1.150894841 3 0
0.875166114
N4BP3 ENSG00000145911.5 0.041666225 3 3 0.030238548
Brain_Cerebellar_Hemisphere 0.107452936 Brain_Spinal_cord_cervical_c-1
1.882462605 0.152345997 0.152345997 -2.16695156 1.609747749
-0.82343948 2.111020693 3 0 0.876028842
SSTR1 ENSG00000139874.5 0.041700751 1 1 0.041700751 Brain_Cortex
0.041700751 Brain_Cortex 1 1 1 2.036494238 2.036494238
2.036494238 NA 1 0 0.876028842
NTRK1 ENSG00000198400.11 0.04186069 2 2 0.022258868 Brain_Cerebellum
0.721508607 Brain_Cortex 1.287729861 0.712270139 0.712270139
-0.3564433 2.285921208 0.964738954 1.868433862 2 0
0.877113826
STK33 ENSG00000130413.15 0.041974819 7 5 0.031015599
Brain_Frontal_Cortex_BA9 0.833605371 Brain_Cerebellum 4.502909389
0.054573556 0.222636807 -2.156872511 0.210079826 -1.30996547
0.906712301 5 0 0.877113826
RNASEL ENSG00000135828.11 0.041991329 10 6 0.003265788
Brain_Spinal_cord_cervical_c-1 0.421146069 Brain_Hippocampus 6.42822677
0.001545948 0.311866153 -2.941545517 -0.804434525 -1.902373217
0.588492959 6 0 0.877113826
NEDD4 ENSG00000069869.15 0.042230891 3 3 0.038097367
Brain_Anterior_cingulate_cortex_BA24 0.197635208
Brain_Spinal_cord_cervical_c-1 1.192800076 0.883612799 0.883612799
-1.411116798 2.073805622 0.650335686 1.827959892 3 0
0.877113826
KB-1507C5.4 ENSG00000254281.1 0.042261223 2 2 0.015682837
Brain_Hypothalamus 0.184195703 Brain_Caudate_basal_ganglia 1.270804966
0.729195034 0.729195034 -2.4162141 -1.327946737 -1.872080418
0.769521232 2 0 0.877113826
LYSMD1 ENSG00000163155.11 0.042437842 3 3 0.164244587
Brain_Cortex 0.936722503 Brain_Nucleus_accumbens_basal_ganglia
2.663457894 0.124052161 0.124052161 -0.079389899 1.390936819
0.68431642 0.736823769 3 0 0.877113826
RP11-426C22.5 ENSG00000260517.2 0.042461125 2 2 0.016358242
Brain_Spinal_cord_cervical_c-1 0.554641782 Brain_Cerebellar_Hemisphere
1.063415813 0.936584187 0.936584187 -2.400823104 0.590818882
-0.905002111 2.115410335 2 0 0.877113826
SF3B3 ENSG00000189091.12 0.042517042 5 5 0.005525021
Brain_Frontal_Cortex_BA9 0.674985084 Brain_Cerebellar_Hemisphere
2.284361618 0.135960945 0.135960945 -0.419316165 2.774714348
1.06222738 1.177620719 5 0 0.877113826
FRA10AC1 ENSG00000148690.11 0.0425489 13 2 0.035729542
Brain_Substantia_nigra 0.72509275 Brain_Amygdala 11.95185166

0.001620066	0.43453841 -2.099992038	-0.351660682	-0.659952422
0.455445028	2	0	0.877113826
FADS3 ENSG00000221968.8	0.042569833	2	2	0.012022955	Brain_Cerebellum
0.040320606	Brain_Cerebellar_Hemisphere 1.833116083	0.166883917
0.166883917	2.050449308	2.511469875	2.280959591	0.325990769
2	0	0.877113826
RBM24 ENSG00000112183.14	0.042605749	1	1	0.042605749
Brain_Frontal_Cortex_BA9	0.042605749	Brain_Frontal_Cortex_BA9	1	1
1	2.027554383	2.027554383	2.027554383	NA	1	0
0.877113826
ETFDH ENSG00000171503.11	0.042625272	13	3	0.014883662
Brain_Nucleus_accumbens_basal_ganglia 0.758318384	Brain_Substantia_nigra
10.609172 0.002416122	0.438654215	0.307689797	2.435197531
1.520522482	0.54271008 3	0	0.877113826
CXCL1 ENSG00000163739.4	0.042629877	1	1	0.042629877	Brain_Cortex
0.042629877	Brain_Cortex	1	1	1	2.027318241	2.027318241
2.027318241	NA	1	0	0.877113826
ZBTB1 ENSG00000126804.13	0.042662949	2	2	0.012013723
Brain_Frontal_Cortex_BA9	0.944354667	Brain_Cerebellum 1.026804295
0.973195705	0.973195705	-2.511740984	0.069797714	-1.220971635
1.825423519	2	0	0.877113826
PAIP1 ENSG00000172239.13	0.042760492	2	2	0.016892318
Brain_Cerebellar_Hemisphere 0.465912705	Brain_Frontal_Cortex_BA9	1.01809374
0.98190626 0.98190626 -2.389043163	0.729145434	-0.829948864
2.204892302	2	0	0.877113826
RP11-805F19.1	ENSG00000264880.1	0.042818506	5	4	0.023249098
Brain_Amygdala	0.065529383	Brain_Hippocampus	3.542401528	0.06669702
0.172967369	-2.269316296	2.161011457	-1.179672826	1.874562724
4	0	0.877113826
GPALPP1	ENSG00000133114.17	0.042822108	3	3	0.00533564 Brain_Cortex
0.312223144	Brain_Cerebellar_Hemisphere 2.242961271	0.155480658
0.155480658	1.010568197	2.786039336	2.034054632	0.918345977
3	0	0.877113826
SLC19A2	ENSG00000117479.12	0.042880842	1	1	0.042880842
Brain_Anterior_cingulate_cortex_BA24	0.042880842
Brain_Anterior_cingulate_cortex_BA24	1	1	1	2.024868764
2.024868764	2.024868764	NA	1	0	0.877113826
SMN1 ENSG00000172062.16	0.043042083	1	1	0.043042083	Brain_Cortex
0.043042083	Brain_Cortex	1	1	1	2.023301392	2.023301392
2.023301392	NA	1	0	0.8791267
DCAF4L1	ENSG00000182308.6	0.043141688	4	4	0.01297989
Brain_Anterior_cingulate_cortex_BA24	0.437261655	Brain_Cerebellum
1.953156322	0.33465302 0.33465302 -2.48432059	1.591552253
0.365886898	1.938009472	4	0	0.879876616
MYB	ENSG00000118513.18	0.043312943	1	1	0.043312943	Brain_Cortex
0.043312943	Brain_Cortex	1	1	1	2.020679604	2.020679604
2.020679604	NA	1	0	0.88018129
FAM135B	ENSG00000147724.11	0.043313002	1	1	0.043313002
Brain_Spinal_cord_cervical_c-1	0.043313002	Brain_Spinal_cord_cervical_c-1
1	1	1	-2.020679028	-2.020679028	-2.020679028	NA	1	0
0.88018129
SMYD1 ENSG00000115593.14	0.043363359	1	1	0.043363359
Brain_Hippocampus	0.043363359	Brain_Hippocampus	1	1	1
-2.020193129	-2.020193129	-2.020193129	NA	1	0	0.88018129
TMEM8A	ENSG00000129925.10	0.043430826	7	5	0.010816189
Brain_Putamen_basal_ganglia 0.615317439	Brain_Anterior_cingulate_cortex_BA24
4.750585299	0.000831779	0.16844866 -2.548581898	2.46237467
-0.404003038	2.042107348	5	0	0.88018129
RGS5 ENSG00000143248.12	0.043471179	1	1	0.043471179
Brain_Hippocampus	0.043471179	Brain_Hippocampus	1	1	1
2.019154349	2.019154349	2.019154349	NA	1	0	0.88018129
RPL21 ENSG00000122026.10	0.043553182	2	2	0.061609275

Brain_Putamen_basal_ganglia 0.093105808 Brain_Frontal_Cortex_BA9
1.007352906 0.992647094 0.992647094 -1.679237286 1.869097323
0.094930018 2.509051464 2 0 0.880567306
CHMP4B ENSG00000101421.3 0.04388756 3 3 0.031949433
Brain_Substantia_nigra 0.762245568 Brain_Hypothalamus 2.109182879
0.265371843 0.265371843 -2.1450427 0.739083409 -0.569497524
1.460478786 3 0 0.884436129
DAB1 ENSG00000173406.15 0.043943646 2 2 0.012432876 Brain_Cerebellum
0.052647488 Brain_Cerebellar_Hemisphere 1.764850135 0.235149865
0.235149865 -2.499613806 -1.937801147 -2.218707477 0.397261541
2 0 0.884436129
CPSF4L ENSG00000187959.9 0.044020132 3 3 0.018166781
Brain_Cerebellum 0.567287701 Brain_Substantia_nigra 1.191869326
0.836328364 0.836328364 -0.572050591 2.362201163 1.045537223
1.490092186 3 0 0.884436129
COQ8B ENSG00000123815.11 0.04404138 2 1 0.015338223 Brain_Cerebellum
0.116199473 Brain_Cerebellar_Hemisphere 1.968173554 0.031826446
1.968173554 -2.424293049 -1.570927574 -1.997610311 0.603420514
1 0 0.884436129
CXCL16 ENSG00000161921.14 0.044060609 4 3 0.14947838
Brain_Frontal_Cortex_BA9 0.832229225 Brain_Putamen_basal_ganglia 3.36421308
0.060807726 0.211742697 -0.211843379 1.441376377 0.710309682
0.734012009 3 0 0.884436129
ZMYND11 ENSG00000015171.19 0.044137596 1 1 0.044137596
Brain_Hippocampus 0.044137596 Brain_Hippocampus 1 1 1
-2.012781735 -2.012781735 -2.012781735 NA 1 0 0.884712192
AHNAK2 ENSG00000185567.6 0.044217954 2 2 0.01412112 Brain_Cerebellum
0.721449872 Brain_Cerebellar_Hemisphere 1.04318512 0.95681488 0.95681488
-0.356521743 2.454167463 1.04882286 1.987457398 2 0
0.885054933
CNMD ENSG00000136110.12 0.044297122 5 5 0.043181558
Brain_Nucleus_accumbens_basal_ganglia 0.625994159 Brain_Frontal_Cortex_BA9
2.027176599 0.337032846 0.337032846 -2.021949611 1.952430706
0.634153311 1.595533353 5 0 0.885332493
GPR142 ENSG00000257008.6 0.044358379 2 2 0.028862199
Brain_Cerebellar_Hemisphere 0.661976667 Brain_Cerebellum 1.322444248
0.677555752 0.677555752 -0.437185717 2.185363577 0.87408893
1.85442239 2 0 0.885332493
STX4 ENSG00000103496.14 0.044528449 7 4 0.001329978
Brain_Nucleus_accumbens_basal_ganglia 0.455279959 Brain_Cerebellar_Hemisphere
5.033270699 0.00133462 0.237874378 -3.209431256 2.774101387
-1.5130044 2.342278066 4 0 0.885347965
FFAR4 ENSG00000186188.10 0.044623103 4 4 0.005158807 Brain_Cortex
0.733040606 Brain_Caudate_basal_ganglia 1.894112213 0.14052278 0.14052278
-0.678447664 2.79694653 0.698150217 1.579080555 4 0
0.885347965
STK19 ENSG00000204344.14 0.044736439 6 6 0.004500964 Brain_Cerebellum
0.644132223 Brain_Cerebellar_Hemisphere 2.609797698 0.366273941
0.366273941 -2.840735432 0.667479138 -1.308871527 1.231937589
6 0 0.885347965
APOD ENSG00000189058.8 0.04475277 1 1 0.04475277 Brain_Putamen_basal_ganglia
0.04475277 Brain_Putamen_basal_ganglia 1 1 1 -2.006970821
-2.006970821 -2.006970821 NA 1 0 0.885347965
LINC00675 ENSG00000263429.3 0.044771847 1 1 0.044771847
Brain_Amygdala 0.044771847 Brain_Amygdala 1 1 1 -2.006791701
-2.006791701 -2.006791701 NA 1 0 0.885347965
TRAP1 ENSG00000126602.10 0.044872454 7 6 0.035352237
Brain_Substantia_nigra 0.98039661 Brain_Cerebellum 1.970299194
0.054250501 0.365862937 -2.104300562 2.073314667 -0.060405031
1.37518706 6 0 0.885347965
MT1G ENSG00000125144.13 0.044877712 3 3 0.024824207 Brain_Amygdala
0.369034228 Brain_Cerebellar_Hemisphere 1.372614994 0.629047631

0.629047631 -0.898283873 2.24412737 0.874520261 1.609538128 3
0 0.885347965
EIF2B5 ENSG00000145191.12 0.044971213 2 2 0.019481659
Brain_Hypothalamus 0.652201086 Brain_Frontal_Cortex_BA9 1.510720377
0.489279623 0.489279623 -2.336183909 -0.450706516 -1.393445212
1.333233851 2 0 0.885347965
KB-1980E6.3 ENSG00000253633.1 0.045168734 2 2 0.022740458
Brain_Nucleus_accumbens_basal_ganglia 0.172572954 Brain_Cortex
1.838693265 0.161306735 0.161306735 1.363982879 2.277767091
1.820874985 0.646143013 2 0 0.885347965
PPIL6 ENSG00000185250.15 0.045211545 8 6 0.09202039 Brain_Hippocampus
0.641313797 Brain_Amygdala 5.298110464 0.086271763 0.209157191
-1.502486987 1.684835105 -0.734175567 1.024451115 6 0
0.885347965
ANXA8L1 ENSG00000264230.7 0.045255988 3 3 0.02180999
Brain_Anterior_cingulate_cortex_BA24 0.174680739 Brain_Frontal_Cortex_BA9
1.491330343 0.516583968 0.516583968 -2.293660804 1.764127382
-0.628949899 2.124688077 3 0 0.885347965
NSUN5 ENSG00000130305.16 0.045265594 2 2 0.057524574 Brain_Cerebellum
0.178995016 Brain_Cerebellar_Hemisphere 1.131320069 0.868679931
0.868679931 -1.343855816 1.899303572 0.277723878 2.293259996
2 0 0.885347965
CCDC93 ENSG00000125633.10 0.045312727 8 5 0.065905565
Brain_Nucleus_accumbens_basal_ganglia 0.358809223 Brain_Substantia_nigra
5.480653342 0.02861948 0.186530902 -1.746033329 1.839065418
0.832269151 1.377498178 5 0 0.885347965
UNC119B ENSG00000175970.10 0.045317077 5 5 0.017060968
Brain_Anterior_cingulate_cortex_BA24 0.894082057 Brain_Putamen_basal_ganglia
2.94417735 0.141781507 0.141781507 -0.158568273 2.385391201
0.925054754 1.120622941 5 0 0.885347965
CDIP1 ENSG00000089486.16 0.045321177 6 4 0.106619385 Brain_Cerebellum
0.625795506 Brain_Nucleus_accumbens_basal_ganglia 3.672717427
0.012732413 0.454905153 -1.046792919 1.613576188 0.361290728
1.235677479 4 0 0.885347965
CRH ENSG00000147571.4 0.045371631 1 1 0.045371631
Brain_Caudate_basal_ganglia 0.045371631 Brain_Caudate_basal_ganglia 1 1
1 -2.001192665 -2.001192665 -2.001192665 NA 1 0
0.885347965
TGFA ENSG00000163235.15 0.045470536 6 3 0.067349049
Brain_Caudate_basal_ganglia 0.475130454 Brain_Cortex 4.551535205
0.005384487 0.325003057 -1.829337602 -0.714156431 -1.566400591
0.421310592 3 0 0.885363885
RXFP3 ENSG00000182631.6 0.045514445 1 1 0.045514445
Brain_Hypothalamus 0.045514445 Brain_Hypothalamus 1 1 1
1.999868692 1.999868692 1.999868692 NA 1 0 0.885363885
PLAC8L1 ENSG00000173261.8 0.045614971 2 2 0.013012314
Brain_Cerebellar_Hemisphere 0.042233709 Brain_Spinal_cord_cervical_c-1
1.79668254 0.20331746 0.20331746 -2.483432088 2.031209877 -0.226111106
3.192333948 2 0 0.885363885
GRM1 ENSG00000152822.13 0.045735682 2 2 0.023812584 Brain_Cerebellum
0.357406147 Brain_Spinal_cord_cervical_c-1 1.048133601 0.951866399
0.951866399 -0.920318877 2.260139662 0.669910392 2.2489238 2
0 0.885363885
TMEM126B ENSG00000171204.12 0.045921357 1 1 0.045921357
Brain_Substantia_nigra 0.045921357 Brain_Substantia_nigra 1 1
1 -1.996115455 -1.996115455 -1.996115455 NA 1 0
0.885363885
GNL3 ENSG00000163938.16 0.045973062 8 3 0.003643684 Brain_Cortex
0.854222918 Brain_Anterior_cingulate_cortex_BA24 6.290843036 0.0122212
0.384343568 -0.183733001 2.90746735 1.945857266 0.922596367 3
0 0.885363885
RAF1 ENSG00000132155.11 0.045995576 3 3 0.007418343

Brain_Frontal_Cortex_BA9 0.868081769 Brain_Caudate_basal_ganglia
1.222557641 0.793563196 0.793563196 -0.74144421 2.677456748
0.700702691 1.771037704 3 0 0.885363885
CLCA4 ENSG00000016602.9 0.045998327 3 3 0.294032224
Brain_Caudate_basal_ganglia 0.770702953 Brain_Nucleus_accumbens_basal_ganglia
1.93995144 0.192654169 0.192654169 -1.049317045 0.713900394
-0.208957392 0.884498949 3 0 0.885363885
PCDHA12 ENSG00000251664.3 0.046001829 2 2 0.016321299
Brain_Frontal_Cortex_BA9 0.472672146 Brain_Cerebellar_Hemisphere
1.521027692 0.478972308 0.478972308 0.718138091 2.401650381
1.559894236 1.190422957 2 0 0.885363885
ARID5B ENSG00000150347.14 0.046005257 2 2 0.03059038
Brain_Caudate_basal_ganglia 0.604151463 Brain_Anterior_cingulate_cortex_BA24
1.297731392 0.702268608 0.702268608 -0.518439784 2.16236075
0.821960483 1.895612236 2 0 0.885363885
SARDH ENSG00000123453.17 0.046168735 8 7 0.003797874
Brain_Nucleus_accumbens_basal_ganglia 0.841521533 Brain_Hippocampus
3.686736998 0.097667936 0.144115663 -2.894479747 -0.199947638
-1.753479759 1.151805105 7 0 0.887085993
TUBB8 ENSG00000261456.5 0.04624578 1 1 0.04624578 Brain_Hypothalamus
0.04624578 Brain_Hypothalamus 1 1 1 1.993143093 1.993143093
1.993143093 NA 1 0 0.887085993
TDRD10 ENSG00000163239.12 0.046284953 3 2 0.013103292
Brain_Cerebellum 0.125428196 Brain_Cortex 2.727560011 0.03358549
0.2388545 -2.480949536 -1.532382003 -2.074546321 0.488639334 2
0 0.887085993
SPC24 ENSG00000161888.11 0.046393163 5 4 0.134237102
Brain_Frontal_Cortex_BA9 0.969334848 Brain_Nucleus_accumbens_basal_ganglia
3.472490668 0.014964362 0.16804996 0.038442535 1.497600403
0.493127326 0.639660232 4 0 0.887943571
RAB33B ENSG00000172007.5 0.04660691 2 2 0.143155597 Brain_Cerebellum
0.677800569 Brain_Cerebellar_Hemisphere 1.695845503 0.304154497
0.304154497 -0.415466316 1.464140377 0.524337031 1.329082639
2 0 0.890815963
ANAPC7 ENSG00000196510.12 0.046683118 5 4 0.039449333
Brain_Frontal_Cortex_BA9 0.531251304 Brain_Hippocampus 1.998848945
0.009627799 0.131188624 -2.059468985 1.844507558 -0.04000813
1.764945227 4 0 0.891055255
BPI ENSG00000101425.12 0.046784433 2 2 0.02072363 Brain_Hypothalamus
0.448623064 Brain_Substantia_nigra 1.052378152 0.947621848
0.947621848 -2.312981986 0.757712585 -0.777634701 2.171308954
2 0 0.891772489
DBP ENSG00000105516.10 0.046948811 2 2 0.023347767
Brain_Putamen_basal_ganglia 0.323131104 Brain_Cerebellum 1.000285615
0.999714385 0.999714385 -0.988043991 2.267695585 0.639825797
2.302155532 2 0 0.893688179
OLIG2 ENSG00000205927.4 0.047051935 1 1 0.047051935
Brain_Caudate_basal_ganglia 0.047051935 Brain_Caudate_basal_ganglia 1 1
1 -1.985832417 -1.985832417 -1.985832417 NA 1 0 0.89443427
GLIS1 ENSG00000174332.5 0.047168553 4 3 0.007439767
Brain_Cerebellar_Hemisphere 0.683241403 Brain_Spinal_cord_cervical_c-1
2.797978001 0.002920369 0.19808217 -2.676490503 -0.408043957
-1.890208353 1.065354412 3 0 0.895434489
PDE1A ENSG00000115252.18 0.047271599 9 4 0.040952571
Brain_Hypothalamus 0.951294996 Brain_Substantia_nigra 7.25701203
0.011337351 0.251111534 -0.061080629 2.044009881 0.798657467
0.556729123 4 0 0.896174718
EMCN ENSG00000164035.9 0.047382789 2 2 0.014303746
Brain_Nucleus_accumbens_basal_ganglia 0.864307862 Brain_Hypothalamus
1.05829053 0.94170947 0.94170947 -0.170893049 2.449543415 1.139325183
1.852928394 2 0 0.897067117
SERPINF2 ENSG00000167711.13 0.047498354 12 3 0.098523959

Brain_Cortex 0.966662056 Brain_Anterior_cingulate_cortex_BA24 10.05769484 0.001423548 0.449556834 -0.495307824 1.652051962 0.383683116 0.595236266 3 0 0.89803982
MED27 ENSG00000160563.13 0.047564989 1 1 0.047564989 Brain_Substantia_nigra 0.047564989 Brain_Substantia_nigra 1 1 1 -1.981234438 -1.981234438 -1.981234438 NA 1 0 0.898086044
NBEAL1 ENSG00000144426.18 0.047745288 1 1 0.047745288 Brain_Substantia_nigra 0.047745288 Brain_Substantia_nigra 1 1 1 1.979628504 1.979628504 1.979628504 NA 1 0 0.898625231
CHMP1A ENSG00000131165.14 0.047747001 5 5 0.047193492 Brain_Spinal_cord_cervical_c-1 0.951465654 Brain_Hippocampus 2.142657185 0.220035664 0.220035664 -1.984559589 0.257477598 -0.688522971 0.965663432 5 0 0.898625231
ARFGEF1 ENSG00000066777.8 0.04784635 2 2 0.03019013 Brain_Caudate_basal_ganglia 0.043245325 Brain_Cerebellum 1.447739301 0.552260699 0.552260699 2.021332809 2.167586923 2.094459866 0.103417276 2 0 0.898625231
SSR2 ENSG00000163479.13 0.047986837 2 2 0.018953538 Brain_Putamen_basal_ganglia 0.444315613 Brain_Spinal_cord_cervical_c-1 1.00484567 0.99515433 0.99515433 -2.346443531 0.764925996 -0.790758767 2.200070491 2 0 0.898625231
GNE ENSG00000159921.14 0.048010839 2 2 0.025205688 Brain_Substantia_nigra 0.321733153 Brain_Cerebellum 1.01784075 0.98215925 0.98215925 -0.990902579 2.238235712 0.623666566 2.283345583 2 0 0.898625231
INAFM1 ENSG00000257704.3 0.048030735 2 2 0.066636883 Brain_Cerebellum 0.254086377 Brain_Cerebellar_Hemisphere 1.247470834 0.752529166 0.752529166 -1.834115284 1.140480008 -0.346817638 2.103356502 2 0 0.898625231
WDR43 ENSG00000163811.11 0.048070852 2 1 0.024545366 Brain_Cerebellar_Hemisphere 0.095424628 Brain_Cerebellum 1.962190841 0.037809159 1.962190841 1.667451666 2.248483657 1.957967661 0.410851661 1 0 0.898625231
DMXL1 ENSG00000172869.14 0.048107376 1 1 0.048107376 Brain_Cortex 0.048107376 Brain_Cortex 1 1 1 -1.976418694 -1.976418694 -1.976418694 NA 1 0 0.898625231
LINC01124 ENSG00000222033.1 0.048297144 5 4 0.016698235 Brain_Cortex 0.722685298 Brain_Cerebellum 3.355127139 0.097962693 0.228461704 -2.393285651 -0.354872251 -1.073010075 0.853834062 4 0 0.900875989
UBAP1L ENSG00000246922.8 0.048442744 2 2 0.041081631 Brain_Nucleus_accumbens_basal_ganglia 0.184844635 Brain_Cerebellar_Hemisphere 1.022308809 0.977691191 0.977691191 -2.042705191 -1.325985094 -1.684345143 0.506797641 2 0 0.900875989
SLC16A9 ENSG00000165449.11 0.048443845 3 2 0.021070353 Brain_Spinal_cord_cervical_c-1 0.43492437 Brain_Cerebellar_Hemisphere 1.967660159 0.032471902 0.999867939 0.7807928 2.306721455 1.307330817 0.865913833 2 0 0.900875989
NEMF ENSG00000165525.17 0.048595985 1 1 0.048595985 Brain_Substantia_nigra 0.048595985 Brain_Substantia_nigra 1 1 1 -1.972119336 -1.972119336 -1.972119336 NA 1 0 0.900875989
CNPY4 ENSG00000166997.7 0.048600968 5 5 0.095817486 Brain_Anterior_cingulate_cortex_BA24 0.9750369 Brain_Nucleus_accumbens_basal_ganglia 1.8783548 0.419094394 0.419094394 -1.556374704 1.665477717 0.327673087 1.326245065 5 0 0.900875989
SGF29 ENSG00000176476.8 0.048614207 1 1 0.048614207 Brain_Cerebellum 0.048614207 Brain_Cerebellum 1 1 1 1.971959701 1.971959701 1.971959701 NA 1 0 0.900875989

DSG2 ENSG00000046604.12 0.048797565 1 1 0.048797565
Brain_Nucleus_accumbens_basal_ganglia 0.048797565
Brain_Nucleus_accumbens_basal_ganglia 1 1 1 -1.970356168
-1.970356168 -1.970356168 NA 1 0 0.901991096
LYSMD3 ENSG00000176018.12 0.048803321 1 1 0.048803321
Brain_Nucleus_accumbens_basal_ganglia 0.048803321
Brain_Nucleus_accumbens_basal_ganglia 1 1 1 1.970305917
1.970305917 1.970305917 NA 1 0 0.901991096
RP11-343L5.2 ENSG00000271862.1 0.049096794 1 1 0.049096794
Brain_Cerebellum 0.049096794 Brain_Cerebellum 1 1 1 -1.967750135
-1.967750135 -1.967750135 NA 1 0 0.906218004
FAM200B ENSG00000237765.6 0.049177857 11 3 0.062196961
Brain_Putamen_basal_ganglia 0.891273969 Brain_Nucleus_accumbens_basal_ganglia
8.895214997 0.014768716 0.398525291 -1.864889024 0.13669236
-1.015001634 0.595053506 3 0 0.906518302
SLC22A8 ENSG00000149452.15 0.049539894 1 1 0.049539894
Brain_Anterior_cingulate_cortex_BA24 0.049539894
Brain_Anterior_cingulate_cortex_BA24 1 1 1 -1.963915483
-1.963915483 -1.963915483 NA 1 0 0.911990338
MIR646HG ENSG00000228340.5 0.049656074 2 2 0.055045637
Brain_Nucleus_accumbens_basal_ganglia 0.221705296 Brain_Caudate_basal_ganglia
1.92968588 0.07031412 0.07031412 -1.91851583 -1.222006165 -1.570260997
0.492506708 2 0 0.912927892
GPRIN1 ENSG00000169258.6 0.049757195 1 1 0.049757195
Brain_Cortex 0.049757195 Brain_Cortex 1 1 1 1.962045429
1.962045429 1.962045429 NA 1 0 0.91323095
PTTG1IP ENSG00000183255.11 0.04983381 2 2 0.021807068 Brain_Cerebellum
0.155506679 Brain_Cerebellar_Hemisphere 1.257583857 0.742416143
0.742416143 1.420347018 2.293711635 1.857029327 0.617562043
2 0 0.91323095
C1orf229 ENSG00000221953.5 0.049988244 12 7 0.014213949
Brain_Caudate_basal_ganglia 0.98764799 Brain_Hypothalamus 7.892698268
0.001083766 0.263839629 -2.451810504 1.687007838 -0.521780272
1.007936181 7 0 0.91323095
MYLK4 ENSG00000145949.9 0.049991466 10 5 0.002945623
Brain_Hippocampus 0.911524687 Brain_Anterior_cingulate_cortex_BA24
6.730464363 0.002526534 0.281784108 -0.37455687 2.97335629
0.59839434 0.938165911 5 0 0.91323095
SPOCK2 ENSG00000107742.12 0.050038108 4 3 0.056212303
Brain_Frontal_Cortex_BA9 0.33719559 Brain_Caudate_basal_ganglia 1.979501897
0.023843232 0.947711446 -1.909386099 1.787631954 0.585331134
1.698242397 3 0 0.91323095
RP11-536I6.2 ENSG00000255021.1 0.050104092 2 2 0.06814299
Brain_Putamen_basal_ganglia 0.964544868 Brain_Caudate_basal_ganglia
1.679984323 0.320015677 0.320015677 -1.824060079 -0.044451053
-0.934255566 1.258373611 2 0 0.91323095
ANKRD6 ENSG00000135299.16 0.050129467 2 2 0.014949705
Brain_Nucleus_accumbens_basal_ganglia 0.701385248 Brain_Caudate_basal_ganglia
1.257929548 0.742070452 0.742070452 -2.43359516 -0.383451198
-1.408523179 1.449670698 2 0 0.91323095
SMYD4 ENSG00000186532.11 0.050208143 13 2 0.014865195 Brain_Cortex
0.56765551 Brain_Cerebellar_Hemisphere 11.63768346 0.002690157
0.532601346 -2.435646714 -0.571507748 -1.96357772 0.462375098
2 0 0.913474813
CNN3 ENSG00000117519.15 0.050317275 5 5 0.232077539 Brain_Cortex
0.701634298 Brain_Frontal_Cortex_BA9 2.624955938 0.087690047
0.087690047 -1.195024293 1.172383137 -0.127627882 0.918367498
5 0 0.914271422
DECR2 ENSG00000242612.6 0.050567988 13 2 0.014453384 Brain_Cortex
0.136475451 Brain_Frontal_Cortex_BA9 11.97046972 0.002281669
0.57348175 1.489045259 2.445793286 2.111672623 0.297911382 2
0 0.91489611

CTD-2162K18.3 ENSG00000267353.1 0.050589901 3 3 0.095253417
Brain_Caudate_basal_ganglia 0.553869421 Brain_Cerebellum 1.805706852
0.350843029 0.350843029 -1.416372567 1.668313964 -0.113343494
1.597071256 3 0 0.91489611
VN1R1 ENSG00000178201.4 0.05081646 9 7 0.017321932
Brain_Nucleus_accumbens_basal_ganglia 0.775478145 Brain_Hypothalamus
5.066220262 0.038684252 0.200275612 -2.379802229 0.71349936
-0.451801076 0.924580816 7 0 0.91489611
RP5-944M2.3 ENSG00000214043.7 0.050822208 3 3 0.0724402
Brain_Cortex 0.922305812 Brain_Frontal_Cortex_BA9 1.860871545
0.49898136 0.49898136 -1.097848664 1.796341638 0.265340865 1.45437438
3 0 0.91489611
TMPO ENSG00000120802.13 0.050983033 1 1 0.050983033
Brain_Nucleus_accumbens_basal_ganglia 0.050983033
Brain_Nucleus_accumbens_basal_ganglia 1 1 1 1.951622558
1.951622558 1.951622558 NA 1 0 0.91489611
RND1 ENSG00000172602.9 0.051004219 2 2 0.033832473
Brain_Cerebellar_Hemisphere 0.273341336 Brain_Caudate_basal_ganglia
1.050250118 0.949749882 0.949749882 -2.122062705 1.095400225
-0.51333124 2.275089856 2 0 0.91489611
TMEM117 ENSG00000139173.9 0.051012311 1 1 0.051012311
Brain_Hippocampus 0.051012311 Brain_Hippocampus 1 1 1
1.9513762 1.9513762 1.9513762 NA 1 0 0.91489611
SLC35D3 ENSG00000182747.4 0.051373108 1 1 0.051373108
Brain_Substantia_nigra 0.051373108 Brain_Substantia_nigra 1 1
1 -1.948349914 -1.948349914 -1.948349914 NA 1 0 0.91489611
NTNG2 ENSG00000196358.10 0.051385013 1 1 0.051385013
Brain_Spinal_cord_cervical_c-1 0.051385013 Brain_Spinal_cord_cervical_c-1
1 1 1 -1.948250362 -1.948250362 -1.948250362 NA 1 0
0.91489611
RPRD2 ENSG00000163125.15 0.05147799 11 3 0.013864496
Brain_Frontal_Cortex_BA9 0.970579799 Brain_Substantia_nigra
8.880700168 0.000408903 0.464120423 0.036881114 2.46075512
1.717502924 0.828494118 3 0 0.91489611
THEMIS2 ENSG00000130775.15 0.051495461 1 1 0.051495461
Brain_Cerebellar_Hemisphere 0.051495461 Brain_Cerebellar_Hemisphere 1 1
1 1.947327687 1.947327687 1.947327687 NA 1 0 0.91489611
NCOA7 ENSG00000111912.19 0.05151695 7 5 0.002928209
Brain_Anterior_cingulate_cortex_BA24 0.857286887 Brain_Cerebellum
4.262854399 0.071004654 0.246145726 -0.17982891 2.975175617
1.363675455 1.055617382 5 0 0.91489611
GPT2 ENSG00000166123.13 0.05157863 1 1 0.05157863 Brain_Cortex 0.05157863
Brain_Cortex 1 1 1 -1.946633995 -1.946633995 -1.946633995
NA 1 0 0.91489611
IL1R2 ENSG00000115590.13 0.051600057 1 1 0.051600057
Brain_Frontal_Cortex_BA9 0.051600057 Brain_Frontal_Cortex_BA9 1 1
1 1.946455432 1.946455432 1.946455432 NA 1 0 0.91489611
CRABP2 ENSG00000143320.8 0.051679349 2 2 0.017383418
Brain_Cerebellar_Hemisphere 0.56230209 Brain_Cerebellum 1.025969372
0.974030628 0.974030628 -0.579425517 2.378496154 0.899535318
2.091566472 2 0 0.91489611
SNAPIN ENSG00000143553.10 0.051693438 1 1 0.051693438
Brain_Cerebellar_Hemisphere 0.051693438 Brain_Cerebellar_Hemisphere 1 1
1 1.945677942 1.945677942 1.945677942 NA 1 0 0.91489611
UBAC1 ENSG00000130560.8 0.051709428 2 2 0.030812824
Brain_Cerebellar_Hemisphere 0.693361541 Brain_Cerebellum 1.31151615 0.68848385
0.68848385 -2.159481593 0.394297321 -0.882592136 1.805794388 2
0 0.91489611
SORBS3 ENSG00000120896.13 0.051747346 1 1 0.051747346
Brain_Hippocampus 0.051747346 Brain_Hippocampus 1 1 1
-1.945229637 -1.945229637 -1.945229637 NA 1 0 0.91489611
SCTR ENSG00000080293.9 0.051853519 1 1 0.051853519

Brain_Anterior_cingulate_cortex_BA24 0.051853519
Brain_Anterior_cingulate_cortex_BA24 1 1 1 1.944347842
1.944347842 1.944347842 NA 1 0 0.91489611
GLYCTK ENSG00000168237.17 0.051937376 6 5 0.002814629
Brain_Cerebellum 0.274988327 Brain_Cortex 3.498626192 0.111677205
0.347984998 1.091646913 2.98728964 1.807823431 0.703577822 5
0 0.91489611
DUSP13 ENSG00000079393.20 0.051961549 1 1 0.051961549
Brain_Anterior_cingulate_cortex_BA24 0.051961549
Brain_Anterior_cingulate_cortex_BA24 1 1 1 1.943452167
1.943452167 1.943452167 NA 1 0 0.91489611
SNAI3 ENSG00000185669.5 0.052140086 3 3 0.034357059
Brain_Nucleus_accumbens_basal_ganglia 0.290666245
Brain_Anterior_cingulate_cortex_BA24 1.057333422 0.933120505
0.933120505 -2.115855974 -1.056661186 -1.51841217 0.542478392
3 0 0.91489611
ATXN1L ENSG00000224470.7 0.052154647 1 1 0.052154647
Brain_Cortex 0.052154647 Brain_Cortex 1 1 1 -1.941855077
-1.941855077 -1.941855077 NA 1 0 0.91489611
ATM ENSG00000149311.18 0.0522961 2 2 0.030848468
Brain_Spinal_cord_cervical_c-1 0.809816711
Brain_Nucleus_accumbens_basal_ganglia 1.36813434 0.63186566 0.63186566
-2.159021904 0.240662492 -0.959179706 1.696833109 2 0
0.91489611
KL ENSG00000133116.7 0.052310499 1 1 0.052310499
Brain_Putamen_basal_ganglia 0.052310499 Brain_Putamen_basal_ganglia 1 1
1 1.940569643 1.940569643 1.940569643 NA 1 0 0.91489611
CDC37L1 ENSG00000106993.11 0.05232551 1 1 0.05232551
Brain_Putamen_basal_ganglia 0.05232551 Brain_Putamen_basal_ganglia 1 1 1
1.940446007 1.940446007 1.940446007 NA 1 0 0.91489611
BPGM ENSG00000172331.11 0.052360062 1 1 0.052360062 Brain_Cortex
0.052360062 Brain_Cortex 1 1 1 1.940161535 1.940161535
1.940161535 NA 1 0 0.91489611
C20orf197 ENSG00000176659.7 0.052395038 4 1 0.045735537
Brain_Nucleus_accumbens_basal_ganglia 0.062048003 Brain_Cerebellar_Hemisphere
3.975704335 1.73E-05 3.975704335 -1.99782591 -1.865952557
-1.933892586 0.068875856 1 0 0.91489611
ACAA1 ENSG00000060971.17 0.05239945 1 1 0.05239945 Brain_Frontal_Cortex_BA9
0.05239945 Brain_Frontal_Cortex_BA9 1 1 1 1.939837432
1.939837432 1.939837432 NA 1 0 0.91489611
SLK ENSG00000065613.13 0.052459348 2 2 0.015238136
Brain_Frontal_Cortex_BA9 0.964381811 Brain_Cerebellar_Hemisphere
1.015348268 0.984651732 0.984651732 -2.426669356 -0.044655617
-1.235662487 1.684338068 2 0 0.91489611
LINC02175 ENSG00000262155.1 0.052541831 2 1 0.053031928
Brain_Cerebellar_Hemisphere 0.057947859 Brain_Cerebellum 1.95223286 0.04776714
1.95223286 1.896092161 1.934660849 1.915376505 0.027272181 1
0 0.91489611
TRIM33 ENSG00000197323.11 0.052556765 1 1 0.052556765
Brain_Nucleus_accumbens_basal_ganglia 0.052556765
Brain_Nucleus_accumbens_basal_ganglia 1 1 1 -1.938545012
-1.938545012 -1.938545012 NA 1 0 0.91489611
PLEKHO1 ENSG00000023902.13 0.052637229 2 2 0.025936677
Brain_Anterior_cingulate_cortex_BA24 0.065022439 Brain_Cerebellum 1.43993653
0.56006347 0.56006347 1.845103811 2.227158591 2.036131201
0.270153526 2 0 0.91489611
NUDT18 ENSG00000275074.1 0.052644292 9 4 0.001934723
Brain_Hypothalamus 0.90690559 Brain_Frontal_Cortex_BA9 6.561759145
0.007093693 0.324013908 -3.100073949 -0.116942536 -2.401575995
0.919293213 4 0 0.91489611
PCSK4 ENSG00000115257.15 0.052728497 2 2 0.017152072
Brain_Cerebellar_Hemisphere 0.273273905 Brain_Cerebellum 1.609984011

0.390015989 0.390015989 -2.383431598 -1.095554221 -1.739492909
0.910666826 2 0 0.91489611
PCDHB2 ENSG00000112852.6 0.052749907 6 5 0.002179889
Brain_Cortex 0.428000967 Brain_Cerebellum 3.81161473 0.100995666
0.267118377 -3.064562107 -0.792617058 -1.871106452 0.74173846 5
0 0.91489611
PIDD1 ENSG00000177595.17 0.052792578 7 6 0.004009685 Brain_Cerebellum
0.447184889 Brain_Hippocampus 4.007722475 0.002484983
0.220444529 -1.792437317 2.877398831 1.500765814 1.622883358
6 0 0.91489611
EWSAT1 ENSG00000212766.9 0.052958865 1 1 0.052958865
Brain_Frontal_Cortex_BA9 0.052958865 Brain_Frontal_Cortex_BA9 1 1
1 1.935256196 1.935256196 1.935256196 NA 1 0 0.91489611
MTERF1 ENSG00000127989.13 0.053005951 2 2 0.047256395
Brain_Frontal_Cortex_BA9 0.114622929 Brain_Cerebellar_Hemisphere
1.096773062 0.903226938 0.903226938 -1.983995022 -1.577750545
-1.780872783 0.287258224 2 0 0.91489611
LILRA6 ENSG00000244482.10 0.053009702 3 1 0.044598158
Brain_Cortex 0.065465022 Brain_Caudate_basal_ganglia 2.899812675
0.024706867 2.899812675 -2.008424907 -1.842069245 -1.902449482
0.092074657 1 0 0.91489611
ADH1B ENSG00000196616.13 0.053032717 2 2 0.049323541
Brain_Caudate_basal_ganglia 0.174000381 Brain_Hypothalamus 1.029230313
0.970769687 0.970769687 -1.965784227 1.359461541 -0.303161343
2.351303832 2 0 0.91489611
EMG1 ENSG00000126749.14 0.053202608 3 3 0.042609535 Brain_Cortex
0.175047582 Brain_Cerebellum 1.703433123 0.296778924 0.296778924
1.356162149 2.027517319 1.750831166 0.350883753 3 0
0.916696663
ZMIZ1 ENSG00000108175.16 0.053371367 1 1 0.053371367 Brain_Cerebellum
0.053371367 Brain_Cerebellum 1 1 1 -1.931903914 -1.931903914
-1.931903914 NA 1 0 0.917505849
RAB25 ENSG00000132698.14 0.05339887 2 2 0.040059377 Brain_Cerebellum
0.147370783 Brain_Putamen_basal_ganglia 1.083251966 0.916748034
0.916748034 -1.448881125 2.053136126 0.302127501 2.476300146
2 0 0.917505849
FRMPD1 ENSG00000070601.9 0.053513165 1 1 0.053513165
Brain_Cerebellum 0.053513165 Brain_Cerebellum 1 1 1 1.930756554
1.930756554 1.930756554 NA 1 0 0.917505849
ELSPBP1 ENSG00000169393.8 0.053568375 1 1 0.053568375
Brain_Spinal_cord_cervical_c-1 0.053568375 Brain_Spinal_cord_cervical_c-1
1 1 1 -1.930310515 -1.930310515 -1.930310515 NA 1 0
0.917505849
UBP1 ENSG00000153560.11 0.053577463 3 1 0.043503833 Brain_Cerebellum
0.073466527 Brain_Cerebellar_Hemisphere 2.910389945 0.023242223
2.910389945 -2.018840188 -1.789921532 -1.900726602 0.114634194
1 0 0.917505849
GALM ENSG00000143891.16 0.053817493 3 1 0.030780203
Brain_Hippocampus 0.082278054 Brain_Cortex 2.885685743
0.031235366 2.885685743 -2.159902693 -1.737618519 -1.892342905
0.232658229 1 0 0.920240629
RP11-403A3.3 ENSG00000275830.1 0.054025805 2 1 0.053080798
Brain_Hypothalamus 0.057727546 Brain_Caudate_basal_ganglia 1.978017061
0.021982939 1.978017061 -1.934263017 -1.897761205 -1.916012111
0.025810678 1 0 0.920240629
CWC25 ENSG00000273559.4 0.054050039 4 3 0.037271436
Brain_Spinal_cord_cervical_c-1 0.401298147 Brain_Substantia_nigra
2.090592848 0.035848555 0.848697237 -1.593721571 2.082778302
-0.440050202 1.712282703 3 0 0.920240629
GNG7 ENSG00000176533.12 0.054102111 3 3 0.062123069
Brain_Nucleus_accumbens_basal_ganglia 0.18582163 Brain_Cortex 1.062140893
0.935791222 0.935791222 -1.865416338 1.323041342 -0.646552095

1.721703034 3 0 0.920240629
KCTD5 ENSG00000167977.8 0.054156513 2 2 0.01578531 Brain_Hippocampus
0.589351789 Brain_Putamen_basal_ganglia 1.194536167 0.805463833
0.805463833 -2.413841909 0.53977561 -0.93703315 2.088522977 2
0 0.920240629
CD34 ENSG00000174059.16 0.0542222 2 2 0.338557207 Brain_Cortex
0.36132912 Brain_Caudate_basal_ganglia 1.700076731 0.299923269
0.299923269 -0.957019908 0.912835394 -0.022092257 1.322187364
2 0 0.920240629
SLC26A6 ENSG00000225697.12 0.054251961 2 2 0.039852545
Brain_Hypothalamus 0.265342395 Brain_Cerebellum 1.072653751
0.927346249 0.927346249 -1.113852674 2.055274025 0.470710676
2.240910979 2 0 0.920240629
C5orf34 ENSG00000172244.8 0.054284802 5 2 0.00915779
Brain_Nucleus_accumbens_basal_ganglia 0.028788082 Brain_Caudate_basal_ganglia
4.583509743 0.020261086 0.224533666 -2.606106826 -2.186376361
-2.306953627 0.173708328 2 0 0.920240629
RRP1B ENSG00000160208.12 0.054329123 1 1 0.054329123 Brain_Cerebellum
0.054329123 Brain_Cerebellum 1 1 1 1.924203162 1.924203162
1.924203162 NA 1 0 0.920240629
ZNF112 ENSG00000062370.16 0.054604702 2 2 0.019683209
Brain_Cerebellum 0.829370809 Brain_Caudate_basal_ganglia 1.339524947
0.660475053 0.660475053 -2.332332444 -0.215508598 -1.273920521
1.496820496 2 0 0.92196835
ALDH1B1 ENSG00000137124.7 0.054630827 3 2 0.017948293
Brain_Nucleus_accumbens_basal_ganglia 0.033311294 Brain_Caudate_basal_ganglia
2.648464196 0.005039431 0.346496372 -2.366683114 -2.128311151
-2.262520189 0.121993339 2 0 0.92196835
DBNDD1 ENSG00000003249.13 0.054633266 5 5 0.022613672
Brain_Amygdala 0.561602399 Brain_Cortex 1.626186098 0.376571691
0.376571691 -1.843961121 2.27989912 -0.271080305 1.72537812 5 0
0.92196835
TNFSF18 ENSG00000120337.8 0.054694713 5 3 0.033803827
Brain_Cerebellum 0.977327068 Brain_Cerebellar_Hemisphere 3.633874067
0.001694963 0.470833337 -0.028420132 2.12240399 1.328240252
0.995733701 3 0 0.92196835
LINC02062 ENSG00000248489.1 0.054890786 2 2 0.019428077
Brain_Nucleus_accumbens_basal_ganglia 0.578520555 Brain_Cerebellar_Hemisphere
1.012591725 0.987408275 0.987408275 -0.555547024 2.337213658
0.890833317 2.045490694 2 0 0.924128356
RP11-472N13.2 ENSG00000223834.2 0.054986916 1 1 0.054986916
Brain_Caudate_basal_ganglia 0.054986916 Brain_Caudate_basal_ganglia 1 1
1 1.918979598 1.918979598 1.918979598 NA 1 0
0.924128356
CSF1 ENSG00000184371.13 0.055021008 2 2 0.061308857
Brain_Cerebellar_Hemisphere 0.167343383 Brain_Amygdala 1.070288153
0.929711847 0.929711847 1.380790506 1.871261409 1.626025958
0.346815301 2 0 0.924128356
RP11-469N6.1 ENSG00000251226.1 0.05527054 11 6 0.034703644
Brain_Caudate_basal_ganglia 0.718311738 Brain_Putamen_basal_ganglia
6.744659167 0.010751726 0.252995254 -0.970865705 2.111799576
0.934593737 1.227871095 6 0 0.92441438
SLC6A16 ENSG00000063127.15 0.055273787 2 2 0.016696263
Brain_Cerebellum 0.090694728 Brain_Nucleus_accumbens_basal_ganglia
1.773294871 0.226705129 0.226705129 -2.393328976 -1.691744353
-2.042536665 0.496095244 2 0 0.92441438
UCHL1 ENSG00000154277.12 0.05529625 2 2 0.016172338
Brain_Cerebellar_Hemisphere 0.487475729 Brain_Cerebellum 1.319378958
0.680621042 0.680621042 0.694329287 2.405002888 1.549666087
1.209628904 2 0 0.92441438
CEP57 ENSG00000166037.10 0.055302325 2 2 0.021859965 Brain_Cortex
0.849354983 Brain_Cerebellum 1.227241117 0.772758883 0.772758883

-0.189941487 2.292792316 1.051425415 1.755557908 2 0
0.92441438
RANBP1 ENSG00000099901.16 0.055429134 1 1 0.055429134
Brain_Amygdala 0.055429134 Brain_Amygdala 1 1 1 1.915497136
1.915497136 1.915497136 NA 1 0 0.925428419
CKAP2 ENSG00000136108.14 0.055615936 13 1 0.035493716
Brain_Frontal_Cortex_BA9 0.163905963 Brain_Spinal_cord_cervical_c-1
12.33290831 8.73E-05 12.33290831 -2.102680412 -1.392054276
-1.86155743 0.189923098 1 0 0.926896684
RP11-390F4.3 ENSG00000225489.6 0.05570428 3 3 0.051712261
Brain_Cerebellar_Hemisphere 0.40685787 Brain_Hippocampus 1.507617744
0.557382803 0.557382803 -1.945521362 0.829435766 -0.948704119
1.543679231 3 0 0.926896684
GTF3C3 ENSG00000119041.10 0.055715825 1 1 0.055715825
Brain_Putamen_basal_ganglia 0.055715825 Brain_Putamen_basal_ganglia 1 1
1 1.913251798 1.913251798 1.913251798 NA 1 0
0.926896684
IL12RB2 ENSG00000081985.10 0.055882819 3 3 0.012006363
Brain_Cerebellum 0.952396768 Brain_Cerebellar_Hemisphere 1.308701284
0.743871307 0.743871307 -0.059697243 2.511957253 1.210292497
1.28611982 3 0 0.928019729
DHRS11 ENSG00000278535.4 0.055946434 12 1 0.039833079
Brain_Cerebellum 0.120790322 Brain_Nucleus_accumbens_basal_ganglia
11.62016064 4.60E-05 11.62016064 1.551464861 2.055475717
1.878836387 0.14327835 1 0 0.928019729
GS1-259H13.2 ENSG00000244219.6 0.055982321 7 4 0.003210233
Brain_Putamen_basal_ganglia 0.314956587 Brain_Spinal_cord_cervical_c-1
5.531574638 0.038779534 0.190910834 1.004875949 2.946855357
1.944089897 0.630343866 4 0 0.928019729
ZNF646 ENSG00000167395.10 0.056106954 5 5 0.004236359
Brain_Hippocampus 0.278183809 Brain_Frontal_Cortex_BA9 2.832395033
0.125137815 0.125137815 1.084408291 2.860003801 1.984420865
0.833511454 5 0 0.928853939
POM121 ENSG00000196313.11 0.056173185 9 8 0.02085347
Brain_Caudate_basal_ganglia 0.724180122 Brain_Hippocampus 4.329189743
0.12118092 0.259814456 -0.870677741 2.310626939 0.655476506
1.002819081 8 0 0.928853939
METRNL ENSG00000176845.12 0.056379223 3 3 0.015070934
Brain_Cortex 0.439408046 Brain_Substantia_nigra 1.435287038
0.467039999 0.467039999 -2.43067 -0.773193207 -1.375826082
0.916612487 3 0 0.928853939
CUL5 ENSG00000166266.13 0.05638782 1 1 0.05638782 Brain_Cerebellar_Hemisphere
0.05638782 Brain_Cerebellar_Hemisphere 1 1 1 1.908026276
1.908026276 1.908026276 NA 1 0 0.928853939
RPS18 ENSG00000231500.6 0.056439418 10 4 0.115082658
Brain_Frontal_Cortex_BA9 0.995835812 Brain_Putamen_basal_ganglia
7.784707686 0.027444961 0.376340076 -1.500349353 1.575753346
0.45679286 0.899854173 4 0 0.928853939
MSL2 ENSG00000174579.3 0.056490046 1 1 0.056490046 Brain_Cerebellum
0.056490046 Brain_Cerebellum 1 1 1 -1.907235906 -1.907235906
-1.907235906 NA 1 0 0.928853939
DYNC1LI1 ENSG00000144635.8 0.056746258 1 1 0.056746258
Brain_Spinal_cord_cervical_c-1 0.056746258 Brain_Spinal_cord_cervical_c-1
1 1 1 1.905260184 1.905260184 1.905260184 NA 1 0
0.928853939
SAMD14 ENSG00000167100.14 0.05682789 3 3 0.010598372
Brain_Cerebellar_Hemisphere 0.694641898 Brain_Putamen_basal_ganglia
1.585970321 0.416414778 0.416414778 0.392563506 2.555669515
1.738568567 1.174547391 3 0 0.928853939
COPG2 ENSG00000158623.14 0.056884191 3 3 0.202033434
Brain_Nucleus_accumbens_basal_ganglia 0.963110588 Brain_Cerebellar_Hemisphere
2.110770585 0.114825004 0.114825004 0.046250505 1.275779619

0.811453482 0.667705485 3 0 0.928853939
AP001258.4 ENSG00000245571.6 0.05688441 2 2 0.017667335
Brain_Putamen_basal_ganglia 0.151261857 Brain_Hypothalamus 1.485703681
0.514296319 0.514296319 -2.372517325 -1.435088581 -1.903802953
0.662862222 2 0 0.928853939
PCDH10 ENSG00000138650.8 0.056909698 2 2 0.035574735
Brain_Cortex 0.462325266 Brain_Putamen_basal_ganglia 1.186371492
0.813628508 0.813628508 -0.735023362 2.101755089 0.683365863
2.005905279 2 0 0.928853939
ABHD8 ENSG00000127220.5 0.056924711 1 1 0.056924711
Brain_Caudate_basal_ganglia 0.056924711 Brain_Caudate_basal_ganglia 1 1
1 1.903888468 1.903888468 1.903888468 NA 1 0
0.928853939
GAP43 ENSG00000172020.12 0.056989802 1 1 0.056989802
Brain_Hippocampus 0.056989802 Brain_Hippocampus 1 1 1
-1.903389024 -1.903389024 -1.903389024 NA 1 0 0.928853939
ST13 ENSG00000100380.13 0.057069898 1 1 0.057069898
Brain_Substantia_nigra 0.057069898 Brain_Substantia_nigra 1 1
1 1.902775098 1.902775098 1.902775098 NA 1 0
0.928853939
SHTN1 ENSG00000187164.19 0.05714056 1 1 0.05714056 Brain_Cerebellar_Hemisphere
0.05714056 Brain_Cerebellar_Hemisphere 1 1 1 -1.902234077
-1.902234077 -1.902234077 NA 1 0 0.928853939
LINC01422 ENSG00000235271.5 0.057155156 3 3 0.023453494
Brain_Caudate_basal_ganglia 0.377622885 Brain_Nucleus_accumbens_basal_ganglia
2.291163263 0.236208038 0.236208038 0.882284669 2.265965535
1.663171871 0.70882379 3 0 0.928853939
PDCD4 ENSG00000150593.17 0.057161264 2 2 0.06258757
Brain_Putamen_basal_ganglia 0.209214877 Brain_Hippocampus 1.125890502
0.874109498 0.874109498 -1.255727246 1.862110116 0.303191435
2.204643941 2 0 0.928853939
ADAMTS5 ENSG00000154736.5 0.057361729 2 2 0.016893016
Brain_Cerebellar_Hemisphere 0.036492144 Brain_Cerebellum 1.893543864
0.106456136 0.106456136 2.091400877 2.389027983 2.24021443
0.210454145 2 0 0.928971634
AKIRIN1 ENSG00000174574.15 0.057523475 3 2 0.039396871
Brain_Hypothalamus 0.222585621 Brain_Hippocampus 2.785793882
0.068691243 0.145514875 -2.060017466 -1.219681544 -1.517064514
0.470924173 2 0 0.928971634
MRPS15 ENSG00000116898.11 0.057601661 4 4 0.015022323
Brain_Putamen_basal_ganglia 0.999475746 Brain_Frontal_Cortex_BA9
1.467842534 0.644735279 0.644735279 -2.43184044 0.000657055
-1.188565268 1.029293237 4 0 0.928971634
ARHGEF35 ENSG00000213214.4 0.057626694 5 2 0.082886126
Brain_Substantia_nigra 0.640124582 Brain_Caudate_basal_ganglia
4.708788414 0.00324667 0.177511574 -1.734180137 -0.46752462
-1.065014191 0.452769732 2 0 0.928971634
RP11-545G3.1 ENSG00000241449.5 0.057638615 3 3 0.051461657
Brain_Cerebellum 0.790978556 Brain_Caudate_basal_ganglia 1.149488753
0.850089305 0.850089305 -1.947609909 0.26504012 -1.098068046
1.19237905 3 0 0.928971634
MED19 ENSG00000156603.15 0.057643481 3 3 0.025539876
Brain_Cerebellar_Hemisphere 0.545721371 Brain_Cerebellum 2.539198696
0.170268046 0.170268046 -2.233137542 -0.604183919 -1.422551184
0.814504686 3 0 0.928971634
MYH10 ENSG00000133026.12 0.057859174 1 1 0.057859174
Brain_Hippocampus 0.057859174 Brain_Hippocampus 1 1 1
1.896763384 1.896763384 1.896763384 NA 1 0 0.928971634
NGEF ENSG00000066248.14 0.057869497 1 1 0.057869497
Brain_Spinal_cord_cervical_c-1 0.057869497 Brain_Spinal_cord_cervical_c-1
1 1 1 -1.896685207 -1.896685207 -1.896685207 NA 1 0
0.928971634

MATN2 ENSG00000132561.13 0.057955709 2 2 0.017049126 Brain_Cortex
0.085108231 Brain_Putamen_basal_ganglia 1.700922129 0.299077871
0.299077871 -2.385646603 -1.721786334 -2.053716468 0.469420098
2 0 0.928971634
FBXW8 ENSG00000174989.12 0.057969149 1 1 0.057969149
Brain_Hypothalamus 0.057969149 Brain_Hypothalamus 1 1 1
1.895931152 1.895931152 1.895931152 NA 1 0 0.928971634
BGLAP ENSG00000242252.1 0.058002261 7 4 0.005416007
Brain_Caudate_basal_ganglia 0.685268402 Brain_Substantia_nigra
5.050264012 5.89E-05 0.290359998 -2.781189764 0.405284495
-1.703886136 1.171570875 4 0 0.928971634
NSG1 ENSG00000168824.14 0.058023684 3 3 0.024237336 Brain_Amygdala
0.380365465 Brain_Nucleus_accumbens_basal_ganglia 1.197864485
0.814014524 0.814014524 -1.299294403 2.253346085 0.025609523
1.940784296 3 0 0.928971634
LINC01060 ENSG00000249378.5 0.058067512 2 1 0.056522682
Brain_Substantia_nigra 0.060521775 Brain_Spinal_cord_cervical_c-1
1.993231904 0.006768096 1.993231904 1.876973144 1.906983824
1.891978484 0.021220756 1 0 0.928971634
ATP5J ENSG00000154723.12 0.058333158 1 1 0.058333158
Brain_Frontal_Cortex_BA9 0.058333158 Brain_Frontal_Cortex_BA9 1 1
1 -1.893185854 -1.893185854 -1.893185854 NA 1 0
0.928971634
LINC01102 ENSG00000235597.1 0.058378503 1 1 0.058378503
Brain_Cortex 0.058378503 Brain_Cortex 1 1 1 -1.892844867
-1.892844867 -1.892844867 NA 1 0 0.928971634
MEPCE ENSG00000146834.13 0.058549579 6 3 0.009392115
Brain_Caudate_basal_ganglia 0.814580575 Brain_Spinal_cord_cervical_c-1
4.702078395 0.003183362 0.373134964 0.234520945 2.59744139
0.835127849 0.920984137 3 0 0.928971634
ABI2 ENSG00000138443.15 0.058564867 1 1 0.058564867
Brain_Putamen_basal_ganglia 0.058564867 Brain_Putamen_basal_ganglia 1 1
1 1.891445743 1.891445743 1.891445743 NA 1 0
0.928971634
OPCML ENSG00000183715.13 0.058576003 2 2 0.022769982
Brain_Caudate_basal_ganglia 0.454831254 Brain_Cerebellar_Hemisphere
1.021224605 0.978775395 0.978775395 0.747384906 2.277272099
1.512328502 1.081793608 2 0 0.928971634
SPRR3 ENSG00000163209.14 0.058632825 1 1 0.058632825
Brain_Anterior_cingulate_cortex_BA24 0.058632825
Brain_Anterior_cingulate_cortex_BA24 1 1 1 -1.890936472
-1.890936472 -1.890936472 NA 1 0 0.928971634
RFXANK ENSG00000064490.13 0.058654625 1 1 0.058654625
Brain_Nucleus_accumbens_basal_ganglia 0.058654625
Brain_Nucleus_accumbens_basal_ganglia 1 1 1 -1.890773208
-1.890773208 -1.890773208 NA 1 0 0.928971634
NMUR2 ENSG00000132911.4 0.058703658 2 2 0.020501971
Brain_Frontal_Cortex_BA9 0.105448051 Brain_Cortex 1.830736205
0.169263795 0.169263795 1.618996338 2.317032324 1.968014331
0.493585979 2 0 0.928971634
TEX15 ENSG00000133863.7 0.058780835 2 2 0.091308335
Brain_Caudate_basal_ganglia 0.211997303 Brain_Cortex 1.228493126
0.771506874 0.771506874 -1.248092177 1.688536256 0.22022204
2.076509879 2 0 0.928971634
ADRB3 ENSG00000188778.5 0.05887007 2 2 0.027091589 Brain_Hippocampus
0.354223017 Brain_Frontal_Cortex_BA9 1.019677808 0.980322192
0.980322192 0.926429119 2.210195612 1.568312365 0.907759993
2 0 0.928971634
DPP8 ENSG00000074603.18 0.058881492 2 2 0.017588745
Brain_Spinal_cord_cervical_c-1 0.935235721 Brain_Cerebellum 1.035960206
0.964039794 0.964039794 -2.374163822 -0.081259325 -1.227711574
1.621328319 2 0 0.928971634

RBBP5 ENSG00000117222.13 0.058882761 6 4 0.003331324 Brain_Cerebellum
0.406001184 Brain_Spinal_cord_cervical_c-1 3.733719889 0.027474165
0.267919351 0.830951225 2.935386549 2.023150748 0.8104669 4
0 0.928971634
TAOK2 ENSG00000149930.17 0.05900236 2 2 0.03079282
Brain_Anterior_cingulate_cortex_BA24 0.409875004 Brain_Frontal_Cortex_BA9
1.079038303 0.920961697 0.920961697 -0.824113617 2.159739781
0.667813082 2.109902972 2 0 0.928971634
HIRA ENSG00000100084.14 0.059248198 3 3 0.071474881 Brain_Cortex
0.443960592 Brain_Spinal_cord_cervical_c-1 1.435414567 0.556873198
0.556873198 -0.860587252 1.802448433 0.058779627 1.510809391
3 0 0.928971634
ENGASE ENSG00000167280.16 0.059427285 7 6 0.112505233
Brain_Frontal_Cortex_BA9 0.573144887 Brain_Putamen_basal_ganglia
4.608960154 0.021337473 0.157529117 -1.587032724 1.068888339
-0.699937691 0.860914141 6 0 0.928971634
C16orf89 ENSG00000153446.15 0.059454918 1 1 0.059454918
Brain_Cerebellar_Hemisphere 0.059454918 Brain_Cerebellar_Hemisphere 1 1
1 -1.884814264 -1.884814264 -1.884814264 NA 1 0
0.928971634
C17orf62 ENSG00000178927.17 0.059629391 10 3 0.075129343
Brain_Frontal_Cortex_BA9 0.614146339 Brain_Hippocampus 8.435954867
0.000114251 0.471594692 -0.504163711 1.77967391 1.097265589
0.628648167 3 0 0.928971634
EXOC3 ENSG00000180104.15 0.059669979 13 6 0.015585455
Brain_Caudate_basal_ganglia 0.963621892 Brain_Putamen_basal_ganglia
8.186669927 0.014664041 0.353395492 -0.584383831 2.418481114
0.465047624 0.899846824 6 0 0.928971634
NPTXR ENSG00000221890.3 0.05979547 2 2 0.135391413 Brain_Cerebellum
0.197446655 Brain_Caudate_basal_ganglia 1.311134771 0.688865229
0.688865229 -1.288860308 1.493174892 0.102157292 1.967195956
2 0 0.928971634
RP11-77K12.10 ENSG00000273971.1 0.059852831 1 1 0.059852831
Brain_Putamen_basal_ganglia 0.059852831 Brain_Putamen_basal_ganglia 1 1
1 -1.88187617 -1.88187617 -1.88187617 NA 1 0
0.928971634
VPS13B ENSG00000132549.18 0.05996898 1 1 0.05996898
Brain_Cerebellar_Hemisphere 0.05996898 Brain_Cerebellar_Hemisphere 1 1 1
-1.881021603 -1.881021603 -1.881021603 NA 1 0 0.928971634
RP11-567C2.1 ENSG00000257194.2 0.059975998 4 4 0.05702996
Brain_Cerebellar_Hemisphere 0.435880445 Brain_Nucleus_accumbens_basal_ganglia
2.490283103 0.084358565 0.084358565 -1.119679451 1.903081129
0.328102628 1.501468851 4 0 0.928971634
CEP70 ENSG00000114107.8 0.060209666 1 1 0.060209666 Brain_Cortex
0.060209666 Brain_Cortex 1 1 1 1.879255121 1.879255121
1.879255121 NA 1 0 0.928971634
VKORC1 ENSG00000167397.14 0.060245332 3 3 0.030144832
Brain_Cortex 0.71480625 Brain_Frontal_Cortex_BA9 1.961192862
0.415917561 0.415917561 -2.168182124 0.365408832 -1.142826742
1.33403551 3 0 0.928971634
SEMA5B ENSG00000082684.14 0.060322315 6 6 0.048088683
Brain_Cortex 0.865955015 Brain_Frontal_Cortex_BA9 2.034878091
0.390203204 0.390203204 -1.976583904 0.714577984 -0.457258675
1.142802977 6 0 0.928971634
RP11-259K5.1 ENSG00000271653.1 0.060341966 1 1 0.060341966
Brain_Hippocampus 0.060341966 Brain_Hippocampus 1 1 1
-1.87828662 -1.87828662 -1.87828662 NA 1 0 0.928971634
KATNAL2 ENSG00000167216.16 0.060347369 3 3 0.020435269
Brain_Putamen_basal_ganglia 0.885153187 Brain_Nucleus_accumbens_basal_ganglia
1.59470131 0.565999011 0.565999011 -0.144439804 2.318258643
1.197901467 1.246265868 3 0 0.928971634
LIN7B ENSG00000104863.11 0.060451594 3 3 0.014666965 Brain_Cortex

0.792080244	Brain_Cerebellar_Hemisphere 1.67019507 0.648565171
0.648565171	-0.26361027	2.440499536	0.928730401	1.380064625
3	0	0.928971634
ZNF417	ENSG00000173480.10	0.060561399	11	7	0.033868459
Brain_Hippocampus	0.97608986 Brain_Frontal_Cortex_BA9	6.443779275
0.051429114	0.397885511	-2.12163431	1.296340427	-0.550028038
1.014867958	7	0	0.928971634
PPM1F ENSG00000100034.13	0.060589003	7	5	0.013765227
Brain_Nucleus_accumbens_basal_ganglia 0.831858019	Brain_Cerebellum
3.873063577	0.006683771	0.271942957	0.212319198	2.463332319
1.243740261	0.947833325	5	0	0.928971634
KCND3 ENSG00000171385.9	0.060608929	1	1	0.060608929
Brain_Substantia_nigra	0.060608929	Brain_Substantia_nigra	1	1
1	1.87633766 1.87633766 1.87633766 NA	1	0	0.928971634
FOXS1 ENSG00000179772.7	0.060782688	2	2	0.460002975	Brain_Cerebellum
0.747149332	Brain_Caudate_basal_ganglia 1.898632867	0.101367133
0.101367133	-0.738841951	0.322400465	-0.208220743	0.750411708
2	0	0.928971634
EFCAB13	ENSG00000178852.15	0.06109238 13	6	0.002471741
Brain_Hypothalamus	0.507144571	Brain_Caudate_basal_ganglia 8.293727747
0.007847353	0.28763634 0.66329042 3.026779561	2.000762221
0.718369379	6	0	0.928971634
KCNJ3 ENSG00000162989.4	0.061095501	7	4	0.072117776
Brain_Frontal_Cortex_BA9	0.642937705	Brain_Amygdala	5.239643981
0.012616425	0.187860257	-0.733588502	1.798373902	0.129145042
0.944354752	4	0	0.928971634
RBM18 ENSG00000119446.13	0.061160878	2	2	0.023669161	Brain_Amygdala
0.45649753 Brain_Caudate_basal_ganglia 1.026279805	0.973720195
0.973720195	-0.744626522	2.262457357	0.758915418	2.126329403
2	0	0.928971634
RAC3	ENSG00000169750.8	0.061160941	3	3	0.040629587
Brain_Putamen_basal_ganglia 0.228820699	Brain_Nucleus_accumbens_basal_ganglia
1.399784104	0.46689659 0.46689659 -2.047290325	1.289305926
0.148472616	1.90207151 3	0	0.928971634
STRA6 ENSG00000137868.18	0.061337106	2	2	0.019333842
Brain_Hippocampus	0.500267881	Brain_Cortex	1.146861096
0.853138904	0.853138904	-2.339030758	-0.674068317	-1.506549538
1.177306233	2	0	0.928971634
CLIP3 ENSG00000105270.14	0.061341901	1	1	0.061341901
Brain_Caudate_basal_ganglia 0.061341901	Brain_Caudate_basal_ganglia 1	1
1	1.87102294 1.87102294 1.87102294 NA	1	0	0.928971634
STIP1 ENSG00000168439.16	0.061362165	4	4	0.012652436
Brain_Nucleus_accumbens_basal_ganglia 0.856873629	Brain_Amygdala
1.446871255	0.604018179	0.604018179	0.18035532 2.49340522
1.450280666	1.051094413	4	0	0.928971634
NPY1R ENSG00000164128.6	0.061410815	1	1	0.061410815
Brain_Hypothalamus	0.061410815	Brain_Hypothalamus	1	1	1
-1.870525961	-1.870525961	-1.870525961	NA	1	0	0.928971634
RP11-1055B8.10	ENSG00000274833.1	0.061467621	2	2	0.062144232
Brain_Cerebellar_Hemisphere 0.472598427	Brain_Cerebellum 1.824554508
0.175445492	0.175445492	0.718257666	1.865265263	1.291761465
0.81105685 2	0	0.928971634
BRD2	ENSG00000204256.12	0.06150519 7	5	0.046870799
Brain_Anterior_cingulate_cortex_BA24	0.871624656	Brain_Frontal_Cortex_BA9
4.056581207	0.001757917	0.173300044	-1.987465832	1.293918747
0.108700931	1.418184582	5	0	0.928971634
ANKRD31	ENSG00000145700.9	0.061510284	2	2	0.04630218 Brain_Cortex
0.15216121 Brain_Hypothalamus	1.084820341	0.915179659	0.915179659
-1.431939244	1.99262815 0.280344453	2.421534827	2	0
0.928971634
KCNK7 ENSG00000173338.12	0.061532303	3	2	0.03589808 Brain_Hypothalamus
0.589835925	Brain_Caudate_basal_ganglia 2.640683726	0.046761718

0.312554556 -2.098079989 -0.539073811 -1.249155997 0.78872229 2
0 0.928971634
L3MBTL3 ENSG00000198945.7 0.061744943 5 5 0.012479795
Brain_Cortex 0.805273351 Brain_Spinal_cord_cervical_c-1 2.282602235
0.312711608 0.312711608 -0.246528234 2.498278955 1.511553591
1.208242755 5 0 0.928971634
TSPAN4 ENSG00000214063.10 0.061791547 6 6 0.11109676
Brain_Nucleus_accumbens_basal_ganglia 0.930765091 Brain_Cerebellum
2.613530759 0.285128199 0.285128199 -1.201729461 1.593282817
0.18227495 1.123053694 6 0 0.928971634
ENPP4 ENSG00000001561.6 0.061829339 7 5 0.203293273 Brain_Cerebellum
0.988785354 Brain_Caudate_basal_ganglia 5.255912758 0.021520502
0.228767392 -1.272224759 0.429015882 -0.287232299 0.645060919
5 0 0.928971634
RGS22 ENSG00000132554.19 0.061849117 1 1 0.061849117
Brain_Hippocampus 0.061849117 Brain_Hippocampus 1 1 1
-1.867375874 -1.867375874 -1.867375874 NA 1 0 0.928971634
RP11-347I19.7 ENSG00000274292.1 0.061869973 8 2 0.010169088
Brain_Cerebellar_Hemisphere 0.170251127 Brain_Putamen_basal_ganglia
7.187980498 0.003093209 0.600807401 1.371397336 2.570025993
2.202801419 0.363351752 2 0 0.928971634
RP6-102O10.1 ENSG00000227935.1 0.061900345 2 2 0.020182254
Brain_Cerebellum 0.027719433 Brain_Cerebellar_Hemisphere 1.856394292
0.143605708 0.143605708 -2.322942266 -2.201234417 -2.262088342
0.086060446 2 0 0.928971634
SAA4 ENSG00000148965.9 0.061921775 1 1 0.061921775
Brain_Anterior_cingulate_cortex_BA24 0.061921775
Brain_Anterior_cingulate_cortex_BA24 1 1 1 -1.866855462
-1.866855462 -1.866855462 NA 1 0 0.928971634
SYPL2 ENSG00000143028.8 0.06201333 3 2 0.037749138 Brain_Cortex
0.205892931 Brain_Cerebellum 2.818799897 0.030216421 0.150983682
-2.077568308 -1.264939737 -1.538329711 0.467009546 2 0
0.928971634
LCK ENSG00000182866.16 0.062104519 1 1 0.062104519
Brain_Cerebellar_Hemisphere 0.062104519 Brain_Cerebellar_Hemisphere 1 1
1 -1.865548794 -1.865548794 -1.865548794 NA 1 0
0.928971634
RP11-16L9.2 ENSG00000225172.5 0.062136537 1 1 0.062136537
Brain_Nucleus_accumbens_basal_ganglia 0.062136537
Brain_Nucleus_accumbens_basal_ganglia 1 1 1 1.86532019 1.86532019
1.86532019 NA 1 0 0.928971634
RIMS3 ENSG00000117016.9 0.062193503 1 1 0.062193503
Brain_Nucleus_accumbens_basal_ganglia 0.062193503
Brain_Nucleus_accumbens_basal_ganglia 1 1 1 -1.864913689
-1.864913689 -1.864913689 NA 1 0 0.928971634
SEC23IP ENSG00000107651.12 0.062269615 1 1 0.062269615
Brain_Caudate_basal_ganglia 0.062269615 Brain_Caudate_basal_ganglia 1 1
1 -1.864371053 -1.864371053 -1.864371053 NA 1 0
0.928971634
GUCY2D ENSG00000132518.6 0.062270005 2 2 0.038660763
Brain_Caudate_basal_ganglia 0.271260873 Brain_Putamen_basal_ganglia
1.014359792 0.985640208 0.985640208 -2.067779494 -1.10016354
-1.583971517 0.684207803 2 0 0.928971634
LRP12 ENSG00000147650.11 0.062299786 3 3 0.007595366 Brain_Cerebellum
0.073610329 Brain_Cerebellar_Hemisphere 2.562242733 0.190306011
0.190306011 -2.669546902 -1.789027857 -2.204243057 0.442391344
3 0 0.928971634
ARPC4 ENSG00000241553.12 0.062348133 1 1 0.062348133
Brain_Cerebellar_Hemisphere 0.062348133 Brain_Cerebellar_Hemisphere 1 1
1 1.863811838 1.863811838 1.863811838 NA 1 0
0.928971634
RP11-486G15.2 ENSG00000271576.1 0.062432714 2 2 0.021892531

Brain_Cerebellar_Hemisphere 0.5836914 Brain_Cortex 1.449965456
0.550034544 0.550034544 0.548000733 2.292227293 1.420114013
1.233354429 2 0 0.928971634
LINC00290 ENSG00000248197.1 0.06252893 6 5 0.02813745 Brain_Cortex
0.434382857 Brain_Hippocampus 3.158142634 0.040998346
0.306914936 -0.850186263 2.195364654 1.1002467 1.060621511 5
0 0.928971634
COG4 ENSG00000103051.18 0.06259392 8 7 0.003604341
Brain_Caudate_basal_ganglia 0.687449574 Brain_Cortex 3.204270796
0.000134377 0.200608461 -2.910861217 0.879893437 -1.567892827
1.498890868 7 0 0.928971634
ALAD ENSG00000148218.15 0.062604004 7 7 0.011020783
Brain_Spinal_cord_cervical_c-1 0.824088909 Brain_Cerebellum 1.904313371
0.302014054 0.302014054 -1.285391235 2.542039146 0.711548972
1.249843304 7 0 0.928971634
OAZ1 ENSG00000104904.12 0.062644454 2 2 0.022201667
Brain_Spinal_cord_cervical_c-1 0.561782365 Brain_Cortex 1.009998224
0.990001776 0.990001776 -0.580196127 2.286899894 0.853351884
2.027343039 2 0 0.928971634
ATF7IP2 ENSG00000166669.13 0.06279937 4 4 0.030080186
Brain_Caudate_basal_ganglia 0.78544043 Brain_Nucleus_accumbens_basal_ganglia
2.872180519 0.103488506 0.103488506 -0.462727798 2.169032902
0.843135525 1.167998504 4 0 0.928971634
RP11-157J24.2 ENSG00000272279.1 0.062835043 13 4 0.183216407
Brain_Spinal_cord_cervical_c-1 0.937014442 Brain_Cerebellar_Hemisphere
10.04017144 8.43E-05 0.376917998 -1.330916744 0.806360019
-0.13628609 0.716443393 4 0 0.928971634
PCYT1A ENSG00000161217.11 0.062990401 8 6 0.057199846
Brain_Frontal_Cortex_BA9 0.939546623 Brain_Cerebellar_Hemisphere
4.131891803 8.08E-05 0.172487989 -0.426295045 1.901780583
0.797768569 1.137517068 6 0 0.928971634
C8orf48 ENSG00000164743.4 0.06300209 1 1 0.06300209
Brain_Nucleus_accumbens_basal_ganglia 0.06300209
Brain_Nucleus_accumbens_basal_ganglia 1 1 1 1.859176743
1.859176743 1.859176743 NA 1 0 0.928971634
NDUFS3 ENSG00000213619.9 0.063010372 3 2 0.186477147
Brain_Cerebellar_Hemisphere 0.837329039 Brain_Cerebellum 2.578011675
0.066693704 0.355294621 -0.519946726 1.321072633 0.198604922
0.984733065 2 0 0.928971634
LINC00320 ENSG00000224924.6 0.063076779 12 2 0.035832988
Brain_Anterior_cingulate_cortex_BA24 0.860220646 Brain_Cerebellum
10.49825193 0.002214505 0.520465185 -2.098817539 -0.176093298
-1.312125014 0.464007011 2 0 0.928971634
STRADA ENSG00000266173.6 0.063201647 5 5 0.018137744
Brain_Hippocampus 0.868939792 Brain_Substantia_nigra 2.189957073
0.194565456 0.194565456 -2.36279409 0.665783538 -0.553740568
1.121753866 5 0 0.928971634
IMPACT ENSG00000154059.9 0.063232719 2 2 0.020582341
Brain_Hypothalamus 0.140254625 Brain_Frontal_Cortex_BA9 1.485924326
0.514075674 0.514075674 -2.315559345 -1.474843491 -1.895201418
0.594475881 2 0 0.928971634
LINC00294 ENSG00000280798.1 0.063323245 6 3 0.064066076
Brain_Hippocampus 0.692124884 Brain_Spinal_cord_cervical_c-1
4.127968873 0.01946189 0.674852682 -1.671182718 1.851719751
0.658776669 1.40586402 3 0 0.928971634
HPX ENSG00000110169.10 0.063430093 4 4 0.044935845
Brain_Hypothalamus 0.656843167 Brain_Cerebellum 1.471383491
0.611748633 0.611748633 -2.005254511 1.841126445 0.429321763
1.725907864 4 0 0.928971634
LA16c-325D7.2 ENSG00000263280.1 0.063507122 13 1 0.035611208
Brain_Cerebellar_Hemisphere 0.168638896 Brain_Cortex 11.9810621
0.002972111 11.9810621 1.376590479 2.101339114 1.782504022

0.239600279 1 0 0.928971634
SH3YL1 ENSG00000035115.21 0.063535429 4 4 0.004702836
Brain_Nucleus_accumbens_basal_ganglia 0.997859785 Brain_Substantia_nigra
2.077516689 0.226284251 0.226284251 0.002682365 2.826713629
1.818905625 1.262877808 4 0 0.928971634
ZNF787 ENSG00000142409.5 0.063731409 1 1 0.063731409
Brain_Cortex 0.063731409 Brain_Cortex 1 1 1 1.85405418
1.85405418 1.85405418 NA 1 0 0.928971634
CTB-75G16.3 ENSG00000275720.1 0.063732635 10 4 0.05006158
Brain_Cerebellar_Hemisphere 0.757993552 Brain_Caudate_basal_ganglia
5.709139444 3.50E-21 0.708319163 -1.959437434 1.643620234
-0.737782583 1.212765824 4 0 0.928971634
TFAP2E ENSG00000116819.6 0.063744535 1 1 0.063744535
Brain_Anterior_cingulate_cortex_BA24 0.063744535
Brain_Anterior_cingulate_cortex_BA24 1 1 1 -1.853962431
-1.853962431 -1.853962431 NA 1 0 0.928971634
FLT3LG ENSG00000090554.12 0.063904776 1 1 0.063904776
Brain_Amygdala 0.063904776 Brain_Amygdala 1 1 1 1.852843619
1.852843619 1.852843619 NA 1 0 0.928971634
GPER1 ENSG00000164850.14 0.063927896 2 2 0.020356553
Brain_Cerebellar_Hemisphere 0.651303653 Brain_Anterior_cingulate_cortex_BA24
1.334918981 0.665081019 0.665081019 -2.319710317 -0.451951874
-1.385831096 1.320704661 2 0 0.928971634
LGALS8 ENSG00000116977.18 0.063956798 5 4 0.006603953
Brain_Caudate_basal_ganglia 0.82638993 Brain_Hippocampus 2.718525153
0.080729082 0.347298786 -2.716182365 0.487163401 -0.887023731
1.334667254 4 0 0.928971634
LINC01945 ENSG00000236485.1 0.064094409 10 10 0.018122252
Brain_Substantia_nigra 0.607478939 Brain_Cortex 2.333999474
0.376114385 0.376114385 -2.36311076 1.335791483 0.12844303
1.370293051 10 0 0.928971634
HIST1H2BD ENSG00000158373.8 0.064098073 2 2 0.025747131
Brain_Cortex 0.320981502 Brain_Hippocampus 1.123610672
0.876389328 0.876389328 0.992442938 2.230004698 1.611223818
0.875088313 2 0 0.928971634
MARCKSL1 ENSG00000175130.6 0.064236887 2 2 0.0249619
Brain_Putamen_basal_ganglia 0.624654348 Brain_Amygdala 1.084456686
0.915543314 0.915543314 -2.241991841 0.489264644 -0.876363598
1.931289982 2 0 0.928971634
KLRC2 ENSG00000205809.9 0.064420143 13 1 0.034139973 Brain_Cerebellum
0.155722965 Brain_Frontal_Cortex_BA9 12.46430078 0.00129551
12.46430078 1.41960412 2.118414572 1.809733626 0.210019571 1
0 0.928971634
TOR2A ENSG00000160404.17 0.064501056 2 2 0.37038712 Brain_Cerebellum
0.985340975 Brain_Cerebellar_Hemisphere 1.92690023 0.07309977 0.07309977
-0.895748464 -0.018373396 -0.45706093 0.62039786 2 0
0.928971634
TBC1D30 ENSG00000111490.12 0.064536144 1 1 0.064536144
Brain_Amygdala 0.064536144 Brain_Amygdala 1 1 1 -1.848457797
-1.848457797 -1.848457797 NA 1 0 0.928971634
NUDC ENSG00000090273.13 0.064605102 10 4 0.025706669
Brain_Hypothalamus 0.995337693 Brain_Putamen_basal_ganglia 7.021944036
0.00412324 0.235688958 -0.231417206 2.230614598 0.546927943
0.711895689 4 0 0.928971634
TIA1 ENSG00000116001.15 0.06468102 2 2 0.035146195 Brain_Amygdala
0.169778798 Brain_Hippocampus 1.175466076 0.824533924
0.824533924 -2.106669996 -1.37291492 -1.739792458 0.51884319 2
0 0.928971634
PPIF ENSG00000108179.13 0.064690569 2 2 0.019417584
Brain_Substantia_nigra 0.999368468 Brain_Spinal_cord_cervical_c-1
1.048623504 0.951376496 0.951376496 -2.337415606 -0.000791509
-1.169103557 1.652242744 2 0 0.928971634

AP2A2 ENSG00000183020.13 0.064789625 11 6 0.008055672
Brain_Hypothalamus 0.629817105 Brain_Cerebellum 7.038686237
0.001430457 0.25822377 -2.649727476 2.036265361 -1.597048834
1.477856957 6 0 0.928971634
C19orf12 ENSG00000131943.17 0.064792796 2 2 0.06597937
Brain_Cerebellar_Hemisphere 0.067192552 Brain_Substantia_nigra
1.229762911 0.770237089 0.770237089 1.830383911 1.8385638
1.834473855 0.005784055 2 0 0.928971634
SULT1A4 ENSG00000213648.10 0.064848962 2 2 0.056371545
Brain_Spinal_cord_cervical_c-1 0.144193497 Brain_Hippocampus
1.057483115 0.942516885 0.942516885 1.460351493 1.908152221
1.684251857 0.316642931 2 0 0.928971634
CCDC148 ENSG00000153237.17 0.06485878 5 5 0.005377565
Brain_Frontal_Cortex_BA9 0.725223044 Brain_Hypothalamus 2.529881604
0.193132403 0.193132403 -2.783501294 -0.351486973 -1.319425221
0.95713863 5 0 0.928971634
MFSD6L ENSG00000185156.5 0.064864615 1 1 0.064864615
Brain_Caudate_basal_ganglia 0.064864615 Brain_Caudate_basal_ganglia 1 1
1 -1.846190049 -1.846190049 -1.846190049 NA 1 0
0.928971634
KCTD17 ENSG00000100379.17 0.064873524 1 1 0.064873524
Brain_Putamen_basal_ganglia 0.064873524 Brain_Putamen_basal_ganglia 1 1
1 1.846128675 1.846128675 1.846128675 NA 1 0
0.928971634
DEF8 ENSG00000140995.16 0.065012541 5 5 0.013771675
Brain_Nucleus_accumbens_basal_ganglia 0.333515664 Brain_Putamen_basal_ganglia
2.88754707 0.145202249 0.145202249 -2.4631644 1.914798917
-1.178860256 1.825421599 5 0 0.928971634
PDGFRL ENSG00000104213.12 0.065106497 5 5 0.003019447
Brain_Cortex 0.907324921 Brain_Cerebellum 1.738589695 0.390300991
0.390300991 -2.965751159 0.626440428 -0.881974364 1.632672116
5 0 0.928971634
ZBTB41 ENSG00000177888.7 0.065144227 3 3 0.031603933
Brain_Nucleus_accumbens_basal_ganglia 0.130524448 Brain_Hypothalamus
1.556444633 0.446963307 0.446963307 1.512037009 2.149384499
1.853881212 0.321190856 3 0 0.928971634
GPR176 ENSG00000166073.10 0.065240699 1 1 0.065240699
Brain_Cerebellum 0.065240699 Brain_Cerebellum 1 1 1 1.843605187
1.843605187 1.843605187 NA 1 0 0.928971634
YJEFN3 ENSG00000250067.11 0.065244111 3 3 0.009357394
Brain_Cerebellar_Hemisphere 0.730801383 Brain_Cortex 1.767547522
0.248548904 0.248548904 -2.598713132 -0.344059708 -1.476192795
1.12735745 3 0 0.928971634
RTP4 ENSG00000136514.2 0.065257207 6 6 0.052621908
Brain_Spinal_cord_cervical_c-1 0.994137755 Brain_Cerebellar_Hemisphere
2.65993018 0.182085538 0.182085538 -1.938010773 1.308680504
-0.053521679 1.096634968 6 0 0.928971634
CENPX ENSG00000169689.14 0.065342222 13 1 0.044783737
Brain_Hypothalamus 0.157095404 Brain_Frontal_Cortex_BA9 12.45558793
0.000204001 12.45558793 -2.006680093 -1.414908231 -1.803488628
0.186848007 1 0 0.928971634
TMEM161A ENSG00000064545.14 0.065419091 5 4 0.011529813
Brain_Cortex 0.370625119 Brain_Caudate_basal_ganglia 2.756768889
0.080322743 0.274391281 -2.526217342 -0.895303033 -1.900105507
0.614542607 4 0 0.928971634
TMC6 ENSG00000141524.15 0.065461489 4 4 0.034103097
Brain_Hippocampus 0.991463289 Brain_Caudate_basal_ganglia 2.624522345
0.147645775 0.147645775 -1.183088073 2.118850581 0.451950976
1.392145942 4 0 0.928971634
SCARA3 ENSG00000168077.13 0.065548639 1 1 0.065548639
Brain_Frontal_Cortex_BA9 0.065548639 Brain_Frontal_Cortex_BA9 1 1
1 -1.841497822 -1.841497822 -1.841497822 NA 1 0

0.928971634
B3GNTL1 ENSG00000175711.8 0.06559904 12 8 0.003010386
Brain_Putamen_basal_ganglia 0.506319525 Brain_Spinal_cord_cervical_c-1
7.274949454 0.08447498 0.281800339 -2.966675428 -0.664579437
-1.970897738 0.711003617 8 0 0.928971634
CLEC4G ENSG00000182566.13 0.065616876 6 6 0.008717647
Brain_Nucleus_accumbens_basal_ganglia 0.990742094 Brain_Cerebellum
1.884272317 0.173645362 0.173645362 -2.622932766 0.659169465
-0.878664945 1.355568671 6 0 0.928971634
ACMSD ENSG00000153086.13 0.065698323 1 1 0.065698323
Brain_Cerebellar_Hemisphere 0.065698323 Brain_Cerebellar_Hemisphere 1 1
1 1.840476419 1.840476419 1.840476419 NA 1 0
0.928971634
COPS8 ENSG00000198612.10 0.065760018 1 1 0.065760018
Brain_Frontal_Cortex_BA9 0.065760018 Brain_Frontal_Cortex_BA9 1 1
1 1.840055988 1.840055988 1.840055988 NA 1 0
0.928971634
LINC01976 ENSG00000261514.1 0.065923482 1 1 0.065923482
Brain_Frontal_Cortex_BA9 0.065923482 Brain_Frontal_Cortex_BA9 1 1
1 -1.838943598 -1.838943598 -1.838943598 NA 1 0
0.928971634
PRSS36 ENSG00000178226.10 0.065925162 11 1 0.028904304
Brain_Amygdala 0.172947449 Brain_Hippocampus 10.7381063 0.00033989
10.7381063 1.36279398 2.184789228 1.817644257 0.227461194 1 0
0.928971634
CCT6B ENSG00000132141.13 0.06600554 11 5 0.127729072
Brain_Putamen_basal_ganglia 0.888915022 Brain_Anterior_cingulate_cortex_BA24
8.063293904 0.003094608 0.328812397 -1.523118455 1.065099639
-0.474222676 0.665485225 5 0 0.928971634
YAF2 ENSG00000015153.14 0.066091379 2 2 0.030353973
Brain_Caudate_basal_ganglia 0.370852857 Brain_Anterior_cingulate_cortex_BA24
1.014890007 0.985109993 0.985109993 -2.16544043 0.894876974
-0.635281728 2.163971189 2 0 0.928971634
LINC00184 ENSG00000224939.1 0.066122905 11 9 0.011105585
Brain_Putamen_basal_ganglia 0.896581335 Brain_Hippocampus 4.703515057
0.02440998 0.176230187 -2.539358854 0.915095706 -0.334230458
1.08510219 9 0 0.928971634
RXFP4 ENSG00000173080.5 0.066139894 1 1 0.066139894 Brain_Cerebellum
0.066139894 Brain_Cerebellum 1 1 1 1.837474391 1.837474391
1.837474391 NA 1 0 0.928971634
LINC02082 ENSG00000242268.2 0.066263192 2 2 0.022404675
Brain_Nucleus_accumbens_basal_ganglia 0.119360846 Brain_Caudate_basal_ganglia
1.507434392 0.492565608 0.492565608 -2.283436396 -1.557461956
-1.920449176 0.51334145 2 0 0.928971634
RHCE ENSG00000188672.17 0.066326025 13 2 0.05211985
Brain_Substantia_nigra 0.978899031 Brain_Cortex 12.13062655
0.000738591 0.557223412 -1.942142515 0.026449227 -1.550757335
0.487945293 2 0 0.928971634
ZC3H13 ENSG00000123200.16 0.066398397 1 1 0.066398397
Brain_Cerebellum 0.066398397 Brain_Cerebellum 1 1 1 -1.835724605
-1.835724605 -1.835724605 NA 1 0 0.928971634
RSF1 ENSG00000048649.13 0.066463865 1 1 0.066463865
Brain_Cerebellar_Hemisphere 0.066463865 Brain_Cerebellar_Hemisphere 1 1
1 -1.835282346 -1.835282346 -1.835282346 NA 1 0
0.928971634
SUCLA2 ENSG00000136143.14 0.066484899 6 3 0.007738429
Brain_Cerebellar_Hemisphere 0.090416538 Brain_Frontal_Cortex_BA9
4.795065141 0.020899419 0.212987374 -2.663274365 -1.693204556
-2.297666265 0.351911693 3 0 0.928971634
C8orf58 ENSG00000241852.9 0.066518621 8 4 0.066803836
Brain_Cerebellum 0.963169776 Brain_Hypothalamus 6.524162622
0.004956169 0.233880484 -0.046176245 1.832991495 1.239722492

0.586599338 4 0 0.928971634
CERKL ENSG00000188452.13 0.066530047 1 1 0.066530047
Brain_Cerebellar_Hemisphere 0.066530047 Brain_Cerebellar_Hemisphere 1 1
1 -1.834835634 -1.834835634 -1.834835634 NA 1 0
0.928971634
RGS12 ENSG00000159788.18 0.066709424 6 6 0.011499206 Brain_Cortex
0.788734437 Brain_Cerebellar_Hemisphere 2.746888985 0.096666363
0.096666363 -2.527150959 0.75842761 -0.380394585 1.357612153 6
0 0.928971634
SLC51A ENSG00000163959.9 0.06680607 12 4 0.005920781
Brain_Spinal_cord_cervical_c-1 0.19803667 Brain_Substantia_nigra
9.870671717 1.59E-05 0.347089992 -2.752136633 -1.287165326
-2.05218542 0.394495411 4 0 0.928971634
APOE ENSG00000130203.9 0.066829707 1 1 0.066829707
Brain_Spinal_cord_cervical_c-1 0.066829707 Brain_Spinal_cord_cervical_c-1
1 1 1 -1.832817559 -1.832817559 -1.832817559 NA 1 0
0.928971634
MIER2 ENSG00000105556.11 0.066832631 2 2 0.065655462
Brain_Spinal_cord_cervical_c-1 0.764268825 Brain_Cortex 1.708306743
0.291693257 0.291693257 -1.840768694 -0.299879823 -1.070324259
1.08957297 2 0 0.928971634
RP11-700H6.1 ENSG00000247011.2 0.06692527 2 1 0.057872331
Brain_Spinal_cord_cervical_c-1 0.084534783 Brain_Caudate_basal_ganglia
1.953630204 0.046369796 1.953630204 -1.896663751 -1.724959437
-1.810811594 0.121413285 1 0 0.928971634
CYP20A1 ENSG00000119004.15 0.066934601 1 1 0.066934601
Brain_Frontal_Cortex_BA9 0.066934601 Brain_Frontal_Cortex_BA9 1 1
1 1.832112908 1.832112908 1.832112908 NA 1 0
0.928971634
SAMD1 ENSG00000141858.11 0.066940487 2 2 0.032844525
Brain_Cerebellar_Hemisphere 0.3136945 Brain_Nucleus_accumbens_basal_ganglia
1.039398774 0.960601226 0.960601226 1.00750012 2.133978696
1.570739408 0.79654064 2 0 0.928971634
ZFP91 ENSG00000186660.14 0.066964231 2 2 0.068224837
Brain_Caudate_basal_ganglia 0.083521478 Brain_Spinal_cord_cervical_c-1
1.169180941 0.830819059 0.830819059 1.730609266 1.823518898
1.777064082 0.065697031 2 0 0.928971634
ABLIM1 ENSG00000099204.18 0.066964424 2 2 0.092070508
Brain_Substantia_nigra 0.108670862 Brain_Cerebellum 1.000750203
0.999249797 0.999249797 -1.604195974 1.684575465 0.040189745
2.325512587 2 0 0.928971634
BACH1 ENSG00000156273.15 0.067000978 2 2 0.033000607
Brain_Anterior_cingulate_cortex_BA24 0.365541235
Brain_Spinal_cord_cervical_c-1 1.010619021 0.989380979 0.989380979
-0.904856839 2.132075908 0.613609534 2.14743574 2 0
0.928971634
ARHGEF10 ENSG00000104728.15 0.067118887 7 6 0.131424863
Brain_Hypothalamus 0.948312796 Brain_Caudate_basal_ganglia 2.93193654
0.000125453 0.254442641 -0.87275149 1.508506819 0.40202576
0.884056456 6 0 0.928971634
NUDT17 ENSG00000186364.11 0.067127589 3 2 0.058830573
Brain_Anterior_cingulate_cortex_BA24 0.174658634 Brain_Hypothalamus
2.000038479 1.70E-16 0.999961521 1.357385874 1.889457345
1.540580924 0.302262717 2 0 0.928971634
ARAP2 ENSG00000047365.11 0.067128177 2 2 0.1526499 Brain_Cortex
0.244619588 Brain_Spinal_cord_cervical_c-1 1.374347278 0.625652722
0.625652722 -1.430233895 1.163517535 -0.13335818 1.834059225
2 0 0.928971634
PSMA6 ENSG00000100902.10 0.067320923 1 1 0.067320923
Brain_Cerebellar_Hemisphere 0.067320923 Brain_Cerebellar_Hemisphere 1 1
1 1.829525497 1.829525497 1.829525497 NA 1 0
0.930718416

PARK7 ENSG00000116288.12 0.067527724 4 4 0.06933594
Brain_Putamen_basal_ganglia 0.636388444 Brain_Hippocampus 1.471117583
0.518495358 0.518495358 -1.816224508 0.758828401 -0.770208598
1.173275217 4 0 0.931227594
TNFSF4 ENSG00000117586.10 0.067557794 2 2 0.035173718
Brain_Nucleus_accumbens_basal_ganglia 0.616666615 Brain_Cerebellum
1.214936296 0.785063704 0.785063704 -0.500580179 2.106352803
0.802886312 1.84337999 2 0 0.931227594
TUBAL3 ENSG00000178462.11 0.067624796 2 2 0.530095468
Brain_Frontal_Cortex_BA9 0.53016973 Brain_Cerebellar_Hemisphere 1.85368616
0.14631384 0.14631384 -0.62774694 0.627860289 5.67E-05 0.887848386
2 0 0.931227594
RASL10A ENSG00000100276.9 0.067824426 4 3 0.033732114
Brain_Putamen_basal_ganglia 0.3124456 Brain_Cerebellum 3.60374835 0.057937147
0.122900874 -2.123259483 -1.01010372 -1.547238124 0.552773559
3 0 0.931227594
RP11-111M22.4 ENSG00000272301.1 0.067938337 1 1 0.067938337
Brain_Substantia_nigra 0.067938337 Brain_Substantia_nigra 1 1
1 -1.825415597 -1.825415597 -1.825415597 NA 1 0
0.931227594
SLC25A29 ENSG00000197119.12 0.067946175 3 2 0.015356404
Brain_Cerebellar_Hemisphere 0.211393016 Brain_Cortex 2.304050117
0.033389288 0.662560595 -2.423862849 -1.249744171 -1.945344263
0.616426761 2 0 0.931227594
CENPBD1 ENSG00000177946.5 0.067960505 4 4 0.020031282
Brain_Cerebellar_Hemisphere 0.159273198 Brain_Frontal_Cortex_BA9
2.239967721 0.301828315 0.301828315 -2.325761422 -1.407520178
-1.866242843 0.446437635 4 0 0.931227594
RP11-111M22.2 ENSG00000179240.9 0.067974088 1 1 0.067974088
Brain_Cortex 0.067974088 Brain_Cortex 1 1 1 1.825178561
1.825178561 1.825178561 NA 1 0 0.931227594
EVA1B ENSG00000142694.6 0.068053402 3 3 0.022116884
Brain_Caudate_basal_ganglia 0.942402121 Brain_Cerebellum 1.517836781
0.514970837 0.514970837 -2.288354515 -0.072251048 -1.090907624
1.118817736 3 0 0.931227594
GPR27 ENSG00000170837.2 0.068074846 4 4 0.018344724 Brain_Cortex
0.697079641 Brain_Nucleus_accumbens_basal_ganglia 1.997304332 0.46894492
0.46894492 -1.235504615 2.358585682 0.514700192 1.469679409 4
0 0.931227594
11-Mar ENSG00000183654.8 0.068089903 1 1 0.068089903
Brain_Hypothalamus 0.068089903 Brain_Hypothalamus 1 1 1
1.824411381 1.824411381 1.824411381 NA 1 0 0.931227594
USP42 ENSG00000106346.11 0.068163809 1 1 0.068163809
Brain_Spinal_cord_cervical_c-1 0.068163809 Brain_Spinal_cord_cervical_c-1
1 1 1 -1.823922368 -1.823922368 -1.823922368 NA 1 0
0.931327987
LNPEP ENSG00000113441.15 0.068233454 2 2 0.034503441 Brain_Cerebellum
0.42170988 Brain_Substantia_nigra 1.067678124 0.932321876
0.932321876 -0.803458316 2.114138482 0.655340083 2.063052481
2 0 0.93137
DTX2 ENSG00000091073.19 0.068371528 4 4 0.011217559
Brain_Putamen_basal_ganglia 0.286181039 Brain_Cortex 2.442263347
0.186314251 0.186314251 1.066536829 2.535847443 1.611504377
0.641293606 4 0 0.932345077
IL32 ENSG00000008517.16 0.068507722 6 6 0.002667754
Brain_Substantia_nigra 0.898930697 Brain_Cortex 1.248024128
0.718440164 0.718440164 0.127012257 3.003634691 1.093149624
1.07543934 6 0 0.933292638
LMO2 ENSG00000135363.11 0.068837315 1 1 0.068837315
Brain_Hippocampus 0.068837315 Brain_Hippocampus 1 1 1
-1.819486009 -1.819486009 -1.819486009 NA 1 0 0.936870505
CCDC112 ENSG00000164221.12 0.068946921 1 1 0.068946921

Brain_Cerebellum 0.068946921 Brain_Cerebellum 1 1 1 1.818767417
1.818767417 1.818767417 NA 1 0 0.937289328
RP11-543C4.1 ENSG00000247970.2 0.06901057 2 2 0.024199708
Brain_Hypothalamus 0.46732449 Brain_Cortex 1.094361532 0.905638468
0.905638468 -0.726839162 2.253943726 0.763552282 2.107731793
2 0 0.937289328
PPP1CC ENSG00000186298.11 0.069114266 2 2 0.276569305
Brain_Putamen_basal_ganglia 0.494430709 Brain_Caudate_basal_ganglia
1.703767746 0.296232254 0.296232254 -0.683278766 1.088058451
0.202389843 1.252524557 2 0 0.937289328
SLC25A12 ENSG00000115840.13 0.069136058 8 4 0.017012211
Brain_Hippocampus 0.524526133 Brain_Hypothalamus 6.388198911
0.007560682 0.219445034 -2.386443713 -0.636384053 -1.566323168
0.512998677 4 0 0.937289328
KLHDC8B ENSG00000185909.14 0.069287312 1 1 0.069287312
Brain_Cerebellum 0.069287312 Brain_Cerebellum 1 1 1 1.816541734
1.816541734 1.816541734 NA 1 0 0.937965237
RANBP2 ENSG00000153201.15 0.069320476 1 1 0.069320476
Brain_Amygdala 0.069320476 Brain_Amygdala 1 1 1 1.816325366
1.816325366 1.816325366 NA 1 0 0.937965237
TMC8 ENSG00000167895.14 0.069606539 6 5 0.016969656
Brain_Anterior_cingulate_cortex_BA24 0.969670222 Brain_Amygdala
2.880869477 0.058641393 0.196057756 -0.038021899 2.387364524
0.875102873 0.947624812 5 0 0.937965237
NUDT5 ENSG00000165609.12 0.069759213 5 4 0.016824379 Brain_Cerebellum
0.698319843 Brain_Nucleus_accumbens_basal_ganglia 3.020190449
0.033555942 0.213993095 -2.390523365 -0.387589496 -1.183936593
0.782879132 4 0 0.937965237
IQCG ENSG00000114473.13 0.069985999 8 5 0.039782365
Brain_Spinal_cord_cervical_c-1 0.795137216 Brain_Cortex 5.509720881
0.040708827 0.201282871 -0.496137701 2.056001573 0.412057021
0.85624368 5 0 0.937965237
ASCC1 ENSG00000138303.17 0.069997737 1 1 0.069997737
Brain_Caudate_basal_ganglia 0.069997737 Brain_Caudate_basal_ganglia 1 1
1 1.811925315 1.811925315 1.811925315 NA 1 0
0.937965237
TNFRSF1A ENSG00000067182.7 0.070008813 2 2 0.157776969
Brain_Caudate_basal_ganglia 0.162601864 Brain_Cerebellum 1.258177528
0.741822472 0.741822472 -1.412587762 1.396374121 -0.00810682
1.986235996 2 0 0.937965237
C9orf142 ENSG00000148362.10 0.070121782 4 4 0.004207461
Brain_Cortex 0.814471084 Brain_Cerebellum 1.115511963 0.883064658
0.883064658 -0.694013178 2.862173819 0.752239 1.509161259 4
0 0.937965237
KMT5C ENSG00000133247.13 0.070210487 3 3 0.072222718
Brain_Cerebellar_Hemisphere 0.722258033 Brain_Cerebellum 1.505577944
0.47376833 0.47376833 -1.615846014 1.797711626 0.17910274 1.713597313
3 0 0.937965237
SLC26A2 ENSG00000155850.7 0.070291333 2 2 0.045749617
Brain_Spinal_cord_cervical_c-1 0.77079603 Brain_Frontal_Cortex_BA9
1.384867636 0.615132364 0.615132364 -0.291333813 1.997696099
0.853181143 1.618588573 2 0 0.937965237
KDM6B ENSG00000132510.10 0.070331373 1 1 0.070331373 Brain_Cortex
0.070331373 Brain_Cortex 1 1 1 -1.809770574 -1.809770574
-1.809770574 NA 1 0 0.937965237
SCMH1 ENSG00000010803.16 0.070375204 2 2 0.042570944
Brain_Anterior_cingulate_cortex_BA24 0.271358352 Brain_Cerebellum
1.003239923 0.996760077 0.996760077 -2.027895216 -1.099939801
-1.563917508 0.656163566 2 0 0.937965237
MAP6D1 ENSG00000180834.7 0.070425238 9 5 0.010248106
Brain_Cerebellum 0.28454033 Brain_Frontal_Cortex_BA9 5.782371603
0.019037076 0.244793701 -1.070175477 2.56734343 1.858514837

1.126485322	5	0	0.937965237
DOCK4	ENSG00000128512.20	0.070440868	2	2	0.025197964
Brain_Cerebellar_Hemisphere	0.026385802	Brain_Cerebellum	1.873929383
0.126070617	0.126070617	2.220485904	2.23835424	2.229420072
0.012634822	2	0	0.937965237
ZNF205	ENSG00000122386.10	0.070505691	4	4	0.046163272
Brain_Cerebellum	0.792068868	Brain_Cerebellar_Hemisphere	1.741562153
0.407296579	0.407296579	-1.993897363	1.233157549	-0.501191613
1.509309314	4	0	0.937965237
YIPF7	ENSG00000177752.14	0.070518092	9	4	0.005179572
Brain_Nucleus_accumbens_basal_ganglia	0.600661049	Brain_Cortex	6.74589441
0.023344191	0.442975191	0.523450127	2.795648351	1.714118341
0.61602484	4	0	0.937965237
GLTSCR2	ENSG00000105373.18	0.070628491	1	1	0.070628491
Brain_Hippocampus	0.070628491	Brain_Hippocampus	1	1	1
1.807858723	1.807858723	1.807858723	NA	1	0	0.937965237
PDCD11	ENSG00000148843.13	0.070721382	4	4	0.01431507	Brain_Cerebellum
0.449511984	Brain_Cerebellar_Hemisphere	2.454385788	0.26922575	0.26922575
0.756228875	2.44925842	1.390517825	0.733356113	4	0
0.937965237
FAM189A2	ENSG00000135063.17	0.070737634	1	1	0.070737634
Brain_Cerebellar_Hemisphere	0.070737634	Brain_Cerebellar_Hemisphere	1	1
1	-1.807158084	-1.807158084	-1.807158084	NA	1	0
0.937965237
PDZD7	ENSG00000186862.17	0.070833122	7	7	0.048750863
Brain_Cerebellar_Hemisphere	0.543971631	Brain_Hippocampus	1.914885635
0.307454041	0.307454041	-1.323211063	1.970764117	0.22334923
1.256217871	7	0	0.937965237
ALOX5	ENSG00000012779.10	0.070840151	5	3	0.020977711
Brain_Anterior_cingulate_cortex_BA24	0.530058644	Brain_Cerebellum
4.275767183	0.010336568	0.150760655	-2.308385387	1.188656309
-0.9313838	1.694689391	3	0	0.937965237
SHE	ENSG00000169291.9	0.070932307	1	1	0.070932307
Brain_Hippocampus	0.070932307	Brain_Hippocampus	1	1	1
-1.805910587	-1.805910587	-1.805910587	NA	1	0	0.937965237
ATP2A2	ENSG00000174437.16	0.070966789	3	3	0.062927143
Brain_Cerebellum	0.500694458	Brain_Spinal_cord_cervical_c-1	1.548257627
0.406518192	0.406518192	-0.67339747	1.859705925	0.928172782
1.393149489	3	0	0.937965237
LINC01010	ENSG00000236700.5	0.07099381	2	2	0.024607478	Brain_Amygdala
0.323167491	Brain_Caudate_basal_ganglia	1.227771223	0.772228777
0.772228777	0.987969693	2.247509595	1.617739644	0.890629206
2	0	0.937965237
UBR4	ENSG00000127481.14	0.071068827	2	2	0.021586101	Brain_Cerebellum
0.821421943	Brain_Nucleus_accumbens_basal_ganglia	1.056174635
0.943825365	0.943825365	-2.297573057	0.225716476	-1.035928291
1.784235139	2	0	0.937965237
CGNL1	ENSG00000128849.10	0.071142038	1	1	0.071142038	Brain_Cerebellum
0.071142038	Brain_Cerebellum	1	1	1	1.804569734	1.804569734
1.804569734	NA	1	0	0.937965237
ACP2	ENSG00000134575.9	0.071142675	4	3	0.010286339	Brain_Cerebellum
0.395396734	Brain_Putamen_basal_ganglia	2.514247703	0.010946902
0.305779967	-2.566052058	-0.849871137	-1.730873709	0.909302519
3	0	0.937965237
RP11-268P4.5	ENSG00000250166.2	0.071176028	1	1	0.071176028
Brain_Frontal_Cortex_BA9	0.071176028	Brain_Frontal_Cortex_BA9	1	1
1	1.804352731	1.804352731	1.804352731	NA	1	0
0.937965237
R3HCC1L	ENSG00000166024.13	0.071227532	1	1	0.071227532
Brain_Cerebellum	0.071227532	Brain_Cerebellum	1	1	1	-1.804024083
-1.804024083	-1.804024083	NA	1	0	0.937965237
POLR3G	ENSG00000113356.11	0.071232909	3	2	0.04823922

Brain_Spinal_cord_cervical_c-1 0.838994018 Brain_Cortex 2.352335966
0.054046132 0.593617903 -0.203180418 1.975254977 1.139824905
1.174572122 2 0 0.937965237
PRPF6 ENSG00000101161.7 0.071266994 3 2 0.324725347
Brain_Nucleus_accumbens_basal_ganglia 0.771506459 Brain_Caudate_basal_ganglia
2.788881775 0.034441526 0.176676699 -0.98479384 0.290404948
-0.513937029 0.699958751 2 0 0.937965237
RP11-395A13.2 ENSG00000272667.1 0.071344269 2 2 0.024709565
Brain_Anterior_cingulate_cortex_BA24 0.660126728 Brain_Cortex
1.020622802 0.979377198 0.979377198 -0.439738206 2.245913244
0.903087519 1.899042352 2 0 0.937965237
CDK18 ENSG00000117266.15 0.071451822 4 4 0.015230848
Brain_Hypothalamus 0.352764044 Brain_Hippocampus 1.806137074
0.38203208 0.38203208 -2.426842937 0.980440894 -1.088824346
1.515607322 4 0 0.937965237
NOL12 ENSG00000273899.4 0.071638405 3 3 0.067239142
Brain_Putamen_basal_ganglia 0.598494687 Brain_Hippocampus 2.265598707
0.139067974 0.139067974 0.526566463 1.830072211 1.056651405
0.68497523 3 0 0.937965237
CDC25A ENSG00000164045.11 0.071643411 6 2 0.025490065
Brain_Spinal_cord_cervical_c-1 0.156875822 Brain_Cerebellum 5.552280218
0.008471511 0.273039726 -2.23389375 -1.415657449 -1.689420974
0.315375772 2 0 0.937965237
RP5-1198O20.4 ENSG00000230615.6 0.071646171 2 2 0.029395292
Brain_Putamen_basal_ganglia 0.328657171 Brain_Spinal_cord_cervical_c-1
1.117251541 0.882748459 0.882748459 0.976822225 2.178144339
1.577483282 0.849463014 2 0 0.937965237
CBLB ENSG00000114423.18 0.071756871 3 3 0.008260307
Brain_Nucleus_accumbens_basal_ganglia 0.898803171 Brain_Cerebellum
1.911266901 0.089172103 0.089172103 0.127173383 2.641240469
0.999026555 1.423104031 3 0 0.937965237
P2RY2 ENSG00000175591.11 0.071794222 1 1 0.071794222
Brain_Cerebellar_Hemisphere 0.071794222 Brain_Cerebellar_Hemisphere 1 1
1 -1.80042078 -1.80042078 -1.80042078 NA 1 0
0.937965237
HSPA9 ENSG00000113013.12 0.071919685 2 2 0.024407621
Brain_Cerebellar_Hemisphere 0.487947878 Brain_Caudate_basal_ganglia
1.111200205 0.888799795 0.888799795 -2.250651471 -0.693576432
-1.472113952 1.101018319 2 0 0.937965237
KIF13A ENSG00000137177.19 0.071994776 5 5 0.085302837
Brain_Nucleus_accumbens_basal_ganglia 0.988525339 Brain_Caudate_basal_ganglia
1.964041984 0.316029138 0.316029138 -1.720713435 1.304152571
-0.385797951 1.172391165 5 0 0.937965237
ZNF442 ENSG00000198342.9 0.072170147 1 1 0.072170147
Brain_Substantia_nigra 0.072170147 Brain_Substantia_nigra 1 1
1 1.798043293 1.798043293 1.798043293 NA 1 0
0.937965237
UBAC2-AS1 ENSG00000228889.6 0.072229465 1 1 0.072229465
Brain_Cortex 0.072229465 Brain_Cortex 1 1 1 1.797669074
1.797669074 1.797669074 NA 1 0 0.937965237
RP5-984P4.6 ENSG00000278041.1 0.0723341 3 3 0.025248617
Brain_Spinal_cord_cervical_c-1 0.3045049 Brain_Cerebellum 1.29563093
0.761245997 0.761245997 -2.23757755 -1.026820701 -1.626696967
0.605453432 3 0 0.937965237
GID8 ENSG00000101193.7 0.072356312 1 1 0.072356312
Brain_Nucleus_accumbens_basal_ganglia 0.072356312
Brain_Nucleus_accumbens_basal_ganglia 1 1 1 1.796869675
1.796869675 1.796869675 NA 1 0 0.937965237
TRPV6 ENSG00000165125.19 0.072365416 3 3 0.02465072 Brain_Cortex
0.253053796 Brain_Putamen_basal_ganglia 2.233339242 0.213569262
0.213569262 -2.246832722 -1.142963379 -1.830366143 0.599738855
3 0 0.937965237

FAM81B ENSG00000153347.9 0.072485319 2 2 0.036714225
Brain_Cerebellar_Hemisphere 0.112227058 Brain_Cerebellum 1.928059638
0.071940362 0.071940362 1.588262215 2.088927775 1.838594995
0.354024012 2 0 0.937965237
BMPR1A ENSG00000107779.13 0.072495466 6 5 0.008269079
Brain_Caudate_basal_ganglia 0.867625289 Brain_Hypothalamus 3.244390261
0.070443423 0.228020451 0.166675621 2.64088085 1.712583758
1.021127702 5 0 0.937965237
TPMT ENSG00000137364.4 0.072522809 10 7 0.015602519 Brain_Amygdala
0.693060669 Brain_Cerebellum 6.485025681 0.025664457 0.231321237
0.394704923 2.418082952 1.378771781 0.646334374 7 0
0.937965237
SEMA3F ENSG00000001617.11 0.072537794 3 3 0.017904534
Brain_Substantia_nigra 0.922858575 Brain_Anterior_cingulate_cortex_BA24
1.891839478 0.348168853 0.348168853 -0.300221655 2.367586494
0.656843761 1.485032729 3 0 0.937965237
DEFB1 ENSG00000164825.3 0.072559509 3 3 0.030539254
Brain_Cerebellar_Hemisphere 0.342361605 Brain_Caudate_basal_ganglia
1.926856045 0.076302447 0.076302447 -2.163025042 -0.949509378
-1.55763247 0.60676244 3 0 0.937965237
RP5-1017F8.2 ENSG00000276093.1 0.072699516 13 1 0.0466267
Brain_Substantia_nigra 0.122502278 Brain_Cortex 12.48899588
0.002478483 12.48899588 -1.98967544 -1.54435532 -1.758038051
0.125016215 1 0 0.937965237
CD93 ENSG00000125810.9 0.07275634 1 1 0.07275634
Brain_Nucleus_accumbens_basal_ganglia 0.07275634
Brain_Nucleus_accumbens_basal_ganglia 1 1 1 1.794356159
1.794356159 1.794356159 NA 1 0 0.937965237
ARHGEF40 ENSG00000165801.9 0.072797632 2 2 0.023796178
Brain_Nucleus_accumbens_basal_ganglia 0.493147153 Brain_Hypothalamus
1.144921452 0.855078548 0.855078548 0.685311854 2.260404173
1.472858014 1.11375846 2 0 0.937965237
ERI2 ENSG00000196678.13 0.073076736 3 3 0.010438168
Brain_Hippocampus 0.585756124 Brain_Cortex 1.451847445
0.529520314 0.529520314 0.54499621 2.560965642 1.407763308
1.038892515 3 0 0.937965237
ATP6V0A2 ENSG00000185344.13 0.073097161 4 3 0.009665476
Brain_Hypothalamus 0.862023835 Brain_Putamen_basal_ganglia 2.834686604
0.032202381 0.16882563 -2.587572832 0.583597799 -0.334560252
1.512239376 3 0 0.937965237
RNF216 ENSG00000011275.18 0.07313061 2 2 0.036369771
Brain_Putamen_basal_ganglia 0.243442898 Brain_Caudate_basal_ganglia
1.119637564 0.880362436 0.880362436 -2.092769107 -1.166424399
-1.629596753 0.655024625 2 0 0.937965237
ADAM12 ENSG00000148848.14 0.073229948 5 5 0.074952197
Brain_Frontal_Cortex_BA9 0.800845201 Brain_Caudate_basal_ganglia
2.423752905 0.202910093 0.202910093 -1.780756754 1.632820547
0.214450096 1.426718894 5 0 0.937965237
LINC01700 ENSG00000232837.1 0.073249186 3 3 0.037176581
Brain_Caudate_basal_ganglia 0.379241456 Brain_Frontal_Cortex_BA9
1.191608307 0.797819706 0.797819706 -2.083819584 -0.879294797
-1.486119965 0.602314243 3 0 0.937965237
RP4-565E6.1 ENSG00000227733.9 0.073260223 11 2 0.216488628
Brain_Cerebellar_Hemisphere 0.939915949 Brain_Cortex 10.08850285
0.000226251 0.5188683 -0.864310853 1.235919124 0.517573338
0.579302142 2 0 0.937965237
TAC1 ENSG00000006128.11 0.07338151 3 3 0.055463183 Brain_Cerebellum
0.546458408 Brain_Putamen_basal_ganglia 1.212248599 0.766079258
0.766079258 0.603075585 1.915229963 1.334983717 0.669095116
3 0 0.937965237
TBCA ENSG00000171530.13 0.073383154 1 1 0.073383154 Brain_Cerebellum
0.073383154 Brain_Cerebellum 1 1 1 -1.790440318 -1.790440318

-1.790440318 NA 1 0 0.937965237
MPND ENSG00000008382.15 0.073494922 1 1 0.073494922
Brain_Caudate_basal_ganglia 0.073494922 Brain_Caudate_basal_ganglia 1 1
1 1.789744957 1.789744957 1.789744957 NA 1 0
0.937965237
RBM22 ENSG00000086589.11 0.073547424 1 1 0.073547424 Brain_Cerebellum
0.073547424 Brain_Cerebellum 1 1 1 1.78941861 1.78941861 1.78941861
NA 1 0 0.937965237
ATP1B1 ENSG00000143153.12 0.073619718 7 3 0.024618668
Brain_Cerebellum 0.541499694 Brain_Caudate_basal_ganglia 5.638173439
0.016291098 0.327722563 -2.24733434 -0.610546735 -1.15427385
0.548221036 3 0 0.937965237
PSMG1 ENSG00000183527.11 0.073660449 8 2 0.401368959
Brain_Frontal_Cortex_BA9 0.986520851 Brain_Caudate_basal_ganglia
7.449541196 0.00463999 0.274812584 -0.654969545 0.839178842
0.214824104 0.558009002 2 0 0.937965237
CERS4 ENSG00000090661.11 0.073809451 3 3 0.016881769
Brain_Nucleus_accumbens_basal_ganglia 0.961294528 Brain_Frontal_Cortex_BA9
1.087317245 0.949699155 0.949699155 0.048529157 2.389272655
1.149628927 1.176505781 3 0 0.937965237
CTD-2532N20.1 ENSG00000272192.1 0.073835515 2 2 0.073457105
Brain_Putamen_basal_ganglia 0.141769739 Brain_Frontal_Cortex_BA9
1.029543182 0.970456818 0.970456818 -1.789980138 1.469232496
-0.160373821 2.304611355 2 0 0.937965237
WDR6 ENSG00000178252.17 0.073874924 11 2 0.012384326
Brain_Putamen_basal_ganglia 0.130202731 Brain_Amygdala 10.10740147
0.000718718 0.397024313 1.513302923 2.500999767 1.9473577
0.276453509 2 0 0.937965237
PKIG ENSG00000168734.13 0.07395222 2 2 0.042150996
Brain_Cerebellar_Hemisphere 0.549341827 Brain_Cerebellum 1.672895471
0.327104529 0.327104529 -2.032026271 -0.598746675 -1.315386473
1.013481722 2 0 0.937965237
TRIM29 ENSG00000137699.16 0.07397264 4 4 0.004706038
Brain_Putamen_basal_ganglia 0.051199965 Brain_Cortex 3.040170999
0.109002991 0.109002991 1.949799968 2.826495691 2.251379792
0.393295726 4 0 0.937965237
BTN3A3 ENSG00000111801.15 0.073973255 4 4 0.051486155
Brain_Cerebellar_Hemisphere 0.608173746 Brain_Hippocampus 1.953540658
0.275036402 0.275036402 -1.947405371 0.933104146 -0.458589977
1.397723473 4 0 0.937965237
ZMYM6 ENSG00000163867.16 0.074100271 1 1 0.074100271
Brain_Hypothalamus 0.074100271 Brain_Hypothalamus 1 1 1
-1.785993744 -1.785993744 -1.785993744 NA 1 0 0.937965237
ZBED5 ENSG00000236287.7 0.074119057 1 1 0.074119057 Brain_Amygdala
0.074119057 Brain_Amygdala 1 1 1 1.785877732 1.785877732
1.785877732 NA 1 0 0.937965237
ZDHHC13 ENSG00000177054.13 0.07414692 2 2 0.068236883
Brain_Putamen_basal_ganglia 0.091245563 Brain_Frontal_Cortex_BA9
1.187947668 0.812052332 0.812052332 -1.823439295 1.688863649
-0.067287823 2.483573229 2 0 0.937965237
CYGB ENSG00000161544.9 0.074216575 1 1 0.074216575
Brain_Hypothalamus 0.074216575 Brain_Hypothalamus 1 1 1
-1.785275904 -1.785275904 -1.785275904 NA 1 0 0.937998289
H2AFV ENSG00000105968.18 0.074286141 3 3 0.019236442
Brain_Caudate_basal_ganglia 0.185079507 Brain_Spinal_cord_cervical_c-1
2.483582628 0.098465556 0.098465556 1.325276359 2.340917019
1.871035125 0.512054159 3 0 0.938030147
CARD14 ENSG00000141527.16 0.074387297 6 6 0.022437246
Brain_Nucleus_accumbens_basal_ganglia 0.79411208 Brain_Cerebellum 1.880888673
0.307809664 0.307809664 -0.379699949 2.282883253 0.615207636
0.9912822 6 0 0.938460481
SS18L2 ENSG00000008324.11 0.074597671 1 1 0.074597671

Brain_Cortex 0.074597671 Brain_Cortex 1 1 1 1.782930173
1.782930173 1.782930173 NA 1 0 0.939695092
LLNLR-268E12.1 ENSG00000276445.1 0.074619487 5 3 0.156513008
Brain_Frontal_Cortex_BA9 0.945460804 Brain_Cortex 4.436126007
0.018991556 0.199360958 -1.416897122 0.169906631 -0.366732253
0.623412324 3 0 0.939695092
FMN1 ENSG00000248905.8 0.074767524 5 4 0.041529417
Brain_Putamen_basal_ganglia 0.936464691 Brain_Cerebellum 3.387628976
0.08074772 0.336281106 -2.03820519 0.079714042 -1.362553223
0.877775524 4 0 0.94019081
WBP1L ENSG00000166272.16 0.074793251 2 2 0.025219738
Brain_Nucleus_accumbens_basal_ganglia 0.462854535 Brain_Cortex
1.141783231 0.858216769 0.858216769 0.734154575 2.238020192
1.486087383 1.063393576 2 0 0.94019081
APH1B ENSG00000138613.13 0.074984272 2 2 0.032850125 Brain_Cortex
0.3520558 Brain_Anterior_cingulate_cortex_BA24 1.065723494 0.934276506
0.934276506 -2.133910296 0.930609111 -0.601650593 2.166942453
2 0 0.940563256
ANKS4B ENSG00000175311.6 0.075079253 1 1 0.075079253
Brain_Cerebellum 0.075079253 Brain_Cerebellum 1 1 1 1.779979885
1.779979885 1.779979885 NA 1 0 0.940563256
HTATIP2 ENSG00000109854.13 0.075079598 8 8 0.069686806
Brain_Frontal_Cortex_BA9 0.810238689 Brain_Caudate_basal_ganglia 2.36793881
0.189617236 0.189617236 -1.773438801 1.813941023 0.0164128
1.227327756 8 0 0.940563256
INSIG1 ENSG00000186480.12 0.075193447 1 1 0.075193447
Brain_Cortex 0.075193447 Brain_Cortex 1 1 1 1.77928257
1.77928257 1.77928257 NA 1 0 0.940563256
TP53INP1 ENSG00000164938.13 0.075219765 2 2 0.023211331
Brain_Cerebellum 0.944959664 Brain_Hypothalamus 1.095550663
0.904449337 0.904449337 -0.069037633 2.269938225 1.100450296
1.65390569 2 0 0.940563256
FAM102A ENSG00000167106.11 0.075254178 1 1 0.075254178
Brain_Cerebellar_Hemisphere 0.075254178 Brain_Cerebellar_Hemisphere 1 1
1 1.778912074 1.778912074 1.778912074 NA 1 0
0.940563256
SYN3 ENSG00000185666.14 0.075293463 5 5 0.049428024 Brain_Cerebellum
0.947904343 Brain_Frontal_Cortex_BA9 2.709445112 0.258786726
0.258786726 -0.681238456 1.964880897 0.474182375 1.144690946
5 0 0.940563256
TMPRSS13 ENSG00000137747.15 0.075399231 1 1 0.075399231
Brain_Cerebellar_Hemisphere 0.075399231 Brain_Cerebellar_Hemisphere 1 1
1 -1.778028152 -1.778028152 -1.778028152 NA 1 0
0.941044288
LINC00877 ENSG00000241163.7 0.075639632 4 4 0.06355378
Brain_Putamen_basal_ganglia 0.122104193 Brain_Cerebellum 1.688604008
0.458600869 0.458600869 -1.855297327 -1.546001554 -1.7406397
0.143545122 4 0 0.941300256
MAT2A ENSG00000168906.12 0.075713497 2 2 0.028736624
Brain_Cerebellar_Hemisphere 0.216655223 Brain_Cerebellum 1.750683382
0.249316618 0.249316618 -2.187080833 -1.235471106 -1.71127597
0.672889691 2 0 0.941300256
ZNF574 ENSG00000105732.12 0.075792068 1 1 0.075792068
Brain_Putamen_basal_ganglia 0.075792068 Brain_Putamen_basal_ganglia 1 1
1 1.775641243 1.775641243 1.775641243 NA 1 0
0.941300256
LBR ENSG00000143815.14 0.075800268 1 1 0.075800268 Brain_Amygdala
0.075800268 Brain_Amygdala 1 1 1 1.775591525 1.775591525
1.775591525 NA 1 0 0.941300256
RNF44 ENSG00000146083.11 0.075884102 1 1 0.075884102 Brain_Cortex
0.075884102 Brain_Cortex 1 1 1 1.775083493 1.775083493
1.775083493 NA 1 0 0.941300256

STBD1 ENSG00000118804.8 0.076006212 11 4 0.019522233
Brain_Caudate_basal_ganglia 0.772385004 Brain_Cerebellum 7.774681927
0.023522874 0.346689601 0.289256623 2.335405776 1.602171238
0.713501757 4 0 0.941300256
HIVEP2 ENSG00000010818.9 0.076028276 2 2 0.027306101
Brain_Nucleus_accumbens_basal_ganglia 0.049128901 Brain_Putamen_basal_ganglia
1.725969091 0.274030909 0.274030909 -2.207113925 -1.967471306
-2.087292615 0.169452921 2 0 0.941300256
FAM180B ENSG00000196666.4 0.076065803 5 3 0.312764467
Brain_Cerebellum 0.428167478 Brain_Frontal_Cortex_BA9 3.627917913
0.041452073 0.366219533 -0.855844567 1.009438322 -0.45325921
0.81801158 3 0 0.941300256
LINC00528 ENSG00000269220.1 0.076130868 2 2 0.067871015
Brain_Cerebellar_Hemisphere 0.220245714 Brain_Cerebellum 1.934389772
0.065610228 0.065610228 -1.825862245 -1.225875011 -1.525868628
0.424255042 2 0 0.941300256
FAM213B ENSG00000157870.14 0.076150801 6 6 0.014579254
Brain_Frontal_Cortex_BA9 0.900662315 Brain_Caudate_basal_ganglia
2.067910874 0.251549141 0.251549141 -2.361179184 2.442665222
-0.391715604 1.625812186 6 0 0.941300256
CRB1 ENSG00000134376.15 0.076159809 4 4 0.031803219 Brain_Cerebellum
0.678143326 Brain_Cortex 2.466579566 0.180553546 0.180553546
-2.14687519 -0.414998056 -0.960111348 0.802573984 4 0
0.941300256
GRM4 ENSG00000124493.13 0.076351961 2 2 0.058663657 Brain_Cerebellum
0.370847617 Brain_Cerebellar_Hemisphere 1.178740491 0.821259509
0.821259509 -1.890705583 0.894886775 -0.497909404 1.969711246
2 0 0.942842265
MT1M ENSG00000205364.3 0.076450543 1 1 0.076450543 Brain_Cerebellum
0.076450543 Brain_Cerebellum 1 1 1 1.771662831 1.771662831
1.771662831 NA 1 0 0.943061045
PIGH ENSG00000100564.8 0.076555013 9 6 0.027356938 Brain_Cortex
0.977725673 Brain_Cerebellum 5.038283315 0.030085914 0.31676318
-2.206386665 1.208002767 -0.95939773 1.086256388 6 0
0.943061045
TMEM181 ENSG00000146433.8 0.076571892 6 6 0.008309212
Brain_Frontal_Cortex_BA9 0.898418526 Brain_Cerebellum 3.213020304
0.220186109 0.220186109 -2.639240046 -0.127659394 -1.286847895
0.857317806 6 0 0.943061045
ZFYVE28 ENSG00000159733.13 0.076780182 3 3 0.034426356
Brain_Caudate_basal_ganglia 0.548749723 Brain_Cerebellum 1.844297884
0.414907635 0.414907635 -2.115042145 0.59963469 -0.746492146
1.357477321 3 0 0.944228234
KLHL17 ENSG00000187961.13 0.076967968 1 1 0.076967968
Brain_Cerebellum 0.076967968 Brain_Cerebellum 1 1 1 -1.768556187
-1.768556187 -1.768556187 NA 1 0 0.944228234
HAX1 ENSG00000143575.14 0.077155716 4 4 0.027590204
Brain_Nucleus_accumbens_basal_ganglia 0.809544419 Brain_Putamen_basal_ganglia
1.604047243 0.460104101 0.460104101 -2.203064489 0.539584261
-0.513909869 1.231154833 4 0 0.944228234
LZTS1 ENSG00000061337.15 0.077239292 1 1 0.077239292
Brain_Hippocampus 0.077239292 Brain_Hippocampus 1 1 1
-1.766933939 -1.766933939 -1.766933939 NA 1 0 0.944228234
NCBP1 ENSG00000136937.12 0.077288527 2 2 0.040506443
Brain_Frontal_Cortex_BA9 0.137864106 Brain_Spinal_cord_cervical_c-1
1.272924527 0.727075473 0.727075473 -1.483792017 2.048546882
0.282377433 2.497740789 2 0 0.944228234
VHL ENSG00000134086.7 0.077313579 1 1 0.077313579
Brain_Frontal_Cortex_BA9 0.077313579 Brain_Frontal_Cortex_BA9 1 1
1 -1.766490588 -1.766490588 -1.766490588 NA 1 0
0.944228234
AP000473.5 ENSG00000228798.1 0.077360514 1 1 0.077360514

Brain_Hypothalamus 0.077360514 Brain_Hypothalamus 1 1 1
1.766210658 1.766210658 1.766210658 NA 1 0 0.944228234
PDIA3 ENSG00000167004.12 0.077758411 1 1 0.077758411
Brain_Hippocampus 0.077758411 Brain_Hippocampus 1 1 1
1.763843025 1.763843025 1.763843025 NA 1 0 0.944228234
NDUFB5 ENSG00000136521.12 0.07785367 2 2 0.030994494 Brain_Cerebellum
0.042876415 Brain_Cerebellar_Hemisphere 1.723368406 0.276631594
0.276631594 -2.157143391 -2.024911868 -2.091027629 0.093501806
2 0 0.944228234
BAK1 ENSG00000030110.12 0.077870614 12 2 0.026890097
Brain_Anterior_cingulate_cortex_BA24 0.750166482 Brain_Frontal_Cortex_BA9
10.43298198 1.51E-10 0.648288628 -2.21310951 -0.318419852
-1.795243588 0.528534485 2 0 0.944228234
AXIN2 ENSG00000168646.12 0.077874985 1 1 0.077874985 Brain_Cerebellum
0.077874985 Brain_Cerebellum 1 1 1 1.763151238 1.763151238
1.763151238 NA 1 0 0.944228234
SCRN2 ENSG00000141295.13 0.077896077 13 11 0.000323472 Brain_Cerebellum
0.876741726 Brain_Cortex 4.77634321 0.018156417 0.239483059
0.155100959 3.595738626 1.900547056 1.066791466 11 0
0.944228234
BBS12 ENSG00000181004.9 0.077926563 3 3 0.03450694
Brain_Caudate_basal_ganglia 0.577778687 Brain_Cerebellum 2.005447433
0.194557407 0.194557407 -0.754917897 2.114097512 0.267515775
1.602256964 3 0 0.944228234
UPF1 ENSG00000005007.12 0.077996561 1 1 0.077996561 Brain_Cerebellum
0.077996561 Brain_Cerebellum 1 1 1 -1.762430668 -1.762430668
-1.762430668 NA 1 0 0.944228234
ZFPM2 ENSG00000169946.13 0.078069471 2 2 0.023922469
Brain_Hypothalamus 0.94199513 Brain_Cerebellum 1.028576456 0.971423544
0.971423544 0.072762478 2.258372126 1.165567302 1.545459404
2 0 0.944228234
CNTN3 ENSG00000113805.8 0.078135081 2 2 0.023969863
Brain_Nucleus_accumbens_basal_ganglia 0.051733508 Brain_Caudate_basal_ganglia
1.870968713 0.129031287 0.129031287 -2.257611944 -1.945344683
-2.101478314 0.220806298 2 0 0.944228234
RP11-542F9.1 ENSG00000223504.1 0.078208166 2 2 0.024007262
Brain_Hypothalamus 0.847666292 Brain_Substantia_nigra 1.062327221
0.937672779 0.937672779 0.192096914 2.25701301 1.224554962
1.460116174 2 0 0.944228234
GPR26 ENSG00000154478.3 0.078341929 2 2 0.028313497
Brain_Caudate_basal_ganglia 0.588871229 Brain_Cortex 1.003246756
0.996753244 0.996753244 -0.540472488 2.1929151 0.826221306
1.932796899 2 0 0.944228234
KDM1B ENSG00000165097.13 0.078373336 5 5 0.006237869
Brain_Substantia_nigra 0.747215066 Brain_Cerebellar_Hemisphere
2.487869527 0.400600328 0.400600328 -2.73500835 -0.322313685
-1.255863333 0.994890834 5 0 0.944228234
PPP4R2 ENSG00000163605.14 0.078413682 3 3 0.011481591
Brain_Spinal_cord_cervical_c-1 0.69041744 Brain_Cerebellum 1.368590922
0.704738515 0.704738515 -2.527689311 0.398288631 -0.977198141
1.470818355 3 0 0.944228234
TEP1 ENSG00000129566.12 0.078441808 3 3 0.010721848 Brain_Cerebellum
0.889233085 Brain_Cerebellar_Hemisphere 1.290813413 0.710592677
0.710592677 0.139274695 2.551635957 1.091113572 1.284110936
3 0 0.944228234
ADGRD1 ENSG00000111452.12 0.078453343 3 3 0.009201996
Brain_Spinal_cord_cervical_c-1 0.928839465 Brain_Putamen_basal_ganglia
1.139099198 0.86090381 0.86090381 -0.129865705 2.604457018
0.795095414 1.567084347 3 0 0.944228234
ACSBG2 ENSG00000130377.13 0.078558645 1 1 0.078558645
Brain_Nucleus_accumbens_basal_ganglia 0.078558645
Brain_Nucleus_accumbens_basal_ganglia 1 1 1 -1.759111069

-1.759111069 -1.759111069 NA 1 0 0.944228234
PXMP4 ENSG00000101417.11 0.078697143 3 3 0.009152108 Brain_Cerebellum 0.94512531 Brain_Nucleus_accumbens_basal_ganglia 1.189500543 0.79664112 0.79664112 -0.068829532 2.606319373 0.906634124 1.47732126 3 0 0.944228234
NME4 ENSG00000103202.12 0.078870486 9 5 0.00333977 Brain_Nucleus_accumbens_basal_ganglia 0.710168476 Brain_Putamen_basal_ganglia 5.750266783 0.007064221 0.298679326 -2.934600886 -0.37162983 -1.968509885 0.763122657 5 0 0.944228234
KCTD10 ENSG00000110906.12 0.078974586 2 2 0.024976445 Brain_Cerebellum 0.187100339 Brain_Cerebellar_Hemisphere 1.664308775 0.335691225 0.335691225 1.319205743 2.241766848 1.780486295 0.652349213 2 0 0.944228234
SULT1A3 ENSG00000261052.5 0.07900607 1 1 0.07900607 Brain_Frontal_Cortex_BA9 0.07900607 Brain_Frontal_Cortex_BA9 1 1 1 -1.756482422 -1.756482422 -1.756482422 NA 1 0 0.944228234
NCOA6 ENSG00000198646.13 0.079098867 5 2 0.053922393 Brain_Frontal_Cortex_BA9 0.790417926 Brain_Cortex 4.190937546 0.018510208 0.676980551 -1.927459478 0.265767953 -1.343352045 0.907464418 2 0 0.944228234
SEC22B ENSG00000265808.3 0.079202187 1 1 0.079202187 Brain_Cerebellar_Hemisphere 0.079202187 Brain_Cerebellar_Hemisphere 1 1 1 -1.755334039 -1.755334039 -1.755334039 NA 1 0 0.944228234
CSMD1 ENSG00000183117.18 0.079264845 7 7 0.008387284 Brain_Hippocampus 0.690219966 Brain_Anterior_cingulate_cortex_BA24 2.328086875 0.307486002 0.307486002 -1.887631434 2.636068344 -0.720868828 1.566800823 7 0 0.944228234
HMGN2 ENSG00000198830.10 0.079323706 1 1 0.079323706 Brain_Putamen_basal_ganglia 0.079323706 Brain_Putamen_basal_ganglia 1 1 1 -1.754623636 -1.754623636 -1.754623636 NA 1 0 0.944228234
MFSD9 ENSG00000135953.10 0.079330089 1 1 0.079330089 Brain_Cerebellar_Hemisphere 0.079330089 Brain_Cerebellar_Hemisphere 1 1 1 1.754586344 1.754586344 1.754586344 NA 1 0 0.944228234
TRPM3 ENSG00000083067.22 0.079477421 6 6 0.110687696 Brain_Caudate_basal_ganglia 0.77682841 Brain_Cortex 3.309269544 0.149335681 0.149335681 -0.788379237 1.59510975 0.767661128 0.885790559 6 0 0.944228234
FANCI ENSG00000140525.17 0.079610892 3 3 0.079895546 Brain_Cortex 0.662173393 Brain_Hippocampus 1.247546036 0.759949364 0.759949364 -1.430311999 1.751292457 0.252631635 1.598787617 3 0 0.944228234
NECTIN3 ENSG00000177707.10 0.079642582 5 3 0.028012012 Brain_Nucleus_accumbens_basal_ganglia 0.606434482 Brain_Cortex 3.832745 0.04647375 0.15689053 0.51516964 2.197118106 1.392557671 0.632428142 3 0 0.944228234
ATP6V1A ENSG00000114573.9 0.079645102 2 2 0.03353509 Brain_Cortex 0.355445053 Brain_Spinal_cord_cervical_c-1 1.089751593 0.910248407 0.910248407 0.924079256 2.125617877 1.524848567 0.849616106 2 0 0.944228234
CYP2A6 ENSG00000255974.7 0.079690451 1 1 0.079690451 Brain_Caudate_basal_ganglia 0.079690451 Brain_Caudate_basal_ganglia 1 1 1 1.752484975 1.752484975 1.752484975 NA 1 0 0.944228234
TTC32 ENSG00000183891.5 0.0797409 13 2 0.058067307 Brain_Putamen_basal_ganglia 0.498188779 Brain_Cerebellum 12.03749026 4.86E-08 0.417700658 -1.895189453 -0.67734233 -0.987181376 0.338477595 2 0 0.944228234
NEK4 ENSG00000114904.12 0.079752018 3 3 0.032145894 Brain_Cerebellar_Hemisphere 0.864544707 Brain_Cortex 2.345299897

0.081280903	0.081280903	-0.17059185	2.142591751	1.213119296
1.221660421	3	0	0.944228234
DIDO1 ENSG00000101191.16	0.07979459 9	4	0.002368624
Brain_Anterior_cingulate_cortex_BA24	0.049770772	Brain_Cerebellum
7.364858714	0.007650992	0.296862213	1.961928817	3.039638325
2.436797349	0.354840626	4	0	0.944228234
NME5	ENSG00000112981.4	0.079883602	5	5	0.012309688
Brain_Nucleus_accumbens_basal_ganglia 0.490976076	Brain_Caudate_basal_ganglia
1.475742789	0.595961817	0.595961817	-2.503139887	0.913655639
-0.787932414	1.526112433	5	0	0.944228234
AC005330.2 ENSG00000267372.2	0.080005497	1	1	0.080005497
Brain_Hypothalamus	0.080005497	Brain_Hypothalamus	1	1	1
1.750654178	1.750654178	1.750654178	NA	1	0	0.944228234
PDRG1 ENSG00000088356.5	0.080036456	1	1	0.080036456
Brain_Caudate_basal_ganglia 0.080036456	Brain_Caudate_basal_ganglia 1	1
1	-1.750474586	-1.750474586	-1.750474586	NA	1	0
0.944228234
TAT	ENSG00000198650.10	0.080097134	1	1	0.080097134
Brain_Nucleus_accumbens_basal_ganglia 0.080097134
Brain_Nucleus_accumbens_basal_ganglia 1	1	1	-1.750122753
-1.750122753	-1.750122753	NA	1	0	0.944228234
KMT2D ENSG00000167548.14	0.080100603	1	1	0.080100603	Brain_Cortex
0.080100603	Brain_Cortex	1	1	1	1.75010265 1.75010265 1.75010265
NA	1	0	0.944228234
LILRB1	ENSG00000104972.15	0.080110941	2	2	0.156870564
Brain_Caudate_basal_ganglia 0.303895961	Brain_Putamen_basal_ganglia
1.402206354	0.597793646	0.597793646	-1.02811452	1.415675402
0.193780441	1.728020425	2	0	0.944228234
FAM206A	ENSG00000119328.11	0.080169661	3	3	0.086759912
Brain_Frontal_Cortex_BA9	0.650119289	Brain_Hippocampus	1.261919278
0.739049018	0.739049018	-1.383324053	1.712742559	-0.041392657
1.588660162	3	0	0.944228234
AC019118.3 ENSG00000236760.1	0.080190127	1	1	0.080190127
Brain_Cerebellar_Hemisphere 0.080190127	Brain_Cerebellar_Hemisphere 1	1
1	1.749583976	1.749583976	1.749583976	NA	1	0
0.944228234
CCDC192	ENSG00000230561.4	0.080255897	2	2	0.064216624
Brain_Putamen_basal_ganglia 0.353499886	Brain_Cortex	1.175718268
0.824281732	0.824281732	0.927822047	1.850672904	1.389247475
0.652554099	2	0	0.944228234
RPS6KA4	ENSG00000162302.12	0.080313279	2	2	0.052214485
Brain_Cerebellar_Hemisphere 0.11985646 Brain_Caudate_basal_ganglia 1.236033488
0.763966512	0.763966512	-1.94136117	1.555376363	-0.192992403
2.472566822	2	0	0.944228234
ACTR3C	ENSG00000106526.10	0.080645792	9	3	0.012118856
Brain_Caudate_basal_ganglia 0.350559671	Brain_Cerebellar_Hemisphere
7.292559641	0.002426978	0.30760778 0.933504263	2.508664415
2.029079344	0.471833088	3	0	0.944228234
PPP2R5A	ENSG00000066027.11	0.08074714 2	2	0.074752161
Brain_Cerebellar_Hemisphere 0.15933945 Brain_Putamen_basal_ganglia 1.025175306
0.974824694	0.974824694	-1.407296625	1.781982037	0.187342706
2.255160569	2	0	0.944228234
PCDHB11	ENSG00000197479.6	0.080957596	4	4	0.004952377
Brain_Substantia_nigra	0.327426866	Brain_Amygdala	2.453045542
0.219290727	0.219290727	-2.810115188	-0.979309929	-1.689979098
0.84767458 4	0	0.944228234
XBP1	ENSG00000100219.16	0.080984356	3	3	0.054823071
Brain_Hippocampus	0.563201607	Brain_Cerebellar_Hemisphere 1.804130256
0.318636941	0.318636941	-1.920275805	1.083073374	-0.471765008
1.504495178	3	0	0.944228234
RP11-134K13.4	ENSG00000271967.1	0.081036331	13	7	0.081777357
Brain_Substantia_nigra	0.949618947	Brain_Cerebellum 8.145680915

0.004411222 0.3406286 -1.740465249 1.432526453 0.484615489
0.99609023 7 0 0.944228234
PCDHA3 ENSG00000255408.3 0.081161257 5 5 0.003358132
Brain_Spinal_cord_cervical_c-1 0.820864517 Brain_Cortex 2.079561587
0.094965508 0.094965508 -0.280147679 2.932898955 0.55147757
1.380042643 5 0 0.944228234
CRYGS ENSG00000213139.7 0.081181325 3 3 0.060520247
Brain_Nucleus_accumbens_basal_ganglia 0.605118795 Brain_Cerebellum
1.669543301 0.361700648 0.361700648 0.517053526 1.876984288
1.336190807 0.721428703 3 0 0.944228234
RP11-195F19.5 ENSG00000187186.14 0.081251965 2 2 0.031268378
Brain_Substantia_nigra 0.514570646 Brain_Cerebellum 1.015583948
0.984416052 0.984416052 -0.651737315 2.153640471 0.750951578
1.983701656 2 0 0.944228234
TSC2 ENSG00000103197.16 0.081269057 7 5 0.020830517
Brain_Cerebellar_Hemisphere 0.9175699 Brain_Cortex 3.7580785 0.006426668
0.483379113 -0.972703445 2.31104232 1.13946181 1.340897217 5 0
0.944228234
MED17 ENSG00000042429.11 0.08129003 7 3 0.014181322 Brain_Hippocampus
0.332460609 Brain_Frontal_Cortex_BA9 5.419693171 0.001289509
0.516399762 -2.45263738 -0.969169534 -1.352429017 0.507546618
3 0 0.944228234
RP11-318C24.2 ENSG00000227740.1 0.081377361 2 2 0.065886041
Brain_Substantia_nigra 0.384142744 Brain_Cerebellum 1.844970605
0.155029395 0.155029395 -1.839198189 -0.870288533 -1.354743361
0.685122588 2 0 0.944228234
ACSL5 ENSG00000197142.10 0.081408297 3 3 0.027805941 Brain_Cerebellum
0.441178802 Brain_Cerebellar_Hemisphere 1.788073777 0.244575566
0.244575566 -1.157283902 2.200013443 0.090841797 1.83682102 3
0 0.944228234
TCF7L2 ENSG00000148737.16 0.081432027 2 2 0.060746989
Brain_Frontal_Cortex_BA9 0.236682929 Brain_Anterior_cingulate_cortex_BA24
1.021740115 0.978259885 0.978259885 1.183318537 1.875332541
1.529325539 0.489327795 2 0 0.944228234
ZNF655 ENSG00000197343.10 0.081441245 1 1 0.081441245
Brain_Caudate_basal_ganglia 0.081441245 Brain_Caudate_basal_ganglia 1 1
1 -1.742384162 -1.742384162 -1.742384162 NA 1 0
0.944228234
FAM71F1 ENSG00000135248.15 0.081444419 7 5 0.007290212
Brain_Frontal_Cortex_BA9 0.979393775 Brain_Amygdala 2.75087558
0.075378424 0.331146999 -0.078981372 2.683288265 1.062252712
1.091161024 5 0 0.944228234
RP11-491F9.6 ENSG00000259839.1 0.081501137 2 2 0.026121732
Brain_Cerebellar_Hemisphere 0.086330579 Brain_Cerebellum 1.686881902
0.313118098 0.313118098 -2.224397209 -1.715079901 -1.969738555
0.360141722 2 0 0.944228234
SHOC2 ENSG00000108061.11 0.08156057 2 2 0.026529489
Brain_Spinal_cord_cervical_c-1 0.789397527 Brain_Amygdala 1.016046593
0.983953407 0.983953407 -2.218371846 0.267093039 -0.975639403
1.757489075 2 0 0.944228234
RGS9 ENSG00000108370.16 0.081632909 2 2 0.028076864
Brain_Hypothalamus 0.295429532 Brain_Cortex 1.287460068
0.712539932 0.712539932 -2.196210728 -1.046284863 -1.621247796
0.813120377 2 0 0.944228234
CD2BP2 ENSG00000169217.8 0.081707264 1 1 0.081707264
Brain_Cortex 0.081707264 Brain_Cortex 1 1 1 1.74086489
1.74086489 1.74086489 NA 1 0 0.944228234
NAALADL1 ENSG00000168060.15 0.081732964 1 1 0.081732964
Brain_Spinal_cord_cervical_c-1 0.081732964 Brain_Spinal_cord_cervical_c-1
1 1 1 -1.740718327 -1.740718327 -1.740718327 NA 1 0
0.944228234
CCDC184 ENSG00000177875.4 0.081746622 4 3 0.207911895

Brain_Frontal_Cortex_BA9 0.989788851 Brain_Nucleus_accumbens_basal_ganglia 3.401435919 0.061639859 0.250183962 -1.259327969 0.385005712 -0.121601254 0.778003216 3 0 0.944228234
UPK3A ENSG00000100373.9 0.081773712 7 7 0.014516733 Brain_Cerebellum 0.706607093 Brain_Hypothalamus 2.69840773 0.205880288 0.205880288 -0.928748341 2.444215986 0.918928112 1.108144849 7 0 0.944228234
ABC7-42404400C24.1 ENSG00000277758.4 0.081842346 8 7 0.006974256 Brain_Cortex 0.980959004 Brain_Amygdala 2.281470367 0.006612654 0.446198588 -2.698071003 1.260979354 -0.492095864 1.477352192 7 0 0.944228234
ALPK3 ENSG00000136383.6 0.082050522 4 3 0.014873374 Brain_Cerebellum 0.654471976 Brain_Cortex 3.05701828 0.01195639 0.104353686 0.447558339 2.435447705 1.08893418 0.917274123 3 0 0.944228234
BIN3 ENSG00000147439.12 0.082143747 10 7 0.068380054 Brain_Hippocampus 0.712134438 Brain_Hypothalamus 6.091560702 0.031470606 0.240803016 -1.073462793 1.82249406 0.531715609 1.027722613 7 0 0.944228234
HLCS ENSG00000159267.14 0.082229758 8 5 0.015914753 Brain_Hypothalamus 0.802104054 Brain_Cortex 5.127608603 0.015274583 0.256334137 -2.410864651 0.926771318 0.217039994 1.101579361 5 0 0.944228234
EN1 ENSG00000163064.6 0.082255172 2 2 0.03926682 Brain_Cerebellum 0.442170707 Brain_Hippocampus 1.045891807 0.954108193 0.954108193 -2.0613798 0.768532808 -0.646423496 2.001050395 2 0 0.944228234
MAP2K6 ENSG00000108984.13 0.082349985 1 1 0.082349985 Brain_Spinal_cord_cervical_c-1 0.082349985 Brain_Spinal_cord_cervical_c-1 1 1 1 1.737210714 1.737210714 1.737210714 NA 1 0 0.944228234
GPANK1 ENSG00000204438.10 0.082429473 2 2 0.10481142 Brain_Spinal_cord_cervical_c-1 0.207518032 Brain_Caudate_basal_ganglia 1.158278547 0.841721453 0.841721453 -1.621962302 1.260419607 -0.180771348 2.038151794 2 0 0.944228234
RAD21 ENSG00000164754.14 0.08267652 2 2 0.027401186 Brain_Cortex 0.131540866 Brain_Cerebellum 1.78174261 0.21825739 0.21825739 1.50805338 2.205754604 1.856903992 0.493349267 2 0 0.944228234
RP11-158I9.8 ENSG00000278376.1 0.082708416 13 2 0.016717824 Brain_Spinal_cord_cervical_c-1 0.152234324 Brain_Amygdala 11.98031136 0.001741811 0.44756934 -2.392855484 -1.431683837 -1.940571622 0.281105313 2 0 0.944228234
LINC00492 ENSG00000250958.1 0.082711155 1 1 0.082711155 Brain_Cerebellum 0.082711155 Brain_Cerebellum 1 1 1 -1.735167422 -1.735167422 -1.735167422 NA 1 0 0.944228234
RBFOX2 ENSG00000100320.22 0.08278063 1 1 0.08278063 Brain_Cerebellum 0.08278063 Brain_Cerebellum 1 1 1 1.734775204 1.734775204 1.734775204 NA 1 0 0.944228234
ALX4 ENSG00000052850.5 0.082816755 1 1 0.082816755 Brain_Cortex 0.082816755 Brain_Cortex 1 1 1 -1.734571367 -1.734571367 -1.734571367 NA 1 0 0.944228234
MERTK ENSG00000153208.16 0.082841298 7 3 0.139033757 Brain_Nucleus_accumbens_basal_ganglia 0.830457405 Brain_Cortex 6.236069031 0.014924268 0.222424632 -0.214114966 1.479398816 0.763659469 0.532918644 3 0 0.944228234
HOXD1 ENSG00000128645.14 0.082874856 1 1 0.082874856 Brain_Spinal_cord_cervical_c-1 0.082874856 Brain_Spinal_cord_cervical_c-1 1 1 1 1.734243676 1.734243676 1.734243676 NA 1 0 0.944228234
OR7D2 ENSG00000188000.3 0.0828972 11 11 0.062325495 Brain_Anterior_cingulate_cortex_BA24 0.973138651 Brain_Putamen_basal_ganglia 3.383197795 0.112794503 0.112794503 -1.099615499 1.863973007

0.08348866 0.976634137 11 0 0.944228234
BCAN ENSG00000132692.18 0.083009784 1 1 0.083009784 Brain_Cerebellum
0.083009784 Brain_Cerebellum 1 1 1 1.73348341 1.73348341 1.73348341
NA 1 0 0.944228234
FAM212B ENSG00000197852.9 0.083015822 1 1 0.083015822
Brain_Putamen_basal_ganglia 0.083015822 Brain_Putamen_basal_ganglia 1 1
1 1.73344941 1.73344941 1.73344941 NA 1 0 0.944228234
TREX1 ENSG00000213689.11 0.083084897 13 5 0.037594475
Brain_Hypothalamus 0.675653452 Brain_Cortex 8.660615241
0.003351634 0.443155702 -1.603245109 2.079248944 1.094284717
1.029915676 5 0 0.944228234
MORF4L1 ENSG00000185787.14 0.083106268 1 1 0.083106268
Brain_Cerebellum 0.083106268 Brain_Cerebellum 1 1 1 -1.732940369
-1.732940369 -1.732940369 NA 1 0 0.944228234
CEBPZOS ENSG00000218739.9 0.083294553 13 6 0.001608011
Brain_Hippocampus 0.182609382 Brain_Caudate_basal_ganglia 8.715464419
0.008160741 0.464604678 1.332763638 3.154449778 2.53781253
0.456057913 6 0 0.944228234
GPAA1 ENSG00000197858.10 0.083305475 7 2 0.056984321
Brain_Anterior_cingulate_cortex_BA24 0.984944548 Brain_Amygdala
6.114352056 0.008527913 0.586961112 -0.018870331 1.903431063
0.487376408 0.662921652 2 0 0.944228234
RP11-734I18.1 ENSG00000251129.1 0.083427798 5 5 0.041180859
Brain_Spinal_cord_cervical_c-1 0.583665836 Brain_Amygdala 3.12229104
0.17550622 0.17550622 -0.548037964 2.041704436 0.885950931
0.968242268 5 0 0.944228234
AQP7 ENSG00000165269.12 0.083451781 10 10 0.002389957
Brain_Frontal_Cortex_BA9 0.930540712 Brain_Cerebellum 2.83495106
0.130877275 0.130877275 -3.036936613 0.682979101 -0.771994194
1.011326673 10 0 0.944228234
MAPK8IP2 ENSG00000008735.13 0.083560561 3 3 0.145688133
Brain_Cerebellum 0.595090898 Brain_Cerebellar_Hemisphere 1.758791284
0.301907776 0.301907776 -0.531473215 1.454931838 0.639417474
1.039793493 3 0 0.944228234
SFT2D1 ENSG00000198818.9 0.083609873 2 2 0.112337787
Brain_Cerebellum 0.132192152 Brain_Anterior_cingulate_cortex_BA24
1.035426202 0.964573798 0.964573798 -1.587772524 -1.505513331
-1.546642927 0.058166033 2 0 0.944228234
NFXL1 ENSG00000170448.11 0.083631917 11 8 0.06613533
Brain_Frontal_Cortex_BA9 0.81660875 Brain_Cerebellum 5.070261896
0.005968219 0.275538317 -1.717227817 1.83750533 -0.052623999
1.077727235 8 0 0.944228234
RPGRIP1 ENSG00000092200.12 0.083755599 3 3 0.036031354
Brain_Cerebellum 0.520296763 Brain_Cortex 1.14646161 0.822386867
0.822386867 -2.096573423 0.64288802 -0.922928912 1.411209288 3
0 0.944228234
LINC01856 ENSG00000237574.1 0.083856536 1 1 0.083856536
Brain_Anterior_cingulate_cortex_BA24 0.083856536
Brain_Anterior_cingulate_cortex_BA24 1 1 1 1.728734992
1.728734992 1.728734992 NA 1 0 0.944228234
ID4 ENSG00000172201.11 0.083881657 3 3 0.014423795
Brain_Caudate_basal_ganglia 0.904121423 Brain_Nucleus_accumbens_basal_ganglia
1.272666693 0.720125289 0.720125289 -2.446532101 0.774108519
-0.597626741 1.662498114 3 0 0.944228234
CCDC126 ENSG00000169193.11 0.08388868 1 1 0.08388868
Brain_Spinal_cord_cervical_c-1 0.08388868 Brain_Spinal_cord_cervical_c-1 1
1 1 -1.728555498 -1.728555498 -1.728555498 NA 1 0
0.944228234
RP11-419I17.2 ENSG00000270154.1 0.083895697 2 2 0.026177514
Brain_Amygdala 0.788256912 Brain_Cortex 1.071428375 0.928571625
0.928571625 -2.223568148 -0.268574793 -1.24607147 1.382389058
2 0 0.944228234

RBM26 ENSG00000139746.15 0.083903191 2 2 0.026108694
Brain_Cerebellar_Hemisphere 0.080747014 Brain_Cerebellum 1.759932245
0.240067755 0.240067755 -2.224591193 -1.746368075 -1.985479634
0.338154809 2 0 0.944228234
VAMP8 ENSG00000118640.10 0.083972812 1 1 0.083972812
Brain_Nucleus_accumbens_basal_ganglia 0.083972812
Brain_Nucleus_accumbens_basal_ganglia 1 1 1 -1.728085972
-1.728085972 -1.728085972 NA 1 0 0.944228234
EIF4E2 ENSG00000135930.13 0.083975676 2 2 0.072778077
Brain_Caudate_basal_ganglia 0.24633083 Brain_Cortex 1.085366127
0.914633873 0.914633873 -1.159307588 1.794219897 0.317456155
2.088459313 2 0 0.944228234
PSMA5 ENSG00000143106.12 0.083980171 1 1 0.083980171
Brain_Spinal_cord_cervical_c-1 0.083980171 Brain_Spinal_cord_cervical_c-1
1 1 1 -1.728044922 -1.728044922 -1.728044922 NA 1 0
0.944228234
SNTB2 ENSG00000168807.16 0.08407488 2 2 0.047376659
Brain_Cerebellar_Hemisphere 0.340555248 Brain_Cerebellum 1.791767546
0.208232454 0.208232454 0.953068732 1.982917378 1.467993055
0.728212961 2 0 0.944228234
PENK ENSG00000181195.10 0.084123314 2 2 0.05710087 Brain_Cerebellum
0.54216405 Brain_Cerebellar_Hemisphere 1.732949961 0.267050039
0.267050039 -1.90253789 -0.609543799 -1.256040844 0.914284889
2 0 0.944228234
ZNF721 ENSG00000182903.15 0.084148752 4 3 0.033927043
Brain_Cerebellum 0.33878167 Brain_Anterior_cingulate_cortex_BA24 3.514569989
0.032850819 0.122012997 -2.120937725 -0.956575283 -1.485249744
0.511850449 3 0 0.944228234
ARNT ENSG00000143437.20 0.084157859 11 5 0.034821143
Brain_Cerebellar_Hemisphere 0.946326489 Brain_Anterior_cingulate_cortex_BA24
8.234466198 0.002914546 0.44543404 -0.449680577 2.110432222
0.004605501 0.719926944 5 0 0.944228234
MCOLN1 ENSG00000090674.15 0.084298858 1 1 0.084298858
Brain_Anterior_cingulate_cortex_BA24 0.084298858
Brain_Anterior_cingulate_cortex_BA24 1 1 1 -1.726269953
-1.726269953 -1.726269953 NA 1 0 0.945052344
SHH ENSG00000164690.7 0.084388323 3 3 0.010868028 Brain_Amygdala
0.745418158 Brain_Caudate_basal_ganglia 1.357534484 0.624574286
0.624574286 -2.546913786 -0.324686758 -1.104709608 1.250374473
3 0 0.945153761
ZNF322 ENSG00000181315.10 0.084443013 13 3 0.006806277
Brain_Spinal_cord_cervical_c-1 0.158330235 Brain_Substantia_nigra
11.02623981 0.002800557 0.420448483 -2.706176924 -1.410709683
-2.14868189 0.399421644 3 0 0.945153761
OLFML1 ENSG00000183801.7 0.084544585 1 1 0.084544585
Brain_Hippocampus 0.084544585 Brain_Hippocampus 1 1 1
-1.724905054 -1.724905054 -1.724905054 NA 1 0 0.945534199
KNDC1 ENSG00000171798.17 0.084668757 3 3 0.02742416 Brain_Cerebellum
0.285136065 Brain_Caudate_basal_ganglia 1.464292795 0.424925226
0.424925226 -1.548045784 2.205426793 0.575411224 1.924773347
3 0 0.945562561
MRPL28 ENSG00000086504.15 0.084682288 7 7 0.020485605
Brain_Caudate_basal_ganglia 0.771578541 Brain_Hippocampus 3.931539233
0.167818085 0.167818085 -2.317332886 0.290310716 -1.326787057
1.039320595 7 0 0.945562561
RNF185 ENSG00000138942.15 0.084966809 4 3 0.053736676
Brain_Cerebellum 0.931368091 Brain_Frontal_Cortex_BA9 3.278043998
0.047980912 0.185164966 -0.086123691 1.928953173 1.327435311
0.950966239 3 0 0.946087088
STARD5 ENSG00000172345.13 0.085073026 2 2 0.047609148
Brain_Cortex 0.387148448 Brain_Cerebellum 1.068273293 0.931726707
0.931726707 -1.980840636 0.864800225 -0.558020205 2.01217195 2

0 0.946087088
OAZ2 ENSG00000180304.14 0.085087363 1 1 0.085087363
Brain_Frontal_Cortex_BA9 0.085087363 Brain_Frontal_Cortex_BA9 1 1
1 1.721901499 1.721901499 1.721901499 NA 1 0
0.946087088
TMEM33 ENSG00000109133.12 0.085095907 1 1 0.085095907
Brain_Cerebellum 0.085095907 Brain_Cerebellum 1 1 1 -1.721854347
-1.721854347 -1.721854347 NA 1 0 0.946087088
TMEM198 ENSG00000188760.10 0.08511998 1 1 0.08511998
Brain_Putamen_basal_ganglia 0.08511998 Brain_Putamen_basal_ganglia 1 1 1
-1.721721502 -1.721721502 -1.721721502 NA 1 0 0.946087088
NT5C1A ENSG00000116981.3 0.085288716 1 1 0.085288716
Brain_Cerebellar_Hemisphere 0.085288716 Brain_Cerebellar_Hemisphere 1 1
1 1.720791219 1.720791219 1.720791219 NA 1 0
0.946087088
EHMT1 ENSG00000181090.19 0.085391174 2 2 0.06402517 Brain_Hypothalamus
0.201184635 Brain_Frontal_Cortex_BA9 1.031138219 0.968861781
0.968861781 1.278183776 1.852004547 1.565094162 0.405752558
2 0 0.946087088
ROR1 ENSG00000185483.11 0.085402559 5 5 0.043220874
Brain_Hippocampus 0.909502653 Brain_Cortex 1.334056997
0.649231912 0.649231912 -2.021569223 0.800833176 -0.320242132
1.275211781 5 0 0.946087088
CTC-360G5.9 ENSG00000269486.2 0.085424269 13 4 0.176190759
Brain_Hippocampus 0.905757126 Brain_Nucleus_accumbens_basal_ganglia
10.09499733 0.00379803 0.407083185 -1.335991577 1.352577142
0.02643534 0.830118614 4 0 0.946087088
GLIPR1 ENSG00000139278.9 0.085454937 5 5 0.025360658
Brain_Caudate_basal_ganglia 0.680921151 Brain_Cortex 3.21686824
0.193545106 0.193545106 -2.235864355 0.411206461 -1.048017029
1.053206933 5 0 0.946087088
CCDC187 ENSG00000260220.6 0.085473096 1 1 0.085473096
Brain_Caudate_basal_ganglia 0.085473096 Brain_Caudate_basal_ganglia 1 1
1 -1.719776392 -1.719776392 -1.719776392 NA 1 0
0.946087088
SLC43A3 ENSG00000134802.17 0.085880827 2 2 0.036584836
Brain_Cerebellar_Hemisphere 0.037414551 Brain_Cerebellum 1.772319364
0.227680636 0.227680636 2.081211502 2.090367099 2.085789301
0.006473984 2 0 0.948453588
RP11-1293J14.1 ENSG00000271868.1 0.085896208 7 6 0.036538812
Brain_Cerebellar_Hemisphere 0.618733468 Brain_Nucleus_accumbens_basal_ganglia
3.782018733 0.051047472 0.170436975 -0.903836606 2.090880114
0.963220404 1.221767512 6 0 0.948453588
NRSN1 ENSG00000152954.11 0.08592797 4 4 0.022407469
Brain_Putamen_basal_ganglia 0.979835123 Brain_Spinal_cord_cervical_c-1
1.869423251 0.175301897 0.175301897 -2.283388925 0.08357648
-0.75641879 1.104120734 4 0 0.948453588
FAM220A ENSG00000178397.12 0.086034728 1 1 0.086034728
Brain_Frontal_Cortex_BA9 0.086034728 Brain_Frontal_Cortex_BA9 1 1
1 -1.716696021 -1.716696021 -1.716696021 NA 1 0
0.948453588
GBGT1 ENSG00000148288.11 0.086117833 5 5 0.003359403
Brain_Anterior_cingulate_cortex_BA24 0.812817615
Brain_Nucleus_accumbens_basal_ganglia 1.257840685 0.743528686
0.743528686 -0.864977347 2.932781421 0.194405126 1.553146021
5 0 0.948453588
FBXO18 ENSG00000134452.19 0.086129409 1 1 0.086129409
Brain_Cortex 0.086129409 Brain_Cortex 1 1 1 1.716178326
1.716178326 1.716178326 NA 1 0 0.948453588
C1QB ENSG00000173369.15 0.086161426 2 2 0.068897278
Brain_Anterior_cingulate_cortex_BA24 0.270111705 Brain_Hypothalamus
1.085273304 0.914726696 0.914726696 1.102805349 1.819092767

1.460949058	0.50649169 2	0	0.948453588
MGST2 ENSG00000085871.8	0.086327937	4	4	0.007334133
Brain_Nucleus_accumbens_basal_ganglia 0.580072121	Brain_Amygdala
1.841904461	0.324352956	0.324352956	-2.681279034	0.553279376
-1.299275154	1.482582078	4	0	0.949539443
TUBGCP3	ENSG00000126216.13	0.086600037	2	2	0.031511599
Brain_Cerebellum 0.733248779	Brain_Cortex	1.381051135	0.618948865
0.618948865	-2.150551733	-0.340807134	-1.245679434	1.279682678
2	0	0.950309951
XRCC4 ENSG00000152422.15	0.086636209	4	4	0.118170473
Brain_Caudate_basal_ganglia 0.862170658	Brain_Putamen_basal_ganglia
2.570666346	0.121428431	0.121428431	-0.173611673	1.562499035
0.978436901	0.786652238	4	0	0.950309951
LINC00957	ENSG00000235314.1	0.086696823	10	4	0.01604256
Brain_Hippocampus	0.892334359	Brain_Nucleus_accumbens_basal_ganglia
8.318130721	0.010944358	0.284879723	-0.135351006	2.40794586
1.915932226	0.755278495	4	0	0.950309951
PAFAH1B2	ENSG00000168092.13	0.086903357	3	3	0.042431026
Brain_Cortex	0.46069512 Brain_Cerebellum 1.84597148 0.160204694
0.160204694	0.737702719	2.029267749	1.431252594	0.651060841
3	0	0.950309951
NDRG4 ENSG00000103034.14	0.086934637	3	3	0.018602137
Brain_Anterior_cingulate_cortex_BA24	0.839780698	Brain_Cerebellum 1.79678464
0.299501498	0.299501498	-0.202174	2.35340946 0.912231831	1.30875382
3	0	0.950309951
IL11RA	ENSG00000137070.17	0.08695638 10	10	0.004348323	Brain_Amygdala
0.880121433	Brain_Cerebellum 2.788493496	0.214984116	0.214984116
-0.798717587	2.851721107	0.802119267	1.190413522	10	0
0.950309951
SPPL2B	ENSG00000005206.16	0.087008777	4	4	0.016836204
Brain_Hippocampus	0.880562955	Brain_Cerebellum 1.500081779
0.548559521	0.548559521	-2.390265342	1.253585171	-0.212479661
1.561816729	4	0	0.950309951
RGL1	ENSG00000143344.15	0.087010896	2	2	0.033919364
Brain_Caudate_basal_ganglia 0.811807593	Brain_Amygdala	1.175618353
0.824381647	0.824381647	-2.121028974	0.238094767	-0.941467103
1.668152395	2	0	0.950309951
NDST1-AS1	ENSG00000254333.1	0.087143162	11	3	0.012967519
Brain_Spinal_cord_cervical_c-1	0.187931729	Brain_Hippocampus
9.150181632	0.004251785	0.345465906	1.316722291	2.484660078
2.10403351 0.381559136	3	0	0.950309951
TAS1R3	ENSG00000169962.4	0.08728398 2	2	0.028815498
Brain_Cerebellar_Hemisphere 0.087072307	Brain_Cerebellum 1.856791709
0.143208291	0.143208291	-2.186001466	-1.711047705	-1.948524585
0.335843025	2	0	0.950309951
SLC9A3R2	ENSG00000065054.13	0.087424769	1	1	0.087424769
Brain_Cerebellar_Hemisphere 0.087424769	Brain_Cerebellar_Hemisphere 1	1
1	1.709141356	1.709141356	1.709141356	NA	1	0
0.950309951
DKFZP434A062	ENSG00000262075.3	0.087542867	1	1	0.087542867
Brain_Cortex	0.087542867	Brain_Cortex	1	1	1	-1.70850399
-1.70850399	-1.70850399	NA	1	0	0.950309951
NUP50-AS1	ENSG00000226328.6	0.087567663	4	3	0.045326824
Brain_Cortex	0.356267667	Brain_Frontal_Cortex_BA9	2.546584584
0.058102879	0.420414202	-1.462248076	2.00160878 0.679684473
1.497494521	3	0	0.950309951
FMO1	ENSG00000010932.16	0.087600296	2	2	0.027331612
Brain_Hypothalamus	0.27863377 Brain_Hippocampus	1.496528038
0.503471962	0.503471962	-2.206748829	-1.083393567	-1.645071198
0.794332124	2	0	0.950309951
ZNF484	ENSG00000127081.13	0.087617231	2	2	0.033644649
Brain_Cerebellar_Hemisphere 0.051027624	Brain_Cerebellum 1.724779071

0.275220929	0.275220929	-2.124304984	-1.951247395	-2.037776189
0.122370195	2	0	0.950309951
DDX41	ENSG00000183258.11	0.087661972	1	1	0.087661972
Brain_Frontal_Cortex_BA9	0.087661972	Brain_Frontal_Cortex_BA9	1	1
1	-1.707861889	-1.707861889	-1.707861889	NA	1	0
0.950309951
RIPK3	ENSG00000129465.15	0.087668207	2	2	0.050769562
Brain_Anterior_cingulate_cortex_BA24	0.067555458	Brain_Frontal_Cortex_BA9
1.473059382	0.526940618	0.526940618	1.827960646	1.953422419
1.890691533	0.08871487	2	0	0.950309951
FXR2	ENSG00000129245.11	0.087683848	1	1	0.087683848	Brain_Amygdala
0.087683848	Brain_Amygdala	1	1	1	-1.707744035	-1.707744035
-1.707744035	NA	1	0	0.950309951
FAM181B	ENSG00000182103.4	0.08777586	1	1	0.08777586
Brain_Caudate_basal_ganglia	0.08777586	Brain_Caudate_basal_ganglia	1	1	1
-1.707248575	-1.707248575	-1.707248575	NA	1	0	0.950309951
ZNF668	ENSG00000167394.12	0.087851043	2	2	0.042879754
Brain_Cerebellar_Hemisphere	0.081249255	Brain_Cortex	1.483043853
0.516956147	0.516956147	-2.024879355	-1.743483153	-1.884181254
0.198977163	2	0	0.950309951
BRI3	ENSG00000164713.9	0.088013723	1	1	0.088013723	Brain_Cerebellum
0.088013723	Brain_Cerebellum	1	1	1	-1.705969691	-1.705969691
-1.705969691	NA	1	0	0.950309951
JAZF1	ENSG00000153814.11	0.088087172	1	1	0.088087172
Brain_Spinal_cord_cervical_c-1	0.088087172	Brain_Spinal_cord_cervical_c-1
1	1	1	1.70557535	1.70557535	1.70557535	NA	1	0	0.950309951
PLEKHH1	ENSG00000054690.13	0.088272525	2	2	0.289939532
Brain_Cerebellum	0.984082541	Brain_Cerebellar_Hemisphere	1.872720261
0.127279739	0.127279739	-1.058254276	0.0199509	-0.519151688
0.762406192	2	0	0.950309951
ARL4A	ENSG00000122644.12	0.0883378	1	1	0.0883378	Brain_Cerebellar_Hemisphere
0.0883378	Brain_Cerebellar_Hemisphere	1	1	1	1.704231747
1.704231747	1.704231747	NA	1	0	0.950309951
PPIAL4A	ENSG00000263353.3	0.088338931	2	2	0.028087444
Brain_Cerebellum	0.229295067	Brain_Cerebellar_Hemisphere	1.610371989
0.389628011	0.389628011	-1.202176721	2.196062865	0.496943072
2.402918256	2	0	0.950309951
PRR29-AS1	ENSG00000264954.1	0.088395317	1	1	0.088395317
Brain_Cortex	0.088395317	Brain_Cortex	1	1	1	1.703923836
1.703923836	1.703923836	NA	1	0	0.950309951
SEPHS2	ENSG00000179918.18	0.088469802	2	2	0.05713908
Brain_Spinal_cord_cervical_c-1	0.225887593	Brain_Putamen_basal_ganglia
1.05406564	0.94593436	0.94593436	-1.211020383	1.902245402	0.34561251
2.201411348	2	0	0.950309951
CPM	ENSG00000135678.11	0.088485755	4	4	0.06726681	Brain_Cortex
0.353788794	Brain_Spinal_cord_cervical_c-1	2.468148059	0.102429572
0.102429572	-0.931226575	1.829887186	0.850344177	1.247022492
4	0	0.950309951
RP11-17E3.1	ENSG00000278532.1	0.088563213	1	1	0.088563213
Brain_Substantia_nigra	0.088563213	Brain_Substantia_nigra	1	1
1	-1.703025948	-1.703025948	-1.703025948	NA	1	0
0.950309951
MC1R	ENSG00000258839.3	0.088587992	11	5	0.015647103
Brain_Putamen_basal_ganglia	0.433020867	Brain_Anterior_cingulate_cortex_BA24
8.192879312	0.011249257	0.317893258	0.784032795	2.417044521
1.756136101	0.512242804	5	0	0.950309951
AC005498.4	ENSG00000267224.1	0.088657402	4	4	0.056340722
Brain_Cerebellum	0.660332619	Brain_Frontal_Cortex_BA9	2.384323599
0.100308633	0.100308633	-0.439453983	1.908390823	0.917530139
1.009582161	4	0	0.950309951
PVALB	ENSG00000100362.12	0.088683904	1	1	0.088683904	Brain_Cerebellum
0.088683904	Brain_Cerebellum	1	1	1	1.702381349	1.702381349

1.702381349 NA 1 0 0.950309951
PSEN1 ENSG00000080815.18 0.088796594 1 1 0.088796594
Brain_Cerebellar_Hemisphere 0.088796594 Brain_Cerebellar_Hemisphere 1 1
1 1.701780122 1.701780122 1.701780122 NA 1 0
0.950309951
PCBP3 ENSG00000183570.16 0.088903028 6 4 0.006209366
Brain_Frontal_Cortex_BA9 0.082663147 Brain_Cerebellum 4.687873198
0.034522468 0.185679956 -2.73651554 -1.735438606 -2.262617119
0.389736708 4 0 0.950309951
LINC01451 ENSG00000279141.3 0.089056533 1 1 0.089056533
Brain_Cerebellum 0.089056533 Brain_Cerebellum 1 1 1 1.700395632
1.700395632 1.700395632 NA 1 0 0.950309951
C1GALT1C1L ENSG00000223658.7 0.089064575 1 1 0.089064575
Brain_Spinal_cord_cervical_c-1 0.089064575 Brain_Spinal_cord_cervical_c-1
1 1 1 1.70035285 1.70035285 1.70035285 NA 1 0 0.950309951
RP11-758H9.2 ENSG00000273702.1 0.08910603 2 2 0.030585417
Brain_Cortex 0.058453872 Brain_Cerebellar_Hemisphere 1.753557502
0.246442498 0.246442498 1.892278587 2.162425197 2.027351892
0.1910225 2 0 0.950309951
DNAJC21 ENSG00000168724.14 0.089108486 1 1 0.089108486
Brain_Frontal_Cortex_BA9 0.089108486 Brain_Frontal_Cortex_BA9 1 1
1 -1.700119309 -1.700119309 -1.700119309 NA 1 0
0.950309951
TNFSF14 ENSG00000125735.10 0.089114833 1 1 0.089114833
Brain_Substantia_nigra 0.089114833 Brain_Substantia_nigra 1 1
1 -1.70008556 -1.70008556 -1.70008556 NA 1 0
0.950309951
C1orf27 ENSG00000157181.15 0.089114906 6 5 0.096335827
Brain_Hypothalamus 0.650430181 Brain_Cerebellar_Hemisphere 3.449317712
0.042582805 0.300114295 -1.662883153 0.907996876 -0.39929381
0.982115665 5 0 0.950309951
AP5S1 ENSG00000125843.10 0.089286229 2 2 0.028684462 Brain_Cerebellum
0.393237703 Brain_Cerebellar_Hemisphere 1.475861992 0.524138008
0.524138008 0.853760503 2.187796041 1.520778272 0.943305575
2 0 0.951411752
KIF9 ENSG00000088727.12 0.089464069 1 1 0.089464069
Brain_Hypothalamus 0.089464069 Brain_Hypothalamus 1 1 1
1.698231546 1.698231546 1.698231546 NA 1 0 0.952099339
AC011995.1 ENSG00000237720.1 0.089486858 4 4 0.038205488
Brain_Caudate_basal_ganglia 0.517519739 Brain_Nucleus_accumbens_basal_ganglia
1.409363882 0.59998248 0.59998248 -2.072643297 1.647344 -0.580262295
1.595885526 4 0 0.952099339
ST8SIA6 ENSG00000148488.15 0.089594123 1 1 0.089594123
Brain_Cerebellar_Hemisphere 0.089594123 Brain_Cerebellar_Hemisphere 1 1
1 1.697542609 1.697542609 1.697542609 NA 1 0
0.952420243
RAB17 ENSG00000124839.12 0.089813549 3 2 0.023881641 Brain_Cortex
0.11289235 Brain_Anterior_cingulate_cortex_BA24 2.592689124 0.012607052
0.394703824 1.585325714 2.259028029 1.98614668 0.354605572 2
0 0.952420243
HLA-DQA1 ENSG00000196735.11 0.08988419 9 7 0.007975966
Brain_Anterior_cingulate_cortex_BA24 0.847472721 Brain_Cortex
4.059890872 0.092429676 0.217651193 -1.29930358 2.65308552
0.046952195 1.140938789 7 0 0.952420243
NMB ENSG00000197696.9 0.089892054 1 1 0.089892054
Brain_Caudate_basal_ganglia 0.089892054 Brain_Caudate_basal_ganglia 1 1
1 -1.695967401 -1.695967401 -1.695967401 NA 1 0
0.952420243
GPD1 ENSG00000167588.12 0.089924308 2 2 0.039524027 Brain_Cerebellum
0.172438179 Brain_Cerebellar_Hemisphere 1.854696251 0.145303749
0.145303749 1.364411217 2.058689139 1.711550178 0.490928627
2 0 0.952420243

SPAG9 ENSG00000008294.20 0.089925462 2 1 0.071547433 Brain_Cerebellum
0.114727175 Brain_Putamen_basal_ganglia 1.985522352 0.014477648
1.985522352 -1.577297117 1.801987111 0.112344997 2.389514793
1 0 0.952420243
AKAP8L ENSG00000011243.17 0.090057514 2 2 0.0329849
Brain_Substantia_nigra 0.610019471 Brain_Cerebellum 1.003605183
0.996394817 0.996394817 -2.13226704 0.510045663 -0.811110688
1.86839723 2 0 0.95259636
RNPS1 ENSG00000205937.11 0.090146052 11 3 0.015569622
Brain_Hippocampus 0.916936926 Brain_Frontal_Cortex_BA9 8.435016572
0.000323129 0.848873555 -2.418850878 0.757234388 0.10433538
0.900773096 3 0 0.95259636
SHROOM3 ENSG00000138771.14 0.090194422 4 4 0.044759431
Brain_Cerebellar_Hemisphere 0.74929592 Brain_Cerebellum 1.726020587
0.315631424 0.315631424 -2.00690827 1.050648503 -0.546686001
1.273835805 4 0 0.95259636
ENKUR ENSG00000151023.16 0.090214436 2 2 0.081491226 Brain_Amygdala
0.21245362 Brain_Putamen_basal_ganglia 1.048413489 0.951586511
0.951586511 1.246846954 1.74209841 1.494472682 0.350195663 2
0 0.95259636
THAP3 ENSG00000041988.15 0.090287921 3 3 0.014902212 Brain_Cerebellum
0.315393239 Brain_Cortex 2.376057945 0.101018659 0.101018659
-2.434746837 -1.003969657 -1.644766494 0.726961225 3 0
0.952653319
HMOX2 ENSG00000103415.11 0.090445378 2 1 0.082283897 Brain_Cerebellum
0.102139479 Brain_Nucleus_accumbens_basal_ganglia 1.983552653
0.016447347 1.983552653 -1.63456877 1.737585386 0.051508308
2.384473071 1 0 0.953595542
REEP2 ENSG00000132563.15 0.090516819 3 3 0.175781806 Brain_Cerebellum
0.289783178 Brain_Cortex 1.465197411 0.592114889 0.592114889
-1.058597386 1.353857621 0.495810542 1.348582076 3 0
0.953630131
AMPD3 ENSG00000133805.15 0.090763073 13 8 0.057082376
Brain_Frontal_Cortex_BA9 0.954063394 Brain_Cerebellum 6.882868544
0.058448404 0.268434677 -0.740009369 1.902679518 0.493351588
0.678818347 8 0 0.955505003
AKR1C2 ENSG00000151632.17 0.090832845 12 1 0.016302379
Brain_Hypothalamus 0.295772923 Brain_Caudate_basal_ganglia 11.00517619
0.00074031 11.00517619 1.045541169 2.402074707 1.619291452
0.353298999 1 0 0.955520549
SPACA6 ENSG00000182310.13 0.091131079 1 1 0.091131079
Brain_Frontal_Cortex_BA9 0.091131079 Brain_Frontal_Cortex_BA9 1 1
1 -1.68946121 -1.68946121 -1.68946121 NA 1 0
0.957814531
SEMA3B ENSG00000012171.19 0.091187832 1 1 0.091187832
Brain_Nucleus_accumbens_basal_ganglia 0.091187832
Brain_Nucleus_accumbens_basal_ganglia 1 1 1 -1.689164908
-1.689164908 -1.689164908 NA 1 0 0.957814531
C21orf91 ENSG00000154642.10 0.091259422 2 2 0.043391012
Brain_Cerebellum 0.055746059 Brain_Cerebellar_Hemisphere 1.620352874
0.379647126 0.379647126 1.913015574 2.019926505 1.966471039
0.075597444 2 0 0.957847387
PSME4 ENSG00000068878.14 0.091394192 1 1 0.091394192
Brain_Substantia_nigra 0.091394192 Brain_Substantia_nigra 1 1
1 -1.688088758 -1.688088758 -1.688088758 NA 1 0
0.958542836
HIST1H2BJ ENSG00000124635.8 0.091575448 1 1 0.091575448
Brain_Cerebellar_Hemisphere 0.091575448 Brain_Cerebellar_Hemisphere 1 1
1 -1.687145138 -1.687145138 -1.687145138 NA 1 0
0.958592126
KPNB1 ENSG00000108424.9 0.091637038 1 1 0.091637038
Brain_Cerebellar_Hemisphere 0.091637038 Brain_Cerebellar_Hemisphere 1 1

1 1.68682484 1.68682484 1.68682484 NA 1 0 0.958592126
CCDC127 ENSG00000164366.3 0.091825839 11 4 0.041386339
Brain_Hypothalamus 0.898034552 Brain_Frontal_Cortex_BA9 9.121795342
0.002723471 0.31965708 0.128144587 2.039638557 1.141047525
0.52415788 4 0 0.958592126
ZNF596 ENSG00000172748.13 0.091859236 1 1 0.091859236
Brain_Cerebellum 0.091859236 Brain_Cerebellum 1 1 1 -1.685670738
-1.685670738 -1.685670738 NA 1 0 0.958592126
MT1F ENSG00000198417.6 0.091996854 1 1 0.091996854 Brain_Cerebellum
0.091996854 Brain_Cerebellum 1 1 1 1.684957073 1.684957073
1.684957073 NA 1 0 0.958592126
FTCD ENSG00000160282.13 0.092059044 12 3 0.080251072 Brain_Cerebellum
0.670041639 Brain_Anterior_cingulate_cortex_BA24 8.36707897 0.015381579
0.523622152 -1.749231147 0.76737811 -1.00850732 0.692227245 3
0 0.958592126
KCNJ12 ENSG00000184185.9 0.092067481 4 4 0.0689058
Brain_Nucleus_accumbens_basal_ganglia 0.448043731 Brain_Hypothalamus
1.621910508 0.455555987 0.455555987 -1.273199904 1.819036905
0.258029471 1.504342734 4 0 0.958592126
RP11-379F4.7 ENSG00000272087.1 0.092129116 1 1 0.092129116
Brain_Hippocampus 0.092129116 Brain_Hippocampus 1 1 1
-1.684271991 -1.684271991 -1.684271991 NA 1 0 0.958592126
RGS16 ENSG00000143333.6 0.092169526 2 2 0.031150708
Brain_Nucleus_accumbens_basal_ganglia 0.681656733 Brain_Cerebellar_Hemisphere
1.343546465 0.656453535 0.656453535 -2.155142201 -0.41020342
-1.28267281 1.233858045 2 0 0.958592126
B4GALT5 ENSG00000158470.5 0.092229716 3 3 0.013617352
Brain_Cerebellar_Hemisphere 0.698039171 Brain_Spinal_cord_cervical_c-1
1.813576656 0.174336611 0.174336611 -2.467201988 0.387968735
-1.367402546 1.53633681 3 0 0.958592126
LINC01151 ENSG00000253819.1 0.092277051 1 1 0.092277051
Brain_Cerebellum 0.092277051 Brain_Cerebellum 1 1 1 -1.683506659
-1.683506659 -1.683506659 NA 1 0 0.958592126
NALCN ENSG00000102452.15 0.092350663 3 3 0.015574307
Brain_Cerebellar_Hemisphere 0.762226944 Brain_Caudate_basal_ganglia
1.169342636 0.837110535 0.837110535 0.302557714 2.418741411
1.218861906 1.086218016 3 0 0.958592126
LYAR ENSG00000145220.13 0.092514405 1 1 0.092514405
Brain_Putamen_basal_ganglia 0.092514405 Brain_Putamen_basal_ganglia 1 1
1 -1.682280784 -1.682280784 -1.682280784 NA 1 0
0.958592126
MBD5 ENSG00000204406.12 0.092540247 1 1 0.092540247 Brain_Cerebellum
0.092540247 Brain_Cerebellum 1 1 1 -1.682147473 -1.682147473
-1.682147473 NA 1 0 0.958592126
LINC00472 ENSG00000233237.6 0.092588611 1 1 0.092588611
Brain_Amygdala 0.092588611 Brain_Amygdala 1 1 1 1.681898051
1.681898051 1.681898051 NA 1 0 0.958592126
GXYLT2 ENSG00000172986.12 0.092598553 9 3 0.106074025
Brain_Hippocampus 0.997644263 Brain_Putamen_basal_ganglia 7.450730629
0.014613336 0.408852588 -1.616093846 0.473402767 -0.200609533
0.717215043 3 0 0.958592126
LINC01504 ENSG00000225434.2 0.092836042 2 2 0.029605695
Brain_Spinal_cord_cervical_c-1 0.910796373 Brain_Substantia_nigra
1.118890947 0.881109053 0.881109053 -0.112034095 2.175326
1.031645953 1.617407835 2 0 0.958592126
SMAD4 ENSG00000141646.13 0.092869953 2 2 0.03843264
Brain_Substantia_nigra 0.710685419 Brain_Cerebellum 1.147432193
0.852567807 0.852567807 0.370935707 2.070210464 1.220573086
1.201568704 2 0 0.958592126
PSG8-AS1 ENSG00000225877.1 0.092931349 2 2 0.029344843
Brain_Caudate_basal_ganglia 0.945348853 Brain_Cerebellar_Hemisphere
1.000604229 0.999395771 0.999395771 -2.178822689 0.068548702

-1.055136994 1.58913155 2 0 0.958592126
SLC35F4 ENSG00000151812.14 0.092993468 3 3 0.06453311
Brain_Putamen_basal_ganglia 0.18035051 Brain_Cerebellar_Hemisphere 1.614910562
0.399849432 0.399849432 1.339676595 1.848478789 1.517289846
0.287069744 3 0 0.958592126
ZNF528 ENSG00000167555.13 0.093024648 13 1 0.0413572
Brain_Hippocampus 0.416235919 Brain_Cerebellar_Hemisphere 11.97452156
0.0004555 11.97452156 -2.039930983 -0.812968848 -1.608012512
0.33493106 1 0 0.958592126
CORO6 ENSG00000167549.18 0.093253239 4 4 0.047200394 Brain_Cerebellum
0.988328796 Brain_Cortex 2.153221447 0.280863166 0.280863166
-1.984497616 1.149529271 -0.071452634 1.357100131 4 0
0.958592126
ANXA9 ENSG00000143412.9 0.09336059 4 4 0.105330988
Brain_Frontal_Cortex_BA9 0.369580731 Brain_Caudate_basal_ganglia
1.960320119 0.32950995 0.32950995 -1.081563204 1.61954065 0.677493177
1.209232417 4 0 0.958592126
TPP1 ENSG00000166340.15 0.093411958 1 1 0.093411958 Brain_Cerebellum
0.093411958 Brain_Cerebellum 1 1 1 1.677667856 1.677667856
1.677667856 NA 1 0 0.958592126
RP11-496H1.2 ENSG00000273308.1 0.093420632 2 2 0.038369781
Brain_Cortex 0.285561757 Brain_Cerebellum 1.197142979 0.802857021
0.802857021 1.067908569 2.070882472 1.56939552 0.709209648 2
0 0.958592126
AQP11 ENSG00000178301.3 0.093433133 6 5 0.047025565
Brain_Hypothalamus 0.877636759 Brain_Anterior_cingulate_cortex_BA24
3.435267322 0.056032628 0.247272738 -1.343581417 1.986069882
0.253940953 1.122308882 5 0 0.958592126
PRKAR2A ENSG00000114302.15 0.093468976 2 2 0.104733156
Brain_Substantia_nigra 0.173043316 Brain_Anterior_cingulate_cortex_BA24
1.05387396 0.94612604 0.94612604 -1.362489943 1.62232791 0.129918983
2.110584944 2 0 0.958592126
HSD11B2 ENSG00000176387.6 0.093551324 1 1 0.093551324
Brain_Frontal_Cortex_BA9 0.093551324 Brain_Frontal_Cortex_BA9 1 1
1 1.676954783 1.676954783 1.676954783 NA 1 0
0.958592126
CYTH4 ENSG00000100055.20 0.093693137 1 1 0.093693137
Brain_Spinal_cord_cervical_c-1 0.093693137 Brain_Spinal_cord_cervical_c-1
1 1 1 -1.676230064 -1.676230064 -1.676230064 NA 1 0
0.958592126
ARRDC1 ENSG00000197070.13 0.093708684 4 3 0.056644334
Brain_Hypothalamus 0.982277195 Brain_Cerebellar_Hemisphere 2.885609519
0.041492454 0.251738324 -0.022214069 1.906045256 0.764225214
0.86949778 3 0 0.958592126
BICDL2 ENSG00000162069.14 0.093776221 2 2 0.041822061
Brain_Amygdala 0.802342439 Brain_Frontal_Cortex_BA9 1.243457158
0.756542842 0.756542842 -0.250316705 2.035286427 0.892484861
1.616165473 2 0 0.958592126
CRYBB2 ENSG00000244752.2 0.093807433 4 3 0.16600354
Brain_Caudate_basal_ganglia 0.474350339 Brain_Nucleus_accumbens_basal_ganglia
2.094284825 0.004832048 0.478639845 -0.715418735 1.385160029
0.689160966 0.951419997 3 0 0.958592126
ANKMY2 ENSG00000106524.8 0.093811208 1 1 0.093811208
Brain_Cerebellum 0.093811208 Brain_Cerebellum 1 1 1 -1.675627351
-1.675627351 -1.675627351 NA 1 0 0.958592126
HFE ENSG00000010704.18 0.093867648 1 1 0.093867648
Brain_Nucleus_accumbens_basal_ganglia 0.093867648
Brain_Nucleus_accumbens_basal_ganglia 1 1 1 -1.675339457
-1.675339457 -1.675339457 NA 1 0 0.958592126
PPP2R2A ENSG00000221914.9 0.09392554 1 1 0.09392554 Brain_Hippocampus
0.09392554 Brain_Hippocampus 1 1 1 1.675044301 1.675044301
1.675044301 NA 1 0 0.958592126

CTD-2547L24.4 ENSG00000260810.1 0.093948532 2 2 0.045086256
Brain_Hypothalamus 0.339970896 Brain_Cerebellar_Hemisphere 1.054972807
0.945027193 0.945027193 0.954222761 2.003848829 1.479035795
0.742197711 2 0 0.958592126
EPB41L4A-AS2 ENSG00000278921.2 0.094058511 1 1 0.094058511
Brain_Nucleus_accumbens_basal_ganglia 0.094058511
Brain_Nucleus_accumbens_basal_ganglia 1 1 1 -1.674366916
-1.674366916 -1.674366916 NA 1 0 0.958592126
ZNF471 ENSG00000196263.7 0.094171404 9 4 0.022534254
Brain_Amygdala 0.196851307 Brain_Cerebellar_Hemisphere 6.561342542
0.007020493 0.39609464 1.290574372 2.281239915 1.743357478
0.329423116 4 0 0.958592126
CIDEC ENSG00000187288.10 0.094263884 4 4 0.023176194
Brain_Hippocampus 0.84204362 Brain_Caudate_basal_ganglia 1.572461187
0.452161898 0.452161898 -0.199280132 2.270517639 0.964956474
1.148223554 4 0 0.958592126
CSTF2T ENSG00000177613.8 0.094315817 2 2 0.03283565
Brain_Substantia_nigra 0.633413968 Brain_Putamen_basal_ganglia
1.031579761 0.968420239 0.968420239 -2.134087123 0.476927192
-0.828579965 1.846265928 2 0 0.958592126
RP11-571I18.4 ENSG00000272650.1 0.094338259 1 1 0.094338259
Brain_Cortex 0.094338259 Brain_Cortex 1 1 1 1.672944313
1.672944313 1.672944313 NA 1 0 0.958592126
RP11-390F4.6 ENSG00000236924.1 0.094411302 1 1 0.094411302
Brain_Cortex 0.094411302 Brain_Cortex 1 1 1 1.672573426
1.672573426 1.672573426 NA 1 0 0.958592126
DUSP14 ENSG00000276023.4 0.094493425 4 4 0.011023358
Brain_Substantia_nigra 0.369397782 Brain_Nucleus_accumbens_basal_ganglia
2.975394245 0.152076618 0.152076618 -2.541957499 -0.897601981
-1.527769343 0.779063433 4 0 0.958592126
WIPF2 ENSG00000171475.13 0.09452434 1 1 0.09452434 Brain_Substantia_nigra
0.09452434 Brain_Substantia_nigra 1 1 1 -1.671999908
-1.671999908 -1.671999908 NA 1 0 0.958592126
PTCD1 ENSG00000106246.17 0.094527517 1 1 0.094527517
Brain_Frontal_Cortex_BA9 0.094527517 Brain_Frontal_Cortex_BA9 1 1
1 -1.671983798 -1.671983798 -1.671983798 NA 1 0
0.958592126
HES7 ENSG00000179111.8 0.094554384 3 1 0.088484674 Brain_Cortex
0.103765758 Brain_Cerebellum 2.964720439 0.000121098 2.964720439
1.626865072 1.703445794 1.662125591 0.038648306 1 0
0.958592126
RP11-284F21.11 ENSG00000272971.1 0.094671486 1 1 0.094671486
Brain_Nucleus_accumbens_basal_ganglia 0.094671486
Brain_Nucleus_accumbens_basal_ganglia 1 1 1 -1.671254161
-1.671254161 -1.671254161 NA 1 0 0.958592126
LRRC10B ENSG00000204950.3 0.094687608 1 1 0.094687608
Brain_Cerebellar_Hemisphere 0.094687608 Brain_Cerebellar_Hemisphere 1 1
1 -1.671172514 -1.671172514 -1.671172514 NA 1 0
0.958592126
AJAP1 ENSG00000196581.10 0.09482909 2 2 0.042733094
Brain_Spinal_cord_cervical_c-1 0.522509372 Brain_Hippocampus 1.06752901
0.93247099 0.93247099 -2.026309349 0.639482074 -0.693413638
1.884999193 2 0 0.9593303
ABCC4 ENSG00000125257.13 0.094951186 2 2 0.050507931
Brain_Putamen_basal_ganglia 0.42453148 Brain_Frontal_Cortex_BA9 1.071270864
0.928729136 0.928729136 -0.798584324 1.955637001 0.578526338
1.947528576 2 0 0.959871415
RP4-621F18.2 ENSG00000282057.1 0.095035178 1 1 0.095035178
Brain_Caudate_basal_ganglia 0.095035178 Brain_Caudate_basal_ganglia 1 1
1 1.669414923 1.669414923 1.669414923 NA 1 0
0.960026845
CFDP1 ENSG00000153774.8 0.095120141 6 4 0.039521501 Brain_Amygdala

0.955165986 Brain_Nucleus_accumbens_basal_ganglia 3.992414076
0.009182672 0.148281653 -0.056220706 2.058715494 0.92829023
0.735253953 4 0 0.960191841
SOD2 ENSG00000112096.16 0.09526714 1 1 0.09526714 Brain_Cerebellum 0.09526714
Brain_Cerebellum 1 1 1 -1.668244805 -1.668244805 -1.668244805
NA 1 0 0.960424307
INMT ENSG00000241644.2 0.095306682 1 1 0.095306682
Brain_Caudate_basal_ganglia 0.095306682 Brain_Caudate_basal_ganglia 1 1
1 -1.668045566 -1.668045566 -1.668045566 NA 1 0
0.960424307
OPTN ENSG00000123240.16 0.095349107 5 5 0.031664013
Brain_Putamen_basal_ganglia 0.625925284 Brain_Cerebellar_Hemisphere
1.451834601 0.56772549 0.56772549 -2.148626582 1.760004027
0.087380711 1.524484087 5 0 0.960424307
GALNT10 ENSG00000164574.15 0.095429276 4 4 0.040771707
Brain_Hypothalamus 0.829919634 Brain_Cerebellum 2.201365473
0.270856116 0.270856116 -2.045844145 -0.214804642 -1.257788714
0.762185707 4 0 0.960528064
FBXO3 ENSG00000110429.13 0.095701514 1 1 0.095701514
Brain_Putamen_basal_ganglia 0.095701514 Brain_Putamen_basal_ganglia 1 1
1 -1.666059751 -1.666059751 -1.666059751 NA 1 0
0.960528064
PPIE ENSG00000084072.16 0.095740798 13 1 0.043987767
Brain_Nucleus_accumbens_basal_ganglia 0.148147671 Brain_Cerebellar_Hemisphere
12.60598054 1.04E-05 12.60598054 1.446105293 2.014207361
1.641643147 0.137956255 1 0 0.960528064
PTRH2 ENSG00000141378.14 0.095787934 1 1 0.095787934 Brain_Cerebellum
0.095787934 Brain_Cerebellum 1 1 1 1.665625978 1.665625978
1.665625978 NA 1 0 0.960528064
CAMKMT ENSG00000143919.14 0.095799149 1 1 0.095799149
Brain_Hippocampus 0.095799149 Brain_Hippocampus 1 1 1
-1.665569708 -1.665569708 -1.665569708 NA 1 0 0.960528064
AK2 ENSG00000004455.16 0.095823647 1 1 0.095823647
Brain_Nucleus_accumbens_basal_ganglia 0.095823647
Brain_Nucleus_accumbens_basal_ganglia 1 1 1 1.665446813
1.665446813 1.665446813 NA 1 0 0.960528064
ZNF549 ENSG00000121406.8 0.09591864 12 8 0.015945646
Brain_Putamen_basal_ganglia 0.998571884 Brain_Caudate_basal_ganglia 6.09744477
3.67E-05 0.265330879 -1.477292298 2.410157246 0.49137155
1.018735706 8 0 0.960528064
AAK1 ENSG00000115977.18 0.095950148 1 1 0.095950148 Brain_Cerebellum
0.095950148 Brain_Cerebellum 1 1 1 -1.664812606 -1.664812606
-1.664812606 NA 1 0 0.960528064
RP11-238F2.1 ENSG00000271239.1 0.096030579 2 2 0.032635217
Brain_Spinal_cord_cervical_c-1 0.976642878 Brain_Frontal_Cortex_BA9
1.174262753 0.825737247 0.825737247 -2.136542545 -0.029277993
-1.082910269 1.490061055 2 0 0.960528064
AC092835.2 ENSG00000233757.6 0.096096354 1 1 0.096096354
Brain_Cerebellar_Hemisphere 0.096096354 Brain_Cerebellar_Hemisphere 1 1
1 -1.664080446 -1.664080446 -1.664080446 NA 1 0
0.960528064
GFM1 ENSG00000168827.14 0.096114594 8 1 0.07322472
Brain_Spinal_cord_cervical_c-1 0.191058049 Brain_Cerebellar_Hemisphere
7.592010139 0.00876808 7.592010139 1.307455527 1.791427492
1.621810407 0.18766675 1 0 0.960528064
BOD1 ENSG00000145919.10 0.096254204 1 1 0.096254204
Brain_Anterior_cingulate_cortex_BA24 0.096254204
Brain_Anterior_cingulate_cortex_BA24 1 1 1 -1.663290977
-1.663290977 -1.663290977 NA 1 0 0.960902993
RRAS2 ENSG00000133818.13 0.096289472 3 3 0.122566754
Brain_Nucleus_accumbens_basal_ganglia 0.539867642 Brain_Cerebellum
1.816856622 0.191297728 0.191297728 -1.544089081 1.464557488

-0.230848249 1.540300638 3 0 0.960902993
RSBN1 ENSG00000081019.13 0.096605149 1 1 0.096605149
Brain_Hippocampus 0.096605149 Brain_Hippocampus 1 1 1
1.661539468 1.661539468 1.661539468 NA 1 0 0.963272587
IGFBP5 ENSG00000115461.4 0.096731339 3 3 0.013922232
Brain_Cerebellum 0.578408056 Brain_Frontal_Cortex_BA9 1.564446306
0.465446634 0.465446634 0.555711555 2.459263693 1.519147269
0.951990298 3 0 0.963272587
ZNF468 ENSG00000204604.9 0.096860856 7 7 0.028654703
Brain_Cerebellum 0.597947281 Brain_Substantia_nigra 2.046656103
0.112260711 0.112260711 -1.606580257 2.188204592 -0.364103616
1.347131846 7 0 0.963272587
ZNF225 ENSG00000256294.7 0.096993132 1 1 0.096993132
Brain_Hypothalamus 0.096993132 Brain_Hypothalamus 1 1 1
1.659609025 1.659609025 1.659609025 NA 1 0 0.963272587
RP11-255P5.2 ENSG00000272542.1 0.097096418 5 5 0.099226646
Brain_Hippocampus 0.906044814 Brain_Cerebellum 2.562716888 0.1457604
0.1457604 -1.648614451 0.399257302 -0.533019136 1.003313365 5
0 0.963272587
GJB5 ENSG00000189280.3 0.097102691 1 1 0.097102691
Brain_Frontal_Cortex_BA9 0.097102691 Brain_Frontal_Cortex_BA9 1 1
1 1.659065022 1.659065022 1.659065022 NA 1 0
0.963272587
CRYBG3 ENSG00000080200.9 0.097154768 4 4 0.092967807
Brain_Anterior_cingulate_cortex_BA24 0.818708838 Brain_Frontal_Cortex_BA9
2.382473062 0.137788126 0.137788126 -1.679946081 -0.229205963
-1.083601499 0.685886778 4 0 0.963272587
CMTM8 ENSG00000170293.8 0.097157957 1 1 0.097157957 Brain_Cerebellum
0.097157957 Brain_Cerebellum 1 1 1 -1.65879079 -1.65879079
-1.65879079 NA 1 0 0.963272587
GPRC5C ENSG00000170412.16 0.097320531 2 2 0.087872843
Brain_Cerebellum 0.300824667 Brain_Cerebellar_Hemisphere 1.916316032
0.083683968 0.083683968 1.03466654 1.706726802 1.370696671
0.475218368 2 0 0.963272587
CLN6 ENSG00000128973.12 0.097342361 3 3 0.017021507
Brain_Hippocampus 0.688047853 Brain_Cerebellar_Hemisphere 1.777802309
0.335526244 0.335526244 -2.386242845 -0.401505686 -1.22868531
1.032791156 3 0 0.963272587
PDE7B ENSG00000171408.13 0.09743598 1 1 0.09743598 Brain_Hypothalamus
0.09743598 Brain_Hypothalamus 1 1 1 1.657413122 1.657413122
1.657413122 NA 1 0 0.963272587
FAM3B ENSG00000183844.16 0.097566253 4 4 0.177443815
Brain_Nucleus_accumbens_basal_ganglia 0.820554498 Brain_Substantia_nigra
2.072238611 0.156029561 0.156029561 -1.150330088 1.348667419
-0.026124853 1.075706681 4 0 0.963272587
DSTYK ENSG00000133059.16 0.097629749 10 5 0.034526355 Brain_Cortex
0.660901318 Brain_Frontal_Cortex_BA9 7.841439955 0.009881724
0.320961422 -2.1138702 0.438669103 -1.109704939 0.657417207 5
0 0.963272587
ZBTB4 ENSG00000174282.11 0.097658718 1 1 0.097658718 Brain_Cortex
0.097658718 Brain_Cortex 1 1 1 1.656311671 1.656311671
1.656311671 NA 1 0 0.963272587
RRP7A ENSG00000189306.10 0.09777992 12 10 0.030609119
Brain_Putamen_basal_ganglia 0.965823691 Brain_Frontal_Cortex_BA9
4.529027853 0.012807618 0.154392073 -1.020174557 2.162117519
0.448902092 0.907259448 10 0 0.963272587
LETM2 ENSG00000165046.12 0.097810459 1 1 0.097810459 Brain_Cortex
0.097810459 Brain_Cortex 1 1 1 1.655562453 1.655562453
1.655562453 NA 1 0 0.963272587
RWDD3 ENSG00000122481.16 0.097924484 13 2 0.106695301
Brain_Substantia_nigra 0.993493681 Brain_Amygdala 11.54708915
0.003370548 0.388833351 -1.613226531 0.335680945 -0.184885217

0.517552733 2 0 0.963272587
ARRB2 ENSG00000141480.17 0.097981345 2 2 0.074485818 Brain_Cerebellum
0.973018272 Brain_Hippocampus 1.548422359 0.451577641
0.451577641 -0.033823029 1.783617637 0.874897304 1.285124619
2 0 0.963272587
MMP24-AS1 ENSG00000126005.16 0.097999168 13 4 0.038876863
Brain_Anterior_cingulate_cortex_BA24 0.917939468 Brain_Cerebellum
10.00786652 0.004916761 0.545206908 -2.065487866 0.513439459
-0.091877126 0.692708774 4 0 0.963272587
ZNF567 ENSG00000189042.13 0.098014659 3 3 0.020456807
Brain_Cerebellum 0.175671976 Brain_Hypothalamus 2.238687193
0.161510799 0.161510799 -2.317862286 -1.354201891 -1.697193619
0.53850599 3 0 0.963272587
TMEM42 ENSG00000169964.7 0.098078247 2 2 0.037221726
Brain_Hypothalamus 0.981144432 Brain_Nucleus_accumbens_basal_ganglia
1.245127086 0.754872914 0.754872914 -2.083323722 0.023634149
-1.029844786 1.489844198 2 0 0.963272587
OR3A2 ENSG00000221882.2 0.098102222 7 4 0.005396888 Brain_Amygdala
0.555483569 Brain_Hypothalamus 4.116936289 0.002346711
0.340017372 -1.656112279 2.782337511 0.719709678 1.288977855
4 0 0.963272587
DNAJC30 ENSG00000176410.7 0.098110459 7 3 0.009354101
Brain_Nucleus_accumbens_basal_ganglia 0.256470528
Brain_Anterior_cingulate_cortex_BA24 5.571938987 0.0126935 0.281277365
1.134772795 2.598833951 1.998693381 0.547426178 3 0
0.963272587
SF3A1 ENSG00000099995.18 0.098210231 1 1 0.098210231
Brain_Hypothalamus 0.098210231 Brain_Hypothalamus 1 1 1
-1.653593024 -1.653593024 -1.653593024 NA 1 0 0.963575976
EIF3L ENSG00000100129.17 0.098436704 1 1 0.098436704
Brain_Hippocampus 0.098436704 Brain_Hippocampus 1 1 1
1.652480173 1.652480173 1.652480173 NA 1 0 0.964541023
MYBPC3 ENSG00000134571.10 0.098576238 3 3 0.041078535
Brain_Amygdala 0.884100776 Brain_Cortex 1.761046797 0.391106385
0.391106385 -1.506530501 2.042736446 0.130144393 1.790648395
3 0 0.964541023
FAM19A2 ENSG00000198673.10 0.098580662 2 2 0.111788931
Brain_Hippocampus 0.193618787 Brain_Putamen_basal_ganglia 1.091110257
0.908889743 0.908889743 -1.590203548 1.299948015 -0.145127767
2.043645769 2 0 0.964541023
RRP9 ENSG00000114767.6 0.098632366 3 3 0.022536205
Brain_Hippocampus 0.614413642 Brain_Caudate_basal_ganglia 1.152643228
0.813470756 0.813470756 -0.954178514 2.281206932 0.610270583
1.620319227 3 0 0.964541023
LINC00116 ENSG00000175701.10 0.098653292 3 2 0.042840772
Brain_Hypothalamus 0.434539735 Brain_Caudate_basal_ganglia 1.989002181
0.01303525 0.997962569 0.781446834 2.025259038 1.202002724
0.713016776 2 0 0.964541023
LINC00487 ENSG00000205837.7 0.09878338 11 10 0.051494473 Brain_Amygdala
0.767413846 Brain_Anterior_cingulate_cortex_BA24 3.777913476
0.027530435 0.239269029 -1.130201356 1.947335937 0.409226156
1.084622223 10 0 0.965138458
ELL3 ENSG00000128886.11 0.09887263 1 1 0.09887263 Brain_Cerebellar_Hemisphere
0.09887263 Brain_Cerebellar_Hemisphere 1 1 1 -1.650343843
-1.650343843 -1.650343843 NA 1 0 0.965336333
SNAPC1 ENSG00000023608.4 0.098995944 2 2 0.068809669
Brain_Cerebellar_Hemisphere 0.173152971 Brain_Putamen_basal_ganglia
1.123392509 0.876607491 0.876607491 -1.819667408 -1.362142333
-1.590904871 0.323519083 2 0 0.965342597
AP3B2 ENSG00000103723.12 0.099015019 5 2 0.097269698
Brain_Substantia_nigra 0.957082337 Brain_Frontal_Cortex_BA9
4.539853919 0.006442103 0.310266616 -1.658236707 0.053815279

-0.96051835 0.623460386 2 0 0.965342597
SHF ENSG00000138606.19 0.099141831 7 3 0.014136996
Brain_Frontal_Cortex_BA9 0.854488399 Brain_Spinal_cord_cervical_c-1
4.7291914 0.010498899 0.239270479 -2.170416312 2.453763407
0.392506221 1.978917294 3 0 0.965342597
ANKRD44 ENSG00000065413.18 0.099166798 2 2 0.034740851
Brain_Anterior_cingulate_cortex_BA24 0.610395556 Brain_Cerebellum
1.049728586 0.950271414 0.950271414 -2.111366165 -0.509508908
-1.310437536 1.132684129 2 0 0.965342597
SSTR3 ENSG00000278195.1 0.099245483 1 1 0.099245483 Brain_Cerebellum
0.099245483 Brain_Cerebellum 1 1 1 1.648522571 1.648522571
1.648522571 NA 1 0 0.965342597
IFNAR2 ENSG00000159110.19 0.099287256 1 1 0.099287256
Brain_Caudate_basal_ganglia 0.099287256 Brain_Caudate_basal_ganglia 1 1
1 -1.648318863 -1.648318863 -1.648318863 NA 1 0
0.965342597
FAM126A ENSG00000122591.11 0.09949738 4 4 0.032214123 Brain_Cortex
0.871405553 Brain_Cerebellar_Hemisphere 1.923405899 0.10438456 0.10438456
-2.141743569 0.161873396 -0.923337411 0.962745774 4 0
0.966226814
ZNF581 ENSG00000171425.9 0.099531146 3 3 0.034092506
Brain_Hippocampus 0.467595038 Brain_Anterior_cingulate_cortex_BA24
1.204696804 0.775126825 0.775126825 -0.748454687 2.118975878
0.698972943 1.43391199 3 0 0.966226814
SHC4 ENSG00000185634.11 0.099585381 1 1 0.099585381
Brain_Anterior_cingulate_cortex_BA24 0.099585381
Brain_Anterior_cingulate_cortex_BA24 1 1 1 1.646867025
1.646867025 1.646867025 NA 1 0 0.966226814
NDUFA7 ENSG00000267855.5 0.09966541 2 2 0.046266273
Brain_Spinal_cord_cervical_c-1 0.450926447 Brain_Caudate_basal_ganglia
1.023039235 0.976960765 0.976960765 -1.992955926 -0.753871398
-1.373413662 0.876165072 2 0 0.96633316
RP11-134G8.5 ENSG00000223396.2 0.099796989 1 1 0.099796989
Brain_Frontal_Cortex_BA9 0.099796989 Brain_Frontal_Cortex_BA9 1 1
1 -1.645838619 -1.645838619 -1.645838619 NA 1 0
0.966938832
USE1 ENSG00000053501.12 0.10012736 5 5 0.044043 Brain_Cerebellar_Hemisphere
0.690073223 Brain_Caudate_basal_ganglia 2.251243772 0.22899514 0.22899514
-2.013681341 1.089822485 -0.460727724 1.223024095 5 0
0.968172935
ADAMTS16 ENSG00000145536.15 0.100177998 3 3 0.042127432
Brain_Hypothalamus 0.677930039 Brain_Cerebellar_Hemisphere 1.759524383
0.271375223 0.271375223 0.415289429 2.032259109 1.258734073
0.810749221 3 0 0.968172935
EXO5 ENSG00000164002.11 0.100272193 10 6 0.04459324
Brain_Putamen_basal_ganglia 0.876760192 Brain_Cortex 6.404877506
0.023797847 0.31896023 -2.008471229 0.766643714 -0.818722607
0.812430222 6 0 0.968172935
MND1 ENSG00000121211.7 0.100563603 1 1 0.100563603
Brain_Cerebellar_Hemisphere 0.100563603 Brain_Cerebellar_Hemisphere 1 1
1 1.642127407 1.642127407 1.642127407 NA 1 0
0.968172935
GFRA4 ENSG00000125861.14 0.100683127 3 3 0.013953363
Brain_Nucleus_accumbens_basal_ganglia 0.539378114 Brain_Frontal_Cortex_BA9
1.568789813 0.550414386 0.550414386 0.613753674 2.458461782
1.43157453 0.939956685 3 0 0.968172935
MSLNL ENSG00000162006.9 0.100715163 6 3 0.108655001
Brain_Anterior_cingulate_cortex_BA24 0.800096416 Brain_Cortex
4.564155408 0.009466245 0.373426386 -1.604267955 0.253222325
-0.980755064 0.720747025 3 0 0.968172935
EPN1 ENSG00000063245.14 0.100785728 1 1 0.100785728
Brain_Anterior_cingulate_cortex_BA24 0.100785728

Brain_Anterior_cingulate_cortex_BA24 1 1 1 1.641056304
1.641056304 1.641056304 NA 1 0 0.968172935
ATP2B1-AS1 ENSG00000271614.1 0.100788275 3 3 0.055237472
Brain_Nucleus_accumbens_basal_ganglia 0.904717727 Brain_Putamen_basal_ganglia
1.958421202 0.359635966 0.359635966 -1.917003615 0.336104378
-0.487065119 1.243080999 3 0 0.968172935
GOLGA8A ENSG00000175265.17 0.100853571 6 6 0.012792592
Brain_Anterior_cingulate_cortex_BA24 0.768753661 Brain_Cerebellar_Hemisphere
1.65823286 0.422428082 0.422428082 -2.489491668 1.436401161
-0.787969247 1.367251373 6 0 0.968172935
PLEKHG3 ENSG00000126822.16 0.100869316 3 3 0.08880837
Brain_Anterior_cingulate_cortex_BA24 0.218006541 Brain_Putamen_basal_ganglia
1.137439697 0.83638246 0.83638246 -1.393734897 1.701717329
0.513276211 1.668146649 3 0 0.968172935
ADCY5 ENSG00000173175.14 0.100937734 1 1 0.100937734 Brain_Cerebellum
0.100937734 Brain_Cerebellum 1 1 1 -1.640324403 -1.640324403
-1.640324403 NA 1 0 0.968172935
ZNF141 ENSG00000131127.13 0.10107989 3 3 0.081208507
Brain_Substantia_nigra 0.352397423 Brain_Nucleus_accumbens_basal_ganglia
1.413876853 0.585006304 0.585006304 -0.929949131 1.743716668
0.584017152 1.371587003 3 0 0.968172935
RP11-44N21.1 ENSG00000257556.1 0.101168236 2 2 0.042602778
Brain_Amygdala 0.115646399 Brain_Frontal_Cortex_BA9 1.478854392
0.521145608 0.521145608 1.573312833 2.027583464 1.800448148
0.321217844 2 0 0.968172935
DNM1 ENSG00000106976.20 0.101190326 1 1 0.101190326 Brain_Cortex
0.101190326 Brain_Cortex 1 1 1 1.639110131 1.639110131
1.639110131 NA 1 0 0.968172935
TBCEL ENSG00000154114.12 0.101295302 2 2 0.032507452
Brain_Hippocampus 0.130869711 Brain_Substantia_nigra 1.675917993
0.324082007 0.324082007 1.510681133 2.138114496 1.824397815
0.443662385 2 0 0.968172935
TIAF1 ENSG00000221995.5 0.101404539 13 1 0.038274402
Brain_Substantia_nigra 0.478276928 Brain_Hippocampus 11.96306727
0.000759664 11.96306727 -2.071903919 -0.709076625 -1.560310008
0.382079972 1 0 0.968172935
RP11-325L12.7 ENSG00000278737.1 0.101407382 3 3 0.012953334
Brain_Anterior_cingulate_cortex_BA24 0.595707979 Brain_Hypothalamus
1.33004724 0.771330052 0.771330052 -2.485049717 0.592695547
-0.807645627 1.557466492 3 0 0.968172935
ANKRD18A ENSG00000180071.19 0.101415249 10 8 0.087388659
Brain_Anterior_cingulate_cortex_BA24 0.86019525 Brain_Hippocampus
4.164344951 0.07058382 0.290884137 -1.463177652 1.709336376
-0.085295195 1.014424388 8 0 0.968172935
GRPEL1 ENSG00000109519.12 0.101439007 6 6 0.039845137
Brain_Cerebellum 0.919535073 Brain_Spinal_cord_cervical_c-1 2.850164374
0.303537049 0.303537049 -0.473679094 2.055350764 0.475925299
0.877215331 6 0 0.968172935
TBC1D2B ENSG00000167202.11 0.101477125 2 2 0.057490039
Brain_Hypothalamus 0.365067952 Brain_Nucleus_accumbens_basal_ganglia
1.040562561 0.959437439 0.959437439 -1.899566443 -0.905750453
-1.402658448 0.702734026 2 0 0.968172935
MPHOSPH10 ENSG00000124383.8 0.101552399 1 1 0.101552399
Brain_Frontal_Cortex_BA9 0.101552399 Brain_Frontal_Cortex_BA9 1 1
1 -1.637373752 -1.637373752 -1.637373752 NA 1 0
0.968172935
AATF ENSG00000275700.4 0.101565338 5 2 0.051948336
Brain_Caudate_basal_ganglia 0.106789524 Brain_Spinal_cord_cervical_c-1
4.16970953 0.011297673 0.738349231 1.612792832 1.943561637
1.765360152 0.125876706 2 0 0.968172935
LINC01305 ENSG00000231453.1 0.101667093 1 1 0.101667093
Brain_Caudate_basal_ganglia 0.101667093 Brain_Caudate_basal_ganglia 1 1

1 1.636824747 1.636824747 1.636824747 NA 1 0
0.968172935
FAXDC2 ENSG00000170271.10 0.101733116 1 1 0.101733116
Brain_Frontal_Cortex_BA9 0.101733116 Brain_Frontal_Cortex_BA9 1 1
1 -1.636508942 -1.636508942 -1.636508942 NA 1 0
0.968172935
IRX1 ENSG00000170549.3 0.101733254 1 1 0.101733254
Brain_Anterior_cingulate_cortex_BA24 0.101733254
Brain_Anterior_cingulate_cortex_BA24 1 1 1 1.63650828 1.63650828
1.63650828 NA 1 0 0.968172935
MRPL37 ENSG00000116221.15 0.101891969 7 3 0.073823236
Brain_Cortex 0.94852265 Brain_Frontal_Cortex_BA9 6.074027611
0.004217638 0.342340548 -0.064562115 1.787707343 1.247465775
0.605700401 3 0 0.968172935
POC5 ENSG00000152359.14 0.101906015 3 2 0.054792032 Brain_Cortex
0.294139936 Brain_Cerebellum 2.777268029 0.056611125 0.166120845
-1.92052172 -1.049082964 -1.482169095 0.435743248 2 0
0.968172935
SMPDL3A ENSG00000172594.12 0.101919948 3 3 0.024102713
Brain_Cerebellar_Hemisphere 0.124356857 Brain_Cerebellum 1.648230274
0.473447007 0.473447007 -2.255488028 -1.536740548 -1.791723849
0.402295384 3 0 0.968172935
RP11-676J12.9 ENSG00000277491.1 0.101931151 1 1 0.101931151
Brain_Hypothalamus 0.101931151 Brain_Hypothalamus 1 1 1
1.635562658 1.635562658 1.635562658 NA 1 0 0.968172935
RBM43 ENSG00000184898.6 0.102194671 7 4 0.010146059 Brain_Cortex
0.835732342 Brain_Spinal_cord_cervical_c-1 4.951695048 4.70E-06
0.227494725 -2.570811317 -0.20735536 -1.587550291 0.928260624
4 0 0.969383589
RFPL3S ENSG00000205853.10 0.102292985 12 3 0.107735422
Brain_Nucleus_accumbens_basal_ganglia 0.978257186 Brain_Substantia_nigra
10.51561756 0.000522669 0.429019403 -1.608455651 0.118719222
-0.674056471 0.564734998 3 0 0.969383589
TFIP11 ENSG00000100109.16 0.102329285 5 3 0.021394487
Brain_Cerebellar_Hemisphere 0.828897415 Brain_Frontal_Cortex_BA9 3.03365912
0.058952584 0.828965804 -0.24411417 2.300949469 1.204101522
1.312467488 3 0 0.969383589
C8orf34 ENSG00000165084.15 0.102429344 2 2 0.069209316
Brain_Cerebellum 0.169435052 Brain_Hippocampus 1.146152508
0.853847492 0.853847492 -1.374021357 1.817050923 0.221514783
2.256428849 2 0 0.969383589
CBX1 ENSG00000108468.14 0.102728855 5 5 0.026317921
Brain_Putamen_basal_ganglia 0.999258189 Brain_Hippocampus 3.399664636
0.230168059 0.230168059 -0.098235213 2.221488087 0.839729222
1.040051557 5 0 0.969383589
TANGO6 ENSG00000103047.7 0.102748376 1 1 0.102748376
Brain_Cerebellum 0.102748376 Brain_Cerebellum 1 1 1 -1.631673069
-1.631673069 -1.631673069 NA 1 0 0.969383589
PRKD3 ENSG00000115825.9 0.102762806 9 4 0.001600645
Brain_Nucleus_accumbens_basal_ganglia 0.029210223 Brain_Hypothalamus
7.50259792 0.01369812 0.266242755 2.180637724 3.155789212
2.501200565 0.313633456 4 0 0.969383589
IRAK1BP1 ENSG00000146243.13 0.102772812 13 1 0.070790631
Brain_Cerebellum 0.201555642 Brain_Cortex 11.85935101 0.000778681
11.85935101 1.277132021 1.806818193 1.557720978 0.191194264
1 0 0.969383589
TTC17 ENSG00000052841.14 0.102925473 3 3 0.025608691 Brain_Cerebellum
0.668702341 Brain_Cortex 1.640403283 0.367444473 0.367444473
-0.46536416 2.232094911 0.446267034 1.546685566 3 0
0.969383589
FOCAD ENSG00000188352.12 0.102949538 5 4 0.02359354 Brain_Cortex
0.56371717 Brain_Putamen_basal_ganglia 3.830828089 0.075875679

0.146269562 0.577329084 2.263684278 1.626379241 0.632841082
4 0 0.969383589
ODF3L1 ENSG00000182950.2 0.103077088 1 1 0.103077088
Brain_Cerebellar_Hemisphere 0.103077088 Brain_Cerebellar_Hemisphere 1 1
1 1.630115503 1.630115503 1.630115503 NA 1 0
0.969383589
EMILIN3 ENSG00000183798.4 0.103252098 9 7 0.035934608
Brain_Hypothalamus 0.996070864 Brain_Putamen_basal_ganglia 6.102409013
0.099936524 0.237924767 0.004924462 2.097666597 0.935500022
0.700271514 7 0 0.969383589
WDR24 ENSG00000127580.15 0.103357689 1 1 0.103357689
Brain_Hypothalamus 0.103357689 Brain_Hypothalamus 1 1 1
-1.628789023 -1.628789023 -1.628789023 NA 1 0 0.969383589
PTCHD4 ENSG00000244694.7 0.103363872 2 2 0.089151068
Brain_Putamen_basal_ganglia 0.373952994 Brain_Anterior_cingulate_cortex_BA24
1.214834215 0.785165785 0.785165785 -0.889093199 1.699892927
0.405399864 1.830689646 2 0 0.969383589
PACSIN3 ENSG00000165912.15 0.10338936 4 2 0.040467089 Brain_Cerebellum
0.621527792 Brain_Nucleus_accumbens_basal_ganglia 1.976092093
0.028512385 1.932936522 -0.809129935 2.048949136 0.671543272
1.533675769 2 0 0.969383589
SYNGR1 ENSG00000100321.14 0.103390401 3 3 0.018579734
Brain_Cerebellum 0.788227867 Brain_Cerebellar_Hemisphere 1.759438039
0.428014163 0.428014163 -0.268612533 2.353857462 0.923620798
1.327336208 3 0 0.969383589
CNTNAP4 ENSG00000152910.18 0.103415821 7 6 0.118566311
Brain_Nucleus_accumbens_basal_ganglia 0.778102491 Brain_Cerebellar_Hemisphere
3.403205547 0.035027478 0.153686756 -1.560819647 0.961102833
-0.549658125 0.910306288 6 0 0.969383589
RP11-300M24.1 ENSG00000227455.2 0.103516025 2 2 0.037710219
Brain_Nucleus_accumbens_basal_ganglia 0.735892162 Brain_Hippocampus
1.061946017 0.938053983 0.938053983 0.33729814 2.077990662
1.207644401 1.230855486 2 0 0.969383589
HIST1H2BF ENSG00000277224.2 0.103518325 1 1 0.103518325
Brain_Hypothalamus 0.103518325 Brain_Hypothalamus 1 1 1
1.628030943 1.628030943 1.628030943 NA 1 0 0.969383589
LINC01361 ENSG00000236268.5 0.103607356 4 3 0.023097116
Brain_Frontal_Cortex_BA9 0.759170461 Brain_Hypothalamus 2.7275319
0.070468956 0.215008986 -0.306570302 2.271824436 1.184123679
1.084716009 3 0 0.969383589
BMPER ENSG00000164619.8 0.103644097 4 4 0.057418843 Brain_Cortex
0.984988483 Brain_Cerebellum 2.020788309 0.10573877 0.10573877
-1.900108799 -0.018815257 -0.841824293 0.804490825 4 0
0.969383589
NAT10 ENSG00000135372.8 0.103652766 3 2 0.072445196
Brain_Frontal_Cortex_BA9 0.544038966 Brain_Cerebellum 2.728525145
0.001798045 0.269676811 0.606716657 1.796310202 1.348537618
0.647017416 2 0 0.969383589
RP11-736N17.11 ENSG00000283071.1 0.103654813 7 3 0.18950952
Brain_Nucleus_accumbens_basal_ganglia 0.731266968 Brain_Cerebellum
5.875923475 0.006122645 0.315494323 -0.700571337 1.312031461
0.688532271 0.727547398 3 0 0.969383589
ZCCHC8 ENSG00000033030.13 0.10375114 1 1 0.10375114 Brain_Amygdala
0.10375114 Brain_Amygdala 1 1 1 1.626933886 1.626933886
1.626933886 NA 1 0 0.969383589
BDKRB1 ENSG00000100739.10 0.103863712 5 5 0.021759574
Brain_Cortex 0.799304676 Brain_Cerebellar_Hemisphere 1.148874536
0.82390708 0.82390708 -0.597543119 2.294538719 0.713835148
1.180071973 5 0 0.969383589
8-Sep ENSG00000164402.13 0.103883653 1 1 0.103883653
Brain_Cerebellar_Hemisphere 0.103883653 Brain_Cerebellar_Hemisphere 1 1
1 -1.626310341 -1.626310341 -1.626310341 NA 1 0

0.969383589
TCF7L1 ENSG00000152284.4 0.103929339 2 2 0.037494518
Brain_Cerebellar_Hemisphere 0.053290882 Brain_Cerebellum 1.799844465
0.200155535 0.200155535 1.932556289 2.08033826 2.006447274
0.104497634 2 0 0.969383589
RP6-74O6.6 ENSG00000272824.1 0.104244339 2 2 0.098072727
Brain_Nucleus_accumbens_basal_ganglia 0.156246237 Brain_Cerebellar_Hemisphere
1.050358534 0.949641466 0.949641466 -1.654269695 1.417810024
-0.118229836 2.172288402 2 0 0.971401205
ZNF853 ENSG00000236609.3 0.104321396 6 6 0.010893403
Brain_Caudate_basal_ganglia 0.954449019 Brain_Nucleus_accumbens_basal_ganglia
1.978747536 0.32624036 0.32624036 -1.329299839 2.546099825 0.81293348
1.351641358 6 0 0.971401205
RP11-517P14.2 ENSG00000230555.2 0.104353943 9 5 0.140831608
Brain_Cerebellar_Hemisphere 0.950180448 Brain_Anterior_cingulate_cortex_BA24
6.452399229 0.008018277 0.221144173 -1.472701255 1.431046723
-0.466193014 0.803033593 5 0 0.971401205
ACOT11 ENSG00000162390.17 0.104493876 4 3 0.028173977
Brain_Nucleus_accumbens_basal_ganglia 0.590896803 Brain_Cortex
2.703665708 0.01903555 0.459194654 0.537536901 2.194855327
1.369533175 0.690068216 3 0 0.971501595
ZNF235 ENSG00000159917.15 0.104620722 5 2 0.121073381
Brain_Cerebellar_Hemisphere 0.920610167 Brain_Frontal_Cortex_BA9 4.35488111
0.020559407 0.496444015 -1.550283938 0.278017049 -0.254321313
0.744816001 2 0 0.971501595
RCVRN ENSG00000109047.7 0.104675849 13 2 0.028387634
Brain_Substantia_nigra 0.562692245 Brain_Amygdala 11.61050087
0.001379948 0.93124854 -2.191887459 -0.578847252 -1.424931108
0.380095812 2 0 0.971501595
PPP1R37 ENSG00000104866.10 0.104760035 6 6 0.032226365
Brain_Cerebellar_Hemisphere 0.953047615 Brain_Cortex 2.199385831
0.269460882 0.269460882 0.058880092 2.141591539 0.82064672
0.877601935 6 0 0.971501595
EPHB4 ENSG00000196411.9 0.104800264 2 2 0.038871555
Brain_Spinal_cord_cervical_c-1 0.890372384 Brain_Frontal_Cortex_BA9
1.169405153 0.830594847 0.830594847 -0.137833024 2.065544019
0.963855497 1.558022849 2 0 0.971501595
TMEM175 ENSG00000127419.16 0.104807257 6 5 0.030199312
Brain_Caudate_basal_ganglia 0.989283663 Brain_Putamen_basal_ganglia
3.094768333 0.020229227 0.124198102 -2.167466364 0.349093753
-0.525361831 1.057894765 5 0 0.971501595
AK4 ENSG00000162433.14 0.10485079 1 1 0.10485079 Brain_Cerebellum 0.10485079
Brain_Cerebellum 1 1 1 1.621778468 1.621778468 1.621778468
NA 1 0 0.971501595
MCMDC2 ENSG00000178460.17 0.104934984 1 1 0.104934984
Brain_Cerebellar_Hemisphere 0.104934984 Brain_Cerebellar_Hemisphere 1 1
1 1.621385521 1.621385521 1.621385521 NA 1 0
0.971638228
KIRREL ENSG00000183853.17 0.105236638 1 1 0.105236638
Brain_Spinal_cord_cervical_c-1 0.105236638 Brain_Spinal_cord_cervical_c-1
1 1 1 1.619979697 1.619979697 1.619979697 NA 1 0
0.972491774
JUN ENSG00000177606.6 0.105293331 1 1 0.105293331 Brain_Cortex
0.105293331 Brain_Cortex 1 1 1 1.619715843 1.619715843
1.619715843 NA 1 0 0.972491774
JAGN1 ENSG00000171135.13 0.105480149 3 3 0.023442604 Brain_Cerebellum
0.557263346 Brain_Spinal_cord_cervical_c-1 1.38819021 0.611670369
0.611670369 -0.586911201 2.266143406 1.101737174 1.497031234
3 0 0.972491774
NARS ENSG00000134440.11 0.105609212 1 1 0.105609212
Brain_Caudate_basal_ganglia 0.105609212 Brain_Caudate_basal_ganglia 1 1
1 -1.618247769 -1.618247769 -1.618247769 NA 1 0

0.972491774
PRSS8 ENSG00000052344.15 0.105711207 1 1 0.105711207 Brain_Cerebellum 0.105711207 Brain_Cerebellum 1 1 1 -1.617774487 -1.617774487 -1.617774487 NA 1 0 0.972491774
MPPED2 ENSG00000066382.16 0.105918965 3 3 0.013522981 Brain_Caudate_basal_ganglia 0.977850165 Brain_Hippocampus 1.426953946 0.573452544 0.573452544 -2.46969098 0.027764269 -1.203617028 1.249089014 3 0 0.972491774
FZD9 ENSG00000188763.4 0.105923818 1 1 0.105923818 Brain_Hippocampus 0.105923818 Brain_Hippocampus 1 1 1 1.616789078 1.616789078 1.616789078 NA 1 0 0.972491774
EPS15L1 ENSG00000127527.13 0.105932688 3 3 0.031451374 Brain_Cerebellar_Hemisphere 0.37943649 Brain_Anterior_cingulate_cortex_BA24 1.969911075 0.068156242 0.068156242 0.878935055 2.151314628 1.707102104 0.71784626 3 0 0.972491774
ZMPSTE24 ENSG00000084073.8 0.105950174 2 2 0.034105181 Brain_Putamen_basal_ganglia 0.76138348 Brain_Caudate_basal_ganglia 1.149022034 0.850977966 0.850977966 -2.118825919 -0.303664535 -1.211245227 1.283512924 2 0 0.972491774
RP11-982M15.6 ENSG00000256050.2 0.105983822 3 3 0.025451875 Brain_Substantia_nigra 0.574947284 Brain_Putamen_basal_ganglia 1.551970958 0.625891628 0.625891628 -0.560780349 2.234474395 0.754008209 1.404972982 3 0 0.972491774
CELF6 ENSG00000140488.15 0.106056298 1 1 0.106056298 Brain_Amygdala 0.106056298 Brain_Amygdala 1 1 1 -1.616175853 -1.616175853 -1.616175853 NA 1 0 0.972491774
NPIPB12 ENSG00000169203.16 0.106110108 7 4 0.024422111 Brain_Nucleus_accumbens_basal_ganglia 0.612479629 Brain_Anterior_cingulate_cortex_BA24 4.587001907 0.017592548 0.589135864 0.506537133 2.250422922 1.273709843 0.621375716 4 0 0.972491774
CTD-3116E22.7 ENSG00000267612.1 0.106123173 3 3 0.202405244 Brain_Cerebellar_Hemisphere 0.761553423 Brain_Caudate_basal_ganglia 2.215137832 0.258469329 0.258469329 -1.274728817 0.3034415 -0.622130976 0.823736457 3 0 0.972491774
TANC2 ENSG00000170921.15 0.106273936 2 1 0.09963433 Brain_Cerebellar_Hemisphere 0.114732264 Brain_Cerebellum 1.992039045 0.007960955 1.992039045 1.577274991 1.64662898 1.611951985 0.049040676 1 0 0.972491774
KCNG1 ENSG00000026559.13 0.106346812 1 1 0.106346812 Brain_Caudate_basal_ganglia 0.106346812 Brain_Caudate_basal_ganglia 1 1 1 1.614833244 1.614833244 1.614833244 NA 1 0 0.972491774
SCAMP4 ENSG00000227500.9 0.106662398 2 2 0.062532957 Brain_Cerebellar_Hemisphere 0.353378754 Brain_Cerebellum 1.812455235 0.187544765 0.187544765 0.928055553 1.862497791 1.395276672 0.660750443 2 0 0.972491774
CRIPT ENSG00000119878.5 0.106720724 12 2 0.025112469 Brain_Nucleus_accumbens_basal_ganglia 0.196132611 Brain_Anterior_cingulate_cortex_BA24 10.85934414 0.003922323 0.473451832 -2.239668241 -1.292648634 -1.804652368 0.277835052 2 0 0.972491774
AP3S1 ENSG00000177879.15 0.106726846 1 1 0.106726846 Brain_Frontal_Cortex_BA9 0.106726846 Brain_Frontal_Cortex_BA9 1 1 1 1.613081298 1.613081298 1.613081298 NA 1 0 0.972491774
LINC00698 ENSG00000244342.5 0.106779062 1 1 0.106779062 Brain_Putamen_basal_ganglia 0.106779062 Brain_Putamen_basal_ganglia 1 1 1 1.612840971 1.612840971 1.612840971 NA 1 0 0.972491774
POLE4 ENSG00000115350.11 0.107007585 8 7 0.005289158 Brain_Hippocampus 0.579590936 Brain_Amygdala 3.655636179

0.110158094	0.263816687	-2.788874365	1.299900392	-0.842983651
1.524749753	7	0	0.972491774
QPRT	ENSG00000103485.17	0.107077534	12	4	0.022763569
Brain_Hypothalamus	0.975725387	Brain_Amygdala	7.975748941	7.95E-17
0.540973223	0.03042841	2.277379566	1.008563637	0.777217883	4
0	0.972491774
RP5-1177M21.1	ENSG00000234104.1	0.107204498	6	5	0.053083023
Brain_Frontal_Cortex_BA9	0.946578612	Brain_Substantia_nigra
3.477038126	0.022978281	0.450856685	0.067003883	1.93424491
1.324045645	0.735221275	5	0	0.972491774
SCAMP5	ENSG00000198794.11	0.107208645	1	1	0.107208645
Brain_Putamen_basal_ganglia	0.107208645	Brain_Putamen_basal_ganglia	1	1
1	1.610867318	1.610867318	1.610867318	NA	1	0
0.972491774
RBM47	ENSG00000163694.14	0.107219709	1	1	0.107219709
Brain_Cerebellar_Hemisphere	0.107219709	Brain_Cerebellar_Hemisphere	1	1
1	1.61081657	1.61081657	1.61081657	NA	1	0	0.972491774
NPEPL1	ENSG00000215440.11	0.107239809	10	2	0.110364578
Brain_Hypothalamus	0.56455316	Brain_Putamen_basal_ganglia	9.263155628
0.001277195	0.353606234	0.576091761	1.596556607	1.090877067
0.349663014	2	0	0.972491774
STRADB	ENSG00000082146.12	0.107296425	1	1	0.107296425
Brain_Spinal_cord_cervical_c-1	0.107296425	Brain_Spinal_cord_cervical_c-1
1	1	1	-1.610464795	-1.610464795	-1.610464795	NA	1	0
0.972491774
DNASE1	ENSG00000213918.10	0.107336048	1	1	0.107336048
Brain_Cerebellum	0.107336048	Brain_Cerebellum	1	1	1	-1.610283189
-1.610283189	-1.610283189	NA	1	0	0.972491774
OPRL1	ENSG00000125510.15	0.107343687	3	3	0.055437345
Brain_Cerebellar_Hemisphere	0.277563213	Brain_Cerebellum	1.101516818
0.906813423	0.906813423	-1.915432695	-1.085809658	-1.447774911
0.424790277	3	0	0.972491774
MRM3	ENSG00000171861.10	0.107400144	3	3	0.091884336	Brain_Cerebellum
0.369958328	Brain_Substantia_nigra	1.475037466	0.520574516
0.520574516	-1.68554051	1.562983288	0.257998068	1.715820361
3	0	0.972491774
FKBP8	ENSG00000105701.15	0.10740439	2	2	0.040064569
Brain_Cerebellar_Hemisphere	0.416315567	Brain_Substantia_nigra
1.156751332	0.843248668	0.843248668	0.81282994	2.053082581
1.43295626	0.876991053	2	0	0.972491774
CLNS1A	ENSG00000074201.8	0.107417865	1	1	0.107417865
Brain_Cerebellar_Hemisphere	0.107417865	Brain_Cerebellar_Hemisphere	1	1
1	1.609908356	1.609908356	1.609908356	NA	1	0
0.972491774
RP11-305L7.3	ENSG00000237422.1	0.107499697	1	1	0.107499697
Brain_Cerebellum	0.107499697	Brain_Cerebellum	1	1	1	1.609533677
1.609533677	1.609533677	NA	1	0	0.972491774
WLS	ENSG00000116729.13	0.107542412	2	2	0.051754769
Brain_Frontal_Cortex_BA9	0.717654774	Brain_Cerebellum	1.225884596
0.774115404	0.774115404	0.361594903	1.945167945	1.153381424
1.119755236	2	0	0.972491774
WFDC1	ENSG00000103175.10	0.107567323	3	3	0.080261646	Brain_Cortex
0.861544173	Brain_Nucleus_accumbens_basal_ganglia	1.050873939
0.946790944	0.946790944	-1.721123	1.749169954	-0.048787293
1.738553673	3	0	0.972491774
SLC35E2	ENSG00000215790.6	0.107622769	1	1	0.107622769
Brain_Cerebellar_Hemisphere	0.107622769	Brain_Cerebellar_Hemisphere	1	1
1	-1.608970606	-1.608970606	-1.608970606	NA	1	0
0.972491774
MN1	ENSG00000169184.5	0.107674103	2	2	0.051273254
Brain_Nucleus_accumbens_basal_ganglia	0.391855612	Brain_Cerebellum
1.023252989	0.976747011	0.976747011	-0.856257051	1.94918568

0.546464314	1.98374758 2	0	0.972491774
ATXN3 ENSG00000066427.21	0.107713593	2	2	0.092366756
Brain_Anterior_cingulate_cortex_BA24	0.215751209	Brain_Putamen_basal_ganglia
1.021469583	0.978530417	0.978530417	-1.683043059	-1.237905214
-1.460474136	0.314759989	2	0	0.972491774
KIAA1549L	ENSG00000110427.14	0.107835874	1	1	0.107835874
Brain_Cerebellar_Hemisphere 0.107835874	Brain_Cerebellar_Hemisphere 1	1
1	1.607996826	1.607996826	1.607996826	NA	1	0
0.972491774
SNCG	ENSG00000173267.13	0.107839838	6	6	0.015910845
Brain_Caudate_basal_ganglia 0.921578522	Brain_Cortex	3.132588423
0.130030177	0.130030177	-1.220609552	2.410954228	0.408050827
1.253585261	6	0	0.972491774
MTMR3 ENSG00000100330.15	0.107909249	3	3	0.015381458
Brain_Cerebellar_Hemisphere 0.065403116	Brain_Cerebellum 2.383774425
0.147400437	0.147400437	-2.423270752	-1.842492685	-2.075336056
0.307018404	3	0	0.972491774
TARBP2	ENSG00000139546.10	0.107934906	1	1	0.107934906
Brain_Cerebellar_Hemisphere 0.107934906	Brain_Cerebellar_Hemisphere 1	1
1	-1.607544817	-1.607544817	-1.607544817	NA	1	0
0.972491774
NUP133	ENSG00000069248.11	0.108152906	2	2	0.115333231
Brain_Amygdala	0.280642874	Brain_Anterior_cingulate_cortex_BA24
1.189746001	0.810253999	0.810253999	-1.574667424	-1.078876304
-1.326771864	0.350577264	2	0	0.972491774
ANKLE1	ENSG00000160117.14	0.108230712	7	6	0.021923079
Brain_Spinal_cord_cervical_c-1	0.717960103	Brain_Cerebellar_Hemisphere
4.112684772	0.1102363	0.23930817 -2.291697943	0.468012771
-1.034645278	1.135450887	6	0	0.972491774
ARMC2 ENSG00000118690.12	0.108366983	11	4	0.058502043
Brain_Anterior_cingulate_cortex_BA24	0.973007829	Brain_Cerebellum 8.578213
0.001194276	0.351268636	-1.891916978	0.576047896	-0.231614883
0.741654573	4	0	0.972491774
PAPD5 ENSG00000121274.12	0.108431711	1	1	0.108431711
Brain_Hypothalamus	0.108431711	Brain_Hypothalamus	1	1	1
1.605282218	1.605282218	1.605282218	NA	1	0	0.972491774
SEZ6L ENSG00000100095.18	0.108467272	2	2	0.042445753
Brain_Cerebellar_Hemisphere 0.129873216	Brain_Cerebellum 1.879955946
0.120044054	0.120044054	-2.029123109	-1.514602038	-1.771862574
0.363821338	2	0	0.972491774
HNRNPC	ENSG00000092199.17	0.108506376	1	1	0.108506376
Brain_Cerebellar_Hemisphere 0.108506376	Brain_Cerebellar_Hemisphere 1	1
1	1.604942879	1.604942879	1.604942879	NA	1	0
0.972491774
RP11-429B14.4	ENSG00000261478.1	0.108542933	13	8	0.001127746
Brain_Substantia_nigra	0.967923804	Brain_Frontal_Cortex_BA9
6.122690845	0.004782056	0.263016688	-0.577462425	3.256551126
1.314625335	1.466586754	8	0	0.972491774
UAP1 ENSG00000117143.13	0.108606224	1	1	0.108606224
Brain_Putamen_basal_ganglia 0.108606224	Brain_Putamen_basal_ganglia 1	1
1	1.604489376	1.604489376	1.604489376	NA	1	0
0.972491774
SCARF1	ENSG00000074660.15	0.108724276	1	1	0.108724276
Brain_Cerebellar_Hemisphere 0.108724276	Brain_Cerebellar_Hemisphere 1	1
1	1.603953623	1.603953623	1.603953623	NA	1	0
0.972491774
TRPM7 ENSG00000092439.13	0.108866628	3	3	0.117445115
Brain_Caudate_basal_ganglia 0.869433098	Brain_Cerebellum 1.76034981
0.316779114	0.316779114	-1.014353516	1.56558794 0.23853768
1.291568486	3	0	0.972491774
RP11-435J9.2	ENSG00000274340.1	0.108964751	1	1	0.108964751
Brain_Anterior_cingulate_cortex_BA24	0.108964751

Brain_Anterior_cingulate_cortex_BA24 1 1 1 -1.602863693
-1.602863693 -1.602863693 NA 1 0 0.972491774
SSC5D ENSG00000179954.15 0.10896618 8 8 0.00914181
Brain_Nucleus_accumbens_basal_ganglia 0.739252437 Brain_Cerebellum
1.763660892 0.347273218 0.347273218 -2.606704936 1.490132518
-0.385645216 1.294569134 8 0 0.972491774
POLR1A ENSG00000068654.15 0.109012254 12 1 0.040794057
Brain_Cortex 0.319172895 Brain_Putamen_basal_ganglia 11.11453059
0.001753432 11.11453059 -2.045617107 -0.996159003 -1.543669834
0.270032585 1 0 0.972491774
MRPL24 ENSG00000143314.12 0.109137932 2 2 0.035800321
Brain_Nucleus_accumbens_basal_ganglia 0.822087684 Brain_Cerebellum
1.033830997 0.966169003 0.966169003 0.224860648 2.099188116
1.162024382 1.325349662 2 0 0.972491774
CD79A ENSG00000105369.9 0.109191461 1 1 0.109191461
Brain_Hypothalamus 0.109191461 Brain_Hypothalamus 1 1 1
1.601837892 1.601837892 1.601837892 NA 1 0 0.972491774
FUT11 ENSG00000196968.10 0.109229139 6 3 0.074624629 Brain_Cortex
0.964091472 Brain_Cerebellum 4.041161452 0.030958086 0.72261667
-0.045019869 1.782764608 1.342612457 0.702006607 3 0
0.972491774
CISD2 ENSG00000145354.9 0.109246642 3 2 0.02834222 Brain_Cerebellum
0.283518064 Brain_Hypothalamus 2.570687517 0.008618605
0.420693878 1.072449768 2.192516675 1.7250083 0.582511922 2
0 0.972491774
C7orf25 ENSG00000136197.12 0.109256235 11 2 0.221384713
Brain_Anterior_cingulate_cortex_BA24 0.729646826 Brain_Cortex
9.549520348 0.011301662 0.619952973 -1.092171026 1.222854351
-0.457929806 0.613475198 2 0 0.972491774
LMOD3 ENSG00000163380.15 0.109275496 3 3 0.127759023
Brain_Spinal_cord_cervical_c-1 0.195333733 Brain_Cerebellar_Hemisphere
1.10336475 0.884905168 0.884905168 -1.522998729 1.518174109
-0.433261826 1.69383499 3 0 0.972491774
PRDX2 ENSG00000167815.11 0.109483508 2 2 0.036104629
Brain_Hippocampus 0.715155141 Brain_Hypothalamus 1.089376623
0.910623377 0.910623377 -2.095747122 -0.364941412 -1.230344267
1.223864454 2 0 0.972491774
C9orf116 ENSG00000160345.12 0.109602569 1 1 0.109602569
Brain_Nucleus_accumbens_basal_ganglia 0.109602569
Brain_Nucleus_accumbens_basal_ganglia 1 1 1 1.599982033
1.599982033 1.599982033 NA 1 0 0.972491774
RPS9 ENSG00000170889.13 0.109721911 13 3 0.044793739
Brain_Nucleus_accumbens_basal_ganglia 0.789919356 Brain_Hypothalamus
11.2428909 0.005453429 0.420014278 0.266415335 2.006586224
1.575022364 0.440585817 3 0 0.972491774
CERK ENSG00000100422.13 0.109727528 2 2 0.059353744 Brain_Cerebellum
0.706741721 Brain_Cerebellar_Hemisphere 1.274532436 0.725467564
0.725467564 -1.885563914 0.37623564 -0.754664137 1.599333802 2
0 0.972491774
FGD4 ENSG00000139132.14 0.109893303 2 2 0.049742857
Brain_Cerebellar_Hemisphere 0.468397887 Brain_Nucleus_accumbens_basal_ganglia
1.013976271 0.986023729 0.986023729 -0.725088258 1.962168614
0.618540178 1.900177557 2 0 0.972491774
RAB11FIP3 ENSG00000090565.15 0.109993493 5 5 0.012522892
Brain_Putamen_basal_ganglia 0.782247063 Brain_Cerebellum 1.355552692
0.631478878 0.631478878 -0.326149056 2.497056725 0.502897016
1.232789152 5 0 0.972491774
TOGARAM2 ENSG00000189350.12 0.110061603 11 5 0.019436294
Brain_Anterior_cingulate_cortex_BA24 0.328877794 Brain_Cerebellar_Hemisphere
7.740042869 0.015210613 0.296932683 -2.337055577 1.323487547
-1.681192908 1.060060892 5 0 0.972491774
LINC02035 ENSG00000273033.2 0.110089992 6 6 0.077315115

Brain_Anterior_cingulate_cortex_BA24 0.749322323 Brain_Cerebellar_Hemisphere 1.782916287 0.657667455 0.657667455 -1.766481425 1.21166293 -0.698581034 1.078622636 6 0 0.972491774
RP11-72M17.1 ENSG00000258561.1 0.110103845 1 1 0.110103845 Brain_Caudate_basal_ganglia 0.110103845 Brain_Caudate_basal_ganglia 1 1 1 1.59772656 1.59772656 1.59772656 NA 1 0 0.972491774
RP11-347C12.3 ENSG00000258130.7 0.11020816 1 1 0.11020816 Brain_Cortex 0.11020816 Brain_Cortex 1 1 1 1.597258217 1.597258217 1.597258217 NA 1 0 0.972491774
FAM19A1 ENSG00000183662.10 0.110254286 2 2 0.03646525 Brain_Putamen_basal_ganglia 0.791928122 Brain_Substantia_nigra 1.037103335 0.962896665 0.962896665 -2.091701238 -0.263807673 -1.177754455 1.292515935 2 0 0.972491774
ZNF30-AS1 ENSG00000270876.1 0.110300493 1 1 0.110300493 Brain_Anterior_cingulate_cortex_BA24 0.110300493 Brain_Anterior_cingulate_cortex_BA24 1 1 1 1.596843966 1.596843966 1.596843966 NA 1 0 0.972491774
SDC3 ENSG00000162512.15 0.110540426 1 1 0.110540426 Brain_Cerebellum 0.110540426 Brain_Cerebellum 1 1 1 -1.595768781 -1.595768781 -1.595768781 NA 1 0 0.972491774
PHACTR1 ENSG00000112137.17 0.110676912 3 3 0.167404102 Brain_Substantia_nigra 0.335197792 Brain_Hippocampus 1.235889842 0.732264227 0.732264227 -1.380593112 1.263581666 -0.360236193 1.421632475 3 0 0.972491774
TINCR ENSG00000223573.6 0.110776765 2 2 0.141473248 Brain_Cerebellar_Hemisphere 0.613853576 Brain_Cerebellum 1.523711122 0.476288878 0.476288878 -1.470326868 0.504580404 -0.482873232 1.396470324 2 0 0.972491774
C19orf18 ENSG00000177025.3 0.110799843 7 7 0.100597076 Brain_Spinal_cord_cervical_c-1 0.877577711 Brain_Cortex 3.281301124 0.146552359 0.146552359 -0.686960889 1.641965875 0.258005149 0.729606872 7 0 0.972491774
GULP1 ENSG00000144366.15 0.110810246 1 1 0.110810246 Brain_Nucleus_accumbens_basal_ganglia 0.110810246 Brain_Nucleus_accumbens_basal_ganglia 1 1 1 -1.594561864 -1.594561864 -1.594561864 NA 1 0 0.972491774
MTHFD2L ENSG00000163738.18 0.110877794 3 3 0.358550226 Brain_Cerebellar_Hemisphere 0.558357192 Brain_Spinal_cord_cervical_c-1 1.865055312 0.267125323 0.267125323 -0.840234711 0.918131117 0.221059927 0.934053872 3 0 0.972491774
MAN1B1 ENSG00000177239.14 0.110958533 6 6 0.008161483 Brain_Cerebellum 0.904325284 Brain_Hippocampus 1.656644613 0.478610953 0.478610953 -2.645315315 1.487256527 -0.316692014 1.383733685 6 0 0.972491774
ADAT1 ENSG00000065457.10 0.111084896 10 5 0.093077759 Brain_Caudate_basal_ganglia 0.597143649 Brain_Frontal_Cortex_BA9 7.141191342 0.024432427 0.293816339 -1.679381283 1.103948727 -0.66778446 0.902315456 5 0 0.972491774
NCS1 ENSG00000107130.9 0.11108786 1 1 0.11108786 Brain_Cerebellum 0.11108786 Brain_Cerebellum 1 1 1 1.59332251 1.59332251 1.59332251 NA 1 0 0.972491774
TMA7 ENSG00000232112.3 0.111173793 12 1 0.058210244 Brain_Amygdala 0.192130859 Brain_Hypothalamus 11.56923223 0.00033248 11.56923223 1.30430134 1.894111253 1.565927405 0.182816827 1 0 0.972491774
RP11-499E18.1 ENSG00000248161.5 0.111198474 4 4 0.043329214 Brain_Putamen_basal_ganglia 0.899891791 Brain_Frontal_Cortex_BA9 1.909626146 0.31041156 0.31041156 -1.431744456 2.020522547 0.37507569 1.490138697 4 0 0.972491774
RP11-352D13.5 ENSG00000259504.2 0.111247695 1 1 0.111247695 Brain_Cerebellar_Hemisphere 0.111247695 Brain_Cerebellar_Hemisphere 1 1 1 1.592610062 1.592610062 1.592610062 NA 1 0

0.972491774
TTYH3 ENSG00000136295.14 0.111340483 1 1 0.111340483 Brain_Cortex
0.111340483 Brain_Cortex 1 1 1 -1.592196842 -1.592196842
-1.592196842 NA 1 0 0.972491774
PCNX2 ENSG00000135749.18 0.111390703 2 2 0.060423239 Brain_Cerebellum
0.274177499 Brain_Anterior_cingulate_cortex_BA24 1.089398447
0.910601553 0.910601553 -1.877692534 1.093492775 -0.392099879
2.100945281 2 0 0.972491774
BTBD19 ENSG00000222009.8 0.111440904 1 1 0.111440904
Brain_Cortex 0.111440904 Brain_Cortex 1 1 1 -1.591749933
-1.591749933 -1.591749933 NA 1 0 0.972491774
ATOX1 ENSG00000177556.11 0.111465633 1 1 0.111465633
Brain_Frontal_Cortex_BA9 0.111465633 Brain_Frontal_Cortex_BA9 1 1
1 1.59163993 1.59163993 1.59163993 NA 1 0 0.972491774
EFTUD2 ENSG00000108883.12 0.111475302 2 2 0.038050191
Brain_Cerebellar_Hemisphere 0.232077149 Brain_Cerebellum 1.679325382
0.320674618 0.320674618 1.195025291 2.074313654 1.634669473
0.621750764 2 0 0.972491774
C10orf11 ENSG00000148655.14 0.111819484 9 4 0.038660338
Brain_Hypothalamus 0.794964219 Brain_Hippocampus 6.436068908
0.019926624 0.503087856 -2.067784004 0.259869784 -0.921645315
0.650998981 4 0 0.972491774
ERI1 ENSG00000104626.14 0.111915192 3 3 0.332378245
Brain_Putamen_basal_ganglia 0.724699225 Brain_Cortex 2.199360195
0.133294528 0.133294528 -0.969334654 0.556579615 -0.020189879
0.8283122 3 0 0.972491774
PLD5 ENSG00000180287.16 0.112020947 3 3 0.105425636
Brain_Cerebellar_Hemisphere 0.759497841 Brain_Cerebellum 1.878700269
0.332766015 0.332766015 0.306140278 1.619100527 0.963029946
0.656480507 3 0 0.972491774
CUBN ENSG00000107611.14 0.112062278 7 3 0.02310237
Brain_Cerebellar_Hemisphere 0.731534443 Brain_Anterior_cingulate_cortex_BA24
5.08663028 0.024171995 0.669545443 -2.271737492 -0.343085096
-1.262950187 0.743074398 3 0 0.972491774
ATP8A2 ENSG00000132932.16 0.112114768 4 4 0.012699853
Brain_Frontal_Cortex_BA9 0.827811649 Brain_Spinal_cord_cervical_c-1
1.099901518 0.836881228 0.836881228 -0.936202517 2.492076909
0.672991483 1.433684797 4 0 0.972491774
AC104135.2 ENSG00000236209.1 0.112140491 13 1 0.086381258
Brain_Substantia_nigra 0.211838935 Brain_Amygdala 12.45010393
0.000360512 12.45010393 1.248524794 1.714803508 1.552199056
0.147180039 1 0 0.972491774
JMJD7 ENSG00000243789.10 0.11250551 9 3 0.139535416
Brain_Nucleus_accumbens_basal_ganglia 0.952250132 Brain_Cerebellar_Hemisphere
7.128750054 0.006353422 0.568982977 -0.411279793 1.477523306
0.45384312 0.737847084 3 0 0.972491774
PIK3CA ENSG00000121879.3 0.112545985 2 2 0.077411818
Brain_Spinal_cord_cervical_c-1 0.241876216 Brain_Hippocampus
1.029791131 0.970208869 0.970208869 -1.765904821 1.170310058
-0.297797381 2.076217452 2 0 0.972491774
PIGO ENSG00000165282.13 0.112565945 7 5 0.018854953
Brain_Frontal_Cortex_BA9 0.775475892 Brain_Cortex 4.361337391
0.01827284 0.3795768 -2.348386341 0.285219617 -0.804711831 0.91438979
5 0 0.972491774
RSU1 ENSG00000148484.17 0.112598621 8 6 0.016815896 Brain_Cortex
0.937578582 Brain_Hippocampus 4.044887932 0.06432202 0.26770062
-2.390708547 1.144945246 -0.086016045 1.013856318 6 0
0.972491774
MICAL1 ENSG00000135596.17 0.112651836 7 5 0.008743527
Brain_Cerebellum 0.964765678 Brain_Cortex 4.190232724 0.042761758
0.219496885 -2.621922658 0.044174036 -0.691289212 0.934867028
5 0 0.972491774

MUC1 ENSG00000185499.16 0.112724326 1 1 0.112724326
Brain_Nucleus_accumbens_basal_ganglia 0.112724326
Brain_Nucleus_accumbens_basal_ganglia 1 1 1 -1.586066055
-1.586066055 -1.586066055 NA 1 0 0.972491774
REXO4 ENSG00000148300.11 0.112887866 5 2 0.03630539 Brain_Hippocampus
0.407447789 Brain_Cortex 3.94808266 0.053467661 0.794903788
0.828393319 2.093490511 1.189914566 0.512951474 2 0
0.972491774
ABAT ENSG00000183044.11 0.112937222 4 4 0.023686706
Brain_Spinal_cord_cervical_c-1 0.721149157
Brain_Anterior_cingulate_cortex_BA24 1.347862001 0.764575638
0.764575638 -0.780891057 2.26217318 0.566201781 1.399154192 4
0 0.972491774
RNF215 ENSG00000099999.14 0.112958664 4 4 0.029629325
Brain_Putamen_basal_ganglia 0.812962146 Brain_Caudate_basal_ganglia
1.678213926 0.445973247 0.445973247 -2.175010562 1.518996752
-0.065488603 1.578458004 4 0 0.972491774
LRFN5 ENSG00000165379.13 0.11302934 4 4 0.145516819
Brain_Cerebellar_Hemisphere 0.821049446 Brain_Cerebellum 1.906688938
0.118345951 0.118345951 0.226195402 1.455550868 0.67081094
0.557558833 4 0 0.972491774
CRTC3 ENSG00000140577.15 0.113134072 2 2 0.095561826 Brain_Cortex
0.270499099 Brain_Cerebellum 1.090035722 0.909964278 0.909964278
1.101913913 1.666761564 1.384337738 0.399407604 2 0
0.972491774
FAM170A ENSG00000164334.15 0.113187685 3 2 0.27242379
Brain_Spinal_cord_cervical_c-1 0.806909529 Brain_Hippocampus
2.629275057 0.052792336 0.317932607 -1.097497919 0.416026092
-0.145685654 0.828747576 2 0 0.972491774
NEBL ENSG00000078114.18 0.113312851 1 1 0.113312851 Brain_Amygdala
0.113312851 Brain_Amygdala 1 1 1 -1.583476711 -1.583476711
-1.583476711 NA 1 0 0.972491774
LRRC25 ENSG00000175489.9 0.113344572 2 2 0.044651162
Brain_Nucleus_accumbens_basal_ganglia 0.12876068 Brain_Caudate_basal_ganglia
1.513709722 0.486290278 0.486290278 -2.007925941 -1.519007214
-1.763466578 0.345717747 2 0 0.972491774
RPS16 ENSG00000105193.8 0.113395712 3 2 0.081022924 Brain_Cerebellum
0.659396183 Brain_Nucleus_accumbens_basal_ganglia 2.647438225
0.029011057 0.323550719 -1.744781421 0.440746978 -0.667326534
1.093085871 2 0 0.972491774
SATB1 ENSG00000182568.16 0.113568762 1 1 0.113568762 Brain_Cerebellum
0.113568762 Brain_Cerebellum 1 1 1 1.582354077 1.582354077
1.582354077 NA 1 0 0.972491774
FASTKD5 ENSG00000215251.3 0.113712377 4 4 0.021026891
Brain_Spinal_cord_cervical_c-1 0.470355891
Brain_Anterior_cingulate_cortex_BA24 2.946276448 0.119329266
0.119329266 0.721900115 2.307501284 1.456265262 0.650836086
4 0 0.972491774
PISD ENSG00000241878.11 0.113712535 10 6 0.084658746
Brain_Anterior_cingulate_cortex_BA24 0.85131274 Brain_Frontal_Cortex_BA9
6.648210765 0.035608333 0.267595208 -1.72427203 0.829837729
-0.813895682 0.726961728 6 0 0.972491774
SEC61A2 ENSG00000065665.20 0.11387567 9 3 0.017112364 Brain_Cortex
0.460001154 Brain_Substantia_nigra 7.299923484 0.000462325
0.496551374 -2.384284578 -0.738844949 -1.61419778 0.434824885
3 0 0.972491774
SLC5A4 ENSG00000100191.5 0.113882862 8 3 0.093866061
Brain_Frontal_Cortex_BA9 0.924060435 Brain_Amygdala 6.046627773
0.004186035 0.343444865 -1.005272625 1.675347551 0.769800844
0.908901576 3 0 0.972491774
MBD4 ENSG00000129071.9 0.113948392 1 1 0.113948392
Brain_Spinal_cord_cervical_c-1 0.113948392 Brain_Spinal_cord_cervical_c-1

1 1 1 -1.580692379 -1.580692379 -1.580692379 NA 1 0
0.972491774
CDCA8 ENSG00000134690.10 0.114094729 2 2 0.073325266
Brain_Nucleus_accumbens_basal_ganglia 0.246691024 Brain_Substantia_nigra
1.052934621 0.947065379 0.947065379 -1.790800803 -1.158424062
-1.474612433 0.447157882 2 0 0.972491774
CCDC173 ENSG00000154479.12 0.114110947 7 2 0.022675954
Brain_Cerebellar_Hemisphere 0.177088988 Brain_Hippocampus 6.439644727
1.42E-09 0.241464768 1.349772441 2.2788505 1.858740854
0.409381257 2 0 0.972491774
PHRF1 ENSG00000070047.11 0.114119489 3 3 0.150008263
Brain_Caudate_basal_ganglia 0.334576905 Brain_Cerebellar_Hemisphere
1.942928419 0.315631357 0.315631357 -1.150624589 1.439502285
-0.225352743 1.444792985 3 0 0.972491774
RP11-573G6.6 ENSG00000261671.1 0.114185256 1 1 0.114185256
Brain_Frontal_Cortex_BA9 0.114185256 Brain_Frontal_Cortex_BA9 1 1
1 1.579657793 1.579657793 1.579657793 NA 1 0
0.972491774
C5orf56 ENSG00000197536.11 0.114238623 10 2 0.065752126
Brain_Hippocampus 0.95993511 Brain_Anterior_cingulate_cortex_BA24
8.777321552 3.55E-05 0.776740431 -0.050235013 1.840109751
0.457562214 0.560461728 2 0 0.972491774
PIGV ENSG00000060642.10 0.114266995 3 3 0.07437643
Brain_Anterior_cingulate_cortex_BA24 0.556124736 Brain_Cerebellar_Hemisphere
1.547984964 0.450356718 0.450356718 -0.588607299 1.784290768
0.763429389 1.220622433 3 0 0.972491774
LINC01549 ENSG00000232560.6 0.114288658 1 1 0.114288658
Brain_Frontal_Cortex_BA9 0.114288658 Brain_Frontal_Cortex_BA9 1 1
1 -1.579206681 -1.579206681 -1.579206681 NA 1 0
0.972491774
RUSC2 ENSG00000198853.11 0.114291017 1 1 0.114291017
Brain_Caudate_basal_ganglia 0.114291017 Brain_Caudate_basal_ganglia 1 1
1 1.579196392 1.579196392 1.579196392 NA 1 0
0.972491774
CABP7 ENSG00000100314.3 0.114297022 2 2 0.298918734 Brain_Cerebellum
0.88753758 Brain_Cerebellar_Hemisphere 1.830887582 0.169112418
0.169112418 -1.038754915 0.141420729 -0.448667093 0.8345102 2
0 0.972491774
ATP5C1 ENSG00000165629.19 0.114348275 4 4 0.01565118
Brain_Cerebellar_Hemisphere 0.400235112 Brain_Frontal_Cortex_BA9
2.082028679 0.085528122 0.085528122 -2.416949705 -0.841201409
-1.827469117 0.712290925 4 0 0.972491774
ATG4B ENSG00000168397.16 0.114402931 4 4 0.035873027
Brain_Frontal_Cortex_BA9 0.499226245 Brain_Caudate_basal_ganglia
2.566205646 0.092757119 0.092757119 -2.098363722 1.399421367
0.177422265 1.552348103 4 0 0.972491774
TLDC1 ENSG00000140950.15 0.114568505 2 2 0.113052267
Brain_Hypothalamus 0.171751843 Brain_Cerebellar_Hemisphere 1.010605914
0.989394086 0.989394086 1.366596411 1.584621894 1.475609152
0.154167298 2 0 0.972491774
LINC01301 ENSG00000251396.6 0.114600232 10 3 0.005236564
Brain_Hypothalamus 0.187875802 Brain_Caudate_basal_ganglia 8.808946932
0.003380283 0.332482098 1.316889096 2.792109477 2.105867719
0.407510765 3 0 0.972491774
SPHKAP ENSG00000153820.12 0.114627533 2 2 0.186658917
Brain_Cerebellum 0.782394341 Brain_Cerebellar_Hemisphere 1.850320717
0.149679283 0.149679283 0.276200151 1.320527632 0.798363891
0.738451044 2 0 0.972491774
RP11-509J21.4 ENSG00000226669.2 0.114667581 2 2 0.041609418
Brain_Substantia_nigra 0.691725677 Brain_Spinal_cord_cervical_c-1
1.013839814 0.986160186 0.986160186 -0.396514278 2.037405552
0.820445637 1.721041217 2 0 0.972491774

SRSF12 ENSG00000154548.8 0.114811773 4 4 0.042120883
Brain_Frontal_Cortex_BA9 0.506159 Brain_Anterior_cingulate_cortex_BA24
1.625102146 0.555173578 0.555173578 -2.032323832 1.310721028
-0.770660593 1.504635488 4 0 0.972491774
BISPR ENSG00000282851.1 0.114881097 1 1 0.114881097
Brain_Cerebellar_Hemisphere 0.114881097 Brain_Cerebellar_Hemisphere 1 1
1 1.576628214 1.576628214 1.576628214 NA 1 0
0.972491774
CANT1 ENSG00000171302.16 0.114888063 4 4 0.057408646
Brain_Frontal_Cortex_BA9 0.739698658 Brain_Caudate_basal_ganglia
1.417595202 0.680877572 0.680877572 -1.900186529 1.427065034
-0.345623077 1.507914913 4 0 0.972491774
PEX6 ENSG00000124587.13 0.114963372 13 1 0.069608068 Brain_Cerebellum
0.221357501 Brain_Spinal_cord_cervical_c-1 12.60331731 0.000126956
12.60331731 -1.814452638 -1.222926387 -1.551384242 0.146644689
1 0 0.972491774
GUF1 ENSG00000151806.13 0.114983779 13 2 0.032976593 Brain_Amygdala
0.679459475 Brain_Cerebellum 11.37245582 0.002272008 0.845172566
-2.132368159 -0.413200837 -1.431590787 0.445218862 2 0
0.972491774
ZAP70 ENSG00000115085.13 0.114991773 11 3 0.136716275
Brain_Nucleus_accumbens_basal_ganglia 0.595420322 Brain_Putamen_basal_ganglia
9.445155175 0.001129384 0.37332217 -1.488131281 1.408961637
-0.824860479 0.774467034 3 0 0.972491774
CH17-140K24.3 ENSG00000280336.1 0.115002544 1 1 0.115002544
Brain_Cerebellum 0.115002544 Brain_Cerebellum 1 1 1 1.576100934
1.576100934 1.576100934 NA 1 0 0.972491774
PRDX5 ENSG00000126432.13 0.115005422 7 7 0.047602248 Brain_Cerebellum
0.539627063 Brain_Nucleus_accumbens_basal_ganglia 2.276259596 0.26794053
0.26794053 -1.910037994 1.98090215 0.577497575 1.409327869 7 0
0.972491774
AKNA ENSG00000106948.16 0.115081203 7 7 0.053814531
Brain_Putamen_basal_ganglia 0.853686584 Brain_Hippocampus 2.922845148
0.10067224 0.10067224 -1.337370094 1.928326472 0.389646789 1.11452166
7 0 0.972491774
RP11-373D23.3 ENSG00000270210.1 0.115172735 1 1 0.115172735
Brain_Cortex 0.115172735 Brain_Cortex 1 1 1 1.575362762
1.575362762 1.575362762 NA 1 0 0.972491774
EXOSC4 ENSG00000178896.8 0.115302056 1 1 0.115302056
Brain_Nucleus_accumbens_basal_ganglia 0.115302056
Brain_Nucleus_accumbens_basal_ganglia 1 1 1 1.574802426
1.574802426 1.574802426 NA 1 0 0.972491774
SPRY4 ENSG00000187678.8 0.115337559 2 2 0.05372071
Brain_Cerebellar_Hemisphere 0.543837523 Brain_Spinal_cord_cervical_c-1
1.084894436 0.915105564 0.915105564 -1.929081781 0.607020176
-0.661030802 1.793294891 2 0 0.972491774
C11orf70 ENSG00000137691.12 0.115374992 2 2 0.105937166
Brain_Anterior_cingulate_cortex_BA24 0.237417659 Brain_Substantia_nigra
1.074698893 0.925301107 0.925301107 1.181465983 1.616727262
1.399096623 0.307776202 2 0 0.972491774
LINC01291 ENSG00000204792.2 0.115400926 13 1 0.085680538
Brain_Hippocampus 0.212804353 Brain_Cortex 12.53777859
0.001518658 12.53777859 1.245891169 1.718636735 1.546551314
0.128574805 1 0 0.972491774
HN1L ENSG00000206053.12 0.11547385 3 3 0.027147657
Brain_Cerebellar_Hemisphere 0.76213588 Brain_Cortex 1.147013535
0.797260922 0.797260922 0.302677194 2.209388111 1.272669087
0.953790828 3 0 0.972491774
LRP5L ENSG00000100068.11 0.115640717 5 4 0.01294843
Brain_Frontal_Cortex_BA9 0.880768895 Brain_Caudate_basal_ganglia
3.055810775 0.074126795 0.189913198 -2.485184527 0.281567365
-1.308863938 1.279152833 4 0 0.972491774

KCNS3 ENSG00000170745.11 0.115755699 7 6 0.147795461
Brain_Hippocampus 0.903506648 Brain_Amygdala 3.465272259
0.071455106 0.212183223 -1.447362365 0.938491437 -0.307271215
0.829072563 6 0 0.972491774
TMED8 ENSG00000100580.7 0.115772542 3 3 0.02932857
Brain_Cerebellar_Hemisphere 0.081317306 Brain_Cortex 2.052485668
0.28346936 0.28346936 1.743093371 2.179041712 1.943930616
0.219985821 3 0 0.972491774
TMEM98 ENSG00000006042.11 0.1158587 9 5 0.040258086
Brain_Hippocampus 0.983991712 Brain_Cerebellar_Hemisphere 5.978678945
0.019997967 0.249692133 -0.670260807 2.051090994 0.483121917
0.732450867 5 0 0.972491774
TBX5 ENSG00000089225.19 0.115864168 2 2 0.077358751 Brain_Cerebellum
0.609914035 Brain_Hippocampus 1.30050195 0.69949805 0.69949805
-1.766221166 0.510196171 -0.628012498 1.609670136 2 0
0.972491774
RP11-398C13.8 ENSG00000284024.1 0.115945149 1 1 0.115945149
Brain_Frontal_Cortex_BA9 0.115945149 Brain_Frontal_Cortex_BA9 1 1
1 1.572023293 1.572023293 1.572023293 NA 1 0
0.972491774
FAM98B ENSG00000171262.11 0.116116486 2 2 0.042819491
Brain_Cerebellar_Hemisphere 0.88723234 Brain_Cerebellum 1.150342272
0.849657728 0.849657728 -0.141807145 2.025466439 0.941829647
1.532493848 2 0 0.972491774
TAB1 ENSG00000100324.13 0.116162172 2 1 0.115327564
Brain_Cerebellar_Hemisphere 0.118218487 Brain_Cerebellum 1.993399508
0.006600492 1.993399508 1.562295093 1.574691964 1.568493529
0.008765912 1 0 0.972491774
RP11-807H7.2 ENSG00000248479.1 0.116325524 1 1 0.116325524
Brain_Cortex 0.116325524 Brain_Cortex 1 1 1 -1.570385196
-1.570385196 -1.570385196 NA 1 0 0.972491774
BCHE ENSG00000114200.9 0.116348898 5 4 0.07311247 Brain_Cerebellum
0.8522711 Brain_Cortex 2.650708062 0.052328692 0.429759889
-1.561442197 1.792127969 0.149080609 1.221877801 4 0
0.972491774
TST ENSG00000128311.13 0.116510732 7 7 0.033277656 Brain_Cortex
0.843432929 Brain_Cerebellar_Hemisphere 2.568596645 0.179080785
0.179080785 -0.898832717 2.128717313 0.847695254 1.051276346
7 0 0.972491774
RP11-83B20.1 ENSG00000214650.2 0.11652935 1 1 0.11652935 Brain_Cerebellum
0.11652935 Brain_Cerebellum 1 1 1 -1.569509144 -1.569509144
-1.569509144 NA 1 0 0.972491774
RPRML ENSG00000179673.4 0.116529397 1 1 0.116529397 Brain_Cerebellum
0.116529397 Brain_Cerebellum 1 1 1 -1.569508944 -1.569508944
-1.569508944 NA 1 0 0.972491774
CRBN ENSG00000113851.14 0.116622507 2 2 0.065094917
Brain_Frontal_Cortex_BA9 0.139500495 Brain_Hippocampus 1.314145732
0.685854268 0.685854268 -1.8446057 -1.477653693 -1.661129696
0.259474253 2 0 0.972491774
MAP2K1 ENSG00000169032.9 0.116726622 2 2 0.113608594
Brain_Cerebellar_Hemisphere 0.82775541 Brain_Hypothalamus 1.562263144
0.437736856 0.437736856 -0.217581231 1.582179523 0.682299146
1.272623034 2 0 0.972491774
DUSP4 ENSG00000120875.8 0.116799472 1 1 0.116799472
Brain_Caudate_basal_ganglia 0.116799472 Brain_Caudate_basal_ganglia 1 1
1 1.568350004 1.568350004 1.568350004 NA 1 0
0.972491774
PIP5K1C ENSG00000186111.9 0.116805355 2 2 0.061133486
Brain_Cerebellum 0.61409505 Brain_Cerebellar_Hemisphere 1.195655875
0.804344125 0.804344125 -1.872528764 0.504236705 -0.68414603
1.68062698 2 0 0.972491774
THUMPD3 ENSG00000134077.15 0.116912372 12 5 0.04397737 Brain_Cerebellum

0.505906722 Brain_Spinal_cord_cervical_c-1 8.793044452 0.001225868
0.366792478 -0.798199679 2.014306447 1.075492099 0.907500384
5 0 0.972491774
12-Sep ENSG00000140623.13 0.116961495 13 5 0.009666459
Brain_Caudate_basal_ganglia 0.908744732 Brain_Anterior_cingulate_cortex_BA24
9.642845514 0.007114576 0.480008769 -0.114622011 2.587537793
1.046635404 0.646305211 5 0 0.972491774
UFD1L ENSG00000070010.18 0.117000286 1 1 0.117000286
Brain_Frontal_Cortex_BA9 0.117000286 Brain_Frontal_Cortex_BA9 1 1
1 1.56748964 1.56748964 1.56748964 NA 1 0 0.972491774
KLRC3 ENSG00000205810.8 0.117187182 11 3 0.011286246
Brain_Hypothalamus 0.369362215 Brain_Frontal_Cortex_BA9 9.289399496
0.00140662 0.309835169 0.897668674 2.533708872 1.704206504
0.42446909 3 0 0.972491774
FHDC1 ENSG00000137460.8 0.117304322 3 3 0.030326813
Brain_Frontal_Cortex_BA9 0.103407686 Brain_Cerebellar_Hemisphere
2.032582636 0.241529992 0.241529992 1.628552975 2.165795557
1.956830501 0.287808959 3 0 0.972491774
SLC35A1 ENSG00000164414.17 0.117355538 6 6 0.040827945
Brain_Anterior_cingulate_cortex_BA24 0.851047554 Brain_Hypothalamus
3.100797565 0.35320602 0.35320602 -2.045273066 -0.187781991
-1.007949128 0.688788061 6 0 0.972491774
CFAP99 ENSG00000206113.10 0.117367918 3 3 0.023967394
Brain_Cerebellum 0.941725096 Brain_Cortex 1.401297054 0.580472172
0.580472172 -0.628687429 2.257651516 0.567355301 1.505308276
3 0 0.972491774
ZNF212 ENSG00000170260.8 0.117401531 4 4 0.007818692
Brain_Hypothalamus 0.406927409 Brain_Putamen_basal_ganglia 2.006404918
0.48709839 0.48709839 0.829312836 2.659800607 1.465025461
0.837731882 4 0 0.972491774
HIST1H2AG ENSG00000196787.3 0.117526715 2 2 0.083490408
Brain_Hypothalamus 0.376398653 Brain_Cerebellar_Hemisphere 1.138009514
0.861990486 0.861990486 -1.730783375 -0.884551354 -1.307667364
0.5983764 2 0 0.972491774
ZNF490 ENSG00000188033.9 0.117629553 1 1 0.117629553
Brain_Cerebellum 0.117629553 Brain_Cerebellum 1 1 1 1.564801101
1.564801101 1.564801101 NA 1 0 0.972491774
KIF18B ENSG00000186185.13 0.117645175 1 1 0.117645175
Brain_Anterior_cingulate_cortex_BA24 0.117645175
Brain_Anterior_cingulate_cortex_BA24 1 1 1 1.564734501
1.564734501 1.564734501 NA 1 0 0.972491774
KIAA2013 ENSG00000116685.15 0.118172281 1 1 0.118172281
Brain_Nucleus_accumbens_basal_ganglia 0.118172281
Brain_Nucleus_accumbens_basal_ganglia 1 1 1 1.562491353
1.562491353 1.562491353 NA 1 0 0.972491774
TXNL1 ENSG00000091164.12 0.118338464 1 1 0.118338464 Brain_Cerebellum
0.118338464 Brain_Cerebellum 1 1 1 1.561785775 1.561785775
1.561785775 NA 1 0 0.972491774
COX6B2 ENSG00000160471.12 0.118395625 3 3 0.08951319
Brain_Cerebellar_Hemisphere 0.773453821 Brain_Cerebellum 1.173849025
0.832630212 0.832630212 -0.287860112 1.697971242 1.013198804
1.12727029 3 0 0.972491774
USP16 ENSG00000156256.14 0.118517805 2 1 0.102389394 Brain_Cerebellum
0.140813687 Brain_Cerebellar_Hemisphere 1.97967541 0.02032459 1.97967541
1.47276769 1.633378603 1.553073147 0.113569066 1 0
0.972491774
LINC00161 ENSG00000226935.6 0.118543477 1 1 0.118543477
Brain_Cerebellum 0.118543477 Brain_Cerebellum 1 1 1 -1.560916402
-1.560916402 -1.560916402 NA 1 0 0.972491774
RP11-188P17.2 ENSG00000272646.1 0.118767754 1 1 0.118767754
Brain_Amygdala 0.118767754 Brain_Amygdala 1 1 1 -1.559966685
-1.559966685 -1.559966685 NA 1 0 0.972491774

NOB1 ENSG00000141101.12 0.118795497 6 4 0.021024087 Brain_Cerebellum
0.21315722 Brain_Caudate_basal_ganglia 4.196957782 0.017746257
0.172330261 -2.307551647 -1.244930713 -1.768676055 0.40101643 4
0 0.972491774
SNHG19 ENSG00000260260.1 0.118797535 1 1 0.118797535
Brain_Nucleus_accumbens_basal_ganglia 0.118797535
Brain_Nucleus_accumbens_basal_ganglia 1 1 1 1.559840684
1.559840684 1.559840684 NA 1 0 0.972491774
C8orf4 ENSG00000176907.4 0.118930833 2 2 0.101092603
Brain_Spinal_cord_cervical_c-1 0.483004108 Brain_Hippocampus 1.3009868
0.6990132 0.6990132 -0.701479044 1.639579621 0.469050288
1.655378458 2 0 0.972491774
VASH2 ENSG00000143494.15 0.119013725 2 2 0.091879794
Brain_Nucleus_accumbens_basal_ganglia 0.227710387 Brain_Cortex
1.009738864 0.990261136 0.990261136 -1.685564071 1.20627782
-0.239643125 2.044841011 2 0 0.972491774
ARHGEF10L ENSG00000074964.16 0.119061676 3 3 0.079288989
Brain_Cerebellum 0.94937492 Brain_Cerebellar_Hemisphere 1.639124297
0.360767661 0.360767661 -1.754826502 0.563862847 -0.375823965
1.220175727 3 0 0.972491774
MDFI ENSG00000112559.13 0.119158404 1 1 0.119158404 Brain_Amygdala
0.119158404 Brain_Amygdala 1 1 1 1.558315804 1.558315804
1.558315804 NA 1 0 0.972491774
WBP2 ENSG00000132471.11 0.119331591 4 4 0.017886342
Brain_Frontal_Cortex_BA9 0.872368995 Brain_Putamen_basal_ganglia
1.336296299 0.59063519 0.59063519 -2.367962645 -0.160650098
-1.238572729 1.063880234 4 0 0.972491774
RP11-468E2.10 ENSG00000276698.1 0.11937373 2 2 0.094661206
Brain_Cerebellum 0.302904324 Brain_Frontal_Cortex_BA9 1.10220523 0.89779477
0.89779477 1.030225154 1.671306231 1.350765693 0.453312777 2
0 0.972491774
GPR179 ENSG00000277399.4 0.119608882 2 2 0.04192825 Brain_Cortex
0.091015477 Brain_Frontal_Cortex_BA9 1.717841408 0.282158592
0.282158592 1.690065225 2.034231594 1.862148409 0.243362373
2 0 0.972491774
NME1 ENSG00000239672.7 0.119717723 13 4 0.006552328 Brain_Cerebellum
0.698357819 Brain_Substantia_nigra 10.33360498 0.000182721
0.564013621 0.387538188 2.718779441 2.017555409 0.626433407
4 0 0.972491774
GZF1 ENSG00000125812.15 0.119719567 3 3 0.043785475 Brain_Cerebellum
0.850906845 Brain_Cerebellar_Hemisphere 1.260344937 0.777931094
0.777931094 -1.227776202 2.016138695 0.325441326 1.626321461
3 0 0.972491774
DPYSL4 ENSG00000151640.12 0.119890358 5 3 0.081414824
Brain_Cerebellar_Hemisphere 0.417238647 Brain_Anterior_cingulate_cortex_BA24
4.044793524 0.021261092 0.19329042 -0.81122121 1.742535275
1.009396214 1.033944741 3 0 0.972491774
ZBTB46 ENSG00000130584.10 0.119892141 6 2 0.254105583
Brain_Putamen_basal_ganglia 0.789563943 Brain_Cortex 5.549050239
0.000342949 0.293068682 -1.140433885 0.266876899 -0.707262469
0.503101443 2 0 0.972491774
C5AR2 ENSG00000134830.5 0.119933868 1 1 0.119933868 Brain_Cerebellum
0.119933868 Brain_Cerebellum 1 1 1 1.555051231 1.555051231
1.555051231 NA 1 0 0.972491774
SMARCD3 ENSG00000082014.16 0.11997801 1 1 0.11997801
Brain_Frontal_Cortex_BA9 0.11997801 Brain_Frontal_Cortex_BA9 1 1 1
-1.5548659 -1.5548659 -1.5548659 NA 1 0 0.972491774
JKAMP ENSG00000050130.17 0.1200744 1 1 0.1200744
Brain_Nucleus_accumbens_basal_ganglia 0.1200744
Brain_Nucleus_accumbens_basal_ganglia 1 1 1 -1.55446139
-1.55446139 -1.55446139 NA 1 0 0.972491774
KBTBD3 ENSG00000182359.14 0.120182014 3 3 0.025799985

Brain_Anterior_cingulate_cortex_BA24 0.727515549 Brain_Cortex
2.007961645 0.343236526 0.343236526 0.3484323 2.22920926
1.213186068 0.949469495 3 0 0.972491774
GIPC2 ENSG00000137960.5 0.12026289 1 1 0.12026289 Brain_Amygdala 0.12026289
Brain_Amygdala 1 1 1 1.553671102 1.553671102 1.553671102
NA 1 0 0.972491774
ORMDL3 ENSG00000172057.9 0.120285926 3 3 0.018408821
Brain_Caudate_basal_ganglia 0.722994147 Brain_Frontal_Cortex_BA9
1.704596293 0.29872961 0.29872961 -2.357290874 0.403108776
-0.533240687 1.579861067 3 0 0.972491774
RP11-17E13.2 ENSG00000272864.1 0.120397985 13 5 0.006211299
Brain_Nucleus_accumbens_basal_ganglia 0.292107665 Brain_Substantia_nigra
9.411678048 0.008294731 0.48055703 -2.736413134 -1.053509232
-2.235129181 0.433415501 5 0 0.972491774
ALDH6A1 ENSG00000119711.12 0.120504788 5 3 0.032115553
Brain_Cerebellum 0.559701093 Brain_Amygdala 3.220243768 0.027364909
0.773653376 -2.142969433 0.583285543 -1.294186038 1.16971169 3
0 0.972491774
IL17RE ENSG00000163701.18 0.120572221 3 3 0.016170089
Brain_Anterior_cingulate_cortex_BA24 0.924358908 Brain_Hippocampus
1.046015839 0.96057557 0.96057557 -2.405053701 0.152244268
-0.71928831 1.460196744 3 0 0.972491774
PSMC3IP ENSG00000131470.14 0.120626299 3 3 0.036836082
Brain_Putamen_basal_ganglia 0.973276639 Brain_Hippocampus 1.592108457
0.563459473 0.563459473 -0.820614136 2.08757619 0.411154341
1.504220798 3 0 0.972491774
SYNGR4 ENSG00000105467.8 0.120745222 2 2 0.087584486
Brain_Hippocampus 0.12792151 Brain_Hypothalamus 1.240907853
0.759092147 0.759092147 1.522349584 1.70827954 1.615314562
0.131472333 2 0 0.972491774
RP5-1102E8.3 ENSG00000272855.1 0.120781583 1 1 0.120781583
Brain_Spinal_cord_cervical_c-1 0.120781583 Brain_Spinal_cord_cervical_c-1
1 1 1 1.551501355 1.551501355 1.551501355 NA 1 0
0.972491774
RP11-266K4.13 ENSG00000276417.1 0.120951902 2 2 0.074183212
Brain_Nucleus_accumbens_basal_ganglia 0.311860757
Brain_Spinal_cord_cervical_c-1 1.003859383 0.996140617 0.996140617
-1.011325307 1.785481734 0.387078214 1.977641225 2 0
0.972491774
AC068831.16 ENSG00000278514.1 0.120994541 3 3 0.022229716
Brain_Cerebellum 0.710744469 Brain_Caudate_basal_ganglia 1.534122917
0.486162521 0.486162521 -2.28641971 0.370856429 -1.146392594
1.36821102 3 0 0.972491774
SAMD15 ENSG00000100583.4 0.121045994 3 2 0.119909354
Brain_Hypothalamus 0.656424652 Brain_Amygdala 2.731928542
0.022516757 0.245554701 -1.555154179 -0.444854851 -1.165832189
0.625050079 2 0 0.972491774
SNX27 ENSG00000143376.12 0.121105185 11 3 0.055243548
Brain_Frontal_Cortex_BA9 0.359506391 Brain_Putamen_basal_ganglia
9.990949308 3.95E-05 0.340075895 0.916306059 1.916955793
1.664951289 0.384006374 3 0 0.972491774
RP11-314B1.2 ENSG00000273301.1 0.121167515 2 2 0.048109877
Brain_Caudate_basal_ganglia 0.56186397 Brain_Amygdala 1.009529984
0.990470016 0.990470016 0.580075107 1.976396594 1.278235851
0.987348393 2 0 0.972491774
MRPL54 ENSG00000183617.4 0.121212471 6 5 0.023571222
Brain_Spinal_cord_cervical_c-1 0.781788685 Brain_Caudate_basal_ganglia
2.560049936 0.00100569 0.444771453 -0.404002052 2.264047035
1.128588669 1.157518548 5 0 0.972491774
AATK-AS1 ENSG00000225180.7 0.121349289 1 1 0.121349289
Brain_Cortex 0.121349289 Brain_Cortex 1 1 1 -1.549134926
-1.549134926 -1.549134926 NA 1 0 0.972491774

SCAMP1-AS1 ENSG00000245556.2 0.121361241 5 3 0.221995288
Brain_Cerebellum 0.892950176 Brain_Cerebellar_Hemisphere 4.461176658
0.047244083 0.166740659 -0.134572133 1.221239671 0.82212795
0.543785618 3 0 0.972491774
FBXW5 ENSG00000159069.13 0.12138695 2 2 0.05312706 Brain_Caudate_basal_ganglia
0.919432279 Brain_Nucleus_accumbens_basal_ganglia 1.38249433 0.61750567
0.61750567 -1.9338867 -0.101148877 -1.017517788 1.295941343 2 0
0.972491774
RP11-367J11.3 ENSG00000245468.3 0.121428845 7 4 0.059368323
Brain_Cerebellar_Hemisphere 0.600867141 Brain_Hypothalamus 5.061383655
5.85E-17 0.352614915 -1.88545583 -0.523153926 -0.945933949
0.599731199 4 0 0.972491774
RP11-688I9.4 ENSG00000254938.1 0.121566194 1 1 0.121566194
Brain_Hippocampus 0.121566194 Brain_Hippocampus 1 1 1
1.548233061 1.548233061 1.548233061 NA 1 0 0.972491774
SLC38A2 ENSG00000134294.13 0.121613651 2 2 0.046178706
Brain_Anterior_cingulate_cortex_BA24 0.703324618 Brain_Caudate_basal_ganglia
1.053706269 0.946293731 0.946293731 -1.993756189 0.380836439
-0.806459875 1.67909055 2 0 0.972491774
POLR2J3 ENSG00000168255.19 0.121662059 2 2 0.081079258
Brain_Cortex 0.083355638 Brain_Spinal_cord_cervical_c-1 1.43400583
0.56599417 0.56599417 1.73153921 1.744458007 1.737998609 0.009134969
2 0 0.972491774
SMYD3 ENSG00000185420.18 0.121740557 2 2 0.097283167 Brain_Cortex
0.142502813 Brain_Cerebellar_Hemisphere 1.165203429 0.834796571
0.834796571 1.466534195 1.658169956 1.562352075 0.135506946
2 0 0.972491774
SACS ENSG00000151835.14 0.121783182 3 3 0.020983291
Brain_Hippocampus 0.669580346 Brain_Cerebellum 1.771636388
0.241931468 0.241931468 -2.308284988 0.487571084 -0.464663292
1.596913057 3 0 0.972491774
CSNK1A1 ENSG00000113712.17 0.121793485 1 1 0.121793485
Brain_Putamen_basal_ganglia 0.121793485 Brain_Putamen_basal_ganglia 1 1
1 1.547289367 1.547289367 1.547289367 NA 1 0
0.972491774
ITGBL1 ENSG00000198542.13 0.121860384 2 2 0.041589049
Brain_Cerebellum 0.436950069 Brain_Cerebellar_Hemisphere 1.26771411 0.73228589
0.73228589 0.777353789 2.037609021 1.407481405 0.891135021 2
0 0.972491774
TOM1 ENSG00000100284.20 0.121980633 1 1 0.121980633
Brain_Frontal_Cortex_BA9 0.121980633 Brain_Frontal_Cortex_BA9 1 1
1 -1.546513372 -1.546513372 -1.546513372 NA 1 0
0.972491774
ZFP64 ENSG00000020256.19 0.122062992 1 1 0.122062992 Brain_Cortex
0.122062992 Brain_Cortex 1 1 1 1.546172172 1.546172172
1.546172172 NA 1 0 0.972491774
COG8 ENSG00000213380.14 0.122144954 1 1 0.122144954
Brain_Frontal_Cortex_BA9 0.122144954 Brain_Frontal_Cortex_BA9 1 1
1 -1.545832798 -1.545832798 -1.545832798 NA 1 0
0.972491774
SOX7 ENSG00000171056.7 0.122202721 2 2 0.090461617 Brain_Cerebellum
0.387137724 Brain_Cerebellar_Hemisphere 1.163254572 0.836745428
0.836745428 -0.86481976 1.692967693 0.414073967 1.808628853
2 0 0.972491774
RP11-206L10.9 ENSG00000237491.8 0.122265938 11 10 0.007115939
Brain_Substantia_nigra 0.886713064 Brain_Anterior_cingulate_cortex_BA24
2.467739884 0.062939637 0.158309474 -2.002658063 2.691369206
0.17849293 1.227225347 10 0 0.972491774
PIK3R1 ENSG00000145675.14 0.122294285 3 3 0.12162017
Brain_Caudate_basal_ganglia 0.64015985 Brain_Nucleus_accumbens_basal_ganglia
1.45031678 0.548617446 0.548617446 -1.548008831 1.140366206
0.01994423 1.398947154 3 0 0.972491774

ASPHD1 ENSG00000174939.10 0.122418521 6 3 0.00334106
Brain_Hypothalamus 0.667457006 Brain_Nucleus_accumbens_basal_ganglia
3.920765801 0.003132877 0.922202327 -0.44065615 2.934480983
1.397686383 1.457005834 3 0 0.972491774
RUFY3 ENSG00000018189.12 0.122464054 1 1 0.122464054
Brain_Hippocampus 0.122464054 Brain_Hippocampus 1 1 1
1.54451321 1.54451321 1.54451321 NA 1 0 0.972491774
SCO1 ENSG00000133028.10 0.122632411 10 2 0.225400805
Brain_Spinal_cord_cervical_c-1 0.977654361 Brain_Hypothalamus
8.704672694 0.000583942 0.700137553 -1.21229153 0.69980392
-0.70457583 0.59973416 2 0 0.972491774
CYP39A1 ENSG00000146233.7 0.122719991 4 4 0.033459013
Brain_Cerebellum 0.586768916 Brain_Nucleus_accumbens_basal_ganglia
1.255359726 0.791697678 0.791697678 -0.543524226 2.126531702
0.942783574 1.109696926 4 0 0.972491774
SPCS2 ENSG00000118363.11 0.122872225 2 2 0.043987842
Brain_Spinal_cord_cervical_c-1 0.776004296 Brain_Frontal_Cortex_BA9
1.041797976 0.958202024 0.958202024 -0.284529936 2.014206648
0.864838356 1.625452226 2 0 0.972491774
SLC8A3 ENSG00000100678.18 0.122910356 5 2 0.031874931
Brain_Putamen_basal_ganglia 0.175275436 Brain_Cerebellum 4.112324423
0.006126488 0.748477335 -1.824736466 2.145975525 0.560513166
1.972420926 2 0 0.972491774
CDKN1B ENSG00000111276.10 0.12307155 1 1 0.12307155
Brain_Cerebellar_Hemisphere 0.12307155 Brain_Cerebellar_Hemisphere 1 1 1
-1.542008408 -1.542008408 -1.542008408 NA 1 0 0.972491774
SNX15 ENSG00000110025.12 0.123088008 9 4 0.026923906
Brain_Anterior_cingulate_cortex_BA24 0.820108442 Brain_Hippocampus
7.354101789 0.02216733 0.279106437 -2.212619268 -0.227405502
-1.55703067 0.575264082 4 0 0.972491774
PTP4A1 ENSG00000112245.10 0.123224605 1 1 0.123224605
Brain_Spinal_cord_cervical_c-1 0.123224605 Brain_Spinal_cord_cervical_c-1
1 1 1 1.541378864 1.541378864 1.541378864 NA 1 0
0.972491774
PACRG ENSG00000112530.11 0.123295162 9 5 0.152872197
Brain_Hypothalamus 0.752058722 Brain_Frontal_Cortex_BA9 6.122323046
0.00260151 0.226549783 -1.060294555 1.429459537 -0.205456081
0.81475555 5 0 0.972491774
RP11-430H10.2 ENSG00000255267.3 0.123317126 2 2 0.043331849
Brain_Hypothalamus 0.824831672 Brain_Hippocampus 1.04954895 0.95045105
0.95045105 -0.221334907 2.020497112 0.899581102 1.585214623 2
0 0.972491774
SLC43A2 ENSG00000167703.14 0.123366188 2 2 0.137778851
Brain_Hypothalamus 0.4659128 Brain_Putamen_basal_ganglia 1.38450918 0.61549082
0.61549082 -1.484113356 0.729145278 -0.377484039 1.565010189 2
0 0.972491774
PTPN14 ENSG00000152104.11 0.123385575 3 3 0.06138096
Brain_Frontal_Cortex_BA9 0.887022431 Brain_Nucleus_accumbens_basal_ganglia
2.28958473 0.256241771 0.256241771 0.142072891 1.870741206
1.087271423 0.875608746 3 0 0.972491774
CD83 ENSG00000112149.9 0.123680967 3 3 0.089276359 Brain_Amygdala
0.340588663 Brain_Hippocampus 1.169270932 0.83728578 0.83728578
-1.69922733 -0.953002778 -1.33212227 0.373257325 3 0
0.972491774
RP11-472I20.3 ENSG00000283217.1 0.123692645 1 1 0.123692645
Brain_Cortex 0.123692645 Brain_Cortex 1 1 1 1.539457504
1.539457504 1.539457504 NA 1 0 0.972491774
INTS6 ENSG00000102786.14 0.123763353 2 2 0.063059839
Brain_Caudate_basal_ganglia 0.301473274 Brain_Cerebellar_Hemisphere
1.100527258 0.899472742 0.899472742 1.033279167 1.858769346
1.446024256 0.583709703 2 0 0.972491774
MDH2 ENSG00000146701.11 0.123768778 2 2 0.077448839 Brain_Cerebellum

0.281591448 Brain_Hypothalamus 1.026646451 0.973353549
0.973353549 -1.765684238 1.076751163 -0.344466538 2.009905348
2 0 0.972491774
ARMC12 ENSG00000157343.8 0.123919274 1 1 0.123919274
Brain_Caudate_basal_ganglia 0.123919274 Brain_Caudate_basal_ganglia 1 1
1 -1.538529203 -1.538529203 -1.538529203 NA 1 0
0.972491774
CACNA1C ENSG00000151067.21 0.123997847 3 2 0.05296381 Brain_Cortex
0.287974695 Brain_Cerebellar_Hemisphere 2.09603764 0.037213214
0.866749146 -1.935215881 -1.062575075 -1.411631942 0.461757803
2 0 0.972491774
RP11-395N3.2 ENSG00000272622.1 0.124052964 1 1 0.124052964
Brain_Frontal_Cortex_BA9 0.124052964 Brain_Frontal_Cortex_BA9 1 1
1 1.537982213 1.537982213 1.537982213 NA 1 0
0.972491774
ETFBKMT ENSG00000139160.13 0.124084642 2 2 0.171366694
Brain_Cerebellar_Hemisphere 0.210244289 Brain_Frontal_Cortex_BA9
1.176076609 0.823923391 0.823923391 -1.367825533 1.252893992
-0.05746577 1.853128548 2 0 0.972491774
GAREM2 ENSG00000157833.12 0.12414204 1 1 0.12414204
Brain_Caudate_basal_ganglia 0.12414204 Brain_Caudate_basal_ganglia 1 1 1
1.537618016 1.537618016 1.537618016 NA 1 0 0.972491774
RP11-571L19.8 ENSG00000272777.1 0.12414634 8 6 0.065138367
Brain_Hippocampus 0.954317882 Brain_Cerebellar_Hemisphere 5.116709517
0.053615838 0.206206155 -1.844307308 -0.057285361 -0.979498734
0.639448358 6 0 0.972491774
TRIM46 ENSG00000163462.17 0.124213552 2 2 0.073083221
Brain_Hippocampus 0.290581093 Brain_Substantia_nigra 1.043761853
0.956238147 0.956238147 -1.792310631 -1.056847717 -1.424579174
0.520050814 2 0 0.972491774
NAT16 ENSG00000167011.8 0.124328865 5 4 0.076302649
Brain_Caudate_basal_ganglia 0.762604751 Brain_Cerebellum 3.122144659
0.072260543 0.375076199 -1.772553945 0.914778606 -0.233832479
1.141451217 4 0 0.972491774
LINC00882 ENSG00000242759.6 0.124342679 2 2 0.047644423
Brain_Frontal_Cortex_BA9 0.931069814 Brain_Nucleus_accumbens_basal_ganglia
1.202059978 0.797940022 0.797940022 -1.980526285 0.08649892
-0.947013683 1.46160754 2 0 0.972491774
CD3EAP ENSG00000117877.10 0.124503784 7 5 0.020483716
Brain_Frontal_Cortex_BA9 0.611725766 Brain_Cortex 4.564918279
0.007800304 0.247356941 -1.568706393 2.317367593 0.920276244
1.227893771 5 0 0.972491774
GOLGA7 ENSG00000147533.16 0.124509958 8 2 0.032098164
Brain_Substantia_nigra 0.197208796 Brain_Amygdala 6.864377102
0.0136944 0.601474059 1.289544674 2.143186022 1.651601891
0.294383629 2 0 0.972491774
IQSEC3 ENSG00000120645.11 0.124730966 3 3 0.035554892
Brain_Cerebellar_Hemisphere 0.740562745 Brain_Frontal_Cortex_BA9
1.540851035 0.437337553 0.437337553 -0.331108217 2.101981554
1.282761797 1.397706418 3 0 0.972491774
MAMDC4 ENSG00000177943.13 0.124799678 2 2 0.085473096
Brain_Cerebellum 0.091655862 Brain_Hippocampus 1.394344857
0.605655143 0.605655143 -1.719776392 -1.686726976 -1.703251684
0.023369466 2 0 0.972491774
PTPN6 ENSG00000111679.16 0.124822991 1 1 0.124822991 Brain_Cerebellum
0.124822991 Brain_Cerebellum 1 1 1 1.534840584 1.534840584
1.534840584 NA 1 0 0.972491774
ITIH5 ENSG00000123243.14 0.12493999 1 1 0.12493999
Brain_Anterior_cingulate_cortex_BA24 0.12493999
Brain_Anterior_cingulate_cortex_BA24 1 1 1 -1.534364565
-1.534364565 -1.534364565 NA 1 0 0.972491774
S100A3 ENSG00000188015.9 0.124948557 3 3 0.065850278

Brain_Anterior_cingulate_cortex_BA24 0.978572428 Brain_Cerebellar_Hemisphere
1.688444206 0.306381109 0.306381109 0.026858707 1.839441476
1.045048772 0.926783695 3 0 0.972491774
RP11-284F21.9 ENSG00000272068.1 0.125034679 1 1 0.125034679
Brain_Cerebellum 0.125034679 Brain_Cerebellum 1 1 1 1.533979571
1.533979571 1.533979571 NA 1 0 0.972491774
RP11-704J17.5 ENSG00000272428.1 0.125072471 1 1 0.125072471
Brain_Anterior_cingulate_cortex_BA24 0.125072471
Brain_Anterior_cingulate_cortex_BA24 1 1 1 1.533825973
1.533825973 1.533825973 NA 1 0 0.972491774
DALRD3 ENSG00000178149.16 0.125087896 5 3 0.024960108
Brain_Cortex 0.071977879 Brain_Anterior_cingulate_cortex_BA24
3.748776375 0.070458342 0.438205239 -2.242019558 -1.799257997
-2.016071769 0.184298689 3 0 0.972491774
MFSD4A ENSG00000174514.12 0.125093333 5 5 0.058565382
Brain_Nucleus_accumbens_basal_ganglia 0.855555288 Brain_Cortex
2.582676242 0.12639934 0.12639934 -1.891441885 -0.182034962
-0.877097391 0.62982875 5 0 0.972491774
MAN2A2 ENSG00000196547.14 0.125128824 2 2 0.041485252
Brain_Anterior_cingulate_cortex_BA24 0.958838664 Brain_Cerebellum
1.011968021 0.988031979 0.988031979 -0.051610988 2.038647185
0.993518099 1.478035728 2 0 0.972491774
ARPIN ENSG00000242498.7 0.125177092 3 3 0.082145492
Brain_Spinal_cord_cervical_c-1 0.53330594 Brain_Caudate_basal_ganglia
1.255747844 0.769182267 0.769182267 -1.478017395 1.738370839
0.294440284 1.633167435 3 0 0.972491774
CRYZ ENSG00000116791.13 0.125188865 13 6 0.009118226
Brain_Nucleus_accumbens_basal_ganglia 0.49441365 Brain_Spinal_cord_cervical_c-1
9.177633731 0.02991335 0.338788902 -2.607589397 -0.683305768
-2.008344972 0.618367936 6 0 0.972491774
FXYD7 ENSG00000221946.7 0.125305753 1 1 0.125305753 Brain_Cerebellum
0.125305753 Brain_Cerebellum 1 1 1 1.532878666 1.532878666
1.532878666 NA 1 0 0.972491774
FOXO3 ENSG00000118689.14 0.125378396 3 3 0.097908072 Brain_Cerebellum
0.476876076 Brain_Anterior_cingulate_cortex_BA24 1.325731034
0.667287653 0.667287653 -1.397300478 1.655080983 0.32303882
1.562798498 3 0 0.972491774
TGOLN2 ENSG00000152291.13 0.12548401 3 3 0.027181887
Brain_Frontal_Cortex_BA9 0.992009939 Brain_Putamen_basal_ganglia
1.733092958 0.467500299 0.467500299 -0.010014224 2.208895828
1.038597583 1.114448808 3 0 0.972491774
ANGPT1 ENSG00000154188.9 0.125642194 1 1 0.125642194
Brain_Cerebellar_Hemisphere 0.125642194 Brain_Cerebellar_Hemisphere 1 1
1 -1.531514871 -1.531514871 -1.531514871 NA 1 0
0.972491774
TP73 ENSG00000078900.14 0.125707514 2 2 0.089852694 Brain_Amygdala
0.344079927 Brain_Cerebellum 1.103201929 0.896798071 0.896798071
0.946134622 1.696175262 1.321154942 0.530358823 2 0
0.972491774
LIMK2 ENSG00000182541.17 0.125741262 6 5 0.029125684
Brain_Anterior_cingulate_cortex_BA24 0.71283255 Brain_Cortex 3.807246866
0.080804587 0.220067561 -2.181781211 1.179703729 -1.020854296
1.255508453 5 0 0.972491774
LENEP ENSG00000163352.5 0.12579458 1 1 0.12579458 Brain_Cerebellum 0.12579458
Brain_Cerebellum 1 1 1 -1.530898094 -1.530898094 -1.530898094
NA 1 0 0.972491774
ELAVL1 ENSG00000066044.14 0.125846482 1 1 0.125846482
Brain_Cortex 0.125846482 Brain_Cortex 1 1 1 -1.530688156
-1.530688156 -1.530688156 NA 1 0 0.972491774
ENO3 ENSG00000108515.17 0.125873369 9 9 0.118402458 Brain_Amygdala
0.990775038 Brain_Hippocampus 4.412762196 0.16621892 0.16621892
-0.932816941 1.561514275 0.15655534 0.830226405 9 0

0.972491774
LINC02084 ENSG00000272282.1 0.126024661 2 2 0.045397254
Brain_Cerebellum 0.584886154 Brain_Cerebellar_Hemisphere 1.08756869 0.91243131
0.91243131 -0.546261562 2.000954866 0.727346652 1.80115401 2 0
0.972491774
EMP1 ENSG00000134531.9 0.126029568 5 5 0.006278036
Brain_Hippocampus 0.990858746 Brain_Putamen_basal_ganglia 1.385103973
0.62943895 0.62943895 -2.732894886 0.501785627 -0.591207311
1.263591458 5 0 0.972491774
C5orf42 ENSG00000197603.13 0.126121885 2 2 0.0663001 Brain_Cerebellum
0.315769681 Brain_Cortex 1.070024938 0.929975062 0.929975062
-1.836389304 1.003188996 -0.416600154 2.007885072 2 0
0.972491774
TMUB1 ENSG00000164897.12 0.126155788 1 1 0.126155788
Brain_Frontal_Cortex_BA9 0.126155788 Brain_Frontal_Cortex_BA9 1 1
1 -1.529438446 -1.529438446 -1.529438446 NA 1 0
0.972491774
AFG3L2 ENSG00000141385.9 0.126293502 1 1 0.126293502
Brain_Spinal_cord_cervical_c-1 0.126293502 Brain_Spinal_cord_cervical_c-1
1 1 1 -1.528882797 -1.528882797 -1.528882797 NA 1 0
0.972491774
CTA-246H3.12 ENSG00000272942.1 0.126301071 4 4 0.045180125
Brain_Hippocampus 0.9307341 Brain_Amygdala 1.995511653 0.494312993
0.494312993 -2.002973578 0.086921261 -1.224735547 0.966169973
4 0 0.972491774
NDST1 ENSG00000070614.14 0.126301637 6 4 0.013059381
Brain_Putamen_basal_ganglia 0.20690477 Brain_Cerebellar_Hemisphere 5.053757289
0.022747674 0.193943533 1.262122334 2.482145841 1.754253824
0.452072943 4 0 0.972491774
ATXN1 ENSG00000124788.17 0.126346552 2 2 0.042524912
Brain_Hippocampus 0.886696066 Brain_Caudate_basal_ganglia 1.004949495
0.995050505 0.995050505 -2.02834635 0.14248609 -0.94293013
1.535010339 2 0 0.972491774
C16orf54 ENSG00000185905.3 0.126395488 1 1 0.126395488
Brain_Cerebellar_Hemisphere 0.126395488 Brain_Cerebellar_Hemisphere 1 1
1 1.528471606 1.528471606 1.528471606 NA 1 0
0.972491774
BTN3A1 ENSG00000026950.16 0.126451888 7 5 0.070522939
Brain_Hippocampus 0.420401327 Brain_Cortex 4.454830144
0.005507034 0.18657959 -1.529795061 1.808537157 0.377478159
1.312230102 5 0 0.972491774
XYLT1 ENSG00000103489.11 0.126455258 1 1 0.126455258
Brain_Cerebellar_Hemisphere 0.126455258 Brain_Cerebellar_Hemisphere 1 1
1 -1.528230743 -1.528230743 -1.528230743 NA 1 0
0.972491774
CTA-384D8.35 ENSG00000272666.1 0.126495446 1 1 0.126495446
Brain_Cerebellar_Hemisphere 0.126495446 Brain_Cerebellar_Hemisphere 1 1
1 1.528068844 1.528068844 1.528068844 NA 1 0
0.972491774
NDUFAF8 ENSG00000224877.3 0.126513031 6 5 0.020251859
Brain_Cerebellar_Hemisphere 0.823641646 Brain_Cerebellum 3.05405942
0.045156286 0.169220966 -1.48248935 2.321648699 0.864642286
1.514976961 5 0 0.972491774
DOK6 ENSG00000206052.10 0.126671717 1 1 0.126671717 Brain_Amygdala
0.126671717 Brain_Amygdala 1 1 1 -1.527359197 -1.527359197
-1.527359197 NA 1 0 0.972491774
INTS2 ENSG00000108506.11 0.126748324 1 1 0.126748324
Brain_Cerebellar_Hemisphere 0.126748324 Brain_Cerebellar_Hemisphere 1 1
1 1.527051025 1.527051025 1.527051025 NA 1 0
0.972491774
GPN1 ENSG00000198522.13 0.126762599 3 3 0.289906404
Brain_Frontal_Cortex_BA9 0.821064836 Brain_Cerebellar_Hemisphere

2.494152437 0.12725663 0.12725663 -1.058326965 0.841093026
-0.147803184 0.952132215 3 0 0.972491774
NCOA5 ENSG00000124160.11 0.126836364 1 1 0.126836364
Brain_Nucleus_accumbens_basal_ganglia 0.126836364
Brain_Nucleus_accumbens_basal_ganglia 1 1 1 -1.52669704
-1.52669704 -1.52669704 NA 1 0 0.972491774
LIPG ENSG00000101670.11 0.126861713 1 1 0.126861713
Brain_Caudate_basal_ganglia 0.126861713 Brain_Caudate_basal_ganglia 1 1
1 -1.526595154 -1.526595154 -1.526595154 NA 1 0
0.972491774
EIF3A ENSG00000107581.12 0.126981847 1 1 0.126981847 Brain_Amygdala
0.126981847 Brain_Amygdala 1 1 1 1.52611251 1.52611251 1.52611251
NA 1 0 0.972491774
COX19 ENSG00000240230.5 0.127042578 1 1 0.127042578
Brain_Substantia_nigra 0.127042578 Brain_Substantia_nigra 1 1
1 -1.525868655 -1.525868655 -1.525868655 NA 1 0
0.972491774
TMEM8B ENSG00000137103.16 0.127107074 13 1 0.05847603
Brain_Anterior_cingulate_cortex_BA24 0.363171091 Brain_Caudate_basal_ganglia
12.4050553 0.000374583 12.4050553 -1.892112221 -0.909339226
-1.488436137 0.258715229 1 0 0.972491774
MRPS14 ENSG00000120333.4 0.127181033 3 3 0.031203733
Brain_Caudate_basal_ganglia 0.527826088 Brain_Putamen_basal_ganglia
1.173978191 0.859191943 0.859191943 0.631327991 2.154464885
1.279790217 0.786362227 3 0 0.972491774
PSMD2 ENSG00000175166.16 0.127314722 9 5 0.207502911
Brain_Anterior_cingulate_cortex_BA24 0.95852331 Brain_Putamen_basal_ganglia
5.856071075 0.056663616 0.368836403 -0.916915651 1.260461547
0.063572279 0.792129243 5 0 0.972491774
CTD-2062F14.3 ENSG00000259031.1 0.127462032 2 2 0.054558956
Brain_Cerebellum 0.458110767 Brain_Putamen_basal_ganglia 1.047476454
0.952523546 0.952523546 0.741961327 1.922372077 1.332166702
0.834676447 2 0 0.972491774
SUMF2 ENSG00000129103.17 0.127499594 10 3 0.04261149
Brain_Spinal_cord_cervical_c-1 0.978206745 Brain_Cortex 8.266407762
0.015368448 0.564379292 0.027317191 2.027498188 0.432686239
0.605258399 3 0 0.972491774
APOPT1 ENSG00000256053.7 0.127589325 10 7 0.001259522
Brain_Hippocampus 0.550731646 Brain_Spinal_cord_cervical_c-1
6.044570761 0.036217166 0.234297326 -3.225046458 -0.59666413
-1.955571926 0.769912983 7 0 0.972491774
ADRM1 ENSG00000130706.12 0.127640466 1 1 0.127640466
Brain_Caudate_basal_ganglia 0.127640466 Brain_Caudate_basal_ganglia 1 1
1 1.523472781 1.523472781 1.523472781 NA 1 0
0.972491774
SLC1A3 ENSG00000079215.13 0.127645088 1 1 0.127645088
Brain_Cerebellum 0.127645088 Brain_Cerebellum 1 1 1 -1.52345429
-1.52345429 -1.52345429 NA 1 0 0.972491774
ZMAT3 ENSG00000172667.10 0.127669971 3 3 0.160611206 Brain_Amygdala
0.601283513 Brain_Cortex 1.321278954 0.680837204 0.680837204
-1.403018891 1.344863442 0.154800065 1.410371389 3 0
0.972491774
SULT1E1 ENSG00000109193.10 0.127780687 1 1 0.127780687
Brain_Spinal_cord_cervical_c-1 0.127780687 Brain_Spinal_cord_cervical_c-1
1 1 1 1.522912144 1.522912144 1.522912144 NA 1 0
0.972491774
RP11-108E14.1 ENSG00000253300.1 0.127849948 1 1 0.127849948
Brain_Substantia_nigra 0.127849948 Brain_Substantia_nigra 1 1
1 1.522635399 1.522635399 1.522635399 NA 1 0
0.972491774
PTK2 ENSG00000169398.19 0.127924831 10 9 0.001748596
Brain_Nucleus_accumbens_basal_ganglia 0.992426374 Brain_Putamen_basal_ganglia

3.432380103 0.041645396 0.218211038 -3.129910469 1.413741101
-0.137437918 1.21325407 9 0 0.972491774
RP11-214O1.2 ENSG00000266709.1 0.128022541 1 1 0.128022541
Brain_Cerebellar_Hemisphere 0.128022541 Brain_Cerebellar_Hemisphere 1 1
1 -1.521946282 -1.521946282 -1.521946282 NA 1 0
0.972491774
WBP11 ENSG00000084463.7 0.128157411 3 3 0.050354504 Brain_Cerebellum
0.960868648 Brain_Cortex 2.18150752 0.207685151 0.207685151
-1.956940165 0.049063555 -0.791853619 1.041550319 3 0
0.972491774
DNAAF2 ENSG00000165506.14 0.128269838 2 2 0.056272296
Brain_Hypothalamus 0.551115467 Brain_Cerebellum 1.043943452
0.956056548 0.956056548 -1.9089209 -0.596089457 -1.252505179
0.928312016 2 0 0.972491774
C10orf105 ENSG00000214688.5 0.128324818 4 4 0.10167831 Brain_Cerebellum
0.448504357 Brain_Nucleus_accumbens_basal_ganglia 1.703539594
0.383844642 0.383844642 -1.322664456 1.636771083 0.577102605
1.316481111 4 0 0.972491774
GNPDA2 ENSG00000163281.11 0.128348847 12 2 0.017497806
Brain_Caudate_basal_ganglia 0.094071135 Brain_Cerebellum 11.01817992
0.002167064 0.378156135 1.674302643 2.376077107 1.910480359
0.196582614 2 0 0.972491774
TRAM2 ENSG00000065308.4 0.128399098 1 1 0.128399098
Brain_Caudate_basal_ganglia 0.128399098 Brain_Caudate_basal_ganglia 1 1
1 -1.520445293 -1.520445293 -1.520445293 NA 1 0
0.972491774
MIR137HG ENSG00000225206.8 0.1284187 6 4 0.038445232
Brain_Hippocampus 0.435551032 Brain_Anterior_cingulate_cortex_BA24
4.5400746 0.057986541 0.218628067 -2.070075955 -0.779727935
-1.291872532 0.601742201 4 0 0.972491774
GLTPD2 ENSG00000182327.7 0.128533282 3 3 0.181432576
Brain_Frontal_Cortex_BA9 0.871392989 Brain_Caudate_basal_ganglia
1.717151178 0.282228425 0.282228425 -1.336357117 0.404352058
-0.36463147 0.887888159 3 0 0.972491774
HECW1 ENSG00000002746.14 0.128740986 1 1 0.128740986
Brain_Nucleus_accumbens_basal_ganglia 0.128740986
Brain_Nucleus_accumbens_basal_ganglia 1 1 1 -1.519085461
-1.519085461 -1.519085461 NA 1 0 0.972491774
NAGLU ENSG00000108784.9 0.128774045 2 2 0.058125675
Brain_Caudate_basal_ganglia 0.790422646 Brain_Cortex 1.472266534
0.527733466 0.527733466 -1.894748908 -0.265761825 -1.080255366
1.151867813 2 0 0.972491774
FKTN ENSG00000106692.13 0.128780277 3 3 0.129269482 Brain_Cortex
0.624827767 Brain_Nucleus_accumbens_basal_ganglia 1.921169294
0.360978612 0.360978612 -0.489019676 1.516988917 0.752373122
1.084668083 3 0 0.972491774
MDGA1 ENSG00000112139.14 0.128832922 3 3 0.080343992
Brain_Putamen_basal_ganglia 0.618316218 Brain_Cortex 1.100879579
0.904212079 0.904212079 -1.74869363 1.550641831 0.100062107
1.685322439 3 0 0.972491774
MAGI3 ENSG00000081026.18 0.128953201 3 3 0.096287233 Brain_Cerebellum
0.992266965 Brain_Spinal_cord_cervical_c-1 2.203656192 0.077910262
0.077910262 -1.663125917 -0.009692074 -0.851697172 0.827140891
3 0 0.972491774
BICD2 ENSG00000185963.13 0.129030567 7 1 0.081102766
Brain_Hypothalamus 0.371499034 Brain_Spinal_cord_cervical_c-1
6.659266581 0.000513214 6.659266581 -1.744323097 -0.893668966
-1.475697838 0.300687137 1 0 0.972491774
GPR19 ENSG00000183150.7 0.129033838 1 1 0.129033838
Brain_Caudate_basal_ganglia 0.129033838 Brain_Caudate_basal_ganglia 1 1
1 1.517922893 1.517922893 1.517922893 NA 1 0
0.972491774

BRWD1 ENSG00000185658.13 0.129036773 1 1 0.129036773
Brain_Caudate_basal_ganglia 0.129036773 Brain_Caudate_basal_ganglia 1 1
1 1.517911253 1.517911253 1.517911253 NA 1 0
0.972491774
C7orf34 ENSG00000165131.6 0.129071445 1 1 0.129071445
Brain_Caudate_basal_ganglia 0.129071445 Brain_Caudate_basal_ganglia 1 1
1 1.517773747 1.517773747 1.517773747 NA 1 0
0.972491774
C3orf35 ENSG00000198590.11 0.129326981 1 1 0.129326981
Brain_Frontal_Cortex_BA9 0.129326981 Brain_Frontal_Cortex_BA9 1 1
1 1.516761219 1.516761219 1.516761219 NA 1 0
0.972491774
MEN1 ENSG00000133895.14 0.129524013 4 4 0.027530664
Brain_Frontal_Cortex_BA9 0.795539286 Brain_Cerebellar_Hemisphere
1.706383696 0.478021227 0.478021227 -2.203910149 1.1615417
-0.541840165 1.396293734 4 0 0.972491774
TUBG2 ENSG00000037042.8 0.129706385 12 1 0.073447003 Brain_Cortex
0.310887646 Brain_Hypothalamus 10.76806292 0.001513652
10.76806292 -1.790042976 -1.013361241 -1.432691446 0.239297646
1 0 0.972491774
KIF16B ENSG00000089177.18 0.129728595 7 7 0.10285567
Brain_Nucleus_accumbens_basal_ganglia 0.951383023 Brain_Cerebellar_Hemisphere
3.347877139 0.218594541 0.218594541 -0.71952421 1.631164233
0.587570467 0.888614625 7 0 0.972491774
SSC4D ENSG00000146700.8 0.129732965 3 3 0.023730103
Brain_Frontal_Cortex_BA9 0.043386568 Brain_Caudate_basal_ganglia
2.519802055 0.147447179 0.147447179 2.019969334 2.261471062
2.160624928 0.125576136 3 0 0.972491774
KIAA0391 ENSG00000100890.15 0.12984675 6 6 0.089553748 Brain_Cerebellum
0.95700969 Brain_Putamen_basal_ganglia 2.869406037 0.128414446
0.128414446 -0.749290356 1.697756398 0.50586787 0.924296415 6
0 0.972491774
BMP2K ENSG00000138756.17 0.130094075 3 3 0.042700447 Brain_Amygdala
0.561500735 Brain_Cerebellar_Hemisphere 1.08872068 0.879746374
0.879746374 -2.026628231 0.827623474 -0.593206203 1.427167518
3 0 0.972491774
IMPDH2 ENSG00000178035.11 0.130112906 1 1 0.130112906
Brain_Cortex 0.130112906 Brain_Cortex 1 1 1 -1.513656805
-1.513656805 -1.513656805 NA 1 0 0.972491774
UBTF ENSG00000108312.14 0.130307971 1 1 0.130307971
Brain_Nucleus_accumbens_basal_ganglia 0.130307971
Brain_Nucleus_accumbens_basal_ganglia 1 1 1 -1.512888551
-1.512888551 -1.512888551 NA 1 0 0.972491774
CISTR ENSG00000260492.2 0.130361764 2 2 0.053150059
Brain_Hypothalamus 0.124735665 Brain_Cortex 1.54214523 0.45785477
0.45785477 1.535196106 1.933699713 1.73444791 0.281784603 2 0
0.972491774
TUBB6 ENSG00000176014.12 0.130377654 5 5 0.095560684
Brain_Cerebellar_Hemisphere 0.741207361 Brain_Hippocampus 1.543850993
0.520038059 0.520038059 -1.201158445 1.666767302 0.39160432
1.165724765 5 0 0.972491774
BEND7 ENSG00000165626.16 0.130378809 5 4 0.014165233
Brain_Cerebellar_Hemisphere 0.90031975 Brain_Nucleus_accumbens_basal_ganglia
2.68501381 0.085832662 0.204574147 -2.453045716 -0.125257433
-1.373054555 0.833859401 4 0 0.972491774
ZNF398 ENSG00000197024.8 0.130529491 1 1 0.130529491
Brain_Cerebellar_Hemisphere 0.130529491 Brain_Cerebellar_Hemisphere 1 1
1 1.512017183 1.512017183 1.512017183 NA 1 0
0.972491774
SPTBN1 ENSG00000115306.15 0.130560555 12 10 0.124776334
Brain_Anterior_cingulate_cortex_BA24 0.945173445 Brain_Cerebellar_Hemisphere
5.754189911 0.031333587 0.221671943 -1.535030508 0.977020383

-0.497174702 0.799777348 10 0 0.972491774
C22orf23 ENSG00000128346.10 0.130587137 1 1 0.130587137
Brain_Anterior_cingulate_cortex_BA24 0.130587137
Brain_Anterior_cingulate_cortex_BA24 1 1 1 -1.511790616
-1.511790616 -1.511790616 NA 1 0 0.972491774
SDHB ENSG00000117118.9 0.130603698 5 5 0.055385665
Brain_Anterior_cingulate_cortex_BA24 0.900640175 Brain_Cerebellar_Hemisphere
2.310076829 0.400438474 0.400438474 -1.915838426 0.124852688
-0.973015052 0.875677201 5 0 0.972491774
RP4-798A10.7 ENSG00000261135.1 0.130682448 1 1 0.130682448
Brain_Spinal_cord_cervical_c-1 0.130682448 Brain_Spinal_cord_cervical_c-1
1 1 1 -1.511416185 -1.511416185 -1.511416185 NA 1 0
0.972491774
SEC11A ENSG00000140612.13 0.130703201 1 1 0.130703201
Brain_Amygdala 0.130703201 Brain_Amygdala 1 1 1 -1.511334687
-1.511334687 -1.511334687 NA 1 0 0.972491774
MISP3 ENSG00000141854.9 0.130712844 11 11 0.014038891
Brain_Substantia_nigra 0.944014923 Brain_Cerebellum 2.381594541
0.319332544 0.319332544 -2.456266739 1.437243018 -0.520097588
1.141733086 11 0 0.972491774
JAKMIP1 ENSG00000152969.17 0.13076622 2 2 0.043877635
Brain_Spinal_cord_cervical_c-1 0.895141329 Brain_Cortex 1.022657906
0.977342094 0.977342094 -2.015257888 -0.131801463 -1.073529675
1.33180481 2 0 0.972491774
TMEM216 ENSG00000187049.9 0.130952918 12 4 0.009744314
Brain_Amygdala 0.641604165 Brain_Cerebellum 9.003974726 0.000662998
0.433566421 -2.584772943 0.465457082 -0.957528527 0.684151454
4 0 0.972491774
CCL27 ENSG00000213927.3 0.13096273 2 2 0.219800539
Brain_Cerebellar_Hemisphere 0.453400309 Brain_Cerebellum 1.510332739
0.489667261 0.489667261 -1.227058673 0.749758265 -0.238650204
1.397820662 2 0 0.972491774
PNO1 ENSG00000115946.7 0.130986966 4 4 0.0122925 Brain_Hypothalamus
0.365426845 Brain_Cortex 2.588313143 0.164436182 0.164436182
-2.503634356 -0.905072754 -1.948621308 0.745654032 4 0
0.972491774
SYNE3 ENSG00000176438.12 0.131086283 3 3 0.033991631 Brain_Cerebellum
0.240643742 Brain_Cortex 1.567481241 0.413667325 0.413667325
1.173379281 2.120170956 1.657519641 0.473761494 3 0
0.972491774
RP11-700H6.4 ENSG00000262006.1 0.131115974 6 6 0.045410754
Brain_Caudate_basal_ganglia 0.978386226 Brain_Nucleus_accumbens_basal_ganglia
2.092328868 0.425802074 0.425802074 -2.000829619 0.445590242
-0.46535413 1.101242392 6 0 0.972491774
UCN2 ENSG00000145040.3 0.131174335 1 1 0.131174335 Brain_Cerebellum
0.131174335 Brain_Cerebellum 1 1 1 -1.509487158 -1.509487158
-1.509487158 NA 1 0 0.972491774
IPMK ENSG00000151151.5 0.131177198 1 1 0.131177198
Brain_Hippocampus 0.131177198 Brain_Hippocampus 1 1 1
1.509475945 1.509475945 1.509475945 NA 1 0 0.972491774
TTC25 ENSG00000204815.9 0.131250879 2 2 0.057866932
Brain_Hippocampus 0.579920413 Brain_Substantia_nigra 1.066443332
0.933556668 0.933556668 -1.896704632 0.553500975 -0.671601829
1.732557 2 0 0.972491774
RP4-816N1.8 ENSG00000283297.1 0.131277716 2 2 0.088489967
Brain_Spinal_cord_cervical_c-1 0.317434851 Brain_Amygdala 1.044541485
0.955458515 0.955458515 -1.70341749 0.999743095 -0.351837198
1.91142318 2 0 0.972491774
KPTN ENSG00000118162.13 0.131303181 2 2 0.242573056 Brain_Cerebellum
0.919189522 Brain_Cerebellar_Hemisphere 1.842837302 0.157162698
0.157162698 -1.168579588 -0.101454692 -0.63501714 0.75457125 2
0 0.972491774

SFN ENSG00000175793.11 0.131460679 1 1 0.131460679
Brain_Putamen_basal_ganglia 0.131460679 Brain_Putamen_basal_ganglia 1 1
1 1.508366784 1.508366784 1.508366784 NA 1 0
0.972491774
FOXD4L1 ENSG00000184492.6 0.131516496 11 1 0.079963951
Brain_Caudate_basal_ganglia 0.302813899 Brain_Hippocampus 10.42859816
0.000458548 10.42859816 1.030417847 1.750895274 1.466149889
0.215540279 1 0 0.972491774
ZNF277 ENSG00000198839.9 0.131568471 2 2 0.054690287
Brain_Cerebellum 0.611248482 Brain_Frontal_Cortex_BA9 1.049486701
0.950513299 0.950513299 -0.508292139 1.921328656 0.706518258
1.718001339 2 0 0.972491774
TMEM81 ENSG00000174529.7 0.131678952 8 4 0.006847224
Brain_Cortex 0.265570437 Brain_Caudate_basal_ganglia 6.154813741
0.010316975 0.393783011 1.113321357 2.704184596 2.161079999
0.50906621 4 0 0.972491774
ABCB4 ENSG00000005471.15 0.131696859 7 7 0.056926115
Brain_Putamen_basal_ganglia 0.757076218 Brain_Caudate_basal_ganglia
2.791744594 0.163128981 0.163128981 -1.903877693 -0.309322498
-1.105029136 0.584143133 7 0 0.972491774
RCHY1 ENSG00000163743.13 0.131706057 4 4 0.055202738
Brain_Hippocampus 0.856131489 Brain_Frontal_Cortex_BA9 3.131618174
0.121354861 0.121354861 -1.917277097 -0.181300786 -1.118656133
0.799494175 4 0 0.972491774
DAND5 ENSG00000179284.5 0.131866043 1 1 0.131866043
Brain_Cerebellar_Hemisphere 0.131866043 Brain_Cerebellar_Hemisphere 1 1
1 -1.506783956 -1.506783956 -1.506783956 NA 1 0
0.972491774
MYLK2 ENSG00000101306.10 0.131964359 8 7 0.064456797
Brain_Substantia_nigra 0.853488171 Brain_Spinal_cord_cervical_c-1
4.833896876 0.066445329 0.172387277 -1.542470885 1.849007033
0.097644104 0.981753981 7 0 0.972491774
SH3GL2 ENSG00000107295.9 0.131997098 5 5 0.060877263
Brain_Frontal_Cortex_BA9 0.830975088 Brain_Cerebellar_Hemisphere 1.94399194
0.139315378 0.139315378 -1.578669204 1.874385842 -0.238878824
1.298728469 5 0 0.972491774
LINC01800 ENSG00000234572.1 0.132017504 1 1 0.132017504
Brain_Hypothalamus 0.132017504 Brain_Hypothalamus 1 1 1
1.506193515 1.506193515 1.506193515 NA 1 0 0.972491774
SEMA6A-AS2 ENSG00000249167.1 0.132022508 1 1 0.132022508
Brain_Nucleus_accumbens_basal_ganglia 0.132022508
Brain_Nucleus_accumbens_basal_ganglia 1 1 1 1.506174015
1.506174015 1.506174015 NA 1 0 0.972491774
JADE2 ENSG00000043143.20 0.132166668 1 1 0.132166668
Brain_Cerebellar_Hemisphere 0.132166668 Brain_Cerebellar_Hemisphere 1 1
1 1.505612538 1.505612538 1.505612538 NA 1 0
0.972491774
CXCL11 ENSG00000169248.12 0.132338859 1 1 0.132338859
Brain_Cerebellum 0.132338859 Brain_Cerebellum 1 1 1 -1.504942506
-1.504942506 -1.504942506 NA 1 0 0.972491774
UNC5D ENSG00000156687.10 0.13235331 2 2 0.139444575 Brain_Cortex
0.303201275 Brain_Frontal_Cortex_BA9 1.206401029 0.793598971
0.793598971 -1.47786254 1.029592632 -0.224134954 1.773038556
2 0 0.972491774
FAM3C ENSG00000196937.10 0.132402274 8 3 0.088921691
Brain_Caudate_basal_ganglia 0.704317184 Brain_Hippocampus 6.778062547
0.001612337 0.3765218 -0.379499219 1.701113425 0.888404027
0.583797233 3 0 0.972491774
IL27RA ENSG00000104998.3 0.13251489 1 1 0.13251489 Brain_Cortex
0.13251489 Brain_Cortex 1 1 1 -1.50425823 -1.50425823
-1.50425823 NA 1 0 0.972491774
AKTIP ENSG00000166971.16 0.132711564 8 4 0.140302915

Brain_Hippocampus 0.886263081 Brain_Amygdala 5.345627143
0.003774816 0.187174714 -1.474663941 1.174368566 0.225205623
0.776227405 4 0 0.972491774
RP11-665C16.6 ENSG00000258413.1 0.132723601 3 2 0.082291587
Brain_Hypothalamus 0.346116607 Brain_Frontal_Cortex_BA9 2.787486393
0.064599078 0.147914529 -1.737541775 -0.942148519 -1.400908576
0.411520152 2 0 0.972491774
LOR ENSG00000203782.5 0.132767597 1 1 0.132767597
Brain_Spinal_cord_cervical_c-1 0.132767597 Brain_Spinal_cord_cervical_c-1
1 1 1 1.503277121 1.503277121 1.503277121 NA 1 0
0.972491774
MYLK3 ENSG00000140795.12 0.132833598 1 1 0.132833598
Brain_Frontal_Cortex_BA9 0.132833598 Brain_Frontal_Cortex_BA9 1 1
1 1.503021119 1.503021119 1.503021119 NA 1 0
0.972491774
AMT ENSG00000145020.15 0.132865606 12 1 0.072170519 Brain_Cerebellum
0.273502339 Brain_Putamen_basal_ganglia 11.41133464 0.00104653
11.41133464 -1.798040946 -1.095032635 -1.46464599 0.200327815
1 0 0.972491774
GTF2A1L ENSG00000242441.7 0.132999315 12 11 0.010808102
Brain_Cerebellar_Hemisphere 0.938871167 Brain_Anterior_cingulate_cortex_BA24
4.05875208 0.000239044 0.153631212 -1.590228125 2.548842771
0.009492103 1.093770887 11 0 0.972491774
FMOD ENSG00000122176.10 0.133017645 1 1 0.133017645
Brain_Frontal_Cortex_BA9 0.133017645 Brain_Frontal_Cortex_BA9 1 1
1 -1.50230776 -1.50230776 -1.50230776 NA 1 0
0.972491774
DNAH17 ENSG00000187775.16 0.133109148 2 2 0.049953968
Brain_Spinal_cord_cervical_c-1 0.781939232
Brain_Nucleus_accumbens_basal_ganglia 1.080533145 0.919466855
0.919466855 -1.960357945 -0.276792771 -1.118575358 1.190460351
2 0 0.972491774
SQSTM1 ENSG00000161011.19 0.133146609 4 4 0.031195369
Brain_Frontal_Cortex_BA9 0.946491677 Brain_Hippocampus 2.53299727
0.205448842 0.205448842 -2.154571654 0.721880494 -0.264089836
1.288983719 4 0 0.972491774
C1orf162 ENSG00000143110.11 0.133204607 3 3 0.030596101
Brain_Cortex 0.907227988 Brain_Caudate_basal_ganglia 1.412284907
0.65445517 0.65445517 -2.162286482 0.667330098 -0.537164031
1.460951278 3 0 0.972491774
SRGAP3 ENSG00000196220.15 0.133353713 2 2 0.102914718
Brain_Cortex 0.328501258 Brain_Caudate_basal_ganglia 1.113808002
0.886191998 0.886191998 -1.630884382 0.977137151 -0.326873616
1.844149711 2 0 0.972491774
EVI5L ENSG00000142459.8 0.133379076 3 2 0.056224069 Brain_Amygdala
0.374498412 Brain_Cerebellum 2.149172826 0.043300651 0.807526522
0.888078713 1.909294829 1.415701569 0.511457818 2 0
0.972491774
NUDCD2 ENSG00000170584.10 0.1334279 5 5 0.018174126
Brain_Frontal_Cortex_BA9 0.80510435 Brain_Putamen_basal_ganglia 2.768647278
0.353072581 0.353072581 -2.362051328 0.727101503 -0.67446265
1.118653735 5 0 0.972491774
AC062028.1 ENSG00000145063.14 0.133438071 5 5 0.243848147
Brain_Nucleus_accumbens_basal_ganglia 0.984592326
Brain_Spinal_cord_cervical_c-1 2.14015267 0.416695317 0.416695317
-1.165422172 1.103387499 0.037593333 0.997933378 5 0
0.972491774
ECI2 ENSG00000198721.12 0.133485094 11 2 0.032080248
Brain_Frontal_Cortex_BA9 0.098490219 Brain_Cerebellar_Hemisphere
9.881876065 0.000612426 0.459470784 -2.143409284 -1.652217508
-1.899695812 0.171420514 2 0 0.972491774
PLA2G4B ENSG00000243708.10 0.133564052 8 4 0.118131362

Brain_Nucleus_accumbens_basal_ganglia 0.906416176 Brain_Cerebellum 6.671247986 0.014250094 0.231575422 -1.562665206 0.171350586 -0.716317992 0.57387856 4 0 0.972491774
NORAD ENSG00000260032.1 0.133578767 2 2 0.10863812 Brain_Frontal_Cortex_BA9 0.257260088 Brain_Cortex 1.044257101 0.955742899 0.955742899 -1.132890862 1.604344578 0.235726858 1.935517741 2 0 0.972491774
EPM2A ENSG00000112425.14 0.13363056 2 2 0.04861333 Brain_Hypothalamus 0.820823298 Brain_Putamen_basal_ganglia 1.072149337 0.927850663 0.927850663 0.226486191 1.971967384 1.099226788 1.234241588 2 0 0.972491774
BARD1 ENSG00000138376.10 0.133648622 10 6 0.075778077 Brain_Cerebellar_Hemisphere 0.939583639 Brain_Cortex 6.467045455 0.019491182 0.292974444 -1.168607719 1.775726077 0.12940029 0.914752916 6 0 0.972491774
KLK7 ENSG00000169035.11 0.133690243 2 2 0.048693943 Brain_Putamen_basal_ganglia 0.878749187 Brain_Nucleus_accumbens_basal_ganglia 1.259756815 0.740243185 0.740243185 -1.971261763 -0.152555035 -1.061908399 1.286019861 2 0 0.972491774
SNTA1 ENSG00000101400.5 0.133762782 1 1 0.133762782 Brain_Putamen_basal_ganglia 0.133762782 Brain_Putamen_basal_ganglia 1 1 1 1.499427431 1.499427431 1.499427431 NA 1 0 0.972491774
LINC00869 ENSG00000277147.4 0.134367256 4 4 0.009122386 Brain_Frontal_Cortex_BA9 0.926661681 Brain_Cortex 1.397945062 0.618628182 0.618628182 -0.092045762 2.607433228 0.791022516 1.232557418 4 0 0.972491774
PCCB ENSG00000114054.13 0.134374196 10 3 0.038355153 Brain_Cerebellar_Hemisphere 0.478867157 Brain_Caudate_basal_ganglia 8.588551313 0.010216615 0.389372009 0.708125771 2.071038984 1.356422672 0.47732051 3 0 0.972491774
RP11-109D9.4 ENSG00000261447.1 0.134427092 2 2 0.090583832 Brain_Nucleus_accumbens_basal_ganglia 0.226903278 Brain_Substantia_nigra 1.082665487 0.917334513 0.917334513 -1.208374412 1.692326006 0.241975797 2.051104936 2 0 0.972491774
UCN ENSG00000163794.6 0.134504309 1 1 0.134504309 Brain_Substantia_nigra 0.134504309 Brain_Substantia_nigra 1 1 1 1.49657335 1.49657335 1.49657335 NA 1 0 0.972491774
SEMA4D ENSG00000187764.11 0.134594734 8 7 0.025044864 Brain_Frontal_Cortex_BA9 0.710990794 Brain_Amygdala 3.195442349 0.052516277 0.147426387 -2.240710029 0.858242484 -0.632968258 1.085050758 7 0 0.972491774
ZNF69 ENSG00000198429.9 0.134625432 4 4 0.038993812 Brain_Putamen_basal_ganglia 0.390687518 Brain_Cerebellar_Hemisphere 1.442279878 0.585482001 0.585482001 -2.064252185 0.992551927 -0.913016706 1.367962904 4 0 0.972491774
IGSF11 ENSG00000144847.12 0.134640747 4 4 0.027153901 Brain_Caudate_basal_ganglia 0.092100619 Brain_Anterior_cingulate_cortex_BA24 2.440523614 0.158205712 0.158205712 1.684419531 2.209298276 1.977927348 0.242854144 4 0 0.972491774
AGBL1 ENSG00000273540.3 0.134911648 1 1 0.134911648 Brain_Cortex 0.134911648 Brain_Cortex 1 1 1 1.495010705 1.495010705 1.495010705 NA 1 0 0.972491774
POFUT2 ENSG00000186866.16 0.134948306 1 1 0.134948306 Brain_Hypothalamus 0.134948306 Brain_Hypothalamus 1 1 1 -1.494870259 -1.494870259 -1.494870259 NA 1 0 0.972491774
HHIP ENSG00000164161.9 0.134991283 2 2 0.057921733 Brain_Putamen_basal_ganglia 0.148931269 Brain_Cerebellum 1.461950207 0.538049793 0.538049793 1.443316732 1.896289813 1.669803272 0.320300338 2 0 0.972491774
KLHL1 ENSG00000150361.11 0.135091312 8 7 0.195466077 Brain_Cerebellum 0.953749651 Brain_Cortex 4.247397822 0.107107209 0.18016724

-1.294577329 1.012607822 -0.114841214 0.871892199 7 0
0.972491774
PAXIP1-AS1 ENSG00000273344.1 0.135239815 11 8 0.037667051
Brain_Putamen_basal_ganglia 0.734288633 Brain_Frontal_Cortex_BA9 5.60729835
0.003313776 0.238491013 -1.252401194 2.078459567 0.486115813
1.037111647 8 0 0.972491774
BTBD9 ENSG00000183826.16 0.135328058 6 6 0.012687373
Brain_Hippocampus 0.889819114 Brain_Frontal_Cortex_BA9 2.255531435
0.203857305 0.203857305 -2.492426089 0.530726671 -0.90075264
1.320961259 6 0 0.972491774
SYNC ENSG00000162520.14 0.135455603 2 2 0.061802621
Brain_Nucleus_accumbens_basal_ganglia 0.398284568 Brain_Amygdala
1.070901562 0.929098438 0.929098438 -1.86770916 -0.844688882
-1.356199021 0.723384576 2 0 0.972491774
FAM19A3 ENSG00000184599.13 0.13554183 1 1 0.13554183 Brain_Cerebellum
0.13554183 Brain_Cerebellum 1 1 1 1.492600357 1.492600357
1.492600357 NA 1 0 0.972491774
CENPU ENSG00000151725.11 0.135579181 7 5 0.042685488
Brain_Nucleus_accumbens_basal_ganglia 0.830380082
Brain_Anterior_cingulate_cortex_BA24 4.525936974 0.093613259
0.266107705 -2.02677442 0.763701805 -0.14562567 1.140454185
5 0 0.972491774
LYPD3 ENSG00000124466.8 0.135609399 1 1 0.135609399
Brain_Hippocampus 0.135609399 Brain_Hippocampus 1 1 1
1.49234243 1.49234243 1.49234243 NA 1 0 0.972491774
RP4-644L1.2 ENSG00000229771.2 0.135800649 2 2 0.355780218
Brain_Caudate_basal_ganglia 0.401974165 Brain_Nucleus_accumbens_basal_ganglia
1.611236835 0.388763165 0.388763165 -0.923435654 0.838100673
-0.04266749 1.245594283 2 0 0.972491774
MPG ENSG00000103152.11 0.135866257 1 1 0.135866257
Brain_Spinal_cord_cervical_c-1 0.135866257 Brain_Spinal_cord_cervical_c-1
1 1 1 -1.491362847 -1.491362847 -1.491362847 NA 1 0
0.972491774
GHITM ENSG00000165678.20 0.135925323 1 1 0.135925323 Brain_Amygdala
0.135925323 Brain_Amygdala 1 1 1 -1.491137786 -1.491137786
-1.491137786 NA 1 0 0.972491774
IQCC ENSG00000160051.11 0.135996259 1 1 0.135996259 Brain_Cortex
0.135996259 Brain_Cortex 1 1 1 -1.490867603 -1.490867603
-1.490867603 NA 1 0 0.972491774
ATP6V1E1 ENSG00000131100.12 0.136125102 3 3 0.115985567
Brain_Cortex 0.765891154 Brain_Cerebellum 1.331616628 0.689783387
0.689783387 -1.571849033 0.2977537 -0.942621966 1.074234869 3
0 0.972491774
E2F6 ENSG00000169016.16 0.136177477 2 2 0.085972028
Brain_Nucleus_accumbens_basal_ganglia 0.23110381
Brain_Anterior_cingulate_cortex_BA24 1.107096703 0.892903297
0.892903297 1.197520326 1.717039099 1.457279712 0.367355247
2 0 0.972491774
C19orf47 ENSG00000160392.13 0.136214352 8 4 0.000262977
Brain_Amygdala 0.632401349 Brain_Nucleus_accumbens_basal_ganglia
4.695953524 0.035296455 0.449469735 0.478349671 3.649279111
1.738621409 0.936465705 4 0 0.972491774
POLR1B ENSG00000125630.15 0.136430336 13 1 0.078361761
Brain_Hypothalamus 0.333822567 Brain_Substantia_nigra 12.53051409
4.31E-05 12.53051409 0.966442979 1.760271635 1.4599862
0.263057338 1 0 0.972491774
CHD4 ENSG00000111642.14 0.136482129 1 1 0.136482129 Brain_Cerebellum
0.136482129 Brain_Cerebellum 1 1 1 -1.489019898 -1.489019898
-1.489019898 NA 1 0 0.972491774
RP11-245D16.4 ENSG00000278238.1 0.136507014 7 3 0.026913328
Brain_Hypothalamus 0.989322333 Brain_Spinal_cord_cervical_c-1 4.11326301
0.031557667 0.662265753 -2.212772592 0.244124404 -1.09376167

1.025094682 3 0 0.972491774
AC093159.1 ENSG00000228541.1 0.136533299 2 2 0.046015376
Brain_Substantia_nigra 0.974007147 Brain_Cortex 1.034679331
0.965320669 0.965320669 -1.995252242 0.032582975 -0.981334634
1.433896033 2 0 0.972491774
RP11-767I20.1 ENSG00000257259.1 0.136541784 1 1 0.136541784
Brain_Hippocampus 0.136541784 Brain_Hippocampus 1 1 1
-1.48879339 -1.48879339 -1.48879339 NA 1 0 0.972491774
ATAD2B ENSG00000119778.14 0.136698148 2 2 0.069123131
Brain_Cerebellum 0.263237792 Brain_Cerebellar_Hemisphere 1.852219217
0.147780783 0.147780783 -1.81761413 -1.118771128 -1.468192629
0.494156626 2 0 0.972491774
MMP21 ENSG00000154485.4 0.13675507 1 1 0.13675507 Brain_Cerebellum 0.13675507
Brain_Cerebellum 1 1 1 -1.487984164 -1.487984164 -1.487984164
NA 1 0 0.972491774
TBC1D3B ENSG00000274808.5 0.136757182 1 1 0.136757182
Brain_Cortex 0.136757182 Brain_Cortex 1 1 1 -1.487976156
-1.487976156 -1.487976156 NA 1 0 0.972491774
BTBD10 ENSG00000148925.10 0.136806528 3 3 0.036648988
Brain_Putamen_basal_ganglia 0.473183867 Brain_Cerebellar_Hemisphere
1.255982964 0.72963476 0.72963476 -0.968156338 2.089652934
0.612934976 1.531574267 3 0 0.972491774
P4HA2 ENSG00000072682.18 0.136828292 4 3 0.100902412
Brain_Anterior_cingulate_cortex_BA24 0.948722307 Brain_Putamen_basal_ganglia
2.530053952 0.01154059 0.501753907 -1.640494397 -0.064311362
-1.154720128 0.732309215 3 0 0.972491774
RBFADN ENSG00000261126.7 0.136829393 13 1 0.098000971
Brain_Cortex 0.262641921 Brain_Anterior_cingulate_cortex_BA24 12.2250501
0.00186199 12.2250501 -1.654623117 -1.120168603 -1.441949728
0.180686267 1 0 0.972491774
FHIT ENSG00000189283.9 0.136832607 2 2 0.126441897
Brain_Frontal_Cortex_BA9 0.165595584 Brain_Cerebellar_Hemisphere 1.0707798
0.9292202 0.9292202 1.386495729 1.528284579 1.457390154
0.100259857 2 0 0.972491774
8-Mar ENSG00000165406.15 0.136899468 10 7 0.061413882 Brain_Cortex
0.832425021 Brain_Frontal_Cortex_BA9 4.250925497 0.028753743
0.170276848 -1.870503858 1.467066511 -0.001418305 0.93560062 7
0 0.972491774
ITGB4 ENSG00000132470.13 0.136904897 4 4 0.030878584
Brain_Caudate_basal_ganglia 0.938096377 Brain_Nucleus_accumbens_basal_ganglia
3.178838906 0.128644383 0.128644383 -2.158633854 0.077662685
-1.199746569 0.939752771 4 0 0.972491774
ULK1 ENSG00000177169.9 0.137133234 2 2 0.118256059 Brain_Cerebellum
0.294597991 Brain_Cerebellar_Hemisphere 1.116365948 0.883634052
0.883634052 -1.048088159 1.562135554 0.257023698 1.845706888
2 0 0.972491774
MOK ENSG00000080823.22 0.137247811 2 2 0.052175788 Brain_Cerebellum
0.694064519 Brain_Frontal_Cortex_BA9 1.028688398 0.971311602
0.971311602 -1.941680525 -0.393345226 -1.167512875 1.094838389
2 0 0.972491774
CLIP4 ENSG00000115295.19 0.137297072 1 1 0.137297072
Brain_Spinal_cord_cervical_c-1 0.137297072 Brain_Spinal_cord_cervical_c-1
1 1 1 1.485932136 1.485932136 1.485932136 NA 1 0
0.972491774
RP11-584P21.2 ENSG00000250075.5 0.137346784 6 5 0.138650571
Brain_Frontal_Cortex_BA9 0.877332176 Brain_Hypothalamus 3.243103683
0.067852723 0.219124261 -1.128057218 1.480834911 0.196909885
0.915091636 5 0 0.972491774
NUP43 ENSG00000120253.13 0.137535563 2 1 0.09577862 Brain_Cerebellum
0.198175356 Brain_Cerebellar_Hemisphere 1.976337288 0.023662712
1.976337288 -1.665672712 -1.286767447 -1.47622008 0.267926482
1 0 0.972491774

AMZ2 ENSG00000196704.11 0.137562209 4 4 0.028676905
Brain_Caudate_basal_ganglia 0.48531517 Brain_Cerebellum 2.831139471
0.097726265 0.097726265 0.697779391 2.187899754 1.527626073
0.633342681 4 0 0.972491774
RNF32 ENSG00000105982.16 0.137575756 4 4 0.018216266 Brain_Cerebellum
0.761874504 Brain_Hypothalamus 1.22665319 0.774777416 0.774777416
-1.128322949 2.361192628 0.279944352 1.505527449 4 0
0.972491774
MAN1C1 ENSG00000117643.14 0.137603491 2 2 0.095221743
Brain_Amygdala 0.215078013 Brain_Cerebellum 1.094041618 0.905958382
0.905958382 -1.668473626 1.239722607 -0.214375509 2.056405277
2 0 0.972491774
GRIN1 ENSG00000176884.14 0.137662351 1 1 0.137662351
Brain_Cerebellar_Hemisphere 0.137662351 Brain_Cerebellar_Hemisphere 1 1
1 -1.484552706 -1.484552706 -1.484552706 NA 1 0
0.972491774
PGPEP1L ENSG00000183571.10 0.137674394 10 10 0.011279916
Brain_Amygdala 0.797395179 Brain_Putamen_basal_ganglia 3.24544519
0.168773892 0.168773892 -1.853883277 2.533905473 0.07742878
1.27439857 10 0 0.972491774
ADAM15 ENSG00000143537.13 0.137699272 9 9 0.232439873
Brain_Amygdala 0.952941244 Brain_Hippocampus 3.16891491 0.11506421
0.11506421 -1.194097394 1.140331964 -0.028940119 0.783593277 9
0 0.972491774
PTPN22 ENSG00000134242.15 0.137828994 2 2 0.046920192
Brain_Cerebellum 0.666209798 Brain_Cerebellar_Hemisphere 1.276778195
0.723221805 0.723221805 0.431355642 1.9870199 1.209187771
1.100020746 2 0 0.972491774
PRSS35 ENSG00000146250.6 0.137899507 2 2 0.05450855 Brain_Cerebellum
0.570306443 Brain_Hypothalamus 1.027366587 0.972633413
0.972633413 -0.567600242 1.922773113 0.677586436 1.760959887
2 0 0.972491774
METTL21B ENSG00000123427.16 0.137905388 13 2 0.032366276
Brain_Spinal_cord_cervical_c-1 0.969653878
Brain_Anterior_cingulate_cortex_BA24 11.95572878 0.000138939
0.685485577 0.038042398 2.13985761 1.54928121 0.48506145 2 0
0.972491774
TAF4B ENSG00000141384.12 0.13796677 2 2 0.076534776
Brain_Frontal_Cortex_BA9 0.572402524 Brain_Putamen_basal_ganglia
1.185082846 0.814917154 0.814917154 -1.771155928 0.564516721
-0.603319604 1.651569969 2 0 0.972491774
DUS2 ENSG00000167264.17 0.138067518 8 5 0.00793084
Brain_Frontal_Cortex_BA9 0.747007955 Brain_Cerebellum 5.400789844
0.012888156 0.216863889 -2.655000028 -0.322587112 -1.262332775
0.73073225 5 0 0.972491774
RBM4B ENSG00000173914.11 0.138117698 1 1 0.138117698 Brain_Cerebellum
0.138117698 Brain_Cerebellum 1 1 1 -1.482837095 -1.482837095
-1.482837095 NA 1 0 0.972491774
TBC1D22A ENSG00000054611.13 0.138173719 6 5 0.048274522
Brain_Cerebellar_Hemisphere 0.691653778 Brain_Putamen_basal_ganglia
3.295802389 5.69E-17 0.264574661 -1.974943837 -0.396611762
-1.113525175 0.742862289 5 0 0.972491774
GMCL1 ENSG00000087338.4 0.138228984 9 6 0.021036529 Brain_Amygdala
0.999594185 Brain_Caudate_basal_ganglia 4.74888606 0.023491973 0.4042315
-2.307328225 1.381913041 -0.086295421 1.088372293 6 0
0.972491774
WDR66 ENSG00000158023.9 0.138278608 6 6 0.016771654 Brain_Cerebellum
0.990942688 Brain_Hippocampus 2.409868209 0.161497489
0.161497489 -0.643051401 2.391675702 0.926017931 1.084795263
6 0 0.972491774
CCL2 ENSG00000108691.9 0.138341664 1 1 0.138341664
Brain_Substantia_nigra 0.138341664 Brain_Substantia_nigra 1 1

1 1.481994853 1.481994853 1.481994853 NA 1 0
0.972491774
RAD52 ENSG00000002016.17 0.138362897 10 5 0.304796715
Brain_Nucleus_accumbens_basal_ganglia 0.677148816 Brain_Putamen_basal_ganglia
7.20624735 0.042891731 0.301730903 -0.983632178 1.026201287
-0.066545477 0.74417604 5 0 0.972491774
NRDE2 ENSG00000119720.17 0.138381012 1 1 0.138381012
Brain_Hypothalamus 0.138381012 Brain_Hypothalamus 1 1 1
1.481846993 1.481846993 1.481846993 NA 1 0 0.972491774
DOHH ENSG00000129932.7 0.138474049 5 5 0.056050115 Brain_Cortex
0.944843708 Brain_Frontal_Cortex_BA9 3.300200842 0.15317676 0.15317676
-0.06918331 1.910645794 0.623857872 0.772865546 5 0
0.972491774
C11orf1 ENSG00000137720.7 0.138546488 5 4 0.095206295
Brain_Cortex 0.63205827 Brain_Caudate_basal_ganglia 3.788026766
0.000925141 0.151632209 -0.478831832 1.668551512 0.887012804
0.816206869 4 0 0.972491774
KCNG2 ENSG00000178342.4 0.138596486 1 1 0.138596486 Brain_Cerebellum
0.138596486 Brain_Cerebellum 1 1 1 -1.481037855 -1.481037855
-1.481037855 NA 1 0 0.972491774
ATF2 ENSG00000115966.16 0.138684484 2 2 0.07073726 Brain_Hypothalamus
0.645343432 Brain_Frontal_Cortex_BA9 1.194638445 0.805361555
0.805361555 -0.460240739 1.807160486 0.673459873 1.603294782
2 0 0.972491774
BMPR1B ENSG00000138696.10 0.138891802 2 2 0.173213071
Brain_Caudate_basal_ganglia 0.654840242 Brain_Spinal_cord_cervical_c-1
1.555250449 0.444749551 0.444749551 -1.361951883 0.447048223
-0.45745183 1.279156242 2 0 0.972491774
HMGN1 ENSG00000205581.10 0.138990075 6 6 0.126437639
Brain_Nucleus_accumbens_basal_ganglia 0.860390131
Brain_Anterior_cingulate_cortex_BA24 3.591458129 0.182438374
0.182438374 -1.164600945 1.528301738 -0.234606367 0.998089943
6 0 0.972491774
CD200 ENSG00000091972.18 0.13902005 2 2 0.12611551
Brain_Anterior_cingulate_cortex_BA24 0.242799896 Brain_Cerebellar_Hemisphere
1.063382302 0.936617698 0.936617698 -1.529601048 1.16801703
-0.180792009 1.907504036 2 0 0.972491774
JMJD4 ENSG00000081692.12 0.139038288 1 1 0.139038288
Brain_Anterior_cingulate_cortex_BA24 0.139038288
Brain_Anterior_cingulate_cortex_BA24 1 1 1 1.479381849
1.479381849 1.479381849 NA 1 0 0.972491774
EFS ENSG00000100842.12 0.139104215 1 1 0.139104215
Brain_Nucleus_accumbens_basal_ganglia 0.139104215
Brain_Nucleus_accumbens_basal_ganglia 1 1 1 1.479135083
1.479135083 1.479135083 NA 1 0 0.972491774
RP11-70F11.11 ENSG00000258135.5 0.139180408 1 1 0.139180408
Brain_Cerebellum 0.139180408 Brain_Cerebellum 1 1 1 1.478850004
1.478850004 1.478850004 NA 1 0 0.972491774
CCDC177 ENSG00000267909.2 0.139192784 1 1 0.139192784
Brain_Frontal_Cortex_BA9 0.139192784 Brain_Frontal_Cortex_BA9 1 1
1 1.478803709 1.478803709 1.478803709 NA 1 0
0.972491774
CTD-2228A4.1 ENSG00000271892.1 0.139321106 12 2 0.039252328
Brain_Cerebellar_Hemisphere 0.885111132 Brain_Nucleus_accumbens_basal_ganglia
10.52800788 0.00049185 0.817054109 -2.061531853 -0.144493065
-0.626422072 0.525660218 2 0 0.972491774
NRAP ENSG00000197893.13 0.139321763 3 3 0.043992749
Brain_Caudate_basal_ganglia 0.926608084 Brain_Cortex 1.135918371
0.861405076 0.861405076 -1.190061855 2.014159897 0.24399494
1.628338349 3 0 0.972491774
RAB40C ENSG00000197562.9 0.139370065 5 3 0.024144085
Brain_Anterior_cingulate_cortex_BA24 0.308440366 Brain_Caudate_basal_ganglia

3.982924092 0.033410375 0.336559455 -2.254828677 -1.018500073
-1.619695564 0.509690087 3 0 0.972491774
WWTR1 ENSG00000018408.14 0.139372568 1 1 0.139372568
Brain_Putamen_basal_ganglia 0.139372568 Brain_Putamen_basal_ganglia 1 1
1 -1.47813156 -1.47813156 -1.47813156 NA 1 0
0.972491774
HDGF ENSG00000143321.18 0.139389847 1 1 0.139389847
Brain_Hippocampus 0.139389847 Brain_Hippocampus 1 1 1
1.478066997 1.478066997 1.478066997 NA 1 0 0.972491774
SMIM5 ENSG00000204323.5 0.139397607 1 1 0.139397607
Brain_Cerebellar_Hemisphere 0.139397607 Brain_Cerebellar_Hemisphere 1 1
1 1.478038003 1.478038003 1.478038003 NA 1 0
0.972491774
ZNF285 ENSG00000267508.5 0.139533161 4 3 0.263253413
Brain_Cerebellum 0.934956568 Brain_Cortex 3.23459286 0.0960722
0.187275995 -1.118734522 0.081610353 -0.731889823 0.563920234
3 0 0.972491774
PDS5B ENSG00000083642.18 0.139702807 2 2 0.050179982 Brain_Cerebellum
0.899353495 Brain_Cortex 1.096962553 0.903037447 0.903037447
-1.958426553 0.126478086 -0.915974233 1.474250208 2 0
0.972491774
RP11-58C22.2 ENSG00000273659.1 0.139800804 1 1 0.139800804
Brain_Cerebellar_Hemisphere 0.139800804 Brain_Cerebellar_Hemisphere 1 1
1 -1.476533221 -1.476533221 -1.476533221 NA 1 0
0.972491774
TMEM189 ENSG00000240849.10 0.139980915 1 1 0.139980915
Brain_Putamen_basal_ganglia 0.139980915 Brain_Putamen_basal_ganglia 1 1
1 1.475862101 1.475862101 1.475862101 NA 1 0
0.972491774
RP11-182L21.6 ENSG00000272447.1 0.139981014 4 4 0.129960265
Brain_Amygdala 0.786629274 Brain_Frontal_Cortex_BA9 2.036194283
0.287532099 0.287532099 -1.077267289 1.514258596 -0.010661249
1.167517653 4 0 0.972491774
CATSPER2 ENSG00000166762.16 0.140037186 2 2 0.124338816
Brain_Putamen_basal_ganglia 0.331215602 Brain_Cerebellum 1.930629199
0.069370801 0.069370801 0.971668281 1.536814195 1.254241238
0.399618509 2 0 0.972491774
IZUMO1 ENSG00000182264.8 0.140153697 4 4 0.016119101
Brain_Cerebellar_Hemisphere 0.938404766 Brain_Hippocampus 1.297149188
0.653385667 0.653385667 -0.653508835 2.406207623 0.669368966
1.310078985 4 0 0.972491774
AHSA1 ENSG00000100591.7 0.140175548 3 1 0.13972601
Brain_Caudate_basal_ganglia 0.146910098 Brain_Cortex 2.969716506
1.62E-05 2.969716506 1.450532447 1.47681211 1.467644144
0.014831801 1 0 0.972491774
TECPR1 ENSG00000205356.9 0.140180212 3 3 0.089872934
Brain_Cortex 0.222962944 Brain_Nucleus_accumbens_basal_ganglia
1.125688872 0.861136654 0.861136654 -1.696068365 1.273851949
-0.546967867 1.594839114 3 0 0.972491774
HCN2 ENSG00000099822.2 0.140249062 1 1 0.140249062
Brain_Cerebellar_Hemisphere 0.140249062 Brain_Cerebellar_Hemisphere 1 1
1 1.474864182 1.474864182 1.474864182 NA 1 0
0.972491774
RRM2B ENSG00000048392.11 0.140306818 3 3 0.020966739
Brain_Cerebellar_Hemisphere 0.854237899 Brain_Spinal_cord_cervical_c-1
1.230268392 0.757558917 0.757558917 -2.30858287 0.313682016
-0.603728983 1.477876182 3 0 0.972491774
DLL1 ENSG00000198719.8 0.140393738 2 2 0.196514956
Brain_Caudate_basal_ganglia 0.623962075 Brain_Cerebellum 1.573580893
0.426419107 0.426419107 -0.490242833 1.291544439 0.400650803
1.259913863 2 0 0.972491774
TIMP4 ENSG00000157150.4 0.140457985 8 6 0.159054834

Brain_Frontal_Cortex_BA9 0.987834764 Brain_Anterior_cingulate_cortex_BA24 5.152885602 0.076393505 0.212659989 -0.816842347 1.408257505 0.437400137 0.780086501 6 0 0.972491774
TADA1 ENSG00000152382.5 0.140479052 4 4 0.032363152 Brain_Caudate_basal_ganglia 0.38186723 Brain_Cerebellum 1.447281187 0.67743567 0.67743567 -1.200462894 2.139896258 0.735612102 1.401675515 4 0 0.972491774
SETD3 ENSG00000183576.12 0.140484618 1 1 0.140484618 Brain_Cerebellum 0.140484618 Brain_Cerebellum 1 1 1 1.473988758 1.473988758 1.473988758 NA 1 0 0.972491774
IGSF6 ENSG00000140749.8 0.140541633 2 2 0.114269095 Brain_Cortex 0.320351437 Brain_Caudate_basal_ganglia 1.122361885 0.877638115 0.877638115 0.993735948 1.579292006 1.286513977 0.414050659 2 0 0.972491774
MYT1 ENSG00000196132.11 0.140558956 1 1 0.140558956 Brain_Nucleus_accumbens_basal_ganglia 0.140558956 Brain_Nucleus_accumbens_basal_ganglia 1 1 1 1.473712723 1.473712723 1.473712723 NA 1 0 0.972491774
TIGD6 ENSG00000164296.6 0.140629628 8 5 0.025769272 Brain_Putamen_basal_ganglia 0.936534999 Brain_Nucleus_accumbens_basal_ganglia 5.552280076 0.004397677 0.306994332 -0.105960235 2.229671312 0.762016752 0.79011251 5 0 0.972491774
DCP1A ENSG00000272886.5 0.140692075 1 1 0.140692075 Brain_Hippocampus 0.140692075 Brain_Hippocampus 1 1 1 1.473218697 1.473218697 1.473218697 NA 1 0 0.972491774
RP11-119J18.1 ENSG00000250378.2 0.140755153 4 4 0.08078428 Brain_Cortex 0.538484555 Brain_Nucleus_accumbens_basal_ganglia 2.311043983 0.313984869 0.313984869 -0.979516467 1.746153511 0.210531869 1.249865245 4 0 0.972491774
SH3BP1 ENSG00000100092.20 0.140792173 3 3 0.025430238 Brain_Cerebellar_Hemisphere 0.853150359 Brain_Nucleus_accumbens_basal_ganglia 1.751232708 0.257861579 0.257861579 -0.18510031 2.234803718 1.098897788 1.216730185 3 0 0.972491774
CCDC181 ENSG00000117477.12 0.140895255 7 3 0.029739276 Brain_Nucleus_accumbens_basal_ganglia 0.576717009 Brain_Caudate_basal_ganglia 5.963417248 0.005409802 0.411986139 -2.173545629 -0.558186543 -0.915706882 0.56205894 3 0 0.972491774
RNF139 ENSG00000170881.4 0.140999376 2 2 0.048614846 Brain_Cerebellar_Hemisphere 0.979840435 Brain_Hypothalamus 1.09933067 0.90066933 0.90066933 -1.971954101 -0.025268957 -0.998611529 1.376514266 2 0 0.972491774
TUBA1B ENSG00000123416.15 0.141009037 5 5 0.013559758 Brain_Anterior_cingulate_cortex_BA24 0.819467421 Brain_Hypothalamus 2.573270566 0.16503489 0.16503489 -0.228230025 2.468719197 1.445916768 1.037172304 5 0 0.972491774
LINC02076 ENSG00000220161.4 0.141290492 2 2 0.197916476 Brain_Caudate_basal_ganglia 0.569312752 Brain_Putamen_basal_ganglia 1.914360942 0.085639058 0.085639058 0.569063935 1.287510318 0.928287126 0.508018309 2 0 0.972491774
SRRM5 ENSG00000226763.4 0.141298055 4 4 0.013094115 Brain_Nucleus_accumbens_basal_ganglia 0.888975658 Brain_Cortex 2.076985808 0.249844367 0.249844367 -0.148637437 2.481199279 0.642888963 1.250072019 4 0 0.972491774
LANCL1 ENSG00000115365.11 0.14132077 4 4 0.286637653 Brain_Anterior_cingulate_cortex_BA24 0.792426644 Brain_Cerebellar_Hemisphere 2.45433169 0.110866098 0.110866098 -0.886942009 1.065526692 0.209838769 0.9431837 4 0 0.972491774
AUNIP ENSG00000127423.10 0.141558588 2 2 0.052592415 Brain_Frontal_Cortex_BA9 0.946512261 Brain_Substantia_nigra 1.164610598 0.835389402 0.835389402 0.067087229 1.93825258 1.002669904 1.323113708 2 0 0.972491774
MSANTD4 ENSG00000170903.10 0.14179147 2 2 0.175976585 Brain_Cortex

0.335908311 Brain_Nucleus_accumbens_basal_ganglia 1.303345147
0.696654853 0.696654853 -1.353247469 0.962281317 -0.195483076
1.637326107 2 0 0.972491774
ATPAF2 ENSG00000171953.15 0.141850216 2 2 0.054517086
Brain_Cerebellum 0.119573886 Brain_Substantia_nigra 1.623620997
0.376379003 0.376379003 1.556564634 1.922705177 1.739634905
0.258900461 2 0 0.972491774
CDK14 ENSG00000058091.16 0.141931326 4 4 0.025219323
Brain_Caudate_basal_ganglia 0.561925877 Brain_Cerebellum 2.075440597
0.104047372 0.104047372 0.579983304 2.238026555 1.12430632
0.756211994 4 0 0.972491774
CD47 ENSG00000196776.14 0.142000304 2 2 0.161039586
Brain_Frontal_Cortex_BA9 0.162520842 Brain_Cerebellum 1.002868344
0.997131656 0.997131656 -1.401583739 1.396643368 -0.002470186
1.978645363 2 0 0.972491774
HERC1 ENSG00000103657.13 0.142011145 1 1 0.142011145
Brain_Caudate_basal_ganglia 0.142011145 Brain_Caudate_basal_ganglia 1 1
1 -1.468342748 -1.468342748 -1.468342748 NA 1 0
0.972491774
ATP6V0A4 ENSG00000105929.15 0.142090784 1 1 0.142090784
Brain_Hippocampus 0.142090784 Brain_Hippocampus 1 1 1
-1.468049476 -1.468049476 -1.468049476 NA 1 0 0.972491774
C1QC ENSG00000159189.11 0.142252905 1 1 0.142252905
Brain_Anterior_cingulate_cortex_BA24 0.142252905
Brain_Anterior_cingulate_cortex_BA24 1 1 1 1.467452854
1.467452854 1.467452854 NA 1 0 0.972491774
PANK1 ENSG00000152782.16 0.142283378 1 1 0.142283378
Brain_Putamen_basal_ganglia 0.142283378 Brain_Putamen_basal_ganglia 1 1
1 1.46734077 1.46734077 1.46734077 NA 1 0 0.972491774
MC4R ENSG00000166603.4 0.142287883 2 1 0.125759472 Brain_Cerebellum
0.174626274 Brain_Cerebellar_Hemisphere 1.937630594 0.062369406
1.937630594 -1.531040143 -1.357487779 -1.444263961 0.122720054
1 0 0.972491774
NUDT7 ENSG00000140876.11 0.142383903 9 7 0.022277809 Brain_Cortex
0.999698804 Brain_Spinal_cord_cervical_c-1 4.722269498 0.063398745
0.308245865 0.000377493 2.285597633 0.933967066 0.83539652 7
0 0.972491774
PEPD ENSG00000124299.13 0.142469473 13 3 0.023931483
Brain_Putamen_basal_ganglia 0.44882581 Brain_Cerebellar_Hemisphere 10.44457408
8.40E-07 0.557383545 -2.258227439 -0.75737403 -1.64112907
0.44168 3 0 0.972491774
EMP2 ENSG00000213853.9 0.142493661 6 6 0.089329828
Brain_Substantia_nigra 0.751298104 Brain_Cerebellum 2.961035645
0.205843966 0.205843966 -1.698943509 0.748818611 -0.40952103
0.977572204 6 0 0.972491774
GDPGP1 ENSG00000183208.12 0.142511362 5 5 0.059913675
Brain_Putamen_basal_ganglia 0.722041051 Brain_Cortex 2.788509605
0.214124699 0.214124699 -1.092832059 1.881428337 0.079045591
1.15533135 5 0 0.972491774
RP11-569D9.5 ENSG00000283361.1 0.142722537 8 4 0.229861199
Brain_Caudate_basal_ganglia 0.948830129 Brain_Cortex 5.768197697
0.052458794 0.235560684 -0.772033597 1.200716481 0.421279112
0.618461019 4 0 0.972491774
POU3F3 ENSG00000198914.3 0.142769839 2 2 0.048568185
Brain_Cerebellar_Hemisphere 0.068690278 Brain_Cerebellum 1.93270237 0.06729763
0.06729763 1.820451486 1.972362977 1.896407232 0.107417646 2
0 0.972491774
ZFAND2A ENSG00000178381.11 0.142770927 3 3 0.074049935
Brain_Cerebellar_Hemisphere 0.628161991 Brain_Hypothalamus 1.347820528
0.657335948 0.657335948 -0.724808763 1.786304705 0.192393487
1.385595173 3 0 0.972491774
TNNI3K ENSG00000116783.14 0.142851388 13 2 0.017846836

Brain_Spinal_cord_cervical_c-1 0.148024345 Brain_Cerebellar_Hemisphere 10.61170571 0.019363045 1.199893224 1.446545196 2.368780614 1.783108314 0.270885401 2 0 0.972491774
PMAIP1 ENSG00000141682.11 0.143025858 1 1 0.143025858 Brain_Hippocampus 0.143025858 Brain_Hippocampus 1 1 1 1.464615472 1.464615472 1.464615472 NA 1 0 0.972491774
LCNL1 ENSG00000214402.6 0.14306774 7 7 0.002203189 Brain_Spinal_cord_cervical_c-1 0.706496984 Brain_Hypothalamus 1.99941381 0.120616993 0.120616993 -3.061380465 0.694013178 -0.796389281 1.130399076 7 0 0.972491774
RP11-770J1.4 ENSG00000255384.1 0.143108725 1 1 0.143108725 Brain_Hypothalamus 0.143108725 Brain_Hypothalamus 1 1 1 -1.464311981 -1.464311981 -1.464311981 NA 1 0 0.972491774
LINC01600 ENSG00000164385.8 0.143123667 10 9 0.012699832 Brain_Anterior_cingulate_cortex_BA24 0.935296065 Brain_Nucleus_accumbens_basal_ganglia 4.38489316 0.069867172 0.198290956 -1.887652032 2.492077499 0.189400405 1.223556336 9 0 0.972491774
SCN2B ENSG00000149575.5 0.143201335 1 1 0.143201335 Brain_Spinal_cord_cervical_c-1 0.143201335 Brain_Spinal_cord_cervical_c-1 1 1 1 1.463972966 1.463972966 1.463972966 NA 1 0 0.972491774
EEF1E1 ENSG00000124802.11 0.14323376 1 1 0.14323376 Brain_Spinal_cord_cervical_c-1 0.14323376 Brain_Spinal_cord_cervical_c-1 1 1 1 -1.463854307 -1.463854307 -1.463854307 NA 1 0 0.972491774
NOM1 ENSG00000146909.7 0.143254615 9 7 0.059889028 Brain_Hippocampus 0.955658601 Brain_Anterior_cingulate_cortex_BA24 5.370649879 0.010778554 0.194263456 -1.881609703 0.055602339 -1.075547114 0.573216053 7 0 0.972491774
LLNLF-187D8.1 ENSG00000277531.2 0.143385133 11 4 0.014396652 Brain_Caudate_basal_ganglia 0.49874634 Brain_Substantia_nigra 8.962454686 0.00480081 0.356433696 0.676463615 2.447211029 1.70281941 0.653055498 4 0 0.972491774
KDELR3 ENSG00000100196.10 0.143462426 2 2 0.056501252 Brain_Substantia_nigra 0.337185218 Brain_Hypothalamus 1.251449381 0.748550619 0.748550619 -1.907149338 -0.959741724 -1.433445531 0.669918349 2 0 0.972491774
SSB ENSG00000138385.15 0.143482376 1 1 0.143482376 Brain_Hypothalamus 0.143482376 Brain_Hypothalamus 1 1 1 1.462945195 1.462945195 1.462945195 NA 1 0 0.972491774
MATN1 ENSG00000162510.5 0.143512453 1 1 0.143512453 Brain_Hypothalamus 0.143512453 Brain_Hypothalamus 1 1 1 -1.462835295 -1.462835295 -1.462835295 NA 1 0 0.972491774
RP11-651P23.5 ENSG00000242791.2 0.143590398 1 1 0.143590398 Brain_Caudate_basal_ganglia 0.143590398 Brain_Caudate_basal_ganglia 1 1 1 -1.462550568 -1.462550568 -1.462550568 NA 1 0 0.972491774
GNPTG ENSG00000090581.9 0.143649635 2 2 0.049588117 Brain_Nucleus_accumbens_basal_ganglia 0.099102858 Brain_Putamen_basal_ganglia 1.790167202 0.209832798 0.209832798 -1.963499895 -1.649218603 -1.806359249 0.222230432 2 0 0.972491774
MVD ENSG00000167508.11 0.143672697 1 1 0.143672697 Brain_Frontal_Cortex_BA9 0.143672697 Brain_Frontal_Cortex_BA9 1 1 1 1.462250065 1.462250065 1.462250065 NA 1 0 0.972491774
PLXND1 ENSG00000004399.12 0.143681082 1 1 0.143681082 Brain_Spinal_cord_cervical_c-1 0.143681082 Brain_Spinal_cord_cervical_c-1 1 1 1 -1.462219456 -1.462219456 -1.462219456 NA 1 0 0.972491774
DHX37 ENSG00000150990.7 0.143696378 1 1 0.143696378 Brain_Cerebellum 0.143696378 Brain_Cerebellum 1 1 1 1.46216362 1.46216362 1.46216362

NA 1 0 0.972491774
CD79B ENSG00000007312.12 0.14375893 1 1 0.14375893 Brain_Cerebellar_Hemisphere
0.14375893 Brain_Cerebellar_Hemisphere 1 1 1 -1.461935338
-1.461935338 -1.461935338 NA 1 0 0.972491774
C17orf102 ENSG00000197322.3 0.143764879 5 5 0.027227896
Brain_Frontal_Cortex_BA9 0.920911672 Brain_Hypothalamus 2.464666043
0.158470246 0.158470246 -2.208235002 0.885598527 -0.565073593
1.196148121 5 0 0.972491774
PBOV1 ENSG00000254440.3 0.14383525 1 1 0.14383525 Brain_Cerebellar_Hemisphere
0.14383525 Brain_Cerebellar_Hemisphere 1 1 1 -1.461656913
-1.461656913 -1.461656913 NA 1 0 0.972491774
DPP7 ENSG00000176978.13 0.143967928 7 7 0.021140437
Brain_Spinal_cord_cervical_c-1 0.989852193 Brain_Cortex 1.846692522
0.298167759 0.298167759 -2.305466921 1.487256527 -0.061069538
1.150866468 7 0 0.972491774
RP11-54O7.17 ENSG00000272512.1 0.144235948 12 4 0.041270584
Brain_Substantia_nigra 0.449660061 Brain_Hypothalamus 9.282693409
1.09E-16 0.414370695 0.755981878 2.040801275 1.392884494
0.374032618 4 0 0.972491774
TCTE3 ENSG00000184786.5 0.144237188 2 2 0.08813909
Brain_Spinal_cord_cervical_c-1 0.537363857 Brain_Amygdala 1.202313279
0.797686721 0.797686721 0.616804237 1.705296769 1.161050503
0.76968045 2 0 0.972491774
BRIP1 ENSG00000136492.8 0.144261659 6 3 0.090843542
Brain_Caudate_basal_ganglia 0.3366547 Brain_Frontal_Cortex_BA9 4.618632062
0.028530565 0.179995625 -1.690964712 1.513282369 -0.90773401
1.218523041 3 0 0.972491774
VRK1 ENSG00000100749.7 0.144280104 4 2 0.150126604
Brain_Caudate_basal_ganglia 0.597149538 Brain_Frontal_Cortex_BA9
3.775547114 0.007186753 0.165838099 0.528504048 1.439084426
0.847809275 0.405314253 2 0 0.972491774
FRS2 ENSG00000166225.8 0.144304135 1 1 0.144304135 Brain_Cerebellum
0.144304135 Brain_Cerebellum 1 1 1 -1.459948838 -1.459948838
-1.459948838 NA 1 0 0.972491774
TMEM104 ENSG00000109066.13 0.144322724 1 1 0.144322724
Brain_Cerebellum 0.144322724 Brain_Cerebellum 1 1 1 -1.45988121
-1.45988121 -1.45988121 NA 1 0 0.972491774
FZD8 ENSG00000177283.7 0.144382967 1 1 0.144382967
Brain_Frontal_Cortex_BA9 0.144382967 Brain_Frontal_Cortex_BA9 1 1
1 -1.459662086 -1.459662086 -1.459662086 NA 1 0
0.972491774
MAPK6 ENSG00000069956.11 0.144416564 2 2 0.139573689
Brain_Putamen_basal_ganglia 0.162723316 Brain_Substantia_nigra
1.067636698 0.932363302 0.932363302 -1.477380431 -1.395970708
-1.436675569 0.057565367 2 0 0.972491774
KRT74 ENSG00000170484.9 0.144485519 1 1 0.144485519
Brain_Spinal_cord_cervical_c-1 0.144485519 Brain_Spinal_cord_cervical_c-1
1 1 1 1.459289226 1.459289226 1.459289226 NA 1 0
0.972491774
OR3A3 ENSG00000159961.1 0.144523414 1 1 0.144523414
Brain_Caudate_basal_ganglia 0.144523414 Brain_Caudate_basal_ganglia 1 1
1 1.459151501 1.459151501 1.459151501 NA 1 0
0.972491774
PROC ENSG00000115718.17 0.144818342 4 3 0.213007541
Brain_Putamen_basal_ganglia 0.767306317 Brain_Nucleus_accumbens_basal_ganglia
2.953361715 0.031946206 0.331685298 0.295900176 1.245337977
0.745247624 0.474474546 3 0 0.972491774
FAIM ENSG00000158234.12 0.144961465 9 5 0.019453946 Brain_Cortex
0.922850339 Brain_Amygdala 6.382813138 0.000272631 0.307481507
-2.3367162 0.096843927 -1.479352543 0.816982292 5 0
0.972491774
ACTR8 ENSG00000113812.13 0.144988592 13 2 0.120975912

Brain_Cerebellar_Hemisphere 0.913510518 Brain_Cortex 11.28387354 0.003262932 0.515475656 -1.550690334 0.635022064 -0.570907132 0.570230961 2 0 0.972491774
RP11-451G4.3 ENSG00000242790.1 0.145027947 1 1 0.145027947 Brain_Putamen_basal_ganglia 0.145027947 Brain_Putamen_basal_ganglia 1 1 1 1.457320439 1.457320439 1.457320439 NA 1 0 0.972491774
DIRAS1 ENSG00000176490.4 0.145109566 1 1 0.145109566 Brain_Frontal_Cortex_BA9 0.145109566 Brain_Frontal_Cortex_BA9 1 1 1 -1.457024685 -1.457024685 -1.457024685 NA 1 0 0.972491774
PARP14 ENSG00000173193.13 0.14518216 7 6 0.026566712 Brain_Hippocampus 0.546384847 Brain_Substantia_nigra 4.78583255 0.073377348 0.173983482 -2.217825807 -0.60318617 -1.512656654 0.617721716 6 0 0.972491774
RERE ENSG00000142599.17 0.145189495 6 3 0.073833462 Brain_Anterior_cingulate_cortex_BA24 0.715347801 Brain_Hippocampus 5.167431049 0.002927019 0.175271399 -1.787644 0.364683333 -1.162327983 0.766535138 3 0 0.972491774
MIR3976HG ENSG00000261738.6 0.145409505 2 1 0.099090001 Brain_Frontal_Cortex_BA9 0.217046618 Brain_Cortex 1.961481073 0.038518927 1.961481073 -1.649281389 -1.234419524 -1.441850456 0.293351638 1 0 0.972491774
LAG3 ENSG00000089692.8 0.145509741 1 1 0.145509741 Brain_Spinal_cord_cervical_c-1 0.145509741 Brain_Spinal_cord_cervical_c-1 1 1 1 -1.455576455 -1.455576455 -1.455576455 NA 1 0 0.972491774
RP11-474P2.6 ENSG00000275481.1 0.145721926 1 1 0.145721926 Brain_Anterior_cingulate_cortex_BA24 0.145721926 Brain_Anterior_cingulate_cortex_BA24 1 1 1 1.454809795 1.454809795 1.454809795 NA 1 0 0.972491774
PITPNC1 ENSG00000154217.14 0.145814692 3 3 0.062057175 Brain_Hippocampus 0.42509021 Brain_Cerebellum 1.567208181 0.562527747 0.562527747 -0.797621431 1.865887014 0.803676323 1.411215949 3 0 0.972491774
ADCY7 ENSG00000121281.12 0.145893592 1 1 0.145893592 Brain_Nucleus_accumbens_basal_ganglia 0.145893592 Brain_Nucleus_accumbens_basal_ganglia 1 1 1 -1.454190162 -1.454190162 -1.454190162 NA 1 0 0.972491774
CCK ENSG00000187094.11 0.145978275 1 1 0.145978275 Brain_Frontal_Cortex_BA9 0.145978275 Brain_Frontal_Cortex_BA9 1 1 1 1.453884702 1.453884702 1.453884702 NA 1 0 0.972491774
NEK8 ENSG00000160602.13 0.146152981 1 1 0.146152981 Brain_Putamen_basal_ganglia 0.146152981 Brain_Putamen_basal_ganglia 1 1 1 1.453254949 1.453254949 1.453254949 NA 1 0 0.972491774
LLGL1 ENSG00000131899.10 0.146201429 10 5 0.147788356 Brain_Spinal_cord_cervical_c-1 0.867512323 Brain_Cortex 6.716181354 0.012236027 0.274330703 -1.345645724 1.447387746 0.460988875 0.929189447 5 0 0.972491774
CPEB2 ENSG00000137449.15 0.146302271 1 1 0.146302271 Brain_Amygdala 0.146302271 Brain_Amygdala 1 1 1 -1.452717268 -1.452717268 -1.452717268 NA 1 0 0.972491774
OGDHL ENSG00000197444.9 0.146590891 2 2 0.051003107 Brain_Cerebellar_Hemisphere 0.537241323 Brain_Cerebellum 1.400237788 0.599762212 0.599762212 0.616989998 1.951453633 1.284221816 0.943608285 2 0 0.972491774
RNPEP ENSG00000176393.10 0.146635657 1 1 0.146635657 Brain_Anterior_cingulate_cortex_BA24 0.146635657 Brain_Anterior_cingulate_cortex_BA24 1 1 1 1.451518059 1.451518059 1.451518059 NA 1 0 0.972491774

SLC2A6 ENSG00000160326.13 0.146746886 6 3 0.048435162
Brain_Cerebellar_Hemisphere 0.440169144 Brain_Frontal_Cortex_BA9
5.086209708 0.030525694 0.24941155 -1.973530423 -0.77190762
-1.391758473 0.406599202 3 0 0.972491774
PRR33 ENSG00000283787.1 0.146819308 1 1 0.146819308
Brain_Cerebellar_Hemisphere 0.146819308 Brain_Cerebellar_Hemisphere 1 1
1 1.45085835 1.45085835 1.45085835 NA 1 0 0.972491774
CLP1 ENSG00000172409.5 0.146902889 2 2 0.090952991
Brain_Spinal_cord_cervical_c-1 0.233351026 Brain_Caudate_basal_ganglia
1.124372876 0.875627124 0.875627124 -1.690391967 -1.191771062
-1.441081515 0.352578224 2 0 0.972491774
GLTSCR1 ENSG00000063169.10 0.146974788 1 1 0.146974788
Brain_Cerebellar_Hemisphere 0.146974788 Brain_Cerebellar_Hemisphere 1 1
1 1.450300325 1.450300325 1.450300325 NA 1 0
0.972491774
TXNL4A ENSG00000141759.14 0.146977078 7 5 0.053207585
Brain_Cerebellum 0.693051168 Brain_Hypothalamus 5.031517883
0.047271449 0.236028752 -1.933232323 0.394717796 -1.003888787
0.785753289 5 0 0.972491774
RAD51AP2 ENSG00000214842.5 0.147045294 2 2 0.057494956
Brain_Cerebellum 0.956536572 Brain_Cortex 1.215594619 0.784405381
0.784405381 -1.899529013 0.054500297 -0.922514358 1.381707375
2 0 0.972491774
UTP11 ENSG00000183520.11 0.147085171 1 1 0.147085171
Brain_Spinal_cord_cervical_c-1 0.147085171 Brain_Spinal_cord_cervical_c-1
1 1 1 -1.449904431 -1.449904431 -1.449904431 NA 1 0
0.972491774
PGM2L1 ENSG00000165434.7 0.147262408 2 2 0.143235357
Brain_Anterior_cingulate_cortex_BA24 0.215127439 Brain_Frontal_Cortex_BA9
1.040088853 0.959911147 0.959911147 -1.463848466 -1.239589032
-1.351718749 0.158575366 2 0 0.972491774
COLGALT2 ENSG00000198756.10 0.14736122 11 3 0.018510177 Brain_Amygdala
0.849118906 Brain_Cerebellum 8.47187738 0.00113449 0.429544295
-2.35525142 1.025738398 -1.538028783 1.059382537 3 0
0.972491774
ZNF66 ENSG00000160229.11 0.147403795 2 2 0.106461416
Brain_Cerebellar_Hemisphere 0.340526706 Brain_Cortex 1.091792215
0.908207785 0.908207785 0.953125069 1.6143044 1.283714734
0.467524389 2 0 0.972491774
RP11-326G21.1 ENSG00000272755.1 0.147609499 5 5 0.01835282
Brain_Anterior_cingulate_cortex_BA24 0.797017267 Brain_Cerebellum
2.161259522 0.123741462 0.123741462 -2.015207957 2.358421916
0.199292797 1.920371089 5 0 0.972491774
ZNF534 ENSG00000198633.10 0.147619028 13 4 0.027994449
Brain_Cerebellar_Hemisphere 0.893327789 Brain_Cortex 9.464344845
0.013600565 0.662446165 -1.251790061 2.197364151 1.00635628
1.006136955 4 0 0.972491774
CDC42 ENSG00000070831.15 0.147771347 2 2 0.103802739
Brain_Frontal_Cortex_BA9 0.15647293 Brain_Caudate_basal_ganglia 1.22026916
0.77973084 0.77973084 1.417034195 1.626691008 1.521862602
0.148249755 2 0 0.972491774
RP11-16D22.2 ENSG00000271850.1 0.147792631 2 2 0.110162893
Brain_Spinal_cord_cervical_c-1 0.249011951 Brain_Substantia_nigra
1.015686352 0.984313648 0.984313648 -1.152752585 1.597461412
0.222354414 1.944694967 2 0 0.972491774
RP4-761J14.10 ENSG00000269892.1 0.147830444 1 1 0.147830444
Brain_Putamen_basal_ganglia 0.147830444 Brain_Putamen_basal_ganglia 1 1
1 1.447237403 1.447237403 1.447237403 NA 1 0
0.972491774
LEPROT ENSG00000213625.8 0.147863577 9 2 0.037784128
Brain_Anterior_cingulate_cortex_BA24 0.24464803 Brain_Substantia_nigra
7.911184937 0.01175345 0.5230723 -2.077188905 -1.163447395

-1.793411288 0.25242027 2 0 0.972491774
LINC01579 ENSG00000258754.7 0.147976659 1 1 0.147976659
Brain_Anterior_cingulate_cortex_BA24 0.147976659
Brain_Anterior_cingulate_cortex_BA24 1 1 1 -1.446715366
-1.446715366 -1.446715366 NA 1 0 0.972491774
NDRG3 ENSG00000101079.20 0.148026795 2 2 0.075782655 Brain_Cerebellum
0.307303298 Brain_Hippocampus 1.117995888 0.882004112
0.882004112 -1.775698315 1.020896878 -0.377400718 1.977491425
2 0 0.972491774
RP11-188D8.1 ENSG00000271427.1 0.148118874 1 1 0.148118874
Brain_Nucleus_accumbens_basal_ganglia 0.148118874
Brain_Nucleus_accumbens_basal_ganglia 1 1 1 1.446207986
1.446207986 1.446207986 NA 1 0 0.972491774
LONRF1 ENSG00000154359.12 0.148174112 1 1 0.148174112
Brain_Cerebellar_Hemisphere 0.148174112 Brain_Cerebellar_Hemisphere 1 1
1 1.446011017 1.446011017 1.446011017 NA 1 0
0.972491774
COX5A ENSG00000178741.11 0.148213071 2 2 0.130241044
Brain_Caudate_basal_ganglia 0.254907886 Brain_Substantia_nigra
1.063039949 0.936960051 0.936960051 -1.513152038 -1.13850928
-1.325830659 0.264912435 2 0 0.972491774
QRFP ENSG00000188710.2 0.148275748 1 1 0.148275748 Brain_Amygdala
0.148275748 Brain_Amygdala 1 1 1 1.445648743 1.445648743
1.445648743 NA 1 0 0.972491774
VGLL4 ENSG00000144560.14 0.148297861 1 1 0.148297861
Brain_Nucleus_accumbens_basal_ganglia 0.148297861
Brain_Nucleus_accumbens_basal_ganglia 1 1 1 -1.445569948
-1.445569948 -1.445569948 NA 1 0 0.972491774
RALGAPA2 ENSG00000188559.13 0.148300036 1 1 0.148300036
Brain_Anterior_cingulate_cortex_BA24 0.148300036
Brain_Anterior_cingulate_cortex_BA24 1 1 1 -1.445562198
-1.445562198 -1.445562198 NA 1 0 0.972491774
ALKBH5 ENSG00000091542.8 0.14862839 8 3 0.071068743
Brain_Hippocampus 0.890590499 Brain_Spinal_cord_cervical_c-1
6.701444441 0.000648378 0.309056941 -0.255306271 1.805037953
0.425778559 0.654616206 3 0 0.972491774
NDUFAF2 ENSG00000164182.10 0.148663037 3 3 0.024558383
Brain_Cortex 0.263138142 Brain_Caudate_basal_ganglia 1.914431339
0.313860736 0.313860736 -2.24827935 -1.119004682 -1.73915152
0.572764553 3 0 0.972491774
GPR132 ENSG00000183484.11 0.148708076 3 3 0.023723192
Brain_Putamen_basal_ganglia 0.116123772 Brain_Substantia_nigra
2.394963247 0.126942075 0.126942075 -2.261582801 -1.571253523
-2.006195349 0.378574244 3 0 0.972491774
LINC00663 ENSG00000266904.5 0.148727813 13 11 0.045789551
Brain_Anterior_cingulate_cortex_BA24 0.865804121 Brain_Caudate_basal_ganglia
5.17361488 1.25E-16 0.230647486 -0.94701312 1.997328112
0.241728166 0.892467424 11 0 0.972491774
TMEM237 ENSG00000155755.18 0.148778757 2 2 0.279390359
Brain_Putamen_basal_ganglia 0.662693495 Brain_Caudate_basal_ganglia
1.687565154 0.312434846 0.312434846 -0.436197426 1.081689863
0.322746218 1.073308395 2 0 0.972491774
ZC3H7A ENSG00000122299.11 0.148859929 1 1 0.148859929
Brain_Cerebellar_Hemisphere 0.148859929 Brain_Cerebellar_Hemisphere 1 1
1 -1.443570143 -1.443570143 -1.443570143 NA 1 0
0.972491774
RP11-586D19.2 ENSG00000251408.1 0.148919634 1 1 0.148919634
Brain_Nucleus_accumbens_basal_ganglia 0.148919634
Brain_Nucleus_accumbens_basal_ganglia 1 1 1 1.443358055
1.443358055 1.443358055 NA 1 0 0.972491774
RAMP3 ENSG00000122679.8 0.149088979 1 1 0.149088979
Brain_Nucleus_accumbens_basal_ganglia 0.149088979

Brain_Nucleus_accumbens_basal_ganglia 1 1 1 -1.442756847
-1.442756847 -1.442756847 NA 1 0 0.972491774
GMFB ENSG00000197045.12 0.149162295 2 2 0.104836497
Brain_Spinal_cord_cervical_c-1 0.300218189 Brain_Hippocampus
1.029330375 0.970669625 0.970669625 1.035965606 1.621845201
1.328905403 0.414279435 2 0 0.972491774
PPIP5K2 ENSG00000145725.19 0.149173096 6 5 0.104074413
Brain_Spinal_cord_cervical_c-1 0.984787419 Brain_Hypothalamus 3.61015902
0.055734295 0.195758257 -0.174526264 1.625413823 0.678171048
0.769586982 5 0 0.972491774
TTC21B ENSG00000123607.14 0.14920517 2 2 0.082710437
Brain_Cerebellar_Hemisphere 0.152700234 Brain_Cerebellum 1.341482878
0.658517122 0.658517122 -1.735171475 -1.430058484 -1.58261498
0.215747465 2 0 0.972491774
CPNE1 ENSG00000214078.12 0.149215174 13 1 0.11152484
Brain_Cerebellar_Hemisphere 0.20573852 Brain_Anterior_cingulate_cortex_BA24
12.49621181 9.63E-05 12.49621181 -1.591376633 -1.26537057
-1.413419334 0.106380866 1 0 0.972491774
TCF7 ENSG00000081059.19 0.149345594 3 3 0.314975346 Brain_Cerebellum
0.977975255 Brain_Cerebellar_Hemisphere 1.894482423 0.095632192
0.095632192 -1.004836997 0.810239663 -0.055663301 0.910398996
3 0 0.972491774
RYR1 ENSG00000196218.12 0.149407692 5 3 0.048557988
Brain_Nucleus_accumbens_basal_ganglia 0.51502224 Brain_Cortex 4.360812456
0.038644304 0.226514035 0.651037562 1.972452379 1.479630383
0.544778475 3 0 0.972491774
PTPRM ENSG00000173482.16 0.149417692 2 2 0.092638096
Brain_Frontal_Cortex_BA9 0.335564918 Brain_Cerebellum 1.014211162
0.985788838 0.985788838 -0.962965339 1.681642952 0.359338806
1.870020456 2 0 0.972491774
TBCK ENSG00000145348.16 0.149437205 2 2 0.208064266
Brain_Cerebellar_Hemisphere 0.752625346 Brain_Cerebellum 1.857953744
0.142046256 0.142046256 -1.258906057 -0.315179532 -0.787042795
0.667315426 2 0 0.972491774
SOCS7 ENSG00000274211.4 0.149557438 1 1 0.149557438
Brain_Putamen_basal_ganglia 0.149557438 Brain_Putamen_basal_ganglia 1 1
1 -1.441096445 -1.441096445 -1.441096445 NA 1 0
0.972491774
EFNA4 ENSG00000243364.7 0.1496466 1 1 0.1496466 Brain_Cortex 0.1496466
Brain_Cortex 1 1 1 1.440780868 1.440780868 1.440780868
NA 1 0 0.972491774
TLR6 ENSG00000174130.12 0.14967149 3 2 0.048545262
Brain_Putamen_basal_ganglia 0.079986283 Brain_Cerebellum 2.819819307
0.059047718 0.121132975 -1.972563965 -1.750765664 -1.866457032
0.111209342 2 0 0.972491774
COQ6 ENSG00000119723.16 0.149687671 7 5 0.006139939
Brain_Hypothalamus 0.328449204 Brain_Cerebellar_Hemisphere 5.249033164
0.073446817 0.223354342 0.977242315 2.740212925 1.672708783
0.577277223 5 0 0.972491774
LRRC3 ENSG00000160233.7 0.149694844 4 4 0.03173205
Brain_Nucleus_accumbens_basal_ganglia 0.871997126 Brain_Putamen_basal_ganglia
2.008384387 0.348054433 0.348054433 0.161122238 2.147769767
1.192614846 1.079460844 4 0 0.972491774
CBX2 ENSG00000173894.10 0.149721945 2 2 0.081522831
Brain_Caudate_basal_ganglia 0.262991283 Brain_Cortex 1.146315619
0.853684381 0.853684381 1.119348991 1.741917789 1.43063339
0.440222619 2 0 0.972491774
MMS22L ENSG00000146263.11 0.149725086 1 1 0.149725086
Brain_Amygdala 0.149725086 Brain_Amygdala 1 1 1 1.440503198
1.440503198 1.440503198 NA 1 0 0.972491774
SLC45A4 ENSG00000022567.9 0.149784027 1 1 0.149784027
Brain_Cerebellum 0.149784027 Brain_Cerebellum 1 1 1 1.440294746

1.440294746 1.440294746 NA 1 0 0.972491774
MAP3K7 ENSG00000135341.17 0.149853061 1 1 0.149853061
Brain_Frontal_Cortex_BA9 0.149853061 Brain_Frontal_Cortex_BA9 1 1
1 -1.440050681 -1.440050681 -1.440050681 NA 1 0
0.972491774
NPIPB9 ENSG00000196993.8 0.149856171 10 2 0.020848054
Brain_Frontal_Cortex_BA9 0.184512534 Brain_Nucleus_accumbens_basal_ganglia
9.037386483 0.000619522 0.3239629 -2.310724921 -1.326988358
-1.718511102 0.296035548 2 0 0.972491774
MAPK4 ENSG00000141639.11 0.149856204 1 1 0.149856204
Brain_Cerebellar_Hemisphere 0.149856204 Brain_Cerebellar_Hemisphere 1 1
1 1.440039572 1.440039572 1.440039572 NA 1 0
0.972491774
RP11-329B9.3 ENSG00000273403.1 0.149867123 9 7 0.065344277
Brain_Hypothalamus 0.656122915 Brain_Anterior_cingulate_cortex_BA24
3.910752795 0.046666421 0.448891466 -1.687518429 1.842895449
-0.389745034 1.3230449 7 0 0.972491774
RP5-890E16.5 ENSG00000278765.1 0.149923271 4 4 0.060889211
Brain_Hippocampus 0.850704155 Brain_Cerebellar_Hemisphere 2.397655407
0.28596296 0.28596296 -1.061776835 1.874299103 0.503365788
1.250051863 4 0 0.972491774
PDPN ENSG00000162493.16 0.149963344 2 2 0.059391811
Brain_Nucleus_accumbens_basal_ganglia 0.84073167 Brain_Frontal_Cortex_BA9
1.150536105 0.849463895 0.849463895 -1.885281735 0.200957675
-0.84216203 1.475194034 2 0 0.972491774
SNX9 ENSG00000130340.15 0.150096013 1 1 0.150096013 Brain_Cerebellum
0.150096013 Brain_Cerebellum 1 1 1 -1.439192419 -1.439192419
-1.439192419 NA 1 0 0.972491774
ZNF142 ENSG00000115568.15 0.150217075 4 3 0.173479081
Brain_Spinal_cord_cervical_c-1 0.841845642
Brain_Nucleus_accumbens_basal_ganglia 2.936651073 0.022411063
0.284484521 0.199533242 1.361109523 0.778267575 0.630844212
3 0 0.972491774
TMEM102 ENSG00000181284.2 0.150233195 1 1 0.150233195
Brain_Frontal_Cortex_BA9 0.150233195 Brain_Frontal_Cortex_BA9 1 1
1 -1.43870827 -1.43870827 -1.43870827 NA 1 0
0.972491774
HS3ST3B1 ENSG00000125430.8 0.150482949 2 2 0.056397987
Brain_Frontal_Cortex_BA9 0.803638375 Brain_Cortex 1.070505721
0.929494279 0.929494279 -0.24864115 1.907947621 0.829653236
1.524938544 2 0 0.972491774
AK9 ENSG00000155085.15 0.150490706 13 1 0.054798279
Brain_Anterior_cingulate_cortex_BA24 0.397943016 Brain_Amygdala
11.89873663 0.009149878 11.89873663 -1.920472221 -0.845300619
-1.382742355 0.321677698 1 0 0.972491774
NAGA ENSG00000198951.11 0.150595698 13 3 0.043147152
Brain_Hippocampus 0.757973159 Brain_Substantia_nigra 11.16957949
0.000429491 0.571709814 -0.308143479 2.022282724 1.28337613
0.577420967 3 0 0.972491774
C21orf62 ENSG00000205929.10 0.150656763 1 1 0.150656763
Brain_Spinal_cord_cervical_c-1 0.150656763 Brain_Spinal_cord_cervical_c-1
1 1 1 -1.437215526 -1.437215526 -1.437215526 NA 1 0
0.972491774
ZNF616 ENSG00000204611.6 0.150670615 1 1 0.150670615
Brain_Substantia_nigra 0.150670615 Brain_Substantia_nigra 1 1
1 -1.437166763 -1.437166763 -1.437166763 NA 1 0
0.972491774
LINC00886 ENSG00000240875.5 0.150703804 9 8 0.021723911
Brain_Putamen_basal_ganglia 0.830940271 Brain_Spinal_cord_cervical_c-1
4.042561341 0.114296064 0.182893619 -2.295160796 1.181341146
-0.514960496 1.029430957 8 0 0.972491774
TP53TG3D ENSG00000205456.11 0.150713253 1 1 0.150713253

Brain_Nucleus_accumbens_basal_ganglia 0.150713253
Brain_Nucleus_accumbens_basal_ganglia 1 1 1 -1.437016684
-1.437016684 -1.437016684 NA 1 0 0.972491774
CCDC88A ENSG00000115355.15 0.150945025 2 1 0.110303066
Brain_Cerebellum 0.204864643 Brain_Cerebellar_Hemisphere 1.989212176
0.010787824 1.989212176 1.267813266 1.596832424 1.432322845
0.232651677 1 0 0.972491774
SPA17 ENSG00000064199.6 0.150976522 4 4 0.011139101
Brain_Frontal_Cortex_BA9 0.755933105 Brain_Putamen_basal_ganglia
2.677206823 0.107299481 0.107299481 -2.538304514 -0.310825734
-1.881043371 1.05346344 4 0 0.972491774
BCKDHA ENSG00000248098.11 0.150988827 2 2 0.059528727
Brain_Cerebellum 0.593479497 Brain_Cortex 1.028527583 0.971472417
0.971472417 -1.884268045 0.533800602 -0.675233722 1.709832738
2 0 0.972491774
GPM6A ENSG00000150625.16 0.150988939 1 1 0.150988939
Brain_Nucleus_accumbens_basal_ganglia 0.150988939
Brain_Nucleus_accumbens_basal_ganglia 1 1 1 1.4360471 1.4360471
1.4360471 NA 1 0 0.972491774
RP11-873E20.1 ENSG00000267397.1 0.15103699 3 3 0.050842489
Brain_Caudate_basal_ganglia 0.765282477 Brain_Frontal_Cortex_BA9
1.683660739 0.569055201 0.569055201 -1.952806827 -0.298551237
-1.241792753 0.851227101 3 0 0.972491774
ZBTB14 ENSG00000198081.10 0.151096177 1 1 0.151096177
Brain_Amygdala 0.151096177 Brain_Amygdala 1 1 1 -1.435670313
-1.435670313 -1.435670313 NA 1 0 0.972491774
SELL ENSG00000188404.8 0.151177501 8 3 0.177862691
Brain_Anterior_cingulate_cortex_BA24 0.75943808
Brain_Nucleus_accumbens_basal_ganglia 7.056545898 0.002665709 0.2688395
-1.347365049 0.476093382 -0.490153248 0.512908906 3 0
0.972491774
FGF17 ENSG00000158815.10 0.151334209 2 2 0.066377595 Brain_Cerebellum
0.530443493 Brain_Cortex 1.005416144 0.994583856 0.994583856
-0.62732916 1.835865205 0.604268022 1.741741439 2 0
0.972491774
TULP3 ENSG00000078246.16 0.151352935 10 2 0.080292983 Brain_Amygdala
0.958877807 Brain_Cerebellar_Hemisphere 8.144139816 0.001341591
1.499406458 -1.748988638 0.187477476 -1.185146964 0.71627163 2
0 0.972491774
ARHGAP24 ENSG00000138639.17 0.151353421 1 1 0.151353421
Brain_Substantia_nigra 0.151353421 Brain_Substantia_nigra 1 1
1 -1.434767292 -1.434767292 -1.434767292 NA 1 0
0.972491774
ALPK1 ENSG00000073331.17 0.1513788 3 3 0.068718763 Brain_Cerebellum
0.725581664 Brain_Anterior_cingulate_cortex_BA24 1.412555218
0.645329813 0.645329813 -1.820264316 0.817898002 -0.217119135
1.40785376 3 0 0.972491774
ZDHHC19 ENSG00000163958.13 0.151496748 1 1 0.151496748
Brain_Caudate_basal_ganglia 0.151496748 Brain_Caudate_basal_ganglia 1 1
1 -1.43426467 -1.43426467 -1.43426467 NA 1 0
0.972491774
MUM1 ENSG00000160953.15 0.151516821 12 7 0.040284697 Brain_Cerebellum
0.8683988 Brain_Putamen_basal_ganglia 7.27149742 0.000698368 0.288228087
-1.866259401 2.050817768 0.911499559 1.248907756 7 0
0.972491774
NSMCE4A ENSG00000107672.14 0.151619299 4 4 0.389456784
Brain_Cortex 0.974623636 Brain_Frontal_Cortex_BA9 2.575632564
0.216300162 0.216300162 -0.855528251 0.860602908 0.133305955
0.756317854 4 0 0.972491774
CRLS1 ENSG00000088766.11 0.151627062 2 2 0.124489915 Brain_Cerebellum
0.438303771 Brain_Cerebellar_Hemisphere 1.245512649 0.754487351
0.754487351 -0.775060737 1.53619764 0.380568452 1.634306471 2

0 0.972491774
EPB41L3 ENSG00000082397.17 0.15171293 1 1 0.15171293 Brain_Cerebellum
0.15171293 Brain_Cerebellum 1 1 1 1.433507246 1.433507246
1.433507246 NA 1 0 0.972491774
IGSF5 ENSG00000183067.5 0.151755851 5 5 0.027609255
Brain_Hippocampus 0.797382419 Brain_Nucleus_accumbens_basal_ganglia
2.871751679 0.267343255 0.267343255 -0.439075054 2.20279423
0.532878557 1.044887857 5 0 0.972491774
APOBEC3B ENSG00000179750.15 0.151788257 1 1 0.151788257
Brain_Cortex 0.151788257 Brain_Cortex 1 1 1 1.433243517
1.433243517 1.433243517 NA 1 0 0.972491774
RN7SL832P ENSG00000243819.4 0.151798827 1 1 0.151798827
Brain_Nucleus_accumbens_basal_ganglia 0.151798827
Brain_Nucleus_accumbens_basal_ganglia 1 1 1 -1.433206519
-1.433206519 -1.433206519 NA 1 0 0.972491774
CACNG2 ENSG00000166862.6 0.151859862 1 1 0.151859862
Brain_Anterior_cingulate_cortex_BA24 0.151859862
Brain_Anterior_cingulate_cortex_BA24 1 1 1 1.432992916
1.432992916 1.432992916 NA 1 0 0.972491774
TMOD4 ENSG00000163157.14 0.151864255 2 2 0.128372795 Brain_Cerebellum
0.231725411 Brain_Frontal_Cortex_BA9 1.007428479 0.992571521
0.992571521 -1.19592607 1.52055003 0.16231198 1.920838672 2 0
0.972491774
SIRT4 ENSG00000089163.4 0.151961106 3 3 0.054221596
Brain_Frontal_Cortex_BA9 0.749032384 Brain_Cerebellum 1.904566965
0.344822915 0.344822915 -1.925062048 -0.319915505 -0.861236576
0.921347922 3 0 0.972491774
WIPI1 ENSG00000070540.12 0.152109662 4 4 0.053458303
Brain_Nucleus_accumbens_basal_ganglia 0.991523459 Brain_Cerebellar_Hemisphere
1.97084289 0.069368653 0.069368653 -1.931200166 0.010623969
-1.0620788 0.819729924 4 0 0.972491774
CNTLN ENSG00000044459.14 0.152174417 5 4 0.028012752
Brain_Hippocampus 0.725941114 Brain_Cortex 3.397434516
0.033886941 0.303453704 -2.197107753 -0.350529821 -0.833789267
0.776205139 4 0 0.972491774
EXOC6B ENSG00000144036.15 0.152255183 2 2 0.054535936
Brain_Frontal_Cortex_BA9 0.283838896 Brain_Cerebellum 1.435176099
0.564823901 0.564823901 -1.922555191 -1.0717354 -1.497145295
0.601620444 2 0 0.972491774
CASC15 ENSG00000272168.6 0.152278462 6 2 0.072407768
Brain_Cortex 0.838479262 Brain_Cerebellum 5.43512629 0.000460475
0.426888982 -1.796545723 -0.203839068 -0.9475308 0.546898109 2
0 0.972491774
ELOVL7 ENSG00000164181.13 0.15230909 3 3 0.048955
Brain_Cerebellar_Hemisphere 0.753353727 Brain_Substantia_nigra
1.626988808 0.487460974 0.487460974 -0.314220298 1.968983374
1.134186584 1.259223078 3 0 0.972491774
SERPINF1 ENSG00000132386.10 0.152315276 1 1 0.152315276
Brain_Putamen_basal_ganglia 0.152315276 Brain_Putamen_basal_ganglia 1 1
1 1.431401162 1.431401162 1.431401162 NA 1 0
0.972491774
SLC4A2 ENSG00000164889.13 0.152403762 3 3 0.0422675
Brain_Cerebellar_Hemisphere 0.411595032 Brain_Cortex 1.065725263
0.940133018 0.940133018 -2.030876743 -0.821089953 -1.239312753
0.685881734 3 0 0.972491774
RP11-542G1.1 ENSG00000251185.1 0.152404382 5 5 0.043247932
Brain_Hypothalamus 0.263054696 Brain_Amygdala 2.692127918
0.139436691 0.139436691 1.119200302 2.02130761 1.698109261
0.409500081 5 0 0.972491774
SPRED2 ENSG00000198369.9 0.152445805 2 2 0.063103056
Brain_Substantia_nigra 0.951755259 Brain_Amygdala 1.256107371
0.743892629 0.743892629 0.060502708 1.858464667 0.959483688

1.271351094	2	0	0.972491774
DMRT2 ENSG00000173253.15	0.152474663	1	1	0.152474663
Brain_Hippocampus	0.152474663	Brain_Hippocampus	1	1	1
-1.43084493	-1.43084493	-1.43084493	NA	1	0	0.972491774
DSEL ENSG00000171451.13	0.152484739	2	2	0.061625091
Brain_Spinal_cord_cervical_c-1	0.746778881
Brain_Anterior_cingulate_cortex_BA24	1.102900887	0.897099113
0.897099113	-1.868983636	0.322889563	-0.773047036	1.549888402
2	0	0.972491774
RP11-131L23.2	ENSG00000273264.1	0.152562451	1	1	0.152562451
Brain_Hippocampus	0.152562451	Brain_Hippocampus	1	1	1
1.430538757	1.430538757	1.430538757	NA	1	0	0.972491774
KCNK1 ENSG00000135750.14	0.152582757	3	3	0.022801682
Brain_Substantia_nigra	0.969076012	Brain_Cortex	1.11235599 0.87153977
0.87153977 0.03876718 2.276741242	0.89045511 1.210982413	3	0
0.972491774
IGSF9 ENSG00000085552.16	0.152610131	1	1	0.152610131
Brain_Nucleus_accumbens_basal_ganglia 0.152610131
Brain_Nucleus_accumbens_basal_ganglia 1	1	1	-1.430372521
-1.430372521	-1.430372521	NA	1	0	0.972491774
CUTA ENSG00000112514.15	0.152720549	2	2	0.090971218	Brain_Cerebellum
0.692935114	Brain_Frontal_Cortex_BA9	1.30181894 0.69818106 0.69818106
-0.394875037	1.690296639	0.647710801	1.474439032	2	0
0.972491774
RP11-575F12.3	ENSG00000278266.1	0.15272259 2	2	0.053358303
Brain_Caudate_basal_ganglia 0.390539465	Brain_Amygdala	1.367400731
0.632599269	0.632599269	0.858639453	1.932009747	1.3953246
0.758987414	2	0	0.972491774
PEF1 ENSG00000162517.12	0.152747811	7	5	0.027480679
Brain_Frontal_Cortex_BA9	0.52225647 Brain_Spinal_cord_cervical_c-1
4.425827003	0.006137611	0.2148637 -2.204621311	1.537312692
-1.168392321	1.308863962	5	0	0.972491774
CIITA ENSG00000179583.18	0.152864719	1	1	0.152864719
Brain_Caudate_basal_ganglia 0.152864719	Brain_Caudate_basal_ganglia 1	1
1	1.429485573	1.429485573	1.429485573	NA	1	0
0.972491774
HSPA13	ENSG00000155304.5	0.1529829 2	2	0.116859769	Brain_Amygdala
0.168541318	Brain_Nucleus_accumbens_basal_ganglia 1.161649461
0.838350539	0.838350539	1.376905982	1.568091546	1.472498764
0.135188609	2	0	0.972491774
ING2 ENSG00000168556.6	0.15301448 1	1	0.15301448 Brain_Hypothalamus
0.15301448 Brain_Hypothalamus	1	1	1	-1.42896435	-1.42896435
-1.42896435	NA	1	0	0.972491774
RHBDD1	ENSG00000144468.16	0.153122947	6	5	0.060619591
Brain_Frontal_Cortex_BA9	0.242120958	Brain_Anterior_cingulate_cortex_BA24
3.42397897 0.025614641	0.236741936	-1.876259972	1.169701887
-1.119772856	1.148452451	5	0	0.972491774
KCNJ10	ENSG00000177807.8	0.153178093	3	3	0.107384574
Brain_Cerebellum 0.719971424	Brain_Spinal_cord_cervical_c-1	1.021541746
0.975400352	0.975400352	-1.610060844	0.358496984	-0.950946263
1.134019625	3	0	0.972491774
RP11-347I19.8	ENSG00000272849.1	0.153219748	10	3	0.009689614
Brain_Cerebellar_Hemisphere 0.643799326	Brain_Anterior_cingulate_cortex_BA24
8.33299523 0.000239568	0.28646648 0.462393268	2.586713421
2.005783275	0.599935158	3	0	0.972491774
WNT6 ENSG00000115596.3	0.153256157	1	1	0.153256157
Brain_Substantia_nigra	0.153256157	Brain_Substantia_nigra	1	1
1	-1.428124048	-1.428124048	-1.428124048	NA	1	0
0.972491774
CHD1L ENSG00000131778.18	0.153347045	7	4	0.008983612
Brain_Nucleus_accumbens_basal_ganglia 0.877223349	Brain_Cerebellum
4.051020075	0.03017911 0.330556689	-0.172901083	2.61267715 1.08821574

0.996852685 4 0 0.972491774
RPS17 ENSG00000182774.10 0.153372955 4 3 0.026341371
Brain_Spinal_cord_cervical_c-1 0.977011635 Brain_Frontal_Cortex_BA9
2.849225723 0.05078984 0.177363316 -2.221141621 -0.02881563
-0.635063427 1.059905859 3 0 0.972491774
RWDD2B ENSG00000156253.6 0.153397941 13 1 0.108675783
Brain_Substantia_nigra 0.1888123 Brain_Caudate_basal_ganglia 12.58793791
0.000169558 12.58793791 -1.604173644 -1.314100755 -1.405700963
0.073698402 1 0 0.972491774
C22orf34 ENSG00000188511.12 0.153609762 8 3 0.158718213
Brain_Cerebellar_Hemisphere 0.915409758 Brain_Hypothalamus 6.715826654
0.024655935 0.2790929 -1.409395639 0.448945064 -0.051932479
0.605242515 3 0 0.972491774
RASA4 ENSG00000105808.17 0.153616812 6 6 0.028104498
Brain_Caudate_basal_ganglia 0.749190303 Brain_Cerebellar_Hemisphere
3.439668002 0.13481766 0.13481766 0.319707198 2.195824625
1.611519391 0.682661542 6 0 0.972491774
TMEM132E ENSG00000181291.6 0.153817895 3 3 0.057678775
Brain_Frontal_Cortex_BA9 0.426306359 Brain_Cerebellum 1.134492632
0.868317649 0.868317649 -0.795528122 1.898131397 0.633394395
1.354314611 3 0 0.972491774
TIMM22 ENSG00000177370.4 0.153865159 10 10 0.008373145
Brain_Putamen_basal_ganglia 0.997180643 Brain_Cerebellum 1.994003629
0.347068172 0.347068172 0.003533547 2.636640787 1.004098115
0.923823844 10 0 0.972491774
PRICKLE2 ENSG00000163637.12 0.153872925 2 2 0.161648343
Brain_Caudate_basal_ganglia 0.222328892 Brain_Putamen_basal_ganglia 1.07950498
0.92049502 0.92049502 -1.220358791 1.399549242 0.089595225
1.852554736 2 0 0.972491774
S100PBP ENSG00000116497.17 0.154096078 2 2 0.055411014
Brain_Frontal_Cortex_BA9 0.575104565 Brain_Nucleus_accumbens_basal_ganglia
1.155520055 0.844479945 0.844479945 0.560549677 1.915639375
1.238094526 0.958193114 2 0 0.972491774
GGA1 ENSG00000100083.18 0.154134451 2 2 0.066799336
Brain_Caudate_basal_ganglia 0.533942976 Brain_Cortex 1.003081284
0.996918716 0.996918716 -1.833021754 -0.621998316 -1.227510035
0.856322885 2 0 0.972491774
WNT3 ENSG00000108379.9 0.154163321 12 12 0.003049231
Brain_Frontal_Cortex_BA9 0.930590165 Brain_Substantia_nigra
2.396709333 0.30213477 0.30213477 -2.962730786 1.643996477
-0.60898777 1.188231663 12 0 0.972491774
JMJD6 ENSG00000070495.14 0.154202626 1 1 0.154202626 Brain_Cerebellum
0.154202626 Brain_Cerebellum 1 1 1 -1.424842875 -1.424842875
-1.424842875 NA 1 0 0.972491774
TSC1 ENSG00000165699.13 0.154246017 9 2 0.099436424
Brain_Substantia_nigra 0.394691653 Brain_Hippocampus 7.708994566
1.75E-05 1.000150204 0.851139883 1.647591996 1.041340432
0.254551338 2 0 0.972491774
PCDHGA6 ENSG00000253731.2 0.154261064 8 5 0.165674889
Brain_Cerebellar_Hemisphere 0.980381232 Brain_Hypothalamus 6.192468283
0.008355499 0.274989586 -1.386235879 0.425841291 -0.774626561
0.705997509 5 0 0.972491774
RAB3B ENSG00000169213.6 0.154321076 3 3 0.025140862
Brain_Caudate_basal_ganglia 0.726654179 Brain_Frontal_Cortex_BA9
1.905648746 0.10526835 0.10526835 -2.239231421 0.349579658
-1.30895152 1.439877822 3 0 0.972491774
SELENOW ENSG00000178980.14 0.154327081 3 3 0.037111588
Brain_Spinal_cord_cervical_c-1 0.664013423 Brain_Cerebellum 1.555520727
0.547024286 0.547024286 -2.084534365 -0.434378755 -1.296587858
0.82758056 3 0 0.972491774
MON2 ENSG00000061987.14 0.154392335 1 1 0.154392335
Brain_Hypothalamus 0.154392335 Brain_Hypothalamus 1 1 1

1.424187042 1.424187042 1.424187042 NA 1 0 0.972491774
ANKRD27 ENSG00000105186.15 0.154399311 10 4 0.093432427
Brain_Cerebellar_Hemisphere 0.963038485 Brain_Anterior_cingulate_cortex_BA24
6.387096378 0.021257773 0.223358004 -1.677563072 0.321083482
-0.700723515 0.792027999 4 0 0.972491774
OTUD3 ENSG00000169914.5 0.154486856 13 1 0.101703008 Brain_Cortex
0.402919343 Brain_Substantia_nigra 11.34635091 0.008272082
11.34635091 0.836418795 1.636652936 1.327168249 0.210345448
1 0 0.972491774
MASTL ENSG00000120539.14 0.154527068 11 3 0.104189363
Brain_Substantia_nigra 0.98556661 Brain_Amygdala 9.307290269
0.007588403 0.502517423 -1.624874219 0.147023018 -0.539149401
0.513267298 3 0 0.972491774
RP11-1263C18.2 ENSG00000283183.1 0.154578146 4 4 0.048347396
Brain_Cortex 0.907747407 Brain_Cerebellum 1.474858316 0.565356695
0.565356695 -1.069511669 1.974302153 -0.003319552 1.428770904
4 0 0.972491774
LEF1 ENSG00000138795.9 0.154598792 1 1 0.154598792
Brain_Hypothalamus 0.154598792 Brain_Hypothalamus 1 1 1
1.423474005 1.423474005 1.423474005 NA 1 0 0.972491774
RP11-783K16.13 ENSG00000257086.1 0.154638654 10 2 0.048050747
Brain_Anterior_cingulate_cortex_BA24 0.862534254 Brain_Cerebellum
8.503586772 0.004820919 0.736416935 -1.976919355 -0.173149072
-1.481218488 0.599629043 2 0 0.972491774
KIAA1324L ENSG00000164659.14 0.154652669 3 3 0.037708865
Brain_Hippocampus 0.954892083 Brain_Cerebellum 1.229889922
0.844598635 0.844598635 -2.078005362 0.056564539 -1.079401035
1.073893966 3 0 0.972491774
SECISBP2L ENSG00000138593.8 0.154766886 1 1 0.154766886
Brain_Putamen_basal_ganglia 0.154766886 Brain_Putamen_basal_ganglia 1 1
1 1.422893999 1.422893999 1.422893999 NA 1 0
0.972491774
ANO4 ENSG00000151572.16 0.154846711 2 2 0.121564361
Brain_Putamen_basal_ganglia 0.26135031 Brain_Nucleus_accumbens_basal_ganglia
1.020261268 0.979738732 0.979738732 -1.548240679 -1.123205304
-1.335722992 0.300545396 2 0 0.972491774
SAFB2 ENSG00000130254.11 0.154965666 1 1 0.154965666
Brain_Nucleus_accumbens_basal_ganglia 0.154965666
Brain_Nucleus_accumbens_basal_ganglia 1 1 1 1.422208727
1.422208727 1.422208727 NA 1 0 0.972491774
LINC01671 ENSG00000225431.1 0.155007816 2 1 0.149783671
Brain_Nucleus_accumbens_basal_ganglia 0.160761757 Brain_Putamen_basal_ganglia
1.99814845 0.00185155 1.99814845 -1.440296006 -1.402514188 -1.421405097
0.026715779 1 0 0.972491774
EMILIN1 ENSG00000138080.13 0.155136306 1 1 0.155136306
Brain_Amygdala 0.155136306 Brain_Amygdala 1 1 1 1.421620997
1.421620997 1.421620997 NA 1 0 0.972491774
PLEKHF2 ENSG00000175895.3 0.15534015 1 1 0.15534015 Brain_Hippocampus
0.15534015 Brain_Hippocampus 1 1 1 -1.420919547 -1.420919547
-1.420919547 NA 1 0 0.972491774
KIAA1024 ENSG00000169330.8 0.155352495 3 3 0.119471114
Brain_Cerebellar_Hemisphere 0.936616531 Brain_Cerebellum 1.685646967
0.389636671 0.389636671 -1.556997354 1.152263348 -0.108403624
1.364371921 3 0 0.972491774
PDZD9 ENSG00000155714.13 0.15536101 2 2 0.247571867
Brain_Nucleus_accumbens_basal_ganglia 0.424669559 Brain_Frontal_Cortex_BA9
1.481015275 0.518984725 0.518984725 -1.156267232 0.798346295
-0.178960468 1.38212048 2 0 0.972491774
ANXA3 ENSG00000138772.12 0.155365338 9 5 0.037452985
Brain_Caudate_basal_ganglia 0.867650382 Brain_Hippocampus 6.632464019
0.004343408 0.221664503 -2.080791602 -0.166643731 -0.755316485
0.567831243 5 0 0.972491774

PWAR6 ENSG00000257151.1 0.155507443 1 1 0.155507443
Brain_Nucleus_accumbens_basal_ganglia 0.155507443
Brain_Nucleus_accumbens_basal_ganglia 1 1 1 -1.420344392
-1.420344392 -1.420344392 NA 1 0 0.972491774
BATF3 ENSG00000123685.8 0.155515224 6 2 0.076806262
Brain_Putamen_basal_ganglia 0.938153437 Brain_Cortex 5.165328707
0.004129925 0.516852878 0.077590956 1.769525244 1.232898416
0.602023042 2 0 0.972491774
MICA ENSG00000204520.12 0.155583387 13 2 0.064166044
Brain_Nucleus_accumbens_basal_ganglia 0.377982479 Brain_Cerebellum
10.66255347 0.003606476 1.724513045 -1.851024395 -0.881619735
-1.203824595 0.253730118 2 0 0.972491774
AC012354.6 ENSG00000225156.2 0.155638534 3 3 0.083269139
Brain_Caudate_basal_ganglia 0.264662857 Brain_Nucleus_accumbens_basal_ganglia
1.204108738 0.778068461 0.778068461 -1.115437805 1.732024846
0.674454015 1.558599181 3 0 0.972491774
SUSD5 ENSG00000173705.8 0.155706649 6 6 0.030916562
Brain_Putamen_basal_ganglia 0.899536711 Brain_Caudate_basal_ganglia
1.914595063 0.225732658 0.225732658 -1.282117776 2.158144979
0.202819454 1.283131743 6 0 0.972491774
CTC-461F20.1 ENSG00000250415.1 0.155732513 2 2 0.062380944
Brain_Cerebellar_Hemisphere 0.619912017 Brain_Cerebellum 1.00017055 0.99982945
0.99982945 -0.495975045 1.863578327 0.683801641 1.66845619 2 0
0.972491774
AP001505.10 ENSG00000276529.1 0.155816454 12 2 0.119863959
Brain_Putamen_basal_ganglia 0.732651599 Brain_Nucleus_accumbens_basal_ganglia
11.2360993 3.32E-05 0.415105135 0.341600449 1.555344861 0.49756384
0.336919538 2 0 0.972491774
HSDL2 ENSG00000119471.14 0.155903141 4 4 0.033613321 Brain_Amygdala
0.878298902 Brain_Cortex 2.635031889 0.139568701 0.139568701
0.153126014 2.124680035 1.238431733 1.007973137 4 0
0.972491774
BTBD6 ENSG00000184887.13 0.155929958 4 3 0.075102658
Brain_Frontal_Cortex_BA9 0.982161426 Brain_Cerebellum 2.796886165
0.085780607 0.201169202 -0.0223592 1.779836893 0.680323629
0.77795133 3 0 0.972491774
ECHDC3 ENSG00000134463.14 0.15596912 11 4 0.097214393
Brain_Spinal_cord_cervical_c-1 0.970913337 Brain_Cortex 8.687139612
0.0080629 0.291283372 -1.65851088 1.521391452 -0.244764023
0.809883728 4 0 0.972491774
SOX21-AS1 ENSG00000227640.2 0.156019224 1 1 0.156019224
Brain_Nucleus_accumbens_basal_ganglia 0.156019224
Brain_Nucleus_accumbens_basal_ganglia 1 1 1 -1.418587804
-1.418587804 -1.418587804 NA 1 0 0.972491774
AACS ENSG00000081760.16 0.156069634 7 7 0.030840388
Brain_Putamen_basal_ganglia 0.869086084 Brain_Caudate_basal_ganglia
2.969935519 0.20664197 0.20664197 -1.052780488 2.159126068
0.582660557 0.980144489 7 0 0.972491774
LINC01607 ENSG00000272138.1 0.156092921 8 8 0.043005242
Brain_Caudate_basal_ganglia 0.903078913 Brain_Hypothalamus 1.434204661
0.575088398 0.575088398 -1.080638379 2.023659079 0.56448543
0.961508556 8 0 0.972491774
C7orf61 ENSG00000185955.4 0.156096374 2 2 0.387590005
Brain_Cortex 0.39521019 Brain_Spinal_cord_cervical_c-1 1.604457116
0.395542884 0.395542884 -0.863996152 0.850206677 -0.006894737
1.212124444 2 0 0.972491774
EXOC6 ENSG00000138190.16 0.156122032 2 2 0.117375127
Brain_Cerebellar_Hemisphere 0.622385496 Brain_Nucleus_accumbens_basal_ganglia
1.355979475 0.644020525 0.644020525 -1.565886774 -0.492472304
-1.029179539 0.759018651 2 0 0.972491774
POLD2 ENSG00000106628.10 0.15612938 7 3 0.098487499
Brain_Nucleus_accumbens_basal_ganglia 0.987016902 Brain_Hippocampus

5.302232729 0.006966312 0.647228122 -0.503269199 1.652230854
0.303080742 0.802650405 3 0 0.972491774
TRRAP ENSG00000196367.12 0.156206981 1 1 0.156206981
Brain_Nucleus_accumbens_basal_ganglia 0.156206981
Brain_Nucleus_accumbens_basal_ganglia 1 1 1 -1.417944459
-1.417944459 -1.417944459 NA 1 0 0.972491774
RP5-999L4.2 ENSG00000275142.1 0.156499829 4 3 0.041824063
Brain_Hippocampus 0.133651145 Brain_Substantia_nigra 2.818405365
0.012204188 0.214843385 -2.035266517 -1.499858171 -1.733095274
0.261513364 3 0 0.972491774
SIX5 ENSG00000177045.7 0.156515606 1 1 0.156515606
Brain_Nucleus_accumbens_basal_ganglia 0.156515606
Brain_Nucleus_accumbens_basal_ganglia 1 1 1 -1.416888237
-1.416888237 -1.416888237 NA 1 0 0.972491774
LPCAT1 ENSG00000153395.9 0.156579065 2 2 0.059329589
Brain_Cerebellar_Hemisphere 0.156730357 Brain_Cerebellum 1.857480573
0.142519427 0.142519427 -1.885743051 -1.416154218 -1.650948634
0.332049448 2 0 0.972491774
CTD-2315E11.1 ENSG00000259685.2 0.156630119 7 6 0.005269608
Brain_Caudate_basal_ganglia 0.660437803 Brain_Cerebellar_Hemisphere
4.210449152 0.094402089 0.215034047 -2.790073458 0.439308794
-1.861576624 1.173611478 6 0 0.972491774
APEX1 ENSG00000100823.11 0.156731666 2 2 0.153517781
Brain_Nucleus_accumbens_basal_ganglia 0.275858506 Brain_Cerebellar_Hemisphere
1.133700259 0.866299741 0.866299741 1.089670067 1.427215526
1.258442797 0.238680683 2 0 0.972491774
XRCC2 ENSG00000196584.2 0.156818114 13 3 0.158794015
Brain_Hippocampus 0.900534785 Brain_Amygdala 11.06176618 2.63E-17
0.395459271 -1.119798181 1.40913919 0.839312486 0.716934733 3
0 0.972491774
GPLD1 ENSG00000112293.14 0.156823625 2 2 0.17094916
Brain_Putamen_basal_ganglia 0.307190804 Brain_Frontal_Cortex_BA9
1.218519403 0.781480597 0.781480597 -1.369160342 1.021134321
-0.17401301 1.690193565 2 0 0.972491774
SRPRB ENSG00000144867.11 0.15683411 4 4 0.074812161
Brain_Putamen_basal_ganglia 0.945121031 Brain_Hippocampus 1.582589979
0.43441675 0.43441675 -1.781614239 0.740317384 -0.629640799
1.172582098 4 0 0.972491774
ZNRF1 ENSG00000186187.11 0.15683766 1 1 0.15683766 Brain_Hypothalamus
0.15683766 Brain_Hypothalamus 1 1 1 -1.415787742 -1.415787742
-1.415787742 NA 1 0 0.972491774
MTMR6 ENSG00000139505.11 0.156883988 3 1 0.129466365
Brain_Frontal_Cortex_BA9 0.214678253 Brain_Cerebellum 2.957701924
0.009439358 2.957701924 -1.516209587 -1.240803756 -1.405620707
0.145490913 1 0 0.972491774
IFT43 ENSG00000119650.12 0.156965988 1 1 0.156965988 Brain_Cerebellum
0.156965988 Brain_Cerebellum 1 1 1 1.415349705 1.415349705
1.415349705 NA 1 0 0.972491774
ETAA1 ENSG00000143971.7 0.157100388 4 4 0.036416289 Brain_Cortex
0.810139877 Brain_Cerebellar_Hemisphere 1.589430217 0.444523616
0.444523616 -2.092248533 1.56978631 -0.40267205 1.524242167 4
0 0.972491774
PTGER3 ENSG00000050628.20 0.157176552 3 3 0.100664183
Brain_Anterior_cingulate_cortex_BA24 0.940139318 Brain_Frontal_Cortex_BA9
2.164448585 0.301060337 0.301060337 -0.532398056 1.641642168
0.344716451 1.146208451 3 0 0.972491774
LRTM1 ENSG00000144771.7 0.157237418 1 1 0.157237418 Brain_Cortex
0.157237418 Brain_Cortex 1 1 1 -1.4144241 -1.4144241 -1.4144241
NA 1 0 0.972491774
PARP10 ENSG00000178685.13 0.157301711 2 2 0.054504597
Brain_Spinal_cord_cervical_c-1 0.956226557 Brain_Substantia_nigra
1.005278446 0.994721554 0.994721554 0.054889424 1.922804581

0.988847003 1.320815474 2 0 0.972491774
PFN2 ENSG00000070087.13 0.15757611 1 1 0.15757611 Brain_Cerebellum 0.15757611 Brain_Cerebellum 1 1 1 1.413270822 1.413270822 1.413270822 NA 1 0 0.972491774
UBN1 ENSG00000118900.14 0.15757879 1 1 0.15757879 Brain_Cerebellum 0.15757879 Brain_Cerebellum 1 1 1 -1.413261704 -1.413261704 -1.413261704 NA 1 0 0.972491774
KAT8 ENSG00000103510.19 0.15770392 11 2 0.007802196 Brain_Caudate_basal_ganglia 0.296742588 Brain_Frontal_Cortex_BA9 9.618986962 0.002869283 0.756016019 1.04344425 2.660511933 1.788195844 0.424784441 2 0 0.972491774
HNRNPA1L2 ENSG00000139675.11 0.157714433 12 3 0.039995847 Brain_Amygdala 0.537970397 Brain_Substantia_nigra 10.40447491 0.002325084 0.409499146 -2.053791801 -0.615885038 -1.091273682 0.363609695 3 0 0.972491774
BBOF1 ENSG00000119636.15 0.157771403 9 4 0.187375902 Brain_Cerebellar_Hemisphere 0.630526859 Brain_Nucleus_accumbens_basal_ganglia 6.25760275 0.020035774 0.291937209 -1.199519464 1.318381706 0.717462942 0.770340163 4 0 0.972491774
MAPK1 ENSG00000100030.14 0.157882009 2 2 0.057617359 Brain_Nucleus_accumbens_basal_ganglia 0.854414252 Brain_Hippocampus 1.05856939 0.94143061 0.94143061 0.183489123 1.898597947 1.041043535 1.21276508 2 0 0.972491774
LKAAEAR1 ENSG00000171695.10 0.157885998 4 4 0.036187864 Brain_Nucleus_accumbens_basal_ganglia 0.5578333 Brain_Caudate_basal_ganglia 1.192809462 0.789552104 0.789552104 -2.094810247 1.057477093 -0.161956872 1.494073979 4 0 0.972491774
SYCE3 ENSG00000217442.3 0.157918227 12 3 0.025137934 Brain_Putamen_basal_ganglia 0.739666347 Brain_Amygdala 9.791099082 1.18E-17 0.356442074 0.332295222 2.239276446 1.834819956 0.557624757 3 0 0.972491774
CCDC50 ENSG00000152492.13 0.157962948 3 3 0.087390906 Brain_Hippocampus 0.205546037 Brain_Caudate_basal_ganglia 1.757120167 0.246128862 0.246128862 -1.413812245 1.709324241 -0.323465321 1.761999992 3 0 0.972491774
CSNK1E ENSG00000213923.11 0.157978159 3 3 0.087383579 Brain_Hippocampus 0.994351347 Brain_Nucleus_accumbens_basal_ganglia 1.275422042 0.739592684 0.739592684 -1.360795975 1.709363817 0.118549146 1.538112279 3 0 0.972491774
HAUS4 ENSG00000092036.18 0.158031423 13 1 0.117743045 Brain_Hypothalamus 0.237822449 Brain_Amygdala 12.3665029 0.000789305 12.3665029 -1.564317412 -1.180447074 -1.377515108 0.105827191 1 0 0.972491774
RMDN3 ENSG00000137824.15 0.158039952 10 3 0.027349305 Brain_Substantia_nigra 0.835992713 Brain_Cerebellum 7.885959685 0.00161022 0.310186144 -0.207021954 2.20649578 1.780425446 0.728097619 3 0 0.972491774
SCIMP ENSG00000161929.14 0.15811572 4 4 0.042535871 Brain_Nucleus_accumbens_basal_ganglia 0.719898884 Brain_Cerebellum 1.691594018 0.566936308 0.566936308 -2.028238907 1.146954767 -0.152828079 1.397559438 4 0 0.972491774
LINC01740 ENSG00000228067.1 0.15835619 4 2 0.048841076 Brain_Putamen_basal_ganglia 0.094741246 Brain_Amygdala 3.742693875 0.04721698 0.161635062 -1.969976391 -1.670900941 -1.85625244 0.129133432 2 0 0.972491774
LITAF ENSG00000189067.12 0.158373569 3 3 0.044472752 Brain_Cerebellum 0.766830321 Brain_Cerebellar_Hemisphere 1.444746197 0.635433056 0.635433056 -0.296523503 2.009607452 0.802537359 1.156853271 3 0 0.972491774
RP11-85E16.1 ENSG00000272984.1 0.158549426 1 1 0.158549426 Brain_Anterior_cingulate_cortex_BA24 0.158549426 Brain_Anterior_cingulate_cortex_BA24 1 1 1 -1.409967003

-1.409967003 -1.409967003 NA 1 0 0.972491774
MVB12A ENSG00000141971.12 0.158584062 6 5 0.284600716
Brain_Putamen_basal_ganglia 0.968113099 Brain_Caudate_basal_ganglia
4.114555035 0.055790615 0.209148684 -1.070041308 0.963851292
-0.245617314 0.689165159 5 0 0.972491774
DHODH ENSG00000102967.11 0.158622833 12 4 0.025726358 Brain_Amygdala
0.600016116 Brain_Caudate_basal_ganglia 9.633607806 0.002984373
0.335127185 -2.230317711 -0.524377337 -1.418460247 0.548554788
4 0 0.972491774
HGSNAT ENSG00000165102.14 0.158777963 1 1 0.158777963
Brain_Caudate_basal_ganglia 0.158777963 Brain_Caudate_basal_ganglia 1 1
1 1.409193486 1.409193486 1.409193486 NA 1 0
0.972491774
RP4-737E23.2 ENSG00000230387.2 0.158836203 6 5 0.072304938
Brain_Frontal_Cortex_BA9 0.857703366 Brain_Nucleus_accumbens_basal_ganglia
2.842137532 0.072165928 0.215286905 -1.797193298 -0.179298447
-1.261799069 0.666258762 5 0 0.972491774
AFTPH ENSG00000119844.15 0.15887089 1 1 0.15887089 Brain_Caudate_basal_ganglia
0.15887089 Brain_Caudate_basal_ganglia 1 1 1 1.408879203
1.408879203 1.408879203 NA 1 0 0.972491774
PLCB2 ENSG00000137841.11 0.158878553 1 1 0.158878553
Brain_Caudate_basal_ganglia 0.158878553 Brain_Caudate_basal_ganglia 1 1
1 -1.408853292 -1.408853292 -1.408853292 NA 1 0
0.972491774
BORCS8 ENSG00000254901.7 0.15895361 8 4 0.082726011
Brain_Cerebellar_Hemisphere 0.683395729 Brain_Substantia_nigra
6.317686711 0.011812242 0.262154147 -0.407833757 1.735083533
0.984287427 0.676042775 4 0 0.972491774
SLC24A3 ENSG00000185052.11 0.159021254 1 1 0.159021254
Brain_Putamen_basal_ganglia 0.159021254 Brain_Putamen_basal_ganglia 1 1
1 1.408370958 1.408370958 1.408370958 NA 1 0
0.972491774
ZDBF2 ENSG00000204186.7 0.159239043 4 3 0.027131897
Brain_Putamen_basal_ganglia 0.11967874 Brain_Hippocampus 3.007132454
0.067715926 0.348012216 1.55612345 2.209614955 1.897949597
0.309492442 3 0 0.972491774
COX7A1 ENSG00000161281.10 0.159474111 10 8 0.018702786
Brain_Cerebellar_Hemisphere 0.550972219 Brain_Frontal_Cortex_BA9 5.38012229
0.091105319 0.227750896 0.596303911 2.351402591 1.139627784
0.587011262 8 0 0.972491774
PRSS48 ENSG00000189099.11 0.159560015 1 1 0.159560015
Brain_Putamen_basal_ganglia 0.159560015 Brain_Putamen_basal_ganglia 1 1
1 1.406552875 1.406552875 1.406552875 NA 1 0
0.972491774
RPUSD2 ENSG00000166133.17 0.159608208 1 1 0.159608208
Brain_Cerebellar_Hemisphere 0.159608208 Brain_Cerebellar_Hemisphere 1 1
1 -1.406390474 -1.406390474 -1.406390474 NA 1 0
0.972491774
WHAMM ENSG00000156232.7 0.159720704 4 4 0.052208823 Brain_Cortex
0.842941609 Brain_Hippocampus 1.595128861 0.461440158
0.461440158 -0.19813223 1.94140788 1.036179676 0.955174105 4
0 0.972491774
ADH5 ENSG00000197894.10 0.159758349 9 6 0.004539593
Brain_Spinal_cord_cervical_c-1 0.338829607 Brain_Cortex 5.045075156
0.007879834 0.189295101 0.956480351 2.83800883 2.039446711
0.724023053 6 0 0.972491774
RP5-1125A11.6 ENSG00000275784.1 0.159832621 2 2 0.110660894
Brain_Cortex 0.237480338 Brain_Cerebellum 1.078415112 0.921584888
0.921584888 -1.595229635 1.181308132 -0.206960752 1.963308683
2 0 0.972491774
ERGIC2 ENSG00000087502.17 0.159970995 1 1 0.159970995
Brain_Hypothalamus 0.159970995 Brain_Hypothalamus 1 1 1

1.405169116 1.405169116 1.405169116 NA 1 0 0.972491774
ZNF77 ENSG00000175691.8 0.160032151 1 1 0.160032151
Brain_Substantia_nigra 0.160032151 Brain_Substantia_nigra 1 1
1 -1.404963436 -1.404963436 -1.404963436 NA 1 0
0.972491774
RHBDL1 ENSG00000103269.13 0.160186836 1 1 0.160186836
Brain_Spinal_cord_cervical_c-1 0.160186836 Brain_Spinal_cord_cervical_c-1
1 1 1 -1.404443464 -1.404443464 -1.404443464 NA 1 0
0.972491774
STRN4 ENSG00000090372.14 0.160310696 4 2 0.062926389 Brain_Cortex
0.079046982 Brain_Spinal_cord_cervical_c-1 3.596766637 0.049511343
0.263898896 -1.859711251 -1.75624267 -1.801550463 0.04290423 2
0 0.972491774
RP11-13K12.2 ENSG00000267665.1 0.160390308 5 5 0.163404388
Brain_Nucleus_accumbens_basal_ganglia 0.865752558
Brain_Spinal_cord_cervical_c-1 1.788270447 0.235231041 0.235231041
-0.503965515 1.393712671 0.112604228 0.765495328 5 0
0.972491774
SNRPA1 ENSG00000131876.16 0.160458604 1 1 0.160458604
Brain_Caudate_basal_ganglia 0.160458604 Brain_Caudate_basal_ganglia 1 1
1 1.403530833 1.403530833 1.403530833 NA 1 0
0.972491774
SPON1 ENSG00000262655.3 0.160542024 3 3 0.50299175 Brain_Cerebellum
0.615672178 Brain_Cerebellar_Hemisphere 1.864870016 0.143085582
0.143085582 -0.669789873 0.50199338 -0.275561337 0.673404201 3
0 0.972491774
EIF3CL ENSG00000205609.12 0.160553485 1 1 0.160553485
Brain_Amygdala 0.160553485 Brain_Amygdala 1 1 1 1.403212488
1.403212488 1.403212488 NA 1 0 0.972491774
RP11-108L7.14 ENSG00000273476.1 0.160553814 1 1 0.160553814
Brain_Hypothalamus 0.160553814 Brain_Hypothalamus 1 1 1
1.403211384 1.403211384 1.403211384 NA 1 0 0.972491774
CAPZA1 ENSG00000116489.12 0.160585054 6 5 0.058717167
Brain_Nucleus_accumbens_basal_ganglia 0.883793658
Brain_Spinal_cord_cervical_c-1 4.184774928 0.074724313 0.163522316
-1.8903051 0.347587165 -0.476055255 0.797343547 5 0
0.972491774
ADCY3 ENSG00000138031.14 0.160593319 6 3 0.133568826 Brain_Cortex
0.826260719 Brain_Spinal_cord_cervical_c-1 5.117478209 0.060244001
0.197817928 -1.500175972 0.502874637 -0.643262073 0.745762704
3 0 0.972491774
SLC10A5 ENSG00000253598.1 0.160627353 2 2 0.294104002
Brain_Cerebellar_Hemisphere 0.77126232 Brain_Cerebellum 1.742965861
0.257034139 0.257034139 -1.04916105 0.290724124 -0.379218463
0.947441893 2 0 0.972491774
TRIM63 ENSG00000158022.6 0.160645875 3 3 0.254736877
Brain_Substantia_nigra 0.583196713 Brain_Cerebellar_Hemisphere
2.506732144 0.124717592 0.124717592 -1.138919152 0.54872132
-0.386816057 0.858643519 3 0 0.972491774
PIGM ENSG00000143315.6 0.160651578 1 1 0.160651578
Brain_Spinal_cord_cervical_c-1 0.160651578 Brain_Spinal_cord_cervical_c-1
1 1 1 -1.402883511 -1.402883511 -1.402883511 NA 1 0
0.972491774
UBXN2A ENSG00000173960.13 0.160654268 2 2 0.057735635
Brain_Cerebellum 0.877031767 Brain_Hippocampus 1.042755464
0.957244536 0.957244536 -0.154733058 1.897699833 0.871483387
1.451289216 2 0 0.972491774
PPM1K ENSG00000163644.14 0.160657677 3 3 0.129398142
Brain_Substantia_nigra 0.504064507 Brain_Spinal_cord_cervical_c-1
1.090129567 0.927471771 0.927471771 -1.506986016 1.516479532
-0.219538239 1.56084825 3 0 0.972491774
BSN-AS2 ENSG00000226913.1 0.16067837 8 1 0.084852143 Brain_Cortex

0.284310597 Brain_Nucleus_accumbens_basal_ganglia 7.503597808
0.006711218 7.503597808 1.070686095 1.723201223 1.358809206
0.244473807 1 0 0.972491774
OGFRL1 ENSG00000119900.7 0.160762659 3 2 0.063235071
Brain_Cerebellum 0.850287548 Brain_Nucleus_accumbens_basal_ganglia
2.221684745 0.002950839 0.775364416 -1.857535042 -0.188751549
-1.283826044 0.948723392 2 0 0.972491774
FOXD4 ENSG00000170122.5 0.160831972 5 5 0.268145843 Brain_Amygdala
0.876921782 Brain_Cerebellar_Hemisphere 3.510848069 0.182327472
0.182327472 -0.58111148 1.107342577 0.294996859 0.772590861
5 0 0.972491774
PCDHGB2 ENSG00000253910.2 0.160856729 4 4 0.012619248
Brain_Cortex 0.590834224 Brain_Cerebellum 2.193569589 0.239780895
0.239780895 0.537627524 2.494337529 1.625978171 0.847574696
4 0 0.972491774
C8orf37 ENSG00000156172.5 0.160952088 2 2 0.103186655
Brain_Caudate_basal_ganglia 0.95570105 Brain_Nucleus_accumbens_basal_ganglia
1.54714587 0.45285413 0.45285413 -1.62959721 -0.055549055 -0.842573132
1.113020124 2 0 0.972491774
MRPL49 ENSG00000149792.8 0.161011631 2 2 0.067878929
Brain_Frontal_Cortex_BA9 0.796702482 Brain_Cortex 1.421594146
0.578405854 0.578405854 0.257617035 1.825809716 1.041713375
1.10887968 2 0 0.972491774
FGL1 ENSG00000104760.16 0.161287478 2 2 0.083815483
Brain_Putamen_basal_ganglia 0.191031346 Brain_Nucleus_accumbens_basal_ganglia
1.309508823 0.690491177 0.690491177 -1.72896431 -1.307534204
-1.518249257 0.297996086 2 0 0.972491774
DCBLD1 ENSG00000164465.18 0.161346489 2 2 0.065153178
Brain_Frontal_Cortex_BA9 0.10543963 Brain_Cortex 1.680252281
0.319747719 0.319747719 -1.844205629 -1.619035476 -1.731620552
0.159219342 2 0 0.972491774
RXRB ENSG00000204231.10 0.161385446 3 3 0.342877546 Brain_Cerebellum
0.638051171 Brain_Caudate_basal_ganglia 1.786514869 0.348709716
0.348709716 -0.852974326 0.948494944 0.188648649 0.933204938
3 0 0.972491774
ZNF133 ENSG00000125846.15 0.161423313 1 1 0.161423313
Brain_Cerebellum 0.161423313 Brain_Cerebellum 1 1 1 1.400300628
1.400300628 1.400300628 NA 1 0 0.972491774
DPT ENSG00000143196.4 0.16143117 1 1 0.16143117 Brain_Hypothalamus
0.16143117 Brain_Hypothalamus 1 1 1 1.400274381 1.400274381
1.400274381 NA 1 0 0.972491774
RP11-88E10.4 ENSG00000277159.1 0.161451605 9 4 0.009664281
Brain_Frontal_Cortex_BA9 0.702796726 Brain_Anterior_cingulate_cortex_BA24
6.911833465 0.002612227 0.307187548 0.381547912 2.587615422
2.03755032 0.697459423 4 0 0.972491774
CARD9 ENSG00000187796.13 0.161471939 2 2 0.116299933 Brain_Cortex
0.256523815 Brain_Anterior_cingulate_cortex_BA24 1.031015502
0.968984498 0.968984498 1.134645657 1.570495275 1.352570466
0.308192221 2 0 0.972491774
AP001432.14 ENSG00000242553.1 0.161519037 1 1 0.161519037
Brain_Anterior_cingulate_cortex_BA24 0.161519037
Brain_Anterior_cingulate_cortex_BA24 1 1 1 -1.399980904
-1.399980904 -1.399980904 NA 1 0 0.972491774
TSEN54 ENSG00000182173.12 0.161628875 4 4 0.037851656
Brain_Hypothalamus 0.385121998 Brain_Cerebellar_Hemisphere 2.583570964
0.189788882 0.189788882 -1.069633132 2.076457516 0.789331633
1.336841664 4 0 0.972491774
TMEM204 ENSG00000131634.13 0.161733202 1 1 0.161733202
Brain_Frontal_Cortex_BA9 0.161733202 Brain_Frontal_Cortex_BA9 1 1
1 1.399266099 1.399266099 1.399266099 NA 1 0
0.972491774
AQP5 ENSG00000161798.6 0.161739599 13 7 0.021455571

Brain_Hypothalamus 0.853763305 Brain_Spinal_cord_cervical_c-1
7.780472862 0.005652473 0.279092918 0.184318879 2.299870268
1.170170537 0.724016625 7 0 0.972491774
RP1-149A16.17 ENSG00000241954.1 0.161759303 5 3 0.043617746
Brain_Nucleus_accumbens_basal_ganglia 0.881442555 Brain_Caudate_basal_ganglia
3.582796492 0.076584617 0.450940409 -2.017745766 0.149140771
-1.039695408 0.882548335 3 0 0.972491774
RP11-238K6.1 ENSG00000253288.1 0.161773257 4 4 0.26899737
Brain_Anterior_cingulate_cortex_BA24 0.658771403 Brain_Hippocampus
1.935252177 0.256954574 0.256954574 -0.851593259 1.10537445
-0.22910194 0.906083073 4 0 0.972491774
GOLGA6L10 ENSG00000278662.4 0.161839115 13 2 0.013543412
Brain_Caudate_basal_ganglia 0.293359908 Brain_Putamen_basal_ganglia
11.10774795 0.004280452 0.56441519 1.050779423 2.469150832
1.647164992 0.379398924 2 0 0.972491774
CPSF1 ENSG00000071894.16 0.161879401 13 1 0.113908725 Brain_Amygdala
0.234558786 Brain_Anterior_cingulate_cortex_BA24 12.63303091
0.000872659 12.63303091 -1.580865802 -1.188697345 -1.380279156
0.093594545 1 0 0.972491774
TMEM8C ENSG00000187616.4 0.161932454 1 1 0.161932454
Brain_Caudate_basal_ganglia 0.161932454 Brain_Caudate_basal_ganglia 1 1
1 -1.398601708 -1.398601708 -1.398601708 NA 1 0
0.972491774
RABGAP1 ENSG00000011454.16 0.161965048 3 2 0.071144203
Brain_Frontal_Cortex_BA9 0.914383519 Brain_Caudate_basal_ganglia 2.35409829
0.029564973 0.616336737 0.1075111 1.80455591 0.864794478
0.863112942 2 0 0.972491774
COA6 ENSG00000168275.14 0.162011443 8 5 0.051128956
Brain_Nucleus_accumbens_basal_ganglia 0.95652885 Brain_Frontal_Cortex_BA9
5.702229713 0.018033945 0.259365191 -1.05960047 1.950395852
0.108478626 0.915428954 5 0 0.972491774
GLT1D1 ENSG00000151948.11 0.162125743 4 4 0.029373109
Brain_Frontal_Cortex_BA9 0.527206351 Brain_Caudate_basal_ganglia
1.936011491 0.209989782 0.209989782 -2.178442496 -0.632276294
-1.125327845 0.718527879 4 0 0.972491774
CTTN ENSG00000085733.15 0.162130987 3 3 0.094116193 Brain_Cerebellum
0.558457275 Brain_Cerebellar_Hemisphere 2.473014587 0.176953971
0.176953971 0.585134511 1.674073308 1.180757255 0.551631145
3 0 0.972491774
PPP1R21 ENSG00000162869.15 0.162179004 9 6 0.108646726
Brain_Cerebellar_Hemisphere 0.960625998 Brain_Nucleus_accumbens_basal_ganglia
5.682654896 0.02767101 0.208022899 -1.604305514 1.452149046
-0.212155549 0.846174088 6 0 0.972491774
SLC18B1 ENSG00000146409.10 0.162253124 1 1 0.162253124
Brain_Caudate_basal_ganglia 0.162253124 Brain_Caudate_basal_ganglia 1 1
1 1.397533752 1.397533752 1.397533752 NA 1 0
0.972491774
UBR3 ENSG00000144357.16 0.162305669 1 1 0.162305669
Brain_Spinal_cord_cervical_c-1 0.162305669 Brain_Spinal_cord_cervical_c-1
1 1 1 -1.39735891 -1.39735891 -1.39735891 NA 1 0
0.972491774
PRDM4 ENSG00000110851.11 0.162307048 4 3 0.107166861 Brain_Cerebellum
0.548970482 Brain_Frontal_Cortex_BA9 2.737638671 0.00274357
0.213900235 -1.611059014 -0.599303549 -1.354486271 0.503486538
3 0 0.972491774
PPWD1 ENSG00000113593.11 0.162354792 13 2 0.067633755 Brain_Cortex
0.645058384 Brain_Substantia_nigra 10.93050483 0.006461624
1.124464754 0.460637943 1.82743923 1.225082482 0.457103949 2
0 0.972491774
TRIM21 ENSG00000132109.9 0.162510653 3 3 0.031548871
Brain_Nucleus_accumbens_basal_ganglia 0.815650858 Brain_Cerebellum
1.585385528 0.414744538 0.414744538 -0.890882205 2.150080204

0.342018544	1.599990827	3	0	0.972491774
DNPEP	ENSG00000123992.18	0.162549679	6	3	0.025705134
Brain_Nucleus_accumbens_basal_ganglia	0.366676741	Brain_Amygdala
4.515489027	0.048172179	0.266158236	0.902715815	2.230637746
1.682997533	0.46250574	3	0	0.972491774
CENPV	ENSG00000166582.9	0.162572976	13	1	0.1275609
Brain_Anterior_cingulate_cortex_BA24	0.214269435	Brain_Putamen_basal_ganglia
12.58167177	2.29E-06	12.58167177	-1.523791115	-1.241910909
-1.37457501	0.070878642	1	0	0.972491774
TRIM72	ENSG00000177238.13	0.162669633	9	4	0.015374356
Brain_Nucleus_accumbens_basal_ganglia	0.535283041	Brain_Amygdala
6.223517118	0.011065213	0.328593699	-2.423438508	-0.619961642
-1.839288794	0.536135516	4	0	0.972491774
STAMBP	ENSG00000124356.15	0.162735144	1	1	0.162735144
Brain_Putamen_basal_ganglia	0.162735144	Brain_Putamen_basal_ganglia	1	1
1	1.395931434	1.395931434	1.395931434	NA	1	0
0.972491774
HBS1L	ENSG00000112339.14	0.162768481	12	2	0.058741319
Brain_Cerebellar_Hemisphere	0.987340283	Brain_Caudate_basal_ganglia
10.33111396	8.58E-06	1.004957338	-1.890124441	-0.015867268
-0.670254137	0.476188404	2	0	0.972491774
KIF26A	ENSG00000066735.14	0.16280391	2	2	0.060066843
Brain_Caudate_basal_ganglia	0.698257337	Brain_Hippocampus	1.042507554
0.957492446	0.957492446	-1.880302645	-0.387673949	-1.133988297
1.055447873	2	0	0.972491774
RP11-560I19.1	ENSG00000234418.1	0.162858947	2	2	0.058529108
Brain_Cortex	0.244116706	Brain_Spinal_cord_cervical_c-1	1.513087673
0.486912327	0.486912327	-1.89171392	-1.164758638	-1.528236279
0.51403501	2	0	0.972491774
PRR29	ENSG00000224383.7	0.163080065	1	1	0.163080065
Brain_Nucleus_accumbens_basal_ganglia	0.163080065
Brain_Nucleus_accumbens_basal_ganglia	1	1	1	1.394787052
1.394787052	1.394787052	NA	1	0	0.972491774
TIMM50	ENSG00000105197.10	0.163104675	1	1	0.163104675
Brain_Substantia_nigra	0.163104675	Brain_Substantia_nigra	1	1
1	1.39470547	1.39470547	1.39470547	NA	1	0	0.972491774
HMGCLL1	ENSG00000146151.12	0.163127997	8	4	0.016079316
Brain_Hypothalamus	0.507665	Brain_Cerebellar_Hemisphere	6.540175915
0.028588031	0.223915285	-2.407110214	-0.66247789	-1.51171619
0.567116939	4	0	0.972491774
UBOX5	ENSG00000185019.16	0.163151309	6	4	0.019492897
Brain_Hippocampus	0.340178115	Brain_Cortex	4.867309651	0.07334389
0.230948925	-2.33596823	-0.953813382	-1.631216028	0.562015318
4	0	0.972491774
RP11-347C12.10	ENSG00000260219.2	0.163192646	2	2	0.05713908
Brain_Hippocampus	0.831427297	Brain_Hypothalamus	1.155692469
0.844307531	0.844307531	-1.902245402	-0.212871366	-1.057558384
1.194567837	2	0	0.972491774
PRKAG2	ENSG00000106617.13	0.163315298	3	3	0.085419506
Brain_Cerebellum	0.579818726	Brain_Hippocampus	1.098009918
0.857259247	0.857259247	-1.720071167	1.144282985	-0.376479235
1.440372577	3	0	0.972491774
CCDC13	ENSG00000244607.5	0.163325096	4	4	0.254296199
Brain_Cortex	0.994310089	Brain_Putamen_basal_ganglia	1.965207377
0.108464366	0.108464366	-1.139976241	0.968130586	-0.219833017
0.922721659	4	0	0.972491774
ACSL6	ENSG00000164398.12	0.163382899	9	8	0.100272647	Brain_Cerebellum
0.932831671	Brain_Cerebellar_Hemisphere	3.815519072	0.060116084
0.199752078	-1.182278641	1.643533272	0.092505718	0.982809123
8	0	0.972491774
IQCE	ENSG00000106012.17	0.16348006	4	4	0.158940427
Brain_Cerebellar_Hemisphere	0.952857571	Brain_Hippocampus	1.462657054

0.539029009 0.539029009 -1.408644115 1.203326499 0.11890738
1.144102195 4 0 0.972491774
CYP4B1 ENSG00000142973.13 0.163622464 1 1 0.163622464
Brain_Caudate_basal_ganglia 0.163622464 Brain_Caudate_basal_ganglia 1 1
1 1.392991159 1.392991159 1.392991159 NA 1 0
0.972491774
RANBP3L ENSG00000164188.8 0.16365675 1 1 0.16365675
Brain_Nucleus_accumbens_basal_ganglia 0.16365675
Brain_Nucleus_accumbens_basal_ganglia 1 1 1 -1.39287779
-1.39287779 -1.39287779 NA 1 0 0.972491774
RBM23 ENSG00000100461.17 0.163789932 11 2 0.043014067
Brain_Putamen_basal_ganglia 0.192298555 Brain_Spinal_cord_cervical_c-1
9.469016086 0.004894277 0.79292012 1.303809464 2.023573375
1.757361369 0.258875692 2 0 0.972491774
NFATC2 ENSG00000101096.19 0.163810566 2 2 0.058545034
Brain_Caudate_basal_ganglia 0.421764817 Brain_Putamen_basal_ganglia
1.515270185 0.484729815 0.484729815 0.803363238 1.891594462
1.34747885 0.769495678 2 0 0.972491774
KRT2 ENSG00000172867.3 0.163817203 2 2 0.088715015
Brain_Caudate_basal_ganglia 0.338332271 Brain_Putamen_basal_ganglia
1.064885178 0.935114822 0.935114822 -0.95746566 1.702215304
0.372374822 1.880678445 2 0 0.972491774
ARPC5L ENSG00000136950.13 0.163819103 4 1 0.120413502
Brain_Cerebellum 0.322009204 Brain_Putamen_basal_ganglia 3.859323969
0.004392575 3.859323969 -1.553040323 -0.990337458 -1.364205387
0.253383584 1 0 0.972491774
GPBP1 ENSG00000062194.15 0.163837463 1 1 0.163837463
Brain_Hippocampus 0.163837463 Brain_Hippocampus 1 1 1
-1.392280535 -1.392280535 -1.392280535 NA 1 0 0.972491774
LINC01132 ENSG00000227630.2 0.16389963 2 2 0.127165634 Brain_Cortex
0.258255575 Brain_Cerebellar_Hemisphere 1.003509803 0.996490197
0.996490197 -1.130523801 1.525374828 0.197425514 1.878003931
2 0 0.972491774
TUBA3D ENSG00000075886.10 0.163922471 6 5 0.012905508
Brain_Hippocampus 0.950879295 Brain_Frontal_Cortex_BA9 2.935848662
0.067422962 0.127073991 -2.48636622 0.533194715 -0.63548877
1.01960487 5 0 0.972491774
SLC9B2 ENSG00000164038.14 0.163947471 1 1 0.163947471
Brain_Hypothalamus 0.163947471 Brain_Hypothalamus 1 1 1
1.391917206 1.391917206 1.391917206 NA 1 0 0.972491774
PAX8 ENSG00000125618.16 0.163996573 10 2 0.020952042 Brain_Amygdala
0.299436962 Brain_Nucleus_accumbens_basal_ganglia 8.955667582
0.000322046 0.308761505 1.037641559 2.30884756 1.796165591
0.369383327 2 0 0.972491774
ANKRD17 ENSG00000132466.18 0.1640123 1 1 0.1640123 Brain_Hippocampus
0.1640123 Brain_Hippocampus 1 1 1 -1.391703178 -1.391703178
-1.391703178 NA 1 0 0.972491774
IL12B ENSG00000113302.4 0.164014605 1 1 0.164014605 Brain_Cerebellum
0.164014605 Brain_Cerebellum 1 1 1 -1.391695568 -1.391695568
-1.391695568 NA 1 0 0.972491774
PLXNA2 ENSG00000076356.6 0.164173055 1 1 0.164173055
Brain_Substantia_nigra 0.164173055 Brain_Substantia_nigra 1 1
1 1.391172727 1.391172727 1.391172727 NA 1 0
0.972491774
ATG3 ENSG00000144848.10 0.16438443 1 1 0.16438443
Brain_Anterior_cingulate_cortex_BA24 0.16438443
Brain_Anterior_cingulate_cortex_BA24 1 1 1 -1.390475843
-1.390475843 -1.390475843 NA 1 0 0.972491774
FNDC5 ENSG00000160097.16 0.164467546 2 2 0.147161094
Brain_Frontal_Cortex_BA9 0.468568138 Brain_Cerebellum 1.306517534
0.693482466 0.693482466 -0.724810753 1.449632262 0.362410754
1.537563402 2 0 0.972491774

VSIG8 ENSG00000243284.1 0.164472466 7 6 0.185596893
Brain_Putamen_basal_ganglia 0.811543514 Brain_Frontal_Cortex_BA9 2.86622111
0.012111241 0.28919421 -1.323717475 1.16617153 -0.414013727
0.859704838 6 0 0.972491774
ZNF358 ENSG00000198816.6 0.164499548 2 2 0.127620652
Brain_Hypothalamus 0.195957718 Brain_Amygdala 1.108097051
0.891902949 0.891902949 -1.293154241 1.523552037 0.115198898
1.99171211 2 0 0.972491774
NAA38 ENSG00000183011.13 0.16460205 3 3 0.038819752
Brain_Frontal_Cortex_BA9 0.112697322 Brain_Hypothalamus 2.072913537
0.296929245 0.296929245 1.586185122 2.066092439 1.821667122
0.240078623 3 0 0.972491774
MAFTRR ENSG00000261390.5 0.165067223 13 2 0.032696994
Brain_Cerebellum 0.151250253 Brain_Frontal_Cortex_BA9 12.12358596
0.000167693 0.557606028 -2.135784368 -1.43512931 -1.779718718
0.18777485 2 0 0.972491774
EID3 ENSG00000255150.2 0.165139376 3 3 0.189053759 Brain_Cerebellum
0.644943778 Brain_Anterior_cingulate_cortex_BA24 1.665630134
0.346863223 0.346863223 -1.313383487 0.460797664 -0.62958927
0.9544202 3 0 0.972491774
ACSL1 ENSG00000151726.13 0.165174326 3 3 0.030674537
Brain_Anterior_cingulate_cortex_BA24 0.755351892 Brain_Hippocampus
1.402488069 0.609077003 0.609077003 -2.161269383 0.510736412
-0.446314217 1.488528873 3 0 0.972491774
SLC1A4 ENSG00000115902.10 0.165219777 5 5 0.029803984
Brain_Cerebellum 0.794689743 Brain_Putamen_basal_ganglia 2.096463926
0.222588687 0.222588687 -2.172685652 0.983065756 -0.649487437
1.260182312 5 0 0.972491774
AP000255.6 ENSG00000273091.1 0.1652611 2 2 0.059472203
Brain_Caudate_basal_ganglia 0.876250124 Brain_Amygdala 1.034054182
0.965945818 0.965945818 0.155724577 1.8846863 1.020205438
1.222560559 2 0 0.972491774
TUFM ENSG00000178952.10 0.165303447 9 1 0.103166571
Brain_Putamen_basal_ganglia 0.582187398 Brain_Hypothalamus 8.226491105
0.009515852 8.226491105 0.550192426 1.62969218 1.321941078
0.347128591 1 0 0.972491774
RP5-894D12.4 ENSG00000271234.1 0.165414502 2 2 0.08992523
Brain_Nucleus_accumbens_basal_ganglia 0.583755382 Brain_Hippocampus
1.68728956 0.31271044 0.31271044 -1.695792253 -0.547907553 -1.121849903
0.811677055 2 0 0.972491774
TRMT2A ENSG00000099899.14 0.165472843 3 3 0.06916384 Brain_Cerebellum
0.339154438 Brain_Nucleus_accumbens_basal_ganglia 1.442063401
0.644282743 0.644282743 0.955837304 1.81734803 1.282031131
0.467278854 3 0 0.972491774
ADAMTS19 ENSG00000145808.9 0.165491289 3 3 0.078225162
Brain_Cortex 0.90641116 Brain_Amygdala 1.488812713 0.496256422
0.496256422 -0.366550538 1.761078238 0.504031396 1.115220784
3 0 0.972491774
SMUG1 ENSG00000123415.15 0.165572005 1 1 0.165572005
Brain_Nucleus_accumbens_basal_ganglia 0.165572005
Brain_Nucleus_accumbens_basal_ganglia 1 1 1 -1.386573004
-1.386573004 -1.386573004 NA 1 0 0.972491774
PKP1 ENSG00000081277.12 0.165635671 1 1 0.165635671
Brain_Anterior_cingulate_cortex_BA24 0.165635671
Brain_Anterior_cingulate_cortex_BA24 1 1 1 1.386364367
1.386364367 1.386364367 NA 1 0 0.972491774
MMP16 ENSG00000156103.15 0.165700363 2 2 0.060248355 Brain_Cerebellum
0.373125046 Brain_Cerebellar_Hemisphere 1.583833551 0.416166449
0.416166449 0.890634945 1.878971718 1.384803332 0.698859634
2 0 0.972491774
S100A2 ENSG00000196754.10 0.165714097 2 2 0.177281324
Brain_Cerebellar_Hemisphere 0.490562476 Brain_Cerebellum 1.395787635

0.604212365 0.604212365 -0.689414477 1.349173251 0.329879387
1.441499207 2 0 0.972491774
USP38 ENSG00000170185.9 0.165724994 2 2 0.069995291
Brain_Hypothalamus 0.429150029 Brain_Anterior_cingulate_cortex_BA24
1.130870479 0.869129521 0.869129521 0.79064697 1.811941147
1.301294059 0.722164038 2 0 0.972491774
CNPY1 ENSG00000146910.12 0.165748967 2 2 0.105585341
Brain_Hypothalamus 0.279060721 Brain_Cerebellum 1.059645406
0.940354594 0.940354594 -1.61835859 -1.082431763 -1.350395177
0.378957494 2 0 0.972491774
HSDL1 ENSG00000103160.11 0.165773852 4 4 0.124576198 Brain_Cortex
0.780596174 Brain_Cerebellum 2.266262214 0.201160603 0.201160603
-1.235914129 1.535845824 0.268042914 1.143130402 4 0
0.972491774
RASSF2 ENSG00000101265.15 0.166145096 2 2 0.122098301
Brain_Cerebellar_Hemisphere 0.561758486 Brain_Cerebellum 1.300407657
0.699592343 0.699592343 -0.580231542 1.546025951 0.482897205
1.503491091 2 0 0.972491774
ADGRV1 ENSG00000164199.16 0.166320732 4 4 0.057121337
Brain_Cerebellar_Hemisphere 0.299353031 Brain_Cerebellum 2.251306739
0.12259802 0.12259802 -1.446746392 1.902381198 0.640461461
1.447975076 4 0 0.972491774
CYP17A1 ENSG00000148795.6 0.166392613 2 2 0.075060083
Brain_Frontal_Cortex_BA9 0.097459729 Brain_Cortex 1.657264513
0.342735487 0.342735487 1.657295587 1.780097029 1.718696308
0.086833732 2 0 0.972491774
ERVH48-1 ENSG00000233056.2 0.166566781 5 5 0.058122074
Brain_Hypothalamus 0.894400042 Brain_Frontal_Cortex_BA9 2.695224038
0.131407113 0.131407113 -1.033067293 1.894776075 0.14959003
1.260411813 5 0 0.972491774
SP3 ENSG00000172845.14 0.166602036 1 1 0.166602036 Brain_Cortex
0.166602036 Brain_Cortex 1 1 1 -1.383204937 -1.383204937
-1.383204937 NA 1 0 0.972491774
PRR36 ENSG00000183248.11 0.16662749 3 3 0.062831517 Brain_Cerebellum
0.314089719 Brain_Caudate_basal_ganglia 1.824389622 0.175469656
0.175469656 -1.006677623 1.860381874 0.658071717 1.488420316
3 0 0.972491774
CPT1C ENSG00000169169.14 0.166659718 9 9 0.007714256
Brain_Cerebellar_Hemisphere 0.997715039 Brain_Caudate_basal_ganglia
1.411201249 0.673724177 0.673724177 -1.246744574 2.664326906
0.086375433 1.19672127 9 0 0.972491774
OR1F1 ENSG00000168124.2 0.166718197 1 1 0.166718197
Brain_Cerebellar_Hemisphere 0.166718197 Brain_Cerebellar_Hemisphere 1 1
1 -1.38282609 -1.38282609 -1.38282609 NA 1 0
0.972491774
AL022344.7 ENSG00000259869.1 0.1667302 5 4 0.066873081
Brain_Caudate_basal_ganglia 0.599057976 Brain_Nucleus_accumbens_basal_ganglia
3.516827101 0.025904264 0.199563501 -1.832526069 -0.525755674
-1.314738231 0.566524767 4 0 0.972491774
PBLD ENSG00000108187.15 0.166738823 12 2 0.047136841
Brain_Hypothalamus 0.867212714 Brain_Cerebellum 10.04153719 0.00181235
0.875618052 0.167199962 1.985068593 0.850849514 0.496752088
2 0 0.972491774
GNB1L ENSG00000185838.13 0.166786803 9 9 0.006320892
Brain_Hypothalamus 0.958894095 Brain_Anterior_cingulate_cortex_BA24
3.297916735 0.148969788 0.148969788 -2.730653355 0.620345322
-0.653870398 1.023487384 9 0 0.972491774
RIF1 ENSG00000080345.17 0.166796372 11 5 0.135765121
Brain_Hypothalamus 0.938514623 Brain_Cerebellar_Hemisphere 7.718492018
0.008633535 0.266408302 -1.491748378 1.23093135 -0.85622677
0.782933218 5 0 0.972491774
HYPK ENSG00000242028.6 0.166797062 1 1 0.166797062

Brain_Cerebellar_Hemisphere 0.166797062 Brain_Cerebellar_Hemisphere 1 1
1 1.382568993 1.382568993 1.382568993 NA 1 0
0.972491774
LINC01238 ENSG00000237940.3 0.166798699 9 9 0.011970414
Brain_Hypothalamus 0.844824911 Brain_Putamen_basal_ganglia 2.552253434
0.152963299 0.152963299 -1.53359984 2.51301531 0.452847301
1.240667121 9 0 0.972491774
BACE2 ENSG00000182240.15 0.166836939 7 7 0.013008531 Brain_Cortex
0.59118536 Brain_Cerebellum 2.071379629 0.069637998 0.069637998
-2.483535652 1.55130262 0.227483796 1.38054519 7 0 0.972491774
BRAT1 ENSG00000106009.15 0.166972119 3 3 0.024502543
Brain_Nucleus_accumbens_basal_ganglia 0.743845523 Brain_Cerebellar_Hemisphere
1.591608825 0.415682939 0.415682939 -0.326765145 2.24915646
1.064645052 1.300364703 3 0 0.972491774
CTD-2376I4.1 ENSG00000271926.1 0.167026581 1 1 0.167026581
Brain_Hippocampus 0.167026581 Brain_Hippocampus 1 1 1
-1.381821287 -1.381821287 -1.381821287 NA 1 0 0.972491774
CTC-501O10.1 ENSG00000267440.1 0.167231819 1 1 0.167231819
Brain_Cerebellum 0.167231819 Brain_Cerebellum 1 1 1 -1.381153336
-1.381153336 -1.381153336 NA 1 0 0.972491774
INSR ENSG00000171105.13 0.167280604 2 2 0.060515088 Brain_Cortex
0.901689635 Brain_Cerebellum 1.056590276 0.943409724 0.943409724
0.123527202 1.877021929 1.000274566 1.239908012 2 0
0.972491774
LRRC45 ENSG00000169683.7 0.167298067 3 3 0.073892957
Brain_Anterior_cingulate_cortex_BA24 0.788117315 Brain_Putamen_basal_ganglia
1.407275045 0.589968002 0.589968002 -1.787275594 0.499598932
-0.518810948 1.163762997 3 0 0.972491774
ERICH2 ENSG00000204334.7 0.167326523 3 3 0.051385888
Brain_Amygdala 0.314821999 Brain_Substantia_nigra 1.366083843
0.630809027 0.630809027 -1.948243053 1.005155458 -0.68597631
1.522689922 3 0 0.972491774
TPRKB ENSG00000144034.14 0.167461288 1 1 0.167461288
Brain_Spinal_cord_cervical_c-1 0.167461288 Brain_Spinal_cord_cervical_c-1
1 1 1 1.380407255 1.380407255 1.380407255 NA 1 0
0.972491774
C11orf52 ENSG00000149300.9 0.167486523 2 2 0.100909316
Brain_Cerebellar_Hemisphere 0.904536491 Brain_Cerebellum 1.440904499
0.559095501 0.559095501 -0.119932661 1.640461169 0.760264254
1.244786415 2 0 0.972491774
LIPE ENSG00000079435.9 0.167660545 1 1 0.167660545
Brain_Frontal_Cortex_BA9 0.167660545 Brain_Frontal_Cortex_BA9 1 1
1 -1.379760023 -1.379760023 -1.379760023 NA 1 0
0.972491774
CELA2A ENSG00000142615.7 0.167660943 1 1 0.167660943
Brain_Cortex 0.167660943 Brain_Cortex 1 1 1 -1.379758732
-1.379758732 -1.379758732 NA 1 0 0.972491774
FCHO2 ENSG00000157107.13 0.167830813 1 1 0.167830813 Brain_Cerebellum
0.167830813 Brain_Cerebellum 1 1 1 -1.379207413 -1.379207413
-1.379207413 NA 1 0 0.972491774
SLC5A9 ENSG00000117834.12 0.167897225 6 4 0.058708647
Brain_Nucleus_accumbens_basal_ganglia 0.521982505 Brain_Cortex 4.8258799
0.032942828 0.168948336 -0.640292424 1.890368846 1.068664089
0.87695086 4 0 0.972491774
RP1-90G24.11 ENSG00000273325.1 0.16793605 3 3 0.029442312
Brain_Cerebellum 0.124115717 Brain_Cerebellar_Hemisphere 2.36129769
0.188895075 0.188895075 -2.177513013 -1.537725617 -1.760415766
0.361493524 3 0 0.972491774
VEGFA ENSG00000112715.21 0.167946814 1 1 0.167946814
Brain_Substantia_nigra 0.167946814 Brain_Substantia_nigra 1 1
1 1.378831169 1.378831169 1.378831169 NA 1 0
0.972491774

CPA4 ENSG00000128510.10 0.167964488 3 3 0.056355523
Brain_Nucleus_accumbens_basal_ganglia 0.895942369 Brain_Caudate_basal_ganglia
1.816686235 0.195838357 0.195838357 -0.664833838 1.908276236
0.370884527 1.357932609 3 0 0.972491774
NKX2-3 ENSG00000119919.10 0.168016148 1 1 0.168016148
Brain_Nucleus_accumbens_basal_ganglia 0.168016148
Brain_Nucleus_accumbens_basal_ganglia 1 1 1 -1.37860638
-1.37860638 -1.37860638 NA 1 0 0.972491774
KATNA1 ENSG00000186625.13 0.168019534 1 1 0.168019534
Brain_Frontal_Cortex_BA9 0.168019534 Brain_Frontal_Cortex_BA9 1 1
1 1.378595406 1.378595406 1.378595406 NA 1 0
0.972491774
GTF2IRD2B ENSG00000174428.16 0.168081871 2 2 0.06986754
Brain_Frontal_Cortex_BA9 0.484257163 Brain_Cortex 1.09502938 0.90497062
0.90497062 -0.699471908 1.812768463 0.556648277 1.776422202 2
0 0.972491774
ST14 ENSG00000149418.10 0.168093005 3 3 0.069375787
Brain_Cerebellar_Hemisphere 0.848194783 Brain_Cerebellum 1.072220297
0.925781252 0.925781252 0.191422259 1.815964699 1.092940322
0.826849159 3 0 0.972491774
RP6-91H8.5 ENSG00000269945.1 0.168097093 2 2 0.125784003
Brain_Frontal_Cortex_BA9 0.591569758 Brain_Cortex 1.331052257
0.668947743 0.668947743 -0.536562638 1.530940888 0.497189125
1.461945763 2 0 0.972491774
STMND1 ENSG00000230873.8 0.168221074 2 2 0.141605835
Brain_Hypothalamus 0.222238865 Brain_Anterior_cingulate_cortex_BA24
1.024369567 0.975630433 0.975630433 -1.469837259 -1.220596412
-1.345216835 0.176239893 2 0 0.972491774
RP11-521O16.1 ENSG00000260634.1 0.168245359 8 5 0.067435629
Brain_Substantia_nigra 0.611376799 Brain_Frontal_Cortex_BA9
5.184227221 0.002683114 0.200212425 -1.828759601 1.298137633
-0.346842043 0.935741 5 0 0.972491774
STXBP3 ENSG00000116266.10 0.168354922 2 2 0.061229589
Brain_Spinal_cord_cervical_c-1 0.258543806 Brain_Hypothalamus
1.489900244 0.510099756 0.510099756 1.12983963 1.871833881
1.500836756 0.524669167 2 0 0.972491774
JPH2 ENSG00000149596.6 0.168586103 1 1 0.168586103
Brain_Caudate_basal_ganglia 0.168586103 Brain_Caudate_basal_ganglia 1 1
1 1.376761158 1.376761158 1.376761158 NA 1 0
0.972491774
SBNO2 ENSG00000064932.15 0.168591814 2 2 0.076490905 Brain_Cerebellum
0.585590691 Brain_Cerebellar_Hemisphere 1.058563574 0.941436426
0.941436426 -0.545236762 1.771419882 0.61309156 1.638123623 2
0 0.972491774
CHRNA6 ENSG00000147434.8 0.168747203 2 2 0.121052516
Brain_Cerebellum 0.294611129 Brain_Cerebellar_Hemisphere 1.017104901
0.982895099 0.982895099 -1.550370914 1.048059641 -0.251155636
1.837367866 2 0 0.972491774
MRNIP ENSG00000161010.14 0.168760423 12 4 0.022905967
Brain_Cerebellar_Hemisphere 0.740444594 Brain_Putamen_basal_ganglia
8.246775852 0.005165147 0.47480087 -2.274999378 -0.331264645
-1.092341912 0.718774857 4 0 0.972491774
SLFN5 ENSG00000166750.9 0.16900792 13 3 0.025547401
Brain_Anterior_cingulate_cortex_BA24 0.405613621 Brain_Cerebellar_Hemisphere
10.96381695 8.49E-05 0.389332847 0.831637442 2.233023404
1.651229781 0.36545427 3 0 0.972491774
C19orf70 ENSG00000174917.8 0.169098488 1 1 0.169098488
Brain_Caudate_basal_ganglia 0.169098488 Brain_Caudate_basal_ganglia 1 1
1 -1.375106312 -1.375106312 -1.375106312 NA 1 0
0.972491774
NPFFR1 ENSG00000148734.7 0.169115709 2 2 0.218426843
Brain_Cerebellum 0.636155486 Brain_Cerebellar_Hemisphere 1.897101132

0.102898868 0.102898868 0.473080868 1.230722038 0.851901453
0.535733209 2 0 0.972491774
BOC ENSG00000144857.14 0.169292257 8 6 0.012917205 Brain_Cerebellum
0.981851956 Brain_Cortex 3.770121076 0.021913032 0.145572445
-2.486043833 0.659737818 -0.553706627 1.235686164 6 0
0.972491774
ECM2 ENSG00000106823.12 0.169325496 4 4 0.013490934 Brain_Cortex
0.364460429 Brain_Hippocampus 2.736754412 0.212149273
0.212149273 -2.470539706 -0.906898587 -1.597317291 0.680745395
4 0 0.972491774
GALNS ENSG00000141012.12 0.169342926 4 4 0.038946996 Brain_Amygdala
0.288502716 Brain_Nucleus_accumbens_basal_ganglia 2.472733478
0.111376517 0.111376517 -2.064746458 -1.061411977 -1.475641176
0.421909004 4 0 0.972491774
C6orf120 ENSG00000185127.6 0.16945719 3 3 0.037578752
Brain_Putamen_basal_ganglia 0.795283856 Brain_Frontal_Cortex_BA9
1.379025705 0.726602325 0.726602325 -0.577468882 2.079420129
0.587135563 1.358416541 3 0 0.972491774
RP11-645C24.5 ENSG00000260306.1 0.169492822 11 1 0.084871704
Brain_Amygdala 0.337690831 Brain_Caudate_basal_ganglia 10.01414771
0.010292504 10.01414771 -1.723093024 -0.958737839 -1.312133564
0.26062007 1 0 0.972491774
PEBP4 ENSG00000134020.7 0.169582413 12 2 0.185316145
Brain_Substantia_nigra 0.679904972 Brain_Nucleus_accumbens_basal_ganglia
10.44940912 0.007681541 0.598088622 -1.324562968 0.748145443
-0.742203871 0.57415654 2 0 0.972491774
ENOX1-AS1 ENSG00000233821.1 0.169675761 3 3 0.147357469
Brain_Spinal_cord_cervical_c-1 0.789064316
Brain_Nucleus_accumbens_basal_ganglia 1.59789336 0.429653833 0.429653833
-0.559185649 1.448928794 0.207405766 1.085034991 3 0
0.972491774
PKN2 ENSG00000065243.19 0.1697073 2 2 0.138669803 Brain_Hippocampus
0.29476233 Brain_Cerebellar_Hemisphere 1.076178938 0.923821062
0.923821062 -1.480762762 1.047731501 -0.21651563 1.787915439
2 0 0.972491774
SCT ENSG00000070031.3 0.169716366 6 4 0.057866283
Brain_Spinal_cord_cervical_c-1 0.771799586 Brain_Hippocampus 3.5309627
0.017590316 0.481153371 0.290021766 1.896709549 1.283606554
0.534083337 4 0 0.972491774
LINC01730 ENSG00000275491.1 0.169779048 10 9 0.006656262
Brain_Hypothalamus 0.915094377 Brain_Frontal_Cortex_BA9 2.576818978
0.02630227 0.44977505 -2.037945018 2.713569401 0.819854141
1.221316591 9 0 0.972491774
WDR59 ENSG00000103091.14 0.169880167 3 3 0.042792923 Brain_Cerebellum
0.478771654 Brain_Spinal_cord_cervical_c-1 1.880813676 0.146043978
0.146043978 -2.025725488 0.708279581 -1.044879536 1.521856317
3 0 0.972491774
RP11-16E12.1 ENSG00000259448.2 0.16997534 1 1 0.16997534
Brain_Caudate_basal_ganglia 0.16997534 Brain_Caudate_basal_ganglia 1 1 1
1.372283051 1.372283051 1.372283051 NA 1 0 0.972491774
AGBL2 ENSG00000165923.15 0.170093422 2 2 0.122244523 Brain_Cortex
0.662741761 Brain_Cerebellum 1.365067028 0.634932972 0.634932972
-0.436130897 1.545420757 0.55464493 1.401168612 2 0
0.972491774
ZNF48 ENSG00000180035.12 0.170218497 1 1 0.170218497
Brain_Frontal_Cortex_BA9 0.170218497 Brain_Frontal_Cortex_BA9 1 1
1 -1.371502076 -1.371502076 -1.371502076 NA 1 0
0.972491774
RP11-159D12.2 ENSG00000264112.1 0.170307094 1 1 0.170307094
Brain_Spinal_cord_cervical_c-1 0.170307094 Brain_Spinal_cord_cervical_c-1
1 1 1 -1.371217726 -1.371217726 -1.371217726 NA 1 0
0.972491774

MAP3K3 ENSG00000198909.7 0.170435647 7 4 0.0792371 Brain_Cerebellum
0.556440756 Brain_Hypothalamus 5.225407628 0.007016563
0.289466242 -1.75512985 1.185887317 -0.772154335 0.93229638 4
0 0.972491774
RAD23B ENSG00000119318.12 0.17047684 2 2 0.185889993 Brain_Amygdala
0.216575194 Brain_Cerebellar_Hemisphere 1.074052315 0.925947685
0.925947685 -1.322835787 -1.235686293 -1.27926104 0.061623998
2 0 0.972491774
SLC6A6 ENSG00000131389.16 0.170581064 7 7 0.072438518
Brain_Cortex 0.741595507 Brain_Cerebellar_Hemisphere 1.453388629
0.649861607 0.649861607 -1.54928932 1.796352221 0.224189142
1.329353037 7 0 0.972491774
DGKG ENSG00000058866.14 0.170591504 2 2 0.104276843
Brain_Hippocampus 0.36649298 Brain_Cerebellar_Hemisphere 1.027245371
0.972754629 0.972754629 -1.624463879 -0.903062019 -1.263762949
0.510108147 2 0 0.972491774
IFNGR1 ENSG00000027697.13 0.170642751 1 1 0.170642751
Brain_Frontal_Cortex_BA9 0.170642751 Brain_Frontal_Cortex_BA9 1 1
1 1.370141451 1.370141451 1.370141451 NA 1 0
0.972491774
RFT1 ENSG00000163933.9 0.170662245 11 1 0.110873673
Brain_Hypothalamus 0.475315793 Brain_Anterior_cingulate_cortex_BA24
10.36553088 0.000741077 10.36553088 -1.594278491 -0.713856703
-1.322861663 0.252662833 1 0 0.972491774
ANGPTL6 ENSG00000130812.10 0.171096285 3 2 0.197706564
Brain_Hippocampus 0.834591923 Brain_Hypothalamus 2.608657289
0.053889117 0.337453595 -0.208815946 1.288113191 0.481671644
0.755170974 2 0 0.972491774
TRIM65 ENSG00000141569.10 0.171216936 13 2 0.020505836
Brain_Nucleus_accumbens_basal_ganglia 0.41523674 Brain_Substantia_nigra
11.08972753 0.00532892 1.141370574 0.814712774 2.316961366
1.712121833 0.395274367 2 0 0.972491774
PSENEN ENSG00000205155.7 0.171225939 1 1 0.171225939
Brain_Frontal_Cortex_BA9 0.171225939 Brain_Frontal_Cortex_BA9 1 1
1 1.368275238 1.368275238 1.368275238 NA 1 0
0.972491774
LINC00623 ENSG00000226067.6 0.171310903 5 3 0.057932792
Brain_Spinal_cord_cervical_c-1 0.900327875 Brain_Cerebellar_Hemisphere
2.174949477 0.000390344 1.33721551 -1.896206136 0.125247169
-1.119148251 0.845234377 3 0 0.972491774
MIMT1 ENSG00000268654.1 0.171315077 1 1 0.171315077
Brain_Hippocampus 0.171315077 Brain_Hippocampus 1 1 1
-1.367990414 -1.367990414 -1.367990414 NA 1 0 0.972491774
PATE2 ENSG00000196844.8 0.171536368 1 1 0.171536368
Brain_Caudate_basal_ganglia 0.171536368 Brain_Caudate_basal_ganglia 1 1
1 1.367283799 1.367283799 1.367283799 NA 1 0
0.972491774
SCRN3 ENSG00000144306.14 0.171655695 3 3 0.07831716 Brain_Cortex
0.783201928 Brain_Spinal_cord_cervical_c-1 1.04506643 0.9630365 0.9630365
-1.288182735 1.760534873 0.249166976 1.524524862 3 0
0.972491774
PQLC3 ENSG00000162976.12 0.171663504 3 3 0.046375468
Brain_Nucleus_accumbens_basal_ganglia 0.595736557 Brain_Hypothalamus
1.116003728 0.883076273 0.883076273 -1.99195981 0.731196995
-0.597101432 1.36279801 3 0 0.972491774
MXRA8 ENSG00000162576.16 0.171670403 4 4 0.020025608 Brain_Cerebellum
0.185672116 Brain_Hippocampus 3.066980765 0.15545494 0.15545494
1.323491094 2.325867723 1.872928632 0.424958636 4 0
0.972491774
RP1-122P22.4 ENSG00000268628.2 0.171754092 2 2 0.101551363
Brain_Cerebellum 0.324702321 Brain_Hypothalamus 1.041419238
0.958580762 0.958580762 0.984840709 1.637378713 1.311109711

0.461414048 2 0 0.972491774
CLPTM1L ENSG00000049656.13 0.171781644 1 1 0.171781644 Brain_Cerebellar_Hemisphere 0.171781644 Brain_Cerebellar_Hemisphere 1 1 1 -1.366501392 -1.366501392 -1.366501392 NA 1 0 0.972491774
BET1 ENSG00000105829.11 0.171802285 1 1 0.171802285 Brain_Cortex 0.171802285 Brain_Cortex 1 1 1 1.366435589 1.366435589 1.366435589 NA 1 0 0.972491774
LINC01152 ENSG00000256124.5 0.171862138 7 2 0.087866092 Brain_Cerebellar_Hemisphere 0.696554355 Brain_Frontal_Cortex_BA9 5.837853165 6.32E-05 0.981136397 -1.70676311 0.960564332 0.217794321 0.871452649 2 0 0.972491774
DAPK3 ENSG00000167657.13 0.17186246 1 1 0.17186246 Brain_Cortex 0.17186246 Brain_Cortex 1 1 1 1.366243781 1.366243781 1.366243781 NA 1 0 0.972491774
DDB1 ENSG00000167986.13 0.171962839 1 1 0.171962839 Brain_Spinal_cord_cervical_c-1 0.171962839 Brain_Spinal_cord_cervical_c-1 1 1 1 1.365923936 1.365923936 1.365923936 NA 1 0 0.972491774
IL20RB ENSG00000174564.12 0.171963121 3 3 0.040105919 Brain_Substantia_nigra 0.431772424 Brain_Anterior_cingulate_cortex_BA24 1.714108562 0.31037395 0.31037395 -2.05265634 1.532585446 -0.435411056 1.818174766 3 0 0.972491774
RP11-403A3.2 ENSG00000276269.1 0.171972201 5 4 0.025329293 Brain_Anterior_cingulate_cortex_BA24 0.547159526 Brain_Caudate_basal_ganglia 3.833276472 0.021982729 0.146426681 -2.236343285 -0.602021951 -1.622875936 0.659260133 4 0 0.972491774
AJ011932.1 ENSG00000274248.1 0.172017832 2 2 0.063810733 Brain_Anterior_cingulate_cortex_BA24 0.090493137 Brain_Hypothalamus 1.824331532 0.175668468 0.175668468 -1.85349995 -1.69280213 -1.77315104 0.113630518 2 0 0.972491774
GLRB ENSG00000109738.10 0.172041725 2 2 0.06228538 Brain_Amygdala 0.801695962 Brain_Cerebellar_Hemisphere 1.02149464 0.97850536 0.97850536 -0.251152817 1.864258727 0.806552955 1.495821847 2 0 0.972491774
LINC01785 ENSG00000268038.1 0.172246426 5 5 0.073512373 Brain_Putamen_basal_ganglia 0.799792365 Brain_Anterior_cingulate_cortex_BA24 2.918908195 0.209608788 0.209608788 -1.78963646 0.950966206 -0.612697073 1.029239749 5 0 0.972491774
RP11-277B15.3 ENSG00000272205.1 0.17237882 1 1 0.17237882 Brain_Nucleus_accumbens_basal_ganglia 0.17237882 Brain_Nucleus_accumbens_basal_ganglia 1 1 1 -1.364599952 -1.364599952 -1.364599952 NA 1 0 0.972491774
MICU3 ENSG00000155970.11 0.172383958 2 2 0.172751643 Brain_Frontal_Cortex_BA9 0.574510142 Brain_Hypothalamus 1.437326457 0.562673543 0.562673543 -1.36341536 0.56142163 -0.400996865 1.361065288 2 0 0.972491774
PDE1B ENSG00000123360.11 0.172399785 4 3 0.262039779 Brain_Cerebellar_Hemisphere 0.929350787 Brain_Hippocampus 2.870256874 0.025597621 0.10295167 -1.121583009 -0.088661681 -0.63428775 0.539841512 3 0 0.972491774
ITGB8 ENSG00000105855.9 0.172504142 1 1 0.172504142 Brain_Hippocampus 0.172504142 Brain_Hippocampus 1 1 1 1.364201546 1.364201546 1.364201546 NA 1 0 0.972491774
GDNF ENSG00000168621.14 0.172594329 2 2 0.09387092 Brain_Cerebellum 0.994600716 Brain_Cerebellar_Hemisphere 1.445315932 0.554684068 0.554684068 -0.00676705 1.675322772 0.834277861 1.18941712 2 0 0.972491774
TM6SF2 ENSG00000213996.12 0.172649571 2 1 0.154099131 Brain_Cerebellum 0.207718491 Brain_Cerebellar_Hemisphere 1.938280811 0.061719189 1.938280811 1.259863824 1.425200917 1.342532371 0.11691098 1 0 0.972491774

ATP23 ENSG00000166896.7 0.172703073 13 1 0.042158808
Brain_Substantia_nigra 0.484840398 Brain_Cortex 12.37439694
1.85E-05 12.37439694 -2.031949107 -0.698538647 -1.333047478
0.288632885 1 0 0.972491774
PMFBP1 ENSG00000118557.15 0.172800097 2 2 0.066811473
Brain_Cortex 0.579989952 Brain_Spinal_cord_cervical_c-1 1.090386391
0.909613609 0.909613609 -1.832940142 0.553399397 -0.639770373
1.687396871 2 0 0.972491774
RP11-360I2.1 ENSG00000272130.1 0.172802032 11 10 0.055814709
Brain_Substantia_nigra 0.96440705 Brain_Hippocampus 3.15494573
0.084783387 0.186336573 -1.912479579 0.708787453 -0.511798284
0.811214715 10 0 0.972491774
RPS15A ENSG00000134419.15 0.172813599 2 2 0.078973415
Brain_Spinal_cord_cervical_c-1 0.526538572 Brain_Putamen_basal_ganglia
1.010658098 0.989341902 0.989341902 -1.756673861 0.633298745
-0.561687558 1.689965837 2 0 0.972491774
NICN1 ENSG00000145029.13 0.173030868 6 5 0.099722034
Brain_Frontal_Cortex_BA9 0.758471885 Brain_Cerebellum 3.907568414
0.078965224 0.199361656 -0.936005563 1.646202698 0.529645428
1.012306334 5 0 0.972491774
TWSG1 ENSG00000128791.11 0.173248174 10 3 0.186562703
Brain_Hippocampus 0.926558043 Brain_Hypothalamus 8.568688302
0.004632013 0.307626194 -1.32081606 0.400098414 -0.230229078
0.606398874 3 0 0.972491774
ATP2C2 ENSG00000064270.12 0.173318629 3 3 0.108062329
Brain_Nucleus_accumbens_basal_ganglia 0.362200607 Brain_Frontal_Cortex_BA9
1.749520401 0.294201806 0.294201806 -1.350521591 1.606963711
0.389207331 1.546292769 3 0 0.972491774
PLEKHG4B ENSG00000153404.14 0.173482433 1 1 0.173482433
Brain_Anterior_cingulate_cortex_BA24 0.173482433
Brain_Anterior_cingulate_cortex_BA24 1 1 1 -1.361098915
-1.361098915 -1.361098915 NA 1 0 0.972491774
RSBN1L ENSG00000187257.15 0.173485076 3 3 0.101896192
Brain_Hypothalamus 0.157327635 Brain_Cerebellum 1.901813528
0.098173662 0.098173662 -1.514296875 1.635729599 -0.430894665
1.790449916 3 0 0.972491774
RP11-953B20.1 ENSG00000261710.1 0.173515102 1 1 0.173515102
Brain_Caudate_basal_ganglia 0.173515102 Brain_Caudate_basal_ganglia 1 1
1 -1.36099553 -1.36099553 -1.36099553 NA 1 0
0.972491774
ITGB1 ENSG00000150093.18 0.173646385 1 1 0.173646385
Brain_Cerebellar_Hemisphere 0.173646385 Brain_Cerebellar_Hemisphere 1 1
1 1.360580224 1.360580224 1.360580224 NA 1 0
0.972491774
DDAH1 ENSG00000153904.18 0.173712402 1 1 0.173712402
Brain_Cerebellar_Hemisphere 0.173712402 Brain_Cerebellar_Hemisphere 1 1
1 -1.360371469 -1.360371469 -1.360371469 NA 1 0
0.972491774
TTLL11 ENSG00000175764.14 0.173802015 1 1 0.173802015
Brain_Cerebellum 0.173802015 Brain_Cerebellum 1 1 1 1.3600882
1.3600882 1.3600882 NA 1 0 0.972491774
KDM3A ENSG00000115548.16 0.173928363 3 3 0.101727731 Brain_Cortex
0.486543902 Brain_Cerebellum 1.740950888 0.272321363 0.272321363
0.695816264 1.636534692 1.246880694 0.490691131 3 0
0.972491774
A1BG ENSG00000121410.11 0.173953952 2 2 0.16096179 Brain_Amygdala
0.167627764 Brain_Nucleus_accumbens_basal_ganglia 1.1061561 0.8938439
0.8938439 1.379866464 1.401844153 1.390855309 0.015540573 2
0 0.972491774
NHLRC4 ENSG00000257108.1 0.174076969 2 2 0.074943843
Brain_Cerebellum 0.402730629 Brain_Cortex 1.153418507 0.846581493
0.846581493 0.836754408 1.780807871 1.30878114 0.667546606 2

0	0.972491774
MCRS1	ENSG00000187778.13	0.174117727	1	1	0.174117727
Brain_Hippocampus	0.174117727	Brain_Hippocampus	1	1	1
1.359091087	1.359091087	1.359091087	NA	1	0	0.972491774
ARFRP1	ENSG00000101246.19	0.174166655	3	3	0.041649619
Brain_Anterior_cingulate_cortex_BA24	0.12425167	Brain_Cortex	2.180589095
0.074847054	0.074847054	1.537170059	2.03700422	1.737953476
0.264009276	3	0	0.972491774
R3HDM4	ENSG00000198858.9	0.17425893	1	1	0.17425893
Brain_Substantia_nigra	0.17425893	Brain_Substantia_nigra	1	1	1
1.358645561	1.358645561	1.358645561	NA	1	0	0.972491774
HPS4	ENSG00000100099.20	0.174279797	10	7	0.038122908
Brain_Cerebellar_Hemisphere	0.968720732	Brain_Cortex	5.726627234
0.038779206	0.332368836	-2.073530799	1.655369319	0.465958724
1.196053552	7	0	0.972491774
CH17-264L24.1	ENSG00000279278.1	0.174353084	1	1	0.174353084
Brain_Cerebellum	0.174353084	Brain_Cerebellum	1	1	1	1.358348638
1.358348638	1.358348638	NA	1	0	0.972491774
SPINK8	ENSG00000229453.2	0.174372021	2	2	0.065585194
Brain_Hypothalamus	0.221198245	Brain_Hippocampus	1.515055111
0.484944889	0.484944889	1.223348106	1.841248207	1.532298157
0.436921351	2	0	0.972491774
FNDC3A	ENSG00000102531.16	0.174406851	1	1	0.174406851
Brain_Cerebellum	0.174406851	Brain_Cerebellum	1	1	1	1.358179131
1.358179131	1.358179131	NA	1	0	0.972491774
GSTA1	ENSG00000243955.5	0.174412377	4	4	0.052789831
Brain_Substantia_nigra	0.926251755	Brain_Cortex	2.006483465
0.248884039	0.248884039	-1.387387184	1.936636191	0.06453574
1.379218499	4	0	0.972491774
STPG1	ENSG00000001460.17	0.174431848	10	4	0.022039602
Brain_Substantia_nigra	0.118420404	Brain_Hypothalamus	8.416785102
0.000340863	0.288684847	1.561438161	2.289684684	1.870530304
0.216213363	4	0	0.972491774
CCM2	ENSG00000136280.15	0.1745049	1	1	0.1745049	Brain_Cerebellum	0.1745049
Brain_Cerebellum	1	1	1	1.35787012	1.35787012	1.35787012	NA	1	0
0.972491774
ZDHHC11B	ENSG00000206077.10	0.174558567	8	6	0.065175687
Brain_Hypothalamus	0.811995741	Brain_Cerebellar_Hemisphere	3.844969078
0.004181262	0.219163436	-0.923962444	1.844051142	0.749143533
1.035732232	6	0	0.972491774
PTPRE	ENSG00000132334.16	0.174620124	1	1	0.174620124	Brain_Cerebellum
0.174620124	Brain_Cerebellum	1	1	1	1.357507148	1.357507148
1.357507148	NA	1	0	0.972491774
ZFP62	ENSG00000196670.13	0.174641659	5	2	0.064690273
Brain_Cerebellar_Hemisphere	0.280035899	Brain_Frontal_Cortex_BA9
4.597576922	0.006498834	0.262723682	1.080238699	1.84739252
1.578256088	0.327988059	2	0	0.972491774
ANKRD34A	ENSG00000272031.2	0.174658634	2	1	0.174658634
Brain_Frontal_Cortex_BA9	0.174658634	Brain_Frontal_Cortex_BA9	2
3.35E-17	2	1.357385874	1.357385874	1.357385874	0	1	0
0.972491774
NUCB1	ENSG00000104805.15	0.174730392	2	2	0.155627404	Brain_Cerebellum
0.943488603	Brain_Cerebellar_Hemisphere	1.678094418	0.321905582
0.321905582	0.070885853	1.419932258	0.745409055	0.953919861
2	0	0.972491774
MYO9B	ENSG00000099331.13	0.174733594	4	4	0.046558182	Brain_Cortex
0.451716986	Brain_Spinal_cord_cervical_c-1	1.665766418	0.35844087
0.35844087	-0.756265067	1.990297421	0.844516089	1.180810095	4
0	0.972491774
METTL16	ENSG00000127804.12	0.174795645	10	4	0.030603649
Brain_Hippocampus	0.990482777	Brain_Caudate_basal_ganglia	7.554390912
0.0001561	0.401246516	-2.162188502	0.011928354	-0.580526815

0.652280325	4	0	0.972491774
GSTZ1	ENSG00000100577.18	0.174890944	12	2	0.060000418
Brain_Substantia_nigra	0.355792605	Brain_Amygdala	10.72897032
0.002729854	0.641735746	0.923411876	1.880790533	1.349139284
0.230511748	2	0	0.972491774
WDR62	ENSG00000075702.16	0.174935218	4	4	0.016554959	Brain_Cerebellum
0.995578266	Brain_Frontal_Cortex_BA9	1.921912209	0.458290074
0.458290074	-2.396445418	0.020702065	-0.669700866	1.161127053
4	0	0.972491774
BBS1	ENSG00000174483.19	0.175043008	4	3	0.280218979
Brain_Nucleus_accumbens_basal_ganglia	0.569733628	Brain_Putamen_basal_ganglia
2.847668944	0.046653347	0.220571714	-0.806686535	1.079827553
0.369234645	0.816136542	3	0	0.972491774
BSG	ENSG00000172270.18	0.175066207	5	5	0.025297873
Brain_Frontal_Cortex_BA9	0.825957353	Brain_Nucleus_accumbens_basal_ganglia
1.589898012	0.438259772	0.438259772	-2.236823569	1.417304594
0.083661706	1.45275526	5	0	0.972491774
RP11-439E19.7	ENSG00000235021.1	0.175082933	5	5	0.076174902
Brain_Frontal_Cortex_BA9	0.76784046	Brain_Putamen_basal_ganglia	1.364636499
0.764088002	0.764088002	-1.773324801	0.295200841	-0.995181873
0.780920252	5	0	0.972491774
TEX40	ENSG00000219435.5	0.175204813	11	3	0.02299041	Brain_Amygdala
0.059588132	Brain_Anterior_cingulate_cortex_BA24	9.753490221
0.002084812	0.329811176	1.883828832	2.273593973	2.087259004
0.13770637	3	0	0.972491774
DENND1B	ENSG00000213047.12	0.175305448	1	1	0.175305448
Brain_Cerebellum	0.175305448	Brain_Cerebellum	1	1	1	-1.355351958
-1.355351958	-1.355351958	NA	1	0	0.972491774
NGB	ENSG00000165553.4	0.175332387	3	3	0.121332572	Brain_Cerebellum
0.54075778	Brain_Cerebellar_Hemisphere	1.139396079	0.864260814
0.864260814	-1.35288142	1.549204484	-0.13844814	1.50780571	3
0	0.972491774
RP11-59H7.4	ENSG00000277144.1	0.175334853	3	3	0.027590162
Brain_Hippocampus	0.983873589	Brain_Hypothalamus	1.383688656
0.660219936	0.660219936	-0.020212835	2.203065081	0.934994872
1.144182407	3	0	0.972491774
ENTPD4	ENSG00000197217.12	0.175371746	6	6	0.011804511
Brain_Cerebellar_Hemisphere	0.946953549	Brain_Frontal_Cortex_BA9
3.256212512	0.216380368	0.216380368	-2.51793498	0.06653292
-1.195297857	0.865641709	6	0	0.972491774
GDPD4	ENSG00000178795.9	0.175454374	1	1	0.175454374
Brain_Spinal_cord_cervical_c-1	0.175454374	Brain_Spinal_cord_cervical_c-1
1	1	1	-1.354884453	-1.354884453	-1.354884453	NA	1	0
0.972491774
NLRP12	ENSG00000142405.21	0.175586889	1	1	0.175586889
Brain_Hippocampus	0.175586889	Brain_Hippocampus	1	1	1
-1.354468712	-1.354468712	-1.354468712	NA	1	0	0.972491774
AGK	ENSG00000006530.15	0.175701002	1	1	0.175701002
Brain_Spinal_cord_cervical_c-1	0.175701002	Brain_Spinal_cord_cervical_c-1
1	1	1	1.354110892	1.354110892	1.354110892	NA	1	0
0.972491774
BDKRB2	ENSG00000168398.6	0.175751482	5	5	0.126785909
Brain_Frontal_Cortex_BA9	0.469423468	Brain_Putamen_basal_ganglia	1.68173422
0.332024354	0.332024354	-0.964127584	1.526899883	0.772901972
1.015393798	5	0	0.972491774
APLP1	ENSG00000105290.11	0.175815827	1	1	0.175815827
Brain_Putamen_basal_ganglia	0.175815827	Brain_Putamen_basal_ganglia	1	1
1	-1.353751014	-1.353751014	-1.353751014	NA	1	0
0.972491774
RP11-285E23.2	ENSG00000274560.1	0.175818271	3	3	0.126693555
Brain_Cerebellar_Hemisphere	0.659050567	Brain_Nucleus_accumbens_basal_ganglia
1.318285314	0.662527403	0.662527403	-0.441224377	1.527271331

0.699894345 1.021062802 3 0 0.972491774
LINC01160 ENSG00000231346.5 0.175821285 1 1 0.175821285
Brain_Cerebellar_Hemisphere 0.175821285 Brain_Cerebellar_Hemisphere 1 1
1 -1.353733911 -1.353733911 -1.353733911 NA 1 0
0.972491774
POLR3H ENSG00000100413.16 0.175826088 12 2 0.05209402
Brain_Nucleus_accumbens_basal_ganglia 0.506512165 Brain_Hippocampus
10.36947985 0.006205491 0.79315834 -1.942355982 -0.664278366
-1.092303299 0.395263284 2 0 0.972491774
PPM1A ENSG00000100614.17 0.175921184 8 2 0.098576228
Brain_Anterior_cingulate_cortex_BA24 0.665858307 Brain_Cerebellum
6.965474984 2.21E-05 0.528037598 0.431839173 1.651795592
0.981133904 0.522456887 2 0 0.972491774
SLC25A20 ENSG00000178537.9 0.175957007 1 1 0.175957007
Brain_Cerebellum 0.175957007 Brain_Cerebellum 1 1 1 1.353308774
1.353308774 1.353308774 NA 1 0 0.972491774
FAM166A ENSG00000188163.7 0.176019369 3 3 0.038627384
Brain_Hippocampus 0.967811828 Brain_Spinal_cord_cervical_c-1
1.147897873 0.879014937 0.879014937 -0.733009449 2.068134428
0.458492606 1.446627942 3 0 0.972491774
GGA3 ENSG00000125447.16 0.17606125 2 2 0.063321251 Brain_Cerebellum
0.352028688 Brain_Cerebellar_Hemisphere 1.571946936 0.428053064
0.428053064 -1.856929033 -0.930661505 -1.393795269 0.65497005 2
0 0.972491774
STMN3 ENSG00000197457.9 0.176064039 6 6 0.103681907
Brain_Cerebellar_Hemisphere 0.993377548 Brain_Cerebellum 3.559983871
0.184535428 0.184535428 -1.627259915 0.958169941 -0.296126425
0.892298629 6 0 0.972491774
RND3 ENSG00000115963.13 0.176220387 2 2 0.145931042
Brain_Nucleus_accumbens_basal_ganglia 0.310331604 Brain_Caudate_basal_ganglia
1.099655226 0.900344774 0.900344774 -1.014526472 1.454055057
0.219764292 1.745550739 2 0 0.972491774
RP1-40E16.9 ENSG00000230269.6 0.176235574 2 2 0.1452227
Brain_Nucleus_accumbens_basal_ganglia 0.221102236 Brain_Cortex 1.04306211
0.95693789 0.95693789 -1.456614946 -1.223602448 -1.340108697
0.164764718 2 0 0.972491774
FAM92B ENSG00000153789.12 0.176253546 2 2 0.316078692
Brain_Cerebellum 0.675901157 Brain_Cerebellar_Hemisphere 1.700682053
0.299317947 0.299317947 -1.002548631 0.418062861 -0.292242885
1.004524019 2 0 0.972491774
SDHAF1 ENSG00000205138.3 0.176267473 1 1 0.176267473
Brain_Putamen_basal_ganglia 0.176267473 Brain_Putamen_basal_ganglia 1 1
1 -1.352337189 -1.352337189 -1.352337189 NA 1 0
0.972491774
MB21D1 ENSG00000164430.15 0.176272119 1 1 0.176272119
Brain_Substantia_nigra 0.176272119 Brain_Substantia_nigra 1 1
1 -1.352322657 -1.352322657 -1.352322657 NA 1 0
0.972491774
RP11-522N14.2 ENSG00000256596.1 0.176316267 1 1 0.176316267
Brain_Hypothalamus 0.176316267 Brain_Hypothalamus 1 1 1
-1.352184605 -1.352184605 -1.352184605 NA 1 0 0.972491774
C10orf82 ENSG00000165863.16 0.176325977 3 3 0.080867492
Brain_Caudate_basal_ganglia 0.804759593 Brain_Cortex 1.101927456
0.887341179 0.887341179 -1.745674712 1.255206753 -0.245886672
1.500441158 3 0 0.972491774
RP11-96L14.7 ENSG00000236782.6 0.176446251 2 2 0.134714568
Brain_Cerebellum 0.869703688 Brain_Cerebellar_Hemisphere 1.522904496
0.477095504 0.477095504 -0.164034877 1.495766293 0.665865708
1.173656662 2 0 0.972491774
CFTR ENSG00000001626.14 0.176448753 5 5 0.067741612
Brain_Hippocampus 0.611117681 Brain_Frontal_Cortex_BA9 2.007979237
0.362751752 0.362751752 -1.826721779 1.319537239 -0.425136242

1.351021401 5 0 0.972491774
FBLN7 ENSG00000144152.12 0.17646944 5 5 0.01913208 Brain_Cortex
0.909262751 Brain_Cerebellum 1.984810076 0.240687789 0.240687789
-2.3429474 0.113968516 -0.920600585 0.952093918 5 0
0.972491774
PAPD7 ENSG00000112941.13 0.176481782 3 3 0.051110242 Brain_Cerebellum
0.920704067 Brain_Cerebellar_Hemisphere 2.119209386 0.203370083
0.203370083 -1.950553007 -0.099546881 -1.098611361 0.934232179
3 0 0.972491774
C12orf43 ENSG00000157895.11 0.17661117 11 4 0.15211279
Brain_Putamen_basal_ganglia 0.961362612 Brain_Cerebellum 8.078236294
4.12E-05 0.309917492 -1.432108439 1.199563044 -0.482309029
0.943243604 4 0 0.972530552
SPPL3 ENSG00000157837.15 0.176743135 9 4 0.016929209
Brain_Hypothalamus 0.23669979 Brain_Cerebellar_Hemisphere 7.313203716
0.018639202 0.263480288 -2.3882416 -1.183275977 -1.592940997
0.428218954 4 0 0.972530552
RP11-131L12.4 ENSG00000275409.1 0.176777384 10 5 0.367321983
Brain_Putamen_basal_ganglia 0.763322811 Brain_Cerebellum 7.266898388
0.020716448 0.349198809 -0.901501036 0.866438091 -0.377109771
0.587893983 5 0 0.972530552
EIF4A3 ENSG00000141543.9 0.176826978 1 1 0.176826978
Brain_Spinal_cord_cervical_c-1 0.176826978 Brain_Spinal_cord_cervical_c-1
1 1 1 1.350589464 1.350589464 1.350589464 NA 1 0
0.972530552
NPIPB13 ENSG00000198064.13 0.176889956 1 1 0.176889956
Brain_Hippocampus 0.176889956 Brain_Hippocampus 1 1 1
1.350392997 1.350392997 1.350392997 NA 1 0 0.972530552
EPS8L2 ENSG00000177106.14 0.177019982 5 4 0.227658187
Brain_Nucleus_accumbens_basal_ganglia 0.824091211 Brain_Cerebellar_Hemisphere
2.912827357 0.062316799 0.152050596 -1.035319363 1.206413256
0.455448004 0.923145613 4 0 0.972530552
ID3 ENSG00000117318.8 0.177043266 1 1 0.177043266
Brain_Nucleus_accumbens_basal_ganglia 0.177043266
Brain_Nucleus_accumbens_basal_ganglia 1 1 1 -1.349914948
-1.349914948 -1.349914948 NA 1 0 0.972530552
TMEM218 ENSG00000150433.9 0.177102498 5 4 0.142109231
Brain_Putamen_basal_ganglia 0.926234273 Brain_Frontal_Cortex_BA9
2.154578741 0.046913754 0.79805857 -1.332231509 1.467981563
0.192192001 1.181054558 4 0 0.972530552
MMAB ENSG00000139428.11 0.17711442 7 3 0.136672158
Brain_Nucleus_accumbens_basal_ganglia 0.852259329 Brain_Cerebellar_Hemisphere
5.513234248 0.022781443 0.492723867 -1.488298623 0.477216043
-0.399614752 0.652351636 3 0 0.972530552
BLOC1S2 ENSG00000196072.11 0.177270387 12 2 0.03425703
Brain_Hypothalamus 0.198414139 Brain_Cerebellum 10.95669682
0.001700931 0.408734561 -2.117033205 -1.286082877 -1.682604234
0.239810858 2 0 0.973005094
RHOF ENSG00000139725.7 0.17741456 4 3 0.018020414
Brain_Cerebellar_Hemisphere 0.661294811 Brain_Cortex 2.135032982
0.030023071 0.861384036 0.438126189 2.365198402 1.329682629
1.007165822 3 0 0.973414554
BLNK ENSG00000095585.16 0.177573406 1 1 0.177573406 Brain_Amygdala
0.177573406 Brain_Amygdala 1 1 1 -1.34826425 -1.34826425
-1.34826425 NA 1 0 0.973741196
CDKL2 ENSG00000138769.10 0.177613289 5 3 0.058048302 Brain_Cortex
0.602562025 Brain_Frontal_Cortex_BA9 3.999405128 0.03195613
0.392788042 -1.895332975 -0.520719734 -0.900455166 0.577538919
3 0 0.973741196
RANBP6 ENSG00000137040.9 0.177927973 2 2 0.111457578
Brain_Amygdala 0.389687072 Brain_Frontal_Cortex_BA9 1.060848283
0.939151717 0.939151717 0.860184999 1.591675758 1.225930379

0.517242076 2 0 0.9738951
CRCT1 ENSG00000169509.5 0.178191181 1 1 0.178191181
Brain_Cerebellar_Hemisphere 0.178191181 Brain_Cerebellar_Hemisphere 1 1
1 1.346345305 1.346345305 1.346345305 NA 1 0 0.9738951
RP11-297M9.1 ENSG00000260362.1 0.178196325 1 1 0.178196325
Brain_Cortex 0.178196325 Brain_Cortex 1 1 1 -1.346329348
-1.346329348 -1.346329348 NA 1 0 0.9738951
CTD-2325M2.1 ENSG00000267939.1 0.178227836 11 6 0.017705038
Brain_Hypothalamus 0.517197826 Brain_Spinal_cord_cervical_c-1
8.323342703 0.030500341 0.340655892 0.647670905 2.37172969
1.30482314 0.524840727 6 0 0.9738951
LINC01068 ENSG00000227676.3 0.178249823 2 1 0.158248536
Brain_Cerebellar_Hemisphere 0.201453173 Brain_Cerebellum 1.994183614
0.005816386 1.994183614 1.277422365 1.410986698 1.344204531
0.094444245 1 0 0.9738951
KPRP ENSG00000203786.6 0.178284418 1 1 0.178284418
Brain_Putamen_basal_ganglia 0.178284418 Brain_Putamen_basal_ganglia 1 1
1 -1.346056122 -1.346056122 -1.346056122 NA 1 0 0.9738951
RBBP8NL ENSG00000130701.3 0.178398835 3 1 0.119342862
Brain_Frontal_Cortex_BA9 0.234700021 Brain_Anterior_cingulate_cortex_BA24
2.891752941 0.025945762 2.891752941 -1.557537763 -1.188338637
-1.322338858 0.204347446 1 0 0.9738951
SETD1B ENSG00000139718.10 0.178531402 1 1 0.178531402
Brain_Anterior_cingulate_cortex_BA24 0.178531402
Brain_Anterior_cingulate_cortex_BA24 1 1 1 1.345290619
1.345290619 1.345290619 NA 1 0 0.9738951
CTC-543D15.8 ENSG00000278611.1 0.178614539 13 2 0.029046866
Brain_Hippocampus 0.195547243 Brain_Substantia_nigra 10.9578459
0.001481638 0.810310956 1.294342208 2.182849887 1.696534705
0.269460359 2 0 0.9738951
MYO6 ENSG00000196586.13 0.178627371 5 3 0.127925014
Brain_Nucleus_accumbens_basal_ganglia 0.957926838
Brain_Anterior_cingulate_cortex_BA24 2.959345682 0.023817305
0.131737053 -0.05275535 1.522335592 0.747014183 0.57314951 3
0 0.9738951
RP11-817O13.8 ENSG00000260274.1 0.178668278 2 2 0.070832259
Brain_Cortex 0.285395321 Brain_Cerebellum 1.372933819 0.627066181
0.627066181 1.068277576 1.806551361 1.437414469 0.5220384 2
0 0.9738951
GOLGA8B ENSG00000215252.11 0.178860508 2 2 0.365145716
Brain_Spinal_cord_cervical_c-1 0.813699526 Brain_Caudate_basal_ganglia
1.803719007 0.196280993 0.196280993 -0.235656115 0.905603575
0.33497373 0.806992466 2 0 0.9738951
SUGCT ENSG00000175600.15 0.178889241 3 3 0.037331052 Brain_Amygdala
0.58426971 Brain_Hypothalamus 1.03229135 0.968340783 0.968340783
-0.547158696 2.082125016 0.706890429 1.318824342 3 0
0.9738951
TOMM34 ENSG00000025772.7 0.179027351 3 3 0.031410006
Brain_Nucleus_accumbens_basal_ganglia 0.937746219
Brain_Anterior_cingulate_cortex_BA24 1.183451056 0.817496088
0.817496088 0.078102877 2.151839399 0.787246873 1.182076721
3 0 0.9738951
CHRM5 ENSG00000184984.9 0.179123992 12 6 0.03304743 Brain_Amygdala
0.746336485 Brain_Substantia_nigra 7.662689623 0.007839532
0.287827329 -2.131506593 -0.32347375 -1.112261175 0.516481728
6 0 0.9738951
SEC11C ENSG00000166562.8 0.179323203 1 1 0.179323203
Brain_Cerebellar_Hemisphere 0.179323203 Brain_Cerebellar_Hemisphere 1 1
1 1.342841806 1.342841806 1.342841806 NA 1 0 0.9738951
KANK3 ENSG00000186994.11 0.179378681 2 1 0.147696768
Brain_Cerebellar_Hemisphere 0.224469987 Brain_Cerebellum 1.966036678
0.033963322 1.966036678 1.21472764 1.447715017 1.331221328

0.164746955	1	0	0.9738951
DACT2 ENSG00000164488.11	0.179390292	1	1	0.179390292	Brain_Cortex
0.179390292	Brain_Cortex	1	1	1	1.342634691	1.342634691
1.342634691	NA	1	0	0.9738951
TMEM179B	ENSG00000185475.10	0.17946801 4	4	0.03142269 Brain_Amygdala
0.840800389	Brain_Frontal_Cortex_BA9	2.079633286	0.293285155
0.293285155	-0.490772041	2.151678428	0.542665723	1.188552525
4	0	0.9738951
CPA5 ENSG00000158525.15	0.17950551 1	1	0.17950551 Brain_Hypothalamus
0.17950551 Brain_Hypothalamus	1	1	1	1.342279124	1.342279124
1.342279124	NA	1	0	0.9738951
CTNNA1	ENSG00000044115.20	0.179551088	1	1	0.179551088
Brain_Anterior_cingulate_cortex_BA24	0.179551088
Brain_Anterior_cingulate_cortex_BA24	1	1	1	1.342138514
1.342138514	1.342138514	NA	1	0	0.9738951
SLC35G2	ENSG00000168917.8	0.179626107	4	3	0.218496731
Brain_Cerebellum 0.54167229 Brain_Substantia_nigra	2.276726183
0.058178723	0.217872315	-1.230535261	1.112072805	-0.089043476
1.127054718	3	0	0.9738951
ISYNA1	ENSG00000105655.18	0.179640191	1	1	0.179640191
Brain_Amygdala	0.179640191	Brain_Amygdala	1	1	1	-1.341863708
-1.341863708	-1.341863708	NA	1	0	0.9738951
PFKP ENSG00000067057.16	0.179664654	3	3	0.050674778
Brain_Anterior_cingulate_cortex_BA24	0.503943659	Brain_Caudate_basal_ganglia
1.317240362	0.797646304	0.797646304	-1.954223612	-0.668297577
-1.304170601	0.643080279	3	0	0.9738951
RP11-489O18.1	ENSG00000253988.1	0.179735284	2	2	0.132283001
Brain_Amygdala	0.378380032	Brain_Cortex	1.126699285	0.873300715
0.873300715	0.880885065	1.505159786	1.193022425	0.441428889
2	0	0.9738951
DPEP1 ENSG00000015413.9	0.17979881 2	2	0.069260454	Brain_Cortex
0.515068925	Brain_Hypothalamus	1.161425799	0.838574201
0.838574201	-1.81671702	-0.650965242	-1.233841131	0.824310988
2	0	0.9738951
RP11-332H14.2	ENSG00000272994.1	0.179826707	2	1	0.179375145
Brain_Cerebellum 0.18027943 Brain_Cerebellar_Hemisphere 1.999998549	1.45E-06
1.999998549	-1.342681445	-1.339895167	-1.341288306	0.001970196
1	0	0.9738951
C1orf54	ENSG00000118292.8	0.180016148	1	1	0.180016148
Brain_Hypothalamus	0.180016148	Brain_Hypothalamus	1	1	1
1.340705315	1.340705315	1.340705315	NA	1	0	0.9738951
NOP10 ENSG00000182117.5	0.180041225	1	1	0.180041225
Brain_Anterior_cingulate_cortex_BA24	0.180041225
Brain_Anterior_cingulate_cortex_BA24	1	1	1	1.340628112
1.340628112	1.340628112	NA	1	0	0.9738951
C12orf54	ENSG00000177627.9	0.180169812	6	6	0.043902812
Brain_Caudate_basal_ganglia 0.925829045	Brain_Spinal_cord_cervical_c-1
3.471176469	0.234323548	0.234323548	-0.093093798	2.015017534
1.2419279 0.820115675	6	0	0.9738951
UTP4 ENSG00000141076.17	0.180225549	13	2	0.02546921
Brain_Anterior_cingulate_cortex_BA24	0.493694805	Brain_Amygdala
11.59341508	9.75E-05	0.551414387	0.684444055	2.234210743
1.701781808	0.356906861	2	0	0.9738951
DIS3L ENSG00000166938.12	0.180238364	12	6	0.00856136
Brain_Spinal_cord_cervical_c-1	0.613152323	Brain_Frontal_Cortex_BA9
8.071723019	0.028102573	0.269558511	-2.629090389	-0.505578864
-1.854891215	0.718604304	6	0	0.9738951
MTCH1 ENSG00000137409.19	0.180275003	4	4	0.027510818
Brain_Spinal_cord_cervical_c-1	0.771452885	Brain_Putamen_basal_ganglia
1.680217618	0.297928399	0.297928399	0.290474985	2.204192376
1.219626694	0.803859237	4	0	0.9738951
MMP19 ENSG00000123342.15	0.180275269	1	1	0.180275269

Brain_Hypothalamus 0.180275269 Brain_Hypothalamus 1 1 1
1.339907962 1.339907962 1.339907962 NA 1 0 0.9738951
RP11-282I1.1 ENSG00000230131.5 0.18028233 2 2 0.079711292
Brain_Hippocampus 0.298109649 Brain_Amygdala 1.7986016 0.2013984
0.2013984 1.040495725 1.752363682 1.396429703 0.50336666 2 0
0.9738951
TADA2B ENSG00000173011.11 0.180331638 1 1 0.180331638
Brain_Hippocampus 0.180331638 Brain_Hippocampus 1 1 1
-1.33973462 -1.33973462 -1.33973462 NA 1 0 0.9738951
PMS1 ENSG00000064933.17 0.180418624 1 1 0.180418624 Brain_Cerebellum
0.180418624 Brain_Cerebellum 1 1 1 1.339467204 1.339467204
1.339467204 NA 1 0 0.9738951
FGF2 ENSG00000138685.13 0.180660136 1 1 0.180660136 Brain_Cortex
0.180660136 Brain_Cortex 1 1 1 -1.338725241 -1.338725241
-1.338725241 NA 1 0 0.9738951
ZNF589 ENSG00000164048.13 0.180715461 13 2 0.085742979
Brain_Nucleus_accumbens_basal_ganglia 0.946894417 Brain_Hippocampus
11.61839315 1.60E-06 0.776262799 -1.718294134 0.066607196
-1.421701511 0.464779762 2 0 0.9738951
ZNF23 ENSG00000167377.17 0.180739497 5 5 0.067022476
Brain_Caudate_basal_ganglia 0.838872835 Brain_Anterior_cingulate_cortex_BA24
3.072941892 0.147893409 0.147893409 -1.831523279 0.203335468
-0.941019325 0.863832102 5 0 0.9738951
CALML5 ENSG00000178372.7 0.18078007 2 2 0.113914688
Brain_Hippocampus 0.319748937 Brain_Frontal_Cortex_BA9 1.022176196
0.977823804 0.977823804 -1.58083973 0.994973944 -0.292932893
1.821375316 2 0 0.9738951
OXT ENSG00000101405.3 0.180827023 1 1 0.180827023
Brain_Spinal_cord_cervical_c-1 0.180827023 Brain_Spinal_cord_cervical_c-1
1 1 1 -1.338212967 -1.338212967 -1.338212967 NA 1 0
0.9738951
TNPO2 ENSG00000105576.15 0.180832329 5 5 0.022631619
Brain_Substantia_nigra 0.798182583 Brain_Hypothalamus 1.939404958
0.135696106 0.135696106 -0.672431327 2.279596692 0.692894348
1.233788742 5 0 0.9738951
PRR5 ENSG00000186654.20 0.180972515 2 2 0.064821447
Brain_Caudate_basal_ganglia 0.864388289 Brain_Putamen_basal_ganglia 1.04029923
0.95970077 0.95970077 -1.846487543 -0.170790767 -1.008639155
1.184896553 2 0 0.9738951
SLC13A4 ENSG00000164707.15 0.180983691 1 1 0.180983691
Brain_Cortex 0.180983691 Brain_Cortex 1 1 1 1.337732381
1.337732381 1.337732381 NA 1 0 0.9738951
OR2T33 ENSG00000177212.3 0.181129788 1 1 0.181129788
Brain_Caudate_basal_ganglia 0.181129788 Brain_Caudate_basal_ganglia 1 1
1 -1.337284502 -1.337284502 -1.337284502 NA 1 0 0.9738951
TTC1 ENSG00000113312.10 0.181144364 2 2 0.17671289 Brain_Cortex
0.364311318 Brain_Cerebellum 1.236030121 0.763969879 0.763969879
-0.907180568 1.350945505 0.221882469 1.596736259 2 0
0.9738951
APOA1 ENSG00000118137.9 0.181166457 1 1 0.181166457
Brain_Anterior_cingulate_cortex_BA24 0.181166457
Brain_Anterior_cingulate_cortex_BA24 1 1 1 1.337172131
1.337172131 1.337172131 NA 1 0 0.9738951
GSTM2 ENSG00000213366.12 0.181175165 8 7 0.057941527
Brain_Putamen_basal_ganglia 0.82282494 Brain_Cortex 3.776951933
0.104526425 0.215852534 -1.896140058 0.899800734 0.028698511
0.90171023 7 0 0.9738951
RP6-74O6.2 ENSG00000215859.8 0.181196356 4 4 0.03289925 Brain_Cerebellum
0.889720484 Brain_Cerebellar_Hemisphere 1.552662596 0.554377872
0.554377872 -0.898184573 2.133310659 0.389350281 1.265479698
4 0 0.9738951
RP11-546B8.6 ENSG00000275038.1 0.181315405 1 1 0.181315405

Brain_Cerebellum 0.181315405 Brain_Cerebellum 1 1 1 1.336715854
1.336715854 1.336715854 NA 1 0 0.9738951
KCNJ5 ENSG00000120457.11 0.181330819 1 1 0.181330819
Brain_Putamen_basal_ganglia 0.181330819 Brain_Putamen_basal_ganglia 1 1
1 1.336668652 1.336668652 1.336668652 NA 1 0 0.9738951
AP006621.6 ENSG00000255142.1 0.181333768 8 5 0.004194312
Brain_Cerebellar_Hemisphere 0.114185191 Brain_Cortex 5.68491321 0.0115943
0.237597491 1.57965808 2.863165672 2.119473559 0.509344416 5
0 0.9738951
ARRDC4 ENSG00000140450.8 0.181405874 1 1 0.181405874
Brain_Hippocampus 0.181405874 Brain_Hippocampus 1 1 1
1.336438853 1.336438853 1.336438853 NA 1 0 0.9738951
XXyac-YX155B6.5 ENSG00000232265.7 0.18149889 2 2 0.135230061
Brain_Cerebellar_Hemisphere 0.431784858 Brain_Frontal_Cortex_BA9
1.876535348 0.123464652 0.123464652 0.786141049 1.493791741
1.139966395 0.500384603 2 0 0.9738951
NME7 ENSG00000143156.13 0.181558163 9 5 0.018274988
Brain_Putamen_basal_ganglia 0.790779659 Brain_Cerebellar_Hemisphere
5.691017814 0.000972966 0.307403854 -2.359998921 -0.26529832
-1.53347232 0.655281373 5 0 0.9738951
SEC22C ENSG00000093183.13 0.181834872 12 4 0.030079481
Brain_Putamen_basal_ganglia 0.818960397 Brain_Cerebellar_Hemisphere
9.177895886 1.03E-09 0.309726896 -2.169042187 -0.228882301
-1.6199216 0.732206104 4 0 0.9738951
NBPF14 ENSG00000270629.5 0.1819174 4 4 0.142118573
Brain_Frontal_Cortex_BA9 0.788786187 Brain_Substantia_nigra
1.275797904 0.674201781 0.674201781 -1.336892982 1.467947173
-0.232116181 1.214507368 4 0 0.9738951
NDUFS2 ENSG00000158864.12 0.181939713 11 7 0.050426318
Brain_Hypothalamus 0.781746799 Brain_Nucleus_accumbens_basal_ganglia
6.314711866 0.002390627 0.287242508 -1.73063801 1.956329784
-0.369605033 1.260436479 7 0 0.9738951
MALRD1 ENSG00000204740.10 0.181946449 8 4 0.111774464
Brain_Nucleus_accumbens_basal_ganglia 0.89148742 Brain_Putamen_basal_ganglia
6.262629124 0.013664221 0.269312355 0.136422332 1.590267751
0.765247455 0.575135323 4 0 0.9738951
EIF2B1 ENSG00000111361.12 0.181998625 1 1 0.181998625
Brain_Cortex 0.181998625 Brain_Cortex 1 1 1 -1.334626485
-1.334626485 -1.334626485 NA 1 0 0.9738951
NACC1 ENSG00000160877.5 0.18204092 1 1 0.18204092 Brain_Putamen_basal_ganglia
0.18204092 Brain_Putamen_basal_ganglia 1 1 1 -1.334497333
-1.334497333 -1.334497333 NA 1 0 0.9738951
PRKAG1 ENSG00000181929.11 0.182061023 1 1 0.182061023
Brain_Anterior_cingulate_cortex_BA24 0.182061023
Brain_Anterior_cingulate_cortex_BA24 1 1 1 -1.334435955
-1.334435955 -1.334435955 NA 1 0 0.9738951
BTBD17 ENSG00000204347.3 0.182096318 2 2 0.078172583
Brain_Cerebellum 0.561286944 Brain_Cerebellar_Hemisphere 1.016858742
0.983141258 0.983141258 -1.761389017 0.580931022 -0.590228998
1.656270383 2 0 0.9738951
MIR4458HG ENSG00000247516.6 0.182366489 2 2 0.065116102
Brain_Cerebellar_Hemisphere 0.599125684 Brain_Cerebellum 1.304592222
0.695407778 0.695407778 -1.844460196 -0.525658239 -1.185059217
0.932533807 2 0 0.973897072
PLD4 ENSG00000166428.12 0.182377482 2 2 0.068687131
Brain_Putamen_basal_ganglia 0.913455722 Brain_Cerebellum 1.103509391
0.896490609 0.896490609 -1.820472168 0.108680736 -0.855895716
1.3641171 2 0 0.973897072
INTS11 ENSG00000127054.20 0.182525477 7 5 0.009198098
Brain_Putamen_basal_ganglia 0.36704756 Brain_Anterior_cingulate_cortex_BA24
4.990371084 0.009520725 0.284337848 -2.604602213 -0.902017523
-1.869476693 0.666609219 5 0 0.973897072

NAT1 ENSG00000171428.13 0.182579044 2 2 0.159178136 Brain_Cerebellum
0.616110338 Brain_Cerebellar_Hemisphere 1.414087233 0.585912767
0.585912767 -1.40784107 0.501370585 -0.453235243 1.350016508
2 0 0.973897072
IL7 ENSG00000104432.13 0.182702073 4 3 0.037337825 Brain_Cerebellum
0.154798893 Brain_Cortex 3.641195117 0.011267012 0.154561793
-2.082050855 -1.422783612 -1.766363335 0.274912634 3 0
0.973897072
COX20 ENSG00000203667.9 0.182749498 2 2 0.067056793
Brain_Spinal_cord_cervical_c-1 0.874462256 Brain_Cerebellum 1.030334341
0.969665659 0.969665659 -0.157993075 1.831293191 0.836650058
1.406637808 2 0 0.973897072
SIX1 ENSG00000126778.8 0.182769722 1 1 0.182769722
Brain_Caudate_basal_ganglia 0.182769722 Brain_Caudate_basal_ganglia 1 1
1 1.332275357 1.332275357 1.332275357 NA 1 0
0.973897072
C14orf159 ENSG00000133943.20 0.18282564 1 1 0.18282564 Brain_Cerebellum
0.18282564 Brain_Cerebellum 1 1 1 1.332105145 1.332105145
1.332105145 NA 1 0 0.973897072
SEZ6L2 ENSG00000174938.14 0.183000231 2 1 0.158602779
Brain_Cerebellar_Hemisphere 0.221413743 Brain_Cerebellum 1.954327099
0.045672901 1.954327099 -1.409786346 -1.222777508 -1.316281927
0.132235218 1 0 0.973897072
LINC02067 ENSG00000240567.1 0.183052381 1 1 0.183052381
Brain_Cerebellar_Hemisphere 0.183052381 Brain_Cerebellar_Hemisphere 1 1
1 -1.331415352 -1.331415352 -1.331415352 NA 1 0
0.973897072
RP11-25O3.1 ENSG00000266335.1 0.183110594 2 2 0.070798901
Brain_Nucleus_accumbens_basal_ganglia 0.684406914 Brain_Frontal_Cortex_BA9
1.024754697 0.975245303 0.975245303 -1.806765176 -0.406456907
-1.106611042 0.990167473 2 0 0.973897072
CHPF2 ENSG00000033100.16 0.183160146 3 3 0.092905155
Brain_Nucleus_accumbens_basal_ganglia 0.591490289 Brain_Cortex
2.325059038 0.197938587 0.197938587 -1.680268149 -0.536677662
-1.058697398 0.57825825 3 0 0.973897072
SLC2A3 ENSG00000059804.15 0.183203173 1 1 0.183203173
Brain_Cerebellum 0.183203173 Brain_Cerebellum 1 1 1 1.33095696
1.33095696 1.33095696 NA 1 0 0.973897072
NCKAP5L ENSG00000167566.16 0.183252932 1 1 0.183252932
Brain_Putamen_basal_ganglia 0.183252932 Brain_Putamen_basal_ganglia 1 1
1 -1.330805759 -1.330805759 -1.330805759 NA 1 0
0.973897072
RHD ENSG00000187010.19 0.183279638 13 4 0.073215607
Brain_Caudate_basal_ganglia 0.833523763 Brain_Amygdala 9.887914048
0.011983401 0.4698271 -0.642717996 1.791484329 1.272224184
0.716093518 4 0 0.973897072
RP11-51G5.1 ENSG00000230852.1 0.183312053 1 1 0.183312053
Brain_Hypothalamus 0.183312053 Brain_Hypothalamus 1 1 1
-1.330626152 -1.330626152 -1.330626152 NA 1 0 0.973897072
RP4-668E10.4 ENSG00000278254.4 0.183365242 3 3 0.038927928
Brain_Caudate_basal_ganglia 0.273793064 Brain_Nucleus_accumbens_basal_ganglia
2.378347915 0.122456896 0.122456896 1.094369251 2.064947919
1.598375414 0.486370946 3 0 0.973897072
OCEL1 ENSG00000099330.8 0.183401309 12 4 0.024977751
Brain_Putamen_basal_ganglia 0.539573209 Brain_Anterior_cingulate_cortex_BA24
9.674742261 0.009398621 0.422305235 -2.241746651 -0.61345851
-1.412213414 0.409772312 4 0 0.973897072
C10orf142 ENSG00000277288.3 0.183419254 7 3 0.107006802
Brain_Putamen_basal_ganglia 0.886772563 Brain_Anterior_cingulate_cortex_BA24
5.564067438 0.032508438 0.330233846 -1.611793873 -0.142389237
-0.98939773 0.576071199 3 0 0.973897072
C7orf57 ENSG00000164746.13 0.183617823 1 1 0.183617823

Brain_Nucleus_accumbens_basal_ganglia 0.183617823
Brain_Nucleus_accumbens_basal_ganglia 1 1 1 -1.329697911
-1.329697911 -1.329697911 NA 1 0 0.974222538
PBX4 ENSG00000105717.13 0.183619815 1 1 0.183619815 Brain_Cerebellum
0.183619815 Brain_Cerebellum 1 1 1 1.329691868 1.329691868
1.329691868 NA 1 0 0.974222538
ETNK1 ENSG00000139163.15 0.183876793 1 1 0.183876793 Brain_Amygdala
0.183876793 Brain_Amygdala 1 1 1 -1.328912641 -1.328912641
-1.328912641 NA 1 0 0.974933956
TMEM177 ENSG00000144120.12 0.183893268 6 3 0.129778449
Brain_Amygdala 0.988603987 Brain_Putamen_basal_ganglia 4.400391779
0.005157264 0.462310024 0.01428327 1.514976129 1.014216346
0.535921941 3 0 0.974933956
RP11-446H18.6 ENSG00000272597.1 0.184151826 2 2 0.104190005
Brain_Amygdala 0.306400763 Brain_Cerebellar_Hemisphere 1.101437859
0.898562141 0.898562141 1.022803499 1.624871204 1.323837351
0.425726157 2 0 0.975276323
NT5DC3 ENSG00000111696.11 0.184177707 6 6 0.05916232 Brain_Cortex
0.780755591 Brain_Amygdala 2.112046654 0.366133037 0.366133037
-1.530001082 1.886985173 0.201881667 1.375252299 6 0
0.975276323
LINC00165 ENSG00000261706.1 0.184453833 4 4 0.091159929
Brain_Caudate_basal_ganglia 0.622782465 Brain_Substantia_nigra
1.974945653 0.398131857 0.398131857 -0.813168803 1.68931057
0.512853573 1.02825063 4 0 0.975276323
RP11-734K23.9 ENSG00000277701.4 0.184562845 1 1 0.184562845
Brain_Nucleus_accumbens_basal_ganglia 0.184562845
Brain_Nucleus_accumbens_basal_ganglia 1 1 1 1.326836283
1.326836283 1.326836283 NA 1 0 0.975276323
MAP1B ENSG00000131711.14 0.184598795 10 5 0.027560675
Brain_Hypothalamus 0.820510751 Brain_Frontal_Cortex_BA9 6.547097454
0.023971899 0.407248865 -0.232792093 2.203483701 0.776233542
0.692444276 5 0 0.975276323
DDTL ENSG00000099974.7 0.184614098 4 4 0.292395426 Brain_Cerebellum
0.962281982 Brain_Putamen_basal_ganglia 2.148424572 0.123603292
0.123603292 -1.052881238 0.047290145 -0.589098327 0.467317362
4 0 0.975276323
TC2N ENSG00000165929.12 0.18477102 12 2 0.13328204
Brain_Nucleus_accumbens_basal_ganglia 0.853632937 Brain_Putamen_basal_ganglia
10.52768891 0.001136415 0.497014873 0.184485072 1.501284313
0.995967197 0.391473821 2 0 0.975276323
RHOA ENSG00000067560.10 0.184775973 3 3 0.05232267 Brain_Hippocampus
0.650373887 Brain_Caudate_basal_ganglia 1.504996587 0.533774631
0.533774631 0.453242841 1.940469395 1.24719339 0.748707038 3
0 0.975276323
PCDHB9 ENSG00000177839.6 0.18514416 1 1 0.18514416
Brain_Cerebellar_Hemisphere 0.18514416 Brain_Cerebellar_Hemisphere 1 1 1
1.325081383 1.325081383 1.325081383 NA 1 0 0.975276323
KDELR2 ENSG00000136240.9 0.185166558 3 3 0.352787034
Brain_Cerebellar_Hemisphere 0.840407646 Brain_Cerebellum 1.861232058
0.140788878 0.140788878 -0.929196939 0.201372079 -0.449317518
0.584318974 3 0 0.975276323
RP11-303E16.7 ENSG00000245059.2 0.185211515 1 1 0.185211515
Brain_Putamen_basal_ganglia 0.185211515 Brain_Putamen_basal_ganglia 1 1
1 -1.324878313 -1.324878313 -1.324878313 NA 1 0
0.975276323
IFITM3 ENSG00000142089.15 0.185211764 5 3 0.155320235
Brain_Hypothalamus 0.281481796 Brain_Amygdala 3.000907921 1.04E-16
0.96404637 -1.420988044 1.076996574 -0.843777814 1.075205498 3
0 0.975276323
GLIS3 ENSG00000107249.21 0.185345006 1 1 0.185345006
Brain_Putamen_basal_ganglia 0.185345006 Brain_Putamen_basal_ganglia 1 1

1 1.324476008 1.324476008 1.324476008 NA 1 0
0.975276323
LDB3 ENSG00000122367.19 0.185362407 4 4 0.056942543
Brain_Cerebellar_Hemisphere 0.263009559 Brain_Nucleus_accumbens_basal_ganglia
2.451478392 0.137826331 0.137826331 -1.903751598 1.738377167
0.574152464 1.671651076 4 0 0.975276323
TTF2 ENSG00000116830.11 0.185364873 13 2 0.054893693 Brain_Cerebellum
0.119146413 Brain_Spinal_cord_cervical_c-1 12.07897888 2.91E-05
0.453604648 -1.919716703 -1.558366415 -1.714507125 0.110505142
2 0 0.975276323
AP001628.7 ENSG00000233754.2 0.185496945 1 1 0.185496945
Brain_Cerebellar_Hemisphere 0.185496945 Brain_Cerebellar_Hemisphere 1 1
1 1.324018366 1.324018366 1.324018366 NA 1 0
0.975276323
RP11-266A24.1 ENSG00000255418.5 0.185596161 2 2 0.43506426
Brain_Cerebellar_Hemisphere 0.556903158 Brain_Cerebellum 1.721308554
0.278691446 0.278691446 -0.780555012 0.587447562 -0.096553725
0.967323897 2 0 0.975276323
C1orf56 ENSG00000143443.9 0.1857074 1 1 0.1857074
Brain_Spinal_cord_cervical_c-1 0.1857074 Brain_Spinal_cord_cervical_c-1 1
1 1 1.323384931 1.323384931 1.323384931 NA 1 0
0.975276323
CIPC ENSG00000198894.7 0.185816563 1 1 0.185816563 Brain_Amygdala
0.185816563 Brain_Amygdala 1 1 1 -1.323056579 -1.323056579
-1.323056579 NA 1 0 0.975276323
CSPG5 ENSG00000114646.9 0.186109709 1 1 0.186109709
Brain_Frontal_Cortex_BA9 0.186109709 Brain_Frontal_Cortex_BA9 1 1
1 -1.322175525 -1.322175525 -1.322175525 NA 1 0
0.975276323
MAPKBP1 ENSG00000137802.13 0.186114218 6 4 0.117261275
Brain_Caudate_basal_ganglia 0.992927442 Brain_Nucleus_accumbens_basal_ganglia
4.344735893 0.04166316 0.180051225 -1.566373192 1.247462258
-0.217092246 1.02976993 4 0 0.975276323
NDFIP1 ENSG00000131507.10 0.186204536 1 1 0.186204536
Brain_Cerebellum 0.186204536 Brain_Cerebellum 1 1 1 -1.321890743
-1.321890743 -1.321890743 NA 1 0 0.975276323
AMDHD1 ENSG00000139344.7 0.18635719 8 6 0.099399237
Brain_Hippocampus 0.853012342 Brain_Caudate_basal_ganglia 5.29629018
0.010805701 0.193165532 -1.647773119 0.812829637 -0.840285513
0.827631269 6 0 0.975276323
CEMIP ENSG00000103888.16 0.186363613 3 3 0.037694101
Brain_Cerebellar_Hemisphere 0.986523385 Brain_Cerebellum 1.095819644
0.940743239 0.940743239 -0.016891236 2.078165686 0.893708652
1.07404105 3 0 0.975276323
C1QA ENSG00000173372.16 0.186391722 2 2 0.11220583 Brain_Cortex
0.391927229 Brain_Nucleus_accumbens_basal_ganglia 1.03657955 0.96342045
0.96342045 -0.856127555 1.588356136 0.36611429 1.728510994 2 0
0.975276323
CRIP1 ENSG00000213145.9 0.186424677 2 2 0.100017399
Brain_Frontal_Cortex_BA9 0.544142764 Brain_Cortex 1.119450674
0.880549326 0.880549326 -1.644769284 -0.606560284 -1.125664784
0.734124624 2 0 0.975276323
CRISPLD1 ENSG00000121005.8 0.186447217 3 3 0.18797595 Brain_Amygdala
0.618165304 Brain_Cortex 1.905999519 0.131495976 0.131495976
-1.041362047 1.316590426 0.257893549 1.197241137 3 0
0.975276323
MTHFSD ENSG00000103248.18 0.186461984 7 7 0.062540224
Brain_Nucleus_accumbens_basal_ganglia 0.399864979 Brain_Frontal_Cortex_BA9
2.5505842 0.086846903 0.086846903 -1.831865628 1.862446189
-0.575482214 1.372324329 7 0 0.975276323
LINC01411 ENSG00000249306.5 0.186553532 2 2 0.246039182
Brain_Cerebellar_Hemisphere 0.968036116 Brain_Cerebellum 1.760102818

0.239897182	0.239897182	-1.160023639	0.040071509	-0.559976065
0.848595417	2	0	0.975276323
HID1	ENSG00000167861.15	0.186641403	1	1	0.186641403
Brain_Anterior_cingulate_cortex_BA24	0.186641403
Brain_Anterior_cingulate_cortex_BA24	1	1	1	1.320580128
1.320580128	1.320580128	NA	1	0	0.975276323
MTPN	ENSG00000105887.10	0.186652764	2	2	0.123604397
Brain_Spinal_cord_cervical_c-1	0.490676044	Brain_Cerebellum	1.185994749
0.814005251	0.814005251	0.689233969	1.539819339	1.114526654
0.601454684	2	0	0.975276323
LINC00638	ENSG00000258701.1	0.186689518	9	4	0.042706492
Brain_Caudate_basal_ganglia	0.878098963	Brain_Hypothalamus	6.522108656
0.002817444	0.284458864	-1.07786689	2.026569171	0.790819984
0.857805888	4	0	0.975276323
PYCR2	ENSG00000143811.18	0.186698983	3	3	0.105441212	Brain_Cerebellum
0.295819393	Brain_Cortex	1.164307112	0.888343591	0.888343591
-1.349492669	1.619028126	-0.258635039	1.633195071	3	0
0.975276323
CD2	ENSG00000116824.4	0.186831995	1	1	0.186831995
Brain_Substantia_nigra	0.186831995	Brain_Substantia_nigra	1	1
1	1.320009054	1.320009054	1.320009054	NA	1	0
0.975276323
ACP5	ENSG00000102575.10	0.186860104	4	3	0.033931589
Brain_Hypothalamus	0.096967934	Brain_Cerebellum	3.540945875	0.04947553
0.139026801	-2.120883723	-1.659734211	-1.882837661	0.20549638	3
0	0.975276323
RPP25L	ENSG00000164967.9	0.187054894	6	6	0.295991307
Brain_Cerebellum	0.897484205	Brain_Cortex	3.776086298	0.21634124
0.21634124	-1.023370385	1.045068509	-0.066809227	0.728978372	6
0	0.975276323
PITPNM3	ENSG00000091622.15	0.187119542	6	6	0.032933258
Brain_Putamen_basal_ganglia	0.740369903	Brain_Cerebellar_Hemisphere
1.800924915	0.30347401	0.30347401	-2.13289601	1.550736673
0.394477759	1.332284155	6	0	0.975276323
BTNL2	ENSG00000204290.10	0.187183522	5	5	0.006896939
Brain_Hippocampus	0.788871057	Brain_Cerebellar_Hemisphere	2.406916966
0.13343824	0.13343824	-2.701779965	-0.267776894	-1.414744839
0.884633858	5	0	0.975276323
CELF1	ENSG00000149187.17	0.187192724	1	1	0.187192724
Brain_Frontal_Cortex_BA9	0.187192724	Brain_Frontal_Cortex_BA9	1	1
1	1.318929377	1.318929377	1.318929377	NA	1	0
0.975276323
ABRACL	ENSG00000146386.7	0.187235636	4	4	0.025254303
Brain_Caudate_basal_ganglia	0.658031331	Brain_Spinal_cord_cervical_c-1
2.632521667	0.197497638	0.197497638	0.442632835	2.237490442
1.136059178	0.826632543	4	0	0.975276323
HRASLS	ENSG00000127252.5	0.187295462	3	3	0.083616207
Brain_Nucleus_accumbens_basal_ganglia	0.195765684	Brain_Cortex
1.681973726	0.302893976	0.302893976	-1.293709787	1.730078745
0.628072118	1.670283779	3	0	0.975276323
NT5C2	ENSG00000076685.18	0.187359609	4	4	0.01528108	Brain_Cerebellum
0.554762852	Brain_Substantia_nigra	2.502774018	0.264885668
0.264885668	-2.425648077	-0.590638217	-1.625801912	0.765408933
4	0	0.975276323
DYNC1I2	ENSG00000077380.15	0.187375125	4	4	0.090033457
Brain_Cerebellar_Hemisphere	0.466825358	Brain_Cerebellum	2.08769732
0.124040789	0.124040789	0.727654094	1.695221251	1.229049101
0.440127279	4	0	0.975276323
STRAP	ENSG00000023734.10	0.187418148	2	2	0.172294523
Brain_Cerebellar_Hemisphere	0.226416538	Brain_Nucleus_accumbens_basal_ganglia
1.006836036	0.993163964	0.993163964	1.209641367	1.364868058
1.287254712	0.109761846	2	0	0.975276323

VPS37D ENSG00000176428.5 0.187662605 2 2 0.075206202
Brain_Putamen_basal_ganglia 0.494964325 Brain_Amygdala 1.147279467
0.852720533 0.852720533 0.682434376 1.779204736 1.230819556
0.775533759 2 0 0.975276323
ZNF746 ENSG00000181220.15 0.187668642 3 3 0.034181547
Brain_Cortex 0.850017766 Brain_Caudate_basal_ganglia 1.375924428
0.63187135 0.63187135 -2.117923498 0.658459074 -0.423456222
1.486098918 3 0 0.975276323
SNTG1 ENSG00000147481.14 0.187735915 2 2 0.352670185
Brain_Cerebellar_Hemisphere 0.930169002 Brain_Cerebellum 1.88462892 0.11537108
0.11537108 0.087632209 0.929422475 0.508527342 0.595235605 2
0 0.975276323
C14orf180 ENSG00000184601.10 0.187923009 7 7 0.028384107
Brain_Hypothalamus 0.821759486 Brain_Nucleus_accumbens_basal_ganglia
2.15008257 0.139481939 0.139481939 -2.191936288 0.514349818
-1.001295386 1.139696011 7 0 0.975276323
UBE2J1 ENSG00000198833.6 0.187946998 2 2 0.073105814
Brain_Cerebellar_Hemisphere 0.194459979 Brain_Cerebellum 1.55590194 0.44409806
0.44409806 -1.79216953 -1.297497749 -1.54483364 0.349785771 2
0 0.975276323
IGFBPL1 ENSG00000137142.4 0.188013366 7 7 0.027925043
Brain_Nucleus_accumbens_basal_ganglia 0.890036717 Brain_Putamen_basal_ganglia
2.095889868 0.36436656 0.36436656 -1.575601159 2.198337793
0.188563467 1.177955226 7 0 0.975276323
RP1-90K10.4 ENSG00000232598.2 0.188021937 1 1 0.188021937
Brain_Spinal_cord_cervical_c-1 0.188021937 Brain_Spinal_cord_cervical_c-1
1 1 1 1.316453317 1.316453317 1.316453317 NA 1 0
0.975276323
WDR64 ENSG00000162843.17 0.188027729 2 2 0.105309751 Brain_Cortex
0.36943307 Brain_Frontal_Cortex_BA9 1.839027464 0.160972536
0.160972536 0.897535818 1.619639446 1.258587632 0.510604372
2 0 0.975276323
RNF170 ENSG00000120925.15 0.188070401 4 4 0.05164262
Brain_Spinal_cord_cervical_c-1 0.880484835 Brain_Cerebellum 1.401024277
0.585732301 0.585732301 -0.277303904 1.946100908 0.667342636
1.069277573 4 0 0.975276323
RP11-44N11.2 ENSG00000272384.1 0.188098545 2 2 0.126674613
Brain_Caudate_basal_ganglia 0.266046165 Brain_Cerebellar_Hemisphere
1.072321225 0.927678775 0.927678775 -1.112213964 1.527347545
0.207566791 1.866451842 2 0 0.975276323
PLCH1 ENSG00000114805.16 0.188099683 6 6 0.038712148
Brain_Nucleus_accumbens_basal_ganglia 0.758053389 Brain_Amygdala
1.487836945 0.55825791 0.55825791 -1.463611023 2.067233595
-0.109875878 1.370115992 6 0 0.975276323
HBM ENSG00000206177.6 0.188269516 1 1 0.188269516
Brain_Hippocampus 0.188269516 Brain_Hippocampus 1 1 1
1.315715602 1.315715602 1.315715602 NA 1 0 0.975276323
BMP8B ENSG00000116985.11 0.188654574 4 4 0.202496028 Brain_Cerebellum
0.71154448 Brain_Frontal_Cortex_BA9 1.831938605 0.333141824
0.333141824 -0.905746351 1.274472456 0.298181006 0.900381321
4 0 0.975276323
TAMM41 ENSG00000144559.10 0.188718592 13 3 0.11843513
Brain_Nucleus_accumbens_basal_ganglia 0.939637936 Brain_Hippocampus
10.43543567 0.006172024 0.502791359 -1.561375707 0.638376888
-0.062954552 0.585884461 3 0 0.975276323
GNB3 ENSG00000111664.10 0.188736701 13 5 0.061914124
Brain_Spinal_cord_cervical_c-1 0.875614189 Brain_Hippocampus
9.365287545 4.93E-17 0.537263734 -1.866910237 0.156531376
-1.015654974 0.536122098 5 0 0.975276323
AKR7A2 ENSG00000053371.12 0.188943941 1 1 0.188943941
Brain_Cerebellar_Hemisphere 0.188943941 Brain_Cerebellar_Hemisphere 1 1
1 -1.313709625 -1.313709625 -1.313709625 NA 1 0

0.975276323
FKBP15 ENSG00000119321.8 0.189006359 4 4 0.111442809
Brain_Frontal_Cortex_BA9 0.891200879 Brain_Nucleus_accumbens_basal_ganglia
2.223176587 0.329125256 0.329125256 -1.591741458 1.067433643
-0.325486595 1.107353145 4 0 0.975276323
FAM163B ENSG00000196990.8 0.189017258 2 2 0.119752574
Brain_Cerebellar_Hemisphere 0.174564618 Brain_Cerebellum 1.284414833
0.715585167 0.715585167 -1.555812967 -1.357681977 -1.456747472
0.140099767 2 0 0.975276323
GBAS ENSG00000146729.9 0.189026747 8 2 0.159212104 Brain_Cortex
0.907748199 Brain_Cerebellar_Hemisphere 6.28123398 0.00400854 1.314812645
-1.407726391 0.708773617 -0.654023811 0.764222105 2 0
0.975276323
NSMCE3 ENSG00000185115.5 0.189227462 1 1 0.189227462
Brain_Hypothalamus 0.189227462 Brain_Hypothalamus 1 1 1
-1.312867909 -1.312867909 -1.312867909 NA 1 0 0.975276323
FSTL5 ENSG00000168843.13 0.189257011 4 4 0.063939719
Brain_Anterior_cingulate_cortex_BA24 0.765230372 Brain_Hippocampus
1.270490851 0.7691825 0.7691825 -1.357081267 1.852599952
-0.122998682 1.387687508 4 0 0.975276323
SMPD2 ENSG00000135587.8 0.189275153 9 3 0.097330367
Brain_Anterior_cingulate_cortex_BA24 0.718236243 Brain_Cortex
7.163140082 0.005042656 0.449233623 -1.417526143 1.65793609
0.593220599 0.843012685 3 0 0.975276323
VTI1B ENSG00000100568.10 0.189279434 3 3 0.208695033 Brain_Cortex
0.919610691 Brain_Cerebellum 2.143406337 0.136209689 0.136209689
0.100924126 1.257161855 0.759129503 0.594527518 3 0
0.975276323
TIGD2 ENSG00000180346.3 0.189305273 1 1 0.189305273
Brain_Nucleus_accumbens_basal_ganglia 0.189305273
Brain_Nucleus_accumbens_basal_ganglia 1 1 1 1.312637068
1.312637068 1.312637068 NA 1 0 0.975276323
MIR7-3HG ENSG00000176840.11 0.189328189 11 5 0.018968441
Brain_Cerebellar_Hemisphere 0.899729835 Brain_Substantia_nigra
8.054805819 1.34E-09 0.287489166 -0.659534305 2.346150601
1.573369745 0.958912808 5 0 0.975276323
CCT6A ENSG00000146731.10 0.189373889 2 2 0.09693879 Brain_Hypothalamus
0.303666572 Brain_Cortex 1.170366992 0.829633008 0.829633008
-1.659879034 -1.028602353 -1.344240693 0.446380022 2 0
0.975276323
PDCD6 ENSG00000249915.7 0.189401605 3 2 0.189005147 Brain_Cerebellum
0.851666 Brain_Nucleus_accumbens_basal_ganglia 2.700316827 0.070337301
0.229345872 0.186993155 1.313527839 0.796260384 0.568874386
2 0 0.975276323
CDC23 ENSG00000094880.10 0.189435589 2 2 0.19788957 Brain_Cerebellum
0.284829332 Brain_Cortex 1.159939559 0.840060441 0.840060441
-1.287587566 1.069533522 -0.109027022 1.666736306 2 0
0.975276323
STRN3 ENSG00000196792.11 0.189562514 4 4 0.319362674 Brain_Cerebellum
0.940790957 Brain_Caudate_basal_ganglia 2.533930464 0.130817606
0.130817606 -0.995768428 0.53410146 -0.36406427 0.730101212 4
0 0.975276323
VILL ENSG00000136059.14 0.18975867 12 1 0.096419034
Brain_Anterior_cingulate_cortex_BA24 0.780347623 Brain_Cerebellum
11.20456165 7.13E-05 11.20456165 0.278866051 1.662467702
1.253850777 0.381148277 1 0 0.975276323
BAX ENSG00000087088.19 0.189849025 1 1 0.189849025
Brain_Anterior_cingulate_cortex_BA24 0.189849025
Brain_Anterior_cingulate_cortex_BA24 1 1 1 -1.311025865
-1.311025865 -1.311025865 NA 1 0 0.975276323
COMP ENSG00000105664.10 0.189881359 3 2 0.0563875 Brain_Cerebellum
0.561857588 Brain_Amygdala 1.971723232 0.029745872 0.998530896

-1.908028755 0.580084571 -0.939687685 1.332566788 2 0
0.975276323
CEP85L ENSG00000111860.13 0.190080395 3 3 0.171279847
Brain_Cerebellar_Hemisphere 0.551150294 Brain_Cerebellum 1.927079905
0.101353827 0.101353827 0.596037323 1.368102972 0.974202365
0.386273281 3 0 0.975276323
MGAT2 ENSG00000168282.5 0.190085625 1 1 0.190085625 Brain_Cortex
0.190085625 Brain_Cortex 1 1 1 -1.310325854 -1.310325854
-1.310325854 NA 1 0 0.975276323
STIM1 ENSG00000167323.11 0.190102376 2 2 0.484656711 Brain_Cerebellum
0.701916291 Brain_Cerebellar_Hemisphere 1.822381039 0.177618961
0.177618961 -0.382734959 0.698832507 0.158048774 0.764783689
2 0 0.975276323
DFFA ENSG00000160049.11 0.190138303 1 1 0.190138303 Brain_Cerebellum
0.190138303 Brain_Cerebellum 1 1 1 -1.310170087 -1.310170087
-1.310170087 NA 1 0 0.975276323
WDR78 ENSG00000152763.16 0.19023732 2 2 0.163862308
Brain_Caudate_basal_ganglia 0.257778057 Brain_Nucleus_accumbens_basal_ganglia
1.030770072 0.969229928 0.969229928 -1.392198463 -1.131658449
-1.261928456 0.18422961 2 0 0.975276323
NANS ENSG00000095380.10 0.19028966 2 2 0.102857112
Brain_Anterior_cingulate_cortex_BA24 0.512299571 Brain_Cerebellar_Hemisphere
1.093315606 0.906684394 0.906684394 0.655261241 1.631157396
1.143209318 0.690062789 2 0 0.975276323
L3MBTL1 ENSG00000185513.14 0.190310826 2 2 0.07521896 Brain_Cerebellum
0.272732709 Brain_Cerebellar_Hemisphere 1.41668112 0.58331888 0.58331888
-1.779126896 -1.096791129 -1.437959012 0.482484248 2 0
0.975276323
AHCTF1 ENSG00000153207.14 0.190338396 3 3 0.091129424
Brain_Caudate_basal_ganglia 0.62390589 Brain_Amygdala 1.123387715
0.922785403 0.922785403 -1.689469857 0.490322242 -0.799408771
1.143536374 3 0 0.975276323
ZNF579 ENSG00000218891.4 0.190404676 1 1 0.190404676
Brain_Spinal_cord_cervical_c-1 0.190404676 Brain_Spinal_cord_cervical_c-1
1 1 1 1.309382914 1.309382914 1.309382914 NA 1 0
0.975276323
ZNF18 ENSG00000154957.13 0.190465867 2 1 0.164968275
Brain_Caudate_basal_ganglia 0.227394455 Brain_Putamen_basal_ganglia
1.965393847 0.034606153 1.965393847 1.207097869 1.388554454
1.297826161 0.128309182 1 0 0.975276323
SLC35A3 ENSG00000117620.14 0.190564489 2 1 0.184890609
Brain_Cortex 0.205865019 Brain_Cerebellar_Hemisphere 1.95902037 0.04097963
1.95902037 -1.325846314 -1.265017599 -1.295431956 0.043012397 1
0 0.975276323
RERG ENSG00000134533.6 0.190587632 1 1 0.190587632 Brain_Cerebellum
0.190587632 Brain_Cerebellum 1 1 1 -1.308842722 -1.308842722
-1.308842722 NA 1 0 0.975276323
ERICH4 ENSG00000204978.2 0.190680513 1 1 0.190680513
Brain_Cerebellum 0.190680513 Brain_Cerebellum 1 1 1 -1.308568631
-1.308568631 -1.308568631 NA 1 0 0.975276323
FAM26F ENSG00000188820.12 0.190702504 9 6 0.197152013
Brain_Cortex 0.97247575 Brain_Nucleus_accumbens_basal_ganglia 5.74646336
0.041189578 0.258955095 -1.289708138 1.164996977 -0.00360919
0.893452895 6 0 0.975276323
RP11-480A16.1 ENSG00000260261.1 0.190745208 6 6 0.073338649
Brain_Cortex 0.815417391 Brain_Cerebellum 2.168446221 0.208613878
0.208613878 -1.790717442 1.263097453 0.108230554 1.133601829
6 0 0.975276323
MEIS1 ENSG00000143995.19 0.190759784 4 3 0.096407185 Brain_Cortex
0.788807087 Brain_Amygdala 1.984655814 0.048600833 0.934469164
0.267859996 1.662526848 1.200620086 0.631330206 3 0
0.975276323

TNFRSF17 ENSG00000048462.10 0.190775243 5 5 0.028929593
Brain_Nucleus_accumbens_basal_ganglia 0.495641429 Brain_Cerebellum
2.675556917 0.105337121 0.105337121 -1.245990879 2.184444606
1.080927429 1.423951346 5 0 0.975276323
ARHGEF37 ENSG00000183111.11 0.190802379 3 3 0.423326731
Brain_Nucleus_accumbens_basal_ganglia 0.918958797
Brain_Anterior_cingulate_cortex_BA24 2.644466772 0.111118313
0.111118313 -0.306441231 0.800663063 0.198655731 0.559878285
3 0 0.975276323
SEMA3C ENSG00000075223.13 0.190835765 4 4 0.048169596
Brain_Nucleus_accumbens_basal_ganglia 0.552635012 Brain_Amygdala
1.775091151 0.538668286 0.538668286 -1.975869178 0.593816261
-1.179555414 1.211271337 4 0 0.975276323
FER1L5 ENSG00000249715.10 0.190916649 1 1 0.190916649
Brain_Nucleus_accumbens_basal_ganglia 0.190916649
Brain_Nucleus_accumbens_basal_ganglia 1 1 1 1.307872234
1.307872234 1.307872234 NA 1 0 0.975276323
C1orf109 ENSG00000116922.14 0.190932644 5 3 0.031348028
Brain_Cortex 0.179071026 Brain_Amygdala 4.484746479 0.009821323
0.153078659 -2.152626712 -1.343620844 -1.812230492 0.317855803
3 0 0.975276323
STK17B ENSG00000081320.10 0.1909885 1 1 0.1909885 Brain_Hypothalamus
0.1909885 Brain_Hypothalamus 1 1 1 1.30766046 1.30766046 1.30766046
NA 1 0 0.975276323
GPR153 ENSG00000158292.6 0.19113734 3 2 0.161951039
Brain_Putamen_basal_ganglia 0.209079721 Brain_Cortex 1.98595211
0.020323145 0.993724745 1.256099987 1.398539769 1.338283202
0.073707912 2 0 0.975276323
SLC35B3 ENSG00000124786.9 0.191200654 3 3 0.096693114
Brain_Cerebellum 0.938929259 Brain_Cerebellar_Hemisphere 1.866213044
0.51714689 0.51714689 -0.586928479 1.661101246 0.332519018
1.178538431 3 0 0.975276323
SMAD5 ENSG00000113658.16 0.191305478 1 1 0.191305478
Brain_Spinal_cord_cervical_c-1 0.191305478 Brain_Spinal_cord_cervical_c-1
1 1 1 -1.306726905 -1.306726905 -1.306726905 NA 1 0
0.975276323
PDE4DIP ENSG00000178104.19 0.191337936 5 5 0.09222856
Brain_Anterior_cingulate_cortex_BA24 0.410881952
Brain_Nucleus_accumbens_basal_ganglia 1.896006679 0.162898564
0.162898564 -1.683757417 1.156918435 -0.416861436 1.30911001 5
0 0.975276323
TGS1 ENSG00000137574.10 0.191384757 2 2 0.069494006
Brain_Nucleus_accumbens_basal_ganglia 0.887850766 Brain_Hippocampus
1.017332919 0.982667081 0.982667081 -1.815194623 -0.141024275
-0.978109449 1.183817206 2 0 0.975276323
SIGLEC10 ENSG00000142512.14 0.191388097 6 5 0.029517854
Brain_Cortex 0.677957774 Brain_Substantia_nigra 2.399251307
0.002338775 0.741274854 -0.415251538 2.176500536 1.065957852
1.012156464 5 0 0.975276323
DLC1 ENSG00000164741.14 0.191447451 1 1 0.191447451
Brain_Nucleus_accumbens_basal_ganglia 0.191447451
Brain_Nucleus_accumbens_basal_ganglia 1 1 1 1.306309137
1.306309137 1.306309137 NA 1 0 0.975276323
RP11-326C3.13 ENSG00000270030.1 0.191534367 4 4 0.10227563
Brain_Putamen_basal_ganglia 0.780707097 Brain_Amygdala 1.314636055
0.725516336 0.725516336 -0.278397684 1.633920091 0.987091884
0.862308614 4 0 0.975276323
CELSR3 ENSG00000008300.16 0.191587381 1 1 0.191587381
Brain_Amygdala 0.191587381 Brain_Amygdala 1 1 1 1.305897605
1.305897605 1.305897605 NA 1 0 0.975276323
SLC25A35 ENSG00000125434.10 0.19169886 2 2 0.167563126 Brain_Cerebellum
0.815702972 Brain_Cerebellar_Hemisphere 1.548018998 0.451981002

0.451981002 -0.233075252 1.380076389 0.573500569 1.140670464
2 0 0.975276323
STAC ENSG00000144681.10 0.191717393 3 3 0.032009679
Brain_Putamen_basal_ganglia 0.981904367 Brain_Cerebellum 1.622025242
0.421667727 0.421667727 -2.144289729 -0.022681458 -0.740813017
1.215557031 3 0 0.975276323
ATAD3C ENSG00000215915.9 0.191726996 13 4 0.010084451
Brain_Cerebellum 0.984951971 Brain_Anterior_cingulate_cortex_BA24
9.889987337 0.004769962 0.370825083 0.018861026 2.572920024
1.357817344 0.570740903 4 0 0.975276323
CACNG5 ENSG00000075429.8 0.191927336 1 1 0.191927336
Brain_Cerebellum 0.191927336 Brain_Cerebellum 1 1 1 1.304898724
1.304898724 1.304898724 NA 1 0 0.975276323
ONECUT2 ENSG00000119547.5 0.192128397 2 2 0.069698007
Brain_Frontal_Cortex_BA9 0.700910423 Brain_Cortex 1.262402711
0.737597289 0.737597289 -1.813868284 -0.38409178 -1.098980032
1.011004661 2 0 0.975276323
MED28 ENSG00000118579.12 0.192144377 10 4 0.062307145
Brain_Anterior_cingulate_cortex_BA24 0.999675486 Brain_Caudate_basal_ganglia
7.636998262 0.00774045 0.292325662 -1.864103683 0.709591587
-0.1939048 0.718971217 4 0 0.975276323
PTPRC ENSG00000081237.18 0.192156317 3 3 0.040480518
Brain_Anterior_cingulate_cortex_BA24 0.77920158 Brain_Frontal_Cortex_BA9
1.384318145 0.609018777 0.609018777 -0.36635426 2.048811838
0.654272414 1.250245927 3 0 0.975276323
DOPEY2 ENSG00000142197.12 0.192194163 4 4 0.086313898
Brain_Frontal_Cortex_BA9 0.838498323 Brain_Cortex 2.075839563
0.206896982 0.206896982 -1.715170905 1.086003975 0.022808198
1.214843686 4 0 0.975276323
RP11-1055B8.3 ENSG00000262223.6 0.192207206 10 1 0.117603656
Brain_Putamen_basal_ganglia 0.387659965 Brain_Hippocampus 9.403819158
8.60E-17 9.403819158 -1.564911524 -0.863868805 -1.262825675
0.231563104 1 0 0.975276323
SPATC1L ENSG00000160284.14 0.19226166 13 4 0.048855664
Brain_Putamen_basal_ganglia 0.566015759 Brain_Anterior_cingulate_cortex_BA24
10.14017545 0.005324886 0.369772 -1.969849127 -0.573929131
-1.249775969 0.479842481 4 0 0.975276323
MRPL38 ENSG00000204316.12 0.192539872 3 3 0.038976969
Brain_Cerebellum 0.198864602 Brain_Cerebellar_Hemisphere 1.823124409
0.504912834 0.504912834 -2.064429956 -1.284793078 -1.61221979
0.404520207 3 0 0.975276323
RP11-250B2.5 ENSG00000260645.1 0.192557734 6 4 0.061740567
Brain_Hippocampus 0.896922048 Brain_Putamen_basal_ganglia 4.306822641
0.051656553 0.289839681 -1.868154298 0.542098653 -0.402920466
0.798932959 4 0 0.975276323
HAVCR2 ENSG00000135077.8 0.192704209 1 1 0.192704209
Brain_Nucleus_accumbens_basal_ganglia 0.192704209
Brain_Nucleus_accumbens_basal_ganglia 1 1 1 -1.302620929
-1.302620929 -1.302620929 NA 1 0 0.975276323
GOLGA1 ENSG00000136935.13 0.192830691 1 1 0.192830691
Brain_Cerebellum 0.192830691 Brain_Cerebellum 1 1 1 -1.302250723
-1.302250723 -1.302250723 NA 1 0 0.975276323
LSM11 ENSG00000155858.5 0.192910132 1 1 0.192910132
Brain_Spinal_cord_cervical_c-1 0.192910132 Brain_Spinal_cord_cervical_c-1
1 1 1 1.302018294 1.302018294 1.302018294 NA 1 0
0.975276323
ITGB3 ENSG00000259207.7 0.192968195 2 2 0.086707172
Brain_Substantia_nigra 0.101501939 Brain_Anterior_cingulate_cortex_BA24
1.711083284 0.288916716 0.288916716 1.637615447 1.713029176
1.675322312 0.053325559 2 0 0.975276323
CHID1 ENSG00000177830.17 0.192996083 1 1 0.192996083 Brain_Cerebellum
0.192996083 Brain_Cerebellum 1 1 1 -1.301766896 -1.301766896

-1.301766896 NA 1 0 0.975276323
FBXO45 ENSG00000174013.7 0.193024191 1 1 0.193024191
Brain_Cerebellum 0.193024191 Brain_Cerebellum 1 1 1 -1.301684702
-1.301684702 -1.301684702 NA 1 0 0.975276323
SLC7A2 ENSG00000003989.17 0.1930274 1 1 0.1930274 Brain_Hippocampus
0.1930274 Brain_Hippocampus 1 1 1 1.301675321 1.301675321
1.301675321 NA 1 0 0.975276323
CDK11B ENSG00000248333.8 0.193158505 3 3 0.041325382
Brain_Cerebellar_Hemisphere 0.87209276 Brain_Substantia_nigra 1.48637822
0.542716744 0.542716744 0.161000813 2.040250499 1.200806736
0.955512214 3 0 0.975276323
NUDT21 ENSG00000167005.13 0.193160665 1 1 0.193160665
Brain_Hypothalamus 0.193160665 Brain_Hypothalamus 1 1 1
-1.301285744 -1.301285744 -1.301285744 NA 1 0 0.975276323
BBS9 ENSG00000122507.20 0.193244755 10 4 0.288804444
Brain_Anterior_cingulate_cortex_BA24 0.996036652 Brain_Putamen_basal_ganglia
7.986736541 1.15E-16 0.320900718 -1.023940652 1.06074799 0.22398386
0.574353608 4 0 0.975276323
RRP1 ENSG00000160214.12 0.193257438 3 3 0.056960597 Brain_Cortex
0.495125865 Brain_Anterior_cingulate_cortex_BA24 1.455029559
0.545854742 0.545854742 -0.917357853 1.903613054 0.101358783
1.565221274 3 0 0.975276323
ERLIN1 ENSG00000107566.13 0.193394195 2 2 0.070146684
Brain_Cerebellar_Hemisphere 0.34471354 Brain_Hippocampus 1.482883539
0.517116461 0.517116461 -1.810962323 -0.944892933 -1.377927628
0.612403538 2 0 0.975276323
BRD9 ENSG00000028310.17 0.193493225 4 2 0.080855714 Brain_Cerebellum
0.601692072 Brain_Anterior_cingulate_cortex_BA24 3.467935208
0.045364521 0.382657598 -1.745742458 -0.521968775 -1.295541614
0.538764257 2 0 0.975276323
KCNA1 ENSG00000111262.5 0.193526815 2 2 0.150880876 Brain_Amygdala
0.281994721 Brain_Spinal_cord_cervical_c-1 1.0202406 0.9797594 0.9797594
-1.436426996 1.075849163 -0.180288916 1.776447508 2 0
0.975276323
LSM2 ENSG00000204392.10 0.193538529 1 1 0.193538529
Brain_Hippocampus 0.193538529 Brain_Hippocampus 1 1 1
-1.300182201 -1.300182201 -1.300182201 NA 1 0 0.975276323
RP11-399E6.1 ENSG00000235358.1 0.193653822 4 2 0.036304707
Brain_Cerebellar_Hemisphere 0.163641963 Brain_Putamen_basal_ganglia
3.645872736 0.036148554 0.257058589 -2.093498177 -1.39292668
-1.673926757 0.303475171 2 0 0.975276323
PPAN ENSG00000130810.19 0.193694052 1 1 0.193694052
Brain_Frontal_Cortex_BA9 0.193694052 Brain_Frontal_Cortex_BA9 1 1
1 1.299728461 1.299728461 1.299728461 NA 1 0
0.975276323
SCN3B ENSG00000166257.8 0.193757852 1 1 0.193757852
Brain_Cerebellar_Hemisphere 0.193757852 Brain_Cerebellar_Hemisphere 1 1
1 1.299542402 1.299542402 1.299542402 NA 1 0
0.975276323
CCDC174 ENSG00000154781.15 0.193855345 13 1 0.132748521
Brain_Nucleus_accumbens_basal_ganglia 0.323098458 Brain_Hypothalamus
12.42651931 0.001020078 12.42651931 0.988110654 1.503351132
1.267684082 0.196073483 1 0 0.975276323
CLIP2 ENSG00000106665.15 0.193908362 1 1 0.193908362 Brain_Cerebellum
0.193908362 Brain_Cerebellum 1 1 1 1.299103646 1.299103646
1.299103646 NA 1 0 0.975276323
NUDT15 ENSG00000136159.3 0.19395256 3 2 0.107665303
Brain_Spinal_cord_cervical_c-1 0.364475656 Brain_Cerebellum 1.987026917
0.035873195 0.977099888 -1.608776126 1.087193717 0.128429129
1.507163147 2 0 0.975276323
STK32C ENSG00000165752.16 0.194147652 4 4 0.015658137
Brain_Putamen_basal_ganglia 0.614323252 Brain_Nucleus_accumbens_basal_ganglia

2.415635217 0.245460288 0.245460288 0.503911951 2.416787931
1.169665707 0.850213849 4 0 0.975276323
BCDIN3D ENSG00000186666.5 0.194267551 1 1 0.194267551
Brain_Cerebellum 0.194267551 Brain_Cerebellum 1 1 1 1.298057576
1.298057576 1.298057576 NA 1 0 0.975276323
LRRC41 ENSG00000132128.16 0.194310472 1 1 0.194310472
Brain_Putamen_basal_ganglia 0.194310472 Brain_Putamen_basal_ganglia 1 1
1 1.297932673 1.297932673 1.297932673 NA 1 0
0.975276323
ATG16L2 ENSG00000168010.10 0.19438282 1 1 0.19438282 Brain_Cortex
0.19438282 Brain_Cortex 1 1 1 1.297722177 1.297722177
1.297722177 NA 1 0 0.975276323
RP11-863P13.6 ENSG00000260166.1 0.194568085 1 1 0.194568085
Brain_Amygdala 0.194568085 Brain_Amygdala 1 1 1 -1.297183418
-1.297183418 -1.297183418 NA 1 0 0.975276323
PNPLA2 ENSG00000177666.16 0.194598387 7 7 0.009309263
Brain_Nucleus_accumbens_basal_ganglia 0.960811101 Brain_Cerebellar_Hemisphere
3.116976967 0.172258292 0.172258292 -2.600483023 1.109023124
-0.905929284 1.330099286 7 0 0.975276323
HAPLN4 ENSG00000187664.8 0.1948024 4 3 0.074550897
Brain_Caudate_basal_ganglia 0.94855291 Brain_Cerebellum 2.661947708
0.049739238 0.40089682 -0.06452411 1.78321755 0.83270782 0.799262671
3 0 0.975276323
CLDN11 ENSG00000013297.10 0.194844174 1 1 0.194844174
Brain_Cerebellar_Hemisphere 0.194844174 Brain_Cerebellar_Hemisphere 1 1
1 -1.296381235 -1.296381235 -1.296381235 NA 1 0
0.975276323
ACYP2 ENSG00000170634.12 0.194922416 4 3 0.035548111 Brain_Cortex
0.631975784 Brain_Caudate_basal_ganglia 2.788687333 0.043895535
0.254647683 0.478947772 2.102058967 1.299587837 0.720999951
3 0 0.975276323
RP11-693J15.6 ENSG00000277738.1 0.195079404 1 1 0.195079404
Brain_Cerebellum 0.195079404 Brain_Cerebellum 1 1 1 1.295698427
1.295698427 1.295698427 NA 1 0 0.975276323
CEP131 ENSG00000141577.13 0.195186245 4 4 0.043340832
Brain_Cerebellar_Hemisphere 0.775528973 Brain_Caudate_basal_ganglia 1.4714581
0.559768767 0.559768767 -2.020410437 0.733312738 -0.47380447
1.141265882 4 0 0.975276323
TAGAP ENSG00000164691.16 0.195197675 1 1 0.195197675
Brain_Cerebellar_Hemisphere 0.195197675 Brain_Cerebellar_Hemisphere 1 1
1 1.295355345 1.295355345 1.295355345 NA 1 0
0.975276323
FBXL8 ENSG00000135722.8 0.195212871 1 1 0.195212871 Brain_Cerebellum
0.195212871 Brain_Cerebellum 1 1 1 1.295311275 1.295311275
1.295311275 NA 1 0 0.975276323
SOX18 ENSG00000203883.6 0.195213379 2 2 0.070721992
Brain_Frontal_Cortex_BA9 0.946184928 Brain_Hippocampus 1.018660701
0.981339299 0.981339299 -1.807258442 -0.06749841 -0.937378426
1.230196117 2 0 0.975276323
PEX5L ENSG00000114757.18 0.195249034 3 3 0.083928778
Brain_Caudate_basal_ganglia 0.172541538 Brain_Putamen_basal_ganglia
1.617407491 0.511920963 0.511920963 -1.728331673 -1.364082703
-1.53525265 0.183110169 3 0 0.975276323
C6orf48 ENSG00000204387.12 0.195273423 3 3 0.070468244
Brain_Caudate_basal_ganglia 0.579145331 Brain_Cerebellum 1.350401802
0.698372269 0.698372269 -1.808889034 0.554633558 -0.884691092
1.263161274 3 0 0.975276323
KCNH4 ENSG00000089558.8 0.1953142 1 1 0.1953142 Brain_Substantia_nigra
0.1953142 Brain_Substantia_nigra 1 1 1 1.29501748 1.29501748
1.29501748 NA 1 0 0.975276323
GMPPB ENSG00000173540.12 0.195331697 13 1 0.123131488 Brain_Amygdala
0.399617724 Brain_Caudate_basal_ganglia 12.09189657 0.000203054

12.09189657 0.842304159 1.541761801 1.247732268 0.186131943
1 0 0.975276323
TBX15 ENSG00000092607.13 0.195405295 1 1 0.195405295
Brain_Nucleus_accumbens_basal_ganglia 0.195405295
Brain_Nucleus_accumbens_basal_ganglia 1 1 1 1.294753449
1.294753449 1.294753449 NA 1 0 0.975276323
DLEU1 ENSG00000176124.11 0.195468616 1 1 0.195468616
Brain_Putamen_basal_ganglia 0.195468616 Brain_Putamen_basal_ganglia 1 1
1 -1.294569973 -1.294569973 -1.294569973 NA 1 0
0.975276323
RP11-192H23.4 ENSG00000258472.8 0.195474845 5 4 0.045176846
Brain_Hypothalamus 0.825025324 Brain_Cortex 3.398076102
0.089804944 0.256004066 0.221086189 2.00300412 0.889702042
0.777045256 4 0 0.975276323
TOMM6 ENSG00000214736.7 0.195582517 1 1 0.195582517
Brain_Spinal_cord_cervical_c-1 0.195582517 Brain_Spinal_cord_cervical_c-1
1 1 1 1.294240049 1.294240049 1.294240049 NA 1 0
0.975276323
POLE3 ENSG00000148229.12 0.195628146 2 2 0.110001053
Brain_Putamen_basal_ganglia 0.452934854 Brain_Cerebellum 1.056361624
0.943638376 0.943638376 0.750531176 1.598188407 1.174359792
0.599384176 2 0 0.975276323
PPM1M ENSG00000164088.17 0.195703077 12 3 0.037562053 Brain_Cerebellum
0.953488971 Brain_Substantia_nigra 8.513668325 0.012460817
0.383703517 -0.910324873 2.079602003 0.457913058 0.962866412
3 0 0.975276323
UBE3B ENSG00000151148.13 0.195750124 3 3 0.058183811 Brain_Cortex
0.543386913 Brain_Cerebellum 2.581879154 0.098400958 0.098400958
0.607699322 1.894310477 1.364725187 0.672784495 3 0
0.975276323
LTA ENSG00000226979.8 0.195893894 4 4 0.098047289
Brain_Cerebellar_Hemisphere 0.810976229 Brain_Cortex 2.175712809
0.295633549 0.295633549 -0.239166821 1.654394965 0.818596332
0.824064797 4 0 0.975276323
SELENOT ENSG00000198843.12 0.196029892 1 1 0.196029892
Brain_Frontal_Cortex_BA9 0.196029892 Brain_Frontal_Cortex_BA9 1 1
1 1.29294555 1.29294555 1.29294555 NA 1 0 0.975276323
PRPF31 ENSG00000105618.13 0.196034565 2 2 0.208298558
Brain_Cortex 0.479964814 Brain_Cerebellum 1.387178839 0.612821161
0.612821161 -0.706359157 1.258257744 0.275949294 1.389193933
2 0 0.975276323
L3MBTL2 ENSG00000100395.14 0.196062885 10 4 0.12055827 Brain_Amygdala
0.749519285 Brain_Frontal_Cortex_BA9 7.625632673 0.002583853
0.293827075 -0.858561712 1.552434601 0.683315799 0.766501676
4 0 0.975276323
ANO9 ENSG00000185101.12 0.196107496 1 1 0.196107496
Brain_Putamen_basal_ganglia 0.196107496 Brain_Putamen_basal_ganglia 1 1
1 1.29272122 1.29272122 1.29272122 NA 1 0 0.975276323
GPN3 ENSG00000111231.8 0.196240304 1 1 0.196240304
Brain_Spinal_cord_cervical_c-1 0.196240304 Brain_Spinal_cord_cervical_c-1
1 1 1 1.292337463 1.292337463 1.292337463 NA 1 0
0.975276323
BRF1 ENSG00000185024.16 0.196335373 1 1 0.196335373
Brain_Putamen_basal_ganglia 0.196335373 Brain_Putamen_basal_ganglia 1 1
1 -1.292062869 -1.292062869 -1.292062869 NA 1 0
0.975276323
SBSPON ENSG00000164764.10 0.196380406 3 3 0.084362561
Brain_Hippocampus 0.487622891 Brain_Hypothalamus 1.342856896
0.689290365 0.689290365 -1.725915803 0.96358568 -0.485474905
1.356833146 3 0 0.975276323
SLC46A3 ENSG00000139508.14 0.196399152 7 4 0.242060757
Brain_Substantia_nigra 0.912366549 Brain_Cerebellum 5.562286758

0.0136991 0.274543412 -0.8383331 1.169851443 0.219162559
0.659711684 4 0 0.975276323
C2orf80 ENSG00000188674.10 0.196465132 7 7 0.139531613
Brain_Hippocampus 0.971534497 Brain_Cortex 4.113503079
0.175719874 0.175719874 -1.477537502 0.497455471 -0.454195873
0.805572559 7 0 0.975276323
FLI1 ENSG00000151702.16 0.196483862 1 1 0.196483862
Brain_Putamen_basal_ganglia 0.196483862 Brain_Putamen_basal_ganglia 1 1
1 -1.291634177 -1.291634177 -1.291634177 NA 1 0
0.975276323
TMEM263 ENSG00000151135.9 0.196526736 1 1 0.196526736
Brain_Spinal_cord_cervical_c-1 0.196526736 Brain_Spinal_cord_cervical_c-1
1 1 1 1.291510445 1.291510445 1.291510445 NA 1 0
0.975276323
PLB1 ENSG00000163803.12 0.196645266 4 4 0.168236366
Brain_Hippocampus 0.929568954 Brain_Putamen_basal_ganglia 1.368245126
0.637054002 0.637054002 -1.175201161 1.377892872 0.381399784
1.187388717 4 0 0.975276323
HPCA ENSG00000121905.9 0.19665436 1 1 0.19665436 Brain_Cortex 0.19665436
Brain_Cortex 1 1 1 1.291142238 1.291142238 1.291142238
NA 1 0 0.975276323
RP11-30K9.6 ENSG00000245975.2 0.196670436 10 9 0.009309072
Brain_Spinal_cord_cervical_c-1 0.601522885 Brain_Caudate_basal_ganglia
4.981739347 0.092402653 0.183093011 -2.600490045 -0.52221178
-1.771332182 0.719132926 9 0 0.975276323
KHDRBS2 ENSG00000112232.8 0.196770298 3 3 0.031875892
Brain_Frontal_Cortex_BA9 0.698169306 Brain_Cerebellar_Hemisphere
1.916584644 0.181229077 0.181229077 -2.145963487 -0.387792892
-1.338879541 0.887887124 3 0 0.975276323
COQ3 ENSG00000132423.11 0.196841575 3 3 0.074171124
Brain_Nucleus_accumbens_basal_ganglia 0.692398554 Brain_Putamen_basal_ganglia
1.692968262 0.425069077 0.425069077 -1.785556326 -0.395602145
-1.067701387 0.696105842 3 0 0.975276323
ACTG2 ENSG00000163017.13 0.19688945 1 1 0.19688945 Brain_Cerebellar_Hemisphere
0.19688945 Brain_Cerebellar_Hemisphere 1 1 1 -1.290464444
-1.290464444 -1.290464444 NA 1 0 0.975276323
HELZ2 ENSG00000130589.16 0.196941941 1 1 0.196941941 Brain_Cerebellum
0.196941941 Brain_Cerebellum 1 1 1 1.290313185 1.290313185
1.290313185 NA 1 0 0.975276323
DOCK5 ENSG00000147459.17 0.196975514 1 1 0.196975514
Brain_Anterior_cingulate_cortex_BA24 0.196975514
Brain_Anterior_cingulate_cortex_BA24 1 1 1 -1.290216455
-1.290216455 -1.290216455 NA 1 0 0.975276323
PLPP7 ENSG00000160539.4 0.197012171 1 1 0.197012171
Brain_Putamen_basal_ganglia 0.197012171 Brain_Putamen_basal_ganglia 1 1
1 1.290110855 1.290110855 1.290110855 NA 1 0
0.975276323
SIX3-AS1 ENSG00000236502.1 0.197034419 4 2 0.141709644
Brain_Frontal_Cortex_BA9 0.419120099 Brain_Nucleus_accumbens_basal_ganglia
2.955216038 0.006847787 0.995044072 -1.469454169 -0.807948727
-1.079922214 0.287235886 2 0 0.975276323
ALOX12B ENSG00000179477.9 0.197062838 1 1 0.197062838
Brain_Putamen_basal_ganglia 0.197062838 Brain_Putamen_basal_ganglia 1 1
1 -1.289964921 -1.289964921 -1.289964921 NA 1 0
0.975276323
DSC1 ENSG00000134765.9 0.197396939 3 3 0.040930492 Brain_Cortex
0.563278582 Brain_Anterior_cingulate_cortex_BA24 1.688480307
0.298166275 0.298166275 -0.577978578 2.04423343 0.813400829
1.318457588 3 0 0.976584361
PLPPR3 ENSG00000129951.18 0.197561124 3 3 0.085865632
Brain_Cerebellum 0.6690569 Brain_Cortex 1.162395168 0.81235428 0.81235428
0.427442717 1.717621743 1.020088029 0.6514535 3 0

0.976995006
UGP2 ENSG00000169764.15 0.197619603 2 2 0.148378355
Brain_Nucleus_accumbens_basal_ganglia 0.316637404 Brain_Cortex
1.049489276 0.950510724 0.950510724 -1.001391847 1.445283199
0.221945676 1.730060516 2 0 0.976995006
ST8SIA5 ENSG00000101638.13 0.197733576 2 1 0.18462316 Brain_Cerebellum
0.222994094 Brain_Cerebellar_Hemisphere 1.952442704 0.047557296
1.952442704 -1.326654011 -1.218605149 -1.27262958 0.076402083
1 0 0.977086887
LINC00237 ENSG00000225127.2 0.197903119 1 1 0.197903119
Brain_Cortex 0.197903119 Brain_Cortex 1 1 1 1.287548666
1.287548666 1.287548666 NA 1 0 0.977086887
YWHAZ ENSG00000164924.17 0.197968036 9 2 0.038067004
Brain_Spinal_cord_cervical_c-1 0.375218892
Brain_Anterior_cingulate_cortex_BA24 7.621813724 0.018490906
0.671534872 -2.074132528 -0.886740011 -1.6284672 0.346146934 2
0 0.977086887
BAZ1A ENSG00000198604.10 0.197975065 1 1 0.197975065
Brain_Cerebellar_Hemisphere 0.197975065 Brain_Cerebellar_Hemisphere 1 1
1 1.287342131 1.287342131 1.287342131 NA 1 0
0.977086887
PPFIA2 ENSG00000139220.16 0.198042089 5 5 0.061474106
Brain_Substantia_nigra 0.990104195 Brain_Caudate_basal_ganglia
3.167932444 0.227007101 0.227007101 -0.20905962 1.87006994
0.758825433 0.861446109 5 0 0.977086887
ZEB1 ENSG00000148516.21 0.198115385 1 1 0.198115385
Brain_Putamen_basal_ganglia 0.198115385 Brain_Putamen_basal_ganglia 1 1
1 -1.286939475 -1.286939475 -1.286939475 NA 1 0
0.977086887
APOBR ENSG00000184730.10 0.198174884 1 1 0.198174884 Brain_Cerebellum
0.198174884 Brain_Cerebellum 1 1 1 1.286768802 1.286768802
1.286768802 NA 1 0 0.977086887
ACADM ENSG00000117054.13 0.198259538 3 2 0.057894596
Brain_Nucleus_accumbens_basal_ganglia 0.987092141 Brain_Cerebellum 1.92061011
0.051034553 1.028355337 -1.896495187 -0.016178308 -0.720178511
1.025285341 2 0 0.977086887
MPDU1 ENSG00000129255.15 0.198266719 1 1 0.198266719
Brain_Cerebellar_Hemisphere 0.198266719 Brain_Cerebellar_Hemisphere 1 1
1 -1.286505446 -1.286505446 -1.286505446 NA 1 0
0.977086887
CXCR1 ENSG00000163464.7 0.198432559 1 1 0.198432559
Brain_Substantia_nigra 0.198432559 Brain_Substantia_nigra 1 1
1 -1.286030094 -1.286030094 -1.286030094 NA 1 0
0.977559837
ARFGAP3 ENSG00000242247.10 0.198604801 11 5 0.089429639
Brain_Nucleus_accumbens_basal_ganglia 0.726665052 Brain_Hypothalamus
8.348229066 0.010178435 0.338773024 -1.698414069 -0.349565173
-0.848758976 0.500271123 5 0 0.977766913
RP11-44M6.1 ENSG00000225807.1 0.198614364 1 1 0.198614364
Brain_Nucleus_accumbens_basal_ganglia 0.198614364
Brain_Nucleus_accumbens_basal_ganglia 1 1 1 -1.285509315
-1.285509315 -1.285509315 NA 1 0 0.977766913
TYW3 ENSG00000162623.15 0.198856248 10 6 0.006441526 Brain_Cerebellum
0.652302844 Brain_Putamen_basal_ganglia 6.339964159 0.042709835
0.255876201 -2.724416265 -0.450565351 -1.570759869 0.780292805
6 0 0.978208199
C3orf18 ENSG00000088543.14 0.198888482 2 2 0.107371661
Brain_Cortex 0.637953957 Brain_Nucleus_accumbens_basal_ganglia
1.663342253 0.336657747 0.336657747 -1.610120005 -0.470561429
-1.040340717 0.805789596 2 0 0.978208199
RP11-713P17.3 ENSG00000204241.7 0.198913754 10 10 0.030269159
Brain_Frontal_Cortex_BA9 0.96430127 Brain_Cerebellar_Hemisphere 2.787475564

0.127312385 0.127312385 -1.129521234 2.166550321 0.128647395
1.198801929 10 0 0.978208199
ACACB ENSG00000076555.15 0.198994954 1 1 0.198994954
Brain_Cerebellar_Hemisphere 0.198994954 Brain_Cerebellar_Hemisphere 1 1
1 -1.284420243 -1.284420243 -1.284420243 NA 1 0
0.978263667
MAN1A2 ENSG00000198162.12 0.199109528 2 2 0.140331782
Brain_Putamen_basal_ganglia 0.196471864 Brain_Cerebellum 1.192987089
0.807012911 0.807012911 1.291668809 1.474556631 1.38311272
0.129321219 2 0 0.978435021
SUSD6 ENSG00000100647.7 0.199303902 1 1 0.199303902
Brain_Caudate_basal_ganglia 0.199303902 Brain_Caudate_basal_ganglia 1 1
1 1.283537295 1.283537295 1.283537295 NA 1 0
0.978435021
MSTO1 ENSG00000125459.14 0.199624871 2 1 0.185172532 Brain_Cerebellum
0.218011848 Brain_Cerebellar_Hemisphere 1.986901092 0.013098908
1.986901092 -1.324995837 -1.231831997 -1.278413917 0.065876783
1 0 0.978435021
NCLN ENSG00000125912.10 0.199703865 12 5 0.051617222
Brain_Frontal_Cortex_BA9 0.957086365 Brain_Cerebellar_Hemisphere
8.577484622 0.006262787 0.33467304 -0.919912641 1.946312428
0.020402962 0.74990941 5 0 0.978435021
LINC02193 ENSG00000260923.6 0.199709516 1 1 0.199709516
Brain_Cortex 0.199709516 Brain_Cortex 1 1 1 1.282379603
1.282379603 1.282379603 NA 1 0 0.978435021
ZNF251 ENSG00000198169.8 0.199712873 8 4 0.128637184
Brain_Nucleus_accumbens_basal_ganglia 0.977514478 Brain_Hypothalamus
5.649186276 0.002567504 0.53871088 -0.30253809 1.519498028
0.252047263 0.764889364 4 0 0.978435021
KRT17 ENSG00000128422.15 0.199719166 3 3 0.045982255 Brain_Cerebellum
0.827979134 Brain_Caudate_basal_ganglia 1.232865293 0.7733085 0.7733085
0.217294126 1.995556162 1.011441159 0.904223335 3 0
0.978435021
ASB13 ENSG00000196372.12 0.199750521 6 6 0.067344976
Brain_Cerebellar_Hemisphere 0.847420734 Brain_Nucleus_accumbens_basal_ganglia
1.416872617 0.611293726 0.611293726 -1.829364806 1.652013815
-0.112457444 1.268030502 6 0 0.978435021
ERC1 ENSG00000082805.19 0.199863317 2 2 0.159991649
Brain_Anterior_cingulate_cortex_BA24 0.225599036 Brain_Caudate_basal_ganglia
1.069953867 0.930046133 0.930046133 1.211773653 1.405099648
1.308436651 0.136702122 2 0 0.978435021
PWP2 ENSG00000241945.7 0.19986637 13 1 0.101059797
Brain_Spinal_cord_cervical_c-1 0.515494026 Brain_Caudate_basal_ganglia
12.51995628 0.001176948 12.51995628 0.650306861 1.639737313
1.256079782 0.240976976 1 0 0.978435021
DEFA3 ENSG00000239839.6 0.199920356 1 1 0.199920356
Brain_Hypothalamus 0.199920356 Brain_Hypothalamus 1 1 1
-1.281778505 -1.281778505 -1.281778505 NA 1 0 0.978435021
SF3A3 ENSG00000183431.11 0.200015628 4 2 0.0938151
Brain_Putamen_basal_ganglia 0.354845258 Brain_Cerebellar_Hemisphere
2.997877084 0.003402129 0.987859651 -1.675607495 0.925231968
-0.976344371 1.268971445 2 0 0.978435021
SIRT5 ENSG00000124523.15 0.200047002 8 4 0.01708108
Brain_Putamen_basal_ganglia 0.481884406 Brain_Cerebellum 6.251162327
0.00303376 0.288359733 -2.384957811 -0.703274971 -1.271000961
0.507430733 4 0 0.978435021
ISCA1 ENSG00000135070.14 0.200048054 3 3 0.145238861
Brain_Anterior_cingulate_cortex_BA24 0.298716946 Brain_Substantia_nigra
1.09345867 0.900969314 0.900969314 1.039188783 1.456556435
1.260999775 0.20991881 3 0 0.978435021
LINC01011 ENSG00000244041.7 0.200197547 9 6 0.0282091 Brain_Cerebellum
0.786637984 Brain_Nucleus_accumbens_basal_ganglia 4.941037226

0.039270521 0.438156426 -2.194366116 -0.27067893 -1.484097848 0.588452477 6 0 0.978435021
DRD4 ENSG00000069696.6 0.200268408 9 6 0.011167538 Brain_Hippocampus 0.75606781 Brain_Putamen_basal_ganglia 4.863923107 0.078524775 0.175652173 0.310648555 2.537412182 1.346812017 0.659934994 6 0 0.978435021
LRRC34 ENSG00000171757.15 0.200302167 3 3 0.11170483 Brain_Cerebellar_Hemisphere 0.886038636 Brain_Cerebellum 1.395827803 0.7504027 0.7504027 -1.590576885 1.111142639 -0.207584253 1.352005792 3 0 0.978435021
RP11-259O2.1 ENSG00000248994.1 0.200350482 2 2 0.143842922 Brain_Cortex 0.394412092 Brain_Amygdala 1.122651077 0.877348923 0.877348923 -1.461628932 0.851643313 -0.30499281 1.635730491 2 0 0.978435021
ATP2B1 ENSG00000070961.15 0.20036703 3 3 0.083724473 Brain_Cerebellum 0.968983101 Brain_Spinal_cord_cervical_c-1 1.897319208 0.2076576 0.2076576 -0.854392354 1.729473011 0.278732314 1.320889856 3 0 0.978435021
FNDC3B ENSG00000075420.12 0.200450904 2 2 0.082101052 Brain_Caudate_basal_ganglia 0.610799537 Brain_Cerebellum 1.041197683 0.958802317 0.958802317 -1.738623261 -0.508932501 -1.123777881 0.869522675 2 0 0.978435021
ZNF32 ENSG00000169740.13 0.200542566 2 2 0.073053568 Brain_Cortex 0.951880181 Brain_Hypothalamus 1.022289966 0.977710034 0.977710034 -1.792495886 0.060345855 -0.866075015 1.31015696 2 0 0.978435021
RP11-88E10.5 ENSG00000274922.1 0.200585829 1 1 0.200585829 Brain_Putamen_basal_ganglia 0.200585829 Brain_Putamen_basal_ganglia 1 1 1 1.279884303 1.279884303 1.279884303 NA 1 0 0.978435021
PGM2 ENSG00000169299.13 0.200684624 11 2 0.026992724 Brain_Cortex 0.336979296 Brain_Hypothalamus 9.700876024 0.005878884 0.651261725 -2.211623014 -0.960150856 -1.655649777 0.348457901 2 0 0.978435021
RNF4 ENSG00000063978.15 0.200756047 5 5 0.028440165 Brain_Putamen_basal_ganglia 0.728715109 Brain_Amygdala 1.629076311 0.419006717 0.419006717 -0.831012591 2.191160707 0.936523325 1.189030854 5 0 0.978435021
RP11-354K4.2 ENSG00000271945.1 0.200843036 1 1 0.200843036 Brain_Hypothalamus 0.200843036 Brain_Hypothalamus 1 1 1 1.279153417 1.279153417 1.279153417 NA 1 0 0.978435021
PPIH ENSG00000171960.10 0.200882891 1 1 0.200882891 Brain_Hypothalamus 0.200882891 Brain_Hypothalamus 1 1 1 -1.279040227 -1.279040227 -1.279040227 NA 1 0 0.978435021
MPV17 ENSG00000115204.14 0.201007202 1 1 0.201007202 Brain_Spinal_cord_cervical_c-1 0.201007202 Brain_Spinal_cord_cervical_c-1 1 1 1 -1.278687277 -1.278687277 -1.278687277 NA 1 0 0.978435021
RP11-56F10.3 ENSG00000254396.1 0.201052301 1 1 0.201052301 Brain_Cerebellar_Hemisphere 0.201052301 Brain_Cerebellar_Hemisphere 1 1 1 1.27855927 1.27855927 1.27855927 NA 1 0 0.978435021
H2AFY ENSG00000113648.16 0.201101178 1 1 0.201101178 Brain_Nucleus_accumbens_basal_ganglia 0.201101178 Brain_Nucleus_accumbens_basal_ganglia 1 1 1 1.278420561 1.278420561 1.278420561 NA 1 0 0.978435021
ZNF414 ENSG00000133250.13 0.201127805 3 3 0.104982236 Brain_Cerebellum 0.62329697 Brain_Cortex 2.223681069 0.262981301 0.262981301 -1.621165098 -0.491183072 -1.141714965 0.584094659 3 0 0.978435021
GIPR ENSG00000010310.8 0.201353438 1 1 0.201353438 Brain_Hippocampus 0.201353438 Brain_Hippocampus 1 1 1 -1.277705068 -1.277705068 -1.277705068 NA 1 0 0.978731946

VSNL1 ENSG00000163032.11 0.201427574 1 1 0.201427574
Brain_Frontal_Cortex_BA9 0.201427574 Brain_Frontal_Cortex_BA9 1 1
1 1.277494916 1.277494916 1.277494916 NA 1 0
0.978731946
ATP2C1 ENSG00000017260.19 0.201462089 4 3 0.152981045
Brain_Nucleus_accumbens_basal_ganglia 0.803975068 Brain_Frontal_Cortex_BA9
2.544157539 0.069480072 0.392444549 -0.248205943 1.429080682
0.680008024 0.801598369 3 0 0.978731946
TPSB2 ENSG00000197253.13 0.201672492 12 3 0.135513745
Brain_Spinal_cord_cervical_c-1 0.970288583 Brain_Substantia_nigra
9.631990819 0.003611193 0.673891639 -0.682423125 1.492707594
-0.173727973 0.618579213 3 0 0.978731946
DIRC2 ENSG00000138463.8 0.201728198 2 2 0.073726193 Brain_Cerebellum
0.896503286 Brain_Nucleus_accumbens_basal_ganglia 1.039122588
0.960877412 0.960877412 -1.78830885 -0.130079807 -0.959194328
1.172545001 2 0 0.978731946
RBM17 ENSG00000134453.15 0.201738672 2 2 0.076183469
Brain_Putamen_basal_ganglia 0.799707318 Brain_Frontal_Cortex_BA9
1.272692942 0.727307058 0.727307058 0.253725907 1.773273073
1.01349949 1.074482106 2 0 0.978731946
C6orf58 ENSG00000184530.8 0.201750169 1 1 0.201750169
Brain_Frontal_Cortex_BA9 0.201750169 Brain_Frontal_Cortex_BA9 1 1
1 -1.276581125 -1.276581125 -1.276581125 NA 1 0
0.978731946
HYLS1 ENSG00000198331.10 0.201771586 2 2 0.145030634
Brain_Substantia_nigra 0.317348831 Brain_Anterior_cingulate_cortex_BA24
1.025903804 0.974096196 0.974096196 -0.999920814 1.457310701
0.228694944 1.737525067 2 0 0.978731946
RP11-234B24.2 ENSG00000255474.1 0.201818431 3 3 0.100733041
Brain_Cerebellum 0.750700591 Brain_Cerebellar_Hemisphere 1.161106234
0.872514212 0.872514212 -1.266998208 1.641310194 0.018865424
1.483081113 3 0 0.978731946
THOC5 ENSG00000100296.13 0.202095176 2 2 0.305017894
Brain_Caudate_basal_ganglia 0.939884891 Brain_Cortex 1.794233923
0.205766077 0.205766077 -1.025732069 0.07541454 -0.475158765
0.778628234 2 0 0.979476941
KIF12 ENSG00000136883.13 0.20217207 7 4 0.015514908
Brain_Caudate_basal_ganglia 0.646187159 Brain_Putamen_basal_ganglia
4.680746848 0.003384181 0.517328919 -1.313048895 2.420131207
0.832932562 1.612701094 4 0 0.979476941
CTC-304I17.6 ENSG00000279668.2 0.202182075 4 3 0.092730423
Brain_Amygdala 0.675384825 Brain_Cerebellar_Hemisphere 2.060167408
0.018799049 0.907482463 -1.681167303 0.441355059 -0.507602898
1.099759803 3 0 0.979476941
LRRN2 ENSG00000170382.11 0.202407918 2 2 0.197291507
Brain_Caudate_basal_ganglia 0.204115466 Brain_Putamen_basal_ganglia
1.025058666 0.974941334 0.974941334 -1.289306629 1.269913437
-0.009696596 1.809641864 2 0 0.979511814
LINC02043 ENSG00000232233.1 0.202517917 1 1 0.202517917
Brain_Cerebellum 0.202517917 Brain_Cerebellum 1 1 1 -1.274410658
-1.274410658 -1.274410658 NA 1 0 0.979511814
NDUFAF5 ENSG00000101247.17 0.202529624 4 4 0.103707188
Brain_Cortex 0.70177093 Brain_Spinal_cord_cervical_c-1 2.120431552
0.272092867 0.272092867 -0.382930993 1.627140844 0.795247562
0.879107399 4 0 0.979511814
CECR2 ENSG00000099954.18 0.202544128 3 2 0.073214309 Brain_Cerebellum
0.912929756 Brain_Caudate_basal_ganglia 1.978107327 0.021859553
1.00003312 -0.109343864 1.791492424 1.146089403 1.087380948 2
0 0.979511814
EPHA5 ENSG00000145242.13 0.202556904 3 3 0.123421672
Brain_Nucleus_accumbens_basal_ganglia 0.669337383 Brain_Cerebellar_Hemisphere
2.006442444 0.082110544 0.082110544 -1.540569189 0.830236272

-0.094425109 1.268517949 3 0 0.979511814
CYB5R4 ENSG00000065615.13 0.202609334 1 1 0.202609334
Brain_Cerebellum 0.202609334 Brain_Cerebellum 1 1 1 -1.274152618
-1.274152618 -1.274152618 NA 1 0 0.979511814
CD300C ENSG00000167850.3 0.202806538 1 1 0.202806538
Brain_Frontal_Cortex_BA9 0.202806538 Brain_Frontal_Cortex_BA9 1 1
1 1.273596262 1.273596262 1.273596262 NA 1 0
0.979708282
NOD2 ENSG00000167207.11 0.203006593 1 1 0.203006593 Brain_Cerebellum
0.203006593 Brain_Cerebellum 1 1 1 -1.273032265 -1.273032265
-1.273032265 NA 1 0 0.979708282
NEUROD2 ENSG00000171532.4 0.203072082 1 1 0.203072082
Brain_Hypothalamus 0.203072082 Brain_Hypothalamus 1 1 1
-1.272847726 -1.272847726 -1.272847726 NA 1 0 0.979708282
RNF8 ENSG00000112130.16 0.203082325 8 8 0.098613882
Brain_Hypothalamus 0.936971464 Brain_Cerebellum 2.439385083
0.260969558 0.260969558 -1.198018312 1.65161097 -0.391377756
0.988459197 8 0 0.979708282
ACAP2 ENSG00000114331.13 0.203125597 11 6 0.071868064 Brain_Amygdala
0.857835596 Brain_Anterior_cingulate_cortex_BA24 6.838293435 0.012766
0.291507207 -1.799952972 0.955275116 0.191566057 0.752833015
6 0 0.979708282
RNF187 ENSG00000168159.11 0.203158321 1 1 0.203158321
Brain_Cerebellum 0.203158321 Brain_Cerebellum 1 1 1 1.272604781
1.272604781 1.272604781 NA 1 0 0.979708282
DUOXA1 ENSG00000140254.12 0.203266954 4 4 0.01895869
Brain_Hippocampus 0.96844073 Brain_Substantia_nigra 1.670483975
0.36066569 0.36066569 0.039563999 2.346342236 1.03498969 1.156322217
4 0 0.979708282
MRPL45 ENSG00000278845.4 0.203428271 6 5 0.186725297
Brain_Hypothalamus 0.939914418 Brain_Frontal_Cortex_BA9 2.880193058
0.043882929 0.28625541 -0.527523743 1.320328701 0.192114083
0.799417939 5 0 0.979708282
NADK ENSG00000008130.15 0.203581188 8 8 0.034923763
Brain_Caudate_basal_ganglia 0.990858719 Brain_Cerebellum 2.021759294
0.209694109 0.209694109 -2.109241247 0.011457148 -0.854602387
0.825075869 8 0 0.979708282
ADRA2B ENSG00000274286.1 0.203647116 3 2 0.111082845
Brain_Cerebellum 0.982762136 Brain_Spinal_cord_cervical_c-1 2.308667459
0.001292637 0.690039904 -1.593344876 0.02160614 -1.047709967
0.926119967 2 0 0.979708282
DHDDS ENSG00000117682.16 0.20368681 5 5 0.171727043 Brain_Hippocampus
0.781133603 Brain_Cerebellum 1.945148393 0.129291464 0.129291464
-0.755719267 1.366675494 0.136461803 0.841188598 5 0
0.979708282
IFNE ENSG00000184995.7 0.203792261 3 3 0.035307767
Brain_Anterior_cingulate_cortex_BA24 0.680832485 Brain_Cerebellum
1.905894079 0.094218128 0.094218128 -0.431584863 2.104810953
0.420632899 1.458576148 3 0 0.979708282
C12orf76 ENSG00000174456.13 0.203824004 2 2 0.358150559
Brain_Cerebellar_Hemisphere 0.549102374 Brain_Hippocampus 1.634200047
0.365799953 0.365799953 -0.918894879 0.59910574 -0.159894569
1.073388531 2 0 0.979708282
PRKAR1B ENSG00000188191.14 0.203836475 2 2 0.094069025
Brain_Cerebellum 0.805994173 Brain_Putamen_basal_ganglia 1.211962426
0.788037574 0.788037574 0.24559705 1.674313387 0.959955218
1.010255011 2 0 0.979708282
CCDC102A ENSG00000135736.5 0.203992027 1 1 0.203992027
Brain_Substantia_nigra 0.203992027 Brain_Substantia_nigra 1 1
1 1.270260013 1.270260013 1.270260013 NA 1 0
0.979708282
ARHGAP27 ENSG00000159314.11 0.204089089 6 5 0.086320344

Brain_Cortex 0.968492812 Brain_Caudate_basal_ganglia 2.894595362
0.069623054 0.141851224 -1.715135732 0.81395936 -0.473373919
0.853163082 5 0 0.979708282
FAM151A ENSG00000162391.11 0.204135955 2 2 0.173478706
Brain_Substantia_nigra 0.817457177 Brain_Anterior_cingulate_cortex_BA24
1.541488752 0.458511248 0.458511248 -0.230816739 1.361110709
0.565146985 1.125662694 2 0 0.979708282
LARGE2 ENSG00000165905.17 0.204172399 1 1 0.204172399
Brain_Cortex 0.204172399 Brain_Cortex 1 1 1 1.269753642
1.269753642 1.269753642 NA 1 0 0.979708282
MKX ENSG00000150051.13 0.204279765 3 3 0.084215187
Brain_Nucleus_accumbens_basal_ganglia 0.948645384 Brain_Cerebellum
1.162611208 0.816865141 0.816865141 -0.06440797 1.726735445
0.987293436 0.935509557 3 0 0.979708282
C21orf33 ENSG00000160221.16 0.204311203 13 1 0.118538737
Brain_Substantia_nigra 0.365363201 Brain_Cerebellar_Hemisphere
12.63346301 0.000298515 12.63346301 0.905192903 1.560936488
1.250375726 0.187681218 1 0 0.979708282
RP11-253E3.3 ENSG00000250899.3 0.2043941 1 1 0.2043941
Brain_Hippocampus 0.2043941 Brain_Hippocampus 1 1 1
1.269131689 1.269131689 1.269131689 NA 1 0 0.979708282
CTD-2587M2.1 ENSG00000249476.1 0.204413884 11 5 0.022067144
Brain_Spinal_cord_cervical_c-1 0.976345616 Brain_Frontal_Cortex_BA9
7.582333372 0.008628105 0.379930026 -0.039136603 2.289210175
1.2855947 0.750885635 5 0 0.979708282
RRS1 ENSG00000179041.3 0.204461908 2 2 0.084542724
Brain_Spinal_cord_cervical_c-1 0.618093514 Brain_Putamen_basal_ganglia
1.030262696 0.969737304 0.969737304 0.498554148 1.724915376
1.111734762 0.86716834 2 0 0.979708282
KIAA1257 ENSG00000114656.10 0.204534454 2 2 0.075751923
Brain_Cortex 0.966219005 Brain_Substantia_nigra 1.103588857
0.896411143 0.896411143 0.042350855 1.775884702 0.909117779
1.225793539 2 0 0.979708282
PHLDA2 ENSG00000181649.5 0.20456243 1 1 0.20456243
Brain_Frontal_Cortex_BA9 0.20456243 Brain_Frontal_Cortex_BA9 1 1 1
-1.268659788 -1.268659788 -1.268659788 NA 1 0 0.979708282
PTK2B ENSG00000120899.17 0.204580726 2 2 0.092751682 Brain_Cerebellum
0.753201612 Brain_Putamen_basal_ganglia 1.159220745 0.840779255
0.840779255 -0.314420601 1.681057831 0.683318615 1.411016331
2 0 0.979708282
TRAPPC2L ENSG00000167515.10 0.204736112 4 4 0.076895149
Brain_Cerebellum 0.591283357 Brain_Cerebellar_Hemisphere 2.692437342
0.114213368 0.114213368 -0.536977209 1.768992361 1.075516656
1.080781966 4 0 0.979708282
ACPT ENSG00000142513.5 0.204979002 3 3 0.120385384
Brain_Hippocampus 0.557853166 Brain_Cerebellar_Hemisphere 1.483423153
0.505118174 0.505118174 0.586033258 1.553158034 1.178118537
0.518810405 3 0 0.979708282
SLC38A6 ENSG00000139974.15 0.205034261 1 1 0.205034261
Brain_Cortex 0.205034261 Brain_Cortex 1 1 1 -1.267338553
-1.267338553 -1.267338553 NA 1 0 0.979708282
DNAL1 ENSG00000119661.14 0.205080301 5 4 0.169964533
Brain_Hippocampus 0.963393654 Brain_Frontal_Cortex_BA9 3.743324897
0.039284345 0.208513143 -1.372317781 0.045895358 -0.609748187
0.630014946 4 0 0.979708282
NUP54 ENSG00000138750.14 0.205122201 13 4 0.048662071
Brain_Frontal_Cortex_BA9 0.807869649 Brain_Cerebellar_Hemisphere
10.03048119 0.001611634 0.548538248 -0.709381099 1.971540624
0.627150764 0.722396017 4 0 0.979708282
PHF2 ENSG00000197724.10 0.205159594 1 1 0.205159594 Brain_Cerebellum
0.205159594 Brain_Cerebellum 1 1 1 1.266987963 1.266987963
1.266987963 NA 1 0 0.979708282

BCCIP ENSG00000107949.16 0.205179302 1 1 0.205179302 Brain_Cerebellum
0.205179302 Brain_Cerebellum 1 1 1 1.266932849 1.266932849
1.266932849 NA 1 0 0.979708282
TBC1D4 ENSG00000136111.12 0.205197616 9 5 0.028351185
Brain_Hypothalamus 0.904372344 Brain_Putamen_basal_ganglia 6.077618004
0.044976596 0.24960495 0.120139877 2.192392395 0.716387649
0.62960865 5 0 0.979708282
TNFAIP6 ENSG00000123610.4 0.205199929 7 5 0.013392368
Brain_Nucleus_accumbens_basal_ganglia 0.251167794 Brain_Putamen_basal_ganglia
4.956498574 0.03283248 0.173794728 -2.473161302 -1.147517526
-1.793879845 0.455218035 5 0 0.979708282
HIC1 ENSG00000177374.12 0.205243531 1 1 0.205243531
Brain_Hypothalamus 0.205243531 Brain_Hypothalamus 1 1 1
-1.266753256 -1.266753256 -1.266753256 NA 1 0 0.979708282
PPIL1 ENSG00000137168.7 0.205244731 1 1 0.205244731
Brain_Anterior_cingulate_cortex_BA24 0.205244731
Brain_Anterior_cingulate_cortex_BA24 1 1 1 1.266749901
1.266749901 1.266749901 NA 1 0 0.979708282
CS ENSG00000062485.18 0.205424185 1 1 0.205424185
Brain_Spinal_cord_cervical_c-1 0.205424185 Brain_Spinal_cord_cervical_c-1
1 1 1 -1.266248343 -1.266248343 -1.266248343 NA 1 0
0.979708282
INTS8 ENSG00000164941.13 0.205521895 5 4 0.046186894
Brain_Cerebellar_Hemisphere 0.976394234 Brain_Anterior_cingulate_cortex_BA24
2.301964868 0.021294939 0.738325838 0.029589757 1.993681304
1.260749204 0.868920564 4 0 0.979708282
SFXN5 ENSG00000144040.12 0.205550613 6 4 0.036445143 Brain_Cerebellum
0.510114782 Brain_Anterior_cingulate_cortex_BA24 4.554038333
0.022243339 0.202215405 0.658658976 2.09192592 1.440967728
0.595882726 4 0 0.979708282
LINC00667 ENSG00000263753.6 0.205729433 12 7 0.015257178
Brain_Amygdala 0.936500453 Brain_Anterior_cingulate_cortex_BA24
6.741825229 0.024411152 0.274148896 -2.426216203 -0.079669079
-0.89962192 0.612197771 7 0 0.979708282
PSMF1 ENSG00000125818.17 0.205789877 10 3 0.079014217
Brain_Caudate_basal_ganglia 0.872203079 Brain_Anterior_cingulate_cortex_BA24
8.261989488 0.000569405 0.622754678 0.160860747 1.756434672
1.359561906 0.456822979 3 0 0.979708282
RP11-70P17.1 ENSG00000225643.1 0.20581839 1 1 0.20581839
Brain_Hippocampus 0.20581839 Brain_Hippocampus 1 1 1
1.265147691 1.265147691 1.265147691 NA 1 0 0.979708282
ZNF692 ENSG00000171163.15 0.205870763 1 1 0.205870763
Brain_Cerebellum 0.205870763 Brain_Cerebellum 1 1 1 1.265001577
1.265001577 1.265001577 NA 1 0 0.979708282
RP11-732A21.3 ENSG00000277459.1 0.205887347 1 1 0.205887347
Brain_Amygdala 0.205887347 Brain_Amygdala 1 1 1 1.264955313
1.264955313 1.264955313 NA 1 0 0.979708282
PAH ENSG00000171759.9 0.205918515 8 8 0.126158156
Brain_Hypothalamus 0.708684794 Brain_Frontal_Cortex_BA9 2.81809963
0.196442098 0.196442098 -1.4755987 1.529428888 0.08865564
1.107209248 8 0 0.979708282
CYP7B1 ENSG00000172817.3 0.205941109 2 2 0.081521214
Brain_Cerebellar_Hemisphere 0.722546456 Brain_Cerebellum 1.000036865
0.999963135 0.999963135 -1.741927028 -0.355057579 -1.048492303
0.980664792 2 0 0.979708282
RGS7BP ENSG00000186479.4 0.206196972 1 1 0.206196972
Brain_Cerebellum 0.206196972 Brain_Cerebellum 1 1 1 1.264092099
1.264092099 1.264092099 NA 1 0 0.980246923
ASPRV1 ENSG00000244617.2 0.206320433 4 4 0.031591798
Brain_Nucleus_accumbens_basal_ganglia 0.98932207 Brain_Putamen_basal_ganglia
1.915964736 0.199194651 0.199194651 -2.149537735 -0.0133832
-1.429698095 0.988912527 4 0 0.980246923

C9orf72 ENSG00000147894.14 0.206359321 12 9 0.052312818
Brain_Cortex 0.999457707 Brain_Cerebellar_Hemisphere 5.752224033
0.071863426 0.261512476 -1.94055054 0.620778567 -0.515061941
0.733923287 9 0 0.980246923
HCRTR1 ENSG00000121764.11 0.206386097 1 1 0.206386097
Brain_Cerebellum 0.206386097 Brain_Cerebellum 1 1 1 -1.263565294
-1.263565294 -1.263565294 NA 1 0 0.980246923
LPCAT3 ENSG00000111684.10 0.206420377 2 2 0.216553276
Brain_Cerebellum 0.535383327 Brain_Cerebellar_Hemisphere 1.915961227
0.084038773 0.084038773 0.619809328 1.235745239 0.927777283
0.435532459 2 0 0.980246923
RP5-998H6.2 ENSG00000229957.1 0.206767983 1 1 0.206767983
Brain_Cerebellar_Hemisphere 0.206767983 Brain_Cerebellar_Hemisphere 1 1
1 1.262502623 1.262502623 1.262502623 NA 1 0
0.980246923
BMP4 ENSG00000125378.15 0.206769416 2 2 0.191898627 Brain_Cerebellum
0.509061617 Brain_Cerebellar_Hemisphere 1.356075543 0.643924457
0.643924457 -1.304983029 0.660299553 -0.322341738 1.389664641
2 0 0.980246923
PKN3 ENSG00000160447.6 0.206820178 2 2 0.092054561
Brain_Frontal_Cortex_BA9 0.747555725 Brain_Cerebellar_Hemisphere
1.138236871 0.861763129 0.861763129 -0.321864003 1.684658068
0.681397033 1.418825363 2 0 0.980246923
CPTP ENSG00000224051.6 0.206899668 3 3 0.081070332 Brain_Cerebellum
0.732865993 Brain_Hypothalamus 1.381089399 0.530478214
0.530478214 -1.744509237 0.466670981 -0.539717957 1.118861969
3 0 0.980246923
CHCHD5 ENSG00000125611.15 0.206933447 7 6 0.006301567
Brain_Cerebellar_Hemisphere 0.515685808 Brain_Hippocampus 5.207766179
0.060034023 0.178468302 -2.731662449 -0.650009929 -1.600262298
0.62928061 6 0 0.980246923
POGLUT1 ENSG00000163389.10 0.206962766 10 2 0.083180405
Brain_Cortex 0.320289557 Brain_Cerebellar_Hemisphere 9.069014237
0.003146101 0.374551919 0.993863027 1.732523453 1.271202422
0.205790179 2 0 0.980246923
SELENOP ENSG00000250722.5 0.207037069 1 1 0.207037069
Brain_Caudate_basal_ganglia 0.207037069 Brain_Caudate_basal_ganglia 1 1
1 1.261754696 1.261754696 1.261754696 NA 1 0
0.980246923
PPP2R2B ENSG00000156475.18 0.207043489 4 4 0.091969385
Brain_Frontal_Cortex_BA9 0.741014431 Brain_Putamen_basal_ganglia
2.113115848 0.174716211 0.174716211 -1.022772641 1.685099452
0.546214775 1.186363526 4 0 0.980246923
ZNF860 ENSG00000197385.5 0.207052875 3 3 0.13162694
Brain_Anterior_cingulate_cortex_BA24 0.63247309 Brain_Cerebellum 1.28998035
0.728809618 0.728809618 -0.927394537 1.507717126 0.034024576
1.295862747 3 0 0.980246923
PLAC9 ENSG00000189129.13 0.207116643 10 7 0.196237773
Brain_Hippocampus 0.964271904 Brain_Anterior_cingulate_cortex_BA24
5.920945201 0.044468447 0.214206449 -1.037952593 1.292344773
0.109045247 0.857975286 7 0 0.980246923
VPS33A ENSG00000139719.9 0.207176478 4 4 0.160373055
Brain_Cerebellar_Hemisphere 0.787801342 Brain_Cerebellum 3.292878577
0.124541831 0.124541831 0.269166781 1.403817991 0.808721184
0.46750526 4 0 0.980246923
NSUN3 ENSG00000178694.9 0.207361532 1 1 0.207361532
Brain_Substantia_nigra 0.207361532 Brain_Substantia_nigra 1 1
1 1.260853783 1.260853783 1.260853783 NA 1 0
0.980246923
RP11-121C2.2 ENSG00000259959.1 0.20738364 9 4 0.100522382
Brain_Amygdala 0.579556048 Brain_Cerebellar_Hemisphere 6.719427516
0.015653779 0.282920615 -1.642326384 -0.554033312 -1.246218598

0.351254924	4	0	0.980246923
LINC00299	ENSG00000236790.5	0.207418905	8	5	0.143373162	Brain_Nucleus_accumbens_basal_ganglia	0.951966427	Brain_Amygdala	5.521729978	0.084650276	0.32247827	-1.463344409	0.905820116	-0.124540022	0.870707135	5	0	0.980246923
EFR3A	ENSG00000132294.14	0.207607559	7	5	0.004393494	Brain_Amygdala	0.843233083	Brain_Hypothalamus	3.850200006	0.045298315	0.18125706	0.197759694	2.848434188	1.694185343	1.010391498	5	0	0.980246923
AHI1	ENSG00000135541.20	0.207656902	11	8	0.107022759	Brain_Cortex	0.86246318	Brain_Nucleus_accumbens_basal_ganglia	5.036337581	0.042805032	0.315000482	-1.611720571	1.56452678	0.188369031	0.969426764	8	0	0.980246923
HOMER2	ENSG00000103942.12	0.207748554	4	4	0.07925963	Brain_Spinal_cord_cervical_c-1	0.585641587	Brain_Cerebellum	1.92729797	0.10747872	0.10747872	-0.990291834	1.754998114	0.557652646	1.149167501	4	0	0.980246923
ZNF566	ENSG00000186017.14	0.207819339	2	2	0.121300885	Brain_Cerebellum	0.727884234	Brain_Cerebellar_Hemisphere	1.647966791	0.352033209	0.352033209	-1.549336353	-0.347941346	-0.94863885	0.849514557	2	0	0.980246923
SPTB	ENSG00000070182.19	0.207826119	1	1	0.207826119	Brain_Cerebellar_Hemisphere	0.207826119	Brain_Cerebellar_Hemisphere	1	1	1	-1.259565579	-1.259565579	-1.259565579	NA	1	0	0.980246923
HAUS6	ENSG00000147874.10	0.207936993	2	2	0.104086404	Brain_Frontal_Cortex_BA9	0.422392441	Brain_Cerebellum	1.059652863	0.940347137	0.940347137	-1.625357509	0.802277523	-0.411539993	1.716597193	2	0	0.980246923
LINC01170	ENSG00000253807.5	0.207941584	2	2	0.206710052	Brain_Caudate_basal_ganglia	0.251195746	Brain_Putamen_basal_ganglia	1.073525857	0.926474143	0.926474143	-1.147449857	1.262663736	0.057606939	1.704207665	2	0	0.980246923
FAM208A	ENSG00000163946.13	0.208038924	4	4	0.178444459	Brain_Spinal_cord_cervical_c-1	0.865742567	Brain_Nucleus_accumbens_basal_ganglia	2.298900518	0.280298075	0.280298075	-0.451024622	1.345560001	0.315134645	0.749240275	4	0	0.980246923
RIC8A	ENSG00000177963.14	0.20811585	10	5	0.158688324	Brain_Putamen_basal_ganglia	0.954102165	Brain_Anterior_cingulate_cortex_BA24	6.987915569	6.94E-18	0.379766904	-0.767562368	1.409496785	-0.186382945	0.713613013	5	0	0.980246923
ACY1	ENSG00000243989.8	0.208137118	1	1	0.208137118	Brain_Cerebellum	0.208137118	Brain_Cerebellum	1	1	1	-1.25870441	-1.25870441	-1.25870441	NA	1	0	0.980246923
RP11-367F23.1	ENSG00000228216.1	0.208204046	1	1	0.208204046	Brain_Frontal_Cortex_BA9	0.208204046	Brain_Frontal_Cortex_BA9	1	1	1	1.258519205	1.258519205	1.258519205	NA	1	0	0.980246923
PFKM	ENSG00000152556.15	0.208226278	3	3	0.368886665	Brain_Cerebellar_Hemisphere	0.874887484	Brain_Cerebellum	2.595144332	0.101567764	0.101567764	-0.898560766	0.330284227	-0.24191	0.618760604	3	0	0.980246923
PKP2	ENSG00000057294.14	0.208539869	1	1	0.208539869	Brain_Caudate_basal_ganglia	0.208539869	Brain_Caudate_basal_ganglia	1	1	1	1.257590559	1.257590559	1.257590559	NA	1	0	0.980935746
ADAMTS4	ENSG00000158859.9	0.208544329	3	3	0.174669064	Brain_Substantia_nigra	0.623252521	Brain_Frontal_Cortex_BA9	1.569665542	0.56786734	0.56786734	-0.491245925	1.357353033	0.735710158	1.062606506	3	0	0.980935746
COX6B1	ENSG00000126267.8	0.208582935	7	5	0.065266217

Brain_Anterior_cingulate_cortex_BA24 0.956403344 Brain_Cortex 4.505548007 0.000676875 0.175086283 -1.843430249 0.355833866 -0.635608478 0.805895172 5 0 0.980935746
P3H3 ENSG00000110811.19 0.208848041 2 2 0.077620722 Brain_Cerebellum 0.930774967 Brain_Caudate_basal_ganglia 1.027335109 0.972664891 0.972664891 -1.764661207 0.086869848 -0.838895679 1.309230164 2 0 0.981194841
GS1-279B7.2 ENSG00000273004.1 0.209000123 1 1 0.209000123 Brain_Anterior_cingulate_cortex_BA24 0.209000123 Brain_Anterior_cingulate_cortex_BA24 1 1 1 1.256319586 1.256319586 1.256319586 NA 1 0 0.981194841
VIPR2 ENSG00000106018.13 0.209029134 3 3 0.125745269 Brain_Frontal_Cortex_BA9 0.715832439 Brain_Cortex 1.682915313 0.309539799 0.309539799 -1.531097617 1.350910066 -0.181407264 1.449657384 3 0 0.981194841
STARD3NL ENSG00000010270.13 0.209039499 5 3 0.079832843 Brain_Cortex 0.522172715 Brain_Putamen_basal_ganglia 4.310096753 0.001673833 0.232466253 0.639999822 1.751656778 1.115850594 0.481408292 3 0 0.981194841
LARP1 ENSG00000155506.16 0.209073609 3 3 0.050575931 Brain_Nucleus_accumbens_basal_ganglia 0.773660928 Brain_Hippocampus 1.214846915 0.803969668 0.803969668 -1.95506049 0.338177277 -0.44309788 1.309642309 3 0 0.981194841
RECQL5 ENSG00000108469.14 0.209132072 4 4 0.106281898 Brain_Cerebellum 0.831417239 Brain_Caudate_basal_ganglia 2.307166987 0.3272425 0.3272425 -0.212884261 1.615132993 0.817310577 0.786512094 4 0 0.981194841
RP1-79C4.4 ENSG00000271811.1 0.209144906 11 9 0.012006269 Brain_Cerebellum 0.942897944 Brain_Amygdala 4.476877462 0.024455701 0.179016883 -2.51196003 0.859522502 -0.495124012 1.016930367 9 0 0.981194841
NSMAF ENSG00000035681.7 0.209292908 3 3 0.135494882 Brain_Cerebellum 0.728650897 Brain_Caudate_basal_ganglia 1.464038636 0.73911982 0.73911982 -1.492779628 1.059511186 -0.260063046 1.278360391 3 0 0.981194841
BNIPL ENSG00000163141.18 0.209326556 2 2 0.079041288 Brain_Caudate_basal_ganglia 0.940861704 Brain_Cerebellum 1.159097428 0.840902572 0.840902572 0.074186856 1.756276028 0.915231442 1.18941666 2 0 0.981194841
NDUFA11 ENSG00000174886.12 0.209527278 2 1 0.143566727 Brain_Cerebellum 0.311580935 Brain_Cerebellar_Hemisphere 1.944314813 0.055685187 1.944314813 1.011910319 1.462637021 1.23727367 0.318711907 1 0 0.981194841
ATP6V1F ENSG00000128524.4 0.209609209 1 1 0.209609209 Brain_Caudate_basal_ganglia 0.209609209 Brain_Caudate_basal_ganglia 1 1 1 -1.254640734 -1.254640734 -1.254640734 NA 1 0 0.981194841
CDT1 ENSG00000167513.8 0.209731542 1 1 0.209731542 Brain_Anterior_cingulate_cortex_BA24 0.209731542 Brain_Anterior_cingulate_cortex_BA24 1 1 1 1.25430397 1.25430397 1.25430397 NA 1 0 0.981194841
STK4-AS1 ENSG00000227477.1 0.209757658 2 2 0.079668808 Brain_Hypothalamus 0.624382557 Brain_Frontal_Cortex_BA9 1.398661247 0.601338753 0.601338753 -1.752610963 -0.489648632 -1.121129798 0.893049229 2 0 0.981194841
DFNA5 ENSG00000105928.13 0.209781529 12 3 0.050379223 Brain_Cortex 0.331615285 Brain_Cerebellum 9.556682919 0.000556216 0.787622173 -1.956729977 0.97086545 -1.246164872 0.738157564 3 0 0.981194841
RANGAP1 ENSG00000100401.19 0.209975123 2 1 0.194958389 Brain_Cerebellum 0.241365695 Brain_Cerebellar_Hemisphere 1.937262892 0.062737108 1.937262892 -1.296049623 -1.171580067 -1.233814845

0.088013267	1	0	0.981194841
KIAA0825	ENSG00000185261.14	0.210007552	2	2	0.403004036
Brain_Cerebellar_Hemisphere 0.853569532	Brain_Cerebellum 1.925504609
0.074495391	0.074495391	0.184565902	0.836268205	0.510417054
0.460823117	2	0	0.981194841
SLC16A14	ENSG00000163053.10	0.210149675	1	1	0.210149675
Brain_Cerebellum 0.210149675	Brain_Cerebellum 1	1	1	1.253153977
1.253153977	1.253153977	NA	1	0	0.981194841
PLXNB2	ENSG00000196576.14	0.210175098	1	1	0.210175098
Brain_Cerebellum 0.210175098	Brain_Cerebellum 1	1	1	1.25308411
1.25308411 1.25308411 NA	1	0	0.981194841
JRKL	ENSG00000183340.6	0.210182014	1	1	0.210182014
Brain_Caudate_basal_ganglia 0.210182014	Brain_Caudate_basal_ganglia 1	1
1	-1.253065106	-1.253065106	-1.253065106	NA	1	0
0.981194841
CBFA2T3	ENSG00000129993.14	0.210265665	1	1	0.210265665
Brain_Putamen_basal_ganglia 0.210265665	Brain_Putamen_basal_ganglia 1	1
1	-1.252835265	-1.252835265	-1.252835265	NA	1	0
0.981194841
CPLX1 ENSG00000168993.14	0.210283137	3	3	0.141996995	Brain_Cortex
0.236792699	Brain_Cerebellar_Hemisphere 1.800699495	0.206228798
0.206228798	-1.468394866	1.427418866	0.380688501	1.60600811 3
0	0.981194841
PABPC1	ENSG00000070756.15	0.210458733	6	6	0.078246223
Brain_Spinal_cord_cervical_c-1	0.907450572
Brain_Anterior_cingulate_cortex_BA24	1.781396926	0.514668441
0.514668441	-1.463101942	1.760953799	0.365838558	1.152958552
6	0	0.981194841
MRPL36	ENSG00000171421.12	0.210498747	6	1	0.151024854
Brain_Cerebellum 0.371507233	Brain_Cortex	5.732907242	7.79E-05
5.732907242	0.893653649	1.435920886	1.219835468	0.250959736
1	0	0.981194841
BRAP	ENSG00000089234.15	0.21059505 1	1	0.21059505 Brain_Caudate_basal_ganglia
0.21059505 Brain_Caudate_basal_ganglia 1	1	1	1.251930884
1.251930884	1.251930884	NA	1	0	0.981194841
PLCG1 ENSG00000124181.14	0.210609219	2	2	0.098572996
Brain_Hippocampus	0.308908772	Brain_Cerebellar_Hemisphere 1.251480007
0.748519993	0.748519993	-1.651811441	-1.017514429	-1.334662935
0.448515719	2	0	0.981194841
NOTCH2	ENSG00000134250.19	0.210699655	2	2	0.100022242
Brain_Hippocampus	0.39675952 Brain_Frontal_Cortex_BA9	1.129455253
0.870544747	0.870544747	0.847422766	1.644745808	1.246084287
0.56379253 2	0	0.981194841
WSCD2 ENSG00000075035.9	0.210747864	4	4	0.071156659
Brain_Putamen_basal_ganglia 0.716762757	Brain_Nucleus_accumbens_basal_ganglia
1.8004567 0.436722644	0.436722644	-1.804476377	0.709970056
-0.233303713	1.147278751	4	0	0.981194841
ALG9	ENSG00000086848.14	0.210765078	3	3	0.04729291
Brain_Cerebellar_Hemisphere 0.885143349	Brain_Cerebellum 1.23186885
0.827146519	0.827146519	-0.144452264	1.98366758 0.846548945
1.071557858	3	0	0.981194841
VKORC1L1	ENSG00000196715.5	0.210840272	3	3	0.123573054
Brain_Spinal_cord_cervical_c-1	0.78304691 Brain_Anterior_cingulate_cortex_BA24
1.744107958	0.358227074	0.358227074	-1.539947897	0.275350579
-0.707959322	0.917060928	3	0	0.981194841
KIAA1586	ENSG00000168116.13	0.210971254	8	2	0.093525286
Brain_Caudate_basal_ganglia 0.378926177	Brain_Cortex	7.222567751
0.013284251	0.253543361	0.879876572	1.677087945	1.356540407
0.275282229	2	0	0.981194841
LRRC71	ENSG00000160838.13	0.210997678	2	2	0.12351085
Brain_Hypothalamus	0.470801173	Brain_Frontal_Cortex_BA9	1.087965485
0.912034515	0.912034515	-1.54020312	-0.721176103	-1.130689612

0.579139558 2 0 0.981194841
RP11-589P10.5 ENSG00000262089.1 0.211092688 1 1 0.211092688 Brain_Cerebellum 0.211092688 Brain_Cerebellum 1 1 1 -1.250566475 -1.250566475 -1.250566475 NA 1 0 0.981194841
VLDLR ENSG00000147852.15 0.211142364 1 1 0.211142364 Brain_Cortex 0.211142364 Brain_Cortex 1 1 1 -1.250430404 -1.250430404 -1.250430404 NA 1 0 0.981194841
DNAJB9 ENSG00000128590.4 0.211154095 1 1 0.211154095 Brain_Hypothalamus 0.211154095 Brain_Hypothalamus 1 1 1 -1.250398273 -1.250398273 -1.250398273 NA 1 0 0.981194841
SOD3 ENSG00000109610.5 0.211324006 3 3 0.035254437 Brain_Cortex 0.977513123 Brain_Cerebellum 1.16173421 0.836197868 0.836197868 0.028186853 2.105423768 0.916595774 1.070708651 3 0 0.981194841
TMEM165 ENSG00000134851.12 0.211327165 2 2 0.091485791 Brain_Putamen_basal_ganglia 0.855310941 Brain_Cerebellar_Hemisphere 1.387004909 0.612995091 0.612995091 -1.687611704 -0.182346332 -0.934979018 1.064383352 2 0 0.981194841
NBPF9 ENSG00000269713.7 0.211332193 9 8 0.01987136 Brain_Putamen_basal_ganglia 0.762422339 Brain_Nucleus_accumbens_basal_ganglia 2.775388676 0.006682182 0.122864544 -1.599187247 2.328767998 0.023537564 1.566218574 8 0 0.981194841
HEATR1 ENSG00000119285.10 0.211358074 2 2 0.1161779 Brain_Cerebellum 0.302722128 Brain_Cerebellar_Hemisphere 1.889123922 0.110876078 0.110876078 1.030613445 1.571020445 1.300816945 0.382125454 2 0 0.981194841
AXDND1 ENSG00000162779.21 0.211373115 3 2 0.469790731 Brain_Caudate_basal_ganglia 0.9306913 Brain_Nucleus_accumbens_basal_ganglia 2.845097359 0.001567441 0.1533352 -0.722819588 0.137352472 -0.166164004 0.482735486 2 0 0.981194841
GPAM ENSG00000119927.13 0.211469139 5 5 0.010358555 Brain_Cerebellar_Hemisphere 0.207436884 Brain_Hypothalamus 3.20783365 0.164152425 0.164152425 1.260644707 2.563624484 1.796400262 0.550566926 5 0 0.981298973
CFI ENSG00000205403.12 0.211633491 1 1 0.211633491 Brain_Hippocampus 0.211633491 Brain_Hippocampus 1 1 1 1.249086354 1.249086354 1.249086354 NA 1 0 0.981298973
ELN ENSG00000049540.16 0.211657999 2 2 0.078263216 Brain_Cortex 0.818458773 Brain_Cerebellar_Hemisphere 1.09050915 0.90949085 0.90949085 0.229527727 1.760853415 0.995190571 1.082810778 2 0 0.981298973
IGSF9B ENSG00000080854.14 0.21176315 2 2 0.122458777 Brain_Frontal_Cortex_BA9 0.424284705 Brain_Cerebellum 1.031402324 0.968597676 0.968597676 0.799009842 1.544535007 1.171772425 0.5271659 2 0 0.981298973
LINGO4 ENSG00000213171.2 0.211832814 5 5 0.016249802 Brain_Caudate_basal_ganglia 0.899230326 Brain_Hypothalamus 2.428297005 0.107152716 0.107152716 -0.126633696 2.403256111 1.064810174 0.997650764 5 0 0.981298973
ZNF385B ENSG00000144331.18 0.211840611 1 1 0.211840611 Brain_Frontal_Cortex_BA9 0.211840611 Brain_Frontal_Cortex_BA9 1 1 1 1.248520212 1.248520212 1.248520212 NA 1 0 0.981298973
RP11-428O18.6 ENSG00000275294.4 0.211886513 1 1 0.211886513 Brain_Frontal_Cortex_BA9 0.211886513 Brain_Frontal_Cortex_BA9 1 1 1 1.248394799 1.248394799 1.248394799 NA 1 0 0.981298973
NUPR1 ENSG00000176046.8 0.211991289 2 1 0.153485939 Brain_Cerebellum 0.303195968 Brain_Cerebellar_Hemisphere 1.937540583 0.062459417 1.937540583 1.029603933 1.427326038 1.228464985 0.281231997 1 0 0.981459338
FAHD1 ENSG00000180185.11 0.212132761 13 1 0.149985379

Brain_Substantia_nigra 0.248722 Brain_Cerebellum 12.75898708
0.000293433 12.75898708 1.15345909 1.439583117 1.236640028
0.072591767 1 0 0.981527124
LINC01055 ENSG00000235366.2 0.212151275 2 2 0.105819151
Brain_Caudate_basal_ganglia 0.559647541 Brain_Amygdala 1.069026049
0.930973951 0.930973951 -0.583365109 1.61727399 0.516954441
1.55608683 2 0 0.981527124
OCM ENSG00000122543.10 0.212275633 2 2 0.078308557 Brain_Cerebellum
0.980062501 Brain_Cortex 1.019193773 0.980806227 0.980806227
-0.02499055 1.760585666 0.867797558 1.262593051 2 0
0.981527124
CMTR2 ENSG00000180917.17 0.212331025 2 2 0.315116866
Brain_Substantia_nigra 0.729875085 Brain_Amygdala 1.917185992
0.082814008 0.082814008 0.345291701 1.004543186 0.674917444
0.466161196 2 0 0.981527124
LINC00506 ENSG00000281392.1 0.212356701 4 4 0.045730503
Brain_Amygdala 0.921874386 Brain_Frontal_Cortex_BA9 1.324357808
0.698110868 0.698110868 -0.668111411 1.99787233 0.426598701
1.146717889 4 0 0.981527124
PHLPP2 ENSG00000040199.18 0.212442757 5 3 0.166832728
Brain_Spinal_cord_cervical_c-1 0.957403161 Brain_Hippocampus 3.75093883
0.075800011 0.235102975 -1.382452754 0.311981486 -0.566771625
0.75619126 3 0 0.981600599
RCN2 ENSG00000117906.13 0.212836575 6 5 0.035368066
Brain_Hippocampus 0.982438835 Brain_Putamen_basal_ganglia 3.566391547
0.115009064 0.158637425 -2.104119017 0.803597093 -0.356585
1.026369057 5 0 0.983095584
EVC2 ENSG00000173040.12 0.212908537 2 2 0.08027183 Brain_Cortex
0.84616775 Brain_Spinal_cord_cervical_c-1 1.004889003 0.995110997
0.995110997 -1.749111022 0.194010385 -0.777550319 1.373994324
2 0 0.983103412
LRRC4 ENSG00000128594.7 0.213132266 1 1 0.213132266
Brain_Hypothalamus 0.213132266 Brain_Hypothalamus 1 1 1
-1.244998597 -1.244998597 -1.244998597 NA 1 0 0.983811789
LRGUK ENSG00000155530.2 0.213712692 6 6 0.155922043
Brain_Substantia_nigra 0.501578603 Brain_Cortex 1.896936435
0.455620604 0.455620604 -1.156670381 1.418921022 0.560724912
1.157053783 6 0 0.985417674
TUBA1C ENSG00000167553.15 0.213763094 12 3 0.016570134
Brain_Frontal_Cortex_BA9 0.156315479 Brain_Cortex 10.41495394
0.00843878 0.358794761 -2.396109609 -1.417572961 -1.878387803
0.37694708 3 0 0.985417674
PCDHA5 ENSG00000204965.8 0.213827434 2 2 0.214041898
Brain_Cerebellar_Hemisphere 0.236020363 Brain_Putamen_basal_ganglia
1.044480542 0.955519458 0.955519458 -1.184992625 1.242527779
0.028767577 1.716516139 2 0 0.985417674
ATP13A1 ENSG00000105726.16 0.213832427 2 2 0.103697567
Brain_Cortex 0.20772586 Brain_Cerebellar_Hemisphere 1.402113466
0.597886534 0.597886534 1.259843401 1.627186156 1.443514778
0.259750553 2 0 0.985417674
LINC02208 ENSG00000250891.1 0.213901006 1 1 0.213901006
Brain_Frontal_Cortex_BA9 0.213901006 Brain_Frontal_Cortex_BA9 1 1
1 -1.242909984 -1.242909984 -1.242909984 NA 1 0
0.985417674
SHISA2 ENSG00000180730.4 0.213902757 3 3 0.050174296
Brain_Substantia_nigra 0.814034605 Brain_Spinal_cord_cervical_c-1
1.074240686 0.908551306 0.908551306 -0.71322242 1.958475046
0.336676091 1.424708541 3 0 0.985417674
ACADSB ENSG00000196177.12 0.214025301 2 2 0.228517715
Brain_Cerebellum 0.429573092 Brain_Cerebellar_Hemisphere 1.341504526
0.658495474 0.658495474 -1.204185952 0.789922394 -0.207131779
1.410047534 2 0 0.985518871

FN3KRP ENSG00000141560.14 0.214085225 13 4 0.010735554
Brain_Cerebellum 0.559016593 Brain_Hippocampus 9.772883303
0.003828333 0.337260211 0.584302824 2.551190781 1.18294889
0.493080387 4 0 0.985518871
RP11-10C24.3 ENSG00000271643.1 0.214136042 6 3 0.18782913
Brain_Caudate_basal_ganglia 0.863554834 Brain_Putamen_basal_ganglia
5.261062405 0.019892378 0.231266221 0.171850791 1.317028325
0.556423428 0.532178206 3 0 0.985518871
TMEM11 ENSG00000178307.9 0.214207331 1 1 0.214207331
Brain_Caudate_basal_ganglia 0.214207331 Brain_Caudate_basal_ganglia 1 1
1 -1.24207923 -1.24207923 -1.24207923 NA 1 0
0.985522778
TMEM199 ENSG00000244045.12 0.214399135 7 3 0.349552728
Brain_Amygdala 0.981096624 Brain_Caudate_basal_ganglia 5.764132027
0.00372374 0.45951216 -0.935457205 0.905490533 0.325236887
0.632665663 3 0 0.985711621
LYPLAL1 ENSG00000143353.11 0.214422794 2 2 0.089066163
Brain_Anterior_cingulate_cortex_BA24 0.106824152 Brain_Hypothalamus
1.792823223 0.207176777 0.207176777 -1.700344403 -1.61263352
-1.656488961 0.06202096 2 0 0.985711621
RP3-449M8.9 ENSG00000270000.1 0.214550271 3 3 0.057966069
Brain_Putamen_basal_ganglia 0.771188153 Brain_Substantia_nigra
1.295532047 0.74821992 0.74821992 0.290821093 1.895954444
1.073753172 0.803286886 3 0 0.985711621
RBP7 ENSG00000162444.11 0.214590729 1 1 0.214590729
Brain_Spinal_cord_cervical_c-1 0.214590729 Brain_Spinal_cord_cervical_c-1
1 1 1 1.241040659 1.241040659 1.241040659 NA 1 0
0.985711621
TTC39B ENSG00000155158.20 0.214600643 1 1 0.214600643
Brain_Nucleus_accumbens_basal_ganglia 0.214600643
Brain_Nucleus_accumbens_basal_ganglia 1 1 1 -1.241013821
-1.241013821 -1.241013821 NA 1 0 0.985711621
MAP3K1 ENSG00000095015.5 0.214710762 2 2 0.092750224
Brain_Nucleus_accumbens_basal_ganglia 0.633905301 Brain_Cerebellar_Hemisphere
1.014665775 0.985334225 0.985334225 -0.476237338 1.681065337
0.602413999 1.525443351 2 0 0.985893753
NDUFA9 ENSG00000139180.10 0.214935476 4 3 0.037763781
Brain_Frontal_Cortex_BA9 0.901292242 Brain_Amygdala 2.15256132
0.004416908 0.770279944 0.12402909 2.077409485 1.239014009
0.990360145 3 0 0.986019991
UGCG ENSG00000148154.9 0.214943931 2 2 0.122998425
Brain_Caudate_basal_ganglia 0.949402795 Brain_Cerebellar_Hemisphere
1.443643575 0.556356425 0.556356425 -0.063456754 1.542309403
0.739426325 1.135448139 2 0 0.986019991
PPP1R14B ENSG00000173457.10 0.21494968 5 4 0.105521921
Brain_Hypothalamus 0.906655966 Brain_Cerebellum 3.787322335
0.083363426 0.186177715 -1.618653109 -0.117257547 -0.85357094
0.560617371 4 0 0.986019991
HEMK1 ENSG00000114735.9 0.215109193 6 5 0.012923563
Brain_Nucleus_accumbens_basal_ganglia 0.420500088 Brain_Cerebellum
3.665236432 0.097787834 0.287413797 0.805553949 2.485868721
1.721202268 0.648192167 5 0 0.98622433
HECTD4 ENSG00000173064.12 0.215199073 2 2 0.137887425
Brain_Cortex 0.638385627 Brain_Cerebellum 1.285998235 0.714001765
0.714001765 -0.469957157 1.483704148 0.506873496 1.381447157
2 0 0.98622433
CDKN1A ENSG00000124762.13 0.215249263 1 1 0.215249263
Brain_Frontal_Cortex_BA9 0.215249263 Brain_Frontal_Cortex_BA9 1 1
1 -1.239259902 -1.239259902 -1.239259902 NA 1 0 0.98622433
RP11-370I10.12 ENSG00000269514.1 0.215276185 3 3 0.088338572
Brain_Amygdala 0.665489563 Brain_Cerebellar_Hemisphere 1.764532918
0.282158191 0.282158191 -1.704227616 1.013778871 -0.086034067

1.431233265 3 0 0.98622433
HAGH ENSG00000063854.12 0.215387605 13 4 0.077979855
Brain_Frontal_Cortex_BA9 0.994670689 Brain_Putamen_basal_ganglia
9.254067979 0.001399945 0.628306443 -1.762529628 0.66334518
-0.362387044 0.73121876 4 0 0.986382004
TAS2R50 ENSG00000212126.3 0.215451605 1 1 0.215451605
Brain_Cerebellar_Hemisphere 0.215451605 Brain_Cerebellar_Hemisphere 1 1
1 1.238713534 1.238713534 1.238713534 NA 1 0
0.986382004
ABLIM3 ENSG00000173210.19 0.215557306 10 2 0.045632229
Brain_Frontal_Cortex_BA9 0.232877881 Brain_Caudate_basal_ganglia
8.865740133 0.001095927 0.464361273 -1.998779384 -1.192978275
-1.628022519 0.253484617 2 0 0.986543104
DCD ENSG00000161634.11 0.215664122 2 2 0.088755707
Brain_Anterior_cingulate_cortex_BA24 0.44197765 Brain_Frontal_Cortex_BA9
1.214230797 0.785769203 0.785769203 -0.768857938 1.701998194
0.466570128 1.747159126 2 0 0.986709198
RP11-482M8.1 ENSG00000260750.5 0.215780529 3 3 0.059136139
Brain_Anterior_cingulate_cortex_BA24 0.582357812 Brain_Substantia_nigra
1.088496577 0.901705608 0.901705608 -1.887179855 0.931974991
-0.135086969 1.529332874 3 0 0.98671628
GCN1 ENSG00000089154.10 0.215818743 4 3 0.398060107 Brain_Cerebellum
0.772857601 Brain_Cortex 3.517330481 0.047583304 0.139335663
-0.569079656 0.845090868 -0.093471998 0.636894437 3 0
0.98671628
GAS2L1 ENSG00000185340.15 0.215888368 10 6 0.115831032
Brain_Hypothalamus 0.836641337 Brain_Caudate_basal_ganglia 5.956094179
0.020815826 0.237348464 -1.034664331 1.572515565 0.125602062
0.744995088 6 0 0.98671628
TOP3A ENSG00000177302.14 0.21594777 10 2 0.390967003
Brain_Spinal_cord_cervical_c-1 0.920381915 Brain_Hippocampus
8.877849169 0.002176524 0.551799708 -0.857865018 0.856892271
0.16443341 0.466683791 2 0 0.98671628
RHOBTB1 ENSG00000072422.16 0.216189567 4 3 0.133574722
Brain_Hippocampus 0.951992098 Brain_Frontal_Cortex_BA9 2.974494922
0.020539764 0.271180425 -1.500153202 0.060205333 -0.897507653
0.696670925 3 0 0.987147804
KLK6 ENSG00000167755.14 0.216265361 1 1 0.216265361
Brain_Substantia_nigra 0.216265361 Brain_Substantia_nigra 1 1
1 1.236519932 1.236519932 1.236519932 NA 1 0
0.987147804
IL17RC ENSG00000163702.18 0.216312688 7 4 0.020104016
Brain_Cortex 0.179606 Brain_Putamen_basal_ganglia 5.434768047
0.013666832 0.27391043 -2.324400928 2.152574121 -0.762599596
1.96248175 4 0 0.987147804
SSBP2 ENSG00000145687.16 0.216493057 2 2 0.099117924
Brain_Cerebellar_Hemisphere 0.653638043 Brain_Cerebellum 1.564296339
0.435703661 0.435703661 0.448713918 1.649145044 1.048929481
0.848832989 2 0 0.987147804
PFDN6 ENSG00000204220.11 0.216696645 2 2 0.138751374 Brain_Cortex
0.340034324 Brain_Spinal_cord_cervical_c-1 1.015678757 0.984321243
0.984321243 -1.480456829 -0.954097436 -1.217277132 0.372192296
2 0 0.987147804
GBX2 ENSG00000168505.6 0.216813289 3 2 0.104279329
Brain_Caudate_basal_ganglia 0.970687521 Brain_Putamen_basal_ganglia 2.33561958
0.049872114 0.614508306 -1.624452224 0.036746012 -0.963017563
0.880763723 2 0 0.987147804
MS4A8 ENSG00000166959.7 0.216824479 1 1 0.216824479 Brain_Amygdala
0.216824479 Brain_Amygdala 1 1 1 -1.235016189 -1.235016189
-1.235016189 NA 1 0 0.987147804
EZH1 ENSG00000108799.12 0.216957142 3 2 0.263424669 Brain_Cortex
0.689078554 Brain_Caudate_basal_ganglia 2.462438126 0.006650082

0.530911792 -1.118333303 0.463436436 -0.084930338 0.895513236
2 0 0.987147804
HAPLN1 ENSG00000145681.10 0.217034133 1 1 0.217034133
Brain_Nucleus_accumbens_basal_ganglia 0.217034133
Brain_Nucleus_accumbens_basal_ganglia 1 1 1 -1.234453045
-1.234453045 -1.234453045 NA 1 0 0.987147804
KIAA1671 ENSG00000197077.13 0.217086185 4 3 0.43267707 Brain_Cortex
0.935313883 Brain_Putamen_basal_ganglia 3.689889365 0.012111081
0.144731405 -0.328549353 0.784618858 0.150501994 0.479975462
3 0 0.987147804
RDH12 ENSG00000139988.9 0.217106673 1 1 0.217106673
Brain_Substantia_nigra 0.217106673 Brain_Substantia_nigra 1 1
1 1.234258291 1.234258291 1.234258291 NA 1 0
0.987147804
WFDC2 ENSG00000101443.17 0.217126857 6 6 0.043457503 Brain_Cortex
0.802378828 Brain_Cerebellum 2.86427875 0.132896261 0.132896261
-2.01928599 0.323397561 -0.902037622 0.832139689 6 0
0.987147804
ZNF571 ENSG00000180479.13 0.217162658 3 3 0.039179501
Brain_Nucleus_accumbens_basal_ganglia 0.796551032 Brain_Frontal_Cortex_BA9
1.386695096 0.599369031 0.599369031 -0.297710232 2.062296657
0.502257722 1.351180614 3 0 0.987147804
LINC01532 ENSG00000267014.5 0.217310311 7 5 0.094610944
Brain_Nucleus_accumbens_basal_ganglia 0.98867557 Brain_Hypothalamus
4.708444433 0.02326193 0.278680457 -1.671560884 0.729994807
-0.153099308 0.793320824 5 0 0.987147804
LMBRD2 ENSG00000164187.6 0.217418164 2 2 0.096684 Brain_Cortex
0.552241707 Brain_Spinal_cord_cervical_c-1 1.032444926 0.967555074
0.967555074 -1.661146636 -0.59440434 -1.127775488 0.754300711
2 0 0.987147804
BDH2 ENSG00000164039.14 0.217455943 10 9 0.011252102
Brain_Substantia_nigra 0.797617091 Brain_Putamen_basal_ganglia
4.312368428 0.102622644 0.199630923 -2.534770493 0.417919992
-0.803605301 0.861168629 9 0 0.987147804
GPCPD1 ENSG00000125772.12 0.217551667 2 2 0.090784135
Brain_Anterior_cingulate_cortex_BA24 0.933592412 Brain_Cerebellum 1.29562234
0.70437766 0.70437766 -1.691275824 -0.083325893 -0.887300859 1.1369923
2 0 0.987147804
ACOT7 ENSG00000097021.19 0.217562883 1 1 0.217562883 Brain_Cerebellum
0.217562883 Brain_Cerebellum 1 1 1 1.233034526 1.233034526
1.233034526 NA 1 0 0.987147804
RASSF7 ENSG00000099849.14 0.217761867 1 1 0.217761867
Brain_Cerebellum 0.217761867 Brain_Cerebellum 1 1 1 -1.232501337
-1.232501337 -1.232501337 NA 1 0 0.987147804
MKRN1 ENSG00000133606.10 0.217771482 2 2 0.161349735
Brain_Frontal_Cortex_BA9 0.268229635 Brain_Nucleus_accumbens_basal_ganglia
1.046881863 0.953118137 0.953118137 1.107148718 1.400546478
1.253847598 0.207463546 2 0 0.987147804
SLC12A2 ENSG00000064651.13 0.21806908 3 3 0.049899737
Brain_Spinal_cord_cervical_c-1 0.739817599 Brain_Cerebellar_Hemisphere
1.702005687 0.346720882 0.346720882 -1.960822463 -0.332094902
-0.901510621 0.918252158 3 0 0.987147804
ACVR1 ENSG00000115170.13 0.2180927 1 1 0.2180927 Brain_Cerebellar_Hemisphere
0.2180927 Brain_Cerebellar_Hemisphere 1 1 1 1.23161563 1.23161563
1.23161563 NA 1 0 0.987147804
HEBP2 ENSG00000051620.10 0.218150345 4 2 0.530961518
Brain_Hypothalamus 0.785271937 Brain_Cerebellar_Hemisphere 3.685893837
0.075740327 0.144036307 -0.626538919 0.272455327 -0.329702882
0.412062257 2 0 0.987147804
GEMIN2 ENSG00000092208.16 0.218163513 1 1 0.218163513
Brain_Frontal_Cortex_BA9 0.218163513 Brain_Frontal_Cortex_BA9 1 1
1 1.231426172 1.231426172 1.231426172 NA 1 0

0.987147804
SMPDL3B ENSG00000130768.14 0.218189069 4 1 0.187734732
Brain_Cerebellar_Hemisphere 0.29024482 Brain_Cerebellum 3.951291255
0.000972841 3.951291255 -1.317310008 -1.057584702 -1.224115577
0.121049743 1 0 0.987147804
LRTOMT ENSG00000184154.13 0.218194455 1 1 0.218194455
Brain_Hippocampus 0.218194455 Brain_Hippocampus 1 1 1
1.231343404 1.231343404 1.231343404 NA 1 0 0.987147804
RP11-448A19.1 ENSG00000273329.1 0.218432402 7 4 0.01274595
Brain_Spinal_cord_cervical_c-1 0.617804985 Brain_Cerebellar_Hemisphere
4.639909353 0.028799712 0.503788654 -2.490789809 0.49896366
-1.532598716 0.990718678 4 0 0.987147804
NCALD ENSG00000104490.17 0.218553284 3 3 0.075444836
Brain_Nucleus_accumbens_basal_ganglia 0.646173499 Brain_Cerebellum
1.104788396 0.882705821 0.882705821 -1.777750532 1.121491173
-0.065724959 1.519200227 3 0 0.987147804
RP11-360K13.1 ENSG00000254587.1 0.218681066 1 1 0.218681066
Brain_Cortex 0.218681066 Brain_Cortex 1 1 1 -1.230042833
-1.230042833 -1.230042833 NA 1 0 0.987147804
NOS2 ENSG00000007171.16 0.218703285 12 5 0.057366904
Brain_Hypothalamus 0.386325371 Brain_Putamen_basal_ganglia 8.230790907
0.001208197 0.522376468 -1.900504814 1.117163062 -1.073404065
0.754828887 5 0 0.987147804
RP13-516M14.1 ENSG00000260563.3 0.218738201 7 2 0.093298157
Brain_Cortex 0.572904313 Brain_Putamen_basal_ganglia 5.851082569
0.000410449 0.99525624 0.563779341 1.678250756 1.442871526
0.397509511 2 0 0.987147804
DNAJC5G ENSG00000163793.12 0.218746078 1 1 0.218746078
Brain_Cortex 0.218746078 Brain_Cortex 1 1 1 1.229869232
1.229869232 1.229869232 NA 1 0 0.987147804
ALDOA ENSG00000149925.17 0.21876987 1 1 0.21876987 Brain_Cerebellum 0.21876987
Brain_Cerebellum 1 1 1 -1.229805708 -1.229805708 -1.229805708
NA 1 0 0.987147804
FCGRT ENSG00000104870.12 0.218886538 7 7 0.015036003
Brain_Hippocampus 0.979896317 Brain_Cerebellar_Hemisphere 3.475833487
0.119912498 0.119912498 -2.431510724 -0.025198897 -1.602332534
0.823975621 7 0 0.987147804
SERINC5 ENSG00000164300.16 0.218911365 12 11 0.030549662
Brain_Caudate_basal_ganglia 0.824442841 Brain_Cerebellar_Hemisphere
2.947707893 0.010045377 0.283720764 -2.162889732 1.40807695
-0.359692185 1.21002858 11 0 0.987147804
C16orf74 ENSG00000154102.10 0.219005326 4 4 0.114615163
Brain_Nucleus_accumbens_basal_ganglia 0.899275473
Brain_Spinal_cord_cervical_c-1 2.770791297 0.094786621 0.094786621
0.126576657 1.57778434 0.827468556 0.593295705 4 0
0.987147804
PLA2G2A ENSG00000188257.10 0.219051808 1 1 0.219051808
Brain_Spinal_cord_cervical_c-1 0.219051808 Brain_Spinal_cord_cervical_c-1
1 1 1 1.229053339 1.229053339 1.229053339 NA 1 0
0.987147804
SNRPG ENSG00000143977.13 0.219111955 1 1 0.219111955 Brain_Cortex
0.219111955 Brain_Cortex 1 1 1 -1.228892922 -1.228892922
-1.228892922 NA 1 0 0.987147804
LGALS9 ENSG00000168961.16 0.219133752 3 3 0.069808422
Brain_Cortex 0.666814095 Brain_Putamen_basal_ganglia 1.719700446
0.330885649 0.330885649 -0.430524573 1.813151734 1.04757947
1.280350341 3 0 0.987147804
RP11-285F16.1 ENSG00000273030.1 0.219149805 1 1 0.219149805
Brain_Hypothalamus 0.219149805 Brain_Hypothalamus 1 1 1
1.228791991 1.228791991 1.228791991 NA 1 0 0.987147804
CACNA2D4 ENSG00000151062.14 0.21917192 2 2 0.099237783
Brain_Putamen_basal_ganglia 0.18621227 Brain_Caudate_basal_ganglia 1.928694407

0.071305593 0.071305593 1.321867519 1.648560126 1.485213822
0.231006558 2 0 0.987147804
SLC1A2 ENSG00000110436.11 0.219186915 1 1 0.219186915
Brain_Cerebellar_Hemisphere 0.219186915 Brain_Cerebellar_Hemisphere 1 1
1 1.228693044 1.228693044 1.228693044 NA 1 0
0.987147804
MGAT3 ENSG00000128268.11 0.219482041 5 3 0.065518849
Brain_Nucleus_accumbens_basal_ganglia 0.433964644 Brain_Hypothalamus
4.126125479 0.005439261 0.284548955 0.782425345 1.841701335
1.445650151 0.432695004 3 0 0.987147804
GRHPR ENSG00000137106.17 0.219577589 8 7 0.130444052
Brain_Nucleus_accumbens_basal_ganglia 0.788238728 Brain_Cerebellar_Hemisphere
2.846250279 0.061483463 0.129350916 -1.512353128 1.08172124
-0.315002863 0.909885218 7 0 0.987147804
WDHD1 ENSG00000198554.11 0.219755709 2 2 0.110869745
Brain_Frontal_Cortex_BA9 0.114958812 Brain_Nucleus_accumbens_basal_ganglia
1.658846976 0.341153024 0.341153024 -1.594296036 -1.576290753
-1.585293395 0.012731658 2 0 0.987147804
RP5-1085F17.3 ENSG00000260257.2 0.219868713 4 4 0.040216199
Brain_Hypothalamus 0.390399755 Brain_Caudate_basal_ganglia 2.475591948
0.218074707 0.218074707 0.858892632 2.051521385 1.339365491
0.506210634 4 0 0.987147804
LINC00940 ENSG00000235049.1 0.219967081 8 8 0.01892352
Brain_Caudate_basal_ganglia 0.987461673 Brain_Cortex 1.879361398
0.162479315 0.162479315 -2.347034155 0.180303913 -0.719707387
0.896234091 8 0 0.987147804
OPN1SW ENSG00000128617.2 0.22002358 3 3 0.038743051 Brain_Amygdala
0.765885329 Brain_Putamen_basal_ganglia 1.46382015 0.703742949
0.703742949 0.297761332 2.066905588 1.029246114 0.923458205
3 0 0.987147804
SLC12A7 ENSG00000113504.20 0.220137645 1 1 0.220137645
Brain_Cerebellum 0.220137645 Brain_Cerebellum 1 1 1 -1.226162195
-1.226162195 -1.226162195 NA 1 0 0.987147804
TXN ENSG00000136810.12 0.220220514 9 4 0.290269947
Brain_Spinal_cord_cervical_c-1 0.811953098 Brain_Hypothalamus
6.957679893 0.022437083 0.241409862 -1.057529612 0.622681132
-0.410899919 0.481534224 4 0 0.987147804
EME1 ENSG00000154920.14 0.220227225 5 3 0.052482522
Brain_Substantia_nigra 0.685284915 Brain_Spinal_cord_cervical_c-1
2.989795642 0.00060841 0.911227846 -0.747763547 1.939154547
0.058567073 1.153226512 3 0 0.987147804
SIK2 ENSG00000170145.4 0.22023841 2 2 0.109531831
Brain_Spinal_cord_cervical_c-1 0.572033498 Brain_Cerebellar_Hemisphere
1.071970053 0.928029947 0.928029947 0.565059201 1.600300975
1.082680088 0.732026478 2 0 0.987147804
INTS4 ENSG00000149262.16 0.220252167 4 3 0.033032175
Brain_Hypothalamus 0.87604214 Brain_Cerebellum 2.431349516 0.009425608
0.615061839 0.155988431 2.131691997 1.30697603 0.944975187 3
0 0.987147804
RP4-669L17.10 ENSG00000237094.11 0.220389633 1 1 0.220389633
Brain_Hippocampus 0.220389633 Brain_Hippocampus 1 1 1
-1.225492716 -1.225492716 -1.225492716 NA 1 0 0.987147804
ACTB ENSG00000075624.13 0.220411096 1 1 0.220411096
Brain_Putamen_basal_ganglia 0.220411096 Brain_Putamen_basal_ganglia 1 1
1 1.225435719 1.225435719 1.225435719 NA 1 0
0.987147804
LYPD6 ENSG00000187123.14 0.220574561 5 5 0.056882477 Brain_Cerebellum
0.999872435 Brain_Substantia_nigra 1.670569549 0.364366642
0.364366642 -0.557456359 1.904212784 0.790321519 1.02681405 5
0 0.987147804
FAM189B ENSG00000160767.20 0.220579505 1 1 0.220579505
Brain_Cortex 0.220579505 Brain_Cortex 1 1 1 -1.22498863

-1.22498863 -1.22498863 NA 1 0 0.987147804
UBL4B ENSG00000186150.4 0.22066876 1 1 0.22066876 Brain_Cortex 0.22066876
Brain_Cortex 1 1 1 -1.224751776 -1.224751776 -1.224751776
NA 1 0 0.987147804
SLC22A14 ENSG00000144671.10 0.220724446 3 3 0.041069168
Brain_Cortex 0.721338296 Brain_Cerebellum 1.295934973 0.702567007
0.702567007 -2.042831026 0.440774112 -0.652909225 1.268026632
3 0 0.987147804
AMPD2 ENSG00000116337.15 0.220727321 3 3 0.035837806
Brain_Cerebellar_Hemisphere 0.960015553 Brain_Spinal_cord_cervical_c-1
1.397203431 0.60101593 0.60101593 0.050134066 2.098762906
0.980179495 1.037246335 3 0 0.987147804
FAIM2 ENSG00000135472.8 0.22089877 3 3 0.133876118
Brain_Cerebellar_Hemisphere 0.564298199 Brain_Frontal_Cortex_BA9
1.721560054 0.29174257 0.29174257 0.576469027 1.498990417
1.135129463 0.491143112 3 0 0.987147804
ZNF175 ENSG00000105497.7 0.221046574 7 6 0.016037677
Brain_Cerebellar_Hemisphere 0.624953445 Brain_Substantia_nigra
4.778035732 0.122014606 0.164714046 -2.408057003 0.488842163
-1.352777231 0.904616426 6 0 0.987147804
PARP2 ENSG00000129484.13 0.221147676 8 3 0.062329333
Brain_Nucleus_accumbens_basal_ganglia 0.99563097 Brain_Spinal_cord_cervical_c-1
6.44161565 0.009995123 0.35761013 -0.941711251 1.863945678
-0.019156005 0.818974648 3 0 0.987147804
NACA ENSG00000196531.10 0.221149379 3 3 0.075034372
Brain_Caudate_basal_ganglia 0.475502465 Brain_Cortex 1.140902629
0.844883419 0.844883419 -1.780254182 -0.713554882 -1.143702179
0.562506913 3 0 0.987147804
IRF8 ENSG00000140968.10 0.221257495 1 1 0.221257495 Brain_Cerebellum
0.221257495 Brain_Cerebellum 1 1 1 1.223191184 1.223191184
1.223191184 NA 1 0 0.987147804
NEK3 ENSG00000136098.16 0.221329769 3 2 0.09470982
Brain_Anterior_cingulate_cortex_BA24 0.144295695 Brain_Putamen_basal_ganglia
2.609638883 0.071461411 0.318899707 1.459979547 1.671060036
1.586456474 0.111596493 2 0 0.987147804
TREM2 ENSG00000095970.16 0.221349972 1 1 0.221349972
Brain_Hypothalamus 0.221349972 Brain_Hypothalamus 1 1 1
-1.22294632 -1.22294632 -1.22294632 NA 1 0 0.987147804
DUSP28 ENSG00000188542.9 0.221518889 2 2 0.098878806
Brain_Hypothalamus 0.643467158 Brain_Cerebellar_Hemisphere 1.046038706
0.953961294 0.953961294 -0.4628566 1.650313628 0.593728514
1.494236998 2 0 0.987147804
CYP24A1 ENSG00000019186.9 0.221529574 5 3 0.098804384
Brain_Amygdala 0.968156887 Brain_Anterior_cingulate_cortex_BA24
3.792139676 1.10E-18 0.163452057 -1.650677796 0.063040614
-0.482769575 0.779792614 3 0 0.987147804
GINM1 ENSG00000055211.12 0.221532651 8 6 0.069357133
Brain_Frontal_Cortex_BA9 0.915601873 Brain_Cortex 5.710329917
0.006444174 0.235814494 -0.105975396 1.816086311 1.218020608
0.713312979 6 0 0.987147804
ZPR1 ENSG00000109917.10 0.221550062 8 4 0.068646655
Brain_Anterior_cingulate_cortex_BA24 0.844314485 Brain_Caudate_basal_ganglia
6.050953334 0.007415431 0.362235479 -1.820738246 0.196377782
-1.122322717 0.68121068 4 0 0.987147804
PPM1L ENSG00000163590.13 0.221695434 2 2 0.185731462
Brain_Cerebellar_Hemisphere 0.818950957 Brain_Cerebellum 1.74201542 0.25798458
0.25798458 0.228894446 1.323312544 0.776103495 0.773870458 2
0 0.987147804
LIN37 ENSG00000267796.7 0.221847583 2 2 0.181901232 Brain_Cerebellum
0.254550662 Brain_Nucleus_accumbens_basal_ganglia 1.02309487 0.97690513
0.97690513 1.139365686 1.33492397 1.237144828 0.138280589 2 0
0.987147804

ALG1 ENSG00000033011.11 0.22185762 2 2 0.108780946 Brain_Cerebellum
0.517780386 Brain_Cerebellar_Hemisphere 1.010111578 0.989888422
0.989888422 -1.6036966 -0.646770656 -1.125233628 0.676648824 2
0 0.987147804
MYO15B ENSG00000266714.6 0.221866013 8 8 0.037287078
Brain_Cerebellum 0.577406871 Brain_Hypothalamus 2.6350294 0.088514893
0.088514893 -1.094939828 2.082606807 0.577928478 1.06797357 8
0 0.987147804
RBM8A ENSG00000265241.6 0.22189923 6 1 0.21408736 Brain_Cerebellum 0.23793172
Brain_Hypothalamus 5.968702191 6.56E-18 5.968702191 1.180172234
1.242404488 1.218250718 0.021136701 1 0 0.987147804
DHX57 ENSG00000163214.20 0.221986006 2 2 0.090598223 Brain_Cerebellum
0.647990877 Brain_Nucleus_accumbens_basal_ganglia 1.043774402
0.956225598 0.956225598 -1.692250493 0.456555073 -0.61784771
1.519434987 2 0 0.987147804
PAGR1 ENSG00000280789.1 0.222022835 1 1 0.222022835 Brain_Cerebellum
0.222022835 Brain_Cerebellum 1 1 1 -1.221166898 -1.221166898
-1.221166898 NA 1 0 0.987147804
DYNC2LI1 ENSG00000138036.18 0.22210417 4 2 0.260850805
Brain_Hypothalamus 0.983422702 Brain_Caudate_basal_ganglia 3.627677591
0.017742018 0.246594434 -1.124382471 -0.020778056 -0.513769119
0.530510466 2 0 0.987147804
MYO10 ENSG00000145555.14 0.222112192 1 1 0.222112192
Brain_Anterior_cingulate_cortex_BA24 0.222112192
Brain_Anterior_cingulate_cortex_BA24 1 1 1 1.220930877
1.220930877 1.220930877 NA 1 0 0.987147804
ZNF384 ENSG00000126746.17 0.222223464 2 2 0.210691098
Brain_Cerebellum 0.222561316 Brain_Hypothalamus 1.015399171
0.984600829 0.984600829 -1.25166736 -1.219745634 -1.235706497
0.022572069 2 0 0.987147804
DISP2 ENSG00000140323.5 0.222382081 11 4 0.034575439
Brain_Frontal_Cortex_BA9 0.314549719 Brain_Nucleus_accumbens_basal_ganglia
8.443061139 0.016477784 0.401049719 1.005721165 2.113296023
1.624458629 0.330607606 4 0 0.987147804
COPE ENSG00000105669.13 0.222399234 11 5 0.054402933
Brain_Putamen_basal_ganglia 0.89826874 Brain_Hypothalamus 7.868489735
0.002896048 0.299278543 -1.923614415 0.267928258 -1.12019152
0.742771953 5 0 0.987147804
FETUB ENSG00000090512.11 0.222503289 1 1 0.222503289
Brain_Spinal_cord_cervical_c-1 0.222503289 Brain_Spinal_cord_cervical_c-1
1 1 1 1.219898672 1.219898672 1.219898672 NA 1 0
0.987147804
R3HCC1 ENSG00000104679.10 0.22261536 1 1 0.22261536
Brain_Spinal_cord_cervical_c-1 0.22261536 Brain_Spinal_cord_cervical_c-1 1
1 1 1.219603128 1.219603128 1.219603128 NA 1 0
0.987147804
RRAS ENSG00000126458.3 0.222937447 1 1 0.222937447
Brain_Spinal_cord_cervical_c-1 0.222937447 Brain_Spinal_cord_cervical_c-1
1 1 1 1.218754338 1.218754338 1.218754338 NA 1 0
0.987147804
NPIPB3 ENSG00000169246.16 0.222990787 4 4 0.639428143
Brain_Amygdala 0.843123107 Brain_Cortex 2.340428475 0.087109053
0.087109053 -0.388288726 0.468498501 -0.017593236 0.420168927
4 0 0.987147804
GRK7 ENSG00000114124.2 0.223129801 6 4 0.080453675
Brain_Spinal_cord_cervical_c-1 0.817947416
Brain_Nucleus_accumbens_basal_ganglia 4.400468402 0.023964301
0.386890778 -0.230185774 1.748059791 0.996168364 0.758804612
4 0 0.987147804
AGGF1 ENSG00000164252.12 0.223173216 3 3 0.156944722
Brain_Caudate_basal_ganglia 0.285522154 Brain_Nucleus_accumbens_basal_ganglia
1.20485807 0.808099211 0.808099211 1.06799636 1.415422278

1.289055676 0.192096915 3 0 0.987147804
RFC4 ENSG00000163918.10 0.223196298 9 3 0.129649358
Brain_Anterior_cingulate_cortex_BA24 0.920761133 Brain_Substantia_nigra
7.236141999 9.72E-19 0.573723098 -1.515486061 0.364462428
-0.212021988 0.619379925 3 0 0.987147804
ARSB ENSG00000113273.15 0.223212223 11 6 0.238876863
Brain_Anterior_cingulate_cortex_BA24 0.997375449 Brain_Putamen_basal_ganglia
7.707231181 0.027415973 0.288282422 -1.177798711 0.853076986
-0.284329316 0.578838333 6 0 0.987147804
UNG ENSG00000076248.10 0.223230661 2 2 0.182420028
Brain_Substantia_nigra 0.237219781 Brain_Nucleus_accumbens_basal_ganglia
1.059076682 0.940923318 0.940923318 -1.181964515 1.333340684
0.075688085 1.778589363 2 0 0.987147804
NXPH4 ENSG00000182379.9 0.223232011 2 2 0.145549356
Brain_Cerebellar_Hemisphere 0.472299038 Brain_Cerebellum 1.144253076
0.855746924 0.855746924 -1.455433253 0.718743399 -0.368344927
1.537375054 2 0 0.987147804
AKR1E2 ENSG00000165568.17 0.223248375 7 7 0.019365375
Brain_Spinal_cord_cervical_c-1 0.587644586 Brain_Cortex 1.749773117
0.46566274 0.46566274 -1.392323825 2.338421856 0.700782148
1.107721185 7 0 0.987147804
SPICE1 ENSG00000163611.11 0.22325768 1 1 0.22325768 Brain_Cerebellum
0.22325768 Brain_Cerebellum 1 1 1 -1.217911305 -1.217911305
-1.217911305 NA 1 0 0.987147804
THSD7B ENSG00000144229.11 0.223278774 3 3 0.063345786
Brain_Caudate_basal_ganglia 0.902300371 Brain_Cerebellum 1.597847556
0.543000383 0.543000383 -1.856756634 0.122755933 -0.841547521
0.990737624 3 0 0.987147804
IFITM1 ENSG00000185885.15 0.223326028 4 4 0.175799871
Brain_Hippocampus 0.274099357 Brain_Spinal_cord_cervical_c-1
1.144605873 0.794043659 0.794043659 1.093670864 1.353801012
1.219057124 0.136273466 4 0 0.987147804
SPRTN ENSG00000010072.15 0.223413406 1 1 0.223413406 Brain_Amygdala
0.223413406 Brain_Amygdala 1 1 1 1.217501659 1.217501659
1.217501659 NA 1 0 0.987147804
VAV2 ENSG00000160293.16 0.22364266 2 2 0.084600199
Brain_Spinal_cord_cervical_c-1 0.862349881 Brain_Cerebellum 1.016163485
0.983836515 0.983836515 -1.724596585 -0.173383644 -0.948990115
1.09687319 2 0 0.987147804
AC133633.2 ENSG00000253444.1 0.223909558 6 6 0.097244896
Brain_Hypothalamus 0.595223337 Brain_Frontal_Cortex_BA9 1.773859133
0.44947651 0.44947651 -1.658359651 0.531282057 -0.904580989
0.846718226 6 0 0.987147804
TSPAN2 ENSG00000134198.9 0.223921716 2 2 0.084131599
Brain_Cerebellum 0.426749064 Brain_Cerebellar_Hemisphere 1.509209832
0.490790168 0.490790168 0.794766977 1.727200843 1.26098391
0.65933031 2 0 0.987147804
RTEL1 ENSG00000258366.7 0.223939883 1 1 0.223939883 Brain_Cortex
0.223939883 Brain_Cortex 1 1 1 -1.21611824 -1.21611824
-1.21611824 NA 1 0 0.987147804
FAM222A ENSG00000139438.5 0.224012841 2 2 0.103196863
Brain_Cortex 0.674446587 Brain_Cerebellar_Hemisphere 1.096632607
0.903367393 0.903367393 0.420053205 1.629548941 1.024801073
0.855242637 2 0 0.987147804
OSTM1 ENSG00000081087.14 0.224061782 6 2 0.0695276 Brain_Cortex
0.241756774 Brain_Nucleus_accumbens_basal_ganglia 5.461149134
0.000196206 0.229348349 -1.814975988 -1.17060702 -1.439761773
0.293765727 2 0 0.987147804
PRDM16 ENSG00000142611.16 0.224066838 1 1 0.224066838
Brain_Spinal_cord_cervical_c-1 0.224066838 Brain_Spinal_cord_cervical_c-1
1 1 1 -1.215784991 -1.215784991 -1.215784991 NA 1 0
0.987147804

NBPF26 ENSG00000273136.6 0.224188435 7 7 0.003891824
Brain_Nucleus_accumbens_basal_ganglia 0.783396523 Brain_Caudate_basal_ganglia
2.099918864 0.074044475 0.074044475 -2.886799302 0.343560606
-0.982362861 1.056031513 7 0 0.987147804
CNIH4 ENSG00000143771.11 0.224261155 5 5 0.014599402
Brain_Hypothalamus 0.407248429 Brain_Cerebellar_Hemisphere 2.375244291
0.354432505 0.354432505 0.828745508 2.442166738 1.499083995
0.589098779 5 0 0.987147804
MAGOHB ENSG00000111196.9 0.224296652 1 1 0.224296652
Brain_Frontal_Cortex_BA9 0.224296652 Brain_Frontal_Cortex_BA9 1 1
1 1.215182084 1.215182084 1.215182084 NA 1 0
0.987147804
STAT6 ENSG00000166888.11 0.224313474 4 3 0.075117486
Brain_Frontal_Cortex_BA9 0.264764229 Brain_Cerebellum 2.621198465
0.046576509 0.292855402 -1.473445506 1.779746322 0.666943858
1.455581596 3 0 0.987147804
ABHD14A ENSG00000248487.8 0.224327723 4 4 0.212654693
Brain_Frontal_Cortex_BA9 0.834535031 Brain_Hippocampus 1.754603511
0.280718923 0.280718923 -0.671060879 1.246298868 0.138579251
0.821221412 4 0 0.987147804
TRIM38 ENSG00000112343.10 0.224390119 1 1 0.224390119
Brain_Substantia_nigra 0.224390119 Brain_Substantia_nigra 1 1
1 1.214937003 1.214937003 1.214937003 NA 1 0
0.987147804
ADRA1D ENSG00000171873.7 0.224519841 3 3 0.180679846
Brain_Frontal_Cortex_BA9 0.321136439 Brain_Cerebellum 1.062213601
0.937647272 0.937647272 -1.338664722 1.176391363 0.276617291
1.40190602 3 0 0.987147804
MT1A ENSG00000205362.11 0.224640124 2 2 0.201216546 Brain_Amygdala
0.255933317 Brain_Cerebellum 1.021021854 0.978978146 0.978978146
-1.278093258 1.136055548 -0.071018855 1.707060992 2 0
0.987147804
HPS5 ENSG00000110756.17 0.224671676 4 3 0.089056345
Brain_Cerebellar_Hemisphere 0.544893404 Brain_Cortex 2.393547643
0.060187688 0.503261183 -1.571000928 1.700396631 0.607455584
1.540881004 3 0 0.987147804
ESPN ENSG00000187017.15 0.224694135 1 1 0.224694135
Brain_Hypothalamus 0.224694135 Brain_Hypothalamus 1 1 1
-1.214140347 -1.214140347 -1.214140347 NA 1 0 0.987147804
ARMC10 ENSG00000170632.13 0.224722892 9 4 0.141945851
Brain_Hippocampus 0.578294122 Brain_Cortex 6.620343611
0.023763209 0.347822465 -1.468583285 1.367051585 0.641114143
0.831670736 4 0 0.987147804
XRCC5 ENSG00000079246.15 0.224851462 11 4 0.129755765 Brain_Cortex
0.924954172 Brain_Cerebellum 8.232822902 0.000546548 0.317294896
-1.515065708 0.997361085 0.303627647 0.679790275 4 0
0.987147804
COX4I2 ENSG00000131055.4 0.224874193 3 2 0.315233574
Brain_Caudate_basal_ganglia 0.491471099 Brain_Anterior_cingulate_cortex_BA24
2.358893888 0.045579214 0.595526898 -0.687970912 1.004300955
0.415328855 0.95621427 2 0 0.987147804
GALNT13 ENSG00000144278.14 0.224989294 3 2 0.164548185
Brain_Nucleus_accumbens_basal_ganglia 0.66606952 Brain_Cerebellum 2.184337408
0.005989159 0.809673433 -1.38993642 -0.431548604 -0.791933411
0.52151089 2 0 0.987147804
GJC1 ENSG00000182963.9 0.22503019 2 1 0.220198171 Brain_Cerebellum
0.236689638 Brain_Cerebellar_Hemisphere 1.971717154 0.028282846
1.971717154 -1.226001339 -1.183301602 -1.20465147 0.030193274
1 0 0.987147804
CENPQ ENSG00000031691.6 0.225079382 13 4 0.038512235 Brain_Amygdala
0.904984278 Brain_Substantia_nigra 10.15036897 0.014099284
0.350857741 -2.069360871 -0.119367412 -0.609690985 0.604968993

4 0 0.987147804
CAPN8 ENSG00000203697.11 0.225079769 11 5 0.040745489
Brain_Substantia_nigra 0.917212737 Brain_Hypothalamus 8.354310408
0.010094317 0.426311818 0.103945327 2.046110616 1.36970316
0.563626209 5 0 0.987147804
LINC01569 ENSG00000262468.5 0.22514249 3 3 0.133588818 Brain_Cortex
0.619866984 Brain_Putamen_basal_ganglia 1.805715776 0.495975092
0.495975092 -1.500098775 0.496038874 -0.76105018 1.094264966
3 0 0.987147804
TGM1 ENSG00000092295.11 0.225313469 10 6 0.050428343
Brain_Hypothalamus 0.959897184 Brain_Cerebellum 6.55751735 0.026461755
0.258921371 -0.593294209 1.956312578 0.42404291 0.782030618 6
0 0.987147804
LYN ENSG00000254087.7 0.225328849 4 4 0.089781977 Brain_Cerebellum
0.955813418 Brain_Hippocampus 1.831723326 0.21847241 0.21847241
-1.696548902 1.668306418 -0.201118873 1.436944224 4 0
0.987147804
CMYA5 ENSG00000164309.14 0.225340366 1 1 0.225340366 Brain_Amygdala
0.225340366 Brain_Amygdala 1 1 1 1.212449491 1.212449491
1.212449491 NA 1 0 0.987147804
DPF2 ENSG00000133884.9 0.225403813 4 4 0.07883651 Brain_Cerebellum
0.935466714 Brain_Cortex 2.25589108 0.414964725 0.414964725
-1.757477168 0.151915988 -0.636331903 0.863369469 4 0
0.987147804
MSX1 ENSG00000163132.6 0.225491392 1 1 0.225491392 Brain_Cerebellum
0.225491392 Brain_Cerebellum 1 1 1 1.212054832 1.212054832
1.212054832 NA 1 0 0.987147804
LYZL4 ENSG00000157093.8 0.225530834 2 2 0.204150509
Brain_Frontal_Cortex_BA9 0.349599652 Brain_Anterior_cingulate_cortex_BA24
1.170396223 0.829603777 0.829603777 -1.269815077 0.935366118
-0.16722448 1.559298577 2 0 0.987147804
ARHGAP28 ENSG00000088756.12 0.225534513 7 5 0.010961906
Brain_Spinal_cord_cervical_c-1 0.604613287 Brain_Hippocampus
4.515771145 0.03929875 0.152120608 0.517777832 2.54391086
1.862838118 0.722033946 5 0 0.987147804
CACNB2 ENSG00000165995.18 0.225682268 5 5 0.024135648
Brain_Putamen_basal_ganglia 0.625564219 Brain_Caudate_basal_ganglia
2.038058541 0.124026674 0.124026674 -0.848087361 2.254963056
0.382632009 1.280531627 5 0 0.987147804
TSNAXIP1 ENSG00000102904.14 0.225707224 4 2 0.069982563
Brain_Cerebellum 0.458466298 Brain_Cortex 3.472653485 0.01012452
0.465696358 -1.812023513 -0.741374671 -1.409556296 0.463630937
2 0 0.987147804
GRIN2C ENSG00000161509.13 0.225772433 1 1 0.225772433
Brain_Cerebellum 0.225772433 Brain_Cerebellum 1 1 1 -1.211320922
-1.211320922 -1.211320922 NA 1 0 0.987147804
TMEM95 ENSG00000182896.12 0.225799945 1 1 0.225799945
Brain_Spinal_cord_cervical_c-1 0.225799945 Brain_Spinal_cord_cervical_c-1
1 1 1 -1.211249113 -1.211249113 -1.211249113 NA 1 0
0.987147804
RIT1 ENSG00000143622.10 0.225910632 2 2 0.327417648 Brain_Cerebellum
0.770620568 Brain_Cerebellar_Hemisphere 1.715374655 0.284625345
0.284625345 -0.291563262 0.979328591 0.343882665 0.898656247
2 0 0.987147804
ENDOV ENSG00000173818.16 0.225966264 1 1 0.225966264 Brain_Cerebellum
0.225966264 Brain_Cerebellum 1 1 1 -1.210815132 -1.210815132
-1.210815132 NA 1 0 0.987147804
HPSE2 ENSG00000172987.12 0.226096977 3 3 0.039705607
Brain_Hippocampus 0.427565352 Brain_Anterior_cingulate_cortex_BA24
1.897693071 0.287429709 0.287429709 -2.05679856 -0.793364733
-1.49651714 0.64371993 3 0 0.987147804
CNOT1 ENSG00000125107.17 0.226105632 2 2 0.085149184 Brain_Cerebellum

0.911186313 Brain_Caudate_basal_ganglia 1.007478589 0.992521411
0.992521411 0.111542314 1.721560388 0.916551351 1.138454698
2 0 0.987147804
RP11-364L4.3 ENSG00000250969.1 0.226137113 1 1 0.226137113
Brain_Amygdala 0.226137113 Brain_Amygdala 1 1 1 1.210369571
1.210369571 1.210369571 NA 1 0 0.987147804
RP3-395P12.2 ENSG00000226375.1 0.226430508 3 3 0.038951031
Brain_Anterior_cingulate_cortex_BA24 0.240421675 Brain_Cerebellum
2.142510287 0.181336355 0.181336355 -2.064703846 -1.17393347
-1.679301476 0.457342083 3 0 0.987147804
LINC00271 ENSG00000231028.8 0.226533129 3 3 0.103161746
Brain_Frontal_Cortex_BA9 0.579011629 Brain_Anterior_cingulate_cortex_BA24
1.819818246 0.422008762 0.422008762 0.554829 1.629714999
1.241702088 0.596517419 3 0 0.987147804
FDFT1 ENSG00000079459.12 0.226547959 2 2 0.106100965 Brain_Cerebellum
0.783632328 Brain_Cerebellar_Hemisphere 1.193466182 0.806533818
0.806533818 -1.615969233 0.274588597 -0.670690318 1.336826262
2 0 0.987147804
ZC3H14 ENSG00000100722.18 0.226581571 2 2 0.12660844 Brain_Cortex
0.574815477 Brain_Frontal_Cortex_BA9 1.148889189 0.851110811
0.851110811 -1.527613855 0.560973684 -0.483320086 1.476854412
2 0 0.987147804
LPXN ENSG00000110031.12 0.226689009 1 1 0.226689009 Brain_Cerebellum
0.226689009 Brain_Cerebellum 1 1 1 -1.208931902 -1.208931902
-1.208931902 NA 1 0 0.987147804
POR ENSG00000127948.15 0.226883119 3 3 0.05456471
Brain_Caudate_basal_ganglia 0.391551287 Brain_Cerebellar_Hemisphere
2.263672063 0.14273954 0.14273954 0.856807488 1.922326322
1.412298472 0.534212288 3 0 0.987147804
NMRAL1 ENSG00000153406.13 0.226922807 13 2 0.051307147
Brain_Cortex 0.233319821 Brain_Anterior_cingulate_cortex_BA24
11.34682166 0.007585048 0.678851687 1.191850626 1.948901853
1.583078501 0.220919777 2 0 0.987147804
RP11-234K24.6 ENSG00000278035.1 0.227120062 1 1 0.227120062
Brain_Nucleus_accumbens_basal_ganglia 0.227120062
Brain_Nucleus_accumbens_basal_ganglia 1 1 1 -1.20781076
-1.20781076 -1.20781076 NA 1 0 0.987147804
TESMIN ENSG00000132749.10 0.227121149 7 5 0.021131387
Brain_Nucleus_accumbens_basal_ganglia 0.765294974 Brain_Cerebellum
4.401569502 0.089826503 0.359175404 -0.29853486 2.305628725
1.110183573 0.862421927 5 0 0.987147804
ZBED9 ENSG00000232040.2 0.227340627 1 1 0.227340627
Brain_Hippocampus 0.227340627 Brain_Hippocampus 1 1 1
-1.20723767 -1.20723767 -1.20723767 NA 1 0 0.987147804
LINC00334 ENSG00000182586.8 0.227426933 4 3 0.198245049
Brain_Putamen_basal_ganglia 0.895101922 Brain_Caudate_basal_ganglia
2.484983418 0.020291619 0.227752307 0.131851284 1.286567582
0.976029723 0.563197458 3 0 0.987147804
MIER3 ENSG00000155545.19 0.227478348 1 1 0.227478348
Brain_Nucleus_accumbens_basal_ganglia 0.227478348
Brain_Nucleus_accumbens_basal_ganglia 1 1 1 -1.206880035
-1.206880035 -1.206880035 NA 1 0 0.987147804
FBXO17 ENSG00000269190.5 0.227497275 8 2 0.0575599
Brain_Frontal_Cortex_BA9 0.275146668 Brain_Caudate_basal_ganglia
7.005697532 0.007566922 0.566419335 -1.899034805 -1.091286882
-1.543350357 0.236782776 2 0 0.987147804
ESRRB ENSG00000119715.14 0.227568864 6 5 0.224462839
Brain_Caudate_basal_ganglia 0.917660053 Brain_Cortex 3.812136972
0.112579208 0.220350719 -1.214746376 1.099836677 -0.064415397
0.775484067 5 0 0.987147804
KLF16 ENSG00000129911.8 0.227610759 1 1 0.227610759 Brain_Cortex
0.227610759 Brain_Cortex 1 1 1 -1.206536331 -1.206536331

-1.206536331	NA	1	0	0.987147804
AC073283.4 ENSG00000234690.6	0.227613498	4	4	0.041685059
Brain_Nucleus_accumbens_basal_ganglia 0.899812047
Brain_Anterior_cingulate_cortex_BA24	1.22921	0.806347715	0.806347715
-0.220458056	2.036650689	0.723378007	1.090417245	4	0
0.987147804
SLC35D1	ENSG00000116704.7	0.227721053	2	2	0.112377438
Brain_Putamen_basal_ganglia 0.377557055	Brain_Caudate_basal_ganglia
1.142843257	0.857156743	0.857156743	0.882406438	1.587597263
1.23500185 0.498645214	2	0	0.987147804
RP11-114F10.2	ENSG00000257890.1	0.227779988	3	2	0.067819296
Brain_Cerebellar_Hemisphere 0.157713064	Brain_Cerebellum 2.6025263
0.042217604	0.355256097	-1.826205611	-1.412805013	-1.59283422
0.211799564	2	0	0.987147804
POLM	ENSG00000122678.16	0.227796944	12	7	0.069781055
Brain_Substantia_nigra	0.997419091	Brain_Cerebellar_Hemisphere
8.074269359	0.011141792	0.294914409	-1.813329244	0.696357341
-1.128166327	0.790285127	7	0	0.987147804
PEX7	ENSG00000112357.12	0.227876604	8	5	0.008106358	Brain_Cortex
0.60682455 Brain_Spinal_cord_cervical_c-1	5.658694227	0.001168467
0.254441331	-2.647607527	0.514611468	-1.441960958	0.955725892
5	0	0.987147804
OR5K2 ENSG00000231861.2	0.227922588	6	1	0.184465675
Brain_Caudate_basal_ganglia 0.345250926	Brain_Frontal_Cortex_BA9
5.906196757	3.11E-10	5.906196757	-1.327130025	-0.943840961
-1.195310211	0.130481567	1	0	0.987147804
SLC16A8	ENSG00000100156.10	0.228026214	4	4	0.024229991
Brain_Spinal_cord_cervical_c-1	0.100116064	Brain_Amygdala	3.265637266
0.135262596	0.135262596	1.644291208	2.253462692	1.912459097
0.313066288	4	0	0.987147804
ZNF419	ENSG00000105136.20	0.228117277	12	3	0.049407235
Brain_Spinal_cord_cervical_c-1	0.312700035
Brain_Anterior_cingulate_cortex_BA24	9.396472209	0.005143258
0.543359132	1.00957274 1.965060509	1.588075637	0.24082675 3	0
0.987147804
DNAJC5	ENSG00000101152.10	0.22825327 2	2	0.087803046
Brain_Hippocampus	0.804010653	Brain_Putamen_basal_ganglia 1.02767122
0.97232878 0.97232878 -0.24815995	1.707102266	0.729471158
1.382579171	2	0	0.987147804
RAB38 ENSG00000123892.11	0.228421964	3	3	0.158330986
Brain_Nucleus_accumbens_basal_ganglia 0.684066564	Brain_Caudate_basal_ganglia
1.589639651	0.443881327	0.443881327	-0.489257969	1.410707136
0.17150964 1.073965876	3	0	0.987147804
RSRC2 ENSG00000111011.17	0.228462831	1	1	0.228462831
Brain_Substantia_nigra	0.228462831	Brain_Substantia_nigra	1	1
1	-1.204327995	-1.204327995	-1.204327995	NA	1	0
0.987147804
CEP120	ENSG00000168944.15	0.228463266	2	2	0.095692134
Brain_Hypothalamus	0.630848091	Brain_Spinal_cord_cervical_c-1
1.036177881	0.963822119	0.963822119	0.480533496	1.666106851
1.073320173	0.838326959	2	0	0.987147804
BORA	ENSG00000136122.15	0.228464425	1	1	0.228464425	Brain_Cerebellum
0.228464425	Brain_Cerebellum 1	1	1	-1.204323867	-1.204323867
-1.204323867	NA	1	0	0.987147804
G3BP1 ENSG00000145907.14	0.228597166	4	4	0.193605788
Brain_Putamen_basal_ganglia 0.668501472	Brain_Cerebellum 2.536113989
0.2957912 0.2957912 -0.428205556	1.299985942	0.633326317	0.74761501
4	0	0.987147804
ZNF211	ENSG00000121417.13	0.228805336	4	3	0.450923807
Brain_Putamen_basal_ganglia 0.649934154	Brain_Frontal_Cortex_BA9
3.160198634	0.075773746	0.188554479	-0.512817768	0.753875793
0.313070137	0.564476844	3	0	0.987147804

HEPACAM2 ENSG00000188175.9 0.228999942 1 1 0.228999942
Brain_Cerebellum 0.228999942 Brain_Cerebellum 1 1 1 1.202938962
1.202938962 1.202938962 NA 1 0 0.987147804
CDCP2 ENSG00000157211.11 0.229004882 1 1 0.229004882 Brain_Cortex
0.229004882 Brain_Cortex 1 1 1 -1.202926196 -1.202926196
-1.202926196 NA 1 0 0.987147804
COLQ ENSG00000206561.12 0.229095276 2 1 0.172349961
Brain_Cerebellar_Hemisphere 0.302711708 Brain_Cerebellum 1.983309282
0.016690718 1.983309282 -1.364691726 -1.030635658 -1.197663692
0.236213311 1 0 0.987147804
SMIM20 ENSG00000250317.8 0.229136768 1 1 0.229136768
Brain_Cerebellum 0.229136768 Brain_Cerebellum 1 1 1 -1.202585484
-1.202585484 -1.202585484 NA 1 0 0.987147804
RPS6KA5 ENSG00000100784.10 0.229229001 2 2 0.086331846
Brain_Frontal_Cortex_BA9 0.652131577 Brain_Spinal_cord_cervical_c-1
1.300532954 0.699467046 0.699467046 0.450802947 1.715072986
1.082937967 0.893973918 2 0 0.987147804
RP11-307C19.1 ENSG00000259362.2 0.229323567 13 6 0.036723444
Brain_Cerebellum 0.786470542 Brain_Hypothalamus 8.599751556
0.008104205 0.386859964 -0.270896624 2.088825393 1.502680821
0.587020911 6 0 0.987147804
RPL27 ENSG00000131469.12 0.229338672 1 1 0.229338672
Brain_Anterior_cingulate_cortex_BA24 0.229338672
Brain_Anterior_cingulate_cortex_BA24 1 1 1 1.202064157
1.202064157 1.202064157 NA 1 0 0.987147804
MAP1LC3A ENSG00000101460.12 0.229640339 11 4 0.026437678
Brain_Amygdala 0.625524485 Brain_Frontal_Cortex_BA9 8.451147739
0.002689021 0.316636231 0.488035798 2.219721503 1.356060124
0.525297578 4 0 0.987147804
PCDHB5 ENSG00000113209.8 0.229655353 2 2 0.135190983
Brain_Cerebellum 0.17780945 Brain_Caudate_basal_ganglia 1.380135954
0.619864046 0.619864046 -1.493941222 -1.34753046 -1.420735841
0.103528043 2 0 0.987147804
TRIP13 ENSG00000071539.13 0.22977588 1 1 0.22977588
Brain_Cerebellar_Hemisphere 0.22977588 Brain_Cerebellar_Hemisphere 1 1 1
-1.200936383 -1.200936383 -1.200936383 NA 1 0 0.987147804
FEN1 ENSG00000168496.3 0.229776581 1 1 0.229776581
Brain_Cerebellar_Hemisphere 0.229776581 Brain_Cerebellar_Hemisphere 1 1
1 -1.200934576 -1.200934576 -1.200934576 NA 1 0
0.987147804
CALML6 ENSG00000169885.9 0.22991216 4 3 0.087039126
Brain_Hypothalamus 0.619124404 Brain_Caudate_basal_ganglia 1.95139833
0.050171905 0.980984713 -1.71122749 0.954075952 -0.535079161
1.114382918 3 0 0.987147804
BOLA2B ENSG00000169627.7 0.229974637 1 1 0.229974637
Brain_Hypothalamus 0.229974637 Brain_Hypothalamus 1 1 1
-1.200424194 -1.200424194 -1.200424194 NA 1 0 0.987147804
CENPE ENSG00000138778.11 0.230105021 4 4 0.045872586 Brain_Cerebellum
0.955598962 Brain_Substantia_nigra 2.403731675 0.3059495 0.3059495
-1.996563824 -0.055677202 -1.043434785 0.813478493 4 0
0.987147804
GRWD1 ENSG00000105447.12 0.230123862 7 4 0.041458961
Brain_Frontal_Cortex_BA9 0.891654196 Brain_Nucleus_accumbens_basal_ganglia
5.52082403 0.001830674 0.254487602 -2.038910493 -0.136211358
-1.037391775 0.599075164 4 0 0.987147804
KIAA1549 ENSG00000122778.9 0.230291271 1 1 0.230291271
Brain_Frontal_Cortex_BA9 0.230291271 Brain_Frontal_Cortex_BA9 1 1
1 -1.199608893 -1.199608893 -1.199608893 NA 1 0
0.987147804
ARL6IP5 ENSG00000144746.6 0.230294308 8 8 0.03425039
Brain_Caudate_basal_ganglia 0.968842248 Brain_Putamen_basal_ganglia
1.684045296 0.497391516 0.497391516 -2.117111448 1.64750099

-0.174444231 1.166005105 8 0 0.987147804
STAT5A ENSG00000126561.16 0.230549457 1 1 0.230549457
Brain_Anterior_cingulate_cortex_BA24 0.230549457
Brain_Anterior_cingulate_cortex_BA24 1 1 1 -1.19894468
-1.19894468 -1.19894468 NA 1 0 0.987147804
RAP1GDS1 ENSG00000138698.14 0.230560385 1 1 0.230560385
Brain_Cerebellar_Hemisphere 0.230560385 Brain_Cerebellar_Hemisphere 1 1
1 1.198916577 1.198916577 1.198916577 NA 1 0
0.987147804
CUL4A ENSG00000139842.14 0.230563057 1 1 0.230563057 Brain_Cerebellum
0.230563057 Brain_Cerebellum 1 1 1 1.198909707 1.198909707
1.198909707 NA 1 0 0.987147804
FAM69A ENSG00000154511.11 0.230605578 4 4 0.217115242
Brain_Cerebellar_Hemisphere 0.908479214 Brain_Cerebellum 2.220341744
0.099001018 0.099001018 -1.234235287 1.050718911 0.078576663
0.959592156 4 0 0.987147804
CST3 ENSG00000101439.8 0.230763302 3 3 0.086655657
Brain_Nucleus_accumbens_basal_ganglia 0.513585662 Brain_Cerebellum
1.392514367 0.609932541 0.609932541 0.653264675 1.713309276
1.180596024 0.530042794 3 0 0.987147804
METTL4 ENSG00000101574.14 0.23088279 10 4 0.227213426 Brain_Amygdala
0.884394138 Brain_Frontal_Cortex_BA9 7.644239905 0.015470839
0.292113348 -0.997446576 1.207568125 0.115954991 0.74547015 4
0 0.987147804
MFSD1 ENSG00000118855.18 0.230882873 5 5 0.1191967
Brain_Anterior_cingulate_cortex_BA24 0.309562429 Brain_Frontal_Cortex_BA9
1.794616325 0.431785928 0.431785928 -1.136020314 1.558154198
-0.156673807 1.331940643 5 0 0.987147804
GPX4 ENSG00000167468.16 0.231021052 8 5 0.047313411
Brain_Putamen_basal_ganglia 0.71915938 Brain_Nucleus_accumbens_basal_ganglia
4.169323704 0.019901015 0.404706693 -1.507483462 1.983483831
0.009608482 1.131960903 5 0 0.987147804
SFRP5 ENSG00000120057.4 0.231134324 2 2 0.087826924 Brain_Cerebellum
0.401022305 Brain_Frontal_Cortex_BA9 1.553231036 0.446768964
0.446768964 -1.706973791 -0.839796841 -1.273385316 0.613186702
2 0 0.987147804
HEBP1 ENSG00000013583.8 0.231248771 5 5 0.034208907
Brain_Substantia_nigra 0.748185144 Brain_Hypothalamus 1.811153981
0.201125757 0.201125757 -2.117600607 -0.321033315 -1.134342972
0.680259129 5 0 0.987147804
TSGA10IP ENSG00000175513.9 0.231333705 1 1 0.231333705
Brain_Caudate_basal_ganglia 0.231333705 Brain_Caudate_basal_ganglia 1 1
1 1.196930346 1.196930346 1.196930346 NA 1 0
0.987147804
EXOC8 ENSG00000116903.7 0.231446895 4 4 0.064646165 Brain_Cortex
0.767572299 Brain_Hippocampus 2.119935094 0.265448184
0.265448184 -0.406435231 1.847697164 0.840375848 1.077220991
4 0 0.987147804
FAXC ENSG00000146267.11 0.231467873 1 1 0.231467873
Brain_Hippocampus 0.231467873 Brain_Hippocampus 1 1 1
-1.196586223 -1.196586223 -1.196586223 NA 1 0 0.987147804
RP11-63G10.4 ENSG00000283445.1 0.231514029 1 1 0.231514029
Brain_Frontal_Cortex_BA9 0.231514029 Brain_Frontal_Cortex_BA9 1 1
1 1.196467873 1.196467873 1.196467873 NA 1 0
0.987147804
CENPS ENSG00000175279.21 0.231555368 4 4 0.040825652
Brain_Nucleus_accumbens_basal_ganglia 0.363884484 Brain_Frontal_Cortex_BA9
2.140020449 0.153012605 0.153012605 0.907988147 2.04529633
1.487833903 0.574284795 4 0 0.987147804
RP11-371M22.1 ENSG00000251628.1 0.231619656 1 1 0.231619656
Brain_Frontal_Cortex_BA9 0.231619656 Brain_Frontal_Cortex_BA9 1 1
1 -1.19619709 -1.19619709 -1.19619709 NA 1 0

0.987147804
CENPK ENSG00000123219.12 0.231622365 7 4 0.14848048
Brain_Anterior_cingulate_cortex_BA24 0.404256985 Brain_Cerebellum
5.400482175 4.06E-17 0.189472664 -1.444919566 1.41518977
0.735283269 0.97920467 4 0 0.987147804
ALLC ENSG00000151360.9 0.231704109 12 11 0.081801132
Brain_Putamen_basal_ganglia 0.895852313 Brain_Amygdala 4.766750811
0.073692936 0.180748444 -1.740329755 0.538538414 -0.781332508
0.88827711 11 0 0.987147804
SCRT1 ENSG00000261678.2 0.231705004 1 1 0.231705004
Brain_Cerebellar_Hemisphere 0.231705004 Brain_Cerebellar_Hemisphere 1 1
1 -1.195978361 -1.195978361 -1.195978361 NA 1 0
0.987147804
DEFA4 ENSG00000164821.4 0.231826766 1 1 0.231826766 Brain_Amygdala
0.231826766 Brain_Amygdala 1 1 1 -1.195666405 -1.195666405
-1.195666405 NA 1 0 0.987147804
SNAP47 ENSG00000143740.14 0.231937225 11 6 0.260282487
Brain_Hypothalamus 0.908083902 Brain_Caudate_basal_ganglia 7.760558327
0.013794481 0.272937687 -1.125723704 0.522265633 -0.506998336
0.569090287 6 0 0.987147804
PHLDB2 ENSG00000144824.20 0.231939803 2 2 0.103141831
Brain_Cerebellum 0.717506647 Brain_Caudate_basal_ganglia 1.093249366
0.906750634 0.906750634 -1.629809188 0.361793103 -0.634008043
1.408275485 2 0 0.987147804
AUTS2 ENSG00000158321.15 0.232192763 2 2 0.135577185
Brain_Frontal_Cortex_BA9 0.317740293 Brain_Caudate_basal_ganglia
1.115417083 0.884582917 0.884582917 -1.492465386 -0.999112302
-1.245788844 0.348853312 2 0 0.987147804
MRPS36 ENSG00000134056.11 0.232267081 1 1 0.232267081
Brain_Cerebellar_Hemisphere 0.232267081 Brain_Cerebellar_Hemisphere 1 1
1 -1.19453929 -1.19453929 -1.19453929 NA 1 0
0.987147804
EEF2KMT ENSG00000118894.14 0.232293227 1 1 0.232293227
Brain_Cerebellum 0.232293227 Brain_Cerebellum 1 1 1 -1.194472409
-1.194472409 -1.194472409 NA 1 0 0.987147804
TNIP2 ENSG00000168884.14 0.232450562 2 2 0.100776727 Brain_Cortex
0.557035536 Brain_Nucleus_accumbens_basal_ganglia 1.590976673
0.409023327 0.409023327 -1.641099672 -0.587250418 -1.114175045
0.745183954 2 0 0.987147804
CTD-2199O4.6 ENSG00000272417.1 0.232609853 1 1 0.232609853
Brain_Spinal_cord_cervical_c-1 0.232609853 Brain_Spinal_cord_cervical_c-1
1 1 1 1.193662915 1.193662915 1.193662915 NA 1 0
0.987147804
FAM53A ENSG00000174137.12 0.232613236 4 4 0.096501601
Brain_Putamen_basal_ganglia 0.535216286 Brain_Cerebellar_Hemisphere 1.44101648
0.435445464 0.435445464 -1.662055727 0.620063037 -0.916329453
1.048570282 4 0 0.987147804
SLC22A3 ENSG00000146477.5 0.232657169 9 4 0.122142971
Brain_Spinal_cord_cervical_c-1 0.901194671
Brain_Anterior_cingulate_cortex_BA24 6.914284072 0.016976751 0.35953405
-1.545841008 1.063442541 0.439767267 0.831067167 4 0
0.987147804
VCPKMT ENSG00000100483.13 0.232686977 8 5 0.080536728
Brain_Putamen_basal_ganglia 0.854514094 Brain_Hypothalamus 5.599136334
0.044779149 0.226376933 -1.747580306 -0.183361867 -1.363130339
0.491202306 5 0 0.987147804
PPP1R12C ENSG00000125503.12 0.232716569 2 2 0.158624611
Brain_Cerebellum 0.334814067 Brain_Hippocampus 1.000592157
0.999407843 0.999407843 -1.409712438 -0.964462568 -1.187087503
0.314839203 2 0 0.987147804
TMEM132B ENSG00000139364.10 0.232778492 3 3 0.148789377
Brain_Putamen_basal_ganglia 0.753260737 Brain_Cerebellum 1.728777056

0.271159111 0.271159111 -1.443820845 1.209743334 0.026755078
1.349955819 3 0 0.987147804
MEF2B ENSG00000213999.15 0.23283307 2 2 0.110757651 Brain_Cerebellum
0.245894876 Brain_Cerebellar_Hemisphere 1.896739547 0.103260453
0.103260453 -1.594796944 -1.160378158 -1.377587551 0.30718047 2
0 0.987147804
KITLG ENSG00000049130.14 0.232909325 1 1 0.232909325 Brain_Cerebellum
0.232909325 Brain_Cerebellum 1 1 1 -1.192897995 -1.192897995
-1.192897995 NA 1 0 0.987147804
CTD-2006K23.1 ENSG00000261222.2 0.232920678 2 2 0.342154796
Brain_Cerebellar_Hemisphere 0.916859525 Brain_Cerebellum 1.863349303
0.136650697 0.136650697 0.10439042 0.949916276 0.527153348
0.597877066 2 0 0.987147804
EGR2 ENSG00000122877.15 0.233091859 1 1 0.233091859
Brain_Caudate_basal_ganglia 0.233091859 Brain_Caudate_basal_ganglia 1 1
1 1.192432103 1.192432103 1.192432103 NA 1 0
0.987147804
LRRC14 ENSG00000160959.7 0.23317174 1 1 0.23317174
Brain_Caudate_basal_ganglia 0.23317174 Brain_Caudate_basal_ganglia 1 1 1
1.1922283 1.1922283 1.1922283 NA 1 0 0.987147804
NCF1 ENSG00000158517.13 0.233221055 2 2 0.088951726
Brain_Nucleus_accumbens_basal_ganglia 0.910141312 Brain_Amygdala
1.013160116 0.986839884 0.986839884 -1.700953467 0.112860299
-0.794046584 1.282560013 2 0 0.987147804
SNX16 ENSG00000104497.13 0.233221515 13 4 0.018601568 Brain_Cerebellum
0.908608093 Brain_Substantia_nigra 9.786116685 0.004748135
0.470243435 -0.114794393 2.353420831 1.432593091 0.671884437
4 0 0.987147804
EEF2 ENSG00000167658.15 0.233330921 2 2 0.097690761
Brain_Cerebellar_Hemisphere 0.654009123 Brain_Amygdala 1.023389974
0.976610026 0.976610026 -1.656153383 -0.44819964 -1.052176511
0.854152284 2 0 0.987147804
TMEM110 ENSG00000213533.11 0.233345787 3 3 0.09935326
Brain_Hippocampus 0.184996611 Brain_Caudate_basal_ganglia 1.653889487
0.397821179 0.397821179 -1.647997131 -1.325526425 -1.491252071
0.161422821 3 0 0.987147804
TMEM59 ENSG00000116209.11 0.233346408 1 1 0.233346408
Brain_Cortex 0.233346408 Brain_Cortex 1 1 1 1.191782835
1.191782835 1.191782835 NA 1 0 0.987147804
STT3A ENSG00000134910.12 0.233351418 1 1 0.233351418
Brain_Hypothalamus 0.233351418 Brain_Hypothalamus 1 1 1
-1.19177006 -1.19177006 -1.19177006 NA 1 0 0.987147804
AF127936.5 ENSG00000226751.2 0.233387617 2 2 0.089431298
Brain_Frontal_Cortex_BA9 0.787189097 Brain_Hippocampus 1.065075137
0.934924863 0.934924863 -1.698405272 -0.269962509 -0.984183891
1.010061564 2 0 0.987147804
UFSP2 ENSG00000109775.10 0.233421228 13 1 0.177410045 Brain_Cerebellum
0.347040055 Brain_Hypothalamus 12.45201661 1.02E-16 12.45201661
-1.348772517 -0.94034611 -1.168314382 0.09819025 1 0
0.987147804
GALNT2 ENSG00000143641.9 0.233668962 4 4 0.051594851
Brain_Putamen_basal_ganglia 0.650673633 Brain_Cerebellar_Hemisphere
1.760768942 0.209562742 0.209562742 0.452826565 1.946498811
1.286952401 0.742026733 4 0 0.987147804
KCTD6 ENSG00000168301.12 0.233720764 3 3 0.098815416
Brain_Cerebellar_Hemisphere 0.43237615 Brain_Cortex 1.038478913
0.968493578 0.968493578 -0.95261062 1.650623798 0.494381743
1.32574853 3 0 0.987147804
AC109826.1 ENSG00000226791.6 0.233790344 3 3 0.09544643 Brain_Cortex
0.678216682 Brain_Spinal_cord_cervical_c-1 1.206604891 0.799273111
0.799273111 -1.66734195 0.785131456 -0.155770881 1.322083036
3 0 0.987147804

RP11-190P13.2 ENSG00000242880.1 0.233852623 1 1 0.233852623
Brain_Spinal_cord_cervical_c-1 0.233852623 Brain_Spinal_cord_cervical_c-1
1 1 1 1.190493144 1.190493144 1.190493144 NA 1 0
0.987147804
CPEB2-AS1 ENSG00000247624.6 0.234009968 2 2 0.094751891
Brain_Cerebellum 0.704721337 Brain_Caudate_basal_ganglia 1.02563461 0.97436539
0.97436539 -0.378954925 1.670847058 0.645946067 1.449428882 2
0 0.987147804
BID ENSG00000015475.18 0.234059251 5 2 0.287734994 Brain_Cortex
0.619843409 Brain_Caudate_basal_ganglia 4.343929802 0.013954409
0.543416183 -1.063103552 0.49607229 -0.660093556 0.657809449 2
0 0.987147804
DZANK1 ENSG00000089091.16 0.23409765 5 5 0.059294878
Brain_Frontal_Cortex_BA9 0.674909515 Brain_Cerebellum 3.063288295
0.12276426 0.12276426 -1.886000566 -0.419419583 -1.095492661 0.52837391
5 0 0.987147804
CCDC69 ENSG00000198624.12 0.234207848 2 2 0.160916766
Brain_Hypothalamus 0.392044798 Brain_Cerebellum 1.078018157
0.921981843 0.921981843 0.855914998 1.401994913 1.128954956
0.386136811 2 0 0.987147804
MAFF ENSG00000185022.11 0.234288866 1 1 0.234288866
Brain_Caudate_basal_ganglia 0.234288866 Brain_Caudate_basal_ganglia 1 1
1 1.189383309 1.189383309 1.189383309 NA 1 0
0.987147804
DESI1 ENSG00000100418.7 0.234333818 3 3 0.115741608 Brain_Cortex
0.78993242 Brain_Putamen_basal_ganglia 1.495286781 0.496414247
0.496414247 -0.266398371 1.572901585 0.688565132 0.921681733
3 0 0.987147804
ZNF266 ENSG00000174652.17 0.234335984 13 1 0.201353251
Brain_Substantia_nigra 0.375286989 Brain_Spinal_cord_cervical_c-1
12.79494339 2.86E-05 12.79494339 -1.277705597 -0.886613564
-1.179164524 0.098880238 1 0 0.987147804
CENPL ENSG00000120334.15 0.234364929 1 1 0.234364929
Brain_Frontal_Cortex_BA9 0.234364929 Brain_Frontal_Cortex_BA9 1 1
1 1.18918995 1.18918995 1.18918995 NA 1 0 0.987147804
TYW1B ENSG00000277149.4 0.234406696 13 1 0.058362561
Brain_Spinal_cord_cervical_c-1 0.370777025 Brain_Amygdala 12.52034533
0.000338584 12.52034533 0.895018825 1.892964726 1.167539391
0.302184625 1 0 0.987147804
ZHX2 ENSG00000178764.7 0.234429041 1 1 0.234429041
Brain_Caudate_basal_ganglia 0.234429041 Brain_Caudate_basal_ganglia 1 1
1 1.189027004 1.189027004 1.189027004 NA 1 0
0.987147804
TMED9 ENSG00000184840.11 0.23451457 4 2 0.118133134
Brain_Cerebellar_Hemisphere 0.555564384 Brain_Putamen_basal_ganglia 3.70856114
0.017783952 0.242919569 0.589442633 1.562657678 0.995941352
0.444055476 2 0 0.987147804
FAM219B ENSG00000178761.14 0.234553113 1 1 0.234553113
Brain_Hypothalamus 0.234553113 Brain_Hypothalamus 1 1 1
1.188711755 1.188711755 1.188711755 NA 1 0 0.987147804
NDST2 ENSG00000166507.17 0.234556222 1 1 0.234556222
Brain_Nucleus_accumbens_basal_ganglia 0.234556222
Brain_Nucleus_accumbens_basal_ganglia 1 1 1 1.188703858
1.188703858 1.188703858 NA 1 0 0.987147804
KIAA0922 ENSG00000121210.15 0.234603816 1 1 0.234603816
Brain_Caudate_basal_ganglia 0.234603816 Brain_Caudate_basal_ganglia 1 1
1 -1.188582961 -1.188582961 -1.188582961 NA 1 0
0.987147804
GALNT15 ENSG00000131386.17 0.23461869 2 2 0.197976582
Brain_Frontal_Cortex_BA9 0.347975772 Brain_Spinal_cord_cervical_c-1
1.129491817 0.870508183 0.870508183 0.938522865 1.287337778
1.112930321 0.24664939 2 0 0.987147804

POLR1D ENSG00000186184.16 0.234619443 11 8 0.01817217 Brain_Cortex
0.797656828 Brain_Cerebellum 4.859504134 0.066341983 0.177807091
-0.362248397 2.362091218 1.066099586 1.014939257 8 0
0.987147804
ERN1 ENSG00000178607.15 0.234638695 4 3 0.13245472
Brain_Nucleus_accumbens_basal_ganglia 0.739752879 Brain_Hypothalamus
1.977816133 0.036151748 0.976596285 -1.504492044 -0.332180617
-1.10519484 0.526988499 3 0 0.987147804
LINC02010 ENSG00000242671.1 0.234698116 1 1 0.234698116
Brain_Cerebellum 0.234698116 Brain_Cerebellum 1 1 1 -1.188343475
-1.188343475 -1.188343475 NA 1 0 0.987147804
RP11-354P17.15 ENSG00000261696.1 0.234769675 1 1 0.234769675
Brain_Frontal_Cortex_BA9 0.234769675 Brain_Frontal_Cortex_BA9 1 1
1 1.188161787 1.188161787 1.188161787 NA 1 0
0.987147804
SDC1 ENSG00000115884.10 0.234852178 2 2 0.158704797 Brain_Cerebellum
0.289507815 Brain_Putamen_basal_ganglia 1.076256696 0.923743304
0.923743304 -1.409441035 1.059201956 -0.17511954 1.745594199
2 0 0.987147804
PRPF19 ENSG00000110107.8 0.234870052 1 1 0.234870052
Brain_Cerebellum 0.234870052 Brain_Cerebellum 1 1 1 1.187906999
1.187906999 1.187906999 NA 1 0 0.987147804
ITIH2 ENSG00000151655.18 0.234879364 4 4 0.055283395
Brain_Caudate_basal_ganglia 0.498218193 Brain_Cerebellum 1.82148446
0.197659824 0.197659824 -1.051169289 1.916642264 0.221178244
1.357287411 4 0 0.987147804
DGCR6 ENSG00000183628.12 0.234950775 4 4 0.057734936
Brain_Nucleus_accumbens_basal_ganglia 0.897758711 Brain_Caudate_basal_ganglia
1.944508877 0.266925431 0.266925431 -1.897705129 -0.12849316
-1.408820765 0.854761877 4 0 0.987147804
KIF22 ENSG00000079616.12 0.235079606 1 1 0.235079606
Brain_Hypothalamus 0.235079606 Brain_Hypothalamus 1 1 1
1.187375331 1.187375331 1.187375331 NA 1 0 0.987147804
MMS19 ENSG00000155229.20 0.235149966 2 2 0.112161245 Brain_Cerebellum
0.635769228 Brain_Substantia_nigra 1.084209346 0.915790654
0.915790654 -1.588553447 -0.47362236 -1.031087903 0.788375332
2 0 0.987147804
KCNJ9 ENSG00000162728.4 0.235235635 3 3 0.133964093
Brain_Putamen_basal_ganglia 0.223240915 Brain_Spinal_cord_cervical_c-1
1.502412398 0.491933067 0.491933067 -1.217955418 1.498651393
0.535935211 1.521333591 3 0 0.987147804
DUSP8 ENSG00000184545.10 0.235278061 1 1 0.235278061
Brain_Frontal_Cortex_BA9 0.235278061 Brain_Frontal_Cortex_BA9 1 1
1 -1.186872133 -1.186872133 -1.186872133 NA 1 0
0.987147804
C12orf71 ENSG00000214700.5 0.23531222 1 1 0.23531222 Brain_Hypothalamus
0.23531222 Brain_Hypothalamus 1 1 1 1.186785551 1.186785551
1.186785551 NA 1 0 0.987147804
GNMT ENSG00000124713.5 0.235352616 12 1 0.16687529
Brain_Cerebellar_Hemisphere 0.310275426 Brain_Putamen_basal_ganglia
11.59661449 0.000166649 11.59661449 1.014644274 1.382314061
1.166816738 0.112301196 1 0 0.987147804
SETD6 ENSG00000103037.11 0.235370516 3 3 0.057525688
Brain_Cerebellar_Hemisphere 0.401040815 Brain_Anterior_cingulate_cortex_BA24
1.824025943 0.17754882 0.17754882 -0.839763833 1.899295091
0.878070256 1.496515716 3 0 0.987147804
PCDHGA1 ENSG00000204956.5 0.23538649 3 3 0.072443954
Brain_Cerebellar_Hemisphere 0.53428444 Brain_Caudate_basal_ganglia 1.745476363
0.254902411 0.254902411 -1.034302504 1.796318016 0.04684547
1.529083527 3 0 0.987147804
ABRA ENSG00000174429.3 0.235397647 1 1 0.235397647
Brain_Frontal_Cortex_BA9 0.235397647 Brain_Frontal_Cortex_BA9 1 1

1 -1.186569057 -1.186569057 -1.186569057 NA 1 0
0.987147804
OLFM1 ENSG00000130558.19 0.235444479 2 2 0.089246182
Brain_Putamen_basal_ganglia 0.76310269 Brain_Substantia_nigra 1.137651889
0.862348111 0.862348111 -0.301408924 1.699387571 0.698989323
1.414776769 2 0 0.987147804
RPS6KA2 ENSG00000071242.11 0.235481797 1 1 0.235481797
Brain_Cerebellar_Hemisphere 0.235481797 Brain_Cerebellar_Hemisphere 1 1
1 -1.186355856 -1.186355856 -1.186355856 NA 1 0
0.987147804
QRFPR ENSG00000186867.10 0.235677484 1 1 0.235677484
Brain_Cerebellar_Hemisphere 0.235677484 Brain_Cerebellar_Hemisphere 1 1
1 -1.185860273 -1.185860273 -1.185860273 NA 1 0
0.987147804
IARS ENSG00000196305.17 0.235739909 2 2 0.323197898
Brain_Cerebellar_Hemisphere 0.728102888 Brain_Cerebellum 1.677784203
0.322215797 0.322215797 -0.347650217 0.987907611 0.320128697
0.944381997 2 0 0.987147804
THUMPD2 ENSG00000138050.14 0.235850007 3 3 0.153126815
Brain_Cerebellar_Hemisphere 0.547092965 Brain_Cortex 1.798604535
0.208622751 0.208622751 -1.428573639 1.377388313 0.183645541
1.449033298 3 0 0.987147804
TAPBP ENSG00000231925.11 0.235851501 6 5 0.05417687 Brain_Hypothalamus
0.978856426 Brain_Anterior_cingulate_cortex_BA24 2.878980225
0.021485946 0.420879871 -0.395267722 1.925419727 0.565452675
0.806464797 5 0 0.987147804
RP11-521C20.2 ENSG00000259584.1 0.235868153 5 3 0.093924597
Brain_Cerebellum 0.333560307 Brain_Anterior_cingulate_cortex_BA24
3.451855617 0.025608267 0.403681898 0.966967447 1.675049107
1.19462256 0.286885082 3 0 0.987147804
CSE1L ENSG00000124207.16 0.235949953 1 1 0.235949953 Brain_Cortex
0.235949953 Brain_Cortex 1 1 1 -1.185170723 -1.185170723
-1.185170723 NA 1 0 0.987147804
RP11-572O17.1 ENSG00000270195.1 0.235958182 2 2 0.179535898
Brain_Cerebellum 0.886098666 Brain_Putamen_basal_ganglia 1.544657997
0.455342003 0.455342003 -1.342185374 0.1432425 -0.599471437
1.050356123 2 0 0.987147804
FAM228A ENSG00000186453.12 0.235967796 7 6 0.276358588
Brain_Substantia_nigra 0.816330008 Brain_Putamen_basal_ganglia
4.479645229 0.127660906 0.164388479 -0.926254054 1.08853592
0.327415349 0.774370632 6 0 0.987147804
RAET1G ENSG00000203722.7 0.236016035 11 1 0.139172063
Brain_Cerebellum 0.370807139 Brain_Cortex 10.42702206 0.00319541
10.42702206 -1.478881222 -0.894962491 -1.152534133 0.171399056
1 0 0.987147804
KBTBD8 ENSG00000163376.11 0.236367124 1 1 0.236367124
Brain_Substantia_nigra 0.236367124 Brain_Substantia_nigra 1 1
1 -1.184116059 -1.184116059 -1.184116059 NA 1 0
0.987147804
HIBADH ENSG00000106049.8 0.236454416 3 3 0.21723293
Brain_Caudate_basal_ganglia 0.922163235 Brain_Hippocampus 1.783242358
0.375449548 0.375449548 -1.233919427 1.060041794 -0.025389488
1.151924251 3 0 0.987147804
ADD1 ENSG00000087274.16 0.23653785 1 1 0.23653785 Brain_Frontal_Cortex_BA9
0.23653785 Brain_Frontal_Cortex_BA9 1 1 1 -1.183684821
-1.183684821 -1.183684821 NA 1 0 0.987147804
GNA12 ENSG00000146535.13 0.236701838 5 4 0.091934212
Brain_Hypothalamus 0.952851491 Brain_Putamen_basal_ganglia 3.368380382
0.102190475 0.14719142 -1.68528182 -0.059126325 -0.984227762
0.637675817 4 0 0.987147804
ATF6B ENSG00000213676.10 0.236871056 9 9 0.051951651
Brain_Spinal_cord_cervical_c-1 0.504841509 Brain_Hippocampus

1.987563202 0.395822121 0.395822121 -1.943534173 0.951814024
-0.661969303 1.166845033 9 0 0.987147804
ZNF276 ENSG00000158805.11 0.236889351 6 5 0.061528248
Brain_Nucleus_accumbens_basal_ganglia 0.787097284 Brain_Cerebellum
4.308747353 0.096312347 0.167234967 -1.869680151 0.386888588
-0.911443567 0.832435707 5 0 0.987147804
GNG8 ENSG00000167414.4 0.236896973 1 1 0.236896973
Brain_Nucleus_accumbens_basal_ganglia 0.236896973
Brain_Nucleus_accumbens_basal_ganglia 1 1 1 1.182778425
1.182778425 1.182778425 NA 1 0 0.987147804
ZNF507 ENSG00000168813.16 0.236929433 6 6 0.065867508
Brain_Frontal_Cortex_BA9 0.966098363 Brain_Hippocampus 2.859668187
0.141326956 0.141326956 -0.988448273 1.839324255 -0.006002752
1.049365718 6 0 0.987147804
PDLIM4 ENSG00000131435.12 0.236976592 4 3 0.206267378
Brain_Cortex 0.891910923 Brain_Frontal_Cortex_BA9 3.489033248
0.085039094 0.166466498 -0.135886608 1.263895943 0.568796365
0.573630222 3 0 0.987147804
ZGRF1 ENSG00000138658.15 0.237178099 13 5 0.047180694
Brain_Spinal_cord_cervical_c-1 0.964973514 Brain_Hippocampus
8.794103636 0.003603806 0.456653816 0.0439133 1.984674531
1.110572822 0.544022028 5 0 0.987147804
NEGR1 ENSG00000172260.14 0.237213604 5 5 0.047003093
Brain_Putamen_basal_ganglia 0.964093532 Brain_Nucleus_accumbens_basal_ganglia
1.647254598 0.392911668 0.392911668 -0.261759971 1.986272336
0.908459813 0.995059667 5 0 0.987147804
PHLDB3 ENSG00000176531.10 0.237256065 10 1 0.173545751
Brain_Spinal_cord_cervical_c-1 0.398220396 Brain_Hypothalamus
9.217841656 0.001552066 9.217841656 0.844803794 1.360898553
1.132898103 0.159281237 1 0 0.987147804
CMTM3 ENSG00000140931.19 0.237413104 3 3 0.074181999
Brain_Spinal_cord_cervical_c-1 0.666680662 Brain_Hippocampus
1.322823765 0.802998845 0.802998845 -1.785489217 0.430708053
-0.602049094 1.115756069 3 0 0.987147804
PAAF1 ENSG00000175575.12 0.237510258 12 3 0.088458041
Brain_Nucleus_accumbens_basal_ganglia 0.976644832 Brain_Cerebellum 9.43914757
0.004677707 0.524647105 -1.703588233 0.543970517 -0.534855991
0.569758428 3 0 0.987147804
MAGEF1 ENSG00000177383.4 0.237604009 1 1 0.237604009
Brain_Frontal_Cortex_BA9 0.237604009 Brain_Frontal_Cortex_BA9 1 1
1 -1.180996764 -1.180996764 -1.180996764 NA 1 0
0.987147804
LINC00987 ENSG00000237248.4 0.237697766 3 3 0.040173083
Brain_Putamen_basal_ganglia 0.66897293 Brain_Caudate_basal_ganglia 1.66600622
0.535678401 0.535678401 0.427558027 2.051964801 1.069242635
0.864236255 3 0 0.987147804
CTA-228A9.3 ENSG00000272720.1 0.237792119 6 4 0.019346881
Brain_Caudate_basal_ganglia 0.95391294 Brain_Hypothalamus 3.930288295
0.029352713 0.188434213 -0.057793721 2.338778859 1.409375695
1.074799843 4 0 0.987147804
NBEAL2 ENSG00000160796.16 0.237792576 6 4 0.098056613
Brain_Anterior_cingulate_cortex_BA24 0.997589299 Brain_Cerebellar_Hemisphere
4.379227102 0.029197115 0.151326194 -0.631573505 1.654349047
0.46131437 0.885583287 4 0 0.987147804
FGF12 ENSG00000114279.13 0.237812829 1 1 0.237812829 Brain_Cortex
0.237812829 Brain_Cortex 1 1 1 -1.180471273 -1.180471273
-1.180471273 NA 1 0 0.987147804
LINC01914 ENSG00000234362.5 0.23793191 1 1 0.23793191
Brain_Spinal_cord_cervical_c-1 0.23793191 Brain_Spinal_cord_cervical_c-1 1
1 1 1.180171757 1.180171757 1.180171757 NA 1 0
0.987147804
ABHD12 ENSG00000100997.18 0.23804275 12 2 0.112022367

Brain_Substantia_nigra 0.792563293 Brain_Anterior_cingulate_cortex_BA24 10.10266244 0.008517663 0.903170287 -0.262983505 1.589168452 0.999958563 0.479050292 2 0 0.987147804
MEIOB ENSG00000162039.14 0.23806839 7 5 0.099561017 Brain_Nucleus_accumbens_basal_ganglia 0.924455956 Brain_Spinal_cord_cervical_c-1 5.118291963 0.018424994 0.182016653 -1.646985543 0.174282309 -0.728517427 0.617076755 5 0 0.987147804
CFD ENSG00000197766.7 0.238165807 8 8 0.007716038 Brain_Hypothalamus 0.976747587 Brain_Putamen_basal_ganglia 2.022181798 0.266978438 0.266978438 -2.664249186 1.166396634 -0.142429841 1.171613857 8 0 0.987147804
RP11-298J23.8 ENSG00000269387.1 0.238217884 1 1 0.238217884 Brain_Cortex 0.238217884 Brain_Cortex 1 1 1 -1.179452893 -1.179452893 -1.179452893 NA 1 0 0.987147804
SLC19A3 ENSG00000135917.13 0.23822348 3 3 0.060720593 Brain_Substantia_nigra 0.414724392 Brain_Cortex 1.400043243 0.61194241 0.61194241 0.815607966 1.875524564 1.306400317 0.534282428 3 0 0.987147804
TTLL11-IT1 ENSG00000237548.1 0.238463354 1 1 0.238463354 Brain_Cerebellum 0.238463354 Brain_Cerebellum 1 1 1 1.178836331 1.178836331 1.178836331 NA 1 0 0.987147804
FHL3 ENSG00000183386.9 0.238517877 5 5 0.059562506 Brain_Hypothalamus 0.713378457 Brain_Cortex 2.206197678 0.388711357 0.388711357 -1.884018254 -0.367322521 -1.136442551 0.657490727 5 0 0.987147804
RHOBTB2 ENSG00000008853.16 0.238618987 2 2 0.307685599 Brain_Cerebellum 0.941525768 Brain_Cerebellar_Hemisphere 1.823416082 0.176583918 0.176583918 0.073352307 1.020090379 0.546721343 0.66944491 2 0 0.987147804
RP11-401N18.1 ENSG00000271148.1 0.238624101 3 3 0.115485409 Brain_Cerebellar_Hemisphere 0.392333567 Brain_Cerebellum 1.828344775 0.194250743 0.194250743 -1.204015625 1.574008826 0.408462098 1.441931152 3 0 0.987147804
MYO7A ENSG00000137474.19 0.238654299 2 2 0.104913233 Brain_Substantia_nigra 0.601869306 Brain_Hypothalamus 1.022059023 0.977940977 0.977940977 -0.521714243 1.621487015 0.549886386 1.515472143 2 0 0.987147804
RASSF5 ENSG00000266094.7 0.238658334 1 1 0.238658334 Brain_Nucleus_accumbens_basal_ganglia 0.238658334 Brain_Nucleus_accumbens_basal_ganglia 1 1 1 -1.17834691 -1.17834691 -1.17834691 NA 1 0 0.987147804
TM2D3 ENSG00000184277.12 0.238726598 10 3 0.06924133 Brain_Substantia_nigra 0.835801204 Brain_Putamen_basal_ganglia 8.29568985 0.002369803 0.306400828 0.207267181 1.816841865 1.127423728 0.434721875 3 0 0.987147804
LRFN2 ENSG00000156564.8 0.238760984 2 2 0.6818361 Brain_Cerebellar_Hemisphere 0.771854745 Brain_Cerebellum 1.914382681 0.085617319 0.085617319 -0.289949665 0.409958896 0.060004615 0.49491009 2 0 0.987147804
GALC ENSG00000054983.16 0.23888233 5 5 0.035185238 Brain_Nucleus_accumbens_basal_ganglia 0.996566146 Brain_Cerebellum 2.014054854 0.112127047 0.112127047 -2.106220098 0.835537999 -0.165173171 1.191501365 5 0 0.987147804
LTBP4 ENSG00000090006.17 0.23933714 3 3 0.057415706 Brain_Putamen_basal_ganglia 0.190737649 Brain_Cerebellum 2.117054605 0.167694991 0.167694991 -1.900132714 -1.308400069 -1.608505536 0.295957415 3 0 0.987147804
ZNF493 ENSG00000196268.11 0.239352647 9 5 0.105230127 Brain_Hippocampus 0.841690931 Brain_Cortex 6.11750647 0.045916976 0.265408381 -1.620010009 0.608216341 -0.573217469 0.669382453 5 0 0.987147804

C15orf65 ENSG00000261652.2 0.239352965 2 2 0.272854867
Brain_Cerebellar_Hemisphere 0.742861361 Brain_Cerebellum 1.872612455
0.127387545 0.127387545 -1.09651179 -0.328066529 -0.712289159
0.543372855 2 0 0.987147804
VPS39 ENSG00000166887.15 0.239423865 2 2 0.4532212
Brain_Nucleus_accumbens_basal_ganglia 0.609704732 Brain_Frontal_Cortex_BA9
1.720481027 0.279518973 0.279518973 -0.750055632 0.510494979
-0.119780327 0.891343885 2 0 0.987147804
ALDH3A2 ENSG00000072210.18 0.239434101 2 1 0.163436673
Brain_Substantia_nigra 0.349090502 Brain_Cerebellum 1.961351202
0.038648798 1.961351202 -1.39360581 -0.936354878 -1.164980344
0.323325235 1 0 0.987147804
ZNF257 ENSG00000197134.11 0.239463276 13 4 0.126704481
Brain_Putamen_basal_ganglia 0.714712046 Brain_Hippocampus 10.67404495
0.005331465 0.423525845 -1.527227377 0.365535053 -0.995783874
0.509035201 4 0 0.987147804
PRSS23 ENSG00000150687.11 0.2395195 4 4 0.122217469 Brain_Cortex
0.841997636 Brain_Hippocampus 3.223792433 0.148022349
0.148022349 0.199338921 1.545532687 0.653478272 0.605112782
4 0 0.987147804
SPIRE1 ENSG00000134278.15 0.239655601 1 1 0.239655601
Brain_Nucleus_accumbens_basal_ganglia 0.239655601
Brain_Nucleus_accumbens_basal_ganglia 1 1 1 -1.17584805
-1.17584805 -1.17584805 NA 1 0 0.987147804
PCDHGA4 ENSG00000262576.2 0.239759784 1 1 0.239759784
Brain_Cortex 0.239759784 Brain_Cortex 1 1 1 1.175587422
1.175587422 1.175587422 NA 1 0 0.987147804
C11orf68 ENSG00000175573.6 0.239768745 1 1 0.239768745
Brain_Substantia_nigra 0.239768745 Brain_Substantia_nigra 1 1
1 -1.175565009 -1.175565009 -1.175565009 NA 1 0
0.987147804
AGAP9 ENSG00000204172.12 0.239816354 11 8 0.066335644
Brain_Nucleus_accumbens_basal_ganglia 0.923606972 Brain_Cerebellum
4.054783475 0.006373013 0.416609071 -1.460000693 1.836148858
0.209358013 1.164265624 8 0 0.987147804
RALGPS2 ENSG00000116191.17 0.239847514 2 2 0.097924438
Brain_Frontal_Cortex_BA9 0.739613211 Brain_Substantia_nigra
1.390655128 0.609344872 0.609344872 0.3323656 1.655000295
0.993682948 0.935243962 2 0 0.987147804
PRPSAP1 ENSG00000161542.16 0.239850556 11 9 0.079245229
Brain_Nucleus_accumbens_basal_ganglia 0.999448542 Brain_Substantia_nigra
4.905900458 0.045666683 0.19391009 -1.755082313 1.509897351
0.067874858 0.891178355 9 0 0.987147804
ELOVL5 ENSG00000012660.13 0.239941131 5 3 0.47464745 Brain_Cortex
0.992449782 Brain_Frontal_Cortex_BA9 4.228755166 0.04354467
0.142471858 -0.714937846 0.457038667 -0.198175844 0.493233037
3 0 0.987147804
EMB ENSG00000170571.11 0.24007078 2 2 0.134623702
Brain_Putamen_basal_ganglia 0.936691899 Brain_Hippocampus 1.422185183
0.577814817 0.577814817 -1.496114951 0.079428376 -0.708343287
1.114077371 2 0 0.987147804
EIF4G3 ENSG00000075151.20 0.240083595 2 2 0.092487031
Brain_Cerebellum 0.791944396 Brain_Spinal_cord_cervical_c-1 1.066864185
0.933135815 0.933135815 -1.682422037 -0.263786554 -0.973104295
1.00312677 2 0 0.987147804
DCDC1 ENSG00000170959.14 0.240125864 2 2 0.093518221
Brain_Nucleus_accumbens_basal_ganglia 0.977233337 Brain_Substantia_nigra
1.102259888 0.897740112 0.897740112 -1.67712408 -0.028537654
-0.852830867 1.165726641 2 0 0.987147804
PTGDR ENSG00000168229.3 0.240257751 1 1 0.240257751
Brain_Hippocampus 0.240257751 Brain_Hippocampus 1 1 1
1.174342788 1.174342788 1.174342788 NA 1 0 0.987147804

HAND2 ENSG00000164107.8 0.240308173 2 2 0.180217385 Brain_Cerebellum
0.530148539 Brain_Cerebellar_Hemisphere 1.859886753 0.140113247
0.140113247 -1.340086006 -0.627779284 -0.983932645 0.503676913
2 0 0.987147804
PIBF1 ENSG00000083535.15 0.24035296 10 4 0.125277223
Brain_Caudate_basal_ganglia 0.747135022 Brain_Anterior_cingulate_cortex_BA24
7.606584592 0.013031558 0.390018931 -1.532994447 0.929513241
-0.828786318 0.73076852 4 0 0.987147804
AF064858.6 ENSG00000205622.9 0.240377381 1 1 0.240377381
Brain_Cortex 0.240377381 Brain_Cortex 1 1 1 -1.174044054
-1.174044054 -1.174044054 NA 1 0 0.987147804
RP11-744I24.3 ENSG00000261797.1 0.24081923 1 1 0.24081923
Brain_Caudate_basal_ganglia 0.24081923 Brain_Caudate_basal_ganglia 1 1 1
-1.172941588 -1.172941588 -1.172941588 NA 1 0 0.987147804
CD300LG ENSG00000161649.12 0.240902811 1 1 0.240902811
Brain_Hippocampus 0.240902811 Brain_Hippocampus 1 1 1
-1.172733206 -1.172733206 -1.172733206 NA 1 0 0.987147804
MICALL2 ENSG00000164877.18 0.241080823 3 3 0.04558935
Brain_Substantia_nigra 0.787454878 Brain_Cerebellum 1.050268114
0.954010613 0.954010613 -1.999175671 -0.269617055 -0.862358661
0.984813071 3 0 0.987147804
PCDH1 ENSG00000156453.13 0.241098594 1 1 0.241098594
Brain_Anterior_cingulate_cortex_BA24 0.241098594
Brain_Anterior_cingulate_cortex_BA24 1 1 1 -1.172245281
-1.172245281 -1.172245281 NA 1 0 0.987147804
GNG12 ENSG00000172380.5 0.241104877 1 1 0.241104877 Brain_Cortex
0.241104877 Brain_Cortex 1 1 1 -1.172229626 -1.172229626
-1.172229626 NA 1 0 0.987147804
ARHGEF3 ENSG00000163947.11 0.241133475 2 2 0.100927056
Brain_Cortex 0.700181772 Brain_Putamen_basal_ganglia 1.00446208 0.99553792
0.99553792 -1.640375791 -0.385075104 -1.012725448 0.887631628 2
0 0.987147804
CDKN1C ENSG00000129757.12 0.241135372 2 2 0.092944496
Brain_Spinal_cord_cervical_c-1 0.765311606 Brain_Frontal_Cortex_BA9
1.263184219 0.736815781 0.736815781 -1.680065893 -0.298513065
-0.989289479 0.976905374 2 0 0.987147804
ZNF662 ENSG00000182983.14 0.241149765 1 1 0.241149765
Brain_Cerebellum 0.241149765 Brain_Cerebellum 1 1 1 1.1721178
1.1721178 1.1721178 NA 1 0 0.987147804
RP11-1060G2.2 ENSG00000271382.1 0.241180741 1 1 0.241180741
Brain_Nucleus_accumbens_basal_ganglia 0.241180741
Brain_Nucleus_accumbens_basal_ganglia 1 1 1 -1.172040637
-1.172040637 -1.172040637 NA 1 0 0.987147804
AP4B1 ENSG00000134262.12 0.24122396 3 3 0.11004355 Brain_Cerebellum
0.898029689 Brain_Cortex 1.375912317 0.639538669 0.639538669
-1.014728268 1.597997427 0.151706143 1.328654881 3 0
0.987147804
PIP4K2B ENSG00000276293.4 0.241246991 2 2 0.109498993
Brain_Frontal_Cortex_BA9 0.727505545 Brain_Cerebellar_Hemisphere
1.112242407 0.887757593 0.887757593 0.348445622 1.600449085
0.974447354 0.885300139 2 0 0.987147804
C2orf71 ENSG00000179270.6 0.241275818 10 2 0.110901878
Brain_Anterior_cingulate_cortex_BA24 0.749668231 Brain_Amygdala
8.633595393 0.000958983 0.710803508 0.31907686 1.594152522
1.172485555 0.426559611 2 0 0.987147804
COL5A2 ENSG00000204262.11 0.2413436 1 1 0.2413436 Brain_Cerebellum
0.2413436 Brain_Cerebellum 1 1 1 1.171635075 1.171635075
1.171635075 NA 1 0 0.987147804
GNG10 ENSG00000242616.3 0.241481778 13 1 0.062872704 Brain_Amygdala
0.810670694 Brain_Substantia_nigra 11.98569333 0.003657292
11.98569333 -1.860090633 -0.239560881 -1.122008084 0.408474382
1 0 0.987147804

RIC3 ENSG00000166405.14 0.241519361 5 2 0.047578799
Brain_Nucleus_accumbens_basal_ganglia 0.21288958 Brain_Cortex 4.243067872
0.000217828 0.682174856 -1.981111253 -1.245659087 -1.480031805
0.288395291 2 0 0.987147804
ADAP2 ENSG00000184060.10 0.241622579 1 1 0.241622579
Brain_Spinal_cord_cervical_c-1 0.241622579 Brain_Spinal_cord_cervical_c-1
1 1 1 1.170940787 1.170940787 1.170940787 NA 1 0
0.987147804
METTL2B ENSG00000165055.15 0.241650295 13 7 0.146430372
Brain_Putamen_basal_ganglia 0.838836394 Brain_Cortex 8.067050214
0.011764042 0.275832572 -0.926798851 1.452256233 0.614893757
0.742323188 7 0 0.987147804
RP11-112J3.16 ENSG00000227388.2 0.241770168 5 4 0.154733707
Brain_Putamen_basal_ganglia 0.885745064 Brain_Anterior_cingulate_cortex_BA24
3.021168888 0.012076638 0.338988862 -1.423008447 0.608690687
-0.453781995 0.78173715 4 0 0.987147804
DKFZp779M0652 ENSG00000205106.4 0.241802323 13 9 0.043584482
Brain_Hippocampus 0.899360703 Brain_Substantia_nigra 7.170369485
0.088655794 0.327224182 -0.351667424 2.018065096 0.755079216
0.752961147 9 0 0.987147804
MECOM ENSG00000085276.17 0.241839421 2 2 0.169611001 Brain_Cortex
0.329572462 Brain_Putamen_basal_ganglia 1.000748982 0.999251018
0.999251018 0.974975402 1.373454809 1.174215106 0.281767491
2 0 0.987147804
ERCC5 ENSG00000134899.18 0.24185063 2 2 0.0945673 Brain_Amygdala
0.912283022 Brain_Putamen_basal_ganglia 1.059378 0.940622 0.940622
-1.671782088 -0.110159323 -0.890970706 1.104234047 2 0
0.987147804
HIVEP3 ENSG00000127124.14 0.241853518 2 1 0.218458298
Brain_Cerebellum 0.281958421 Brain_Cerebellar_Hemisphere 1.942043201
0.057956799 1.942043201 1.075930319 1.230637969 1.153284144
0.109394829 1 0 0.987147804
SOX21 ENSG00000125285.5 0.241882851 2 2 0.107835161
Brain_Frontal_Cortex_BA9 0.777616501 Brain_Cerebellar_Hemisphere
1.134296161 0.865703839 0.865703839 -1.608000083 0.282426495
-0.662786794 1.336733453 2 0 0.987147804
GPR55 ENSG00000135898.9 0.241913075 6 6 0.036151302
Brain_Frontal_Cortex_BA9 0.847979931 Brain_Nucleus_accumbens_basal_ganglia
2.436621847 0.112264718 0.112264718 -0.525529265 2.09522155
0.814940307 0.956680551 6 0 0.987147804
TMEM249 ENSG00000261587.2 0.241922792 5 5 0.034169714
Brain_Cerebellum 0.49829457 Brain_Caudate_basal_ganglia 1.614666736
0.372305811 0.372305811 -0.677175564 2.118063224 1.021588554
1.027432326 5 0 0.987147804
LA16c-OS12.2 ENSG00000268836.1 0.241993785 10 10 0.017064086
Brain_Hippocampus 0.48648503 Brain_Cerebellum 3.575188988 0.148498475
0.148498475 -0.983423638 2.385323981 1.097024491 0.916373102
10 0 0.987147804
MBLAC2 ENSG00000176055.9 0.24216699 1 1 0.24216699
Brain_Substantia_nigra 0.24216699 Brain_Substantia_nigra 1 1 1
1.169587551 1.169587551 1.169587551 NA 1 0 0.987147804
RP11-335L23.5 ENSG00000272784.1 0.242186854 1 1 0.242186854
Brain_Anterior_cingulate_cortex_BA24 0.242186854
Brain_Anterior_cingulate_cortex_BA24 1 1 1 -1.169538216
-1.169538216 -1.169538216 NA 1 0 0.987147804
ARID3B ENSG00000179361.17 0.242208625 1 1 0.242208625
Brain_Cerebellum 0.242208625 Brain_Cerebellum 1 1 1 -1.169484147
-1.169484147 -1.169484147 NA 1 0 0.987147804
ZNF701 ENSG00000167562.12 0.242275135 3 2 0.234777124
Brain_Cerebellar_Hemisphere 0.260427564 Brain_Caudate_basal_ganglia
2.006132213 1.99E-08 0.993867767 -1.12538113 1.188142878
0.416946206 1.335694654 2 0 0.987147804

RAPGEF4 ENSG00000091428.17 0.242306273 2 2 0.099121612
Brain_Spinal_cord_cervical_c-1 0.721096832 Brain_Cerebellum 1.010417686
0.989582314 0.989582314 -1.649127037 0.356993286 -0.646066875
1.418541285 2 0 0.987147804
LINC01621 ENSG00000235357.1 0.242440334 1 1 0.242440334
Brain_Cerebellum 0.242440334 Brain_Cerebellum 1 1 1 -1.168908908
-1.168908908 -1.168908908 NA 1 0 0.987147804
FTCDNL1 ENSG00000226124.6 0.242479952 13 1 0.144062879
Brain_Anterior_cingulate_cortex_BA24 0.393612601 Brain_Hypothalamus
12.02923438 0.003264557 12.02923438 0.85308422 1.460827163
1.122453886 0.171986186 1 0 0.987147804
CAMK2A ENSG00000070808.15 0.242539838 3 3 0.059244627
Brain_Caudate_basal_ganglia 0.606378312 Brain_Nucleus_accumbens_basal_ganglia
1.188913214 0.772910214 0.772910214 -1.886373604 0.901668047
-0.499985196 1.394083506 3 0 0.987147804
MICALL1 ENSG00000100139.13 0.242593741 3 2 0.101032637
Brain_Cerebellum 0.297326374 Brain_Anterior_cingulate_cortex_BA24
2.665469335 0.04578911 0.288741556 -1.639867899 -1.042184013
-1.428313624 0.33490882 2 0 0.987147804
PDE1C ENSG00000154678.16 0.242599149 2 2 0.092535106
Brain_Hippocampus 0.963174378 Brain_Anterior_cingulate_cortex_BA24
1.059841758 0.940158242 0.940158242 -0.046170471 1.682173992
0.81800176 1.22212409 2 0 0.987147804
MYBPC2 ENSG00000086967.9 0.242599801 2 2 0.159335031
Brain_Cortex 0.564187125 Brain_Frontal_Cortex_BA9 1.228806588
0.771193412 0.771193412 -0.57663341 1.407311532 0.415339061
1.402860922 2 0 0.987147804
SLC24A4 ENSG00000140090.17 0.242616349 2 1 0.230734994
Brain_Cerebellum 0.26750412 Brain_Cerebellar_Hemisphere 1.949575169
0.050424831 1.949575169 1.108828624 1.198467691 1.153648158
0.063384392 1 0 0.987147804
PCDHA9 ENSG00000204961.6 0.242754196 7 4 0.104397152
Brain_Spinal_cord_cervical_c-1 0.969117997
Brain_Anterior_cingulate_cortex_BA24 5.113056191 0.047453069 0.17256053
-1.6239 0.191243436 -0.422686801 0.659632308 4 0
0.987147804
PIGC ENSG00000135845.9 0.242781131 7 7 0.071626702
Brain_Nucleus_accumbens_basal_ganglia 0.808736729 Brain_Amygdala
2.259427533 0.485987427 0.485987427 -1.788523393 1.801483526
0.521070735 1.146520539 7 0 0.987147804
GPATCH2L ENSG00000089916.17 0.242898033 4 4 0.16470052 Brain_Cerebellum
0.776640412 Brain_Cortex 2.696920732 0.261986326 0.261986326
-0.290620522 1.389434977 0.362532618 0.810966707 4 0
0.987147804
RP11-388K12.2 ENSG00000254089.1 0.242982493 1 1 0.242982493
Brain_Spinal_cord_cervical_c-1 0.242982493 Brain_Spinal_cord_cervical_c-1
1 1 1 1.167564459 1.167564459 1.167564459 NA 1 0
0.987147804
RPP30 ENSG00000148688.13 0.243047767 1 1 0.243047767 Brain_Cortex
0.243047767 Brain_Cortex 1 1 1 -1.167402736 -1.167402736
-1.167402736 NA 1 0 0.987147804
EIF3G ENSG00000130811.11 0.243086904 1 1 0.243086904
Brain_Spinal_cord_cervical_c-1 0.243086904 Brain_Spinal_cord_cervical_c-1
1 1 1 1.167305784 1.167305784 1.167305784 NA 1 0
0.987147804
RBM34 ENSG00000188739.14 0.243289442 1 1 0.243289442
Brain_Hypothalamus 0.243289442 Brain_Hypothalamus 1 1 1
1.16680422 1.16680422 1.16680422 NA 1 0 0.987147804
CYTH3 ENSG00000008256.15 0.243319675 2 2 0.1804682 Brain_Hypothalamus
0.284556642 Brain_Cerebellum 1.040783287 0.959216713 0.959216713
-1.33931484 -1.070139232 -1.204727036 0.190335898 2 0
0.987147804

MYOM1 ENSG00000101605.12 0.24337049 7 7 0.071975588
Brain_Putamen_basal_ganglia 0.938062973 Brain_Caudate_basal_ganglia
2.036777831 0.310502144 0.310502144 -1.799272489 1.304342783
0.353247164 1.049042375 7 0 0.987147804
RIOK2 ENSG00000058729.10 0.24338048 8 5 0.011116479 Brain_Cerebellum
0.410964365 Brain_Nucleus_accumbens_basal_ganglia 5.727998572
0.016255596 0.301467067 0.82219774 2.53901583 1.920227811
0.536039408 5 0 0.987147804
DDX39B ENSG00000198563.13 0.243461485 2 2 0.113558093
Brain_Cortex 0.433729005 Brain_Cerebellum 1.139851574 0.860148426
0.860148426 -1.582400841 -0.782826498 -1.182613669 0.56538444 2
0 0.987147804
RP4-633O19__A.1 ENSG00000223695.1 0.243464986 1 1 0.243464986
Brain_Cerebellum 0.243464986 Brain_Cerebellum 1 1 1 1.166369744
1.166369744 1.166369744 NA 1 0 0.987147804
POC1B ENSG00000139323.13 0.243466728 2 2 0.093675235
Brain_Hypothalamus 0.233776669 Brain_Cerebellum 1.654128164
0.345871836 0.345871836 1.190686528 1.676321507 1.433504017
0.343395787 2 0 0.987147804
ZC3H18 ENSG00000158545.15 0.243477107 1 1 0.243477107
Brain_Cortex 0.243477107 Brain_Cortex 1 1 1 -1.166339753
-1.166339753 -1.166339753 NA 1 0 0.987147804
SLC25A23 ENSG00000125648.14 0.243522209 6 6 0.027432906
Brain_Cortex 0.730622703 Brain_Spinal_cord_cervical_c-1 1.857322786
0.218479263 0.218479263 -2.205302048 0.690381662 -0.324431161
1.180706722 6 0 0.987147804
USP1 ENSG00000162607.12 0.243732951 1 1 0.243732951
Brain_Putamen_basal_ganglia 0.243732951 Brain_Putamen_basal_ganglia 1 1
1 -1.165706947 -1.165706947 -1.165706947 NA 1 0
0.987147804
FOPNL ENSG00000133393.12 0.243803148 2 2 0.094196465
Brain_Caudate_basal_ganglia 0.110880707 Brain_Cortex 1.91761888 0.08238112
0.08238112 -1.673664953 -1.594247073 -1.633956013 0.056156921 2
0 0.987147804
FAT1 ENSG00000083857.13 0.24395051 2 2 0.248989142
Brain_Putamen_basal_ganglia 0.261119962 Brain_Hippocampus 1.081467594
0.918532406 0.918532406 -1.152808142 1.123747965 -0.014530089
1.609768261 2 0 0.987147804
U91328.22 ENSG00000272810.1 0.243995596 1 1 0.243995596
Brain_Putamen_basal_ganglia 0.243995596 Brain_Putamen_basal_ganglia 1 1
1 -1.165057803 -1.165057803 -1.165057803 NA 1 0
0.987147804
FAM20B ENSG00000116199.11 0.244030284 10 2 0.093395185
Brain_Cerebellum 0.205127958 Brain_Putamen_basal_ganglia 8.459642595
0.003212948 0.774693865 1.267076444 1.677753734 1.471596801
0.147913648 2 0 0.987147804
GOLIM4 ENSG00000173905.8 0.244095137 4 4 0.324210604
Brain_Frontal_Cortex_BA9 0.478689794 Brain_Nucleus_accumbens_basal_ganglia
1.929913974 0.503314506 0.503314506 -0.985842098 0.982879494
-0.038115669 1.027940406 4 0 0.987147804
FGF5 ENSG00000138675.16 0.244146909 2 2 0.095682841
Brain_Cerebellar_Hemisphere 0.929984463 Brain_Cerebellum 1.176516812
0.823483188 0.823483188 -1.666153518 -0.087864385 -0.877008952
1.116018948 2 0 0.987147804
C20orf27 ENSG00000101220.17 0.244163766 2 2 0.122282436
Brain_Putamen_basal_ganglia 0.590976456 Brain_Nucleus_accumbens_basal_ganglia
1.076312224 0.923687776 0.923687776 -1.545263931 -0.537421558
-1.041342744 0.712652176 2 0 0.987147804
LINC00706 ENSG00000281186.1 0.244188931 5 5 0.104269928
Brain_Caudate_basal_ganglia 0.605751435 Brain_Substantia_nigra
2.708683117 0.150898367 0.150898367 -0.82769745 1.624496307
0.601784829 0.910585769 5 0 0.987147804

SLC39A7 ENSG00000112473.17 0.244189651 1 1 0.244189651
Brain_Cerebellar_Hemisphere 0.244189651 Brain_Cerebellar_Hemisphere 1 1
1 -1.1645785 -1.1645785 -1.1645785 NA 1 0 0.987147804
NELFCD ENSG00000101158.13 0.24423921 12 4 0.081345959
Brain_Nucleus_accumbens_basal_ganglia 0.901735869 Brain_Substantia_nigra
10.02883523 0.010088116 0.351613919 -1.742929337 0.289141415
-0.655184046 0.621062497 4 0 0.987147804
ZNF2 ENSG00000275111.4 0.244278892 1 1 0.244278892 Brain_Cerebellum
0.244278892 Brain_Cerebellum 1 1 1 -1.164358171 -1.164358171
-1.164358171 NA 1 0 0.987147804
CAST ENSG00000153113.23 0.244287418 13 2 0.091900807
Brain_Substantia_nigra 0.662898025 Brain_Cerebellum 11.17259534
0.00146659 0.991528336 -0.593817529 1.685455067 0.988648254
0.562163375 2 0 0.987147804
PINK1 ENSG00000158828.7 0.244301077 2 2 0.129053164
Brain_Substantia_nigra 0.386677324 Brain_Nucleus_accumbens_basal_ganglia
1.099958442 0.900041558 0.900041558 0.865658756 1.517846244
1.1917525 0.461166196 2 0 0.987147804
SLC25A10 ENSG00000183048.11 0.244337113 1 1 0.244337113
Brain_Spinal_cord_cervical_c-1 0.244337113 Brain_Spinal_cord_cervical_c-1
1 1 1 -1.164214457 -1.164214457 -1.164214457 NA 1 0
0.987147804
UBAC2 ENSG00000134882.15 0.244405689 1 1 0.244405689
Brain_Caudate_basal_ganglia 0.244405689 Brain_Caudate_basal_ganglia 1 1
1 1.164045215 1.164045215 1.164045215 NA 1 0
0.987147804
SAMD11 ENSG00000187634.11 0.244661359 4 4 0.06737967
Brain_Putamen_basal_ganglia 0.321034049 Brain_Cerebellum 1.473395268
0.517405094 0.517405094 -1.258755656 1.829133108 0.150763181
1.513786764 4 0 0.98789526
NCL ENSG00000115053.15 0.244820619 4 3 0.076000597 Brain_Cerebellum
0.539422847 Brain_Spinal_cord_cervical_c-1 3.044924464 5.10E-17
0.316613916 -1.7743783 -0.613685992 -1.220120088 0.641418559 3
0 0.988253109
OLR1 ENSG00000173391.8 0.245015483 1 1 0.245015483 Brain_Cortex
0.245015483 Brain_Cortex 1 1 1 -1.162541734 -1.162541734
-1.162541734 NA 1 0 0.988377671
DDX51 ENSG00000185163.9 0.245141538 12 3 0.138049741
Brain_Putamen_basal_ganglia 0.85225564 Brain_Cerebellar_Hemisphere 9.63236303
0.004906646 0.658909426 -1.483092858 0.702081301 -0.492793416
0.568599996 3 0 0.988377671
CTD-2541M15.3 ENSG00000271743.1 0.245155973 12 11 0.027552134
Brain_Hippocampus 0.981443418 Brain_Substantia_nigra 4.872346192
0.072145499 0.225997768 -1.720291022 2.20360502 -0.049437206
1.058901494 11 0 0.988377671
ZMAT2 ENSG00000146007.10 0.245222269 9 5 0.183025721
Brain_Frontal_Cortex_BA9 0.91215299 Brain_Cortex 6.839086386
0.047381212 0.228946112 -1.331496424 0.664099297 -0.066801146
0.730077349 5 0 0.988377671
IKBIP ENSG00000166130.14 0.245428279 11 5 0.112684754
Brain_Cerebellar_Hemisphere 0.890130592 Brain_Nucleus_accumbens_basal_ganglia
8.679669372 0.009194293 0.313956616 -1.586240541 0.384184358
-0.849590225 0.680683005 5 0 0.988377671
HRH2 ENSG00000113749.7 0.24577601 1 1 0.24577601 Brain_Cerebellum 0.24577601
Brain_Cerebellum 1 1 1 -1.160670286 -1.160670286 -1.160670286
NA 1 0 0.988377671
FAM196A ENSG00000188916.8 0.245790759 3 3 0.068695904
Brain_Cortex 0.485201621 Brain_Caudate_basal_ganglia 2.174068516
0.091248948 0.091248948 -1.82041451 -0.697960942 -1.161051025
0.58640254 3 0 0.988377671
SLC2A14 ENSG00000173262.11 0.246025152 8 4 0.097325612
Brain_Putamen_basal_ganglia 0.602203939 Brain_Hypothalamus 6.623091885

0.006841998 0.249298852 -0.52123376 1.657959644 0.9953542
0.657940356 4 0 0.988377671
ISCU ENSG00000136003.15 0.246123853 3 3 0.084178733
Brain_Cerebellar_Hemisphere 0.477767369 Brain_Amygdala 1.334217264
0.674590148 0.674590148 -0.709898035 1.726938365 0.716231448
1.270423095 3 0 0.988377671
PIK3CB ENSG00000051382.8 0.246139277 2 2 0.103477975
Brain_Cerebellar_Hemisphere 0.634556189 Brain_Hypothalamus 1.052329841
0.947670159 0.947670159 -1.628221277 -0.475323813 -1.051772545
0.815221615 2 0 0.988377671
ROPN1 ENSG00000065371.17 0.246163128 1 1 0.246163128
Brain_Nucleus_accumbens_basal_ganglia 0.246163128
Brain_Nucleus_accumbens_basal_ganglia 1 1 1 1.159719256
1.159719256 1.159719256 NA 1 0 0.988377671
NELFE ENSG00000204356.13 0.246175337 2 2 0.115545079
Brain_Hypothalamus 0.956852087 Brain_Nucleus_accumbens_basal_ganglia
1.310839404 0.689160596 0.689160596 -0.054104275 1.573750771
0.759823248 1.151067342 2 0 0.988377671
LINC01511 ENSG00000250584.2 0.246177503 3 3 0.070160938
Brain_Frontal_Cortex_BA9 0.743643627 Brain_Caudate_basal_ganglia
1.056104159 0.956048011 0.956048011 -0.327032073 1.810870255
0.778525601 1.070829912 3 0 0.988377671
CX3CL1 ENSG00000006210.6 0.246245477 2 2 0.113280079
Brain_Frontal_Cortex_BA9 0.606495303 Brain_Cerebellar_Hemisphere
1.017662244 0.982337756 0.982337756 -1.583620619 0.515082598
-0.534269011 1.484007276 2 0 0.988377671
RWDD2A ENSG00000013392.7 0.246267126 2 2 0.239561248
Brain_Cortex 0.383315458 Brain_Cerebellum 1.241727861 0.758272139
0.758272139 -1.176084158 0.871803732 -0.152140213 1.448075414
2 0 0.988377671
ZNF587B ENSG00000269343.6 0.246339863 5 5 0.1127417 Brain_Cerebellum
0.876259628 Brain_Cerebellar_Hemisphere 2.084066067 0.226101446
0.226101446 0.155712521 1.585989462 0.884171854 0.588293554
5 0 0.988377671
AKIRIN2 ENSG00000135334.8 0.246348216 4 4 0.078582584
Brain_Cortex 0.64773165 Brain_Anterior_cingulate_cortex_BA24 2.824899218
0.290439788 0.290439788 0.456915684 1.758970122 1.140204202
0.647103363 4 0 0.988377671
FDX1 ENSG00000137714.2 0.246363617 4 4 0.045073704
Brain_Spinal_cord_cervical_c-1 0.501711917 Brain_Frontal_Cortex_BA9
1.51663635 0.445301874 0.445301874 -1.245734909 2.003965988
0.671485088 1.389579219 4 0 0.988377671
AGFG2 ENSG00000106351.12 0.246390388 1 1 0.246390388 Brain_Cerebellum
0.246390388 Brain_Cerebellum 1 1 1 1.159161434 1.159161434
1.159161434 NA 1 0 0.988377671
ANKRD34B ENSG00000189127.7 0.246413789 2 2 0.106443209
Brain_Spinal_cord_cervical_c-1 0.706287522 Brain_Cortex 1.040048807
0.959951193 0.959951193 -1.614388386 0.376846715 -0.618770835
1.408015843 2 0 0.988377671
P4HA3 ENSG00000149380.11 0.246415106 5 5 0.041701557 Brain_Cerebellum
0.331749313 Brain_Spinal_cord_cervical_c-1 2.52836783 0.313447942
0.313447942 0.970596373 2.036486202 1.557736362 0.475706857
5 0 0.988377671
RFC5 ENSG00000111445.13 0.246517219 1 1 0.246517219
Brain_Cerebellar_Hemisphere 0.246517219 Brain_Cerebellar_Hemisphere 1 1
1 1.158850278 1.158850278 1.158850278 NA 1 0
0.988377671
C7orf73 ENSG00000243317.7 0.246540275 13 3 0.045099646
Brain_Hippocampus 0.372873582 Brain_Cerebellum 10.35046882
0.008680776 0.495770901 0.891103623 2.003723891 1.398630528
0.321448256 3 0 0.988377671
CKMT1A ENSG00000223572.9 0.246635315 8 2 0.150243692

Brain_Cerebellum 0.738573792 Brain_Amygdala 6.59054399 0.025742428
0.913678313 0.333742612 1.438671239 0.756937209 0.34013709 2
0 0.988377671
FBXO41 ENSG00000163013.11 0.246646191 2 2 0.114257858
Brain_Hippocampus 0.343870965 Brain_Cerebellum 1.261673976
0.738326024 0.738326024 -1.579341019 -0.946544443 -1.262942731
0.44745475 2 0 0.988377671
LEFTY2 ENSG00000143768.12 0.24688982 7 6 0.109090505
Brain_Caudate_basal_ganglia 0.83728464 Brain_Cortex 4.030114701
0.059854605 0.202095321 -1.602294485 1.011143405 -0.659275493
0.936836559 6 0 0.988377671
DERL1 ENSG00000136986.9 0.24689248 1 1 0.24689248 Brain_Cerebellum 0.24689248
Brain_Cerebellum 1 1 1 -1.157930304 -1.157930304 -1.157930304
NA 1 0 0.988377671
ZNF518B ENSG00000178163.7 0.246998529 1 1 0.246998529
Brain_Putamen_basal_ganglia 0.246998529 Brain_Putamen_basal_ganglia 1 1
1 -1.157670494 -1.157670494 -1.157670494 NA 1 0
0.988377671
LINC01140 ENSG00000267272.5 0.247098539 8 6 0.225252607
Brain_Nucleus_accumbens_basal_ganglia 0.821233907 Brain_Putamen_basal_ganglia
4.334172421 0.007963444 0.19719469 -1.212678908 0.824527389
-0.578440839 0.66283539 6 0 0.988377671
UFSP1 ENSG00000176125.4 0.247217399 4 4 0.249429346 Brain_Amygdala
0.906237178 Brain_Spinal_cord_cervical_c-1 2.148543662 0.301280747
0.301280747 -1.151736555 0.902136671 0.090678165 0.888047962
4 0 0.988377671
PLCD1 ENSG00000187091.13 0.247222595 3 3 0.23859167
Brain_Putamen_basal_ganglia 0.439404872 Brain_Spinal_cord_cervical_c-1
1.517251254 0.437567737 0.437567737 -1.178514212 0.800897312
0.131860557 1.134902344 3 0 0.988377671
RBM44 ENSG00000177483.11 0.247243629 8 5 0.160014943
Brain_Hypothalamus 0.658345299 Brain_Putamen_basal_ganglia 5.213331769
0.001837329 0.364271107 -0.581818531 1.405021307 0.617449189
0.780614729 5 0 0.988377671
CTD-3025N20.3 ENSG00000272010.1 0.247244595 11 1 0.134014635
Brain_Cerebellar_Hemisphere 0.445826756 Brain_Amygdala 10.30726122
0.000873833 10.30726122 0.762390865 1.498456699 1.121652157
0.221973188 1 0 0.988377671
RB1 ENSG00000139687.13 0.247280854 1 1 0.247280854 Brain_Cortex
0.247280854 Brain_Cortex 1 1 1 -1.156979211 -1.156979211
-1.156979211 NA 1 0 0.988377671
MTMR11 ENSG00000014914.20 0.24732401 2 2 0.180488853
Brain_Frontal_Cortex_BA9 0.263096525 Brain_Cerebellar_Hemisphere
1.091899219 0.908100781 0.908100781 -1.11910224 1.339251372
0.110074566 1.73831851 2 0 0.988377671
SUGT1 ENSG00000165416.14 0.247592818 3 3 0.073389552
Brain_Nucleus_accumbens_basal_ganglia 0.898143991 Brain_Substantia_nigra
1.038777485 0.94698752 0.94698752 -1.790400489 -0.128006296
-0.937293893 0.832062914 3 0 0.98846115
SLC39A8 ENSG00000138821.12 0.247613058 1 1 0.247613058
Brain_Frontal_Cortex_BA9 0.247613058 Brain_Frontal_Cortex_BA9 1 1
1 -1.156166504 -1.156166504 -1.156166504 NA 1 0 0.98846115
LINC01358 ENSG00000237352.3 0.247636656 5 4 0.067069666
Brain_Spinal_cord_cervical_c-1 0.941771288 Brain_Cerebellar_Hemisphere
2.043820384 0.004073624 0.623961318 -1.831206907 0.807932266
-0.091316521 1.018064157 4 0 0.98846115
EYA1 ENSG00000104313.17 0.247652079 1 1 0.247652079
Brain_Anterior_cingulate_cortex_BA24 0.247652079
Brain_Anterior_cingulate_cortex_BA24 1 1 1 1.156071092
1.156071092 1.156071092 NA 1 0 0.98846115
GPR37L1 ENSG00000170075.8 0.247732935 1 1 0.247732935
Brain_Putamen_basal_ganglia 0.247732935 Brain_Putamen_basal_ganglia 1 1

1 -1.155873424 -1.155873424 -1.155873424 NA 1 0 0.98846115
SLITRK3 ENSG00000121871.3 0.247768798 1 1 0.247768798
Brain_Substantia_nigra 0.247768798 Brain_Substantia_nigra 1 1
1 1.155785763 1.155785763 1.155785763 NA 1 0 0.98846115
RIOX2 ENSG00000170854.17 0.247867107 2 2 0.398308963 Brain_Cortex
0.522491923 Brain_Frontal_Cortex_BA9 1.60334372 0.39665628 0.39665628
-0.639508904 0.844645202 0.102568149 1.049455433 2 0
0.988571465
TTC7A ENSG00000068724.15 0.24804572 3 3 0.083675844
Brain_Caudate_basal_ganglia 0.982949897 Brain_Cerebellar_Hemisphere 1.16494698
0.84729401 0.84729401 -1.207311174 1.729745008 0.181268199
1.475042422 3 0 0.988929228
KLRK1 ENSG00000213809.8 0.24829104 6 4 0.039681826 Brain_Hypothalamus
0.439266218 Brain_Nucleus_accumbens_basal_ganglia 4.449165036 0.00441364
0.157822758 0.773432912 2.057045738 1.269333045 0.447817858
4 0 0.988929228
SP140 ENSG00000079263.18 0.248329348 1 1 0.248329348
Brain_Caudate_basal_ganglia 0.248329348 Brain_Caudate_basal_ganglia 1 1
1 1.154416759 1.154416759 1.154416759 NA 1 0
0.988929228
PROZ ENSG00000126231.13 0.248346298 2 1 0.228745725 Brain_Cerebellum
0.273898275 Brain_Cerebellar_Hemisphere 1.980943702 0.019056298
1.980943702 1.094129296 1.203596108 1.148862702 0.077404725
1 0 0.988929228
CTSK ENSG00000143387.12 0.248475242 4 2 0.307628058
Brain_Caudate_basal_ganglia 0.943396096 Brain_Cerebellar_Hemisphere
3.743011431 0.007837064 0.222214913 -1.020211725 -0.071002086
-0.355187843 0.446033902 2 0 0.988929228
HPCAL4 ENSG00000116983.12 0.248476768 4 4 0.086131369
Brain_Caudate_basal_ganglia 0.587152636 Brain_Nucleus_accumbens_basal_ganglia
2.513826141 0.152081527 0.152081527 -0.805419535 1.716167611
0.683288541 1.104260953 4 0 0.988929228
SS18L1 ENSG00000184402.14 0.24858476 1 1 0.24858476
Brain_Spinal_cord_cervical_c-1 0.24858476 Brain_Spinal_cord_cervical_c-1 1
1 1 -1.153793693 -1.153793693 -1.153793693 NA 1 0
0.988929228
PAQR7 ENSG00000182749.5 0.248639054 1 1 0.248639054 Brain_Cerebellum
0.248639054 Brain_Cerebellum 1 1 1 -1.153661305 -1.153661305
-1.153661305 NA 1 0 0.988929228
PSMD14 ENSG00000115233.11 0.248649267 1 1 0.248649267
Brain_Frontal_Cortex_BA9 0.248649267 Brain_Frontal_Cortex_BA9 1 1
1 -1.153636404 -1.153636404 -1.153636404 NA 1 0
0.988929228
TBPL1 ENSG00000028839.9 0.248732689 3 3 0.111910151 Brain_Cerebellum
0.606832629 Brain_Cerebellar_Hemisphere 1.165715315 0.874808653
0.874808653 -1.589665824 0.514599909 -0.71273914 1.095022916
3 0 0.988929228
TXLNA ENSG00000084652.15 0.248756636 4 3 0.096698059 Brain_Cortex
0.426750727 Brain_Cerebellum 3.292546962 0.008193868 0.223961247
-1.661076622 -0.794764118 -1.201206756 0.387808894 3 0
0.988929228
GAD1 ENSG00000128683.13 0.248805009 1 1 0.248805009
Brain_Anterior_cingulate_cortex_BA24 0.248805009
Brain_Anterior_cingulate_cortex_BA24 1 1 1 -1.153256768
-1.153256768 -1.153256768 NA 1 0 0.988929228
SLC12A8 ENSG00000221955.10 0.249031841 7 5 0.01689016
Brain_Hippocampus 0.668396055 Brain_Caudate_basal_ganglia 3.751273188
0.08644981 0.129894922 -2.389090102 0.531424312 -0.952472282
1.07702454 5 0 0.989089975
UBLCP1 ENSG00000164332.7 0.249040337 1 1 0.249040337
Brain_Cerebellum 0.249040337 Brain_Cerebellum 1 1 1 1.15268345
1.15268345 1.15268345 NA 1 0 0.989089975

DRD5 ENSG00000169676.5 0.24908243 4 4 0.269358916
Brain_Frontal_Cortex_BA9 0.947355891 Brain_Hippocampus 2.908335382
0.131565959 0.131565959 -0.066027551 1.104540106 0.624145522
0.578429688 4 0 0.989089975
SCP2 ENSG00000116171.17 0.2491326 3 2 0.120662096 Brain_Cerebellum
0.332098164 Brain_Cortex 2.159985298 0.047340175 0.792674527
0.969896342 1.552000535 1.266451266 0.291208115 2 0
0.989089975
SNX29 ENSG00000048471.13 0.249357741 3 3 0.096419741
Brain_Putamen_basal_ganglia 0.717286627 Brain_Hippocampus 1.894138425
0.300476134 0.300476134 -1.662464175 0.362087523 -0.708827072
1.017358296 3 0 0.989089975
RP11-493L12.4 ENSG00000258181.1 0.249391481 5 4 0.261731508
Brain_Nucleus_accumbens_basal_ganglia 0.803966765 Brain_Hypothalamus
3.785514354 0.011521163 0.252796844 -0.577109168 1.122307994
0.071995207 0.713010977 4 0 0.989089975
GPATCH1 ENSG00000076650.6 0.249532771 4 3 0.093408616
Brain_Putamen_basal_ganglia 0.894034018 Brain_Cerebellum 2.326191936
0.040905316 0.65161209 -0.751833492 1.677684963 0.320872417
1.006008362 3 0 0.989089975
MRPS6 ENSG00000243927.5 0.249582864 1 1 0.249582864
Brain_Anterior_cingulate_cortex_BA24 0.249582864
Brain_Anterior_cingulate_cortex_BA24 1 1 1 -1.151363157
-1.151363157 -1.151363157 NA 1 0 0.989089975
MED22 ENSG00000148297.15 0.249599569 2 2 0.129648264 Brain_Amygdala
0.565582031 Brain_Putamen_basal_ganglia 1.076261036 0.923738964
0.923738964 -1.515490382 0.574570171 -0.470460105 1.47789599 2
0 0.989089975
CERS5 ENSG00000139624.12 0.24968481 13 4 0.028370369
Brain_Anterior_cingulate_cortex_BA24 0.311819336
Brain_Spinal_cord_cervical_c-1 10.43624188 0.008665677 0.433026495
1.011411882 2.192126571 1.591155203 0.313270621 4 0
0.989089975
GGT7 ENSG00000131067.16 0.249706752 3 3 0.076154089
Brain_Nucleus_accumbens_basal_ganglia 0.847176611 Brain_Amygdala
1.629119649 0.494974027 0.494974027 -1.773450488 0.192722105
-0.734339186 0.987863892 3 0 0.989089975
MFSD13A ENSG00000138111.14 0.249737652 12 3 0.169810285
Brain_Anterior_cingulate_cortex_BA24 0.964658032 Brain_Frontal_Cortex_BA9
10.33273428 0.004248982 0.398920144 -1.372813653 0.08297396
-0.517619421 0.472276626 3 0 0.989089975
LYPD1 ENSG00000150551.10 0.249764483 4 4 0.092498478
Brain_Cerebellar_Hemisphere 0.882307133 Brain_Cortex 1.494201739
0.655130463 0.655130463 -0.924448245 1.682362969 0.241806216
1.095549924 4 0 0.989089975
BCL2 ENSG00000171791.12 0.250016027 2 2 0.105012682 Brain_Amygdala
0.810802986 Brain_Caudate_basal_ganglia 1.08614835 0.91385165 0.91385165
-1.621023113 0.239390255 -0.690816429 1.315510908 2 0
0.989147565
ZFR2 ENSG00000105278.10 0.250025245 3 3 0.066841985
Brain_Putamen_basal_ganglia 0.635386045 Brain_Amygdala 1.262857485
0.779427366 0.779427366 -1.832735032 0.570309034 -0.578861892
1.204938634 3 0 0.989147565
CYP4F26P ENSG00000226562.3 0.250028699 8 5 0.098626244
Brain_Hypothalamus 0.935283308 Brain_Anterior_cingulate_cortex_BA24
5.988356574 0.038416453 0.202025136 -0.702109008 1.65155037
0.090093185 0.755714479 5 0 0.989147565
LETMD1 ENSG00000050426.15 0.250061821 10 5 0.173677861
Brain_Amygdala 0.995331342 Brain_Hypothalamus 7.40909364 0.002104129
0.259256337 -1.360480685 0.722297348 -0.459499346 0.589407819
5 0 0.989147565
PPP1R3D ENSG00000132825.6 0.250160111 1 1 0.250160111

Brain_Putamen_basal_ganglia 0.250160111 Brain_Putamen_basal_ganglia 1 1
1 -1.149960571 -1.149960571 -1.149960571 NA 1 0
0.989256676
CTNND1 ENSG00000198561.13 0.250257833 1 1 0.250257833
Brain_Nucleus_accumbens_basal_ganglia 0.250257833
Brain_Nucleus_accumbens_basal_ganglia 1 1 1 -1.149723352
-1.149723352 -1.149723352 NA 1 0 0.989275155
RBM19 ENSG00000122965.10 0.250340024 8 5 0.019465954
Brain_Cerebellar_Hemisphere 0.129699385 Brain_Nucleus_accumbens_basal_ganglia
6.096048634 0.019150583 0.229996929 -2.336485478 -1.515288399
-1.953698556 0.303480707 5 0 0.989275155
GRAMD4 ENSG00000075240.16 0.250483982 1 1 0.250483982
Brain_Cerebellum 0.250483982 Brain_Cerebellum 1 1 1 1.149174626
1.149174626 1.149174626 NA 1 0 0.989275155
SERGEF ENSG00000129158.10 0.250592958 2 2 0.192050436
Brain_Nucleus_accumbens_basal_ganglia 0.963366066 Brain_Caudate_basal_ganglia
1.642014247 0.357985753 0.357985753 0.045929971 1.304537341
0.675233656 0.889969806 2 0 0.989275155
CTA-150C2.13 ENSG00000235209.1 0.250616294 13 2 0.091501918
Brain_Hippocampus 0.90945668 Brain_Putamen_basal_ganglia 11.25123735
0.000572835 0.726401992 -1.687527751 -0.113723882 -0.843768136
0.334307375 2 0 0.989275155
SEC14L1 ENSG00000129657.14 0.250620828 2 1 0.245805051
Brain_Nucleus_accumbens_basal_ganglia 0.255570746 Brain_Cortex
1.999714089 0.000285911 1.999714089 1.136922354 1.160598906
1.14876063 0.01674185 1 0 0.989275155
C5orf67 ENSG00000225940.5 0.250663855 1 1 0.250663855
Brain_Nucleus_accumbens_basal_ganglia 0.250663855
Brain_Nucleus_accumbens_basal_ganglia 1 1 1 1.148738428
1.148738428 1.148738428 NA 1 0 0.989275155
HVCN1 ENSG00000122986.13 0.250730448 1 1 0.250730448
Brain_Nucleus_accumbens_basal_ganglia 0.250730448
Brain_Nucleus_accumbens_basal_ganglia 1 1 1 -1.148576994
-1.148576994 -1.148576994 NA 1 0 0.989275155
TMEM57 ENSG00000204178.9 0.250812456 3 2 0.07722363 Brain_Cerebellum
0.610072365 Brain_Frontal_Cortex_BA9 2.252154929 0.021509442
0.726335629 -1.76702746 -0.509970163 -1.264707109 0.665457636
2 0 0.989319727
TCTE1 ENSG00000146221.9 0.250957074 5 3 0.116296518
Brain_Frontal_Cortex_BA9 0.918214204 Brain_Cortex 3.452568617
0.040711973 0.574181254 -0.584983593 1.570509964 0.257092143
0.805103922 3 0 0.989573785
LINC01679 ENSG00000237989.1 0.251053989 1 1 0.251053989
Brain_Hypothalamus 0.251053989 Brain_Hypothalamus 1 1 1
1.147793095 1.147793095 1.147793095 NA 1 0 0.989573785
ABHD11 ENSG00000106077.18 0.251216438 10 4 0.029495476
Brain_Putamen_basal_ganglia 0.88241889 Brain_Caudate_basal_ganglia 7.437259748
0.000797276 0.419325636 -0.216106989 2.176800222 0.774590817
0.643178659 4 0 0.989573785
ZNF37A ENSG00000075407.18 0.251243209 3 3 0.253390539
Brain_Amygdala 0.636381181 Brain_Nucleus_accumbens_basal_ganglia
2.256861557 0.349242251 0.349242251 -1.142152733 0.546230051
-0.356229071 0.850202618 3 0 0.989573785
STXBP6 ENSG00000168952.15 0.251297075 6 6 0.210393493
Brain_Substantia_nigra 0.830490697 Brain_Nucleus_accumbens_basal_ganglia
2.417558607 0.147117645 0.147117645 -1.226323267 1.25248417
0.201265691 0.938121078 6 0 0.989573785
DHX30 ENSG00000132153.14 0.251307247 2 2 0.105220621
Brain_Anterior_cingulate_cortex_BA24 0.873280685 Brain_Amygdala
1.315696036 0.684303964 0.684303964 -1.620054262 -0.159492731
-0.889773497 1.032772963 2 0 0.989573785
RP11-687F6.5 ENSG00000274776.1 0.251458524 3 2 0.101970781

Brain_Cortex 0.204330465 Brain_Cerebellum 2.80538096 0.074581628
0.120037412 -1.635373469 -1.269310158 -1.48869321 0.193558412
2 0 0.989573785
BBOX1 ENSG00000129151.8 0.251462561 4 4 0.070245092 Brain_Cortex
0.8314721 Brain_Putamen_basal_ganglia 2.329418897 0.256975211
0.256975211 -1.810327001 0.601496519 -0.313756789 1.057935865
4 0 0.989573785
HYAL3 ENSG00000186792.16 0.251560685 10 9 0.014818761
Brain_Anterior_cingulate_cortex_BA24 0.585767371 Brain_Cortex
3.067365861 0.095460634 0.186334573 -2.436778307 0.949120015
-1.181953049 1.029174616 9 0 0.989573785
EIF4EBP1 ENSG00000187840.4 0.251640053 1 1 0.251640053
Brain_Spinal_cord_cervical_c-1 0.251640053 Brain_Spinal_cord_cervical_c-1
1 1 1 -1.146374925 -1.146374925 -1.146374925 NA 1 0
0.989573785
MPIG6B ENSG00000204420.8 0.251654887 3 3 0.08039602 Brain_Cerebellum
0.713846131 Brain_Nucleus_accumbens_basal_ganglia 1.39002313 0.612424969
0.612424969 -1.748392881 0.900260177 -0.160479053 1.40081246 3
0 0.989573785
DEK ENSG00000124795.14 0.251768309 1 1 0.251768309
Brain_Putamen_basal_ganglia 0.251768309 Brain_Putamen_basal_ganglia 1 1
1 1.146064878 1.146064878 1.146064878 NA 1 0
0.989590105
ZNF678 ENSG00000181450.17 0.251800498 3 3 0.082366281
Brain_Spinal_cord_cervical_c-1 0.822544984 Brain_Cortex 1.276249668
0.76119342 0.76119342 -0.830813006 1.737118363 0.37685941 1.290747794
3 0 0.989590105
YPEL4 ENSG00000166793.10 0.251881808 1 1 0.251881808
Brain_Frontal_Cortex_BA9 0.251881808 Brain_Frontal_Cortex_BA9 1 1
1 -1.145790594 -1.145790594 -1.145790594 NA 1 0 0.98963167
CHSY1 ENSG00000131873.5 0.252010222 1 1 0.252010222
Brain_Hypothalamus 0.252010222 Brain_Hypothalamus 1 1 1
1.145480371 1.145480371 1.145480371 NA 1 0 0.989788891
TNFAIP2 ENSG00000185215.8 0.252087754 1 1 0.252087754
Brain_Frontal_Cortex_BA9 0.252087754 Brain_Frontal_Cortex_BA9 1 1
1 -1.145293122 -1.145293122 -1.145293122 NA 1 0
0.989788891
PFKFB2 ENSG00000123836.14 0.252196216 1 1 0.252196216
Brain_Anterior_cingulate_cortex_BA24 0.252196216
Brain_Anterior_cingulate_cortex_BA24 1 1 1 1.145031243
1.145031243 1.145031243 NA 1 0 0.989788891
PRAG1 ENSG00000275342.4 0.252204803 3 3 0.280685546
Brain_Putamen_basal_ganglia 0.454956605 Brain_Cortex 1.463095025
0.597737092 0.597737092 -1.001931545 1.078780598 0.274675418
1.117937479 3 0 0.989788891
STK16 ENSG00000115661.13 0.252292771 4 2 0.109532388
Brain_Frontal_Cortex_BA9 0.607509109 Brain_Cortex 3.331500204
0.010127499 0.582362753 0.513632276 1.600298463 0.818189887
0.522366512 2 0 0.989856467
DGKQ ENSG00000145214.13 0.252522981 8 7 0.013844863
Brain_Frontal_Cortex_BA9 0.834805523 Brain_Anterior_cingulate_cortex_BA24
3.13580671 0.082240643 0.134786208 -0.714141314 2.461263544
0.737208377 1.064290263 7 0 0.989943081
SIAH2 ENSG00000181788.3 0.252638602 1 1 0.252638602
Brain_Substantia_nigra 0.252638602 Brain_Substantia_nigra 1 1
1 1.143963916 1.143963916 1.143963916 NA 1 0
0.989943081
LSM5 ENSG00000106355.9 0.252721967 3 3 0.290242968
Brain_Cerebellar_Hemisphere 0.865292371 Brain_Frontal_Cortex_BA9
2.474630159 0.20473858 0.20473858 -0.169641135 1.057588763
0.501439779 0.621635196 3 0 0.989943081
DDX1 ENSG00000079785.14 0.252790022 5 5 0.078169246

Brain_Hippocampus 0.989241776 Brain_Cerebellum 1.214634725
0.849789423 0.849789423 -1.761408743 0.449458569 -0.731404152
0.923702936 5 0 0.989943081
NEIL1 ENSG00000140398.13 0.252800667 2 2 0.107991623
Brain_Nucleus_accumbens_basal_ganglia 0.169801725 Brain_Caudate_basal_ganglia
1.664074768 0.335925232 0.335925232 -1.607286096 -1.372841182
-1.490063639 0.165777589 2 0 0.989943081
TMIGD3 ENSG00000121933.18 0.252809207 2 2 0.125980768
Brain_Hippocampus 0.190103642 Brain_Caudate_basal_ganglia 1.492632757
0.507367243 0.507367243 -1.530145297 -1.310272574 -1.420208936
0.155473493 2 0 0.989943081
ZGLP1 ENSG00000220201.7 0.252810137 4 4 0.151794964 Brain_Cerebellum
0.909937583 Brain_Cerebellar_Hemisphere 1.713004961 0.305219188
0.305219188 -1.43322004 0.359809862 -0.288433927 0.796036418
4 0 0.989943081
LINC01694 ENSG00000233922.2 0.252884994 2 2 0.110286017
Brain_Cerebellar_Hemisphere 0.418277704 Brain_Cerebellum 1.705186531
0.294813469 0.294813469 -1.596908895 -0.809412866 -1.203160881
0.556843783 2 0 0.989959136
CD55 ENSG00000196352.14 0.252981287 5 5 0.190671364
Brain_Cerebellar_Hemisphere 0.906178177 Brain_Hippocampus 2.849365008
0.223120664 0.223120664 -1.308595623 0.250027698 -0.462558121
0.645030839 5 0 0.990059072
USH1C ENSG00000006611.15 0.253102165 2 2 0.185384688
Brain_Cerebellar_Hemisphere 0.389259523 Brain_Caudate_basal_ganglia 1.09901927
0.90098073 0.90098073 -0.860960999 1.324356458 0.231697729
1.545252793 2 0 0.990255142
CASD1 ENSG00000127995.16 0.253208726 9 9 0.153156441
Brain_Frontal_Cortex_BA9 0.886508908 Brain_Cerebellum 2.631717953
0.204336174 0.204336174 -1.413543019 1.428470635 0.188863644
0.947525736 9 0 0.990395103
UBA2 ENSG00000126261.12 0.253327371 1 1 0.253327371 Brain_Amygdala
0.253327371 Brain_Amygdala 1 1 1 1.142304742 1.142304742
1.142304742 NA 1 0 0.990582238
CD48 ENSG00000117091.9 0.253444756 1 1 0.253444756
Brain_Hypothalamus 0.253444756 Brain_Hypothalamus 1 1 1
-1.142022287 -1.142022287 -1.142022287 NA 1 0 0.990645409
GDF5 ENSG00000125965.8 0.253694567 3 3 0.274247023
Brain_Putamen_basal_ganglia 0.941816046 Brain_Frontal_Cortex_BA9 2.12300591
0.18262087 0.18262087 -0.163180804 1.093334358 0.334380359
0.667796563 3 0 0.990645409
VEZT ENSG00000028203.17 0.253791636 5 5 0.035290451
Brain_Cerebellar_Hemisphere 0.84646576 Brain_Caudate_basal_ganglia 2.015989189
0.491717787 0.491717787 0.193629803 2.10500984 1.340028642
0.710143834 5 0 0.990645409
BCAS4 ENSG00000124243.17 0.253797513 2 2 0.117993363 Brain_Cortex
0.548609252 Brain_Frontal_Cortex_BA9 1.034319857 0.965680143
0.965680143 0.599845432 1.563251877 1.081548654 0.68123123 2
0 0.990645409
ELOB ENSG00000103363.14 0.253848737 1 1 0.253848737
Brain_Putamen_basal_ganglia 0.253848737 Brain_Putamen_basal_ganglia 1 1
1 -1.141050912 -1.141050912 -1.141050912 NA 1 0
0.990645409
SIGLEC14 ENSG00000254415.3 0.253928987 12 2 0.088567863
Brain_Hippocampus 0.513862741 Brain_Substantia_nigra 10.40825034
0.006633439 0.472320601 0.652834869 1.703001099 1.262622033
0.333250516 2 0 0.990645409
FAM182B ENSG00000175170.14 0.253988569 13 3 0.037657501
Brain_Anterior_cingulate_cortex_BA24 0.540503097 Brain_Cortex
10.61119493 0.013170163 0.558770025 0.612052388 2.078563364
1.412978216 0.404328695 3 0 0.990645409
INPP5F ENSG00000198825.12 0.2541471 1 1 0.2541471 Brain_Cerebellum

0.2541471 Brain_Cerebellum 1 1 1 1.140334187 1.140334187
1.140334187 NA 1 0 0.990645409
FAM95C ENSG00000273036.2 0.254202872 6 4 0.206319278
Brain_Cerebellar_Hemisphere 0.976752729 Brain_Amygdala 4.099814682
0.032991651 0.270976701 -1.263751379 0.824329352 -0.287638009
0.704256669 4 0 0.990645409
DNASE2B ENSG00000137976.7 0.25421295 1 1 0.25421295
Brain_Anterior_cingulate_cortex_BA24 0.25421295
Brain_Anterior_cingulate_cortex_BA24 1 1 1 -1.140176082
-1.140176082 -1.140176082 NA 1 0 0.990645409
SLC52A1 ENSG00000132517.14 0.254362841 1 1 0.254362841
Brain_Hippocampus 0.254362841 Brain_Hippocampus 1 1 1
-1.139816301 -1.139816301 -1.139816301 NA 1 0 0.990645409
CCDC136 ENSG00000128596.16 0.254368792 2 2 0.162761696
Brain_Cerebellar_Hemisphere 0.585276321 Brain_Cerebellum 1.228762128
0.771237872 0.771237872 -1.395843274 0.545693965 -0.425074654
1.372874148 2 0 0.990645409
C2orf81 ENSG00000284308.1 0.25441693 1 1 0.25441693
Brain_Anterior_cingulate_cortex_BA24 0.25441693
Brain_Anterior_cingulate_cortex_BA24 1 1 1 1.139686508
1.139686508 1.139686508 NA 1 0 0.990645409
AP000230.1 ENSG00000273492.5 0.254545761 13 7 0.014822474
Brain_Amygdala 0.867451955 Brain_Substantia_nigra 7.888113462
0.024681788 0.337352956 -0.166895905 2.436687719 1.283073013
0.723199669 7 0 0.990645409
CTD-2186M15.3 ENSG00000272086.1 0.254556026 1 1 0.254556026
Brain_Cerebellum 0.254556026 Brain_Cerebellum 1 1 1 1.139352819
1.139352819 1.139352819 NA 1 0 0.990645409
HOXB3 ENSG00000120093.11 0.254561702 2 2 0.110023935 Brain_Cerebellum
0.11227703 Brain_Cerebellar_Hemisphere 1.855102692 0.144897308
0.144897308 1.588041169 1.598085568 1.593063368 0.007102463
2 0 0.990645409
PTGIR ENSG00000160013.8 0.254613657 1 1 0.254613657
Brain_Hypothalamus 0.254613657 Brain_Hypothalamus 1 1 1
1.139214602 1.139214602 1.139214602 NA 1 0 0.990645409
LINC00500 ENSG00000249381.1 0.254618032 11 11 0.009307395
Brain_Nucleus_accumbens_basal_ganglia 0.891613502 Brain_Amygdala
2.334892576 0.442615363 0.442615363 -2.600551851 0.310999857
-0.746242105 1.03168238 11 0 0.990645409
RNF130 ENSG00000113269.13 0.254833297 1 1 0.254833297
Brain_Cerebellum 0.254833297 Brain_Cerebellum 1 1 1 -1.13868803
-1.13868803 -1.13868803 NA 1 0 0.991040625
PIGQ ENSG00000007541.16 0.255021117 9 1 0.016889853
Brain_Hippocampus 0.545128795 Brain_Hypothalamus 8.640766364
0.001392783 8.640766364 0.605075556 2.389096762 1.12637081
0.528682451 1 0 0.991040625
RFC1 ENSG00000035928.15 0.255110855 2 2 0.216954946 Brain_Cerebellum
0.616362223 Brain_Cerebellar_Hemisphere 1.859997822 0.140002178
0.140002178 0.501012646 1.2346657 0.867839173 0.518771049 2
0 0.991040625
KRT7 ENSG00000135480.15 0.255118598 2 2 0.103983662
Brain_Hippocampus 0.813609501 Brain_Spinal_cord_cervical_c-1
1.038031583 0.961968417 0.961968417 -0.235772123 1.625840164
0.69503402 1.316358672 2 0 0.991040625
GUCA1B ENSG00000112599.8 0.255177304 12 3 0.418252995
Brain_Substantia_nigra 0.959264291 Brain_Nucleus_accumbens_basal_ganglia
10.17524954 0.000704885 0.342828431 -0.809455838 0.737736151
0.10855973 0.458510221 3 0 0.991040625
CDKL3 ENSG00000006837.11 0.255282077 1 1 0.255282077
Brain_Anterior_cingulate_cortex_BA24 0.255282077
Brain_Anterior_cingulate_cortex_BA24 1 1 1 1.137613092
1.137613092 1.137613092 NA 1 0 0.991040625

DDX18 ENSG00000088205.12 0.255284973 2 2 0.100699579 Brain_Cerebellum
0.231221754 Brain_Hypothalamus 1.640160857 0.359839143
0.359839143 -1.641471498 1.197217594 -0.222126952 2.007256306
2 0 0.991040625
GPRIN3 ENSG00000185477.4 0.255373739 3 2 0.187774435
Brain_Cortex 0.264837906 Brain_Cerebellum 2.013221821 0.002229292
0.984548887 -1.115029207 1.317191523 0.503349386 1.401564697
2 0 0.991040625
CTB-61M7.2 ENSG00000268734.1 0.25542945 2 2 0.16751764 Brain_Hypothalamus
0.418629508 Brain_Amygdala 1.070297858 0.929702142 0.929702142
-1.38022415 0.808801197 -0.285711477 1.547874667 2 0
0.991040625
HPD ENSG00000158104.11 0.255655304 1 1 0.255655304
Brain_Nucleus_accumbens_basal_ganglia 0.255655304
Brain_Nucleus_accumbens_basal_ganglia 1 1 1 1.136720125
1.136720125 1.136720125 NA 1 0 0.991040625
RP1-223B1.1 ENSG00000228793.1 0.255663306 13 6 0.036787061
Brain_Anterior_cingulate_cortex_BA24 0.7197972 Brain_Substantia_nigra
9.032404228 0.016459367 0.329141657 -2.088119449 0.358729845
-0.824438864 0.599639197 6 0 0.991040625
RDH13 ENSG00000160439.15 0.255703304 5 4 0.05806547
Brain_Spinal_cord_cervical_c-1 0.975850929 Brain_Putamen_basal_ganglia
2.949889902 0.026802608 0.231246066 0.030270995 1.895203321
1.327060391 0.779806383 4 0 0.991040625
RP11-365P13.5 ENSG00000277767.1 0.255726043 13 4 0.028049061
Brain_Hypothalamus 0.209706267 Brain_Amygdala 10.74215667
0.000957131 0.386825293 1.254373536 2.196599517 1.926200035
0.272832404 4 0 0.991040625
RP11-282A11.4 ENSG00000277863.1 0.255748966 9 9 0.041530847
Brain_Hypothalamus 0.922771822 Brain_Amygdala 2.479395084
0.278760681 0.278760681 -1.206839497 2.038190878 0.581668576
1.035985487 9 0 0.991040625
CROT ENSG00000005469.11 0.255782124 3 3 0.126648005
Brain_Spinal_cord_cervical_c-1 0.363398388 Brain_Substantia_nigra
1.114401841 0.886894965 0.886894965 -1.527454615 -0.908908572
-1.178838917 0.316691238 3 0 0.991040625
CTA-929C8.8 ENSG00000230051.1 0.255977905 6 6 0.028820655
Brain_Putamen_basal_ganglia 0.791327337 Brain_Cortex 2.158696071
0.354968371 0.354968371 -2.185930987 -0.264587388 -1.101570424
0.654596476 6 0 0.991226735
RP1-193H18.2 ENSG00000267194.1 0.255978546 1 1 0.255978546
Brain_Frontal_Cortex_BA9 0.255978546 Brain_Frontal_Cortex_BA9 1 1
1 -1.135947479 -1.135947479 -1.135947479 NA 1 0
0.991226735
DNAJC4 ENSG00000110011.13 0.2560427 3 3 0.12464399
Brain_Nucleus_accumbens_basal_ganglia 0.820764111 Brain_Hippocampus
1.23324823 0.82141564 0.82141564 -1.476940317 1.535569539 -0.055977692
1.513482157 3 0 0.991226735
SLC39A9 ENSG00000029364.11 0.256154486 1 1 0.256154486
Brain_Cerebellar_Hemisphere 0.256154486 Brain_Cerebellar_Hemisphere 1 1
1 -1.135527213 -1.135527213 -1.135527213 NA 1 0
0.991382948
TTC39C ENSG00000168234.12 0.256274568 2 2 0.331469675
Brain_Cerebellar_Hemisphere 0.871437328 Brain_Cerebellum 1.746863994
0.253136006 0.253136006 -0.161833048 0.97115786 0.404662406
0.801145554 2 0 0.991382948
CGA ENSG00000135346.8 0.256295627 1 1 0.256295627
Brain_Spinal_cord_cervical_c-1 0.256295627 Brain_Spinal_cord_cervical_c-1
1 1 1 1.135190217 1.135190217 1.135190217 NA 1 0
0.991382948
RP11-433A10.3 ENSG00000272774.1 0.256415343 1 1 0.256415343
Brain_Hypothalamus 0.256415343 Brain_Hypothalamus 1 1 1

1.134904478 1.134904478 1.134904478 NA 1 0 0.991571882
FAM13C ENSG00000148541.12 0.256552796 1 1 0.256552796
Brain_Putamen_basal_ganglia 0.256552796 Brain_Putamen_basal_ganglia 1 1
1 -1.134576518 -1.134576518 -1.134576518 NA 1 0 0.99177351
RP11-51B23.3 ENSG00000254847.1 0.256656214 5 5 0.103322077
Brain_Cerebellar_Hemisphere 0.916018908 Brain_Caudate_basal_ganglia
1.681204351 0.393728476 0.393728476 -1.469001515 1.628957215
0.56824402 1.331531977 5 0 0.99177351
HOMER3 ENSG00000051128.18 0.256680143 5 5 0.05402307 Brain_Cortex
0.631837097 Brain_Frontal_Cortex_BA9 1.265003951 0.763593213
0.763593213 -1.926651545 1.143968111 -0.630888163 1.124962532
5 0 0.99177351
TNNI2 ENSG00000130598.15 0.256849298 2 2 0.18970348
Brain_Caudate_basal_ganglia 0.342715454 Brain_Frontal_Cortex_BA9
1.037952118 0.962047882 0.962047882 0.948813541 1.3114568
1.13013517 0.256427507 2 0 0.992153101
PNPLA7 ENSG00000130653.15 0.257176709 9 8 0.012186875
Brain_Hippocampus 0.782551869 Brain_Nucleus_accumbens_basal_ganglia
2.583999373 0.034867195 0.103487437 -2.506686493 1.568183899
-0.300782779 1.392938462 8 0 0.992493858
TCEA3 ENSG00000204219.9 0.257550347 13 2 0.035218979
Brain_Nucleus_accumbens_basal_ganglia 0.504678789 Brain_Cerebellum
11.81644306 0.000589567 0.580797268 -2.105831647 -0.667146142
-1.3723942 0.312018095 2 0 0.992493858
NF2 ENSG00000186575.17 0.257651185 1 1 0.257651185
Brain_Spinal_cord_cervical_c-1 0.257651185 Brain_Spinal_cord_cervical_c-1
1 1 1 1.131960159 1.131960159 1.131960159 NA 1 0
0.992493858
ADPRHL2 ENSG00000116863.10 0.257682809 3 3 0.072303872
Brain_Hippocampus 0.844390656 Brain_Cortex 1.106914622
0.877854119 0.877854119 0.196280458 1.797200019 1.017580512
0.801273244 3 0 0.992493858
NHLH1 ENSG00000171786.5 0.257736824 2 2 0.100806878 Brain_Cerebellum
0.215536142 Brain_Cerebellar_Hemisphere 1.694450667 0.305549333
0.305549333 -1.640954414 -1.238485373 -1.439719893 0.284588588
2 0 0.992493858
PIK3R4 ENSG00000196455.7 0.257780186 6 3 0.065810922
Brain_Cortex 0.480180253 Brain_Spinal_cord_cervical_c-1 4.880432889
0.003486776 0.5368537 -1.839709337 -0.706012679 -1.501916658
0.404005329 3 0 0.992493858
NEMP2 ENSG00000189362.11 0.257807104 1 1 0.257807104 Brain_Cerebellum
0.257807104 Brain_Cerebellum 1 1 1 1.131589387 1.131589387
1.131589387 NA 1 0 0.992493858
ITGA6 ENSG00000091409.14 0.257947601 3 3 0.04567341 Brain_Cortex
0.816504321 Brain_Caudate_basal_ganglia 1.276791931 0.638153195
0.638153195 -0.389553814 1.998399095 0.613629554 1.238864714
3 0 0.992493858
NPEPPS ENSG00000141279.15 0.257980184 5 4 0.056531574
Brain_Nucleus_accumbens_basal_ganglia 0.986976179 Brain_Cerebellum
3.212878817 0.062342628 0.192168121 -1.906915164 0.928611159
-0.656764579 1.128776451 4 0 0.992493858
ZC3H4 ENSG00000130749.9 0.258098138 1 1 0.258098138
Brain_Nucleus_accumbens_basal_ganglia 0.258098138
Brain_Nucleus_accumbens_basal_ganglia 1 1 1 -1.130897731
-1.130897731 -1.130897731 NA 1 0 0.992493858
RBM48 ENSG00000127993.15 0.258162392 8 5 0.066938285
Brain_Anterior_cingulate_cortex_BA24 0.839156566 Brain_Cortex
5.675670771 0.00843137 0.252683886 -1.832088177 -0.202972449
-1.22395268 0.670793359 5 0 0.992493858
ACR ENSG00000100312.10 0.258180132 1 1 0.258180132 Brain_Amygdala
0.258180132 Brain_Amygdala 1 1 1 1.130702968 1.130702968
1.130702968 NA 1 0 0.992493858

RAI14 ENSG00000039560.13 0.258219405 5 5 0.054776178
Brain_Putamen_basal_ganglia 0.658061457 Brain_Amygdala 1.253624701
0.626192469 0.626192469 -1.920647378 0.812610806 -0.365392628
1.160059621 5 0 0.992493858
SPPL2C ENSG00000185294.6 0.258250808 4 3 0.170197839
Brain_Cerebellum 0.523314444 Brain_Cerebellar_Hemisphere 2.823170558
0.086500668 0.191972663 -1.371568391 1.329723731 -0.390027487
1.186405194 3 0 0.992493858
SUSD4 ENSG00000143502.14 0.25841516 5 4 0.031922782 Brain_Cerebellum
0.547117682 Brain_Putamen_basal_ganglia 3.123558505 0.082064587
0.121375736 0.602084816 2.145376181 1.334260239 0.59327793 4
0 0.992493858
BCAT2 ENSG00000105552.14 0.258424544 3 3 0.059652134
Brain_Frontal_Cortex_BA9 0.798829123 Brain_Cerebellum 1.55390644
0.550855142 0.550855142 -0.255612623 1.88335604 0.627535382
1.117121486 3 0 0.992493858
RP4-535B20.1 ENSG00000231485.1 0.258492911 1 1 0.258492911
Brain_Cortex 0.258492911 Brain_Cortex 1 1 1 -1.129960401
-1.129960401 -1.129960401 NA 1 0 0.992493858
USP6NL ENSG00000148429.14 0.258610273 4 4 0.084559972
Brain_Nucleus_accumbens_basal_ganglia 0.530664293
Brain_Spinal_cord_cervical_c-1 1.887310954 0.408651443 0.408651443
-1.72481969 -0.626992283 -1.326792618 0.49404095 4 0
0.992493858
RAB37 ENSG00000172794.19 0.258671978 5 4 0.16865654
Brain_Caudate_basal_ganglia 0.717305606 Brain_Frontal_Cortex_BA9
2.162646337 0.012478244 0.443015293 0.362062125 1.376533446
0.93122544 0.459701168 4 0 0.992493858
SNRPE ENSG00000182004.12 0.258675923 8 2 0.294976945 Brain_Amygdala
0.974999127 Brain_Frontal_Cortex_BA9 7.170809183 6.21E-05
0.479578429 -1.047265931 0.380640198 0.006011585 0.447160087
2 0 0.992493858
NKX1-2 ENSG00000229544.8 0.258717464 1 1 0.258717464
Brain_Cerebellum 0.258717464 Brain_Cerebellum 1 1 1 1.129427674
1.129427674 1.129427674 NA 1 0 0.992493858
DENND3 ENSG00000105339.10 0.258739486 2 2 0.188650679
Brain_Anterior_cingulate_cortex_BA24 0.404276521 Brain_Cortex
1.112239241 0.887760759 0.887760759 -0.83400792 1.314581238
0.240286659 1.519281964 2 0 0.992493858
SPHK1 ENSG00000176170.13 0.258800306 1 1 0.258800306 Brain_Cortex
0.258800306 Brain_Cortex 1 1 1 -1.129231221 -1.129231221
-1.129231221 NA 1 0 0.992493858
ROBO2 ENSG00000185008.17 0.258807662 2 2 0.179019709 Brain_Cerebellum
0.746028123 Brain_Cerebellar_Hemisphere 1.40393719 0.59606281 0.59606281
-1.343779475 0.323881008 -0.509949233 1.179214037 2 0
0.992493858
ASIC4 ENSG00000072182.12 0.258860848 1 1 0.258860848 Brain_Cerebellum
0.258860848 Brain_Cerebellum 1 1 1 1.129087679 1.129087679
1.129087679 NA 1 0 0.992493858
TLR10 ENSG00000174123.10 0.259028891 1 1 0.259028891
Brain_Substantia_nigra 0.259028891 Brain_Substantia_nigra 1 1
1 1.12868938 1.12868938 1.12868938 NA 1 0 0.992493858
KLHL7 ENSG00000122550.17 0.259253642 2 2 0.133394164
Brain_Caudate_basal_ganglia 0.33969131 Brain_Amygdala 1.199472977
0.800527023 0.800527023 0.954775361 1.50085077 1.227813066
0.386133624 2 0 0.992493858
HEY2 ENSG00000135547.8 0.25932316 3 3 0.080464998
Brain_Anterior_cingulate_cortex_BA24 0.24587521 Brain_Spinal_cord_cervical_c-1
2.012679493 0.475565948 0.475565948 -1.747994395 -1.160426483
-1.546584224 0.334526174 3 0 0.992493858
TPH1 ENSG00000129167.9 0.259355863 3 3 0.107662781
Brain_Frontal_Cortex_BA9 0.875940364 Brain_Cerebellum 1.392453386

0.596926311 0.596926311 -1.608787657 0.882241161 -0.190142982
1.281104372 3 0 0.992493858
FXYD5 ENSG00000089327.14 0.259493261 1 1 0.259493261 Brain_Cortex
0.259493261 Brain_Cortex 1 1 1 -1.127589646 -1.127589646
-1.127589646 NA 1 0 0.992493858
STAG1 ENSG00000118007.12 0.259663015 1 1 0.259663015
Brain_Substantia_nigra 0.259663015 Brain_Substantia_nigra 1 1
1 -1.127187971 -1.127187971 -1.127187971 NA 1 0
0.992493858
RP11-124N19.4 ENSG00000283075.1 0.259675968 2 2 0.128433186
Brain_Cerebellar_Hemisphere 0.274846741 Brain_Cerebellum 1.897945232
0.102054768 0.102054768 1.091968968 1.520309587 1.306139278
0.302882557 2 0 0.992493858
PIGF ENSG00000151665.12 0.259765512 5 5 0.126224698
Brain_Spinal_cord_cervical_c-1 0.937692359 Brain_Cortex 2.77775844
0.143826359 0.143826359 -0.084903178 1.529160348 0.666129328
0.692371969 5 0 0.992493858
RP11-411K7.1 ENSG00000236740.6 0.25985539 1 1 0.25985539
Brain_Frontal_Cortex_BA9 0.25985539 Brain_Frontal_Cortex_BA9 1 1 1
1.126732989 1.126732989 1.126732989 NA 1 0 0.992493858
GLCE ENSG00000138604.9 0.25990434 2 2 0.484202989 Brain_Hypothalamus
0.772205779 Brain_Amygdala 1.815330601 0.184669399 0.184669399
-0.289490852 0.699558625 0.205033886 0.699363592 2 0
0.992493858
OSER1-AS1 ENSG00000223891.5 0.260089529 3 3 0.056507949
Brain_Cortex 0.786604965 Brain_Putamen_basal_ganglia 1.773035352
0.410885295 0.410885295 -1.907097606 -0.270721857 -1.116328512
0.819564986 3 0 0.992493858
AF146191.4 ENSG00000245685.6 0.260091904 13 5 0.060157776
Brain_Anterior_cingulate_cortex_BA24 0.948306665 Brain_Caudate_basal_ganglia
10.07495021 9.51E-06 0.363529942 -1.879635471 0.466545791
-0.304572924 0.601575278 5 0 0.992493858
NEFL ENSG00000277586.2 0.260112706 2 1 0.184344839
Brain_Cerebellar_Hemisphere 0.36501105 Brain_Cerebellum 1.966581167
0.033418833 1.966581167 0.905857939 1.327495465 1.116676702
0.298142754 1 0 0.992493858
PHF21B ENSG00000056487.15 0.26012253 1 1 0.26012253 Brain_Cortex
0.26012253 Brain_Cortex 1 1 1 1.126101568 1.126101568
1.126101568 NA 1 0 0.992493858
GRIN3B ENSG00000116032.5 0.26017408 6 4 0.267352206 Brain_Cortex
0.878604413 Brain_Cerebellum 4.306345191 0.026794253 0.22860767
-1.109180772 1.066044955 0.067342415 0.776255233 4 0
0.992493858
ACAT1 ENSG00000075239.13 0.260305423 10 9 0.06314791
Brain_Anterior_cingulate_cortex_BA24 0.780357857 Brain_Cerebellum 2.76293395
0.091631182 0.284889507 -1.833444002 1.858148633 -0.255685411
1.230747467 9 0 0.992493858
SPATA21 ENSG00000187144.11 0.260311417 2 2 0.147284654
Brain_Cerebellar_Hemisphere 0.576314584 Brain_Cerebellum 1.741617521
0.258382479 0.258382479 -1.449189555 -0.558776029 -1.003982792
0.629617442 2 0 0.992493858
AC159540.1 ENSG00000230606.10 0.260354946 11 4 0.134843128
Brain_Cerebellar_Hemisphere 0.987511468 Brain_Putamen_basal_ganglia
9.058572571 0.006087637 0.41024154 -1.495273311 0.124573793
-0.667177452 0.512913292 4 0 0.992493858
HRH3 ENSG00000101180.15 0.260355405 2 2 0.18879815 Brain_Cerebellum
0.741553709 Brain_Cerebellar_Hemisphere 1.425364931 0.574635069
0.574635069 -1.314142811 0.329796531 -0.49217314 1.162440657
2 0 0.992493858
HS3ST5 ENSG00000249853.7 0.260362174 1 1 0.260362174
Brain_Cerebellar_Hemisphere 0.260362174 Brain_Cerebellar_Hemisphere 1 1
1 -1.125535521 -1.125535521 -1.125535521 NA 1 0

0.992493858
TSFM ENSG00000123297.17 0.260637923 10 3 0.065080731
Brain_Caudate_basal_ganglia 0.844977886 Brain_Amygdala 8.027760833
0.006529596 0.57079867 -1.84470316 -0.195530214 -1.329902792
0.565714742 3 0 0.992493858
ZNF239 ENSG00000196793.13 0.26073803 6 3 0.046688027
Brain_Nucleus_accumbens_basal_ganglia 0.186211393 Brain_Cerebellar_Hemisphere
5.198403509 0.014680022 0.228111439 -1.989119385 -1.321870152
-1.654169328 0.274231077 3 0 0.992493858
HIF3A ENSG00000124440.15 0.260846998 1 1 0.260846998
Brain_Caudate_basal_ganglia 0.260846998 Brain_Caudate_basal_ganglia 1 1
1 1.124391448 1.124391448 1.124391448 NA 1 0
0.992493858
DZIP1 ENSG00000134874.17 0.261031153 1 1 0.261031153
Brain_Caudate_basal_ganglia 0.261031153 Brain_Caudate_basal_ganglia 1 1
1 -1.123957271 -1.123957271 -1.123957271 NA 1 0
0.992493858
POLL ENSG00000166169.16 0.261074379 2 2 0.145798381
Brain_Cerebellar_Hemisphere 0.249080121 Brain_Cortex 1.296612258
0.703387742 0.703387742 -1.152586564 1.454533759 0.150973597
1.84351246 2 0 0.992493858
ASH1L ENSG00000116539.12 0.261215906 1 1 0.261215906
Brain_Caudate_basal_ganglia 0.261215906 Brain_Caudate_basal_ganglia 1 1
1 1.123521898 1.123521898 1.123521898 NA 1 0
0.992493858
GLB1L3 ENSG00000166105.15 0.261218594 4 4 0.034656057
Brain_Cerebellar_Hemisphere 0.520431598 Brain_Amygdala 2.114703598
0.103859925 0.103859925 -2.112354474 1.03175507 -0.589209731
1.65662091 4 0 0.992493858
WIPF1 ENSG00000115935.17 0.261226178 12 9 0.027948001
Brain_Putamen_basal_ganglia 0.924998072 Brain_Spinal_cord_cervical_c-1
6.448860504 0.016756952 0.226147034 -1.20893468 2.1980155
0.352276806 0.977926323 9 0 0.992493858
KLK10 ENSG00000129451.11 0.261463831 9 9 0.03285255 Brain_Cortex
0.979912867 Brain_Cerebellar_Hemisphere 1.51133712 0.560289554
0.560289554 -2.133880667 1.725433668 0.052608963 1.106288817
9 0 0.992493858
ANXA11 ENSG00000122359.17 0.261503812 12 3 0.04067752
Brain_Cerebellar_Hemisphere 0.62169206 Brain_Cerebellum 9.563347167
0.000641183 0.527330154 -2.046802098 -0.493453681 -0.942029824
0.492557915 3 0 0.992493858
ZBTB25 ENSG00000089775.11 0.261534942 2 2 0.108314575
Brain_Cerebellar_Hemisphere 0.329461423 Brain_Cerebellum 1.741798414
0.258201586 0.258201586 -1.605814952 -0.975199272 -1.290507112
0.445912623 2 0 0.992493858
RP11-1275H24.2 ENSG00000272953.1 0.261537419 1 1 0.261537419
Brain_Frontal_Cortex_BA9 0.261537419 Brain_Frontal_Cortex_BA9 1 1
1 1.122764752 1.122764752 1.122764752 NA 1 0
0.992493858
HDHD3 ENSG00000119431.9 0.261552807 5 5 0.063328739
Brain_Cerebellar_Hemisphere 0.756836691 Brain_Amygdala 2.278549679
0.305601394 0.305601394 -0.332033629 1.856876411 0.704789486
1.080045756 5 0 0.992493858
CYP46A1 ENSG00000036530.8 0.261649623 2 1 0.219243301
Brain_Cerebellar_Hemisphere 0.312132744 Brain_Cerebellum 1.989854147
0.010145853 1.989854147 1.01075701 1.228542726 1.119649868
0.153997757 1 0 0.992493858
GCAT ENSG00000100116.16 0.261690612 4 4 0.108422197 Brain_Amygdala
0.980974386 Brain_Hypothalamus 2.81353615 0.114334694 0.114334694
-1.605325469 0.058127326 -0.644881954 0.823212461 4 0
0.992493858
LINC01337 ENSG00000251221.1 0.261735336 1 1 0.261735336

Brain_Cerebellum 0.261735336 Brain_Cerebellum 1 1 1 -1.122298989
-1.122298989 -1.122298989 NA 1 0 0.992493858
EFHD1 ENSG00000115468.11 0.26174964 5 4 0.058373014
Brain_Cerebellar_Hemisphere 0.83076614 Brain_Cortex 2.711289885
0.017282334 0.384003926 -0.706254981 1.892886131 0.786494493
1.185539608 4 0 0.992493858
UNC5C ENSG00000182168.14 0.261785323 2 2 0.207563991
Brain_Cerebellar_Hemisphere 0.329030855 Brain_Caudate_basal_ganglia
1.053543736 0.946456264 0.946456264 -0.976067825 1.26029215
0.142112163 1.581345304 2 0 0.992493858
SPARCL1 ENSG00000152583.12 0.261948879 6 4 0.11224241
Brain_Anterior_cingulate_cortex_BA24 0.936548153 Brain_Cortex
4.054514712 0.058288053 0.371729854 -1.588194299 1.166889907
-0.077817431 0.882927457 4 0 0.992493858
CTC-529P8.1 ENSG00000250072.5 0.262102236 1 1 0.262102236
Brain_Spinal_cord_cervical_c-1 0.262102236 Brain_Spinal_cord_cervical_c-1
1 1 1 -1.121436197 -1.121436197 -1.121436197 NA 1 0
0.992493858
SH2D5 ENSG00000189410.11 0.262159815 1 1 0.262159815
Brain_Cerebellar_Hemisphere 0.262159815 Brain_Cerebellar_Hemisphere 1 1
1 -1.121300872 -1.121300872 -1.121300872 NA 1 0
0.992493858
CTD-2544H17.1 ENSG00000249621.1 0.26228804 1 1 0.26228804
Brain_Hypothalamus 0.26228804 Brain_Hypothalamus 1 1 1
-1.120999582 -1.120999582 -1.120999582 NA 1 0 0.992493858
COL15A1 ENSG00000204291.10 0.262332564 3 3 0.151227313
Brain_Frontal_Cortex_BA9 0.730456127 Brain_Cerebellar_Hemisphere
1.092517079 0.927244443 0.927244443 -1.323099825 1.435209831
-0.077469613 1.398411423 3 0 0.992493858
CUL9 ENSG00000112659.13 0.262483359 4 4 0.050566828
Brain_Frontal_Cortex_BA9 0.216498917 Brain_Cortex 2.328507733
0.228720015 0.228720015 -1.955137629 -1.235891446 -1.668583926
0.314664173 4 0 0.992493858
TP53I3 ENSG00000115129.13 0.262484856 9 3 0.147159066
Brain_Substantia_nigra 0.783598367 Brain_Cerebellum 8.079919737
4.54E-05 0.310695402 0.274632797 1.449639532 0.476969516
0.368807832 3 0 0.992493858
HINFP ENSG00000172273.12 0.262653214 1 1 0.262653214 Brain_Cortex
0.262653214 Brain_Cortex 1 1 1 -1.120142096 -1.120142096
-1.120142096 NA 1 0 0.992493858
KLHL26 ENSG00000167487.11 0.262865224 2 1 0.241783331
Brain_Cerebellum 0.295943944 Brain_Cerebellar_Hemisphere 1.958106038
0.041893962 1.958106038 -1.170540985 -1.045171001 -1.107855993
0.088649965 1 0 0.992493858
PRAME ENSG00000185686.17 0.262986769 2 2 0.597597453
Brain_Substantia_nigra 0.740382078 Brain_Hypothalamus 1.861282483
0.138717517 0.138717517 -0.52785864 0.331347417 -0.098255611
0.60755043 2 0 0.992493858
NOTUM ENSG00000185269.11 0.262997025 2 2 0.106611614
Brain_Cerebellar_Hemisphere 0.781508412 Brain_Hypothalamus 1.010905577
0.989094423 0.989094423 -0.277353853 1.613611993 0.66812907
1.337114773 2 0 0.992493858
LA16c-360A4.1 ENSG00000275438.1 0.263179167 4 2 0.132984001
Brain_Nucleus_accumbens_basal_ganglia 0.74860528 Brain_Frontal_Cortex_BA9
3.538814203 0.018210279 0.358821232 0.320478957 1.502438106
0.824713366 0.528653167 2 0 0.992493858
UIMC1 ENSG00000087206.16 0.263513294 4 4 0.137082671
Brain_Putamen_basal_ganglia 0.838244389 Brain_Cerebellum 2.152110987
0.280558049 0.280558049 -1.486743112 -0.204139626 -0.977968352
0.566401854 4 0 0.992493858
EPRS ENSG00000136628.17 0.263524896 1 1 0.263524896 Brain_Cerebellum
0.263524896 Brain_Cerebellum 1 1 1 -1.118098573 -1.118098573

-1.118098573 NA 1 0 0.992493858
AIM2 ENSG00000163568.14 0.263541847 1 1 0.263541847
Brain_Putamen_basal_ganglia 0.263541847 Brain_Putamen_basal_ganglia 1 1
1 1.118058879 1.118058879 1.118058879 NA 1 0
0.992493858
OR11H4 ENSG00000176198.2 0.263616686 1 1 0.263616686
Brain_Cerebellar_Hemisphere 0.263616686 Brain_Cerebellar_Hemisphere 1 1
1 1.117883657 1.117883657 1.117883657 NA 1 0
0.992493858
GSTO2 ENSG00000065621.14 0.263716139 13 2 0.068934916
Brain_Caudate_basal_ganglia 0.308468845 Brain_Hippocampus 11.82377401
0.000194505 0.630798732 1.018440118 1.81884608 1.551030809
0.207283868 2 0 0.992493858
STON1 ENSG00000243244.5 0.26372562 10 4 0.231952515
Brain_Caudate_basal_ganglia 0.996012434 Brain_Anterior_cingulate_cortex_BA24
7.437247113 0.053034565 0.324940155 -0.795251578 1.19534436
0.461162199 0.615450805 4 0 0.992493858
CD63 ENSG00000135404.11 0.263932977 1 1 0.263932977
Brain_Substantia_nigra 0.263932977 Brain_Substantia_nigra 1 1
1 1.117143493 1.117143493 1.117143493 NA 1 0
0.992493858
PPID ENSG00000171497.4 0.264157217 4 4 0.14935245
Brain_Cerebellar_Hemisphere 0.950270148 Brain_Cerebellum 1.898477305
0.309751685 0.309751685 -1.176714457 1.441822513 0.05656017
1.074686168 4 0 0.992493858
ZNF439 ENSG00000171291.8 0.264196789 1 1 0.264196789
Brain_Cortex 0.264196789 Brain_Cortex 1 1 1 1.116526604
1.116526604 1.116526604 NA 1 0 0.992493858
OSBP ENSG00000110048.11 0.264245327 1 1 0.264245327
Brain_Frontal_Cortex_BA9 0.264245327 Brain_Frontal_Cortex_BA9 1 1
1 -1.116413151 -1.116413151 -1.116413151 NA 1 0
0.992493858
ZDHHC23 ENSG00000184307.14 0.264348196 1 1 0.264348196
Brain_Spinal_cord_cervical_c-1 0.264348196 Brain_Spinal_cord_cervical_c-1
1 1 1 -1.11617275 -1.11617275 -1.11617275 NA 1 0
0.992493858
P2RY11 ENSG00000244165.1 0.264418324 5 3 0.094920169
Brain_Cortex 0.86841313 Brain_Cerebellar_Hemisphere 3.6347244 0.009005008
0.320488271 -1.669995931 1.115530739 0.07835644 1.041012044 3
0 0.992493858
MRPS27 ENSG00000113048.16 0.264501688 9 7 0.149232172
Brain_Nucleus_accumbens_basal_ganglia 0.926987554 Brain_Hippocampus
5.573041493 0.018655594 0.208823513 -1.071384661 1.442248892
-0.031893561 0.750060646 7 0 0.992493858
NAT2 ENSG00000156006.4 0.264528762 1 1 0.264528762
Brain_Cerebellar_Hemisphere 0.264528762 Brain_Cerebellar_Hemisphere 1 1
1 1.115750933 1.115750933 1.115750933 NA 1 0
0.992493858
RP11-290F5.1 ENSG00000249096.6 0.264603482 4 4 0.082004403
Brain_Spinal_cord_cervical_c-1 0.626093522
Brain_Nucleus_accumbens_basal_ganglia 1.210392853 0.769810675
0.769810675 -0.926413402 1.739172624 -0.114874105 1.24951134 4
0 0.992493858
ATP1B2 ENSG00000129244.8 0.264661216 1 1 0.264661216
Brain_Caudate_basal_ganglia 0.264661216 Brain_Caudate_basal_ganglia 1 1
1 1.115441637 1.115441637 1.115441637 NA 1 0
0.992493858
KISS1 ENSG00000170498.8 0.26474143 3 3 0.054795875
Brain_Putamen_basal_ganglia 0.825498313 Brain_Nucleus_accumbens_basal_ganglia
1.208263016 0.789222607 0.789222607 0.220478759 1.920491272
1.002680791 0.858080925 3 0 0.992493858
TMEM215 ENSG00000188133.5 0.264783092 6 1 0.218769839

Brain_Caudate_basal_ganglia 0.351396728 Brain_Cortex 5.908035413
3.22E-05 5.908035413 -1.22980579 -0.931883515 -1.107065783
0.098430145 1 0 0.992493858
CKMT1B ENSG00000237289.9 0.264787053 1 1 0.264787053
Brain_Cerebellum 0.264787053 Brain_Cerebellum 1 1 1 1.115147888
1.115147888 1.115147888 NA 1 0 0.992493858
ZSCAN22 ENSG00000182318.5 0.264792719 2 2 0.196014804
Brain_Cerebellar_Hemisphere 0.576908492 Brain_Anterior_cingulate_cortex_BA24
1.300832464 0.699167536 0.699167536 -1.292989173 0.55790612
-0.367541526 1.308780613 2 0 0.992493858
RP3-460G2.2 ENSG00000234147.1 0.264824072 7 3 0.113856553
Brain_Cerebellar_Hemisphere 0.692737056 Brain_Hippocampus 5.663950133
0.015708747 0.288963026 -1.581093969 0.525158705 -0.885087416
0.721684688 3 0 0.992493858
LINC01356 ENSG00000215866.7 0.26485077 6 5 0.165798682
Brain_Nucleus_accumbens_basal_ganglia 0.964317755
Brain_Anterior_cingulate_cortex_BA24 4.05297237 9.90E-05 0.142747715
-0.938063359 1.385830452 0.189404182 0.738386822 5 0
0.992493858
CCDC170 ENSG00000120262.9 0.265003738 10 8 0.166684418
Brain_Spinal_cord_cervical_c-1 0.940040876 Brain_Frontal_Cortex_BA9
5.189181143 0.102899934 0.30652708 -1.382936238 0.739767897
-0.479044402 0.755956033 8 0 0.992493858
ERCC4 ENSG00000175595.14 0.26519757 3 3 0.194727739
Brain_Spinal_cord_cervical_c-1 0.71866656 Brain_Putamen_basal_ganglia
1.340023491 0.65776692 0.65776692 -1.296719436 0.920611588
-0.005288791 1.152973764 3 0 0.992493858
PURG ENSG00000172733.11 0.265331665 1 1 0.265331665
Brain_Substantia_nigra 0.265331665 Brain_Substantia_nigra 1 1
1 -1.113877682 -1.113877682 -1.113877682 NA 1 0
0.992493858
CLCN2 ENSG00000114859.15 0.265356051 4 4 0.048781627 Brain_Cerebellum
0.839242131 Brain_Nucleus_accumbens_basal_ganglia 2.843123298
0.225283316 0.225283316 0.202862979 1.970495357 0.956455836
0.772018305 4 0 0.992493858
SLC38A7 ENSG00000103042.8 0.265394717 2 2 0.606600077
Brain_Cerebellar_Hemisphere 0.778856635 Brain_Cerebellum 1.880006388
0.119993612 0.119993612 -0.280809346 0.514932661 0.117061657
0.562674569 2 0 0.992493858
LINC00235 ENSG00000277142.1 0.265456169 2 2 0.149300995
Brain_Cortex 0.761659136 Brain_Cerebellum 1.291854574 0.708145426
0.708145426 -0.30330277 1.442004887 0.569351058 1.234118879
2 0 0.992493858
COBLL1 ENSG00000082438.15 0.265495498 5 5 0.018636067
Brain_Caudate_basal_ganglia 0.631631911 Brain_Cerebellum 1.7252435
0.353128546 0.353128546 -2.352731874 -0.479431188 -1.139593548
0.749856842 5 0 0.992493858
C5orf58 ENSG00000234511.8 0.265535964 2 2 0.127872972
Brain_Anterior_cingulate_cortex_BA24 0.568860283 Brain_Cortex
1.005305058 0.994694942 0.994694942 -1.52254343 -0.56973082
-1.046137125 0.673740258 2 0 0.992493858
PLXNC1 ENSG00000136040.8 0.265542035 2 2 0.138120294
Brain_Putamen_basal_ganglia 0.545299298 Brain_Cerebellum 1.045636857
0.954363143 0.954363143 0.604818954 1.482827325 1.043823139
0.620845673 2 0 0.992493858
PEX11G ENSG00000104883.7 0.265596219 10 6 0.016894555
Brain_Substantia_nigra 0.955748201 Brain_Cerebellar_Hemisphere
6.251965216 4.17E-18 0.468785191 -2.3889945 0.055489869
-1.249123987 0.849123756 6 0 0.992493858
UBE2Z ENSG00000159202.17 0.265703961 3 3 0.06043182
Brain_Spinal_cord_cervical_c-1 0.878560752 Brain_Cerebellar_Hemisphere
1.526433937 0.472562593 0.472562593 -0.152793972 1.877629848

1.073621087 1.079108675 3 0 0.992493858
ANKRD33 ENSG00000167612.12 0.265770857 1 1 0.265770857
Brain_Cortex 0.265770857 Brain_Cortex 1 1 1 -1.112854656
-1.112854656 -1.112854656 NA 1 0 0.992493858
S100A1 ENSG00000160678.11 0.265857511 4 3 0.137195582
Brain_Cortex 0.873949143 Brain_Substantia_nigra 2.463959503
0.041133046 0.502859837 -1.486315903 0.256171822 -0.59841288
0.933741618 3 0 0.992493858
SPOCD1 ENSG00000134668.12 0.265920107 3 2 0.105401625
Brain_Putamen_basal_ganglia 0.120777982 Brain_Caudate_basal_ganglia
2.837720082 0.002126566 0.160153352 -1.619212149 -1.551516395
-1.582296123 0.034262507 2 0 0.992493858
SUCLG1 ENSG00000163541.11 0.265946182 4 2 0.155806259
Brain_Frontal_Cortex_BA9 0.588676157 Brain_Cortex 3.662751954
0.060660311 0.187421103 -1.419318233 -0.540755444 -0.929953931
0.405537578 2 0 0.992493858
CEBPZ ENSG00000115816.14 0.266017328 7 6 0.009474281
Brain_Hypothalamus 0.283249562 Brain_Nucleus_accumbens_basal_ganglia
4.924327739 0.065085029 0.166016678 1.073048041 2.594448497
1.842464552 0.604144788 6 0 0.992493858
ITGA5 ENSG00000161638.10 0.26616514 2 2 0.579743625
Brain_Anterior_cingulate_cortex_BA24 0.617699803 Brain_Cerebellum 1.79056039
0.20943961 0.20943961 -0.499112967 0.553759243 0.027323138
0.744493079 2 0 0.992493858
LY6E ENSG00000160932.10 0.26630056 3 3 0.228728136 Brain_Amygdala
0.385318017 Brain_Putamen_basal_ganglia 1.239866653 0.769525848
0.769525848 -1.203641594 1.006529848 0.22367589 1.238028423 3
0 0.992493858
CCNE1 ENSG00000105173.13 0.266464906 1 1 0.266464906
Brain_Frontal_Cortex_BA9 0.266464906 Brain_Frontal_Cortex_BA9 1 1
1 1.111240352 1.111240352 1.111240352 NA 1 0
0.992493858
TM2D1 ENSG00000162604.12 0.266581291 5 5 0.13245527
Brain_Nucleus_accumbens_basal_ganglia 0.719381654 Brain_Putamen_basal_ganglia
1.337544136 0.709390008 0.709390008 -1.504489908 0.540749438
-0.446965675 0.95442252 5 0 0.992493858
VWA5B2 ENSG00000145198.14 0.266680508 9 2 0.191070878
Brain_Amygdala 0.910900828 Brain_Substantia_nigra 8.041589
0.002126497 0.392152682 -1.307417735 0.111902356 -0.448182724
0.395255513 2 0 0.992493858
RP11-80H5.9 ENSG00000235100.3 0.266727703 6 5 0.119481551
Brain_Cortex 0.573474361 Brain_Caudate_basal_ganglia 4.054136001
0.022731782 0.149813818 -1.556953394 -0.562942027 -0.887102661
0.456398916 5 0 0.992493858
GPX2 ENSG00000176153.11 0.266871956 1 1 0.266871956
Brain_Cerebellar_Hemisphere 0.266871956 Brain_Cerebellar_Hemisphere 1 1
1 -1.110294932 -1.110294932 -1.110294932 NA 1 0
0.992493858
RP11-676J15.1 ENSG00000263958.1 0.266982875 6 2 0.085474743
Brain_Putamen_basal_ganglia 0.579338387 Brain_Anterior_cingulate_cortex_BA24
5.268453143 0.039698041 0.281785971 -1.71976733 -0.554351389
-0.989780427 0.415577948 2 0 0.992493858
LGALS1 ENSG00000100097.11 0.267063654 2 2 0.399161414
Brain_Caudate_basal_ganglia 0.50816818 Brain_Anterior_cingulate_cortex_BA24
1.569502388 0.430497612 0.430497612 -0.661692706 0.843119858
0.090713576 1.064063168 2 0 0.992493858
CHST12 ENSG00000136213.9 0.267091324 3 3 0.24644867
Brain_Frontal_Cortex_BA9 0.763172596 Brain_Cortex 2.228358952
0.299271025 0.299271025 -0.30131724 1.159018437 0.549719052
0.759587139 3 0 0.992493858
TMEM221 ENSG00000188051.6 0.267117033 1 1 0.267117033
Brain_Cerebellar_Hemisphere 0.267117033 Brain_Cerebellar_Hemisphere 1 1

1 1.109726192 1.109726192 1.109726192 NA 1 0
0.992493858
BNIP1 ENSG00000113734.17 0.26713404 12 4 0.123917379 Brain_Cerebellum
0.949978339 Brain_Caudate_basal_ganglia 9.875273551 0.000617367
0.382949386 -1.538536957 0.062733979 -0.390349594 0.430872269
4 0 0.992493858
RUNDC3B ENSG00000105784.15 0.267178582 3 3 0.191858715
Brain_Cerebellum 0.967791447 Brain_Cerebellar_Hemisphere 1.512435597
0.493922399 0.493922399 -1.305100246 0.040378405 -0.834039848
0.75802465 3 0 0.992493858
TEAD2 ENSG00000074219.13 0.267207364 9 3 0.086248576 Brain_Cerebellum
0.755420501 Brain_Putamen_basal_ganglia 7.706869696 0.00194651
0.346251382 0.311500055 1.715527402 0.899039136 0.406259281
3 0 0.992493858
ZNF558 ENSG00000167785.8 0.267313188 1 1 0.267313188
Brain_Cortex 0.267313188 Brain_Cortex 1 1 1 -1.109271241
-1.109271241 -1.109271241 NA 1 0 0.992493858
AK3 ENSG00000147853.16 0.267421113 4 4 0.031397019 Brain_Cortex
0.674100825 Brain_Cerebellum 2.380179853 0.166119542 0.166119542
-2.15200426 0.995929947 -0.470923274 1.44118056 4 0
0.992493858
CUX1 ENSG00000257923.9 0.267523094 2 2 0.202550181 Brain_Cerebellum
0.622730634 Brain_Cerebellar_Hemisphere 1.35297778 0.64702222 0.64702222
-1.274319578 0.491984029 -0.391167775 1.248965258 2 0
0.992493858
C12orf65 ENSG00000130921.7 0.267592667 3 3 0.06387413
Brain_Caudate_basal_ganglia 0.643416593 Brain_Nucleus_accumbens_basal_ganglia
1.634775211 0.465979306 0.465979306 -1.853057412 -0.46292714
-1.298516407 0.736448666 3 0 0.992493858
TAF1C ENSG00000103168.16 0.26761079 13 3 0.187144168
Brain_Caudate_basal_ganglia 0.965635671 Brain_Hypothalamus 10.69018686
0.00272083 0.577234457 -0.807955882 1.319074618 -0.135570649
0.509312356 3 0 0.992493858
FBXO9 ENSG00000112146.16 0.267612377 1 1 0.267612377
Brain_Cerebellar_Hemisphere 0.267612377 Brain_Cerebellar_Hemisphere 1 1
1 -1.108577759 -1.108577759 -1.108577759 NA 1 0
0.992493858
CEP72 ENSG00000112877.7 0.267803997 3 3 0.253396589 Brain_Cortex
0.614498152 Brain_Nucleus_accumbens_basal_ganglia 1.920159809
0.123813624 0.123813624 0.503663086 1.142138177 0.926162062
0.365925374 3 0 0.992493858
GALK1 ENSG00000108479.11 0.267814029 1 1 0.267814029 Brain_Cerebellum
0.267814029 Brain_Cerebellum 1 1 1 1.108110657 1.108110657
1.108110657 NA 1 0 0.992493858
MFAP3 ENSG00000037749.11 0.267859524 1 1 0.267859524
Brain_Hypothalamus 0.267859524 Brain_Hypothalamus 1 1 1
-1.108005307 -1.108005307 -1.108005307 NA 1 0 0.992493858
KLHDC3 ENSG00000124702.17 0.267982878 1 1 0.267982878
Brain_Caudate_basal_ganglia 0.267982878 Brain_Caudate_basal_ganglia 1 1
1 -1.107719725 -1.107719725 -1.107719725 NA 1 0
0.992493858
TMEM80 ENSG00000177042.14 0.267999738 13 1 0.120753913
Brain_Anterior_cingulate_cortex_BA24 0.453174048 Brain_Substantia_nigra
12.3788854 0.001107417 12.3788854 0.750133927 1.551616917
1.081105717 0.194805644 1 0 0.992493858
LYSMD2 ENSG00000140280.13 0.268028944 2 2 0.173273984
Brain_Anterior_cingulate_cortex_BA24 0.386872866 Brain_Frontal_Cortex_BA9
1.01263016 0.98736984 0.98736984 -1.361758907 -0.865302342 -1.113530624
0.351047803 2 0 0.992493858
OSCP1 ENSG00000116885.18 0.268029135 10 3 0.067491969 Brain_Amygdala
0.80636937 Brain_Hypothalamus 8.26482952 2.78E-17 0.550900781
-0.245112442 1.828383812 0.613134201 0.512086617 3 0

0.992493858
DIP2C ENSG00000151240.16 0.268060492 3 2 0.112112574
Brain_Putamen_basal_ganglia 0.56208457 Brain_Cerebellar_Hemisphere 2.055007622
0.064057499 0.880934879 0.579747998 1.588768913 1.011962507
0.519818155 2 0 0.992493858
RP11-54O7.18 ENSG00000273443.1 0.268094817 3 3 0.079758389
Brain_Cortex 0.558593576 Brain_Frontal_Cortex_BA9 1.083802805
0.920538497 0.920538497 -0.585582474 1.752089676 0.193858468
1.34946785 3 0 0.992493858
ADGRA1 ENSG00000197177.15 0.268151429 4 4 0.045529518
Brain_Cerebellum 0.599804008 Brain_Spinal_cord_cervical_c-1 1.555431609
0.60057363 0.60057363 -0.636820386 1.999729153 0.621384327
1.079014083 4 0 0.992493858
CHURC1 ENSG00000258289.8 0.268246515 11 1 0.207139143
Brain_Cortex 0.387592377 Brain_Hippocampus 10.58281843
0.002013018 10.58281843 -1.261471164 -0.863991833 -1.087144199
0.106772025 1 0 0.992493858
PLCD3 ENSG00000161714.11 0.268300357 2 2 0.142360489 Brain_Cortex
0.427226602 Brain_Hippocampus 1.070580608 0.929419392
0.929419392 0.793946458 1.467057225 1.130501842 0.475961188
2 0 0.992493858
DMKN ENSG00000161249.20 0.268399091 2 2 0.266875394
Brain_Nucleus_accumbens_basal_ganglia 0.803750084 Brain_Caudate_basal_ganglia
1.825234077 0.174765923 0.174765923 -1.110286951 -0.248496751
-0.679391851 0.609377695 2 0 0.992493858
BCAM ENSG00000187244.10 0.268459702 2 2 0.18179046
Brain_Cerebellar_Hemisphere 0.441667865 Brain_Frontal_Cortex_BA9 1.10901249
0.89098751 0.89098751 -0.76937982 1.335262461 0.282941321
1.488206829 2 0 0.992493858
IFIT1 ENSG00000185745.9 0.268669691 7 2 0.39720397
Brain_Spinal_cord_cervical_c-1 0.947111234 Brain_Hypothalamus
6.325630349 0.00275048 0.466246704 -0.709143384 0.846625368
-0.16930393 0.500069931 2 0 0.992493858
PLEKHA6 ENSG00000143850.13 0.268956761 5 4 0.058180903
Brain_Cerebellum 0.704137612 Brain_Spinal_cord_cervical_c-1 3.135157489
0.034852684 0.149325921 0.379741095 1.894332401 1.374064337
0.590332872 4 0 0.992493858
AZIN2 ENSG00000142920.16 0.268985243 1 1 0.268985243 Brain_Cerebellum
0.268985243 Brain_Cerebellum 1 1 1 -1.10540245 -1.10540245
-1.10540245 NA 1 0 0.992493858
CCDC71 ENSG00000177352.9 0.269002512 9 3 0.034769357
Brain_Cerebellar_Hemisphere 0.115072087 Brain_Frontal_Cortex_BA9
7.588426405 0.012511757 0.366277277 1.5757992 2.111034384
1.800041699 0.189154403 3 0 0.992493858
EIF4E ENSG00000151247.12 0.269087093 4 3 0.195357743
Brain_Frontal_Cortex_BA9 0.804482681 Brain_Cerebellar_Hemisphere
2.788895977 0.021866399 0.272183558 -0.374839507 1.294891264
0.489557477 0.717624771 3 0 0.992493858
VIP ENSG00000146469.12 0.269131299 2 2 0.105815368
Brain_Cerebellar_Hemisphere 0.223657419 Brain_Cerebellum 1.710012203
0.289987797 0.289987797 -1.617291526 -1.216860178 -1.417075852
0.283147722 2 0 0.992493858
DOK7 ENSG00000175920.16 0.269216104 6 6 0.126856166
Brain_Hippocampus 0.832840766 Brain_Cerebellar_Hemisphere 1.837916403
0.286201093 0.286201093 -1.417769309 1.526617446 0.195348428
1.141581882 6 0 0.992493858
MINDY2 ENSG00000128923.10 0.269309756 1 1 0.269309756
Brain_Cortex 0.269309756 Brain_Cortex 1 1 1 1.104653509
1.104653509 1.104653509 NA 1 0 0.992493858
TAF1D ENSG00000166012.16 0.269417576 1 1 0.269417576 Brain_Cerebellum
0.269417576 Brain_Cerebellum 1 1 1 1.104404808 1.104404808
1.104404808 NA 1 0 0.992493858

WBSCR17 ENSG00000185274.11 0.269672114 1 1 0.269672114
Brain_Cerebellum 0.269672114 Brain_Cerebellum 1 1 1 -1.103817956
-1.103817956 -1.103817956 NA 1 0 0.992493858
CTD-2620I22.3 ENSG00000267943.1 0.26976923 1 1 0.26976923 Brain_Cerebellum
0.26976923 Brain_Cerebellum 1 1 1 1.10359415 1.10359415 1.10359415 NA
1 0 0.992493858
RRBP1 ENSG00000125844.15 0.269784407 3 3 0.113070807
Brain_Putamen_basal_ganglia 0.548134052 Brain_Cerebellar_Hemisphere
1.402585461 0.600772697 0.600772697 -1.584540349 1.261133218
-0.30798856 1.445220541 3 0 0.992493858
CLCN6 ENSG00000011021.21 0.269892025 4 3 0.207401275 Brain_Cerebellum
0.88328444 Brain_Hippocampus 2.538809058 0.080593688 0.217257177
-1.260743502 -0.146806897 -0.896143623 0.515996577 3 0
0.992493858
ITIH4 ENSG00000055955.15 0.269929643 13 2 0.04519631 Brain_Hippocampus
0.660748193 Brain_Frontal_Cortex_BA9 11.92728446 0.000843018
0.481559416 -2.002822819 -0.438880409 -1.412714112 0.353201647
2 0 0.992493858
CDS2 ENSG00000101290.13 0.270069544 13 10 0.157477878 Brain_Amygdala
0.873330468 Brain_Cerebellum 4.748278117 0.00350426 0.219618449
-1.413605117 1.154375655 -0.173074777 0.942167751 10 0
0.992493858
CLOCK ENSG00000134852.14 0.270131945 1 1 0.270131945
Brain_Hypothalamus 0.270131945 Brain_Hypothalamus 1 1 1
-1.102758753 -1.102758753 -1.102758753 NA 1 0 0.992493858
IGF1 ENSG00000017427.15 0.270136311 1 1 0.270136311
Brain_Cerebellar_Hemisphere 0.270136311 Brain_Cerebellar_Hemisphere 1 1
1 1.102748702 1.102748702 1.102748702 NA 1 0
0.992493858
RP5-1021I20.2 ENSG00000258586.1 0.270145997 10 3 0.195731018
Brain_Spinal_cord_cervical_c-1 0.938207207
Brain_Anterior_cingulate_cortex_BA24 9.199210923 0.002260483 0.30686335
0.077523361 1.293810114 0.846050466 0.467272053 3 0
0.992493858
KARS ENSG00000065427.14 0.270237605 1 1 0.270237605
Brain_Cerebellar_Hemisphere 0.270237605 Brain_Cerebellar_Hemisphere 1 1
1 -1.102515542 -1.102515542 -1.102515542 NA 1 0
0.992493858
RP5-1065P14.2 ENSG00000236975.1 0.270289023 2 2 0.154749945
Brain_Hippocampus 0.39952343 Brain_Putamen_basal_ganglia 1.052200944
0.947799056 0.947799056 -0.842472672 1.422952432 0.29023988
1.601897454 2 0 0.992493858
HSPA4 ENSG00000170606.14 0.270414251 6 1 0.238391711
Brain_Cerebellar_Hemisphere 0.365729799 Brain_Putamen_basal_ganglia
5.861733516 0.000806856 5.861733516 -1.179016236 -0.90450101
-1.088451816 0.096176706 1 0 0.992493858
SMAD6 ENSG00000137834.14 0.270453715 1 1 0.270453715 Brain_Cortex
0.270453715 Brain_Cortex 1 1 1 1.102018302 1.102018302
1.102018302 NA 1 0 0.992493858
KDM5A ENSG00000073614.12 0.270523727 1 1 0.270523727
Brain_Frontal_Cortex_BA9 0.270523727 Brain_Frontal_Cortex_BA9 1 1
1 -1.101857271 -1.101857271 -1.101857271 NA 1 0
0.992493858
DOPEY1 ENSG00000083097.14 0.270583456 1 1 0.270583456
Brain_Anterior_cingulate_cortex_BA24 0.270583456
Brain_Anterior_cingulate_cortex_BA24 1 1 1 1.101719916
1.101719916 1.101719916 NA 1 0 0.992493858
BLOC1S4 ENSG00000186222.4 0.270761028 1 1 0.270761028
Brain_Cortex 0.270761028 Brain_Cortex 1 1 1 -1.101311684
-1.101311684 -1.101311684 NA 1 0 0.992493858
KCNC4 ENSG00000116396.14 0.270778895 3 3 0.101326471
Brain_Cerebellar_Hemisphere 0.859519596 Brain_Cortex 1.464369934

0.535521955 0.535521955 -1.638456646 0.907392494 -0.302683295
1.27757069 3 0 0.992493858
RBM11 ENSG00000185272.13 0.270942008 2 2 0.313004359 Brain_Cerebellum
0.43546779 Brain_Putamen_basal_ganglia 1.382922141 0.617077859
0.617077859 -0.779869334 1.008938023 0.114534344 1.264877812
2 0 0.992493858
MYT1L ENSG00000186487.17 0.271107769 1 1 0.271107769 Brain_Cerebellum
0.271107769 Brain_Cerebellum 1 1 1 1.100515065 1.100515065
1.100515065 NA 1 0 0.992493858
RP11-798G7.6 ENSG00000267198.1 0.2712393 4 4 0.133757513
Brain_Cerebellar_Hemisphere 0.542139636 Brain_Cerebellum 1.296378129
0.726671517 0.726671517 -0.818257987 1.499447753 0.391478214
1.279244719 4 0 0.992493858
ARHGEF25 ENSG00000240771.6 0.271295609 2 2 0.148528166
Brain_Amygdala 0.484936205 Brain_Caudate_basal_ganglia 1.016519564
0.983480436 0.983480436 0.698385399 1.444749836 1.071567618
0.527759355 2 0 0.992493858
NDUFAF3 ENSG00000178057.14 0.271416918 2 2 0.106751825
Brain_Nucleus_accumbens_basal_ganglia 0.584782501 Brain_Amygdala 1.38502776
0.61497224 0.61497224 -1.61296632 -0.546412381 -1.07968935
0.754167523 2 0 0.992493858
KLHDC4 ENSG00000104731.13 0.271561234 10 10 0.043691662
Brain_Putamen_basal_ganglia 0.907944736 Brain_Frontal_Cortex_BA9
2.041876382 0.317974434 0.317974434 -2.017036904 1.842358688
-0.275818705 1.048048785 10 0 0.992493858
KHNYN ENSG00000100441.9 0.271568226 8 8 0.10522812 Brain_Cortex
0.734776455 Brain_Hippocampus 2.776393006 0.140883035
0.140883035 -1.620019351 1.539861767 -0.217284494 0.9930888 8
0 0.992493858
AP001056.1 ENSG00000237604.1 0.27157628 3 3 0.133476276
Brain_Substantia_nigra 0.535082516 Brain_Spinal_cord_cervical_c-1
1.293022125 0.674058842 0.674058842 -0.620266242 1.500533447
0.733707963 1.176012561 3 0 0.992493858
ZHX1 ENSG00000165156.14 0.271602231 1 1 0.271602231 Brain_Cortex
0.271602231 Brain_Cortex 1 1 1 -1.099380273 -1.099380273
-1.099380273 NA 1 0 0.992493858
CD53 ENSG00000143119.12 0.271689622 3 3 0.425065479 Brain_Cortex
0.997873137 Brain_Frontal_Cortex_BA9 1.921040279 0.095261657
0.095261657 -0.562276091 0.797664036 0.079351192 0.683205528
3 0 0.992493858
RP11-1029J19.4 ENSG00000258919.1 0.271766932 1 1 0.271766932
Brain_Amygdala 0.271766932 Brain_Amygdala 1 1 1 1.099002599
1.099002599 1.099002599 NA 1 0 0.992493858
SAMD4A ENSG00000020577.13 0.27181675 1 1 0.27181675
Brain_Cerebellar_Hemisphere 0.27181675 Brain_Cerebellar_Hemisphere 1 1 1
1.098888393 1.098888393 1.098888393 NA 1 0 0.992493858
LINC00421 ENSG00000236834.1 0.271829852 4 4 0.271189255
Brain_Hippocampus 0.809827012 Brain_Anterior_cingulate_cortex_BA24
2.937868719 0.152764747 0.152764747 -0.240649203 1.100327957
0.475294692 0.577630116 4 0 0.992493858
TMEM167B ENSG00000215717.5 0.272151149 2 2 0.119652699
Brain_Cerebellum 0.681168557 Brain_Cortex 1.00930206 0.99069794 0.99069794
-0.410869051 1.556232992 0.572681971 1.390951194 2 0
0.992493858
MXD3 ENSG00000213347.10 0.272362181 13 7 0.094582525
Brain_Frontal_Cortex_BA9 0.901160041 Brain_Putamen_basal_ganglia
7.801498004 0.029702963 0.302075736 -1.671704913 0.907315591
-0.224715738 0.7759832 7 0 0.992493858
CXCL3 ENSG00000163734.4 0.272404388 1 1 0.272404388
Brain_Substantia_nigra 0.272404388 Brain_Substantia_nigra 1 1
1 -1.097542329 -1.097542329 -1.097542329 NA 1 0
0.992493858

ACADL ENSG00000115361.7 0.272558078 2 2 0.150843075
Brain_Cerebellar_Hemisphere 0.258647749 Brain_Frontal_Cortex_BA9
1.300132643 0.699867357 0.699867357 -1.436559935 1.129593031
-0.153483452 1.814544164 2 0 0.992493858
CCNI2 ENSG00000205089.7 0.272584173 1 1 0.272584173 Brain_Amygdala
0.272584173 Brain_Amygdala 1 1 1 1.097130902 1.097130902
1.097130902 NA 1 0 0.992493858
DMGDH ENSG00000132837.14 0.272612018 4 4 0.195118112
Brain_Caudate_basal_ganglia 0.744380193 Brain_Putamen_basal_ganglia
2.792080128 0.112788274 0.112788274 0.326058371 1.295586125
0.754469185 0.407523224 4 0 0.992493858
ANKHD1 ENSG00000131503.20 0.272752198 1 1 0.272752198
Brain_Frontal_Cortex_BA9 0.272752198 Brain_Frontal_Cortex_BA9 1 1
1 1.096746558 1.096746558 1.096746558 NA 1 0
0.992493858
SRXN1 ENSG00000271303.1 0.272789827 2 2 0.130142002
Brain_Caudate_basal_ganglia 0.507218931 Brain_Nucleus_accumbens_basal_ganglia
1.072878202 0.927121798 0.927121798 -1.513542153 -0.663174298
-1.088358225 0.601300877 2 0 0.992493858
PIF1 ENSG00000140451.12 0.272836052 11 1 0.155116777
Brain_Cerebellar_Hemisphere 0.447611932 Brain_Cortex 10.53547138
0.000823964 10.53547138 -1.421688234 -0.75940231 -1.072646359
0.184312106 1 0 0.992493858
RNU12 ENSG00000270022.3 0.272890564 2 2 0.126579976
Brain_Cerebellar_Hemisphere 0.687615367 Brain_Cerebellum 1.071883434
0.928116566 0.928116566 -1.527728438 -0.402093295 -0.964910867
0.795944243 2 0 0.992493858
IP6K2 ENSG00000068745.14 0.272966632 11 8 0.029962706 Brain_Amygdala
0.8660229 Brain_Cerebellum 6.026243918 0.037221316 0.262798414
-2.170583034 1.167488669 -0.573833492 1.103889324 8 0
0.992493858
SPATA25 ENSG00000149634.4 0.272976323 1 1 0.272976323
Brain_Hippocampus 0.272976323 Brain_Hippocampus 1 1 1
1.096234139 1.096234139 1.096234139 NA 1 0 0.992493858
FCN2 ENSG00000160339.15 0.273005045 1 1 0.273005045
Brain_Anterior_cingulate_cortex_BA24 0.273005045
Brain_Anterior_cingulate_cortex_BA24 1 1 1 1.096168494
1.096168494 1.096168494 NA 1 0 0.992493858
HLA-DRA ENSG00000204287.13 0.273096166 4 3 0.114430838
Brain_Caudate_basal_ganglia 0.966230327 Brain_Frontal_Cortex_BA9
3.007110417 0.063427206 0.314268285 -1.578586916 0.042336652
-0.660553695 0.682635279 3 0 0.992493858
KIAA0319 ENSG00000137261.13 0.273131408 3 3 0.058575713
Brain_Cortex 0.616409477 Brain_Nucleus_accumbens_basal_ganglia
1.496276102 0.518608887 0.518608887 0.500945503 1.891364431
1.018278141 0.760418763 3 0 0.992493858
MLXIPL ENSG00000009950.15 0.273206118 4 4 0.111587366
Brain_Nucleus_accumbens_basal_ganglia 0.8259291 Brain_Cerebellum 1.267119692
0.791798175 0.791798175 -1.586282227 1.591098697 -0.159679775
1.313228657 4 0 0.992493858
DOCK2 ENSG00000134516.15 0.273264331 2 2 0.113100338
Brain_Spinal_cord_cervical_c-1 0.822805115 Brain_Hypothalamus
1.041725235 0.958274765 0.958274765 -1.584410484 -0.223938555
-0.904174519 0.961998927 2 0 0.992493858
KIAA1109 ENSG00000138688.15 0.273327066 5 4 0.063578513
Brain_Nucleus_accumbens_basal_ganglia 0.424287933 Brain_Caudate_basal_ganglia
3.896449065 0.055276469 0.185196385 -1.855124059 -0.799004276
-1.275933514 0.437170369 4 0 0.992493858
CCDC190 ENSG00000185860.13 0.273380862 9 4 0.053279103
Brain_Nucleus_accumbens_basal_ganglia 0.919441297 Brain_Substantia_nigra
6.599655664 0.010005537 0.302259467 -1.932651833 -0.101137516
-1.404420718 0.569455675 4 0 0.992493858

RABAC1 ENSG00000105404.10 0.273497736 3 2 0.59081022 Brain_Cortex
0.792674108 Brain_Nucleus_accumbens_basal_ganglia 2.777775825
0.020932874 0.201291301 -0.452787194 0.537662288 -0.059321547
0.525654148 2 0 0.992493858
SLC14A1 ENSG00000141469.16 0.273635814 4 4 0.133384441
Brain_Cerebellum 0.573218931 Brain_Nucleus_accumbens_basal_ganglia
1.952350282 0.454322238 0.454322238 -1.500888355 0.92683626
-0.196436354 1.136560576 4 0 0.992493858
YARS ENSG00000134684.10 0.273649469 1 1 0.273649469 Brain_Cortex
0.273649469 Brain_Cortex 1 1 1 1.094696851 1.094696851
1.094696851 NA 1 0 0.992493858
AAMDC ENSG00000087884.14 0.273687903 4 4 0.03203148 Brain_Amygdala
0.800578423 Brain_Hypothalamus 1.8306117 0.405384365 0.405384365
-2.144017558 -0.252598586 -1.298650413 0.826478891 4 0
0.992493858
UHRF2 ENSG00000147854.16 0.2737733 1 1 0.2737733 Brain_Cerebellum 0.2737733
Brain_Cerebellum 1 1 1 1.094414333 1.094414333 1.094414333
NA 1 0 0.992493858
AP1M2 ENSG00000129354.11 0.273808158 2 2 0.154299178
Brain_Frontal_Cortex_BA9 0.915439968 Brain_Cortex 1.406126322
0.593873678 0.593873678 -1.424509012 0.106179459 -0.659164776
1.082360198 2 0 0.992493858
ZFAT ENSG00000066827.15 0.273850941 2 2 0.107517079 Brain_Cortex
0.33524783 Brain_Frontal_Cortex_BA9 1.598557415 0.401442585
0.401442585 -1.609454121 -0.963597362 -1.286525741 0.456689693
2 0 0.992493858
RASGRP3 ENSG00000152689.17 0.273880653 7 6 0.25298885 Brain_Cortex
0.743823268 Brain_Spinal_cord_cervical_c-1 4.426303465 0.069002335
0.219714061 -1.143119811 0.751798204 -0.393223525 0.748676823
6 0 0.992493858
TFAP2B ENSG00000008196.12 0.274103643 1 1 0.274103643
Brain_Cerebellum 0.274103643 Brain_Cerebellum 1 1 1 -1.093661095
-1.093661095 -1.093661095 NA 1 0 0.992493858
LINC01197 ENSG00000248441.6 0.274186056 5 5 0.085695228
Brain_Frontal_Cortex_BA9 0.417705468 Brain_Cortex 1.804319655
0.210211533 0.210211533 -1.19443411 1.718556116 -0.134890946
1.322752986 5 0 0.992493858
C21orf59 ENSG00000159079.18 0.274249961 2 2 0.117791689
Brain_Frontal_Cortex_BA9 0.205434779 Brain_Caudate_basal_ganglia
1.621470799 0.378529201 0.378529201 -1.564110208 -1.266218742
-1.415164475 0.210641076 2 0 0.992493858
RP11-475B2.1 ENSG00000261672.1 0.274312194 2 2 0.223282707
Brain_Cerebellum 0.479059583 Brain_Cerebellar_Hemisphere 1.919771976
0.080228024 0.080228024 -1.217845454 -0.707815913 -0.962830684
0.360645347 2 0 0.992493858
ZNF17 ENSG00000186272.12 0.274340987 11 2 0.022142048
Brain_Anterior_cingulate_cortex_BA24 0.345086864 Brain_Cortex
9.353176115 0.001418119 0.60345954 0.944162013 2.287922264
1.457751149 0.369268923 2 0 0.992493858
CD72 ENSG00000137101.12 0.27434236 5 5 0.037242624
Brain_Frontal_Cortex_BA9 0.989139227 Brain_Cerebellum 2.559286141
0.168870321 0.168870321 -0.013612381 2.083094353 0.980845741
0.866657786 5 0 0.992493858
GOLM1 ENSG00000135052.16 0.274432551 4 4 0.038998834
Brain_Hippocampus 0.848024429 Brain_Cerebellum 1.812325249
0.230231123 0.230231123 0.191639717 2.064199192 1.289892961
0.912223856 4 0 0.992493858
GATS ENSG00000239521.7 0.274447263 6 5 0.108044779 Brain_Amygdala
0.806729923 Brain_Cortex 3.3584374 0.091206709 0.208918141
-0.821487441 1.607043713 0.617067412 0.867996543 5 0
0.992493858
C5orf22 ENSG00000082213.17 0.274655747 1 1 0.274655747

Brain_Cerebellar_Hemisphere 0.274655747 Brain_Cerebellar_Hemisphere 1 1 1 -1.092403587 -1.092403587 -1.092403587 NA 1 0 0.992493858
SRGAP1 ENSG00000196935.8 0.274704152 2 2 0.192374814 Brain_Spinal_cord_cervical_c-1 0.376678901 Brain_Anterior_cingulate_cortex_BA24 1.042757296 0.957242704 0.957242704 0.884032071 1.303585889 1.09380898 0.29666935 2 0 0.992493858
ZRANB3 ENSG00000121988.17 0.274825056 3 3 0.153461673 Brain_Hippocampus 0.39416765 Brain_Frontal_Cortex_BA9 1.865243753 0.134230845 0.134230845 -1.427410266 1.013838257 -0.421885229 1.276215741 3 0 0.992493858
C20orf96 ENSG00000196476.11 0.274857542 2 2 0.34488901 Brain_Cerebellum 0.531977882 Brain_Cerebellar_Hemisphere 1.516845675 0.483154325 0.483154325 -0.944549322 0.624989603 -0.159779859 1.109831617 2 0 0.992493858
LATS1 ENSG00000131023.12 0.275059396 4 3 0.070996272 Brain_Spinal_cord_cervical_c-1 0.107692945 Brain_Cerebellum 3.491386269 0.026746529 0.150192633 -1.8055013 -1.608649768 -1.730379126 0.086708845 3 0 0.992493858
TLL2 ENSG00000095587.8 0.275089294 3 3 0.185916015 Brain_Frontal_Cortex_BA9 0.690467696 Brain_Cerebellar_Hemisphere 1.119056897 0.874214691 0.874214691 0.398220446 1.322757558 1.007512737 0.527769034 3 0 0.992493858
PQLC1 ENSG00000122490.18 0.275159225 8 8 0.093999068 Brain_Cortex 0.972868355 Brain_Spinal_cord_cervical_c-1 3.730809873 0.176965878 0.176965878 -0.207920839 1.674669635 0.487965919 0.696328851 8 0 0.992493858
CEP89 ENSG00000121289.17 0.27519849 12 5 0.133817416 Brain_Cerebellar_Hemisphere 0.961548573 Brain_Hypothalamus 8.21279069 0.004441615 0.333670498 -1.072672853 1.49921673 0.319326437 0.782084443 5 0 0.992493858
USP53 ENSG00000145390.11 0.275245878 3 3 0.054782375 Brain_Cortex 0.597140577 Brain_Anterior_cingulate_cortex_BA24 2.091566292 0.279234415 0.279234415 -1.920598254 -0.528516962 -1.120301536 0.719083143 3 0 0.992493858
RP11-47I22.2 ENSG00000250548.6 0.275252342 2 2 0.384376973 Brain_Cerebellar_Hemisphere 0.958939033 Brain_Cerebellum 1.822887401 0.177112599 0.177112599 -0.869859898 0.051485027 -0.409187435 0.651489244 2 0 0.992493858
GSAP ENSG00000186088.15 0.275448424 11 7 0.06323694 Brain_Putamen_basal_ganglia 0.999243274 Brain_Nucleus_accumbens_basal_ganglia 7.248147228 0.024524172 0.272176651 -1.857521888 0.260072722 -0.701923736 0.682928637 7 0 0.992493858
PCGF3 ENSG00000185619.18 0.275460099 12 3 0.034482614 Brain_Anterior_cingulate_cortex_BA24 0.770192606 Brain_Nucleus_accumbens_basal_ganglia 9.876754029 0.011098661 0.647390412 0.292122968 2.114382466 1.276846906 0.50243476 3 0 0.992493858
TSPAN33 ENSG00000158457.5 0.27550311 2 2 0.146668929 Brain_Cerebellum 0.674018465 Brain_Cerebellar_Hemisphere 1.649601101 0.350398899 0.350398899 -1.451398494 -0.420639336 -0.936018915 0.72885679 2 0 0.992493858
SURF4 ENSG00000148248.13 0.275544777 1 1 0.275544777 Brain_Amygdala 0.275544777 Brain_Amygdala 1 1 1 -1.090382297 -1.090382297 -1.090382297 NA 1 0 0.992493858
CSMD3 ENSG00000164796.17 0.275587505 4 4 0.028956338 Brain_Caudate_basal_ganglia 0.84103017 Brain_Spinal_cord_cervical_c-1 1.819235431 0.387198342 0.387198342 -2.184080439 -0.200575943 -0.974626531 0.847949613 4 0 0.992493858
ABHD3 ENSG00000158201.9 0.275589168 1 1 0.275589168 Brain_Spinal_cord_cervical_c-1 0.275589168 Brain_Spinal_cord_cervical_c-1

1 1 1 -1.090281487 -1.090281487 -1.090281487 NA 1 0
0.992493858
WDR45B ENSG00000141580.15 0.275599492 6 5 0.035045509
Brain_Hippocampus 0.421369656 Brain_Putamen_basal_ganglia 3.962281535
0.115634124 0.200326617 -2.107832172 -0.804047304 -1.721051064
0.462221939 5 0 0.992493858
SELENON ENSG00000162430.16 0.275693564 3 3 0.066838175
Brain_Cerebellar_Hemisphere 0.684747227 Brain_Amygdala 1.341002378
0.782028642 0.782028642 0.405993703 1.83276064 0.927235948
0.787191668 3 0 0.992493858
CCKBR ENSG00000110148.9 0.275706289 3 3 0.107113854
Brain_Frontal_Cortex_BA9 0.595363647 Brain_Hippocampus 1.067259184
0.923544864 0.923544864 -1.611302282 -0.531079561 -1.050425404
0.541307585 3 0 0.992493858
OSBPL3 ENSG00000070882.12 0.275750599 3 3 0.057162101
Brain_Cortex 0.866730804 Brain_Caudate_basal_ganglia 1.26659578
0.673556049 0.673556049 -1.902069255 -0.16781248 -0.976913243
0.872933706 3 0 0.992493858
RP11-326C3.15 ENSG00000270972.1 0.275858876 8 8 0.024588523
Brain_Putamen_basal_ganglia 0.866811186 Brain_Amygdala 1.733383458
0.297426553 0.297426553 -1.717509944 2.247806637 -0.329604658
1.23774101 8 0 0.992493858
TULP4 ENSG00000130338.12 0.275869505 3 2 0.103717872 Brain_Cerebellum
0.424499934 Brain_Hypothalamus 2.227384993 0.030398001
0.742217006 -0.798638712 1.627090531 0.789316442 1.375906724
2 0 0.992493858
TMEM141 ENSG00000244187.7 0.275903096 3 3 0.079875933
Brain_Nucleus_accumbens_basal_ganglia 0.760783782 Brain_Substantia_nigra
1.464439766 0.536582085 0.536582085 -1.751406392 0.304451704
-1.005090303 1.137785438 3 0 0.992493858
ARHGDIB ENSG00000111348.8 0.275912619 3 3 0.108960799
Brain_Substantia_nigra 0.740729375 Brain_Cortex 1.208015539
0.845653397 0.845653397 -0.330887618 1.602881592 0.769947847
0.994331101 3 0 0.992493858
TRIO ENSG00000038382.18 0.27593977 3 3 0.052357144
Brain_Frontal_Cortex_BA9 0.944149878 Brain_Substantia_nigra
1.103734425 0.940334872 0.940334872 0.070055007 1.940185551
0.726177371 1.05250867 3 0 0.992493858
BRK1 ENSG00000254999.3 0.275984579 1 1 0.275984579
Brain_Caudate_basal_ganglia 0.275984579 Brain_Caudate_basal_ganglia 1 1
1 1.089384012 1.089384012 1.089384012 NA 1 0
0.992493858
TMEM214 ENSG00000119777.18 0.276131457 1 1 0.276131457
Brain_Nucleus_accumbens_basal_ganglia 0.276131457
Brain_Nucleus_accumbens_basal_ganglia 1 1 1 -1.089050862
-1.089050862 -1.089050862 NA 1 0 0.992493858
IMPAD1 ENSG00000104331.8 0.276271592 3 3 0.058185123
Brain_Cerebellum 0.975477116 Brain_Cerebellar_Hemisphere 1.211989677
0.731671091 0.731671091 -1.894300587 0.568809537 -0.452076922
1.284473297 3 0 0.992493858
ZDHHC21 ENSG00000175893.11 0.276272433 5 4 0.025463143
Brain_Cerebellum 0.731863562 Brain_Hypothalamus 3.623943383
0.028351882 0.132679926 -2.234303 -0.342647634 -1.615388743
0.732950638 4 0 0.992493858
RTN4RL2 ENSG00000186907.7 0.276476596 2 2 0.121793792
Brain_Cerebellar_Hemisphere 0.365092886 Brain_Cerebellum 1.76162578 0.23837422
0.23837422 0.905703356 1.54728809 1.226495723 0.453668916 2 0
0.992493858
NPPA ENSG00000175206.10 0.276520428 1 1 0.276520428 Brain_Amygdala
0.276520428 Brain_Amygdala 1 1 1 -1.08816918 -1.08816918
-1.08816918 NA 1 0 0.992493858
CORO2B ENSG00000103647.12 0.276587819 1 1 0.276587819

Brain_Caudate_basal_ganglia 0.276587819 Brain_Caudate_basal_ganglia 1 1
1 1.08801651 1.08801651 1.08801651 NA 1 0 0.992493858
VSIG2 ENSG00000019102.11 0.276588797 3 3 0.089932301
Brain_Nucleus_accumbens_basal_ganglia 0.835167591 Brain_Frontal_Cortex_BA9
1.022389272 0.967535657 0.967535657 -1.695754931 0.960145837
-0.314562567 1.331148507 3 0 0.992493858
NET1 ENSG00000173848.18 0.276643135 2 1 0.272067422
Brain_Cerebellar_Hemisphere 0.296355406 Brain_Cerebellum 1.939452799
0.060547201 1.939452799 1.044280988 1.098313952 1.07129747
0.038207075 1 0 0.992493858
CERCAM ENSG00000167123.18 0.276650577 4 3 0.159517918
Brain_Cerebellum 0.683595435 Brain_Cortex 2.410925602 0.000220547
0.322572668 -1.146751068 1.406694767 0.31210326 1.29347128 3 0
0.992493858
C9orf43 ENSG00000157653.11 0.276733898 2 2 0.23258194
Brain_Spinal_cord_cervical_c-1 0.301394001 Brain_Hypothalamus
1.030018323 0.969981677 0.969981677 -1.033448625 1.193734245
0.08014281 1.57485611 2 0 0.992493858
STXBP5 ENSG00000164506.14 0.276892236 1 1 0.276892236
Brain_Cortex 0.276892236 Brain_Cortex 1 1 1 -1.08732719
-1.08732719 -1.08732719 NA 1 0 0.992493858
LINC01224 ENSG00000269416.5 0.276929014 12 1 0.209819469
Brain_Hippocampus 0.559705489 Brain_Substantia_nigra 11.44166425
0.001885692 11.44166425 -1.254062004 -0.583279012 -1.058508471
0.182527822 1 0 0.992493858
CLEC4M ENSG00000104938.16 0.276933988 5 5 0.022950241
Brain_Cerebellar_Hemisphere 0.916967262 Brain_Cerebellum 1.282984443
0.662827682 0.662827682 -2.274261964 0.402465761 -0.738333434
1.016198044 5 0 0.992493858
UPP2 ENSG00000007001.12 0.276953143 3 3 0.096631278
Brain_Putamen_basal_ganglia 0.982193072 Brain_Hypothalamus 1.416866172
0.571419721 0.571419721 0.022319527 1.661409267 1.051783173
0.896577643 3 0 0.992493858
HSD17B1 ENSG00000108786.10 0.27701154 8 3 0.051271282
Brain_Substantia_nigra 0.320999821 Brain_Amygdala 6.704396861
0.000626012 0.426608663 -1.949202203 -0.992405368 -1.240897117
0.329968122 3 0 0.992493858
RUBCN ENSG00000145016.15 0.277210474 1 1 0.277210474 Brain_Cerebellum
0.277210474 Brain_Cerebellum 1 1 1 -1.086607128 -1.086607128
-1.086607128 NA 1 0 0.992493858
RTP1 ENSG00000175077.5 0.27725899 2 2 0.120690698 Brain_Cortex
0.866265879 Brain_Putamen_basal_ganglia 1.144352564 0.855647436
0.855647436 -1.551881009 0.16840347 -0.69173877 1.21642482 2 0
0.992493858
ZNF473 ENSG00000142528.15 0.277305295 2 2 0.123098096
Brain_Nucleus_accumbens_basal_ganglia 0.713059485 Brain_Cortex
1.042313385 0.957686615 0.957686615 0.367750227 1.541899177
0.954824702 0.830248684 2 0 0.992493858
COPB2 ENSG00000184432.9 0.277413154 3 3 0.098206655
Brain_Hippocampus 0.513681202 Brain_Cerebellum 1.04538949 0.951306996
0.951306996 -1.653610614 0.790613367 -0.069960263 1.373203436
3 0 0.992493858
GPR62 ENSG00000180929.5 0.27742019 2 2 0.197291113
Brain_Caudate_basal_ganglia 0.38786518 Brain_Substantia_nigra 1.065367063
0.934632937 0.934632937 -0.863495341 1.289307765 0.212906212
1.522261675 2 0 0.992493858
VAV3 ENSG00000134215.15 0.277505513 4 4 0.024297669
Brain_Nucleus_accumbens_basal_ganglia 0.878038511 Brain_Cortex
2.058238962 0.1836516 0.1836516 -2.252389491 -0.153456222
-1.137323028 1.033424807 4 0 0.992493858
G2E3 ENSG00000092140.14 0.277591593 1 1 0.277591593 Brain_Cortex
0.277591593 Brain_Cortex 1 1 1 1.085745525 1.085745525

1.085745525	NA	1	0	0.992493858
RP11-352G9.1	ENSG00000273009.1	0.277642203	3	3	0.080561979
Brain_Cerebellum	0.227717446	Brain_Cerebellar_Hemisphere	2.161829638
0.136329833	0.136329833	1.206259506	1.747434606	1.545430644
0.295514379	3	0	0.992493858
RPL34-AS1	ENSG00000234492.4	0.277669731	2	2	0.172449682
Brain_Cerebellum	0.425834507	Brain_Cerebellar_Hemisphere	1.877730133
0.122269867	0.122269867	-1.364374649	-0.796339888	-1.080357268
0.401661232	2	0	0.992493858
CGB2	ENSG00000104818.14	0.277762535	3	3	0.062910306
Brain_Nucleus_accumbens_basal_ganglia	0.731463185	Brain_Caudate_basal_ganglia
1.142458214	0.826772648	0.826772648	-0.665141742	1.859824882
0.512620987	1.270982629	3	0	0.992493858
CTB-32O4.4	ENSG00000276631.1	0.277800326	1	1	0.277800326
Brain_Substantia_nigra	0.277800326	Brain_Substantia_nigra	1	1
1	-1.085273981	-1.085273981	-1.085273981	NA	1	0
0.992493858
AC138969.4	ENSG00000183889.12	0.277804065	1	1	0.277804065
Brain_Hippocampus	0.277804065	Brain_Hippocampus	1	1	1
-1.085265536	-1.085265536	-1.085265536	NA	1	0	0.992493858
LINC01990	ENSG00000273125.1	0.277892281	4	4	0.102772349
Brain_Nucleus_accumbens_basal_ganglia	0.809022875	Brain_Caudate_basal_ganglia
1.276689136	0.760105647	0.760105647	-0.241686779	1.631559341
0.831735027	0.842956939	4	0	0.992493858
DLEU7	ENSG00000186047.9	0.27800486	4	4	0.127556703	Brain_Cortex
0.850520231	Brain_Hypothalamus	1.897483795	0.266309647
0.266309647	-1.523807911	-0.18845469	-0.906823014	0.705823336
4	0	0.992493858
POLR2L	ENSG00000177700.5	0.278086369	2	1	0.208561749
Brain_Nucleus_accumbens_basal_ganglia	0.378726468	Brain_Hippocampus
1.94237179	0.05762821	1.94237179	0.880245244	1.257530093	1.068887669
0.266780675	1	0	0.992493858
LINC01703	ENSG00000225518.2	0.278214771	1	1	0.278214771
Brain_Cerebellar_Hemisphere	0.278214771	Brain_Cerebellar_Hemisphere	1	1
1	-1.084338433	-1.084338433	-1.084338433	NA	1	0
0.992493858
SERPINI2	ENSG00000114204.14	0.278453019	5	4	0.250730098
Brain_Cerebellum	0.894074281	Brain_Caudate_basal_ganglia	3.085542308
0.060231275	0.171230414	-0.443208867	1.148577843	0.445450978
0.65109065	4	0	0.992493858
RHOU	ENSG00000116574.5	0.278459401	1	1	0.278459401
Brain_Putamen_basal_ganglia	0.278459401	Brain_Putamen_basal_ganglia	1	1
1	1.08378666	1.08378666	1.08378666	NA	1	0	0.992493858
SAP30BP	ENSG00000161526.14	0.278459878	1	1	0.278459878
Brain_Hippocampus	0.278459878	Brain_Hippocampus	1	1	1
1.083785585	1.083785585	1.083785585	NA	1	0	0.992493858
SERTAD4	ENSG00000082497.11	0.278485258	1	1	0.278485258
Brain_Spinal_cord_cervical_c-1	0.278485258	Brain_Spinal_cord_cervical_c-1
1	1	1	1.083728358	1.083728358	1.083728358	NA	1	0
0.992493858
SH3BGRL3	ENSG00000142669.14	0.278597063	1	1	0.278597063
Brain_Cerebellum	0.278597063	Brain_Cerebellum	1	1	1	-1.083476305
-1.083476305	-1.083476305	NA	1	0	0.992493858
PTPMT1	ENSG00000110536.13	0.278600406	2	2	0.216951877
Brain_Nucleus_accumbens_basal_ganglia	0.345736536	Brain_Caudate_basal_ganglia
1.057561199	0.942438801	0.942438801	-1.234673943	0.94289124
-0.145891351	1.539771108	2	0	0.992493858
C17orf80	ENSG00000141219.15	0.278602981	2	2	0.11336142
Brain_Hypothalamus	0.164459463	Brain_Cerebellar_Hemisphere	1.792654475
0.207345525	0.207345525	1.390228627	1.583263494	1.48674606
0.136496264	2	0	0.992493858
PLCB3	ENSG00000149782.11	0.278694567	1	1	0.278694567

Brain_Frontal_Cortex_BA9 0.278694567 Brain_Frontal_Cortex_BA9 1 1 1 -1.083256547 -1.083256547 -1.083256547 NA 1 0 0.992493858
RNF31 ENSG00000092098.16 0.278954829 2 2 0.278506384 Brain_Nucleus_accumbens_basal_ganglia 0.384686457 Brain_Cerebellar_Hemisphere 1.247707892 0.752292108 0.752292108 -0.869293794 1.083680726 0.107193466 1.380961527 2 0 0.992493858
GMEB1 ENSG00000162419.12 0.279007903 1 1 0.279007903 Brain_Spinal_cord_cervical_c-1 0.279007903 Brain_Spinal_cord_cervical_c-1 1 1 1 1.082550694 1.082550694 1.082550694 NA 1 0 0.992493858
THAP8 ENSG00000161277.10 0.279025408 3 3 0.181238419 Brain_Hippocampus 0.958299936 Brain_Substantia_nigra 2.435947284 0.165547609 0.165547609 -1.336951652 0.145555142 -0.379703138 0.830312152 3 0 0.992493858
THNSL2 ENSG00000144115.16 0.279097963 13 1 0.211852687 Brain_Cerebellar_Hemisphere 0.433877854 Brain_Putamen_basal_ganglia 12.68227632 0.000556669 12.68227632 0.782573082 1.248487215 1.068348686 0.150174829 1 0 0.992493858
YWHAE ENSG00000108953.16 0.279181977 2 2 0.142794109 Brain_Cortex 0.282229989 Brain_Frontal_Cortex_BA9 1.323233989 0.676766011 0.676766011 -1.465464949 -1.075323343 -1.270394146 0.275871775 2 0 0.992493858
MANSC1 ENSG00000111261.13 0.279252891 1 1 0.279252891 Brain_Amygdala 0.279252891 Brain_Amygdala 1 1 1 1.081999183 1.081999183 1.081999183 NA 1 0 0.992493858
INPP5B ENSG00000204084.12 0.279412824 12 5 0.022690416 Brain_Putamen_basal_ganglia 0.745802787 Brain_Cerebellum 8.669328572 0.000667026 0.333540489 -2.278607362 -0.324178647 -1.158535864 0.600646548 5 0 0.992493858
ZNF343 ENSG00000088876.11 0.279436143 1 1 0.279436143 Brain_Cerebellar_Hemisphere 0.279436143 Brain_Cerebellar_Hemisphere 1 1 1 -1.081586866 -1.081586866 -1.081586866 NA 1 0 0.992493858
RP11-245J9.5 ENSG00000271843.1 0.279438 2 2 0.183914641 Brain_Cortex 0.376924363 Brain_Cerebellum 1.001503647 0.998496353 0.998496353 -1.328797942 -0.883577437 -1.106187689 0.314818438 2 0 0.992493858
RP11-433J8.1 ENSG00000258702.1 0.279621225 9 9 0.024181392 Brain_Hippocampus 0.931227138 Brain_Amygdala 2.439230851 0.233217206 0.233217206 -2.254234941 1.356353045 -0.555338121 1.03147776 9 0 0.992493858
TTLL10 ENSG00000162571.13 0.279679746 1 1 0.279679746 Brain_Putamen_basal_ganglia 0.279679746 Brain_Putamen_basal_ganglia 1 1 1 -1.081039044 -1.081039044 -1.081039044 NA 1 0 0.992493858
GRB7 ENSG00000141738.13 0.27968691 2 2 0.139828932 Brain_Spinal_cord_cervical_c-1 0.427096946 Brain_Hypothalamus 1.130345905 0.869654095 0.869654095 -0.794169184 1.476428367 0.341129592 1.605554925 2 0 0.992493858
NCAPG ENSG00000109805.9 0.279830548 1 1 0.279830548 Brain_Nucleus_accumbens_basal_ganglia 0.279830548 Brain_Nucleus_accumbens_basal_ganglia 1 1 1 1.08070008 1.08070008 1.08070008 NA 1 0 0.992493858
CHML ENSG00000203668.2 0.279873467 3 3 0.062025379 Brain_Nucleus_accumbens_basal_ganglia 0.741774746 Brain_Cortex 1.343999004 0.648852198 0.648852198 -0.329504033 1.866114279 0.890326713 1.117968134 3 0 0.992493858
DNAJC6 ENSG00000116675.15 0.280026593 3 3 0.132678391 Brain_Caudate_basal_ganglia 0.796342304 Brain_Cortex 1.298085312 0.770098611 0.770098611 -1.503623289 1.117328442 -0.042737046 1.336120069 3 0 0.992493858

WWC2 ENSG00000151718.15 0.280139614 1 1 0.280139614 Brain_Cortex
0.280139614 Brain_Cortex 1 1 1 -1.080005762 -1.080005762
-1.080005762 NA 1 0 0.992493858
TMCO3 ENSG00000150403.17 0.280238101 11 4 0.131061923
Brain_Frontal_Cortex_BA9 0.805233085 Brain_Cerebellum 8.617246083
0.009033336 0.376540646 -0.433681307 1.509927508 0.915160415
0.581072104 4 0 0.992493858
SAT2 ENSG00000141504.11 0.280275613 8 2 0.44761733 Brain_Cortex
0.738791669 Brain_Putamen_basal_ganglia 7.37892256 0.002003255
0.327734312 -0.333453918 0.759393285 0.520955734 0.360631746
2 0 0.992493858
SCN4A ENSG00000007314.12 0.280283044 1 1 0.280283044
Brain_Anterior_cingulate_cortex_BA24 0.280283044
Brain_Anterior_cingulate_cortex_BA24 1 1 1 1.079683725
1.079683725 1.079683725 NA 1 0 0.992493858
TICAM2 ENSG00000243414.5 0.280365083 2 2 0.155887029
Brain_Caudate_basal_ganglia 0.31925771 Brain_Nucleus_accumbens_basal_ganglia
1.912112788 0.087887212 0.087887212 -1.419041116 -0.995984431
-1.207512773 0.299146251 2 0 0.992493858
SOCS1 ENSG00000185338.4 0.280370935 1 1 0.280370935
Brain_Caudate_basal_ganglia 0.280370935 Brain_Caudate_basal_ganglia 1 1
1 -1.079486441 -1.079486441 -1.079486441 NA 1 0
0.992493858
OR51E2 ENSG00000167332.7 0.28041652 2 2 0.113417526 Brain_Cortex
0.190870167 Brain_Frontal_Cortex_BA9 1.88326149 0.11673851 0.11673851
-1.58301728 -1.308009265 -1.445513273 0.194460033 2 0
0.992493858
ZNF880 ENSG00000221923.8 0.280483709 13 1 0.191997094
Brain_Cerebellum 0.508266196 Brain_Cortex 12.6264254 8.69E-06 12.6264254
0.661539805 1.304693916 1.061810577 0.225481007 1 0
0.992493858
KANK1 ENSG00000107104.18 0.280527805 3 3 0.145631127
Brain_Caudate_basal_ganglia 0.906593295 Brain_Cerebellum 1.888447789
0.115104107 0.115104107 -0.117336636 1.455137762 0.628579837
0.789332766 3 0 0.992493858
PAIP2B ENSG00000124374.8 0.280707326 3 3 0.089055729
Brain_Caudate_basal_ganglia 0.98114205 Brain_Putamen_basal_ganglia 2.151382629
0.222717824 0.222717824 -1.700399909 -0.023637136 -0.601932227
0.95174471 3 0 0.992493858
UBL7 ENSG00000138629.15 0.280710457 1 1 0.280710457 Brain_Cerebellum
0.280710457 Brain_Cerebellum 1 1 1 1.078724732 1.078724732
1.078724732 NA 1 0 0.992493858
MSRB3 ENSG00000174099.10 0.280783896 3 3 0.158690513 Brain_Amygdala
0.5076848 Brain_Caudate_basal_ganglia 1.064486902 0.932093621
0.932093621 -1.237466601 1.409489374 -0.163474737 1.392237035
3 0 0.992493858
LRRIQ3 ENSG00000162620.15 0.280802814 11 1 0.164105791
Brain_Anterior_cingulate_cortex_BA24 0.579820119
Brain_Nucleus_accumbens_basal_ganglia 10.68069527 0.001120855
10.68069527 0.553647489 1.391394632 1.062081588 0.269127502
1 0 0.992493858
KPNA5 ENSG00000196911.10 0.280933584 2 1 0.240843814 Brain_Cerebellum
0.34069779 Brain_Cerebellar_Hemisphere 1.943311271 0.056688729
1.943311271 -1.172880292 -0.95278742 -1.062833856 0.155629162
1 0 0.992493858
GPR63 ENSG00000112218.8 0.28098523 2 2 0.245929407 Brain_Cortex
0.267811653 Brain_Cerebellar_Hemisphere 1.013917041 0.986082959
0.986082959 -1.108116159 1.16029331 0.026088575 1.604007718 2
0 0.992493858
ZNF559-ZNF177 ENSG00000270011.6 0.281072433 2 2 0.111953305
Brain_Putamen_basal_ganglia 0.824404121 Brain_Cerebellar_Hemisphere
1.071264545 0.928735455 0.928735455 -0.221884084 1.589474506

0.683795211 1.280823942 2 0 0.992493858
POMZP3 ENSG00000146707.14 0.281102186 13 2 0.083655195
Brain_Frontal_Cortex_BA9 0.726404404 Brain_Spinal_cord_cervical_c-1
11.88606221 0.000954102 0.560434934 0.349912449 1.72986054
1.226055161 0.348669269 2 0 0.992493858
KCNAB1 ENSG00000169282.17 0.281155838 3 3 0.067868258
Brain_Cerebellar_Hemisphere 0.827281257 Brain_Spinal_cord_cervical_c-1
1.915756282 0.128601497 0.128601497 0.218189768 1.825880543
1.286803022 0.925458522 3 0 0.992493858
CEP128 ENSG00000100629.16 0.281195838 4 4 0.199145604
Brain_Hypothalamus 0.996322116 Brain_Cerebellar_Hemisphere 2.153540794
0.362894844 0.362894844 0.004609561 1.283989573 0.873411592
0.585953622 4 0 0.992493858
TNKS1BP1 ENSG00000149115.13 0.281197878 1 1 0.281197878
Brain_Frontal_Cortex_BA9 0.281197878 Brain_Frontal_Cortex_BA9 1 1
1 1.077632308 1.077632308 1.077632308 NA 1 0
0.992493858
RP11-212P7.2 ENSG00000273270.1 0.281206693 13 2 0.083421978
Brain_Cerebellar_Hemisphere 0.941401351 Brain_Putamen_basal_ganglia
11.25384355 4.29E-05 0.651392758 -1.731167029 -0.073508662
-0.811218863 0.433684247 2 0 0.992493858
AMIGO1 ENSG00000181754.6 0.281310892 10 8 0.20753219 Brain_Cerebellum
0.988207344 Brain_Putamen_basal_ganglia 5.488130499 0.017887575
0.220247996 -1.260380341 1.151051443 0.262100544 0.776709793
8 0 0.992493858
CCT3 ENSG00000163468.14 0.281332976 2 1 0.282558074
Brain_Cerebellar_Hemisphere 0.284435569 Brain_Cerebellum 1.982105798
0.017894202 1.982105798 1.070408291 1.074590575 1.072499433
0.002957321 1 0 0.992493858
ABCG8 ENSG00000143921.6 0.281339973 3 3 0.157707731
Brain_Frontal_Cortex_BA9 0.919003838 Brain_Spinal_cord_cervical_c-1
1.551272761 0.364225337 0.364225337 0.101688616 1.412823144
0.704983237 0.661789789 3 0 0.992493858
CRLF1 ENSG00000006016.10 0.28136087 6 6 0.085626858 Brain_Amygdala
0.469773272 Brain_Hypothalamus 2.192966616 0.184732999
0.184732999 -1.718931431 1.282357483 -0.069355675 1.287082518
6 0 0.992493858
PDCD7 ENSG00000090470.14 0.281383338 3 3 0.072603525
Brain_Caudate_basal_ganglia 0.63910624 Brain_Cerebellar_Hemisphere 1.255845872
0.67213796 0.67213796 -1.795315012 0.873607839 -0.463551988 1.33446961
3 0 0.992493858
ULBP1 ENSG00000111981.4 0.281412073 4 4 0.067997105
Brain_Cerebellar_Hemisphere 0.82376363 Brain_Cortex 1.911146647
0.112623914 0.112623914 -1.825026009 -0.222706901 -0.860020557
0.743345377 4 0 0.992493858
TIGD3 ENSG00000173825.6 0.28141391 2 2 0.284027408 Brain_Cerebellum
0.650369127 Brain_Hippocampus 1.517782967 0.482217033
0.482217033 -1.07131591 0.453249452 -0.309033229 1.078030506
2 0 0.992493858
ATP2B4 ENSG00000058668.14 0.281491144 2 2 0.428773419
Brain_Cerebellum 0.710642154 Brain_Cerebellar_Hemisphere 1.728099482
0.271900518 0.271900518 -0.791292337 0.370993794 -0.210149271
0.821860405 2 0 0.992493858
ENPEP ENSG00000138792.9 0.281532011 1 1 0.281532011
Brain_Frontal_Cortex_BA9 0.281532011 Brain_Frontal_Cortex_BA9 1 1
1 -1.076884182 -1.076884182 -1.076884182 NA 1 0
0.992493858
ACSS2 ENSG00000131069.19 0.281542936 6 4 0.151210467
Brain_Cerebellar_Hemisphere 0.969903048 Brain_Hippocampus 4.455476035
0.058320566 0.294198784 -0.512173822 1.435268968 0.71100151
0.774032657 4 0 0.992493858
RBPMS ENSG00000157110.15 0.28163273 1 1 0.28163273 Brain_Cerebellum 0.28163273

Brain_Cerebellum 1 1 1 1.076658788 1.076658788 1.076658788
NA 1 0 0.992493858
SGSH ENSG00000181523.12 0.28176912 5 4 0.029296507
Brain_Nucleus_accumbens_basal_ganglia 0.751118604 Brain_Cerebellum
2.443215341 0.078991219 0.540031178 -0.950639374 2.179473559
0.726259542 1.178596337 4 0 0.992493858
MAP2K4 ENSG00000065559.14 0.281836754 1 1 0.281836754
Brain_Caudate_basal_ganglia 0.281836754 Brain_Caudate_basal_ganglia 1 1
1 -1.076202384 -1.076202384 -1.076202384 NA 1 0
0.992493858
TBC1D22B ENSG00000065491.8 0.282019108 1 1 0.282019108
Brain_Cerebellar_Hemisphere 0.282019108 Brain_Cerebellar_Hemisphere 1 1
1 1.075794645 1.075794645 1.075794645 NA 1 0
0.992493858
CETN3 ENSG00000153140.8 0.282035082 3 3 0.070415403
Brain_Putamen_basal_ganglia 0.968884528 Brain_Hippocampus 1.325862632
0.668452712 0.668452712 -0.102939293 1.809229201 0.555760852
1.086005985 3 0 0.992493858
SLC6A13 ENSG00000010379.15 0.282039176 1 1 0.282039176
Brain_Cerebellum 0.282039176 Brain_Cerebellum 1 1 1 -1.075749785
-1.075749785 -1.075749785 NA 1 0 0.992493858
LINC01119 ENSG00000239332.5 0.282200004 6 5 0.101405566
Brain_Cortex 0.718840698 Brain_Putamen_basal_ganglia 3.007398938
0.06654925 0.273845965 -1.638077319 1.261625067 -0.380327279
1.039065146 5 0 0.992493858
CWC15 ENSG00000150316.11 0.282203206 5 3 0.058008937
Brain_Nucleus_accumbens_basal_ganglia 0.29092887 Brain_Cerebellum 4.364718609
0.033544119 0.160401064 -1.89563038 -1.056086123 -1.487900801
0.333131941 3 0 0.992493858
ENO2 ENSG00000111674.8 0.282249762 1 1 0.282249762
Brain_Hippocampus 0.282249762 Brain_Hippocampus 1 1 1
1.075279165 1.075279165 1.075279165 NA 1 0 0.992493858
AHCYL2 ENSG00000158467.16 0.282291663 1 1 0.282291663
Brain_Cerebellar_Hemisphere 0.282291663 Brain_Cerebellar_Hemisphere 1 1
1 1.075185552 1.075185552 1.075185552 NA 1 0
0.992493858
SLC23A2 ENSG00000089057.14 0.282385929 1 1 0.282385929
Brain_Cerebellar_Hemisphere 0.282385929 Brain_Cerebellar_Hemisphere 1 1
1 1.074974982 1.074974982 1.074974982 NA 1 0
0.992493858
CTA-390C10.9 ENSG00000272798.1 0.282407213 2 2 0.143731314
Brain_Cerebellum 0.70517297 Brain_Cerebellar_Hemisphere 1.163309347
0.836690653 0.836690653 -1.462036114 0.378346817 -0.541844648
1.301347251 2 0 0.992493858
LINC02036 ENSG00000225742.5 0.28244684 3 3 0.056772126
Brain_Caudate_basal_ganglia 0.804243483 Brain_Nucleus_accumbens_basal_ganglia
1.091431218 0.940705361 0.940705361 -0.491065425 1.905061126
0.553951576 1.227039159 3 0 0.992493858
SGCD ENSG00000170624.13 0.282473814 3 3 0.155932114
Brain_Caudate_basal_ganglia 0.353069804 Brain_Spinal_cord_cervical_c-1
1.587521848 0.365276185 0.365276185 -1.41888648 -0.928651345
-1.105224773 0.272355501 3 0 0.992493858
CASC18 ENSG00000257859.1 0.282499084 2 2 0.112299917
Brain_Cortex 0.973994831 Brain_Nucleus_accumbens_basal_ganglia
1.030633769 0.969366231 0.969366231 -1.587939959 -0.032598419
-0.810269189 1.09979255 2 0 0.992493858
RAD9B ENSG00000151164.18 0.282499121 3 3 0.066459234
Brain_Spinal_cord_cervical_c-1 0.466038748 Brain_Frontal_Cortex_BA9
1.413245016 0.612491531 0.612491531 -1.835313618 -0.728939374
-1.188008833 0.576706507 3 0 0.992493858
C1orf74 ENSG00000162757.4 0.282536512 1 1 0.282536512
Brain_Cerebellar_Hemisphere 0.282536512 Brain_Cerebellar_Hemisphere 1 1

1 -1.074638714 -1.074638714 -1.074638714 NA 1 0
0.992493858
SPTBN4 ENSG00000160460.15 0.282564064 6 3 0.009750068
Brain_Cerebellum 0.860053251 Brain_Cortex 4.120293791 0.026763284
0.421562299 -0.230438294 2.584569407 0.634926589 1.037825278
3 0 0.992493858
ACSF3 ENSG00000176715.15 0.282680566 11 3 0.107642119
Brain_Spinal_cord_cervical_c-1 0.848296601 Brain_Cortex 8.695087222
0.003780684 0.404282035 -1.608882123 0.196497185 -0.582375512
0.532529989 3 0 0.992493858
ME3 ENSG00000151376.16 0.282688389 6 6 0.127195135
Brain_Spinal_cord_cervical_c-1 0.988312425 Brain_Hippocampus
2.376750238 0.255678299 0.255678299 -1.52525649 0.830382256
-0.186021484 0.89825006 6 0 0.992493858
SLC40A1 ENSG00000138449.10 0.282720006 6 5 0.185127086
Brain_Hypothalamus 0.794419701 Brain_Hippocampus 2.500857555
0.010960424 0.66437099 -1.32513287 1.203501917 0.397931304
0.895544164 5 0 0.992493858
LINC00493 ENSG00000232388.4 0.282731026 11 4 0.229648424
Brain_Amygdala 0.952653125 Brain_Nucleus_accumbens_basal_ganglia
7.884735182 0.036004077 0.32935744 -1.201264995 0.775024031
-0.242916705 0.656709594 4 0 0.992493858
ELMOD1 ENSG00000110675.12 0.282762797 1 1 0.282762797
Brain_Amygdala 0.282762797 Brain_Amygdala 1 1 1 -1.074133622
-1.074133622 -1.074133622 NA 1 0 0.992493858
LINC01534 ENSG00000225975.6 0.282799624 2 2 0.219789192
Brain_Cerebellar_Hemisphere 0.284605275 Brain_Caudate_basal_ganglia
1.049870757 0.950129243 0.950129243 -1.070031178 1.227088867
0.078528844 1.624309161 2 0 0.992493858
PNMA2 ENSG00000240694.8 0.282859125 2 2 0.12040201 Brain_Cerebellum
0.279335693 Brain_Cerebellar_Hemisphere 1.509933592 0.490066408
0.490066408 -1.553088428 -1.081812855 -1.317450642 0.333242154
2 0 0.992493858
HMOX1 ENSG00000100292.16 0.28288649 1 1 0.28288649 Brain_Cortex 0.28288649
Brain_Cortex 1 1 1 -1.073857644 -1.073857644 -1.073857644
NA 1 0 0.992493858
BTNL3 ENSG00000168903.8 0.282894147 7 2 0.402513235 Brain_Cortex
0.912028448 Brain_Anterior_cingulate_cortex_BA24 6.255182211
0.014681509 0.415221513 -0.837141146 0.354715612 -0.087231607
0.457542832 2 0 0.992493858
FNBP1L ENSG00000137942.16 0.282896325 1 1 0.282896325
Brain_Cerebellum 0.282896325 Brain_Cerebellum 1 1 1 -1.073835702
-1.073835702 -1.073835702 NA 1 0 0.992493858
RRAGC ENSG00000116954.7 0.282903888 1 1 0.282903888
Brain_Hippocampus 0.282903888 Brain_Hippocampus 1 1 1
-1.073818831 -1.073818831 -1.073818831 NA 1 0 0.992493858
ZNF783 ENSG00000204946.9 0.282953304 2 2 0.216447026
Brain_Cerebellar_Hemisphere 0.77576857 Brain_Putamen_basal_ganglia 1.478818757
0.521181243 0.521181243 -1.236031039 0.284837592 -0.475596723
1.075416522 2 0 0.992493858
DPH7 ENSG00000148399.12 0.282960708 2 2 0.17245652 Brain_Cortex
0.652486656 Brain_Cerebellum 1.248254429 0.751745571 0.751745571
-1.364352914 0.450310379 -0.457021267 1.28316072 2 0
0.992493858
PGS1 ENSG00000087157.18 0.28301569 6 5 0.11260685 Brain_Caudate_basal_ganglia
0.974260816 Brain_Anterior_cingulate_cortex_BA24 3.792194221
0.116120989 0.185214718 -1.586584193 0.424577437 -0.502482748
0.708446153 5 0 0.992493858
APLNR ENSG00000134817.10 0.283088631 1 1 0.283088631
Brain_Nucleus_accumbens_basal_ganglia 0.283088631
Brain_Nucleus_accumbens_basal_ganglia 1 1 1 -1.073406808
-1.073406808 -1.073406808 NA 1 0 0.992493858

AC006946.17 ENSG00000273442.1 0.283162731 4 2 0.206892245
Brain_Spinal_cord_cervical_c-1 0.303042509 Brain_Cortex 2.925448886
0.004091549 0.998128533 -1.262157147 1.172028518 0.520005382
1.189667567 2 0 0.992493858
ARHGDIG ENSG00000242173.8 0.283185881 1 1 0.283185881
Brain_Spinal_cord_cervical_c-1 0.283185881 Brain_Spinal_cord_cervical_c-1
1 1 1 -1.07318999 -1.07318999 -1.07318999 NA 1 0
0.992493858
TRIM50 ENSG00000146755.10 0.28324693 2 2 0.117395196 Brain_Cerebellum
0.18920208 Brain_Cerebellar_Hemisphere 1.909371393 0.090628607
0.090628607 1.312943224 1.56580107 1.439372147 0.178797498 2
0 0.992493858
RP11-571M6.18 ENSG00000269903.1 0.283252439 1 1 0.283252439
Brain_Anterior_cingulate_cortex_BA24 0.283252439
Brain_Anterior_cingulate_cortex_BA24 1 1 1 1.073041629
1.073041629 1.073041629 NA 1 0 0.992493858
C9orf78 ENSG00000136819.15 0.283477431 1 1 0.283477431
Brain_Frontal_Cortex_BA9 0.283477431 Brain_Frontal_Cortex_BA9 1 1
1 1.072540282 1.072540282 1.072540282 NA 1 0
0.992493858
RBP3 ENSG00000265203.1 0.283506633 4 4 0.155145734
Brain_Caudate_basal_ganglia 0.985688104 Brain_Cortex 1.63241724 0.35621978
0.35621978 -1.185924696 1.42158854 0.083462754 1.066657417 4 0
0.992493858
C3orf67 ENSG00000163689.19 0.283534825 1 1 0.283534825
Brain_Frontal_Cortex_BA9 0.283534825 Brain_Frontal_Cortex_BA9 1 1
1 -1.072412435 -1.072412435 -1.072412435 NA 1 0
0.992493858
RP11-442O1.3 ENSG00000275088.1 0.283550007 2 2 0.11260379
Brain_Cerebellar_Hemisphere 0.906346807 Brain_Cerebellum 1.037202617
0.962797383 0.962797383 -1.586597695 -0.117647701 -0.852122698
1.038704502 2 0 0.992493858
FAH ENSG00000103876.11 0.283564516 7 6 0.098036261
Brain_Frontal_Cortex_BA9 0.972523758 Brain_Cerebellar_Hemisphere
2.957975732 0.033746336 0.188615986 -1.654449278 0.611165723
-0.279533765 0.740116749 6 0 0.992493858
ZMYND15 ENSG00000141497.13 0.283643262 2 2 0.118990883
Brain_Hippocampus 0.810756457 Brain_Cerebellar_Hemisphere 1.334454785
0.665545215 0.665545215 0.239450266 1.559023225 0.899236745
0.933078988 2 0 0.992493858
FGL2 ENSG00000127951.6 0.283682077 5 3 0.130078354
Brain_Nucleus_accumbens_basal_ganglia 0.763012481
Brain_Spinal_cord_cervical_c-1 3.915750491 0.046204882 0.250347817
0.30152724 1.51379298 1.009922933 0.515930099 3 0 0.992493858
RNF175 ENSG00000145428.14 0.283727567 5 3 0.143531654
Brain_Putamen_basal_ganglia 0.702927675 Brain_Cortex 3.924912409
0.031506341 0.198186725 -1.462765145 0.435885826 -0.635371775
0.721699018 3 0 0.992493858
HMGXB3 ENSG00000113716.12 0.283752086 7 4 0.050949197
Brain_Spinal_cord_cervical_c-1 0.991317013 Brain_Cerebellum 5.32703838
0.017415129 0.272281274 0.010882725 1.951907425 0.667806991
0.684601142 4 0 0.992493858
SYT6 ENSG00000134207.14 0.283856884 2 2 0.118903418
Brain_Putamen_basal_ganglia 0.469399368 Brain_Caudate_basal_ganglia
1.282640026 0.717359974 0.717359974 -1.55939289 -0.723456666
-1.141424778 0.591096172 2 0 0.992612265
RP11-1084I9.1 ENSG00000206187.3 0.2839484 7 4 0.090809438
Brain_Amygdala 0.703104767 Brain_Nucleus_accumbens_basal_ganglia
5.312510593 0.00302842 0.18061676 -1.691143294 1.370884139
0.486356321 1.012624087 4 0 0.992684172
CARD18 ENSG00000255501.1 0.284233765 1 1 0.284233765
Brain_Nucleus_accumbens_basal_ganglia 0.284233765

Brain_Nucleus_accumbens_basal_ganglia 1 1 1 -1.070856929
-1.070856929 -1.070856929 NA 1 0 0.993193183
TES ENSG00000135269.17 0.284235974 3 3 0.243259389 Brain_Amygdala
0.850094835 Brain_Cortex 1.60636651 0.485619983 0.485619983
-1.137629188 1.166878625 0.072748954 1.156643569 3 0
0.993193183
AC005537.2 ENSG00000232006.8 0.284465379 5 5 0.131713296
Brain_Cerebellum 0.822783305 Brain_Anterior_cingulate_cortex_BA24
2.425235919 0.119730791 0.119730791 -1.420165338 1.507379947
0.303452579 1.088034152 5 0 0.993501203
CBX6 ENSG00000183741.11 0.284565476 2 2 0.114454089 Brain_Cortex
0.962001222 Brain_Substantia_nigra 1.123360595 0.876639405
0.876639405 -1.57848562 0.047642422 -0.765421599 1.149846166
2 0 0.993501203
WARS2 ENSG00000116874.11 0.284677701 13 1 0.13462302 Brain_Hippocampus
0.455145916 Brain_Substantia_nigra 12.35480834 0.000777965
12.35480834 -1.496117569 -0.746863573 -1.043895722 0.248601417
1 0 0.993501203
ENKD1 ENSG00000124074.11 0.284687886 2 2 0.11364435
Brain_Cerebellar_Hemisphere 0.132522937 Brain_Cerebellum 1.927241249
0.072758751 0.072758751 -1.58202287 -1.504226963 -1.543124917
0.055010013 2 0 0.993501203
MGME1 ENSG00000125871.13 0.284690727 6 5 0.339403169
Brain_Caudate_basal_ganglia 0.965546663 Brain_Hypothalamus 4.39414212
0.070567124 0.180107112 -0.769405908 0.955345175 0.291500611
0.601739302 5 0 0.993501203
1-Sep ENSG00000180096.11 0.284750184 9 4 0.094728935
Brain_Frontal_Cortex_BA9 0.607535861 Brain_Putamen_basal_ganglia
7.360298074 0.005079379 0.309665587 -1.67096326 0.689116348
-0.813911439 0.663245506 4 0 0.993501203
CDKL1 ENSG00000100490.9 0.284981416 4 3 0.152819816
Brain_Frontal_Cortex_BA9 0.901854904 Brain_Cortex 2.486439365
0.034162928 0.479115353 -1.429641925 0.700178867 -0.171298535
0.905547111 3 0 0.993575862
CFAP53 ENSG00000172361.5 0.285141233 3 3 0.192188483
Brain_Cerebellar_Hemisphere 0.799768134 Brain_Putamen_basal_ganglia
1.502966855 0.496328773 0.496328773 0.253647192 1.304132285
0.83097325 0.532933213 3 0 0.993575862
NR3C2 ENSG00000151623.14 0.285177638 1 1 0.285177638
Brain_Cerebellar_Hemisphere 0.285177638 Brain_Cerebellar_Hemisphere 1 1
1 1.068760421 1.068760421 1.068760421 NA 1 0
0.993575862
SLC31A1 ENSG00000136868.10 0.285219651 5 5 0.071613245
Brain_Cortex 0.671491167 Brain_Putamen_basal_ganglia 1.219275356
0.865376806 0.865376806 -1.055226723 1.801568985 0.587817172
1.046098988 5 0 0.993575862
USP40 ENSG00000085982.13 0.285230991 9 6 0.04967455
Brain_Nucleus_accumbens_basal_ganglia 0.91990776 Brain_Cerebellar_Hemisphere
5.442731518 0.059639664 0.195207938 -0.894653157 1.962755855
0.618294123 0.796093837 6 0 0.993575862
CCDC146 ENSG00000135205.14 0.2853026 2 2 0.193908579
Brain_Putamen_basal_ganglia 0.361847429 Brain_Cerebellum 1.004550988
0.995449012 0.995449012 -1.299103014 -0.911850487 -1.10547675
0.273828888 2 0 0.993575862
STK11 ENSG00000118046.14 0.285338923 1 1 0.285338923
Brain_Cerebellar_Hemisphere 0.285338923 Brain_Cerebellar_Hemisphere 1 1
1 -1.068402648 -1.068402648 -1.068402648 NA 1 0
0.993575862
TMEM56 ENSG00000152078.9 0.285395572 1 1 0.285395572
Brain_Amygdala 0.285395572 Brain_Amygdala 1 1 1 -1.068277019
-1.068277019 -1.068277019 NA 1 0 0.993575862
LURAP1 ENSG00000171357.5 0.285510798 1 1 0.285510798

Brain_Hypothalamus 0.285510798 Brain_Hypothalamus 1 1 1
-1.068021535 -1.068021535 -1.068021535 NA 1 0 0.993575862
LECT2 ENSG00000145826.8 0.285636644 1 1 0.285636644
Brain_Caudate_basal_ganglia 0.285636644 Brain_Caudate_basal_ganglia 1 1
1 -1.067742583 -1.067742583 -1.067742583 NA 1 0
0.993575862
DCLRE1C ENSG00000152457.17 0.285737676 5 4 0.265631365
Brain_Caudate_basal_ganglia 0.821377261 Brain_Frontal_Cortex_BA9
3.842152857 0.0356048 0.244965814 -0.225773921 1.113179455
0.823368286 0.587308571 4 0 0.993575862
SLC2A12 ENSG00000146411.5 0.285759424 4 4 0.328567719
Brain_Cortex 0.981315212 Brain_Amygdala 2.027898335 0.135081618
0.135081618 -0.977002895 0.864389111 -0.218797651 0.828073018
4 0 0.993575862
UQCC1 ENSG00000101019.21 0.285774801 6 4 0.351423716 Brain_Cortex
0.915974782 Brain_Putamen_basal_ganglia 4.873906357 0.006378338
0.216456067 -0.715873979 0.9318313 -0.104718193 0.611393481 4
0 0.993575862
PPARG ENSG00000132170.19 0.285784158 4 3 0.056145962
Brain_Spinal_cord_cervical_c-1 0.902337955 Brain_Hypothalamus 2.00099068
0.00332815 0.940417056 -1.909900991 0.122708472 -0.619124314
0.88827753 3 0 0.993575862
AGO3 ENSG00000126070.19 0.285854975 2 2 0.245664627
Brain_Putamen_basal_ganglia 0.28566058 Brain_Hippocampus 1.006726692
0.993273308 0.993273308 -1.160944116 1.067689537 -0.04662729
1.575881968 2 0 0.993575862
RP11-6O2.2 ENSG00000278090.1 0.285907828 7 7 0.057074554
Brain_Cerebellar_Hemisphere 0.873983002 Brain_Frontal_Cortex_BA9
2.524396946 0.188001383 0.188001383 -1.90273943 1.507365946
-0.241393693 1.088728616 7 0 0.993575862
CTNS ENSG00000040531.14 0.28603658 12 3 0.105860205
Brain_Spinal_cord_cervical_c-1 0.924101541 Brain_Hippocampus
10.46436311 6.85E-17 0.398910259 -0.389970584 1.617083746
0.516610896 0.484329293 3 0 0.99365189
RP11-480D4.1 ENSG00000245729.2 0.286112723 2 2 0.152020687
Brain_Spinal_cord_cervical_c-1 0.472143098 Brain_Substantia_nigra
1.033435781 0.966564219 0.966564219 0.718996465 1.432430387
1.075713426 0.504473964 2 0 0.99365189
ORM1 ENSG00000229314.5 0.286142768 1 1 0.286142768
Brain_Cerebellar_Hemisphere 0.286142768 Brain_Cerebellar_Hemisphere 1 1
1 -1.066621544 -1.066621544 -1.066621544 NA 1 0 0.99365189
RP5-1159O4.1 ENSG00000272894.5 0.286330138 13 5 0.062264052
Brain_Substantia_nigra 0.962028468 Brain_Nucleus_accumbens_basal_ganglia
9.263713124 0.002085678 0.316062244 -0.047608236 1.864410697
1.026465125 0.512079792 5 0 0.994055822
ZNF292 ENSG00000188994.12 0.286519784 3 3 0.23393469
Brain_Substantia_nigra 0.528752513 Brain_Cerebellar_Hemisphere
1.933274629 0.374872043 0.374872043 -1.190284247 0.763651566
0.067759594 1.091548136 3 0 0.994213519
RNF41 ENSG00000181852.17 0.286608454 1 1 0.286608454 Brain_Cerebellum
0.286608454 Brain_Cerebellum 1 1 1 -1.065591255 -1.065591255
-1.065591255 NA 1 0 0.994213519
ARFIP1 ENSG00000164144.15 0.286628145 12 3 0.353676106
Brain_Hypothalamus 0.981795869 Brain_Spinal_cord_cervical_c-1
9.555517327 0.002760463 0.608093431 -0.864630388 0.927482437
-0.091867743 0.546213536 3 0 0.994213519
GHRL ENSG00000157017.15 0.286683875 3 2 0.115889984 Brain_Cortex
0.221057875 Brain_Cerebellum 2.755154566 0.021788254 0.22305718
1.223719994 1.572261214 1.451232671 0.197165917 2 0
0.994213519
ZNF57 ENSG00000171970.12 0.286747305 2 2 0.276725089
Brain_Cerebellar_Hemisphere 0.991329876 Brain_Cerebellum 1.730274438

0.269725562 0.269725562 0.010866603 1.087705613 0.549286108
0.761440166 2 0 0.994213519
EBI3 ENSG00000105246.5 0.286801927 2 2 0.117616093
Brain_Caudate_basal_ganglia 0.812429727 Brain_Cerebellum 1.009980113
0.990019887 0.990019887 -0.237292697 1.564858492 0.663782897
1.274313326 2 0 0.994213519
FNDC10 ENSG00000228594.3 0.28722162 1 1 0.28722162
Brain_Putamen_basal_ganglia 0.28722162 Brain_Putamen_basal_ganglia 1 1 1
1.064236403 1.064236403 1.064236403 NA 1 0 0.994554841
HEPACAM ENSG00000165478.6 0.287312565 1 1 0.287312565
Brain_Putamen_basal_ganglia 0.287312565 Brain_Putamen_basal_ganglia 1 1
1 -1.064035618 -1.064035618 -1.064035618 NA 1 0
0.994554841
MST1 ENSG00000173531.15 0.287353936 7 3 0.33910618 Brain_Cortex
0.583026312 Brain_Cerebellum 6.011050435 0.010906764 0.267482359
-0.796377677 0.955932812 0.507356576 0.593216266 3 0
0.994554841
LRP6 ENSG00000070018.8 0.287397479 3 3 0.117598532 Brain_Cerebellum
0.303394632 Brain_Cerebellar_Hemisphere 1.432454568 0.583790592
0.583790592 -1.564933375 1.181509985 -0.470868128 1.455858071
3 0 0.994554841
LINC01299 ENSG00000254081.1 0.287407574 11 2 0.178364616
Brain_Cerebellum 0.991201535 Brain_Anterior_cingulate_cortex_BA24
9.687214005 0.01143485 0.395932627 -0.056194784 1.34580747
0.714849606 0.491229121 2 0 0.994554841
FREM1 ENSG00000164946.19 0.287410301 10 9 0.088257921 Brain_Cerebellum
0.993457084 Brain_Nucleus_accumbens_basal_ganglia 4.359832632
0.074824508 0.156079632 -0.787550845 1.704659643 0.038891726
0.726016701 9 0 0.994554841
TMEM64 ENSG00000180694.13 0.287450111 1 1 0.287450111
Brain_Hypothalamus 0.287450111 Brain_Hypothalamus 1 1 1
1.063732028 1.063732028 1.063732028 NA 1 0 0.994554841
SENP3 ENSG00000161956.12 0.287469071 2 2 0.115195394
Brain_Nucleus_accumbens_basal_ganglia 0.985578339 Brain_Hypothalamus
1.080273634 0.919726366 0.919726366 -1.575264548 -0.018075856
-0.796670202 1.101098684 2 0 0.994554841
LINC01562 ENSG00000203356.2 0.287574666 2 2 0.114391483
Brain_Cerebellum 0.848742688 Brain_Substantia_nigra 1.125964017
0.874035983 0.874035983 -1.578758405 -0.19072291 -0.884740658
0.98148931 2 0 0.994674202
SRGAP2 ENSG00000266028.7 0.287715909 1 1 0.287715909
Brain_Substantia_nigra 0.287715909 Brain_Substantia_nigra 1 1
1 -1.063145642 -1.063145642 -1.063145642 NA 1 0
0.994725712
SRFBP1 ENSG00000151304.5 0.287885777 2 2 0.115743315
Brain_Putamen_basal_ganglia 0.654731783 Brain_Cerebellum 1.204652709
0.795347291 0.795347291 -1.572894216 -0.447198447 -1.010046331
0.795987112 2 0 0.994725712
GYPA ENSG00000170180.19 0.287895132 1 1 0.287895132
Brain_Frontal_Cortex_BA9 0.287895132 Brain_Frontal_Cortex_BA9 1 1
1 -1.062750459 -1.062750459 -1.062750459 NA 1 0
0.994725712
FBLIM1 ENSG00000162458.12 0.287916441 1 1 0.287916441
Brain_Caudate_basal_ganglia 0.287916441 Brain_Caudate_basal_ganglia 1 1
1 1.062703483 1.062703483 1.062703483 NA 1 0
0.994725712
MRPS11 ENSG00000181991.15 0.287945046 1 1 0.287945046
Brain_Nucleus_accumbens_basal_ganglia 0.287945046
Brain_Nucleus_accumbens_basal_ganglia 1 1 1 -1.062640429
-1.062640429 -1.062640429 NA 1 0 0.994725712
NUP85 ENSG00000125450.10 0.288071663 13 2 0.067618314
Brain_Hippocampus 0.430613688 Brain_Substantia_nigra 11.81805067

0.000215623	0.434848719	-1.827542024	-0.788141936	-1.457087588
0.358544325	2	0	0.994917463
TDO2	ENSG00000151790.8	0.288146267	3	3	0.452312911
Brain_Frontal_Cortex_BA9	0.982325355	Brain_Cortex	2.016714565
0.076183302	0.076183302	0.022153694	0.751564646	0.323656849
0.380780362	3	0	0.994929521
DNAJC25	ENSG00000059769.19	0.288481185	3	3	0.054101079
Brain_Cerebellum	0.935137372	Brain_Anterior_cingulate_cortex_BA24
1.182959385	0.794656084	0.794656084	-1.926026388	0.194907222
-0.604167387	1.153068725	3	0	0.995840183
LINC01771	ENSG00000225675.2	0.288795993	1	1	0.288795993
Brain_Putamen_basal_ganglia	0.288795993	Brain_Putamen_basal_ganglia	1	1
1	1.060766581	1.060766581	1.060766581	NA	1	0
0.996489356
PIK3C2B	ENSG00000133056.13	0.288875862	1	1	0.288875862
Brain_Frontal_Cortex_BA9	0.288875862	Brain_Frontal_Cortex_BA9	1	1
1	-1.060590896	-1.060590896	-1.060590896	NA	1	0
0.996489356
CCHCR1	ENSG00000204536.13	0.288889506	6	5	0.271793922
Brain_Putamen_basal_ganglia	0.963018228	Brain_Cerebellar_Hemisphere
4.251036094	0.09536014	0.153692604	-0.88971875	1.098940724
0.151584437	0.647193692	5	0	0.996489356
OCIAD2	ENSG00000145247.11	0.288954136	4	3	0.084330685
Brain_Putamen_basal_ganglia	0.997686873	Brain_Cerebellum	2.419378407
0.071884599	0.466049784	0.002899079	1.726092991	0.640352616
0.749886869	3	0	0.996489356
PSMB1	ENSG00000008018.8	0.289092931	3	3	0.24459742
Brain_Frontal_Cortex_BA9	0.668896504	Brain_Cerebellum	2.530474303
0.095080575	0.095080575	-1.163572207	-0.427662982	-0.854072344
0.381630017	3	0	0.996722327
SIAH1	ENSG00000196470.11	0.289269995	1	1	0.289269995
Brain_Cerebellar_Hemisphere	0.289269995	Brain_Cerebellar_Hemisphere	1	1
1	-1.059724409	-1.059724409	-1.059724409	NA	1	0	0.99708709
SFI1	ENSG00000198089.15	0.28941212	11	8	0.018440902
Brain_Spinal_cord_cervical_c-1	0.952846598
Brain_Nucleus_accumbens_basal_ganglia	5.403533894	0.057455239
0.206438145	-2.356644294	0.360440474	-0.803856384	0.834207629
8	0	0.997148584
TCEA2	ENSG00000171703.16	0.289528764	5	5	0.046759678
Brain_Hippocampus	0.569333316	Brain_Cerebellar_Hemisphere	1.298012545
0.609476934	0.609476934	-1.988470501	0.980301323	-0.11410284
1.262842484	5	0	0.997148584
TACR2	ENSG00000075073.14	0.289530735	3	3	0.1079016
Brain_Caudate_basal_ganglia	0.908174256	Brain_Nucleus_accumbens_basal_ganglia
1.052416211	0.931031792	0.931031792	-1.607696798	0.115341739
-0.840267065	0.876797566	3	0	0.997148584
RP11-452F19.3	ENSG00000228106.5	0.289598673	7	4	0.212828155
Brain_Cerebellar_Hemisphere	0.775099703	Brain_Cortex	4.701169317
0.021736346	0.305561018	-0.419658131	1.245826345	0.24338095
0.670912051	4	0	0.997148584
RP11-20D14.6	ENSG00000249790.2	0.289644925	2	2	0.141714322
Brain_Cerebellum	0.556561194	Brain_Putamen_basal_ganglia	1.015834279
0.984165721	0.984165721	-0.587956943	1.469436912	0.440739985
1.454797146	2	0	0.997148584
RP11-45M22.5	ENSG00000266498.1	0.289956229	1	1	0.289956229
Brain_Hippocampus	0.289956229	Brain_Hippocampus	1	1	1
1.058217645	1.058217645	1.058217645	NA	1	0	0.997148584
BIRC7	ENSG00000101197.12	0.289982615	7	6	0.0586197	Brain_Hypothalamus
0.873849558	Brain_Caudate_basal_ganglia	3.179016371	0.034859358
0.358087519	-1.891034791	0.525906739	-0.506719546	0.892746195
6	0	0.997148584
MAP3K7CL	ENSG00000156265.15	0.290042084	4	4	0.359304258

Brain_Hippocampus 0.712226638 Brain_Hypothalamus 2.488368852
0.159697619 0.159697619 -0.897125482 0.916691622 0.114587388
0.890879591 4 0 0.997148584
GPR137B ENSG00000077585.13 0.290055718 6 6 0.044253962
Brain_Putamen_basal_ganglia 0.637526761 Brain_Cerebellar_Hemisphere
2.852475053 0.195561739 0.195561739 -2.011677329 0.853352212
-0.885179513 1.045576436 6 0 0.997148584
ZNF460 ENSG00000197714.8 0.290101009 1 1 0.290101009
Brain_Amygdala 0.290101009 Brain_Amygdala 1 1 1 1.057900057
1.057900057 1.057900057 NA 1 0 0.997148584
CRP ENSG00000132693.12 0.290277254 1 1 0.290277254 Brain_Cortex
0.290277254 Brain_Cortex 1 1 1 -1.057513595 -1.057513595
-1.057513595 NA 1 0 0.997148584
TUBGCP5 ENSG00000275835.4 0.290312896 4 4 0.081285128
Brain_Nucleus_accumbens_basal_ganglia 0.921566923 Brain_Frontal_Cortex_BA9
1.960381073 0.244132581 0.244132581 -0.098460139 1.743277646
0.780875548 0.768347228 4 0 0.997148584
NT5C ENSG00000125458.6 0.290365179 3 3 0.079983789
Brain_Putamen_basal_ganglia 0.640326972 Brain_Cerebellum 1.462582872
0.624978396 0.624978396 -1.75078014 0.467241686 -0.842910702
1.162445218 3 0 0.997148584
SZT2 ENSG00000198198.15 0.290391736 4 4 0.111223901 Brain_Cortex
0.990599444 Brain_Cerebellar_Hemisphere 1.482727569 0.540771368
0.540771368 -1.304497741 1.592716071 -0.107404742 1.254963351
4 0 0.997148584
GRAMD1B ENSG00000023171.16 0.290407244 4 4 0.048478548
Brain_Cerebellum 0.999738692 Brain_Amygdala 1.701912325 0.502525595
0.502525595 -0.000327501 1.97314936 0.584773896 0.929695103 4
0 0.997148584
NDFIP2 ENSG00000102471.13 0.290428167 1 1 0.290428167
Brain_Amygdala 0.290428167 Brain_Amygdala 1 1 1 -1.057182803
-1.057182803 -1.057182803 NA 1 0 0.997148584
UGGT2 ENSG00000102595.19 0.290575616 2 2 0.130621388
Brain_Cerebellar_Hemisphere 0.22428293 Brain_Spinal_cord_cervical_c-1
1.568821665 0.431178335 0.431178335 1.215218072 1.511656037
1.363437055 0.209613295 2 0 0.997306517
RP11-160H22.5 ENSG00000227373.5 0.29070929 5 5 0.064141236
Brain_Cortex 0.547520691 Brain_Spinal_cord_cervical_c-1 2.847490156
0.195286321 0.195286321 -1.851196871 -0.601479456 -1.225719604
0.519740322 5 0 0.997306517
RP11-1055B8.9 ENSG00000275966.1 0.29077415 1 1 0.29077415
Brain_Nucleus_accumbens_basal_ganglia 0.29077415
Brain_Nucleus_accumbens_basal_ganglia 1 1 1 -1.056424867
-1.056424867 -1.056424867 NA 1 0 0.997306517
PALM2 ENSG00000243444.7 0.290783671 1 1 0.290783671
Brain_Hypothalamus 0.290783671 Brain_Hypothalamus 1 1 1
-1.056404019 -1.056404019 -1.056404019 NA 1 0 0.997306517
ZNF391 ENSG00000124613.8 0.290841749 13 2 0.239702486
Brain_Anterior_cingulate_cortex_BA24 0.99766832 Brain_Spinal_cord_cervical_c-1
11.90924496 0.003439555 0.471308523 -1.175730751 0.017586029
-0.352983281 0.355252605 2 0 0.997306517
ZNF618 ENSG00000157657.14 0.290901858 2 2 0.13135706
Brain_Cerebellar_Hemisphere 0.230964538 Brain_Cerebellum 1.912863795
0.087136205 0.087136205 1.197877942 1.508771994 1.353324968
0.219835292 2 0 0.997306517
ASNS ENSG00000070669.16 0.291020956 3 3 0.155933775 Brain_Cerebellum
0.817402288 Brain_Frontal_Cortex_BA9 1.775831973 0.348632664
0.348632664 -0.376743559 1.418880786 0.270416613 0.997269258
3 0 0.997319833
LRRC20 ENSG00000172731.13 0.291118611 2 2 0.144955801
Brain_Putamen_basal_ganglia 0.763871379 Brain_Frontal_Cortex_BA9 1.18877392
0.81122608 0.81122608 -1.457581975 0.300400898 -0.578590539 1.24308161

2 0 0.997319833
ESD ENSG00000139684.13 0.291299695 3 2 0.118014771 Brain_Cortex
0.994539864 Brain_Anterior_cingulate_cortex_BA24 2.005437444
0.005442725 0.989119831 -1.563160831 0.067436093 -0.496293806
0.924430531 2 0 0.997319833
CTD-2201E18.5 ENSG00000271788.1 0.291508978 1 1 0.291508978
Brain_Caudate_basal_ganglia 0.291508978 Brain_Caudate_basal_ganglia 1 1
1 1.054817109 1.054817109 1.054817109 NA 1 0
0.997319833
HCRT ENSG00000161610.1 0.29154907 1 1 0.29154907
Brain_Nucleus_accumbens_basal_ganglia 0.29154907
Brain_Nucleus_accumbens_basal_ganglia 1 1 1 1.05472947 1.05472947
1.05472947 NA 1 0 0.997319833
GPSM3 ENSG00000213654.9 0.291593205 1 1 0.291593205 Brain_Cerebellum
0.291593205 Brain_Cerebellum 1 1 1 1.054633 1.054633 1.054633
NA 1 0 0.997319833
PREP ENSG00000085377.13 0.291668434 1 1 0.291668434 Brain_Amygdala
0.291668434 Brain_Amygdala 1 1 1 -1.05446859 -1.05446859
-1.05446859 NA 1 0 0.997319833
PRMT6 ENSG00000198890.7 0.291901038 7 6 0.00976791 Brain_Hippocampus
0.889812876 Brain_Spinal_cord_cervical_c-1 3.197721886 0.001495747
0.333670692 -2.58393889 -0.138540991 -1.478413174 1.039738616
6 0 0.997319833
RP11-431K24.1 ENSG00000238290.1 0.292061511 11 2 0.063381027
Brain_Nucleus_accumbens_basal_ganglia 0.818789218 Brain_Cerebellum
9.474483829 5.06E-05 0.997571468 0.229102542 1.856509099
1.375653105 0.474561231 2 0 0.997319833
ZNF195 ENSG00000005801.16 0.292070156 11 2 0.110867352
Brain_Anterior_cingulate_cortex_BA24 0.23342381 Brain_Hypothalamus
10.05065344 0.001280164 0.395113129 1.191585508 1.594306727
1.381820927 0.133578942 2 0 0.997319833
CTD-2540B15.9 ENSG00000267130.1 0.292317228 1 1 0.292317228
Brain_Nucleus_accumbens_basal_ganglia 0.292317228
Brain_Nucleus_accumbens_basal_ganglia 1 1 1 1.053051852
1.053051852 1.053051852 NA 1 0 0.997319833
EDEM3 ENSG00000116406.18 0.292325918 5 5 0.078862439
Brain_Nucleus_accumbens_basal_ganglia 0.668067609 Brain_Cortex
3.231375908 0.109237712 0.109237712 -1.75732494 0.428801607
-0.889125866 0.799798245 5 0 0.997319833
OR2L3 ENSG00000198128.3 0.292488298 3 2 0.288479619 Brain_Cerebellum
0.745883356 Brain_Cerebellar_Hemisphere 2.401860339 0.03242993
0.565709732 -1.061462825 0.406756798 -0.110211268 0.824844729
2 0 0.997319833
NUTM2G ENSG00000188152.12 0.292513927 2 2 0.135767826
Brain_Hippocampus 0.145553812 Brain_Cerebellar_Hemisphere 1.768528089
0.231471911 0.231471911 -1.491738065 -1.455417149 -1.473577607
0.025682766 2 0 0.997319833
ZMYM5 ENSG00000132950.18 0.292536523 1 1 0.292536523 Brain_Cortex
0.292536523 Brain_Cortex 1 1 1 1.052573466 1.052573466
1.052573466 NA 1 0 0.997319833
ARL15 ENSG00000185305.10 0.292594839 4 3 0.179777741
Brain_Nucleus_accumbens_basal_ganglia 0.787351375 Brain_Putamen_basal_ganglia
2.651502845 0.06307438 0.206249417 -1.341439684 0.914850937
-0.223714442 1.008903758 3 0 0.997319833
RASSF9 ENSG00000198774.4 0.292644707 2 2 0.120582173
Brain_Cerebellum 0.894452984 Brain_Substantia_nigra 1.05523222 0.94476778
0.94476778 0.132671752 1.552334643 0.842503197 1.003853257 2
0 0.997319833
TMEM106B ENSG00000106460.18 0.292648963 2 2 0.117720523
Brain_Cerebellum 0.179994612 Brain_Cortex 1.886836937 0.113163063
0.113163063 1.340771624 1.56441337 1.452592497 0.158138596 2
0 0.997319833

REV1 ENSG00000135945.9 0.292693142 1 1 0.292693142
Brain_Hypothalamus 0.292693142 Brain_Hypothalamus 1 1 1
-1.052231955 -1.052231955 -1.052231955 NA 1 0 0.997319833
SYTL2 ENSG00000137501.17 0.292697603 1 1 0.292697603
Brain_Putamen_basal_ganglia 0.292697603 Brain_Putamen_basal_ganglia 1 1
1 1.052222229 1.052222229 1.052222229 NA 1 0
0.997319833
ABHD2 ENSG00000140526.17 0.292774783 2 2 0.18516964
Brain_Nucleus_accumbens_basal_ganglia 0.393802155 Brain_Cerebellar_Hemisphere
1.011700183 0.988299817 0.988299817 -1.325004556 -0.852742431
-1.088873493 0.333939751 2 0 0.997319833
ITGB3BP ENSG00000142856.16 0.292835293 13 1 0.223814522
Brain_Cerebellar_Hemisphere 0.488782989 Brain_Cerebellum 12.81599335
0.000100788 12.81599335 -1.21644744 -0.692245788 -1.044650124
0.134024222 1 0 0.997319833
LINC00326 ENSG00000231023.6 0.292978014 4 2 0.248313229
Brain_Spinal_cord_cervical_c-1 0.555068416
Brain_Anterior_cingulate_cortex_BA24 2.985819673 0.000656614
0.928629927 0.590182332 1.154456095 0.869943202 0.269089436
2 0 0.997319833
ASXL3 ENSG00000141431.10 0.292982014 1 1 0.292982014 Brain_Cerebellum
0.292982014 Brain_Cerebellum 1 1 1 1.051602382 1.051602382
1.051602382 NA 1 0 0.997319833
ANKRD65 ENSG00000235098.8 0.293169473 13 7 0.00593631 Brain_Cortex
0.935021099 Brain_Hypothalamus 8.136996408 0.007160242
0.424296288 0.081529206 2.75127876 1.66047555 0.827189159 7 0
0.997319833
TMEM59L ENSG00000105696.8 0.293233939 4 4 0.103529415
Brain_Nucleus_accumbens_basal_ganglia 0.705952227 Brain_Caudate_basal_ganglia
1.361868361 0.698812124 0.698812124 -1.627978639 0.377297908
-0.76937651 0.848476979 4 0 0.997319833
NME9 ENSG00000181322.13 0.293289792 7 7 0.011572645
Brain_Caudate_basal_ganglia 0.832954017 Brain_Spinal_cord_cervical_c-1
3.966230288 0.143939665 0.143939665 -2.524914479 0.210914465
-1.317553759 0.972008647 7 0 0.997319833
ALG11 ENSG00000253710.2 0.293325692 1 1 0.293325692 Brain_Cerebellum
0.293325692 Brain_Cerebellum 1 1 1 -1.05085391 -1.05085391
-1.05085391 NA 1 0 0.997319833
KLKB1 ENSG00000164344.15 0.293359888 11 3 0.052358979
Brain_Substantia_nigra 0.295319559 Brain_Hippocampus 8.840742547
0.007538113 0.511521747 1.046523158 1.940170449 1.404314524
0.33524881 3 0 0.997319833
CTD-2561B21.10 ENSG00000262098.1 0.293418497 2 2 0.295116234
Brain_Putamen_basal_ganglia 0.314971744 Brain_Caudate_basal_ganglia
1.141370613 0.858629387 0.858629387 -1.046963887 1.004844476
-0.021059706 1.450847608 2 0 0.997319833
PANX2 ENSG00000073150.13 0.293611118 1 1 0.293611118
Brain_Frontal_Cortex_BA9 0.293611118 Brain_Frontal_Cortex_BA9 1 1
1 -1.050232746 -1.050232746 -1.050232746 NA 1 0
0.997319833
RP11-424M24.5 ENSG00000262890.1 0.293631951 1 1 0.293631951
Brain_Amygdala 0.293631951 Brain_Amygdala 1 1 1 1.050187422
1.050187422 1.050187422 NA 1 0 0.997319833
SLC25A30 ENSG00000174032.16 0.293638856 3 3 0.20483275
Brain_Caudate_basal_ganglia 0.474207674 Brain_Hippocampus 1.137033692
0.881389302 0.881389302 -1.26790256 1.239233709 -0.248106185
1.31733852 3 0 0.997319833
RAPGEF1 ENSG00000107263.18 0.293693094 2 2 0.571238257
Brain_Cerebellum 0.890106949 Brain_Cerebellar_Hemisphere 1.896721932
0.103278068 0.103278068 -0.138168881 0.566228796 0.214029958
0.498084374 2 0 0.997319833
PREX1 ENSG00000124126.13 0.293736274 3 3 0.098391204

Brain_Nucleus_accumbens_basal_ganglia 0.456481625 Brain_Cerebellum
1.914116599 0.088921031 0.088921031 -0.962039544 1.652703587
-0.01799626 1.450945461 3 0 0.997319833
C19orf67 ENSG00000188032.9 0.293797403 1 1 0.293797403
Brain_Hypothalamus 0.293797403 Brain_Hypothalamus 1 1 1
-1.049827556 -1.049827556 -1.049827556 NA 1 0 0.997319833
TECRL ENSG00000205678.7 0.293803828 1 1 0.293803828
Brain_Hypothalamus 0.293803828 Brain_Hypothalamus 1 1 1
-1.049813585 -1.049813585 -1.049813585 NA 1 0 0.997319833
ACTR3B ENSG00000133627.17 0.293822822 1 1 0.293822822
Brain_Caudate_basal_ganglia 0.293822822 Brain_Caudate_basal_ganglia 1 1
1 1.049772281 1.049772281 1.049772281 NA 1 0
0.997319833
AC005224.2 ENSG00000231595.1 0.293893927 1 1 0.293893927
Brain_Spinal_cord_cervical_c-1 0.293893927 Brain_Spinal_cord_cervical_c-1
1 1 1 1.049617676 1.049617676 1.049617676 NA 1 0
0.997319833
GNS ENSG00000135677.10 0.293903958 1 1 0.293903958
Brain_Spinal_cord_cervical_c-1 0.293903958 Brain_Spinal_cord_cervical_c-1
1 1 1 1.049595867 1.049595867 1.049595867 NA 1 0
0.997319833
SIRPB1 ENSG00000101307.15 0.294054784 13 2 0.48364558
Brain_Substantia_nigra 0.971342617 Brain_Hippocampus 12.43188409
4.31E-05 0.474496595 -0.177818243 0.700451186 0.104619101
0.313078225 2 0 0.997319833
LIG4 ENSG00000174405.13 0.294102966 4 3 0.090214228
Brain_Hippocampus 0.55021813 Brain_Cerebellum 3.248956806 0.073824647
0.123922125 0.597433295 1.694268738 1.032720091 0.470669051
3 0 0.997319833
ZNF814 ENSG00000204514.9 0.294152996 10 3 0.033881731
Brain_Spinal_cord_cervical_c-1 0.959511612
Brain_Anterior_cingulate_cortex_BA24 8.363561561 0.013844396
0.333784459 0.050766467 2.121476406 1.248611259 0.677730351
3 0 0.997319833
CCDC88B ENSG00000168071.21 0.294156509 7 3 0.188251008
Brain_Frontal_Cortex_BA9 0.89529206 Brain_Putamen_basal_ganglia 5.803259489
0.00370512 0.3710505 -1.315770725 0.504448183 -0.610574285
0.624859643 3 0 0.997319833
DVL1 ENSG00000107404.19 0.294183069 1 1 0.294183069
Brain_Spinal_cord_cervical_c-1 0.294183069 Brain_Spinal_cord_cervical_c-1
1 1 1 1.048989244 1.048989244 1.048989244 NA 1 0
0.997319833
C19orf35 ENSG00000188305.5 0.294354992 1 1 0.294354992
Brain_Hypothalamus 0.294354992 Brain_Hypothalamus 1 1 1
-1.048615776 -1.048615776 -1.048615776 NA 1 0 0.997319833
SMC6 ENSG00000163029.15 0.294358374 3 3 0.06799178 Brain_Amygdala
0.624081321 Brain_Cerebellum 1.881948115 0.118835888 0.118835888
0.490074303 1.825061298 0.969126502 0.743019473 3 0
0.997319833
C1orf194 ENSG00000179902.12 0.29444545 9 3 0.042737388
Brain_Caudate_basal_ganglia 0.705197535 Brain_Anterior_cingulate_cortex_BA24
7.254225782 0.017998848 0.315984454 0.378313746 2.026267421
1.363165201 0.484121964 3 0 0.997319833
RCE1 ENSG00000173653.7 0.294477686 1 1 0.294477686
Brain_Cerebellar_Hemisphere 0.294477686 Brain_Cerebellar_Hemisphere 1 1
1 1.048349337 1.048349337 1.048349337 NA 1 0
0.997319833
RAC1 ENSG00000136238.17 0.294575996 10 7 0.038053568 Brain_Cortex
0.951764906 Brain_Spinal_cord_cervical_c-1 6.149873384 0.03118222
0.319588028 0.060490596 2.074277267 1.227125653 0.670628979
7 0 0.997319833
USP45 ENSG00000123552.17 0.29495143 2 2 0.317016719

Brain_Caudate_basal_ganglia 0.684222686 Brain_Nucleus_accumbens_basal_ganglia 1.57497671 0.42502329 0.42502329 -1.000607259 0.406707698 -0.296949781 0.995121949 2 0 0.997319833
TLR9 ENSG00000239732.3 0.294991074 3 3 0.082701891 Brain_Cerebellar_Hemisphere 0.45389703 Brain_Amygdala 2.101577507 0.130769769 0.130769769 0.748933928 1.735219743 1.186066154 0.502594784 3 0 0.997319833
GBA ENSG00000177628.15 0.294999008 2 2 0.161909363 Brain_Nucleus_accumbens_basal_ganglia 0.584682488 Brain_Cerebellum 1.104551273 0.895448727 0.895448727 -0.546557916 1.398678672 0.426060378 1.375489983 2 0 0.997319833
COLEC11 ENSG00000118004.17 0.295237229 13 13 0.061947991 Brain_Amygdala 0.979216575 Brain_Nucleus_accumbens_basal_ganglia 2.952990297 0.163676754 0.163676754 -1.733238908 1.866667812 0.071167465 1.006317149 13 0 0.997319833
RP11-357P18.2 ENSG00000274245.1 0.295245993 1 1 0.295245993 Brain_Hypothalamus 0.295245993 Brain_Hypothalamus 1 1 1 -1.046682596 -1.046682596 -1.046682596 NA 1 0 0.997319833
C6orf89 ENSG00000198663.16 0.295254302 6 5 0.181952408 Brain_Hippocampus 0.502427133 Brain_Putamen_basal_ganglia 3.082285946 0.002085501 0.138721842 -1.334767638 1.122393187 -0.352940847 0.997928103 5 0 0.997319833
CTBP1 ENSG00000159692.15 0.295373241 3 3 0.069448799 Brain_Spinal_cord_cervical_c-1 0.758682353 Brain_Cerebellum 1.273161241 0.725719788 0.725719788 -0.500620187 1.815488976 0.540693447 1.175574651 3 0 0.997319833
EPB41L4A-AS1 ENSG00000224032.6 0.295380466 1 1 0.295380466 Brain_Cortex 0.295380466 Brain_Cortex 1 1 1 -1.046391174 -1.046391174 -1.046391174 NA 1 0 0.997319833
CYMP-AS1 ENSG00000235407.1 0.29540574 1 1 0.29540574 Brain_Cerebellum 0.29540574 Brain_Cerebellum 1 1 1 -1.046336411 -1.046336411 -1.046336411 NA 1 0 0.997319833
GRAMD2 ENSG00000175318.11 0.29551396 4 4 0.042136705 Brain_Cortex 0.787781891 Brain_Cerebellum 1.36568505 0.636824563 0.636824563 -0.269192058 2.032167472 0.789177811 0.988728026 4 0 0.997319833
PSMG4 ENSG00000180822.11 0.295616827 10 1 0.211176897 Brain_Cerebellar_Hemisphere 0.479733215 Brain_Hippocampus 9.483297277 0.001611009 9.483297277 -1.250335826 -0.706731718 -1.01612507 0.142419256 1 0 0.997319833
ZBTB24 ENSG00000112365.4 0.295717321 2 2 0.142277966 Brain_Hippocampus 0.482392671 Brain_Nucleus_accumbens_basal_ganglia 1.118379025 0.881620975 0.881620975 -1.467360675 -0.702459467 -1.084910071 0.540866831 2 0 0.997319833
PSMD13 ENSG00000185627.17 0.295728968 13 2 0.123515261 Brain_Substantia_nigra 0.980361586 Brain_Hippocampus 11.55674 1.48E-16 0.879803923 -1.540185017 0.111006328 -0.442942891 0.412487407 2 0 0.997319833
GUCA1A ENSG00000048545.13 0.295754875 4 4 0.293505365 Brain_Hypothalamus 0.933345289 Brain_Cerebellum 2.457383149 0.216369172 0.216369172 -1.050462844 0.083636697 -0.67088727 0.528646021 4 0 0.997319833
FAM129A ENSG00000135842.16 0.295763256 1 1 0.295763256 Brain_Putamen_basal_ganglia 0.295763256 Brain_Putamen_basal_ganglia 1 1 1 1.045562098 1.045562098 1.045562098 NA 1 0 0.997319833
HIST1H4C ENSG00000197061.4 0.295847259 1 1 0.295847259 Brain_Anterior_cingulate_cortex_BA24 0.295847259 Brain_Anterior_cingulate_cortex_BA24 1 1 1 1.045380255 1.045380255 1.045380255 NA 1 0 0.997319833
CYBA ENSG00000051523.10 0.295866229 4 4 0.249990394 Brain_Putamen_basal_ganglia 0.418088887 Brain_Nucleus_accumbens_basal_ganglia

1.28074755 0.780614486 0.780614486 -1.150372714 1.059428573
0.430100659 1.059039578 4 0 0.997319833
CTC-498J12.1 ENSG00000250237.1 0.295893323 1 1 0.295893323
Brain_Substantia_nigra 0.295893323 Brain_Substantia_nigra 1 1
1 1.045280554 1.045280554 1.045280554 NA 1 0
0.997319833
MSH4 ENSG00000057468.6 0.295943546 13 3 0.075236944
Brain_Hippocampus 0.958862654 Brain_Substantia_nigra 10.33447738
0.0157946 0.759259124 -1.779017189 -0.051580881 -1.241380613
0.47290589 3 0 0.997319833
SULT2B1 ENSG00000088002.11 0.295974837 6 6 0.080724134
Brain_Anterior_cingulate_cortex_BA24 0.988671786 Brain_Amygdala 2.8172652
0.152542342 0.152542342 -0.351286356 1.746499847 0.479880922
0.863075186 6 0 0.997319833
CTD-3088G3.8 ENSG00000188897.8 0.296012742 6 6 0.012898681
Brain_Cerebellum 0.80144556 Brain_Nucleus_accumbens_basal_ganglia 2.385947426
0.165908625 0.165908625 -0.776965365 2.486554497 0.375292021
1.155815914 6 0 0.997319833
POLR2F ENSG00000100142.14 0.296146847 2 2 0.182902274
Brain_Hypothalamus 0.559293585 Brain_Cerebellum 1.163342431
0.836657569 0.836657569 -0.583891095 1.331871937 0.373990421
1.354649031 2 0 0.997319833
LGI4 ENSG00000153902.13 0.296160726 1 1 0.296160726
Brain_Spinal_cord_cervical_c-1 0.296160726 Brain_Spinal_cord_cervical_c-1
1 1 1 1.044701988 1.044701988 1.044701988 NA 1 0
0.997319833
AEBP2 ENSG00000139154.14 0.296207961 1 1 0.296207961
Brain_Anterior_cingulate_cortex_BA24 0.296207961
Brain_Anterior_cingulate_cortex_BA24 1 1 1 -1.044599824
-1.044599824 -1.044599824 NA 1 0 0.997319833
NME2 ENSG00000243678.11 0.296251964 12 4 0.021720781
Brain_Putamen_basal_ganglia 0.190952648 Brain_Substantia_nigra
9.871776829 0.000210281 0.350128172 -2.295215444 -1.307766122
-1.84974915 0.234293695 4 0 0.997319833
AC069513.4 ENSG00000229178.1 0.296519246 13 1 0.180822178
Brain_Hypothalamus 0.533949196 Brain_Cerebellar_Hemisphere 12.41687914
0.001303049 12.41687914 -1.338227836 -0.621988855 -1.01725618
0.23525862 1 0 0.9973828
SKIV2L ENSG00000204351.11 0.296701889 12 4 0.02060559
Brain_Cerebellar_Hemisphere 0.354034607 Brain_Spinal_cord_cervical_c-1
9.657162964 0.001871761 0.423632569 -2.315134176 -0.926791869
-1.828523049 0.470782807 4 0 0.9973828
PPP2R1A ENSG00000105568.17 0.296710339 1 1 0.296710339
Brain_Anterior_cingulate_cortex_BA24 0.296710339
Brain_Anterior_cingulate_cortex_BA24 1 1 1 -1.043513916
-1.043513916 -1.043513916 NA 1 0 0.9973828
RAP1B ENSG00000127314.17 0.296770685 2 2 0.162399641
Brain_Substantia_nigra 0.423109719 Brain_Hypothalamus 1.080164673
0.919835327 0.919835327 -1.397046325 0.801037876 -0.298004224
1.554280244 2 0 0.9973828
ZNF524 ENSG00000171443.6 0.296792661 1 1 0.296792661
Brain_Cerebellar_Hemisphere 0.296792661 Brain_Cerebellar_Hemisphere 1 1
1 1.043336092 1.043336092 1.043336092 NA 1 0 0.9973828
LRRC8D ENSG00000171492.14 0.296895063 2 2 0.245943928
Brain_Cortex 0.341137872 Brain_Caudate_basal_ganglia 1.073861028
0.926138972 0.926138972 -1.160257634 -0.951919377 -1.056088505
0.147317394 2 0 0.9973828
ARMC1 ENSG00000104442.9 0.29690579 13 2 0.160658721 Brain_Hippocampus
0.85865017 Brain_Hypothalamus 11.76085747 0.004070798 0.467752493
-1.402859564 0.178092707 -0.995583932 0.415778577 2 0
0.9973828
ATP5S ENSG00000125375.14 0.296910032 4 4 0.349163703

Brain_Caudate_basal_ganglia 0.998441058 Brain_Frontal_Cortex_BA9
3.143229955 0.12141028 0.12141028 -0.936212666 0.751112479
-0.123575355 0.700948266 4 0 0.9973828
PPIC ENSG00000168938.5 0.296912255 2 2 0.284648647 Brain_Amygdala
0.290249529 Brain_Frontal_Cortex_BA9 1.068113465 0.931886535
0.931886535 1.057574377 1.069934823 1.0637546 0.008740155 2
0 0.9973828
LINC02033 ENSG00000279727.1 0.297334726 1 1 0.297334726
Brain_Cerebellum 0.297334726 Brain_Cerebellum 1 1 1 -1.042165995
-1.042165995 -1.042165995 NA 1 0 0.997539919
CTD-2145A24.5 ENSG00000265478.2 0.297488696 4 4 0.244934124
Brain_Nucleus_accumbens_basal_ganglia 0.496260343 Brain_Hypothalamus
2.084425054 0.445551768 0.445551768 -1.162742177 0.977622688
-0.039095273 1.043612812 4 0 0.997539919
GNG4 ENSG00000168243.10 0.297652746 2 2 0.24695895
Brain_Spinal_cord_cervical_c-1 0.341240516 Brain_Cortex 1.074435541
0.925564459 0.925564459 -0.951717019 1.157767449 0.103025215
1.491630773 2 0 0.997539919
SEL1L2 ENSG00000101251.11 0.29777374 2 2 0.198034621
Brain_Nucleus_accumbens_basal_ganglia 0.808076625 Brain_Caudate_basal_ganglia
1.684450472 0.315549528 0.315549528 -1.287171205 -0.242908056
-0.76503963 0.738405554 2 0 0.997539919
PRELID3B ENSG00000101166.15 0.297827948 3 3 0.170934476
Brain_Hippocampus 0.955267563 Brain_Substantia_nigra 2.201070626
0.127036495 0.127036495 -1.369207328 -0.056093198 -0.753772036
0.660409101 3 0 0.997539919
ATAD5 ENSG00000176208.8 0.297863803 4 2 0.165780865
Brain_Nucleus_accumbens_basal_ganglia 0.532433302 Brain_Hippocampus
3.71775102 0.016196219 0.171283965 -1.385888788 -0.62429586
-0.963511706 0.328887061 2 0 0.997539919
RP11-418H16.1 ENSG00000281920.1 0.29788626 7 7 0.081332736
Brain_Cerebellar_Hemisphere 0.951312357 Brain_Frontal_Cortex_BA9
3.830698839 0.204387105 0.204387105 -1.74300503 1.373812409
-0.14773858 0.937703522 7 0 0.997539919
PSMD4 ENSG00000159352.15 0.29792418 8 6 0.078644419
Brain_Nucleus_accumbens_basal_ganglia 0.921712288 Brain_Cerebellum
5.515714681 0.116025701 0.19567784 0.098277067 1.7586062
0.891621365 0.645051061 6 0 0.997539919
CPNE2 ENSG00000140848.16 0.297930024 4 4 0.103710057
Brain_Cerebellar_Hemisphere 0.973672819 Brain_Putamen_basal_ganglia
1.892290823 0.164566949 0.164566949 -1.627127332 0.033002218
-0.623870121 0.711060123 4 0 0.997539919
USP54 ENSG00000166348.18 0.297932758 1 1 0.297932758 Brain_Amygdala
0.297932758 Brain_Amygdala 1 1 1 -1.040876741 -1.040876741
-1.040876741 NA 1 0 0.997539919
COA4 ENSG00000181924.6 0.297967634 1 1 0.297967634
Brain_Putamen_basal_ganglia 0.297967634 Brain_Putamen_basal_ganglia 1 1
1 1.040801608 1.040801608 1.040801608 NA 1 0
0.997539919
RASAL2-AS1 ENSG00000224687.1 0.298101576 2 2 0.126456735
Brain_Nucleus_accumbens_basal_ganglia 0.884598172 Brain_Cortex
1.278392403 0.721607597 0.721607597 -1.528224794 -0.145142742
-0.836683768 0.977986698 2 0 0.997539919
AF067845.1 ENSG00000260721.1 0.29811535 2 2 0.142422193 Brain_Cortex
0.547275633 Brain_Frontal_Cortex_BA9 1.057326802 0.942673198
0.942673198 -1.466830417 -0.601847531 -1.034338974 0.611635264
2 0 0.997539919
GOLGB1 ENSG00000173230.15 0.298156864 1 1 0.298156864
Brain_Caudate_basal_ganglia 0.298156864 Brain_Caudate_basal_ganglia 1 1
1 -1.040394052 -1.040394052 -1.040394052 NA 1 0
0.997539919
NAV2 ENSG00000166833.19 0.298223177 1 1 0.298223177

Brain_Anterior_cingulate_cortex_BA24 0.298223177
Brain_Anterior_cingulate_cortex_BA24 1 1 1 -1.040251271
-1.040251271 -1.040251271 NA 1 0 0.997539919
C20orf202 ENSG00000215595.1 0.298311066 2 2 0.153869406
Brain_Amygdala 0.757775144 Brain_Caudate_basal_ganglia 1.209610279
0.790389721 0.790389721 -1.425996315 0.30840373 -0.558796292
1.226406033 2 0 0.997539919
RP3-393E18.2 ENSG00000237927.1 0.298659932 2 2 0.133554722
Brain_Frontal_Cortex_BA9 0.626105588 Brain_Putamen_basal_ganglia
1.053399127 0.946600873 0.946600873 0.487215549 1.500230434
0.993722991 0.716309695 2 0 0.997539919
CYB561 ENSG00000008283.15 0.298679216 11 4 0.06019354 Brain_Amygdala
0.183341493 Brain_Cerebellum 8.674602296 0.001652429 0.432786588
-1.879373294 -1.330536729 -1.600131806 0.152600399 4 0
0.997539919
GNAT1 ENSG00000114349.9 0.298706122 1 1 0.298706122
Brain_Hippocampus 0.298706122 Brain_Hippocampus 1 1 1
1.039212062 1.039212062 1.039212062 NA 1 0 0.997539919
WDR20 ENSG00000140153.17 0.2988101 1 1 0.2988101 Brain_Cortex 0.2988101
Brain_Cortex 1 1 1 -1.038988468 -1.038988468 -1.038988468
NA 1 0 0.997539919
NMBR ENSG00000135577.4 0.298863659 1 1 0.298863659
Brain_Substantia_nigra 0.298863659 Brain_Substantia_nigra 1 1
1 -1.038873315 -1.038873315 -1.038873315 NA 1 0
0.997539919
DNAJB14 ENSG00000164031.16 0.298929308 7 6 0.047211581
Brain_Substantia_nigra 0.993228678 Brain_Caudate_basal_ganglia
3.825127672 0.050160697 0.252161948 -1.494203127 1.984397176
-0.192378223 1.169411145 6 0 0.997539919
PCGF1 ENSG00000115289.13 0.299113737 1 1 0.299113737 Brain_Cortex
0.299113737 Brain_Cortex 1 1 1 1.038335823 1.038335823
1.038335823 NA 1 0 0.997539919
TXNL4B ENSG00000140830.8 0.299200076 9 6 0.13004414
Brain_Caudate_basal_ganglia 0.992989646 Brain_Frontal_Cortex_BA9
4.805500075 0.062799395 0.223182828 -1.416500242 1.51392785
-0.171758355 0.895711377 6 0 0.997539919
OSTM1-AS1 ENSG00000225174.1 0.299215091 2 2 0.167686212
Brain_Anterior_cingulate_cortex_BA24 0.41451668 Brain_Caudate_basal_ganglia
1.075444322 0.924555678 0.924555678 -1.379676694 -0.815971074
-1.097823884 0.398600066 2 0 0.997539919
RTN2 ENSG00000125744.11 0.299246122 1 1 0.299246122 Brain_Cerebellum
0.299246122 Brain_Cerebellum 1 1 1 -1.038051409 -1.038051409
-1.038051409 NA 1 0 0.997539919
IVL ENSG00000163207.6 0.29927968 2 2 0.183869813 Brain_Cerebellum
0.530648265 Brain_Substantia_nigra 1.128303044 0.871696956
0.871696956 -1.328933794 0.627016736 -0.350958529 1.383065884
2 0 0.997539919
SH3BGR ENSG00000185437.13 0.299420492 7 5 0.062558671
Brain_Hippocampus 0.492842983 Brain_Frontal_Cortex_BA9 4.2099157
0.040275294 0.196528529 -0.750936087 1.862315222 1.059662146
0.906821521 5 0 0.997539919
MASP1 ENSG00000127241.16 0.299702588 3 3 0.05833498
Brain_Caudate_basal_ganglia 0.788243325 Brain_Nucleus_accumbens_basal_ganglia
1.472275676 0.66669627 0.66669627 0.268592447 1.893172149
0.893502542 0.874727569 3 0 0.997539919
TBCD ENSG00000141556.20 0.299759481 6 5 0.090949824
Brain_Hypothalamus 0.977794752 Brain_Cerebellar_Hemisphere 3.20894874
0.049924639 0.164015234 -1.690408531 0.268142477 -0.432097698
0.70409062 5 0 0.997539919
RP1-232L24.3 ENSG00000231533.1 0.299779752 6 6 0.061339481
Brain_Nucleus_accumbens_basal_ganglia 0.949206381 Brain_Frontal_Cortex_BA9
3.467921593 0.17260317 0.17260317 -1.6631984 1.871040406 0.285846887

1.219867479 6 0 0.997539919
AKR7A3 ENSG00000162482.4 0.299808855 3 3 0.062800689
Brain_Substantia_nigra 0.907174616 Brain_Cortex 1.351091087
0.758454513 0.758454513 -0.116603056 1.860599971 0.784735219
1.000088782 3 0 0.997539919
SLC25A40 ENSG00000075303.12 0.299871515 1 1 0.299871515
Brain_Cerebellum 0.299871515 Brain_Cerebellum 1 1 1 1.03670896
1.03670896 1.03670896 NA 1 0 0.997539919
RP11-432J3.4 ENSG00000279797.1 0.299881572 1 1 0.299881572
Brain_Nucleus_accumbens_basal_ganglia 0.299881572
Brain_Nucleus_accumbens_basal_ganglia 1 1 1 1.036687388
1.036687388 1.036687388 NA 1 0 0.997539919
TRAF3 ENSG00000131323.14 0.299903878 4 4 0.146165179 Brain_Cerebellum
0.571102928 Brain_Hypothalamus 1.993291657 0.205004418
0.205004418 -0.842038026 1.453211 0.633325244 1.060693187 4
0 0.997539919
GPR12 ENSG00000132975.7 0.299935092 3 3 0.161129108
Brain_Putamen_basal_ganglia 0.763039274 Brain_Cerebellum 1.847676581
0.2822032 0.2822032 -1.401284186 0.301492098 -0.578400319
0.852818416 3 0 0.997539919
FIG4 ENSG00000112367.10 0.299984443 1 1 0.299984443
Brain_Putamen_basal_ganglia 0.299984443 Brain_Putamen_basal_ganglia 1 1
1 -1.036466752 -1.036466752 -1.036466752 NA 1 0
0.997539919
MLKL ENSG00000168404.12 0.300013267 4 4 0.101163936
Brain_Spinal_cord_cervical_c-1 0.764258003 Brain_Cortex 1.532262281
0.463385679 0.463385679 -1.63923688 -0.299894011 -0.941511536
0.597846685 4 0 0.997539919
TSTD1 ENSG00000215845.10 0.300122816 11 8 0.009885702
Brain_Frontal_Cortex_BA9 0.95058371 Brain_Hypothalamus 5.312486123
0.019751221 0.187760829 -2.579801861 -0.061973783 -1.028425458
0.676903065 8 0 0.997539919
FLT4 ENSG00000037280.15 0.300146537 4 3 0.226347262
Brain_Caudate_basal_ganglia 0.458884598 Brain_Putamen_basal_ganglia
2.041874568 0.057904123 0.844505599 -0.954018295 1.209821845
0.112856969 1.117759912 3 0 0.997539919
RUVBL2 ENSG00000183207.13 0.300164901 12 3 0.066699916
Brain_Cerebellum 0.736151426 Brain_Hippocampus 9.838358788
0.001565852 0.502285489 0.3369542 1.833690725 1.454442951
0.451786934 3 0 0.997539919
DTX4 ENSG00000110042.7 0.30017075 6 5 0.132906465
Brain_Caudate_basal_ganglia 0.844077732 Brain_Frontal_Cortex_BA9 3.43917375
0.114092734 0.238054721 -1.5027386 1.035380747 -0.391652873
0.829036987 5 0 0.997539919
RPN1 ENSG00000163902.11 0.300309055 4 4 0.18518508 Brain_Hippocampus
0.962110057 Brain_Spinal_cord_cervical_c-1 2.045062681 0.394814761
0.394814761 -0.475916688 1.324958006 0.519560407 0.919143407
4 0 0.997539919
KRT72 ENSG00000170486.10 0.30034072 1 1 0.30034072 Brain_Cerebellum 0.30034072
Brain_Cerebellum 1 1 1 -1.035703005 -1.035703005 -1.035703005
NA 1 0 0.997539919
GPR37 ENSG00000170775.2 0.300502547 1 1 0.300502547 Brain_Cortex
0.300502547 Brain_Cortex 1 1 1 1.035356299 1.035356299
1.035356299 NA 1 0 0.997539919
RCAN1 ENSG00000159200.17 0.300503348 4 4 0.083910926
Brain_Putamen_basal_ganglia 0.389832941 Brain_Hippocampus 1.976496139
0.285561245 0.285561245 -1.728431309 1.447888131 -0.021358627
1.520370499 4 0 0.997539919
MPP7 ENSG00000150054.18 0.300646018 3 3 0.160251895 Brain_Cortex
0.928794467 Brain_Cerebellar_Hemisphere 1.772511307 0.343695437
0.343695437 -0.089361692 1.404224881 0.73975797 0.760284887 3
0 0.997539919

SMCO3 ENSG00000179256.2 0.300770237 2 2 0.269325549 Brain_Cerebellum 0.276876411 Brain_Caudate_basal_ganglia 1.000116576 0.999883424 0.999883424 -1.104617074 -1.087363011 -1.095990043 0.012200465 2 0 0.997539919
LCN10 ENSG00000187922.13 0.300936729 2 2 0.127798051 Brain_Cerebellum 0.346721231 Brain_Cerebellar_Hemisphere 1.74404316 0.25595684 0.25595684 0.940968053 1.522842752 1.231905402 0.411447546 2 0 0.997539919
GIN1 ENSG00000145723.16 0.301102266 1 1 0.301102266 Brain_Cerebellum 0.301102266 Brain_Cerebellum 1 1 1 -1.034072511 -1.034072511 -1.034072511 NA 1 0 0.997539919
TMEM2 ENSG00000135048.13 0.301179386 1 1 0.301179386 Brain_Caudate_basal_ganglia 0.301179386 Brain_Caudate_basal_ganglia 1 1 1 1.033907549 1.033907549 1.033907549 NA 1 0 0.997539919
SPG21 ENSG00000090487.10 0.301391382 13 2 0.181317136 Brain_Spinal_cord_cervical_c-1 0.509408416 Brain_Amygdala 10.86303468 0.000344981 1.1405768 -1.246823546 1.336710552 0.901210184 0.668618787 2 0 0.997539919
DUSP2 ENSG00000158050.4 0.301397804 2 2 0.143714323 Brain_Cerebellar_Hemisphere 0.794613044 Brain_Putamen_basal_ganglia 1.166439639 0.833560361 0.833560361 -1.462098122 -0.260325059 -0.861211591 0.849781882 2 0 0.997539919
OSBPL6 ENSG00000079156.16 0.301455801 4 4 0.073734339 Brain_Putamen_basal_ganglia 0.560834714 Brain_Nucleus_accumbens_basal_ganglia 1.467681536 0.586805536 0.586805536 -1.788258335 0.758248728 -0.592432957 1.04593003 4 0 0.997539919
DEPTOR ENSG00000155792.9 0.301496643 3 3 0.309272835 Brain_Cerebellum 0.873507469 Brain_Cerebellar_Hemisphere 2.135852635 0.32967834 0.32967834 -1.01674903 -0.159204868 -0.66795746 0.450595322 3 0 0.997539919
FAM126B ENSG00000155744.9 0.301539083 2 2 0.122425491 Brain_Nucleus_accumbens_basal_ganglia 0.659818911 Brain_Cerebellar_Hemisphere 1.216598178 0.783401822 0.783401822 0.440163199 1.544672535 0.992417867 0.781006041 2 0 0.997539919
SYT7 ENSG00000011347.9 0.30164242 2 2 0.181747174 Brain_Cerebellum 0.604765288 Brain_Cerebellar_Hemisphere 1.765233496 0.234766504 0.234766504 -1.335394774 -0.517560012 -0.926477393 0.578296506 2 0 0.997539919
EDC4 ENSG00000038358.14 0.301722104 1 1 0.301722104 Brain_Spinal_cord_cervical_c-1 0.301722104 Brain_Spinal_cord_cervical_c-1 1 1 1 -1.032747445 -1.032747445 -1.032747445 NA 1 0 0.997539919
UBAP1 ENSG00000165006.13 0.301806751 4 4 0.263876106 Brain_Spinal_cord_cervical_c-1 0.42042983 Brain_Nucleus_accumbens_basal_ganglia 2.573347496 0.221292249 0.221292249 -1.117276534 0.973778929 0.385956267 1.0045095 4 0 0.997539919
BCL2A1 ENSG00000140379.7 0.301904905 2 2 0.121730616 Brain_Nucleus_accumbens_basal_ganglia 0.89235847 Brain_Caudate_basal_ganglia 1.099885724 0.900114276 0.900114276 -1.547550255 -0.13532051 -0.841435383 0.99859723 2 0 0.997539919
C16orf71 ENSG00000166246.13 0.301949782 1 1 0.301949782 Brain_Putamen_basal_ganglia 0.301949782 Brain_Putamen_basal_ganglia 1 1 1 1.032261179 1.032261179 1.032261179 NA 1 0 0.997539919
GTF3C5 ENSG00000148308.17 0.301950216 3 3 0.098488195 Brain_Cerebellum 0.665364644 Brain_Nucleus_accumbens_basal_ganglia 1.871185628 0.276202378 0.276202378 -1.652227439 -0.432518452 -0.866047736 0.682051495 3 0 0.997539919
TNFRSF6B ENSG00000243509.4 0.302015023 1 1 0.302015023 Brain_Cerebellum 0.302015023 Brain_Cerebellum 1 1 1 -1.032121884 -1.032121884 -1.032121884 NA 1 0 0.997539919

RP11-384P7.7 ENSG00000260947.1 0.30202136 13 1 0.188635134
Brain_Substantia_nigra 0.539679972 Brain_Caudate_basal_ganglia
12.07818056 0.000486183 12.07818056 -1.314627467 -0.613297007
-0.994941019 0.199321515 1 0 0.997539919
LSM10 ENSG00000181817.5 0.30203367 5 5 0.107534368 Brain_Cerebellum
0.868933756 Brain_Caudate_basal_ganglia 1.276915522 0.78276065 0.78276065
-1.574357238 1.609375 0.363442685 1.231325444 5 0
0.997539919
TMEM26 ENSG00000196932.11 0.302056732 7 3 0.072037728
Brain_Hippocampus 0.60764883 Brain_Frontal_Cortex_BA9 5.478605181
0.010691398 0.255912072 0.51343248 1.798879602 1.201583283
0.403698168 3 0 0.997539919
SPATA24 ENSG00000170469.10 0.302111211 1 1 0.302111211
Brain_Hippocampus 0.302111211 Brain_Hippocampus 1 1 1
-1.031916552 -1.031916552 -1.031916552 NA 1 0 0.997539919
RP11-275H4.1 ENSG00000241231.1 0.30253831 7 7 0.051380702
Brain_Anterior_cingulate_cortex_BA24 0.859631973 Brain_Amygdala 1.49551576
0.633584611 0.633584611 -1.948286414 1.218824148 -0.512759205
1.02769574 7 0 0.997539919
SULT1A1 ENSG00000196502.11 0.302618563 12 4 0.05711455
Brain_Cerebellar_Hemisphere 0.333158143 Brain_Frontal_Cortex_BA9
9.739133678 0.001721961 0.418202418 -1.902433153 -0.967772215
-1.271638531 0.29486149 4 0 0.997539919
LRP1 ENSG00000123384.13 0.302664403 1 1 0.302664403
Brain_Hippocampus 0.302664403 Brain_Hippocampus 1 1 1
1.030736501 1.030736501 1.030736501 NA 1 0 0.997539919
6-Mar ENSG00000145495.15 0.302690647 1 1 0.302690647
Brain_Cerebellar_Hemisphere 0.302690647 Brain_Cerebellar_Hemisphere 1 1
1 -1.030680555 -1.030680555 -1.030680555 NA 1 0
0.997539919
PRUNE2 ENSG00000106772.17 0.302749217 2 2 0.200889932
Brain_Cerebellar_Hemisphere 0.382599995 Brain_Cerebellum 1.930789588
0.069210412 0.069210412 0.873115741 1.279020232 1.076067986
0.287017818 2 0 0.997539919
MACROD2 ENSG00000172264.16 0.302793366 1 1 0.302793366
Brain_Cerebellum 0.302793366 Brain_Cerebellum 1 1 1 1.030461606
1.030461606 1.030461606 NA 1 0 0.997539919
SKA3 ENSG00000165480.15 0.302857612 2 2 0.126295191
Brain_Caudate_basal_ganglia 0.736138812 Brain_Hippocampus 1.07232638
0.92767362 0.92767362 -1.528875982 -0.336970933 -0.932923458
0.842804143 2 0 0.997539919
DLGAP1 ENSG00000170579.16 0.303086622 1 1 0.303086622
Brain_Putamen_basal_ganglia 0.303086622 Brain_Putamen_basal_ganglia 1 1
1 1.029836801 1.029836801 1.029836801 NA 1 0
0.997539919
TOR1AIP2 ENSG00000169905.12 0.303104622 1 1 0.303104622
Brain_Anterior_cingulate_cortex_BA24 0.303104622
Brain_Anterior_cingulate_cortex_BA24 1 1 1 -1.029798463
-1.029798463 -1.029798463 NA 1 0 0.997539919
C5orf49 ENSG00000215217.6 0.303120745 2 2 0.254592908
Brain_Caudate_basal_ganglia 0.272449826 Brain_Putamen_basal_ganglia
1.048229051 0.951770949 0.951770949 -1.139264362 -1.097438329
-1.118351346 0.029575472 2 0 0.997539919
CRNKL1 ENSG00000101343.14 0.303248663 1 1 0.303248663
Brain_Cerebellar_Hemisphere 0.303248663 Brain_Cerebellar_Hemisphere 1 1
1 -1.029491732 -1.029491732 -1.029491732 NA 1 0
0.997539919
LRR1 ENSG00000165501.16 0.303361728 1 1 0.303361728 Brain_Cortex
0.303361728 Brain_Cortex 1 1 1 1.02925103 1.02925103 1.02925103
NA 1 0 0.997539919
NPAP1 ENSG00000185823.3 0.303470457 4 4 0.367321792
Brain_Spinal_cord_cervical_c-1 0.725388082 Brain_Hippocampus

2.477478318 0.29783164 0.29783164 -0.568017889 0.901501397
0.150242934 0.719097534 4 0 0.997539919
PARD6A ENSG00000102981.9 0.303493577 1 1 0.303493577
Brain_Nucleus_accumbens_basal_ganglia 0.303493577
Brain_Nucleus_accumbens_basal_ganglia 1 1 1 1.028970417
1.028970417 1.028970417 NA 1 0 0.997539919
AC009404.2 ENSG00000236255.1 0.303541916 10 6 0.095539009
Brain_Anterior_cingulate_cortex_BA24 0.970384381 Brain_Putamen_basal_ganglia
6.668775896 0.016779898 0.268574644 -1.666876276 0.037126201
-0.820428282 0.510965194 6 0 0.997539919
APBB1 ENSG00000166313.18 0.303552622 1 1 0.303552622 Brain_Cerebellum
0.303552622 Brain_Cerebellum 1 1 1 -1.028844777 -1.028844777
-1.028844777 NA 1 0 0.997539919
KCNH1 ENSG00000143473.12 0.303705151 2 2 0.162961168
Brain_Putamen_basal_ganglia 0.40568225 Brain_Spinal_cord_cervical_c-1
1.126025251 0.873974749 0.873974749 0.831515899 1.395181322
1.113348611 0.398571643 2 0 0.997539919
HELB ENSG00000127311.9 0.303715913 12 3 0.121767151
Brain_Nucleus_accumbens_basal_ganglia 0.964930871
Brain_Spinal_cord_cervical_c-1 10.3692722 0.00126799 0.467816451
-1.547398631 -0.043966797 -0.693122464 0.414476599 3 0
0.997539919
TUBD1 ENSG00000108423.14 0.303780017 3 3 0.093809623
Brain_Nucleus_accumbens_basal_ganglia 0.741861795 Brain_Caudate_basal_ganglia
1.345943871 0.801397355 0.801397355 -1.675635438 -0.329388849
-1.054122163 0.679032953 3 0 0.997539919
PSMD7 ENSG00000103035.10 0.303831176 1 1 0.303831176
Brain_Nucleus_accumbens_basal_ganglia 0.303831176
Brain_Nucleus_accumbens_basal_ganglia 1 1 1 1.02825227 1.02825227
1.02825227 NA 1 0 0.997539919
INPP5A ENSG00000068383.18 0.30387239 3 3 0.09687205
Brain_Caudate_basal_ganglia 0.425819899 Brain_Spinal_cord_cervical_c-1
1.676651332 0.320245218 0.320245218 -1.660210814 0.796365028
-0.573173519 1.252416239 3 0 0.997539919
RP11-862G15.2 ENSG00000258499.1 0.303992892 2 2 0.212591515
Brain_Cerebellum 0.330878997 Brain_Cerebellar_Hemisphere 1.051097684
0.948902316 0.948902316 -1.24647104 -0.972344893 -1.109407966
0.193836458 2 0 0.997539919
SCFD2 ENSG00000184178.15 0.304086439 1 1 0.304086439
Brain_Hippocampus 0.304086439 Brain_Hippocampus 1 1 1
-1.027709624 -1.027709624 -1.027709624 NA 1 0 0.997539919
LSMEM1 ENSG00000181016.9 0.304109062 10 2 0.434000536
Brain_Hippocampus 0.988086227 Brain_Frontal_Cortex_BA9 8.896033659
0.00244208 0.419910437 -0.782364252 0.532768317 0.000480484
0.448786152 2 0 0.997539919
ACADS ENSG00000122971.8 0.304188035 12 3 0.31862645
Brain_Spinal_cord_cervical_c-1 0.95928824 Brain_Caudate_basal_ganglia
10.20887142 0.014568831 0.391006586 -0.765880959 0.997284469
0.263228611 0.489624056 3 0 0.997539919
INCENP ENSG00000149503.12 0.304311976 1 1 0.304311976
Brain_Putamen_basal_ganglia 0.304311976 Brain_Putamen_basal_ganglia 1 1
1 -1.027230421 -1.027230421 -1.027230421 NA 1 0
0.997539919
IRF3 ENSG00000126456.15 0.30436206 5 4 0.08664625 Brain_Cerebellum
0.436196702 Brain_Cerebellar_Hemisphere 3.847752166 0.033221708
0.155057765 0.778631696 1.713360438 1.285051023 0.46595969 4
0 0.997539919
MCEE ENSG00000124370.10 0.304391264 1 1 0.304391264 Brain_Cerebellum
0.304391264 Brain_Cerebellum 1 1 1 1.027062014 1.027062014
1.027062014 NA 1 0 0.997539919
ZNF91 ENSG00000167232.13 0.304414064 4 4 0.095566687 Brain_Cortex
0.756707378 Brain_Amygdala 2.14482159 0.398962054 0.398962054

-1.666737127	-0.30980746	-1.009875044	0.554527342	4	0
0.997539919
PAX6	ENSG00000007372.21	0.30453225	1	1	0.30453225	Brain_Cerebellum	0.30453225
Brain_Cerebellum	1	1	1	1.02676263	1.02676263	1.02676263	NA	1	0
0.997539919
PRSS54	ENSG00000103023.11	0.304643164	3	3	0.371861469
Brain_Frontal_Cortex_BA9	0.8876097	Brain_Cortex	2.19394066	0.083234405
0.083234405	-0.892991978	0.341784985	-0.230845475	0.622236594
3	0	0.997539919
CTD-2501M5.1	ENSG00000253507.5	0.304787873	8	4	0.145464504
Brain_Hypothalamus	0.82018867	Brain_Cerebellum	5.883143787	0.002852093
0.338397418	-1.029631531	1.455740015	-0.185763433	0.763906568
4	0	0.997539919
RICTOR	ENSG00000164327.12	0.304882048	1	1	0.304882048
Brain_Frontal_Cortex_BA9	0.304882048	Brain_Frontal_Cortex_BA9	1	1
1	1.02602023	1.02602023	1.02602023	NA	1	0	0.997539919
RP11-173M1.4	ENSG00000266313.1	0.30492369	1	1	0.30492369
Brain_Anterior_cingulate_cortex_BA24	0.30492369
Brain_Anterior_cingulate_cortex_BA24	1	1	1	1.025931889
1.025931889	1.025931889	NA	1	0	0.997539919
PRODH2	ENSG00000250799.9	0.305108662	2	2	0.127254992
Brain_Cerebellum	0.825500124	Brain_Cerebellar_Hemisphere	1.000164364
0.999835636	0.999835636	0.220476434	1.525016459	0.872746446
0.922449098	2	0	0.997539919
IRGC	ENSG00000124449.6	0.305130786	1	1	0.305130786	Brain_Cerebellum
0.305130786	Brain_Cerebellum	1	1	1	1.025492662	1.025492662
1.025492662	NA	1	0	0.997539919
ADAM23	ENSG00000114948.12	0.305190946	2	2	0.153871938
Brain_Cerebellum	0.294280103	Brain_Hypothalamus	1.352743866
0.647256134	0.647256134	-1.425987542	-1.048778439	-1.23738299
0.266727115	2	0	0.997539919
AC108868.6	ENSG00000232001.1	0.305232298	2	2	0.124952996
Brain_Substantia_nigra	0.474921562	Brain_Caudate_basal_ganglia
1.541692863	0.458307137	0.458307137	0.714494328	1.534311671
1.124402999	0.579698403	2	0	0.997539919
RP11-83N9.5	ENSG00000260193.1	0.30528886	1	1	0.30528886
Brain_Hypothalamus	0.30528886	Brain_Hypothalamus	1	1	1
-1.025157537	-1.025157537	-1.025157537	NA	1	0	0.997539919
MYH7B	ENSG00000078814.15	0.305301479	3	3	0.091157352
Brain_Nucleus_accumbens_basal_ganglia	0.460854324	Brain_Cerebellar_Hemisphere
1.937662078	0.086640781	0.086640781	-1.689324023	0.923913966
-0.009323082	1.457907895	3	0	0.997539919
SNX4	ENSG00000114520.10	0.305446856	1	1	0.305446856
Brain_Caudate_basal_ganglia	0.305446856	Brain_Caudate_basal_ganglia	1	1
1	1.024822695	1.024822695	1.024822695	NA	1	0
0.997539919
SVEP1	ENSG00000165124.17	0.305464291	4	4	0.030260987
Brain_Cerebellar_Hemisphere	0.927346888	Brain_Spinal_cord_cervical_c-1
2.02624063	0.369148131	0.369148131	-2.166657397	0.091183371
-1.005435206	0.957220919	4	0	0.997539919
MTERF2	ENSG00000120832.9	0.305477771	1	1	0.305477771
Brain_Hypothalamus	0.305477771	Brain_Hypothalamus	1	1	1
1.024757189	1.024757189	1.024757189	NA	1	0	0.997539919
FUT7	ENSG00000180549.7	0.305532055	2	2	0.213411421
Brain_Nucleus_accumbens_basal_ganglia	0.32140368	Brain_Cortex	1.069461912
0.930538088	0.930538088	0.991577479	1.244239525	1.117908502
0.178659045	2	0	0.997539919
CMTM5	ENSG00000166091.19	0.305614036	1	1	0.305614036	Brain_Cerebellum
0.305614036	Brain_Cerebellum	1	1	1	-1.024468512	-1.024468512
-1.024468512	NA	1	0	0.997539919
CISD1	ENSG00000122873.11	0.305761881	1	1	0.305761881	Brain_Cerebellum
0.305761881	Brain_Cerebellum	1	1	1	1.0241554	1.0241554	1.0241554

NA 1 0 0.997539919
NFATC1 ENSG00000131196.17 0.305811353 3 3 0.121093481
Brain_Hippocampus 0.591636341 Brain_Caudate_basal_ganglia 1.114729878
0.895021563 0.895021563 -1.550200161 1.084452556 -0.334071292
1.32893624 3 0 0.997539919
NFRKB ENSG00000170322.14 0.305874199 11 2 0.153258378
Brain_Hypothalamus 0.857569408 Brain_Frontal_Cortex_BA9 9.323677553
0.000890941 1.024214868 0.179469062 1.42811633 0.861712963
0.39701773 2 0 0.997539919
RNH1 ENSG00000023191.16 0.305889997 8 5 0.243130772 Brain_Cortex
0.97060736 Brain_Anterior_cingulate_cortex_BA24 4.938387739 0.004489364
0.280510846 -0.762772896 1.167197124 -0.159091306 0.66322025 5
0 0.997539919
VWA5B1 ENSG00000158816.15 0.305964401 3 3 0.058722848
Brain_Hypothalamus 0.993748699 Brain_Spinal_cord_cervical_c-1
1.105891858 0.894142732 0.894142732 -1.890262602 0.077816578
-0.606760316 1.112370277 3 0 0.997539919
SIGLEC1 ENSG00000088827.12 0.306035796 2 2 0.123877678
Brain_Cerebellar_Hemisphere 0.980877296 Brain_Hippocampus 1.000896463
0.999103537 0.999103537 -1.538699487 0.02396905 -0.757365218
1.10497352 2 0 0.997539919
AANAT ENSG00000129673.9 0.306300842 8 5 0.057841212
Brain_Frontal_Cortex_BA9 0.594020524 Brain_Spinal_cord_cervical_c-1
5.777351512 0.011894849 0.230743643 -1.896899434 -0.533018862
-1.140938914 0.480914551 5 0 0.997539919
MRPL46 ENSG00000259494.1 0.306331795 1 1 0.306331795
Brain_Substantia_nigra 0.306331795 Brain_Substantia_nigra 1 1
1 -1.022949348 -1.022949348 -1.022949348 NA 1 0
0.997539919
AVPR1A ENSG00000166148.3 0.3063859 2 2 0.206277459
Brain_Cerebellar_Hemisphere 0.308261332 Brain_Caudate_basal_ganglia
1.117351583 0.882648417 0.882648417 1.018877068 1.263867861
1.141372465 0.173234651 2 0 0.997539919
SMARCAL1 ENSG00000138375.12 0.306419472 3 3 0.057604073
Brain_Cortex 0.980665334 Brain_Spinal_cord_cervical_c-1 1.189504669
0.813485551 0.813485551 -0.024234783 1.898698925 0.747768076
1.015927324 3 0 0.997539919
GDA ENSG00000119125.16 0.306422633 1 1 0.306422633
Brain_Caudate_basal_ganglia 0.306422633 Brain_Caudate_basal_ganglia 1 1
1 -1.022757254 -1.022757254 -1.022757254 NA 1 0
0.997539919
RP11-461O7.1 ENSG00000246379.6 0.306440139 5 4 0.120788681
Brain_Substantia_nigra 0.702316011 Brain_Cortex 3.555201408
0.04169419 0.343989906 -1.551471712 0.723276523 -0.457868814
0.814404685 4 0 0.997539919
CCDC114 ENSG00000105479.15 0.306443234 10 4 0.021247545
Brain_Hypothalamus 0.715697192 Brain_Caudate_basal_ganglia 7.028704652
0.009256375 0.662833585 0.364215368 2.303556618 1.365544288
0.610239345 4 0 0.997539919
ZNF334 ENSG00000198185.11 0.306474323 7 3 0.061435925
Brain_Cerebellar_Hemisphere 0.952484305 Brain_Substantia_nigra
5.440193081 0.002748318 0.379533027 -1.870344998 -0.059587336
-1.059274869 0.543090632 3 0 0.997539919
NADSYN1 ENSG00000172890.11 0.306542008 8 5 0.12703568 Brain_Amygdala
0.698454802 Brain_Spinal_cord_cervical_c-1 5.512776358 0.001472847
0.272976076 -1.525896349 1.1165098 0.220233635 0.81694275 5 0
0.997539919
STEAP4 ENSG00000127954.12 0.306571335 1 1 0.306571335
Brain_Spinal_cord_cervical_c-1 0.306571335 Brain_Spinal_cord_cervical_c-1
1 1 1 -1.022442878 -1.022442878 -1.022442878 NA 1 0
0.997539919
RP13-977J11.9 ENSG00000276693.1 0.306578486 1 1 0.306578486

Brain_Cerebellum 0.306578486 Brain_Cerebellum 1 1 1 1.022427763
1.022427763 1.022427763 NA 1 0 0.997539919
CCS ENSG00000173992.8 0.306623138 6 6 0.069532485
Brain_Nucleus_accumbens_basal_ganglia 0.720096391 Brain_Putamen_basal_ganglia
2.834916202 0.239884756 0.239884756 -0.609908847 1.814944202
0.5679816 0.815158062 6 0 0.997539919
CBS ENSG00000160200.17 0.306643378 13 1 0.168811989
Brain_Substantia_nigra 0.697303588 Brain_Amygdala 11.86861627
0.005478743 11.86861627 0.388962904 1.376031153 0.972144845
0.297520429 1 0 0.997539919
OPALIN ENSG00000197430.10 0.306651545 1 1 0.306651545
Brain_Cortex 0.306651545 Brain_Cortex 1 1 1 1.022273345
1.022273345 1.022273345 NA 1 0 0.997539919
FAM83E ENSG00000105523.3 0.306654347 5 4 0.098330401
Brain_Anterior_cingulate_cortex_BA24 0.586247447 Brain_Cerebellum
2.069206331 0.063553543 0.848889557 -0.981506419 1.653002272
0.627468827 1.001052634 4 0 0.997539919
TAP2 ENSG00000204267.13 0.306680179 10 9 0.105254468
Brain_Frontal_Cortex_BA9 0.8565615 Brain_Anterior_cingulate_cortex_BA24
5.556317822 0.160158856 0.193719291 -1.619896702 1.284043996
-0.609926549 0.850004615 9 0 0.997539919
PRKAR2B ENSG00000005249.12 0.306698626 3 3 0.162786176
Brain_Cerebellum 0.956722833 Brain_Spinal_cord_cervical_c-1 2.084266631
0.349344996 0.349344996 0.054266508 1.395762002 0.77081924
0.675423445 3 0 0.997539919
PIGP ENSG00000185808.13 0.306934177 7 5 0.182305754
Brain_Hypothalamus 0.949108086 Brain_Nucleus_accumbens_basal_ganglia
4.55002802 0.01846582 0.192896286 -0.906195958 1.333689145
-0.259907206 0.784345467 5 0 0.997539919
TRPM5 ENSG00000070985.13 0.306985489 1 1 0.306985489
Brain_Caudate_basal_ganglia 0.306985489 Brain_Caudate_basal_ganglia 1 1
1 -1.021567833 -1.021567833 -1.021567833 NA 1 0
0.997539919
CD4 ENSG00000010610.9 0.307091373 4 4 0.229869197
Brain_Putamen_basal_ganglia 0.739751183 Brain_Nucleus_accumbens_basal_ganglia
1.733864803 0.469311482 0.469311482 -1.200695868 0.98734785
-0.223843101 0.903481776 4 0 0.997539919
SSTR4 ENSG00000132671.5 0.30715031 4 4 0.044733506
Brain_Nucleus_accumbens_basal_ganglia 0.517256648 Brain_Cerebellar_Hemisphere
2.255769336 0.096367694 0.096367694 -2.007151761 -0.647579982
-1.095474192 0.624361082 4 0 0.997539919
XXbac-BPGBPG55C20.3 ENSG00000271761.1 0.307178014 4 4 0.205118855
Brain_Cortex 0.452130347 Brain_Nucleus_accumbens_basal_ganglia
2.243222361 0.227395268 0.227395268 -1.11919899 1.267101904
0.015463974 1.172547747 4 0 0.997539919
PPIAL4C ENSG00000263464.2 0.30724298 1 1 0.30724298 Brain_Cerebellum
0.30724298 Brain_Cerebellum 1 1 1 -1.021024185 -1.021024185
-1.021024185 NA 1 0 0.997539919
LRRC32 ENSG00000137507.11 0.307244536 1 1 0.307244536
Brain_Putamen_basal_ganglia 0.307244536 Brain_Putamen_basal_ganglia 1 1
1 -1.021020902 -1.021020902 -1.021020902 NA 1 0
0.997539919
TTC37 ENSG00000198677.10 0.307332663 9 3 0.066998947
Brain_Substantia_nigra 0.52727231 Brain_Anterior_cingulate_cortex_BA24
7.387953947 0.002524596 0.383858434 0.632175339 1.831681094
1.485865399 0.391557135 3 0 0.997539919
CAPN10 ENSG00000142330.19 0.307337378 5 3 0.518242847
Brain_Nucleus_accumbens_basal_ganglia 0.806272037 Brain_Putamen_basal_ganglia
4.32041627 0.037729214 0.254043929 -0.646056375 0.412166363
-0.213815193 0.501460265 3 0 0.997539919
LINC01864 ENSG00000267522.1 0.307340129 2 2 0.148814033
Brain_Nucleus_accumbens_basal_ganglia 0.602494147 Brain_Hypothalamus

1.002638515 0.997361485 0.997361485 0.520817161 1.443733221
0.982275191 0.652600205 2 0 0.997539919
NDNF ENSG00000173376.13 0.307368636 1 1 0.307368636
Brain_Substantia_nigra 0.307368636 Brain_Substantia_nigra 1 1
1 1.020758995 1.020758995 1.020758995 NA 1 0
0.997539919
CYP4A22 ENSG00000162365.11 0.307637166 7 4 0.105466359
Brain_Hippocampus 0.998657225 Brain_Cortex 5.19838747 0.018369577
0.267331354 -1.618911255 -0.001682919 -0.952533646 0.648556811
4 0 0.998179868
UBQLN4 ENSG00000160803.7 0.307823281 4 4 0.177936293
Brain_Hypothalamus 0.742728804 Brain_Anterior_cingulate_cortex_BA24
1.30687461 0.641879421 0.641879421 -1.314168243 1.347136444
-0.111303496 1.190283348 4 0 0.998207054
DIRAS2 ENSG00000165023.6 0.307882801 1 1 0.307882801
Brain_Spinal_cord_cervical_c-1 0.307882801 Brain_Spinal_cord_cervical_c-1
1 1 1 -1.019674622 -1.019674622 -1.019674622 NA 1 0
0.998207054
TPST2 ENSG00000128294.15 0.307923122 3 3 0.128423803 Brain_Cortex
0.665085899 Brain_Anterior_cingulate_cortex_BA24 1.311516831
0.662861789 0.662861789 -0.808442075 1.520346938 0.093000923
1.250297986 3 0 0.998207054
RP11-838N2.5 ENSG00000266578.1 0.308101475 1 1 0.308101475
Brain_Hypothalamus 0.308101475 Brain_Hypothalamus 1 1 1
1.019213803 1.019213803 1.019213803 NA 1 0 0.998207054
SNX6 ENSG00000129515.18 0.308184301 1 1 0.308184301 Brain_Amygdala
0.308184301 Brain_Amygdala 1 1 1 -1.019039318 -1.019039318
-1.019039318 NA 1 0 0.998207054
CNGB3 ENSG00000170289.12 0.308255602 1 1 0.308255602 Brain_Cortex
0.308255602 Brain_Cortex 1 1 1 -1.018889135 -1.018889135
-1.018889135 NA 1 0 0.998207054
MTMR4 ENSG00000108389.9 0.30854063 1 1 0.30854063 Brain_Cerebellum 0.30854063
Brain_Cerebellum 1 1 1 -1.01828901 -1.01828901 -1.01828901
NA 1 0 0.998207054
CTD-2054N24.2 ENSG00000259363.5 0.308692569 13 9 0.005406037
Brain_Frontal_Cortex_BA9 0.972500407 Brain_Hippocampus 7.199054234
0.022017273 0.275939269 -2.78178784 1.735435317 0.358330915
1.061663429 9 0 0.998207054
KDM7A ENSG00000006459.10 0.308735956 8 2 0.138812821
Brain_Frontal_Cortex_BA9 0.672630395 Brain_Spinal_cord_cervical_c-1
7.39068625 0.000203802 0.329359535 -1.480226461 -0.422540706
-0.791354407 0.359671121 2 0 0.998207054
ANAPC5 ENSG00000089053.12 0.30880248 4 4 0.10750238 Brain_Cortex
0.770566278 Brain_Cerebellum 1.18573892 0.792287319 0.792287319
-0.536691476 1.609521396 0.454596337 1.034855632 4 0
0.998207054
WDR41 ENSG00000164253.13 0.308935054 11 4 0.070331489
Brain_Frontal_Cortex_BA9 0.606034131 Brain_Substantia_nigra
8.057234931 0.007905571 0.366461161 -1.809769824 -0.515742694
-1.212608278 0.441237059 4 0 0.998207054
NFATC3 ENSG00000072736.18 0.30900516 8 7 0.025439636 Brain_Cerebellum
0.876039382 Brain_Caudate_basal_ganglia 3.851207656 0.038407971
0.216400508 0.15599193 2.234660648 1.304102605 0.822701118 7
0 0.998207054
H6PD ENSG00000049239.12 0.309132805 8 8 0.010815327
Brain_Spinal_cord_cervical_c-1 0.899601683 Brain_Hippocampus
2.252464464 0.14891827 0.14891827 -0.972527408 2.548609688
0.423355597 1.029662559 8 0 0.998207054
EFCC1 ENSG00000114654.7 0.309233802 2 2 0.125504623
Brain_Cerebellar_Hemisphere 0.16773342 Brain_Cerebellum 1.898025214
0.101974786 0.101974786 1.379523455 1.532072183 1.455797819
0.10786824 2 0 0.998207054

RASIP1 ENSG00000105538.9 0.309287199 2 2 0.154975165
Brain_Cerebellar_Hemisphere 0.195048569 Brain_Cerebellum 1.586872805
0.413127195 0.413127195 1.295787896 1.422175997 1.358981947
0.089369883 2 0 0.998207054
MFSD12 ENSG00000161091.12 0.309409986 6 4 0.116383057
Brain_Spinal_cord_cervical_c-1 0.88320967 Brain_Hippocampus 3.580885337
0.010700375 0.521135224 -0.295849031 1.570137799 0.507396528
0.675329534 4 0 0.998207054
TAS1R1 ENSG00000173662.20 0.309438263 1 1 0.309438263
Brain_Cerebellar_Hemisphere 0.309438263 Brain_Cerebellar_Hemisphere 1 1
1 1.016401435 1.016401435 1.016401435 NA 1 0
0.998207054
MIAT ENSG00000225783.6 0.309554242 4 4 0.04487002
Brain_Caudate_basal_ganglia 0.678487958 Brain_Cerebellar_Hemisphere
1.489335806 0.55957612 0.55957612 -0.414527327 2.005870936
0.705817473 0.994825792 4 0 0.998207054
C7orf72 ENSG00000164500.6 0.309577652 1 1 0.309577652
Brain_Substantia_nigra 0.309577652 Brain_Substantia_nigra 1 1
1 1.016108647 1.016108647 1.016108647 NA 1 0
0.998207054
ZRANB2 ENSG00000132485.13 0.309778184 1 1 0.309778184
Brain_Amygdala 0.309778184 Brain_Amygdala 1 1 1 -1.015687581
-1.015687581 -1.015687581 NA 1 0 0.998207054
TMEM136 ENSG00000181264.8 0.309931923 4 4 0.370121804
Brain_Hippocampus 0.877362311 Brain_Amygdala 2.559593125
0.144177875 0.144177875 -0.89624523 0.232054248 -0.284251613
0.563031275 4 0 0.998207054
GINS2 ENSG00000131153.8 0.309992922 1 1 0.309992922
Brain_Spinal_cord_cervical_c-1 0.309992922 Brain_Spinal_cord_cervical_c-1
1 1 1 -1.015236885 -1.015236885 -1.015236885 NA 1 0
0.998207054
TMEM143 ENSG00000161558.10 0.310023589 3 3 0.17937873
Brain_Nucleus_accumbens_basal_ganglia 0.812377138 Brain_Substantia_nigra
1.878322692 0.171979608 0.171979608 0.237360491 1.342670381
0.842399573 0.560053374 3 0 0.998207054
C2orf91 ENSG00000205086.6 0.310026032 1 1 0.310026032
Brain_Spinal_cord_cervical_c-1 0.310026032 Brain_Spinal_cord_cervical_c-1
1 1 1 1.015167411 1.015167411 1.015167411 NA 1 0
0.998207054
RP11-568N6.1 ENSG00000260101.1 0.310074845 1 1 0.310074845
Brain_Hypothalamus 0.310074845 Brain_Hypothalamus 1 1 1
-1.015064997 -1.015064997 -1.015064997 NA 1 0 0.998207054
RP11-408A13.4 ENSG00000272871.1 0.310104555 11 7 0.035827481
Brain_Spinal_cord_cervical_c-1 0.903362009 Brain_Frontal_Cortex_BA9
5.759293861 0.019490928 0.413054132 -2.098879988 0.158018827
-1.167969172 0.82598524 7 0 0.998207054
LILRB4 ENSG00000186818.12 0.31018261 1 1 0.31018261
Brain_Nucleus_accumbens_basal_ganglia 0.31018261
Brain_Nucleus_accumbens_basal_ganglia 1 1 1 -1.014838937
-1.014838937 -1.014838937 NA 1 0 0.998207054
RUNX1T1 ENSG00000079102.16 0.310209445 1 1 0.310209445
Brain_Hypothalamus 0.310209445 Brain_Hypothalamus 1 1 1
-1.014782651 -1.014782651 -1.014782651 NA 1 0 0.998207054
TNNT3 ENSG00000130595.17 0.310215549 2 2 0.137167357
Brain_Spinal_cord_cervical_c-1 0.6977126 Brain_Frontal_Cortex_BA9
1.017359189 0.982640811 0.982640811 -1.486422672 -0.388410062
-0.937416367 0.776412163 2 0 0.998207054
TNFRSF10C ENSG00000173535.13 0.310252914 12 5 0.018096991
Brain_Hypothalamus 0.711043544 Brain_Substantia_nigra 9.10422794
0.016542909 0.326204531 -2.363627641 -0.37045494 -1.70546978
0.500571381 5 0 0.998207054
OSBPL5 ENSG0000021762.19 0.310312076 3 3 0.112571096

Brain_Cerebellum 0.735534082 Brain_Frontal_Cortex_BA9 1.190044182
0.810045463 0.810045463 -1.586741971 -0.337773234 -0.985624301
0.625794483 3 0 0.998207054
LRIT2 ENSG00000204033.9 0.310357857 7 3 0.047974365
Brain_Caudate_basal_ganglia 0.40003537 Brain_Amygdala 5.548824687
0.012164994 0.373304575 0.841558066 1.977595431 1.360375422
0.414342637 3 0 0.998207054
RP11-407N8.6 ENSG00000274156.1 0.310676074 1 1 0.310676074
Brain_Caudate_basal_ganglia 0.310676074 Brain_Caudate_basal_ganglia 1 1
1 1.013804446 1.013804446 1.013804446 NA 1 0
0.998207054
TCFL5 ENSG00000101190.12 0.310699507 13 2 0.050321537 Brain_Cortex
0.33079356 Brain_Putamen_basal_ganglia 11.29268264 0.012298391
0.459413274 0.972516701 1.957220607 1.354091631 0.274163021
2 0 0.998207054
RP11-30P6.6 ENSG00000234155.1 0.310775478 5 4 0.489631022
Brain_Anterior_cingulate_cortex_BA24 0.816632938 Brain_Cortex
4.100656872 0.071230869 0.143091639 -0.690895806 0.6774696
-0.073895118 0.533211939 4 0 0.998207054
SATB2 ENSG00000119042.16 0.310793215 1 1 0.310793215
Brain_Spinal_cord_cervical_c-1 0.310793215 Brain_Spinal_cord_cervical_c-1
1 1 1 1.013559031 1.013559031 1.013559031 NA 1 0
0.998207054
OSER1 ENSG00000132823.10 0.311314045 1 1 0.311314045 Brain_Cerebellum
0.311314045 Brain_Cerebellum 1 1 1 -1.012468616 -1.012468616
-1.012468616 NA 1 0 0.998207054
TFAP4 ENSG00000090447.11 0.311564834 1 1 0.311564834
Brain_Spinal_cord_cervical_c-1 0.311564834 Brain_Spinal_cord_cervical_c-1
1 1 1 1.01194399 1.01194399 1.01194399 NA 1 0 0.998207054
TRMT12 ENSG00000183665.5 0.311592855 1 1 0.311592855
Brain_Cerebellum 0.311592855 Brain_Cerebellum 1 1 1 1.011885392
1.011885392 1.011885392 NA 1 0 0.998207054
NPAT ENSG00000149308.16 0.311637714 1 1 0.311637714 Brain_Cerebellum
0.311637714 Brain_Cerebellum 1 1 1 1.011791586 1.011791586
1.011791586 NA 1 0 0.998207054
AC079779.5 ENSG00000235779.7 0.311828005 1 1 0.311828005
Brain_Cerebellum 0.311828005 Brain_Cerebellum 1 1 1 -1.011393763
-1.011393763 -1.011393763 NA 1 0 0.998207054
OVOS2 ENSG00000177359.17 0.311832466 4 4 0.058980457 Brain_Cortex
0.402807656 Brain_Frontal_Cortex_BA9 3.203501509 0.124762907
0.124762907 0.83661741 1.888338982 1.312083667 0.454690504 4
0 0.998207054
SNRPD1 ENSG00000167088.10 0.311852832 5 3 0.155113767
Brain_Cerebellar_Hemisphere 0.537720971 Brain_Caudate_basal_ganglia
3.113938066 0.00177754 0.59215831 -0.616262975 1.421698598
0.799961976 0.850986971 3 0 0.998207054
DECR1 ENSG00000104325.6 0.3119046 2 2 0.144834706 Brain_Cortex
0.386784673 Brain_Hypothalamus 1.29763836 0.70236164 0.70236164
0.865463078 1.458021178 1.161742128 0.419001851 2 0
0.998207054
GATM ENSG00000171766.15 0.312003869 2 2 0.276630936
Brain_Hippocampus 0.375359654 Brain_Hypothalamus 1.156932842
0.843067158 0.843067158 -0.88647865 1.087918844 0.100720097
1.396109857 2 0 0.998207054
L3MBTL4 ENSG00000154655.15 0.312011753 1 1 0.312011753
Brain_Substantia_nigra 0.312011753 Brain_Substantia_nigra 1 1
1 1.011009772 1.011009772 1.011009772 NA 1 0
0.998207054
AC073133.1 ENSG00000233878.1 0.312107324 9 8 0.043784287
Brain_Putamen_basal_ganglia 0.973007416 Brain_Substantia_nigra
3.904585847 0.043356124 0.205432032 -2.01615006 0.787880063
-0.636168285 0.90686588 8 0 0.998207054

GPATCH2 ENSG00000092978.10 0.312225437 1 1 0.312225437
Brain_Putamen_basal_ganglia 0.312225437 Brain_Putamen_basal_ganglia 1 1
1 1.010563407 1.010563407 1.010563407 NA 1 0
0.998207054
CACNA2D2 ENSG00000007402.11 0.312294446 1 1 0.312294446
Brain_Cerebellum 0.312294446 Brain_Cerebellum 1 1 1 1.010419298
1.010419298 1.010419298 NA 1 0 0.998207054
ASAP3 ENSG00000088280.18 0.312326493 4 3 0.149026252
Brain_Cerebellar_Hemisphere 0.699157801 Brain_Cortex 3.355361365
0.063640757 0.216354517 -1.442979479 -0.386457596 -1.045550042
0.490816558 3 0 0.998207054
CASP8AP2 ENSG00000118412.12 0.312454194 1 1 0.312454194
Brain_Cerebellum 0.312454194 Brain_Cerebellum 1 1 1 -1.01008578
-1.01008578 -1.01008578 NA 1 0 0.998207054
RIIAD1 ENSG00000178796.12 0.312576669 8 8 0.007177446
Brain_Substantia_nigra 0.693678657 Brain_Putamen_basal_ganglia 3.9389762
0.155772885 0.155772885 0.393867783 2.688497079 0.917440793
0.74375282 8 0 0.998207054
SCUBE2 ENSG00000175356.12 0.312710347 8 8 0.0809235
Brain_Caudate_basal_ganglia 0.806912506 Brain_Spinal_cord_cervical_c-1
2.925716505 0.193391688 0.193391688 -1.745352663 0.787834272
-0.210867177 0.816376284 8 0 0.998207054
ETV3L ENSG00000253831.1 0.312940678 1 1 0.312940678 Brain_Amygdala
0.312940678 Brain_Amygdala 1 1 1 1.009070806 1.009070806
1.009070806 NA 1 0 0.998207054
NRG1 ENSG00000157168.18 0.312996105 3 3 0.169167137 Brain_Cerebellum
0.629540249 Brain_Caudate_basal_ganglia 1.136037521 0.872616243
0.872616243 -1.374884881 0.482374054 -0.649127575 0.992886715
3 0 0.998207054
LXN ENSG00000079257.7 0.312999832 10 3 0.065712067
Brain_Cerebellar_Hemisphere 0.438309715 Brain_Anterior_cingulate_cortex_BA24
8.22888571 0.009244197 0.441250188 -1.84038273 -0.775050676
-1.531297715 0.31974444 3 0 0.998207054
LINC00540 ENSG00000276476.2 0.313068834 1 1 0.313068834
Brain_Spinal_cord_cervical_c-1 0.313068834 Brain_Spinal_cord_cervical_c-1
1 1 1 1.008803601 1.008803601 1.008803601 NA 1 0
0.998207054
NSMCE1 ENSG00000169189.16 0.313077961 2 2 0.140602032
Brain_Hypothalamus 0.671816212 Brain_Spinal_cord_cervical_c-1
1.023656532 0.976343468 0.976343468 -1.473552823 -0.423656681
-0.948604752 0.742388682 2 0 0.998207054
GABRG3 ENSG00000182256.12 0.313203776 2 2 0.129634503
Brain_Amygdala 0.869105484 Brain_Cerebellar_Hemisphere 1.00456266 0.99543734
0.99543734 0.164794816 1.515544765 0.840169791 0.955124448 2
0 0.998207054
FGFR4 ENSG00000160867.14 0.313229453 10 6 0.055461212
Brain_Hippocampus 0.761461202 Brain_Cerebellar_Hemisphere 6.439786483
0.023451393 0.279322873 -1.915245427 1.167629191 -0.700137079
0.807745774 6 0 0.998207054
NXNL1 ENSG00000171773.2 0.313329751 2 2 0.218633403
Brain_Anterior_cingulate_cortex_BA24 0.36538997 Brain_Caudate_basal_ganglia
1.005238849 0.994761151 0.994761151 -1.230170129 -0.905142367
-1.067656248 0.229829335 2 0 0.998207054
PVRIG ENSG00000213413.2 0.313381167 13 2 0.066375522 Brain_Cortex
0.85733496 Brain_Anterior_cingulate_cortex_BA24 11.36649061 0.000604804
0.612413216 0.179767677 1.835879217 1.108982167 0.422531849
2 0 0.998207054
SLC39A4 ENSG00000147804.9 0.313383398 5 2 0.27012328 Brain_Cortex
0.863013567 Brain_Cerebellum 4.241747503 0.004726335 0.613940338
-1.102778701 0.333844255 -0.178056717 0.575219242 2 0
0.998207054
SLC2A9 ENSG00000109667.11 0.313810098 2 2 0.185935923

Brain_Spinal_cord_cervical_c-1 0.838832662 Brain_Caudate_basal_ganglia 1.606818889 0.393181111 0.393181111 0.20338687 1.322697719 0.763042294 0.791472292 2 0 0.998207054
AARS2 ENSG00000124608.4 0.313844712 5 5 0.049190441 Brain_Amygdala 0.568420579 Brain_Cerebellum 1.636964457 0.366554978 0.366554978 -0.583299813 1.96693729 0.687069109 0.904694679 5 0 0.998207054
ASIC3 ENSG00000213199.7 0.313869893 1 1 0.313869893 Brain_Amygdala 0.313869893 Brain_Amygdala 1 1 1 -1.007135023 -1.007135023 -1.007135023 NA 1 0 0.998207054
TSPAN31 ENSG00000135452.9 0.313923844 3 3 0.166991048 Brain_Cerebellar_Hemisphere 0.36260786 Brain_Hypothalamus 1.631690643 0.403564083 0.403564083 -1.381936994 0.91040709 -0.605342456 1.312811085 3 0 0.998207054
VEPH1 ENSG00000197415.11 0.31394143 2 2 0.140227508 Brain_Cerebellum 0.736212836 Brain_Cerebellar_Hemisphere 1.45554374 0.54445626 0.54445626 0.336872739 1.474944339 0.905908539 0.804738146 2 0 0.998207054
BTBD3 ENSG00000132640.14 0.314295572 3 3 0.162872975 Brain_Putamen_basal_ganglia 0.48874129 Brain_Anterior_cingulate_cortex_BA24 1.281636686 0.732971232 0.732971232 -1.395473916 -0.6923122 -1.069094888 0.354280252 3 0 0.998207054
PIP4K2C ENSG00000166908.17 0.314326865 2 2 0.154665101 Brain_Nucleus_accumbens_basal_ganglia 0.67602401 Brain_Amygdala 1.08281187 0.91718813 0.91718813 -1.42324515 0.417894834 -0.502675158 1.301882567 2 0 0.998207054
PYGO1 ENSG00000171016.11 0.314585873 3 3 0.347274466 Brain_Cerebellar_Hemisphere 0.864970803 Brain_Nucleus_accumbens_basal_ganglia 2.389141693 0.224975244 0.224975244 -0.939889067 0.669571339 -0.033422571 0.823797014 3 0 0.998207054
C9orf135 ENSG00000204711.8 0.314673525 1 1 0.314673525 Brain_Nucleus_accumbens_basal_ganglia 0.314673525 Brain_Nucleus_accumbens_basal_ganglia 1 1 1 -1.005463897 -1.005463897 -1.005463897 NA 1 0 0.998207054
LINC00967 ENSG00000253138.5 0.314886642 1 1 0.314886642 Brain_Putamen_basal_ganglia 0.314886642 Brain_Putamen_basal_ganglia 1 1 1 1.0050212 1.0050212 1.0050212 NA 1 0 0.998207054
MAP3K21 ENSG00000143674.10 0.314931241 10 2 0.114473716 Brain_Amygdala 0.313655011 Brain_Putamen_basal_ganglia 9.174906272 0.000662852 0.333709855 -1.578400129 -1.007582338 -1.436957645 0.189462228 2 0 0.998207054
MGLL ENSG00000074416.13 0.314991696 2 2 0.13293918 Brain_Spinal_cord_cervical_c-1 0.861611196 Brain_Cerebellum 1.036481777 0.963518223 0.963518223 0.174323546 1.502611794 0.83846767 0.939241628 2 0 0.998207054
UCP2 ENSG00000175567.8 0.315060544 9 8 0.008936766 Brain_Spinal_cord_cervical_c-1 0.808857346 Brain_Cortex 4.589183255 0.054714298 0.198821964 -2.614463692 0.550641342 -0.752496029 1.010460181 8 0 0.998207054
DHX8 ENSG00000067596.10 0.315117728 5 4 0.155529724 Brain_Spinal_cord_cervical_c-1 0.759962313 Brain_Cerebellum 2.460449223 1.71E-09 0.252798763 -1.420267825 0.464769509 -0.075335933 0.820089781 4 0 0.998207054
ZNF763 ENSG00000197054.11 0.315195887 4 1 0.25224877 Brain_Cerebellum 0.353452504 Brain_Hippocampus 3.901683803 0.001176412 3.901683803 -1.144904379 -0.927913379 -0.992071599 0.102480787 1 0 0.998207054
FAM114A1 ENSG00000197712.11 0.315257203 5 5 0.175982819 Brain_Spinal_cord_cervical_c-1 0.811653693 Brain_Caudate_basal_ganglia 2.80509961 0.094962516 0.094962516 -0.238293202 1.353227949 0.761238385 0.638822324 5 0 0.998207054
RFWD3 ENSG00000168411.13 0.315331629 11 4 0.116425798

Brain_Cerebellar_Hemisphere 0.696101834 Brain_Nucleus_accumbens_basal_ganglia 7.853015175 0.010963603 0.29520145 -1.569954065 -0.390587951 -0.940655965 0.397286708 4 0 0.998207054
HRNR ENSG00000197915.5 0.315379658 6 3 0.098080696 Brain_Cortex 0.691804067 Brain_Spinal_cord_cervical_c-1 4.92651603 0.055276317 0.282713269 0.396407998 1.65423046 0.969759267 0.499350631 3 0 0.998207054
FKBP5 ENSG00000096060.14 0.315461577 2 2 0.177967985 Brain_Substantia_nigra 0.525819923 Brain_Cerebellar_Hemisphere 1.049606758 0.950393242 0.950393242 -1.347038031 0.634399827 -0.356319102 1.401088146 2 0 0.998207054
LRRC58 ENSG00000163428.3 0.315480099 2 2 0.128838355 Brain_Caudate_basal_ganglia 0.984944875 Brain_Hippocampus 1.007036639 0.992963361 0.992963361 0.01886992 1.518698696 0.768784308 1.060539098 2 0 0.998207054
CNTF ENSG00000242689.2 0.315504891 2 2 0.214040219 Brain_Hippocampus 0.303346093 Brain_Cortex 1.131341615 0.868658385 0.868658385 -1.242532332 -1.029284312 -1.135908322 0.150789122 2 0 0.998207054
PIANP ENSG00000139200.13 0.315701334 1 1 0.315701334 Brain_Spinal_cord_cervical_c-1 0.315701334 Brain_Spinal_cord_cervical_c-1 1 1 1 -1.003330689 -1.003330689 -1.003330689 NA 1 0 0.998207054
RNF114 ENSG00000124226.10 0.315734897 6 3 0.141654038 Brain_Cortex 0.863417545 Brain_Hypothalamus 5.260175584 0.010237104 0.219996098 -1.469659348 -0.172025419 -0.496089071 0.487303515 3 0 0.998207054
SCNM1 ENSG00000163156.11 0.315791467 2 2 0.139919484 Brain_Frontal_Cortex_BA9 0.96641574 Brain_Amygdala 1.261119249 0.738880751 0.738880751 -1.476090929 -0.042104064 -0.759097497 1.013981836 2 0 0.998207054
MARVELD1 ENSG00000155254.12 0.315874241 1 1 0.315874241 Brain_Caudate_basal_ganglia 0.315874241 Brain_Caudate_basal_ganglia 1 1 1 -1.002972269 -1.002972269 -1.002972269 NA 1 0 0.998207054
CADPS2 ENSG00000081803.15 0.316126095 2 2 0.173263407 Brain_Nucleus_accumbens_basal_ganglia 0.561796567 Brain_Frontal_Cortex_BA9 1.06484316 0.93515684 0.93515684 -1.361792411 -0.580175065 -0.970983738 0.552686925 2 0 0.998207054
BLVRB ENSG00000090013.9 0.316179314 1 1 0.316179314 Brain_Hypothalamus 0.316179314 Brain_Hypothalamus 1 1 1 1.002340198 1.002340198 1.002340198 NA 1 0 0.998207054
LA16c-380H5.5 ENSG00000272079.2 0.316218897 13 2 0.054083491 Brain_Amygdala 0.413569899 Brain_Cerebellum 11.39555707 2.67E-05 1.007211028 -0.817627536 1.926167272 1.124158339 0.629358852 2 0 0.998207054
CAMK2G ENSG00000148660.20 0.316303471 2 2 0.129767933 Brain_Nucleus_accumbens_basal_ganglia 0.638222504 Brain_Anterior_cingulate_cortex_BA24 1.257650068 0.742349932 0.742349932 -1.515017654 -0.470185484 -0.992601569 0.738807913 2 0 0.998207054
CPNE4 ENSG00000196353.11 0.3163219 1 1 0.3163219 Brain_Caudate_basal_ganglia 0.3163219 Brain_Caudate_basal_ganglia 1 1 1 1.002044916 1.002044916 1.002044916 NA 1 0 0.998207054
C16orf59 ENSG00000162062.14 0.316349297 1 1 0.316349297 Brain_Hippocampus 0.316349297 Brain_Hippocampus 1 1 1 -1.00198819 -1.00198819 -1.00198819 NA 1 0 0.998207054
MSRB1 ENSG00000198736.11 0.316422547 1 1 0.316422547 Brain_Hypothalamus 0.316422547 Brain_Hypothalamus 1 1 1 1.001836538 1.001836538 1.001836538 NA 1 0 0.998207054
SYNE2 ENSG00000054654.16 0.316652834 5 4 0.089890388 Brain_Hippocampus 0.958108904 Brain_Cortex 2.594211252

0.064711999 0.357220386 -0.930470822 1.695976195 0.48195728
0.98371944 4 0 0.998207054
MFAP2 ENSG00000117122.13 0.316661207 3 3 0.117481828
Brain_Spinal_cord_cervical_c-1 0.867094452 Brain_Hippocampus 1.23887308
0.760579278 0.760579278 -1.565431239 -0.16735027 -0.883056791
0.699636241 3 0 0.998207054
TMEM220 ENSG00000187824.8 0.316689905 13 2 0.188241938
Brain_Substantia_nigra 0.951468411 Brain_Amygdala 11.96566272
0.001222938 0.435341643 0.060862881 1.315797741 1.008809535
0.297299756 2 0 0.998207054
VDAC2 ENSG00000165637.13 0.316705061 1 1 0.316705061
Brain_Caudate_basal_ganglia 0.316705061 Brain_Caudate_basal_ganglia 1 1
1 -1.001251858 -1.001251858 -1.001251858 NA 1 0
0.998207054
GTSF1 ENSG00000170627.10 0.316754419 5 3 0.462958686
Brain_Caudate_basal_ganglia 0.979079437 Brain_Spinal_cord_cervical_c-1
4.242981027 0.000635426 0.197456373 -0.129067909 0.733983677
0.243436676 0.440220498 3 0 0.998207054
ACY3 ENSG00000132744.7 0.316784987 8 5 0.232416521 Brain_Amygdala
0.906948507 Brain_Cortex 6.066145637 0.009845877 0.237025943
-0.380072688 1.194157099 0.240007118 0.500401985 5 0
0.998207054
RP11-403A21.1 ENSG00000265752.2 0.316785709 5 5 0.103428936
Brain_Hypothalamus 0.929780226 Brain_Spinal_cord_cervical_c-1
2.046016158 0.520405207 0.520405207 -1.628452676 0.150539216
-0.595907384 0.814279469 5 0 0.998207054
NOTCH4 ENSG00000204301.6 0.316849039 10 9 0.148834173
Brain_Cortex 0.993799862 Brain_Anterior_cingulate_cortex_BA24
4.629434704 0.068804583 0.180452119 -1.443661654 1.07153279
-0.232995232 0.741383622 9 0 0.998207054
AGPS ENSG00000018510.13 0.316874661 5 4 0.054289803 Brain_Cerebellum
0.418963095 Brain_Putamen_basal_ganglia 2.829804577 0.065671815
0.363252966 -0.808221479 1.924517067 1.126778736 1.099229682
4 0 0.998207054
C11orf58 ENSG00000110696.9 0.31688354 1 1 0.31688354 Brain_Amygdala
0.31688354 Brain_Amygdala 1 1 1 1.000882662 1.000882662
1.000882662 NA 1 0 0.998207054
PRPF40A ENSG00000196504.15 0.316923323 1 1 0.316923323
Brain_Nucleus_accumbens_basal_ganglia 0.316923323
Brain_Nucleus_accumbens_basal_ganglia 1 1 1 1.000800385
1.000800385 1.000800385 NA 1 0 0.998207054
TMCC2 ENSG00000133069.15 0.316938065 6 2 0.080290453
Brain_Putamen_basal_ganglia 0.556119993 Brain_Cerebellar_Hemisphere
5.276560127 0.029914728 0.455681215 0.588614368 1.749003277
1.25151094 0.396656751 2 0 0.998207054
HDAC7 ENSG00000061273.17 0.316961517 2 2 0.132651737 Brain_Cerebellum
0.402282813 Brain_Cerebellar_Hemisphere 1.442660203 0.557339797
0.557339797 0.837551195 1.503726757 1.170638976 0.471057258
2 0 0.998207054
LOXL4 ENSG00000138131.3 0.316991479 2 2 0.137391317
Brain_Caudate_basal_ganglia 0.763464465 Brain_Putamen_basal_ganglia
1.000294224 0.999705776 0.999705776 0.30093447 1.485575964
0.893255217 0.837668034 2 0 0.998207054
MZF1 ENSG00000099326.8 0.316997646 1 1 0.316997646 Brain_Amygdala
0.316997646 Brain_Amygdala 1 1 1 -1.000646695 -1.000646695
-1.000646695 NA 1 0 0.998207054
SEC23B ENSG00000101310.14 0.317161609 12 2 0.095656816
Brain_Nucleus_accumbens_basal_ganglia 0.515407443 Brain_Hypothalamus
10.98226993 0.000913754 0.397823896 0.650440934 1.666284231
1.314826761 0.256066701 2 0 0.998207054
JAM3 ENSG00000166086.12 0.317287109 1 1 0.317287109
Brain_Spinal_cord_cervical_c-1 0.317287109 Brain_Spinal_cord_cervical_c-1

1 1 1 1.000048352 1.000048352 1.000048352 NA 1 0
0.998207054
SMDT1 ENSG00000183172.8 0.317540735 13 2 0.156388191
Brain_Substantia_nigra 0.596677504 Brain_Cortex 11.71385913
0.000452119 0.790132638 0.529184448 1.417324105 0.922317699
0.324479177 2 0 0.998207054
TMEM159 ENSG00000011638.10 0.317550467 7 7 0.195003068
Brain_Cerebellar_Hemisphere 0.99541091 Brain_Frontal_Cortex_BA9 2.596462114
0.132416254 0.132416254 -0.780864463 1.295919941 0.259811665
0.671121245 7 0 0.998207054
RP11-148L24.1 ENSG00000248174.5 0.317740707 11 4 0.074763246
Brain_Nucleus_accumbens_basal_ganglia 0.429322588 Brain_Cerebellum 8.17251272
2.01E-16 0.443221726 -1.21079221 1.78191407 1.244919603
0.873097885 4 0 0.998207054
ZDHHC12 ENSG00000160446.18 0.317763638 1 1 0.317763638
Brain_Cerebellum 0.317763638 Brain_Cerebellum 1 1 1 0.999064105
0.999064105 0.999064105 NA 1 0 0.998207054
SAE1 ENSG00000142230.11 0.317805552 2 2 0.192570796
Brain_Caudate_basal_ganglia 0.588295163 Brain_Hypothalamus 1.16803616
0.83196384 0.83196384 -1.303011615 0.541308209 -0.380851703
1.304131054 2 0 0.998207054
LIMD1 ENSG00000144791.9 0.317826539 11 5 0.02439832 Brain_Hypothalamus
0.743594105 Brain_Spinal_cord_cervical_c-1 7.776745107 2.50E-16
0.297311606 0.32709755 2.25079822 1.712887081 0.626198004 5 0
0.998207054
TMEM88B ENSG00000205116.3 0.317855686 1 1 0.317855686
Brain_Hippocampus 0.317855686 Brain_Hippocampus 1 1 1
0.998874096 0.998874096 0.998874096 NA 1 0 0.998207054
NLRC3 ENSG00000167984.17 0.317959256 11 11 0.024494874 Brain_Cerebellum
0.973057707 Brain_Cerebellar_Hemisphere 1.922214508 0.245549648
0.245549648 -2.249277066 1.513978379 0.138718165 1.042788618
11 0 0.998207054
RP11-430H10.1 ENSG00000254427.1 0.317969266 13 2 0.145777165
Brain_Cerebellar_Hemisphere 0.835608861 Brain_Spinal_cord_cervical_c-1
11.32754037 0.001682361 1.090036451 0.207513486 1.454610348
0.710306087 0.347192776 2 0 0.998207054
PARP15 ENSG00000173200.12 0.31801411 2 2 0.233060364 Brain_Cortex
0.649738075 Brain_Spinal_cord_cervical_c-1 1.351200937 0.648799063
0.648799063 -0.454126091 1.19251247 0.36919319 1.164349293 2 0
0.998207054
RP11-297N6.4 ENSG00000255046.1 0.31802262 3 3 0.06269053
Brain_Nucleus_accumbens_basal_ganglia 0.838803938 Brain_Frontal_Cortex_BA9
1.694210714 0.382831926 0.382831926 -1.861380023 -0.203423622
-1.009438451 0.829931808 3 0 0.998207054
KCNMA1 ENSG00000156113.22 0.318083643 4 4 0.140467565
Brain_Caudate_basal_ganglia 0.747359918 Brain_Putamen_basal_ganglia
2.285619224 0.220548929 0.220548929 -1.474052098 0.818980251
-0.150123604 0.999845914 4 0 0.998207054
FAM174B ENSG00000185442.12 0.318228475 1 1 0.318228475
Brain_Cerebellar_Hemisphere 0.318228475 Brain_Cerebellar_Hemisphere 1 1
1 0.99810494 0.99810494 0.99810494 NA 1 0 0.998207054
RPIA ENSG00000153574.8 0.318245353 4 1 0.301767351
Brain_Nucleus_accumbens_basal_ganglia 0.367020548
Brain_Anterior_cingulate_cortex_BA24 3.910874632 0.007513144
3.910874632 0.902068374 1.032650789 0.987067049 0.059111768
1 0 0.998207054
LRSAM1 ENSG00000148356.13 0.318280311 3 3 0.145573693
Brain_Cerebellar_Hemisphere 0.877955606 Brain_Cerebellum 1.659862767
0.340527091 0.340527091 -1.455345295 -0.153561359 -0.691880454
0.679469138 3 0 0.998207054
ARHGEF7 ENSG00000102606.17 0.318332128 11 5 0.104899086
Brain_Frontal_Cortex_BA9 0.500298779 Brain_Hippocampus 7.477874177

0.002274311	0.481363424	-1.260576198	1.621553031	0.883468617
0.766969967	5	0	0.998207054
CDK7	ENSG00000134058.11	0.318476504	3	3	0.229683752
Brain_Spinal_cord_cervical_c-1	0.756287117	Brain_Caudate_basal_ganglia
1.88958337 0.247936352	0.247936352	-1.201173899	0.310360121
-0.57050378	0.786213374	3	0	0.998207054
SLC25A18	ENSG00000182902.13	0.318532246	3	3	0.158893239
Brain_Nucleus_accumbens_basal_ganglia 0.94396798 Brain_Caudate_basal_ganglia
1.415493687	0.598684121	0.598684121	-1.408803638	0.070283544
-0.785116629	0.766285172	3	0	0.998207054
RP4-781K5.4	ENSG00000228044.2	0.318549184	2	2	0.253262997
Brain_Substantia_nigra	0.666482367	Brain_Spinal_cord_cervical_c-1
1.843422307	0.156577693	0.156577693	0.430980751	1.142459679
0.786720215	0.503091574	2	0	0.998207054
LINC01886	ENSG00000224568.1	0.318613308	1	1	0.318613308
Brain_Anterior_cingulate_cortex_BA24	0.318613308
Brain_Anterior_cingulate_cortex_BA24	1	1	1	0.997311553
0.997311553	0.997311553	NA	1	0	0.998207054
CAMTA2	ENSG00000108509.20	0.318742763	3	3	0.103871444
Brain_Putamen_basal_ganglia 0.809725389	Brain_Cerebellar_Hemisphere
1.315539474	0.630299422	0.630299422	-1.626367764	-0.240780312
-1.057062546	0.725059571	3	0	0.998207054
C1QTNF8	ENSG00000184471.7	0.31884787 1	1	0.31884787
Brain_Anterior_cingulate_cortex_BA24	0.31884787
Brain_Anterior_cingulate_cortex_BA24	1	1	1	-0.996828278
-0.996828278	-0.996828278	NA	1	0	0.998207054
RP11-61A14.4	ENSG00000246777.1	0.318923991	2	2	0.140807004
Brain_Cortex	0.746842557	Brain_Hypothalamus	1.42855803 0.57144197
0.57144197 0.322805487	1.472792468	0.897798977	0.813163592	2
0	0.998207054
SLC33A1	ENSG00000169359.13	0.318933589	5	5	0.025546508
Brain_Frontal_Cortex_BA9	0.840337144	Brain_Cerebellar_Hemisphere
2.439563841	0.181265554	0.181265554	-0.201462251	2.233036953
1.092207172	1.057171478	5	0	0.998207054
CASC1 ENSG00000118307.18	0.319024396	1	1	0.319024396	Brain_Cerebellum
0.319024396	Brain_Cerebellum 1	1	1	0.996464729	0.996464729
0.996464729	NA	1	0	0.998207054
IFT140	ENSG00000187535.13	0.319075536	7	4	0.480188938
Brain_Cerebellum 0.972262933	Brain_Cerebellar_Hemisphere 4.985389409
0.03497073 0.252802921	-0.65899307	0.705998713	0.290449175
0.474825045	4	0	0.998207054
FABP3 ENSG00000121769.7	0.319161235	4	3	0.148933595
Brain_Cerebellar_Hemisphere 0.889614629	Brain_Putamen_basal_ganglia
3.436768369	0.020796394	0.150295827	-1.443308471	-0.138791857
-0.739972916	0.6368891 3	0	0.998207054
ANKRD16	ENSG00000134461.15	0.319213338	1	1	0.319213338
Brain_Cerebellum 0.319213338	Brain_Cerebellum 1	1	1	0.996075758
0.996075758	0.996075758	NA	1	0	0.998207054
PPP4C ENSG00000149923.13	0.31949363 1	1	0.31949363 Brain_Frontal_Cortex_BA9
0.31949363 Brain_Frontal_Cortex_BA9	1	1	1	0.995499002
0.995499002	0.995499002	NA	1	0	0.998207054
LINC00443	ENSG00000230156.2	0.319495014	10	9	0.154878481
Brain_Putamen_basal_ganglia 0.991073478	Brain_Nucleus_accumbens_basal_ganglia
4.617993332	0.065778071	0.232704415	-1.212668849	1.422509206
0.010455551	0.872303929	9	0	0.998207054
DDX5	ENSG00000108654.13	0.319570922	3	3	0.073883472
Brain_Spinal_cord_cervical_c-1	0.641604406	Brain_Cerebellar_Hemisphere
1.418032544	0.58048748 0.58048748 -0.477783991	1.787334308
0.281364524	1.304222655	3	0	0.998207054
HERC6 ENSG00000138642.14	0.319622749	1	1	0.319622749
Brain_Nucleus_accumbens_basal_ganglia 0.319622749
Brain_Nucleus_accumbens_basal_ganglia 1	1	1	0.995233425

0.995233425	0.995233425	NA	1	0	0.998207054
PAF1	ENSG00000006712.14	0.319731653	1	1	0.319731653
Brain_Hippocampus	0.319731653	Brain_Hippocampus	1	1	1
-0.995009482	-0.995009482	-0.995009482	NA	1	0	0.998207054
DHCR7	ENSG00000172893.15	0.319918777	1	1	0.319918777	Brain_Cerebellum
0.319918777	Brain_Cerebellum	1	1	1	-0.994624809	-0.994624809
-0.994624809	NA	1	0	0.998207054
RP11-738E22.3	ENSG00000269949.1	0.319931699	11	2	0.068351176
Brain_Putamen_basal_ganglia	0.935166572	Brain_Spinal_cord_cervical_c-1
9.181140518	0.001457831	0.899833401	-0.081346277	1.822684586
1.230691166	0.482823437	2	0	0.998207054
FAM171B	ENSG00000144369.12	0.320027643	3	3	0.171751716
Brain_Putamen_basal_ganglia	0.399818757	Brain_Spinal_cord_cervical_c-1
1.997336485	0.180905367	0.180905367	-1.366596814	-0.841944969
-1.158314454	0.278526497	3	0	0.998207054
NME6	ENSG00000172113.8	0.320151515	7	3	0.083224557
Brain_Cerebellar_Hemisphere	0.198446236	Brain_Frontal_Cortex_BA9
5.790792561	0.003908394	0.366620717	1.285990906	1.732275306
1.443228102	0.168848808	3	0	0.998207054
NKAIN2	ENSG00000188580.14	0.320168824	4	3	0.108689529
Brain_Substantia_nigra	0.552475776	Brain_Frontal_Cortex_BA9
2.175743856	0.043548266	0.663660076	-1.604111264	1.097586214
-0.007113216	1.326652662	3	0	0.998207054
B4GALNT1	ENSG00000135454.13	0.320195088	3	3	0.133349824
Brain_Cortex	0.586988125	Brain_Cerebellar_Hemisphere	1.2368044
0.696047894	0.696047894	-1.095794157	1.501022183	0.316144603
1.313214133	3	0	0.998207054
F11	ENSG00000088926.13	0.320249674	6	3	0.05352275
Brain_Caudate_basal_ganglia	0.198506607	Brain_Nucleus_accumbens_basal_ganglia
5.005227939	0.040769408	0.19308669	-1.930679096	-1.285817943
-1.62982073	0.311942708	3	0	0.998207054
PSCA	ENSG00000167653.4	0.320253071	10	3	0.225731036
Brain_Cerebellar_Hemisphere	0.984715863	Brain_Hypothalamus	8.163132053
0.013603217	0.528453296	-1.211428986	0.203953895	-0.490792465
0.498120041	3	0	0.998207054
SETBP1	ENSG00000152217.16	0.320311063	1	1	0.320311063
Brain_Hypothalamus	0.320311063	Brain_Hypothalamus	1	1	1
-0.993818859	-0.993818859	-0.993818859	NA	1	0	0.998207054
ITPRIPL1	ENSG00000198885.9	0.320335464	7	4	0.09460369	Brain_Cortex
0.992694337	Brain_Cerebellum	4.340284967	0.008766134	0.265819903
-0.299552002	1.67159764	0.634996469	0.805506486	4	0
0.998207054
ZBTB34	ENSG00000177125.5	0.320381583	1	1	0.320381583
Brain_Amygdala	0.320381583	Brain_Amygdala	1	1	1	-0.993674045
-0.993674045	-0.993674045	NA	1	0	0.998207054
LIN52	ENSG00000205659.10	0.320396638	7	4	0.307717277
Brain_Frontal_Cortex_BA9	0.963437498	Brain_Hypothalamus	4.999594012
0.000568794	0.176603389	-0.292730559	1.020023582	0.515595192
0.540571729	4	0	0.998207054
CYP11B1	ENSG00000160882.11	0.320498903	1	1	0.320498903
Brain_Nucleus_accumbens_basal_ganglia	0.320498903
Brain_Nucleus_accumbens_basal_ganglia	1	1	1	-0.993433171
-0.993433171	-0.993433171	NA	1	0	0.998207054
RP1-111D6.5	ENSG00000283608.1	0.320727311	8	2	0.061637994
Brain_Anterior_cingulate_cortex_BA24	0.342472843
Brain_Nucleus_accumbens_basal_ganglia	6.901030285	0.000595527
0.497553623	0.949290581	1.868890903	1.398682521	0.332568624
2	0	0.998207054
CCDC102B	ENSG00000150636.17	0.320823402	2	2	0.373685013
Brain_Hypothalamus	0.392217552	Brain_Nucleus_accumbens_basal_ganglia
1.330112212	0.669887788	0.669887788	-0.855602744	0.889591982
0.016994619	1.234039026	2	0	0.998207054

TIMM23B ENSG00000204152.10 0.320838357 12 1 0.154842963 Brain_Cerebellum 0.475613644 Brain_Caudate_basal_ganglia 11.00249809 0.000322541 11.00249809 0.713375154 1.422631654 0.958102751 0.196956645 1 0 0.998207054
CUEDC2 ENSG00000107874.10 0.320842287 4 3 0.407138932 Brain_Cerebellar_Hemisphere 0.916413929 Brain_Putamen_basal_ganglia 3.5841886 0.009825822 0.131081335 -0.828938989 0.384097526 -0.001280165 0.565021915 3 0 0.998207054
CACNB3 ENSG00000167535.7 0.321003991 6 3 0.075907258 Brain_Hypothalamus 0.609300521 Brain_Nucleus_accumbens_basal_ganglia 4.458681293 0.033501155 0.275077692 0.511072172 1.774943252 0.819154229 0.482048309 3 0 0.998207054
PCAT18 ENSG00000265369.3 0.321030842 3 3 0.196042766 Brain_Putamen_basal_ganglia 0.959611482 Brain_Caudate_basal_ganglia 2.041639695 0.270781736 0.270781736 -0.163863973 1.292908331 0.393228499 0.786492823 3 0 0.998207054
CEP41 ENSG00000106477.18 0.321209508 6 6 0.096860772 Brain_Nucleus_accumbens_basal_ganglia 0.693094424 Brain_Cerebellum 1.952323448 0.200030111 0.200030111 -0.508572555 1.660266898 0.850345499 0.799433708 6 0 0.998207054
HNRNPAB ENSG00000197451.11 0.321256353 2 2 0.136899219 Brain_Hippocampus 0.795060948 Brain_Putamen_basal_ganglia 1.01194594 0.98805406 0.98805406 -1.487437802 0.259744392 -0.613846705 1.235444377 2 0 0.998207054
MED21 ENSG00000152944.8 0.32125682 2 2 0.132340095 Brain_Cerebellar_Hemisphere 0.691807242 Brain_Frontal_Cortex_BA9 1.313058555 0.686941445 0.686941445 0.396403694 1.504937699 0.950670697 0.783851913 2 0 0.998207054
FASTKD3 ENSG00000124279.11 0.321276828 2 2 0.141344797 Brain_Cerebellum 0.857371968 Brain_Putamen_basal_ganglia 1.332567831 0.667432169 0.667432169 0.179720539 1.470801538 0.825261038 0.912932129 2 0 0.998207054
CYP2D6 ENSG00000100197.20 0.321279704 13 2 0.125440512 Brain_Substantia_nigra 0.430663587 Brain_Cerebellar_Hemisphere 12.30722078 0.000224855 0.483102817 -1.532332067 -0.788056622 -1.243621244 0.21458282 2 0 0.998207054
TRAF5 ENSG00000082512.14 0.321342493 1 1 0.321342493 Brain_Putamen_basal_ganglia 0.321342493 Brain_Putamen_basal_ganglia 1 1 1 0.991702864 0.991702864 0.991702864 NA 1 0 0.998207054
RP13-870H17.3 ENSG00000254872.3 0.321386792 7 7 0.097992099 Brain_Hypothalamus 0.965897906 Brain_Nucleus_accumbens_basal_ganglia 2.335229119 0.159091964 0.159091964 -1.654666831 1.509148466 0.424693698 1.235608501 7 0 0.998207054
SAMD4B ENSG00000179134.15 0.321398377 2 2 0.179699702 Brain_Caudate_basal_ganglia 0.606585706 Brain_Hippocampus 1.123287495 0.876712505 0.876712505 -0.514953225 1.341680225 0.4133635 1.312838103 2 0 0.998207054
P4HTM ENSG00000178467.17 0.321538459 12 4 0.024722774 Brain_Anterior_cingulate_cortex_BA24 0.493292035 Brain_Caudate_basal_ganglia 9.033607968 0.010769883 0.390089231 -2.245707122 -0.685082227 -1.633570741 0.435853405 4 0 0.998207054
CRIP3 ENSG00000146215.13 0.321545742 1 1 0.321545742 Brain_Putamen_basal_ganglia 0.321545742 Brain_Putamen_basal_ganglia 1 1 1 0.991286421 0.991286421 0.991286421 NA 1 0 0.998207054
RUFY1 ENSG00000176783.14 0.32159526 4 3 0.263873134 Brain_Hippocampus 0.971891481 Brain_Caudate_basal_ganglia 2.609011574 0.002865464 0.401719786 -1.117283487 0.347526352 -0.447883744 0.749492629 3 0 0.998207054
PRR19 ENSG00000188368.9 0.321636087 1 1 0.321636087 Brain_Cerebellum 0.321636087 Brain_Cerebellum 1 1 1 0.991101365 0.991101365

0.991101365 NA 1 0 0.998207054
FLVCR1 ENSG00000162769.12 0.321748124 12 4 0.144712666
Brain_Frontal_Cortex_BA9 0.983596118 Brain_Substantia_nigra
9.033040542 0.01980622 0.388146144 -1.45846409 1.081204217
-0.156979951 0.670619411 4 0 0.998207054
RP11-462G22.2 ENSG00000272566.1 0.321904445 1 1 0.321904445
Brain_Putamen_basal_ganglia 0.321904445 Brain_Putamen_basal_ganglia 1 1
1 -0.990551879 -0.990551879 -0.990551879 NA 1 0
0.998207054
ELP5 ENSG00000170291.14 0.321914314 5 5 0.088260052
Brain_Putamen_basal_ganglia 0.773990557 Brain_Cortex 3.279939215
0.129282805 0.129282805 -1.704648224 0.287159028 -0.727843602
0.716061424 5 0 0.998207054
DENND1C ENSG00000205744.9 0.321935632 2 2 0.165259293
Brain_Caudate_basal_ganglia 0.54481337 Brain_Frontal_Cortex_BA9 1.015382312
0.984617688 0.984617688 0.605550366 1.387598649 0.996574507
0.552991644 2 0 0.998207054
FEZF1 ENSG00000128610.11 0.321986564 2 2 0.232563841 Brain_Cortex
0.248202965 Brain_Cerebellum 1.21726745 0.78273255 0.78273255 -1.193780501
-1.154725228 -1.174252864 0.027616249 2 0 0.998207054
ESRRA ENSG00000173153.13 0.322014361 1 1 0.322014361 Brain_Amygdala
0.322014361 Brain_Amygdala 1 1 1 0.990326903 0.990326903
0.990326903 NA 1 0 0.998207054
MYO19 ENSG00000278259.4 0.322182927 7 5 0.067842265
Brain_Hippocampus 0.535415984 Brain_Spinal_cord_cervical_c-1
5.333028083 0.000494811 0.195584903 0.61975973 1.826053094
1.169989169 0.416023677 5 0 0.998207054
PCLAF ENSG00000166803.11 0.322188225 1 1 0.322188225 Brain_Amygdala
0.322188225 Brain_Amygdala 1 1 1 0.989971141 0.989971141
0.989971141 NA 1 0 0.998207054
CADPS ENSG00000163618.17 0.322201861 1 1 0.322201861
Brain_Spinal_cord_cervical_c-1 0.322201861 Brain_Spinal_cord_cervical_c-1
1 1 1 -0.989943244 -0.989943244 -0.989943244 NA 1 0
0.998207054
TSPYL6 ENSG00000178021.10 0.322315248 4 4 0.246503749
Brain_Frontal_Cortex_BA9 0.966084757 Brain_Putamen_basal_ganglia
3.289647757 0.153912843 0.153912843 -0.388377033 1.15888332
0.274613741 0.652041482 4 0 0.998207054
RP11-141B14.1 ENSG00000270019.1 0.322480394 1 1 0.322480394
Brain_Cortex 0.322480394 Brain_Cortex 1 1 1 0.989373584
0.989373584 0.989373584 NA 1 0 0.998207054
CDK5R2 ENSG00000171450.5 0.322517123 1 1 0.322517123
Brain_Anterior_cingulate_cortex_BA24 0.322517123
Brain_Anterior_cingulate_cortex_BA24 1 1 1 -0.98929849
-0.98929849 -0.98929849 NA 1 0 0.998207054
RP5-968J1.1 ENSG00000230839.1 0.322621094 8 7 0.132068635
Brain_Putamen_basal_ganglia 0.799090724 Brain_Caudate_basal_ganglia
2.070121285 0.002057015 0.694919561 -1.505994309 1.269155203
-0.067041043 1.115442317 7 0 0.998207054
ERCC1 ENSG00000012061.15 0.322673234 2 2 0.481217692
Brain_Caudate_basal_ganglia 0.86701678 Brain_Cortex 1.82594542 0.17405458
0.17405458 -0.167448991 0.704345412 0.268448211 0.616451734 2
0 0.998207054
HCG9 ENSG00000204625.10 0.322711716 1 1 0.322711716
Brain_Nucleus_accumbens_basal_ganglia 0.322711716
Brain_Nucleus_accumbens_basal_ganglia 1 1 1 0.988900725
0.988900725 0.988900725 NA 1 0 0.998207054
SLC22A9 ENSG00000149742.9 0.322770169 3 3 0.21542051 Brain_Cortex
0.920663452 Brain_Amygdala 2.513421711 0.131748654 0.131748654
-0.099598038 1.238797474 0.423541757 0.715419053 3 0
0.998207054
FBXO46 ENSG00000177051.5 0.322770801 2 2 0.13266972

Brain_Substantia_nigra 0.88547946 Brain_Cortex 1.078994116
0.921005884 0.921005884 -1.503656947 0.144026607 -0.67981517
1.165088214 2 0 0.998207054
BTRC ENSG00000166167.17 0.322813786 2 2 0.246386909
Brain_Frontal_Cortex_BA9 0.594969369 Brain_Cortex 1.323370223
0.676629777 0.676629777 -0.531648642 1.159169972 0.313760665
1.195589307 2 0 0.998207054
CLEC18B ENSG00000140839.11 0.322998017 3 3 0.12516157
Brain_Spinal_cord_cervical_c-1 0.29870793 Brain_Cerebellar_Hemisphere
2.090620343 0.138348452 0.138348452 1.039208172 1.533464002
1.298015552 0.247954504 3 0 0.998207054
EFL1 ENSG00000140598.13 0.323125228 3 3 0.141214245
Brain_Spinal_cord_cervical_c-1 0.583718231 Brain_Caudate_basal_ganglia
1.311904253 0.773912683 0.773912683 -1.471284308 0.547961656
-0.683799445 1.080451109 3 0 0.998207054
RP11-474D1.3 ENSG00000214039.8 0.323142739 2 2 0.151804235
Brain_Putamen_basal_ganglia 0.581219015 Brain_Cerebellum 1.069498917
0.930501083 0.930501083 0.551604993 1.433187589 0.992396291
0.623373032 2 0 0.998207054
METTL18 ENSG00000171806.11 0.323298646 13 1 0.152479379
Brain_Substantia_nigra 0.517625633 Brain_Amygdala 12.6418991 9.86E-05
12.6418991 0.64700975 1.430828478 0.973672064 0.198329486 1 0
0.998207054
SYCE1L ENSG00000205078.5 0.323331904 12 1 0.237867182
Brain_Cortex 0.383670342 Brain_Nucleus_accumbens_basal_ganglia
11.80400713 0.000286013 11.80400713 0.871153507 1.180334549
0.979620728 0.087571967 1 0 0.998207054
PIM3 ENSG00000198355.4 0.323366438 1 1 0.323366438 Brain_Cerebellum
0.323366438 Brain_Cerebellum 1 1 1 0.987563564 0.987563564
0.987563564 NA 1 0 0.998207054
GNA11 ENSG00000088256.8 0.323371031 1 1 0.323371031
Brain_Spinal_cord_cervical_c-1 0.323371031 Brain_Spinal_cord_cervical_c-1
1 1 1 -0.98755419 -0.98755419 -0.98755419 NA 1 0
0.998207054
PPP1R8 ENSG00000117751.17 0.32340824 1 1 0.32340824 Brain_Cortex
0.32340824 Brain_Cortex 1 1 1 -0.987478252 -0.987478252
-0.987478252 NA 1 0 0.998207054
RP11-826N14.4 ENSG00000251623.1 0.323455861 7 5 0.112507797
Brain_Cerebellum 0.978765828 Brain_Anterior_cingulate_cortex_BA24
5.137669022 0.043389381 0.182118385 -0.639078054 1.587021405
0.126013688 0.731491974 5 0 0.998207054
MRPL34 ENSG00000130312.6 0.323548027 13 1 0.16577109
Brain_Anterior_cingulate_cortex_BA24 0.599089737 Brain_Cerebellar_Hemisphere
12.51261842 3.75E-05 12.51261842 0.525709968 1.385920796
0.970713146 0.213691066 1 0 0.998207054
SWAP70 ENSG00000133789.14 0.323631187 2 2 0.198641988
Brain_Cerebellum 0.448683777 Brain_Caudate_basal_ganglia 1.015135204
0.984864796 0.984864796 -0.757611193 1.285430216 0.263909512
1.444648434 2 0 0.998207054
TP53 ENSG00000141510.16 0.323853043 1 1 0.323853043
Brain_Putamen_basal_ganglia 0.323853043 Brain_Putamen_basal_ganglia 1 1
1 -0.986570897 -0.986570897 -0.986570897 NA 1 0
0.998207054
RAB15 ENSG00000139998.14 0.323924375 5 4 0.066339527
Brain_Hippocampus 0.838342011 Brain_Substantia_nigra 3.209132633
0.072732486 0.225423981 -1.836122596 -0.204014701 -1.046270379
0.579692687 4 0 0.998207054
TLL1 ENSG00000038295.7 0.323989198 2 2 0.133631022
Brain_Hypothalamus 0.884258388 Brain_Cerebellum 1.05348541 0.94651459
0.94651459 0.145573121 1.499935845 0.822754483 0.957679066 2
0 0.998207054
CNKSR3 ENSG00000153721.18 0.32399897 3 3 0.071638098 Brain_Cerebellum

0.917989761 Brain_Spinal_cord_cervical_c-1 1.049427588 0.933178972
0.933178972 -0.102966245 1.801411164 0.686983202 0.992788024
3 0 0.998207054
LINC00265 ENSG00000188185.11 0.324091657 4 4 0.201807618
Brain_Nucleus_accumbens_basal_ganglia 0.754803397 Brain_Putamen_basal_ganglia
2.174083845 0.209072648 0.209072648 -1.276418504 1.216324458
0.259772252 1.088648658 4 0 0.998207054
RBAK ENSG00000146587.17 0.324115753 11 6 0.07022171
Brain_Caudate_basal_ganglia 0.811951631 Brain_Cerebellum 8.163680625
0.00955286 0.287827839 -0.237909056 1.810477889 0.929151551
0.596547446 6 0 0.998207054
ZBED3 ENSG00000132846.5 0.324139442 1 1 0.324139442 Brain_Amygdala
0.324139442 Brain_Amygdala 1 1 1 -0.985987101 -0.985987101
-0.985987101 NA 1 0 0.998207054
ENPP5 ENSG00000112796.9 0.324147543 13 1 0.155455948
Brain_Spinal_cord_cervical_c-1 0.720311383 Brain_Cortex 12.06796587
0.005029295 12.06796587 -0.358042668 1.420521383 0.940114071
0.486482574 1 0 0.998207054
AC114730.2 ENSG00000235151.1 0.324288023 1 1 0.324288023
Brain_Cerebellum 0.324288023 Brain_Cerebellum 1 1 1 -0.985684368
-0.985684368 -0.985684368 NA 1 0 0.998207054
DTX1 ENSG00000135144.7 0.324391684 2 2 0.171076751
Brain_Cerebellar_Hemisphere 0.489272783 Brain_Cortex 1.056646421
0.943353579 0.943353579 0.691465931 1.368752189 1.03010906
0.478913705 2 0 0.998207054
CDA ENSG00000158825.5 0.324401554 8 3 0.041332025
Brain_Frontal_Cortex_BA9 0.28433918 Brain_Cortex 6.826256919 3.88E-16
0.320634981 1.070622549 2.040183778 1.555771641 0.308591159
3 0 0.998207054
CDCA7 ENSG00000144354.13 0.324457299 2 2 0.310871771 Brain_Cerebellum
0.357280743 Brain_Cortex 1.167930109 0.832069891 0.832069891
-1.013394488 0.920558949 -0.04641777 1.36751159 2 0
0.998207054
RNFT1 ENSG00000189050.15 0.324815484 1 1 0.324815484 Brain_Cortex
0.324815484 Brain_Cortex 1 1 1 0.98461039 0.98461039 0.98461039
NA 1 0 0.998207054
ALDH18A1 ENSG00000059573.8 0.324865563 3 2 0.264810372
Brain_Cerebellar_Hemisphere 0.766508799 Brain_Nucleus_accumbens_basal_ganglia
2.387701379 0.018987959 0.593310662 -0.296944608 1.115093463
0.613799982 0.790063629 2 0 0.998207054
CHN2 ENSG00000106069.21 0.324879256 2 2 0.274722026
Brain_Cerebellar_Hemisphere 0.345416353 Brain_Nucleus_accumbens_basal_ganglia
1.074219767 0.925780233 0.925780233 -1.092252743 -0.943517335
-1.017885039 0.105171816 2 0 0.998207054
BSND ENSG00000162399.6 0.324889469 5 2 0.16644341
Brain_Caudate_basal_ganglia 0.403290206 Brain_Nucleus_accumbens_basal_ganglia
4.655952334 0.003036614 0.196647883 0.835759516 1.383722603
1.1981726 0.22672908 2 0 0.998207054
ADRA1B ENSG00000170214.3 0.324896376 1 1 0.324896376
Brain_Caudate_basal_ganglia 0.324896376 Brain_Caudate_basal_ganglia 1 1
1 0.984445784 0.984445784 0.984445784 NA 1 0
0.998207054
ABI3 ENSG00000108798.8 0.325022272 1 1 0.325022272 Brain_Cortex
0.325022272 Brain_Cortex 1 1 1 0.984189654 0.984189654
0.984189654 NA 1 0 0.998207054
TTLL3 ENSG00000214021.15 0.325123322 8 3 0.076584331
Brain_Spinal_cord_cervical_c-1 0.595431293 Brain_Cortex 6.721220744
0.004055825 0.266356841 0.530981941 1.770857921 1.146766167
0.432461675 3 0 0.998207054
TRAPPC1 ENSG00000170043.11 0.32513679 1 1 0.32513679
Brain_Caudate_basal_ganglia 0.32513679 Brain_Caudate_basal_ganglia 1 1 1
-0.983956727 -0.983956727 -0.983956727 NA 1 0 0.998207054

PPP1R2 ENSG00000184203.7 0.325221458 1 1 0.325221458 Brain_Spinal_cord_cervical_c-1 0.325221458 Brain_Spinal_cord_cervical_c-1 1 1 1 -0.983784549 -0.983784549 -0.983784549 NA 1 0 0.998207054
IQCA1 ENSG00000132321.16 0.325233187 4 4 0.204523901 Brain_Nucleus_accumbens_basal_ganglia 0.841133177 Brain_Cerebellar_Hemisphere 2.097312698 0.252488624 0.252488624 -1.268767776 1.070225978 -0.213166781 0.969432727 4 0 0.998207054
CR2 ENSG00000117322.17 0.325300687 1 1 0.325300687 Brain_Hypothalamus 0.325300687 Brain_Hypothalamus 1 1 1 -0.983623457 -0.983623457 -0.983623457 NA 1 0 0.998207054
NFKBIB ENSG00000104825.16 0.325391747 6 4 0.187067112 Brain_Putamen_basal_ganglia 0.951785914 Brain_Cerebellum 4.026507823 0.047217302 0.259309059 -1.319305164 -0.060464218 -0.587131281 0.566779811 4 0 0.998207054
A3GALT2 ENSG00000184389.9 0.32541259 2 1 0.205372907 Brain_Cerebellum 0.496175025 Brain_Cerebellar_Hemisphere 1.959773113 0.040226887 1.959773113 -1.266391627 -0.680520375 -0.973456001 0.414273535 1 0 0.998207054
FANCA ENSG00000187741.14 0.325415706 6 6 0.041410502 Brain_Hippocampus 0.943551733 Brain_Frontal_Cortex_BA9 2.069348186 0.383349175 0.383349175 -1.01738037 2.039396191 0.760925027 1.202920841 6 0 0.998207054
DTNBP1 ENSG00000047579.19 0.325503401 5 5 0.055174538 Brain_Nucleus_accumbens_basal_ganglia 0.930546343 Brain_Hippocampus 3.057177168 0.163687091 0.163687091 -0.087157473 1.917499236 0.914025603 0.831418266 5 0 0.998207054
HELLS ENSG00000119969.14 0.325579917 3 1 0.283834801 Brain_Cerebellum 0.380032227 Brain_Cerebellar_Hemisphere 2.905777048 0.025099605 2.905777048 -1.071744515 -0.877836918 -0.967556101 0.097760207 1 0 0.998207054
ARF1 ENSG00000143761.15 0.325684529 1 1 0.325684529 Brain_Caudate_basal_ganglia 0.325684529 Brain_Caudate_basal_ganglia 1 1 1 -0.982843379 -0.982843379 -0.982843379 NA 1 0 0.998207054
NBL1 ENSG00000158747.13 0.325902838 7 7 0.1258078 Brain_Cerebellum 0.905013687 Brain_Frontal_Cortex_BA9 3.66314681 0.149059598 0.149059598 -0.795941136 1.530844615 0.57774943 0.786511447 7 0 0.998207054
GEMIN6 ENSG00000152147.10 0.325910106 1 1 0.325910106 Brain_Spinal_cord_cervical_c-1 0.325910106 Brain_Spinal_cord_cervical_c-1 1 1 1 0.982385218 0.982385218 0.982385218 NA 1 0 0.998207054
RP11-321G12.1 ENSG00000259459.5 0.326016167 8 6 0.06630504 Brain_Nucleus_accumbens_basal_ganglia 0.996884094 Brain_Substantia_nigra 5.27170801 0.048239308 0.229819342 -0.003905219 1.836355879 1.223377941 0.683216146 6 0 0.998207054
MECR ENSG00000116353.15 0.326036632 7 4 0.059534492 Brain_Substantia_nigra 0.900701232 Brain_Nucleus_accumbens_basal_ganglia 4.499626627 0.040620934 0.413828822 -0.3545511 1.884225412 0.490311563 0.708848936 4 0 0.998207054
C2CD4D ENSG00000225556.1 0.326172607 2 2 0.280472625 Brain_Cerebellar_Hemisphere 0.387594867 Brain_Amygdala 1.149696556 0.850303444 0.850303444 -1.079258236 0.863987301 -0.107635468 1.374082097 2 0 0.998207054
DCC ENSG00000187323.11 0.326418572 1 1 0.326418572 Brain_Cerebellar_Hemisphere 0.326418572 Brain_Cerebellar_Hemisphere 1 1 1 -0.981353249 -0.981353249 -0.981353249 NA 1 0 0.998207054
NDUFS5 ENSG00000168653.10 0.326529533 10 3 0.250758153 Brain_Anterior_cingulate_cortex_BA24 0.951348804 Brain_Cerebellar_Hemisphere 8.426549026 0.001969027 0.464280844 -1.14850984 0.279762857

-0.542040284 0.521979424 3 0 0.998207054
ANKS1B ENSG00000185046.18 0.32653272 2 2 0.235001662
Brain_Caudate_basal_ganglia 0.612377731 Brain_Hippocampus 1.303484154
0.696515846 0.696515846 -0.50668233 1.187573048 0.340445359
1.198019467 2 0 0.998207054
HAVCR1 ENSG00000113249.12 0.326559256 1 1 0.326559256
Brain_Frontal_Cortex_BA9 0.326559256 Brain_Frontal_Cortex_BA9 1 1
1 0.981067905 0.981067905 0.981067905 NA 1 0
0.998207054
GZMK ENSG00000113088.5 0.326691078 1 1 0.326691078
Brain_Hippocampus 0.326691078 Brain_Hippocampus 1 1 1
-0.98080061 -0.98080061 -0.98080061 NA 1 0 0.998207054
WTH3DI ENSG00000233087.7 0.326775325 5 4 0.088908311
Brain_Amygdala 0.961515713 Brain_Caudate_basal_ganglia 2.752664067
0.088542701 0.290284488 -1.701184696 -0.048251618 -0.764488518
0.62812265 4 0 0.998207054
CLRN3 ENSG00000180745.4 0.326832959 3 3 0.143020101 Brain_Cortex
0.442813813 Brain_Frontal_Cortex_BA9 1.797091427 0.209541472
0.209541472 0.767450333 1.464636564 1.071989558 0.35684646 3
0 0.998207054
PTGIS ENSG00000124212.5 0.326898574 1 1 0.326898574 Brain_Cortex
0.326898574 Brain_Cortex 1 1 1 -0.980380009 -0.980380009
-0.980380009 NA 1 0 0.998207054
RP1-35C21.2 ENSG00000227579.5 0.326919202 3 3 0.096471102
Brain_Anterior_cingulate_cortex_BA24 0.703125159 Brain_Amygdala
1.128545852 0.884839585 0.884839585 -1.662207873 0.955121899
-0.362730404 1.308761632 3 0 0.998207054
MFNG ENSG00000100060.17 0.326964558 1 1 0.326964558
Brain_Nucleus_accumbens_basal_ganglia 0.326964558
Brain_Nucleus_accumbens_basal_ganglia 1 1 1 -0.980246295
-0.980246295 -0.980246295 NA 1 0 0.998207054
CDH4 ENSG00000179242.15 0.326965448 1 1 0.326965448 Brain_Cerebellum
0.326965448 Brain_Cerebellum 1 1 1 -0.98024449 -0.98024449
-0.98024449 NA 1 0 0.998207054
RSPH1 ENSG00000160188.9 0.326991105 6 3 0.136477738
Brain_Frontal_Cortex_BA9 0.852061529 Brain_Cortex 4.731014113
0.050340526 0.184722329 -1.489036575 0.533233532 -0.009330568
0.771081989 3 0 0.998207054
C15orf39 ENSG00000167173.18 0.327019729 1 1 0.327019729
Brain_Frontal_Cortex_BA9 0.327019729 Brain_Frontal_Cortex_BA9 1 1
1 -0.980134504 -0.980134504 -0.980134504 NA 1 0
0.998207054
NFIB ENSG00000147862.15 0.327026875 1 1 0.327026875
Brain_Spinal_cord_cervical_c-1 0.327026875 Brain_Spinal_cord_cervical_c-1
1 1 1 0.980120026 0.980120026 0.980120026 NA 1 0
0.998207054
LAMC2 ENSG00000058085.14 0.327195041 10 10 0.053672484
Brain_Hypothalamus 0.913702184 Brain_Caudate_basal_ganglia 2.018545242
0.366653633 0.366653633 -1.929470463 1.223581337 -0.528750258
0.961625978 10 0 0.998207054
ZDHHC3 ENSG00000163812.13 0.32729874 3 2 0.171926099
Brain_Putamen_basal_ganglia 0.622397649 Brain_Hippocampus 2.706705833
0.032638834 0.260655333 -1.366040988 -0.492455109 -0.913317633
0.437663577 2 0 0.998207054
TANC1 ENSG00000115183.14 0.327387629 3 3 0.158386624
Brain_Cerebellar_Hemisphere 0.245326429 Brain_Spinal_cord_cervical_c-1
1.920565245 0.07853208 0.07853208 1.161776088 1.410518546
1.310567102 0.131366587 3 0 0.998207054
SAMD9L ENSG00000177409.11 0.327422181 10 3 0.310964611
Brain_Anterior_cingulate_cortex_BA24 0.847314076 Brain_Cerebellum
8.228136504 0.021876926 0.289773349 -1.013200062 0.498139313
-0.443303939 0.425924191 3 0 0.998207054

SLN ENSG00000170290.3 0.327460491 6 3 0.24608719
Brain_Nucleus_accumbens_basal_ganglia 0.968641223 Brain_Frontal_Cortex_BA9
4.768450691 0.014824049 0.186453631 -1.159905729 -0.039312523
-0.563625868 0.410288076 3 0 0.998207054
OSBP2 ENSG00000184792.15 0.327545903 4 2 0.114406819 Brain_Cerebellum
0.493362015 Brain_Hypothalamus 3.018435671 0.023435523
0.873124715 -1.578691572 -0.684971326 -1.245312175 0.402711366
2 0 0.998207054
MRGPRF ENSG00000172935.8 0.327728603 4 4 0.173082914
Brain_Cortex 0.692919482 Brain_Cerebellum 1.85847885 0.201883964
0.201883964 -1.362364396 0.394896217 -0.656620124 0.768069331
4 0 0.998207054
FSIP1 ENSG00000150667.6 0.327737713 1 1 0.327737713
Brain_Caudate_basal_ganglia 0.327737713 Brain_Caudate_basal_ganglia 1 1
1 -0.978680818 -0.978680818 -0.978680818 NA 1 0
0.998207054
SUMO2 ENSG00000188612.11 0.32796348 1 1 0.32796348 Brain_Cerebellum 0.32796348
Brain_Cerebellum 1 1 1 0.978224139 0.978224139 0.978224139
NA 1 0 0.998207054
CDC42BPA ENSG00000143776.18 0.328203794 3 3 0.447179179
Brain_Cerebellar_Hemisphere 0.725780734 Brain_Putamen_basal_ganglia
1.752086814 0.261110132 0.261110132 -0.550562699 0.760126156
-0.047060038 0.706147354 3 0 0.998207054
KIAA1755 ENSG00000149633.11 0.328364223 2 2 0.19016656
Brain_Frontal_Cortex_BA9 0.39607266 Brain_Putamen_basal_ganglia 1.105202309
0.894797691 0.894797691 0.848656137 1.310086544 1.079371341
0.32628057 2 0 0.998207054
NISCH ENSG00000010322.15 0.328643467 2 2 0.17821729
Brain_Caudate_basal_ganglia 0.595097213 Brain_Nucleus_accumbens_basal_ganglia
1.081095118 0.918904882 0.918904882 -0.5314641 1.346264314
0.407400107 1.327754495 2 0 0.998207054
SLC50A1 ENSG00000169241.17 0.32875864 1 1 0.32875864 Brain_Cerebellum
0.32875864 Brain_Cerebellum 1 1 1 -0.976617323 -0.976617323
-0.976617323 NA 1 0 0.998207054
TRIM27 ENSG00000204713.10 0.328781671 1 1 0.328781671
Brain_Nucleus_accumbens_basal_ganglia 0.328781671
Brain_Nucleus_accumbens_basal_ganglia 1 1 1 -0.976570821
-0.976570821 -0.976570821 NA 1 0 0.998207054
TTC23 ENSG00000103852.12 0.328797447 13 7 0.013337575
Brain_Anterior_cingulate_cortex_BA24 0.692153569 Brain_Cerebellar_Hemisphere
8.226470502 0.001892429 0.363384624 -2.47462605 0.458596727
-1.017253303 0.674128055 7 0 0.998207054
TBX3 ENSG00000135111.15 0.328835878 1 1 0.328835878
Brain_Putamen_basal_ganglia 0.328835878 Brain_Putamen_basal_ganglia 1 1
1 -0.976461379 -0.976461379 -0.976461379 NA 1 0
0.998207054
SPATA5 ENSG00000145375.7 0.328854381 2 2 0.209731599
Brain_Caudate_basal_ganglia 0.398433981 Brain_Cerebellum 1.030250585
0.969749415 0.969749415 0.844421377 1.254303813 1.049362595
0.28983065 2 0 0.998207054
DKKL1 ENSG00000104901.6 0.328993055 1 1 0.328993055
Brain_Nucleus_accumbens_basal_ganglia 0.328993055
Brain_Nucleus_accumbens_basal_ganglia 1 1 1 0.97614411 0.97614411
0.97614411 NA 1 0 0.998207054
EFCAB5 ENSG00000176927.15 0.32900835 9 5 0.193834674
Brain_Anterior_cingulate_cortex_BA24 0.983757765 Brain_Frontal_Cortex_BA9
6.974673658 0.000411037 0.246226448 -1.299318424 0.725772276
-0.314995473 0.556176081 5 0 0.998207054
DDX6 ENSG00000110367.11 0.329420362 5 4 0.035556441 Brain_Amygdala
0.780376259 Brain_Substantia_nigra 3.279242935 0.083589306
0.224609391 0.278828737 2.101963863 1.482996996 0.721854113
4 0 0.998207054

TWIST2 ENSG00000233608.3 0.329463399 2 2 0.193840011
Brain_Spinal_cord_cervical_c-1 0.786485642 Brain_Putamen_basal_ganglia
1.63998399 0.36001601 0.36001601 0.270876991 1.299302866 0.785089929
0.72720691 2 0 0.998207054
C5orf38 ENSG00000186493.11 0.329514518 1 1 0.329514518
Brain_Cortex 0.329514518 Brain_Cortex 1 1 1 -0.975092219
-0.975092219 -0.975092219 NA 1 0 0.998207054
MARCKS ENSG00000277443.2 0.329527748 1 1 0.329527748
Brain_Substantia_nigra 0.329527748 Brain_Substantia_nigra 1 1
1 -0.975065546 -0.975065546 -0.975065546 NA 1 0
0.998207054
MYADML2 ENSG00000185105.5 0.329579203 11 10 0.091487257
Brain_Hippocampus 0.794470749 Brain_Amygdala 2.358402458 0.05932234
0.274490704 -0.662162573 1.68760407 0.346499798 0.887760147 10
0 0.998207054
SOS2 ENSG00000100485.11 0.329793136 3 1 0.286044814 Brain_Cerebellum
0.37829001 Brain_Cerebellar_Hemisphere 2.987921876 0.00136423 2.987921876
-1.066838402 -0.881051382 -0.972640262 0.09292099 1 0
0.998207054
VMAC ENSG00000187650.3 0.329886419 1 1 0.329886419 Brain_Cortex
0.329886419 Brain_Cortex 1 1 1 0.974342682 0.974342682
0.974342682 NA 1 0 0.998207054
CTU1 ENSG00000142544.6 0.329899008 3 3 0.166798207 Brain_Cerebellum
0.978218225 Brain_Cerebellar_Hemisphere 1.060023128 0.945916511
0.945916511 -1.167876961 1.38256526 0.080663699 1.276058157 3
0 0.998207054
HLTF ENSG00000071794.15 0.329943311 11 4 0.034099968
Brain_Caudate_basal_ganglia 0.461930297 Brain_Hypothalamus 8.54966622
0.011695469 0.382982039 0.73567206 2.118887586 1.58725991
0.432606329 4 0 0.998207054
CALML4 ENSG00000129007.14 0.329958923 1 1 0.329958923
Brain_Cerebellum 0.329958923 Brain_Cerebellum 1 1 1 -0.974196619
-0.974196619 -0.974196619 NA 1 0 0.998207054
HERC3 ENSG00000138641.15 0.330176444 2 2 0.174315696
Brain_Substantia_nigra 0.419004601 Brain_Nucleus_accumbens_basal_ganglia
1.151829459 0.848170541 0.848170541 -1.35846653 -0.808149368
-1.083307949 0.389132997 2 0 0.998207054
CATSPERG ENSG00000099338.22 0.330239076 2 2 0.461735418
Brain_Anterior_cingulate_cortex_BA24 0.584795526
Brain_Spinal_cord_cervical_c-1 1.627065809 0.372934191 0.372934191
-0.735992244 0.546393429 -0.094799407 0.906783605 2 0
0.998207054
SCN4B ENSG00000177098.8 0.330243219 5 5 0.051471782
Brain_Frontal_Cortex_BA9 0.776009939 Brain_Cerebellar_Hemisphere
2.085739648 0.301657052 0.301657052 -1.947525368 0.326454498
-0.657658011 1.096554759 5 0 0.998207054
KREMEN2 ENSG00000131650.13 0.330376884 1 1 0.330376884
Brain_Cortex 0.330376884 Brain_Cortex 1 1 1 -0.973355025
-0.973355025 -0.973355025 NA 1 0 0.998207054
WDSUB1 ENSG00000196151.10 0.330417642 13 3 0.043808032
Brain_Putamen_basal_ganglia 0.455292386 Brain_Substantia_nigra
10.78603702 0.01380394 0.365169142 -2.015922963 -0.746620972
-1.542652683 0.34761188 3 0 0.998207054
COX5B ENSG00000135940.6 0.330546625 2 2 0.252054374
Brain_Caudate_basal_ganglia 0.87669457 Brain_Putamen_basal_ganglia 1.554580839
0.445419161 0.445419161 -0.155160776 1.145373735 0.49510648
0.919616772 2 0 0.998207054
DYDC2 ENSG00000133665.12 0.330596683 4 2 0.100300064
Brain_Putamen_basal_ganglia 0.753200417 Brain_Cortex 3.179219358
0.002514627 0.790090791 -0.314422174 1.643400658 0.988284275
0.885888839 2 0 0.998207054
ARL3 ENSG00000138175.8 0.330639572 6 6 0.148426213

Brain_Cerebellar_Hemisphere 0.950634859 Brain_Cortex 2.206643159
0.094856215 0.094856215 -0.612567827 1.445112769 0.502655373
0.749183348 6 0 0.998207054
ZNF813 ENSG00000198346.10 0.330647147 1 1 0.330647147
Brain_Cortex 0.330647147 Brain_Cortex 1 1 1 -0.972811195
-0.972811195 -0.972811195 NA 1 0 0.998207054
BCL6 ENSG00000113916.17 0.330651547 1 1 0.330651547 Brain_Amygdala
0.330651547 Brain_Amygdala 1 1 1 -0.972802344 -0.972802344
-0.972802344 NA 1 0 0.998207054
GLO1 ENSG00000124767.6 0.330673293 10 3 0.319543766
Brain_Hippocampus 0.988925909 Brain_Caudate_basal_ganglia 8.498860184
0.012478464 0.372550173 -0.995395871 0.37413612 0.071484122
0.398931794 3 0 0.998207054
XPOT ENSG00000184575.11 0.330735271 1 1 0.330735271
Brain_Hippocampus 0.330735271 Brain_Hippocampus 1 1 1
-0.972633934 -0.972633934 -0.972633934 NA 1 0 0.998207054
YIPF5 ENSG00000145817.16 0.330743912 1 1 0.330743912
Brain_Nucleus_accumbens_basal_ganglia 0.330743912
Brain_Nucleus_accumbens_basal_ganglia 1 1 1 -0.972616555
-0.972616555 -0.972616555 NA 1 0 0.998207054
TNFRSF14 ENSG00000157873.17 0.330778389 5 4 0.093061992
Brain_Amygdala 0.886854638 Brain_Caudate_basal_ganglia 3.311586828
0.068820783 0.190602629 0.142285325 1.679462242 0.818558745
0.61501236 4 0 0.998207054
CFAP157 ENSG00000160401.14 0.330898861 3 3 0.424964114
Brain_Cerebellar_Hemisphere 0.482789563 Brain_Putamen_basal_ganglia
1.560385487 0.488988173 0.488988173 -0.797838676 0.784906749
-0.238251637 0.887380733 3 0 0.998207054
RP11-108M9.6 ENSG00000272426.1 0.330921183 4 4 0.071387856
Brain_Hippocampus 0.340351891 Brain_Cortex 1.919006667
0.238105283 0.238105283 0.953470193 1.803002284 1.423762332
0.375334749 4 0 0.998207054
TRIM26 ENSG00000234127.8 0.330924744 7 6 0.23284772
Brain_Cerebellar_Hemisphere 0.895418142 Brain_Frontal_Cortex_BA9
3.844384587 0.095459948 0.337778629 -0.95503315 1.19305529
0.264411761 0.840147317 6 0 0.998207054
CRLF3 ENSG00000176390.11 0.330953604 11 4 0.29466578
Brain_Spinal_cord_cervical_c-1 0.954225026
Brain_Nucleus_accumbens_basal_ganglia 7.588266132 0.014072277
0.358358652 -0.925004268 1.047941024 0.307086792 0.54513015 4
0 0.998207054
POLR2H ENSG00000163882.9 0.33099727 1 1 0.33099727
Brain_Caudate_basal_ganglia 0.33099727 Brain_Caudate_basal_ganglia 1 1 1
0.972107101 0.972107101 0.972107101 NA 1 0 0.998207054
FGD6 ENSG00000180263.13 0.33105803 1 1 0.33105803 Brain_Frontal_Cortex_BA9
0.33105803 Brain_Frontal_Cortex_BA9 1 1 1 -0.971984962
-0.971984962 -0.971984962 NA 1 0 0.998207054
CNTRL ENSG00000119397.16 0.331072502 3 3 0.101031894
Brain_Spinal_cord_cervical_c-1 0.971646493
Brain_Nucleus_accumbens_basal_ganglia 1.675545046 0.392238438
0.392238438 -0.035543334 1.639871471 0.63338697 0.887249018 3
0 0.998207054
PPIL2 ENSG00000100023.18 0.331302494 9 8 0.205219724
Brain_Frontal_Cortex_BA9 0.997786095 Brain_Spinal_cord_cervical_c-1
3.997130105 0.073206871 0.264948892 -0.87571376 1.266819818
0.184539346 0.815872614 8 0 0.998207054
GRAP2 ENSG00000100351.16 0.331339227 1 1 0.331339227 Brain_Amygdala
0.331339227 Brain_Amygdala 1 1 1 0.971419892 0.971419892
0.971419892 NA 1 0 0.998207054
TEX26 ENSG00000175664.9 0.331386775 7 4 0.184597957
Brain_Hippocampus 0.987891099 Brain_Nucleus_accumbens_basal_ganglia
5.339701887 0.026639726 0.237993478 -0.290825118 1.326730169

0.270398728 0.556639037 4 0 0.998207054
CREBBP ENSG00000005339.14 0.331403586 1 1 0.331403586
Brain_Substantia_nigra 0.331403586 Brain_Substantia_nigra 1 1
1 0.971290606 0.971290606 0.971290606 NA 1 0
0.998207054
VPS13C ENSG00000129003.15 0.331477597 1 1 0.331477597
Brain_Cerebellum 0.331477597 Brain_Cerebellum 1 1 1 -0.971141949
-0.971141949 -0.971141949 NA 1 0 0.998207054
SEMA6A ENSG00000092421.16 0.3314872 1 1 0.3314872 Brain_Cerebellum
0.3314872 Brain_Cerebellum 1 1 1 0.971122662 0.971122662
0.971122662 NA 1 0 0.998207054
TJP2 ENSG00000119139.17 0.3314911 1 1 0.3314911 Brain_Caudate_basal_ganglia
0.3314911 Brain_Caudate_basal_ganglia 1 1 1 0.97111483 0.97111483
0.97111483 NA 1 0 0.998207054
APLP2 ENSG00000084234.16 0.331693333 1 1 0.331693333
Brain_Caudate_basal_ganglia 0.331693333 Brain_Caudate_basal_ganglia 1 1
1 0.970708751 0.970708751 0.970708751 NA 1 0
0.998207054
PRMT7 ENSG00000132600.16 0.331881887 12 5 0.013946936 Brain_Cortex
0.725905094 Brain_Amygdala 7.565589007 0.006068463 0.370871098
-2.458627208 -0.350577826 -1.522358876 0.819803819 5 0
0.998207054
TOMM5 ENSG00000175768.12 0.33193266 1 1 0.33193266
Brain_Anterior_cingulate_cortex_BA24 0.33193266
Brain_Anterior_cingulate_cortex_BA24 1 1 1 -0.970228394
-0.970228394 -0.970228394 NA 1 0 0.998207054
NKIRAS1 ENSG00000197885.10 0.331939866 5 5 0.034858888
Brain_Hypothalamus 0.884016139 Brain_Anterior_cingulate_cortex_BA24
2.521314785 0.326610165 0.326610165 -2.109993826 -0.145879976
-1.034998708 0.72560815 5 0 0.998207054
ERI3 ENSG00000117419.14 0.331941096 2 2 0.162935985
Brain_Caudate_basal_ganglia 0.60256765 Brain_Spinal_cord_cervical_c-1
1.008583017 0.991416983 0.991416983 -1.39526486 0.52071166
-0.4372766 1.35479999 2 0 0.998207054
EPHB1 ENSG00000154928.16 0.331968614 5 5 0.076886533
Brain_Hypothalamus 0.996414388 Brain_Putamen_basal_ganglia 1.38854056
0.584100478 0.584100478 -1.372519338 1.769043996 -0.064985652
1.17927253 5 0 0.998207054
BMP7 ENSG00000101144.12 0.332008097 4 4 0.05415294
Brain_Substantia_nigra 0.902175215 Brain_Putamen_basal_ganglia
1.519448103 0.494204647 0.494204647 -1.925611195 0.706386209
-0.459243602 1.144119942 4 0 0.998207054
BBS5 ENSG00000163093.11 0.332017005 2 2 0.139420406 Brain_Cerebellum
0.385113776 Brain_Cerebellar_Hemisphere 1.503338777 0.496661223
0.496661223 0.868512604 1.477952824 1.173232714 0.430939312
2 0 0.998207054
RP11-686O6.2 ENSG00000273456.1 0.332195908 8 2 0.126901374
Brain_Nucleus_accumbens_basal_ganglia 0.986758411
Brain_Spinal_cord_cervical_c-1 7.20516994 0.004002599 0.274499988
0.016596633 1.526435773 0.756990564 0.424895013 2 0
0.998207054
GALK2 ENSG00000156958.14 0.332200274 8 4 0.087836513 Brain_Cortex
0.826727132 Brain_Anterior_cingulate_cortex_BA24 6.429862712
0.001149641 0.218892735 -0.218901045 1.706922204 1.102929967
0.63357543 4 0 0.998207054
H3F3A ENSG00000163041.9 0.332302043 13 4 0.132307314 Brain_Cortex
0.76694882 Brain_Cerebellum 10.39282636 0.002920286 0.415939006
-1.505065203 0.474225855 -0.45066023 0.534872149 4 0
0.998207054
LYRM1 ENSG00000102897.9 0.332314814 1 1 0.332314814
Brain_Cerebellar_Hemisphere 0.332314814 Brain_Cerebellar_Hemisphere 1 1
1 -0.969461834 -0.969461834 -0.969461834 NA 1 0

0.998207054
ISOC1 ENSG00000066583.11 0.332367981 1 1 0.332367981
Brain_Caudate_basal_ganglia 0.332367981 Brain_Caudate_basal_ganglia 1 1
1 0.969355232 0.969355232 0.969355232 NA 1 0
0.998207054
KLC3 ENSG00000104892.16 0.332593785 6 2 0.153993998
Brain_Frontal_Cortex_BA9 0.322779029 Brain_Cortex 5.402282914
0.010870571 0.348467433 -1.425564816 -0.988763167 -1.179591888
0.201707102 2 0 0.998207054
ERBB4 ENSG00000178568.14 0.33264315 2 2 0.192570729
Brain_Cerebellar_Hemisphere 0.490073095 Brain_Cerebellum 1.83189639 0.16810361
0.16810361 -1.303011813 -0.69019257 -0.996602191 0.433328642 2
0 0.998207054
TTC12 ENSG00000149292.16 0.332665005 13 2 0.128517329
Brain_Frontal_Cortex_BA9 0.832034856 Brain_Amygdala 11.00459718
0.007141539 0.717338417 0.212092519 1.519974722 0.968514221
0.363633017 2 0 0.998207054
PTGES3L ENSG00000267060.5 0.332718538 3 3 0.417501086
Brain_Cortex 0.591777234 Brain_Cerebellar_Hemisphere 2.271802768
0.288570892 0.288570892 -0.810764219 0.536262369 -0.313304541
0.739327251 3 0 0.998207054
IFNAR1 ENSG00000142166.12 0.332746814 2 2 0.138102211
Brain_Cerebellum 0.456663288 Brain_Cerebellar_Hemisphere 1.526073809
0.473926191 0.473926191 -1.482895373 -0.744352434 -1.113623903
0.522228721 2 0 0.998207054
SHISA3 ENSG00000178343.4 0.332883706 6 6 0.0634147
Brain_Hippocampus 0.936278595 Brain_Frontal_Cortex_BA9 2.562328415
0.316581903 0.316581903 -0.517961429 1.856272691 0.386690758
0.858295491 6 0 0.998207054
RP11-138E2.1 ENSG00000270755.1 0.33296216 1 1 0.33296216 Brain_Cerebellum
0.33296216 Brain_Cerebellum 1 1 1 0.968164623 0.968164623
0.968164623 NA 1 0 0.998207054
ZNF282 ENSG00000170265.11 0.333070377 1 1 0.333070377
Brain_Amygdala 0.333070377 Brain_Amygdala 1 1 1 0.967947926
0.967947926 0.967947926 NA 1 0 0.998207054
TP53RK ENSG00000172315.5 0.333097087 1 1 0.333097087
Brain_Amygdala 0.333097087 Brain_Amygdala 1 1 1 0.967894449
0.967894449 0.967894449 NA 1 0 0.998207054
MAN2B1 ENSG00000104774.12 0.333191257 4 3 0.172276578
Brain_Cerebellum 0.536037606 Brain_Spinal_cord_cervical_c-1 2.059685677
0.011006719 0.448258457 -1.364925146 1.208410868 -0.210598292
1.320483846 3 0 0.998207054
ZCCHC7 ENSG00000147905.17 0.333193051 1 1 0.333193051
Brain_Hippocampus 0.333193051 Brain_Hippocampus 1 1 1
-0.967702337 -0.967702337 -0.967702337 NA 1 0 0.998207054
TMEM120B ENSG00000188735.12 0.333219834 2 2 0.139819138
Brain_Cerebellum 0.854343323 Brain_Cerebellar_Hemisphere 1.213014435
0.786985565 0.786985565 -1.476464875 -0.18357953 -0.830022203
0.914207995 2 0 0.998207054
CFAP44 ENSG00000206530.10 0.333280634 3 3 0.087440801
Brain_Cerebellar_Hemisphere 0.636750379 Brain_Frontal_Cortex_BA9
1.594743233 0.402075337 0.402075337 -0.472247164 1.709054791
0.791590365 1.13114999 3 0 0.998207054
DYNLL1 ENSG00000088986.10 0.333546734 6 4 0.04107307
Brain_Frontal_Cortex_BA9 0.759899676 Brain_Caudate_basal_ganglia 3.57928161
0.078577602 0.495148828 -2.042791628 -0.305612534 -0.81684808
0.620427799 4 0 0.998207054
SERPINB9P1 ENSG00000230438.6 0.333630012 13 8 0.06425588
Brain_Spinal_cord_cervical_c-1 0.921467993
Brain_Anterior_cingulate_cortex_BA24 7.221989968 0.008223991
0.278754174 -1.850400268 1.126619842 -0.420673493 0.76298062 8
0 0.998207054

PPT1 ENSG00000131238.16 0.333654793 2 1 0.337960589 Brain_Amygdala
0.338780447 Brain_Spinal_cord_cervical_c-1 1.96134498 0.03865502 1.96134498
0.956577704 0.958202634 0.957390169 0.001149 1 0
0.998207054
G0S2 ENSG00000123689.5 0.333744237 3 3 0.092617455 Brain_Cortex
0.862708393 Brain_Cerebellar_Hemisphere 1.168896723 0.808546059
0.808546059 -0.5377973 1.681749344 0.323674838 1.19019211 3 0
0.998207054
LRPPRC ENSG00000138095.18 0.333833914 13 2 0.131951877
Brain_Substantia_nigra 0.383767158 Brain_Amygdala 11.49589719
0.000257643 0.800008015 0.870976183 1.506449286 1.118272511
0.2208377 2 0 0.998207054
FLYWCH2 ENSG00000162076.12 0.33386018 2 2 0.156912374
Brain_Anterior_cingulate_cortex_BA24 0.875934507 Brain_Cerebellum
1.393617596 0.606382404 0.606382404 -1.41553268 -0.156124982
-0.785828831 0.890535724 2 0 0.998207054
MDM2 ENSG00000135679.22 0.333888602 1 1 0.333888602
Brain_Spinal_cord_cervical_c-1 0.333888602 Brain_Spinal_cord_cervical_c-1
1 1 1 -0.966310963 -0.966310963 -0.966310963 NA 1 0
0.998207054
BHLHA15 ENSG00000180535.3 0.333960199 10 2 0.092108525
Brain_Hypothalamus 0.527257158 Brain_Nucleus_accumbens_basal_ganglia
8.759547116 0.008060384 0.562687392 -1.684378595 -0.632198529
-1.191379557 0.306010547 2 0 0.998207054
ZNF784 ENSG00000179922.5 0.334217905 2 2 0.259421215
Brain_Cortex 0.352001143 Brain_Cerebellar_Hemisphere 1.025000058
0.974999942 0.974999942 -1.127760178 0.930714741 -0.098522719
1.455561574 2 0 0.998207054
LINC01833 ENSG00000259439.2 0.334263548 2 2 0.141476498
Brain_Putamen_basal_ganglia 0.34894976 Brain_Cerebellum 1.718696871
0.281303129 0.281303129 0.936628358 1.470314861 1.203471609
0.377373346 2 0 0.998207054
SLC35B4 ENSG00000205060.10 0.334325993 3 3 0.104139813
Brain_Spinal_cord_cervical_c-1 0.66989963 Brain_Cerebellum 2.332132874
0.253760675 0.253760675 -0.491574168 1.625106759 0.235748942
1.203661913 3 0 0.998207054
ZNF107 ENSG00000196247.11 0.334548647 10 3 0.088585617
Brain_Spinal_cord_cervical_c-1 0.889965676 Brain_Substantia_nigra
6.998440131 0.010332937 0.669412382 -1.702906233 0.13834764
-0.960086772 0.543784192 3 0 0.998207054
THBS4 ENSG00000113296.14 0.334565703 1 1 0.334565703
Brain_Putamen_basal_ganglia 0.334565703 Brain_Putamen_basal_ganglia 1 1
1 -0.964958292 -0.964958292 -0.964958292 NA 1 0
0.998207054
KCNS2 ENSG00000156486.7 0.334647068 3 3 0.184176348
Brain_Putamen_basal_ganglia 0.908774442 Brain_Amygdala 1.234779932
0.665796164 0.665796164 -0.947898003 1.328005324 0.164897284
1.138785545 3 0 0.998207054
PDE4D ENSG00000113448.18 0.334718762 1 1 0.334718762
Brain_Spinal_cord_cervical_c-1 0.334718762 Brain_Spinal_cord_cervical_c-1
1 1 1 0.964652763 0.964652763 0.964652763 NA 1 0
0.998207054
TBC1D12 ENSG00000108239.8 0.33481242 1 1 0.33481242
Brain_Frontal_Cortex_BA9 0.33481242 Brain_Frontal_Cortex_BA9 1 1 1
-0.964465854 -0.964465854 -0.964465854 NA 1 0 0.998207054
PIWIL4 ENSG00000134627.11 0.334856506 2 2 0.145221158
Brain_Caudate_basal_ganglia 0.558227031 Brain_Hypothalamus 1.229821223
0.770178777 0.770178777 0.585476994 1.456620527 1.021048761
0.615991499 2 0 0.998207054
SETX ENSG00000107290.13 0.334964727 1 1 0.334964727
Brain_Hippocampus 0.334964727 Brain_Hippocampus 1 1 1
0.96416197 0.96416197 0.96416197 NA 1 0 0.998207054

TTC30A ENSG00000197557.6 0.334975328 6 6 0.062609471
Brain_Cerebellum 0.968638078 Brain_Frontal_Cortex_BA9 3.544285956
0.127491932 0.127491932 -0.471941126 1.861954731 0.535708534
0.940551778 6 0 0.998207054
LLPH ENSG00000139233.6 0.335090266 1 1 0.335090266
Brain_Putamen_basal_ganglia 0.335090266 Brain_Putamen_basal_ganglia 1 1
1 0.963911563 0.963911563 0.963911563 NA 1 0
0.998207054
ERBB2 ENSG00000141736.13 0.335107235 2 2 0.140882606
Brain_Frontal_Cortex_BA9 0.915001734 Brain_Hypothalamus 1.163162559
0.836837441 0.836837441 0.106731825 1.472512234 0.78962203
0.965752589 2 0 0.998207054
CYP26A1 ENSG00000095596.11 0.335141733 1 1 0.335141733
Brain_Frontal_Cortex_BA9 0.335141733 Brain_Frontal_Cortex_BA9 1 1
1 -0.963808922 -0.963808922 -0.963808922 NA 1 0
0.998207054
POU6F1 ENSG00000184271.16 0.335159271 3 3 0.37085131
Brain_Cerebellar_Hemisphere 0.977306181 Brain_Cerebellum 1.927308234
0.134111719 0.134111719 -0.894879868 0.02844632 -0.350904248
0.483175815 3 0 0.998207054
CLDN18 ENSG00000066405.12 0.335178863 1 1 0.335178863
Brain_Hippocampus 0.335178863 Brain_Hippocampus 1 1 1
-0.963734878 -0.963734878 -0.963734878 NA 1 0 0.998207054
ALG6 ENSG00000088035.15 0.335247414 7 5 0.086638507 Brain_Cortex
0.384756237 Brain_Cerebellum 4.626288788 0.041374177 0.233630291
-1.336315724 1.713402555 0.914829141 1.038342222 5 0
0.998207054
FGF1 ENSG00000113578.17 0.335347142 2 2 0.540604551
Brain_Putamen_basal_ganglia 0.624190908 Brain_Spinal_cord_cervical_c-1
1.721668135 0.278331865 0.278331865 0.489919438 0.611899049
0.550909243 0.08625261 2 0 0.998207054
UROD ENSG00000126088.13 0.335364781 5 4 0.04371649 Brain_Hippocampus
0.993158499 Brain_Cerebellar_Hemisphere 2.960802049 0.03506407
0.159189607 0.008574654 2.016799031 0.568124976 0.829454615
4 0 0.998207054
CDV3 ENSG00000091527.15 0.33539036 1 1 0.33539036 Brain_Substantia_nigra
0.33539036 Brain_Substantia_nigra 1 1 1 -0.963313222
-0.963313222 -0.963313222 NA 1 0 0.998207054
TAL1 ENSG00000162367.11 0.335482106 3 3 0.068876475 Brain_Cortex
0.95752206 Brain_Amygdala 1.545894061 0.491273506 0.491273506
0.053263376 1.819229167 0.852341118 0.894862569 3 0
0.998207054
SLC4A9 ENSG00000113073.14 0.335563591 1 1 0.335563591
Brain_Anterior_cingulate_cortex_BA24 0.335563591
Brain_Anterior_cingulate_cortex_BA24 1 1 1 -0.962967983
-0.962967983 -0.962967983 NA 1 0 0.998207054
HSD17B4 ENSG00000133835.14 0.33563627 4 4 0.129505381
Brain_Spinal_cord_cervical_c-1 0.831653711
Brain_Nucleus_accumbens_basal_ganglia 1.935851763 0.222275792
0.222275792 -1.516055254 -0.212581104 -0.835707116 0.589577726
4 0 0.998207054
FAM160A2 ENSG00000051009.10 0.335658935 2 2 0.197008169
Brain_Caudate_basal_ganglia 0.872530684 Brain_Cortex 1.389822038
0.610177962 0.610177962 -0.160444822 1.290122385 0.564838782
1.025705909 2 0 0.998207054
UTF1 ENSG00000171794.3 0.335667303 2 2 0.160579714 Brain_Cerebellum
0.645396398 Brain_Cerebellar_Hemisphere 1.002127767 0.997872233
0.997872233 -0.460166941 1.403124507 0.471478783 1.317546018
2 0 0.998207054
MUC4 ENSG00000145113.21 0.335735214 9 4 0.159176379
Brain_Anterior_cingulate_cortex_BA24 0.859979134 Brain_Putamen_basal_ganglia
6.38064972 0.04127152 0.397363255 -1.407847003 0.958238312

-0.157193075 0.788749374 4 0 0.998207054
DNAI1 ENSG00000122735.15 0.335793632 2 2 0.224433683
Brain_Cerebellar_Hemisphere 0.594856356 Brain_Amygdala 1.234880242
0.765119758 0.765119758 -0.531811791 1.214822801 0.341505505
1.235057164 2 0 0.998207054
ARSG ENSG00000141337.12 0.335938974 1 1 0.335938974
Brain_Cerebellar_Hemisphere 0.335938974 Brain_Cerebellar_Hemisphere 1 1
1 -0.96222026 -0.96222026 -0.96222026 NA 1 0
0.998207054
9-Sep ENSG00000184640.17 0.336000673 3 2 0.077898639
Brain_Cerebellar_Hemisphere 0.87461366 Brain_Spinal_cord_cervical_c-1
1.947853649 0.051240971 1.00090538 -0.157800937 1.763010975
0.91208937 0.978948451 2 0 0.998207054
IFITM10 ENSG00000244242.1 0.336055062 1 1 0.336055062
Brain_Putamen_basal_ganglia 0.336055062 Brain_Putamen_basal_ganglia 1 1
1 0.961989135 0.961989135 0.961989135 NA 1 0
0.998207054
RP11-493E12.1 ENSG00000271889.1 0.336094403 3 3 0.20704678
Brain_Cerebellum 0.892125056 Brain_Cortex 2.19945705 0.316903475
0.316903475 -0.135615747 1.261727718 0.436077231 0.732470479
3 0 0.998207054
EXD2 ENSG00000081177.18 0.336135572 1 1 0.336135572
Brain_Frontal_Cortex_BA9 0.336135572 Brain_Frontal_Cortex_BA9 1 1
1 -0.961828872 -0.961828872 -0.961828872 NA 1 0
0.998207054
NTPCR ENSG00000135778.11 0.336142904 13 3 0.029215894
Brain_Frontal_Cortex_BA9 0.204093677 Brain_Substantia_nigra
10.74203876 0.009091655 0.51030809 1.269974603 2.180561118
1.540111832 0.254035507 3 0 0.998207054
SLC26A1 ENSG00000145217.13 0.336274877 11 4 0.053311444
Brain_Hypothalamus 0.938234331 Brain_Caudate_basal_ganglia 7.775595782
5.74E-18 0.693556808 -1.932389538 0.788483756 -0.07733162
0.679428732 4 0 0.998207054
NTSR2 ENSG00000169006.6 0.33627955 7 3 0.29186839 Brain_Hippocampus
0.823555216 Brain_Nucleus_accumbens_basal_ganglia 5.729290047
0.015288383 0.302315138 -0.985067496 1.054031731 0.284441595
0.639682256 3 0 0.998207054
USP36 ENSG00000055483.19 0.336551762 11 2 0.129130358 Brain_Cortex
0.865991546 Brain_Hypothalamus 9.813868103 5.38E-07 0.980968989
-1.517540178 0.168752215 -1.262816235 0.485268052 2 0
0.998207054
FMC1 ENSG00000164898.12 0.336634956 3 3 0.282548499
Brain_Anterior_cingulate_cortex_BA24 0.513864236 Brain_Putamen_basal_ganglia
1.481768582 0.584923592 0.584923592 -1.074611952 0.764556856
-0.320962549 0.963451478 3 0 0.998207054
SFXN2 ENSG00000156398.12 0.336738039 4 4 0.034557392 Brain_Cortex
0.500809023 Brain_Spinal_cord_cervical_c-1 2.618924951 0.100900953
0.100900953 0.673217353 2.11350705 1.589798181 0.633486634 4
0 0.998207054
CCDC47 ENSG00000108588.13 0.336867651 1 1 0.336867651
Brain_Spinal_cord_cervical_c-1 0.336867651 Brain_Spinal_cord_cervical_c-1
1 1 1 -0.960372742 -0.960372742 -0.960372742 NA 1 0
0.998207054
ZNF446 ENSG00000083838.15 0.336885955 1 1 0.336885955
Brain_Anterior_cingulate_cortex_BA24 0.336885955
Brain_Anterior_cingulate_cortex_BA24 1 1 1 0.960336363
0.960336363 0.960336363 NA 1 0 0.998207054
GRASP ENSG00000161835.10 0.336920509 1 1 0.336920509
Brain_Nucleus_accumbens_basal_ganglia 0.336920509
Brain_Nucleus_accumbens_basal_ganglia 1 1 1 0.960267686
0.960267686 0.960267686 NA 1 0 0.998207054
DAW1 ENSG00000123977.9 0.336973192 4 4 0.066000629

Brain_Frontal_Cortex_BA9 0.245004929 Brain_Putamen_basal_ganglia 2.690911482 0.151329915 0.151329915 -1.838419397 1.162567733 -1.005606824 1.452971399 4 0 0.998207054
ELMSAN1 ENSG00000156030.13 0.336975307 5 2 0.081936013 Brain_Frontal_Cortex_BA9 0.35916179 Brain_Putamen_basal_ganglia 4.255027229 0.018215226 0.554899883 -1.739561679 -0.916963456 -1.325322751 0.306431116 2 0 0.998207054
ATP7B ENSG00000123191.14 0.336984295 2 2 0.268054293 Brain_Cerebellum 0.519914788 Brain_Cerebellar_Hemisphere 1.266628759 0.733371241 0.733371241 -1.107554429 0.643476762 -0.232038833 1.23816603 2 0 0.998207054
UTRN ENSG00000152818.18 0.337105591 3 3 0.13030622 Brain_Putamen_basal_ganglia 0.802355544 Brain_Caudate_basal_ganglia 1.515946482 0.661927948 0.661927948 -1.51289544 0.625444501 -0.379250232 1.074986323 3 0 0.998207054
CHL1 ENSG00000134121.9 0.337113944 4 4 0.213297799 Brain_Frontal_Cortex_BA9 0.68614147 Brain_Substantia_nigra 1.980137958 0.105620893 0.105620893 -1.244548396 0.404096892 -0.575903443 0.732476412 4 0 0.998207054
RP11-359E10.1 ENSG00000270607.1 0.337123173 2 2 0.302890119 Brain_Cerebellar_Hemisphere 0.332788245 Brain_Caudate_basal_ganglia 1.080683217 0.919316783 0.919316783 -1.030255423 0.968512969 -0.030871227 1.413342684 2 0 0.998207054
PARD3 ENSG00000148498.15 0.337343317 1 1 0.337343317 Brain_Spinal_cord_cervical_c-1 0.337343317 Brain_Spinal_cord_cervical_c-1 1 1 1 -0.959427718 -0.959427718 -0.959427718 NA 1 0 0.998207054
TRAF4 ENSG00000076604.14 0.337487629 5 2 0.134888415 Brain_Putamen_basal_ganglia 0.479832362 Brain_Nucleus_accumbens_basal_ganglia 4.599160781 0.008468591 0.292040189 -1.495099736 -0.706572213 -1.138010564 0.348360775 2 0 0.998207054
C14orf178 ENSG00000197734.8 0.33773993 2 2 0.211929736 Brain_Nucleus_accumbens_basal_ganglia 0.462053303 Brain_Caudate_basal_ganglia 1.883243631 0.116756369 0.116756369 -1.248276723 -0.735470003 -0.991873363 0.362609109 2 0 0.998207054
AC114877.3 ENSG00000234165.1 0.337818641 1 1 0.337818641 Brain_Cerebellum 0.337818641 Brain_Cerebellum 1 1 1 0.958484228 0.958484228 0.958484228 NA 1 0 0.998207054
EIF4B ENSG00000063046.17 0.337869158 7 6 0.06590608 Brain_Frontal_Cortex_BA9 0.926155719 Brain_Hippocampus 4.556567418 0.070248978 0.229112146 -0.092682602 1.839061913 0.664660779 0.627851525 6 0 0.998207054
CFB ENSG00000243649.8 0.337941018 1 1 0.337941018 Brain_Putamen_basal_ganglia 0.337941018 Brain_Putamen_basal_ganglia 1 1 1 0.958241454 0.958241454 0.958241454 NA 1 0 0.998207054
N4BP2L2 ENSG00000244754.8 0.338012499 2 2 0.143332779 Brain_Caudate_basal_ganglia 0.75117626 Brain_Frontal_Cortex_BA9 1.323937555 0.676062445 0.676062445 -1.463492079 -0.317088752 -0.890290415 0.810629567 2 0 0.998207054
HAS3 ENSG00000103044.10 0.338015672 4 4 0.071197255 Brain_Amygdala 0.996416599 Brain_Nucleus_accumbens_basal_ganglia 2.136049008 0.278523277 0.278523277 -0.949644821 1.804217259 0.081185239 1.211976853 4 0 0.998207054
ABCA7 ENSG00000064687.12 0.338045967 8 6 0.025770856 Brain_Frontal_Cortex_BA9 0.960379248 Brain_Putamen_basal_ganglia 4.576017464 0.003195433 0.269095298 -2.229647471 0.187677387 -0.595174595 0.849290013 6 0 0.998207054
RP11-98O2.1 ENSG00000250501.2 0.338156509 1 1 0.338156509 Brain_Nucleus_accumbens_basal_ganglia 0.338156509 Brain_Nucleus_accumbens_basal_ganglia 1 1 1 0.957814097 0.957814097 0.957814097 NA 1 0 0.998207054

LINC00950 ENSG00000281205.1 0.338244016 10 3 0.095665761
Brain_Anterior_cingulate_cortex_BA24 0.9114974 Brain_Frontal_Cortex_BA9
8.026559021 0.006224138 0.423019909 -1.666239303 0.724064356
-0.484273016 0.667522502 3 0 0.998207054
DRICH1 ENSG00000189269.12 0.338264045 12 1 0.148036285
Brain_Anterior_cingulate_cortex_BA24 0.574105911 Brain_Putamen_basal_ganglia
11.23464348 0.001793339 11.23464348 0.562014836 1.446502594
0.925473031 0.225763672 1 0 0.998207054
MYRFL ENSG00000166268.10 0.338303586 13 1 0.2426095 Brain_Amygdala
0.547686966 Brain_Spinal_cord_cervical_c-1 12.47097451 0.002736953
12.47097451 0.601229759 1.168489183 0.936884315 0.162678597
1 0 0.998207054
FAM178B ENSG00000168754.14 0.338324263 7 7 0.270826295
Brain_Frontal_Cortex_BA9 0.662277919 Brain_Cortex 2.668641201
0.110382276 0.110382276 -1.101161682 1.011129718 0.179671056
0.833601141 7 0 0.998207054
MAPK12 ENSG00000188130.13 0.338387137 13 2 0.104196004
Brain_Cerebellar_Hemisphere 0.535441154 Brain_Amygdala 11.72936142
4.46E-05 0.431352093 -1.624843058 -0.619721506 -1.253119095
0.282009709 2 0 0.998207054
RP11-321M21.4 ENSG00000276993.1 0.33842501 2 2 0.245571164
Brain_Cortex 0.550998813 Brain_Frontal_Cortex_BA9 1.242427905
0.757572095 0.757572095 -1.161173954 0.596264096 -0.282454929
1.242696363 2 0 0.998207054
PEBP1 ENSG00000089220.4 0.338484936 11 8 0.077950645
Brain_Frontal_Cortex_BA9 0.953570876 Brain_Anterior_cingulate_cortex_BA24
6.236043332 0.006947812 0.216680883 -0.181994215 1.762702701
0.574661219 0.614745957 8 0 0.998207054
DYDC1 ENSG00000170788.13 0.338558109 1 1 0.338558109
Brain_Nucleus_accumbens_basal_ganglia 0.338558109
Brain_Nucleus_accumbens_basal_ganglia 1 1 1 0.95701812 0.95701812
0.95701812 NA 1 0 0.998207054
RP11-795F19.5 ENSG00000267127.7 0.338660893 10 2 0.27666982
Brain_Cerebellum 0.991780544 Brain_Cortex 8.435098619 0.004851767
0.776531427 -1.087830777 0.202710029 -0.393036698 0.472629208
2 0 0.998207054
RP11-718O11.1 ENSG00000259200.1 0.338667284 2 2 0.285473291
Brain_Putamen_basal_ganglia 0.405798104 Brain_Caudate_basal_ganglia
1.157554828 0.842445172 0.842445172 -0.831310749 1.06810469
0.118396971 1.343089537 2 0 0.998207054
EEPD1 ENSG00000122547.10 0.338781419 2 2 0.232257159 Brain_Cerebellum
0.504077843 Brain_Caudate_basal_ganglia 1.15148737 0.84851263 0.84851263
0.668087339 1.194564671 0.931326005 0.372275691 2 0
0.998207054
SDC4 ENSG00000124145.6 0.338866834 2 2 0.152765612 Brain_Cerebellum
0.263800261 Brain_Cerebellar_Hemisphere 1.891316462 0.108683538
0.108683538 1.117453991 1.429830711 1.273642351 0.220883697
2 0 0.998207054
APC2 ENSG00000115266.11 0.338907498 11 1 0.283686962 Brain_Cortex
0.609764879 Brain_Putamen_basal_ganglia 10.8039522 7.38E-17 10.8039522
-1.07207363 -0.510409106 -0.946354328 0.159594177 1 0
0.998207054
SPATA13 ENSG00000182957.15 0.338928298 1 1 0.338928298
Brain_Nucleus_accumbens_basal_ganglia 0.338928298
Brain_Nucleus_accumbens_basal_ganglia 1 1 1 0.956284937
0.956284937 0.956284937 NA 1 0 0.998207054
RP1-111D6.3 ENSG00000228408.6 0.339004071 13 3 0.028579544
Brain_Putamen_basal_ganglia 0.367037171 Brain_Frontal_Cortex_BA9
11.00560796 0.0036198 0.528351395 0.902037079 2.189238031
1.528735539 0.311142985 3 0 0.998207054
EHD3 ENSG00000013016.15 0.339134969 1 1 0.339134969 Brain_Cortex
0.339134969 Brain_Cortex 1 1 1 0.955875835 0.955875835

0.955875835 NA 1 0 0.998207054
ZNF84 ENSG00000198040.10 0.3392663 3 3 0.242780685
Brain_Cerebellar_Hemisphere 0.826749783 Brain_Spinal_cord_cervical_c-1
1.437644927 0.650342622 0.650342622 -1.017157447 1.168064656
0.123259726 1.095744124 3 0 0.998207054
ZNF304 ENSG00000131845.14 0.339299018 13 2 0.150783792
Brain_Amygdala 0.664841568 Brain_Spinal_cord_cervical_c-1 11.5680097
0.000232879 0.556173378 0.433238423 1.436768471 0.914136408
0.265296048 2 0 0.998207054
RP11-723O4.9 ENSG00000261159.1 0.339570039 12 1 0.186334289
Brain_Putamen_basal_ganglia 0.699497113 Brain_Frontal_Cortex_BA9
10.97042178 0.003903775 10.97042178 -1.32150124 -0.385999401
-0.908485914 0.242661244 1 0 0.998207054
RP11-707A18.1 ENSG00000250125.1 0.339765889 5 4 0.046909897
Brain_Frontal_Cortex_BA9 0.737498585 Brain_Hippocampus 3.242078995
0.068750256 0.238735888 -1.987112812 -0.335167701 -1.437044174
0.644217101 4 0 0.998207054
MTX1 ENSG00000173171.14 0.339940312 1 1 0.339940312 Brain_Cerebellum
0.339940312 Brain_Cerebellum 1 1 1 0.954283195 0.954283195
0.954283195 NA 1 0 0.998207054
ROPN1B ENSG00000114547.9 0.339954938 11 2 0.086966049
Brain_Spinal_cord_cervical_c-1 0.40423903 Brain_Substantia_nigra
10.13925033 0.001717693 0.437164953 0.834074453 1.71162364
1.195273757 0.267311244 2 0 0.998207054
LRRC57 ENSG00000180979.9 0.340037742 3 2 0.248684683
Brain_Hypothalamus 0.79517687 Brain_Caudate_basal_ganglia 2.697801636
0.028934643 0.27326372 0.259594123 1.15355006 0.612580146
0.475702388 2 0 0.998207054
DPM2 ENSG00000136908.17 0.340113447 11 2 0.28894211
Brain_Frontal_Cortex_BA9 0.86241482 Brain_Cerebellum 10.12663654
0.001943264 0.338247421 0.173301023 1.060445197 0.702491277
0.323421477 2 0 0.998207054
SLC4A3 ENSG00000114923.16 0.340240303 1 1 0.340240303
Brain_Frontal_Cortex_BA9 0.340240303 Brain_Frontal_Cortex_BA9 1 1
1 -0.953690555 -0.953690555 -0.953690555 NA 1 0
0.998207054
GOLGA8N ENSG00000232653.8 0.340431167 13 12 0.057000972
Brain_Cerebellum 0.896392825 Brain_Nucleus_accumbens_basal_ganglia
3.592437032 0.060503928 0.299167008 -1.903303363 1.518731534
-0.361952617 1.003633099 12 0 0.998207054
CWF19L2 ENSG00000152404.15 0.340492334 5 3 0.271492221
Brain_Frontal_Cortex_BA9 0.504533719 Brain_Anterior_cingulate_cortex_BA24
3.592518226 0.00018823 0.602823402 -0.882039979 1.099632625
0.531020362 0.817797712 3 0 0.998207054
BCAR1 ENSG00000050820.16 0.340571971 5 5 0.192609326
Brain_Putamen_basal_ganglia 0.841875169 Brain_Hippocampus 2.420852017
0.307999268 0.307999268 -0.538762724 1.302898765 0.498664335
0.827455968 5 0 0.998207054
TIRAP ENSG00000150455.13 0.340636948 6 6 0.034930572
Brain_Caudate_basal_ganglia 0.71373282 Brain_Hypothalamus 3.106811808
0.201909689 0.201909689 -2.109162331 0.366847439 -0.780679677
0.839821178 6 0 0.998207054
RP11-20E24.1 ENSG00000257526.2 0.340755207 1 1 0.340755207
Brain_Hypothalamus 0.340755207 Brain_Hypothalamus 1 1 1
0.952674125 0.952674125 0.952674125 NA 1 0 0.998207054
SYNPO ENSG00000171992.12 0.340827948 1 1 0.340827948
Brain_Substantia_nigra 0.340827948 Brain_Substantia_nigra 1 1
1 -0.952530612 -0.952530612 -0.952530612 NA 1 0
0.998207054
RAD1 ENSG00000113456.18 0.34090823 3 3 0.149370278
Brain_Putamen_basal_ganglia 0.725431411 Brain_Frontal_Cortex_BA9
1.228980563 0.773782189 0.773782189 -1.441759335 -0.351209196

-0.975329293 0.562116105 3 0 0.998207054
TSHZ1 ENSG00000179981.9 0.340962341 1 1 0.340962341
Brain_Cerebellar_Hemisphere 0.340962341 Brain_Cerebellar_Hemisphere 1 1
1 -0.952265518 -0.952265518 -0.952265518 NA 1 0
0.998207054
ITIH3 ENSG00000162267.12 0.341063272 1 1 0.341063272
Brain_Caudate_basal_ganglia 0.341063272 Brain_Caudate_basal_ganglia 1 1
1 -0.952066471 -0.952066471 -0.952066471 NA 1 0
0.998207054
RP11-598F7.6 ENSG00000256540.1 0.341272411 4 4 0.131570757
Brain_Cerebellum 0.428630454 Brain_Cortex 1.654168842 0.527289242
0.527289242 -1.507936588 -0.791537411 -1.278997161 0.333048061
4 0 0.998207054
ZSCAN18 ENSG00000121413.12 0.341398219 10 8 0.032241088
Brain_Cerebellar_Hemisphere 0.775312457 Brain_Hippocampus 4.986709823
0.051441241 0.199860909 -2.141408774 1.013850057 -0.747800107
0.993507948 8 0 0.998207054
ZNF233 ENSG00000159915.12 0.341448638 5 4 0.208288553
Brain_Hippocampus 0.839836025 Brain_Cerebellum 3.123370654
0.084885242 0.129629578 0.202103227 1.258285417 0.730519742
0.414985054 4 0 0.998207054
INTS14 ENSG00000138614.14 0.341616893 1 1 0.341616893
Brain_Hypothalamus 0.341616893 Brain_Hypothalamus 1 1 1
0.950975342 0.950975342 0.950975342 NA 1 0 0.998207054
CENPN ENSG00000166451.13 0.341726925 2 2 0.143096156
Brain_Hippocampus 0.462252076 Brain_Putamen_basal_ganglia 1.534380651
0.465619349 0.465619349 0.735143547 1.464358006 1.099750777
0.515632489 2 0 0.998207054
LRAT ENSG00000121207.11 0.341734415 1 1 0.341734415
Brain_Spinal_cord_cervical_c-1 0.341734415 Brain_Spinal_cord_cervical_c-1
1 1 1 0.950743864 0.950743864 0.950743864 NA 1 0
0.998207054
SEMA3G ENSG00000010319.6 0.341762408 3 3 0.16007187 Brain_Cerebellum
0.309947726 Brain_Putamen_basal_ganglia 1.268391008 0.668626195
0.668626195 1.015331726 1.404829886 1.147484397 0.222894898
3 0 0.998207054
STX7 ENSG00000079950.13 0.341844384 1 1 0.341844384
Brain_Frontal_Cortex_BA9 0.341844384 Brain_Frontal_Cortex_BA9 1 1
1 0.95052731 0.95052731 0.95052731 NA 1 0 0.998207054
DR1 ENSG00000117505.12 0.342094033 3 2 0.163619647 Brain_Cortex
0.536318468 Brain_Substantia_nigra 2.508550499 0.069865111
0.42158439 0.618389727 1.393000477 1.122097975 0.436639828 2
0 0.998207054
MTSS1 ENSG00000170873.18 0.342109255 1 1 0.342109255
Brain_Hippocampus 0.342109255 Brain_Hippocampus 1 1 1
-0.950005899 -0.950005899 -0.950005899 NA 1 0 0.998207054
LAMA5 ENSG00000130702.15 0.342236502 3 3 0.133505051
Brain_Frontal_Cortex_BA9 0.704963659 Brain_Amygdala 1.101030165
0.876745448 0.876745448 -1.500422283 0.37862863 -0.728834093
0.983522678 3 0 0.998207054
STX3 ENSG00000166900.15 0.342338905 3 3 0.073258131
Brain_Hypothalamus 0.246732703 Brain_Caudate_basal_ganglia 2.596747085
0.175832734 0.175832734 1.158321886 1.791219172 1.437677931
0.322904515 3 0 0.998207054
TLE2 ENSG00000065717.14 0.342379984 1 1 0.342379984 Brain_Cortex
0.342379984 Brain_Cortex 1 1 1 0.949473223 0.949473223
0.949473223 NA 1 0 0.998207054
TXN2 ENSG00000100348.9 0.342412034 10 4 0.176474938
Brain_Putamen_basal_ganglia 0.938241514 Brain_Hippocampus 7.525963904
0.022556144 0.252421313 -1.351688647 0.569603756 0.126170761
0.57478989 4 0 0.998207054
BRD3 ENSG00000169925.16 0.342424011 4 4 0.119747092 Brain_Cortex

0.555825183	Brain_Hippocampus	3.219575983	0.172135389
0.172135389	0.589053801	1.555836013	1.157769978	0.45073455 4
0	0.998207054
FSBP ENSG00000265817.2	0.342446717	1	1	0.342446717
Brain_Hippocampus	0.342446717	Brain_Hippocampus	1	1	1
0.949341964	0.949341964	0.949341964	NA	1	0	0.998207054
RP11-359I18.5	ENSG00000272360.1	0.342491545	1	1	0.342491545
Brain_Putamen_basal_ganglia 0.342491545	Brain_Putamen_basal_ganglia 1	1
1	-0.949253799	-0.949253799	-0.949253799	NA	1	0
0.998207054
RELN ENSG00000189056.13	0.342515119	5	5	0.057968524
Brain_Putamen_basal_ganglia 0.945016122	Brain_Cerebellum 1.518965872
0.612610602	0.612610602	-0.068966705	1.895935875	0.93097554
0.723297201	5	0	0.998207054
ERMAP ENSG00000164010.13	0.342521203	12	8	0.182624258	Brain_Amygdala
0.483499538	Brain_Frontal_Cortex_BA9	7.784227159	0.001438823
0.324393668	-1.332718322	0.897748739	-0.682475095	0.716732221
8	0	0.998207054
CCDC51	ENSG00000164051.13	0.342526062	1	1	0.342526062
Brain_Cerebellar_Hemisphere 0.342526062	Brain_Cerebellar_Hemisphere 1	1
1	-0.949185919	-0.949185919	-0.949185919	NA	1	0
0.998207054
TRIM14	ENSG00000106785.14	0.3425748 4	4	0.053511052
Brain_Nucleus_accumbens_basal_ganglia 0.585210999	Brain_Cerebellum
2.614695861	0.131321283	0.131321283	-1.930773638	0.54578898
-0.992804364	1.078175418	4	0	0.998207054
KNSTRN	ENSG00000128944.13	0.342650595	13	1	0.293279919
Brain_Hippocampus	0.456182431	Brain_Cerebellum 12.56795978
0.000269521	12.56795978	-1.050953561	-0.745147708	-0.932286299
0.108666892	1	0	0.998207054
MTFMT ENSG00000103707.9	0.342767388	1	1	0.342767388
Brain_Cerebellar_Hemisphere 0.342767388	Brain_Cerebellar_Hemisphere 1	1
1	-0.948711451	-0.948711451	-0.948711451	NA	1	0
0.998207054
SLC22A18	ENSG00000110628.13	0.342781371	8	5	0.140918054
Brain_Hypothalamus	0.842324729	Brain_Putamen_basal_ganglia 5.244092163
0.004039073	0.287201471	-1.472380877	1.4161126 0.403421007
0.954852603	5	0	0.998207054
TTLL9 ENSG00000131044.16	0.34289527 3	3	0.219435128
Brain_Nucleus_accumbens_basal_ganglia 0.488201715	Brain_Hippocampus
1.143675953	0.912724705	0.912724705	-1.228031542	1.097846206
0.18766217 1.2426109	3	0	0.998207054
SPATA3	ENSG00000173699.15	0.342932694	4	4	0.095137638
Brain_Substantia_nigra	0.950667621	Brain_Hypothalamus	1.231465205
0.789712436	0.789712436	-1.668897788	-0.061868414	-0.905501194
0.849738679	4	0	0.998207054
LRRC29	ENSG00000125122.15	0.342970199	1	1	0.342970199
Brain_Amygdala	0.342970199	Brain_Amygdala	1	1	1	-0.948312873
-0.948312873	-0.948312873	NA	1	0	0.998207054
CELF3 ENSG00000159409.14	0.342973973	1	1	0.342973973
Brain_Spinal_cord_cervical_c-1	0.342973973	Brain_Spinal_cord_cervical_c-1
1	1	1	0.948305457	0.948305457	0.948305457	NA	1	0
0.998207054
PCDHB3	ENSG00000113205.5	0.342986093	4	3	0.160113618
Brain_Cortex	0.233073589	Brain_Nucleus_accumbens_basal_ganglia 2.25939802
0.008653113	0.509778501	-1.404689539	-1.192478722	-1.253151762
0.101282815	3	0	0.998207054
IL6R ENSG00000160712.12	0.343007639	2	2	0.157734448
Brain_Cerebellar_Hemisphere 0.436032701	Brain_Cerebellum 1.294479771
0.705520229	0.705520229	-0.778910055	1.41273231 0.316911128
1.549725178	2	0	0.998207054
ADAMTS8	ENSG00000134917.9	0.343181663	5	5	0.359756143

Brain_Caudate_basal_ganglia 0.747323302 Brain_Putamen_basal_ganglia 2.380669862 0.220528425 0.220528425 -0.915829852 0.821908176 -0.114100213 0.782326432 5 0 0.998207054
FAHD2B ENSG00000144199.11 0.343270539 8 4 0.082266149 Brain_Cerebellar_Hemisphere 0.991239351 Brain_Frontal_Cortex_BA9 5.761319998 0.008180378 0.236145449 -0.227438243 1.737686047 0.797316044 0.683070276 4 0 0.998207054
IL16 ENSG00000172349.17 0.343312205 1 1 0.343312205 Brain_Cerebellum 0.343312205 Brain_Cerebellum 1 1 1 -0.947641081 -0.947641081 -0.947641081 NA 1 0 0.998207054
PGR ENSG00000082175.14 0.343315851 1 1 0.343315851 Brain_Cortex 0.343315851 Brain_Cortex 1 1 1 -0.947633921 -0.947633921 -0.947633921 NA 1 0 0.998207054
SIL1 ENSG00000120725.12 0.343321952 9 9 0.092677383 Brain_Nucleus_accumbens_basal_ganglia 0.983209381 Brain_Spinal_cord_cervical_c-1 2.921539563 0.215056009 0.215056009 -1.681440507 0.86323947 -0.02426927 0.867666418 9 0 0.998207054
STEAP2 ENSG00000157214.13 0.343327357 12 2 0.253190477 Brain_Cerebellum 0.96993869 Brain_Hypothalamus 10.39210803 8.29E-05 1.114469067 -1.142634254 0.354435729 -0.318423913 0.480810172 2 0 0.998207054
ABHD5 ENSG00000011198.7 0.343441711 1 1 0.343441711 Brain_Hippocampus 0.343441711 Brain_Hippocampus 1 1 1 -0.947386809 -0.947386809 -0.947386809 NA 1 0 0.998207054
LINC01080 ENSG00000281721.1 0.343557091 12 11 0.122757109 Brain_Hypothalamus 0.905204606 Brain_Substantia_nigra 2.405067546 0.000879868 0.133245132 -1.541282415 1.543303687 -0.35068663 1.066051603 11 0 0.998207054
RAD50 ENSG00000113522.13 0.343581319 2 2 0.193070573 Brain_Cerebellum 0.896045027 Brain_Cerebellar_Hemisphere 1.532918314 0.467081686 0.467081686 -1.301549089 -0.130659051 -0.71610407 0.827944286 2 0 0.998207054
LINC00856 ENSG00000230417.11 0.343631818 1 1 0.343631818 Brain_Cerebellum 0.343631818 Brain_Cerebellum 1 1 1 -0.94701366 -0.94701366 -0.94701366 NA 1 0 0.998207054
NAAA ENSG00000138744.14 0.343710454 13 4 0.223271145 Brain_Frontal_Cortex_BA9 0.967140737 Brain_Cerebellar_Hemisphere 10.09745853 0.010809988 0.362556987 -0.739043821 1.217875876 0.177029199 0.607861084 4 0 0.998207054
FBXO34 ENSG00000178974.9 0.343784522 3 3 0.102019181 Brain_Cerebellum 0.903124374 Brain_Putamen_basal_ganglia 1.949038058 0.289835473 0.289835473 0.121715454 1.63514249 0.827125832 0.761912977 3 0 0.998207054
FBXL6 ENSG00000182325.10 0.343897126 2 2 0.166998213 Brain_Cerebellum 0.278143898 Brain_Cortex 1.484394667 0.515605333 0.515605333 -1.084498351 1.381913662 0.148707656 1.744016659 2 0 0.998207054
RP11-268F1.3 ENSG00000236164.1 0.344101207 2 2 0.21277234 Brain_Hypothalamus 0.5031159 Brain_Amygdala 1.072818686 0.927181314 0.927181314 -1.245978359 0.669595161 -0.288191599 1.354515025 2 0 0.998207054
MTMR10 ENSG00000166912.16 0.344114303 4 4 0.240970425 Brain_Cerebellum 0.701966011 Brain_Amygdala 1.989792666 0.081384671 0.081384671 -1.172564668 0.38266791 -0.718324573 0.736582978 4 0 0.998207054
SERPINB6 ENSG00000124570.17 0.344229732 3 3 0.08346029 Brain_Cerebellum 0.772819253 Brain_Caudate_basal_ganglia 1.821605183 0.179177943 0.179177943 -1.7309522 -0.288689168 -1.050233936 0.724520777 3 0 0.998207054
PTPRN ENSG00000054356.13 0.34425747 4 4 0.056220537 Brain_Caudate_basal_ganglia 0.666848545 Brain_Cortex 1.866617611

0.136562944	0.136562944	-1.909322226	0.61994814 -0.68448382
1.399031879	4	0	0.998207054
CPB1	ENSG00000153002.11	0.344378206	2	2	0.251633363
Brain_Substantia_nigra	0.385070553	Brain_Hippocampus	1.030802447
0.969197553	0.969197553	-0.868591596	1.146391102	0.138899753
1.42480793 2	0	0.998207054
RYR2	ENSG00000198626.15	0.344529207	1	1	0.344529207
Brain_Nucleus_accumbens_basal_ganglia 0.344529207
Brain_Nucleus_accumbens_basal_ganglia 1	1	1	-0.94525402
-0.94525402	-0.94525402	NA	1	0	0.998207054
SCRN1 ENSG00000136193.16	0.344534153	2	2	0.172417373
Brain_Hypothalamus	0.613752761	Brain_Spinal_cord_cervical_c-1
1.010417065	0.989582935	0.989582935	-0.504723916	1.364477365
0.429876725	1.321724901	2	0	0.998207054
TRIM74	ENSG00000155428.12	0.344535147	2	2	0.144478606
Brain_Anterior_cingulate_cortex_BA24	0.846019521
Brain_Nucleus_accumbens_basal_ganglia 1.106512295	0.893487705
0.893487705	0.194199695	1.459314355	0.826757025	0.894571155
2	0	0.998207054
GCNT1 ENSG00000187210.12	0.344573354	1	1	0.344573354
Brain_Hypothalamus	0.344573354	Brain_Hypothalamus	1	1	1
-0.945167529	-0.945167529	-0.945167529	NA	1	0	0.998207054
SERTAD2	ENSG00000179833.4	0.344592817	1	1	0.344592817
Brain_Hypothalamus	0.344592817	Brain_Hypothalamus	1	1	1
0.9451294 0.9451294 0.9451294 NA	1	0	0.998207054
ENOSF1	ENSG00000132199.18	0.344828189	10	5	0.087110056
Brain_Nucleus_accumbens_basal_ganglia 0.681258767	Brain_Cortex
5.974173877	0.030474067	0.292226967	-1.710843236	-0.410746035
-0.946122067	0.529957734	5	0	0.998207054
PSMB8 ENSG00000204264.8	0.344879767	1	1	0.344879767	Brain_Cortex
0.344879767	Brain_Cortex	1	1	1	-0.944567419	-0.944567419
-0.944567419	NA	1	0	0.998207054
IMPA1 ENSG00000133731.9	0.344948459	7	1	0.307648233
Brain_Putamen_basal_ganglia 0.431146688	Brain_Cortex	6.707896411
0.0001264 6.707896411	0.787230941	1.020169177	0.924062425
0.085724912	1	0	0.998207054
CRTAC1	ENSG00000095713.13	0.344999208	2	2	0.241136819
Brain_Cortex	0.330143893	Brain_Cerebellum 1.092967407	0.907032593
0.907032593	-1.17215005	-0.973824084	-1.072987067	0.140237636
2	0	0.998207054
RP11-1263C18.1	ENSG00000273238.1	0.345401646	1	1	0.345401646
Brain_Nucleus_accumbens_basal_ganglia 0.345401646
Brain_Nucleus_accumbens_basal_ganglia 1	1	1	0.943546102
0.943546102	0.943546102	NA	1	0	0.998207054
KRT86 ENSG00000170442.11	0.345420196	1	1	0.345420196
Brain_Cerebellar_Hemisphere 0.345420196	Brain_Cerebellar_Hemisphere 1	1
1	-0.943509817	-0.943509817	-0.943509817	NA	1	0
0.998207054
PLA2G5	ENSG00000127472.10	0.345431605	10	5	0.097707727
Brain_Amygdala	0.795396117	Brain_Frontal_Cortex_BA9	6.160370652
0.012455085	0.280588028	-1.656069592	0.365041311	-0.91080075
0.619919037	5	0	0.998207054
TTC9C ENSG00000162222.13	0.345619622	6	2	0.273322949	Brain_Cortex
0.781042486	Brain_Nucleus_accumbens_basal_ganglia 5.593730009	0.01567004
0.200624442	-1.095442212	-0.277960754	-0.815167235	0.29723003 2
0	0.998207054
CCDC116	ENSG00000161180.10	0.345656415	7	3	0.359866334
Brain_Cerebellum 0.563417011	Brain_Hippocampus	6.09826382 0.001989349
0.267160192	-0.577773556	0.915619816	0.608582362	0.530291902
3	0	0.998207054
CTNND2	ENSG00000169862.18	0.345831102	1	1	0.345831102
Brain_Spinal_cord_cervical_c-1	0.345831102	Brain_Spinal_cord_cervical_c-1

1 1 1 -0.942706395 -0.942706395 -0.942706395 NA 1 0
0.998207054
UBASH3B ENSG00000154127.9 0.346277183 2 2 0.183040959
Brain_Hypothalamus 0.715885555 Brain_Cortex 1.163884254
0.836115746 0.836115746 -0.363963112 1.331450083 0.483743486
1.198838167 2 0 0.998207054
TNFRSF25 ENSG00000215788.9 0.346292586 4 4 0.093261802
Brain_Putamen_basal_ganglia 0.873132531 Brain_Cortex 1.905431899
0.379952373 0.379952373 0.159680793 1.67843709 1.098330244
0.691966374 4 0 0.998207054
TTC31 ENSG00000115282.19 0.346355974 1 1 0.346355974
Brain_Nucleus_accumbens_basal_ganglia 0.346355974
Brain_Nucleus_accumbens_basal_ganglia 1 1 1 0.941681023
0.941681023 0.941681023 NA 1 0 0.998207054
RP11-197K6.1 ENSG00000223812.5 0.346381008 3 3 0.076008099
Brain_Nucleus_accumbens_basal_ganglia 0.65598686 Brain_Cortex 2.171138658
0.239149217 0.239149217 -1.774332919 -0.445460693 -0.973126381
0.705403285 3 0 0.998207054
RNF166 ENSG00000158717.10 0.34649692 8 6 0.01504048
Brain_Anterior_cingulate_cortex_BA24 0.790111174
Brain_Spinal_cord_cervical_c-1 4.566626385 0.013569799 0.233777552
-2.431402874 0.266166251 -0.987072787 0.783322809 6 0
0.998207054
RUBCNL ENSG00000102445.18 0.346562901 1 1 0.346562901
Brain_Hypothalamus 0.346562901 Brain_Hypothalamus 1 1 1
-0.94127705 -0.94127705 -0.94127705 NA 1 0 0.998207054
LAMC1 ENSG00000135862.5 0.346564338 4 3 0.138514578 Brain_Cerebellum
0.477547257 Brain_Nucleus_accumbens_basal_ganglia 2.0031241 0.019178332
0.889861255 -1.481345319 0.710253004 -0.807219786 1.025904032
3 0 0.998207054
RP11-369E15.3 ENSG00000254092.1 0.346594316 1 1 0.346594316
Brain_Amygdala 0.346594316 Brain_Amygdala 1 1 1 -0.941215732
-0.941215732 -0.941215732 NA 1 0 0.998207054
ZNF138 ENSG00000197008.9 0.346620051 11 6 0.013707238
Brain_Caudate_basal_ganglia 0.641950731 Brain_Cerebellum 8.053476687
0.004741046 0.285012158 -2.464845405 -0.464973085 -1.583344972
0.57018015 6 0 0.998207054
SELENOO ENSG00000073169.13 0.346628026 3 3 0.130937308
Brain_Cerebellar_Hemisphere 0.765502542 Brain_Substantia_nigra
1.957733266 0.11783968 0.11783968 -0.298262868 1.510416002
0.871306542 1.014332605 3 0 0.998207054
RMDN1 ENSG00000176623.11 0.346832858 13 1 0.226724117 Brain_Amygdala
0.529774049 Brain_Substantia_nigra 12.31859638 0.001054355
12.31859638 -1.20884053 -0.62835097 -0.914860336 0.177011999
1 0 0.998207054
AC005498.3 ENSG00000267421.6 0.347179627 2 2 0.203293956
Brain_Putamen_basal_ganglia 0.41027959 Brain_Cerebellum 1.095458661
0.904541339 0.904541339 -0.823401711 1.272222836 0.224410562
1.481830328 2 0 0.998207054
RPL8 ENSG00000161016.17 0.347243636 6 2 0.41713712
Brain_Frontal_Cortex_BA9 0.987355758 Brain_Nucleus_accumbens_basal_ganglia
5.64869207 0.001721909 0.201472757 0.01584787 0.811398048
0.234352222 0.290793812 2 0 0.998207054
LINC01176 ENSG00000281404.1 0.3474356 13 13 0.009187377
Brain_Nucleus_accumbens_basal_ganglia 0.895192378 Brain_Amygdala
1.585100596 0.495350308 0.495350308 -2.605001821 1.299569136
-0.473667139 1.08794395 13 0 0.998207054
TMEM63B ENSG00000137216.18 0.347448566 4 4 0.038862389
Brain_Cerebellum 0.923362047 Brain_Spinal_cord_cervical_c-1 1.826443478
0.287522898 0.287522898 -0.096199602 2.065641016 1.018428267
1.029602544 4 0 0.998207054
ECSIT ENSG00000130159.13 0.347551812 1 1 0.347551812

Brain_Hippocampus 0.347551812 Brain_Hippocampus 1 1 1
0.939348565 0.939348565 0.939348565 NA 1 0 0.998207054
PGPEP1 ENSG00000130517.13 0.347559032 11 11 0.032041869
Brain_Cerebellum 0.827054973 Brain_Substantia_nigra 2.508399996
0.172564628 0.172564628 -1.468693654 2.143887918 0.178117554
1.117836622 11 0 0.998207054
MAJIN ENSG00000168070.11 0.347569596 1 1 0.347569596 Brain_Cerebellum
0.347569596 Brain_Cerebellum 1 1 1 -0.939313916 -0.939313916
-0.939313916 NA 1 0 0.998207054
SCAI ENSG00000173611.17 0.347617098 4 4 0.089254927
Brain_Hippocampus 0.863385212 Brain_Spinal_cord_cervical_c-1
1.959884663 0.302823969 0.302823969 -0.748042302 1.699341133
0.649706792 1.149702324 4 0 0.998207054
ADCY2 ENSG00000078295.16 0.347802871 1 1 0.347802871
Brain_Nucleus_accumbens_basal_ganglia 0.347802871
Brain_Nucleus_accumbens_basal_ganglia 1 1 1 0.938859528
0.938859528 0.938859528 NA 1 0 0.998207054
MAPRE1 ENSG00000101367.8 0.347819837 1 1 0.347819837
Brain_Spinal_cord_cervical_c-1 0.347819837 Brain_Spinal_cord_cervical_c-1
1 1 1 0.938826488 0.938826488 0.938826488 NA 1 0
0.998207054
MCPH1 ENSG00000147316.12 0.347855383 6 5 0.37973361 Brain_Cortex
0.891489261 Brain_Nucleus_accumbens_basal_ganglia 4.087868851
0.109170555 0.185008401 -0.878387233 0.543266004 -0.207561327
0.592834989 5 0 0.998207054
H2AFY2 ENSG00000099284.13 0.347878573 1 1 0.347878573
Brain_Hippocampus 0.347878573 Brain_Hippocampus 1 1 1
-0.938712113 -0.938712113 -0.938712113 NA 1 0 0.998207054
PCDHB16 ENSG00000272674.3 0.347928647 3 3 0.183813771
Brain_Cerebellum 0.984995757 Brain_Hippocampus 2.356908349
0.274006991 0.274006991 0.018806138 1.329103666 0.797711168
0.689323395 3 0 0.998207054
PRDM11 ENSG00000019485.13 0.347996415 4 4 0.059169723
Brain_Putamen_basal_ganglia 0.796964114 Brain_Anterior_cingulate_cortex_BA24
1.8208391 0.587810445 0.587810445 -1.886930134 0.565162996
-0.335088687 1.091340264 4 0 0.998207054
ZNF559 ENSG00000188321.13 0.348032862 13 9 0.038650159
Brain_Cerebellar_Hemisphere 0.892912914 Brain_Substantia_nigra
5.740926632 0.039878561 0.210848625 -1.153114102 2.067892218
0.852152764 0.882508352 9 0 0.998207054
DDX42 ENSG00000198231.12 0.3481936 13 1 0.218445082
Brain_Caudate_basal_ganglia 0.996220201 Brain_Hypothalamus 12.50955572
0.000115856 12.50955572 -0.004737293 1.230673289 0.914285383
0.296038621 1 0 0.998207054
WRNIP1 ENSG00000124535.15 0.348403253 1 1 0.348403253
Brain_Cerebellum 0.348403253 Brain_Cerebellum 1 1 1 -0.937690956
-0.937690956 -0.937690956 NA 1 0 0.998207054
RP11-563N4.1 ENSG00000272716.1 0.348482284 2 2 0.157595982
Brain_Caudate_basal_ganglia 0.46729281 Brain_Hippocampus 1.288427617
0.711572383 0.711572383 -0.726890871 1.413203214 0.343156171
1.51327504 2 0 0.998207054
SPRN ENSG00000203772.7 0.34849092 2 2 0.153798523
Brain_Cerebellar_Hemisphere 0.499791286 Brain_Anterior_cingulate_cortex_BA24
1.622531055 0.377468945 0.377468945 -1.42624192 -0.674818184
-1.050530052 0.531336819 2 0 0.998207054
SSFA2 ENSG00000138434.16 0.348552021 2 2 0.219615765 Brain_Amygdala
0.354159457 Brain_Nucleus_accumbens_basal_ganglia 1.128465428
0.871534572 0.871534572 0.926551479 1.227550469 1.077050974
0.212838427 2 0 0.998207054
MEI4 ENSG00000269964.2 0.348580539 1 1 0.348580539 Brain_Cortex
0.348580539 Brain_Cortex 1 1 1 -0.937346134 -0.937346134
-0.937346134 NA 1 0 0.998207054

TSPAN9 ENSG00000011105.12 0.348580611 4 4 0.107442193
Brain_Cerebellar_Hemisphere 0.733003776 Brain_Caudate_basal_ganglia
1.940948659 0.072638942 0.072638942 -1.609796944 1.30841758
-0.347583309 1.412051088 4 0 0.998207054
LGSN ENSG00000146166.16 0.348703539 2 2 0.158858016
Brain_Spinal_cord_cervical_c-1 0.325698429 Brain_Substantia_nigra
1.83425762 0.16574238 0.16574238 -1.408922734 -0.98281514 -1.195868937
0.301303569 2 0 0.998207054
STARD3 ENSG00000131748.15 0.348762567 1 1 0.348762567
Brain_Substantia_nigra 0.348762567 Brain_Substantia_nigra 1 1
1 0.936992206 0.936992206 0.936992206 NA 1 0
0.998207054
WWP2 ENSG00000198373.12 0.349011622 4 3 0.073479237
Brain_Spinal_cord_cervical_c-1 0.241377882 Brain_Cerebellum 3.463109366
0.018311511 0.203673987 1.171549727 1.789842486 1.542947954
0.262656695 3 0 0.998207054
MTUS2 ENSG00000132938.19 0.349026012 1 1 0.349026012 Brain_Amygdala
0.349026012 Brain_Amygdala 1 1 1 -0.936480181 -0.936480181
-0.936480181 NA 1 0 0.998207054
RASL12 ENSG00000103710.10 0.349081894 3 3 0.138550971
Brain_Anterior_cingulate_cortex_BA24 0.965721023 Brain_Caudate_basal_ganglia
1.906306271 0.093781028 0.093781028 -1.48120869 0.042975552
-0.779472383 0.769228752 3 0 0.998207054
XIRP2 ENSG00000163092.19 0.349083401 1 1 0.349083401 Brain_Cortex
0.349083401 Brain_Cortex 1 1 1 0.936368675 0.936368675
0.936368675 NA 1 0 0.998207054
DOCK10 ENSG00000135905.18 0.349276746 1 1 0.349276746
Brain_Cerebellum 0.349276746 Brain_Cerebellum 1 1 1 -0.935993089
-0.935993089 -0.935993089 NA 1 0 0.998207054
KLHL32 ENSG00000186231.16 0.349310836 2 1 0.32408167
Brain_Cerebellar_Hemisphere 0.379475086 Brain_Cerebellum 1.985314275
0.014685725 1.985314275 0.878863879 0.986104838 0.932484359
0.075830809 1 0 0.998207054
SLC39A2 ENSG00000165794.9 0.349362773 3 2 0.397015671
Brain_Cortex 0.985106522 Brain_Frontal_Cortex_BA9 2.740518988
0.083043644 0.176437367 -0.846963134 -0.018667291 -0.378448831
0.424718273 2 0 0.998207054
TEKT4 ENSG00000163060.7 0.349451572 2 2 0.163825448
Brain_Frontal_Cortex_BA9 0.493453314 Brain_Anterior_cingulate_cortex_BA24
1.699780144 0.300219856 0.300219856 0.684826654 1.392320231
1.038573443 0.500273506 2 0 0.998207054
SGK2 ENSG00000101049.14 0.349518835 4 4 0.072766008 Brain_Cortex
0.616639018 Brain_Frontal_Cortex_BA9 1.305141363 0.66730237 0.66730237
0.500619384 1.794295549 0.979385003 0.569266385 4 0
0.998207054
DBR1 ENSG00000138231.12 0.349525937 1 1 0.349525937
Brain_Hippocampus 0.349525937 Brain_Hippocampus 1 1 1
0.935509213 0.935509213 0.935509213 NA 1 0 0.998207054
ARPP19 ENSG00000128989.10 0.349558453 3 3 0.104320149
Brain_Substantia_nigra 0.594491163 Brain_Cerebellar_Hemisphere 1.27176453
0.758112682 0.758112682 -1.624260848 0.53233909 -0.644923408
1.091838619 3 0 0.998207054
RP11-329B9.4 ENSG00000272970.1 0.34966696 9 8 0.077280878
Brain_Frontal_Cortex_BA9 0.744240865 Brain_Cortex 4.691649074
0.103101581 0.232671336 -1.766685709 0.506445069 -0.465830509
0.781212012 8 0 0.998207054
EAF1 ENSG00000144597.13 0.349679762 3 3 0.166123345
Brain_Cerebellar_Hemisphere 0.354955485 Brain_Frontal_Cortex_BA9
1.896567427 0.103423557 0.103423557 -1.38476824 0.925020037
-0.464581695 1.224354293 3 0 0.998207054
LINC00906 ENSG00000267339.5 0.349916993 3 3 0.181457133
Brain_Cortex 0.898904452 Brain_Cerebellum 1.911897002 0.398230826

0.398230826 0.127045417 1.336281952 0.83179083 0.628998875 3
0 0.998207054
2-Mar ENSG00000099785.10 0.349945184 13 1 0.209798897 Brain_Cortex
0.82116987 Brain_Substantia_nigra 12.11476243 0.000163584
12.11476243 0.226040565 1.254118611 0.895431018 0.253454835
1 0 0.998207054
RPLP2 ENSG00000177600.8 0.349950591 1 1 0.349950591
Brain_Hippocampus 0.349950591 Brain_Hippocampus 1 1 1
-0.934685133 -0.934685133 -0.934685133 NA 1 0 0.998207054
M6PR ENSG00000003056.7 0.35005218 4 4 0.263143051 Brain_Cerebellum
0.548138699 Brain_Cortex 2.075034918 0.286230223 0.286230223
-1.118993174 0.864500246 -0.081861857 0.963841891 4 0
0.998207054
KLRC4 ENSG00000183542.5 0.350065365 8 3 0.04847563 Brain_Amygdala
0.459677191 Brain_Substantia_nigra 6.950549006 0.008334998
0.248247313 0.739378505 1.973174983 1.462172728 0.382882784
3 0 0.998207054
CAMK1 ENSG00000134072.10 0.350186305 1 1 0.350186305
Brain_Hippocampus 0.350186305 Brain_Hippocampus 1 1 1
-0.934227981 -0.934227981 -0.934227981 NA 1 0 0.998207054
PEX3 ENSG00000034693.14 0.350227782 1 1 0.350227782
Brain_Caudate_basal_ganglia 0.350227782 Brain_Caudate_basal_ganglia 1 1
1 0.934147559 0.934147559 0.934147559 NA 1 0
0.998207054
P3H4 ENSG00000141696.12 0.35022927 2 2 0.147589785
Brain_Putamen_basal_ganglia 0.786992768 Brain_Cortex 1.161579154
0.838420846 0.838420846 -1.448097499 -0.270217711 -0.859157605
0.832886786 2 0 0.998207054
KIAA0040 ENSG00000235750.9 0.350304127 3 3 0.122653053
Brain_Frontal_Cortex_BA9 0.393089164 Brain_Cerebellum 1.869683855
0.186244921 0.186244921 0.854028562 1.543732897 1.099955282
0.385073694 3 0 0.998207054
CDC42EP5 ENSG00000167617.2 0.350470357 1 1 0.350470357
Brain_Frontal_Cortex_BA9 0.350470357 Brain_Frontal_Cortex_BA9 1 1
1 -0.933677341 -0.933677341 -0.933677341 NA 1 0
0.998207054
RP11-157E21.1 ENSG00000253593.2 0.350586762 2 1 0.340878182
Brain_Caudate_basal_ganglia 0.361195641 Brain_Nucleus_accumbens_basal_ganglia
1.997038242 0.002961758 1.997038242 -0.952431516 -0.913089179
-0.932760347 0.027819234 1 0 0.998207054
LINC01551 ENSG00000186960.10 0.350596232 2 2 0.168982636
Brain_Anterior_cingulate_cortex_BA24 0.816195408 Brain_Cortex
1.155612754 0.844387246 0.844387246 -0.232441129 1.37548015
0.571519511 1.136972039 2 0 0.998207054
EIF3H ENSG00000147677.10 0.350680573 2 2 0.154596227 Brain_Cerebellum
0.322164915 Brain_Cortex 1.53961904 0.46038096 0.46038096 -1.42348286
-0.990018831 -1.206750846 0.306505354 2 0 0.998207054
POLG ENSG00000140521.12 0.350714704 3 3 0.131445896 Brain_Cerebellum
0.329286943 Brain_Caudate_basal_ganglia 1.921251385 0.078690162
0.078690162 0.97555115 1.508424581 1.32412815 0.302042385 3 0
0.998207054
ASAH1 ENSG00000104763.18 0.350781911 3 3 0.101662579
Brain_Cerebellar_Hemisphere 0.660814076 Brain_Putamen_basal_ganglia
2.453393017 0.151426957 0.151426957 0.43878949 1.636846345
1.100094447 0.608662595 3 0 0.998207054
MRPL30 ENSG00000185414.19 0.350814723 6 2 0.16759006
Brain_Caudate_basal_ganglia 0.45363391 Brain_Cerebellar_Hemisphere 5.671289504
0.004459767 0.216905181 0.749370527 1.379988909 1.014855283
0.210043219 2 0 0.998207054
AC012360.4 ENSG00000235319.1 0.350888792 5 5 0.198961967
Brain_Cortex 0.825405104 Brain_Nucleus_accumbens_basal_ganglia
2.628923989 0.24830434 0.24830434 -1.284514574 0.220598456

-0.63050631 0.585096835 5 0 0.998207054
FAM215A ENSG00000267496.4 0.351087933 2 2 0.332819026
Brain_Cortex 0.566973863 Brain_Frontal_Cortex_BA9 1.417465187
0.582534813 0.582534813 -0.968451308 0.572513913 -0.197968697
1.089626957 2 0 0.998207054
ARHGAP19 ENSG00000213390.10 0.351166517 4 4 0.095634719
Brain_Anterior_cingulate_cortex_BA24 0.820684628 Brain_Cerebellum
1.796102909 0.375908387 0.375908387 -1.666395234 0.226664508
-0.741598864 0.792260445 4 0 0.998207054
RGS17 ENSG00000091844.7 0.351190519 2 2 0.156343538 Brain_Cerebellum
0.281942677 Brain_Cerebellar_Hemisphere 1.86235437 0.13764563 0.13764563
-1.41747692 -1.075965521 -1.246721221 0.241485026 2 0
0.998207054
RFPL2 ENSG00000128253.13 0.351236565 2 2 0.172308236
Brain_Cerebellar_Hemisphere 0.174977284 Brain_Cerebellum 1.76946167 0.23053833
0.23053833 -1.364824438 -1.356383174 -1.360603806 0.005968875 2
0 0.998207054
MAPT ENSG00000186868.15 0.351277751 2 2 0.151490701
Brain_Caudate_basal_ganglia 0.955237674 Brain_Cerebellum 1.168046768
0.831953232 0.831953232 -1.434285871 0.056130717 -0.689077577
1.053883676 2 0 0.998207054
GOLGA4 ENSG00000144674.16 0.351437313 3 3 0.205740831
Brain_Anterior_cingulate_cortex_BA24 0.890430079 Brain_Cortex
1.133700264 0.868990462 0.868990462 -1.265364121 1.239544006
0.037313303 1.255471361 3 0 0.998207054
EDRF1 ENSG00000107938.17 0.351473076 1 1 0.351473076
Brain_Spinal_cord_cervical_c-1 0.351473076 Brain_Spinal_cord_cervical_c-1
1 1 1 0.931735809 0.931735809 0.931735809 NA 1 0
0.998207054
PLPP3 ENSG00000162407.8 0.351559476 3 3 0.158823589 Brain_Cerebellum
0.62622757 Brain_Anterior_cingulate_cortex_BA24 1.091731148 0.92959601
0.92959601 -1.409039162 -0.487043407 -1.003093801 0.470756181 3
0 0.998207054
TMEM185B ENSG00000226479.3 0.351711718 4 4 0.164912293
Brain_Cortex 0.960096831 Brain_Putamen_basal_ganglia 2.436190359
0.252323959 0.252323959 -1.388738464 0.050032072 -0.741231288
0.624215743 4 0 0.998207054
ANKRA2 ENSG00000164331.9 0.351714689 3 3 0.464837947
Brain_Cerebellar_Hemisphere 0.543584359 Brain_Frontal_Cortex_BA9
1.884397921 0.311592308 0.311592308 -0.630454478 0.730903747
-0.168984145 0.779411009 3 0 0.998207054
GAD2 ENSG00000136750.11 0.351723597 1 1 0.351723597 Brain_Cerebellum
0.351723597 Brain_Cerebellum 1 1 1 -0.93125128 -0.93125128
-0.93125128 NA 1 0 0.998207054
ARL9 ENSG00000196503.3 0.351774141 3 3 0.194734943
Brain_Anterior_cingulate_cortex_BA24 0.956761131 Brain_Cortex
1.710135232 0.435897344 0.435897344 -1.296698508 0.992721401
-0.119398515 1.146100873 3 0 0.998207054
RP11-367H1.1 ENSG00000273113.1 0.351819068 2 2 0.149053747
Brain_Cerebellar_Hemisphere 0.858912232 Brain_Spinal_cord_cervical_c-1
1.06382705 0.93617295 0.93617295 -0.17775902 1.442881885 0.632561432
1.145966174 2 0 0.998207054
APOBEC3F ENSG00000128394.16 0.351860521 1 1 0.351860521
Brain_Cerebellar_Hemisphere 0.351860521 Brain_Cerebellar_Hemisphere 1 1
1 0.93098655 0.93098655 0.93098655 NA 1 0 0.998207054
MYBBP1A ENSG00000132382.14 0.35189668 1 1 0.35189668 Brain_Hippocampus
0.35189668 Brain_Hippocampus 1 1 1 0.930916652 0.930916652
0.930916652 NA 1 0 0.998207054
MYNN ENSG00000085274.15 0.35195915 2 2 0.180099599
Brain_Frontal_Cortex_BA9 0.700550936 Brain_Cerebellum 1.113403578
0.886596422 0.886596422 -0.384576867 1.340448432 0.477935783
1.219777086 2 0 0.998207054

DENND4C ENSG00000137145.20 0.351966404 1 1 0.351966404
Brain_Spinal_cord_cervical_c-1 0.351966404 Brain_Spinal_cord_cervical_c-1
1 1 1 -0.930781882 -0.930781882 -0.930781882 NA 1 0
0.998207054
XKR4 ENSG00000206579.8 0.352001198 1 1 0.352001198 Brain_Cerebellum
0.352001198 Brain_Cerebellum 1 1 1 -0.930714633 -0.930714633
-0.930714633 NA 1 0 0.998207054
ARL17A ENSG00000185829.17 0.352078252 11 3 0.10838315
Brain_Nucleus_accumbens_basal_ganglia 0.889625337 Brain_Caudate_basal_ganglia
8.984230898 0.00785473 0.45268475 -1.605503014 -0.138778306
-0.901838303 0.441359454 3 0 0.998207054
LEMD2 ENSG00000161904.11 0.352104174 9 2 0.108726308
Brain_Frontal_Cortex_BA9 0.949439607 Brain_Cerebellar_Hemisphere
7.731805273 0.00316427 0.827447658 -1.603944403 -0.063410524
-0.99185417 0.448834839 2 0 0.998207054
CUL3 ENSG00000036257.12 0.352248787 3 2 0.216609131
Brain_Hippocampus 0.529048197 Brain_Cortex 1.9521384 0.054336925
0.993524674 -1.235595037 -0.629459619 -0.947892316 0.304233931
2 0 0.998207054
HAUS8 ENSG00000131351.14 0.35234317 3 2 0.148598515
Brain_Caudate_basal_ganglia 0.185823904 Brain_Cerebellum 2.892433765
0.001702658 0.105863577 1.323034503 1.444499518 1.3639712
0.069742812 2 0 0.998207054
ANGPT4 ENSG00000101280.7 0.35239414 2 2 0.150688555
Brain_Caudate_basal_ganglia 0.989286299 Brain_Anterior_cingulate_cortex_BA24
1.090464272 0.909535728 0.909535728 -1.437103614 0.013428037
-0.711837788 1.025680767 2 0 0.998207054
ZNF226 ENSG00000167380.16 0.352461479 1 1 0.352461479
Brain_Cerebellum 0.352461479 Brain_Cerebellum 1 1 1 -0.929825426
-0.929825426 -0.929825426 NA 1 0 0.998207054
AP000462.3 ENSG00000256972.1 0.352491853 2 2 0.150046661
Brain_Cerebellum 0.270067915 Brain_Cerebellar_Hemisphere 1.708936152
0.291063848 0.291063848 1.10290617 1.439366674 1.271136422
0.237913504 2 0 0.998207054
KRT1 ENSG00000167768.4 0.352613533 3 3 0.248725601
Brain_Caudate_basal_ganglia 0.401751396 Brain_Anterior_cingulate_cortex_BA24
1.042461179 0.957277129 0.957277129 -1.15031176 1.153450312
-0.278452956 1.249826897 3 0 0.998207054
MSH5 ENSG00000204410.14 0.352671954 2 2 0.14901033
Brain_Putamen_basal_ganglia 0.770460132 Brain_Cerebellar_Hemisphere
1.171047064 0.828952936 0.828952936 -1.443036 -0.291773076
-0.867404538 0.814065821 2 0 0.998207054
FAM221B ENSG00000204930.9 0.352674379 1 1 0.352674379
Brain_Cerebellar_Hemisphere 0.352674379 Brain_Cerebellar_Hemisphere 1 1
1 0.929414379 0.929414379 0.929414379 NA 1 0
0.998207054
PLA2G15 ENSG00000103066.12 0.352715607 2 2 0.185898254
Brain_Hippocampus 0.45756373 Brain_Frontal_Cortex_BA9 1.128051719
0.871948281 0.871948281 -1.322810951 -0.742864483 -1.032837717
0.41008408 2 0 0.998207054
LURAP1L ENSG00000153714.5 0.352758691 10 2 0.184466422
Brain_Cerebellar_Hemisphere 0.999307337 Brain_Substantia_nigra
8.708334408 0.000492904 0.991578827 0.000868125 1.327127767
0.603298575 0.356116327 2 0 0.998207054
YTHDF3-AS1 ENSG00000270673.1 0.352758922 2 2 0.375908988
Brain_Putamen_basal_ganglia 0.724734784 Brain_Cerebellum 1.61631187 0.38368813
0.38368813 -0.88545925 0.352137983 -0.266660633 0.875113397 2
0 0.998207054
NOMO2 ENSG00000185164.14 0.352902737 1 1 0.352902737 Brain_Cerebellum
0.352902737 Brain_Cerebellum 1 1 1 -0.928973659 -0.928973659
-0.928973659 NA 1 0 0.998207054
REM2 ENSG00000139890.9 0.352914497 2 2 0.158595274

Brain_Caudate_basal_ganglia 0.840290636 Brain_Cerebellum 1.075618371
0.924381629 0.924381629 0.201521735 1.409811756 0.805666746
0.854390068 2 0 0.998207054
RCAN2 ENSG00000172348.14 0.35294271 1 1 0.35294271 Brain_Cerebellum 0.35294271
Brain_Cerebellum 1 1 1 -0.928896533 -0.928896533 -0.928896533
NA 1 0 0.998207054
MTRNR2L12 ENSG00000269028.3 0.353086155 1 1 0.353086155
Brain_Anterior_cingulate_cortex_BA24 0.353086155
Brain_Anterior_cingulate_cortex_BA24 1 1 1 -0.928619803
-0.928619803 -0.928619803 NA 1 0 0.998207054
PADI4 ENSG00000159339.13 0.353148321 5 2 0.203832108
Brain_Caudate_basal_ganglia 0.708620639 Brain_Anterior_cingulate_cortex_BA24
3.888592065 0.002542688 0.993470348 0.373709255 1.270709238
0.853122301 0.329948834 2 0 0.998207054
EPHB3 ENSG00000182580.2 0.353198436 1 1 0.353198436 Brain_Cerebellum
0.353198436 Brain_Cerebellum 1 1 1 0.928403244 0.928403244
0.928403244 NA 1 0 0.998207054
BET1L ENSG00000177951.17 0.353324161 6 5 0.054915758
Brain_Spinal_cord_cervical_c-1 0.766181212
Brain_Anterior_cingulate_cortex_BA24 3.029645112 0.033471289
0.152806764 -1.879045268 1.919542141 -0.299309435 1.364144615
5 0 0.998207054
HEATR5A ENSG00000129493.14 0.353341767 5 4 0.201212262
Brain_Spinal_cord_cervical_c-1 0.935216743
Brain_Nucleus_accumbens_basal_ganglia 3.593483376 0.058250528
0.252725137 -1.278105409 0.386443842 -0.198972228 0.698261813
4 0 0.998207054
B4GALT3 ENSG00000158850.14 0.353380812 1 1 0.353380812
Brain_Cerebellum 0.353380812 Brain_Cerebellum 1 1 1 -0.928051586
-0.928051586 -0.928051586 NA 1 0 0.998207054
GPC1 ENSG00000063660.8 0.353467709 4 4 0.141936272
Brain_Cerebellar_Hemisphere 0.647036416 Brain_Putamen_basal_ganglia
1.921000578 0.134520586 0.134520586 -0.457883118 1.468618583
0.884856876 0.911582531 4 0 0.998207054
4-Mar ENSG00000144583.4 0.353473959 1 1 0.353473959 Brain_Cerebellum
0.353473959 Brain_Cerebellum 1 1 1 -0.927872021 -0.927872021
-0.927872021 NA 1 0 0.998207054
NHP2 ENSG00000145912.8 0.353589322 2 2 0.26461721 Brain_Amygdala
0.354883731 Brain_Anterior_cingulate_cortex_BA24 1.010338862
0.989661138 0.989661138 -1.115544384 0.925157993 -0.095193196
1.442994489 2 0 0.998207054
ITGA9 ENSG00000144668.11 0.353614918 3 3 0.123437523 Brain_Cerebellum
0.568068283 Brain_Cerebellar_Hemisphere 2.227444438 0.227707611
0.227707611 -1.450838565 1.540504105 0.220188094 1.526198287
3 0 0.998207054
DLX1 ENSG00000144355.14 0.353663995 1 1 0.353663995
Brain_Caudate_basal_ganglia 0.353663995 Brain_Caudate_basal_ganglia 1 1
1 -0.927505775 -0.927505775 -0.927505775 NA 1 0
0.998207054
SRP68 ENSG00000167881.14 0.353770658 1 1 0.353770658
Brain_Cerebellar_Hemisphere 0.353770658 Brain_Cerebellar_Hemisphere 1 1
1 -0.927300263 -0.927300263 -0.927300263 NA 1 0
0.998207054
LIX1 ENSG00000145721.11 0.35386954 2 2 0.163994174 Brain_Cortex
0.683203743 Brain_Anterior_cingulate_cortex_BA24 1.0238323 0.9761677
0.9761677 -1.391763012 -0.408095255 -0.899929134 0.695558142 2
0 0.998207054
NKTR ENSG00000114857.17 0.353896373 8 4 0.251136935
Brain_Frontal_Cortex_BA9 0.98789641 Brain_Cerebellar_Hemisphere 6.51517425
1.39E-05 0.219391805 -1.035597805 1.147592239 0.398631733
0.770800779 4 0 0.998207054
EIF5A ENSG00000132507.17 0.354067884 12 4 0.026502666

Brain_Caudate_basal_ganglia 0.483474105 Brain_Cerebellar_Hemisphere 9.806084707 0.003507555 0.366619058 0.700725874 2.218765746 1.631197071 0.421000531 4 0 0.998207054
BZW1 ENSG00000082153.17 0.354146997 1 1 0.354146997 Brain_Cerebellar_Hemisphere 0.354146997 Brain_Cerebellar_Hemisphere 1 1 1 0.926575468 0.926575468 0.926575468 NA 1 0 0.998207054
RP11-299L17.3 ENSG00000258675.1 0.354165853 1 1 0.354165853 Brain_Hypothalamus 0.354165853 Brain_Hypothalamus 1 1 1 0.926539165 0.926539165 0.926539165 NA 1 0 0.998207054
TMEM43 ENSG00000170876.7 0.354236635 1 1 0.354236635 Brain_Cerebellar_Hemisphere 0.354236635 Brain_Cerebellar_Hemisphere 1 1 1 -0.926402905 -0.926402905 -0.926402905 NA 1 0 0.998207054
BHLHE40 ENSG00000134107.4 0.354253587 1 1 0.354253587 Brain_Substantia_nigra 0.354253587 Brain_Substantia_nigra 1 1 1 -0.926370273 -0.926370273 -0.926370273 NA 1 0 0.998207054
SYCP3 ENSG00000139351.14 0.354257922 2 2 0.647378748 Brain_Cerebellum 0.849615533 Brain_Cerebellar_Hemisphere 1.898221469 0.101778531 0.101778531 -0.457406701 0.189609001 -0.13389885 0.457509191 2 0 0.998207054
ZBED6 ENSG00000257315.2 0.354267169 1 1 0.354267169 Brain_Hippocampus 0.354267169 Brain_Hippocampus 1 1 1 -0.926344129 -0.926344129 -0.926344129 NA 1 0 0.998207054
RP11-325E5.1 ENSG00000259656.1 0.354308258 1 1 0.354308258 Brain_Caudate_basal_ganglia 0.354308258 Brain_Caudate_basal_ganglia 1 1 1 -0.926265042 -0.926265042 -0.926265042 NA 1 0 0.998207054
SMC2-AS1 ENSG00000270332.1 0.354345416 2 2 0.208412818 Brain_Nucleus_accumbens_basal_ganglia 0.443672305 Brain_Cerebellum 1.051774953 0.948225047 0.948225047 0.766006718 1.257941764 1.011974241 0.347850607 2 0 0.998207054
HIPK1 ENSG00000163349.21 0.354421946 1 1 0.354421946 Brain_Cortex 0.354421946 Brain_Cortex 1 1 1 0.926046249 0.926046249 0.926046249 NA 1 0 0.998207054
RP11-619A14.3 ENSG00000255362.1 0.354460325 3 3 0.211439596 Brain_Nucleus_accumbens_basal_ganglia 0.33562502 Brain_Caudate_basal_ganglia 1.555551229 0.457176587 0.457176587 0.962845586 1.249616709 1.082220719 0.149294746 3 0 0.998207054
GRIN2B ENSG00000273079.5 0.354592787 1 1 0.354592787 Brain_Putamen_basal_ganglia 0.354592787 Brain_Putamen_basal_ganglia 1 1 1 -0.925717547 -0.925717547 -0.925717547 NA 1 0 0.998207054
AC000403.4 ENSG00000278727.1 0.354776311 1 1 0.354776311 Brain_Cerebellum 0.354776311 Brain_Cerebellum 1 1 1 0.925364553 0.925364553 0.925364553 NA 1 0 0.998207054
ARHGAP12 ENSG00000165322.17 0.354780909 3 3 0.233041044 Brain_Cerebellar_Hemisphere 0.647883197 Brain_Anterior_cingulate_cortex_BA24 2.067662576 0.226407399 0.226407399 -0.729969721 1.192561774 0.306432305 0.970035163 3 0 0.998207054
GPR162 ENSG00000250510.7 0.354862793 2 2 0.150605876 Brain_Spinal_cord_cervical_c-1 0.726506065 Brain_Cerebellum 1.190685519 0.809314481 0.809314481 0.349776996 1.437394694 0.893585845 0.76906185 2 0 0.998207054
SENP5 ENSG00000119231.10 0.354876463 5 3 0.242135761 Brain_Caudate_basal_ganglia 0.934636499 Brain_Nucleus_accumbens_basal_ganglia 3.785012166 0.00844193 0.368923948 -1.169665119 0.23995369 -0.342284022 0.716224642 3 0 0.998207054
ZNF90 ENSG00000213988.10 0.35494682 1 1 0.35494682 Brain_Hypothalamus 0.35494682 Brain_Hypothalamus 1 1 1 -0.925036696 -0.925036696 -0.925036696 NA 1 0 0.998207054

EOGT ENSG00000163378.13 0.354964268 4 4 0.373000531
Brain_Spinal_cord_cervical_c-1 0.715833802 Brain_Caudate_basal_ganglia
2.416172201 0.202370218 0.202370218 -0.575433914 0.890866991
0.09289401 0.682963627 4 0 0.998207054
IKZF1 ENSG00000185811.16 0.354978432 2 2 0.177407681
Brain_Caudate_basal_ganglia 0.632468125 Brain_Hypothalamus 1.017682807
0.982317193 0.982317193 -1.348779874 -0.478255836 -0.913517855
0.615553451 2 0 0.998207054
NDC1 ENSG00000058804.11 0.355045207 1 1 0.355045207
Brain_Caudate_basal_ganglia 0.355045207 Brain_Caudate_basal_ganglia 1 1
1 0.92484756 0.92484756 0.92484756 NA 1 0 0.998207054
ZKSCAN1 ENSG00000106261.16 0.355196845 2 2 0.175089149
Brain_Caudate_basal_ganglia 0.996448249 Brain_Amygdala 1.337207321
0.662792679 0.662792679 -1.35603149 -0.004451474 -0.680241482
0.955711394 2 0 0.998207054
LRRC27 ENSG00000148814.17 0.355198734 10 2 0.299575436
Brain_Caudate_basal_ganglia 0.924466198 Brain_Cerebellar_Hemisphere
8.969649881 0.002134272 0.575068426 -0.094809428 1.03734428
0.675439662 0.346637567 2 0 0.998207054
PMP2 ENSG00000147588.6 0.3552278 2 2 0.166148161
Brain_Frontal_Cortex_BA9 0.430584731 Brain_Caudate_basal_ganglia
1.300076869 0.699923131 0.699923131 -1.384687113 -0.788191447
-1.08643928 0.42178613 2 0 0.998207054
B4GALNT4 ENSG00000182272.11 0.355287504 6 6 0.041579811
Brain_Putamen_basal_ganglia 0.648483151 Brain_Cortex 3.760707314
0.165588541 0.165588541 0.455870434 2.037701331 0.945772613
0.565912823 6 0 0.998207054
CCSER1 ENSG00000184305.14 0.355305275 3 3 0.19117298
Brain_Cerebellar_Hemisphere 0.962046945 Brain_Spinal_cord_cervical_c-1
1.077231588 0.944063672 0.944063672 -1.307116996 1.305035047
0.015167701 1.306377716 3 0 0.998207054
C2orf40 ENSG00000119147.9 0.355424593 9 5 0.027901444
Brain_Hippocampus 0.279634346 Brain_Cortex 6.875516309 3.23E-18
0.349399493 -2.198669324 -1.081141116 -1.793841074 0.300787282
5 0 0.998207054
RP11-430G17.3 ENSG00000271200.1 0.355436364 1 1 0.355436364
Brain_Cerebellum 0.355436364 Brain_Cerebellum 1 1 1 -0.924095946
-0.924095946 -0.924095946 NA 1 0 0.998207054
CPEB1 ENSG00000214575.9 0.355507783 3 3 0.416471627 Brain_Cerebellum
0.710260007 Brain_Hippocampus 1.881085138 0.338706471
0.338706471 -0.682810717 0.812557814 -0.080586606 0.78899159 3
0 0.998207054
LINC01544 ENSG00000260440.2 0.355511887 2 2 0.199542311
Brain_Cerebellar_Hemisphere 0.493447687 Brain_Cerebellum 1.027239577
0.972760423 0.972760423 0.684835569 1.282856628 0.983846098
0.422864746 2 0 0.998207054
SLFN12 ENSG00000172123.12 0.355529658 2 2 0.205955562
Brain_Caudate_basal_ganglia 0.302374114 Brain_Cerebellum 1.298923156
0.701076844 0.701076844 1.031355557 1.264765053 1.148060305
0.165045438 2 0 0.998207054
ZNF600 ENSG00000189190.9 0.355792446 6 6 0.055448683
Brain_Cerebellum 0.67641353 Brain_Cerebellar_Hemisphere 2.111674921
0.385789326 0.385789326 -1.915343721 -0.417362158 -1.074080402
0.572289952 6 0 0.998207054
NPR1 ENSG00000169418.9 0.355876103 2 2 0.181272906 Brain_Cerebellum
0.655937083 Brain_Frontal_Cortex_BA9 1.061234644 0.938765356
0.938765356 -0.445529587 1.336846013 0.445658213 1.260329873
2 0 0.998207054
NGFR ENSG00000064300.8 0.355940508 5 4 0.07828156 Brain_Hippocampus
0.942478238 Brain_Cerebellum 3.433042342 0.080733733 0.240313387
0.0721554 1.760745073 0.794191066 0.611303904 4 0
0.998207054

KIRREL3 ENSG00000149571.11 0.35610293 2 2 0.310610168
Brain_Spinal_cord_cervical_c-1 0.352485432 Brain_Hippocampus
1.083798485 0.916201515 0.916201515 -0.929779172 1.013942546
0.042081687 1.374418807 2 0 0.998207054
ZNF644 ENSG00000122482.20 0.356133202 1 1 0.356133202
Brain_Anterior_cingulate_cortex_BA24 0.356133202
Brain_Anterior_cingulate_cortex_BA24 1 1 1 -0.922758251
-0.922758251 -0.922758251 NA 1 0 0.998207054
NPM1 ENSG00000181163.13 0.356172894 2 2 0.243150657 Brain_Cerebellum
0.375837244 Brain_Putamen_basal_ganglia 1.041286738 0.958713262
0.958713262 -1.167147875 -0.885592334 -1.026370105 0.199089832
2 0 0.998207054
CCNB2 ENSG00000157456.7 0.356208748 3 3 0.094844514
Brain_Spinal_cord_cervical_c-1 0.861724676 Brain_Cortex 1.144890915
0.852229137 0.852229137 -0.174179143 1.670378431 0.633278979
0.943477354 3 0 0.998207054
TPGS2 ENSG00000134779.14 0.356216115 10 4 0.03569221 Brain_Cerebellum
0.947446729 Brain_Cortex 7.230997967 0.011797007 0.261300341
-2.100416597 0.570936041 -0.374862039 0.763194807 4 0
0.998207054
CTD-3220F14.1 ENSG00000267605.5 0.356272788 6 6 0.068962772
Brain_Hippocampus 0.982146418 Brain_Hypothalamus 3.038211961
0.171371887 0.171371887 -1.331081593 1.818663575 0.178457285
1.042718971 6 0 0.998207054
GTPBP10 ENSG00000105793.15 0.356411558 8 6 0.292764632
Brain_Nucleus_accumbens_basal_ganglia 0.618447556 Brain_Frontal_Cortex_BA9
4.878796494 0.064358037 0.212444633 -1.009162418 1.052076111
0.612996124 0.684128876 6 0 0.998207054
SENP2 ENSG00000163904.12 0.356457431 2 2 0.207522066 Brain_Cerebellum
0.354447496 Brain_Substantia_nigra 1.199131934 0.800868066
0.800868066 0.925997083 1.260408418 1.093202751 0.236464523
2 0 0.998207054
P2RX7 ENSG00000089041.16 0.356491715 7 4 0.29244121
Brain_Cerebellar_Hemisphere 0.862301405 Brain_Caudate_basal_ganglia
5.246842351 0.026001958 0.345917968 -0.622431138 1.052781359
0.287980786 0.632474785 4 0 0.998207054
SPG7 ENSG00000197912.14 0.3566184 2 2 0.155095149
Brain_Caudate_basal_ganglia 0.983570541 Brain_Amygdala 1.154788103
0.845211897 0.845211897 0.020592728 1.421762708 0.721177718
0.990776794 2 0 0.998207054
PCNP ENSG00000081154.11 0.356674858 1 1 0.356674858
Brain_Caudate_basal_ganglia 0.356674858 Brain_Caudate_basal_ganglia 1 1
1 -0.921719593 -0.921719593 -0.921719593 NA 1 0
0.998207054
RP3-340N1.2 ENSG00000227066.1 0.356773605 9 2 0.133701917
Brain_Substantia_nigra 0.897182983 Brain_Caudate_basal_ganglia
7.776508289 7.32E-06 0.586007391 0.129220744 1.499662236
0.395990053 0.4522163 2 0 0.998207054
POLR3K ENSG00000161980.5 0.357041732 3 3 0.152893516
Brain_Cerebellar_Hemisphere 0.712508257 Brain_Cerebellum 1.060717884
0.945455541 0.945455541 -0.94127373 1.429385319 0.285533704
1.187504666 3 0 0.998207054
CDPF1 ENSG00000205643.10 0.357150936 1 1 0.357150936
Brain_Putamen_basal_ganglia 0.357150936 Brain_Putamen_basal_ganglia 1 1
1 -0.920807505 -0.920807505 -0.920807505 NA 1 0
0.998207054
ATP6AP1L ENSG00000205464.11 0.357164095 13 2 0.332367697
Brain_Hypothalamus 0.973458384 Brain_Nucleus_accumbens_basal_ganglia
11.82185184 0.002722757 0.479623671 -0.9693558 0.226410705
-0.081093053 0.306694085 2 0 0.998207054
CARD19 ENSG00000165233.17 0.357164938 12 6 0.178658301
Brain_Anterior_cingulate_cortex_BA24 0.966986539 Brain_Cerebellum

8.327637284 0.009218269 0.295544017 -1.344897615 1.078843881 -0.295589836 0.673674918 6 0 0.998207054
C1QTNF1 ENSG00000173918.14 0.357236068 1 1 0.357236068 Brain_Putamen_basal_ganglia 0.357236068 Brain_Putamen_basal_ganglia 1 1 1 0.920644488 0.920644488 0.920644488 NA 1 0 0.998207054
NAE1 ENSG00000159593.14 0.35757963 1 1 0.35757963 Brain_Cortex 0.35757963 Brain_Cortex 1 1 1 -0.919986853 -0.919986853 -0.919986853 NA 1 0 0.998207054
TMEM51 ENSG00000171729.13 0.357584543 5 5 0.038643042 Brain_Amygdala 0.944468076 Brain_Cerebellum 1.770688472 0.145680689 0.145680689 -0.283345924 2.067967897 0.515407792 0.971780754 5 0 0.998207054
TMED5 ENSG00000117500.12 0.357595007 6 4 0.088225312 Brain_Spinal_cord_cervical_c-1 0.907557874 Brain_Cerebellum 4.734390437 0.027000817 0.247356422 -1.704834408 -0.11611945 -1.125924116 0.534595267 4 0 0.998207054
UBE4B ENSG00000130939.18 0.357645454 1 1 0.357645454 Brain_Cerebellar_Hemisphere 0.357645454 Brain_Cerebellar_Hemisphere 1 1 1 0.9198609 0.9198609 0.9198609 NA 1 0 0.998207054
UBR5 ENSG00000104517.12 0.357654043 2 2 0.306448903 Brain_Cerebellar_Hemisphere 0.312814619 Brain_Anterior_cingulate_cortex_BA24 1.004034185 0.995965815 0.995965815 -1.02270171 1.00933371 -0.006684 1.436866025 2 0 0.998207054
SCNN1A ENSG00000111319.12 0.357657077 1 1 0.357657077 Brain_Cerebellum 0.357657077 Brain_Cerebellum 1 1 1 0.91983866 0.91983866 0.91983866 NA 1 0 0.998207054
COLCA2 ENSG00000214290.8 0.357670825 5 4 0.049790772 Brain_Spinal_cord_cervical_c-1 0.764592075 Brain_Frontal_Cortex_BA9 2.861420938 0.068679035 0.340155097 0.299456085 1.961757089 1.115500275 0.600841967 4 0 0.998207054
MIS12 ENSG00000167842.15 0.357679639 11 7 0.113695312 Brain_Spinal_cord_cervical_c-1 0.924515811 Brain_Hypothalamus 6.346608969 0.035910728 0.236444841 -1.581799661 0.993957805 -0.327085275 0.838025646 7 0 0.998207054
PGAP3 ENSG00000161395.13 0.357707633 6 4 0.36272934 Brain_Spinal_cord_cervical_c-1 0.950111361 Brain_Nucleus_accumbens_basal_ganglia 4.463171715 0.029268684 0.223852183 -0.910176682 0.526008667 -0.249187894 0.528995081 4 0 0.998207054
MORN3 ENSG00000139714.12 0.357724729 6 6 0.048628308 Brain_Spinal_cord_cervical_c-1 0.991658669 Brain_Cortex 3.572181509 0.192831034 0.192831034 -1.220320443 1.971836203 -0.338345446 1.211533821 6 0 0.998207054
ZNF764 ENSG00000169951.9 0.357742122 1 1 0.357742122 Brain_Frontal_Cortex_BA9 0.357742122 Brain_Frontal_Cortex_BA9 1 1 1 -0.919675953 -0.919675953 -0.919675953 NA 1 0 0.998207054
ATP2A1 ENSG00000196296.13 0.357832697 1 1 0.357832697 Brain_Hypothalamus 0.357832697 Brain_Hypothalamus 1 1 1 0.919502694 0.919502694 0.919502694 NA 1 0 0.998207054
SCPEP1 ENSG00000121064.12 0.357900831 2 2 0.228304412 Brain_Cerebellum 0.501178209 Brain_Substantia_nigra 1.084266951 0.915733049 0.915733049 -1.204738128 0.672637075 -0.266050526 1.327504737 2 0 0.998207054
RP11-429J17.7 ENSG00000254973.1 0.35801597 4 2 0.486356744 Brain_Nucleus_accumbens_basal_ganglia 0.939306767 Brain_Cerebellar_Hemisphere 3.521497114 0.009997773 0.44029575 -0.696115111 0.542148197 -0.213284296 0.574602141 2 0 0.998207054
ZNF436 ENSG00000125945.14 0.358108549 3 3 0.102313329 Brain_Putamen_basal_ganglia 0.773549859 Brain_Cortex 1.160350683 0.837793015 0.837793015 -0.939510745 1.6337406 0.135498399

1.337815491	3	0	0.998207054
DIP2B	ENSG00000066084.12	0.35812939	3	3	0.138237585
Brain_Putamen_basal_ganglia	0.416736175	Brain_Nucleus_accumbens_basal_ganglia
2.23146879	0.133388209	0.133388209	0.81209665	1.48238612	1.125508687
0.337252019	3	0	0.998207054
CSRNP2	ENSG00000110925.6	0.35813388	2	1	0.27248734
Brain_Cerebellar_Hemisphere	0.474510754	Brain_Cerebellum	1.945224667
0.054775333	1.945224667	0.715159074	1.097352476	0.906255775
0.270251546	1	0	0.998207054
LANCL2	ENSG00000132434.9	0.358195069	3	3	0.159825228
Brain_Caudate_basal_ganglia	0.622741312	Brain_Cortex	1.528009516
0.681254908	0.681254908	-1.405659603	0.54700252	-0.450208669
0.977000655	3	0	0.998207054
LCOR	ENSG00000196233.12	0.358239209	2	2	0.318400586	Brain_Amygdala
0.363904187	Brain_Hypothalamus	1.11399981	0.88600019	0.88600019
-0.907950855	0.997750031	0.044899588	1.34753402	2	0
0.998207054
FARP2	ENSG00000006607.13	0.358281921	10	4	0.230467242	Brain_Amygdala
0.796801353	Brain_Cerebellar_Hemisphere	6.984585523	0.006794043
0.26193264	-1.19915613	0.827123029	-0.17674793	0.664042231	4
0	0.998207054
FLVCR1-AS1	ENSG00000198468.7	0.358304341	13	1	0.251435676
Brain_Amygdala	0.51051328	Brain_Spinal_cord_cervical_c-1	12.33299561
3.61E-05	12.33299561	0.658038675	1.146869219	0.893891788
0.153474035	1	0	0.998207054
NUDT19	ENSG00000213965.3	0.358345278	2	2	0.175161713
Brain_Substantia_nigra	0.806198312	Brain_Hippocampus	1.156303493
0.843696507	0.843696507	-0.245333375	1.355803452	0.555235039
1.132174708	2	0	0.998207054
PLA2G6	ENSG00000184381.18	0.358549008	6	6	0.15863103
Brain_Spinal_cord_cervical_c-1	0.81539187	Brain_Hippocampus	2.952148131
0.19923203	0.19923203	-1.223040211	1.409690708	-0.124128874
0.994713749	6	0	0.998207054
NR2C2	ENSG00000177463.15	0.358684185	2	2	0.285036858
Brain_Spinal_cord_cervical_c-1	0.356901474	Brain_Cortex	1.02904433
0.97095567	0.97095567	-0.921285339	1.06907282	0.07389374	1.407395751	2
0	0.998207054
ASB3	ENSG00000115239.21	0.358793239	1	1	0.358793239
Brain_Caudate_basal_ganglia	0.358793239	Brain_Caudate_basal_ganglia	1	1
1	0.917666982	0.917666982	0.917666982	NA	1	0
0.998207054
ELAVL4	ENSG00000162374.16	0.358826929	1	1	0.358826929
Brain_Cerebellum	0.358826929	Brain_Cerebellum	1	1	1	-0.917602652
-0.917602652	-0.917602652	NA	1	0	0.998207054
PERP	ENSG00000112378.11	0.358905037	9	5	0.025641659
Brain_Hypothalamus	0.282409266	Brain_Cortex	6.62025591	0.067348827
0.310423133	-2.231596266	-1.07492286	-1.612856985	0.38331732	5
0	0.998207054
CHMP4C	ENSG00000164695.4	0.359048488	5	3	0.315360057
Brain_Caudate_basal_ganglia	0.921764186	Brain_Putamen_basal_ganglia
4.439568814	0.012109091	0.193341877	0.098211708	1.0040385
0.714721691	0.387033717	3	0	0.998207054
MOB3B	ENSG00000120162.9	0.359090346	4	3	0.162146006
Brain_Cerebellar_Hemisphere	0.428091967	Brain_Substantia_nigra
1.992858184	0.005891183	0.90531802	-1.397890319	0.93015986
-0.258140891	1.293958942	3	0	0.998207054
CTD-2012J19.3	ENSG00000271119.1	0.359155633	6	6	0.109312118
Brain_Hypothalamus	0.659911989	Brain_Putamen_basal_ganglia	1.994664098
0.310365944	0.310365944	-0.83733587	1.601292643	0.457938585
0.95316453	6	0	0.998207054
MAGEL2	ENSG00000254585.2	0.359208918	1	1	0.359208918
Brain_Putamen_basal_ganglia	0.359208918	Brain_Putamen_basal_ganglia	1	1

1	-0.916873526	-0.916873526	-0.916873526	NA	1	0
0.998207054
PROSC	ENSG00000147471.11	0.359262571	1	1	0.359262571
Brain_Nucleus_accumbens_basal_ganglia	0.359262571
Brain_Nucleus_accumbens_basal_ganglia	1	1	1	-0.916771154
-0.916771154	-0.916771154	NA	1	0	0.998207054
SLC17A6	ENSG00000091664.7	0.359331139	2	2	0.160735238
Brain_Cortex	0.735752685	Brain_Hypothalamus	1.039458256
0.960541744	0.960541744	0.337483186	1.402603062	0.870043124
0.753153487	2	0	0.998207054
SCAMP2	ENSG00000140497.16	0.359378058	3	3	0.148901843
Brain_Cerebellum	0.795900249	Brain_Nucleus_accumbens_basal_ganglia
1.519128323	0.403125139	0.403125139	-1.443421246	0.502376101
-0.227462866	1.060078248	3	0	0.998207054
LINC00648	ENSG00000259129.5	0.359387519	10	4	0.163017365
Brain_Hippocampus	0.645673536	Brain_Anterior_cingulate_cortex_BA24
7.683892777	0.016660827	0.392120567	-1.394994943	0.459780842
-0.935166532	0.537206793	4	0	0.998207054
MIPOL1	ENSG00000151338.18	0.359400111	1	1	0.359400111
Brain_Caudate_basal_ganglia	0.359400111	Brain_Caudate_basal_ganglia	1	1
1	-0.916508768	-0.916508768	-0.916508768	NA	1	0
0.998207054
RP11-575H3.1	ENSG00000261600.1	0.359427162	3	3	0.145544608
Brain_Cortex	0.705762098	Brain_Cerebellar_Hemisphere	1.324282894
0.637228504	0.637228504	-1.455450416	1.356135058	-0.158956383
1.418482231	3	0	0.998207054
C4orf3	ENSG00000164096.12	0.35946639	1	1	0.35946639	Brain_Cerebellum
0.35946639	Brain_Cerebellum	1	1	1	0.916382349	0.916382349
0.916382349	NA	1	0	0.998207054
RP3-486I3.7	ENSG00000237021.2	0.359471699	2	2	0.230105115
Brain_Cerebellar_Hemisphere	0.537741939	Brain_Cortex	1.129715265
0.870284735	0.870284735	-0.616231201	1.20008813	0.291928465
1.284331715	2	0	0.998207054
QRSL1	ENSG00000130348.11	0.359592096	13	2	0.429113538	Brain_Amygdala
0.984154871	Brain_Cerebellum	11.49284484	4.36E-05	0.456992382
-0.720340644	0.790709488	0.070267311	0.356231303	2	0
0.998207054
PPP2R2D	ENSG00000175470.18	0.359602059	3	2	0.275702558
Brain_Nucleus_accumbens_basal_ganglia	0.413903499	Brain_Cerebellar_Hemisphere
1.994122389	0.010919383	0.994958228	-0.91832973	1.090024032
-0.215116441	1.131418781	2	0	0.998207054
BYSL	ENSG00000112578.9	0.35964237	1	1	0.35964237	Brain_Cerebellar_Hemisphere
0.35964237	Brain_Cerebellar_Hemisphere	1	1	1	-0.916046759
-0.916046759	-0.916046759	NA	1	0	0.998207054
SNN	ENSG00000184602.5	0.359767012	1	1	0.359767012	Brain_Cortex
0.359767012	Brain_Cortex	1	1	1	0.915809134	0.915809134
0.915809134	NA	1	0	0.998207054
C1QL2	ENSG00000144119.3	0.359779048	4	4	0.16630285
Brain_Putamen_basal_ganglia	0.801326644	Brain_Cortex	2.016575623
0.17318912	0.17318912	-1.384181621	0.977446692	-0.357570405
1.161798798	4	0	0.998207054
RP11-1348G14.4	ENSG00000251417.2	0.359796526	12	4	0.044179351
Brain_Cerebellum	0.650841166	Brain_Caudate_basal_ganglia	9.589297621
0.000750398	0.380745047	-2.012385165	0.591431023	-1.097943834
0.667136511	4	0	0.998207054
MPP3	ENSG00000161647.18	0.359856975	2	2	0.197101464
Brain_Frontal_Cortex_BA9	0.57119165	Brain_Cerebellar_Hemisphere	1.038752161
0.961247839	0.961247839	-1.289853686	0.566297368	-0.361778159
1.312496997	2	0	0.998207054
NARF	ENSG00000141562.17	0.360053369	8	2	0.118327134
Brain_Anterior_cingulate_cortex_BA24	0.404162408	Brain_Frontal_Cortex_BA9
7.091322956	0.001015721	0.566117133	0.834210442	1.561833857

1.267142458 0.276965865 2 0 0.998207054
PTPA ENSG00000119383.19 0.360074563 7 7 0.024085875
Brain_Frontal_Cortex_BA9 0.974087265 Brain_Cerebellum 2.15394589
0.235092613 0.235092613 -0.644203602 2.255756654 0.303316289
1.014404428 7 0 0.998207054
CDCA3 ENSG00000111665.11 0.360185388 12 3 0.13666506 Brain_Hypothalamus
0.73412505 Brain_Hippocampus 9.666130518 0.002198182 0.632573698
-1.488325551 0.339643454 -0.823990108 0.45385703 3 0
0.998207054
TTI1 ENSG00000101407.12 0.360236291 4 4 0.047754978
Brain_Hypothalamus 0.96546878 Brain_Amygdala 2.182135099 0.364334875
0.364334875 -1.979542334 -0.043291985 -0.664418499 0.890228875
4 0 0.998207054
TFPI ENSG00000003436.15 0.360321484 1 1 0.360321484
Brain_Anterior_cingulate_cortex_BA24 0.360321484
Brain_Anterior_cingulate_cortex_BA24 1 1 1 -0.914752675
-0.914752675 -0.914752675 NA 1 0 0.998207054
LINC01772 ENSG00000226029.1 0.360327189 8 5 0.033211696
Brain_Caudate_basal_ganglia 0.774131737 Brain_Anterior_cingulate_cortex_BA24
5.173471941 0.000169016 0.18494384 -2.129514744 0.286974641
-1.530558106 0.756053476 5 0 0.998207054
MYH1 ENSG00000109061.9 0.360489388 2 2 0.295478633
Brain_Substantia_nigra 0.32499359 Brain_Cerebellar_Hemisphere 1.01111993
0.98888007 0.98888007 -1.046178489 -0.984248001 -1.015213245
0.043791468 2 0 0.998207054
SNHG15 ENSG00000232956.8 0.360498997 7 6 0.150174699
Brain_Caudate_basal_ganglia 0.881357747 Brain_Cerebellum 4.079745129
0.093293274 0.146332923 -0.624078204 1.438914675 0.677839825
0.768307149 6 0 0.998207054
CALHM3 ENSG00000183128.7 0.360555992 2 2 0.236681336
Brain_Cerebellum 0.398550114 Brain_Anterior_cingulate_cortex_BA24
1.037867892 0.962132108 0.962132108 -1.183322558 0.844213497
-0.169554531 1.433684494 2 0 0.998207054
SNX20 ENSG00000167208.14 0.360603994 1 1 0.360603994 Brain_Cerebellum
0.360603994 Brain_Cerebellum 1 1 1 -0.91421479 -0.91421479
-0.91421479 NA 1 0 0.998207054
TFRC ENSG00000072274.12 0.360680825 2 2 0.178377577
Brain_Cerebellar_Hemisphere 0.620170249 Brain_Substantia_nigra
1.013154301 0.986845699 0.986845699 -1.345767292 -0.495609075
-0.920688183 0.60115264 2 0 0.998207054
CARD6 ENSG00000132357.13 0.360726082 2 2 0.30059004
Brain_Spinal_cord_cervical_c-1 0.857835282 Brain_Cerebellar_Hemisphere
1.592491424 0.407508576 0.407508576 -1.0351689 0.179130439
-0.428019231 0.858639297 2 0 0.998207054
GSK3B ENSG00000082701.14 0.360787326 1 1 0.360787326
Brain_Nucleus_accumbens_basal_ganglia 0.360787326
Brain_Nucleus_accumbens_basal_ganglia 1 1 1 -0.913865876
-0.913865876 -0.913865876 NA 1 0 0.998207054
RHBDL2 ENSG00000158315.10 0.360809382 1 1 0.360809382
Brain_Nucleus_accumbens_basal_ganglia 0.360809382
Brain_Nucleus_accumbens_basal_ganglia 1 1 1 -0.913823907
-0.913823907 -0.913823907 NA 1 0 0.998207054
ZFYVE16 ENSG00000039319.16 0.360910137 2 2 0.204991338
Brain_Anterior_cingulate_cortex_BA24 0.722221623 Brain_Cortex
1.224716073 0.775283927 0.775283927 -0.355491217 1.267458655
0.455983719 1.14759886 2 0 0.998207054
ADCY6 ENSG00000174233.11 0.36106523 3 3 0.200921481 Brain_Cortex
0.481350794 Brain_Cerebellar_Hemisphere 1.218148772 0.765364914
0.765364914 -1.278930641 0.704131646 -0.562744006 1.100262607
3 0 0.998207054
BAP1 ENSG00000163930.9 0.361078664 2 2 0.159119825
Brain_Hippocampus 0.270884083 Brain_Cortex 1.865266059

0.134733941 0.134733941 1.101028891 1.408037978 1.254533435
0.217088207 2 0 0.998207054
ARL1 ENSG00000120805.13 0.361096013 6 5 0.131587963 Brain_Cerebellum
0.935859507 Brain_Hippocampus 3.083242688 0.096508797
0.341124421 -1.127823525 1.507869373 -0.013878262 0.909365564
5 0 0.998207054
RC3H1 ENSG00000135870.11 0.361097952 2 2 0.333947688 Brain_Cerebellum
0.857258872 Brain_Nucleus_accumbens_basal_ganglia 1.815481732
0.184518268 0.184518268 0.179864594 0.966192855 0.573028724
0.556018045 2 0 0.998207054
IDH3B ENSG00000101365.20 0.361177587 6 5 0.29755078
Brain_Cerebellar_Hemisphere 0.860822212 Brain_Cortex 3.666503553
0.096430086 0.150696722 -1.041700018 0.79470998 -0.264003523
0.805944062 5 0 0.998207054
PAK1 ENSG00000149269.9 0.361349061 3 3 0.078849161
Brain_Cerebellar_Hemisphere 0.971246722 Brain_Cortex 1.67816174
0.350983294 0.350983294 -0.110730602 1.75740289 0.56090566 1.03879255
3 0 0.998207054
CBLN2 ENSG00000141668.9 0.361373367 1 1 0.361373367 Brain_Cerebellum
0.361373367 Brain_Cerebellum 1 1 1 -0.912751281 -0.912751281
-0.912751281 NA 1 0 0.998207054
LINC00621 ENSG00000262619.1 0.361589353 1 1 0.361589353
Brain_Nucleus_accumbens_basal_ganglia 0.361589353
Brain_Nucleus_accumbens_basal_ganglia 1 1 1 -0.91234078
-0.91234078 -0.91234078 NA 1 0 0.998207054
NSL1 ENSG00000117697.14 0.361610399 3 3 0.15516591
Brain_Cerebellar_Hemisphere 0.687785808 Brain_Nucleus_accumbens_basal_ganglia
1.423061005 0.592281046 0.592281046 -1.092302831 1.421519082
0.243692652 1.264352894 3 0 0.998207054
RP11-822E23.7 ENSG00000265265.1 0.361662371 1 1 0.361662371
Brain_Cerebellar_Hemisphere 0.361662371 Brain_Cerebellar_Hemisphere 1 1
1 -0.912202039 -0.912202039 -0.912202039 NA 1 0
0.998207054
SEC61G ENSG00000132432.13 0.361901918 7 4 0.187496047
Brain_Nucleus_accumbens_basal_ganglia 0.740120383 Brain_Putamen_basal_ganglia
5.525254838 0.044230743 0.243406402 -0.459425427 1.318022705
0.479082344 0.525908819 4 0 0.998207054
MR1 ENSG00000153029.14 0.362084772 2 2 0.232728125 Brain_Cerebellum
0.377299829 Brain_Nucleus_accumbens_basal_ganglia 1.089223784
0.910776216 0.910776216 0.882882371 1.193360737 1.038121554
0.219541358 2 0 0.998207054
CLK4 ENSG00000113240.12 0.36210808 1 1 0.36210808 Brain_Cerebellum 0.36210808
Brain_Cerebellum 1 1 1 0.911355523 0.911355523 0.911355523
NA 1 0 0.998207054
ANKS3 ENSG00000168096.14 0.362144839 1 1 0.362144839
Brain_Spinal_cord_cervical_c-1 0.362144839 Brain_Spinal_cord_cervical_c-1
1 1 1 0.911285738 0.911285738 0.911285738 NA 1 0
0.998207054
PCNX1 ENSG00000100731.15 0.36217782 7 3 0.071675859
Brain_Spinal_cord_cervical_c-1 0.819191122 Brain_Substantia_nigra
5.588947275 0.013885531 0.379706291 0.228585466 1.801171463
1.211204289 0.5440302 3 0 0.998207054
CAPG ENSG00000042493.15 0.362213646 11 8 0.006940325
Brain_Cerebellar_Hemisphere 0.903058938 Brain_Spinal_cord_cervical_c-1
6.282117998 0.050857146 0.250395384 0.121798075 2.699694144
1.573622779 0.747412638 8 0 0.998207054
AC007383.3 ENSG00000227946.1 0.362217176 2 2 0.172841887
Brain_Nucleus_accumbens_basal_ganglia 0.816467264 Brain_Hypothalamus
1.132107505 0.867892495 0.867892495 -1.36312891 0.232091091
-0.565518909 1.12799088 2 0 0.998207054
PGM3 ENSG00000013375.15 0.362220424 3 3 0.129062098
Brain_Anterior_cingulate_cortex_BA24 0.668214503 Brain_Frontal_Cortex_BA9

1.049372287 0.933697158 0.933697158 -1.517810813 0.882086901
-0.354774565 1.201650898 3 0 0.998207054
CXXC1 ENSG00000154832.14 0.362278569 1 1 0.362278569
Brain_Hypothalamus 0.362278569 Brain_Hypothalamus 1 1 1
-0.911031894 -0.911031894 -0.911031894 NA 1 0 0.998207054
SBK1 ENSG00000188322.4 0.362453268 1 1 0.362453268
Brain_Nucleus_accumbens_basal_ganglia 0.362453268
Brain_Nucleus_accumbens_basal_ganglia 1 1 1 -0.910700373
-0.910700373 -0.910700373 NA 1 0 0.998207054
ILKAP ENSG00000132323.8 0.36259571 1 1 0.36259571 Brain_Putamen_basal_ganglia
0.36259571 Brain_Putamen_basal_ganglia 1 1 1 -0.910430138
-0.910430138 -0.910430138 NA 1 0 0.998207054
ATP6V1G2 ENSG00000213760.10 0.362658749 4 4 0.175586212
Brain_Caudate_basal_ganglia 0.629962328 Brain_Nucleus_accumbens_basal_ganglia
1.967249372 0.415403115 0.415403115 -0.740438883 1.354470836
0.178837161 0.970742587 4 0 0.998207054
ASCL5 ENSG00000232237.3 0.362740862 1 1 0.362740862 Brain_Cerebellum
0.362740862 Brain_Cerebellum 1 1 1 -0.91015483 -0.91015483
-0.91015483 NA 1 0 0.998207054
ZBTB43 ENSG00000169155.9 0.362900545 1 1 0.362900545
Brain_Cortex 0.362900545 Brain_Cortex 1 1 1 0.909852041
0.909852041 0.909852041 NA 1 0 0.998207054
SLC43A1 ENSG00000149150.8 0.36290329 7 4 0.176604074
Brain_Nucleus_accumbens_basal_ganglia 0.886988337 Brain_Hypothalamus
5.545871065 0.004373842 0.212299326 -0.390264489 1.351285253
0.735637136 0.660998491 4 0 0.998207054
GNGT2 ENSG00000167083.6 0.362957638 2 2 0.159738583
Brain_Frontal_Cortex_BA9 0.936462587 Brain_Putamen_basal_ganglia
1.101893832 0.898106168 0.898106168 -0.079716688 1.405951313
0.663117313 1.050525918 2 0 0.998207054
LINC01715 ENSG00000229388.1 0.363060647 1 1 0.363060647
Brain_Spinal_cord_cervical_c-1 0.363060647 Brain_Spinal_cord_cervical_c-1
1 1 1 -0.909548541 -0.909548541 -0.909548541 NA 1 0
0.998207054
MYO16 ENSG00000041515.15 0.363143075 1 1 0.363143075
Brain_Putamen_basal_ganglia 0.363143075 Brain_Putamen_basal_ganglia 1 1
1 0.909392319 0.909392319 0.909392319 NA 1 0
0.998207054
NPIPB8 ENSG00000255524.7 0.363144778 1 1 0.363144778
Brain_Cerebellum 0.363144778 Brain_Cerebellum 1 1 1 -0.909389091
-0.909389091 -0.909389091 NA 1 0 0.998207054
CYBRD1 ENSG00000071967.11 0.363199509 13 3 0.195330409
Brain_Caudate_basal_ganglia 0.969768259 Brain_Amygdala 11.1010979
0.013787815 0.398091555 -1.294970491 0.453970559 -0.498555456
0.498868617 3 0 0.998207054
TLR1 ENSG00000174125.7 0.363239634 1 1 0.363239634
Brain_Caudate_basal_ganglia 0.363239634 Brain_Caudate_basal_ganglia 1 1
1 -0.909209342 -0.909209342 -0.909209342 NA 1 0
0.998207054
AC058791.1 ENSG00000226380.9 0.363251145 7 2 0.188099779
Brain_Cerebellar_Hemisphere 0.69458131 Brain_Cerebellum 6.298820388
0.007326403 0.314214562 0.392645525 1.316221293 0.954740001
0.313092551 2 0 0.998207054
TXNDC17 ENSG00000129235.10 0.363254647 1 1 0.363254647
Brain_Substantia_nigra 0.363254647 Brain_Substantia_nigra 1 1
1 0.909180895 0.909180895 0.909180895 NA 1 0
0.998207054
THOC3 ENSG00000051596.9 0.363395324 11 11 0.022084518
Brain_Putamen_basal_ganglia 0.930870876 Brain_Spinal_cord_cervical_c-1
2.343104247 0.220551715 0.220551715 -1.238364577 2.288911104
0.082039998 1.152504658 11 0 0.998207054
NOC4L ENSG00000184967.6 0.363528224 4 4 0.159515142

Brain_Cerebellar_Hemisphere 0.977353505 Brain_Caudate_basal_ganglia 2.276243071 0.408150374 0.408150374 -1.406704125 1.206056467 0.067292589 1.097108731 4 0 0.998207054
ALDH1L1 ENSG00000144908.13 0.36371373 3 2 0.171298383 Brain_Cerebellum 0.46014325 Brain_Spinal_cord_cervical_c-1 1.97706323 0.045326713 0.977610057 0.738610988 1.368043748 1.093811814 0.322433514 2 0 0.998207054
TLE6 ENSG00000104953.19 0.363725027 1 1 0.363725027 Brain_Cerebellum 0.363725027 Brain_Cerebellum 1 1 1 -0.908289994 -0.908289994 -0.908289994 NA 1 0 0.998207054
MTUS1 ENSG00000129422.14 0.363751438 10 6 0.021655784 Brain_Caudate_basal_ganglia 0.699759432 Brain_Hippocampus 6.649721465 0.016315015 0.22486267 -2.29635164 0.60209098 -1.247904005 0.93333285 6 0 0.998207054
CC2D1B ENSG00000154222.14 0.36376032 6 6 0.071213293 Brain_Cerebellum 0.897122457 Brain_Cerebellar_Hemisphere 2.105513204 0.32725665 0.32725665 -1.804114921 0.863824504 -0.23029541 1.135932074 6 0 0.998207054
TELO2 ENSG00000100726.14 0.363776026 11 9 0.163365446 Brain_Hippocampus 0.962646297 Brain_Frontal_Cortex_BA9 3.509570413 0.049162622 0.425995209 -0.730042702 1.393841591 0.24299714 0.704838819 9 0 0.998207054
PRRX2 ENSG00000167157.10 0.363880688 1 1 0.363880688 Brain_Anterior_cingulate_cortex_BA24 0.363880688 Brain_Anterior_cingulate_cortex_BA24 1 1 1 0.907995332 0.907995332 0.907995332 NA 1 0 0.998207054
SSH2 ENSG00000141298.17 0.364113852 1 1 0.364113852 Brain_Cerebellum 0.364113852 Brain_Cerebellum 1 1 1 -0.907554104 -0.907554104 -0.907554104 NA 1 0 0.998207054
ZNF44 ENSG00000197857.13 0.364115845 6 4 0.116511512 Brain_Caudate_basal_ganglia 0.53892928 Brain_Hippocampus 3.7381468 0.001954315 0.212347402 -0.627412556 1.569585767 0.460381297 0.893211278 4 0 0.998207054
MCUB ENSG00000005059.15 0.36414946 1 1 0.36414946 Brain_Caudate_basal_ganglia 0.36414946 Brain_Caudate_basal_ganglia 1 1 1 0.907486737 0.907486737 0.907486737 NA 1 0 0.998207054
ABCA1 ENSG00000165029.15 0.364280619 1 1 0.364280619 Brain_Spinal_cord_cervical_c-1 0.364280619 Brain_Spinal_cord_cervical_c-1 1 1 1 0.907238631 0.907238631 0.907238631 NA 1 0 0.998207054
PCDHGC4 ENSG00000242419.5 0.364290942 1 1 0.364290942 Brain_Cerebellar_Hemisphere 0.364290942 Brain_Cerebellar_Hemisphere 1 1 1 -0.907219106 -0.907219106 -0.907219106 NA 1 0 0.998207054
COL11A2 ENSG00000204248.10 0.364317593 7 6 0.180498908 Brain_Nucleus_accumbens_basal_ganglia 0.901583199 Brain_Putamen_basal_ganglia 3.208707474 0.089199032 0.165692477 -0.611299142 1.339220477 0.275827497 0.653332904 6 0 0.998207054
RP11-521D12.1 ENSG00000240687.1 0.364321312 1 1 0.364321312 Brain_Substantia_nigra 0.364321312 Brain_Substantia_nigra 1 1 1 -0.907161667 -0.907161667 -0.907161667 NA 1 0 0.998207054
ZNF638 ENSG00000075292.18 0.364524728 1 1 0.364524728 Brain_Frontal_Cortex_BA9 0.364524728 Brain_Frontal_Cortex_BA9 1 1 1 0.906777013 0.906777013 0.906777013 NA 1 0 0.998207054
PMEPA1 ENSG00000124225.15 0.364548355 1 1 0.364548355 Brain_Putamen_basal_ganglia 0.364548355 Brain_Putamen_basal_ganglia 1 1 1 0.906732344 0.906732344 0.906732344 NA 1 0 0.998207054
TH ENSG00000180176.14 0.364631196 1 1 0.364631196 Brain_Hippocampus 0.364631196 Brain_Hippocampus 1 1 1

-0.906575739	-0.906575739	-0.906575739	NA	1	0	0.998207054
PCDHGA9	ENSG00000261934.2	0.364634859	2	2	0.15962619
Brain_Nucleus_accumbens_basal_ganglia 0.392213816	Brain_Caudate_basal_ganglia
1.484025587	0.515974413	0.515974413	-1.406329885	-0.855609496
-1.130969691	0.389418122	2	0	0.998207054
DRAP1 ENSG00000175550.7	0.364646378	1	1	0.364646378	Brain_Cerebellum
0.364646378	Brain_Cerebellum 1	1	1	0.906547042	0.906547042
0.906547042	NA	1	0	0.998207054
FRZB ENSG00000162998.4	0.364780178	2	2	0.178332747
Brain_Caudate_basal_ganglia 0.531118933	Brain_Cortex	1.131480287
0.868519713	0.868519713	0.626298862	1.345906269	0.986102565
0.508839277	2	0	0.998207054
FOLR2 ENSG00000165457.13	0.364871892	1	1	0.364871892
Brain_Putamen_basal_ganglia 0.364871892	Brain_Putamen_basal_ganglia 1	1
1	-0.906120847	-0.906120847	-0.906120847	NA	1	0
0.998207054
TRAPPC2B	ENSG00000256060.2	0.364961135	2	2	0.156946529
Brain_Hippocampus	0.923533501	Brain_Amygdala	1.011253987
0.988746013	0.988746013	-0.095983721	1.415416109	0.659716194
1.068721069	2	0	0.998207054
ZNF365	ENSG00000138311.15	0.364967873	4	3	0.075376247
Brain_Putamen_basal_ganglia 0.962100392	Brain_Amygdala	2.565327721
0.025076613	0.396815881	-1.778168123	-0.047517991	-1.037212225
0.725423921	3	0	0.998207054
R3HDM2	ENSG00000179912.20	0.365010949	1	1	0.365010949
Brain_Cerebellum 0.365010949	Brain_Cerebellum 1	1	1	0.905858129
0.905858129	0.905858129	NA	1	0	0.998207054
LGI3 ENSG00000168481.8	0.365072643	1	1	0.365072643
Brain_Hippocampus	0.365072643	Brain_Hippocampus	1	1	1
0.905741591	0.905741591	0.905741591	NA	1	0	0.998207054
CTA-223H9.9	ENSG00000273424.1	0.3650818 1	1	0.3650818
Brain_Caudate_basal_ganglia 0.3650818 Brain_Caudate_basal_ganglia 1	1	1
0.905724294	0.905724294	0.905724294	NA	1	0	0.998207054
CYP2R1	ENSG00000186104.10	0.365115592	1	1	0.365115592
Brain_Cortex	0.365115592	Brain_Cortex	1	1	1	-0.905660469
-0.905660469	-0.905660469	NA	1	0	0.998207054
SERPINI1	ENSG00000163536.12	0.365182324	6	5	0.110802656
Brain_Spinal_cord_cervical_c-1	0.563654951	Brain_Cerebellum 2.516671377
0.078703902	0.330213389	0.577421207	1.594595783	1.110944396
0.357019313	5	0	0.998207054
CNNM3 ENSG00000168763.15	0.365261588	1	1	0.365261588
Brain_Substantia_nigra	0.365261588	Brain_Substantia_nigra	1	1
1	0.905384757	0.905384757	0.905384757	NA	1	0
0.998207054
PPP1R32	ENSG00000162148.10	0.365263881	2	2	0.157401872
Brain_Cerebellum 0.984496288	Brain_Caudate_basal_ganglia 1.073390001
0.926609999	0.926609999	-1.413863882	0.019432245	-0.697215819
1.013493411	2	0	0.998207054
FAM120AOS	ENSG00000188938.15	0.365267569	4	4	0.115734044
Brain_Hypothalamus	0.833667451	Brain_Cortex	1.173858697
0.745934645	0.745934645	-1.572934248	1.271501781	-0.14420826
1.195459319	4	0	0.998207054
RNF7 ENSG00000114125.13	0.365317466	8	7	0.10950966
Brain_Frontal_Cortex_BA9	0.914659518	Brain_Hippocampus	5.036014434
0.078076983	0.226127985	0.107163189	1.600400969	0.731179889
0.586285029	7	0	0.998207054
ARHGEF1	ENSG00000076928.17	0.365425117	1	1	0.365425117
Brain_Cortex	0.365425117	Brain_Cortex	1	1	1	-0.905076016
-0.905076016	-0.905076016	NA	1	0	0.998207054
CHST13	ENSG00000180767.9	0.365474618	7	4	0.064343532
Brain_Nucleus_accumbens_basal_ganglia 0.79080256 Brain_Caudate_basal_ganglia
4.951219224	0.044012921	0.378996089	-1.849792017	-0.26526859

-1.259034104 0.65362008 4 0 0.998207054
HIGD1A ENSG00000181061.13 0.365547886 12 2 0.12860969 Brain_Cortex
0.705905685 Brain_Amygdala 11.06212284 0.000292822 0.548670648
-1.519607348 -0.377360543 -1.200127038 0.333472039 2 0
0.998207054
ZNF843 ENSG00000176723.9 0.365561239 2 2 0.514262059
Brain_Cerebellar_Hemisphere 0.882526027 Brain_Cerebellum 1.835340035
0.164659965 0.164659965 -0.147767794 0.65221566 0.252223933
0.565673725 2 0 0.998207054
SYT5 ENSG00000129990.14 0.365658578 4 4 0.054452706 Brain_Cortex
0.769005423 Brain_Substantia_nigra 1.074731721 0.883007277
0.883007277 -1.923217773 0.355753683 -0.521366919 1.077513028
4 0 0.998207054
SPAG7 ENSG00000091640.7 0.365759304 10 1 0.150258904
Brain_Cerebellar_Hemisphere 0.559238077 Brain_Nucleus_accumbens_basal_ganglia
9.624312298 0.001616288 9.624312298 -1.438617574 -0.583973595
-0.889261227 0.240376615 1 0 0.998207054
HYAL2 ENSG00000068001.13 0.365965946 2 2 0.256704036 Brain_Cortex
0.327846207 Brain_Frontal_Cortex_BA9 1.117705416 0.882294584
0.882294584 -1.134215805 -0.978461332 -1.056338569 0.110135044
2 0 0.998207054
GRB2 ENSG00000177885.13 0.365994882 2 2 0.175917031 Brain_Cerebellum
0.54088904 Brain_Amygdala 1.142838463 0.857161537 0.857161537
0.611469144 1.353433972 0.982451558 0.524648361 2 0
0.998207054
TEKT5 ENSG00000153060.7 0.366293246 7 7 0.031053944
Brain_Hypothalamus 0.771352984 Brain_Caudate_basal_ganglia 1.570145826
0.490268671 0.490268671 -2.156380783 0.410577783 -0.883509236
0.829154532 7 0 0.998207054
VPS36 ENSG00000136100.12 0.366392612 4 4 0.039951656
Brain_Nucleus_accumbens_basal_ganglia 0.419728143 Brain_Substantia_nigra
2.571267867 0.261613037 0.261613037 0.806892981 2.054248403
1.335238169 0.552450728 4 0 0.998207054
LINC01909 ENSG00000266258.1 0.366468945 2 2 0.15699419
Brain_Cerebellar_Hemisphere 0.944853464 Brain_Frontal_Cortex_BA9
1.000225496 0.999774504 0.999774504 -1.415253476 0.069171053
-0.673041212 1.049646651 2 0 0.998207054
MRAS ENSG00000158186.12 0.366484294 1 1 0.366484294
Brain_Hypothalamus 0.366484294 Brain_Hypothalamus 1 1 1
0.903078386 0.903078386 0.903078386 NA 1 0 0.998207054
ZNF623 ENSG00000183309.11 0.366500154 7 7 0.170696046
Brain_Putamen_basal_ganglia 0.966741659 Brain_Cortex 3.076648464
0.114589473 0.114589473 -1.304694316 1.369970708 0.116811823
0.966153975 7 0 0.998207054
FSD1 ENSG00000105255.10 0.366639803 6 6 0.03336098 Brain_Hypothalamus
0.902782564 Brain_Substantia_nigra 1.288528903 0.724721498
0.724721498 -0.299435329 2.127711876 0.474638929 0.939248801
6 0 0.998207054
ANXA5 ENSG00000164111.14 0.366670698 13 2 0.093996128
Brain_Nucleus_accumbens_basal_ganglia 0.568879494
Brain_Spinal_cord_cervical_c-1 11.71571255 0.000985317 0.619588776
0.5697025 1.674684613 1.335253838 0.287925512 2 0
0.998207054
SCARA5 ENSG00000168079.16 0.366872576 2 2 0.197489147
Brain_Hippocampus 0.607692191 Brain_Anterior_cingulate_cortex_BA24
1.056450572 0.943549428 0.943549428 -1.288738114 -0.51337048
-0.901054297 0.548267712 2 0 0.998207054
ZNF717 ENSG00000227124.8 0.366884527 9 9 0.105728236
Brain_Spinal_cord_cervical_c-1 0.793328545 Brain_Cerebellar_Hemisphere
2.340169848 0.248582197 0.248582197 -0.869566671 1.617695502
0.084979498 0.842549996 9 0 0.998207054
TMEM245 ENSG00000106771.12 0.366963825 9 4 0.076784777

Brain_Cortex 0.913906924 Brain_Putamen_basal_ganglia 6.474323771
0.015712476 0.3156351 -0.192258944 1.769654123 0.946996037
0.709872396 4 0 0.998207054
GUCY1B3 ENSG00000061918.12 0.366974056 1 1 0.366974056
Brain_Anterior_cingulate_cortex_BA24 0.366974056
Brain_Anterior_cingulate_cortex_BA24 1 1 1 -0.902155904
-0.902155904 -0.902155904 NA 1 0 0.998207054
BTBD1 ENSG00000064726.9 0.367150265 9 7 0.029887053
Brain_Spinal_cord_cervical_c-1 0.979680818 Brain_Caudate_basal_ganglia
5.565548216 0.082454975 0.195614482 -0.588399935 2.171584031
0.533511174 0.781194462 7 0 0.998207054
BCL2L2-PABPN1 ENSG00000258643.5 0.367186691 1 1 0.367186691
Brain_Substantia_nigra 0.367186691 Brain_Substantia_nigra 1 1
1 0.901755636 0.901755636 0.901755636 NA 1 0
0.998207054
MYH9 ENSG00000100345.20 0.367237438 1 1 0.367237438
Brain_Nucleus_accumbens_basal_ganglia 0.367237438
Brain_Nucleus_accumbens_basal_ganglia 1 1 1 -0.901660132
-0.901660132 -0.901660132 NA 1 0 0.998207054
KCNQ2 ENSG00000075043.18 0.367471304 4 4 0.079210215 Brain_Cerebellum
0.373962083 Brain_Hippocampus 2.325869057 0.26469516 0.26469516
-1.755287083 0.889076285 -0.678552151 1.116487591 4 0
0.998207054
DST ENSG00000151914.19 0.367537677 2 2 0.306655781 Brain_Cerebellum
0.322504236 Brain_Caudate_basal_ganglia 1.010960444 0.989039556
0.989039556 -0.989324838 1.022264393 0.016469778 1.422408386
2 0 0.998207054
LSM12 ENSG00000161654.9 0.367662166 1 1 0.367662166 Brain_Amygdala
0.367662166 Brain_Amygdala 1 1 1 -0.90086112 -0.90086112
-0.90086112 NA 1 0 0.998207054
CHRNE ENSG00000108556.7 0.367763649 13 1 0.113617498
Brain_Hypothalamus 0.781634488 Brain_Nucleus_accumbens_basal_ganglia
11.49465933 0.00970351 11.49465933 0.277189648 1.582140506
0.851956525 0.367054801 1 0 0.998207054
AP1M1 ENSG00000072958.8 0.367801886 9 4 0.144668327
Brain_Putamen_basal_ganglia 0.945869251 Brain_Cerebellar_Hemisphere
7.627489073 3.79E-17 0.302720387 -0.06789496 1.45862508
0.338292584 0.43244914 4 0 0.998207054
VASN ENSG00000168140.4 0.367845999 3 3 0.251630627 Brain_Cerebellum
0.467666481 Brain_Caudate_basal_ganglia 1.2358559 0.742245969
0.742245969 -1.146397716 0.93446603 -0.312737586 1.100346374 3
0 0.998207054
B4GALT6 ENSG00000118276.11 0.367933149 1 1 0.367933149
Brain_Substantia_nigra 0.367933149 Brain_Substantia_nigra 1 1
1 0.900351639 0.900351639 0.900351639 NA 1 0
0.998207054
ECHDC1 ENSG00000093144.18 0.368018185 2 2 0.157556692
Brain_Anterior_cingulate_cortex_BA24 0.418263432
Brain_Nucleus_accumbens_basal_ganglia 1.596302136 0.403697864
0.403697864 -1.41333689 -0.809437687 -1.111387289 0.427021221
2 0 0.998207054
RP11-37B2.1 ENSG00000251136.8 0.368064834 2 2 0.204876589
Brain_Cerebellar_Hemisphere 0.507419001 Brain_Cerebellum 1.811403354
0.188596646 0.188596646 -1.267779825 -0.662861906 -0.965320866
0.427741563 2 0 0.998207054
LAMP1 ENSG00000185896.10 0.36814198 1 1 0.36814198 Brain_Putamen_basal_ganglia
0.36814198 Brain_Putamen_basal_ganglia 1 1 1 -0.899959169
-0.899959169 -0.899959169 NA 1 0 0.998207054
BEST1 ENSG00000167995.15 0.368304292 5 2 0.120438645
Brain_Caudate_basal_ganglia 0.364352055 Brain_Cortex 4.370685974
0.009043363 0.545074522 0.907103523 1.552935082 1.21788772
0.248163639 2 0 0.998207054

CCNJL ENSG00000135083.14 0.368487954 2 2 0.188394801 Brain_Cortex 0.651859377 Brain_Spinal_cord_cervical_c-1 1.049037364 0.950962636 0.950962636 -0.451180619 1.315342558 0.432080969 1.249120518 2 0 0.998207054

MRS2 ENSG00000124532.14 0.36849908 12 3 0.424149481 Brain_Putamen_basal_ganglia 0.948008032 Brain_Cortex 9.652840728 0.007434547 0.479272945 -0.799243073 0.683354016 -0.25084467 0.533123251 3 0 0.998207054

SIM2 ENSG00000159263.15 0.368504835 3 2 0.373172757 Brain_Hippocampus 0.480768881 Brain_Cerebellar_Hemisphere 2.142056937 0.062976473 0.79496659 0.705066455 0.890546043 0.776934093 0.099536978 2 0 0.998207054

TMEM65 ENSG00000164983.7 0.368510471 3 3 0.090923682 Brain_Hypothalamus 0.770258355 Brain_Cortex 1.62429132 0.506771995 0.506771995 -0.292036973 1.690545285 0.567580781 1.017188152 3 0 0.998207054

SPATA45 ENSG00000185523.6 0.368579459 6 6 0.072515987 Brain_Cortex 0.80108662 Brain_Hypothalamus 3.043430194 0.225256785 0.225256785 -0.251941062 1.795865018 0.881383288 0.765618963 6 0 0.998207054

ST7 ENSG00000004866.20 0.368659035 3 3 0.118528789 Brain_Cerebellar_Hemisphere 0.476302363 Brain_Caudate_basal_ganglia 1.537783155 0.673311522 0.673311522 -0.712262303 1.560978648 0.705945018 1.236842577 3 0 0.998207054

TDRKH ENSG00000182134.15 0.368689348 5 4 0.238750993 Brain_Hippocampus 0.874601812 Brain_Putamen_basal_ganglia 3.767400054 0.070318541 0.149062839 -0.157815973 1.178114423 0.477971698 0.516678898 4 0 0.998207054

LIPT1 ENSG00000144182.16 0.36872392 11 1 0.199464965 Brain_Hypothalamus 0.650400986 Brain_Cerebellar_Hemisphere 10.1467848 0.002121974 10.1467848 0.453205203 1.283077388 0.858826762 0.249125159 1 0 0.998207054

MGARP ENSG00000137463.4 0.368733967 3 2 0.128846048 Brain_Cerebellum 0.577450021 Brain_Amygdala 1.951690115 0.048009879 1.000300007 0.557113301 1.518668149 1.039773441 0.480788483 2 0 0.998207054

CEP104 ENSG00000116198.12 0.368738083 5 3 0.138582181 Brain_Cerebellum 0.987411087 Brain_Nucleus_accumbens_basal_ganglia 3.865486971 0.026656597 0.289334685 -0.175687698 1.481091545 0.368291233 0.657018532 3 0 0.998207054

FOXP1 ENSG00000114861.18 0.368752793 1 1 0.368752793 Brain_Caudate_basal_ganglia 0.368752793 Brain_Caudate_basal_ganglia 1 1 1 0.898812028 0.898812028 0.898812028 NA 1 0 0.998207054

DAAM2 ENSG00000146122.16 0.368780995 1 1 0.368780995 Brain_Putamen_basal_ganglia 0.368780995 Brain_Putamen_basal_ganglia 1 1 1 -0.89875909 -0.89875909 -0.89875909 NA 1 0 0.998207054

KCNN3 ENSG00000143603.18 0.368940068 9 4 0.168049324 Brain_Cerebellum 0.901019048 Brain_Hypothalamus 6.027138323 0.050336294 0.201106176 -0.751524251 1.378498846 0.227746118 0.718334341 4 0 0.998207054

SCN7A ENSG00000136546.13 0.368968618 6 5 0.118194473 Brain_Substantia_nigra 0.959508973 Brain_Hypothalamus 3.591311182 0.099867955 0.195458441 -0.604952322 1.562397087 0.576046244 0.91841666 5 0 0.998207054

SEC24D ENSG00000150961.14 0.369047229 1 1 0.369047229 Brain_Cerebellum 0.369047229 Brain_Cerebellum 1 1 1 0.898259481 0.898259481 0.898259481 NA 1 0 0.998207054

POLH ENSG00000170734.11 0.36905593 1 1 0.36905593 Brain_Cerebellum 0.36905593 Brain_Cerebellum 1 1 1 0.898243157 0.898243157 0.898243157 NA 1 0 0.998207054

TRIM3 ENSG00000110171.19 0.369089837 1 1 0.369089837 Brain_Cortex
0.369089837 Brain_Cortex 1 1 1 0.898179544 0.898179544
0.898179544 NA 1 0 0.998207054
CRTC2 ENSG00000160741.16 0.36927321 3 3 0.228433847 Brain_Hippocampus
0.926059317 Brain_Cerebellum 1.599445142 0.406917791 0.406917791
-1.204403015 0.092803945 -0.687256944 0.687407904 3 0
0.998207054
ITGB5 ENSG00000082781.11 0.369318162 4 4 0.07904821
Brain_Anterior_cingulate_cortex_BA24 0.944821235 Brain_Cerebellum
1.464056612 0.556852604 0.556852604 -0.862594411 1.756235474
0.287340466 1.096744463 4 0 0.998207054
AGL ENSG00000162688.16 0.369488 7 4 0.046369628
Brain_Putamen_basal_ganglia 0.544102928 Brain_Cerebellum 5.461062807
0.047654589 0.197776491 -0.606620296 1.992013033 1.348819622
0.887452363 4 0 0.998207054
TRIM59 ENSG00000213186.7 0.369591396 1 1 0.369591396
Brain_Cortex 0.369591396 Brain_Cortex 1 1 1 -0.897239005
-0.897239005 -0.897239005 NA 1 0 0.998207054
ZCWPW1 ENSG00000078487.17 0.369607671 13 3 0.379916195
Brain_Cerebellum 0.904492004 Brain_Frontal_Cortex_BA9 11.31029699
0.000749125 0.447630989 -0.87805072 0.857821504 -0.281011912
0.390264995 3 0 0.998207054
DBF4B ENSG00000161692.17 0.369709666 13 3 0.042931748
Brain_Frontal_Cortex_BA9 0.45552201 Brain_Putamen_basal_ganglia 11.49283556
0.001341471 0.395569618 0.746240726 2.024373389 1.491005855
0.367053634 3 0 0.998207054
SPAG6 ENSG00000077327.15 0.369775958 1 1 0.369775958
Brain_Nucleus_accumbens_basal_ganglia 0.369775958
Brain_Nucleus_accumbens_basal_ganglia 1 1 1 -0.896893108
-0.896893108 -0.896893108 NA 1 0 0.998207054
QRICH1 ENSG00000198218.10 0.369805606 7 2 0.046212474
Brain_Hippocampus 0.419660612 Brain_Caudate_basal_ganglia 6.057882266
0.002131355 0.627761027 0.807010191 1.993447432 1.307639994
0.388264976 2 0 0.998207054
OSGIN2 ENSG00000164823.9 0.370063232 8 6 0.074010663
Brain_Amygdala 0.618367496 Brain_Frontal_Cortex_BA9 5.381418552
0.059768269 0.205871732 -1.492681281 1.786547442 0.665935393
0.971482957 6 0 0.998207054
F7 ENSG00000057593.13 0.370120038 9 7 0.034237082
Brain_Cerebellar_Hemisphere 0.939442794 Brain_Cortex 5.723202776
0.041630854 0.202169492 -2.117268324 1.131785942 0.317774114
0.983064765 7 0 0.998207054
C16orf95 ENSG00000260456.6 0.370137669 1 1 0.370137669
Brain_Cortex 0.370137669 Brain_Cortex 1 1 1 -0.896215519
-0.896215519 -0.896215519 NA 1 0 0.998207054
IQCH-AS1 ENSG00000259673.5 0.370225211 1 1 0.370225211
Brain_Cerebellum 0.370225211 Brain_Cerebellum 1 1 1 0.896051588
0.896051588 0.896051588 NA 1 0 0.998207054
QDPR ENSG00000151552.11 0.37025221 5 3 0.268832311 Brain_Cerebellum
0.852690293 Brain_Putamen_basal_ganglia 3.794513116 0.022711537
0.450990274 -0.493117791 1.105755615 -0.052914142 0.657411072
3 0 0.998207054
KRT15 ENSG00000171346.15 0.370364472 1 1 0.370364472
Brain_Spinal_cord_cervical_c-1 0.370364472 Brain_Spinal_cord_cervical_c-1
1 1 1 0.89579086 0.89579086 0.89579086 NA 1 0 0.998207054
APBB3 ENSG00000113108.19 0.370401642 1 1 0.370401642
Brain_Nucleus_accumbens_basal_ganglia 0.370401642
Brain_Nucleus_accumbens_basal_ganglia 1 1 1 -0.89572128
-0.89572128 -0.89572128 NA 1 0 0.998207054
HSPA6 ENSG00000173110.7 0.370601036 2 2 0.188029558
Brain_Cerebellar_Hemisphere 0.570758782 Brain_Anterior_cingulate_cortex_BA24
1.049595281 0.950404719 0.950404719 -0.566934354 1.316430598

0.374748122 1.331740129 2 0 0.998207054
TMEM18 ENSG00000151353.14 0.370691102 1 1 0.370691102
Brain_Cerebellum 0.370691102 Brain_Cerebellum 1 1 1 0.895179573
0.895179573 0.895179573 NA 1 0 0.998207054
EPHA10 ENSG00000183317.16 0.370950549 5 5 0.041240174
Brain_Frontal_Cortex_BA9 0.874807793 Brain_Cerebellar_Hemisphere
2.195186821 0.138362756 0.138362756 -2.041107191 0.390409708
-0.815166584 0.974219504 5 0 0.998207054
LL22NC03-2H8.5 ENSG00000274422.1 0.370975866 4 3 0.07637528
Brain_Cortex 0.989348053 Brain_Frontal_Cortex_BA9 2.339477
0.025702573 0.621294214 -1.772116141 -0.013350632 -1.095396178
0.834726037 3 0 0.998207054
CACNA1D ENSG00000157388.15 0.37098961 1 1 0.37098961
Brain_Cerebellar_Hemisphere 0.37098961 Brain_Cerebellar_Hemisphere 1 1 1
-0.894621209 -0.894621209 -0.894621209 NA 1 0 0.998207054
SNX17 ENSG00000115234.10 0.371052843 3 3 0.136071104
Brain_Spinal_cord_cervical_c-1 0.847902202 Brain_Putamen_basal_ganglia
1.830967975 0.222572558 0.222572558 -0.689042462 1.490582642
0.203248144 1.142250478 3 0 0.998207054
PLOD2 ENSG00000152952.11 0.371311436 7 2 0.15894809 Brain_Hippocampus
0.680728419 Brain_Anterior_cingulate_cortex_BA24 5.812757977
0.007185679 0.921428051 0.411469339 1.408618214 0.901919639
0.361329024 2 0 0.998207054
TEN1 ENSG00000257949.6 0.371342897 13 1 0.201615052
Brain_Frontal_Cortex_BA9 0.970926624 Brain_Hippocampus 12.31680364
0.000786613 12.31680364 -1.276963732 0.03644614 -0.873424824
0.331123639 1 0 0.998207054
NELFB ENSG00000188986.6 0.371444727 5 5 0.038712847 Brain_Cortex
0.859204315 Brain_Hypothalamus 1.339372089 0.764521565
0.764521565 -2.067226171 0.414930671 -0.572401565 0.989847609
5 0 0.998207054
ZNF765 ENSG00000196417.12 0.37148076 5 5 0.134767034 Brain_Cortex
0.826485503 Brain_Hypothalamus 2.711106084 0.217286106
0.217286106 -1.495565059 0.952448375 0.147959656 1.034470596
5 0 0.998207054
RP11-161M6.2 ENSG00000260807.6 0.371494439 10 4 0.177325184
Brain_Putamen_basal_ganglia 0.95477268 Brain_Nucleus_accumbens_basal_ganglia
7.33891234 0.002552725 0.343674643 -0.25008097 1.349036682
0.536811515 0.511516321 4 0 0.998207054
KHDC3L ENSG00000203908.3 0.371707531 7 3 0.132579888
Brain_Putamen_basal_ganglia 0.888968041 Brain_Amygdala 5.823743082
0.003205846 0.213385671 -1.504005744 0.254310667 -0.476199273
0.576198485 3 0 0.998207054
SPATS1 ENSG00000249481.6 0.371725255 1 1 0.371725255
Brain_Cortex 0.371725255 Brain_Cortex 1 1 1 -0.893246363
-0.893246363 -0.893246363 NA 1 0 0.998207054
CCL3L3 ENSG00000276085.1 0.371730896 8 6 0.059455587
Brain_Cerebellar_Hemisphere 0.822613688 Brain_Cerebellum 5.613586069
0.037500193 0.199410458 -0.365342868 1.884809313 0.518163978
0.747154357 6 0 0.998207054
PNPT1 ENSG00000138035.14 0.371818302 3 3 0.51770329
Brain_Spinal_cord_cervical_c-1 0.722906651 Brain_Cerebellum 1.895533788
0.276916365 0.276916365 -0.609312572 0.646889766 0.130718002
0.657339831 3 0 0.998207054
C2orf88 ENSG00000187699.10 0.371918154 2 2 0.171838618
Brain_Amygdala 0.584149729 Brain_Caudate_basal_ganglia 1.159508119
0.840491881 0.840491881 -1.366319771 -0.54733336 -0.956826565
0.579110845 2 0 0.998207054
THEG ENSG00000105549.10 0.371979657 1 1 0.371979657 Brain_Cortex
0.371979657 Brain_Cortex 1 1 1 -0.892771304 -0.892771304
-0.892771304 NA 1 0 0.998207054
CYP27A1 ENSG00000135929.8 0.371996465 1 1 0.371996465

Brain_Hypothalamus 0.371996465 Brain_Hypothalamus 1 1 1
0.892739924 0.892739924 0.892739924 NA 1 0 0.998207054
DBF4 ENSG00000006634.7 0.372077498 2 2 0.252427545
Brain_Putamen_basal_ganglia 0.469705319 Brain_Hypothalamus 1.079887834
0.920112166 0.920112166 -0.722958601 1.144472962 0.210757181
1.320473522 2 0 0.998207054
UBE3D ENSG00000118420.16 0.372083754 3 2 0.158095527 Brain_Cortex
0.437018858 Brain_Putamen_basal_ganglia 2.53440623 0.037125981
0.428467789 -1.4115058 -0.777237168 -1.028145199 0.337241659 2
0 0.998207054
TRDMT1 ENSG00000107614.21 0.372175871 5 5 0.026207421
Brain_Hypothalamus 0.933225709 Brain_Cerebellum 3.156293722
0.107248261 0.107248261 -0.316706594 2.223124278 0.964301439
1.106450799 5 0 0.998207054
PLAGL2 ENSG00000126003.6 0.372253782 2 2 0.193808719
Brain_Cortex 0.463621767 Brain_Putamen_basal_ganglia 1.156136222
0.843863778 0.843863778 -1.299394088 -0.732896157 -1.016145123
0.400574529 2 0 0.998207054
CNTN4 ENSG00000144619.14 0.372260484 2 2 0.208052188 Brain_Amygdala
0.452851763 Brain_Cerebellum 1.10192069 0.89807931 0.89807931 0.750669201
1.258939491 1.004804346 0.359401368 2 0 0.998207054
ZNF254 ENSG00000213096.10 0.37232968 5 5 0.091965972 Brain_Cortex
0.725045155 Brain_Hypothalamus 1.817430083 0.255066786
0.255066786 -1.21213637 1.685117147 -0.019588664 1.223052886
5 0 0.998207054
ECSCR ENSG00000249751.3 0.372380388 1 1 0.372380388
Brain_Hypothalamus 0.372380388 Brain_Hypothalamus 1 1 1
0.892023404 0.892023404 0.892023404 NA 1 0 0.998207054
FAM210A ENSG00000177150.12 0.372448389 1 1 0.372448389
Brain_Cerebellum 0.372448389 Brain_Cerebellum 1 1 1 0.89189654
0.89189654 0.89189654 NA 1 0 0.998207054
FGFR1 ENSG00000077782.19 0.372505287 1 1 0.372505287 Brain_Cerebellum
0.372505287 Brain_Cerebellum 1 1 1 0.891790401 0.891790401
0.891790401 NA 1 0 0.998207054
E2F1 ENSG00000101412.12 0.372523529 8 4 0.07268067
Brain_Caudate_basal_ganglia 0.575995526 Brain_Nucleus_accumbens_basal_ganglia
5.663375007 0.011010278 0.194046489 0.559243535 1.794830752
1.422052911 0.416411888 4 0 0.998207054
TPM4 ENSG00000167460.14 0.372552133 2 2 0.235852922
Brain_Hippocampus 0.426830315 Brain_Cerebellar_Hemisphere 1.034066804
0.965933196 0.965933196 0.794627332 1.185416219 0.990021775
0.276329472 2 0 0.998207054
ANKRD42 ENSG00000137494.13 0.372624047 9 5 0.071154485
Brain_Cerebellar_Hemisphere 0.431562463 Brain_Hippocampus 6.508925858
0.006338908 0.276741522 -1.804490255 -0.786520757 -1.319552833
0.345307441 5 0 0.998207054
SLC48A1 ENSG00000211584.13 0.372669417 3 3 0.094497622
Brain_Cerebellum 0.732675938 Brain_Cortex 1.63687817 0.363119011
0.363119011 0.341568112 1.672135419 1.154757238 0.712902816
3 0 0.998207054
PECR ENSG00000115425.13 0.372920725 3 3 0.141664638 Brain_Cortex
0.525076163 Brain_Nucleus_accumbens_basal_ganglia 1.436794901
0.659096122 0.659096122 -1.46962023 -0.635540191 -1.084198982
0.420620515 3 0 0.998207054
CKM ENSG00000104879.4 0.372981612 1 1 0.372981612
Brain_Spinal_cord_cervical_c-1 0.372981612 Brain_Spinal_cord_cervical_c-1
1 1 1 0.890902252 0.890902252 0.890902252 NA 1 0
0.998207054
PGGT1B ENSG00000164219.9 0.373000687 4 4 0.162527619
Brain_Hippocampus 0.767288464 Brain_Frontal_Cortex_BA9 2.92495955
0.124182849 0.124182849 -1.396620843 0.295923552 -0.511525307
0.694359093 4 0 0.998207054

CALHM1 ENSG00000185933.6 0.373346576 1 1 0.373346576
Brain_Frontal_Cortex_BA9 0.373346576 Brain_Frontal_Cortex_BA9 1 1
1 -0.89022222 -0.89022222 -0.89022222 NA 1 0
0.998207054
CHRNB2 ENSG00000160716.5 0.373429945 6 5 0.280605726
Brain_Cerebellar_Hemisphere 0.976149051 Brain_Cerebellum 3.032222569
0.064525916 0.204989424 -1.078959626 0.892898207 -0.056455556
0.757529834 5 0 0.998207054
AC004221.2 ENSG00000267778.1 0.373527903 1 1 0.373527903
Brain_Hypothalamus 0.373527903 Brain_Hypothalamus 1 1 1
-0.889884508 -0.889884508 -0.889884508 NA 1 0 0.998207054
CTNNBL1 ENSG00000132792.18 0.373564902 1 1 0.373564902
Brain_Cerebellum 0.373564902 Brain_Cerebellum 1 1 1 -0.889815613
-0.889815613 -0.889815613 NA 1 0 0.998207054
FAM111A ENSG00000166801.15 0.373618996 1 1 0.373618996
Brain_Amygdala 0.373618996 Brain_Amygdala 1 1 1 -0.889714892
-0.889714892 -0.889714892 NA 1 0 0.998207054
FBXO16 ENSG00000214050.7 0.373635321 1 1 0.373635321
Brain_Cerebellum 0.373635321 Brain_Cerebellum 1 1 1 -0.889684497
-0.889684497 -0.889684497 NA 1 0 0.998207054
TRAK2 ENSG00000115993.12 0.373651618 1 1 0.373651618 Brain_Cerebellum
0.373651618 Brain_Cerebellum 1 1 1 0.889654155 0.889654155
0.889654155 NA 1 0 0.998207054
PPHLN1 ENSG00000134283.17 0.373807797 2 2 0.562900676
Brain_Cerebellum 0.85891459 Brain_Cerebellar_Hemisphere 1.851406226
0.148593774 0.148593774 -0.177756018 0.578538405 0.200391193
0.534780915 2 0 0.998207054
C4orf19 ENSG00000154274.14 0.373857027 2 2 0.19255045 Brain_Cerebellum
0.822638195 Brain_Cerebellar_Hemisphere 1.513940951 0.486059049
0.486059049 0.224153076 1.303071215 0.763612145 0.762910333
2 0 0.998207054
UPK3B ENSG00000243566.6 0.373884756 13 2 0.138834367
Brain_Substantia_nigra 0.401216683 Brain_Cerebellar_Hemisphere
11.55215114 0.003017286 0.435554239 -1.480145702 -0.839450272
-1.07874019 0.229314458 2 0 0.998207054
PIP5K1A ENSG00000143398.19 0.373917526 7 7 0.091853102
Brain_Substantia_nigra 0.781751112 Brain_Nucleus_accumbens_basal_ganglia
2.262673211 0.344348492 0.344348492 -1.437132197 1.685702567
-0.095720973 1.058791547 7 0 0.998207054
CTD-3051D23.1 ENSG00000258811.1 0.37404678 3 3 0.143076138
Brain_Putamen_basal_ganglia 0.297160137 Brain_Hypothalamus 1.99567458
0.06819306 0.06819306 -1.166796207 1.464431312 -0.248302534
1.484571539 3 0 0.998207054
NRG3 ENSG00000185737.12 0.374114299 2 2 0.253119572
Brain_Nucleus_accumbens_basal_ganglia 0.356988397 Brain_Substantia_nigra
1.098371835 0.901628165 0.901628165 0.921118817 1.142804978
1.031961897 0.156755788 2 0 0.998207054
FAM218A ENSG00000250486.3 0.374185692 11 4 0.101921532
Brain_Anterior_cingulate_cortex_BA24 0.416882362 Brain_Cerebellum
8.467463369 0.026472353 0.299164025 -1.635608588 -0.81184189
-1.221595988 0.269600552 4 0 0.998207054
P3H2 ENSG00000090530.9 0.374379453 1 1 0.374379453 Brain_Cerebellum
0.374379453 Brain_Cerebellum 1 1 1 0.888299901 0.888299901
0.888299901 NA 1 0 0.998207054
KCNQ5 ENSG00000185760.15 0.374404599 2 2 0.25783046
Brain_Anterior_cingulate_cortex_BA24 0.407153718 Brain_Cerebellar_Hemisphere
1.001403484 0.998596516 0.998596516 -1.13153386 -0.828912859
-0.98022336 0.213985362 2 0 0.998207054
CRTC1 ENSG00000105662.15 0.374416302 2 1 0.357719711 Brain_Cerebellum
0.406390896 Brain_Cerebellar_Hemisphere 1.940815094 0.059184906
1.940815094 -0.919718829 -0.830261597 -0.874990213 0.063255815
1 0 0.998207054

RP11-236B18.5	ENSG00000273124.1	0.374418251	1	1	0.374418251
Brain_Anterior_cingulate_cortex_BA24	0.374418251
Brain_Anterior_cingulate_cortex_BA24	1	1	1	0.888227757
0.888227757	0.888227757	NA	1	0	0.998207054
RP5-832C2.5	ENSG00000215014.4	0.374435148	2	2	0.206767494
Brain_Nucleus_accumbens_basal_ganglia	0.453585056	Brain_Putamen_basal_ganglia
1.11450107	0.88549893	0.88549893	-1.262503982	-0.749451607	-1.005977795
0.362782813	2	0	0.998207054
RP11-222K16.1	ENSG00000271943.1	0.37448499	5	5	0.102067193
Brain_Cortex	0.971968562	Brain_Hypothalamus	1.801821126
0.490321356	0.490321356	-1.063452783	1.634913446	0.346309108
0.980951399	5	0	0.998207054
RCBTB1	ENSG00000136144.11	0.37466923	10	5	0.056773849	Brain_Amygdala
0.828702292	Brain_Nucleus_accumbens_basal_ganglia	7.015189468
0.037960654	0.277842599	-1.905047869	0.822723875	-0.703015176
0.736656615	5	0	0.998207054
C5	ENSG00000106804.7	0.374763431	3	3	0.291730053
Brain_Caudate_basal_ganglia	0.881211586	Brain_Cerebellum	2.446100199
0.141617618	0.141617618	-1.054333944	-0.149433492	-0.574664402
0.454899858	3	0	0.998207054
HRASLS2	ENSG00000133328.3	0.374842877	3	3	0.176104525
Brain_Cortex	0.807751635	Brain_Hippocampus	1.328150968
0.817363214	0.817363214	0.243327587	1.352846966	0.829428578
0.557409315	3	0	0.998207054
ASIC1	ENSG00000110881.11	0.374846569	2	2	0.198639467	Brain_Cortex
0.467626987	Brain_Frontal_Cortex_BA9	1.815681551	0.184318449
0.184318449	-1.285437435	-0.72634551	-1.005891473	0.395337692
2	0	0.998207054
POP5	ENSG00000167272.10	0.374894305	10	7	0.078829727
Brain_Cerebellar_Hemisphere	0.953135639	Brain_Hippocampus	6.336784842
0.042751287	0.220949757	-0.548879661	1.757517	0.451772888
0.684703814	7	0	0.998207054
SRD5A2	ENSG00000277893.1	0.37503128	1	1	0.37503128	Brain_Cerebellum
0.37503128	Brain_Cerebellum	1	1	1	-0.887088454	-0.887088454
-0.887088454	NA	1	0	0.998207054
AC092171.4	ENSG00000230733.2	0.375056723	2	2	0.163509761
Brain_Caudate_basal_ganglia	0.887525744	Brain_Hypothalamus	1.000282418
0.999717582	0.999717582	-1.393363953	0.141435711	-0.625964121
1.08526725	2	0	0.998207054
SPCS1	ENSG00000114902.13	0.375061879	4	4	0.071676094
Brain_Frontal_Cortex_BA9	0.627230998	Brain_Cortex	1.918049774
0.31111223	0.31111223	-1.80116997	1.139685223	-0.603259274
1.281380479	4	0	0.998207054
MPZL3	ENSG00000160588.9	0.375145231	1	1	0.375145231
Brain_Hypothalamus	0.375145231	Brain_Hypothalamus	1	1	1
-0.886876805	-0.886876805	-0.886876805	NA	1	0	0.998207054
DNLZ	ENSG00000213221.4	0.375150914	13	3	0.283466017
Brain_Spinal_cord_cervical_c-1	0.948367517
Brain_Nucleus_accumbens_basal_ganglia	10.40914806	0.021651273
0.546379427	-0.984096566	1.07256571	-0.165015078	0.529432004	3
0	0.998207054
CDK13	ENSG00000065883.14	0.37519722	2	2	0.184932594	Brain_Hippocampus
0.747940381	Brain_Putamen_basal_ganglia	1.095949701	0.904050299
0.904050299	-0.321356319	1.325719595	0.502181638	1.164658548
2	0	0.998207054
RAB2A	ENSG00000104388.14	0.375225119	2	2	0.172644468
Brain_Cerebellar_Hemisphere	0.776079305	Brain_Spinal_cord_cervical_c-1
1.023975652	0.976024348	0.976024348	-1.363755696	-0.284432045
-0.82409387	0.763197073	2	0	0.998207054
POT1	ENSG00000128513.14	0.375355046	3	3	0.2430407	Brain_Cortex
0.725270532	Brain_Hippocampus	1.938723051	0.506822518
0.506822518	-0.351423664	1.167420244	0.590565955	0.822626623

3	0	0.998207054
PLXNA1	ENSG00000114554.11	0.375498109	2	2	0.347596635
Brain_Cerebellum	0.64746896	Brain_Cerebellar_Hemisphere	1.919955253
0.080044747	0.080044747	-0.939261237	-0.457281174	-0.698271205
0.340811371	2	0	0.998207054
CTD-3162L10.1	ENSG00000267053.6	0.375595968	4	2	0.437116368
Brain_Cortex	0.883569483	Brain_Hypothalamus	3.603815607
0.026260609	0.297355241	-0.777071873	0.2944517	-0.226996055
0.441087832	2	0	0.998207054
APP	ENSG00000142192.20	0.375650496	1	1	0.375650496
Brain_Cerebellar_Hemisphere	0.375650496	Brain_Cerebellar_Hemisphere	1	1
1	0.885938819	0.885938819	0.885938819	NA	1	0
0.998207054
LINC00205	ENSG00000223768.1	0.375654184	13	3	0.21477515	Brain_Cerebellum
0.970753163	Brain_Amygdala	10.87606263	0.000801602	0.563631865
-1.240541564	0.46159295	-0.715408951	0.509236212	3	0
0.998207054
RFX1	ENSG00000132005.8	0.375670184	1	1	0.375670184
Brain_Hippocampus	0.375670184	Brain_Hippocampus	1	1	1
-0.885902287	-0.885902287	-0.885902287	NA	1	0	0.998207054
7-Mar	ENSG00000136536.14	0.375720144	1	1	0.375720144	Brain_Amygdala
0.375720144	Brain_Amygdala	1	1	1	0.885809585	0.885809585
0.885809585	NA	1	0	0.998207054
RPL9	ENSG00000163682.15	0.375907456	13	1	0.32231109
Brain_Anterior_cingulate_cortex_BA24	0.440687401	Brain_Hypothalamus
12.83560606	4.55E-05	12.83560606	-0.989719809	-0.771032951
-0.880104099	0.052665594	1	0	0.998207054
PSMD3	ENSG00000108344.14	0.375937941	1	1	0.375937941	Brain_Cerebellum
0.375937941	Brain_Cerebellum	1	1	1	-0.885405548	-0.885405548
-0.885405548	NA	1	0	0.998207054
RTTN	ENSG00000176225.13	0.37597893	2	2	0.211393044
Brain_Substantia_nigra	0.490622211	Brain_Cerebellum	1.049555791
0.950444209	0.950444209	-1.249744094	0.68931953	-0.280212282
1.371125037	2	0	0.998207054
AC008060.8	ENSG00000236544.1	0.376018053	2	2	0.170066843
Brain_Cerebellum	0.875341954	Brain_Cerebellar_Hemisphere	1.083088311
0.916911689	0.916911689	-1.371989061	0.156876788	-0.607556137
1.081071409	2	0	0.998207054
RP9	ENSG00000164610.8	0.376030728	10	1	0.218295067	Brain_Amygdala
0.860878649	Brain_Caudate_basal_ganglia	9.160560664	0.003493031
9.160560664	0.175255789	1.231074325	0.84899713	0.277924862	1
0	0.998207054
TICAM1	ENSG00000127666.9	0.376104769	2	1	0.375578287
Brain_Caudate_basal_ganglia	0.376658915	Brain_Cortex	1.999886098
0.000113902	1.999886098	0.884069095	0.886072823	0.885070959
0.00141685	1	0	0.998207054
KIF1B	ENSG00000054523.17	0.376118563	2	2	0.445729838
Brain_Hippocampus	0.959262334	Brain_Spinal_cord_cervical_c-1
1.857611649	0.142388351	0.142388351	0.051079296	0.762553312
0.406816304	0.503088101	2	0	0.998207054
PDE5A	ENSG00000138735.15	0.376127082	1	1	0.376127082	Brain_Cortex
0.376127082	Brain_Cortex	1	1	1	0.88505479	0.88505479	0.88505479
NA	1	0	0.998207054
DGCR6L	ENSG00000128185.9	0.376233581	3	3	0.151972652
Brain_Cerebellum	0.562626418	Brain_Cerebellar_Hemisphere	1.249262677
0.766909176	0.766909176	-1.432598356	0.627352328	-0.075433742
1.175588221	3	0	0.998207054
SPDYE6	ENSG00000260097.2	0.376271902	2	2	0.162259053
Brain_Cortex	0.666449998	Brain_Cerebellar_Hemisphere	1.337326104
0.662673896	0.662673896	0.431025268	1.397514023	0.914269645
0.683410753	2	0	0.998207054
RNF38	ENSG00000137075.17	0.376339473	1	1	0.376339473

Brain_Frontal_Cortex_BA9 0.376339473 Brain_Frontal_Cortex_BA9 1 1
1 -0.884661043 -0.884661043 -0.884661043 NA 1 0
0.998207054
STX6 ENSG00000135823.13 0.376374069 11 4 0.14339777
Brain_Frontal_Cortex_BA9 0.998960237 Brain_Cerebellar_Hemisphere 7.87236183
0.005356496 0.555493751 -0.910618697 1.463254436 0.249804766
0.565543828 4 0 0.998207054
CXCL14 ENSG00000145824.12 0.376398816 2 2 0.224637765
Brain_Cerebellum 0.254188152 Brain_Hippocampus 1.421869348
0.578130652 0.578130652 -1.214288004 -1.140235618 -1.177261811
0.052362945 2 0 0.998207054
RNF112 ENSG00000128482.15 0.376418527 3 3 0.225807423
Brain_Caudate_basal_ganglia 0.729684122 Brain_Cerebellum 1.75439822
0.417488584 0.417488584 -0.977011383 1.211229597 -0.037109179
1.126254627 3 0 0.998207054
CRNDE ENSG00000245694.9 0.376474376 7 2 0.629064736
Brain_Anterior_cingulate_cortex_BA24 0.878266974 Brain_Putamen_basal_ganglia
6.507877729 0.020851138 0.26766814 -0.461383594 0.483043661
-0.189210523 0.317178468 2 0 0.998207054
ZSCAN12 ENSG00000158691.14 0.376758008 1 1 0.376758008
Brain_Cerebellum 0.376758008 Brain_Cerebellum 1 1 1 -0.883885533
-0.883885533 -0.883885533 NA 1 0 0.998207054
ERCC8 ENSG00000049167.13 0.376784557 1 1 0.376784557
Brain_Putamen_basal_ganglia 0.376784557 Brain_Putamen_basal_ganglia 1 1
1 0.883836358 0.883836358 0.883836358 NA 1 0
0.998207054
BUB1 ENSG00000169679.14 0.376865977 2 2 0.169588428
Brain_Caudate_basal_ganglia 0.592818541 Brain_Frontal_Cortex_BA9
1.207539903 0.792460097 0.792460097 0.534756075 1.373527468
0.954141771 0.59310094 2 0 0.998207054
IL10RB ENSG00000243646.9 0.377039499 13 5 0.341786246
Brain_Caudate_basal_ganglia 0.827371173 Brain_Nucleus_accumbens_basal_ganglia
10.0394323 0.004810005 0.377437831 -0.448761182 0.950641791
0.375176561 0.536774651 5 0 0.998207054
PHLDA3 ENSG00000174307.6 0.377046214 7 6 0.046746523
Brain_Putamen_basal_ganglia 0.791500682 Brain_Hypothalamus 2.283355606
0.008854142 0.696951939 -1.988589572 -0.2643624 -1.121658799
0.752025528 6 0 0.998207054
RAB3C ENSG00000152932.7 0.37705296 1 1 0.37705296 Brain_Caudate_basal_ganglia
0.37705296 Brain_Caudate_basal_ganglia 1 1 1 -0.88333933
-0.88333933 -0.88333933 NA 1 0 0.998207054
TYW1 ENSG00000198874.12 0.377133979 12 1 0.104660191 Brain_Cerebellum
0.866046372 Brain_Hypothalamus 11.20778812 0.001627914
11.20778812 -1.622668955 -0.168682516 -0.854807108 0.329058312
1 0 0.998207054
COL24A1 ENSG00000171502.14 0.377149352 4 3 0.416763899
Brain_Cortex 0.80097741 Brain_Caudate_basal_ganglia 3.277071556
0.061128675 0.223260105 -0.812048333 0.481990699 0.058613834
0.58854442 3 0 0.998207054
PTGR2 ENSG00000140043.11 0.377378428 13 3 0.140072036
Brain_Cerebellar_Hemisphere 0.92490818 Brain_Frontal_Cortex_BA9 9.330705287
0.003870274 0.573574731 -1.475522826 1.358175785 0.453299654
0.876916751 3 0 0.998207054
MAP3K14 ENSG00000006062.14 0.377400058 2 2 0.287517369
Brain_Cerebellar_Hemisphere 0.295854739 Brain_Cerebellum 1.141183159
0.858816841 0.858816841 1.045364065 1.063583612 1.054473839
0.012883166 2 0 0.998207054
PVT1 ENSG00000249859.9 0.377405722 2 2 0.231569633
Brain_Caudate_basal_ganglia 0.674088732 Brain_Nucleus_accumbens_basal_ganglia
1.233296854 0.766703146 0.766703146 -1.196325315 0.420543125
-0.387891095 1.143298638 2 0 0.998207054
LINC00645 ENSG00000258548.5 0.377433829 2 2 0.175821754

Brain_Putamen_basal_ganglia 0.756220617 Brain_Hypothalamus 1.022340961
0.977659039 0.977659039 -1.353732443 -0.31044758 -0.832090012
0.737713802 2 0 0.998207054
POLR2E ENSG00000099817.11 0.377433854 9 7 0.132147179
Brain_Frontal_Cortex_BA9 0.786042486 Brain_Putamen_basal_ganglia 3.02125202
0.025787305 0.35292812 -1.427938665 1.505688415 -0.369435361
1.105776599 7 0 0.998207054
FLCN ENSG00000154803.12 0.377457976 10 7 0.154582789
Brain_Frontal_Cortex_BA9 0.940897475 Brain_Nucleus_accumbens_basal_ganglia
5.783749875 0.012982705 0.329552136 -1.42352925 1.129069604
-0.277265104 0.834444916 7 0 0.998207054
NXNL2 ENSG00000130045.15 0.377483581 1 1 0.377483581
Brain_Substantia_nigra 0.377483581 Brain_Substantia_nigra 1 1
1 -0.882542363 -0.882542363 -0.882542363 NA 1 0
0.998207054
MOB3A ENSG00000172081.13 0.377496644 2 2 0.280455609 Brain_Cerebellum
0.390918512 Brain_Caudate_basal_ganglia 1.024951969 0.975048031
0.975048031 -1.079296419 0.857952826 -0.110671796 1.369842078
2 0 0.998207054
FALEC ENSG00000228126.1 0.377567864 2 2 0.21347323 Brain_Hypothalamus
0.398991685 Brain_Frontal_Cortex_BA9 1.177462745 0.822537255
0.822537255 -1.244071552 -0.843423407 -1.04374748 0.28330102 2
0 0.998207054
RPS25 ENSG00000118181.10 0.377569461 9 2 0.213790632
Brain_Nucleus_accumbens_basal_ganglia 0.688057067
Brain_Spinal_cord_cervical_c-1 8.316053832 7.17E-06 0.461754678
-1.243209529 -0.401493169 -0.703635935 0.273540404 2 0
0.998207054
CLDN16 ENSG00000113946.3 0.377616058 3 3 0.153344979
Brain_Amygdala 0.743157374 Brain_Frontal_Cortex_BA9 1.214271069
0.786250163 0.786250163 -0.998984601 1.427815468 0.033718607
1.253113592 3 0 0.998207054
COG7 ENSG00000168434.12 0.377649736 2 2 0.162863996
Brain_Caudate_basal_ganglia 0.964766478 Brain_Cerebellum 1.023007596
0.976992404 0.976992404 -1.395503712 -0.044173032 -0.719838372
0.955535088 2 0 0.998207054
MLF2 ENSG00000089693.10 0.377681057 1 1 0.377681057 Brain_Cerebellum
0.377681057 Brain_Cerebellum 1 1 1 0.882177075 0.882177075
0.882177075 NA 1 0 0.998207054
CTD-2017D11.1 ENSG00000268362.5 0.377697981 11 2 0.113907198
Brain_Hippocampus 0.517190943 Brain_Cerebellum 9.701963863
0.003947373 0.81348959 0.647681545 1.580872478 1.287705168
0.276402465 2 0 0.998207054
MAF ENSG00000178573.6 0.377752784 1 1 0.377752784
Brain_Caudate_basal_ganglia 0.377752784 Brain_Caudate_basal_ganglia 1 1
1 -0.882044425 -0.882044425 -0.882044425 NA 1 0
0.998207054
CTC-591M7.1 ENSG00000283213.1 0.377885491 2 2 0.206047012
Brain_Cortex 0.85698241 Brain_Frontal_Cortex_BA9 1.535991262
0.464008738 0.464008738 -1.264510059 -0.180216749 -0.722363404
0.766711153 2 0 0.998207054
PHF7 ENSG00000010318.20 0.378013033 1 1 0.378013033
Brain_Putamen_basal_ganglia 0.378013033 Brain_Putamen_basal_ganglia 1 1
1 0.881563256 0.881563256 0.881563256 NA 1 0
0.998207054
EXTL3 ENSG00000012232.8 0.37803009 1 1 0.37803009 Brain_Substantia_nigra
0.37803009 Brain_Substantia_nigra 1 1 1 -0.881531725
-0.881531725 -0.881531725 NA 1 0 0.998207054
LIPC ENSG00000166035.10 0.378222756 2 2 0.206320801
Brain_Putamen_basal_ganglia 0.566921559 Brain_Caudate_basal_ganglia
1.013322992 0.986677008 0.986677008 0.572591142 1.263747134
0.918169138 0.488721089 2 0 0.998207054

PKD2L1 ENSG00000107593.16 0.378380227 6 6 0.097204483 Brain_Hypothalamus 0.747763536 Brain_Caudate_basal_ganglia 2.400099162 0.098147029 0.098147029 -1.658560021 1.140293291 -0.591301404 0.964141316 6 0 0.998207054
TWIST1 ENSG00000122691.12 0.378458999 1 1 0.378458999 Brain_Hippocampus 0.378458999 Brain_Hippocampus 1 1 1 0.880739192 0.880739192 0.880739192 NA 1 0 0.998207054
EEF1A2 ENSG00000101210.10 0.378560994 1 1 0.378560994 Brain_Nucleus_accumbens_basal_ganglia 0.378560994 Brain_Nucleus_accumbens_basal_ganglia 1 1 1 -0.880550808 -0.880550808 -0.880550808 NA 1 0 0.998207054
BANF1 ENSG00000175334.7 0.378695668 1 1 0.378695668 Brain_Cortex 0.378695668 Brain_Cortex 1 1 1 -0.880302114 -0.880302114 -0.880302114 NA 1 0 0.998207054
RBCK1 ENSG00000125826.20 0.378830539 1 1 0.378830539 Brain_Cerebellum 0.378830539 Brain_Cerebellum 1 1 1 -0.880053111 -0.880053111 -0.880053111 NA 1 0 0.998207054
CA5A ENSG00000174990.4 0.378861115 9 5 0.034083446 Brain_Cerebellum 0.885893367 Brain_Nucleus_accumbens_basal_ganglia 5.448255124 0.014734801 0.310058395 -0.143502462 2.11908308 0.671510399 0.640135599 5 0 0.998207054
TPT1 ENSG00000133112.16 0.37887289 1 1 0.37887289 Brain_Putamen_basal_ganglia 0.37887289 Brain_Putamen_basal_ganglia 1 1 1 -0.879974932 -0.879974932 -0.879974932 NA 1 0 0.998207054
CPA1 ENSG00000091704.9 0.378943765 1 1 0.378943765 Brain_Hippocampus 0.378943765 Brain_Hippocampus 1 1 1 -0.879844111 -0.879844111 -0.879844111 NA 1 0 0.998207054
RP4-794H19.1 ENSG00000232453.6 0.378979645 1 1 0.378979645 Brain_Anterior_cingulate_cortex_BA24 0.378979645 Brain_Anterior_cingulate_cortex_BA24 1 1 1 0.879777888 0.879777888 0.879777888 NA 1 0 0.998207054
CMTM1 ENSG00000089505.17 0.379005832 1 1 0.379005832 Brain_Anterior_cingulate_cortex_BA24 0.379005832 Brain_Anterior_cingulate_cortex_BA24 1 1 1 0.879729561 0.879729561 0.879729561 NA 1 0 0.998207054
RD3 ENSG00000198570.5 0.379053373 1 1 0.379053373 Brain_Nucleus_accumbens_basal_ganglia 0.379053373 Brain_Nucleus_accumbens_basal_ganglia 1 1 1 0.879641825 0.879641825 0.879641825 NA 1 0 0.998207054
MADD ENSG00000110514.19 0.379315011 6 5 0.219173555 Brain_Cerebellum 0.99603966 Brain_Cortex 3.626202225 0.029083884 0.150642218 -1.228728666 -0.00496357 -0.571986318 0.400044051 5 0 0.998207054
LINC01588 ENSG00000214900.9 0.379379017 1 1 0.379379017 Brain_Nucleus_accumbens_basal_ganglia 0.379379017 Brain_Nucleus_accumbens_basal_ganglia 1 1 1 0.879041052 0.879041052 0.879041052 NA 1 0 0.998207054
CCNL1 ENSG00000163660.11 0.379380396 1 1 0.379380396 Brain_Nucleus_accumbens_basal_ganglia 0.379380396 Brain_Nucleus_accumbens_basal_ganglia 1 1 1 0.879038509 0.879038509 0.879038509 NA 1 0 0.998207054
CCDC90B ENSG00000137500.9 0.379413114 11 3 0.097750341 Brain_Frontal_Cortex_BA9 0.63763725 Brain_Cerebellum 9.059443409 0.006950409 0.435019593 0.47100488 1.655859176 1.240869181 0.290205501 3 0 0.998207054
IMMP1L ENSG00000148950.9 0.379439057 1 1 0.379439057 Brain_Spinal_cord_cervical_c-1 0.379439057 Brain_Spinal_cord_cervical_c-1 1 1 1 -0.878930321 -0.878930321 -0.878930321 NA 1 0 0.998207054
INTS10 ENSG00000104613.11 0.379575885 7 4 0.094728295 Brain_Spinal_cord_cervical_c-1 0.980750818 Brain_Cortex 5.188619417 0.029264277 0.198904766 -1.670966502 0.139462819 -0.482191593

0.659821098 4 0 0.998207054
LINC01829 ENSG00000236780.5 0.379601465 4 3 0.081396184
Brain_Nucleus_accumbens_basal_ganglia 0.325236762
Brain_Anterior_cingulate_cortex_BA24 3.05281745 0.097830753 0.39102274
0.98375343 1.742641915 1.399399755 0.331646052 3 0
0.998207054
LPGAT1 ENSG00000123684.12 0.379607435 1 1 0.379607435
Brain_Cerebellar_Hemisphere 0.379607435 Brain_Cerebellar_Hemisphere 1 1
1 -0.87861984 -0.87861984 -0.87861984 NA 1 0
0.998207054
ILF3 ENSG00000129351.17 0.379681142 1 1 0.379681142
Brain_Caudate_basal_ganglia 0.379681142 Brain_Caudate_basal_ganglia 1 1
1 0.878483953 0.878483953 0.878483953 NA 1 0
0.998207054
ACTR3 ENSG00000115091.11 0.379685126 2 2 0.167415964 Brain_Cerebellum
0.653044551 Brain_Hippocampus 1.200897725 0.799102275
0.799102275 -1.380554557 -0.449536685 -0.915045621 0.658329051
2 0 0.998207054
FXYD6 ENSG00000137726.16 0.379766737 2 2 0.221107677
Brain_Substantia_nigra 0.536447975 Brain_Cerebellar_Hemisphere
1.036057027 0.963942973 0.963942973 -0.618193226 1.223588032
0.302697403 1.302336017 2 0 0.998207054
LDHC ENSG00000166796.11 0.379824387 13 1 0.266405469 Brain_Cerebellum
0.565766971 Brain_Frontal_Cortex_BA9 12.6208127 0.001205439 12.6208127
0.574296805 1.111378485 0.865139597 0.142070285 1 0
0.998207054
SLC1A6 ENSG00000105143.12 0.379936795 8 4 0.134797221
Brain_Hypothalamus 0.938481912 Brain_Cortex 5.905212513 0.00229031
0.303107432 0.077178039 1.495449305 0.645130114 0.476461662
4 0 0.998207054
RP5-1092A3.5 ENSG00000271398.1 0.379978251 1 1 0.379978251
Brain_Cerebellar_Hemisphere 0.379978251 Brain_Cerebellar_Hemisphere 1 1
1 0.877936369 0.877936369 0.877936369 NA 1 0
0.998207054
KB-208E9.1 ENSG00000272733.1 0.38006924 12 1 0.294057999
Brain_Cerebellar_Hemisphere 0.638899492 Brain_Frontal_Cortex_BA9
11.13460297 0.009559437 11.13460297 0.469238048 1.049261024
0.844310111 0.177143569 1 0 0.998207054
URM1 ENSG00000167118.10 0.380126329 1 1 0.380126329
Brain_Frontal_Cortex_BA9 0.380126329 Brain_Frontal_Cortex_BA9 1 1
1 0.877663553 0.877663553 0.877663553 NA 1 0
0.998207054
COX6A1 ENSG00000111775.2 0.380273223 7 3 0.111934133
Brain_Spinal_cord_cervical_c-1 0.5974544 Brain_Anterior_cingulate_cortex_BA24
5.436321906 0.001096572 0.690672229 0.528064744 1.589559498
0.815887589 0.365982582 3 0 0.998207054
C10orf62 ENSG00000203942.4 0.380295641 2 2 0.293211875
Brain_Hypothalamus 0.382702872 Brain_Cerebellar_Hemisphere 1.035101574
0.964898426 0.964898426 -1.051101718 0.872926994 -0.089087362
1.360493749 2 0 0.998207054
SNUPN ENSG00000169371.13 0.380339279 12 2 0.113730453
Brain_Anterior_cingulate_cortex_BA24 0.67921752 Brain_Cerebellum 10.73839775
1.13E-16 0.446475923 -1.581645797 -0.41353113 -1.092959302
0.276880937 2 0 0.998207054
CYHR1 ENSG00000187954.12 0.380626953 6 5 0.128859516 Brain_Cerebellum
0.977279889 Brain_Nucleus_accumbens_basal_ganglia 2.084558263
0.015235343 0.705376382 -1.51861467 0.877781858 -0.498637157
0.987100471 5 0 0.998207054
FBXO28 ENSG00000143756.11 0.380662994 1 1 0.380662994
Brain_Nucleus_accumbens_basal_ganglia 0.380662994
Brain_Nucleus_accumbens_basal_ganglia 1 1 1 0.87667536 0.87667536
0.87667536 NA 1 0 0.998207054

WDR12 ENSG00000138442.9 0.380737888 3 2 0.164227377 Brain_Amygdala
0.202395867 Brain_Putamen_basal_ganglia 2.886597782 0.006785698
0.10661652 1.2747553 1.390993568 1.347426114 0.063348937 2 0
0.998207054
RP11-436K8.1 ENSG00000231252.2 0.380960917 1 1 0.380960917
Brain_Cortex 0.380960917 Brain_Cortex 1 1 1 0.876127145
0.876127145 0.876127145 NA 1 0 0.998207054
PLAC8 ENSG00000145287.10 0.380965012 10 4 0.085943322
Brain_Substantia_nigra 0.602510469 Brain_Amygdala 8.288348233
0.007287163 0.289382512 0.520793733 1.717196241 1.035836315
0.379622217 4 0 0.998207054
FLYWCH1 ENSG00000059122.16 0.380985932 13 4 0.058852053
Brain_Cortex 0.957975027 Brain_Cerebellum 9.298838289 0.004590549
0.529401467 -1.889296929 0.754278392 0.154330336 0.734949017
4 0 0.998207054
TBC1D15 ENSG00000121749.15 0.381023952 1 1 0.381023952
Brain_Cortex 0.381023952 Brain_Cortex 1 1 1 -0.876011187
-0.876011187 -0.876011187 NA 1 0 0.998207054
PKN1 ENSG00000123143.12 0.381302771 2 1 0.263437976
Brain_Cerebellar_Hemisphere 0.541193425 Brain_Cerebellum 1.950768336
0.049231664 1.950768336 0.611009297 1.118302134 0.864655716
0.358710205 1 0 0.998207054
RP11-180M15.6 ENSG00000275963.1 0.381323883 2 2 0.194037141
Brain_Cerebellar_Hemisphere 0.355864586 Brain_Cerebellum 1.35751141 0.64248859
0.64248859 0.923273709 1.298728439 1.111001074 0.265486586 2
0 0.998207054
CCDC92 ENSG00000119242.8 0.38140213 1 1 0.38140213
Brain_Caudate_basal_ganglia 0.38140213 Brain_Caudate_basal_ganglia 1 1 1
0.875315743 0.875315743 0.875315743 NA 1 0 0.998207054
HSPB7 ENSG00000173641.17 0.381409736 2 2 0.28940775
Brain_Cerebellar_Hemisphere 0.735406576 Brain_Cerebellum 1.812643437
0.187356563 0.187356563 0.337942425 1.059421746 0.698682086
0.51016292 2 0 0.998207054
ACAD8 ENSG00000151498.11 0.381428531 5 5 0.032096379
Brain_Spinal_cord_cervical_c-1 0.835981997 Brain_Substantia_nigra
1.375910906 0.600746181 0.600746181 -2.143208265 0.664114804
-0.224271163 1.139567973 5 0 0.998207054
ABCA10 ENSG00000154263.17 0.381463008 5 5 0.060598767
Brain_Cerebellum 0.959975167 Brain_Hippocampus 2.997477196
0.128918294 0.128918294 -1.876411719 0.050184746 -0.496385004
0.790245315 5 0 0.998207054
GORAB ENSG00000120370.12 0.381463531 11 5 0.226295359 Brain_Cerebellum
0.976974465 Brain_Hippocampus 7.8568559 0.008819248 0.280719063
-0.141549418 1.20995709 0.649544301 0.469556172 5 0
0.998207054
ANKRD61 ENSG00000157999.5 0.381474172 1 1 0.381474172
Brain_Hypothalamus 0.381474172 Brain_Hypothalamus 1 1 1
0.875183312 0.875183312 0.875183312 NA 1 0 0.998207054
THAP4 ENSG00000176946.11 0.381591036 4 4 0.184182534
Brain_Cerebellar_Hemisphere 0.965841984 Brain_Putamen_basal_ganglia
2.476784155 0.357941025 0.357941025 -0.583002768 1.327986598
0.241491408 0.796605796 4 0 0.998207054
ZNF532 ENSG00000074657.13 0.381591219 2 2 0.223623558
Brain_Hypothalamus 0.621917183 Brain_Cerebellum 1.138193895
0.861806105 0.861806105 0.493135026 1.216949165 0.855042095
0.511813886 2 0 0.998207054
ADCK1 ENSG00000063761.15 0.381756183 9 9 0.095358046
Brain_Hippocampus 0.810260498 Brain_Cortex 3.080745851
0.160683202 0.160683202 -1.074130868 1.667786858 -0.189388882
0.884274253 9 0 0.998207054
ADA ENSG00000196839.12 0.381927316 6 6 0.0845789
Brain_Putamen_basal_ganglia 0.735454031 Brain_Spinal_cord_cervical_c-1

2.298680491 0.139150819 0.139150819 -1.724714702 1.41234786
-0.274174093 1.137708178 6 0 0.998207054
ENY2 ENSG00000120533.12 0.38203916 2 2 0.284429329
Brain_Nucleus_accumbens_basal_ganglia 0.427077444 Brain_Cerebellum
1.079783391 0.920216609 0.920216609 -0.794202687 1.070422159
0.138109736 1.318488873 2 0 0.998207054
PRRC1 ENSG00000164244.20 0.382048392 1 1 0.382048392 Brain_Cerebellum
0.382048392 Brain_Cerebellum 1 1 1 0.874128292 0.874128292
0.874128292 NA 1 0 0.998207054
GLB1L2 ENSG00000149328.14 0.382063326 1 1 0.382063326
Brain_Hippocampus 0.382063326 Brain_Hippocampus 1 1 1
-0.874100866 -0.874100866 -0.874100866 NA 1 0 0.998207054
NEK9 ENSG00000119638.12 0.382089481 1 1 0.382089481
Brain_Hypothalamus 0.382089481 Brain_Hypothalamus 1 1 1
-0.874052835 -0.874052835 -0.874052835 NA 1 0 0.998207054
RP11-77K12.9 ENSG00000274220.1 0.382162767 1 1 0.382162767
Brain_Caudate_basal_ganglia 0.382162767 Brain_Caudate_basal_ganglia 1 1
1 0.873918265 0.873918265 0.873918265 NA 1 0
0.998207054
HCAR1 ENSG00000196917.5 0.382318423 1 1 0.382318423 Brain_Cortex
0.382318423 Brain_Cortex 1 1 1 0.873632497 0.873632497
0.873632497 NA 1 0 0.998207054
DHX36 ENSG00000174953.13 0.382323 5 3 0.146475927
Brain_Caudate_basal_ganglia 0.975800026 Brain_Cortex 4.234520641
0.025445357 0.218913222 -1.452092355 -0.030334822 -0.546703894
0.538185106 3 0 0.998207054
DGCR8 ENSG00000128191.15 0.382349755 2 2 0.231016719 Brain_Cerebellum
0.717565954 Brain_Anterior_cingulate_cortex_BA24 1.261146699
0.738853301 0.738853301 0.361713746 1.197743937 0.779728842
0.591162617 2 0 0.998207054
RPL36AL ENSG00000165502.6 0.382359998 13 1 0.27250544
Brain_Caudate_basal_ganglia 0.719841427 Brain_Frontal_Cortex_BA9
12.43868366 0.000183173 12.43868366 -1.097311056 -0.358670731
-0.851258014 0.204871247 1 0 0.998207054
BLOC1S5 ENSG00000188428.19 0.382369783 1 1 0.382369783
Brain_Nucleus_accumbens_basal_ganglia 0.382369783
Brain_Nucleus_accumbens_basal_ganglia 1 1 1 0.87353822 0.87353822
0.87353822 NA 1 0 0.998207054
CTD-2130F23.1 ENSG00000248533.1 0.382450267 2 2 0.165636382
Brain_Cerebellum 0.767187355 Brain_Cerebellar_Hemisphere 1.225992093
0.774007907 0.774007907 0.296055949 1.386362038 0.841208993
0.770962829 2 0 0.998207054
HAP1 ENSG00000173805.15 0.382533462 6 3 0.106522853
Brain_Putamen_basal_ganglia 0.362272802 Brain_Cerebellum 4.376150769
0.002577945 0.360470049 -1.614021086 0.91104284 -1.096397785
0.988117616 3 0 0.998207054
PCDH17 ENSG00000118946.11 0.382689428 1 1 0.382689428
Brain_Substantia_nigra 0.382689428 Brain_Substantia_nigra 1 1
1 0.872951658 0.872951658 0.872951658 NA 1 0
0.998207054
GREB1 ENSG00000196208.13 0.382960078 11 6 0.113500217
Brain_Hippocampus 0.983652272 Brain_Nucleus_accumbens_basal_ganglia
5.850752804 0.00822963 0.295210772 -0.918666328 1.582654574
0.065696602 0.833249987 6 0 0.998207054
HAS1 ENSG00000105509.10 0.383047715 5 5 0.055426722
Brain_Cerebellar_Hemisphere 0.993544316 Brain_Cortex 1.702365256
0.494083522 0.494083522 -1.915516069 -0.008091088 -1.077772917
0.695971019 5 0 0.998207054
CTD-3116E22.8 ENSG00000277587.1 0.383203248 12 2 0.317348519
Brain_Nucleus_accumbens_basal_ganglia 0.985413896 Brain_Cortex
10.94158866 0.000146978 0.477527324 -0.124897168 0.999921459
0.379171102 0.352494253 2 0 0.998207054

MAP4K5 ENSG00000012983.11 0.383237768 2 2 0.407299666
Brain_Cortex 0.499165442 Brain_Putamen_basal_ganglia 1.40745697 0.59254303
0.59254303 -0.828654983 0.675803452 -0.076425765 1.063812762 2
0 0.998207054
FAM131B ENSG00000159784.17 0.383242248 1 1 0.383242248
Brain_Putamen_basal_ganglia 0.383242248 Brain_Putamen_basal_ganglia 1 1
1 -0.871937916 -0.871937916 -0.871937916 NA 1 0
0.998207054
TBX6 ENSG00000149922.10 0.383380566 13 4 0.091474986
Brain_Spinal_cord_cervical_c-1 0.837243003 Brain_Hippocampus
10.02415763 0.009905712 0.392157481 -1.687667959 0.205421271
-0.89173308 0.557862168 4 0 0.998207054
RP11-563J2.3 ENSG00000212743.2 0.38351163 1 1 0.38351163
Brain_Spinal_cord_cervical_c-1 0.38351163 Brain_Spinal_cord_cervical_c-1 1
1 1 0.871444256 0.871444256 0.871444256 NA 1 0
0.998207054
LIFR ENSG00000113594.9 0.383647061 3 3 0.103761787
Brain_Putamen_basal_ganglia 0.970750897 Brain_Cerebellum 1.316689058
0.672130483 0.672130483 -1.626883763 -0.036666528 -0.729640059
0.814550453 3 0 0.998207054
CDHR2 ENSG00000074276.10 0.383768386 3 2 0.187824653
Brain_Caudate_basal_ganglia 0.368755141 Brain_Cortex 2.133005282
0.039942729 0.827051989 -1.317041683 -0.898807619 -1.145918012
0.219226867 2 0 0.998207054
POLR2I ENSG00000105258.8 0.38387434 10 6 0.048596289
Brain_Nucleus_accumbens_basal_ganglia 0.667904372 Brain_Cortex
6.646734947 0.00191434 0.365067971 -1.972116673 0.486468463
-1.411848497 0.805020619 6 0 0.998207054
C4orf45 ENSG00000164123.6 0.383874682 1 1 0.383874682
Brain_Hippocampus 0.383874682 Brain_Hippocampus 1 1 1
0.870779277 0.870779277 0.870779277 NA 1 0 0.998207054
SYVN1 ENSG00000162298.18 0.383888812 2 2 0.170066779
Brain_Cerebellar_Hemisphere 0.895746181 Brain_Amygdala 1.035705391
0.964294609 0.964294609 -0.131036819 1.371989267 0.620476224
1.062799938 2 0 0.998207054
ATP5G2 ENSG00000135390.17 0.383917116 13 1 0.219395218
Brain_Spinal_cord_cervical_c-1 0.954400078 Brain_Amygdala 12.29671525
5.41E-05 12.29671525 -1.228137868 0.057182175 -0.841999835
0.329862896 1 0 0.998207054
DEUP1 ENSG00000165325.13 0.383958428 2 2 0.323664143
Brain_Spinal_cord_cervical_c-1 0.772966305
Brain_Nucleus_accumbens_basal_ganglia 1.834176663 0.165823337
0.165823337 0.288497029 0.986956136 0.637726582 0.493885171
2 0 0.998207054
ZNF700 ENSG00000196757.7 0.383959182 1 1 0.383959182
Brain_Cerebellar_Hemisphere 0.383959182 Brain_Cerebellar_Hemisphere 1 1
1 0.87062456 0.87062456 0.87062456 NA 1 0 0.998207054
AFF4 ENSG00000072364.12 0.384077859 3 2 0.292069463
Brain_Hippocampus 0.923527203 Brain_Cerebellar_Hemisphere 2.612950213
0.08134523 0.305704556 -1.053592634 -0.095991651 -0.595193711
0.480102679 2 0 0.998207054
ARGLU1 ENSG00000134884.13 0.384131298 1 1 0.384131298
Brain_Putamen_basal_ganglia 0.384131298 Brain_Putamen_basal_ganglia 1 1
1 0.870309484 0.870309484 0.870309484 NA 1 0
0.998207054
TNNI3 ENSG00000129991.12 0.38428281 4 4 0.258046331
Brain_Anterior_cingulate_cortex_BA24 0.828629338 Brain_Amygdala
2.108457898 0.271729177 0.271729177 -0.216459825 1.131020813
0.644542483 0.602982436 4 0 0.998207054
NXN ENSG00000167693.16 0.384337397 4 4 0.104963289
Brain_Putamen_basal_ganglia 0.983037134 Brain_Cerebellum 1.732960852
0.351054712 0.351054712 -1.621253473 0.021261401 -0.80567285

0.728480769	4	0	0.998207054
FAM209B	ENSG00000213714.1	0.384407793	4	4	0.065737507
Brain_Substantia_nigra	0.879022031	Brain_Hippocampus	1.060614015
0.953211459	0.953211459	-1.840209358	0.817328197	-0.12457619
1.176738994	4	0	0.998207054
TCERG1L	ENSG00000176769.9	0.384535742	5	5	0.200028723
Brain_Nucleus_accumbens_basal_ganglia	0.975172669	Brain_Putamen_basal_ganglia
1.980023909	0.074509999	0.074509999	-1.281469738	1.247532901
0.078969157	0.981087826	5	0	0.998207054
RSPRY1	ENSG00000159579.13	0.384536985	3	3	0.180685265
Brain_Caudate_basal_ganglia	0.382561948	Brain_Frontal_Cortex_BA9
1.432304628	0.618975721	0.618975721	-1.338648084	0.873185553
-0.447906142	1.167027289	3	0	0.998207054
SLC9A3R1	ENSG00000109062.11	0.384575938	2	1	0.296982782
Brain_Cerebellar_Hemisphere	0.488006955	Brain_Cerebellum	1.994060634
0.005939366	1.994060634	0.693482259	1.042925536	0.868203898
0.247093711	1	0	0.998207054
ERICH5	ENSG00000177459.10	0.384594935	2	2	0.197783928
Brain_Substantia_nigra	0.48545577	Brain_Cerebellar_Hemisphere	1.156277317
0.843722683	0.843722683	-0.69755462	1.287890944	0.295168162
1.403922022	2	0	0.998207054
ULK3	ENSG00000140474.12	0.384599558	2	2	0.223124793
Brain_Caudate_basal_ganglia	0.267794761	Brain_Putamen_basal_ganglia
1.423828416	0.576171584	0.576171584	1.10815528	1.218261032
1.163208156	0.077856524	2	0	0.998207054
HLA-F	ENSG00000204642.13	0.384660255	4	3	0.233876584	Brain_Cerebellum
0.967043396	Brain_Nucleus_accumbens_basal_ganglia	2.073528301
0.057497067	0.255664629	-1.190432148	0.523713044	-0.442402605
0.860124587	3	0	0.998207054
RP11-118K6.3	ENSG00000273001.1	0.384665191	2	2	0.283196571
Brain_Amygdala	0.287595939	Brain_Nucleus_accumbens_basal_ganglia
1.194574576	0.805425424	0.805425424	-1.063410267	1.073166159
0.004877946	1.510787679	2	0	0.998207054
NSMCE2	ENSG00000156831.7	0.385014139	1	1	0.385014139
Brain_Cortex	0.385014139	Brain_Cortex	1	1	1	-0.868694703
-0.868694703	-0.868694703	NA	1	0	0.998207054
FOXRED1	ENSG00000110074.10	0.385138266	13	2	0.243016081
Brain_Spinal_cord_cervical_c-1	0.608968983	Brain_Hypothalamus
10.98568681	9.79E-06	1.019800571	-1.111725622	1.167481237
0.625222874	0.553901805	2	0	0.998207054
ZIK1	ENSG00000171649.11	0.3853612	4	3	0.419118026	Brain_Amygdala
0.95286542	Brain_Cerebellum	3.362536698	0.072525135	0.215894539
-0.766432653	0.807952328	0.077934979	0.665297434	3	0
0.998207054
LA16c-380H5.6	ENSG00000274367.1	0.385381543	4	4	0.141912104
Brain_Hypothalamus	0.808352685	Brain_Cerebellar_Hemisphere	2.407912979
0.183407382	0.183407382	0.242551722	1.468707642	0.958013293
0.5576382	4	0	0.998207054
PRCD	ENSG00000214140.10	0.385492194	4	4	0.066382825
Brain_Anterior_cingulate_cortex_BA24	0.844499407	Brain_Cortex
1.783083691	0.443372184	0.443372184	-0.701924786	1.835829854
0.264263764	1.110048673	4	0	0.998207054
RANBP17	ENSG00000204764.13	0.385537665	6	2	0.144352649
Brain_Cerebellum	0.748395301	Brain_Cortex	5.398730216	0.02974315
0.251038676	0.320756007	1.459772354	0.746455055	0.411352601
2	0	0.998207054
ZNF229	ENSG00000278318.4	0.38554418	6	6	0.066043062
Brain_Cerebellar_Hemisphere	0.917926669	Brain_Nucleus_accumbens_basal_ganglia
3.481897727	0.218652494	0.218652494	-1.838131286	-0.10304574
-0.844595093	0.603133735	6	0	0.998207054
EXOSC8	ENSG00000120699.12	0.385609386	4	3	0.106881618
Brain_Putamen_basal_ganglia	0.522903636	Brain_Cerebellar_Hemisphere	3.26164126

0.07003354 0.23411909 -1.612369224 -0.638875947 -1.236723662 0.44195627 3 0 0.998207054
ZDHHC20 ENSG00000180776.15 0.385631064 3 3 0.46962116 Brain_Putamen_basal_ganglia 0.737831614 Brain_Cerebellum 1.785654879 0.301895372 0.301895372 -0.723095588 0.62785226 0.079827634 0.710629997 3 0 0.998207054
PHYKPL ENSG00000175309.14 0.38568959 1 1 0.38568959 Brain_Cerebellar_Hemisphere 0.38568959 Brain_Cerebellar_Hemisphere 1 1 1 0.867460785 0.867460785 0.867460785 NA 1 0 0.998207054
DPYSL3 ENSG00000113657.12 0.385967301 2 2 0.173906852 Brain_Nucleus_accumbens_basal_ganglia 0.696532335 Brain_Cerebellum 1.11539654 0.88460346 0.88460346 0.390005696 1.359756944 0.87488132 0.685717683 2 0 0.998207054
SRC ENSG00000197122.11 0.386160155 1 1 0.386160155 Brain_Cerebellar_Hemisphere 0.386160155 Brain_Cerebellar_Hemisphere 1 1 1 0.866601932 0.866601932 0.866601932 NA 1 0 0.998207054
AC012074.2 ENSG00000235072.2 0.38631067 3 3 0.085154988 Brain_Hypothalamus 0.974061144 Brain_Nucleus_accumbens_basal_ganglia 1.442784442 0.502300132 0.502300132 -0.032515264 1.721528376 0.7195682 0.903324997 3 0 0.998207054
SEMA6D ENSG00000137872.16 0.386376278 2 2 0.168223466 Brain_Anterior_cingulate_cortex_BA24 0.958104258 Brain_Cerebellum 1.002821149 0.997178851 0.997178851 0.052532679 1.377934648 0.715233663 0.937200721 2 0 0.998207054
C6orf141 ENSG00000197261.11 0.386431612 5 5 0.108564462 Brain_Cortex 0.952862188 Brain_Nucleus_accumbens_basal_ganglia 2.502927847 0.196341446 0.196341446 -0.516725615 1.604679017 0.483491825 0.81253699 5 0 0.998207054
LARP6 ENSG00000166173.10 0.386432628 5 2 0.393187578 Brain_Cerebellar_Hemisphere 0.984693476 Brain_Cerebellum 4.404738606 0.007821087 0.457118263 -0.529847138 0.853850953 0.004719872 0.516168962 2 0 0.998207054
EPB42 ENSG00000166947.11 0.38643341 2 2 0.171081308 Brain_Caudate_basal_ganglia 0.81286492 Brain_Nucleus_accumbens_basal_ganglia 1.05053572 0.94946428 0.94946428 0.236731727 1.368737616 0.802734671 0.80044904 2 0 0.998207054
SLC5A11 ENSG00000158865.12 0.38649476 6 3 0.254326692 Brain_Nucleus_accumbens_basal_ganglia 0.925402637 Brain_Substantia_nigra 4.607920136 0.000656064 0.529109021 -1.139903056 0.287299521 -0.113017923 0.561549987 3 0 0.998207054
ATP1B3 ENSG00000069849.10 0.386521798 2 2 0.175701235 Brain_Cerebellum 0.961504246 Brain_Anterior_cingulate_cortex_BA24 1.222718184 0.777281816 0.777281816 -1.354110162 -0.048266006 -0.701188084 0.923371258 2 0 0.998207054
ATP9A ENSG00000054793.13 0.386598021 3 3 0.131203576 Brain_Anterior_cingulate_cortex_BA24 0.565430797 Brain_Putamen_basal_ganglia 1.372049029 0.632502467 0.632502467 -0.725281779 1.509372658 0.452961542 1.122297833 3 0 0.998207054
MAP3K13 ENSG00000073803.13 0.386742346 1 1 0.386742346 Brain_Cortex 0.386742346 Brain_Cortex 1 1 1 0.865540229 0.865540229 0.865540229 NA 1 0 0.998207054
CFAP61 ENSG00000089101.17 0.38686775 3 3 0.190196448 Brain_Anterior_cingulate_cortex_BA24 0.568905304 Brain_Cortex 2.7019902 0.11936365 0.11936365 0.569664454 1.30999819 0.868036108 0.390496054 3 0 0.998207054
LINC01876 ENSG00000226383.6 0.386904393 1 1 0.386904393 Brain_Spinal_cord_cervical_c-1 0.386904393 Brain_Spinal_cord_cervical_c-1 1 1 1 0.865244887 0.865244887 0.865244887 NA 1 0 0.998207054
ABCC12 ENSG00000140798.15 0.38698396 1 1 0.38698396 Brain_Amygdala 0.38698396 Brain_Amygdala 1 1 1 -0.8650999 -0.8650999 -0.8650999 NA

1 0 0.998207054
EFCAB7 ENSG00000203965.12 0.38726946 10 5 0.184673586
Brain_Nucleus_accumbens_basal_ganglia 0.906885882 Brain_Hippocampus
7.072087102 0.043018908 0.277765933 -0.910169237 1.326501658
0.205984451 0.723245093 5 0 0.998207054
AC131097.4 ENSG00000216921.7 0.387283335 4 4 0.287928551
Brain_Nucleus_accumbens_basal_ganglia 0.919696157 Brain_Cortex
2.593683738 0.145255426 0.145255426 -1.062676787 1.055614752
0.187599013 0.92090048 4 0 0.998207054
TMEM176A ENSG00000002933.7 0.387335545 13 4 0.405334558
Brain_Caudate_basal_ganglia 0.92183785 Brain_Cortex 10.42374548
0.007930469 0.384041885 -0.832131794 0.71707859 -0.267677601
0.437124965 4 0 0.998207054
TMEM156 ENSG00000121895.7 0.387348342 1 1 0.387348342
Brain_Cortex 0.387348342 Brain_Cortex 1 1 1 0.86443615
0.86443615 0.86443615 NA 1 0 0.998207054
SYT16 ENSG00000139973.16 0.38744856 2 2 0.220115499 Brain_Cerebellum
0.510275641 Brain_Substantia_nigra 1.026049492 0.973950508
0.973950508 0.658408553 1.226221058 0.942314806 0.401504073
2 0 0.998207054
FAM229A ENSG00000225828.1 0.387472378 1 1 0.387472378
Brain_Anterior_cingulate_cortex_BA24 0.387472378
Brain_Anterior_cingulate_cortex_BA24 1 1 1 -0.864210294
-0.864210294 -0.864210294 NA 1 0 0.998207054
NMRK1 ENSG00000106733.20 0.387516223 13 1 0.193797346 Brain_Cerebellum
0.632089419 Brain_Hippocampus 12.37693151 0.000243913
12.37693151 0.47878805 1.299427248 0.843789251 0.226231215 1
0 0.998207054
STRC ENSG00000242866.9 0.387562385 9 3 0.110767076
Brain_Caudate_basal_ganglia 0.60144203 Brain_Amygdala 7.178922678
0.008348012 0.48293952 -1.594754809 -0.522327923 -1.026152898
0.384330865 3 0 0.998207054
PROSER1 ENSG00000120685.19 0.387637742 1 1 0.387637742
Brain_Caudate_basal_ganglia 0.387637742 Brain_Caudate_basal_ganglia 1 1
1 0.863909255 0.863909255 0.863909255 NA 1 0
0.998207054
TFPT ENSG00000105619.13 0.387656434 3 2 0.13797329 Brain_Cortex
0.925524524 Brain_Putamen_basal_ganglia 1.971256332 0.030179545
0.998564123 -1.483380708 -0.093477122 -0.937937101 0.741630695
2 0 0.998207054
PSMD8 ENSG00000099341.11 0.387663723 1 1 0.387663723 Brain_Cerebellum
0.387663723 Brain_Cerebellum 1 1 1 -0.863861964 -0.863861964
-0.863861964 NA 1 0 0.998207054
RP11-269G24.6 ENSG00000265971.1 0.387674395 2 2 0.171506183
Brain_Hippocampus 0.833941341 Brain_Caudate_basal_ganglia 1.265795193
0.734204807 0.734204807 -1.367380142 -0.209649374 -0.788514758
0.818639277 2 0 0.998207054
LAIR2 ENSG00000167618.9 0.38789908 1 1 0.38789908 Brain_Frontal_Cortex_BA9
0.38789908 Brain_Frontal_Cortex_BA9 1 1 1 -0.863433658
-0.863433658 -0.863433658 NA 1 0 0.998207054
SLC25A33 ENSG00000171612.6 0.387999002 1 1 0.387999002
Brain_Cortex 0.387999002 Brain_Cortex 1 1 1 -0.863251866
-0.863251866 -0.863251866 NA 1 0 0.998207054
VAMP5 ENSG00000168899.4 0.388052138 1 1 0.388052138
Brain_Hypothalamus 0.388052138 Brain_Hypothalamus 1 1 1
-0.863155207 -0.863155207 -0.863155207 NA 1 0 0.998207054
CCDC153 ENSG00000248712.7 0.388373673 7 5 0.138799148
Brain_Caudate_basal_ganglia 0.675529904 Brain_Hippocampus 4.622729684
0.011818659 0.185426449 0.418570703 1.480277714 0.97488451
0.416729426 5 0 0.998207054
PTPN3 ENSG00000070159.13 0.388459668 1 1 0.388459668 Brain_Cerebellum
0.388459668 Brain_Cerebellum 1 1 1 0.86241413 0.86241413 0.86241413

NA 1 0 0.998207054
GLYATL2 ENSG00000156689.6 0.388562141 5 4 0.146571715
Brain_Anterior_cingulate_cortex_BA24 0.6356097 Brain_Cortex 2.960663116
0.060394435 0.401340522 -0.720905869 1.451747903 0.613137129
0.822474503 4 0 0.998207054
CBLN3 ENSG00000139899.10 0.388629664 10 7 0.028320807
Brain_Anterior_cingulate_cortex_BA24 0.911281474 Brain_Putamen_basal_ganglia
5.342144732 0.032291002 0.33396997 -2.192813663 -0.111422304
-1.315486377 0.713872706 7 0 0.998207054
SOX15 ENSG00000129194.7 0.388691514 4 3 0.120823457
Brain_Nucleus_accumbens_basal_ganglia 0.842818429 Brain_Caudate_basal_ganglia
3.070215551 7.66E-05 0.219977996 0.198289676 1.551326508
0.670897401 0.638077569 3 0 0.998207054
CEP295NL ENSG00000178404.9 0.388793651 1 1 0.388793651
Brain_Caudate_basal_ganglia 0.388793651 Brain_Caudate_basal_ganglia 1 1
1 0.861807147 0.861807147 0.861807147 NA 1 0
0.998207054
RNF182 ENSG00000180537.12 0.388857769 3 3 0.2882582
Brain_Caudate_basal_ganglia 0.857233523 Brain_Hippocampus 1.927734879
0.076946513 0.076946513 -1.061950408 0.179896882 -0.444676863
0.62095583 3 0 0.998207054
ARRDC3 ENSG00000113369.8 0.388929507 2 2 0.180732542
Brain_Frontal_Cortex_BA9 0.74576528 Brain_Hypothalamus 1.009409329
0.990590671 0.990590671 0.324228191 1.338502942 0.831365567
0.717200555 2 0 0.998207054
ALDH4A1 ENSG00000159423.16 0.388946981 8 4 0.050971493
Brain_Putamen_basal_ganglia 0.476776741 Brain_Hypothalamus 6.553561885
9.92E-09 0.219050343 -1.951719695 -0.711496303 -1.367792723
0.462345102 4 0 0.998207054
LATS2 ENSG00000150457.8 0.389035101 2 2 0.169412361 Brain_Cerebellum
0.235288281 Brain_Cerebellar_Hemisphere 1.861517524 0.138482476
0.138482476 -1.374094453 -1.186846227 -1.28047034 0.132404491
2 0 0.998207054
TMX4 ENSG00000125827.8 0.389132562 7 5 0.078339112
Brain_Frontal_Cortex_BA9 0.856830145 Brain_Cerebellar_Hemisphere
4.446985559 0.036611187 0.264313342 -1.760405296 0.180410713
-0.95043613 0.708223268 5 0 0.998207054
SKP2 ENSG00000145604.15 0.38920104 1 1 0.38920104 Brain_Cerebellum 0.38920104
Brain_Cerebellum 1 1 1 -0.861067187 -0.861067187 -0.861067187
NA 1 0 0.998207054
PPP2R2C ENSG00000074211.13 0.389214404 3 3 0.190594026
Brain_Hypothalamus 0.769835112 Brain_Frontal_Cortex_BA9 1.121996626
0.863849335 0.863849335 -0.958129838 1.30882385 0.019367809
1.165228855 3 0 0.998207054
RP5-1121E10.2 ENSG00000238358.2 0.389260141 1 1 0.389260141
Brain_Cerebellum 0.389260141 Brain_Cerebellum 1 1 1 -0.860959879
-0.860959879 -0.860959879 NA 1 0 0.998207054
JMY ENSG00000152409.8 0.389272315 3 3 0.123595381
Brain_Nucleus_accumbens_basal_ganglia 0.690954669 Brain_Cerebellum
2.361012718 0.280736453 0.280736453 -0.397559839 1.539856318
0.708240291 0.997385599 3 0 0.998207054
COG6 ENSG00000133103.16 0.389344622 9 2 0.475478059
Brain_Putamen_basal_ganglia 0.894513291 Brain_Substantia_nigra
7.899731849 0.004912953 0.731636881 -0.520499734 0.713594339
0.237560913 0.447467629 2 0 0.998207054
CPSF7 ENSG00000149532.15 0.389368449 2 2 0.170010975 Brain_Cortex
0.97451857 Brain_Cerebellum 1.020511213 0.979488787 0.979488787
0.031941667 1.372168546 0.702055107 0.947683514 2 0
0.998207054
RP11-480D4.2 ENSG00000249159.6 0.389402518 3 3 0.230520273
Brain_Putamen_basal_ganglia 0.692942309 Brain_Substantia_nigra
1.155705035 0.839826505 0.839826505 0.394865287 1.199019732

0.876948526 0.425286939 3 0 0.998207054
KCNC1 ENSG00000129159.7 0.38946924 1 1 0.38946924 Brain_Frontal_Cortex_BA9
0.38946924 Brain_Frontal_Cortex_BA9 1 1 1 0.8605803 0.8605803
0.8605803 NA 1 0 0.998207054
GJB3 ENSG00000188910.7 0.389478112 2 2 0.171972148
Brain_Cerebellar_Hemisphere 0.88764564 Brain_Spinal_cord_cervical_c-1
1.000689159 0.999310841 0.999310841 -0.141283935 1.365894282
0.612305174 1.065735938 2 0 0.998207054
MPDZ ENSG00000107186.16 0.389524673 1 1 0.389524673
Brain_Cerebellar_Hemisphere 0.389524673 Brain_Cerebellar_Hemisphere 1 1
1 0.860479693 0.860479693 0.860479693 NA 1 0
0.998207054
PWWP2B ENSG00000171813.13 0.389574617 2 2 0.337209218
Brain_Amygdala 0.345583278 Brain_Substantia_nigra 1.039667135
0.960332865 0.960332865 -0.95969405 -0.94319088 -0.951442465
0.011669504 2 0 0.998207054
KIAA1841 ENSG00000162929.13 0.389619924 2 2 0.389107077
Brain_Nucleus_accumbens_basal_ganglia 0.785943197 Brain_Cerebellum
1.639349572 0.360650428 0.360650428 -0.271582317 0.861237815
0.294827749 0.801024797 2 0 0.998207054
SEC22A ENSG00000121542.11 0.389628124 3 3 0.271742506
Brain_Anterior_cingulate_cortex_BA24 0.632725111
Brain_Nucleus_accumbens_basal_ganglia 1.497788131 0.59224239 0.59224239
-1.099058601 0.477894761 -0.507541639 0.859112838 3 0
0.998207054
SPATA7 ENSG00000042317.16 0.389655984 13 2 0.059427999
Brain_Caudate_basal_ganglia 0.451526278 Brain_Cerebellum 11.59646553
0.006803119 0.46535328 0.752872927 1.885013616 1.210525871
0.33002482 2 0 0.998207054
PPP6C ENSG00000119414.11 0.38972898 6 3 0.097683344 Brain_Cortex
0.665798674 Brain_Anterior_cingulate_cortex_BA24 4.594756275
0.065689377 0.399583234 -0.431921217 1.656190018 0.812573064
0.687016033 3 0 0.998207054
BCL10 ENSG00000142867.12 0.390092816 3 3 0.169848359
Brain_Putamen_basal_ganglia 0.852976537 Brain_Cerebellar_Hemisphere
1.261995638 0.729130995 0.729130995 -1.372691223 -0.185321931
-0.856474623 0.608658651 3 0 0.998207054
RP11-266K4.9 ENSG00000215241.3 0.390323877 5 5 0.129760439
Brain_Nucleus_accumbens_basal_ganglia 0.612040062 Brain_Cerebellum
2.222740663 0.263548087 0.263548087 -1.040077122 1.51504725
0.278869987 1.041877569 5 0 0.998207054
SKI ENSG00000157933.9 0.390364653 2 2 0.30907336
Brain_Substantia_nigra 0.360722165 Brain_Cerebellum 1.00606117 0.99393883
0.99393883 -1.017168328 0.913989878 -0.051589225 1.365535063 2
0 0.998207054
WDR7 ENSG00000091157.13 0.390413047 7 6 0.069551894
Brain_Cerebellar_Hemisphere 0.967934736 Brain_Spinal_cord_cervical_c-1
4.773815511 0.049742989 0.234902653 -0.139652985 1.814817932
0.726905961 0.68951607 6 0 0.998207054
IRS1 ENSG00000169047.5 0.390453014 4 4 0.080222099
Brain_Cerebellar_Hemisphere 0.866538821 Brain_Substantia_nigra
1.402427452 0.717306181 0.717306181 -0.895548284 1.749398852
0.258538005 1.117233449 4 0 0.998207054
EPHA7 ENSG00000135333.13 0.390480931 1 1 0.390480931
Brain_Nucleus_accumbens_basal_ganglia 0.390480931
Brain_Nucleus_accumbens_basal_ganglia 1 1 1 -0.858745519
-0.858745519 -0.858745519 NA 1 0 0.998207054
MANEA ENSG00000172469.15 0.390508367 5 4 0.030607041 Brain_Cerebellum
0.990017228 Brain_Anterior_cingulate_cortex_BA24 3.070410117 0.09251168
0.294548941 0.012511876 2.162144482 1.358328759 0.816881042
4 0 0.998207054
CBR4 ENSG00000145439.11 0.390527975 4 4 0.184733413

Brain_Hippocampus 0.736084787 Brain_Putamen_basal_ganglia 2.785778328
0.224880874 0.224880874 0.3370426 1.326320943 0.886415374
0.480239053 4 0 0.998207054
MEOX2 ENSG00000106511.5 0.390733232 4 4 0.143465632
Brain_Anterior_cingulate_cortex_BA24 0.846887783
Brain_Nucleus_accumbens_basal_ganglia 1.218722058 0.78125139 0.78125139
-1.463006383 -0.193090895 -0.857903002 0.663317732 4 0
0.998207054
NDE1 ENSG00000072864.14 0.390757242 13 5 0.102825481
Brain_Substantia_nigra 0.901453587 Brain_Nucleus_accumbens_basal_ganglia
8.990084934 0.00057101 0.428593406 -0.722243905 1.631307361
-0.126563625 0.634395889 5 0 0.998207054
RP11-388C12.1 ENSG00000263063.1 0.390794277 13 4 0.052714289
Brain_Cortex 0.940668237 Brain_Putamen_basal_ganglia 10.18178847
0.000137379 0.344229924 -0.549421381 1.937254108 0.750538543
0.619688929 4 0 0.998207054
RP11-329B9.5 ENSG00000272922.1 0.390931853 4 4 0.11821698
Brain_Frontal_Cortex_BA9 0.747961917 Brain_Cerebellar_Hemisphere
1.763832773 0.440102088 0.440102088 -1.562301495 1.170880295
-0.325132124 1.131023028 4 0 0.998207054
TFCP2 ENSG00000135457.9 0.391025954 1 1 0.391025954
Brain_Cerebellar_Hemisphere 0.391025954 Brain_Cerebellar_Hemisphere 1 1
1 -0.857758274 -0.857758274 -0.857758274 NA 1 0
0.998207054
RSPO1 ENSG00000169218.13 0.391035771 1 1 0.391035771
Brain_Frontal_Cortex_BA9 0.391035771 Brain_Frontal_Cortex_BA9 1 1
1 -0.8577405 -0.8577405 -0.8577405 NA 1 0 0.998207054
SMIM7 ENSG00000214046.8 0.391038551 7 4 0.102662093
Brain_Putamen_basal_ganglia 0.630778717 Brain_Cortex 5.172076249
0.022561252 0.210119151 0.480631086 1.632082572 1.114568974
0.473893802 4 0 0.998207054
TMEM14C ENSG00000111843.13 0.391237953 1 1 0.391237953
Brain_Cerebellum 0.391237953 Brain_Cerebellum 1 1 1 0.857374489
0.857374489 0.857374489 NA 1 0 0.998207054
EME2 ENSG00000197774.12 0.391249241 7 5 0.468194262
Brain_Nucleus_accumbens_basal_ganglia 0.650985574 Brain_Putamen_basal_ganglia
4.168345478 0.006371815 0.439047779 -0.725420237 0.705079861
0.087831933 0.641251272 5 0 0.998207054
GLIS2-AS1 ENSG00000262686.1 0.39133654 2 2 0.297344286 Brain_Cortex
0.375868102 Brain_Cerebellum 1.003312965 0.996687035 0.996687035
-1.042145371 -0.885535091 -0.963840231 0.110740191 2 0
0.998207054
MYO1B ENSG00000128641.18 0.391342355 1 1 0.391342355
Brain_Nucleus_accumbens_basal_ganglia 0.391342355
Brain_Nucleus_accumbens_basal_ganglia 1 1 1 -0.857185536
-0.857185536 -0.857185536 NA 1 0 0.998207054
PDIA4 ENSG00000155660.10 0.391422217 3 3 0.138998929
Brain_Hippocampus 0.853824153 Brain_Cortex 1.723146967
0.293071798 0.293071798 0.18424131 1.479529214 0.771616192
0.656002874 3 0 0.998207054
NUDT16 ENSG00000198585.11 0.391533298 1 1 0.391533298
Brain_Cerebellar_Hemisphere 0.391533298 Brain_Cerebellar_Hemisphere 1 1
1 -0.856840033 -0.856840033 -0.856840033 NA 1 0
0.998207054
LINC02156 ENSG00000257906.1 0.39155503 3 3 0.130401312
Brain_Caudate_basal_ganglia 0.837462292 Brain_Spinal_cord_cervical_c-1
2.209081047 0.214296974 0.214296974 -1.512521247 -0.20514058
-1.05101338 0.733562099 3 0 0.998207054
FLG ENSG00000143631.10 0.391622748 10 2 0.325239912
Brain_Anterior_cingulate_cortex_BA24 0.952656059 Brain_Frontal_Cortex_BA9
8.494360757 0.000967095 0.854058232 -0.666161833 0.983747025
0.259403233 0.494892897 2 0 0.998207054

SHCBP1 ENSG00000171241.8 0.391826269 2 1 0.386790316
Brain_Frontal_Cortex_BA9 0.396997279 Brain_Cortex 1.999599916
0.000400084 1.999599916 -0.865452791 -0.846996129 -0.85622446
0.013050831 1 0 0.998207054
PRTFDC1 ENSG00000099256.18 0.391889851 5 4 0.110381296
Brain_Caudate_basal_ganglia 0.879946521 Brain_Nucleus_accumbens_basal_ganglia
2.566410815 0.056812506 0.397008235 -1.596481667 0.187111351
-0.506595027 0.733480223 4 0 0.998207054
SBK3 ENSG00000231274.5 0.391914925 2 2 0.175301842 Brain_Cerebellum
0.297191351 Brain_Cerebellar_Hemisphere 1.844538929 0.155461071
0.155461071 -1.355363282 -1.042475343 -1.198919312 0.221245183
2 0 0.998207054
THAP5 ENSG00000177683.13 0.391947201 12 2 0.070831596
Brain_Anterior_cingulate_cortex_BA24 0.501921671 Brain_Cerebellum
10.71865313 0.005097378 0.504831252 0.671469204 1.806555613
1.26772175 0.332252371 2 0 0.998207054
CCDC84 ENSG00000186166.8 0.391996907 2 2 0.593137386
Brain_Hippocampus 0.884572848 Brain_Nucleus_accumbens_basal_ganglia
1.874037385 0.125962615 0.125962615 -0.145174818 0.534295093
0.194560138 0.480457782 2 0 0.998207054
HIST1H2AE ENSG00000277075.2 0.392013738 1 1 0.392013738
Brain_Spinal_cord_cervical_c-1 0.392013738 Brain_Spinal_cord_cervical_c-1
1 1 1 0.855971149 0.855971149 0.855971149 NA 1 0
0.998207054
RBFOX1 ENSG00000078328.19 0.392080508 1 1 0.392080508
Brain_Cerebellar_Hemisphere 0.392080508 Brain_Cerebellar_Hemisphere 1 1
1 0.855850446 0.855850446 0.855850446 NA 1 0
0.998207054
C2 ENSG00000166278.14 0.392089563 2 2 0.215473628
Brain_Cerebellar_Hemisphere 0.355048557 Brain_Nucleus_accumbens_basal_ganglia
1.298511456 0.701488544 0.701488544 0.924841121 1.238654089
1.081747605 0.221899277 2 0 0.998207054
RNF146 ENSG00000118518.15 0.392135538 2 2 0.219981066
Brain_Putamen_basal_ganglia 0.660757122 Brain_Frontal_Cortex_BA9
1.132292623 0.867707377 0.867707377 -1.22657847 -0.438868086
-0.832723278 0.556995354 2 0 0.998207054
UNC13B ENSG00000198722.14 0.39216921 1 1 0.39216921 Brain_Cerebellum
0.39216921 Brain_Cerebellum 1 1 1 0.855690114 0.855690114
0.855690114 NA 1 0 0.998207054
VASH1 ENSG00000071246.10 0.392218324 3 3 0.144864289 Brain_Cerebellum
0.993790889 Brain_Cerebellar_Hemisphere 1.372319311 0.644684489
0.644684489 -1.457913856 0.351913385 -0.371260839 0.958100353
3 0 0.998207054
STIM2 ENSG00000109689.15 0.392337627 1 1 0.392337627 Brain_Cerebellum
0.392337627 Brain_Cerebellum 1 1 1 -0.855385755 -0.855385755
-0.855385755 NA 1 0 0.998207054
TESK2 ENSG00000070759.16 0.392397315 8 7 0.401357485
Brain_Spinal_cord_cervical_c-1 0.806207878 Brain_Putamen_basal_ganglia
4.072458135 0.082030021 0.206956957 -0.740854065 0.839199291
-0.058551891 0.61412523 7 0 0.998207054
HIST1H4K ENSG00000273542.1 0.392464878 2 2 0.315420223
Brain_Anterior_cingulate_cortex_BA24 0.734901023
Brain_Nucleus_accumbens_basal_ganglia 1.83971758 0.16028242 0.16028242
0.338613351 1.003913678 0.671263515 0.470438373 2 0
0.998207054
TM7SF3 ENSG00000064115.10 0.392523576 10 7 0.141603931
Brain_Cerebellum 0.916289696 Brain_Cerebellar_Hemisphere 5.779024014
0.035642046 0.216143768 -1.295690855 1.469844289 0.201841224
0.745842822 7 0 0.998207054
PAXBP1 ENSG00000159086.14 0.392530039 1 1 0.392530039
Brain_Hippocampus 0.392530039 Brain_Hippocampus 1 1 1
0.855038129 0.855038129 0.855038129 NA 1 0 0.998207054

RETN ENSG00000104918.7 0.39253314 1 1 0.39253314 Brain_Caudate_basal_ganglia
0.39253314 Brain_Caudate_basal_ganglia 1 1 1 0.85503253 0.85503253
0.85503253 NA 1 0 0.998207054
TOMM20 ENSG00000173726.10 0.392665883 1 1 0.392665883
Brain_Putamen_basal_ganglia 0.392665883 Brain_Putamen_basal_ganglia 1 1
1 0.854792768 0.854792768 0.854792768 NA 1 0
0.998207054
ZNF839 ENSG00000022976.15 0.392689592 3 2 0.380240513
Brain_Cerebellum 0.799003189 Brain_Cerebellar_Hemisphere 2.706234023
0.041730385 0.252035592 -0.877453228 0.271380283 -0.28690344
0.575096021 2 0 0.998207054
CTDNEP1 ENSG00000175826.11 0.392707158 5 4 0.103852693
Brain_Putamen_basal_ganglia 0.757459826 Brain_Cerebellum 4.002571158
0.025136894 0.156119672 0.308818197 1.626455969 0.846420199
0.483971462 4 0 0.998207054
ZNF329 ENSG00000181894.14 0.392711358 5 4 0.247125049
Brain_Cerebellar_Hemisphere 0.69672325 Brain_Caudate_basal_ganglia 2.804027944
0.090092404 0.381365454 -1.157360638 0.443269293 -0.523359688
0.640028098 4 0 0.998207054
LAMA1 ENSG00000101680.14 0.392834805 2 2 0.172911838
Brain_Putamen_basal_ganglia 0.973788612 Brain_Cortex 1.053489319
0.946510681 0.946510681 -1.36290695 0.032857014 -0.665024968
0.986954164 2 0 0.998207054
CAPS2 ENSG00000180881.19 0.392842404 4 4 0.222098901
Brain_Caudate_basal_ganglia 0.702027826 Brain_Anterior_cingulate_cortex_BA24
1.943882245 0.113960774 0.113960774 -1.092313929 1.220965979
-0.00657536 1.019649088 4 0 0.998207054
LINC01902 ENSG00000283503.1 0.392848161 1 1 0.392848161
Brain_Caudate_basal_ganglia 0.392848161 Brain_Caudate_basal_ganglia 1 1
1 -0.854463615 -0.854463615 -0.854463615 NA 1 0
0.998207054
PQLC2L ENSG00000174899.10 0.392899079 5 5 0.089645593
Brain_Frontal_Cortex_BA9 0.922602463 Brain_Caudate_basal_ganglia
2.675781328 0.134651074 0.134651074 -1.074019361 1.697270179
0.18216587 1.040508215 5 0 0.998207054
RPUSD4 ENSG00000165526.8 0.392923051 5 5 0.084016025
Brain_Caudate_basal_ganglia 0.9823954 Brain_Nucleus_accumbens_basal_ganglia
1.945912963 0.09004889 0.09004889 0.022065885 1.727844954
1.119316102 0.683006018 5 0 0.998207054
DAPK2 ENSG00000035664.11 0.392947226 2 1 0.276002232 Brain_Cortex
0.551909289 Brain_Frontal_Cortex_BA9 1.9434594 0.0565406 1.9434594
-1.089343965 -0.594901538 -0.842122751 0.349623593 1 0
0.998207054
BTN2A1 ENSG00000112763.15 0.393079791 5 5 0.196157088
Brain_Nucleus_accumbens_basal_ganglia 0.905734177 Brain_Frontal_Cortex_BA9
3.355880645 0.164407385 0.164407385 -0.185803971 1.292577898
0.623394772 0.66684234 5 0 0.998207054
SCN1B ENSG00000105711.11 0.393147434 1 1 0.393147434 Brain_Cerebellum
0.393147434 Brain_Cerebellum 1 1 1 -0.853923398 -0.853923398
-0.853923398 NA 1 0 0.998207054
FZD4 ENSG00000174804.3 0.393153323 1 1 0.393153323
Brain_Nucleus_accumbens_basal_ganglia 0.393153323
Brain_Nucleus_accumbens_basal_ganglia 1 1 1 0.85391277 0.85391277
0.85391277 NA 1 0 0.998207054
OPN4 ENSG00000122375.11 0.393164695 8 2 0.154456513 Brain_Amygdala
0.641221906 Brain_Hippocampus 7.08551696 0.005467893 0.523340433
0.465991051 1.423965315 1.146839945 0.315662033 2 0
0.998207054
NAGPA-AS1 ENSG00000267072.2 0.393253824 6 6 0.099583756
Brain_Putamen_basal_ganglia 0.831075472 Brain_Substantia_nigra
1.659113298 0.587876229 0.587876229 -1.64687493 1.001158218
-0.481612719 0.952143182 6 0 0.998207054

RHOG ENSG00000177105.9 0.393273658 1 1 0.393273658
Brain_Putamen_basal_ganglia 0.393273658 Brain_Putamen_basal_ganglia 1 1
1 0.853695627 0.853695627 0.853695627 NA 1 0
0.998207054
DNAJC9 ENSG00000213551.4 0.393317004 1 1 0.393317004
Brain_Cortex 0.393317004 Brain_Cortex 1 1 1 0.853617419
0.853617419 0.853617419 NA 1 0 0.998207054
DQX1 ENSG00000144045.13 0.393533011 1 1 0.393533011 Brain_Amygdala
0.393533011 Brain_Amygdala 1 1 1 -0.853227762 -0.853227762
-0.853227762 NA 1 0 0.998207054
CDC42EP4 ENSG00000179604.9 0.393537066 2 2 0.252489228
Brain_Substantia_nigra 0.468690645 Brain_Anterior_cingulate_cortex_BA24
1.018096108 0.981903892 0.981903892 -0.724611104 1.144324158
0.209856527 1.321536798 2 0 0.998207054
FMNL3 ENSG00000161791.13 0.393596505 1 1 0.393596505
Brain_Cerebellar_Hemisphere 0.393596505 Brain_Cerebellar_Hemisphere 1 1
1 0.853113249 0.853113249 0.853113249 NA 1 0
0.998207054
SOBP ENSG00000112320.11 0.393624344 1 1 0.393624344
Brain_Putamen_basal_ganglia 0.393624344 Brain_Putamen_basal_ganglia 1 1
1 0.853063044 0.853063044 0.853063044 NA 1 0
0.998207054
C15orf40 ENSG00000169609.13 0.393653493 13 2 0.131781979
Brain_Cerebellar_Hemisphere 0.554341543 Brain_Nucleus_accumbens_basal_ganglia
11.96671902 0.005377997 0.461532786 0.59126699 1.507111894
1.063319748 0.273035248 2 0 0.998207054
C19orf81 ENSG00000235034.6 0.393819687 1 1 0.393819687
Brain_Hypothalamus 0.393819687 Brain_Hypothalamus 1 1 1
0.852710824 0.852710824 0.852710824 NA 1 0 0.998207054
TOX4 ENSG00000092203.13 0.393955389 11 3 0.090334053
Brain_Hypothalamus 0.829170978 Brain_Cortex 9.106409837
0.001947893 0.634420328 -1.693638209 -0.215764941 -0.758510551
0.460766436 3 0 0.998207054
RP11-167B3.3 ENSG00000277987.1 0.393956849 2 2 0.261583471
Brain_Caudate_basal_ganglia 0.988135262 Brain_Putamen_basal_ganglia
1.559766781 0.440233219 0.440233219 -0.014870792 1.122656355
0.553892782 0.804353159 2 0 0.998207054
PAWR ENSG00000177425.10 0.394071109 2 2 0.172712964
Brain_Caudate_basal_ganglia 0.977803959 Brain_Cerebellum 1.061885658
0.938114342 0.938114342 0.027822201 1.363538168 0.695680185
0.944493818 2 0 0.998207054
RPL10A ENSG00000198755.10 0.394207716 8 2 0.289031962
Brain_Hippocampus 0.93572883 Brain_Nucleus_accumbens_basal_ganglia
7.342509595 0.000148519 0.250447635 0.080639276 1.060247621
0.637827507 0.383058745 2 0 0.998207054
SLCO5A1 ENSG00000137571.10 0.394212598 2 2 0.24338467 Brain_Cortex
0.861701952 Brain_Frontal_Cortex_BA9 1.405269631 0.594730369
0.594730369 -1.166568502 0.17420806 -0.496180221 0.948072199 2
0 0.998207054
ZNF883 ENSG00000228623.5 0.394214798 4 4 0.070686985
Brain_Nucleus_accumbens_basal_ganglia 0.875957164
Brain_Spinal_cord_cervical_c-1 2.225702012 0.432589322 0.432589322
-1.807483112 -0.156096237 -0.688467067 0.762969448 4 0
0.998207054
RASD2 ENSG00000100302.6 0.394251382 4 4 0.075908509
Brain_Frontal_Cortex_BA9 0.941093559 Brain_Amygdala 1.404246119
0.66531213 0.66531213 0.073895472 1.774935673 0.86626949 0.697689724
4 0 0.998207054
PKM ENSG00000067225.17 0.394375851 1 1 0.394375851
Brain_Spinal_cord_cervical_c-1 0.394375851 Brain_Spinal_cord_cervical_c-1
1 1 1 0.851708591 0.851708591 0.851708591 NA 1 0
0.998207054

ADAM11 ENSG00000073670.13 0.394394976 3 3 0.16654338
Brain_Hypothalamus 0.670986922 Brain_Cerebellar_Hemisphere 1.588342898
0.392973447 0.392973447 0.424793904 1.383396315 0.970854082
0.493051647 3 0 0.998207054
OR2A7 ENSG00000243896.3 0.394399956 3 3 0.239042864
Brain_Substantia_nigra 0.955903043 Brain_Spinal_cord_cervical_c-1
1.688869969 0.327263812 0.327263812 -1.177382519 0.871726273
-0.120317251 1.026100668 3 0 0.998207054
SAR1A ENSG00000079332.14 0.39443778 12 5 0.202363462 Brain_Cortex
0.934815028 Brain_Cerebellum 9.550172093 0.00291124 0.349363871
-0.174277521 1.27484683 0.883137204 0.470650227 5 0
0.998207054
RSG1 ENSG00000132881.11 0.394512438 7 4 0.15225027
Brain_Nucleus_accumbens_basal_ganglia 0.917418659 Brain_Frontal_Cortex_BA9
4.890021835 0.035007701 0.197288526 -1.431628145 0.80103969
-0.12015267 0.710419732 4 0 0.998207054
SLC25A48 ENSG00000145832.12 0.394514647 2 2 0.204783006
Brain_Nucleus_accumbens_basal_ganglia 0.251071408 Brain_Cerebellar_Hemisphere
1.583449621 0.416550379 0.416550379 -1.26804185 -1.14775091
-1.20789638 0.085058539 2 0 0.998207054
ADPRHL1 ENSG00000153531.13 0.394620064 4 4 0.121970047
Brain_Cerebellar_Hemisphere 0.661552406 Brain_Hypothalamus 1.833868595
0.412411736 0.412411736 -0.437770847 1.546557242 0.580801909
0.820131283 4 0 0.998207054
UMPS ENSG00000114491.13 0.394663768 8 7 0.026453822
Brain_Caudate_basal_ganglia 0.939676822 Brain_Putamen_basal_ganglia
3.555451494 0.048921298 0.298603501 -2.219483888 0.075676061
-0.824709337 0.708685473 7 0 0.998207054
SCAMP3 ENSG00000116521.10 0.394692299 1 1 0.394692299
Brain_Cerebellar_Hemisphere 0.394692299 Brain_Cerebellar_Hemisphere 1 1
1 0.85113872 0.85113872 0.85113872 NA 1 0 0.998207054
LINC02073 ENSG00000267452.1 0.394693755 4 3 0.281468529
Brain_Nucleus_accumbens_basal_ganglia 0.892753379 Brain_Frontal_Cortex_BA9
3.006419184 0.033965455 0.112532231 -0.134821029 1.077026272
0.491208646 0.496925434 3 0 0.998207054
STK32B ENSG00000152953.12 0.39478107 1 1 0.39478107
Brain_Anterior_cingulate_cortex_BA24 0.39478107
Brain_Anterior_cingulate_cortex_BA24 1 1 1 -0.850978908
-0.850978908 -0.850978908 NA 1 0 0.998207054
TMC7 ENSG00000170537.12 0.39485854 1 1 0.39485854 Brain_Hypothalamus
0.39485854 Brain_Hypothalamus 1 1 1 0.850839458 0.850839458
0.850839458 NA 1 0 0.998207054
ZNF124 ENSG00000196418.12 0.394878528 13 7 0.190897635
Brain_Amygdala 0.96321967 Brain_Spinal_cord_cervical_c-1 7.37372665
0.000591663 0.445554284 -1.307928285 0.8196239 -0.39131795
0.634679083 7 0 0.998207054
LINC00958 ENSG00000251381.7 0.394928908 2 2 0.327595382
Brain_Cerebellum 0.469018779 Brain_Cortex 1.208074219 0.791925781
0.791925781 -0.978968829 0.724076486 -0.127446171 1.204234891
2 0 0.998207054
SEC31B ENSG00000075826.16 0.395169251 10 1 0.192655918
Brain_Caudate_basal_ganglia 0.831166205 Brain_Putamen_basal_ganglia
9.492029693 0.000455183 9.492029693 0.213206107 1.302762322
0.824025412 0.334564635 1 0 0.998207054
IGDCC3 ENSG00000174498.13 0.39519494 3 3 0.240808581 Brain_Cerebellum
0.770975851 Brain_Amygdala 1.80517923 0.264055936 0.264055936
-0.291098677 1.172968144 0.472189684 0.734032377 3 0
0.998207054
ADAM9 ENSG00000168615.11 0.395302385 2 2 0.27364534
Brain_Caudate_basal_ganglia 0.308928625 Brain_Cortex 1.204154914
0.795845086 0.795845086 -1.094706271 -1.017472675 -1.056089473
0.054612399 2 0 0.998207054

SPTLC1 ENSG00000090054.13 0.395380842 6 4 0.107434991
Brain_Hypothalamus 0.861018899 Brain_Nucleus_accumbens_basal_ganglia
4.162713498 0.011488164 0.228549785 -1.609829923 -0.175077294
-0.882512659 0.515709371 4 0 0.998207054
ZNF154 ENSG00000179909.15 0.395483948 2 2 0.261722125
Brain_Frontal_Cortex_BA9 0.41614473 Brain_Putamen_basal_ganglia 1.03730774
0.96269226 0.96269226 0.813127902 1.122330071 0.967728986 0.21863895
2 0 0.998207054
PRB2 ENSG00000121335.11 0.395518467 9 8 0.056121689 Brain_Amygdala
0.976434343 Brain_Hippocampus 3.65345125 0.107655147 0.139948439
-1.296422907 1.91008951 0.436099052 1.012441681 8 0
0.998207054
BNIP3 ENSG00000176171.11 0.395573449 1 1 0.395573449 Brain_Cerebellum
0.395573449 Brain_Cerebellum 1 1 1 -0.849553365 -0.849553365
-0.849553365 NA 1 0 0.998207054
SNHG8 ENSG00000269893.6 0.395633847 9 5 0.070107496
Brain_Spinal_cord_cervical_c-1 0.982450718 Brain_Cerebellar_Hemisphere
6.121620173 0.022735521 0.277897922 -1.811215527 0.047035916
-0.95051157 0.68422624 5 0 0.998207054
HIST1H2AC ENSG00000180573.9 0.395637993 1 1 0.395637993
Brain_Hypothalamus 0.395637993 Brain_Hypothalamus 1 1 1
0.849437322 0.849437322 0.849437322 NA 1 0 0.998207054
GGCX ENSG00000115486.11 0.395695198 12 2 0.222066458 Brain_Amygdala
0.943373591 Brain_Putamen_basal_ganglia 11.21413852 0.000337163
0.510714515 0.071030362 1.221051667 0.937175143 0.31231719 2
0 0.998207054
CLK3 ENSG00000179335.18 0.395715202 1 1 0.395715202
Brain_Cerebellar_Hemisphere 0.395715202 Brain_Cerebellar_Hemisphere 1 1
1 0.849298523 0.849298523 0.849298523 NA 1 0
0.998207054
VSTM4 ENSG00000165633.12 0.395777129 3 2 0.20527607 Brain_Hypothalamus
0.279446912 Brain_Cortex 2.423276293 0.080294857 0.496428849
1.081562642 1.266662288 1.183955301 0.094106929 2 0
0.998207054
TRIM55 ENSG00000147573.16 0.39586766 5 5 0.049437126
Brain_Cerebellar_Hemisphere 0.870806608 Brain_Frontal_Cortex_BA9
1.909794475 0.201161266 0.201161266 -1.964802282 0.994406841
-0.410031743 1.269972734 5 0 0.998207054
THAP10 ENSG00000129028.8 0.395941647 2 2 0.30887164 Brain_Cerebellum
0.347324204 Brain_Caudate_basal_ganglia 1.035591531 0.964408469
0.964408469 -1.017592528 -0.939792115 -0.978692321 0.0550132 2
0 0.998207054
ERC2 ENSG00000187672.12 0.395980956 4 3 0.089605087 Brain_Amygdala
0.861287495 Brain_Caudate_basal_ganglia 1.977157482 0.039510105
0.980942132 -0.364420957 1.697484564 0.304561867 0.958748729
3 0 0.998207054
LSMEM2 ENSG00000179564.3 0.396003649 1 1 0.396003649
Brain_Amygdala 0.396003649 Brain_Amygdala 1 1 1 0.848780129
0.848780129 0.848780129 NA 1 0 0.998207054
FLJ12825 ENSG00000248265.1 0.396113126 10 10 0.119373832
Brain_Frontal_Cortex_BA9 0.967461236 Brain_Cerebellar_Hemisphere
2.352835469 0.186028181 0.186028181 -0.089529356 1.557407224
0.745562778 0.644458022 10 0 0.998207054
SAP18 ENSG00000150459.12 0.396127789 2 2 0.340005252
Brain_Putamen_basal_ganglia 0.342686826 Brain_Nucleus_accumbens_basal_ganglia
1.022284645 0.977715355 0.977715355 -0.954154876 0.94886982
-0.002642528 1.345641667 2 0 0.998207054
LINC01007 ENSG00000233123.1 0.39625182 1 1 0.39625182
Brain_Frontal_Cortex_BA9 0.39625182 Brain_Frontal_Cortex_BA9 1 1 1
-0.848334301 -0.848334301 -0.848334301 NA 1 0 0.998207054
CACNG8 ENSG00000142408.4 0.396295419 1 1 0.396295419
Brain_Cortex 0.396295419 Brain_Cortex 1 1 1 0.848255994

0.848255994	0.848255994	NA	1	0	0.998207054
TBC1D5	ENSG00000131374.14	0.396326654	1	1	0.396326654
Brain_Frontal_Cortex_BA9	0.396326654	Brain_Frontal_Cortex_BA9	1	1
1	-0.848199899	-0.848199899	-0.848199899	NA	1	0
0.998207054
SLC2A5	ENSG00000142583.17	0.396339784	3	3	0.290639078
Brain_Cerebellum	0.421790341	Brain_Caudate_basal_ganglia	1.098707124
0.86563301	0.86563301	-0.803319066	1.056720693	0.384772075
1.031862057	3	0	0.998207054
SIDT1	ENSG00000072858.10	0.39636487	7	7	0.108585213
Brain_Anterior_cingulate_cortex_BA24	0.847018552	Brain_Cerebellar_Hemisphere
2.50712683	0.249783652	0.249783652	-1.604584778	0.537589115
-0.428557424	0.768368785	7	0	0.998207054
TRAF3IP1	ENSG00000204104.11	0.396400641	3	3	0.376375036
Brain_Frontal_Cortex_BA9	0.585241439	Brain_Nucleus_accumbens_basal_ganglia
1.773255321	0.456239323	0.456239323	-0.545744703	0.884595127
0.346511952	0.7781691	3	0	0.998207054
CTD-3051D23.3	ENSG00000259067.1	0.396439632	1	1	0.396439632
Brain_Cerebellum	0.396439632	Brain_Cerebellum	1	1	1	0.847997017
0.847997017	0.847997017	NA	1	0	0.998207054
SH3D19	ENSG00000109686.17	0.396532645	3	2	0.245890934
Brain_Caudate_basal_ganglia	0.931745942	Brain_Cerebellum	2.209230544
0.012748978	0.778020477	-1.160387843	-0.085648374	-0.528890372
0.561557053	2	0	0.998207054
RP11-629O1.2	ENSG00000261220.2	0.396619054	1	1	0.396619054
Brain_Putamen_basal_ganglia	0.396619054	Brain_Putamen_basal_ganglia	1	1
1	0.84767489	0.84767489	0.84767489	NA	1	0	0.998207054
ROM1	ENSG00000149489.8	0.396767235	1	1	0.396767235
Brain_Spinal_cord_cervical_c-1	0.396767235	Brain_Spinal_cord_cervical_c-1
1	1	1	-0.847408919	-0.847408919	-0.847408919	NA	1	0
0.998207054
QSER1	ENSG00000060749.14	0.396806152	6	5	0.13489369	Brain_Hippocampus
0.992399729	Brain_Caudate_basal_ganglia	4.16161894	0.130686924
0.194881875	-0.323613914	1.495079522	0.402745	0.739924918	5
0	0.998207054
DRD2	ENSG00000149295.13	0.396864822	3	3	0.237621942
Brain_Frontal_Cortex_BA9	0.969164495	Brain_Cerebellar_Hemisphere
1.553432806	0.542240423	0.542240423	-1.180951622	0.702087652
-0.172506723	0.948628589	3	0	0.998207054
HAGHL	ENSG00000103253.17	0.396871199	2	2	0.174164543
Brain_Cerebellar_Hemisphere	0.897549	Brain_Cerebellum	1.065747615
0.934252385	0.934252385	-0.128758177	1.358943343	0.615092583
1.051963833	2	0	0.998207054
SPR	ENSG00000116096.5	0.397072037	1	1	0.397072037	Brain_Cortex
0.397072037	Brain_Cortex	1	1	1	-0.846862015	-0.846862015
-0.846862015	NA	1	0	0.998207054
VAMP2	ENSG00000220205.8	0.397193409	9	2	0.148478411	Brain_Cerebellum
0.397631702	Brain_Substantia_nigra	8.399059196	0.000130936
0.306980976	0.845858471	1.444926931	1.186686322	0.164397368
2	0	0.998207054
KCNA2	ENSG00000177301.14	0.397345525	2	2	0.217633893	Brain_Cerebellum
0.501699575	Brain_Hippocampus	1.083425104	0.916574896
0.916574896	-1.232844212	-0.671817989	-0.9523311	0.396705447	2
0	0.998207054
EYA3	ENSG00000158161.15	0.397393137	12	3	0.165092593
Brain_Hypothalamus	0.872880197	Brain_Spinal_cord_cervical_c-1
9.872518505	0.002038667	0.513611061	-1.388145997	0.380832153
-0.393760503	0.434486472	3	0	0.998207054
PLXNB1	ENSG00000164050.12	0.39743938	1	1	0.39743938
Brain_Spinal_cord_cervical_c-1	0.39743938	Brain_Spinal_cord_cervical_c-1	1
1	1	-0.846203231	-0.846203231	-0.846203231	NA	1	0
0.998207054

CTDSPL ENSG00000144677.14 0.397475917 1 1 0.397475917
Brain_Cerebellar_Hemisphere 0.397475917 Brain_Cerebellar_Hemisphere 1 1
1 0.846137726 0.846137726 0.846137726 NA 1 0
0.998207054
RPL28 ENSG00000108107.14 0.397493659 10 10 0.032576919
Brain_Hypothalamus 0.954651359 Brain_Putamen_basal_ganglia 2.423136114
0.458292245 0.458292245 -2.137259154 0.48676656 -0.96094812
0.813415996 10 0 0.998207054
TCF12 ENSG00000140262.17 0.39759418 1 1 0.39759418
Brain_Spinal_cord_cervical_c-1 0.39759418 Brain_Spinal_cord_cervical_c-1 1
1 1 -0.845925727 -0.845925727 -0.845925727 NA 1 0
0.998207054
ADAMTSL4 ENSG00000143382.14 0.397677506 3 3 0.135947222
Brain_Cerebellum 0.867007624 Brain_Cerebellar_Hemisphere 1.621819492
0.420501264 0.420501264 -0.176856391 1.491054366 0.493886201
0.88056619 3 0 0.998207054
PRRT2 ENSG00000167371.17 0.39769691 3 3 0.175292476 Brain_Amygdala
0.435230201 Brain_Cerebellar_Hemisphere 1.894950219 0.106088274
0.106088274 -1.032386846 1.355392694 -0.152422384 1.31187655 3
0 0.998207054
CD40 ENSG00000101017.13 0.397798133 5 5 0.064714394
Brain_Frontal_Cortex_BA9 0.967652375 Brain_Amygdala 3.224189809
0.285552352 0.285552352 -1.847225991 -0.040552849 -1.054156652
0.712482309 5 0 0.998207054
SIAE ENSG00000110013.12 0.39781906 2 2 0.212470733
Brain_Caudate_basal_ganglia 0.494563594 Brain_Cerebellum 1.119784433
0.880215567 0.880215567 0.683068445 1.246800292 0.964934369
0.398618612 2 0 0.998207054
LINC01497 ENSG00000237560.5 0.398039832 6 5 0.035767539
Brain_Amygdala 0.780790099 Brain_Substantia_nigra 2.834891495
0.090292197 0.155727633 -0.278289547 2.099560288 1.066436138
0.84013251 5 0 0.998207054
CACNG7 ENSG00000105605.7 0.398108716 1 1 0.398108716
Brain_Caudate_basal_ganglia 0.398108716 Brain_Caudate_basal_ganglia 1 1
1 0.845003803 0.845003803 0.845003803 NA 1 0
0.998207054
RP11-398E10.1 ENSG00000258998.1 0.398132662 1 1 0.398132662
Brain_Substantia_nigra 0.398132662 Brain_Substantia_nigra 1 1
1 -0.844960914 -0.844960914 -0.844960914 NA 1 0
0.998207054
RAD18 ENSG00000070950.9 0.398153488 5 5 0.173340267
Brain_Nucleus_accumbens_basal_ganglia 0.870846011 Brain_Hippocampus
2.356407523 0.333388857 0.333388857 -1.361548977 0.511517195
-0.346186506 0.881824595 5 0 0.998207054
LINC01736 ENSG00000228058.1 0.398156062 2 2 0.175799464
Brain_Substantia_nigra 0.725796168 Brain_Amygdala 1.190107926
0.809892074 0.809892074 -1.353802287 0.350723001 -0.501539643
1.20528139 2 0 0.998207054
CD164 ENSG00000135535.15 0.39824576 4 4 0.246016393 Brain_Cortex
0.795131553 Brain_Spinal_cord_cervical_c-1 2.106897665 0.1695038
0.1695038 -0.789986286 1.160079616 -0.144795425 0.899814522 4
0 0.998207054
PODNL1 ENSG00000132000.11 0.398266813 1 1 0.398266813
Brain_Cerebellum 0.398266813 Brain_Cerebellum 1 1 1 0.844720676
0.844720676 0.844720676 NA 1 0 0.998207054
SBDS ENSG00000126524.9 0.398297853 1 1 0.398297853
Brain_Caudate_basal_ganglia 0.398297853 Brain_Caudate_basal_ganglia 1 1
1 -0.844665095 -0.844665095 -0.844665095 NA 1 0
0.998207054
MKRN3 ENSG00000179455.7 0.398314794 1 1 0.398314794
Brain_Frontal_Cortex_BA9 0.398314794 Brain_Frontal_Cortex_BA9 1 1
1 -0.844634762 -0.844634762 -0.844634762 NA 1 0

0.998207054
TNNT2 ENSG00000118194.18 0.398346156 1 1 0.398346156
Brain_Spinal_cord_cervical_c-1 0.398346156 Brain_Spinal_cord_cervical_c-1
1 1 1 0.84457861 0.84457861 0.84457861 NA 1 0 0.998207054
AC093609.1 ENSG00000230587.1 0.398482573 1 1 0.398482573
Brain_Putamen_basal_ganglia 0.398482573 Brain_Putamen_basal_ganglia 1 1
1 -0.844334392 -0.844334392 -0.844334392 NA 1 0
0.998207054
FCGBP ENSG00000275395.4 0.398538003 11 5 0.083672845 Brain_Cerebellum
0.790560183 Brain_Anterior_cingulate_cortex_BA24 7.713219532
0.000756008 0.296910052 -0.75203462 1.729761784 0.662334279
0.747382483 5 0 0.998207054
RSRC1 ENSG00000174891.12 0.398640644 11 5 0.242957629
Brain_Substantia_nigra 0.974276419 Brain_Hypothalamus 8.034814468
0.046758631 0.279692312 -1.01658977 1.167626072 0.223588459
0.591980094 5 0 0.998207054
ZNF260 ENSG00000254004.6 0.398678065 8 5 0.036309009
Brain_Anterior_cingulate_cortex_BA24 0.953462495 Brain_Substantia_nigra
5.774215662 0.012185694 0.197658573 -2.093449936 0.058359222
-0.971868402 0.654875219 5 0 0.998207054
AC024704.2 ENSG00000244063.1 0.398739207 2 2 0.213338333
Brain_Hypothalamus 0.601744104 Brain_Cortex 1.01351845 0.98648155
0.98648155 -1.244438194 0.521894046 -0.361272074 1.248985505 2
0 0.998207054
ZP3 ENSG00000188372.14 0.398787577 13 3 0.069657984
Brain_Frontal_Cortex_BA9 0.502644835 Brain_Substantia_nigra
11.21350641 0.006461946 0.393441494 0.670334099 1.814128247
1.408951326 0.313895596 3 0 0.998207054
PRDM8 ENSG00000152784.15 0.398805352 3 3 0.277671461
Brain_Frontal_Cortex_BA9 0.660057264 Brain_Cerebellum 2.448761222
0.12084552 0.12084552 -1.085565069 -0.439834105 -0.845466041
0.353260495 3 0 0.998207054
SART3 ENSG00000075856.11 0.398893786 1 1 0.398893786
Brain_Caudate_basal_ganglia 0.398893786 Brain_Caudate_basal_ganglia 1 1
1 -0.84359853 -0.84359853 -0.84359853 NA 1 0
0.998207054
SPNS1 ENSG00000169682.17 0.398903887 2 2 0.175292476
Brain_Nucleus_accumbens_basal_ganglia 0.908205814 Brain_Putamen_basal_ganglia
1.06201456 0.93798544 0.93798544 -0.115301924 1.355392694 0.620045385
1.039938137 2 0 0.998207054
RHEBL1 ENSG00000167550.10 0.399285969 6 3 0.09411248
Brain_Frontal_Cortex_BA9 0.170037457 Brain_Putamen_basal_ganglia
5.124820799 7.09E-07 0.316644232 -1.6740922 -1.372083464
-1.567701831 0.138623603 3 0 0.998207054
THSD1 ENSG00000136114.15 0.399340559 1 1 0.399340559 Brain_Amygdala
0.399340559 Brain_Amygdala 1 1 1 -0.842799552 -0.842799552
-0.842799552 NA 1 0 0.998207054
GHET1 ENSG00000281189.1 0.399349697 1 1 0.399349697 Brain_Cerebellum
0.399349697 Brain_Cerebellum 1 1 1 0.842783215 0.842783215
0.842783215 NA 1 0 0.998207054
RP11-84A19.2 ENSG00000203620.2 0.399378645 1 1 0.399378645
Brain_Spinal_cord_cervical_c-1 0.399378645 Brain_Spinal_cord_cervical_c-1
1 1 1 0.842731466 0.842731466 0.842731466 NA 1 0
0.998207054
ZNF667 ENSG00000198046.11 0.399477608 8 7 0.026200928
Brain_Cerebellum 0.606836654 Brain_Putamen_basal_ganglia 3.57417636
0.059714025 0.210287379 -2.223220608 0.51459415 -1.35592646
0.912252319 7 0 0.998207054
ZNF253 ENSG00000256771.3 0.399491982 5 3 0.209435603
Brain_Frontal_Cortex_BA9 0.980606322 Brain_Cerebellar_Hemisphere
4.207522879 0.038099521 0.28930125 0.024308764 1.255118892
0.494878272 0.478721981 3 0 0.998207054

NFE2L1 ENSG00000082641.15 0.399558535 1 1 0.399558535
Brain_Cortex 0.399558535 Brain_Cortex 1 1 1 -0.842409932
-0.842409932 -0.842409932 NA 1 0 0.998207054
TRPC1 ENSG00000144935.14 0.399684697 1 1 0.399684697
Brain_Frontal_Cortex_BA9 0.399684697 Brain_Frontal_Cortex_BA9 1 1
1 -0.842184485 -0.842184485 -0.842184485 NA 1 0
0.998207054
VPS33B ENSG00000184056.14 0.39969858 4 4 0.40549687 Brain_Cerebellum
0.850936134 Brain_Cerebellar_Hemisphere 2.258432166 0.210263425
0.210263425 -0.831844238 0.80031642 0.104662787 0.759636983 4
0 0.998207054
DGKD ENSG00000077044.10 0.399709705 1 1 0.399709705
Brain_Cerebellar_Hemisphere 0.399709705 Brain_Cerebellar_Hemisphere 1 1
1 0.842139801 0.842139801 0.842139801 NA 1 0
0.998207054
FKBPL ENSG00000204315.3 0.399896048 1 1 0.399896048
Brain_Spinal_cord_cervical_c-1 0.399896048 Brain_Spinal_cord_cervical_c-1
1 1 1 -0.841806901 -0.841806901 -0.841806901 NA 1 0
0.998207054
GPX1 ENSG00000233276.3 0.399985814 11 3 0.099922735
Brain_Caudate_basal_ganglia 0.905437144 Brain_Hippocampus 8.579233833
0.000374802 0.774414887 0.118795791 1.645228322 1.145286411
0.478745275 3 0 0.998207054
GIPC1 ENSG00000123159.15 0.400029051 1 1 0.400029051
Brain_Caudate_basal_ganglia 0.400029051 Brain_Caudate_basal_ganglia 1 1
1 0.841569352 0.841569352 0.841569352 NA 1 0
0.998207054
C11orf54 ENSG00000182919.14 0.40020164 12 1 0.113163455 Brain_Amygdala
0.524012601 Brain_Putamen_basal_ganglia 11.41843664 2.30E-09
11.41843664 -1.584133007 -0.637172327 -0.827507641 0.247401854
1 0 0.998207054
RAB30-AS1 ENSG00000246067.7 0.400210563 13 8 0.126226589
Brain_Hippocampus 0.979764834 Brain_Frontal_Cortex_BA9 7.800828319
0.085472275 0.32205684 -0.516843609 1.529152716 0.407371215
0.670367199 8 0 0.998207054
GCSAML ENSG00000169224.12 0.40024832 2 2 0.422605993
Brain_Frontal_Cortex_BA9 0.659322309 Brain_Hypothalamus 1.568538601
0.431461399 0.431461399 -0.801908321 0.440849012 -0.180529654
0.878762137 2 0 0.998207054
KLB ENSG00000134962.6 0.400319143 10 2 0.338053983
Brain_Putamen_basal_ganglia 0.668238284 Brain_Spinal_cord_cervical_c-1
8.394897211 0.002344606 0.945501289 -0.958017404 0.865260258
0.520398629 0.536608753 2 0 0.998207054
PDE8B ENSG00000113231.13 0.400360472 6 6 0.137963803 Brain_Cerebellum
0.89411233 Brain_Cerebellar_Hemisphere 2.745320563 0.199976808
0.199976808 -0.733028288 1.483416438 0.52423386 0.811196598 6
0 0.998207054
DDT ENSG00000099977.13 0.400375539 11 3 0.256247137
Brain_Hippocampus 0.982292707 Brain_Nucleus_accumbens_basal_ganglia
8.601919902 0.00161795 0.62231396 -1.135305982 0.731345534
-0.640971621 0.564801847 3 0 0.998207054
DNAAF5 ENSG00000164818.15 0.400428667 9 6 0.258453909
Brain_Substantia_nigra 0.680813992 Brain_Cerebellum 5.602183872
0.02636191 0.291313472 -1.130052961 0.887019316 0.351664513
0.710738892 6 0 0.998207054
ZBTB48 ENSG00000204859.12 0.400480112 1 1 0.400480112
Brain_Nucleus_accumbens_basal_ganglia 0.400480112
Brain_Nucleus_accumbens_basal_ganglia 1 1 1 -0.840764084
-0.840764084 -0.840764084 NA 1 0 0.998207054
GMFG ENSG00000130755.12 0.400528529 1 1 0.400528529 Brain_Cortex
0.400528529 Brain_Cortex 1 1 1 0.840677677 0.840677677
0.840677677 NA 1 0 0.998207054

ZNF354B ENSG00000178338.10 0.400697945 2 2 0.178342293
Brain_Nucleus_accumbens_basal_ganglia 0.879926697 Brain_Putamen_basal_ganglia
1.013368407 0.986631593 0.986631593 0.151062141 1.345876671
0.748469406 0.844861457 2 0 0.998207054
CYP2E1 ENSG00000130649.9 0.40082811 3 3 0.094448526
Brain_Frontal_Cortex_BA9 0.862296609 Brain_Spinal_cord_cervical_c-1
1.746333356 0.268814698 0.268814698 0.173451422 1.672384501
1.106275874 0.813978173 3 0 0.998207054
ALMS1 ENSG00000116127.17 0.400875257 4 3 0.091761307
Brain_Frontal_Cortex_BA9 0.967525705 Brain_Nucleus_accumbens_basal_ganglia
2.003629072 0.044204274 0.943769126 -1.686179106 0.254787773
-0.432648926 0.8711251 3 0 0.998207054
RAD51C ENSG00000108384.14 0.400886109 11 2 0.429907312
Brain_Caudate_basal_ganglia 0.997590253 Brain_Cerebellum 10.35054273
0.001873874 0.372992508 -0.789350272 0.213620565 -0.039359127
0.291248192 2 0 0.998207054
ZNF333 ENSG00000160961.11 0.400992342 5 5 0.077191407
Brain_Amygdala 0.802540221 Brain_Cerebellum 1.217755416 0.765132607
0.765132607 -1.19384861 1.767219908 -0.157601741 1.130102279
5 0 0.998207054
GIPC3 ENSG00000179855.5 0.40101171 4 4 0.143379733
Brain_Spinal_cord_cervical_c-1 0.77428694 Brain_Anterior_cingulate_cortex_BA24
1.093507858 0.918220792 0.918220792 -1.292694211 1.463320381
0.260971743 1.149803923 4 0 0.998207054
CCDC89 ENSG00000179071.4 0.401176766 2 2 0.240255975
Brain_Cerebellum 0.928377906 Brain_Cerebellar_Hemisphere 1.551704087
0.448295913 0.448295913 0.089885874 1.174347225 0.63211655
0.766829975 2 0 0.998207054
HIC2 ENSG00000169635.9 0.401213159 1 1 0.401213159 Brain_Cerebellum
0.401213159 Brain_Cerebellum 1 1 1 0.839456555 0.839456555
0.839456555 NA 1 0 0.998207054
RP11-819C21.1 ENSG00000261098.1 0.401303574 9 5 0.23487323
Brain_Cortex 0.738841536 Brain_Putamen_basal_ganglia 5.82607876
0.016510121 0.385104633 -1.187898932 0.546047533 -0.496485279
0.587643121 5 0 0.998207054
LINC00652 ENSG00000179935.9 0.40151444 2 2 0.249483952 Brain_Amygdala
0.504866774 Brain_Cortex 1.033886003 0.966113997 0.966113997
-1.151603721 -0.666851843 -0.909227782 0.34277134 2 0
0.998207054
NPC1 ENSG00000141458.12 0.401739721 11 6 0.030110651
Brain_Frontal_Cortex_BA9 0.968883105 Brain_Anterior_cingulate_cortex_BA24
6.967664198 0.008272759 0.277809273 -2.168631767 -0.039009136
-1.034392815 0.655033458 6 0 0.998207054
ANKRD10 ENSG00000088448.14 0.401756452 5 4 0.111574127
Brain_Cortex 0.874064497 Brain_Cerebellar_Hemisphere 2.7059863
0.017226558 0.391561974 -1.591157536 0.664014948 -0.343027911
0.925014102 4 0 0.998207054
RP11-445H22.3 ENSG00000283440.1 0.401765802 10 7 0.283269552
Brain_Cerebellar_Hemisphere 0.931229718 Brain_Nucleus_accumbens_basal_ganglia
5.84856789 0.071418747 0.235025869 -1.073003487 1.015544445
0.013068565 0.624175398 7 0 0.998207054
C11orf42 ENSG00000180878.2 0.401989601 1 1 0.401989601
Brain_Amygdala 0.401989601 Brain_Amygdala 1 1 1 0.838073186
0.838073186 0.838073186 NA 1 0 0.998207054
SLC26A5 ENSG00000170615.14 0.402116867 3 3 0.091709567
Brain_Spinal_cord_cervical_c-1 0.880361861 Brain_Cerebellum 1.182004732
0.828460543 0.828460543 -0.160415122 1.686447875 0.558847754
0.988827696 3 0 0.998207054
STEAP3 ENSG00000115107.19 0.402320588 5 5 0.083943833
Brain_Caudate_basal_ganglia 0.918042265 Brain_Nucleus_accumbens_basal_ganglia
1.750822429 0.300225499 0.300225499 -0.102900092 1.728247656
0.883040373 0.6775918 5 0 0.998207054

LINC00937 ENSG00000226091.7 0.402427544 13 4 0.070422501
Brain_Cortex 0.446234998 Brain_Substantia_nigra 9.535329755
0.003671297 0.457902983 -1.238602616 1.809183494 0.999237236
0.737564601 4 0 0.998207054
MMRN1 ENSG00000138722.9 0.402457042 12 5 0.062757738
Brain_Cerebellar_Hemisphere 0.993714847 Brain_Cerebellum 8.854143733
1.50E-18 0.311908447 -0.063192218 1.860903977 0.637218064
0.588425854 5 0 0.998207054
LINC00282 ENSG00000281106.2 0.402481708 6 5 0.051222659
Brain_Cortex 0.768516435 Brain_Caudate_basal_ganglia 2.999903344
0.076075179 0.144662304 -0.315234678 1.949609674 1.173087171
0.952081104 5 0 0.998207054
JAK2 ENSG00000096968.13 0.402499498 1 1 0.402499498
Brain_Frontal_Cortex_BA9 0.402499498 Brain_Frontal_Cortex_BA9 1 1
1 0.837165587 0.837165587 0.837165587 NA 1 0
0.998207054
ABCA6 ENSG00000154262.12 0.402513569 13 6 0.15381622
Brain_Cerebellar_Hemisphere 0.866192854 Brain_Amygdala 8.571818758
0.00144043 0.327944202 -0.963041693 1.426180592 0.455024332
0.66525495 6 0 0.998207054
LRRN4CL ENSG00000177363.4 0.402684065 1 1 0.402684065
Brain_Hypothalamus 0.402684065 Brain_Hypothalamus 1 1 1
-0.836837235 -0.836837235 -0.836837235 NA 1 0 0.998207054
RP11-281O15.8 ENSG00000272431.1 0.402900873 1 1 0.402900873
Brain_Putamen_basal_ganglia 0.402900873 Brain_Putamen_basal_ganglia 1 1
1 -0.836451637 -0.836451637 -0.836451637 NA 1 0
0.998207054
TRMT10A ENSG00000145331.13 0.40296574 1 1 0.40296574 Brain_Hypothalamus
0.40296574 Brain_Hypothalamus 1 1 1 -0.836336296 -0.836336296
-0.836336296 NA 1 0 0.998207054
APOL4 ENSG00000100336.17 0.403117367 10 9 0.044395315
Brain_Nucleus_accumbens_basal_ganglia 0.716269128 Brain_Amygdala
4.527162772 4.53E-17 0.196364516 -2.010339065 1.861820698
-0.847190064 1.048275942 9 0 0.998207054
TMEM236 ENSG00000148483.8 0.403170599 2 2 0.19250598 Brain_Cerebellum
0.760487048 Brain_Spinal_cord_cervical_c-1 1.030063457 0.969936543
0.969936543 0.30484127 1.303201495 0.804021382 0.705947285 2
0 0.998207054
DCST1 ENSG00000163357.10 0.403208756 2 2 0.236262296 Brain_Cerebellum
0.240327525 Brain_Cerebellar_Hemisphere 1.531182198 0.468817802
0.468817802 1.174168539 1.184380955 1.179274747 0.007221269
2 0 0.998207054
STAP2 ENSG00000178078.11 0.403414428 13 9 0.165828726
Brain_Hypothalamus 0.936925337 Brain_Cerebellum 6.376417441
0.001803168 0.408551078 -0.995440437 1.385732087 0.096847456
0.769416335 9 0 0.998207054
WFDC13 ENSG00000168634.4 0.403558459 1 1 0.403558459
Brain_Frontal_Cortex_BA9 0.403558459 Brain_Frontal_Cortex_BA9 1 1
1 -0.835282872 -0.835282872 -0.835282872 NA 1 0
0.998207054
LCN1 ENSG00000160349.9 0.403591906 4 4 0.05537688
Brain_Frontal_Cortex_BA9 0.649038041 Brain_Anterior_cingulate_cortex_BA24
1.777955483 0.402078089 0.402078089 -0.455098967 1.91590743
0.749046057 0.980217112 4 0 0.998207054
C4orf47 ENSG00000205129.8 0.403667931 2 2 0.237638845
Brain_Cerebellum 0.274345623 Brain_Cerebellar_Hemisphere 1.430427342
0.569572658 0.569572658 1.093109734 1.180909077 1.137009406
0.06208351 2 0 0.998207054
PDYN ENSG00000101327.8 0.403738893 1 1 0.403738893
Brain_Anterior_cingulate_cortex_BA24 0.403738893
Brain_Anterior_cingulate_cortex_BA24 1 1 1 -0.834962376
-0.834962376 -0.834962376 NA 1 0 0.998207054

TNFRSF10D ENSG00000173530.5 0.403753798 1 1 0.403753798
Brain_Frontal_Cortex_BA9 0.403753798 Brain_Frontal_Cortex_BA9 1 1
1 0.834935903 0.834935903 0.834935903 NA 1 0
0.998207054
UBE2QL1 ENSG00000215218.3 0.404181287 1 1 0.404181287
Brain_Cerebellum 0.404181287 Brain_Cerebellum 1 1 1 0.834176935
0.834176935 0.834176935 NA 1 0 0.998207054
NEK11 ENSG00000114670.13 0.404205082 1 1 0.404205082 Brain_Cortex
0.404205082 Brain_Cortex 1 1 1 -0.834134703 -0.834134703
-0.834134703 NA 1 0 0.998207054
JAKMIP3 ENSG00000188385.11 0.40431459 13 3 0.133279147 Brain_Cerebellum
0.732926982 Brain_Caudate_basal_ganglia 10.50913665 0.012372616
0.473837936 0.341234594 1.501295504 1.02167891 0.386719243 3
0 0.998207054
ACTN1 ENSG00000072110.13 0.404346669 1 1 0.404346669 Brain_Cerebellum
0.404346669 Brain_Cerebellum 1 1 1 0.833883442 0.833883442
0.833883442 NA 1 0 0.998207054
TBC1D10C ENSG00000175463.11 0.404381884 3 2 0.331998531
Brain_Caudate_basal_ganglia 0.92297701 Brain_Nucleus_accumbens_basal_ganglia
2.611249606 0.048070961 0.340679434 -0.096684424 0.970096225
0.408486293 0.535625127 2 0 0.998207054
LINC01668 ENSG00000283051.1 0.404470179 7 7 0.21589861
Brain_Putamen_basal_ganglia 0.983082575 Brain_Frontal_Cortex_BA9
1.724952742 0.254728002 0.254728002 -0.821606423 1.237507828
0.058770574 0.776613737 7 0 0.998207054
RP11-108M9.4 ENSG00000238142.1 0.404473911 12 9 0.03319682
Brain_Putamen_basal_ganglia 0.805257538 Brain_Cerebellum 6.611522844
0.070237141 0.246568697 -0.634320549 2.129694785 1.378651363
0.907849987 9 0 0.998207054
NAXE ENSG00000163382.11 0.404479564 13 2 0.116495293
Brain_Anterior_cingulate_cortex_BA24 0.687687295 Brain_Cerebellar_Hemisphere
11.74692291 0.001324538 0.398574353 -1.569655439 -0.401995559
-0.851583778 0.367255242 2 0 0.998207054
RP11-12D24.10 ENSG00000272866.1 0.404489804 1 1 0.404489804
Brain_Substantia_nigra 0.404489804 Brain_Substantia_nigra 1 1
1 0.833629486 0.833629486 0.833629486 NA 1 0
0.998207054
DCST2 ENSG00000163354.14 0.404537613 12 5 0.045488174
Brain_Spinal_cord_cervical_c-1 0.512158082 Brain_Frontal_Cortex_BA9
9.132831944 0.004983826 0.390820069 0.655481052 2.000111978
1.350299538 0.336482103 5 0 0.998207054
PTPRH ENSG00000080031.9 0.404593716 7 2 0.233378586
Brain_Frontal_Cortex_BA9 0.658158519 Brain_Cerebellum 5.752276585
6.47E-17 0.99897412 -1.191700796 0.791106224 0.386934272
0.706802907 2 0 0.998207054
IFT122 ENSG00000163913.11 0.404702845 12 2 0.138223448
Brain_Anterior_cingulate_cortex_BA24 0.880538194 Brain_Cerebellum
10.64324579 0.004434686 0.593514153 -1.482439283 0.273777094
-0.792758728 0.488580562 2 0 0.998207054
STMN4 ENSG00000015592.16 0.404930803 9 6 0.056891254
Brain_Hypothalamus 0.650961333 Brain_Caudate_basal_ganglia 4.791239646
0.000647603 0.212239405 -1.904145374 0.980106568 -1.033360288
0.901637393 6 0 0.998207054
EHBP1 ENSG00000115504.14 0.404941358 1 1 0.404941358
Brain_Hypothalamus 0.404941358 Brain_Hypothalamus 1 1 1
-0.832828678 -0.832828678 -0.832828678 NA 1 0 0.998207054
SF1 ENSG00000168066.20 0.405097324 1 1 0.405097324
Brain_Caudate_basal_ganglia 0.405097324 Brain_Caudate_basal_ganglia 1 1
1 -0.832552204 -0.832552204 -0.832552204 NA 1 0
0.998207054
SDS ENSG00000135094.10 0.405114203 2 2 0.207498817
Brain_Frontal_Cortex_BA9 0.653025087 Brain_Nucleus_accumbens_basal_ganglia

1.014044203 0.985955797 0.985955797 0.449563673 1.260472903
0.855018288 0.573399416 2 0 0.998207054
GALNT3 ENSG00000115339.13 0.405144633 9 8 0.008289912
Brain_Frontal_Cortex_BA9 0.733158929 Brain_Putamen_basal_ganglia
4.086072984 0.028923733 0.162593212 -0.34092648 2.64002822
1.309673738 0.842386016 8 0 0.998207054
CTDSP2 ENSG00000175215.10 0.40524329 1 1 0.40524329 Brain_Cerebellum
0.40524329 Brain_Cerebellum 1 1 1 0.832293516 0.832293516
0.832293516 NA 1 0 0.998207054
NINL ENSG00000101004.14 0.405465921 8 4 0.106444937
Brain_Nucleus_accumbens_basal_ganglia 0.9362855 Brain_Putamen_basal_ganglia
6.109283155 0.005435289 0.273786383 -0.079939341 1.614380417
0.988545167 0.613959745 4 0 0.998207054
SMG9 ENSG00000105771.13 0.405529504 1 1 0.405529504
Brain_Cerebellar_Hemisphere 0.405529504 Brain_Cerebellar_Hemisphere 1 1
1 0.831786432 0.831786432 0.831786432 NA 1 0
0.998207054
OSBPL7 ENSG00000006025.11 0.405699897 2 2 0.255214574
Brain_Nucleus_accumbens_basal_ganglia 0.516672574 Brain_Cerebellum
1.056552017 0.943447983 0.943447983 -1.137774695 0.648483044
-0.244645825 1.26307496 2 0 0.998207054
LINC00921 ENSG00000281005.1 0.405764726 10 4 0.062505385
Brain_Putamen_basal_ganglia 0.533975492 Brain_Frontal_Cortex_BA9
7.731250512 1.77E-05 0.420622851 -1.862693611 -0.621948865
-1.489278633 0.377089957 4 0 0.998207054
BABAM1 ENSG00000105393.15 0.405833055 1 1 0.405833055
Brain_Anterior_cingulate_cortex_BA24 0.405833055
Brain_Anterior_cingulate_cortex_BA24 1 1 1 0.831248865
0.831248865 0.831248865 NA 1 0 0.998207054
EYA4 ENSG00000112319.17 0.405843622 4 4 0.175845602
Brain_Spinal_cord_cervical_c-1 0.727037633 Brain_Cortex 2.217073021
0.278435126 0.278435126 -1.353657722 0.349068834 -0.5581641
0.729486323 4 0 0.998207054
RAB36 ENSG00000100228.12 0.405850159 10 2 0.179310846 Brain_Cortex
0.990633645 Brain_Cerebellar_Hemisphere 8.369605551 0.001819907
0.937188063 -0.641950433 1.342879961 0.230734909 0.583407442
2 0 0.998207054
C11orf96 ENSG00000187479.5 0.405995127 1 1 0.405995127
Brain_Cortex 0.405995127 Brain_Cortex 1 1 1 -0.830961946
-0.830961946 -0.830961946 NA 1 0 0.998207054
GRK3 ENSG00000100077.14 0.406078379 4 4 0.182889167
Brain_Caudate_basal_ganglia 0.548668105 Brain_Hippocampus 1.896349401
0.140393704 0.140393704 -1.33191182 0.599757135 -0.58685315
0.8387658 4 0 0.998207054
SDK1 ENSG00000146555.18 0.406359293 2 2 0.259478845 Brain_Cerebellum
0.567578527 Brain_Hypothalamus 1.132679591 0.867320409
0.867320409 0.571621352 1.127623767 0.849622559 0.393153078
2 0 0.998207054
HSD17B14 ENSG00000087076.8 0.406435641 5 3 0.09221383 Brain_Cerebellum
0.935113979 Brain_Nucleus_accumbens_basal_ganglia 3.09826042 0.013915485
0.578131486 -1.68383361 -0.081412411 -0.619435072 0.657050156
3 0 0.998207054
TSPYL1 ENSG00000189241.6 0.406514601 3 2 0.460869514
Brain_Cerebellum 0.926217886 Brain_Cortex 2.789131708 0.084257193
0.1266111 -0.737415826 -0.092604353 -0.400141188 0.323432701 2
0 0.998207054
DLL4 ENSG00000128917.6 0.406529964 1 1 0.406529964
Brain_Substantia_nigra 0.406529964 Brain_Substantia_nigra 1 1
1 -0.8300156 -0.8300156 -0.8300156 NA 1 0 0.998207054
TACR3 ENSG00000169836.4 0.406530018 2 2 0.293113526
Brain_Spinal_cord_cervical_c-1 0.331812128 Brain_Cortex 1.137722088
0.862277912 0.862277912 -1.051315902 -0.970470288 -1.010893095

0.057166482 2 0 0.998207054
CNOT11 ENSG00000158435.7 0.406566668 2 2 0.179720892
Brain_Anterior_cingulate_cortex_BA24 0.935376346 Brain_Hypothalamus
1.067259529 0.932740471 0.932740471 -1.341614901 -0.081082496
-0.711348698 0.891331012 2 0 0.998207054
CCDC107 ENSG00000159884.11 0.406569861 1 1 0.406569861
Brain_Cortex 0.406569861 Brain_Cortex 1 1 1 0.829945035
0.829945035 0.829945035 NA 1 0 0.998207054
CD109 ENSG00000156535.13 0.406840372 1 1 0.406840372
Brain_Nucleus_accumbens_basal_ganglia 0.406840372
Brain_Nucleus_accumbens_basal_ganglia 1 1 1 0.829466701
0.829466701 0.829466701 NA 1 0 0.998207054
NBPF11 ENSG00000263956.6 0.406878057 10 4 0.18183748
Brain_Cerebellar_Hemisphere 0.975814639 Brain_Putamen_basal_ganglia 7.02304961
7.43E-07 0.272621913 -1.335118762 0.89944913 0.456619448
0.699381631 4 0 0.998207054
CLEC12B ENSG00000256660.5 0.4069064 2 2 0.225652015
Brain_Spinal_cord_cervical_c-1 0.525293072 Brain_Putamen_basal_ganglia
1.050542074 0.949457926 0.949457926 -1.211635301 -0.635207531
-0.923421416 0.407595985 2 0 0.998207054
PIGBOS1 ENSG00000225973.3 0.406914944 9 4 0.075741447
Brain_Anterior_cingulate_cortex_BA24 0.980134766 Brain_Hypothalamus
6.637751811 0.000731491 0.355236717 -0.50660726 1.775948252
0.093856919 0.663409006 4 0 0.998207054
GPR45 ENSG00000135973.2 0.406929372 1 1 0.406929372
Brain_Cerebellar_Hemisphere 0.406929372 Brain_Cerebellar_Hemisphere 1 1
1 0.829309367 0.829309367 0.829309367 NA 1 0
0.998207054
DCAF1 ENSG00000145041.15 0.406932601 1 1 0.406932601
Brain_Hippocampus 0.406932601 Brain_Hippocampus 1 1 1
-0.829303659 -0.829303659 -0.829303659 NA 1 0 0.998207054
CD320 ENSG00000167775.10 0.406938936 1 1 0.406938936
Brain_Cerebellar_Hemisphere 0.406938936 Brain_Cerebellar_Hemisphere 1 1
1 -0.82929246 -0.82929246 -0.82929246 NA 1 0
0.998207054
FAM120B ENSG00000112584.13 0.407225943 8 5 0.04942707 Brain_Cortex
0.775278191 Brain_Cerebellum 4.5726752 0.031404799 0.190756767
-0.285477695 1.964889143 1.159273777 0.79204214 5 0
0.998207054
ICK ENSG00000112144.15 0.407336984 5 4 0.053590965
Brain_Hippocampus 0.307716871 Brain_Amygdala 3.554268852
0.036397434 0.135316564 -1.930128118 -1.020024437 -1.439115827
0.43561697 4 0 0.998207054
DNM3 ENSG00000197959.13 0.407382141 3 2 0.196462119
Brain_Substantia_nigra 0.503711175 Brain_Cerebellar_Hemisphere
2.104140844 0.013118165 0.882740991 -0.67823321 1.291696936
-0.018399392 1.134586795 2 0 0.998207054
CYP27C1 ENSG00000186684.12 0.407510535 1 1 0.407510535
Brain_Cortex 0.407510535 Brain_Cortex 1 1 1 -0.828282494
-0.828282494 -0.828282494 NA 1 0 0.998207054
NDUFA12 ENSG00000184752.12 0.407530356 2 2 0.208241248
Brain_Substantia_nigra 0.680936251 Brain_Spinal_cord_cervical_c-1
1.038576885 0.961423115 0.961423115 -1.258416277 -0.411185866
-0.834801072 0.599082369 2 0 0.998207054
LDAH ENSG00000118961.14 0.407741957 13 4 0.30175357 Brain_Hippocampus
0.937079463 Brain_Cerebellum 9.876618544 0.007845376 0.474648061
-0.976560969 1.032680226 -0.297877102 0.533227309 4 0
0.998207054
EXOSC5 ENSG00000077348.8 0.407749761 6 5 0.140136168
Brain_Substantia_nigra 0.763911695 Brain_Cerebellum 3.097557714
0.006642593 0.155155245 -0.596033584 1.475284142 0.577318707
0.728355827 5 0 0.998207054

TMOD2 ENSG00000128872.9 0.407863313 3 3 0.208352302 Brain_Cerebellum 0.607020311 Brain_Putamen_basal_ganglia 1.214104669 0.77561254 0.77561254 -0.514331401 1.258109101 0.509571326 0.917744914 3 0 0.998207054
C17orf53 ENSG00000125319.14 0.407915245 1 1 0.407915245 Brain_Cerebellum 0.407915245 Brain_Cerebellum 1 1 1 0.827567915 0.827567915 0.827567915 NA 1 0 0.998207054
SLX1B ENSG00000181625.17 0.407957799 3 3 0.206964424 Brain_Cerebellar_Hemisphere 0.558286681 Brain_Cortex 1.24453609 0.792115226 0.792115226 -1.261956544 0.688878628 -0.386155392 0.990560331 3 0 0.998207054
MMP17 ENSG00000198598.6 0.408079264 1 1 0.408079264 Brain_Cerebellum 0.408079264 Brain_Cerebellum 1 1 1 -0.827278435 -0.827278435 -0.827278435 NA 1 0 0.998207054
ORMDL1 ENSG00000128699.13 0.408114086 13 1 0.222158937 Brain_Putamen_basal_ganglia 0.700112693 Brain_Anterior_cingulate_cortex_BA24 12.0608239 0.001607455 12.0608239 0.385168347 1.220807437 0.796473968 0.209841256 1 0 0.998207054
RP11-105C19.2 ENSG00000260973.1 0.40812187 1 1 0.40812187 Brain_Cerebellar_Hemisphere 0.40812187 Brain_Cerebellar_Hemisphere 1 1 1 0.82720325 0.82720325 0.82720325 NA 1 0 0.998207054
ZNF131 ENSG00000172262.11 0.408131743 1 1 0.408131743 Brain_Cerebellum 0.408131743 Brain_Cerebellum 1 1 1 0.827185828 0.827185828 0.827185828 NA 1 0 0.998207054
PDLIM3 ENSG00000154553.13 0.408306909 2 2 0.214396332 Brain_Hypothalamus 0.623979762 Brain_Cortex 1.007853171 0.992146829 0.992146829 -1.241567085 0.490217835 -0.375674625 1.22455686 2 0 0.998207054
SYT10 ENSG00000110975.8 0.408372221 3 3 0.099321082 Brain_Frontal_Cortex_BA9 0.417884617 Brain_Cortex 1.798159749 0.248410632 0.248410632 0.810096672 1.648153958 1.103681387 0.472010106 3 0 0.998207054
PCDHA7 ENSG00000204963.5 0.408510796 8 6 0.331190723 Brain_Cerebellar_Hemisphere 0.811407829 Brain_Putamen_basal_ganglia 5.23931369 0.091377721 0.273490742 -0.959909464 0.971718274 0.101232707 0.675678852 6 0 0.998207054
RASA4B ENSG00000170667.14 0.408664203 3 3 0.483030513 Brain_Cortex 0.70652879 Brain_Spinal_cord_cervical_c-1 2.027653312 0.342747359 0.342747359 -0.64397615 0.70143672 -0.106353843 0.712233889 3 0 0.998207054
LRRC73 ENSG00000204052.4 0.408924209 4 4 0.116741245 Brain_Cortex 0.968178747 Brain_Caudate_basal_ganglia 1.860849454 0.284729375 0.284729375 -0.039892604 1.56859969 0.673700334 0.66909889 4 0 0.998207054
LSM7 ENSG00000130332.14 0.409003204 13 3 0.181175261 Brain_Hypothalamus 0.86908877 Brain_Spinal_cord_cervical_c-1 10.73841379 1.23E-16 0.422586628 -1.337145153 -0.164816051 -0.349201994 0.3151055 3 0 0.998207054
ERFE ENSG00000178752.15 0.409041241 2 1 0.313387169 Brain_Cerebellar_Hemisphere 0.531127667 Brain_Cerebellum 1.959656432 0.040343568 1.959656432 -1.008140184 -0.626285543 -0.817212864 0.270012006 1 0 0.998207054
FBXW10 ENSG00000171931.12 0.409115779 5 5 0.10883395 Brain_Nucleus_accumbens_basal_ganglia 0.969061625 Brain_Cerebellum 1.941466917 0.179656199 0.179656199 -1.603456301 0.35454022 -0.30032541 0.753523541 5 0 0.998207054
CSMD2 ENSG00000121904.17 0.409169314 7 4 0.127746646 Brain_Cerebellum 0.998675467 Brain_Substantia_nigra 3.933030066 4.19E-07 0.684574349 -1.091036849 1.523048203 -0.203245123 1.097335901 4 0 0.998207054
RP11-480D4.6 ENSG00000272049.1 0.4093027 7 7 0.086116634 Brain_Caudate_basal_ganglia 0.979515014 Brain_Cerebellum 1.425544639

0.53537284 0.53537284 -1.71624815 1.62573369 -0.107235695 1.054822594
7 0 0.998207054
TSPYL5 ENSG00000180543.4 0.409326773 4 4 0.134917956
Brain_Cerebellar_Hemisphere 0.346451315 Brain_Anterior_cingulate_cortex_BA24
2.191291829 0.128257166 0.128257166 -1.494986535 0.941494875
-0.783864676 1.159459866 4 0 0.998207054
FGFRL1 ENSG00000127418.14 0.409337469 3 3 0.195168689
Brain_Cerebellum 0.876200085 Brain_Anterior_cingulate_cortex_BA24
1.639573257 0.60005809 0.60005809 -1.295439415 0.429769134
-0.236627408 0.927134595 3 0 0.998207054
LINC00900 ENSG00000246100.3 0.409374139 9 5 0.057064596
Brain_Substantia_nigra 0.574464796 Brain_Anterior_cingulate_cortex_BA24
6.441095145 1.05E-19 0.348864461 0.561488165 1.902815717
1.276791788 0.408521257 5 0 0.998207054
GPX7 ENSG00000116157.5 0.409399235 2 2 0.491672382
Brain_Cerebellar_Hemisphere 0.711086176 Brain_Spinal_cord_cervical_c-1
1.6812806 0.3187194 0.3187194 -0.687651319 0.370397715 -0.158626802
0.748153647 2 0 0.998207054
TEX101 ENSG00000131126.18 0.409441816 4 4 0.051625551
Brain_Cerebellum 0.807394032 Brain_Cerebellar_Hemisphere 1.146738355
0.865599488 0.865599488 -0.348756939 1.946243048 0.528253056
0.987684558 4 0 0.998207054
BCAS3 ENSG00000141376.22 0.409479013 1 1 0.409479013
Brain_Spinal_cord_cervical_c-1 0.409479013 Brain_Spinal_cord_cervical_c-1
1 1 1 0.824810803 0.824810803 0.824810803 NA 1 0
0.998207054
ELOVL1 ENSG00000066322.14 0.409496385 1 1 0.409496385
Brain_Cerebellum 0.409496385 Brain_Cerebellum 1 1 1 -0.824780208
-0.824780208 -0.824780208 NA 1 0 0.998207054
EEF1G ENSG00000254772.9 0.409642344 2 2 0.720414898 Brain_Cortex
0.730763232 Brain_Nucleus_accumbens_basal_ganglia 1.861945385
0.138054615 0.138054615 -0.357904346 0.344110439 -0.006896954
0.496399415 2 0 0.998207054
EBAG9 ENSG00000147654.14 0.409653644 4 4 0.192859419
Brain_Hypothalamus 0.533885669 Brain_Spinal_cord_cervical_c-1
1.872522945 0.170275233 0.170275233 -1.077869079 1.302166661
0.427411157 1.042261877 4 0 0.998207054
PCCA ENSG00000175198.15 0.409709767 1 1 0.409709767
Brain_Nucleus_accumbens_basal_ganglia 0.409709767
Brain_Nucleus_accumbens_basal_ganglia 1 1 1 -0.824404486
-0.824404486 -0.824404486 NA 1 0 0.998207054
LINC00499 ENSG00000251372.5 0.409717135 13 12 0.048198375
Brain_Amygdala 0.919770547 Brain_Putamen_basal_ganglia 2.858988205
0.05316579 0.25760344 -1.975615207 1.43692811 -0.32807627 0.901771435
12 0 0.998207054
EIF3B ENSG00000106263.17 0.409726026 4 4 0.201028156
Brain_Hypothalamus 0.76840386 Brain_Cortex 2.691057296 0.113956544
0.113956544 -1.278627799 0.452520252 -0.189691583 0.798611974
4 0 0.998207054
NPY4R ENSG00000204174.7 0.409999579 1 1 0.409999579 Brain_Cerebellum
0.409999579 Brain_Cerebellum 1 1 1 0.823894371 0.823894371
0.823894371 NA 1 0 0.998207054
CBWD2 ENSG00000136682.14 0.410046819 8 3 0.095071948
Brain_Cerebellar_Hemisphere 0.298579394 Brain_Frontal_Cortex_BA9
6.805470857 0.000272253 0.323594099 1.039484649 1.669229286
1.440254817 0.19454514 3 0 0.998207054
BSPRY ENSG00000119411.10 0.410073792 3 2 0.292907048
Brain_Frontal_Cortex_BA9 0.75720241 Brain_Anterior_cingulate_cortex_BA24
2.766800563 0.009991724 0.223207713 -1.051765724 -0.309156595
-0.75859774 0.395200019 2 0 0.998207054
DHRS4 ENSG00000157326.18 0.410149259 5 5 0.09578144 Brain_Cortex
0.498962318 Brain_Cerebellum 1.777900227 0.538310401 0.538310401

-0.915702522 1.66565856 0.391062003 1.127874931 5 0
0.998207054
LAT ENSG00000213658.11 0.410188374 3 2 0.182731608
Brain_Putamen_basal_ganglia 0.313446916 Brain_Frontal_Cortex_BA9
2.730073747 7.55E-08 0.269926177 1.008015719 1.332391397
1.116144118 0.187275637 2 0 0.998207054
RP11-285A1.1 ENSG00000260988.1 0.410261368 2 2 0.349078672
Brain_Putamen_basal_ganglia 0.371851335 Brain_Nucleus_accumbens_basal_ganglia
1.060472331 0.939527669 0.939527669 -0.8930109 0.936377861
0.021683481 1.293573199 2 0 0.998207054
CTLA4 ENSG00000163599.14 0.41026857 1 1 0.41026857 Brain_Cerebellum 0.41026857
Brain_Cerebellum 1 1 1 -0.823421096 -0.823421096 -0.823421096
NA 1 0 0.998207054
SIGLEC9 ENSG00000129450.8 0.41027093 1 1 0.41027093 Brain_Cerebellum
0.41027093 Brain_Cerebellum 1 1 1 0.823416946 0.823416946
0.823416946 NA 1 0 0.998207054
PDIA5 ENSG00000065485.19 0.410312033 1 1 0.410312033 Brain_Cortex
0.410312033 Brain_Cortex 1 1 1 0.823344644 0.823344644
0.823344644 NA 1 0 0.998207054
PDXP ENSG00000241360.1 0.410340946 2 2 0.181961179
Brain_Hippocampus 0.945748181 Brain_Spinal_cord_cervical_c-1
1.050379882 0.949620118 0.949620118 -0.06804705 1.334740848
0.633346899 0.991920836 2 0 0.998207054
MCM2 ENSG00000073111.13 0.410609855 2 2 0.241898971
Brain_Anterior_cingulate_cortex_BA24 0.552189431 Brain_Hippocampus
1.039227936 0.960772064 0.960772064 -0.594482519 1.170253495
0.287885488 1.247856802 2 0 0.998207054
SIRT3 ENSG00000142082.14 0.410676929 10 5 0.048533198
Brain_Substantia_nigra 0.473131695 Brain_Anterior_cingulate_cortex_BA24
7.100330893 0.00523817 0.303516071 0.717392907 1.972669772
1.355303923 0.408584488 5 0 0.998207054
RP11-255C15.3 ENSG00000260743.1 0.410738228 2 2 0.430405585
Brain_Cerebellar_Hemisphere 0.696151295 Brain_Cerebellum 1.59825253 0.40174747
0.40174747 -0.7884978 0.390521047 -0.198988377 0.833692222 2 0
0.998207054
KYAT3 ENSG00000137944.17 0.41076702 12 1 0.30195243
Brain_Anterior_cingulate_cortex_BA24 0.598346906 Brain_Putamen_basal_ganglia
11.12916745 0.002718457 11.12916745 0.526779233 1.032255524
0.791467134 0.145995535 1 0 0.998207054
PBX1 ENSG00000185630.18 0.410781509 1 1 0.410781509
Brain_Putamen_basal_ganglia 0.410781509 Brain_Putamen_basal_ganglia 1 1
1 0.82251912 0.82251912 0.82251912 NA 1 0 0.998207054
TMTC3 ENSG00000139324.11 0.410864165 3 2 0.146014141 Brain_Cerebellum
0.821105238 Brain_Caudate_basal_ganglia 1.96618631 0.03385199 0.9999617
-0.226123667 1.453755369 0.791036809 0.894270763 2 0
0.998207054
TMEM17 ENSG00000186889.9 0.411012971 8 5 0.029338925
Brain_Cerebellum 0.779073104 Brain_Spinal_cord_cervical_c-1 4.58748739
0.033788257 0.577685763 -2.178902325 0.280527143 -1.173161736
0.911804116 5 0 0.998207054
KSR2 ENSG00000171435.13 0.411093226 1 1 0.411093226
Brain_Caudate_basal_ganglia 0.411093226 Brain_Caudate_basal_ganglia 1 1
1 -0.821971309 -0.821971309 -0.821971309 NA 1 0
0.998207054
LPAR2 ENSG00000064547.13 0.411102725 3 3 0.105194291
Brain_Spinal_cord_cervical_c-1 0.527480228 Brain_Amygdala 1.914447786
0.096511054 0.096511054 -1.620176856 1.516621855 -0.245137383
1.603758296 3 0 0.998207054
CACNA1G ENSG00000006283.17 0.411176251 2 2 0.280900637
Brain_Hippocampus 0.436549293 Brain_Frontal_Cortex_BA9 1.005571299
0.994428701 0.994428701 0.778033451 1.078298341 0.928165896
0.21231934 2 0 0.998207054

EBNA1BP2 ENSG00000117395.12 0.411177121 3 3 0.140716054
Brain_Substantia_nigra 0.918296792 Brain_Nucleus_accumbens_basal_ganglia
1.744186775 0.44007608 0.44007608 -0.469711194 1.473129748
0.300279718 1.032172143 3 0 0.998207054
PIP5KL1 ENSG00000167103.11 0.411286351 4 3 0.330151102
Brain_Caudate_basal_ganglia 0.749541804 Brain_Spinal_cord_cervical_c-1
2.817623767 0.070215987 0.200491773 -0.971465753 0.973809568
0.230850684 0.845178639 3 0 0.998207054
PEX5 ENSG00000139197.10 0.411343538 11 2 0.562878765
Brain_Nucleus_accumbens_basal_ganglia 0.997512458 Brain_Caudate_basal_ganglia
10.25960716 0.000917717 0.342409233 -0.278166582 0.57857087
0.150143572 0.267566396 2 0 0.998207054
PTPN12 ENSG00000127947.15 0.411421403 9 6 0.373764815
Brain_Frontal_Cortex_BA9 0.882370114 Brain_Caudate_basal_ganglia
5.880216274 0.029512641 0.213414414 -0.642144309 0.889443427
0.179224038 0.604437689 6 0 0.998207054
B3GAT2 ENSG00000112309.10 0.41145428 1 1 0.41145428
Brain_Putamen_basal_ganglia 0.41145428 Brain_Putamen_basal_ganglia 1 1 1
0.821337101 0.821337101 0.821337101 NA 1 0 0.998207054
ZDHHC18 ENSG00000204160.11 0.411469843 1 1 0.411469843
Brain_Cortex 0.411469843 Brain_Cortex 1 1 1 -0.821309772
-0.821309772 -0.821309772 NA 1 0 0.998207054
CXADR ENSG00000154639.18 0.41149316 2 2 0.182655389 Brain_Cerebellum
0.256424233 Brain_Cerebellar_Hemisphere 1.849097393 0.150902607
0.150902607 1.134883264 1.332623501 1.233753383 0.139823463
2 0 0.998207054
LIPJ ENSG00000204022.9 0.411507435 2 2 0.184440883
Brain_Anterior_cingulate_cortex_BA24 0.873674263 Brain_Cerebellar_Hemisphere
1.02947158 0.97052842 0.97052842 0.158993159 1.327204987 0.743099073
0.826050505 2 0 0.998207054
TAPBPL ENSG00000139192.11 0.411599646 13 2 0.336353441
Brain_Hippocampus 0.984190444 Brain_Hypothalamus 11.91532855
3.71E-05 0.447843768 -0.961395311 0.037134102 -0.216879972
0.298460402 2 0 0.998207054
TKT ENSG00000163931.15 0.411692324 8 3 0.131034349
Brain_Frontal_Cortex_BA9 0.720257674 Brain_Spinal_cord_cervical_c-1
6.827876589 0.005000454 0.35133746 -1.510035568 -0.358114439
-0.752727887 0.36663209 3 0 0.998207054
MED7 ENSG00000155868.7 0.411704491 2 2 0.247637688
Brain_Hippocampus 0.305647678 Brain_Hypothalamus 1.349537916
0.650462084 0.650462084 -1.156106278 -1.024397255 -1.090251766
0.093132343 2 0 0.998207054
IQCD ENSG00000166578.9 0.411818076 1 1 0.411818076
Brain_Hypothalamus 0.411818076 Brain_Hypothalamus 1 1 1
0.82069841 0.82069841 0.82069841 NA 1 0 0.998207054
IGSF8 ENSG00000162729.13 0.4118753 4 3 0.076844101
Brain_Cerebellar_Hemisphere 0.770771275 Brain_Caudate_basal_ganglia
1.981222648 0.022160653 0.89962281 -1.769298333 0.291366183
-0.841180058 0.955450944 3 0 0.998207054
MSH6 ENSG00000116062.14 0.411998902 3 3 0.198181197
Brain_Nucleus_accumbens_basal_ganglia 0.987112177 Brain_Cerebellar_Hemisphere
1.74358813 0.469035838 0.469035838 -0.727114268 1.286750695
0.191929873 1.018374245 3 0 0.998207054
CDC16 ENSG00000130177.14 0.412018559 5 4 0.215241828 Brain_Cerebellum
0.874249927 Brain_Putamen_basal_ganglia 3.758069756 0.065509377
0.166301061 -1.239279986 0.324276512 -0.515830963 0.7165995 4
0 0.998207054
C3orf58 ENSG00000181744.8 0.412088871 1 1 0.412088871
Brain_Hypothalamus 0.412088871 Brain_Hypothalamus 1 1 1
-0.820223214 -0.820223214 -0.820223214 NA 1 0 0.998207054
SLC26A3 ENSG00000091138.12 0.412127074 1 1 0.412127074
Brain_Cerebellar_Hemisphere 0.412127074 Brain_Cerebellar_Hemisphere 1 1

1	0.820156188	0.820156188	0.820156188	NA	1	0
0.998207054
MCFD2	ENSG00000180398.11	0.412469159	13	1	0.287108838
Brain_Nucleus_accumbens_basal_ganglia	0.650665822
Brain_Anterior_cingulate_cortex_BA24	12.17347533	0.000263705
12.17347533	-1.06448546	-0.452837412	-0.792485842	0.165548348
1	0	0.998207054
PPFIA4	ENSG00000143847.15	0.412812486	4	4	0.103730901
Brain_Frontal_Cortex_BA9	0.653000329	Brain_Cerebellar_Hemisphere
1.859787776	0.153924045	0.153924045	-1.627029176	-0.449598002
-0.947769893	0.498396421	4	0	0.998207054
LINC01354	ENSG00000231768.1	0.412843335	5	4	0.077286167
Brain_Cerebellum	0.845891121	Brain_Putamen_basal_ganglia	2.768184937
0.073830313	0.199629427	-1.766654145	-0.194363687	-0.787848544
0.624013072	4	0	0.998207054
FPGT	ENSG00000254685.6	0.412889073	5	4	0.180787637
Brain_Spinal_cord_cervical_c-1	0.923574461	Brain_Cortex	3.110949877
0.091451905	0.141555378	-1.048045424	1.338333835	0.132244821
0.885781732	4	0	0.998207054
EXOC2	ENSG00000112685.13	0.412910054	3	3	0.101452937	Brain_Cerebellum
0.969830189	Brain_Spinal_cord_cervical_c-1	1.394737808	0.761721174
0.761721174	-0.037821266	1.637850244	0.711608336	0.851713389
3	0	0.998207054
AMBRA1	ENSG00000110497.14	0.412981432	1	1	0.412981432
Brain_Hypothalamus	0.412981432	Brain_Hypothalamus	1	1	1
0.818658234	0.818658234	0.818658234	NA	1	0	0.998207054
ANKRD36B	ENSG00000196912.12	0.412987513	11	5	0.141890888
Brain_Substantia_nigra	0.863401025	Brain_Frontal_Cortex_BA9
7.220345004	6.73E-05	0.269082122	-0.645198464	1.468785834
-0.015719762	0.780222068	5	0	0.998207054
PLPP5	ENSG00000147535.16	0.413075667	6	1	0.271125556
Brain_Hypothalamus	0.782722164	Brain_Cerebellar_Hemisphere	5.670790894
0.005336881	5.670790894	-1.100474221	-0.275773337	-0.795337887
0.315532138	1	0	0.998207054
PTGFRN	ENSG00000134247.9	0.413094936	2	2	0.212311525
Brain_Cerebellum	0.481903799	Brain_Frontal_Cortex_BA9	1.201793818
0.798206182	0.798206182	-1.247234504	-0.703243846	-0.975239175
0.384659483	2	0	0.998207054
STARD9	ENSG00000159433.11	0.413216684	1	1	0.413216684
Brain_Cerebellar_Hemisphere	0.413216684	Brain_Cerebellar_Hemisphere	1	1
1	0.818246086	0.818246086	0.818246086	NA	1	0
0.998207054
ERVV-1	ENSG00000269526.1	0.413424279	1	1	0.413424279
Brain_Cerebellar_Hemisphere	0.413424279	Brain_Cerebellar_Hemisphere	1	1
1	0.817882508	0.817882508	0.817882508	NA	1	0
0.998207054
DCP2	ENSG00000172795.15	0.413522809	4	3	0.602524157	Brain_Cerebellum
0.902351046	Brain_Putamen_basal_ganglia	3.224590292	0.044471515
0.237139943	-0.520774086	0.518076159	-0.080079031	0.49087822	3
0	0.998207054
RPL29	ENSG00000162244.10	0.413602227	6	5	0.148561594
Brain_Anterior_cingulate_cortex_BA24	0.93639021	Brain_Cerebellar_Hemisphere
3.824235389	0.061181276	0.194676858	-1.444630882	0.734678207
0.150741703	0.816079327	5	0	0.998207054
CABLES2	ENSG00000149679.11	0.413645523	5	4	0.128649214
Brain_Hippocampus	0.944421107	Brain_Hypothalamus	2.587036621
0.057284116	0.58255538	-1.519450199	0.320534868	-0.707545002
0.824446566	4	0	0.998207054
COQ8A	ENSG00000163050.16	0.413751884	3	3	0.14905012	Brain_Cortex
0.681970093	Brain_Cerebellum	1.848879929	0.154679177	0.154679177
-1.442894759	0.64935637	-0.127920714	1.145083933	3	0
0.998207054

TDP1 ENSG00000042088.13 0.413804548 2 2 0.475280674
Brain_Cerebellar_Hemisphere 0.788553454 Brain_Cerebellum 1.717460045
0.282539955 0.282539955 -0.713913491 0.268189503 -0.222861994
0.694451687 2 0 0.998207054
NEK2 ENSG00000117650.12 0.413886106 1 1 0.413886106
Brain_Hypothalamus 0.413886106 Brain_Hypothalamus 1 1 1
-0.81707406 -0.81707406 -0.81707406 NA 1 0 0.998207054
C17orf100 ENSG00000256806.5 0.413933068 7 3 0.114763201
Brain_Cerebellar_Hemisphere 0.616422665 Brain_Frontal_Cortex_BA9
5.474689798 0.021174086 0.311592777 -1.577140498 -0.500926765
-1.15474573 0.445124372 3 0 0.998207054
TICRR ENSG00000140534.13 0.414009952 2 2 0.299003955
Brain_Substantia_nigra 0.304486052 Brain_Spinal_cord_cervical_c-1
1.209431084 0.790568916 0.790568916 1.026860721 1.038571739
1.03271623 0.00828094 2 0 0.998207054
ARSK ENSG00000164291.16 0.414081192 1 1 0.414081192
Brain_Nucleus_accumbens_basal_ganglia 0.414081192
Brain_Nucleus_accumbens_basal_ganglia 1 1 1 0.816732714
0.816732714 0.816732714 NA 1 0 0.998207054
CDHR1 ENSG00000148600.14 0.414238346 12 4 0.095903073
Brain_Cerebellar_Hemisphere 0.965841709 Brain_Anterior_cingulate_cortex_BA24
9.289537037 0.004536712 0.320431641 0.042824155 1.665048535
1.054263977 0.479049932 4 0 0.998207054
ZNF516 ENSG00000101493.10 0.41434461 3 3 0.196670723 Brain_Cortex
0.709075629 Brain_Cerebellum 1.068939586 0.886669884 0.886669884
-1.291095042 -0.373097837 -0.921104988 0.484197566 3 0
0.998207054
PCDHB14 ENSG00000120327.6 0.414364072 3 3 0.101083371
Brain_Hippocampus 0.478516495 Brain_Cerebellar_Hemisphere 1.967884036
0.158365022 0.158365022 0.708690606 1.639623995 1.03104623
0.527353372 3 0 0.998207054
CRIM1 ENSG00000150938.9 0.414364924 2 2 0.190666926
Brain_Cerebellar_Hemisphere 0.328159761 Brain_Cerebellum 1.839628895
0.160371105 0.160371105 0.977827271 1.308608718 1.143217995
0.233897805 2 0 0.998207054
SEC31A ENSG00000138674.16 0.414369516 1 1 0.414369516
Brain_Cortex 0.414369516 Brain_Cortex 1 1 1 0.816228401
0.816228401 0.816228401 NA 1 0 0.998207054
RP11-396O20.2 ENSG00000254645.1 0.414427297 1 1 0.414427297
Brain_Amygdala 0.414427297 Brain_Amygdala 1 1 1 0.816127359
0.816127359 0.816127359 NA 1 0 0.998207054
RBAKDN ENSG00000273313.1 0.414494313 4 2 0.759410567
Brain_Hypothalamus 0.830917496 Brain_Nucleus_accumbens_basal_ganglia
3.841881891 0.002692458 0.133159733 -0.304673919 0.306254911
-0.1158472 0.283874022 2 0 0.998207054
ZGPAT ENSG00000197114.11 0.414794168 2 2 0.285538074
Brain_Cerebellar_Hemisphere 0.911256742 Brain_Cerebellum 1.523520958
0.476479042 0.476479042 -0.111453495 1.067961067 0.478253786
0.833972034 2 0 0.998207054
EPM2AIP1 ENSG00000178567.7 0.4148415 2 2 0.191482953
Brain_Putamen_basal_ganglia 0.602812332 Brain_Amygdala 1.225795177
0.774204823 0.774204823 0.520360506 1.306204707 0.913282606
0.555675764 2 0 0.998207054
RPS14 ENSG00000164587.11 0.414915675 4 2 0.191810652 Brain_Amygdala
0.478449054 Brain_Hippocampus 3.2278841 1.51E-07 0.729479864
-1.305241425 -0.708799265 -0.915751812 0.281536572 2 0
0.998207054
OR13A1 ENSG00000256574.6 0.414959317 5 4 0.127810287
Brain_Hippocampus 0.86842351 Brain_Amygdala 2.985744354 0.021497149
0.27431127 -1.522793859 1.504670108 0.230900548 1.273717585 4
0 0.998207054
LINC01574 ENSG00000248859.2 0.415031295 3 3 0.126747272

Brain_Frontal_Cortex_BA9 0.783145989 Brain_Substantia_nigra
1.175389933 0.777324161 0.777324161 -0.275221606 1.527055256
0.718209435 0.915207216 3 0 0.998207054
TSPAN19 ENSG00000231738.10 0.415226788 2 2 0.320001152
Brain_Cerebellum 0.634927183 Brain_Frontal_Cortex_BA9 1.348906279
0.651093721 0.651093721 0.474803297 0.994455516 0.734629407
0.367449608 2 0 0.998207054
RPS6 ENSG00000137154.12 0.415288595 3 3 0.148992626 Brain_Cortex
0.728655639 Brain_Cerebellar_Hemisphere 1.741455288 0.40065205 0.40065205
-1.443098854 0.491212369 -0.201657367 1.077538029 3 0
0.998207054
ALKBH4 ENSG00000160993.3 0.415385456 1 1 0.415385456
Brain_Cerebellum 0.415385456 Brain_Cerebellum 1 1 1 -0.814453054
-0.814453054 -0.814453054 NA 1 0 0.998207054
PARPBP ENSG00000185480.11 0.415431616 1 1 0.415431616
Brain_Nucleus_accumbens_basal_ganglia 0.415431616
Brain_Nucleus_accumbens_basal_ganglia 1 1 1 0.81437245 0.81437245
0.81437245 NA 1 0 0.998207054
CHTF18 ENSG00000127586.16 0.415458549 4 4 0.130564948
Brain_Amygdala 0.924830841 Brain_Nucleus_accumbens_basal_ganglia
2.383772516 0.374691914 0.374691914 -1.511877819 0.825238476
-0.158145868 0.982798559 4 0 0.998207054
SMARCA4 ENSG00000127616.17 0.415587369 2 2 0.395067054
Brain_Caudate_basal_ganglia 0.521636441 Brain_Frontal_Cortex_BA9
1.360927862 0.639072138 0.639072138 -0.850464204 0.640824919
-0.104819642 1.054500652 2 0 0.998207054
RP11-108M9.3 ENSG00000228549.3 0.415604572 7 7 0.052580285
Brain_Hippocampus 0.891913863 Brain_Hypothalamus 1.92798719 0.28912425
0.28912425 -0.932702618 1.938352061 0.365135782 0.989343039 7
0 0.998207054
PRSS51 ENSG00000253649.3 0.415624399 6 6 0.09113531
Brain_Frontal_Cortex_BA9 0.958301568 Brain_Hypothalamus 1.704826975
0.389389662 0.389389662 -1.391990418 1.68943912 0.021993072
1.022018899 6 0 0.998207054
PERM1 ENSG00000187642.9 0.415699587 3 3 0.098373505
Brain_Anterior_cingulate_cortex_BA24 0.731934347 Brain_Cortex
1.797254748 0.266272823 0.266272823 0.342553556 1.652790515
1.141065446 0.700617751 3 0 0.998207054
LINC01352 ENSG00000238078.1 0.41578734 11 9 0.049889861
Brain_Hypothalamus 0.892150921 Brain_Caudate_basal_ganglia 4.688364131
1.71E-07 0.271383441 -0.778259276 1.960907101 0.7676221
0.868504901 9 0 0.998207054
RP11-256L6.5 ENSG00000283422.1 0.415860962 1 1 0.415860962
Brain_Cerebellar_Hemisphere 0.415860962 Brain_Cerebellar_Hemisphere 1 1
1 -0.813622992 -0.813622992 -0.813622992 NA 1 0
0.998207054
CATSPER1 ENSG00000175294.5 0.415886795 1 1 0.415886795
Brain_Anterior_cingulate_cortex_BA24 0.415886795
Brain_Anterior_cingulate_cortex_BA24 1 1 1 0.813577914
0.813577914 0.813577914 NA 1 0 0.998207054
CTSC ENSG00000109861.15 0.415894294 4 4 0.099671155
Brain_Nucleus_accumbens_basal_ganglia 0.945441304 Brain_Cortex
2.813128371 0.143386685 0.143386685 0.068432559 1.646449957
0.727640927 0.677288184 4 0 0.998207054
RCOR3 ENSG00000117625.13 0.415918188 1 1 0.415918188
Brain_Caudate_basal_ganglia 0.415918188 Brain_Caudate_basal_ganglia 1 1
1 0.813523135 0.813523135 0.813523135 NA 1 0
0.998207054
KCTD21 ENSG00000188997.7 0.416045259 13 3 0.189341289
Brain_Spinal_cord_cervical_c-1 0.999274842 Brain_Cerebellum 11.34784888
0.000270102 0.651741282 -1.312530242 0.035874143 -0.392924502
0.362123751 3 0 0.998207054

DNER ENSG00000187957.7 0.416232185 3 3 0.091988206
Brain_Hypothalamus 0.938140634 Brain_Putamen_basal_ganglia 1.07667606
0.948988147 0.948988147 0.077607051 1.685001897 0.627273181
0.9162561 3 0 0.998207054
N4BP2 ENSG00000078177.13 0.41625505 1 1 0.41625505 Brain_Cerebellum 0.41625505
Brain_Cerebellum 1 1 1 0.812935483 0.812935483 0.812935483
NA 1 0 0.998207054
FBXO31 ENSG00000103264.17 0.416444555 2 2 0.186618387
Brain_Cerebellum 0.27247475 Brain_Cerebellar_Hemisphere 1.864721418
0.135278582 0.135278582 -1.320649118 -1.097381287 -1.209015203
0.157874197 2 0 0.998207054
CLDN9 ENSG00000213937.3 0.416674457 9 2 0.167017222
Brain_Anterior_cingulate_cortex_BA24 0.658046062
Brain_Nucleus_accumbens_basal_ganglia 7.943205384 0.00465445 0.401261347
0.442612472 1.381851761 1.144954533 0.279168692 2 0
0.998207054
AC099552.3 ENSG00000228521.2 0.41681203 4 4 0.053988914
Brain_Spinal_cord_cervical_c-1 0.442730922 Brain_Caudate_basal_ganglia
2.570640478 0.198316486 0.198316486 -1.926925507 0.876320377
-0.804676651 1.216770083 4 0 0.998207054
CRAMP1 ENSG00000007545.15 0.416833817 3 3 0.119467424
Brain_Cortex 0.912152627 Brain_Cerebellum 1.886826163 0.136310062
0.136310062 -1.557012894 0.110323744 -0.561549698 0.879508734
3 0 0.998207054
RP11-266K4.14 ENSG00000275367.1 0.416843923 2 2 0.193092051
Brain_Cerebellar_Hemisphere 0.463718112 Brain_Cerebellum 1.395687337
0.604312663 0.604312663 -1.3014863 -0.732738214 -1.017112257
0.402165628 2 0 0.998207054
FAM208B ENSG00000108021.19 0.417070361 2 2 0.327824802
Brain_Cerebellum 0.378008244 Brain_Putamen_basal_ganglia 1.008254376
0.991745624 0.991745624 -0.978504631 -0.881572108 -0.930038369
0.068541644 2 0 0.998207054
WASHC3 ENSG00000120860.10 0.417139105 10 4 0.275830752
Brain_Putamen_basal_ganglia 0.951933824 Brain_Nucleus_accumbens_basal_ganglia
7.1149613 0.033635418 0.376920169 -1.089733052 0.374078321
-0.38717975 0.50798691 4 0 0.998207054
SLC13A3 ENSG00000158296.13 0.417247783 10 3 0.169157951
Brain_Hippocampus 0.980180344 Brain_Caudate_basal_ganglia 8.325878638
0.000543714 0.398786227 -0.661196173 1.374914506 -0.089410704
0.571247325 3 0 0.998207054
TSEN34 ENSG00000170892.10 0.417274841 1 1 0.417274841
Brain_Hypothalamus 0.417274841 Brain_Hypothalamus 1 1 1
-0.811158176 -0.811158176 -0.811158176 NA 1 0 0.998207054
CSNK1G3 ENSG00000151292.17 0.417397244 2 2 0.21536638 Brain_Cerebellum
0.909138718 Brain_Cerebellar_Hemisphere 1.428296401 0.571703599
0.571703599 0.114124984 1.238943616 0.6765343 0.795366883 2
0 0.998207054
GALNT8 ENSG00000130035.6 0.417625381 1 1 0.417625381
Brain_Cerebellar_Hemisphere 0.417625381 Brain_Cerebellar_Hemisphere 1 1
1 0.810547841 0.810547841 0.810547841 NA 1 0
0.998207054
SYT14 ENSG00000143469.18 0.417854892 4 4 0.184037091
Brain_Spinal_cord_cervical_c-1 0.717908907 Brain_Amygdala 2.943149319
0.165807104 0.165807104 -1.328426981 0.361254897 -0.555708883
0.702239595 4 0 0.998207054
APBA3 ENSG00000011132.11 0.417856889 1 1 0.417856889 Brain_Cerebellum
0.417856889 Brain_Cerebellum 1 1 1 0.810144922 0.810144922
0.810144922 NA 1 0 0.998207054
PDIK1L ENSG00000175087.9 0.417979327 5 4 0.360336637
Brain_Cerebellum 0.531087461 Brain_Anterior_cingulate_cortex_BA24
3.644428095 0.007097674 0.163323339 -0.872122063 0.914723819
0.465614878 0.756266012 4 0 0.998207054

ZNF470 ENSG00000197016.11 0.417988093 12 6 0.064048073
Brain_Cortex 0.9559218 Brain_Amygdala 8.832972035 0.001967614
0.352839744 -1.851845077 -0.055271961 -0.916707404 0.59323327 6
0 0.998207054
RP11-471B22.3 ENSG00000275198.1 0.418002105 6 4 0.131484134
Brain_Cerebellar_Hemisphere 0.765482159 Brain_Nucleus_accumbens_basal_ganglia
4.539178346 0.061810762 0.255458279 -0.298289576 1.508275098
0.705488114 0.601438694 4 0 0.998207054
ZNF227 ENSG00000131115.15 0.418002209 2 2 0.217242924
Brain_Spinal_cord_cervical_c-1 0.684891376 Brain_Cerebellar_Hemisphere
1.052492849 0.947507151 0.947507151 -1.23389261 0.405797526
-0.414047542 1.159436014 2 0 0.998207054
RP3-395M20.9 ENSG00000228037.1 0.418012682 4 3 0.115904506
Brain_Nucleus_accumbens_basal_ganglia 0.725439676 Brain_Substantia_nigra
1.999528573 0.02278048 0.973518877 -1.572198575 0.677153625
-0.012853413 1.048095906 3 0 0.998207054
KCMF1 ENSG00000176407.17 0.418135587 1 1 0.418135587
Brain_Substantia_nigra 0.418135587 Brain_Substantia_nigra 1 1
1 -0.809660047 -0.809660047 -0.809660047 NA 1 0
0.998207054
CCDC59 ENSG00000133773.11 0.418278078 1 1 0.418278078
Brain_Substantia_nigra 0.418278078 Brain_Substantia_nigra 1 1
1 0.809412216 0.809412216 0.809412216 NA 1 0
0.998207054
QSOX2 ENSG00000165661.16 0.418396582 5 4 0.143606852
Brain_Frontal_Cortex_BA9 0.929245376 Brain_Cerebellum 3.0767891
0.060769023 0.504437163 -0.088794315 1.462490477 0.676272449
0.618821923 4 0 0.998207054
PTK6 ENSG00000101213.6 0.418408109 7 6 0.16428421
Brain_Putamen_basal_ganglia 0.822137175 Brain_Spinal_cord_cervical_c-1
2.574185531 0.080075214 0.518526017 -1.265847315 1.390806176
-0.173367322 1.044960391 6 0 0.998207054
TTC13 ENSG00000143643.12 0.418488506 2 2 0.240251353 Brain_Cerebellum
0.364710007 Brain_Nucleus_accumbens_basal_ganglia 1.279136113
0.720863887 0.720863887 -1.174358769 0.906426774 -0.133965998
1.471337568 2 0 0.998207054
CCZ1 ENSG00000122674.11 0.41851243 13 6 0.052454221
Brain_Nucleus_accumbens_basal_ganglia 0.926343967 Brain_Cerebellar_Hemisphere
9.362345296 0.041730069 0.35155454 -1.939387094 0.153430891
-0.622853784 0.555663228 6 0 0.998207054
YBX3 ENSG00000060138.12 0.418548092 1 1 0.418548092
Brain_Spinal_cord_cervical_c-1 0.418548092 Brain_Spinal_cord_cervical_c-1
1 1 1 -0.808942725 -0.808942725 -0.808942725 NA 1 0
0.998207054
HCG11 ENSG00000228223.2 0.418558788 1 1 0.418558788 Brain_Cerebellum
0.418558788 Brain_Cerebellum 1 1 1 0.80892413 0.80892413 0.80892413
NA 1 0 0.998207054
RP11-320M2.1 ENSG00000257135.5 0.418562305 1 1 0.418562305
Brain_Caudate_basal_ganglia 0.418562305 Brain_Caudate_basal_ganglia 1 1
1 -0.808918016 -0.808918016 -0.808918016 NA 1 0
0.998207054
ACCS ENSG00000110455.13 0.4186554 13 5 0.03548542 Brain_Hippocampus
0.517627027 Brain_Substantia_nigra 9.830309633 0.032634601
0.367168753 -2.102775261 -0.647007596 -1.49818217 0.395535485
5 0 0.998207054
BORCS7 ENSG00000166275.15 0.418731461 13 3 0.042980252
Brain_Caudate_basal_ganglia 0.367116892 Brain_Substantia_nigra 10.5013104
0.00764522 0.433385302 -2.023901849 -0.901887011 -1.486681641
0.336758611 3 0 0.998207054
MADCAM1 ENSG00000099866.14 0.418736709 8 5 0.392143535
Brain_Spinal_cord_cervical_c-1 0.87396157 Brain_Cortex 4.746023044
0.035729848 0.306302386 -0.799235733 0.85573652 0.087422971

0.594976591 5 0 0.998207054
SDCCAG3 ENSG00000165689.16 0.418774201 1 1 0.418774201 Brain_Spinal_cord_cervical_c-1 0.418774201 Brain_Spinal_cord_cervical_c-1 1 1 1 -0.808549712 -0.808549712 -0.808549712 NA 1 0 0.998207054
HLX ENSG00000136630.12 0.418891185 2 2 0.268181645 Brain_Cerebellar_Hemisphere 0.734325841 Brain_Cerebellum 1.309378571 0.690621429 0.690621429 -0.33937687 1.107259741 0.383941435 1.022926558 2 0 0.998207054
FAM47E ENSG00000189157.13 0.419044688 13 2 0.080047134 Brain_Frontal_Cortex_BA9 0.457827883 Brain_Cortex 11.98856914 0.000719261 0.527181088 -1.750412654 -0.742428293 -1.261402042 0.270495104 2 0 0.998207054
SMARCD2 ENSG00000108604.15 0.419058457 1 1 0.419058457 Brain_Nucleus_accumbens_basal_ganglia 0.419058457 Brain_Nucleus_accumbens_basal_ganglia 1 1 1 -0.808055805 -0.808055805 -0.808055805 NA 1 0 0.998207054
RP11-670E13.6 ENSG00000274213.1 0.419065493 1 1 0.419065493 Brain_Hippocampus 0.419065493 Brain_Hippocampus 1 1 1 -0.808043584 -0.808043584 -0.808043584 NA 1 0 0.998207054
TINAG ENSG00000137251.15 0.419195337 1 1 0.419195337 Brain_Frontal_Cortex_BA9 0.419195337 Brain_Frontal_Cortex_BA9 1 1 1 -0.807818042 -0.807818042 -0.807818042 NA 1 0 0.998207054
PCDH20 ENSG00000280165.1 0.419209633 2 2 0.237097968 Brain_Substantia_nigra 0.494433674 Brain_Putamen_basal_ganglia 1.085830524 0.914169476 0.914169476 -1.182271556 0.683274073 -0.249498742 1.319139965 2 0 0.998207054
HIST1H3H ENSG00000278828.1 0.419284815 1 1 0.419284815 Brain_Cerebellar_Hemisphere 0.419284815 Brain_Cerebellar_Hemisphere 1 1 1 0.807662643 0.807662643 0.807662643 NA 1 0 0.998207054
DDR2 ENSG00000162733.16 0.419324457 1 1 0.419324457 Brain_Amygdala 0.419324457 Brain_Amygdala 1 1 1 0.8075938 0.8075938 0.8075938 NA 1 0 0.998207054
CTB-58E17.1 ENSG00000277969.1 0.419472314 2 2 0.290953131 Brain_Cerebellum 0.414979536 Brain_Anterior_cingulate_cortex_BA24 1.025136729 0.974863271 0.974863271 -1.056033015 -0.815162087 -0.935597551 0.170321467 2 0 0.998207054
ENTPD3 ENSG00000168032.8 0.419474732 5 3 0.208381607 Brain_Nucleus_accumbens_basal_ganglia 0.832953336 Brain_Caudate_basal_ganglia 4.423364922 0.01283269 0.227196201 0.210915338 1.258028065 0.827603032 0.381117278 3 0 0.998207054
PUS10 ENSG00000162927.13 0.419486641 2 2 0.189121185 Brain_Nucleus_accumbens_basal_ganglia 0.870146605 Brain_Cerebellum 1.037732764 0.962267236 0.962267236 -0.163472269 1.313183314 0.574855522 1.044153176 2 0 0.998207054
FKRP ENSG00000181027.10 0.419524188 10 5 0.311683443 Brain_Amygdala 0.988714093 Brain_Cerebellum 6.951891387 0.006602788 0.260624574 -0.947951513 1.011695969 0.152902395 0.682809315 5 0 0.998207054
MED20 ENSG00000124641.15 0.419533349 5 4 0.249972778 Brain_Cortex 0.997734291 Brain_Cerebellar_Hemisphere 3.465726141 0.066773126 0.138275621 -1.150415503 0.159919033 -0.175777545 0.552214055 4 0 0.998207054
ZNF775 ENSG00000196456.11 0.419546408 1 1 0.419546408 Brain_Spinal_cord_cervical_c-1 0.419546408 Brain_Spinal_cord_cervical_c-1 1 1 1 0.807208433 0.807208433 0.807208433 NA 1 0 0.998207054
CYB5R2 ENSG00000166394.14 0.419764152 1 1 0.419764152 Brain_Putamen_basal_ganglia 0.419764152 Brain_Putamen_basal_ganglia 1 1 1 0.806830487 0.806830487 0.806830487 NA 1 0

0.998207054
SPAG1 ENSG00000104450.12 0.419862655 3 2 0.363710335
Brain_Nucleus_accumbens_basal_ganglia 0.676289638 Brain_Cerebellum
2.130475043 0.019090978 0.850433979 -0.822169826 0.908317812
-0.110461195 0.905188393 2 0 0.998207054
TRIM2 ENSG00000109654.14 0.420089691 3 3 0.123965635
Brain_Hypothalamus 0.77024643 Brain_Putamen_basal_ganglia 1.758283002
0.243418009 0.243418009 -1.538339464 -0.29205257 -0.806693695
0.650865591 3 0 0.998207054
IL21R ENSG00000103522.15 0.420313498 7 4 0.068906653
Brain_Substantia_nigra 0.742517099 Brain_Cerebellum 4.738820074
0.040726043 0.283094391 -1.819031315 -0.328521886 -0.738534469
0.508582954 4 0 0.998207054
ESYT3 ENSG00000158220.13 0.420380368 4 4 0.072678821
Brain_Cerebellar_Hemisphere 0.759997831 Brain_Hippocampus 2.131657653
0.233298397 0.233298397 -1.79484235 0.367415274 -0.649450733
0.914816344 4 0 0.998207054
UGGT1 ENSG00000136731.12 0.420391593 2 2 0.257517726
Brain_Hippocampus 0.488577159 Brain_Spinal_cord_cervical_c-1 1.01860101
0.98139899 0.98139899 0.692573638 1.132277643 0.912425641
0.310917684 2 0 0.998207054
AP000350.5 ENSG00000273295.1 0.42039608 13 1 0.305618112
Brain_Hippocampus 0.683567576 Brain_Cerebellar_Hemisphere 12.04380331
0.00080472 12.04380331 -1.024459878 -0.407599712 -0.775508198
0.226213097 1 0 0.998207054
ZNF428 ENSG00000131116.11 0.420399211 2 2 0.283828996
Brain_Substantia_nigra 0.431737667 Brain_Nucleus_accumbens_basal_ganglia
1.020430333 0.979569667 0.979569667 -0.786221612 1.071757436
0.142767912 1.313789584 2 0 0.998207054
SLC5A5 ENSG00000105641.3 0.420401619 3 3 0.166874695
Brain_Nucleus_accumbens_basal_ganglia 0.831326893 Brain_Cerebellum
1.171325794 0.823916648 0.823916648 -0.213000089 1.382315999
0.695735702 0.820532238 3 0 0.998207054
LRRK2 ENSG00000188906.14 0.42084069 4 4 0.177872462
Brain_Frontal_Cortex_BA9 0.963907605 Brain_Hippocampus 2.257584821
0.158102232 0.158102232 -1.347334698 0.045250547 -0.812896194
0.609313527 4 0 0.998207054
RPS6KA1 ENSG00000117676.13 0.420904544 3 3 0.130005709
Brain_Cerebellar_Hemisphere 0.642301177 Brain_Frontal_Cortex_BA9
1.133233517 0.864057022 0.864057022 -1.514079375 -0.46448378
-0.882498381 0.556435694 3 0 0.998207054
APTR ENSG00000214293.8 0.420973545 13 3 0.236041131
Brain_Anterior_cingulate_cortex_BA24 0.979822195 Brain_Cerebellum
11.23541805 0.007522336 0.488687417 0.025291825 1.184940101
0.614221295 0.378586408 3 0 0.998207054
NDUFAF6 ENSG00000156170.12 0.421083523 8 4 0.039693193
Brain_Hypothalamus 0.373700163 Brain_Nucleus_accumbens_basal_ganglia
6.082507134 0.038786107 0.414494706 -2.056927575 -0.889563778
-1.51048574 0.453640216 4 0 0.998207054
RCOR2 ENSG00000167771.5 0.421098153 1 1 0.421098153
Brain_Anterior_cingulate_cortex_BA24 0.421098153
Brain_Anterior_cingulate_cortex_BA24 1 1 1 0.804517524
0.804517524 0.804517524 NA 1 0 0.998207054
OSGEPL1 ENSG00000128694.11 0.421121723 1 1 0.421121723
Brain_Caudate_basal_ganglia 0.421121723 Brain_Caudate_basal_ganglia 1 1
1 -0.804476695 -0.804476695 -0.804476695 NA 1 0
0.998207054
ADM2 ENSG00000128165.8 0.421192207 1 1 0.421192207 Brain_Cerebellum
0.421192207 Brain_Cerebellum 1 1 1 -0.80435461 -0.80435461
-0.80435461 NA 1 0 0.998207054
LCN6 ENSG00000267206.5 0.421259179 1 1 0.421259179
Brain_Cerebellar_Hemisphere 0.421259179 Brain_Cerebellar_Hemisphere 1 1

1 0.80423862 0.80423862 0.80423862 NA 1 0 0.998207054
RP11-180M15.3 ENSG00000257004.1 0.421315457 1 1 0.421315457 Brain_Cerebellar_Hemisphere 0.421315457 Brain_Cerebellar_Hemisphere 1 1 1 -0.804141158 -0.804141158 -0.804141158 NA 1 0 0.998207054
TCAF1 ENSG00000198420.9 0.42137743 2 2 0.20981623 Brain_Cerebellum 0.388248516 Brain_Hippocampus 1.399496414 0.600503586 0.600503586 0.862798041 1.254070917 1.058434479 0.276671704 2 0 0.998207054
EPDR1 ENSG00000086289.11 0.421468816 3 2 0.181703338 Brain_Caudate_basal_ganglia 0.216863324 Brain_Putamen_basal_ganglia 2.85760104 0.035511316 0.106887644 1.234911821 1.335528793 1.279626926 0.051232817 2 0 0.998207054
MYZAP ENSG00000263155.5 0.421530008 5 4 0.134310524 Brain_Cerebellum 0.710073922 Brain_Hippocampus 2.456825624 0.036243859 0.342532271 -0.995650048 1.497318038 0.096791147 1.080988088 4 0 0.998207054
COPB1 ENSG00000129083.12 0.421570204 1 1 0.421570204 Brain_Cerebellum 0.421570204 Brain_Cerebellum 1 1 1 0.803700086 0.803700086 0.803700086 NA 1 0 0.998207054
LCA5L ENSG00000157578.13 0.421583965 13 2 0.089285077 Brain_Spinal_cord_cervical_c-1 0.749178509 Brain_Substantia_nigra 11.64931078 0.000927889 0.708364918 0.319722754 1.699181044 1.159159689 0.383341707 2 0 0.998207054
ZC3H12D ENSG00000178199.13 0.421749354 4 4 0.162469939 Brain_Amygdala 0.926781968 Brain_Cortex 1.19475783 0.79422186 0.79422186 -1.396812578 1.259160161 0.146358805 1.133980543 4 0 0.998207054
FMO5 ENSG00000131781.12 0.421857582 5 4 0.097195222 Brain_Spinal_cord_cervical_c-1 0.55320833 Brain_Caudate_basal_ganglia 3.53857186 0.062368511 0.13336017 -1.658605948 -0.59295939 -1.106157284 0.392727729 4 0 0.998207054
HLA-E ENSG00000204592.8 0.421871854 1 1 0.421871854 Brain_Anterior_cingulate_cortex_BA24 0.421871854 Brain_Anterior_cingulate_cortex_BA24 1 1 1 0.80317801 0.80317801 0.80317801 NA 1 0 0.998207054
PTPRD-AS2 ENSG00000226717.2 0.421973082 1 1 0.421973082 Brain_Nucleus_accumbens_basal_ganglia 0.421973082 Brain_Nucleus_accumbens_basal_ganglia 1 1 1 -0.803002859 -0.803002859 -0.803002859 NA 1 0 0.998207054
ZNF585B ENSG00000245680.9 0.422015612 7 4 0.331980864 Brain_Nucleus_accumbens_basal_ganglia 0.99359273 Brain_Cerebellum 5.377373357 0.046564548 0.356953849 -0.845379358 0.970131672 0.217369008 0.588807026 4 0 0.998207054
RAMP1 ENSG00000132329.10 0.422071652 2 2 0.193064283 Brain_Putamen_basal_ganglia 0.682813513 Brain_Cerebellum 1.180726497 0.819273503 0.819273503 -1.301567478 -0.408626864 -0.855097171 0.631404363 2 0 0.998207054
HOXB2 ENSG00000173917.10 0.422101665 5 5 0.085972512 Brain_Cerebellum 0.774441948 Brain_Anterior_cingulate_cortex_BA24 2.959828401 0.23763183 0.23763183 -0.397148585 1.717036449 0.952659298 0.957279025 5 0 0.998207054
MFSD2A ENSG00000168389.17 0.422208156 1 1 0.422208156 Brain_Cortex 0.422208156 Brain_Cortex 1 1 1 0.802596217 0.802596217 0.802596217 NA 1 0 0.998207054
ZNF749 ENSG00000186230.6 0.422214551 13 5 0.244426206 Brain_Nucleus_accumbens_basal_ganglia 0.77432776 Brain_Anterior_cingulate_cortex_BA24 9.374965907 5.67E-05 0.363162389 -0.702785003 1.163994586 0.627535939 0.580144953 5 0 0.998207054
EEF2K ENSG00000103319.11 0.422253773 2 2 0.233742081 Brain_Anterior_cingulate_cortex_BA24 0.832857243 Brain_Cortex

1.270288156 0.729711844 0.729711844 -0.211038483 1.190774605
0.489868061 0.99123154 2 0 0.998207054
SIPA1L3 ENSG00000105738.10 0.422271419 5 5 0.150174963
Brain_Spinal_cord_cervical_c-1 0.899034795 Brain_Cerebellum 1.649638167
0.454258824 0.454258824 -0.720565071 1.438913745 0.271429451
0.91299197 5 0 0.998207054
CCNF ENSG00000162063.12 0.42229684 1 1 0.42229684
Brain_Nucleus_accumbens_basal_ganglia 0.42229684
Brain_Nucleus_accumbens_basal_ganglia 1 1 1 -0.80244284
-0.80244284 -0.80244284 NA 1 0 0.998207054
MUS81 ENSG00000172732.11 0.42230259 1 1 0.42230259
Brain_Nucleus_accumbens_basal_ganglia 0.42230259
Brain_Nucleus_accumbens_basal_ganglia 1 1 1 0.802432897
0.802432897 0.802432897 NA 1 0 0.998207054
LAMA3 ENSG00000053747.15 0.422445043 1 1 0.422445043
Brain_Putamen_basal_ganglia 0.422445043 Brain_Putamen_basal_ganglia 1 1
1 0.802186571 0.802186571 0.802186571 NA 1 0
0.998207054
FAM177A1 ENSG00000151327.12 0.42261958 9 7 0.04896533 Brain_Cerebellum
0.839573248 Brain_Anterior_cingulate_cortex_BA24 4.265503196
0.118357622 0.282762041 -0.202439376 1.968893429 1.060446438
0.620971289 7 0 0.998207054
PSMC5 ENSG00000087191.12 0.422650875 11 5 0.253426178
Brain_Frontal_Cortex_BA9 0.978140866 Brain_Putamen_basal_ganglia
7.290379106 0.002694047 0.426280947 -1.030783073 1.142066985
0.019920025 0.68699788 5 0 0.998207054
RP11-405M12.3 ENSG00000278607.1 0.422698673 1 1 0.422698673
Brain_Spinal_cord_cervical_c-1 0.422698673 Brain_Spinal_cord_cervical_c-1
1 1 1 0.801748122 0.801748122 0.801748122 NA 1 0
0.998207054
CTH ENSG00000116761.11 0.422752646 1 1 0.422752646
Brain_Hippocampus 0.422752646 Brain_Hippocampus 1 1 1
0.801654839 0.801654839 0.801654839 NA 1 0 0.998207054
MCCC2 ENSG00000131844.15 0.422753929 3 2 0.407660205
Brain_Caudate_basal_ganglia 0.911565646 Brain_Cerebellar_Hemisphere
2.620185656 0.043776247 0.336038097 -0.828018178 0.202346985
-0.245578377 0.528189069 2 0 0.998207054
MGST3 ENSG00000143198.12 0.422978991 7 6 0.122741686
Brain_Spinal_cord_cervical_c-1 0.973798179 Brain_Cerebellar_Hemisphere
3.435081536 0.040493092 0.171078529 -1.543367289 0.189814137
-0.552483613 0.638039468 6 0 0.998207054
AS3MT ENSG00000214435.7 0.42308192 13 3 0.069036007 Brain_Hippocampus
0.270587647 Brain_Cerebellar_Hemisphere 11.35286888 0.006408375
0.391189054 -1.818184052 -1.101710278 -1.477294387 0.262832771
3 0 0.998207054
LILRB3 ENSG00000204577.11 0.423142488 3 3 0.431826615
Brain_Cerebellum 0.960765645 Brain_Cerebellar_Hemisphere 1.847925795
0.152419407 0.152419407 -0.786069767 0.049192805 -0.483845237
0.463003292 3 0 0.998207054
UGT3A2 ENSG00000168671.9 0.423155317 1 1 0.423155317
Brain_Substantia_nigra 0.423155317 Brain_Substantia_nigra 1 1
1 -0.800959111 -0.800959111 -0.800959111 NA 1 0
0.998207054
CCDC106 ENSG00000173581.7 0.423173542 2 2 0.224094811
Brain_Caudate_basal_ganglia 0.670965013 Brain_Amygdala 1.054592268
0.945407732 0.945407732 -1.21571158 0.424823956 -0.395443812
1.160033802 2 0 0.998207054
ZNF30 ENSG00000168661.14 0.423305935 6 5 0.093645463
Brain_Cerebellar_Hemisphere 0.994191027 Brain_Hippocampus 2.646325253
0.018867928 0.363603088 -0.007280532 1.676473602 0.67774337
0.704616916 5 0 0.998207054
FAM3D ENSG00000198643.6 0.423601114 2 2 0.292453525 Brain_Amygdala

0.379960083 Brain_Hippocampus 1.09571457 0.90428543 0.90428543
-1.052754496 -0.877969846 -0.965362171 0.123591411 2 0
0.998207054
MAGI2 ENSG00000187391.19 0.423724091 6 5 0.068436559 Brain_Cerebellum
0.791274964 Brain_Caudate_basal_ganglia 2.879145953 0.017563495
0.249781658 0.264655367 1.822121452 0.775337378 0.576337354
5 0 0.998207054
CARMIL1 ENSG00000079691.17 0.42373243 4 4 0.228762489
Brain_Putamen_basal_ganglia 0.916833169 Brain_Anterior_cingulate_cortex_BA24
1.431229927 0.708649888 0.708649888 -1.203552756 1.055904554
0.146908003 1.031689984 4 0 0.998207054
RAB11B ENSG00000185236.11 0.423746068 1 1 0.423746068
Brain_Frontal_Cortex_BA9 0.423746068 Brain_Frontal_Cortex_BA9 1 1
1 0.799939125 0.799939125 0.799939125 NA 1 0
0.998207054
CTD-2270P14.5 ENSG00000276791.1 0.423754494 10 10 0.096979663
Brain_Nucleus_accumbens_basal_ganglia 0.958664774 Brain_Putamen_basal_ganglia
1.707160976 0.375204321 0.375204321 -1.659675938 1.461046915
-0.235544975 0.959157901 10 0 0.998207054
SUGP2 ENSG00000064607.16 0.423767622 2 2 0.205307237
Brain_Hippocampus 0.315995203 Brain_Cerebellum 1.574517812
0.425482188 0.425482188 -1.266575165 -1.002721604 -1.134648384
0.186572642 2 0 0.998207054
INTS13 ENSG00000064102.14 0.423820847 3 2 0.256241215
Brain_Spinal_cord_cervical_c-1 0.471389575 Brain_Frontal_Cortex_BA9
1.97925872 0.021806919 0.998934362 0.720219961 1.13532012
0.954179554 0.212530992 2 0 0.998207054
ABCF3 ENSG00000161204.11 0.423889512 5 4 0.068475972
Brain_Caudate_basal_ganglia 0.792383644 Brain_Amygdala 3.200740859
0.090135153 0.273968269 -1.821861709 0.263216591 -1.081136391
0.834169833 4 0 0.998207054
EFNB2 ENSG00000125266.6 0.423978871 1 1 0.423978871 Brain_Amygdala
0.423978871 Brain_Amygdala 1 1 1 -0.799537398 -0.799537398
-0.799537398 NA 1 0 0.998207054
BBS4 ENSG00000140463.13 0.424075582 5 5 0.112056749 Brain_Cortex
0.807545141 Brain_Putamen_basal_ganglia 2.354712338 0.163778488
0.163778488 -0.867872501 1.589016138 0.591965285 1.084036197
5 0 0.998207054
FAM8A1 ENSG00000137414.5 0.424292673 3 3 0.189164121
Brain_Caudate_basal_ganglia 0.74547007 Brain_Hippocampus 1.286011586
0.736573279 0.736573279 -1.313055874 0.324618175 -0.657306697
0.866191093 3 0 0.998207054
C5orf47 ENSG00000185056.9 0.424345136 3 3 0.138405004
Brain_Cortex 0.912256789 Brain_Nucleus_accumbens_basal_ganglia
1.419120057 0.568339977 0.568339977 -1.481756851 0.182512639
-0.396350604 0.940684642 3 0 0.998207054
NTS ENSG00000133636.10 0.424360132 1 1 0.424360132 Brain_Cerebellum
0.424360132 Brain_Cerebellum 1 1 1 -0.798879767 -0.798879767
-0.798879767 NA 1 0 0.998207054
CDAN1 ENSG00000140326.12 0.424365912 4 2 0.266509293
Brain_Anterior_cingulate_cortex_BA24 0.718167762 Brain_Cerebellum
3.586329659 0.013968145 0.334850771 -1.111137211 -0.360908616
-0.567522503 0.363541914 2 0 0.998207054
CDR2 ENSG00000140743.7 0.424402671 2 2 0.193460633
Brain_Caudate_basal_ganglia 0.770766807 Brain_Nucleus_accumbens_basal_ganglia
1.334171513 0.665828487 0.665828487 0.291372026 1.300409566
0.795890796 0.713497287 2 0 0.998207054
NT5C3A ENSG00000122643.18 0.424449728 2 2 0.563717857
Brain_Cerebellum 0.919499787 Brain_Cerebellar_Hemisphere 1.92887754 0.07112246
0.07112246 0.101063834 0.577328068 0.339195951 0.336769669 2
0 0.998207054
HIP1R ENSG00000130787.13 0.424562002 1 1 0.424562002 Brain_Cerebellum

0.424562002 Brain_Cerebellum 1 1 1 0.798531706 0.798531706
0.798531706 NA 1 0 0.998207054
ACOT4 ENSG00000177465.4 0.424573004 10 6 0.207263929
Brain_Hypothalamus 0.903550778 Brain_Nucleus_accumbens_basal_ganglia
6.241693881 0.034097521 0.246072442 -0.646987826 1.261124682
0.563449965 0.608500877 6 0 0.998207054
ITGB6 ENSG00000115221.10 0.424605305 3 1 0.386423208
Brain_Hypothalamus 0.49704125 Brain_Spinal_cord_cervical_c-1 2.988680718
0.000286262 2.988680718 0.679152484 0.866122098 0.796848212
0.102459334 1 0 0.998207054
DPY30 ENSG00000162961.13 0.424618269 1 1 0.424618269
Brain_Cerebellar_Hemisphere 0.424618269 Brain_Cerebellar_Hemisphere 1 1
1 0.798434707 0.798434707 0.798434707 NA 1 0
0.998207054
FARP1 ENSG00000152767.16 0.424879112 3 3 0.187142093
Brain_Nucleus_accumbens_basal_ganglia 0.752611624 Brain_Hypothalamus
1.137808352 0.880053997 0.880053997 0.315197605 1.319080826
0.866558991 0.509187905 3 0 0.998207054
FDXACB1 ENSG00000255561.6 0.424902502 1 1 0.424902502
Brain_Amygdala 0.424902502 Brain_Amygdala 1 1 1 0.797944839
0.797944839 0.797944839 NA 1 0 0.998207054
LINC00599 ENSG00000253230.8 0.424973251 1 1 0.424973251
Brain_Frontal_Cortex_BA9 0.424973251 Brain_Frontal_Cortex_BA9 1 1
1 0.797822933 0.797822933 0.797822933 NA 1 0
0.998207054
PDHB ENSG00000168291.12 0.425138867 12 5 0.15036958 Brain_Amygdala
0.94924102 Brain_Cortex 8.639366035 0.004513724 0.329318536
-1.438227269 0.797202115 -0.261515677 0.563216175 5 0
0.998207054
RP11-490M8.1 ENSG00000260025.1 0.425390708 4 4 0.205074001
Brain_Cortex 0.397681439 Brain_Cerebellum 1.797237091 0.207578545
0.207578545 -1.267227373 1.264184242 -0.035821204 1.276249434
4 0 0.998207054
MICU1 ENSG00000107745.17 0.425473536 3 3 0.098504243
Brain_Putamen_basal_ganglia 0.976258305 Brain_Cortex 1.569194977
0.429189043 0.429189043 -1.65214869 0.118415426 -0.501324357
0.997628399 3 0 0.998207054
LMBR1 ENSG00000105983.20 0.425586956 1 1 0.425586956
Brain_Cerebellar_Hemisphere 0.425586956 Brain_Cerebellar_Hemisphere 1 1
1 -0.79676598 -0.79676598 -0.79676598 NA 1 0
0.998207054
CTD-2292M16.8 ENSG00000258768.2 0.425840353 3 3 0.130944696
Brain_Hippocampus 0.870019686 Brain_Caudate_basal_ganglia 1.583163413
0.46714534 0.46714534 -0.411649806 1.510387032 0.311701248
1.045472969 3 0 0.998207054
PFDN2 ENSG00000143256.4 0.426165287 1 1 0.426165287
Brain_Caudate_basal_ganglia 0.426165287 Brain_Caudate_basal_ganglia 1 1
1 0.795770766 0.795770766 0.795770766 NA 1 0
0.998207054
RP11-140I16.3 ENSG00000272343.1 0.426268157 1 1 0.426268157
Brain_Hypothalamus 0.426268157 Brain_Hypothalamus 1 1 1
-0.795593825 -0.795593825 -0.795593825 NA 1 0 0.998207054
DOCK7 ENSG00000116641.17 0.426284892 1 1 0.426284892
Brain_Anterior_cingulate_cortex_BA24 0.426284892
Brain_Anterior_cingulate_cortex_BA24 1 1 1 -0.795565042
-0.795565042 -0.795565042 NA 1 0 0.998207054
C19orf84 ENSG00000262874.1 0.426419716 9 6 0.034577465
Brain_Hippocampus 0.844210996 Brain_Frontal_Cortex_BA9 5.957894214
0.001268705 0.23935443 -2.113272336 -0.196510013 -1.345824832
0.552868879 6 0 0.998207054
STRIP2 ENSG00000128578.9 0.426531972 1 1 0.426531972
Brain_Cerebellar_Hemisphere 0.426531972 Brain_Cerebellar_Hemisphere 1 1

1 0.795140166 0.795140166 0.795140166 NA 1 0
0.998207054
BCL7C ENSG00000099385.11 0.426591564 2 2 0.191908499
Brain_Cerebellar_Hemisphere 0.495007682 Brain_Nucleus_accumbens_basal_ganglia
1.542756869 0.457243131 0.457243131 0.682365789 1.304954037
0.993659913 0.440236372 2 0 0.998207054
UGT2B17 ENSG00000197888.2 0.426729818 2 2 0.201824642
Brain_Spinal_cord_cervical_c-1 0.616894748 Brain_Substantia_nigra
1.567520285 0.432479715 0.432479715 0.500256118 1.276370323
0.888313221 0.548795617 2 0 0.998207054
RNF111 ENSG00000157450.15 0.426832056 2 2 0.238192568
Brain_Cerebellar_Hemisphere 0.460428443 Brain_Cerebellum 1.158635782
0.841364218 0.841364218 -1.179516506 -0.738141541 -0.958829024
0.312099231 2 0 0.998207054
ASXL1 ENSG00000171456.16 0.426845534 1 1 0.426845534
Brain_Anterior_cingulate_cortex_BA24 0.426845534
Brain_Anterior_cingulate_cortex_BA24 1 1 1 0.794601177
0.794601177 0.794601177 NA 1 0 0.998207054
RP11-527D7.1 ENSG00000225554.1 0.42693863 10 6 0.029792814
Brain_Spinal_cord_cervical_c-1 0.789560365 Brain_Hippocampus 5.90751458
0.021361646 0.231928625 0.266881546 2.172833988 0.963374117
0.579534063 6 0 0.998207054
ZNF514 ENSG00000144026.11 0.427010355 13 6 0.093505704
Brain_Hippocampus 0.992954885 Brain_Frontal_Cortex_BA9 7.884906365
0.011699668 0.368436345 -1.677188109 0.048596024 -0.807114334
0.543150014 6 0 0.998207054
DCAF10 ENSG00000122741.15 0.427054573 4 3 0.391265709
Brain_Anterior_cingulate_cortex_BA24 0.880069389 Brain_Putamen_basal_ganglia
3.516166541 0.001123556 0.143128445 0.150881253 0.857324252
0.644909032 0.335107659 3 0 0.998207054
OSR2 ENSG00000164920.9 0.427190308 1 1 0.427190308
Brain_Spinal_cord_cervical_c-1 0.427190308 Brain_Spinal_cord_cervical_c-1
1 1 1 -0.794008801 -0.794008801 -0.794008801 NA 1 0
0.998207054
MYL3 ENSG00000160808.9 0.427190547 2 2 0.249753293 Brain_Amygdala
0.633896574 Brain_Nucleus_accumbens_basal_ganglia 1.115661709
0.884338291 0.884338291 -1.150948817 0.47624959 -0.337349613
1.150603028 2 0 0.998207054
OXGR1 ENSG00000165621.8 0.427202306 9 8 0.038063769
Brain_Hypothalamus 0.888232658 Brain_Cortex 4.091713999
0.052261829 0.164004204 -0.746930511 2.074167373 1.243302547
0.959097376 8 0 0.998207054
AP000797.4 ENSG00000257067.5 0.427341373 3 3 0.096567267
Brain_Nucleus_accumbens_basal_ganglia 0.817864146 Brain_Cerebellar_Hemisphere
1.755076055 0.305740947 0.305740947 0.23029294 1.661728286
0.758642251 0.785857809 3 0 0.998207054
ING5 ENSG00000168395.15 0.427357892 4 4 0.237599673 Brain_Cerebellum
0.858818567 Brain_Frontal_Cortex_BA9 1.987791199 0.26289851 0.26289851
-1.18100768 1.150698632 0.085500717 0.959008188 4 0
0.998207054
GPR35 ENSG00000178623.11 0.427366377 5 5 0.044699314
Brain_Hypothalamus 0.946919684 Brain_Nucleus_accumbens_basal_ganglia
1.648132202 0.522850602 0.522850602 -2.007473086 0.066575458
-0.698412517 0.822243424 5 0 0.998207054
TOGARAM1 ENSG00000198718.12 0.427393275 1 1 0.427393275
Brain_Anterior_cingulate_cortex_BA24 0.427393275
Brain_Anterior_cingulate_cortex_BA24 1 1 1 -0.793660202
-0.793660202 -0.793660202 NA 1 0 0.998207054
CTBP2 ENSG00000175029.16 0.427540218 2 2 0.22217973 Brain_Hypothalamus
0.585108469 Brain_Cerebellum 1.073576716 0.926423284 0.926423284
0.545938127 1.220752536 0.883345331 0.477165845 2 0
0.998207054

C4orf22 ENSG00000197826.11 0.427565846 1 1 0.427565846
Brain_Cerebellum 0.427565846 Brain_Cerebellum 1 1 1 0.793363885
0.793363885 0.793363885 NA 1 0 0.998207054
SCARB1 ENSG00000073060.15 0.427663045 1 1 0.427663045
Brain_Caudate_basal_ganglia 0.427663045 Brain_Caudate_basal_ganglia 1 1
1 0.793197017 0.793197017 0.793197017 NA 1 0
0.998207054
RPRD1B ENSG00000101413.11 0.427887879 6 6 0.125944994
Brain_Hypothalamus 0.697659878 Brain_Cerebellar_Hemisphere 2.836394886
0.100954693 0.100954693 -1.126059637 1.530289872 -0.026151698
1.175268416 6 0 0.998207054
ROBO3 ENSG00000154134.14 0.427889113 1 1 0.427889113
Brain_Spinal_cord_cervical_c-1 0.427889113 Brain_Spinal_cord_cervical_c-1
1 1 1 0.792808999 0.792808999 0.792808999 NA 1 0
0.998207054
FKBP1B ENSG00000119782.13 0.427908282 1 1 0.427908282
Brain_Caudate_basal_ganglia 0.427908282 Brain_Caudate_basal_ganglia 1 1
1 0.792776102 0.792776102 0.792776102 NA 1 0
0.998207054
VGLL2 ENSG00000170162.13 0.427922172 1 1 0.427922172
Brain_Spinal_cord_cervical_c-1 0.427922172 Brain_Spinal_cord_cervical_c-1
1 1 1 0.792752267 0.792752267 0.792752267 NA 1 0
0.998207054
NUTM2D ENSG00000214562.14 0.427996164 10 9 0.152939342
Brain_Putamen_basal_ganglia 0.975124635 Brain_Nucleus_accumbens_basal_ganglia
3.173516808 0.087364848 0.270970075 -1.429225808 1.38242363
-0.008786736 0.930178461 9 0 0.998207054
PRB1 ENSG00000251655.6 0.42813076 2 2 0.192726374
Brain_Nucleus_accumbens_basal_ganglia 0.680179993 Brain_Caudate_basal_ganglia
1.317195006 0.682804994 0.682804994 -1.302556039 -0.412217525
-0.857386782 0.629564401 2 0 0.998207054
CAPN11 ENSG00000137225.12 0.428142761 3 3 0.172956505
Brain_Frontal_Cortex_BA9 0.64610517 Brain_Cortex 1.122614178
0.917261469 0.917261469 -1.362765252 0.45917964 -0.574816522
0.93556149 3 0 0.998207054
TRMT112 ENSG00000173113.6 0.42817296 7 5 0.148290298 Brain_Cortex
0.708479188 Brain_Putamen_basal_ganglia 4.572647588 0.003933855
0.201732699 -0.373899366 1.445596895 0.750054735 0.580918523
5 0 0.998207054
ERICD ENSG00000280303.2 0.428188748 1 1 0.428188748
Brain_Frontal_Cortex_BA9 0.428188748 Brain_Frontal_Cortex_BA9 1 1
1 -0.792294894 -0.792294894 -0.792294894 NA 1 0
0.998207054
GSDMD ENSG00000104518.10 0.428220218 11 3 0.157229892
Brain_Anterior_cingulate_cortex_BA24 0.601872206 Brain_Frontal_Cortex_BA9
8.818398985 0.002894012 0.534629625 0.521710079 1.414449748
1.073471863 0.297083429 3 0 0.998207054
DEPDC7 ENSG00000121690.10 0.428249148 2 2 0.196982574
Brain_Caudate_basal_ganglia 0.8373977 Brain_Cerebellar_Hemisphere 1.021545774
0.978454226 0.978454226 -0.205223256 1.290196117 0.54248643
1.057421179 2 0 0.998207054
LINC01470 ENSG00000249484.8 0.428300295 2 2 0.196108004
Brain_Putamen_basal_ganglia 0.231683518 Brain_Caudate_basal_ganglia
1.863935746 0.136064254 0.136064254 1.19603342 1.292719753
1.244376586 0.068367562 2 0 0.998207054
TRABD ENSG00000170638.9 0.428319707 3 3 0.097243694 Brain_Cerebellum
0.915123595 Brain_Hypothalamus 1.179295578 0.821059831
0.821059831 0.106578223 1.658365607 0.715645654 0.827951705
3 0 0.998207054
TRIP12 ENSG00000153827.13 0.428325468 2 2 0.192919604
Brain_Cortex 0.970263438 Brain_Putamen_basal_ganglia 1.010385994
0.989614006 0.989614006 -1.301990585 -0.037277885 -0.669634235

0.894286926	2	0	0.998207054
DDO	ENSG00000203797.9	0.428378468	11	6	0.388172148	Brain_Cerebellum
0.973727234	Brain_Substantia_nigra	6.966582313	0.002519269
0.267783729	-0.581100134	0.862936923	0.181502818	0.436649732
6	0	0.998207054
FAM134B	ENSG00000154153.13	0.428411096	2	2	0.210505635
Brain_Cerebellum 0.765135459	Brain_Cerebellar_Hemisphere 1.046163649
0.953836351	0.953836351	0.298743899	1.252176285	0.775460092
0.674178505	2	0	0.998207054
FAM192A	ENSG00000172775.16	0.428451239	2	2	0.309331654
Brain_Spinal_cord_cervical_c-1	0.390303941	Brain_Cerebellar_Hemisphere
1.045751343	0.954248657	0.954248657	-1.016625426	0.859066295
-0.078779565	1.326314335	2	0	0.998207054
PCDHA1	ENSG00000204970.9	0.428463911	3	2	0.19457714
Brain_Substantia_nigra	0.965912477	Brain_Cerebellum 1.988024917
0.016102645	0.995872438	-1.297157094	-0.042735378	-0.541469891
0.665516321	2	0	0.998207054
ELOVL4	ENSG00000118402.5	0.428525457	1	1	0.428525457
Brain_Cerebellar_Hemisphere 0.428525457	Brain_Cerebellar_Hemisphere 1	1
1	0.79171743 0.79171743 0.79171743 NA	1	0	0.998207054
EBPL	ENSG00000123179.13	0.428557219	13	3	0.069523521
Brain_Spinal_cord_cervical_c-1	0.839601136	Brain_Putamen_basal_ganglia
10.59558064	0.013043589	0.475541174	0.2024037 1.815002529
0.951078389	0.541833565	3	0	0.998207054
PIAS2	ENSG00000078043.15	0.428589475	9	5	0.060061557
Brain_Hippocampus	0.490726921	Brain_Hypothalamus	6.466938062
0.011840362	0.258748715	-1.880341455	-0.689153109	-1.267006241
0.361257597	5	0	0.998207054
AASDH	ENSG00000157426.13	0.428739554	2	2	0.204793431	Brain_Cerebellum
0.386557083	Brain_Cerebellar_Hemisphere 1.816388528	0.183611472
0.183611472	0.865877973	1.268012656	1.066945315	0.284352161
2	0	0.998207054
CCDC163	ENSG00000280670.2	0.428827416	11	2	0.16846056
Brain_Caudate_basal_ganglia 0.25104049 Brain_Amygdala	10.32952752	0.00077682
0.427723562	1.147825787	1.3771672 1.234982978	0.075684428	2
0	0.998207054
FBXO21	ENSG00000135108.14	0.428874221	3	3	0.318233287
Brain_Putamen_basal_ganglia 0.928977909	Brain_Nucleus_accumbens_basal_ganglia
2.638489771	0.089635876	0.089635876	-0.998095015	-0.089130865
-0.530136521	0.45508109 3	0	0.998207054
TMEM259	ENSG00000182087.12	0.428891714	9	8	0.208285744
Brain_Nucleus_accumbens_basal_ganglia 0.95741915 Brain_Cortex	2.890171365
0.062429874	0.12007793 -1.258293188	1.236836008	-0.158548093
0.939566998	8	0	0.998207054
ZBED4	ENSG00000100426.6	0.428923225	3	2	0.235489153
Brain_Cerebellar_Hemisphere 0.401053922	Brain_Cerebellum 1.945777056
0.057432963	0.996789981	-1.186337222	0.84671774 -0.393119981
1.087626089	2	0	0.998207054
AGFG1	ENSG00000173744.17	0.429000105	3	3	0.195068229
Brain_Cerebellar_Hemisphere 0.368804728	Brain_Putamen_basal_ganglia 1.86309668
0.143939489	0.143939489	-0.898714543	1.295730849	0.433031307
1.170000416	3	0	0.998207054
RNF123	ENSG00000164068.15	0.429021142	10	4	0.269619307
Brain_Putamen_basal_ganglia 0.980126607	Brain_Cortex	8.34597223
0.001832492	0.280450766	-1.103939675	0.603066659	0.011349083
0.499941747	4	0	0.998207054
PLA2G7	ENSG00000146070.16	0.429052337	5	5	0.067680987
Brain_Cortex	0.774785582	Brain_Cerebellar_Hemisphere 2.286595
0.280891491	0.280891491	-1.827124938	0.584332848	-0.210725995
0.990898946	5	0	0.998207054
GLIPR1L2	ENSG00000180481.10	0.42909066 13	1	0.326614251	Brain_Cerebellum
0.54812942 Brain_Cerebellar_Hemisphere 12.9023734 3.46E-05	12.9023734

-0.980956383 -0.600565503 -0.787919787 0.099051032 1 0
0.998207054
MYO1D ENSG00000176658.16 0.429143294 2 2 0.196996662
Brain_Caudate_basal_ganglia 0.818941414 Brain_Cerebellum 1.049214946
0.950785054 0.950785054 -0.228906725 1.290155533 0.530624404
1.074139224 2 0 0.998207054
CCDC40 ENSG00000141519.14 0.429276483 11 3 0.049007464
Brain_Caudate_basal_ganglia 0.489426932 Brain_Amygdala 9.274522239
2.18E-08 0.416825445 -1.968526728 -0.691220582 -1.444477397
0.311244034 3 0 0.998207054
TRAF7 ENSG00000131653.12 0.429424397 2 2 0.210281472
Brain_Hypothalamus 0.835478689 Brain_Hippocampus 1.110341148
0.889658852 0.889658852 -0.207680187 1.252791839 0.522555826
1.032709674 2 0 0.998207054
C14orf37 ENSG00000139971.15 0.429446752 4 4 0.103034933
Brain_Anterior_cingulate_cortex_BA24 0.852712741 Brain_Hippocampus
2.014277672 0.496519809 0.496519809 0.185658287 1.63031503
1.014520304 0.625599058 4 0 0.998207054
FUT2 ENSG00000176920.11 0.429706104 4 3 0.302450993
Brain_Nucleus_accumbens_basal_ganglia 0.997735955 Brain_Caudate_basal_ganglia
3.262603101 0.004617895 0.125521875 -0.002837563 1.03119157
0.508294562 0.4679761 3 0 0.998207054
TP53I11 ENSG00000175274.18 0.429748379 1 1 0.429748379
Brain_Cerebellar_Hemisphere 0.429748379 Brain_Cerebellar_Hemisphere 1 1
1 -0.789622304 -0.789622304 -0.789622304 NA 1 0
0.998207054
KAT14 ENSG00000149474.13 0.429948063 4 4 0.339266978
Brain_Caudate_basal_ganglia 0.849235226 Brain_Cerebellum 2.98383597
0.139511038 0.139511038 -0.955614609 0.788804284 -0.282136386
0.784978397 4 0 0.998207054
FAM216A ENSG00000204856.11 0.43013574 1 1 0.43013574 Brain_Hypothalamus
0.43013574 Brain_Hypothalamus 1 1 1 -0.788959393 -0.788959393
-0.788959393 NA 1 0 0.998207054
IAH1 ENSG00000134330.18 0.430202442 5 3 0.09401435
Brain_Frontal_Cortex_BA9 0.894983571 Brain_Nucleus_accumbens_basal_ganglia
3.266749111 0.012235245 0.781028055 0.132000912 1.674591798
1.040612548 0.711386444 3 0 0.998207054
FAM13A ENSG00000138640.14 0.430226381 2 2 0.195340007
Brain_Amygdala 0.975895559 Brain_Cerebellar_Hemisphere 1.100209981
0.899790019 0.899790019 -1.294942672 -0.030215033 -0.662578852
0.89429749 2 0 0.998207054
NEDD9 ENSG00000111859.16 0.430252714 4 4 0.098747252 Brain_Cortex
0.821785285 Brain_Hypothalamus 1.793778419 0.473563674
0.473563674 -1.650957505 0.369293106 -0.486041578 0.958459186
4 0 0.998207054
DNAJC15 ENSG00000120675.5 0.430300317 13 1 0.35339782
Brain_Frontal_Cortex_BA9 0.486461263 Brain_Cortex 12.90758228
6.83E-05 12.90758228 0.695948212 0.928018795 0.785851138
0.075150943 1 0 0.998207054
PRH2 ENSG00000134551.12 0.430343607 2 2 0.612705782
Brain_Hippocampus 0.72646772 Brain_Spinal_cord_cervical_c-1 1.78183434
0.21816566 0.21816566 -0.349828086 0.50621492 0.078193417 0.605313815
2 0 0.998207054
SFMBT1 ENSG00000163935.13 0.430378537 3 3 0.176349311
Brain_Cerebellar_Hemisphere 0.654480757 Brain_Cerebellum 1.885505204
0.277746466 0.277746466 -0.447546174 1.352081294 0.687536358
0.987784884 3 0 0.998207054
RP11-305L7.7 ENSG00000273381.1 0.430479331 3 3 0.332876381
Brain_Hippocampus 0.450208053 Brain_Cortex 1.311275168
0.688424606 0.688424606 -0.866097939 0.968336422 0.285768899
1.003229157 3 0 0.998207054
ASNSD1 ENSG00000138381.9 0.430483672 13 1 0.330395856

Brain_Hippocampus 0.854402684 Brain_Substantia_nigra 12.21471578
0.000648955 12.21471578 -0.97331684 -0.183503868 -0.761186444
0.201165077 1 0 0.998207054
RBL2 ENSG00000103479.15 0.430781981 13 1 0.178901572
Brain_Caudate_basal_ganglia 0.797657922 Brain_Substantia_nigra
12.18645447 1.82E-05 12.18645447 -0.259559131 1.344144787
0.749516682 0.413085651 1 0 0.998207054
LRP1B ENSG00000168702.17 0.430850974 6 6 0.110318701
Brain_Putamen_basal_ganglia 0.743501155 Brain_Substantia_nigra
2.603885484 0.191748641 0.191748641 -1.596762309 0.48474829
-0.840857761 0.811295104 6 0 0.998207054
LRRC28 ENSG00000168904.14 0.43089525 1 1 0.43089525 Brain_Cortex
0.43089525 Brain_Cortex 1 1 1 -0.787660614 -0.787660614
-0.787660614 NA 1 0 0.998207054
RP11-823E8.3 ENSG00000274021.1 0.43110394 2 2 0.211055847
Brain_Substantia_nigra 0.768123821 Brain_Cortex 1.039457344
0.960542656 0.960542656 -1.250667407 0.294829905 -0.477918751
1.092831629 2 0 0.998207054
ZNF177 ENSG00000188629.11 0.431211201 11 11 0.192422598
Brain_Amygdala 0.678576273 Brain_Nucleus_accumbens_basal_ganglia
2.114146913 0.27427101 0.27427101 -1.303445832 1.216664756
-0.518776215 0.964188104 11 0 0.998207054
RP11-548P2.2 ENSG00000272564.1 0.431221801 7 2 0.468806859
Brain_Caudate_basal_ganglia 0.982716508 Brain_Spinal_cord_cervical_c-1
6.425094953 0.000164028 0.29455195 -0.724421737 0.244079017
-0.196766658 0.396527732 2 0 0.998207054
AC093802.1 ENSG00000220256.3 0.431340161 1 1 0.431340161
Brain_Anterior_cingulate_cortex_BA24 0.431340161
Brain_Anterior_cingulate_cortex_BA24 1 1 1 -0.786900422
-0.786900422 -0.786900422 NA 1 0 0.998207054
IL12A ENSG00000168811.6 0.431386149 4 4 0.082215435
Brain_Nucleus_accumbens_basal_ganglia 0.937740422 Brain_Amygdala
1.523984096 0.533058614 0.533058614 -0.205402332 1.737973777
0.506063719 0.85935733 4 0 0.998207054
ALG10B ENSG00000175548.8 0.431396136 4 4 0.129002561
Brain_Caudate_basal_ganglia 0.798272934 Brain_Spinal_cord_cervical_c-1
2.865409624 0.145313838 0.145313838 -0.344627952 1.518046959
0.45244222 0.777758586 4 0 0.998207054
EMX2 ENSG00000170370.11 0.431437113 1 1 0.431437113 Brain_Amygdala
0.431437113 Brain_Amygdala 1 1 1 0.786734826 0.786734826
0.786734826 NA 1 0 0.998207054
CD300A ENSG00000167851.13 0.431440424 5 5 0.323133828
Brain_Cerebellar_Hemisphere 0.963591443 Brain_Caudate_basal_ganglia
2.953063836 0.137748252 0.137748252 -0.86007162 0.988038429
-0.067784737 0.676642086 5 0 0.998207054
PSMA3 ENSG00000100567.12 0.431476806 3 3 0.111288892
Brain_Anterior_cingulate_cortex_BA24 0.905875727 Brain_Putamen_basal_ganglia
1.213938844 0.795860358 0.795860358 -0.118242234 1.592426562
0.551972029 0.913457894 3 0 0.998207054
GATAD2A ENSG00000167491.17 0.431655763 3 3 0.122875978
Brain_Frontal_Cortex_BA9 0.258616139 Brain_Caudate_basal_ganglia
2.144068735 0.20859178 0.20859178 -1.542813728 -1.129668016
-1.388439071 0.225491243 3 0 0.998207054
PSPC1 ENSG00000121390.18 0.431929309 1 1 0.431929309
Brain_Nucleus_accumbens_basal_ganglia 0.431929309
Brain_Nucleus_accumbens_basal_ganglia 1 1 1 -0.78589448
-0.78589448 -0.78589448 NA 1 0 0.998207054
TNFRSF18 ENSG00000186891.13 0.432091962 6 4 0.066558141
Brain_Hippocampus 0.457652852 Brain_Cerebellum 3.73905268 0.002947375
0.207432192 0.742717302 1.834646114 1.24571482 0.446006425 4
0 0.998207054
POPDC3 ENSG00000132429.9 0.432178209 4 3 0.298616497

Brain_Caudate_basal_ganglia 0.915823663 Brain_Hypothalamus 3.215554187
0.105293787 0.225828323 -1.039404833 0.105695862 -0.334824462
0.491992844 3 0 0.998207054
RHBDD2 ENSG00000005486.16 0.432191321 2 2 0.293372189
Brain_Cerebellum 0.385515443 Brain_Anterior_cingulate_cortex_BA24
1.109345902 0.890654098 0.890654098 -1.05075269 -0.86777879
-0.95926574 0.129382086 2 0 0.998207054
GLB1L ENSG00000163521.15 0.432218852 1 1 0.432218852
Brain_Hypothalamus 0.432218852 Brain_Hypothalamus 1 1 1
-0.785400391 -0.785400391 -0.785400391 NA 1 0 0.998207054
DGCR9 ENSG00000273032.1 0.432304185 11 6 0.065048858
Brain_Hippocampus 0.652772709 Brain_Hypothalamus 6.799067107
0.006019813 0.291132249 -0.506197038 1.844922195 0.862381099
0.648638735 6 0 0.998207054
PTPRN2 ENSG00000155093.17 0.432419541 8 7 0.036042765
Brain_Nucleus_accumbens_basal_ganglia 0.988190234 Brain_Amygdala
3.872733812 0.070958875 0.369924817 -2.09644465 0.014801888
-0.888507077 0.636883336 7 0 0.998207054
LINC01954 ENSG00000271952.1 0.432446133 10 5 0.11244833
Brain_Hippocampus 0.800090162 Brain_Cerebellar_Hemisphere 6.588977412
0.001574039 0.276275875 0.253230418 1.587284033 0.699459296
0.385523096 5 0 0.998207054
EVL ENSG00000196405.12 0.432472419 1 1 0.432472419
Brain_Anterior_cingulate_cortex_BA24 0.432472419
Brain_Anterior_cingulate_cortex_BA24 1 1 1 0.78496785 0.78496785
0.78496785 NA 1 0 0.998207054
AMY2B ENSG00000240038.6 0.432475154 7 5 0.39589998
Brain_Putamen_basal_ganglia 0.984991592 Brain_Frontal_Cortex_BA9
4.947231214 0.035768379 0.260719311 -0.514685319 0.848966416
0.335422594 0.485512711 5 0 0.998207054
PM20D2 ENSG00000146281.5 0.43247956 5 5 0.144018319 Brain_Cerebellum
0.763388645 Brain_Spinal_cord_cervical_c-1 2.198238771 0.18412412
0.18412412 -1.287152344 1.460989515 0.621192263 1.167004944 5
0 0.998207054
GTF3C1 ENSG00000077235.17 0.432774825 3 3 0.105508798
Brain_Spinal_cord_cervical_c-1 0.861572691 Brain_Hypothalamus 1.23629416
0.801286508 0.801286508 -0.174372543 1.618714072 0.644359838
0.906616525 3 0 0.998207054
COL27A1 ENSG00000196739.14 0.432786241 1 1 0.432786241
Brain_Hypothalamus 0.432786241 Brain_Hypothalamus 1 1 1
0.784432729 0.784432729 0.784432729 NA 1 0 0.998207054
PLXNA4 ENSG00000221866.9 0.432803626 1 1 0.432803626
Brain_Caudate_basal_ganglia 0.432803626 Brain_Caudate_basal_ganglia 1 1
1 -0.78440309 -0.78440309 -0.78440309 NA 1 0
0.998207054
NEK10 ENSG00000163491.16 0.432854361 2 2 0.460080254
Brain_Frontal_Cortex_BA9 0.949605734 Brain_Cortex 1.847966506
0.152033494 0.152033494 -0.738714705 -0.063201897 -0.400958301
0.477659687 2 0 0.998207054
MYO1A ENSG00000166866.12 0.433011204 1 1 0.433011204
Brain_Spinal_cord_cervical_c-1 0.433011204 Brain_Spinal_cord_cervical_c-1
1 1 1 0.784049264 0.784049264 0.784049264 NA 1 0
0.998207054
LINC02078 ENSG00000267719.1 0.433043608 1 1 0.433043608
Brain_Cerebellar_Hemisphere 0.433043608 Brain_Cerebellar_Hemisphere 1 1
1 0.78399404 0.78399404 0.78399404 NA 1 0 0.998207054
LINC01783 ENSG00000233421.4 0.433074574 1 1 0.433074574
Brain_Frontal_Cortex_BA9 0.433074574 Brain_Frontal_Cortex_BA9 1 1
1 0.783941266 0.783941266 0.783941266 NA 1 0
0.998207054
PTBP2 ENSG00000117569.18 0.433155001 5 5 0.116128428 Brain_Cerebellum
0.639799841 Brain_Spinal_cord_cervical_c-1 2.651796418 0.143400566

0.143400566 -1.57123347 0.467978674 -0.779164461 0.762301501
5 0 0.998207054
FCHO1 ENSG00000130475.14 0.433202826 3 3 0.159372544
Brain_Cerebellar_Hemisphere 0.92338418 Brain_Cerebellum 1.962862969
0.368286909 0.368286909 -0.096171733 1.407184979 0.545009586
0.775660452 3 0 0.998207054
PI15 ENSG00000137558.7 0.433220105 3 3 0.184396372
Brain_Cerebellar_Hemisphere 0.636768192 Brain_Nucleus_accumbens_basal_ganglia
1.450472913 0.597357657 0.597357657 -0.472222205 1.327339593
0.532037368 0.917797933 3 0 0.998207054
SYNGR2 ENSG00000108639.7 0.433285812 2 2 0.298468837
Brain_Caudate_basal_ganglia 0.464408717 Brain_Spinal_cord_cervical_c-1
1.035700437 0.964299563 0.964299563 -1.039722515 0.731606603
-0.154057956 1.252518831 2 0 0.998207054
FAM109B ENSG00000177096.8 0.433427026 4 4 0.158519853
Brain_Frontal_Cortex_BA9 0.997131012 Brain_Hypothalamus 2.435617296
0.104065516 0.104065516 0.003595751 1.410067161 0.922173961
0.626966116 4 0 0.998207054
NHSL1 ENSG00000135540.11 0.433509028 4 3 0.174401661 Brain_Amygdala
0.904015351 Brain_Caudate_basal_ganglia 1.957916208 0.048765662
0.939311127 -0.873694923 1.358195491 -0.116692566 1.042135787
3 0 0.998207054
TMEM155 ENSG00000164112.12 0.433617896 3 3 0.28236633 Brain_Cerebellum
0.933582991 Brain_Cerebellar_Hemisphere 1.869411359 0.267146707
0.267146707 -1.075018758 0.606638243 -0.128347591 0.860581534
3 0 0.998207054
RP11-70D24.4 ENSG00000275040.1 0.433628253 2 2 0.20865709
Brain_Cerebellar_Hemisphere 0.85395259 Brain_Cerebellum 1.368722841
0.631277159 0.631277159 -1.257266667 -0.184077585 -0.720672126
0.758859277 2 0 0.998207054
PGGHG ENSG00000142102.15 0.433656088 13 4 0.329535601
Brain_Hippocampus 0.975206704 Brain_Anterior_cingulate_cortex_BA24
10.63560776 0.0001358 0.420162559 -0.849545194 0.975049712
-0.170808667 0.471670717 4 0 0.998207054
PDE11A ENSG00000128655.16 0.433672583 3 3 0.126268114
Brain_Hypothalamus 0.632829371 Brain_Anterior_cingulate_cortex_BA24
2.255311796 0.300021351 0.300021351 0.477748289 1.528985196
0.975146624 0.527886242 3 0 0.998207054
PRODH ENSG00000100033.16 0.433676993 8 8 0.01882825
Brain_Frontal_Cortex_BA9 0.940873257 Brain_Amygdala 3.89563024
0.153150729 0.153150729 -2.348914101 -0.074172337 -1.302778854
0.745766081 8 0 0.998207054
SLC15A2 ENSG00000163406.10 0.43371561 7 5 0.118876708
Brain_Nucleus_accumbens_basal_ganglia 0.79612075 Brain_Hypothalamus
4.404170395 0.036683914 0.163609998 -0.845266359 1.55950582
0.681696023 0.846319425 5 0 0.998207054
SLC25A41 ENSG00000181240.13 0.433741621 9 7 0.28954429
Brain_Cerebellar_Hemisphere 0.947634836 Brain_Caudate_basal_ganglia
3.956840305 1.14E-16 0.273046133 -0.258952705 1.05912185
0.393412561 0.397815469 7 0 0.998207054
APCDD1 ENSG00000154856.12 0.43405942 4 4 0.080199929
Brain_Nucleus_accumbens_basal_ganglia 0.633061232
Brain_Spinal_cord_cervical_c-1 2.131175166 0.285723008 0.285723008
-1.749527212 0.477422589 -0.84697944 1.04507394 4 0
0.998207054
ASPG ENSG00000166183.15 0.434062381 5 4 0.100906325 Brain_Cortex
0.823248788 Brain_Putamen_basal_ganglia 3.309750833 0.047767379
0.171523444 0.223368411 1.640475566 1.028595052 0.559427591
4 0 0.998207054
PRKCD ENSG00000163932.13 0.434073124 6 6 0.231336415
Brain_Frontal_Cortex_BA9 0.657533453 Brain_Anterior_cingulate_cortex_BA24
2.555536169 0.101349276 0.101349276 -1.196923394 1.000723874

-0.563697308 0.815957238 6 0 0.998207054
GOT2 ENSG00000125166.12 0.434124818 2 2 0.20430939
Brain_Caudate_basal_ganglia 0.609615208 Brain_Nucleus_accumbens_basal_ganglia
1.217817652 0.782182348 0.782182348 0.5106228 1.269369273
0.889996036 0.536514776 2 0 0.998207054
PNKP ENSG00000039650.11 0.434142196 8 3 0.058641707
Brain_Anterior_cingulate_cortex_BA24 0.685213101 Brain_Caudate_basal_ganglia
6.839458686 0.042916109 0.228085125 -1.890869949 -0.405359739
-1.023141051 0.446249725 3 0 0.998207054
RIN2 ENSG00000132669.12 0.434151158 1 1 0.434151158
Brain_Hippocampus 0.434151158 Brain_Hippocampus 1 1 1
0.78210791 0.78210791 0.78210791 NA 1 0 0.998207054
PREPL ENSG00000138078.15 0.434151885 2 2 0.614308685 Brain_Cerebellum
0.926800375 Brain_Cerebellar_Hemisphere 1.890695248 0.109304752
0.109304752 -0.50393268 0.091871199 -0.206030741 0.421296963
2 0 0.998207054
MCM8 ENSG00000125885.13 0.434179413 13 3 0.157444965
Brain_Nucleus_accumbens_basal_ganglia 0.85355537 Brain_Putamen_basal_ganglia
10.70989831 0.007270279 0.369247194 -1.413717161 -0.184583957
-0.802442301 0.327131703 3 0 0.998207054
KMT2B ENSG00000272333.5 0.434252147 2 2 0.196495161
Brain_Anterior_cingulate_cortex_BA24 0.54475798 Brain_Spinal_cord_cervical_c-1
1.465458185 0.534541815 0.534541815 -1.291601568 -0.605633759
-0.948617663 0.485052489 2 0 0.998207054
ZNF570 ENSG00000171827.10 0.434281715 1 1 0.434281715
Brain_Substantia_nigra 0.434281715 Brain_Substantia_nigra 1 1
1 -0.781885759 -0.781885759 -0.781885759 NA 1 0
0.998207054
F2R ENSG00000181104.6 0.43430024 2 2 0.279024953
Brain_Nucleus_accumbens_basal_ganglia 0.740121572 Brain_Putamen_basal_ganglia
1.74238137 0.25761863 0.25761863 -1.082512299 -0.331692357 -0.707102328
0.530909872 2 0 0.998207054
DDX20 ENSG00000064703.11 0.434351826 2 2 0.20455534
Brain_Cerebellar_Hemisphere 0.273178884 Brain_Cerebellum 1.734638712
0.265361288 0.265361288 1.095771271 1.268679658 1.182225465
0.122264693 2 0 0.998207054
ZNF341 ENSG00000131061.13 0.434371832 1 1 0.434371832
Brain_Nucleus_accumbens_basal_ganglia 0.434371832
Brain_Nucleus_accumbens_basal_ganglia 1 1 1 0.781732441
0.781732441 0.781732441 NA 1 0 0.998207054
ZKSCAN4 ENSG00000187626.8 0.434466921 1 1 0.434466921
Brain_Cortex 0.434466921 Brain_Cortex 1 1 1 -0.781570685
-0.781570685 -0.781570685 NA 1 0 0.998207054
CREB3L3 ENSG00000060566.13 0.434552623 1 1 0.434552623
Brain_Amygdala 0.434552623 Brain_Amygdala 1 1 1 0.781424914
0.781424914 0.781424914 NA 1 0 0.998207054
AGPAT5 ENSG00000155189.11 0.43456863 2 2 0.196835033
Brain_Spinal_cord_cervical_c-1 0.967616609 Brain_Caudate_basal_ganglia
1.005764329 0.994235671 0.994235671 -1.290621281 -0.040597711
-0.665609496 0.883900143 2 0 0.998207054
SLC15A5 ENSG00000188991.3 0.43471421 3 3 0.255948117
Brain_Anterior_cingulate_cortex_BA24 0.746771828 Brain_Hypothalamus
1.617376817 0.439568451 0.439568451 -1.136020184 0.322898875
-0.583579798 0.791276524 3 0 0.998207054
DNPH1 ENSG00000112667.12 0.434739149 8 5 0.063242041 Brain_Cerebellum
0.866650089 Brain_Caudate_basal_ganglia 5.042279744 0.030078444
0.258208956 -0.632430289 1.857485999 0.206386054 0.872638601
5 0 0.998207054
MED10 ENSG00000133398.3 0.434743687 8 2 0.226117091
Brain_Nucleus_accumbens_basal_ganglia 0.537293812 Brain_Caudate_basal_ganglia
7.416920826 3.22E-05 0.257226347 -1.210421774 -0.616910422
-1.054560619 0.215036546 2 0 0.998207054

PEX16 ENSG00000121680.15 0.4347967 1 1 0.4347967
Brain_Anterior_cingulate_cortex_BA24 0.4347967
Brain_Anterior_cingulate_cortex_BA24 1 1 1 0.781009853
0.781009853 0.781009853 NA 1 0 0.998207054
SYT9 ENSG00000170743.16 0.434880935 2 2 0.303772817
Brain_Cerebellar_Hemisphere 0.505655538 Brain_Putamen_basal_ganglia
1.106542085 0.893457915 0.893457915 -0.665617626 1.028376375
0.181379374 1.197834645 2 0 0.998207054
LINC01376 ENSG00000236204.5 0.434992169 1 1 0.434992169
Brain_Frontal_Cortex_BA9 0.434992169 Brain_Frontal_Cortex_BA9 1 1
1 -0.780677547 -0.780677547 -0.780677547 NA 1 0
0.998207054
EFHC1 ENSG00000096093.15 0.435000092 2 1 0.426208392 Brain_Cortex
0.454435144 Brain_Putamen_basal_ganglia 1.955189477 0.044810523
1.955189477 0.74804147 0.79569662 0.771869045 0.03369728 1 0
0.998207054
FAM71F2 ENSG00000205085.10 0.435005447 6 4 0.118464293
Brain_Nucleus_accumbens_basal_ganglia 0.804529793 Brain_Frontal_Cortex_BA9
3.951830802 0.019353873 0.215011831 -0.608313703 1.561252049
0.775505718 0.95525422 4 0 0.998207054
BNIP2 ENSG00000140299.11 0.435056632 2 2 0.263386899 Brain_Cerebellum
0.501991524 Brain_Cerebellar_Hemisphere 1.876803341 0.123196659
0.123196659 0.671359523 1.118421775 0.894890649 0.31612075 2
0 0.998207054
PYDC1 ENSG00000169900.7 0.435078575 3 3 0.149122271 Brain_Cortex
0.855776594 Brain_Frontal_Cortex_BA9 1.568364718 0.440100792
0.440100792 -0.450961292 1.442638716 0.391143465 0.964009093
3 0 0.998207054
ETNK2 ENSG00000143845.14 0.435215051 1 1 0.435215051
Brain_Substantia_nigra 0.435215051 Brain_Substantia_nigra 1 1
1 -0.780298745 -0.780298745 -0.780298745 NA 1 0
0.998207054
FAM110D ENSG00000197245.5 0.435223984 11 3 0.13463237
Brain_Substantia_nigra 0.524360798 Brain_Cerebellum 8.699154211
0.008130369 0.456113798 0.636637799 1.496081686 1.200766371
0.250401884 3 0 0.998207054
PDGFC ENSG00000145431.10 0.435252937 3 3 0.219112864 Brain_Cerebellum
0.665491139 Brain_Caudate_basal_ganglia 1.127301044 0.852183077
0.852183077 -1.228890498 0.805668907 -0.285188656 1.025231241
3 0 0.998207054
AC078842.3 ENSG00000228031.2 0.435392343 1 1 0.435392343
Brain_Putamen_basal_ganglia 0.435392343 Brain_Putamen_basal_ganglia 1 1
1 0.779997507 0.779997507 0.779997507 NA 1 0
0.998207054
CEP162 ENSG00000135315.11 0.435408241 3 3 0.500606874
Brain_Cortex 0.751926205 Brain_Nucleus_accumbens_basal_ganglia 2.45909282
0.189427485 0.189427485 -0.673535183 0.31610053 -0.294158608
0.533689778 3 0 0.998207054
IL17RA ENSG00000177663.13 0.435619095 11 5 0.078408349
Brain_Frontal_Cortex_BA9 0.935055448 Brain_Spinal_cord_cervical_c-1
5.718670805 0.008616351 0.826129527 -1.759996803 0.843830049
-0.720150676 0.839349355 5 0 0.998207054
GLMP ENSG00000198715.11 0.435763598 2 2 0.200820416
Brain_Putamen_basal_ganglia 0.308088587 Brain_Nucleus_accumbens_basal_ganglia
1.859778471 0.140221529 0.140221529 1.019240956 1.279217668
1.149229312 0.183831295 2 0 0.998207054
MICB ENSG00000204516.9 0.435778031 11 5 0.109998928
Brain_Hippocampus 0.995176545 Brain_Substantia_nigra 7.645821294
0.006758951 0.508695992 -1.598197958 0.006045341 -0.93049193
0.599986235 5 0 0.998207054
LINC01905 ENSG00000267057.5 0.43584084 1 1 0.43584084 Brain_Hippocampus
0.43584084 Brain_Hippocampus 1 1 1 -0.779235775 -0.779235775

-0.779235775 NA 1 0 0.998207054
XPO5 ENSG00000124571.17 0.435876977 1 1 0.435876977 Brain_Cerebellum
0.435876977 Brain_Cerebellum 1 1 1 -0.779174419 -0.779174419
-0.779174419 NA 1 0 0.998207054
LINC01923 ENSG00000236653.2 0.435936411 1 1 0.435936411
Brain_Cortex 0.435936411 Brain_Cortex 1 1 1 0.779073514
0.779073514 0.779073514 NA 1 0 0.998207054
EIF2B3 ENSG00000070785.16 0.4360153 5 4 0.151020058 Brain_Amygdala
0.904787854 Brain_Frontal_Cortex_BA9 3.184236667 0.011094036
0.283779705 -1.435937742 0.988197475 -0.003465915 0.880485557
4 0 0.998207054
USP8 ENSG00000138592.13 0.436074684 1 1 0.436074684 Brain_Cerebellum
0.436074684 Brain_Cerebellum 1 1 1 -0.778838791 -0.778838791
-0.778838791 NA 1 0 0.998207054
AMH ENSG00000104899.7 0.436136482 11 9 0.129108388
Brain_Hippocampus 0.929835617 Brain_Cortex 3.959392303 4.99E-17
0.449178851 -1.517627271 1.300812483 0.41082554 0.939501911 9
0 0.998207054
NEDD8 ENSG00000129559.12 0.436436894 3 3 0.176182524 Brain_Cerebellum
0.913061239 Brain_Hippocampus 1.325539675 0.648431343
0.648431343 0.109178089 1.352602906 0.929217291 0.710300549
3 0 0.998207054
RASSF1 ENSG00000068028.17 0.436525994 3 2 0.156715929
Brain_Cerebellar_Hemisphere 0.955344287 Brain_Hippocampus 2.016563072
0.036477118 0.94695981 -1.41620351 -0.055996887 -0.861255649
0.713815224 2 0 0.998207054
MAN2C1 ENSG00000140400.16 0.436841751 13 4 0.235579949
Brain_Hypothalamus 0.963212835 Brain_Cerebellar_Hemisphere 10.63606304
0.009160314 0.429161951 -0.667414254 1.186107247 0.329465052
0.522940825 4 0 0.998207054
MAL2 ENSG00000147676.13 0.436891579 4 3 0.260661381 Brain_Cortex
0.91571107 Brain_Cerebellar_Hemisphere 1.989600057 0.010891294
0.923561268 -1.124829289 1.111875744 -0.077518239 0.917471632
3 0 0.998207054
RP11-344N10.5 ENSG00000272630.1 0.436990503 4 4 0.425811234
Brain_Cerebellum 0.886776897 Brain_Hippocampus 2.62192748 0.100743065
0.100743065 -0.297325326 0.79637994 0.143893605 0.485796178 4
0 0.998207054
TRIM5 ENSG00000132256.18 0.437059379 1 1 0.437059379
Brain_Putamen_basal_ganglia 0.437059379 Brain_Putamen_basal_ganglia 1 1
1 0.777168474 0.777168474 0.777168474 NA 1 0
0.998207054
SCGB3A1 ENSG00000161055.3 0.437091082 5 3 0.203636487
Brain_Putamen_basal_ganglia 0.926918867 Brain_Frontal_Cortex_BA9
3.317253302 0.055014694 0.50311288 -0.60277846 1.271259103
0.205809878 0.740417555 3 0 0.998207054
CARD8 ENSG00000105483.17 0.437092637 9 7 0.113783876
Brain_Spinal_cord_cervical_c-1 0.961390285 Brain_Cerebellar_Hemisphere
4.132161535 0.066269924 0.177704692 -0.657195732 1.581411954
0.616298412 0.662465322 7 0 0.998207054
BCL2L11 ENSG00000153094.22 0.437165128 6 6 0.061848787
Brain_Nucleus_accumbens_basal_ganglia 0.989638524
Brain_Anterior_cingulate_cortex_BA24 2.074776914 0.21885919 0.21885919
-1.867378234 0.012986549 -0.860466607 0.761128296 6 0
0.998207054
PLAGL1 ENSG00000118495.18 0.437209485 3 3 0.269701727
Brain_Cerebellum 0.586637198 Brain_Cerebellar_Hemisphere 1.036100832
0.959307671 0.959307671 -1.103749706 1.092870904 0.177612265
1.143157585 3 0 0.998207054
UCHL3 ENSG00000118939.17 0.437231086 10 2 0.184735928 Brain_Cerebellum
0.687329726 Brain_Amygdala 8.359379443 0.00502972 0.952426482
0.402481465 1.326313345 0.81971516 0.302305038 2 0

0.998207054
CCDC171 ENSG00000164989.16 0.437271641 12 2 0.179538018
Brain_Putamen_basal_ganglia 0.570006506 Brain_Amygdala 10.65606044
0.007543226 0.382404088 -1.342178834 -0.568041916 -0.994351828
0.26377304 2 0 0.998207054
RP11-473L15.3 ENSG00000249203.1 0.437314381 2 2 0.272287867
Brain_Hippocampus 0.805600441 Brain_Anterior_cingulate_cortex_BA24
1.348046666 0.651953334 0.651953334 -1.097809076 0.246105661
-0.425851707 0.950291224 2 0 0.998207054
ELP2 ENSG00000134759.13 0.437361519 3 2 0.217492231
Brain_Hypothalamus 0.69138532 Brain_Cerebellar_Hemisphere 1.970128764
0.045007045 0.98486419 -1.233223927 -0.396975781 -0.727020197
0.445086304 2 0 0.998207054
ENTPD6 ENSG00000197586.12 0.437444809 4 3 0.22380921
Brain_Cerebellar_Hemisphere 0.385448835 Brain_Cortex 2.854464776
0.030139803 0.129476569 -1.21646139 -0.867900445 -1.064137668
0.147733325 3 0 0.998207054
MTRF1 ENSG00000120662.15 0.437563455 3 3 0.14094759
Brain_Cerebellar_Hemisphere 0.713015125 Brain_Cortex 2.274697641
0.178054465 0.178054465 0.367809714 1.472271448 0.931913739
0.55261365 3 0 0.998207054
RP4-568F9.6 ENSG00000230010.1 0.437621795 1 1 0.437621795
Brain_Cerebellum 0.437621795 Brain_Cerebellum 1 1 1 0.776215434
0.776215434 0.776215434 NA 1 0 0.998207054
NKAIN1 ENSG00000084628.9 0.437624499 11 1 0.20217752
Brain_Substantia_nigra 0.842743645 Brain_Hypothalamus 10.36397014
1.88E-05 10.36397014 -1.275372239 0.198385266 -0.756036509
0.426727344 1 0 0.998207054
GRID2 ENSG00000152208.12 0.437679873 4 2 0.133876379
Brain_Substantia_nigra 0.395899098 Brain_Hippocampus 3.586174602
0.028120841 0.279207041 -1.498989411 -0.848968002 -1.111448121
0.288019771 2 0 0.998207054
LPIN3 ENSG00000132793.11 0.437734337 8 5 0.098905898
Brain_Nucleus_accumbens_basal_ganglia 0.932010781 Brain_Cortex
5.273443896 0.028094196 0.249005739 -1.650181117 0.394078852
-0.254644904 0.683408383 5 0 0.998207054
TMEM133 ENSG00000170647.3 0.437802158 1 1 0.437802158
Brain_Cerebellum 0.437802158 Brain_Cerebellum 1 1 1 -0.775909948
-0.775909948 -0.775909948 NA 1 0 0.998207054
CTD-3222D19.12 ENSG00000269399.2 0.437822235 2 2 0.327439205
Brain_Nucleus_accumbens_basal_ganglia 0.361392377 Brain_Frontal_Cortex_BA9
1.085100094 0.914899906 0.914899906 -0.97928495 -0.912715144
-0.946000047 0.047071961 2 0 0.998207054
UBXN10 ENSG00000162543.5 0.438066508 2 2 0.212747472
Brain_Hypothalamus 0.746161969 Brain_Caudate_basal_ganglia 1.008382572
0.991617428 0.991617428 -1.246046098 -0.323704228 -0.784875163
0.65219419 2 0 0.998207054
B3GNT2 ENSG00000170340.10 0.43810539 1 1 0.43810539
Brain_Caudate_basal_ganglia 0.43810539 Brain_Caudate_basal_ganglia 1 1 1
0.775396522 0.775396522 0.775396522 NA 1 0 0.998207054
HTRA2 ENSG00000115317.11 0.438121987 1 1 0.438121987
Brain_Nucleus_accumbens_basal_ganglia 0.438121987
Brain_Nucleus_accumbens_basal_ganglia 1 1 1 0.775368426
0.775368426 0.775368426 NA 1 0 0.998207054
MTMR7 ENSG00000003987.13 0.438183531 2 2 0.232676364
Brain_Hippocampus 0.510225864 Brain_Cerebellar_Hemisphere 1.158616612
0.841383388 0.841383388 -0.658486041 1.19349297 0.267503465
1.309546917 2 0 0.998207054
FAM35A ENSG00000122376.11 0.438300222 10 3 0.147796127
Brain_Cerebellum 0.958543165 Brain_Frontal_Cortex_BA9 7.677302889
0.00054943 0.704125829 -1.447359983 -0.051981838 -0.617717982
0.462274727 3 0 0.998207054

ATP10D ENSG00000145246.13 0.438301231 2 2 0.326162356
Brain_Anterior_cingulate_cortex_BA24 0.98238515 Brain_Cortex 1.657720108
0.342279892 0.342279892 -0.981873127 -0.022078735 -0.501975931
0.678677124 2 0 0.998207054
GNA15 ENSG00000060558.3 0.438393964 6 5 0.163097715 Brain_Cortex
0.996533942 Brain_Hippocampus 2.014222248 1.26E-17 0.834620043
-1.394728541 1.083858101 -0.51513469 0.994925174 5 0
0.998207054
ATRIP ENSG00000164053.19 0.438400758 4 4 0.102904715
Brain_Putamen_basal_ganglia 0.739961114 Brain_Nucleus_accumbens_basal_ganglia
1.647547681 0.414601032 0.414601032 -1.270031907 1.630931782
0.106148788 1.223997486 4 0 0.998207054
MIR210HG ENSG00000247095.2 0.438421009 1 1 0.438421009
Brain_Nucleus_accumbens_basal_ganglia 0.438421009
Brain_Nucleus_accumbens_basal_ganglia 1 1 1 0.774862337
0.774862337 0.774862337 NA 1 0 0.998207054
AF186192.1 ENSG00000254533.1 0.438570584 2 2 0.199315754
Brain_Spinal_cord_cervical_c-1 0.744701597 Brain_Hippocampus
1.228689502 0.771310498 0.771310498 0.325633588 1.283503444
0.804568516 0.67731627 2 0 0.998207054
TLN2 ENSG00000171914.16 0.438575327 1 1 0.438575327 Brain_Cerebellum
0.438575327 Brain_Cerebellum 1 1 1 0.774601235 0.774601235
0.774601235 NA 1 0 0.998207054
YBX2 ENSG00000006047.12 0.438741548 3 3 0.195086334 Brain_Amygdala
0.892280616 Brain_Cerebellar_Hemisphere 1.021176549 0.985043945
0.985043945 -1.295678319 1.021861828 -0.046132503 1.169388212
3 0 0.998207054
TCF19 ENSG00000137310.11 0.438824603 12 2 0.126135093 Brain_Amygdala
0.779157795 Brain_Cerebellum 10.55103612 0.01351822 0.715875283
0.280416741 1.529521986 0.625864158 0.364415397 2 0
0.998207054
PBX3 ENSG00000167081.16 0.438927369 3 3 0.104521595
Brain_Frontal_Cortex_BA9 0.515632026 Brain_Cortex 1.963508604
0.346930633 0.346930633 0.650093193 1.623317293 1.041559465
0.513760104 3 0 0.998207054
GATA2 ENSG00000179348.11 0.439071078 2 2 0.249033896
Brain_Cerebellar_Hemisphere 0.654033432 Brain_Frontal_Cortex_BA9
1.097672572 0.902327428 0.902327428 -1.152699136 0.448165954
-0.352266591 1.131982561 2 0 0.998207054
MFSD14C ENSG00000196312.12 0.439107307 9 2 0.111431894
Brain_Hippocampus 0.765442291 Brain_Cortex 7.517133005
0.002623723 0.696426242 0.298341818 1.591790017 1.157690992
0.44852273 2 0 0.998207054
CES3 ENSG00000172828.12 0.43911563 2 1 0.424414232
Brain_Cerebellar_Hemisphere 0.456396545 Brain_Cerebellum 1.99020204 0.00979796
1.99020204 0.744793533 0.798786479 0.771790006 0.038178778 1
0 0.998207054
LAMB3 ENSG00000196878.14 0.439149238 3 2 0.166691061
Brain_Nucleus_accumbens_basal_ganglia 0.397679477 Brain_Caudate_basal_ganglia
2.550341241 0.054947855 0.394710903 -1.382914575 -0.845772845
-1.119887692 0.268742473 2 0 0.998207054
URAD ENSG00000183463.5 0.439286837 2 2 0.703431067 Brain_Cerebellum
0.82112166 Brain_Cerebellar_Hemisphere 1.88766661 0.11233339 0.11233339
-0.380692995 0.226102551 -0.077295222 0.429069246 2 0
0.998207054
AC005301.9 ENSG00000283633.1 0.439328822 1 1 0.439328822
Brain_Spinal_cord_cervical_c-1 0.439328822 Brain_Spinal_cord_cervical_c-1
1 1 1 -0.7733271 -0.7733271 -0.7733271 NA 1 0 0.998207054
C4orf46 ENSG00000205208.4 0.43937966 1 1 0.43937966
Brain_Caudate_basal_ganglia 0.43937966 Brain_Caudate_basal_ganglia 1 1 1
-0.773241178 -0.773241178 -0.773241178 NA 1 0 0.998207054
TMEM35B ENSG00000243749.1 0.439392959 1 1 0.439392959

Brain_Hippocampus 0.439392959 Brain_Hippocampus 1 1 1
0.773218703 0.773218703 0.773218703 NA 1 0 0.998207054
INTS1 ENSG00000164880.15 0.439462273 13 1 0.319807304
Brain_Hypothalamus 0.673064366 Brain_Frontal_Cortex_BA9 12.15598126
0.000199083 12.15598126 -0.994853946 -0.421946092 -0.747737308
0.175054373 1 0 0.998207054
RP11-351J23.1 ENSG00000203688.4 0.439465046 4 4 0.114690528
Brain_Nucleus_accumbens_basal_ganglia 0.680669693 Brain_Putamen_basal_ganglia
1.20767225 0.779804807 0.779804807 -0.999455452 1.57745648 0.08571182
1.161248185 4 0 0.998207054
NKD1 ENSG00000140807.6 0.439549891 2 2 0.214813777 Brain_Cerebellum
0.378290212 Brain_Cerebellar_Hemisphere 1.848660935 0.151339065
0.151339065 -1.240437071 -0.881051009 -1.06074404 0.254124321
2 0 0.998207054
C19orf33 ENSG00000167644.11 0.439578824 7 3 0.482504252
Brain_Substantia_nigra 0.900691604 Brain_Caudate_basal_ganglia
5.691593868 0.018136132 0.263900699 -0.702280499 0.441441836
-0.150424914 0.420036093 3 0 0.998207054
RP11-535M15.1 ENSG00000224848.1 0.439669818 12 1 0.337467835
Brain_Frontal_Cortex_BA9 0.77346207 Brain_Amygdala 11.67399745 3.48E-06
11.67399745 -0.959180474 -0.287849336 -0.761599692 0.166624408
1 0 0.998207054
DHRS3 ENSG00000162496.8 0.439681554 1 1 0.439681554
Brain_Caudate_basal_ganglia 0.439681554 Brain_Caudate_basal_ganglia 1 1
1 0.772731071 0.772731071 0.772731071 NA 1 0
0.998207054
ADAL ENSG00000168803.14 0.439802281 13 1 0.273081389
Brain_Putamen_basal_ganglia 0.634993459 Brain_Cerebellar_Hemisphere
12.51688451 0.000234867 12.51688451 -1.095994026 -0.474710323
-0.757725778 0.203360244 1 0 0.998207054
DDX54 ENSG00000123064.12 0.439834404 1 1 0.439834404
Brain_Caudate_basal_ganglia 0.439834404 Brain_Caudate_basal_ganglia 1 1
1 0.77247288 0.77247288 0.77247288 NA 1 0 0.998207054
RP11-121A8.1 ENSG00000272908.1 0.439848456 1 1 0.439848456
Brain_Anterior_cingulate_cortex_BA24 0.439848456
Brain_Anterior_cingulate_cortex_BA24 1 1 1 -0.772449146
-0.772449146 -0.772449146 NA 1 0 0.998207054
ATG7 ENSG00000197548.12 0.439906712 3 2 0.191410427 Brain_Cerebellum
0.919760606 Brain_Hypothalamus 2.041546285 0.035418017
0.923035698 -0.100735278 1.306418063 0.80648419 0.787039578 2
0 0.998207054
NFKB1 ENSG00000109320.11 0.439922106 1 1 0.439922106
Brain_Frontal_Cortex_BA9 0.439922106 Brain_Frontal_Cortex_BA9 1 1
1 -0.772324758 -0.772324758 -0.772324758 NA 1 0
0.998207054
TMEM116 ENSG00000198270.12 0.44001348 4 4 0.117112008
Brain_Spinal_cord_cervical_c-1 0.844008778 Brain_Frontal_Cortex_BA9
2.296864526 0.086611937 0.086611937 -1.56701148 0.196768404
-0.49366394 0.757660148 4 0 0.998207054
ANKRD36C ENSG00000174501.14 0.440016796 3 3 0.200697061
Brain_Spinal_cord_cervical_c-1 0.78361342 Brain_Putamen_basal_ganglia 1.147368
0.756816556 0.756816556 0.274613206 1.279568138 0.885680372
0.536524718 3 0 0.998207054
SIN3B ENSG00000127511.9 0.440036579 2 2 0.254480333 Brain_Cortex
0.276190015 Brain_Frontal_Cortex_BA9 1.514805322 0.485194678
0.485194678 1.088918074 1.13953439 1.114226232 0.03579114 2 0
0.998207054
MTR ENSG00000116984.12 0.440045331 4 4 0.094694616 Brain_Cortex
0.673394358 Brain_Nucleus_accumbens_basal_ganglia 2.349012922
0.291402579 0.291402579 0.421494047 1.671137024 1.067139179
0.619752778 4 0 0.998207054
MRPL1 ENSG00000169288.17 0.440110502 1 1 0.440110502 Brain_Amygdala

0.440110502 Brain_Amygdala 1 1 1 -0.772006628 -0.772006628 -0.772006628 NA 1 0 0.998207054
UACA ENSG00000137831.14 0.440125572 2 2 0.200533096 Brain_Putamen_basal_ganglia 0.469758387 Brain_Caudate_basal_ganglia 1.598450051 0.401549949 0.401549949 0.722872228 1.280034235 1.001453232 0.393973034 2 0 0.998207054
LINC01719 ENSG00000233396.7 0.440418192 9 5 0.174658634 Brain_Amygdala 0.99037029 Brain_Cortex 5.781932739 1.07E-16 0.363664855 0.012069345 1.357385874 1.055168622 0.408316615 5 0 0.998207054
SCN8A ENSG00000196876.15 0.44043225 1 1 0.44043225 Brain_Caudate_basal_ganglia 0.44043225 Brain_Caudate_basal_ganglia 1 1 1 -0.771463499 -0.771463499 -0.771463499 NA 1 0 0.998207054
ARHGEF26 ENSG00000114790.12 0.440490953 1 1 0.440490953 Brain_Amygdala 0.440490953 Brain_Amygdala 1 1 1 0.771364429 0.771364429 0.771364429 NA 1 0 0.998207054
COX15 ENSG00000014919.12 0.440563018 4 4 0.117719171 Brain_Cerebellar_Hemisphere 0.755107096 Brain_Cortex 2.455159458 0.102077907 0.102077907 -1.564419131 -0.311912403 -0.987230076 0.521865837 4 0 0.998207054
RANGRF ENSG00000108961.13 0.440641309 1 1 0.440641309 Brain_Hypothalamus 0.440641309 Brain_Hypothalamus 1 1 1 0.771110717 0.771110717 0.771110717 NA 1 0 0.998207054
HPGDS ENSG00000163106.10 0.440828045 4 4 0.0862179 Brain_Substantia_nigra 0.679843156 Brain_Caudate_basal_ganglia 1.313372695 0.5964128 0.5964128 -1.715694892 0.422386795 -0.46308383 1.061248463 4 0 0.998207054
CHODL ENSG00000154645.13 0.440841885 1 1 0.440841885 Brain_Nucleus_accumbens_basal_ganglia 0.440841885 Brain_Nucleus_accumbens_basal_ganglia 1 1 1 -0.770772339 -0.770772339 -0.770772339 NA 1 0 0.998207054
GYG1 ENSG00000163754.17 0.440959724 1 1 0.440959724 Brain_Caudate_basal_ganglia 0.440959724 Brain_Caudate_basal_ganglia 1 1 1 -0.770573583 -0.770573583 -0.770573583 NA 1 0 0.998207054
NLRP1 ENSG00000091592.15 0.440965697 4 3 0.098288508 Brain_Cortex 0.405352803 Brain_Spinal_cord_cervical_c-1 2.987316853 0.000924586 0.180228112 0.832099467 1.653208151 1.363392973 0.374842329 3 0 0.998207054
RP11-474O21.5 ENSG00000272482.1 0.441074177 1 1 0.441074177 Brain_Substantia_nigra 0.441074177 Brain_Substantia_nigra 1 1 1 0.770380568 0.770380568 0.770380568 NA 1 0 0.998207054
PPDPF ENSG00000125534.9 0.441098008 1 1 0.441098008 Brain_Cortex 0.441098008 Brain_Cortex 1 1 1 0.770340382 0.770340382 0.770340382 NA 1 0 0.998207054
ANGPTL4 ENSG00000167772.11 0.44110258 1 1 0.44110258 Brain_Spinal_cord_cervical_c-1 0.44110258 Brain_Spinal_cord_cervical_c-1 1 1 1 -0.770332673 -0.770332673 -0.770332673 NA 1 0 0.998207054
SYTL1 ENSG00000142765.17 0.441113627 2 2 0.241234306 Brain_Cerebellar_Hemisphere 0.437860194 Brain_Cerebellum 1.23636473 0.76363527 0.76363527 0.775811667 1.171907226 0.973859446 0.280081856 2 0 0.998207054
F13A1 ENSG00000124491.15 0.441171712 2 2 0.237563722 Brain_Hypothalamus 0.704950763 Brain_Spinal_cord_cervical_c-1 1.093782108 0.906217892 0.906217892 -0.378645994 1.181098182 0.401226094 1.102905684 2 0 0.998207054
LLGL2 ENSG00000073350.13 0.441379107 1 1 0.441379107 Brain_Cerebellum 0.441379107 Brain_Cerebellum 1 1 1 -0.769866467 -0.769866467 -0.769866467 NA 1 0 0.998207054
STAT4 ENSG00000138378.17 0.441495231 1 1 0.441495231

Brain_Putamen_basal_ganglia 0.441495231 Brain_Putamen_basal_ganglia 1 1
1 -0.76967074 -0.76967074 -0.76967074 NA 1 0
0.998207054
JPH1 ENSG00000104369.4 0.441572853 5 2 0.120968316
Brain_Substantia_nigra 0.5802846 Brain_Cerebellar_Hemisphere 3.870307161
0.008564151 0.999992818 -1.550722016 0.552969055 -0.792155571
0.815049159 2 0 0.998207054
RP11-675F6.3 ENSG00000253361.1 0.441631811 3 2 0.315953106
Brain_Cerebellum 0.852836735 Brain_Cortex 2.712034507 0.044546493
0.243419 0.185500186 1.002808833 0.542792953 0.41822528 2 0
0.998207054
FGF18 ENSG00000156427.7 0.441780447 1 1 0.441780447
Brain_Caudate_basal_ganglia 0.441780447 Brain_Caudate_basal_ganglia 1 1
1 -0.769190133 -0.769190133 -0.769190133 NA 1 0
0.998207054
RP11-249C24.12 ENSG00000275691.1 0.441882926 1 1 0.441882926
Brain_Nucleus_accumbens_basal_ganglia 0.441882926
Brain_Nucleus_accumbens_basal_ganglia 1 1 1 0.769017492
0.769017492 0.769017492 NA 1 0 0.998207054
KRT33B ENSG00000131738.10 0.441981453 1 1 0.441981453
Brain_Hippocampus 0.441981453 Brain_Hippocampus 1 1 1
0.768851532 0.768851532 0.768851532 NA 1 0 0.998207054
WBSCR27 ENSG00000165171.10 0.441990822 13 1 0.225270994
Brain_Cortex 0.715375494 Brain_Substantia_nigra 12.62293824
0.00035262 12.62293824 -1.212630835 -0.364646239 -0.757075162
0.218844515 1 0 0.998207054
RP11-120K24.3 ENSG00000267868.1 0.442004572 6 6 0.161162027
Brain_Anterior_cingulate_cortex_BA24 0.997569292 Brain_Frontal_Cortex_BA9
2.479322418 0.257161888 0.257161888 -1.401174066 0.476538332
-0.37882194 0.671785223 6 0 0.998207054
AADAT ENSG00000109576.13 0.442013266 8 6 0.212881406
Brain_Anterior_cingulate_cortex_BA24 0.982226472 Brain_Putamen_basal_ganglia
4.956235535 0.032665545 0.211912723 -1.245681343 0.821294222
-0.060103932 0.729665453 6 0 0.998207054
IPO7 ENSG00000205339.9 0.442014196 1 1 0.442014196
Brain_Nucleus_accumbens_basal_ganglia 0.442014196
Brain_Nucleus_accumbens_basal_ganglia 1 1 1 0.768796384
0.768796384 0.768796384 NA 1 0 0.998207054
TSLP ENSG00000145777.14 0.442075883 12 2 0.309213244 Brain_Cortex
0.959177027 Brain_Nucleus_accumbens_basal_ganglia 10.87561498
0.003015814 0.444842675 -0.26812692 1.016874273 0.235545659
0.298076713 2 0 0.998207054
RBBP4 ENSG00000162521.18 0.442140856 1 1 0.442140856 Brain_Cortex
0.442140856 Brain_Cortex 1 1 1 -0.768583076 -0.768583076
-0.768583076 NA 1 0 0.998207054
PRSS37 ENSG00000165076.13 0.442211591 5 2 0.602010952
Brain_Nucleus_accumbens_basal_ganglia 0.958540033 Brain_Cerebellar_Hemisphere
4.681553269 0.001639686 0.202763452 -0.521510846 0.422500725
0.037591182 0.344145741 2 0 0.998207054
KCNAB3 ENSG00000170049.9 0.44221957 6 2 0.090518457
Brain_Frontal_Cortex_BA9 0.346734005 Brain_Cerebellum 5.406361462
0.021683129 0.270521977 -1.692669169 -0.940943127 -1.192671651
0.280689919 2 0 0.998207054
NPIPB6 ENSG00000198156.10 0.442284032 4 4 0.090268123
Brain_Nucleus_accumbens_basal_ganglia 0.877619911 Brain_Cerebellum
1.727814854 0.39578263 0.39578263 0.153987095 1.693985054
0.979954038 0.63243056 4 0 0.998207054
ZBTB38 ENSG00000177311.11 0.442372832 1 1 0.442372832
Brain_Cerebellum 0.442372832 Brain_Cerebellum 1 1 1 0.768192494
0.768192494 0.768192494 NA 1 0 0.998207054
DDR1 ENSG00000204580.13 0.442469146 1 1 0.442469146 Brain_Cerebellum
0.442469146 Brain_Cerebellum 1 1 1 0.768030364 0.768030364

0.768030364 NA 1 0 0.998207054
TRPV1 ENSG00000196689.11 0.442586728 1 1 0.442586728 Brain_Cerebellum 0.442586728 Brain_Cerebellum 1 1 1 -0.767832459 -0.767832459 -0.767832459 NA 1 0 0.998207054
LINC02199 ENSG00000250198.1 0.442638886 2 2 0.323538581 Brain_Substantia_nigra 0.360672319 Brain_Nucleus_accumbens_basal_ganglia 1.110922684 0.889077316 0.889077316 0.914084743 0.987212284 0.950648513 0.05170898 2 0 0.998207054
ANGPTL1 ENSG00000116194.12 0.442698712 1 1 0.442698712 Brain_Cortex 0.442698712 Brain_Cortex 1 1 1 -0.767644005 -0.767644005 -0.767644005 NA 1 0 0.998207054
KPNA2 ENSG00000182481.8 0.442852177 3 3 0.254916406 Brain_Caudate_basal_ganglia 0.795364082 Brain_Amygdala 2.15974691 0.165953901 0.165953901 -1.138488864 0.265017776 -0.377607514 0.709186925 3 0 0.998207054
AC017074.2 ENSG00000227359.1 0.442912718 1 1 0.442912718 Brain_Cerebellum 0.442912718 Brain_Cerebellum 1 1 1 -0.767283934 -0.767283934 -0.767283934 NA 1 0 0.998207054
HS6ST3 ENSG00000185352.8 0.443159096 2 2 0.258807745 Brain_Nucleus_accumbens_basal_ganglia 0.926576222 Brain_Caudate_basal_ganglia 1.528107183 0.471892817 0.471892817 0.092153324 1.129213583 0.610683453 0.733312342 2 0 0.998207054
FEM1C ENSG00000145780.7 0.443168702 1 1 0.443168702 Brain_Cerebellum 0.443168702 Brain_Cerebellum 1 1 1 -0.766853367 -0.766853367 -0.766853367 NA 1 0 0.998207054
C20orf194 ENSG00000088854.12 0.443177193 4 3 0.559156585 Brain_Hippocampus 0.914294059 Brain_Cortex 3.416517267 0.029447349 0.190037311 -0.584094722 0.340888272 -0.172883828 0.468502055 3 0 0.998207054
ADAMTS10 ENSG00000142303.13 0.44322945 1 1 0.44322945 Brain_Cerebellar_Hemisphere 0.44322945 Brain_Cerebellar_Hemisphere 1 1 1 0.76675121 0.76675121 0.76675121 NA 1 0 0.998207054
FBXL4 ENSG00000112234.8 0.443471031 1 1 0.443471031 Brain_Spinal_cord_cervical_c-1 0.443471031 Brain_Spinal_cord_cervical_c-1 1 1 1 -0.76634503 -0.76634503 -0.76634503 NA 1 0 0.998207054
ZNF208 ENSG00000160321.14 0.443494154 10 4 0.04137289 Brain_Amygdala 0.561930082 Brain_Cerebellum 8.105726131 0.013158309 0.289572824 -2.039773506 -0.579977068 -1.209794954 0.442465256 4 0 0.998207054
MT1E ENSG00000169715.14 0.443561985 8 7 0.133813273 Brain_Cortex 0.945330438 Brain_Frontal_Cortex_BA9 3.012144884 0.03706576 0.18517776 -1.499232705 0.704862454 -0.431355552 0.714238886 7 0 0.998207054
CHAC2 ENSG00000143942.4 0.443594112 1 1 0.443594112 Brain_Cerebellar_Hemisphere 0.443594112 Brain_Cerebellar_Hemisphere 1 1 1 -0.766138139 -0.766138139 -0.766138139 NA 1 0 0.998207054
DFFB ENSG00000169598.15 0.443694335 10 3 0.146864853 Brain_Frontal_Cortex_BA9 0.542662596 Brain_Anterior_cingulate_cortex_BA24 7.721562916 0.002728874 0.655460726 -0.750269666 1.45069484 0.778631166 0.579130496 3 0 0.998207054
ITGA4 ENSG00000115232.13 0.443787878 1 1 0.443787878 Brain_Substantia_nigra 0.443787878 Brain_Substantia_nigra 1 1 1 0.765812496 0.765812496 0.765812496 NA 1 0 0.998207054
C11orf74 ENSG00000166352.15 0.443808 2 2 0.212690352 Brain_Cortex 0.305226729 Brain_Hypothalamus 1.661603147 0.338396853 0.338396853 1.025289246 1.246201709 1.135745477 0.1562087 2 0 0.998207054
TBC1D9B ENSG00000197226.12 0.44402658 11 11 0.163679946 Brain_Nucleus_accumbens_basal_ganglia 0.911902193 Brain_Caudate_basal_ganglia

3.615215769	0.28533291 0.28533291 -1.292467734	1.3928011	-0.111672792
0.914596368	11	0	0.998207054
PYURF ENSG00000145337.4	0.444057276	3	3	0.144631645
Brain_Spinal_cord_cervical_c-1	0.60622396 Brain_Hypothalamus	1.149094415
0.848028009	0.848028009	-0.515470956	1.458758294	0.547758159
0.995879274	3	0	0.998207054
UBE2W ENSG00000104343.19	0.444149582	5	3	0.106355986
Brain_Caudate_basal_ganglia 0.254628267	Brain_Cerebellum 4.42176694
0.021825619	0.197088229	1.139179566	1.614790893	1.361389807
0.188171003	3	0	0.998207054
MRPS22	ENSG00000175110.11	0.444157371	4	3	0.324215368
Brain_Putamen_basal_ganglia 0.893475874	Brain_Cortex	3.095347506
0.018967237	0.231910288	-0.449901371	0.985832392	0.282384845
0.601267598	3	0	0.998207054
ID2	ENSG00000115738.9	0.444189785	1	1	0.444189785
Brain_Spinal_cord_cervical_c-1	0.444189785	Brain_Spinal_cord_cervical_c-1
1	1	1	0.76513731 0.76513731 0.76513731 NA	1	0	0.998207054
PRR34 ENSG00000182257.6	0.444237867	3	3	0.130236462
Brain_Hypothalamus	0.991448009	Brain_Cerebellar_Hemisphere 1.202345515
0.81816444 0.81816444 -1.513170082	-0.010718537	-0.629987544
0.785224761	3	0	0.998207054
MROH7 ENSG00000184313.19	0.444241906	9	8	0.119520287	Brain_Cerebellum
0.833842582	Brain_Frontal_Cortex_BA9	2.624538814	0.008119607
0.38714613 -1.556790275	0.898201286	-0.334059149	0.892797811	8
0	0.998207054
MRPS18A	ENSG00000096080.11	0.444279284	1	1	0.444279284
Brain_Amygdala	0.444279284	Brain_Amygdala	1	1	1	0.764987003
0.764987003	0.764987003	NA	1	0	0.998207054
MIS18A	ENSG00000159055.3	0.444353953	1	1	0.444353953
Brain_Hypothalamus	0.444353953	Brain_Hypothalamus	1	1	1
-0.764861615	-0.764861615	-0.764861615	NA	1	0	0.998207054
CALM2 ENSG00000143933.16	0.444367958	1	1	0.444367958	Brain_Cerebellum
0.444367958	Brain_Cerebellum 1	1	1	0.764838099	0.764838099
0.764838099	NA	1	0	0.998207054
XCL2	ENSG00000143185.3	0.444538255	1	1	0.444538255
Brain_Spinal_cord_cervical_c-1	0.444538255	Brain_Spinal_cord_cervical_c-1
1	1	1	-0.764552178	-0.764552178	-0.764552178	NA	1	0
0.998207054
CHD8	ENSG00000100888.12	0.444588683	1	1	0.444588683
Brain_Cerebellar_Hemisphere 0.444588683	Brain_Cerebellar_Hemisphere 1	1
1	-0.764467524	-0.764467524	-0.764467524	NA	1	0
0.998207054
SLC18A1	ENSG00000036565.14	0.444647342	2	2	0.625773364
Brain_Spinal_cord_cervical_c-1	0.782853472	Brain_Substantia_nigra
1.818906864	0.181093136	0.181093136	-0.487684455	0.275602392
-0.106041031	0.539725306	2	0	0.998207054
FAM66B	ENSG00000215374.5	0.444721343	1	1	0.444721343
Brain_Spinal_cord_cervical_c-1	0.444721343	Brain_Spinal_cord_cervical_c-1
1	1	1	-0.764244852	-0.764244852	-0.764244852	NA	1	0
0.998207054
COX6C ENSG00000164919.10	0.444740172	3	3	0.57407948
Brain_Nucleus_accumbens_basal_ganglia	0.850928365	Brain_Cortex
1.940718981	0.142542776	0.142542776	-0.243306224	0.56205363
0.043604458	0.449842947	3	0	0.998207054
FAM168A	ENSG00000054965.10	0.444822964	1	1	0.444822964
Brain_Caudate_basal_ganglia 0.444822964	Brain_Caudate_basal_ganglia 1	1
1	-0.764074304	-0.764074304	-0.764074304	NA	1	0
0.998207054
SLC37A1	ENSG00000160190.13	0.444841634	3	3	0.191460506
Brain_Anterior_cingulate_cortex_BA24	0.885659735	Brain_Frontal_Cortex_BA9
1.275301834	0.723677638	0.723677638	-0.862976795	1.306270733
0.099831875	1.104954959	3	0	0.998207054

RAB13 ENSG00000143545.8 0.444882313 2 2 0.206939646 Brain_Cortex
0.97075025 Brain_Nucleus_accumbens_basal_ganglia 1.158053311 0.841946689
0.841946689 -1.262025402 -0.03666734 -0.649346371 0.866458995
2 0 0.998207054
RCCD1 ENSG00000166965.12 0.444895927 12 1 0.150523968
Brain_Substantia_nigra 0.588358851 Brain_Cerebellar_Hemisphere
11.49644532 2.07E-05 11.49644532 0.541215796 1.437683179
0.75659648 0.233501752 1 0 0.998207054
SP110 ENSG00000135899.17 0.444968837 9 2 0.340576137
Brain_Caudate_basal_ganglia 0.910678597 Brain_Hippocampus 8.134050109
0.000151205 0.642573174 -0.953027501 0.411839313 -0.446812533
0.438219113 2 0 0.998207054
LINC01220 ENSG00000259687.1 0.445044784 1 1 0.445044784
Brain_Hypothalamus 0.445044784 Brain_Hypothalamus 1 1 1
-0.763702109 -0.763702109 -0.763702109 NA 1 0 0.998207054
GSKIP ENSG00000100744.14 0.445058321 8 5 0.041753432 Brain_Cerebellum
0.860897726 Brain_Frontal_Cortex_BA9 4.398162048 0.024045369
0.275046771 -2.035969356 1.117802633 -0.358132935 1.070258412
5 0 0.998207054
COPS9 ENSG00000172428.10 0.445126159 2 2 0.20424317
Brain_Nucleus_accumbens_basal_ganglia 0.340575734 Brain_Caudate_basal_ganglia
1.703777284 0.296222716 0.296222716 0.953028297 1.269555049
1.111291673 0.223818213 2 0 0.998207054
LRPAP1 ENSG00000163956.10 0.445165942 11 4 0.164187762
Brain_Cerebellum 0.968811968 Brain_Spinal_cord_cervical_c-1 8.59455418
0.009574214 0.416365993 0.039098361 1.39112422 0.72941883
0.472837919 4 0 0.998207054
TDRD5 ENSG00000162782.15 0.445201284 10 4 0.124852601
Brain_Substantia_nigra 0.933764476 Brain_Nucleus_accumbens_basal_ganglia
7.697845052 0.002760436 0.305470215 -1.53472008 -0.083109495
-1.033434113 0.402946152 4 0 0.998207054
RP11-675F6.4 ENSG00000254100.1 0.445234971 3 3 0.358656308
Brain_Cerebellum 0.757535688 Brain_Cerebellar_Hemisphere 2.248849874
0.170890863 0.170890863 -0.556944045 0.917928484 0.223234305
0.74114297 3 0 0.998207054
SFTA1P ENSG00000225383.7 0.445347691 9 6 0.06860485
Brain_Spinal_cord_cervical_c-1 0.901110667 Brain_Putamen_basal_ganglia
6.070693162 0.024821923 0.30951692 -0.124258422 1.821013203
0.915972025 0.605825496 6 0 0.998207054
C5orf64-AS1 ENSG00000251575.2 0.445430387 8 8 0.063243094
Brain_Caudate_basal_ganglia 0.913947669 Brain_Hippocampus 1.757288498
0.350579815 0.350579815 -1.857478596 1.004403472 -0.355768037
0.863872692 8 0 0.998207054
TTC22 ENSG00000006555.10 0.445497136 2 2 0.32656628
Brain_Caudate_basal_ganglia 0.454562027 Brain_Spinal_cord_cervical_c-1
1.060932168 0.939067832 0.939067832 -0.747831122 0.98105366
0.116611269 1.222506154 2 0 0.998207054
RP11-191L17.1 ENSG00000272663.1 0.445550711 4 4 0.230222963
Brain_Cortex 0.920709693 Brain_Caudate_basal_ganglia 2.513281199
0.093396553 0.093396553 -1.199784711 0.143428602 -0.495917536
0.624883101 4 0 0.998207054
ANKRD30B ENSG00000180777.13 0.44578662 8 7 0.114785362 Brain_Amygdala
0.925091617 Brain_Nucleus_accumbens_basal_ganglia 3.726963017
0.070057888 0.198587294 0.094022081 1.577044169 0.633789662
0.467421904 7 0 0.998207054
PCDH18 ENSG00000189184.11 0.445792158 2 2 0.32516783 Brain_Amygdala
0.423323669 Brain_Putamen_basal_ganglia 1.004226855 0.995773145
0.995773145 -0.800668352 0.983893602 0.091612625 1.261875859
2 0 0.998207054
IGFBP7 ENSG00000163453.11 0.445881823 7 5 0.080521308
Brain_Putamen_basal_ganglia 0.956713837 Brain_Nucleus_accumbens_basal_ganglia
4.283786213 0.071053229 0.327171124 -0.098836576 1.747669299

0.680079458 0.721615325 5 0 0.998207054
ATL3 ENSG00000184743.12 0.445925815 3 3 0.179109815
Brain_Caudate_basal_ganglia 0.51997312 Brain_Nucleus_accumbens_basal_ganglia
1.296970308 0.688349192 0.688349192 -1.343500961 0.669266937
-0.010282394 1.154673657 3 0 0.998207054
NAP1L5 ENSG00000177432.6 0.44600656 6 4 0.259469423
Brain_Caudate_basal_ganglia 0.679300401 Brain_Nucleus_accumbens_basal_ganglia
3.741153985 0.050884305 0.246867757 -1.127646067 0.974737778
0.144149717 0.842238601 4 0 0.998207054
KCTD18 ENSG00000155729.12 0.446129355 1 1 0.446129355
Brain_Cerebellar_Hemisphere 0.446129355 Brain_Cerebellar_Hemisphere 1 1
1 -0.761883808 -0.761883808 -0.761883808 NA 1 0
0.998207054
FCRL6 ENSG00000181036.13 0.446159101 2 2 0.293783442
Brain_Caudate_basal_ganglia 0.519030384 Brain_Cerebellar_Hemisphere
1.065836578 0.934163422 0.934163422 0.644840761 1.049857918
0.84734934 0.286390378 2 0 0.998207054
NPIPA2 ENSG00000254852.8 0.446182126 3 3 0.193636768
Brain_Cerebellar_Hemisphere 0.657755163 Brain_Caudate_basal_ganglia
1.187120541 0.828796211 0.828796211 0.443014616 1.299895558
0.883110567 0.428915828 3 0 0.998207054
HS3ST1 ENSG00000002587.9 0.446257558 1 1 0.446257558
Brain_Cerebellum 0.446257558 Brain_Cerebellum 1 1 1 0.761669039
0.761669039 0.761669039 NA 1 0 0.998207054
LINC00440 ENSG00000234660.1 0.4462772 1 1 0.4462772
Brain_Anterior_cingulate_cortex_BA24 0.4462772
Brain_Anterior_cingulate_cortex_BA24 1 1 1 0.761636139
0.761636139 0.761636139 NA 1 0 0.998207054
GCLC ENSG00000001084.10 0.446348508 6 6 0.164756937
Brain_Frontal_Cortex_BA9 0.947896054 Brain_Hippocampus 3.167222674
0.266527147 0.266527147 -1.088003082 1.389249359 0.188863993
0.796573407 6 0 0.998207054
LINC02032 ENSG00000241131.1 0.446350688 2 1 0.394431362
Brain_Cerebellum 0.511482202 Brain_Cerebellar_Hemisphere 1.961095251
0.038904749 1.961095251 -0.851608605 -0.656531507 -0.754070056
0.137940339 1 0 0.998207054
FAAP24 ENSG00000131944.9 0.44646211 5 4 0.433328274
Brain_Hypothalamus 0.944550983 Brain_Frontal_Cortex_BA9 4.078260471
0.064443299 0.150005607 -0.783508994 0.494489957 0.063881695
0.519699122 4 0 0.998207054
CAMKK2 ENSG00000110931.18 0.446465476 3 3 0.133069773
Brain_Cortex 0.973441277 Brain_Nucleus_accumbens_basal_ganglia
1.577117626 0.434398507 0.434398507 -1.502105857 -0.033292573
-0.967920526 0.812169217 3 0 0.998207054
ZNF552 ENSG00000178935.5 0.446474777 5 3 0.141603877
Brain_Caudate_basal_ganglia 0.487749053 Brain_Hypothalamus 4.157698623
0.044701812 0.259673944 -1.469844487 -0.693893416 -0.978849795
0.322248813 3 0 0.998207054
WISP3 ENSG00000112761.20 0.446722741 2 2 0.205324386 Brain_Cerebellum
0.995827382 Brain_Anterior_cingulate_cortex_BA24 1.064302915
0.935697085 0.935697085 -0.005229624 1.266527232 0.630648804
0.899267897 2 0 0.998207054
LRRC39 ENSG00000122477.12 0.44679356 8 4 0.123336178
Brain_Putamen_basal_ganglia 0.812708791 Brain_Amygdala 6.232629849
0.014256672 0.217317487 0.236932971 1.540920327 1.048729047
0.394081433 4 0 0.998207054
ZNRF3 ENSG00000183579.15 0.446804689 3 3 0.282195628 Brain_Amygdala
0.548857717 Brain_Cerebellum 1.160408106 0.849666223 0.849666223
-1.075400119 -0.599472689 -0.869161488 0.244225659 3 0
0.998207054
SRSF6 ENSG00000124193.14 0.446862279 3 3 0.346108157
Brain_Cerebellar_Hemisphere 0.87501317 Brain_Caudate_basal_ganglia 1.549141171

0.587333701 0.587333701 -0.942165026 0.85899987 0.024709606
0.907872627 3 0 0.998207054
PAK4 ENSG00000130669.17 0.446958272 11 8 0.128666498
Brain_Anterior_cingulate_cortex_BA24 0.891438988 Brain_Hippocampus
5.7505651 0.02103768 0.232226409 -1.519381491 0.825242281
-0.184830815 0.797927163 8 0 0.998207054
VAMP3 ENSG00000049245.12 0.447006178 6 4 0.055695401
Brain_Putamen_basal_ganglia 0.357259617 Brain_Cerebellar_Hemisphere 4.49925708
0.044205213 0.185213255 -1.913411443 -0.920599398 -1.439371197
0.335467525 4 0 0.998207054
VPS8 ENSG00000156931.15 0.447016604 11 3 0.187410323
Brain_Frontal_Cortex_BA9 0.939106322 Brain_Putamen_basal_ganglia
9.435602693 0.007702327 0.35411653 -0.076393146 1.318278838
0.451419619 0.368370321 3 0 0.998207054
LINC02137 ENSG00000260186.5 0.447277065 5 3 0.293169058
Brain_Cerebellum 0.435003467 Brain_Putamen_basal_ganglia 2.991488062
0.00354746 0.913309489 -1.051194959 0.894330797 0.107707367
1.000567809 3 0 0.998207054
MRPL20 ENSG00000242485.5 0.447348543 11 2 0.136081899
Brain_Hypothalamus 0.450418753 Brain_Hippocampus 9.99719844 8.56E-05
0.457743062 -1.490541553 -0.754717084 -1.141580608 0.210325362
2 0 0.998207054
PIGU ENSG00000101464.10 0.447349507 4 4 0.136784077
Brain_Caudate_basal_ganglia 0.527665209 Brain_Cortex 1.805542394
0.317235252 0.317235252 -0.781967987 1.487874184 0.256373188
1.135183853 4 0 0.998207054
GSTM5 ENSG00000134201.10 0.447413849 13 5 0.144867422 Brain_Cortex
0.986807399 Brain_Cerebellar_Hemisphere 9.950215276 0.003054471
0.400041813 -1.109527538 1.457902489 0.76876035 0.722752254 5
0 0.998207054
GLE1 ENSG00000119392.14 0.447513555 1 1 0.447513555
Brain_Spinal_cord_cervical_c-1 0.447513555 Brain_Spinal_cord_cervical_c-1
1 1 1 0.759566826 0.759566826 0.759566826 NA 1 0
0.998207054
TNFAIP8 ENSG00000145779.7 0.447548263 2 2 0.206118993
Brain_Cerebellar_Hemisphere 0.298466956 Brain_Cerebellum 1.861443288
0.138556712 0.138556712 1.039726563 1.264309411 1.152017987
0.158804055 2 0 0.998207054
RP11-449D8.5 ENSG00000266573.5 0.447673782 1 1 0.447673782
Brain_Cortex 0.447673782 Brain_Cortex 1 1 1 -0.759298889
-0.759298889 -0.759298889 NA 1 0 0.998207054
ZNF503 ENSG00000165655.16 0.447906195 3 3 0.131962322
Brain_Amygdala 0.824970493 Brain_Cerebellar_Hemisphere 1.834092132
0.175531447 0.175531447 -0.22115661 1.506408568 0.503858547
0.896598886 3 0 0.998207054
ACOX2 ENSG00000168306.12 0.44797686 3 3 0.140163649
Brain_Caudate_basal_ganglia 0.901255839 Brain_Nucleus_accumbens_basal_ganglia
1.225924538 0.738159679 0.738159679 -0.124075067 1.475181891
0.602280345 0.809637284 3 0 0.998207054
C16orf45 ENSG00000166780.10 0.447986377 1 1 0.447986377
Brain_Amygdala 0.447986377 Brain_Amygdala 1 1 1 0.758776314
0.758776314 0.758776314 NA 1 0 0.998207054
MCCC1 ENSG00000078070.12 0.447997007 8 6 0.269069797
Brain_Caudate_basal_ganglia 0.989107892 Brain_Nucleus_accumbens_basal_ganglia
5.596593415 0.067444691 0.202595311 -0.892625244 1.105207247
-0.030573964 0.594445026 6 0 0.998207054
TTYH1 ENSG00000167614.13 0.448041641 1 1 0.448041641
Brain_Putamen_basal_ganglia 0.448041641 Brain_Putamen_basal_ganglia 1 1
1 0.758683948 0.758683948 0.758683948 NA 1 0
0.998207054
PARP3 ENSG00000041880.14 0.448181056 1 1 0.448181056
Brain_Frontal_Cortex_BA9 0.448181056 Brain_Frontal_Cortex_BA9 1 1

1 0.758450967 0.758450967 0.758450967 NA 1 0
0.998207054
FBXO15 ENSG00000141665.11 0.448183465 3 3 0.279792268
Brain_Nucleus_accumbens_basal_ganglia 0.975628389 Brain_Caudate_basal_ganglia
2.035048283 0.369764046 0.369764046 -1.08078611 0.342047405
-0.25642958 0.737821103 3 0 0.998207054
TMEM50A ENSG00000183726.10 0.448271248 6 5 0.086704907
Brain_Cerebellum 0.262791622 Brain_Nucleus_accumbens_basal_ganglia
4.064242715 0.025987618 0.150311847 -1.713041489 -1.119817308
-1.389822634 0.257382258 5 0 0.998207054
CDKN2D ENSG00000129355.6 0.448342117 3 2 0.172738044
Brain_Caudate_basal_ganglia 0.29724762 Brain_Cerebellum 2.59198172 0.053986279
0.354032001 -1.363458534 -1.042353925 -1.176750143 0.166821656
2 0 0.998207054
LINC02119 ENSG00000248461.1 0.448348263 1 1 0.448348263
Brain_Nucleus_accumbens_basal_ganglia 0.448348263
Brain_Nucleus_accumbens_basal_ganglia 1 1 1 -0.758171597
-0.758171597 -0.758171597 NA 1 0 0.998207054
MYOM3 ENSG00000142661.18 0.448488052 2 2 0.532247345
Brain_Cerebellar_Hemisphere 0.858279643 Brain_Cerebellum 1.930768988
0.069231012 0.069231012 -0.624579093 -0.178564536 -0.401571815
0.315379918 2 0 0.998207054
CA14 ENSG00000118298.10 0.448644141 5 2 0.158659126
Brain_Frontal_Cortex_BA9 0.395982604 Brain_Nucleus_accumbens_basal_ganglia
4.545962578 0.035520563 0.262788203 0.848817943 1.409595605
1.193575029 0.227470249 2 0 0.998207054
KB-1471A8.1 ENSG00000245330.4 0.448701641 2 2 0.222853137
Brain_Hypothalamus 0.376593184 Brain_Nucleus_accumbens_basal_ganglia
1.8657342 0.1342658 0.1342658 0.884190875 1.218976435 1.051583655
0.23672914 2 0 0.998207054
SDHAF4 ENSG00000154079.5 0.448715741 9 5 0.060670345
Brain_Hypothalamus 0.842710615 Brain_Nucleus_accumbens_basal_ganglia
6.201445258 0.024372857 0.308243874 0.198427485 1.875890301
1.265426133 0.529138936 5 0 0.998207054
SH2D6 ENSG00000152292.16 0.448759199 10 8 0.062139284
Brain_Cerebellar_Hemisphere 0.482494782 Brain_Nucleus_accumbens_basal_ganglia
4.401886961 0.058719448 0.150177819 -1.865300581 0.823899664
-1.083893087 0.746234755 8 0 0.998207054
SF3B2 ENSG00000087365.15 0.448799171 8 1 0.305425504
Brain_Frontal_Cortex_BA9 0.751821766 Brain_Hippocampus 7.701692294
0.000198249 7.701692294 0.316238133 1.02486794 0.741370766
0.231274177 1 0 0.998207054
MRPL14 ENSG00000180992.6 0.448840771 10 4 0.179145042
Brain_Caudate_basal_ganglia 0.985830778 Brain_Nucleus_accumbens_basal_ganglia
7.796953901 3.80E-05 0.282765031 -1.343392106 0.231555254
-0.381178839 0.537530572 4 0 0.998207054
OSBPL10 ENSG00000144645.13 0.448973667 3 3 0.181941314
Brain_Cerebellar_Hemisphere 0.371810951 Brain_Spinal_cord_cervical_c-1
1.620751631 0.37971409 0.37971409 -1.334801524 0.893086314
-0.507149499 1.219327766 3 0 0.998207054
DRG2 ENSG00000108591.9 0.449075148 6 3 0.112631202
Brain_Putamen_basal_ganglia 0.315465779 Brain_Cerebellar_Hemisphere
4.938154291 0.002672023 0.325143442 1.003819176 1.58647675
1.362492292 0.221076915 3 0 0.998207054
NMD3 ENSG00000169251.12 0.449112847 4 4 0.092183621
Brain_Hypothalamus 0.922603584 Brain_Nucleus_accumbens_basal_ganglia
2.003016546 0.321475654 0.321475654 -0.19706723 1.6839899
0.538546493 0.877173871 4 0 0.998207054
SNF8 ENSG00000159210.9 0.449167715 12 2 0.127050028
Brain_Hippocampus 0.614142143 Brain_Frontal_Cortex_BA9 10.60332795
0.007890014 0.409140339 0.504169682 1.525838749 1.132748251
0.300737147 2 0 0.998207054

SIRPA ENSG00000198053.11 0.449171138 2 2 0.562412739 Brain_Cortex
0.94937487 Brain_Cerebellum 1.910887782 0.089112218 0.089112218
0.063491824 0.5792615 0.321376662 0.364704236 2 0
0.998207054
RABGAP1L ENSG00000152061.23 0.449258334 5 5 0.112631244
Brain_Cerebellar_Hemisphere 0.971411122 Brain_Cerebellum 1.601152477
0.477984618 0.477984618 -0.89153615 1.586476564 0.337266816
1.059780851 5 0 0.998207054
ACOX1 ENSG00000161533.11 0.449506972 4 4 0.102340569
Brain_Putamen_basal_ganglia 0.807983153 Brain_Cerebellar_Hemisphere
1.302628477 0.606272502 0.606272502 -1.633610941 0.514750719
-0.508345731 0.896280948 4 0 0.998207054
RMND5B ENSG00000145916.18 0.449629763 1 1 0.449629763
Brain_Putamen_basal_ganglia 0.449629763 Brain_Putamen_basal_ganglia 1 1
1 0.756032412 0.756032412 0.756032412 NA 1 0
0.998207054
CCNL2 ENSG00000221978.11 0.44975887 1 1 0.44975887 Brain_Substantia_nigra
0.44975887 Brain_Substantia_nigra 1 1 1 0.755817088
0.755817088 0.755817088 NA 1 0 0.998207054
PTGFR ENSG00000122420.9 0.44986503 1 1 0.44986503
Brain_Spinal_cord_cervical_c-1 0.44986503 Brain_Spinal_cord_cervical_c-1 1
1 1 0.755640061 0.755640061 0.755640061 NA 1 0
0.998207054
STK17A ENSG00000164543.5 0.449884303 3 3 0.172808328
Brain_Cortex 0.562076071 Brain_Cerebellum 1.850840196 0.12203107
0.12203107 0.579760599 1.363235418 0.959359742 0.392301173 3
0 0.998207054
TRIM35 ENSG00000104228.12 0.449943804 11 8 0.041786231
Brain_Substantia_nigra 0.958572262 Brain_Frontal_Cortex_BA9
4.599101339 0.034099502 0.206426044 -2.035642856 1.298094277
-0.437064828 0.897957289 8 0 0.998207054
COL7A1 ENSG00000114270.17 0.450028782 2 2 0.206481325
Brain_Putamen_basal_ganglia 0.894694978 Brain_Cortex 1.129100635
0.870899365 0.870899365 0.132365782 1.263300171 0.697832976
0.799691375 2 0 0.998207054
LMO3 ENSG00000048540.14 0.450066262 1 1 0.450066262
Brain_Nucleus_accumbens_basal_ganglia 0.450066262
Brain_Nucleus_accumbens_basal_ganglia 1 1 1 -0.755304562
-0.755304562 -0.755304562 NA 1 0 0.998207054
SRRT ENSG00000087087.18 0.450094899 1 1 0.450094899 Brain_Cerebellum
0.450094899 Brain_Cerebellum 1 1 1 -0.755256824 -0.755256824
-0.755256824 NA 1 0 0.998207054
RNF168 ENSG00000163961.4 0.450110622 3 3 0.20902366
Brain_Cerebellar_Hemisphere 0.593472942 Brain_Hypothalamus 1.059752066
0.924929194 0.924929194 0.533810076 1.256254646 0.888679586
0.361389834 3 0 0.998207054
SLC35C1 ENSG00000181830.8 0.450130373 1 1 0.450130373
Brain_Hippocampus 0.450130373 Brain_Hippocampus 1 1 1
-0.755197692 -0.755197692 -0.755197692 NA 1 0 0.998207054
ARL6IP6 ENSG00000177917.10 0.450174247 7 6 0.017989075
Brain_Frontal_Cortex_BA9 0.932853112 Brain_Hypothalamus 2.572958707
0.074923121 0.478438918 -0.38263566 2.365842925 0.882062266
0.953866001 6 0 0.998207054
AC005618.6 ENSG00000272070.1 0.450187072 2 2 0.263804982
Brain_Cerebellum 0.514862549 Brain_Frontal_Cortex_BA9 1.056126651
0.943873349 0.943873349 0.65128497 1.117442943 0.884363957
0.329623464 2 0 0.998207054
GGCT ENSG00000006625.17 0.450258066 9 6 0.204510077 Brain_Cortex
0.942119243 Brain_Caudate_basal_ganglia 4.907608242 0.021386281
0.398290059 -0.764187452 1.268806527 0.3839862 0.62681326 6 0
0.998207054
AGO4 ENSG00000134698.10 0.450351135 2 2 0.293658892

Brain_Anterior_cingulate_cortex_BA24 0.46338749 Brain_Cerebellum 1.030172222 0.969827778 0.969827778 0.733280296 1.050128815 0.891704555 0.224045736 2 0 0.998207054
SIRPB2 ENSG00000196209.12 0.450355913 8 8 0.090509883 Brain_Amygdala 0.952171311 Brain_Nucleus_accumbens_basal_ganglia 2.720612673 0.144446746 0.144446746 -1.585578289 1.692714189 -0.644068141 1.093814463 8 0 0.998207054
TP53AIP1 ENSG00000120471.15 0.450393381 1 1 0.450393381 Brain_Cortex 0.450393381 Brain_Cortex 1 1 1 0.754759363 0.754759363 0.754759363 NA 1 0 0.998207054
MAP1LC3B ENSG00000140941.12 0.450568977 3 3 0.288467206 Brain_Anterior_cingulate_cortex_BA24 0.784296034 Brain_Cerebellum 1.785860854 0.217023696 0.217023696 -1.061490152 0.273724909 -0.441572479 0.672698136 3 0 0.998207054
SRSF9 ENSG00000111786.8 0.450572419 1 1 0.450572419 Brain_Nucleus_accumbens_basal_ganglia 0.450572419 Brain_Nucleus_accumbens_basal_ganglia 1 1 1 -0.754461059 -0.754461059 -0.754461059 NA 1 0 0.998207054
CAMK1G ENSG00000008118.9 0.450602491 1 1 0.450602491 Brain_Cerebellum 0.450602491 Brain_Cerebellum 1 1 1 -0.754410962 -0.754410962 -0.754410962 NA 1 0 0.998207054
ITFG2 ENSG00000111203.11 0.450755129 1 1 0.450755129 Brain_Nucleus_accumbens_basal_ganglia 0.450755129 Brain_Nucleus_accumbens_basal_ganglia 1 1 1 -0.754156709 -0.754156709 -0.754156709 NA 1 0 0.998207054
HEATR4 ENSG00000187105.8 0.450938462 3 3 0.163441591 Brain_Substantia_nigra 0.822291865 Brain_Anterior_cingulate_cortex_BA24 1.336774727 0.661654986 0.661654986 -1.393589533 0.224598201 -0.508000104 0.819870477 3 0 0.998207054
ZNF132 ENSG00000131849.11 0.450994532 3 2 0.299818946 Brain_Cerebellar_Hemisphere 0.395995049 Brain_Spinal_cord_cervical_c-1 1.993094292 0.033518555 0.973387153 -1.03682173 -0.848795583 -0.961184396 0.099254529 2 0 0.998207054
GOLGA8O ENSG00000206127.10 0.451105954 6 6 0.184159576 Brain_Anterior_cingulate_cortex_BA24 0.937187453 Brain_Caudate_basal_ganglia 1.813739305 0.32446887 0.32446887 -1.328056097 1.327447882 -0.32756818 1.167905189 6 0 0.998207054
EMC7 ENSG00000134153.9 0.451144328 1 1 0.451144328 Brain_Hippocampus 0.451144328 Brain_Hippocampus 1 1 1 -0.753508629 -0.753508629 -0.753508629 NA 1 0 0.998207054
LENG8 ENSG00000167615.16 0.451163935 2 2 0.422002938 Brain_Cerebellum 0.897645794 Brain_Cerebellar_Hemisphere 1.702444201 0.297555799 0.297555799 -0.802951205 0.128635855 -0.337157675 0.658731527 2 0 0.998207054
ARHGAP31 ENSG00000031081.10 0.451179081 2 2 0.259713043 Brain_Hippocampus 0.630727135 Brain_Cerebellum 1.07514985 0.92485015 0.92485015 0.480703652 1.127069629 0.803886641 0.457049766 2 0 0.998207054
ZNF512B ENSG00000196700.8 0.451218724 3 3 0.184954832 Brain_Hypothalamus 0.849654965 Brain_Caudate_basal_ganglia 1.334323813 0.665239645 0.665239645 -1.325652488 0.189558685 -0.652433846 0.77157611 3 0 0.998207054
MYH3 ENSG00000109063.14 0.451239943 8 7 0.129833189 Brain_Caudate_basal_ganglia 0.967261856 Brain_Nucleus_accumbens_basal_ganglia 4.866458433 0.10108743 0.172682703 -1.514760017 0.390580412 -0.582517418 0.596639894 7 0 0.998207054
LRRC37A3 ENSG00000176809.10 0.451312597 7 7 0.062369956 Brain_Caudate_basal_ganglia 0.92620682 Brain_Hypothalamus 2.095814098 0.45510627 0.45510627 -1.863656515 0.470170686 -0.811638795 0.955480916 7 0 0.998207054
RP11-497E19.1 ENSG00000205562.2 0.4513981 1 1 0.4513981 Brain_Frontal_Cortex_BA9 0.4513981 Brain_Frontal_Cortex_BA9 1 1 1

0.753086227	0.753086227	0.753086227	NA	1	0	0.998207054
NFS1	ENSG00000244005.12	0.451442258	3	3	0.116946823
Brain_Substantia_nigra	0.736078103	Brain_Cerebellar_Hemisphere
1.647675385	0.436152753	0.436152753	0.337051467	1.567718583
1.044793069	0.635809034	3	0	0.998207054
PRDM5	ENSG00000138738.10	0.451456563	1	1	0.451456563
Brain_Frontal_Cortex_BA9	0.451456563	Brain_Frontal_Cortex_BA9	1	1
1	0.752988936	0.752988936	0.752988936	NA	1	0
0.998207054
THAP9	ENSG00000168152.12	0.451492075	2	2	0.210693861
Brain_Anterior_cingulate_cortex_BA24	0.713056925	Brain_Putamen_basal_ganglia
1.172553476	0.827446524	0.827446524	-0.36775366	1.251659782
0.441953061	1.145098227	2	0	0.998207054
RP11-43F13.4	ENSG00000272347.1	0.451496304	3	3	0.201358859
Brain_Frontal_Cortex_BA9	0.951184905	Brain_Anterior_cingulate_cortex_BA24
2.303019592	0.078401637	0.078401637	-0.061218866	1.277689698
0.731613488	0.70273429	3	0	0.998207054
TCAIM	ENSG00000179152.19	0.451614971	3	3	0.142711939
Brain_Hippocampus	0.694832594	Brain_Cortex	1.080298027
0.918471976	0.918471976	-1.465766394	0.490868846	-0.194197392
1.102313243	3	0	0.998207054
SGCA	ENSG00000108823.15	0.451639753	13	5	0.104616124
Brain_Substantia_nigra	0.998148436	Brain_Spinal_cord_cervical_c-1
9.267312272	0.006096051	0.321346889	-1.091480914	1.622875023
0.162900742	0.618033166	5	0	0.998207054
C1orf141	ENSG00000203963.11	0.451671634	3	3	0.11816654
Brain_Frontal_Cortex_BA9	0.852212142	Brain_Hypothalamus	1.67778755
0.333444977	0.333444977	-0.18629664	1.562515741	0.733198076
0.877886731	3	0	0.998207054
AMMECR1L	ENSG00000144233.9	0.451996943	1	1	0.451996943
Brain_Substantia_nigra	0.451996943	Brain_Substantia_nigra	1	1
1	0.75208999	0.75208999	0.75208999	NA	1	0	0.998207054
RP5-828H9.1	ENSG00000230563.2	0.452045862	12	12	0.027644478
Brain_Cerebellum	0.959605415	Brain_Cerebellar_Hemisphere	1.646910375
0.452135522	0.452135522	-2.20229499	0.52485692	-0.518610746
0.812157249	12	0	0.998207054
NFIA	ENSG00000162599.15	0.452066129	1	1	0.452066129	Brain_Cerebellum
0.452066129	Brain_Cerebellum	1	1	1	0.75197494	0.75197494	0.75197494
NA	1	0	0.998207054
ZBTB20-AS4	ENSG00000242767.1	0.452110864	2	2	0.285319457
Brain_Cortex	0.473281712	Brain_Hypothalamus	1.046787134
0.953212866	0.953212866	-1.068445821	-0.717149733	-0.892797777
0.248403846	2	0	0.998207054
HPF1	ENSG00000056050.6	0.452117083	1	1	0.452117083
Brain_Nucleus_accumbens_basal_ganglia	0.452117083
Brain_Nucleus_accumbens_basal_ganglia	1	1	1	-0.751890215
-0.751890215	-0.751890215	NA	1	0	0.998207054
FASTKD1	ENSG00000138399.17	0.45217775	12	3	0.297167934
Brain_Substantia_nigra	0.966610333	Brain_Hippocampus	9.898615156
0.005487703	0.37639245	-1.042525879	0.773220499	-0.000301978
0.485433769	3	0	0.998207054
ZSCAN21	ENSG00000166529.14	0.452182666	11	4	0.118988738
Brain_Hypothalamus	0.79690377	Brain_Substantia_nigra	8.735617053
0.003349617	0.429773991	-1.559032288	-0.257356254	-0.919293639
0.425494488	4	0	0.998207054
FAM227B	ENSG00000166262.15	0.452257992	5	4	0.416165835
Brain_Spinal_cord_cervical_c-1	0.975420892	Brain_Frontal_Cortex_BA9
3.768072904	0.057476895	0.202575042	-0.81309109	0.673740296
0.102199823	0.567884174	4	0	0.998207054
BIRC5	ENSG00000089685.14	0.452483935	3	3	0.194276319	Brain_Cerebellum
0.815560293	Brain_Cerebellar_Hemisphere	1.165175656	0.857402842
0.857402842	-1.298032058	1.068130136	-0.154386975	1.185051256

3	0	0.998207054
KRTAP5-4	ENSG00000241598.5	0.452498536	2	2	0.21780199	Brain_Cerebellum	0.873993061	Brain_Cerebellar_Hemisphere	1.3273609	0.6726391	0.6726391	-1.23239387	-0.158588537	-0.695491203	0.759295033	2	0	0.998207054
IFT27	ENSG00000100360.14	0.452571026	12	4	0.258494562	Brain_Cerebellum	0.943083648	Brain_Amygdala	8.945225597	0.005243144	0.549031816	-1.06617466	1.129956483	0.1950605	0.573007275	4	0	0.998207054
THSD4	ENSG00000187720.14	0.452592722	2	2	0.413775728	Brain_Caudate_basal_ganglia	0.662692242	Brain_Cerebellum	1.488800721	0.511199279	0.511199279	0.436199154	0.817267233	0.626733194	0.269455823	2	0	0.998207054
C2CD2L	ENSG00000172375.12	0.452611624	1	1	0.452611624	Brain_Caudate_basal_ganglia	0.452611624	Brain_Caudate_basal_ganglia	1	1	1	0.75106818	0.75106818	0.75106818	NA	1	0	0.998207054
LINC01444	ENSG00000264301.1	0.452781932	1	1	0.452781932	Brain_Cortex	0.452781932	Brain_Cortex	1	1	1	0.750785209	0.750785209	0.750785209	NA	1	0	0.998207054
PCDH8	ENSG00000136099.13	0.452793793	3	3	0.115170467	Brain_Caudate_basal_ganglia	0.708296766	Brain_Cerebellar_Hemisphere	1.730978039	0.26940498	0.26940498	-0.374144563	1.575372591	0.753291538	1.009992853	3	0	0.998207054
RIPPLY2	ENSG00000203877.8	0.452929855	2	2	0.231875762	Brain_Putamen_basal_ganglia	0.693203141	Brain_Amygdala	1.000978693	0.999021307	0.999021307	-1.195540912	0.394511903	-0.400514505	1.124337128	2	0	0.998207054
PRRC2B	ENSG00000130723.18	0.453122838	1	1	0.453122838	Brain_Amygdala	0.453122838	Brain_Amygdala	1	1	1	0.750218966	0.750218966	0.750218966	NA	1	0	0.998207054
CD180	ENSG00000134061.5	0.453128475	1	1	0.453128475	Brain_Caudate_basal_ganglia	0.453128475	Brain_Caudate_basal_ganglia	1	1	1	-0.750209605	-0.750209605	-0.750209605	NA	1	0	0.998207054
WNT8A	ENSG00000061492.11	0.453163048	2	2	0.4001935	Brain_Cerebellum	0.677330171	Brain_Cerebellar_Hemisphere	1.480599029	0.519400971	0.519400971	-0.4161091	0.841275701	0.2125833	0.889105319	2	0	0.998207054
WASF1	ENSG00000112290.12	0.453225116	1	1	0.453225116	Brain_Spinal_cord_cervical_c-1	0.453225116	Brain_Spinal_cord_cervical_c-1	1	1	1	-0.75004913	-0.75004913	-0.75004913	NA	1	0	0.998207054
CTB-133G6.1	ENSG00000263264.1	0.453232314	1	1	0.453232314	Brain_Substantia_nigra	0.453232314	Brain_Substantia_nigra	1	1	1	0.750037178	0.750037178	0.750037178	NA	1	0	0.998207054
PCSK6	ENSG00000140479.16	0.45331652	2	2	0.282830481	Brain_Cerebellum	0.536094133	Brain_Hippocampus	1.033336966	0.966663034	0.966663034	-0.618730167	1.073982597	0.227626215	1.196928674	2	0	0.998207054
ANKK1	ENSG00000170209.4	0.453671349	7	4	0.131968855	Brain_Cortex	0.601962362	Brain_Nucleus_accumbens_basal_ganglia	4.226170665	0.013858553	0.632543899	-1.506383104	-0.521580616	-1.122378122	0.391682948	4	0	0.998207054
CD24	ENSG00000272398.5	0.45368555	3	3	0.119441993	Brain_Cerebellar_Hemisphere	0.830135329	Brain_Hypothalamus	1.453698591	0.524816386	0.524816386	0.214528007	1.557120018	0.937144769	0.67715566	3	0	0.998207054
EPHA8	ENSG00000070886.11	0.453686669	4	3	0.38456162	Brain_Cerebellum	0.836041446	Brain_Cortex	2.990193597	0.049419243	0.218596873	-0.478379044	0.86952211	0.105857669	0.588830278	3	0	0.998207054

PYGM ENSG00000068976.13 0.453711319 4 3 0.321964381 Brain_Cerebellum
0.882904846 Brain_Hippocampus 3.531908374 0.004228422
0.185089759 -0.147287819 0.990429195 0.433292281 0.464769023
3 0 0.998207054
AP4M1 ENSG00000221838.9 0.453723034 6 5 0.091893842
Brain_Nucleus_accumbens_basal_ganglia 0.738913591 Brain_Frontal_Cortex_BA9
2.867541811 0.015715782 0.139641896 -1.459765847 1.685491197
0.033576838 1.286318679 5 0 0.998207054
GRAMD1C ENSG00000178075.19 0.453737114 2 2 0.304606479
Brain_Cerebellar_Hemisphere 0.533175191 Brain_Cerebellum 1.09651996 0.90348004
0.90348004 -0.623166382 1.026605042 0.20171933 1.166564561 2 0
0.998207054
BNC2 ENSG00000173068.17 0.453746369 1 1 0.453746369
Brain_Hypothalamus 0.453746369 Brain_Hypothalamus 1 1 1
-0.749183905 -0.749183905 -0.749183905 NA 1 0 0.998207054
CHST1 ENSG00000175264.7 0.453779038 4 4 0.143792292
Brain_Frontal_Cortex_BA9 0.904087755 Brain_Nucleus_accumbens_basal_ganglia
1.51068019 0.51531674 0.51531674 -1.138269749 1.461813616 -0.021757909
1.084639384 4 0 0.998207054
NECTIN1 ENSG00000110400.10 0.453803934 2 2 0.260079883
Brain_Hippocampus 0.712237892 Brain_Cerebellum 1.161803236
0.838196764 0.838196764 0.368852202 1.126202342 0.747527272
0.535527419 2 0 0.998207054
ASAH2 ENSG00000188611.14 0.453870716 13 2 0.184880479
Brain_Putamen_basal_ganglia 0.573833411 Brain_Substantia_nigra
11.99823807 9.87E-05 0.539844507 -1.325876891 -0.56241484
-1.163746699 0.193355096 2 0 0.998207054
MRI1 ENSG00000037757.13 0.454023204 12 2 0.21874932
Brain_Nucleus_accumbens_basal_ganglia 0.76416889 Brain_Caudate_basal_ganglia
10.15439512 1.17E-17 0.442076919 -1.229860575 -0.300010836
-0.647851759 0.281078556 2 0 0.998207054
ANAPC13 ENSG00000129055.12 0.454091036 9 5 0.136568156
Brain_Frontal_Cortex_BA9 0.948532759 Brain_Cerebellum 5.938809194
0.00716193 0.385638805 -1.488693278 0.324282112 -0.352661959
0.498809393 5 0 0.998207054
PPA2 ENSG00000138777.19 0.454154404 12 3 0.072722266
Brain_Anterior_cingulate_cortex_BA24 0.550049486 Brain_Cerebellar_Hemisphere
10.57978948 0.00335991 0.380328028 -1.794569814 -0.597685974
-1.243971254 0.273212197 3 0 0.998207054
WDFY2 ENSG00000139668.8 0.454433408 6 3 0.537877213
Brain_Hypothalamus 0.981222524 Brain_Cerebellum 4.770318604
0.001210863 0.1902836 -0.616026223 0.352871503 -0.086860845
0.411626653 3 0 0.998207054
PTPRU ENSG00000060656.19 0.454460366 5 5 0.106613661
Brain_Spinal_cord_cervical_c-1 0.868859526 Brain_Cortex 1.77084052
0.412774775 0.412774775 -1.280010585 1.613602562 0.059948245
1.105573094 5 0 0.998207054
RPS6KB2 ENSG00000175634.14 0.454490185 2 2 0.219455579
Brain_Frontal_Cortex_BA9 0.677838115 Brain_Nucleus_accumbens_basal_ganglia
1.521161143 0.478838857 0.478838857 0.415415018 1.22797706
0.821696039 0.57456813 2 0 0.998207054
CENPC ENSG00000145241.10 0.45449256 8 5 0.136999539 Brain_Cerebellum
0.830854984 Brain_Hypothalamus 4.720967134 0.021835375
0.250167998 -1.487057825 -0.213605149 -0.776660581 0.519494568
5 0 0.998207054
TCTEX1D2 ENSG00000213123.10 0.45456716 5 4 0.154405604 Brain_Cerebellum
0.616494885 Brain_Frontal_Cortex_BA9 3.54738716 0.090398124
0.240913635 0.500824153 1.424141192 0.859755425 0.425923309
4 0 0.998207054
DNAH7 ENSG00000118997.13 0.454610682 9 2 0.164641024
Brain_Frontal_Cortex_BA9 0.837516859 Brain_Amygdala 7.390892169
0.001355508 0.809699679 -1.38963078 0.205507783 -0.705593744

0.48738859 2 0 0.998207054
GATSL3 ENSG00000239282.7 0.454613372 5 4 0.304574946
Brain_Caudate_basal_ganglia 0.954867796 Brain_Cerebellar_Hemisphere
3.253704554 0.10613711 0.292162282 -1.026671984 0.301384855
-0.174842328 0.569159164 4 0 0.998207054
AC073133.2 ENSG00000228569.1 0.454705959 2 2 0.321357049
Brain_Nucleus_accumbens_basal_ganglia 0.482872413 Brain_Hippocampus
1.067065512 0.932934488 0.932934488 -0.701690156 0.991673034
0.144991439 1.197388595 2 0 0.998207054
LOX ENSG00000113083.13 0.454751409 1 1 0.454751409
Brain_Spinal_cord_cervical_c-1 0.454751409 Brain_Spinal_cord_cervical_c-1
1 1 1 -0.747517225 -0.747517225 -0.747517225 NA 1 0
0.998207054
ANGEL1 ENSG00000013523.9 0.454805262 6 6 0.048347137
Brain_Frontal_Cortex_BA9 0.922147924 Brain_Substantia_nigra
3.216836755 0.107324779 0.107324779 -0.097728449 1.974304436
0.871319594 0.795433323 6 0 0.998207054
SPX ENSG00000134548.9 0.454912842 7 5 0.445763531
Brain_Caudate_basal_ganglia 0.789961865 Brain_Putamen_basal_ganglia
4.090817426 0.044581089 0.222957706 -0.762496837 0.582398132
-0.207962614 0.569313192 5 0 0.998207054
TMEM178A ENSG00000152154.10 0.455115514 2 2 0.21548564 Brain_Cerebellum
0.796451808 Brain_Caudate_basal_ganglia 1.046502164 0.953497836
0.953497836 -1.238621668 0.257941821 -0.490339924 1.058230191
2 0 0.998207054
GAS2 ENSG00000148935.10 0.455121103 4 4 0.112665721
Brain_Putamen_basal_ganglia 0.887619289 Brain_Caudate_basal_ganglia
2.290169772 0.192478861 0.192478861 -0.141317293 1.586324485
1.02034046 0.789514355 4 0 0.998207054
PYCR1 ENSG00000183010.16 0.455307783 3 3 0.127872629
Brain_Substantia_nigra 0.658920396 Brain_Cerebellar_Hemisphere 1.22689914
0.787300527 0.787300527 -1.5225448 -0.441404208 -0.886120164
0.565491298 3 0 0.998207054
TFB1M ENSG00000029639.10 0.455356288 4 3 0.425102358
Brain_Hypothalamus 0.924126382 Brain_Frontal_Cortex_BA9 3.312794381
0.103633394 0.140521415 -0.797600505 0.463682437 -0.170514782
0.518940354 3 0 0.998207054
RP11-416I2.1 ENSG00000260328.1 0.455384622 4 4 0.061361981
Brain_Putamen_basal_ganglia 0.935503412 Brain_Cerebellum 1.933597561
0.305604165 0.305604165 -1.870878085 0.080922718 -0.608010028
0.868388219 4 0 0.998207054
MMP3 ENSG00000149968.11 0.455498619 3 3 0.171288557
Brain_Cerebellar_Hemisphere 0.642610714 Brain_Spinal_cord_cervical_c-1
1.123600438 0.842425973 0.842425973 -0.843343106 1.36807514
0.020226782 1.182576379 3 0 0.998207054
PPIA ENSG00000196262.13 0.455627712 2 2 0.280745469
Brain_Hypothalamus 0.438527791 Brain_Cortex 1.130604385
0.869395615 0.869395615 0.77468166 1.078646218 0.926663939
0.2149354 2 0 0.998207054
RP11-147L13.13 ENSG00000277476.1 0.455712814 1 1 0.455712814
Brain_Putamen_basal_ganglia 0.455712814 Brain_Putamen_basal_ganglia 1 1
1 0.745924847 0.745924847 0.745924847 NA 1 0
0.998207054
ANG ENSG00000214274.9 0.455847549 1 1 0.455847549
Brain_Hypothalamus 0.455847549 Brain_Hypothalamus 1 1 1
-0.745701838 -0.745701838 -0.745701838 NA 1 0 0.998207054
C22orf39 ENSG00000242259.8 0.455900814 4 4 0.11526733
Brain_Substantia_nigra 0.845357792 Brain_Amygdala 2.066330012
0.140524407 0.140524407 0.195044907 1.574952841 0.957574536
0.570849204 4 0 0.998207054
SRM ENSG00000116649.9 0.455930969 1 1 0.455930969
Brain_Hippocampus 0.455930969 Brain_Hippocampus 1 1 1

0.74556378 0.74556378 0.74556378 NA 1 0 0.998207054
FZD6 ENSG00000164930.11 0.455944073 7 6 0.036909168 Brain_Cortex
0.490551689 Brain_Putamen_basal_ganglia 3.622828791 0.079152094
0.167976394 0.689431622 2.086767368 1.148698327 0.495545219
6 0 0.998207054
RNF212 ENSG00000178222.12 0.455994678 10 6 0.03195237
Brain_Hypothalamus 0.561921862 Brain_Cerebellar_Hemisphere 6.287071085
0.006040245 0.214364897 -2.145005962 -0.579989257 -1.406912272
0.403552892 6 0 0.998207054
BTG4 ENSG00000137707.13 0.456054874 1 1 0.456054874
Brain_Hypothalamus 0.456054874 Brain_Hypothalamus 1 1 1
-0.74535875 -0.74535875 -0.74535875 NA 1 0 0.998207054
CTD-2534I21.8 ENSG00000267334.1 0.45606238 1 1 0.45606238
Brain_Substantia_nigra 0.45606238 Brain_Substantia_nigra 1 1 1
-0.74534633 -0.74534633 -0.74534633 NA 1 0 0.998207054
SSR3 ENSG00000114850.6 0.45608017 2 2 0.276244475
Brain_Caudate_basal_ganglia 0.84849128 Brain_Hippocampus 1.356718611
0.643281389 0.643281389 -0.191043797 1.088794597 0.4488754
0.904982407 2 0 0.998207054
PECAM1 ENSG00000261371.5 0.456116731 1 1 0.456116731
Brain_Nucleus_accumbens_basal_ganglia 0.456116731
Brain_Nucleus_accumbens_basal_ganglia 1 1 1 0.745256403
0.745256403 0.745256403 NA 1 0 0.998207054
RRAD ENSG00000166592.11 0.456125283 1 1 0.456125283
Brain_Caudate_basal_ganglia 0.456125283 Brain_Caudate_basal_ganglia 1 1
1 -0.745242254 -0.745242254 -0.745242254 NA 1 0
0.998207054
PPARGC1B ENSG00000155846.16 0.456231345 1 1 0.456231345
Brain_Cortex 0.456231345 Brain_Cortex 1 1 1 -0.74506679
-0.74506679 -0.74506679 NA 1 0 0.998207054
S100A16 ENSG00000188643.10 0.456442305 4 3 0.210122724
Brain_Cerebellar_Hemisphere 0.77820421 Brain_Anterior_cingulate_cortex_BA24
3.316942498 0.019324472 0.252056159 0.281660024 1.253228051
0.744577872 0.511013873 3 0 0.998207054
IKBKE ENSG00000263528.7 0.456443108 5 5 0.280245058
Brain_Caudate_basal_ganglia 0.942600919 Brain_Anterior_cingulate_cortex_BA24
2.282243057 0.145502036 0.145502036 -1.079769001 -0.072001243
-0.618842637 0.466285373 5 0 0.998207054
ANK3 ENSG00000151150.21 0.456466359 1 1 0.456466359
Brain_Hypothalamus 0.456466359 Brain_Hypothalamus 1 1 1
-0.744678071 -0.744678071 -0.744678071 NA 1 0 0.998207054
AC106869.2 ENSG00000226087.1 0.456469165 1 1 0.456469165
Brain_Cortex 0.456469165 Brain_Cortex 1 1 1 -0.74467343
-0.74467343 -0.74467343 NA 1 0 0.998207054
ZNF519 ENSG00000175322.11 0.456469673 12 3 0.130740626
Brain_Anterior_cingulate_cortex_BA24 0.539928776 Brain_Frontal_Cortex_BA9
10.43191513 8.83E-18 0.405506641 -1.511187738 -0.612920698
-1.151579656 0.252147629 3 0 0.998207054
SUPT4H1 ENSG00000213246.6 0.456499876 9 5 0.061873291
Brain_Hypothalamus 0.88529599 Brain_Spinal_cord_cervical_c-1 6.103800068
0.004986775 0.483179499 -0.635389171 1.867202673 -0.119816731
0.819870737 5 0 0.998207054
TMED7 ENSG00000134970.13 0.45657667 1 1 0.45657667
Brain_Anterior_cingulate_cortex_BA24 0.45657667
Brain_Anterior_cingulate_cortex_BA24 1 1 1 0.744495654
0.744495654 0.744495654 NA 1 0 0.998207054
NFATC2IP ENSG00000176953.12 0.456582585 2 2 0.229537446
Brain_Cortex 0.529048806 Brain_Caudate_basal_ganglia 1.239015831
0.760984169 0.760984169 0.629458688 1.201551231 0.91550496
0.404530516 2 0 0.998207054
SULT1A2 ENSG00000197165.10 0.456676281 13 3 0.141969453
Brain_Cortex 0.777563258 Brain_Cerebellar_Hemisphere 11.29694198

0.001919424 0.490175558 0.282495941 1.468496327 1.066877131
0.351817887 3 0 0.998207054
BMPR1B-AS1 ENSG00000249599.1 0.456698326 1 1 0.456698326
Brain_Caudate_basal_ganglia 0.456698326 Brain_Caudate_basal_ganglia 1 1
1 0.744294504 0.744294504 0.744294504 NA 1 0
0.998207054
FOS ENSG00000170345.9 0.456833264 1 1 0.456833264
Brain_Substantia_nigra 0.456833264 Brain_Substantia_nigra 1 1
1 -0.744071428 -0.744071428 -0.744071428 NA 1 0
0.998207054
SLC29A2 ENSG00000174669.11 0.456917664 1 1 0.456917664
Brain_Nucleus_accumbens_basal_ganglia 0.456917664
Brain_Nucleus_accumbens_basal_ganglia 1 1 1 0.743931919
0.743931919 0.743931919 NA 1 0 0.998207054
ARHGEF33 ENSG00000214694.10 0.456977555 3 3 0.126412969
Brain_Caudate_basal_ganglia 0.879253367 Brain_Hippocampus 1.04637076
0.955938298 0.955938298 -1.528401151 0.513608733 -0.388902731
1.041428444 3 0 0.998207054
GSTM1 ENSG00000134184.12 0.457022433 13 12 0.041039997 Brain_Cerebellum
0.765284572 Brain_Putamen_basal_ganglia 5.027312003 0.109699377
0.17129474 -0.448826683 2.043125693 0.782537003 0.670703868 12
0 0.998207054
IRF7 ENSG00000185507.19 0.457241992 3 3 0.188093529 Brain_Cerebellum
0.284082628 Brain_Cerebellar_Hemisphere 1.929627517 0.22424807 0.22424807
-1.31623992 -1.071193067 -1.162227837 0.134114117 3 0
0.998207054
SLCO4A1 ENSG00000101187.15 0.457300802 2 2 0.257234229
Brain_Amygdala 0.597685351 Brain_Cerebellum 1.002289111 0.997710889
0.997710889 -1.132952433 -0.527732012 -0.830342222 0.427955464
2 0 0.998207054
FAM71E2 ENSG00000180043.11 0.457392314 1 1 0.457392314
Brain_Cerebellum 0.457392314 Brain_Cerebellum 1 1 1 -0.743147616
-0.743147616 -0.743147616 NA 1 0 0.998207054
MYO5C ENSG00000128833.12 0.457413983 2 2 0.263272043
Brain_Cerebellar_Hemisphere 0.467244115 Brain_Hippocampus 1.155961627
0.844038373 0.844038373 -1.118690866 -0.726970357 -0.922830611
0.276988228 2 0 0.998207054
C1orf52 ENSG00000162642.13 0.457421046 3 3 0.171882805
Brain_Cerebellar_Hemisphere 0.595814587 Brain_Caudate_basal_ganglia
1.884151084 0.185098029 0.185098029 -1.366178942 0.530428908
-0.51268647 0.962417773 3 0 0.998207054
FAM60A ENSG00000139146.13 0.457510731 2 2 0.26868458 Brain_Cerebellum
0.525831778 Brain_Cerebellar_Hemisphere 1.046583621 0.953416379
0.953416379 -1.1060969 0.634381657 -0.235857622 1.23070419 2 0
0.998207054
PAK5 ENSG00000101349.16 0.457548298 2 2 0.215014074 Brain_Cerebellum
0.340887823 Brain_Cerebellar_Hemisphere 1.839375324 0.160624676
0.160624676 -1.23989543 -0.952412499 -1.096153964 0.20328113 2
0 0.998207054
EEFSEC ENSG00000132394.10 0.457593839 7 3 0.4832329
Brain_Anterior_cingulate_cortex_BA24 0.940854569 Brain_Cortex
6.066265796 0.007753024 0.234246777 -0.701112355 0.483750051
-0.081642643 0.471153769 3 0 0.998207054
TNS3 ENSG00000136205.16 0.457673407 5 4 0.184261158
Brain_Hypothalamus 0.971295301 Brain_Putamen_basal_ganglia 2.55561185
0.047807883 0.511214839 -1.327748642 1.027402199 -0.072711106
0.88686843 4 0 0.998207054
TECR ENSG00000099797.13 0.45770314 4 2 0.267082983
Brain_Putamen_basal_ganglia 0.756006039 Brain_Nucleus_accumbens_basal_ganglia
3.153578811 0.088262724 0.657655294 -0.631401405 1.109805188
0.351382052 0.733246944 2 0 0.998207054
ADCY8 ENSG00000155897.9 0.457715243 2 2 0.211327269 Brain_Amygdala

0.99931417 Brain_Cortex 1.020562043 0.979437957 0.979437957
-1.249924116 -0.000859561 -0.625391838 0.883222017 2 0
0.998207054
LINC00941 ENSG00000235884.3 0.457740687 3 3 0.107359751
Brain_Putamen_basal_ganglia 0.956511272 Brain_Cortex 1.217377007
0.84602638 0.84602638 0.054532053 1.610174572 0.617266153 0.86243456
3 0 0.998207054
AK7 ENSG00000140057.8 0.457767594 2 2 0.211898566
Brain_Frontal_Cortex_BA9 0.950955047 Brain_Putamen_basal_ganglia
1.102008496 0.897991504 0.897991504 -1.248361871 0.061507493
-0.593427189 0.92621751 2 0 0.998207054
CTD-3234P18.6 ENSG00000276071.1 0.457808856 2 2 0.321087371
Brain_Cerebellum 0.664350359 Brain_Nucleus_accumbens_basal_ganglia
1.294892101 0.705107899 0.705107899 -0.992225838 0.433914735
-0.279155551 1.00843367 2 0 0.998207054
GPR20 ENSG00000204882.3 0.457816942 2 2 0.225409187
Brain_Substantia_nigra 0.732948925 Brain_Anterior_cingulate_cortex_BA24
1.030048275 0.969951725 0.969951725 -1.212269626 -0.341205444
-0.776737535 0.615935391 2 0 0.998207054
HADH ENSG00000138796.16 0.457913418 1 1 0.457913418
Brain_Cerebellar_Hemisphere 0.457913418 Brain_Cerebellar_Hemisphere 1 1
1 -0.74228708 -0.74228708 -0.74228708 NA 1 0
0.998207054
IGLL1 ENSG00000128322.6 0.45796597 2 2 0.228017113
Brain_Cerebellar_Hemisphere 0.350374975 Brain_Cerebellum 1.545406409
0.454593591 0.454593591 0.933862209 1.20548244 1.069672325
0.192064507 2 0 0.998207054
SELENOS ENSG00000131871.14 0.458123177 1 1 0.458123177
Brain_Caudate_basal_ganglia 0.458123177 Brain_Caudate_basal_ganglia 1 1
1 -0.741940845 -0.741940845 -0.741940845 NA 1 0
0.998207054
ECH1 ENSG00000104823.8 0.458168723 7 6 0.089840563
Brain_Nucleus_accumbens_basal_ganglia 0.954667303 Brain_Hippocampus
4.332431265 0.142273706 0.222354936 -0.608232505 1.696239339
0.589797876 0.717949436 6 0 0.998207054
LINC01977 ENSG00000262772.1 0.458240728 1 1 0.458240728
Brain_Amygdala 0.458240728 Brain_Amygdala 1 1 1 -0.741746851
-0.741746851 -0.741746851 NA 1 0 0.998207054
ZNF697 ENSG00000143067.4 0.458368459 8 8 0.18711097
Brain_Cerebellar_Hemisphere 0.789601453 Brain_Amygdala 2.92831883
0.106240079 0.106240079 -1.230684218 1.319173934 0.180396377
0.924658977 8 0 0.998207054
RP11-143M1.3 ENSG00000227917.2 0.458376664 1 1 0.458376664
Brain_Putamen_basal_ganglia 0.458376664 Brain_Putamen_basal_ganglia 1 1
1 -0.741522551 -0.741522551 -0.741522551 NA 1 0
0.998207054
C1GALT1 ENSG00000106392.10 0.458405352 13 2 0.172015067
Brain_Frontal_Cortex_BA9 0.725215059 Brain_Putamen_basal_ganglia
11.62833155 0.001678234 0.73194239 -1.365757573 -0.351497619
-0.899472868 0.260922555 2 0 0.998207054
RP11-212I21.2 ENSG00000260135.6 0.45843236 1 1 0.45843236
Brain_Spinal_cord_cervical_c-1 0.45843236 Brain_Spinal_cord_cervical_c-1 1
1 1 0.74143066 0.74143066 0.74143066 NA 1 0 0.998207054
WNT10B ENSG00000169884.13 0.458454365 4 4 0.251066062
Brain_Anterior_cingulate_cortex_BA24 0.87218283 Brain_Frontal_Cortex_BA9
2.887823602 0.106966293 0.106966293 -1.147763856 0.428377422
-0.288112521 0.650332905 4 0 0.998207054
RP11-48B3.3 ENSG00000254162.1 0.458578706 1 1 0.458578706
Brain_Cerebellum 0.458578706 Brain_Cerebellum 1 1 1 0.74118924
0.74118924 0.74118924 NA 1 0 0.998207054
TMC1 ENSG00000165091.15 0.458836915 3 3 0.128698449 Brain_Cortex
0.884604057 Brain_Hippocampus 1.330205474 0.475473735

0.475473735 -1.519254494 0.440646849 -0.311157452 1.056624634
3 0 0.998207054
TRIAP1 ENSG00000170855.3 0.458873233 1 1 0.458873233
Brain_Substantia_nigra 0.458873233 Brain_Substantia_nigra 1 1
1 0.740703507 0.740703507 0.740703507 NA 1 0
0.998207054
RP11-589B3.6 ENSG00000226676.1 0.45914576 5 5 0.050363416
Brain_Frontal_Cortex_BA9 0.902296684 Brain_Cerebellum 1.379554568
0.695560044 0.695560044 -0.459761092 1.956864374 0.574374006
0.963215412 5 0 0.998207054
ENG ENSG00000106991.13 0.459189456 1 1 0.459189456
Brain_Nucleus_accumbens_basal_ganglia 0.459189456
Brain_Nucleus_accumbens_basal_ganglia 1 1 1 -0.740182185
-0.740182185 -0.740182185 NA 1 0 0.998207054
DLST ENSG00000119689.14 0.459293993 2 2 0.221065113
Brain_Caudate_basal_ganglia 0.610542037 Brain_Cerebellum 1.22322533 0.77677467
0.77677467 -0.509299888 1.223700812 0.357200462 1.225416547 2
0 0.998207054
LCN8 ENSG00000204001.9 0.459446682 5 1 0.437461706 Brain_Cerebellum
0.495156605 Brain_Frontal_Cortex_BA9 4.976728064 0.000325738
4.976728064 0.682130233 0.77648664 0.738019176 0.047944589 1
0 0.998207054
EGFR ENSG00000146648.17 0.459452115 5 5 0.23204009
Brain_Caudate_basal_ganglia 0.524826372 Brain_Cortex 3.00654255
0.181784251 0.181784251 -1.195120151 1.149823284 0.450077627
0.94166963 5 0 0.998207054
NECAB2 ENSG00000103154.9 0.459496514 4 4 0.130858379
Brain_Caudate_basal_ganglia 0.896865953 Brain_Putamen_basal_ganglia
1.515800141 0.487838648 0.487838648 -1.510725593 0.403242005
-0.493318081 0.822635813 4 0 0.998207054
XKR6 ENSG00000171044.10 0.459602504 1 1 0.459602504
Brain_Anterior_cingulate_cortex_BA24 0.459602504
Brain_Anterior_cingulate_cortex_BA24 1 1 1 -0.739501541
-0.739501541 -0.739501541 NA 1 0 0.998207054
NPR2 ENSG00000159899.14 0.459625765 1 1 0.459625765 Brain_Cerebellum
0.459625765 Brain_Cerebellum 1 1 1 0.739463221 0.739463221
0.739463221 NA 1 0 0.998207054
KCNMB4 ENSG00000135643.4 0.459778312 7 5 0.121917592
Brain_Caudate_basal_ganglia 0.936983108 Brain_Hypothalamus 3.798126952
0.07829936 0.206912252 -1.546774661 0.923094869 -0.494913693
1.034886276 5 0 0.998207054
ANKRD13D ENSG00000172932.14 0.459932365 1 1 0.459932365
Brain_Cerebellar_Hemisphere 0.459932365 Brain_Cerebellar_Hemisphere 1 1
1 0.738958224 0.738958224 0.738958224 NA 1 0
0.998207054
ACTR2 ENSG00000138071.13 0.459940178 4 4 0.442554678
Brain_Nucleus_accumbens_basal_ganglia 0.854833281 Brain_Caudate_basal_ganglia
3.259877019 0.16743304 0.16743304 -0.7678864 0.338732958 -0.180169805
0.526988148 4 0 0.998207054
CEP152 ENSG00000103995.13 0.46003784 3 2 0.24221131 Brain_Cortex
0.692948881 Brain_Cerebellar_Hemisphere 1.940416289 0.059157558
1.000426153 -1.169477478 -0.394856383 -0.676078585 0.428688315
2 0 0.998207054
SNRPN ENSG00000128739.21 0.460078595 2 2 0.212740772 Brain_Cortex
0.980983399 Brain_Caudate_basal_ganglia 1.014084093 0.985915907
0.985915907 -1.24606435 -0.023836032 -0.634950191 0.864245932
2 0 0.998207054
RFESD ENSG00000175449.13 0.460164778 2 2 0.245557824
Brain_Caudate_basal_ganglia 0.72196739 Brain_Cerebellar_Hemisphere 1.613535921
0.386464079 0.386464079 -0.355830656 1.161206765 0.402688055
1.072707448 2 0 0.998207054
HIGD2A ENSG00000146066.2 0.46036684 1 1 0.46036684

Brain_Frontal_Cortex_BA9 0.46036684 Brain_Frontal_Cortex_BA9 1 1 1
0.738242929 0.738242929 0.738242929 NA 1 0 0.998207054
ZADH2 ENSG00000180011.6 0.460465447 2 1 0.383618555 Brain_Cortex
0.557519908 Brain_Spinal_cord_cervical_c-1 1.950495426 0.049504574
1.950495426 -0.871248369 -0.586529255 -0.728888812 0.201326817
1 0 0.998207054
CTC-534A2.2 ENSG00000253251.2 0.460519312 3 3 0.166845591
Brain_Cerebellum 0.77356276 Brain_Nucleus_accumbens_basal_ganglia 1.757867846
0.24774644 0.24774644 -1.382410837 -0.287717805 -0.780582908
0.555421355 3 0 0.998207054
GTF3C4 ENSG00000125484.11 0.46059517 2 2 0.227203817
Brain_Nucleus_accumbens_basal_ganglia 0.799413444 Brain_Cerebellum
1.040799948 0.959200052 0.959200052 -0.25410629 1.207593094
0.476743402 1.033577547 2 0 0.998207054
CARF ENSG00000138380.17 0.460598896 3 3 0.132533709
Brain_Spinal_cord_cervical_c-1 0.678471047 Brain_Cerebellum 2.258600846
0.250627509 0.250627509 0.414550424 1.504185114 0.821535207
0.594827189 3 0 0.998207054
SYNE4 ENSG00000181392.14 0.460672207 1 1 0.460672207
Brain_Caudate_basal_ganglia 0.460672207 Brain_Caudate_basal_ganglia 1 1
1 0.737740417 0.737740417 0.737740417 NA 1 0
0.998207054
EMC6 ENSG00000127774.6 0.460761647 1 1 0.460761647
Brain_Cerebellar_Hemisphere 0.460761647 Brain_Cerebellar_Hemisphere 1 1
1 0.737593268 0.737593268 0.737593268 NA 1 0
0.998207054
CISH ENSG00000114737.15 0.460789206 1 1 0.460789206 Brain_Amygdala
0.460789206 Brain_Amygdala 1 1 1 -0.737547932 -0.737547932
-0.737547932 NA 1 0 0.998207054
ILDR2 ENSG00000143195.12 0.460820514 1 1 0.460820514
Brain_Frontal_Cortex_BA9 0.460820514 Brain_Frontal_Cortex_BA9 1 1
1 -0.737496429 -0.737496429 -0.737496429 NA 1 0
0.998207054
ANTXR2 ENSG00000163297.16 0.460860695 2 2 0.255895904
Brain_Frontal_Cortex_BA9 0.56283845 Brain_Anterior_cingulate_cortex_BA24
1.062674686 0.937325314 0.937325314 0.578630603 1.136144954
0.857387779 0.394222178 2 0 0.998207054
GNRH1 ENSG00000147437.9 0.460876244 1 1 0.460876244
Brain_Anterior_cingulate_cortex_BA24 0.460876244
Brain_Anterior_cingulate_cortex_BA24 1 1 1 -0.737404756
-0.737404756 -0.737404756 NA 1 0 0.998207054
DCLK2 ENSG00000170390.15 0.460893701 5 4 0.111914762
Brain_Nucleus_accumbens_basal_ganglia 0.576145783 Brain_Cortex
3.257435902 0.09972231 0.298567297 -1.589645381 0.559340601
-0.853089198 0.882196111 4 0 0.998207054
RIT2 ENSG00000152214.12 0.460907066 3 3 0.170746016
Brain_Caudate_basal_ganglia 0.985763289 Brain_Substantia_nigra
1.718784342 0.34436888 0.34436888 0.017844018 1.369810651
0.527395968 0.734882061 3 0 0.998207054
PDZD8 ENSG00000165650.11 0.460977027 13 3 0.068113932 Brain_Cortex
0.325585765 Brain_Anterior_cingulate_cortex_BA24 10.8865449 0.003440412
0.476758878 0.983044037 1.824252338 1.384806117 0.24994627 3
0 0.998207054
MUC5B ENSG00000117983.17 0.461074771 2 2 0.234302179 Brain_Cortex
0.351503598 Brain_Frontal_Cortex_BA9 1.910525919 0.089474081
0.089474081 -1.189349463 -0.931676765 -1.060513114 0.182202112
2 0 0.998207054
USP31 ENSG00000103404.14 0.461221866 5 4 0.120289054
Brain_Frontal_Cortex_BA9 0.892555705 Brain_Substantia_nigra
2.119273151 0.041089184 0.609566138 -0.746080034 1.553561478
0.47599319 0.966078023 4 0 0.998207054
GATB ENSG00000059691.11 0.461277862 6 3 0.167102539

Brain_Hippocampus 0.803881175 Brain_Frontal_Cortex_BA9 4.599620391
0.010192483 0.465741965 0.248327304 1.381574009 1.004356049
0.395699347 3 0 0.998207054
ZNF324 ENSG00000083812.11 0.461447341 6 6 0.093865312
Brain_Cerebellar_Hemisphere 0.909085732 Brain_Cerebellum 2.402999637
0.393727715 0.393727715 -0.274359538 1.675351372 0.570372656
0.771398315 6 0 0.998207054
TRIM47 ENSG00000132481.6 0.462003357 6 5 0.054038321
Brain_Cerebellum 0.575698608 Brain_Putamen_basal_ganglia 3.406241588
0.02610169 0.197378138 -1.92652927 -0.559678707 -1.275890797
0.512935491 5 0 0.998207054
SFRP1 ENSG00000104332.11 0.462014868 4 4 0.107801554 Brain_Amygdala
0.893377629 Brain_Cerebellum 1.899751854 0.098431956 0.098431956
-0.574687708 1.60815355 0.552943282 0.966422093 4 0
0.998207054
TREH ENSG00000118094.11 0.462118844 1 1 0.462118844
Brain_Cerebellar_Hemisphere 0.462118844 Brain_Cerebellar_Hemisphere 1 1
1 -0.735362351 -0.735362351 -0.735362351 NA 1 0
0.998207054
MPP2 ENSG00000108852.14 0.462139956 7 6 0.093068935
Brain_Frontal_Cortex_BA9 0.919848361 Brain_Cerebellar_Hemisphere
2.917099832 0.073535017 0.286293823 -1.679426589 0.131879023
-0.845436786 0.750545439 6 0 0.998207054
PHKB ENSG00000102893.15 0.462239866 1 1 0.462239866 Brain_Cerebellum
0.462239866 Brain_Cerebellum 1 1 1 -0.735163598 -0.735163598
-0.735163598 NA 1 0 0.998207054
LCE1A ENSG00000186844.5 0.462296101 1 1 0.462296101
Brain_Spinal_cord_cervical_c-1 0.462296101 Brain_Spinal_cord_cervical_c-1
1 1 1 0.735071253 0.735071253 0.735071253 NA 1 0
0.998207054
DENND5B ENSG00000170456.15 0.462305018 2 2 0.32971708
Brain_Nucleus_accumbens_basal_ganglia 0.388601335 Brain_Frontal_Cortex_BA9
1.098273486 0.901726514 0.901726514 0.862156624 0.974683903
0.918420264 0.079568802 2 0 0.998207054
CHMP7 ENSG00000147457.13 0.462307404 3 3 0.332419245
Brain_Cerebellar_Hemisphere 0.874090583 Brain_Cerebellum 1.869782316
0.155136128 0.155136128 -0.969252455 0.299110555 -0.276202222
0.642326057 3 0 0.998207054
SF3B1 ENSG00000115524.15 0.46233296 4 3 0.131229262
Brain_Caudate_basal_ganglia 0.625414663 Brain_Cortex 3.380364636
0.063350364 0.130485592 -1.509272098 -0.488190853 -0.984288314
0.439560359 3 0 0.998207054
WIPI2 ENSG00000157954.14 0.462467103 2 2 0.244421871
Brain_Putamen_basal_ganglia 0.662359387 Brain_Cortex 1.00318178 0.99681822
0.99681822 -1.164005282 0.436658008 -0.363673637 1.131839867 2
0 0.998207054
LEPR ENSG00000116678.19 0.462547656 4 4 0.099589059 Brain_Cerebellum
0.982050358 Brain_Cortex 2.654461175 0.233019134 0.233019134
-0.022498439 1.646849133 0.963440773 0.742950167 4 0
0.998207054
UBE2N ENSG00000177889.9 0.462578552 2 2 0.278066753
Brain_Frontal_Cortex_BA9 0.41280672 Brain_Cerebellar_Hemisphere 1.210218516
0.789781484 0.789781484 -1.084672452 -0.818964409 -0.95181843
0.187883959 2 0 0.998207054
NAT6 ENSG00000243477.5 0.462645618 7 7 0.095689857
Brain_Frontal_Cortex_BA9 0.954456575 Brain_Hypothalamus 1.706566217
0.427272302 0.427272302 -1.597655654 1.666118285 -0.066268512
1.065643266 7 0 0.998207054
PDDC1 ENSG00000177225.16 0.462724095 2 2 0.214948185
Brain_Frontal_Cortex_BA9 0.965468193 Brain_Spinal_cord_cervical_c-1
1.012561221 0.987438779 0.987438779 0.043292722 1.240073566
0.641683144 0.84625185 2 0 0.998207054

PPP1R1B ENSG00000131771.13 0.463013686 1 1 0.463013686
Brain_Cerebellar_Hemisphere 0.463013686 Brain_Cerebellar_Hemisphere 1 1
1 -0.733893438 -0.733893438 -0.733893438 NA 1 0
0.998207054
G6PC3 ENSG00000141349.8 0.463039771 6 3 0.117597073
Brain_Caudate_basal_ganglia 0.63721194 Brain_Cerebellar_Hemisphere 4.667267872
0.004316314 0.405043791 0.471600542 1.564939598 0.935856316
0.490423871 3 0 0.998207054
LARP4B ENSG00000107929.14 0.463051592 1 1 0.463051592
Brain_Cerebellar_Hemisphere 0.463051592 Brain_Cerebellar_Hemisphere 1 1
1 0.73383125 0.73383125 0.73383125 NA 1 0 0.998207054
LRFN1 ENSG00000128011.4 0.463058802 2 2 0.228797448 Brain_Cerebellum
0.991707339 Brain_Putamen_basal_ganglia 1.235611395 0.764388605
0.764388605 0.010393496 1.203462362 0.606927929 0.843627085
2 0 0.998207054
CCDC167 ENSG00000198937.8 0.46309153 13 3 0.066096354
Brain_Putamen_basal_ganglia 0.68930772 Brain_Cortex 11.05868414
0.000970279 0.545606679 -1.837769659 -0.39979472 -1.316305197
0.333234244 3 0 0.998207054
TNS2 ENSG00000111077.17 0.463194249 12 7 0.06540244 Brain_Cerebellum
0.993153681 Brain_Amygdala 5.679855582 0.000967508 0.290226905
-0.782068237 1.84249731 0.483619501 0.881801676 7 0
0.998207054
RP3-395M20.12 ENSG00000272449.1 0.46319941 2 2 0.217172329
Brain_Cerebellum 0.840884052 Brain_Spinal_cord_cervical_c-1 1.059611894
0.940388106 0.940388106 -1.234082056 0.2007628 -0.516659628
1.014588527 2 0 0.998207054
CIB4 ENSG00000157884.10 0.463275458 1 1 0.463275458 Brain_Cerebellum
0.463275458 Brain_Cerebellum 1 1 1 -0.733464031 -0.733464031
-0.733464031 NA 1 0 0.998207054
AC007879.4 ENSG00000225916.1 0.463328794 2 2 0.217860207
Brain_Hippocampus 0.872620291 Brain_Caudate_basal_ganglia 1.014710872
0.985289128 0.985289128 -1.23223796 0.160331062 -0.535953449
0.984694999 2 0 0.998207054
CHRNA4 ENSG00000101204.16 0.463332233 7 7 0.049026039
Brain_Nucleus_accumbens_basal_ganglia 0.812679866 Brain_Cerebellar_Hemisphere
2.193000678 0.450048225 0.450048225 -1.968365148 1.751992119
-0.453756605 1.128020218 7 0 0.998207054
CYB5D2 ENSG00000167740.9 0.463335297 1 1 0.463335297
Brain_Spinal_cord_cervical_c-1 0.463335297 Brain_Spinal_cord_cervical_c-1
1 1 1 0.73336589 0.73336589 0.73336589 NA 1 0 0.998207054
CD59 ENSG00000085063.14 0.46353922 9 6 0.213597697
Brain_Putamen_basal_ganglia 0.77616814 Brain_Substantia_nigra 4.675349336
1.12E-06 0.481227649 -0.876344858 1.243733406 0.041240465
0.679740372 6 0 0.998207054
ARHGAP8 ENSG00000241484.9 0.463653077 1 1 0.463653077
Brain_Hypothalamus 0.463653077 Brain_Hypothalamus 1 1 1
-0.732844827 -0.732844827 -0.732844827 NA 1 0 0.998207054
NSA2 ENSG00000164346.9 0.463690531 13 1 0.358465358
Brain_Substantia_nigra 0.532270462 Brain_Cortex 12.91233147
7.42E-06 12.91233147 0.624543881 0.918293254 0.730606083
0.070994463 1 0 0.998207054
HPR ENSG00000261701.6 0.463763229 13 7 0.258679184
Brain_Hippocampus 0.95936811 Brain_Anterior_cingulate_cortex_BA24
7.591525452 0.021878735 0.281679588 -1.129518466 0.820848855
-0.491284376 0.580440197 7 0 0.998207054
RP4-614C10.2 ENSG00000283196.1 0.463788504 3 2 0.217081391
Brain_Anterior_cingulate_cortex_BA24 0.942330195 Brain_Frontal_Cortex_BA9
2.001629923 3.25E-06 0.99836683 -0.085801575 1.234326161
0.358727719 0.75832036 2 0 0.998207054
ZNF155 ENSG00000204920.10 0.464064941 6 6 0.105030734
Brain_Nucleus_accumbens_basal_ganglia 0.828579215 Brain_Cortex

2.431104336 0.344923622 0.344923622 -1.620938942 0.376258651
-0.464494874 0.653413453 6 0 0.998207054
RHOBTB3 ENSG00000164292.12 0.464168676 1 1 0.464168676
Brain_Caudate_basal_ganglia 0.464168676 Brain_Caudate_basal_ganglia 1 1
1 0.731999822 0.731999822 0.731999822 NA 1 0
0.998207054
TEX30 ENSG00000151287.16 0.464174068 1 1 0.464174068 Brain_Cerebellum
0.464174068 Brain_Cerebellum 1 1 1 -0.731990989 -0.731990989
-0.731990989 NA 1 0 0.998207054
KLHL36 ENSG00000135686.12 0.464213019 11 6 0.086527576
Brain_Cortex 0.561263197 Brain_Hippocampus 6.677751024
0.011352881 0.313640662 -0.872146368 1.714006261 0.64863763
0.776040588 6 0 0.998207054
SRI ENSG00000075142.13 0.464224826 1 1 0.464224826
Brain_Spinal_cord_cervical_c-1 0.464224826 Brain_Spinal_cord_cervical_c-1
1 1 1 0.731907831 0.731907831 0.731907831 NA 1 0
0.998207054
GNPAT ENSG00000116906.11 0.464383364 6 3 0.298440519
Brain_Hippocampus 0.912655329 Brain_Spinal_cord_cervical_c-1 5.09296971
0.010542383 0.253806848 -0.109689877 1.039783452 0.405698542
0.369396983 3 0 0.998207054
KDM4D ENSG00000186280.6 0.464412266 1 1 0.464412266
Brain_Hypothalamus 0.464412266 Brain_Hypothalamus 1 1 1
-0.73160079 -0.73160079 -0.73160079 NA 1 0 0.998207054
PPP1R16B ENSG00000101445.9 0.464482077 2 2 0.289056558
Brain_Cerebellum 0.477439374 Brain_Anterior_cingulate_cortex_BA24
1.067776671 0.932223329 0.932223329 -1.060193543 -0.710427017
-0.88531028 0.247322282 2 0 0.998207054
KLF9 ENSG00000119138.4 0.464525381 1 1 0.464525381 Brain_Cerebellum
0.464525381 Brain_Cerebellum 1 1 1 -0.731415532 -0.731415532
-0.731415532 NA 1 0 0.998207054
FHAD1 ENSG00000142621.19 0.464774833 1 1 0.464774833
Brain_Spinal_cord_cervical_c-1 0.464774833 Brain_Spinal_cord_cervical_c-1
1 1 1 -0.731007072 -0.731007072 -0.731007072 NA 1 0
0.998207054
LEKR1 ENSG00000197980.12 0.464853801 6 5 0.152934768
Brain_Cerebellar_Hemisphere 0.833810323 Brain_Cortex 4.355752809
0.102387889 0.145283045 -0.209817233 1.429241727 0.806526187
0.556654566 5 0 0.998207054
POLR2J ENSG00000005075.15 0.464950614 2 2 0.632993192
Brain_Hippocampus 0.708451178 Brain_Anterior_cingulate_cortex_BA24
1.762863569 0.237136431 0.237136431 -0.373937013 0.47751816
0.051790574 0.602069727 2 0 0.998207054
C14orf2 ENSG00000156411.9 0.465051851 3 3 0.161354535
Brain_Amygdala 0.614272766 Brain_Cerebellum 1.698895257 0.243963368
0.243963368 -0.509238507 1.40053044 0.129102713 1.101091845 3
0 0.998207054
PIGG ENSG00000174227.15 0.465196941 5 4 0.097114063
Brain_Anterior_cingulate_cortex_BA24 0.556771419 Brain_Hippocampus
3.394385668 0.048226522 0.275506533 0.587643779 1.65900858
1.16573164 0.423377465 4 0 0.998207054
LCMT1-AS1 ENSG00000260448.5 0.465217242 1 1 0.465217242
Brain_Putamen_basal_ganglia 0.465217242 Brain_Putamen_basal_ganglia 1 1
1 0.73028296 0.73028296 0.73028296 NA 1 0 0.998207054
C2orf72 ENSG00000204128.5 0.46561862 3 3 0.214460489 Brain_Cerebellum
0.774668605 Brain_Hippocampus 1.171114126 0.834290213
0.834290213 -0.945020883 1.241393311 0.19421533 1.096110303 3
0 0.998207054
CAP2 ENSG00000112186.11 0.465686792 2 2 0.269815663 Brain_Cerebellum
0.530137524 Brain_Cerebellar_Hemisphere 1.064769437 0.935230563
0.935230563 0.627796096 1.103487163 0.86564163 0.336364379 2
0 0.998207054

TFB2M ENSG00000162851.7 0.465708199 2 2 0.227379622
Brain_Caudate_basal_ganglia 0.756561961 Brain_Spinal_cord_cervical_c-1
1.03587946 0.96412054 0.96412054 -1.207136391 -0.309998679 -0.758567535
0.63437216 2 0 0.998207054
STK39 ENSG00000198648.10 0.465713129 3 3 0.200170686
Brain_Putamen_basal_ganglia 0.789604332 Brain_Spinal_cord_cervical_c-1
2.037388771 0.219359304 0.219359304 -0.266824445 1.281065428
0.485914377 0.774816018 3 0 0.998207054
RALY ENSG00000125970.11 0.46571748 1 1 0.46571748 Brain_Hippocampus
0.46571748 Brain_Hippocampus 1 1 1 0.729464656 0.729464656
0.729464656 NA 1 0 0.998207054
RP11-326A19.4 ENSG00000260123.1 0.465821602 1 1 0.465821602
Brain_Spinal_cord_cervical_c-1 0.465821602 Brain_Spinal_cord_cervical_c-1
1 1 1 -0.729294391 -0.729294391 -0.729294391 NA 1 0
0.998207054
RP11-574K11.29 ENSG00000272140.2 0.465873894 3 3 0.219694065
Brain_Cerebellar_Hemisphere 0.709525393 Brain_Substantia_nigra
1.637424497 0.268367115 0.268367115 -0.372493577 1.227342029
0.430454145 0.799935018 3 0 0.998207054
LINC00504 ENSG00000248360.7 0.466087233 2 2 0.21672028 Brain_Cerebellum
0.710179201 Brain_Cerebellar_Hemisphere 1.322614055 0.677385945
0.677385945 0.371615427 1.23529622 0.803455823 0.610714545 2
0 0.998207054
POLR2D ENSG00000144231.10 0.46626094 11 2 0.422655351
Brain_Anterior_cingulate_cortex_BA24 0.99593502 Brain_Cortex 9.837189829
0.000945764 0.471822102 -0.801823002 0.1050594 -0.205635806
0.286341145 2 0 0.998207054
ANGPTL7 ENSG00000171819.4 0.466524859 1 1 0.466524859
Brain_Substantia_nigra 0.466524859 Brain_Substantia_nigra 1 1
1 0.728144952 0.728144952 0.728144952 NA 1 0
0.998207054
LUC7L ENSG00000007392.16 0.466555218 2 2 0.25170747
Brain_Caudate_basal_ganglia 0.998790419 Brain_Nucleus_accumbens_basal_ganglia
1.370819472 0.629180528 0.629180528 -1.146211937 0.001515985
-0.572347976 0.811566197 2 0 0.998207054
RP11-506M13.3 ENSG00000244733.5 0.466697483 8 8 0.103551957
Brain_Hippocampus 0.937432302 Brain_Putamen_basal_ganglia 1.679567747
0.510334386 0.510334386 -1.627872341 0.426874181 -0.443885571
0.815114307 8 0 0.998207054
C1orf101 ENSG00000179397.17 0.466848624 1 1 0.466848624
Brain_Cerebellum 0.466848624 Brain_Cerebellum 1 1 1 0.727616097
0.727616097 0.727616097 NA 1 0 0.998207054
CD7 ENSG00000173762.7 0.466915332 1 1 0.466915332
Brain_Frontal_Cortex_BA9 0.466915332 Brain_Frontal_Cortex_BA9 1 1
1 0.727507157 0.727507157 0.727507157 NA 1 0
0.998207054
CDH3 ENSG00000062038.13 0.467033748 6 3 0.274286664
Brain_Caudate_basal_ganglia 0.976953168 Brain_Putamen_basal_ganglia
4.344157646 0.087180189 0.517542652 -1.093244044 0.494640306
-0.246647113 0.642441637 3 0 0.998207054
LA16c-312E8.5 ENSG00000284395.1 0.467093484 5 5 0.077830388
Brain_Cerebellar_Hemisphere 0.645035093 Brain_Hypothalamus 1.49328293
0.607468152 0.607468152 -1.763415792 0.737939314 -0.349413715
0.994384096 5 0 0.998207054
PYCRL ENSG00000104524.13 0.467163881 10 6 0.287390623
Brain_Hypothalamus 0.912047958 Brain_Caudate_basal_ganglia 6.32607503
0.028562834 0.24047067 -0.719759216 1.063863317 0.384781665
0.55565281 6 0 0.998207054
ARHGAP32 ENSG00000134909.18 0.467175168 2 2 0.326537254
Brain_Cortex 0.463219737 Brain_Cerebellum 1.014651854 0.985348146
0.985348146 0.733555424 0.981112526 0.857333975 0.175049305
2 0 0.998207054

RP11-587P21.2 ENSG00000257893.2 0.467197922 1 1 0.467197922
Brain_Cerebellum 0.467197922 Brain_Cerebellum 1 1 1 0.727045764
0.727045764 0.727045764 NA 1 0 0.998207054
EPHA4 ENSG00000116106.11 0.467268487 3 3 0.231042371
Brain_Cerebellar_Hemisphere 0.777138152 Brain_Putamen_basal_ganglia
1.348819052 0.626422571 0.626422571 -1.197678068 0.893369871
-0.195786221 1.048251715 3 0 0.998207054
HDLBP ENSG00000115677.16 0.467294056 11 3 0.268008258
Brain_Hippocampus 0.886315719 Brain_Cerebellum 8.855416023
0.001619801 0.432785933 -0.388304015 1.107660978 0.625587632
0.51879979 3 0 0.998207054
SSH3 ENSG00000172830.12 0.467489467 6 2 0.51628918
Brain_Frontal_Cortex_BA9 0.995634083 Brain_Hypothalamus 5.428742896
0.014730546 0.25846069 -0.649076114 0.196963303 -0.285686194
0.31628631 2 0 0.998207054
ATG2B ENSG00000066739.11 0.467519511 1 1 0.467519511
Brain_Substantia_nigra 0.467519511 Brain_Substantia_nigra 1 1
1 -0.726520881 -0.726520881 -0.726520881 NA 1 0
0.998207054
AC004471.10 ENSG00000272682.1 0.467599995 2 2 0.246752773
Brain_Cerebellum 0.550465555 Brain_Cerebellar_Hemisphere 1.754849155
0.245150845 0.245150845 -1.15827269 -0.597062647 -0.877667669
0.396835427 2 0 0.998207054
AC010731.4 ENSG00000235118.7 0.467651701 1 1 0.467651701
Brain_Putamen_basal_ganglia 0.467651701 Brain_Putamen_basal_ganglia 1 1
1 0.726305186 0.726305186 0.726305186 NA 1 0
0.998207054
RP11-213G21.2 ENSG00000251635.1 0.467699208 1 1 0.467699208
Brain_Hippocampus 0.467699208 Brain_Hippocampus 1 1 1
-0.726227676 -0.726227676 -0.726227676 NA 1 0 0.998207054
KBTBD11 ENSG00000176595.3 0.467817482 3 3 0.124307401
Brain_Nucleus_accumbens_basal_ganglia 0.988979306 Brain_Cerebellum
2.019696172 0.067463808 0.067463808 -1.536942458 0.134667904
-0.462820574 0.932177479 3 0 0.998207054
AC083843.1 ENSG00000259820.1 0.467823496 10 7 0.084903803
Brain_Cerebellum 0.981296741 Brain_Anterior_cingulate_cortex_BA24
5.867538683 0.013156371 0.217447697 -1.381701853 1.72291552
0.533768552 0.966245569 7 0 0.998207054
PTPN18 ENSG00000072135.12 0.46791322 3 2 0.147302186 Brain_Cerebellum
0.411697494 Brain_Substantia_nigra 2.434947397 0.037764487
0.527288116 0.820910071 1.44912676 1.084094645 0.326257173 2
0 0.998207054
EHMT2 ENSG00000204371.11 0.468011444 3 3 0.163203684
Brain_Putamen_basal_ganglia 0.774358365 Brain_Cerebellar_Hemisphere
1.141886975 0.868744717 0.868744717 -0.614622659 1.394377351
0.35547779 1.006265496 3 0 0.998207054
DGCR14 ENSG00000100056.11 0.468068264 2 2 0.498508871
Brain_Hippocampus 0.591871261 Brain_Cerebellar_Hemisphere 1.513381522
0.486618478 0.486618478 -0.676837802 -0.536126306 -0.606482054
0.099498053 2 0 0.998207054
GOLGA8H ENSG00000261794.1 0.468162078 8 8 0.10029145
Brain_Caudate_basal_ganglia 0.982313428 Brain_Nucleus_accumbens_basal_ganglia
2.437224395 0.453149219 0.453149219 -0.725327084 1.643442321
0.282180382 0.875548828 8 0 0.998207054
LINC00571 ENSG00000223685.5 0.4682126 6 6 0.248963451 Brain_Amygdala
0.90485029 Brain_Frontal_Cortex_BA9 3.350808794 0.177981339
0.177981339 -1.152870721 0.721372564 -0.464721367 0.745348917
6 0 0.998207054
CTD-2509G16.2 ENSG00000255002.1 0.46822152 2 2 0.408106302
Brain_Nucleus_accumbens_basal_ganglia 0.565388977 Brain_Putamen_basal_ganglia
1.342041232 0.657958768 0.657958768 -0.574855575 0.827230721
0.126187573 0.991424728 2 0 0.998207054

EFCAB11 ENSG00000140025.15 0.46824688 9 1 0.315477663
Brain_Putamen_basal_ganglia 0.896517854 Brain_Hypothalamus 8.226967256
0.004235053 8.226967256 0.130061393 1.003794528 0.693722142
0.278403232 1 0 0.998207054
NOC2L ENSG00000188976.10 0.468251923 4 4 0.110483303
Brain_Hypothalamus 0.941213005 Brain_Cerebellar_Hemisphere 2.593430489
0.096244505 0.096244505 -1.596024592 0.07374536 -0.738043174
0.68938309 4 0 0.998207054
HPGD ENSG00000164120.13 0.468323254 2 2 0.293959856
Brain_Hypothalamus 0.508512036 Brain_Cortex 1.015638589
0.984361411 0.984361411 -0.661156373 1.049474346 0.194158987
1.209598582 2 0 0.998207054
PLA2G2C ENSG00000187980.6 0.468334178 1 1 0.468334178
Brain_Cerebellum 0.468334178 Brain_Cerebellum 1 1 1 -0.725192118
-0.725192118 -0.725192118 NA 1 0 0.998207054
RASGRP4 ENSG00000171777.15 0.468521974 3 3 0.246827166
Brain_Hippocampus 0.596344421 Brain_Cortex 1.189405129
0.893687967 0.893687967 -0.828727274 1.158090353 0.286342596
1.015512338 3 0 0.998207054
SYNDIG1L ENSG00000183379.8 0.468540196 4 4 0.216634833
Brain_Cortex 0.983412172 Brain_Putamen_basal_ganglia 1.363607644
0.770575909 0.770575909 -1.235525928 0.020791258 -0.815468434
0.586704755 4 0 0.998207054
FCGR3B ENSG00000162747.9 0.468585859 1 1 0.468585859
Brain_Amygdala 0.468585859 Brain_Amygdala 1 1 1 0.724781871
0.724781871 0.724781871 NA 1 0 0.998207054
CCDC155 ENSG00000161609.9 0.468680609 2 1 0.432639512
Brain_Cerebellar_Hemisphere 0.51100667 Brain_Cerebellum 1.979899709
0.020100291 1.979899709 0.657271016 0.784682899 0.720976957
0.090093807 1 0 0.998207054
KB-1460A1.1 ENSG00000253395.1 0.468681998 1 1 0.468681998
Brain_Anterior_cingulate_cortex_BA24 0.468681998
Brain_Anterior_cingulate_cortex_BA24 1 1 1 -0.724625195
-0.724625195 -0.724625195 NA 1 0 0.998207054
PQLC2 ENSG00000040487.12 0.4687181 4 3 0.121028295 Brain_Hypothalamus
0.651462049 Brain_Nucleus_accumbens_basal_ganglia 2.892324137
0.048500051 0.271626489 -1.550471891 -0.451732018 -0.756536864
0.530223577 3 0 0.998207054
ZNF217 ENSG00000171940.13 0.468824252 1 1 0.468824252
Brain_Cerebellum 0.468824252 Brain_Cerebellum 1 1 1 -0.724393397
-0.724393397 -0.724393397 NA 1 0 0.998207054
ELMO2 ENSG00000062598.17 0.468848577 1 1 0.468848577 Brain_Cortex
0.468848577 Brain_Cortex 1 1 1 0.724353765 0.724353765
0.724353765 NA 1 0 0.998207054
NIN ENSG00000100503.23 0.468938449 2 2 0.346725822 Brain_Cerebellum
0.417535137 Brain_Frontal_Cortex_BA9 1.018753373 0.981246627
0.981246627 -0.940959095 -0.810704938 -0.875832016 0.092103598
2 0 0.998207054
TP63 ENSG00000073282.12 0.469067342 1 1 0.469067342
Brain_Frontal_Cortex_BA9 0.469067342 Brain_Frontal_Cortex_BA9 1 1
1 -0.723997382 -0.723997382 -0.723997382 NA 1 0
0.998207054
UCHL5 ENSG00000116750.13 0.469081381 1 1 0.469081381
Brain_Hippocampus 0.469081381 Brain_Hippocampus 1 1 1
0.723974515 0.723974515 0.723974515 NA 1 0 0.998207054
RP11-196G11.6 ENSG00000232748.3 0.469085286 1 1 0.469085286
Brain_Hypothalamus 0.469085286 Brain_Hypothalamus 1 1 1
-0.723968155 -0.723968155 -0.723968155 NA 1 0 0.998207054
PPP1R1A ENSG00000135447.16 0.469218657 2 2 0.231856303
Brain_Spinal_cord_cervical_c-1 0.705655438 Brain_Hypothalamus
1.074256911 0.925743089 0.925743089 -0.377697348 1.195590751
0.408946701 1.112482683 2 0 0.998207054

PEX2 ENSG00000164751.14 0.469376234 2 2 0.250144224 Brain_Cerebellum
0.339998082 Brain_Cerebellar_Hemisphere 1.501644204 0.498355796
0.498355796 0.954169043 1.149999143 1.052084093 0.138472792
2 0 0.998207054
CTD-2132N18.2 ENSG00000267221.2 0.469396392 1 1 0.469396392
Brain_Spinal_cord_cervical_c-1 0.469396392 Brain_Spinal_cord_cervical_c-1
1 1 1 0.723461511 0.723461511 0.723461511 NA 1 0
0.998207054
ARL14EP ENSG00000152219.4 0.469407139 10 6 0.012921928
Brain_Cerebellar_Hemisphere 0.885730253 Brain_Hypothalamus 6.77664298
1.29E-06 0.238959366 -2.485913753 -0.143709014 -1.614462509
0.711722689 6 0 0.998207054
MAPRE2 ENSG00000166974.12 0.469594097 5 4 0.162551764
Brain_Cerebellar_Hemisphere 0.742386392 Brain_Spinal_cord_cervical_c-1
2.035456689 0.023157212 0.631971193 0.328694791 1.396540598
0.938661673 0.414024225 4 0 0.998207054
DEFA5 ENSG00000164816.7 0.469657048 2 2 0.380274099
Brain_Hypothalamus 0.427788368 Brain_Hippocampus 1.07500224 0.92499776
0.92499776 -0.87739137 0.7929819 -0.042204735 1.181132266 2 0
0.998207054
ADAMTS13 ENSG00000160323.18 0.469690536 13 7 0.135487896
Brain_Hippocampus 0.914193537 Brain_Cerebellum 7.919252833
0.003570341 0.316191985 -1.492806308 0.450537424 -0.415061908
0.609524664 7 0 0.998207054
APOBEC3D ENSG00000243811.8 0.469702522 1 1 0.469702522
Brain_Amygdala 0.469702522 Brain_Amygdala 1 1 1 -0.722963153
-0.722963153 -0.722963153 NA 1 0 0.998207054
RALA ENSG00000006451.7 0.469820841 2 2 0.248431339
Brain_Nucleus_accumbens_basal_ganglia 0.583872522 Brain_Putamen_basal_ganglia
1.110550636 0.889449364 0.889449364 -1.154167903 -0.547736971
-0.850952437 0.428811424 2 0 0.998207054
TMEM53 ENSG00000126106.13 0.46983692 3 3 0.121574774 Brain_Cerebellum
0.906599282 Brain_Cortex 1.478815217 0.494901648 0.494901648
-0.136778562 1.548197412 0.431363257 0.967255638 3 0
0.998207054
NAIP ENSG00000249437.7 0.46986779 2 2 0.251035208 Brain_Hippocampus
0.876728001 Brain_Cerebellum 1.236782886 0.763217114 0.763217114
-0.155118369 1.14783858 0.496360105 0.921329694 2 0
0.998207054
RAB22A ENSG00000124209.3 0.46988655 1 1 0.46988655
Brain_Putamen_basal_ganglia 0.46988655 Brain_Putamen_basal_ganglia 1 1 1
-0.722663655 -0.722663655 -0.722663655 NA 1 0 0.998207054
FAM83H-AS1 ENSG00000203499.11 0.470095998 8 7 0.03632383
Brain_Anterior_cingulate_cortex_BA24 0.812618355 Brain_Cerebellum 2.41221715
0.055225818 0.358404282 -0.639764158 2.093283773 0.680344427
1.061380041 7 0 0.998207054
C9orf62 ENSG00000178243.4 0.470229814 1 1 0.470229814
Brain_Frontal_Cortex_BA9 0.470229814 Brain_Frontal_Cortex_BA9 1 1
1 0.722105179 0.722105179 0.722105179 NA 1 0
0.998207054
LDHAL6A ENSG00000166800.9 0.470230182 12 4 0.036859815
Brain_Cerebellum 0.753114239 Brain_Substantia_nigra 9.084643122
0.016657369 0.499139885 -0.314535657 2.087313395 1.231749338
0.730906061 4 0 0.998207054
PKMYT1 ENSG00000127564.16 0.470315314 5 2 0.180443645
Brain_Frontal_Cortex_BA9 0.373678821 Brain_Cerebellar_Hemisphere
4.626753828 0.005864456 0.247125094 0.88960351 1.339390303
1.099667211 0.180937435 2 0 0.998207054
EQTN ENSG00000120160.10 0.470331907 1 1 0.470331907
Brain_Caudate_basal_ganglia 0.470331907 Brain_Caudate_basal_ganglia 1 1
1 0.721939122 0.721939122 0.721939122 NA 1 0
0.998207054

CYP21A2 ENSG00000231852.6 0.470370989 8 8 0.063168021
Brain_Cerebellar_Hemisphere 0.927347145 Brain_Nucleus_accumbens_basal_ganglia
3.209394223 0.204847754 0.204847754 0.091183047 1.858006994
1.029523467 0.563459444 8 0 0.998207054
ZNF667-AS1 ENSG00000166770.10 0.470374279 12 6 0.113332708
Brain_Hypothalamus 0.936229083 Brain_Cerebellum 7.98993837 0.010683274
0.290014392 -1.284515851 1.583389529 0.708778839 0.791591804
6 0 0.998207054
OVGP1 ENSG00000085465.12 0.470423711 6 4 0.129779478
Brain_Nucleus_accumbens_basal_ganglia 0.537829658 Brain_Putamen_basal_ganglia
5.013528416 0.039790143 0.180574151 0.616098279 1.514972066
1.087228195 0.313428693 4 0 0.998207054
ESRP2 ENSG00000103067.13 0.470425127 1 1 0.470425127
Brain_Hippocampus 0.470425127 Brain_Hippocampus 1 1 1
0.721787514 0.721787514 0.721787514 NA 1 0 0.998207054
CLDN3 ENSG00000165215.6 0.470462878 3 3 0.156901797
Brain_Putamen_basal_ganglia 0.691758749 Brain_Cerebellum 1.24433964
0.756177696 0.756177696 -0.715784442 1.415568783 0.365417927
1.066015849 3 0 0.998207054
RP11-445N20.3 ENSG00000273293.1 0.470470376 2 2 0.358295812
Brain_Hippocampus 0.397051006 Brain_Caudate_basal_ganglia 1.035304304
0.964695696 0.964695696 0.846899742 0.918617239 0.882758491
0.050711928 2 0 0.998207054
ITPR2 ENSG00000123104.11 0.47050716 3 3 0.392401334 Brain_Hippocampus
0.922340138 Brain_Cerebellar_Hemisphere 2.077204054 0.080027443
0.080027443 -0.552115587 0.855270646 0.068556221 0.71823507 3
0 0.998207054
USP37 ENSG00000135913.10 0.47083772 2 1 0.455646233
Brain_Cerebellar_Hemisphere 0.489316094 Brain_Cerebellum 1.986705777
0.013294223 1.986705777 -0.746035065 -0.691396992 -0.718716028
0.038634952 1 0 0.998207054
FLJ36000 ENSG00000276399.1 0.471025944 2 1 0.343960315
Brain_Cerebellar_Hemisphere 0.633755456 Brain_Cerebellum 1.948163876
0.051836124 1.948163876 0.476447703 0.946369188 0.711408446
0.332284668 1 0 0.998207054
C15orf41 ENSG00000186073.11 0.471084906 2 2 0.344929379
Brain_Anterior_cingulate_cortex_BA24 0.400944094 Brain_Cerebellum
1.061644108 0.938355892 0.938355892 0.839936317 0.944470286
0.892203302 0.073916678 2 0 0.998207054
NAXD ENSG00000213995.11 0.471085546 7 2 0.419618052
Brain_Frontal_Cortex_BA9 0.994419954 Brain_Cerebellar_Hemisphere
6.016575844 0.008295833 0.477168261 -0.807084066 0.242588683
-0.302851472 0.389449074 2 0 0.998207054
SLC9A9 ENSG00000181804.14 0.471155347 1 1 0.471155347
Brain_Anterior_cingulate_cortex_BA24 0.471155347
Brain_Anterior_cingulate_cortex_BA24 1 1 1 -0.720600498
-0.720600498 -0.720600498 NA 1 0 0.998207054
CFAP161 ENSG00000156206.13 0.471246083 1 1 0.471246083
Brain_Cerebellar_Hemisphere 0.471246083 Brain_Cerebellar_Hemisphere 1 1
1 -0.720453073 -0.720453073 -0.720453073 NA 1 0
0.998207054
LBHD1 ENSG00000162194.12 0.471259039 7 2 0.664455207
Brain_Cerebellar_Hemisphere 0.961933536 Brain_Nucleus_accumbens_basal_ganglia
6.415963989 0.000525868 0.29967305 -0.414911541 0.43377036
0.099100502 0.346403032 2 0 0.998207054
RRM1 ENSG00000167325.14 0.4713855 13 7 0.022654996
Brain_Frontal_Cortex_BA9 0.857265732 Brain_Nucleus_accumbens_basal_ganglia
8.689464436 0.007125138 0.347562543 -2.27920308 -0.179855855
-1.296744476 0.556024975 7 0 0.998207054
LINC01749 ENSG00000272259.5 0.471646358 3 2 0.571609025
Brain_Cerebellar_Hemisphere 0.668356351 Brain_Cerebellum 2.484142476
0.049701004 0.46615652 -0.565683396 0.552496807 -0.147197166

0.609827916 2 0 0.998207054
PSTPIP1 ENSG00000140368.12 0.472059036 4 4 0.09862928
Brain_Frontal_Cortex_BA9 0.751445129 Brain_Caudate_basal_ganglia
1.583938854 0.312589113 0.312589113 -1.651535492 -0.31673442
-0.737230138 0.627019515 4 0 0.998207054
ZNF846 ENSG00000196605.7 0.472133151 1 1 0.472133151
Brain_Anterior_cingulate_cortex_BA24 0.472133151
Brain_Anterior_cingulate_cortex_BA24 1 1 1 -0.719012609
-0.719012609 -0.719012609 NA 1 0 0.998207054
DNAJA1 ENSG00000086061.15 0.472542872 3 2 0.42835974
Brain_Anterior_cingulate_cortex_BA24 0.994112159 Brain_Frontal_Cortex_BA9
2.244861476 0.032402537 0.722735988 -0.792001605 0.727746709
-0.018958505 0.760216415 2 0 0.998207054
FKBP9 ENSG00000122642.10 0.472697575 3 2 0.301751133
Brain_Cerebellar_Hemisphere 0.512936041 Brain_Cerebellum 2.054936263
0.052968667 0.89209507 -1.032685433 0.661658984 -0.341766429
0.889351417 2 0 0.998207054
WNT7B ENSG00000188064.9 0.472749823 2 2 0.228507127
Brain_Cerebellar_Hemisphere 0.286575704 Brain_Cerebellum 1.768159594
0.231840406 0.231840406 1.065663676 1.204213351 1.134938513
0.097969415 2 0 0.998207054
TMEM61 ENSG00000143001.4 0.472844929 3 2 0.275067508
Brain_Cerebellum 0.452919948 Brain_Cerebellar_Hemisphere 1.937522233
0.064058419 0.998419349 -0.832001788 1.091466857 0.336673668
1.026356082 2 0 0.998207054
RP11-219B17.3 ENSG00000273958.1 0.473071114 2 2 0.324256638
Brain_Amygdala 0.510490869 Brain_Putamen_basal_ganglia 1.066073469
0.933926531 0.933926531 -0.985748307 0.658073553 -0.163837377
1.162357584 2 0 0.998207054
SMIM18 ENSG00000253457.2 0.473157618 1 1 0.473157618
Brain_Hypothalamus 0.473157618 Brain_Hypothalamus 1 1 1
-0.717350883 -0.717350883 -0.717350883 NA 1 0 0.998207054
KLK14 ENSG00000129437.9 0.47318012 3 3 0.167625159 Brain_Cerebellum
0.59900196 Brain_Hippocampus 1.16798853 0.894180432 0.894180432
-1.379874923 -0.525836287 -0.900920413 0.436391213 3 0
0.998207054
FEM1A ENSG00000141965.4 0.47321723 4 4 0.188321887 Brain_Cerebellum
0.440138428 Brain_Anterior_cingulate_cortex_BA24 1.478611445
0.533139458 0.533139458 -0.830351212 1.315559642 0.627793449
1.002020301 4 0 0.998207054
WDR25 ENSG00000176473.13 0.473229051 5 5 0.131091698 Brain_Cerebellum
0.72272755 Brain_Amygdala 2.941928695 0.213698111 0.213698111
0.354815855 1.509810839 0.906258869 0.515369541 5 0
0.998207054
CCDC65 ENSG00000139537.10 0.473265534 7 1 0.398354244
Brain_Frontal_Cortex_BA9 0.578280202 Brain_Nucleus_accumbens_basal_ganglia
6.834796184 0.001741123 6.834796184 -0.844564129 -0.555898561
-0.708939787 0.092423669 1 0 0.998207054
ZNF418 ENSG00000196724.12 0.47327703 10 10 0.070408675 Brain_Cerebellum
0.9699192 Brain_Anterior_cingulate_cortex_BA24 3.709067448 0.130467399
0.130467399 -0.657460645 1.809272528 0.460483331 0.678289935
10 0 0.998207054
ITLN1 ENSG00000179914.4 0.473325191 2 2 0.254520224
Brain_Anterior_cingulate_cortex_BA24 0.642464971 Brain_Frontal_Cortex_BA9
1.01735246 0.98264754 0.98264754 -0.464255125 1.139438697 0.337591786
1.133982776 2 0 0.998207054
LDLRAD4 ENSG00000168675.18 0.473344417 1 1 0.473344417
Brain_Hippocampus 0.473344417 Brain_Hippocampus 1 1 1
-0.717048102 -0.717048102 -0.717048102 NA 1 0 0.998207054
TLE4 ENSG00000106829.18 0.473378663 1 1 0.473378663 Brain_Cerebellum
0.473378663 Brain_Cerebellum 1 1 1 0.7169926 0.7169926 0.7169926
NA 1 0 0.998207054

NT5DC2 ENSG00000168268.10 0.473408333 1 1 0.473408333
Brain_Caudate_basal_ganglia 0.473408333 Brain_Caudate_basal_ganglia 1 1
1 -0.716944516 -0.716944516 -0.716944516 NA 1 0
0.998207054
LIMA1 ENSG00000050405.13 0.47349858 1 1 0.47349858
Brain_Spinal_cord_cervical_c-1 0.47349858 Brain_Spinal_cord_cervical_c-1 1
1 1 0.716798269 0.716798269 0.716798269 NA 1 0
0.998207054
NR1H2 ENSG00000131408.13 0.473656721 1 1 0.473656721
Brain_Hippocampus 0.473656721 Brain_Hippocampus 1 1 1
0.716542037 0.716542037 0.716542037 NA 1 0 0.998207054
PUM3 ENSG00000080608.9 0.473747328 3 3 0.226812335
Brain_Putamen_basal_ganglia 0.540220616 Brain_Caudate_basal_ganglia
1.534635065 0.485029074 0.485029074 -1.208610984 -0.61247941
-0.97965935 0.321206338 3 0 0.998207054
RP11-61K9.3 ENSG00000275964.1 0.473842045 1 1 0.473842045
Brain_Amygdala 0.473842045 Brain_Amygdala 1 1 1 0.71624182
0.71624182 0.71624182 NA 1 0 0.998207054
SYK ENSG00000165025.14 0.473855522 1 1 0.473855522 Brain_Cortex
0.473855522 Brain_Cortex 1 1 1 0.716219989 0.716219989
0.716219989 NA 1 0 0.998207054
IL12RB1 ENSG00000096996.15 0.473947886 4 4 0.157575939
Brain_Cortex 0.919823559 Brain_Frontal_Cortex_BA9 1.527216519
0.562755932 0.562755932 -1.413271402 0.100655977 -0.802490638
0.676437566 4 0 0.998207054
MIATNB ENSG00000244625.5 0.474005306 5 5 0.15257831 Brain_Cerebellum
0.593274668 Brain_Cerebellar_Hemisphere 2.294370101 0.284672468
0.284672468 0.534096649 1.430483457 1.13926396 0.388271531 5
0 0.998207054
TMPRSS4-AS1 ENSG00000255274.9 0.474068591 10 4 0.097595424
Brain_Spinal_cord_cervical_c-1 0.802210313
Brain_Anterior_cingulate_cortex_BA24 8.115369856 0.000841739
0.300021201 0.250487574 1.65662446 0.99326713 0.413304606 4 0
0.998207054
MCM7 ENSG00000166508.17 0.474244242 2 2 0.500409221 Brain_Cerebellum
0.611089363 Brain_Cerebellar_Hemisphere 1.528702387 0.471297613
0.471297613 -0.673846008 0.508519078 -0.082663465 0.83605837 2
0 0.998207054
OSMR-AS1 ENSG00000249740.2 0.474393252 1 1 0.474393252
Brain_Putamen_basal_ganglia 0.474393252 Brain_Putamen_basal_ganglia 1 1
1 0.715349269 0.715349269 0.715349269 NA 1 0
0.998207054
ANP32B ENSG00000136938.8 0.474446838 1 1 0.474446838
Brain_Hippocampus 0.474446838 Brain_Hippocampus 1 1 1
0.715262529 0.715262529 0.715262529 NA 1 0 0.998207054
SHISA9 ENSG00000237515.8 0.474447947 1 1 0.474447947
Brain_Cortex 0.474447947 Brain_Cortex 1 1 1 0.715260733
0.715260733 0.715260733 NA 1 0 0.998207054
RHOQ ENSG00000119729.11 0.474489416 1 1 0.474489416
Brain_Caudate_basal_ganglia 0.474489416 Brain_Caudate_basal_ganglia 1 1
1 -0.715193611 -0.715193611 -0.715193611 NA 1 0
0.998207054
PRELID3A ENSG00000141391.13 0.474495443 6 4 0.321246764
Brain_Hippocampus 0.899674374 Brain_Spinal_cord_cervical_c-1
4.478060204 0.024452268 0.177271159 -0.991899069 0.726310975
-0.20449999 0.618863994 4 0 0.998207054
SH3RF1 ENSG00000154447.14 0.474496363 3 2 0.250091503
Brain_Spinal_cord_cervical_c-1 0.429335771 Brain_Substantia_nigra
2.793168584 0.055702159 0.151129257 -1.150127157 -0.790328801
-1.016110229 0.196670427 2 0 0.998207054
GBP4 ENSG00000162654.8 0.474669653 1 1 0.474669653
Brain_Cerebellar_Hemisphere 0.474669653 Brain_Cerebellar_Hemisphere 1 1

1 -0.714901916 -0.714901916 -0.714901916 NA 1 0
0.998207054
AKR1C1 ENSG00000187134.13 0.474819178 13 3 0.075439469
Brain_Spinal_cord_cervical_c-1 0.96011207 Brain_Frontal_Cortex_BA9
10.61587202 0.001348494 0.362413968 -1.777783197 -0.050012948
-1.200558296 0.403497474 3 0 0.998207054
AC007405.6 ENSG00000239467.5 0.474825948 9 9 0.033825231
Brain_Anterior_cingulate_cortex_BA24 0.936085466
Brain_Nucleus_accumbens_basal_ganglia 3.738645162 0.129431772
0.129431772 -2.122148957 -0.080190852 -1.078044352 0.660839722
9 0 0.998207054
ZNF888 ENSG00000213793.5 0.474866042 3 3 0.117773073
Brain_Cerebellar_Hemisphere 0.944491752 Brain_Caudate_basal_ganglia
1.107232173 0.898650198 0.898650198 -1.564189499 0.088369056
-0.468731653 0.948740613 3 0 0.998207054
ATRAID ENSG00000138085.16 0.475016599 7 2 0.209397598
Brain_Cortex 0.994484663 Brain_Anterior_cingulate_cortex_BA24
5.817451117 0.003272095 0.803343057 -1.255223607 0.006912505
-0.901992653 0.427872403 2 0 0.998207054
PLS1 ENSG00000120756.12 0.475167424 2 2 0.26852702
Brain_Anterior_cingulate_cortex_BA24 0.744463416 Brain_Putamen_basal_ganglia
1.163469522 0.836530478 0.836530478 -1.106461036 0.325948373
-0.390256332 1.012866407 2 0 0.998207054
AJUBA ENSG00000129474.15 0.475236886 1 1 0.475236886
Brain_Caudate_basal_ganglia 0.475236886 Brain_Caudate_basal_ganglia 1 1
1 -0.713984303 -0.713984303 -0.713984303 NA 1 0
0.998207054
CIDEA ENSG00000176194.17 0.47528071 4 4 0.367535297
Brain_Caudate_basal_ganglia 0.995171605 Brain_Anterior_cingulate_cortex_BA24
1.778480519 0.362764783 0.362764783 -0.715928204 0.901099728
0.100431498 0.66677783 4 0 0.998207054
ETV7 ENSG00000010030.13 0.475294356 4 4 0.282263114
Brain_Caudate_basal_ganglia 0.676549602 Brain_Substantia_nigra
1.751482957 0.341297251 0.341297251 -0.417176104 1.075249332
0.595334204 0.691722969 4 0 0.998207054
AC108488.4 ENSG00000242282.6 0.475301109 1 1 0.475301109
Brain_Nucleus_accumbens_basal_ganglia 0.475301109
Brain_Nucleus_accumbens_basal_ganglia 1 1 1 -0.713880446
-0.713880446 -0.713880446 NA 1 0 0.998207054
ACAD9 ENSG00000177646.18 0.475424164 10 10 0.179587742
Brain_Cerebellar_Hemisphere 0.848281527 Brain_Anterior_cingulate_cortex_BA24
2.092388099 0.14782681 0.14782681 -1.294468971 1.342025457
0.371060292 0.925213093 10 0 0.998207054
ZNF287 ENSG00000141040.14 0.475567458 1 1 0.475567458
Brain_Caudate_basal_ganglia 0.475567458 Brain_Caudate_basal_ganglia 1 1
1 -0.713449813 -0.713449813 -0.713449813 NA 1 0
0.998207054
KCNF1 ENSG00000162975.4 0.475604974 1 1 0.475604974
Brain_Frontal_Cortex_BA9 0.475604974 Brain_Frontal_Cortex_BA9 1 1
1 0.713389168 0.713389168 0.713389168 NA 1 0
0.998207054
MILR1 ENSG00000271605.5 0.475660582 3 3 0.144419269
Brain_Spinal_cord_cervical_c-1 0.759113094 Brain_Cerebellum 2.31623929
0.179572262 0.179572262 0.306645661 1.459530076 0.755519501
0.617338386 3 0 0.998207054
C1QBP ENSG00000108561.8 0.475831126 2 2 0.222950907
Brain_Frontal_Cortex_BA9 0.775117246 Brain_Amygdala 1.24271945 0.75728055
0.75728055 -1.218718887 -0.285687805 -0.752203346 0.659752605 2
0 0.998207054
SPRED1 ENSG00000166068.12 0.475848471 2 2 0.295447534
Brain_Hippocampus 0.462903023 Brain_Frontal_Cortex_BA9 1.107684636
0.892315364 0.892315364 0.73407501 1.046245862 0.890160436

0.220738126 2 0 0.998207054
SHQ1 ENSG00000144736.13 0.475898759 2 2 0.324094593
Brain_Frontal_Cortex_BA9 0.615088105 Brain_Cerebellum 1.201375599
0.798624401 0.798624401 0.502823877 0.986078501 0.744451189
0.341712622 2 0 0.998207054
SPTLC2 ENSG00000100596.6 0.476015944 1 1 0.476015944
Brain_Cerebellum 0.476015944 Brain_Cerebellum 1 1 1 -0.712724999
-0.712724999 -0.712724999 NA 1 0 0.998207054
TUBGCP4 ENSG00000137822.12 0.476053059 3 3 0.143717397
Brain_Anterior_cingulate_cortex_BA24 0.85032951 Brain_Hypothalamus
1.909976322 0.510963523 0.510963523 0.188698012 1.462086905
0.707395666 0.668692416 3 0 0.998207054
SULF2 ENSG00000196562.14 0.476098589 1 1 0.476098589
Brain_Hypothalamus 0.476098589 Brain_Hypothalamus 1 1 1
0.712591474 0.712591474 0.712591474 NA 1 0 0.998207054
HSPA14 ENSG00000187522.14 0.476195647 1 1 0.476195647
Brain_Nucleus_accumbens_basal_ganglia 0.476195647
Brain_Nucleus_accumbens_basal_ganglia 1 1 1 0.712434679
0.712434679 0.712434679 NA 1 0 0.998207054
NT5DC1 ENSG00000178425.13 0.476212066 3 3 0.211919928
Brain_Spinal_cord_cervical_c-1 0.972230785 Brain_Caudate_basal_ganglia
1.844113113 0.466408749 0.466408749 -0.034810578 1.248303514
0.627477001 0.642561054 3 0 0.998207054
CASQ1 ENSG00000143318.12 0.476344316 3 3 0.286959494
Brain_Anterior_cingulate_cortex_BA24 0.865537006 Brain_Frontal_Cortex_BA9
2.269915412 0.175053461 0.175053461 -0.466315968 1.064815357
0.143056431 0.81196046 3 0 0.998207054
CBR3 ENSG00000159231.5 0.476362683 8 4 0.374752663 Brain_Cerebellum
0.91206978 Brain_Nucleus_accumbens_basal_ganglia 6.283334141 0.001938362
0.235163425 -0.734612727 0.887606115 0.272399591 0.492760445
4 0 0.998207054
RP11-539L10.3 ENSG00000251580.1 0.476513607 12 4 0.074603267
Brain_Caudate_basal_ganglia 0.961210622 Brain_Cortex 8.72015604
0.017858125 0.516762569 0.048634442 1.782895802 0.847813674
0.569095625 4 0 0.998207054
KCTD3 ENSG00000136636.12 0.476526488 1 1 0.476526488
Brain_Substantia_nigra 0.476526488 Brain_Substantia_nigra 1 1
1 -0.711900346 -0.711900346 -0.711900346 NA 1 0
0.998207054
MAK16 ENSG00000198042.10 0.476581828 2 2 0.412631357
Brain_Substantia_nigra 0.430613771 Brain_Caudate_basal_ganglia
1.128163504 0.871836496 0.871836496 -0.819271801 0.788141794
-0.015565003 1.136613053 2 0 0.998207054
SLC41A1 ENSG00000133065.10 0.476582247 5 4 0.096758156
Brain_Spinal_cord_cervical_c-1 0.783366407 Brain_Cerebellum 3.577403339
0.001172959 0.120498689 -1.66077743 -0.2749347 -0.676554719
0.576962808 4 0 0.998207054
LAMTOR4 ENSG00000188186.10 0.476603251 4 4 0.141441443
Brain_Cerebellar_Hemisphere 0.956778051 Brain_Cortex 2.439403284
0.11789936 0.11789936 -1.470444366 0.626587082 -0.545988047
1.050300596 4 0 0.998207054
ZNF649 ENSG00000198093.10 0.47660376 11 7 0.392722851
Brain_Hippocampus 0.985826353 Brain_Spinal_cord_cervical_c-1
7.140740856 0.042645103 0.288765384 -0.854689886 0.713891214
-0.017579255 0.527115249 7 0 0.998207054
HACD1 ENSG00000165996.13 0.47670537 3 3 0.39400655
Brain_Spinal_cord_cervical_c-1 0.794655576 Brain_Hippocampus
2.218528753 0.326167936 0.326167936 -0.852373993 0.449123378
-0.047660233 0.703270634 3 0 0.998207054
SMPD4 ENSG00000136699.19 0.476839853 1 1 0.476839853
Brain_Spinal_cord_cervical_c-1 0.476839853 Brain_Spinal_cord_cervical_c-1
1 1 1 0.711394425 0.711394425 0.711394425 NA 1 0

0.998207054
MPST ENSG00000128309.16 0.476954496 3 3 0.197333928
Brain_Caudate_basal_ganglia 0.95986418 Brain_Spinal_cord_cervical_c-1
1.472532748 0.526300631 0.526300631 -1.28918457 0.808305829
-0.177067588 1.054473538 3 0 0.998207054
RP11-1000B6.3 ENSG00000261064.1 0.477115459 5 5 0.045154472
Brain_Frontal_Cortex_BA9 0.925049948 Brain_Putamen_basal_ganglia
1.129895786 0.787885032 0.787885032 -0.62596744 2.003212616
0.398333284 0.972113765 5 0 0.998207054
FTSJ3 ENSG00000108592.16 0.477220562 13 1 0.222178579
Brain_Hippocampus 0.840970055 Brain_Substantia_nigra 12.41012936
0.002679057 12.41012936 -1.220755573 -0.200652818 -0.692553942
0.23775067 1 0 0.998207054
PLEKHG5 ENSG00000171680.21 0.477304128 5 5 0.116570403
Brain_Anterior_cingulate_cortex_BA24 0.770095008 Brain_Cortex
1.856188244 0.444518096 0.444518096 0.292250623 1.569332844
1.012554138 0.476720826 5 0 0.998207054
C18orf8 ENSG00000141452.9 0.477308721 8 3 0.115099237
Brain_Cerebellum 0.361003175 Brain_Spinal_cord_cervical_c-1 6.92339771
0.001673748 0.338871643 -1.575681439 -0.91345522 -1.385494977
0.209102437 3 0 0.998207054
GPD1L ENSG00000152642.10 0.477346592 8 6 0.304894142
Brain_Frontal_Cortex_BA9 0.972375593 Brain_Cerebellum 3.732438014
0.017015729 0.387288521 -1.025994573 0.886770725 -0.14537625
0.639548276 6 0 0.998207054
MROH8 ENSG00000101353.14 0.47736606 7 4 0.06646297 Brain_Cortex
0.596343067 Brain_Caudate_basal_ganglia 3.752205302 0.00845525
0.302698812 -1.835288393 -0.529666661 -1.206060883 0.411671297
4 0 0.998207054
SLC25A1 ENSG00000100075.9 0.477411989 3 3 0.175626239
Brain_Spinal_cord_cervical_c-1 0.97693533 Brain_Cerebellum 1.216499607
0.784621593 0.784621593 -0.820019274 1.354345302 0.168471575
1.100538653 3 0 0.998207054
UBE2S ENSG00000108106.13 0.477416368 1 1 0.477416368
Brain_Substantia_nigra 0.477416368 Brain_Substantia_nigra 1 1
1 -0.710464128 -0.710464128 -0.710464128 NA 1 0
0.998207054
ATP6V0E2 ENSG00000171130.17 0.477434914 13 9 0.073068811
Brain_Putamen_basal_ganglia 0.917742118 Brain_Hippocampus 5.132684537
0.00417984 0.243336279 -1.792400651 1.762728569 0.29388595
0.813915221 9 0 0.998207054
NPDC1 ENSG00000107281.9 0.477478011 1 1 0.477478011
Brain_Putamen_basal_ganglia 0.477478011 Brain_Putamen_basal_ganglia 1 1
1 0.710364695 0.710364695 0.710364695 NA 1 0
0.998207054
RP3-454B23.1 ENSG00000255775.1 0.477529595 1 1 0.477529595
Brain_Hippocampus 0.477529595 Brain_Hippocampus 1 1 1
-0.710281491 -0.710281491 -0.710281491 NA 1 0 0.998207054
CFAP74 ENSG00000142609.17 0.477602417 4 3 0.156163081
Brain_Anterior_cingulate_cortex_BA24 0.559596826 Brain_Amygdala
1.993657255 0.008900831 0.443213245 0.583440462 1.418094827
0.900046891 0.363302803 3 0 0.998207054
SBNO1 ENSG00000139697.13 0.477629166 2 2 0.22773798
Brain_Spinal_cord_cervical_c-1 0.93451885 Brain_Hippocampus 1.057158589
0.942841411 0.942841411 -0.082160793 1.206206236 0.562022721
0.911013063 2 0 0.998207054
TMEM74B ENSG00000125895.5 0.477647 3 3 0.164260016
Brain_Caudate_basal_ganglia 0.934588301 Brain_Nucleus_accumbens_basal_ganglia
1.084026648 0.916025091 0.916025091 -1.390885944 -0.082073456
-0.698563607 0.657693258 3 0 0.998207054
LIAS ENSG00000121897.14 0.477682645 3 3 0.292640999
Brain_Anterior_cingulate_cortex_BA24 0.539470751 Brain_Cerebellum

1.931142026 0.092031178 0.092031178 0.613613514 1.05234564
0.814405931 0.22171245 3 0 0.998207054
SHISA6 ENSG00000188803.14 0.47768335 3 3 0.250312379
Brain_Anterior_cingulate_cortex_BA24 0.681200621 Brain_Cerebellum
1.141003552 0.82858168 0.82858168 -1.014229351 1.149590969
0.182062315 1.099899532 3 0 0.998207054
SLC26A7 ENSG00000147606.8 0.477791706 1 1 0.477791706
Brain_Cortex 0.477791706 Brain_Cortex 1 1 1 0.709858793
0.709858793 0.709858793 NA 1 0 0.998207054
NID2 ENSG00000087303.17 0.478030919 2 2 0.227128888
Brain_Spinal_cord_cervical_c-1 0.672604045 Brain_Cerebellum 1.447566016
0.552433984 0.552433984 -0.422576815 1.20778782 0.392605502
1.152841889 2 0 0.998207054
TTC27 ENSG00000018699.11 0.478238711 11 6 0.241878223
Brain_Cerebellar_Hemisphere 0.94974899 Brain_Frontal_Cortex_BA9 6.987685381
0.057797612 0.293484394 -1.170305067 0.752639127 -0.339433442
0.559842726 6 0 0.998207054
PALD1 ENSG00000107719.8 0.478285271 4 4 0.091609557 Brain_Amygdala
0.98196607 Brain_Nucleus_accumbens_basal_ganglia 1.183392941 0.871059294
0.871059294 -1.686967731 -0.022604105 -0.631162109 0.747686478
4 0 0.998207054
CNGA3 ENSG00000144191.11 0.478394516 1 1 0.478394516 Brain_Cerebellum
0.478394516 Brain_Cerebellum 1 1 1 0.70888714 0.70888714 0.70888714
NA 1 0 0.998207054
ACTN2 ENSG00000077522.12 0.478416843 3 3 0.225038479
Brain_Spinal_cord_cervical_c-1 0.648445872
Brain_Nucleus_accumbens_basal_ganglia 1.059617821 0.924411066
0.924411066 -0.813025672 1.213238944 0.285378515 1.023841273
3 0 0.998207054
RP11-48G14.1 ENSG00000261823.1 0.478497794 1 1 0.478497794
Brain_Anterior_cingulate_cortex_BA24 0.478497794
Brain_Anterior_cingulate_cortex_BA24 1 1 1 -0.708720736
-0.708720736 -0.708720736 NA 1 0 0.998207054
RPAIN ENSG00000129197.14 0.478504799 1 1 0.478504799
Brain_Anterior_cingulate_cortex_BA24 0.478504799
Brain_Anterior_cingulate_cortex_BA24 1 1 1 -0.70870945
-0.70870945 -0.70870945 NA 1 0 0.998207054
AC019186.1 ENSG00000234584.1 0.478565915 2 2 0.251981129
Brain_Hippocampus 0.655759002 Brain_Cortex 1.038438231
0.961561769 0.961561769 0.44577608 1.145550645 0.795663362
0.494815341 2 0 0.998207054
KLF11 ENSG00000172059.10 0.478772331 2 2 0.235532095 Brain_Cerebellum
0.840002111 Brain_Cerebellar_Hemisphere 1.371178136 0.628821864
0.628821864 -1.186228446 -0.201890778 -0.694059612 0.69603184 2
0 0.998207054
TTC24 ENSG00000187862.11 0.478830477 1 1 0.478830477
Brain_Nucleus_accumbens_basal_ganglia 0.478830477
Brain_Nucleus_accumbens_basal_ganglia 1 1 1 0.708184844
0.708184844 0.708184844 NA 1 0 0.998207054
LINC00519 ENSG00000258955.1 0.478909694 1 1 0.478909694
Brain_Hypothalamus 0.478909694 Brain_Hypothalamus 1 1 1
-0.70805727 -0.70805727 -0.70805727 NA 1 0 0.998207054
ZNF586 ENSG00000083828.15 0.478912077 9 6 0.097320543
Brain_Substantia_nigra 0.683553974 Brain_Nucleus_accumbens_basal_ganglia
5.309924794 0.030474235 0.255145877 -0.9695021 1.657984759
-0.319511581 0.890161737 6 0 0.998207054
CACNG1 ENSG00000108878.4 0.47897278 1 1 0.47897278
Brain_Spinal_cord_cervical_c-1 0.47897278 Brain_Spinal_cord_cervical_c-1 1
1 1 0.707955681 0.707955681 0.707955681 NA 1 0
0.998207054
ATR ENSG00000175054.14 0.478992171 2 2 0.481989923
Brain_Cerebellar_Hemisphere 0.646384338 Brain_Caudate_basal_ganglia

1.535347899 0.464652101 0.464652101 -0.458790889 0.703105631
0.122157371 0.821584908 2 0 0.998207054
EPX ENSG00000121053.5 0.478996078 4 4 0.195177631
Brain_Substantia_nigra 0.801680576 Brain_Cerebellar_Hemisphere
2.017872209 0.305428232 0.305428232 -1.295413478 0.567931617
-0.354710394 0.764682259 4 0 0.998207054
LINC01993 ENSG00000204277.1 0.479009546 1 1 0.479009546
Brain_Nucleus_accumbens_basal_ganglia 0.479009546
Brain_Nucleus_accumbens_basal_ganglia 1 1 1 0.707896479
0.707896479 0.707896479 NA 1 0 0.998207054
GJD2 ENSG00000159248.4 0.479042741 1 1 0.479042741 Brain_Cerebellum
0.479042741 Brain_Cerebellum 1 1 1 0.70784303 0.70784303 0.70784303
NA 1 0 0.998207054
EBF2 ENSG00000221818.8 0.47915996 1 1 0.47915996 Brain_Putamen_basal_ganglia
0.47915996 Brain_Putamen_basal_ganglia 1 1 1 0.707654306
0.707654306 0.707654306 NA 1 0 0.998207054
ENSA ENSG00000143420.18 0.479383214 1 1 0.479383214
Brain_Caudate_basal_ganglia 0.479383214 Brain_Caudate_basal_ganglia 1 1
1 0.707294932 0.707294932 0.707294932 NA 1 0
0.998207054
CA11 ENSG00000063180.8 0.479457389 1 1 0.479457389 Brain_Cerebellum
0.479457389 Brain_Cerebellum 1 1 1 0.707175552 0.707175552
0.707175552 NA 1 0 0.998207054
RHOT1 ENSG00000126858.16 0.479629423 2 2 0.412308183
Brain_Cerebellar_Hemisphere 0.882660176 Brain_Caudate_basal_ganglia 1.64879003
0.35120997 0.35120997 -0.14759782 0.819838495 0.336120338
0.684080778 2 0 0.998207054
AC009120.11 ENSG00000275236.1 0.479649044 1 1 0.479649044
Brain_Cortex 0.479649044 Brain_Cortex 1 1 1 -0.706867143
-0.706867143 -0.706867143 NA 1 0 0.998207054
RP5-1159O4.2 ENSG00000272732.1 0.479692257 6 4 0.354659789
Brain_Nucleus_accumbens_basal_ganglia 0.81331617 Brain_Putamen_basal_ganglia
4.493886841 0.023054829 0.22992418 -0.236150137 0.925588659
0.493195942 0.422058988 4 0 0.998207054
OCIAD1 ENSG00000109180.14 0.479720121 7 5 0.05837688
Brain_Hypothalamus 0.956431506 Brain_Cerebellum 4.033744485
0.089607982 0.194891595 -1.892857068 -0.054632173 -0.673540453
0.630785396 5 0 0.998207054
TMEM39B ENSG00000121775.17 0.479720545 1 1 0.479720545
Brain_Cerebellum 0.479720545 Brain_Cerebellum 1 1 1 -0.706752101
-0.706752101 -0.706752101 NA 1 0 0.998207054
ACO2 ENSG00000100412.15 0.479772033 1 1 0.479772033 Brain_Cerebellum
0.479772033 Brain_Cerebellum 1 1 1 -0.706669266 -0.706669266
-0.706669266 NA 1 0 0.998207054
ODF2 ENSG00000136811.16 0.47988924 8 5 0.130745159
Brain_Caudate_basal_ganglia 0.763319115 Brain_Substantia_nigra
4.450967683 0.024034402 0.299813289 -1.511169942 1.011822623
-0.342035712 0.840485916 5 0 0.998207054
ATIC ENSG00000138363.14 0.479978527 13 2 0.24249807
Brain_Putamen_basal_ganglia 0.797515153 Brain_Hippocampus 10.6683932
0.00358462 1.055880014 -1.168765633 0.329235865 -0.55722257
0.492251249 2 0 0.998207054
WDR77 ENSG00000116455.13 0.480158298 2 2 0.331243428 Brain_Cortex
0.478615226 Brain_Hypothalamus 1.015174972 0.984825028
0.984825028 0.708531552 0.971612366 0.840071959 0.186026228
2 0 0.998207054
FUZ ENSG00000010361.13 0.480339905 4 4 0.481224747 Brain_Cortex
0.935602433 Brain_Cerebellum 2.28902123 0.115924492 0.115924492
-0.151070687 0.70433408 0.227564974 0.412293641 4 0
0.998207054
ZNHIT3 ENSG00000273611.4 0.480494733 1 1 0.480494733
Brain_Spinal_cord_cervical_c-1 0.480494733 Brain_Spinal_cord_cervical_c-1

1 1 1 0.705507071 0.705507071 0.705507071 NA 1 0
0.998207054
HTR1B ENSG00000135312.6 0.480523489 3 3 0.166106641
Brain_Anterior_cingulate_cortex_BA24 0.566066818 Brain_Amygdala
1.711480827 0.269360516 0.269360516 0.573853683 1.384822852
0.96855069 0.405914848 3 0 0.998207054
CD274 ENSG00000120217.13 0.480549581 5 3 0.210662395
Brain_Caudate_basal_ganglia 0.971219217 Brain_Cortex 3.739184955
0.004667479 0.439694672 -0.065978717 1.251746104 0.259672051
0.561000002 3 0 0.998207054
CCDC8 ENSG00000169515.5 0.480590846 11 2 0.0928072 Brain_Cerebellum
0.979312585 Brain_Frontal_Cortex_BA9 9.39498945 5.86E-05 0.813272996
-0.025930735 1.68077205 0.882213951 0.542672891 2 0
0.998207054
CLVS2 ENSG00000146352.12 0.480628593 4 3 0.166688967
Brain_Caudate_basal_ganglia 0.673764551 Brain_Anterior_cingulate_cortex_BA24
2.568244892 0.054752201 0.396679323 -0.723493604 1.382921404
0.520393357 0.918735768 3 0 0.998207054
NXPE3 ENSG00000144815.15 0.480710283 1 1 0.480710283 Brain_Cortex
0.480710283 Brain_Cortex 1 1 1 0.705160624 0.705160624
0.705160624 NA 1 0 0.998207054
SRGAP3-AS4 ENSG00000235830.1 0.480735041 1 1 0.480735041
Brain_Hippocampus 0.480735041 Brain_Hippocampus 1 1 1
0.705120837 0.705120837 0.705120837 NA 1 0 0.998207054
RASGRP1 ENSG00000172575.11 0.480763275 1 1 0.480763275
Brain_Caudate_basal_ganglia 0.480763275 Brain_Caudate_basal_ganglia 1 1
1 -0.705075464 -0.705075464 -0.705075464 NA 1 0
0.998207054
AP001372.2 ENSG00000254837.2 0.480791403 1 1 0.480791403
Brain_Hypothalamus 0.480791403 Brain_Hypothalamus 1 1 1
-0.705030262 -0.705030262 -0.705030262 NA 1 0 0.998207054
MDGA2 ENSG00000139915.18 0.480807519 4 4 0.199771676
Brain_Cerebellar_Hemisphere 0.65597315 Brain_Caudate_basal_ganglia 1.49798024
0.635734738 0.635734738 -1.28220234 1.122448949 -0.375604936
1.055622138 4 0 0.998207054
ZNF582-AS1 ENSG00000267454.5 0.480975461 12 9 0.085671035
Brain_Anterior_cingulate_cortex_BA24 0.844195784
Brain_Nucleus_accumbens_basal_ganglia 4.833328857 0.005766133
0.573668855 -1.449452684 1.718688898 -0.291201299 0.957284832
9 0 0.998207054
SORBS2 ENSG00000154556.17 0.481000468 1 1 0.481000468
Brain_Frontal_Cortex_BA9 0.481000468 Brain_Frontal_Cortex_BA9 1 1
1 -0.70469435 -0.70469435 -0.70469435 NA 1 0
0.998207054
EIF2B4 ENSG00000115211.15 0.481125812 2 2 0.261210531
Brain_Spinal_cord_cervical_c-1 0.58485716 Brain_Hypothalamus 1.088855622
0.911144378 0.911144378 -1.12353456 0.546303748 -0.288615406
1.180753991 2 0 0.998207054
MPP4 ENSG00000082126.17 0.481245992 4 4 0.197799872
Brain_Hypothalamus 0.715812955 Brain_Caudate_basal_ganglia 1.455985032
0.544160971 0.544160971 -1.28784515 0.47405994 -0.269990578
0.840919628 4 0 0.998207054
TEX29 ENSG00000153495.10 0.48130193 7 7 0.197001382 Brain_Cerebellum
0.710117672 Brain_Putamen_basal_ganglia 2.331474081 0.329257193
0.329257193 -0.959656122 1.290141935 0.0606669 0.813987889 7
0 0.998207054
RP11-126K15.1 ENSG00000265380.1 0.481313942 1 1 0.481313942
Brain_Nucleus_accumbens_basal_ganglia 0.481313942
Brain_Nucleus_accumbens_basal_ganglia 1 1 1 0.704190828
0.704190828 0.704190828 NA 1 0 0.998207054
FAM171A2 ENSG00000161682.14 0.481363879 2 2 0.233285404
Brain_Frontal_Cortex_BA9 0.751993744 Brain_Substantia_nigra

1.094888942 0.905111058 0.905111058 -1.191938389 0.316011547 -0.437963421 1.066281625 2 0 0.998207054
CAPN9 ENSG00000135773.12 0.481452342 12 6 0.06705067 Brain_Cortex 0.972859522 Brain_Cerebellar_Hemisphere 7.093633808 0.002396023 0.293962999 -1.831334239 -0.034022108 -1.247246767 0.538341119 6 0 0.998207054
MYO5A ENSG00000197535.14 0.481470164 7 3 0.132597066 Brain_Cortex 0.486590906 Brain_Putamen_basal_ganglia 5.845751899 0.012262853 0.375982318 -1.503939031 -0.69574122 -1.250550275 0.270630214 3 0 0.998207054
RP11-554D14.7 ENSG00000257141.1 0.481504391 1 1 0.481504391 Brain_Hypothalamus 0.481504391 Brain_Hypothalamus 1 1 1 0.703885005 0.703885005 0.703885005 NA 1 0 0.998207054
RP11-153F10.2 ENSG00000282860.1 0.481607894 6 4 0.259443691 Brain_Cortex 0.976281305 Brain_Spinal_cord_cervical_c-1 4.257788414 0.023471779 0.314342993 -1.127706975 0.193529531 -0.361470793 0.475889886 4 0 0.998207054
STARD10 ENSG00000214530.8 0.481639841 13 3 0.160060185 Brain_Hypothalamus 0.961051529 Brain_Cerebellum 10.72026354 0.004026873 0.417031663 -1.404869174 0.19671434 -0.886698341 0.467881656 3 0 0.998207054
RNMT ENSG00000101654.17 0.481676287 3 3 0.575907319 Brain_Cerebellar_Hemisphere 0.945152008 Brain_Cerebellum 2.181380568 0.127128863 0.127128863 -0.264252505 0.559372802 0.075441435 0.430340549 3 0 0.998207054
FAF1 ENSG00000185104.19 0.481768684 1 1 0.481768684 Brain_Frontal_Cortex_BA9 0.481768684 Brain_Frontal_Cortex_BA9 1 1 1 0.70346071 0.70346071 0.70346071 NA 1 0 0.998207054
LETM1 ENSG00000168924.14 0.481884618 1 1 0.481884618 Brain_Cerebellum 0.481884618 Brain_Cerebellum 1 1 1 0.703274629 0.703274629 0.703274629 NA 1 0 0.998207054
GNB2 ENSG00000172354.9 0.481912928 2 2 0.263386223 Brain_Cerebellum 0.788423506 Brain_Cerebellar_Hemisphere 1.574581225 0.425418775 0.425418775 0.268358337 1.118423359 0.693390848 0.601086742 2 0 0.998207054
RP11-722E23.2 ENSG00000272338.2 0.481927065 4 4 0.113015452 Brain_Cerebellar_Hemisphere 0.530387233 Brain_Cerebellum 3.171261258 0.176289096 0.176289096 -1.584783854 -0.627415007 -0.914937342 0.449762351 4 0 0.998207054
OTULIN ENSG00000154124.4 0.482080987 1 1 0.482080987 Brain_Hippocampus 0.482080987 Brain_Hippocampus 1 1 1 0.702959503 0.702959503 0.702959503 NA 1 0 0.998207054
GUCY1A2 ENSG00000152402.10 0.482151995 3 3 0.374730181 Brain_Cerebellar_Hemisphere 0.650199458 Brain_Spinal_cord_cervical_c-1 1.542338538 0.489745146 0.489745146 -0.540857891 0.887647896 -0.035565037 0.800718484 3 0 0.998207054
LAMA4 ENSG00000112769.18 0.482198758 1 1 0.482198758 Brain_Frontal_Cortex_BA9 0.482198758 Brain_Frontal_Cortex_BA9 1 1 1 0.702770543 0.702770543 0.702770543 NA 1 0 0.998207054
ANKDD1B ENSG00000189045.13 0.4823345 1 1 0.4823345 Brain_Cerebellum 0.4823345 Brain_Cerebellum 1 1 1 0.702552778 0.702552778 0.702552778 NA 1 0 0.998207054
SLC20A1 ENSG00000144136.10 0.482424779 1 1 0.482424779 Brain_Cerebellar_Hemisphere 0.482424779 Brain_Cerebellar_Hemisphere 1 1 1 -0.702407966 -0.702407966 -0.702407966 NA 1 0 0.998207054
LINC00581 ENSG00000280989.1 0.482450202 1 1 0.482450202 Brain_Putamen_basal_ganglia 0.482450202 Brain_Putamen_basal_ganglia 1 1 1 -0.702367189 -0.702367189 -0.702367189 NA 1 0 0.998207054
C3orf33 ENSG00000174928.15 0.482512015 2 2 0.233208898

Brain_Cerebellar_Hemisphere 0.843438952 Brain_Cerebellum 1.317263493
0.682736507 0.682736507 -1.192133513 -0.197496587 -0.69481505
0.703314515 2 0 0.998207054
SLAMF6 ENSG00000162739.13 0.482555738 2 2 0.314314077
Brain_Nucleus_accumbens_basal_ganglia 0.553763421 Brain_Cortex
1.072633117 0.927366883 0.927366883 -1.006211009 0.592130176
-0.207040417 1.130197891 2 0 0.998207054
SLC7A5 ENSG00000103257.8 0.482603276 1 1 0.482603276
Brain_Anterior_cingulate_cortex_BA24 0.482603276
Brain_Anterior_cingulate_cortex_BA24 1 1 1 -0.702121691
-0.702121691 -0.702121691 NA 1 0 0.998207054
SPTAN1 ENSG00000197694.15 0.482695098 1 1 0.482695098
Brain_Frontal_Cortex_BA9 0.482695098 Brain_Frontal_Cortex_BA9 1 1
1 -0.70197445 -0.70197445 -0.70197445 NA 1 0
0.998207054
SPATA41 ENSG00000189419.6 0.483029706 3 3 0.146880567
Brain_Substantia_nigra 0.925858744 Brain_Cerebellum 1.791262359
0.225030411 0.225030411 0.093056414 1.450638434 0.832304002
0.686820376 3 0 0.998207054
CMTR1 ENSG00000137200.12 0.483079686 2 2 0.230526295 Brain_Cerebellum
0.852254725 Brain_Caudate_basal_ganglia 1.044997661 0.955002339
0.955002339 -1.199004244 -0.186242336 -0.69262329 0.716130813
2 0 0.998207054
C9orf64 ENSG00000165118.14 0.483092733 2 2 0.23106403
Brain_Nucleus_accumbens_basal_ganglia 0.957887604 Brain_Cerebellar_Hemisphere
1.07596644 0.92403356 0.92403356 0.052804591 1.197622457 0.625213524
0.809508476 2 0 0.998207054
EPB41L4B ENSG00000095203.14 0.48309986 2 2 0.261411279 Brain_Cerebellum
0.664919987 Brain_Putamen_basal_ganglia 1.006307415 0.993692585
0.993692585 -0.433130471 1.123061727 0.344965628 1.100394056
2 0 0.998207054
CCL21 ENSG00000137077.7 0.483164924 1 1 0.483164924 Brain_Cerebellum
0.483164924 Brain_Cerebellum 1 1 1 0.701221292 0.701221292
0.701221292 NA 1 0 0.998207054
SPON2 ENSG00000159674.11 0.483428722 7 7 0.315808519 Brain_Cerebellum
0.965084714 Brain_Cortex 3.129380348 0.116016384 0.116016384
-0.653962295 1.003108491 0.314407728 0.726959853 7 0
0.998207054
HIST1H4H ENSG00000158406.4 0.483442825 1 1 0.483442825
Brain_Hypothalamus 0.483442825 Brain_Hypothalamus 1 1 1
0.70077599 0.70077599 0.70077599 NA 1 0 0.998207054
NUTM2B ENSG00000188199.10 0.48346008 13 13 0.066253388 Brain_Amygdala
0.907485936 Brain_Frontal_Cortex_BA9 3.101100654 0.134935035
0.134935035 -1.836705467 1.797463769 -0.481378286 0.89444063 13
0 0.998207054
N6AMT1 ENSG00000156239.11 0.483461817 10 5 0.191811091
Brain_Substantia_nigra 0.976097556 Brain_Cortex 7.304340522
0.004204428 0.260523553 -0.394587331 1.305240137 0.207282766
0.611500663 5 0 0.998207054
RP4-555D20.3 ENSG00000271192.1 0.483486695 1 1 0.483486695
Brain_Anterior_cingulate_cortex_BA24 0.483486695
Brain_Anterior_cingulate_cortex_BA24 1 1 1 0.700705706
0.700705706 0.700705706 NA 1 0 0.998207054
LIG1 ENSG00000105486.13 0.483589959 9 4 0.143517165
Brain_Nucleus_accumbens_basal_ganglia 0.743121148 Brain_Cerebellum
6.526798003 0.028143712 0.303568838 0.327722952 1.462818079
0.962039277 0.449990342 4 0 0.998207054
P2RX2 ENSG00000187848.12 0.483716831 5 4 0.444174277 Brain_Cortex
0.93324513 Brain_Frontal_Cortex_BA9 3.984107673 0.037299763
0.168754876 -0.444310945 0.765163356 0.206684324 0.507111782
4 0 0.998207054
SOS1 ENSG00000115904.12 0.483787835 2 2 0.238938238

Brain_Hypothalamus 0.811399288 Brain_Cerebellum 1.014459617
0.985540383 0.985540383 -0.238621247 1.17764481 0.469511781
1.001451333 2 0 0.998207054
PODN ENSG00000174348.13 0.483797016 4 4 0.345612795
Brain_Caudate_basal_ganglia 0.890287889 Brain_Cerebellar_Hemisphere
2.211195222 0.240231769 0.240231769 -0.943133162 0.141150544
-0.327409779 0.552707627 4 0 0.998207054
CABYR ENSG00000154040.20 0.483928983 9 7 0.190144504
Brain_Nucleus_accumbens_basal_ganglia 0.637584534 Brain_Putamen_basal_ganglia
3.957913185 0.021471327 0.451795583 -1.310151751 0.672325061
-0.726878243 0.574944268 7 0 0.998207054
CTD-2192J16.11 ENSG00000230310.1 0.483933756 5 5 0.061347881
Brain_Nucleus_accumbens_basal_ganglia 0.627842524 Brain_Hypothalamus
2.064271459 0.374467486 0.374467486 0.484765746 1.870979799
1.083365403 0.592850076 5 0 0.998207054
GUCA1C ENSG00000138472.10 0.483967761 2 2 0.24709384 Brain_Cortex
0.544619098 Brain_Frontal_Cortex_BA9 1.723016711 0.276983289
0.276983289 -1.15743706 -0.605842871 -0.881639966 0.390035991
2 0 0.998207054
FEZ1 ENSG00000149557.12 0.484163904 3 3 0.214782956 Brain_Cortex
0.563768366 Brain_Cerebellum 1.942035851 0.086663675 0.086663675
-1.240520449 -0.577253284 -0.903503224 0.331764651 3 0
0.998207054
RP11-118B22.4 ENSG00000256427.1 0.484203487 13 6 0.250833654
Brain_Cerebellar_Hemisphere 0.987493816 Brain_Putamen_basal_ganglia
8.808488708 0.005604174 0.356086226 -1.148326863 0.968333123
0.063900652 0.625265094 6 0 0.998207054
CEP170B ENSG00000099814.15 0.484346149 3 3 0.126002587
Brain_Hypothalamus 0.96823154 Brain_Substantia_nigra 1.273650477
0.786290779 0.786290779 -0.161165437 1.530057134 0.469572694
0.923888448 3 0 0.998207054
MCOLN2 ENSG00000153898.12 0.484356241 9 2 0.22087287
Brain_Hypothalamus 0.906691113 Brain_Nucleus_accumbens_basal_ganglia
7.894245118 0.012620978 0.585039291 -0.233489055 1.224210392
0.292496358 0.432136497 2 0 0.998207054
TNK2 ENSG00000061938.16 0.484410906 3 3 0.129917042 Brain_Cerebellum
0.939785919 Brain_Nucleus_accumbens_basal_ganglia 1.265783108
0.738751591 0.738751591 -1.514429106 -0.075538937 -0.777988793
0.720047042 3 0 0.998207054
SH3RF3 ENSG00000172985.10 0.484506216 1 1 0.484506216
Brain_Cerebellum 0.484506216 Brain_Cerebellum 1 1 1 -0.699073312
-0.699073312 -0.699073312 NA 1 0 0.998207054
MDM4 ENSG00000198625.12 0.484570013 1 1 0.484570013 Brain_Cortex
0.484570013 Brain_Cortex 1 1 1 0.698971226 0.698971226
0.698971226 NA 1 0 0.998207054
CLEC19A ENSG00000261210.7 0.484593099 5 3 0.170903512
Brain_Substantia_nigra 0.808596231 Brain_Spinal_cord_cervical_c-1
3.297330017 0.025284546 0.658130423 -1.36930642 -0.242237381
-0.585424065 0.476317207 3 0 0.998207054
PPP2R5E ENSG00000154001.13 0.484778695 1 1 0.484778695
Brain_Putamen_basal_ganglia 0.484778695 Brain_Putamen_basal_ganglia 1 1
1 -0.698637351 -0.698637351 -0.698637351 NA 1 0
0.998207054
OPN5 ENSG00000124818.15 0.484822405 1 1 0.484822405
Brain_Frontal_Cortex_BA9 0.484822405 Brain_Frontal_Cortex_BA9 1 1
1 0.698567428 0.698567428 0.698567428 NA 1 0
0.998207054
ASB4 ENSG00000005981.12 0.484909271 4 4 0.366632538
Brain_Hypothalamus 0.665301494 Brain_Caudate_basal_ganglia 2.296988488
0.215970315 0.215970315 -0.902799083 0.845782061 0.226956742
0.773472987 4 0 0.998207054
PHF20 ENSG00000025293.16 0.484944673 1 1 0.484944673

Brain_Cerebellar_Hemisphere 0.484944673 Brain_Cerebellar_Hemisphere 1 1
1 0.698371855 0.698371855 0.698371855 NA 1 0
0.998207054
RALBP1 ENSG00000017797.12 0.485011892 1 1 0.485011892
Brain_Cerebellum 0.485011892 Brain_Cerebellum 1 1 1 0.698264346
0.698264346 0.698264346 NA 1 0 0.998207054
CFAP221 ENSG00000163075.12 0.485088489 4 4 0.183272938
Brain_Cerebellar_Hemisphere 0.734950565 Brain_Frontal_Cortex_BA9
1.242855521 0.813695399 0.813695399 -0.409169199 1.330744977
0.600103797 0.798474223 4 0 0.998207054
RAD54L2 ENSG00000164080.13 0.485093984 4 3 0.170840707
Brain_Caudate_basal_ganglia 0.972717833 Brain_Hippocampus 2.279971056
0.027776283 0.680012082 -0.034199792 1.369507453 0.75512919
0.582189984 3 0 0.998207054
WSCD1 ENSG00000179314.13 0.485261813 2 2 0.430256469
Brain_Cerebellar_Hemisphere 0.925781119 Brain_Cerebellum 1.803389047
0.196610953 0.196610953 -0.788752855 -0.093154125 -0.44095349
0.491862579 2 0 0.998207054
DNAJC16 ENSG00000116138.12 0.485343992 5 5 0.203775277
Brain_Nucleus_accumbens_basal_ganglia 0.934727846 Brain_Cerebellar_Hemisphere
3.383325574 0.118567646 0.118567646 -1.270868943 0.089428588
-0.542762724 0.590170267 5 0 0.998207054
SPDYE3 ENSG00000214300.7 0.485376948 2 2 0.259228424
Brain_Nucleus_accumbens_basal_ganglia 0.59457717 Brain_Cerebellar_Hemisphere
1.105345344 0.894654656 0.894654656 0.532214892 1.128216674
0.830215783 0.421436902 2 0 0.998207054
EXD1 ENSG00000178997.11 0.48549764 1 1 0.48549764 Brain_Cortex 0.48549764
Brain_Cortex 1 1 1 -0.69748769 -0.69748769 -0.69748769
NA 1 0 0.998207054
RP11-444D3.1 ENSG00000255864.5 0.48565584 2 2 0.230149914
Brain_Caudate_basal_ganglia 0.910193805 Brain_Hippocampus 1.037667556
0.962332444 0.962332444 -1.199972775 -0.112794089 -0.656383432
0.768751421 2 0 0.998207054
FGA ENSG00000171560.14 0.485904327 2 2 0.246576423
Brain_Anterior_cingulate_cortex_BA24 0.817230585 Brain_Hippocampus
1.073922033 0.926077967 0.926077967 -1.158705071 0.231108405
-0.463798333 0.982746534 2 0 0.998207054
OSTN ENSG00000188729.6 0.485940151 4 4 0.384946926
Brain_Nucleus_accumbens_basal_ganglia 0.801556501 Brain_Putamen_basal_ganglia
1.762511414 0.240416813 0.240416813 -0.868817561 0.25133321
-0.52421434 0.529954313 4 0 0.998207054
TSPAN10 ENSG00000182612.10 0.486062886 5 3 0.153165147
Brain_Frontal_Cortex_BA9 0.503985911 Brain_Nucleus_accumbens_basal_ganglia
4.296179219 0.044585025 0.165694907 0.668231374 1.428440367
1.169636414 0.309455514 3 0 0.998207054
COX14 ENSG00000178449.8 0.486089788 7 5 0.112938802 Brain_Cortex
0.857560053 Brain_Substantia_nigra 5.071371286 0.057491757
0.222292416 -1.585121192 -0.179480977 -0.766778523 0.544430657
5 0 0.998207054
KCTD13 ENSG00000174943.9 0.48609726 2 2 0.231210833
Brain_Frontal_Cortex_BA9 0.790217132 Brain_Cortex 1.299046758
0.700953242 0.700953242 -1.197245622 -0.266028666 -0.731637144
0.658469825 2 0 0.998207054
FUT3 ENSG00000171124.12 0.486120023 2 2 0.303585233
Brain_Cerebellar_Hemisphere 0.603649822 Brain_Spinal_cord_cervical_c-1
1.095146306 0.904853694 0.904853694 -1.028775391 0.519159066
-0.254808163 1.094554951 2 0 0.998207054
ADGRL2 ENSG00000117114.19 0.486134748 2 2 0.231523722
Brain_Hippocampus 0.863580474 Brain_Cerebellum 1.055833497
0.944166503 0.944166503 -1.196443019 -0.171818178 -0.684130599
0.724519173 2 0 0.998207054
CRKL ENSG00000099942.12 0.486190774 1 1 0.486190774

Brain_Caudate_basal_ganglia 0.486190774 Brain_Caudate_basal_ganglia 1 1
1 0.696380177 0.696380177 0.696380177 NA 1 0
0.998207054
CASS4 ENSG00000087589.16 0.486266167 2 2 0.231129433
Brain_Cerebellar_Hemisphere 0.987755718 Brain_Cortex 1.062201987
0.937798013 0.937798013 -0.015346534 1.197454548 0.591054007
0.857579869 2 0 0.998207054
VSTM2B ENSG00000187135.7 0.486328581 2 2 0.229894158
Brain_Cerebellum 0.263179353 Brain_Cerebellar_Hemisphere 1.927249996
0.072750004 0.072750004 -1.200631547 -1.118908086 -1.159769816
0.057787214 2 0 0.998207054
COPA ENSG00000122218.14 0.486421939 12 4 0.079090845 Brain_Cortex
0.548183591 Brain_Substantia_nigra 9.620482043 0.00140203
0.410609624 -1.755985729 -0.600484194 -0.98897553 0.344488484
4 0 0.998207054
WDR73 ENSG00000177082.12 0.486500015 7 6 0.067082841
Brain_Nucleus_accumbens_basal_ganglia 0.957332005 Brain_Cerebellum
3.272114029 0.019817424 0.362569632 -1.83111861 0.682456944
-0.642532518 0.893814039 6 0 0.998207054
KLHL33 ENSG00000185271.7 0.486518537 2 2 0.238905144
Brain_Nucleus_accumbens_basal_ganglia 0.680093393 Brain_Caudate_basal_ganglia
1.155343568 0.844656432 0.844656432 0.41233569 1.177727791
0.79503174 0.541213945 2 0 0.998207054
SLC25A30-AS1 ENSG00000251015.1 0.486678145 2 2 0.242325745
Brain_Cerebellar_Hemisphere 0.462725237 Brain_Caudate_basal_ganglia
1.774552949 0.225447051 0.225447051 -1.169193336 -0.734366765
-0.951780051 0.307468817 2 0 0.998207054
PGD ENSG00000142657.20 0.48678761 2 2 0.446697274 Brain_Amygdala
0.500807454 Brain_Nucleus_accumbens_basal_ganglia 1.28427467 0.71572533
0.71572533 -0.673219819 0.760932687 0.043856434 1.014098962 2
0 0.998207054
EFCAB12 ENSG00000172771.11 0.486799056 3 3 0.21774545 Brain_Cerebellum
0.914234177 Brain_Cerebellar_Hemisphere 1.300759203 0.68048735 0.68048735
-0.10769936 1.232545315 0.723277736 0.725731843 3 0
0.998207054
RP11-107N15.1 ENSG00000273209.1 0.48688019 3 3 0.253450913
Brain_Cerebellar_Hemisphere 0.623999135 Brain_Cerebellum 1.617976703
0.584291727 0.584291727 -0.590504107 1.142007475 0.347231274
0.875058336 3 0 0.998207054
CCDC62 ENSG00000130783.13 0.487103856 2 2 0.382800398
Brain_Amygdala 0.440121348 Brain_Hippocampus 1.05661556 0.94338444
0.94338444 -0.771988316 0.872748092 0.050379888 1.163004267 2
0 0.998207054
SMIM13 ENSG00000224531.5 0.487148118 2 2 0.384059568
Brain_Cerebellar_Hemisphere 0.405673052 Brain_Hippocampus 1.007473868
0.992526132 0.992526132 -0.870440781 -0.831532189 -0.850986485
0.027512529 2 0 0.998207054
ABCA8 ENSG00000141338.13 0.48719356 3 3 0.196400204
Brain_Spinal_cord_cervical_c-1 0.424006737 Brain_Putamen_basal_ganglia
2.480174337 0.090028148 0.090028148 0.79948932 1.291875671
1.108396673 0.26909159 3 0 0.998207054
MROH6 ENSG00000204839.8 0.487225663 7 4 0.398496668
Brain_Putamen_basal_ganglia 0.881375866 Brain_Cortex 5.590292752
0.001502513 0.251482441 -0.844309161 0.815719078 0.017939822
0.568961848 4 0 0.998207054
FECH ENSG00000066926.10 0.487239033 5 5 0.129595636
Brain_Frontal_Cortex_BA9 0.74503807 Brain_Nucleus_accumbens_basal_ganglia
1.666875 0.35902106 0.35902106 -0.623870755 1.515698388 0.570398855
0.791009643 5 0 0.998207054
PPP3CB ENSG00000107758.15 0.487243676 1 1 0.487243676
Brain_Caudate_basal_ganglia 0.487243676 Brain_Caudate_basal_ganglia 1 1
1 -0.694699445 -0.694699445 -0.694699445 NA 1 0

0.998207054
PCDHA2 ENSG00000204969.6 0.48742349 5 5 0.2863169
Brain_Frontal_Cortex_BA9 0.993972522 Brain_Putamen_basal_ganglia
3.311225633 0.115447144 0.115447144 -0.129361492 1.06623616
0.14742045 0.516423774 5 0 0.998207054
PRRT4 ENSG00000224940.8 0.487501418 1 1 0.487501418
Brain_Putamen_basal_ganglia 0.487501418 Brain_Putamen_basal_ganglia 1 1
1 -0.694288313 -0.694288313 -0.694288313 NA 1 0
0.998207054
TEKT2 ENSG00000092850.11 0.48750333 1 1 0.48750333
Brain_Anterior_cingulate_cortex_BA24 0.48750333
Brain_Anterior_cingulate_cortex_BA24 1 1 1 0.694285265
0.694285265 0.694285265 NA 1 0 0.998207054
RSL24D1 ENSG00000137876.9 0.487541478 2 2 0.288523947
Brain_Amygdala 0.520393071 Brain_Cerebellum 1.08256172 0.91743828 0.91743828
0.642739616 1.061365241 0.852052428 0.296013018 2 0
0.998207054
MLX ENSG00000108788.11 0.487669927 1 1 0.487669927
Brain_Nucleus_accumbens_basal_ganglia 0.487669927
Brain_Nucleus_accumbens_basal_ganglia 1 1 1 -0.694019585
-0.694019585 -0.694019585 NA 1 0 0.998207054
C11orf49 ENSG00000149179.13 0.487781562 1 1 0.487781562
Brain_Cerebellum 0.487781562 Brain_Cerebellum 1 1 1 0.693841582
0.693841582 0.693841582 NA 1 0 0.998207054
CTC-444N24.8 ENSG00000268713.1 0.48779695 1 1 0.48779695
Brain_Putamen_basal_ganglia 0.48779695 Brain_Putamen_basal_ganglia 1 1 1
-0.693817048 -0.693817048 -0.693817048 NA 1 0 0.998207054
SCO2 ENSG00000130489.14 0.488069787 3 3 0.144841988
Brain_Substantia_nigra 0.955229621 Brain_Hippocampus 1.327455533
0.68289661 0.68289661 -0.056140826 1.457994759 0.488729724
0.841566784 3 0 0.998207054
ZRSR1 ENSG00000212643.2 0.488298035 1 1 0.488298035 Brain_Cerebellum
0.488298035 Brain_Cerebellum 1 1 1 0.693018351 0.693018351
0.693018351 NA 1 0 0.998207054
IGFALS ENSG00000099769.5 0.488386704 3 2 0.251642212
Brain_Cerebellum 0.330143005 Brain_Frontal_Cortex_BA9 2.335445169
0.04049918 0.624055651 0.973825871 1.146369707 1.04664351
0.089363844 2 0 0.998207054
UTP23 ENSG00000147679.11 0.48848317 6 2 0.216784549
Brain_Frontal_Cortex_BA9 0.907731649 Brain_Cortex 4.777871253
1.10E-05 1.006285974 0.115900185 1.235123489 0.635361914
0.428639095 2 0 0.998207054
RIC1 ENSG00000107036.11 0.48850037 2 2 0.231519742 Brain_Cerebellum
0.277972289 Brain_Cerebellar_Hemisphere 1.893036572 0.106963428
0.106963428 -1.196453225 -1.084885684 -1.140669454 0.078890165
2 0 0.998207054
CHST5 ENSG00000135702.14 0.488530703 4 4 0.073441935
Brain_Cerebellar_Hemisphere 0.259381524 Brain_Cerebellum 3.223113817
0.15323999 0.15323999 -1.790074504 -1.127854142 -1.410006141
0.277451176 4 0 0.998207054
FBL ENSG00000105202.8 0.488792559 1 1 0.488792559 Brain_Cerebellum
0.488792559 Brain_Cerebellum 1 1 1 0.692230546 0.692230546
0.692230546 NA 1 0 0.998207054
SSTR5 ENSG00000162009.8 0.488807357 1 1 0.488807357
Brain_Hypothalamus 0.488807357 Brain_Hypothalamus 1 1 1
0.692206979 0.692206979 0.692206979 NA 1 0 0.998207054
LARS2 ENSG00000011376.9 0.488820459 13 4 0.017614637
Brain_Anterior_cingulate_cortex_BA24 0.942831856
Brain_Spinal_cord_cervical_c-1 10.55686658 0.00111007 0.46329186
0.071711057 2.373620653 1.427923726 0.796218432 4 0
0.998207054
ITCH ENSG00000078747.14 0.488875927 1 1 0.488875927 Brain_Cortex

0.488875927 Brain_Cortex 1 1 1 -0.692097778 -0.692097778
-0.692097778 NA 1 0 0.998207054
MTHFD1 ENSG00000100714.15 0.488915315 3 3 0.181285869
Brain_Cerebellar_Hemisphere 0.877462049 Brain_Hypothalamus 1.126360048
0.843544549 0.843544549 -1.336806311 0.154187309 -0.695244832
0.766924476 3 0 0.998207054
UBTD2 ENSG00000168246.5 0.488959008 1 1 0.488959008
Brain_Nucleus_accumbens_basal_ganglia 0.488959008
Brain_Nucleus_accumbens_basal_ganglia 1 1 1 -0.691965479
-0.691965479 -0.691965479 NA 1 0 0.998207054
HNRNPDL ENSG00000152795.17 0.489104176 1 1 0.489104176
Brain_Nucleus_accumbens_basal_ganglia 0.489104176
Brain_Nucleus_accumbens_basal_ganglia 1 1 1 -0.691734343
-0.691734343 -0.691734343 NA 1 0 0.998207054
LRRC37B ENSG00000185158.12 0.489186635 5 4 0.283209647
Brain_Nucleus_accumbens_basal_ganglia 0.982595994 Brain_Amygdala
3.456528493 0.010231558 0.117483382 -1.07313701 -0.021814416
-0.435195419 0.450817222 4 0 0.998207054
NAGPA ENSG00000103174.11 0.489235462 6 6 0.120921937
Brain_Anterior_cingulate_cortex_BA24 0.646813268 Brain_Putamen_basal_ganglia
1.717322117 0.299024264 0.299024264 -1.550915492 1.324773566
-0.042510816 1.022474794 6 0 0.998207054
IL15RA ENSG00000134470.20 0.489244809 1 1 0.489244809
Brain_Nucleus_accumbens_basal_ganglia 0.489244809
Brain_Nucleus_accumbens_basal_ganglia 1 1 1 -0.691510461
-0.691510461 -0.691510461 NA 1 0 0.998207054
DGKH ENSG00000102780.16 0.489260598 2 1 0.485219929
Brain_Cerebellar_Hemisphere 0.498078092 Brain_Cerebellum 1.978302659
0.021697341 1.978302659 0.677516835 0.697931667 0.687724251
0.014435466 1 0 0.998207054
LINC02021 ENSG00000249846.6 0.489281157 11 4 0.192429293
Brain_Spinal_cord_cervical_c-1 0.989674368 Brain_Cerebellar_Hemisphere
8.287052312 0.006534983 0.276565206 -1.30342621 0.86425561
-0.616402559 0.630054854 4 0 0.998207054
RP11-428J1.5 ENSG00000272142.1 0.489313591 1 1 0.489313591
Brain_Hypothalamus 0.489313591 Brain_Hypothalamus 1 1 1
0.691400975 0.691400975 0.691400975 NA 1 0 0.998207054
GK5 ENSG00000175066.15 0.489354333 1 1 0.489354333
Brain_Nucleus_accumbens_basal_ganglia 0.489354333
Brain_Nucleus_accumbens_basal_ganglia 1 1 1 0.691336128
0.691336128 0.691336128 NA 1 0 0.998207054
C19orf71 ENSG00000183397.5 0.489433181 8 5 0.368677455
Brain_Caudate_basal_ganglia 0.923095565 Brain_Nucleus_accumbens_basal_ganglia
5.069531881 0.032817439 0.23309969 -0.675857374 0.898953452
0.166948949 0.54566461 5 0 0.998207054
RP11-10L7.1 ENSG00000246375.2 0.489449586 13 1 0.349000619
Brain_Hypothalamus 0.687478848 Brain_Substantia_nigra 12.68608361
3.31E-05 12.68608361 0.402278809 0.936529524 0.682739773
0.197263758 1 0 0.998207054
CSRP2 ENSG00000175183.9 0.4894609 10 7 0.041972157 Brain_Hypothalamus
0.982403054 Brain_Frontal_Cortex_BA9 5.289140869 0.005477261
0.197850022 0.02205629 2.033796107 0.824184366 0.631189457 7
0 0.998207054
PANK2 ENSG00000125779.21 0.489468872 1 1 0.489468872
Brain_Hypothalamus 0.489468872 Brain_Hypothalamus 1 1 1
0.691153835 0.691153835 0.691153835 NA 1 0 0.998207054
PLEKHB1 ENSG00000021300.13 0.489508477 5 5 0.048029816
Brain_Putamen_basal_ganglia 0.399590151 Brain_Cerebellar_Hemisphere
3.214183005 0.169381014 0.169381014 -1.97710453 -0.842353431
-1.131562877 0.488990483 5 0 0.998207054
PCNT ENSG00000160299.16 0.4895417 6 4 0.343526064 Brain_Hypothalamus
0.940827558 Brain_Nucleus_accumbens_basal_ganglia 4.633877161

0.034328594 0.176375979 -0.94722122 0.941778084 0.007124788 0.614729229 4 0 0.998207054
TTC9 ENSG00000133985.2 0.489612373 1 1 0.489612373 Brain_Cerebellar_Hemisphere 0.489612373 Brain_Cerebellar_Hemisphere 1 1 1 0.69092548 0.69092548 0.69092548 NA 1 0 0.998207054
HSPE1 ENSG00000115541.10 0.489645324 1 1 0.489645324 Brain_Anterior_cingulate_cortex_BA24 0.489645324 Brain_Anterior_cingulate_cortex_BA24 1 1 1 -0.690873049 -0.690873049 -0.690873049 NA 1 0 0.998207054
SNRNP200 ENSG00000144028.14 0.489725062 1 1 0.489725062 Brain_Caudate_basal_ganglia 0.489725062 Brain_Caudate_basal_ganglia 1 1 1 0.690746181 0.690746181 0.690746181 NA 1 0 0.998207054
C15orf57 ENSG00000128891.15 0.489756364 13 2 0.280400131 Brain_Nucleus_accumbens_basal_ganglia 0.986090875 Brain_Cerebellar_Hemisphere 11.55230891 0.000953425 0.693482078 -1.048131027 1.079420917 -0.196735875 0.537962814 2 0 0.998207054
SCLT1 ENSG00000151466.11 0.489823137 2 2 0.569226608 Brain_Cerebellum 0.801491674 Brain_Cerebellar_Hemisphere 1.759281274 0.240718726 0.240718726 -0.569190882 0.251417067 -0.158886908 0.580257445 2 0 0.998207054
DPP3 ENSG00000254986.7 0.489835844 3 3 0.19831176 Brain_Spinal_cord_cervical_c-1 0.467970786 Brain_Caudate_basal_ganglia 1.762036354 0.342999223 0.342999223 0.72578467 1.286376315 1.058112672 0.294427905 3 0 0.998207054
TJP3 ENSG00000105289.14 0.489848958 2 2 0.353911235 Brain_Putamen_basal_ganglia 0.467445999 Brain_Nucleus_accumbens_basal_ganglia 1.028759126 0.971240874 0.971240874 -0.726640847 0.927029468 0.10019431 1.169321493 2 0 0.998207054
SOX9 ENSG00000125398.5 0.489870571 1 1 0.489870571 Brain_Putamen_basal_ganglia 0.489870571 Brain_Putamen_basal_ganglia 1 1 1 0.690514696 0.690514696 0.690514696 NA 1 0 0.998207054
KIF2C ENSG00000142945.12 0.489961088 3 3 0.380888439 Brain_Anterior_cingulate_cortex_BA24 0.755238335 Brain_Hypothalamus 1.887670687 0.238101332 0.238101332 -0.620597625 0.876260489 0.189134196 0.755923369 3 0 0.998207054
RPL13 ENSG00000167526.13 0.489977621 8 4 0.260022315 Brain_Amygdala 0.62858495 Brain_Caudate_basal_ganglia 5.489652752 0.006060814 0.428373463 -1.049783772 1.126338388 -0.512433979 0.692221177 4 0 0.998207054
LRRC42 ENSG00000116212.14 0.490043635 1 1 0.490043635 Brain_Spinal_cord_cervical_c-1 0.490043635 Brain_Spinal_cord_cervical_c-1 1 1 1 -0.690239423 -0.690239423 -0.690239423 NA 1 0 0.998207054
LPIN1 ENSG00000134324.11 0.490099366 12 10 0.081559575 Brain_Amygdala 0.96173702 Brain_Putamen_basal_ganglia 4.179212904 0.004192097 0.264017994 -1.741707867 1.272773504 -0.264358801 0.866190941 10 0 0.998207054
ETFRF1 ENSG00000205707.10 0.490106622 2 2 0.267822912 Brain_Cerebellum 0.576545211 Brain_Nucleus_accumbens_basal_ganglia 1.104178933 0.895821067 0.895821067 -1.108090086 0.558438174 -0.274825956 1.178413434 2 0 0.998207054
TEX14 ENSG00000121101.15 0.490134937 13 7 0.13265593 Brain_Cortex 0.923587069 Brain_Cerebellum 8.340635217 0.034667537 0.35241706 -1.503710478 1.146105878 0.457367924 0.69124718 7 0 0.998207054
PPP1R17 ENSG00000106341.10 0.490135216 2 2 0.298302705 Brain_Nucleus_accumbens_basal_ganglia 0.625625358 Brain_Caudate_basal_ganglia 1.092704107 0.907295893 0.907295893 -0.487893388 1.040080064 0.276093338 1.08044039 2 0 0.998207054
TMEM244 ENSG00000203756.7 0.49015142 5 4 0.096928891 Brain_Cortex

0.973391772 Brain_Spinal_cord_cervical_c-1 2.88006556 0.030249982
0.360639712 -1.659928235 -0.033354652 -0.96361266 0.724157354
4 0 0.998207054
LINC01842 ENSG00000267147.2 0.490244581 2 2 0.232537193
Brain_Putamen_basal_ganglia 0.970929948 Brain_Spinal_cord_cervical_c-1
1.013212932 0.986787068 0.986787068 -0.036441971 1.19384861
0.578703319 0.869946813 2 0 0.998207054
SEMA7A ENSG00000138623.9 0.490249053 1 1 0.490249053
Brain_Cerebellum 0.490249053 Brain_Cerebellum 1 1 1 0.689912756
0.689912756 0.689912756 NA 1 0 0.998207054
LINC01748 ENSG00000226476.3 0.490356524 4 3 0.22063153
Brain_Putamen_basal_ganglia 0.915787428 Brain_Spinal_cord_cervical_c-1
2.742023273 0.066502892 0.211313269 -1.224850564 0.105741531
-0.523274591 0.55266736 3 0 0.998207054
C2orf15 ENSG00000273045.5 0.490500327 1 1 0.490500327
Brain_Spinal_cord_cervical_c-1 0.490500327 Brain_Spinal_cord_cervical_c-1
1 1 1 -0.689513268 -0.689513268 -0.689513268 NA 1 0
0.998207054
RP11-304L19.13 ENSG00000260778.5 0.490502412 12 3 0.272829508
Brain_Substantia_nigra 0.853782114 Brain_Putamen_basal_ganglia
9.419355448 0.007299483 0.48843035 -1.09656977 -0.184294901
-0.634333828 0.359013495 3 0 0.998207054
SLC22A18AS ENSG00000254827.5 0.490517157 2 2 0.258006986
Brain_Frontal_Cortex_BA9 0.403560981 Brain_Cortex 1.435177894
0.564822106 0.564822106 0.835278392 1.131114301 0.983196346
0.209187577 2 0 0.998207054
NBPF3 ENSG00000142794.18 0.490602731 13 1 0.312243951
Brain_Anterior_cingulate_cortex_BA24 0.617144046 Brain_Cortex
12.82675093 0.000150849 12.82675093 -1.010524744 -0.499902054
-0.684840356 0.139834767 1 0 0.998207054
ACTR10 ENSG00000131966.13 0.490640321 4 4 0.192004914
Brain_Hypothalamus 0.68273117 Brain_Frontal_Cortex_BA9 1.941276074
0.29789237 0.29789237 -0.903168752 1.304670961 0.238540959
1.061701753 4 0 0.998207054
CDCA5 ENSG00000146670.9 0.490689562 1 1 0.490689562
Brain_Putamen_basal_ganglia 0.490689562 Brain_Putamen_basal_ganglia 1 1
1 -0.689212484 -0.689212484 -0.689212484 NA 1 0
0.998207054
ZNF273 ENSG00000198039.11 0.490767607 13 8 0.131494652
Brain_Putamen_basal_ganglia 0.91447461 Brain_Hippocampus 6.495123977
0.023723909 0.219660404 -0.919968531 1.508233985 0.42284677
0.694295541 8 0 0.998207054
LINC01915 ENSG00000265485.5 0.490778452 2 1 0.465723838
Brain_Cerebellum 0.521459924 Brain_Cerebellar_Hemisphere 1.978026463
0.021973537 1.978026463 -0.729454259 -0.641096599 -0.685275429
0.0624783 1 0 0.998207054
SRSF4 ENSG00000116350.16 0.490804367 2 2 0.274207959
Brain_Cerebellar_Hemisphere 0.66899703 Brain_Cortex 1.045106071
0.954893929 0.954893929 0.427524932 1.093423366 0.760474149
0.470861298 2 0 0.998207054
GPRC5D ENSG00000111291.8 0.49082912 1 1 0.49082912
Brain_Substantia_nigra 0.49082912 Brain_Substantia_nigra 1 1 1
0.688990701 0.688990701 0.688990701 NA 1 0 0.998207054
CHRD ENSG00000090539.15 0.490838021 2 2 0.256280956 Brain_Cerebellum
0.503459574 Brain_Cerebellar_Hemisphere 1.788206566 0.211793434
0.211793434 0.669056285 1.135225241 0.902140763 0.32963123 2
0 0.998207054
AC008088.4 ENSG00000260907.1 0.490892746 1 1 0.490892746
Brain_Cerebellum 0.490892746 Brain_Cerebellum 1 1 1 -0.688889597
-0.688889597 -0.688889597 NA 1 0 0.998207054
CNTNAP2 ENSG00000174469.19 0.490893469 4 4 0.157456471
Brain_Cerebellum 0.819531089 Brain_Caudate_basal_ganglia 1.646518778

0.354100342	0.354100342	-1.413677987	0.228148124	-0.669704837
0.68774038 4	0	0.998207054
PRELID2	ENSG00000186314.11	0.490924333	1	1	0.490924333
Brain_Spinal_cord_cervical_c-1	0.490924333	Brain_Spinal_cord_cervical_c-1
1	1	1	-0.688839408	-0.688839408	-0.688839408	NA	1	0
0.998207054
SPINT1	ENSG00000166145.14	0.490927259	1	1	0.490927259
Brain_Hypothalamus	0.490927259	Brain_Hypothalamus	1	1	1
0.688834759	0.688834759	0.688834759	NA	1	0	0.998207054
TIMMDC1	ENSG00000113845.9	0.491008135	1	1	0.491008135
Brain_Nucleus_accumbens_basal_ganglia 0.491008135
Brain_Nucleus_accumbens_basal_ganglia 1	1	1	-0.688706261
-0.688706261	-0.688706261	NA	1	0	0.998207054
LSINCT5	ENSG00000281560.1	0.491042775	1	1	0.491042775
Brain_Cerebellum 0.491042775	Brain_Cerebellum 1	1	1	0.688651228
0.688651228	0.688651228	NA	1	0	0.998207054
SLC23A1	ENSG00000170482.16	0.491287396	2	2	0.249210915
Brain_Hippocampus	0.710950594	Brain_Cortex	1.056334152
0.943665848	0.943665848	-1.152268116	-0.370579714	-0.761423915
0.55273717 2	0	0.998207054
RP11-326I11.4	ENSG00000271538.5	0.491337805	12	1	0.292050654
Brain_Nucleus_accumbens_basal_ganglia 0.934090693	Brain_Putamen_basal_ganglia
11.27116526	6.93E-17	11.27116526	-1.053633699	-0.082699235
-0.665061658	0.257554542	1	0	0.998207054
SHISA5	ENSG00000164054.15	0.491406027	2	2	0.235481813
Brain_Frontal_Cortex_BA9	0.411515573	Brain_Cerebellum 1.614912162
0.385087838	0.385087838	0.821229471	1.186355814	1.003792642
0.258183314	2	0	0.998207054
DUOX1 ENSG00000137857.17	0.491544567	9	6	0.038806367
Brain_Substantia_nigra	0.939458533	Brain_Amygdala	5.520533032
0.018140707	0.216234403	-1.749215114	2.066234243	0.77053663
1.494415088	6	0	0.998207054
MYL12A	ENSG00000101608.12	0.491568154	1	1	0.491568154
Brain_Cerebellum 0.491568154	Brain_Cerebellum 1	1	1	-0.687816803
-0.687816803	-0.687816803	NA	1	0	0.998207054
CENPF ENSG00000117724.12	0.491611099	2	2	0.328976393
Brain_Hypothalamus	0.427124057	Brain_Cortex	1.118201374
0.881798626	0.881798626	-0.976177739	0.794122609	-0.091027565
1.25179138 2	0	0.998207054
FAM118A	ENSG00000100376.11	0.492011162	13	1	0.317302302
Brain_Amygdala	0.652167468	Brain_Cerebellum 12.76477676	0.000254112
12.76477676	0.450753154	1.000016956	0.680859711	0.154717895
1	0	0.998207054
GCC1	ENSG00000179562.2	0.492056487	1	1	0.492056487	Brain_Cerebellum
0.492056487	Brain_Cerebellum 1	1	1	-0.687041643	-0.687041643
-0.687041643	NA	1	0	0.998207054
PSIP1 ENSG00000164985.14	0.492210522	2	2	0.239501388
Brain_Putamen_basal_ganglia 0.895245239	Brain_Frontal_Cortex_BA9
1.263543986	0.736456014	0.736456014	-1.176233984	-0.131670097
-0.65395204	0.738618208	2	0	0.998207054
DNAJC8	ENSG00000126698.10	0.4922716	4	2	0.245817384
Brain_Spinal_cord_cervical_c-1	0.581300768	Brain_Caudate_basal_ganglia
3.306418809	0.027988918	0.622882996	-1.160568592	-0.551485698
-0.814933337	0.28776812 2	0	0.998207054
HHAT	ENSG00000054392.12	0.492389338	5	5	0.178128795
Brain_Caudate_basal_ganglia 0.868671956	Brain_Frontal_Cortex_BA9	2.40449513
0.184444681	0.184444681	-0.284413384	1.346538866	0.440461224
0.79684367 5	0	0.998207054
RUNX3 ENSG00000020633.18	0.492453942	1	1	0.492453942
Brain_Substantia_nigra	0.492453942	Brain_Substantia_nigra	1	1
1	0.686411044	0.686411044	0.686411044	NA	1	0
0.998207054

PCP4 ENSG00000183036.10 0.49248013 1 1 0.49248013 Brain_Cortex 0.49248013
Brain_Cortex 1 1 1 -0.686369504 -0.686369504 -0.686369504
NA 1 0 0.998207054
CRACR2A ENSG00000130038.9 0.492622946 1 1 0.492622946
Brain_Spinal_cord_cervical_c-1 0.492622946 Brain_Spinal_cord_cervical_c-1
1 1 1 -0.686142987 -0.686142987 -0.686142987 NA 1 0
0.998207054
LINC00437 ENSG00000236354.1 0.492650209 7 5 0.317635522
Brain_Cortex 0.960173802 Brain_Nucleus_accumbens_basal_ganglia
5.139566374 0.008428592 0.253568441 -0.628549493 0.999328627
0.063493802 0.574648885 5 0 0.998207054
TMC5 ENSG00000103534.16 0.492667244 2 2 0.403469203
Brain_Cerebellar_Hemisphere 0.748428886 Brain_Cortex 1.4964698 0.5035302
0.5035302 -0.835441445 0.320711692 -0.257364877 0.817523724 2
0 0.998207054
CELF5 ENSG00000161082.12 0.492955702 1 1 0.492955702
Brain_Cerebellar_Hemisphere 0.492955702 Brain_Cerebellar_Hemisphere 1 1
1 0.685615345 0.685615345 0.685615345 NA 1 0
0.998207054
APOBEC3G ENSG00000239713.8 0.493115964 12 9 0.09048961
Brain_Hypothalamus 0.94312356 Brain_Anterior_cingulate_cortex_BA24
5.836495889 0.00769134 0.206486932 -1.549510792 1.692820656
0.357535813 1.014486809 9 0 0.998207054
NAMA ENSG00000271086.5 0.493219665 2 2 0.401100013 Brain_Cerebellum
0.885097382 Brain_Cerebellar_Hemisphere 1.616907731 0.383092269
0.383092269 -0.144510479 0.839658279 0.3475739 0.695912403 2
0 0.998207054
ACTL6B ENSG00000077080.9 0.493231053 2 2 0.364551923
Brain_Spinal_cord_cervical_c-1 0.462191241 Brain_Hypothalamus
1.036688733 0.963311267 0.963311267 -0.906725598 -0.735243451
-0.820984525 0.121256189 2 0 0.998207054
MSANTD1 ENSG00000188981.10 0.493293855 4 4 0.084204934
Brain_Anterior_cingulate_cortex_BA24 0.908027814 Brain_Cerebellum
1.819670724 0.185416193 0.185416193 -0.136314086 1.726792509
0.475857927 0.873989347 4 0 0.998207054
GPHN ENSG00000171723.15 0.493434268 1 1 0.493434268
Brain_Cerebellar_Hemisphere 0.493434268 Brain_Cerebellar_Hemisphere 1 1
1 -0.684856833 -0.684856833 -0.684856833 NA 1 0
0.998207054
ODF1 ENSG00000155087.3 0.49350936 4 3 0.309349758
Brain_Frontal_Cortex_BA9 0.984189553 Brain_Cortex 3.136801654
0.029433965 0.153963254 -0.019816753 1.016587384 0.520670963
0.459468797 3 0 0.998207054
NSF ENSG00000073969.18 0.493566574 3 3 0.342225791
Brain_Cerebellar_Hemisphere 0.923857916 Brain_Putamen_basal_ganglia 1.50984215
0.499528551 0.499528551 -0.949776576 0.600958315 -0.084414334
0.790880437 3 0 0.998207054
FGF14-AS2 ENSG00000272143.1 0.493643217 12 5 0.120536939
Brain_Putamen_basal_ganglia 0.951012572 Brain_Amygdala 8.681929906
0.00034377 0.419375063 -0.078517567 1.552523816 0.979988171
0.504335946 5 0 0.998207054
TECPR2 ENSG00000196663.15 0.493992783 2 2 0.458918571
Brain_Cerebellum 0.694833627 Brain_Cerebellar_Hemisphere 1.531852029
0.468147971 0.468147971 -0.74062875 0.392303974 -0.174162388
0.801104412 2 0 0.998207054
GGT2 ENSG00000133475.16 0.49402172 2 2 0.236983448
Brain_Caudate_basal_ganglia 0.884894022 Brain_Cerebellum 1.030204259
0.969795741 0.969795741 0.144768033 1.182560315 0.663664174
0.73382996 2 0 0.998207054
APOLD1 ENSG00000178878.12 0.494097738 1 1 0.494097738
Brain_Spinal_cord_cervical_c-1 0.494097738 Brain_Spinal_cord_cervical_c-1
1 1 1 0.683805903 0.683805903 0.683805903 NA 1 0

0.998207054
WDR75 ENSG00000115368.9 0.494098344 8 2 0.442641365
Brain_Putamen_basal_ganglia 0.938917955 Brain_Hippocampus 7.012193475
0.004448608 0.731951913 -0.767740509 0.693337102 0.212463582
0.465414015 2 0 0.998207054
LGALS7 ENSG00000205076.4 0.494206573 1 1 0.494206573
Brain_Hypothalamus 0.494206573 Brain_Hypothalamus 1 1 1
0.683633583 0.683633583 0.683633583 NA 1 0 0.998207054
LAMA2 ENSG00000196569.11 0.494307939 4 4 0.112213543
Brain_Putamen_basal_ganglia 0.8147483 Brain_Caudate_basal_ganglia 2.3493532
0.149801326 0.149801326 -0.234304877 1.588322011 0.761767628
0.900236148 4 0 0.998207054
VPS51 ENSG00000149823.8 0.494339035 1 1 0.494339035
Brain_Nucleus_accumbens_basal_ganglia 0.494339035
Brain_Nucleus_accumbens_basal_ganglia 1 1 1 0.68342388 0.68342388
0.68342388 NA 1 0 0.998207054
TEX261 ENSG00000144043.11 0.494551394 8 5 0.091386149
Brain_Cerebellar_Hemisphere 0.996544196 Brain_Nucleus_accumbens_basal_ganglia
5.768177951 0.001934404 0.197748014 -1.688130665 0.482423041
-0.059714375 0.721086326 5 0 0.998207054
METTL15 ENSG00000169519.20 0.494680917 8 4 0.068081119
Brain_Anterior_cingulate_cortex_BA24 0.843270018 Brain_Hypothalamus
5.790163776 0.028598261 0.372870635 -1.824469525 0.197712489
-0.980880153 0.603791994 4 0 0.998207054
ZFP3 ENSG00000180787.5 0.49469666 1 1 0.49469666 Brain_Cerebellar_Hemisphere
0.49469666 Brain_Cerebellar_Hemisphere 1 1 1 -0.682857866
-0.682857866 -0.682857866 NA 1 0 0.998207054
CTD-2240J17.4 ENSG00000275995.1 0.494729117 1 1 0.494729117
Brain_Frontal_Cortex_BA9 0.494729117 Brain_Frontal_Cortex_BA9 1 1
1 0.682806507 0.682806507 0.682806507 NA 1 0
0.998207054
ARID3A ENSG00000116017.10 0.49473139 5 5 0.229946019
Brain_Hippocampus 0.558095872 Brain_Cerebellar_Hemisphere 1.256234753
0.730889245 0.730889245 -1.200497921 1.058698297 0.476769239
0.956928399 5 0 0.998207054
RP11-203M5.8 ENSG00000258908.1 0.494778224 1 1 0.494778224
Brain_Substantia_nigra 0.494778224 Brain_Substantia_nigra 1 1
1 -0.682728805 -0.682728805 -0.682728805 NA 1 0
0.998207054
SCIN ENSG00000006747.14 0.494844174 4 4 0.222389939
Brain_Nucleus_accumbens_basal_ganglia 0.933709237 Brain_Frontal_Cortex_BA9
1.930779558 0.113135583 0.113135583 0.083178967 1.2201977
0.656425165 0.483869577 4 0 0.998207054
RP1-60O19.2 ENSG00000229654.1 0.494958083 12 6 0.215478576
Brain_Hippocampus 0.907236832 Brain_Cerebellum 8.42272021 0.01049511
0.322627054 -1.221446362 1.238640732 -0.10938137 0.61369532 6
0 0.998207054
NOSTRIN ENSG00000163072.15 0.494967009 4 4 0.106700413
Brain_Substantia_nigra 0.690083181 Brain_Cortex 1.859534337
0.259022828 0.259022828 -1.613202995 -0.398742185 -0.994363051
0.508864709 4 0 0.998207054
BIVM ENSG00000134897.13 0.495017601 10 7 0.124152156
Brain_Anterior_cingulate_cortex_BA24 0.990902038 Brain_Cortex
5.971148532 0.015971428 0.299789413 -1.537576669 0.521849478
-0.22193498 0.753754274 7 0 0.998207054
DPY19L2 ENSG00000177990.11 0.495036345 10 4 0.173854852
Brain_Amygdala 0.957932061 Brain_Anterior_cingulate_cortex_BA24
7.890652201 0.000974734 0.404762107 -0.563501672 1.359921231
-0.035863068 0.527870386 4 0 0.998207054
TMEM5 ENSG00000118600.11 0.495063289 5 5 0.387120669
Brain_Substantia_nigra 0.979333133 Brain_Anterior_cingulate_cortex_BA24
2.845267511 0.289639705 0.289639705 -0.842079206 0.864850829

-0.111584752 0.623348771 5 0 0.998207054
RAPGEF2 ENSG00000109756.8 0.495069338 1 1 0.495069338
Brain_Hippocampus 0.495069338 Brain_Hippocampus 1 1 1
0.682268262 0.682268262 0.682268262 NA 1 0 0.998207054
ABCC2 ENSG00000023839.10 0.495101905 2 2 0.236880728
Brain_Spinal_cord_cervical_c-1 0.400577083 Brain_Cerebellum 1.657682649
0.342317351 0.342317351 0.840591034 1.182819405 1.01170522
0.241992002 2 0 0.998207054
GNRHR ENSG00000109163.6 0.495120137 5 3 0.219329117
Brain_Hypothalamus 0.967842756 Brain_Cerebellum 4.276389144
0.006735212 0.185847894 -0.040314045 1.228314001 0.410028535
0.531229463 3 0 0.998207054
TONSL ENSG00000160949.16 0.495128781 5 5 0.073384412 Brain_Cortex
0.740371119 Brain_Cerebellum 2.27097943 0.117310921 0.117310921
-1.790432482 0.641051889 -0.197576774 1.028553865 5 0
0.998207054
KIAA1324 ENSG00000116299.16 0.495164395 1 1 0.495164395
Brain_Nucleus_accumbens_basal_ganglia 0.495164395
Brain_Nucleus_accumbens_basal_ganglia 1 1 1 0.682117912
0.682117912 0.682117912 NA 1 0 0.998207054
NIPSNAP3A ENSG00000136783.9 0.495197439 13 4 0.432566765
Brain_Nucleus_accumbens_basal_ganglia 0.997941064 Brain_Cerebellum
9.844221812 0.000874351 0.398431495 -0.78480695 0.731819544
-0.00932738 0.44643455 4 0 0.998207054
LL22NC03-N95F10.1 ENSG00000282012.1 0.495237794 1 1 0.495237794
Brain_Hypothalamus 0.495237794 Brain_Hypothalamus 1 1 1
-0.682001829 -0.682001829 -0.682001829 NA 1 0 0.998207054
HPYR1 ENSG00000253521.1 0.495244487 1 1 0.495244487 Brain_Cortex
0.495244487 Brain_Cortex 1 1 1 0.681991244 0.681991244
0.681991244 NA 1 0 0.998207054
GNPTAB ENSG00000111670.14 0.495327526 3 3 0.274400359
Brain_Nucleus_accumbens_basal_ganglia 0.529823248
Brain_Spinal_cord_cervical_c-1 1.369308276 0.587002622 0.587002622
-1.09298506 1.041607649 -0.226551088 1.122567859 3 0
0.998207054
KLHL22 ENSG00000099910.16 0.495372775 2 2 0.235928854
Brain_Cerebellar_Hemisphere 0.898278138 Brain_Cortex 1.098165566
0.901834434 0.901834434 0.127836786 1.1852241 0.656530443
0.74768574 2 0 0.998207054
C1orf131 ENSG00000143633.12 0.495419226 1 1 0.495419226
Brain_Cerebellum 0.495419226 Brain_Cerebellum 1 1 1 0.681714928
0.681714928 0.681714928 NA 1 0 0.998207054
FAM138B ENSG00000226516.7 0.495420152 7 6 0.112524126
Brain_Caudate_basal_ganglia 0.636662861 Brain_Nucleus_accumbens_basal_ganglia
4.598360273 0.103103931 0.176368828 -1.58694931 0.486619659
-0.659653214 0.664509335 6 0 0.998207054
NOG ENSG00000183691.4 0.495474519 2 2 0.23915135
Brain_Caudate_basal_ganglia 0.901820357 Brain_Spinal_cord_cervical_c-1
1.011949805 0.988050195 0.988050195 -1.177110638 -0.123362113
-0.650236375 0.745112728 2 0 0.998207054
GABPB2 ENSG00000143458.11 0.49557828 4 4 0.104758241
Brain_Hippocampus 0.913634184 Brain_Cerebellar_Hemisphere 1.734174135
0.474863388 0.474863388 -0.108455745 1.622210703 0.723701474
0.711002208 4 0 0.998207054
GFRA1 ENSG00000151892.14 0.495827162 7 4 0.08865285 Brain_Amygdala
0.909590401 Brain_Anterior_cingulate_cortex_BA24 4.37534464 0.000177205
0.716806267 -1.70254714 0.119216082 -0.775761951 0.606353171
4 0 0.998207054
RP11-90P13.1 ENSG00000261795.1 0.495965366 2 2 0.270754575
Brain_Caudate_basal_ganglia 0.649513982 Brain_Putamen_basal_ganglia
1.017340068 0.982659932 0.982659932 -1.101326516 -0.454437478
-0.777881997 0.457419625 2 0 0.998207054

TNFSF11 ENSG00000120659.14 0.495980019 1 1 0.495980019
Brain_Hypothalamus 0.495980019 Brain_Hypothalamus 1 1 1
0.680828492 0.680828492 0.680828492 NA 1 0 0.998207054
C1orf204 ENSG00000188004.9 0.495986374 10 8 0.248336925
Brain_Anterior_cingulate_cortex_BA24 0.840644719 Brain_Frontal_Cortex_BA9
4.574495551 0.029029444 0.221873144 -1.102456447 1.154398268
-0.146366264 0.762775193 8 0 0.998207054
ZNF485 ENSG00000198298.12 0.496062571 1 1 0.496062571
Brain_Substantia_nigra 0.496062571 Brain_Substantia_nigra 1 1
1 -0.68069805 -0.68069805 -0.68069805 NA 1 0
0.998207054
PHC1 ENSG00000111752.10 0.496097397 3 3 0.348132303
Brain_Cerebellar_Hemisphere 0.931502202 Brain_Cerebellum 2.24152072
0.179274519 0.179274519 -0.938218169 -0.085954984 -0.485581275
0.428597395 3 0 0.998207054
FST ENSG00000134363.11 0.496218123 1 1 0.496218123
Brain_Caudate_basal_ganglia 0.496218123 Brain_Caudate_basal_ganglia 1 1
1 0.680452287 0.680452287 0.680452287 NA 1 0
0.998207054
RTKN2 ENSG00000182010.10 0.496241541 1 1 0.496241541 Brain_Cortex
0.496241541 Brain_Cortex 1 1 1 0.680415292 0.680415292
0.680415292 NA 1 0 0.998207054
SKAP2 ENSG00000005020.12 0.496248702 4 3 0.110073311 Brain_Cerebellum
0.75857428 Brain_Cortex 2.967183858 0.084299422 0.383307341
0.30735355 1.597863716 1.045713515 0.5406851 3 0 0.998207054
RPS2 ENSG00000140988.15 0.496332446 1 1 0.496332446
Brain_Hippocampus 0.496332446 Brain_Hippocampus 1 1 1
-0.68027169 -0.68027169 -0.68027169 NA 1 0 0.998207054
VPS13D ENSG00000048707.13 0.496391882 1 1 0.496391882
Brain_Cortex 0.496391882 Brain_Cortex 1 1 1 -0.680177809
-0.680177809 -0.680177809 NA 1 0 0.998207054
RP11-568A7.4 ENSG00000280707.2 0.496527841 1 1 0.496527841
Brain_Caudate_basal_ganglia 0.496527841 Brain_Caudate_basal_ganglia 1 1
1 -0.679963075 -0.679963075 -0.679963075 NA 1 0
0.998207054
PEAK1 ENSG00000173517.10 0.496528687 13 4 0.048398722 Brain_Cortex
0.432690804 Brain_Anterior_cingulate_cortex_BA24 9.97286326 0.011091118
0.417194352 0.784595441 1.973850695 1.329668737 0.357170197
4 0 0.998207054
RP11-11N7.4 ENSG00000273175.1 0.496642526 1 1 0.496642526
Brain_Hypothalamus 0.496642526 Brain_Hypothalamus 1 1 1
-0.679781968 -0.679781968 -0.679781968 NA 1 0 0.998207054
NCAM2 ENSG00000154654.14 0.496653413 2 2 0.238530595
Brain_Hypothalamus 0.962106404 Brain_Cortex 1.0464045 0.9535955
0.9535955 -1.178667517 -0.047510447 -0.613088982 0.799848835 2
0 0.998207054
PARD6G ENSG00000178184.15 0.49667102 1 1 0.49667102
Brain_Cerebellar_Hemisphere 0.49667102 Brain_Cerebellar_Hemisphere 1 1 1
-0.679736975 -0.679736975 -0.679736975 NA 1 0 0.998207054
ETF1 ENSG00000120705.12 0.496755946 1 1 0.496755946
Brain_Nucleus_accumbens_basal_ganglia 0.496755946
Brain_Nucleus_accumbens_basal_ganglia 1 1 1 0.67960288 0.67960288
0.67960288 NA 1 0 0.998207054
RP1-93H18.1 ENSG00000240050.5 0.496877599 3 3 0.124225247
Brain_Anterior_cingulate_cortex_BA24 0.882024107
Brain_Spinal_cord_cervical_c-1 1.4957862 0.551666761 0.551666761
0.148403793 1.537277999 0.74795601 0.713619167 3 0
0.998207054
KCTD1 ENSG00000134504.12 0.496907549 3 3 0.222293376
Brain_Caudate_basal_ganglia 0.778726715 Brain_Cerebellum 1.136529899
0.87907303 0.87907303 -1.220452524 0.736716338 -0.067585819
1.024085163 3 0 0.998207054

SCYL3 ENSG00000000457.13 0.496994699 10 6 0.256065345
Brain_Nucleus_accumbens_basal_ganglia 0.964806015 Brain_Cerebellum
6.225463377 0.004532388 0.275830924 -0.886483522 1.135740118
0.472844376 0.712648736 6 0 0.998207054
CASQ2 ENSG00000118729.11 0.497129623 2 2 0.241012593
Brain_Nucleus_accumbens_basal_ganglia 0.914758456 Brain_Cerebellum
1.217938721 0.782061279 0.782061279 -1.172459576 -0.107038475
-0.639749025 0.753366485 2 0 0.998207054
AJ003147.8 ENSG00000262521.1 0.497167412 2 2 0.280266484
Brain_Cerebellum 0.745519137 Brain_Cerebellar_Hemisphere 1.64237402 0.35762598
0.35762598 -1.0797209 -0.324553352 -0.702137126 0.533984094 2 0
0.998207054
GLI1 ENSG00000111087.9 0.497393156 1 1 0.497393156
Brain_Nucleus_accumbens_basal_ganglia 0.497393156
Brain_Nucleus_accumbens_basal_ganglia 1 1 1 -0.678597139
-0.678597139 -0.678597139 NA 1 0 0.998207054
DUSP22 ENSG00000112679.14 0.497412503 1 1 0.497412503
Brain_Putamen_basal_ganglia 0.497412503 Brain_Putamen_basal_ganglia 1 1
1 0.678566613 0.678566613 0.678566613 NA 1 0
0.998207054
ZNF578 ENSG00000258405.9 0.497427253 1 1 0.497427253
Brain_Hypothalamus 0.497427253 Brain_Hypothalamus 1 1 1
0.678543341 0.678543341 0.678543341 NA 1 0 0.998207054
IER2 ENSG00000160888.6 0.497458373 2 2 0.24426173
Brain_Frontal_Cortex_BA9 0.834293393 Brain_Hypothalamus 1.00649593
0.99350407 0.99350407 0.20919836 1.164400537 0.686799449 0.675429937
2 0 0.998207054
PART1 ENSG00000152931.7 0.497686981 5 4 0.106221591
Brain_Anterior_cingulate_cortex_BA24 0.441604249 Brain_Caudate_basal_ganglia
2.911447828 0.046331028 0.415500458 -1.615411594 -0.769487016
-1.099001599 0.329893433 4 0 0.998207054
CYTH1 ENSG00000108669.16 0.497714756 2 2 0.274743629
Brain_Nucleus_accumbens_basal_ganglia 0.489921699 Brain_Cerebellar_Hemisphere
1.231257453 0.768742547 0.768742547 -1.09220358 -0.690433368
-0.891318474 0.284094441 2 0 0.998207054
RP11-1055B8.4 ENSG00000262877.4 0.497747801 13 7 0.091581622
Brain_Cerebellum 0.988933976 Brain_Putamen_basal_ganglia 8.045859233
0.014602774 0.360441579 -0.024966911 1.687113018 0.879233162
0.568792922 7 0 0.998207054
TVP23B ENSG00000171928.13 0.497777092 13 4 0.201058047
Brain_Nucleus_accumbens_basal_ganglia 0.98102508 Brain_Amygdala 10.74447588
0.0007883 0.395874402 -0.495527211 1.278542962 0.094178675
0.424178529 4 0 0.998207054
AC005013.5 ENSG00000228421.2 0.497870097 10 3 0.239091221
Brain_Nucleus_accumbens_basal_ganglia 0.848830706 Brain_Hypothalamus
8.43047376 0.005450738 0.399148717 -1.177261319 0.452215381
-0.284332466 0.517275702 3 0 0.998207054
LRRC40 ENSG00000066557.5 0.497949448 3 3 0.220903634
Brain_Spinal_cord_cervical_c-1 0.690737097 Brain_Hippocampus
1.126664489 0.898911078 0.898911078 -0.71655818 1.224128823
0.301808537 0.973902048 3 0 0.998207054
ATP11A ENSG00000068650.18 0.49796111 1 1 0.49796111
Brain_Spinal_cord_cervical_c-1 0.49796111 Brain_Spinal_cord_cervical_c-1 1
1 1 0.677701288 0.677701288 0.677701288 NA 1 0
0.998207054
NMT2 ENSG00000152465.17 0.498028208 6 6 0.15827535
Brain_Frontal_Cortex_BA9 0.95818295 Brain_Caudate_basal_ganglia 2.425695301
0.106706363 0.106706363 -0.743777829 1.410895767 0.441050619
0.76010661 6 0 0.998207054
ANKRD66 ENSG00000230062.5 0.498116439 2 2 0.238008268
Brain_Amygdala 0.789765611 Brain_Anterior_cingulate_cortex_BA24 1.25756444
0.74243556 0.74243556 -0.266614993 1.179979754 0.45668238 1.022896955

2 0 0.998207054
AGT ENSG00000135744.7 0.498335206 9 8 0.02721936
Brain_Putamen_basal_ganglia 0.899575861 Brain_Frontal_Cortex_BA9
3.409862819 0.023350083 0.246510379 -2.208357531 0.888020428
-0.365293736 1.125759896 8 0 0.998207054
CCDC134 ENSG00000100147.13 0.498394622 1 1 0.498394622
Brain_Cerebellum 0.498394622 Brain_Cerebellum 1 1 1 0.677017861
0.677017861 0.677017861 NA 1 0 0.998207054
SMARCA5 ENSG00000153147.5 0.498520309 1 1 0.498520309
Brain_Frontal_Cortex_BA9 0.498520309 Brain_Frontal_Cortex_BA9 1 1
1 0.676819776 0.676819776 0.676819776 NA 1 0
0.998207054
LIPA ENSG00000107798.17 0.498592079 3 3 0.126372842 Brain_Cortex
0.577965705 Brain_Cerebellar_Hemisphere 1.927639894 0.478205424
0.478205424 0.556358643 1.528562887 0.900471237 0.54477367 3
0 0.998207054
PAM ENSG00000145730.20 0.498655322 5 5 0.101093536
Brain_Cerebellar_Hemisphere 0.730790233 Brain_Putamen_basal_ganglia
3.180268496 0.219501909 0.219501909 -1.639575138 0.344074536
-0.757574639 0.727291445 5 0 0.998207054
RTCB ENSG00000100220.11 0.498738379 1 1 0.498738379
Brain_Putamen_basal_ganglia 0.498738379 Brain_Putamen_basal_ganglia 1 1
1 0.676476157 0.676476157 0.676476157 NA 1 0
0.998207054
ATG12 ENSG00000145782.12 0.498829161 8 4 0.315823684
Brain_Hippocampus 0.94444475 Brain_Caudate_basal_ganglia 5.691427759
5.05E-17 0.212309231 -0.502757627 1.003077056 0.180616948
0.466895761 4 0 0.998207054
ALKBH2 ENSG00000189046.10 0.498836832 1 1 0.498836832
Brain_Cerebellar_Hemisphere 0.498836832 Brain_Cerebellar_Hemisphere 1 1
1 -0.676321049 -0.676321049 -0.676321049 NA 1 0
0.998207054
TMEM248 ENSG00000106609.16 0.498848446 2 2 0.271492468
Brain_Cerebellum 0.347705699 Brain_Cerebellar_Hemisphere 1.523726006
0.476273994 0.476273994 -1.099632059 -0.939048782 -1.019340421
0.113549524 2 0 0.998207054
ATPIF1 ENSG00000130770.17 0.49887251 10 2 0.185420186 Brain_Amygdala
0.696648488 Brain_Cerebellum 8.973817748 0.001379505 0.311592433
-1.324249529 -0.389848621 -0.850228939 0.273349031 2 0
0.998207054
RP11-49P4.7 ENSG00000282881.1 0.498918938 2 2 0.245624686
Brain_Anterior_cingulate_cortex_BA24 0.812668609 Brain_Putamen_basal_ganglia
1.026398117 0.973601883 0.973601883 -1.161042329 0.236984765
-0.462028782 0.988554439 2 0 0.998207054
RFC3 ENSG00000133119.12 0.498994614 1 1 0.498994614 Brain_Cerebellum
0.498994614 Brain_Cerebellum 1 1 1 -0.676072502 -0.676072502
-0.676072502 NA 1 0 0.998207054
LINC01739 ENSG00000229537.5 0.499001559 1 1 0.499001559
Brain_Amygdala 0.499001559 Brain_Amygdala 1 1 1 -0.676061563
-0.676061563 -0.676061563 NA 1 0 0.998207054
INTS12 ENSG00000138785.14 0.499043343 2 2 0.239266187
Brain_Anterior_cingulate_cortex_BA24 0.881335932 Brain_Substantia_nigra
1.065629551 0.934370449 0.934370449 -0.149275899 1.176822935
0.513773518 0.937693478 2 0 0.998207054
U2AF2 ENSG00000063244.12 0.499080118 2 2 0.297233363
Brain_Frontal_Cortex_BA9 0.594039927 Brain_Substantia_nigra
1.017035168 0.982964832 0.982964832 -1.042384687 -0.532990832
-0.78768776 0.360195849 2 0 0.998207054
TAF13 ENSG00000197780.9 0.499119911 1 1 0.499119911
Brain_Cerebellar_Hemisphere 0.499119911 Brain_Cerebellar_Hemisphere 1 1
1 0.675875159 0.675875159 0.675875159 NA 1 0
0.998207054

KRBA1 ENSG00000133619.17 0.499240563 2 2 0.252282846
Brain_Substantia_nigra 0.702944708 Brain_Cerebellar_Hemisphere
1.089006868 0.910993132 0.910993132 0.381348449 1.144822131
0.76308529 0.539857418 2 0 0.998207054
TLR2 ENSG00000137462.6 0.499259885 2 2 0.292766638 Brain_Cerebellum
0.614169042 Brain_Cerebellar_Hemisphere 1.789313949 0.210686051
0.210686051 -1.052071737 -0.504131401 -0.778101569 0.387452327
2 0 0.998207054
RHBDF2 ENSG00000129667.12 0.499289022 1 1 0.499289022
Brain_Caudate_basal_ganglia 0.499289022 Brain_Caudate_basal_ganglia 1 1
1 -0.675608849 -0.675608849 -0.675608849 NA 1 0
0.998207054
FAM96B ENSG00000166595.11 0.499312179 1 1 0.499312179
Brain_Substantia_nigra 0.499312179 Brain_Substantia_nigra 1 1
1 -0.675572386 -0.675572386 -0.675572386 NA 1 0
0.998207054
MCRIP1 ENSG00000225663.7 0.499324657 1 1 0.499324657
Brain_Spinal_cord_cervical_c-1 0.499324657 Brain_Spinal_cord_cervical_c-1
1 1 1 0.675552738 0.675552738 0.675552738 NA 1 0
0.998207054
TMTC2 ENSG00000179104.8 0.49950884 3 3 0.158287678 Brain_Cortex
0.732721959 Brain_Spinal_cord_cervical_c-1 1.06463516 0.946483411
0.946483411 -0.557274375 1.410853965 0.398362187 0.985295224
3 0 0.998207054
CHADL ENSG00000100399.15 0.499509682 2 2 0.243675585 Brain_Cortex
0.309335059 Brain_Nucleus_accumbens_basal_ganglia 1.78062121 0.21937879
0.21937879 1.016618271 1.165848794 1.091233533 0.105521914 2
0 0.998207054
TOR3A ENSG00000186283.13 0.499510478 9 8 0.043988649
Brain_Cerebellar_Hemisphere 0.742258703 Brain_Nucleus_accumbens_basal_ganglia
2.668255493 0.064086874 0.182518321 -1.13984815 2.014198959
0.185415385 0.961635966 8 0 0.998207054
SLC30A2 ENSG00000158014.14 0.499565782 1 1 0.499565782
Brain_Frontal_Cortex_BA9 0.499565782 Brain_Frontal_Cortex_BA9 1 1
1 -0.67517312 -0.67517312 -0.67517312 NA 1 0
0.998207054
SNX3 ENSG00000112335.14 0.499650074 2 2 0.404023869 Brain_Cortex
0.445306322 Brain_Caudate_basal_ganglia 1.078674217 0.921325783
0.921325783 0.763263405 0.834456361 0.798859883 0.050341022
2 0 0.998207054
TAX1BP3 ENSG00000213977.7 0.499681187 5 5 0.221529376
Brain_Cerebellum 0.939063055 Brain_Cerebellar_Hemisphere 2.97294002
0.220584443 0.220584443 -1.222471497 0.076447532 -0.573678835
0.494226268 5 0 0.998207054
BCL9L ENSG00000186174.12 0.499746084 1 1 0.499746084
Brain_Putamen_basal_ganglia 0.499746084 Brain_Putamen_basal_ganglia 1 1
1 0.674889324 0.674889324 0.674889324 NA 1 0
0.998207054
RP11-621L6.3 ENSG00000273321.1 0.499789608 1 1 0.499789608
Brain_Anterior_cingulate_cortex_BA24 0.499789608
Brain_Anterior_cingulate_cortex_BA24 1 1 1 0.674820824
0.674820824 0.674820824 NA 1 0 0.998207054
AGO2 ENSG00000123908.11 0.499805693 1 1 0.499805693
Brain_Nucleus_accumbens_basal_ganglia 0.499805693
Brain_Nucleus_accumbens_basal_ganglia 1 1 1 -0.67479551
-0.67479551 -0.67479551 NA 1 0 0.998207054
GGNBP2 ENSG00000278311.4 0.499932051 7 6 0.114192789
Brain_Caudate_basal_ganglia 0.955831588 Brain_Cortex 4.031741796
0.053147103 0.207663476 -1.57962492 -0.055385198 -0.967949107
0.590965566 6 0 0.998207054
UBR7 ENSG00000012963.14 0.500008769 1 1 0.500008769
Brain_Putamen_basal_ganglia 0.500008769 Brain_Putamen_basal_ganglia 1 1

1 -0.674475953 -0.674475953 -0.674475953 NA 1 0
0.998207054
AC007228.9 ENSG00000268568.1 0.500025275 1 1 0.500025275
Brain_Nucleus_accumbens_basal_ganglia 0.500025275
Brain_Nucleus_accumbens_basal_ganglia 1 1 1 0.674449982
0.674449982 0.674449982 NA 1 0 0.998207054
LONP2 ENSG00000102910.13 0.500054294 1 1 0.500054294
Brain_Cerebellar_Hemisphere 0.500054294 Brain_Cerebellar_Hemisphere 1 1
1 0.674404325 0.674404325 0.674404325 NA 1 0
0.998207054
PARVA ENSG00000197702.11 0.500098555 5 4 0.133755984 Brain_Cerebellum
0.598463778 Brain_Amygdala 3.435820097 0.101103211 0.167270373
0.526610962 1.499453652 0.984349535 0.454514849 4 0
0.998207054
PPP1R11 ENSG00000204619.7 0.50011505 2 2 0.571470801
Brain_Cerebellar_Hemisphere 0.963668854 Brain_Cerebellum 1.894828383
0.105171617 0.105171617 0.045550085 0.565886703 0.305718394
0.367933551 2 0 0.998207054
PIP4K2A ENSG00000150867.13 0.500216609 9 5 0.109283828
Brain_Caudate_basal_ganglia 0.984413608 Brain_Cortex 5.681034458
0.023514122 0.266724134 -0.298443933 1.601420443 0.528728562
0.671510651 5 0 0.998207054
INSL3 ENSG00000248099.3 0.500281821 1 1 0.500281821
Brain_Cerebellar_Hemisphere 0.500281821 Brain_Cerebellar_Hemisphere 1 1
1 0.67404639 0.67404639 0.67404639 NA 1 0 0.998207054
CLUAP1 ENSG00000103351.12 0.500325277 13 9 0.104858132
Brain_Anterior_cingulate_cortex_BA24 0.957466645 Brain_Cortex
5.727653718 0.001041874 0.209074553 -0.732943009 1.621744194
0.254041949 0.607654587 9 0 0.998207054
SLC35E1 ENSG00000127526.14 0.500368055 3 3 0.147302525
Brain_Cerebellar_Hemisphere 0.619499823 Brain_Substantia_nigra 1.84099849
0.158796403 0.158796403 0.496559352 1.449125546 1.016847785
0.482343236 3 0 0.998207054
ZNF823 ENSG00000197933.12 0.500372385 1 1 0.500372385
Brain_Cortex 0.500372385 Brain_Cortex 1 1 1 -0.673903943
-0.673903943 -0.673903943 NA 1 0 0.998207054
UCK1 ENSG00000130717.12 0.500420173 9 5 0.034238818
Brain_Hippocampus 0.832564156 Brain_Cortex 5.327616058
0.016365027 0.277997699 0.211414099 2.117247859 1.006573105
0.577800988 5 0 0.998207054
CNDP2 ENSG00000133313.14 0.500482609 11 1 0.249055226
Brain_Hippocampus 0.987780996 Brain_Cerebellar_Hemisphere 9.946766828
0.002237055 9.946766828 -0.015314849 1.152647189 0.646036951
0.33382473 1 0 0.998207054
CDKAL1 ENSG00000145996.11 0.500787677 1 1 0.500787677
Brain_Cerebellum 0.500787677 Brain_Cerebellum 1 1 1 -0.673250912
-0.673250912 -0.673250912 NA 1 0 0.998207054
NPTN ENSG00000156642.16 0.500866073 3 3 0.247404484
Brain_Hypothalamus 0.928924561 Brain_Caudate_basal_ganglia 1.280747276
0.719988202 0.719988202 -1.156676671 -0.089197992 -0.726732015
0.563203064 3 0 0.998207054
CHCHD6 ENSG00000159685.10 0.500902976 2 2 0.516394871
Brain_Cortex 0.744629715 Brain_Hippocampus 1.648437439
0.351562561 0.351562561 -0.325728585 0.648912599 0.161592007
0.68917539 2 0 0.998207054
FOXL1 ENSG00000176678.5 0.500929458 1 1 0.500929458 Brain_Cortex
0.500929458 Brain_Cortex 1 1 1 0.673028031 0.673028031
0.673028031 NA 1 0 0.998207054
SMOC1 ENSG00000198732.10 0.500965984 4 4 0.161334037
Brain_Nucleus_accumbens_basal_ganglia 0.427248888 Brain_Caudate_basal_ganglia
2.265689614 0.168423152 0.168423152 -1.400598943 -0.793908179
-1.083896192 0.311208434 4 0 0.998207054

OPRK1 ENSG00000082556.10 0.501157908 4 4 0.259924959 Brain_Cerebellar_Hemisphere 0.681452883 Brain_Cerebellum 1.594782485 0.536626647 0.536626647 -1.12656851 -0.410481349 -0.830563035 0.320766912 4 0 0.998207054
PMS2 ENSG00000122512.14 0.501278962 13 8 0.067677625 Brain_Hypothalamus 0.867445641 Brain_Caudate_basal_ganglia 7.522704039 0.013947257 0.294242876 -0.955885326 1.827147303 0.357460977 0.813756037 8 0 0.998207054
EXD3 ENSG00000187609.15 0.501316298 3 3 0.179929257 Brain_Cortex 0.845802287 Brain_Amygdala 1.264911072 0.720037125 0.720037125 -1.340972883 0.37656489 -0.386295047 0.874688276 3 0 0.998207054
IFI27L2 ENSG00000119632.3 0.501326689 9 8 0.077019444 Brain_Hypothalamus 0.876984843 Brain_Cerebellum 3.714708542 0.084854207 0.236900481 -0.154792577 1.768248054 0.836079599 0.570056794 8 0 0.998207054
KMO ENSG00000117009.11 0.501326908 2 2 0.332747976 Brain_Cortex 0.58650914 Brain_Frontal_Cortex_BA9 1.120482089 0.879517911 0.879517911 -0.54390167 0.968593643 0.212345987 1.069495692 2 0 0.998207054
BAIAP2L1 ENSG00000006453.13 0.50148825 1 1 0.50148825 Brain_Caudate_basal_ganglia 0.50148825 Brain_Caudate_basal_ganglia 1 1 1 -0.672149935 -0.672149935 -0.672149935 NA 1 0 0.998207054
FIBP ENSG00000172500.12 0.501644977 8 5 0.300171011 Brain_Cerebellum 0.918106903 Brain_Caudate_basal_ganglia 6.247320597 0.004270983 0.218496346 -0.10281865 1.036066733 0.739752752 0.387492985 5 0 0.998207054
CRIP2 ENSG00000182809.10 0.50165046 2 2 0.245221674 Brain_Cortex 0.835284573 Brain_Caudate_basal_ganglia 1.031502074 0.968497926 0.968497926 0.207928785 1.162033952 0.684981368 0.674654233 2 0 0.998207054
ASCC3 ENSG00000112249.13 0.50182757 4 4 0.229822713 Brain_Frontal_Cortex_BA9 0.930620012 Brain_Cortex 2.434028973 0.246392688 0.246392688 -1.200815666 0.213173575 -0.195853876 0.672129312 4 0 0.998207054
CLIC4 ENSG00000169504.14 0.50183933 1 1 0.50183933 Brain_Nucleus_accumbens_basal_ganglia 0.50183933 Brain_Nucleus_accumbens_basal_ganglia 1 1 1 0.671598505 0.671598505 0.671598505 NA 1 0 0.998207054
INHBE ENSG00000139269.2 0.501899445 1 1 0.501899445 Brain_Spinal_cord_cervical_c-1 0.501899445 Brain_Spinal_cord_cervical_c-1 1 1 1 -0.671504105 -0.671504105 -0.671504105 NA 1 0 0.998207054
DDX31 ENSG00000125485.17 0.502088099 8 2 0.051690619 Brain_Frontal_Cortex_BA9 0.643036596 Brain_Putamen_basal_ganglia 6.770160839 0.018033292 0.603435591 -1.945701397 0.650901781 -0.848523644 0.761196869 2 0 0.998207054
ZNF517 ENSG00000197363.9 0.502133695 11 6 0.13618844 Brain_Hippocampus 0.985349403 Brain_Frontal_Cortex_BA9 5.463714364 0.001563293 0.443447867 -1.490136153 1.190088117 -0.071799225 0.680256487 6 0 0.998207054
GRIA1 ENSG00000155511.17 0.50216758 3 2 0.197828743 Brain_Cerebellar_Hemisphere 0.611834312 Brain_Nucleus_accumbens_basal_ganglia 1.995254053 0.010046808 0.994699139 -1.287762233 0.507456841 -0.551372257 0.940041553 2 0 0.998207054
DHX33 ENSG00000005100.12 0.502354872 1 1 0.502354872 Brain_Hypothalamus 0.502354872 Brain_Hypothalamus 1 1 1 -0.670789132 -0.670789132 -0.670789132 NA 1 0 0.998207054
VPS16 ENSG00000215305.9 0.502456531 4 3 0.195520851 Brain_Cortex 0.623255706 Brain_Caudate_basal_ganglia 3.453346627 0.02818886 0.116567692 0.491241421 1.294418653 0.929872049 0.348239302 3 0 0.998207054

TFAP2A ENSG00000137203.10 0.50269126 4 4 0.09671048 Brain_Hippocampus 0.846783995 Brain_Frontal_Cortex_BA9 1.102917947 0.92455271 0.92455271 -0.729138331 1.661014773 0.202146118 1.04318069 4 0 0.998207054
ATXN7 ENSG00000163635.17 0.50269515 1 1 0.50269515 Brain_Hippocampus 0.50269515 Brain_Hippocampus 1 1 1 0.670255154 0.670255154 0.670255154 NA 1 0 0.998207054
RP11-158H5.8 ENSG00000274184.1 0.502715582 8 4 0.35192503 Brain_Caudate_basal_ganglia 0.983237982 Brain_Spinal_cord_cervical_c-1 5.639800043 0.025283953 0.199066781 -0.930861853 0.02100962 -0.598332444 0.372511033 4 0 0.998207054
BLMH ENSG00000108578.14 0.502988524 4 4 0.308544767 Brain_Amygdala 0.51232504 Brain_Putamen_basal_ganglia 2.303878766 0.34487492 0.34487492 -0.767344935 1.0182803 0.419738489 0.805713336 4 0 0.998207054
CAND2 ENSG00000144712.11 0.502992759 4 4 0.360956502 Brain_Cerebellar_Hemisphere 0.813794026 Brain_Hypothalamus 2.597084705 0.292383211 0.292383211 -0.652480161 0.913544002 0.0649978 0.672025158 4 0 0.998207054
SERINC2 ENSG00000168528.11 0.503057132 2 2 0.534153234 Brain_Cortex 0.660253735 Brain_Substantia_nigra 1.58705311 0.41294689 0.41294689 -0.621678588 -0.439562873 -0.530620731 0.128775257 2 0 0.998207054
RPS10 ENSG00000124614.13 0.503062535 2 2 0.253502767 Brain_Cerebellum 0.624876692 Brain_Anterior_cingulate_cortex_BA24 1.200920594 0.799079406 0.799079406 0.488950571 1.141882732 0.815416651 0.461692759 2 0 0.998207054
AGAP3 ENSG00000133612.18 0.503176481 1 1 0.503176481 Brain_Cerebellar_Hemisphere 0.503176481 Brain_Cerebellar_Hemisphere 1 1 1 -0.669500156 -0.669500156 -0.669500156 NA 1 0 0.998207054
ZNF395 ENSG00000186918.13 0.503278649 1 1 0.503278649 Brain_Hippocampus 0.503278649 Brain_Hippocampus 1 1 1 0.669339948 0.669339948 0.669339948 NA 1 0 0.998207054
DAAM1 ENSG00000100592.15 0.50348118 2 2 0.705871941 Brain_Cerebellar_Hemisphere 0.802502598 Brain_Cerebellum 1.856081652 0.143918348 0.143918348 -0.250109592 0.377405957 0.063648182 0.4437205 2 0 0.998207054
CDK20 ENSG00000156345.17 0.503682205 2 2 0.356920231 Brain_Nucleus_accumbens_basal_ganglia 0.460884214 Brain_Amygdala 1.015574431 0.984425569 0.984425569 -0.921249402 -0.737391646 -0.829320524 0.130007066 2 0 0.998207054
SHBG ENSG00000129214.14 0.503884662 3 3 0.235470043 Brain_Cerebellar_Hemisphere 0.742482997 Brain_Cortex 1.405819494 0.63611299 0.63611299 -1.186385632 -0.328566997 -0.832692709 0.448258542 3 0 0.998207054
KRBA2 ENSG00000184619.3 0.50390499 8 3 0.382185608 Brain_Cerebellar_Hemisphere 0.991299392 Brain_Nucleus_accumbens_basal_ganglia 7.061220822 0.001617026 0.313435975 -0.873876326 0.63996017 -0.362014185 0.477335117 3 0 0.998207054
CDH7 ENSG00000081138.13 0.504091899 3 3 0.163960407 Brain_Putamen_basal_ganglia 0.889872959 Brain_Cerebellum 1.573650767 0.413375662 0.413375662 -1.391874492 0.138464963 -0.506537648 0.792972332 3 0 0.998207054
CREG2 ENSG00000175874.9 0.504149454 3 3 0.229596295 Brain_Nucleus_accumbens_basal_ganglia 0.37748273 Brain_Frontal_Cortex_BA9 1.431806983 0.435631417 0.435631417 -1.201399435 0.882543939 -0.416411534 1.133060896 3 0 0.998207054
IGSF21 ENSG00000117154.11 0.504174036 3 2 0.370848808 Brain_Cerebellum 0.824897202 Brain_Frontal_Cortex_BA9 2.707780723 0.036633721 0.255585556 -0.894884548 -0.221250741 -0.480840593 0.362405407 2 0 0.998207054

RP11-753A21.1 ENSG00000259478.2 0.504174363 1 1 0.504174363
Brain_Hippocampus 0.504174363 Brain_Hippocampus 1 1 1
-0.66793613 -0.66793613 -0.66793613 NA 1 0 0.998207054
FBXL18 ENSG00000155034.18 0.504201063 6 3 0.143728343
Brain_Cerebellar_Hemisphere 0.967857908 Brain_Hippocampus 4.66349773
0.047085886 0.296138244 -0.040295041 1.462046957 0.956943602
0.522328033 3 0 0.998207054
CITED4 ENSG00000179862.6 0.504545564 3 3 0.176534235
Brain_Caudate_basal_ganglia 0.610502297 Brain_Cerebellum 1.136540271
0.841534487 0.841534487 -1.35150339 0.509356592 -0.466606235
0.933766398 3 0 0.998207054
LINC01271 ENSG00000233077.1 0.50459441 1 1 0.50459441
Brain_Anterior_cingulate_cortex_BA24 0.50459441
Brain_Anterior_cingulate_cortex_BA24 1 1 1 0.667278261
0.667278261 0.667278261 NA 1 0 0.998207054
RP1-197B17.4 ENSG00000274902.1 0.504596872 2 2 0.298114291
Brain_Substantia_nigra 0.514810265 Brain_Hypothalamus 1.116067446
0.883932554 0.883932554 -1.040485728 -0.651365981 -0.845925855
0.275149212 2 0 0.998207054
S1PR2 ENSG00000267534.2 0.504883702 1 1 0.504883702
Brain_Hypothalamus 0.504883702 Brain_Hypothalamus 1 1 1
-0.666825344 -0.666825344 -0.666825344 NA 1 0 0.998207054
RP11-351I21.11 ENSG00000270074.1 0.504957746 11 10 0.035833165
Brain_Spinal_cord_cervical_c-1 0.854210855 Brain_Putamen_basal_ganglia
2.959565549 0.074855387 0.197129229 -1.28200523 2.098815535
-0.13480046 0.997673903 10 0 0.998207054
RIOX1 ENSG00000170468.7 0.504971506 1 1 0.504971506
Brain_Anterior_cingulate_cortex_BA24 0.504971506
Brain_Anterior_cingulate_cortex_BA24 1 1 1 -0.666687906
-0.666687906 -0.666687906 NA 1 0 0.998207054
GLRA1 ENSG00000145888.10 0.504978552 2 2 0.48141295
Brain_Cerebellar_Hemisphere 0.684473115 Brain_Cerebellum 1.539138126
0.460861874 0.460861874 -0.406366793 0.704031833 0.14883252
0.785170399 2 0 0.998207054
INPP4A ENSG00000040933.15 0.505080088 1 1 0.505080088
Brain_Cerebellar_Hemisphere 0.505080088 Brain_Cerebellar_Hemisphere 1 1
1 -0.66651796 -0.66651796 -0.66651796 NA 1 0
0.998207054
TTK ENSG00000112742.9 0.505149232 2 2 0.242692004 Brain_Cerebellum
0.953269096 Brain_Cortex 1.02429113 0.97570887 0.97570887 -1.168284553
0.058602027 -0.554841263 0.867539821 2 0 0.998207054
MORC2 ENSG00000133422.12 0.505340935 2 2 0.346107322
Brain_Hypothalamus 0.872656447 Brain_Anterior_cingulate_cortex_BA24
1.475138819 0.524861181 0.524861181 -0.160285161 0.942166658
0.390940748 0.779551157 2 0 0.998207054
DFNB59 ENSG00000204311.12 0.505384501 1 1 0.505384501
Brain_Substantia_nigra 0.505384501 Brain_Substantia_nigra 1 1
1 -0.666041615 -0.666041615 -0.666041615 NA 1 0
0.998207054
ZNF500 ENSG00000103199.13 0.505469953 13 5 0.152846
Brain_Anterior_cingulate_cortex_BA24 0.98847831 Brain_Hippocampus
9.740333472 0.002777579 0.329139694 -0.317907851 1.429550748
0.588016801 0.491129494 5 0 0.998207054
VWDE ENSG00000146530.11 0.505534633 9 3 0.383514067
Brain_Anterior_cingulate_cortex_BA24 0.968564061
Brain_Nucleus_accumbens_basal_ganglia 7.525805443 0.014054676
0.281571308 -0.154852439 0.871439792 0.3077778 0.353534197 3
0 0.998207054
SERINC3 ENSG00000132824.13 0.505559734 1 1 0.505559734
Brain_Hypothalamus 0.505559734 Brain_Hypothalamus 1 1 1
0.66576748 0.66576748 0.66576748 NA 1 0 0.998207054
UTP15 ENSG00000164338.9 0.505571443 8 4 0.221946273

Brain_Hypothalamus 0.685515334 Brain_Cerebellar_Hemisphere 6.354007109 0.000336742 0.300062486 0.404948545 1.221369173 0.711408099 0.310003287 4 0 0.998207054
SUCO ENSG00000094975.13 0.505779682 6 4 0.199184427 Brain_Cerebellar_Hemisphere 0.890646462 Brain_Frontal_Cortex_BA9 4.249531485 0.076689413 0.191687438 -0.93209702 1.283878625 0.047284743 0.761648622 4 0 0.998207054
TYSND1 ENSG00000156521.13 0.505805497 10 7 0.090989677 Brain_Cerebellum 0.923353906 Brain_Frontal_Cortex_BA9 6.408289811 0.056967585 0.215580339 -0.88476032 1.690200113 0.648081435 0.74897766 7 0 0.998207054
CD2AP ENSG00000198087.7 0.505845111 3 3 0.150152942 Brain_Spinal_cord_cervical_c-1 0.999855272 Brain_Cerebellar_Hemisphere 1.266601623 0.693187365 0.693187365 -1.43899146 0.345156769 -0.364551101 0.946344846 3 0 0.998207054
SERINC1 ENSG00000111897.6 0.505963309 3 3 0.149222594 Brain_Hippocampus 0.986690169 Brain_Amygdala 1.244960613 0.716550617 0.716550617 -0.655316603 1.442282858 0.267882809 1.071124266 3 0 0.998207054
AMBP ENSG00000106927.11 0.506095584 3 3 0.150793761 Brain_Cerebellum 0.754387653 Brain_Amygdala 1.937546412 0.076631836 0.076631836 -1.436733398 -0.312859177 -0.990255313 0.596460959 3 0 0.998207054
RP11-54A9.1 ENSG00000257219.5 0.506214986 1 1 0.506214986 Brain_Putamen_basal_ganglia 0.506214986 Brain_Putamen_basal_ganglia 1 1 1 0.664742843 0.664742843 0.664742843 NA 1 0 0.998207054
TRAPPC10 ENSG00000160218.12 0.506326339 4 4 0.178877257 Brain_Cerebellum 0.93138824 Brain_Cerebellar_Hemisphere 2.755050197 0.152237493 0.152237493 -1.344219997 1.304527165 0.188443508 1.136435634 4 0 0.998207054
CTC-526N19.1 ENSG00000277013.1 0.506391397 1 1 0.506391397 Brain_Caudate_basal_ganglia 0.506391397 Brain_Caudate_basal_ganglia 1 1 1 0.664467104 0.664467104 0.664467104 NA 1 0 0.998207054
MORN4 ENSG00000171160.17 0.506393304 13 3 0.320460608 Brain_Substantia_nigra 0.993410588 Brain_Caudate_basal_ganglia 11.10830446 6.90E-05 0.417572058 -0.314058825 0.99351179 0.047236686 0.40128777 3 0 0.998207054
SUZ12 ENSG00000178691.10 0.50650536 2 2 0.30614326 Brain_Caudate_basal_ganglia 0.527912402 Brain_Amygdala 1.071313696 0.928686304 0.928686304 0.631195962 1.02334816 0.827272061 0.277293478 2 0 0.998207054
GAK ENSG00000178950.16 0.506649351 7 7 0.069132778 Brain_Hippocampus 0.874320337 Brain_Cerebellar_Hemisphere 2.515912965 0.219223763 0.219223763 -1.817551059 1.198022934 -0.573865213 1.101194253 7 0 0.998207054
WNT7A ENSG00000154764.5 0.506656206 3 3 0.18756806 Brain_Spinal_cord_cervical_c-1 0.726766317 Brain_Substantia_nigra 1.246901176 0.754717176 0.754717176 -0.625901685 1.317807609 0.347112062 0.97185672 3 0 0.998207054
ZNF704 ENSG00000164684.13 0.506732264 1 1 0.506732264 Brain_Caudate_basal_ganglia 0.506732264 Brain_Caudate_basal_ganglia 1 1 1 0.663934453 0.663934453 0.663934453 NA 1 0 0.998207054
NBAS ENSG00000151779.12 0.506767062 6 2 0.487523926 Brain_Nucleus_accumbens_basal_ganglia 0.922536782 Brain_Cortex 5.339649514 0.001768864 0.366809312 -0.694252417 0.643806501 0.028860278 0.451002952 2 0 0.998207054
LINC01971 ENSG00000278200.1 0.506874714 1 1 0.506874714 Brain_Cerebellar_Hemisphere 0.506874714 Brain_Cerebellar_Hemisphere 1 1 1 0.663711912 0.663711912 0.663711912 NA 1 0

0.998207054
RP11-724N1.1 ENSG00000272912.1 0.507097316 4 4 0.108207273 Brain_Hypothalamus 0.481690982 Brain_Cortex 2.630349173 0.113658989 0.113658989 -1.606303356 -0.703585439 -1.24485212 0.388404299 4 0 0.998207054
FANCF ENSG00000183161.4 0.50718562 2 2 0.282297919 Brain_Nucleus_accumbens_basal_ganglia 0.381370678 Brain_Anterior_cingulate_cortex_BA24 1.43875573 0.56124427 0.56124427 0.875373566 1.075171574 0.97527257 0.141278526 2 0 0.998207054
GLRX2 ENSG00000023572.8 0.507245112 5 5 0.068094781 Brain_Caudate_basal_ganglia 0.791022526 Brain_Cerebellum 1.456906781 0.602854054 0.602854054 -1.824379092 0.879776004 -0.379237409 1.053785968 5 0 0.998207054
THNSL1 ENSG00000185875.12 0.507319622 2 2 0.325200262 Brain_Frontal_Cortex_BA9 0.497958788 Brain_Putamen_basal_ganglia 1.055622313 0.944377687 0.944377687 -0.98382765 -0.67770495 -0.8307663 0.216461438 2 0 0.998207054
ATP8B1 ENSG00000081923.11 0.507338151 1 1 0.507338151 Brain_Hypothalamus 0.507338151 Brain_Hypothalamus 1 1 1 -0.662988138 -0.662988138 -0.662988138 NA 1 0 0.998207054
TRAPPC13 ENSG00000113597.17 0.507423622 4 4 0.087798577 Brain_Anterior_cingulate_cortex_BA24 0.792376482 Brain_Cerebellar_Hemisphere 1.960807643 0.330925571 0.330925571 -0.263225884 1.707126316 0.97184403 0.89070135 4 0 0.998207054
SRPK1 ENSG00000096063.15 0.507518845 11 3 0.107781139 Brain_Cortex 0.688063455 Brain_Hypothalamus 9.106423213 0.004002833 0.36560499 0.401484489 1.608246791 1.25821267 0.32546961 3 0 0.998207054
SMC5 ENSG00000198887.8 0.507615596 2 2 0.395243659 Brain_Cerebellum 0.576178062 Brain_Cerebellar_Hemisphere 1.24907742 0.75092258 0.75092258 -0.850146469 0.558976056 -0.145585207 0.996400093 2 0 0.998207054
RP3-522J7.6 ENSG00000260613.1 0.507634371 3 2 0.250240051 Brain_Cerebellar_Hemisphere 0.384238268 Brain_Hypothalamus 2.889863585 0.006237484 0.103898931 -1.149766513 -0.870113707 -1.014006692 0.140003694 2 0 0.998207054
AF064858.8 ENSG00000235888.1 0.507673181 1 1 0.507673181 Brain_Anterior_cingulate_cortex_BA24 0.507673181 Brain_Anterior_cingulate_cortex_BA24 1 1 1 -0.662465121 -0.662465121 -0.662465121 NA 1 0 0.998207054
NIT1 ENSG00000158793.13 0.507761101 2 2 0.310688762 Brain_Cortex 0.533983137 Brain_Nucleus_accumbens_basal_ganglia 1.049468246 0.950531754 0.950531754 -1.01377786 -0.62193724 -0.81785755 0.27707316 2 0 0.998207054
MYBPHL ENSG00000221986.6 0.508043839 1 1 0.508043839 Brain_Spinal_cord_cervical_c-1 0.508043839 Brain_Spinal_cord_cervical_c-1 1 1 1 0.661886696 0.661886696 0.661886696 NA 1 0 0.998207054
LINC01231 ENSG00000236511.1 0.508057815 2 2 0.363136636 Brain_Hypothalamus 0.458368839 Brain_Cerebellar_Hemisphere 1.017026617 0.982973383 0.982973383 -0.909404521 -0.741535461 -0.825469991 0.118701351 2 0 0.998207054
GRP ENSG00000134443.9 0.508174311 1 1 0.508174311 Brain_Caudate_basal_ganglia 0.508174311 Brain_Caudate_basal_ganglia 1 1 1 0.661683142 0.661683142 0.661683142 NA 1 0 0.998207054
EFCAB1 ENSG00000034239.10 0.508175729 13 7 0.20177548 Brain_Amygdala 0.978266464 Brain_Cortex 8.1114182 0.00171457 0.303407825 -1.276509473 1.041638692 0.22547073 0.602471246 7 0 0.998207054
XPC ENSG00000154767.14 0.508292793 4 4 0.267335976 Brain_Cerebellar_Hemisphere 0.441049272 Brain_Hypothalamus 2.66043328

0.092150951 0.092150951 0.770422566 1.109218402 0.899950519
0.153066928 4 0 0.998207054
RAB11A ENSG00000103769.9 0.508351298 1 1 0.508351298
Brain_Substantia_nigra 0.508351298 Brain_Substantia_nigra 1 1
1 0.661407062 0.661407062 0.661407062 NA 1 0
0.998207054
EIF3D ENSG00000100353.17 0.508376271 1 1 0.508376271 Brain_Cortex
0.508376271 Brain_Cortex 1 1 1 -0.661368111 -0.661368111
-0.661368111 NA 1 0 0.998207054
DGCR2 ENSG00000070413.19 0.508398379 11 3 0.146897993 Brain_Cerebellum
0.531491821 Brain_Spinal_cord_cervical_c-1 9.303609096 0.009566784
0.406245272 0.625730353 1.45057589 1.102772662 0.268205624 3
0 0.998207054
HSF2 ENSG00000025156.12 0.508514012 3 3 0.37719564
Brain_Putamen_basal_ganglia 0.838596725 Brain_Cerebellum 1.956949305
0.109673249 0.109673249 -0.883075204 0.75200497 0.024206176
0.832185252 3 0 0.998207054
EPG5 ENSG00000152223.12 0.508622698 11 3 0.094512443
Brain_Nucleus_accumbens_basal_ganglia 0.550807832
Brain_Anterior_cingulate_cortex_BA24 9.248929439 0.001951513
0.397504513 -1.672060244 -0.596550045 -1.135298644 0.404975711
3 0 0.998207054
HS2ST1 ENSG00000153936.16 0.508646239 4 4 0.338034564
Brain_Frontal_Cortex_BA9 0.81910064 Brain_Anterior_cingulate_cortex_BA24
2.495933196 0.305192718 0.305192718 -0.958055914 0.530939601
0.027739713 0.669503898 4 0 0.998207054
NOTCH2NL ENSG00000264343.5 0.508743955 1 1 0.508743955
Brain_Nucleus_accumbens_basal_ganglia 0.508743955
Brain_Nucleus_accumbens_basal_ganglia 1 1 1 0.660794743
0.660794743 0.660794743 NA 1 0 0.998207054
DDHD1 ENSG00000100523.14 0.508857979 6 4 0.124687647
Brain_Spinal_cord_cervical_c-1 0.837039617 Brain_Hippocampus
5.027841749 0.037734134 0.188251544 -1.535391678 -0.205681619
-0.911065756 0.42244288 4 0 0.998207054
AC004012.1 ENSG00000233942.1 0.508988962 2 2 0.290380204
Brain_Caudate_basal_ganglia 0.334571297 Brain_Putamen_basal_ganglia
1.523973143 0.476026857 0.476026857 -1.057287922 -0.964947123
-1.011117522 0.065294805 2 0 0.998207054
BRMS1 ENSG00000174744.13 0.50904886 2 2 0.282194814 Brain_Cortex
0.820978569 Brain_Amygdala 1.191501924 0.808498076 0.808498076
-1.075401938 0.226286537 -0.424557701 0.920432748 2 0
0.998207054
SEMA4A ENSG00000196189.12 0.509260752 3 3 0.151847761
Brain_Cerebellum 0.559172603 Brain_Cortex 1.889650787 0.222971229
0.222971229 -1.433035259 -0.584070913 -1.136334782 0.478716618
3 0 0.998207054
HCFC1R1 ENSG00000103145.10 0.509301764 2 2 0.403156197
Brain_Anterior_cingulate_cortex_BA24 0.408676006 Brain_Cerebellum
1.023798521 0.976201479 0.976201479 -0.8359977 0.826225817
-0.004885941 1.175369521 2 0 0.998207054
VSX2 ENSG00000119614.2 0.509313047 1 1 0.509313047
Brain_Nucleus_accumbens_basal_ganglia 0.509313047
Brain_Nucleus_accumbens_basal_ganglia 1 1 1 -0.659907724
-0.659907724 -0.659907724 NA 1 0 0.998207054
RSPH10B ENSG00000155026.16 0.509407706 5 5 0.281177763
Brain_Cerebellar_Hemisphere 0.79751497 Brain_Cerebellum 2.441860817
0.189917091 0.189917091 -1.077677365 1.017780678 -0.234230331
0.873564852 5 0 0.998207054
RP11-1152H14.1 ENSG00000271320.1 0.509452635 3 3 0.192975045
Brain_Cerebellum 0.919954909 Brain_Caudate_basal_ganglia 1.335009252
0.712398198 0.712398198 -1.301828422 0.725185255 -0.225711229
1.019292049 3 0 0.998207054

CSTB ENSG00000160213.6 0.509475046 6 5 0.300543246
Brain_Nucleus_accumbens_basal_ganglia 0.904885864 Brain_Cerebellum
4.179587924 0.100660128 0.20429186 -0.798411786 1.035269122
-0.148465025 0.693474127 5 0 0.998207054
DEGS2 ENSG00000168350.7 0.509486087 6 6 0.206236882 Brain_Cortex
0.906146679 Brain_Cerebellar_Hemisphere 1.687120246 0.387767751
0.387767751 -1.263980903 0.975225505 -0.147210412 0.957793971
6 0 0.998207054
ACOT8 ENSG00000101473.16 0.509632631 1 1 0.509632631 Brain_Amygdala
0.509632631 Brain_Amygdala 1 1 1 -0.659409831 -0.659409831
-0.659409831 NA 1 0 0.998207054
GPRC5B ENSG00000167191.11 0.509669069 3 3 0.219993106
Brain_Caudate_basal_ganglia 0.756613922 Brain_Nucleus_accumbens_basal_ganglia
1.503296431 0.525666196 0.525666196 -1.226546451 0.309930351
-0.518631004 0.775310787 3 0 0.998207054
MAP3K10 ENSG00000130758.7 0.509771163 1 1 0.509771163
Brain_Cerebellar_Hemisphere 0.509771163 Brain_Cerebellar_Hemisphere 1 1
1 0.659194057 0.659194057 0.659194057 NA 1 0
0.998207054
PPP1R14C ENSG00000198729.4 0.509777326 2 2 0.271442487
Brain_Cortex 0.648719959 Brain_Cerebellum 1.077298343 0.922701657
0.922701657 -1.099746733 0.455541165 -0.322102784 1.099754619
2 0 0.998207054
ARPC2 ENSG00000163466.15 0.509826521 2 1 0.492597818
Brain_Cerebellar_Hemisphere 0.530191961 Brain_Cerebellum 1.98691336 0.01308664
1.98691336 0.62771301 0.68618284 0.656947925 0.041344413 1 0
0.998207054
ZNF575 ENSG00000176472.10 0.509864825 5 5 0.256055488
Brain_Amygdala 0.95332453 Brain_Cerebellum 3.229184745 0.152982608
0.152982608 -1.135763665 0.599893144 -0.22687807 0.622660393
5 0 0.998207054
ARHGEF16 ENSG00000130762.14 0.50994338 3 3 0.178249298
Brain_Cerebellar_Hemisphere 0.790241563 Brain_Cerebellum 1.495224507
0.504964067 0.504964067 -1.346165037 0.537781888 -0.358126697
0.945346456 3 0 0.998207054
NID1 ENSG00000116962.14 0.509982274 1 1 0.509982274
Brain_Cerebellar_Hemisphere 0.509982274 Brain_Cerebellar_Hemisphere 1 1
1 0.658865294 0.658865294 0.658865294 NA 1 0
0.998207054
TRAPPC9 ENSG00000167632.14 0.510056252 3 3 0.136086243
Brain_Cortex 0.290819097 Brain_Cerebellum 2.366099759 0.138592211
0.138592211 1.056326448 1.49052502 1.318290457 0.230587576 3
0 0.998207054
AQP1 ENSG00000240583.11 0.510280612 1 1 0.510280612 Brain_Cortex
0.510280612 Brain_Cortex 1 1 1 0.658400815 0.658400815
0.658400815 NA 1 0 0.998207054
YKT6 ENSG00000106636.7 0.510306519 2 2 0.248988212
Brain_Frontal_Cortex_BA9 0.89350622 Brain_Cortex 1.004647164
0.995352836 0.995352836 -0.13386893 1.152810407 0.509470739
0.909819684 2 0 0.998207054
C8orf33 ENSG00000182307.12 0.510454309 3 2 0.364628285
Brain_Hippocampus 0.463342782 Brain_Frontal_Cortex_BA9 1.953240357
0.047635707 0.999123936 -0.733353615 0.906581242 0.336916163
0.92752379 2 0 0.998207054
PRKCSH ENSG00000130175.9 0.510575561 2 2 0.254314069
Brain_Cerebellum 0.898944842 Brain_Nucleus_accumbens_basal_ganglia
1.289498743 0.710501257 0.710501257 -1.13993335 -0.126994386
-0.633463868 0.716256011 2 0 0.998207054
GJA5 ENSG00000265107.2 0.510594193 1 1 0.510594193
Brain_Putamen_basal_ganglia 0.510594193 Brain_Putamen_basal_ganglia 1 1
1 0.657912757 0.657912757 0.657912757 NA 1 0
0.998207054

PRKD2 ENSG00000105287.12 0.510650339 10 4 0.083950134
Brain_Putamen_basal_ganglia 0.796970211 Brain_Amygdala 7.03637915
0.019229832 0.368655628 0.25727018 1.728212497 1.08710057 0.4591101
4 0 0.998207054
ATP5G3 ENSG00000154518.9 0.510663533 9 5 0.24229447
Brain_Nucleus_accumbens_basal_ganglia 0.92698839 Brain_Hypothalamus
6.920411492 0.022475458 0.254734359 -0.091634562 1.169270984
0.545262901 0.380377266 5 0 0.998207054
STK38L ENSG00000211455.7 0.510825489 3 3 0.520875029
Brain_Nucleus_accumbens_basal_ganglia 0.883064047 Brain_Cortex
2.114903906 0.177268254 0.177268254 -0.318381295 0.641997157
0.058843248 0.512237124 3 0 0.998207054
PAX7 ENSG00000009709.11 0.510853224 2 2 0.401419593
Brain_Anterior_cingulate_cortex_BA24 0.978081561 Brain_Frontal_Cortex_BA9
1.706810367 0.293189633 0.293189633 0.027474145 0.8390886
0.433281372 0.573898085 2 0 0.998207054
OR2L2 ENSG00000203663.3 0.51096665 10 7 0.047085089 Brain_Cerebellum
0.97504598 Brain_Hippocampus 5.497860483 0.06374501 0.307071276
-1.98553402 0.733479211 0.154671107 0.795869822 7 0
0.998207054
TMEM168 ENSG00000146802.12 0.510977973 4 3 0.179903163
Brain_Putamen_basal_ganglia 0.813648573 Brain_Cerebellum 2.588160531
0.02393599 0.394949557 -1.341053253 -0.235721773 -0.639633368
0.486631866 3 0 0.998207054
PEX12 ENSG00000108733.9 0.511015612 4 4 0.178495428
Brain_Hypothalamus 0.705148312 Brain_Caudate_basal_ganglia 2.973773824
0.14742378 0.14742378 0.378380015 1.345402069 0.837081436
0.405675201 4 0 0.998207054
RP11-314C16.1 ENSG00000230939.1 0.511109857 6 3 0.157693578
Brain_Nucleus_accumbens_basal_ganglia 0.713456005 Brain_Caudate_basal_ganglia
5.147196433 0.021418955 0.208998417 0.367218548 1.41287127
0.806904778 0.386171018 3 0 0.998207054
RDH10 ENSG00000121039.9 0.511148416 3 3 0.388506252 Brain_Cortex
0.762254623 Brain_Cerebellar_Hemisphere 2.542301128 0.104774529
0.104774529 -0.862329449 -0.302521399 -0.630510913 0.292032371
3 0 0.998207054
RP11-461L13.3 ENSG00000270750.1 0.511171345 1 1 0.511171345
Brain_Hippocampus 0.511171345 Brain_Hippocampus 1 1 1
-0.657014886 -0.657014886 -0.657014886 NA 1 0 0.998207054
RP11-92G12.3 ENSG00000260088.1 0.511194859 13 1 0.417156308
Brain_Cerebellum 0.634157844 Brain_Hippocampus 12.69417599
0.000793637 12.69417599 -0.811364625 -0.475882846 -0.648988041
0.084128863 1 0 0.998207054
DYNLT1 ENSG00000146425.10 0.511590929 2 2 0.304985196
Brain_Caudate_basal_ganglia 0.592869941 Brain_Hypothalamus 1.002097486
0.997902514 0.997902514 -1.025801422 -0.534681754 -0.780241588
0.347274048 2 0 0.998207054
LINC02054 ENSG00000224153.2 0.511717421 1 1 0.511717421
Brain_Cerebellar_Hemisphere 0.511717421 Brain_Cerebellar_Hemisphere 1 1
1 -0.656165847 -0.656165847 -0.656165847 NA 1 0
0.998207054
PDLIM5 ENSG00000163110.14 0.511732664 4 2 0.264655678
Brain_Nucleus_accumbens_basal_ganglia 0.39458683 Brain_Caudate_basal_ganglia
3.196334148 0.0002205 0.74070543 -1.115454566 -0.851328621
-0.999980755 0.135954161 2 0 0.998207054
RP11-643A5.2 ENSG00000259669.5 0.511732946 2 2 0.325864245
Brain_Cerebellar_Hemisphere 0.491817443 Brain_Cerebellum 1.929482491
0.070517509 0.070517509 -0.982478347 -0.687421039 -0.834949693
0.208637023 2 0 0.998207054
TJP1 ENSG00000104067.16 0.511736817 2 2 0.26134271 Brain_Hypothalamus
0.780121122 Brain_Cerebellar_Hemisphere 1.000522909 0.999477091
0.999477091 -1.123223204 0.279161195 -0.422031004 0.991635518

2	0	0.998207054
RP11-281A20.2	ENSG00000228512.2	0.511772088	1	1	0.511772088
Brain_Cerebellum	0.511772088	Brain_Cerebellum	1	1	1	0.656080876
0.656080876	0.656080876	NA	1	0	0.998207054
ATP8B3	ENSG00000130270.16	0.511799056	4	4	0.193218678
Brain_Caudate_basal_ganglia	0.980808405	Brain_Spinal_cord_cervical_c-1
1.49255894	0.384888209	0.384888209	-1.301116215	1.00348568
-0.248211739	0.995218785	4	0	0.998207054
FLJ37505	ENSG00000256597.2	0.511814914	1	1	0.511814914
Brain_Putamen_basal_ganglia	0.511814914	Brain_Putamen_basal_ganglia	1	1
1	0.656014315	0.656014315	0.656014315	NA	1	0
0.998207054
GPR137C	ENSG00000180998.11	0.511957316	3	3	0.156494327
Brain_Cerebellar_Hemisphere	0.381340988	Brain_Frontal_Cortex_BA9
1.891596683	0.535669877	0.535669877	-1.41696101	-0.875428151
-1.12753607	0.272688246	3	0	0.998207054
HLF	ENSG00000108924.13	0.51218822	1	1	0.51218822
Brain_Spinal_cord_cervical_c-1	0.51218822	Brain_Spinal_cord_cervical_c-1	1
1	1	0.655434228	0.655434228	0.655434228	NA	1	0
0.998207054
RP11-127L20.3	ENSG00000228261.1	0.512191666	8	7	0.1031591
Brain_Putamen_basal_ganglia	0.813198976	Brain_Cortex	4.294975728
0.038069784	0.165875023	0.236301174	1.629727512	1.128903483
0.491895163	7	0	0.998207054
BCAT1	ENSG00000060982.14	0.512246969	2	2	0.267115948
Brain_Frontal_Cortex_BA9	0.730943584	Brain_Cortex	1.554481098
0.445518902	0.445518902	0.343870625	1.109728708	0.726799666
0.541543443	2	0	0.998207054
RP11-525A16.4	ENSG00000273143.1	0.512267194	1	1	0.512267194
Brain_Cerebellum	0.512267194	Brain_Cerebellum	1	1	1	0.655311538
0.655311538	0.655311538	NA	1	0	0.998207054
RP13-942N8.1	ENSG00000256092.2	0.512513643	10	3	0.118403724
Brain_Cerebellar_Hemisphere	0.440984361	Brain_Hippocampus	8.848507489
0.000810474	0.314860773	0.770532033	1.561508906	1.113429929
0.240311805	3	0	0.998207054
PHTF2	ENSG00000006576.16	0.512675065	2	2	0.301977063	Brain_Amygdala
0.492261321	Brain_Cerebellum	1.168354249	0.831645751	0.831645751
0.68671662	1.032202929	0.859459775	0.244295712	2	0
0.998207054
SNTB1	ENSG00000172164.13	0.512706938	2	2	0.252241158
Brain_Putamen_basal_ganglia	0.836356976	Brain_Cerebellum	1.041732154
0.958267846	0.958267846	-0.206555552	1.144922752	0.4691836
0.955639474	2	0	0.998207054
MBLAC1	ENSG00000214309.4	0.51288943	8	4	0.243148172
Brain_Nucleus_accumbens_basal_ganglia	0.874733241	Brain_Cerebellum
5.867525343	0.000157294	0.232380976	-1.16715403	0.24775084
-0.593104405	0.464269834	4	0	0.998207054
GPR180	ENSG00000152749.7	0.512969323	7	3	0.165566653
Brain_Spinal_cord_cervical_c-1	0.724341239	Brain_Caudate_basal_ganglia
5.963591574	0.007469207	0.307007335	-1.386590544	-0.352662816
-0.969217562	0.368736227	3	0	0.998207054
COQ9	ENSG00000088682.13	0.513036957	9	2	0.28429697	Brain_Hypothalamus
0.439177146	Brain_Nucleus_accumbens_basal_ganglia	7.805365186
0.002330158	0.823154207	0.773583477	1.070716392	0.926967965
0.096350096	2	0	0.998207054
NKAPL	ENSG00000189134.3	0.513212508	1	1	0.513212508
Brain_Spinal_cord_cervical_c-1	0.513212508	Brain_Spinal_cord_cervical_c-1
1	1	1	-0.653843701	-0.653843701	-0.653843701	NA	1	0
0.998207054
PRX	ENSG00000105227.14	0.513268297	1	1	0.513268297
Brain_Nucleus_accumbens_basal_ganglia	0.513268297
Brain_Nucleus_accumbens_basal_ganglia	1	1	1	-0.653757119

-0.653757119 -0.653757119 NA 1 0 0.998207054
GABRR2 ENSG00000111886.10 0.513431098 3 3 0.132681147
Brain_Cerebellum 0.94069887 Brain_Putamen_basal_ganglia 1.067687718
0.919364621 0.919364621 -0.093446057 1.503612591 0.445258343
0.916611179 3 0 0.998207054
IST1 ENSG00000182149.20 0.513698145 5 4 0.145472288
Brain_Hippocampus 0.563833329 Brain_Cerebellar_Hemisphere 3.0346093
0.056110364 0.48103176 -1.455711865 0.577157107 -0.771750325
0.800931347 4 0 0.998207054
ISY1 ENSG00000240682.9 0.513706425 1 1 0.513706425
Brain_Hippocampus 0.513706425 Brain_Hippocampus 1 1 1
-0.653077332 -0.653077332 -0.653077332 NA 1 0 0.998207054
CHMP3 ENSG00000115561.15 0.513915575 8 7 0.073116891
Brain_Spinal_cord_cervical_c-1 0.534732189
Brain_Nucleus_accumbens_basal_ganglia 4.389686499 0.084389088 0.18697928
-1.79210036 -0.620798532 -1.409157318 0.437377112 7 0
0.998207054
NYNRIN ENSG00000205978.5 0.513922227 6 6 0.09707943
Brain_Spinal_cord_cervical_c-1 0.859432351 Brain_Frontal_Cortex_BA9
1.491338938 0.605374695 0.605374695 -1.659180481 1.010715362
-0.095406403 1.017189122 6 0 0.998207054
CEP290 ENSG00000198707.14 0.514015338 1 1 0.514015338
Brain_Cerebellum 0.514015338 Brain_Cerebellum 1 1 1 -0.652598213
-0.652598213 -0.652598213 NA 1 0 0.998207054
NAP1L4 ENSG00000205531.12 0.514141844 4 4 0.267929699
Brain_Hippocampus 0.506665829 Brain_Substantia_nigra 1.86576057
0.484026045 0.484026045 -1.107842831 0.664038251 -0.577340316
0.837217803 4 0 0.998207054
ARAP3 ENSG00000120318.15 0.514312973 1 1 0.514312973
Brain_Caudate_basal_ganglia 0.514312973 Brain_Caudate_basal_ganglia 1 1
1 0.652136728 0.652136728 0.652136728 NA 1 0
0.998207054
LINC01659 ENSG00000234928.1 0.514342388 5 2 0.640258179
Brain_Caudate_basal_ganglia 0.962016099 Brain_Anterior_cingulate_cortex_BA24
4.636594868 1.16E-16 0.247098045 -0.190159558 0.467337853
0.199264122 0.296760636 2 0 0.998207054
ONECUT1 ENSG00000169856.8 0.514564337 1 1 0.514564337
Brain_Cerebellum 0.514564337 Brain_Cerebellum 1 1 1 0.651747093
0.651747093 0.651747093 NA 1 0 0.998207054
DOT1L ENSG00000104885.17 0.514613172 4 4 0.170197491 Brain_Cerebellum
0.696482858 Brain_Putamen_basal_ganglia 3.462787893 0.120339699
0.120339699 -1.371569509 -0.390072608 -0.832714309 0.404575346
4 0 0.998207054
BOLA1 ENSG00000178096.8 0.514677835 1 1 0.514677835
Brain_Spinal_cord_cervical_c-1 0.514677835 Brain_Spinal_cord_cervical_c-1
1 1 1 -0.651571195 -0.651571195 -0.651571195 NA 1 0
0.998207054
THSD7A ENSG00000005108.15 0.514692945 3 3 0.200370703
Brain_Cortex 0.916986704 Brain_Hippocampus 1.645322047
0.374693326 0.374693326 -0.521234442 1.280496136 0.287830616
0.914789657 3 0 0.998207054
AFDN ENSG00000130396.20 0.514762587 3 3 0.138927006
Brain_Putamen_basal_ganglia 0.716622183 Brain_Cerebellar_Hemisphere
1.554056118 0.639792864 0.639792864 0.362976842 1.479798585
0.888733657 0.561267818 3 0 0.998207054
TSC22D2 ENSG00000196428.12 0.514876893 1 1 0.514876893
Brain_Cerebellum 0.514876893 Brain_Cerebellum 1 1 1 0.651262745
0.651262745 0.651262745 NA 1 0 0.998207054
GNAQ ENSG00000156052.10 0.514885622 7 4 0.100339016
Brain_Anterior_cingulate_cortex_BA24 0.990046922 Brain_Putamen_basal_ganglia
4.891877878 0.008340199 0.264647812 -0.603865055 1.643212301
0.048576059 0.738479923 4 0 0.998207054

ABHD12B ENSG00000131969.14 0.514941225 3 3 0.131861891
Brain_Caudate_basal_ganglia 0.974381229 Brain_Substantia_nigra 1.88822042
0.117000258 0.117000258 -1.506800149 -0.032113887 -0.938269355
0.793200926 3 0 0.998207054
RP1-104O17.3 ENSG00000226207.1 0.514979989 1 1 0.514979989
Brain_Spinal_cord_cervical_c-1 0.514979989 Brain_Spinal_cord_cervical_c-1
1 1 1 0.651103017 0.651103017 0.651103017 NA 1 0
0.998207054
LINC00284 ENSG00000233725.7 0.515060237 1 1 0.515060237
Brain_Hippocampus 0.515060237 Brain_Hippocampus 1 1 1
-0.650978699 -0.650978699 -0.650978699 NA 1 0 0.998207054
MARVELD3 ENSG00000140832.9 0.515066989 3 2 0.210687536
Brain_Nucleus_accumbens_basal_ganglia 0.473816234 Brain_Cerebellum
2.570473968 0.010200615 0.419325417 -1.251677132 -0.716283628
-1.001923893 0.269494824 2 0 0.998207054
UBE2Q2 ENSG00000140367.11 0.515118695 1 1 0.515118695
Brain_Cortex 0.515118695 Brain_Cortex 1 1 1 0.650888145
0.650888145 0.650888145 NA 1 0 0.998207054
TMEM92 ENSG00000167105.7 0.515216453 1 1 0.515216453
Brain_Cortex 0.515216453 Brain_Cortex 1 1 1 0.650736723
0.650736723 0.650736723 NA 1 0 0.998207054
TRAPPC6B ENSG00000182400.14 0.51529491 5 4 0.138576427 Brain_Cortex
0.95268066 Brain_Cerebellar_Hemisphere 3.286350927 0.097734486
0.216395356 -1.48111314 -0.059340806 -0.758489728 0.657165168
4 0 0.998207054
FADD ENSG00000168040.4 0.515308723 1 1 0.515308723
Brain_Caudate_basal_ganglia 0.515308723 Brain_Caudate_basal_ganglia 1 1
1 -0.650593817 -0.650593817 -0.650593817 NA 1 0
0.998207054
ZNF568 ENSG00000198453.12 0.515383489 9 6 0.124328794
Brain_Putamen_basal_ganglia 0.941402656 Brain_Caudate_basal_ganglia
6.240297961 0.06434661 0.219850539 -0.073507023 1.536855113
0.699755244 0.537029011 6 0 0.998207054
BCAS1 ENSG00000064787.13 0.51538747 1 1 0.51538747
Brain_Spinal_cord_cervical_c-1 0.51538747 Brain_Spinal_cord_cervical_c-1 1
1 1 0.650471864 0.650471864 0.650471864 NA 1 0
0.998207054
RIMS1 ENSG00000079841.18 0.515478421 2 2 0.278788244 Brain_Cerebellum
0.672808289 Brain_Cerebellar_Hemisphere 1.029704751 0.970295249
0.970295249 -1.083045463 0.422296943 -0.33037426 1.064437823
2 0 0.998207054
DNAJC11 ENSG00000007923.15 0.515488732 1 1 0.515488732
Brain_Frontal_Cortex_BA9 0.515488732 Brain_Frontal_Cortex_BA9 1 1
1 -0.650315059 -0.650315059 -0.650315059 NA 1 0
0.998207054
RSPH4A ENSG00000111834.12 0.515579289 5 5 0.274838199
Brain_Cerebellum 0.931781029 Brain_Cerebellar_Hemisphere 2.423413501
0.171640865 0.171640865 -1.089462377 1.091988402 -0.226041995
0.859738188 5 0 0.998207054
PHF14 ENSG00000106443.15 0.515641221 1 1 0.515641221
Brain_Putamen_basal_ganglia 0.515641221 Brain_Putamen_basal_ganglia 1 1
1 -0.650078957 -0.650078957 -0.650078957 NA 1 0
0.998207054
GABPB1 ENSG00000104064.16 0.515721967 8 3 0.242125116
Brain_Caudate_basal_ganglia 0.910202156 Brain_Hippocampus 6.216175396
0.011127616 0.322304209 -1.169691559 0.300089904 -0.148353493
0.582952425 3 0 0.998207054
RP11-111M22.3 ENSG00000255135.3 0.515791694 4 4 0.131522844
Brain_Caudate_basal_ganglia 0.858738207 Brain_Amygdala 1.533208563
0.516102448 0.516102448 -0.177980606 1.508123803 0.751596512
0.70621545 4 0 0.998207054
RP11-342K6.4 ENSG00000270996.1 0.515825326 5 5 0.177510078

Brain_Putamen_basal_ganglia 0.813645707 Brain_Caudate_basal_ganglia
1.691366158 0.367255395 0.367255395 -1.348461241 0.553045841
-0.610417187 0.926421455 5 0 0.998207054
UVSSA ENSG00000163945.15 0.515852125 13 3 0.071519566
Brain_Nucleus_accumbens_basal_ganglia 0.681252448 Brain_Amygdala
9.953626368 0.015337623 0.540060334 -1.802164262 -0.410754652
-1.05585419 0.407730544 3 0 0.998207054
FAT2 ENSG00000086570.12 0.516015131 3 3 0.205950425
Brain_Nucleus_accumbens_basal_ganglia 0.985588125 Brain_Cerebellar_Hemisphere
1.281435501 0.758978552 0.758978552 -1.264779382 -0.018063589
-0.778439869 0.667006131 3 0 0.998207054
ZNF106 ENSG00000103994.16 0.516055252 3 2 0.481543452
Brain_Anterior_cingulate_cortex_BA24 0.918753696 Brain_Substantia_nigra
2.633134429 0.038288136 0.328577435 -0.102003753 0.703822287
0.419317666 0.452106789 2 0 0.998207054
SPRR2G ENSG00000159516.8 0.516083829 1 1 0.516083829
Brain_Frontal_Cortex_BA9 0.516083829 Brain_Frontal_Cortex_BA9 1 1
1 -0.649393864 -0.649393864 -0.649393864 NA 1 0
0.998207054
SCG5 ENSG00000166922.8 0.516084014 3 3 0.304085847
Brain_Substantia_nigra 0.542010347 Brain_Cortex 1.364075481
0.632983902 0.632983902 0.609775782 1.027710882 0.875157009
0.230683649 3 0 0.998207054
RP11-378J18.9 ENSG00000276997.4 0.516150411 1 1 0.516150411
Brain_Hypothalamus 0.516150411 Brain_Hypothalamus 1 1 1
-0.649290831 -0.649290831 -0.649290831 NA 1 0 0.998207054
TSTA3 ENSG00000104522.15 0.516213272 2 2 0.344696139
Brain_Nucleus_accumbens_basal_ganglia 0.667381406 Brain_Cortex
1.221591128 0.778408872 0.778408872 -0.429744642 0.944927013
0.257591186 0.972039649 2 0 0.998207054
TMEM132A ENSG00000006118.14 0.51627853 1 1 0.51627853 Brain_Amygdala
0.51627853 Brain_Amygdala 1 1 1 -0.649092592 -0.649092592
-0.649092592 NA 1 0 0.998207054
RP11-468N14.13 ENSG00000272626.1 0.516307399 3 2 0.794895207
Brain_Cerebellum 0.919655021 Brain_Cerebellar_Hemisphere 2.874527672
0.024108411 0.101363916 -0.25995925 0.242797863 0.027902299
0.25919916 2 0 0.998207054
CA9 ENSG00000107159.12 0.516315587 2 2 0.464836999 Brain_Cerebellum
0.964098744 Brain_Cerebellar_Hemisphere 1.796251495 0.203748505
0.203748505 -0.730905299 -0.045010746 -0.387958022 0.48500069 2
0 0.998207054
SPATA9 ENSG00000145757.15 0.516381807 2 2 0.251089862
Brain_Frontal_Cortex_BA9 0.567776584 Brain_Caudate_basal_ganglia
1.444946494 0.555053506 0.555053506 0.571329093 1.147706221
0.859517657 0.407560176 2 0 0.998207054
FAM49A ENSG00000197872.11 0.516420871 1 1 0.516420871
Brain_Frontal_Cortex_BA9 0.516420871 Brain_Frontal_Cortex_BA9 1 1
1 -0.648872376 -0.648872376 -0.648872376 NA 1 0
0.998207054
ZBTB7C ENSG00000184828.9 0.51642924 1 1 0.51642924
Brain_Nucleus_accumbens_basal_ganglia 0.51642924
Brain_Nucleus_accumbens_basal_ganglia 1 1 1 -0.648859429
-0.648859429 -0.648859429 NA 1 0 0.998207054
RP11-96A15.1 ENSG00000278464.1 0.516452055 1 1 0.516452055
Brain_Hypothalamus 0.516452055 Brain_Hypothalamus 1 1 1
-0.648824136 -0.648824136 -0.648824136 NA 1 0 0.998207054
OLFML3 ENSG00000116774.11 0.516526828 4 3 0.162401017
Brain_Putamen_basal_ganglia 0.710643964 Brain_Cortex 2.723252248
0.079654465 0.219418778 0.370991363 1.39704175 0.767463268
0.445642366 3 0 0.998207054
PIK3CD ENSG00000171608.15 0.516585775 4 4 0.179416424
Brain_Cortex 0.752474058 Brain_Frontal_Cortex_BA9 1.356149415

0.769194117 0.769194117 -1.342554031 0.927314187 -0.138101429
0.981552407 4 0 0.998207054
MTHFR ENSG00000177000.10 0.516641619 8 4 0.093856034
Brain_Nucleus_accumbens_basal_ganglia 0.871732215
Brain_Anterior_cingulate_cortex_BA24 6.154406336 5.37E-18 0.248168968
-0.161458601 1.675398687 1.183708732 0.570925351 4 0
0.998207054
TPCN2 ENSG00000162341.16 0.516652096 6 6 0.225522734 Brain_Cerebellum
0.942444857 Brain_Frontal_Cortex_BA9 2.427245554 0.360149359
0.360149359 -1.211972953 0.618876571 -0.316523305 0.786778215
6 0 0.998207054
EAPP ENSG00000129518.8 0.516698748 1 1 0.516698748
Brain_Hypothalamus 0.516698748 Brain_Hypothalamus 1 1 1
-0.648442564 -0.648442564 -0.648442564 NA 1 0 0.998207054
HACD4 ENSG00000188921.13 0.516704941 6 6 0.26389482
Brain_Anterior_cingulate_cortex_BA24 0.963021717 Brain_Caudate_basal_ganglia
2.920021091 0.11767196 0.11767196 -1.117232752 0.265228924
-0.495567025 0.546619352 6 0 0.998207054
FANCD2 ENSG00000144554.10 0.516772398 4 4 0.105949555
Brain_Hippocampus 0.889617286 Brain_Spinal_cord_cervical_c-1
2.051355223 0.362219985 0.362219985 -0.384776241 1.616669896
0.410077176 0.852639406 4 0 0.998207054
ZFP57 ENSG00000204644.9 0.516775793 2 2 0.251327634
Brain_Frontal_Cortex_BA9 0.95433083 Brain_Caudate_basal_ganglia 1.007791572
0.992208428 0.992208428 0.057269106 1.147130635 0.602199871
0.770648478 2 0 0.998207054
CBFA2T2 ENSG00000078699.21 0.517215293 4 4 0.296354819
Brain_Cerebellum 0.965186695 Brain_Caudate_basal_ganglia 1.744254456
0.437255156 0.437255156 -0.619073363 1.044282258 0.041738866
0.721077175 4 0 0.998207054
KCNJ11 ENSG00000187486.5 0.517264761 2 2 0.336534076
Brain_Caudate_basal_ganglia 0.367616394 Brain_Nucleus_accumbens_basal_ganglia
1.317486709 0.682513291 0.682513291 -0.961035983 -0.900947199
-0.930991591 0.042489186 2 0 0.998207054
ENDOG ENSG00000167136.6 0.517317852 6 3 0.201217422 Brain_Amygdala
0.88776246 Brain_Cortex 5.164945142 0.01448703 0.198187688
-1.278090773 -0.141136056 -0.569186312 0.407270285 3 0
0.998207054
NEFM ENSG00000104722.13 0.517421366 1 1 0.517421366
Brain_Putamen_basal_ganglia 0.517421366 Brain_Putamen_basal_ganglia 1 1
1 -0.6473254 -0.6473254 -0.6473254 NA 1 0 0.998207054
GALNT6 ENSG00000139629.15 0.517499587 12 1 0.27057358
Brain_Cerebellar_Hemisphere 0.797958653 Brain_Cortex 10.85241926
0.000205356 10.85241926 -1.101742625 -0.255989878 -0.616943374
0.213195855 1 0 0.998207054
WRAP73 ENSG00000116213.15 0.517534551 1 1 0.517534551
Brain_Hypothalamus 0.517534551 Brain_Hypothalamus 1 1 1
-0.64715049 -0.64715049 -0.64715049 NA 1 0 0.998207054
FIGN ENSG00000182263.13 0.517543349 3 3 0.166524122
Brain_Nucleus_accumbens_basal_ganglia 0.970600997 Brain_Putamen_basal_ganglia
1.311210631 0.691618322 0.691618322 -0.518656511 1.38345916
0.275982707 0.988894007 3 0 0.998207054
POLE ENSG00000177084.16 0.517682299 8 1 0.475737689 Brain_Cerebellum
0.564997114 Brain_Caudate_basal_ganglia 7.904108533 0.000876989
7.904108533 0.575435037 0.713174653 0.642874635 0.048031871
1 0 0.998207054
MYCN ENSG00000134323.11 0.517800474 1 1 0.517800474
Brain_Spinal_cord_cervical_c-1 0.517800474 Brain_Spinal_cord_cervical_c-1
1 1 1 -0.646739623 -0.646739623 -0.646739623 NA 1 0
0.998207054
SDCBP ENSG00000137575.11 0.517901119 1 1 0.517901119
Brain_Hypothalamus 0.517901119 Brain_Hypothalamus 1 1 1

0.64658415 0.64658415 0.64658415 NA 1 0 0.998207054
CASKIN1 ENSG00000167971.15 0.517937658 1 1 0.517937658
Brain_Hypothalamus 0.517937658 Brain_Hypothalamus 1 1 1
0.646527709 0.646527709 0.646527709 NA 1 0 0.998207054
SAXO2 ENSG00000188659.9 0.51800862 13 2 0.170411895 Brain_Hypothalamus
0.903326033 Brain_Putamen_basal_ganglia 11.87492695 0.000551696
0.49244367 0.121460836 1.370881515 1.077461984 0.340989069 2
0 0.998207054
FSCN1 ENSG00000075618.17 0.518108902 2 2 0.603734806 Brain_Cerebellum
0.773275397 Brain_Cerebellar_Hemisphere 1.749418614 0.250581386
0.250581386 -0.288093201 0.519037192 0.115471995 0.570727374
2 0 0.998207054
MRGPRE ENSG00000184350.9 0.518122793 3 3 0.227449945
Brain_Cerebellum 0.641770858 Brain_Nucleus_accumbens_basal_ganglia
1.054622567 0.943608067 0.943608067 -1.20695378 0.465224273
-0.523661975 0.876975389 3 0 0.998207054
P2RY1 ENSG00000169860.6 0.518173289 3 3 0.201137452 Brain_Cerebellum
0.909682282 Brain_Caudate_basal_ganglia 1.874219514 0.177190676
0.177190676 -0.113439303 1.278317636 0.589294265 0.695979756
3 0 0.998207054
CREB5 ENSG00000146592.16 0.518197468 1 1 0.518197468
Brain_Nucleus_accumbens_basal_ganglia 0.518197468
Brain_Nucleus_accumbens_basal_ganglia 1 1 1 0.646126449
0.646126449 0.646126449 NA 1 0 0.998207054
CCDC189 ENSG00000196118.11 0.518282328 7 2 0.480286571
Brain_Frontal_Cortex_BA9 0.946522721 Brain_Caudate_basal_ganglia 6.44199165
0.001207439 0.280749248 -0.410204991 0.705841725 0.070168154
0.41494709 2 0 0.998207054
TRIML2 ENSG00000179046.8 0.518354333 2 2 0.275383862
Brain_Caudate_basal_ganglia 0.930853473 Brain_Nucleus_accumbens_basal_ganglia
1.378878151 0.621121849 0.621121849 0.086771084 1.090747821
0.588759452 0.709918759 2 0 0.998207054
SNX30 ENSG00000148158.16 0.518505331 2 2 0.267557872 Brain_Amygdala
0.875809552 Brain_Cerebellar_Hemisphere 1.119181853 0.880818147
0.880818147 -1.108704054 -0.156283513 -0.632493783 0.673463023
2 0 0.998207054
H1F0 ENSG00000189060.5 0.518600308 1 1 0.518600308
Brain_Putamen_basal_ganglia 0.518600308 Brain_Putamen_basal_ganglia 1 1
1 0.645504493 0.645504493 0.645504493 NA 1 0
0.998207054
AGTRAP ENSG00000177674.15 0.518639825 2 2 0.257932314
Brain_Spinal_cord_cervical_c-1 0.992444565 Brain_Substantia_nigra
1.167329953 0.832670047 0.832670047 -1.131291754 -0.009469476
-0.570380615 0.79324814 2 0 0.998207054
ZBBX ENSG00000169064.12 0.518656201 2 2 0.298568047 Brain_Cortex
0.891300867 Brain_Frontal_Cortex_BA9 1.525940487 0.474059513
0.474059513 0.136658331 1.03950906 0.588083696 0.638411873 2
0 0.998207054
FAM227A ENSG00000184949.15 0.518684552 10 2 0.131052833
Brain_Cortex 0.450600961 Brain_Cerebellum 9.371959931 0.006111483
0.317404747 0.754413511 1.509963126 1.106461289 0.24426636 2
0 0.998207054
NNT ENSG00000112992.16 0.518776624 2 2 0.251931319
Brain_Nucleus_accumbens_basal_ganglia 0.481832877 Brain_Caudate_basal_ganglia
1.614849105 0.385150895 0.385150895 -1.145670971 -0.703357674
-0.924514323 0.312762732 2 0 0.998207054
CPAMD8 ENSG00000160111.12 0.518782175 2 2 0.392003964
Brain_Caudate_basal_ganglia 0.91046519 Brain_Nucleus_accumbens_basal_ganglia
1.588102328 0.411897672 0.411897672 -0.112451794 0.85598882
0.371768513 0.684790925 2 0 0.998207054
AP3S2 ENSG00000157823.16 0.519173258 12 2 0.135068855 Brain_Cortex
0.545612039 Brain_Putamen_basal_ganglia 10.81299921 0.001566261

0.503522006 -1.494408602 -0.604348393 -0.981159306 0.228776893
2 0 0.998207054
RP3-446N13.5 ENSG00000275212.1 0.519290812 2 2 0.456039493
Brain_Frontal_Cortex_BA9 0.849239397 Brain_Cortex 1.64034369 0.35965631
0.35965631 -0.19008899 0.745384199 0.277647604 0.661479436 2
0 0.998207054
FBXO7 ENSG00000100225.17 0.519305176 10 4 0.170465624
Brain_Cerebellar_Hemisphere 0.87992119 Brain_Spinal_cord_cervical_c-1
7.262872601 0.031368003 0.494516715 0.151069122 1.370709206
0.758354523 0.483268789 4 0 0.998207054
RGS14 ENSG00000169220.17 0.519372836 7 7 0.069998419
Brain_Substantia_nigra 0.825628658 Brain_Hypothalamus 1.917414866
0.373895512 0.373895512 -1.811920901 0.265638156 -0.804031753
0.85288988 7 0 0.998207054
C15orf52 ENSG00000188549.12 0.519401015 2 2 0.266448276
Brain_Cerebellum 0.547612135 Brain_Cerebellar_Hemisphere 1.713934543
0.286065457 0.286065457 0.601342129 1.111279 0.856310565
0.360579819 2 0 0.998207054
MAN2B2 ENSG00000013288.8 0.519460667 8 1 0.435970403
Brain_Cerebellum 0.746519155 Brain_Anterior_cingulate_cortex_BA24
7.564397828 3.18E-05 7.564397828 0.323232519 0.779015808
0.628411551 0.162295815 1 0 0.998207054
FAM129C ENSG00000167483.17 0.51946587 2 2 0.275216988
Brain_Hypothalamus 0.748631858 Brain_Cerebellum 1.022854125
0.977145875 0.977145875 0.320443891 1.091127036 0.705785464
0.544955278 2 0 0.998207054
CTSO ENSG00000256043.2 0.519523415 6 5 0.202847918
Brain_Hypothalamus 0.958155727 Brain_Nucleus_accumbens_basal_ganglia
4.442977523 0.075386411 0.186153932 -1.273479568 0.219160605
-0.206684137 0.55463105 5 0 0.998207054
NDUFA2 ENSG00000131495.8 0.519659128 1 1 0.519659128
Brain_Anterior_cingulate_cortex_BA24 0.519659128
Brain_Anterior_cingulate_cortex_BA24 1 1 1 0.643870938
0.643870938 0.643870938 NA 1 0 0.998207054
ZNF774 ENSG00000196391.10 0.519708748 3 3 0.216474749
Brain_Nucleus_accumbens_basal_ganglia 0.909980424 Brain_Frontal_Cortex_BA9
1.141692155 0.872786102 0.872786102 -1.235956458 0.917010436
-0.068627596 1.08792246 3 0 0.998207054
TDRD9 ENSG00000156414.18 0.51981337 13 3 0.198514329 Brain_Cerebellum
0.982761525 Brain_Anterior_cingulate_cortex_BA24 10.99781905 0.0002291
0.433909241 -1.285795821 -0.021606906 -0.660679522 0.387069466
3 0 0.998207054
BLVRA ENSG00000106605.10 0.519824949 8 5 0.134539075
Brain_Anterior_cingulate_cortex_BA24 0.93439482 Brain_Substantia_nigra
6.075429028 0.07100881 0.227843393 -0.320833159 1.496439835
0.582354568 0.54340987 5 0 0.998207054
SLC2A4RG ENSG00000125520.13 0.519879714 2 2 0.260762493
Brain_Cerebellar_Hemisphere 0.297172354 Brain_Cerebellum 1.820998339
0.179001661 0.179001661 1.04251634 1.124590756 1.083553548
0.058035376 2 0 0.998207054
ANO7 ENSG00000146205.13 0.519921389 13 5 0.023547997 Brain_Amygdala
0.551222108 Brain_Cerebellar_Hemisphere 9.799774939 0.000814856
0.349664148 -2.264424856 -0.595929825 -1.336733868 0.503068071
5 0 0.998207054
PAM16 ENSG00000217930.7 0.519933838 2 2 0.274431046
Brain_Caudate_basal_ganglia 0.337802844 Brain_Cerebellar_Hemisphere
1.640027344 0.359972656 0.359972656 0.95851557 1.092915173
1.025715372 0.095034871 2 0 0.998207054
RP11-95D17.1 ENSG00000269973.1 0.520204507 3 3 0.144745346
Brain_Cerebellum 0.796524006 Brain_Hippocampus 1.905602871
0.095177634 0.095177634 0.257848275 1.458345459 0.970636962
0.631105531 3 0 0.998207054

STON1-GTF2A1L ENSG00000068781.21 0.520301687 4 4 0.2735304
Brain_Cerebellar_Hemisphere 0.902396666 Brain_Spinal_cord_cervical_c-1
2.012236382 0.304230298 0.304230298 -0.95595697 1.094968585
-0.165806563 0.909164966 4 0 0.998207054
MEGF10 ENSG00000145794.16 0.520426851 1 1 0.520426851
Brain_Frontal_Cortex_BA9 0.520426851 Brain_Frontal_Cortex_BA9 1 1
1 0.642687565 0.642687565 0.642687565 NA 1 0
0.998207054
CHRAC1 ENSG00000104472.9 0.52054802 1 1 0.52054802 Brain_Amygdala
0.52054802 Brain_Amygdala 1 1 1 0.642500877 0.642500877
0.642500877 NA 1 0 0.998207054
RP11-446N19.1 ENSG00000272369.1 0.52056705 1 1 0.52056705 Brain_Amygdala
0.52056705 Brain_Amygdala 1 1 1 0.642471559 0.642471559
0.642471559 NA 1 0 0.998207054
OTOA ENSG00000155719.16 0.520604102 1 1 0.520604102 Brain_Cortex
0.520604102 Brain_Cortex 1 1 1 -0.642414478 -0.642414478
-0.642414478 NA 1 0 0.998207054
NOL11 ENSG00000130935.9 0.520663975 1 1 0.520663975
Brain_Nucleus_accumbens_basal_ganglia 0.520663975
Brain_Nucleus_accumbens_basal_ganglia 1 1 1 -0.642322242
-0.642322242 -0.642322242 NA 1 0 0.998207054
CAND1 ENSG00000111530.12 0.520737911 1 1 0.520737911 Brain_Cortex
0.520737911 Brain_Cortex 1 1 1 0.642208352 0.642208352
0.642208352 NA 1 0 0.998207054
PURA ENSG00000185129.5 0.520740513 1 1 0.520740513
Brain_Putamen_basal_ganglia 0.520740513 Brain_Putamen_basal_ganglia 1 1
1 -0.642204344 -0.642204344 -0.642204344 NA 1 0
0.998207054
NKG7 ENSG00000105374.9 0.521130837 1 1 0.521130837
Brain_Hypothalamus 0.521130837 Brain_Hypothalamus 1 1 1
-0.641603227 -0.641603227 -0.641603227 NA 1 0 0.998207054
ZNF469 ENSG00000225614.2 0.521306651 1 1 0.521306651
Brain_Frontal_Cortex_BA9 0.521306651 Brain_Frontal_Cortex_BA9 1 1
1 -0.641332541 -0.641332541 -0.641332541 NA 1 0
0.998207054
ANKRD50 ENSG00000151458.11 0.52140439 2 2 0.402233308 Brain_Cerebellum
0.503969004 Brain_Cerebellar_Hemisphere 1.120804482 0.879195518
0.879195518 -0.668257865 0.83763931 0.084690723 1.064830105 2
0 0.998207054
GPX3 ENSG00000211445.11 0.521410523 5 5 0.185469988
Brain_Anterior_cingulate_cortex_BA24 0.945334074 Brain_Cortex
1.896346001 0.461154908 0.461154908 -1.168867215 1.32409954
0.381028728 1.022923299 5 0 0.998207054
SLC4A1AP ENSG00000163798.13 0.521653091 2 2 0.262987389
Brain_Substantia_nigra 0.841698092 Brain_Caudate_basal_ganglia
1.018371469 0.981628531 0.981628531 -1.119358123 -0.199721891
-0.659540007 0.650281016 2 0 0.998207054
SYT4 ENSG00000132872.11 0.521671424 1 1 0.521671424 Brain_Cerebellum
0.521671424 Brain_Cerebellum 1 1 1 0.640771082 0.640771082
0.640771082 NA 1 0 0.998207054
CASC3 ENSG00000108349.16 0.521672531 7 3 0.077350549 Brain_Cortex
0.494524509 Brain_Nucleus_accumbens_basal_ganglia 5.607595979
0.004038336 0.366118859 0.683130303 1.766270078 1.210163073
0.333406175 3 0 0.998207054
ME1 ENSG00000065833.8 0.521722303 4 3 0.29611615
Brain_Substantia_nigra 0.840204819 Brain_Cerebellar_Hemisphere
2.889683465 1.25E-16 0.235861431 -1.044798409 -0.201631498
-0.509031282 0.400945354 3 0 0.998207054
PFDN5 ENSG00000123349.13 0.521739465 1 1 0.521739465 Brain_Cerebellum
0.521739465 Brain_Cerebellum 1 1 1 -0.640666375 -0.640666375
-0.640666375 NA 1 0 0.998207054
CA13 ENSG00000185015.7 0.521756577 4 2 0.40829047

Brain_Cerebellar_Hemisphere 0.99698746 Brain_Cerebellum 3.51735812 0.009744231
0.457884866 -0.826905774 0.307993132 -0.172307254 0.478756665
2 0 0.998207054
RARRES1 ENSG00000118849.9 0.521860113 9 9 0.133469961
Brain_Hippocampus 0.567278392 Brain_Hypothalamus 4.066371416
0.184158008 0.184158008 -0.738443814 1.500557847 0.763086874
0.640928234 9 0 0.998207054
AC011288.2 ENSG00000229618.2 0.521963858 2 2 0.25416764 Brain_Cortex
0.416420416 Brain_Frontal_Cortex_BA9 1.717567443 0.282432557
0.282432557 -1.140284868 -0.812647105 -0.976465986 0.231674884
2 0 0.998207054
TMEM255B ENSG00000184497.12 0.5220116 10 6 0.170320654 Brain_Cerebellum
0.648320901 Brain_Cerebellar_Hemisphere 6.310565245 0.019335946
0.357302199 0.456096063 1.371174216 0.920334566 0.352533441
6 0 0.998207054
HLA-DQA2 ENSG00000237541.3 0.522060291 13 1 0.119245443
Brain_Putamen_basal_ganglia 0.848600442 Brain_Cortex 12.02740554
0.008549707 12.02740554 -0.319639278 1.55794856 0.615689547
0.524211634 1 0 0.998207054
DNAI2 ENSG00000171595.13 0.522116568 4 4 0.281347455
Brain_Spinal_cord_cervical_c-1 0.559620876
Brain_Anterior_cingulate_cortex_BA24 1.33848617 0.688512591 0.688512591
-0.851658028 1.077297329 0.162357639 1.022294111 4 0
0.998207054
CAV2 ENSG00000105971.14 0.522234889 4 4 0.241485668
Brain_Nucleus_accumbens_basal_ganglia 0.971748931 Brain_Caudate_basal_ganglia
1.435965199 0.652129748 0.652129748 -1.171281444 0.850718028
0.076697869 0.898839685 4 0 0.998207054
TRPC4AP ENSG00000100991.11 0.522277773 3 3 0.151642911
Brain_Putamen_basal_ganglia 0.96255417 Brain_Cerebellar_Hemisphere 1.772583588
0.242436555 0.242436555 0.04694863 1.433752477 0.529144957
0.783983188 3 0 0.998207054
5-Sep ENSG00000184702.17 0.52230683 2 2 0.270363431
Brain_Frontal_Cortex_BA9 0.79058518 Brain_Substantia_nigra 1.022114157
0.977885843 0.977885843 -1.102225999 0.265550802 -0.418337599
0.967164252 2 0 0.998207054
MIR22HG ENSG00000186594.14 0.522339763 2 2 0.473390014
Brain_Caudate_basal_ganglia 0.656613748 Brain_Hypothalamus 1.470323559
0.529676441 0.529676441 -0.716974203 0.44459322 -0.136190491
0.821352202 2 0 0.998207054
CDH15 ENSG00000129910.7 0.522392822 2 2 0.254760453 Brain_Cerebellum
0.438189601 Brain_Cerebellar_Hemisphere 1.653628525 0.346371475
0.346371475 0.775253974 1.138862633 0.957058303 0.257110149
2 0 0.998207054
ZNF215 ENSG00000149054.15 0.522442475 1 1 0.522442475
Brain_Hippocampus 0.522442475 Brain_Hippocampus 1 1 1
0.639584942 0.639584942 0.639584942 NA 1 0 0.998207054
TPRA1 ENSG00000163870.14 0.522455922 6 3 0.149969935
Brain_Putamen_basal_ganglia 0.828260773 Brain_Cerebellum 4.365140812
0.010718069 0.675385706 -1.439637675 -0.216932726 -0.611759668
0.431005954 3 0 0.998207054
RP11-538C21.2 ENSG00000277448.1 0.522496463 1 1 0.522496463
Brain_Spinal_cord_cervical_c-1 0.522496463 Brain_Spinal_cord_cervical_c-1
1 1 1 -0.639501924 -0.639501924 -0.639501924 NA 1 0
0.998207054
DLD ENSG00000091140.13 0.522602115 12 7 0.223898638 Brain_Cerebellum
0.974435528 Brain_Amygdala 7.407889015 0.001461016 0.356817575
-1.216226534 0.755540897 -0.141906806 0.563882414 7 0
0.998207054
TRIM54 ENSG00000138100.13 0.522641007 1 1 0.522641007
Brain_Anterior_cingulate_cortex_BA24 0.522641007
Brain_Anterior_cingulate_cortex_BA24 1 1 1 0.639279676

0.639279676 0.639279676 NA 1 0 0.998207054
10-Sep ENSG00000186522.14 0.522720209 7 4 0.07351195
Brain_Hippocampus 0.763645894 Brain_Cerebellar_Hemisphere 4.762126596
0.035422519 0.242184278 -1.789639091 0.300696558 -1.167426984
0.751469768 4 0 0.998207054
LAPTM4B ENSG00000104341.16 0.522746845 8 7 0.076364689
Brain_Nucleus_accumbens_basal_ganglia 0.941381108 Brain_Putamen_basal_ganglia
2.291756947 0.050967651 0.571594075 -1.772179961 0.349590416
-0.408608016 0.800737582 7 0 0.998207054
ITGB2 ENSG00000160255.17 0.522824746 1 1 0.522824746
Brain_Spinal_cord_cervical_c-1 0.522824746 Brain_Spinal_cord_cervical_c-1
1 1 1 -0.638997212 -0.638997212 -0.638997212 NA 1 0
0.998207054
GALNT1 ENSG00000141429.13 0.522890414 3 3 0.154140829
Brain_Frontal_Cortex_BA9 0.938733984 Brain_Cerebellum 1.661179003
0.411463273 0.411463273 -0.076861175 1.42505664 0.627787657
0.755230528 3 0 0.998207054
PTER ENSG00000165983.14 0.523151652 1 1 0.523151652
Brain_Caudate_basal_ganglia 0.523151652 Brain_Caudate_basal_ganglia 1 1
1 -0.638494778 -0.638494778 -0.638494778 NA 1 0
0.998207054
SRD5A3 ENSG00000128039.10 0.523162362 10 10 0.119992787
Brain_Caudate_basal_ganglia 0.950040239 Brain_Cerebellum 2.91857756
0.279392805 0.279392805 -0.666246431 1.554803871 0.645091798
0.761817739 10 0 0.998207054
AKAP11 ENSG00000023516.8 0.523459515 10 3 0.130442213
Brain_Putamen_basal_ganglia 0.783113816 Brain_Anterior_cingulate_cortex_BA24
8.582614115 1.39E-05 0.420613772 0.275263485 1.512360363
1.099293086 0.33910247 3 0 0.998207054
TMEM30A ENSG00000112697.15 0.523672067 3 2 0.255724777
Brain_Caudate_basal_ganglia 0.343757472 Brain_Cortex 2.762516232
0.019645738 0.21783803 0.946767097 1.136554006 1.068419173
0.105607004 2 0 0.998207054
CDK2AP2 ENSG00000167797.7 0.523742683 1 1 0.523742683
Brain_Hippocampus 0.523742683 Brain_Hippocampus 1 1 1
-0.637586811 -0.637586811 -0.637586811 NA 1 0 0.998207054
RP11-775C24.5 ENSG00000277782.1 0.523798625 9 5 0.236250884
Brain_Substantia_nigra 0.931942484 Brain_Putamen_basal_ganglia
6.260324779 0.004336671 0.399896679 -1.184409797 0.704852203
-0.370549255 0.571593524 5 0 0.998207054
ANO2 ENSG00000047617.14 0.523849797 2 2 0.277069769 Brain_Cortex
0.760835883 Brain_Cerebellum 1.027322914 0.972677086 0.972677086
-1.086925425 0.304383308 -0.391271059 0.98380384 2 0
0.998207054
GPHA2 ENSG00000149735.6 0.523971686 2 2 0.659340743
Brain_Cerebellar_Hemisphere 0.779339478 Brain_Cerebellum 1.79779737 0.20220263
0.20220263 -0.280179911 0.440823551 0.08032182 0.509826437 2 0
0.998207054
CA1 ENSG00000133742.13 0.523974509 1 1 0.523974509
Brain_Spinal_cord_cervical_c-1 0.523974509 Brain_Spinal_cord_cervical_c-1
1 1 1 0.637230813 0.637230813 0.637230813 NA 1 0
0.998207054
PTK7 ENSG00000112655.15 0.524208827 3 3 0.145433465
Brain_Putamen_basal_ganglia 0.893616569 Brain_Spinal_cord_cervical_c-1
1.574208764 0.430581118 0.430581118 -0.237929037 1.455852261
0.361397947 0.949256065 3 0 0.998207054
FAM83D ENSG00000101447.14 0.524257826 1 1 0.524257826
Brain_Cortex 0.524257826 Brain_Cortex 1 1 1 0.636795856
0.636795856 0.636795856 NA 1 0 0.998207054
LCN12 ENSG00000184925.11 0.524258703 10 8 0.033185279
Brain_Frontal_Cortex_BA9 0.853249402 Brain_Nucleus_accumbens_basal_ganglia
3.188860472 0.005243857 0.240654494 -2.1298345 0.336485893

-0.806089226 0.943882888 8 0 0.998207054
UBXN4 ENSG00000144224.16 0.524333546 1 1 0.524333546
Brain_Cerebellar_Hemisphere 0.524333546 Brain_Cerebellar_Hemisphere 1 1
1 0.636679627 0.636679627 0.636679627 NA 1 0
0.998207054
RAB27A ENSG00000069974.15 0.524362827 11 8 0.04715168
Brain_Substantia_nigra 0.809372608 Brain_Cortex 5.102022019
0.029191682 0.240517731 -1.984935212 -0.241235488 -1.109709744
0.586015479 8 0 0.998207054
NEURL2 ENSG00000124257.6 0.524386846 1 1 0.524386846
Brain_Cortex 0.524386846 Brain_Cortex 1 1 1 0.636597819
0.636597819 0.636597819 NA 1 0 0.998207054
ATP9B ENSG00000166377.19 0.524416147 2 2 0.579640797 Brain_Cortex
0.700781174 Brain_Frontal_Cortex_BA9 1.656786667 0.343213333
0.343213333 -0.384266178 0.553909481 0.084821651 0.66339037 2
0 0.998207054
RP11-726G1.1 ENSG00000214776.10 0.524627081 13 4 0.147151399
Brain_Putamen_basal_ganglia 0.977423621 Brain_Hippocampus 10.05609252
7.58E-05 0.366637337 -0.537002397 1.449667012 0.06788072
0.526992587 4 0 0.998207054
RNF10 ENSG00000022840.15 0.524698342 1 1 0.524698342
Brain_Cerebellar_Hemisphere 0.524698342 Brain_Cerebellar_Hemisphere 1 1
1 -0.636119799 -0.636119799 -0.636119799 NA 1 0
0.998207054
ANGEL2 ENSG00000174606.13 0.524731612 7 4 0.034850149
Brain_Putamen_basal_ganglia 0.850182926 Brain_Frontal_Cortex_BA9
5.250000368 0.001724691 0.220957026 -2.110095286 -0.188885031
-1.420488212 0.616103155 4 0 0.998207054
KLHL23 ENSG00000213160.9 0.524786467 2 2 0.50896516
Brain_Cerebellar_Hemisphere 0.819895219 Brain_Cerebellum 1.913565935
0.086434065 0.086434065 0.227679746 0.660449899 0.444064822
0.30601471 2 0 0.998207054
BTBD8 ENSG00000284413.1 0.524843897 1 1 0.524843897
Brain_Nucleus_accumbens_basal_ganglia 0.524843897
Brain_Nucleus_accumbens_basal_ganglia 1 1 1 -0.63589648
-0.63589648 -0.63589648 NA 1 0 0.998207054
HTT ENSG00000197386.10 0.524864721 1 1 0.524864721
Brain_Nucleus_accumbens_basal_ganglia 0.524864721
Brain_Nucleus_accumbens_basal_ganglia 1 1 1 0.635864534
0.635864534 0.635864534 NA 1 0 0.998207054
TBKBP1 ENSG00000198933.9 0.525096765 2 2 0.314588208
Brain_Cortex 0.373927979 Brain_Frontal_Cortex_BA9 1.405797479
0.594202521 0.594202521 -1.005641179 -0.889139749 -0.947390464
0.082378951 2 0 0.998207054
FER ENSG00000151422.12 0.525314541 1 1 0.525314541
Brain_Nucleus_accumbens_basal_ganglia 0.525314541
Brain_Nucleus_accumbens_basal_ganglia 1 1 1 -0.635174609
-0.635174609 -0.635174609 NA 1 0 0.998207054
RLBP1 ENSG00000140522.11 0.525351752 6 6 0.215241562
Brain_Caudate_basal_ganglia 0.841006378 Brain_Anterior_cingulate_cortex_BA24
1.632260624 0.383238567 0.383238567 -0.360810935 1.239280703
0.469333678 0.633483101 6 0 0.998207054
PDE6H ENSG00000139053.2 0.525360703 5 5 0.183882153 Brain_Cortex
0.608037833 Brain_Putamen_basal_ganglia 2.062910983 0.226627566
0.226627566 -1.328896397 1.004053877 -0.456050826 0.880697255
5 0 0.998207054
FAM32A ENSG00000105058.11 0.525453815 2 1 0.441534214
Brain_Nucleus_accumbens_basal_ganglia 0.625247999 Brain_Caudate_basal_ganglia
1.962721446 0.037278554 1.962721446 0.488426184 0.769605041
0.629015612 0.198823476 1 0 0.998207054
GSTM3 ENSG00000134202.10 0.525495096 13 7 0.163408196
Brain_Anterior_cingulate_cortex_BA24 0.959483463 Brain_Cerebellar_Hemisphere

6.765969304 0.0001931 0.286948375 -0.885144938 1.393700068
0.259764504 0.745513686 7 0 0.998207054
GOLGA8M ENSG00000188626.6 0.525546319 1 1 0.525546319
Brain_Amygdala 0.525546319 Brain_Amygdala 1 1 1 -0.634819231
-0.634819231 -0.634819231 NA 1 0 0.998207054
CTB-25B13.5 ENSG00000268798.1 0.525624142 1 1 0.525624142
Brain_Cerebellum 0.525624142 Brain_Cerebellum 1 1 1 -0.634699926
-0.634699926 -0.634699926 NA 1 0 0.998207054
MUT ENSG00000146085.7 0.525637786 11 6 0.31575275
Brain_Putamen_basal_ganglia 0.943752409 Brain_Cerebellum 7.251374681
0.061893609 0.329368628 -0.798234148 1.003224095 0.093373127
0.553528399 6 0 0.998207054
ST3GAL4 ENSG00000110080.18 0.525755501 3 3 0.314756244
Brain_Spinal_cord_cervical_c-1 0.466959355 Brain_Caudate_basal_ganglia
1.197878635 0.779833677 0.779833677 -0.850067867 1.005292045
-0.190737031 1.037604863 3 0 0.998207054
RP11-267M23.4 ENSG00000253704.1 0.525842835 5 3 0.214111731
Brain_Cerebellum 0.962478503 Brain_Frontal_Cortex_BA9 3.546868212
0.024483566 0.588623239 -1.242338406 0.047043569 -0.787295603
0.500061047 3 0 0.998207054
ACIN1 ENSG00000100813.14 0.526037237 2 2 0.277186657 Brain_Cerebellum
0.72240427 Brain_Nucleus_accumbens_basal_ganglia 1.030375168 0.969624832
0.969624832 0.355247383 1.086660997 0.72095419 0.517187526 2
0 0.998207054
BTBD2 ENSG00000133243.8 0.526039224 2 2 0.3595808 Brain_Cerebellum
0.533901843 Brain_Cerebellar_Hemisphere 1.048610054 0.951389946
0.951389946 -0.91616416 0.62206087 -0.147051645 1.08768935 2 0
0.998207054
RP11-396B14.2 ENSG00000263033.2 0.526149012 6 5 0.075304229
Brain_Spinal_cord_cervical_c-1 0.815746789 Brain_Hippocampus
2.866460655 0.061742448 0.494808585 -1.778606917 0.430501753
-0.348671103 0.791800234 5 0 0.998207054
SYT8 ENSG00000149043.16 0.526190262 1 1 0.526190262 Brain_Cortex
0.526190262 Brain_Cortex 1 1 1 -0.633832314 -0.633832314
-0.633832314 NA 1 0 0.998207054
RIPK2 ENSG00000104312.7 0.526287331 3 3 0.268719669 Brain_Cortex
0.722269775 Brain_Frontal_Cortex_BA9 1.796182801 0.211209715
0.211209715 -1.106015828 -0.355426932 -0.776347853 0.383524768
3 0 0.998207054
PLD2 ENSG00000129219.13 0.526392438 3 3 0.422878577
Brain_Cerebellar_Hemisphere 0.913992772 Brain_Cerebellum 1.87693898
0.123493366 0.123493366 -0.801437217 0.287584527 -0.207285457
0.55125742 3 0 0.998207054
LINC01150 ENSG00000229671.1 0.526430619 1 1 0.526430619
Brain_Substantia_nigra 0.526430619 Brain_Substantia_nigra 1 1
1 -0.633464098 -0.633464098 -0.633464098 NA 1 0
0.998207054
SRMS ENSG00000125508.3 0.526447735 1 1 0.526447735 Brain_Cortex
0.526447735 Brain_Cortex 1 1 1 0.63343788 0.63343788 0.63343788
NA 1 0 0.998207054
MTERF4 ENSG00000122085.16 0.526475601 11 2 0.173003781
Brain_Nucleus_accumbens_basal_ganglia 0.481853746
Brain_Anterior_cingulate_cortex_BA24 9.822531861 0.001948223
0.480732273 0.703324178 1.36261531 1.028588882 0.191889308 2
0 0.998207054
MOV10 ENSG00000155363.18 0.52651029 1 1 0.52651029
Brain_Nucleus_accumbens_basal_ganglia 0.52651029
Brain_Nucleus_accumbens_basal_ganglia 1 1 1 0.633342064
0.633342064 0.633342064 NA 1 0 0.998207054
DOK4 ENSG00000125170.10 0.526703654 1 1 0.526703654
Brain_Spinal_cord_cervical_c-1 0.526703654 Brain_Spinal_cord_cervical_c-1
1 1 1 0.633045924 0.633045924 0.633045924 NA 1 0

0.998207054
LTBR ENSG00000111321.10 0.526760804 13 3 0.148723954 Brain_Cortex
0.881828151 Brain_Putamen_basal_ganglia 10.76571746 0.003722054
0.441510774 -1.444053404 0.479715076 -0.969057061 0.542442748
3 0 0.998207054
FCGR2B ENSG00000072694.20 0.526775736 1 1 0.526775736
Brain_Nucleus_accumbens_basal_ganglia 0.526775736
Brain_Nucleus_accumbens_basal_ganglia 1 1 1 -0.632935543
-0.632935543 -0.632935543 NA 1 0 0.998207054
CREBL2 ENSG00000111269.2 0.526820651 1 1 0.526820651
Brain_Substantia_nigra 0.526820651 Brain_Substantia_nigra 1 1
1 -0.632866768 -0.632866768 -0.632866768 NA 1 0
0.998207054
RP11-527H14.4 ENSG00000263618.5 0.526887041 1 1 0.526887041
Brain_Nucleus_accumbens_basal_ganglia 0.526887041
Brain_Nucleus_accumbens_basal_ganglia 1 1 1 -0.632765114
-0.632765114 -0.632765114 NA 1 0 0.998207054
PON2 ENSG00000105854.12 0.526910848 1 1 0.526910848 Brain_Cerebellum
0.526910848 Brain_Cerebellum 1 1 1 -0.632728664 -0.632728664
-0.632728664 NA 1 0 0.998207054
TCIRG1 ENSG00000110719.9 0.52695502 1 1 0.52695502
Brain_Frontal_Cortex_BA9 0.52695502 Brain_Frontal_Cortex_BA9 1 1 1
0.632661035 0.632661035 0.632661035 NA 1 0 0.998207054
EAF2 ENSG00000145088.8 0.526991549 4 4 0.159138766
Brain_Hippocampus 0.901713171 Brain_Amygdala 1.888629226
0.230067248 0.230067248 0.123497479 1.407974012 0.891541989
0.598410933 4 0 0.998207054
PLGLB2 ENSG00000125551.18 0.527036002 2 2 0.282804409
Brain_Cerebellum 0.865539944 Brain_Cerebellar_Hemisphere 1.453800819
0.546199181 0.546199181 0.169326358 1.074040771 0.621683564
0.639729696 2 0 0.998207054
CLEC7A ENSG00000172243.17 0.527133874 2 2 0.257982868
Brain_Hypothalamus 0.516467388 Brain_Hippocampus 1.549607693
0.450392307 0.450392307 -1.131171611 -0.648800416 -0.889986014
0.341087943 2 0 0.998207054
PGAM1 ENSG00000171314.8 0.527180159 9 3 0.410759645 Brain_Cortex
0.932101682 Brain_Frontal_Cortex_BA9 7.556906289 0.010063382
0.256732714 -0.822557554 0.507170567 -0.142603229 0.42008677 3
0 0.998207054
ACPP ENSG00000014257.15 0.527188422 5 2 0.574854536
Brain_Nucleus_accumbens_basal_ganglia 0.80038814 Brain_Amygdala 4.546010034
0.009575074 0.313884098 -0.56091639 0.252844809 -0.309903506
0.332280089 2 0 0.998207054
METAP1D ENSG00000172878.13 0.527521379 5 5 0.189914376
Brain_Cortex 0.942374583 Brain_Putamen_basal_ganglia 2.384319617
0.135770864 0.135770864 -0.466433705 1.310832451 0.538514026
0.778262579 5 0 0.998207054
C15orf59-AS1 ENSG00000260469.2 0.527528447 9 6 0.298009109
Brain_Spinal_cord_cervical_c-1 0.962895843
Brain_Nucleus_accumbens_basal_ganglia 5.855733 0.006090937 0.274156895
-0.812703513 1.040712264 0.208421761 0.633864401 6 0
0.998207054
TRAM1L1 ENSG00000174599.5 0.527533969 1 1 0.527533969
Brain_Cerebellum 0.527533969 Brain_Cerebellum 1 1 1 0.631774913
0.631774913 0.631774913 NA 1 0 0.998207054
CCDC124 ENSG00000007080.10 0.527580179 4 4 0.127966461
Brain_Cortex 0.975375298 Brain_Cerebellum 1.077024535 0.93318363
0.93318363 0.030867388 1.522170116 0.738120797 0.631299512 4
0 0.998207054
RP3-355L5.5 ENSG00000272102.1 0.527602419 1 1 0.527602419
Brain_Putamen_basal_ganglia 0.527602419 Brain_Putamen_basal_ganglia 1 1
1 0.631670177 0.631670177 0.631670177 NA 1 0

0.998207054
GIMAP1 ENSG00000213203.2 0.527618807 2 2 0.291340066
Brain_Cortex 0.727374901 Brain_Anterior_cingulate_cortex_BA24
1.054401174 0.945598826 0.945598826 -0.348619613 1.055186438
0.353283412 0.992640778 2 0 0.998207054
CAPN5 ENSG00000149260.15 0.52762964 4 4 0.189537308 Brain_Cerebellum
0.653904254 Brain_Cortex 1.099498708 0.910449058 0.910449058
-0.87006877 1.311949102 -0.156450577 0.99413536 4 0
0.998207054
CAPN12 ENSG00000182472.8 0.52774176 4 4 0.152945077
Brain_Putamen_basal_ganglia 0.976589496 Brain_Nucleus_accumbens_basal_ganglia
1.848160953 0.20633389 0.20633389 -1.429205849 -0.029344926
-0.800831754 0.577435391 4 0 0.998207054
RCOR1 ENSG00000089902.9 0.527884324 1 1 0.527884324
Brain_Nucleus_accumbens_basal_ganglia 0.527884324
Brain_Nucleus_accumbens_basal_ganglia 1 1 1 -0.63123891
-0.63123891 -0.63123891 NA 1 0 0.998207054
RPSA ENSG00000168028.13 0.527888248 9 4 0.208109737
Brain_Frontal_Cortex_BA9 0.9978552 Brain_Cerebellar_Hemisphere 6.467818617
0.011713372 0.393051869 -1.258780192 1.134409235 -0.011145604
0.65369236 4 0 0.998207054
KCNJ14 ENSG00000182324.6 0.527981957 5 4 0.30598239
Brain_Substantia_nigra 0.994406759 Brain_Caudate_basal_ganglia 3.90474191
0.071705027 0.143756098 -0.111029726 1.023688583 0.504198613
0.524118807 4 0 0.998207054
CCDC96 ENSG00000173013.5 0.528022752 2 2 0.315103831
Brain_Cerebellar_Hemisphere 0.66874395 Brain_Nucleus_accumbens_basal_ganglia
1.087497097 0.912502903 0.912502903 -0.427872499 1.004570245
0.288348873 1.012889978 2 0 0.998207054
RETSAT ENSG00000042445.13 0.528309503 7 6 0.034620426
Brain_Caudate_basal_ganglia 0.865379763 Brain_Spinal_cord_cervical_c-1
4.502734447 0.023901939 0.164379018 -2.112770385 -0.169530018
-1.424211257 0.69757629 6 0 0.998207054
FAM210B ENSG00000124098.9 0.528774235 4 3 0.346204675
Brain_Cerebellar_Hemisphere 0.940908584 Brain_Caudate_basal_ganglia
2.347839688 0.050405578 0.684808889 -0.941976494 0.155145036
-0.447512108 0.57126256 3 0 0.998207054
NSRP1 ENSG00000126653.17 0.528781816 2 2 0.444757061
Brain_Cerebellar_Hemisphere 0.449993983 Brain_Hypothalamus 1.093945101
0.906054899 0.906054899 -0.764184904 0.755425058 -0.004379923
1.074526509 2 0 0.998207054
GINS4 ENSG00000147536.11 0.528927474 1 1 0.528927474
Brain_Putamen_basal_ganglia 0.528927474 Brain_Putamen_basal_ganglia 1 1
1 0.629644084 0.629644084 0.629644084 NA 1 0
0.998207054
TMEM231 ENSG00000205084.10 0.528958296 4 4 0.198908815
Brain_Cerebellum 0.639092699 Brain_Anterior_cingulate_cortex_BA24
2.367191491 0.262280937 0.262280937 -0.468967734 1.284666599
0.73903816 0.811839091 4 0 0.998207054
FAM49B ENSG00000153310.19 0.528980896 3 3 0.237253804
Brain_Hippocampus 0.704437616 Brain_Nucleus_accumbens_basal_ganglia
1.237026146 0.749918551 0.749918551 -1.181878778 -0.379337015
-0.694331476 0.428192927 3 0 0.998207054
DCP1B ENSG00000151065.13 0.5290995 1 1 0.5290995 Brain_Cortex 0.5290995
Brain_Cortex 1 1 1 0.629381236 0.629381236 0.629381236
NA 1 0 0.998207054
UTS2R ENSG00000181408.3 0.529102263 1 1 0.529102263 Brain_Amygdala
0.529102263 Brain_Amygdala 1 1 1 0.629377013 0.629377013
0.629377013 NA 1 0 0.998207054
ZNF101 ENSG00000181896.11 0.529180442 8 5 0.31439293
Brain_Anterior_cingulate_cortex_BA24 0.824835356 Brain_Putamen_basal_ganglia
6.264179897 0.035878829 0.223507122 -1.006047065 1.002999222

0.353301471 0.623142363 5 0 0.998207054
AURKC ENSG00000105146.12 0.529226897 11 4 0.186275356
Brain_Spinal_cord_cervical_c-1 0.913632138 Brain_Cerebellar_Hemisphere
8.338810227 0.006567703 0.377080827 -0.108458324 1.321678125
0.58847828 0.398546037 4 0 0.998207054
IL7R ENSG00000168685.14 0.529249257 1 1 0.529249257
Brain_Substantia_nigra 0.529249257 Brain_Substantia_nigra 1 1
1 -0.629152447 -0.629152447 -0.629152447 NA 1 0
0.998207054
PMVK ENSG00000163344.5 0.529307404 1 1 0.529307404
Brain_Nucleus_accumbens_basal_ganglia 0.529307404
Brain_Nucleus_accumbens_basal_ganglia 1 1 1 -0.629063624
-0.629063624 -0.629063624 NA 1 0 0.998207054
LAMP5 ENSG00000125869.9 0.529328758 2 2 0.317708787 Brain_Cerebellum
0.569728163 Brain_Cerebellar_Hemisphere 1.045646257 0.954353743
0.954353743 -0.568451893 0.999177348 0.215362728 1.108481266
2 0 0.998207054
CDYL ENSG00000153046.17 0.529431375 1 1 0.529431375 Brain_Cortex
0.529431375 Brain_Cortex 1 1 1 -0.628874265 -0.628874265
-0.628874265 NA 1 0 0.998207054
RP11-381K20.4 ENSG00000270697.1 0.529445687 1 1 0.529445687
Brain_Hippocampus 0.529445687 Brain_Hippocampus 1 1 1
-0.628852407 -0.628852407 -0.628852407 NA 1 0 0.998207054
AP003774.6 ENSG00000231680.1 0.529449717 7 5 0.16290987 Brain_Cerebellum
0.950487952 Brain_Nucleus_accumbens_basal_ganglia 3.512119831
0.004645494 0.437533236 -1.395351496 -0.062094028 -0.721544889
0.581532277 5 0 0.998207054
ICA1L ENSG00000163596.16 0.529453941 3 3 0.476974415
Brain_Caudate_basal_ganglia 0.734055989 Brain_Frontal_Cortex_BA9
2.220252124 0.228375188 0.228375188 -0.711177233 0.702187854
0.11024859 0.734096913 3 0 0.998207054
RPL22L1 ENSG00000163584.17 0.529467824 3 3 0.182893761
Brain_Cerebellum 0.702662967 Brain_Amygdala 1.386771719 0.60354244
0.60354244 -0.381728219 1.33189784 0.505528413 0.858434043 3 0
0.998207054
DGKA ENSG00000065357.19 0.529728566 2 2 0.296635277
Brain_Nucleus_accumbens_basal_ganglia 0.390357097 Brain_Cerebellar_Hemisphere
1.460702861 0.539297139 0.539297139 0.858969947 1.043676087
0.951323017 0.130606964 2 0 0.998207054
TMEM229B ENSG00000198133.8 0.52973504 2 2 0.357283496
Brain_Nucleus_accumbens_basal_ganglia 0.471171852
Brain_Anterior_cingulate_cortex_BA24 1.078125123 0.921874877
0.921874877 -0.920553678 0.720573681 -0.099989999 1.160452284
2 0 0.998207054
PDCL3 ENSG00000115539.13 0.529753177 2 2 0.259659947 Brain_Cerebellum
0.851554708 Brain_Spinal_cord_cervical_c-1 1.175549282 0.824450718
0.824450718 -0.1871351 1.127195229 0.470030064 0.929371888 2
0 0.998207054
EIF2A ENSG00000144895.11 0.529756017 12 1 0.381940692
Brain_Frontal_Cortex_BA9 0.727845519 Brain_Anterior_cingulate_cortex_BA24
11.59239012 1.57E-05 11.59239012 -0.874326096 -0.347992895
-0.618322965 0.137350618 1 0 0.998207054
COX11 ENSG00000166260.10 0.529780597 12 2 0.212304959
Brain_Frontal_Cortex_BA9 0.48203009 Brain_Cerebellar_Hemisphere 11.01093685
0.000813452 0.698892712 0.703041175 1.247252416 1.073663894
0.141594793 2 0 0.998207054
NOL8 ENSG00000198000.11 0.529804207 1 1 0.529804207
Brain_Nucleus_accumbens_basal_ganglia 0.529804207
Brain_Nucleus_accumbens_basal_ganglia 1 1 1 0.628304923
0.628304923 0.628304923 NA 1 0 0.998207054
FLG2 ENSG00000143520.6 0.529945843 1 1 0.529945843
Brain_Frontal_Cortex_BA9 0.529945843 Brain_Frontal_Cortex_BA9 1 1

1 -0.628088687 -0.628088687 -0.628088687 NA 1 0
0.998207054
GCDH ENSG00000105607.12 0.529946056 12 6 0.161190472
Brain_Hypothalamus 0.989666938 Brain_Substantia_nigra 7.368820805
3.70E-17 0.479942292 -0.418632624 1.401078928 0.21097782 0.51443542
6 0 0.998207054
B4GAT1 ENSG00000174684.6 0.529958892 1 1 0.529958892
Brain_Cortex 0.529958892 Brain_Cortex 1 1 1 -0.628068768
-0.628068768 -0.628068768 NA 1 0 0.998207054
PSORS1C1 ENSG00000204540.10 0.530087101 12 11 0.012320844
Brain_Anterior_cingulate_cortex_BA24 0.992486894 Brain_Cerebellar_Hemisphere
3.945574958 0.115982839 0.263018186 -0.720865387 2.502819269
0.194332553 0.846553315 11 0 0.998207054
WDR90 ENSG00000161996.18 0.530141203 5 5 0.345861274
Brain_Caudate_basal_ganglia 0.986910694 Brain_Hippocampus 2.556328211
0.095869952 0.095869952 -0.942647425 0.016405748 -0.463684099
0.428419721 5 0 0.998207054
ZNF143 ENSG00000166478.9 0.530208291 8 6 0.066413766
Brain_Amygdala 0.993911876 Brain_Cerebellar_Hemisphere 4.00636946
0.020702322 0.197523351 -0.258367691 1.83562075 1.080284768
0.850741467 6 0 0.998207054
GOLGA6L9 ENSG00000197978.9 0.530331765 12 3 0.118023913
Brain_Nucleus_accumbens_basal_ganglia 0.911588751 Brain_Cortex
9.776732339 0.016191453 0.438755047 -1.563121952 0.111034799
-0.878955791 0.464614473 3 0 0.998207054
RP11-46H11.3 ENSG00000250790.4 0.530387517 7 5 0.246917529
Brain_Substantia_nigra 0.953834818 Brain_Frontal_Cortex_BA9
4.853987804 0.027417429 0.269614576 -1.157868928 0.863439305
0.033553154 0.633548419 5 0 0.998207054
UBE4A ENSG00000110344.9 0.530398195 2 2 0.320900161
Brain_Frontal_Cortex_BA9 0.582611905 Brain_Cerebellar_Hemisphere
1.017830944 0.982169056 0.982169056 -0.992609772 0.549573549
-0.221518111 1.090488284 2 0 0.998207054
LTBP2 ENSG00000119681.11 0.530423803 1 1 0.530423803
Brain_Putamen_basal_ganglia 0.530423803 Brain_Putamen_basal_ganglia 1 1
1 -0.627359205 -0.627359205 -0.627359205 NA 1 0
0.998207054
TEFM ENSG00000172171.10 0.530470397 5 4 0.388361706
Brain_Cerebellar_Hemisphere 0.820792582 Brain_Nucleus_accumbens_basal_ganglia
3.20909852 0.0789549 0.198605567 -0.550865369 0.862592226
0.271246561 0.627027922 4 0 0.998207054
ARL6IP1 ENSG00000170540.14 0.53060967 3 2 0.253504722 Brain_Cortex
0.497464517 Brain_Cerebellum 2.648092807 0.03904053 0.312866663
-1.141878029 0.91651261 0.15103971 1.126006905 2 0 0.998207054
C6orf62 ENSG00000112308.12 0.530750384 6 3 0.154641274
Brain_Cerebellum 0.696324623 Brain_Caudate_basal_ganglia 4.848042447
0.003088726 0.22205102 -1.423327376 -0.390286611 -0.904280038
0.43204283 3 0 0.998207054
PRSS55 ENSG00000184647.10 0.530812831 8 3 0.170582875
Brain_Cerebellum 0.609390819 Brain_Anterior_cingulate_cortex_BA24
6.958480058 0.001211358 0.270482176 0.510943216 1.370333326
1.104501781 0.307349935 3 0 0.998207054
AC093627.10 ENSG00000240859.1 0.530871286 8 7 0.216273509
Brain_Anterior_cingulate_cortex_BA24 0.935276272 Brain_Frontal_Cortex_BA9
3.518345474 7.06E-17 0.19184998 -1.236497998 0.76447739 -0.705395929
0.742758752 7 0 0.998207054
RP11-47A8.5 ENSG00000272933.1 0.530961773 4 4 0.43682247
Brain_Anterior_cingulate_cortex_BA24 0.74137993 Brain_Spinal_cord_cervical_c-1
2.447313658 0.203645135 0.203645135 -0.354356531 0.77757014
0.195048969 0.621531987 4 0 0.998207054
KCNN4 ENSG00000104783.11 0.53103127 13 5 0.042553438
Brain_Frontal_Cortex_BA9 0.744515146 Brain_Spinal_cord_cervical_c-1

7.505731874 0.006441308 0.388540696 -2.028066725 0.325880002
-1.122335504 0.590275958 5 0 0.998207054
PARD3B ENSG00000116117.17 0.53107428 1 1 0.53107428
Brain_Spinal_cord_cervical_c-1 0.53107428 Brain_Spinal_cord_cervical_c-1 1
1 1 -0.626366954 -0.626366954 -0.626366954 NA 1 0
0.998207054
PROCA1 ENSG00000167525.13 0.531077758 11 2 0.117725738
Brain_Caudate_basal_ganglia 0.683142019 Brain_Cortex 8.969270537
0.007792538 1.146181631 0.408179335 1.564391149 1.011817832
0.286998928 2 0 0.998207054
CALU ENSG00000128595.16 0.531155313 4 4 0.25666933 Brain_Cortex
0.794236885 Brain_Cerebellum 1.403171318 0.64677067 0.64677067
-1.134298567 0.601393553 -0.476676756 0.825871833 4 0
0.998207054
IFI16 ENSG00000163565.18 0.531156858 1 1 0.531156858 Brain_Cortex
0.531156858 Brain_Cortex 1 1 1 0.626241031 0.626241031
0.626241031 NA 1 0 0.998207054
VWA1 ENSG00000179403.11 0.531171448 1 1 0.531171448
Brain_Cerebellar_Hemisphere 0.531171448 Brain_Cerebellar_Hemisphere 1 1
1 0.626218784 0.626218784 0.626218784 NA 1 0
0.998207054
RAB8A ENSG00000167461.11 0.53118742 2 2 0.274494153 Brain_Cortex
0.742579085 Brain_Cerebellum 1.056889664 0.943110336 0.943110336
0.328439892 1.092771465 0.710605679 0.540464039 2 0
0.998207054
RP11-177J6.1 ENSG00000273257.1 0.531303814 3 3 0.171397833
Brain_Putamen_basal_ganglia 0.741065802 Brain_Nucleus_accumbens_basal_ganglia
1.657028524 0.352289553 0.352289553 -0.330442277 1.367726081
0.742564869 0.933471172 3 0 0.998207054
RP11-1017G21.6 ENSG00000272444.1 0.531371469 8 7 0.212193961
Brain_Cortex 0.884204946 Brain_Substantia_nigra 3.413274577
0.111551503 0.254413975 -0.88833725 1.247555288 0.175150565
0.709325127 7 0 0.998207054
DNA2 ENSG00000138346.14 0.531477805 13 1 0.228973849
Brain_Anterior_cingulate_cortex_BA24 0.831793793
Brain_Spinal_cord_cervical_c-1 12.36771987 0.000487174 12.36771987
-1.203006388 -0.212401529 -0.611690568 0.228707306 1 0
0.998207054
TTC34 ENSG00000215912.12 0.53153649 2 1 0.508797488 Brain_Cerebellum
0.562460169 Brain_Nucleus_accumbens_basal_ganglia 1.963654772
0.036345228 1.963654772 0.579191199 0.66071128 0.619951239
0.057643403 1 0 0.998207054
PLA2G12A ENSG00000123739.10 0.531569587 10 8 0.029919531
Brain_Frontal_Cortex_BA9 0.903070688 Brain_Caudate_basal_ganglia
5.278367072 0.013829802 0.236798174 -2.171154029 0.366205496
-0.318177877 0.728581091 8 0 0.998207054
C7orf50 ENSG00000146540.14 0.531574328 10 4 0.225809651
Brain_Cortex 0.935939713 Brain_Frontal_Cortex_BA9 8.168359844
0.012484213 0.296082033 -0.122223068 1.211223782 0.623170812
0.436515806 4 0 0.998207054
LDLRAD3 ENSG00000179241.12 0.531585604 3 3 0.15701275
Brain_Putamen_basal_ganglia 0.986408841 Brain_Cerebellum 1.292694256
0.712406305 0.712406305 -1.415190155 0.389050562 -0.336368259
0.952623452 3 0 0.998207054
NOL9 ENSG00000162408.10 0.531592563 1 1 0.531592563
Brain_Cerebellar_Hemisphere 0.531592563 Brain_Cerebellar_Hemisphere 1 1
1 -0.625576794 -0.625576794 -0.625576794 NA 1 0
0.998207054
RP11-458F8.4 ENSG00000273142.1 0.531890742 13 3 0.192453521
Brain_Substantia_nigra 0.914553 Brain_Spinal_cord_cervical_c-1
10.41037172 0.018736496 0.531193489 -0.340090621 1.303355209
0.521970932 0.501518541 3 0 0.998207054

EXT2 ENSG00000151348.13 0.53190453 8 7 0.09229 Brain_Putamen_basal_ganglia 0.864405894 Brain_Frontal_Cortex_BA9 3.293003532 0.044897084 0.238544517 -1.683439717 1.068790051 -0.057269502 0.932644602 7 0 0.998207054
MITD1 ENSG00000158411.11 0.53193362 8 4 0.46987089 Brain_Anterior_cingulate_cortex_BA24 0.964105366 Brain_Cerebellum 6.232744534 0.016103898 0.260556385 -0.703362346 0.722689139 -0.288263866 0.479507854 4 0 0.998207054
ASPA ENSG00000108381.10 0.532025256 3 3 0.264646142 Brain_Cerebellum 0.672101447 Brain_Amygdala 1.182692807 0.821134962 0.821134962 -0.423265658 1.115476829 0.482454975 0.804801577 3 0 0.998207054
LNX2 ENSG00000139517.7 0.532026272 3 3 0.562275221 Brain_Cerebellar_Hemisphere 0.952777389 Brain_Spinal_cord_cervical_c-1 1.90422164 0.099833973 0.099833973 -0.070500838 0.579465349 0.189394624 0.34398141 3 0 0.998207054
ZNF608 ENSG00000168916.15 0.532070346 1 1 0.532070346 Brain_Cortex 0.532070346 Brain_Cortex 1 1 1 -0.624848727 -0.624848727 -0.624848727 NA 1 0 0.998207054
ASPH ENSG00000198363.17 0.532092263 3 3 0.228656304 Brain_Cortex 0.713486714 Brain_Nucleus_accumbens_basal_ganglia 1.184848645 0.824055961 0.824055961 -0.791807147 1.203827382 0.01494762 1.051263016 3 0 0.998207054
EED ENSG00000074266.19 0.53212583 2 1 0.413977761 Brain_Cerebellar_Hemisphere 0.67770208 Brain_Cerebellum 1.945905966 0.054094034 1.945905966 -0.816913677 -0.415600884 -0.616257281 0.283770997 1 0 0.998207054
TNFRSF1B ENSG00000028137.18 0.532188925 1 1 0.532188925 Brain_Cerebellum 0.532188925 Brain_Cerebellum 1 1 1 0.624668083 0.624668083 0.624668083 NA 1 0 0.998207054
TPPP3 ENSG00000159713.10 0.532300406 2 2 0.266804893 Brain_Cortex 0.929815299 Brain_Putamen_basal_ganglia 1.070975491 0.929024509 0.929024509 -0.088077223 1.110450626 0.511186701 0.847487169 2 0 0.998207054
PCDHGA10 ENSG00000253846.2 0.532344914 1 1 0.532344914 Brain_Nucleus_accumbens_basal_ganglia 0.532344914 Brain_Nucleus_accumbens_basal_ganglia 1 1 1 0.624430478 0.624430478 0.624430478 NA 1 0 0.998207054
PDP2 ENSG00000172840.6 0.532438009 2 2 0.270796365 Brain_Cortex 0.857204322 Brain_Cerebellum 1.035161244 0.964838756 0.964838756 -1.101230467 0.179934078 -0.460648195 0.905920138 2 0 0.998207054
PPP1R3G ENSG00000219607.3 0.532446014 1 1 0.532446014 Brain_Spinal_cord_cervical_c-1 0.532446014 Brain_Spinal_cord_cervical_c-1 1 1 1 0.6242765 0.6242765 0.6242765 NA 1 0 0.998207054
WNK2 ENSG00000165238.16 0.532506631 2 2 0.307791714 Brain_Nucleus_accumbens_basal_ganglia 0.830656658 Brain_Cerebellum 1.237272619 0.762727381 0.762727381 -1.019866638 0.213859456 -0.403003591 0.872376088 2 0 0.998207054
SLC19A1 ENSG00000173638.18 0.532590422 8 8 0.028605564 Brain_Anterior_cingulate_cortex_BA24 0.981464518 Brain_Cerebellum 2.003033411 0.474775849 0.474775849 -0.892968908 2.18887999 0.439543923 0.915390779 8 0 0.998207054
GABRA1 ENSG00000022355.16 0.532658416 1 1 0.532658416 Brain_Hippocampus 0.532658416 Brain_Hippocampus 1 1 1 0.623953054 0.623953054 0.623953054 NA 1 0 0.998207054
FGF11 ENSG00000161958.10 0.532692963 3 3 0.269062694 Brain_Amygdala 0.873987899 Brain_Hypothalamus 2.444674957 0.272524808 0.272524808 0.158595088 1.105223645 0.534657943 0.502394192 3 0 0.998207054
GCK ENSG00000106633.15 0.53275265 1 1 0.53275265 Brain_Hypothalamus 0.53275265 Brain_Hypothalamus 1 1 1 0.623809576 0.623809576

0.623809576 NA 1 0 0.998207054
LHX2 ENSG00000106689.10 0.532866494 1 1 0.532866494 Brain_Amygdala
0.532866494 Brain_Amygdala 1 1 1 -0.623636257 -0.623636257
-0.623636257 NA 1 0 0.998207054
PPP2R5C ENSG00000078304.19 0.532886161 3 3 0.30565127
Brain_Hypothalamus 0.695358432 Brain_Amygdala 1.757672201 0.24193693
0.24193693 -0.987799621 1.024389647 -0.118334563 1.033551806 3
0 0.998207054
XYLB ENSG00000093217.9 0.532939215 1 1 0.532939215
Brain_Cerebellar_Hemisphere 0.532939215 Brain_Cerebellar_Hemisphere 1 1
1 0.623525554 0.623525554 0.623525554 NA 1 0
0.998207054
NUDT8 ENSG00000167799.9 0.533029629 3 3 0.575031641
Brain_Putamen_basal_ganglia 0.769286197 Brain_Caudate_basal_ganglia
2.372237803 0.285227116 0.285227116 -0.560656627 0.543294773
0.091982285 0.578857858 3 0 0.998207054
GLI4 ENSG00000250571.6 0.533099077 1 1 0.533099077
Brain_Spinal_cord_cervical_c-1 0.533099077 Brain_Spinal_cord_cervical_c-1
1 1 1 0.623282224 0.623282224 0.623282224 NA 1 0
0.998207054
ZFYVE9 ENSG00000157077.14 0.533369756 1 1 0.533369756
Brain_Anterior_cingulate_cortex_BA24 0.533369756
Brain_Anterior_cingulate_cortex_BA24 1 1 1 -0.622870301
-0.622870301 -0.622870301 NA 1 0 0.998207054
MX1 ENSG00000157601.13 0.533416261 3 3 0.254507805 Brain_Cortex
0.53239656 Brain_Nucleus_accumbens_basal_ganglia 1.050725463 0.953369635
0.953369635 -1.139468486 0.651517197 -0.370767703 0.922028257
3 0 0.998207054
RAB4A ENSG00000168118.11 0.533459707 1 1 0.533459707
Brain_Cerebellar_Hemisphere 0.533459707 Brain_Cerebellar_Hemisphere 1 1
1 0.622733436 0.622733436 0.622733436 NA 1 0
0.998207054
ZNF443 ENSG00000180855.15 0.533475588 10 2 0.206293562
Brain_Nucleus_accumbens_basal_ganglia 0.55873098 Brain_Cerebellum 8.601608497
7.69E-05 0.875717546 0.58472747 1.263823006 0.920303617
0.237024139 2 0 0.998207054
TMEM72 ENSG00000187783.11 0.533480371 6 6 0.213642645
Brain_Nucleus_accumbens_basal_ganglia 0.954632509 Brain_Hypothalamus
1.554927234 0.452394536 0.452394536 -0.590725057 1.243611328
0.402589685 0.895987974 6 0 0.998207054
C4orf50 ENSG00000181215.14 0.533494427 1 1 0.533494427
Brain_Substantia_nigra 0.533494427 Brain_Substantia_nigra 1 1
1 -0.622680611 -0.622680611 -0.622680611 NA 1 0
0.998207054
BAG5 ENSG00000166170.9 0.533510076 3 3 0.153337016
Brain_Cerebellar_Hemisphere 0.25640299 Brain_Anterior_cingulate_cortex_BA24
2.409757658 0.150508876 0.150508876 1.13493396 1.427843129
1.233346166 0.168443331 3 0 0.998207054
CNTROB ENSG00000170037.13 0.533571459 5 4 0.210185819
Brain_Cortex 0.384288704 Brain_Cerebellum 3.483228273 0.095699827
0.128537375 -1.253054649 -0.870021411 -1.071607423 0.168120166
4 0 0.998207054
ASAP1 ENSG00000153317.14 0.533598108 6 4 0.251911148
Brain_Putamen_basal_ganglia 0.893009097 Brain_Cerebellar_Hemisphere
4.047134299 0.081282056 0.221796969 -0.436761173 1.145719704
0.010761807 0.604545885 4 0 0.998207054
BBS2 ENSG00000125124.11 0.53364612 8 3 0.248566325
Brain_Nucleus_accumbens_basal_ganglia 0.729432051 Brain_Cortex
6.018994488 0.001074992 0.654446404 -0.345881129 1.15383865
0.755804403 0.481236175 3 0 0.998207054
BRINP1 ENSG00000078725.12 0.533651512 2 2 0.324899161
Brain_Caudate_basal_ganglia 0.548616264 Brain_Anterior_cingulate_cortex_BA24

1.066418655	0.933581345	0.933581345	0.599834912	0.984440118
0.792137515	0.27195695 2	0	0.998207054
FAM229B	ENSG00000203778.7	0.533681786	12	4	0.052203568
Brain_Nucleus_accumbens_basal_ganglia 0.540490703	Brain_Caudate_basal_ganglia
9.344746715	0.018897541	0.371042026	-1.941451239	-0.612071122
-1.17857998	0.37017277 4	0	0.998207054
KRTAP16-1	ENSG00000212657.1	0.533779268	1	1	0.533779268
Brain_Cerebellar_Hemisphere 0.533779268	Brain_Cerebellar_Hemisphere 1	1
1	0.622247301	0.622247301	0.622247301	NA	1	0
0.998207054
FAM20C	ENSG00000177706.8	0.534032727	2	2	0.348008336
Brain_Spinal_cord_cervical_c-1	0.532330332	Brain_Hippocampus
1.013901736	0.986098264	0.986098264	-0.938459471	0.624452688
-0.157003391	1.105145786	2	0	0.998207054
RAB11FIP4	ENSG00000131242.17	0.534326137	3	3	0.155319266
Brain_Hippocampus	0.961169205	Brain_Putamen_basal_ganglia 1.426701014
0.569599662	0.569599662	0.048686412	1.420991377	0.601319093
0.724077234	3	0	0.998207054
RP11-90C4.1	ENSG00000234810.2	0.534545736	1	1	0.534545736
Brain_Spinal_cord_cervical_c-1	0.534545736	Brain_Spinal_cord_cervical_c-1
1	1	1	0.621081902	0.621081902	0.621081902	NA	1	0
0.998207054
AP1S1 ENSG00000106367.13	0.534556188	2	2	0.299689253
Brain_Hypothalamus	0.730302576	Brain_Cerebellar_Hemisphere 1.643095764
0.356904236	0.356904236	0.344723066	1.037100004	0.690911535
0.489584428	2	0	0.998207054
RP11-554D14.6	ENSG00000257398.6	0.53456008 3	3	0.393330227
Brain_Cerebellum 0.763737956	Brain_Cerebellar_Hemisphere 1.919003032
0.094839715	0.094839715	0.300575841	0.853593562	0.521244903
0.292935828	3	0	0.998207054
FOXE3 ENSG00000186790.5	0.534581862	3	2	0.541692188
Brain_Frontal_Cortex_BA9	0.844243971	Brain_Cerebellum 2.571245459
0.048904829	0.379849712	-0.427665231	0.610256077	0.126352909
0.52250095 2	0	0.998207054
ASPSCR1	ENSG00000169696.15	0.534583419	2	2	0.328795094
Brain_Cerebellum 0.652936874	Brain_Frontal_Cortex_BA9	1.096734131
0.903265869	0.903265869	-0.449685991	0.976543718	0.263428864
1.008496699	2	0	0.998207054
OXER1 ENSG00000162881.6	0.534724649	8	3	0.086859344
Brain_Substantia_nigra	0.994981986	Brain_Putamen_basal_ganglia
5.893787929	0.000622962	0.761199609	-0.006289189	1.712202571
1.014407914	0.52988864 3	0	0.998207054
YME1L1	ENSG00000136758.18	0.534849156	1	1	0.534849156
Brain_Anterior_cingulate_cortex_BA24	0.534849156
Brain_Anterior_cingulate_cortex_BA24	1	1	1	-0.620620792
-0.620620792	-0.620620792	NA	1	0	0.998207054
LRRC37A2	ENSG00000238083.7	0.534857099	13	1	0.359747475
Brain_Amygdala	0.687621775	Brain_Frontal_Cortex_BA9	12.3399279
0.000798693	12.3399279 -0.915846378	-0.402084588	-0.604585095
0.183192915	1	0	0.998207054
DENND4A	ENSG00000174485.15	0.534964759	3	2	0.48103403 Brain_Cortex
0.751648784	Brain_Cerebellum 2.456437385	0.018628527	0.524934088
-0.704640433	0.316466058	-0.34544077	0.573923634	2	0
0.998207054
AC007228.5 ENSG00000268352.1	0.535159417	1	1	0.535159417
Brain_Cerebellar_Hemisphere 0.535159417	Brain_Cerebellar_Hemisphere 1	1
1	-0.620149422	-0.620149422	-0.620149422	NA	1	0
0.998207054
RP11-121M22.1	ENSG00000175773.12	0.535227215	10	10	0.028630721
Brain_Cortex	0.931384243	Brain_Cerebellar_Hemisphere 1.254091188
0.7939715 0.7939715 -2.188534089	1.551010544	-0.112023423
0.975225499	10	0	0.998207054

LRRC49	ENSG00000137821.11	0.535520817	2	2	0.381441932
Brain_Cerebellar_Hemisphere 0.644381759	Brain_Cerebellum 1.242878826
0.757121174	0.757121174	-0.461581089	0.875242576	0.206830744
0.945277079	2	0	0.998207054
TRAPPC12	ENSG00000171853.15	0.535552788	11	7	0.10466438
Brain_Cerebellar_Hemisphere 0.936259874	Brain_Hippocampus	6.517673818
0.086213943	0.243170742	-1.622649372	0.243331215	-0.706509697
0.614861319	7	0	0.998207054
KRR1 ENSG00000111615.13	0.53556014 13	2	0.14845965 Brain_Hypothalamus
0.569878022	Brain_Anterior_cingulate_cortex_BA24	11.27644624
0.000535878	0.848501987	0.568231152	1.444993718	1.015634852
0.30321912 2	0	0.998207054
PHACTR4	ENSG00000204138.12	0.53581147 12	3	0.10962823 Brain_Cortex
0.44008068 Brain_Anterior_cingulate_cortex_BA24	10.18201286	0.00225253
0.405548135	-1.599866374	-0.772056981	-1.252062454	0.278583277
3	0	0.998207054
TMEM129	ENSG00000168936.10	0.535925412	12	4	0.280072512
Brain_Frontal_Cortex_BA9	0.809715047	Brain_Caudate_basal_ganglia
9.201422909	0.00053555 0.358397051	-1.080156464	0.745005938
-0.391158537	0.547133265	4	0	0.998207054
ZNF486	ENSG00000256229.7	0.535932617	1	1	0.535932617
Brain_Hippocampus	0.535932617	Brain_Hippocampus	1	1	1
-0.618975321	-0.618975321	-0.618975321	NA	1	0	0.998207054
RP13-20L14.1	ENSG00000260011.2	0.535992996	2	2	0.264367719
Brain_Cerebellar_Hemisphere 0.480487714	Brain_Cerebellum 1.604200399
0.395799601	0.395799601	0.705518354	1.116127135	0.910822744
0.290344253	2	0	0.998207054
WNT8B ENSG00000075290.7	0.535999289	4	2	0.654699676	Brain_Cortex
0.952663897	Brain_Spinal_cord_cervical_c-1	3.850991497	0.000559052
0.140790901	-0.44724292	0.059361853	-0.295124892	0.240148933
2	0	0.998207054
MUSTN1	ENSG00000272573.5	0.536028159	1	1	0.536028159
Brain_Cerebellum 0.536028159	Brain_Cerebellum 1	1	1	-0.6188303
-0.6188303 -0.6188303 NA	1	0	0.998207054
RNF139-AS1 ENSG00000245149.3	0.536036685	7	3	0.089175319
Brain_Cortex	0.83158741 Brain_Cerebellar_Hemisphere 4.967302059
0.038509841	0.795312129	-0.2126661 1.699764038	0.99883252
0.626679471	3	0	0.998207054
CCDC78	ENSG00000162004.16	0.536046378	6	4	0.09615616 Brain_Amygdala
0.967463999	Brain_Cerebellum 3.310709475	0.000844257	0.620988581
-0.090626395	1.66378121 0.341527648	0.712725725	4	0
0.998207054
PTP4A2	ENSG00000184007.18	0.536123821	2	2	0.275854283
Brain_Cerebellum 0.844085288	Brain_Caudate_basal_ganglia 1.042931694
0.957068306	0.957068306	-1.08967965	-0.19667064	-0.643175145
0.631452727	2	0	0.998207054
MCM9 ENSG00000111877.17	0.536199125	5	5	0.150672858	Brain_Amygdala
0.957110486	Brain_Anterior_cingulate_cortex_BA24	2.19678054 0.085974527
0.085974527	-1.437158867	0.053779948	-0.661691602	0.562358263
5	0	0.998207054
UROS ENSG00000188690.12	0.536229529	12	2	0.22645098
Brain_Putamen_basal_ganglia 0.386280556	Brain_Substantia_nigra
11.11936134	0.000139689	0.41794288 0.866382284	1.209551651
1.070664653	0.122743658	2	0	0.998207054
LHB	ENSG00000104826.12	0.536278757	1	1	0.536278757
Brain_Cerebellar_Hemisphere 0.536278757	Brain_Cerebellar_Hemisphere 1	1
1	-0.618449986	-0.618449986	-0.618449986	NA	1	0
0.998207054
IQGAP3	ENSG00000183856.10	0.536290754	1	1	0.536290754
Brain_Frontal_Cortex_BA9	0.536290754	Brain_Frontal_Cortex_BA9	1	1
1	-0.61843178	-0.61843178	-0.61843178	NA	1	0
0.998207054

C2orf54 ENSG00000172478.17 0.536322798 4 4 0.205532028
Brain_Cerebellum 0.990175289 Brain_Hippocampus 2.135242358
0.200268392 0.200268392 -1.265947083 0.01231376 -0.688454897
0.535439223 4 0 0.998207054
COX4I1 ENSG00000131143.8 0.536323074 1 1 0.536323074
Brain_Substantia_nigra 0.536323074 Brain_Substantia_nigra 1 1
1 0.618382738 0.618382738 0.618382738 NA 1 0
0.998207054
BECN1 ENSG00000126581.12 0.536520703 1 1 0.536520703
Brain_Hippocampus 0.536520703 Brain_Hippocampus 1 1 1
0.618082886 0.618082886 0.618082886 NA 1 0 0.998207054
CLNK ENSG00000109684.14 0.536672941 2 2 0.264570035 Brain_Cerebellum
0.577582635 Brain_Cerebellar_Hemisphere 1.493798683 0.506201317
0.506201317 0.556919201 1.115654544 0.836286873 0.395085551
2 0 0.998207054
UPK3BL ENSG00000267368.1 0.536692746 13 5 0.112543019
Brain_Anterior_cingulate_cortex_BA24 0.983218011 Brain_Hypothalamus
8.547786131 0.010051917 0.386257002 -1.089887665 1.586865904
0.282712653 0.672985713 5 0 0.998207054
TMEM132D ENSG00000151952.15 0.536694407 1 1 0.536694407
Brain_Hippocampus 0.536694407 Brain_Hippocampus 1 1 1
-0.61781938 -0.61781938 -0.61781938 NA 1 0 0.998207054
C8orf82 ENSG00000213563.6 0.536746422 1 1 0.536746422
Brain_Substantia_nigra 0.536746422 Brain_Substantia_nigra 1 1
1 -0.617740482 -0.617740482 -0.617740482 NA 1 0
0.998207054
TMEM229A ENSG00000234224.2 0.536796043 9 6 0.096858422
Brain_Putamen_basal_ganglia 0.551579277 Brain_Nucleus_accumbens_basal_ganglia
5.691532184 0.037604654 0.253803368 -0.615104732 1.660278583
0.841174916 0.660368262 6 0 0.998207054
PLEKHA1 ENSG00000107679.14 0.536907508 5 4 0.245120416
Brain_Caudate_basal_ganglia 0.982634919 Brain_Spinal_cord_cervical_c-1
3.465080884 0.043069629 0.208075996 -1.16228328 0.233236023
-0.341115758 0.576958337 4 0 0.998207054
LCORL ENSG00000178177.15 0.537146258 1 1 0.537146258 Brain_Cerebellum
0.537146258 Brain_Cerebellum 1 1 1 -0.61713413 -0.61713413
-0.61713413 NA 1 0 0.998207054
HLA-DOA ENSG00000204252.13 0.537303549 3 3 0.222106911
Brain_Cerebellum 0.923217022 Brain_Cerebellar_Hemisphere 1.454642589
0.57341113 0.57341113 -0.096382209 1.220944824 0.593745779
0.660914266 3 0 0.998207054
RP11-673E1.1 ENSG00000251600.5 0.537322374 11 1 0.375934704
Brain_Spinal_cord_cervical_c-1 0.73959255 Brain_Nucleus_accumbens_basal_ganglia
10.49145905 0.000425698 10.49145905 0.332392965 0.885411552
0.604181678 0.16357764 1 0 0.998207054
PSMG3-AS1 ENSG00000230487.7 0.537336099 12 4 0.40186392
Brain_Putamen_basal_ganglia 0.969375364 Brain_Cerebellar_Hemisphere
8.625165312 2.48E-16 0.447280929 -0.838297001 0.695131576
0.106337046 0.462067954 4 0 0.998207054
STAP1 ENSG00000035720.7 0.537360122 1 1 0.537360122
Brain_Hippocampus 0.537360122 Brain_Hippocampus 1 1 1
0.616809898 0.616809898 0.616809898 NA 1 0 0.998207054
SLC8B1 ENSG00000089060.11 0.537440608 4 4 0.151494749
Brain_Caudate_basal_ganglia 0.766072024 Brain_Putamen_basal_ganglia
2.864831425 0.178488923 0.178488923 0.297516748 1.434271679
1.126154993 0.553177771 4 0 0.998207054
TAS2R31 ENSG00000256436.1 0.537536652 1 1 0.537536652
Brain_Cortex 0.537536652 Brain_Cortex 1 1 1 0.616542317
0.616542317 0.616542317 NA 1 0 0.998207054
IPPK ENSG00000127080.9 0.537537243 4 3 0.232420067
Brain_Cerebellar_Hemisphere 0.681518251 Brain_Hypothalamus 2.158810585
0.044472999 0.616776806 -1.194148033 0.734494253 -0.365719255

0.806295941 3 0 0.998207054
KIN ENSG00000151657.11 0.537594732 7 2 0.318392042
Brain_Nucleus_accumbens_basal_ganglia 0.969573941 Brain_Cerebellar_Hemisphere
6.352917426 0.001656234 0.282229283 -0.038142657 0.997767646
0.525294487 0.334175851 2 0 0.998207054
RP11-426C22.6 ENSG00000260953.1 0.537686715 7 1 0.326730213
Brain_Amygdala 0.715553627 Brain_Nucleus_accumbens_basal_ganglia
6.806680172 0.000302205 6.806680172 -0.980721268 -0.364407645
-0.609610794 0.247771704 1 0 0.998207054
NEURL1 ENSG00000107954.10 0.537785617 4 4 0.092372726
Brain_Cortex 0.906178667 Brain_Amygdala 1.552414374 0.437292986
0.437292986 -1.683012218 0.1178599 -0.789885343 0.808874508 4
0 0.998207054
PTCH2 ENSG00000117425.13 0.537859818 1 1 0.537859818
Brain_Cerebellar_Hemisphere 0.537859818 Brain_Cerebellar_Hemisphere 1 1
1 0.61605258 0.61605258 0.61605258 NA 1 0 0.998207054
RPS23 ENSG00000186468.12 0.537948407 13 2 0.437573652
Brain_Spinal_cord_cervical_c-1 0.998914912 Brain_Cerebellar_Hemisphere
12.0387936 0.000103118 0.492125319 -0.776296986 0.151139871
-0.030714684 0.237463209 2 0 0.998207054
EMC2 ENSG00000104412.7 0.538086942 8 6 0.199337912 Brain_Cortex
0.945116492 Brain_Caudate_basal_ganglia 4.93477796 0.070960879
0.243293802 -1.283440159 0.068840611 -0.731219672 0.52410234 6
0 0.998207054
CTSZ ENSG00000101160.13 0.538101315 2 2 0.298089097
Brain_Hippocampus 0.484641885 Brain_Substantia_nigra 1.863496502
0.136503498 0.136503498 0.698856228 1.040539985 0.869698106
0.241606902 2 0 0.998207054
DCXR ENSG00000169738.7 0.538210094 9 3 0.212379722
Brain_Nucleus_accumbens_basal_ganglia 0.701133913
Brain_Anterior_cingulate_cortex_BA24 7.450705028 6.34E-08 0.26538416
0.383790253 1.247048481 0.968543926 0.31937651 3 0
0.998207054
OTOF ENSG00000115155.16 0.538355036 4 4 0.159814839 Brain_Cerebellum
0.372961465 Brain_Cerebellar_Hemisphere 2.038104692 0.268787164
0.268787164 -1.405694575 -0.890939804 -1.178100208 0.217236677
4 0 0.998207054
CACNA1E ENSG00000198216.10 0.538415861 1 1 0.538415861
Brain_Cerebellar_Hemisphere 0.538415861 Brain_Cerebellar_Hemisphere 1 1
1 -0.615210279 -0.615210279 -0.615210279 NA 1 0
0.998207054
MRPS5 ENSG00000144029.11 0.538440535 2 2 0.269788649 Brain_Cortex
0.663232475 Brain_Nucleus_accumbens_basal_ganglia 1.270179678
0.729820322 0.729820322 -1.103549404 -0.435454615 -0.769502009
0.472414356 2 0 0.998207054
C3orf80 ENSG00000180044.5 0.53845129 1 1 0.53845129
Brain_Anterior_cingulate_cortex_BA24 0.53845129
Brain_Anterior_cingulate_cortex_BA24 1 1 1 0.615156625
0.615156625 0.615156625 NA 1 0 0.998207054
UBIAD1 ENSG00000120942.13 0.538509653 1 1 0.538509653
Brain_Frontal_Cortex_BA9 0.538509653 Brain_Frontal_Cortex_BA9 1 1
1 -0.615068243 -0.615068243 -0.615068243 NA 1 0
0.998207054
VPS29 ENSG00000111237.18 0.538539485 9 5 0.221687112
Brain_Nucleus_accumbens_basal_ganglia 0.887300432 Brain_Cerebellum
6.888629501 0.020236166 0.318650798 -1.222054251 0.204764746
-0.580009591 0.550430751 5 0 0.998207054
RP5-933K21.3 ENSG00000271040.1 0.538699232 6 6 0.068835966
Brain_Nucleus_accumbens_basal_ganglia 0.963464505 Brain_Amygdala
1.396064299 0.537607582 0.537607582 -0.045806467 1.81949486
0.839830735 0.606376616 6 0 0.998207054
HPS1 ENSG00000107521.18 0.538775376 12 5 0.353556908

Brain_Spinal_cord_cervical_c-1 0.973916514 Brain_Hippocampus
5.861544502 0.010454896 0.232576851 -0.416713993 0.927712143
0.246985284 0.451903632 5 0 0.998207054
IL22RA1 ENSG00000142677.3 0.538847238 2 1 0.510793087
Brain_Cerebellar_Hemisphere 0.571222342 Brain_Cerebellum 1.982922645
0.017077355 1.982922645 -0.65760328 -0.566252211 -0.611927745
0.06459496 1 0 0.998207054
TRAPPC6A ENSG00000007255.10 0.538890541 1 1 0.538890541
Brain_Amygdala 0.538890541 Brain_Amygdala 1 1 1 -0.614491572
-0.614491572 -0.614491572 NA 1 0 0.998207054
PCDHA11 ENSG00000249158.6 0.538947934 6 4 0.325288134
Brain_Nucleus_accumbens_basal_ganglia 0.893163811 Brain_Caudate_basal_ganglia
3.928966213 0.003440246 0.227709164 -0.385241082 0.983648979
0.347174731 0.510523383 4 0 0.998207054
GGPS1 ENSG00000152904.11 0.538954211 4 4 0.283312647
Brain_Spinal_cord_cervical_c-1 0.979026911 Brain_Frontal_Cortex_BA9
1.641682981 0.371626823 0.371626823 -0.888470816 1.072907442
-0.018613909 0.820801287 4 0 0.998207054
ABCA3 ENSG00000167972.13 0.539073973 5 4 0.210910604 Brain_Cerebellum
0.757877509 Brain_Frontal_Cortex_BA9 2.002954217 0.021161369
0.9210965 -0.821920058 1.251065438 0.554530286 0.847125059 4
0 0.998207054
KIAA0232 ENSG00000170871.11 0.539092324 2 2 0.303231587
Brain_Anterior_cingulate_cortex_BA24 0.499334377
Brain_Spinal_cord_cervical_c-1 1.263258878 0.736741122 0.736741122
-1.029528089 -0.675537433 -0.852532761 0.250309193 2 0
0.998207054
SHB ENSG00000107338.9 0.539110151 3 3 0.302224352
Brain_Caudate_basal_ganglia 0.911025035 Brain_Cerebellar_Hemisphere
1.467051783 0.519119315 0.519119315 -1.031675087 0.682177119
-0.153747893 0.857697781 3 0 0.998207054
GS1-57L11.1 ENSG00000253853.1 0.539134757 4 3 0.257029857
Brain_Cerebellar_Hemisphere 0.478503267 Brain_Cerebellum 2.502160434
0.041635867 0.46119013 -0.833126316 1.133439208 -0.298198275
0.955796648 3 0 0.998207054
TMEM251 ENSG00000153485.5 0.53923222 2 2 0.298103713 Brain_Cortex
0.719757749 Brain_Anterior_cingulate_cortex_BA24 1.030394969
0.969605031 0.969605031 -1.040508509 0.358782575 -0.340862967
0.989448214 2 0 0.998207054
CLDN1 ENSG00000163347.5 0.539264376 1 1 0.539264376
Brain_Hippocampus 0.539264376 Brain_Hippocampus 1 1 1
-0.613925776 -0.613925776 -0.613925776 NA 1 0 0.998207054
TMEM232 ENSG00000186952.14 0.539326806 4 2 0.460374742
Brain_Cerebellum 0.645927735 Brain_Cerebellar_Hemisphere 3.039006157
0.042722339 0.84267669 -0.738229923 0.713030205 -0.241937289
0.64903553 2 0 0.998207054
PHF23 ENSG00000040633.12 0.539356366 2 2 0.36814602
Brain_Spinal_cord_cervical_c-1 0.490483315
Brain_Anterior_cingulate_cortex_BA24 1.042591931 0.957408069
0.957408069 0.689540311 0.899951577 0.794745944 0.148783233
2 0 0.998207054
FAM215B ENSG00000232300.1 0.539364281 2 2 0.320085033
Brain_Cerebellum 0.461449387 Brain_Cerebellar_Hemisphere 1.927375767
0.072624233 0.072624233 -0.994283158 -0.736462326 -0.865372742
0.182306858 2 0 0.998207054
AMPH ENSG00000078053.16 0.539375362 1 1 0.539375362 Brain_Cortex
0.539375362 Brain_Cortex 1 1 1 0.613757837 0.613757837
0.613757837 NA 1 0 0.998207054
VPS41 ENSG00000006715.15 0.539472614 7 5 0.344544719
Brain_Cerebellar_Hemisphere 0.953001349 Brain_Nucleus_accumbens_basal_ganglia
3.764587455 0.063871706 0.327380452 -0.67952622 0.945223629
0.099332853 0.64644892 5 0 0.998207054

EPCAM ENSG00000119888.10 0.539615106 12 1 0.368652844 Brain_Cortex 0.756212446 Brain_Hypothalamus 11.25059744 0.001149496 11.25059744 0.310458326 0.898999656 0.593568628 0.198893737 1 0 0.998207054
RGMA ENSG00000182175.13 0.539725789 5 5 0.10018252 Brain_Hypothalamus 0.990657616 Brain_Cerebellar_Hemisphere 1.248414758 0.811522743 0.811522743 -0.40502173 1.643969417 0.51736456 0.837322292 5 0 0.998207054
CUL2 ENSG00000108094.14 0.53975013 1 1 0.53975013 Brain_Cerebellum 0.53975013 Brain_Cerebellum 1 1 1 0.613190886 0.613190886 0.613190886 NA 1 0 0.998207054
ZBTB18 ENSG00000179456.10 0.539772196 2 2 0.42793639 Brain_Amygdala 0.458705166 Brain_Hippocampus 1.045298145 0.954701855 0.954701855 -0.740980661 0.792727868 0.025873604 1.084495702 2 0 0.998207054
KLF2 ENSG00000127528.5 0.539785197 1 1 0.539785197 Brain_Cortex 0.539785197 Brain_Cortex 1 1 1 0.613137847 0.613137847 0.613137847 NA 1 0 0.998207054
C10orf107 ENSG00000183346.6 0.539823583 13 2 0.126574605 Brain_Substantia_nigra 0.411456391 Brain_Nucleus_accumbens_basal_ganglia 12.01610516 0.000374715 0.432457482 0.821333394 1.527750063 1.070945653 0.193012243 2 0 0.998207054
TANGO2 ENSG00000183597.15 0.539884113 3 3 0.303026567 Brain_Cerebellum 0.754035632 Brain_Cerebellar_Hemisphere 1.55540693 0.420622482 0.420622482 -0.452897295 1.029964721 0.087914964 0.818818409 3 0 0.998207054
NRARP ENSG00000198435.3 0.53993314 1 1 0.53993314 Brain_Anterior_cingulate_cortex_BA24 0.53993314 Brain_Anterior_cingulate_cortex_BA24 1 1 1 -0.612914099 -0.612914099 -0.612914099 NA 1 0 0.998207054
HARBI1 ENSG00000180423.4 0.53998757 1 1 0.53998757 Brain_Hippocampus 0.53998757 Brain_Hippocampus 1 1 1 0.612831788 0.612831788 0.612831788 NA 1 0 0.998207054
AC142472.6 ENSG00000276728.1 0.539999014 1 1 0.539999014 Brain_Hypothalamus 0.539999014 Brain_Hypothalamus 1 1 1 -0.612814482 -0.612814482 -0.612814482 NA 1 0 0.998207054
CLC ENSG00000105205.6 0.540000761 1 1 0.540000761 Brain_Substantia_nigra 0.540000761 Brain_Substantia_nigra 1 1 1 0.61281184 0.61281184 0.61281184 NA 1 0 0.998207054
OS9 ENSG00000135506.15 0.540003439 1 1 0.540003439 Brain_Caudate_basal_ganglia 0.540003439 Brain_Caudate_basal_ganglia 1 1 1 -0.61280779 -0.61280779 -0.61280779 NA 1 0 0.998207054
SCNN1G ENSG00000166828.2 0.540029512 3 2 0.239011003 Brain_Cerebellar_Hemisphere 0.725787901 Brain_Frontal_Cortex_BA9 2.003581049 0.02188641 0.974532541 -1.177462384 0.350734019 -0.522637949 0.787190148 2 0 0.998207054
KCTD4 ENSG00000180332.6 0.540188422 1 1 0.540188422 Brain_Nucleus_accumbens_basal_ganglia 0.540188422 Brain_Nucleus_accumbens_basal_ganglia 1 1 1 -0.612528086 -0.612528086 -0.612528086 NA 1 0 0.998207054
RP11-758N13.1 ENSG00000259721.1 0.540218071 1 1 0.540218071 Brain_Anterior_cingulate_cortex_BA24 0.540218071 Brain_Anterior_cingulate_cortex_BA24 1 1 1 0.612483258 0.612483258 0.612483258 NA 1 0 0.998207054
OTX1 ENSG00000115507.9 0.540273745 1 1 0.540273745 Brain_Caudate_basal_ganglia 0.540273745 Brain_Caudate_basal_ganglia 1 1 1 -0.612399088 -0.612399088 -0.612399088 NA 1 0 0.998207054
GRM8 ENSG00000179603.17 0.540280337 2 2 0.271760445 Brain_Putamen_basal_ganglia 0.406825843 Brain_Caudate_basal_ganglia 1.832109889 0.167890111 0.167890111 -1.099017473 -0.829492386

-0.96425493 0.190583017 2 0 0.998207054
MANSC4 ENSG00000205693.3 0.540360076 1 1 0.540360076
Brain_Cerebellar_Hemisphere 0.540360076 Brain_Cerebellar_Hemisphere 1 1
1 0.612268578 0.612268578 0.612268578 NA 1 0
0.998207054
CLSTN1 ENSG00000171603.16 0.540502334 1 1 0.540502334
Brain_Cerebellum 0.540502334 Brain_Cerebellum 1 1 1 0.612053541
0.612053541 0.612053541 NA 1 0 0.998207054
CCDC58 ENSG00000160124.9 0.54056698 4 4 0.292278996 Brain_Cerebellum
0.971761179 Brain_Frontal_Cortex_BA9 2.476360896 0.157357345
0.157357345 -1.053135279 0.447571454 -0.037067004 0.703128222
4 0 0.998207054
FBXL2 ENSG00000153558.13 0.54057305 2 2 0.269244885 Brain_Cerebellum
0.485847764 Brain_Cerebellar_Hemisphere 1.694801193 0.305198807
0.305198807 -1.104803174 -0.696928144 -0.900865659 0.288411199
2 0 0.998207054
VAMP4 ENSG00000117533.14 0.540577567 7 7 0.128595068 Brain_Amygdala
0.881533719 Brain_Nucleus_accumbens_basal_ganglia 3.063930147
0.119469578 0.119469578 -1.519665496 0.549684042 -0.313036946
0.765557243 7 0 0.998207054
PRKAB2 ENSG00000131791.7 0.540671917 3 3 0.223384691
Brain_Cortex 0.50324271 Brain_Cerebellar_Hemisphere 2.192457496 0.10633524
0.10633524 0.669396303 1.217577179 0.947529157 0.274179853 3
0 0.998207054
CTTNBP2 ENSG00000077063.10 0.540712987 2 2 0.297378948
Brain_Putamen_basal_ganglia 0.712361921 Brain_Cerebellum 1.010108499
0.989891501 0.989891501 -1.042070601 -0.368685818 -0.705378209
0.476154947 2 0 0.998207054
LINC00533 ENSG00000235570.1 0.540730857 2 2 0.378530413
Brain_Cerebellum 0.732054314 Brain_Cerebellar_Hemisphere 1.823259138
0.176740862 0.176740862 0.342394119 0.880607286 0.611500703
0.38057418 2 0 0.998207054
PDS5A ENSG00000121892.14 0.540749169 2 2 0.291152169 Brain_Cerebellum
0.738966497 Brain_Amygdala 1.006015595 0.993984405 0.993984405
-1.055597443 -0.333222285 -0.694409864 0.510796373 2 0
0.998207054
SLC37A3 ENSG00000157800.17 0.540964517 6 6 0.055715176
Brain_Cortex 0.961570178 Brain_Putamen_basal_ganglia 2.071318199
0.237499949 0.237499949 -0.752109279 1.913256876 0.446183498
0.87907534 6 0 0.998207054
RAB6C ENSG00000222014.5 0.540972173 10 9 0.066906307
Brain_Frontal_Cortex_BA9 0.960704746 Brain_Amygdala 2.776701895
1.34E-16 0.122188809 -0.715395143 1.832302886 0.29093471
0.969501022 9 0 0.998207054
RAB11FIP5 ENSG00000135631.16 0.541003869 2 2 0.278537812
Brain_Hippocampus 0.683725315 Brain_Cerebellum 1.163514466
0.836485534 0.836485534 -1.083609871 -0.407384901 -0.745497386
0.478163262 2 0 0.998207054
HMGXB4 ENSG00000100281.13 0.54112359 1 1 0.54112359
Brain_Anterior_cingulate_cortex_BA24 0.54112359
Brain_Anterior_cingulate_cortex_BA24 1 1 1 0.611114789
0.611114789 0.611114789 NA 1 0 0.998207054
LINC01085 ENSG00000248698.5 0.541210509 5 5 0.076730069
Brain_Nucleus_accumbens_basal_ganglia 0.744475463
Brain_Anterior_cingulate_cortex_BA24 2.937774276 0.192420834
0.192420834 0.325932451 1.769982424 0.955556159 0.550807117
5 0 0.998207054
HPS3 ENSG00000163755.8 0.541379968 2 2 0.499857083
Brain_Cerebellar_Hemisphere 0.616552043 Brain_Cerebellum 1.432194096
0.567805904 0.567805904 -0.500742947 0.674714637 0.086985845
0.831174029 2 0 0.998207054
WDR38 ENSG00000136918.7 0.541409583 1 1 0.541409583

Brain_Anterior_cingulate_cortex_BA24 0.541409583
Brain_Anterior_cingulate_cortex_BA24 1 1 1 0.610682818
0.610682818 0.610682818 NA 1 0 0.998207054
FDX2 ENSG00000267673.6 0.541473989 2 2 0.30279133 Brain_Cerebellum
0.653922627 Brain_Nucleus_accumbens_basal_ganglia 1.041133873
0.958866127 0.958866127 -0.448319503 1.030465946 0.291073221
1.045659219 2 0 0.998207054
UBE2B ENSG00000119048.7 0.54155512 2 2 0.288437035 Brain_Cortex
0.688325467 Brain_Nucleus_accumbens_basal_ganglia 1.081384021
0.918615979 0.918615979 -1.061556578 -0.401128571 -0.731342574
0.466993122 2 0 0.998207054
KY ENSG00000174611.11 0.541563834 3 3 0.342093022
Brain_Spinal_cord_cervical_c-1 0.937879489 Brain_Cerebellum 2.242770204
0.124373514 0.124373514 -0.950037848 -0.077935339 -0.509074375
0.436134252 3 0 0.998207054
ASB1 ENSG00000065802.11 0.541637205 1 1 0.541637205 Brain_Cerebellum
0.541637205 Brain_Cerebellum 1 1 1 -0.610339094 -0.610339094
-0.610339094 NA 1 0 0.998207054
SKIV2L2 ENSG00000039123.15 0.542032117 3 3 0.454217272
Brain_Cerebellar_Hemisphere 0.628826033 Brain_Spinal_cord_cervical_c-1
1.909638809 0.447458453 0.447458453 -0.748402737 0.556938281
-0.224948111 0.689977555 3 0 0.998207054
ZUFSP ENSG00000153975.9 0.542128263 3 3 0.271833981 Brain_Cortex
0.678012598 Brain_Nucleus_accumbens_basal_ganglia 2.524447244
0.224432963 0.224432963 -1.098848896 -0.415176641 -0.848859699
0.377040467 3 0 0.998207054
SCFD1 ENSG00000092108.20 0.542153028 4 1 0.466024699
Brain_Cerebellar_Hemisphere 0.676234126 Brain_Anterior_cingulate_cortex_BA24
3.820664508 0.005340352 3.820664508 -0.728962341 -0.417607482
-0.595182352 0.142004367 1 0 0.998207054
OMA1 ENSG00000162600.11 0.542202723 9 2 0.218788653
Brain_Frontal_Cortex_BA9 0.537077271 Brain_Caudate_basal_ganglia
8.171778443 0.001697956 0.488661042 -1.229755563 -0.617238733
-1.018627885 0.182980075 2 0 0.998207054
LNPK ENSG00000144320.13 0.542250949 8 6 0.205605938
Brain_Caudate_basal_ganglia 0.826735517 Brain_Cortex 5.304283868
0.016930989 0.194871184 -1.265740683 1.019853451 0.390502053
0.72055816 6 0 0.998207054
MTBP ENSG00000172167.7 0.542256995 12 2 0.137317056
Brain_Spinal_cord_cervical_c-1 0.566518749 Brain_Substantia_nigra
10.85438471 0.001900221 0.844787562 0.57318602 1.485856596
1.02931156 0.26006135 2 0 0.998207054
PICSAR ENSG00000275874.1 0.542338407 6 6 0.445685524
Brain_Hypothalamus 0.962314179 Brain_Substantia_nigra 3.822663947
0.154322835 0.154322835 -0.585819428 0.762627592 -0.055173115
0.473518654 6 0 0.998207054
TRPC4 ENSG00000133107.14 0.542406823 2 2 0.269706166
Brain_Caudate_basal_ganglia 0.986821006 Brain_Cortex 1.050516636
0.949483364 0.949483364 -1.103739476 0.01651817 -0.543610653
0.792141778 2 0 0.998207054
PTGER4P2-CDK2AP2P2 ENSG00000275450.1 0.54261395 1 1 0.54261395
Brain_Cerebellum 0.54261395 Brain_Cerebellum 1 1 1 -0.608864963
-0.608864963 -0.608864963 NA 1 0 0.998207054
CNTNAP3B ENSG00000154529.14 0.54261395 10 1 0.54261395 Brain_Cerebellum
0.54261395 Brain_Cortex 10 1.63E-17 10 -0.608864963 0.608864963
-0.243545985 0.588219438 1 0 0.998207054
FAM95B1 ENSG00000223839.7 0.54261395 1 1 0.54261395
Brain_Nucleus_accumbens_basal_ganglia 0.54261395
Brain_Nucleus_accumbens_basal_ganglia 1 1 1 0.608864963
0.608864963 0.608864963 NA 1 0 0.998207054
RP11-58A12.3 ENSG00000225655.5 0.54261395 1 1 0.54261395
Brain_Caudate_basal_ganglia 0.54261395 Brain_Caudate_basal_ganglia 1 1 1

-0.608864963 -0.608864963 -0.608864963 NA 1 0 0.998207054
BX255923.3 ENSG00000276462.1 0.54261395 1 1 0.54261395
Brain_Frontal_Cortex_BA9 0.54261395 Brain_Frontal_Cortex_BA9 1 1 1
-0.608864963 -0.608864963 -0.608864963 NA 1 0 0.998207054
CBWD7 ENSG00000215126.10 0.54261395 2 1 0.54261395 Brain_Cerebellar_Hemisphere
0.54261395 Brain_Cerebellar_Hemisphere 2 3.35E-17 2 -0.608864963
-0.608864963 -0.608864963 0 1 0 0.998207054
FOXD4L6 ENSG00000273514.1 0.54261395 3 1 0.54261395
Brain_Cerebellar_Hemisphere 0.54261395 Brain_Cortex 3 2.11E-48 3
-0.608864963 0.608864963 -0.202954988 0.703056701 1 0
0.998207054
MRPL11 ENSG00000174547.13 0.542796443 1 1 0.542796443
Brain_Cortex 0.542796443 Brain_Cortex 1 1 1 -0.608589686
-0.608589686 -0.608589686 NA 1 0 0.998207054
CLEC4E ENSG00000166523.7 0.54283038 1 1 0.54283038
Brain_Substantia_nigra 0.54283038 Brain_Substantia_nigra 1 1 1
-0.608538499 -0.608538499 -0.608538499 NA 1 0 0.998207054
SS18 ENSG00000141380.13 0.542896848 2 2 0.343894766
Brain_Cerebellar_Hemisphere 0.468330916 Brain_Frontal_Cortex_BA9 1.17212448
0.82787552 0.82787552 -0.946497756 -0.725197435 -0.835847596
0.156482957 2 0 0.998207054
MALSU1 ENSG00000156928.4 0.542943647 1 1 0.542943647
Brain_Amygdala 0.542943647 Brain_Amygdala 1 1 1 0.608367674
0.608367674 0.608367674 NA 1 0 0.998207054
SELENOI ENSG00000138018.17 0.543030657 2 2 0.275220651
Brain_Nucleus_accumbens_basal_ganglia 0.992560744 Brain_Cerebellum
1.150060684 0.849939316 0.849939316 0.00932386 1.091118711
0.550221285 0.764944475 2 0 0.998207054
SIVA1 ENSG00000184990.12 0.543101523 6 4 0.36031562
Brain_Cerebellar_Hemisphere 0.90884826 Brain_Anterior_cingulate_cortex_BA24
4.464436194 0.019819208 0.222718542 -0.386354816 0.914763843
0.333106847 0.483890665 4 0 0.998207054
EHD4 ENSG00000103966.10 0.543317333 9 5 0.091841679 Brain_Cerebellum
0.942218874 Brain_Nucleus_accumbens_basal_ganglia 5.1443692 0.005868094
0.282106712 -0.151056036 1.685761849 0.48555887 0.665350352 5
0 0.998207054
DDI2 ENSG00000197312.11 0.543329086 4 3 0.223352542
Brain_Anterior_cingulate_cortex_BA24 0.878179885 Brain_Hippocampus
1.983328181 0.043119044 0.897898933 -1.21766174 -0.15327694
-0.722010819 0.458905456 3 0 0.998207054
SMIM19 ENSG00000176209.11 0.543331772 13 1 0.314955142
Brain_Hypothalamus 0.750300972 Brain_Cerebellum 12.26488556
0.000364645 12.26488556 0.318242533 1.00487895 0.58900809
0.195253948 1 0 0.998207054
TNIK ENSG00000154310.16 0.543438193 1 1 0.543438193
Brain_Frontal_Cortex_BA9 0.543438193 Brain_Frontal_Cortex_BA9 1 1
1 -0.607622019 -0.607622019 -0.607622019 NA 1 0
0.998207054
XXbac-BPG181B23.7 ENSG00000272221.1 0.543520144 13 5 0.064596865
Brain_Frontal_Cortex_BA9 0.676853746 Brain_Cerebellum 9.543701614
0.009288364 0.389849899 -1.84803787 -0.416760296 -1.062524103
0.39450113 5 0 0.998207054
PARP4 ENSG00000102699.5 0.543713635 1 1 0.543713635
Brain_Caudate_basal_ganglia 0.543713635 Brain_Caudate_basal_ganglia 1 1
1 0.607206868 0.607206868 0.607206868 NA 1 0
0.998207054
NSUN7 ENSG00000179299.16 0.543760063 2 2 0.278397373
Brain_Hypothalamus 0.844266541 Brain_Anterior_cingulate_cortex_BA24
1.011391826 0.988608174 0.988608174 0.196439041 1.083926536
0.640182789 0.627548426 2 0 0.998207054
DRC1 ENSG00000157856.10 0.543766263 3 3 0.270080018 Brain_Cortex
0.721799046 Brain_Cerebellum 1.805796037 0.356842981 0.356842981

-0.539483348 1.102878302 0.306483465 0.822302246 3 0
0.998207054
LRRC43 ENSG00000158113.12 0.543956381 6 6 0.261639543
Brain_Cerebellum 0.92780442 Brain_Caudate_basal_ganglia 2.067017771
0.335980878 0.335980878 -0.923413872 1.122524391 -0.149375094
0.705275292 6 0 0.998207054
DDX55 ENSG00000111364.15 0.544095835 13 2 0.580188205
Brain_Anterior_cingulate_cortex_BA24 0.971546814
Brain_Nucleus_accumbens_basal_ganglia 11.63824816 0.000258687
0.551601043 -0.540737136 0.553109832 0.084249295 0.274352249
2 0 0.998207054
HLA-B ENSG00000234745.10 0.544110271 13 9 0.191424328
Brain_Caudate_basal_ganglia 0.991706739 Brain_Hypothalamus 6.59258334
0.064059682 0.303625113 -1.306377162 1.292704634 -0.249801632
0.73013317 9 0 0.998207054
RP11-569G13.2 ENSG00000237212.1 0.544136485 3 3 0.14737079
Brain_Cerebellar_Hemisphere 0.367960409 Brain_Hypothalamus 2.463846524
0.166417756 0.166417756 0.900300399 1.4488811 1.13108292
0.284453846 3 0 0.998207054
IRF1 ENSG00000125347.13 0.544250266 1 1 0.544250266
Brain_Nucleus_accumbens_basal_ganglia 0.544250266
Brain_Nucleus_accumbens_basal_ganglia 1 1 1 0.606398347
0.606398347 0.606398347 NA 1 0 0.998207054
PPOX ENSG00000143224.17 0.544344129 6 5 0.490966118
Brain_Cerebellar_Hemisphere 0.979523975 Brain_Caudate_basal_ganglia
4.016382843 0.025840323 0.17674211 -0.688773017 0.655188408
0.055498471 0.488907544 5 0 0.998207054
SCGN ENSG00000079689.13 0.544369849 2 2 0.271353782
Brain_Putamen_basal_ganglia 0.721111255 Brain_Cortex 1.391270455
0.608729545 0.608729545 -0.35697402 1.099950288 0.371488134
1.030201058 2 0 0.998207054
TNKS2 ENSG00000107854.5 0.544455921 2 2 0.611625769
Brain_Frontal_Cortex_BA9 0.709661264 Brain_Cerebellar_Hemisphere
1.680078709 0.319921291 0.319921291 -0.507754148 0.372311061
-0.067721543 0.622300078 2 0 0.998207054
DLG2 ENSG00000150672.16 0.544536847 1 1 0.544536847
Brain_Cerebellar_Hemisphere 0.544536847 Brain_Cerebellar_Hemisphere 1 1
1 0.605966729 0.605966729 0.605966729 NA 1 0
0.998207054
MCAM ENSG00000076706.16 0.544554271 2 1 0.509202941 Brain_Cerebellum
0.581532114 Brain_Cerebellar_Hemisphere 1.99784354 0.00215646 1.99784354
-0.660079302 -0.55114816 -0.605613731 0.077025949 1 0
0.998207054
L2HGDH ENSG00000087299.11 0.544655292 3 3 0.187467594
Brain_Caudate_basal_ganglia 0.700761473 Brain_Cerebellum 2.336897554
0.081474564 0.081474564 -1.31810771 -0.38429276 -0.757108103
0.494532687 3 0 0.998207054
ZNF331 ENSG00000130844.16 0.544678397 2 1 0.538342203
Brain_Cerebellum 0.555736575 Brain_Cerebellar_Hemisphere 1.97695692 0.02304308
1.97695692 -0.615321831 -0.589185899 -0.602253865 0.018480895 1
0 0.998207054
PGLS ENSG00000130313.6 0.544713023 1 1 0.544713023
Brain_Hypothalamus 0.544713023 Brain_Hypothalamus 1 1 1
0.605701447 0.605701447 0.605701447 NA 1 0 0.998207054
EEF1B2 ENSG00000114942.13 0.544820423 1 1 0.544820423
Brain_Hypothalamus 0.544820423 Brain_Hypothalamus 1 1 1
0.605539747 0.605539747 0.605539747 NA 1 0 0.998207054
SMTNL2 ENSG00000188176.11 0.544826862 1 1 0.544826862
Brain_Hippocampus 0.544826862 Brain_Hippocampus 1 1 1
0.605530053 0.605530053 0.605530053 NA 1 0 0.998207054
ADSL ENSG00000239900.12 0.544827737 5 5 0.167770022
Brain_Cerebellar_Hemisphere 0.937672692 Brain_Nucleus_accumbens_basal_ganglia

2.048403932	0.189847863	0.189847863	-1.379404664	0.555887981
-0.227872611	0.774050113	5	0	0.998207054
IQUB	ENSG00000164675.10	0.544866441	1	1	0.544866441
Brain_Substantia_nigra	0.544866441	Brain_Substantia_nigra	1	1
1	-0.605470469	-0.605470469	-0.605470469	NA	1	0
0.998207054
NEO1	ENSG00000067141.16	0.5449932	1	1	0.5449932	Brain_Amygdala	0.5449932
Brain_Amygdala	1	1	1	0.60527965	0.60527965	0.60527965	NA	1	0
0.998207054
URB1-AS1	ENSG00000256073.3	0.5451517	7	4	0.106547716
Brain_Hypothalamus	0.660502936	Brain_Amygdala	5.104613693
0.002638111	0.292209315	-0.979270664	1.613906467	-0.293895916
0.860130949	4	0	0.998207054
RP11-231C18.1	ENSG00000248184.1	0.545318584	10	3	0.141361709
Brain_Anterior_cingulate_cortex_BA24	0.949700497	Brain_Cerebellum
7.649668265	0.009880893	0.310748991	-1.47073902	-0.063082893
-0.754821223	0.453334775	3	0	0.998207054
KIAA1211	ENSG00000109265.13	0.545356089	3	3	0.194608577
Brain_Cerebellar_Hemisphere	0.382279275	Brain_Spinal_cord_cervical_c-1
2.781086094	0.102156194	0.102156194	-1.297065715	-0.873704362
-1.105406289	0.214502355	3	0	0.998207054
BARX2	ENSG00000043039.6	0.545356903	1	1	0.545356903
Brain_Hippocampus	0.545356903	Brain_Hippocampus	1	1	1
-0.60473227	-0.60473227	-0.60473227	NA	1	0	0.998207054
RGS8	ENSG00000135824.12	0.545501012	1	1	0.545501012	Brain_Cortex
0.545501012	Brain_Cortex	1	1	1	-0.604515433	-0.604515433
-0.604515433	NA	1	0	0.998207054
MUC20	ENSG00000176945.16	0.545639038	13	7	0.077112279
Brain_Nucleus_accumbens_basal_ganglia	0.757785723	Brain_Frontal_Cortex_BA9
7.158230303	0.016266842	0.270030766	-1.351728775	1.767692769
0.799997218	0.978255916	7	0	0.998207054
AKAIN1	ENSG00000231824.3	0.545759742	1	1	0.545759742
Brain_Cerebellum	0.545759742	Brain_Cerebellum	1	1	1	0.6041262
0.6041262	0.6041262	NA	1	0	0.998207054
PTX4	ENSG00000251692.7	0.545791711	1	1	0.545791711
Brain_Hypothalamus	0.545791711	Brain_Hypothalamus	1	1	1
0.604078112	0.604078112	0.604078112	NA	1	0	0.998207054
TPK1	ENSG00000196511.14	0.545833878	1	1	0.545833878
Brain_Frontal_Cortex_BA9	0.545833878	Brain_Frontal_Cortex_BA9	1	1
1	0.604014686	0.604014686	0.604014686	NA	1	0
0.998207054
APOBEC3C	ENSG00000244509.3	0.545835235	7	7	0.057942106
Brain_Hippocampus	0.873978872	Brain_Putamen_basal_ganglia	1.617922644
0.539830574	0.539830574	-1.896135674	0.235987171	-0.57170393
0.810896604	7	0	0.998207054
LYRM9	ENSG00000232859.9	0.545860279	13	2	0.180801598	Brain_Amygdala
0.599228789	Brain_Spinal_cord_cervical_c-1	11.42755876	6.39E-05
0.573215631	0.525509877	1.338290988	0.985294542	0.209402622
2	0	0.998207054
LRRC7	ENSG00000033122.18	0.545948581	1	1	0.545948581
Brain_Frontal_Cortex_BA9	0.545948581	Brain_Frontal_Cortex_BA9	1	1
1	0.603842168	0.603842168	0.603842168	NA	1	0
0.998207054
IL2RA	ENSG00000134460.15	0.54596685	2	2	0.326724094	Brain_Hypothalamus
0.626903606	Brain_Spinal_cord_cervical_c-1	1.012667817	0.987332183
0.987332183	-0.486089649	0.980733673	0.247322012	1.037200718
2	0	0.998207054
MTFR1L	ENSG00000117640.17	0.546039961	5	5	0.074235952
Brain_Caudate_basal_ganglia	0.936680837	Brain_Anterior_cingulate_cortex_BA24
2.828316997	0.136630204	0.136630204	0.079442284	1.785156401
1.125653189	0.645958291	5	0	0.998207054
CHAC1	ENSG00000128965.11	0.546092902	1	1	0.546092902

Brain_Hypothalamus 0.546092902 Brain_Hypothalamus 1 1 1
-0.603625129 -0.603625129 -0.603625129 NA 1 0 0.998207054
LARP1B ENSG00000138709.17 0.546322472 2 2 0.476885576
Brain_Frontal_Cortex_BA9 0.86125648 Brain_Cortex 1.650060338
0.349939662 0.349939662 -0.17477494 0.711320621 0.26827284
0.62656418 2 0 0.998207054
UNC50 ENSG00000115446.11 0.54632986 1 1 0.54632986
Brain_Anterior_cingulate_cortex_BA24 0.54632986
Brain_Anterior_cingulate_cortex_BA24 1 1 1 -0.603268838
-0.603268838 -0.603268838 NA 1 0 0.998207054
KLHL29 ENSG00000119771.14 0.546361125 1 1 0.546361125
Brain_Cortex 0.546361125 Brain_Cortex 1 1 1 0.603221834
0.603221834 0.603221834 NA 1 0 0.998207054
OXCT1 ENSG00000083720.12 0.546363426 1 1 0.546363426
Brain_Spinal_cord_cervical_c-1 0.546363426 Brain_Spinal_cord_cervical_c-1
1 1 1 -0.603218374 -0.603218374 -0.603218374 NA 1 0
0.998207054
GAB2 ENSG00000033327.12 0.546396058 2 2 0.278144327 Brain_Cerebellum
0.319070735 Brain_Cerebellar_Hemisphere 1.824371765 0.175628235
0.175628235 0.996369317 1.084497382 1.04043335 0.062315952 2
0 0.998207054
NOS1 ENSG00000089250.18 0.546477499 7 5 0.119708021 Brain_Amygdala
0.933322816 Brain_Nucleus_accumbens_basal_ganglia 3.818170027
0.062411981 0.205170473 -1.556000298 0.426901576 -0.687870155
0.677385027 5 0 0.998207054
ACTL8 ENSG00000117148.7 0.54650061 1 1 0.54650061 Brain_Frontal_Cortex_BA9
0.54650061 Brain_Frontal_Cortex_BA9 1 1 1 0.603012145
0.603012145 0.603012145 NA 1 0 0.998207054
CPEB3 ENSG00000107864.14 0.546504527 2 2 0.279509398
Brain_Anterior_cingulate_cortex_BA24 0.888010323 Brain_Cerebellar_Hemisphere
1.052035179 0.947964821 0.947964821 -1.081422094 0.140822304
-0.470299895 0.864257302 2 0 0.998207054
NLGN1 ENSG00000169760.17 0.546606114 1 1 0.546606114
Brain_Substantia_nigra 0.546606114 Brain_Substantia_nigra 1 1
1 0.602853557 0.602853557 0.602853557 NA 1 0
0.998207054
BEST2 ENSG00000039987.6 0.546764326 1 1 0.546764326
Brain_Frontal_Cortex_BA9 0.546764326 Brain_Frontal_Cortex_BA9 1 1
1 0.602615772 0.602615772 0.602615772 NA 1 0
0.998207054
C1orf112 ENSG00000000460.16 0.547035472 6 6 0.317114036
Brain_Putamen_basal_ganglia 0.854085628 Brain_Cortex 2.915489623
0.152122933 0.152122933 -0.942329832 1.000406065 -0.189973027
0.731812452 6 0 0.998207054
C19orf60 ENSG00000006015.17 0.547039996 1 1 0.547039996
Brain_Amygdala 0.547039996 Brain_Amygdala 1 1 1 0.602201532
0.602201532 0.602201532 NA 1 0 0.998207054
SCUBE3 ENSG00000146197.8 0.547230617 1 1 0.547230617
Brain_Nucleus_accumbens_basal_ganglia 0.547230617
Brain_Nucleus_accumbens_basal_ganglia 1 1 1 -0.601915153
-0.601915153 -0.601915153 NA 1 0 0.998207054
RP11-479G22.8 ENSG00000273038.2 0.547318839 12 2 0.299339642
Brain_Putamen_basal_ganglia 0.983764376 Brain_Hippocampus 10.44026824
0.001549392 0.619753192 -0.894762296 1.037850542 0.285395081
0.46179748 2 0 0.998207054
KCNJ16 ENSG00000153822.13 0.547403902 5 5 0.139107158
Brain_Substantia_nigra 0.943281023 Brain_Cerebellum 1.538035957
0.600269119 0.600269119 -0.380913015 1.479124069 0.639258474
0.771362804 5 0 0.998207054
ILF3-AS1 ENSG00000267100.1 0.547585642 5 3 0.326183673
Brain_Frontal_Cortex_BA9 0.598114018 Brain_Putamen_basal_ganglia
3.528213419 0.021830901 0.225027469 -0.698811052 0.981829864

-0.058723188 0.772908368 3 0 0.998207054
RTN4R ENSG00000040608.13 0.547675859 1 1 0.547675859
Brain_Cerebellar_Hemisphere 0.547675859 Brain_Cerebellar_Hemisphere 1 1
1 -0.601246438 -0.601246438 -0.601246438 NA 1 0
0.998207054
SLC25A45 ENSG00000162241.12 0.54768632 1 1 0.54768632 Brain_Amygdala
0.54768632 Brain_Amygdala 1 1 1 0.601230729 0.601230729
0.601230729 NA 1 0 0.998207054
ALG2 ENSG00000119523.9 0.547734343 1 1 0.547734343
Brain_Spinal_cord_cervical_c-1 0.547734343 Brain_Spinal_cord_cervical_c-1
1 1 1 0.601158619 0.601158619 0.601158619 NA 1 0
0.998207054
PFKFB4 ENSG00000114268.11 0.547747388 3 2 0.069245779
Brain_Hypothalamus 0.589137477 Brain_Cerebellar_Hemisphere 2.813427293
0.074399039 0.112173668 0.54008636 1.816812821 1.042705656
0.680284397 2 0 0.998207054
RP11-332J15.4 ENSG00000272071.1 0.547851477 1 1 0.547851477
Brain_Frontal_Cortex_BA9 0.547851477 Brain_Frontal_Cortex_BA9 1 1
1 0.600982748 0.600982748 0.600982748 NA 1 0
0.998207054
1-Mar ENSG00000186205.12 0.547979552 2 2 0.492541972
Brain_Caudate_basal_ganglia 0.708217126 Brain_Putamen_basal_ganglia
1.519767429 0.480232571 0.480232571 -0.686271413 0.374251615
-0.156009899 0.749903025 2 0 0.998207054
ZNF200 ENSG00000010539.11 0.548038929 6 6 0.208678283
Brain_Cerebellum 0.849238898 Brain_Spinal_cord_cervical_c-1 2.49178714
0.188954484 0.188954484 -0.224162289 1.257208123 0.536008599
0.603484368 6 0 0.998207054
EIF2AK1 ENSG00000086232.12 0.5480906 12 7 0.168939151
Brain_Putamen_basal_ganglia 0.800851732 Brain_Spinal_cord_cervical_c-1
6.824002252 0.008186504 0.284961423 -1.013756871 1.375620518
0.698038141 0.701214952 7 0 0.998207054
LINC01819 ENSG00000231826.5 0.548140437 2 2 0.408869858
Brain_Cortex 0.430309594 Brain_Caudate_basal_ganglia 1.084621402
0.915378598 0.915378598 -0.825884069 -0.788661982 -0.807273026
0.02631999 2 0 0.998207054
RASGEF1C ENSG00000146090.15 0.548289266 4 4 0.156832286
Brain_Cortex 0.90738577 Brain_Cerebellum 2.012078002 0.068408273
0.068408273 -1.415806089 0.212122872 -0.398111491 0.707879972
4 0 0.998207054
EPPK1 ENSG00000261150.2 0.548335394 3 3 0.314677111
Brain_Caudate_basal_ganglia 0.615345832 Brain_Cerebellar_Hemisphere
1.297175948 0.820223727 0.820223727 -0.50245737 1.005456448
0.43785445 0.820143572 3 0 0.998207054
RAP2A ENSG00000125249.6 0.548370724 1 1 0.548370724
Brain_Frontal_Cortex_BA9 0.548370724 Brain_Frontal_Cortex_BA9 1 1
1 0.600203346 0.600203346 0.600203346 NA 1 0
0.998207054
GNA14 ENSG00000156049.6 0.548394343 3 3 0.173351694
Brain_Hypothalamus 0.88963198 Brain_Substantia_nigra 1.26936273
0.777416408 0.777416408 -0.1387699 1.361512792 0.562629711
0.754877016 3 0 0.998207054
SLC38A8 ENSG00000166558.10 0.548572198 4 4 0.28478238
Brain_Hippocampus 0.621881318 Brain_Cortex 2.270843702
0.356337494 0.356337494 -0.63780442 1.069637786 -0.152512743
0.816941705 4 0 0.998207054
PROK1 ENSG00000143125.5 0.548700795 1 1 0.548700795
Brain_Putamen_basal_ganglia 0.548700795 Brain_Putamen_basal_ganglia 1 1
1 -0.599708091 -0.599708091 -0.599708091 NA 1 0
0.998207054
ZNF213 ENSG00000085644.13 0.548735404 5 4 0.195093427
Brain_Caudate_basal_ganglia 0.907286984 Brain_Hippocampus 3.239001548

0.003539039 0.222038702 -0.176873603 1.295657739 0.644598048
0.731597348 4 0 0.998207054
ZNF24 ENSG00000172466.15 0.54875958 1 1 0.54875958 Brain_Cortex 0.54875958
Brain_Cortex 1 1 1 -0.599619902 -0.599619902 -0.599619902
NA 1 0 0.998207054
RFK ENSG00000135002.11 0.548760526 4 4 0.10242761
Brain_Putamen_basal_ganglia 0.824393876 Brain_Cerebellar_Hemisphere
2.416985884 0.19111993 0.19111993 -0.814318332 1.633196815
0.029200416 1.096486257 4 0 0.998207054
PAFAH2 ENSG00000158006.13 0.548780202 2 2 0.305906927
Brain_Cerebellum 0.471100556 Brain_Cerebellar_Hemisphere 1.882735795
0.117264205 0.117264205 -1.023848312 -0.72068953 -0.872268921
0.214365631 2 0 0.998207054
SGSM1 ENSG00000167037.18 0.548851213 2 2 0.281410751 Brain_Cerebellum
0.456487279 Brain_Cerebellar_Hemisphere 1.801715799 0.198284201
0.198284201 -1.077155615 -0.744643475 -0.910899545 0.235121589
2 0 0.998207054
TREML4 ENSG00000188056.11 0.548855813 1 1 0.548855813
Brain_Spinal_cord_cervical_c-1 0.548855813 Brain_Spinal_cord_cervical_c-1
1 1 1 0.599475545 0.599475545 0.599475545 NA 1 0
0.998207054
SERF1B ENSG00000205572.9 0.548934778 12 2 0.540698268
Brain_Hippocampus 0.984972203 Brain_Putamen_basal_ganglia 10.64773277
0.002642978 0.476247939 -0.284964556 0.611757415 0.114256853
0.276704929 2 0 0.998207054
EP300 ENSG00000100393.12 0.548955028 3 3 0.269004595 Brain_Cerebellum
0.67719171 Brain_Substantia_nigra 1.542096408 0.327570142
0.327570142 -1.105357769 0.416298336 -0.573853453 0.858270035
3 0 0.998207054
PIGN ENSG00000197563.10 0.549152391 11 4 0.105356478 Brain_Cerebellum
0.935998328 Brain_Hypothalamus 8.049245404 0.003149409
0.642402041 -1.619422087 0.080300415 -0.467804761 0.429096743
4 0 0.998207054
RP11-114G11.5 ENSG00000260653.5 0.549215446 6 4 0.115996062
Brain_Anterior_cingulate_cortex_BA24 0.306252229 Brain_Cerebellar_Hemisphere
4.094643144 0.004600551 0.144599726 -1.571803792 -1.023117636
-1.190413126 0.208861178 4 0 0.998207054
PNPLA5 ENSG00000100341.11 0.549227025 8 6 0.107489325
Brain_Anterior_cingulate_cortex_BA24 0.985415203 Brain_Cerebellum
4.651950886 0.046693688 0.254349119 -1.609581155 -0.01828035
-0.723853694 0.536253262 6 0 0.998207054
CLEC5A ENSG00000258227.6 0.549254161 2 2 0.499823493
Brain_Frontal_Cortex_BA9 0.642184515 Brain_Nucleus_accumbens_basal_ganglia
1.451365425 0.548634575 0.548634575 -0.674767498 0.464646655
-0.105060421 0.805687474 2 0 0.998207054
CDH8 ENSG00000150394.13 0.549402355 2 2 0.276618022
Brain_Nucleus_accumbens_basal_ganglia 0.679168681 Brain_Amygdala
1.274784298 0.725215702 0.725215702 -0.413597805 1.087948095
0.337175145 1.061753289 2 0 0.998207054
WDTC1 ENSG00000142784.15 0.549433131 1 1 0.549433131 Brain_Cortex
0.549433131 Brain_Cortex 1 1 1 0.598609783 0.598609783
0.598609783 NA 1 0 0.998207054
IBSP ENSG00000029559.6 0.549505328 4 4 0.339831968 Brain_Amygdala
0.984741554 Brain_Frontal_Cortex_BA9 2.613263631 0.204441914
0.204441914 0.019124792 0.954497315 0.512202906 0.490086286
4 0 0.998207054
WRB ENSG00000182093.14 0.549881381 8 5 0.182867532
Brain_Caudate_basal_ganglia 0.963924779 Brain_Putamen_basal_ganglia
5.721296841 0.038235516 0.265717121 -0.45163051 1.331977651
0.2221833 0.589443773 5 0 0.998207054
GEMIN7 ENSG00000142252.10 0.549939433 12 4 0.188677527
Brain_Frontal_Cortex_BA9 0.981348708 Brain_Anterior_cingulate_cortex_BA24

8.580683582 0.000796965 0.348939789 -1.3145014 0.34020262
-0.380096174 0.45190621 4 0 0.998207054
PSTPIP2 ENSG00000152229.18 0.550085747 1 1 0.550085747
Brain_Caudate_basal_ganglia 0.550085747 Brain_Caudate_basal_ganglia 1 1
1 -0.597631641 -0.597631641 -0.597631641 NA 1 0
0.998207054
CD247 ENSG00000198821.10 0.550218398 4 4 0.155625311
Brain_Anterior_cingulate_cortex_BA24 0.921151877 Brain_Caudate_basal_ganglia
1.21004585 0.829227485 0.829227485 0.098982863 1.419939445
0.785274737 0.603302676 4 0 0.998207054
F2 ENSG00000180210.14 0.55025477 2 2 0.279144801
Brain_Cerebellar_Hemisphere 0.547717387 Brain_Cerebellum 1.427445934
0.572554066 0.572554066 -1.082242471 -0.601184079 -0.841713275
0.340159651 2 0 0.998207054
SNRPC ENSG00000124562.9 0.550289078 10 2 0.541479145
Brain_Hypothalamus 0.930006521 Brain_Cerebellum 8.794967212 0.00220946
0.669700704 -0.610577767 0.418484719 0.202579177 0.323864617
2 0 0.998207054
RP5-978I12.1 ENSG00000260951.1 0.550388137 1 1 0.550388137
Brain_Nucleus_accumbens_basal_ganglia 0.550388137
Brain_Nucleus_accumbens_basal_ganglia 1 1 1 -0.597178613
-0.597178613 -0.597178613 NA 1 0 0.998207054
AKAP1 ENSG00000121057.12 0.550448362 4 3 0.217702344 Brain_Cerebellum
0.791247938 Brain_Frontal_Cortex_BA9 2.63016514 0.078338876
0.337960811 -1.232660797 0.578869438 0.0099703 0.838298182 3
0 0.998207054
SPEF1 ENSG00000101222.12 0.550455968 2 2 0.343843849
Brain_Nucleus_accumbens_basal_ganglia 0.58873648
Brain_Anterior_cingulate_cortex_BA24 1.005482496 0.994517504
0.994517504 -0.54066794 0.946597636 0.202964848 1.051655574
2 0 0.998207054
FOXH1 ENSG00000160973.7 0.550648062 1 1 0.550648062
Brain_Caudate_basal_ganglia 0.550648062 Brain_Caudate_basal_ganglia 1 1
1 0.5967893 0.5967893 0.5967893 NA 1 0 0.998207054
RP11-110I1.12 ENSG00000255121.2 0.550664436 1 1 0.550664436
Brain_Anterior_cingulate_cortex_BA24 0.550664436
Brain_Anterior_cingulate_cortex_BA24 1 1 1 0.596764779
0.596764779 0.596764779 NA 1 0 0.998207054
LINC00551 ENSG00000272274.1 0.550687316 6 5 0.124516363
Brain_Nucleus_accumbens_basal_ganglia 0.98271657 Brain_Cortex 2.001375664
0.005754208 0.895463024 -1.314837537 1.536089778 -0.003929095
0.920217508 5 0 0.998207054
SLC17A5 ENSG00000119899.12 0.550711891 1 1 0.550711891
Brain_Spinal_cord_cervical_c-1 0.550711891 Brain_Spinal_cord_cervical_c-1
1 1 1 -0.596693713 -0.596693713 -0.596693713 NA 1 0
0.998207054
RP11-274B21.10 ENSG00000271553.1 0.550745148 12 5 0.056602297
Brain_Frontal_Cortex_BA9 0.747133866 Brain_Hippocampus 8.607821668
0.01318933 0.332090361 -1.90636939 -0.322420883 -1.020749777
0.532952346 5 0 0.998207054
INO80B ENSG00000115274.14 0.550773161 3 3 0.265424793
Brain_Putamen_basal_ganglia 0.816145898 Brain_Cortex 2.464178595
0.198042135 0.198042135 -1.113660658 -0.232504879 -0.799605997
0.49207038 3 0 0.998207054
KIF25 ENSG00000125337.16 0.550794754 13 10 0.147214277
Brain_Caudate_basal_ganglia 0.983458897 Brain_Hippocampus 5.733461375
9.38E-06 0.264367793 -0.293567313 1.449441676 0.510466174
0.599749108 10 0 0.998207054
ABCA5 ENSG00000154265.15 0.550927467 3 3 0.31484529
Brain_Putamen_basal_ganglia 0.921014047 Brain_Cerebellum 2.466351923
0.180669193 0.180669193 -1.005107082 0.099156457 -0.473864332
0.553315953 3 0 0.998207054

ACSS1 ENSG00000154930.14 0.550970908 4 4 0.481568321
Brain_Cerebellar_Hemisphere 0.832361046 Brain_Cerebellum 1.941180988
0.186949021 0.186949021 -0.70378236 0.211674419 -0.371427846
0.416570643 4 0 0.998207054
GPR108 ENSG00000125734.15 0.551026618 13 1 0.316232276
Brain_Hippocampus 0.851061155 Brain_Nucleus_accumbens_basal_ganglia
12.22500816 2.22E-16 12.22500816 0.187764641 1.002230509
0.577038851 0.197302417 1 0 0.998207054
MS4A7 ENSG00000166927.12 0.551094813 2 2 0.331751628
Brain_Hippocampus 0.660298506 Brain_Hypothalamus 1.061010756
0.938989244 0.938989244 0.439501071 0.970591726 0.705046399
0.375537804 2 0 0.998207054
RABL2A ENSG00000144134.18 0.551231867 8 5 0.219688301
Brain_Cortex 0.977717172 Brain_Caudate_basal_ganglia 5.684293013
0.010608616 0.25072347 -0.335867548 1.227357372 0.224336044
0.577307353 5 0 0.998207054
MRPL35 ENSG00000132313.14 0.551280837 10 4 0.148475222
Brain_Caudate_basal_ganglia 0.885521848 Brain_Spinal_cord_cervical_c-1
6.152048233 0.000733106 0.398560437 -1.444938284 0.838854141
-0.384213173 0.690042304 4 0 0.998207054
ZNF496 ENSG00000162714.12 0.551413277 10 2 0.142732544
Brain_Putamen_basal_ganglia 0.642294197 Brain_Hypothalamus 8.604516162
0.003385687 0.908821274 0.464493525 1.46569079 0.984211214
0.309722344 2 0 0.998207054
EIF6 ENSG00000242372.6 0.551417633 4 4 0.170611237
Brain_Caudate_basal_ganglia 0.967304227 Brain_Cerebellum 2.224132695
0.350768559 0.350768559 -1.370242431 0.344691569 -0.278173884
0.750702826 4 0 0.998207054
CTA-268H5.14 ENSG00000273287.2 0.551506399 2 2 0.301480629
Brain_Spinal_cord_cervical_c-1 0.744458035 Brain_Cortex 1.023275882
0.976724118 0.976724118 -0.325955485 1.033263445 0.35365398
0.961112923 2 0 0.998207054
THEGL ENSG00000249693.2 0.551546168 1 1 0.551546168
Brain_Hypothalamus 0.551546168 Brain_Hypothalamus 1 1 1
-0.595444828 -0.595444828 -0.595444828 NA 1 0 0.998207054
UBAP2L ENSG00000143569.18 0.551579792 2 2 0.322214167
Brain_Cerebellum 0.758949725 Brain_Nucleus_accumbens_basal_ganglia
1.147851222 0.852148778 0.852148778 -0.989918069 0.306860278
-0.341528895 0.916960763 2 0 0.998207054
RP11-147L13.15 ENSG00000274712.1 0.551744411 8 7 0.290916293
Brain_Caudate_basal_ganglia 0.967225286 Brain_Cerebellar_Hemisphere
4.975928156 0.06494897 0.217679478 -0.393275822 1.056113654
0.422917227 0.499125948 7 0 0.998207054
FAS ENSG00000026103.21 0.551763175 9 3 0.095500793
Brain_Anterior_cingulate_cortex_BA24 0.708350982 Brain_Cerebellum
7.786346742 0.007067734 0.339265723 -1.66706846 0.374071687
-1.108101416 0.585430183 3 0 0.998207054
IKBKAP ENSG00000070061.14 0.551808323 5 4 0.147330485
Brain_Spinal_cord_cervical_c-1 0.921494182 Brain_Cerebellum 2.850680103
0.052464628 0.16341626 -1.449025416 0.09855175 -0.671719221
0.555376903 4 0 0.998207054
CLDN4 ENSG00000189143.9 0.551907258 11 3 0.162546046 Brain_Amygdala
0.916676052 Brain_Frontal_Cortex_BA9 9.238801019 0.000178595
0.495062329 -1.3965596 -0.104621628 -0.877082909 0.337422512 3
0 0.998207054
CXXC4 ENSG00000168772.10 0.551949482 2 2 0.285148634
Brain_Caudate_basal_ganglia 0.855757361 Brain_Cerebellar_Hemisphere
1.030956612 0.969043388 0.969043388 -1.068824775 -0.181777477
-0.625301126 0.62723716 2 0 0.998207054
AF131217.1 ENSG00000232855.6 0.551960853 7 4 0.125338814
Brain_Frontal_Cortex_BA9 0.923487692 Brain_Nucleus_accumbens_basal_ganglia
4.669936581 0.005725109 0.194895939 -1.532744524 -0.096041401

-0.815648446 0.539350489 4 0 0.998207054
MB ENSG00000198125.12 0.55209869 1 1 0.55209869
Brain_Nucleus_accumbens_basal_ganglia 0.55209869
Brain_Nucleus_accumbens_basal_ganglia 1 1 1 0.594618233
0.594618233 0.594618233 NA 1 0 0.998207054
SCNN1D ENSG00000162572.20 0.552103457 3 3 0.535721088
Brain_Cerebellar_Hemisphere 0.800220348 Brain_Cortex 2.310552585
0.299539873 0.299539873 -0.585668702 0.619296443 -0.073144734
0.622304508 3 0 0.998207054
HAGLROS ENSG00000226363.3 0.552117588 7 7 0.048533488
Brain_Hippocampus 0.945100424 Brain_Cerebellum 1.789019298
0.316216726 0.316216726 -0.722653329 1.972667229 0.251134987
0.907064008 7 0 0.998207054
WSB1 ENSG00000109046.14 0.552166936 1 1 0.552166936
Brain_Spinal_cord_cervical_c-1 0.552166936 Brain_Spinal_cord_cervical_c-1
1 1 1 -0.594516162 -0.594516162 -0.594516162 NA 1 0
0.998207054
ZNF420 ENSG00000197050.10 0.552190191 2 2 0.602885525
Brain_Nucleus_accumbens_basal_ganglia 0.638679949 Brain_Cerebellum
1.587289274 0.412710726 0.412710726 -0.46954525 0.520255475
0.025355112 0.699894804 2 0 0.998207054
PDGFA ENSG00000197461.13 0.552329511 1 1 0.552329511
Brain_Cerebellar_Hemisphere 0.552329511 Brain_Cerebellar_Hemisphere 1 1
1 0.594273035 0.594273035 0.594273035 NA 1 0
0.998207054
ACTL6A ENSG00000136518.16 0.552340882 1 1 0.552340882
Brain_Nucleus_accumbens_basal_ganglia 0.552340882
Brain_Nucleus_accumbens_basal_ganglia 1 1 1 0.594256031
0.594256031 0.594256031 NA 1 0 0.998207054
PDZK1IP1 ENSG00000162366.7 0.552385189 7 3 0.178948259
Brain_Frontal_Cortex_BA9 0.771479562 Brain_Amygdala 5.640211329
0.003020461 0.466524984 -1.344000397 -0.29044011 -0.597121571
0.350547452 3 0 0.998207054
RP11-338N10.2 ENSG00000270035.1 0.552627755 1 1 0.552627755
Brain_Cerebellum 0.552627755 Brain_Cerebellum 1 1 1 -0.593827109
-0.593827109 -0.593827109 NA 1 0 0.998207054
CCDC191 ENSG00000163617.10 0.552661872 1 1 0.552661872
Brain_Substantia_nigra 0.552661872 Brain_Substantia_nigra 1 1
1 -0.593776107 -0.593776107 -0.593776107 NA 1 0
0.998207054
SKOR1 ENSG00000188779.10 0.552726098 2 2 0.277946488 Brain_Cerebellum
0.99444963 Brain_Nucleus_accumbens_basal_ganglia 1.091930507 0.908069493
0.908069493 0.006956414 1.084943934 0.545950174 0.762252286
2 0 0.998207054
CTD-3157E16.2 ENSG00000276855.1 0.552847335 12 4 0.258180719
Brain_Nucleus_accumbens_basal_ganglia 0.893376169 Brain_Hypothalamus
9.496988882 0.001623506 0.332213525 -1.130701574 0.928134594
0.175031464 0.695108536 4 0 0.998207054
CTC-542B22.2 ENSG00000263990.1 0.55317522 2 2 0.42221631
Brain_Anterior_cingulate_cortex_BA24 0.44864973
Brain_Nucleus_accumbens_basal_ganglia 1.028794889 0.971205111
0.971205111 -0.802582113 -0.75766805 -0.780125081 0.031759038
2 0 0.998207054
ZNF727 ENSG00000214652.5 0.55318048 3 3 0.178334642 Brain_Cerebellum
0.932683621 Brain_Amygdala 1.73995735 0.247865743 0.247865743
-0.084468909 1.345900394 0.712990858 0.729243903 3 0
0.998207054
ARMC5 ENSG00000140691.16 0.553216296 1 1 0.553216296
Brain_Nucleus_accumbens_basal_ganglia 0.553216296
Brain_Nucleus_accumbens_basal_ganglia 1 1 1 0.592947488
0.592947488 0.592947488 NA 1 0 0.998207054
GH1 ENSG00000259384.6 0.553218697 1 1 0.553218697

Brain_Hippocampus 0.553218697 Brain_Hippocampus 1 1 1
0.592943901 0.592943901 0.592943901 NA 1 0 0.998207054
ADAMTS12 ENSG00000151388.10 0.553309978 3 3 0.265957659
Brain_Putamen_basal_ganglia 0.669879413 Brain_Caudate_basal_ganglia
2.666895826 0.160141675 0.160141675 0.426313512 1.112419885
0.7140938 0.356161155 3 0 0.998207054
MYO1E ENSG00000157483.8 0.553365682 1 1 0.553365682
Brain_Frontal_Cortex_BA9 0.553365682 Brain_Frontal_Cortex_BA9 1 1
1 0.592724291 0.592724291 0.592724291 NA 1 0
0.998207054
FHL5 ENSG00000112214.10 0.553410766 1 1 0.553410766
Brain_Putamen_basal_ganglia 0.553410766 Brain_Putamen_basal_ganglia 1 1
1 0.592656937 0.592656937 0.592656937 NA 1 0
0.998207054
SAMD10 ENSG00000130590.13 0.553452363 3 3 0.397866927
Brain_Hypothalamus 0.991161581 Brain_Putamen_basal_ganglia 2.64106611
0.118153673 0.118153673 -0.845436941 0.011077542 -0.383604292
0.432187677 3 0 0.998207054
FUT9 ENSG00000172461.10 0.553453849 3 3 0.157226728
Brain_Hippocampus 0.876119221 Brain_Cerebellum 1.514498765
0.558673336 0.558673336 -1.414460531 0.155890643 -0.610067077
0.785880832 3 0 0.998207054
SWT1 ENSG00000116668.12 0.553667578 2 2 0.303483545 Brain_Cerebellum
0.871026477 Brain_Substantia_nigra 1.178346731 0.821653269
0.821653269 -1.028991765 0.162354782 -0.433318491 0.842409222
2 0 0.998207054
FFAR3 ENSG00000185897.6 0.553676978 2 2 0.310678741
Brain_Hippocampus 0.875919103 Brain_Substantia_nigra 1.223923119
0.776076881 0.776076881 -1.013798856 0.156144525 -0.428827166
0.827274898 2 0 0.998207054
YTHDF2 ENSG00000198492.15 0.553682443 2 2 0.28712478
Brain_Putamen_basal_ganglia 0.905885813 Brain_Amygdala 1.096492293
0.903507707 0.903507707 0.118229504 1.064450251 0.591339877
0.669079107 2 0 0.998207054
GAPVD1 ENSG00000165219.21 0.553795656 3 3 0.489705035
Brain_Cortex 0.742620815 Brain_Cerebellum 2.313931746 0.314650473
0.314650473 -0.414797529 0.690778045 -0.017468059 0.614879015
3 0 0.998207054
POLR2M ENSG00000255529.7 0.553836119 9 7 0.154314278
Brain_Hippocampus 0.955080284 Brain_Nucleus_accumbens_basal_ganglia
3.892051586 0.035912393 0.250666253 -1.424456815 0.43369725
-0.560159963 0.633757809 7 0 0.998207054
FBXO27 ENSG00000161243.8 0.553853573 11 4 0.330222499
Brain_Cerebellar_Hemisphere 0.990580026 Brain_Nucleus_accumbens_basal_ganglia
7.638545806 0.006369542 0.414791666 -0.973665808 0.845816442
-0.069952344 0.457610653 4 0 0.998207054
TERF2IP ENSG00000166848.5 0.553887308 1 1 0.553887308
Brain_Cortex 0.553887308 Brain_Cortex 1 1 1 0.591945166
0.591945166 0.591945166 NA 1 0 0.998207054
WNT10A ENSG00000135925.8 0.553926492 1 1 0.553926492
Brain_Hypothalamus 0.553926492 Brain_Hypothalamus 1 1 1
0.591886653 0.591886653 0.591886653 NA 1 0 0.998207054
ERAP1 ENSG00000164307.12 0.554034752 12 2 0.164867649
Brain_Anterior_cingulate_cortex_BA24 0.730063308 Brain_Substantia_nigra
10.7172057 0.002900064 0.695021656 0.345041319 1.388885243
0.861730912 0.35042542 2 0 0.998207054
ZNF280B ENSG00000275004.3 0.554117352 1 1 0.554117352
Brain_Cerebellum 0.554117352 Brain_Cerebellum 1 1 1 -0.591601675
-0.591601675 -0.591601675 NA 1 0 0.998207054
TACC3 ENSG00000013810.18 0.554157875 10 4 0.261764806
Brain_Substantia_nigra 0.964632445 Brain_Anterior_cingulate_cortex_BA24
7.47469651 0.00184995 0.289626661 -1.122229658 0.74527489 -0.12040218

0.519836374 4 0 0.998207054
ADAM10 ENSG00000137845.14 0.554260568 3 3 0.172970607
Brain_Cortex 0.975378537 Brain_Cerebellum 1.220410129 0.846381929
0.846381929 0.030863327 1.362720523 0.660303996 0.668920776
3 0 0.998207054
POLD4 ENSG00000175482.8 0.554342428 1 1 0.554342428 Brain_Cortex
0.554342428 Brain_Cortex 1 1 1 0.59126567 0.59126567 0.59126567
NA 1 0 0.998207054
AC007193.6 ENSG00000267922.1 0.554346168 2 2 0.396792168
Brain_Amygdala 0.742662631 Brain_Frontal_Cortex_BA9 1.360619884
0.639380116 0.639380116 -0.328329382 0.847364172 0.259517395
0.831340885 2 0 0.998207054
IL13 ENSG00000169194.9 0.554418684 1 1 0.554418684
Brain_Nucleus_accumbens_basal_ganglia 0.554418684
Brain_Nucleus_accumbens_basal_ganglia 1 1 1 -0.591151846
-0.591151846 -0.591151846 NA 1 0 0.998207054
RAMP2 ENSG00000131477.10 0.554560025 1 1 0.554560025 Brain_Amygdala
0.554560025 Brain_Amygdala 1 1 1 -0.590940893 -0.590940893
-0.590940893 NA 1 0 0.998207054
YTHDF3 ENSG00000185728.16 0.55471405 1 1 0.55471405
Brain_Cerebellar_Hemisphere 0.55471405 Brain_Cerebellar_Hemisphere 1 1 1
-0.59071104 -0.59071104 -0.59071104 NA 1 0 0.998207054
MIIP ENSG00000116691.10 0.554748227 3 3 0.212243127
Brain_Frontal_Cortex_BA9 0.8179146 Brain_Cerebellar_Hemisphere 1.241965356
0.752928269 0.752928269 -0.414715034 1.247421118 0.200826026
0.91105973 3 0 0.998207054
CCL25 ENSG00000131142.13 0.554768081 3 3 0.182746291 Brain_Cortex
0.99412654 Brain_Hippocampus 1.92178865 0.178955535 0.178955535
-0.007361357 1.332346691 0.725913138 0.678801063 3 0
0.998207054
SCGB1D2 ENSG00000124935.3 0.554779913 1 1 0.554779913
Brain_Anterior_cingulate_cortex_BA24 0.554779913
Brain_Anterior_cingulate_cortex_BA24 1 1 1 0.59061276 0.59061276
0.59061276 NA 1 0 0.998207054
RIPK1 ENSG00000137275.13 0.554801091 6 6 0.236272566
Brain_Nucleus_accumbens_basal_ganglia 0.924505814 Brain_Cortex
3.083839998 0.151928441 0.151928441 -1.184355 0.277299202
-0.617853731 0.573103466 6 0 0.998207054
CCDC3 ENSG00000151468.10 0.554817898 6 6 0.090571838 Brain_Cortex
0.824519758 Brain_Caudate_basal_ganglia 2.673209589 0.244817922
0.244817922 -1.692388948 0.613377868 -0.885174301 0.906826035
6 0 0.998207054
RP11-473M20.9 ENSG00000262370.5 0.554818059 1 1 0.554818059
Brain_Hippocampus 0.554818059 Brain_Hippocampus 1 1 1
0.590555843 0.590555843 0.590555843 NA 1 0 0.998207054
MLH3 ENSG00000119684.15 0.554896084 1 1 0.554896084
Brain_Nucleus_accumbens_basal_ganglia 0.554896084
Brain_Nucleus_accumbens_basal_ganglia 1 1 1 0.590439427
0.590439427 0.590439427 NA 1 0 0.998207054
PCDHB7 ENSG00000113212.6 0.554921496 5 4 0.104008443
Brain_Frontal_Cortex_BA9 0.398107754 Brain_Amygdala 3.709816938
0.028762444 0.127165278 0.845005525 1.625723713 1.230764352
0.287470977 4 0 0.998207054
RP11-36B6.1 ENSG00000271522.1 0.555055728 1 1 0.555055728
Brain_Putamen_basal_ganglia 0.555055728 Brain_Putamen_basal_ganglia 1 1
1 -0.590201259 -0.590201259 -0.590201259 NA 1 0
0.998207054
XRN2 ENSG00000088930.7 0.555107583 3 3 0.374000169
Brain_Caudate_basal_ganglia 0.670569925 Brain_Cerebellum 1.266938677
0.759254755 0.759254755 -0.80415521 0.889005416 -0.113505247
0.888617493 3 0 0.998207054
CFL2 ENSG00000165410.14 0.55516547 1 1 0.55516547 Brain_Hypothalamus

0.55516547 Brain_Hypothalamus 1 1 1 0.590037559 0.590037559
0.590037559 NA 1 0 0.998207054
RBBP9 ENSG00000089050.15 0.555176857 5 5 0.156754194
Brain_Nucleus_accumbens_basal_ganglia 0.896119978 Brain_Caudate_basal_ganglia
3.504707361 0.127170071 0.127170071 -0.130564309 1.41607279
0.709980143 0.566249851 5 0 0.998207054
SKAP1 ENSG00000141293.15 0.55518497 3 3 0.299685489
Brain_Caudate_basal_ganglia 0.535776746 Brain_Cortex 1.681669387
0.459136623 0.459136623 -1.037108083 0.624806738 0.068970199
0.957895975 3 0 0.998207054
LAMTOR5 ENSG00000134248.13 0.555195732 1 1 0.555195732
Brain_Frontal_Cortex_BA9 0.555195732 Brain_Frontal_Cortex_BA9 1 1
1 -0.589992419 -0.589992419 -0.589992419 NA 1 0
0.998207054
ZNF425 ENSG00000204947.8 0.555246783 10 8 0.138094312
Brain_Caudate_basal_ganglia 0.901006611 Brain_Cerebellum 4.938452931
0.018279478 0.189747904 -1.482925101 0.941775608 0.118142079
0.723363014 8 0 0.998207054
ZNF595 ENSG00000272602.5 0.555329681 4 4 0.22772294
Brain_Cerebellar_Hemisphere 0.835062225 Brain_Cerebellum 1.808592451
0.38606941 0.38606941 -1.206245252 1.192137718 0.111189314
1.051207892 4 0 0.998207054
LINC02123 ENSG00000250668.1 0.555363435 1 1 0.555363435
Brain_Spinal_cord_cervical_c-1 0.555363435 Brain_Spinal_cord_cervical_c-1
1 1 1 -0.589742295 -0.589742295 -0.589742295 NA 1 0
0.998207054
FAM118B ENSG00000197798.8 0.5555237 1 1 0.5555237 Brain_Cerebellum
0.5555237 Brain_Cerebellum 1 1 1 0.589503299 0.589503299
0.589503299 NA 1 0 0.998207054
ZNF85 ENSG00000105750.14 0.555528164 11 2 0.184150972
Brain_Hypothalamus 0.968684199 Brain_Cerebellum 9.550496013
0.014226459 0.623978415 -1.328082143 -0.039258618 -0.763657181
0.357015388 2 0 0.998207054
CASC10 ENSG00000204682.5 0.555726242 2 2 0.430612817
Brain_Cerebellum 0.475847224 Brain_Amygdala 1.038839142 0.961160858
0.961160858 -0.788143425 0.712997629 -0.037572898 1.061467019
2 0 0.998207054
PIGR ENSG00000162896.5 0.555804234 1 1 0.555804234
Brain_Caudate_basal_ganglia 0.555804234 Brain_Caudate_basal_ganglia 1 1
1 0.589085032 0.589085032 0.589085032 NA 1 0
0.998207054
NME8 ENSG00000086288.11 0.555888306 3 3 0.187230002
Brain_Hippocampus 0.914837766 Brain_Cerebellum 2.149513286
0.227398359 0.227398359 -1.318817891 -0.106938504 -0.821313088
0.634379451 3 0 0.998207054
AP4S1 ENSG00000100478.14 0.555949204 2 2 0.505580749
Brain_Hippocampus 0.582520826 Brain_Hypothalamus 1.36826474 0.63173526
0.63173526 -0.665734608 0.549706313 -0.058014147 0.859446517 2
0 0.998207054
RELT ENSG00000054967.12 0.555966039 1 1 0.555966039
Brain_Cerebellar_Hemisphere 0.555966039 Brain_Cerebellar_Hemisphere 1 1
1 -0.588843833 -0.588843833 -0.588843833 NA 1 0
0.998207054
ADORA2A ENSG00000128271.21 0.555980013 1 1 0.555980013
Brain_Caudate_basal_ganglia 0.555980013 Brain_Caudate_basal_ganglia 1 1
1 0.588823004 0.588823004 0.588823004 NA 1 0
0.998207054
VPS53 ENSG00000141252.19 0.55598779 12 4 0.094720928
Brain_Frontal_Cortex_BA9 0.578021623 Brain_Cerebellum 8.594764497
0.010609905 0.306591386 -1.671003795 -0.556276831 -1.117939828
0.306160292 4 0 0.998207054
C19orf68 ENSG00000185453.12 0.556267902 1 1 0.556267902

Brain_Cerebellar_Hemisphere 0.556267902 Brain_Cerebellar_Hemisphere 1 1
1 -0.588393944 -0.588393944 -0.588393944 NA 1 0
0.998207054
C1orf127 ENSG00000175262.14 0.556273407 2 2 0.279198124
Brain_Cerebellar_Hemisphere 0.937796777 Brain_Cerebellum 1.030791014
0.969208986 0.969208986 -0.078039318 1.082122445 0.502041563
0.82035825 2 0 0.998207054
NRF1 ENSG00000106459.14 0.556294975 6 5 0.025731813
Brain_Hypothalamus 0.717775854 Brain_Frontal_Cortex_BA9 3.659162729
0.088272696 0.240064962 0.361432905 2.230235499 1.183029005
0.656311009 5 0 0.998207054
RFLNA ENSG00000178882.14 0.55631294 3 3 0.346614157 Brain_Cerebellum
0.409019571 Brain_Hippocampus 1.231563577 0.688614717
0.688614717 -0.941177009 0.825620203 -0.318424864 0.992069972
3 0 0.998207054
ATXN7L3B ENSG00000253719.3 0.556362665 3 3 0.346329076
Brain_Putamen_basal_ganglia 0.765545242 Brain_Cerebellum 2.386631732
0.100777262 0.100777262 -0.941733545 -0.298206918 -0.6404906
0.323720383 3 0 0.998207054
SULT1C2 ENSG00000198203.9 0.556373249 12 7 0.143097663
Brain_Hippocampus 0.988210374 Brain_Frontal_Cortex_BA9 6.913208869
0.00941475 0.256314554 -1.464352489 1.03815773 -0.144853066
0.959508324 7 0 0.998207054
VPS25 ENSG00000131475.6 0.556592244 1 1 0.556592244
Brain_Hippocampus 0.556592244 Brain_Hippocampus 1 1 1
-0.587910685 -0.587910685 -0.587910685 NA 1 0 0.998207054
CTD-2553L13.10 ENSG00000274104.1 0.556612601 4 2 0.308810625
Brain_Cortex 0.971276361 Brain_Anterior_cingulate_cortex_BA24
3.204467262 8.86E-08 0.762526722 -1.017720873 -0.036007523
-0.37172127 0.463055666 2 0 0.998207054
AKAP9 ENSG00000127914.16 0.556843455 1 1 0.556843455 Brain_Cerebellum
0.556843455 Brain_Cerebellum 1 1 1 -0.587536484 -0.587536484
-0.587536484 NA 1 0 0.998207054
TTLL6 ENSG00000170703.15 0.556865221 10 7 0.046551979
Brain_Caudate_basal_ganglia 0.790908814 Brain_Cerebellum 5.226658163
0.005204615 0.181475433 -1.990353771 -0.265130653 -1.154076955
0.536802532 7 0 0.998207054
RORC ENSG00000143365.17 0.55690621 1 1 0.55690621 Brain_Putamen_basal_ganglia
0.55690621 Brain_Putamen_basal_ganglia 1 1 1 -0.587443017
-0.587443017 -0.587443017 NA 1 0 0.998207054
IBA57 ENSG00000181873.12 0.557079426 1 1 0.557079426
Brain_Cerebellar_Hemisphere 0.557079426 Brain_Cerebellar_Hemisphere 1 1
1 -0.587185058 -0.587185058 -0.587185058 NA 1 0
0.998207054
VAT1 ENSG00000108828.15 0.557099377 2 2 0.413625613
Brain_Cerebellar_Hemisphere 0.757004401 Brain_Cerebellum 1.843640422
0.156359578 0.156359578 0.30941692 0.817529998 0.563473459
0.359290203 2 0 0.998207054
KLK4 ENSG00000167749.11 0.557133022 1 1 0.557133022 Brain_Cerebellum
0.557133022 Brain_Cerebellum 1 1 1 -0.587105249 -0.587105249
-0.587105249 NA 1 0 0.998207054
FAM198A ENSG00000144649.8 0.557134784 8 4 0.167979634
Brain_Frontal_Cortex_BA9 0.995283734 Brain_Putamen_basal_ganglia
6.331893426 0.024725172 0.25405219 -0.005910997 1.378724754
0.573969547 0.539174167 4 0 0.998207054
RCBTB2 ENSG00000136161.12 0.557185188 1 1 0.557185188
Brain_Substantia_nigra 0.557185188 Brain_Substantia_nigra 1 1
1 -0.587027574 -0.587027574 -0.587027574 NA 1 0
0.998207054
TMEM266 ENSG00000169758.12 0.55735295 1 1 0.55735295 Brain_Cerebellum
0.55735295 Brain_Cerebellum 1 1 1 -0.586777798 -0.586777798
-0.586777798 NA 1 0 0.998207054

CEP192 ENSG00000101639.18 0.557436943 13 2 0.2895769
Brain_Frontal_Cortex_BA9 0.996901027 Brain_Hypothalamus 12.20461092
0.000447381 0.45264097 -1.05905024 0.293145853 -0.062695095
0.349537554 2 0 0.998207054
RP11-47L3.1 ENSG00000267364.1 0.557486376 1 1 0.557486376
Brain_Spinal_cord_cervical_c-1 0.557486376 Brain_Spinal_cord_cervical_c-1
1 1 1 0.586579169 0.586579169 0.586579169 NA 1 0
0.998207054
NARS2 ENSG00000137513.9 0.557603651 13 2 0.075970116
Brain_Hippocampus 0.914851285 Brain_Cortex 11.26708117 5.93E-05
0.809106492 -0.236551609 1.774562729 0.798785201 0.507988133
2 0 0.998207054
INPP5K ENSG00000132376.19 0.557608094 4 4 0.130665751
Brain_Putamen_basal_ganglia 0.904735444 Brain_Cerebellum 1.593478155
0.234973788 0.234973788 -1.511481764 0.427824469 -0.296537586
0.852361023 4 0 0.998207054
CBX5 ENSG00000094916.15 0.557688306 2 2 0.300663782
Brain_Anterior_cingulate_cortex_BA24 0.778243868 Brain_Hippocampus
1.028187059 0.971812941 0.971812941 -1.035010984 0.28160831
-0.376701337 0.930990431 2 0 0.998207054
RP11-218C14.8 ENSG00000270001.1 0.557860188 9 6 0.103472661
Brain_Amygdala 0.922984711 Brain_Cerebellar_Hemisphere 4.75326299
0.002266704 0.267780034 -0.889376593 1.628246349 -0.179864803
0.722674852 6 0 0.998207054
AP3M2 ENSG00000070718.11 0.557918836 3 3 0.51873295
Brain_Putamen_basal_ganglia 0.839888002 Brain_Cerebellar_Hemisphere
2.487822987 0.084031499 0.084031499 -0.248784592 0.645299757
0.199517302 0.447047499 3 0 0.998207054
GUCD1 ENSG00000138867.16 0.558020346 1 1 0.558020346 Brain_Cortex
0.558020346 Brain_Cortex 1 1 1 0.585784492 0.585784492
0.585784492 NA 1 0 0.998207054
DSN1 ENSG00000149636.15 0.558088823 1 1 0.558088823 Brain_Cortex
0.558088823 Brain_Cortex 1 1 1 -0.585682609 -0.585682609
-0.585682609 NA 1 0 0.998207054
MALT1 ENSG00000172175.12 0.558203408 3 3 0.200328268
Brain_Hypothalamus 0.658229178 Brain_Nucleus_accumbens_basal_ganglia
1.365582765 0.634717121 0.634717121 -0.562741924 1.280616879
0.091838529 1.031270316 3 0 0.998207054
PIK3C3 ENSG00000078142.12 0.55821608 6 2 0.216545722 Brain_Cerebellum
0.56990374 Brain_Cerebellar_Hemisphere 5.262832464 0.003095026
0.542165915 -1.235765556 -0.568193272 -0.971771781 0.226354332
2 0 0.998207054
RSPH14 ENSG00000100218.11 0.558287903 4 4 0.146780933
Brain_Cerebellar_Hemisphere 0.781661954 Brain_Cortex 2.505882867
0.101593398 0.101593398 -1.450996148 1.099496007 -0.344374945
1.24028602 4 0 0.998207054
RPGRIP1L ENSG00000103494.12 0.558457095 7 5 0.182441835
Brain_Substantia_nigra 0.927339865 Brain_Cerebellar_Hemisphere
3.790943691 0.044090764 0.335113426 -1.333274208 1.152307534
0.523698001 0.90552593 5 0 0.998207054
LRRC66 ENSG00000188993.3 0.558491802 1 1 0.558491802
Brain_Cortex 0.558491802 Brain_Cortex 1 1 1 -0.585083159
-0.585083159 -0.585083159 NA 1 0 0.998207054
FGF19 ENSG00000162344.3 0.558522188 1 1 0.558522188 Brain_Cerebellum
0.558522188 Brain_Cerebellum 1 1 1 0.585037968 0.585037968
0.585037968 NA 1 0 0.998207054
CTD-2639E6.9 ENSG00000267898.1 0.558548526 1 1 0.558548526
Brain_Substantia_nigra 0.558548526 Brain_Substantia_nigra 1 1
1 0.584998797 0.584998797 0.584998797 NA 1 0
0.998207054
TCTEX1D1 ENSG00000152760.9 0.558607598 8 4 0.153561182
Brain_Frontal_Cortex_BA9 0.297939776 Brain_Nucleus_accumbens_basal_ganglia

6.069691891 0.030345129 0.21197359 1.040861621 1.427064924
1.232736847 0.147606302 4 0 0.998207054
PHACTR3 ENSG00000087495.16 0.558634508 2 2 0.459417619
Brain_Cerebellar_Hemisphere 0.493006717 Brain_Cerebellum 1.126730639
0.873269361 0.873269361 -0.739806163 0.685534469 -0.027135847
1.007868027 2 0 0.998207054
PSME2 ENSG00000100911.15 0.558662614 1 1 0.558662614
Brain_Anterior_cingulate_cortex_BA24 0.558662614
Brain_Anterior_cingulate_cortex_BA24 1 1 1 0.584829131
0.584829131 0.584829131 NA 1 0 0.998207054
GTPBP3 ENSG00000130299.16 0.558884962 3 3 0.312844571
Brain_Cerebellar_Hemisphere 0.64065825 Brain_Cortex 1.688525191
0.525284737 0.525284737 -0.466778653 1.009271236 0.375006326
0.759591504 3 0 0.998207054
RP11-757G1.6 ENSG00000250508.1 0.558981429 7 4 0.116147968
Brain_Substantia_nigra 0.826158947 Brain_Hypothalamus 5.854342291
0.000205899 0.201523911 0.219630485 1.571149323 0.87244042
0.40809092 4 0 0.998207054
FAM86B1 ENSG00000186523.14 0.559063396 4 4 0.331653324
Brain_Caudate_basal_ganglia 0.706440109 Brain_Cerebellar_Hemisphere
2.606948576 0.174304547 0.174304547 -0.376641411 0.970789075
0.560883804 0.63146389 4 0 0.998207054
LINC02003 ENSG00000264026.1 0.55942583 1 1 0.55942583 Brain_Cortex
0.55942583 Brain_Cortex 1 1 1 0.583694557 0.583694557
0.583694557 NA 1 0 0.998207054
USP21 ENSG00000143258.15 0.559452067 2 2 0.295472671 Brain_Cerebellum
0.858505793 Brain_Caudate_basal_ganglia 1.397560278 0.602439722
0.602439722 -0.178276552 1.046191403 0.433957425 0.865829594
2 0 0.998207054
ELL ENSG00000105656.12 0.559469197 1 1 0.559469197 Brain_Cerebellum
0.559469197 Brain_Cerebellum 1 1 1 0.583630112 0.583630112
0.583630112 NA 1 0 0.998207054
LINC00662 ENSG00000261824.6 0.559470026 11 7 0.08167356
Brain_Anterior_cingulate_cortex_BA24 0.668055148 Brain_Frontal_Cortex_BA9
6.420759263 0.008455826 0.256968524 -1.741057159 0.438568668
-0.919587436 0.583297851 7 0 0.998207054
LEPROTL1 ENSG00000104660.17 0.559572794 1 1 0.559572794
Brain_Hippocampus 0.559572794 Brain_Hippocampus 1 1 1
0.58347617 0.58347617 0.58347617 NA 1 0 0.998207054
SAMD3 ENSG00000164483.16 0.55969616 1 1 0.55969616 Brain_Cortex 0.55969616
Brain_Cortex 1 1 1 -0.583292873 -0.583292873 -0.583292873
NA 1 0 0.998207054
STX19 ENSG00000178750.2 0.559807456 9 4 0.317508685
Brain_Hypothalamus 0.947068892 Brain_Substantia_nigra 7.014113008
0.001324033 0.265587716 -0.514377122 0.999590578 -0.058055619
0.527917246 4 0 0.998207054
LINC01422 ENSG00000223704.1 0.559839331 7 4 0.257613802
Brain_Hippocampus 0.82486638 Brain_Hypothalamus 4.999507005
0.036139094 0.355757126 -1.132049076 0.486488743 -0.477859988
0.508510656 4 0 0.998207054
KCNQ4 ENSG00000117013.14 0.559843412 2 2 0.390107074
Brain_Spinal_cord_cervical_c-1 0.517006981 Brain_Cerebellum 1.001549204
0.998450796 0.998450796 -0.8594232 0.647965938 -0.105728631
1.065885081 2 0 0.998207054
RESP18 ENSG00000182698.11 0.559958566 12 2 0.125722953
Brain_Hippocampus 0.609999135 Brain_Hypothalamus 11.14330941
0.000683977 0.515152167 0.510074692 1.531187929 0.974298068
0.280416282 2 0 0.998207054
SP2 ENSG00000167182.13 0.560076393 1 1 0.560076393
Brain_Caudate_basal_ganglia 0.560076393 Brain_Caudate_basal_ganglia 1 1
1 -0.582728042 -0.582728042 -0.582728042 NA 1 0
0.998207054

SGMS2 ENSG00000164023.14 0.560086191 1 1 0.560086191 Brain_Cortex
0.560086191 Brain_Cortex 1 1 1 -0.582713489 -0.582713489
-0.582713489 NA 1 0 0.998207054
LRRC14B ENSG00000185028.3 0.560087849 1 1 0.560087849
Brain_Cerebellum 0.560087849 Brain_Cerebellum 1 1 1 0.582711027
0.582711027 0.582711027 NA 1 0 0.998207054
FCF1 ENSG00000119616.11 0.560230462 2 2 0.330013302 Brain_Cerebellum
0.718686484 Brain_Cortex 1.098335592 0.901664408 0.901664408
-0.974087084 0.360214832 -0.306936126 0.943493932 2 0
0.998207054
PHF19 ENSG00000119403.13 0.560277358 1 1 0.560277358
Brain_Putamen_basal_ganglia 0.560277358 Brain_Putamen_basal_ganglia 1 1
1 0.582429586 0.582429586 0.582429586 NA 1 0
0.998207054
SLC7A6OS ENSG00000103061.12 0.560281015 1 1 0.560281015
Brain_Cortex 0.560281015 Brain_Cortex 1 1 1 0.582424156
0.582424156 0.582424156 NA 1 0 0.998207054
DDX60L ENSG00000181381.13 0.560291928 3 3 0.298135318
Brain_Hippocampus 0.720193782 Brain_Caudate_basal_ganglia 1.788673161
0.442367753 0.442367753 -1.040440447 0.35819982 -0.573975748
0.807287811 3 0 0.998207054
APAF1 ENSG00000120868.13 0.560332423 1 1 0.560332423
Brain_Hippocampus 0.560332423 Brain_Hippocampus 1 1 1
-0.582347817 -0.582347817 -0.582347817 NA 1 0 0.998207054
ZNF736 ENSG00000234444.9 0.560337792 6 5 0.19947883
Brain_Caudate_basal_ganglia 0.929203879 Brain_Cortex 2.953316879
0.064778079 0.462298355 -0.27175084 1.283037809 0.437120771
0.683816809 5 0 0.998207054
LPAR3 ENSG00000171517.5 0.56056505 5 5 0.309816173
Brain_Anterior_cingulate_cortex_BA24 0.765667848 Brain_Hypothalamus
1.428273156 0.759979763 0.759979763 -1.015607834 0.984791422
0.313067421 0.803412118 5 0 0.998207054
ATP5O ENSG00000241837.6 0.56072722 12 10 0.131883351 Brain_Cerebellum
0.952660722 Brain_Anterior_cingulate_cortex_BA24 4.122685824
0.084310378 0.27329756 -1.177043965 1.506716458 0.25673335
0.975035642 10 0 0.998207054
PCDHGC3 ENSG00000240184.6 0.560765226 1 1 0.560765226
Brain_Nucleus_accumbens_basal_ganglia 0.560765226
Brain_Nucleus_accumbens_basal_ganglia 1 1 1 0.581705262
0.581705262 0.581705262 NA 1 0 0.998207054
RP11-430H10.3 ENSG00000254651.1 0.560770839 2 2 0.322339125
Brain_Cerebellar_Hemisphere 0.355971968 Brain_Cerebellum 1.588601588
0.411398412 0.411398412 0.92306762 0.989662469 0.956365044
0.047089669 2 0 0.998207054
PDE4C ENSG00000105650.21 0.560804603 6 4 0.080438643
Brain_Nucleus_accumbens_basal_ganglia 0.390715741 Brain_Frontal_Cortex_BA9
4.066818489 0.053580499 0.325353216 0.858320086 1.748146612
1.210986758 0.309095314 4 0 0.998207054
GSTT2B ENSG00000133433.10 0.56090719 13 1 0.301097558
Brain_Cerebellar_Hemisphere 0.815385767 Brain_Substantia_nigra
11.75768224 0.002423761 11.75768224 0.233483775 1.034082582
0.554600234 0.223798982 1 0 0.998207054
ZNF480 ENSG00000198464.13 0.560921079 1 1 0.560921079
Brain_Amygdala 0.560921079 Brain_Amygdala 1 1 1 0.581473937
0.581473937 0.581473937 NA 1 0 0.998207054
AC195454.1 ENSG00000249241.1 0.561010214 1 1 0.561010214
Brain_Anterior_cingulate_cortex_BA24 0.561010214
Brain_Anterior_cingulate_cortex_BA24 1 1 1 0.581341651
0.581341651 0.581341651 NA 1 0 0.998207054
ATMIN ENSG00000166454.9 0.561011802 1 1 0.561011802
Brain_Nucleus_accumbens_basal_ganglia 0.561011802
Brain_Nucleus_accumbens_basal_ganglia 1 1 1 -0.581339295

-0.581339295 -0.581339295 NA 1 0 0.998207054
NDUFA6 ENSG00000184983.9 0.561057504 13 8 0.128658071
Brain_Cortex 0.993935053 Brain_Amygdala 7.313206606 0.075289982
0.275956232 -0.259449433 1.519414991 0.554544767 0.519022104
8 0 0.998207054
RP11-122G18.12 ENSG00000283696.1 0.5613281 3 3 0.176637248
Brain_Cerebellar_Hemisphere 0.756863412 Brain_Cortex 1.285488925
0.776724635 0.776724635 -1.35118166 0.309602294 -0.450467137
0.839277438 3 0 0.998207054
NKX2-2 ENSG00000125820.5 0.561357347 2 2 0.350373438
Brain_Frontal_Cortex_BA9 0.630070123 Brain_Cerebellum 1.052809414
0.947190586 0.947190586 -0.481628154 0.933865188 0.226118517
1.000904941 2 0 0.998207054
MUC6 ENSG00000184956.15 0.561367376 6 6 0.075765999
Brain_Putamen_basal_ganglia 0.559941592 Brain_Cerebellum 2.509986829
0.167814826 0.167814826 0.582928266 1.775799325 0.965729248
0.438841829 6 0 0.998207054
GFPT1 ENSG00000198380.12 0.561510693 1 1 0.561510693 Brain_Cortex
0.561510693 Brain_Cortex 1 1 1 0.580599079 0.580599079
0.580599079 NA 1 0 0.998207054
AC144835.1 ENSG00000258483.2 0.561521717 1 1 0.561521717
Brain_Hypothalamus 0.561521717 Brain_Hypothalamus 1 1 1
0.580582726 0.580582726 0.580582726 NA 1 0 0.998207054
GCKR ENSG00000084734.8 0.561535163 1 1 0.561535163
Brain_Caudate_basal_ganglia 0.561535163 Brain_Caudate_basal_ganglia 1 1
1 0.58056278 0.58056278 0.58056278 NA 1 0 0.998207054
CDC20 ENSG00000117399.13 0.561647103 2 2 0.627088476
Brain_Hypothalamus 0.684642893 Brain_Substantia_nigra 1.654640344
0.345359656 0.345359656 -0.406135704 0.485828909 0.039846602
0.630714226 2 0 0.998207054
RP11-131L12.3 ENSG00000275759.1 0.561734995 13 5 0.132166566
Brain_Caudate_basal_ganglia 0.908386797 Brain_Substantia_nigra
10.15182857 0.002939921 0.368969285 -1.505612934 -0.115073585
-0.838815444 0.398850099 5 0 0.998207054
CECR6 ENSG00000183307.3 0.56175389 3 3 0.300433479
Brain_Cerebellar_Hemisphere 0.643138292 Brain_Cerebellum 1.791512042
0.207935982 0.207935982 -1.035504258 -0.463315423 -0.751127487
0.286109885 3 0 0.998207054
OR51Q1 ENSG00000167360.4 0.561753956 1 1 0.561753956
Brain_Cerebellum 0.561753956 Brain_Cerebellum 1 1 1 0.58023826
0.58023826 0.58023826 NA 1 0 0.998207054
UROC1 ENSG00000159650.8 0.561895789 5 5 0.120345369
Brain_Caudate_basal_ganglia 0.765131991 Brain_Spinal_cord_cervical_c-1
1.288184139 0.712564481 0.712564481 -1.553325595 0.753064167
-0.438414621 0.839099394 5 0 0.998207054
AGPAT2 ENSG00000169692.12 0.562592625 1 1 0.562592625
Brain_Putamen_basal_ganglia 0.562592625 Brain_Putamen_basal_ganglia 1 1
1 0.578994884 0.578994884 0.578994884 NA 1 0
0.998207054
HIF1A-AS2 ENSG00000258667.1 0.562606593 1 1 0.562606593
Brain_Cerebellar_Hemisphere 0.562606593 Brain_Cerebellar_Hemisphere 1 1
1 0.578974183 0.578974183 0.578974183 NA 1 0
0.998207054
RP11-368I23.3 ENSG00000272797.1 0.56263295 1 1 0.56263295 Brain_Amygdala
0.56263295 Brain_Amygdala 1 1 1 -0.578935123 -0.578935123
-0.578935123 NA 1 0 0.998207054
KB-1000E4.2 ENSG00000260640.1 0.562737724 1 1 0.562737724
Brain_Caudate_basal_ganglia 0.562737724 Brain_Caudate_basal_ganglia 1 1
1 -0.578779857 -0.578779857 -0.578779857 NA 1 0
0.998207054
XYLT2 ENSG00000015532.9 0.562771886 1 1 0.562771886 Brain_Amygdala
0.562771886 Brain_Amygdala 1 1 1 0.578729236 0.578729236

0.578729236 NA 1 0 0.998207054
RP11-2L8.2 ENSG00000270321.1 0.562928986 2 2 0.304095371
Brain_Cortex 0.813907615 Brain_Frontal_Cortex_BA9 1.488113447
0.511886553 0.511886553 -1.027690641 -0.235387979 -0.63153931
0.560242585 2 0 0.998207054
TTLL7 ENSG00000137941.16 0.563012963 1 1 0.563012963
Brain_Cerebellar_Hemisphere 0.563012963 Brain_Cerebellar_Hemisphere 1 1
1 0.578372045 0.578372045 0.578372045 NA 1 0
0.998207054
ANKFY1 ENSG00000185722.16 0.563190087 1 1 0.563190087
Brain_Substantia_nigra 0.563190087 Brain_Substantia_nigra 1 1
1 -0.578109658 -0.578109658 -0.578109658 NA 1 0
0.998207054
LINC00943 ENSG00000189238.5 0.563206262 1 1 0.563206262
Brain_Cortex 0.563206262 Brain_Cortex 1 1 1 0.578085698
0.578085698 0.578085698 NA 1 0 0.998207054
EIF3F ENSG00000175390.13 0.563229388 11 4 0.056491448
Brain_Caudate_basal_ganglia 0.596471274 Brain_Frontal_Cortex_BA9
8.410147305 0.040317767 0.418667595 -1.907225073 -0.52948179
-1.201298521 0.50891214 4 0 0.998207054
HBE1 ENSG00000213931.5 0.563357691 2 2 0.350544168
Brain_Frontal_Cortex_BA9 0.621726381 Brain_Hypothalamus 1.034097982
0.965902018 0.965902018 -0.493405098 0.933534302 0.220064602
1.008998527 2 0 0.998207054
CEBPA ENSG00000245848.2 0.563382267 1 1 0.563382267 Brain_Cerebellum
0.563382267 Brain_Cerebellum 1 1 1 0.577825012 0.577825012
0.577825012 NA 1 0 0.998207054
RP11-80H5.7 ENSG00000240996.1 0.563587986 1 1 0.563587986
Brain_Cerebellum 0.563587986 Brain_Cerebellum 1 1 1 -0.577520364
-0.577520364 -0.577520364 NA 1 0 0.998207054
LINC01445 ENSG00000231427.5 0.563802727 2 2 0.583903353
Brain_Cortex 0.81130248 Brain_Frontal_Cortex_BA9 1.723498506
0.276501494 0.276501494 -0.547692078 0.238746084 -0.154472997
0.556095757 2 0 0.998207054
PLGLB1 ENSG00000183281.14 0.564092266 8 8 0.100789911
Brain_Frontal_Cortex_BA9 0.998367509 Brain_Hippocampus 3.344285965
0.301196625 0.301196625 -1.641036153 0.409261595 -0.289129953
0.694717957 8 0 0.998207054
ERCC2 ENSG00000104884.14 0.564096768 1 1 0.564096768
Brain_Substantia_nigra 0.564096768 Brain_Substantia_nigra 1 1
1 -0.576767144 -0.576767144 -0.576767144 NA 1 0
0.998207054
DTNB ENSG00000138101.18 0.564207302 2 2 0.327518233
Brain_Frontal_Cortex_BA9 0.643958398 Brain_Hypothalamus 1.031797705
0.968202295 0.968202295 -0.979124974 0.462171419 -0.258476778
1.019150453 2 0 0.998207054
M1AP ENSG00000159374.17 0.564301026 1 1 0.564301026
Brain_Hypothalamus 0.564301026 Brain_Hypothalamus 1 1 1
0.576464843 0.576464843 0.576464843 NA 1 0 0.998207054
CNN2 ENSG00000064666.14 0.564335337 4 3 0.180327025
Brain_Hippocampus 0.393893699 Brain_Hypothalamus 2.856833588
0.008964815 0.273946199 -1.339748804 -0.852577402 -1.173450402
0.221393663 3 0 0.998207054
PXDN ENSG00000130508.10 0.564371896 5 5 0.145032073
Brain_Hypothalamus 0.809265783 Brain_Cerebellar_Hemisphere 1.688543161
0.525786836 0.525786836 -1.457305488 0.748275164 -0.474918422
0.816897471 5 0 0.998207054
AC001226.7 ENSG00000274898.1 0.56453107 2 2 0.337542198
Brain_Frontal_Cortex_BA9 0.579526794 Brain_Substantia_nigra
1.086329415 0.913670585 0.913670585 -0.554076059 0.959032847
0.202478394 1.069929568 2 0 0.998207054
GPI ENSG00000105220.14 0.564587272 1 1 0.564587272 Brain_Cortex

0.564587272 Brain_Cortex 1 1 1 -0.576041291 -0.576041291
-0.576041291 NA 1 0 0.998207054
ZNF426 ENSG00000130818.11 0.564601058 2 2 0.28666837
Brain_Anterior_cingulate_cortex_BA24 0.506426561 Brain_Cerebellar_Hemisphere
1.685015359 0.314984641 0.314984641 -1.065458779 -0.664412146
-0.864935463 0.283582793 2 0 0.998207054
TJAP1 ENSG00000137221.14 0.564752793 1 1 0.564752793
Brain_Anterior_cingulate_cortex_BA24 0.564752793
Brain_Anterior_cingulate_cortex_BA24 1 1 1 -0.575796422
-0.575796422 -0.575796422 NA 1 0 0.998207054
C5orf46 ENSG00000178776.4 0.564857954 2 2 0.285274151
Brain_Cerebellum 0.950189339 Brain_Cerebellar_Hemisphere 1.036118312
0.963881688 0.963881688 -1.068546312 0.062469012 -0.50303865
0.799748605 2 0 0.998207054
CXCL10 ENSG00000169245.5 0.564937304 1 1 0.564937304
Brain_Hippocampus 0.564937304 Brain_Hippocampus 1 1 1
-0.575523497 -0.575523497 -0.575523497 NA 1 0 0.998207054
TUFT1 ENSG00000143367.15 0.565082379 3 3 0.382414972
Brain_Frontal_Cortex_BA9 0.967165973 Brain_Cerebellar_Hemisphere
2.220551231 0.220840418 0.220840418 -0.873455278 0.041162971
-0.524830009 0.494538347 3 0 0.998207054
PLA2G16 ENSG00000176485.11 0.565131071 7 7 0.074080409
Brain_Cerebellar_Hemisphere 0.824685163 Brain_Anterior_cingulate_cortex_BA24
2.646276363 0.091908821 0.091908821 -1.786116428 0.68606643
-0.417838861 0.81755301 7 0 0.998207054
S100Z ENSG00000171643.13 0.565229009 1 1 0.565229009
Brain_Hypothalamus 0.565229009 Brain_Hypothalamus 1 1 1
0.575092102 0.575092102 0.575092102 NA 1 0 0.998207054
TAP1 ENSG00000168394.10 0.565346431 2 2 0.291837448 Brain_Cerebellum
0.749552393 Brain_Cerebellar_Hemisphere 1.141523934 0.858476066
0.858476066 0.319229626 1.05409932 0.686664473 0.519631344 2
0 0.998207054
KIAA2012 ENSG00000182329.12 0.565352389 1 1 0.565352389
Brain_Nucleus_accumbens_basal_ganglia 0.565352389
Brain_Nucleus_accumbens_basal_ganglia 1 1 1 0.574909672
0.574909672 0.574909672 NA 1 0 0.998207054
RTCA ENSG00000137996.12 0.565355239 4 3 0.246200523 Brain_Amygdala
0.796761619 Brain_Nucleus_accumbens_basal_ganglia 2.347302818 0.07306821
0.587837932 -1.159627443 0.730338676 -0.100444973 0.813501441
3 0 0.998207054
ZNF548 ENSG00000188785.11 0.565361927 1 1 0.565361927
Brain_Spinal_cord_cervical_c-1 0.565361927 Brain_Spinal_cord_cervical_c-1
1 1 1 0.574895568 0.574895568 0.574895568 NA 1 0
0.998207054
MMP9 ENSG00000100985.7 0.565368335 1 1 0.565368335
Brain_Spinal_cord_cervical_c-1 0.565368335 Brain_Spinal_cord_cervical_c-1
1 1 1 0.574886094 0.574886094 0.574886094 NA 1 0
0.998207054
CHD6 ENSG00000124177.14 0.565401073 1 1 0.565401073 Brain_Cortex
0.565401073 Brain_Cortex 1 1 1 -0.574837692 -0.574837692
-0.574837692 NA 1 0 0.998207054
CENPP ENSG00000188312.13 0.56557801 13 1 0.388845074 Brain_Hippocampus
0.764919387 Brain_Putamen_basal_ganglia 12.61309849 0.00167555
12.61309849 -0.86171372 -0.299027076 -0.566993565 0.160756506
1 0 0.998207054
PDK2 ENSG00000005882.11 0.565658325 2 2 0.285897606
Brain_Caudate_basal_ganglia 0.64418214 Brain_Spinal_cord_cervical_c-1
1.454717822 0.545282178 0.545282178 0.461859415 1.067164398
0.764511907 0.428015258 2 0 0.998207054
FAM53B ENSG00000189319.13 0.56566479 1 1 0.56566479
Brain_Frontal_Cortex_BA9 0.56566479 Brain_Frontal_Cortex_BA9 1 1 1
-0.574447837 -0.574447837 -0.574447837 NA 1 0 0.998207054

ATP5D ENSG00000099624.7 0.56571675 1 1 0.56571675 Brain_Caudate_basal_ganglia 0.56571675 Brain_Caudate_basal_ganglia 1 1 1 -0.574371033 -0.574371033 -0.574371033 NA 1 0 0.998207054
FAM149A ENSG00000109794.13 0.565828191 11 5 0.092328503 Brain_Hippocampus 0.613144365 Brain_Cerebellar_Hemisphere 8.189924545 0.011825339 0.323389485 -0.505590196 1.683240711 1.043725924 0.592120211 5 0 0.998207054
TULP2 ENSG00000104804.7 0.565974021 4 4 0.106195025 Brain_Cerebellum 0.409939787 Brain_Cortex 2.313155919 0.24147154 0.24147154 -1.615534365 -0.823999598 -1.101894837 0.369654224 4 0 0.998207054
IFI6 ENSG00000126709.14 0.565975323 1 1 0.565975323 Brain_Cerebellar_Hemisphere 0.565975323 Brain_Cerebellar_Hemisphere 1 1 1 0.573988885 0.573988885 0.573988885 NA 1 0 0.998207054
LINC00526 ENSG00000264575.1 0.566096455 2 2 0.301611221 Brain_Cerebellar_Hemisphere 0.91188274 Brain_Cerebellum 1.147849677 0.852150323 0.852150323 -1.032984353 0.110664069 -0.461160142 0.808681555 2 0 0.998207054
CTB-32O4.3 ENSG00000276251.1 0.56611715 3 3 0.208649982 Brain_Cerebellar_Hemisphere 0.556209435 Brain_Spinal_cord_cervical_c-1 1.595611272 0.401584325 0.401584325 -1.257286303 0.601740637 -0.022354865 1.069502546 3 0 0.998207054
GNA13 ENSG00000120063.9 0.566537588 4 4 0.244018128 Brain_Anterior_cingulate_cortex_BA24 0.814782322 Brain_Spinal_cord_cervical_c-1 1.216327585 0.751786975 0.751786975 -0.385909223 1.165002136 0.520099049 0.734302659 4 0 0.998207054
DCSTAMP ENSG00000164935.6 0.566758205 2 2 0.347424371 Brain_Caudate_basal_ganglia 0.742164854 Brain_Hypothalamus 1.176572455 0.823427545 0.823427545 -0.939596891 -0.328987873 -0.634292382 0.431765778 2 0 0.998207054
GLIPR2 ENSG00000122694.15 0.567034459 7 7 0.139646068 Brain_Hippocampus 0.930314916 Brain_Caudate_basal_ganglia 2.281674018 0.225877713 0.225877713 -1.477110318 0.590051001 -0.059255841 0.753317622 7 0 0.998207054
ZNF385D ENSG00000151789.10 0.567099165 1 1 0.567099165 Brain_Putamen_basal_ganglia 0.567099165 Brain_Putamen_basal_ganglia 1 1 1 0.572328914 0.572328914 0.572328914 NA 1 0 0.998207054
FES ENSG00000182511.11 0.567140137 1 1 0.567140137 Brain_Amygdala 0.567140137 Brain_Amygdala 1 1 1 0.572268426 0.572268426 0.572268426 NA 1 0 0.998207054
SLC28A2 ENSG00000137860.11 0.567259508 10 4 0.155658533 Brain_Anterior_cingulate_cortex_BA24 0.976418728 Brain_Hippocampus 7.736517777 0.003201934 0.279288644 -1.419825349 0.199809689 -0.320209016 0.494940553 4 0 0.998207054
METAP2 ENSG00000111142.13 0.567549045 1 1 0.567549045 Brain_Hippocampus 0.567549045 Brain_Hippocampus 1 1 1 0.571664861 0.571664861 0.571664861 NA 1 0 0.998207054
SYN2 ENSG00000157152.16 0.567649001 2 2 0.512501325 Brain_Cerebellum 0.991675192 Brain_Cerebellar_Hemisphere 1.794178654 0.205821346 0.205821346 -0.65494786 -0.010433789 -0.332690824 0.45574027 2 0 0.998207054
MANEAL ENSG00000185090.14 0.567678523 1 1 0.567678523 Brain_Hypothalamus 0.567678523 Brain_Hypothalamus 1 1 1 0.571473788 0.571473788 0.571473788 NA 1 0 0.998207054
ZNF75A ENSG00000162086.14 0.567712647 2 2 0.339762336 Brain_Frontal_Cortex_BA9 0.917105466 Brain_Cerebellar_Hemisphere 1.351875963 0.648124037 0.648124037 -0.954634951 0.1040805 -0.425277225 0.748624875 2 0 0.998207054
MTFP1 ENSG00000242114.5 0.567724764 1 1 0.567724764 Brain_Cortex

0.567724764 Brain_Cortex 1 1 1 -0.571405556 -0.571405556
-0.571405556 NA 1 0 0.998207054
CEP250 ENSG00000126001.15 0.567727928 2 2 0.316442173
Brain_Frontal_Cortex_BA9 0.400506848 Brain_Cortex 1.53725849 0.46274151
0.46274151 -1.001795909 -0.84071637 -0.921256139 0.113900434 2
0 0.998207054
ZNF431 ENSG00000196705.8 0.567755875 2 2 0.295701935
Brain_Putamen_basal_ganglia 0.600290638 Brain_Cerebellar_Hemisphere
1.323169094 0.676830906 0.676830906 -1.045694863 -0.523982606
-0.784838734 0.368906275 2 0 0.998207054
NRCAM ENSG00000091129.19 0.567843645 3 3 0.157012238
Brain_Caudate_basal_ganglia 0.784468006 Brain_Cerebellum 2.040676681
0.129942492 0.129942492 -1.415191904 0.426324583 -0.23845539
1.021944406 3 0 0.998207054
STC1 ENSG00000159167.11 0.567870365 1 1 0.567870365 Brain_Cortex
0.567870365 Brain_Cortex 1 1 1 0.571190725 0.571190725
0.571190725 NA 1 0 0.998207054
KLF12 ENSG00000118922.17 0.567928703 1 1 0.567928703
Brain_Putamen_basal_ganglia 0.567928703 Brain_Putamen_basal_ganglia 1 1
1 -0.571104656 -0.571104656 -0.571104656 NA 1 0
0.998207054
TESC ENSG00000088992.17 0.567977825 4 3 0.099709175 Brain_Cortex
0.603779505 Brain_Nucleus_accumbens_basal_ganglia 2.424690905
0.063747924 0.556618146 -1.646265181 -0.518973092 -0.942957585
0.514996703 3 0 0.998207054
ABCD4 ENSG00000119688.20 0.568054929 2 2 0.340867472
Brain_Anterior_cingulate_cortex_BA24 0.542175135 Brain_Cerebellum
1.148664418 0.851335582 0.851335582 -0.952452644 -0.609527071
-0.780989858 0.242484999 2 0 0.998207054
FABP5 ENSG00000164687.10 0.56809007 2 2 0.290493047 Brain_Hypothalamus
0.808033164 Brain_Putamen_basal_ganglia 1.12026471 0.87973529 0.87973529
-1.057040625 -0.242964157 -0.650002391 0.575638991 2 0
0.998207054
PRLHR ENSG00000119973.5 0.56810497 4 4 0.195917002
Brain_Cerebellar_Hemisphere 0.921838869 Brain_Cortex 1.960633039
0.358865564 0.358865564 -0.455190259 1.293271998 0.364627358
0.737195822 4 0 0.998207054
USP24 ENSG00000162402.13 0.568106533 2 2 0.331548923 Brain_Cerebellum
0.794872357 Brain_Cerebellar_Hemisphere 1.618639752 0.381360248
0.381360248 0.259988873 0.970998706 0.615493789 0.502759874
2 0 0.998207054
ZEB2 ENSG00000169554.18 0.56811106 1 1 0.56811106 Brain_Cerebellar_Hemisphere
0.56811106 Brain_Cerebellar_Hemisphere 1 1 1 -0.570835642
-0.570835642 -0.570835642 NA 1 0 0.998207054
KRT16 ENSG00000186832.8 0.568281292 2 2 0.397517792
Brain_Hypothalamus 0.505266919 Brain_Cortex 1.026800211
0.973199789 0.973199789 -0.846062655 -0.666225589 -0.756144122
0.127164009 2 0 0.998207054
TIMM13 ENSG00000099800.7 0.568324213 12 4 0.202815978
Brain_Putamen_basal_ganglia 0.965544917 Brain_Hypothalamus 8.704150908
1.38E-05 0.402879718 -0.590248364 1.273569638 0.388168614
0.586753056 4 0 0.998207054
SLC4A8 ENSG00000050438.16 0.568339227 5 4 0.177751247
Brain_Hippocampus 0.762474545 Brain_Cerebellum 2.669897314
0.065987893 0.417324648 -1.347711328 -0.302232874 -0.792442963
0.417519966 4 0 0.998207054
MAU2 ENSG00000129933.20 0.568443384 5 5 0.138515727 Brain_Cerebellum
0.822733395 Brain_Frontal_Cortex_BA9 2.576337297 0.101555669
0.101555669 -1.481341004 0.224030725 -1.087904851 0.736704894
5 0 0.998207054
AGAP5 ENSG00000172650.13 0.568451336 1 1 0.568451336 Brain_Cortex
0.568451336 Brain_Cortex 1 1 1 0.570333776 0.570333776

0.570333776 NA 1 0 0.998207054
FAAP20 ENSG00000162585.16 0.56851826 9 9 0.154565396 Brain_Cerebellum 0.96102571 Brain_Frontal_Cortex_BA9 3.42330802 0.131590474 0.131590474 -0.822173268 1.423589297 0.175629199 0.706891412 9 0 0.998207054
DCAF4 ENSG00000119599.16 0.568535788 5 5 0.341480682 Brain_Cerebellar_Hemisphere 0.967844224 Brain_Hippocampus 2.933197252 0.129199504 0.129199504 -0.729567023 0.951243695 0.249536413 0.686009448 5 0 0.998207054
PDE6C ENSG00000095464.9 0.568582935 1 1 0.568582935 Brain_Hippocampus 0.568582935 Brain_Hippocampus 1 1 1 0.570139722 0.570139722 0.570139722 NA 1 0 0.998207054
ZNF202 ENSG00000166261.10 0.568828898 10 4 0.168838862 Brain_Frontal_Cortex_BA9 0.965073649 Brain_Hypothalamus 7.822288001 0.023570893 0.295477486 -1.375944352 0.773842759 0.110978774 0.640823841 4 0 0.998207054
GSTA4 ENSG00000170899.10 0.568863724 2 2 0.418268337 Brain_Spinal_cord_cervical_c-1 0.709689021 Brain_Caudate_basal_ganglia 1.339370623 0.660629377 0.660629377 -0.809429157 0.372273776 -0.218577691 0.835590157 2 0 0.998207054
KIAA1468 ENSG00000134444.13 0.568940857 3 3 0.255069116 Brain_Cerebellum 0.506441417 Brain_Cerebellar_Hemisphere 2.498698443 0.085944919 0.085944919 -1.138123022 -0.664388929 -0.851701223 0.251938521 3 0 0.998207054
CTC-459F4.3 ENSG00000267575.6 0.568973484 5 5 0.142090337 Brain_Cortex 0.878672594 Brain_Nucleus_accumbens_basal_ganglia 2.472243528 0.196136512 0.196136512 -1.468051122 -0.152652154 -0.672819461 0.660964049 5 0 0.998207054
FAM163A ENSG00000143340.6 0.568981495 5 5 0.331892375 Brain_Nucleus_accumbens_basal_ganglia 0.890049574 Brain_Frontal_Cortex_BA9 1.887894369 0.179976503 0.179976503 -0.970309235 0.832811001 -0.012834519 0.794642565 5 0 0.998207054
EFCAB6 ENSG00000186976.14 0.569028784 5 5 0.361343678 Brain_Nucleus_accumbens_basal_ganglia 0.810616138 Brain_Putamen_basal_ganglia 3.153587723 0.17102411 0.17102411 -0.91280772 0.577515282 0.023863914 0.6046591 5 0 0.998207054
PDZD3 ENSG00000172367.15 0.569064825 1 1 0.569064825 Brain_Cerebellar_Hemisphere 0.569064825 Brain_Cerebellar_Hemisphere 1 1 1 -0.569429318 -0.569429318 -0.569429318 NA 1 0 0.998207054
CLCNKB ENSG00000184908.17 0.569443346 4 4 0.414764872 Brain_Substantia_nigra 0.775668199 Brain_Cerebellar_Hemisphere 2.458858098 0.144052236 0.144052236 -0.815537214 0.337655731 -0.195277191 0.592666604 4 0 0.998207054
C11orf57 ENSG00000150776.17 0.569579701 1 1 0.569579701 Brain_Hypothalamus 0.569579701 Brain_Hypothalamus 1 1 1 0.568670604 0.568670604 0.568670604 NA 1 0 0.998207054
CST7 ENSG00000077984.5 0.569584069 1 1 0.569584069 Brain_Cortex 0.569584069 Brain_Cortex 1 1 1 -0.568664168 -0.568664168 -0.568664168 NA 1 0 0.998207054
LINC01116 ENSG00000163364.9 0.569728458 4 4 0.201112748 Brain_Cortex 0.803529533 Brain_Frontal_Cortex_BA9 1.273984282 0.741853347 0.741853347 -1.278387731 -0.248781847 -0.726539918 0.538439353 4 0 0.998207054
PTPN21 ENSG00000070778.12 0.569740376 3 3 0.16779864 Brain_Cerebellum 0.785337955 Brain_Hippocampus 1.444637047 0.558412088 0.558412088 -1.3793118 0.272369457 -0.582188768 0.827337201 3 0 0.998207054
AC144652.1 ENSG00000273117.1 0.569809959 7 2 0.161602852 Brain_Cerebellum 0.454798705 Brain_Anterior_cingulate_cortex_BA24 6.21866927 0.006603691 0.619661625 0.747438845 1.399701075 0.995762302 0.23783389 2 0 0.998207054

LILRB2 ENSG00000131042.14 0.569837124 1 1 0.569837124
Brain_Caudate_basal_ganglia 0.569837124 Brain_Caudate_basal_ganglia 1 1
1 0.568291391 0.568291391 0.568291391 NA 1 0
0.998207054
SLITRK6 ENSG00000184564.8 0.569869089 3 2 0.33126169
Brain_Putamen_basal_ganglia 0.69612357 Brain_Cerebellar_Hemisphere 1.939412058
0.059749205 1.000838737 0.390558548 0.971575673 0.601478995
0.321555639 2 0 0.998207054
LENG9 ENSG00000275183.1 0.569911122 3 3 0.270432496
Brain_Putamen_basal_ganglia 0.795271326 Brain_Amygdala 1.08012251
0.886932542 0.886932542 -0.765308161 1.102067113 0.198743546
0.93516765 3 0 0.998207054
CCNG1 ENSG00000113328.18 0.570087545 3 3 0.178596177
Brain_Putamen_basal_ganglia 0.583666456 Brain_Spinal_cord_cervical_c-1
1.595350208 0.59079182 0.59079182 0.54803706 1.345089984 0.863548011
0.423672121 3 0 0.998207054
RP11-403A3.1 ENSG00000274331.1 0.570150027 4 4 0.110202224
Brain_Cortex 0.567568126 Brain_Hypothalamus 2.132684452
0.265669878 0.265669878 -1.59728486 -0.571636701 -1.053966347
0.529709632 4 0 0.998207054
RP11-324I22.2 ENSG00000233200.1 0.570198229 13 2 0.340584444
Brain_Amygdala 0.97083318 Brain_Hypothalamus 11.33808937 0.005824628
0.724510696 -0.113062377 0.953011106 0.383519263 0.341809857
2 0 0.998207054
RP11-807H17.1 ENSG00000227869.1 0.57034707 4 4 0.145188745
Brain_Cortex 0.39985788 Brain_Putamen_basal_ganglia 3.095010863
0.111151027 0.111151027 -1.456737896 -0.841875081 -1.229791911
0.269069213 4 0 0.998207054
IFIH1 ENSG00000115267.5 0.570383439 1 1 0.570383439 Brain_Cerebellum
0.570383439 Brain_Cerebellum 1 1 1 -0.567486878 -0.567486878
-0.567486878 NA 1 0 0.998207054
PRKCA ENSG00000154229.11 0.570777508 9 9 0.110957041 Brain_Cerebellum
0.86984628 Brain_Putamen_basal_ganglia 2.191710299 0.397027478
0.397027478 -1.593906222 0.970436386 -0.057178298 0.861128749
9 0 0.998207054
HSD17B11 ENSG00000198189.10 0.570786018 3 3 0.199394548
Brain_Substantia_nigra 0.480838052 Brain_Nucleus_accumbens_basal_ganglia
1.778636925 0.416982092 0.416982092 -1.283278425 -0.704955304
-1.009128461 0.29032818 3 0 0.998207054
NKX6-1 ENSG00000163623.9 0.570798761 5 4 0.276923926
Brain_Nucleus_accumbens_basal_ganglia 0.76259349 Brain_Amygdala 2.654325058
0.041281034 0.507284989 -0.302076836 1.087255461 0.523296507
0.559814507 4 0 0.998207054
GRIK4 ENSG00000149403.12 0.570847014 2 2 0.390823009
Brain_Putamen_basal_ganglia 0.505997146 Brain_Caudate_basal_ganglia
1.053098302 0.946901698 0.946901698 -0.665083408 0.858125787
0.09652119 1.077071551 2 0 0.998207054
NUP35 ENSG00000163002.12 0.570880543 7 5 0.119585739
Brain_Putamen_basal_ganglia 0.684677432 Brain_Cerebellar_Hemisphere
5.145201989 0.012841309 0.178178448 -1.556514744 -0.406088695
-1.07288926 0.39084983 5 0 0.998207054
DENND2C ENSG00000175984.14 0.570938164 1 1 0.570938164
Brain_Cerebellum 0.570938164 Brain_Cerebellum 1 1 1 0.566670357
0.566670357 0.566670357 NA 1 0 0.998207054
SPECC1 ENSG00000128487.16 0.57095408 5 4 0.278908739 Brain_Cerebellum
0.976896481 Brain_Cortex 3.756455878 0.034188409 0.144182355
-0.144177026 1.082774023 0.417891606 0.491126972 4 0
0.998207054
NPIPB7 ENSG00000233232.6 0.570977706 11 3 0.048010719
Brain_Amygdala 0.296693712 Brain_Caudate_basal_ganglia 9.419604453
0.004933964 0.364824466 -1.977273535 -1.043549837 -1.274691984
0.266697697 3 0 0.998207054

RAB43 ENSG00000172780.16 0.571005211 6 5 0.288708451 Brain_Cerebellum
0.983640153 Brain_Putamen_basal_ganglia 3.949449625 0.095281938
0.155587322 -0.770633011 1.060959184 0.136548807 0.612276395
5 0 0.998207054
TBC1D3L ENSG00000274512.5 0.571070919 13 1 0.352063411
Brain_Nucleus_accumbens_basal_ganglia 0.973816822 Brain_Caudate_basal_ganglia
12.25166943 1.15E-16 12.25166943 -0.032821639 0.930594403
0.544666788 0.296384814 1 0 0.998207054
RNF115 ENSG00000265491.4 0.571328882 1 1 0.571328882
Brain_Cerebellar_Hemisphere 0.571328882 Brain_Cerebellar_Hemisphere 1 1
1 -0.566095471 -0.566095471 -0.566095471 NA 1 0
0.998207054
ATP10B ENSG00000118322.12 0.571460066 8 2 0.224906209
Brain_Amygdala 0.46997074 Brain_Cerebellum 7.137905144 0.005480185
0.368863108 -1.213585077 -0.722526661 -0.938932308 0.151387746
2 0 0.998207054
RP11-327J17.9 ENSG00000277135.1 0.571500532 3 3 0.360668637
Brain_Cortex 0.770100489 Brain_Hypothalamus 1.586477116
0.622668986 0.622668986 -0.914091751 0.292243455 -0.49820472
0.684859983 3 0 0.998207054
TBC1D19 ENSG00000109680.10 0.571551097 1 1 0.571551097
Brain_Spinal_cord_cervical_c-1 0.571551097 Brain_Spinal_cord_cervical_c-1
1 1 1 0.565768596 0.565768596 0.565768596 NA 1 0
0.998207054
TXNRD2 ENSG00000184470.20 0.571563958 10 10 0.135778625
Brain_Nucleus_accumbens_basal_ganglia 0.770066293 Brain_Frontal_Cortex_BA9
2.163888775 0.117185509 0.117185509 -1.49169689 0.806373836
-0.577602389 0.709190931 10 0 0.998207054
DHRS2 ENSG00000100867.14 0.571690789 2 2 0.290928182
Brain_Spinal_cord_cervical_c-1 0.977556566 Brain_Cortex 1.025147681
0.974852319 0.974852319 -0.028132383 1.056087628 0.513977622
0.766659322 2 0 0.998207054
CAMSAP1 ENSG00000130559.18 0.571720461 1 1 0.571720461
Brain_Putamen_basal_ganglia 0.571720461 Brain_Putamen_basal_ganglia 1 1
1 -0.565519505 -0.565519505 -0.565519505 NA 1 0
0.998207054
MLIP ENSG00000146147.14 0.571728468 2 1 0.565026182
Brain_Cerebellar_Hemisphere 0.579623926 Brain_Cerebellum 1.994030068
0.005969932 1.994030068 0.553934131 0.575392046 0.564663089
0.015173038 1 0 0.998207054
DRC3 ENSG00000171962.17 0.571766798 4 4 0.249246583
Brain_Nucleus_accumbens_basal_ganglia 0.684864444 Brain_Substantia_nigra
2.366715095 0.198734331 0.198734331 -1.079656317 1.152181292
0.330348948 0.988613497 4 0 0.998207054
TIGAR ENSG00000078237.6 0.571855603 3 3 0.261360392
Brain_Hypothalamus 0.927196191 Brain_Nucleus_accumbens_basal_ganglia
1.28560679 0.686997823 0.686997823 -0.709242906 1.12318156
0.168437228 0.918639769 3 0 0.998207054
C9orf153 ENSG00000187753.12 0.571906863 1 1 0.571906863
Brain_Substantia_nigra 0.571906863 Brain_Substantia_nigra 1 1
1 -0.565245397 -0.565245397 -0.565245397 NA 1 0
0.998207054
CCDC97 ENSG00000142039.3 0.571923022 1 1 0.571923022
Brain_Cerebellum 0.571923022 Brain_Cerebellum 1 1 1 -0.565221636
-0.565221636 -0.565221636 NA 1 0 0.998207054
CFAP206 ENSG00000272514.5 0.572015425 8 3 0.143475622
Brain_Nucleus_accumbens_basal_ganglia 0.898938093 Brain_Caudate_basal_ganglia
6.327557036 0.013283452 0.330745335 -1.462969876 -0.127002913
-0.807748044 0.486464811 3 0 0.998207054
USP47 ENSG00000170242.17 0.572083267 4 4 0.249627698
Brain_Cerebellar_Hemisphere 0.74026239 Brain_Cerebellum 1.127129363
0.827969096 0.827969096 -0.738440122 1.15125414 0.454206238

0.876666014 4 0 0.998207054
ERICH1 ENSG00000104714.13 0.572150422 9 8 0.023858646
Brain_Cerebellar_Hemisphere 0.6594089 Brain_Putamen_basal_ganglia 3.758796355
0.106195125 0.185218476 0.440729414 2.259397887 1.08036291
0.564216846 8 0 0.998207054
COL13A1 ENSG00000197467.13 0.572221723 3 3 0.169765693
Brain_Cortex 0.961959177 Brain_Cerebellum 1.724371946 0.274446175
0.274446175 -1.372957071 0.21818555 -0.369025448 0.873599319 3
0 0.998207054
MPP6 ENSG00000105926.15 0.572241654 2 2 0.39294563
Brain_Putamen_basal_ganglia 0.522878919 Brain_Cerebellum 1.020233586
0.979766414 0.979766414 -0.854287646 0.638913941 -0.107686853
1.055852968 2 0 0.998207054
BUB1B ENSG00000156970.12 0.572386604 2 2 0.330682181
Brain_Cerebellar_Hemisphere 0.784126559 Brain_Cerebellum 1.137776282
0.862223718 0.862223718 -0.273945429 0.972740721 0.349397646
0.881540231 2 0 0.998207054
MSX2 ENSG00000120149.8 0.572406339 1 1 0.572406339
Brain_Substantia_nigra 0.572406339 Brain_Substantia_nigra 1 1
1 0.564511113 0.564511113 0.564511113 NA 1 0
0.998207054
IGF2BP2 ENSG00000073792.15 0.57241692 3 3 0.172989664 Brain_Cerebellum
0.955307464 Brain_Spinal_cord_cervical_c-1 1.071952338 0.938551252
0.938551252 -1.362660083 0.343198889 -0.358501435 0.892244073
3 0 0.998207054
RP11-9G1.3 ENSG00000250241.5 0.572442673 5 5 0.218917614
Brain_Caudate_basal_ganglia 0.97080572 Brain_Hippocampus 2.215329559
0.234688002 0.234688002 -1.229411358 0.106019864 -0.603676747
0.648493177 5 0 0.998207054
KDM2A ENSG00000173120.14 0.572461901 1 1 0.572461901
Brain_Nucleus_accumbens_basal_ganglia 0.572461901
Brain_Nucleus_accumbens_basal_ganglia 1 1 1 0.56442945 0.56442945
0.56442945 NA 1 0 0.998207054
DNAH5 ENSG00000039139.9 0.572694985 6 5 0.161333636 Brain_Cortex
0.54465267 Brain_Caudate_basal_ganglia 3.157267382 0.054112671
0.138017923 -1.196832801 1.400600283 0.465885049 1.101796062
5 0 0.998207054
GRIA4 ENSG00000152578.12 0.572738103 1 1 0.572738103
Brain_Cerebellar_Hemisphere 0.572738103 Brain_Cerebellar_Hemisphere 1 1
1 -0.564023553 -0.564023553 -0.564023553 NA 1 0
0.998207054
MEP1A ENSG00000112818.9 0.572910697 1 1 0.572910697
Brain_Caudate_basal_ganglia 0.572910697 Brain_Caudate_basal_ganglia 1 1
1 0.563769962 0.563769962 0.563769962 NA 1 0
0.998207054
TDRD3 ENSG00000083544.13 0.572943926 4 4 0.126121772
Brain_Cerebellar_Hemisphere 0.765608607 Brain_Caudate_basal_ganglia
1.915945607 0.427176853 0.427176853 -1.529575767 0.658415313
-0.021265182 1.01625164 4 0 0.998207054
RNF11 ENSG00000123091.4 0.572989018 1 1 0.572989018
Brain_Caudate_basal_ganglia 0.572989018 Brain_Caudate_basal_ganglia 1 1
1 -0.563654898 -0.563654898 -0.563654898 NA 1 0
0.998207054
SLX4IP ENSG00000149346.14 0.573013962 1 1 0.573013962
Brain_Nucleus_accumbens_basal_ganglia 0.573013962
Brain_Nucleus_accumbens_basal_ganglia 1 1 1 -0.563618253
-0.563618253 -0.563618253 NA 1 0 0.998207054
FANCG ENSG00000221829.9 0.573288377 4 3 0.623156983
Brain_Hypothalamus 0.947070483 Brain_Cortex 3.363853806
0.106610399 0.175658421 -0.491381023 0.302253475 -0.173190135
0.368973885 3 0 0.998207054
GRID1 ENSG00000182771.18 0.573311027 1 1 0.573311027

Brain_Putamen_basal_ganglia 0.573311027 Brain_Putamen_basal_ganglia 1 1
1 -0.563181899 -0.563181899 -0.563181899 NA 1 0
0.998207054
USP33 ENSG00000077254.14 0.573394994 2 2 0.328825918
Brain_Frontal_Cortex_BA9 0.677325094 Brain_Putamen_basal_ganglia
1.712519398 0.287480602 0.287480602 0.416116039 0.976481486
0.696298762 0.396238207 2 0 0.998207054
NRTN ENSG00000171119.2 0.573441842 1 1 0.573441842
Brain_Caudate_basal_ganglia 0.573441842 Brain_Caudate_basal_ganglia 1 1
1 -0.562989781 -0.562989781 -0.562989781 NA 1 0
0.998207054
WDR18 ENSG00000065268.10 0.573444061 13 2 0.183928442
Brain_Spinal_cord_cervical_c-1 0.898148274 Brain_Hypothalamus
11.85071225 0.000869014 0.643660155 0.128000883 1.328756124
0.994878125 0.319536207 2 0 0.998207054
ADO ENSG00000181915.4 0.573451824 2 2 0.359943291
Brain_Nucleus_accumbens_basal_ganglia 0.724868168 Brain_Caudate_basal_ganglia
1.178241338 0.821758662 0.821758662 -0.351960123 0.915473152
0.281756514 0.896210664 2 0 0.998207054
THBS2 ENSG00000186340.14 0.573527535 10 8 0.15759716 Brain_Cerebellum
0.972832029 Brain_Caudate_basal_ganglia 5.367936727 0.069915959
0.20238739 -1.413199207 0.65178138 -0.628973986 0.652226286 8 0
0.998207054
CNKSR1 ENSG00000142675.17 0.573670022 13 4 0.143717752
Brain_Frontal_Cortex_BA9 0.998018857 Brain_Cortex 10.13840383
0.011473563 0.352850222 -0.002482997 1.46208561 0.699861627
0.454556232 4 0 0.998207054
SORCS2 ENSG00000184985.16 0.573739503 3 2 0.37396481
Brain_Spinal_cord_cervical_c-1 0.612876587 Brain_Hippocampus
2.047294451 0.046067354 0.906638195 -0.889071211 0.5059716
-0.351644316 0.750617657 2 0 0.998207054
RP11-138A9.2 ENSG00000273319.1 0.573885558 1 1 0.573885558
Brain_Hypothalamus 0.573885558 Brain_Hypothalamus 1 1 1
0.562338286 0.562338286 0.562338286 NA 1 0 0.998207054
CHRM4 ENSG00000180720.7 0.57392839 1 1 0.57392839
Brain_Spinal_cord_cervical_c-1 0.57392839 Brain_Spinal_cord_cervical_c-1 1
1 1 -0.56227541 -0.56227541 -0.56227541 NA 1 0
0.998207054
DNAJB5 ENSG00000137094.14 0.573996049 1 1 0.573996049
Brain_Substantia_nigra 0.573996049 Brain_Substantia_nigra 1 1
1 0.562176092 0.562176092 0.562176092 NA 1 0
0.998207054
RP4-760C5.3 ENSG00000231081.1 0.573997796 6 3 0.211888565
Brain_Nucleus_accumbens_basal_ganglia 0.43238758 Brain_Amygdala 5.064931454
0.016306431 0.253721227 -1.248389192 -0.785112554 -1.021541928
0.188439056 3 0 0.998207054
KPNA6 ENSG00000025800.13 0.574087138 5 4 0.249752191
Brain_Putamen_basal_ganglia 0.972440908 Brain_Amygdala 2.416616393
0.063085466 0.578805894 -0.8152332 1.150951495 0.132066789
0.890542486 4 0 0.998207054
MARK1 ENSG00000116141.15 0.574150071 3 2 0.318349754 Brain_Amygdala
0.975274049 Brain_Putamen_basal_ganglia 2.165314698 0.001756503
0.832928799 -0.997854837 0.047557152 -0.327097344 0.582219285
2 0 0.998207054
NAT14 ENSG00000090971.4 0.574163566 6 6 0.089840177 Brain_Cortex
0.835413714 Brain_Caudate_basal_ganglia 1.357749785 0.708100638
0.708100638 -1.696241377 0.671656078 -0.217665953 0.913049286
6 0 0.998207054
LINC01322 ENSG00000244128.5 0.574181201 2 2 0.448501249
Brain_Substantia_nigra 0.57768107 Brain_Anterior_cingulate_cortex_BA24
1.209248128 0.790751872 0.790751872 -0.757916038 0.556775142
-0.100570448 0.929627048 2 0 0.998207054

CHIT1 ENSG00000133063.15 0.57418952 2 2 0.344869376
Brain_Frontal_Cortex_BA9 0.50047403 Brain_Cortex 1.913767379
0.086232621 0.086232621 -0.944587764 -0.673744083 -0.809165924
0.191515403 2 0 0.998207054
BAHCC1 ENSG00000266074.8 0.574286072 1 1 0.574286072
Brain_Cerebellar_Hemisphere 0.574286072 Brain_Cerebellar_Hemisphere 1 1
1 0.561750426 0.561750426 0.561750426 NA 1 0
0.998207054
PCDHB8 ENSG00000120322.3 0.574341757 13 3 0.346556579
Brain_Frontal_Cortex_BA9 0.964144155 Brain_Nucleus_accumbens_basal_ganglia
10.40703654 0.001707215 0.482062719 -0.274945542 0.94128939
0.301730762 0.426952524 3 0 0.998207054
CHMP2A ENSG00000130724.8 0.574420179 2 2 0.435390087
Brain_Cerebellar_Hemisphere 0.863758848 Brain_Cerebellum 1.783512106
0.216487894 0.216487894 0.171591299 0.78000134 0.47579632
0.430210865 2 0 0.998207054
ZWINT ENSG00000122952.16 0.574439558 2 2 0.326491111
Brain_Hippocampus 0.555886665 Brain_Nucleus_accumbens_basal_ganglia
1.220464627 0.779535373 0.779535373 0.58896215 0.981206112
0.785084131 0.277358366 2 0 0.998207054
ADRB1 ENSG00000043591.5 0.574478025 1 1 0.574478025 Brain_Cortex
0.574478025 Brain_Cortex 1 1 1 0.561468754 0.561468754
0.561468754 NA 1 0 0.998207054
DHRS9 ENSG00000073737.16 0.574544168 1 1 0.574544168
Brain_Substantia_nigra 0.574544168 Brain_Substantia_nigra 1 1
1 -0.561371706 -0.561371706 -0.561371706 NA 1 0
0.998207054
HCG27 ENSG00000206344.7 0.574576513 13 5 0.066496364
Brain_Frontal_Cortex_BA9 0.452139878 Brain_Spinal_cord_cervical_c-1
9.690257973 0.005108533 0.364613595 0.751852313 1.835062944
1.397057699 0.351312225 5 0 0.998207054
ZNF337 ENSG00000130684.13 0.574582401 6 4 0.237733261
Brain_Cortex 0.941702413 Brain_Nucleus_accumbens_basal_ganglia
4.370174297 0.033860325 0.236713166 -0.165551695 1.180671466
0.674191107 0.621589535 4 0 0.998207054
KLHL25 ENSG00000183655.12 0.574601796 4 4 0.110455898
Brain_Anterior_cingulate_cortex_BA24 0.900667905 Brain_Cerebellum
1.807472281 0.35747267 0.35747267 0.124817661 1.596147354
0.738335536 0.625283554 4 0 0.998207054
ADPGK ENSG00000159322.17 0.574605195 2 2 0.294008682
Brain_Hippocampus 0.55361297 Brain_Caudate_basal_ganglia 1.626499456
0.373500544 0.373500544 0.592354885 1.049368213 0.820861549
0.323157224 2 0 0.998207054
KLF7 ENSG00000118263.14 0.574628061 3 3 0.31550094
Brain_Cerebellar_Hemisphere 0.480458772 Brain_Anterior_cingulate_cortex_BA24
1.131316313 0.85103092 0.85103092 -0.851180673 1.003746245
-0.184333102 1.03147971 3 0 0.998207054
PHLDB1 ENSG00000019144.18 0.574812868 2 1 0.433707839
Brain_Cerebellar_Hemisphere 0.739787799 Brain_Cerebellum 1.975269811
0.024730189 1.975269811 0.332134369 0.782862537 0.557498453
0.318712944 1 0 0.998207054
SOCS5 ENSG00000171150.7 0.574866049 3 3 0.336532035
Brain_Putamen_basal_ganglia 0.663938956 Brain_Frontal_Cortex_BA9
1.315545139 0.682239501 0.682239501 -0.434481321 0.961040043
0.436057635 0.759227874 3 0 0.998207054
RP4-564F22.7 ENSG00000275401.1 0.574898555 6 3 0.190725167
Brain_Cerebellar_Hemisphere 0.993122868 Brain_Nucleus_accumbens_basal_ganglia
5.073817553 0.012742128 0.309543732 -1.30843689 0.008619314
-0.447787836 0.447904343 3 0 0.998207054
RP4-811H24.9 ENSG00000233775.1 0.574921937 1 1 0.574921937
Brain_Putamen_basal_ganglia 0.574921937 Brain_Putamen_basal_ganglia 1 1
1 0.560817528 0.560817528 0.560817528 NA 1 0

0.998207054
APRT ENSG00000198931.10 0.574952066 6 4 0.095430485 Brain_Cortex
0.337145483 Brain_Nucleus_accumbens_basal_ganglia 4.224501986
0.066655949 0.235313069 0.959820659 1.667422185 1.322793136
0.250036534 4 0 0.998207054
MOV10L1 ENSG00000073146.15 0.574953292 6 6 0.400404884
Brain_Nucleus_accumbens_basal_ganglia 0.900804078 Brain_Hypothalamus
3.1419631 0.168606464 0.168606464 -0.519015091 0.840898347
0.200300433 0.563022641 6 0 0.998207054
TFR2 ENSG00000106327.12 0.574954497 1 1 0.574954497
Brain_Nucleus_accumbens_basal_ganglia 0.574954497
Brain_Nucleus_accumbens_basal_ganglia 1 1 1 0.56076977 0.56076977
0.56076977 NA 1 0 0.998207054
NECTIN4 ENSG00000143217.8 0.574964805 1 1 0.574964805
Brain_Cortex 0.574964805 Brain_Cortex 1 1 1 0.560754651
0.560754651 0.560754651 NA 1 0 0.998207054
NKD2 ENSG00000145506.13 0.57501007 10 3 0.038227134
Brain_Caudate_basal_ganglia 0.658852745 Brain_Cortex 7.688272001
0.01365243 0.544877841 0.441497674 2.072410935 1.161446946
0.462026877 3 0 0.998207054
EIF2B2 ENSG00000119718.10 0.575037384 11 6 0.220405769
Brain_Frontal_Cortex_BA9 0.853428288 Brain_Hippocampus 7.182931425
0.007997591 0.259959739 -0.890312447 1.225449864 0.474554846
0.64670111 6 0 0.998207054
KLF5 ENSG00000102554.13 0.575297977 2 2 0.295921203
Brain_Cerebellar_Hemisphere 0.408998208 Brain_Cerebellum 1.712686301
0.287313699 0.287313699 -1.045220213 -0.825657851 -0.935439032
0.155254036 2 0 0.998207054
ARPC5 ENSG00000162704.15 0.575454874 2 2 0.2966822 Brain_Hypothalamus
0.461608955 Brain_Cerebellar_Hemisphere 1.781426235 0.218573765
0.218573765 -1.043574707 -0.736200062 -0.889887384 0.217346696
2 0 0.998207054
CTA-14H9.5 ENSG00000261353.1 0.575463634 1 1 0.575463634
Brain_Frontal_Cortex_BA9 0.575463634 Brain_Frontal_Cortex_BA9 1 1
1 -0.56002317 -0.56002317 -0.56002317 NA 1 0
0.998207054
FAM27C ENSG00000154537.4 0.575528102 1 1 0.575528102
Brain_Cerebellum 0.575528102 Brain_Cerebellum 1 1 1 -0.559928656
-0.559928656 -0.559928656 NA 1 0 0.998207054
RP11-374M1.5 ENSG00000235659.1 0.575528102 1 1 0.575528102
Brain_Nucleus_accumbens_basal_ganglia 0.575528102
Brain_Nucleus_accumbens_basal_ganglia 1 1 1 -0.559928656
-0.559928656 -0.559928656 NA 1 0 0.998207054
RP11-211N8.2 ENSG00000226007.2 0.575528102 2 1 0.575528102
Brain_Cerebellar_Hemisphere 0.575528102 Brain_Cerebellar_Hemisphere 2
3.53E-16 2 -0.559928656 -0.559928656 -0.559928656 0 1 0
0.998207054
SMU1 ENSG00000122692.8 0.575553693 2 2 0.343213145
Brain_Nucleus_accumbens_basal_ganglia 0.584582349 Brain_Hippocampus
1.099807675 0.900192325 0.900192325 0.546703646 0.947835618
0.747269632 0.283643138 2 0 0.998207054
SPATA6 ENSG00000132122.11 0.575562299 8 6 0.115176568
Brain_Caudate_basal_ganglia 0.974225892 Brain_Cerebellum 4.144855173
0.005689458 0.253701625 -1.575346147 0.305111719 -0.707702623
0.634213189 6 0 0.998207054
DNTTIP2 ENSG00000067334.13 0.575593679 1 1 0.575593679
Brain_Amygdala 0.575593679 Brain_Amygdala 1 1 1 0.559832521
0.559832521 0.559832521 NA 1 0 0.998207054
POLR2C ENSG00000102978.12 0.575651495 7 6 0.239609079
Brain_Substantia_nigra 0.678656117 Brain_Caudate_basal_ganglia
3.892935914 0.017536088 0.248796651 -1.175964459 1.104793294
0.448105304 0.884929584 6 0 0.998207054

CDH11 ENSG00000140937.13 0.575792577 4 4 0.192938762 Brain_Cerebellum
0.95661696 Brain_Anterior_cingulate_cortex_BA24 1.497364091 0.438120673
0.438120673 -1.301934546 0.197269146 -0.458250228 0.709367987
4 0 0.998207054
BVES ENSG00000112276.13 0.57580188 4 4 0.285212902
Brain_Cerebellar_Hemisphere 0.545849669 Brain_Hippocampus 1.2248553
0.813611547 0.813611547 -1.068682185 0.88082567 0.297058008
0.917587672 4 0 0.998207054
RAB26 ENSG00000167964.12 0.575889202 1 1 0.575889202 Brain_Cerebellum
0.575889202 Brain_Cerebellum 1 1 1 -0.559399354 -0.559399354
-0.559399354 NA 1 0 0.998207054
CILP ENSG00000138615.5 0.575985361 2 2 0.340629477 Brain_Cerebellum
0.758400106 Brain_Hypothalamus 1.13661679 0.86338321 0.86338321
-0.952922228 -0.30758241 -0.630252319 0.456324161 2 0
0.998207054
C1orf198 ENSG00000119280.16 0.576016157 1 1 0.576016157
Brain_Hippocampus 0.576016157 Brain_Hippocampus 1 1 1
-0.559213301 -0.559213301 -0.559213301 NA 1 0 0.998207054
SLC6A15 ENSG00000072041.16 0.576024597 2 2 0.382619282
Brain_Hippocampus 0.578780262 Brain_Cortex 1.032556391
0.967443609 0.967443609 -0.555167258 0.873080353 0.158956548
1.009923571 2 0 0.998207054
FOXL2 ENSG00000183770.5 0.576261019 1 1 0.576261019
Brain_Cerebellar_Hemisphere 0.576261019 Brain_Cerebellar_Hemisphere 1 1
1 -0.558854508 -0.558854508 -0.558854508 NA 1 0
0.998207054
CFAP57 ENSG00000243710.7 0.576270675 2 2 0.552566607
Brain_Frontal_Cortex_BA9 0.95829265 Brain_Cortex 1.851781332
0.148218668 0.148218668 0.052296239 0.593918527 0.323107383
0.382984793 2 0 0.998207054
CHST11 ENSG00000171310.10 0.576377707 2 2 0.300692355
Brain_Cerebellar_Hemisphere 0.803177727 Brain_Cerebellum 1.071508255
0.928491745 0.928491745 -1.034949802 0.249236655 -0.392856573
0.908056953 2 0 0.998207054
CYP4F12 ENSG00000186204.14 0.5764221 8 7 0.139330284
Brain_Putamen_basal_ganglia 0.892896821 Brain_Anterior_cingulate_cortex_BA24
4.707164527 0.066520245 0.244790752 -0.161937316 1.478289587
0.54796366 0.648458061 7 0 0.998207054
MYO1F ENSG00000142347.16 0.5764575 2 2 0.299235944
Brain_Caudate_basal_ganglia 0.924965861 Brain_Frontal_Cortex_BA9
1.236080698 0.763919302 0.763919302 0.094180391 1.038073271
0.566126831 0.667433056 2 0 0.998207054
RP11-760H22.2 ENSG00000254343.2 0.576460061 2 2 0.301245584
Brain_Spinal_cord_cervical_c-1 0.835139401 Brain_Cerebellar_Hemisphere
1.364985138 0.635014862 0.635014862 -1.03376597 0.208114711
-0.41282563 0.87814225 2 0 0.998207054
HMCES ENSG00000183624.13 0.576492339 1 1 0.576492339 Brain_Cortex
0.576492339 Brain_Cortex 1 1 1 0.558515624 0.558515624
0.558515624 NA 1 0 0.998207054
LINC00578 ENSG00000228221.5 0.576554138 1 1 0.576554138
Brain_Cortex 0.576554138 Brain_Cortex 1 1 1 -0.558425099
-0.558425099 -0.558425099 NA 1 0 0.998207054
LNX1 ENSG00000072201.13 0.576859347 2 2 0.301869424
Brain_Frontal_Cortex_BA9 0.773995404 Brain_Cerebellum 1.099385512
0.900614488 0.900614488 0.287152698 1.032432778 0.659792738
0.526992598 2 0 0.998207054
ZC3H7B ENSG00000100403.11 0.576885384 2 2 0.313651916
Brain_Cerebellum 0.483323384 Brain_Frontal_Cortex_BA9 1.435191695
0.564808305 0.564808305 -1.007588784 -0.700967361 -0.854278073
0.216814087 2 0 0.998207054
CBL ENSG00000110395.6 0.577064475 9 4 0.146073244
Brain_Substantia_nigra 0.884397125 Brain_Amygdala 5.607815759

0.004175789 0.359674413 -0.655434783 1.4535423 0.28478689
0.808142942 4 0 0.998207054
LINC02139 ENSG00000278214.1 0.57711698 2 2 0.329279512
Brain_Putamen_basal_ganglia 0.488566698 Brain_Caudate_basal_ganglia
1.339386249 0.660613751 0.660613751 -0.975566137 -0.692590302
-0.83407822 0.200094132 2 0 0.998207054
RHOJ ENSG00000126785.12 0.577255836 1 1 0.577255836 Brain_Cortex
0.577255836 Brain_Cortex 1 1 1 -0.557397555 -0.557397555
-0.557397555 NA 1 0 0.998207054
SUMO3 ENSG00000184900.15 0.577294416 1 1 0.577294416
Brain_Anterior_cingulate_cortex_BA24 0.577294416
Brain_Anterior_cingulate_cortex_BA24 1 1 1 0.557341076
0.557341076 0.557341076 NA 1 0 0.998207054
COX10 ENSG00000006695.10 0.577299049 3 3 0.187635949
Brain_Hippocampus 0.833031626 Brain_Substantia_nigra 1.141687989
0.841202233 0.841202233 -1.317604885 -0.21081501 -0.620493379
0.606802532 3 0 0.998207054
SCAPER ENSG00000140386.12 0.577309211 8 6 0.079846152
Brain_Anterior_cingulate_cortex_BA24 0.983422186 Brain_Hippocampus
4.164104886 0.046946673 0.139753159 -1.751579431 0.288124984
-0.340066432 0.753926688 6 0 0.998207054
ESPL1 ENSG00000135476.11 0.577333241 5 2 0.295581574
Brain_Substantia_nigra 0.669366258 Brain_Nucleus_accumbens_basal_ganglia
4.148705703 0.024107804 0.739704045 0.427017944 1.04595551
0.652002765 0.233276943 2 0 0.998207054
UTS2 ENSG00000049247.13 0.577362321 3 3 0.208845387 Brain_Cerebellum
0.458736073 Brain_Spinal_cord_cervical_c-1 1.881589028 0.143894416
0.143894416 -1.256746657 0.74092969 -0.559068799 1.126840851 3
0 0.998207054
GM2A ENSG00000196743.8 0.577423675 5 4 0.265847228
Brain_Spinal_cord_cervical_c-1 0.988079412 Brain_Cerebellum 2.892746405
0.088708717 0.461264304 -0.963921623 1.112676882 0.008460016
0.769352052 4 0 0.998207054
ARID5A ENSG00000196843.15 0.57743134 2 2 0.296255756
Brain_Nucleus_accumbens_basal_ganglia 0.830374273 Brain_Caudate_basal_ganglia
1.123597294 0.876402706 0.876402706 0.214221573 1.04449646
0.629359017 0.587093003 2 0 0.998207054
CHMP1B ENSG00000255112.2 0.577597206 2 2 0.325985757
Brain_Spinal_cord_cervical_c-1 0.63783749 Brain_Nucleus_accumbens_basal_ganglia
1.110244515 0.889755485 0.889755485 -0.470724495 0.982231613
0.255753559 1.027395117 2 0 0.998207054
HDAC2 ENSG00000196591.11 0.577612727 1 1 0.577612727
Brain_Substantia_nigra 0.577612727 Brain_Substantia_nigra 1 1
1 0.556875161 0.556875161 0.556875161 NA 1 0
0.998207054
TXK ENSG00000074966.10 0.577639443 1 1 0.577639443
Brain_Anterior_cingulate_cortex_BA24 0.577639443
Brain_Anterior_cingulate_cortex_BA24 1 1 1 -0.556836061
-0.556836061 -0.556836061 NA 1 0 0.998207054
ZDHHC14 ENSG00000175048.16 0.577703707 4 4 0.326407705
Brain_Cerebellum 0.574925292 Brain_Substantia_nigra 1.546700641
0.683099574 0.683099574 -0.981375294 0.58903195 -0.438264355
0.706188518 4 0 0.998207054
BBX ENSG00000114439.18 0.577745354 2 2 0.300880342 Brain_Amygdala
0.949352899 Brain_Cerebellum 1.096559938 0.903440062 0.903440062
-1.034547374 0.063519416 -0.485513979 0.776450473 2 0
0.998207054
AC008060.7 ENSG00000218672.1 0.577770861 10 10 0.116444683
Brain_Caudate_basal_ganglia 0.795341425 Brain_Hypothalamus 1.611219862
0.426600401 0.426600401 -1.412697451 1.569872902 0.378359254
0.993761627 10 0 0.998207054
PLCH2 ENSG00000149527.17 0.577787061 4 4 0.289473062

Brain_Nucleus_accumbens_basal_ganglia 0.663574132 Brain_Cerebellum
1.168440238 0.830628186 0.830628186 -0.434983875 1.059278284
0.612072007 0.701489641 4 0 0.998207054
ZNF584 ENSG00000171574.17 0.577940726 12 3 0.40701172
Brain_Spinal_cord_cervical_c-1 0.924939359
Brain_Nucleus_accumbens_basal_ganglia 9.305738578 0.003953623
0.339614996 -0.82916381 0.34256969 -0.172353957 0.429314687 3
0 0.998207054
CH17-189H20.1 ENSG00000274265.4 0.577962539 6 5 0.228641569
Brain_Frontal_Cortex_BA9 0.926625809 Brain_Hippocampus 2.987366576
8.13E-17 0.190542051 -0.770849228 1.203865498 -0.038919816
0.912768655 5 0 0.998207054
ZC3H6 ENSG00000188177.13 0.578011419 2 2 0.308150402
Brain_Hippocampus 0.689077497 Brain_Hypothalamus 1.159857849
0.840142151 0.840142151 0.400107287 1.019110727 0.709609007
0.43770153 2 0 0.998207054
PSMD5 ENSG00000095261.13 0.578017351 2 2 0.296511987
Brain_Caudate_basal_ganglia 0.424403292 Brain_Putamen_basal_ganglia
1.710923742 0.289076258 0.289076258 0.798805343 1.043942513
0.921373928 0.173338155 2 0 0.998207054
TCTN3 ENSG00000119977.20 0.5780974 8 5 0.357007501
Brain_Spinal_cord_cervical_c-1 0.913588677 Brain_Cerebellar_Hemisphere
4.348072468 0.05734012 0.257845335 -0.921082223 0.461669743
-0.253615055 0.456439942 5 0 0.998207054
ZNF324B ENSG00000249471.7 0.578192764 3 3 0.485212338
Brain_Cerebellum 0.871866796 Brain_Cerebellar_Hemisphere 2.630429236
0.118770611 0.118770611 -0.198265462 0.697943806 0.220322021
0.451011695 3 0 0.998207054
CDH12 ENSG00000154162.14 0.578315548 4 4 0.091816726
Brain_Putamen_basal_ganglia 0.946002473 Brain_Spinal_cord_cervical_c-1
1.857361203 0.176778619 0.176778619 -1.68589136 0.102147467
-0.398108354 0.861999486 4 0 0.998207054
TBC1D16 ENSG00000167291.15 0.578469394 4 4 0.324519356
Brain_Cerebellum 0.899816738 Brain_Frontal_Cortex_BA9 1.644455002
0.398778681 0.398778681 -0.342833668 0.985213204 0.294291596
0.630897902 4 0 0.998207054
SDF4 ENSG00000078808.16 0.578516697 2 2 0.296953253 Brain_Cortex
0.391862583 Brain_Hypothalamus 1.770768877 0.229231123
0.229231123 -1.04298929 -0.856244445 -0.949616868 0.132048546
2 0 0.998207054
RBMS3 ENSG00000144642.21 0.578600219 1 1 0.578600219
Brain_Anterior_cingulate_cortex_BA24 0.578600219
Brain_Anterior_cingulate_cortex_BA24 1 1 1 -0.555430524
-0.555430524 -0.555430524 NA 1 0 0.998207054
SHD ENSG00000105251.10 0.57869301 7 6 0.172102586
Brain_Cerebellar_Hemisphere 0.948955488 Brain_Nucleus_accumbens_basal_ganglia
2.467313741 0.009742184 0.14284737 -1.096537299 1.365478881
0.535438806 0.883343395 6 0 0.998207054
EXOSC3 ENSG00000107371.12 0.579049859 4 4 0.280944352
Brain_Frontal_Cortex_BA9 0.717544509 Brain_Nucleus_accumbens_basal_ganglia
2.485885001 0.298148191 0.298148191 -0.779396545 1.07820036
-0.199513465 0.872162924 4 0 0.998207054
LINC01115 ENSG00000272342.1 0.579077226 2 1 0.556185302
Brain_Anterior_cingulate_cortex_BA24 0.604958221 Brain_Hypothalamus
1.986806067 0.013193933 1.986806067 -0.588517037 -0.517283571
-0.552900304 0.050369667 1 0 0.998207054
YTHDC2 ENSG00000047188.15 0.579200866 3 2 0.262728898
Brain_Cerebellum 0.606384042 Brain_Cerebellar_Hemisphere 2.57443152
0.066169812 0.359398668 -1.119964481 -0.51524183 -0.884368561
0.323720913 2 0 0.998207054
ITGB1BP1 ENSG00000119185.12 0.579297706 1 1 0.579297706
Brain_Anterior_cingulate_cortex_BA24 0.579297706

Brain_Anterior_cingulate_cortex_BA24 1 1 1 0.554410844
0.554410844 0.554410844 NA 1 0 0.998207054
TMEM179 ENSG00000258986.6 0.579299982 1 1 0.579299982
Brain_Caudate_basal_ganglia 0.579299982 Brain_Caudate_basal_ganglia 1 1
1 0.554407518 0.554407518 0.554407518 NA 1 0
0.998207054
RP11-400K9.4 ENSG00000237807.3 0.579385335 2 2 0.415405611
Brain_Nucleus_accumbens_basal_ganglia 0.501521601 Brain_Hippocampus
1.021866901 0.978133099 0.978133099 -0.81441786 0.672097542
-0.071160159 1.051125121 2 0 0.998207054
PCNA ENSG00000132646.10 0.579514528 1 1 0.579514528
Brain_Anterior_cingulate_cortex_BA24 0.579514528
Brain_Anterior_cingulate_cortex_BA24 1 1 1 -0.554093983
-0.554093983 -0.554093983 NA 1 0 0.998207054
COL6A6 ENSG00000206384.10 0.579514627 1 1 0.579514627
Brain_Putamen_basal_ganglia 0.579514627 Brain_Putamen_basal_ganglia 1 1
1 -0.554093838 -0.554093838 -0.554093838 NA 1 0
0.998207054
RP11-57A19.2 ENSG00000246465.1 0.579570004 1 1 0.579570004
Brain_Cerebellum 0.579570004 Brain_Cerebellum 1 1 1 -0.55401292
-0.55401292 -0.55401292 NA 1 0 0.998207054
PPCDC ENSG00000138621.11 0.579597321 10 2 0.349266095 Brain_Amygdala
0.851344833 Brain_Putamen_basal_ganglia 8.93343852 0.011563565
0.312278704 0.187402793 0.936013775 0.565786187 0.294487304
2 0 0.998207054
OR6B3 ENSG00000178586.4 0.579624541 1 1 0.579624541
Brain_Hippocampus 0.579624541 Brain_Hippocampus 1 1 1
-0.553933232 -0.553933232 -0.553933232 NA 1 0 0.998207054
ABL1 ENSG00000097007.17 0.579645048 4 4 0.179933421
Brain_Hypothalamus 0.706976955 Brain_Spinal_cord_cervical_c-1 1.24008321
0.769285403 0.769285403 -0.424430827 1.340960058 0.540630229
0.754438552 4 0 0.998207054
MOXD1 ENSG00000079931.14 0.579668656 2 2 0.30874018
Brain_Cerebellar_Hemisphere 0.721026215 Brain_Hippocampus 1.123126902
0.876873098 0.876873098 -0.357087617 1.017869076 0.330390729
0.972241201 2 0 0.998207054
ABHD15 ENSG00000168792.4 0.579709715 4 2 0.398044274
Brain_Cerebellum 0.77749997 Brain_Putamen_basal_ganglia 3.539826319
0.026537633 0.383127182 -0.845119227 0.282578491 -0.286281698
0.460447275 2 0 0.998207054
SYNJ2BP ENSG00000213463.4 0.579722311 2 2 0.315143363
Brain_Frontal_Cortex_BA9 0.723094392 Brain_Cerebellum 1.069368041
0.930631959 0.930631959 -1.004488186 -0.354326258 -0.679407222
0.459733908 2 0 0.998207054
SP5 ENSG00000204335.3 0.579762833 2 2 0.333158762 Brain_Amygdala
0.474772184 Brain_Anterior_cingulate_cortex_BA24 1.360720578
0.639279422 0.639279422 -0.967770975 -0.714736008 -0.841253492
0.178922742 2 0 0.998207054
FCER1A ENSG00000179639.10 0.579898207 4 4 0.126104829
Brain_Anterior_cingulate_cortex_BA24 0.783929974 Brain_Caudate_basal_ganglia
1.409541434 0.606015723 0.606015723 -1.529644172 0.480081045
-0.539804423 0.852037249 4 0 0.998207054
TLE3 ENSG00000140332.15 0.579898461 1 1 0.579898461
Brain_Hippocampus 0.579898461 Brain_Hippocampus 1 1 1
0.553533042 0.553533042 0.553533042 NA 1 0 0.998207054
TAF1B ENSG00000115750.16 0.579898683 2 2 0.313549591 Brain_Cerebellum
0.469806137 Brain_Cerebellar_Hemisphere 1.856603679 0.143396321
0.143396321 0.722794516 1.007801863 0.86529819 0.201530628 2
0 0.998207054
RP11-756G20.1 ENSG00000277851.1 0.580181524 2 2 0.419987634
Brain_Substantia_nigra 0.530687488 Brain_Spinal_cord_cervical_c-1
1.042917142 0.957082858 0.957082858 -0.6269569 0.806442701

0.089742901	1.013566578	2	0	0.998207054
THG1L	ENSG00000113272.13	0.580186187	6	5	0.146336367
Brain_Caudate_basal_ganglia	0.903512098	Brain_Spinal_cord_cervical_c-1
2.2175651	0.022829845	0.760186255	-0.391936559	1.452594527
0.473655499	0.695100078	5	0	0.998207054
UQCRH	ENSG00000173660.11	0.580298192	2	2	0.29925572
Brain_Nucleus_accumbens_basal_ganglia	0.401515132	Brain_Cortex
1.859615468	0.140384532	0.140384532	0.838918345	1.038030791
0.938474568	0.14079376	2	0	0.998207054
EPHB2	ENSG00000133216.16	0.580337496	3	3	0.20020935	Brain_Cortex
0.728848851	Brain_Putamen_basal_ganglia	1.856561352	0.177846269
0.177846269	-0.369367162	1.280955349	0.188310321	0.946326478
3	0	0.998207054
PUF60	ENSG00000179950.13	0.580407679	1	1	0.580407679	Brain_Cerebellum
0.580407679	Brain_Cerebellum	1	1	1	-0.552789325	-0.552789325
-0.552789325	NA	1	0	0.998207054
ACCSL	ENSG00000205126.2	0.580434729	1	1	0.580434729
Brain_Cerebellar_Hemisphere	0.580434729	Brain_Cerebellar_Hemisphere	1	1
1	0.552749827	0.552749827	0.552749827	NA	1	0
0.998207054
BSN	ENSG00000164061.4	0.580441462	1	1	0.580441462	Brain_Cortex
0.580441462	Brain_Cortex	1	1	1	-0.552739995	-0.552739995
-0.552739995	NA	1	0	0.998207054
PRKRIP1	ENSG00000128563.13	0.580715524	8	6	0.191464161
Brain_Cortex	0.891723146	Brain_Spinal_cord_cervical_c-1	4.910852059
0.095926353	0.221681724	-0.319653218	1.306259983	0.64598707
0.604978303	6	0	0.998207054
RP11-351J23.2	ENSG00000272549.1	0.580728514	2	2	0.324732864
Brain_Caudate_basal_ganglia	0.725255468	Brain_Nucleus_accumbens_basal_ganglia
1.009321322	0.990678678	0.990678678	0.351443746	0.98477854
0.668111143	0.447835328	2	0	0.998207054
CD5	ENSG00000110448.10	0.580743161	3	2	0.314851564	Brain_Amygdala
0.769686374	Brain_Cerebellum	2.000947365	0.006739522	0.992313113
0.292785157	1.005094051	0.560985331	0.387367852	2	0
0.998207054
SSU72	ENSG00000160075.11	0.580802531	6	3	0.164476431	Brain_Cortex
0.980201802	Brain_Caudate_basal_ganglia	4.295613974	0.024346656
0.558811478	-1.390172733	0.024815909	-0.439274801	0.504299662
3	0	0.998207054
CNOT8	ENSG00000155508.13	0.580819538	7	4	0.084760591
Brain_Nucleus_accumbens_basal_ganglia	0.230436812	Brain_Substantia_nigra
5.548669974	0.032217631	0.237040825	-1.723707887	-1.199234406
-1.439301369	0.183838938	4	0	0.998207054
APOC2	ENSG00000234906.9	0.580984767	5	2	0.246850257
Brain_Nucleus_accumbens_basal_ganglia	0.584815838	Brain_Cerebellum
3.859996616	0.002585323	0.951167277	-0.561989283	1.158033766
0.474915825	0.630175946	2	0	0.998207054
VAPB	ENSG00000124164.15	0.580989831	13	2	0.283202783
Brain_Hypothalamus	0.974498099	Brain_Substantia_nigra	11.24319722
0.002691594	0.96573925	-1.073152312	0.237557866	-0.46537504
0.368057623	2	0	0.998207054
NPM3	ENSG00000107833.10	0.581026482	6	4	0.307833408
Brain_Hippocampus	0.809029259	Brain_Amygdala	4.179567116
0.080347242	0.174060555	-1.01977874	0.688562159	0.246995117
0.641982895	4	0	0.998207054
LINS1	ENSG00000140471.16	0.581048982	3	3	0.300550695
Brain_Anterior_cingulate_cortex_BA24	0.979561394	Brain_Cerebellum
2.250872395	0.232923385	0.232923385	-0.025618796	1.035253167
0.585278809	0.548438282	3	0	0.998207054
SLC25A15	ENSG00000102743.14	0.58115412	1	1	0.58115412
Brain_Substantia_nigra	0.58115412	Brain_Substantia_nigra	1	1	1
0.551699693	0.551699693	0.551699693	NA	1	0	0.998207054

NAALAD2 ENSG00000077616.10 0.581172139 9 5 0.320070158 Brain_Substantia_nigra 0.936182473 Brain_Caudate_basal_ganglia 5.754004797 0.027780438 0.240286163 -0.809885872 0.994313721 -0.131224327 0.602182372 5 0 0.998207054
NUPL2 ENSG00000136243.16 0.581191517 13 1 0.277408751 Brain_Putamen_basal_ganglia 0.779397649 Brain_Spinal_cord_cervical_c-1 12.23274307 0.005991825 12.23274307 0.280104087 1.086158778 0.53755879 0.225839905 1 0 0.998207054
HNRNPA3 ENSG00000170144.20 0.581338978 1 1 0.581338978 Brain_Hippocampus 0.581338978 Brain_Hippocampus 1 1 1 -0.551429945 -0.551429945 -0.551429945 NA 1 0 0.998207054
CCDC74A ENSG00000163040.14 0.581423061 9 4 0.125952653 Brain_Cortex 0.598698929 Brain_Nucleus_accumbens_basal_ganglia 7.344801133 0.033522532 0.292308298 -1.530258916 -0.52627244 -0.853300691 0.343629804 4 0 0.998207054
ABHD16A ENSG00000204427.11 0.581481857 2 2 0.407323301 Brain_Caudate_basal_ganglia 0.471076684 Brain_Cerebellar_Hemisphere 1.113607069 0.886392931 0.886392931 -0.828613227 0.720728321 -0.053942453 1.095549915 2 0 0.998207054
DNAJC18 ENSG00000170464.9 0.581562724 6 3 0.213429452 Brain_Spinal_cord_cervical_c-1 0.786695777 Brain_Cerebellar_Hemisphere 4.184858946 0.021438616 0.64613176 -1.244190521 0.270603794 -0.927666225 0.590384466 3 0 0.998207054
INPP5D ENSG00000168918.13 0.581637344 1 1 0.581637344 Brain_Cerebellar_Hemisphere 0.581637344 Brain_Cerebellar_Hemisphere 1 1 1 -0.550994647 -0.550994647 -0.550994647 NA 1 0 0.998207054
CANX ENSG00000127022.14 0.581648314 13 7 0.071914448 Brain_Spinal_cord_cervical_c-1 0.923622487 Brain_Hypothalamus 8.58967953 0.004380389 0.342338469 0.09587168 1.799659323 1.177276777 0.600296916 7 0 0.998207054
C2orf74 ENSG00000237651.6 0.581725274 13 1 0.485074685 Brain_Amygdala 0.652481244 Brain_Spinal_cord_cervical_c-1 12.85197267 6.60E-05 12.85197267 0.450317887 0.698163924 0.548035831 0.08607805 1 0 0.998207054
HPSE ENSG00000173083.14 0.581786023 3 3 0.279194208 Brain_Hippocampus 0.404828492 Brain_Cerebellum 1.807094609 0.192624722 0.192624722 0.833028792 1.082131259 0.980465184 0.130706546 3 0 0.998207054
ATXN2L ENSG00000168488.18 0.582006509 1 1 0.582006509 Brain_Caudate_basal_ganglia 0.582006509 Brain_Caudate_basal_ganglia 1 1 1 0.550456202 0.550456202 0.550456202 NA 1 0 0.998207054
AKT1 ENSG00000142208.15 0.582067604 4 4 0.114586289 Brain_Substantia_nigra 0.697673371 Brain_Cerebellar_Hemisphere 2.275087524 0.091739949 0.091739949 0.38846308 1.577909992 0.779408034 0.543063976 4 0 0.998207054
MLLT11 ENSG00000213190.3 0.58210384 2 2 0.333414039 Brain_Anterior_cingulate_cortex_BA24 0.641098049 Brain_Cerebellum 1.087592665 0.912407335 0.912407335 -0.466164093 0.967260071 0.250547989 1.013583946 2 0 0.998207054
RP11-162A12.4 ENSG00000278107.1 0.582152183 2 2 0.298244225 Brain_Caudate_basal_ganglia 0.402490605 Brain_Nucleus_accumbens_basal_ganglia 1.807252205 0.192747795 0.192747795 0.837181411 1.040205956 0.938693683 0.143560032 2 0 0.998207054
YWHAH ENSG00000128245.14 0.582211655 3 3 0.291485509 Brain_Cerebellar_Hemisphere 0.732539723 Brain_Cerebellum 1.469584598 0.614963851 0.614963851 -1.054868417 0.341749093 -0.390427903 0.700768357 3 0 0.998207054
OGFR ENSG00000060491.16 0.582326396 11 10 0.13290153 Brain_Amygdala 0.900460591 Brain_Frontal_Cortex_BA9 3.885868063 0.121522857 0.295784339 -0.957682389 1.50275773 0.384855673 0.783049037 10

0 0.998207054
ZDHHC11 ENSG00000188818.12 0.58233373 10 9 0.051151112
Brain_Frontal_Cortex_BA9 0.883123006 Brain_Spinal_cord_cervical_c-1
3.857060056 0.035644803 0.20251534 -0.79542837 1.95020985 0.46643299
0.885255699 9 0 0.998207054
GLIDR ENSG00000278175.3 0.582381326 1 1 0.582381326
Brain_Cerebellar_Hemisphere 0.582381326 Brain_Cerebellar_Hemisphere 1 1
1 0.549909678 0.549909678 0.549909678 NA 1 0
0.998207054
OR51E1 ENSG00000180785.8 0.582488006 1 1 0.582488006
Brain_Caudate_basal_ganglia 0.582488006 Brain_Caudate_basal_ganglia 1 1
1 0.549754157 0.549754157 0.549754157 NA 1 0
0.998207054
SAP25 ENSG00000205307.11 0.582490675 12 3 0.265778628
Brain_Hypothalamus 0.982281998 Brain_Nucleus_accumbens_basal_ganglia
9.847630419 3.33E-09 0.496891219 -0.503391699 1.112836566
-0.020163485 0.507179908 3 0 0.998207054
MSRB2 ENSG00000148450.12 0.582499394 4 4 0.272226332
Brain_Frontal_Cortex_BA9 0.913049493 Brain_Nucleus_accumbens_basal_ganglia
2.469439006 0.168895272 0.168895272 -0.580170765 1.09794998
0.064086249 0.72128695 4 0 0.998207054
FAM86B2 ENSG00000145002.12 0.58250052 7 7 0.199009875
Brain_Hippocampus 0.990550677 Brain_Nucleus_accumbens_basal_ganglia
1.869532743 0.239112091 0.239112091 -0.195960701 1.284377575
0.496598418 0.577706854 7 0 0.998207054
LINC01616 ENSG00000261340.1 0.582524364 3 3 0.225928893
Brain_Cortex 0.88468763 Brain_Caudate_basal_ganglia 1.358801879
0.636029544 0.636029544 -0.560849708 1.210912625 0.168344494
0.926519215 3 0 0.998207054
FBXL19 ENSG00000099364.16 0.582526164 1 1 0.582526164
Brain_Nucleus_accumbens_basal_ganglia 0.582526164
Brain_Nucleus_accumbens_basal_ganglia 1 1 1 0.549698532
0.549698532 0.549698532 NA 1 0 0.998207054
ZFP1 ENSG00000184517.11 0.582714074 13 2 0.15914358 Brain_Cerebellum
0.685465894 Brain_Amygdala 11.05740721 0.001267669 1.002769029
-1.407957755 -0.405015804 -0.918442088 0.287623698 2 0
0.998207054
NDUFS7 ENSG00000115286.19 0.582828747 2 1 0.511598462
Brain_Cerebellar_Hemisphere 0.662939325 Brain_Cerebellum 1.977105289
0.022894711 1.977105289 -0.656350765 -0.435858599 -0.546104682
0.155911505 1 0 0.998207054
NDUFAF1 ENSG00000137806.8 0.582832994 12 2 0.160838177
Brain_Hypothalamus 0.596719006 Brain_Frontal_Cortex_BA9 10.51189992
0.000477133 0.68592365 -0.557973581 1.402258136 0.835354828
0.488039935 2 0 0.998207054
STEAP1B ENSG00000105889.14 0.58305629 6 5 0.366672416
Brain_Substantia_nigra 0.793823841 Brain_Putamen_basal_ganglia
3.659343325 0.119754624 0.243750361 -0.902723962 0.509437497
-0.128480009 0.563743299 5 0 0.998207054
PDE4A ENSG00000065989.15 0.583069682 2 2 0.414084539
Brain_Cerebellar_Hemisphere 0.481125537 Brain_Cerebellum 1.079285893
0.920714107 0.920714107 -0.816726859 -0.704493435 -0.760610147
0.079361015 2 0 0.998207054
CRYZL1 ENSG00000205758.11 0.583071701 1 1 0.583071701
Brain_Spinal_cord_cervical_c-1 0.583071701 Brain_Spinal_cord_cervical_c-1
1 1 1 -0.548903464 -0.548903464 -0.548903464 NA 1 0
0.998207054
BMS1 ENSG00000165733.7 0.583080894 7 6 0.171463927
Brain_Caudate_basal_ganglia 0.750498896 Brain_Frontal_Cortex_BA9
4.439600602 0.013636766 0.1966973 0.317981597 1.367515039
0.931065548 0.355034536 6 0 0.998207054
LA16c-381G6.1 ENSG00000260532.1 0.583146866 1 1 0.583146866

Brain_Substantia_nigra 0.583146866 Brain_Substantia_nigra 1 1
1 0.548793945 0.548793945 0.548793945 NA 1 0
0.998207054
CEP112 ENSG00000154240.16 0.583324879 4 4 0.315029621
Brain_Spinal_cord_cervical_c-1 0.947490584 Brain_Cortex 1.498497449
0.437606581 0.437606581 -1.004724307 0.689904604 -0.333276945
0.806764087 4 0 0.998207054
HAR1A ENSG00000225978.3 0.583439921 11 11 0.021721977
Brain_Caudate_basal_ganglia 0.943467169 Brain_Nucleus_accumbens_basal_ganglia
2.090515895 0.348622753 0.348622753 -2.29519456 1.188175461
-0.295703636 1.025581274 11 0 0.998207054
RP4-798A10.4 ENSG00000224174.2 0.583500146 1 1 0.583500146
Brain_Cerebellar_Hemisphere 0.583500146 Brain_Cerebellar_Hemisphere 1 1
1 -0.54827929 -0.54827929 -0.54827929 NA 1 0
0.998207054
PCDHB10 ENSG00000120324.8 0.58366782 8 7 0.065162189
Brain_Caudate_basal_ganglia 0.959930833 Brain_Cerebellum 5.069711001
0.082491932 0.185128434 0.050240381 1.844143784 0.741713828
0.547357705 7 0 0.998207054
NECAB3 ENSG00000125967.16 0.583742837 1 1 0.583742837
Brain_Cerebellum 0.583742837 Brain_Cerebellum 1 1 1 0.547925823
0.547925823 0.547925823 NA 1 0 0.998207054
WDR31 ENSG00000148225.15 0.583784404 4 3 0.462845282
Brain_Cerebellar_Hemisphere 0.919212161 Brain_Cerebellum 2.792483826
0.023594309 0.358775386 -0.367129579 0.734169759 0.020090143
0.4888016 3 0 0.998207054
CDHR5 ENSG00000099834.18 0.583942253 1 1 0.583942253 Brain_Cerebellum
0.583942253 Brain_Cerebellum 1 1 1 0.547635435 0.547635435
0.547635435 NA 1 0 0.998207054
CHIC2 ENSG00000109220.10 0.583946077 1 1 0.583946077
Brain_Putamen_basal_ganglia 0.583946077 Brain_Putamen_basal_ganglia 1 1
1 -0.547629867 -0.547629867 -0.547629867 NA 1 0
0.998207054
SIGLEC12 ENSG00000254521.6 0.583982518 2 2 0.451370405
Brain_Hypothalamus 0.804770458 Brain_Amygdala 1.494705541
0.505294459 0.505294459 -0.753132319 0.247178016 -0.252977151
0.707326221 2 0 0.998207054
SAP30 ENSG00000164105.3 0.583995386 1 1 0.583995386
Brain_Hippocampus 0.583995386 Brain_Hippocampus 1 1 1
0.547558071 0.547558071 0.547558071 NA 1 0 0.998207054
HAAO ENSG00000162882.14 0.584037697 9 7 0.027621865
Brain_Caudate_basal_ganglia 0.840356972 Brain_Frontal_Cortex_BA9
4.564105108 0.00138308 0.33483163 0.20143689 2.202615436 1.236188027
0.561551789 7 0 0.998207054
NHLRC1 ENSG00000187566.5 0.584106688 3 3 0.168290885
Brain_Nucleus_accumbens_basal_ganglia 0.952090947 Brain_Cerebellar_Hemisphere
1.563383299 0.428464762 0.428464762 -0.06008122 1.37771634
0.752079712 0.736823549 3 0 0.998207054
HLA-DPA1 ENSG00000231389.7 0.584198265 3 2 0.309700007
Brain_Nucleus_accumbens_basal_ganglia 0.771190629 Brain_Cerebellar_Hemisphere
2.349233426 0.008173883 0.64259269 0.290817855 1.01585171
0.552890543 0.402101553 2 0 0.998207054
GCC2 ENSG00000135968.20 0.584300234 4 4 0.19579986 Brain_Cortex
0.680933211 Brain_Cerebellar_Hemisphere 2.421239012 0.082970116
0.082970116 0.411190013 1.293610888 0.89534762 0.363663121 4
0 0.998207054
RARS ENSG00000113643.8 0.584352636 5 4 0.129643803
Brain_Frontal_Cortex_BA9 0.40111108 Brain_Cerebellar_Hemisphere 3.875591241
0.024334138 0.140215667 -1.515508011 -0.839638545 -1.117406029
0.263476461 4 0 0.998207054
ZNF25 ENSG00000175395.15 0.584387092 12 3 0.175056838
Brain_Cerebellar_Hemisphere 0.688035351 Brain_Caudate_basal_ganglia

9.464197499 0.005180713 0.48593517 0.40152267 1.356133053
0.753381367 0.309626427 3 0 0.998207054
ENHO ENSG00000168913.6 0.584593892 1 1 0.584593892
Brain_Hypothalamus 0.584593892 Brain_Hypothalamus 1 1 1
0.546686847 0.546686847 0.546686847 NA 1 0 0.998207054
SCCPDH ENSG00000143653.9 0.584613701 2 2 0.325422053
Brain_Nucleus_accumbens_basal_ganglia 0.362251527 Brain_Frontal_Cortex_BA9
1.684449819 0.315550181 0.315550181 -0.983376742 -0.911083219
-0.947229981 0.05111924 2 0 0.998207054
DCUN1D4 ENSG00000109184.14 0.584671029 3 3 0.201062938
Brain_Spinal_cord_cervical_c-1 0.7922502 Brain_Nucleus_accumbens_basal_ganglia
1.851761151 0.175869689 0.175869689 -0.263389737 1.27852908
0.688994303 0.832540149 3 0 0.998207054
PRR4 ENSG00000111215.11 0.5846963 13 4 0.199190649
Brain_Substantia_nigra 0.973378297 Brain_Spinal_cord_cervical_c-1
9.911328976 0.002575267 0.373458168 -0.18385551 1.283860846
0.24832101 0.388259022 4 0 0.998207054
MTG2 ENSG00000101181.17 0.584717553 9 6 0.192531684
Brain_Cerebellar_Hemisphere 0.890611712 Brain_Spinal_cord_cervical_c-1
4.694115143 0.070002447 0.246908692 -1.303126188 0.896004116
-0.429675206 0.82490048 6 0 0.998207054
TMEFF2 ENSG00000144339.11 0.58479258 1 1 0.58479258 Brain_Cerebellum
0.58479258 Brain_Cerebellum 1 1 1 0.546397715 0.546397715
0.546397715 NA 1 0 0.998207054
MTHFD1L ENSG00000120254.15 0.584795768 4 4 0.23737588
Brain_Hypothalamus 0.606306803 Brain_Putamen_basal_ganglia 1.464023667
0.672161811 0.672161811 -1.181571218 0.565040307 -0.196909295
0.875043153 4 0 0.998207054
POLR3E ENSG00000058600.15 0.584801249 1 1 0.584801249
Brain_Caudate_basal_ganglia 0.584801249 Brain_Caudate_basal_ganglia 1 1
1 -0.546385101 -0.546385101 -0.546385101 NA 1 0
0.998207054
HHIPL2 ENSG00000143512.12 0.584875984 3 3 0.375303733
Brain_Amygdala 0.589354478 Brain_Cerebellar_Hemisphere 2.084694145
0.292983222 0.292983222 -0.886582474 0.539771712 -0.329427323
0.762659888 3 0 0.998207054
NR1I2 ENSG00000144852.17 0.584881277 1 1 0.584881277
Brain_Hypothalamus 0.584881277 Brain_Hypothalamus 1 1 1
0.546268659 0.546268659 0.546268659 NA 1 0 0.998207054
EP400NL ENSG00000185684.12 0.58493154 13 5 0.111632039
Brain_Frontal_Cortex_BA9 0.971223539 Brain_Cortex 9.148359269
0.007556495 0.324895829 -1.590900192 -0.036073768 -0.751248613
0.555700093 5 0 0.998207054
BCL11B ENSG00000127152.17 0.585002999 1 1 0.585002999
Brain_Caudate_basal_ganglia 0.585002999 Brain_Caudate_basal_ganglia 1 1
1 -0.546091563 -0.546091563 -0.546091563 NA 1 0
0.998207054
SLC25A25 ENSG00000148339.12 0.585055014 1 1 0.585055014
Brain_Caudate_basal_ganglia 0.585055014 Brain_Caudate_basal_ganglia 1 1
1 0.54601589 0.54601589 0.54601589 NA 1 0 0.998207054
RBPJ ENSG00000168214.20 0.585059023 2 2 0.348021917
Brain_Putamen_basal_ganglia 0.557495634 Brain_Caudate_basal_ganglia
1.165225832 0.834774168 0.834774168 0.586565388 0.938433032
0.76249921 0.248807997 2 0 0.998207054
TRIP11 ENSG00000100815.12 0.585078287 2 2 0.378065663
Brain_Cerebellum 0.674125593 Brain_Amygdala 1.133649424 0.866350576
0.866350576 -0.881465973 -0.420492656 -0.650979315 0.325957358
2 0 0.998207054
DSCAM ENSG00000171587.14 0.585160564 2 2 0.332245166
Brain_Cerebellar_Hemisphere 0.6808626 Brain_Hypothalamus 1.050502226
0.949497774 0.949497774 -0.969601499 -0.41128632 -0.690443909
0.394788449 2 0 0.998207054

UHRF1BP1 ENSG00000065060.16 0.585226498 12 2 0.339900677
Brain_Amygdala 0.935282699 Brain_Anterior_cingulate_cortex_BA24
10.84225336 0.000261953 0.603253845 -0.95436152 0.245893231
-0.363045064 0.308548967 2 0 0.998207054
LINC00327 ENSG00000232977.6 0.585240328 3 3 0.166740505
Brain_Frontal_Cortex_BA9 0.886683347 Brain_Putamen_basal_ganglia
1.113418263 0.89225726 0.89225726 -1.382753358 -0.142502193
-0.601274344 0.680191537 3 0 0.998207054
TMIE ENSG00000181585.3 0.585248813 2 2 0.356598364
Brain_Nucleus_accumbens_basal_ganglia 0.571709568 Brain_Frontal_Cortex_BA9
1.105686489 0.894313511 0.894313511 -0.565535525 0.921866215
0.178165345 1.051751857 2 0 0.998207054
TBL1XR1 ENSG00000177565.16 0.585483794 4 4 0.265354333
Brain_Amygdala 0.760600819 Brain_Caudate_basal_ganglia 1.202582147
0.874274993 0.874274993 -1.113824852 0.955830145 0.221876054
0.930758353 4 0 0.998207054
FAM193A ENSG00000125386.15 0.585570479 1 1 0.585570479
Brain_Hypothalamus 0.585570479 Brain_Hypothalamus 1 1 1
0.545266153 0.545266153 0.545266153 NA 1 0 0.998207054
SCART1 ENSG00000214279.13 0.585612003 12 5 0.301412377
Brain_Cerebellum 0.84762407 Brain_Caudate_basal_ganglia 8.343480749
0.007289603 0.30146297 -1.033409341 0.368028326 -0.259373809
0.440866618 5 0 0.998207054
DMAP1 ENSG00000178028.13 0.585643803 2 2 0.318244545
Brain_Frontal_Cortex_BA9 0.467501252 Brain_Cortex 1.86476795 0.13523205
0.13523205 0.726550678 0.998071796 0.862311237 0.191994423 2
0 0.998207054
CTD-2561B21.11 ENSG00000262873.1 0.585672838 4 4 0.220916633
Brain_Putamen_basal_ganglia 0.69545824 Brain_Frontal_Cortex_BA9 1.256365695
0.808461721 0.808461721 -1.22409436 0.690843046 -0.220433841
0.909589999 4 0 0.998207054
GTF3A ENSG00000122034.14 0.585733316 5 5 0.208596608
Brain_Spinal_cord_cervical_c-1 0.747143145 Brain_Putamen_basal_ganglia
2.171309254 0.445645293 0.445645293 -0.678257804 1.257433769
0.034618266 0.829654915 5 0 0.998207054
RP11-546K22.3 ENSG00000272024.1 0.585734773 1 1 0.585734773
Brain_Hippocampus 0.585734773 Brain_Hippocampus 1 1 1
0.545027254 0.545027254 0.545027254 NA 1 0 0.998207054
SPATA2 ENSG00000158480.10 0.58573691 2 2 0.322223199 Brain_Cerebellum
0.379069041 Brain_Cerebellar_Hemisphere 1.661466865 0.338533135
0.338533135 -0.989899591 -0.879612914 -0.934756253 0.077984457
2 0 0.998207054
HACD2 ENSG00000206527.9 0.585747915 2 2 0.361550787
Brain_Hypothalamus 0.730682159 Brain_Substantia_nigra 1.737673543
0.262326457 0.262326457 0.344218249 0.912414068 0.628316158
0.401775116 2 0 0.998207054
SLC35G6 ENSG00000259224.2 0.585767927 1 1 0.585767927
Brain_Caudate_basal_ganglia 0.585767927 Brain_Caudate_basal_ganglia 1 1
1 -0.54497905 -0.54497905 -0.54497905 NA 1 0
0.998207054
RP11-834C11.4 ENSG00000250742.2 0.585782076 1 1 0.585782076
Brain_Cerebellum 0.585782076 Brain_Cerebellum 1 1 1 0.544958477
0.544958477 0.544958477 NA 1 0 0.998207054
RP11-576D8.4 ENSG00000224717.1 0.585919703 1 1 0.585919703
Brain_Cerebellum 0.585919703 Brain_Cerebellum 1 1 1 -0.544758386
-0.544758386 -0.544758386 NA 1 0 0.998207054
AF064860.7 ENSG00000231713.2 0.585932865 3 3 0.382819343
Brain_Nucleus_accumbens_basal_ganglia 0.917117256 Brain_Caudate_basal_ganglia
2.000020305 0.341077689 0.341077689 -0.872713343 0.36623636
-0.134137113 0.652919984 3 0 0.998207054
FRMD5 ENSG00000171877.20 0.586026907 1 1 0.586026907
Brain_Caudate_basal_ganglia 0.586026907 Brain_Caudate_basal_ganglia 1 1

1 -0.544602541 -0.544602541 -0.544602541 NA 1 0
0.998207054
SPATA22 ENSG00000141255.12 0.586185461 4 4 0.133359526
Brain_Nucleus_accumbens_basal_ganglia 0.945066323 Brain_Substantia_nigra
1.34336772 0.713578004 0.713578004 0.068903638 1.500984673
0.616657281 0.631877968 4 0 0.998207054
METTL5 ENSG00000138382.14 0.58621873 2 2 0.469673486
Brain_Anterior_cingulate_cortex_BA24 0.797597817 Brain_Cerebellum
1.518615918 0.481384082 0.481384082 -0.256457211 0.723010415
0.233276602 0.6925882 2 0 0.998207054
DLG4 ENSG00000132535.18 0.586330175 3 3 0.209254424
Brain_Hippocampus 0.846730138 Brain_Cortex 1.205814275
0.790013693 0.790013693 -1.255618215 -0.193292196 -0.753890244
0.533604167 3 0 0.998207054
SDR16C5 ENSG00000170786.12 0.586330487 5 5 0.20837236
Brain_Frontal_Cortex_BA9 0.87947513 Brain_Putamen_basal_ganglia 2.332489173
0.357512987 0.357512987 -1.001112224 1.258053634 0.071266695
0.846601994 5 0 0.998207054
PLEKHD1 ENSG00000175985.9 0.58633867 3 3 0.254957733
Brain_Caudate_basal_ganglia 0.579198226 Brain_Frontal_Cortex_BA9
1.064090798 0.946897295 0.946897295 -1.138389843 -0.554556242
-0.750120242 0.336254376 3 0 0.998207054
C19orf53 ENSG00000104979.8 0.586347353 10 2 0.302385997
Brain_Cerebellum 0.5920125 Brain_Anterior_cingulate_cortex_BA24 9.279525572
5.48E-08 0.484966125 -1.031330209 -0.535921941 -0.692643408
0.134699554 2 0 0.998207054
DMXL2 ENSG00000104093.13 0.586556351 2 2 0.632272136 Brain_Cerebellum
0.735157541 Brain_Cerebellar_Hemisphere 1.685635055 0.314364945
0.314364945 -0.338272904 0.478531252 0.070129174 0.577567757
2 0 0.998207054
RBFA ENSG00000101546.12 0.58658852 5 3 0.20700607 Brain_Cerebellar_Hemisphere
0.896928321 Brain_Nucleus_accumbens_basal_ganglia 2.713061217
0.019967909 0.487885466 0.129542597 1.26184082 0.798743254
0.443626031 3 0 0.998207054
RP4-669P10.20 ENSG00000281538.1 0.586728006 11 3 0.159469388
Brain_Caudate_basal_ganglia 0.787543977 Brain_Hippocampus 9.442917497
0.003024301 0.439435558 0.269501254 1.406858378 0.957678058
0.359257338 3 0 0.998207054
BRDT ENSG00000137948.18 0.586748962 1 1 0.586748962
Brain_Putamen_basal_ganglia 0.586748962 Brain_Putamen_basal_ganglia 1 1
1 0.543553215 0.543553215 0.543553215 NA 1 0
0.998207054
GLT8D1 ENSG00000016864.18 0.586862186 1 1 0.586862186
Brain_Amygdala 0.586862186 Brain_Amygdala 1 1 1 0.543388727
0.543388727 0.543388727 NA 1 0 0.998207054
ACAT2 ENSG00000120437.8 0.586894603 2 2 0.368446121
Brain_Putamen_basal_ganglia 0.593171361 Brain_Spinal_cord_cervical_c-1
1.030699271 0.969300729 0.969300729 -0.53424598 0.899387826
0.182570923 1.013732186 2 0 0.998207054
NTAN1 ENSG00000157045.8 0.586998546 5 5 0.401887385
Brain_Frontal_Cortex_BA9 0.932842566 Brain_Cerebellum 2.764542021
0.173137663 0.173137663 -0.497200792 0.83825521 0.070554356
0.503442708 5 0 0.998207054
CAMKK1 ENSG00000004660.14 0.587182185 2 2 0.335137102
Brain_Cerebellar_Hemisphere 0.51373057 Brain_Cerebellum 1.314482857
0.685517143 0.685517143 -0.963818156 -0.653039878 -0.808429017
0.219753428 2 0 0.998207054
EDIL3 ENSG00000164176.12 0.587206931 1 1 0.587206931 Brain_Cerebellum
0.587206931 Brain_Cerebellum 1 1 1 -0.542887981 -0.542887981
-0.542887981 NA 1 0 0.998207054
LINC01664 ENSG00000235478.4 0.587266249 10 4 0.203101765
Brain_Cerebellar_Hemisphere 0.960344705 Brain_Hypothalamus 6.801871533

0.000389867 0.845892477 -1.272764098 0.417659651 -0.179544495
0.553859662 4 0 0.998207054
ALKAL2 ENSG00000189292.15 0.587358615 2 2 0.310615947
Brain_Caudate_basal_ganglia 0.983559597 Brain_Spinal_cord_cervical_c-1
1.16472269 0.83527731 0.83527731 -1.013930436 0.020606448 -0.496661994
0.731528046 2 0 0.998207054
RP11-108L7.15 ENSG00000273162.1 0.5875374 1 1 0.5875374
Brain_Substantia_nigra 0.5875374 Brain_Substantia_nigra 1 1 1
0.542408102 0.542408102 0.542408102 NA 1 0 0.998207054
PABPC1L ENSG00000101104.12 0.587579933 9 1 0.516408918
Brain_Hippocampus 0.743535837 Brain_Amygdala 8.688645385 5.00E-05
8.688645385 -0.648890867 -0.327174592 -0.530791407 0.117335628
1 0 0.998207054
HHEX ENSG00000152804.10 0.587627732 1 1 0.587627732
Brain_Nucleus_accumbens_basal_ganglia 0.587627732
Brain_Nucleus_accumbens_basal_ganglia 1 1 1 0.542276951
0.542276951 0.542276951 NA 1 0 0.998207054
ALOX15B ENSG00000179593.15 0.587662198 2 2 0.313788791
Brain_Spinal_cord_cervical_c-1 0.696617337 Brain_Cortex 1.171575614
0.828424386 0.828424386 -1.007303828 -0.389890747 -0.698597287
0.436576977 2 0 0.998207054
SAMD12 ENSG00000177570.13 0.587775756 1 1 0.587775756
Brain_Cerebellar_Hemisphere 0.587775756 Brain_Cerebellar_Hemisphere 1 1
1 0.542062058 0.542062058 0.542062058 NA 1 0
0.998207054
MRPS9 ENSG00000135972.8 0.587796889 13 1 0.133163291
Brain_Anterior_cingulate_cortex_BA24 0.989560151
Brain_Nucleus_accumbens_basal_ganglia 11.38712525 2.70E-05 11.38712525
-1.501743784 0.186664339 -0.499735566 0.420303988 1 0
0.998207054
SESTD1 ENSG00000187231.13 0.587925776 3 3 0.234697187
Brain_Cerebellar_Hemisphere 0.668524788 Brain_Spinal_cord_cervical_c-1
1.786729661 0.251284847 0.251284847 -1.188345835 0.428173528
-0.441943216 0.81532975 3 0 0.998207054
HSPA12B ENSG00000132622.10 0.587949947 3 3 0.176659242
Brain_Substantia_nigra 0.861613749 Brain_Caudate_basal_ganglia
1.155235876 0.845451373 0.845451373 -1.351112988 0.295830141
-0.409867714 0.848361572 3 0 0.998207054
AXL ENSG00000167601.11 0.588194189 5 5 0.240871189
Brain_Hypothalamus 0.892440142 Brain_Hippocampus 2.668812949
0.135115462 0.135115462 -1.025307688 1.17281204 0.220524831
0.870554902 5 0 0.998207054
PDK1 ENSG00000152256.13 0.588238663 3 3 0.338974539 Brain_Cerebellum
0.922341405 Brain_Cortex 2.380511741 0.25780152 0.25780152
-0.097484799 0.95619339 0.399327644 0.529399878 3 0
0.998207054
SGMS1 ENSG00000198964.13 0.588359066 1 1 0.588359066
Brain_Hippocampus 0.588359066 Brain_Hippocampus 1 1 1
-0.541215484 -0.541215484 -0.541215484 NA 1 0 0.998207054
TECTA ENSG00000109927.9 0.588554917 1 1 0.588554917
Brain_Substantia_nigra 0.588554917 Brain_Substantia_nigra 1 1
1 -0.540931328 -0.540931328 -0.540931328 NA 1 0
0.998207054
ABCC8 ENSG00000006071.12 0.588647976 9 7 0.057082786
Brain_Cerebellar_Hemisphere 0.713489051 Brain_Anterior_cingulate_cortex_BA24
5.170466865 0.060287083 0.192064043 -0.945325195 1.902676381
0.768579997 0.833558366 7 0 0.998207054
RP11-462B18.2 ENSG00000231193.1 0.588979341 2 2 0.31204198
Brain_Frontal_Cortex_BA9 0.93436473 Brain_Substantia_nigra 1.106986784
0.893013216 0.893013216 -1.010946618 0.082354609 -0.464296005
0.773080712 2 0 0.998207054
ASGR2 ENSG00000161944.16 0.589020587 2 2 0.308826248

Brain_Putamen_basal_ganglia 0.87535149 Brain_Hypothalamus 1.005445118
0.994554882 0.994554882 -1.017688008 -0.156864688 -0.587276348
0.608694007 2 0 0.998207054
LSG1 ENSG00000041802.10 0.589250423 13 1 0.238861946
Brain_Nucleus_accumbens_basal_ganglia 0.949756566 Brain_Caudate_basal_ganglia
12.10634973 0.000628164 12.10634973 -0.063012481 1.177836118
0.526019159 0.301078536 1 0 0.998207054
LRIT3 ENSG00000183423.11 0.589316181 3 3 0.169262231 Brain_Cerebellum
0.363963068 Brain_Frontal_Cortex_BA9 2.268167783 0.186998501
0.186998501 -1.374578263 -0.907839419 -1.144852196 0.233454726
3 0 0.998207054
CCRL2 ENSG00000121797.9 0.589422762 1 1 0.589422762
Brain_Hypothalamus 0.589422762 Brain_Hypothalamus 1 1 1
-0.539672712 -0.539672712 -0.539672712 NA 1 0 0.998207054
DNAJC27 ENSG00000115137.11 0.589496096 2 2 0.305315532
Brain_Frontal_Cortex_BA9 0.446474076 Brain_Anterior_cingulate_cortex_BA24
1.789580396 0.210419604 0.210419604 -1.025101003 -0.761306404
-0.893203703 0.18653095 2 0 0.998207054
ABCB6 ENSG00000115657.12 0.589513861 2 2 0.339849212
Brain_Cerebellar_Hemisphere 0.593621719 Brain_Cerebellum 1.799464673
0.200535327 0.200535327 -0.954463233 -0.533595071 -0.744029152
0.297598731 2 0 0.998207054
FIBIN ENSG00000176971.3 0.589649321 1 1 0.589649321
Brain_Cerebellar_Hemisphere 0.589649321 Brain_Cerebellar_Hemisphere 1 1
1 -0.53934428 -0.53934428 -0.53934428 NA 1 0
0.998207054
PRDX3 ENSG00000165672.6 0.589723751 2 2 0.315986471
Brain_Nucleus_accumbens_basal_ganglia 0.838654985 Brain_Cerebellar_Hemisphere
1.021475458 0.978524542 0.978524542 -1.002739698 0.203614214
-0.399562742 0.853021032 2 0 0.998207054
EFR3B ENSG00000084710.13 0.589885186 1 1 0.589885186
Brain_Caudate_basal_ganglia 0.589885186 Brain_Caudate_basal_ganglia 1 1
1 0.539002419 0.539002419 0.539002419 NA 1 0
0.998207054
RP11-408A13.3 ENSG00000273399.2 0.589891074 5 5 0.069519302
Brain_Cerebellar_Hemisphere 0.683338747 Brain_Cerebellum 2.719391973
0.159991362 0.159991362 -1.815029981 -0.407911367 -1.130846449
0.51632765 5 0 0.998207054
BMF ENSG00000104081.13 0.590163741 3 3 0.180237322
Brain_Caudate_basal_ganglia 0.785075554 Brain_Cortex 1.578722158
0.509975857 0.509975857 -1.340024677 -0.272710772 -0.66190603
0.58940418 3 0 0.998207054
LINC00342 ENSG00000232931.5 0.590242144 2 2 0.351273347
Brain_Putamen_basal_ganglia 0.664612005 Brain_Anterior_cingulate_cortex_BA24
1.002951728 0.997048272 0.997048272 -0.932122256 0.433554468
-0.249283894 0.965679272 2 0 0.998207054
ANKRD26 ENSG00000107890.16 0.590247388 1 1 0.590247388
Brain_Cerebellum 0.590247388 Brain_Cerebellum 1 1 1 -0.538477567
-0.538477567 -0.538477567 NA 1 0 0.998207054
ADARB1 ENSG00000197381.15 0.590259123 3 3 0.229110658
Brain_Cerebellar_Hemisphere 0.625610976 Brain_Cerebellum 2.549818162
0.087354025 0.087354025 -0.500876283 1.202652924 0.396563444
0.855430646 3 0 0.998207054
RP11-433J8.2 ENSG00000258979.1 0.590431139 3 3 0.268005882
Brain_Nucleus_accumbens_basal_ganglia 0.852934124 Brain_Putamen_basal_ganglia
1.335668797 0.663655751 0.663655751 -1.107666477 0.835835542
-0.028818308 0.989297442 3 0 0.998207054
ASB16 ENSG00000161664.6 0.590648893 3 3 0.183982058 Brain_Cerebellum
0.919237995 Brain_Spinal_cord_cervical_c-1 1.782643604 0.31153879
0.31153879 -0.101393628 1.32859368 0.572770613 0.718482462 3 0
0.998207054
PHYHD1 ENSG00000175287.18 0.590652087 10 4 0.280776881

Brain_Cerebellum 0.909980491 Brain_Putamen_basal_ganglia 7.387948921
0.00100067 0.609024394 -1.078575785 0.867258727 0.34644742
0.547641988 4 0 0.998207054
SCUBE1 ENSG00000159307.18 0.590718799 8 8 0.102826252
Brain_Caudate_basal_ganglia 0.841377541 Brain_Frontal_Cortex_BA9
2.424836846 0.215777571 0.215777571 -1.098882486 1.631303704
0.454123194 0.768348424 8 0 0.998207054
WNT2B ENSG00000134245.17 0.590820415 4 2 0.225608925 Brain_Cerebellum
0.980810154 Brain_Cortex 2.939502585 0.020542465 0.988740519
-0.024053225 1.211747827 0.452732915 0.557561701 2 0
0.998207054
PARVB ENSG00000188677.14 0.590853171 6 6 0.11039327 Brain_Cerebellum
0.515809072 Brain_Cerebellar_Hemisphere 1.795777912 0.435517688
0.435517688 -1.596427993 0.658228688 -0.733622215 0.767224501
6 0 0.998207054
NAA40 ENSG00000110583.12 0.590876735 1 1 0.590876735
Brain_Cerebellar_Hemisphere 0.590876735 Brain_Cerebellar_Hemisphere 1 1
1 -0.537565962 -0.537565962 -0.537565962 NA 1 0
0.998207054
MSANTD2 ENSG00000120458.10 0.590902499 2 2 0.393817442
Brain_Substantia_nigra 0.525562426 Brain_Nucleus_accumbens_basal_ganglia
1.077822879 0.922177121 0.922177121 0.634794538 0.852714872
0.743754705 0.154092946 2 0 0.998207054
FILIP1L ENSG00000168386.18 0.59092689 1 1 0.59092689
Brain_Anterior_cingulate_cortex_BA24 0.59092689
Brain_Anterior_cingulate_cortex_BA24 1 1 1 0.537493331
0.537493331 0.537493331 NA 1 0 0.998207054
KIAA1143 ENSG00000163807.5 0.590957567 3 3 0.307483249
Brain_Cerebellar_Hemisphere 0.733067629 Brain_Cortex 2.534478013
0.190863117 0.190863117 0.341047757 1.020517173 0.570378666
0.389854676 3 0 0.998207054
HSD17B6 ENSG00000025423.11 0.591029685 9 3 0.149484925
Brain_Amygdala 0.835271583 Brain_Hypothalamus 7.565458791 0.00048013
0.428073941 -1.441353198 -0.207945422 -0.561887312 0.368457628
3 0 0.998207054
EML4 ENSG00000143924.18 0.591052782 3 3 0.250660387 Brain_Cerebellum
0.833129297 Brain_Frontal_Cortex_BA9 1.74165617 0.261047261
0.261047261 -0.392472369 1.148746836 0.322321438 0.776650094
3 0 0.998207054
SNX19 ENSG00000120451.10 0.591106669 13 8 0.220120309
Brain_Anterior_cingulate_cortex_BA24 0.924407161 Brain_Cerebellar_Hemisphere
7.673282746 0.011533767 0.267837887 -1.226208273 0.867309808
0.025597836 0.617738387 8 0 0.998207054
CMKLR1 ENSG00000174600.13 0.591135368 3 3 0.19997801 Brain_Cerebellum
0.671040264 Brain_Amygdala 1.427989284 0.665345783 0.665345783
-1.281614219 -0.424720738 -0.823573884 0.431501972 3 0
0.998207054
CPD ENSG00000108582.11 0.591193105 1 1 0.591193105
Brain_Caudate_basal_ganglia 0.591193105 Brain_Caudate_basal_ganglia 1 1
1 -0.537107869 -0.537107869 -0.537107869 NA 1 0
0.998207054
BRD7 ENSG00000166164.15 0.591293024 1 1 0.591293024
Brain_Putamen_basal_ganglia 0.591293024 Brain_Putamen_basal_ganglia 1 1
1 -0.536963214 -0.536963214 -0.536963214 NA 1 0
0.998207054
CTD-2619J13.19 ENSG00000269473.1 0.591418552 13 3 0.136157286
Brain_Spinal_cord_cervical_c-1 0.713593114
Brain_Nucleus_accumbens_basal_ganglia 10.83752107 0.007365815
0.461069305 0.367034728 1.490254674 0.998527521 0.308101738
3 0 0.998207054
ZDHHC2 ENSG00000104219.12 0.591664368 3 3 0.282098441
Brain_Cerebellar_Hemisphere 0.977123877 Brain_Nucleus_accumbens_basal_ganglia

1.04291616 0.960190331 0.960190331 -1.075617313 0.908710272
-0.046077381 0.994273563 3 0 0.998207054
KIF25-AS1 ENSG00000229921.6 0.591679718 13 8 0.118518343
Brain_Anterior_cingulate_cortex_BA24 0.919194295 Brain_Hippocampus
6.930385111 0.073823989 0.307184108 -1.244916844 1.561022918
0.254814068 0.757677867 8 0 0.998207054
EPC2 ENSG00000135999.11 0.591699543 1 1 0.591699543
Brain_Hippocampus 0.591699543 Brain_Hippocampus 1 1 1
-0.536374801 -0.536374801 -0.536374801 NA 1 0 0.998207054
COQ10B ENSG00000115520.8 0.591720576 1 1 0.591720576
Brain_Substantia_nigra 0.591720576 Brain_Substantia_nigra 1 1
1 -0.536344362 -0.536344362 -0.536344362 NA 1 0
0.998207054
GPR4 ENSG00000177464.4 0.591754641 1 1 0.591754641
Brain_Anterior_cingulate_cortex_BA24 0.591754641
Brain_Anterior_cingulate_cortex_BA24 1 1 1 0.536295065
0.536295065 0.536295065 NA 1 0 0.998207054
SCN1A ENSG00000144285.17 0.591767183 8 5 0.140680708
Brain_Nucleus_accumbens_basal_ganglia 0.870516663 Brain_Cerebellar_Hemisphere
5.566732573 0.017968086 0.256290079 -1.47326087 0.627721905
-0.388264356 0.581228931 5 0 0.998207054
ATF3 ENSG00000162772.16 0.591807384 2 2 0.322835212
Brain_Spinal_cord_cervical_c-1 0.819511238 Brain_Frontal_Cortex_BA9
1.03987661 0.96012339 0.96012339 -0.22817366 0.988648368 0.380237354
0.860423108 2 0 0.998207054
OTUD6B ENSG00000155100.10 0.592127408 2 2 0.306881097
Brain_Frontal_Cortex_BA9 0.785399181 Brain_Cerebellum 1.32513108 0.67486892
0.67486892 -1.021788323 0.272289822 -0.37474925 0.915051432 2
0 0.998207054
ZNF432 ENSG00000256087.6 0.592142147 5 3 0.182650289
Brain_Substantia_nigra 0.662139975 Brain_Amygdala 4.108731584
0.048989465 0.280691018 0.436960526 1.332639034 0.877214356
0.319889747 3 0 0.998207054
MARVELD2 ENSG00000152939.14 0.592154132 1 1 0.592154132
Brain_Anterior_cingulate_cortex_BA24 0.592154132
Brain_Anterior_cingulate_cortex_BA24 1 1 1 0.53571703 0.53571703
0.53571703 NA 1 0 0.998207054
RP11-96H19.1 ENSG00000257261.5 0.592272036 13 3 0.16310887
Brain_Cerebellar_Hemisphere 0.743031976 Brain_Frontal_Cortex_BA9
11.41314492 0.003184195 0.407486233 0.32784088 1.394691562
0.795653396 0.28793238 3 0 0.998207054
JAM2 ENSG00000154721.14 0.592303291 3 3 0.208340366
Brain_Nucleus_accumbens_basal_ganglia 0.964853445 Brain_Caudate_basal_ganglia
1.540334892 0.705239441 0.705239441 0.04406393 1.258142111
0.692401577 0.611239045 3 0 0.998207054
MRPS26 ENSG00000125901.5 0.592381889 3 3 0.232164719
Brain_Spinal_cord_cervical_c-1 0.710095884
Brain_Anterior_cingulate_cortex_BA24 1.226693607 0.684894611
0.684894611 -0.441905548 1.194801181 0.127056105 0.92535988 3
0 0.998207054
RP11-420A23.1 ENSG00000251432.6 0.592391808 3 3 0.263645815
Brain_Hippocampus 0.679116275 Brain_Nucleus_accumbens_basal_ganglia
1.665110177 0.410229216 0.410229216 -1.117815465 0.427446935
-0.368012628 0.773642278 3 0 0.998207054
NPSR1 ENSG00000187258.13 0.592413023 3 3 0.348980222 Brain_Cerebellum
0.584661657 Brain_Cerebellar_Hemisphere 1.155218851 0.844687726
0.844687726 0.54658823 0.93656916 0.803625969 0.222645175 3 0
0.998207054
IMP4 ENSG00000136718.9 0.592498297 2 1 0.55185447
Brain_Cerebellar_Hemisphere 0.635964041 Brain_Cerebellum 1.992139552
0.007860448 1.992139552 -0.594983545 -0.473349236 -0.53416639
0.086008445 1 0 0.998207054

ZNF569 ENSG00000196437.10 0.59269786 3 3 0.190629889
Brain_Frontal_Cortex_BA9 0.871964521 Brain_Hypothalamus 1.152178073
0.835092802 0.835092802 -0.423592211 1.308718008 0.24132072
0.933659422 3 0 0.998207054
TACSTD2 ENSG00000184292.6 0.592701982 1 1 0.592701982
Brain_Anterior_cingulate_cortex_BA24 0.592701982
Brain_Anterior_cingulate_cortex_BA24 1 1 1 -0.534924622
-0.534924622 -0.534924622 NA 1 0 0.998207054
AIF1L ENSG00000126878.12 0.592811842 3 3 0.17926728 Brain_Hippocampus
0.934618532 Brain_Caudate_basal_ganglia 1.505412684 0.688434322
0.688434322 -1.343014497 -0.08203544 -0.504769923 0.725950083
3 0 0.998207054
CMTM6 ENSG00000091317.7 0.592876842 3 3 0.375635691
Brain_Spinal_cord_cervical_c-1 0.914729977 Brain_Cerebellar_Hemisphere
2.363322254 0.087257957 0.087257957 -0.885966293 0.299258241
-0.231260809 0.602292289 3 0 0.998207054
TRAM2-AS1 ENSG00000225791.6 0.593010756 3 3 0.401230133
Brain_Caudate_basal_ganglia 0.873684079 Brain_Hypothalamus 1.815554736
0.192352549 0.192352549 -0.595145668 0.839426295 0.134420442
0.717601271 3 0 0.998207054
DRG1 ENSG00000185721.12 0.593099308 1 1 0.593099308
Brain_Putamen_basal_ganglia 0.593099308 Brain_Putamen_basal_ganglia 1 1
1 0.53435014 0.53435014 0.53435014 NA 1 0 0.998207054
WDR55 ENSG00000120314.18 0.593126966 5 2 0.307675651
Brain_Hypothalamus 0.915789285 Brain_Caudate_basal_ganglia 4.20147428
0.028241322 0.559601995 0.105739191 1.020111358 0.636822483
0.348010788 2 0 0.998207054
HSPB2 ENSG00000170276.5 0.593145355 2 2 0.340973661
Brain_Nucleus_accumbens_basal_ganglia 0.600576958 Brain_Caudate_basal_ganglia
1.165268528 0.834731472 0.834731472 -0.952243192 -0.523570997
-0.737907095 0.303117016 2 0 0.998207054
MIR4300HG ENSG00000245832.6 0.593153427 9 4 0.203104386
Brain_Hippocampus 0.99393044 Brain_Amygdala 7.043601606 0.017415173
0.27414394 0.007607139 1.272756713 0.674182173 0.452766942 4
0 0.998207054
DBI ENSG00000155368.16 0.593281865 2 2 0.378983599 Brain_Amygdala
0.592003639 Brain_Cerebellum 1.018458974 0.981541026 0.981541026
-0.879770591 0.535934761 -0.171917915 1.001054855 2 0
0.998207054
FBN2 ENSG00000138829.11 0.593387481 7 4 0.121489132 Brain_Cortex
0.60874571 Brain_Frontal_Cortex_BA9 5.820507798 0.010516282
0.253213575 -1.548553332 -0.511864693 -1.178202744 0.339586286
4 0 0.998207054
PEX13 ENSG00000162928.8 0.59345277 3 3 0.177018056 Brain_Hypothalamus
0.805141778 Brain_Cortex 1.878093547 0.284489254 0.284489254
0.246698228 1.349993536 0.81014969 0.55202638 3 0 0.998207054
OLFML2A ENSG00000185585.19 0.593465575 6 4 0.099345073
Brain_Putamen_basal_ganglia 0.971939295 Brain_Anterior_cingulate_cortex_BA24
3.803891102 0.00518439 0.255728333 -0.035176131 1.648037029
0.991709406 0.63619566 4 0 0.998207054
ARHGAP20 ENSG00000137727.12 0.593532148 4 4 0.218862632
Brain_Cortex 0.959782725 Brain_Cerebellum 1.703547669 0.320798449
0.320798449 0.050426241 1.22955809 0.685215231 0.519487789 4
0 0.998207054
ABL2 ENSG00000143322.19 0.593874782 12 4 0.052757357
Brain_Cerebellar_Hemisphere 0.874380946 Brain_Hypothalamus 9.105841171
0.023873414 0.401884335 -1.936901727 -0.158096262 -1.131470595
0.494267276 4 0 0.998207054
LINC01126 ENSG00000279873.2 0.59396311 1 1 0.59396311
Brain_Nucleus_accumbens_basal_ganglia 0.59396311
Brain_Nucleus_accumbens_basal_ganglia 1 1 1 -0.533101804
-0.533101804 -0.533101804 NA 1 0 0.998207054

AFF3 ENSG00000144218.18 0.594009522 10 4 0.311799745
Brain_Hypothalamus 0.97738342 Brain_Hippocampus 8.25455082 0.002339129
0.318220094 -0.309892406 1.011452833 0.237339114 0.386402554
4 0 0.998207054
ANKRD34C ENSG00000235711.4 0.594166866 1 1 0.594166866
Brain_Cerebellum 0.594166866 Brain_Cerebellum 1 1 1 -0.532807465
-0.532807465 -0.532807465 NA 1 0 0.998207054
SCRIB ENSG00000180900.18 0.594235042 3 3 0.342867392 Brain_Amygdala
0.648309041 Brain_Cerebellum 1.211554006 0.850696786 0.850696786
-0.948514899 0.746080282 -0.219515725 0.87172057 3 0
0.998207054
SUDS3 ENSG00000111707.11 0.594517381 2 1 0.580580337
Brain_Putamen_basal_ganglia 0.617132485 Brain_Nucleus_accumbens_basal_ganglia
1.954621689 0.045378311 1.954621689 -0.552537225 -0.499918472
-0.526227849 0.037207077 1 0 0.998207054
CDH24 ENSG00000139880.19 0.594558941 1 1 0.594558941 Brain_Cerebellum
0.594558941 Brain_Cerebellum 1 1 1 -0.532241215 -0.532241215
-0.532241215 NA 1 0 0.998207054
LINC01735 ENSG00000261453.1 0.594608149 2 2 0.448786118
Brain_Caudate_basal_ganglia 0.507944907 Brain_Putamen_basal_ganglia
1.026862639 0.973137361 0.973137361 0.662041061 0.757440303
0.709740682 0.067457451 2 0 0.998207054
RNF126 ENSG00000070423.17 0.594643719 1 1 0.594643719
Brain_Putamen_basal_ganglia 0.594643719 Brain_Putamen_basal_ganglia 1 1
1 0.532118797 0.532118797 0.532118797 NA 1 0
0.998207054
ADCY1 ENSG00000164742.14 0.59492503 2 2 0.312192364
Brain_Substantia_nigra 0.647020395 Brain_Spinal_cord_cervical_c-1
1.560729315 0.439270685 0.439270685 0.457905416 1.01063248
0.734268948 0.390837055 2 0 0.998207054
LRRC4B ENSG00000131409.12 0.595037994 3 3 0.201827508
Brain_Hypothalamus 0.705015179 Brain_Spinal_cord_cervical_c-1
1.663107444 0.342913442 0.342913442 -0.434099793 1.276362213
0.154567719 0.971899353 3 0 0.998207054
PLK5 ENSG00000185988.11 0.595072704 7 7 0.136774167
Brain_Putamen_basal_ganglia 0.920970675 Brain_Cerebellum 1.874085691
0.222499071 0.222499071 -0.656388197 1.487911756 0.215460455
0.855498013 7 0 0.998207054
NAA25 ENSG00000111300.9 0.595101816 6 2 0.725834136 Brain_Cortex
0.986507362 Brain_Cerebellar_Hemisphere 5.592838648 0.000969519
0.228473757 -0.343608465 0.350672398 -0.035690493 0.262546025
2 0 0.998207054
SLITRK5 ENSG00000165300.7 0.595209879 5 5 0.219178277
Brain_Cortex 0.996456136 Brain_Cerebellum 2.130169127 0.130834273
0.130834273 0.00444159 1.228716075 0.500093365 0.484190466 5
0 0.998207054
CTD-2014B16.3 ENSG00000258847.1 0.595366032 2 2 0.30958951
Brain_Nucleus_accumbens_basal_ganglia 0.955171399 Brain_Hippocampus
1.012368465 0.987631535 0.987631535 -1.016083743 0.056213911
-0.479934916 0.758228943 2 0 0.998207054
LRRC9 ENSG00000131951.10 0.595380624 2 2 0.30850601
Brain_Spinal_cord_cervical_c-1 0.884037635 Brain_Substantia_nigra
1.13649578 0.86350422 0.86350422 -1.018361882 -0.145852746 -0.582107314
0.616957127 2 0 0.998207054
CCL18 ENSG00000275385.1 0.595388021 2 2 0.324523799
Brain_Substantia_nigra 0.835022558 Brain_Spinal_cord_cervical_c-1
1.049944797 0.950055203 0.950055203 -0.20826436 0.985204157
0.388469899 0.843909682 2 0 0.998207054
DACT3 ENSG00000197380.10 0.595525367 1 1 0.595525367
Brain_Hypothalamus 0.595525367 Brain_Hypothalamus 1 1 1
0.530846193 0.530846193 0.530846193 NA 1 0 0.998207054
ANXA1 ENSG00000135046.13 0.595619646 2 2 0.339937235

Brain_Nucleus_accumbens_basal_ganglia 0.752132057
Brain_Anterior_cingulate_cortex_BA24 1.04017541 0.95982459 0.95982459
-0.315829326 0.954289275 0.319229974 0.898109476 2 0
0.998207054
PCMTD1 ENSG00000168300.13 0.595785182 4 3 0.582603307
Brain_Hippocampus 0.841020206 Brain_Cerebellar_Hemisphere 2.703916074
0.034639478 0.248909377 -0.549586082 0.478382352 0.124074105
0.463384953 3 0 0.998207054
ANKRD63 ENSG00000230778.1 0.595947523 2 2 0.309460956
Brain_Cerebellum 0.933099626 Brain_Cerebellar_Hemisphere 1.036158549
0.963841451 0.963841451 -1.016353762 -0.083945672 -0.550149717
0.659312083 2 0 0.998207054
PRMT1 ENSG00000126457.21 0.596060093 8 4 0.10386111
Brain_Putamen_basal_ganglia 0.557139457 Brain_Nucleus_accumbens_basal_ganglia
6.511178827 0.001395203 0.238317564 -1.626416372 -0.587095668
-1.257576863 0.32666559 4 0 0.998207054
LINC00391 ENSG00000238230.1 0.596103247 2 2 0.309595823
Brain_Cerebellum 0.915316768 Brain_Substantia_nigra 1.057700487
0.942299513 0.942299513 -1.016070484 0.106334741 -0.454867871
0.793660346 2 0 0.998207054
LRRC61 ENSG00000127399.14 0.596119018 13 2 0.080799895
Brain_Substantia_nigra 0.712127625 Brain_Amygdala 11.45527445
0.00042158 0.783552559 -1.746063633 -0.369000133 -0.949210248
0.307721593 2 0 0.998207054
FAM189A1 ENSG00000104059.4 0.596320789 1 1 0.596320789
Brain_Putamen_basal_ganglia 0.596320789 Brain_Putamen_basal_ganglia 1 1
1 0.529698788 0.529698788 0.529698788 NA 1 0
0.998207054
GSTO1 ENSG00000148834.12 0.596578378 5 5 0.544892938
Brain_Anterior_cingulate_cortex_BA24 0.959661956 Brain_Caudate_basal_ganglia
3.029261445 0.119921285 0.119921285 -0.605430579 0.566699295
0.034149647 0.417808453 5 0 0.998207054
C8orf86 ENSG00000196166.3 0.596594564 1 1 0.596594564
Brain_Anterior_cingulate_cortex_BA24 0.596594564
Brain_Anterior_cingulate_cortex_BA24 1 1 1 0.529304024
0.529304024 0.529304024 NA 1 0 0.998207054
FSHB ENSG00000131808.10 0.596792612 2 2 0.379505955
Brain_Anterior_cingulate_cortex_BA24 0.384161496 Brain_Hypothalamus
1.481746122 0.518253878 0.518253878 -0.878806954 -0.870254212
-0.874530583 0.006047702 2 0 0.998207054
LILRA4 ENSG00000239961.2 0.596844347 3 3 0.489024205
Brain_Caudate_basal_ganglia 0.82674166 Brain_Hippocampus 1.763763914
0.241077375 0.241077375 -0.218882396 0.691861667 0.270010916
0.459058511 3 0 0.998207054
RP11-723O4.6 ENSG00000187695.8 0.596990127 2 2 0.370013437
Brain_Cerebellum 0.575386192 Brain_Putamen_basal_ganglia 1.09446698 0.90553302
0.90553302 -0.56013671 0.896448195 0.168155742 1.029961064 2
0 0.998207054
STAM ENSG00000136738.14 0.596994092 1 1 0.596994092 Brain_Cerebellum
0.596994092 Brain_Cerebellum 1 1 1 0.528728084 0.528728084
0.528728084 NA 1 0 0.998207054
COL9A1 ENSG00000112280.15 0.597091493 5 5 0.131039486
Brain_Cortex 0.948077061 Brain_Nucleus_accumbens_basal_ganglia
1.665814689 0.58614242 0.58614242 -0.249116688 1.510015432
0.421529381 0.780890251 5 0 0.998207054
HCP5B ENSG00000281831.1 0.597210344 13 4 0.11161784 Brain_Cerebellum
0.979293375 Brain_Spinal_cord_cervical_c-1 9.281733662 0.008309867
0.44373479 -1.590963278 0.059232809 -0.561319494 0.469180552 4
0 0.998207054
CCR6 ENSG00000112486.15 0.597211234 6 6 0.128020202
Brain_Nucleus_accumbens_basal_ganglia 0.55413016 Brain_Amygdala 2.916759812
0.254170379 0.254170379 -1.521955616 0.788673718 -0.536200342

1.006455661 6 0 0.998207054
MGAT5B ENSG00000167889.12 0.597278727 1 1 0.597278727
Brain_Nucleus_accumbens_basal_ganglia 0.597278727
Brain_Nucleus_accumbens_basal_ganglia 1 1 1 0.528317876
0.528317876 0.528317876 NA 1 0 0.998207054
KLHL24 ENSG00000114796.15 0.597343358 8 4 0.37397521 Brain_Cortex
0.949059772 Brain_Frontal_Cortex_BA9 6.466580154 0.013029923
0.220580269 -0.889051858 0.063887543 -0.393890449 0.344362284
4 0 0.998207054
RP11-357K6.1 ENSG00000255618.1 0.597418109 4 3 0.422827657
Brain_Nucleus_accumbens_basal_ganglia 0.987680083 Brain_Frontal_Cortex_BA9
3.299797971 0.07094069 0.286146048 -0.801525208 -0.015441339
-0.381535845 0.424436531 3 0 0.998207054
RBL1 ENSG00000080839.11 0.59742541 1 1 0.59742541 Brain_Caudate_basal_ganglia
0.59742541 Brain_Caudate_basal_ganglia 1 1 1 0.528106514
0.528106514 0.528106514 NA 1 0 0.998207054
TAF9 ENSG00000273841.4 0.597456067 4 4 0.3644059
Brain_Frontal_Cortex_BA9 0.870221067 Brain_Nucleus_accumbens_basal_ganglia
2.769512348 0.204899919 0.204899919 -0.907001696 0.528092461
-0.062651242 0.631823722 4 0 0.998207054
ZNF577 ENSG00000161551.14 0.597507589 11 5 0.248576945
Brain_Hippocampus 0.988088406 Brain_Cerebellar_Hemisphere 7.497428619
0.021485322 0.389181307 -1.153812751 0.545275141 -0.090597346
0.624277893 5 0 0.998207054
AC005281.1 ENSG00000226690.7 0.597574473 1 1 0.597574473
Brain_Hypothalamus 0.597574473 Brain_Hypothalamus 1 1 1
-0.527891747 -0.527891747 -0.527891747 NA 1 0 0.998207054
RP11-618L22.1 ENSG00000274723.1 0.59757547 3 2 0.392632869
Brain_Caudate_basal_ganglia 0.78173168 Brain_Putamen_basal_ganglia 2.694836925
0.046001868 0.259161207 0.277063068 0.854852393 0.616990654
0.302114554 2 0 0.998207054
RPS26 ENSG00000197728.9 0.597760855 13 1 0.185140134
Brain_Spinal_cord_cervical_c-1 0.879774809 Brain_Hippocampus
12.17759187 0.00251205 12.17759187 0.151254691 1.325093524
0.51393156 0.335137586 1 0 0.998207054
EBF3 ENSG00000108001.13 0.597784436 5 5 0.087809162
Brain_Caudate_basal_ganglia 0.847118542 Brain_Cerebellum 2.124927374
0.314754089 0.314754089 -0.666406176 1.707069354 0.565935667
0.929702914 5 0 0.998207054
PPP1R7 ENSG00000115685.14 0.597949741 2 2 0.348973881
Brain_Frontal_Cortex_BA9 0.655947696 Brain_Substantia_nigra
1.061130405 0.938869595 0.938869595 -0.445514897 0.936581482
0.245533292 0.977289722 2 0 0.998207054
BAIAP2L2 ENSG00000128298.16 0.597952611 4 4 0.240708388
Brain_Anterior_cingulate_cortex_BA24 0.865622694 Brain_Cerebellum
1.319689202 0.798597444 0.798597444 -1.173218018 1.063961707
0.228359709 1.009441441 4 0 0.998207054
HNF4G ENSG00000164749.11 0.597981757 4 4 0.157881231 Brain_Cerebellum
0.538710004 Brain_Hypothalamus 1.762499535 0.301635109
0.301635109 0.614764882 1.412233461 0.917094809 0.357401619
4 0 0.998207054
SLC6A4 ENSG00000108576.9 0.598005663 1 1 0.598005663
Brain_Cortex 0.598005663 Brain_Cortex 1 1 1 0.527270635
0.527270635 0.527270635 NA 1 0 0.998207054
SNRNP48 ENSG00000168566.12 0.598062749 1 1 0.598062749
Brain_Putamen_basal_ganglia 0.598062749 Brain_Putamen_basal_ganglia 1 1
1 0.52718842 0.52718842 0.52718842 NA 1 0 0.998207054
GBP3 ENSG00000117226.11 0.598104441 13 1 0.215505967
Brain_Putamen_basal_ganglia 0.86968294 Brain_Anterior_cingulate_cortex_BA24
12.3311697 7.32E-06 12.3311697 -1.238566807 0.619231966 -0.509706994
0.443472597 1 0 0.998207054
ST20 ENSG00000180953.11 0.598110715 13 3 0.15185232 Brain_Cortex

0.707048447 Brain_Substantia_nigra 11.13058885 0.009813875
0.445977131 0.375823055 1.433019307 0.943989321 0.31937585 3
0 0.998207054
ANAPC1 ENSG00000153107.11 0.59816614 3 3 0.241813846 Brain_Amygdala
0.7534123 Brain_Spinal_cord_cervical_c-1 1.109284826 0.890715174
0.890715174 -1.170465112 0.314143174 -0.514714621 0.757291255
3 0 0.998207054
PCDHGA5 ENSG00000253485.2 0.59830256 2 2 0.364539762
Brain_Caudate_basal_ganglia 0.494135861 Brain_Anterior_cingulate_cortex_BA24
1.273643478 0.726356522 0.726356522 -0.906748591 -0.68374554
-0.795247065 0.157686969 2 0 0.998207054
LEMD1 ENSG00000186007.9 0.598354944 1 1 0.598354944 Brain_Cortex
0.598354944 Brain_Cortex 1 1 1 0.526767659 0.526767659
0.526767659 NA 1 0 0.998207054
SLC25A51 ENSG00000122696.13 0.59844491 13 1 0.366080334
Brain_Hippocampus 0.968092149 Brain_Substantia_nigra 12.58111807
0.000450389 12.58111807 0.040001225 0.903839838 0.51709868
0.196433448 1 0 0.998207054
ADGRA3 ENSG00000152990.13 0.598462903 2 2 0.366443967
Brain_Hippocampus 0.573116967 Brain_Caudate_basal_ganglia 1.118772455
0.881227545 0.881227545 -0.903154378 -0.563466939 -0.733310659
0.240195292 2 0 0.998207054
RP11-569G13.3 ENSG00000275465.5 0.598632124 2 2 0.744864665
Brain_Substantia_nigra 0.765501948 Brain_Putamen_basal_ganglia
1.810441419 0.189558581 0.189558581 -0.325418091 0.298263646
-0.013577222 0.441009586 2 0 0.998207054
HEXIM1 ENSG00000186834.3 0.598692109 3 3 0.290756334
Brain_Putamen_basal_ganglia 0.942143923 Brain_Cortex 1.234503626
0.77142066 0.77142066 -1.056463881 -0.0725755 -0.658566308 0.518211718
3 0 0.998207054
CCT8 ENSG00000156261.12 0.598798127 6 5 0.09343193
Brain_Cerebellar_Hemisphere 0.94312578 Brain_Anterior_cingulate_cortex_BA24
3.425972098 0.033282717 0.117586516 -0.870024622 1.677565616
0.446598469 0.872817206 5 0 0.998207054
SCGB1B2P ENSG00000268751.1 0.598897055 7 5 0.124474125
Brain_Cerebellum 0.78267469 Brain_Putamen_basal_ganglia 4.262654742
0.060371355 0.260874441 -1.536262043 1.049764372 0.147729802
0.953159019 5 0 0.998207054
MCAT ENSG00000100294.12 0.598952417 6 5 0.186130844 Brain_Amygdala
0.728961702 Brain_Cortex 2.498132509 0.067252231 0.422618213
-1.322112042 -0.34650703 -0.8020568 0.342980325 5 0
0.998207054
VCL ENSG00000035403.17 0.599000072 2 2 0.314536443
Brain_Frontal_Cortex_BA9 0.897839755 Brain_Nucleus_accumbens_basal_ganglia
1.012326276 0.987673724 0.987673724 -1.005748756 0.128390745
-0.438679005 0.801957732 2 0 0.998207054
FIGNL1 ENSG00000132436.11 0.5990356 12 3 0.109837876
Brain_Hippocampus 0.945147123 Brain_Putamen_basal_ganglia 10.00830978
0.01058853 0.410051347 0.068802129 1.598922265 0.642073979
0.425378104 3 0 0.998207054
PI16 ENSG00000164530.13 0.599120333 10 6 0.11604908
Brain_Anterior_cingulate_cortex_BA24 0.969480661 Brain_Caudate_basal_ganglia
6.029900802 0.014691806 0.251479177 -0.708129481 1.571575296
0.130003129 0.729907227 6 0 0.998207054
LINC02150 ENSG00000248150.1 0.599435104 6 4 0.126436142
Brain_Cerebellum 0.893684129 Brain_Frontal_Cortex_BA9 3.415928011
0.045672286 0.184008105 -1.528307769 0.370726332 -0.606836442
0.784742099 4 0 0.998207054
ZNF768 ENSG00000169957.9 0.599607031 1 1 0.599607031
Brain_Putamen_basal_ganglia 0.599607031 Brain_Putamen_basal_ganglia 1 1
1 0.524965706 0.524965706 0.524965706 NA 1 0
0.998207054

CTD-2245E15.3 ENSG00000251532.1 0.599609428 5 5 0.098907874
Brain_Cerebellum 0.867061308 Brain_Cortex 2.376451712 0.238828059
0.238828059 -0.300939583 1.65017145 0.497125031 0.720350614 5
0 0.998207054
ELAC2 ENSG00000006744.18 0.599632655 13 1 0.392118165
Brain_Cerebellar_Hemisphere 0.980028321 Brain_Caudate_basal_ganglia
12.11637606 8.05E-05 12.11637606 -0.064858248 0.855782377
0.500555766 0.288472307 1 0 0.998207054
LINC01198 ENSG00000231817.7 0.599751223 3 2 0.2961862 Brain_Cerebellum
0.610895574 Brain_Substantia_nigra 2.391420459 0.012795451
0.59578409 -1.044646888 -0.5087955 -0.841308453 0.290342418 2 0
0.998207054
ALG1L ENSG00000189366.9 0.599920398 13 1 0.350450674
Brain_Hypothalamus 0.97136834 Brain_Putamen_basal_ganglia 11.9621893 0.00413438
11.9621893 -0.209579807 0.933715487 0.499410444 0.322076677 1
0 0.998207054
CFAP43 ENSG00000197748.12 0.59993711 5 5 0.118741394
Brain_Frontal_Cortex_BA9 0.576471713 Brain_Nucleus_accumbens_basal_ganglia
2.82126172 0.140569216 0.140569216 -1.560078236 -0.558545838
-1.060422105 0.386402042 5 0 0.998207054
TBC1D2 ENSG00000095383.19 0.599952807 13 3 0.342505422
Brain_Amygdala 0.837285536 Brain_Substantia_nigra 11.06166065
0.004117994 0.570002261 -0.402795225 0.949226508 0.527313464
0.355825944 3 0 0.998207054
COX6A2 ENSG00000156885.5 0.599970527 1 1 0.599970527
Brain_Substantia_nigra 0.599970527 Brain_Substantia_nigra 1 1
1 0.524442898 0.524442898 0.524442898 NA 1 0
0.998207054
ST8SIA4 ENSG00000113532.12 0.600121825 1 1 0.600121825
Brain_Caudate_basal_ganglia 0.600121825 Brain_Caudate_basal_ganglia 1 1
1 -0.52422533 -0.52422533 -0.52422533 NA 1 0
0.998207054
CCDC94 ENSG00000105248.15 0.600306961 8 4 0.274911565
Brain_Cerebellar_Hemisphere 0.749774979 Brain_Anterior_cingulate_cortex_BA24
6.540556188 0.003479536 0.231408232 -0.780963302 1.091821505
0.610343927 0.614312701 4 0 0.998207054
PFKL ENSG00000141959.16 0.600432743 11 9 0.18001336 Brain_Cerebellum
0.934372133 Brain_Putamen_basal_ganglia 5.179667062 0.088161699
0.184784926 -0.600416797 1.3407139 0.249595678 0.541460008 9
0 0.998207054
RP11-301G7.1 ENSG00000272479.1 0.600651042 3 2 0.331977909
Brain_Caudate_basal_ganglia 0.821264649 Brain_Spinal_cord_cervical_c-1
2.006943726 0.027555999 0.965500275 -0.9701376 0.225918705
-0.55042493 0.673072734 2 0 0.998207054
ELF1 ENSG00000120690.15 0.600676913 1 1 0.600676913
Brain_Substantia_nigra 0.600676913 Brain_Substantia_nigra 1 1
1 0.523427325 0.523427325 0.523427325 NA 1 0
0.998207054
KANSL2 ENSG00000139620.12 0.600968922 4 4 0.278269146
Brain_Cortex 0.468817495 Brain_Substantia_nigra 1.92838555
0.072800504 0.072800504 -0.909493594 1.084215759 0.015282214
1.037427932 4 0 0.998207054
TOP1MT ENSG00000184428.12 0.600981608 3 3 0.179045555
Brain_Cerebellum 0.870882875 Brain_Hippocampus 2.028419138
0.138916967 0.138916967 -1.343699574 0.162537151 -0.726357597
0.788982223 3 0 0.998207054
LINC00403 ENSG00000224243.1 0.601061661 3 3 0.257992935
Brain_Cerebellum 0.690366227 Brain_Cortex 1.297157351 0.742929989
0.742929989 -0.506968305 1.13114769 0.075273756 0.916024763 3
0 0.998207054
JMJD8 ENSG00000161999.11 0.601112516 6 6 0.173741827
Brain_Hypothalamus 0.93579757 Brain_Cortex 1.973358203 0.432797521

0.432797521 -1.360278443 0.435443848 -0.657670832 0.693064351
6 0 0.998207054
TTLL1 ENSG00000100271.16 0.601142424 11 11 0.246569105
Brain_Substantia_nigra 0.94921707 Brain_Nucleus_accumbens_basal_ganglia
4.028953523 0.142095633 0.142095633 -1.085622801 1.158723018
-0.00494685 0.698708551 11 0 0.998207054
LRRN3 ENSG00000173114.12 0.601151714 1 1 0.601151714 Brain_Cortex
0.601151714 Brain_Cortex 1 1 1 0.522745006 0.522745006
0.522745006 NA 1 0 0.998207054
VGLL3 ENSG00000206538.8 0.601238158 1 1 0.601238158
Brain_Caudate_basal_ganglia 0.601238158 Brain_Caudate_basal_ganglia 1 1
1 0.522620807 0.522620807 0.522620807 NA 1 0
0.998207054
APIP ENSG00000149089.12 0.60125293 13 1 0.491574198 Brain_Cerebellum
0.752655603 Brain_Cerebellar_Hemisphere 12.86681538 0.000497302
12.86681538 0.315139679 0.687807206 0.519827327 0.102269218
1 0 0.998207054
PLK2 ENSG00000145632.14 0.601344238 1 1 0.601344238
Brain_Frontal_Cortex_BA9 0.601344238 Brain_Frontal_Cortex_BA9 1 1
1 0.522468407 0.522468407 0.522468407 NA 1 0
0.998207054
MYH14 ENSG00000105357.15 0.601373728 2 2 0.331974257
Brain_Spinal_cord_cervical_c-1 0.782011594 Brain_Caudate_basal_ganglia
1.001243203 0.998756797 0.998756797 -0.970144928 0.276698539
-0.346723194 0.88165147 2 0 0.998207054
PIAS1 ENSG00000033800.13 0.601374005 1 1 0.601374005
Brain_Hippocampus 0.601374005 Brain_Hippocampus 1 1 1
0.522425645 0.522425645 0.522425645 NA 1 0 0.998207054
KRT23 ENSG00000108244.16 0.601425995 3 3 0.208428167
Brain_Nucleus_accumbens_basal_ganglia 0.242525168 Brain_Putamen_basal_ganglia
2.41700609 0.131957042 0.131957042 1.168698396 1.257899327
1.221905663 0.047025872 3 0 0.998207054
RP11-624D11.2 ENSG00000254532.1 0.601432238 3 1 0.544066226
Brain_Caudate_basal_ganglia 0.71309184 Brain_Putamen_basal_ganglia 2.96002293
0.010421946 2.96002293 -0.606675589 -0.367706839 -0.518773966
0.131411287 1 0 0.998207054
TAS2R20 ENSG00000255837.1 0.601457082 4 4 0.287352537
Brain_Cerebellum 0.990865052 Brain_Caudate_basal_ganglia 3.130765881
0.142231231 0.142231231 -1.063947381 0.163939207 -0.345360368
0.555226443 4 0 0.998207054
ASB14 ENSG00000239388.8 0.601500947 4 4 0.158550361 Brain_Amygdala
0.981225736 Brain_Cerebellar_Hemisphere 2.575178413 0.180683791
0.180683791 -0.09565781 1.409963839 0.434057109 0.69342381 4
0 0.998207054
SDK2 ENSG00000069188.16 0.601514551 1 1 0.601514551 Brain_Cerebellum
0.601514551 Brain_Cerebellum 1 1 1 0.52222375 0.52222375 0.52222375
NA 1 0 0.998207054
CDX2 ENSG00000165556.9 0.601744757 2 2 0.654887227
Brain_Cerebellar_Hemisphere 0.93756919 Brain_Cerebellum 1.859107819
0.140892181 0.140892181 -0.078325429 0.446983148 0.184328859
0.371449257 2 0 0.998207054
VEZF1 ENSG00000136451.8 0.601781781 1 1 0.601781781
Brain_Hypothalamus 0.601781781 Brain_Hypothalamus 1 1 1
0.521839936 0.521839936 0.521839936 NA 1 0 0.998207054
LONP1 ENSG00000196365.11 0.601815849 11 10 0.034604167 Brain_Cortex
0.969629274 Brain_Hippocampus 2.353268592 9.57E-18 0.476966718
-2.112960289 0.047171712 -0.594426643 0.622978783 10 0
0.998207054
AHSA2 ENSG00000173209.22 0.601835247 13 1 0.456654524
Brain_Caudate_basal_ganglia 0.745250164 Brain_Cerebellum 12.13118863
0.006646081 12.13118863 -0.744366923 -0.324908711 -0.50611933
0.140812606 1 0 0.998207054

MRPS12 ENSG00000128626.11 0.601917072 2 2 0.5366702 Brain_Cerebellum 0.559817388 Brain_Hippocampus 1.289356607 0.710643393 0.710643393 -0.617856099 0.58311277 -0.017371665 0.849213231 2 0 0.998207054
LA16c-352F7.1 ENSG00000278716.1 0.602033514 1 1 0.602033514 Brain_Hypothalamus 0.602033514 Brain_Hypothalamus 1 1 1 -0.52147845 -0.52147845 -0.52147845 NA 1 0 0.998207054
PHF24 ENSG00000122733.12 0.602045138 6 6 0.136500469 Brain_Hippocampus 0.71025113 Brain_Frontal_Cortex_BA9 1.506637966 0.672832521 0.672832521 -0.585284687 1.488950252 0.369169157 0.919870783 6 0 0.998207054
MGEA5 ENSG00000198408.13 0.602129606 1 1 0.602129606 Brain_Nucleus_accumbens_basal_ganglia 0.602129606 Brain_Nucleus_accumbens_basal_ganglia 1 1 1 -0.521340481 -0.521340481 -0.521340481 NA 1 0 0.998207054
MSS51 ENSG00000166343.9 0.602144016 1 1 0.602144016 Brain_Substantia_nigra 0.602144016 Brain_Substantia_nigra 1 1 1 0.521319791 0.521319791 0.521319791 NA 1 0 0.998207054
FSTL4 ENSG00000053108.16 0.602255401 4 4 0.366017901 Brain_Putamen_basal_ganglia 0.83432356 Brain_Cerebellum 1.876852852 0.359657867 0.359657867 -0.90395757 0.444826277 -0.390529731 0.644885507 4 0 0.998207054
MCHR1 ENSG00000128285.4 0.602260427 5 3 0.370762557 Brain_Frontal_Cortex_BA9 0.808375749 Brain_Cerebellum 3.364175953 0.041154061 0.440601861 -0.827286623 0.895045891 0.120125905 0.675594809 3 0 0.998207054
MEFV ENSG00000103313.12 0.60249649 1 1 0.60249649 Brain_Putamen_basal_ganglia 0.60249649 Brain_Putamen_basal_ganglia 1 1 1 0.520813799 0.520813799 0.520813799 NA 1 0 0.998207054
HEY1 ENSG00000164683.16 0.602679916 2 2 0.324924794 Brain_Hypothalamus 0.769176249 Brain_Putamen_basal_ganglia 1.0864971 0.9135029 0.9135029 -0.293452569 0.984387963 0.345467697 0.903569705 2 0 0.998207054
DMBT1 ENSG00000187908.16 0.602785614 3 2 0.312404012 Brain_Amygdala 0.988763358 Brain_Hippocampus 1.95127757 0.06310147 0.985620961 -0.014083507 1.010190538 0.40245366 0.538240027 2 0 0.998207054
RGS7 ENSG00000182901.16 0.602805343 1 1 0.602805343 Brain_Spinal_cord_cervical_c-1 0.602805343 Brain_Spinal_cord_cervical_c-1 1 1 1 0.520370535 0.520370535 0.520370535 NA 1 0 0.998207054
ZCRB1 ENSG00000139168.7 0.602829116 6 4 0.527727589 Brain_Cortex 0.899591103 Brain_Cerebellum 4.398384738 0.018596645 0.164947485 -0.35458824 0.631478674 0.115760469 0.377314821 4 0 0.998207054
SMIM22 ENSG00000267795.5 0.603136656 11 8 0.137676621 Brain_Hippocampus 0.972942118 Brain_Caudate_basal_ganglia 5.098188957 0.022082654 0.230130315 -0.134668156 1.484498877 0.722571216 0.577075055 8 0 0.998207054
NOTO ENSG00000214513.3 0.603197723 2 2 0.317474908 Brain_Cerebellum 0.35502825 Brain_Cerebellar_Hemisphere 1.872283742 0.127716258 0.127716258 -0.999660346 -0.924880155 -0.962270251 0.05287758 2 0 0.998207054
ZNF529 ENSG00000186020.12 0.60324274 10 6 0.05381124 Brain_Hippocampus 0.66739303 Brain_Frontal_Cortex_BA9 6.809986212 0.043070315 0.261154585 -1.92835295 -0.429728664 -1.228104889 0.491469104 6 0 0.998207054
WDR33 ENSG00000136709.11 0.603718477 1 1 0.603718477 Brain_Substantia_nigra 0.603718477 Brain_Substantia_nigra 1 1 1 -0.519060607 -0.519060607 -0.519060607 NA 1 0 0.998207054

ACOT6 ENSG00000205669.2 0.603795694 8 2 0.216831379 Brain_Cortex
0.988730065 Brain_Caudate_basal_ganglia 6.613118733 0.002790968
0.935469968 -1.234997648 0.014125238 -0.889464442 0.468579802
2 0 0.998207054
ENC1 ENSG00000171617.13 0.603864299 1 1 0.603864299 Brain_Cerebellum
0.603864299 Brain_Cerebellum 1 1 1 -0.518851503 -0.518851503
-0.518851503 NA 1 0 0.998207054
INA ENSG00000148798.10 0.603889359 6 4 0.129449967
Brain_Anterior_cingulate_cortex_BA24 0.454320696 Brain_Putamen_basal_ganglia
3.490667397 0.030012753 0.474239579 -1.516274462 -0.748231231
-0.920921701 0.295969404 4 0 0.998207054
GDI2 ENSG00000057608.16 0.603940365 6 3 0.146983292
Brain_Caudate_basal_ganglia 0.754445677 Brain_Frontal_Cortex_BA9
4.608308748 0.011722518 0.291677359 -1.450269819 -0.312782808
-0.972715262 0.392410751 3 0 0.998207054
DNASE2 ENSG00000105612.8 0.604068451 4 3 0.271302889
Brain_Nucleus_accumbens_basal_ganglia 0.932610535 Brain_Cerebellar_Hemisphere
2.230680611 0.032926492 0.732164348 -1.100067095 0.360488209
-0.306816106 0.61460419 3 0 0.998207054
CALCRL ENSG00000064989.12 0.604102052 3 3 0.359720751
Brain_Cerebellum 0.534673144 Brain_Cerebellar_Hemisphere 1.634208464
0.332819359 0.332819359 -0.915897324 -0.620888264 -0.799045715
0.156768591 3 0 0.998207054
TMEM184B ENSG00000198792.12 0.604271847 2 2 0.445374693
Brain_Cerebellar_Hemisphere 0.987258473 Brain_Nucleus_accumbens_basal_ganglia
1.661566826 0.338433174 0.338433174 -0.763148743 -0.015969815
-0.389559279 0.528335287 2 0 0.998207054
CYP51A1 ENSG00000001630.15 0.604450537 2 2 0.447321752
Brain_Spinal_cord_cervical_c-1 0.527200008 Brain_Hypothalamus
1.029923527 0.970076473 0.970076473 -0.632286002 0.759887637
0.063800817 0.984415421 2 0 0.998207054
VPS37A ENSG00000155975.9 0.604580678 3 3 0.266986675
Brain_Spinal_cord_cervical_c-1 0.7361644 Brain_Amygdala 1.095036428
0.899764238 0.899764238 -0.732838773 1.110028663 0.238042293
0.925405434 3 0 0.998207054
GOLGA8T ENSG00000261247.1 0.604582022 2 2 0.388223255
Brain_Putamen_basal_ganglia 0.705413111 Brain_Caudate_basal_ganglia
1.148464354 0.851535646 0.851535646 -0.862843979 0.378023534
-0.242410222 0.877425833 2 0 0.998207054
LACTB2 ENSG00000147592.8 0.604605851 8 3 0.373752958
Brain_Caudate_basal_ganglia 0.930876839 Brain_Anterior_cingulate_cortex_BA24
6.323787987 0.00398324 0.339127995 0.086741688 0.889465497
0.530163012 0.262359643 3 0 0.998207054
NCMAP ENSG00000184454.6 0.60462718 7 6 0.141490614
Brain_Nucleus_accumbens_basal_ganglia 0.958539792 Brain_Cortex
3.136390634 0.017471199 0.289432932 -1.145140065 1.470262718
-0.108920866 0.868053111 6 0 0.998207054
HDAC4 ENSG00000068024.16 0.604645673 2 2 0.316758509
Brain_Hippocampus 0.937604345 Brain_Cortex 1.015490131
0.984509869 0.984509869 -0.078281233 1.001141282 0.461430024
0.76326698 2 0 0.998207054
POPDC2 ENSG00000121577.13 0.604718529 3 3 0.182245017
Brain_Cerebellar_Hemisphere 0.966174803 Brain_Amygdala 1.153777145
0.865136181 0.865136181 -1.333874417 -0.042406304 -0.603532315
0.662154088 3 0 0.998207054
SLC37A2 ENSG00000134955.11 0.604736121 2 2 0.325743985
Brain_Cerebellar_Hemisphere 0.552821363 Brain_Cerebellum 1.740956142
0.259043858 0.259043858 0.593537696 0.9827226 0.788130148
0.275195285 2 0 0.998207054
ATAD3A ENSG00000197785.13 0.604767306 3 3 0.348496514
Brain_Anterior_cingulate_cortex_BA24 0.659484226 Brain_Cortex
1.404391685 0.608339484 0.608339484 -0.937509549 0.538606316

0.013907382 0.825406384 3 0 0.998207054
LCE1C ENSG00000197084.5 0.604778699 2 2 0.32031121 Brain_Cerebellum
0.396859715 Brain_Cerebellar_Hemisphere 1.765362591 0.234637409
0.234637409 0.847242956 0.993818557 0.920530757 0.103644601
2 0 0.998207054
PC ENSG00000173599.13 0.604830116 2 2 0.335642021 Brain_Cerebellum
0.766342194 Brain_Caudate_basal_ganglia 1.01748302 0.98251698 0.98251698
-0.962811714 -0.297162835 -0.629987274 0.470684836 2 0
0.998207054
NCK2 ENSG00000071051.13 0.604957953 2 2 0.316058275
Brain_Cerebellar_Hemisphere 0.955386544 Brain_Caudate_basal_ganglia
1.053788666 0.946211334 0.946211334 0.055943843 1.002590928
0.529267386 0.669380573 2 0 0.998207054
QRICH2 ENSG00000129646.14 0.604998862 6 6 0.082096045
Brain_Anterior_cingulate_cortex_BA24 0.816874924 Brain_Cerebellum
3.822419813 0.145037357 0.145037357 -1.738651707 -0.231566249
-0.783830933 0.62518223 6 0 0.998207054
FANCE ENSG00000112039.3 0.605084823 1 1 0.605084823
Brain_Caudate_basal_ganglia 0.605084823 Brain_Caudate_basal_ganglia 1 1
1 0.517102193 0.517102193 0.517102193 NA 1 0
0.998207054
RAB2B ENSG00000129472.13 0.605152382 1 1 0.605152382
Brain_Spinal_cord_cervical_c-1 0.605152382 Brain_Spinal_cord_cervical_c-1
1 1 1 0.517005411 0.517005411 0.517005411 NA 1 0
0.998207054
CHCHD2 ENSG00000106153.12 0.605326128 11 2 0.186268496
Brain_Hypothalamus 0.699189872 Brain_Anterior_cingulate_cortex_BA24
9.640494126 0.00043181 0.760344318 0.386414286 1.321698716
0.937425903 0.276795008 2 0 0.998207054
DTX3 ENSG00000178498.15 0.605330353 2 2 0.435736599 Brain_Amygdala
0.571172762 Brain_Caudate_basal_ganglia 1.078756342 0.921243658
0.921243658 -0.566325156 0.779412779 0.106543811 0.95158042 2
0 0.998207054
SLC27A1 ENSG00000130304.16 0.605356723 6 2 0.496009024
Brain_Substantia_nigra 0.846776459 Brain_Cerebellum 5.484541935
0.005218484 0.294758597 -0.21793265 0.68078266 0.243859582
0.287356945 2 0 0.998207054
FBXO22 ENSG00000167196.13 0.605404842 5 4 0.138735383
Brain_Cerebellar_Hemisphere 0.974352579 Brain_Anterior_cingulate_cortex_BA24
3.252882309 0.059975016 0.186268423 -1.480516791 -0.032149813
-0.798174996 0.636430935 4 0 0.998207054
PPP1CB ENSG00000213639.9 0.605502163 9 4 0.468932286
Brain_Caudate_basal_ganglia 0.960737607 Brain_Hippocampus 7.12621986
0.008360137 0.250625177 -0.724217387 0.584918335 -0.176129312
0.458858339 4 0 0.998207054
DIS3L2 ENSG00000144535.19 0.605533929 1 1 0.605533929
Brain_Hippocampus 0.605533929 Brain_Hippocampus 1 1 1
0.516458911 0.516458911 0.516458911 NA 1 0 0.998207054
RP11-861E21.1 ENSG00000267108.1 0.605555902 13 2 0.188032061
Brain_Spinal_cord_cervical_c-1 0.967930612 Brain_Amygdala 11.0847111
0.005182862 0.748862896 -0.866349665 1.316423138 -0.090491378
0.508417625 2 0 0.998207054
PCYOX1 ENSG00000116005.11 0.605605754 1 1 0.605605754
Brain_Caudate_basal_ganglia 0.605605754 Brain_Caudate_basal_ganglia 1 1
1 -0.516356052 -0.516356052 -0.516356052 NA 1 0
0.998207054
NRIP2 ENSG00000053702.14 0.605697333 4 4 0.240108968
Brain_Caudate_basal_ganglia 0.5373702 Brain_Putamen_basal_ganglia 2.387439659
0.206251671 0.206251671 -1.174714471 0.683349871 -0.446019769
0.793408228 4 0 0.998207054
LYPD8 ENSG00000259823.5 0.60574967 4 4 0.153941124 Brain_Cortex
0.735788792 Brain_Caudate_basal_ganglia 1.995288844 0.09020112 0.09020112

-0.97217065 1.425747902 -0.088555977 1.045882536 4 0
0.998207054
LRRC4C ENSG00000148948.7 0.605790561 3 3 0.191627447
Brain_Spinal_cord_cervical_c-1 0.938765192 Brain_Caudate_basal_ganglia
1.154467167 0.875395096 0.875395096 -0.076821945 1.305779814
0.550826906 0.700036891 3 0 0.998207054
CAHM ENSG00000270419.1 0.605794444 1 1 0.605794444
Brain_Nucleus_accumbens_basal_ganglia 0.605794444
Brain_Nucleus_accumbens_basal_ganglia 1 1 1 -0.51608586
-0.51608586 -0.51608586 NA 1 0 0.998207054
TRAPPC8 ENSG00000153339.13 0.605825702 1 1 0.605825702
Brain_Cerebellum 0.605825702 Brain_Cerebellum 1 1 1 -0.516041103
-0.516041103 -0.516041103 NA 1 0 0.998207054
POMGNT2 ENSG00000144647.5 0.605895121 10 4 0.218623744
Brain_Spinal_cord_cervical_c-1 0.819354223 Brain_Cerebellar_Hemisphere
7.597509332 0.001382161 0.396138819 -0.633681746 1.23019593
-0.268080507 0.539385263 4 0 0.998207054
ZBTB49 ENSG00000168826.15 0.606102984 4 4 0.168190486
Brain_Hippocampus 0.511880025 Brain_Spinal_cord_cervical_c-1
1.842583583 0.373361399 0.373361399 -1.188446394 1.378041466
0.38303131 1.099426543 4 0 0.998207054
CENPJ ENSG00000151849.14 0.606107373 8 4 0.106495711
Brain_Anterior_cingulate_cortex_BA24 0.557954173 Brain_Putamen_basal_ganglia
5.977707244 0.03402641 0.3330081 0.585882955 1.614146234
1.185402668 0.32743429 4 0 0.998207054
TSGA10 ENSG00000135951.14 0.606228295 12 3 0.29511602 Brain_Cerebellum
0.782338377 Brain_Amygdala 9.564375491 0.001363727 0.707041859
-0.464032825 1.046964352 0.493070483 0.459406599 3 0
0.998207054
GFPT2 ENSG00000131459.12 0.606288343 7 7 0.097548686
Brain_Caudate_basal_ganglia 0.862249113 Brain_Putamen_basal_ganglia
2.107852049 0.210843729 0.210843729 -0.477740153 1.656855533
0.367000691 0.785404568 7 0 0.998207054
LINC00339 ENSG00000218510.6 0.606302981 13 1 0.411571899
Brain_Nucleus_accumbens_basal_ganglia 0.942718978
Brain_Spinal_cord_cervical_c-1 12.49107273 0.000460029 12.49107273
-0.82113057 0.204418793 -0.499033049 0.317189472 1 0
0.998207054
C1orf220 ENSG00000213057.5 0.606360074 3 3 0.585057807
Brain_Caudate_basal_ganglia 0.778739948 Brain_Cerebellum 2.102703203
0.121715457 0.121715457 -0.546011828 0.280961475 -0.183470268
0.422797136 3 0 0.998207054
HIPK2 ENSG00000064393.15 0.606385121 2 2 0.341065372
Brain_Hypothalamus 0.752195336 Brain_Putamen_basal_ganglia 1.009529091
0.990470909 0.990470909 -0.95206233 -0.315745963 -0.633904147
0.449943618 2 0 0.998207054
ASRGL1 ENSG00000162174.12 0.606435109 10 3 0.343074325
Brain_Hippocampus 0.906249579 Brain_Caudate_basal_ganglia 7.520339737
0.000263359 0.934262697 -0.355195167 0.948108297 0.323750101
0.520245116 3 0 0.998207054
SH2B1 ENSG00000178188.14 0.606440489 4 3 0.211727629 Brain_Cerebellum
0.741492178 Brain_Hypothalamus 1.979147882 0.038463229 0.97089311
-1.248828987 0.352496762 -0.433893846 0.895625663 3 0
0.998207054
WAC ENSG00000095787.21 0.606478654 4 4 0.289889627
Brain_Substantia_nigra 0.930977917 Brain_Cerebellar_Hemisphere
2.594303088 0.127966548 0.127966548 -0.38724801 1.058363777
0.144688745 0.63924169 4 0 0.998207054
UTP18 ENSG00000011260.13 0.606606133 11 3 0.257476261 Brain_Cerebellum
0.970984458 Brain_Spinal_cord_cervical_c-1 8.92754264 0.000470483
0.433852814 -0.826896042 1.132376309 -0.079430803 0.526351168
3 0 0.998207054

KYNU ENSG00000115919.14 0.606610789 2 2 0.336892461
Brain_Substantia_nigra 0.577611472 Brain_Putamen_basal_ganglia
1.303656748 0.696343252 0.696343252 0.556876998 0.96032343
0.758600214 0.285279708 2 0 0.998207054
PIGK ENSG00000142892.14 0.606779245 2 2 0.4320058
Brain_Nucleus_accumbens_basal_ganglia 0.533118165 Brain_Putamen_basal_ganglia
1.006877529 0.993122471 0.993122471 -0.785763933 0.623253172
-0.081255381 0.99632555 2 0 0.998207054
ZNF781 ENSG00000196381.10 0.60701034 10 3 0.344467641 Brain_Cortex
0.880673325 Brain_Spinal_cord_cervical_c-1 8.166701072 0.015363892
0.339051576 -0.473938251 0.945374647 0.178192224 0.503996509
3 0 0.998207054
RAB8B ENSG00000166128.12 0.607012062 2 2 0.353870778
Brain_Substantia_nigra 0.73250373 Brain_Cerebellum 1.033083558
0.966916442 0.966916442 -0.927107394 0.341796916 -0.292655239
0.897250842 2 0 0.998207054
TMEM45A ENSG00000181458.10 0.607029285 4 4 0.430984452
Brain_Spinal_cord_cervical_c-1 0.884985856 Brain_Substantia_nigra
2.435875249 0.136393638 0.136393638 -0.594167223 0.787508162
0.220737788 0.604485176 4 0 0.998207054
OGFOD3 ENSG00000181396.12 0.607064507 12 9 0.139398448
Brain_Frontal_Cortex_BA9 0.861795892 Brain_Anterior_cingulate_cortex_BA24
5.042143231 0.006476779 0.1982555 -1.069670476 1.478034858
-0.032581797 0.847224226 9 0 0.998207054
RARS2 ENSG00000146282.17 0.607152414 2 2 0.459305617 Brain_Cortex
0.95547426 Brain_Hippocampus 1.629329351 0.370670649 0.370670649
-0.055833736 0.739990733 0.342078499 0.562732879 2 0
0.998207054
LINC02019 ENSG00000273356.1 0.607266205 2 2 0.387627255
Brain_Caudate_basal_ganglia 0.510829595 Brain_Substantia_nigra
1.191731997 0.808268003 0.808268003 0.657546479 0.863928344
0.760737412 0.145934016 2 0 0.998207054
EFEMP2 ENSG00000172638.12 0.607328454 4 3 0.183808834
Brain_Cerebellar_Hemisphere 0.802003753 Brain_Hypothalamus 2.687234065
0.059375523 0.434847096 0.250754718 1.329118634 0.824822104
0.508105303 3 0 0.998207054
TSEN15 ENSG00000198860.11 0.607526101 9 4 0.114864679
Brain_Cerebellum 0.979419457 Brain_Nucleus_accumbens_basal_ganglia 7.07117774
0.006572691 0.306817227 -1.576699529 0.025796747 -1.088989006
0.45054089 4 0 0.998207054
DNAJC3-AS1 ENSG00000247400.3 0.607589449 6 6 0.07978137
Brain_Nucleus_accumbens_basal_ganglia 0.760887695 Brain_Caudate_basal_ganglia
3.381822394 0.12920723 0.12920723 0.304315293 1.751956026
1.273341991 0.517445008 6 0 0.998207054
UBE2F ENSG00000184182.18 0.607656972 4 4 0.20303574
Brain_Nucleus_accumbens_basal_ganglia 0.851991684
Brain_Spinal_cord_cervical_c-1 2.638938326 0.187184852 0.187184852
-1.272950126 -0.186577788 -0.781660146 0.462753025 4 0
0.998207054
AC003090.1 ENSG00000223561.6 0.607792954 4 3 0.188961432
Brain_Caudate_basal_ganglia 0.552049753 Brain_Hypothalamus 2.818408846
0.093455676 0.406243434 0.594691427 1.313657671 0.841587704
0.332295467 3 0 0.998207054
AP001065.15 ENSG00000228709.1 0.607801715 1 1 0.607801715
Brain_Spinal_cord_cervical_c-1 0.607801715 Brain_Spinal_cord_cervical_c-1
1 1 1 0.513213883 0.513213883 0.513213883 NA 1 0
0.998207054
ZFP69 ENSG00000187815.9 0.607826491 12 3 0.547291836 Brain_Cerebellum
0.963468496 Brain_Cortex 10.3296247 0.01291083 0.52708695 -0.601823192
0.557420474 0.117494739 0.408505084 3 0 0.998207054
ZCCHC2 ENSG00000141664.9 0.607854251 1 1 0.607854251
Brain_Cerebellum 0.607854251 Brain_Cerebellum 1 1 1 0.513138773

0.513138773 0.513138773 NA 1 0 0.998207054
RASGRP2 ENSG00000068831.18 0.607876889 1 1 0.607876889
Brain_Hippocampus 0.607876889 Brain_Hippocampus 1 1 1
-0.513106409 -0.513106409 -0.513106409 NA 1 0 0.998207054
FAM124A ENSG00000150510.16 0.6080096 1 1 0.6080096 Brain_Cortex
0.6080096 Brain_Cortex 1 1 1 -0.512916687 -0.512916687
-0.512916687 NA 1 0 0.998207054
LAYN ENSG00000204381.11 0.608113631 10 5 0.100965632 Brain_Amygdala
0.93479934 Brain_Putamen_basal_ganglia 6.643794968 0.014956186
0.455798741 -1.640190174 0.396091736 -0.051779258 0.590796379
5 0 0.998207054
TPM1 ENSG00000140416.19 0.608160045 3 3 0.219086141
Brain_Cerebellar_Hemisphere 0.980131451 Brain_Cerebellum 1.409219126
0.509202383 0.509202383 -1.228961768 0.024904107 -0.571690339
0.62913129 3 0 0.998207054
ANXA13 ENSG00000104537.16 0.608236231 4 4 0.129947291
Brain_Spinal_cord_cervical_c-1 0.868812059 Brain_Hypothalamus
1.479112986 0.522442918 0.522442918 -0.16516761 1.514309772
0.645464733 0.68822552 4 0 0.998207054
PLPP2 ENSG00000141934.9 0.608418218 1 1 0.608418218 Brain_Cortex
0.608418218 Brain_Cortex 1 1 1 -0.51233265 -0.51233265
-0.51233265 NA 1 0 0.998207054
VTA1 ENSG00000009844.15 0.608434988 7 6 0.171700921 Brain_Cortex
0.953866667 Brain_Cerebellum 4.924282482 0.037209361 0.168657782
-0.24082681 1.3667588 0.48375908 0.601752905 6 0 0.998207054
RAB42 ENSG00000188060.7 0.608489262 1 1 0.608489262 Brain_Cortex
0.608489262 Brain_Cortex 1 1 1 -0.512231126 -0.512231126
-0.512231126 NA 1 0 0.998207054
RP1-182D15.2 ENSG00000236173.1 0.608541426 10 3 0.216272597
Brain_Nucleus_accumbens_basal_ganglia 0.925883178 Brain_Hypothalamus
7.947524271 0.007403018 0.576385623 -1.236500455 0.327080739
-0.449260584 0.460097806 3 0 0.998207054
ELK4 ENSG00000158711.13 0.608614709 1 1 0.608614709 Brain_Cerebellum
0.608614709 Brain_Cerebellum 1 1 1 -0.512051869 -0.512051869
-0.512051869 NA 1 0 0.998207054
CA12 ENSG00000074410.13 0.60862218 9 7 0.069144437
Brain_Substantia_nigra 0.984749698 Brain_Cerebellum 5.524154078
0.019248921 0.233360464 -1.817474841 0.628106866 -0.413510579
0.70151004 7 0 0.998207054
GFM2 ENSG00000164347.17 0.608747829 8 5 0.280451269
Brain_Caudate_basal_ganglia 0.973854673 Brain_Cerebellum 5.514577771
0.001305738 0.267588869 -1.079306158 0.60052948 -0.37686803
0.512636202 5 0 0.998207054
TMTC4 ENSG00000125247.15 0.608842933 6 6 0.096217015 Brain_Amygdala
0.974167439 Brain_Putamen_basal_ganglia 3.280904958 0.116623861
0.116623861 -1.663476882 0.230549766 -0.272187534 0.696173523
6 0 0.998207054
PRR15 ENSG00000176532.3 0.608879904 1 1 0.608879904 Brain_Amygdala
0.608879904 Brain_Amygdala 1 1 1 -0.511672974 -0.511672974
-0.511672974 NA 1 0 0.998207054
DSE ENSG00000111817.16 0.609024512 3 3 0.294743496
Brain_Substantia_nigra 0.998103885 Brain_Cerebellar_Hemisphere
1.536745576 0.459766881 0.459766881 -0.00237643 1.047772369
0.571708996 0.531892252 3 0 0.998207054
AURKA ENSG00000087586.17 0.609111625 1 1 0.609111625 Brain_Cortex
0.609111625 Brain_Cortex 1 1 1 0.511341965 0.511341965
0.511341965 NA 1 0 0.998207054
MYOM2 ENSG00000036448.9 0.609186503 13 5 0.328657071
Brain_Hypothalamus 0.885020529 Brain_Nucleus_accumbens_basal_ganglia
7.914367307 0.01059561 0.342844021 -0.976822427 0.886884575
-0.228000283 0.629187398 5 0 0.998207054
PITRM1 ENSG00000107959.15 0.609281423 11 5 0.164864152

Brain_Amygdala 0.79174526 Brain_Cerebellum 7.279857644 0.021780651
0.321241881 0.264044979 1.388896741 0.784510716 0.40383425 5
0 0.998207054
UCK2 ENSG00000143179.14 0.609335861 1 1 0.609335861 Brain_Cerebellum
0.609335861 Brain_Cerebellum 1 1 1 -0.511021702 -0.511021702
-0.511021702 NA 1 0 0.998207054
NBR1 ENSG00000188554.13 0.60939093 7 3 0.301887017
Brain_Caudate_basal_ganglia 0.959915138 Brain_Cerebellum 5.460052436
0.001685989 0.430968903 -0.327804024 1.032395206 0.278125998
0.42353205 3 0 0.998207054
TPM2 ENSG00000198467.13 0.609878066 12 2 0.418423556
Brain_Caudate_basal_ganglia 0.886907025 Brain_Nucleus_accumbens_basal_ganglia
10.84155913 0.004047949 0.403829604 -0.809159242 0.597855507
-0.267285786 0.368039502 2 0 0.998207054
ADGRE2 ENSG00000127507.17 0.609933777 2 2 0.339259074
Brain_Spinal_cord_cervical_c-1 0.65468864 Brain_Amygdala 1.679176889
0.320823111 0.320823111 0.447258205 0.955630248 0.701444226
0.359473319 2 0 0.998207054
ICAM5 ENSG00000105376.4 0.610076141 2 2 0.328541323
Brain_Cerebellar_Hemisphere 0.892584785 Brain_Putamen_basal_ganglia
1.049462845 0.950537155 0.950537155 0.135034262 0.977056214
0.556045238 0.595399433 2 0 0.998207054
ODF3B ENSG00000177989.13 0.610239159 1 1 0.610239159 Brain_Cortex
0.610239159 Brain_Cortex 1 1 1 -0.509732103 -0.509732103
-0.509732103 NA 1 0 0.998207054
GGT1 ENSG00000100031.18 0.610371367 2 2 0.320421232
Brain_Nucleus_accumbens_basal_ganglia 0.827786184 Brain_Cerebellar_Hemisphere
1.206921359 0.793078641 0.793078641 -0.993592634 -0.217541737
-0.605567186 0.548750852 2 0 0.998207054
FAM184A ENSG00000111879.18 0.610647313 2 2 0.333491182
Brain_Cortex 0.337176737 Brain_Frontal_Cortex_BA9 1.88229947 0.11770053
0.11770053 -0.967105728 -0.959758571 -0.96343215 0.005195224 2
0 0.998207054
C1QTNF4 ENSG00000172247.3 0.610665082 4 2 0.255997407
Brain_Caudate_basal_ganglia 0.815594201 Brain_Cerebellum 3.486333439
0.029088463 0.382270553 0.233215332 1.135902415 0.63747486
0.427881222 2 0 0.998207054
LINC00664 ENSG00000268658.5 0.610699046 9 3 0.299459624
Brain_Frontal_Cortex_BA9 0.825106736 Brain_Cortex 7.370917486
0.004271595 0.27475913 -0.500594103 1.037592901 0.425703134
0.41744848 3 0 0.998207054
PON1 ENSG00000005421.8 0.610738744 2 2 0.321041862
Brain_Anterior_cingulate_cortex_BA24 0.828501744 Brain_Hypothalamus
1.25571262 0.74428738 0.74428738 0.216623534 0.992319155 0.604471344
0.548499633 2 0 0.998207054
KDELC2 ENSG00000178202.12 0.610926048 6 2 0.267939475
Brain_Cortex 0.531471539 Brain_Nucleus_accumbens_basal_ganglia
5.499815795 0.024388684 0.244327894 0.62576127 1.107820198
0.932333324 0.168845165 2 0 0.998207054
SPOPL ENSG00000144228.8 0.610990768 1 1 0.610990768 Brain_Cerebellum
0.610990768 Brain_Cerebellum 1 1 1 0.508659709 0.508659709
0.508659709 NA 1 0 0.998207054
UPP1 ENSG00000183696.13 0.611070536 2 2 0.384957557
Brain_Cerebellar_Hemisphere 0.595768291 Brain_Cerebellum 1.876502658
0.123497342 0.123497342 0.530495698 0.868798128 0.699646913
0.239215942 2 0 0.998207054
CPSF2 ENSG00000165934.12 0.611150065 10 2 0.260920264
Brain_Nucleus_accumbens_basal_ganglia 0.789417128 Brain_Putamen_basal_ganglia
9.042745067 0.001735753 0.47298064 0.267067581 1.124218688
0.732513141 0.250758682 2 0 0.998207054
IZUMO4 ENSG00000099840.13 0.611234554 1 1 0.611234554
Brain_Hippocampus 0.611234554 Brain_Hippocampus 1 1 1

-0.508312003	-0.508312003	-0.508312003	NA	1	0	0.998207054
CNOT7 ENSG00000198791.11	0.61127219 11	2	0.270431014
Brain_Frontal_Cortex_BA9	0.590172682	Brain_Spinal_cord_cervical_c-1
9.79797696 0.008323644	0.402363316	0.538585809	1.102070521
0.872395114	0.159270961	2	0	0.998207054
MFSD5 ENSG00000182544.8	0.611327029	2	2	0.322276879
Brain_Nucleus_accumbens_basal_ganglia 0.844193769	Brain_Frontal_Cortex_BA9
1.130902494	0.869097506	0.869097506	-0.989789784	-0.196532025
-0.593160904	0.560917941	2	0	0.998207054
SLC7A14	ENSG00000013293.5	0.611345786	2	2	0.346599161
Brain_Caudate_basal_ganglia 0.767647867	Brain_Hypothalamus	1.019170043
0.980829957	0.980829957	-0.295452979	0.941206276	0.322876648
0.874450145	2	0	0.998207054
PLCXD3	ENSG00000182836.9	0.611448867	1	1	0.611448867
Brain_Cortex	0.611448867	Brain_Cortex	1	1	1	-0.508006383
-0.508006383	-0.508006383	NA	1	0	0.998207054
NR6A1 ENSG00000148200.16	0.611556853	1	1	0.611556853
Brain_Caudate_basal_ganglia 0.611556853	Brain_Caudate_basal_ganglia 1	1
1	-0.507852407	-0.507852407	-0.507852407	NA	1	0
0.998207054
SENP1 ENSG00000079387.13	0.611680324	2	2	0.568294928
Brain_Hypothalamus	0.974490785	Brain_Cortex	1.798852838
0.201147162	0.201147162	-0.031976508	0.570564442	0.269293967
0.426060792	2	0	0.998207054
ZNF573	ENSG00000189144.13	0.611776566	1	1	0.611776566
Brain_Cerebellar_Hemisphere 0.611776566	Brain_Cerebellar_Hemisphere 1	1
1	0.507539162	0.507539162	0.507539162	NA	1	0
0.998207054
LINC01099	ENSG00000251504.1	0.611785964	8	7	0.261128075
Brain_Putamen_basal_ganglia 0.93843272 Brain_Substantia_nigra	4.578689676
0.00965019 0.261097531	-0.685875214	1.123728845	0.292913792
0.555715388	7	0	0.998207054
CTTNBP2NL	ENSG00000143079.14	0.611787258	4	4	0.142356415
Brain_Substantia_nigra	0.732550118	Brain_Hypothalamus	1.210577519
0.810885063	0.810885063	-1.467072202	0.372204242	-0.271356953
0.863405205	4	0	0.998207054
GRM3	ENSG00000198822.10	0.611817387	2	2	0.322434413	Brain_Amygdala
0.970511928	Brain_Cerebellum 1.023234921	0.976765079	0.976765079
-0.989467604	0.036966234	-0.476250685	0.725798327	2	0
0.998207054
SOHLH1	ENSG00000165643.10	0.611879115	3	3	0.25227661
Brain_Nucleus_accumbens_basal_ganglia 0.809574313	Brain_Putamen_basal_ganglia
1.040155575	0.96614825 0.96614825 -1.144837181	0.240975231
-0.519944574	0.702848888	3	0	0.998207054
CDC26 ENSG00000176386.8	0.611951494	12	2	0.08909165
Brain_Cerebellar_Hemisphere 0.88830003 Brain_Hypothalamus	10.7218531
0.002699234	0.494514781	0.1404556 1.70020884 0.819566814
0.429857956	2	0	0.998207054
DBH	ENSG00000123454.10	0.611973647	3	3	0.325767528
Brain_Frontal_Cortex_BA9	0.551944448	Brain_Cortex	1.180580653
0.855303866	0.855303866	-0.635150607	0.982674778	-0.082441591
0.922637911	3	0	0.998207054
LINC00909	ENSG00000264247.1	0.612018226	13	3	0.327904988
Brain_Cortex	0.697571157	Brain_Caudate_basal_ganglia 10.8310006
0.004428864	0.402145939	-0.978342436	0.429288536	-0.653770555
0.377135485	3	0	0.998207054
TRABD2A	ENSG00000186854.10	0.612062158	4	4	0.221844816
Brain_Caudate_basal_ganglia 0.906717967	Brain_Cerebellar_Hemisphere
1.887383083	0.36130427 0.36130427 -0.117179304	1.221637303
0.555749991	0.558786018	4	0	0.998207054
HIPK4 ENSG00000160396.8	0.612122861	1	1	0.612122861	Brain_Cortex
0.612122861	Brain_Cortex	1	1	1	-0.507045547	-0.507045547

-0.507045547 NA 1 0 0.998207054
FBXW7 ENSG00000109670.13 0.612160721 1 1 0.612160721
Brain_Putamen_basal_ganglia 0.612160721 Brain_Putamen_basal_ganglia 1 1
1 -0.506991588 -0.506991588 -0.506991588 NA 1 0
0.998207054
NAA50 ENSG00000121579.12 0.612167638 2 2 0.32339962
Brain_Frontal_Cortex_BA9 0.951301691 Brain_Spinal_cord_cervical_c-1
1.018837273 0.981162727 0.981162727 0.061072222 0.987495843
0.524284033 0.655080425 2 0 0.998207054
LINC01678 ENSG00000225331.1 0.612219636 8 6 0.179518083
Brain_Hypothalamus 0.92115926 Brain_Spinal_cord_cervical_c-1 4.867375999
1.54E-16 0.189352162 -0.147319132 1.342240334 0.604942377
0.52903295 6 0 0.998207054
CLYBL ENSG00000125246.15 0.612285597 3 3 0.180008612
Brain_Caudate_basal_ganglia 0.968391654 Brain_Frontal_Cortex_BA9
1.136508292 0.806584762 0.806584762 0.039625555 1.340728517
0.509548207 0.721870869 3 0 0.998207054
CAPZA2 ENSG00000198898.13 0.612553059 1 1 0.612553059
Brain_Anterior_cingulate_cortex_BA24 0.612553059
Brain_Anterior_cingulate_cortex_BA24 1 1 1 0.506432508
0.506432508 0.506432508 NA 1 0 0.998207054
DMC1 ENSG00000100206.9 0.612682723 1 1 0.612682723
Brain_Substantia_nigra 0.612682723 Brain_Substantia_nigra 1 1
1 -0.506247771 -0.506247771 -0.506247771 NA 1 0
0.998207054
ATG5 ENSG00000057663.14 0.612717196 1 1 0.612717196
Brain_Hypothalamus 0.612717196 Brain_Hypothalamus 1 1 1
-0.506198659 -0.506198659 -0.506198659 NA 1 0 0.998207054
SLC7A11 ENSG00000151012.13 0.612738118 3 3 0.193115321
Brain_Nucleus_accumbens_basal_ganglia 0.96232683 Brain_Caudate_basal_ganglia
1.171991774 0.809621153 0.809621153 -0.047233874 1.301418277
0.450133542 0.740715031 3 0 0.998207054
GLTP ENSG00000139433.9 0.612782552 13 6 0.215461047
Brain_Anterior_cingulate_cortex_BA24 0.992765796 Brain_Caudate_basal_ganglia
9.506515216 0.003279573 0.326689368 -0.754468529 1.238688047
-0.286638023 0.548078009 6 0 0.998207054
SDR42E1 ENSG00000184860.9 0.612795279 1 1 0.612795279
Brain_Nucleus_accumbens_basal_ganglia 0.612795279
Brain_Nucleus_accumbens_basal_ganglia 1 1 1 -0.506087424
-0.506087424 -0.506087424 NA 1 0 0.998207054
HSD17B7 ENSG00000132196.13 0.612858999 9 8 0.078695869
Brain_Hypothalamus 0.784845369 Brain_Cortex 4.308029784
0.076887919 0.18633189 -1.758303579 0.665549633 0.14175397
0.769593159 8 0 0.998207054
RRH ENSG00000180245.4 0.612860003 4 4 0.108605287
Brain_Hypothalamus 0.292782935 Brain_Frontal_Cortex_BA9 3.275326859
0.149039398 0.149039398 -1.604493632 -1.052036215 -1.245357402
0.248371736 4 0 0.998207054
PDE8A ENSG00000073417.14 0.612910536 2 2 0.323660907 Brain_Amygdala
0.885880425 Brain_Spinal_cord_cervical_c-1 1.074130451 0.925869549
0.925869549 -0.986962735 0.14351885 -0.421721943 0.799371195 2
0 0.998207054
MAPK10 ENSG00000109339.19 0.612912364 5 5 0.387165701
Brain_Hypothalamus 0.912955667 Brain_Putamen_basal_ganglia 3.634561732
0.130380691 0.130380691 -0.864768795 0.843660784 0.082319311
0.610541007 5 0 0.998207054
HGS ENSG00000185359.12 0.612950615 1 1 0.612950615 Brain_Amygdala
0.612950615 Brain_Amygdala 1 1 1 0.505866153 0.505866153
0.505866153 NA 1 0 0.998207054
NCAPD3 ENSG00000151503.12 0.613054519 1 1 0.613054519
Brain_Hippocampus 0.613054519 Brain_Hippocampus 1 1 1
-0.505718159 -0.505718159 -0.505718159 NA 1 0 0.998207054

MEOX2-AS1 ENSG00000229108.1 0.61317305 1 1 0.61317305
Brain_Nucleus_accumbens_basal_ganglia 0.61317305
Brain_Nucleus_accumbens_basal_ganglia 1 1 1 -0.505549344
-0.505549344 -0.505549344 NA 1 0 0.998207054
NUDT9 ENSG00000170502.12 0.613247269 3 3 0.432130941
Brain_Substantia_nigra 0.82530254 Brain_Caudate_basal_ganglia 1.864624875
0.463304091 0.463304091 -0.428856152 0.785550386 0.045321355
0.649448512 3 0 0.998207054
KIAA1456 ENSG00000250305.8 0.613427571 3 3 0.210272825
Brain_Nucleus_accumbens_basal_ganglia 0.836933155 Brain_Cerebellum
2.036418996 0.30925475 0.30925475 -0.205817904 1.252815595
0.564319507 0.732735901 3 0 0.998207054
ATG4C ENSG00000125703.14 0.613481098 3 3 0.341995032 Brain_Cerebellum
0.760422793 Brain_Cortex 2.669230138 0.095013626 0.095013626
-0.95023072 -0.304925633 -0.731930486 0.369828173 3 0
0.998207054
ZNF317 ENSG00000130803.14 0.613494912 9 8 0.116854847
Brain_Putamen_basal_ganglia 0.969248809 Brain_Cerebellar_Hemisphere 4.70663075
0.147996793 0.217241554 -1.568112642 -0.038550448 -0.768805934
0.529567823 8 0 0.998207054
DDX43 ENSG00000080007.7 0.613627806 7 7 0.069014464 Brain_Amygdala
0.883577562 Brain_Spinal_cord_cervical_c-1 4.10395941 0.176130234
0.176130234 -1.818325064 0.364433825 -0.452731257 0.775856473
7 0 0.998207054
SPTBN5 ENSG00000137877.9 0.613644618 13 5 0.101063408
Brain_Cerebellum 0.987670453 Brain_Amygdala 8.432155362 0.00320254
0.38050625 -0.324200593 1.639719953 0.266949383 0.498290012 5
0 0.998207054
SMIM1 ENSG00000235169.7 0.613763675 2 2 0.338398895
Brain_Cerebellar_Hemisphere 0.799459447 Brain_Spinal_cord_cervical_c-1
1.009936257 0.990063743 0.990063743 -0.957333613 0.254046742
-0.351643436 0.856575264 2 0 0.998207054
SH3TC1 ENSG00000125089.16 0.613821748 2 2 0.414116147
Brain_Nucleus_accumbens_basal_ganglia 0.803215423 Brain_Cerebellum
1.336095252 0.663904748 0.663904748 -0.249187921 0.816671562
0.283741821 0.753676468 2 0 0.998207054
CDH23 ENSG00000107736.19 0.613870331 5 5 0.118138388
Brain_Caudate_basal_ganglia 0.877381116 Brain_Cerebellum 3.080206523
0.15381358 0.15381358 -0.154289956 1.562635355 0.809447032
0.656863694 5 0 0.998207054
RP11-390P24.1 ENSG00000273576.1 0.613957161 1 1 0.613957161
Brain_Putamen_basal_ganglia 0.613957161 Brain_Putamen_basal_ganglia 1 1
1 0.504432961 0.504432961 0.504432961 NA 1 0
0.998207054
SERF2 ENSG00000140264.19 0.614000251 1 1 0.614000251
Brain_Spinal_cord_cervical_c-1 0.614000251 Brain_Spinal_cord_cervical_c-1
1 1 1 0.504371629 0.504371629 0.504371629 NA 1 0
0.998207054
KLF13 ENSG00000169926.10 0.614155736 2 2 0.336205786 Brain_Cerebellum
0.343973509 Brain_Cerebellar_Hemisphere 1.868660483 0.131339517
0.131339517 0.946343312 0.961689127 0.95401622 0.01085113 2 0
0.998207054
RP3-522D1.1 ENSG00000224167.1 0.614166868 1 1 0.614166868
Brain_Spinal_cord_cervical_c-1 0.614166868 Brain_Spinal_cord_cervical_c-1
1 1 1 0.504134495 0.504134495 0.504134495 NA 1 0
0.998207054
CERS1 ENSG00000223802.7 0.614241493 12 2 0.207987405 Brain_Cortex
0.527976339 Brain_Hypothalamus 10.63658846 0.000750625
0.657995816 -1.259118856 -0.631098168 -0.877217768 0.194119081
2 0 0.998207054
KIAA1147 ENSG00000257093.6 0.614472829 2 2 0.517545681
Brain_Caudate_basal_ganglia 0.602226986 Brain_Anterior_cingulate_cortex_BA24

1.29483177 0.70516823 0.70516823 -0.521200672 0.647133291 0.062966309
0.826136868 2 0 0.998207054
AC115522.3 ENSG00000268601.1 0.614503026 6 4 0.197051406
Brain_Frontal_Cortex_BA9 0.995550059 Brain_Cerebellum 3.453477277
0.034257666 0.416084334 -1.289997845 0.005577203 -0.66033154
0.600304632 4 0 0.998207054
BCL2L10 ENSG00000137875.4 0.614635417 2 2 0.345482585
Brain_Cerebellum 0.474587705 Brain_Cerebellar_Hemisphere 1.491180207
0.508819793 0.508819793 0.715034534 0.943387792 0.829211163
0.161470137 2 0 0.998207054
MAPK9 ENSG00000050748.17 0.614687492 7 7 0.159756193 Brain_Cortex
0.944224384 Brain_Nucleus_accumbens_basal_ganglia 3.264604382
0.114795961 0.114795961 -1.405892015 1.052882436 -0.185347127
0.907313209 7 0 0.998207054
PPFIA3 ENSG00000177380.13 0.614691465 2 2 0.370768533
Brain_Caudate_basal_ganglia 0.66550725 Brain_Spinal_cord_cervical_c-1
1.019499022 0.980500978 0.980500978 0.432322206 0.895034712
0.663678459 0.327187151 2 0 0.998207054
NAIF1 ENSG00000171169.8 0.61473001 1 1 0.61473001 Brain_Cortex 0.61473001
Brain_Cortex 1 1 1 0.503333226 0.503333226 0.503333226
NA 1 0 0.998207054
TRIB1 ENSG00000173334.3 0.614798912 1 1 0.614798912
Brain_Spinal_cord_cervical_c-1 0.614798912 Brain_Spinal_cord_cervical_c-1
1 1 1 -0.50323521 -0.50323521 -0.50323521 NA 1 0
0.998207054
LIME1 ENSG00000203896.9 0.614920841 2 2 0.328056867 Brain_Cortex
0.866515455 Brain_Caudate_basal_ganglia 1.042767051 0.957232949
0.957232949 -0.978035296 -0.168086213 -0.573060755 0.572720489
2 0 0.998207054
FAM153B ENSG00000182230.11 0.615336898 3 3 0.283980741
Brain_Spinal_cord_cervical_c-1 0.778956057 Brain_Cerebellum 1.5404458
0.472148908 0.472148908 -1.07141974 0.687647036 -0.221484145
0.881026145 3 0 0.998207054
LINC01515 ENSG00000228065.10 0.615425023 6 4 0.188375161
Brain_Spinal_cord_cervical_c-1 0.992012776 Brain_Hypothalamus
4.662686087 0.105406568 0.282408703 -0.320741966 1.315401025
0.42453914 0.617470319 4 0 0.998207054
C4A ENSG00000244731.7 0.615497333 12 4 0.078682748
Brain_Frontal_Cortex_BA9 0.560691809 Brain_Amygdala 9.199976336
0.048964298 0.369245088 -1.758380742 -0.581814243 -1.221217416
0.404290536 4 0 0.998207054
LINC02170 ENSG00000260896.5 0.615691387 2 2 0.338608411
Brain_Cortex 0.410571884 Brain_Nucleus_accumbens_basal_ganglia
1.681738064 0.318261936 0.318261936 -0.956918464 -0.822887654
-0.889903059 0.094774094 2 0 0.998207054
MMADHC ENSG00000168288.12 0.615706489 1 1 0.615706489
Brain_Hippocampus 0.615706489 Brain_Hippocampus 1 1 1
-0.501944603 -0.501944603 -0.501944603 NA 1 0 0.998207054
DNAJC2 ENSG00000105821.14 0.615716118 1 1 0.615716118
Brain_Cerebellar_Hemisphere 0.615716118 Brain_Cerebellar_Hemisphere 1 1
1 -0.501930915 -0.501930915 -0.501930915 NA 1 0
0.998207054
RP11-265E18.1 ENSG00000272905.1 0.615757149 7 5 0.079132893
Brain_Hypothalamus 0.601035569 Brain_Cerebellar_Hemisphere 5.106131738
0.080755795 0.242647744 -1.755739533 -0.522911891 -0.949162483
0.384079781 5 0 0.998207054
KCNT1 ENSG00000107147.12 0.615760931 11 8 0.115868542
Brain_Nucleus_accumbens_basal_ganglia 0.935005908 Brain_Putamen_basal_ganglia
5.226643059 0.00860419 0.288373361 -1.572353714 0.104886106
-0.766883797 0.563585057 8 0 0.998207054
FAM86C1 ENSG00000158483.15 0.6158742 5 4 0.31586006
Brain_Caudate_basal_ganglia 0.765988317 Brain_Cerebellum 3.068089067

0.029068828	0.107124715	-1.00300166	0.743229687	-0.315025803
0.64620834 4	0	0.998207054
KRTAP5-8	ENSG00000241233.3	0.615904706	3	2	0.328315335
Brain_Nucleus_accumbens_basal_ganglia 0.467843923	Brain_Caudate_basal_ganglia
2.764911335	0.035989746	0.199098919	0.725991596	0.977512819
0.866732235	0.128409249	2	0	0.998207054
ANKH	ENSG00000154122.12	0.615954376	4	4	0.209127291
Brain_Hypothalamus	0.878601566	Brain_Cerebellum 1.572087533
0.571355611	0.571355611	-1.255968775	0.444470496	-0.335094578
0.777559678	4	0	0.998207054
LINC01006	ENSG00000182648.11	0.615960579	11	4	0.199860104
Brain_Hypothalamus	0.918706592	Brain_Nucleus_accumbens_basal_ganglia
8.852130695	0.008641181	0.296070604	-1.281950236	-0.102063098
-0.518753955	0.366000697	4	0	0.998207054
LINC00240	ENSG00000224843.6	0.616037041	5	5	0.105115166
Brain_Cerebellar_Hemisphere 0.583981576	Brain_Cerebellum 3.370077748
0.135922565	0.135922565	-1.62054542	-0.547578179	-1.022393388
0.472327758	5	0	0.998207054
IFNGR2	ENSG00000159128.14	0.61605466 3	3	0.526498496
Brain_Nucleus_accumbens_basal_ganglia 0.824326475	Brain_Cerebellum
1.886273538	0.119370618	0.119370618	-0.221983824	0.633360128
0.25385273 0.435732486	3	0	0.998207054
SLC12A5	ENSG00000124140.13	0.616092628	5	5	0.387052735
Brain_Hippocampus	0.957783653	Brain_Cortex	2.686872311
0.231398327	0.231398327	-0.841125973	0.864974591	-0.029164065
0.756313825	5	0	0.998207054
LINC01125	ENSG00000228486.10	0.616201657	4	4	0.149100145
Brain_Anterior_cingulate_cortex_BA24	0.950813283
Brain_Nucleus_accumbens_basal_ganglia 1.88316539 0.457844883	0.457844883
-0.939520927	1.442717224	0.217258327	0.978372982	4	0
0.998207054
B2M	ENSG00000166710.17	0.616269898	2	2	0.32522499
Brain_Spinal_cord_cervical_c-1	0.964521027	Brain_Cerebellar_Hemisphere
1.026963119	0.973036881	0.973036881	0.044480962	0.983777366
0.514129164	0.664182857	2	0	0.998207054
GALE	ENSG00000117308.14	0.616317452	1	1	0.616317452
Brain_Hippocampus	0.616317452	Brain_Hippocampus	1	1	1
0.501076263	0.501076263	0.501076263	NA	1	0	0.998207054
MCTP2	ENSG00000140563.14	0.616381604	1	1	0.616381604
Brain_Hippocampus	0.616381604	Brain_Hippocampus	1	1	1
-0.500985107	-0.500985107	-0.500985107	NA	1	0	0.998207054
C14orf132	ENSG00000227051.6	0.616469199	4	4	0.177892405
Brain_Cerebellum 0.93730316 Brain_Putamen_basal_ganglia 1.855655528
0.146893393	0.146893393	-1.347272751	-0.078659877	-0.86165009
0.545681542	4	0	0.998207054
CACHD1	ENSG00000158966.13	0.616644901	1	1	0.616644901
Brain_Caudate_basal_ganglia 0.616644901	Brain_Caudate_basal_ganglia 1	1
1	-0.500611027	-0.500611027	-0.500611027	NA	1	0
0.998207054
VPREB3	ENSG00000128218.7	0.616727625	12	8	0.033506649
Brain_Frontal_Cortex_BA9	0.99110664 Brain_Putamen_basal_ganglia 6.534240721
0.012835257	0.266538668	-0.035763652	2.125959292	0.757141232
0.739702643	8	0	0.998207054
THUMPD1	ENSG00000066654.13	0.616814534	4	4	0.451033521
Brain_Cerebellar_Hemisphere 0.661414909	Brain_Frontal_Cortex_BA9
3.075017553	0.114260928	0.114260928	-0.437960511	0.753693109
0.365012838	0.54230842 4	0	0.998207054
DAGLB ENSG00000164535.14	0.616838749	4	3	0.43251176 Brain_Cerebellum
0.890338356	Brain_Hippocampus	3.331087266	0.033151363
0.209509745	-0.2511401 0.784900754	0.235184973	0.427909251	3
0	0.998207054
LRRC74B	ENSG00000187905.10	0.616868274	9	1	0.311633907

Brain_Cerebellum 0.841639261 Brain_Hippocampus 8.449636261
0.001808135 8.449636261 -1.011799546 -0.199797111 -0.491173958
0.264243673 1 0 0.998207054
B3GAT3 ENSG00000149541.9 0.617010828 8 4 0.355281733
Brain_Substantia_nigra 0.869699767 Brain_Spinal_cord_cervical_c-1
5.570907765 0.016522642 0.379737248 -0.92439301 0.561329324
-0.123683497 0.569203372 4 0 0.998207054
FIZ1 ENSG00000179943.7 0.617081918 1 1 0.617081918
Brain_Frontal_Cortex_BA9 0.617081918 Brain_Frontal_Cortex_BA9 1 1
1 -0.499990285 -0.499990285 -0.499990285 NA 1 0
0.998207054
RNF40 ENSG00000103549.21 0.617295799 7 2 0.372379012
Brain_Cerebellar_Hemisphere 0.854005443 Brain_Cerebellum 6.264627742
0.00074908 0.438924314 -0.196940942 0.892025972 0.358971504
0.381736213 2 0 0.998207054
D2HGDH ENSG00000180902.17 0.617415137 13 6 0.110213294
Brain_Hypothalamus 0.594113814 Brain_Amygdala 8.503044022 2.67E-18
0.522309717 0.532884097 1.597235177 1.255234768 0.420940578
6 0 0.998207054
ROGDI ENSG00000067836.12 0.617647594 9 8 0.176775267
Brain_Substantia_nigra 0.905292863 Brain_Amygdala 3.325724172
0.089774234 0.120470541 -1.35075082 -0.118977901 -0.908929016
0.489057503 8 0 0.998207054
COPRS ENSG00000172301.10 0.617669503 1 1 0.617669503 Brain_Cortex
0.617669503 Brain_Cortex 1 1 1 -0.49915598 -0.49915598
-0.49915598 NA 1 0 0.998207054
HIF1AN ENSG00000166135.13 0.617711851 4 4 0.110538641
Brain_Putamen_basal_ganglia 0.931132321 Brain_Cerebellar_Hemisphere
2.288692485 0.298102343 0.298102343 -1.595776772 0.117838513
-0.442765171 0.780260571 4 0 0.998207054
C16orf96 ENSG00000205832.7 0.617728939 1 1 0.617728939
Brain_Frontal_Cortex_BA9 0.617728939 Brain_Frontal_Cortex_BA9 1 1
1 -0.499071607 -0.499071607 -0.499071607 NA 1 0
0.998207054
ANKRD45 ENSG00000183831.6 0.617906916 3 2 0.728607352
Brain_Nucleus_accumbens_basal_ganglia 0.934756976 Brain_Cortex
2.727876433 0.043438634 0.228684932 -0.335446476 0.346978659
-0.023443053 0.344942805 2 0 0.998207054
RP11-166O4.6 ENSG00000273448.1 0.617947047 9 3 0.3864032
Brain_Nucleus_accumbens_basal_ganglia 0.949334599 Brain_Cerebellum
7.339310373 0.013674143 0.336290284 0.063542398 0.866158587
0.534444661 0.314366136 3 0 0.998207054
RP11-143E21.6 ENSG00000259862.1 0.618111782 1 1 0.618111782
Brain_Spinal_cord_cervical_c-1 0.618111782 Brain_Spinal_cord_cervical_c-1
1 1 1 0.498528223 0.498528223 0.498528223 NA 1 0
0.998207054
PACS1 ENSG00000175115.11 0.618139696 5 3 0.094821887 Brain_Cortex
0.383499585 Brain_Nucleus_accumbens_basal_ganglia 4.226273583
0.050475032 0.229795469 -1.670492882 -0.871466324 -1.172921254
0.310779481 3 0 0.998207054
TMEM241 ENSG00000134490.13 0.618198303 7 6 0.074427602
Brain_Cerebellum 0.966530669 Brain_Cerebellar_Hemisphere 2.340149165
0.054854263 0.595456677 -1.783975771 -0.041959895 -0.593892747
0.608722576 6 0 0.998207054
SPOCK3 ENSG00000196104.10 0.618201357 1 1 0.618201357
Brain_Amygdala 0.618201357 Brain_Amygdala 1 1 1 -0.498401106
-0.498401106 -0.498401106 NA 1 0 0.998207054
PARG ENSG00000227345.8 0.618242114 3 3 0.304861842
Brain_Spinal_cord_cervical_c-1 0.640155999 Brain_Cerebellar_Hemisphere
1.369311225 0.691822666 0.691822666 -1.0260631 0.467480698
-0.370491004 0.763295802 3 0 0.998207054
RMI1 ENSG00000178966.16 0.618328453 5 3 0.350812784

Brain_Hypothalamus 0.994212476 Brain_Cerebellum 3.430209779
0.024600441 0.545635488 -0.308351835 0.933013915 0.196348593
0.533240295 3 0 0.998207054
FAM92A ENSG00000188343.12 0.618375501 2 2 0.443765756
Brain_Frontal_Cortex_BA9 0.488723448 Brain_Hippocampus 1.109439749
0.890560251 0.890560251 -0.76584967 -0.692340618 -0.729095144
0.051978749 2 0 0.998207054
LRRC53 ENSG00000162621.6 0.618392407 3 3 0.348277713
Brain_Cerebellar_Hemisphere 0.966072671 Brain_Cerebellum 1.863006771
0.393144941 0.393144941 -0.937935199 0.27690704 -0.206164578
0.64447561 3 0 0.998207054
TMEM37 ENSG00000171227.6 0.618415914 3 3 0.22475581
Brain_Putamen_basal_ganglia 0.581316238 Brain_Hippocampus 1.393688993
0.605677475 0.605677475 0.551463125 1.213978825 0.782569196
0.373929004 3 0 0.998207054
CALD1 ENSG00000122786.19 0.61842754 1 1 0.61842754 Brain_Putamen_basal_ganglia
0.61842754 Brain_Putamen_basal_ganglia 1 1 1 0.498080165
0.498080165 0.498080165 NA 1 0 0.998207054
FAM96A ENSG00000166797.10 0.618500813 2 2 0.386636649
Brain_Amygdala 0.640437953 Brain_Cortex 1.008430434 0.991569566
0.991569566 0.467086555 0.865732908 0.666409732 0.281885539
2 0 0.998207054
MRO ENSG00000134042.12 0.618504388 4 4 0.446049678 Brain_Cerebellum
0.986149292 Brain_Nucleus_accumbens_basal_ganglia 2.678782415
0.168966237 0.168966237 -0.618616507 0.762017302 0.054557624
0.564226531 4 0 0.998207054
DNAJB13 ENSG00000187726.8 0.618510853 4 4 0.301839279
Brain_Cerebellum 0.871214743 Brain_Cerebellar_Hemisphere 2.525090055
0.313193861 0.313193861 -0.557284251 1.032497157 0.178285124
0.691806791 4 0 0.998207054
LINC00609 ENSG00000257585.1 0.618544667 2 2 0.342937121
Brain_Hippocampus 0.992116887 Brain_Spinal_cord_cervical_c-1
1.242932122 0.757067878 0.757067878 -0.948377871 0.009880178
-0.469248847 0.677590765 2 0 0.998207054
LINC01094 ENSG00000251442.5 0.618556479 1 1 0.618556479
Brain_Substantia_nigra 0.618556479 Brain_Substantia_nigra 1 1
1 0.497897231 0.497897231 0.497897231 NA 1 0
0.998207054
PSAP ENSG00000197746.13 0.618570894 2 2 0.337891873 Brain_Amygdala
0.754796578 Brain_Putamen_basal_ganglia 1.112722575 0.887277425
0.887277425 0.312321005 0.958338942 0.635329974 0.456803664
2 0 0.998207054
HCST ENSG00000126264.9 0.618610356 1 1 0.618610356 Brain_Amygdala
0.618610356 Brain_Amygdala 1 1 1 -0.497820797 -0.497820797
-0.497820797 NA 1 0 0.998207054
NBN ENSG00000104320.13 0.618612384 4 4 0.623540777 Brain_Amygdala
0.743527561 Brain_Spinal_cord_cervical_c-1 2.679130528 0.110932614
0.110932614 -0.490838359 0.372127816 -0.027864855 0.44541885 4
0 0.998207054
XPO7 ENSG00000130227.16 0.618681613 2 2 0.328353115 Brain_Amygdala
0.956078858 Brain_Nucleus_accumbens_basal_ganglia 1.015603168
0.984396832 0.984396832 0.055074818 0.97743647 0.516255644
0.652208179 2 0 0.998207054
NCR3LG1 ENSG00000188211.8 0.61877346 1 1 0.61877346 Brain_Cerebellum
0.61877346 Brain_Cerebellum 1 1 1 -0.497589424 -0.497589424
-0.497589424 NA 1 0 0.998207054
RCL1 ENSG00000120158.11 0.618793374 5 5 0.117501752
Brain_Spinal_cord_cervical_c-1 0.919458919 Brain_Cerebellum 3.086047096
0.170437804 0.170437804 -0.101115317 1.565346216 0.698286895
0.603699558 5 0 0.998207054
RPUSD1 ENSG00000007376.7 0.618852506 3 3 0.262951796
Brain_Cortex 0.976517618 Brain_Cerebellum 1.425822983 0.575981201

0.575981201 -0.521284223 1.119441591 0.209197473 0.83500371 3
0 0.998207054
EHF ENSG00000135373.12 0.618948361 2 2 0.341231811
Brain_Spinal_cord_cervical_c-1 0.72629409 Brain_Substantia_nigra
1.126348828 0.873651172 0.873651172 -0.951734178 -0.35005944
-0.650896809 0.425448287 2 0 0.998207054
RP1-122P22.2 ENSG00000233895.1 0.61897871 1 1 0.61897871
Brain_Hippocampus 0.61897871 Brain_Hippocampus 1 1 1 0.4972983
0.4972983 0.4972983 NA 1 0 0.998207054
CTC-429P9.2 ENSG00000268087.1 0.61914543 3 3 0.191802901
Brain_Cortex 0.763304079 Brain_Hippocampus 1.767665425
0.233888131 0.233888131 0.301144802 1.305264196 0.883207505
0.520831434 3 0 0.998207054
LINC01750 ENSG00000231437.3 0.619231263 11 2 0.240509991
Brain_Nucleus_accumbens_basal_ganglia 0.947127091 Brain_Cerebellum
9.684614542 0.003696186 0.484561288 -1.173713027 -0.066314937
-0.854658538 0.399133866 2 0 0.998207054
CHST8 ENSG00000124302.12 0.619276495 3 3 0.286139119
Brain_Putamen_basal_ganglia 0.928757604 Brain_Caudate_basal_ganglia
1.415317527 0.578476251 0.578476251 -1.066629621 0.273234037
-0.234662502 0.726343556 3 0 0.998207054
RP11-117D22.2 ENSG00000230138.1 0.61932663 1 1 0.61932663
Brain_Spinal_cord_cervical_c-1 0.61932663 Brain_Spinal_cord_cervical_c-1 1
1 1 0.496804913 0.496804913 0.496804913 NA 1 0
0.998207054
FTO ENSG00000140718.20 0.619431955 9 9 0.108294765 Brain_Cortex
0.954999941 Brain_Cerebellum 3.20323385 0.256111185 0.256111185
-0.420227072 1.60590509 0.670752301 0.71217798 9 0 0.998207054
TAS2R10 ENSG00000121318.2 0.619432203 1 1 0.619432203
Brain_Cerebellum 0.619432203 Brain_Cerebellum 1 1 1 -0.496655224
-0.496655224 -0.496655224 NA 1 0 0.998207054
LINC01139 ENSG00000215808.2 0.619448884 3 3 0.347837056
Brain_Nucleus_accumbens_basal_ganglia 0.656711467 Brain_Hypothalamus
1.054483426 0.972271074 0.972271074 0.444458029 0.938792957
0.730768175 0.2562971 3 0 0.998207054
CRYGB ENSG00000182187.3 0.619517063 2 2 0.336260023
Brain_Spinal_cord_cervical_c-1 0.689898364 Brain_Substantia_nigra
1.231156992 0.768843008 0.768843008 -0.961581191 -0.398992997
-0.680287094 0.397809927 2 0 0.998207054
ZYG11B ENSG00000162378.12 0.619542437 7 4 0.111569735
Brain_Cortex 0.762702012 Brain_Amygdala 5.120315339 0.003142687
0.199192993 -1.591177056 0.301934478 -1.012495638 0.645163473
4 0 0.998207054
RNF167 ENSG00000108523.15 0.619609928 1 1 0.619609928
Brain_Nucleus_accumbens_basal_ganglia 0.619609928
Brain_Nucleus_accumbens_basal_ganglia 1 1 1 -0.496403256
-0.496403256 -0.496403256 NA 1 0 0.998207054
IRS2 ENSG00000185950.8 0.619784674 1 1 0.619784674
Brain_Caudate_basal_ganglia 0.619784674 Brain_Caudate_basal_ganglia 1 1
1 0.496155544 0.496155544 0.496155544 NA 1 0
0.998207054
PDZK1 ENSG00000174827.13 0.619818366 1 1 0.619818366 Brain_Cerebellum
0.619818366 Brain_Cerebellum 1 1 1 0.496107787 0.496107787
0.496107787 NA 1 0 0.998207054
KRT18 ENSG00000111057.10 0.61983058 2 2 0.38046675 Brain_Cerebellum
0.692241309 Brain_Putamen_basal_ganglia 1.041880736 0.958119264
0.958119264 -0.395815272 0.877036616 0.240610672 0.900042202
2 0 0.998207054
LACC1 ENSG00000179630.10 0.620088317 13 4 0.303958435
Brain_Spinal_cord_cervical_c-1 0.974469312 Brain_Frontal_Cortex_BA9
9.787092209 0.010103442 0.33009172 -0.60871364 1.027981702
0.017672575 0.475933664 4 0 0.998207054

AC009495.3 ENSG00000232411.1 0.620218529 2 2 0.350157475
Brain_Cerebellar_Hemisphere 0.948491922 Brain_Cerebellum 1.23000853 0.76999147
0.76999147 -0.934283884 0.064600707 -0.434841589 0.706318068 2
0 0.998207054
DIEXF ENSG00000117597.17 0.620375852 6 3 0.377159119
Brain_Putamen_basal_ganglia 0.890384716 Brain_Hypothalamus 4.001156812
0.003249917 0.892323503 -0.883142805 0.869795621 0.17029724
0.606777352 3 0 0.998207054
CAMK2N1 ENSG00000162545.5 0.620427112 4 4 0.421934472
Brain_Substantia_nigra 0.970833987 Brain_Cerebellum 1.824181293
0.186140603 0.186140603 -0.803069661 0.600865158 -0.032906845
0.581504956 4 0 0.998207054
MESP2 ENSG00000188095.4 0.620453883 1 1 0.620453883
Brain_Nucleus_accumbens_basal_ganglia 0.620453883
Brain_Nucleus_accumbens_basal_ganglia 1 1 1 -0.495207179
-0.495207179 -0.495207179 NA 1 0 0.998207054
TENM3 ENSG00000218336.8 0.620593157 3 3 0.231215892
Brain_Hypothalamus 0.803419696 Brain_Nucleus_accumbens_basal_ganglia
1.255102237 0.756251597 0.756251597 -0.248923838 1.197232638
0.489720177 0.723580692 3 0 0.998207054
HDAC11 ENSG00000163517.14 0.620762902 3 3 0.256100411
Brain_Cerebellum 0.490909106 Brain_Cortex 1.717291975 0.298756628
0.298756628 -1.13565636 -0.688863603 -0.958623051 0.237391122
3 0 0.998207054
WDR48 ENSG00000114742.13 0.620780933 2 2 0.454809969 Brain_Cerebellum
0.96448009 Brain_Hippocampus 1.607971958 0.392028042 0.392028042
-0.747420178 0.04453232 -0.351443929 0.559994982 2 0
0.998207054
RP11-360F5.3 ENSG00000249685.1 0.620781756 10 3 0.289556884
Brain_Nucleus_accumbens_basal_ganglia 0.823407126
Brain_Spinal_cord_cervical_c-1 8.808041692 8.15E-07 0.302291527
-1.059094194 0.223164955 -0.56584368 0.344702052 3 0
0.998207054
ADAM17 ENSG00000151694.12 0.62080202 6 3 0.190975888 Brain_Cortex
0.553358688 Brain_Frontal_Cortex_BA9 5.045121963 0.018807818
0.270585045 -1.307697629 -0.59273474 -0.995199119 0.30771241 3
0 0.998207054
SLITRK1 ENSG00000178235.7 0.620848281 1 1 0.620848281
Brain_Cerebellum 0.620848281 Brain_Cerebellum 1 1 1 -0.494648471
-0.494648471 -0.494648471 NA 1 0 0.998207054
GYPB ENSG00000250361.7 0.620914903 1 1 0.620914903
Brain_Anterior_cingulate_cortex_BA24 0.620914903
Brain_Anterior_cingulate_cortex_BA24 1 1 1 -0.494554108
-0.494554108 -0.494554108 NA 1 0 0.998207054
TRIM16L ENSG00000108448.20 0.620941413 8 5 0.18752488
Brain_Frontal_Cortex_BA9 0.994858614 Brain_Cerebellum 4.533763195
5.65E-16 0.221692246 -0.737936556 1.317936576 0.005843932
0.697674327 5 0 0.998207054
CPNE7 ENSG00000178773.14 0.621219388 2 2 0.356802389
Brain_Anterior_cingulate_cortex_BA24 0.977674863 Brain_Cortex
1.301725499 0.698274501 0.698274501 -0.027984062 0.921475189
0.446745564 0.671369075 2 0 0.998207054
HABP4 ENSG00000130956.13 0.621380917 2 2 0.466721754
Brain_Frontal_Cortex_BA9 0.520153586 Brain_Substantia_nigra
1.008794469 0.991205531 0.991205531 -0.643108675 0.727823309
0.042357317 0.969395303 2 0 0.998207054
C4orf32 ENSG00000174749.5 0.621449598 7 6 0.381383955
Brain_Caudate_basal_ganglia 0.921651534 Brain_Cortex 3.735562792
0.031660204 0.200929266 -0.875349157 0.227664164 -0.361116751
0.485474124 6 0 0.998207054
SVBP ENSG00000177868.11 0.621547445 13 1 0.479615939
Brain_Caudate_basal_ganglia 0.911017376 Brain_Spinal_cord_cervical_c-1

12.11455493 0.000338133 12.11455493 0.111755369 0.706920412
0.475812868 0.186994488 1 0 0.998207054
EEF1D ENSG00000104529.17 0.621547796 1 1 0.621547796
Brain_Substantia_nigra 0.621547796 Brain_Substantia_nigra 1 1
1 -0.493657908 -0.493657908 -0.493657908 NA 1 0
0.998207054
ZNF845 ENSG00000213799.11 0.621555673 1 1 0.621555673
Brain_Amygdala 0.621555673 Brain_Amygdala 1 1 1 0.493646758
0.493646758 0.493646758 NA 1 0 0.998207054
MRM1 ENSG00000278619.4 0.621669524 6 6 0.06101951
Brain_Frontal_Cortex_BA9 0.948261446 Brain_Hypothalamus 2.037467762
0.385076888 0.385076888 -0.064890172 1.873354056 0.80746421
0.675639718 6 0 0.998207054
PNPO ENSG00000108439.9 0.62174103 8 6 0.010001104
Brain_Substantia_nigra 0.73356804 Brain_Anterior_cingulate_cortex_BA24
5.45627228 0.028092399 0.223901211 -2.575791147 -0.340383105
-1.285139117 0.740138038 6 0 0.998207054
OBP2A ENSG00000122136.13 0.621751808 4 4 0.165897236 Brain_Cortex
0.311372159 Brain_Anterior_cingulate_cortex_BA24 2.525438129
0.180840855 0.180840855 1.012347024 1.385507843 1.169590044
0.15953383 4 0 0.998207054
LINC01597 ENSG00000205611.4 0.622005254 13 3 0.3626997
Brain_Spinal_cord_cervical_c-1 0.984727855 Brain_Amygdala 10.99173934
0.000116602 0.562969904 -0.527990563 0.910232894 -0.088598012
0.346734116 3 0 0.998207054
KLHL35 ENSG00000149243.15 0.622010165 1 1 0.622010165
Brain_Caudate_basal_ganglia 0.622010165 Brain_Caudate_basal_ganglia 1 1
1 0.493003428 0.493003428 0.493003428 NA 1 0
0.998207054
PRPH ENSG00000135406.13 0.62204571 3 2 0.43546029 Brain_Cerebellum
0.803388214 Brain_Spinal_cord_cervical_c-1 2.016204819 0.029739919
0.954055262 -0.779882075 0.667892907 0.045658456 0.744992028
2 0 0.998207054
COX7A2 ENSG00000112695.11 0.622062896 1 1 0.622062896
Brain_Amygdala 0.622062896 Brain_Amygdala 1 1 1 0.492928801
0.492928801 0.492928801 NA 1 0 0.998207054
YWHAB ENSG00000166913.12 0.622106941 1 1 0.622106941
Brain_Cerebellar_Hemisphere 0.622106941 Brain_Cerebellar_Hemisphere 1 1
1 -0.49286647 -0.49286647 -0.49286647 NA 1 0
0.998207054
FIBCD1 ENSG00000130720.12 0.622134995 2 2 0.369422459
Brain_Nucleus_accumbens_basal_ganglia 0.705569809 Brain_Frontal_Cortex_BA9
1.001247764 0.998752236 0.998752236 -0.377812605 0.897555712
0.259871554 0.901821585 2 0 0.998207054
RP11-497H16.9 ENSG00000269983.1 0.622150955 4 2 0.689926429
Brain_Cerebellum 0.922748752 Brain_Spinal_cord_cervical_c-1 3.815243818
0.001646347 0.155917291 -0.39895491 0.096971847 -0.247308023
0.233747696 2 0 0.998207054
RP11-963H4.3 ENSG00000263508.5 0.622253379 7 7 0.233729977
Brain_Substantia_nigra 0.733803963 Brain_Hippocampus 3.204823167
0.268750932 0.268750932 -1.190805429 0.80962866 0.031353217
0.840751962 7 0 0.998207054
AP000343.2 ENSG00000236611.1 0.622387819 3 3 0.21726614
Brain_Nucleus_accumbens_basal_ganglia 0.684866676 Brain_Cerebellum
1.432217105 0.726468101 0.726468101 -1.233830315 -0.40583114
-0.812450441 0.414196891 3 0 0.998207054
TRMO ENSG00000136932.13 0.622442049 13 1 0.090075586
Brain_Spinal_cord_cervical_c-1 0.973595859 Brain_Frontal_Cortex_BA9
12.37085705 0.00042042 12.37085705 -0.033098726 1.694999129
0.47970802 0.420381747 1 0 0.998207054
NECAP2 ENSG00000157191.19 0.622454205 2 2 0.341377475
Brain_Anterior_cingulate_cortex_BA24 0.819407016 Brain_Hypothalamus

1.435921733	0.564078267	0.564078267	0.228307729	0.95144707
0.5898774	0.511336732	2	0	0.998207054
SCN5A	ENSG00000183873.15	0.622541462	2	2	0.331000157	Brain_Cortex
0.969265982	Brain_Cerebellar_Hemisphere	1.015842998	0.984157002
0.984157002	-0.972101296	-0.03852891	-0.505315103	0.660135365
2	0	0.998207054
SLC13A5	ENSG00000141485.16	0.622710206	3	3	0.244277987
Brain_Cerebellar_Hemisphere	0.549373226	Brain_Hypothalamus	1.636904917
0.3632996	0.3632996	0.598699596	1.164360404	0.834560335
0.294298294	3	0	0.998207054
BRD4	ENSG00000141867.17	0.62286434	1	1	0.62286434
Brain_Spinal_cord_cervical_c-1	0.62286434	Brain_Spinal_cord_cervical_c-1	1
1	1	0.491794903	0.491794903	0.491794903	NA	1	0
0.998207054
UBE2G2	ENSG00000184787.18	0.622885287	10	6	0.137298479
Brain_Spinal_cord_cervical_c-1	0.810735732	Brain_Amygdala	5.375737408
0.040779973	0.185489002	-0.638704523	1.485926818	0.20711804
0.680116511	6	0	0.998207054
C3orf52	ENSG00000114529.12	0.623093416	2	2	0.331139845
Brain_Cerebellum	0.962175825	Brain_Cerebellar_Hemisphere	1.090831969
0.909168031	0.909168031	-0.971820522	-0.047423343	-0.509621932
0.653647514	2	0	0.998207054
BEND5	ENSG00000162373.12	0.623140167	1	1	0.623140167	Brain_Cerebellum
0.623140167	Brain_Cerebellum	1	1	1	-0.491404804	-0.491404804
-0.491404804	NA	1	0	0.998207054
AMACR	ENSG00000242110.7	0.623195548	13	2	0.285112289
Brain_Anterior_cingulate_cortex_BA24	0.942789387	Brain_Cerebellum
11.55957447	0.000331126	0.773431342	0.071764422	1.068905422
0.620293939	0.336272423	2	0	0.998207054
PPT2	ENSG00000221988.12	0.623284887	1	1	0.623284887
Brain_Caudate_basal_ganglia	0.623284887	Brain_Caudate_basal_ganglia	1	1
1	-0.491200159	-0.491200159	-0.491200159	NA	1	0
0.998207054
CTSW	ENSG00000172543.7	0.623310062	5	5	0.285612704
Brain_Putamen_basal_ganglia	0.64009298	Brain_Nucleus_accumbens_basal_ganglia
2.805853708	0.134701187	0.134701187	-0.727099827	1.067795642
-0.260589655	0.752896668	5	0	0.998207054
LINC02072	ENSG00000225860.2	0.623386939	1	1	0.623386939
Brain_Amygdala	0.623386939	Brain_Amygdala	1	1	1	0.49105586
0.49105586	0.49105586	NA	1	0	0.998207054
RP11-26J3.1	ENSG00000249328.2	0.623411005	7	7	0.056106186
Brain_Amygdala	0.977799402	Brain_Substantia_nigra	2.675302327
0.105053746	0.105053746	-1.910209954	0.08774088	-0.726985412
0.733009515	7	0	0.998207054
EPB41	ENSG00000159023.19	0.623435795	2	1	0.610594265
Brain_Cerebellar_Hemisphere	0.63699136	Brain_Cerebellum	1.996585214
0.003414786	1.996585214	0.471909537	0.509225365	0.490567451
0.026386275	1	0	0.998207054
DNAJB11	ENSG00000090520.11	0.623448571	1	1	0.623448571
Brain_Cortex	0.623448571	Brain_Cortex	1	1	1	-0.490968721
-0.490968721	-0.490968721	NA	1	0	0.998207054
C1orf159	ENSG00000131591.17	0.62366399	12	3	0.436943491
Brain_Spinal_cord_cervical_c-1	0.996814604	Brain_Cerebellar_Hemisphere
9.630161375	0.001790298	0.377565846	-0.738920338	0.77736494
0.002956165	0.373784226	3	0	0.998207054
C9orf3	ENSG00000148120.16	0.623746801	3	3	0.334202992
Brain_Cortex	0.929242292	Brain_Cerebellum	1.399506238	0.713456493
0.713456493	-0.965682675	0.725522987	-0.109652628	0.845795678
3	0	0.998207054
KREMEN1	ENSG00000183762.12	0.623762448	4	4	0.206088038
Brain_Hypothalamus	0.308381538	Brain_Cerebellum	2.284441915
0.332993819	0.332993819	-1.264395694	-1.018623933	-1.122630822

0.102725099 4 0 0.998207054
MAVS ENSG00000088888.17 0.623774951 8 4 0.510221482 Brain_Amygdala 0.989011993 Brain_Putamen_basal_ganglia 5.521872308 0.017444559 0.351514232 -0.658492862 0.482501147 -0.096077852 0.469616179 4 0 0.998207054
RP11-170M17.1 ENSG00000228566.1 0.62405172 2 2 0.399859242 Brain_Cerebellar_Hemisphere 0.568840192 Brain_Nucleus_accumbens_basal_ganglia 1.104999618 0.895000382 0.895000382 -0.569760438 0.841872648 0.136056105 0.998175327 2 0 0.998207054
ZDHHC24 ENSG00000174165.7 0.624113878 12 4 0.147735995 Brain_Cerebellum 0.532140405 Brain_Hypothalamus 10.13304573 6.17E-05 0.342043314 0.624741996 1.44757483 0.941117487 0.231103223 4 0 0.998207054
RP11-294N21.3 ENSG00000274400.1 0.624228236 3 3 0.198012752 Brain_Anterior_cingulate_cortex_BA24 0.390600237 Brain_Hypothalamus 2.382830413 0.127044993 0.127044993 -1.287233965 -0.858529339 -1.026545797 0.228884137 3 0 0.998207054
RAB6B ENSG00000154917.10 0.624273512 3 3 0.381198175 Brain_Hypothalamus 0.798083972 Brain_Cerebellar_Hemisphere 1.464843515 0.537348596 0.537348596 -0.795074329 0.875690751 0.112148003 0.844598636 3 0 0.998207054
AP000265.1 ENSG00000261610.1 0.624275204 2 2 0.372250965 Brain_Cerebellar_Hemisphere 0.757373813 Brain_Cerebellum 1.080879385 0.919120615 0.919120615 -0.892264897 0.308931265 -0.291666816 0.849373951 2 0 0.998207054
RABGEF1 ENSG00000154710.16 0.62439954 4 4 0.524875217 Brain_Cerebellar_Hemisphere 0.921772488 Brain_Frontal_Cortex_BA9 3.174345111 0.117287939 0.117287939 -0.635848431 0.318785253 -0.129387175 0.423406966 4 0 0.998207054
HEATR6 ENSG00000068097.14 0.62440242 10 3 0.366925296 Brain_Anterior_cingulate_cortex_BA24 0.996710612 Brain_Substantia_nigra 8.431109516 0.001198613 0.494449175 -0.352191646 0.90224771 0.072073573 0.335350528 3 0 0.998207054
RBM12 ENSG00000244462.7 0.624496124 2 2 0.427261492 Brain_Putamen_basal_ganglia 0.511248348 Brain_Cerebellar_Hemisphere 1.130589324 0.869410676 0.869410676 -0.656895134 0.79388653 0.068495698 1.025857552 2 0 0.998207054
ZBED8 ENSG00000221886.3 0.624561352 2 2 0.421611437 Brain_Cerebellum 0.575809068 Brain_Cerebellar_Hemisphere 1.935419835 0.064580165 0.064580165 -0.80362871 -0.559516801 -0.681572755 0.172613187 2 0 0.998207054
CDH2 ENSG00000170558.8 0.624580173 2 2 0.341768172 Brain_Cerebellar_Hemisphere 0.468039875 Brain_Cerebellum 1.865456142 0.134543858 0.134543858 0.725671992 0.950677384 0.838174688 0.159102839 2 0 0.998207054
RAB27B ENSG00000041353.9 0.624599716 2 2 0.44792715 Brain_Cerebellum 0.725515024 Brain_Amygdala 1.297761486 0.702238514 0.702238514 -0.758875309 -0.351097739 -0.554986524 0.288342285 2 0 0.998207054
TMEM127 ENSG00000135956.8 0.62469672 3 3 0.196930837 Brain_Caudate_basal_ganglia 0.39479538 Brain_Cerebellar_Hemisphere 2.15302611 0.137158612 0.137158612 0.850953148 1.290345178 1.028586696 0.231460779 3 0 0.998207054
ABCG4 ENSG00000172350.9 0.624701287 2 2 0.332037323 Brain_Cerebellum 0.683060279 Brain_Caudate_basal_ganglia 1.425817786 0.574182214 0.574182214 -0.970018396 -0.408290683 -0.689154539 0.397201475 2 0 0.998207054
PIEZO1 ENSG00000103335.21 0.624719209 4 4 0.213916716 Brain_Cerebellar_Hemisphere 0.80478826 Brain_Cerebellum 1.647922444 0.427616791 0.427616791 -0.667915845 1.242867357 -0.035257086 0.869241226 4 0 0.998207054
MYCBP2 ENSG00000005810.17 0.624764802 1 1 0.624764802

Brain_Nucleus_accumbens_basal_ganglia 0.624764802
Brain_Nucleus_accumbens_basal_ganglia 1 1 1 0.489108615
0.489108615 0.489108615 NA 1 0 0.998207054
LINC01786 ENSG00000230415.1 0.624818455 1 1 0.624818455
Brain_Cortex 0.624818455 Brain_Cortex 1 1 1 0.489032829
0.489032829 0.489032829 NA 1 0 0.998207054
ZNF45 ENSG00000124459.11 0.624836564 6 5 0.268314066 Brain_Cerebellum
0.985798593 Brain_Hypothalamus 3.855069956 0.061986483
0.144050849 -0.205790708 1.106953424 0.346830423 0.509609691
5 0 0.998207054
PROS1 ENSG00000184500.14 0.624838359 4 4 0.154486876
Brain_Substantia_nigra 0.860956538 Brain_Cortex 2.254121275
0.133362831 0.133362831 -1.423860437 0.813437857 -0.260606762
0.915431921 4 0 0.998207054
TEPSIN ENSG00000167302.10 0.62483951 2 2 0.437304495
Brain_Spinal_cord_cervical_c-1 0.560510111 Brain_Cerebellar_Hemisphere
1.001823867 0.998176133 0.998176133 -0.776753028 -0.582083987
-0.679418507 0.137651799 2 0 0.998207054
RING1 ENSG00000204227.4 0.624911833 1 1 0.624911833
Brain_Cerebellar_Hemisphere 0.624911833 Brain_Cerebellar_Hemisphere 1 1
1 -0.488900936 -0.488900936 -0.488900936 NA 1 0
0.998207054
RP11-766N7.3 ENSG00000255693.1 0.624922247 11 7 0.129136534
Brain_Cerebellum 0.851786102 Brain_Caudate_basal_ganglia 6.889739236
0.000989634 0.251697298 -0.186839977 1.517515696 0.846895033
0.473815595 7 0 0.998207054
RECQL ENSG00000004700.15 0.624938886 3 3 0.245357724
Brain_Caudate_basal_ganglia 0.490085587 Brain_Cerebellar_Hemisphere
1.875012284 0.13172858 0.13172858 -1.161699069 0.690172703
-0.520338902 1.04895533 3 0 0.998207054
IGFLR1 ENSG00000126246.9 0.624970871 2 2 0.375469072
Brain_Cerebellum 0.654837585 Brain_Caudate_basal_ganglia 1.061590097
0.938409903 0.938409903 0.447051904 0.88627553 0.666663717
0.310578005 2 0 0.998207054
SLC34A2 ENSG00000157765.11 0.624971303 1 1 0.624971303
Brain_Hypothalamus 0.624971303 Brain_Hypothalamus 1 1 1
-0.488816941 -0.488816941 -0.488816941 NA 1 0 0.998207054
PREX2 ENSG00000046889.18 0.625018113 5 5 0.110830271
Brain_Nucleus_accumbens_basal_ganglia 0.885820233 Brain_Cortex 2.42456133
0.224647475 0.224647475 -0.870668936 1.594472386 0.012337971
0.929390989 5 0 0.998207054
ZDHHC22 ENSG00000177108.5 0.625070019 2 2 0.340041784
Brain_Cerebellum 0.901031482 Brain_Putamen_basal_ganglia 1.049486441
0.950513559 0.950513559 -0.954082696 0.124358435 -0.414862131
0.762573036 2 0 0.998207054
VSTM2L ENSG00000132821.11 0.625152811 2 2 0.334909767
Brain_Caudate_basal_ganglia 0.862194784 Brain_Cortex 1.07819497 0.92180503
0.92180503 -0.964271616 -0.173580976 -0.568926296 0.559102713 2
0 0.998207054
INHBB ENSG00000163083.5 0.625312469 1 1 0.625312469 Brain_Cortex
0.625312469 Brain_Cortex 1 1 1 0.488335149 0.488335149
0.488335149 NA 1 0 0.998207054
TRMT10B ENSG00000165275.9 0.625405272 4 4 0.596409405
Brain_Nucleus_accumbens_basal_ganglia 0.894343286
Brain_Spinal_cord_cervical_c-1 2.322807753 0.118335311 0.118335311
-0.529571001 0.19629804 -0.069941212 0.34242493 4 0 0.998207054
NDUFAF4 ENSG00000123545.5 0.625689451 13 1 0.409027283
Brain_Substantia_nigra 0.975006747 Brain_Hippocampus 11.88314875
0.000412232 11.88314875 0.031329522 0.825606613 0.464390016
0.242093277 1 0 0.998207054
ARC ENSG00000198576.3 0.625829473 1 1 0.625829473 Brain_Cerebellum
0.625829473 Brain_Cerebellum 1 1 1 0.487605255 0.487605255

0.487605255 NA 1 0 0.998207054
ZNF330 ENSG00000109445.10 0.625835494 3 3 0.194854114
Brain_Frontal_Cortex_BA9 0.825998305 Brain_Substantia_nigra
1.300130263 0.729314952 0.729314952 0.21983674 1.296352368
0.666363695 0.561217539 3 0 0.998207054
NLRP14 ENSG00000158077.4 0.62587862 1 1 0.62587862
Brain_Spinal_cord_cervical_c-1 0.62587862 Brain_Spinal_cord_cervical_c-1 1
1 1 -0.487535883 -0.487535883 -0.487535883 NA 1 0
0.998207054
GCH1 ENSG00000131979.18 0.625907289 5 4 0.136963171
Brain_Putamen_basal_ganglia 0.96744704 Brain_Hippocampus 3.381087053
0.083109349 0.200612274 0.04081041 1.48719555 0.439191613
0.619393482 4 0 0.998207054
CFAP69 ENSG00000105792.19 0.626021598 7 5 0.209754039
Brain_Caudate_basal_ganglia 0.914149905 Brain_Hippocampus 5.017886979
0.023087245 0.177133211 0.107805594 1.254242053 0.704706082
0.381404472 5 0 0.998207054
CCND3 ENSG00000112576.12 0.626062103 1 1 0.626062103 Brain_Cortex
0.626062103 Brain_Cortex 1 1 1 0.487276917 0.487276917
0.487276917 NA 1 0 0.998207054
SLC20A2 ENSG00000168575.9 0.626074264 1 1 0.626074264
Brain_Cortex 0.626074264 Brain_Cortex 1 1 1 0.487259755
0.487259755 0.487259755 NA 1 0 0.998207054
CUTC ENSG00000119929.12 0.62609259 1 1 0.62609259 Brain_Cortex 0.62609259
Brain_Cortex 1 1 1 -0.487233891 -0.487233891 -0.487233891
NA 1 0 0.998207054
PKLR ENSG00000143627.17 0.626136219 1 1 0.626136219
Brain_Frontal_Cortex_BA9 0.626136219 Brain_Frontal_Cortex_BA9 1 1
1 0.487172321 0.487172321 0.487172321 NA 1 0
0.998207054
CASP1 ENSG00000137752.23 0.626288057 1 1 0.626288057
Brain_Anterior_cingulate_cortex_BA24 0.626288057
Brain_Anterior_cingulate_cortex_BA24 1 1 1 -0.486958053
-0.486958053 -0.486958053 NA 1 0 0.998207054
FZD1 ENSG00000157240.3 0.626300165 4 4 0.249655313 Brain_Cerebellum
0.637016068 Brain_Frontal_Cortex_BA9 1.339048062 0.723946983
0.723946983 -1.151186998 0.790334612 -0.131752625 0.914727171
4 0 0.998207054
LOXL1 ENSG00000129038.15 0.626319935 3 3 0.256462321
Brain_Caudate_basal_ganglia 0.963583572 Brain_Hippocampus 1.320676751
0.705636618 0.705636618 -0.636801689 1.134792377 0.150777869
0.901984701 3 0 0.998207054
ADORA1 ENSG00000163485.16 0.626345008 2 2 0.437015581
Brain_Cerebellar_Hemisphere 0.690666084 Brain_Cerebellum 1.212036147
0.787963853 0.787963853 -0.3979513 0.777242723 0.189645711
0.830987662 2 0 0.998207054
ZRANB1 ENSG00000019995.6 0.626507393 1 1 0.626507393
Brain_Amygdala 0.626507393 Brain_Amygdala 1 1 1 0.486648577
0.486648577 0.486648577 NA 1 0 0.998207054
CERNA1 ENSG00000259577.1 0.6265469 13 4 0.117291397
Brain_Hypothalamus 0.909115865 Brain_Hippocampus 10.61463315
0.004910803 0.363213554 -1.566244464 -0.114153812 -1.183204502
0.421741108 4 0 0.998207054
GLRA3 ENSG00000145451.12 0.626578027 3 2 0.337084807
Brain_Caudate_basal_ganglia 0.481701935 Brain_Nucleus_accumbens_basal_ganglia
2.887395387 0.011595688 0.101008925 -0.959941204 -0.703567856
-0.865153762 0.140635519 2 0 0.998207054
ZNF831 ENSG00000124203.6 0.626820877 1 1 0.626820877
Brain_Cerebellar_Hemisphere 0.626820877 Brain_Cerebellar_Hemisphere 1 1
1 -0.48620634 -0.48620634 -0.48620634 NA 1 0
0.998207054
CASKIN2 ENSG00000177303.9 0.626845887 1 1 0.626845887

Brain_Nucleus_accumbens_basal_ganglia 0.626845887
Brain_Nucleus_accumbens_basal_ganglia 1 1 1 -0.486171062
-0.486171062 -0.486171062 NA 1 0 0.998207054
PANK4 ENSG00000157881.13 0.626953685 2 2 0.50051771
Brain_Frontal_Cortex_BA9 0.518915006 Brain_Substantia_nigra
1.068464809 0.931535191 0.931535191 -0.645018795 0.673675392
0.014328298 0.932457602 2 0 0.998207054
ZNF302 ENSG00000089335.20 0.627061775 12 3 0.137425505
Brain_Substantia_nigra 0.521851743 Brain_Caudate_basal_ganglia 10.0754618
0.000140713 0.496443264 -1.485446805 -0.640493608 -1.124705277
0.263248876 3 0 0.998207054
RP1-102E24.8 ENSG00000256433.2 0.627079964 6 6 0.125347721
Brain_Cerebellum 0.94391178 Brain_Anterior_cingulate_cortex_BA24 2.450565999
0.089504469 0.089504469 -0.61268517 1.532708389 0.504869746
0.758890422 6 0 0.998207054
SIGLEC11 ENSG00000161640.15 0.627178118 8 7 0.274010619
Brain_Hippocampus 0.988983716 Brain_Cerebellum 3.978255657
0.006029161 0.138282312 -1.093873143 0.751472719 -0.215100363
0.640178582 7 0 0.998207054
KIAA1217 ENSG00000120549.17 0.627220597 2 2 0.481391199
Brain_Frontal_Cortex_BA9 0.503406302 Brain_Hippocampus 1.011317335
0.988682665 0.988682665 -0.669139802 0.704066761 0.017463479
0.971003673 2 0 0.998207054
RP11-38G5.2 ENSG00000259548.1 0.627229026 2 1 0.567883393
Brain_Cerebellum 0.692489452 Brain_Cerebellar_Hemisphere 1.981055565
0.018944435 1.981055565 -0.571171504 -0.395478953 -0.483325228
0.124233394 1 0 0.998207054
SPHK2 ENSG00000063176.15 0.627260762 3 2 0.347873315
Brain_Frontal_Cortex_BA9 0.485197616 Brain_Putamen_basal_ganglia 2.37336951
0.037407366 0.589223125 -0.93872235 -0.697967346 -0.781651992
0.136124355 2 0 0.998207054
RP11-162A12.2 ENSG00000264278.1 0.627337154 2 2 0.475057913
Brain_Nucleus_accumbens_basal_ganglia 0.511050758 Brain_Hippocampus
1.010287569 0.989712431 0.989712431 -0.714273762 -0.657202438
-0.6857381 0.04035552 2 0 0.998207054
ASTE1 ENSG00000034533.11 0.627447239 9 6 0.113287072
Brain_Anterior_cingulate_cortex_BA24 0.993630569 Brain_Cortex
5.900583457 0.048458639 0.312152944 -1.583589908 0.782841343
0.079239788 0.68109071 6 0 0.998207054
SLC2A11 ENSG00000133460.19 0.627448953 11 3 0.346396698
Brain_Cerebellar_Hemisphere 0.952415379 Brain_Hippocampus 9.333010183
2.66E-08 0.462638798 -0.941601507 0.686164762 -0.217193351
0.398755736 3 0 0.998207054
UBE2U ENSG00000177414.13 0.627480912 4 4 0.189478968 Brain_Cortex
0.773237031 Brain_Amygdala 2.291926192 0.183468789 0.183468789
0.288143323 1.312122017 0.728825923 0.428125933 4 0
0.998207054
MEIKIN ENSG00000239642.5 0.627488548 3 2 0.335404014
Brain_Caudate_basal_ganglia 0.429561224 Brain_Cerebellar_Hemisphere 2.68291551
0.065981637 0.251102853 0.789942714 0.963286005 0.86631789
0.088487434 2 0 0.998207054
PCTP ENSG00000141179.13 0.62749665 1 1 0.62749665
Brain_Nucleus_accumbens_basal_ganglia 0.62749665
Brain_Nucleus_accumbens_basal_ganglia 1 1 1 -0.485253339
-0.485253339 -0.485253339 NA 1 0 0.998207054
FABP1 ENSG00000163586.9 0.627503068 13 2 0.467256113
Brain_Frontal_Cortex_BA9 0.942002745 Brain_Spinal_cord_cervical_c-1
11.4616218 5.26E-18 0.748472809 -0.275687595 0.726950773 0.20304724
0.275184336 2 0 0.998207054
COL14A1 ENSG00000187955.11 0.627658796 1 1 0.627658796
Brain_Putamen_basal_ganglia 0.627658796 Brain_Putamen_basal_ganglia 1 1
1 0.48502474 0.48502474 0.48502474 NA 1 0 0.998207054

RP11-452H21.4 ENSG00000251323.2 0.62766401 1 1 0.62766401 Brain_Cerebellum
0.62766401 Brain_Cerebellum 1 1 1 -0.485017391 -0.485017391
-0.485017391 NA 1 0 0.998207054
APLF ENSG00000169621.9 0.627686437 1 1 0.627686437 Brain_Cortex
0.627686437 Brain_Cortex 1 1 1 -0.484985773 -0.484985773
-0.484985773 NA 1 0 0.998207054
ADAD2 ENSG00000140955.10 0.62774101 10 6 0.231834287
Brain_Substantia_nigra 0.91526064 Brain_Cortex 4.588907955
0.011623109 0.233033215 -1.19564714 0.853488913 0.094964999
0.677012799 6 0 0.998207054
AP1S3 ENSG00000152056.16 0.627800998 5 5 0.143927345 Brain_Cortex
0.772745333 Brain_Spinal_cord_cervical_c-1 1.462023576 0.693644883
0.693644883 -1.46132109 0.674778663 -0.30326764 0.838455379
5 0 0.998207054
FKBP11 ENSG00000134285.10 0.62787237 11 3 0.304741795
Brain_Caudate_basal_ganglia 0.965322572 Brain_Frontal_Cortex_BA9
9.336583876 0.000187992 0.37557286 -1.026317831 0.043475402
-0.566354525 0.280271685 3 0 0.998207054
CTC-281F24.1 ENSG00000261996.1 0.627936086 3 3 0.219472004
Brain_Cerebellum 0.573128563 Brain_Amygdala 1.644426897 0.47952993
0.47952993 -1.227933309 0.782179807 0.039232134 1.102833572 3
0 0.998207054
SGPP2 ENSG00000163082.9 0.628025574 3 2 0.284721205
Brain_Nucleus_accumbens_basal_ganglia 0.470760757 Brain_Putamen_basal_ganglia
2.623947571 0.011848479 0.36420395 0.721241801 1.069773651
0.885356741 0.175150621 2 0 0.998207054
TMEM100 ENSG00000166292.11 0.628054163 1 1 0.628054163
Brain_Nucleus_accumbens_basal_ganglia 0.628054163
Brain_Nucleus_accumbens_basal_ganglia 1 1 1 -0.484467445
-0.484467445 -0.484467445 NA 1 0 0.998207054
B4GALT2 ENSG00000117411.16 0.628061353 1 1 0.628061353
Brain_Hippocampus 0.628061353 Brain_Hippocampus 1 1 1
-0.484457312 -0.484457312 -0.484457312 NA 1 0 0.998207054
RUFY2 ENSG00000204130.12 0.628068379 3 1 0.557338859
Brain_Putamen_basal_ganglia 0.682260837 Brain_Nucleus_accumbens_basal_ganglia
2.882184616 0.02669208 2.882184616 0.40937997 0.586798776
0.475400406 0.097024357 1 0 0.998207054
CENPM ENSG00000100162.14 0.628084704 10 5 0.408631766
Brain_Anterior_cingulate_cortex_BA24 0.97761366 Brain_Putamen_basal_ganglia
6.954872899 0.001935931 0.337709223 -0.826303823 0.794739288
-0.042720602 0.497836357 5 0 0.998207054
POMT1 ENSG00000130714.15 0.628096935 7 3 0.578431483
Brain_Cerebellar_Hemisphere 0.981209302 Brain_Spinal_cord_cervical_c-1
6.195161593 0.006158539 0.274388677 -0.555677292 0.419546422
-0.12262814 0.380182299 3 0 0.998207054
GSDMC ENSG00000147697.8 0.628187386 1 1 0.628187386
Brain_Putamen_basal_ganglia 0.628187386 Brain_Putamen_basal_ganglia 1 1
1 0.484279692 0.484279692 0.484279692 NA 1 0
0.998207054
HDAC1 ENSG00000116478.11 0.628360031 1 1 0.628360031 Brain_Cortex
0.628360031 Brain_Cortex 1 1 1 0.484036408 0.484036408
0.484036408 NA 1 0 0.998207054
FRAT2 ENSG00000181274.6 0.628373056 2 2 0.550753769 Brain_Cortex
0.691128943 Brain_Substantia_nigra 1.460518422 0.539481578
0.539481578 -0.397323469 0.596631 0.099653766 0.702831945 2
0 0.998207054
MRPL21 ENSG00000197345.12 0.628560018 13 1 0.447114261
Brain_Spinal_cord_cervical_c-1 0.793159645 Brain_Cerebellum 12.800172
2.02E-05 12.800172 0.26220987 0.760234776 0.48081247 0.145090352 1
0 0.998207054
FAM45A ENSG00000119979.16 0.628588944 1 1 0.628588944
Brain_Putamen_basal_ganglia 0.628588944 Brain_Putamen_basal_ganglia 1 1

1 0.483713876 0.483713876 0.483713876 NA 1 0
0.998207054
TUBA8 ENSG00000183785.14 0.628634894 2 2 0.338493521
Brain_Frontal_Cortex_BA9 0.865647637 Brain_Amygdala 1.061201848
0.938798152 0.938798152 -0.957146095 0.169189439 -0.393978328
0.796439493 2 0 0.998207054
MCTP1 ENSG00000175471.19 0.628651766 3 3 0.253101745 Brain_Cortex
0.689021966 Brain_Cerebellar_Hemisphere 1.312649952 0.691176783
0.691176783 -0.411549302 1.142847905 0.37716043 0.777454299 3
0 0.998207054
B3GAT1 ENSG00000109956.12 0.628707736 2 2 0.358269635
Brain_Frontal_Cortex_BA9 0.50133325 Brain_Cortex 1.449764243
0.550235757 0.550235757 0.672393453 0.918667269 0.795530361
0.174141886 2 0 0.998207054
SDHC ENSG00000143252.14 0.628795121 8 5 0.195599509
Brain_Hypothalamus 0.932530564 Brain_Anterior_cingulate_cortex_BA24
5.254223664 0.033544709 0.420202113 -0.331658242 1.294190844
0.553801172 0.583986629 5 0 0.998207054
GACAT3 ENSG00000236289.2 0.628803108 1 1 0.628803108
Brain_Spinal_cord_cervical_c-1 0.628803108 Brain_Spinal_cord_cervical_c-1
1 1 1 0.483412172 0.483412172 0.483412172 NA 1 0
0.998207054
DACT1 ENSG00000165617.14 0.628821205 1 1 0.628821205 Brain_Cortex
0.628821205 Brain_Cortex 1 1 1 0.483386679 0.483386679
0.483386679 NA 1 0 0.998207054
RP11-399K21.11 ENSG00000270087.5 0.629065818 2 2 0.41242858
Brain_Hypothalamus 0.558548265 Brain_Hippocampus 1.100450598
0.899549402 0.899549402 -0.819627344 -0.584999185 -0.702313265
0.165907162 2 0 0.998207054
SCAF1 ENSG00000126461.14 0.629100341 1 1 0.629100341 Brain_Cerebellum
0.629100341 Brain_Cerebellum 1 1 1 -0.482993514 -0.482993514
-0.482993514 NA 1 0 0.998207054
CLU ENSG00000120885.21 0.629111026 1 1 0.629111026
Brain_Putamen_basal_ganglia 0.629111026 Brain_Putamen_basal_ganglia 1 1
1 -0.482978466 -0.482978466 -0.482978466 NA 1 0
0.998207054
CTC-471C19.1 ENSG00000261037.1 0.629301776 8 7 0.163848521
Brain_Nucleus_accumbens_basal_ganglia 0.925746785 Brain_Hypothalamus
4.042320171 0.076818572 0.165940725 -0.892046779 1.392244006
0.57465246 0.776926076 7 0 0.998207054
MYEOV ENSG00000172927.7 0.629316924 2 2 0.382249672 Brain_Cerebellum
0.387331535 Brain_Cerebellar_Hemisphere 1.631222875 0.368777125
0.368777125 0.864466756 0.873758708 0.869112732 0.006570402
2 0 0.998207054
ALDH5A1 ENSG00000112294.12 0.629320809 6 4 0.084610688
Brain_Cerebellum 0.964943795 Brain_Anterior_cingulate_cortex_BA24
4.097737481 0.047472147 0.409625689 0.043950583 1.724538427
0.822504295 0.636260124 4 0 0.998207054
APBA1 ENSG00000107282.7 0.62936419 1 1 0.62936419 Brain_Putamen_basal_ganglia
0.62936419 Brain_Putamen_basal_ganglia 1 1 1 0.482621952
0.482621952 0.482621952 NA 1 0 0.998207054
SLAIN1 ENSG00000139737.21 0.629399063 3 3 0.341222439
Brain_Cerebellar_Hemisphere 0.734446142 Brain_Cerebellum 1.171797853
0.847257863 0.847257863 -0.951752653 0.796202992 -0.164922274
0.886916718 3 0 0.998207054
RITA1 ENSG00000139405.15 0.629406112 6 3 0.133032824 Brain_Cortex
0.393887523 Brain_Cerebellar_Hemisphere 4.735937597 0.039300201
0.453208873 -1.502248965 -0.852588535 -1.145611356 0.311747472
3 0 0.998207054
SLIT1 ENSG00000187122.16 0.629651623 1 1 0.629651623 Brain_Cerebellum
0.629651623 Brain_Cerebellum 1 1 1 -0.482217251 -0.482217251
-0.482217251 NA 1 0 0.998207054

LRTM2 ENSG00000166159.10 0.629719389 3 3 0.272930613
Brain_Frontal_Cortex_BA9 0.840862234 Brain_Cerebellum 1.107478455
0.892957622 0.892957622 -1.096338624 0.728606657 -0.055647088
0.939109459 3 0 0.998207054
OAF ENSG00000184232.8 0.629845027 7 3 0.211414827 Brain_Cortex
0.894980124 Brain_Putamen_basal_ganglia 5.227947425 0.023673889
0.435569407 -0.267177409 1.249684486 0.641700109 0.551446003
3 0 0.998207054
MGST1 ENSG00000008394.12 0.629848091 6 6 0.057900042 Brain_Cerebellum
0.838904084 Brain_Nucleus_accumbens_basal_ganglia 3.133165753 0.1215729
0.1215729 -0.66662358 1.896453965 0.363461708 0.942713085 6
0 0.998207054
DARS ENSG00000115866.10 0.629915288 2 2 0.356314408 Brain_Cerebellum
0.936864977 Brain_Cerebellar_Hemisphere 1.201979044 0.798020956
0.798020956 0.079210772 0.922410667 0.500810719 0.596232364
2 0 0.998207054
RP11-672A2.5 ENSG00000255363.2 0.630113619 1 1 0.630113619
Brain_Nucleus_accumbens_basal_ganglia 0.630113619
Brain_Nucleus_accumbens_basal_ganglia 1 1 1 0.481566936
0.481566936 0.481566936 NA 1 0 0.998207054
PLEKHJ1 ENSG00000104886.11 0.630236775 4 4 0.129326541
Brain_Hypothalamus 0.893614843 Brain_Hippocampus 2.311357214
0.199930951 0.199930951 -0.445526625 1.516762963 0.186449624
0.897152057 4 0 0.998207054
JCHAIN ENSG00000132465.10 0.630250559 1 1 0.630250559
Brain_Substantia_nigra 0.630250559 Brain_Substantia_nigra 1 1
1 0.481374216 0.481374216 0.481374216 NA 1 0
0.998207054
SART1 ENSG00000175467.14 0.63028949 1 1 0.63028949 Brain_Cerebellum 0.63028949
Brain_Cerebellum 1 1 1 0.48131943 0.48131943 0.48131943 NA 1 0
0.998207054
ARHGEF11 ENSG00000132694.18 0.63046697 1 1 0.63046697 Brain_Hypothalamus
0.63046697 Brain_Hypothalamus 1 1 1 -0.481069689 -0.481069689
-0.481069689 NA 1 0 0.998207054
LPL ENSG00000175445.14 0.630558638 1 1 0.630558638
Brain_Anterior_cingulate_cortex_BA24 0.630558638
Brain_Anterior_cingulate_cortex_BA24 1 1 1 -0.48094071
-0.48094071 -0.48094071 NA 1 0 0.998207054
BROX ENSG00000162819.11 0.630704403 6 5 0.556949045
Brain_Frontal_Cortex_BA9 0.895715308 Brain_Putamen_basal_ganglia
4.710739025 0.08999191 0.164348259 -0.587379222 0.515607545
-0.140142608 0.425559324 5 0 0.998207054
MOAP1 ENSG00000165943.4 0.630751453 1 1 0.630751453
Brain_Frontal_Cortex_BA9 0.630751453 Brain_Frontal_Cortex_BA9 1 1
1 -0.480669442 -0.480669442 -0.480669442 NA 1 0
0.998207054
RP11-382D12.2 ENSG00000271387.1 0.630783954 1 1 0.630783954
Brain_Hippocampus 0.630783954 Brain_Hippocampus 1 1 1
0.48062372 0.48062372 0.48062372 NA 1 0 0.998207054
CHI3L1 ENSG00000133048.12 0.63086549 1 1 0.63086549
Brain_Frontal_Cortex_BA9 0.63086549 Brain_Frontal_Cortex_BA9 1 1 1
0.480509022 0.480509022 0.480509022 NA 1 0 0.998207054
EPHA5-AS1 ENSG00000250846.6 0.631021901 1 1 0.631021901
Brain_Anterior_cingulate_cortex_BA24 0.631021901
Brain_Anterior_cingulate_cortex_BA24 1 1 1 -0.480289012
-0.480289012 -0.480289012 NA 1 0 0.998207054
FMO4 ENSG00000076258.9 0.631076811 6 6 0.082699797
Brain_Hippocampus 0.862315064 Brain_Cortex 1.738186582
0.382555646 0.382555646 -1.735231568 0.489794548 -0.025264088
0.847573661 6 0 0.998207054
RP11-392O17.2 ENSG00000277007.1 0.63108896 1 1 0.63108896
Brain_Frontal_Cortex_BA9 0.63108896 Brain_Frontal_Cortex_BA9 1 1 1

-0.480194693 -0.480194693 -0.480194693 NA 1 0 0.998207054
NCOA1 ENSG00000084676.15 0.631151641 4 4 0.218182096
Brain_Substantia_nigra 0.867415939 Brain_Cortex 2.278140585
0.314346546 0.314346546 -0.241674547 1.231376463 0.417797137
0.624260315 4 0 0.998207054
RP11-2C24.7 ENSG00000274678.1 0.631300865 1 1 0.631300865
Brain_Spinal_cord_cervical_c-1 0.631300865 Brain_Spinal_cord_cervical_c-1
1 1 1 0.479896675 0.479896675 0.479896675 NA 1 0
0.998207054
RABEP1 ENSG00000029725.16 0.631389391 5 5 0.12406455
Brain_Substantia_nigra 0.933321261 Brain_Nucleus_accumbens_basal_ganglia
2.8165375 0.198879727 0.198879727 -0.318841752 1.537934831
0.238193535 0.757215352 5 0 0.998207054
PNP ENSG00000198805.11 0.631391063 3 3 0.372573849
Brain_Putamen_basal_ganglia 0.577651416 Brain_Amygdala 1.072451988
0.929885217 0.929885217 -0.891662519 -0.55681854 -0.756984513
0.176767158 3 0 0.998207054
CSDC2 ENSG00000172346.14 0.6313998 7 3 0.298755824
Brain_Nucleus_accumbens_basal_ganglia 0.768384445 Brain_Putamen_basal_ganglia
6.164087574 0.010545191 0.262556141 -1.039105177 -0.294488767
-0.70204828 0.272347882 3 0 0.998207054
CSGALNACT2 ENSG00000169826.7 0.631481025 13 1 0.452601312
Brain_Frontal_Cortex_BA9 0.8015441 Brain_Putamen_basal_ganglia 12.55630588
1.27E-05 12.55630588 0.251349252 0.751085317 0.470346206
0.131247653 1 0 0.998207054
KLHL28 ENSG00000179454.13 0.631633172 1 1 0.631633172
Brain_Putamen_basal_ganglia 0.631633172 Brain_Putamen_basal_ganglia 1 1
1 0.479429415 0.479429415 0.479429415 NA 1 0
0.998207054
RP11-663P9.1 ENSG00000250863.1 0.631697755 4 4 0.239689966
Brain_Cerebellum 0.781481821 Brain_Cortex 2.334709371 0.33956556
0.33956556 0.277388487 1.175762073 0.702929337 0.463584223 4
0 0.998207054
DPYD ENSG00000188641.12 0.631812196 1 1 0.631812196 Brain_Cortex
0.631812196 Brain_Cortex 1 1 1 0.479177729 0.479177729
0.479177729 NA 1 0 0.998207054
TNFSF13 ENSG00000161955.16 0.63184221 3 3 0.31446374
Brain_Caudate_basal_ganglia 0.76718902 Brain_Amygdala 1.19115358 0.822939202
0.822939202 -0.623699439 1.005899867 0.028715554 0.861977267
3 0 0.998207054
LYVE1 ENSG00000133800.8 0.631890755 2 2 0.34511517
Brain_Anterior_cingulate_cortex_BA24 0.841393117 Brain_Cerebellar_Hemisphere
1.038884552 0.961115448 0.961115448 -0.944106614 0.200111836
-0.371997389 0.809084625 2 0 0.998207054
ITGAM ENSG00000169896.16 0.631970986 2 2 0.338552999
Brain_Hippocampus 0.991396063 Brain_Anterior_cingulate_cortex_BA24
1.034496488 0.965503512 0.965503512 -0.957028244 0.010783645
-0.473122299 0.68434635 2 0 0.998207054
SOHLH2 ENSG00000120669.15 0.631971834 13 1 0.359682798
Brain_Substantia_nigra 0.9000928 Brain_Amygdala 12.54388754
0.000559503 12.54388754 0.125544118 0.91596968 0.471874377
0.211805086 1 0 0.998207054
RP11-20G13.5 ENSG00000274294.1 0.632015387 1 1 0.632015387
Brain_Amygdala 0.632015387 Brain_Amygdala 1 1 1 -0.478892106
-0.478892106 -0.478892106 NA 1 0 0.998207054
TRIB2 ENSG00000071575.11 0.632016031 1 1 0.632016031 Brain_Cortex
0.632016031 Brain_Cortex 1 1 1 -0.478891201 -0.478891201
-0.478891201 NA 1 0 0.998207054
DISP3 ENSG00000204624.7 0.632316148 2 2 0.469432477 Brain_Cortex
0.813185674 Brain_Frontal_Cortex_BA9 1.448350445 0.551649555
0.551649555 -0.723402759 0.236318318 -0.24354222 0.678625282
2 0 0.998207054

LINC00665 ENSG00000232677.6 0.632708523 12 3 0.157128354
Brain_Amygdala 0.748435299 Brain_Substantia_nigra 9.449917428
0.004196016 0.497319575 -1.414795873 -0.320703231 -0.743774498
0.361220157 3 0 0.998207054
PSMB3 ENSG00000277791.4 0.632802396 1 1 0.632802396
Brain_Cerebellar_Hemisphere 0.632802396 Brain_Cerebellar_Hemisphere 1 1
1 0.477786183 0.477786183 0.477786183 NA 1 0
0.998207054
RP11-1017G21.5 ENSG00000271780.1 0.632965631 11 7 0.286174457
Brain_Cerebellar_Hemisphere 0.998260875 Brain_Cerebellum 7.115968215
0.006746458 0.271074338 -1.066551399 1.00051973 0.107695269
0.654804817 7 0 0.998207054
RP11-307L14.2 ENSG00000272354.1 0.632986821 1 1 0.632986821
Brain_Hippocampus 0.632986821 Brain_Hippocampus 1 1 1
0.47752711 0.47752711 0.47752711 NA 1 0 0.998207054
ANTXR1 ENSG00000169604.19 0.633056701 2 2 0.467508055
Brain_Caudate_basal_ganglia 0.992750388 Brain_Nucleus_accumbens_basal_ganglia
1.64292003 0.35707997 0.35707997 -0.009086166 0.726539576 0.358726705
0.520165951 2 0 0.998207054
RP11-173P15.9 ENSG00000277423.1 0.633131461 1 1 0.633131461
Brain_Caudate_basal_ganglia 0.633131461 Brain_Caudate_basal_ganglia 1 1
1 0.477323947 0.477323947 0.477323947 NA 1 0
0.998207054
HCG20 ENSG00000228022.5 0.633145743 11 5 0.161279564
Brain_Putamen_basal_ganglia 0.995230791 Brain_Caudate_basal_ganglia
7.954365616 0.011750268 0.387368686 -0.191454758 1.400781028
0.250430458 0.446564307 5 0 0.998207054
DES ENSG00000175084.11 0.633159065 2 2 0.559341901
Brain_Caudate_basal_ganglia 0.690041478 Brain_Amygdala 1.465006861
0.534993139 0.534993139 -0.583819287 0.398798777 -0.092510255
0.694815896 2 0 0.998207054
RP1-111D6.4 ENSG00000225924.2 0.633164405 2 2 0.339335108
Brain_Hippocampus 0.975265756 Brain_Caudate_basal_ganglia 1.067218467
0.932781533 0.932781533 0.031004745 0.955479816 0.49324228
0.653702592 2 0 0.998207054
SENP7 ENSG00000138468.15 0.633182248 5 3 0.394814312
Brain_Caudate_basal_ganglia 0.949328467 Brain_Cerebellar_Hemisphere 4.12084542
0.066858243 0.230857725 -0.850919068 -0.063550099 -0.467849919
0.375118271 3 0 0.998207054
LRRC37A ENSG00000176681.14 0.633362483 13 3 0.450360354
Brain_Hippocampus 0.992762684 Brain_Cerebellar_Hemisphere 10.87835631
0.010517638 0.415270164 -0.329179257 0.754814398 0.130421343
0.301859662 3 0 0.998207054
FUT8 ENSG00000033170.16 0.633394512 2 2 0.362631415
Brain_Cerebellar_Hemisphere 0.650604742 Brain_Amygdala 1.183511503
0.816488497 0.816488497 -0.91036241 0.452922232 -0.228720089
0.963987815 2 0 0.998207054
BLOC1S1 ENSG00000135441.7 0.633395578 10 1 0.419213267
Brain_Caudate_basal_ganglia 0.921677385 Brain_Hypothalamus 9.689509629
6.88E-17 9.689509629 -0.098321023 0.807786901 0.468991893
0.229868108 1 0 0.998207054
PPP6R3 ENSG00000110075.14 0.633571751 1 1 0.633571751
Brain_Frontal_Cortex_BA9 0.633571751 Brain_Frontal_Cortex_BA9 1 1
1 -0.476705633 -0.476705633 -0.476705633 NA 1 0
0.998207054
CFLAR ENSG00000003402.19 0.633587766 1 1 0.633587766
Brain_Cerebellar_Hemisphere 0.633587766 Brain_Cerebellar_Hemisphere 1 1
1 -0.476683146 -0.476683146 -0.476683146 NA 1 0
0.998207054
MYLIP ENSG00000007944.14 0.633631719 3 3 0.259134081 Brain_Cerebellum
0.737071418 Brain_Cerebellar_Hemisphere 1.646397569 0.356081927
0.356081927 -0.481208526 1.128440147 0.327655228 0.804854747

3	0	0.998207054
MED31	ENSG00000108590.10	0.633766618	4	4	0.202208437
Brain_Caudate_basal_ganglia	0.974924079	Brain_Cerebellum	2.382919562
0.180255422	0.180255422	-1.275284854	0.231564468	-0.462685104
0.689489314	4	0	0.998207054
CDH5	ENSG00000179776.17	0.633770746	1	1	0.633770746
Brain_Hippocampus	0.633770746	Brain_Hippocampus	1	1	1
-0.476426237	-0.476426237	-0.476426237	NA	1	0	0.998207054
PARP8	ENSG00000151883.17	0.633856447	3	3	0.43285466	Brain_Hippocampus
0.563536435	Brain_Cerebellum	1.787201967	0.393287742	0.393287742
-0.620642897	0.784316092	0.247089965	0.758553536	3	0
0.998207054
DNAJC17	ENSG00000104129.9	0.633886484	1	1	0.633886484
Brain_Hippocampus	0.633886484	Brain_Hippocampus	1	1	1
0.476263754	0.476263754	0.476263754	NA	1	0	0.998207054
SLX4	ENSG00000188827.10	0.633998098	2	2	0.344732854
Brain_Cerebellar_Hemisphere	0.463920849	Brain_Cerebellum	1.851074659
0.148925341	0.148925341	-0.944855105	-0.732405916	-0.838630511
0.150224263	2	0	0.998207054
FBXO38	ENSG00000145868.16	0.634060836	8	2	0.232965683
Brain_Caudate_basal_ganglia	0.630041855	Brain_Anterior_cingulate_cortex_BA24
6.796457066	0.005771769	0.637171072	0.481667939	1.19275412
0.78689506	0.268432747	2	0	0.998207054
SCGB2B2	ENSG00000205209.7	0.634355789	3	3	0.266895817
Brain_Frontal_Cortex_BA9	0.786315362	Brain_Caudate_basal_ganglia
2.344103315	0.263865874	0.263865874	0.271098387	1.110239544
0.670816249	0.420977269	3	0	0.998207054
ULBP2	ENSG00000131015.4	0.634484084	6	5	0.105021119
Brain_Substantia_nigra	0.525415632	Brain_Cerebellar_Hemisphere
3.381316902	0.05411767	0.237678398	-1.620983775	-0.6350196
-1.111713726	0.325760364	5	0	0.998207054
GDAP1	ENSG00000104381.12	0.634505234	2	2	0.468450042	Brain_Cortex
0.853898297	Brain_Cerebellum	1.490858364	0.509141636	0.509141636
-0.184146795	0.725003241	0.270428223	0.642866155	2	0
0.998207054
DDX10	ENSG00000178105.9	0.634569685	2	2	0.390458748	Brain_Cortex
0.666347284	Brain_Cerebellum	1.019083433	0.980916567	0.980916567
-0.858785719	0.431166536	-0.213809592	0.912133987	2	0
0.998207054
LHFP	ENSG00000183722.7	0.634593761	4	4	0.26938367
Brain_Nucleus_accumbens_basal_ganglia	0.739982537
Brain_Spinal_cord_cervical_c-1	1.30282392	0.690133788	0.690133788
-1.063671655	1.104483008	-0.207075827	0.927096791	4	0
0.998207054
AZU1	ENSG00000172232.9	0.634654769	1	1	0.634654769
Brain_Putamen_basal_ganglia	0.634654769	Brain_Putamen_basal_ganglia	1	1
1	0.475185489	0.475185489	0.475185489	NA	1	0
0.998207054
NAV3	ENSG00000067798.14	0.634683398	1	1	0.634683398	Brain_Cerebellum
0.634683398	Brain_Cerebellum	1	1	1	0.47514532	0.47514532	0.47514532
NA	1	0	0.998207054
FOXL2NB	ENSG00000206262.8	0.63474785	1	1	0.63474785	Brain_Cerebellum
0.63474785	Brain_Cerebellum	1	1	1	0.47505489	0.47505489	0.47505489	NA
1	0	0.998207054
PTHLH	ENSG00000087494.15	0.634752502	2	2	0.361587555	Brain_Cerebellum
0.775572512	Brain_Anterior_cingulate_cortex_BA24	1.00900352	0.99099648
0.99099648	0.285093497	0.912344196	0.598718847	0.443533223	2
0	0.998207054
RP11-3B12.2	ENSG00000237764.2	0.634796133	1	1	0.634796133
Brain_Cerebellar_Hemisphere	0.634796133	Brain_Cerebellar_Hemisphere	1	1
1	-0.47498715	-0.47498715	-0.47498715	NA	1	0
0.998207054

C3orf70 ENSG00000187068.2 0.634886112 9 4 0.389485846
Brain_Nucleus_accumbens_basal_ganglia 0.998223067 Brain_Frontal_Cortex_BA9
6.959304163 2.28E-06 0.236502592 -0.380796881 0.86055016
0.372590292 0.467167853 4 0 0.998207054
FAM222B ENSG00000173065.13 0.63495261 1 1 0.63495261 Brain_Cerebellum
0.63495261 Brain_Cerebellum 1 1 1 0.474767627 0.474767627
0.474767627 NA 1 0 0.998207054
APMAP ENSG00000101474.11 0.635065827 6 5 0.530237025
Brain_Substantia_nigra 0.79182322 Brain_Caudate_basal_ganglia 4.051094001
0.036297361 0.198632458 -0.577757303 0.627644233 -0.235417308
0.444344697 5 0 0.998207054
ELAC1 ENSG00000141642.8 0.635155296 1 1 0.635155296
Brain_Caudate_basal_ganglia 0.635155296 Brain_Caudate_basal_ganglia 1 1
1 -0.474483312 -0.474483312 -0.474483312 NA 1 0
0.998207054
RP11-426C22.7 ENSG00000273582.1 0.635173831 2 2 0.383132319
Brain_Hypothalamus 0.403687559 Brain_Spinal_cord_cervical_c-1
1.608525287 0.391474713 0.391474713 -0.872139429 -0.835053548
-0.853596488 0.026223678 2 0 0.998207054
REPS1 ENSG00000135597.18 0.635187721 6 6 0.26335499
Brain_Frontal_Cortex_BA9 0.934707653 Brain_Nucleus_accumbens_basal_ganglia
1.944636753 0.461000468 0.461000468 -0.727048067 1.118496525
0.390492447 0.690286856 6 0 0.998207054
AQP3 ENSG00000165272.14 0.635231159 4 4 0.187783961 Brain_Cerebellum
0.579427454 Brain_Anterior_cingulate_cortex_BA24 2.333367419
0.244610332 0.244610332 0.554221225 1.317163095 0.91738247
0.414136886 4 0 0.998207054
RPRD1A ENSG00000141425.17 0.635261455 2 2 0.397259229
Brain_Cortex 0.749159762 Brain_Substantia_nigra 1.133690581
0.866309419 0.866309419 -0.319747483 0.846526264 0.263389391
0.824680075 2 0 0.998207054
CLPB ENSG00000162129.12 0.635400315 1 1 0.635400315
Brain_Anterior_cingulate_cortex_BA24 0.635400315
Brain_Anterior_cingulate_cortex_BA24 1 1 1 -0.474139665
-0.474139665 -0.474139665 NA 1 0 0.998207054
SYMPK ENSG00000125755.18 0.635442073 7 3 0.43960891
Brain_Nucleus_accumbens_basal_ganglia 0.793923769 Brain_Cortex
5.979473355 0.027589655 0.311623473 -0.261218816 0.7728538
0.418264364 0.326709759 3 0 0.998207054
C11orf21 ENSG00000110665.11 0.6354485 6 6 0.15899275 Brain_Cortex
0.934873829 Brain_Spinal_cord_cervical_c-1 1.637105496 0.371215812
0.371215812 -1.408467278 -0.081714398 -0.743375581 0.443182288
6 0 0.998207054
DAZAP2 ENSG00000183283.15 0.635598112 1 1 0.635598112
Brain_Cerebellar_Hemisphere 0.635598112 Brain_Cerebellar_Hemisphere 1 1
1 0.473862289 0.473862289 0.473862289 NA 1 0
0.998207054
MOBP ENSG00000168314.17 0.635601892 1 1 0.635601892
Brain_Substantia_nigra 0.635601892 Brain_Substantia_nigra 1 1
1 0.473856989 0.473856989 0.473856989 NA 1 0
0.998207054
LINC00452 ENSG00000229373.8 0.635607369 1 1 0.635607369
Brain_Cortex 0.635607369 Brain_Cortex 1 1 1 -0.473849309
-0.473849309 -0.473849309 NA 1 0 0.998207054
RAB3GAP2 ENSG00000118873.15 0.635640791 3 3 0.292351356
Brain_Anterior_cingulate_cortex_BA24 0.561643726
Brain_Nucleus_accumbens_basal_ganglia 1.270374033 0.729545536
0.729545536 -1.052977388 0.666485802 0.064636721 0.968838784
3 0 0.998207054
LINC01476 ENSG00000265313.5 0.635663946 1 1 0.635663946
Brain_Anterior_cingulate_cortex_BA24 0.635663946
Brain_Anterior_cingulate_cortex_BA24 1 1 1 0.473769977

0.473769977 0.473769977 NA 1 0 0.998207054
MTIF3 ENSG00000122033.14 0.636044316 6 6 0.094714788
Brain_Cerebellar_Hemisphere 0.901550232 Brain_Nucleus_accumbens_basal_ganglia
2.590354657 0.12371216 0.12371216 -0.2381147 1.671034885 0.50401233
0.850607733 6 0 0.998207054
AC004775.5 ENSG00000272203.1 0.636045998 2 2 0.354186448
Brain_Spinal_cord_cervical_c-1 0.795979855 Brain_Cerebellar_Hemisphere
1.046401461 0.953598539 0.953598539 -0.926499517 -0.258553383
-0.59252645 0.472309241 2 0 0.998207054
PSMA2 ENSG00000106588.10 0.636079655 1 1 0.636079655 Brain_Cortex
0.636079655 Brain_Cortex 1 1 1 0.473187163 0.473187163
0.473187163 NA 1 0 0.998207054
CCDC85A ENSG00000055813.5 0.636114562 4 4 0.181032641
Brain_Cerebellar_Hemisphere 0.939567734 Brain_Putamen_basal_ganglia
1.974067203 0.077283604 0.077283604 -1.337582288 -0.075813175
-0.804920083 0.549500428 4 0 0.998207054
EBF4 ENSG00000088881.20 0.636116704 3 3 0.222280291
Brain_Putamen_basal_ganglia 0.806326872 Brain_Cerebellum 1.082851547
0.906213169 0.906213169 -1.220487061 -0.24516733 -0.58859083
0.547927488 3 0 0.998207054
BMP8A ENSG00000183682.7 0.63618868 6 6 0.145976122
Brain_Cerebellar_Hemisphere 0.863610855 Brain_Cortex 2.535668591
0.224434216 0.224434216 -0.171779535 1.453892467 0.600625489
0.58646241 6 0 0.998207054
PP2D1 ENSG00000183977.13 0.636219593 2 2 0.352018098 Brain_Cerebellum
0.804225575 Brain_Cerebellar_Hemisphere 1.453631606 0.546368394
0.546368394 0.247882171 0.930681971 0.589282071 0.482812368
2 0 0.998207054
PYROXD1 ENSG00000121350.15 0.636267631 11 5 0.100826199
Brain_Spinal_cord_cervical_c-1 0.477848455 Brain_Caudate_basal_ganglia
8.046741088 0.005220104 0.292946084 0.709767292 1.640861352
1.111809581 0.244784449 5 0 0.998207054
TTLL12 ENSG00000100304.12 0.63648282 8 8 0.211103162 Brain_Cortex
0.940548358 Brain_Anterior_cingulate_cortex_BA24 2.766196133
0.251053432 0.251053432 -0.30210459 1.250537783 0.27426628
0.564552906 8 0 0.998207054
LINC00348 ENSG00000226846.1 0.636502133 1 1 0.636502133
Brain_Caudate_basal_ganglia 0.636502133 Brain_Caudate_basal_ganglia 1 1
1 -0.472595024 -0.472595024 -0.472595024 NA 1 0
0.998207054
MAPRE3 ENSG00000084764.10 0.636567804 2 2 0.39286675 Brain_Cerebellum
0.686977519 Brain_Spinal_cord_cervical_c-1 1.015514923 0.984485077
0.984485077 -0.40296018 0.854430054 0.225734937 0.889109161
2 0 0.998207054
RIBC2 ENSG00000128408.8 0.636611679 5 2 0.390292264
Brain_Hypothalamus 0.952926202 Brain_Cortex 4.403546978
0.004583462 0.501784146 -0.859087462 0.059032525 -0.331562917
0.330618993 2 0 0.998207054
LINC01164 ENSG00000189275.3 0.636612629 2 2 0.45611209 Brain_Cortex
0.955098241 Brain_Frontal_Cortex_BA9 1.572970509 0.427029491
0.427029491 -0.056305746 0.745264083 0.344479168 0.566795462
2 0 0.998207054
RP11-986G18.2 ENSG00000274659.1 0.636717498 3 3 0.429037789
Brain_Hypothalamus 0.89574344 Brain_Frontal_Cortex_BA9 2.058735316
0.384794826 0.384794826 -0.790839272 0.372752478 -0.09568217
0.614034807 3 0 0.998207054
FAM129B ENSG00000136830.11 0.636862149 4 4 0.283235071
Brain_Frontal_Cortex_BA9 0.934709578 Brain_Amygdala 1.474453914
0.531149228 0.531149228 -1.073080339 0.673762773 0.04022398
0.82016939 4 0 0.998207054
LMOD1 ENSG00000163431.12 0.636872987 6 6 0.310972962
Brain_Hypothalamus 0.737836533 Brain_Cortex 3.51557124 0.120292417

0.120292417 -0.334719709 1.013182576 0.583366556 0.516521361
6 0 0.998207054
CFAP20 ENSG00000070761.7 0.637128372 2 2 0.362587734
Brain_Spinal_cord_cervical_c-1 0.780927777 Brain_Hippocampus
1.009414404 0.990585596 0.990585596 -0.278110186 0.910445268
0.316167541 0.840435621 2 0 0.998207054
DHFR ENSG00000228716.6 0.637148838 13 1 0.407070767
Brain_Hippocampus 0.907140581 Brain_Putamen_basal_ganglia 11.77617812
0.008302598 11.77617812 -0.829059451 0.27119991 -0.447726341
0.326571169 1 0 0.998207054
ZNF256 ENSG00000152454.3 0.637205422 1 1 0.637205422
Brain_Anterior_cingulate_cortex_BA24 0.637205422
Brain_Anterior_cingulate_cortex_BA24 1 1 1 0.471609672
0.471609672 0.471609672 NA 1 0 0.998207054
SPSB2 ENSG00000111671.9 0.637470786 11 8 0.167965667
Brain_Hippocampus 0.875414174 Brain_Cortex 5.37986697 0.003229967
0.215409243 -1.107805328 1.378770041 0.398941572 0.700708805
8 0 0.998207054
KIFC2 ENSG00000167702.11 0.637504363 5 5 0.258820752
Brain_Hypothalamus 0.739231768 Brain_Cerebellar_Hemisphere 2.335442484
0.25287788 0.25287788 -1.129182743 0.332870858 -0.575933587
0.586998609 5 0 0.998207054
KCTD14 ENSG00000151364.16 0.637710156 3 3 0.284685432
Brain_Caudate_basal_ganglia 0.632334805 Brain_Cortex 1.382258245
0.653650169 0.653650169 -0.478443182 1.069853109 0.431186358
0.808931997 3 0 0.998207054
TCP11L2 ENSG00000166046.10 0.637798727 1 1 0.637798727
Brain_Hypothalamus 0.637798727 Brain_Hypothalamus 1 1 1
-0.470778769 -0.470778769 -0.470778769 NA 1 0 0.998207054
RP11-488L18.10 ENSG00000259865.1 0.63789488 10 7 0.121145225
Brain_Spinal_cord_cervical_c-1 0.973408358 Brain_Cerebellar_Hemisphere
4.668569621 0.019946018 0.251753429 -1.549984547 0.61800205
-0.596515765 0.66195011 7 0 0.998207054
GAS1RR ENSG00000226237.1 0.637917316 11 5 0.193537239
Brain_Cerebellar_Hemisphere 0.959941717 Brain_Anterior_cingulate_cortex_BA24
7.928370314 0.01541049 0.350369419 -1.300185966 0.219741143
-0.696600228 0.46354149 5 0 0.998207054
TARSL2 ENSG00000185418.15 0.637948564 8 6 0.104620908
Brain_Cerebellar_Hemisphere 0.872967342 Brain_Nucleus_accumbens_basal_ganglia
3.278385002 0.027569799 0.188789772 -1.075283552 1.622852647
-0.015350658 1.090963773 6 0 0.998207054
RALGAPA1 ENSG00000174373.16 0.637973487 10 1 0.342404595
Brain_Caudate_basal_ganglia 0.999892854 Brain_Putamen_basal_ganglia
9.356667545 0.001296384 9.356667545 -0.949424814 -0.000134288
-0.452741917 0.271010896 1 0 0.998207054
C2CD3 ENSG00000168014.16 0.638009616 7 6 0.273921702 Brain_Cerebellum
0.81646577 Brain_Cortex 3.364328081 0.102863133 0.23608335
-1.094075874 0.771211029 -0.310184655 0.636024382 6 0
0.998207054
ADGRL3 ENSG00000150471.16 0.638052347 1 1 0.638052347
Brain_Hippocampus 0.638052347 Brain_Hippocampus 1 1 1
-0.470423683 -0.470423683 -0.470423683 NA 1 0 0.998207054
C2orf73 ENSG00000177994.15 0.638108335 1 1 0.638108335
Brain_Nucleus_accumbens_basal_ganglia 0.638108335
Brain_Nucleus_accumbens_basal_ganglia 1 1 1 0.470345304
0.470345304 0.470345304 NA 1 0 0.998207054
THBS3 ENSG00000169231.13 0.638134702 8 7 0.216415784
Brain_Anterior_cingulate_cortex_BA24 0.959551885 Brain_Cortex
4.374364057 0.035663281 0.159737193 -1.236115097 0.895705158
0.209906074 0.695725761 7 0 0.998207054
FAM167A ENSG00000154319.14 0.638267465 6 6 0.212111938
Brain_Caudate_basal_ganglia 0.98749197 Brain_Cortex 3.078718516

0.123068431	0.123068431	-1.247779173	0.641379073	-0.074067179
0.735036484	6	0	0.998207054
OXSR1 ENSG00000172939.8	0.638289803	1	1	0.638289803
Brain_Substantia_nigra	0.638289803	Brain_Substantia_nigra	1	1
1	-0.470091281	-0.470091281	-0.470091281	NA	1	0
0.998207054
GAA	ENSG00000171298.12	0.638290854	13	9	0.235417902
Brain_Spinal_cord_cervical_c-1	0.892126427	Brain_Cerebellar_Hemisphere
7.104490527	0.020358816	0.275242938	-1.186517736	0.509855916
-0.550349999	0.510813959	9	0	0.998207054
CARM1 ENSG00000142453.11	0.638310117	3	3	0.200673008
Brain_Anterior_cingulate_cortex_BA24	0.879235698	Brain_Substantia_nigra
1.73420407 0.341652057	0.341652057	0.151938175	1.279636495
0.682214175	0.566839789	3	0	0.998207054
C16orf70	ENSG00000125149.11	0.638315969	1	1	0.638315969
Brain_Cortex	0.638315969	Brain_Cortex	1	1	1	0.470054656
0.470054656	0.470054656	NA	1	0	0.998207054
RP11-54O7.16	ENSG00000272438.1	0.638344616	1	1	0.638344616
Brain_Putamen_basal_ganglia 0.638344616	Brain_Putamen_basal_ganglia 1	1
1	-0.470014558	-0.470014558	-0.470014558	NA	1	0
0.998207054
SNX21 ENSG00000124104.18	0.638433943	2	2	0.632201295	Brain_Cerebellum
0.878623732	Brain_Cerebellar_Hemisphere 1.933141646	0.066858354
0.066858354	0.152714112	0.478630811	0.315672461	0.230457908
2	0	0.998207054
COL28A1	ENSG00000215018.9	0.638459122	13	7	0.195140348
Brain_Spinal_cord_cervical_c-1	0.780114741	Brain_Putamen_basal_ganglia
7.530779727	0.035709973	0.481116651	-1.29552162	1.257186936
-0.092587766	0.819397673	7	0	0.998207054
SFTPB ENSG00000168878.16	0.638535317	1	1	0.638535317	Brain_Cerebellum
0.638535317	Brain_Cerebellum 1	1	1	-0.469747653	-0.469747653
-0.469747653	NA	1	0	0.998207054
RP11-439E19.9	ENSG00000260698.1	0.638571545	2	2	0.447781827
Brain_Putamen_basal_ganglia 0.528244553	Brain_Nucleus_accumbens_basal_ganglia
1.087585619	0.912414381	0.912414381	0.630687991	0.759118243
0.694903117	0.090813903	2	0	0.998207054
SLC9A2	ENSG00000115616.2	0.638730619	3	3	0.425779935
Brain_Hippocampus	0.712754891	Brain_Cortex	1.525738505
0.504489579	0.504489579	-0.796433806	0.549636723	0.040453878
0.730424064	3	0	0.998207054
RP5-1028K7.2	ENSG00000266088.5	0.638760192	2	2	0.397616344
Brain_Substantia_nigra	0.834768123	Brain_Spinal_cord_cervical_c-1
1.241390535	0.758609465	0.758609465	0.208590249	0.845885999
0.527238124	0.450636146	2	0	0.998207054
AC009495.4 ENSG00000229195.1	0.638778388	2	2	0.346082607
Brain_Cerebellum 0.947759108	Brain_Cerebellar_Hemisphere 1.028993681
0.971006319	0.971006319	-0.942214938	0.065521098	-0.43834692
0.712576985	2	0	0.998207054
SLC2A8	ENSG00000136856.17	0.638786602	13	3	0.253282019
Brain_Hypothalamus	0.823661091	Brain_Cerebellar_Hemisphere 10.7710738
0.000371061	0.869365081	-0.495379799	1.142413892	-0.25546477
0.427365	3	0	0.998207054
TADA3 ENSG00000171148.13	0.638789639	1	1	0.638789639	Brain_Cerebellum
0.638789639	Brain_Cerebellum 1	1	1	-0.469391758	-0.469391758
-0.469391758	NA	1	0	0.998207054
DSTN	ENSG00000125868.15	0.638914035	3	3	0.281136163
Brain_Hippocampus	0.799977371	Brain_Hypothalamus	1.131040965
0.843791751	0.843791751	-1.077770555	0.253376389	-0.443098772
0.667722091	3	0	0.998207054
LINC00222	ENSG00000203801.8	0.638923906	1	1	0.638923906
Brain_Substantia_nigra	0.638923906	Brain_Substantia_nigra	1	1
1	-0.469203888	-0.469203888	-0.469203888	NA	1	0

0.998207054
LUZP1 ENSG00000169641.13 0.639061785 2 2 0.348507202 Brain_Cortex 0.549992578 Brain_Nucleus_accumbens_basal_ganglia 1.520173374 0.479826626 0.479826626 0.597771247 0.937488762 0.767630005 0.240216558 2 0 0.998207054
MXD4 ENSG00000123933.16 0.63907666 1 1 0.63907666 Brain_Cerebellum 0.63907666 Brain_Cerebellum 1 1 1 0.468990174 0.468990174 0.468990174 NA 1 0 0.998207054
ZDHHC8 ENSG00000099904.15 0.639128323 1 1 0.639128323 Brain_Nucleus_accumbens_basal_ganglia 0.639128323 Brain_Nucleus_accumbens_basal_ganglia 1 1 1 -0.468917897 -0.468917897 -0.468917897 NA 1 0 0.998207054
RORB ENSG00000198963.10 0.639154819 1 1 0.639154819 Brain_Cerebellum 0.639154819 Brain_Cerebellum 1 1 1 -0.468880831 -0.468880831 -0.468880831 NA 1 0 0.998207054
CXCL12 ENSG00000107562.16 0.639221765 1 1 0.639221765 Brain_Cerebellum 0.639221765 Brain_Cerebellum 1 1 1 -0.46878718 -0.46878718 -0.46878718 NA 1 0 0.998207054
MCIDAS ENSG00000234602.7 0.639280822 2 2 0.482511248 Brain_Putamen_basal_ganglia 0.550767171 Brain_Nucleus_accumbens_basal_ganglia 1.051680335 0.948319665 0.948319665 -0.70226928 -0.596610932 -0.649440106 0.074711734 2 0 0.998207054
FAM167B ENSG00000183615.5 0.639292838 1 1 0.639292838 Brain_Cerebellar_Hemisphere 0.639292838 Brain_Cerebellar_Hemisphere 1 1 1 0.468687758 0.468687758 0.468687758 NA 1 0 0.998207054
RP11-250B2.3 ENSG00000233967.6 0.639352243 5 4 0.240562991 Brain_Anterior_cingulate_cortex_BA24 0.985238648 Brain_Cerebellar_Hemisphere 3.301664572 0.097348168 0.366956172 -1.17358076 0.018501666 -0.525106134 0.479323494 4 0 0.998207054
AGRN ENSG00000188157.14 0.639639916 1 1 0.639639916 Brain_Hippocampus 0.639639916 Brain_Hippocampus 1 1 1 -0.468202316 -0.468202316 -0.468202316 NA 1 0 0.998207054
RP11-622C24.2 ENSG00000277152.1 0.639654139 2 2 0.546347811 Brain_Cerebellar_Hemisphere 0.920366987 Brain_Cerebellum 1.698131059 0.301868941 0.301868941 -0.603241849 0.099971456 -0.251635196 0.497246897 2 0 0.998207054
G3BP2 ENSG00000138757.14 0.639772972 9 4 0.165546513 Brain_Nucleus_accumbens_basal_ganglia 0.881227523 Brain_Spinal_cord_cervical_c-1 7.467044822 0.011247478 0.249886959 0.149413293 1.386656557 0.910348333 0.386299025 4 0 0.998207054
TPBG ENSG00000146242.8 0.639904404 3 3 0.289896352 Brain_Cortex 0.59186021 Brain_Cerebellum 1.207620884 0.783820519 0.783820519 -1.058349021 0.678885665 -0.305201884 0.89134516 3 0 0.998207054
UBAP2 ENSG00000137073.21 0.639975549 2 2 0.410280166 Brain_Caudate_basal_ganglia 0.416658157 Brain_Cortex 1.498721482 0.501278518 0.501278518 0.812232635 0.823400699 0.817816667 0.007897014 2 0 0.998207054
MCM3AP ENSG00000160294.10 0.640004734 8 5 0.254953298 Brain_Caudate_basal_ganglia 0.907085266 Brain_Cerebellar_Hemisphere 5.459210439 0.076540372 0.251513594 -0.874221866 1.138400469 0.024307226 0.682082372 5 0 0.998207054
SLC16A7 ENSG00000118596.11 0.640481529 2 1 0.557135011 Brain_Cerebellar_Hemisphere 0.730611502 Brain_Cerebellum 1.988724072 0.011275928 1.988724072 0.344312211 0.587102288 0.465707249 0.17167851 1 0 0.998207054
ZNF136 ENSG00000196646.11 0.640553167 2 2 0.444155779 Brain_Putamen_basal_ganglia 0.585187332 Brain_Hippocampus 1.008777419 0.991222581 0.991222581 0.545823406 0.765194426 0.655508916 0.155118735 2 0 0.998207054

RNF19A	ENSG00000034677.12	0.640557081	4	3	0.186169121
Brain_Cerebellum	0.917162665	Brain_Anterior_cingulate_cortex_BA24
2.006527667	0.013070327	0.955527276	-1.321997086	0.104008423
-0.656503108	0.738571726	3	0	0.998207054
GSTCD	ENSG00000138780.14	0.640565069	3	2	0.422261456	Brain_Cortex
0.791808287	Brain_Cerebellum	2.171130001	0.048528024	0.780341975
-0.802504034	0.263963185	-0.405503021	0.583108947	2	0
0.998207054
GGH	ENSG00000137563.11	0.640597863	11	3	0.22672709	Brain_Amygdala
0.979008407	Brain_Cerebellum	9.19839304	0.003437766	0.529237229
-0.026312096	1.208832794	0.485763521	0.347897176	3	0
0.998207054
ALKBH7	ENSG00000125652.7	0.640650666	1	1	0.640650666
Brain_Cerebellum	0.640650666	Brain_Cerebellum	1	1	1	-0.466789253
-0.466789253	-0.466789253	NA	1	0	0.998207054
MSH2	ENSG00000095002.13	0.640657643	3	2	0.364623409
Brain_Spinal_cord_cervical_c-1	0.444052909	Brain_Hippocampus
2.402009031	0.077047759	0.52094321	0.765367216	0.906590459
0.83392323	0.070701327	2	0	0.998207054
OR8S1	ENSG00000197376.2	0.640659855	1	1	0.640659855
Brain_Anterior_cingulate_cortex_BA24	0.640659855
Brain_Anterior_cingulate_cortex_BA24	1	1	1	0.46677641	0.46677641
0.46677641	NA	1	0	0.998207054
MRPS33	ENSG00000090263.15	0.640725082	4	4	0.247968612
Brain_Substantia_nigra	0.992296312	Brain_Frontal_Cortex_BA9
2.341619984	0.27218012	0.27218012	-1.155297518	-0.009655291
-0.526426776	0.496990196	4	0	0.998207054
LINC02080	ENSG00000267065.2	0.640999563	10	4	0.199834179
Brain_Cerebellar_Hemisphere	0.83995913	Brain_Anterior_cingulate_cortex_BA24
7.864204693	0.019370818	0.302633442	-1.282024138	-0.201945757
-0.903333874	0.359672859	4	0	0.998207054
RPL26	ENSG00000161970.12	0.641018512	1	1	0.641018512
Brain_Caudate_basal_ganglia	0.641018512	Brain_Caudate_basal_ganglia	1	1
1	0.466275222	0.466275222	0.466275222	NA	1	0
0.998207054
NATD1	ENSG00000274180.1	0.641154693	4	4	0.293075214
Brain_Cerebellar_Hemisphere	0.940916183	Brain_Spinal_cord_cervical_c-1
1.612879201	0.452795256	0.452795256	-1.05139935	0.516884625
-0.376030015	0.733577178	4	0	0.998207054
CCAR2	ENSG00000158941.16	0.641213741	1	1	0.641213741	Brain_Cerebellum
0.641213741	Brain_Cerebellum	1	1	1	0.466002458	0.466002458
0.466002458	NA	1	0	0.998207054
LRCH3	ENSG00000186001.12	0.641246141	1	1	0.641246141
Brain_Putamen_basal_ganglia	0.641246141	Brain_Putamen_basal_ganglia	1	1
1	0.465957194	0.465957194	0.465957194	NA	1	0
0.998207054
RP11-563K23.1	ENSG00000272619.1	0.641275797	4	4	0.248188504
Brain_Cortex	0.936974361	Brain_Frontal_Cortex_BA9	1.28921342
0.783471923	0.783471923	-0.914258234	1.154760532	-0.109288581
0.94876353	4	0	0.998207054
RP11-74E22.8	ENSG00000277200.1	0.641283256	2	2	0.348138291
Brain_Cerebellar_Hemisphere	0.496696713	Brain_Cerebellum	1.650554001
0.349445999	0.349445999	0.679696405	0.938206515	0.80895146
0.182794252	2	0	0.998207054
RP5-965G21.4	ENSG00000274414.1	0.641299258	5	4	0.11816477
Brain_Frontal_Cortex_BA9	0.467394142	Brain_Cerebellum	3.06016139
0.016276327	0.111108564	-1.562523262	-0.726725479	-1.034542119
0.3391602	4	0	0.998207054
DDIT3	ENSG00000175197.11	0.641303198	2	2	0.430930585
Brain_Nucleus_accumbens_basal_ganglia	0.868116108	Brain_Caudate_basal_ganglia
1.688006591	0.311993409	0.311993409	0.166051899	0.787600221
0.47682606	0.439501033	2	0	0.998207054

PRR16 ENSG00000184838.14 0.641660851 1 1 0.641660851 Brain_Cortex
0.641660851 Brain_Cortex 1 1 1 -0.46537791 -0.46537791
-0.46537791 NA 1 0 0.998207054
LINC01018 ENSG00000250056.5 0.641820644 2 2 0.446147285
Brain_Cerebellum 0.553488875 Brain_Caudate_basal_ganglia 1.052101027
0.947898973 0.947898973 -0.761853769 -0.592540251 -0.67719701
0.119722737 2 0 0.998207054
DCAKD ENSG00000172992.11 0.641885304 10 7 0.148192157 Brain_Cerebellum
0.837298251 Brain_Caudate_basal_ganglia 5.013365739 0.059067374
0.208556153 -1.445946682 1.354090452 -0.200862098 0.864480564
7 0 0.998207054
LASP1 ENSG00000002834.17 0.641944158 3 3 0.250497633
Brain_Cerebellar_Hemisphere 0.905141551 Brain_Cortex 1.322875782
0.682187973 0.682187973 0.119168892 1.149141514 0.722509334
0.537243201 3 0 0.998207054
LSM8 ENSG00000128534.7 0.642024379 6 4 0.267872673 Brain_Cortex
0.97375282 Brain_Frontal_Cortex_BA9 4.320374526 0.036470184
0.283674896 -0.268296101 1.107974861 0.132413933 0.502041134
4 0 0.998207054
SLCO1A2 ENSG00000084453.16 0.642124528 3 3 0.254420469
Brain_Frontal_Cortex_BA9 0.312892553 Brain_Cerebellum 2.324239737
0.119919904 0.119919904 -1.139678016 -1.009171165 -1.094682596
0.074088933 3 0 0.998207054
PHOSPHO2 ENSG00000144362.11 0.642190125 9 3 0.373504212
Brain_Anterior_cingulate_cortex_BA24 0.960370801 Brain_Hypothalamus
7.947068385 0.004973541 0.300744445 -0.889928625 0.10136709
-0.372056235 0.325107912 3 0 0.998207054
PTS ENSG00000150787.7 0.642244191 1 1 0.642244191 Brain_Cortex
0.642244191 Brain_Cortex 1 1 1 0.46456334 0.46456334 0.46456334
NA 1 0 0.998207054
GFY ENSG00000261949.5 0.642246042 1 1 0.642246042
Brain_Cerebellar_Hemisphere 0.642246042 Brain_Cerebellar_Hemisphere 1 1
1 -0.464560755 -0.464560755 -0.464560755 NA 1 0
0.998207054
CD14 ENSG00000170458.13 0.642373776 1 1 0.642373776 Brain_Cerebellum
0.642373776 Brain_Cerebellum 1 1 1 -0.46438243 -0.46438243
-0.46438243 NA 1 0 0.998207054
VPS4A ENSG00000132612.15 0.64243329 7 2 0.251548578
Brain_Caudate_basal_ganglia 0.490509199 Brain_Anterior_cingulate_cortex_BA24
6.335576519 0.005008926 0.265193866 -1.146596127 -0.689499165
-0.901217144 0.193262021 2 0 0.998207054
PITHD1 ENSG00000057757.9 0.642476423 1 1 0.642476423
Brain_Spinal_cord_cervical_c-1 0.642476423 Brain_Spinal_cord_cervical_c-1
1 1 1 0.464239138 0.464239138 0.464239138 NA 1 0
0.998207054
PPM1N ENSG00000213889.10 0.642480144 3 3 0.19869169 Brain_Hypothalamus
0.82706325 Brain_Anterior_cingulate_cortex_BA24 1.178165456 0.741221553
0.741221553 -1.28528792 0.237214556 -0.422180985 0.781426386
3 0 0.998207054
KCNH5 ENSG00000140015.19 0.642647698 1 1 0.642647698
Brain_Frontal_Cortex_BA9 0.642647698 Brain_Frontal_Cortex_BA9 1 1
1 -0.464000067 -0.464000067 -0.464000067 NA 1 0
0.998207054
EID2 ENSG00000176396.10 0.642773757 1 1 0.642773757 Brain_Amygdala
0.642773757 Brain_Amygdala 1 1 1 0.463824126 0.463824126
0.463824126 NA 1 0 0.998207054
RP11-867G23.3 ENSG00000245156.1 0.642797567 2 2 0.35270492
Brain_Hypothalamus 0.82155862 Brain_Caudate_basal_ganglia 1.090574375
0.909425625 0.909425625 -0.22554076 0.929355425 0.351907332
0.816634924 2 0 0.998207054
ZP1 ENSG00000149506.11 0.64285751 2 2 0.34869036 Brain_Cerebellar_Hemisphere
0.454523588 Brain_Cerebellum 1.840711268 0.159288732 0.159288732

0.747894844 0.937132589 0.842513716 0.133811293 2 0
0.998207054
RAB40B ENSG00000141542.10 0.643071489 2 2 0.413287828
Brain_Hippocampus 0.604654878 Brain_Cerebellar_Hemisphere 1.069150766
0.930849234 0.930849234 -0.818121475 -0.517718229 -0.667919852
0.212417173 2 0 0.998207054
GUK1 ENSG00000143774.16 0.643187024 4 3 0.585626191 Brain_Cortex
0.763377755 Brain_Putamen_basal_ganglia 3.552206764 0.048846041
0.167053528 -0.301048181 0.545185139 0.237360292 0.370740898
3 0 0.998207054
CAPN7 ENSG00000131375.9 0.643208294 1 1 0.643208294 Brain_Amygdala
0.643208294 Brain_Amygdala 1 1 1 -0.46321775 -0.46321775
-0.46321775 NA 1 0 0.998207054
ADPRH ENSG00000144843.11 0.643291568 4 4 0.140032431
Brain_Cerebellar_Hemisphere 0.98702304 Brain_Hippocampus 1.636482367
0.455752093 0.455752093 -1.47567027 0.016264925 -0.557113466
0.641916713 4 0 0.998207054
TAGLN ENSG00000149591.16 0.643370351 2 2 0.380294891 Brain_Cerebellum
0.926687316 Brain_Frontal_Cortex_BA9 1.263606464 0.736393536
0.736393536 -0.877353078 0.092013497 -0.392669791 0.685445679
2 0 0.998207054
ITGAD ENSG00000156886.11 0.643430054 2 2 0.391587135
Brain_Spinal_cord_cervical_c-1 0.651928896 Brain_Cortex 1.079068873
0.920931127 0.920931127 -0.856742636 -0.451084157 -0.653913396
0.286843861 2 0 0.998207054
JAK3 ENSG00000105639.18 0.643437574 4 4 0.261756774
Brain_Spinal_cord_cervical_c-1 0.924176433 Brain_Cerebellar_Hemisphere
1.311958855 0.713896317 0.713896317 -0.996959729 1.122248553
0.013395204 0.873605672 4 0 0.998207054
ABHD13 ENSG00000139826.5 0.643518735 1 1 0.643518735
Brain_Cerebellum 0.643518735 Brain_Cerebellum 1 1 1 -0.462784651
-0.462784651 -0.462784651 NA 1 0 0.998207054
IFT80 ENSG00000068885.14 0.64353376 4 4 0.136256184 Brain_Hippocampus
0.243654888 Brain_Frontal_Cortex_BA9 3.096145714 0.111866013
0.111866013 -1.489878504 -1.165899978 -1.378841533 0.144776542
4 0 0.998207054
CLHC1 ENSG00000162994.15 0.64356924 13 8 0.269250578
Brain_Putamen_basal_ganglia 0.918556462 Brain_Hypothalamus 7.790626073
0.015786805 0.269739351 -0.730558832 1.104790037 0.447449537
0.603638406 8 0 0.998207054
C10orf76 ENSG00000120029.12 0.643607296 1 1 0.643607296
Brain_Hypothalamus 0.643607296 Brain_Hypothalamus 1 1 1
-0.462661113 -0.462661113 -0.462661113 NA 1 0 0.998207054
USP32 ENSG00000170832.12 0.643646646 1 1 0.643646646 Brain_Cortex
0.643646646 Brain_Cortex 1 1 1 0.462606224 0.462606224
0.462606224 NA 1 0 0.998207054
TOMM7 ENSG00000196683.10 0.643706734 12 1 0.342504031
Brain_Substantia_nigra 0.795732421 Brain_Putamen_basal_ganglia
11.06501433 4.12E-05 11.06501433 -0.949229245 0.666113739
0.428785774 0.447613849 1 0 0.998207054
ADORA2B ENSG00000170425.3 0.643748781 9 3 0.373777455
Brain_Hypothalamus 0.978570414 Brain_Frontal_Cortex_BA9 8.030400791
0.006160105 0.309163308 -0.889419897 -0.026861233 -0.315896083
0.27403973 3 0 0.998207054
C21orf58 ENSG00000160298.17 0.644017225 2 2 0.34904065 Brain_Cerebellum
0.385278542 Brain_Cerebellar_Hemisphere 1.901299151 0.098700849
0.098700849 -0.936451737 -0.868211532 -0.902331635 0.048253112
2 0 0.998207054
SIX4 ENSG00000100625.8 0.644030357 1 1 0.644030357
Brain_Cerebellar_Hemisphere 0.644030357 Brain_Cerebellar_Hemisphere 1 1
1 -0.462071069 -0.462071069 -0.462071069 NA 1 0
0.998207054

ALDH9A1 ENSG00000143149.12 0.644152571 4 4 0.165721481
Brain_Nucleus_accumbens_basal_ganglia 0.97373697 Brain_Frontal_Cortex_BA9
1.872537756 0.243034003 0.243034003 0.032921773 1.386083261
0.825014578 0.664642988 4 0 0.998207054
LMF1 ENSG00000103227.18 0.644289507 12 4 0.212950856
Brain_Spinal_cord_cervical_c-1 0.883112309 Brain_Hippocampus
8.646019842 0.002022722 0.298669791 -1.245492267 1.071200911
0.213300515 0.828572944 4 0 0.998207054
RAG1 ENSG00000166349.9 0.644308906 2 2 0.378829332
Brain_Caudate_basal_ganglia 0.734110737 Brain_Substantia_nigra
1.018419998 0.981580002 0.981580002 0.339662458 0.880055338
0.609858898 0.38211547 2 0 0.998207054
CSK ENSG00000103653.16 0.644324822 2 2 0.421655058
Brain_Caudate_basal_ganglia 0.63590012 Brain_Cerebellum 1.012007046
0.987992954 0.987992954 -0.803553204 -0.473438848 -0.638496026
0.2334261 2 0 0.998207054
PRDM1 ENSG00000057657.15 0.644347101 2 2 0.406144516
Brain_Frontal_Cortex_BA9 0.638288401 Brain_Cerebellum 1.043074431
0.956925569 0.956925569 -0.83069754 0.470093244 -0.180302148
0.919797984 2 0 0.998207054
RP11-202K23.1 ENSG00000233359.1 0.644361385 1 1 0.644361385
Brain_Spinal_cord_cervical_c-1 0.644361385 Brain_Spinal_cord_cervical_c-1
1 1 1 0.461609495 0.461609495 0.461609495 NA 1 0
0.998207054
MAP3K5 ENSG00000197442.9 0.644472584 2 2 0.348574489
Brain_Cerebellum 0.487729332 Brain_Cerebellar_Hemisphere 1.738513667
0.261486333 0.261486333 0.69392486 0.9373579 0.81564138 0.172133153
2 0 0.998207054
CPNE3 ENSG00000085719.12 0.644540016 10 7 0.064906086
Brain_Nucleus_accumbens_basal_ganglia 0.962008109 Brain_Caudate_basal_ganglia
5.190699236 0.014862059 0.255414136 -1.845904408 0.560627337
-0.66199768 0.82138548 7 0 0.998207054
CARHSP1 ENSG00000153048.10 0.644584265 2 2 0.453109788
Brain_Substantia_nigra 0.659751481 Brain_Frontal_Cortex_BA9
1.152980046 0.847019954 0.847019954 -0.440256309 0.750240637
0.154992164 0.841808464 2 0 0.998207054
CTHRC1 ENSG00000164932.12 0.644650828 2 2 0.49357372
Brain_Anterior_cingulate_cortex_BA24 0.760003045
Brain_Nucleus_accumbens_basal_ganglia 1.407313314 0.592686686
0.592686686 -0.68463588 0.30547679 -0.189579545 0.700115383 2
0 0.998207054
C18orf25 ENSG00000152242.10 0.644679108 1 1 0.644679108
Brain_Cerebellum 0.644679108 Brain_Cerebellum 1 1 1 0.461166564
0.461166564 0.461166564 NA 1 0 0.998207054
DNAH9 ENSG00000007174.17 0.644742802 4 4 0.301635567
Brain_Hypothalamus 0.883547183 Brain_Caudate_basal_ganglia 2.304635616
0.239205223 0.239205223 -0.334644325 1.032932332 0.317823529
0.661814469 4 0 0.998207054
BCL2L15 ENSG00000188761.11 0.64502831 3 3 0.443771973
Brain_Spinal_cord_cervical_c-1 0.845030473 Brain_Cerebellar_Hemisphere
1.731139486 0.288520571 0.288520571 -0.765839223 0.580230295
-0.127023988 0.675639485 3 0 0.998207054
SLC30A9 ENSG00000014824.13 0.64503814 10 7 0.078929652 Brain_Cerebellum
0.785611775 Brain_Hippocampus 6.937925909 0.021341038
0.272405034 0.272013323 1.756930525 1.292670891 0.470128099
7 0 0.998207054
MED8 ENSG00000159479.16 0.645055481 4 3 0.273053995
Brain_Hippocampus 0.556590873 Brain_Spinal_cord_cervical_c-1
2.748657185 0.012694912 0.269804844 0.587912728 1.096056625
0.79660673 0.215392384 3 0 0.998207054
SETD9 ENSG00000155542.11 0.645344042 13 2 0.122985335
Brain_Nucleus_accumbens_basal_ganglia 0.728530188

Brain_Spinal_cord_cervical_c-1 11.56589815 0.00282168 0.471601436
-1.542363297 -0.347081371 -0.743261439 0.339863791 2 0
0.998207054
DCAF13 ENSG00000164934.13 0.645404614 1 1 0.645404614
Brain_Caudate_basal_ganglia 0.645404614 Brain_Caudate_basal_ganglia 1 1
1 -0.460155494 -0.460155494 -0.460155494 NA 1 0
0.998207054
ITGA11 ENSG00000137809.16 0.645424567 1 1 0.645424567
Brain_Frontal_Cortex_BA9 0.645424567 Brain_Frontal_Cortex_BA9 1 1
1 -0.460127693 -0.460127693 -0.460127693 NA 1 0
0.998207054
DCTN3 ENSG00000137100.15 0.645752915 1 1 0.645752915 Brain_Amygdala
0.645752915 Brain_Amygdala 1 1 1 -0.459670266 -0.459670266
-0.459670266 NA 1 0 0.998207054
PFN4 ENSG00000176732.6 0.645808407 4 4 0.430280464
Brain_Cerebellar_Hemisphere 0.684116856 Brain_Caudate_basal_ganglia 2.38794623
0.114418379 0.114418379 -0.732183869 0.788711809 0.091644215
0.775465422 4 0 0.998207054
CCDC74B ENSG00000152076.18 0.645939575 7 3 0.280379848
Brain_Cortex 0.976140228 Brain_Hippocampus 5.727317957
0.013940603 0.223240384 -1.079466438 0.769222268 -0.212863231
0.558061052 3 0 0.998207054
DDRGK1 ENSG00000198171.12 0.646118776 1 1 0.646118776
Brain_Cerebellum 0.646118776 Brain_Cerebellum 1 1 1 0.459160692
0.459160692 0.459160692 NA 1 0 0.998207054
PDIA2 ENSG00000185615.15 0.646126709 2 2 0.439143322 Brain_Cerebellum
0.878229485 Brain_Spinal_cord_cervical_c-1 1.417800284 0.582199716
0.582199716 0.153214041 0.773640657 0.463427349 0.438707867
2 0 0.998207054
ZNF738 ENSG00000172687.13 0.646187144 11 1 0.460703207
Brain_Caudate_basal_ganglia 0.951620912 Brain_Cerebellar_Hemisphere
10.04209839 0.000439897 10.04209839 -0.737689414 -0.060671396
-0.435270844 0.200810538 1 0 0.998207054
CCDC144A ENSG00000170160.17 0.646247698 1 1 0.646247698
Brain_Cerebellar_Hemisphere 0.646247698 Brain_Cerebellar_Hemisphere 1 1
1 -0.458981156 -0.458981156 -0.458981156 NA 1 0
0.998207054
GABBR1 ENSG00000204681.10 0.646270193 8 7 0.100994487
Brain_Cerebellar_Hemisphere 0.823022859 Brain_Cortex 4.520677313
0.14883043 0.269097806 -0.634052568 1.640051368 0.830306999
0.720059238 7 0 0.998207054
CRNN ENSG00000143536.7 0.646301562 2 2 0.356194312
Brain_Anterior_cingulate_cortex_BA24 0.853937445 Brain_Frontal_Cortex_BA9
1.039956705 0.960043295 0.960043295 -0.92264102 -0.184096891
-0.553368955 0.522229561 2 0 0.998207054
USP48 ENSG00000090686.15 0.646605073 3 3 0.270600342
Brain_Nucleus_accumbens_basal_ganglia 0.563348726 Brain_Caudate_basal_ganglia
2.436597771 0.246060439 0.246060439 -1.101681088 -0.577874687
-0.909189894 0.2881794 3 0 0.998207054
ARMT1 ENSG00000146476.10 0.646905029 3 3 0.292248594
Brain_Hypothalamus 0.376269664 Brain_Putamen_basal_ganglia 1.848435403
0.414572047 0.414572047 -1.053201625 -0.884790445 -0.978775054
0.085892194 3 0 0.998207054
NKX6-2 ENSG00000148826.8 0.647000681 2 2 0.367083977
Brain_Substantia_nigra 0.838637426 Brain_Cerebellar_Hemisphere
1.039425276 0.960574724 0.960574724 -0.203636681 0.901948968
0.349156143 0.781767109 2 0 0.998207054
CRELD2 ENSG00000184164.14 0.647028695 2 2 0.353396734
Brain_Hypothalamus 0.524856144 Brain_Hippocampus 1.754188492
0.245811508 0.245811508 0.635877691 0.92802089 0.781949291
0.206576436 2 0 0.998207054
LACTB ENSG00000103642.11 0.647049071 12 2 0.182391413

Brain_Nucleus_accumbens_basal_ganglia 0.781274131
Brain_Anterior_cingulate_cortex_BA24 10.43032709 0.000125687
0.717120156 -1.333427927 -0.277659007 -0.75652752 0.285130475
2 0 0.998207054
GPA33 ENSG00000143167.11 0.64705897 5 3 0.185702485 Brain_Cortex
0.502876091 Brain_Anterior_cingulate_cortex_BA24 3.812216154
0.036664664 0.4172206 0.669971292 1.323399718 1.035994775
0.281536615 3 0 0.998207054
CHRNB3 ENSG00000147432.6 0.647063484 2 2 0.391370067
Brain_Cerebellum 0.550397585 Brain_Cerebellar_Hemisphere 1.284291803
0.715708197 0.715708197 0.59716446 0.857135386 0.727149923
0.183827204 2 0 0.998207054
SMCHD1 ENSG00000101596.14 0.647137624 1 1 0.647137624
Brain_Cerebellum 0.647137624 Brain_Cerebellum 1 1 1 0.457742258
0.457742258 0.457742258 NA 1 0 0.998207054
SHMT1 ENSG00000176974.19 0.647299526 9 8 0.193526462
Brain_Anterior_cingulate_cortex_BA24 0.947303093 Brain_Hypothalamus
3.762474937 0.02572162 0.175596644 -0.403426756 1.300217419
0.280716207 0.616853328 8 0 0.998207054
MS4A14 ENSG00000166928.10 0.647311247 12 2 0.369653221
Brain_Spinal_cord_cervical_c-1 0.95941422 Brain_Nucleus_accumbens_basal_ganglia
10.70697796 0.004935359 0.424185123 -0.897123123 -0.050888687
-0.349550554 0.272375013 2 0 0.998207054
PPP4R1-AS1 ENSG00000263627.1 0.647318834 13 1 0.488562981
Brain_Putamen_basal_ganglia 0.969924443 Brain_Hippocampus 12.32244888
0.001496516 12.32244888 0.037703052 0.692596224 0.442719497
0.198570562 1 0 0.998207054
SIPA1 ENSG00000213445.9 0.647339539 3 3 0.293814334
Brain_Hypothalamus 0.774579702 Brain_Cerebellar_Hemisphere 1.349752074
0.763137204 0.763137204 -1.04979074 0.434460264 -0.109646942
0.81754755 3 0 0.998207054
SLC26A8 ENSG00000112053.13 0.647508589 11 3 0.318557031
Brain_Hippocampus 0.981185817 Brain_Putamen_basal_ganglia 8.779122525
0.005572356 0.617439531 -0.056437096 0.997427537 0.343310196
0.359642198 3 0 0.998207054
SPDL1 ENSG00000040275.16 0.647828368 2 2 0.35197577 Brain_Hippocampus
0.967455109 Brain_Frontal_Cortex_BA9 1.003458848 0.996541152
0.996541152 -0.930763778 0.040800289 -0.444981745 0.68699954 2
0 0.998207054
GPR65 ENSG00000140030.5 0.647863944 1 1 0.647863944
Brain_Hypothalamus 0.647863944 Brain_Hypothalamus 1 1 1
0.456731643 0.456731643 0.456731643 NA 1 0 0.998207054
RP11-258F22.1 ENSG00000226578.1 0.647906434 13 3 0.279109733
Brain_Anterior_cingulate_cortex_BA24 0.787404953 Brain_Cortex
10.06136709 0.008107662 0.780677301 -1.082321416 0.653478555
-0.469553131 0.480504681 3 0 0.998207054
ZNF554 ENSG00000172006.11 0.647928839 9 8 0.054188147
Brain_Cortex 0.863266024 Brain_Cerebellum 3.626746776 0.061885807
0.171009569 -0.172218157 1.925329521 0.798273175 0.666912384
8 0 0.998207054
PKIA ENSG00000171033.12 0.647938755 2 2 0.35758002 Brain_Cortex
0.993414303 Brain_Hypothalamus 1.148795699 0.851204301
0.851204301 -0.00825404 0.919986106 0.455866033 0.656364902
2 0 0.998207054
RP11-521C20.5 ENSG00000275636.1 0.648043611 8 3 0.173473268
Brain_Cortex 0.349396398 Brain_Caudate_basal_ganglia 7.322797879
3.41E-09 0.303835557 0.935760724 1.361127919 1.199441638
0.132601335 3 0 0.998207054
ARHGEF19 ENSG00000142632.16 0.648048124 13 1 0.324017665
Brain_Substantia_nigra 0.928344465 Brain_Cerebellar_Hemisphere
11.86793333 0.002021344 11.86793333 -0.312592028 0.986235292
0.430394122 0.344419839 1 0 0.998207054

RBBP8 ENSG00000101773.18 0.648111936 3 3 0.399449486 Brain_Cortex
0.996558114 Brain_Cerebellar_Hemisphere 2.044720893 0.104099565
0.104099565 -0.388547249 0.842604836 0.152790455 0.628862351
3 0 0.998207054
OSGEP ENSG00000092094.10 0.648119796 13 1 0.425003298 Brain_Cerebellum
0.762317273 Brain_Frontal_Cortex_BA9 12.31922635 0.000266699
12.31922635 -0.302439203 0.797771163 0.435124621 0.257244913
1 0 0.998207054
LBH ENSG00000213626.11 0.648280873 2 2 0.505247337
Brain_Substantia_nigra 0.51431626 Brain_Putamen_basal_ganglia 1.002666053
0.997333947 0.997333947 -0.652131631 0.66625623 0.0070623
0.932240997 2 0 0.998207054
FLNC ENSG00000128591.15 0.648416829 2 2 0.692287879 Brain_Cerebellum
0.716230984 Brain_Substantia_nigra 1.667218574 0.332781426
0.332781426 -0.395752151 0.363500574 -0.016125788 0.536872751
2 0 0.998207054
RP11-706J10.2 ENSG00000270866.1 0.64847137 2 2 0.35283652
Brain_Nucleus_accumbens_basal_ganglia 0.830968528 Brain_Cortex 1.17122967
0.82877033 0.82877033 -0.213459561 0.929101438 0.357820938 0.80791263
2 0 0.998207054
NAT8 ENSG00000144035.3 0.648722343 4 1 0.499135404
Brain_Spinal_cord_cervical_c-1 0.774231618 Brain_Cerebellar_Hemisphere
3.878330444 0.011878395 3.878330444 0.2868442 0.675850759
0.449625807 0.173230804 1 0 0.998207054
PRSS22 ENSG00000005001.9 0.648842641 4 3 0.304375582
Brain_Cerebellum 0.969752032 Brain_Frontal_Cortex_BA9 2.556771706
0.057211692 0.308079589 -1.027095321 0.330365635 -0.380768756
0.634344904 3 0 0.998207054
SOST ENSG00000167941.2 0.6489646 2 2 0.39796639 Brain_Cortex
0.678446203 Brain_Frontal_Cortex_BA9 1.032071635 0.967928365
0.967928365 -0.845258744 -0.414584356 -0.62992155 0.304532781
2 0 0.998207054
API5 ENSG00000166181.12 0.648971593 1 1 0.648971593
Brain_Cerebellar_Hemisphere 0.648971593 Brain_Cerebellar_Hemisphere 1 1
1 0.455191336 0.455191336 0.455191336 NA 1 0
0.998207054
CCT4 ENSG00000115484.14 0.649171067 1 1 0.649171067
Brain_Cerebellar_Hemisphere 0.649171067 Brain_Cerebellar_Hemisphere 1 1
1 -0.45491406 -0.45491406 -0.45491406 NA 1 0
0.998207054
SNX2 ENSG00000205302.6 0.649194353 2 2 0.35634859 Brain_Cerebellum
0.984710114 Brain_Cortex 1.103703815 0.896296185 0.896296185
-0.922345113 -0.019164203 -0.470754658 0.638645346 2 0
0.998207054
SRL ENSG00000185739.13 0.649211645 1 1 0.649211645 Brain_Amygdala
0.649211645 Brain_Amygdala 1 1 1 0.45485766 0.45485766 0.45485766
NA 1 0 0.998207054
RPS11 ENSG00000142534.6 0.649257568 5 2 0.581041972 Brain_Cerebellum
0.803266185 Brain_Cerebellar_Hemisphere 4.238745656 0.000204975
0.723555935 -0.551863362 0.548198005 -0.212345036 0.438427371
2 0 0.998207054
ZNF625 ENSG00000257591.5 0.64949209 1 1 0.64949209
Brain_Substantia_nigra 0.64949209 Brain_Substantia_nigra 1 1 1
0.454467901 0.454467901 0.454467901 NA 1 0 0.998207054
ZMYND10 ENSG00000004838.13 0.649716179 1 1 0.649716179
Brain_Amygdala 0.649716179 Brain_Amygdala 1 1 1 0.454156514
0.454156514 0.454156514 NA 1 0 0.998207054
TMEM99 ENSG00000167920.8 0.649765732 6 5 0.169220286
Brain_Amygdala 0.872231916 Brain_Hypothalamus 3.190073767
0.008679966 0.195279283 -0.89419514 1.374713495 0.174750641
0.821475679 5 0 0.998207054
SLC27A3 ENSG00000143554.13 0.649765907 2 2 0.803359528

Brain_Cerebellar_Hemisphere 0.837231849 Brain_Cerebellum 1.880182426
0.119817574 0.119817574 -0.205435548 0.24900162 0.021783036
0.321335603 2 0 0.998207054
HSD17B12 ENSG00000149084.12 0.649848903 13 1 0.384844635
Brain_Amygdala 0.823806899 Brain_Nucleus_accumbens_basal_ganglia
12.66142343 0.001659124 12.66142343 0.222651309 0.869004563
0.448361136 0.180157886 1 0 0.998207054
ZNF263 ENSG00000006194.9 0.649859385 4 3 0.390546429
Brain_Hippocampus 0.955683079 Brain_Nucleus_accumbens_basal_ganglia
3.025122496 0.097559972 0.226063834 -0.858626833 0.267539863
-0.190982529 0.476011599 3 0 0.998207054
RRP8 ENSG00000132275.10 0.6498726 4 4 0.132241788
Brain_Substantia_nigra 0.990076969 Brain_Caudate_basal_ganglia
1.336052353 0.797194529 0.797194529 -1.505320147 0.012436996
-0.570521024 0.677101702 4 0 0.998207054
ZMYND12 ENSG00000066185.12 0.649881247 6 2 0.361462702
Brain_Cerebellar_Hemisphere 0.850606242 Brain_Cortex 5.40222317
0.034148114 0.259063253 0.188344962 0.912581473 0.541322459
0.293149494 2 0 0.998207054
CRYBB1 ENSG00000100122.5 0.649915604 1 1 0.649915604
Brain_Cortex 0.649915604 Brain_Cortex 1 1 1 0.453879437
0.453879437 0.453879437 NA 1 0 0.998207054
CA3 ENSG00000164879.6 0.650013459 2 2 0.531640454
Brain_Cerebellar_Hemisphere 0.968077261 Brain_Cerebellum 1.767190685
0.232809315 0.232809315 -0.625503802 -0.040019901 -0.332761852
0.413999637 2 0 0.998207054
SENP8 ENSG00000166192.14 0.650034839 2 2 0.42906823
Brain_Anterior_cingulate_cortex_BA24 0.535226647 Brain_Frontal_Cortex_BA9
1.179610035 0.820389965 0.820389965 0.620047299 0.790787116
0.705417207 0.120731282 2 0 0.998207054
PGM1 ENSG00000079739.15 0.650050632 3 3 0.256434111
Brain_Spinal_cord_cervical_c-1 0.618417534 Brain_Cerebellum 1.760120732
0.258204028 0.258204028 -1.134859691 0.919976161 -0.237659297
1.051882794 3 0 0.998207054
LINC02018 ENSG00000272690.5 0.650131804 11 6 0.075299294
Brain_Substantia_nigra 0.799369758 Brain_Frontal_Cortex_BA9
7.426118515 0.009265697 0.269989948 -0.563621885 1.778636997
0.608113443 0.689635981 6 0 0.998207054
SUN1 ENSG00000164828.17 0.650153291 3 3 0.578229895
Brain_Cerebellar_Hemisphere 0.932588156 Brain_Cerebellum 2.176908961
0.128709793 0.128709793 -0.555972149 0.084588986 -0.217091827
0.321896714 3 0 0.998207054
UNC93B1 ENSG00000110057.7 0.650159687 5 4 0.434926149
Brain_Spinal_cord_cervical_c-1 0.901054313 Brain_Frontal_Cortex_BA9
2.814537169 0.023593082 0.227243615 0.124329599 0.780789774
0.487453226 0.300885839 4 0 0.998207054
CASC6 ENSG00000224944.1 0.650329627 1 1 0.650329627
Brain_Hippocampus 0.650329627 Brain_Hippocampus 1 1 1
-0.453304313 -0.453304313 -0.453304313 NA 1 0 0.998207054
LIN28B-AS1 ENSG00000203809.6 0.650440205 2 2 0.40982506
Brain_Putamen_basal_ganglia 0.452533385 Brain_Cortex 1.450783611
0.549216389 0.549216389 -0.824201526 -0.751198197 -0.787699862
0.051621149 2 0 0.998207054
CC2D2A ENSG00000048342.15 0.65067859 1 1 0.65067859
Brain_Putamen_basal_ganglia 0.65067859 Brain_Putamen_basal_ganglia 1 1 1
-0.452819682 -0.452819682 -0.452819682 NA 1 0 0.998207054
LINC00636 ENSG00000240423.1 0.650873516 1 1 0.650873516
Brain_Anterior_cingulate_cortex_BA24 0.650873516
Brain_Anterior_cingulate_cortex_BA24 1 1 1 -0.452549019
-0.452549019 -0.452549019 NA 1 0 0.998207054
TPSAB1 ENSG00000172236.16 0.650972981 3 3 0.23125904
Brain_Putamen_basal_ganglia 0.727524363 Brain_Anterior_cingulate_cortex_BA24

2.001248145 0.400878768 0.400878768 0.348420562 1.197121912
0.642098242 0.480936369 3 0 0.998207054
LINC01992 ENSG00000260019.1 0.651000983 1 1 0.651000983
Brain_Hippocampus 0.651000983 Brain_Hippocampus 1 1 1
-0.452372044 -0.452372044 -0.452372044 NA 1 0 0.998207054
KIAA0895 ENSG00000164542.12 0.651051081 2 2 0.357565208
Brain_Putamen_basal_ganglia 0.936467517 Brain_Cerebellar_Hemisphere
1.031908886 0.968091114 0.968091114 -0.92001445 -0.079710489
-0.499862469 0.594184629 2 0 0.998207054
RASGEF1B ENSG00000138670.17 0.651190689 4 4 0.293878083
Brain_Cerebellum 0.985417936 Brain_Cerebellar_Hemisphere 1.992843453
0.272706654 0.272706654 -0.018276925 1.049652124 0.388374911
0.460115508 4 0 0.998207054
ATOH8 ENSG00000168874.12 0.651197444 1 1 0.651197444
Brain_Substantia_nigra 0.651197444 Brain_Substantia_nigra 1 1
1 -0.452099306 -0.452099306 -0.452099306 NA 1 0
0.998207054
ZNF223 ENSG00000178386.12 0.651520535 3 3 0.390037528
Brain_Cerebellum 0.500414296 Brain_Amygdala 1.510769862 0.517819323
0.517819323 -0.783753176 0.859549309 0.249878053 0.899954059
3 0 0.998207054
RP11-370I10.11 ENSG00000273765.1 0.65155175 3 3 0.205116625
Brain_Caudate_basal_ganglia 0.856940696 Brain_Frontal_Cortex_BA9
1.644258253 0.609423991 0.609423991 0.180269886 1.267108141
0.60645419 0.58011754 3 0 0.998207054
CCNG2 ENSG00000138764.14 0.651588497 3 3 0.278148817
Brain_Putamen_basal_ganglia 0.835858265 Brain_Cerebellum 1.4694968
0.738336145 0.738336145 0.207194113 1.084487251 0.732578325
0.46366051 3 0 0.998207054
PCGF2 ENSG00000277258.4 0.651599261 2 2 0.367196908 Brain_Cerebellum
0.383749485 Brain_Cerebellar_Hemisphere 1.840614335 0.159385665
0.159385665 -0.901736408 -0.871008549 -0.886372478 0.021727878
2 0 0.998207054
TAF2 ENSG00000064313.11 0.651787506 1 1 0.651787506
Brain_Cerebellar_Hemisphere 0.651787506 Brain_Cerebellar_Hemisphere 1 1
1 0.45128035 0.45128035 0.45128035 NA 1 0 0.998207054
NKX3-1 ENSG00000167034.9 0.652025072 2 1 0.556123368
Brain_Putamen_basal_ganglia 0.762240461 Brain_Caudate_basal_ganglia
1.952597851 0.047402149 1.952597851 -0.588609338 -0.30253998
-0.445574659 0.202281583 1 0 0.998207054
TOMM70 ENSG00000154174.7 0.65206795 2 2 0.372672142 Brain_Cerebellum
0.702272257 Brain_Cortex 1.156319732 0.843680268 0.843680268
-0.891479206 0.382254963 -0.254612121 0.900666068 2 0
0.998207054
MYH2 ENSG00000125414.18 0.652096718 2 2 0.374424277
Brain_Caudate_basal_ganglia 0.689598324 Brain_Frontal_Cortex_BA9
1.163917437 0.836082563 0.836082563 -0.888216553 0.399400231
-0.244408161 0.910482559 2 0 0.998207054
FAM65A ENSG00000039523.19 0.652176964 1 1 0.652176964
Brain_Cerebellum 0.652176964 Brain_Cerebellum 1 1 1 0.450739981
0.450739981 0.450739981 NA 1 0 0.998207054
ZSCAN29 ENSG00000140265.12 0.652205971 4 4 0.36723292
Brain_Anterior_cingulate_cortex_BA24 0.915323616
Brain_Nucleus_accumbens_basal_ganglia 2.503433839 0.142236518
0.142236518 -0.887021285 0.901668633 -0.102670448 0.746260986
4 0 0.998207054
WDR37 ENSG00000047056.14 0.652240631 2 2 0.477897832 Brain_Cerebellum
0.846825807 Brain_Cerebellar_Hemisphere 1.466321457 0.533678543
0.533678543 -0.709687683 0.193170033 -0.258258825 0.638416813
2 0 0.998207054
RP4-535B20.4 ENSG00000272506.1 0.652256609 1 1 0.652256609
Brain_Anterior_cingulate_cortex_BA24 0.652256609

Brain_Anterior_cingulate_cortex_BA24 1 1 1 -0.450629489
-0.450629489 -0.450629489 NA 1 0 0.998207054
RP11-463O9.9 ENSG00000270020.1 0.652329594 13 4 0.129400638
Brain_Hypothalamus 0.846452577 Brain_Amygdala 10.63215042
0.001293236 0.398757822 0.193646638 1.516469652 0.922871356
0.340524111 4 0 0.998207054
EEF1AKMT1 ENSG00000150456.10 0.652409635 3 3 0.235071797
Brain_Spinal_cord_cervical_c-1 0.977234765 Brain_Frontal_Cortex_BA9
1.281940689 0.75579386 0.75579386 -1.187395137 0.028535863
-0.516797263 0.617568208 3 0 0.998207054
LRIG1 ENSG00000144749.13 0.652433782 3 3 0.228146226
Brain_Hypothalamus 0.929380184 Brain_Caudate_basal_ganglia 1.250279284
0.754969928 0.754969928 -1.205147862 0.12365645 -0.390038701
0.713840339 3 0 0.998207054
SLC25A3 ENSG00000075415.12 0.652472374 3 3 0.32342171
Brain_Nucleus_accumbens_basal_ganglia 0.774278844 Brain_Amygdala
2.145093534 0.261481721 0.261481721 0.286782525 0.987450761
0.687099576 0.36087238 3 0 0.998207054
MAPK14 ENSG00000112062.10 0.652507506 6 5 0.206632888
Brain_Hypothalamus 0.665996464 Brain_Amygdala 2.887166587
0.084533261 0.379695509 -0.431649104 1.262878388 0.716975026
0.598781987 5 0 0.998207054
IRF2BP2 ENSG00000168264.10 0.65251119 1 1 0.65251119
Brain_Putamen_basal_ganglia 0.65251119 Brain_Putamen_basal_ganglia 1 1 1
-0.45027635 -0.45027635 -0.45027635 NA 1 0 0.998207054
LTV1 ENSG00000135521.8 0.652755058 1 1 0.652755058
Brain_Nucleus_accumbens_basal_ganglia 0.652755058
Brain_Nucleus_accumbens_basal_ganglia 1 1 1 0.449938123
0.449938123 0.449938123 NA 1 0 0.998207054
CTB-16J4.1 ENSG00000283267.1 0.652807457 5 5 0.192508204
Brain_Cortex 0.880000118 Brain_Caudate_basal_ganglia 3.017956237
0.188383517 0.188383517 -1.303194978 0.150969066 -0.658137014
0.530071418 5 0 0.998207054
TMEM252 ENSG00000181778.4 0.652842838 1 1 0.652842838
Brain_Cerebellum 0.652842838 Brain_Cerebellum 1 1 1 -0.449816391
-0.449816391 -0.449816391 NA 1 0 0.998207054
ST6GAL1 ENSG00000073849.14 0.652859629 2 2 0.360263931
Brain_Cortex 0.862632161 Brain_Cerebellar_Hemisphere 1.051857955
0.948142045 0.948142045 0.173024511 0.914862286 0.543943399
0.524558521 2 0 0.998207054
SGCG ENSG00000102683.7 0.652867256 2 2 0.394499836
Brain_Spinal_cord_cervical_c-1 0.826391279 Brain_Cerebellum 1.156949001
0.843050999 0.843050999 -0.851485282 -0.219332201 -0.535408742
0.44699973 2 0 0.998207054
SLC38A11 ENSG00000169507.9 0.652928819 3 3 0.357262087
Brain_Frontal_Cortex_BA9 0.681274341 Brain_Cortex 1.649775192
0.356903176 0.356903176 -0.809935604 0.920594669 0.173794621
0.889261316 3 0 0.998207054
RAC2 ENSG00000128340.14 0.652934789 1 1 0.652934789
Brain_Caudate_basal_ganglia 0.652934789 Brain_Caudate_basal_ganglia 1 1
1 -0.449688882 -0.449688882 -0.449688882 NA 1 0
0.998207054
RP11-626P14.2 ENSG00000257185.1 0.653174086 1 1 0.653174086
Brain_Nucleus_accumbens_basal_ganglia 0.653174086
Brain_Nucleus_accumbens_basal_ganglia 1 1 1 0.449357082
0.449357082 0.449357082 NA 1 0 0.998207054
CNGB1 ENSG00000070729.13 0.6532563 1 1 0.6532563
Brain_Anterior_cingulate_cortex_BA24 0.6532563
Brain_Anterior_cingulate_cortex_BA24 1 1 1 -0.449243099
-0.449243099 -0.449243099 NA 1 0 0.998207054
RP11-287D1.4 ENSG00000217702.2 0.653307924 13 1 0.44183484
Brain_Hippocampus 0.864043456 Brain_Cerebellum 12.23101323

0.001574385 12.23101323 0.171229317 0.769098498 0.436141472
0.151760612 1 0 0.998207054
SLC27A2 ENSG00000140284.10 0.653324204 6 6 0.107276872
Brain_Nucleus_accumbens_basal_ganglia 0.84111308 Brain_Frontal_Cortex_BA9
1.734346268 0.287536534 0.287536534 -1.014632029 1.610554433
0.382154188 0.845805862 6 0 0.998207054
CSNK1G2 ENSG00000133275.15 0.653499409 3 3 0.369018465
Brain_Cerebellar_Hemisphere 0.744578056 Brain_Nucleus_accumbens_basal_ganglia
1.262249785 0.739666158 0.739666158 -0.889403378 0.898313448
0.111568976 0.912909115 3 0 0.998207054
RP11-662J14.2 ENSG00000258480.1 0.653644675 1 1 0.653644675
Brain_Cortex 0.653644675 Brain_Cortex 1 1 1 0.448704726
0.448704726 0.448704726 NA 1 0 0.998207054
PTPN7 ENSG00000143851.15 0.653795908 3 3 0.246971255
Brain_Putamen_basal_ganglia 0.613602064 Brain_Hypothalamus 1.684150359
0.315955098 0.315955098 -0.504938455 1.157737306 0.527765418
0.901536167 3 0 0.998207054
CDRT4 ENSG00000239704.10 0.653872256 12 3 0.206129017
Brain_Anterior_cingulate_cortex_BA24 0.969497251 Brain_Cerebellum
9.024632022 0.019457759 0.873556157 -0.197435139 1.264281472
0.331542336 0.426662138 3 0 0.998207054
WDR11 ENSG00000120008.15 0.653930043 11 2 0.243246815
Brain_Caudate_basal_ganglia 0.817669969 Brain_Putamen_basal_ganglia
9.358160417 0.001829607 0.775639483 -1.166909756 -0.230542852
-0.827521511 0.264364743 2 0 0.998207054
TIMM44 ENSG00000104980.7 0.65397455 10 1 0.373806003
Brain_Putamen_basal_ganglia 0.899715836 Brain_Nucleus_accumbens_basal_ganglia
9.498075249 0.000619809 9.498075249 -0.889366761 -0.126020325
-0.442132033 0.277025645 1 0 0.998207054
TRADD ENSG00000102871.15 0.654030888 1 1 0.654030888 Brain_Cerebellum
0.654030888 Brain_Cerebellum 1 1 1 0.448169479 0.448169479
0.448169479 NA 1 0 0.998207054
LARGE1 ENSG00000133424.20 0.654275459 1 1 0.654275459
Brain_Caudate_basal_ganglia 0.654275459 Brain_Caudate_basal_ganglia 1 1
1 -0.447830598 -0.447830598 -0.447830598 NA 1 0
0.998207054
RP11-230C9.4 ENSG00000271265.1 0.654299853 7 4 0.225751067
Brain_Cerebellar_Hemisphere 0.903948191 Brain_Anterior_cingulate_cortex_BA24
4.494090488 1.98E-07 0.67570568 -0.406302212 1.211376696
0.291926884 0.512300076 4 0 0.998207054
HELZ ENSG00000198265.11 0.654375926 2 2 0.483698886 Brain_Amygdala
0.509199438 Brain_Cerebellum 1.092251129 0.907748871 0.907748871
-0.66008476 0.700365804 0.020140522 0.961983819 2 0
0.998207054
OIT3 ENSG00000138315.12 0.654464345 2 2 0.445155279 Brain_Cerebellum
0.613993292 Brain_Cerebellar_Hemisphere 1.92185247 0.07814753 0.07814753
-0.763516747 -0.504381533 -0.63394914 0.183236267 2 0
0.998207054
ZNF675 ENSG00000197372.9 0.654524694 1 1 0.654524694
Brain_Hippocampus 0.654524694 Brain_Hippocampus 1 1 1
-0.447485307 -0.447485307 -0.447485307 NA 1 0 0.998207054
AC010729.2 ENSG00000236106.1 0.654621471 1 1 0.654621471
Brain_Caudate_basal_ganglia 0.654621471 Brain_Caudate_basal_ganglia 1 1
1 0.447351247 0.447351247 0.447351247 NA 1 0
0.998207054
CHST14 ENSG00000169105.7 0.654776361 2 2 0.37321618
Brain_Caudate_basal_ganglia 0.945055401 Brain_Amygdala 1.324247494
0.675752506 0.675752506 -0.890465138 -0.068917358 -0.479691248
0.580922006 2 0 0.998207054
USP43 ENSG00000154914.16 0.654778899 1 1 0.654778899 Brain_Cerebellum
0.654778899 Brain_Cerebellum 1 1 1 0.447133186 0.447133186
0.447133186 NA 1 0 0.998207054

SLPI ENSG00000124107.5 0.65483518 4 3 0.245454738 Brain_Amygdala
0.968622157 Brain_Hippocampus 2.659727087 0.050186802
0.417329827 -0.328808088 1.161460352 0.221791319 0.650818568
3 0 0.998207054
STAG3 ENSG00000066923.17 0.654926525 13 3 0.125434359
Brain_Caudate_basal_ganglia 0.448073858 Brain_Cerebellar_Hemisphere
10.79106922 0.006616443 0.39843322 0.758630105 1.532357012
1.117752084 0.250078333 3 0 0.998207054
FLRT1 ENSG00000126500.3 0.654973845 2 2 0.38786423
Brain_Cerebellar_Hemisphere 0.911969904 Brain_Spinal_cord_cervical_c-1
1.229632966 0.770367034 0.770367034 -0.110554155 0.863497069
0.376471457 0.688758226 2 0 0.998207054
MEAF6 ENSG00000163875.15 0.654986807 3 3 0.434466276
Brain_Cerebellar_Hemisphere 0.925614987 Brain_Caudate_basal_ganglia 2.43278865
0.214793734 0.214793734 -0.781571781 0.123145105 -0.188354476
0.51395702 3 0 0.998207054
ZNF100 ENSG00000197020.10 0.6551245 13 9 0.162570895 Brain_Cortex
0.941655147 Brain_Substantia_nigra 6.211995852 0.019758993
0.207705633 -0.773287292 1.396477022 0.499571352 0.696767004
9 0 0.998207054
LINC01503 ENSG00000233901.5 0.655193661 1 1 0.655193661
Brain_Caudate_basal_ganglia 0.655193661 Brain_Caudate_basal_ganglia 1 1
1 0.446558782 0.446558782 0.446558782 NA 1 0
0.998207054
MSC ENSG00000178860.8 0.65521265 1 1 0.65521265 Brain_Frontal_Cortex_BA9
0.65521265 Brain_Frontal_Cortex_BA9 1 1 1 -0.446532488
-0.446532488 -0.446532488 NA 1 0 0.998207054
EIF3E ENSG00000104408.9 0.655291399 4 4 0.345751767
Brain_Putamen_basal_ganglia 0.902936857 Brain_Nucleus_accumbens_basal_ganglia
2.589624673 0.300710603 0.300710603 -0.138735553 0.942861465
0.226865549 0.505961343 4 0 0.998207054
PLEKHG6 ENSG00000008323.15 0.655293582 1 1 0.655293582
Brain_Spinal_cord_cervical_c-1 0.655293582 Brain_Spinal_cord_cervical_c-1
1 1 1 0.446420424 0.446420424 0.446420424 NA 1 0
0.998207054
ASL ENSG00000126522.16 0.655306776 6 4 0.167494011
Brain_Caudate_basal_ganglia 0.947094133 Brain_Cerebellar_Hemisphere
3.353619379 0.027805771 0.328923537 -1.380300924 0.203764957
-0.21012853 0.594435736 4 0 0.998207054
NDUFB10 ENSG00000140990.14 0.65535878 3 3 0.224623394
Brain_Putamen_basal_ganglia 0.950506147 Brain_Cerebellar_Hemisphere
1.453297533 0.665230527 0.665230527 0.06207118 1.21432565
0.454069405 0.658508214 3 0 0.998207054
IDUA ENSG00000127415.12 0.655386006 3 3 0.235114152
Brain_Cerebellar_Hemisphere 0.577294645 Brain_Nucleus_accumbens_basal_ganglia
1.89689166 0.124545876 0.124545876 -1.187287715 0.55734074
-0.555493874 0.966686237 3 0 0.998207054
ATP13A2 ENSG00000159363.17 0.655437943 1 1 0.655437943
Brain_Cerebellum 0.655437943 Brain_Cerebellum 1 1 1 -0.446220546
-0.446220546 -0.446220546 NA 1 0 0.998207054
LRRC63 ENSG00000173988.12 0.655501937 3 3 0.406520174
Brain_Anterior_cingulate_cortex_BA24 0.880097499 Brain_Caudate_basal_ganglia
1.787696012 0.21232559 0.21232559 -0.150845619 0.830032915
0.395027699 0.499749414 3 0 0.998207054
FSHR ENSG00000170820.11 0.6556331 1 1 0.6556331 Brain_Frontal_Cortex_BA9
0.6556331 Brain_Frontal_Cortex_BA9 1 1 1 -0.445950365
-0.445950365 -0.445950365 NA 1 0 0.998207054
BTN3A2 ENSG00000186470.13 0.655654045 13 1 0.239155867
Brain_Cerebellar_Hemisphere 0.923816601 Brain_Substantia_nigra 11.6482688
0.014688969 11.6482688 -1.177099319 0.220760435 -0.422802436
0.39078919 1 0 0.998207054
LINC01649 ENSG00000228127.2 0.655748245 2 2 0.40019717 Brain_Cerebellum

0.467584751 Brain_Cortex 1.47815512 0.52184488 0.52184488 -0.841269148
-0.726414425 -0.783841786 0.081214554 2 0 0.998207054
LINC00539 ENSG00000224429.7 0.655829142 4 2 0.386801361
Brain_Putamen_basal_ganglia 0.669140955 Brain_Substantia_nigra 3.74118584
0.009054385 0.219125751 -0.865432661 -0.427327294 -0.619404068
0.18252882 2 0 0.998207054
STAM2 ENSG00000115145.9 0.655832655 2 2 0.411503211
Brain_Spinal_cord_cervical_c-1 0.696821886 Brain_Putamen_basal_ganglia
1.026347494 0.973652506 0.973652506 -0.821251177 0.389614152
-0.215818512 0.856211085 2 0 0.998207054
APOB ENSG00000084674.14 0.655911131 2 2 0.389396286
Brain_Spinal_cord_cervical_c-1 0.695574191 Brain_Hypothalamus 1.08372345
0.91627655 0.91627655 -0.860712719 -0.39130177 -0.626007245
0.331923665 2 0 0.998207054
NCOR2 ENSG00000196498.13 0.655964419 2 2 0.49259474
Brain_Anterior_cingulate_cortex_BA24 0.548937263 Brain_Amygdala 1.01581506
0.98418494 0.98418494 -0.599353373 0.686187721 0.043417174
0.909014825 2 0 0.998207054
MFSD3 ENSG00000167700.8 0.655987988 1 1 0.655987988
Brain_Caudate_basal_ganglia 0.655987988 Brain_Caudate_basal_ganglia 1 1
1 0.445459132 0.445459132 0.445459132 NA 1 0
0.998207054
DIO3OS ENSG00000258498.8 0.656050292 2 2 0.523689368
Brain_Amygdala 0.534092256 Brain_Caudate_basal_ganglia 1.059106441
0.940893559 0.940893559 -0.637668695 0.621771307 -0.007948694
0.890558566 2 0 0.998207054
TMBIM4 ENSG00000155957.17 0.656076425 3 2 0.694536374
Brain_Cerebellum 0.932598475 Brain_Frontal_Cortex_BA9 2.802519827
0.039571329 0.157908843 -0.084576006 0.392706358 0.205510374
0.254734055 2 0 0.998207054
RP4-669P10.16 ENSG00000232710.1 0.656166265 2 2 0.393034124
Brain_Cerebellar_Hemisphere 0.491921286 Brain_Cortex 1.453666212
0.546333788 0.546333788 0.687256214 0.854127904 0.770692059
0.117996104 2 0 0.998207054
ERMARD ENSG00000130023.15 0.656226032 1 1 0.656226032
Brain_Cerebellum 0.656226032 Brain_Cerebellum 1 1 1 0.445129692
0.445129692 0.445129692 NA 1 0 0.998207054
RALGAPB ENSG00000170471.14 0.656306939 2 2 0.489263732
Brain_Frontal_Cortex_BA9 0.539383828 Brain_Spinal_cord_cervical_c-1
1.015039269 0.984960731 0.984960731 0.613745029 0.691480339
0.652612684 0.054967165 2 0 0.998207054
IFT57 ENSG00000114446.4 0.656367164 4 4 0.278492322 Brain_Cortex
0.865761578 Brain_Cerebellar_Hemisphere 3.03218388 0.196281314
0.196281314 -0.169044577 1.083712432 0.473767943 0.517441771
4 0 0.998207054
RBP1 ENSG00000114115.9 0.656426957 7 7 0.130553753 Brain_Cerebellum
0.996607754 Brain_Nucleus_accumbens_basal_ganglia 2.716600914
0.199937174 0.199937174 -1.511921818 1.27246244 -0.478618814
0.940243648 7 0 0.998207054
TMEM135 ENSG00000166575.16 0.656446595 7 6 0.156877384
Brain_Cerebellum 0.820529605 Brain_Cortex 4.130438498 0.019564631
0.142570492 -1.415652116 0.423908916 -0.299037751 0.645656627
6 0 0.998207054
RP11-317N12.1 ENSG00000253642.5 0.656690688 1 1 0.656690688
Brain_Cerebellum 0.656690688 Brain_Cerebellum 1 1 1 0.444486775
0.444486775 0.444486775 NA 1 0 0.998207054
NOC3L ENSG00000173145.11 0.656812143 9 6 0.134077275
Brain_Hypothalamus 0.883804876 Brain_Hippocampus 6.398872018
0.029180165 0.215182354 -1.49821548 -0.146147593 -0.593489349
0.461583465 6 0 0.998207054
KIAA0930 ENSG00000100364.18 0.656954591 5 5 0.258883375
Brain_Cerebellum 0.970085837 Brain_Hippocampus 2.493629916

0.196528603 0.196528603 -1.129034276 0.149062544 -0.262880185
0.505566434 5 0 0.998207054
GBP7 ENSG00000213512.1 0.656959701 8 4 0.189802082
Brain_Spinal_cord_cervical_c-1 0.99019544 Brain_Putamen_basal_ganglia
5.742553192 0.031715908 0.304745866 -0.523979235 1.31116483
0.371417598 0.600207293 4 0 0.998207054
RSPO2 ENSG00000147655.10 0.656976831 1 1 0.656976831 Brain_Cortex
0.656976831 Brain_Cortex 1 1 1 0.444090948 0.444090948
0.444090948 NA 1 0 0.998207054
RRN3 ENSG00000085721.12 0.657020149 8 4 0.444183705
Brain_Cerebellar_Hemisphere 0.870321124 Brain_Putamen_basal_ganglia
5.682378511 0.006272931 0.306542341 -0.231785053 0.765147522
0.426424475 0.401187622 4 0 0.998207054
CYSTM1 ENSG00000120306.9 0.657033373 5 4 0.150482074
Brain_Nucleus_accumbens_basal_ganglia 0.789128793
Brain_Spinal_cord_cervical_c-1 3.544683842 0.077371578 0.141160783
-1.437830779 -0.267442094 -0.72563924 0.440804226 4 0
0.998207054
RP11-1228E12.1 ENSG00000280279.1 0.657034778 1 1 0.657034778
Brain_Cerebellum 0.657034778 Brain_Cerebellum 1 1 1 0.444010796
0.444010796 0.444010796 NA 1 0 0.998207054
COBL ENSG00000106078.17 0.657127907 1 1 0.657127907
Brain_Cerebellar_Hemisphere 0.657127907 Brain_Cerebellar_Hemisphere 1 1
1 0.443881989 0.443881989 0.443881989 NA 1 0
0.998207054
DPH1 ENSG00000108963.17 0.657272416 8 7 0.118119498 Brain_Cerebellum
0.935927806 Brain_Caudate_basal_ganglia 3.159524941 0.000196122
0.372404673 -0.249210552 1.562715623 0.737592299 0.740525894
7 0 0.998207054
NUP214 ENSG00000126883.16 0.65731123 1 1 0.65731123
Brain_Caudate_basal_ganglia 0.65731123 Brain_Caudate_basal_ganglia 1 1 1
0.443628454 0.443628454 0.443628454 NA 1 0 0.998207054
C5orf63 ENSG00000164241.13 0.657317666 5 4 0.499725346
Brain_Frontal_Cortex_BA9 0.984832353 Brain_Hypothalamus 3.897010242
0.084478924 0.15933974 -0.674921963 0.179135711 -0.173977757
0.395660987 4 0 0.998207054
RPA3 ENSG00000106399.11 0.657435039 9 7 0.047288459
Brain_Anterior_cingulate_cortex_BA24 0.9062916 Brain_Cerebellum 4.248390995
0.011697628 0.166940411 -1.360942884 1.983707478 0.682370233
1.017263544 7 0 0.998207054
SLC29A1 ENSG00000112759.16 0.657492808 2 2 0.359796545
Brain_Cerebellum 0.952431773 Brain_Cerebellar_Hemisphere 1.07199919 0.92800081
0.92800081 -0.915752837 0.059653292 -0.428049773 0.689716289 2
0 0.998207054
BTD ENSG00000169814.12 0.657616653 1 1 0.657616653
Brain_Nucleus_accumbens_basal_ganglia 0.657616653
Brain_Nucleus_accumbens_basal_ganglia 1 1 1 0.44320612 0.44320612
0.44320612 NA 1 0 0.998207054
PCDHA4 ENSG00000204967.10 0.657654509 3 3 0.208658766
Brain_Cerebellum 0.832332406 Brain_Spinal_cord_cervical_c-1 1.542513734
0.532246279 0.532246279 0.211711128 1.257262037 0.585940569
0.58265936 3 0 0.998207054
WDR35 ENSG00000118965.14 0.657714924 2 2 0.364186543
Brain_Putamen_basal_ganglia 0.608882886 Brain_Anterior_cingulate_cortex_BA24
1.444763933 0.555236067 0.555236067 0.511668714 0.907416583
0.709542648 0.279836002 2 0 0.998207054
CHFR ENSG00000072609.17 0.657868502 11 5 0.186121265
Brain_Hippocampus 0.974537642 Brain_Cortex 7.696243645
0.019063945 0.393353989 -1.322140814 0.139167879 -0.214628274
0.396303299 5 0 0.998207054
HERC2 ENSG00000128731.15 0.657881523 2 2 0.482581218
Brain_Spinal_cord_cervical_c-1 0.573264462 Brain_Cortex 1.033202098

0.966797902 0.966797902 0.563250291 0.702157064 0.632703678
0.098221922 2 0 0.998207054
CAPN1 ENSG00000014216.15 0.657956417 3 3 0.329235517
Brain_Putamen_basal_ganglia 0.680895237 Brain_Hippocampus 1.117852191
0.934842429 0.934842429 -0.975654883 0.411241806 -0.414880941
0.730532749 3 0 0.998207054
TTC14 ENSG00000163728.10 0.658095322 6 4 0.137307144 Brain_Cortex
0.747253395 Brain_Substantia_nigra 3.588285044 0.018236847
0.173702068 -0.322263087 1.485894064 0.899788644 0.665396409
4 0 0.998207054
ADGRD2 ENSG00000180264.11 0.658141752 2 2 0.363045963
Brain_Cerebellar_Hemisphere 0.99763034 Brain_Cerebellum 1.102431246
0.897568754 0.897568754 -0.909576373 0.002969932 -0.45330322
0.645267681 2 0 0.998207054
C2CD4C ENSG00000183186.7 0.65831613 3 3 0.388278726
Brain_Nucleus_accumbens_basal_ganglia 0.585648993 Brain_Cerebellum 1.05939506
0.920003382 0.920003382 -0.862743107 0.666273517 0.116227465
0.849973612 3 0 0.998207054
MNT ENSG00000070444.14 0.658406687 2 2 0.370842829 Brain_Cerebellum
0.845195202 Brain_Cortex 1.409025138 0.590974862 0.590974862
0.195252601 0.894895731 0.545074166 0.494722402 2 0
0.998207054
EXOC4 ENSG00000131558.14 0.658433575 8 1 0.482702499
Brain_Frontal_Cortex_BA9 0.979797698 Brain_Nucleus_accumbens_basal_ganglia
7.50083995 0.001051049 7.50083995 -0.701962581 -0.025322537
-0.427591946 0.214109299 1 0 0.998207054
HMGCL ENSG00000117305.14 0.658437633 10 5 0.262517394 Brain_Amygdala
0.85404425 Brain_Spinal_cord_cervical_c-1 7.592136752 0.013020803
0.286616513 -0.726812988 1.120460927 0.25907279 0.522373734 5
0 0.998207054
ODF3L2 ENSG00000181781.9 0.658488476 3 3 0.337257754
Brain_Putamen_basal_ganglia 0.780070591 Brain_Hippocampus 1.043841974
0.970578959 0.970578959 -0.279227043 0.959597647 0.491148484
0.672340167 3 0 0.998207054
MAEA ENSG00000090316.15 0.658497284 9 6 0.5301028
Brain_Cerebellar_Hemisphere 0.883658395 Brain_Frontal_Cortex_BA9
5.538775244 0.09532092 0.236926734 -0.627849096 0.583127112
0.052044309 0.429967197 6 0 0.998207054
STON2 ENSG00000140022.9 0.658503096 4 4 0.120891377
Brain_Hypothalamus 0.90061518 Brain_Cerebellum 2.142038328 0.303505925
0.303505925 -1.551043011 -0.124884259 -0.62622183 0.63675135 4
0 0.998207054
ADGRF3 ENSG00000173567.14 0.658610392 2 2 0.37147519
Brain_Cerebellar_Hemisphere 0.719696635 Brain_Putamen_basal_ganglia
1.576298267 0.423701733 0.423701733 -0.358864263 0.893713519
0.267424628 0.885706244 2 0 0.998207054
RGL2 ENSG00000237441.9 0.658724343 1 1 0.658724343 Brain_Cerebellum
0.658724343 Brain_Cerebellum 1 1 1 0.441675083 0.441675083
0.441675083 NA 1 0 0.998207054
SLC9A3 ENSG00000066230.10 0.6587644 13 4 0.233692139
Brain_Hypothalamus 0.862175397 Brain_Cerebellum 10.16173865
0.004522765 0.57130849 -0.960062817 1.190901797 0.584970381
0.54815371 4 0 0.998207054
SFTPA2 ENSG00000185303.16 0.658948366 3 3 0.46510017 Brain_Cerebellum
0.577076238 Brain_Cortex 1.956827259 0.249106414 0.249106414
-0.730474539 0.557660496 -0.28989112 0.734193641 3 0
0.998207054
ILVBL ENSG00000105135.15 0.658971926 4 4 0.259519823
Brain_Caudate_basal_ganglia 0.775351298 Brain_Anterior_cingulate_cortex_BA24
1.878954267 0.136900238 0.136900238 -0.894214009 1.127526784
-0.02601567 0.919691667 4 0 0.998207054
FUCA1 ENSG00000179163.11 0.658980014 1 1 0.658980014

Brain_Hypothalamus 0.658980014 Brain_Hypothalamus 1 1 1
-0.441321844 -0.441321844 -0.441321844 NA 1 0 0.998207054
AHRR ENSG00000063438.16 0.659034898 7 6 0.145956606 Brain_Cortex
0.762755953 Brain_Caudate_basal_ganglia 2.937867034 0.09757648 0.2192072
-1.45396285 0.531208986 -0.561528024 0.638546111 6 0
0.998207054
NWD1 ENSG00000188039.13 0.659056566 9 9 0.072138324 Brain_Cortex
0.986457208 Brain_Putamen_basal_ganglia 1.981325747 0.513053516
0.513053516 -1.230545494 1.798244165 0.425560569 0.879513564
9 0 0.998207054
NUTM2A ENSG00000184923.12 0.659137669 13 8 0.183903589
Brain_Spinal_cord_cervical_c-1 0.984320788 Brain_Frontal_Cortex_BA9
7.81383528 0.016329778 0.263481604 -1.328831433 0.930152615
-0.332751761 0.638767266 8 0 0.998207054
HMCN2 ENSG00000148357.16 0.659234503 4 4 0.254358972
Brain_Cerebellar_Hemisphere 0.786738657 Brain_Cerebellum 1.662430113
0.336179719 0.336179719 -0.727184614 1.139825585 0.264375465
0.766317739 4 0 0.998207054
HCK ENSG00000101336.13 0.659311679 2 2 0.362241745
Brain_Anterior_cingulate_cortex_BA24 0.756083303
Brain_Spinal_cord_cervical_c-1 1.283172199 0.716827801 0.716827801
0.310628178 0.911101788 0.610864983 0.424598961 2 0
0.998207054
PRG4 ENSG00000116690.12 0.659330289 1 1 0.659330289 Brain_Cerebellum
0.659330289 Brain_Cerebellum 1 1 1 -0.44083799 -0.44083799
-0.44083799 NA 1 0 0.998207054
LINC01252 ENSG00000247157.6 0.659391836 13 3 0.328962025
Brain_Frontal_Cortex_BA9 0.998503928 Brain_Hippocampus 10.08543963
0.022514467 0.432214416 -0.633478821 0.976206738 -0.082513308
0.465441535 3 0 0.998207054
ZNF248 ENSG00000198105.13 0.659452686 7 4 0.186591143
Brain_Hippocampus 0.61333244 Brain_Frontal_Cortex_BA9 5.624976253
0.013051697 0.3089147 0.505322361 1.320730792 0.804187253
0.284209359 4 0 0.998207054
MEGF6 ENSG00000162591.15 0.659495121 4 4 0.161985965
Brain_Putamen_basal_ganglia 0.982673246 Brain_Cortex 1.280692111
0.79397035 0.79397035 -0.485875319 1.398423383 0.212934387
0.820077458 4 0 0.998207054
RP11-571M6.17 ENSG00000270039.1 0.659617595 7 5 0.08698623
Brain_Frontal_Cortex_BA9 0.913914063 Brain_Putamen_basal_ganglia
5.030654833 0.035968671 0.186942313 -1.711514213 -0.108102907
-0.940290848 0.509554193 5 0 0.998207054
POSTN ENSG00000133110.14 0.65971571 1 1 0.65971571 Brain_Cortex 0.65971571
Brain_Cortex 1 1 1 -0.440305704 -0.440305704 -0.440305704
NA 1 0 0.998207054
SLC25A39 ENSG00000013306.15 0.659817313 12 7 0.114332021
Brain_Nucleus_accumbens_basal_ganglia 0.911871355 Brain_Amygdala
6.692228982 0.003694711 0.283728152 -0.225311356 1.579017595
0.701036665 0.60521417 7 0 0.998207054
ADRA1A ENSG00000120907.17 0.659872896 2 2 0.362692222
Brain_Frontal_Cortex_BA9 0.890093784 Brain_Substantia_nigra
1.209325321 0.790674679 0.790674679 0.138185538 0.910247076
0.524216307 0.545929949 2 0 0.998207054
FBXO44 ENSG00000132879.13 0.659878461 1 1 0.659878461
Brain_Cerebellum 0.659878461 Brain_Cerebellum 1 1 1 -0.440080974
-0.440080974 -0.440080974 NA 1 0 0.998207054
IGHMBP2 ENSG00000132740.8 0.660089226 13 2 0.293670892
Brain_Putamen_basal_ganglia 0.990582755 Brain_Frontal_Cortex_BA9
10.90979587 0.001939175 1.136062032 -1.050102713 0.181853046
-0.258267472 0.416423286 2 0 0.998207054
CBX3 ENSG00000122565.18 0.660104238 1 1 0.660104238 Brain_Cortex
0.660104238 Brain_Cortex 1 1 1 -0.439769254 -0.439769254

-0.439769254 NA 1 0 0.998207054
CD163L1 ENSG00000177675.8 0.660129332 2 2 0.47952481 Brain_Cerebellum 0.896957399 Brain_Frontal_Cortex_BA9 1.514326486 0.485673514 0.485673514 -0.707067053 0.129505847 -0.288780603 0.59154637 2 0 0.998207054
GAR1 ENSG00000109534.16 0.660196602 11 3 0.133441385 Brain_Hippocampus 0.890264237 Brain_Amygdala 9.128834567 0.009083348 0.310768697 -1.500668266 -0.137969861 -0.865467785 0.458050194 3 0 0.998207054
PPME1 ENSG00000214517.9 0.660215585 3 3 0.302412931 Brain_Cerebellum 0.362454574 Brain_Substantia_nigra 1.871448614 0.21916211 0.21916211 0.910697895 1.031272754 0.955900826 0.065705407 3 0 0.998207054
SLC52A2 ENSG00000185803.8 0.660508214 7 4 0.148013519 Brain_Putamen_basal_ganglia 0.741155665 Brain_Cerebellar_Hemisphere 5.496622515 5.11E-05 0.219547499 0.330323332 1.446583826 0.97084451 0.372390274 4 0 0.998207054
RGPD1 ENSG00000187627.14 0.660622366 1 1 0.660622366 Brain_Cerebellum 0.660622366 Brain_Cerebellum 1 1 1 -0.439054059 -0.439054059 -0.439054059 NA 1 0 0.998207054
DCLK1 ENSG00000133083.14 0.660667322 1 1 0.660667322 Brain_Cerebellum 0.660667322 Brain_Cerebellum 1 1 1 0.438992016 0.438992016 0.438992016 NA 1 0 0.998207054
ZNF146 ENSG00000167635.11 0.660864644 2 2 0.792994725 Brain_Putamen_basal_ganglia 0.863828876 Brain_Hypothalamus 1.886052389 0.113947611 0.113947611 -0.171502231 0.262423801 0.045460785 0.30683204 2 0 0.998207054
GRAMD3 ENSG00000155324.9 0.660907309 5 5 0.319727214 Brain_Putamen_basal_ganglia 0.946113651 Brain_Nucleus_accumbens_basal_ganglia 2.697366819 0.123598203 0.123598203 -0.966805422 0.995018608 0.091093279 0.699607575 5 0 0.998207054
LYPD5 ENSG00000159871.14 0.660919019 6 3 0.096048067 Brain_Anterior_cingulate_cortex_BA24 0.897258349 Brain_Hippocampus 4.090610375 0.011220207 0.321751161 -1.664322155 0.879686338 -0.377845761 1.059303896 3 0 0.998207054
TFAM ENSG00000108064.10 0.66100157 8 4 0.331703854 Brain_Cortex 0.999016596 Brain_Anterior_cingulate_cortex_BA24 6.70514781 0.005776098 0.256266156 -0.97068763 0.012598349 -0.229486186 0.334626077 4 0 0.998207054
LINC00377 ENSG00000229246.1 0.661007103 2 1 0.608138978 Brain_Cerebellum 0.724041366 Brain_Cerebellar_Hemisphere 1.949012702 0.050987298 1.949012702 -0.512731749 -0.353062793 -0.432897271 0.112903002 1 0 0.998207054
MTCL1 ENSG00000168502.17 0.661026428 1 1 0.661026428 Brain_Cerebellum 0.661026428 Brain_Cerebellum 1 1 1 0.43849647 0.43849647 0.43849647 NA 1 0 0.998207054
ITK ENSG00000113263.12 0.66102934 2 2 0.392701659 Brain_Nucleus_accumbens_basal_ganglia 0.69147236 Brain_Caudate_basal_ganglia 1.718288434 0.281711566 0.281711566 -0.854728157 -0.396857753 -0.625792955 0.323763268 2 0 0.998207054
LINC02012 ENSG00000272989.1 0.661031643 11 8 0.283618015 Brain_Cerebellar_Hemisphere 0.999482694 Brain_Hippocampus 5.21071674 0.030625273 0.186784695 -1.072227158 0.764732005 -0.013425365 0.546553066 8 0 0.998207054
CRACR2B ENSG00000177685.16 0.661119467 3 3 0.25953406 Brain_Anterior_cingulate_cortex_BA24 0.707315931 Brain_Caudate_basal_ganglia 1.452186935 0.548538644 0.548538644 -1.127493091 -0.375463307 -0.702272606 0.385552552 3 0 0.998207054
KCNA4 ENSG00000182255.6 0.661346779 1 1 0.661346779 Brain_Cerebellum 0.661346779 Brain_Cerebellum 1 1 1 0.438054497 0.438054497 0.438054497 NA 1 0 0.998207054
PIGZ ENSG00000119227.7 0.661406277 13 6 0.102504528

Brain_Spinal_cord_cervical_c-1 0.971475206 Brain_Cortex 8.417600864 0.006963097 0.379600211 -1.632831084 0.665643662 0.133603711 0.601460864 6 0 0.998207054
LINC00642 ENSG00000233208.5 0.661442922 3 3 0.247510845 Brain_Cerebellum 0.799977341 Brain_Hippocampus 1.112689534 0.892607591 0.892607591 -0.367273606 1.156416478 0.347506433 0.766193978 3 0 0.998207054
LINC02202 ENSG00000245812.2 0.661478542 2 2 0.383689928 Brain_Spinal_cord_cervical_c-1 0.858392309 Brain_Frontal_Cortex_BA9 1.092606698 0.907393302 0.907393302 0.178421062 0.871117631 0.524769347 0.489810441 2 0 0.998207054
PVR ENSG00000073008.14 0.661489692 4 3 0.3299465 Brain_Cerebellum 0.637150191 Brain_Cortex 2.778734854 0.049541709 0.176317394 0.471687038 0.974221644 0.753585934 0.208276617 3 0 0.998207054
LRBA ENSG00000198589.10 0.661508331 2 2 0.363333985 Brain_Cerebellar_Hemisphere 0.526121359 Brain_Cerebellum 1.688321125 0.311678875 0.311678875 0.633937886 0.909030579 0.771484233 0.194519909 2 0 0.998207054
SLC9C2 ENSG00000162753.11 0.661509432 9 1 0.298902788 Brain_Spinal_cord_cervical_c-1 0.982862737 Brain_Cerebellum 8.171333829 0.005809901 8.171333829 -1.038789194 -0.021480025 -0.420357025 0.350246403 1 0 0.998207054
SNCAIP ENSG00000064692.18 0.661520431 3 3 0.312271463 Brain_Cerebellum 0.375209548 Brain_Putamen_basal_ganglia 1.964943118 0.146114512 0.146114512 -1.010467291 -0.886757362 -0.944333712 0.062297321 3 0 0.998207054
SPARC ENSG00000113140.10 0.661609736 3 3 0.230870697 Brain_Cerebellar_Hemisphere 0.915811191 Brain_Putamen_basal_ganglia 1.097729222 0.900622772 0.900622772 -1.19811899 0.255486081 -0.349448164 0.75683391 3 0 0.998207054
LINC00640 ENSG00000258479.5 0.66163302 1 1 0.66163302 Brain_Spinal_cord_cervical_c-1 0.66163302 Brain_Spinal_cord_cervical_c-1 1 1 1 0.437659655 0.437659655 0.437659655 NA 1 0 0.998207054
5-Mar ENSG00000198060.9 0.661736174 1 1 0.661736174 Brain_Spinal_cord_cervical_c-1 0.661736174 Brain_Spinal_cord_cervical_c-1 1 1 1 0.437517381 0.437517381 0.437517381 NA 1 0 0.998207054
LINC02029 ENSG00000241544.1 0.661774481 2 2 0.393939243 Brain_Putamen_basal_ganglia 0.752599115 Brain_Hypothalamus 1.000833305 0.999166695 0.999166695 0.315214081 0.852495307 0.583854694 0.379915198 2 0 0.998207054
DOCK9 ENSG00000088387.18 0.661880758 2 2 0.475096138 Brain_Cerebellum 0.762361817 Brain_Cortex 1.321118747 0.678881253 0.678881253 -0.302380765 0.714211936 0.205915585 0.718839592 2 0 0.998207054
TPD52L2 ENSG00000101150.17 0.662159048 1 1 0.662159048 Brain_Cortex 0.662159048 Brain_Cortex 1 1 1 0.436934228 0.436934228 0.436934228 NA 1 0 0.998207054
ERO1A ENSG00000197930.12 0.662191708 1 1 0.662191708 Brain_Cerebellar_Hemisphere 0.662191708 Brain_Cerebellar_Hemisphere 1 1 1 0.436889195 0.436889195 0.436889195 NA 1 0 0.998207054
ST6GALNAC4 ENSG00000136840.18 0.662214412 1 1 0.662214412 Brain_Cerebellum 0.662214412 Brain_Cerebellum 1 1 1 0.436857892 0.436857892 0.436857892 NA 1 0 0.998207054
GDPD1 ENSG00000153982.10 0.662314412 1 1 0.662314412 Brain_Cerebellum 0.662314412 Brain_Cerebellum 1 1 1 0.436720015 0.436720015 0.436720015 NA 1 0 0.998207054
ELP3 ENSG00000134014.16 0.662327546 13 1 0.510310589 Brain_Nucleus_accumbens_basal_ganglia 0.808415969 Brain_Hippocampus

11.81687703 0.000728759 11.81687703 -0.658354152 -0.24247004
-0.418464144 0.121172971 1 0 0.998207054
NINJ2 ENSG00000171840.11 0.662444287 1 1 0.662444287
Brain_Frontal_Cortex_BA9 0.662444287 Brain_Frontal_Cortex_BA9 1 1
1 0.436540961 0.436540961 0.436540961 NA 1 0
0.998207054
RP3-369A17.4 ENSG00000224164.1 0.662589191 1 1 0.662589191
Brain_Caudate_basal_ganglia 0.662589191 Brain_Caudate_basal_ganglia 1 1
1 -0.436341203 -0.436341203 -0.436341203 NA 1 0
0.998207054
LINC01202 ENSG00000280776.1 0.662605657 1 1 0.662605657
Brain_Frontal_Cortex_BA9 0.662605657 Brain_Frontal_Cortex_BA9 1 1
1 0.436318506 0.436318506 0.436318506 NA 1 0
0.998207054
IL2RB ENSG00000100385.13 0.66284666 2 2 0.366259895 Brain_Hypothalamus
0.912471147 Brain_Caudate_basal_ganglia 1.035210971 0.964789029
0.964789029 -0.903501307 0.10992211 -0.396789599 0.716598571 2
0 0.998207054
RGS4 ENSG00000117152.13 0.662952161 2 2 0.423246014 Brain_Cerebellum
0.578033964 Brain_Cerebellar_Hemisphere 1.171488426 0.828511574
0.828511574 -0.80080246 -0.556258776 -0.678530618 0.172918498
2 0 0.998207054
PPFIBP1 ENSG00000110841.13 0.663088097 1 1 0.663088097
Brain_Spinal_cord_cervical_c-1 0.663088097 Brain_Spinal_cord_cervical_c-1
1 1 1 0.435653571 0.435653571 0.435653571 NA 1 0
0.998207054
ARHGAP30 ENSG00000186517.13 0.663108155 2 2 0.500435559
Brain_Cerebellar_Hemisphere 0.569410664 Brain_Cerebellum 1.054181716
0.945818284 0.945818284 -0.673804586 0.568919658 -0.052442464
0.87873874 2 0 0.998207054
RP11-436D23.1 ENSG00000271860.6 0.663120488 2 2 0.456266009
Brain_Frontal_Cortex_BA9 0.592814261 Brain_Caudate_basal_ganglia
1.025007882 0.974992118 0.974992118 -0.745009447 0.534762263
-0.105123592 0.904935255 2 0 0.998207054
TNRC6C ENSG00000078687.17 0.663257573 1 1 0.663257573
Brain_Frontal_Cortex_BA9 0.663257573 Brain_Frontal_Cortex_BA9 1 1
1 0.435420032 0.435420032 0.435420032 NA 1 0
0.998207054
FBN3 ENSG00000142449.12 0.663292497 2 2 0.365018111 Brain_Cortex
0.401230806 Brain_Frontal_Cortex_BA9 1.916571941 0.083428059
0.083428059 0.839425096 0.905844599 0.872634848 0.046965681
2 0 0.998207054
ACO1 ENSG00000122729.18 0.663303069 11 9 0.085149698
Brain_Putamen_basal_ganglia 0.864685793 Brain_Anterior_cingulate_cortex_BA24
4.030675205 0.004399052 0.219716797 -1.31486918 1.721557554
0.248869743 0.993137218 9 0 0.998207054
FILIP1 ENSG00000118407.14 0.663414473 3 3 0.288481732
Brain_Cerebellar_Hemisphere 0.802905863 Brain_Putamen_basal_ganglia
1.945941279 0.074439587 0.074439587 -1.061458172 -0.24958815
-0.686385615 0.409439502 3 0 0.998207054
RPA2 ENSG00000117748.9 0.663472641 13 1 0.496718564
Brain_Frontal_Cortex_BA9 0.918795764 Brain_Spinal_cord_cervical_c-1
12.79881439 0.000185658 12.79881439 0.101950754 0.679661903
0.432230883 0.134885053 1 0 0.998207054
ZDHHC6 ENSG00000023041.11 0.663546064 10 10 0.077016067
Brain_Caudate_basal_ganglia 0.88219697 Brain_Anterior_cingulate_cortex_BA24
2.744719657 0.173116882 0.173116882 -1.338819453 1.768268264
0.529407827 0.8873894 10 0 0.998207054
HHIPL1 ENSG00000182218.9 0.663552355 5 5 0.150328934
Brain_Cerebellar_Hemisphere 0.98392694 Brain_Putamen_basal_ganglia 2.087588977
0.45143311 0.45143311 -0.362515945 1.438370585 0.415170386
0.702689818 5 0 0.998207054

UPK1A ENSG00000105668.7 0.663682152 12 6 0.167077415
Brain_Frontal_Cortex_BA9 0.997453561 Brain_Nucleus_accumbens_basal_ganglia
7.225198461 0.008655762 0.429158002 -1.381655788 0.194336785
-0.572610777 0.560038243 6 0 0.998207054
HDAC10 ENSG00000100429.17 0.663727667 13 2 0.312448523
Brain_Nucleus_accumbens_basal_ganglia 0.964669329
Brain_Spinal_cord_cervical_c-1 11.07165891 6.73E-17 0.85951011
-1.010097619 -0.04429491 -0.7660155 0.237051248 2 0
0.998207054
VPS50 ENSG00000004766.15 0.663846427 3 3 0.327810795
Brain_Anterior_cingulate_cortex_BA24 0.880483308 Brain_Cerebellum
1.373142239 0.580775059 0.580775059 -0.509705467 0.978532966
0.106156978 0.776571186 3 0 0.998207054
CREB1 ENSG00000118260.14 0.663867798 1 1 0.663867798
Brain_Putamen_basal_ganglia 0.663867798 Brain_Putamen_basal_ganglia 1 1
1 0.434579334 0.434579334 0.434579334 NA 1 0
0.998207054
ERMP1 ENSG00000099219.13 0.663871192 7 6 0.183331655
Brain_Frontal_Cortex_BA9 0.922164905 Brain_Putamen_basal_ganglia
4.262414514 0.131976403 0.290883779 -1.330566612 0.398518722
-0.565091452 0.612959432 6 0 0.998207054
FAM26E ENSG00000178033.5 0.663949024 1 1 0.663949024
Brain_Hippocampus 0.663949024 Brain_Hippocampus 1 1 1
-0.434467453 -0.434467453 -0.434467453 NA 1 0 0.998207054
CTNNA3 ENSG00000183230.16 0.663962352 2 2 0.397483453
Brain_Putamen_basal_ganglia 0.584158906 Brain_Amygdala 1.84800866 0.15199134
0.15199134 0.54732 0.846124215 0.696722108 0.211286487 2 0
0.998207054
CTD-3074O7.2 ENSG00000250105.1 0.663963043 2 1 0.491577452
Brain_Cerebellar_Hemisphere 0.86647125 Brain_Cerebellum 1.940820022
0.059179978 1.940820022 0.168142405 0.687802039 0.427972222
0.367454851 1 0 0.998207054
TDGF1 ENSG00000241186.9 0.664008792 13 1 0.514486814
Brain_Hippocampus 0.836706489 Brain_Hypothalamus 12.57194563
8.35E-05 12.57194563 0.206108078 0.651867249 0.426757069
0.119371976 1 0 0.998207054
ACBD7 ENSG00000176244.6 0.66402505 1 1 0.66402505
Brain_Nucleus_accumbens_basal_ganglia 0.66402505
Brain_Nucleus_accumbens_basal_ganglia 1 1 1 -0.434362741
-0.434362741 -0.434362741 NA 1 0 0.998207054
FAM83H ENSG00000180921.6 0.664062377 9 9 0.120584151
Brain_Cortex 0.925556539 Brain_Cerebellar_Hemisphere 2.333932583
0.160711685 0.160711685 -1.552326373 1.157869726 -0.141615038
0.728112157 9 0 0.998207054
ABHD17B ENSG00000107362.13 0.664064615 1 1 0.664064615
Brain_Putamen_basal_ganglia 0.664064615 Brain_Putamen_basal_ganglia 1 1
1 -0.434308249 -0.434308249 -0.434308249 NA 1 0
0.998207054
INCA1 ENSG00000196388.8 0.664182576 11 7 0.036133492
Brain_Spinal_cord_cervical_c-1 0.94825323 Brain_Putamen_basal_ganglia
6.382855726 0.003091734 0.300680731 -0.06490049 2.095422038
1.173686356 0.587754255 7 0 0.998207054
C11orf80 ENSG00000173715.15 0.664302906 9 6 0.050515756
Brain_Cerebellar_Hemisphere 0.994679176 Brain_Hypothalamus 5.279315083
0.06779574 0.240137779 -1.955570623 -0.006668714 -0.922683751
0.570277428 6 0 0.998207054
ETV5 ENSG00000244405.7 0.664411869 2 2 0.448092979
Brain_Substantia_nigra 0.692595901 Brain_Hypothalamus 1.122748843
0.877251157 0.877251157 -0.75859815 -0.395334691 -0.576966421
0.256866056 2 0 0.998207054
FOXD4L3 ENSG00000187559.5 0.664425069 2 2 0.459342717
Brain_Nucleus_accumbens_basal_ganglia 0.49929874 Brain_Cortex 1.229295915

0.770704085 0.770704085 -0.739929592 -0.675593546 -0.707761569
0.045492454 2 0 0.998207054
WDR5B ENSG00000196981.3 0.664481269 10 6 0.405281806 Brain_Amygdala
0.905990521 Brain_Nucleus_accumbens_basal_ganglia 6.538081132
0.012452733 0.233445688 -0.832225264 0.807367965 0.276514926
0.43830108 6 0 0.998207054
ITGA2B ENSG00000005961.17 0.664506018 1 1 0.664506018
Brain_Cerebellar_Hemisphere 0.664506018 Brain_Cerebellar_Hemisphere 1 1
1 0.433700398 0.433700398 0.433700398 NA 1 0
0.998207054
FAM81A ENSG00000157470.11 0.664517929 1 1 0.664517929
Brain_Frontal_Cortex_BA9 0.664517929 Brain_Frontal_Cortex_BA9 1 1
1 0.433683997 0.433683997 0.433683997 NA 1 0
0.998207054
TTC23L ENSG00000205838.13 0.664538719 1 1 0.664538719
Brain_Spinal_cord_cervical_c-1 0.664538719 Brain_Spinal_cord_cervical_c-1
1 1 1 0.433655372 0.433655372 0.433655372 NA 1 0
0.998207054
STEAP1 ENSG00000164647.8 0.664632957 2 2 0.366064928
Brain_Frontal_Cortex_BA9 0.449586548 Brain_Cortex 1.841219064
0.158780936 0.158780936 0.756104494 0.903868888 0.829986691
0.104485205 2 0 0.998207054
CHRNA1 ENSG00000138435.15 0.664695796 2 2 0.402715831
Brain_Cortex 0.562683331 Brain_Nucleus_accumbens_basal_ganglia 1.88319785
0.11680215 0.11680215 0.578860462 0.83678073 0.707820596 0.182377171
2 0 0.998207054
OBSL1 ENSG00000124006.14 0.664784854 1 1 0.664784854 Brain_Cortex
0.664784854 Brain_Cortex 1 1 1 0.433316498 0.433316498
0.433316498 NA 1 0 0.998207054
ECM1 ENSG00000143369.14 0.664957668 6 3 0.415874648
Brain_Hippocampus 0.967240511 Brain_Nucleus_accumbens_basal_ganglia
4.632740676 0.009796415 0.45893199 -0.81359911 0.352350678
-0.250447263 0.502004072 3 0 0.998207054
CD300LF ENSG00000186074.18 0.664994041 2 2 0.371594499
Brain_Spinal_cord_cervical_c-1 0.714694059 Brain_Hypothalamus
1.265883431 0.734116569 0.734116569 0.365559154 0.89349061
0.629524882 0.373303913 2 0 0.998207054
MRFAP1L1 ENSG00000178988.10 0.66513898 1 1 0.66513898 Brain_Hypothalamus
0.66513898 Brain_Hypothalamus 1 1 1 -0.432829032 -0.432829032
-0.432829032 NA 1 0 0.998207054
KANSL1 ENSG00000120071.13 0.665272872 11 9 0.066973219
Brain_Cerebellum 0.927004286 Brain_Cortex 4.357695237 0.028131799
0.222049583 -1.831853705 1.236326878 -0.088495794 0.835371762
9 0 0.998207054
ACTL10 ENSG00000182584.4 0.665278973 1 1 0.665278973
Brain_Cortex 0.665278973 Brain_Cortex 1 1 1 0.432636355
0.432636355 0.432636355 NA 1 0 0.998207054
MFAP5 ENSG00000197614.10 0.665303053 2 2 0.366992191 Brain_Cerebellum
0.929495248 Brain_Hippocampus 1.136146724 0.863853276
0.863853276 -0.90212176 -0.088479914 -0.495300837 0.575331667
2 0 0.998207054
ATP11B ENSG00000058063.15 0.665440274 2 2 0.375581965
Brain_Cortex 0.512579473 Brain_Cerebellar_Hemisphere 1.5813013 0.4186987
0.4186987 0.65482649 0.886065997 0.770446243 0.163511023 2 0
0.998207054
ATF7IP ENSG00000171681.12 0.665466356 1 1 0.665466356
Brain_Cortex 0.665466356 Brain_Cortex 1 1 1 0.43237848
0.43237848 0.43237848 NA 1 0 0.998207054
TSPAN13 ENSG00000106537.7 0.66548193 1 1 0.66548193
Brain_Frontal_Cortex_BA9 0.66548193 Brain_Frontal_Cortex_BA9 1 1 1
0.432357048 0.432357048 0.432357048 NA 1 0 0.998207054
RP11-491F9.8 ENSG00000259957.2 0.665506195 2 2 0.374638413

Brain_Cerebellum 0.377717871 Brain_Cerebellar_Hemisphere 1.9237532 0.0762468
0.0762468 -0.887818456 -0.882108991 -0.884963724 0.004037202 2
0 0.998207054
TRHDE ENSG00000072657.8 0.665519248 2 2 0.379703573
Brain_Frontal_Cortex_BA9 0.825863887 Brain_Putamen_basal_ganglia 1.01520817
0.98479183 0.98479183 -0.878442603 -0.220009332 -0.549225967 0.46558263
2 0 0.998207054
C18orf54 ENSG00000166845.13 0.665556769 2 1 0.639324131
Brain_Cerebellum 0.694640441 Brain_Cerebellar_Hemisphere 1.9847665 0.0152335
1.9847665 -0.468643986 -0.392565478 -0.430604732 0.053795629 1
0 0.998207054
KLRD1 ENSG00000134539.16 0.665559966 3 3 0.461688055
Brain_Nucleus_accumbens_basal_ganglia 0.890405893 Brain_Caudate_basal_ganglia
1.854964845 0.200713483 0.200713483 -0.736070071 0.646299074
-0.075853874 0.693262746 3 0 0.998207054
MCL1 ENSG00000143384.12 0.665655441 2 2 0.375782791
Brain_Caudate_basal_ganglia 0.97128214 Brain_Cerebellum 1.151093285
0.848906715 0.848906715 -0.036000274 0.885693353 0.42484654
0.651735814 2 0 0.998207054
RAD17 ENSG00000152942.18 0.665685888 3 3 0.215426811
Brain_Spinal_cord_cervical_c-1 0.7901793 Brain_Cortex 1.984579678
0.274460743 0.274460743 -1.238780462 -0.266077789 -0.812899733
0.497501463 3 0 0.998207054
SLC26A10 ENSG00000135502.17 0.665830329 4 4 0.159936107
Brain_Cerebellar_Hemisphere 0.776236438 Brain_Frontal_Cortex_BA9
1.295652742 0.683740744 0.683740744 -1.40528648 0.284226984
-0.524289204 0.693548628 4 0 0.998207054
RP11-358N2.2 ENSG00000277692.1 0.665918872 2 2 0.548058278
Brain_Hypothalamus 0.637954545 Brain_Cerebellar_Hemisphere 1.288661778
0.711338222 0.711338222 -0.600672291 -0.470560606 -0.535616448
0.092002854 2 0 0.998207054
SLC16A6 ENSG00000108932.11 0.665966635 1 1 0.665966635
Brain_Cortex 0.665966635 Brain_Cortex 1 1 1 -0.43169014
-0.43169014 -0.43169014 NA 1 0 0.998207054
FARSA ENSG00000179115.10 0.665990667 4 3 0.550156417
Brain_Hippocampus 0.98102524 Brain_Caudate_basal_ganglia 3.591029041
0.051493035 0.123631412 -0.597525754 0.088306376 -0.278689617
0.362016836 3 0 0.998207054
UAP1L1 ENSG00000197355.10 0.66611518 3 3 0.342175803 Brain_Cortex
0.720214302 Brain_Cerebellum 1.084775897 0.912957839 0.912957839
-0.949874938 -0.358172398 -0.689997826 0.302341519 3 0
0.998207054
CIZ1 ENSG00000148337.20 0.666222208 2 2 0.37858998 Brain_Hypothalamus
0.862264296 Brain_Caudate_basal_ganglia 1.02133776 0.97866224 0.97866224
-0.880497277 -0.173492534 -0.526994906 0.499927848 2 0
0.998207054
KIAA0355 ENSG00000166398.12 0.666293732 2 2 0.493586166
Brain_Substantia_nigra 0.541979005 Brain_Cerebellum 1.035391197
0.964608803 0.964608803 -0.609823089 0.684616162 0.037396536
0.915306772 2 0 0.998207054
POLE2 ENSG00000100479.12 0.666326867 2 2 0.392207292 Brain_Cerebellum
0.766412175 Brain_Amygdala 1.016961957 0.983038043 0.983038043
-0.855621286 0.29707117 -0.279275058 0.815076652 2 0
0.998207054
PROB1 ENSG00000228672.3 0.66638264 5 4 0.169988259 Brain_Cortex
0.577420515 Brain_Amygdala 3.85003533 0.041098741 0.16776995
0.557156489 1.372241537 1.050763809 0.348510521 4 0
0.998207054
LRRTM4 ENSG00000176204.13 0.666529609 1 1 0.666529609
Brain_Frontal_Cortex_BA9 0.666529609 Brain_Frontal_Cortex_BA9 1 1
1 0.43091578 0.43091578 0.43091578 NA 1 0 0.998207054
KLRC1 ENSG00000134545.13 0.666569141 12 1 0.205720579

Brain_Substantia_nigra 0.856362367 Brain_Anterior_cingulate_cortex_BA24 11.49259353 0.000216639 11.49259353 -1.265420643 -0.181006636 -0.431701941 0.293338028 1 0 0.998207054
MFAP3L ENSG00000198948.11 0.666634 3 3 0.211999577 Brain_Cerebellum 0.99329868 Brain_Hippocampus 1.293725119 0.712858765 0.712858765 0.008398958 1.248085965 0.565480271 0.629303779 3 0 0.998207054
C1QTNF3 ENSG00000082196.20 0.666660525 3 2 0.376087737 Brain_Cerebellum 0.865975746 Brain_Frontal_Cortex_BA9 1.969355819 0.032558601 0.99808558 -0.214214436 0.885127746 0.167380336 0.622002617 2 0 0.998207054
CD151 ENSG00000177697.17 0.666690759 12 2 0.279084628 Brain_Hippocampus 0.85570529 Brain_Anterior_cingulate_cortex_BA24 10.89240823 0.002168283 0.465103599 0.181843826 1.082377937 0.840646651 0.264689869 2 0 0.998207054
MEDAG ENSG00000102802.9 0.666731179 1 1 0.666731179 Brain_Caudate_basal_ganglia 0.666731179 Brain_Caudate_basal_ganglia 1 1 1 -0.430638588 -0.430638588 -0.430638588 NA 1 0 0.998207054
PRTG ENSG00000166450.12 0.666763408 3 3 0.254511868 Brain_Nucleus_accumbens_basal_ganglia 0.725284916 Brain_Cerebellum 1.133779166 0.888794197 0.888794197 -0.351404488 1.13945874 0.44028074 0.749724271 3 0 0.998207054
LINC01658 ENSG00000178248.11 0.666810758 7 6 0.320834015 Brain_Hippocampus 0.888833388 Brain_Cortex 3.165335077 0.090195684 0.35048237 -0.768206891 0.992745461 -0.075568139 0.6270221 6 0 0.998207054
FAM136A ENSG00000035141.7 0.666832201 2 2 0.405801998 Brain_Anterior_cingulate_cortex_BA24 0.886932138 Brain_Nucleus_accumbens_basal_ganglia 1.524813487 0.475186513 0.475186513 -0.831303854 -0.142187205 -0.486745529 0.487279055 2 0 0.998207054
ZNF283 ENSG00000167637.16 0.666879495 3 3 0.264554183 Brain_Nucleus_accumbens_basal_ganglia 0.984836447 Brain_Cerebellar_Hemisphere 1.916137526 0.518382052 0.518382052 -1.115691564 -0.019005839 -0.468686386 0.574354117 3 0 0.998207054
ASPDH ENSG00000204653.9 0.66693431 9 6 0.296350106 Brain_Frontal_Cortex_BA9 0.937562411 Brain_Anterior_cingulate_cortex_BA24 4.806548483 0.012899563 0.248337256 -0.809787954 1.044292448 -0.170294828 0.575199453 6 0 0.998207054
RCN3 ENSG00000142552.7 0.666967439 2 2 0.369770982 Brain_Hypothalamus 0.967704691 Brain_Spinal_cord_cervical_c-1 1.127848178 0.872151822 0.872151822 0.040487226 0.896902433 0.468694829 0.605577001 2 0 0.998207054
KAT6B ENSG00000156650.12 0.666996517 1 1 0.666996517 Brain_Caudate_basal_ganglia 0.666996517 Brain_Caudate_basal_ganglia 1 1 1 0.430273754 0.430273754 0.430273754 NA 1 0 0.998207054
HS3ST4 ENSG00000182601.6 0.667020542 2 2 0.443752457 Brain_Caudate_basal_ganglia 0.621864122 Brain_Cerebellum 1.027196699 0.972803301 0.972803301 -0.765872018 0.493210128 -0.136330945 0.890305524 2 0 0.998207054
RPP40 ENSG00000124787.13 0.667043036 1 1 0.667043036 Brain_Cerebellum 0.667043036 Brain_Cerebellum 1 1 1 -0.430209797 -0.430209797 -0.430209797 NA 1 0 0.998207054
STYX ENSG00000198252.11 0.667059216 8 2 0.243709087 Brain_Nucleus_accumbens_basal_ganglia 0.85791349 Brain_Amygdala 6.507104974 0.010605587 0.901565488 -1.165765951 -0.179030835 -0.801179166 0.348778546 2 0 0.998207054
SPINK9 ENSG00000204909.7 0.667166814 8 2 0.295275713 Brain_Hippocampus 0.842976779 Brain_Cortex 7.409655595 0.000639853 0.271080353 -1.046618181 -0.198087277 -0.564599021

0.28035839 2 0 0.998207054
PTPN9 ENSG00000169410.9 0.667179325 1 1 0.667179325
Brain_Hippocampus 0.667179325 Brain_Hippocampus 1 1 1
0.430022429 0.430022429 0.430022429 NA 1 0 0.998207054
TAL2 ENSG00000186051.6 0.667215154 1 1 0.667215154
Brain_Caudate_basal_ganglia 0.667215154 Brain_Caudate_basal_ganglia 1 1
1 -0.429973175 -0.429973175 -0.429973175 NA 1 0
0.998207054
NRIP1 ENSG00000180530.10 0.667229556 1 1 0.667229556
Brain_Caudate_basal_ganglia 0.667229556 Brain_Caudate_basal_ganglia 1 1
1 -0.429953378 -0.429953378 -0.429953378 NA 1 0
0.998207054
PCDHGA2 ENSG00000081853.14 0.667290832 1 1 0.667290832
Brain_Caudate_basal_ganglia 0.667290832 Brain_Caudate_basal_ganglia 1 1
1 -0.429869144 -0.429869144 -0.429869144 NA 1 0
0.998207054
PTRH1 ENSG00000187024.13 0.667363631 7 3 0.300649784
Brain_Cerebellar_Hemisphere 0.83287781 Brain_Frontal_Cortex_BA9 5.159912331
0.000476638 0.538106085 -0.679812879 1.035040957 -0.036744911
0.590511814 3 0 0.998207054
MRPL51 ENSG00000111639.7 0.66738803 2 2 0.63703983
Brain_Caudate_basal_ganglia 0.691190174 Brain_Cortex 1.532001515
0.467998485 0.467998485 -0.471841635 0.397240426 -0.037300604
0.614533819 2 0 0.998207054
HMGB2 ENSG00000164104.11 0.667399673 1 1 0.667399673
Brain_Hippocampus 0.667399673 Brain_Hippocampus 1 1 1
0.429719533 0.429719533 0.429719533 NA 1 0 0.998207054
KLHL3 ENSG00000146021.14 0.667434062 2 2 0.438232548 Brain_Cerebellum
0.819199594 Brain_Cerebellar_Hemisphere 1.709309356 0.290690644
0.290690644 -0.77518128 -0.228574567 -0.501877924 0.386509313
2 0 0.998207054
TRAK1 ENSG00000182606.14 0.667509704 3 3 0.310005252
Brain_Hypothalamus 0.860635672 Brain_Putamen_basal_ganglia 1.911572908
0.334804434 0.334804434 -0.308704463 1.015211013 0.294023862
0.669859998 3 0 0.998207054
MRPL12 ENSG00000262814.7 0.667619527 3 3 0.382647798
Brain_Cerebellar_Hemisphere 0.670005219 Brain_Substantia_nigra
1.161916952 0.81921455 0.81921455 0.426140845 0.873028034
0.656936377 0.22380615 3 0 0.998207054
AKR1A1 ENSG00000117448.13 0.667637022 2 1 0.594794406
Brain_Cerebellum 0.746564747 Brain_Cerebellar_Hemisphere 1.982742867
0.017257133 1.982742867 -0.531901229 -0.323172314 -0.427536771
0.147593631 1 0 0.998207054
NLRP4 ENSG00000160505.15 0.667639656 2 2 0.380728218
Brain_Substantia_nigra 0.440886776 Brain_Spinal_cord_cervical_c-1
1.722040703 0.277959297 0.277959297 0.77069662 0.876555317
0.823625968 0.074853402 2 0 0.998207054
RP11-302M6.5 ENSG00000272672.1 0.667665563 1 1 0.667665563
Brain_Substantia_nigra 0.667665563 Brain_Substantia_nigra 1 1
1 -0.429354084 -0.429354084 -0.429354084 NA 1 0
0.998207054
BCL6B ENSG00000161940.10 0.667729631 1 1 0.667729631
Brain_Hippocampus 0.667729631 Brain_Hippocampus 1 1 1
-0.429266036 -0.429266036 -0.429266036 NA 1 0 0.998207054
LPIN2 ENSG00000101577.9 0.667838936 7 5 0.220222699 Brain_Cortex
0.749046468 Brain_Frontal_Cortex_BA9 4.162807818 0.022395391
0.225244306 -0.704734525 1.225936162 0.632022091 0.822005933
5 0 0.998207054
ZNF268 ENSG00000090612.20 0.667873921 8 4 0.120818574
Brain_Spinal_cord_cervical_c-1 0.838982205 Brain_Cerebellar_Hemisphere
6.243859529 0.018539821 0.220647603 0.203195531 1.551346897
0.511402139 0.439722383 4 0 0.998207054

USP34 ENSG00000115464.14 0.668413921 1 1 0.668413921 Brain_Cerebellum 0.668413921 Brain_Cerebellum 1 1 1 0.428325822 0.428325822 0.428325822 NA 1 0 0.998207054
ALG8 ENSG00000159063.12 0.668419671 13 4 0.328322013 Brain_Caudate_basal_ganglia 0.98061575 Brain_Spinal_cord_cervical_c-1 10.08405925 0.001081505 0.402560955 -0.977499323 0.61706647 -0.091033867 0.486665534 4 0 0.998207054
DDIT4L ENSG00000145358.6 0.668432417 6 6 0.204304814 Brain_Substantia_nigra 0.946234652 Brain_Putamen_basal_ganglia 1.8269104 0.342666118 0.342666118 -1.269382109 0.194345993 -0.517183965 0.590607834 6 0 0.998207054
SLC18A3 ENSG00000187714.6 0.668433967 1 1 0.668433967 Brain_Cerebellar_Hemisphere 0.668433967 Brain_Cerebellar_Hemisphere 1 1 1 -0.428298285 -0.428298285 -0.428298285 NA 1 0 0.998207054
LINC02185 ENSG00000262097.1 0.668695914 1 1 0.668695914 Brain_Putamen_basal_ganglia 0.668695914 Brain_Putamen_basal_ganglia 1 1 1 0.427938476 0.427938476 0.427938476 NA 1 0 0.998207054
NEXN ENSG00000162614.18 0.668768135 3 3 0.324393707 Brain_Nucleus_accumbens_basal_ganglia 0.771377251 Brain_Spinal_cord_cervical_c-1 1.380666597 0.624212587 0.624212587 -0.985469089 0.290573864 -0.489064267 0.683547537 3 0 0.998207054
PARP9 ENSG00000138496.16 0.668783158 3 2 0.431531292 Brain_Amygdala 0.66990851 Brain_Substantia_nigra 2.001201085 0.044026972 0.954771942 -0.786573987 0.426273576 -0.329496442 0.65928954 2 0 0.998207054
TCP1 ENSG00000120438.11 0.668834968 3 2 0.385074866 Brain_Hippocampus 0.444744947 Brain_Cortex 2.590315782 0.077436333 0.332247884 0.764205236 0.868583712 0.831248884 0.058186571 2 0 0.998207054
BCL7A ENSG00000110987.8 0.668859309 1 1 0.668859309 Brain_Spinal_cord_cervical_c-1 0.668859309 Brain_Spinal_cord_cervical_c-1 1 1 1 -0.427714064 -0.427714064 -0.427714064 NA 1 0 0.998207054
CSNK1G1 ENSG00000169118.17 0.668901326 1 1 0.668901326 Brain_Caudate_basal_ganglia 0.668901326 Brain_Caudate_basal_ganglia 1 1 1 0.427656361 0.427656361 0.427656361 NA 1 0 0.998207054
FAM71E1 ENSG00000142530.10 0.668930465 2 2 0.369910045 Brain_Nucleus_accumbens_basal_ganglia 0.748247924 Brain_Cerebellum 1.343753526 0.656246474 0.656246474 0.320950473 0.896641877 0.608796175 0.407075295 2 0 0.998207054
AC005753.1 ENSG00000278925.1 0.668941109 2 2 0.710049165 Brain_Cerebellar_Hemisphere 0.730650511 Brain_Cerebellum 1.681096793 0.318903207 0.318903207 -0.344260335 0.371790059 0.013764862 0.50632409 2 0 0.998207054
POLR2J2 ENSG00000228049.7 0.669001386 13 2 0.626665151 Brain_Cerebellar_Hemisphere 0.95587398 Brain_Putamen_basal_ganglia 11.78397446 0.005664341 0.523431022 -0.486426013 0.382841531 -0.179949973 0.249644423 2 0 0.998207054
ZNF726 ENSG00000213967.10 0.669044286 10 8 0.138977994 Brain_Spinal_cord_cervical_c-1 0.9429358 Brain_Amygdala 3.298010046 0.004256417 0.114109425 -1.479607611 0.770956621 -0.398031385 0.671169587 8 0 0.998207054
CTB-88F18.5 ENSG00000283897.1 0.669048871 1 1 0.669048871 Brain_Putamen_basal_ganglia 0.669048871 Brain_Putamen_basal_ganglia 1 1 1 0.427453741 0.427453741 0.427453741 NA 1 0 0.998207054
LARP7 ENSG00000174720.15 0.669111268 4 4 0.4504599 Brain_Cerebellum 0.85013193 Brain_Substantia_nigra 2.827501337 0.203759196

0.203759196 -0.754648525 0.621034836 0.049283496 0.645820182
4 0 0.998207054
CAPS ENSG00000105519.15 0.669407914 6 4 0.121609573
Brain_Cerebellar_Hemisphere 0.545855544 Brain_Nucleus_accumbens_basal_ganglia
4.668770992 0.030186989 0.218988121 0.603982098 1.548052847
1.103337882 0.310020981 4 0 0.998207054
NOTCH3 ENSG00000074181.8 0.669621331 1 1 0.669621331
Brain_Frontal_Cortex_BA9 0.669621331 Brain_Frontal_Cortex_BA9 1 1
1 -0.426667768 -0.426667768 -0.426667768 NA 1 0
0.998207054
RP11-397O4.1 ENSG00000229206.3 0.669653458 2 2 0.465052717
Brain_Caudate_basal_ganglia 0.626978695 Brain_Cerebellar_Hemisphere
1.043111818 0.956888182 0.956888182 -0.48598374 0.730552202
0.122284231 0.860220814 2 0 0.998207054
PCDHB12 ENSG00000120328.6 0.669656868 7 2 0.289253021
Brain_Hippocampus 0.880044705 Brain_Hypothalamus 6.128276226
4.94E-07 0.642246397 -1.059761709 0.150912544 -0.601119195
0.3880727 2 0 0.998207054
LINC01315 ENSG00000229891.1 0.669836196 2 2 0.440343435
Brain_Cerebellum 0.808794366 Brain_Putamen_basal_ganglia 1.255207802
0.744792198 0.744792198 -0.771613401 0.241981671 -0.264815865
0.716719949 2 0 0.998207054
CYFIP2 ENSG00000055163.19 0.66983813 1 1 0.66983813 Brain_Cerebellum
0.66983813 Brain_Cerebellum 1 1 1 0.426370175 0.426370175
0.426370175 NA 1 0 0.998207054
ASF1A ENSG00000111875.7 0.669872791 3 3 0.294353931 Brain_Cerebellum
0.675732908 Brain_Cerebellar_Hemisphere 1.868536436 0.227351267
0.227351267 -1.04861808 0.460699148 -0.335403977 0.758065014
3 0 0.998207054
TECTB ENSG00000119913.4 0.669923407 9 3 0.152972953
Brain_Putamen_basal_ganglia 0.541560714 Brain_Anterior_cingulate_cortex_BA24
7.742023142 8.77E-05 0.280543154 -1.429108839 -0.610454592
-1.042915031 0.236682762 3 0 0.998207054
SMG1 ENSG00000157106.16 0.669933918 2 1 0.602767564 Brain_Cerebellum
0.740501651 Brain_Cerebellar_Hemisphere 1.995950073 0.004049927
1.995950073 -0.520424749 -0.331189102 -0.425806926 0.133809809
1 0 0.998207054
CDKN3 ENSG00000100526.19 0.670163933 1 1 0.670163933
Brain_Nucleus_accumbens_basal_ganglia 0.670163933
Brain_Nucleus_accumbens_basal_ganglia 1 1 1 0.42592303 0.42592303
0.42592303 NA 1 0 0.998207054
CLINT1 ENSG00000113282.13 0.670210546 1 1 0.670210546
Brain_Amygdala 0.670210546 Brain_Amygdala 1 1 1 -0.425859063
-0.425859063 -0.425859063 NA 1 0 0.998207054
CMBL ENSG00000164237.8 0.6703604 1 1 0.6703604 Brain_Cerebellar_Hemisphere
0.6703604 Brain_Cerebellar_Hemisphere 1 1 1 -0.425653431
-0.425653431 -0.425653431 NA 1 0 0.998207054
CSTF3 ENSG00000176102.11 0.670364789 2 2 0.522065942
Brain_Anterior_cingulate_cortex_BA24 0.560087656
Brain_Spinal_cord_cervical_c-1 1.063386525 0.936613475 0.936613475
-0.640164066 0.582711313 -0.028726377 0.864703473 2 0
0.998207054
FIP1L1 ENSG00000145216.15 0.670421668 1 1 0.670421668
Brain_Hypothalamus 0.670421668 Brain_Hypothalamus 1 1 1
0.425569364 0.425569364 0.425569364 NA 1 0 0.998207054
NMNAT3 ENSG00000163864.15 0.670616671 7 7 0.118255803
Brain_Hippocampus 0.756111646 Brain_Frontal_Cortex_BA9 2.245935396
0.129728166 0.129728166 -1.193455417 1.562136638 0.301467132
0.972422842 7 0 0.998207054
RP11-315D16.4 ENSG00000260657.2 0.670681172 12 5 0.43254523
Brain_Cerebellar_Hemisphere 0.994168872 Brain_Spinal_cord_cervical_c-1
8.307577478 0.002982912 0.402102312 -0.784843674 0.719139623

-0.042718688 0.461344335 5 0 0.998207054
ZNF74 ENSG00000185252.18 0.670746843 1 1 0.670746843
Brain_Anterior_cingulate_cortex_BA24 0.670746843
Brain_Anterior_cingulate_cortex_BA24 1 1 1 0.425123232
0.425123232 0.425123232 NA 1 0 0.998207054
NFYB ENSG00000120837.7 0.670803335 4 4 0.442888138 Brain_Cortex
0.999167301 Brain_Cerebellum 2.525775205 0.19926633 0.19926633
-0.767325285 0.530384637 0.041858196 0.584740266 4 0
0.998207054
RP11-225B17.2 ENSG00000273014.1 0.671139299 4 4 0.19666584
Brain_Anterior_cingulate_cortex_BA24 0.789653716 Brain_Caudate_basal_ganglia
2.559157407 0.34171964 0.34171964 0.266760309 1.291109126
0.699894571 0.438091093 4 0 0.998207054
RP11-273G15.2 ENSG00000247317.3 0.671165113 10 5 0.157981213
Brain_Cerebellar_Hemisphere 0.852590908 Brain_Hypothalamus 7.262691251
0.000755051 0.243945303 -1.411893869 -0.185813639 -1.101451944
0.357225705 5 0 0.998207054
PTPN2 ENSG00000175354.18 0.671176129 1 1 0.671176129
Brain_Nucleus_accumbens_basal_ganglia 0.671176129
Brain_Nucleus_accumbens_basal_ganglia 1 1 1 -0.424534392
-0.424534392 -0.424534392 NA 1 0 0.998207054
C2CD2 ENSG00000157617.16 0.671223646 1 1 0.671223646
Brain_Spinal_cord_cervical_c-1 0.671223646 Brain_Spinal_cord_cervical_c-1
1 1 1 -0.424469224 -0.424469224 -0.424469224 NA 1 0
0.998207054
RP11-286B14.1 ENSG00000228971.2 0.671230727 3 3 0.368634177
Brain_Frontal_Cortex_BA9 0.99600062 Brain_Substantia_nigra 1.943909253
0.158768681 0.158768681 0.0050125 0.899034701 0.396425399
0.457266229 3 0 0.998207054
GLRX5 ENSG00000182512.4 0.67126059 7 7 0.123675176
Brain_Spinal_cord_cervical_c-1 0.989664568 Brain_Putamen_basal_ganglia
3.084721004 0.2214626 0.2214626 -0.868370499 1.539529113 0.10501859
0.755361215 7 0 0.998207054
SLC6A7 ENSG00000011083.8 0.671266622 3 3 0.296632475
Brain_Cerebellar_Hemisphere 0.692292315 Brain_Cerebellum 1.515943027
0.481264063 0.481264063 -0.627903293 1.043682141 0.270508329
0.842800598 3 0 0.998207054
CCDC144NL ENSG00000205212.3 0.671384471 11 5 0.205934423
Brain_Hypothalamus 0.822255251 Brain_Frontal_Cortex_BA9 5.865797878
0.002578499 0.281401113 0.224645262 1.264824007 0.851315127
0.365640869 5 0 0.998207054
CLIC6 ENSG00000159212.12 0.671423257 9 4 0.211170985
Brain_Putamen_basal_ganglia 0.623430661 Brain_Cortex 6.715440963
0.000771335 0.300417927 -1.250352016 -0.490994043 -0.97036535
0.295864475 4 0 0.998207054
HES4 ENSG00000188290.10 0.671487953 1 1 0.671487953 Brain_Cortex
0.671487953 Brain_Cortex 1 1 1 -0.424106763 -0.424106763
-0.424106763 NA 1 0 0.998207054
PSD4 ENSG00000125637.15 0.671488624 7 2 0.37599665
Brain_Spinal_cord_cervical_c-1 0.837321169 Brain_Caudate_basal_ganglia
5.250243706 0.011131345 1.358656629 -0.885296662 0.205321216
-0.584741098 0.394681151 2 0 0.998207054
GGA2 ENSG00000103365.15 0.671494578 1 1 0.671494578
Brain_Nucleus_accumbens_basal_ganglia 0.671494578
Brain_Nucleus_accumbens_basal_ganglia 1 1 1 -0.424097679
-0.424097679 -0.424097679 NA 1 0 0.998207054
PSMD11 ENSG00000108671.9 0.671516557 1 1 0.671516557
Brain_Hypothalamus 0.671516557 Brain_Hypothalamus 1 1 1
-0.42406754 -0.42406754 -0.42406754 NA 1 0 0.998207054
CA4 ENSG00000167434.9 0.671564242 2 2 0.380494764
Brain_Caudate_basal_ganglia 0.89918888 Brain_Frontal_Cortex_BA9 1.043384827
0.956615173 0.956615173 -0.126686059 0.87698504 0.37514949

0.709702641	2	0	0.998207054
CROCC	ENSG00000058453.16	0.671603611	11	9	0.086450274
Brain_Hypothalamus	0.739595457	Brain_Substantia_nigra	4.214702242
0.072715001	0.292631901	-0.713680186	1.714427325	0.946744452
0.677468869	9	0	0.998207054
PLEKHH2	ENSG00000152527.13	0.671774779	11	8	0.057084309
Brain_Spinal_cord_cervical_c-1	0.867687399
Brain_Anterior_cingulate_cortex_BA24	5.253013297	0.05549106	0.175343769
-0.814924815	1.902664711	0.430817795	0.722659474	8	0
0.998207054
KRT31	ENSG00000094796.4	0.671826357	5	5	0.199460114
Brain_Cerebellar_Hemisphere	0.657471028	Brain_Anterior_cingulate_cortex_BA24
2.323662066	0.079791633	0.079791633	0.443407478	1.283091236
0.859454975	0.321662202	5	0	0.998207054
PJA2	ENSG00000198961.9	0.67193368	5	5	0.196051687
Brain_Anterior_cingulate_cortex_BA24	0.817340613	Brain_Hippocampus
1.279244502	0.700981643	0.700981643	-1.292882542	0.71618691
-0.228517597	0.85429306	5	0	0.998207054
RP11-1055B8.2	ENSG00000263154.1	0.671951506	9	8	0.062007789
Brain_Spinal_cord_cervical_c-1	0.834996636	Brain_Putamen_basal_ganglia
3.945604455	0.019577251	0.221853845	-1.866240044	0.208297561
-0.956513604	0.567652135	8	0	0.998207054
RP11-473M20.16	ENSG00000261889.1	0.671978026	1	1	0.671978026
Brain_Hippocampus	0.671978026	Brain_Hippocampus	1	1	1
-0.423434846	-0.423434846	-0.423434846	NA	1	0	0.998207054
LINC01732	ENSG00000237292.1	0.671990929	1	1	0.671990929
Brain_Hippocampus	0.671990929	Brain_Hippocampus	1	1	1
-0.423417157	-0.423417157	-0.423417157	NA	1	0	0.998207054
KLRG1	ENSG00000139187.9	0.672020746	12	1	0.478528593
Brain_Hippocampus	0.833638356	Brain_Anterior_cingulate_cortex_BA24
11.65286407	9.08E-05	11.65286407	0.210037563	0.708671116
0.417964029	0.139573677	1	0	0.998207054
CCDC110	ENSG00000168491.9	0.672126127	9	2	0.450423324
Brain_Hippocampus	0.993279697	Brain_Cortex	7.97732429	0.001210268
0.277626888	-0.382853684	0.754709469	0.292198615	0.3373016	2
0	0.998207054
RP11-65J3.3	ENSG00000230676.1	0.672339417	1	1	0.672339417
Brain_Nucleus_accumbens_basal_ganglia	0.672339417
Brain_Nucleus_accumbens_basal_ganglia	1	1	1	0.422939481
0.422939481	0.422939481	NA	1	0	0.998207054
SNAPC5	ENSG00000174446.12	0.672359792	5	5	0.194922786
Brain_Hippocampus	0.980963619	Brain_Anterior_cingulate_cortex_BA24
1.863722314	0.426531367	0.426531367	-0.375298824	1.296152979
0.317250684	0.685471824	5	0	0.998207054
LINC00159	ENSG00000230323.5	0.672562757	2	2	0.393848456
Brain_Cerebellar_Hemisphere	0.799531015	Brain_Frontal_Cortex_BA9
1.004124527	0.995875473	0.995875473	0.253954105	0.852658961
0.553306533	0.423348263	2	0	0.998207054
RP11-662J14.1	ENSG00000258418.1	0.672624099	1	1	0.672624099
Brain_Cortex	0.672624099	Brain_Cortex	1	1	1	0.422549335
0.422549335	0.422549335	NA	1	0	0.998207054
RP11-503C24.2	ENSG00000237987.1	0.672626614	2	1	0.610704684
Brain_Caudate_basal_ganglia	0.747097602	Brain_Putamen_basal_ganglia	1.93635493
0.06364507	1.93635493	-0.509067824	-0.322468758	-0.415768291
0.131945465	1	0	0.998207054
RP11-28H5.2	ENSG00000271983.1	0.672641296	1	1	0.672641296
Brain_Putamen_basal_ganglia	0.672641296	Brain_Putamen_basal_ganglia	1	1
1	-0.422525768	-0.422525768	-0.422525768	NA	1	0
0.998207054
LTA4H	ENSG00000111144.9	0.672661332	1	1	0.672661332
Brain_Substantia_nigra	0.672661332	Brain_Substantia_nigra	1	1
1	0.422498312	0.422498312	0.422498312	NA	1	0

0.998207054
RP11-534L20.5 ENSG00000261000.1 0.672695726 5 2 0.295776718
Brain_Caudate_basal_ganglia 0.471589351 Brain_Putamen_basal_ganglia
4.636815183 0.013526926 0.254889912 -1.045532953 -0.71989548
-0.842393649 0.129506105 2 0 0.998207054
PTDSS2 ENSG00000174915.11 0.672704457 6 3 0.436600382
Brain_Cortex 0.945910033 Brain_Frontal_Cortex_BA9 5.166030202
0.022907927 0.259697635 -0.777946791 0.210906283 -0.377961366
0.398516083 3 0 0.998207054
SMCP ENSG00000163206.5 0.672735912 3 1 0.535365053
Brain_Cerebellar_Hemisphere 0.823716161 Brain_Cerebellum 2.840492685
0.06907936 2.840492685 0.222767889 0.619837082 0.410603076
0.199397638 1 0 0.998207054
SERPINC1 ENSG00000117601.13 0.672773964 9 1 0.295067666
Brain_Putamen_basal_ganglia 0.836379404 Brain_Nucleus_accumbens_basal_ganglia
8.43774225 0.00035201 8.43774225 -1.047069195 -0.206526837 -0.414805399
0.244619031 1 0 0.998207054
GPIHBP1 ENSG00000277494.1 0.672802202 13 8 0.091516847
Brain_Hypothalamus 0.931066524 Brain_Amygdala 6.810653318
0.006171717 0.341256989 -1.687450052 1.101469601 -0.189728873
0.700244713 8 0 0.998207054
MXD1 ENSG00000059728.10 0.672802784 1 1 0.672802784
Brain_Frontal_Cortex_BA9 0.672802784 Brain_Frontal_Cortex_BA9 1 1
1 -0.422304486 -0.422304486 -0.422304486 NA 1 0
0.998207054
RP11-321L2.1 ENSG00000231460.2 0.672830197 1 1 0.672830197
Brain_Cerebellar_Hemisphere 0.672830197 Brain_Cerebellar_Hemisphere 1 1
1 -0.422266925 -0.422266925 -0.422266925 NA 1 0
0.998207054
DDX50 ENSG00000107625.12 0.672870977 4 4 0.290178029
Brain_Nucleus_accumbens_basal_ganglia 0.762373829 Brain_Substantia_nigra
1.110378193 0.922732267 0.922732267 -1.057731152 0.321782857
-0.343524815 0.758572582 4 0 0.998207054
C2orf49 ENSG00000135974.9 0.672876671 1 1 0.672876671
Brain_Cortex 0.672876671 Brain_Cortex 1 1 1 0.422203248
0.422203248 0.422203248 NA 1 0 0.998207054
TBC1D13 ENSG00000107021.15 0.672978873 1 1 0.672978873
Brain_Anterior_cingulate_cortex_BA24 0.672978873
Brain_Anterior_cingulate_cortex_BA24 1 1 1 0.42206322 0.42206322
0.42206322 NA 1 0 0.998207054
RP11-508N22.12 ENSG00000272983.1 0.673137414 12 2 0.353270543
Brain_Caudate_basal_ganglia 0.669347993 Brain_Cerebellar_Hemisphere
10.75572005 0.001035762 0.430047803 0.427043021 0.928264192
0.749604687 0.138727652 2 0 0.998207054
SUGP1 ENSG00000105705.15 0.673244132 2 2 0.47460579 Brain_Cortex
0.62940241 Brain_Hippocampus 1.06372684 0.93627316 0.93627316 -0.482568134
0.715005265 0.116218565 0.846812271 2 0 0.998207054
FLOT1 ENSG00000137312.14 0.673253136 8 8 0.339201766
Brain_Hippocampus 0.974080606 Brain_Cerebellum 3.925958463
0.160560488 0.160560488 -0.955743646 0.855270728 0.083190754
0.589973053 8 0 0.998207054
AMIGO2 ENSG00000139211.6 0.673268942 3 3 0.244036813
Brain_Spinal_cord_cervical_c-1 0.601277156 Brain_Cerebellum 1.440352185
0.576255255 0.576255255 -0.522564779 1.164955977 0.422137612
0.861684075 3 0 0.998207054
NKX2-5 ENSG00000183072.9 0.673485206 2 2 0.431966066
Brain_Cerebellum 0.681629567 Brain_Cerebellar_Hemisphere 1.007301307
0.992698693 0.992698693 0.410240455 0.785831746 0.5980361
0.265583148 2 0 0.998207054
MYO15A ENSG00000091536.16 0.673553652 8 4 0.364073375
Brain_Caudate_basal_ganglia 0.953854966 Brain_Hypothalamus 5.255262484
0.002708307 0.284496173 -0.907630689 0.821746306 0.356394319

0.56742563 4 0 0.998207054
PFAS ENSG00000178921.13 0.673981698 2 2 0.375911145
Brain_Caudate_basal_ganglia 0.523584312 Brain_Cortex 1.771520219
0.228479781 0.228479781 -0.88545525 -0.637830057 -0.761642653
0.175097453 2 0 0.998207054
KATNAL1 ENSG00000102781.13 0.674003371 5 5 0.19921065
Brain_Putamen_basal_ganglia 0.84300927 Brain_Cerebellum 2.46665589 0.139740556
0.139740556 -1.283803695 0.675476454 -0.182780017 0.801237783
5 0 0.998207054
ZNHIT6 ENSG00000117174.10 0.674096302 6 5 0.342699622
Brain_Cerebellum 0.999927905 Brain_Cortex 4.231251365 0.129071188
0.157342263 -0.948844664 0.212389568 -0.358165006 0.466748687
5 0 0.998207054
RP11-81A22.5 ENSG00000262165.2 0.674197354 13 1 0.502668962
Brain_Putamen_basal_ganglia 0.852288943 Brain_Cerebellum 12.51540519
0.000322191 12.51540519 0.186198701 0.670296242 0.412441915
0.158692295 1 0 0.998207054
LRRC8B ENSG00000197147.13 0.674228852 6 5 0.103595584
Brain_Frontal_Cortex_BA9 0.610849854 Brain_Hippocampus 2.7940721
0.042338032 0.190251146 -1.413104069 1.627666672 -0.083438173
1.206503183 5 0 0.998207054
MED16 ENSG00000175221.14 0.674270668 5 3 0.471421757 Brain_Cortex
0.702430309 Brain_Hypothalamus 3.727896685 0.01187706 0.403842279
-0.382041869 0.720167687 0.355849073 0.430474049 3 0
0.998207054
CABIN1 ENSG00000099991.17 0.674294872 2 2 0.458795875
Brain_Cerebellar_Hemisphere 0.839841186 Brain_Frontal_Cortex_BA9
1.726551276 0.273448724 0.273448724 0.202096625 0.740831068
0.471463847 0.380942778 2 0 0.998207054
MPLKIP ENSG00000168303.6 0.67430233 1 1 0.67430233 Brain_Cortex
0.67430233 Brain_Cortex 1 1 1 0.420250687 0.420250687
0.420250687 NA 1 0 0.998207054
PGBD1 ENSG00000137338.5 0.674307464 7 6 0.170159402
Brain_Anterior_cingulate_cortex_BA24 0.703171467 Brain_Cerebellar_Hemisphere
3.255129289 0.042385517 0.218899885 -0.412999728 1.371691801
0.622586487 0.571410337 6 0 0.998207054
PRKG1 ENSG00000185532.15 0.674412147 12 6 0.094211889
Brain_Hypothalamus 0.964096479 Brain_Spinal_cord_cervical_c-1
7.877035818 0.004009006 0.382044495 -0.325269598 1.673586521
0.303182455 0.535535897 6 0 0.998207054
KRTAP5-10 ENSG00000204572.9 0.674457027 5 2 0.310517524
Brain_Nucleus_accumbens_basal_ganglia 0.797732639 Brain_Hippocampus
4.00634203 0.000659645 0.950629427 -1.014136708 -0.256282591
-0.754217108 0.295450813 2 0 0.998207054
PLA2G4E ENSG00000188089.13 0.674506527 1 1 0.674506527
Brain_Caudate_basal_ganglia 0.674506527 Brain_Caudate_basal_ganglia 1 1
1 -0.419971153 -0.419971153 -0.419971153 NA 1 0
0.998207054
SGO2 ENSG00000163535.17 0.674536224 2 2 0.37906296 Brain_Cortex
0.951681069 Brain_Hypothalamus 1.064006205 0.935993795
0.935993795 0.060595862 0.879624134 0.470109998 0.579140445
2 0 0.998207054
NOL6 ENSG00000165271.16 0.674578092 11 5 0.439532544 Brain_Cortex
0.998015109 Brain_Hippocampus 8.087726835 0.02655086 0.277690766
-0.772982826 0.598546809 -0.119582717 0.44714284 5 0
0.998207054
UBE2E3 ENSG00000170035.15 0.674643031 3 3 0.37604615
Brain_Cerebellar_Hemisphere 0.730086225 Brain_Cerebellum 1.206944733
0.855355901 0.855355901 -0.885204863 0.683784442 0.047863471
0.825623569 3 0 0.998207054
SF3B5 ENSG00000169976.6 0.674802728 1 1 0.674802728
Brain_Caudate_basal_ganglia 0.674802728 Brain_Caudate_basal_ganglia 1 1

1 -0.41956573 -0.41956573 -0.41956573 NA 1 0 0.998207054
MED4 ENSG00000136146.14 0.674870462 2 2 0.397042653 Brain_Cerebellar_Hemisphere 0.40511927 Brain_Cerebellum 1.794277543 0.205722457 0.205722457 -0.846914728 -0.832513307 -0.839714017 0.010183343 2 0 0.998207054
MYOF ENSG00000138119.16 0.674907531 2 2 0.478391705 Brain_Spinal_cord_cervical_c-1 0.752076418 Brain_Caudate_basal_ganglia 1.276589657 0.723410343 0.723410343 -0.708891669 0.315902627 -0.196494521 0.724638997 2 0 0.998207054
LINC02177 ENSG00000261617.1 0.674908462 7 3 0.481547662 Brain_Cortex 0.965754557 Brain_Putamen_basal_ganglia 5.816371794 0.042069436 0.360570223 -0.703815529 -0.042933484 -0.395147094 0.283410286 3 0 0.998207054
PINLYP ENSG00000234465.10 0.674925874 11 5 0.221723934 Brain_Cortex 0.995814275 Brain_Frontal_Cortex_BA9 7.172924868 0.001083669 0.296627897 -1.221956878 0.938323534 0.049279717 0.652270174 5 0 0.998207054
HERPUD1 ENSG00000051108.14 0.67520073 1 1 0.67520073 Brain_Frontal_Cortex_BA9 0.67520073 Brain_Frontal_Cortex_BA9 1 1 1 -0.419021075 -0.419021075 -0.419021075 NA 1 0 0.998207054
TSPAN15 ENSG00000099282.9 0.67523093 10 9 0.154454442 Brain_Frontal_Cortex_BA9 0.963729835 Brain_Substantia_nigra 3.862297216 0.09322913 0.131118679 -1.423972468 0.657546712 -0.234944867 0.655318763 9 0 0.998207054
PLIN1 ENSG00000166819.11 0.675317747 6 5 0.154169304 Brain_Frontal_Cortex_BA9 0.965962229 Brain_Nucleus_accumbens_basal_ganglia 2.752983431 0.040959253 0.298182364 -1.424958132 0.042672967 -0.645005943 0.559060231 5 0 0.998207054
FAM184B ENSG00000047662.4 0.675406079 3 2 0.493066114 Brain_Cerebellum 0.869779385 Brain_Caudate_basal_ganglia 2.769169972 0.06707805 0.163751977 -0.16393872 0.685440312 0.374787134 0.468373829 2 0 0.998207054
PGP ENSG00000184207.8 0.675581996 1 1 0.675581996 Brain_Nucleus_accumbens_basal_ganglia 0.675581996 Brain_Nucleus_accumbens_basal_ganglia 1 1 1 -0.41849944 -0.41849944 -0.41849944 NA 1 0 0.998207054
TM9SF4 ENSG00000101337.15 0.675683937 1 1 0.675683937 Brain_Hippocampus 0.675683937 Brain_Hippocampus 1 1 1 -0.418359987 -0.418359987 -0.418359987 NA 1 0 0.998207054
FRMD3 ENSG00000172159.15 0.67572704 2 2 0.385336388 Brain_Cerebellar_Hemisphere 0.739615883 Brain_Spinal_cord_cervical_c-1 1.185753573 0.814246427 0.814246427 0.332362061 0.868105852 0.600233956 0.378828068 2 0 0.998207054
QSOX1 ENSG00000116260.16 0.675767468 4 3 0.537485793 Brain_Cerebellar_Hemisphere 0.980170784 Brain_Nucleus_accumbens_basal_ganglia 3.456482104 0.03072896 0.173564146 -0.616619403 0.323985433 -0.024144646 0.413433428 3 0 0.998207054
STK35 ENSG00000125834.12 0.675826774 3 3 0.321385834 Brain_Frontal_Cortex_BA9 0.753663544 Brain_Cerebellum 1.247326954 0.804414682 0.804414682 -0.991614048 0.313812377 -0.435536719 0.673832338 3 0 0.998207054
FAM46A ENSG00000112773.15 0.675838229 2 2 0.766576299 Brain_Cerebellar_Hemisphere 0.929436492 Brain_Cerebellum 1.903820618 0.096179382 0.096179382 -0.296856198 0.088553843 -0.104151177 0.272526054 2 0 0.998207054
AC007386.2 ENSG00000237638.1 0.675860439 3 3 0.262930395 Brain_Cerebellum 0.38923038 Brain_Caudate_basal_ganglia 2.007397905 0.411888242 0.411888242 0.861013913 1.119491781 0.981327302 0.130160279 3 0 0.998207054
NLRP6 ENSG00000174885.12 0.676053668 1 1 0.676053668 Brain_Spinal_cord_cervical_c-1 0.676053668 Brain_Spinal_cord_cervical_c-1

1 1 1 0.417854271 0.417854271 0.417854271 NA 1 0
0.998207054
FAM98C ENSG00000130244.12 0.676081382 7 4 0.485135346
Brain_Cerebellum 0.907798976 Brain_Putamen_basal_ganglia 5.552015365
0.0006835 0.262030921 -0.565662511 0.698066917 -0.216806998
0.438523728 4 0 0.998207054
KRTCAP2 ENSG00000163463.11 0.676183828 1 1 0.676183828
Brain_Substantia_nigra 0.676183828 Brain_Substantia_nigra 1 1
1 -0.417676265 -0.417676265 -0.417676265 NA 1 0
0.998207054
LINC01896 ENSG00000263146.2 0.676459423 1 1 0.676459423
Brain_Caudate_basal_ganglia 0.676459423 Brain_Caudate_basal_ganglia 1 1
1 -0.417299406 -0.417299406 -0.417299406 NA 1 0
0.998207054
CDKN2AIPNL ENSG00000237190.3 0.676571097 8 8 0.075341193
Brain_Cortex 0.937122528 Brain_Hypothalamus 1.70392172 0.478493258
0.478493258 -0.827780924 1.778381661 0.355746817 0.846432556
8 0 0.998207054
SSRP1 ENSG00000149136.8 0.676626542 2 2 0.386715929
Brain_Cerebellar_Hemisphere 0.437116083 Brain_Cerebellum 1.764446709
0.235553291 0.235553291 0.777072355 0.865588382 0.821330368
0.062590283 2 0 0.998207054
LINC01537 ENSG00000227467.3 0.676692633 8 7 0.45746627
Brain_Nucleus_accumbens_basal_ganglia 0.713902603
Brain_Anterior_cingulate_cortex_BA24 2.919681946 0.043833216
0.244102593 -0.743025455 0.72080482 -0.093170113 0.691612958 7
0 0.998207054
CCDC138 ENSG00000163006.11 0.676832985 1 1 0.676832985
Brain_Cerebellum 0.676832985 Brain_Cerebellum 1 1 1 0.416788677
0.416788677 0.416788677 NA 1 0 0.998207054
RBM27 ENSG00000091009.7 0.676920918 1 1 0.676920918
Brain_Cerebellar_Hemisphere 0.676920918 Brain_Cerebellar_Hemisphere 1 1
1 0.416668472 0.416668472 0.416668472 NA 1 0
0.998207054
HBEGF ENSG00000113070.7 0.676950123 2 2 0.381319469
Brain_Frontal_Cortex_BA9 0.9343064 Brain_Substantia_nigra 1.040269271
0.959730729 0.959730729 -0.082427963 0.875467715 0.396519876
0.67733453 2 0 0.998207054
ZNF236 ENSG00000130856.15 0.677194321 4 3 0.263743071
Brain_Spinal_cord_cervical_c-1 0.784625771 Brain_Putamen_basal_ganglia
2.124399674 0.056701996 0.827529553 -1.117587826 -0.273295895
-0.644921405 0.349650396 3 0 0.998207054
F11R ENSG00000158769.17 0.677245299 6 4 0.229285773 Brain_Cortex
0.992381099 Brain_Cerebellar_Hemisphere 4.470542862 0.022781899
0.271650241 -0.009549021 1.202200713 0.549649692 0.483452138
4 0 0.998207054
AAAS ENSG00000094914.12 0.677250932 1 1 0.677250932 Brain_Cortex
0.677250932 Brain_Cortex 1 1 1 -0.416217395 -0.416217395
-0.416217395 NA 1 0 0.998207054
NDUFA3 ENSG00000170906.15 0.677290438 11 3 0.443071562
Brain_Hippocampus 0.812495331 Brain_Hypothalamus 9.04445734 0.00236331
0.406627682 -0.767016741 0.694786698 0.16696376 0.481300926 3
0 0.998207054
IGFL4 ENSG00000204869.8 0.677300151 1 1 0.677300151
Brain_Hypothalamus 0.677300151 Brain_Hypothalamus 1 1 1
-0.416150127 -0.416150127 -0.416150127 NA 1 0 0.998207054
DENR ENSG00000139726.10 0.677376829 1 1 0.677376829 Brain_Cerebellum
0.677376829 Brain_Cerebellum 1 1 1 -0.416045336 -0.416045336
-0.416045336 NA 1 0 0.998207054
RP11-1C8.7 ENSG00000271830.1 0.677469316 12 1 0.468762473
Brain_Frontal_Cortex_BA9 0.915933122 Brain_Amygdala 11.37416538
0.004292659 11.37416538 -0.72449406 -0.105557909 -0.40198367

0.238722536 1 0 0.998207054
CACUL1 ENSG00000151893.14 0.677493467 5 4 0.167947313
Brain_Hippocampus 0.605651212 Brain_Cerebellum 3.515127079
0.071695534 0.134862097 0.516290956 1.378829553 0.786310409
0.341840822 4 0 0.998207054
KNG1 ENSG00000113889.11 0.677639764 2 1 0.670733575
Brain_Frontal_Cortex_BA9 0.688817091 Brain_Cortex 1.972336202
0.027663798 1.972336202 -0.425141434 -0.40046088 -0.412801157
0.017451787 1 0 0.998207054
SPSB4 ENSG00000175093.4 0.677718137 1 1 0.677718137
Brain_Hippocampus 0.677718137 Brain_Hippocampus 1 1 1
0.415578944 0.415578944 0.415578944 NA 1 0 0.998207054
SMCO2 ENSG00000165935.9 0.677838903 4 4 0.243934754
Brain_Caudate_basal_ganglia 0.818978523 Brain_Cerebellar_Hemisphere
1.793336748 0.490117905 0.490117905 -1.165208135 0.5359323
-0.398727023 0.731257312 4 0 0.998207054
EPS8L1 ENSG00000131037.14 0.677889729 4 4 0.318178796
Brain_Cerebellum 0.662630241 Brain_Frontal_Cortex_BA9 2.403083203
0.267373098 0.267373098 -0.644824536 0.998207405 0.369776365
0.714363925 4 0 0.998207054
WFS1 ENSG00000109501.13 0.677916001 3 3 0.36541459 Brain_Hippocampus
0.741623871 Brain_Cerebellum 1.899065515 0.102947633 0.102947633
0.329703683 0.905095888 0.622931744 0.287855615 3 0
0.998207054
NIPA1 ENSG00000170113.15 0.677927854 6 6 0.320991196
Brain_Caudate_basal_ganglia 0.810801103 Brain_Hippocampus 1.724701412
0.287035281 0.287035281 -0.992423058 0.882072642 0.307456169
0.756150062 6 0 0.998207054
TSKU ENSG00000182704.7 0.677960327 4 4 0.316297557
Brain_Caudate_basal_ganglia 0.787224482 Brain_Cerebellar_Hemisphere
1.636476853 0.319941686 0.319941686 -0.683438003 1.002095322
0.298204199 0.719616574 4 0 0.998207054
MLXIP ENSG00000175727.13 0.678002459 2 2 0.53205959 Brain_Cerebellum
0.604791284 Brain_Cerebellar_Hemisphere 1.155001885 0.844998115
0.844998115 0.517522762 0.624865115 0.571193938 0.075902505
2 0 0.998207054
TWF1 ENSG00000151239.13 0.678019278 1 1 0.678019278
Brain_Caudate_basal_ganglia 0.678019278 Brain_Caudate_basal_ganglia 1 1
1 -0.415167514 -0.415167514 -0.415167514 NA 1 0
0.998207054
MAML1 ENSG00000161021.11 0.678122528 1 1 0.678122528 Brain_Cortex
0.678122528 Brain_Cortex 1 1 1 0.415026467 0.415026467
0.415026467 NA 1 0 0.998207054
RP5-1125A11.7 ENSG00000276073.1 0.678145062 13 3 0.288685316
Brain_Spinal_cord_cervical_c-1 0.936074933
Brain_Anterior_cingulate_cortex_BA24 10.73416471 0.002354126
0.536833668 -0.19235343 1.061010089 0.379635294 0.366028914
3 0 0.998207054
SGSM2 ENSG00000141258.12 0.678254262 3 1 0.670155609 Brain_Cortex
0.698490303 Brain_Cerebellar_Hemisphere 2.951059036 0.011614275
2.951059036 -0.425934453 -0.387359202 -0.411501071 0.021040522
1 0 0.998207054
GPR151 ENSG00000173250.2 0.678395554 1 1 0.678395554
Brain_Spinal_cord_cervical_c-1 0.678395554 Brain_Spinal_cord_cervical_c-1
1 1 1 -0.414653531 -0.414653531 -0.414653531 NA 1 0
0.998207054
RBM38 ENSG00000132819.16 0.678556233 2 1 0.634070486
Brain_Cerebellar_Hemisphere 0.731048809 Brain_Cerebellum 1.956173098
0.043826902 1.956173098 -0.476005463 -0.343730716 -0.409868089
0.09353237 1 0 0.998207054
SMARCB1 ENSG00000099956.18 0.678596013 6 3 0.365528974
Brain_Hippocampus 0.995847914 Brain_Cerebellum 4.566054585

0.011358638 0.455814384 -0.269484314 0.904879981 0.338598832 0.467470754 3 0 0.998207054
UBE2T ENSG00000077152.9 0.678608762 3 3 0.275672273 Brain_Cortex 0.822605019 Brain_Frontal_Cortex_BA9 1.977849629 0.361582506 0.361582506 0.224195713 1.090092788 0.638791119 0.434113981 3 0 0.998207054
KLHDC1 ENSG00000197776.7 0.678688953 3 3 0.474641944 Brain_Cerebellum 0.92130261 Brain_Amygdala 2.175838949 0.231971922 0.231971922 -0.714946757 0.311259943 -0.167493278 0.51654123 3 0 0.998207054
SUN2 ENSG00000100242.15 0.678800622 3 3 0.266247725 Brain_Hippocampus 0.882286509 Brain_Putamen_basal_ganglia 1.175378264 0.879440155 0.879440155 -1.111745188 0.456363823 -0.267817551 0.790882951 3 0 0.998207054
TRMT44 ENSG00000155275.18 0.67898121 10 4 0.219969614 Brain_Hypothalamus 0.909703838 Brain_Anterior_cingulate_cortex_BA24 7.863612633 0.04340181 0.340346778 -1.226608923 -0.113412111 -0.661524869 0.328657675 4 0 0.998207054
RP11-262I2.2 ENSG00000224222.1 0.679026587 2 2 0.399642492 Brain_Substantia_nigra 0.833905887 Brain_Cerebellum 1.052816596 0.947183404 0.947183404 0.209694796 0.842259899 0.525977348 0.447291074 2 0 0.998207054
PLEKHH3 ENSG00000068137.14 0.679127099 2 2 0.379165693 Brain_Spinal_cord_cervical_c-1 0.95740221 Brain_Frontal_Cortex_BA9 1.085783613 0.914216387 0.914216387 -0.879434573 -0.053413801 -0.466424187 0.584084889 2 0 0.998207054
TOMM40L ENSG00000158882.14 0.679136041 1 1 0.679136041 Brain_Caudate_basal_ganglia 0.679136041 Brain_Caudate_basal_ganglia 1 1 1 -0.413642367 -0.413642367 -0.413642367 NA 1 0 0.998207054
CD177 ENSG00000204936.9 0.679140461 1 1 0.679140461 Brain_Cerebellum 0.679140461 Brain_Cerebellum 1 1 1 0.413636332 0.413636332 0.413636332 NA 1 0 0.998207054
ADGRF4 ENSG00000153294.11 0.679176537 2 2 0.379085354 Brain_Cerebellum 0.414734858 Brain_Cerebellar_Hemisphere 1.93101407 0.06898593 0.06898593 -0.879582811 -0.815589672 -0.847586241 0.045249982 2 0 0.998207054
NANP ENSG00000170191.4 0.679204577 2 2 0.382229309 Brain_Caudate_basal_ganglia 0.842931659 Brain_Hypothalamus 1.112214748 0.887785252 0.887785252 -0.873796092 -0.198144948 -0.53597052 0.477757506 2 0 0.998207054
CACNA1A ENSG00000141837.19 0.679376945 2 2 0.379283675 Brain_Caudate_basal_ganglia 0.926638786 Brain_Cerebellum 1.091518361 0.908481639 0.908481639 -0.879216915 0.092074579 -0.393571168 0.686806802 2 0 0.998207054
UBR2 ENSG00000024048.10 0.679432379 2 2 0.761340141 Brain_Cerebellum 0.936859898 Brain_Cerebellar_Hemisphere 1.903494017 0.096505983 0.096505983 -0.079217157 0.303721416 0.112252129 0.270778462 2 0 0.998207054
WBP2NL ENSG00000183066.14 0.679649495 11 4 0.129958579 Brain_Frontal_Cortex_BA9 0.602275607 Brain_Cerebellum 8.570549173 0.013851524 0.30210163 0.521130871 1.514265247 0.977995576 0.291444673 4 0 0.998207054
TMEM209 ENSG00000146842.16 0.679800267 1 1 0.679800267 Brain_Substantia_nigra 0.679800267 Brain_Substantia_nigra 1 1 1 0.412735698 0.412735698 0.412735698 NA 1 0 0.998207054
NEAT1 ENSG00000245532.7 0.679965581 13 2 0.217414369 Brain_Substantia_nigra 0.671892257 Brain_Cortex 11.9502465 0.00092779 0.586605399 0.423552427 1.233432707 0.809083154 0.208295153 2 0 0.998207054
WNT9B ENSG00000158955.10 0.67998212 1 1 0.67998212 Brain_Cerebellum 0.67998212

Brain_Cerebellum 1 1 1 0.412487528 0.412487528 0.412487528
NA 1 0 0.998207054
NTM ENSG00000182667.14 0.67999561 6 5 0.355634876 Brain_Hippocampus
0.917713294 Brain_Caudate_basal_ganglia 3.87054096 0.112345582
0.261066983 -0.447068279 0.923714701 0.302138312 0.456452554
5 0 0.998207054
ACHE ENSG00000087085.13 0.68000889 1 1 0.68000889 Brain_Cerebellum 0.68000889
Brain_Cerebellum 1 1 1 -0.412450999 -0.412450999 -0.412450999
NA 1 0 0.998207054
DIXDC1 ENSG00000150764.13 0.680068186 1 1 0.680068186
Brain_Hippocampus 0.680068186 Brain_Hippocampus 1 1 1
-0.412370085 -0.412370085 -0.412370085 NA 1 0 0.998207054
CNGA1 ENSG00000198515.13 0.680075829 13 3 0.232783969
Brain_Substantia_nigra 0.843762705 Brain_Amygdala 10.903637 0.01055486
0.447439393 -1.193218097 -0.197082849 -0.758266451 0.26392221 3
0 0.998207054
SHROOM1 ENSG00000164403.14 0.680136041 2 2 0.526684932
Brain_Cerebellum 0.54243436 Brain_Hypothalamus 1.001174934 0.998825066
0.998825066 -0.633074594 -0.609135905 -0.62110525 0.016927209
2 0 0.998207054
HEXA ENSG00000213614.9 0.680227938 4 4 0.443623119
Brain_Spinal_cord_cervical_c-1 0.865413607 Brain_Caudate_basal_ganglia
2.673946625 0.225332275 0.225332275 -0.766089384 0.676996114
-0.042118285 0.613400748 4 0 0.998207054
ATN1 ENSG00000111676.14 0.68036981 4 4 0.179169292 Brain_Cerebellum
0.747854814 Brain_Hypothalamus 1.57901928 0.472694766 0.472694766
-1.343317179 0.519718762 -0.381868656 0.761772388 4 0
0.998207054
11-Sep ENSG00000138758.11 0.680508371 6 4 0.194910959
Brain_Hippocampus 0.929427129 Brain_Cerebellum 3.187334466
0.043563866 0.342296626 -1.296187314 0.088565623 -0.637485799
0.57916804 4 0 0.998207054
ALS2CR11 ENSG00000155754.14 0.680586645 1 1 0.680586645
Brain_Putamen_basal_ganglia 0.680586645 Brain_Putamen_basal_ganglia 1 1
1 0.411662731 0.411662731 0.411662731 NA 1 0
0.998207054
RP11-116D17.3 ENSG00000271579.1 0.680685495 2 2 0.476143069
Brain_Cerebellum 0.630061907 Brain_Nucleus_accumbens_basal_ganglia
1.041286809 0.958713191 0.958713191 -0.481639717 0.712519616
0.11543995 0.844398162 2 0 0.998207054
NUBP2 ENSG00000095906.16 0.68075982 8 4 0.280285839 Brain_Cortex
0.990732372 Brain_Hippocampus 5.987908711 0.024897049
0.249896348 -0.231261412 1.079677449 0.359023919 0.497508068
4 0 0.998207054
LINC02145 ENSG00000250490.1 0.680842373 12 3 0.154417243
Brain_Cortex 0.735562687 Brain_Hypothalamus 9.938759143
0.010893671 0.389189887 0.337735278 1.42410098 0.938757504
0.35025758 3 0 0.998207054
SMKR1 ENSG00000240204.2 0.680979959 2 1 0.672102375
Brain_Cerebellar_Hemisphere 0.69389956 Brain_Cerebellum 1.973722657
0.026277343 1.973722657 -0.423264386 -0.393568611 -0.408416499
0.020998085 1 0 0.998207054
TMEM161B ENSG00000164180.13 0.681030863 2 2 0.383215524
Brain_Cerebellum 0.647788773 Brain_Cerebellar_Hemisphere 1.431764263
0.568235737 0.568235737 0.456836215 0.8719869 0.664411557
0.293555864 2 0 0.998207054
ACVRL1 ENSG00000139567.12 0.681036846 1 1 0.681036846
Brain_Putamen_basal_ganglia 0.681036846 Brain_Putamen_basal_ganglia 1 1
1 0.411048672 0.411048672 0.411048672 NA 1 0
0.998207054
CCDC7 ENSG00000216937.13 0.681273299 6 5 0.165857242
Brain_Hippocampus 0.932256967 Brain_Putamen_basal_ganglia 3.540132574

0.106382522 0.128449514 -0.537523104 1.385638741 0.011607587
0.689323385 5 0 0.998207054
ABCC9 ENSG00000069431.11 0.681394839 3 3 0.271853873
Brain_Nucleus_accumbens_basal_ganglia 0.71545562 Brain_Cortex 2.236157945
0.182608774 0.182608774 0.364538914 1.098803299 0.702283947
0.370643848 3 0 0.998207054
FLNB ENSG00000136068.14 0.681510304 5 4 0.296502181
Brain_Caudate_basal_ganglia 0.89291567 Brain_Cortex 3.102750045
0.101303392 0.195391583 -1.043963707 -0.134615773 -0.639004529
0.356074191 4 0 0.998207054
MED12L ENSG00000144893.12 0.681656404 3 3 0.311293546
Brain_Cortex 0.796571778 Brain_Nucleus_accumbens_basal_ganglia
1.174960997 0.825975966 0.825975966 0.257786379 1.012511511
0.655556148 0.379014338 3 0 0.998207054
EIF4EBP3 ENSG00000243056.1 0.681669722 6 3 0.448157946
Brain_Nucleus_accumbens_basal_ganglia 0.837852347 Brain_Frontal_Cortex_BA9
4.660285669 0.015172768 0.432575474 -0.758489585 0.535672226
-0.358068695 0.477432326 3 0 0.998207054
CRHR1 ENSG00000120088.14 0.681773275 6 6 0.245841605
Brain_Caudate_basal_ganglia 0.773884232 Brain_Nucleus_accumbens_basal_ganglia
2.221093957 0.257539157 0.257539157 -0.746384064 1.160509064
0.192309029 0.797533046 6 0 0.998207054
AC090044.2 ENSG00000224239.1 0.681797758 1 1 0.681797758
Brain_Cerebellum 0.681797758 Brain_Cerebellum 1 1 1 0.410011164
0.410011164 0.410011164 NA 1 0 0.998207054
CASP5 ENSG00000137757.10 0.681823725 1 1 0.681823725 Brain_Amygdala
0.681823725 Brain_Amygdala 1 1 1 -0.409975765 -0.409975765
-0.409975765 NA 1 0 0.998207054
KCNK6 ENSG00000099337.4 0.68191255 2 2 0.389371929
Brain_Nucleus_accumbens_basal_ganglia 0.985090385 Brain_Cerebellar_Hemisphere
1.158938217 0.841061783 0.841061783 -0.018687519 0.860756934
0.421034707 0.621861136 2 0 0.998207054
NPIPA5 ENSG00000183793.13 0.681919149 13 9 0.38384222
Brain_Cerebellar_Hemisphere 0.884164453 Brain_Amygdala 6.345778547
0.025054715 0.293017775 -0.801260181 0.87083872 -0.147251763
0.546377423 9 0 0.998207054
ATP5B ENSG00000110955.8 0.682063582 1 1 0.682063582
Brain_Caudate_basal_ganglia 0.682063582 Brain_Caudate_basal_ganglia 1 1
1 -0.409648815 -0.409648815 -0.409648815 NA 1 0
0.998207054
NUCB2 ENSG00000070081.15 0.682138415 2 1 0.447806903 Brain_Cerebellum
0.960620161 Brain_Cerebellar_Hemisphere 1.948368183 0.051631817
1.948368183 -0.759076321 -0.049375364 -0.404225842 0.501834359
1 0 0.998207054
AQP8 ENSG00000103375.10 0.682226708 1 1 0.682226708 Brain_Cerebellum
0.682226708 Brain_Cerebellum 1 1 1 -0.409426483 -0.409426483
-0.409426483 NA 1 0 0.998207054
SLC17A8 ENSG00000179520.10 0.682296471 1 1 0.682296471
Brain_Hippocampus 0.682296471 Brain_Hippocampus 1 1 1
-0.409331406 -0.409331406 -0.409331406 NA 1 0 0.998207054
GSTP1 ENSG00000084207.15 0.68233463 13 2 0.427007539
Brain_Spinal_cord_cervical_c-1 0.894210359 Brain_Frontal_Cortex_BA9
11.65554623 0.000839997 0.456015286 -0.794322792 -0.132978532
-0.491768351 0.202166589 2 0 0.998207054
RNF24 ENSG00000101236.16 0.682474772 9 4 0.170908788
Brain_Nucleus_accumbens_basal_ganglia 0.677221946 Brain_Amygdala
7.783950163 0.0109251 0.270282927 0.416257011 1.369289534
0.730529091 0.301860338 4 0 0.998207054
HAUS5 ENSG00000249115.8 0.682588708 2 2 0.527153901
Brain_Cerebellar_Hemisphere 0.546290653 Brain_Nucleus_accumbens_basal_ganglia
1.000201452 0.999798548 0.999798548 -0.603327784 0.632356578
0.014514397 0.873760791 2 0 0.998207054

CNPPD1 ENSG00000115649.15 0.682612732 7 3 0.345729386 Brain_Cerebellar_Hemisphere 0.971346198 Brain_Substantia_nigra 5.967906768 0.013274541 0.267982492 -0.942905218 0.035919937 -0.343236042 0.307147259 3 0 0.998207054
TESK1 ENSG00000107140.15 0.682638954 2 2 0.638951272 Brain_Cerebellum 0.948467361 Brain_Cerebellar_Hemisphere 1.80161885 0.19838115 0.19838115 -0.064631554 0.4691656 0.202267023 0.377451587 2 0 0.998207054
FMO2 ENSG00000094963.13 0.682728707 11 7 0.193105198 Brain_Substantia_nigra 0.944577138 Brain_Cerebellar_Hemisphere 6.763009604 0.008798466 0.235440356 -0.772802743 1.301447866 0.29503346 0.65880439 7 0 0.998207054
APOC4 ENSG00000267467.3 0.682803189 1 1 0.682803189 Brain_Hippocampus 0.682803189 Brain_Hippocampus 1 1 1 0.40864093 0.40864093 0.40864093 NA 1 0 0.998207054
PCM1 ENSG00000078674.17 0.682809027 13 1 0.426178133 Brain_Hypothalamus 0.922732426 Brain_Nucleus_accumbens_basal_ganglia 12.2242776 0.000824848 12.2242776 -0.795748669 -0.096992405 -0.394525276 0.15917895 1 0 0.998207054
IGF1R ENSG00000140443.13 0.68285463 1 1 0.68285463 Brain_Cerebellum 0.68285463 Brain_Cerebellum 1 1 1 0.408570845 0.408570845 0.408570845 NA 1 0 0.998207054
E2F4 ENSG00000205250.8 0.6831132 1 1 0.6831132 Brain_Cerebellar_Hemisphere 0.6831132 Brain_Cerebellar_Hemisphere 1 1 1 0.408218591 0.408218591 0.408218591 NA 1 0 0.998207054
TIMM10B ENSG00000132286.11 0.68323027 1 1 0.68323027 Brain_Nucleus_accumbens_basal_ganglia 0.68323027 Brain_Nucleus_accumbens_basal_ganglia 1 1 1 -0.408059122 -0.408059122 -0.408059122 NA 1 0 0.998207054
ABI3BP ENSG00000154175.16 0.683256839 8 7 0.427477611 Brain_Cerebellum 0.836752408 Brain_Substantia_nigra 3.980047136 0.036307184 0.188251056 -0.793515382 0.656007731 -0.062764786 0.51377955 7 0 0.998207054
SFT2D2 ENSG00000213064.9 0.683370115 1 1 0.683370115 Brain_Putamen_basal_ganglia 0.683370115 Brain_Putamen_basal_ganglia 1 1 1 0.407868643 0.407868643 0.407868643 NA 1 0 0.998207054
RP11-176H8.1 ENSG00000203546.7 0.683501349 12 4 0.501711858 Brain_Anterior_cingulate_cortex_BA24 0.94338477 Brain_Frontal_Cortex_BA9 10.05980408 0.001740687 0.379283804 -0.331107556 0.671798697 0.028583799 0.34370314 4 0 0.998207054
LINC02044 ENSG00000243795.1 0.683721048 13 10 0.109485556 Brain_Amygdala 0.960717993 Brain_Cortex 6.004974808 0.00506636 0.24654184 -0.978979286 1.600509704 0.417046387 0.908110265 10 0 0.998207054
GMPR ENSG00000137198.9 0.684026569 1 1 0.684026569 Brain_Cerebellar_Hemisphere 0.684026569 Brain_Cerebellar_Hemisphere 1 1 1 0.406974702 0.406974702 0.406974702 NA 1 0 0.998207054
MGAT4B ENSG00000161013.16 0.684045196 2 2 0.486347772 Brain_Nucleus_accumbens_basal_ganglia 0.590244488 Brain_Cerebellum 1.020564617 0.979435383 0.979435383 -0.696129439 0.53848177 -0.078823834 0.873001958 2 0 0.998207054
RP11-1C8.5 ENSG00000261670.1 0.68437516 4 4 0.359929668 Brain_Hypothalamus 0.976843492 Brain_Frontal_Cortex_BA9 1.462261427 0.581514848 0.581514848 -0.674479946 0.915499112 0.136106567 0.665912015 4 0 0.998207054
HDHD5 ENSG00000069998.12 0.684411851 3 3 0.27770921 Brain_Cerebellum 0.526816593 Brain_Cerebellar_Hemisphere 1.397876747 0.732570189 0.732570189 0.632872981 1.08547979 0.787615045 0.25802385 3 0 0.998207054
TPD52 ENSG00000076554.15 0.684462568 4 4 0.175630773

Brain_Putamen_basal_ganglia 0.769486107 Brain_Caudate_basal_ganglia
1.844833159 0.430139101 0.430139101 -0.318686041 1.354331084
0.656444268 0.812486507 4 0 0.998207054
LINC02091 ENSG00000273172.1 0.684480072 3 3 0.265780694
Brain_Hypothalamus 0.767702021 Brain_Cerebellar_Hemisphere 1.076577871
0.920172194 0.920172194 -0.447878352 1.112831757 0.320111828
0.780648885 3 0 0.998207054
MAFA ENSG00000182759.3 0.684519219 2 2 0.499391499
Brain_Nucleus_accumbens_basal_ganglia 0.584980039
Brain_Spinal_cord_cervical_c-1 1.004849141 0.995150859 0.995150859
-0.546124967 0.675447495 0.064661264 0.863782172 2 0
0.998207054
THTPA ENSG00000259431.5 0.684581067 1 1 0.684581067
Brain_Frontal_Cortex_BA9 0.684581067 Brain_Frontal_Cortex_BA9 1 1
1 -0.406219854 -0.406219854 -0.406219854 NA 1 0
0.998207054
SLC27A5 ENSG00000083807.9 0.684696191 11 9 0.069835627
Brain_Amygdala 0.970217132 Brain_Anterior_cingulate_cortex_BA24
5.818470638 0.040989511 0.195558307 -0.45379415 1.812975328
0.458139719 0.697863018 9 0 0.998207054
POLRMT ENSG00000099821.13 0.684700956 1 1 0.684700956
Brain_Spinal_cord_cervical_c-1 0.684700956 Brain_Spinal_cord_cervical_c-1
1 1 1 -0.406056678 -0.406056678 -0.406056678 NA 1 0
0.998207054
FAM78A ENSG00000126882.12 0.684723425 1 1 0.684723425
Brain_Spinal_cord_cervical_c-1 0.684723425 Brain_Spinal_cord_cervical_c-1
1 1 1 0.406026098 0.406026098 0.406026098 NA 1 0
0.998207054
LINC01331 ENSG00000248673.1 0.684850491 2 2 0.401453389
Brain_Cortex 0.414625864 Brain_Frontal_Cortex_BA9 1.811889472
0.188110528 0.188110528 0.815780192 0.839028371 0.827404282
0.016438945 2 0 0.998207054
MAD2L1BP ENSG00000124688.13 0.684955583 1 1 0.684955583
Brain_Cortex 0.684955583 Brain_Cortex 1 1 1 0.40571015
0.40571015 0.40571015 NA 1 0 0.998207054
ITGAE ENSG00000083457.11 0.685130038 1 1 0.685130038
Brain_Caudate_basal_ganglia 0.685130038 Brain_Caudate_basal_ganglia 1 1
1 0.405472759 0.405472759 0.405472759 NA 1 0
0.998207054
CYB561D2 ENSG00000114395.10 0.685151965 2 2 0.387377011
Brain_Cortex 0.935763482 Brain_Hippocampus 1.016919918
0.983080082 0.983080082 -0.864383942 -0.080595705 -0.472489824
0.554221977 2 0 0.998207054
USP44 ENSG00000136014.11 0.685180355 5 5 0.12126583
Brain_Caudate_basal_ganglia 0.909752737 Brain_Cerebellar_Hemisphere
2.433523992 0.186230291 0.186230291 -1.549482273 0.147229142
-0.438242773 0.656510197 5 0 0.998207054
MEPE ENSG00000152595.16 0.685181509 3 3 0.356174593
Brain_Spinal_cord_cervical_c-1 0.605113698 Brain_Cortex 1.147084962
0.874246396 0.874246396 0.517060826 0.922678847 0.684755741
0.211732127 3 0 0.998207054
CD37 ENSG00000104894.11 0.685208824 1 1 0.685208824
Brain_Hippocampus 0.685208824 Brain_Hippocampus 1 1 1
0.405365558 0.405365558 0.405365558 NA 1 0 0.998207054
SCN11A ENSG00000168356.11 0.685443447 9 4 0.20645501 Brain_Cerebellum
0.944107306 Brain_Hypothalamus 6.785590386 0.024619416
0.279899421 -1.263373426 0.773580234 -0.041454607 0.591098946
4 0 0.998207054
LINC01858 ENSG00000261615.6 0.685482758 2 2 0.39073802
Brain_Caudate_basal_ganglia 0.610954 Brain_Nucleus_accumbens_basal_ganglia
1.45079639 0.54920361 0.54920361 0.508712157 0.858279728 0.683495943
0.2471816 2 0 0.998207054

RP11-1070N10.3 ENSG00000258572.1 0.685500742 3 3 0.270962217
Brain_Caudate_basal_ganglia 0.924458723 Brain_Cerebellum 1.107144628
0.900408096 0.900408096 -1.100849379 -0.094818838 -0.578187026
0.504165038 3 0 0.998207054
RP11-363J20.1 ENSG00000258957.1 0.685651939 1 1 0.685651939
Brain_Anterior_cingulate_cortex_BA24 0.685651939
Brain_Anterior_cingulate_cortex_BA24 1 1 1 0.404762714
0.404762714 0.404762714 NA 1 0 0.998207054
LINC01273 ENSG00000231742.5 0.685700071 13 2 0.494888169
Brain_Cerebellar_Hemisphere 0.965003966 Brain_Nucleus_accumbens_basal_ganglia
11.8818062 2.01E-08 0.740526555 -0.390150843 0.682554855
-0.093220994 0.292188718 2 0 0.998207054
FAM161B ENSG00000156050.8 0.685761988 1 1 0.685761988
Brain_Putamen_basal_ganglia 0.685761988 Brain_Putamen_basal_ganglia 1 1
1 0.404613018 0.404613018 0.404613018 NA 1 0
0.998207054
SRD5A1 ENSG00000145545.11 0.68584182 5 4 0.235338585
Brain_Frontal_Cortex_BA9 0.97117213 Brain_Cortex 3.763258416
0.019333587 0.237195933 -1.186718728 -0.036138242 -0.391515365
0.472385865 4 0 0.998207054
PTPN11 ENSG00000179295.17 0.685870842 1 1 0.685870842
Brain_Hypothalamus 0.685870842 Brain_Hypothalamus 1 1 1
0.404464957 0.404464957 0.404464957 NA 1 0 0.998207054
NNAT ENSG00000053438.8 0.685939636 1 1 0.685939636
Brain_Anterior_cingulate_cortex_BA24 0.685939636
Brain_Anterior_cingulate_cortex_BA24 1 1 1 -0.404371389
-0.404371389 -0.404371389 NA 1 0 0.998207054
GNAL ENSG00000141404.15 0.685985936 2 2 0.445692745 Brain_Cerebellum
0.759001601 Brain_Cerebellar_Hemisphere 1.136760129 0.863239871
0.863239871 -0.762615489 0.306792127 -0.227911681 0.756185377
2 0 0.998207054
RUNX2 ENSG00000124813.20 0.685987626 4 3 0.31614807 Brain_Hippocampus
0.65478937 Brain_Putamen_basal_ganglia 3.106227888 0.053637867 0.27885339
0.447118683 1.002404914 0.834180427 0.259645157 3 0
0.998207054
SLC5A2 ENSG00000140675.12 0.686038734 3 3 0.328563738
Brain_Cerebellar_Hemisphere 0.831894441 Brain_Cerebellum 2.116080198
0.248692379 0.248692379 -0.297002971 0.977010937 0.297426825
0.641261493 3 0 0.998207054
PDE6G ENSG00000185527.11 0.686066539 1 1 0.686066539
Brain_Cerebellar_Hemisphere 0.686066539 Brain_Cerebellar_Hemisphere 1 1
1 0.404198795 0.404198795 0.404198795 NA 1 0
0.998207054
MYCBPAP ENSG00000136449.13 0.68651387 5 5 0.165800784 Brain_Cortex
0.818862217 Brain_Cerebellum 2.68262195 0.295016136 0.295016136
-0.22900862 1.385823567 0.680906951 0.582762931 5 0
0.998207054
HOGA1 ENSG00000241935.8 0.686519481 5 5 0.268225225
Brain_Substantia_nigra 0.997885621 Brain_Caudate_basal_ganglia
1.730912857 0.306108956 0.306108956 -1.005007756 1.10715892
0.183538072 0.819784827 5 0 0.998207054
STX1A ENSG00000106089.11 0.686530567 4 3 0.328922031
Brain_Cerebellar_Hemisphere 0.740748516 Brain_Hypothalamus 1.9941279
0.013041021 0.990873741 0.330862278 0.976287463 0.687571006
0.319653408 3 0 0.998207054
ATF6 ENSG00000118217.5 0.686533592 3 3 0.283188529
Brain_Substantia_nigra 0.592593304 Brain_Cerebellum 1.780215527
0.225361504 0.225361504 -0.618445352 1.073184087 -0.026781017
0.953509199 3 0 0.998207054
PDXDC1 ENSG00000179889.18 0.68660126 10 8 0.177243087
Brain_Putamen_basal_ganglia 0.982129275 Brain_Spinal_cord_cervical_c-1
5.55746424 0.045676362 0.211722561 -1.025389734 1.349292333

-0.018315508 0.730872688 8 0 0.998207054
SLC37A4 ENSG00000137700.17 0.686616796 1 1 0.686616796
Brain_Caudate_basal_ganglia 0.686616796 Brain_Caudate_basal_ganglia 1 1
1 -0.403450564 -0.403450564 -0.403450564 NA 1 0
0.998207054
TIAM1 ENSG00000156299.13 0.686623499 4 3 0.471874006
Brain_Putamen_basal_ganglia 0.924024049 Brain_Caudate_basal_ganglia
2.507843613 0.011594293 0.243735343 -0.224243289 0.719433267
0.056498884 0.445126113 3 0 0.998207054
HOPX ENSG00000171476.21 0.686823038 1 1 0.686823038 Brain_Cerebellum
0.686823038 Brain_Cerebellum 1 1 1 -0.403170176 -0.403170176
-0.403170176 NA 1 0 0.998207054
METTL21A ENSG00000144401.14 0.686866893 2 2 0.398945134
Brain_Frontal_Cortex_BA9 0.861687333 Brain_Anterior_cingulate_cortex_BA24
1.02963771 0.97036229 0.97036229 -0.174226662 0.843506675 0.334640007
0.719646144 2 0 0.998207054
INPP4B ENSG00000109452.12 0.686990845 1 1 0.686990845
Brain_Substantia_nigra 0.686990845 Brain_Substantia_nigra 1 1
1 0.402942066 0.402942066 0.402942066 NA 1 0
0.998207054
TIMD4 ENSG00000145850.8 0.687002811 2 2 0.476344386 Brain_Cortex
0.911142595 Brain_Hypothalamus 1.459012595 0.540987405
0.540987405 -0.111597449 0.712194429 0.30029849 0.582508823 2
0 0.998207054
SPRED3 ENSG00000188766.12 0.687029091 2 2 0.4579251
Brain_Putamen_basal_ganglia 0.660184989 Brain_Nucleus_accumbens_basal_ganglia
1.009842557 0.990157443 0.990157443 -0.439657776 0.742267795
0.15130501 0.835747586 2 0 0.998207054
BDH1 ENSG00000161267.11 0.68703865 4 4 0.374536485
Brain_Putamen_basal_ganglia 0.867918531 Brain_Caudate_basal_ganglia 2.74957286
0.138464088 0.138464088 -0.576459848 0.888007931 0.248561442
0.623979984 4 0 0.998207054
DNAJC12 ENSG00000108176.14 0.687083832 3 3 0.270212071
Brain_Cerebellum 0.678904828 Brain_Cortex 1.472821552 0.548528826
0.548528826 0.413958051 1.102574312 0.652586528 0.389939298
3 0 0.998207054
NCAN ENSG00000130287.13 0.687163737 2 1 0.561086035 Brain_Cerebellum
0.828701116 Brain_Cerebellar_Hemisphere 1.961364847 0.038635153
1.961364847 -0.581229134 -0.216367733 -0.398798433 0.257995971
1 0 0.998207054
KRT13 ENSG00000171401.14 0.687224342 2 2 0.413986138
Brain_Caudate_basal_ganglia 0.75273685 Brain_Cerebellum 1.033457099
0.966542901 0.966542901 -0.81689902 0.315032668 -0.250933176
0.800396573 2 0 0.998207054
WRN ENSG00000165392.9 0.687245251 2 2 0.403251676
Brain_Anterior_cingulate_cortex_BA24 0.754673785 Brain_Cerebellar_Hemisphere
1.103763379 0.896236621 0.896236621 -0.835827993 -0.312482599
-0.574155296 0.370061077 2 0 0.998207054
MTHFS ENSG00000136371.10 0.687322518 9 5 0.393926048
Brain_Cerebellar_Hemisphere 0.847936389 Brain_Putamen_basal_ganglia
5.274837336 0.028824034 0.255222885 -0.191752106 0.852519091
0.492611428 0.319554995 5 0 0.998207054
TMEM128 ENSG00000132406.11 0.687347078 12 5 0.156760618
Brain_Substantia_nigra 0.977046936 Brain_Anterior_cingulate_cortex_BA24
7.267490273 0.014737599 0.344248884 -0.168638765 1.416050847
0.650185906 0.560429523 5 0 0.998207054
WASF2 ENSG00000158195.10 0.687373475 5 5 0.191455586
Brain_Cerebellar_Hemisphere 0.651275558 Brain_Spinal_cord_cervical_c-1
1.875386562 0.127086987 0.127086987 -0.451990873 1.306285208
0.703585497 0.679845949 5 0 0.998207054
KRT19 ENSG00000171345.13 0.687422337 2 1 0.668825488
Brain_Cerebellar_Hemisphere 0.713396201 Brain_Cerebellum 1.95231043 0.04768957

1.95231043 0.36729873 0.427760513 0.397529622 0.042752937 1 0
0.998207054
PCLO ENSG00000186472.19 0.687440759 2 2 0.490560556
Brain_Nucleus_accumbens_basal_ganglia 0.599563236
Brain_Anterior_cingulate_cortex_BA24 1.001927278 0.998072722
0.998072722 -0.689417528 -0.525028706 -0.607223117 0.11624045 2
0 0.998207054
BRD1 ENSG00000100425.18 0.687586924 1 1 0.687586924
Brain_Substantia_nigra 0.687586924 Brain_Substantia_nigra 1 1
1 0.402131945 0.402131945 0.402131945 NA 1 0
0.998207054
FXN ENSG00000165060.11 0.687599837 2 2 0.386779648
Brain_Anterior_cingulate_cortex_BA24 0.909255946 Brain_Putamen_basal_ganglia
1.123244642 0.876755358 0.876755358 0.113977101 0.865472236
0.489724669 0.531387306 2 0 0.998207054
LINC00658 ENSG00000226995.7 0.687770421 9 7 0.254779758
Brain_Cortex 0.825390225 Brain_Nucleus_accumbens_basal_ganglia
3.981794831 0.067165183 0.172850258 -0.871155709 1.138816357
0.447349219 0.636303278 7 0 0.998207054
USP39 ENSG00000168883.19 0.687960457 2 2 0.415201341 Brain_Amygdala
0.698197254 Brain_Cerebellum 1.122468029 0.877531971 0.877531971
-0.814774602 -0.38775513 -0.601264866 0.301948365 2 0
0.998207054
ACYP1 ENSG00000119640.8 0.688177266 2 2 0.420897056
Brain_Nucleus_accumbens_basal_ganglia 0.818370136 Brain_Frontal_Cortex_BA9
1.104654959 0.895345041 0.895345041 -0.804865921 -0.229641784
-0.517253853 0.406744888 2 0 0.998207054
CD8A ENSG00000153563.15 0.688410825 3 3 0.278457056 Brain_Cerebellum
0.832401382 Brain_Cerebellar_Hemisphere 1.353919685 0.666540298
0.666540298 -1.083791949 -0.211622721 -0.6771913 0.439064578 3
0 0.998207054
TNFRSF11B ENSG00000164761.8 0.688602922 1 1 0.688602922
Brain_Cerebellar_Hemisphere 0.688602922 Brain_Cerebellar_Hemisphere 1 1
1 -0.400751729 -0.400751729 -0.400751729 NA 1 0
0.998207054
LIN7C ENSG00000148943.11 0.688945066 1 1 0.688945066
Brain_Spinal_cord_cervical_c-1 0.688945066 Brain_Spinal_cord_cervical_c-1
1 1 1 -0.400287103 -0.400287103 -0.400287103 NA 1 0
0.998207054
SPINT2 ENSG00000167642.12 0.68904481 2 2 0.389093731
Brain_Frontal_Cortex_BA9 0.997018137 Brain_Cerebellar_Hemisphere 1.0690973
0.9309027 0.9309027 -0.861262052 0.00373722 -0.428762416 0.611646851
2 0 0.998207054
GINS3 ENSG00000181938.13 0.689256742 2 2 0.399342357 Brain_Cerebellum
0.87871375 Brain_Caudate_basal_ganglia 1.037493701 0.962506299
0.962506299 -0.842796338 -0.152599968 -0.497698153 0.488042533
2 0 0.998207054
CABLES1 ENSG00000134508.12 0.689499476 3 3 0.236548774
Brain_Hypothalamus 0.981304246 Brain_Cortex 1.240812743
0.757577283 0.757577283 -1.183657234 -0.023433797 -0.519313912
0.598175974 3 0 0.998207054
SCML4 ENSG00000146285.13 0.689536918 3 3 0.353164395
Brain_Frontal_Cortex_BA9 0.782546165 Brain_Cortex 1.532906702
0.511525814 0.511525814 -0.928468896 0.405023538 -0.266482611
0.666797187 3 0 0.998207054
WDR91 ENSG00000105875.13 0.689644521 7 6 0.080444504
Brain_Hippocampus 0.911149404 Brain_Substantia_nigra 3.747669228
0.011643393 0.223202473 -1.748112759 -0.111588862 -1.052130383
0.620519584 6 0 0.998207054
AGPAT3 ENSG00000160216.18 0.689669608 2 2 0.626644871
Brain_Frontal_Cortex_BA9 0.659011474 Brain_Cortex 1.420307758
0.579692242 0.579692242 -0.486454622 0.441278383 -0.02258812

0.656006299	2	0	0.998207054
LRRC8C	ENSG00000171488.14	0.689693953	4	4	0.155024814
Brain_Cerebellar_Hemisphere 0.899582068	Brain_Caudate_basal_ganglia
1.683599558	0.492646574	0.492646574	-1.422004951	-0.126189317
-0.643473632	0.568965909	4	0	0.998207054
RNF6 ENSG00000127870.16	0.68975922 4	4	0.292337295	Brain_Hypothalamus
0.815429356	Brain_Cerebellum 2.501319336	0.355423187	0.355423187
-0.233427636	1.053008066	0.454116612	0.560445843	4	0
0.998207054
WHRN ENSG00000095397.13	0.689795553	7	6	0.119073588
Brain_Spinal_cord_cervical_c-1	0.674592783	Brain_Putamen_basal_ganglia
4.123859055	0.108819951	0.16337391 -1.558673877	0.885554436
-0.313908798	0.988728608	6	0	0.998207054
U2AF1L4	ENSG00000161265.14	0.689828636	12	6	0.219392789
Brain_Amygdala	0.975613494	Brain_Putamen_basal_ganglia 8.519988578
0.003909203	0.317289398	-1.228144338	0.389956224	-0.307563556
0.407547411	6	0	0.998207054
ZNF295-AS1 ENSG00000237232.7	0.690481998	11	11	0.121474695
Brain_Frontal_Cortex_BA9	0.872457683	Brain_Anterior_cingulate_cortex_BA24
2.087039832	0.423332255	0.423332255	-1.277565909	1.548613349
0.240820085	0.945927298	11	0	0.998207054
LY75 ENSG00000054219.10	0.690512544	13	4	0.332131005
Brain_Cerebellar_Hemisphere 0.920247874	Brain_Spinal_cord_cervical_c-1
9.783683961	0.010470883	0.399461473	-0.688896174	0.969830465
-0.06256913	0.461700996	4	0	0.998207054
TRIOBP	ENSG00000100106.19	0.690608677	12	4	0.14889092
Brain_Cerebellar_Hemisphere 0.958587159	Brain_Nucleus_accumbens_basal_ganglia
8.714677466	0.009060677	0.387377604	-1.443460045	0.258764695
-0.218367989	0.524955976	4	0	0.998207054
ELOVL2	ENSG00000197977.3	0.690641802	3	2	0.296731701
Brain_Cortex	0.74566028 Brain_Caudate_basal_ganglia 2.420088657	0.06477668
0.515134662	0.324366895	1.043467768	0.729988401	0.368298991
2	0	0.998207054
TRPM4 ENSG00000130529.15	0.690849816	6	6	0.161443828	Brain_Cerebellum
0.901430927	Brain_Cortex	1.988373998	0.472653762	0.472653762
-0.362617995	1.400232093	0.529705341	0.693113016	6	0
0.998207054
IFIT3 ENSG00000119917.13	0.690864526	1	1	0.690864526
Brain_Spinal_cord_cervical_c-1	0.690864526	Brain_Spinal_cord_cervical_c-1
1	1	1	0.397682111	0.397682111	0.397682111	NA	1	0
0.998207054
NIP7 ENSG00000132603.13	0.690873008	4	3	0.45345148
Brain_Frontal_Cortex_BA9	0.780046053	Brain_Nucleus_accumbens_basal_ganglia
3.115182532	0.034012336	0.35319299 -0.27925902	0.749673319
0.336053001	0.437616865	3	0	0.998207054
PNLDC1	ENSG00000146453.12	0.690895047	11	2	0.308989233
Brain_Substantia_nigra	0.830425365	Brain_Nucleus_accumbens_basal_ganglia
9.680132067	0.000611677	0.989157377	0.214156053	1.017345218
0.698464479	0.233826032	2	0	0.998207054
HIST1H2BE	ENSG00000274290.2	0.690908588	1	1	0.690908588
Brain_Anterior_cingulate_cortex_BA24	0.690908588
Brain_Anterior_cingulate_cortex_BA24	1	1	1	-0.397622344
-0.397622344	-0.397622344	NA	1	0	0.998207054
TDRD6 ENSG00000180113.15	0.691209079	13	6	0.158426521
Brain_Anterior_cingulate_cortex_BA24	0.992473915	Brain_Cerebellum
8.480829807	0.003834275	0.317656867	-1.298014473	1.410383343
-0.41579053	0.655872641	6	0	0.998207054
RAPGEFL1	ENSG00000108352.11	0.691222613	4	4	0.196546725
Brain_Cerebellar_Hemisphere 0.533929602	Brain_Frontal_Cortex_BA9
1.821836728	0.340640198	0.340640198	-1.291452761	-0.622018654
-0.899360795	0.310990412	4	0	0.998207054
ATG9A ENSG00000198925.10	0.691274479	2	2	0.443154602	Brain_Amygdala

0.600999781	Brain_Cerebellum 1.185074652	0.814925348	0.814925348
-0.522963318	0.76687708 0.121956881	0.912054892	2	0
0.998207054
TMC4 ENSG00000167608.11	0.691308188	13	2	0.27381058 Brain_Hypothalamus
0.965150279	Brain_Substantia_nigra	10.78575931	0.000113354
1.386745562	-0.273203899	1.094329298	0.366141165	0.336923813
2	0	0.998207054
L3HYPDH	ENSG00000126790.11	0.691356401	13	1	0.442344361
Brain_Spinal_cord_cervical_c-1	0.972047478	Brain_Cerebellar_Hemisphere
12.39056961	0.001336091	12.39056961	-0.768240426	-0.035040461
-0.38555395	0.243048864	1	0	0.998207054
CASP7 ENSG00000165806.19	0.691707491	12	4	0.161739746	Brain_Amygdala
0.887135219	Brain_Spinal_cord_cervical_c-1	9.289148843	0.009076713
0.366958932	-1.399244266	0.141930099	-0.964303872	0.440096491
4	0	0.998207054
ZFAND6	ENSG00000086666.18	0.691722326	4	4	0.456012404
Brain_Anterior_cingulate_cortex_BA24	0.969896983	Brain_Cerebellum
2.020933331	0.082812501	0.082812501	-0.186425179	0.745429022
0.246484314	0.43387551 4	0	0.998207054
RP11-1079K10.3	ENSG00000248714.6	0.691829704	1	1	0.691829704
Brain_Hypothalamus	0.691829704	Brain_Hypothalamus	1	1	1
-0.39637324	-0.39637324	-0.39637324	NA	1	0	0.998207054
RP11-689P11.2	ENSG00000205959.3	0.691894171	13	2	0.273256418
Brain_Hippocampus	0.692293029	Brain_Cerebellar_Hemisphere 11.16840132
0.011299131	0.557028774	-1.095594162	-0.39574517	-0.622141732
0.246789893	2	0	0.998207054
TPSD1 ENSG00000095917.13	0.691936844	12	3	0.126987085	Brain_Cerebellum
0.835537425	Brain_Hypothalamus	9.558746257	0.009876407
0.457672583	0.207604968	1.526091475	1.023321755	0.408978323
3	0	0.998207054
HINT3 ENSG00000111911.6	0.69193835 1	1	0.69193835 Brain_Cerebellum 0.69193835
Brain_Cerebellum 1	1	1	-0.396225949	-0.396225949	-0.396225949
NA	1	0	0.998207054
TACC1 ENSG00000147526.19	0.691956901	1	1	0.691956901
Brain_Cerebellar_Hemisphere 0.691956901	Brain_Cerebellar_Hemisphere 1	1
1	0.396200801	0.396200801	0.396200801	NA	1	0
0.998207054
NCCRP1	ENSG00000188505.4	0.692159702	1	1	0.692159702
Brain_Cerebellar_Hemisphere 0.692159702	Brain_Cerebellar_Hemisphere 1	1
1	0.395925889	0.395925889	0.395925889	NA	1	0
0.998207054
GPAT4 ENSG00000158669.11	0.692173351	2	2	0.461695574	Brain_Cerebellum
0.68412596 Brain_Cortex	1.045146147	0.954853853	0.954853853
-0.736057717	-0.406839382	-0.571448549	0.232792517	2	0
0.998207054
FOXE1 ENSG00000178919.8	0.692182683	1	1	0.692182683	Brain_Cerebellum
0.692182683	Brain_Cerebellum 1	1	1	0.395894738	0.395894738
0.395894738	NA	1	0	0.998207054
LY6D ENSG00000167656.4	0.692193196	2	2	0.649714761
Brain_Frontal_Cortex_BA9	0.674883372	Brain_Cortex	1.481079445
0.518920555	0.518920555	-0.41945536	0.454158485	0.017351563
0.617738274	2	0	0.998207054
S100A10	ENSG00000197747.8	0.692202868	7	4	0.151976171
Brain_Substantia_nigra	0.979787225	Brain_Nucleus_accumbens_basal_ganglia
4.768883378	0.015208039	0.217760736	-1.43258605	0.294079166
-0.18459588	0.567064539	4	0	0.998207054
ZNF782	ENSG00000196597.11	0.692203441	1	1	0.692203441
Brain_Cerebellum 0.692203441	Brain_Cerebellum 1	1	1	-0.395866601
-0.395866601	-0.395866601	NA	1	0	0.998207054
AC007106.1 ENSG00000251410.1	0.692253652	6	6	0.122057392
Brain_Substantia_nigra	0.792670371	Brain_Hippocampus	3.721331218
0.194900672	0.194900672	-1.546195367	0.262844583	-0.636733668

0.576558351 6 0 0.998207054
PUSL1 ENSG00000169972.11 0.692431663 3 3 0.27217503
Brain_Nucleus_accumbens_basal_ganglia 0.670638259 Brain_Amygdala
1.485021669 0.529326888 0.529326888 0.425272197 1.098067466
0.72022027 0.343973207 3 0 0.998207054
RPP14 ENSG00000163684.11 0.692447266 7 5 0.252460226
Brain_Frontal_Cortex_BA9 0.993206598 Brain_Cerebellum 4.840661933
0.110253864 0.213349483 -1.14439412 0.480353456 -0.43683322
0.595001007 5 0 0.998207054
FGFR2 ENSG00000066468.22 0.692517283 3 3 0.243487938 Brain_Cortex
0.974796817 Brain_Frontal_Cortex_BA9 1.060038318 0.953956376
0.953956376 -1.166312952 0.265631436 -0.310758092 0.755689185
3 0 0.998207054
TWNK ENSG00000107815.7 0.692549255 1 1 0.692549255
Brain_Hippocampus 0.692549255 Brain_Hippocampus 1 1 1
-0.395397905 -0.395397905 -0.395397905 NA 1 0 0.998207054
C1orf61 ENSG00000125462.16 0.692555034 1 1 0.692555034
Brain_Cerebellum 0.692555034 Brain_Cerebellum 1 1 1 0.395390073
0.395390073 0.395390073 NA 1 0 0.998207054
ADAMTSL1 ENSG00000178031.16 0.692648066 3 3 0.263102277
Brain_Cerebellar_Hemisphere 0.685227986 Brain_Spinal_cord_cervical_c-1
1.845631798 0.157931525 0.157931525 0.405339486 1.119088755
0.837890423 0.380184446 3 0 0.998207054
GRTP1 ENSG00000139835.13 0.692714948 1 1 0.692714948
Brain_Anterior_cingulate_cortex_BA24 0.692714948
Brain_Anterior_cingulate_cortex_BA24 1 1 1 0.395173365
0.395173365 0.395173365 NA 1 0 0.998207054
ZNF415 ENSG00000170954.11 0.69281747 11 9 0.111875536 Brain_Cortex
0.898756913 Brain_Anterior_cingulate_cortex_BA24 3.978117537
0.029265456 0.197684656 -1.589819326 0.713977189 -0.272372418
0.643829069 9 0 0.998207054
FANCC ENSG00000158169.12 0.692853613 3 3 0.300425371 Brain_Cerebellum
0.887501759 Brain_Cerebellar_Hemisphere 1.113204332 0.870417723
0.870417723 -0.141466075 1.035521628 0.523470429 0.603204319
3 0 0.998207054
TRIT1 ENSG00000043514.15 0.692900428 12 8 0.295700548
Brain_Frontal_Cortex_BA9 0.86629607 Brain_Putamen_basal_ganglia 5.392502211
3.55E-05 0.377622678 -1.045697866 1.014741129 0.223594787
0.609899559 8 0 0.998207054
RP5-1007H16.1 ENSG00000272745.1 0.692956471 3 3 0.38192068
Brain_Caudate_basal_ganglia 0.712737872 Brain_Cortex 1.331227183
0.626014288 0.626014288 -0.750420351 0.874362853 0.164041348
0.831405454 3 0 0.998207054
TULP1 ENSG00000112041.12 0.693003259 9 3 0.314730745 Brain_Cortex
0.983060114 Brain_Nucleus_accumbens_basal_ganglia 7.512548706
0.002419261 0.459822314 -1.005345018 -0.021232593 -0.589994377
0.383309466 3 0 0.998207054
INF2 ENSG00000203485.12 0.693006347 2 2 0.42068081
Brain_Frontal_Cortex_BA9 0.725035638 Brain_Nucleus_accumbens_basal_ganglia
1.075745184 0.924254816 0.924254816 0.351736829 0.805240673
0.578488751 0.320675644 2 0 0.998207054
SLAMF8 ENSG00000158714.10 0.693008911 8 4 0.169811548
Brain_Substantia_nigra 0.990275701 Brain_Cerebellar_Hemisphere
5.634597704 2.45E-19 0.33892082 -1.372809591 0.415555517
-0.113224018 0.645219127 4 0 0.998207054
CYTIP ENSG00000115165.9 0.69303015 1 1 0.69303015
Brain_Nucleus_accumbens_basal_ganglia 0.69303015
Brain_Nucleus_accumbens_basal_ganglia 1 1 1 0.394746272
0.394746272 0.394746272 NA 1 0 0.998207054
C14orf93 ENSG00000100802.14 0.693087728 1 1 0.693087728
Brain_Cortex 0.693087728 Brain_Cortex 1 1 1 0.394668263
0.394668263 0.394668263 NA 1 0 0.998207054

FAT4 ENSG00000196159.11 0.69310489 1 1 0.69310489 Brain_Cortex 0.69310489 Brain_Cortex 1 1 1 -0.394645012 -0.394645012 -0.394645012 NA 1 0 0.998207054
RP11-989F5.3 ENSG00000256299.1 0.693109745 1 1 0.693109745 Brain_Cerebellum 0.693109745 Brain_Cerebellum 1 1 1 -0.394638434 -0.394638434 -0.394638434 NA 1 0 0.998207054
ZNF710 ENSG00000140548.9 0.693132818 1 1 0.693132818 Brain_Nucleus_accumbens_basal_ganglia 0.693132818 Brain_Nucleus_accumbens_basal_ganglia 1 1 1 0.394607174 0.394607174 0.394607174 NA 1 0 0.998207054
COX16 ENSG00000133983.14 0.693190314 7 3 0.570172771 Brain_Frontal_Cortex_BA9 0.95449615 Brain_Caudate_basal_ganglia 6.150872144 0.024266726 0.249246055 -0.567797069 0.324333928 -0.211158968 0.318644089 3 0 0.998207054
IGFBP3 ENSG00000146674.14 0.693520273 3 3 0.44604991 Brain_Substantia_nigra 0.686374559 Brain_Cerebellum 1.318965593 0.666814368 0.666814368 -0.762016912 0.403779927 -0.303288795 0.621309391 3 0 0.998207054
C7orf31 ENSG00000153790.11 0.69352707 1 1 0.69352707 Brain_Cortex 0.69352707 Brain_Cortex 1 1 1 0.394073101 0.394073101 0.394073101 NA 1 0 0.998207054
DUS3L ENSG00000141994.15 0.693529438 11 3 0.231117251 Brain_Hippocampus 0.79451171 Brain_Cerebellar_Hemisphere 9.716842981 0.001258076 0.32495471 -1.197485822 1.012380848 0.577702945 0.623907089 3 0 0.998207054
LTB4R2 ENSG00000213906.9 0.69356552 1 1 0.69356552 Brain_Putamen_basal_ganglia 0.69356552 Brain_Putamen_basal_ganglia 1 1 1 -0.39402102 -0.39402102 -0.39402102 NA 1 0 0.998207054
TBC1D1 ENSG00000065882.15 0.693615836 4 4 0.15104254 Brain_Hypothalamus 0.947900405 Brain_Cerebellum 1.349842119 0.648733306 0.648733306 -1.435858746 -0.06534363 -0.57341643 0.595757432 4 0 0.998207054
METTL7A ENSG00000185432.11 0.693642058 2 2 0.461565459 Brain_Cerebellum 0.653381553 Brain_Substantia_nigra 1.018780044 0.981219956 0.981219956 -0.449069457 0.736271546 0.143601045 0.838162661 2 0 0.998207054
RP3-467K16.4 ENSG00000228140.1 0.693714631 8 8 0.151430406 Brain_Amygdala 0.938048834 Brain_Cerebellar_Hemisphere 1.999558461 0.420587073 0.420587073 -1.434497275 0.951264119 -0.221938097 0.747041875 8 0 0.998207054
FBXL17 ENSG00000145743.15 0.693773194 3 3 0.260469823 Brain_Spinal_cord_cervical_c-1 0.799551393 Brain_Cerebellar_Hemisphere 1.163301166 0.833604449 0.833604449 -1.125281366 0.357466882 -0.171295585 0.827796317 3 0 0.998207054
LRRN4 ENSG00000125872.7 0.693862463 1 1 0.693862463 Brain_Putamen_basal_ganglia 0.693862463 Brain_Putamen_basal_ganglia 1 1 1 -0.393618849 -0.393618849 -0.393618849 NA 1 0 0.998207054
XPO1 ENSG00000082898.16 0.693869325 1 1 0.693869325 Brain_Amygdala 0.693869325 Brain_Amygdala 1 1 1 -0.393609556 -0.393609556 -0.393609556 NA 1 0 0.998207054
RP11-274B21.9 ENSG00000271344.1 0.693911857 11 6 0.117507178 Brain_Cerebellum 0.980032436 Brain_Hippocampus 7.404312329 0.007596726 0.257690977 -0.025028243 1.565323063 0.59173618 0.438360128 6 0 0.998207054
DLEC1 ENSG00000008226.19 0.693975869 1 1 0.693975869 Brain_Caudate_basal_ganglia 0.693975869 Brain_Caudate_basal_ganglia 1 1 1 0.393465271 0.393465271 0.393465271 NA 1 0 0.998207054
RP11-63G10.3 ENSG00000272226.1 0.694029134 1 1 0.694029134 Brain_Spinal_cord_cervical_c-1 0.694029134 Brain_Spinal_cord_cervical_c-1 1 1 1 0.393393142 0.393393142 0.393393142 NA 1 0

0.998207054
YAE1D1 ENSG00000241127.7 0.694123372 1 1 0.694123372
Brain_Anterior_cingulate_cortex_BA24 0.694123372
Brain_Anterior_cingulate_cortex_BA24 1 1 1 -0.393265533
-0.393265533 -0.393265533 NA 1 0 0.998207054
PRSS56 ENSG00000237412.6 0.694256066 5 5 0.336757009
Brain_Cortex 0.819600071 Brain_Frontal_Cortex_BA9 2.833237574
0.118182733 0.118182733 -0.960592683 0.741912315 -0.191019834
0.692245647 5 0 0.998207054
MANBA ENSG00000109323.8 0.69430308 6 6 0.127921814 Brain_Cerebellum
0.933763362 Brain_Spinal_cord_cervical_c-1 2.170100123 0.178176543
0.178176543 -0.724996869 1.522348371 0.31050569 0.84529262 6 0
0.998207054
PRKCDBP ENSG00000170955.9 0.694335871 4 4 0.265610598
Brain_Cerebellar_Hemisphere 0.980661422 Brain_Caudate_basal_ganglia 2.18479668
0.073245446 0.073245446 -1.11322782 0.024239687 -0.783277469
0.541263127 4 0 0.998207054
RP1-80N2.3 ENSG00000261211.1 0.69467934 1 1 0.69467934
Brain_Cerebellar_Hemisphere 0.69467934 Brain_Cerebellar_Hemisphere 1 1 1
-0.392512821 -0.392512821 -0.392512821 NA 1 0 0.998207054
NEURL4 ENSG00000215041.9 0.694845571 2 2 0.393586072
Brain_Hippocampus 0.931027135 Brain_Cerebellar_Hemisphere 1.121503536
0.878496464 0.878496464 -0.853132064 -0.086552611 -0.469842337
0.542053529 2 0 0.998207054
CECR1 ENSG00000093072.15 0.694918977 10 10 0.15320413 Brain_Cortex
0.957349087 Brain_Substantia_nigra 2.881147652 0.136950287
0.136950287 -1.428304859 1.173030096 -0.346628456 0.906125879
10 0 0.998207054
THOC7 ENSG00000163634.11 0.695049068 10 5 0.236264589
Brain_Putamen_basal_ganglia 0.990372492 Brain_Frontal_Cortex_BA9
7.136232818 0.02231556 0.286517167 -0.810429686 1.184375159
0.494202245 0.595334849 5 0 0.998207054
LINC01167 ENSG00000224758.1 0.695074356 5 4 0.384771985
Brain_Amygdala 0.913650814 Brain_Putamen_basal_ganglia 3.897670571
0.04224354 0.140635591 -0.549493661 0.869137395 -0.015155842
0.526685461 4 0 0.998207054
SEPHS1 ENSG00000086475.14 0.695092739 1 1 0.695092739
Brain_Frontal_Cortex_BA9 0.695092739 Brain_Frontal_Cortex_BA9 1 1
1 0.391953274 0.391953274 0.391953274 NA 1 0
0.998207054
CCDC141 ENSG00000163492.14 0.695097006 3 3 0.357361423
Brain_Caudate_basal_ganglia 0.983120003 Brain_Nucleus_accumbens_basal_ganglia
2.250306986 0.234724071 0.234724071 -0.92040449 0.021157517
-0.56228035 0.509616974 3 0 0.998207054
SOAT1 ENSG00000057252.12 0.695128486 3 3 0.279339918
Brain_Caudate_basal_ganglia 0.677874412 Brain_Nucleus_accumbens_basal_ganglia
2.501346268 0.156738669 0.156738669 0.415365427 1.081803349
0.736915371 0.333831355 3 0 0.998207054
C16orf62 ENSG00000103544.14 0.695144427 7 5 0.104947862
Brain_Hippocampus 0.939428835 Brain_Putamen_basal_ganglia 4.920447528
0.115893627 0.250665461 0.075987762 1.621325438 0.79921691
0.456231196 5 0 0.998207054
PACS2 ENSG00000179364.13 0.695195143 2 2 0.44161694
Brain_Spinal_cord_cervical_c-1 0.645226301 Brain_Cortex 1.124466647
0.875533353 0.875533353 -0.769465631 0.460403949 -0.154530841
0.86964912 2 0 0.998207054
MPV17L2 ENSG00000254858.9 0.695200439 1 1 0.695200439
Brain_Cerebellar_Hemisphere 0.695200439 Brain_Cerebellar_Hemisphere 1 1
1 0.391807519 0.391807519 0.391807519 NA 1 0
0.998207054
ASF1B ENSG00000105011.8 0.695204335 1 1 0.695204335 Brain_Amygdala
0.695204335 Brain_Amygdala 1 1 1 -0.391802247 -0.391802247

-0.391802247	NA	1	0	0.998207054
ARHGEF28	ENSG00000214944.9	0.695245371	1	1	0.695245371
Brain_Hippocampus	0.695245371	Brain_Hippocampus	1	1	1
0.391746714	0.391746714	0.391746714	NA	1	0	0.998207054
SOWAHC	ENSG00000198142.4	0.695421222	1	1	0.695421222
Brain_Anterior_cingulate_cortex_BA24	0.695421222
Brain_Anterior_cingulate_cortex_BA24	1	1	1	0.391508751
0.391508751	0.391508751	NA	1	0	0.998207054
EIF3J-AS1	ENSG00000179523.4	0.695432197	1	1	0.695432197
Brain_Cortex	0.695432197	Brain_Cortex	1	1	1	0.3914939
0.3914939	0.3914939	NA	1	0	0.998207054
SLC5A6	ENSG00000138074.14	0.695461194	3	3	0.312598447
Brain_Cerebellar_Hemisphere	0.998125959	Brain_Frontal_Cortex_BA9
1.887880934	0.218224129	0.218224129	-0.002348764	1.009784709
0.513991211	0.506379463	3	0	0.998207054
TMEM268	ENSG00000157693.14	0.695579891	3	3	0.244231332
Brain_Cerebellum	0.641554547	Brain_Frontal_Cortex_BA9	2.269697543
0.175957941	0.175957941	-1.164475584	-0.465526384	-0.829819322
0.350415818	3	0	0.998207054
RP11-497H17.1	ENSG00000262663.1	0.695605187	2	2	0.47731963
Brain_Cerebellar_Hemisphere	0.665238716	Brain_Cerebellum	1.89882709	0.10117291
0.10117291	0.432691762	0.710620187	0.571655974	0.196525074	2
0	0.998207054
TAS2R43	ENSG00000255374.3	0.695618309	2	1	0.636928455
Brain_Cerebellum	0.759635904	Brain_Cerebellar_Hemisphere	1.976922499
0.023077501	1.976922499	-0.471997666	-0.305958946	-0.388978306
0.117407104	1	0	0.998207054
ELF2	ENSG00000109381.19	0.695627245	2	2	0.422109624
Brain_Hippocampus	0.491903822	Brain_Spinal_cord_cervical_c-1
1.562116697	0.437883303	0.437883303	0.687283932	0.802766645
0.745025289	0.08165861	2	0	0.998207054
AP000318.2	ENSG00000214955.5	0.695693246	1	1	0.695693246
Brain_Putamen_basal_ganglia	0.695693246	Brain_Putamen_basal_ganglia	1	1
1	-0.391140689	-0.391140689	-0.391140689	NA	1	0
0.998207054
CFHR1	ENSG00000244414.6	0.695714817	4	4	0.162211186
Brain_Anterior_cingulate_cortex_BA24	0.956353178	Brain_Hippocampus
2.471293874	0.182240277	0.182240277	-0.05473049	1.397673331
0.487206796	0.632513963	4	0	0.998207054
RP11-981G7.3	ENSG00000253678.1	0.695844491	13	4	0.111237617
Brain_Spinal_cord_cervical_c-1	0.678056754	Brain_Hypothalamus
10.07532029	9.29E-05	0.51078564	-0.48587966	1.592654961
0.823838439	0.495848659	4	0	0.998207054
SRP14-AS1	ENSG00000248508.6	0.695897631	13	11	0.092196619
Brain_Putamen_basal_ganglia	0.981573458	Brain_Cortex	4.455678237
0.003972912	0.173635914	-1.605167134	1.683922648	-0.059010933
0.83265685	11	0	0.998207054
ANKRD30BL	ENSG00000163046.15	0.695920753	12	4	0.212378824
Brain_Amygdala	0.919432439	Brain_Anterior_cingulate_cortex_BA24
9.110970643	0.000755279	0.365861049	-1.063524755	1.247050928
0.580921904	0.615748914	4	0	0.998207054
RP11-244E17.1	ENSG00000283654.1	0.695978438	1	1	0.695978438
Brain_Substantia_nigra	0.695978438	Brain_Substantia_nigra	1	1
1	0.390754869	0.390754869	0.390754869	NA	1	0
0.998207054
PADI1	ENSG00000142623.9	0.696005217	2	2	0.478774764
Brain_Hypothalamus	0.777781383	Brain_Spinal_cord_cervical_c-1
1.247710357	0.752289643	0.752289643	-0.282211445	0.708274573
0.213031564	0.70037938	2	0	0.998207054
ABTB2	ENSG00000166016.5	0.696313596	1	1	0.696313596
Brain_Nucleus_accumbens_basal_ganglia	0.696313596
Brain_Nucleus_accumbens_basal_ganglia	1	1	1	-0.390301524

-0.390301524 -0.390301524 NA 1 0 0.998207054
MAP2 ENSG00000078018.19 0.696417235 1 1 0.696417235
Brain_Nucleus_accumbens_basal_ganglia 0.696417235
Brain_Nucleus_accumbens_basal_ganglia 1 1 1 0.390161356
0.390161356 0.390161356 NA 1 0 0.998207054
TCP10L2 ENSG00000166984.11 0.696482649 2 1 0.633092065
Brain_Nucleus_accumbens_basal_ganglia 0.76701297 Brain_Caudate_basal_ganglia
1.966902282 0.033097718 1.966902282 -0.477379281 -0.296284306
-0.386831794 0.128053485 1 0 0.998207054
ELMOD3 ENSG00000115459.17 0.696487871 1 1 0.696487871
Brain_Cerebellum 0.696487871 Brain_Cerebellum 1 1 1 0.390065828
0.390065828 0.390065828 NA 1 0 0.998207054
RP11-526I2.5 ENSG00000270127.2 0.696688074 13 2 0.237351989
Brain_Substantia_nigra 0.703809104 Brain_Frontal_Cortex_BA9
11.08715976 0.003985505 1.021230835 -1.181631399 -0.380183638
-0.782069139 0.244800053 2 0 0.998207054
CYP1B1 ENSG00000138061.11 0.696866934 1 1 0.696866934
Brain_Caudate_basal_ganglia 0.696866934 Brain_Caudate_basal_ganglia 1 1
1 0.389553241 0.389553241 0.389553241 NA 1 0
0.998207054
SEC14L2 ENSG00000100003.17 0.696895894 3 3 0.289113239
Brain_Caudate_basal_ganglia 0.926074801 Brain_Cerebellar_Hemisphere
2.000416807 0.097701497 0.097701497 -1.060068936 0.092784454
-0.380579486 0.603434776 3 0 0.998207054
C8orf37-AS1 ENSG00000253773.2 0.696934548 2 2 0.414617976
Brain_Amygdala 0.819154964 Brain_Hippocampus 1.01137105 0.98862895
0.98862895 -0.815793982 -0.228631984 -0.522212983 0.415186231 2
0 0.998207054
SPATA13 ENSG00000228741.2 0.696946288 6 5 0.12564049 Brain_Cortex
0.640234301 Brain_Hippocampus 4.037886733 0.002250261
0.213765441 0.467371232 1.531521772 0.828168764 0.392004883
5 0 0.998207054
ANK2 ENSG00000145362.17 0.696968821 1 1 0.696968821
Brain_Cerebellar_Hemisphere 0.696968821 Brain_Cerebellar_Hemisphere 1 1
1 -0.389415482 -0.389415482 -0.389415482 NA 1 0
0.998207054
F2RL1 ENSG00000164251.4 0.697049345 6 4 0.471792382 Brain_Amygdala
0.980826375 Brain_Frontal_Cortex_BA9 4.55988705 0.003841074
0.237201201 -0.236961935 0.719565789 0.249084857 0.366989566
4 0 0.998207054
EIF2D ENSG00000143486.15 0.697064174 3 3 0.323451436 Brain_Cortex
0.682875539 Brain_Anterior_cingulate_cortex_BA24 1.881361387
0.108658981 0.108658981 -0.987390099 0.702321405 0.041157888
0.902778907 3 0 0.998207054
HNF4A ENSG00000101076.16 0.697079762 2 2 0.400058623 Brain_Cortex
0.917995019 Brain_Frontal_Cortex_BA9 1.016014237 0.983985763
0.983985763 -0.10295962 0.841516541 0.36927846 0.667845498 2
0 0.998207054
EIF3C ENSG00000184110.14 0.69709734 8 5 0.089321417
Brain_Substantia_nigra 0.588127533 Brain_Hypothalamus 5.314015397
1.20E-06 0.208138435 0.541551468 1.698988149 1.119488502
0.340513291 5 0 0.998207054
PDCD5 ENSG00000105185.11 0.697107192 12 10 0.064106956 Brain_Cerebellum
0.923910881 Brain_Putamen_basal_ganglia 5.653255572 0.07018626
0.215915422 0.095508573 1.851435293 0.857109224 0.524322952
10 0 0.998207054
FGFR1OP ENSG00000213066.11 0.697348846 3 3 0.395801757
Brain_Frontal_Cortex_BA9 0.965526178 Brain_Caudate_basal_ganglia
1.347351148 0.649378536 0.649378536 -0.04321998 0.849142944
0.507799894 0.481716903 3 0 0.998207054
MRPL58 ENSG00000167862.9 0.697390939 2 2 0.400697512
Brain_Spinal_cord_cervical_c-1 0.769245454 Brain_Substantia_nigra

1.208530209 0.791469791 0.791469791 -0.840376159 -0.293362018
-0.566869088 0.386797409 2 0 0.998207054
FKBP14 ENSG00000106080.10 0.697425584 3 3 0.360098874
Brain_Caudate_basal_ganglia 0.740159888 Brain_Cerebellum 1.854504552
0.419493242 0.419493242 -0.33164162 0.915176702 0.38663818
0.644701711 3 0 0.998207054
C11orf24 ENSG00000171067.10 0.697613906 8 5 0.079954915
Brain_Hypothalamus 0.91881017 Brain_Cerebellar_Hemisphere 5.083422854
0.007651683 0.297480384 -0.101932604 1.750947725 0.774809049
0.548230674 5 0 0.998207054
NIPAL1 ENSG00000163293.11 0.697652323 4 4 0.368725015
Brain_Caudate_basal_ganglia 0.989916284 Brain_Hypothalamus 2.956940389
0.145902812 0.145902812 -0.012638401 0.898864171 0.270854985
0.425655866 4 0 0.998207054
PRSS33 ENSG00000103355.13 0.697663716 3 3 0.257620149
Brain_Anterior_cingulate_cortex_BA24 0.884200871 Brain_Amygdala
1.013659565 0.9869687 0.9869687 -0.366775334 1.132033981
0.206537557 0.809093487 3 0 0.998207054
SOAT2 ENSG00000167780.11 0.697680106 1 1 0.697680106
Brain_Spinal_cord_cervical_c-1 0.697680106 Brain_Spinal_cord_cervical_c-1
1 1 1 0.388453977 0.388453977 0.388453977 NA 1 0
0.998207054
CD248 ENSG00000174807.3 0.697846244 1 1 0.697846244
Brain_Hypothalamus 0.697846244 Brain_Hypothalamus 1 1 1
-0.388229446 -0.388229446 -0.388229446 NA 1 0 0.998207054
FGFBP3 ENSG00000174721.9 0.697886114 2 2 0.546874085
Brain_Hypothalamus 0.561561649 Brain_Cerebellum 1.027045969
0.972954031 0.972954031 -0.602450828 0.580523493 -0.010963668
0.836489164 2 0 0.998207054
RP6-65G23.3 ENSG00000259153.1 0.6979196 2 2 0.479240596
Brain_Hippocampus 0.697317751 Brain_Spinal_cord_cervical_c-1
1.107438838 0.892561162 0.892561162 -0.388943759 0.707524494
0.159290368 0.775320138 2 0 0.998207054
MAP3K9 ENSG00000006432.15 0.698039676 5 5 0.293896033
Brain_Putamen_basal_ganglia 0.690461756 Brain_Anterior_cingulate_cortex_BA24
2.682261177 0.103943185 0.103943185 -1.049613098 0.838587405
0.107235032 0.80078918 5 0 0.998207054
POLR2B ENSG00000047315.15 0.698078511 3 3 0.469673642
Brain_Hypothalamus 0.502271191 Brain_Frontal_Cortex_BA9 1.083304579
0.907958763 0.907958763 -0.670920477 0.72301016 0.241278755
0.790403421 3 0 0.998207054
RP11-213G2.2 ENSG00000230303.5 0.698258843 1 1 0.698258843
Brain_Substantia_nigra 0.698258843 Brain_Substantia_nigra 1 1
1 -0.387671913 -0.387671913 -0.387671913 NA 1 0
0.998207054
LEMD3 ENSG00000174106.2 0.698319118 1 1 0.698319118 Brain_Cerebellum
0.698319118 Brain_Cerebellum 1 1 1 -0.387590476 -0.387590476
-0.387590476 NA 1 0 0.998207054
MTDH ENSG00000147649.9 0.698351319 2 2 0.441556545 Brain_Cortex
0.965088196 Brain_Putamen_basal_ganglia 1.371146115 0.628853885
0.628853885 -0.043769428 0.769567408 0.36289899 0.575115992 2
0 0.998207054
CDK5RAP1 ENSG00000101391.20 0.698412011 1 1 0.698412011
Brain_Cortex 0.698412011 Brain_Cortex 1 1 1 -0.387464973
-0.387464973 -0.387464973 NA 1 0 0.998207054
TMEM147 ENSG00000105677.11 0.69850513 1 1 0.69850513 Brain_Hippocampus
0.69850513 Brain_Hippocampus 1 1 1 -0.387339171 -0.387339171
-0.387339171 NA 1 0 0.998207054
ALKBH8 ENSG00000137760.14 0.698576802 2 2 0.397000843
Brain_Cortex 0.566732842 Brain_Nucleus_accumbens_basal_ganglia
1.671674367 0.328325633 0.328325633 0.572869817 0.846989736
0.709929776 0.193832053 2 0 0.998207054

RP11-498P14.3 ENSG00000242375.1 0.698582545 2 2 0.405239055
Brain_Spinal_cord_cervical_c-1 0.985458328 Brain_Hypothalamus
1.164284763 0.835715237 0.835715237 -0.018226292 0.832301019
0.407037364 0.601413629 2 0 0.998207054
MYH11 ENSG00000133392.17 0.698583423 1 1 0.698583423 Brain_Amygdala
0.698583423 Brain_Amygdala 1 1 1 0.387233404 0.387233404
0.387233404 NA 1 0 0.998207054
PANX1 ENSG00000110218.8 0.698680234 5 4 0.12456796
Brain_Spinal_cord_cervical_c-1 0.883853185 Brain_Cortex 2.884138399
0.095940122 0.215606317 -0.297441627 1.535879407 0.354390524
0.814596344 4 0 0.998207054
LGALS2 ENSG00000100079.6 0.698791508 1 1 0.698791508
Brain_Substantia_nigra 0.698791508 Brain_Substantia_nigra 1 1
1 0.386952318 0.386952318 0.386952318 NA 1 0
0.998207054
MRPL23 ENSG00000214026.10 0.698825376 11 10 0.102486046
Brain_Cerebellar_Hemisphere 0.945733336 Brain_Hypothalamus 4.797639663
0.123750265 0.172801073 -1.562365278 1.632918939 -0.164784469
0.76925475 10 0 0.998207054
ST5 ENSG00000166444.18 0.698917937 1 1 0.698917937
Brain_Frontal_Cortex_BA9 0.698917937 Brain_Frontal_Cortex_BA9 1 1
1 0.38678155 0.38678155 0.38678155 NA 1 0 0.998207054
GSDMA ENSG00000167914.11 0.699208759 3 3 0.257072775
Brain_Spinal_cord_cervical_c-1 0.440972292 Brain_Substantia_nigra
2.642106868 0.131505184 0.131505184 -1.133336964 -0.770552388
-0.963990958 0.182588337 3 0 0.998207054
GOSR2 ENSG00000108433.16 0.699228732 5 3 0.233836827
Brain_Spinal_cord_cervical_c-1 0.834318024 Brain_Cortex 3.945527665
0.034112521 0.184755924 -1.190533359 -0.209166806 -0.645593364
0.419466203 3 0 0.998207054
NUAK2 ENSG00000163545.8 0.699285836 2 2 0.400272848 Brain_Cerebellum
0.601388382 Brain_Cerebellar_Hemisphere 1.53166032 0.46833968 0.46833968
0.52240499 0.841134038 0.681769514 0.225375471 2 0
0.998207054
MAGOH ENSG00000162385.10 0.699320832 1 1 0.699320832 Brain_Cerebellum
0.699320832 Brain_Cerebellum 1 1 1 -0.386237435 -0.386237435
-0.386237435 NA 1 0 0.998207054
UST ENSG00000111962.7 0.699320896 6 5 0.130203886 Brain_Cortex
0.696728651 Brain_Cerebellar_Hemisphere 2.675196382 0.043304684
0.368076138 -1.513298371 0.992982479 -0.58553623 1.0225194 5
0 0.998207054
ABCD3 ENSG00000117528.11 0.699346486 1 1 0.699346486 Brain_Cortex
0.699346486 Brain_Cortex 1 1 1 0.386202792 0.386202792
0.386202792 NA 1 0 0.998207054
CTD-2081C10.7 ENSG00000272416.1 0.699350125 1 1 0.699350125
Brain_Hypothalamus 0.699350125 Brain_Hypothalamus 1 1 1
-0.386197878 -0.386197878 -0.386197878 NA 1 0 0.998207054
KRT81 ENSG00000205426.10 0.69935335 10 4 0.425987773 Brain_Amygdala
0.833593154 Brain_Putamen_basal_ganglia 7.88938153 0.001483516
0.379389079 -0.423941087 0.796076155 0.444032761 0.372799511
4 0 0.998207054
PPP1R9A ENSG00000158528.11 0.699363378 1 1 0.699363378
Brain_Nucleus_accumbens_basal_ganglia 0.699363378
Brain_Nucleus_accumbens_basal_ganglia 1 1 1 0.386179982
0.386179982 0.386179982 NA 1 0 0.998207054
DAO ENSG00000110887.7 0.699501712 2 2 0.660104376 Brain_Cerebellum
0.892778658 Brain_Spinal_cord_cervical_c-1 1.760212455 0.239787545
0.239787545 -0.439769064 0.134789057 -0.152490003 0.406273944
2 0 0.998207054
FAM170B ENSG00000172538.6 0.699639602 1 1 0.699639602
Brain_Spinal_cord_cervical_c-1 0.699639602 Brain_Spinal_cord_cervical_c-1
1 1 1 0.385807012 0.385807012 0.385807012 NA 1 0

0.998207054
ACSM3 ENSG00000005187.11 0.699654986 2 2 0.402034148
Brain_Hypothalamus 0.940015528 Brain_Cerebellum 1.041367037
0.958632963 0.958632963 -0.837993867 0.075250346 -0.38137176
0.645761176 2 0 0.998207054
SLC25A17 ENSG00000100372.14 0.699674006 3 3 0.336751259
Brain_Cerebellum 0.963671219 Brain_Putamen_basal_ganglia 1.829227566
0.41857911 0.41857911 -0.960604114 0.335486595 -0.223554879
0.666129007 3 0 0.998207054
ANLN ENSG00000011426.10 0.699704118 1 1 0.699704118 Brain_Amygdala
0.699704118 Brain_Amygdala 1 1 1 -0.385719907 -0.385719907
-0.385719907 NA 1 0 0.998207054
BAZ1B ENSG00000009954.10 0.699749938 1 1 0.699749938
Brain_Caudate_basal_ganglia 0.699749938 Brain_Caudate_basal_ganglia 1 1
1 0.385658046 0.385658046 0.385658046 NA 1 0
0.998207054
TCP11 ENSG00000124678.17 0.69976684 5 5 0.175888488
Brain_Substantia_nigra 0.679143402 Brain_Cortex 1.846030942
0.416773109 0.416773109 0.413632316 1.353523373 0.853671853
0.380808728 5 0 0.998207054
RP11-568A7.3 ENSG00000231720.1 0.699800212 3 3 0.235570115
Brain_Substantia_nigra 0.446051533 Brain_Hypothalamus 2.385654279
0.107209266 0.107209266 0.762014193 1.186132151 0.974314412
0.212059391 3 0 0.998207054
GPC5 ENSG00000179399.14 0.700019213 5 5 0.229514009
Brain_Hippocampus 0.987641295 Brain_Frontal_Cortex_BA9 1.573668351
0.524072969 0.524072969 -0.882267286 1.201611693 0.345624077
0.854799691 5 0 0.998207054
PPFIA1 ENSG00000131626.16 0.700081814 12 2 0.447732502
Brain_Putamen_basal_ganglia 0.927582751 Brain_Spinal_cord_cervical_c-1
10.46212405 0.000574622 0.98227089 -0.759200709 -0.090886532
-0.503096301 0.253635894 2 0 0.998207054
NR2E3 ENSG00000278570.4 0.700124784 4 4 0.427354088 Brain_Cortex
0.550111912 Brain_Cerebellar_Hemisphere 1.815982748 0.229955659
0.229955659 -0.778297051 0.793727499 0.320617246 0.737075927
4 0 0.998207054
PRMT2 ENSG00000160310.17 0.700152451 9 9 0.114662329
Brain_Spinal_cord_cervical_c-1 0.856588882
Brain_Nucleus_accumbens_basal_ganglia 2.369118279 0.175752676
0.175752676 -1.51915479 1.577579133 -0.31928871 0.840544624
9 0 0.998207054
RBM39 ENSG00000131051.22 0.700181676 3 3 0.250576901
Brain_Hypothalamus 0.85897332 Brain_Nucleus_accumbens_basal_ganglia
1.085892136 0.943960446 0.943960446 -0.177681239 1.148949265
0.397293149 0.680734492 3 0 0.998207054
AC006946.16 ENSG00000273203.1 0.700189194 8 4 0.146909223
Brain_Putamen_basal_ganglia 0.987971573 Brain_Cerebellum 6.379948129
0.038348725 0.26652026 0.015075969 1.450535587 0.86221095
0.434082995 4 0 0.998207054
VSTM5 ENSG00000214376.5 0.700231487 5 5 0.285830584 Brain_Cortex
0.711167439 Brain_Frontal_Cortex_BA9 2.767004586 0.108321365
0.108321365 -0.691314043 1.067312858 0.230598425 0.754243216
5 0 0.998207054
SIKE1 ENSG00000052723.11 0.700329896 1 1 0.700329896
Brain_Caudate_basal_ganglia 0.700329896 Brain_Caudate_basal_ganglia 1 1
1 0.384875179 0.384875179 0.384875179 NA 1 0
0.998207054
RPS24 ENSG00000138326.18 0.70040337 6 6 0.434566657
Brain_Substantia_nigra 0.866969354 Brain_Frontal_Cortex_BA9
2.039120841 0.112569628 0.112569628 -0.627018188 0.781401045
-0.013512486 0.506936691 6 0 0.998207054
RPL30 ENSG00000156482.10 0.700451856 1 1 0.700451856

Brain_Putamen_basal_ganglia 0.700451856 Brain_Putamen_basal_ganglia 1 1
1 -0.38471058 -0.38471058 -0.38471058 NA 1 0
0.998207054
HRCT1 ENSG00000196196.2 0.700694998 1 1 0.700694998 Brain_Cortex
0.700694998 Brain_Cortex 1 1 1 -0.38438246 -0.38438246
-0.38438246 NA 1 0 0.998207054
RP11-254F7.2 ENSG00000260077.1 0.700704807 10 4 0.303895144
Brain_Spinal_cord_cervical_c-1 0.984990266 Brain_Substantia_nigra
7.494265581 0.016881249 0.392525847 -0.028755672 1.028116257
0.340491517 0.375876062 4 0 0.998207054
LDLRAD2 ENSG00000187942.11 0.700722235 1 1 0.700722235
Brain_Amygdala 0.700722235 Brain_Amygdala 1 1 1 -0.384345708
-0.384345708 -0.384345708 NA 1 0 0.998207054
GTF2A1 ENSG00000165417.11 0.700723826 2 2 0.415686524
Brain_Caudate_basal_ganglia 0.877241778 Brain_Anterior_cingulate_cortex_BA24
1.080756993 0.919243007 0.919243007 -0.154466684 0.813927433
0.329730374 0.684758047 2 0 0.998207054
RNASE4 ENSG00000258818.3 0.70082326 3 3 0.373087249 Brain_Amygdala
0.99556539 Brain_Nucleus_accumbens_basal_ganglia 1.779339041 0.479601749
0.479601749 -0.302017085 0.890705378 0.198082094 0.61923006 3
0 0.998207054
LRRC75A ENSG00000181350.11 0.701005094 2 2 0.467429411
Brain_Cortex 0.493977444 Brain_Nucleus_accumbens_basal_ganglia
1.403457479 0.596542521 0.596542521 -0.726667918 -0.683996392
-0.705332155 0.030173326 2 0 0.998207054
C2CD4A ENSG00000198535.5 0.701420394 2 2 0.431910309
Brain_Hypothalamus 0.604314131 Brain_Nucleus_accumbens_basal_ganglia
1.290887325 0.709112675 0.709112675 0.518206599 0.785926909
0.652066754 0.189306847 2 0 0.998207054
DCLRE1B ENSG00000118655.4 0.701444502 2 2 0.457344808
Brain_Spinal_cord_cervical_c-1 0.501282192 Brain_Cerebellum 1.421625381
0.578374619 0.578374619 0.672473677 0.743226093 0.707849885
0.050029513 2 0 0.998207054
SLC17A7 ENSG00000104888.9 0.701567173 1 1 0.701567173
Brain_Cerebellar_Hemisphere 0.701567173 Brain_Cerebellar_Hemisphere 1 1
1 -0.383205806 -0.383205806 -0.383205806 NA 1 0
0.998207054
CFAP97 ENSG00000164323.12 0.701709551 8 7 0.41051404
Brain_Hypothalamus 0.950979253 Brain_Cortex 5.256182498
0.115092302 0.211870503 -0.822989367 0.754382376 0.19226435
0.589726703 7 0 0.998207054
NUDT2 ENSG00000164978.17 0.70178313 13 2 0.29446022 Brain_Amygdala
0.742978647 Brain_Caudate_basal_ganglia 11.42696762 0.002808078
0.764919521 0.327911409 1.048387262 0.733024202 0.225470318
2 0 0.998207054
ABC13-47488600E17.1 ENSG00000283199.1 0.701818307 1 1 0.701818307
Brain_Spinal_cord_cervical_c-1 0.701818307 Brain_Spinal_cord_cervical_c-1
1 1 1 0.382867099 0.382867099 0.382867099 NA 1 0
0.998207054
GLP2R ENSG00000065325.12 0.701927675 3 2 0.452095788 Brain_Cortex
0.662785532 Brain_Nucleus_accumbens_basal_ganglia 2.790629991
0.051219935 0.158150074 -0.751925623 -0.436070566 -0.630990013
0.170429781 2 0 0.998207054
MRPL40 ENSG00000185608.8 0.70195421 3 3 0.406380894
Brain_Hippocampus 0.973291188 Brain_Cerebellum 1.796696934
0.307776692 0.307776692 -0.830279291 0.580907708 -0.094284123
0.707555631 3 0 0.998207054
FAM221A ENSG00000188732.10 0.702275321 13 3 0.515870767
Brain_Caudate_basal_ganglia 0.959514132 Brain_Cerebellar_Hemisphere
10.08572727 0.000713591 0.34944158 -0.621117766 0.649723616
-0.211680915 0.311299599 3 0 0.998207054
ZNF503-AS1 ENSG00000226051.6 0.702370211 1 1 0.702370211

Brain_Substantia_nigra 0.702370211 Brain_Substantia_nigra 1 1
1 0.382122894 0.382122894 0.382122894 NA 1 0
0.998207054
CLN8 ENSG00000182372.9 0.702392142 4 4 0.214453296
Brain_Putamen_basal_ganglia 0.853392429 Brain_Cerebellum 1.408579333
0.654704931 0.654704931 -1.241412792 0.620196454 0.003266064
0.848900257 4 0 0.998207054
MAGI1 ENSG00000151276.23 0.702601205 2 2 0.501371421
Brain_Cerebellar_Hemisphere 0.974500419 Brain_Cerebellum 1.568844015
0.431155985 0.431155985 -0.672333479 0.031964427 -0.320184526
0.498013826 2 0 0.998207054
RNF213 ENSG00000173821.19 0.702685382 5 5 0.228302021
Brain_Cerebellar_Hemisphere 0.98760221 Brain_Cerebellum 2.304043205
0.340455034 0.340455034 0.015538951 1.20474432 0.556461933
0.5765559 5 0 0.998207054
STAU1 ENSG00000124214.19 0.702779246 3 3 0.313769673
Brain_Anterior_cingulate_cortex_BA24 0.703840762 Brain_Hypothalamus
2.23292184 0.235484177 0.235484177 -1.007343624 -0.380140987
-0.65143056 0.322050677 3 0 0.998207054
FGF22 ENSG00000070388.11 0.702908756 2 2 0.502936849
Brain_Cerebellar_Hemisphere 0.598503286 Brain_Nucleus_accumbens_basal_ganglia
1.030615131 0.969384869 0.969384869 0.526554082 0.669875987
0.598215034 0.101343891 2 0 0.998207054
TAF4 ENSG00000130699.18 0.702921202 3 3 0.264470048
Brain_Nucleus_accumbens_basal_ganglia 0.890009756 Brain_Cerebellar_Hemisphere
1.405203804 0.595538546 0.595538546 -0.394956348 1.115888073
0.194213287 0.808444477 3 0 0.998207054
TBCB ENSG00000105254.11 0.702942417 1 1 0.702942417
Brain_Spinal_cord_cervical_c-1 0.702942417 Brain_Spinal_cord_cervical_c-1
1 1 1 -0.381351536 -0.381351536 -0.381351536 NA 1 0
0.998207054
WASHC2C ENSG00000172661.17 0.702947081 13 6 0.079434848
Brain_Substantia_nigra 0.919352307 Brain_Frontal_Cortex_BA9
8.640261882 0.046923866 0.396437796 -0.325704074 1.753974668
0.43580691 0.509695822 6 0 0.998207054
TTC28 ENSG00000100154.14 0.703027857 3 3 0.469006889
Brain_Hippocampus 0.986822032 Brain_Cerebellum 2.221727367
0.152502429 0.152502429 0.016516885 0.724095855 0.458059985
0.385057257 3 0 0.998207054
ARHGAP22 ENSG00000128805.14 0.70313984 3 3 0.347982203
Brain_Anterior_cingulate_cortex_BA24 0.66483426 Brain_Cerebellum 1.23185495
0.758987122 0.758987122 0.433248483 0.938510345 0.67854566
0.252950072 3 0 0.998207054
RP11-479J7.2 ENSG00000229588.1 0.703195062 1 1 0.703195062
Brain_Nucleus_accumbens_basal_ganglia 0.703195062
Brain_Nucleus_accumbens_basal_ganglia 1 1 1 -0.381011033
-0.381011033 -0.381011033 NA 1 0 0.998207054
PTAR1 ENSG00000188647.12 0.703283987 3 3 0.248859047 Brain_Cerebellum
0.790704286 Brain_Hypothalamus 1.902829011 0.098225051
0.098225051 -0.265396171 1.153125085 0.625351327 0.775798356
3 0 0.998207054
GRIP1 ENSG00000155974.11 0.703291842 1 1 0.703291842
Brain_Spinal_cord_cervical_c-1 0.703291842 Brain_Spinal_cord_cervical_c-1
1 1 1 0.380880609 0.380880609 0.380880609 NA 1 0
0.998207054
TPGS1 ENSG00000141933.9 0.703350953 2 2 0.414598502
Brain_Putamen_basal_ganglia 0.84863699 Brain_Cortex 1.005695554
0.994304446 0.994304446 -0.190857817 0.815828025 0.312485104
0.711834385 2 0 0.998207054
RP11-218E20.3 ENSG00000258711.2 0.703449642 10 6 0.326977298
Brain_Cortex 0.856868758 Brain_Anterior_cingulate_cortex_BA24 6.5304242
0.051769334 0.234349337 -0.225466594 0.98022048 0.521640203

0.367053571 6 0 0.998207054
ZNF582 ENSG00000018869.16 0.703669361 3 3 0.342798314
Brain_Cerebellar_Hemisphere 0.992801963 Brain_Amygdala 1.827843583
0.200593886 0.200593886 -0.948650664 0.397801545 -0.186623548
0.690572405 3 0 0.998207054
KCNH3 ENSG00000135519.6 0.703746891 1 1 0.703746891
Brain_Cerebellar_Hemisphere 0.703746891 Brain_Cerebellar_Hemisphere 1 1
1 -0.380267456 -0.380267456 -0.380267456 NA 1 0
0.998207054
SERAC1 ENSG00000122335.13 0.703872772 8 5 0.222862962
Brain_Spinal_cord_cervical_c-1 0.97013305 Brain_Cerebellar_Hemisphere
5.260634877 0.027058822 0.212286343 -1.218950548 0.676237511
0.073561733 0.585648438 5 0 0.998207054
ZNF114 ENSG00000178150.9 0.703873444 3 3 0.353036377
Brain_Cerebellum 0.853702825 Brain_Cortex 1.966273779 0.388728106
0.388728106 -0.184395978 0.928715825 0.485710374 0.590284326
3 0 0.998207054
KBTBD12 ENSG00000187715.13 0.703896318 9 5 0.322576173
Brain_Putamen_basal_ganglia 0.92014874 Brain_Caudate_basal_ganglia 6.52241643
0.006606152 0.252193386 -0.98917777 0.69605959 -0.340390131
0.480097079 5 0 0.998207054
MMP2 ENSG00000087245.12 0.703916087 2 2 0.422844855
Brain_Putamen_basal_ganglia 0.85700095 Brain_Anterior_cingulate_cortex_BA24
1.079315726 0.920684274 0.920684274 -0.801495488 0.180193132
-0.310651178 0.69415868 2 0 0.998207054
CYB5R3 ENSG00000100243.20 0.704023078 9 2 0.335096241
Brain_Nucleus_accumbens_basal_ganglia 0.997300651 Brain_Cortex
8.105805793 3.50E-05 0.402505684 -0.963899647 0.070980663
-0.455516586 0.351037705 2 0 0.998207054
FUBP3 ENSG00000107164.15 0.704092477 2 2 0.416044014 Brain_Cortex
0.867089464 Brain_Cerebellum 1.040634179 0.959365821 0.959365821
-0.813303599 0.167356609 -0.322973495 0.693431483 2 0
0.998207054
ENTPD1 ENSG00000138185.18 0.704223154 3 3 0.382856318
Brain_Cerebellum 0.904484396 Brain_Anterior_cingulate_cortex_BA24
1.920076132 0.336064879 0.336064879 -0.872645525 0.470109269
-0.174178227 0.673015005 3 0 0.998207054
RP11-521O16.2 ENSG00000260163.1 0.704255028 2 2 0.427063033
Brain_Substantia_nigra 0.646861165 Brain_Spinal_cord_cervical_c-1
1.784174313 0.215825687 0.215825687 -0.794227447 -0.458127052
-0.626177249 0.237658869 2 0 0.998207054
SLC35B1 ENSG00000121073.13 0.704272173 1 1 0.704272173
Brain_Amygdala 0.704272173 Brain_Amygdala 1 1 1 -0.379559845
-0.379559845 -0.379559845 NA 1 0 0.998207054
ZFP28 ENSG00000196867.7 0.70440398 8 8 0.178575985
Brain_Nucleus_accumbens_basal_ganglia 0.940972712 Brain_Putamen_basal_ganglia
2.727172139 0.325360326 0.325360326 -0.525449137 1.345152522
0.295012674 0.744390017 8 0 0.998207054
CDCA7L ENSG00000164649.19 0.704446696 4 4 0.23558426
Brain_Putamen_basal_ganglia 0.965383486 Brain_Cerebellar_Hemisphere
1.426533564 0.644267288 0.644267288 -1.186096329 0.421332802
-0.384420259 0.744196699 4 0 0.998207054
ADAMTS3 ENSG00000156140.9 0.704664605 2 2 0.493533102
Brain_Frontal_Cortex_BA9 0.889695017 Brain_Cortex 1.431145797
0.568854203 0.568854203 -0.138690131 0.684700233 0.273005051
0.58222491 2 0 0.998207054
ZNF626 ENSG00000188171.15 0.704823914 13 3 0.304514038
Brain_Cerebellum 0.955701906 Brain_Substantia_nigra 10.87537791
0.000691235 0.683404897 -0.293116051 1.026801299 0.385011101
0.425894765 3 0 0.998207054
KIAA1644 ENSG00000138944.7 0.704826487 1 1 0.704826487
Brain_Spinal_cord_cervical_c-1 0.704826487 Brain_Spinal_cord_cervical_c-1

1 1 1 0.378813332 0.378813332 0.378813332 NA 1 0
0.998207054
RP11-86H7.6 ENSG00000223675.1 0.704846854 6 6 0.139744413
Brain_Cortex 0.895601599 Brain_Caudate_basal_ganglia 2.805190306
0.152114431 0.152114431 -0.89626314 1.476743478 0.527602624
0.86760599 6 0 0.998207054
USP20 ENSG00000136878.12 0.704958977 5 4 0.203946613
Brain_Hypothalamus 0.942404244 Brain_Putamen_basal_ganglia 2.000772555
0.052054849 0.566778184 -1.270387559 0.07224838 -0.544847723
0.478809766 4 0 0.998207054
NADK2 ENSG00000152620.12 0.705014153 3 3 0.260505162
Brain_Anterior_cingulate_cortex_BA24 0.387875407 Brain_Amygdala
2.683020588 0.089770495 0.089770495 0.863476732 1.12519795
1.027733733 0.143075001 3 0 0.998207054
CDC42BPG ENSG00000171219.8 0.705023467 4 4 0.577370344
Brain_Putamen_basal_ganglia 0.898031597 Brain_Cerebellar_Hemisphere
1.916090487 0.108790655 0.108790655 -0.557229929 0.491791739
-0.1360715 0.453767422 4 0 0.998207054
SLC38A3 ENSG00000188338.14 0.705070032 2 2 0.45555933
Brain_Frontal_Cortex_BA9 0.976669165 Brain_Caudate_basal_ganglia 1.41945852
0.58054148 0.58054148 -0.746178937 0.029245034 -0.358466952
0.548307548 2 0 0.998207054
ZNF462 ENSG00000148143.12 0.705131008 10 3 0.211258894
Brain_Cortex 0.965319726 Brain_Substantia_nigra 8.576560062
0.003224178 0.298682716 0.043478973 1.250111295 0.881306037
0.345468473 3 0 0.998207054
SPSB1 ENSG00000171621.13 0.705145816 2 2 0.420408825
Brain_Nucleus_accumbens_basal_ganglia 0.447764782 Brain_Caudate_basal_ganglia
1.763979206 0.236020794 0.236020794 0.75914674 0.80571218 0.78242946
0.032926738 2 0 0.998207054
GTF2A2 ENSG00000140307.10 0.705149917 1 1 0.705149917
Brain_Caudate_basal_ganglia 0.705149917 Brain_Caudate_basal_ganglia 1 1
1 -0.378377855 -0.378377855 -0.378377855 NA 1 0
0.998207054
ZNF709 ENSG00000242852.6 0.705172584 8 3 0.2416976 Brain_Cerebellum
0.582935927 Brain_Hypothalamus 6.455654193 0.001350216
0.226721661 -1.17075418 -0.549101309 -0.889918191 0.234347891
3 0 0.998207054
MBD6 ENSG00000166987.14 0.705528024 4 4 0.264295366
Brain_Hippocampus 0.988902224 Brain_Anterior_cingulate_cortex_BA24
2.101640555 0.186634857 0.186634857 -0.013909449 1.116296203
0.50069212 0.508704127 4 0 0.998207054
GRM6 ENSG00000113262.14 0.705545422 2 2 0.426851629
Brain_Hippocampus 0.978761104 Brain_Frontal_Cortex_BA9 1.338214383
0.661785617 0.661785617 -0.794590702 -0.026622153 -0.410606428
0.543035769 2 0 0.998207054
ALG1L2 ENSG00000251287.8 0.705569219 4 4 0.158100528
Brain_Caudate_basal_ganglia 0.782657631 Brain_Spinal_cord_cervical_c-1
2.372304318 0.210654714 0.210654714 -0.275857353 1.411488828
0.718260272 0.744431073 4 0 0.998207054
TGFB1I1 ENSG00000140682.18 0.705637802 2 2 0.419946309
Brain_Cerebellar_Hemisphere 0.619064463 Brain_Cerebellum 1.783276719
0.216723281 0.216723281 0.497176683 0.806514399 0.651845541
0.218734797 2 0 0.998207054
SEC14L5 ENSG00000103184.11 0.705677383 2 2 0.414272373
Brain_Cerebellum 0.883772619 Brain_Frontal_Cortex_BA9 1.035377267
0.964622733 0.964622733 -0.816398292 -0.146188455 -0.481293374
0.473909921 2 0 0.998207054
CTD-2650P22.1 ENSG00000259438.1 0.705814651 1 1 0.705814651
Brain_Amygdala 0.705814651 Brain_Amygdala 1 1 1 0.37748306
0.37748306 0.37748306 NA 1 0 0.998207054
SLA ENSG00000155926.13 0.705817473 4 4 0.255989874

Brain_Caudate_basal_ganglia 0.971166033 Brain_Putamen_basal_ganglia
1.976420036 0.335286891 0.335286891 -1.135920413 0.036145888
-0.412073138 0.537311233 4 0 0.998207054
SLC16A3 ENSG00000141526.16 0.70595028 1 1 0.70595028 Brain_Cerebellum
0.70595028 Brain_Cerebellum 1 1 1 0.377300528 0.377300528
0.377300528 NA 1 0 0.998207054
PPP4R4 ENSG00000119698.11 0.706090694 8 5 0.224460917
Brain_Cortex 0.975649578 Brain_Caudate_basal_ganglia 5.028323516
0.004228499 0.372049886 -1.214751413 0.56074833 0.10585606
0.562802148 5 0 0.998207054
TMEM260 ENSG00000070269.13 0.706114679 4 4 0.205417319
Brain_Cerebellum 0.920465175 Brain_Spinal_cord_cervical_c-1 1.732594687
0.479240573 0.479240573 -1.266267525 0.09984778 -0.495291412
0.56768033 4 0 0.998207054
AARD ENSG00000205002.3 0.706167656 2 1 0.650676143 Brain_Cortex
0.772167721 Brain_Frontal_Cortex_BA9 1.938662835 0.061337165
1.938662835 -0.452823079 -0.289540593 -0.371181836 0.115458153
1 0 0.998207054
SPCS3 ENSG00000129128.12 0.706305805 4 4 0.329524471
Brain_Caudate_basal_ganglia 0.787614537 Brain_Nucleus_accumbens_basal_ganglia
1.691698509 0.331656224 0.331656224 -0.975072152 0.930197176
0.027203844 0.829591733 4 0 0.998207054
RP11-626G11.1 ENSG00000261357.1 0.706373085 1 1 0.706373085
Brain_Cerebellar_Hemisphere 0.706373085 Brain_Cerebellar_Hemisphere 1 1
1 -0.376731589 -0.376731589 -0.376731589 NA 1 0
0.998207054
IFT46 ENSG00000118096.7 0.706380755 5 2 0.420289007
Brain_Putamen_basal_ganglia 0.800300306 Brain_Cerebellar_Hemisphere
4.458502481 0.011858397 0.355435796 -0.805919951 -0.25295847
-0.521153503 0.203250653 2 0 0.998207054
RSPH3 ENSG00000130363.11 0.706531301 1 1 0.706531301
Brain_Hypothalamus 0.706531301 Brain_Hypothalamus 1 1 1
0.376518721 0.376518721 0.376518721 NA 1 0 0.998207054
ARL4D ENSG00000175906.4 0.706547472 2 1 0.706490844
Brain_Cerebellar_Hemisphere 0.707246332 Brain_Cerebellum 1.995413645
0.004586355 1.995413645 0.375556907 0.376573151 0.376065029
0.000718593 1 0 0.998207054
BOD1L1 ENSG00000038219.12 0.706561567 2 2 0.4136408
Brain_Frontal_Cortex_BA9 0.967024183 Brain_Anterior_cingulate_cortex_BA24
1.243270987 0.756729013 0.756729013 0.04134083 0.817503412
0.429422121 0.548829825 2 0 0.998207054
C1orf115 ENSG00000162817.6 0.706681623 6 6 0.069386565
Brain_Cerebellum 0.88094903 Brain_Nucleus_accumbens_basal_ganglia 2.725010056
0.093730653 0.093730653 -1.815894451 -0.149766259 -0.830759227
0.717564492 6 0 0.998207054
LY6G5B ENSG00000240053.8 0.706718093 10 7 0.380920926
Brain_Cortex 0.971206352 Brain_Putamen_basal_ganglia 6.861502501
0.00546873 0.233737619 -0.876200719 0.557130069 -0.273646352
0.512995899 7 0 0.998207054
PAPSS2 ENSG00000198682.12 0.706747283 1 1 0.706747283
Brain_Nucleus_accumbens_basal_ganglia 0.706747283
Brain_Nucleus_accumbens_basal_ganglia 1 1 1 -0.376228159
-0.376228159 -0.376228159 NA 1 0 0.998207054
ZNF619 ENSG00000177873.12 0.706783124 3 2 0.43643247 Brain_Cerebellum
0.605681432 Brain_Frontal_Cortex_BA9 2.081696691 0.04606991
0.872233399 -0.778231636 -0.516247681 -0.678098213 0.141476675
2 0 0.998207054
RPS5 ENSG00000083845.8 0.706952561 4 2 0.407439083
Brain_Cerebellar_Hemisphere 0.721340078 Brain_Nucleus_accumbens_basal_ganglia
3.418090835 0.010424656 0.544679437 -0.828408697 -0.356668383
-0.624863612 0.196171448 2 0 0.998207054
RP11-567M16.6 ENSG00000274828.1 0.707019484 13 2 0.511676654

Brain_Amygdala 0.843134735 Brain_Spinal_cord_cervical_c-1 11.37243171
0.001795342 0.808318694 -0.295355284 0.656229214 0.42197147
0.253300511 2 0 0.998207054
PROCR ENSG00000101000.5 0.707112544 2 2 0.506929834
Brain_Cerebellar_Hemisphere 0.736663807 Brain_Cerebellum 1.23043191 0.76956809
0.76956809 -0.66362581 0.336274593 -0.163675609 0.707036356 2
0 0.998207054
FRRS1L ENSG00000260230.2 0.707113331 4 4 0.166694726
Brain_Putamen_basal_ganglia 0.722109681 Brain_Cerebellar_Hemisphere
2.107641641 0.258183637 0.258183637 -1.382902624 0.657570983
-0.467306008 0.860066026 4 0 0.998207054
ZNF740 ENSG00000139651.10 0.707249732 3 3 0.470122748
Brain_Putamen_basal_ganglia 0.661113507 Brain_Cortex 1.997462278
0.196990943 0.196990943 -0.668482664 0.722279347 0.164057669
0.734842014 3 0 0.998207054
FUS ENSG00000089280.18 0.707259234 1 1 0.707259234 Brain_Amygdala
0.707259234 Brain_Amygdala 1 1 1 0.375539555 0.375539555
0.375539555 NA 1 0 0.998207054
LRRC59 ENSG00000108829.9 0.707385552 1 1 0.707385552
Brain_Hippocampus 0.707385552 Brain_Hippocampus 1 1 1
0.375369678 0.375369678 0.375369678 NA 1 0 0.998207054
NOXA1 ENSG00000188747.8 0.707520028 3 3 0.366954752
Brain_Hippocampus 0.706496984 Brain_Hypothalamus 1.412448846
0.588238779 0.588238779 -0.37656489 0.902192248 0.429191167
0.701319727 3 0 0.998207054
RPL41 ENSG00000229117.8 0.707525075 2 2 0.501761055
Brain_Hypothalamus 0.623373101 Brain_Cerebellar_Hemisphere 1.000607209
0.999392791 0.999392791 -0.491075426 0.671721432 0.090323003
0.822221543 2 0 0.998207054
AP1G2 ENSG00000213983.11 0.707541799 1 1 0.707541799
Brain_Substantia_nigra 0.707541799 Brain_Substantia_nigra 1 1
1 -0.375159567 -0.375159567 -0.375159567 NA 1 0
0.998207054
CUL7 ENSG00000044090.8 0.707597272 5 5 0.389825853
Brain_Nucleus_accumbens_basal_ganglia 0.823260578
Brain_Anterior_cingulate_cortex_BA24 2.517469159 0.088572543
0.088572543 -0.63597685 0.859933224 -0.114476021 0.646647972
5 0 0.998207054
SMO ENSG00000128602.9 0.707619325 2 2 0.4257561
Brain_Cerebellar_Hemisphere 0.898883822 Brain_Hypothalamus 1.138183639
0.861816361 0.861816361 -0.796474828 -0.127071483 -0.461773155
0.473339645 2 0 0.998207054
INPP5E ENSG00000148384.12 0.707731818 12 1 0.250501418
Brain_Anterior_cingulate_cortex_BA24 0.907104888 Brain_Caudate_basal_ganglia
11.58301549 0.00077233 11.58301549 -1.149132333 -0.116691044
-0.370904539 0.271346189 1 0 0.998207054
KAZALD1 ENSG00000107821.14 0.707748388 3 3 0.306378459
Brain_Nucleus_accumbens_basal_ganglia 0.754771755 Brain_Putamen_basal_ganglia
1.698407144 0.325270706 0.325270706 -1.022850664 0.373222495
-0.320660614 0.69807365 3 0 0.998207054
LINC00989 ENSG00000250334.5 0.707903537 1 1 0.707903537
Brain_Substantia_nigra 0.707903537 Brain_Substantia_nigra 1 1
1 0.374673185 0.374673185 0.374673185 NA 1 0
0.998207054
PDXK ENSG00000160209.18 0.707969793 1 1 0.707969793
Brain_Putamen_basal_ganglia 0.707969793 Brain_Putamen_basal_ganglia 1 1
1 0.374584109 0.374584109 0.374584109 NA 1 0
0.998207054
RP1-234P15.4 ENSG00000225793.2 0.708162725 1 1 0.708162725
Brain_Spinal_cord_cervical_c-1 0.708162725 Brain_Spinal_cord_cervical_c-1
1 1 1 -0.374324745 -0.374324745 -0.374324745 NA 1 0
0.998207054

FAM69B ENSG00000165716.10 0.708418633 4 4 0.337066166
Brain_Cerebellar_Hemisphere 0.967811828 Brain_Cerebellum 1.819180979
0.230964908 0.230964908 -0.95997824 0.201810481 -0.281317725
0.501371836 4 0 0.998207054
AP2A1 ENSG00000196961.12 0.708425097 2 2 0.459613433
Brain_Cerebellar_Hemisphere 0.94694817 Brain_Cerebellum 1.524683014
0.475316986 0.475316986 -0.739483537 -0.066539677 -0.403011607
0.475843166 2 0 0.998207054
CST6 ENSG00000175315.2 0.708485769 3 3 0.396563807
Brain_Nucleus_accumbens_basal_ganglia 0.981975657 Brain_Cerebellar_Hemisphere
2.200523373 0.259250256 0.259250256 -0.84777407 0.058118013
-0.255687991 0.513069164 3 0 0.998207054
IMMP2L ENSG00000184903.9 0.708525118 2 2 0.451936017
Brain_Cerebellar_Hemisphere 0.65257583 Brain_Hypothalamus 1.135918762
0.864081238 0.864081238 -0.450186694 0.752191312 0.151002309
0.850209642 2 0 0.998207054
LINC00951 ENSG00000204092.2 0.708537191 2 1 0.705135602
Brain_Cerebellum 0.713043065 Brain_Cerebellar_Hemisphere 1.992124625
0.007875375 1.992124625 -0.378397128 -0.367772246 -0.373084687
0.007512926 1 0 0.998207054
NUDT14 ENSG00000183828.14 0.708559776 12 2 0.207696871
Brain_Cerebellar_Hemisphere 0.890058176 Brain_Spinal_cord_cervical_c-1
11.17364625 7.62E-05 0.489835641 -1.259923747 0.138230595
-0.5756697 0.310447476 2 0 0.998207054
CHTF8 ENSG00000168802.12 0.708591692 2 2 0.417979023
Brain_Anterior_cingulate_cortex_BA24 0.47856653 Brain_Spinal_cord_cervical_c-1
1.719141712 0.280858288 0.280858288 -0.708609998 0.809932411
0.050661206 1.073771635 2 0 0.998207054
ASTN1 ENSG00000152092.15 0.708624674 6 4 0.262439539
Brain_Frontal_Cortex_BA9 0.979257013 Brain_Hypothalamus 4.862053781
0.011118614 0.196484113 -1.120643738 -0.026000407 -0.761257942
0.404479289 4 0 0.998207054
NAV1 ENSG00000134369.15 0.708945599 1 1 0.708945599 Brain_Cerebellum
0.708945599 Brain_Cerebellum 1 1 1 -0.373272557 -0.373272557
-0.373272557 NA 1 0 0.998207054
LRWD1 ENSG00000161036.12 0.709033284 6 3 0.634645845
Brain_Putamen_basal_ganglia 0.859271372 Brain_Hippocampus 5.065135574
0.001183491 0.241002627 -0.474832142 0.47519801 -0.089430478
0.360290879 3 0 0.998207054
RASA3 ENSG00000185989.10 0.709072973 7 5 0.159726523
Brain_Hippocampus 0.957336471 Brain_Cerebellar_Hemisphere 4.335968834
0.025946603 0.201518716 -1.405991923 -0.05349631 -0.483923774
0.446405767 5 0 0.998207054
RP4-564F22.5 ENSG00000224635.1 0.709245945 13 1 0.546803047
Brain_Substantia_nigra 0.856180722 Brain_Anterior_cingulate_cortex_BA24
12.55004058 1.91E-08 12.55004058 -0.602557581 -0.18123806
-0.36746836 0.146257275 1 0 0.998207054
PRUNE1 ENSG00000143363.15 0.709555069 2 2 0.49805424
Brain_Hippocampus 0.748204713 Brain_Caudate_basal_ganglia 1.21343939
0.78656061 0.78656061 -0.321007492 0.677554443 0.178273475
0.706089916 2 0 0.998207054
OR14I1 ENSG00000189181.4 0.709576805 1 1 0.709576805
Brain_Caudate_basal_ganglia 0.709576805 Brain_Caudate_basal_ganglia 1 1
1 -0.372424515 -0.372424515 -0.372424515 NA 1 0
0.998207054
RP11-421M1.8 ENSG00000272097.1 0.709615258 6 5 0.251246304
Brain_Substantia_nigra 0.897273129 Brain_Cerebellar_Hemisphere 1.97761057
0.065405897 0.745407274 -0.611340539 1.147327473 0.201071718
0.713228079 5 0 0.998207054
LINC00216 ENSG00000279636.2 0.709634328 1 1 0.709634328
Brain_Anterior_cingulate_cortex_BA24 0.709634328
Brain_Anterior_cingulate_cortex_BA24 1 1 1 0.372347244

0.372347244	0.372347244	NA	1	0	0.998207054
ABT1	ENSG00000146109.4	0.709753728	4	4	0.226074536
Brain_Caudate_basal_ganglia	0.796929459	Brain_Cerebellum	2.086980905
0.313448958	0.313448958	-0.257322973	1.210532738	0.801433886
0.707090507	4	0	0.998207054
CCDC86	ENSG00000110104.11	0.709847191	5	5	0.37634439
Brain_Nucleus_accumbens_basal_ganglia	0.962647728	Brain_Substantia_nigra
1.551627814	0.622737661	0.622737661	-0.884651929	0.798764792
-0.037057037	0.774440013	5	0	0.998207054
NPIPA7	ENSG00000214967.5	0.709977544	1	1	0.709977544
Brain_Hypothalamus	0.709977544	Brain_Hypothalamus	1	1	1
-0.371886249	-0.371886249	-0.371886249	NA	1	0	0.998207054
IL18R1	ENSG00000115604.10	0.710003157	13	1	0.535635927
Brain_Cortex	0.77865397	Brain_Substantia_nigra	12.84830442	5.16E-07
12.84830442	-0.619425742	-0.281073573	-0.370211356	0.093058054
1	0	0.998207054
RP11-10C24.1	ENSG00000271020.1	0.71003639	1	1	0.71003639
Brain_Hypothalamus	0.71003639	Brain_Hypothalamus	1	1	1
0.371807217	0.371807217	0.371807217	NA	1	0	0.998207054
TENM4	ENSG00000149256.15	0.71004389	1	1	0.71004389	Brain_Cerebellum	0.71004389
Brain_Cerebellum	1	1	1	-0.371797144	-0.371797144	-0.371797144
NA	1	0	0.998207054
GPD2	ENSG00000115159.15	0.710136072	1	1	0.710136072
Brain_Anterior_cingulate_cortex_BA24	0.710136072
Brain_Anterior_cingulate_cortex_BA24	1	1	1	-0.371673347
-0.371673347	-0.371673347	NA	1	0	0.998207054
POLK	ENSG00000122008.15	0.710145451	5	4	0.375251219
Brain_Nucleus_accumbens_basal_ganglia	0.959224734	Brain_Caudate_basal_ganglia
2.863353385	0.068121985	0.269849787	-0.886679981	0.859459381
-0.165100742	0.686799894	4	0	0.998207054
FOXN2	ENSG00000170802.15	0.71015998	13	1	0.537329069	Brain_Hippocampus
0.927961071	Brain_Substantia_nigra	12.20782304	0.001648062
12.20782304	-0.616856973	-0.090410427	-0.359757716	0.170591727
1	0	0.998207054
CDK5RAP3	ENSG00000108465.14	0.710201923	13	3	0.350087355
Brain_Amygdala	0.932789313	Brain_Hippocampus	10.40904525
0.004820651	0.553792656	-0.934419865	0.397346933	-0.233395026
0.456416396	3	0	0.998207054
NPFF	ENSG00000139574.8	0.710304363	1	1	0.710304363	Brain_Amygdala
0.710304363	Brain_Amygdala	1	1	1	0.371447351	0.371447351
0.371447351	NA	1	0	0.998207054
LIMCH1	ENSG00000064042.17	0.710311645	3	3	0.410509883
Brain_Anterior_cingulate_cortex_BA24	0.924900765	Brain_Cerebellum	1.55517941
0.442281471	0.442281471	-0.822996677	0.689791418	-0.075822533
0.756562605	3	0	0.998207054
ZFR	ENSG00000056097.15	0.710424065	1	1	0.710424065	Brain_Cerebellum
0.710424065	Brain_Cerebellum	1	1	1	-0.371286617	-0.371286617
-0.371286617	NA	1	0	0.998207054
RP11-43A14.2	ENSG00000282375.1	0.710481397	2	2	0.464242417
Brain_Anterior_cingulate_cortex_BA24	0.700979623	Brain_Hippocampus
1.000930058	0.999069942	0.999069942	-0.731879012	-0.383998414
-0.557938713	0.24598873	2	0	0.998207054
C6orf201	ENSG00000185689.15	0.710640208	6	3	0.297646496
Brain_Frontal_Cortex_BA9	0.972207317	Brain_Cerebellar_Hemisphere
4.699254085	0.052792476	0.337830936	-0.403857717	1.041493656
0.351674729	0.54011373	3	0	0.998207054
LINC01219	ENSG00000232987.1	0.710792841	4	4	0.275419983
Brain_Cortex	0.564290426	Brain_Cerebellum	1.254385023	0.754080492
0.754080492	-1.090665757	-0.57648053	-0.751495318	0.23056233	4
0	0.998207054
MYO3A	ENSG00000095777.14	0.710923302	3	3	0.742955455
Brain_Nucleus_accumbens_basal_ganglia	0.778226066	Brain_Caudate_basal_ganglia

1.860413242	0.142629168	0.142629168	-0.281631523	0.327942081
0.117731447	0.346018713	3	0	0.998207054
LINC00954	ENSG00000228784.7	0.710942898	1	1	0.710942898
Brain_Spinal_cord_cervical_c-1	0.710942898	Brain_Spinal_cord_cervical_c-1
1	1	1	-0.370590045	-0.370590045	-0.370590045	NA	1	0
0.998207054
DCUN1D2	ENSG00000150401.14	0.711157085	7	3	0.268625167
Brain_Cerebellar_Hemisphere	0.825150836	Brain_Anterior_cingulate_cortex_BA24
5.74185616	0.001678516	0.252969236	-1.106234193	-0.220924993
-0.59133839	0.291113568	3	0	0.998207054
C8orf22	ENSG00000168333.13	0.711314896	1	1	0.711314896
Brain_Hypothalamus	0.711314896	Brain_Hypothalamus	1	1	1
0.37009072	0.37009072	0.37009072	NA	1	0	0.998207054
USF3	ENSG00000176542.9	0.711403197	1	1	0.711403197	Brain_Cortex
0.711403197	Brain_Cortex	1	1	1	-0.36997221	-0.36997221
-0.36997221	NA	1	0	0.998207054
CPSF3	ENSG00000119203.13	0.711705473	2	2	0.43094407	Brain_Cerebellum
0.923388025	Brain_Hippocampus	1.183392595	0.816607405
0.816607405	-0.787577174	0.096166893	-0.345705141	0.624901423
2	0	0.998207054
ABLIM2	ENSG00000163995.19	0.711721437	3	3	0.328184278
Brain_Spinal_cord_cervical_c-1	0.798691812	Brain_Substantia_nigra
1.081464695	0.91843793	0.91843793	0.255040508	0.977777709
0.618300278	0.361383446	3	0	0.998207054
TSNARE1	ENSG00000171045.14	0.71174073	12	4	0.118394488	Brain_Amygdala
0.876120614	Brain_Substantia_nigra	9.066455114	0.017583148
0.341034813	0.155888876	1.561548084	0.95693145	0.389136336	4
0	0.998207054
DDHD2	ENSG00000085788.13	0.711890325	7	3	0.213862743
Brain_Frontal_Cortex_BA9	0.488979677	Brain_Cortex	5.65605678	0.00055865
0.278015925	-1.243013815	-0.691932568	-0.961887527	0.165913955
3	0	0.998207054
RADIL	ENSG00000157927.16	0.711945584	8	4	0.18886323	Brain_Cerebellum
0.987153869	Brain_Hypothalamus	5.585400872	3.91E-17	0.407329016
0.016100933	1.313949409	0.333687076	0.515486467	4	0
0.998207054
GABRB2	ENSG00000145864.12	0.712006127	1	1	0.712006127
Brain_Anterior_cingulate_cortex_BA24	0.712006127
Brain_Anterior_cingulate_cortex_BA24	1	1	1	-0.369163142
-0.369163142	-0.369163142	NA	1	0	0.998207054
QPCTL	ENSG00000011478.11	0.712017616	3	3	0.315505276
Brain_Frontal_Cortex_BA9	0.765267001	Brain_Putamen_basal_ganglia
1.141585857	0.85820123	0.85820123	-1.003737252	0.383244552
-0.107307061	0.777484852	3	0	0.998207054
GRPEL2	ENSG00000164284.14	0.71208691	1	1	0.71208691
Brain_Frontal_Cortex_BA9	0.71208691	Brain_Frontal_Cortex_BA9	1	1	1
0.369054759	0.369054759	0.369054759	NA	1	0	0.998207054
PEX11A	ENSG00000166821.8	0.712198117	3	3	0.285092759
Brain_Substantia_nigra	0.693663145	Brain_Cortex	1.58376428
0.488082473	0.488082473	0.393888793	1.068948761	0.677516557
0.350203994	3	0	0.998207054
NUTM2E	ENSG00000228570.7	0.712210622	11	10	0.139883226
Brain_Cerebellar_Hemisphere	0.968430656	Brain_Nucleus_accumbens_basal_ganglia
3.745768378	0.078104475	0.235755308	-0.477254175	1.476226022
0.720237726	0.639271429	10	0	0.998207054
GHDC	ENSG00000167925.15	0.71224496	3	3	0.400085267
Brain_Nucleus_accumbens_basal_ganglia	0.828460194	Brain_Hippocampus
1.280936866	0.74136232	0.74136232	-0.84146896	0.558480233
-0.02210396	0.729881251	3	0	0.998207054
ZSCAN1	ENSG00000152467.9	0.712273369	2	2	0.802246405
Brain_Cerebellar_Hemisphere	0.813669541	Brain_Cerebellum	1.825852385
0.174147615	0.174147615	-0.235694754	0.250440899	0.007373072

0.343749816	2	0	0.998207054
CYP4F11	ENSG00000171903.16	0.712381746	7	6	0.234223328
Brain_Caudate_basal_ganglia	0.920770598	Brain_Substantia_nigra
3.635140073	0.096199868	0.206596331	-0.499768658	1.18954995
0.243811177	0.646852528	6	0	0.998207054
REEP1	ENSG00000068615.17	0.71244817	8	6	0.185336569
Brain_Nucleus_accumbens_basal_ganglia	0.861729572	Brain_Caudate_basal_ganglia
4.411149929	0.038992152	0.199257434	-1.324501429	1.121191831
0.217417528	0.722333693	6	0	0.998207054
KRT5	ENSG00000186081.11	0.712602853	1	1	0.712602853
Brain_Caudate_basal_ganglia	0.712602853	Brain_Caudate_basal_ganglia	1	1
1	0.368362637	0.368362637	0.368362637	NA	1	0
0.998207054
TAS2R46	ENSG00000226761.3	0.712706566	2	1	0.690062719
Brain_Cerebellar_Hemisphere	0.736471016	Brain_Cerebellum	1.993775897
0.006224103	1.993775897	-0.398769952	-0.336530287	-0.36765012
0.044010089	1	0	0.998207054
RABL2B	ENSG00000079974.17	0.712787673	3	3	0.339425618
Brain_Substantia_nigra	0.896296269	Brain_Cerebellar_Hemisphere
1.093439842	0.92322227	0.92322227	-0.955300771	0.73672392	-0.029411793
0.857250091	3	0	0.998207054
RP11-667K14.3	ENSG00000263050.1	0.712879722	1	1	0.712879722
Brain_Cerebellum	0.712879722	Brain_Cerebellum	1	1	1	0.367991298
0.367991298	0.367991298	NA	1	0	0.998207054
COPS3	ENSG00000141030.12	0.712985016	1	1	0.712985016	Brain_Cortex
0.712985016	Brain_Cortex	1	1	1	-0.367850092	-0.367850092
-0.367850092	NA	1	0	0.998207054
MAPK8IP3	ENSG00000138834.12	0.713009032	1	1	0.713009032
Brain_Cerebellum	0.713009032	Brain_Cerebellum	1	1	1	0.367817886
0.367817886	0.367817886	NA	1	0	0.998207054
ALDH1A2	ENSG00000128918.14	0.713082331	2	2	0.504806527
Brain_Nucleus_accumbens_basal_ganglia	0.584272324	Brain_Cerebellum
1.102796499	0.897203501	0.897203501	-0.666946156	0.54715489
-0.059895633	0.858499083	2	0	0.998207054
RP11-7F17.3	ENSG00000258819.1	0.713235023	1	1	0.713235023
Brain_Cerebellar_Hemisphere	0.713235023	Brain_Cerebellar_Hemisphere	1	1
1	-0.367514842	-0.367514842	-0.367514842	NA	1	0
0.998207054
ZNF99	ENSG00000213973.8	0.713612994	1	1	0.713612994	Brain_Cerebellum
0.713612994	Brain_Cerebellum	1	1	1	-0.367008076	-0.367008076
-0.367008076	NA	1	0	0.998207054
RP5-994D16.3	ENSG00000234917.2	0.713641339	2	2	0.435808558
Brain_Caudate_basal_ganglia	0.808319315	Brain_Cerebellum	1.021898521
0.978101479	0.978101479	-0.779290587	0.242594793	-0.268347897
0.722582082	2	0	0.998207054
AZGP1	ENSG00000160862.12	0.713756624	7	6	0.250839134
Brain_Frontal_Cortex_BA9	0.999681059	Brain_Cerebellum	4.606238548
0.040743083	0.170801201	-0.412981279	1.148313582	0.529980327
0.568095446	6	0	0.998207054
ZNF467	ENSG00000181444.12	0.713772985	11	4	0.109799584
Brain_Caudate_basal_ganglia	0.711014888	Brain_Hippocampus	8.827332861
0.004501144	0.417610313	-1.5990946	-0.370493407	-1.124839917
0.317101719	4	0	0.998207054
CYB5R1	ENSG00000159348.12	0.713774287	2	1	0.637132728
Brain_Cerebellar_Hemisphere	0.797009321	Brain_Cerebellum	1.974710812
0.025289188	1.974710812	-0.4717115	-0.257219513	-0.364465506
0.151668738	1	0	0.998207054
GOLGA6B	ENSG00000215186.6	0.713844052	1	1	0.713844052
Brain_Cerebellum	0.713844052	Brain_Cerebellum	1	1	1	0.366698331
0.366698331	0.366698331	NA	1	0	0.998207054
GYPE	ENSG00000197465.13	0.713917006	1	1	0.713917006
Brain_Spinal_cord_cervical_c-1	0.713917006	Brain_Spinal_cord_cervical_c-1

1 1 1 0.36660054 0.36660054 0.36660054 NA 1 0 0.998207054
LY6G5C ENSG00000204428.12 0.713942708 13 3 0.172556993
Brain_Amygdala 0.94309968 Brain_Caudate_basal_ganglia 11.30380377
0.001203851 0.40359961 -1.364033594 -0.07137453 -0.892468784
0.366864377 3 0 0.998207054
CNPY2 ENSG00000257727.5 0.714021159 2 2 0.468094125
Brain_Nucleus_accumbens_basal_ganglia 0.645368562 Brain_Cerebellum
1.108588281 0.891411719 0.891411719 -0.460205725 0.725583524
0.132688899 0.838479618 2 0 0.998207054
ZNF454 ENSG00000178187.7 0.714100952 11 5 0.083952796
Brain_Putamen_basal_ganglia 0.99316006 Brain_Spinal_cord_cervical_c-1
8.231894207 7.37E-05 0.275563056 -0.008572698 1.728197644
1.158514024 0.536447389 5 0 0.998207054
JUP ENSG00000173801.16 0.714131373 4 4 0.427444632 Brain_Cerebellum
0.852266462 Brain_Putamen_basal_ganglia 2.798190774 0.107620581
0.107620581 -0.79357201 0.743685963 0.146763626 0.666955797
4 0 0.998207054
SECISBP2 ENSG00000187742.14 0.714144506 2 2 0.433137909
Brain_Cerebellum 0.593189293 Brain_Cerebellar_Hemisphere 1.397301174
0.602698826 0.602698826 0.534220059 0.783833338 0.659026699
0.176503242 2 0 0.998207054
SLC35E2B ENSG00000189339.11 0.714176583 7 6 0.175491903
Brain_Amygdala 0.825521426 Brain_Substantia_nigra 2.331784907
0.067199682 0.522211649 -1.354766689 1.155870606 0.034722458
0.877652698 6 0 0.998207054
ZNF19 ENSG00000157429.15 0.714268943 3 3 0.357592306
Brain_Nucleus_accumbens_basal_ganglia 0.69123911 Brain_Substantia_nigra
1.15317112 0.852081861 0.852081861 0.39717406 0.919962595
0.595509092 0.283295543 3 0 0.998207054
HPDL ENSG00000186603.5 0.714412547 1 1 0.714412547
Brain_Frontal_Cortex_BA9 0.714412547 Brain_Frontal_Cortex_BA9 1 1
1 0.365936383 0.365936383 0.365936383 NA 1 0
0.998207054
TNF ENSG00000232810.3 0.714500772 1 1 0.714500772
Brain_Hippocampus 0.714500772 Brain_Hippocampus 1 1 1
0.365818155 0.365818155 0.365818155 NA 1 0 0.998207054
TMEM97 ENSG00000109084.13 0.714525211 4 2 0.417360292
Brain_Nucleus_accumbens_basal_ganglia 0.576740479
Brain_Spinal_cord_cervical_c-1 3.671914056 0.008041438 0.23134216
[truncated: 412,046 more chars]
