# Supplementary material for: Multi-omic and multi-species meta-analyses of nicotine consumption
Source: Transl Psychiatry. 2021 Feb 4;11:98. doi: 10.1038/s41398-021-01231-y (PMC7862377; doi:10.1038/s41398-021-01231-y)
Supplement: Supplementary file 5 — Supplementary File 3. H-MAGMA Summary Statistics File [file 41398_2021_1231_MOESM5_ESM.pdf]

H-MAGMA Gene Based Associations with Human Cigarettes per Day

hgnc_symbol	GENE	Zneuro	Pneuro	Zastro	Pastro	Zfetal	Pfetal	Zadult	Padult	P_Minimum_of_Neuro_Astro_Fetal_Adult	Padj_of_P_Minimum
CSPG4P13	ENSG00000260139	5.309	5.51E-08	10.061	4.12E-24	-1.5359	0.93772	14.985	4.60E-51	4.60E-51	2.24E-46
NA	ENSG00000279373	14.933	1.00E-50	14.933	1.00E-50	14.933	1.00E-50	14.933	1.00E-50	1.00E-50	2.24E-46
COMMD4P1	ENSG00000214646	14.912	1.38E-50	7.5924	1.57E-14	11.811	1.71E-32	4.4262	4.80E-06	1.38E-50	2.24E-46
NA	ENSG00000261303	11.68	8.11E-32	14.002	7.56E-45	12.408	1.19E-35	8.4461	1.51E-17	7.56E-45	9.21E-41
IREB2	ENSG00000136381	13.684	6.34E-43	13.684	6.34E-43	13.54	4.55E-42	11.049	1.10E-28	6.34E-43	6.18E-39
CHRNA5	ENSG00000169684	13.244	2.44E-40	13.244	2.44E-40	13.244	2.44E-40	13.244	2.44E-40	2.44E-40	1.98E-36
NA	ENSG00000260776	10.936	3.87E-28	13.198	4.48E-40	5.5638	1.32E-08	9.8604	3.09E-23	4.48E-40	3.12E-36
NA	ENSG00000261403	10.297	3.64E-25	7.4374	5.13E-14	13.166	6.88E-40	7.6944	7.11E-15	6.88E-40	4.19E-36
WDR61	ENSG00000140395	9.9277	1.58E-23	10.275	4.58E-25	1.1702	0.12097	12.919	1.77E-38	1.77E-38	9.57E-35
CHRNA3	ENSG00000080644	12.815	6.76E-38	10.724	3.92E-27	12.399	1.33E-35	10.344	2.22E-25	6.76E-38	3.29E-34
ADAMTS7	ENSG00000136378	12.613	8.93E-37	7.3376	1.09E-13	8.2261	9.67E-17	6.8494	3.71E-12	8.93E-37	3.96E-33
DNAJA4	ENSG00000140403	10.55	2.53E-26	1.0328	0.15086	1.0328	0.15086	0.78919	0.215	2.53E-26	1.03E-22
ADAMTS7P3	ENSG00000261143	9.7169	1.28E-22	8.0727	3.44E-16	7.6339	1.14E-14	10.462	6.44E-26	6.44E-26	2.42E-22
NA	ENSG00000269843	10.062	4.08E-24	10.062	4.08E-24	10.062	4.08E-24	10.062	4.08E-24	4.08E-24	1.42E-20
NA	ENSG00000279421	9.109	4.16E-20	7.6956	7.04E-15	7.6956	7.04E-15	7.6956	7.04E-15	4.16E-20	1.35E-16
PRR13P5	ENSG00000187534	9.0402	7.82E-20	4.7344	1.10E-06	1.0665	0.14309	1.0665	0.14309	7.82E-20	2.38E-16
TBC1D2B	ENSG00000167202	8.8624	3.91E-19	5.5957	1.10E-08	4.3093	8.19E-06	0.9392	0.17381	3.91E-19	1.12E-15
ACSBG1	ENSG00000103740	-0.038718	0.51544	-0.15755	0.5626	0.56248	0.2869	8.7694	8.98E-19	8.98E-19	2.43E-15
NA	ENSG00000259474	8.7233	1.35E-18	8.7233	1.35E-18	8.7233	1.35E-18	8.7233	1.35E-18	1.35E-18	3.47E-15
NA	ENSG00000273512	7.9603	8.58E-16	8.4885	1.05E-17	5.6171	9.71E-09	5.6171	9.71E-09	1.05E-17	2.55E-14
RN7SL214P	ENSG00000242066	-0.2212	0.58753	-0.2212	0.58753	7.9522	9.16E-16	8.2286	9.47E-17	9.47E-17	2.20E-13
HYKK	ENSG00000188266	8.0272	4.98E-16	8.0272	4.98E-16	8.0272	4.98E-16	6.1149	4.83E-10	4.98E-16	1.10E-12
SPTBN4	ENSG00000160460	7.6624	9.12E-15	-1.0926	0.86271	-0.017736	0.50708	5.0477	2.24E-07	9.12E-15	1.93E-11
RPL18P11	ENSG00000213307	7.634	1.14E-14	7.634	1.14E-14	7.634	1.14E-14	7.634	1.14E-14	1.14E-14	2.31E-11
NA	ENSG00000279108	7.6109	1.36E-14	7.6109	1.36E-14	7.6109	1.36E-14	7.6109	1.36E-14	1.36E-14	2.65E-11
NA	ENSG00000261762	7.4582	4.38E-14	7.4582	4.38E-14	7.4582	4.38E-14	7.4582	4.38E-14	4.38E-14	8.22E-11
CCDC97	ENSG00000142039	-0.77	0.77935	-0.70444	0.75942	7.402	6.71E-14	1.6103	0.053669	6.71E-14	1.21E-10
CYP2T3P	ENSG00000268529	0.6132	0.26987	7.1799	3.49E-13	-0.86267	0.80584	-0.86267	0.80584	3.49E-13	6.07E-10
FCGBP	ENSG00000275395	2.6727	0.0037617	2.9716	0.0014814	5.6273	9.15E-09	7.1155	5.57E-13	5.57E-13	9.37E-10

SNRPA	ENSG00000077312	1.5016	0.066605	2.0973	0.017983	6.4898	4.30E-11	7.0218	1.10E-12	1.10E-12	1.78E-09
CYP2T1P	ENSG00000233622	6.9893	1.38E-12	6.9893	1.38E-12	6.9893	1.38E-12	6.9893	1.38E-12	1.38E-12	2.11E-09
CIB2	ENSG00000136425	6.9888	1.39E-12	-0.34569	0.63521	-0.20569	0.58148	-0.31071	0.62199	1.39E-12	2.11E-09
PCAT19	ENSG00000267107	6.9359	2.02E-12	2.3127	0.01037	1.556	0.059855	6.4816	4.54E-11	2.02E-12	2.98E-09
CYP2F2P	ENSG00000237118	2.5132	0.0059817	6.8038	5.09E-12	2.0501	0.020177	3.9051	4.71E-05	5.09E-12	7.30E-09
NUMBL	ENSG00000105245	0.80704	0.20982	4.5217	3.07E-06	1.8942	0.029098	6.7521	7.29E-12	7.29E-12	1.01E-08
HNRNPUL1	ENSG00000105323	6.6706	1.27E-11	-0.50821	0.69435	-0.049069	0.51957	0.31326	0.37704	1.27E-11	1.72E-08
NA	ENSG00000259420	0.27463	0.3918	-0.31653	0.6242	0.3584	0.36002	6.6208	1.79E-11	1.79E-11	2.35E-08
ZNF780B	ENSG00000128000	4.6011	2.10E-06	3.9273	4.29E-05	-0.64034	0.73903	6.5958	2.12E-11	2.12E-11	2.71E-08
NA	ENSG00000268034	0.63751	0.2619	6.4709	4.87E-11	0.63751	0.2619	0.63751	0.2619	4.87E-11	6.09E-08
CYP2G2P	ENSG00000268101	2.1779	0.014708	0.99558	0.15973	6.4565	5.36E-11	6.1083	5.04E-10	5.36E-11	6.53E-08
CHRNB3	ENSG00000147432	5.1401	1.37E-07	6.355	1.04E-10	5.2836	6.33E-08	5.2437	7.87E-08	1.04E-10	1.24E-07
MAP3K10	ENSG00000130758	6.3472	1.10E-10	0.36165	0.35881	0.36165	0.35881	-0.47195	0.68152	1.10E-10	1.27E-07
CYP2G1P	ENSG00000130612	3.6244	0.00014482	2.6877	0.0035968	6.2583	1.95E-10	2.5327	0.005659	1.95E-10	2.21E-07
CHRNA6	ENSG00000147434	5.8057	3.21E-09	4.7559	9.88E-07	6.2445	2.13E-10	3.1335	0.00086369	2.13E-10	2.35E-07
MORF4L1	ENSG00000185787	2.3904	0.0084161	1.7631	0.038943	6.1968	2.88E-10	1.5599	0.059389	2.88E-10	3.12E-07
SLC20A2	ENSG00000168575	4.5647	2.50E-06	5.4287	2.84E-08	6.1534	3.79E-10	1.4929	0.067728	3.79E-10	4.02E-07
PSMA4	ENSG00000041357	6.0495	7.27E-10	6.0495	7.27E-10	6.0495	7.27E-10	6.0495	7.27E-10	7.27E-10	7.53E-07
EGLN2	ENSG00000269858	6.014	9.05E-10	6.014	9.05E-10	6.014	9.05E-10	6.014	9.05E-10	9.05E-10	9.19E-07
PSMC4	ENSG00000013275	-1.3932	0.91821	5.9075	1.74E-09	-1.3932	0.91821	-1.3932	0.91821	1.74E-09	1.73E-06
NA	ENSG00000238166	5.2601	7.20E-08	5.2601	7.20E-08	5.2388	8.08E-08	5.8658	2.24E-09	2.24E-09	2.18E-06
CYP2A7	ENSG00000198077	4.1312	1.80E-05	5.8569	2.36E-09	5.0527	2.18E-07	4.5811	2.31E-06	2.36E-09	2.25E-06
CYP2A7P2	ENSG00000198251	1.6282	0.051742	5.8045	3.23E-09	0.84332	0.19953	5.8045	3.23E-09	3.23E-09	3.03E-06
VDAC3	ENSG00000078668	5.6835	6.60E-09	4.4644	4.01E-06	4.3338	7.33E-06	4.0942	2.12E-05	6.60E-09	6.07E-06
NA	ENSG00000253408	0.1303	0.44817	-0.23096	0.59133	0.070766	0.47179	5.6351	8.75E-09	8.75E-09	7.90E-06
RAB4B	ENSG00000167578	5.5952	1.10E-08	5.5952	1.10E-08	4.7403	1.07E-06	5.1681	1.18E-07	1.10E-08	9.76E-06
POLB	ENSG00000070501	-0.41202	0.65984	-0.41202	0.65984	-0.15946	0.56335	5.5532	1.40E-08	1.40E-08	1.22E-05
ZNF546	ENSG00000187187	5.463	2.34E-08	-0.16989	0.56745	-0.16989	0.56745	2.6233	0.0043541	2.34E-08	2.00E-05
RAB4B-EGLN2	ENSG00000171570	5.427	2.87E-08	5.427	2.87E-08	4.8319	6.76E-07	5.0619	2.08E-07	2.87E-08	2.41E-05
NA	ENSG00000255101	5.1764	1.13E-07	4.906	4.65E-07	3.3801	0.00036235	3.1768	0.00074465	1.13E-07	9.28E-05
NA	ENSG00000255361	5.1744	1.14E-07	1.9971	0.022906	1.9971	0.022906	1.9971	0.022906	1.14E-07	9.28E-05

CYP2A6	ENSG00000255974	3.4651	0.00026504	2.6467	0.0040636	5.1389	1.38E-07	3.5048	0.00022845	1.38E-07	0.000110122
NA	ENSG00000268797	5.1363	1.40E-07	5.1363	1.40E-07	5.1363	1.40E-07	5.1363	1.40E-07	1.40E-07	0.000110122
CYP2B6	ENSG00000197408	4.9603	3.52E-07	3.423	0.00030973	3.5131	0.00022146	2.6605	0.0039014	3.52E-07	0.000272169
CEACAM3	ENSG00000170956	-2.0127	0.97793	4.8012	7.89E-07	1.3647	0.086181	-1.2076	0.8864	7.89E-07	0.000600529
MIA-RAB4B	ENSG00000268975	4.7959	8.10E-07	4.7959	8.10E-07	4.7959	8.10E-07	4.7959	8.10E-07	8.10E-07	0.000607033
PEF1	ENSG00000162517	2.1123	0.017332	2.1123	0.017332	4.7829	8.64E-07	2.1123	0.017332	8.64E-07	0.000635076
GTF2I	ENSG00000263001	3.6489	0.00013171	4.7808	8.73E-07	0.30351	0.38075	3.1799	0.00073651	8.73E-07	0.000635076
C19orf54	ENSG00000188493	4.7184	1.19E-06	4.7184	1.19E-06	4.7184	1.19E-06	4.7184	1.19E-06	1.19E-06	0.000851915
TGFB1	ENSG00000105329	4.6947	1.33E-06	-0.41565	0.66117	2.4085	0.0080087	-0.76939	0.77917	1.33E-06	0.000942678
DPY19L1	ENSG00000173852	4.2855	9.11E-06	4.6614	1.57E-06	2.6589	0.0039194	1.5507	0.060483	1.57E-06	0.001093292
HCRTR1	ENSG00000121764	2.1813	0.01458	4.6327	1.81E-06	1.2932	0.097972	1.8392	0.032945	1.81E-06	0.00123884
VN1R96P	ENSG00000230086	4.6107	2.01E-06	-0.14516	0.55771	4.1592	1.60E-05	0.82267	0.20535	2.01E-06	0.001357807
NA	ENSG00000260416	0.074612	0.47026	1.1018	0.13527	4.596	2.15E-06	0.51253	0.30414	2.15E-06	0.001437802
CXXC4	ENSG00000168772	2.7535	0.0029484	4.354	6.68E-06	4.4716	3.88E-06	3.3596	0.00039034	3.88E-06	0.00255681
RPL21P116	ENSG00000243711	4.3477	6.88E-06	4.2387	1.12E-05	4.2387	1.12E-05	4.2387	1.12E-05	6.88E-06	0.004469905
CHMP1A	ENSG00000131165	3.008	0.0013149	3.2993	0.00048466	2.0886	0.01837	4.3172	7.90E-06	7.90E-06	0.005065548
NA	ENSG00000269353	4.3138	8.02E-06	4.3138	8.02E-06	4.3138	8.02E-06	4.3138	8.02E-06	8.02E-06	0.005077666
GTF2IRD1	ENSG00000006704	4.2604	1.02E-05	3.68	0.0001166	2.4747	0.0066678	3.6998	0.00010786	1.02E-05	0.006373634
NA	ENSG00000236212	4.2384	1.13E-05	-0.89231	0.81389	-0.31599	0.624	3.8143	6.83E-05	1.13E-05	0.006943717
COQ8B	ENSG00000123815	0.84376	0.1994	4.1731	1.50E-05	4.1731	1.50E-05	4.1731	1.50E-05	1.50E-05	0.009149667
AXL	ENSG00000167601	4.1663	1.55E-05	-0.36559	0.64267	1.1268	0.12991	1.1268	0.12991	1.55E-05	0.00931104
SHKBP1	ENSG00000160410	2.5004	0.006203	2.5004	0.006203	2.5004	0.006203	4.1472	1.68E-05	1.68E-05	0.009999158
DPY19L2P1	ENSG00000189212	2.9243	0.0017263	3.8404	6.14E-05	3.585	0.00016855	4.1415	1.72E-05	1.72E-05	0.010127033
NDUFS3	ENSG00000213619	4.0927	2.13E-05	4.0927	2.13E-05	4.0927	2.13E-05	4.0927	2.13E-05	2.13E-05	0.012369298
CEACAM6	ENSG00000086548	-0.1313	0.55223	4.0651	2.40E-05	-0.1313	0.55223	-0.1313	0.55223	2.40E-05	0.013759632
GOLGA4	ENSG00000144674	4.0561	2.50E-05	0.78895	0.21507	1.5289	0.063141	-0.40461	0.65712	2.50E-05	0.014137366
DMAC2	ENSG00000105341	4.0172	2.94E-05	-1.345	0.91068	-1.345	0.91068	-1.345	0.91068	2.94E-05	0.016392443
APH1A	ENSG00000117362	4.0133	2.99E-05	4.0133	2.99E-05	0.5575	0.28859	2.5218	0.0058373	2.99E-05	0.016392443
C1orf54	ENSG00000118292	4.0133	2.99E-05	4.0133	2.99E-05	1.5371	0.062139	4.0133	2.99E-05	2.99E-05	0.016392443
MIA	ENSG00000261857	3.9774	3.48E-05	3.9774	3.48E-05	3.9774	3.48E-05	3.9774	3.48E-05	3.48E-05	0.018865007
NA	ENSG00000227902	1.1508	0.12491	3.9498	3.91E-05	-0.71488	0.76266	0.25087	0.40096	3.91E-05	0.020858029

DIDO1	ENSG00000101191	3.9481	3.94E-05	1.6408	0.050424	0.99447	0.16	2.3018	0.010673	3.94E-05	0.020858029
SMG6-IT1	ENSG00000278941	3.9424	4.03E-05	3.9424	4.03E-05	3.9424	4.03E-05	3.9424	4.03E-05	4.03E-05	0.021131005
CYBA	ENSG00000051523	0.75348	0.22558	1.1179	0.13181	3.9269	4.30E-05	-0.12622	0.55022	4.30E-05	0.022307972
THEM7P	ENSG00000227160	2.9195	0.0017531	3.8986	4.84E-05	3.035	0.0012025	2.2927	0.010934	4.84E-05	0.024812803
AP3M2	ENSG00000070718	0.0048395	0.49807	-0.86103	0.80539	3.8839	5.14E-05	-1.3278	0.90787	5.14E-05	0.0260939
NA	ENSG00000230599	0.56318	0.28666	3.8774	5.28E-05	0.76552	0.22198	-1.056	0.85452	5.28E-05	0.026524694
ITPKC	ENSG00000086544	2.3566	0.0092209	3.874	5.35E-05	1.5693	0.058287	3.874	5.35E-05	5.35E-05	0.026618017
SPG7	ENSG00000197912	3.8385	6.19E-05	0.69342	0.24402	0.56119	0.28733	0.21576	0.41459	6.19E-05	0.030463633
PSMC3	ENSG00000165916	3.8229	6.60E-05	3.7568	8.60E-05	2.9283	0.0017042	3.8229	6.60E-05	6.60E-05	0.032137435
ZNF280D	ENSG00000137871	3.8046	7.10E-05	2.5045	0.0061305	2.3681	0.0089388	2.7563	0.0029226	7.10E-05	0.034021662
SLC22A14	ENSG00000144671	3.1543	0.0008045	3.8039	7.12E-05	3.1543	0.0008045	3.3612	0.00038806	7.12E-05	0.034021662
XRCC3	ENSG00000126215	3.79	7.53E-05	3.79	7.53E-05	3.79	7.53E-05	3.79	7.53E-05	7.53E-05	0.035639608
UBL5P3	ENSG00000240359	3.0578	0.0011149	3.7856	7.67E-05	2.9679	0.0014992	-0.68311	0.75273	7.67E-05	0.035922913
LINC02730	ENSG00000255406	-0.7954	0.78681	0.17888	0.42902	3.7831	7.75E-05	1.3204	0.093359	7.75E-05	0.035944176
NA	ENSG00000255525	3.0575	0.0011159	3.4829	0.00024805	3.4469	0.00028353	3.7708	8.14E-05	8.14E-05	0.037406251
NAA20	ENSG00000173418	1.6735	0.047116	3.7325	9.48E-05	0.95773	0.1691	1.369	0.085496	9.48E-05	0.043163394
RNU4-68P	ENSG00000201184	3.722	9.88E-05	2.5802	0.0049366	1.0321	0.15102	3.1152	0.00091908	9.88E-05	0.044584373
NA	ENSG00000269940	3.7197	9.97E-05	3.7197	9.97E-05	3.7197	9.97E-05	3.7197	9.97E-05	9.97E-05	0.044584373
CCNP	ENSG00000105219	-0.027289	0.51089	3.7124	0.00010267	-0.027289	0.51089	0.41804	0.33796	0.00010267	0.04548281
NA	ENSG00000203593	1.2103	0.11309	3.0665	0.0010829	3.7031	0.00010651	0.58803	0.27826	0.00010651	0.046397921
ZNF780A	ENSG00000197782	-0.67953	0.7516	-0.67953	0.7516	-0.67953	0.7516	3.7028	0.00010664	0.00010664	0.046397921
LINC00652	ENSG00000179935	0.47222	0.31838	0.47222	0.31838	0.97528	0.16471	3.7004	0.00010765	0.00010765	0.046422872
RAPSN	ENSG00000165917	1.8039	0.035627	3.5614	0.00018441	3.6967	0.0001092	2.1579	0.015468	0.0001092	0.046678211
SPDYE5	ENSG00000170092	1.6602	0.048442	3.2247	0.00063062	2.866	0.0020781	3.692	0.00011124	0.00011124	0.047136741
GAS8-AS1	ENSG00000221819	3.6883	0.00011287	3.6883	0.00011287	3.6883	0.00011287	3.6883	0.00011287	0.00011287	0.04741513
CLEC18A	ENSG00000157322	2.4414	0.0073143	3.6771	0.00011796	3.0126	0.0012951	2.8191	0.0024082	0.00011796	0.049129836
ZFYVE28	ENSG00000159733	2.497	0.0062624	2.4709	0.006738	2.6546	0.0039697	3.6725	0.0001201	0.0001201	0.049426143
NA	ENSG00000224997	3.6712	0.0001207	3.6712	0.0001207	3.6712	0.0001207	3.6712	0.0001207	0.0001207	0.049426143
RAB42P1	ENSG00000230997	1.2537	0.10497	2.8336	0.0023016	2.4318	0.0075111	3.6634	0.00012445	0.00012445	0.050537071
CDK10	ENSG00000185324	2.4189	0.0077844	2.8724	0.0020368	2.3823	0.0086034	3.6528	0.00012971	0.00012971	0.052237755
TRIM35	ENSG00000104228	2.5475	0.0054245	3.151	0.00081347	3.6504	0.00013094	3.072	0.0010632	0.00013094	0.05230087

LINC02503	ENSG00000249614	-0.60228	0.72651	0.63012	0.26431	0.10818	0.45693	3.6332	0.00013997	0.00013997	0.055453155
GDPD1	ENSG00000153982	3.6216	0.00014641	1.5778	0.057307	1.8082	0.035286	3.4498	0.00028053	0.00014641	0.057536769
DYNLL1P6	ENSG00000248820	3.6148	0.0001503	-0.56359	0.71348	0.030476	0.48784	0.62681	0.26539	0.0001503	0.058226594
PPIAP55	ENSG00000228979	2.974	0.0014699	3.614	0.00015073	2.974	0.0014699	2.974	0.0014699	0.00015073	0.058226594
NTNG2	ENSG00000196358	-0.45072	0.6739	-0.90408	0.81702	3.6123	0.00015175	-0.7212	0.76461	0.00015175	0.058226594
JADRR	ENSG00000280711	0.13625	0.44581	2.5143	0.0059637	3.6087	0.00015385	3.6087	0.00015385	0.00015385	0.058419337
AFG1L	ENSG00000135537	1.2261	0.11007	2.2587	0.01195	1.5892	0.056003	3.6074	0.00015465	0.00015465	0.058419337
AKT2	ENSG00000105221	0.49642	0.3098	3.6026	0.00015751	0.49642	0.3098	0.47014	0.31913	0.00015751	0.059042018
POLN	ENSG00000130997	0.90184	0.18357	1.6502	0.049452	3.594	0.00016279	2.2207	0.013187	0.00016279	0.060185242
SORBS3	ENSG00000120896	3.1186	0.00090854	2.7991	0.0025622	3.5937	0.00016303	1.8626	0.031262	0.00016303	0.060185242
NPEPPSP1	ENSG00000274487	-0.19883	0.5788	0.993	0.16036	3.589	0.00016601	1.1006	0.13553	0.00016601	0.060824566
SLC39A14	ENSG00000104635	1.0929	0.13722	2.2975	0.010795	3.5817	0.00017069	2.6954	0.0035157	0.00017069	0.062072565
NA	ENSG00000260392	1.8776	0.030216	3.5739	0.00017583	0.75205	0.22601	3.5739	0.00017583	0.00017583	0.063468118
NUP50	ENSG00000093000	3.279	0.00052081	3.5696	0.00017875	2.9272	0.0017099	3.279	0.00052081	0.00017875	0.064047702
CYP2B7P	ENSG00000256612	1.9629	0.024827	3.5645	0.00018225	2.8004	0.0025517	3.0799	0.0010354	0.00018225	0.064825128
NUP50-DT	ENSG00000226328	2.7493	0.0029865	3.5607	0.0001849	3.5607	0.0001849	3.5607	0.0001849	0.0001849	0.065291138
NA	ENSG00000234340	0.02334	0.49069	3.5489	0.00019341	0.14544	0.44218	0.14544	0.44218	0.00019341	0.067804815
GALNS	ENSG00000141012	0.56713	0.28531	3.5378	0.00020172	0.23815	0.40588	-0.65039	0.74228	0.00020172	0.070212969
CDH15	ENSG00000129910	0.31328	0.37703	3.534	0.00020466	0.94526	0.17226	1.4129	0.078842	0.00020466	0.070425145
RNA5SP18	ENSG00000212237	3.5333	0.00020522	3.5333	0.00020522	3.5333	0.00020522	3.5333	0.00020522	0.00020522	0.070425145
TUBB8P6	ENSG00000224625	3.5257	0.00021122	1.1686	0.12129	-0.20165	0.57991	1.4205	0.077726	0.00021122	0.071770888
MIR885	ENSG00000216135	3.5229	0.00021343	3.5229	0.00021343	3.5229	0.00021343	3.5229	0.00021343	0.00021343	0.071770888
CLDN3	ENSG00000165215	-0.60204	0.72643	-0.23964	0.59469	-0.67185	0.74916	3.5227	0.00021356	0.00021356	0.071770888
ACAA1	ENSG00000060971	3.5134	0.00022123	2.1599	0.01539	3.5134	0.00022123	3.2335	0.00061152	0.00022123	0.073839301
NPSR1-AS1	ENSG00000197085	3.1587	0.00079241	2.7591	0.0028983	2.3824	0.0086003	3.4889	0.00024247	0.00024247	0.08037798
DPEP1	ENSG00000015413	0.97512	0.16475	0.95293	0.17031	1.2316	0.10906	3.4797	0.00025096	0.00025096	0.082630276
UFSP1	ENSG00000176125	3.4727	0.00025767	3.4727	0.00025767	3.4727	0.00025767	3.4727	0.00025767	0.00025767	0.084270195
RNU6-1274P	ENSG00000207287	3.4519	0.00027836	3.4519	0.00027836	3.4519	0.00027836	3.4519	0.00027836	0.00027836	0.090429885
PHYHIP	ENSG00000168490	3.4466	0.00028382	3.1464	0.00082653	-0.078148	0.53114	1.7857	0.037073	0.00028382	0.091570723
LINC01276	ENSG00000226917	2.2565	0.012019	0.792	0.21418	3.4449	0.00028563	2.9637	0.0015198	0.00028563	0.091570723
PLAT	ENSG00000104368	-0.29413	0.61567	-0.22034	0.5872	-0.29706	0.61679	3.4429	0.00028772	0.00028772	0.09163788

FUBP3	ENSG00000107164	0.90076	0.18386	0.69314	0.24411	3.3922	0.00034667	3.4384	0.00029263	0.00029263	0.092596493
GGTLC2	ENSG00000100121	0.68248	0.24747	-0.50836	0.6944	3.433	0.00029842	0.084342	0.46639	0.00029842	0.093819397
NA	ENSG00000279005	2.3367	0.009728	3.1833	0.00072801	3.4293	0.00030256	2.7657	0.0028402	0.00030256	0.094275479
LCT	ENSG00000115850	3.4283	0.00030374	2.0392	0.020716	1.4725	0.070446	1.3888	0.08244	0.00030374	0.094275479
LINC02731	ENSG00000204241	1.744	0.040575	1.213	0.11256	0.67993	0.24827	3.4224	0.0003103	0.0003103	0.095596614
NA	ENSG00000273691	3.421	0.00031192	3.421	0.00031192	3.421	0.00031192	1.1352	0.12815	0.00031192	0.095596614
LMOD1	ENSG00000163431	-1.316	0.90591	-0.9968	0.84057	3.4181	0.0003153	-1.4411	0.92522	0.0003153	0.095801364
NR2F1-AS1	ENSG00000237187	0.71434	0.23751	3.4171	0.00031652	1.598	0.055026	1.319	0.093587	0.00031652	0.095801364
NA	ENSG00000212569	2.6676	0.0038199	2.6676	0.0038199	3.4073	0.000328	2.6676	0.0038199	0.000328	0.097773053
NA	ENSG00000244470	1.065	0.14343	1.5048	0.066191	3.4072	0.00032812	-0.79546	0.78683	0.00032812	0.097773053
RNA5SP233	ENSG00000238391	2.7291	0.0031756	-0.28998	0.61408	0.51935	0.30176	3.4056	0.00033012	0.00033012	0.097773053
ZNF678	ENSG00000181450	1.4621	0.071856	1.9614	0.024916	3.4048	0.00033106	2.9612	0.0015323	0.00033106	0.097773053
MAPK8IP1	ENSG00000121653	-1.1419	0.87325	0.95405	0.17003	3.3939	0.0003445	0.83193	0.20272	0.0003445	0.101129428
RN7SKP16	ENSG00000222112	3.2374	0.00060318	3.2374	0.00060318	3.3922	0.00034662	3.1166	0.00091481	0.00034662	0.101142471
NA	ENSG00000241026	2.2041	0.013759	1.0281	0.15195	3.3856	0.00035513	2.3609	0.0091151	0.00035513	0.102474288
NA	ENSG00000264078	3.3854	0.00035539	1.5852	0.056463	1.7296	0.041851	1.7296	0.041851	0.00035539	0.102474288
DLK2	ENSG00000171462	1.098	0.1361	3.2233	0.00063371	2.2423	0.012472	3.3803	0.00036208	0.00036208	0.103789167
NA	ENSG00000198211	2.8494	0.0021899	2.6859	0.0036172	3.3749	0.00036927	2.9945	0.0013745	0.00036927	0.104608945
SPI1	ENSG00000066336	0.55306	0.29011	3.3746	0.00036962	1.2997	0.09686	1.6859	0.045907	0.00036962	0.104608945
NA	ENSG00000253633	2.1035	0.017713	2.4058	0.0080689	3.3733	0.00037138	3.3733	0.00037138	0.00037138	0.104608945
NA	ENSG00000265711	3.3691	0.00037706	0.30834	0.37891	3.3691	0.00037706	1.229	0.10953	0.00037706	0.10559847
NA	ENSG00000268593	3.363	0.00038557	3.363	0.00038557	3.363	0.00038557	3.363	0.00038557	0.00038557	0.10627545
SDC3	ENSG00000162512	2.1802	0.014621	0.43878	0.33041	2.244	0.012415	3.3629	0.0003856	0.0003856	0.10627545
CELF1	ENSG00000149187	2.0293	0.021213	2.6049	0.0045948	3.3626	0.00038602	2.7225	0.0032392	0.00038602	0.10627545
B3GALNT2	ENSG00000162885	2.2156	0.01336	2.471	0.0067363	1.2609	0.10368	3.3493	0.00040504	0.00040504	0.110600766
NA	ENSG00000214970	-0.15303	0.56081	1.4927	0.067756	3.3485	0.00040627	0.84481	0.19911	0.00040627	0.110600766
KBTBD4	ENSG00000123444	3.344	0.00041297	3.344	0.00041297	3.344	0.00041297	3.344	0.00041297	0.00041297	0.111800156
NA	ENSG00000250670	1.5219	0.064013	3.3377	0.00042239	1.8513	0.032061	-0.84373	0.80059	0.00042239	0.112361948
NA	ENSG00000268153	1.8004	0.035901	0.69503	0.24352	1.8004	0.035901	3.337	0.00042344	0.00042344	0.112361948
IL6RP1	ENSG00000234910	1.6034	0.054419	0.70491	0.24043	3.3365	0.00042419	2.8025	0.0025355	0.00042419	0.112361948
NA	ENSG00000257239	1.2167	0.11186	3.336	0.00042502	3.336	0.00042502	3.336	0.00042502	0.00042502	0.112361948

RNU7-193P	ENSG00000252532	3.3338	0.0004283	2.0648	0.01947	2.553	0.0053404	3.3338	0.0004283	0.0004283	0.112361948
NA	ENSG00000160828	1.1138	0.13269	3.3335	0.00042888	-0.055924	0.5223	2.5148	0.0059546	0.00042888	0.112361948
RPS17P13	ENSG00000224706	3.3316	0.00043171	3.3316	0.00043171	3.3316	0.00043171	3.3316	0.00043171	0.00043171	0.112498547
NA	ENSG00000273069	2.3452	0.0095085	-0.45849	0.6767	3.3266	0.00043964	-0.45849	0.6767	0.00043964	0.113955623
LINC01413	ENSG00000260172	0.93692	0.1744	3.3231	0.00044505	0.93692	0.1744	0.71439	0.23749	0.00044505	0.114747548
SYPL1P1	ENSG00000243154	2.1411	0.016132	3.3212	0.00044816	-0.45265	0.6746	-0.45265	0.6746	0.00044816	0.114941246
NA	ENSG00000231136	3.3174	0.00045433	-3.2204	0.99936	1.2152	0.11215	-3.2204	0.99936	0.00045433	0.115817505
UCHL5	ENSG00000116750	2.7347	0.0031215	3.3162	0.00045633	0.81233	0.2083	2.7567	0.0029196	0.00045633	0.115817505
RPL36AP45	ENSG00000240366	0.38142	0.35145	-0.052611	0.52098	0.56585	0.28575	3.3126	0.00046225	0.00046225	0.116712137
YPEL2	ENSG00000175155	3.3102	0.00046611	-0.23072	0.59124	-0.35233	0.63771	-0.79626	0.78706	0.00046611	0.117080105
ANKRD24	ENSG00000089847	0.51307	0.30395	2.2316	0.01282	3.1253	0.00088825	3.307	0.00047153	0.00047153	0.117834138
CCDC171	ENSG00000164989	3.3037	0.00047701	1.0372	0.14982	0.89537	0.18529	0.39224	0.34744	0.00047701	0.118595394
PIAS4	ENSG00000105229	2.7802	0.0027159	3.1196	0.00090553	0.7674	0.22142	3.2982	0.00048655	0.00048655	0.119096602
SKINT1L	ENSG00000242267	2.2876	0.011081	3.2955	0.00049118	2.9791	0.0014457	2.9791	0.0014457	0.00049118	0.119096602
DLEC1	ENSG00000008226	-0.005602	0.50223	-0.046059	0.51837	0.73507	0.23115	3.2952	0.00049175	0.00049175	0.119096602
NA	ENSG00000267133	3.2944	0.00049315	0.063094	0.47485	0.063094	0.47485	0.063094	0.47485	0.00049315	0.119096602
PPP3CC	ENSG00000120910	2.1149	0.017221	2.9162	0.0017716	2.4766	0.0066324	3.2942	0.00049353	0.00049353	0.119096602
NA	ENSG00000196696	3.2941	0.00049369	2.8024	0.0025363	3.0807	0.0010324	2.1015	0.017797	0.00049369	0.119096602
NA	ENSG00000272744	3.2883	0.00050392	3.2883	0.00050392	3.2883	0.00050392	3.2883	0.00050392	0.00050392	0.120793071
HHLA1	ENSG00000132297	3.2873	0.00050568	1.4077	0.079604	1.0651	0.14342	0.79663	0.21283	0.00050568	0.120793071
NA	ENSG00000203900	3.2857	0.00050865	2.6707	0.0037846	3.2377	0.00060257	3.2377	0.00060257	0.00050865	0.120909827
NA	ENSG00000206129	-0.70633	0.76001	3.2778	0.00052309	0.37057	0.35548	0.68965	0.24521	0.00052309	0.123738717
NA	ENSG00000282828	-0.89708	0.81516	3.2719	0.00053411	0.62651	0.26549	-0.89708	0.81516	0.00053411	0.125735171
NA	ENSG00000185128	3.2662	0.00054503	-1.5065	0.93403	0.46861	0.31968	0.62025	0.26755	0.00054503	0.126818093
YPEL5P2	ENSG00000271557	3.2659	0.00054555	3.2659	0.00054555	3.2659	0.00054555	3.2659	0.00054555	0.00054555	0.126818093
UBXN4	ENSG00000144224	-0.25736	0.60155	3.2647	0.00054792	0.50225	0.30774	-0.68065	0.75196	0.00054792	0.126818093
NA	ENSG00000279382	3.2641	0.00054912	3.2641	0.00054912	3.2641	0.00054912	3.2641	0.00054912	0.00054912	0.126818093
FOXO3	ENSG00000118689	3.2627	0.00055186	2.6886	0.0035873	1.8303	0.033603	1.4559	0.072715	0.00055186	0.126849707
NA	ENSG00000200754	-0.54907	0.70852	3.2488	0.00057946	-0.54907	0.70852	-0.54907	0.70852	0.00057946	0.131985772
YIPF1	ENSG00000058799	1.7902	0.036711	1.4101	0.079252	3.2486	0.00057989	2.389	0.0084469	0.00057989	0.131985772
GNG12-AS1	ENSG00000232284	-0.081976	0.53267	3.2474	0.00058233	2.9057	0.001832	2.7349	0.0031204	0.00058233	0.131985772

RBBP8NL	ENSG00000130701	0.79527	0.21323	0.064928	0.47412	0.79527	0.21323	3.2446	0.00058814	0.00058814	0.132076265
NA	ENSG00000260285	1.966	0.024648	3.2446	0.00058815	3.2446	0.00058815	3.2446	0.00058815	0.00058815	0.132076265
WWP2	ENSG00000198373	2.2058	0.0137	2.9078	0.00182	2.97	0.001489	3.2399	0.00059785	0.00059785	0.133638672
NA	ENSG00000234427	-0.33636	0.6317	-0.33636	0.6317	3.2303	0.00061837	-0.33636	0.6317	0.00061837	0.135547368
NA	ENSG00000259674	0.39629	0.34595	0.39629	0.34595	0.39629	0.34595	3.2302	0.00061857	0.00061857	0.135547368
PPIAP14	ENSG00000264775	3.23	0.00061885	0.41231	0.34006	0.17729	0.42964	2.6105	0.00452	0.00061885	0.135547368
NA	ENSG00000261145	3.2282	0.00062281	1.3334	0.091202	1.564	0.058911	0.73468	0.23127	0.00062281	0.135547368
PTPMT1	ENSG00000110536	0.99901	0.1589	3.228	0.0006232	0.51717	0.30252	-0.65532	0.74387	0.0006232	0.135547368
DHX34	ENSG00000134815	2.2713	0.011564	3.2272	0.00062502	1.4247	0.077128	0.96086	0.16831	0.00062502	0.135547368
LYST	ENSG00000143669	3.1422	0.00083837	3.2268	0.00062586	2.5803	0.0049362	-0.24698	0.59754	0.00062586	0.135547368
CYB5D1	ENSG00000182224	1.4635	0.071667	3.2244	0.00063128	2.6272	0.004305	2.9367	0.0016584	0.00063128	0.135853659
LINC01300	ENSG00000253595	3.2236	0.00063285	-0.46689	0.67971	-0.80986	0.79099	0.016515	0.49341	0.00063285	0.135853659
STRA6	ENSG00000137868	3.2224	0.00063568	1.4103	0.079225	1.2889	0.098716	1.3278	0.092116	0.00063568	0.13586266
RN7SKP66	ENSG00000252490	3.2207	0.00063936	3.2207	0.00063936	3.2207	0.00063936	3.2207	0.00063936	0.00063936	0.136052458
NA	ENSG00000254627	2.6428	0.0041107	3.0135	0.0012911	3.2159	0.00065017	0.92183	0.17831	0.00065017	0.137513529
BCO2	ENSG00000197580	3.2152	0.00065187	0.86051	0.19475	1.2124	0.11269	1.2124	0.11269	0.00065187	0.137513529
SCAF1	ENSG00000126461	1.7449	0.040501	3.2102	0.00066316	2.0991	0.017905	1.9764	0.024056	0.00066316	0.138959974
ZFYVE21	ENSG00000100711	3.1634	0.00077959	1.1501	0.12505	2.7974	0.0025761	3.2097	0.00066443	0.00066443	0.138959974
KLC1	ENSG00000126214	3.0582	0.0011133	2.5455	0.0054558	3.2075	0.00066937	2.9468	0.0016056	0.00066937	0.139394872
NA	ENSG00000255375	2.8071	0.0024998	3.2055	0.00067422	-0.76512	0.7779	-0.30536	0.61995	0.00067422	0.139528857
GULOP	ENSG00000234770	3.2048	0.00067574	0.8164	0.20714	2.1342	0.016414	-0.018481	0.50737	0.00067574	0.139528857
RNU6-649P	ENSG00000207192	2.1378	0.016267	2.1181	0.017084	3.2028	0.00068041	2.1181	0.017084	0.00068041	0.139556168
HMGB1P51	ENSG00000270986	3.2023	0.0006816	3.2023	0.0006816	3.2023	0.0006816	0.65145	0.25738	0.0006816	0.139556168
PDLIM2	ENSG00000120913	2.3315	0.0098627	3.1973	0.00069358	3.0251	0.0012428	-1.0654	0.85664	0.00069358	0.14116878
NA	ENSG00000261627	2.7476	0.0030016	3.1966	0.00069527	2.7476	0.0030016	2.5051	0.0061209	0.00069527	0.14116878
NA	ENSG00000261238	-0.61811	0.73175	0.23788	0.40599	3.1923	0.00070579	3.1923	0.00070579	0.00070579	0.142383951
C4orf48	ENSG00000243449	0.20358	0.41934	0.15383	0.43887	3.19	0.00071137	0.90729	0.18213	0.00071137	0.142383951
RNA5SP77	ENSG00000202264	3.1891	0.00071353	-1.4996	0.93314	0.9413	0.17328	0.9413	0.17328	0.00071353	0.142383951
TMEM254-AS1	ENSG00000230091	2.1638	0.015241	2.1638	0.015241	2.1638	0.015241	3.189	0.00071375	0.00071375	0.142383951
NA	ENSG00000238390	3.1874	0.00071778	3.1874	0.00071778	3.1874	0.00071778	3.1874	0.00071778	0.00071778	0.142383951
LINC02447	ENSG00000245468	-1.1644	0.87787	3.1861	0.00072109	0.16504	0.43446	-0.23603	0.5933	0.00072109	0.142383951

LINC02337	ENSG00000215881	3.1847	0.00072448	2.9055	0.0018331	-1.472	0.92948	-1.472	0.92948	0.00072448	0.142383951
PGGT1BP2	ENSG00000214584	3.1847	0.00072463	-0.51581	0.69701	-0.031477	0.51256	-0.031477	0.51256	0.00072463	0.142383951
LINC01854	ENSG00000204460	3.1833	0.00072801	1.3207	0.0933	-1.8838	0.97021	-0.16747	0.5665	0.00072801	0.142473604
CYP2F1	ENSG00000197446	0.7472	0.22747	3.1799	0.0007367	0.84877	0.198	1.3499	0.088519	0.0007367	0.143597564
RNU6-195P	ENSG00000223284	3.1765	0.00074527	3.1765	0.00074527	3.1765	0.00074527	3.1765	0.00074527	0.00074527	0.144382769
PRMT8	ENSG00000111218	-0.20328	0.58054	3.1742	0.00075137	-0.44503	0.67185	0.63591	0.26242	0.00075137	0.144382769
GRK4	ENSG00000125388	2.2931	0.010921	3.1738	0.00075238	2.079	0.01881	0.88456	0.1882	0.00075238	0.144382769
DMRTB1	ENSG00000143006	3.1737	0.00075258	1.6059	0.054147	0.63028	0.26425	0.63028	0.26425	0.00075258	0.144382769
COA8	ENSG00000256053	1.751	0.039975	3.1707	0.00076048	2.0099	0.02222	1.3301	0.091739	0.00076048	0.145326237
CD24P2	ENSG00000261333	-0.25455	0.60046	3.165	0.00077532	-0.25455	0.60046	-0.25455	0.60046	0.00077532	0.147583373
MYD88	ENSG00000172936	3.1096	0.0009368	3.1096	0.0009368	3.1626	0.00078172	3.1096	0.0009368	0.00078172	0.148222629
NA	ENSG00000279609	3.1556	0.00080088	1.0473	0.14747	3.1556	0.00080088	3.1556	0.00080088	0.00080088	0.151266986
FNDC5	ENSG00000160097	2.8529	0.0021664	2.2734	0.011501	2.8529	0.0021664	3.1517	0.0008115	0.0008115	0.152601743
NA	ENSG00000228687	3.1508	0.00081421	3.1508	0.00081421	3.1508	0.00081421	3.1508	0.00081421	0.00081421	0.152601743
NOP14-AS1	ENSG00000249673	3.1482	0.00082143	2.7187	0.0032768	2.6812	0.0036678	2.6812	0.0036678	0.00082143	0.152811328
NOP14	ENSG00000087269	3.1481	0.0008216	1.2133	0.1125	2.3054	0.010572	1.3269	0.092276	0.0008216	0.152811328
RN7SL674P	ENSG00000239899	3.146	0.00082757	3.146	0.00082757	3.146	0.00082757	3.146	0.00082757	0.00082757	0.153336449
ADIPOR1P2	ENSG00000258805	3.1434	0.00083496	0.2355	0.40691	1.7131	0.04335	1.7131	0.04335	0.00083496	0.153803153
NA	ENSG00000257941	1.887	0.029578	1.887	0.029578	3.142	0.00083888	3.142	0.00083888	0.00083888	0.153803153
NA	ENSG00000274093	3.1403	0.00084386	3.1403	0.00084386	3.1403	0.00084386	3.1403	0.00084386	0.00084386	0.153803153
NA	ENSG00000224149	3.1399	0.00084509	3.1399	0.00084509	3.1399	0.00084509	3.1399	0.00084509	0.00084509	0.153803153
TMEM91	ENSG00000142046	3.1396	0.00084587	-0.44544	0.672	-0.44544	0.672	-0.44544	0.672	0.00084587	0.153803153
NA	ENSG00000254584	2.9915	0.001388	3.1385	0.00084921	3.0892	0.0010034	1.6165	0.052991	0.00084921	0.153836443
CREB3L1	ENSG00000157613	3.1346	0.00086046	2.6494	0.0040321	2.218	0.013277	2.4275	0.0076008	0.00086046	0.155297096
NA	ENSG00000203648	3.132	0.00086803	3.132	0.00086803	3.132	0.00086803	3.132	0.00086803	0.00086803	0.155907335
PLCH1	ENSG00000114805	3.1313	0.00087024	2.2054	0.013713	2.2404	0.012532	1.5164	0.064714	0.00087024	0.155907335
RNU6-235P	ENSG00000199514	3.1294	0.00087581	3.1294	0.00087581	3.1294	0.00087581	3.1294	0.00087581	0.00087581	0.15628458
GLYCAM1	ENSG00000257780	3.1284	0.00087876	3.1284	0.00087876	1.4961	0.067313	3.1284	0.00087876	0.00087876	0.15628458
NA	ENSG00000269910	3.1224	0.00089679	3.1224	0.00089679	3.1224	0.00089679	3.1224	0.00089679	0.00089679	0.157651992
NA	ENSG00000211571	3.121	0.00090105	3.121	0.00090105	3.121	0.00090105	3.121	0.00090105	0.00090105	0.157651992
PRPSAP1	ENSG00000161542	-0.64663	0.74106	-0.77086	0.77961	0.23299	0.40788	3.1202	0.00090359	0.00090359	0.157651992

NA	ENSG00000229896	2.8467	0.002209	3.12	0.00090422	1.7177	0.042924	1.9928	0.02314	0.00090422	0.157651992
SNAP47-AS1	ENSG00000230005	-0.36843	0.64372	3.1196	0.00090542	0.5077	0.30583	0.5077	0.30583	0.00090542	0.157651992
RAB11A	ENSG00000103769	3.1195	0.00090586	0.38677	0.34946	-0.24091	0.59519	-0.61959	0.73224	0.00090586	0.157651992
NA	ENSG00000228133	1.1748	0.12004	3.1182	0.00090967	1.1748	0.12004	1.1748	0.12004	0.00090967	0.157751669
NA	ENSG00000270859	-0.36408	0.6421	-0.29942	0.61769	-0.61393	0.73037	3.1164	0.00091535	0.00091535	0.15792136
NA	ENSG00000241853	3.1158	0.00091713	3.1158	0.00091713	3.1158	0.00091713	3.1158	0.00091713	0.00091713	0.15792136
COL16A1	ENSG00000084636	3.1112	0.00093168	2.1811	0.014589	2.7523	0.0029592	3.0021	0.0013408	0.00093168	0.15963777
NA	ENSG00000278875	3.1106	0.00093365	3.1106	0.00093365	3.1106	0.00093365	3.1106	0.00093365	0.00093365	0.15963777
NA	ENSG00000251802	2.5777	0.0049726	3.1079	0.00094219	2.6452	0.0040824	2.6452	0.0040824	0.00094219	0.160534681
PITX2	ENSG00000164093	0.43604	0.3314	1.9659	0.024653	1.3369	0.090625	3.1045	0.00095291	0.00095291	0.161795485
NA	ENSG00000269958	3.1009	0.00096458	3.1009	0.00096458	3.1009	0.00096458	3.1009	0.00096458	0.00096458	0.162938619
USP45	ENSG00000123552	1.7246	0.042298	2.4673	0.0068065	1.9946	0.023045	3.1004	0.00096633	0.00096633	0.162938619
LINC01011	ENSG00000244041	1.3042	0.09609	3.099	0.00097096	1.9588	0.02507	1.9588	0.02507	0.00097096	0.163154761
NA	ENSG00000278382	2.74	0.0030719	3.0937	0.00098848	0.50993	0.30505	0.61842	0.26815	0.00098848	0.16552794
NA	ENSG00000251523	-0.058531	0.52334	-0.058531	0.52334	-0.41831	0.66214	3.092	0.00099413	0.00099413	0.165903955
NA	ENSG00000260198	0.91014	0.18138	3.0899	0.001001	0.039933	0.48407	3.0899	0.001001	0.001001	0.16595184
DOC2A	ENSG00000149927	0.41034	0.34078	0.71574	0.23708	0.71574	0.23708	3.0865	0.0010126	0.0010126	0.16595184
LINC01353	ENSG00000231507	3.0862	0.0010136	0.73835	0.23015	1.0277	0.15204	0.71651	0.23684	0.0010136	0.16595184
NA	ENSG00000280182	3.0861	0.0010141	3.0861	0.0010141	3.0861	0.0010141	3.0861	0.0010141	0.0010141	0.16595184
NA	ENSG00000233953	3.084	0.0010211	1.7396	0.040964	1.7396	0.040964	-0.55977	0.71218	0.0010211	0.16595184
LINC00557	ENSG00000260962	3.0834	0.0010233	0.50594	0.30645	0.38406	0.35047	0.4916	0.3115	0.0010233	0.16595184
NA	ENSG00000219867	0.37012	0.35565	-1.0246	0.84722	3.0831	0.0010242	0.37012	0.35565	0.0010242	0.16595184
NA	ENSG00000268670	3.0815	0.0010299	3.0815	0.0010299	3.0815	0.0010299	3.0815	0.0010299	0.0010299	0.16595184
NA	ENSG00000265041	3.0804	0.0010337	3.0804	0.0010337	3.0804	0.0010337	3.0804	0.0010337	0.0010337	0.16595184
B3GNTL1	ENSG00000175711	2.1944	0.014102	2.0192	0.021735	0.93915	0.17383	3.0803	0.001034	0.001034	0.16595184
OR2L9P	ENSG00000237492	2.4814	0.0065425	2.9925	0.0013835	3.0789	0.0010388	2.8724	0.0020368	0.0010388	0.16595184
NA	ENSG00000251034	2.0831	0.01862	3.0779	0.0010424	2.0219	0.021595	2.0219	0.021595	0.0010424	0.16595184
SYNDIG1L	ENSG00000183379	2.9722	0.0014783	2.2413	0.012504	3.0774	0.0010441	2.6987	0.0034809	0.0010441	0.16595184
ACOT11	ENSG00000162390	1.1184	0.1317	1.2779	0.10064	3.0773	0.0010446	0.26716	0.39467	0.0010446	0.16595184
HSD3B7	ENSG00000099377	3.077	0.0010455	2.964	0.0015183	2.8075	0.0024964	2.6506	0.0040174	0.0010455	0.16595184
NA	ENSG00000241400	3.0748	0.0010532	3.0748	0.0010532	3.0748	0.0010532	3.0748	0.0010532	0.0010532	0.166631286

NA	ENSG00000268632	3.0715	0.001065	1.0787	0.14037	1.3613	0.086712	1.8076	0.035337	0.001065	0.167952913
LINC02162	ENSG00000249830	3.0667	0.0010821	-0.013661	0.50545	1.0475	0.14743	1.1412	0.12689	0.0010821	0.169046331
PRSS36	ENSG00000178226	3.0641	0.0010916	1.2208	0.11108	-0.35854	0.64003	1.2208	0.11108	0.0010916	0.169046331
TMEM71	ENSG00000165071	3.0638	0.0010927	2.2035	0.013779	2.2035	0.013779	2.2035	0.013779	0.0010927	0.169046331
BAG5	ENSG00000166170	2.7093	0.0033708	2.7093	0.0033708	2.8342	0.0022974	3.0636	0.0010933	0.0010933	0.169046331
NA	ENSG00000254780	0.23269	0.408	1.3811	0.083624	1.6305	0.051494	3.0629	0.001096	0.001096	0.169046331
IQCF3	ENSG00000229972	2.523	0.0058185	2.3984	0.0082325	2.4153	0.0078616	3.0617	0.0011004	0.0011004	0.169046331
NA	ENSG00000200090	3.0609	0.0011033	3.0609	0.0011033	3.0609	0.0011033	3.0609	0.0011033	0.0011033	0.169046331
SNW1	ENSG00000100603	0.70626	0.24001	2.1221	0.016916	3.0602	0.0011059	0.87227	0.19153	0.0011059	0.169046331
LINC01551	ENSG00000186960	0.70921	0.2391	1.1403	0.12708	3.0587	0.0011114	1.8218	0.034245	0.0011114	0.169046331
NOP53-AS1	ENSG00000269656	-0.80578	0.78982	-0.80578	0.78982	-0.80578	0.78982	3.0586	0.0011117	0.0011117	0.169046331
OXSR1	ENSG00000172939	0.24751	0.40226	3.0584	0.0011124	1.0045	0.15757	1.0842	0.13914	0.0011124	0.169046331
COX6CP11	ENSG00000258668	3.057	0.0011177	0.6779	0.24892	2.3973	0.008258	1.618	0.052832	0.0011177	0.169046331
CCNT2-AS1	ENSG00000224043	3.057	0.001118	3.057	0.001118	3.057	0.001118	3.057	0.001118	0.001118	0.169046331
NA	ENSG00000268863	3.0563	0.0011205	3.0563	0.0011205	3.0563	0.0011205	3.0563	0.0011205	0.0011205	0.169046331
NA	ENSG00000254939	2.8457	0.0022156	2.0765	0.018925	3.0547	0.0011266	3.0547	0.0011266	0.0011266	0.169442031
NA	ENSG00000259828	3.053	0.0011329	2.763	0.0028638	2.1568	0.015509	1.7233	0.042415	0.0011329	0.169865283
TCF4	ENSG00000196628	1.9852	0.023561	1.3049	0.095962	1.5815	0.056882	3.0484	0.0011503	0.0011503	0.17194515
NA	ENSG00000275263	3.0438	0.0011682	3.0438	0.0011682	3.0438	0.0011682	3.0438	0.0011682	0.0011682	0.174086807
SACS	ENSG00000151835	3.0408	0.0011797	1.4604	0.072089	2.7988	0.0025643	2.2051	0.013725	0.0011797	0.175264576
NA	ENSG00000250599	0.35895	0.35982	0.095075	0.46213	0.58943	0.27779	3.0392	0.0011859	0.0011859	0.175650173
CRY2	ENSG00000121671	-0.42695	0.66529	2.1227	0.01689	2.3702	0.0088892	3.0368	0.0011956	0.0011956	0.176134659
NA	ENSG00000248772	3.0366	0.0011964	3.0366	0.0011964	3.0366	0.0011964	3.0366	0.0011964	0.0011964	0.176134659
PPP4R2	ENSG00000163605	1.8156	0.034713	2.065	0.01946	1.7075	0.04386	3.0324	0.0012132	0.0012132	0.177481312
RNY3P15	ENSG00000252106	3.0316	0.0012163	3.0316	0.0012163	3.0316	0.0012163	3.0316	0.0012163	0.0012163	0.177481312
MIR4669	ENSG00000263897	0.075391	0.46995	0.075391	0.46995	0.075391	0.46995	3.0311	0.0012183	0.0012183	0.177481312
ETNK2	ENSG00000143845	3.0305	0.0012206	2.0933	0.018159	2.1037	0.0177	0.039646	0.48419	0.0012206	0.177481312
INTS2	ENSG00000108506	0.41794	0.338	-0.31316	0.62292	0.42809	0.33429	3.0289	0.0012271	0.0012271	0.177481312
NA	ENSG00000261527	0.11113	0.45576	3.0289	0.0012274	0.81012	0.20893	2.3064	0.010546	0.0012274	0.177481312
LINC02218	ENSG00000249662	-0.31754	0.62458	3.0246	0.0012447	-0.31754	0.62458	0.79118	0.21442	0.0012447	0.179450388
CLIP2	ENSG00000106665	0.49188	0.3114	2.1774	0.014726	3.0217	0.0012567	2.7224	0.0032404	0.0012567	0.180114679

MARCHF4	ENSG00000144583	2.5679	0.0051163	1.6842	0.046071	3.0217	0.0012567	0.59967	0.27436	0.0012567	0.180114679
RN7SL480P	ENSG00000266187	2.8087	0.0024868	2.8087	0.0024868	3.02	0.0012638	2.8087	0.0024868	0.0012638	0.180601097
RECQL5	ENSG00000108469	1.8913	0.02929	3.0169	0.0012769	2.3052	0.010577	1.4894	0.068194	0.0012769	0.181939582
AFF1	ENSG00000172493	1.2666	0.10265	3.0144	0.0012874	2.1341	0.016419	1.394	0.081655	0.0012874	0.18290088
NA	ENSG00000265985	2.5241	0.0058004	0.091807	0.46343	3.0132	0.0012925	2.3828	0.0085913	0.0012925	0.183091642
ZNF577	ENSG00000161551	1.4855	0.0687	3.0101	0.0013057	2.8168	0.0024256	2.611	0.0045137	0.0013057	0.183681986
NA	ENSG00000236687	3.0083	0.0013135	-0.37095	0.64466	0.78074	0.21748	-0.11191	0.54455	0.0013135	0.183681986
NA	ENSG00000242073	3.0079	0.0013151	0.692	0.24447	0.2315	0.40846	0.11139	0.45565	0.0013151	0.183681986
NA	ENSG00000249982	3.0073	0.0013178	3.0073	0.0013178	3.0073	0.0013178	3.0073	0.0013178	0.0013178	0.183681986
GLYCTK	ENSG00000168237	0.73618	0.23081	0.31637	0.37586	3.0066	0.001321	0.3211	0.37407	0.001321	0.183681986
TBX20	ENSG00000164532	3.006	0.0013238	0.90029	0.18398	0.81015	0.20893	-0.54928	0.70859	0.0013238	0.183681986
SCGB1B2P	ENSG00000268751	-0.18758	0.5744	3.0045	0.0013301	-0.18758	0.5744	-0.18758	0.5744	0.0013301	0.183681986
LINC01894	ENSG00000264345	1.0419	0.14873	1.1944	0.11616	3.0033	0.0013352	1.9292	0.026855	0.0013352	0.183681986
NSL1	ENSG00000117697	1.3475	0.088913	0.99449	0.15999	3.0031	0.0013361	0.97999	0.16355	0.0013361	0.183681986
YTHDF1	ENSG00000149658	3.0031	0.0013363	0.090065	0.46412	1.5489	0.060707	0.92848	0.17658	0.0013363	0.183681986
NA	ENSG00000261499	2.6523	0.0039972	3.0021	0.0013408	1.5623	0.059113	2.8257	0.0023592	0.0013408	0.183681986
S100PBP	ENSG00000116497	2.72	0.0032646	3.0018	0.0013419	2.8656	0.0020809	2.9984	0.0013571	0.0013419	0.183681986
IBSP	ENSG00000029559	2.9996	0.0013518	0.26824	0.39426	-0.56807	0.71501	0.11881	0.45271	0.0013518	0.184518807
NLRP9	ENSG00000185792	2.9982	0.0013577	2.9982	0.0013577	-0.025958	0.51035	2.9982	0.0013577	0.0013577	0.184806483
NA	ENSG00000263443	1.4021	0.08044	2.9966	0.0013649	-0.87921	0.81036	0.82588	0.20443	0.0013649	0.184903278
GTF3C1	ENSG00000077235	2.9964	0.001366	2.805	0.0025156	1.9098	0.02808	0.21308	0.41563	0.001366	0.184903278
WNT5B	ENSG00000111186	2.546	0.0054477	2.0449	0.020434	2.9889	0.0014001	1.2192	0.11139	0.0014001	0.188382394
PACC1	ENSG00000065600	-0.67148	0.74904	2.9884	0.0014024	0.41942	0.33746	0.75592	0.22485	0.0014024	0.188382394
SMG1P5	ENSG00000183604	2.9882	0.0014033	2.256	0.012036	1.1395	0.12725	0.63792	0.26176	0.0014033	0.188382394
CASKIN1	ENSG00000167971	2.8289	0.0023356	2.5993	0.0046705	2.9862	0.0014125	2.8289	0.0023356	0.0014125	0.189096497
TRMT61A	ENSG00000166166	2.9776	0.0014528	2.7153	0.0033111	2.9776	0.0014528	0.84961	0.19777	0.0014528	0.193958751
SEMA3G	ENSG00000010319	0.95273	0.17036	2.9755	0.0014627	1.9313	0.026724	1.1326	0.12868	0.0014627	0.194734109
TMEM141	ENSG00000244187	2.3659	0.0089938	2.1118	0.017351	2.9747	0.0014666	2.193	0.014153	0.0014666	0.194734109
TENT2	ENSG00000164329	1.3296	0.091829	2.9734	0.0014726	1.3296	0.091829	1.487	0.068508	0.0014726	0.194999451
AFG3L1P	ENSG00000223959	2.8614	0.0021088	2.351	0.0093623	2.9694	0.0014917	1.9081	0.028192	0.0014917	0.19690335
NA	ENSG00000263427	2.9687	0.0014954	-0.33483	0.63112	-0.33483	0.63112	-0.63076	0.7359	0.0014954	0.19690335

NA	ENSG00000267284	0.48226	0.31481	2.9679	0.0014991	0.05537	0.47792	-0.56757	0.71484	0.0014991	0.19690335
MIR124-2	ENSG00000207816	0.037748	0.48494	0.037748	0.48494	2.9636	0.0015205	0.037748	0.48494	0.0015205	0.1982425
NA	ENSG00000250855	2.9634	0.0015211	2.8813	0.0019799	2.9605	0.0015357	-0.23779	0.59398	0.0015211	0.1982425
MRPL2P1	ENSG00000257480	2.9634	0.0015215	2.9634	0.0015215	2.9634	0.0015215	2.9634	0.0015215	0.0015215	0.1982425
ABCC4	ENSG00000125257	2.9599	0.0015389	1.6083	0.053888	1.7026	0.044321	1.8881	0.029505	0.0015389	0.199961481
C11orf49	ENSG00000149179	-0.11047	0.54398	-0.42993	0.66638	-0.30768	0.62084	2.9591	0.0015429	0.0015429	0.199961481
NA	ENSG00000279196	2.9552	0.0015623	2.9552	0.0015623	2.9552	0.0015623	2.9552	0.0015623	0.0015623	0.201938671
REN	ENSG00000143839	2.9531	0.0015732	2.2358	0.012682	2.3984	0.0082329	1.9383	0.026295	0.0015732	0.202809619
MTCYBP14	ENSG00000238236	2.9504	0.0015866	2.2321	0.012804	1.6012	0.054667	2.871	0.002046	0.0015866	0.203820168
NA	ENSG00000267599	2.9495	0.0015915	2.9495	0.0015915	1.3372	0.090574	2.9495	0.0015915	0.0015915	0.203820168
SCRN2	ENSG00000141295	2.949	0.0015942	2.949	0.0015942	2.949	0.0015942	2.949	0.0015942	0.0015942	0.203820168
NA	ENSG00000217455	2.9478	0.0016003	1.4357	0.07554	1.4562	0.072667	1.0024	0.15809	0.0016003	0.203820168
DTD1-AS1	ENSG00000233993	2.9457	0.0016109	2.3452	0.0095077	1.0319	0.15105	1.0319	0.15105	0.0016109	0.203820168
NA	ENSG00000242747	2.9456	0.0016117	2.9456	0.0016117	0.75663	0.22464	1.2059	0.11393	0.0016117	0.203820168
SP6	ENSG00000189120	2.9453	0.0016131	0.68231	0.24752	2.9453	0.0016131	2.9453	0.0016131	0.0016131	0.203820168
NA	ENSG00000279439	2.9451	0.0016145	2.9002	0.0018645	2.9002	0.0018645	2.9002	0.0018645	0.0016145	0.203820168
NELFA	ENSG00000185049	-1.1582	0.87661	-0.065017	0.52592	-0.64699	0.74118	2.9395	0.0016436	0.0016436	0.206957695
MYO18A	ENSG00000196535	2.9381	0.0016512	2.8834	0.0019669	0.954	0.17004	2.066	0.019413	0.0016512	0.207378804
GPT2	ENSG00000166123	2.9369	0.0016574	1.1481	0.12547	0.8815	0.18902	-0.30389	0.6194	0.0016574	0.20762237
VAV3-AS1	ENSG00000230489	2.1012	0.017811	0.63424	0.26296	2.1012	0.017811	2.9345	0.0016704	0.0016704	0.208524822
GALNT17	ENSG00000185274	2.5246	0.0057912	2.2706	0.011586	2.9338	0.0016744	2.8534	0.0021629	0.0016744	0.208524822
SLC24A1	ENSG00000074621	1.2116	0.11283	-0.84585	0.80118	2.9322	0.0016831	1.5374	0.062099	0.0016831	0.208524822
LINC01478	ENSG00000267337	-0.74859	0.77295	2.9319	0.0016844	0.44763	0.32721	-0.16454	0.56535	0.0016844	0.208524822
DEF8	ENSG00000140995	2.5815	0.0049185	2.9316	0.001686	1.5111	0.065382	0.98848	0.16146	0.001686	0.208524822
ANAPC1	ENSG00000153107	1.8216	0.034255	1.4459	0.074109	1.941	0.026127	2.9285	0.001703	0.001703	0.208977201
NA	ENSG00000274756	1.4085	0.079491	2.9283	0.0017039	0.64992	0.25787	0.99481	0.15991	0.0017039	0.208977201
NA	ENSG00000226026	2.2596	0.011924	2.9276	0.0017082	2.9276	0.0017082	1.3817	0.083537	0.0017082	0.208977201
SLC25A51P4	ENSG00000189332	2.9275	0.0017083	1.7013	0.044447	-1.5253	0.9364	1.5792	0.057142	0.0017083	0.208977201
LINC00690	ENSG00000233570	2.927	0.0017111	1.7092	0.043704	0.34387	0.36547	2.0225	0.021564	0.0017111	0.208977201
POLR3D	ENSG00000168495	2.4279	0.0075926	-0.3347	0.63107	-0.52585	0.7005	2.9244	0.0017258	0.0017258	0.210219519
NA	ENSG00000254339	0.41042	0.34075	2.9236	0.0017299	1.6129	0.053384	2.9236	0.0017299	0.0017299	0.210219519

MRPS21P3	ENSG00000221879	2.8899	0.001927	2.8899	0.001927	2.9215	0.0017416	2.8899	0.001927	0.0017416	0.210297813
NA	ENSG00000248991	1.9578	0.02513	2.9207	0.0017461	2.9207	0.0017461	0.49767	0.30936	0.0017461	0.210297813
NA	ENSG00000263501	0.44097	0.32962	0.44097	0.32962	2.9196	0.0017525	0.44097	0.32962	0.0017525	0.210297813
BMPER	ENSG00000164619	2.9185	0.0017585	2.4499	0.0071438	1.2149	0.11221	0.75613	0.22479	0.0017585	0.210297813
NA	ENSG00000255314	2.9179	0.0017621	2.9179	0.0017621	2.9179	0.0017621	2.9179	0.0017621	0.0017621	0.210297813
PHF12	ENSG00000109118	2.7925	0.0026151	2.358	0.0091871	2.9178	0.0017625	2.2955	0.010851	0.0017625	0.210297813
NA	ENSG00000252742	-0.67649	0.75064	-0.47952	0.68421	-0.79772	0.78748	2.9172	0.0017662	0.0017662	0.210297813
SLC25A5P5	ENSG00000226421	-0.95335	0.82979	0.97806	0.16402	-0.51801	0.69778	2.9169	0.0017677	0.0017677	0.210297813
NA	ENSG00000249258	2.9162	0.0017716	0.66536	0.25291	0.35067	0.36292	0.66536	0.25291	0.0017716	0.210297813
MEIS2	ENSG00000134138	-0.096303	0.53836	2.9158	0.0017737	-1.4983	0.93297	1.1901	0.11701	0.0017737	0.210297813
NEK8	ENSG00000160602	2.1846	0.014461	2.9022	0.0018525	2.7889	0.0026445	2.9151	0.0017781	0.0017781	0.210307799
MYBPC3	ENSG00000134571	2.9136	0.0017862	1.4582	0.072399	1.4582	0.072399	1.4582	0.072399	0.0017862	0.210497434
RNA5SP87	ENSG00000206882	1.6519	0.049278	2.9122	0.0017946	0.81835	0.20658	2.9122	0.0017946	0.0017946	0.210497434
EDAR	ENSG00000135960	-1.3234	0.90715	2.9105	0.001804	-0.20482	0.58114	-0.61367	0.73028	0.001804	0.210497434
LINC01289	ENSG00000253734	2.9101	0.0018063	2.0115	0.022136	1.1086	0.13381	0.98372	0.16263	0.0018063	0.210497434
NA	ENSG00000264138	-0.6509	0.74245	0.4603	0.32265	-0.50837	0.6944	2.9099	0.0018076	0.0018076	0.210497434
FAM110C	ENSG00000184731	2.1856	0.014422	2.3411	0.0096147	2.6217	0.0043747	2.9092	0.0018116	0.0018116	0.210497434
RN7SL666P	ENSG00000240639	2.909	0.0018132	2.0033	0.022574	2.909	0.0018132	1.0629	0.14392	0.0018132	0.210497434
C16orf87	ENSG00000155330	-0.20914	0.58283	2.9086	0.0018155	-0.19848	0.57867	0.4297	0.33371	0.0018155	0.210497434
NA	ENSG00000238201	1.4049	0.080019	1.6846	0.046037	1.4049	0.080019	2.9079	0.0018192	0.0018192	0.210497434
SLC12A9	ENSG00000146828	-0.30106	0.61832	2.9073	0.0018229	2.6447	0.0040883	0.6404	0.26096	0.0018229	0.210497434
NA	ENSG00000237612	1.7066	0.043948	2.9058	0.0018315	2.9058	0.0018315	2.9058	0.0018315	0.0018315	0.210990532
MIR4487	ENSG00000264583	1.4337	0.07583	2.9043	0.0018407	2.9043	0.0018407	2.9043	0.0018407	0.0018407	0.21109836
C1QTNF9B	ENSG00000205863	1.6517	0.049293	1.4939	0.067598	2.9042	0.0018411	0.98368	0.16264	0.0018411	0.21109836
NA	ENSG00000248569	0.085741	0.46584	-0.60481	0.72735	0.085741	0.46584	2.9032	0.0018469	0.0018469	0.211266284
NA	ENSG00000237654	2.279	0.011332	-0.20754	0.58221	2.9019	0.0018546	0.36767	0.35656	0.0018546	0.211650253
LINC01206	ENSG00000242512	2.8984	0.0018754	2.819	0.0024084	2.4386	0.0073724	2.819	0.0024084	0.0018754	0.21352393
NA	ENSG00000223945	2.8959	0.0018906	2.4307	0.0075356	2.4519	0.0071044	2.0481	0.020277	0.0018906	0.214752769
VPS4A	ENSG00000132612	1.4233	0.077329	2.8938	0.0019031	0.69196	0.24448	1.7356	0.04132	0.0019031	0.215669914
CRABP1	ENSG00000166426	2.7218	0.0032464	2.7218	0.0032464	2.8896	0.0019286	1.9069	0.028264	0.0019286	0.217717067
TIFAB	ENSG00000255833	2.8894	0.0019301	2.8894	0.0019301	2.8894	0.0019301	2.8894	0.0019301	0.0019301	0.217717067

MIR3161	ENSG00000263693	-0.35122	0.63729	2.6492	0.004034	2.8873	0.0019429	1.8367	0.033128	0.0019429	0.218654774
PNISR	ENSG00000132424	2.8823	0.0019742	2.8823	0.0019742	2.8823	0.0019742	2.296	0.010837	0.0019742	0.220782665
NQO2	ENSG00000124588	2.8822	0.0019746	2.4754	0.0066545	2.3596	0.0091472	2.8482	0.0021985	0.0019746	0.220782665
RBM22P13	ENSG00000261284	1.8399	0.03289	0.70237	0.24122	0.70237	0.24122	2.8821	0.0019754	0.0019754	0.220782665
RPL6P7	ENSG00000239212	2.8808	0.0019833	1.9799	0.023858	1.5878	0.056161	1.5878	0.056161	0.0019833	0.221053966
NA	ENSG00000253355	0.026886	0.48928	-0.69028	0.75499	0.66587	0.25275	2.8802	0.0019869	0.0019869	0.221053966
GATA5	ENSG00000130700	0.60706	0.2719	2.8769	0.0020083	0.96806	0.16651	1.2097	0.11321	0.0020083	0.222255
SPATA33	ENSG00000167523	2.3659	0.0089931	2.8766	0.0020101	2.3659	0.0089931	2.3659	0.0089931	0.0020101	0.222255
FAM133FP	ENSG00000225934	1.3854	0.082966	2.8763	0.0020119	0.93009	0.17616	0.55357	0.28994	0.0020119	0.222255
RNU6-148P	ENSG00000252823	2.8753	0.0020181	2.8753	0.0020181	2.8753	0.0020181	2.8753	0.0020181	0.0020181	0.222255
NA	ENSG00000226771	1.2687	0.10227	2.8749	0.0020205	2.8749	0.0020205	2.8749	0.0020205	0.0020205	0.222255
NA	ENSG00000270739	2.8732	0.0020317	2.8732	0.0020317	2.8732	0.0020317	2.8732	0.0020317	0.0020317	0.222983651
DPP9	ENSG00000142002	2.031	0.02113	1.3041	0.096099	0.18654	0.42601	2.8697	0.0020543	0.0020543	0.224957391
ABCD4	ENSG00000119688	1.7512	0.039956	1.8017	0.0358	2.8688	0.0020603	1.8017	0.0358	0.0020603	0.225108563
BCKDK	ENSG00000103507	0.99474	0.15993	2.8677	0.0020672	2.5064	0.0060987	0.45695	0.32385	0.0020672	0.225357172
NA	ENSG00000234238	-0.022306	0.5089	1.2452	0.10653	2.8623	0.0021031	1.0715	0.14196	0.0021031	0.228759069
BMP4	ENSG00000125378	2.8611	0.0021109	1.8921	0.029238	2.8611	0.0021109	2.8611	0.0021109	0.0021109	0.229031
MREGP1	ENSG00000270395	2.8592	0.0021236	2.8592	0.0021236	2.8592	0.0021236	2.8592	0.0021236	0.0021236	0.229031
PRPF38A	ENSG00000134748	-1.0837	0.86076	2.8586	0.0021274	-0.72312	0.7652	-0.72312	0.7652	0.0021274	0.229031
SETP14	ENSG00000240489	2.8575	0.0021348	2.8575	0.0021348	-0.11977	0.54767	1.8695	0.030778	0.0021348	0.229031
RWDD3	ENSG00000122481	2.8574	0.0021357	2.8574	0.0021357	2.8574	0.0021357	2.1953	0.014073	0.0021357	0.229031
NMI	ENSG00000123609	1.0361	0.15007	2.8572	0.0021372	1.0361	0.15007	1.0361	0.15007	0.0021372	0.229031
NA	ENSG00000253282	2.857	0.0021385	0.036959	0.48526	-1.2383	0.8922	-1.6174	0.9471	0.0021385	0.229031
SNX22	ENSG00000157734	-0.98275	0.83714	-1.4188	0.92203	2.7512	0.0029689	2.8536	0.0021617	0.0021617	0.230737083
EFR3A	ENSG00000132294	2.8532	0.0021639	2.1835	0.0145	2.1778	0.014712	1.8045	0.035577	0.0021639	0.230737083
DCLRE1A	ENSG00000198924	-1.3671	0.91421	-1.3671	0.91421	2.8516	0.0021749	-1.3671	0.91421	0.0021749	0.231149696
KISS1	ENSG00000170498	1.9065	0.028292	1.9065	0.028292	1.9065	0.028292	2.8506	0.0021816	0.0021816	0.231149696
RCN1	ENSG00000049449	2.7216	0.0032483	2.3	0.010725	1.7645	0.03882	2.8506	0.002182	0.002182	0.231149696
NA	ENSG00000265840	2.8467	0.0022087	2.8467	0.0022087	2.8467	0.0022087	2.8467	0.0022087	0.0022087	0.23347061
RPS4XP14	ENSG00000243663	2.8458	0.0022152	2.8458	0.0022152	2.8458	0.0022152	2.8458	0.0022152	0.0022152	0.233536425
NPM1P33	ENSG00000213925	2.8451	0.0022201	2.8451	0.0022201	2.8451	0.0022201	2.8451	0.0022201	0.0022201	0.233536425

LINC01812	ENSG00000237013	-1.4499	0.92645	-1.7324	0.9584	2.8445	0.0022237	-1.4499	0.92645	0.0022237	0.233536425
RNU6-336P	ENSG00000201761	2.8437	0.0022298	2.8437	0.0022298	2.8437	0.0022298	2.8437	0.0022298	0.0022298	0.233673449
H2BC18	ENSG00000203814	2.8427	0.0022368	2.5596	0.005239	1.4995	0.066868	1.4995	0.066868	0.0022368	0.233904
LINC02495	ENSG00000249896	2.8417	0.0022439	0.46148	0.32223	0.58237	0.28016	0.99667	0.15946	0.0022439	0.234143998
TXNDC12	ENSG00000117862	1.2413	0.10724	-0.54787	0.70811	2.8405	0.002252	-0.41389	0.66052	0.002252	0.234487094
NA	ENSG00000259445	0.44074	0.3297	0.36153	0.35885	-0.62233	0.73314	2.8384	0.0022668	0.0022668	0.235262219
PNO1	ENSG00000115946	2.8381	0.0022691	2.8381	0.0022691	2.8381	0.0022691	2.6834	0.0036442	0.0022691	0.235262219
NA	ENSG00000264246	-0.18984	0.57528	-0.2322	0.59181	2.8362	0.0022828	1.1739	0.12021	0.0022828	0.236180136
EVA1A	ENSG00000115363	1.5527	0.060252	2.8328	0.002307	1.054	0.14595	1.7228	0.042461	0.002307	0.237804447
IGLV3-19	ENSG00000211663	0.45353	0.32508	2.8322	0.0023112	2.8322	0.0023112	1.1494	0.1252	0.0023112	0.237804447
FBXL19-AS1	ENSG00000260852	2.8316	0.0023161	2.8316	0.0023161	2.8316	0.0023161	2.8316	0.0023161	0.0023161	0.237804447
SEC16A	ENSG00000148396	2.5981	0.0046875	1.6278	0.051778	2.8311	0.0023197	1.6269	0.051874	0.0023197	0.237804447
LINC01301	ENSG00000251396	2.8306	0.0023229	0.42051	0.33706	0.82099	0.20583	-0.45057	0.67385	0.0023229	0.237804447
NA	ENSG00000262445	0.31325	0.37704	0.19417	0.42302	2.4905	0.0063785	2.8292	0.0023335	0.0023335	0.238388795
NA	ENSG00000221072	0.85299	0.19683	0.21537	0.41474	2.8282	0.0023404	2.298	0.01078	0.0023404	0.238593498
GATA3	ENSG00000107485	2.8259	0.0023575	-0.21162	0.5838	1.5634	0.058983	0.29556	0.38378	0.0023575	0.239019137
NA	ENSG00000243517	0.71705	0.23667	2.8255	0.0023601	2.8255	0.0023601	2.8255	0.0023601	0.0023601	0.239019137
NA	ENSG00000264066	2.7697	0.0028054	2.6007	0.0046517	2.825	0.0023637	2.825	0.0023637	0.0023637	0.239019137
NA	ENSG00000204398	0.74086	0.22939	0.89424	0.1856	2.8248	0.0023651	1.9235	0.027212	0.0023651	0.239019137
PLEKHO2	ENSG00000241839	2.8243	0.0023691	1.5634	0.058978	1.7808	0.037473	1.3842	0.083145	0.0023691	0.239019137
PIANP	ENSG00000139200	-0.50291	0.69249	-0.50291	0.69249	2.823	0.0023788	-0.34751	0.6359	0.0023788	0.239501909
EXOC3L4	ENSG00000205436	1.6618	0.048278	0.98608	0.16205	2.1224	0.0169	2.8212	0.0023921	0.0023921	0.240344398
MARCHF10	ENSG00000173838	0.4641	0.32129	2.8183	0.0024138	2.2746	0.011465	1.2393	0.10761	0.0024138	0.241724619
RNLS	ENSG00000184719	2.8167	0.0024258	0.68839	0.2456	1.2416	0.1072	1.1201	0.13133	0.0024258	0.241724619
A3GALT2	ENSG00000184389	2.2915	0.010969	2.2915	0.010969	2.8167	0.0024261	2.8167	0.0024261	0.0024261	0.241724619
NA	ENSG00000255872	-0.3224	0.62643	-0.3224	0.62643	2.8166	0.0024267	-0.39772	0.65458	0.0024267	0.241724619
ADIPOR2	ENSG00000006831	2.1399	0.016182	2.2818	0.01125	1.9476	0.025729	2.8157	0.0024334	0.0024334	0.241724619
MEGF11	ENSG00000157890	2.8154	0.0024356	2.0021	0.022637	0.13604	0.4459	2.7478	0.0029996	0.0024356	0.241724619
GAS8	ENSG00000141013	2.8129	0.0024551	2.8129	0.0024551	2.0613	0.019637	2.8129	0.0024551	0.0024551	0.241908245
NA	ENSG00000282142	2.8122	0.0024604	2.8122	0.0024604	2.8122	0.0024604	2.8122	0.0024604	0.0024604	0.241908245
NA	ENSG00000233581	2.812	0.0024618	1.6774	0.046736	2.1038	0.017698	2.2894	0.011029	0.0024618	0.241908245

MIR4778	ENSG00000264525	1.8936	0.029138	2.8115	0.0024654	-0.065506	0.52611	-0.46185	0.67791	0.0024654	0.241908245
RNU6-712P	ENSG00000222490	2.8112	0.002468	2.1255	0.016772	-0.33379	0.63073	1.4916	0.067904	0.002468	0.241908245
POLG	ENSG00000140521	2.8106	0.0024722	0.70217	0.24129	2.8106	0.0024722	1.1846	0.11809	0.0024722	0.241908245
NA	ENSG00000261441	2.8106	0.0024722	2.8106	0.0024722	2.8106	0.0024722	2.8106	0.0024722	0.0024722	0.241908245
NT5DC2	ENSG00000168268	2.0379	0.020781	2.6356	0.0041996	2.8085	0.0024885	2.8085	0.0024885	0.0024885	0.242725978
HSPA8P5	ENSG00000256356	0.50303	0.30747	0.39896	0.34496	2.8081	0.0024921	0.88798	0.18728	0.0024921	0.242725978
CAPSL	ENSG00000152611	1.7616	0.03907	2.8076	0.0024955	-0.14295	0.55683	0.431	0.33323	0.0024955	0.242725978
C9orf85	ENSG00000155621	2.8053	0.0025137	-0.10098	0.54022	-0.70875	0.76076	-2.1039	0.98231	0.0025137	0.243766254
DUSP14	ENSG00000276023	2.805	0.0025162	1.5123	0.06523	2.805	0.0025162	2.5935	0.0047503	0.0025162	0.243766254
NA	ENSG00000236555	2.8032	0.0025297	2.1897	0.014272	2.1897	0.014272	2.1897	0.014272	0.0025297	0.244351635
NPIPB6	ENSG00000198156	0.080102	0.46808	2.8023	0.0025368	1.767	0.038612	0.82184	0.20558	0.0025368	0.244351635
RNA5SP124	ENSG00000199609	2.1382	0.01625	1.7015	0.044422	2.8017	0.0025413	2.7963	0.0025846	0.0025413	0.244351635
NA	ENSG00000276110	0.61059	0.27074	2.8016	0.0025423	0.61059	0.27074	0.61059	0.27074	0.0025423	0.244351635
NA	ENSG00000279926	2.0538	0.019996	2.8004	0.0025517	2.8004	0.0025517	2.8004	0.0025517	0.0025517	0.244772325
NA	ENSG00000261318	0.38399	0.35049	2.7991	0.002562	-0.18006	0.57145	0.011113	0.49557	0.002562	0.245277525
NA	ENSG00000282998	2.3294	0.0099177	0.72061	0.23558	1.1834	0.11832	2.7973	0.0025764	0.0025764	0.245767037
NA	ENSG00000279100	2.7972	0.0025772	2.7972	0.0025772	2.7972	0.0025772	2.7972	0.0025772	0.0025772	0.245767037
LHX8	ENSG00000162624	1.5564	0.059803	0.86512	0.19349	0.35372	0.36178	2.7948	0.002597	0.002597	0.247171504
NA	ENSG00000225313	2.7925	0.0026149	2.7925	0.0026149	2.7925	0.0026149	2.7925	0.0026149	0.0026149	0.247994937
PLEKHH2	ENSG00000152527	1.9709	0.024367	2.2625	0.011832	2.792	0.002619	0.39026	0.34817	0.002619	0.247994937
NA	ENSG00000235042	-1.4834	0.93102	2.7914	0.0026237	2.186	0.014409	-1.7877	0.96309	0.0026237	0.247994937
RNU6-334P	ENSG00000252881	1.439	0.075073	2.0008	0.022708	2.7893	0.0026415	2.7893	0.0026415	0.0026415	0.247994937
FBXL19	ENSG00000099364	2.4216	0.0077252	2.7886	0.0026467	1.2341	0.10858	-0.15952	0.56337	0.0026467	0.247994937
LINC02744	ENSG00000254863	1.3845	0.083096	1.8856	0.029672	2.7882	0.00265	0.90333	0.18318	0.00265	0.247994937
NA	ENSG00000200492	2.2051	0.013725	2.7878	0.002653	2.0364	0.020853	0.5228	0.30056	0.002653	0.247994937
NA	ENSG00000254186	1.1733	0.12033	0.43607	0.33139	2.7878	0.0026532	0.55546	0.28929	0.0026532	0.247994937
MIR4266	ENSG00000265965	2.5116	0.0060091	2.7866	0.0026633	2.4808	0.0065541	2.3467	0.0094695	0.0026633	0.247994937
PGM1	ENSG00000079739	2.165	0.015193	2.7149	0.0033146	2.7861	0.0026677	1.5176	0.064554	0.0026677	0.247994937
DYNLT3P1	ENSG00000232241	0.55048	0.29099	0.43164	0.333	0.30899	0.37866	2.7858	0.0026699	0.0026699	0.247994937
SPARCL1	ENSG00000152583	2.7857	0.0026709	2.1652	0.015185	-0.28726	0.61304	-1.1948	0.88391	0.0026709	0.247994937
CDC23	ENSG00000094880	0.95785	0.16907	2.651	0.004013	1.6544	0.049024	2.7853	0.002674	0.002674	0.247994937

NA	ENSG00000246451	2.7849	0.0026769	2.3608	0.0091174	2.3608	0.0091174	2.3608	0.0091174	0.0026769	0.247994937
MIR4486	ENSG00000265210	2.7827	0.0026956	-0.54831	0.70826	-0.81793	0.7933	-1.5908	0.94418	0.0026956	0.248955951
RNU4-13P	ENSG00000200889	-0.96842	0.83358	2.7819	0.0027023	2.7819	0.0027023	2.7819	0.0027023	0.0027023	0.248955951
DBNDD1	ENSG00000003249	2.5294	0.0057127	2.7818	0.0027026	2.7818	0.0027026	2.2286	0.012921	0.0027026	0.248955951
NPM1P22	ENSG00000227969	0.637	0.26206	0.637	0.26206	0.637	0.26206	2.7807	0.0027121	0.0027121	0.249283261
LRP4	ENSG00000134569	1.2409	0.10733	0.98474	0.16238	2.1324	0.016487	2.7801	0.0027171	0.0027171	0.249283261
BSDC1	ENSG00000160058	1.4876	0.068432	0.011478	0.49542	2.7796	0.0027215	-0.89931	0.81576	0.0027215	0.249283261
RNU6-909P	ENSG00000252867	1.3647	0.086176	2.7773	0.0027406	1.3647	0.086176	1.3647	0.086176	0.0027406	0.250561797
PABPN1L	ENSG00000205022	-0.71024	0.76122	-0.47433	0.68237	-0.83681	0.79865	2.7737	0.0027714	0.0027714	0.252903225
HOOK1	ENSG00000134709	2.7713	0.0027918	1.3699	0.085352	2.7301	0.0031656	2.0773	0.018887	0.0027918	0.254288624
PPT1	ENSG00000131238	2.3893	0.0084395	2.4932	0.0063293	2.7693	0.0028092	1.6993	0.044629	0.0028092	0.254938663
COQ3	ENSG00000132423	1.7862	0.037037	1.7862	0.037037	1.7862	0.037037	2.7692	0.0028094	0.0028094	0.254938663
LINC01720	ENSG00000231175	-0.44275	0.67103	2.7679	0.002821	-0.44275	0.67103	-0.37698	0.64691	0.002821	0.255515483
NA	ENSG00000252436	2.7669	0.0028294	2.4562	0.0070213	0.28161	0.38912	0.9899	0.16111	0.0028294	0.255800857
SETD1A	ENSG00000099381	2.2451	0.01238	2.0148	0.021961	1.8984	0.028819	2.7653	0.0028437	0.0028437	0.255984781
HEBP2P1	ENSG00000235548	2.765	0.0028466	2.765	0.0028466	2.765	0.0028466	2.765	0.0028466	0.0028466	0.255984781
NA	ENSG00000199442	1.0481	0.1473	1.6641	0.048046	0.86049	0.19476	2.7642	0.0028528	0.0028528	0.255984781
SPOCD1	ENSG00000134668	2.7642	0.0028535	1.6727	0.047198	1.3396	0.090187	1.358	0.087236	0.0028535	0.255984781
BDH2	ENSG00000164039	0.30068	0.38183	2.7637	0.0028577	0.30068	0.38183	1.618	0.052828	0.0028577	0.255984781
NPM1	ENSG00000181163	1.085	0.13895	2.7624	0.0028689	1.7976	0.036118	1.3505	0.088424	0.0028689	0.256207346
DNASE2B	ENSG00000137976	1.8409	0.032817	2.6319	0.0042452	1.8409	0.032817	2.7622	0.0028707	0.0028707	0.256207346
NA	ENSG00000237321	-0.72204	0.76487	2.7615	0.0028767	-0.42635	0.66507	0.046565	0.48143	0.0028767	0.256273475
MRAP	ENSG00000170262	1.4387	0.075113	1.7492	0.040127	2.7594	0.0028957	1.4732	0.070354	0.0028957	0.256958392
TTLL7	ENSG00000137941	2.7583	0.0029052	2.7134	0.0033299	1.959	0.025054	2.315	0.010307	0.0029052	0.256958392
NA	ENSG00000224127	2.7581	0.0029068	2.7581	0.0029068	2.7581	0.0029068	2.7581	0.0029068	0.0029068	0.256958392
BICDL1	ENSG00000135127	2.757	0.0029164	2.757	0.0029164	2.757	0.0029164	2.4198	0.0077647	0.0029164	0.256958392
NA	ENSG00000234275	1.4019	0.080466	-0.60407	0.7271	2.7568	0.0029188	0.79943	0.21202	0.0029188	0.256958392
OTUD7B	ENSG00000264522	2.7568	0.0029189	2.3512	0.0093573	2.627	0.0043073	2.5073	0.0060823	0.0029189	0.256958392
PODXL	ENSG00000128567	-0.88187	0.81108	-1.6078	0.94606	-0.76122	0.77674	2.7565	0.0029213	0.0029213	0.256958392
KIR2DS4	ENSG00000221957	-0.0016981	0.50068	-0.10829	0.54312	2.7544	0.0029399	-0.79481	0.78664	0.0029399	0.257587828
NA	ENSG00000248100	1.7429	0.040676	1.3753	0.084522	1.7429	0.040676	2.7543	0.0029405	0.0029405	0.257587828

CCDC183	ENSG00000213213	1.8898	0.029392	2.3161	0.010277	2.3639	0.0090431	2.7536	0.0029473	0.0029473	0.257587828
RPL36AP29	ENSG00000224683	0.16033	0.43631	0.16033	0.43631	0.16033	0.43631	2.7533	0.0029496	0.0029496	0.257587828
RBM43	ENSG00000184898	0.59728	0.27516	1.2563	0.1045	0.89321	0.18587	2.7526	0.0029566	0.0029566	0.257677279
RN7SKP255	ENSG00000200312	0.37929	0.35224	1.7779	0.037711	1.7686	0.038479	2.752	0.0029612	0.0029612	0.257677279
NA	ENSG00000261675	2.7515	0.0029665	2.7515	0.0029665	2.7515	0.0029665	2.7515	0.0029665	0.0029665	0.257678333
PDGFA	ENSG00000197461	0.10176	0.45947	0.33161	0.37009	2.7492	0.002987	-0.65102	0.74248	0.002987	0.25816783
LINC00637	ENSG00000258735	2.7487	0.0029912	2.7487	0.0029912	2.7487	0.0029912	2.7487	0.0029912	0.0029912	0.25816783
TCF12	ENSG00000140262	1.2768	0.10084	2.7484	0.0029946	1.0912	0.1376	1.6149	0.053162	0.0029946	0.25816783
TFIP11	ENSG00000100109	0.8218	0.2056	2.2851	0.011154	2.7471	0.0030063	2.7471	0.0030063	0.0030063	0.25816783
FAM8A1	ENSG00000137414	0.77989	0.21773	2.7463	0.0030135	1.1457	0.12597	0.82386	0.20501	0.0030135	0.25816783
MICU3	ENSG00000155970	1.5414	0.061613	1.9048	0.028405	2.7456	0.0030196	0.74103	0.22934	0.0030196	0.25816783
STX1B	ENSG00000099365	2.7455	0.0030211	2.3961	0.0082844	2.6897	0.0035757	2.6312	0.0042537	0.0030211	0.25816783
GLYCTK-AS1	ENSG00000242797	1.542	0.06154	1.542	0.06154	0.18858	0.42521	2.7448	0.0030273	0.0030273	0.25816783
NA	ENSG00000232218	2.7444	0.0030309	2.7444	0.0030309	2.7444	0.0030309	2.7444	0.0030309	0.0030309	0.25816783
NA	ENSG00000234189	2.5233	0.0058134	2.7437	0.0030378	1.7021	0.044367	1.1347	0.12824	0.0030378	0.25816783
TNFRSF10C	ENSG00000173535	2.7433	0.0030408	1.3891	0.0824	-0.19445	0.57709	1.8745	0.030433	0.0030408	0.25816783
MIR320A	ENSG00000208037	-0.28949	0.6139	2.7428	0.0030463	-0.82287	0.79471	-0.82287	0.79471	0.0030463	0.25816783
EPHA10	ENSG00000183317	1.11	0.1335	2.7424	0.0030495	1.11	0.1335	1.0054	0.15735	0.0030495	0.25816783
NA	ENSG00000231092	2.7423	0.0030507	1.9368	0.026383	1.7046	0.044132	1.7046	0.044132	0.0030507	0.25816783
LINC02607	ENSG00000228971	2.7422	0.0030517	2.7422	0.0030517	2.7422	0.0030517	2.7422	0.0030517	0.0030517	0.25816783
MEIS1	ENSG00000143995	-0.077892	0.53104	1.3629	0.08646	2.7415	0.0030575	0.88884	0.18704	0.0030575	0.25816783
KCNQ3	ENSG00000184156	1.621	0.052512	1.5989	0.054916	2.741	0.0030622	2.427	0.0076128	0.0030622	0.25816783
NA	ENSG00000271985	0.66204	0.25397	1.1254	0.13022	0.46799	0.31989	2.74	0.0030717	0.0030717	0.258521487
PAUPAR	ENSG00000281880	1.4707	0.070688	2.7393	0.0030782	1.4707	0.070688	1.4707	0.070688	0.0030782	0.258621872
KCTD10	ENSG00000110906	2.5571	0.0052769	2.5247	0.0057896	2.5247	0.0057896	2.7383	0.0030883	0.0030883	0.258871845
ADGRA1	ENSG00000197177	2.7379	0.0030918	1.3458	0.089188	1.3356	0.090841	1.017	0.15458	0.0030918	0.258871845
XPO7	ENSG00000130227	1.9083	0.028177	1.8293	0.033675	1.0864	0.13865	2.736	0.0031094	0.0031094	0.259586699
NALCN	ENSG00000102452	-0.20191	0.58001	-0.25071	0.59898	0.91768	0.17939	2.7353	0.0031159	0.0031159	0.259586699
TLE3	ENSG00000140332	-0.45665	0.67604	-0.20992	0.58313	-0.76798	0.77875	2.7353	0.0031166	0.0031166	0.259586699
PRKAR1B-AS1	ENSG00000237181	2.4483	0.0071766	2.7345	0.0031241	2.7345	0.0031241	2.7345	0.0031241	0.0031241	0.259586699
NA	ENSG00000271964	2.2277	0.01295	2.6465	0.0040666	2.7338	0.00313	2.7338	0.00313	0.00313	0.259586699

LINC00299	ENSG00000236790	-0.59183	0.72302	2.7336	0.0031323	-0.34955	0.63666	0.86599	0.19325	0.0031323	0.259586699
RNA5SP19	ENSG00000202056	1.5762	0.057487	0.67016	0.25138	2.7328	0.0031402	2.7328	0.0031402	0.0031402	0.259799569
TMEM225B	ENSG00000244219	0.77975	0.21777	2.7295	0.0031718	1.1812	0.11876	0.52618	0.29938	0.0031718	0.261969176
ATP5MPL	ENSG00000156411	2.6634	0.0038679	2.727	0.0031954	2.7143	0.0033213	2.209	0.013586	0.0031954	0.262128154
RPL6P12	ENSG00000214671	2.7266	0.0031995	2.7266	0.0031995	2.7266	0.0031995	2.7266	0.0031995	0.0031995	0.262128154
MYMK	ENSG00000187616	2.7265	0.003201	1.4983	0.067025	2.0728	0.019096	0.62588	0.2657	0.003201	0.262128154
RAB34	ENSG00000109113	1.6673	0.047731	1.6673	0.047731	2.6841	0.0036368	2.7264	0.0032014	0.0032014	0.262128154
VRTN	ENSG00000133980	2.7261	0.003204	2.4762	0.0066396	2.0865	0.018468	1.7807	0.037481	0.003204	0.262128154
OBP2B	ENSG00000171102	-0.14931	0.55934	2.7259	0.003206	-0.40414	0.65695	0.80638	0.21001	0.003206	0.262128154
TP53AIP1	ENSG00000120471	0.65231	0.2571	1.1345	0.1283	1.6683	0.047628	2.7236	0.003229	0.003229	0.263158216
ZNF843	ENSG00000176723	-0.57256	0.71653	2.0755	0.01897	2.7235	0.0032294	0.12648	0.44967	0.0032294	0.263158216
MEIS3	ENSG00000105419	2.7229	0.0032353	-1.155	0.87596	1.0991	0.13585	0.055836	0.47774	0.0032353	0.263158216
SLC25A29	ENSG00000197119	1.4652	0.071434	1.4652	0.071434	1.4652	0.071434	2.7223	0.0032418	0.0032418	0.263158216
NA	ENSG00000207049	2.7219	0.0032456	2.3088	0.010477	2.1492	0.015811	2.032	0.021077	0.0032456	0.263158216
PCNPP5	ENSG00000177197	0.16952	0.43269	2.7211	0.003253	-0.85025	0.80241	0.46103	0.32239	0.003253	0.263320083
NA	ENSG00000241183	0.21807	0.41369	-0.49119	0.68836	2.7194	0.0032697	0.84845	0.19809	0.0032697	0.26398106
NA	ENSG00000262075	2.7192	0.003272	2.3764	0.0087418	0.58723	0.27852	1.8637	0.031185	0.003272	0.26398106
NA	ENSG00000229282	2.7171	0.0032925	0.25286	0.40019	0.25286	0.40019	0.73937	0.22984	0.0032925	0.264734327
DCP1B	ENSG00000151065	2.7157	0.0033071	1.7278	0.042012	1.7201	0.042707	0.35491	0.36133	0.0033071	0.264734327
SHISA3	ENSG00000178343	2.6341	0.0042175	2.6341	0.0042175	2.7156	0.0033075	1.0634	0.1438	0.0033075	0.264734327
NA	ENSG00000261564	0.15869	0.43695	0.15869	0.43695	0.15869	0.43695	2.7156	0.0033079	0.0033079	0.264734327
NA	ENSG00000267689	2.7155	0.0033085	2.7155	0.0033085	2.7155	0.0033085	2.7155	0.0033085	0.0033085	0.264734327
IFT80	ENSG00000068885	2.7144	0.0033198	-0.32519	0.62748	0.12876	0.44877	-0.096473	0.53843	0.0033198	0.265203039
MIR3649	ENSG00000266043	2.2455	0.012368	1.3051	0.095935	1.8089	0.035231	2.7137	0.0033271	0.0033271	0.2653512
FARSBP1	ENSG00000231060	2.7126	0.0033377	2.7126	0.0033377	2.7126	0.0033377	2.7126	0.0033377	0.0033377	0.265761636
KIAA0556	ENSG00000047578	0.18235	0.42765	0.82232	0.20545	2.0375	0.020802	2.7119	0.0033447	0.0033447	0.265884553
KCNJ16	ENSG00000153822	1.9281	0.026922	2.4205	0.0077485	2.7102	0.0033619	1.6083	0.053885	0.0033619	0.266816591
NA	ENSG00000260710	0.59433	0.27615	0.072704	0.47102	2.7086	0.0033786	0.28733	0.38693	0.0033786	0.267250456
MIR4294	ENSG00000264800	2.7069	0.0033953	2.7069	0.0033953	2.7069	0.0033953	2.7069	0.0033953	0.0033953	0.267250456
NAP1L1	ENSG00000187109	2.706	0.0034048	0.029366	0.48829	1.4975	0.067133	1.0816	0.13971	0.0034048	0.267250456
TAL2	ENSG00000186051	2.7056	0.0034092	2.7056	0.0034092	2.7056	0.0034092	2.7056	0.0034092	0.0034092	0.267250456

CEACAMP3	ENSG00000239736	-0.63741	0.73807	-0.63741	0.73807	0.32602	0.3722	2.7054	0.0034107	0.0034107	0.267250456
FAM222B	ENSG00000173065	2.6443	0.0040932	2.6753	0.0037331	2.7051	0.0034144	2.2494	0.012244	0.0034144	0.267250456
KCNJ4	ENSG00000168135	2.7046	0.0034192	1.9638	0.024779	0.96349	0.16765	0.90016	0.18402	0.0034192	0.267250456
LINC00454	ENSG00000226921	2.7038	0.0034274	2.7038	0.0034274	2.7038	0.0034274	2.7038	0.0034274	0.0034274	0.267250456
NA	ENSG00000274766	2.7038	0.0034274	2.7038	0.0034274	2.7038	0.0034274	2.7038	0.0034274	0.0034274	0.267250456
ZNF655	ENSG00000197343	0.96203	0.16802	2.1436	0.016031	2.4422	0.0072993	2.7026	0.00344	0.00344	0.267250456
MIR211	ENSG00000207702	2.7026	0.00344	2.7026	0.00344	2.7026	0.00344	2.7026	0.00344	0.00344	0.267250456
NA	ENSG00000254265	2.7025	0.0034415	2.7025	0.0034415	0.44259	0.32903	0.78865	0.21516	0.0034415	0.267250456
RNA5S17	ENSG00000200370	-0.45285	0.67467	-0.21725	0.586	2.7021	0.0034449	2.7021	0.0034449	0.0034449	0.267250456
FBXW4	ENSG00000107829	-1.9657	0.97533	2.7013	0.0034538	-1.2083	0.88653	-0.63184	0.73625	0.0034538	0.267250456
NQO1	ENSG00000181019	0.78099	0.2174	1.6115	0.053537	0.39514	0.34637	2.7012	0.003455	0.003455	0.267250456
IGLVI-20	ENSG00000254240	2.7007	0.0034601	2.7007	0.0034601	2.7007	0.0034601	2.7007	0.0034601	0.0034601	0.267250456
CDCA2	ENSG00000184661	0.098951	0.46059	2.7006	0.0034606	1.3552	0.08768	-0.16545	0.5657	0.0034606	0.267250456
EXOSC5	ENSG00000077348	-0.090669	0.53612	-0.090669	0.53612	2.6995	0.0034723	-0.090669	0.53612	0.0034723	0.267729714
NA	ENSG00000253776	0.78145	0.21727	2.6989	0.0034784	2.6989	0.0034784	2.6989	0.0034784	0.0034784	0.267776354
FAM53C	ENSG00000120709	2.6982	0.003486	2.6982	0.003486	2.1068	0.017567	2.5365	0.0055991	0.003486	0.267938139
MBTD1	ENSG00000011258	2.6967	0.0035012	2.508	0.0060702	2.508	0.0060702	2.508	0.0060702	0.0035012	0.268283239
RNY1P10	ENSG00000200138	2.6956	0.0035135	2.6956	0.0035135	2.6956	0.0035135	2.6956	0.0035135	0.0035135	0.268283239
MIR491	ENSG00000207609	2.6954	0.0035147	2.6954	0.0035147	2.6954	0.0035147	2.6954	0.0035147	0.0035147	0.268283239
NA	ENSG00000261188	2.6953	0.0035165	2.6953	0.0035165	2.6953	0.0035165	2.6953	0.0035165	0.0035165	0.268283239
ADD1	ENSG00000087274	1.4454	0.074168	2.6492	0.0040345	2.6931	0.003539	1.5273	0.063342	0.003539	0.268283239
ADCK1	ENSG00000063761	1.2635	0.1032	2.6922	0.003549	1.6866	0.045844	1.6262	0.051952	0.003549	0.268283239
NOS2P1	ENSG00000265788	2.6917	0.0035539	2.6917	0.0035539	2.6917	0.0035539	2.6917	0.0035539	0.0035539	0.268283239
PPP1R26-AS1	ENSG00000225361	-0.49269	0.68889	-0.67302	0.74953	-0.31622	0.62408	2.6914	0.0035582	0.0035582	0.268283239
NA	ENSG00000243802	2.3321	0.0098479	2.6909	0.0035625	1.89	0.02938	1.1264	0.13	0.0035625	0.268283239
NA	ENSG00000250258	0.2608	0.39713	-0.41531	0.66104	2.6908	0.003564	1.2523	0.10523	0.003564	0.268283239
ARNTL	ENSG00000133794	1.3663	0.08593	2.4632	0.0068857	2.6901	0.0035711	1.5572	0.05971	0.0035711	0.268283239
EIF4H	ENSG00000106682	0.96611	0.16699	1.2586	0.10409	1.9443	0.025928	2.6894	0.003579	0.003579	0.268283239
DPYD-AS1	ENSG00000232878	2.6887	0.0035861	1.301	0.096632	1.2448	0.10659	1.2448	0.10659	0.0035861	0.268283239
RPL10AP1	ENSG00000244691	2.6886	0.0035873	2.6886	0.0035873	2.6886	0.0035873	2.6886	0.0035873	0.0035873	0.268283239
RNU7-80P	ENSG00000238778	1.2226	0.11074	2.6882	0.0035914	-0.10031	0.53995	-0.2529	0.59983	0.0035914	0.268283239

UNC45A	ENSG00000140553	-0.58225	0.7198	1.1268	0.12991	1.6055	0.054196	2.688	0.0035945	0.0035945	0.268283239
AZIN2	ENSG00000142920	2.6878	0.0035962	2.5838	0.0048863	1.6661	0.047846	1.3827	0.083377	0.0035962	0.268283239
KCNN3	ENSG00000143603	2.6877	0.0035975	2.3233	0.010082	1.5665	0.058613	1.6648	0.047977	0.0035975	0.268283239
CALHM3	ENSG00000183128	0.71954	0.2359	1.8079	0.03531	2.6877	0.0035975	2.2118	0.01349	0.0035975	0.268283239
NA	ENSG00000233082	1.9812	0.023783	2.6874	0.0036006	0.4064	0.34222	1.0744	0.14133	0.0036006	0.268283239
MIR4299	ENSG00000266645	2.6865	0.0036099	0.66027	0.25454	-1.0192	0.84596	2.58	0.0049395	0.0036099	0.268565537
ROR1	ENSG00000185483	2.4686	0.0067817	2.1578	0.01547	2.6841	0.0036364	1.7532	0.039783	0.0036364	0.270124652
ARSB	ENSG00000113273	2.5041	0.0061376	2.6415	0.004127	2.6821	0.0036582	2.4541	0.0070622	0.0036582	0.270624746
NA	ENSG00000279693	2.6818	0.0036609	2.6095	0.0045334	2.6095	0.0045334	2.6095	0.0045334	0.0036609	0.270624746
SLC12A9-AS1	ENSG00000236305	2.6816	0.003663	2.6816	0.003663	2.6816	0.003663	0.59596	0.2756	0.003663	0.270624746
NA	ENSG00000200072	0.52569	0.29955	0.7633	0.22264	-0.54456	0.70697	2.6811	0.0036696	0.0036696	0.270624746
MTND1P21	ENSG00000235940	2.6809	0.0036709	2.6809	0.0036709	2.6809	0.0036709	2.6809	0.0036709	0.0036709	0.270624746
LINC00368	ENSG00000225870	1.5515	0.060392	0.92074	0.17859	-0.21966	0.58693	2.6793	0.0036889	0.0036889	0.271540932
ZNHIT1	ENSG00000106400	2.6787	0.0036953	2.6787	0.0036953	2.6787	0.0036953	2.6787	0.0036953	0.0036953	0.271601763
B9D2	ENSG00000123810	-0.50529	0.69332	2.6769	0.003715	-0.50529	0.69332	-0.50529	0.69332	0.003715	0.27231643
NA	ENSG00000233981	2.6768	0.0037162	2.6768	0.0037162	1.9618	0.024892	2.6768	0.0037162	0.0037162	0.27231643
KCNH6	ENSG00000173826	2.6749	0.0037381	-0.83319	0.79763	0.44294	0.32891	1.1446	0.12618	0.0037381	0.273509929
NA	ENSG00000242687	2.4908	0.0063731	2.6737	0.003751	1.4127	0.078873	1.4127	0.078873	0.003751	0.273989531
LINC00672	ENSG00000263874	2.6733	0.0037559	2.6733	0.0037559	2.0729	0.019091	2.6733	0.0037559	0.0037559	0.273989531
FNBP4	ENSG00000109920	2.6727	0.0037618	0.47042	0.31903	1.949	0.025649	2.0212	0.021632	0.0037618	0.274009737
PIDD1	ENSG00000177595	2.6719	0.0037708	2.5489	0.0054034	2.1035	0.017709	0.91224	0.18082	0.0037708	0.274255349
NA	ENSG00000261268	1.5764	0.05747	2.6707	0.0037847	1.1536	0.12434	0.77158	0.22018	0.0037847	0.27440901
NA	ENSG00000237153	2.6706	0.0037858	1.7884	0.036854	-0.73315	0.76827	-0.32838	0.62869	0.0037858	0.27440901
NA	ENSG00000201668	2.6689	0.0038048	2.6689	0.0038048	2.6689	0.0038048	2.6689	0.0038048	0.0038048	0.27440901
LINC02785	ENSG00000226822	2.6689	0.0038051	2.6689	0.0038051	1.9875	0.023435	2.3307	0.0098844	0.0038051	0.27440901
GGA3	ENSG00000125447	1.2821	0.099912	1.4087	0.079456	2.6689	0.0038053	1.4087	0.079456	0.0038053	0.27440901
MIR4474	ENSG00000264941	2.6687	0.0038067	2.6687	0.0038067	-1.268	0.8976	2.6687	0.0038067	0.0038067	0.27440901
NCAPD2P1	ENSG00000230356	2.6677	0.0038183	2.2167	0.013322	2.6677	0.0038183	1.7794	0.037586	0.0038183	0.27483864
BTBD3	ENSG00000132640	2.3423	0.0095839	2.6669	0.0038279	2.6669	0.0038279	2.412	0.0079322	0.0038279	0.275086185
IFT27	ENSG00000100360	0.55415	0.28974	-0.89052	0.81341	2.6658	0.0038405	0.4381	0.33066	0.0038405	0.275086185
SPIRE2	ENSG00000204991	1.3315	0.091518	1.3315	0.091518	2.6657	0.0038414	1.6886	0.045647	0.0038414	0.275086185

SLC6A6	ENSG00000131389	2.6641	0.0038599	1.8669	0.030959	1.6311	0.051439	0.81077	0.20875	0.0038599	0.275086185
ZKSCAN5	ENSG00000196652	1.6236	0.052234	2.2968	0.010816	2.6638	0.0038634	0.32856	0.37125	0.0038634	0.275086185
NA	ENSG00000232729	2.2632	0.011813	2.3639	0.009043	2.6638	0.0038635	2.4011	0.0081733	0.0038635	0.275086185
LYG2	ENSG00000185674	0.92854	0.17656	2.6635	0.0038665	0.63251	0.26353	0.84457	0.19918	0.0038665	0.275086185
OR2L13	ENSG00000196071	2.6635	0.0038669	0.45389	0.32496	0.93413	0.17512	0.45389	0.32496	0.0038669	0.275086185
CH25H	ENSG00000138135	-0.1155	0.54598	2.1047	0.01766	0.83921	0.20068	2.6616	0.0038882	0.0038882	0.275810381
NHLRC2	ENSG00000196865	-0.86076	0.80531	-0.92834	0.82338	-0.78236	0.783	2.6616	0.0038884	0.0038884	0.275810381
OR7E66P	ENSG00000243674	1.5971	0.055124	1.5971	0.055124	1.5971	0.055124	2.6606	0.0039002	0.0039002	0.27624527
ERAL1	ENSG00000132591	2.6593	0.0039155	2.6123	0.0044965	2.551	0.0053711	2.6593	0.0039155	0.0039155	0.276926437
SV2A	ENSG00000159164	2.3274	0.0099715	2.3274	0.0099715	2.6583	0.0039265	2.3136	0.010344	0.0039265	0.277013478
NA	ENSG00000256500	2.6582	0.0039281	2.6582	0.0039281	2.6582	0.0039281	2.6582	0.0039281	0.0039281	0.277013478
SYNRG	ENSG00000275066	2.6563	0.0039505	2.5195	0.0058764	2.1814	0.014576	2.0844	0.01856	0.0039505	0.278019311
PTK2B	ENSG00000120899	1.5504	0.060524	2.6559	0.0039546	1.5258	0.063525	2.3888	0.0084514	0.0039546	0.278019311
PLPP7	ENSG00000160539	0.82577	0.20447	2.6549	0.0039667	-0.43536	0.66835	0.45205	0.32562	0.0039667	0.278019311
NA	ENSG00000265910	2.6543	0.0039737	2.6543	0.0039737	2.6543	0.0039737	2.6543	0.0039737	0.0039737	0.278019311
DNAAF4	ENSG00000256061	-0.67303	0.74954	-0.28897	0.6137	2.6537	0.0039806	-1.3639	0.91369	0.0039806	0.278019311
C16orf91	ENSG00000174109	2.6536	0.003982	-1.0344	0.84953	1.4573	0.072517	-0.60559	0.72761	0.003982	0.278019311
NA	ENSG00000260772	2.6536	0.0039823	2.6536	0.0039823	2.6536	0.0039823	2.6536	0.0039823	0.0039823	0.278019311
NA	ENSG00000212511	2.6522	0.0039979	2.6522	0.0039979	2.6522	0.0039979	2.6522	0.0039979	0.0039979	0.278467031
NPEPPS	ENSG00000141279	2.652	0.0040013	2.3224	0.010105	0.6892	0.24535	2.0805	0.018742	0.0040013	0.278467031
NA	ENSG00000249359	1.6392	0.050586	0.63423	0.26297	-0.36397	0.64206	2.6511	0.0040111	0.0040111	0.278467031
AGBL4-AS1	ENSG00000230114	2.6507	0.0040166	1.4996	0.06686	1.1893	0.11716	1.8838	0.029799	0.0040166	0.278467031
TRIP6	ENSG00000087077	0.84773	0.19829	2.2261	0.013002	2.6505	0.0040191	0.89067	0.18655	0.0040191	0.278467031
SMARCAL1	ENSG00000138375	2.2487	0.012267	2.6501	0.004023	0.68981	0.24516	-2.5351	0.99438	0.004023	0.278467031
LCN1P2	ENSG00000204031	-0.50727	0.69402	0.28539	0.38767	-0.1367	0.55437	2.6477	0.004052	0.004052	0.279518588
LRRC6	ENSG00000129295	2.0649	0.019467	-0.74551	0.77202	0.5034	0.30734	2.6477	0.0040524	0.0040524	0.279518588
FGFR3P6	ENSG00000226164	2.6474	0.0040554	2.6474	0.0040554	2.6474	0.0040554	2.6474	0.0040554	0.0040554	0.279518588
UBE2U	ENSG00000177414	2.1857	0.014418	2.6454	0.0040799	2.0534	0.020017	0.51205	0.30431	0.0040799	0.280810066
PROS2P	ENSG00000189002	1.7316	0.041672	1.9131	0.027865	1.0025	0.15806	2.644	0.0040971	0.0040971	0.280947983
H1-4	ENSG00000168298	0.94831	0.17149	1.1973	0.11559	2.6439	0.0040973	2.6439	0.0040973	0.0040973	0.280947983
TPRKBP2	ENSG00000260452	0.83109	0.20296	1.7867	0.036996	2.6438	0.0040992	2.6438	0.0040992	0.0040992	0.280947983

PARP15	ENSG00000173200	2.1937	0.01413	2.6037	0.0046118	2.6428	0.0041116	2.2683	0.011655	0.0041116	0.281134615
ZNF649	ENSG00000198093	2.6418	0.0041229	2.6418	0.0041229	2.6418	0.0041229	2.6418	0.0041229	0.0041229	0.281134615
RPS3	ENSG00000149273	-0.8146	0.79235	-0.46909	0.6805	2.6418	0.0041239	-1.9968	0.97708	0.0041239	0.281134615
TSTD3	ENSG00000279170	2.5341	0.0056376	2.5341	0.0056376	1.8012	0.035834	2.6417	0.004125	0.004125	0.281134615
HS6ST1	ENSG00000136720	-0.62134	0.73281	1.4776	0.069761	2.6411	0.0041317	0.65166	0.25731	0.0041317	0.281197962
CCNT2	ENSG00000082258	1.641	0.050402	1.4425	0.074574	1.7375	0.041149	2.6404	0.004141	0.004141	0.281437838
NA	ENSG00000253855	1.2013	0.11482	1.2013	0.11482	1.2013	0.11482	2.6397	0.0041494	0.0041494	0.281615964
GCN1	ENSG00000089154	1.148	0.12548	1.1407	0.127	1.2139	0.1124	2.6378	0.0041718	0.0041718	0.282742439
RN7SKP265	ENSG00000252257	2.6367	0.0041862	2.6367	0.0041862	2.6367	0.0041862	2.6367	0.0041862	0.0041862	0.283254936
RPL32P29	ENSG00000244270	2.6359	0.0041962	2.6359	0.0041962	2.6359	0.0041962	2.6359	0.0041962	0.0041962	0.283254936
LINC00955	ENSG00000216560	0.27568	0.3914	0.68534	0.24656	2.6358	0.0041968	0.46956	0.31933	0.0041968	0.283254936
NA	ENSG00000240634	2.6342	0.0042172	0.75669	0.22462	-0.56818	0.71504	0.50852	0.30554	0.0042172	0.284238113
NA	ENSG00000274667	2.6334	0.0042263	2.6334	0.0042263	2.6334	0.0042263	2.6334	0.0042263	0.0042263	0.28445801
FAM114A2	ENSG00000055147	2.6321	0.0042426	1.3237	0.092794	2.4421	0.0073014	1.0633	0.14382	0.0042426	0.284889273
SIRT6	ENSG00000077463	2.632	0.0042444	0.8771	0.19022	2.632	0.0042444	2.632	0.0042444	0.0042444	0.284889273
KIR3DX1	ENSG00000104970	2.6293	0.0042776	2.6293	0.0042776	0.39194	0.34755	2.6293	0.0042776	0.0042776	0.286722762
ALDH18A1	ENSG00000059573	2.3206	0.010155	2.6275	0.0043013	1.4208	0.077689	1.5055	0.066092	0.0043013	0.287767695
PIPOX	ENSG00000179761	1.5565	0.059793	-1.2401	0.89254	2.6272	0.004305	-0.32926	0.62902	0.004305	0.287767695
TEKT2	ENSG00000092850	2.5295	0.0057114	2.6243	0.004341	0.32013	0.37444	1.8421	0.032729	0.004341	0.289521351
NA	ENSG00000254433	0.40934	0.34115	2.6236	0.0043506	0.69245	0.24433	2.6236	0.0043506	0.0043506	0.289521351
TMEM156	ENSG00000121895	-0.16831	0.56683	-0.019186	0.50765	2.6234	0.0043525	1.0302	0.15145	0.0043525	0.289521351
NA	ENSG00000247416	-0.53298	0.70298	2.6232	0.004355	2.6232	0.004355	2.6232	0.004355	0.004355	0.289521351
UTP18	ENSG00000011260	2.6216	0.0043761	2.4582	0.0069824	2.257	0.012005	2.4582	0.0069824	0.0043761	0.290527729
ARSG	ENSG00000141337	0.21676	0.4142	1.3256	0.092479	2.6194	0.0044043	1.7657	0.038726	0.0044043	0.29187157
ACTR1AP1	ENSG00000233370	2.4101	0.0079744	2.6186	0.0044142	1.6192	0.052702	1.6192	0.052702	0.0044142	0.29187157
GREM1	ENSG00000166923	2.6182	0.0044198	2.6182	0.0044198	2.6182	0.0044198	2.6182	0.0044198	0.0044198	0.29187157
NHLRC1	ENSG00000187566	-0.29197	0.61484	2.6182	0.0044203	-0.29197	0.61484	-0.29197	0.61484	0.0044203	0.29187157
NA	ENSG00000254255	2.6166	0.0044409	2.6166	0.0044409	2.1871	0.014367	2.6166	0.0044409	0.0044409	0.292834989
YBX1P7	ENSG00000232349	2.6145	0.004468	0.45025	0.32627	2.4867	0.0064469	2.4867	0.0064469	0.004468	0.294223838
NA	ENSG00000273243	2.6132	0.0044853	2.6132	0.0044853	2.6132	0.0044853	2.6132	0.0044853	0.0044853	0.294757394
NA	ENSG00000273107	2.613	0.0044882	2.613	0.0044882	2.613	0.0044882	2.613	0.0044882	0.0044882	0.294757394

IQCF5	ENSG00000214681	1.33	0.091763	2.6123	0.0044968	2.6123	0.0044968	1.5918	0.05572	0.0044968	0.294800278
NA	ENSG00000265845	2.6119	0.0045016	2.6119	0.0045016	2.6119	0.0045016	2.6119	0.0045016	0.0045016	0.294800278
PEG13	ENSG00000282164	0.96806	0.16651	2.6108	0.0045168	-1.7298	0.95817	-1.1187	0.86836	0.0045168	0.294800278
RPL7P3	ENSG00000226094	0.014202	0.49433	2.6102	0.0045241	0.53047	0.29789	0.53047	0.29789	0.0045241	0.294800278
LINC01960	ENSG00000260868	2.61	0.0045268	2.3843	0.0085568	2.61	0.0045268	1.2023	0.11463	0.0045268	0.294800278
NA	ENSG00000212422	2.6097	0.0045315	2.6097	0.0045315	2.5407	0.0055316	2.0155	0.021924	0.0045315	0.294800278
SARDH	ENSG00000123453	0.35392	0.3617	1.3091	0.09525	1.2994	0.09691	2.6093	0.0045363	0.0045363	0.294800278
CHD3	ENSG00000170004	2.6091	0.004539	1.4611	0.071988	1.7491	0.040133	2.6091	0.004539	0.004539	0.294800278
RABL6	ENSG00000196642	1.5352	0.062364	2.6088	0.0045433	1.5352	0.062364	1.5036	0.066338	0.0045433	0.294800278
GXYLT2	ENSG00000172986	-0.033133	0.51322	2.6078	0.0045562	-0.16487	0.56548	1.6415	0.05035	0.0045562	0.294868066
NA	ENSG00000258452	-0.094754	0.53774	0.038228	0.48475	2.6075	0.0045609	-0.094754	0.53774	0.0045609	0.294868066
NA	ENSG00000278638	2.607	0.0045669	2.607	0.0045669	2.607	0.0045669	2.607	0.0045669	0.0045669	0.294868066
MALL	ENSG00000144063	2.0596	0.019717	2.6067	0.0045708	2.0596	0.019717	2.0596	0.019717	0.0045708	0.294868066
LINC01570	ENSG00000260338	1.2484	0.10594	1.2885	0.098794	2.6064	0.0045746	1.2484	0.10594	0.0045746	0.294868066
CALCR	ENSG00000004948	2.3576	0.0091963	2.6056	0.0045854	2.3576	0.0091963	1.8682	0.030865	0.0045854	0.295173768
RNU6-1224P	ENSG00000252593	2.6031	0.0046196	2.6031	0.0046196	2.6031	0.0046196	2.5551	0.0053076	0.0046196	0.296439766
RNA5SP131	ENSG00000202322	2.0272	0.021319	2.2548	0.012072	2.6031	0.0046198	2.0717	0.019145	0.0046198	0.296439766
SH2D6	ENSG00000152292	-0.42112	0.66317	-0.0060713	0.50242	2.6025	0.004627	-0.58999	0.7224	0.004627	0.296439766
VPS45	ENSG00000136631	2.1448	0.015986	2.0507	0.020146	2.1727	0.0149	2.6023	0.0046294	0.0046294	0.296439766
NA	ENSG00000273398	2.6018	0.0046362	2.421	0.0077382	2.6018	0.0046362	2.6018	0.0046362	0.0046362	0.296457666
PYY2	ENSG00000237575	0.93394	0.17517	0.93394	0.17517	2.6013	0.0046437	2.0662	0.019406	0.0046437	0.296457666
NA	ENSG00000236478	1.9039	0.028461	1.8944	0.029084	0.39715	0.34563	2.6004	0.0046554	0.0046554	0.296457666
TRAC	ENSG00000277734	2.4039	0.0081109	1.7346	0.041408	2.5997	0.004665	2.5997	0.004665	0.004665	0.296457666
BAK1P1	ENSG00000175730	2.4309	0.0075297	1.7357	0.04131	2.4309	0.0075297	2.5996	0.0046669	0.0046669	0.296457666
RNU7-160P	ENSG00000252469	-0.15352	0.561	2.5993	0.0046701	0.39719	0.34562	1.4667	0.07123	0.0046701	0.296457666
ZC3H8	ENSG00000144161	2.599	0.0046748	2.3714	0.0088612	1.8285	0.033738	1.8285	0.033738	0.0046748	0.296457666
PPM1K	ENSG00000163644	-0.67658	0.75067	2.4571	0.0070024	-0.11507	0.54581	2.5976	0.0046933	0.0046933	0.296457666
TPX2	ENSG00000088325	-0.14162	0.55631	0.1159	0.45387	0.57947	0.28113	2.5976	0.0046934	0.0046934	0.296457666
CYTH1	ENSG00000108669	2.5974	0.0046961	0.96606	0.16701	0.20297	0.41958	1.2111	0.11293	0.0046961	0.296457666
RGS12	ENSG00000159788	-0.073253	0.5292	1.2404	0.10741	2.5974	0.0046966	1.465	0.071458	0.0046966	0.296457666
AFTPH	ENSG00000119844	1.382	0.083487	0.64297	0.26012	2.5958	0.004718	2.4736	0.0066883	0.004718	0.297152266

LINC01474	ENSG00000236849	2.402	0.0081525	2.0435	0.020504	2.5957	0.0047198	1.4109	0.079141	0.0047198	0.297152266
KRTAP10-8	ENSG00000187766	2.5927	0.0047606	1.8696	0.030766	0.15727	0.43752	-0.2358	0.59321	0.0047606	0.298932499
NA	ENSG00000237947	2.5922	0.0047686	2.049	0.020229	2.5922	0.0047686	2.5922	0.0047686	0.0047686	0.298932499
NA	ENSG00000270108	2.5912	0.0047814	2.5912	0.0047814	2.5912	0.0047814	2.5912	0.0047814	0.0047814	0.298932499
NUDT18	ENSG00000275074	2.5911	0.0047841	2.5911	0.0047841	2.5911	0.0047841	2.5911	0.0047841	0.0047841	0.298932499
NA	ENSG00000259336	2.5905	0.0047914	2.5905	0.0047914	2.5905	0.0047914	2.5905	0.0047914	0.0047914	0.298932499
ANKRD34B	ENSG00000189127	0.45427	0.32482	0.45427	0.32482	0.45427	0.32482	2.5898	0.0048014	0.0048014	0.298932499
NA	ENSG00000203620	0.043486	0.48266	0.043486	0.48266	0.043486	0.48266	2.5889	0.0048139	0.0048139	0.298932499
NA	ENSG00000258914	2.4397	0.0073491	2.5883	0.0048225	0.015171	0.49395	0.015171	0.49395	0.0048225	0.298932499
NA	ENSG00000226833	1.4575	0.072491	1.4959	0.067346	2.5881	0.0048258	2.1504	0.01576	0.0048258	0.298932499
NA	ENSG00000279694	2.5874	0.0048358	2.5874	0.0048358	2.5874	0.0048358	2.5874	0.0048358	0.0048358	0.298932499
AUTS2	ENSG00000158321	2.5864	0.0048493	2.488	0.006423	2.2812	0.011267	2.1403	0.016167	0.0048493	0.298932499
NA	ENSG00000275350	1.089	0.13808	2.5862	0.0048526	0.93433	0.17507	1.8729	0.030539	0.0048526	0.298932499
NA	ENSG00000252443	2.5857	0.0048589	2.5181	0.0058999	1.3457	0.089205	1.3457	0.089205	0.0048589	0.298932499
SF3B4	ENSG00000143368	2.2046	0.01374	2.5853	0.0048642	2.3039	0.010615	1.9668	0.024601	0.0048642	0.298932499
TRAPPC3L	ENSG00000173626	2.5852	0.0048658	-0.021445	0.50855	1.2879	0.098888	1.6128	0.053397	0.0048658	0.298932499
NA	ENSG00000269967	0.0084722	0.49662	0.0084722	0.49662	0.0084722	0.49662	2.5851	0.0048678	0.0048678	0.298932499
FAM151B	ENSG00000152380	2.585	0.0048685	2.4619	0.0069109	1.5531	0.060204	-0.46552	0.67922	0.0048685	0.298932499
NPM1P40	ENSG00000236523	-0.94098	0.82664	-0.94098	0.82664	2.585	0.0048696	0.85413	0.19652	0.0048696	0.298932499
NA	ENSG00000270460	1.9446	0.025914	1.9446	0.025914	0.34395	0.36544	2.5849	0.0048707	0.0048707	0.298932499
NA	ENSG00000267716	0.47286	0.31816	2.5846	0.004875	0.47286	0.31816	0.47286	0.31816	0.004875	0.298932499
NA	ENSG00000249072	2.5844	0.0048769	-0.48656	0.68672	2.1924	0.014176	0.26339	0.39613	0.0048769	0.298932499
RPL7P31	ENSG00000235379	1.997	0.022915	2.5838	0.0048852	1.997	0.022915	1.997	0.022915	0.0048852	0.29906507
NA	ENSG00000269605	2.0364	0.020855	2.1695	0.015023	2.5819	0.0049127	1.7849	0.037141	0.0049127	0.300371231
SMURF1	ENSG00000198742	2.5815	0.0049191	0.4535	0.3251	1.0616	0.1442	0.31574	0.3761	0.0049191	0.300385643
DDB2	ENSG00000134574	0.74322	0.22867	1.6349	0.051035	2.5807	0.0049302	-0.047341	0.51888	0.0049302	0.300628947
RN7SL420P	ENSG00000239984	-1.6871	0.95421	2.5803	0.004936	2.0802	0.018753	-0.8429	0.80036	0.004936	0.300628947
ABCG2	ENSG00000118777	1.5277	0.063299	0.85429	0.19647	2.5799	0.0049416	1.6657	0.047891	0.0049416	0.300628947
COX18	ENSG00000163626	2.4885	0.006414	0.87922	0.18964	2.5794	0.0049482	2.5794	0.0049482	0.0049482	0.300628947
ASNSP6	ENSG00000267333	0.81334	0.20801	-0.35811	0.63987	0.67099	0.25111	2.5789	0.0049564	0.0049564	0.300628947
RPL18P1	ENSG00000242417	2.5786	0.0049601	1.9849	0.023576	0.12507	0.45023	1.9243	0.027157	0.0049601	0.300628947

LRP5L	ENSG00000100068	2.0164	0.021878	2.5778	0.0049716	0.30552	0.37998	1.3341	0.091081	0.0049716	0.300951637
SLC39A13	ENSG00000165915	1.9718	0.024317	2.5764	0.0049918	2.1864	0.014395	1.9718	0.024317	0.0049918	0.301799521
NA	ENSG00000234021	-0.74061	0.77054	2.5755	0.0050052	-2.1442	0.98399	-0.059593	0.52376	0.0050052	0.302234691
RPL5P27	ENSG00000228195	2.5721	0.0050545	2.5721	0.0050545	2.3605	0.0091251	1.1859	0.11782	0.0050545	0.304655864
NA	ENSG00000224658	2.2321	0.012804	1.2472	0.10617	2.5719	0.0050578	1.7716	0.038226	0.0050578	0.304655864
H4C15	ENSG00000270276	2.1094	0.017455	2.5705	0.0050771	1.387	0.082721	2.1141	0.017252	0.0050771	0.305440843
ZBTB7A	ENSG00000178951	1.2128	0.1126	1.2128	0.1126	1.2128	0.1126	2.5694	0.0050938	0.0050938	0.306067662
NA	ENSG00000250039	0.42069	0.33699	-0.0031364	0.50125	0.0080463	0.49679	2.5689	0.0051006	0.0051006	0.306098815
NA	ENSG00000229167	1.1398	0.12718	0.044589	0.48222	2.5673	0.005125	0.044589	0.48222	0.005125	0.306846902
RN7SL62P	ENSG00000239640	2.5666	0.0051357	2.5666	0.0051357	2.4418	0.0073062	2.5666	0.0051357	0.0051357	0.306846902
NA	ENSG00000261240	2.565	0.0051582	2.565	0.0051582	2.565	0.0051582	2.565	0.0051582	0.0051582	0.306846902
RPS27AP1	ENSG00000242692	2.565	0.0051584	2.565	0.0051584	2.565	0.0051584	2.565	0.0051584	0.0051584	0.306846902
NA	ENSG00000249818	2.5648	0.005162	-0.039726	0.51584	1.9897	0.023311	0.99318	0.16031	0.005162	0.306846902
ADAMTS10	ENSG00000142303	-0.33281	0.63036	0.88618	0.18776	2.5647	0.0051628	1.4557	0.072737	0.0051628	0.306846902
IQCF2	ENSG00000184345	2.4837	0.0065008	2.4893	0.0063996	2.5644	0.0051675	1.5175	0.06457	0.0051675	0.306846902
EDF1	ENSG00000107223	2.5642	0.0051702	2.086	0.018488	1.3132	0.094559	0.42631	0.33494	0.0051702	0.306846902
NA	ENSG00000106540	2.0914	0.018248	2.0914	0.018248	2.0914	0.018248	2.5638	0.0051759	0.0051759	0.306846902
MTCO1P3	ENSG00000228930	2.5628	0.0051915	-0.12458	0.54957	-0.93565	0.82527	-0.93565	0.82527	0.0051915	0.306846902
LAPTM5	ENSG00000162511	1.2609	0.10368	2.5627	0.0051927	0.89487	0.18543	0.095285	0.46204	0.0051927	0.306846902
RNU6-1287P	ENSG00000199784	-1.4095	0.92066	2.5626	0.0051943	-0.16403	0.56515	2.0587	0.019763	0.0051943	0.306846902
SRR	ENSG00000167720	0.15221	0.43951	2.5624	0.0051981	1.5898	0.055937	2.5624	0.0051981	0.0051981	0.306846902
ADAMTSL4	ENSG00000143382	2.5618	0.0052068	1.6221	0.052386	1.7113	0.043512	0.21482	0.41495	0.0052068	0.306846902
LINC00609	ENSG00000257585	0.91931	0.17897	0.91931	0.17897	2.5617	0.0052088	0.91931	0.17897	0.0052088	0.306846902
MARK3	ENSG00000075413	2.5608	0.0052217	2.5428	0.0054978	2.4364	0.0074164	2.4374	0.0073957	0.0052217	0.306846902
TEN1	ENSG00000257949	2.5603	0.0052286	2.3062	0.01055	2.5603	0.0052286	2.5603	0.0052286	0.0052286	0.306846902
CALY	ENSG00000130643	-0.55166	0.70941	-0.42499	0.66458	2.5601	0.0052329	-0.35269	0.63784	0.0052329	0.306846902
TPRG1-AS1	ENSG00000234076	1.1625	0.12252	2.5599	0.0052355	0.87092	0.1919	-0.8923	0.81388	0.0052355	0.306846902
TRPV4	ENSG00000111199	2.5593	0.0052439	2.0446	0.020447	1.5982	0.055004	0.0078261	0.49688	0.0052439	0.306846902
NA	ENSG00000251361	0.046271	0.48155	0.032926	0.48687	0.046271	0.48155	2.5592	0.0052453	0.0052453	0.306846902
LINC02710	ENSG00000255269	1.8836	0.029811	2.5584	0.005258	0.60029	0.27416	0.7457	0.22793	0.005258	0.307221031
MIS18A-AS1	ENSG00000227256	0.012278	0.4951	2.5552	0.0053064	-1.0618	0.85583	-1.0618	0.85583	0.0053064	0.309677691

OCSTAMP	ENSG00000149635	-1.7435	0.95938	-1.7435	0.95938	0.67061	0.25123	2.5545	0.0053169	0.0053169	0.309919303
ZNF788P	ENSG00000214189	0.36296	0.35832	2.5535	0.005333	2.0102	0.022207	2.0102	0.022207	0.005333	0.310486368
SPAG4	ENSG00000061656	-0.0049891	0.50199	-0.0049891	0.50199	2.553	0.0053403	-0.35023	0.63692	0.0053403	0.310540357
NA	ENSG00000261474	2.5518	0.0053585	2.3625	0.0090771	-0.23817	0.59412	-0.23817	0.59412	0.0053585	0.3112273
NA	ENSG00000283765	2.5511	0.0053693	2.5511	0.0053693	2.5511	0.0053693	2.5511	0.0053693	0.0053693	0.311265735
LINC01839	ENSG00000227509	1.7111	0.043527	0.10623	0.4577	2.5501	0.0053843	1.2915	0.09827	0.0053843	0.311265735
RNF182	ENSG00000180537	-0.01272	0.50507	2.5497	0.0053904	1.6619	0.048268	1.0906	0.13772	0.0053904	0.311265735
NA	ENSG00000201749	2.5489	0.0054037	2.5489	0.0054037	2.5489	0.0054037	2.5489	0.0054037	0.0054037	0.311265735
NA	ENSG00000270090	2.5487	0.0054063	0.35732	0.36043	0.61189	0.2703	-0.90585	0.81749	0.0054063	0.311265735
KIF13A	ENSG00000137177	1.0356	0.1502	1.47	0.070779	2.5479	0.0054185	2.1596	0.015402	0.0054185	0.311265735
NA	ENSG00000229857	2.0616	0.019621	2.5479	0.0054193	1.7629	0.038958	2.0183	0.021782	0.0054193	0.311265735
AGBL4-IT1	ENSG00000225623	1.9565	0.025205	2.5477	0.0054224	1.18	0.119	1.2316	0.10905	0.0054224	0.311265735
NA	ENSG00000259166	2.5476	0.0054233	1.4774	0.06979	0.86616	0.1932	1.69	0.045515	0.0054233	0.311265735
PPP3R1	ENSG00000221823	2.4103	0.0079699	2.3662	0.0089864	1.6591	0.048544	2.5471	0.0054315	0.0054315	0.311265735
SYN2	ENSG00000157152	2.0372	0.020817	0.56333	0.28661	0.52482	0.29985	2.5469	0.0054341	0.0054341	0.311265735
KRTAP10-13P	ENSG00000236382	-0.2182	0.58636	-0.73042	0.76743	-0.2182	0.58636	2.5464	0.0054421	0.0054421	0.311265735
LINC02841	ENSG00000267407	2.5464	0.0054422	2.4289	0.0075732	-0.16684	0.56625	2.5464	0.0054422	0.0054422	0.311265735
RAPH1	ENSG00000173166	2.5457	0.0054529	2.2583	0.011962	1.5199	0.064272	1.2487	0.10589	0.0054529	0.311512095
NA	ENSG00000277301	2.545	0.0054635	2.2352	0.012703	1.6517	0.049301	1.6517	0.049301	0.0054635	0.311752172
NA	ENSG00000261458	2.5446	0.0054704	1.0844	0.13909	1.0844	0.13909	0.39204	0.34751	0.0054704	0.311758278
NFKB1	ENSG00000109320	1.5257	0.063542	1.9881	0.023402	2.5441	0.0054786	1.9679	0.02454	0.0054786	0.311758278
NA	ENSG00000261610	2.4097	0.0079831	1.6901	0.045504	2.5438	0.0054828	1.3856	0.082934	0.0054828	0.311758278
NA	ENSG00000259376	2.543	0.0054951	2.1797	0.014639	2.1797	0.014639	2.1797	0.014639	0.0054951	0.31179258
NA	ENSG00000260082	0.069958	0.47211	1.0784	0.14044	2.5429	0.0054962	-0.42525	0.66467	0.0054962	0.31179258
NA	ENSG00000248766	2.5413	0.0055217	0.48781	0.31284	2.5413	0.0055217	2.5413	0.0055217	0.0055217	0.312516956
NA	ENSG00000259775	-0.37166	0.64493	-0.50971	0.69487	-0.50971	0.69487	2.5411	0.0055253	0.0055253	0.312516956
NA	ENSG00000251249	0.63925	0.26133	2.202	0.013832	2.5404	0.0055355	2.2466	0.012332	0.0055355	0.312516956
COMMD7	ENSG00000149600	0.94714	0.17178	1.6243	0.052157	2.5402	0.0055394	1.6705	0.047406	0.0055394	0.312516956
RPL23AP46	ENSG00000213122	2.5398	0.0055463	2.5398	0.0055463	2.5398	0.0055463	2.5398	0.0055463	0.0055463	0.312516956
GAPDHP35	ENSG00000250982	1.7481	0.040224	1.4723	0.070467	1.5427	0.061457	2.5385	0.0055666	0.0055666	0.312516956
NA	ENSG00000282097	-0.35491	0.63867	1.6934	0.045189	-0.35491	0.63867	2.5382	0.0055708	0.0055708	0.312516956

CEBPZ	ENSG00000115816	2.4452	0.0072387	2.5381	0.0055734	2.5381	0.0055734	2.5381	0.0055734	0.0055734	0.312516956
ARHGAP26-IT1	ENSG00000230789	2.5359	0.0056073	2.5359	0.0056073	1.6975	0.044799	1.2135	0.11246	0.0056073	0.312516956
NA	ENSG00000229486	0.85028	0.19759	2.5239	0.0058031	2.5358	0.0056097	0.94008	0.17359	0.0056097	0.312516956
NA	ENSG00000233292	2.5356	0.0056124	2.1574	0.015486	2.1574	0.015486	2.1574	0.015486	0.0056124	0.312516956
GNA12	ENSG00000146535	2.2918	0.010958	2.5352	0.0056197	2.1982	0.013969	0.88624	0.18775	0.0056197	0.312516956
GFRA3	ENSG00000146013	2.5352	0.0056199	1.1092	0.13367	1.3771	0.084248	1.3089	0.095288	0.0056199	0.312516956
HMGA1P2	ENSG00000248641	2.5349	0.0056236	2.5349	0.0056236	2.5349	0.0056236	2.5349	0.0056236	0.0056236	0.312516956
RPL21P17	ENSG00000214073	-0.4532	0.6748	2.5343	0.005633	1.538	0.062019	1.538	0.062019	0.005633	0.312516956
YWHAG	ENSG00000170027	1.6719	0.047268	1.011	0.156	2.5343	0.0056339	2.1471	0.015893	0.0056339	0.312516956
LINC01694	ENSG00000233922	2.5343	0.0056343	2.2783	0.011353	1.7948	0.036346	1.7427	0.040697	0.0056343	0.312516956
NA	ENSG00000264193	0.78575	0.21601	2.5341	0.0056372	0.5479	0.29188	0.59224	0.27684	0.0056372	0.312516956
CHRNA4	ENSG00000101204	1.4473	0.073909	1.8662	0.031005	1.6549	0.048975	2.5333	0.0056503	0.0056503	0.312516956
NA	ENSG00000264434	-0.23502	0.5929	2.3729	0.0088257	2.5332	0.005652	2.3729	0.0088257	0.005652	0.312516956
KIAA1522	ENSG00000162522	-0.16121	0.56404	1.0809	0.13988	0.11833	0.4529	2.533	0.0056553	0.0056553	0.312516956
RNF157-AS1	ENSG00000267128	2.5328	0.005657	2.5328	0.005657	2.5328	0.005657	2.5328	0.005657	0.005657	0.312516956
RN7SL97P	ENSG00000242216	0.291	0.38552	1.3986	0.080971	0.18337	0.42726	2.5327	0.0056592	0.0056592	0.312516956
DNPEP	ENSG00000123992	2.5324	0.0056644	2.0601	0.019696	2.1553	0.015571	1.7855	0.037092	0.0056644	0.312516956
SOX1	ENSG00000182968	2.5321	0.0056693	1.0011	0.15839	0.43213	0.33282	1.0433	0.1484	0.0056693	0.312516956
MLST8	ENSG00000167965	2.5311	0.0056858	2.5311	0.0056858	2.5311	0.0056858	2.5311	0.0056858	0.0056858	0.313072355
LINC00882	ENSG00000242759	0.66815	0.25202	2.2946	0.010878	2.5301	0.0057021	1.0241	0.15289	0.0057021	0.3136155
NA	ENSG00000261797	0.21351	0.41547	2.5292	0.0057161	0.79279	0.21395	0.7669	0.22157	0.0057161	0.314031063
CA14	ENSG00000118298	0.61059	0.27074	2.5283	0.0057312	1.8998	0.02873	2.1463	0.015924	0.0057312	0.314506054
NA	ENSG00000233895	1.9836	0.023651	2.0512	0.020125	2.527	0.0057521	1.7746	0.03798	0.0057521	0.315117025
IFNAR1	ENSG00000142166	0.0095632	0.49618	0.21447	0.41509	2.5266	0.0057584	0.4343	0.33204	0.0057584	0.315117025
RPS3AP36	ENSG00000214362	2.5264	0.0057624	2.0177	0.021809	2.3768	0.008732	1.886	0.029649	0.0057624	0.315117025
LTBP4	ENSG00000090006	0.53854	0.2951	1.487	0.068512	2.4219	0.007719	2.526	0.0057682	0.0057682	0.315117025
LINC00687	ENSG00000228422	1.6687	0.047589	1.2468	0.10623	2.5238	0.0058045	0.9202	0.17873	0.0058045	0.316347537
TERF2	ENSG00000132604	2.056	0.019891	2.5237	0.0058069	1.4652	0.071429	1.7219	0.042546	0.0058069	0.316347537
SNRPGP9	ENSG00000228551	0.39423	0.34671	0.39423	0.34671	1.4235	0.077299	2.5235	0.0058102	0.0058102	0.316347537
DHX40	ENSG00000108406	1.5806	0.056988	0.74673	0.22761	2.5223	0.00583	0.23509	0.40707	0.00583	0.316750433
CPSF4	ENSG00000160917	2.4602	0.0069424	2.0411	0.02062	2.5222	0.0058306	1.8299	0.033634	0.0058306	0.316750433

MTMR11	ENSG00000014914	2.047	0.020331	2.047	0.020331	2.047	0.020331	2.5214	0.0058452	0.0058452	0.316934716
ZNF844	ENSG00000223547	2.3909	0.0084036	2.3294	0.0099194	1.1704	0.12093	2.5212	0.005847	0.005847	0.316934716
UNC5A	ENSG00000113763	-1.2897	0.90142	0.82931	0.20346	2.5198	0.0058708	-0.868	0.8073	0.0058708	0.31771853
TTLL9	ENSG00000131044	2.5187	0.0058889	1.2561	0.10453	2.1926	0.01417	2.4464	0.0072153	0.0058889	0.31771853
PCGF3	ENSG00000185619	2.5185	0.0058926	1.378	0.084103	-0.36813	0.64361	0.10003	0.46016	0.0058926	0.31771853
RPS3AP52	ENSG00000230405	2.5182	0.0058985	2.5182	0.0058985	0.56802	0.28501	1.3581	0.087223	0.0058985	0.31771853
NA	ENSG00000259621	-0.47379	0.68218	0.069637	0.47224	2.5176	0.0059075	-0.12827	0.55103	0.0059075	0.31771853
ZFHX2	ENSG00000136367	2.5174	0.0059106	0.40192	0.34387	0.40959	0.34105	0.8279	0.20386	0.0059106	0.31771853
FGD5P1	ENSG00000275340	0.51512	0.30323	2.5171	0.0059164	2.0849	0.01854	2.0849	0.01854	0.0059164	0.31771853
PCDH9-AS4	ENSG00000233840	0.75353	0.22557	0.75353	0.22557	0.75353	0.22557	2.5169	0.0059197	0.0059197	0.31771853
C5AR1	ENSG00000197405	1.0304	0.15141	2.0209	0.021645	2.1788	0.014674	2.5169	0.0059203	0.0059203	0.31771853
NA	ENSG00000227253	2.5164	0.0059285	0.043588	0.48262	2.5164	0.0059285	2.5164	0.0059285	0.0059285	0.31771853
PCSK2	ENSG00000125851	1.8836	0.029812	2.5161	0.0059338	2.2661	0.011722	1.6932	0.045211	0.0059338	0.31771853
SPA17	ENSG00000064199	2.1858	0.014416	2.1858	0.014416	2.5157	0.0059397	1.515	0.064889	0.0059397	0.31771853
NA	ENSG00000251285	0.80059	0.21168	2.5148	0.0059555	1.2179	0.11163	1.2179	0.11163	0.0059555	0.317914106
NA	ENSG00000231931	-1.2147	0.88776	2.5134	0.005978	-0.36925	0.64403	-0.99112	0.83919	0.005978	0.317914106
ALG1L6P	ENSG00000238278	-0.39221	0.65255	1.1671	0.12159	2.5129	0.0059873	1.8834	0.029822	0.0059873	0.317914106
RNU7-170P	ENSG00000239082	2.5128	0.0059893	2.5128	0.0059893	2.5128	0.0059893	2.5128	0.0059893	0.0059893	0.317914106
AMZ1	ENSG00000174945	1.1064	0.13427	2.3431	0.0095619	2.2963	0.010831	2.5128	0.0059895	0.0059895	0.317914106
ZNF830	ENSG00000198783	2.5112	0.0060169	-1.3173	0.90613	-1.3173	0.90613	-1.3173	0.90613	0.0060169	0.317914106
NA	ENSG00000250917	-0.39665	0.65419	-0.39665	0.65419	2.5112	0.0060169	0.60142	0.27378	0.0060169	0.317914106
SLC9A8	ENSG00000197818	2.3014	0.010683	2.5109	0.0060211	0.34768	0.36404	2.1421	0.016092	0.0060211	0.317914106
RN7SL568P	ENSG00000239703	2.5103	0.0060318	2.5103	0.0060318	2.5103	0.0060318	2.5103	0.0060318	0.0060318	0.317914106
LINC02055	ENSG00000254101	1.4078	0.079597	0.9522	0.1705	2.5102	0.0060327	1.1457	0.12597	0.0060327	0.317914106
NA	ENSG00000258751	0.90243	0.18341	0.38335	0.35073	1.7762	0.037847	2.5101	0.0060342	0.0060342	0.317914106
SERBP1	ENSG00000142864	2.5094	0.0060468	2.5094	0.0060468	2.5094	0.0060468	2.5094	0.0060468	0.0060468	0.317914106
CYB5B	ENSG00000103018	2.3016	0.010679	1.5726	0.057911	2.5094	0.0060475	2.0571	0.01984	0.0060475	0.317914106
NA	ENSG00000259708	1.259	0.10402	2.5091	0.0060515	1.259	0.10402	1.259	0.10402	0.0060515	0.317914106
SOCAR	ENSG00000240350	-0.060958	0.5243	2.2832	0.011209	2.5089	0.0060551	-0.49998	0.69145	0.0060551	0.317914106
EPN1	ENSG00000063245	2.1405	0.016157	2.5085	0.0060616	2.5085	0.0060616	1.9662	0.024636	0.0060616	0.317914106
TRAF4	ENSG00000076604	2.5084	0.006064	2.069	0.019272	2.2387	0.012587	2.069	0.019272	0.006064	0.317914106

TMEM176B	ENSG00000106565	0.58853	0.27809	0.58853	0.27809	0.58853	0.27809	2.5083	0.0060659	0.0060659	0.317914106
NA	ENSG00000260121	2.5073	0.0060831	2.5073	0.0060831	2.5073	0.0060831	2.5073	0.0060831	0.0060831	0.317914106
PCDHGB5	ENSG00000276547	2.5071	0.0060856	0.76044	0.2235	0.76044	0.2235	0.76044	0.2235	0.0060856	0.317914106
BNC2-AS1	ENSG00000234779	1.8751	0.030392	1.4002	0.080724	2.5066	0.0060951	2.5066	0.0060951	0.0060951	0.317914106
LINC02310	ENSG00000258808	2.5059	0.0061069	1.7253	0.042239	2.5059	0.0061069	1.1361	0.12796	0.0061069	0.317914106
MIR548N	ENSG00000221669	1.2817	0.099966	1.4692	0.070893	2.5056	0.006112	2.5056	0.006112	0.006112	0.317914106
LINC01361	ENSG00000236268	0.51464	0.3034	1.4545	0.072905	0.61043	0.27079	2.5052	0.0061197	0.0061197	0.317914106
GALK1	ENSG00000108479	2.3144	0.010323	1.9559	0.02524	2.1487	0.01583	2.5051	0.006121	0.006121	0.317914106
FAM136BP	ENSG00000232654	0.96589	0.16705	2.505	0.0061226	0.96589	0.16705	0.96589	0.16705	0.0061226	0.317914106
NA	ENSG00000200059	2.5044	0.006133	-0.14447	0.55743	2.5044	0.006133	2.5044	0.006133	0.006133	0.317914106
NA	ENSG00000225625	2.5043	0.0061341	1.921	0.027368	2.5043	0.0061341	2.5043	0.0061341	0.0061341	0.317914106
NA	ENSG00000259353	2.5039	0.006142	2.5039	0.006142	2.5039	0.006142	2.5039	0.006142	0.006142	0.317914106
CDC27	ENSG00000004897	2.5038	0.0061436	2.315	0.010306	1.6357	0.050951	2.2673	0.011686	0.0061436	0.317914106
MYH8	ENSG00000133020	1.6661	0.047851	2.5037	0.0061456	1.6661	0.047851	-0.29377	0.61553	0.0061456	0.317914106
RNA5SP490	ENSG00000252950	2.5026	0.0061637	1.0656	0.1433	-0.82351	0.79489	-0.88896	0.81299	0.0061637	0.318303947
NA	ENSG00000233293	1.04	0.14916	1.04	0.14916	1.95	0.025588	2.5025	0.0061662	0.0061662	0.318303947
PLOD3	ENSG00000106397	2.4999	0.0062106	2.3713	0.008864	1.9264	0.027025	2.4999	0.0062106	0.0062106	0.319724374
LINC00399	ENSG00000229792	2.4999	0.0062107	2.4999	0.0062107	2.4999	0.0062107	2.4999	0.0062107	0.0062107	0.319724374
UBE2V1P5	ENSG00000226582	2.4998	0.0062134	2.4998	0.0062134	0.18143	0.42801	0.0068746	0.49726	0.0062134	0.319724374
CRYM-AS1	ENSG00000189149	2.499	0.0062267	2.499	0.0062267	2.499	0.0062267	1.2001	0.11506	0.0062267	0.320070771
NA	ENSG00000229996	2.4982	0.0062405	0.58291	0.27998	2.4982	0.0062405	2.4982	0.0062405	0.0062405	0.32033698
RTN4RL1	ENSG00000185924	2.4977	0.006251	0.8328	0.20248	0.87977	0.18949	1.9268	0.027	0.006251	0.32033698
CDC42BPB	ENSG00000198752	1.293	0.098012	-0.62003	0.73238	0.55249	0.29031	2.4976	0.0062516	0.0062516	0.32033698
RPSAP24	ENSG00000235414	2.4968	0.0062657	2.4968	0.0062657	2.4968	0.0062657	1.7048	0.044119	0.0062657	0.320363955
NA	ENSG00000255723	2.4959	0.0062811	2.4959	0.0062811	2.4959	0.0062811	2.4959	0.0062811	0.0062811	0.320363955
REC114	ENSG00000183324	2.1544	0.015603	2.4958	0.0062833	2.0034	0.022566	2.1544	0.015603	0.0062833	0.320363955
CARD14	ENSG00000141527	2.0239	0.021489	0.7855	0.21608	2.4956	0.0062876	0.75667	0.22462	0.0062876	0.320363955
RNU6ATAC12P	ENSG00000252351	2.4955	0.0062886	2.4955	0.0062886	2.4955	0.0062886	2.4955	0.0062886	0.0062886	0.320363955
SILC1	ENSG00000232044	1.0761	0.14094	1.7453	0.040463	-0.17616	0.56992	2.4947	0.0063038	0.0063038	0.320363955
RGS2	ENSG00000116741	2.453	0.0070841	2.453	0.0070841	2.453	0.0070841	2.4944	0.0063085	0.0063085	0.320363955
STRN4	ENSG00000090372	1.2008	0.11491	0.8555	0.19614	0.71692	0.23671	2.4941	0.0063132	0.0063132	0.320363955

GRIN2A	ENSG00000183454	0.22381	0.41145	2.4931	0.0063317	1.3304	0.0917	1.1999	0.11509	0.0063317	0.320363955
LHX9	ENSG00000143355	2.0097	0.022234	2.4923	0.0063461	2.3021	0.010665	1.8762	0.030311	0.0063461	0.320363955
TPMT	ENSG00000137364	1.5482	0.060792	0.81113	0.20865	2.3886	0.0084571	2.4918	0.0063543	0.0063543	0.320363955
EDEM3	ENSG00000116406	1.4538	0.072995	1.4144	0.078625	0.60361	0.27305	2.4917	0.0063573	0.0063573	0.320363955
INTS14	ENSG00000138614	1.5725	0.057919	0.13238	0.44734	2.4916	0.0063593	1.5112	0.065365	0.0063593	0.320363955
TEKT1	ENSG00000167858	2.4915	0.0063598	-0.53203	0.70265	-0.53203	0.70265	-0.86885	0.80753	0.0063598	0.320363955
EBLN2	ENSG00000255423	2.4899	0.0063896	2.4899	0.0063896	2.4899	0.0063896	2.4899	0.0063896	0.0063896	0.320363955
SLC26A6	ENSG00000225697	2.1189	0.01705	2.4891	0.0064026	2.1456	0.015953	1.9195	0.027458	0.0064026	0.320363955
NA	ENSG00000233569	0.72863	0.23311	2.489	0.0064055	0.72863	0.23311	0.72863	0.23311	0.0064055	0.320363955
NOXRED1	ENSG00000165555	2.4888	0.0064082	2.4888	0.0064082	2.4888	0.0064082	0.77939	0.21787	0.0064082	0.320363955
KAT8	ENSG00000103510	1.256	0.10455	2.4885	0.006415	0.81875	0.20646	1.9459	0.025832	0.006415	0.320363955
TMEM233	ENSG00000224982	0.64065	0.26088	1.5376	0.062075	2.4884	0.0064166	1.092	0.13742	0.0064166	0.320363955
PRTG	ENSG00000166450	-0.54741	0.70795	2.4883	0.0064184	-0.50318	0.69258	0.22198	0.41217	0.0064184	0.320363955
NA	ENSG00000251054	-0.44557	0.67205	2.4881	0.0064214	0.060915	0.47571	-0.15292	0.56077	0.0064214	0.320363955
KIAA0408	ENSG00000189367	2.4881	0.0064216	-1.2064	0.88617	2.4881	0.0064216	-0.23915	0.59451	0.0064216	0.320363955
NA	ENSG00000270000	1.2001	0.11505	2.1557	0.015555	2.4881	0.0064222	2.4081	0.008018	0.0064222	0.320363955
ROR1-AS1	ENSG00000223949	2.4878	0.0064266	2.4404	0.0073363	0.72853	0.23315	2.2626	0.01183	0.0064266	0.320363955
NA	ENSG00000233577	2.4874	0.0064331	1.611	0.053589	-0.25301	0.59987	-0.52805	0.70127	0.0064331	0.320363955
NA	ENSG00000273271	2.4871	0.0064393	2.4871	0.0064393	2.4871	0.0064393	2.4871	0.0064393	0.0064393	0.320363955
STK40	ENSG00000196182	2.1588	0.015433	0.0046454	0.49815	2.4869	0.0064431	-0.21965	0.58693	0.0064431	0.320363955
PCDH1	ENSG00000156453	2.4861	0.0064578	1.0267	0.15229	-0.023313	0.5093	-0.1727	0.56856	0.0064578	0.320363955
NA	ENSG00000264179	2.3227	0.010097	2.4861	0.0064578	2.2961	0.010835	2.4861	0.0064578	0.0064578	0.320363955
NPTN-IT1	ENSG00000281183	2.4856	0.0064662	2.4856	0.0064662	2.4856	0.0064662	2.4856	0.0064662	0.0064662	0.320363955
NA	ENSG00000228506	2.4853	0.0064727	2.4853	0.0064727	2.4853	0.0064727	2.4853	0.0064727	0.0064727	0.320363955
EYA2	ENSG00000064655	0.78557	0.21606	0.86885	0.19246	2.4849	0.0064787	1.0451	0.14799	0.0064787	0.320363955
TRAJ7	ENSG00000211882	2.4847	0.0064834	2.4847	0.0064834	2.4847	0.0064834	2.4847	0.0064834	0.0064834	0.320363955
SLC22A7	ENSG00000137204	0.16148	0.43586	2.4844	0.0064879	0.19265	0.42362	2.2436	0.012429	0.0064879	0.320363955
NA	ENSG00000254802	1.572	0.057975	1.572	0.057975	2.4844	0.0064888	0.40607	0.34234	0.0064888	0.320363955
H1-6	ENSG00000187475	0.53838	0.29516	0.53838	0.29516	0.28107	0.38933	2.4832	0.0065111	0.0065111	0.320998426
CPSF4L	ENSG00000187959	0.61065	0.27071	2.4824	0.0065241	0.31156	0.37769	-0.052107	0.52078	0.0065241	0.320998426
CKB	ENSG00000166165	0.4449	0.3282	-0.33982	0.633	2.4824	0.0065256	1.3334	0.091196	0.0065256	0.320998426

PMS2P3	ENSG00000127957	2.4822	0.006528	0.13364	0.44684	1.1369	0.12778	0.48575	0.31357	0.006528	0.320998426
XPO4	ENSG00000132953	0.56489	0.28607	1.5322	0.062736	-0.62791	0.73497	2.4811	0.0065482	0.0065482	0.321020507
RNU6-294P	ENSG00000201869	1.3986	0.080969	1.8843	0.029762	2.4087	0.0080046	2.4811	0.0065483	0.0065483	0.321020507
SLC4A1	ENSG00000004939	2.0599	0.019704	1.9558	0.025247	2.4811	0.0065485	2.4811	0.0065485	0.0065485	0.321020507
NA	ENSG00000274549	2.4808	0.0065548	0.4009	0.34425	-0.007518	0.503	-0.007518	0.503	0.0065548	0.321020507
NA	ENSG00000229403	-0.38977	0.65165	-0.38977	0.65165	2.4803	0.0065637	-0.38977	0.65165	0.0065637	0.321133636
RPS6KB1	ENSG00000108443	0.18967	0.42478	2.4793	0.0065823	0.83526	0.20179	1.1473	0.12564	0.0065823	0.321466634
ADAR	ENSG00000160710	0.89025	0.18666	1.2947	0.097708	2.4792	0.0065837	0.32293	0.37337	0.0065837	0.321466634
NA	ENSG00000226576	2.4781	0.0066046	2.4781	0.0066046	2.4781	0.0066046	2.4781	0.0066046	0.0066046	0.322045968
RNU6-409P	ENSG00000206841	-0.51485	0.69667	2.4776	0.0066127	1.7574	0.039425	0.73132	0.23229	0.0066127	0.322045968
LRRC37A12P	ENSG00000229259	2.4774	0.0066179	0.55314	0.29008	1.0706	0.14218	-0.36006	0.6406	0.0066179	0.322045968
LINC01031	ENSG00000232077	2.3842	0.0085584	-0.54029	0.7055	2.4771	0.006622	1.0735	0.14152	0.006622	0.322045968
LMO1	ENSG00000166407	2.3631	0.0090624	2.4766	0.0066324	2.1143	0.017247	2.2477	0.012298	0.0066324	0.322230162
PPIF	ENSG00000108179	2.4754	0.0066537	1.6825	0.046238	1.4082	0.079543	2.1439	0.016022	0.0066537	0.322899096
RASA4DP	ENSG00000233297	2.4751	0.0066604	-0.44717	0.67262	-0.67142	0.74902	-0.67713	0.75084	0.0066604	0.322899096
NA	ENSG00000268861	2.4747	0.0066666	2.4747	0.0066666	2.4747	0.0066666	2.4747	0.0066666	0.0066666	0.322899096
NDUFA8	ENSG00000119421	-1.6243	0.94785	2.4743	0.0066749	-0.57299	0.71667	0.095213	0.46207	0.0066749	0.322899096
COL5A1	ENSG00000130635	2.4741	0.0066793	0.65578	0.25598	2.3898	0.0084284	1.1849	0.11803	0.0066793	0.322899096
NA	ENSG00000238516	2.4726	0.0067058	2.4726	0.0067058	2.4726	0.0067058	2.4726	0.0067058	0.0067058	0.323858904
CFHR5	ENSG00000134389	1.957	0.025175	2.4722	0.0067144	0.93639	0.17454	0.93639	0.17454	0.0067144	0.32395318
NA	ENSG00000206679	2.471	0.0067362	2.471	0.0067362	2.471	0.0067362	2.3347	0.0097798	0.0067362	0.324683507
MIR4269	ENSG00000265215	1.3492	0.088632	2.4701	0.0067536	0.045908	0.48169	-1.4324	0.92399	0.0067536	0.325200522
LINC02762	ENSG00000250303	2.4692	0.0067711	-0.86324	0.806	2.1435	0.016038	1.7731	0.038104	0.0067711	0.325721326
NA	ENSG00000239572	2.4677	0.0067991	-0.45346	0.67489	-1.4305	0.92372	-1.1443	0.87376	0.0067991	0.326078927
NA	ENSG00000207386	2.4676	0.0068009	2.4676	0.0068009	2.4676	0.0068009	2.4676	0.0068009	0.0068009	0.326078927
NA	ENSG00000270279	2.4675	0.0068028	0.79454	0.21344	2.4675	0.0068028	2.4675	0.0068028	0.0068028	0.326078927
NA	ENSG00000244313	1.9729	0.024252	1.1936	0.11632	2.4674	0.0068053	2.4674	0.0068053	0.0068053	0.326078927
JAGN1	ENSG00000171135	2.4668	0.0068164	2.4668	0.0068164	0.97865	0.16388	2.4668	0.0068164	0.0068164	0.326289953
YWHAH	ENSG00000128245	2.4664	0.0068237	2.4664	0.0068237	-0.29484	0.61594	-0.19748	0.57828	0.0068237	0.326318843
ENAM	ENSG00000132464	2.4654	0.0068425	0.67002	0.25142	1.6947	0.045067	1.006	0.1572	0.0068425	0.326897083
NA	ENSG00000249857	-0.80937	0.79085	-0.58747	0.72156	-1.0893	0.862	2.4649	0.0068521	0.0068521	0.326972578

OSER1-DT	ENSG00000223891	2.4646	0.0068575	0.53828	0.29519	0.1851	0.42658	0.026624	0.48938	0.0068575	0.326972578
NA	ENSG00000270702	2.2445	0.012401	1.7617	0.039062	2.4635	0.0068798	2.4635	0.0068798	0.0068798	0.327642627
ALG14	ENSG00000172339	-0.46751	0.67993	-0.54999	0.70884	-0.40464	0.65713	2.4632	0.006885	0.006885	0.327642627
MAMDC4	ENSG00000177943	2.4628	0.0068929	-0.25567	0.6009	-0.25567	0.6009	-0.25567	0.6009	0.0068929	0.327698553
HAUS3	ENSG00000214367	2.1747	0.014827	2.4609	0.0069295	1.1375	0.12767	2.1426	0.016074	0.0069295	0.329117481
MIR548AI	ENSG00000265226	2.4598	0.0069499	1.1955	0.11594	2.4598	0.0069499	2.4598	0.0069499	0.0069499	0.329337135
HMHB1	ENSG00000158497	2.4598	0.0069509	2.4414	0.0073161	1.3817	0.083524	-0.58218	0.71978	0.0069509	0.329337135
EXOSC6	ENSG00000223496	2.4596	0.0069544	2.4596	0.0069544	2.4596	0.0069544	2.4596	0.0069544	0.0069544	0.329337135
TMC3	ENSG00000188869	-0.66369	0.74656	1.2816	0.099985	2.4586	0.0069738	1.6155	0.053106	0.0069738	0.329338674
TREML5P	ENSG00000225690	1.6342	0.051107	2.4584	0.0069786	0.64395	0.2598	-0.47016	0.68088	0.0069786	0.329338674
NR1D2	ENSG00000174738	0.010886	0.49566	-0.93412	0.82488	2.458	0.0069859	-0.95427	0.83003	0.0069859	0.329338674
ZNF695	ENSG00000197472	-0.70814	0.76057	-0.35774	0.63973	0.89175	0.18626	2.4576	0.0069933	0.0069933	0.329338674
RNU7-121P	ENSG00000252413	2.4576	0.0069939	2.4576	0.0069939	2.4576	0.0069939	2.4303	0.0075442	0.0069939	0.329338674
NA	ENSG00000253577	1.5601	0.059365	1.5601	0.059365	2.4574	0.0069972	1.5601	0.059365	0.0069972	0.329338674
IL9RP3	ENSG00000226942	2.457	0.0070045	-0.8017	0.78864	-0.42333	0.66397	0.62327	0.26655	0.0070045	0.329338674
NA	ENSG00000248840	0.15594	0.43804	2.4568	0.0070085	0.83404	0.20213	0.83404	0.20213	0.0070085	0.329338674
HTATSF1P2	ENSG00000271361	2.4543	0.0070574	2.4543	0.0070574	2.4543	0.0070574	2.4543	0.0070574	0.0070574	0.330792052
NA	ENSG00000212598	-0.073336	0.52923	2.4531	0.0070819	2.0568	0.019854	-0.073336	0.52923	0.0070819	0.330792052
NR1H3	ENSG00000025434	2.2611	0.011876	2.4526	0.0070914	2.4526	0.0070914	2.1268	0.016717	0.0070914	0.330792052
HOXA-AS3	ENSG00000254369	1.4783	0.069658	2.169	0.015042	2.4525	0.0070943	2.4525	0.0070943	0.0070943	0.330792052
SNX8	ENSG00000106266	1.618	0.052827	1.1267	0.12993	1.618	0.052827	2.4523	0.0070966	0.0070966	0.330792052
TM9SF4	ENSG00000101337	0.82038	0.206	1.7157	0.04311	0.26049	0.39724	2.4523	0.0070977	0.0070977	0.330792052
WWC1	ENSG00000113645	0.82644	0.20428	2.4519	0.0071043	2.1625	0.01529	1.5754	0.057588	0.0071043	0.330792052
ANKZF1	ENSG00000163516	1.9776	0.023989	2.3409	0.0096198	1.9214	0.027341	2.4517	0.0071094	0.0071094	0.330792052
TIMM17BP1	ENSG00000256756	2.4081	0.008018	0.19502	0.42269	2.4516	0.0071117	1.3308	0.091632	0.0071117	0.330792052
NA	ENSG00000199964	0.71886	0.23611	0.30825	0.37895	0.023271	0.49072	2.4512	0.0071186	0.0071186	0.330792052
LINC02071	ENSG00000249383	0.2634	0.39612	1.1107	0.13335	0.82411	0.20494	2.451	0.0071239	0.0071239	0.330792052
FLOT2	ENSG00000132589	1.8484	0.032276	0.76494	0.22215	1.7295	0.041861	2.4509	0.0071255	0.0071255	0.330792052
NA	ENSG00000261777	2.0301	0.021173	2.0301	0.021173	2.4502	0.0071393	1.6655	0.047906	0.0071393	0.330792052
PCGF6	ENSG00000156374	0.33328	0.36946	2.4501	0.0071409	0.75305	0.22571	-0.011678	0.50466	0.0071409	0.330792052
DHRS13	ENSG00000167536	2.4494	0.0071551	2.4494	0.0071551	2.4494	0.0071551	2.4494	0.0071551	0.0071551	0.330792052

FAM3C	ENSG00000196937	2.4494	0.0071553	1.3696	0.085406	2.2329	0.012776	1.266	0.10276	0.0071553	0.330792052
NA	ENSG00000264081	-0.37031	0.64442	2.4491	0.0071608	0.0077167	0.49692	-0.11573	0.54607	0.0071608	0.330792052
NA	ENSG00000227282	2.4482	0.0071782	0.5293	0.2983	-0.6341	0.73699	-0.30528	0.61992	0.0071782	0.330792052
NA	ENSG00000280242	2.4476	0.0071914	1.6525	0.049216	1.7507	0.039995	1.8673	0.030929	0.0071914	0.330792052
PAQR3	ENSG00000163291	-0.29648	0.61657	2.4473	0.0071965	0.092224	0.46326	1.0342	0.15052	0.0071965	0.330792052
ATP5PD	ENSG00000167863	-0.21045	0.58334	-0.21045	0.58334	-0.21045	0.58334	2.4472	0.007198	0.007198	0.330792052
DVL3	ENSG00000161202	2.4471	0.0071998	0.23735	0.40619	-0.48242	0.68525	-0.068102	0.52715	0.0071998	0.330792052
RNU6-663P	ENSG00000212407	2.4463	0.0072157	2.4463	0.0072157	2.4463	0.0072157	2.4463	0.0072157	0.0072157	0.330792052
REPIN1	ENSG00000214022	2.1489	0.01582	2.4462	0.0072195	2.3951	0.0083072	2.3812	0.0086282	0.0072195	0.330792052
ROBO2P1	ENSG00000229478	-0.20632	0.58173	-0.56034	0.71238	2.4459	0.0072252	2.4459	0.0072252	0.0072252	0.330792052
PRRX2-AS1	ENSG00000236024	-1.1391	0.87267	1.5496	0.060623	2.4458	0.0072275	-0.069721	0.52779	0.0072275	0.330792052
ZBTB42	ENSG00000179627	2.4457	0.0072279	0.94611	0.17205	0.33175	0.37004	-0.71278	0.76201	0.0072279	0.330792052
ITGA9-AS1	ENSG00000235257	2.4457	0.0072295	1.8188	0.034469	2.2652	0.01175	1.7593	0.039261	0.0072295	0.330792052
NA	ENSG00000233665	2.4449	0.0072453	2.4449	0.0072453	2.4449	0.0072453	1.2131	0.11255	0.0072453	0.330939268
NA	ENSG00000249960	-0.81424	0.79224	-0.81424	0.79224	2.4448	0.0072463	-0.81424	0.79224	0.0072463	0.330939268
TEN1-CDK3	ENSG00000261408	2.4436	0.0072709	2.4436	0.0072709	2.4436	0.0072709	2.4436	0.0072709	0.0072709	0.331518988
NA	ENSG00000219314	1.4608	0.072038	1.4608	0.072038	-0.66739	0.74774	2.4435	0.0072726	0.0072726	0.331518988
NUB1	ENSG00000013374	-0.95503	0.83022	0.74034	0.22955	2.4431	0.007281	0.90163	0.18363	0.007281	0.331539969
NA	ENSG00000202508	2.4426	0.0072901	2.4426	0.0072901	0.57092	0.28403	1.4487	0.073705	0.0072901	0.331539969
NME1-NME2	ENSG00000011052	2.2166	0.013324	2.2835	0.011199	2.4419	0.0073048	2.4419	0.0073048	0.0073048	0.331539969
SDHAF4	ENSG00000154079	1.2259	0.11012	1.2259	0.11012	0.97255	0.16539	2.4416	0.0073108	0.0073108	0.331539969
GAPDHP23	ENSG00000235595	1.1449	0.12612	2.4411	0.007321	2.4411	0.007321	2.4411	0.007321	0.007321	0.331539969
HMGB3P26	ENSG00000259428	0.083117	0.46688	2.2205	0.013191	0.25603	0.39896	2.4407	0.0073289	0.0073289	0.331539969
TTPAL	ENSG00000124120	2.4402	0.0073391	2.4402	0.0073391	2.4402	0.0073391	1.7571	0.039451	0.0073391	0.331539969
MRPL43	ENSG00000055950	1.8935	0.029149	1.8935	0.029149	2.4401	0.0073409	1.1402	0.1271	0.0073409	0.331539969
TRIM58	ENSG00000162722	0.87532	0.1907	2.4401	0.007341	-0.70384	0.75923	-0.8553	0.80381	0.007341	0.331539969
NA	ENSG00000267898	2.44	0.0073426	2.44	0.0073426	2.44	0.0073426	2.44	0.0073426	0.0073426	0.331539969
TPRX1	ENSG00000178928	2.4398	0.0073479	2.4398	0.0073479	2.4398	0.0073479	1.9833	0.023665	0.0073479	0.331539969
NA	ENSG00000234360	2.4392	0.0073609	2.4392	0.0073609	2.4392	0.0073609	2.4392	0.0073609	0.0073609	0.331819294
ITPKA	ENSG00000137825	2.4383	0.0073773	-0.51738	0.69755	-0.51738	0.69755	-0.03328	0.51327	0.0073773	0.332251228
CHML	ENSG00000203668	1.63	0.051549	2.4373	0.0073994	2.4373	0.0073994	1.6921	0.045314	0.0073994	0.332761404

NA	ENSG00000224410	0.99247	0.16048	2.4371	0.0074028	0.16489	0.43451	0.16489	0.43451	0.0074028	0.332761404
NPC2	ENSG00000119655	2.2829	0.011217	1.0749	0.14121	1.1985	0.11535	2.4367	0.0074108	0.0074108	0.332761404
FGF12	ENSG00000114279	1.4792	0.069549	2.4364	0.0074179	-0.33773	0.63222	-0.052005	0.52074	0.0074179	0.332761404
LINC01939	ENSG00000228799	-0.78964	0.78513	-0.85431	0.80353	2.436	0.0074248	-0.94333	0.82724	0.0074248	0.332761404
NA	ENSG00000232675	2.4358	0.0074296	2.2547	0.012076	2.4358	0.0074296	2.4358	0.0074296	0.0074296	0.332761404
HSPB11	ENSG00000081870	-0.738	0.76974	2.4349	0.0074485	1.1452	0.12606	1.1072	0.1341	0.0074485	0.333007688
STUM	ENSG00000203685	-0.32241	0.62643	1.0871	0.1385	0.056711	0.47739	2.4348	0.0074497	0.0074497	0.333007688
ABCG5	ENSG00000138075	-0.45564	0.67568	1.1	0.13568	2.4345	0.0074556	2.4345	0.0074556	0.0074556	0.333007688
MIRLET7BHG	ENSG00000197182	0.32462	0.37273	0.49779	0.30932	2.434	0.0074667	1.144	0.1263	0.0074667	0.333198069
OR4K12P	ENSG00000218549	2.4326	0.0074958	1.1734	0.12033	1.4356	0.075559	0.06915	0.47244	0.0074958	0.334190608
KCNJ2	ENSG00000123700	2.4315	0.0075181	0.15384	0.43887	-1.1342	0.87164	-0.69792	0.75738	0.0075181	0.33487844
RPS3AP30	ENSG00000228744	0.51711	0.30254	-0.064774	0.52582	2.4309	0.0075302	0.42778	0.33441	0.0075302	0.335005411
NA	ENSG00000270160	0.26268	0.3964	2.4307	0.0075347	0.89774	0.18466	0.89774	0.18466	0.0075347	0.335005411
OR7E55P	ENSG00000230655	-1.2416	0.89281	2.4299	0.0075511	1.5283	0.063221	1.6211	0.052496	0.0075511	0.335428535
STX4	ENSG00000103496	2.4292	0.0075664	1.4452	0.074203	2.4219	0.0077193	1.8305	0.033588	0.0075664	0.335802069
ZNF299P	ENSG00000219368	2.4286	0.0075785	2.4286	0.0075785	2.4286	0.0075785	2.4286	0.0075785	0.0075785	0.336033035
NA	ENSG00000283913	2.4277	0.0075966	0.32541	0.37244	0.46244	0.32188	0.46244	0.32188	0.0075966	0.33652938
EFCAB13	ENSG00000178852	1.2474	0.10612	2.4263	0.0076264	1.857	0.031653	1.3331	0.091254	0.0076264	0.337445328
NA	ENSG00000253184	2.4256	0.0076421	0.81393	0.20784	0.023336	0.49069	0.11508	0.45419	0.0076421	0.337445328
LINC02212	ENSG00000249396	2.4254	0.0076454	2.2904	0.010999	2.4254	0.0076454	2.4254	0.0076454	0.0076454	0.337445328
AARS1	ENSG00000090861	1.9241	0.027171	1.9272	0.02698	0.72329	0.23475	2.4253	0.0076472	0.0076472	0.337445328
SBSN	ENSG00000189001	2.4251	0.0076519	1.3888	0.082452	2.4251	0.0076519	2.4251	0.0076519	0.0076519	0.337445328
NA	ENSG00000272647	2.4241	0.0076726	2.4241	0.0076726	2.4241	0.0076726	2.4241	0.0076726	0.0076726	0.337693198
USP6	ENSG00000129204	0.13161	0.44765	-0.81578	0.79269	2.4241	0.0076735	1.1052	0.13453	0.0076735	0.337693198
NA	ENSG00000270624	2.4232	0.0076923	0.16922	0.43281	0.033438	0.48666	-0.063663	0.52538	0.0076923	0.337693198
NA	ENSG00000212397	-0.29626	0.61648	-0.29626	0.61648	2.4231	0.0076944	-0.29626	0.61648	0.0076944	0.337693198
NA	ENSG00000133808	0.29501	0.38399	2.4229	0.0076986	0.25639	0.39883	-0.7466	0.77235	0.0076986	0.337693198
TCF4-AS2	ENSG00000267402	2.4229	0.0076991	2.4229	0.0076991	2.4229	0.0076991	2.3799	0.0086588	0.0076991	0.337693198
HOXB-AS2	ENSG00000239552	2.4217	0.007724	2.4217	0.007724	2.4217	0.007724	2.4217	0.007724	0.007724	0.338344714
TRRAP	ENSG00000196367	2.1178	0.017095	2.1325	0.016483	2.1122	0.017336	2.4212	0.007735	0.007735	0.338344714
BRINP1	ENSG00000078725	1.7015	0.044425	0.44077	0.32969	2.4211	0.0077367	2.2904	0.010998	0.0077367	0.338344714

PHC2	ENSG00000134686	2.049	0.02023	2.4204	0.0077509	1.7315	0.04168	1.8071	0.035376	0.0077509	0.338344714
VEZF1P1	ENSG00000236229	0.15868	0.43696	2.4201	0.0077572	1.7423	0.04073	1.7423	0.04073	0.0077572	0.338344714
SH3RF3-AS1	ENSG00000259863	1.0213	0.15355	2.4197	0.0077675	0.6453	0.25937	0.6453	0.25937	0.0077675	0.338344714
NA	ENSG00000237845	2.4196	0.0077688	2.4196	0.0077688	2.4196	0.0077688	2.4196	0.0077688	0.0077688	0.338344714
KHDRBS1	ENSG00000121774	2.4196	0.0077695	0.26521	0.39542	0.26521	0.39542	-0.66144	0.74584	0.0077695	0.338344714
CNRIP1	ENSG00000119865	-0.41221	0.65991	0.1792	0.42889	2.419	0.0077809	2.3754	0.0087646	0.0077809	0.338353995
IQCF4P	ENSG00000224792	2.4189	0.0077836	1.7507	0.039999	1.1007	0.13552	1.8672	0.030939	0.0077836	0.338353995
NOD1	ENSG00000106100	2.4185	0.0077913	2.3564	0.0092275	0.61119	0.27054	0.71023	0.23878	0.0077913	0.338386853
FLI1	ENSG00000151702	2.0448	0.020439	-0.042113	0.5168	2.4175	0.0078129	0.95793	0.16905	0.0078129	0.338781885
OVGP1	ENSG00000085465	2.4175	0.0078143	1.4598	0.072166	1.1486	0.12536	1.1486	0.12536	0.0078143	0.338781885
FILNC1	ENSG00000231426	2.4163	0.0078404	2.3028	0.010646	-0.03027	0.51207	-0.03027	0.51207	0.0078404	0.339611282
DENND4A	ENSG00000174485	0.58349	0.27978	0.0080187	0.4968	0.33121	0.37024	2.4149	0.0078704	0.0078704	0.340409002
CRYBB2P1	ENSG00000100058	0.54798	0.29185	0.35865	0.35993	2.4137	0.0078957	0.23836	0.4058	0.0078957	0.340409002
MFSD1	ENSG00000118855	2.4129	0.0079132	1.3408	0.089995	-0.55194	0.70951	1.3365	0.090689	0.0079132	0.340409002
NA	ENSG00000243969	0.15575	0.43811	0.48823	0.3127	0.81353	0.20796	2.4125	0.0079208	0.0079208	0.340409002
NA	ENSG00000223271	2.4125	0.0079212	2.4125	0.0079212	2.4125	0.0079212	2.4125	0.0079212	0.0079212	0.340409002
SCARNA14	ENSG00000252712	2.4125	0.0079212	2.4125	0.0079212	2.4125	0.0079212	2.4125	0.0079212	0.0079212	0.340409002
GPD1	ENSG00000167588	2.4121	0.0079297	0.45944	0.32296	2.4121	0.0079297	-0.30562	0.62005	0.0079297	0.340409002
RNU2-40P	ENSG00000222765	2.4121	0.0079305	0.51454	0.30344	-0.50631	0.69368	0.423	0.33615	0.0079305	0.340409002
GPR68	ENSG00000119714	0.073905	0.47054	0.073905	0.47054	2.4118	0.0079368	0.94774	0.17163	0.0079368	0.340409002
NFIA-AS1	ENSG00000237853	2.4117	0.0079398	2.4117	0.0079398	2.4117	0.0079398	2.4117	0.0079398	0.0079398	0.340409002
NA	ENSG00000278897	2.0711	0.019173	2.4111	0.0079513	2.0498	0.020194	2.0498	0.020194	0.0079513	0.340409002
NKX2-1	ENSG00000136352	2.4106	0.0079623	2.4106	0.0079623	1.1454	0.12601	2.4106	0.0079623	0.0079623	0.340409002
PNPT1P2	ENSG00000233272	2.4101	0.007974	2.4101	0.007974	1.0379	0.14966	2.4101	0.007974	0.007974	0.340409002
ATP5MC1P7	ENSG00000232734	0.24961	0.40145	2.4099	0.0079778	2.4099	0.0079778	1.219	0.11143	0.0079778	0.340409002
IL17RA	ENSG00000177663	-1.1474	0.8744	-0.78582	0.78401	2.4099	0.007979	-0.2797	0.61015	0.007979	0.340409002
BCL6	ENSG00000113916	0.93862	0.17396	1.367	0.085816	2.4095	0.0079869	-0.055432	0.5221	0.0079869	0.340409002
LINC01312	ENSG00000223586	-0.51543	0.69687	1.9776	0.023985	2.4093	0.0079906	1.487	0.068514	0.0079906	0.340409002
MADD	ENSG00000110514	2.4091	0.0079968	2.1954	0.014066	1.8526	0.031973	2.3767	0.0087337	0.0079968	0.340409002
SPEG	ENSG00000072195	2.2028	0.013803	2.409	0.0079987	2.1639	0.015238	2.131	0.016543	0.0079987	0.340409002
MRPL35P1	ENSG00000220868	2.409	0.0079991	2.028	0.02128	2.409	0.0079991	-0.25959	0.60241	0.0079991	0.340409002

LINC00365	ENSG00000224511	0.7595	0.22378	2.4086	0.0080076	1.4503	0.073486	1.2841	0.099561	0.0080076	0.340409002
MEF2C	ENSG00000081189	0.78605	0.21592	0.77336	0.21965	2.4083	0.0080125	0.75565	0.22493	0.0080125	0.340409002
DIRAS3	ENSG00000162595	-1.5185	0.93555	2.4075	0.0080312	-1.5185	0.93555	-1.5185	0.93555	0.0080312	0.3408725
MTCYBP21	ENSG00000224747	2.4072	0.0080374	2.4072	0.0080374	2.4072	0.0080374	2.4072	0.0080374	0.0080374	0.3408725
LINC01414	ENSG00000253554	2.4067	0.0080478	1.6741	0.047056	2.0942	0.018122	2.2173	0.013301	0.0080478	0.341016777
EEF1A1P8	ENSG00000223529	0.46473	0.32106	0.83562	0.20169	2.4062	0.0080592	2.4062	0.0080592	0.0080592	0.341203142
WFDC1	ENSG00000103175	-1.3096	0.90483	-0.98798	0.83842	-0.98798	0.83842	2.4051	0.0080831	0.0080831	0.341400862
NA	ENSG00000202275	1.1422	0.12669	2.4051	0.0080832	2.4051	0.0080832	2.4051	0.0080832	0.0080832	0.341400862
C10orf71	ENSG00000177354	1.2081	0.11351	2.4047	0.0080939	1.2081	0.11351	1.2081	0.11351	0.0080939	0.341400862
RPS12P18	ENSG00000232965	2.4045	0.0080971	0.55563	0.28923	1.1124	0.13299	0.56573	0.28579	0.0080971	0.341400862
GATA2-AS1	ENSG00000244300	-0.0035817	0.50143	1.0269	0.15223	2.4044	0.0080989	2.4044	0.0080989	0.0080989	0.341400862
LILRB1-AS1	ENSG00000224730	2.4032	0.0081257	0.48063	0.31539	0.48063	0.31539	0.48063	0.31539	0.0081257	0.342023161
NSF	ENSG00000073969	1.2718	0.10172	0.51838	0.3021	2.4031	0.0081277	1.4421	0.074635	0.0081277	0.342023161
RNA5SP169	ENSG00000253077	2.4025	0.0081422	2.4025	0.0081422	2.4025	0.0081422	2.4025	0.0081422	0.0081422	0.34233771
LDHAL6EP	ENSG00000270098	1.0528	0.14621	1.044	0.14825	2.4019	0.0081552	2.4019	0.0081552	0.0081552	0.342588703
USP39	ENSG00000168883	1.6731	0.047154	1.2888	0.098734	0.5466	0.29233	2.4015	0.0081637	0.0081637	0.342650388
GTF2H1	ENSG00000110768	2.3995	0.0082079	2.3995	0.0082079	0.201	0.42035	2.364	0.0090405	0.0082079	0.344209094
NA	ENSG00000237271	2.3989	0.0082214	1.2009	0.11489	0.5754	0.28251	0.5754	0.28251	0.0082214	0.344241447
HMGB1P40	ENSG00000255291	-0.30398	0.61943	1.2137	0.11243	2.3989	0.0082228	2.3989	0.0082228	0.0082228	0.344241447
CST9L	ENSG00000101435	0.64482	0.25952	2.1209	0.016964	1.0209	0.15364	2.3975	0.0082536	0.0082536	0.345118638
EEF1A1P14	ENSG00000233057	2.397	0.0082642	-0.99744	0.84072	-0.99744	0.84072	-0.99744	0.84072	0.0082642	0.345118638
LINC00710	ENSG00000229240	2.397	0.008265	-0.51053	0.69516	2.397	0.008265	0.34456	0.36521	0.008265	0.345118638
OR6S1	ENSG00000181803	2.3956	0.0082974	2.3956	0.0082974	2.3956	0.0082974	2.3956	0.0082974	0.0082974	0.346174916
NA	ENSG00000276054	2.395	0.0083106	2.395	0.0083106	0.65134	0.25741	1.2024	0.1146	0.0083106	0.346212073
HSPA5P1	ENSG00000215895	-0.92462	0.82242	-0.25001	0.59871	0.36186	0.35873	2.3949	0.0083125	0.0083125	0.346212073
NA	ENSG00000249831	0.34179	0.36626	2.3941	0.0083315	1.6412	0.050373	0.16291	0.43529	0.0083315	0.346447859
OR2M1P	ENSG00000177233	-0.52023	0.69855	0.21876	0.41342	2.3932	0.008351	-0.71159	0.76164	0.008351	0.346447859
PROP1	ENSG00000175325	1.5648	0.058814	1.7819	0.037383	2.3926	0.0083641	-0.38152	0.64859	0.0083641	0.346447859
NA	ENSG00000270504	-0.24833	0.59806	-0.14912	0.55927	2.1741	0.014848	2.3923	0.0083716	0.0083716	0.346447859
NPFFR1	ENSG00000148734	2.3922	0.008373	0.93195	0.17568	1.0058	0.15726	1.4157	0.078432	0.008373	0.346447859
NA	ENSG00000275139	1.491	0.067981	2.3922	0.0083732	2.1404	0.016163	2.0175	0.021824	0.0083732	0.346447859

RNU6-326P	ENSG00000202350	0.73799	0.23026	-1.1612	0.87722	1.3036	0.09618	2.3921	0.0083761	0.0083761	0.346447859
MCL1	ENSG00000143384	0.091161	0.46368	1.0018	0.15821	2.3919	0.0083818	-0.4537	0.67498	0.0083818	0.346447859
RIN2	ENSG00000132669	-1.1542	0.87579	2.391	0.0084022	2.1998	0.013909	1.2519	0.10531	0.0084022	0.346447859
PRTFDC1	ENSG00000099256	2.3907	0.0084091	0.68705	0.24603	1.18	0.11899	0.8655	0.19338	0.0084091	0.346447859
MGA	ENSG00000174197	2.3902	0.0084204	1.5085	0.065719	1.9002	0.028703	0.96898	0.16628	0.0084204	0.346447859
RN7SL441P	ENSG00000241413	2.3893	0.0084393	-0.40428	0.657	-0.40428	0.657	-0.40428	0.657	0.0084393	0.346447859
LINC02166	ENSG00000260259	2.3891	0.0084446	2.3891	0.0084446	2.3891	0.0084446	2.3891	0.0084446	0.0084446	0.346447859
NDUFA12P1	ENSG00000225999	2.3887	0.0084545	0.86353	0.19392	1.1486	0.12537	0.69435	0.24373	0.0084545	0.346447859
NA	ENSG00000251675	2.3886	0.0084569	2.3886	0.0084569	2.3886	0.0084569	0.91575	0.1799	0.0084569	0.346447859
FSD1	ENSG00000105255	-0.18516	0.57345	1.1801	0.11897	2.3882	0.0084661	1.1801	0.11897	0.0084661	0.346447859
SULT2A1	ENSG00000105398	1.2335	0.1087	1.3691	0.085482	2.3879	0.0084723	-0.34957	0.63667	0.0084723	0.346447859
NA	ENSG00000273064	2.3879	0.0084725	1.5742	0.057716	2.3879	0.0084725	2.3879	0.0084725	0.0084725	0.346447859
ATP6V1G3	ENSG00000151418	2.3081	0.010497	2.3878	0.0084758	0.97646	0.16442	1.1239	0.13053	0.0084758	0.346447859
NA	ENSG00000268401	-0.47824	0.68376	-1.1136	0.86727	-0.47824	0.68376	2.3877	0.008476	0.008476	0.346447859
CLCP2	ENSG00000226158	2.3877	0.0084782	2.3877	0.0084782	2.3877	0.0084782	2.3877	0.0084782	0.0084782	0.346447859
NA	ENSG00000226739	-0.23159	0.59157	2.3876	0.0084796	0.97115	0.16574	0.81151	0.20854	0.0084796	0.346447859
FAM222A-AS1	ENSG00000255650	2.241	0.012512	1.7273	0.042054	2.3229	0.010091	2.3871	0.0084909	0.0084909	0.346447859
SH3RF3	ENSG00000172985	2.3868	0.008497	-0.86625	0.80682	1.8917	0.029266	0.43537	0.33165	0.008497	0.346447859
APAF1	ENSG00000120868	-0.36138	0.64109	2.3867	0.0084992	-0.46591	0.67936	1.6098	0.053724	0.0084992	0.346447859
NA	ENSG00000251171	-1.3832	0.9167	-1.3832	0.9167	2.3862	0.0085124	0.83992	0.20048	0.0085124	0.346447859
NA	ENSG00000235705	-0.8669	0.807	0.45931	0.32301	2.3858	0.0085213	0.46695	0.32027	0.0085213	0.346447859
LINC02323	ENSG00000259230	1.8994	0.028754	0.63967	0.26119	0.55864	0.28821	2.3857	0.0085223	0.0085223	0.346447859
BRWD1	ENSG00000185658	1.4831	0.069029	1.1828	0.11844	1.6181	0.052825	2.3853	0.0085322	0.0085322	0.346447859
PDCD11	ENSG00000148843	1.7452	0.040475	2.385	0.0085388	0.68562	0.24648	1.3983	0.081015	0.0085388	0.346447859
NA	ENSG00000253923	-0.2104	0.58332	2.3846	0.0085486	-0.2104	0.58332	-0.2104	0.58332	0.0085486	0.346447859
SLIRP	ENSG00000119705	2.3845	0.008552	1.2322	0.10894	1.2322	0.10894	1.2322	0.10894	0.008552	0.346447859
ZDHHC22	ENSG00000177108	0.77262	0.21987	-0.73913	0.77009	0.81258	0.20823	2.3842	0.0085582	0.0085582	0.346447859
NA	ENSG00000261351	2.043	0.020525	1.9347	0.026513	2.3836	0.008571	2.3836	0.008571	0.008571	0.346447859
NA	ENSG00000270442	0.22591	0.41064	2.383	0.0085864	0.22591	0.41064	0.22591	0.41064	0.0085864	0.346447859
NA	ENSG00000236724	-2.566	0.99486	2.3824	0.0086002	-2.566	0.99486	-2.566	0.99486	0.0086002	0.346447859
NA	ENSG00000253259	2.3823	0.0086025	0.83542	0.20174	0.55167	0.29059	0.18398	0.42701	0.0086025	0.346447859

AGAP1	ENSG00000157985	2.1876	0.014348	2.3822	0.0086044	1.4869	0.06852	1.4446	0.074281	0.0086044	0.346447859
CEP164P1	ENSG00000226937	2.0293	0.021212	2.3821	0.0086072	1.8621	0.031291	0.62739	0.2652	0.0086072	0.346447859
C12orf43	ENSG00000157895	1.5413	0.061621	2.1539	0.015625	2.382	0.0086106	2.382	0.0086106	0.0086106	0.346447859
TRAPPC3	ENSG00000054116	2.3816	0.0086192	2.1269	0.016712	1.9489	0.025657	1.6353	0.050991	0.0086192	0.346447859
NA	ENSG00000240449	2.3812	0.0086273	2.3812	0.0086273	2.3812	0.0086273	2.3812	0.0086273	0.0086273	0.346447859
STMN2	ENSG00000104435	2.0728	0.019095	2.0124	0.022088	2.3811	0.0086308	0.4611	0.32236	0.0086308	0.346447859
OPN3	ENSG00000054277	0.12944	0.4485	2.3807	0.0086394	2.3807	0.0086394	2.3807	0.0086394	0.0086394	0.346447859
NOTCH2	ENSG00000134250	2.3805	0.0086452	1.8927	0.0292	0.46441	0.32118	0.3588	0.35987	0.0086452	0.346447859
NA	ENSG00000267081	2.3805	0.0086452	-0.019242	0.50768	2.2952	0.010859	2.2952	0.010859	0.0086452	0.346447859
C9orf153	ENSG00000187753	1.1832	0.11836	1.1832	0.11836	2.3784	0.0086946	1.1832	0.11836	0.0086946	0.347310568
NPIPB10P	ENSG00000196796	0.76161	0.22315	-0.013953	0.50557	0.66649	0.25255	2.3779	0.0087068	0.0087068	0.347310568
RPSAP33	ENSG00000226765	2.3771	0.0087248	2.3771	0.0087248	2.3771	0.0087248	2.3771	0.0087248	0.0087248	0.347310568
DDAH1	ENSG00000153904	2.377	0.0087264	-0.87582	0.80944	-1.602	0.94543	-1.417	0.92176	0.0087264	0.347310568
SLC9B2	ENSG00000164038	1.1986	0.11534	1.1986	0.11534	1.6304	0.051511	2.3769	0.0087299	0.0087299	0.347310568
CMYA5	ENSG00000164309	0.81459	0.20765	0.61176	0.27035	0.15449	0.43861	2.3767	0.008734	0.008734	0.347310568
RPRML	ENSG00000179673	0.29611	0.38357	0.29611	0.38357	0.29611	0.38357	2.3765	0.0087388	0.0087388	0.347310568
TLE6	ENSG00000104953	-0.14662	0.55828	2.376	0.0087513	0.95157	0.17066	0.57704	0.28196	0.0087513	0.347310568
NA	ENSG00000279479	2.3758	0.0087555	2.3758	0.0087555	2.3758	0.0087555	2.3758	0.0087555	0.0087555	0.347310568
NRF1	ENSG00000106459	1.6238	0.052205	0.78014	0.21765	2.3753	0.008767	0.33829	0.36757	0.008767	0.347310568
RAB38	ENSG00000123892	0.041091	0.48361	0.050281	0.47995	1.0464	0.14768	2.3753	0.0087673	0.0087673	0.347310568
NA	ENSG00000213132	1.9804	0.023831	1.9804	0.023831	0.96522	0.16722	2.3752	0.0087701	0.0087701	0.347310568
SH3YL1	ENSG00000035115	2.1561	0.015538	2.2471	0.012315	1.0713	0.14202	2.375	0.0087747	0.0087747	0.347310568
CHD7	ENSG00000171316	0.53766	0.29541	-0.26113	0.603	-0.29846	0.61732	2.3745	0.0087871	0.0087871	0.347310568
NA	ENSG00000254651	0.28059	0.38951	-0.35156	0.63741	-0.14826	0.55893	2.3744	0.0087887	0.0087887	0.347310568
IMMP1LP2	ENSG00000258159	2.3744	0.0087887	2.3744	0.0087887	2.3744	0.0087887	2.3744	0.0087887	0.0087887	0.347310568
ENPP7P2	ENSG00000239959	2.3743	0.00879	-0.26841	0.60581	-0.21809	0.58632	0.22388	0.41142	0.00879	0.347310568
PGAP6	ENSG00000129925	0.86953	0.19228	0.48292	0.31458	2.3739	0.0088015	1.0366	0.14996	0.0088015	0.347310568
SHISA4	ENSG00000198892	2.3738	0.0088029	-1.7523	0.96014	-1.7523	0.96014	-1.7523	0.96014	0.0088029	0.347310568
NA	ENSG00000254197	-1.0466	0.85236	2.3735	0.0088097	-0.73396	0.76851	-1.8441	0.96742	0.0088097	0.347310568
CEP68	ENSG00000011523	0.9984	0.15904	2.3365	0.0097331	1.8819	0.029928	2.3732	0.0088164	0.0088164	0.347310568
NA	ENSG00000237088	2.3723	0.0088395	2.3723	0.0088395	0.092122	0.4633	0.9876	0.16167	0.0088395	0.347679322

FRAS1	ENSG00000138759	2.303	0.01064	2.0787	0.018821	1.9734	0.024226	2.3719	0.0088495	0.0088495	0.347679322
SLC26A1	ENSG00000145217	2.3718	0.0088515	2.3718	0.0088515	1.4484	0.073756	2.3718	0.0088515	0.0088515	0.347679322
ZNF789	ENSG00000198556	2.2506	0.012205	1.7201	0.042711	2.3717	0.0088543	1.1028	0.13505	0.0088543	0.347679322
CSMD2	ENSG00000121904	0.27341	0.39227	1.1674	0.12153	2.0138	0.022014	2.3711	0.0088669	0.0088669	0.34789375
HMGN1P6	ENSG00000224070	2.3708	0.0088741	2.3708	0.0088741	2.3708	0.0088741	2.3708	0.0088741	0.0088741	0.347896133
SMG1P7	ENSG00000261556	1.6998	0.044581	0.84526	0.19898	2.3698	0.0088988	1.6243	0.052151	0.0088988	0.348182456
RNU6-753P	ENSG00000200796	2.3695	0.0089071	2.3695	0.0089071	1.8122	0.034978	2.3695	0.0089071	0.0089071	0.348182456
TAS2R1	ENSG00000169777	2.3693	0.0089104	2.3693	0.0089104	2.3693	0.0089104	2.3693	0.0089104	0.0089104	0.348182456
OR4Q3	ENSG00000182652	-0.15781	0.5627	1.0966	0.13642	2.3689	0.0089205	0.93205	0.17565	0.0089205	0.348182456
NA	ENSG00000249708	2.3688	0.0089236	1.8232	0.034138	-0.14973	0.55951	1.5292	0.063103	0.0089236	0.348182456
NA	ENSG00000259005	2.131	0.016543	2.3686	0.0089269	2.1641	0.015227	2.1641	0.015227	0.0089269	0.348182456
PPP1R8P1	ENSG00000224986	2.3678	0.0089473	2.3678	0.0089473	2.3678	0.0089473	2.3678	0.0089473	0.0089473	0.348182456
NA	ENSG00000283421	2.3678	0.0089477	2.3678	0.0089477	2.3678	0.0089477	2.3678	0.0089477	0.0089477	0.348182456
ANXA9	ENSG00000143412	2.3678	0.0089478	1.6041	0.054348	-0.88168	0.81103	0.74418	0.22838	0.0089478	0.348182456
KCNMB2-AS1	ENSG00000237978	2.3673	0.0089595	2.0823	0.01866	1.2322	0.10893	1.5781	0.057273	0.0089595	0.348182456
MNS1	ENSG00000138587	2.3673	0.00896	-0.084068	0.5335	-1.2982	0.90289	-0.084068	0.5335	0.00896	0.348182456
PRKACB	ENSG00000142875	0.56642	0.28555	1.3897	0.082315	0.12478	0.45035	2.3667	0.0089746	0.0089746	0.348471919
GAPLINC	ENSG00000266835	2.3657	0.0089973	2.3657	0.0089973	2.3657	0.0089973	0.82219	0.20548	0.0089973	0.348812984
RN7SL677P	ENSG00000242863	2.3657	0.0089977	2.3657	0.0089977	2.3657	0.0089977	2.3657	0.0089977	0.0089977	0.348812984
NA	ENSG00000265125	2.3649	0.0090179	1.6037	0.054388	2.3003	0.010715	1.201	0.11487	0.0090179	0.349318177
NA	ENSG00000259345	1.7693	0.038422	1.8608	0.031387	0.28284	0.38865	2.3639	0.0090421	0.0090421	0.34947307
S100A2	ENSG00000196754	2.3637	0.0090471	1.6534	0.049127	1.6354	0.050981	1.3987	0.080957	0.0090471	0.34947307
MYBPC1	ENSG00000196091	0.60581	0.27232	2.3636	0.0090497	0.60581	0.27232	-0.27111	0.60685	0.0090497	0.34947307
HR	ENSG00000168453	1.3356	0.090836	2.3634	0.0090541	1.3356	0.090836	1.3356	0.090836	0.0090541	0.34947307
PRPF38AP1	ENSG00000225053	2.363	0.0090631	0.38869	0.34875	0.38869	0.34875	0.38869	0.34875	0.0090631	0.34947307
CNOT4	ENSG00000080802	2.3594	0.0091519	2.3629	0.0090667	1.9022	0.028575	1.3136	0.094494	0.0090667	0.34947307
RAD1	ENSG00000113456	-0.22119	0.58753	-0.22119	0.58753	0.60282	0.27332	2.3627	0.0090721	0.0090721	0.34947307
NA	ENSG00000271010	2.362	0.0090878	2.362	0.0090878	2.362	0.0090878	2.362	0.0090878	0.0090878	0.349801338
LINC01795	ENSG00000225226	2.3607	0.00912	2.3607	0.00912	2.3607	0.00912	2.3607	0.00912	0.00912	0.350763694
SCFD1	ENSG00000092108	0.17705	0.42973	0.3144	0.37661	2.3603	0.0091305	-0.33638	0.63171	0.0091305	0.350890588
THTPA	ENSG00000259431	2.3584	0.0091765	2.3584	0.0091765	2.3584	0.0091765	2.3584	0.0091765	0.0091765	0.351932504

UBAP2L	ENSG00000143569	0.66366	0.25345	-1.2376	0.89207	-1.2646	0.89699	2.3581	0.0091837	0.0091837	0.351932504
RNU6-1169P	ENSG00000200407	-0.96452	0.83261	2.3581	0.009185	-0.96452	0.83261	-0.96452	0.83261	0.009185	0.351932504
NA	ENSG00000222309	2.358	0.0091865	2.358	0.0091865	2.358	0.0091865	2.358	0.0091865	0.0091865	0.351932504
SLC51A	ENSG00000163959	2.2755	0.011438	1.4427	0.074558	2.3574	0.0092011	2.3574	0.0092011	0.0092011	0.352214928
CDKN2AIP	ENSG00000168564	0.89606	0.18511	0.89606	0.18511	1.9671	0.024588	2.3571	0.0092093	0.0092093	0.352252111
DDX60	ENSG00000137628	0.4355	0.3316	1.0107	0.15608	2.3568	0.0092178	-0.9556	0.83036	0.0092178	0.352300701
SERPINA4	ENSG00000100665	2.0326	0.021047	1.0863	0.13866	2.3562	0.0092308	2.3562	0.0092308	0.0092308	0.352521069
DLG2	ENSG00000150672	1.945	0.025886	1.9654	0.024685	2.3553	0.0092542	-0.17766	0.57051	0.0092542	0.353137953
NA	ENSG00000234913	2.3549	0.0092647	2.171	0.014964	1.2834	0.099671	1.9393	0.026229	0.0092647	0.353261996
DRD1	ENSG00000184845	-0.23933	0.59457	-0.25372	0.60014	2.3544	0.009276	-1.2523	0.89477	0.009276	0.353383869
MYL4	ENSG00000198336	0.056684	0.4774	0.50962	0.30516	-0.013532	0.5054	2.3542	0.0092824	0.0092824	0.353383869
RPL7P40	ENSG00000242991	-0.28069	0.61053	0.7677	0.22133	0.7958	0.21307	2.3537	0.0092946	0.0092946	0.353470873
NGLY1	ENSG00000151092	1.0017	0.15825	2.3534	0.0093012	-0.41362	0.66042	-0.013743	0.50548	0.0093012	0.353470873
NA	ENSG00000260669	2.3531	0.0093085	2.3531	0.0093085	2.3531	0.0093085	2.3531	0.0093085	0.0093085	0.353470873
NA	ENSG00000248376	2.3529	0.0093137	0.88822	0.18721	0.74889	0.22696	0.44107	0.32958	0.0093137	0.353470873
RNU6-1222P	ENSG00000207345	2.3523	0.0093283	0.39245	0.34736	0.39245	0.34736	0.39245	0.34736	0.0093283	0.353749462
HERC5	ENSG00000138646	1.1335	0.12851	2.3508	0.0093676	0.93367	0.17524	0.80533	0.21032	0.0093676	0.353937235
MIR4666A	ENSG00000266174	2.3506	0.0093704	2.3506	0.0093704	2.3506	0.0093704	2.3506	0.0093704	0.0093704	0.353937235
NME1	ENSG00000239672	2.3505	0.0093738	2.3505	0.0093738	2.3505	0.0093738	2.3505	0.0093738	0.0093738	0.353937235
RFTN1	ENSG00000131378	1.4089	0.079439	2.2809	0.011278	2.3501	0.0093844	1.9992	0.022794	0.0093844	0.353937235
NA	ENSG00000238804	2.35	0.009386	2.35	0.009386	2.35	0.009386	2.35	0.009386	0.009386	0.353937235
JAK1	ENSG00000162434	0.86099	0.19462	1.1374	0.12768	0.80738	0.20972	2.3499	0.0093897	0.0093897	0.353937235
MMEL1	ENSG00000142606	2.3494	0.0094015	0.19051	0.42445	0.25674	0.39869	-0.24105	0.59524	0.0094015	0.353937235
HMGB3P7	ENSG00000215399	-1.7095	0.95632	0.65011	0.25781	-0.067276	0.52682	2.3492	0.0094065	0.0094065	0.353937235
EPB41L4A	ENSG00000129595	2.3488	0.0094171	1.8965	0.028944	1.6915	0.045375	1.0564	0.14539	0.0094171	0.353937235
RPL37AP1	ENSG00000226243	2.3487	0.0094195	2.3487	0.0094195	2.3487	0.0094195	2.3487	0.0094195	0.0094195	0.353937235
NA	ENSG00000214331	-0.46702	0.67976	0.26132	0.39692	-0.12875	0.55122	2.3486	0.009422	0.009422	0.353937235
RNU7-154P	ENSG00000252003	2.3485	0.0094247	2.3485	0.0094247	2.3485	0.0094247	2.3485	0.0094247	0.0094247	0.353937235
OR7E22P	ENSG00000179799	1.4144	0.078622	1.1298	0.12929	2.3479	0.0094397	-0.39877	0.65497	0.0094397	0.353937235
NA	ENSG00000261583	2.3479	0.00944	2.3479	0.00944	2.3479	0.00944	2.3479	0.00944	0.00944	0.353937235
NA	ENSG00000253894	2.3478	0.0094422	-0.030295	0.51208	0.69963	0.24208	0.69963	0.24208	0.0094422	0.353937235

OTULINL	ENSG00000145569	2.3473	0.0094542	-0.26136	0.60309	0.70559	0.24022	0.0057813	0.49769	0.0094542	0.354114655
KLHL6	ENSG00000172578	-0.28314	0.61146	0.30565	0.37994	1.8529	0.031949	2.3466	0.0094729	0.0094729	0.354194285
MGP	ENSG00000111341	-0.51144	0.69548	-0.0013626	0.50054	-0.0013626	0.50054	2.3463	0.0094806	0.0094806	0.354194285
NA	ENSG00000249207	2.3462	0.0094821	1.341	0.08996	-0.084441	0.53365	-0.084441	0.53365	0.0094821	0.354194285
RAB26	ENSG00000167964	2.3461	0.0094854	2.3461	0.0094854	0.0059795	0.49761	2.3461	0.0094854	0.0094854	0.354194285
SLC22A11	ENSG00000168065	2.3455	0.0095005	1.4086	0.079471	0.88366	0.18844	1.6957	0.044968	0.0095005	0.354315942
TP53INP2	ENSG00000078804	2.233	0.012773	1.9702	0.024407	2.3454	0.0095032	1.5397	0.061818	0.0095032	0.354315942
TVP23CP1	ENSG00000223584	2.3285	0.0099428	0.78351	0.21666	1.4886	0.068302	2.3445	0.009526	0.009526	0.35489448
FBLN7	ENSG00000144152	2.3438	0.0095437	1.7387	0.041048	2.0507	0.020146	-0.035664	0.51422	0.0095437	0.355282277
LINC01267	ENSG00000251576	2.1177	0.017099	-1.4879	0.93161	2.3428	0.0095692	-1.3949	0.91847	0.0095692	0.355699264
NA	ENSG00000279789	2.3428	0.0095695	2.3428	0.0095695	2.3428	0.0095695	2.3428	0.0095695	0.0095695	0.355699264
NDUFAF2	ENSG00000164182	1.8814	0.029959	0.34513	0.365	2.3412	0.0096102	1.3085	0.09536	0.0096102	0.356315087
ADGRL3	ENSG00000150471	-0.25975	0.60247	0.62876	0.26475	2.0287	0.021245	2.3411	0.009613	0.009613	0.356315087
NA	ENSG00000270182	2.3411	0.009613	2.3411	0.009613	2.3411	0.009613	2.3411	0.009613	0.009613	0.356315087
NA	ENSG00000225798	2.3408	0.0096205	2.3408	0.0096205	2.3408	0.0096205	2.3408	0.0096205	0.0096205	0.356315087
LINC01555	ENSG00000180869	2.3401	0.0096398	-0.58365	0.72027	1.6041	0.054351	-0.58365	0.72027	0.0096398	0.356315087
ANKRD66	ENSG00000230062	0.83346	0.20229	0.15114	0.43993	2.34	0.0096426	0.15114	0.43993	0.0096426	0.356315087
NA	ENSG00000223881	2.3398	0.009647	2.3398	0.009647	2.3398	0.009647	2.3398	0.009647	0.009647	0.356315087
RNU6-469P	ENSG00000252062	2.3395	0.0096556	2.0828	0.018636	2.3395	0.0096556	-0.79812	0.7876	0.0096556	0.356315087
HMGN1P19	ENSG00000224519	2.3393	0.0096603	2.3393	0.0096603	2.3393	0.0096603	2.3393	0.0096603	0.0096603	0.356315087
NA	ENSG00000269686	2.3392	0.0096623	0.27259	0.39258	2.3392	0.0096623	2.3392	0.0096623	0.0096623	0.356315087
SMIM15	ENSG00000188725	0.44238	0.32911	-0.1704	0.56765	2.339	0.0096665	-1.0193	0.84598	0.0096665	0.356315087
IQCJ-SCHIP1	ENSG00000283154	1.9552	0.025279	1.2365	0.10814	2.3373	0.0097129	0.64667	0.25892	0.0097129	0.357386408
EVPL	ENSG00000167880	2.3371	0.0097175	-0.95346	0.82982	0.74395	0.22845	0.74395	0.22845	0.0097175	0.357386408
NA	ENSG00000261317	2.3368	0.0097239	2.3368	0.0097239	2.3368	0.0097239	2.3368	0.0097239	0.0097239	0.357386408
NA	ENSG00000255197	2.0855	0.018511	2.3368	0.0097249	2.0855	0.018511	1.5387	0.061944	0.0097249	0.357386408
NA	ENSG00000271608	2.3075	0.010512	1.8346	0.033283	2.336	0.0097453	2.0864	0.018473	0.0097453	0.357614496
ACTG1P9	ENSG00000229349	-0.32121	0.62597	-0.64654	0.74103	-1.0541	0.85408	2.3359	0.0097484	0.0097484	0.357614496
ZNF507	ENSG00000168813	0.50996	0.30504	0.66217	0.25393	2.3354	0.0097622	1.29	0.098529	0.0097622	0.357614496
FGF12-AS1	ENSG00000231383	-0.45576	0.67572	0.26769	0.39447	-0.56605	0.71432	2.3352	0.0097655	0.0097655	0.357614496
NA	ENSG00000245904	1.0904	0.13776	2.3351	0.0097678	1.8243	0.034052	1.0904	0.13776	0.0097678	0.357614496

NA	ENSG00000250310	1.3289	0.091947	0.5167	0.30268	1.4381	0.075205	2.3345	0.0097841	0.0097841	0.357915462
NA	ENSG00000255605	1.5669	0.058564	1.9942	0.023065	2.3339	0.0098014	1.5896	0.05596	0.0098014	0.357915462
PSMC1P12	ENSG00000226126	1.0958	0.13658	-0.066613	0.52655	2.3338	0.0098043	-0.74369	0.77147	0.0098043	0.357915462
NA	ENSG00000253762	1.0438	0.14828	-0.13884	0.55521	0.25127	0.4008	2.3337	0.0098054	0.0098054	0.357915462
ERICH1	ENSG00000104714	0.36137	0.35891	2.3325	0.0098384	-0.27381	0.60788	-0.25435	0.60039	0.0098384	0.358790571
LINC00877	ENSG00000241163	0.66384	0.2534	2.3322	0.0098441	-0.56555	0.71415	0.69076	0.24486	0.0098441	0.358790571
LINC02220	ENSG00000248457	1.1503	0.12501	-1.5552	0.94005	-1.5552	0.94005	2.3319	0.0098517	0.0098517	0.358799209
PDGFRA	ENSG00000134853	-0.57909	0.71873	2.3314	0.0098668	-1.2522	0.89475	-0.93822	0.82593	0.0098668	0.35908078
PHF21A	ENSG00000135365	2.2606	0.011891	2.3305	0.0098886	1.2372	0.10801	1.8735	0.030501	0.0098886	0.359351118
ADAM21P1	ENSG00000235812	2.3304	0.0098937	1.1772	0.11956	2.3109	0.01042	2.3109	0.01042	0.0098937	0.359351118
OR7E23P	ENSG00000228433	-2.465	0.99315	-0.26002	0.60257	2.33	0.0099023	1.4596	0.072203	0.0099023	0.359351118
WDR92	ENSG00000243667	-0.71853	0.76379	2.3297	0.0099111	0.72942	0.23287	-0.47264	0.68176	0.0099111	0.359351118
NA	ENSG00000265246	1.1269	0.12989	1.3846	0.083089	2.3297	0.0099111	-0.10509	0.54185	0.0099111	0.359351118
PROCA1	ENSG00000167525	1.0562	0.14543	0.72762	0.23342	2.3282	0.0099515	1.0016	0.15827	0.0099515	0.360547654
RPEP1	ENSG00000250732	2.3272	0.0099773	2.3272	0.0099773	2.3272	0.0099773	2.3272	0.0099773	0.0099773	0.36121384
ZNF536	ENSG00000198597	0.26495	0.39552	-0.26645	0.60506	2.3264	0.0099979	1.3242	0.092721	0.0099979	0.361516137
LINC01800	ENSG00000234572	2.2757	0.011433	2.1961	0.014041	2.3261	0.010006	1.6395	0.050551	0.010006	0.361516137
CSRP1	ENSG00000159176	1.3468	0.089019	1.935	0.026495	0.49091	0.31175	2.3257	0.010017	0.010017	0.361516137
NA	ENSG00000231083	2.3257	0.010017	0.020132	0.49197	0.020132	0.49197	0.020132	0.49197	0.010017	0.361516137
TRAF7	ENSG00000131653	1.9786	0.023932	2.3253	0.010027	2.3253	0.010027	1.1513	0.12481	0.010027	0.361516137
ETS1	ENSG00000134954	2.325	0.010035	-1.1548	0.87592	0.67709	0.24917	0.1186	0.45279	0.010035	0.361516137
ST8SIA5	ENSG00000101638	1.1099	0.13352	1.1486	0.12535	0.92602	0.17722	2.3247	0.010044	0.010044	0.361516137
DCANP1	ENSG00000251380	1.3841	0.083163	1.4693	0.070872	2.3247	0.010045	2.149	0.015817	0.010045	0.361516137
FADS1	ENSG00000149485	2.3238	0.010067	2.3238	0.010067	2.3238	0.010067	0.88523	0.18802	0.010067	0.361917279
AKNA	ENSG00000106948	2.3138	0.01034	2.3237	0.010071	0.56727	0.28527	1.3887	0.082457	0.010071	0.361917279
ANKRD20A5P	ENSG00000186481	0.91966	0.17887	0.65549	0.25608	2.323	0.010089	1.882	0.029915	0.010089	0.362296957
GDA	ENSG00000119125	-0.26446	0.60429	2.3223	0.010108	0.78393	0.21654	1.0001	0.15862	0.010108	0.362711959
RHOF	ENSG00000139725	1.9916	0.023208	1.9916	0.023208	1.4984	0.067015	2.322	0.010116	0.010116	0.362731921
NA	ENSG00000206734	2.3217	0.010124	1.4913	0.067939	1.8405	0.032844	1.6657	0.047891	0.010124	0.362751853
PKIG	ENSG00000168734	2.2502	0.012219	2.2656	0.011739	1.5032	0.066388	2.3212	0.010139	0.010139	0.362980109
PRDM7	ENSG00000126856	2.0236	0.021504	2.3198	0.010176	0.48288	0.31459	0.48288	0.31459	0.010176	0.362980109

NUTF2P5	ENSG00000215227	2.3196	0.010182	2.3196	0.010182	-0.30969	0.6216	2.3196	0.010182	0.010182	0.362980109
RN7SKP102	ENSG00000223118	2.3194	0.010185	2.3194	0.010185	-0.88725	0.81253	2.3194	0.010185	0.010185	0.362980109
NA	ENSG00000235881	2.319	0.010197	2.319	0.010197	0.12297	0.45106	-0.45619	0.67587	0.010197	0.362980109
OR13Z3P	ENSG00000272480	-0.84692	0.80148	2.319	0.010198	0.39907	0.34492	0.42457	0.33558	0.010198	0.362980109
FXYD6	ENSG00000137726	2.3189	0.010199	-0.1762	0.56993	-0.0061987	0.50247	1.1816	0.11867	0.010199	0.362980109
NA	ENSG00000202268	1.0084	0.15663	2.2255	0.013024	2.3189	0.010199	0.88159	0.189	0.010199	0.362980109
ZNF485	ENSG00000198298	1.463	0.071739	0.22143	0.41238	2.3185	0.01021	0.85655	0.19585	0.01021	0.362980109
LINC01252	ENSG00000247157	1.8319	0.033481	0.94371	0.17266	0.85539	0.19617	2.3184	0.010214	0.010214	0.362980109
MIR5011	ENSG00000263354	2.3184	0.010215	2.3184	0.010215	2.3184	0.010215	2.3184	0.010215	0.010215	0.362980109
ZNF101P1	ENSG00000237746	2.3177	0.010232	1.977	0.024018	1.0524	0.14631	1.683	0.046192	0.010232	0.362980109
TMEM53	ENSG00000126106	2.3175	0.010239	-1.2407	0.89264	-0.11561	0.54602	-0.063654	0.52538	0.010239	0.362980109
CAMTA2	ENSG00000108509	1.583	0.056707	1.583	0.056707	1.583	0.056707	2.3172	0.010246	0.010246	0.362980109
ZIC1	ENSG00000152977	2.317	0.010252	2.317	0.010252	0.4316	0.33302	-0.12584	0.55007	0.010252	0.362980109
RPS2P24	ENSG00000244019	1.9883	0.02339	1.7208	0.042641	0.93864	0.17396	2.3169	0.010255	0.010255	0.362980109
LINC01634	ENSG00000235295	-0.15121	0.5601	2.3168	0.010257	2.2681	0.011661	-0.15121	0.5601	0.010257	0.362980109
NA	ENSG00000230205	0.049546	0.48024	0.88992	0.18675	-0.82735	0.79598	2.3153	0.010298	0.010298	0.364166575
NA	ENSG00000257879	0.75932	0.22383	0.68455	0.24681	0.75932	0.22383	2.3144	0.010323	0.010323	0.364785925
TRAJ2	ENSG00000211887	2.314	0.010335	2.314	0.010335	2.314	0.010335	2.314	0.010335	0.010335	0.364945326
LINC02277	ENSG00000258487	-0.26047	0.60275	2.3134	0.01035	0.40057	0.34437	0.93368	0.17524	0.01035	0.364981353
NOS2	ENSG00000007171	0.10095	0.4598	2.3134	0.010351	-0.27937	0.61002	0.1804	0.42842	0.010351	0.364981353
PPIAP30	ENSG00000206448	2.313	0.010363	2.313	0.010363	2.313	0.010363	2.313	0.010363	0.010363	0.365140268
TTBK1	ENSG00000146216	2.3121	0.010386	0.45683	0.3239	0.28805	0.38666	1.0635	0.14377	0.010386	0.365141713
NA	ENSG00000235415	1.8429	0.032669	2.3118	0.010394	1.8429	0.032669	0.84439	0.19922	0.010394	0.365141713
LINC00334	ENSG00000182586	0.44507	0.32814	2.3116	0.010401	1.1096	0.1336	0.63552	0.26255	0.010401	0.365141713
C12orf50	ENSG00000165805	-0.17192	0.56825	2.3115	0.010402	-1.1671	0.87842	-1.1671	0.87842	0.010402	0.365141713
SNX33P1	ENSG00000267609	2.3115	0.010403	2.3115	0.010403	2.3115	0.010403	2.3115	0.010403	0.010403	0.365141713
VDAC2P1	ENSG00000214976	1.6333	0.051204	2.3113	0.010408	1.3992	0.080872	2.3113	0.010408	0.010408	0.365141713
LINC01054	ENSG00000229723	2.311	0.010417	2.311	0.010417	2.311	0.010417	2.311	0.010417	0.010417	0.36519454
SOX21	ENSG00000125285	1.522	0.064008	0.97119	0.16573	0.2888	0.38637	2.3103	0.010436	0.010436	0.365597613
NA	ENSG00000244560	2.3095	0.010458	0.008024	0.4968	0.39419	0.34672	0.75235	0.22592	0.010458	0.365788712
GLIS1	ENSG00000174332	1.8685	0.030844	1.3872	0.082695	2.309	0.010471	1.9933	0.023117	0.010471	0.365788712

ZNF646	ENSG00000167395	2.3089	0.010474	1.85	0.032154	2.3089	0.010474	2.3089	0.010474	0.010474	0.365788712
BGLAP	ENSG00000242252	2.3088	0.010477	2.1845	0.014462	2.3088	0.010477	2.3088	0.010477	0.010477	0.365788712
CICP5	ENSG00000233771	2.0767	0.018915	-1.4015	0.91947	0.11336	0.45487	2.3086	0.010482	0.010482	0.365788712
C11orf45	ENSG00000174370	0.2768	0.39097	2.3083	0.010491	2.3083	0.010491	2.3083	0.010491	0.010491	0.365788712
MIR3180-5	ENSG00000264397	2.3082	0.010494	2.3082	0.010494	2.3082	0.010494	2.3082	0.010494	0.010494	0.365788712
NA	ENSG00000237480	1.7184	0.042864	2.2322	0.012801	2.3078	0.010505	2.1866	0.014385	0.010505	0.3659104
RCC2P6	ENSG00000254454	-0.47711	0.68336	2.3071	0.010525	-0.59493	0.72405	0.77587	0.21891	0.010525	0.366345179
MTND6P21	ENSG00000223431	2.3061	0.010551	2.3061	0.010551	2.3061	0.010551	-0.71753	0.76348	0.010551	0.366956888
NA	ENSG00000229616	2.3053	0.010576	-0.24925	0.59842	1.167	0.1216	1.167	0.1216	0.010576	0.366956888
CHCHD5	ENSG00000125611	2.305	0.010585	2.305	0.010585	2.305	0.010585	2.305	0.010585	0.010585	0.366956888
CUEDC1	ENSG00000180891	2.3047	0.010592	1.9655	0.024679	0.82641	0.20429	1.6024	0.054534	0.010592	0.366956888
MAN2B2	ENSG00000013288	0.21341	0.4155	2.3045	0.010597	0.61994	0.26765	-0.16424	0.56523	0.010597	0.366956888
ABCC5	ENSG00000114770	2.3045	0.010598	1.9599	0.025006	2.0077	0.022338	1.5165	0.064698	0.010598	0.366956888
MDGA2	ENSG00000139915	0.86831	0.19261	2.3043	0.010603	-0.058584	0.52336	-0.2252	0.58909	0.010603	0.366956888
NA	ENSG00000250889	2.304	0.010611	0.92009	0.17876	2.304	0.010611	2.304	0.010611	0.010611	0.366956888
LINC01003	ENSG00000261455	-0.88147	0.81097	-0.88147	0.81097	2.3039	0.010614	-0.95721	0.83077	0.010614	0.366956888
CD44-AS1	ENSG00000255443	2.3036	0.010622	2.3036	0.010622	2.3036	0.010622	2.3036	0.010622	0.010622	0.366956888
NA	ENSG00000228681	2.2802	0.011299	2.3034	0.010628	2.1952	0.014073	1.5406	0.061704	0.010628	0.366956888
MIR4512	ENSG00000266589	2.303	0.010639	2.303	0.010639	2.303	0.010639	2.303	0.010639	0.010639	0.366956888
SOGA3	ENSG00000214338	2.3029	0.010642	2.3029	0.010642	0.20852	0.41741	0.011439	0.49544	0.010642	0.366956888
MIR4506	ENSG00000265768	2.3027	0.010648	2.3027	0.010648	2.3027	0.010648	2.3027	0.010648	0.010648	0.366956888
SDHDP2	ENSG00000227073	2.3014	0.010685	2.3014	0.010685	2.3014	0.010685	2.3014	0.010685	0.010685	0.367655659
ALOX12-AS1	ENSG00000215067	2.3013	0.010687	0.52981	0.29812	1.1188	0.13161	1.4559	0.072705	0.010687	0.367655659
COL8A2	ENSG00000171812	-0.46	0.67724	2.3009	0.010699	0.31987	0.37453	2.0856	0.018509	0.010699	0.367655659
SH3GL1	ENSG00000141985	0.71231	0.23814	0.23809	0.4059	0.31064	0.37804	2.3008	0.010701	0.010701	0.367655659
RFX3	ENSG00000080298	0.71401	0.23761	2.3006	0.010706	0.93188	0.1757	-0.49968	0.69135	0.010706	0.367655659
RNU6-297P	ENSG00000207366	2.2997	0.010731	0.89289	0.18596	0.89289	0.18596	0.89289	0.18596	0.010731	0.368182222
GRAP2	ENSG00000100351	1.3265	0.092332	2.2993	0.010743	0.12193	0.45148	-0.30796	0.62095	0.010743	0.368182222
OTOR	ENSG00000125879	2.2993	0.010744	2.2993	0.010744	2.2993	0.010744	2.2993	0.010744	0.010744	0.368182222
SLC19A1	ENSG00000173638	0.1153	0.45411	0.70918	0.23911	1.9132	0.027861	2.2985	0.010766	0.010766	0.368330407
CTAGE14P	ENSG00000214211	2.2985	0.010766	2.2985	0.010766	2.2985	0.010766	2.2985	0.010766	0.010766	0.368330407

YWHAEP7	ENSG00000276715	2.2408	0.012521	1.0485	0.1472	2.2984	0.010771	-1.0334	0.84929	0.010771	0.368330407
SLFNL1-AS1	ENSG00000281207	1.0701	0.14228	2.2972	0.010803	1.0701	0.14228	1.0701	0.14228	0.010803	0.368785923
NA	ENSG00000279705	-0.12793	0.5509	-0.12793	0.5509	-0.12793	0.5509	2.2971	0.010807	0.010807	0.368785923
LINC02018	ENSG00000272690	1.2572	0.10433	2.297	0.01081	-0.40369	0.65678	-0.40369	0.65678	0.01081	0.368785923
FANCG	ENSG00000221829	2.2965	0.010824	-0.21015	0.58322	-0.9143	0.81972	-0.9143	0.81972	0.010824	0.368785923
ARHGAP22-IT1	ENSG00000248682	0.8905	0.1866	-0.074446	0.52967	2.2963	0.010828	2.2377	0.01262	0.010828	0.368785923
H2BC5	ENSG00000158373	2.2958	0.010844	2.2958	0.010844	2.2958	0.010844	1.2004	0.115	0.010844	0.368785923
RLF	ENSG00000117000	1.6959	0.04495	2.2957	0.010848	-0.20773	0.58228	1.8558	0.031745	0.010848	0.368785923
SH3RF1	ENSG00000154447	0.69186	0.24451	0.30942	0.3785	2.2955	0.010852	0.72655	0.23375	0.010852	0.368785923
LINC01122	ENSG00000233723	-0.046132	0.5184	2.2953	0.010858	1.3669	0.085836	1.2072	0.11368	0.010858	0.368785923
NDUFA10	ENSG00000130414	2.2952	0.01086	-0.91657	0.82032	0.42855	0.33413	-1.5607	0.94071	0.01086	0.368785923
WSB1	ENSG00000109046	-0.18765	0.57442	-0.18765	0.57442	-0.77673	0.78134	2.2948	0.010873	0.010873	0.368815226
NA	ENSG00000199285	-0.48418	0.68587	-0.87008	0.80787	2.2947	0.010876	-0.87008	0.80787	0.010876	0.368815226
LINC01596	ENSG00000250666	-0.96504	0.83274	1.9482	0.025697	-0.96133	0.83181	2.2936	0.010907	0.010907	0.369609256
NA	ENSG00000265240	2.2933	0.010916	1.9014	0.028625	2.1017	0.017792	2.1017	0.017792	0.010916	0.369657179
ACOT12	ENSG00000172497	-0.19349	0.57671	2.293	0.010925	0.077195	0.46923	-0.21604	0.58552	0.010925	0.369705035
CHAMP1	ENSG00000198824	2.2918	0.010958	2.2918	0.010958	2.2918	0.010958	2.2918	0.010958	0.010958	0.370510208
FMOD	ENSG00000122176	0.83903	0.20073	2.2916	0.010964	0.18183	0.42786	0.025797	0.48971	0.010964	0.370510208
CAMP	ENSG00000164047	1.4251	0.077058	1.3294	0.091861	0.468	0.31989	2.2912	0.010975	0.010975	0.370624913
ADGRF1	ENSG00000153292	2.2908	0.010988	-1.1733	0.87967	-0.63971	0.73882	-1.3595	0.91301	0.010988	0.370732178
NA	ENSG00000244535	-0.82348	0.79488	0.35066	0.36292	2.2906	0.010995	0.46284	0.32174	0.010995	0.370732178
INSC	ENSG00000188487	1.9024	0.028557	2.1241	0.016831	2.2903	0.011001	1.9567	0.025194	0.011001	0.370732178
TCP1P1	ENSG00000229161	2.29	0.01101	0.58033	0.28085	0.89643	0.18501	0.26788	0.3944	0.01101	0.370744015
LOXL2	ENSG00000134013	1.9122	0.027924	0.79232	0.21409	2.2895	0.011024	1.796	0.036244	0.011024	0.370744015
CHAF1A	ENSG00000167670	1.5461	0.061044	2.2895	0.011026	1.485	0.068772	1.6891	0.045604	0.011026	0.370744015
CMTR1	ENSG00000137200	0.2864	0.38729	0.99772	0.15921	1.6166	0.052979	2.2891	0.011037	0.011037	0.370744015
NA	ENSG00000236935	1.5807	0.056977	1.3555	0.087622	2.2888	0.011044	2.2665	0.011711	0.011044	0.370744015
NA	ENSG00000224647	2.2887	0.011047	2.2887	0.011047	2.2887	0.011047	2.2887	0.011047	0.011047	0.370744015
KIF18A	ENSG00000121621	-0.34152	0.63364	2.2874	0.011085	1.6699	0.04747	-0.34152	0.63364	0.011085	0.371708686
SIRT2	ENSG00000068903	0.47233	0.31834	1.3624	0.08654	0.90859	0.18178	2.2872	0.011091	0.011091	0.371708686
NA	ENSG00000215450	2.1092	0.017463	2.0901	0.018304	2.2764	0.011411	2.2869	0.011102	0.011102	0.371711775

MATN1	ENSG00000162510	0.52806	0.29873	0.51591	0.30296	0.49084	0.31177	2.2863	0.011119	0.011119	0.371711775
MADD-AS1	ENSG00000256746	2.2862	0.011121	2.2862	0.011121	2.2862	0.011121	2.2862	0.011121	0.011121	0.371711775
DUSP22	ENSG00000112679	2.2862	0.011123	-0.95772	0.8309	0.63853	0.26156	-0.060458	0.5241	0.011123	0.371711775
ATRIP	ENSG00000164053	2.2856	0.011139	1.6256	0.052019	1.2947	0.097703	0.52498	0.2998	0.011139	0.371711775
OR6F1	ENSG00000169214	2.2854	0.011146	2.2854	0.011146	0.28621	0.38736	0.92699	0.17697	0.011146	0.371711775
NUS1	ENSG00000153989	-0.47919	0.6841	2.2852	0.01115	2.2852	0.01115	-1.5747	0.94233	0.01115	0.371711775
MIR3692	ENSG00000266617	2.285	0.011155	-0.65663	0.74429	-0.65663	0.74429	-0.65663	0.74429	0.011155	0.371711775
HPCA	ENSG00000121905	0.8479	0.19825	1.3618	0.086632	2.2848	0.011162	2.2848	0.011162	0.011162	0.371711775
NA	ENSG00000259874	2.2844	0.011173	0.043038	0.48284	0.043038	0.48284	0.043038	0.48284	0.011173	0.371711775
TSR1	ENSG00000167721	2.2844	0.011175	2.2844	0.011175	2.2844	0.011175	2.2844	0.011175	0.011175	0.371711775
INPP5E	ENSG00000148384	1.7426	0.040701	1.7426	0.040701	2.284	0.011187	1.7426	0.040701	0.011187	0.371857101
LINC00494	ENSG00000235621	2.1936	0.014133	1.3085	0.095352	2.2834	0.011204	-0.035935	0.51433	0.011204	0.372168316
NA	ENSG00000277757	0.95671	0.16936	2.2825	0.011229	1.8524	0.031987	1.8524	0.031987	0.011229	0.372744666
LINC02241	ENSG00000251629	0.68088	0.24797	1.5063	0.065998	2.2817	0.011254	1.2676	0.10246	0.011254	0.373047905
GPR79	ENSG00000232374	2.1058	0.017611	-0.089091	0.5355	2.2815	0.01126	1.6388	0.050625	0.01126	0.373047905
NA	ENSG00000233430	1.9784	0.023944	2.2807	0.011283	1.9124	0.027914	1.2899	0.098549	0.011283	0.373047905
ZDHHC20	ENSG00000180776	-0.38485	0.64983	-0.37326	0.64552	2.2805	0.01129	-0.56346	0.71344	0.01129	0.373047905
NA	ENSG00000280097	2.0402	0.020663	0.47848	0.31615	2.2804	0.011292	2.0402	0.020663	0.011292	0.373047905
NA	ENSG00000202177	2.28	0.011305	2.28	0.011305	2.28	0.011305	2.28	0.011305	0.011305	0.373047905
NA	ENSG00000236892	-0.1911	0.57578	0.21234	0.41592	0.25482	0.39943	2.2799	0.011308	0.011308	0.373047905
H2AW	ENSG00000181218	2.2797	0.011313	2.2797	0.011313	2.2797	0.011313	1.4349	0.075653	0.011313	0.373047905
REXO1	ENSG00000079313	0.58151	0.28045	2.2796	0.011316	0.017134	0.49316	0.23431	0.40737	0.011316	0.373047905
PCDH18	ENSG00000189184	-0.11316	0.54505	0.20087	0.4204	2.2796	0.011317	1.971	0.024362	0.011317	0.373047905
CDC25C	ENSG00000158402	2.252	0.012161	2.2792	0.011329	2.213	0.013448	1.5471	0.060923	0.011329	0.373047905
NA	ENSG00000219755	2.2791	0.01133	2.2791	0.01133	1.7305	0.041769	2.2791	0.01133	0.01133	0.373047905
DNAJB6P8	ENSG00000264529	-0.80933	0.79084	-0.80933	0.79084	2.2788	0.01134	0.84199	0.1999	0.01134	0.373125051
CXADRP3	ENSG00000265766	0.51494	0.3033	-0.75344	0.77441	1.3475	0.088913	2.2782	0.011357	0.011357	0.37327967
MYO5B	ENSG00000167306	0.78897	0.21506	0.98281	0.16285	2.2777	0.011371	1.6955	0.044987	0.011371	0.37327967
RN7SL668P	ENSG00000239690	2.1712	0.014958	2.2777	0.011373	1.3036	0.096177	1.7257	0.042197	0.011373	0.37327967
YBX1P5	ENSG00000251108	2.2774	0.011383	0.81271	0.20819	1.1547	0.1241	2.2774	0.011383	0.011383	0.37327967
NA	ENSG00000222262	0.81942	0.20627	1.1423	0.12667	0.40371	0.34321	2.2774	0.011383	0.011383	0.37327967

PPFIBP1	ENSG00000110841	0.53979	0.29467	2.2769	0.011396	2.1637	0.015244	1.9494	0.025622	0.011396	0.37345466
ALDH16A1	ENSG00000161618	2.2756	0.011436	2.2756	0.011436	2.2756	0.011436	2.2756	0.011436	0.011436	0.374327562
GGNBP2	ENSG00000278311	1.2612	0.10362	0.74602	0.22783	0.10978	0.45629	2.2755	0.011438	0.011438	0.374327562
HOXA6	ENSG00000106006	2.2746	0.011464	2.2746	0.011464	2.2746	0.011464	2.2746	0.011464	0.011464	0.374926658
LINC01476	ENSG00000265313	2.1895	0.014279	2.274	0.011482	1.1666	0.12168	0.14084	0.444	0.011482	0.375077292
RPL39P29	ENSG00000236860	2.274	0.011484	2.274	0.011484	2.274	0.011484	2.274	0.011484	0.011484	0.375077292
NA	ENSG00000269985	0.35566	0.36105	0.28886	0.38634	2.2733	0.011505	0.43906	0.33031	0.011505	0.375511487
NA	ENSG00000244538	-0.42935	0.66617	2.273	0.011514	0.092257	0.46325	0.092257	0.46325	0.011514	0.375553695
LINC02311	ENSG00000258718	2.2727	0.011523	2.2727	0.011523	2.2727	0.011523	2.2727	0.011523	0.011523	0.375595846
MAP1LC3A	ENSG00000101460	1.414	0.078684	1.8101	0.03514	1.2223	0.1108	2.2724	0.011532	0.011532	0.375637941
LINC02254	ENSG00000259664	-0.30055	0.61812	-0.51619	0.69714	2.2441	0.012414	2.2719	0.011547	0.011547	0.375875291
RNU6-71P	ENSG00000207203	2.2712	0.011567	1.9778	0.023973	-0.29321	0.61532	0.26501	0.3955	0.011567	0.376186498
PLEKHA6	ENSG00000143850	0.97867	0.16387	1.5513	0.060415	1.1397	0.1272	2.271	0.011572	0.011572	0.376186498
KDM3B	ENSG00000120733	2.2707	0.011583	1.751	0.039972	1.9003	0.0287	1.3679	0.085672	0.011583	0.37629306
NA	ENSG00000202536	0.36567	0.35731	0.36567	0.35731	2.2703	0.011595	0.39475	0.34651	0.011595	0.376431945
ALLC	ENSG00000151360	1.8844	0.029753	2.2699	0.011606	1.8844	0.029753	1.6845	0.04604	0.011606	0.376538202
NA	ENSG00000255161	-0.40905	0.65875	-0.40905	0.65875	-0.40905	0.65875	2.2683	0.011655	0.011655	0.377876347
SEMA6C	ENSG00000143434	2.0794	0.018792	1.7565	0.039504	1.6571	0.048754	2.2678	0.011672	0.011672	0.37805414
NA	ENSG00000206849	2.2676	0.011676	2.2676	0.011676	2.2676	0.011676	2.2676	0.011676	0.011676	0.37805414
NA	ENSG00000279985	1.4397	0.074973	2.267	0.011695	1.4397	0.074973	1.4397	0.074973	0.011695	0.378417895
MIR5587	ENSG00000266124	2.2665	0.011712	2.2665	0.011712	2.2665	0.011712	2.2665	0.011712	0.011712	0.378716496
MIR4675	ENSG00000265372	0.47801	0.31632	2.2656	0.011738	0.084516	0.46632	0.084516	0.46632	0.011738	0.379305531
RNU6-1174P	ENSG00000253087	0.93835	0.17403	1.6401	0.050496	2.265	0.011755	2.1844	0.014468	0.011755	0.379416298
NA	ENSG00000272588	2.265	0.011757	-0.01937	0.50773	1.7873	0.036947	1.7873	0.036947	0.011757	0.379416298
NA	ENSG00000238559	2.2645	0.011773	2.2645	0.011773	2.2645	0.011773	2.2645	0.011773	0.011773	0.379681198
ISCA2	ENSG00000165898	2.2641	0.011785	2.2641	0.011785	2.2641	0.011785	2.2641	0.011785	0.011785	0.379816832
ARNTL2-AS1	ENSG00000245311	1.4206	0.07772	0.80638	0.21001	2.2632	0.011811	2.1296	0.016601	0.011811	0.38028068
RNU6-1125P	ENSG00000200471	1.6188	0.052748	2.2631	0.011815	2.2631	0.011815	2.2631	0.011815	0.011815	0.38028068
TACR3	ENSG00000169836	1.1063	0.13429	2.2626	0.011829	0.10535	0.45805	0.10535	0.45805	0.011829	0.380337971
NA	ENSG00000251170	1.5436	0.061338	1.1476	0.12556	2.2624	0.011836	1.9557	0.02525	0.011836	0.380337971
LIN7A	ENSG00000111052	-0.88006	0.81059	2.2621	0.011844	-0.76349	0.77741	-0.76349	0.77741	0.011844	0.380337971

GATD1	ENSG00000177225	0.051393	0.47951	1.2096	0.11322	2.262	0.011848	0.91941	0.17894	0.011848	0.380337971
NA	ENSG00000284048	0.74372	0.22852	1.029	0.15174	0.74372	0.22852	2.2614	0.011868	0.011868	0.380671066
PGP	ENSG00000184207	2.2612	0.011874	2.2612	0.011874	2.2612	0.011874	2.2612	0.011874	0.011874	0.380671066
MIR4719	ENSG00000266426	1.6224	0.052355	2.2606	0.011893	1.0856	0.13884	1.6224	0.052355	0.011893	0.380971268
SRSF3P6	ENSG00000233307	2.2604	0.011899	-0.1679	0.56667	0.36534	0.35743	1.1039	0.13482	0.011899	0.380971268
NA	ENSG00000116883	2.2597	0.011921	-0.16802	0.56672	2.1839	0.014485	1.9438	0.025959	0.011921	0.381206732
NA	ENSG00000233714	0.29932	0.38235	0.16833	0.43316	2.2596	0.011922	0.16833	0.43316	0.011922	0.381206732
BHLHB9P1	ENSG00000258915	1.4196	0.077869	1.4196	0.077869	2.2593	0.011931	1.4196	0.077869	0.011931	0.381244348
RNU6-673P	ENSG00000201954	1.9983	0.022842	2.2585	0.011958	1.9983	0.022842	1.9983	0.022842	0.011958	0.381516355
KIF11	ENSG00000138160	-0.1247	0.54962	-0.1247	0.54962	-0.1247	0.54962	2.2583	0.011963	0.011963	0.381516355
RNA5SP46	ENSG00000199240	-0.62584	0.73429	-0.51503	0.69673	-0.51503	0.69673	2.2583	0.011963	0.011963	0.381516355
TBL2	ENSG00000106638	2.258	0.011974	2.258	0.011974	2.258	0.011974	-0.33507	0.63121	0.011974	0.38161741
TACR1	ENSG00000115353	1.1867	0.11767	2.2575	0.011987	2.2575	0.011987	0.79964	0.21196	0.011987	0.381782033
LCN1	ENSG00000160349	0.32539	0.37244	2.257	0.012004	-1.2957	0.90245	0.018944	0.49244	0.012004	0.382073756
NA	ENSG00000212144	1.5971	0.055119	0.9913	0.16077	2.2568	0.012012	0.31495	0.3764	0.012012	0.382078825
NA	ENSG00000231357	2.1137	0.017271	2.2565	0.012021	2.0441	0.020474	1.7623	0.039005	0.012021	0.382115675
NA	ENSG00000206028	-0.0099212	0.50396	2.2562	0.012029	0.36232	0.35855	0.83135	0.20289	0.012029	0.382120711
NA	ENSG00000238195	2.2554	0.012055	2.2554	0.012055	2.2554	0.012055	2.2554	0.012055	0.012055	0.382674756
ACSS2	ENSG00000131069	0.78516	0.21618	2.2549	0.012068	2.2549	0.012068	-0.47312	0.68194	0.012068	0.382674756
NA	ENSG00000249341	0.6777	0.24898	0.77668	0.21867	-0.49316	0.68905	2.2549	0.01207	0.01207	0.382674756
ACOX1	ENSG00000161533	2.2539	0.012103	2.2539	0.012103	1.0891	0.13805	-0.20086	0.57959	0.012103	0.383471515
LINC00391	ENSG00000238230	1.3426	0.089693	1.3426	0.089693	2.2533	0.012121	1.3426	0.089693	0.012121	0.383792287
LINC02263	ENSG00000228358	2.2523	0.012151	2.2523	0.012151	2.0383	0.020758	2.2523	0.012151	0.012151	0.384467691
SPATA31C1	ENSG00000230246	2.2519	0.012163	-1.7443	0.95944	1.2705	0.10195	0.62974	0.26443	0.012163	0.384467691
B4GALT5	ENSG00000158470	-0.43627	0.66868	-0.41866	0.66227	2.2519	0.012166	-0.83401	0.79786	0.012166	0.384467691
NA	ENSG00000280046	2.251	0.012192	2.251	0.012192	2.251	0.012192	2.251	0.012192	0.012192	0.385039637
NA	ENSG00000263618	1.911	0.028003	2.2506	0.012205	1.6928	0.045251	0.82155	0.20567	0.012205	0.385200551
NA	ENSG00000222001	-0.76069	0.77658	-0.39203	0.65248	2.2503	0.012215	-0.76069	0.77658	0.012215	0.385266634
DGAT2	ENSG00000062282	2.25	0.012224	1.2599	0.10386	-0.89359	0.81423	-0.28413	0.61185	0.012224	0.385301113
RPL5P4	ENSG00000229994	0.1387	0.44485	0.1387	0.44485	0.1387	0.44485	2.2494	0.012242	0.012242	0.385619043
VAT1L	ENSG00000171724	1.6611	0.048343	1.8097	0.035169	2.2484	0.012276	2.1095	0.01745	0.012276	0.386440233

ECE2	ENSG00000145194	1.8957	0.028999	0.34401	0.36542	2.2479	0.012291	-1.7603	0.96082	0.012291	0.38666264
BCL11A	ENSG00000119866	-0.77243	0.78007	0.68909	0.24538	2.2474	0.012308	-0.289	0.61371	0.012308	0.386698156
CCDC190	ENSG00000185860	2.2474	0.012308	1.7678	0.038551	0.51273	0.30407	1.0144	0.15519	0.012308	0.386698156
NME2	ENSG00000243678	2.2468	0.012325	2.2468	0.012325	2.2468	0.012325	2.2468	0.012325	0.012325	0.386982764
SMARCD1	ENSG00000066117	2.2463	0.012342	1.5839	0.05661	1.3628	0.086473	1.8799	0.030058	0.012342	0.386993549
KCNC2	ENSG00000166006	1.741	0.040845	2.2461	0.012349	2.2461	0.012349	2.1332	0.016455	0.012349	0.386993549
ALDH1A2	ENSG00000128918	2.2459	0.012356	1.227	0.10991	0.35137	0.36265	2.1661	0.015154	0.012356	0.386993549
DECR2	ENSG00000242612	0.15619	0.43794	1.1348	0.12824	2.2447	0.012393	0.53695	0.29565	0.012393	0.386993549
NA	ENSG00000251310	1.3982	0.081023	2.1433	0.016044	2.2245	0.013057	2.2445	0.012399	0.012399	0.386993549
NA	ENSG00000277397	0.80489	0.21044	2.2443	0.012406	0.59475	0.27601	1.0833	0.13935	0.012406	0.386993549
HTR3C	ENSG00000178084	2.244	0.012415	-0.27686	0.60906	-0.59695	0.72473	-0.7284	0.76682	0.012415	0.386993549
MFAP3	ENSG00000037749	1.2053	0.11405	1.1668	0.12165	1.2213	0.11098	2.244	0.012417	0.012417	0.386993549
FFAR4	ENSG00000186188	2.2439	0.01242	0.70502	0.2404	0.70502	0.2404	0.70502	0.2404	0.01242	0.386993549
APOE	ENSG00000130203	0.71622	0.23693	-0.21399	0.58472	-0.32259	0.6265	2.2436	0.012428	0.012428	0.386993549
MRM1	ENSG00000278619	2.2427	0.012459	1.6328	0.051255	-0.1703	0.56761	0.17372	0.43104	0.012459	0.386993549
RNA5SP454	ENSG00000253040	2.2423	0.012471	1.5242	0.06373	1.1668	0.12164	2.1309	0.01655	0.012471	0.386993549
DCLK1	ENSG00000133083	-1.0218	0.84657	0.52109	0.30115	2.2418	0.012488	1.3622	0.086571	0.012488	0.386993549
FKBP4P1	ENSG00000251463	2.2418	0.012489	-0.32383	0.62697	-1.2113	0.8871	-0.32383	0.62697	0.012489	0.386993549
PCBP3	ENSG00000183570	1.0555	0.1456	2.0808	0.018728	2.2417	0.012491	0.59093	0.27728	0.012491	0.386993549
ARFGAP2	ENSG00000149182	1.1816	0.11868	1.1816	0.11868	1.1816	0.11868	2.2416	0.012493	0.012493	0.386993549
SEZ6L-AS1	ENSG00000224192	2.1398	0.016186	2.2416	0.012494	2.2416	0.012494	1.482	0.069176	0.012494	0.386993549
DHFR	ENSG00000228716	2.2415	0.012497	0.87593	0.19053	0.084248	0.46643	1.0072	0.15692	0.012497	0.386993549
NA	ENSG00000230339	2.2415	0.012498	0.17976	0.42867	0.77354	0.2196	1.092	0.13742	0.012498	0.386993549
STMND1	ENSG00000230873	-0.62776	0.73492	-0.29239	0.61501	0.50045	0.30838	2.2412	0.012505	0.012505	0.386993549
NA	ENSG00000233947	2.2412	0.012505	-0.73909	0.77007	-0.40383	0.65683	-0.40383	0.65683	0.012505	0.386993549
CCDC51	ENSG00000164051	0.95368	0.17012	0.77957	0.21782	2.2412	0.012507	1.5811	0.05693	0.012507	0.386993549
CACNA1C-AS2	ENSG00000256271	2.2411	0.012508	0.23842	0.40578	2.2411	0.012508	2.2411	0.012508	0.012508	0.386993549
DCAF13P2	ENSG00000250816	0.71099	0.23854	0.15042	0.44022	0.17254	0.43151	2.2406	0.012525	0.012525	0.38702806
NA	ENSG00000258750	0.91718	0.17952	0.91718	0.17952	2.2406	0.012525	-1.0532	0.85387	0.012525	0.38702806
RPL31P58	ENSG00000243053	2.2401	0.012544	2.2401	0.012544	2.2401	0.012544	2.2401	0.012544	0.012544	0.387102014
NA	ENSG00000279089	2.2399	0.012549	2.2399	0.012549	2.2399	0.012549	2.2399	0.012549	0.012549	0.387102014

NA	ENSG00000197320	2.2397	0.012555	0.69355	0.24398	-0.22306	0.58826	-0.47275	0.6818	0.012555	0.387102014
MAPK10	ENSG00000109339	2.2391	0.012576	0.075436	0.46993	-0.17452	0.56927	-1.1806	0.88113	0.012576	0.387102014
RPS7P9	ENSG00000233353	2.239	0.012577	2.239	0.012577	2.239	0.012577	2.239	0.012577	0.012577	0.387102014
TRPV6	ENSG00000165125	2.2389	0.012583	1.9759	0.024081	0.17467	0.43067	1.5782	0.057257	0.012583	0.387102014
LINC02262	ENSG00000224932	2.2388	0.012583	1.8424	0.032709	2.2388	0.012583	2.2388	0.012583	0.012583	0.387102014
PNPLA6	ENSG00000032444	-2.5704	0.99492	-1.1754	0.88008	0.065164	0.47402	2.2381	0.012606	0.012606	0.38753707
NA	ENSG00000231443	1.0472	0.1475	-0.89762	0.81531	2.2378	0.012616	0.13422	0.44661	0.012616	0.38753707
NA	ENSG00000240979	2.2377	0.012621	2.2377	0.012621	2.2377	0.012621	2.2377	0.012621	0.012621	0.38753707
NA	ENSG00000252874	2.2371	0.012639	2.2371	0.012639	2.2371	0.012639	2.2371	0.012639	0.012639	0.387754638
MC1R	ENSG00000258839	2.237	0.012644	2.237	0.012644	2.237	0.012644	2.237	0.012644	0.012644	0.387754638
NA	ENSG00000253342	1.8685	0.030844	1.8685	0.030844	1.1418	0.12677	2.236	0.012675	0.012675	0.388460849
NA	ENSG00000258422	2.2357	0.012684	2.2357	0.012684	2.2357	0.012684	0.15388	0.43885	0.012684	0.388462582
ACP6	ENSG00000162836	2.2355	0.012691	0.3647	0.35767	2.0048	0.022491	-0.35675	0.63936	0.012691	0.388462582
CYP2A13	ENSG00000197838	0.69553	0.24336	0.73835	0.23015	-0.63794	0.73824	2.235	0.012707	0.012707	0.388708167
CYCSP1	ENSG00000271423	-1.166	0.87819	-0.97938	0.8363	0.12973	0.44839	2.2343	0.012732	0.012732	0.388711708
ZNF444P1	ENSG00000259738	2.2343	0.012733	2.2343	0.012733	2.2343	0.012733	2.2343	0.012733	0.012733	0.388711708
ATG9A	ENSG00000198925	1.9229	0.027249	2.2342	0.012737	2.1544	0.015603	2.1544	0.015603	0.012737	0.388711708
RN7SL246P	ENSG00000265093	2.234	0.012742	2.234	0.012742	2.234	0.012742	2.234	0.012742	0.012742	0.388711708
HK3	ENSG00000160883	-1.0466	0.85236	2.2338	0.012747	1.4487	0.073709	1.3507	0.088396	0.012747	0.388711708
SCOC-AS1	ENSG00000196951	0.51511	0.30324	-0.076426	0.53046	2.2335	0.012758	0.0030033	0.4988	0.012758	0.38880384
NA	ENSG00000249658	2.233	0.012775	2.233	0.012775	2.233	0.012775	2.233	0.012775	0.012775	0.388926883
XRCC4	ENSG00000152422	0.23247	0.40809	-0.62659	0.73454	2.2329	0.012778	-1.3064	0.90429	0.012778	0.388926883
MTCO2P34	ENSG00000232198	2.2323	0.012798	2.2323	0.012798	2.2323	0.012798	2.2323	0.012798	0.012798	0.389141253
MYMX	ENSG00000262179	2.2321	0.012804	2.2312	0.012832	1.8253	0.033978	1.8253	0.033978	0.012804	0.389141253
VPS9D1-AS1	ENSG00000261373	2.2319	0.012809	2.2319	0.012809	2.2319	0.012809	2.2319	0.012809	0.012809	0.389141253
GRIK4	ENSG00000149403	0.07296	0.47092	1.6046	0.05429	2.2308	0.012847	0.98333	0.16272	0.012847	0.389567257
NA	ENSG00000278531	2.2308	0.012848	1.2815	0.10001	1.5911	0.055795	1.5152	0.064865	0.012848	0.389567257
ZC3H6	ENSG00000188177	0.21161	0.41621	0.66282	0.25372	1.3833	0.08329	2.2306	0.012852	0.012852	0.389567257
NA	ENSG00000236108	2.2306	0.012855	2.2306	0.012855	2.2306	0.012855	1.6654	0.047917	0.012855	0.389567257
WFIKKN1	ENSG00000127578	2.2296	0.012886	2.0497	0.020197	2.0497	0.020197	2.0497	0.020197	0.012886	0.390264002
FAM200A	ENSG00000221909	2.2292	0.012901	1.989	0.023351	2.2292	0.012901	2.2292	0.012901	0.012901	0.390384132

LINC01549	ENSG00000232560	0.26524	0.39541	0.26524	0.39541	0.19517	0.42263	2.229	0.012908	0.012908	0.390384132
PCDH9-AS2	ENSG00000228842	2.2288	0.012914	2.2288	0.012914	1.6684	0.047616	1.8533	0.031922	0.012914	0.390384132
MIR4799	ENSG00000264274	2.2278	0.012947	2.2278	0.012947	2.2278	0.012947	1.0747	0.14126	0.012947	0.391139064
NA	ENSG00000226197	2.2272	0.012967	-0.95546	0.83033	1.9385	0.026279	0.22332	0.41164	0.012967	0.391500564
EIF2B5	ENSG00000145191	1.8879	0.029523	-1.8109	0.96492	-1.3638	0.91369	2.2262	0.013001	0.013001	0.392284043
ARNTL2	ENSG00000029153	2.2255	0.013024	1.2465	0.10629	0.9501	0.17103	1.2926	0.098067	0.013024	0.392639191
TUBGCP3	ENSG00000126216	1.5234	0.063833	0.57202	0.28365	2.225	0.01304	1.7655	0.038742	0.01304	0.392639191
NA	ENSG00000207351	-0.69026	0.75499	-0.69026	0.75499	2.2249	0.013043	-0.73812	0.76978	0.013043	0.392639191
NA	ENSG00000274678	2.2249	0.013045	2.2249	0.013045	2.2249	0.013045	2.2249	0.013045	0.013045	0.392639191
MRPL45P2	ENSG00000228782	1.7191	0.042801	1.919	0.027491	2.2243	0.013064	0.41141	0.34038	0.013064	0.392968346
ADAMTSL2	ENSG00000197859	1.0958	0.13659	1.2202	0.11119	2.2238	0.013082	1.7792	0.037604	0.013082	0.393267033
BTF3L4P3	ENSG00000213108	2.2232	0.013101	2.2232	0.013101	2.2232	0.013101	2.2232	0.013101	0.013101	0.393320714
NCR1	ENSG00000189430	2.197	0.014011	2.1993	0.013929	0.051842	0.47933	2.223	0.013108	0.013108	0.393320714
PPIAP28	ENSG00000226633	-0.26862	0.60589	0.32463	0.37273	0.52485	0.29985	2.223	0.013108	0.013108	0.393320714
NA	ENSG00000273816	2.2223	0.013132	2.2223	0.013132	2.2223	0.013132	2.2223	0.013132	0.013132	0.393798375
PPP1CB	ENSG00000213639	2.2217	0.013153	0.54273	0.29366	1.2038	0.11434	0.73748	0.23042	0.013153	0.393940743
GPR162	ENSG00000250510	-0.53145	0.70245	2.2216	0.013154	-0.53145	0.70245	-0.53145	0.70245	0.013154	0.393940743
NA	ENSG00000266846	-0.75769	0.77568	-0.0076349	0.50305	-0.0076349	0.50305	2.2214	0.013161	0.013161	0.393940743
AHCY	ENSG00000101444	2.2211	0.013171	1.2892	0.098665	1.1857	0.11786	1.0899	0.13787	0.013171	0.393998054
NA	ENSG00000267662	-0.68663	0.75384	-0.72732	0.76649	1.3739	0.084735	2.2198	0.013216	0.013216	0.395101644
RAB1A	ENSG00000138069	1.9056	0.02835	2.1504	0.01576	1.9056	0.02835	2.219	0.013244	0.013244	0.395657648
IL17RC	ENSG00000163702	-0.17417	0.56913	2.2187	0.013254	0.83705	0.20128	0.87855	0.18982	0.013254	0.395657648
NA	ENSG00000219404	1.2022	0.11463	2.2183	0.013268	2.2183	0.013268	2.2183	0.013268	0.013268	0.395657648
NA	ENSG00000225118	-0.45588	0.67576	0.62756	0.26515	2.0503	0.020167	2.2177	0.013288	0.013288	0.395657648
HELLS	ENSG00000119969	2.2176	0.013292	0.1419	0.44358	-0.18309	0.57264	-0.26737	0.60541	0.013292	0.395657648
SLA	ENSG00000155926	-0.75314	0.77432	-0.75314	0.77432	-1.0433	0.8516	2.2172	0.013304	0.013304	0.395657648
SNAP47	ENSG00000143740	0.18525	0.42652	2.2168	0.013319	-0.00254	0.50101	1.2345	0.1085	0.013319	0.395657648
NDUFB4P6	ENSG00000235258	0.52631	0.29934	2.2165	0.013327	1.3848	0.083052	1.5988	0.054931	0.013327	0.395657648
NA	ENSG00000177788	-0.75133	0.77377	0.40787	0.34168	2.2162	0.013337	-0.47362	0.68212	0.013337	0.395657648
KLHL5	ENSG00000109790	0.034628	0.48619	2.2162	0.01334	-0.31869	0.62502	0.42316	0.33609	0.01334	0.395657648
LRRC10B	ENSG00000204950	2.2161	0.013342	2.2161	0.013342	2.2161	0.013342	0.57557	0.28245	0.013342	0.395657648

MIR4488	ENSG00000266006	0.36554	0.35736	2.2161	0.013342	2.2161	0.013342	2.2161	0.013342	0.013342	0.395657648
ATG13	ENSG00000175224	-0.0019327	0.50077	1.3882	0.082541	1.7713	0.038252	2.216	0.013346	0.013346	0.395657648
WNT8A	ENSG00000061492	0.36718	0.35674	0.33719	0.36799	0.97156	0.16564	2.2157	0.013355	0.013355	0.395657648
NA	ENSG00000229172	2.2149	0.013383	2.2149	0.013383	2.2149	0.013383	2.2149	0.013383	0.013383	0.395657648
NA	ENSG00000280305	1.6283	0.051725	2.2149	0.013383	2.2149	0.013383	2.2149	0.013383	0.013383	0.395657648
NATD1	ENSG00000274180	0.16161	0.43581	2.1921	0.014187	2.2147	0.013389	1.8108	0.035088	0.013389	0.395657648
LINC02586	ENSG00000224965	2.214	0.013414	-0.36251	0.64152	0.5039	0.30717	0.5039	0.30717	0.013414	0.395657648
RPL13	ENSG00000167526	2.2138	0.013422	-0.24394	0.59636	1.0707	0.14215	1.0106	0.1561	0.013422	0.395657648
MIR140	ENSG00000208017	2.2137	0.013424	2.2137	0.013424	2.2137	0.013424	2.2137	0.013424	0.013424	0.395657648
NA	ENSG00000223119	0.74785	0.22728	2.2137	0.013425	1.5136	0.065061	-0.26446	0.60429	0.013425	0.395657648
SPSB3	ENSG00000162032	0.12215	0.45139	2.2136	0.013426	0.12215	0.45139	0.12215	0.45139	0.013426	0.395657648
RNF214	ENSG00000167257	0.36286	0.35835	-0.62477	0.73394	-0.62477	0.73394	2.2135	0.013432	0.013432	0.395657648
CST13P	ENSG00000204663	0.28878	0.38638	0.98743	0.16172	-0.42975	0.66631	2.2125	0.013465	0.013465	0.395657648
ARPC1B	ENSG00000130429	2.2122	0.013476	1.5545	0.060038	1.0597	0.14465	1.3889	0.082437	0.013476	0.395657648
NA	ENSG00000255575	2.2122	0.013476	2.2122	0.013476	2.2122	0.013476	2.2122	0.013476	0.013476	0.395657648
LINC01916	ENSG00000264232	1.1867	0.11767	2.2121	0.01348	-0.24944	0.59849	-0.57833	0.71848	0.01348	0.395657648
NA	ENSG00000232172	0.77032	0.22056	2.2119	0.013487	2.2119	0.013487	0.36554	0.35735	0.013487	0.395657648
PRIMA1	ENSG00000175785	0.87676	0.19031	2.2116	0.013498	0.75614	0.22478	0.61039	0.2708	0.013498	0.395657648
ABCC5-AS1	ENSG00000223882	1.0733	0.14157	1.8757	0.030349	2.211	0.013519	2.0666	0.019384	0.013519	0.395657648
DNAJC24	ENSG00000170946	2.2105	0.013536	2.0781	0.01885	0.42275	0.33624	2.2105	0.013536	0.013536	0.395657648
NA	ENSG00000264312	1.9884	0.023386	0.67668	0.2493	2.2103	0.013543	2.2103	0.013543	0.013543	0.395657648
RNU2-9P	ENSG00000222126	1.5629	0.059039	1.5629	0.059039	1.5629	0.059039	2.2099	0.013557	0.013557	0.395657648
RNU6ATAC3P	ENSG00000210709	0.57145	0.28385	2.2099	0.013558	0.04059	0.48381	0.04059	0.48381	0.013558	0.395657648
LMO3	ENSG00000048540	-0.5234	0.69965	-0.7165	0.76316	0.040472	0.48386	2.2096	0.013565	0.013565	0.395657648
PRKAR2A-AS1	ENSG00000224424	2.2095	0.01357	2.2095	0.01357	2.2095	0.01357	2.2095	0.01357	0.01357	0.395657648
PGLYRP2	ENSG00000161031	2.2093	0.013576	2.1	0.017866	1.129	0.12945	2.2093	0.013576	0.013576	0.395657648
LINC00861	ENSG00000245164	2.2093	0.013577	2.2093	0.013577	0.78042	0.21757	1.9394	0.026225	0.013577	0.395657648
ZMAT5	ENSG00000100319	2.209	0.013587	2.209	0.013587	2.209	0.013587	0.371	0.35532	0.013587	0.395657648
NA	ENSG00000253343	1.389	0.082416	1.9882	0.023393	2.209	0.013587	2.209	0.013587	0.013587	0.395657648
RPL7P23	ENSG00000244363	1.5791	0.057157	1.9641	0.024762	2.209	0.013589	1.1962	0.11581	0.013589	0.395657648
NA	ENSG00000263781	2.2089	0.01359	2.2089	0.01359	2.2089	0.01359	2.2089	0.01359	0.01359	0.395657648

RN7SKP222	ENSG00000222583	-0.18744	0.57434	-0.20664	0.58186	2.2088	0.013593	0.28484	0.38788	0.013593	0.395657648
TRBV7-5	ENSG00000227550	2.2087	0.013599	1.2357	0.10828	2.2087	0.013599	2.2087	0.013599	0.013599	0.395657648
NA	ENSG00000283338	2.2085	0.013605	2.2085	0.013605	2.2085	0.013605	2.2085	0.013605	0.013605	0.395657648
NA	ENSG00000231756	2.2084	0.013609	2.2084	0.013609	-0.23974	0.59473	2.2084	0.013609	0.013609	0.395657648
NA	ENSG00000278291	2.2076	0.013635	1.7266	0.042119	1.7266	0.042119	1.7266	0.042119	0.013635	0.395657648
OR7E38P	ENSG00000183444	0.68454	0.24682	0.68454	0.24682	0.68454	0.24682	2.2076	0.013637	0.013637	0.395657648
NA	ENSG00000237070	2.2072	0.013649	2.2072	0.013649	2.2072	0.013649	0.7948	0.21336	0.013649	0.395657648
TAF4B	ENSG00000141384	0.23127	0.40855	1.3828	0.083366	1.2116	0.11283	2.2071	0.013654	0.013654	0.395657648
NA	ENSG00000258695	-0.66239	0.74614	2.2068	0.013664	-0.52753	0.70109	-0.52753	0.70109	0.013664	0.395657648
NA	ENSG00000278475	2.2066	0.01367	2.2066	0.01367	2.2066	0.01367	2.2066	0.01367	0.01367	0.395657648
CHMP4A	ENSG00000254505	2.206	0.013693	2.206	0.013693	2.206	0.013693	2.206	0.013693	0.013693	0.395657648
NA	ENSG00000254692	2.206	0.013693	2.206	0.013693	2.206	0.013693	2.206	0.013693	0.013693	0.395657648
SLC33A1	ENSG00000169359	2.0601	0.019693	2.1686	0.015057	2.206	0.013694	1.3298	0.091799	0.013694	0.395657648
LINC01587	ENSG00000082929	2.2056	0.013707	0.64795	0.25851	2.0199	0.021695	1.8996	0.028745	0.013707	0.395657648
NA	ENSG00000264869	0.41872	0.33771	0.89652	0.18499	2.2054	0.013713	-0.87525	0.80928	0.013713	0.395657648
MIR26B	ENSG00000199121	2.2052	0.013721	2.2052	0.013721	2.2052	0.013721	2.2052	0.013721	0.013721	0.395657648
C1orf167	ENSG00000215910	2.205	0.013726	1.3949	0.08152	1.3949	0.08152	1.3949	0.08152	0.013726	0.395657648
NA	ENSG00000229498	2.205	0.013728	-0.94337	0.82726	-0.95887	0.83119	-0.6767	0.7507	0.013728	0.395657648
MIS18A	ENSG00000159055	0.59765	0.27504	-1.7998	0.96405	2.2049	0.013731	0.6713	0.25101	0.013731	0.395657648
NA	ENSG00000279676	2.2047	0.013738	2.2047	0.013738	2.2047	0.013738	2.2047	0.013738	0.013738	0.395657648
NA	ENSG00000261227	2.2042	0.013756	-0.7317	0.76782	-1.552	0.93967	-0.7317	0.76782	0.013756	0.395765844
LNX1	ENSG00000072201	0.92498	0.17749	1.7547	0.039658	2.2041	0.013758	-1.2877	0.90108	0.013758	0.395765844
TPD52L2	ENSG00000101150	2.2031	0.013795	1.9127	0.027891	0.81509	0.20751	1.417	0.07824	0.013795	0.396390497
MIR320B2	ENSG00000221406	2.2028	0.013806	2.2028	0.013806	2.2028	0.013806	1.8926	0.029204	0.013806	0.396390497
COX20P2	ENSG00000235013	2.2022	0.013825	-1.0418	0.85124	-1.0418	0.85124	-1.0418	0.85124	0.013825	0.396390497
PHLDA3	ENSG00000174307	1.2761	0.10097	1.288	0.098876	-0.6665	0.74745	2.2017	0.013842	0.013842	0.396390497
NA	ENSG00000283403	1.4123	0.078935	0.88502	0.18807	1.2961	0.097475	2.2015	0.013849	0.013849	0.396390497
RN7SL432P	ENSG00000242769	1.122	0.13093	2.2014	0.013853	2.2014	0.013853	2.2014	0.013853	0.013853	0.396390497
NA	ENSG00000261602	2.2014	0.013854	2.2014	0.013854	2.2014	0.013854	2.2014	0.013854	0.013854	0.396390497
NA	ENSG00000254775	0.92401	0.17774	2.1885	0.014317	2.2011	0.013865	0.57858	0.28144	0.013865	0.396390497
RN7SL117P	ENSG00000264192	2.2011	0.013865	2.2011	0.013865	2.2011	0.013865	2.2011	0.013865	0.013865	0.396390497

GMPR2	ENSG00000100938	2.2006	0.013883	2.2006	0.013883	1.7773	0.037759	2.2006	0.013883	0.013883	0.396390497
GSTZ1	ENSG00000100577	1.4024	0.080397	1.4024	0.080397	1.4024	0.080397	2.2003	0.013892	0.013892	0.396390497
NA	ENSG00000230730	1.4347	0.075687	2.2002	0.013895	1.4347	0.075687	1.4347	0.075687	0.013895	0.396390497
RNA5SP307	ENSG00000199733	2.2002	0.013898	2.2002	0.013898	2.2002	0.013898	2.2002	0.013898	0.013898	0.396390497
PKD1L3	ENSG00000277481	2.1999	0.013906	2.1999	0.013906	2.1999	0.013906	1.5789	0.057185	0.013906	0.396390497
REEP6	ENSG00000115255	2.1996	0.013916	2.133	0.016462	2.1996	0.013916	2.1996	0.013916	0.013916	0.396390497
NA	ENSG00000249787	2.1996	0.013917	2.0196	0.021712	2.1711	0.014963	0.32447	0.37279	0.013917	0.396390497
NA	ENSG00000225860	2.1996	0.013918	2.1996	0.013918	2.1996	0.013918	2.1996	0.013918	0.013918	0.396390497
NA	ENSG00000269529	0.84394	0.19935	2.1992	0.013932	0.74785	0.22727	-0.19319	0.5766	0.013932	0.396411296
RN7SKP172	ENSG00000223273	-0.57669	0.71793	2.1991	0.013935	0.14481	0.44243	-0.65214	0.74284	0.013935	0.396411296
FGF3	ENSG00000186895	2.1985	0.013957	1.7494	0.040109	0.96258	0.16788	0.7556	0.22495	0.013957	0.39680549
NA	ENSG00000255102	-0.19115	0.5758	0.82513	0.20465	2.1977	0.013985	-0.0088794	0.50354	0.013985	0.397138141
NA	ENSG00000255241	2.1977	0.013985	2.1977	0.013985	2.1977	0.013985	2.1977	0.013985	0.013985	0.397138141
NA	ENSG00000249050	2.197	0.014011	0.51916	0.30183	2.197	0.014011	2.197	0.014011	0.014011	0.397437231
BBS10	ENSG00000179941	2.1969	0.014013	2.068	0.019319	-1.0202	0.84619	0.11833	0.4529	0.014013	0.397437231
MTND6P14	ENSG00000235638	2.1967	0.01402	1.489	0.068246	2.1967	0.01402	2.1967	0.01402	0.01402	0.397437231
KCNJ1	ENSG00000151704	1.8187	0.034482	2.1954	0.014068	1.2842	0.099541	1.8123	0.034973	0.014068	0.398220318
NA	ENSG00000212590	2.195	0.014083	2.195	0.014083	2.195	0.014083	2.195	0.014083	0.014083	0.398220318
PPP1R9B	ENSG00000108819	2.1949	0.014085	2.1949	0.014085	2.1779	0.014705	0.7126	0.23805	0.014085	0.398220318
YAE1	ENSG00000241127	1.2216	0.11093	2.1949	0.014086	1.7955	0.036284	1.2577	0.10425	0.014086	0.398220318
NA	ENSG00000232604	2.1944	0.014103	2.1944	0.014103	2.1944	0.014103	2.1944	0.014103	0.014103	0.398220318
FEV	ENSG00000163497	-0.18968	0.57522	0.32944	0.37091	2.1943	0.014106	0.353	0.36204	0.014106	0.398220318
SNN	ENSG00000184602	1.9207	0.027386	2.166	0.015156	2.166	0.015156	2.1941	0.014112	0.014112	0.398220318
COX6CP8	ENSG00000253231	1.6353	0.050997	-0.85122	0.80268	-0.69776	0.75734	2.1941	0.014113	0.014113	0.398220318
BNIP3L	ENSG00000104765	1.02	0.15387	0.86972	0.19223	2.193	0.014152	0.68583	0.24641	0.014152	0.398797306
NA	ENSG00000272896	2.193	0.014153	2.193	0.014153	2.193	0.014153	2.193	0.014153	0.014153	0.398797306
NA	ENSG00000249213	0.41697	0.33835	2.1929	0.014158	2.1929	0.014158	2.1929	0.014158	0.014158	0.398797306
GYPA	ENSG00000170180	2.1922	0.014181	0.14347	0.44296	1.6102	0.053675	1.6292	0.051632	0.014181	0.398866157
AMER3	ENSG00000178171	0.54415	0.29317	-1.0537	0.854	0.89317	0.18588	2.1922	0.014184	0.014184	0.398866157
NA	ENSG00000267787	2.1921	0.014185	1.0315	0.15115	0.53901	0.29494	1.0023	0.15809	0.014185	0.398866157
RNA5SP150	ENSG00000199839	-0.7882	0.78471	-0.7882	0.78471	-0.7882	0.78471	2.1916	0.014205	0.014205	0.399198183

NA ENSG00000222326 2.1905 0.014245 2.1905 0.014245 2.1905 0.014245
2.1905 0.014245 0.014245 0.399759735
PXYLP1 ENSG00000155893 0.33073 0.37042 1.1557 0.12391 2.1904
0.014248 1.4797 0.069473 0.014248 0.399759735
NA ENSG00000225982 0.93725 0.17431 0.67829 0.24879 2.1902 0.014256
1.0273 0.15214 0.014256 0.399759735
NA ENSG00000257520 2.1901 0.01426 2.1901 0.01426 2.1901 0.01426
2.1901 0.01426 0.01426 0.399759735
NREP-AS1 ENSG00000250095 2.1899 0.014266 2.1899 0.014266 2.1899
0.014266 0.49691 0.30963 0.014266 0.399759735
RN7SL587P ENSG00000240233 0.58585 0.27899 2.1887 0.014308 0.58585
0.27899 0.58585 0.27899 0.014308 0.400469966
B3GALT2 ENSG00000162630 -0.28822 0.61341 -0.28822 0.61341 2.1887
0.014311 -0.28822 0.61341 0.014311 0.400469966
MIAT ENSG00000225783 2.1885 0.014316 -0.090834 0.53619 0.35706 0.36052
1.8864 0.029622 0.014316 0.400469966
SMIM5 ENSG00000204323 2.0681 0.019314 2.1875 0.014353 2.1875 0.014353
1.3526 0.08809 0.014353 0.400647281
CLCN2 ENSG00000114859 1.8651 0.031082 2.1873 0.014361 1.9439 0.025955
1.662 0.048254 0.014361 0.400647281
ORC1 ENSG00000085840 1.9386 0.026275 2.1873 0.014362 0.28807 0.38665
-0.33627 0.63166 0.014362 0.400647281
RNU6-750P ENSG00000212248 2.1872 0.014365 2.1872 0.014365 2.1872
0.014365 2.1872 0.014365 0.014365 0.400647281
VEZF1 ENSG00000136451 -0.19763 0.57833 0.54966 0.29128 1.7082 0.043798
2.1871 0.014368 0.014368 0.400647281
TUT1 ENSG00000149016 2.1863 0.014396 2.1863 0.014396 0.80799 0.20955
0.46373 0.32142 0.014396 0.400647281
GNAT3 ENSG00000214415 1.5171 0.064618 0.053769 0.47856 1.4452 0.074199
2.186 0.014407 0.014407 0.400647281
NA ENSG00000272710 0.46999 0.31918 1.9145 0.027777 0.84378 0.19939
2.186 0.014407 0.014407 0.400647281
CCDC9 ENSG00000105321 1.9531 0.025401 1.953 0.025411 2.1561 0.015537
2.1859 0.01441 0.01441 0.400647281
HMGN4 ENSG00000182952 1.6477 0.049702 0.94065 0.17344 1.02 0.15386
2.1859 0.014411 0.014411 0.400647281
GOLM1 ENSG00000135052 1.6832 0.04617 0.88775 0.18734 0.5314 0.29757
2.1858 0.014414 0.014414 0.400647281
NA ENSG00000204003 0.31827 0.37514 2.1856 0.014421 -0.50465 0.6931
-0.50465 0.6931 0.014421 0.400647281
GOSR2 ENSG00000108433 1.3407 0.090016 0.66526 0.25294 2.1853 0.014433
-0.042986 0.51714 0.014433 0.400752188
GUCY2GP ENSG00000243316 0.015155 0.49395 2.1848 0.014451 -1.1471
0.87433 -1.8563 0.96829 0.014451 0.40093391
NA ENSG00000234902 1.5246 0.063682 2.1847 0.014456 2.1847 0.014456
2.1847 0.014456 0.014456 0.40093391
PTCD1 ENSG00000106246 2.0894 0.018338 2.1844 0.014467 1.4723 0.070467
0.98198 0.16305 0.014467 0.400970375
NMB ENSG00000197696 1.7183 0.042867 1.7183 0.042867 1.7183 0.042867
2.184 0.01448 0.01448 0.400970375
STXBP5-AS1 ENSG00000233452 1.9242 0.027164 2.184 0.014482 2.1003 0.017851
2.1049 0.01765 0.014482 0.400970375
NA ENSG00000256557 1.7875 0.036931 2.1837 0.014493 1.7875 0.036931
0.18813 0.42539 0.014493 0.40104707
RNU1-94P ENSG00000199497 2.1833 0.014507 2.1833 0.014507 2.1833
0.014507 2.1833 0.014507 0.014507 0.401112947
ARPC1A ENSG00000241685 2.0012 0.022685 1.5268 0.063411 2.1831
0.014515 1.3961 0.081337 0.014515 0.401112947
RIC3-DT ENSG00000246820 2.1828 0.014525 1.6878 0.045727 2.0752
0.018983 0.085582 0.4659 0.014525 0.401112947
EGLN3 ENSG00000129521 2.1824 0.01454 1.2692 0.10219 2.1824 0.01454
-1.2196 0.88869 0.01454 0.401112947

NA	ENSG00000230958	2.1823	0.014545	2.1823	0.014545	2.1823	0.014545	2.1823	0.014545	0.014545	0.401112947
ATP6V1G1P6	ENSG00000249372	2.1821	0.014552	2.1821	0.014552	2.1821	0.014552	2.1821	0.014552	0.014552	0.401112947
NA	ENSG00000229200	-1.2491	0.89418	2.1821	0.014553	-0.042194	0.51683	-0.042194	0.51683	0.014553	0.401112947
LINC02679	ENSG00000226676	-0.64831	0.74161	2.1808	0.014601	-0.78075	0.78252	0.040639	0.48379	0.014601	0.40220844
NA	ENSG00000233969	-0.24317	0.59606	2.1802	0.014623	1.8187	0.03448	-0.24317	0.59606	0.014623	0.402248992
RN7SKP36	ENSG00000200867	1.6609	0.048363	2.1801	0.014624	0.58537	0.27915	0.33471	0.36892	0.014624	0.402248992
SAMD12	ENSG00000177570	2.1799	0.014631	1.4909	0.067988	2.1569	0.015507	-0.21329	0.58445	0.014631	0.402248992
NA	ENSG00000278962	2.1798	0.014636	2.1798	0.014636	2.1798	0.014636	2.1798	0.014636	0.014636	0.402248992
NA	ENSG00000231951	1.0709	0.1421	2.118	0.017085	2.1795	0.014648	2.1795	0.014648	0.014648	0.402248992
THRAP3	ENSG00000054118	2.1794	0.014652	0.80894	0.20927	1.6607	0.04839	-0.60989	0.72903	0.014652	0.402248992
DEFB125	ENSG00000178591	1.1693	0.12115	2.0637	0.019522	2.0053	0.022465	2.1789	0.01467	0.01467	0.402516385
EFCAB11	ENSG00000140025	2.1785	0.014686	1.5745	0.057681	1.478	0.0697	0.26689	0.39478	0.014686	0.402529533
SLC45A4	ENSG00000022567	0.1753	0.43042	0.75426	0.22535	1.2355	0.10832	2.1784	0.014687	0.014687	0.402529533
DUSP7	ENSG00000164086	2.07	0.019226	2.1775	0.014723	1.0351	0.15031	2.022	0.021591	0.014723	0.40328937
PYGO1	ENSG00000171016	1.2584	0.10412	1.9067	0.028282	1.9175	0.027588	2.1767	0.014751	0.014751	0.403556391
NA	ENSG00000261269	2.1763	0.014768	0.87528	0.19071	2.1763	0.014768	2.1763	0.014768	0.014768	0.403556391
DUXAP7	ENSG00000232136	0.75561	0.22494	-0.038217	0.51524	2.1762	0.014769	0.75561	0.22494	0.014769	0.403556391
MAP3K20-AS1	ENSG00000238133	1.0815	0.13975	0.54726	0.2921	2.1761	0.014775	2.1761	0.014775	0.014775	0.403556391
LINC00942	ENSG00000249628	2.1759	0.014782	1.1521	0.12463	2.0504	0.020164	1.6591	0.048551	0.014782	0.403556391
HOXA7	ENSG00000122592	2.1757	0.014788	2.1757	0.014788	2.1757	0.014788	1.3767	0.084308	0.014788	0.403556391
TLR12P	ENSG00000234512	2.1756	0.014793	1.4539	0.072985	0.51158	0.30447	-0.63369	0.73686	0.014793	0.403556391
IDH2	ENSG00000182054	0.1374	0.44536	2.1754	0.014799	2.1754	0.014799	0.81643	0.20713	0.014799	0.403556391
NA	ENSG00000223942	0.59738	0.27513	2.1749	0.014819	2.1749	0.014819	2.1749	0.014819	0.014819	0.403875766
RNF217	ENSG00000146373	0.51762	0.30236	2.1745	0.014834	0.51639	0.30279	2.0306	0.021147	0.014834	0.404050648
RN7SL228P	ENSG00000244033	2.1743	0.014842	1.3225	0.093007	2.1743	0.014842	2.1743	0.014842	0.014842	0.404050648
RNU6-544P	ENSG00000200146	0.14141	0.44377	2.1737	0.014863	0.14141	0.44377	0.14141	0.44377	0.014863	0.404396421
NA	ENSG00000175604	1.3188	0.093615	2.1727	0.014902	2.1084	0.0175	1.3188	0.093615	0.014902	0.405231283
NA	ENSG00000271243	2.1719	0.014932	0.33318	0.3695	0.33318	0.3695	0.33318	0.3695	0.014932	0.405393257
CELF2-AS2	ENSG00000237986	2.1713	0.014956	2.1713	0.014956	2.1713	0.014956	2.1713	0.014956	0.014956	0.405393257
SCOC	ENSG00000153130	2.1712	0.014959	1.6876	0.045746	-0.98423	0.8375	1.066	0.14321	0.014959	0.405393257
PCDHGB6	ENSG00000253305	0.73851	0.2301	2.1702	0.014997	0.73851	0.2301	0.73851	0.2301	0.014997	0.405393257

PMS2P5	ENSG00000123965	1.301	0.096632	-0.22966	0.59082	2.1697	0.015014	-0.26865	0.6059	0.015014	0.405393257
SCN2A	ENSG00000136531	1.4878	0.0684	1.6365	0.050867	2.1695	0.015024	1.3271	0.092239	0.015024	0.405393257
HSPA9P1	ENSG00000226666	0.72279	0.2349	1.146	0.12589	2.1693	0.015029	0.52513	0.29975	0.015029	0.405393257
IL18	ENSG00000150782	-0.30041	0.61807	2.1693	0.015031	-0.30041	0.61807	-0.30041	0.61807	0.015031	0.405393257
PDPR	ENSG00000090857	1.5472	0.060907	2.1025	0.017756	2.169	0.015041	1.7097	0.043657	0.015041	0.405393257
IPO9	ENSG00000198700	0.83269	0.20251	1.0601	0.14455	2.1688	0.015047	-1.1686	0.87871	0.015047	0.405393257
NA	ENSG00000272362	0.77591	0.2189	2.1688	0.015047	0.77591	0.2189	0.77591	0.2189	0.015047	0.405393257
RNU1-62P	ENSG00000199469	2.1686	0.015057	2.1686	0.015057	2.1686	0.015057	0.20478	0.41887	0.015057	0.405393257
NA	ENSG00000259035	2.1682	0.015071	1.5835	0.056656	2.1682	0.015071	1.4361	0.075481	0.015071	0.405393257
NA	ENSG00000278128	2.1681	0.015076	2.1681	0.015076	2.1681	0.015076	2.1681	0.015076	0.015076	0.405393257
SMOC2	ENSG00000112562	1.1055	0.13448	2.1678	0.015087	1.5289	0.063144	0.85527	0.1962	0.015087	0.405393257
RNU6-442P	ENSG00000206695	0.063646	0.47463	0.063646	0.47463	2.1678	0.015087	-0.8914	0.81364	0.015087	0.405393257
ANTXRL	ENSG00000274209	2.1676	0.015093	0.94196	0.17311	0.34768	0.36404	0.33222	0.36986	0.015093	0.405393257
GPSM1	ENSG00000160360	2.1675	0.015099	0.83041	0.20315	1.675	0.046967	1.9955	0.022993	0.015099	0.405393257
ASS1P10	ENSG00000215325	0.49154	0.31152	0.49154	0.31152	0.24807	0.40204	2.1672	0.015109	0.015109	0.405393257
ADPRHL1	ENSG00000153531	1.0382	0.14959	1.4982	0.067045	-0.03022	0.51205	2.1669	0.015122	0.015122	0.405393257
NA	ENSG00000205682	-0.58656	0.72125	-0.58656	0.72125	-0.58656	0.72125	2.1669	0.015122	0.015122	0.405393257
SNAPC5	ENSG00000174446	0.42343	0.33599	1.6943	0.045107	2.1668	0.015126	2.1668	0.015126	0.015126	0.405393257
MSX2	ENSG00000120149	-0.24635	0.59729	2.1665	0.015136	0.63868	0.26152	-0.10252	0.54083	0.015136	0.405393257
NA	ENSG00000270591	2.1663	0.015145	1.749	0.040141	2.1663	0.015145	2.1663	0.015145	0.015145	0.405393257
BTN2A1	ENSG00000112763	2.1658	0.015163	0.3114	0.37775	1.0776	0.14061	0.9435	0.17271	0.015163	0.405393257
RN7SL132P	ENSG00000242653	2.1657	0.015169	2.1657	0.015169	2.1657	0.015169	2.1657	0.015169	0.015169	0.405393257
LINC02316	ENSG00000258912	0.22735	0.41007	0.86357	0.19391	0.86357	0.19391	2.1655	0.015173	0.015173	0.405393257
FAM180B	ENSG00000196666	2.1652	0.015185	2.1652	0.015185	2.1652	0.015185	2.1652	0.015185	0.015185	0.405393257
THUMPD3P1	ENSG00000248711	2.1651	0.015189	0.42952	0.33377	0.42952	0.33377	-0.70031	0.75813	0.015189	0.405393257
NA	ENSG00000284292	2.1651	0.015189	2.1651	0.015189	2.1651	0.015189	2.1651	0.015189	0.015189	0.405393257
PTCSC3	ENSG00000259104	-0.37878	0.64758	2.1649	0.015199	0.33447	0.36901	-0.50347	0.69268	0.015199	0.405393257
PIGC	ENSG00000135845	-0.36264	0.64156	1.0305	0.15139	2.1645	0.015211	-0.80356	0.78918	0.015211	0.405393257
GAPDHS	ENSG00000105679	2.1644	0.015216	1.8824	0.029893	2.1644	0.015216	2.1644	0.015216	0.015216	0.405393257
OLFM1	ENSG00000130558	0.40924	0.34118	2.1644	0.015217	0.33075	0.37042	0.8037	0.21079	0.015217	0.405393257
BPIFC	ENSG00000184459	-1.1353	0.87188	0.17311	0.43128	-1.0957	0.86339	2.1643	0.01522	0.01522	0.405393257

TBCA	ENSG00000171530	-0.29793	0.61712	2.1642	0.015226	0.83149	0.20285	1.5483	0.060776	0.015226	0.405393257
VN1R20P	ENSG00000233121	0.4213	0.33677	0.77729	0.21849	-0.16525	0.56563	2.1639	0.015235	0.015235	0.405393257
MIR100HG	ENSG00000255248	1.2065	0.11381	1.6976	0.044791	1.5079	0.065795	2.1635	0.01525	0.01525	0.405393257
RNU4-83P	ENSG00000222795	2.1635	0.015252	0.78195	0.21712	-0.069466	0.52769	-0.13384	0.55324	0.015252	0.405393257
COBLL1	ENSG00000082438	2.1633	0.015259	0.74892	0.22695	0.5319	0.2974	1.6377	0.050746	0.015259	0.405393257
NA	ENSG00000228162	0.37602	0.35345	0.94416	0.17254	2.1632	0.015261	0.23759	0.4061	0.015261	0.405393257
LINC02647	ENSG00000223993	2.1631	0.015266	1.6511	0.049361	1.6511	0.049361	0.90317	0.18322	0.015266	0.405393257
RNU6-321P	ENSG00000222844	2.1631	0.015267	0.16165	0.43579	2.1631	0.015267	2.1631	0.015267	0.015267	0.405393257
PTS	ENSG00000150787	2.1629	0.015274	-0.89326	0.81414	2.0955	0.018061	-0.19232	0.57626	0.015274	0.405393257
RGS10	ENSG00000148908	1.3599	0.086932	0.49044	0.31191	2.162	0.01531	1.2382	0.10782	0.01531	0.406127545
CARM1P1	ENSG00000227835	2.161	0.015346	0.60357	0.27307	-1.1071	0.86587	0.23249	0.40808	0.015346	0.406861034
RESP18	ENSG00000182698	1.4487	0.073716	0.89023	0.18667	2.1608	0.015357	0.89023	0.18667	0.015357	0.406912705
PAX6	ENSG00000007372	0.71382	0.23767	1.7095	0.043682	2.1604	0.015371	0.2158	0.41457	0.015371	0.406912705
APOB	ENSG00000084674	0.98256	0.16291	2.1603	0.015373	1.8938	0.029126	0.55035	0.29104	0.015373	0.406912705
NA	ENSG00000274949	2.1601	0.015382	2.1601	0.015382	2.1146	0.017231	0.57667	0.28208	0.015382	0.406929891
ABCF3	ENSG00000161204	-0.39589	0.65391	2.1598	0.015393	1.1382	0.12751	0.26081	0.39712	0.015393	0.406990634
SLC16A12	ENSG00000152779	0.27772	0.39061	1.2856	0.099291	0.27772	0.39061	2.1596	0.015401	0.015401	0.406990634
NA	ENSG00000255860	0.33659	0.36821	-0.94198	0.8269	0.20166	0.42009	2.1592	0.015419	0.015419	0.407077644
CKAP5	ENSG00000175216	0.33039	0.37055	1.9085	0.028165	2.1591	0.015421	0.95478	0.16985	0.015421	0.407077644
NA	ENSG00000280057	-1.1855	0.88208	-0.40031	0.65553	1.8443	0.032572	2.1587	0.015436	0.015436	0.407198416
RNU6-1286P	ENSG00000206598	2.1583	0.015452	-0.39083	0.65204	2.1583	0.015452	2.1583	0.015452	0.015452	0.407198416
NBPF5P	ENSG00000243967	-0.46566	0.67927	0.8197	0.20619	2.1583	0.015453	2.1014	0.017801	0.015453	0.407198416
GPN1	ENSG00000198522	-1.3235	0.90717	-0.47903	0.68404	-0.49889	0.69107	2.1581	0.015459	0.015459	0.407198416
ABHD14A-ACY1	ENSG00000114786	1.3993	0.080855	2.1577	0.015475	1.1271	0.12985	0.83791	0.20104	0.015475	0.407232295
TUBBP9	ENSG00000220586	2.1577	0.015477	-1.0567	0.85468	1.9887	0.023367	0.094316	0.46243	0.015477	0.407232295
NA	ENSG00000236015	2.1571	0.0155	2.1571	0.0155	2.1571	0.0155	2.1571	0.0155	0.0155	0.407333493
RPL39P3	ENSG00000235174	0.95607	0.16952	-0.12014	0.54781	-0.12014	0.54781	2.157	0.015504	0.015504	0.407333493
GATA2	ENSG00000179348	1.6644	0.048013	0.80242	0.21115	1.1866	0.11769	2.1566	0.015517	0.015517	0.407333493
AGAP7P	ENSG00000264204	1.4521	0.073238	-1.0363	0.84996	1.4646	0.071515	2.1565	0.015524	0.015524	0.407333493
OR7E93P	ENSG00000228963	2.0562	0.019882	2.1563	0.015531	0.70921	0.2391	1.3171	0.093909	0.015531	0.407333493
NA	ENSG00000239072	2.1563	0.015531	1.6756	0.046912	2.1563	0.015531	2.1563	0.015531	0.015531	0.407333493

LINC02102	ENSG00000248677	2.1558	0.015551	-0.088155	0.53512	2.1558	0.015551	0.036071	0.48561	0.015551	0.407567099
NA	ENSG00000257178	2.1555	0.015562	2.1555	0.015562	2.1555	0.015562	2.1555	0.015562	0.015562	0.407567099
DCP1A	ENSG00000272886	1.44	0.074938	0.59711	0.27522	2.1551	0.015576	1.6332	0.051212	0.015576	0.407567099
BECN2	ENSG00000196289	2.1345	0.016402	0.70618	0.24004	2.1551	0.015578	0.99518	0.15983	0.015578	0.407567099
NA	ENSG00000266801	-1.5225	0.93606	-1.5225	0.93606	2.1547	0.015592	1.9881	0.023398	0.015592	0.407567099
NA	ENSG00000256897	2.0141	0.022002	2.0141	0.022002	2.0141	0.022002	2.1543	0.015608	0.015608	0.407567099
NEDD9	ENSG00000111859	2.1542	0.01561	0.43611	0.33138	1.0361	0.15008	1.4089	0.079434	0.01561	0.407567099
PAX5	ENSG00000196092	0.073263	0.4708	2.1541	0.015614	-1.0477	0.85262	-0.19617	0.57776	0.015614	0.407567099
NA	ENSG00000223779	2.0297	0.021195	2.1538	0.015627	1.2461	0.10637	1.4631	0.071723	0.015627	0.407567099
POT1-AS1	ENSG00000224897	2.1535	0.01564	1.2344	0.10852	0.87467	0.19088	-0.60732	0.72818	0.01564	0.407567099
NA	ENSG00000226500	2.1534	0.015643	1.7827	0.03732	1.8127	0.034937	2.1448	0.015984	0.015643	0.407567099
GPR132	ENSG00000183484	0.79773	0.21251	1.1811	0.11878	1.1962	0.11581	2.1532	0.01565	0.01565	0.407567099
NDUFA5P6	ENSG00000256310	2.1532	0.015652	2.1532	0.015652	2.1532	0.015652	0.19891	0.42117	0.015652	0.407567099
LYPLAL1	ENSG00000143353	1.0268	0.15225	2.1531	0.015657	0.07138	0.47155	0.93726	0.17431	0.015657	0.407567099
MTCO1P5	ENSG00000233026	2.1518	0.015707	2.1518	0.015707	2.1518	0.015707	2.1518	0.015707	0.015707	0.408650352
NA	ENSG00000231539	0.3378	0.36776	1.6068	0.054045	0.72737	0.2335	2.1514	0.015723	0.015723	0.40884834
FAM209B	ENSG00000213714	2.1508	0.015747	1.0651	0.14342	0.4586	0.32326	0.44498	0.32817	0.015747	0.409083034
SLC29A1	ENSG00000112759	-0.038727	0.51545	1.9423	0.026053	2.1502	0.015769	1.4494	0.073608	0.015769	0.409083034
NA	ENSG00000237031	2.1502	0.015771	1.1324	0.12874	2.1502	0.015771	2.1502	0.015771	0.015771	0.409083034
IQCF5-AS1	ENSG00000235455	2.1501	0.015772	2.1501	0.015772	2.1501	0.015772	2.1501	0.015772	0.015772	0.409083034
RNU6-100P	ENSG00000252414	2.1501	0.015774	2.1501	0.015774	2.1501	0.015774	2.1501	0.015774	0.015774	0.409083034
NA	ENSG00000257826	0.90788	0.18197	2.1496	0.015792	0.90788	0.18197	0.90788	0.18197	0.015792	0.40912147
NA	ENSG00000225416	2.1494	0.0158	2.1494	0.0158	2.1494	0.0158	2.1494	0.0158	0.0158	0.40912147
NA	ENSG00000274855	0.9923	0.16053	2.1491	0.015814	0.9923	0.16053	2.0922	0.018212	0.015814	0.40912147
SLTM	ENSG00000137776	2.1487	0.015829	-0.42641	0.66509	-0.0026548	0.50106	0.94037	0.17351	0.015829	0.40912147
POLR2J4	ENSG00000272655	1.372	0.085026	-1.2795	0.89964	2.1487	0.01583	2.1487	0.01583	0.01583	0.40912147
NA	ENSG00000215428	2.1486	0.015832	2.1486	0.015832	2.1486	0.015832	2.1486	0.015832	0.015832	0.40912147
NA	ENSG00000253130	2.1481	0.015854	2.1436	0.016032	2.1436	0.016032	1.092	0.13742	0.015854	0.40912147
NEURL4	ENSG00000215041	2.1478	0.015864	0.46055	0.32256	2.1478	0.015864	1.9435	0.02598	0.015864	0.40912147
YWHAQP9	ENSG00000230019	2.1478	0.015866	2.1478	0.015866	2.1478	0.015866	2.1478	0.015866	0.015866	0.40912147
TMEM147-AS1	ENSG00000236144	2.1474	0.015881	2.1474	0.015881	2.1474	0.015881	2.1474	0.015881	0.015881	0.40912147

MT1P1	ENSG00000213761	0.48275	0.31464	2.1469	0.015901	1.6751	0.04696	1.7957	0.036272	0.015901	0.40912147
MANBAL	ENSG00000101363	2.1469	0.015902	0.4403	0.32986	0.77574	0.21895	1.3505	0.088422	0.015902	0.40912147
RNA5SP352	ENSG00000200278	2.1468	0.015905	2.1468	0.015905	2.1468	0.015905	2.1468	0.015905	0.015905	0.40912147
NA	ENSG00000267523	2.1465	0.015917	2.1465	0.015917	2.1465	0.015917	2.1465	0.015917	0.015917	0.40912147
OR1N2	ENSG00000171501	1.7113	0.043514	2.1464	0.015922	2.1464	0.015922	2.1464	0.015922	0.015922	0.40912147
RAB40C	ENSG00000197562	1.9781	0.023959	2.1462	0.01593	1.5384	0.061974	1.161	0.12283	0.01593	0.40912147
AKR1C5P	ENSG00000225418	2.1462	0.01593	2.01	0.022214	2.0284	0.021262	2.01	0.022214	0.01593	0.40912147
BRD7P5	ENSG00000235768	0.91262	0.18072	2.1461	0.015931	2.1461	0.015931	2.1461	0.015931	0.015931	0.40912147
VWA2	ENSG00000165816	2.146	0.015935	2.146	0.015935	2.0526	0.020055	1.4844	0.068847	0.015935	0.40912147
SETBP1-DT	ENSG00000267414	2.1451	0.015972	1.5301	0.062998	0.74374	0.22852	1.2177	0.11166	0.015972	0.40969581
NWD1	ENSG00000188039	1.1675	0.12149	2.1448	0.015986	0.4709	0.31886	-0.33562	0.63142	0.015986	0.40969581
MAP7D1	ENSG00000116871	1.9317	0.026698	-1.1094	0.86637	2.1446	0.015991	1.314	0.094418	0.015991	0.40969581
NA	ENSG00000273091	2.1446	0.015991	2.1446	0.015991	2.1446	0.015991	2.1446	0.015991	0.015991	0.40969581
RNU6-832P	ENSG00000200189	1.9719	0.02431	1.9719	0.02431	1.2762	0.10094	2.1437	0.016027	0.016027	0.410224958
MIR4465	ENSG00000264390	0.69171	0.24456	2.1436	0.016033	2.0424	0.020557	2.0424	0.020557	0.016033	0.410224958
NA	ENSG00000275484	2.1429	0.016061	2.1429	0.016061	2.1429	0.016061	2.1429	0.016061	0.016061	0.410224958
ADGRL3-AS1	ENSG00000248692	1.1798	0.11903	2.1427	0.016068	2.1427	0.016068	2.1427	0.016068	0.016068	0.410224958
LINC01909	ENSG00000266258	2.1426	0.016071	1.9338	0.026569	0.98503	0.16231	0.98503	0.16231	0.016071	0.410224958
BPIFB5P	ENSG00000233146	2.1426	0.016072	-1.9391	0.97376	2.0942	0.01812	-1.826	0.96608	0.016072	0.410224958
PPM1AP1	ENSG00000250483	-0.036135	0.51441	2.1426	0.016074	-0.036135	0.51441	-0.036135	0.51441	0.016074	0.410224958
NA	ENSG00000278925	1.0306	0.15137	1.1635	0.12231	1.8266	0.033879	2.1424	0.016079	0.016079	0.410224958
PEBP1P3	ENSG00000236278	2.1421	0.016092	1.2397	0.10754	0.37731	0.35297	0.37731	0.35297	0.016092	0.41034179
NA	ENSG00000232789	2.1417	0.016108	0.90914	0.18164	0.90914	0.18164	0.90914	0.18164	0.016108	0.410534958
RNU6-141P	ENSG00000222431	2.141	0.016136	2.141	0.016136	2.141	0.016136	2.141	0.016136	0.016136	0.41082905
LINC02496	ENSG00000249066	2.141	0.016139	0.4733	0.318	0.4733	0.318	0.4733	0.318	0.016139	0.41082905
CST9LP2	ENSG00000204658	0.43443	0.33199	0.46837	0.31976	2.1407	0.01615	2.1407	0.01615	0.01615	0.41082905
NLE1	ENSG00000073536	-0.045321	0.51807	2.1404	0.01616	0.48933	0.3123	0.48933	0.3123	0.01616	0.41082905
CFL1P7	ENSG00000213877	2.1402	0.016168	2.1402	0.016168	2.1402	0.016168	2.1402	0.016168	0.016168	0.41082905
NA	ENSG00000235271	2	0.02275	1.0703	0.14224	0.25054	0.40108	2.1399	0.016181	0.016181	0.41082905
SPTBN1	ENSG00000115306	2.0502	0.020172	2.1396	0.016195	1.7394	0.040978	1.2257	0.11016	0.016195	0.41082905
CCDC110	ENSG00000168491	1.1023	0.13517	1.8896	0.029404	1.5245	0.063697	2.1392	0.016211	0.016211	0.41082905

NA	ENSG00000233407	1.7839	0.037222	1.9972	0.022904	2.1386	0.016232	1.7839	0.037222	0.016232	0.41082905
NA	ENSG00000230932	2.0303	0.021164	2.0303	0.021164	2.1385	0.016237	2.0303	0.021164	0.016237	0.41082905
NA	ENSG00000254281	2.1385	0.016238	-0.88057	0.81072	2.1385	0.016238	0.72144	0.23532	0.016238	0.41082905
LINC02298	ENSG00000257556	0.43048	0.33342	0.092535	0.46314	-0.13476	0.5536	2.1384	0.016242	0.016242	0.41082905
NA	ENSG00000199580	1.0381	0.14961	2.1384	0.016243	0.56606	0.28567	1.0381	0.14961	0.016243	0.41082905
TMEM220	ENSG00000187824	2.1383	0.016245	0.59625	0.2755	0.25871	0.39793	0.81201	0.20839	0.016245	0.41082905
RN7SL630P	ENSG00000264978	2.1383	0.016246	2.1383	0.016246	2.1383	0.016246	2.1383	0.016246	0.016246	0.41082905
ISCA1	ENSG00000135070	1.8836	0.02981	1.3408	0.089987	2.0857	0.018501	2.1378	0.016265	0.016265	0.410976511
LINC01444	ENSG00000264301	2.1377	0.01627	2.1377	0.01627	2.1377	0.01627	2.1377	0.01627	0.01627	0.410976511
SAMD15	ENSG00000100583	2.1373	0.016285	2.1373	0.016285	-0.51541	0.69687	1.003	0.15794	0.016285	0.410976511
RPL29P3	ENSG00000243730	0.97419	0.16498	0.41905	0.33759	2.1371	0.016293	2.1371	0.016293	0.016293	0.410976511
NA	ENSG00000252459	-0.92867	0.82347	2.1371	0.016294	1.0292	0.15169	-0.92867	0.82347	0.016294	0.410976511
NA	ENSG00000255541	-0.25267	0.59974	2.1368	0.016308	2.1368	0.016308	2.1368	0.016308	0.016308	0.410983182
NA	ENSG00000268149	2.1366	0.016316	2.1366	0.016316	2.1366	0.016316	2.1366	0.016316	0.016316	0.410983182
NA	ENSG00000276687	2.1364	0.016323	-0.82736	0.79598	0.58974	0.27768	-1.0036	0.8422	0.016323	0.410983182
LINC00570	ENSG00000224177	-0.37095	0.64466	2.1363	0.016328	0.11637	0.45368	0.76372	0.22252	0.016328	0.410983182
LINC00524	ENSG00000259023	0.043466	0.48267	0.53177	0.29744	2.1355	0.016359	-0.4936	0.68921	0.016359	0.411439107
NA	ENSG00000212589	1.7264	0.04214	-0.69353	0.75601	-0.69353	0.75601	2.1354	0.016363	0.016363	0.411439107
JAKMIP1	ENSG00000152969	1.1684	0.12132	1.4505	0.073462	2.1348	0.01639	0.32178	0.37381	0.01639	0.411905467
ARL4C	ENSG00000188042	0.89799	0.1846	0.7905	0.21462	1.6034	0.054425	2.1342	0.016413	0.016413	0.412158892
NA	ENSG00000236874	1.3779	0.084111	1.8534	0.031912	2.1341	0.016417	1.263	0.10329	0.016417	0.412158892
NA	ENSG00000249413	0.41168	0.34029	-0.31753	0.62458	2.1339	0.016426	1.1266	0.12996	0.016426	0.412172492
NA	ENSG00000234382	-0.60234	0.72653	-0.7081	0.76056	2.1332	0.016456	0.7148	0.23737	0.016456	0.412712753
RPL31P15	ENSG00000232430	2.1322	0.016495	2.1322	0.016495	2.1322	0.016495	2.1322	0.016495	0.016495	0.413478061
NA	ENSG00000255465	0.47026	0.31908	1.1692	0.12116	-0.088837	0.53539	2.1318	0.01651	0.01651	0.413629054
NA	ENSG00000238565	2.1316	0.016518	1.3883	0.082527	1.2389	0.1077	1.1683	0.12134	0.016518	0.413629054
RNU7-19P	ENSG00000238959	2.1312	0.016535	2.1312	0.016535	2.1312	0.016535	2.1312	0.016535	0.016535	0.41384209
RPL13P6	ENSG00000213176	-1.4106	0.92081	-1.4303	0.92369	0.50843	0.30558	2.1309	0.016551	0.016551	0.414029892
ADPRS	ENSG00000116863	2.1303	0.016574	2.1303	0.016574	2.1303	0.016574	2.1303	0.016574	0.016574	0.414392519
RSL24D1P9	ENSG00000265402	2.0573	0.019831	0.40648	0.3422	2.1294	0.016612	1.9311	0.026735	0.016612	0.415129621
PTK6	ENSG00000101213	2.1278	0.016678	0.99417	0.16007	0.62194	0.26699	1.328	0.092087	0.016678	0.41656532

NA	ENSG00000234084	2.1269	0.016714	2.1269	0.016714	2.1269	0.016714	2.1269	0.016714	0.016714	0.417097876
NA	ENSG00000236838	2.1268	0.01672	2.1268	0.01672	2.1268	0.01672	2.1268	0.01672	0.01672	0.417097876
DISC1	ENSG00000162946	2.1266	0.016725	2.0556	0.01991	1.8771	0.030251	1.8963	0.028962	0.016725	0.417097876
GABRD	ENSG00000187730	2.1261	0.016746	-0.74408	0.77159	-0.51731	0.69753	1.8408	0.032825	0.016746	0.417219484
ZNF415P1	ENSG00000266127	2.1261	0.016747	0.66896	0.25176	2.1261	0.016747	0.46788	0.31994	0.016747	0.417219484
NA	ENSG00000264270	2.1255	0.01677	2.1255	0.01677	2.1255	0.01677	2.1255	0.01677	0.01677	0.417578998
FKBP9	ENSG00000122642	-0.37909	0.64769	1.3945	0.081585	0.085587	0.4659	2.1241	0.016829	0.016829	0.418378691
NA	ENSG00000230365	2.1238	0.016844	-0.9424	0.82701	2.1238	0.016844	1.0648	0.14348	0.016844	0.418378691
NA	ENSG00000258807	2.0587	0.01976	1.3947	0.081546	2.1237	0.016849	2.0587	0.01976	0.016849	0.418378691
PYY	ENSG00000131096	2.123	0.016877	-0.90929	0.8184	-1.1325	0.8713	-1.4642	0.92844	0.016877	0.418378691
COPS7B	ENSG00000144524	2.123	0.016877	-0.11291	0.54495	1.9386	0.026276	1.2469	0.10622	0.016877	0.418378691
NA	ENSG00000272849	2.1229	0.016881	2.1229	0.016881	2.1229	0.016881	2.1229	0.016881	0.016881	0.418378691
UBE2V2P4	ENSG00000231849	0.31683	0.37568	2.1228	0.016886	2.1228	0.016886	2.1228	0.016886	0.016886	0.418378691
ZNF239	ENSG00000196793	-0.67247	0.74936	2.1228	0.016887	0.90849	0.18181	0.90849	0.18181	0.016887	0.418378691
NA	ENSG00000273702	2.1228	0.016887	2.1228	0.016887	2.1228	0.016887	2.1228	0.016887	0.016887	0.418378691
NR1H5P	ENSG00000227970	2.1226	0.016895	1.2561	0.10454	2.1226	0.016895	2.1226	0.016895	0.016895	0.418378691
NA	ENSG00000253927	2.1224	0.016902	1.4655	0.071391	0.10051	0.45997	1.7241	0.042344	0.016902	0.418378691
LPCAT3	ENSG00000111684	2.1223	0.016906	1.298	0.097149	1.8004	0.035901	1.1184	0.13169	0.016906	0.418378691
FCAR	ENSG00000186431	1.0823	0.13956	1.7589	0.039293	1.7589	0.039293	2.1218	0.016928	0.016928	0.418378691
NA	ENSG00000222044	2.1216	0.016937	2.1216	0.016937	2.1216	0.016937	2.1216	0.016937	0.016937	0.418378691
MARK2P16	ENSG00000270670	2.1215	0.016939	2.1215	0.016939	2.1215	0.016939	2.1215	0.016939	0.016939	0.418378691
DMAC1	ENSG00000137038	0.74605	0.22782	0.24854	0.40186	0.085919	0.46577	2.1213	0.016949	0.016949	0.418378691
NA	ENSG00000272885	2.1209	0.016964	0.77944	0.21786	0.77944	0.21786	0.77944	0.21786	0.016964	0.418378691
NA	ENSG00000253543	2.1208	0.016969	0.29555	0.38379	1.4362	0.075469	2.1208	0.016969	0.016969	0.418378691
TAF7	ENSG00000178913	-0.69742	0.75723	-0.69742	0.75723	2.1207	0.016972	-0.69742	0.75723	0.016972	0.418378691
BLID	ENSG00000259571	1.0858	0.13879	0.49726	0.3095	2.1205	0.016981	2.1205	0.016981	0.016981	0.418378691
NA	ENSG00000261400	2.048	0.020278	1.8092	0.035212	2.1204	0.016985	2.048	0.020278	0.016985	0.418378691
DNMT3A	ENSG00000119772	-0.4019	0.65612	0.34598	0.36468	0.2057	0.41851	2.1203	0.016991	0.016991	0.418378691
RPL17P19	ENSG00000241261	-0.01596	0.50637	-0.01596	0.50637	2.1194	0.017027	0.19469	0.42282	0.017027	0.419053389
ETNK1	ENSG00000139163	0.99437	0.16002	0.99437	0.16002	0.99437	0.16002	2.1187	0.017058	0.017058	0.419495335
SMPD3	ENSG00000103056	2.1186	0.017064	1.8963	0.028958	1.4252	0.077044	1.084	0.13918	0.017064	0.419495335

SLC1A4	ENSG00000115902	2.1182	0.01708	0.10255	0.45916	0.028807	0.48851	-0.63571	0.73752	0.01708	0.419495335
RNU6-919P	ENSG00000252540	-0.12571	0.55002	2.1181	0.017085	1.7627	0.038972	-0.055858	0.52227	0.017085	0.419495335
MCCC1	ENSG00000078070	2.118	0.017088	0.66435	0.25323	0.14531	0.44223	1.3407	0.090003	0.017088	0.419495335
CFL1P4	ENSG00000213026	2.1176	0.017104	0.0036185	0.49856	0.76602	0.22183	0.76602	0.22183	0.017104	0.419676697
MTND5P19	ENSG00000231199	2.1172	0.017122	2.1172	0.017122	2.1172	0.017122	2.1172	0.017122	0.017122	0.419818265
ZFAS1	ENSG00000177410	2.1171	0.017127	1.3191	0.093566	2.0261	0.021375	1.0628	0.14393	0.017127	0.419818265
R3HCC1	ENSG00000104679	0.94608	0.17205	1.5889	0.056042	2.1167	0.017142	-0.11131	0.54431	0.017142	0.419929307
RPL23AP44	ENSG00000244088	2.1164	0.017157	2.1164	0.017157	2.1164	0.017157	2.1164	0.017157	0.017157	0.419929307
CERS5	ENSG00000139624	1.5692	0.058298	2.0899	0.018315	1.9656	0.024674	2.1162	0.017162	0.017162	0.419929307
HHLA3	ENSG00000197568	2.1162	0.017166	-1.2767	0.89914	-1.2767	0.89914	-1.2767	0.89914	0.017166	0.419929307
ALDH7A1P1	ENSG00000251400	1.8367	0.033124	1.8367	0.033124	2.1081	0.01751	2.1159	0.017176	0.017176	0.419963111
RANBP9	ENSG00000010017	1.3165	0.094001	2.0904	0.018293	1.6536	0.049105	2.1154	0.017198	0.017198	0.420238585
OR4M1	ENSG00000176299	0.76481	0.22219	1.3153	0.094209	-0.42492	0.66455	2.1151	0.017209	0.017209	0.420238585
MIR3167	ENSG00000266215	0.31394	0.37678	1.5894	0.055983	2.1149	0.017218	0.21491	0.41492	0.017218	0.420238585
RPL23AP66	ENSG00000243141	2.1146	0.017232	2.1146	0.017232	2.1146	0.017232	2.1146	0.017232	0.017232	0.420238585
TAFA4	ENSG00000163377	2.1145	0.017235	1.0757	0.14103	1.9192	0.027476	1.5408	0.061678	0.017235	0.420238585
NA	ENSG00000224750	2.1144	0.017242	-0.27635	0.60886	-0.27635	0.60886	-0.27635	0.60886	0.017242	0.420238585
KCNH2	ENSG00000055118	1.0377	0.14971	2.1134	0.017283	1.2857	0.099278	0.35685	0.3606	0.017283	0.420238585
IGLC3	ENSG00000211679	2.1134	0.017285	2.1134	0.017285	2.1134	0.017285	2.1134	0.017285	0.017285	0.420238585
RPL4	ENSG00000174444	2.1133	0.017286	2.1133	0.017286	2.1133	0.017286	2.1133	0.017286	0.017286	0.420238585
NA	ENSG00000231896	0.81485	0.20758	0.81485	0.20758	2.1131	0.017294	0.81485	0.20758	0.017294	0.420238585
NA	ENSG00000279429	2.1131	0.017298	2.1131	0.017298	2.1131	0.017298	2.1131	0.017298	0.017298	0.420238585
RBMXL1	ENSG00000213516	0.46722	0.32017	1.3661	0.085948	0.17716	0.42969	2.113	0.017301	0.017301	0.420238585
RN7SL434P	ENSG00000241868	2.1129	0.017307	2.1129	0.017307	2.1129	0.017307	2.1129	0.017307	0.017307	0.420238585
LRRC30	ENSG00000206422	0.57388	0.28302	0.62495	0.266	0.49893	0.30892	2.1128	0.017308	0.017308	0.420238585
SPATA31D5P	ENSG00000240632	0.23316	0.40782	0.78353	0.21666	2.1124	0.017326	-0.39671	0.65421	0.017326	0.420241701
NA	ENSG00000269430	2.1123	0.01733	2.1123	0.01733	2.1123	0.01733	2.1123	0.01733	0.01733	0.420241701
SETP22	ENSG00000213701	2.1122	0.017334	0.95185	0.17059	2.1122	0.017334	1.5147	0.064927	0.017334	0.420241701
EN2	ENSG00000164778	0.93438	0.17505	0.7714	0.22023	0.05096	0.47968	2.1115	0.017365	0.017365	0.420783913
STPG2	ENSG00000163116	1.3887	0.082459	1.4464	0.074031	2.1111	0.017381	0.41874	0.3377	0.017381	0.420909301
NA	ENSG00000231138	2.1109	0.017389	2.1109	0.017389	2.1109	0.017389	2.1109	0.017389	0.017389	0.420909301

LINC02608	ENSG00000226251	2.1105	0.017406	2.1105	0.017406	1.483	0.069036	1.7323	0.04161	0.017406	0.420909301
PNMA2	ENSG00000240694	0.46225	0.32195	-0.18519	0.57346	2.1103	0.017417	-0.033828	0.51349	0.017417	0.420909301
RNU6-1053P	ENSG00000251739	2.1103	0.017417	2.1103	0.017417	2.1103	0.017417	2.1103	0.017417	0.017417	0.420909301
NA	ENSG00000270361	2.1102	0.017422	1.6912	0.045396	-0.16005	0.56358	-0.16005	0.56358	0.017422	0.420909301
ALOX12P2	ENSG00000262943	2.1096	0.017446	2.1096	0.017446	2.1096	0.017446	2.1096	0.017446	0.017446	0.421280268
GYG1P1	ENSG00000251633	2.1092	0.017463	2.1092	0.017463	2.1092	0.017463	2.1092	0.017463	0.017463	0.421281774
WLS	ENSG00000116729	2.1086	0.017489	1.8751	0.030388	0.15641	0.43786	1.0497	0.14694	0.017489	0.421281774
NA	ENSG00000250642	1.9781	0.02396	2.1086	0.01749	1.0636	0.14376	-1.1587	0.87672	0.01749	0.421281774
ARMC7	ENSG00000125449	-0.60419	0.72714	-0.42578	0.66487	-1.142	0.87328	2.1084	0.017496	0.017496	0.421281774
NA	ENSG00000263895	2.1083	0.017501	0.59728	0.27516	-0.11046	0.54398	-0.11046	0.54398	0.017501	0.421281774
NA	ENSG00000238802	2.1074	0.017541	1.7305	0.041773	0.3742	0.35413	2.1074	0.017541	0.017541	0.421281774
ASPA	ENSG00000108381	0.97036	0.16593	1.3997	0.080795	2.1074	0.017542	-0.040594	0.51619	0.017542	0.421281774
NA	ENSG00000253617	2.0305	0.021152	1.769	0.03845	2.1073	0.017545	2.1073	0.017545	0.017545	0.421281774
NA	ENSG00000225129	2.1071	0.017553	1.7723	0.038176	1.7723	0.038176	1.7723	0.038176	0.017553	0.421281774
SPATA2L	ENSG00000158792	2.1069	0.017562	2.1069	0.017562	1.653	0.049162	1.6518	0.04929	0.017562	0.421281774
NA	ENSG00000260668	2.1069	0.017563	0.62868	0.26478	0.51864	0.30201	0.51864	0.30201	0.017563	0.421281774
NAA38	ENSG00000183011	2.1068	0.017566	2.1068	0.017566	2.1068	0.017566	2.1068	0.017566	0.017566	0.421281774
LINC01135	ENSG00000234807	0.7493	0.22684	2.1061	0.017599	0.28255	0.38876	-0.4796	0.68425	0.017599	0.421281774
ACSS3	ENSG00000111058	0.5782	0.28156	0.097159	0.4613	-0.089082	0.53549	2.1061	0.0176	0.0176	0.421281774
SPAG9	ENSG00000008294	2.1057	0.017615	2.094	0.01813	1.2633	0.10325	0.95337	0.1702	0.017615	0.421281774
TRHDE-AS1	ENSG00000236333	-0.59436	0.72387	2.1057	0.017615	-0.59436	0.72387	-0.59436	0.72387	0.017615	0.421281774
NA	ENSG00000272904	2.1046	0.017664	2.1046	0.017664	2.1046	0.017664	2.1046	0.017664	0.017664	0.421281774
SCT	ENSG00000070031	0.028038	0.48882	0.028038	0.48882	2.1044	0.017672	0.45144	0.32583	0.017672	0.421281774
RPLP0P2	ENSG00000243742	1.5457	0.061089	1.0403	0.1491	2.1043	0.017678	-0.10679	0.54252	0.017678	0.421281774
NA	ENSG00000280113	2.1042	0.01768	2.1042	0.01768	2.1042	0.01768	2.1042	0.01768	0.01768	0.421281774
NA	ENSG00000249166	2.1042	0.017681	2.1042	0.017681	2.1042	0.017681	2.1042	0.017681	0.017681	0.421281774
RNU6-276P	ENSG00000251923	2.104	0.017691	2.104	0.017691	2.104	0.017691	2.104	0.017691	0.017691	0.421281774
NA	ENSG00000199398	1.5501	0.060562	2.1039	0.017693	-0.17042	0.56766	-0.17042	0.56766	0.017693	0.421281774
FAM222A	ENSG00000139438	2.1039	0.017694	1.8572	0.03164	2.0247	0.021449	1.7873	0.036943	0.017694	0.421281774
NA	ENSG00000279352	-0.14961	0.55946	2.1035	0.017712	0.64788	0.25853	-0.87571	0.80941	0.017712	0.421281774
AKR1C4	ENSG00000198610	0.58282	0.28001	0.88656	0.18766	2.1034	0.017714	1.1098	0.13354	0.017714	0.421281774

NA	ENSG00000237471	1.2804	0.1002	-1.1173	0.86807	1.0571	0.14524	2.1034	0.017714	0.017714	0.421281774
LINC02618	ENSG00000242104	-1.4705	0.92929	-0.5973	0.72485	2.1034	0.017715	-1.9161	0.97232	0.017715	0.421281774
NA	ENSG00000258987	2.1032	0.017725	2.1032	0.017725	2.1032	0.017725	2.1032	0.017725	0.017725	0.421281774
NA	ENSG00000253583	0.56619	0.28563	2.1031	0.017727	1.3482	0.088799	0.43155	0.33303	0.017727	0.421281774
NA	ENSG00000266441	2.103	0.017732	2.103	0.017732	2.103	0.017732	2.103	0.017732	0.017732	0.421281774
NA	ENSG00000252846	0.8363	0.20149	0.8363	0.20149	0.8363	0.20149	2.103	0.017734	0.017734	0.421281774
CKS1BP2	ENSG00000225976	1.596	0.055239	2.1029	0.017735	2.1029	0.017735	2.1029	0.017735	0.017735	0.421281774
PIGW	ENSG00000277161	0.53659	0.29578	-0.37151	0.64487	-0.37151	0.64487	2.1028	0.01774	0.01774	0.421281774
GLB1L	ENSG00000163521	2.1021	0.01777	2.1021	0.01777	2.1021	0.01777	2.1021	0.01777	0.01777	0.421749372
ADAM15	ENSG00000143537	-0.22031	0.58718	-0.051419	0.5205	-0.22031	0.58718	2.102	0.017777	0.017777	0.421749372
COLGALT1	ENSG00000130309	-0.24938	0.59847	-0.24938	0.59847	2.1017	0.017792	-0.14797	0.55882	0.017792	0.421844973
ACKR1	ENSG00000213088	2.1013	0.017806	2.1013	0.017806	2.1013	0.017806	2.1013	0.017806	0.017806	0.421844973
NA	ENSG00000225240	2.1013	0.017807	2.1013	0.017807	2.1013	0.017807	0.39431	0.34668	0.017807	0.421844973
NA	ENSG00000255965	2.101	0.017821	2.101	0.017821	2.101	0.017821	2.101	0.017821	0.017821	0.421971492
SNORD16	ENSG00000199673	2.1005	0.017842	2.1005	0.017842	2.1005	0.017842	2.1005	0.017842	0.017842	0.421972009
C1DP3	ENSG00000224760	0.93739	0.17428	0.93739	0.17428	0.24985	0.40135	2.1005	0.017843	0.017843	0.421972009
NA	ENSG00000260487	2.1004	0.017847	2.1004	0.017847	2.1004	0.017847	2.1004	0.017847	0.017847	0.421972009
NA	ENSG00000151303	2.0999	0.017871	0.37865	0.35247	0.53737	0.29551	0.10563	0.45794	0.017871	0.422295172
LCA5L	ENSG00000157578	2.0997	0.017878	1.5991	0.054899	1.5991	0.054899	1.638	0.050706	0.017878	0.422295172
MROH3P	ENSG00000233217	1.8995	0.02875	2.0993	0.017896	2.0993	0.017896	0.67881	0.24863	0.017896	0.422303295
MIR2276	ENSG00000252695	2.0992	0.0179	2.0992	0.0179	2.0992	0.0179	2.0992	0.0179	0.0179	0.422303295
SHD	ENSG00000105251	0.6863	0.24626	1.1799	0.11903	2.099	0.017907	1.0416	0.1488	0.017907	0.422303295
SLC35G3	ENSG00000164729	-0.03165	0.51262	-1.2209	0.88894	2.0987	0.017923	1.0236	0.15302	0.017923	0.422303295
NA	ENSG00000259372	2.0986	0.017926	1.0196	0.15396	1.1268	0.12992	0.079854	0.46818	0.017926	0.422303295
POLR2H	ENSG00000163882	2.0985	0.017932	2.005	0.022481	1.32	0.093423	1.32	0.093423	0.017932	0.422303295
PTCRA	ENSG00000171611	1.3078	0.095462	1.4376	0.075271	2.0977	0.017967	1.0753	0.14113	0.017967	0.422303295
PARP1P1	ENSG00000227105	1.1559	0.12385	1.0639	0.14369	1.1559	0.12385	2.0976	0.01797	0.01797	0.422303295
TMED6	ENSG00000157315	1.8525	0.031976	2.0975	0.017973	-2.148	0.98414	-2.148	0.98414	0.017973	0.422303295
NA	ENSG00000268055	2.0971	0.017991	2.0971	0.017991	2.0971	0.017991	2.0971	0.017991	0.017991	0.422303295
RASSF7	ENSG00000099849	0.10437	0.45844	0.10437	0.45844	0.545	0.29288	2.0971	0.017994	0.017994	0.422303295
DNAH1	ENSG00000114841	2.097	0.017995	0.48589	0.31352	1.2773	0.10076	1.18	0.119	0.017995	0.422303295

GNAS	ENSG00000087460	1.8524	0.031981	2.097	0.017997	1.1554	0.12397	0.38159	0.35138	0.017997	0.422303295
SCO1	ENSG00000133028	2.0968	0.018005	0.51187	0.30437	0.51187	0.30437	0.78426	0.21644	0.018005	0.422303295
OR2AT4	ENSG00000171561	0.91566	0.17992	0.91566	0.17992	0.91566	0.17992	2.0965	0.01802	0.01802	0.422303295
NME4	ENSG00000103202	1.3468	0.089024	1.5203	0.064213	0.66821	0.252	2.0963	0.018026	0.018026	0.422303295
TSEN15P2	ENSG00000230604	2.0963	0.018028	2.0963	0.018028	2.0963	0.018028	2.0963	0.018028	0.018028	0.422303295
DGKZ	ENSG00000149091	1.5582	0.059596	2.0961	0.018038	1.5793	0.057137	1.9259	0.027056	0.018038	0.422303295
CLRN2	ENSG00000249581	0.9782	0.16399	2.0959	0.018045	2.0959	0.018045	1.3883	0.082524	0.018045	0.422303295
ITIH3	ENSG00000162267	2.0956	0.018058	0.47144	0.31866	1.5214	0.064078	1.5214	0.064078	0.018058	0.422303295
GBP3	ENSG00000117226	2.0955	0.018065	1.734	0.041461	1.4006	0.080663	0.38361	0.35063	0.018065	0.422303295
EEF1GP8	ENSG00000234125	2.0954	0.018069	2.0954	0.018069	2.0954	0.018069	2.0954	0.018069	0.018069	0.422303295
PRKAR1B	ENSG00000188191	1.1655	0.12191	0.54003	0.29459	2.0949	0.018089	0.69651	0.24305	0.018089	0.422311115
NA	ENSG00000260672	2.0947	0.018099	0.95013	0.17102	1.3964	0.081293	0.34471	0.36516	0.018099	0.422311115
MAPK9	ENSG00000050748	-1.4487	0.92629	0.84417	0.19929	0.78981	0.21482	2.0946	0.018104	0.018104	0.422311115
NA	ENSG00000271671	2.0946	0.018104	2.0946	0.018104	2.0946	0.018104	2.0946	0.018104	0.018104	0.422311115
HPS5	ENSG00000110756	0.95949	0.16866	2.0938	0.01814	1.2414	0.10723	1.1	0.13566	0.01814	0.422419221
VIPAS39	ENSG00000151445	-0.030068	0.51199	0.12343	0.45088	2.0936	0.018148	0.75476	0.2252	0.018148	0.422419221
TUBB8P7	ENSG00000261812	2.0936	0.018149	1.5913	0.055768	-0.074376	0.52964	-0.074376	0.52964	0.018149	0.422419221
TRAJ6	ENSG00000211883	2.0932	0.018164	2.0932	0.018164	2.0932	0.018164	2.0932	0.018164	0.018164	0.422419221
NA	ENSG00000279328	2.0931	0.01817	1.2596	0.10391	2.0931	0.01817	2.0931	0.01817	0.01817	0.422419221
MRPS21	ENSG00000266472	0.44359	0.32867	1.5035	0.066352	0.71504	0.23729	2.093	0.018173	0.018173	0.422419221
HMCN2	ENSG00000148357	0.43094	0.33326	1.3474	0.088918	-0.14926	0.55933	2.0929	0.01818	0.01818	0.422419221
RN7SL624P	ENSG00000265777	0.38187	0.35128	2.0921	0.018216	2.0581	0.019792	1.4464	0.074037	0.018216	0.422419221
SNORA77	ENSG00000221643	2.0917	0.018232	2.0917	0.018232	2.0917	0.018232	2.0917	0.018232	0.018232	0.422419221
C12orf57	ENSG00000111678	2.0916	0.018236	2.0916	0.018236	2.0916	0.018236	2.0916	0.018236	0.018236	0.422419221
RNU7-1	ENSG00000238923	2.0916	0.018236	2.0916	0.018236	2.0916	0.018236	2.0916	0.018236	0.018236	0.422419221
NA	ENSG00000272173	2.0916	0.018236	2.0916	0.018236	2.0916	0.018236	2.0916	0.018236	0.018236	0.422419221
CRACR2A	ENSG00000130038	0.79926	0.21207	2.0915	0.018244	0.83509	0.20183	-0.50421	0.69294	0.018244	0.422419221
RPRM	ENSG00000177519	1.802	0.035773	-0.24953	0.59852	0.94081	0.1734	2.0913	0.018251	0.018251	0.422419221
TRAJ20	ENSG00000211869	2.0912	0.018256	2.0912	0.018256	2.0912	0.018256	2.0912	0.018256	0.018256	0.422419221
TRAJ19	ENSG00000211870	2.0912	0.018256	2.0912	0.018256	2.0912	0.018256	2.0912	0.018256	0.018256	0.422419221
TRAJ18	ENSG00000211871	2.0912	0.018256	2.0912	0.018256	2.0912	0.018256	2.0912	0.018256	0.018256	0.422419221

MGAT3	ENSG00000128268	0.95423	0.16998	1.5769	0.057408	1.5769	0.057408	2.0908	0.018271	0.018271	0.422565653
C1QA	ENSG00000173372	2.0904	0.01829	-0.091125	0.5363	1.3815	0.083558	1.0494	0.147	0.01829	0.422804412
KDM6B	ENSG00000132510	2.0899	0.018314	2.0899	0.018314	2.0899	0.018314	1.7084	0.043782	0.018314	0.422919152
PEX16	ENSG00000121680	-1.0056	0.84271	2.0898	0.018316	0.32269	0.37346	-0.41745	0.66183	0.018316	0.422919152
PCDHB4	ENSG00000081818	0.42058	0.33703	0.42058	0.33703	0.42058	0.33703	2.0897	0.018321	0.018321	0.422919152
NA	ENSG00000238851	2.0892	0.018347	2.0892	0.018347	2.0892	0.018347	2.0892	0.018347	0.018347	0.423216712
RPRD2	ENSG00000163125	2.0887	0.018367	1.8866	0.029607	1.7205	0.042675	1.6919	0.045337	0.018367	0.423216712
NA	ENSG00000268734	2.0887	0.018368	2.0887	0.018368	2.0887	0.018368	2.0887	0.018368	0.018368	0.423216712
MYBL2	ENSG00000101057	2.0887	0.018369	1.683	0.046186	1.4194	0.077894	1.0102	0.15619	0.018369	0.423216712
IGBP1P1	ENSG00000226677	2.0884	0.01838	2.0884	0.01838	2.0884	0.01838	2.0884	0.01838	0.01838	0.423216712
IQGAP2	ENSG00000145703	2.0883	0.018386	0.33524	0.36872	0.011016	0.49561	0.24185	0.40445	0.018386	0.423216712
RNA5SP461	ENSG00000252260	2.0879	0.018401	0.12676	0.44956	-0.019615	0.50782	1.9329	0.026622	0.018401	0.423362007
ASCC3	ENSG00000112249	2.0873	0.018428	0.23852	0.40574	1.4067	0.07976	1.3886	0.082474	0.018428	0.423526762
NA	ENSG00000250681	2.0871	0.018438	1.992	0.023184	2.0871	0.018438	2.0586	0.019767	0.018438	0.423526762
NA	ENSG00000188897	1.4846	0.068827	1.2783	0.10058	1.7704	0.038327	2.0871	0.01844	0.01844	0.423526762
LINC01339	ENSG00000248555	2.0869	0.018448	1.6291	0.05165	0.60871	0.27136	-0.011446	0.50457	0.018448	0.423526762
LINC01924	ENSG00000267134	2.0866	0.018464	0.26018	0.39736	-1.7586	0.96068	-0.89353	0.81421	0.018464	0.423526762
NA	ENSG00000231533	2.0865	0.018467	2.0865	0.018467	2.0865	0.018467	2.0865	0.018467	0.018467	0.423526762
NA	ENSG00000276662	-0.6303	0.73575	1.9571	0.025166	0.35095	0.36281	2.0864	0.018469	0.018469	0.423526762
NPTN	ENSG00000156642	1.2378	0.10789	2.0859	0.018494	0.0039999	0.4984	0.62729	0.26524	0.018494	0.423900574
OSBPL8	ENSG00000091039	-0.57983	0.71898	2.0613	0.019637	0.27065	0.39333	2.0856	0.01851	0.01851	0.423914361
ZNF383	ENSG00000188283	0.38597	0.34976	2.0855	0.018512	1.6014	0.054641	2.0855	0.018512	0.018512	0.423914361
ACKR2	ENSG00000144648	1.6658	0.047872	2.0853	0.018523	-1.5596	0.94057	-1.0107	0.84392	0.018523	0.423967022
PHB2P1	ENSG00000232728	2.0848	0.018542	2.0848	0.018542	2.0848	0.018542	2.0848	0.018542	0.018542	0.424140798
RAB3A	ENSG00000105649	2.0847	0.018548	2.0847	0.018548	2.0847	0.018548	1.555	0.059968	0.018548	0.424140798
NUP58	ENSG00000139496	-1.0186	0.84581	-0.26705	0.60528	-0.24734	0.59768	2.0839	0.018585	0.018585	0.424400606
NA	ENSG00000253682	0.12279	0.45114	0.81416	0.20778	2.0838	0.018587	0.71506	0.23729	0.018587	0.424400606
SERPINA5	ENSG00000188488	2.0834	0.018607	1.7827	0.037318	2.0834	0.018607	2.0834	0.018607	0.018607	0.424400606
OASL	ENSG00000135114	-0.039501	0.51575	-0.039501	0.51575	2.0832	0.018617	1.0271	0.15218	0.018617	0.424400606
RNU7-87P	ENSG00000238500	2.083	0.018628	2.083	0.018628	2.083	0.018628	2.083	0.018628	0.018628	0.424400606
NA	ENSG00000228434	2.0828	0.018636	2.0828	0.018636	2.0828	0.018636	2.0828	0.018636	0.018636	0.424400606

POLM	ENSG00000122678	2.0827	0.018641	1.3756	0.084472	1.1723	0.12054	0.75195	0.22604	0.018641	0.424400606
PCBP3-AS1	ENSG00000205424	1.0671	0.14297	-0.36201	0.64133	2.0824	0.018652	1.2339	0.10861	0.018652	0.424400606
NA	ENSG00000253263	-0.77815	0.78176	2.0824	0.018652	-0.77815	0.78176	-1.5432	0.93861	0.018652	0.424400606
NA	ENSG00000279635	0.8327	0.20251	0.8327	0.20251	0.8327	0.20251	2.0823	0.018658	0.018658	0.424400606
NA	ENSG00000236031	2.0822	0.018664	2.0822	0.018664	2.0822	0.018664	2.0822	0.018664	0.018664	0.424400606
LINC00184	ENSG00000224939	-0.50093	0.69179	-0.67731	0.75089	2.0819	0.018677	0.52322	0.30041	0.018677	0.424400606
NA	ENSG00000277738	-0.99991	0.84132	-0.065657	0.52617	2.0817	0.018684	-0.47996	0.68437	0.018684	0.424400606
NA	ENSG00000278428	0.1112	0.45573	-0.72119	0.7646	-0.46305	0.67834	2.0816	0.018688	0.018688	0.424400606
NEDD4	ENSG00000069869	1.3558	0.087582	0.27501	0.39165	2.0816	0.01869	0.33892	0.36734	0.01869	0.424400606
SMAD1	ENSG00000170365	1.2597	0.10388	1.4526	0.073162	0.68299	0.24731	2.0812	0.018706	0.018706	0.424443062
TRBV6-6	ENSG00000211724	1.5827	0.05675	1.7612	0.039102	2.0811	0.018714	2.0811	0.018714	0.018714	0.424443062
NA	ENSG00000227722	2.081	0.018718	0.25482	0.39943	0.13565	0.44605	1.0504	0.14676	0.018718	0.424443062
NA	ENSG00000269469	2.0802	0.018752	2.0802	0.018752	2.0802	0.018752	2.0802	0.018752	0.018752	0.424978793
GPAM	ENSG00000119927	1.8573	0.031632	2.0517	0.0201	-0.13175	0.55241	2.0798	0.018774	0.018774	0.424978793
NA	ENSG00000226814	1.6032	0.054447	0.52471	0.29989	2.0797	0.018775	0.99082	0.16089	0.018775	0.424978793
NA	ENSG00000232036	0.22896	0.40945	2.0793	0.018794	1.7969	0.036177	0.22896	0.40945	0.018794	0.424978793
NA	ENSG00000258506	2.079	0.018808	1.1235	0.13061	1.1235	0.13061	1.1235	0.13061	0.018808	0.424978793
RSF1	ENSG00000048649	1.116	0.13221	2.079	0.018809	0.85107	0.19737	0.9526	0.1704	0.018809	0.424978793
NA	ENSG00000260911	2.0789	0.018812	1.6839	0.046101	2.0789	0.018812	2.0789	0.018812	0.018812	0.424978793
RIF1	ENSG00000080345	1.8477	0.032326	2.0789	0.018813	2.0102	0.022206	2.0525	0.020059	0.018813	0.424978793
LYPLA1P1	ENSG00000223595	2.0785	0.018832	2.0785	0.018832	2.0785	0.018832	2.0785	0.018832	0.018832	0.424978793
SPOCK2	ENSG00000107742	2.0783	0.018839	1.4643	0.071553	0.50917	0.30532	0.0022132	0.49912	0.018839	0.424978793
TRAV25	ENSG00000211806	2.0782	0.018846	2.0782	0.018846	2.0782	0.018846	2.0782	0.018846	0.018846	0.424978793
TMEM267	ENSG00000151881	2.0781	0.018851	2.0781	0.018851	2.0781	0.018851	0.80158	0.2114	0.018851	0.424978793
MIR298	ENSG00000216031	0.57208	0.28364	0.57208	0.28364	0.57208	0.28364	2.078	0.018855	0.018855	0.424978793
NA	ENSG00000254653	1.8891	0.029438	1.8891	0.029438	2.0778	0.018864	1.8891	0.029438	0.018864	0.424985076
RAB11FIP5	ENSG00000135631	1.7469	0.040327	-0.080402	0.53204	2.0542	0.01998	2.077	0.018899	0.018899	0.425407555
ADGRB2	ENSG00000121753	2.0603	0.019684	1.6265	0.05192	2.0766	0.018918	1.2924	0.098102	0.018918	0.425407555
NA	ENSG00000230096	0.53278	0.29709	0.57446	0.28283	2.0766	0.01892	1.177	0.11959	0.01892	0.425407555
RNU6-823P	ENSG00000212344	2.0764	0.018929	0.62223	0.2669	1.7254	0.042225	1.1334	0.12852	0.018929	0.425407555
NA	ENSG00000260834	-0.16899	0.5671	-0.1869	0.57413	2.076	0.018945	0.19622	0.42222	0.018945	0.425407555

NA	ENSG00000259408	2.076	0.018948	2.076	0.018948	2.076	0.018948	2.076	0.018948	0.018948	0.425407555
NA	ENSG00000264677	2.0759	0.018952	2.0759	0.018952	2.0759	0.018952	2.0759	0.018952	0.018952	0.425407555
RPL3P7	ENSG00000225093	2.0752	0.018986	2.0752	0.018986	-0.14696	0.55842	-0.69935	0.75783	0.018986	0.425407555
ESAM	ENSG00000149564	1.8669	0.030956	2.0749	0.018997	-0.6044	0.72721	-0.6044	0.72721	0.018997	0.425407555
SPATA31D1	ENSG00000214929	-0.83007	0.79675	1.0979	0.13612	-0.83007	0.79675	2.0749	0.018999	0.018999	0.425407555
LINC01730	ENSG00000275491	2.0748	0.019001	1.7685	0.038491	2.0748	0.019001	2.0748	0.019001	0.019001	0.425407555
MAP6D1	ENSG00000180834	1.9308	0.026753	1.8869	0.029589	2.0746	0.019014	2.0746	0.019014	0.019014	0.425407555
EPG5	ENSG00000152223	2.0745	0.019018	1.9869	0.023466	-0.20845	0.58256	1.9068	0.028271	0.019018	0.425407555
SNORD18A	ENSG00000200623	2.0741	0.019036	2.0741	0.019036	2.0741	0.019036	2.0741	0.019036	0.019036	0.425407555
RPUSD3	ENSG00000156990	2.074	0.019038	1.4159	0.078406	1.6099	0.053713	1.6886	0.045647	0.019038	0.425407555
CCDC178	ENSG00000166960	1.2201	0.11122	2.0736	0.01906	1.4035	0.080229	1.2547	0.10479	0.01906	0.425407555
RAB17	ENSG00000124839	0.77551	0.21902	0.44344	0.32872	2.0735	0.019063	0.3187	0.37498	0.019063	0.425407555
PPP1R13B	ENSG00000088808	1.7308	0.041742	1.0098	0.1563	2.0734	0.019066	1.8637	0.031181	0.019066	0.425407555
RNU6-637P	ENSG00000206777	1.1104	0.13341	-0.036956	0.51474	0.044633	0.4822	2.0733	0.019071	0.019071	0.425407555
NMBR-AS1	ENSG00000236822	2.0733	0.019073	0.59134	0.27715	0.59134	0.27715	0.59134	0.27715	0.019073	0.425407555
NA	ENSG00000268650	2.0729	0.019088	2.0729	0.019088	2.0729	0.019088	2.0729	0.019088	0.019088	0.425407555
CD96	ENSG00000153283	2.0729	0.019092	1.9971	0.022907	1.9971	0.022907	1.9971	0.022907	0.019092	0.425407555
NA	ENSG00000274267	1.4243	0.077178	1.2902	0.098484	2.0729	0.019092	0.38378	0.35057	0.019092	0.425407555
RNA5SP44	ENSG00000222378	2.0727	0.019101	1.757	0.039455	1.0062	0.15716	2.0727	0.019101	0.019101	0.425407555
NA	ENSG00000267849	2.0727	0.019101	1.3034	0.096222	2.0727	0.019101	2.0727	0.019101	0.019101	0.425407555
SOX5-AS1	ENSG00000256120	2.0721	0.01913	2.0721	0.01913	0.40538	0.3426	1.6768	0.046794	0.01913	0.425775594
NA	ENSG00000276593	0.32867	0.3712	1.7669	0.03862	2.072	0.019135	1.7725	0.038157	0.019135	0.425775594
NA	ENSG00000185495	-0.21723	0.58599	2.0715	0.019155	0.057721	0.47699	-0.79816	0.78761	0.019155	0.425982342
GCOM1	ENSG00000137878	0.39627	0.34595	2.0711	0.019175	-0.42458	0.66443	-0.062551	0.52494	0.019175	0.425982342
TAF9BP2	ENSG00000215760	-0.72198	0.76485	0.099884	0.46022	2.071	0.019179	-0.29356	0.61545	0.019179	0.425982342
NA	ENSG00000220745	2.0708	0.019187	0.35965	0.35955	1.6096	0.053737	1.6096	0.053737	0.019187	0.425982342
E2F3-IT1	ENSG00000224707	2.0708	0.019188	2.0708	0.019188	2.0708	0.019188	2.0708	0.019188	0.019188	0.425982342
NA	ENSG00000266336	2.0705	0.019201	0.97672	0.16435	0.97672	0.16435	0.97672	0.16435	0.019201	0.425983079
NA	ENSG00000264310	2.0703	0.019214	2.0703	0.019214	2.0703	0.019214	2.0703	0.019214	0.019214	0.425983079
VKORC1	ENSG00000167397	2.0702	0.019216	2.0702	0.019216	2.0702	0.019216	2.0702	0.019216	0.019216	0.425983079
GRB14	ENSG00000115290	1.5034	0.066373	2.0701	0.019223	-0.77616	0.78117	-1.064	0.85635	0.019223	0.425983079

NA	ENSG00000259994	2.0695	0.01925	2.0695	0.01925	2.0695	0.01925	2.0695	0.01925	0.01925	0.4263875
BTC	ENSG00000174808	0.15898	0.43684	1.2878	0.09891	2.069	0.019271	1.3239	0.092768	0.019271	0.426641994
EIF5	ENSG00000100664	1.4488	0.073698	2.0689	0.019279	0.18665	0.42597	1.7682	0.038516	0.019279	0.426641994
AURKAIP1	ENSG00000175756	2.0677	0.019335	2.0677	0.019335	2.0677	0.019335	1.9246	0.027139	0.019335	0.426815149
NA	ENSG00000261007	2.0677	0.019336	0.94022	0.17355	2.0677	0.019336	2.0677	0.019336	0.019336	0.426815149
NEDD8P1	ENSG00000226970	2.0673	0.019354	2.0673	0.019354	2.0673	0.019354	2.0673	0.019354	0.019354	0.426815149
TADA2A	ENSG00000276234	2.0669	0.019373	1.6939	0.045138	2.064	0.019509	1.8413	0.032791	0.019373	0.426815149
CHMP1B	ENSG00000255112	2.0666	0.019384	0.21205	0.41603	-0.026875	0.51072	2.0666	0.019384	0.019384	0.426815149
RPS23P5	ENSG00000242608	0.10091	0.45981	2.0664	0.019396	-0.91669	0.82035	-0.91669	0.82035	0.019396	0.426815149
NA	ENSG00000261124	2.0662	0.019404	2.0662	0.019404	2.0662	0.019404	2.0662	0.019404	0.019404	0.426815149
CCDC166	ENSG00000255181	2.0659	0.019417	2.0659	0.019417	2.0659	0.019417	2.0659	0.019417	0.019417	0.426815149
AK2	ENSG00000004455	1.4117	0.079018	1.668	0.04766	-0.25846	0.60198	2.0658	0.019425	0.019425	0.426815149
LCN15	ENSG00000177984	1.9014	0.028623	1.9014	0.028623	1.9014	0.028623	2.0658	0.019425	0.019425	0.426815149
CD28	ENSG00000178562	2.0657	0.019428	1.6988	0.044676	1.5413	0.061623	1.5413	0.061623	0.019428	0.426815149
NA	ENSG00000241776	2.0657	0.019429	2.0657	0.019429	2.0657	0.019429	2.0657	0.019429	0.019429	0.426815149
NA	ENSG00000265474	2.0656	0.019431	2.0656	0.019431	2.0656	0.019431	2.0656	0.019431	0.019431	0.426815149
IQCA1	ENSG00000132321	2.0654	0.01944	1.6259	0.051988	0.78631	0.21584	0.94194	0.17311	0.01944	0.426815149
SHC1	ENSG00000160691	2.0653	0.019449	0.66845	0.25192	2.0279	0.021287	0.66845	0.25192	0.019449	0.426815149
LINC01661	ENSG00000237460	2.0651	0.019455	0.27972	0.38985	-0.41123	0.65955	-0.83982	0.79949	0.019455	0.426815149
NA	ENSG00000249042	2.0651	0.019458	-0.524	0.69986	2.0651	0.019458	2.0651	0.019458	0.019458	0.426815149
SNX3	ENSG00000112335	0.34569	0.36479	0.90299	0.18326	0.34569	0.36479	2.065	0.019462	0.019462	0.426815149
RIC3	ENSG00000166405	2.0256	0.021402	2.0256	0.021402	2.065	0.019462	1.2747	0.10121	0.019462	0.426815149
NA	ENSG00000249492	2.065	0.019462	2.065	0.019462	2.065	0.019462	2.065	0.019462	0.019462	0.426815149
TNRC6B	ENSG00000100354	-0.86222	0.80572	-0.94379	0.82736	-0.45484	0.67539	2.0648	0.019473	0.019473	0.426864278
RN7SL808P	ENSG00000240637	2.0645	0.019486	0.90888	0.18171	2.0645	0.019486	2.0645	0.019486	0.019486	0.426957185
STMN4	ENSG00000015592	1.2805	0.10019	0.71462	0.23742	-0.49632	0.69016	2.064	0.019509	0.019509	0.42726902
NA	ENSG00000265342	1.9619	0.024885	2.0631	0.01955	1.3418	0.089836	1.8896	0.029404	0.01955	0.427343097
FOXQ1	ENSG00000164379	0.10055	0.45996	1.4109	0.07913	2.0631	0.019554	-0.65995	0.74536	0.019554	0.427343097
MARK3P1	ENSG00000271642	1.6902	0.045495	2.0629	0.01956	0.95209	0.17053	0.27332	0.3923	0.01956	0.427343097
TPSB2	ENSG00000197253	0.67278	0.25054	2.0629	0.019563	0.50294	0.3075	0.23001	0.40904	0.019563	0.427343097
NA	ENSG00000255931	1.8315	0.033513	1.882	0.029921	1.4112	0.079096	2.0628	0.019564	0.019564	0.427343097

TCP1P3	ENSG00000256849	2.0628	0.019565	0.033868	0.48649	2.0628	0.019565	2.0628	0.019565	0.019565	0.427343097
FOXJ1	ENSG00000129654	2.0625	0.019582	1.6246	0.052126	2.0625	0.019582	1.6998	0.044584	0.019582	0.427522787
YTHDC1	ENSG00000083896	1.4985	0.067004	1.9876	0.023427	-0.68379	0.75294	2.0622	0.019595	0.019595	0.427615025
NUP160	ENSG00000030066	-0.18435	0.57313	-0.57358	0.71688	1.005	0.15745	2.0616	0.019621	0.019621	0.427787099
NA	ENSG00000264194	2.0614	0.019631	2.0614	0.019631	0.15535	0.43827	0.15535	0.43827	0.019631	0.427787099
NA	ENSG00000207356	0.28415	0.38815	2.0613	0.019637	-0.66964	0.74845	-0.69099	0.75521	0.019637	0.427787099
LMAN2	ENSG00000169223	2.0613	0.019638	-0.29524	0.61609	-1.349	0.91134	1.6195	0.05267	0.019638	0.427787099
NA	ENSG00000268597	-0.16718	0.56638	2.0609	0.019655	-0.27616	0.60879	-1.5183	0.93553	0.019655	0.427966108
ACTR3C	ENSG00000106526	-0.23131	0.59146	2.0606	0.01967	-0.023542	0.50939	-0.43467	0.6681	0.01967	0.428101429
LCN8	ENSG00000204001	0.58181	0.28035	1.6003	0.054769	0.99231	0.16052	2.0596	0.01972	0.01972	0.428109853
NA	ENSG00000251062	0.49818	0.30918	0.1891	0.42501	-1.1459	0.87409	2.0594	0.019728	0.019728	0.428109853
SUPT6H	ENSG00000109111	1.023	0.15315	0.86459	0.19363	0.927	0.17696	2.0591	0.019744	0.019744	0.428109853
NA	ENSG00000254534	-0.12864	0.55118	2.059	0.019749	2.059	0.019749	2.059	0.019749	0.019749	0.428109853
NA	ENSG00000249568	1.751	0.039975	1.751	0.039975	2.0585	0.019769	0.082886	0.46697	0.019769	0.428109853
NA	ENSG00000258973	2.0583	0.01978	2.0583	0.01978	2.0583	0.01978	2.0583	0.01978	0.01978	0.428109853
NA	ENSG00000244662	2.0583	0.019781	-0.26402	0.60412	-0.26402	0.60412	-0.26402	0.60412	0.019781	0.428109853
NRGN	ENSG00000154146	-1.5661	0.94134	-1.5661	0.94134	-1.5661	0.94134	2.0582	0.019784	0.019784	0.428109853
LINC02424	ENSG00000258220	0.2756	0.39143	2.058	0.019793	0.26057	0.39721	0.26057	0.39721	0.019793	0.428109853
IFNWP4	ENSG00000225027	1.3019	0.096479	0.42477	0.3355	2.057	0.019842	2.057	0.019842	0.019842	0.428109853
SLC23A3	ENSG00000213901	1.6891	0.045599	2.0569	0.019848	-0.24594	0.59714	-0.24594	0.59714	0.019848	0.428109853
RAB44	ENSG00000255587	0.29776	0.38294	2.0568	0.019852	1.9574	0.025151	0.37913	0.3523	0.019852	0.428109853
FAM83F	ENSG00000133477	2.0566	0.019861	0.64081	0.26082	0.99961	0.15875	1.674	0.047062	0.019861	0.428109853
NA	ENSG00000272461	-0.51734	0.69754	0.43402	0.33214	-0.29394	0.6156	2.056	0.019892	0.019892	0.428109853
RNU6-1310P	ENSG00000206880	2.0559	0.019895	0.18999	0.42466	0.18999	0.42466	0.18999	0.42466	0.019895	0.428109853
C10orf143	ENSG00000237489	2.0559	0.019896	2.0559	0.019896	2.0559	0.019896	0.13664	0.44566	0.019896	0.428109853
CAPRIN1	ENSG00000135387	2.0558	0.019901	1.6944	0.045095	1.2792	0.10042	2.0335	0.021003	0.019901	0.428109853
DNAAF5	ENSG00000164818	-0.10018	0.5399	-0.47874	0.68394	-0.25749	0.6016	2.0558	0.019901	0.019901	0.428109853
EEF1A1P32	ENSG00000213046	2.0557	0.019908	2.0557	0.019908	2.0557	0.019908	2.0557	0.019908	0.019908	0.428109853
AMD1P4	ENSG00000215124	2.0553	0.019923	-0.3797	0.64791	-0.3797	0.64791	-0.3797	0.64791	0.019923	0.428109853
MTOR-AS1	ENSG00000225602	0.015961	0.49363	2.0549	0.019944	0.015961	0.49363	0.015961	0.49363	0.019944	0.428109853
DYRK3	ENSG00000143479	2.0547	0.019955	0.049703	0.48018	1.5253	0.063593	-0.38523	0.64997	0.019955	0.428109853

NA	ENSG00000247763	-1.1722	0.87944	2.0546	0.019959	-1.1722	0.87944	-1.1722	0.87944	0.019959	0.428109853
ZNF859P	ENSG00000215887	1.8943	0.029094	2.0544	0.019966	0.77554	0.21901	1.067	0.14299	0.019966	0.428109853
NA	ENSG00000201820	0.077105	0.46927	0.3744	0.35405	2.0542	0.019979	2.0542	0.019979	0.019979	0.428109853
RPS4XP12	ENSG00000244400	0.68425	0.24691	0.68425	0.24691	-0.32609	0.62782	2.0539	0.019991	0.019991	0.428109853
NA	ENSG00000236403	2.0538	0.019997	0.1943	0.42297	-0.46093	0.67758	0.0879	0.46498	0.019997	0.428109853
H2BC20P	ENSG00000261716	2.0537	0.020002	2.0461	0.020375	0.76243	0.2229	0.76243	0.2229	0.020002	0.428109853
USF1P1	ENSG00000226501	2.0534	0.020018	2.0534	0.020018	2.0534	0.020018	2.0534	0.020018	0.020018	0.428109853
NA	ENSG00000263766	2.0527	0.02005	2.0527	0.02005	2.0527	0.02005	2.0527	0.02005	0.02005	0.428109853
CA8	ENSG00000178538	0.98744	0.16171	2.0525	0.020059	2.0525	0.020059	2.0525	0.020059	0.020059	0.428109853
FAM238C	ENSG00000283709	2.0525	0.020059	1.7781	0.037697	1.8652	0.031074	1.0143	0.15522	0.020059	0.428109853
NA	ENSG00000219758	-0.20792	0.58235	2.0519	0.020088	0.69716	0.24285	1.2395	0.10758	0.020088	0.428109853
NA	ENSG00000230699	2.0271	0.021326	1.9613	0.02492	1.9163	0.027661	2.0517	0.020097	0.020097	0.428109853
MFSD4A	ENSG00000174514	1.1475	0.12559	1.3001	0.096778	2.0513	0.020119	0.024464	0.49024	0.020119	0.428109853
EIF4EP4	ENSG00000232968	1.3482	0.088793	2.0512	0.020124	0.76955	0.22078	-0.20056	0.57948	0.020124	0.428109853
NA	ENSG00000251964	2.0512	0.020125	2.0512	0.020125	2.0512	0.020125	2.0512	0.020125	0.020125	0.428109853
PPP1R2B	ENSG00000231989	1.0842	0.13913	2.0297	0.021195	2.0297	0.021195	2.0509	0.020137	0.020137	0.428109853
SDK2	ENSG00000069188	1.2645	0.10302	0.32397	0.37298	-0.050551	0.52016	2.0509	0.020138	0.020138	0.428109853
NA	ENSG00000249265	2.0457	0.020395	2.0509	0.020138	2.0509	0.020138	2.0509	0.020138	0.020138	0.428109853
WDR86-AS1	ENSG00000243836	-0.40812	0.65841	2.0507	0.020146	0.54285	0.29362	-1.7562	0.96047	0.020146	0.428109853
NA	ENSG00000240097	2.0507	0.02015	0.86543	0.1934	1.051	0.14662	2.0507	0.02015	0.02015	0.428109853
NA	ENSG00000252123	2.0506	0.020153	-1.1822	0.88143	-1.1822	0.88143	-1.1822	0.88143	0.020153	0.428109853
CFHR4	ENSG00000134365	1.3109	0.094947	0.68313	0.24726	2.0506	0.020155	0.60586	0.27231	0.020155	0.428109853
LINC01710	ENSG00000225561	2.0503	0.020167	-0.56868	0.71521	-0.83811	0.79902	-0.83811	0.79902	0.020167	0.428109853
RN7SKP260	ENSG00000252716	2.0502	0.020172	2.0502	0.020172	2.0502	0.020172	2.0502	0.020172	0.020172	0.428109853
LINC01533	ENSG00000267779	0.49973	0.30863	0.16908	0.43287	2.05	0.020181	-0.074332	0.52963	0.020181	0.428109853
RN7SL549P	ENSG00000239825	0.80962	0.20908	1.9347	0.026514	1.9347	0.026514	2.05	0.020184	0.020184	0.428109853
SOX2-OT	ENSG00000242808	2.0496	0.020202	1.8091	0.035216	1.0014	0.15832	0.61542	0.26914	0.020202	0.428109853
LINC00939	ENSG00000249267	-1.2863	0.90083	-0.19901	0.57887	-0.66241	0.74614	2.0495	0.020208	0.020208	0.428109853
NA	ENSG00000267465	2.0495	0.020209	2.0495	0.020209	2.0495	0.020209	2.0495	0.020209	0.020209	0.428109853
NA	ENSG00000271118	2.0492	0.020219	1.7539	0.039723	1.7539	0.039723	1.7539	0.039723	0.020219	0.428109853
RNA5SP204	ENSG00000201415	-0.030217	0.51205	-0.030217	0.51205	2.0491	0.020226	-0.059411	0.52369	0.020226	0.428109853

PRPF3	ENSG00000117360	-0.33504	0.6312	1.5765	0.057457	2.0491	0.020227	1.627	0.051868	0.020227	0.428109853
RSU1P2	ENSG00000232554	1.1519	0.12468	2.049	0.020233	1.6097	0.053733	1.472	0.070507	0.020233	0.428109853
CCDC102A	ENSG00000135736	2.0489	0.020235	0.99764	0.15923	1.3355	0.090854	0.395	0.34642	0.020235	0.428109853
NA	ENSG00000243979	2.0489	0.020235	2.0489	0.020235	2.0489	0.020235	2.0489	0.020235	0.020235	0.428109853
SERPINE3	ENSG00000253309	1.3921	0.081947	1.1644	0.12213	1.8966	0.028937	2.0489	0.020238	0.020238	0.428109853
MYEOV	ENSG00000172927	1.3704	0.085286	1.4002	0.080726	2.0488	0.020239	1.7588	0.039303	0.020239	0.428109853
LINC01103	ENSG00000234781	2.0488	0.020243	1.7351	0.041363	-0.51385	0.69632	1.6043	0.054321	0.020243	0.428109853
NA	ENSG00000244699	1.0917	0.13748	1.4452	0.074204	1.0201	0.15385	2.0488	0.020243	0.020243	0.428109853
NA	ENSG00000242553	2.0487	0.020245	0.26584	0.39518	2.0487	0.020245	2.0487	0.020245	0.020245	0.428109853
HMGN1P26	ENSG00000259557	2.0485	0.020255	1.1607	0.12287	2.0485	0.020255	2.0485	0.020255	0.020255	0.428109853
RPS3AP42	ENSG00000244244	2.0484	0.020262	-0.12711	0.55057	2.0484	0.020262	1.3708	0.085216	0.020262	0.428109853
NA	ENSG00000229505	1.9714	0.024338	2.0483	0.020267	2.0483	0.020267	2.0483	0.020267	0.020267	0.428109853
NA	ENSG00000228151	-0.48857	0.68743	2.048	0.020279	2.048	0.020279	-0.033342	0.5133	0.020279	0.428109853
ARHGAP30	ENSG00000186517	-0.53755	0.70455	-0.32533	0.62753	-0.53755	0.70455	2.0479	0.020287	0.020287	0.428109853
SLC17A7	ENSG00000104888	2.0476	0.020299	2.0476	0.020299	2.0476	0.020299	2.0476	0.020299	0.020299	0.428109853
RNA5SP38	ENSG00000222682	1.5322	0.062742	2.0476	0.020302	1.5322	0.062742	1.5322	0.062742	0.020302	0.428109853
NA	ENSG00000273987	2.0474	0.02031	2.0474	0.02031	2.0474	0.02031	2.0474	0.02031	0.02031	0.428109853
TRAJ21	ENSG00000211868	2.0472	0.02032	2.0472	0.02032	2.0472	0.02032	2.0472	0.02032	0.02032	0.428109853
SNORA63E	ENSG00000199363	0.42963	0.33373	0.42963	0.33373	2.0272	0.021319	2.0471	0.020322	0.020322	0.428109853
CNTN1	ENSG00000018236	0.17064	0.43225	1.061	0.14435	0.62282	0.2667	2.0471	0.020326	0.020326	0.428109853
NA	ENSG00000264853	1.7477	0.04026	1.9422	0.026055	2.047	0.020331	1.2922	0.098136	0.020331	0.428109853
ZP3	ENSG00000188372	2.0464	0.020359	0.74472	0.22822	1.4927	0.067758	0.82545	0.20456	0.020359	0.428109853
NA	ENSG00000182109	-1.1083	0.86614	1.2879	0.098887	2.0463	0.020364	1.4458	0.074119	0.020364	0.428109853
UTP4	ENSG00000141076	0.51108	0.30465	0.49813	0.3092	1.1471	0.12567	2.0462	0.020368	0.020368	0.428109853
C3orf33	ENSG00000174928	1.6166	0.052982	1.6166	0.052982	1.6166	0.052982	2.0461	0.020373	0.020373	0.428109853
KRT18P3	ENSG00000215553	2.046	0.02038	2.046	0.02038	2.046	0.02038	-0.061609	0.52456	0.02038	0.428109853
NPHP1	ENSG00000144061	2.0459	0.020381	1.6642	0.04804	1.0186	0.15421	1.7699	0.038376	0.020381	0.428109853
LINC02290	ENSG00000258502	2.0459	0.020382	2.0459	0.020382	2.0459	0.020382	2.0459	0.020382	0.020382	0.428109853
NA	ENSG00000242197	2.0454	0.020407	2.0454	0.020407	1.9248	0.027127	2.0454	0.020407	0.020407	0.428333134
DPT	ENSG00000143196	1.5405	0.061718	2.0075	0.022348	1.5405	0.061718	2.0453	0.020414	0.020414	0.428333134
NA	ENSG00000226426	2.0452	0.020419	2.0452	0.020419	2.0452	0.020419	2.0452	0.020419	0.020419	0.428333134

NA ENSG00000240201 2.0448 0.020438 2.0448 0.020438 2.0448 0.020438
2.0448 0.020438 0.020438 0.42854722
BACH1-IT1 ENSG00000248476 2.0445 0.02045 0.054552 0.47825 0.91274
0.18069 0.067686 0.47302 0.02045 0.428614409
NA ENSG00000255356 2.0442 0.020466 2.0442 0.020466 2.0442 0.020466
2.0442 0.020466 0.020466 0.42876534
NA ENSG0000228156 2.0428 0.020536 2.0428 0.020536 2.0428 0.020536
0.82179 0.2056 0.020536 0.429535903
LCEP1 ENSG00000233819 2.0427 0.02054 2.0427 0.02054 2.0427 0.02054
2.0427 0.02054 0.02054 0.429535903
NA ENSG00000253646 2.0427 0.020542 0.79063 0.21458 0.82307 0.20523
0.82307 0.20523 0.020542 0.429535903
MLN ENSG00000096395 -0.49182 0.68858 2.0426 0.020548 0.73516 0.23112
-0.23886 0.59439 0.020548 0.429535903
HSP90AB4P ENSG00000282100 0.97728 0.16422 1.5839 0.056612 2.0423
0.02056 0.93127 0.17586 0.02056 0.429535903
NA ENSG00000227297 1.8784 0.030165 0.31661 0.37577 2.042 0.020575 2.042
0.020575 0.020575 0.429535903
ECT2L ENSG00000203734 2.0419 0.020582 1.0479 0.14735 1.3551 0.08769
0.93665 0.17447 0.020582 0.429535903
ABCB6 ENSG00000115657 0.43774 0.33079 1.7138 0.04328 0.58217 0.28023
2.0418 0.020588 0.020588 0.429535903
NA ENSG00000251556 2.0416 0.020596 2.0416 0.020596 2.0416 0.020596
2.0416 0.020596 0.020596 0.429535903
SLC24A3 ENSG00000185052 0.70564 0.2402 2.0415 0.020602 1.3114
0.094867 1.8301 0.03362 0.020602 0.429535903
NA ENSG00000254933 2.0412 0.020617 2.0412 0.020617 2.0412 0.020617
2.0412 0.020617 0.020617 0.429535903
CYCSP25 ENSG00000255370 2.041 0.020626 2.041 0.020626 2.041 0.020626 1.2299
0.10937 0.020626 0.429535903
MYO3B ENSG00000071909 1.2714 0.10179 1.0521 0.14637 2.0409 0.020629
-0.37518 0.64624 0.020629 0.429535903
NA ENSG00000266771 2.0408 0.020634 2.0408 0.020634 2.0408 0.020634
2.0408 0.020634 0.020634 0.429535903
OTX2P1 ENSG00000234644 2.0408 0.020635 2.0408 0.020635 0.70865
0.23927 2.0408 0.020635 0.020635 0.429535903
PAIP1 ENSG00000172239 2.0228 0.021548 2.0228 0.021548 2.0228 0.021548
2.0406 0.020646 0.020646 0.429581375
NA ENSG00000266456 2.0398 0.020687 2.0398 0.020687 2.0398 0.020687
2.0398 0.020687 0.020687 0.429738074
TNR ENSG00000116147 1.872 0.030606 0.63573 0.26248 0.57923 0.28122
2.0397 0.020689 0.020689 0.429738074
MCOLN2 ENSG00000153898 -0.10685 0.54254 1.6372 0.050796 2.0396
0.020695 0.28957 0.38607 0.020695 0.429738074
TBX19 ENSG00000143178 -0.65437 0.74356 1.9032 0.028506 0.17694 0.42978
2.0395 0.020701 0.020701 0.429738074
ALDH6A1 ENSG00000119711 2.0394 0.020703 1.4446 0.07429 1.4314
0.076153 1.6411 0.050391 0.020703 0.429738074
NA ENSG00000257452 2.0389 0.02073 2.0389 0.02073 2.0389 0.02073
0.63129 0.26392 0.02073 0.429738074
RPL34P20 ENSG00000231508 2.0386 0.020747 2.0386 0.020747 2.0386
0.020747 2.0386 0.020747 0.020747 0.429738074
NA ENSG00000239317 2.0385 0.020748 2.0385 0.020748 2.0385 0.020748
2.0385 0.020748 0.020748 0.429738074
ZNF467 ENSG00000181444 -1.5363 0.93777 -1.885 0.97029 2.0383
0.020761 0.04903 0.48045 0.020761 0.429738074
NA ENSG00000259173 2.0381 0.020771 2.0381 0.020771 2.0381 0.020771
2.0381 0.020771 0.020771 0.429738074
RPL23AP37 ENSG00000236148 -0.42015 0.66281 1.6969 0.044857 2.0381
0.020772 -0.42015 0.66281 0.020772 0.429738074
RPL3P1 ENSG00000228149 2.038 0.020774 1.012 0.15577 1.012 0.15577 1.1666
0.1217 0.020774 0.429738074

LINC00554	ENSG00000260738	0.24817	0.402	-0.21272	0.58423	2.038	0.020777	0.73773	0.23034	0.020777	0.429738074
MIR557	ENSG00000207974	0.92081	0.17857	2.0371	0.020818	0.92081	0.17857	2.0371	0.020818	0.020818	0.429738074
NA	ENSG00000262810	1.3828	0.083359	2.0371	0.020822	0.20102	0.42034	1.3828	0.083359	0.020822	0.429738074
UQCRBP2	ENSG00000227941	2.037	0.020825	2.037	0.020825	2.037	0.020825	2.037	0.020825	0.020825	0.429738074
FBXO9	ENSG00000112146	1.0144	0.15519	2.0369	0.020829	1.8856	0.029671	0.60371	0.27302	0.020829	0.429738074
NA	ENSG00000250378	1.2611	0.10363	2.0368	0.020833	-1.1285	0.87044	0.18271	0.42751	0.020833	0.429738074
NANOGP6	ENSG00000227351	2.0367	0.02084	2.0367	0.02084	2.0367	0.02084	2.0367	0.02084	0.02084	0.429738074
ANKRD13D	ENSG00000172932	2.0366	0.020843	2.0366	0.020843	1.5486	0.060735	1.7426	0.040703	0.020843	0.429738074
NA	ENSG00000263220	2.0366	0.020843	2.0366	0.020843	2.0366	0.020843	2.0366	0.020843	0.020843	0.429738074
NAGLU	ENSG00000108784	-1.4057	0.92009	-0.45274	0.67463	2.0364	0.020855	-0.68702	0.75396	0.020855	0.429738074
ARL13B	ENSG00000169379	1.1495	0.12517	0.5588	0.28815	1.1495	0.12517	2.0364	0.020857	0.020857	0.429738074
NA	ENSG00000264286	2.0361	0.020869	2.0361	0.020869	2.0361	0.020869	2.0361	0.020869	0.020869	0.429738074
NA	ENSG00000258407	2.036	0.020874	2.036	0.020874	2.036	0.020874	2.036	0.020874	0.020874	0.429738074
YEATS2-AS1	ENSG00000233885	2.0356	0.020897	2.0356	0.020897	2.0356	0.020897	2.0356	0.020897	0.020897	0.429911869
NA	ENSG00000271659	2.0354	0.020903	2.0354	0.020903	2.0354	0.020903	2.0354	0.020903	0.020903	0.429911869
NA	ENSG00000261293	2.0344	0.020956	2.0344	0.020956	2.0344	0.020956	1.7662	0.038678	0.020956	0.429911869
IMPDH1P7	ENSG00000226553	2.0341	0.020969	2.0341	0.020969	2.0341	0.020969	1.2282	0.10968	0.020969	0.429911869
CT66	ENSG00000234215	1.8402	0.032873	2.0338	0.020987	2.0338	0.020987	2.0338	0.020987	0.020987	0.429911869
LINC02501	ENSG00000249882	2.0337	0.020989	-0.44145	0.67056	-0.44145	0.67056	-0.44145	0.67056	0.020989	0.429911869
NA	ENSG00000270934	2.0335	0.020999	2.0335	0.020999	2.0335	0.020999	2.0335	0.020999	0.020999	0.429911869
NA	ENSG00000261529	2.0335	0.021004	2.0335	0.021004	2.0335	0.021004	2.0335	0.021004	0.021004	0.429911869
CD19	ENSG00000177455	1.6205	0.052566	0.79807	0.21241	1.2779	0.10065	2.0332	0.021018	0.021018	0.429911869
MICALL2	ENSG00000164877	0.23514	0.40705	0.14747	0.44138	2.0331	0.02102	0.58519	0.27921	0.02102	0.429911869
NA	ENSG00000239602	2.0327	0.021043	2.0327	0.021043	2.0327	0.021043	2.0327	0.021043	0.021043	0.429911869
MED9	ENSG00000141026	2.0326	0.021044	2.0326	0.021044	2.0326	0.021044	2.0326	0.021044	0.021044	0.429911869
CCDC88B	ENSG00000168071	0.79412	0.21356	2.0326	0.021044	0.31762	0.37539	1.7613	0.039092	0.021044	0.429911869
RNU6-91P	ENSG00000272439	0.82329	0.20517	0.82329	0.20517	0.82329	0.20517	2.0325	0.02105	0.02105	0.429911869
NA	ENSG00000280991	2.0324	0.021055	0.65089	0.25756	0.23901	0.40555	0.23901	0.40555	0.021055	0.429911869
COL22A1	ENSG00000169436	2.0324	0.021059	-0.69477	0.7564	-1.0124	0.84432	0.24401	0.40361	0.021059	0.429911869
MMS19	ENSG00000155229	0.33949	0.36712	0.64241	0.2603	2.032	0.021074	-0.026902	0.51073	0.021074	0.429911869
GPR1-AS	ENSG00000279220	2.0321	0.021074	1.4496	0.073582	2.0321	0.021074	1.3902	0.082233	0.021074	0.429911869

NA	ENSG00000280251	2.0321	0.021074	-0.84683	0.80146	-0.84683	0.80146	-0.84683	0.80146	0.021074	0.429911869
GK3P	ENSG00000229894	-0.47169	0.68143	0.17978	0.42866	1.3506	0.088407	2.032	0.021075	0.021075	0.429911869
AGPAT5	ENSG00000155189	2.0319	0.02108	1.08	0.14008	-0.23696	0.59365	0.14623	0.44187	0.02108	0.429911869
PIGO	ENSG00000165282	0.11897	0.45265	2.0319	0.021083	1.8099	0.035156	0.4869	0.31316	0.021083	0.429911869
NA	ENSG00000264895	2.0317	0.021092	2.0317	0.021092	2.0317	0.021092	2.0317	0.021092	0.021092	0.429911869
CLTC	ENSG00000141367	1.96	0.024996	1.7348	0.041389	2.0315	0.021103	1.9415	0.026096	0.021103	0.429911869
YY1P1	ENSG00000230661	1.1064	0.13427	-0.003024	0.50121	-0.11511	0.54582	2.0315	0.021103	0.021103	0.429911869
NA	ENSG00000239775	2.031	0.02113	2.031	0.02113	2.031	0.02113	2.031	0.02113	0.02113	0.429946661
NA	ENSG00000271769	2.0309	0.021132	2.0309	0.021132	2.0309	0.021132	2.0309	0.021132	0.021132	0.429946661
SLC6A11	ENSG00000132164	2.0308	0.021137	-0.46063	0.67747	0.66256	0.25381	-0.33591	0.63153	0.021137	0.429946661
IL17RE	ENSG00000163701	1.4053	0.079964	2.0308	0.02114	0.54578	0.29261	1.8052	0.035522	0.02114	0.429946661
NA	ENSG00000248416	0.4476	0.32722	-0.087221	0.53475	-0.66031	0.74547	2.0302	0.02117	0.02117	0.430238349
PARP3	ENSG00000041880	2.0301	0.021172	0.44224	0.32916	1.0354	0.15023	0.33524	0.36872	0.021172	0.430238349
NA	ENSG00000231704	-0.91864	0.82086	1.5004	0.066757	-0.53221	0.70271	2.0295	0.021205	0.021205	0.43071556
NA	ENSG00000279311	0.36403	0.35792	2.0292	0.021218	-0.04896	0.51952	-0.04896	0.51952	0.021218	0.43071556
NA	ENSG00000272144	0.13105	0.44787	0.017222	0.49313	0.74549	0.22799	2.0291	0.021222	0.021222	0.43071556
H4C8	ENSG00000158406	2.0288	0.021238	1.4203	0.077758	1.345	0.089312	1.9937	0.023093	0.021238	0.430765903
NA	ENSG00000165121	1.7424	0.040721	1.2215	0.11094	2.0287	0.021245	0.82351	0.20511	0.021245	0.430765903
MSH5-SAPCD1	ENSG00000255152	2.0286	0.021251	1.8872	0.029564	1.8872	0.029564	1.8872	0.029564	0.021251	0.430765903
BRICD5	ENSG00000182685	2.0283	0.021267	2.0283	0.021267	2.0283	0.021267	1.231	0.10917	0.021267	0.430910981
DAW1	ENSG00000123977	2.0276	0.021299	0.94183	0.17314	0.98327	0.16274	1.1656	0.12189	0.021299	0.431241267
TNFRSF25	ENSG00000215788	2.0276	0.021301	2.0276	0.021301	2.0276	0.021301	2.0276	0.021301	0.021301	0.431241267
PKN2	ENSG00000065243	0.4212	0.3368	1.5136	0.065064	0.4212	0.3368	2.0274	0.02131	0.02131	0.431244311
SUPT16HP1	ENSG00000256238	1.8369	0.033112	2.0269	0.021337	0.91545	0.17998	0.95066	0.17089	0.021337	0.431326079
NA	ENSG00000228231	2.0267	0.021349	0.27642	0.39111	-0.37399	0.64579	-0.083417	0.53324	0.021349	0.431326079
PRSS53	ENSG00000151006	0.83057	0.20311	0.91472	0.18017	0.26897	0.39397	2.0265	0.021356	0.021356	0.431326079
CCDC183-AS1	ENSG00000228544	2.0263	0.021367	1.0496	0.14695	1.0496	0.14695	1.0496	0.14695	0.021367	0.431326079
NA	ENSG00000253774	2.0263	0.021369	2.0263	0.021369	2.0263	0.021369	2.0263	0.021369	0.021369	0.431326079
NUP153	ENSG00000124789	0.18227	0.42769	0.81925	0.20632	1.3917	0.082012	2.0262	0.021374	0.021374	0.431326079
PCDH17	ENSG00000118946	2.0261	0.021376	-1.0642	0.85638	-0.12286	0.54889	-0.036139	0.51441	0.021376	0.431326079
NA	ENSG00000251009	1.7958	0.036261	2.0258	0.02139	1.7958	0.036261	1.7958	0.036261	0.02139	0.431347015

BPIFB2	ENSG00000078898	2.0257	0.021395	1.4119	0.078991	0.33336	0.36943	1.7401	0.040917	0.021395	0.431347015
NA	ENSG00000265579	2.0254	0.021411	2.0254	0.021411	2.0254	0.021411	2.0254	0.021411	0.021411	0.431347015
FRY	ENSG00000073910	2.0254	0.021414	1.4522	0.073228	1.1677	0.12147	-0.10086	0.54017	0.021414	0.431347015
LHB	ENSG00000104826	1.1247	0.13037	1.8619	0.03131	2.0251	0.021429	-0.34499	0.63495	0.021429	0.431347015
CHMP7	ENSG00000147457	1.6474	0.049742	0.35654	0.36072	0.54522	0.2928	2.025	0.021434	0.021434	0.431347015
LINC02179	ENSG00000261623	2.0249	0.021439	1.1177	0.13185	1.7098	0.043654	1.7098	0.043654	0.021439	0.431347015
SLC9B1	ENSG00000164037	2.0245	0.021457	0.80975	0.20904	0.58827	0.27818	0.7843	0.21643	0.021457	0.431530999
RAD54L2	ENSG00000164080	-0.15138	0.56016	-0.554	0.71021	-0.0826	0.53292	2.0242	0.021475	0.021475	0.431714831
SSBP4	ENSG00000130511	1.8039	0.035621	1.9391	0.026248	2.024	0.021487	0.58001	0.28095	0.021487	0.431763461
NECTIN2	ENSG00000130202	2.0238	0.021497	-0.12644	0.55031	-0.4846	0.68602	-1.7807	0.96252	0.021497	0.431763461
CEP19P1	ENSG00000231032	0.41519	0.339	2.0236	0.021504	1.449	0.07367	-2.0665	0.98061	0.021504	0.431763461
BCDIN3D-AS1	ENSG00000258057	2.0233	0.02152	2.0233	0.02152	2.0233	0.02152	2.0233	0.02152	0.02152	0.431839668
ESRRA	ENSG00000173153	1.2649	0.10296	0.41911	0.33757	2.023	0.021538	-0.49392	0.68932	0.021538	0.431839668
RNU6-1213P	ENSG00000252884	2.0227	0.021553	2.0227	0.021553	2.0227	0.021553	0.42445	0.33562	0.021553	0.431839668
NA	ENSG00000279512	2.0222	0.021576	2.0222	0.021576	2.0222	0.021576	2.0222	0.021576	0.021576	0.431839668
LINC00994	ENSG00000189196	2.0221	0.021581	2.0221	0.021581	0.19765	0.42166	2.0221	0.021581	0.021581	0.431839668
RPL36AP51	ENSG00000242291	0.036284	0.48553	-0.13635	0.55423	-0.035797	0.51428	2.0221	0.021586	0.021586	0.431839668
CTPS1	ENSG00000171793	1.2324	0.1089	2.0219	0.021594	2.0219	0.021594	0.44479	0.32824	0.021594	0.431839668
IFNL3	ENSG00000197110	-0.12496	0.54972	2.0219	0.021594	0.7512	0.22627	0.7512	0.22627	0.021594	0.431839668
PKD2	ENSG00000118762	2.0217	0.021602	1.2135	0.11247	2.0217	0.021602	1.9655	0.02468	0.021602	0.431839668
CDC42EP4	ENSG00000179604	2.0217	0.021606	2.0217	0.021606	1.5716	0.058022	1.2376	0.10794	0.021606	0.431839668
NA	ENSG00000254352	2.0214	0.021617	2.0214	0.021617	2.0214	0.021617	2.0214	0.021617	0.021617	0.431839668
RPS3AP53	ENSG00000227578	-2.0422	0.97944	2.0213	0.021623	1.4161	0.078369	0.49586	0.31	0.021623	0.431839668
MIR4681	ENSG00000265719	2.0213	0.021623	0.064102	0.47444	0.064102	0.47444	1.8478	0.032317	0.021623	0.431839668
DMC1	ENSG00000100206	1.946	0.025828	1.5542	0.060065	2.021	0.021639	0.39747	0.34551	0.021639	0.431982167
PSMD2	ENSG00000175166	-0.99884	0.84106	-1.1315	0.87108	2.0204	0.021673	-0.99884	0.84106	0.021673	0.432483739
NA	ENSG00000250192	-0.38048	0.64821	-1.2702	0.89799	-0.38048	0.64821	2.0201	0.021685	0.021685	0.43254607
GOT1L1	ENSG00000169154	2.0198	0.021701	2.0198	0.021701	2.0198	0.021701	2.0198	0.021701	0.021701	0.432688106
NA	ENSG00000245768	1.2716	0.10175	1.2716	0.10175	2.0195	0.021717	1.1645	0.12211	0.021717	0.432830025
HBA2	ENSG00000188536	0.54915	0.29145	-0.877	0.80976	2.0192	0.021736	0.0051259	0.49796	0.021736	0.433031594
KCTD12	ENSG00000178695	0.20469	0.41891	2.0188	0.021754	0.81925	0.20632	1.7896	0.036761	0.021754	0.433213085

NA	ENSG00000273600	1.9276	0.026952	1.6316	0.051377	1.7449	0.040498	2.0184	0.021775	0.021775	0.433279314
NIFKP1	ENSG00000214434	2.0184	0.021777	2.0184	0.021777	0.83481	0.20191	1.1975	0.11555	0.021777	0.433279314
UBOX5-AS1	ENSG00000235958	1.3235	0.092832	0.91571	0.17991	2.0182	0.021784	2.0182	0.021784	0.021784	0.433279314
ZFYVE16	ENSG00000039319	2.0175	0.021819	0.7649	0.22217	1.4697	0.070816	1.8762	0.030316	0.021819	0.43359391
NA	ENSG00000239804	0.24971	0.40141	1.3484	0.088765	2.0172	0.021836	2.0172	0.021836	0.021836	0.43359391
ZSCAN10	ENSG00000130182	2.017	0.021847	1.4953	0.067424	2.017	0.021847	1.1699	0.12102	0.021847	0.43359391
NA	ENSG00000278953	0.88839	0.18717	0.88839	0.18717	2.0168	0.021859	0.88839	0.18717	0.021859	0.43359391
ESRRAP2	ENSG00000235438	2.0167	0.021865	1.1964	0.11577	-0.98237	0.83704	-1.6336	0.94883	0.021865	0.43359391
MIR4435-2HG	ENSG00000172965	-0.67595	0.75046	-0.68996	0.75489	1.765	0.038782	2.0167	0.021866	0.021866	0.43359391
C5orf66-AS2	ENSG00000249647	1.8014	0.035823	2.0166	0.021869	2.0166	0.021869	0.12881	0.44876	0.021869	0.43359391
TLCD4-RWDD3	ENSG00000271092	2.0166	0.021871	2.0166	0.021871	2.0166	0.021871	2.0166	0.021871	0.021871	0.43359391
FMO4	ENSG00000076258	-0.18881	0.57488	2.016	0.021898	-0.85104	0.80263	-0.57168	0.71623	0.021898	0.433776236
VTI1B	ENSG00000100568	0.13675	0.44562	2.016	0.021898	0.13675	0.44562	0.13675	0.44562	0.021898	0.433776236
NKAIN4	ENSG00000101198	0.33271	0.36968	1.1931	0.11641	2.0156	0.021922	1.5174	0.064584	0.021922	0.433884803
NA	ENSG00000276436	2.0154	0.02193	2.0154	0.02193	2.0154	0.02193	2.0154	0.02193	0.02193	0.433884803
TRAJ22	ENSG00000211867	2.0154	0.021931	2.0154	0.021931	2.0154	0.021931	2.0154	0.021931	0.021931	0.433884803
GYPB	ENSG00000250361	-1.1156	0.8677	2.0152	0.021943	0.054422	0.4783	-0.38393	0.64949	0.021943	0.433884803
NDUFB4P8	ENSG00000223663	2.0151	0.021948	2.0151	0.021948	2.0151	0.021948	2.0151	0.021948	0.021948	0.433884803
LINC02719	ENSG00000254580	0.37931	0.35223	1.805	0.035536	0.77501	0.21917	2.0143	0.02199	0.02199	0.43438242
NA	ENSG00000271904	2.0143	0.021991	0.95449	0.16992	0.21168	0.41618	2.0143	0.021991	0.021991	0.43438242
EEF1A1P19	ENSG00000249855	2.0141	0.022	2.0141	0.022	2.0141	0.022	2.0141	0.022	0.022	0.434384117
C1orf105	ENSG00000180999	0.41661	0.33848	-0.13861	0.55512	2.0136	0.022027	1.1847	0.11807	0.022027	0.434528879
SERINC3	ENSG00000132824	1.4748	0.070138	1.6575	0.048712	2.0136	0.022028	1.3998	0.080782	0.022028	0.434528879
FAM205A	ENSG00000205108	0.94942	0.1712	0.91264	0.18071	2.0133	0.022039	0.77413	0.21943	0.022039	0.434528879
DUXAP4	ENSG00000270258	1.2227	0.11072	1.2227	0.11072	2.0133	0.022043	0.22493	0.41102	0.022043	0.434528879
NR2F2	ENSG00000185551	2.013	0.022057	0.054586	0.47823	1.8965	0.02895	0.054586	0.47823	0.022057	0.434591237
NA	ENSG00000272368	1.6005	0.054748	1.7011	0.04446	1.9255	0.027085	2.0129	0.022064	0.022064	0.434591237
NDUFS5P3	ENSG00000233664	2.0124	0.022089	-0.24308	0.59603	0.32876	0.37117	1.852	0.032012	0.022089	0.434835147
STC2	ENSG00000113739	0.64402	0.25978	-0.54634	0.70758	-0.99373	0.83982	2.0121	0.022105	0.022105	0.434835147
NA	ENSG00000229765	0.44256	0.32904	2.012	0.022109	1.2146	0.11226	2.012	0.022109	0.022109	0.434835147
RDH11	ENSG00000072042	2.0118	0.022121	0.12455	0.45044	0.12455	0.45044	1.6584	0.048619	0.022121	0.434835147

BCL9	ENSG00000116128	1.0382	0.1496	2.0118	0.022121	0.46961	0.31932	2.0008	0.022705	0.022121	0.434835147
KRT16P6	ENSG00000226145	2.0114	0.022141	-0.71021	0.76121	0.27623	0.39118	-1.0909	0.86234	0.022141	0.435052794
IFNWP9	ENSG00000226597	-1.9739	0.9758	2.0111	0.022158	1.7029	0.044297	1.0121	0.15574	0.022158	0.435211342
IFITM8P	ENSG00000215096	0.94788	0.1716	2.0105	0.02219	1.2409	0.10732	1.0921	0.13739	0.02219	0.435437497
NA	ENSG00000213828	2.0102	0.022203	1.8494	0.0322	0.068801	0.47257	0.068801	0.47257	0.022203	0.435437497
DNAJC6	ENSG00000116675	1.4442	0.074338	1.7606	0.039156	1.0517	0.14648	2.0101	0.022213	0.022213	0.435437497
NA	ENSG00000206705	0.39555	0.34622	1.5215	0.064062	2.0097	0.022229	1.2068	0.11376	0.022229	0.435437497
DIPK1B	ENSG00000165716	0.44173	0.32934	-0.30087	0.61824	2.0097	0.02223	0.42772	0.33443	0.02223	0.435437497
SNAPC4	ENSG00000165684	2.0097	0.022232	-0.83906	0.79928	0.92622	0.17717	0.72404	0.23452	0.022232	0.435437497
RNU1-141P	ENSG00000201493	1.0122	0.15573	2.0095	0.02224	2.0095	0.02224	2.0095	0.02224	0.02224	0.435437497
ILDR2	ENSG00000143195	1.5682	0.058421	2.0095	0.022241	0.96618	0.16698	1.1736	0.12028	0.022241	0.435437497
PHF7	ENSG00000010318	2.0091	0.022262	-0.07801	0.53109	1.652	0.049263	0.46335	0.32156	0.022262	0.435673598
RPL35AP28	ENSG00000242041	0.01716	0.49315	0.01716	0.49315	-0.70903	0.76085	2.0088	0.022281	0.022281	0.435794364
RNU6-256P	ENSG00000252556	-1.3382	0.90958	-1.3382	0.90958	2.0086	0.022292	-0.40691	0.65796	0.022292	0.435794364
NA	ENSG00000267295	0.63744	0.26192	0.14836	0.44103	0.65632	0.25581	2.0085	0.022295	0.022295	0.435794364
NA	ENSG00000251412	2.0082	0.022309	0.49919	0.30882	0.35007	0.36314	1.0726	0.14173	0.022309	0.435837841
MYOG	ENSG00000122180	2.008	0.022322	0.20096	0.42036	0.66597	0.25271	2.008	0.022322	0.022322	0.435837841
ELAVL4-AS1	ENSG00000237337	1.8382	0.033014	2.0079	0.022329	1.2561	0.10454	1.2561	0.10454	0.022329	0.435837841
RALA	ENSG00000006451	0.86712	0.19294	2.0078	0.022333	1.7938	0.036426	1.7458	0.040425	0.022333	0.435837841
NA	ENSG00000261643	2.0073	0.022357	-0.30762	0.62081	0.3922	0.34745	0.3922	0.34745	0.022357	0.436016548
AMD1P1	ENSG00000228339	2.0072	0.022362	2.0072	0.022362	2.0072	0.022362	2.0072	0.022362	0.022362	0.436016548
TRAJ17	ENSG00000211872	2.0071	0.022369	2.0071	0.022369	2.0071	0.022369	2.0071	0.022369	0.022369	0.436016548
NA	ENSG00000271860	0.31003	0.37827	2.0069	0.022379	1.5993	0.054882	-0.13048	0.55191	0.022379	0.436037053
COG2	ENSG00000135775	2.0062	0.022418	1.3859	0.082892	0.91693	0.17959	0.97065	0.16586	0.022418	0.436340903
PEX2	ENSG00000164751	0.18108	0.42815	2.0061	0.022423	2.0061	0.022423	0.69475	0.24361	0.022423	0.436340903
NA	ENSG00000221746	1.609	0.053812	1.1068	0.1342	1.4941	0.067572	2.006	0.02243	0.02243	0.436340903
PIGK	ENSG00000142892	-0.74939	0.77319	1.3831	0.083324	0.59569	0.27569	2.0056	0.022447	0.022447	0.436340903
FAM135B	ENSG00000147724	-1.2298	0.89062	-0.82571	0.79551	2.0055	0.022454	1.153	0.12446	0.022454	0.436340903
NA	ENSG00000223829	0.3745	0.35402	2.0054	0.022459	1.3058	0.095814	0.49926	0.3088	0.022459	0.436340903
NA	ENSG00000248576	2.0051	0.022477	1.3187	0.093629	2.0051	0.022477	1.8842	0.029771	0.022477	0.436340903
SOX11	ENSG00000176887	-0.31508	0.62365	0.20649	0.4182	2.005	0.022479	0.20649	0.4182	0.022479	0.436340903

NA	ENSG00000279189	0.81527	0.20746	2.0049	0.022487	-0.64678	0.74111	-0.1965	0.57789	0.022487	0.436340903
RTEL1-TNFRSF6B	ENSG00000026036	0.99862	0.15899	2.0046	0.022501	0.38493	0.35014	1.1306	0.12912	0.022501	0.436340903
H3C13	ENSG00000183598	1.5472	0.060905	1.5472	0.060905	1.7345	0.041411	2.0045	0.022509	0.022509	0.436340903
ZNF408	ENSG00000175213	1.1924	0.11655	1.1924	0.11655	2.0045	0.02251	1.1924	0.11655	0.02251	0.436340903
NA	ENSG00000256514	2.0045	0.022511	2.0045	0.022511	2.0045	0.022511	2.0045	0.022511	0.022511	0.436340903
MOCS1	ENSG00000124615	2.004	0.022533	1.7497	0.040089	0.78973	0.21484	1.4817	0.069214	0.022533	0.436536355
RNU6-1223P	ENSG00000252933	2.0039	0.022539	2.0039	0.022539	2.0039	0.022539	2.0039	0.022539	0.022539	0.436536355
SLC39A8	ENSG00000138821	0.64304	0.2601	1.026	0.15244	2.0034	0.022567	0.44557	0.32795	0.022567	0.436732227
RNU6-518P	ENSG00000251865	0.14573	0.44207	0.14573	0.44207	0.14573	0.44207	2.0033	0.022572	0.022572	0.436732227
NA	ENSG00000225610	2.0032	0.022576	2.0032	0.022576	2.0032	0.022576	2.0032	0.022576	0.022576	0.436732227
MTURN	ENSG00000180354	0.54224	0.29383	1.5462	0.061025	2.0027	0.022605	1.8411	0.0328	0.022605	0.437119702
ZBTB7B	ENSG00000160685	1.8454	0.03249	2.0022	0.02263	-0.86001	0.80511	0.39957	0.34474	0.02263	0.437256106
SNORD18B	ENSG00000202529	2.0022	0.02263	2.0022	0.02263	2.0022	0.02263	2.0022	0.02263	0.02263	0.437256106
NOC2LP1	ENSG00000213225	2.0019	0.022648	-1.293	0.902	0.96303	0.16777	0.045328	0.48192	0.022648	0.437430456
LINC00235	ENSG00000277142	2.0016	0.022665	2.0016	0.022665	2.0016	0.022665	2.0016	0.022665	0.022665	0.437431358
NA	ENSG00000239116	1.6302	0.051533	1.855	0.031801	2.0016	0.022666	1.8778	0.030202	0.022666	0.437431358
NA	ENSG00000266947	2.0012	0.022686	2.0012	0.022686	2.0012	0.022686	2.0012	0.022686	0.022686	0.437644014
NEPNP	ENSG00000218233	-2.0144	0.97801	2.0007	0.022713	-0.97366	0.83489	0.12866	0.44881	0.022713	0.437917452
AMN	ENSG00000166126	0.013737	0.49452	1.096	0.13654	0.28612	0.38739	2.0002	0.022739	0.022739	0.437917452
RNU6-681P	ENSG00000200882	1.9998	0.02276	1.9998	0.02276	1.9998	0.02276	1.9998	0.02276	0.02276	0.437917452
NA	ENSG00000257281	1.9998	0.022762	1.9998	0.022762	1.9998	0.022762	1.9998	0.022762	0.022762	0.437917452
BOLA1	ENSG00000178096	1.9997	0.022766	1.4689	0.070935	1.9774	0.023995	1.4689	0.070935	0.022766	0.437917452
ENTR1P2	ENSG00000181101	1.9996	0.022773	1.6351	0.051016	1.6351	0.051016	1.3186	0.093652	0.022773	0.437917452
LINC01013	ENSG00000228495	0.76908	0.22092	1.0856	0.13882	1.9993	0.022786	0.84711	0.19847	0.022786	0.437917452
CCNK	ENSG00000090061	1.9991	0.022801	-0.473	0.68189	0.41087	0.34058	1.1555	0.12395	0.022801	0.437917452
ARHGAP28	ENSG00000088756	1.1191	0.13154	1.1464	0.12581	1.1322	0.12877	1.999	0.022803	0.022803	0.437917452
RNU6-201P	ENSG00000252909	1.9989	0.022808	1.9989	0.022808	1.9989	0.022808	1.9989	0.022808	0.022808	0.437917452
AXIN1	ENSG00000103126	0.035936	0.48567	0.16897	0.43291	0.33005	0.37068	1.9988	0.022816	0.022816	0.437917452
NA	ENSG00000214773	1.6743	0.047038	1.6422	0.05027	1.9988	0.022817	1.9988	0.022817	0.022817	0.437917452
NA	ENSG00000264596	0.73635	0.23076	1.9988	0.022817	0.73635	0.23076	0.83777	0.20108	0.022817	0.437917452
LINC01134	ENSG00000236423	1.9985	0.022834	-0.16443	0.5653	-0.3916	0.65232	0.45301	0.32527	0.022834	0.437956562

NA	ENSG00000236580	1.9983	0.022844	1.9983	0.022844	1.9983	0.022844	1.9983	0.022844	0.022844	0.437956562
RFPL4AP5	ENSG00000248720	1.9982	0.022846	1.855	0.031802	1.855	0.031802	1.855	0.031802	0.022846	0.437956562
NA	ENSG00000279086	0.97771	0.16411	1.9978	0.02287	0.97771	0.16411	1.3425	0.089715	0.02287	0.438244239
MYH16	ENSG00000002079	0.52632	0.29933	1.3748	0.08459	1.9972	0.0229	0.1623	0.43553	0.0229	0.438646619
NOSIP	ENSG00000142546	1.997	0.022911	1.997	0.022911	1.997	0.022911	1.997	0.022911	0.022911	0.438684884
NA	ENSG00000267755	1.9967	0.022927	1.9967	0.022927	1.9967	0.022927	1.9967	0.022927	0.022927	0.438703874
NA	ENSG00000258549	1.9967	0.022931	1.9967	0.022931	1.9967	0.022931	1.9967	0.022931	0.022931	0.438703874
PTDSS2	ENSG00000174915	1.9965	0.022939	0.56118	0.28734	-0.18807	0.57459	0.80266	0.21109	0.022939	0.438703874
NA	ENSG00000235575	0.20319	0.41949	0.21126	0.41634	-0.39525	0.65367	1.9963	0.022949	0.022949	0.438722938
LINC02475	ENSG00000251350	1.9957	0.022983	-0.87474	0.80914	-1.2076	0.88641	-0.76048	0.77651	0.022983	0.439085157
ANKRD17	ENSG00000132466	1.6085	0.053866	1.7268	0.0421	1.9955	0.022995	0.74251	0.22889	0.022995	0.439085157
EPHB6	ENSG00000106123	1.6771	0.046757	1.9954	0.022999	1.6771	0.046757	0.020855	0.49168	0.022999	0.439085157
LARP7P3	ENSG00000265698	1.0255	0.15256	1.4429	0.074519	1.0946	0.13684	1.9952	0.023011	0.023011	0.439085157
NA	ENSG00000270091	1.9952	0.023013	1.468	0.071056	1.9952	0.023013	0.1387	0.44484	0.023013	0.439085157
NA	ENSG00000279711	0.1258	0.44994	1.6282	0.051739	1.9947	0.023035	1.1238	0.13055	0.023035	0.439332896
RN7SKP135	ENSG00000252358	0.73949	0.22981	1.0324	0.15094	1.9941	0.023071	1.0229	0.15318	0.023071	0.439713002
MIR4701	ENSG00000264201	1.994	0.023077	1.994	0.023077	1.994	0.023077	1.994	0.023077	0.023077	0.439713002
SHOC2	ENSG00000108061	1.9939	0.023082	-0.77161	0.77983	-0.77161	0.77983	-1.3148	0.9057	0.023082	0.439713002
NA	ENSG00000270790	1.9932	0.023122	1.9932	0.023122	1.9932	0.023122	1.9932	0.023122	0.023122	0.440302876
RNF103	ENSG00000239305	0.60804	0.27158	1.9929	0.023134	-0.20945	0.58295	0.48151	0.31508	0.023134	0.440323149
LINC01235	ENSG00000270547	-0.22956	0.59078	1.9923	0.023167	1.7772	0.037766	-0.42918	0.6661	0.023167	0.440323149
NA	ENSG00000248242	1.1107	0.13335	1.9922	0.023173	1.241	0.1073	1.241	0.1073	0.023173	0.440323149
NA	ENSG00000223651	1.1428	0.12657	1.1428	0.12657	1.9917	0.023201	1.1428	0.12657	0.023201	0.440323149
NA	ENSG00000264380	0.0013209	0.49947	0.071698	0.47142	1.9915	0.023212	0.2213	0.41243	0.023212	0.440323149
GPC6-AS1	ENSG00000236520	1.9915	0.023215	0.64996	0.25786	1.9915	0.023215	0.32004	0.37447	0.023215	0.440323149
TATDN2P3	ENSG00000235205	1.9912	0.023228	1.7805	0.0375	1.7805	0.0375	1.7805	0.0375	0.023228	0.440323149
RNU6-1106P	ENSG00000212147	1.6033	0.054432	1.6033	0.054432	1.9912	0.023229	1.6205	0.052559	0.023229	0.440323149
CD160	ENSG00000117281	1.9912	0.02323	1.9912	0.02323	1.9912	0.02323	-0.45187	0.67432	0.02323	0.440323149
NA	ENSG00000231743	1.9911	0.023233	0.099528	0.46036	-0.20422	0.58091	-0.20422	0.58091	0.023233	0.440323149
NA	ENSG00000232271	1.0233	0.15309	1.6275	0.051819	1.991	0.023242	1.551	0.060448	0.023242	0.440323149
NA	ENSG00000272330	1.9909	0.023248	1.9909	0.023248	1.9909	0.023248	1.9909	0.023248	0.023248	0.440323149

C2orf42	ENSG00000115998	-0.82669	0.79579	-0.21804	0.5863	1.9906	0.023263	0.76142	0.2232	0.023263	0.440323149
NA	ENSG00000231970	1.9906	0.023264	1.9906	0.023264	1.211	0.11295	1.263	0.10329	0.023264	0.440323149
SELP	ENSG00000174175	0.65509	0.2562	1.9904	0.023271	1.9904	0.023271	-0.13072	0.552	0.023271	0.440323149
PRELID1P6	ENSG00000228305	-0.48011	0.68443	-0.9665	0.8331	1.9904	0.023273	-0.9665	0.8331	0.023273	0.440323149
WDR89	ENSG00000140006	0.06789	0.47294	0.083211	0.46684	0.06789	0.47294	1.9902	0.023286	0.023286	0.440323149
LHX3	ENSG00000107187	1.3527	0.088077	1.4069	0.079734	1.9896	0.02332	1.9052	0.028378	0.02332	0.440323149
LINC01799	ENSG00000230525	0.405	0.34274	1.9896	0.02332	0.018468	0.49263	1.5225	0.063937	0.02332	0.440323149
CSRNP2	ENSG00000110925	-0.24303	0.59601	1.9894	0.023328	-0.24303	0.59601	-0.12046	0.54794	0.023328	0.440323149
EPHA6	ENSG00000080224	-1.0599	0.8554	-1.5741	0.94227	-0.73839	0.76986	1.9893	0.023334	0.023334	0.440323149
MT1HL1	ENSG00000244020	1.9893	0.023335	1.9893	0.023335	1.9893	0.023335	0.85773	0.19552	0.023335	0.440323149
CABLES1	ENSG00000134508	1.9891	0.023344	1.3685	0.085582	0.35645	0.36075	1.6195	0.052665	0.023344	0.440323149
PCDHGA5	ENSG00000253485	0.8148	0.20759	0.8148	0.20759	0.8148	0.20759	1.989	0.023351	0.023351	0.440323149
NA	ENSG00000284430	-1.4688	0.92905	1.989	0.023352	-1.0173	0.8455	-1.0173	0.8455	0.023352	0.440323149
POFUT1	ENSG00000101346	1.1503	0.12501	0.18876	0.42514	0.94448	0.17246	1.9889	0.023358	0.023358	0.440323149
NA	ENSG00000233656	1.6501	0.049459	1.9884	0.023383	1.8684	0.030851	1.9658	0.02466	0.023383	0.440381732
NA	ENSG00000244036	1.7804	0.037505	1.9883	0.023391	1.4934	0.067661	0.47877	0.31605	0.023391	0.440381732
TREML2	ENSG00000112195	0.82452	0.20482	0.82452	0.20482	0.97385	0.16507	1.9879	0.023409	0.023409	0.440381732
ARHGAP8	ENSG00000241484	1.9878	0.023417	-0.71793	0.7636	-0.82056	0.79405	-0.94353	0.82729	0.023417	0.440381732
FAM193A	ENSG00000125386	0.73913	0.22991	1.9877	0.023422	-0.020301	0.5081	0.43429	0.33204	0.023422	0.440381732
NA	ENSG00000230637	1.8757	0.030345	1.9876	0.023427	1.421	0.077655	1.4836	0.068963	0.023427	0.440381732
NKX2-6	ENSG00000180053	0.68314	0.24726	1.9872	0.023449	-0.049581	0.51977	0.39329	0.34705	0.023449	0.440381732
RN7SL865P	ENSG00000240205	1.9872	0.023452	1.7093	0.043697	1.4644	0.071539	1.9872	0.023452	0.023452	0.440381732
NA	ENSG00000261047	1.987	0.023462	1.987	0.023462	1.987	0.023462	1.987	0.023462	0.023462	0.440381732
RNA5SP141	ENSG00000201413	-0.14906	0.55925	0.039403	0.48428	1.9868	0.023475	1.7591	0.039282	0.023475	0.440381732
GPM6A	ENSG00000150625	0.88253	0.18874	-0.76723	0.77853	1.2663	0.1027	1.9867	0.023476	0.023476	0.440381732
MRGPRX8P	ENSG00000276789	1.9864	0.023495	1.9864	0.023495	1.6788	0.046598	1.9864	0.023495	0.023495	0.440381732
NA	ENSG00000261146	1.9863	0.023498	0.065942	0.47371	1.0939	0.137	-0.041945	0.51673	0.023498	0.440381732
TBC1D1	ENSG00000065882	1.2824	0.099858	0.92978	0.17624	0.78424	0.21645	1.9861	0.023513	0.023513	0.440381732
NA	ENSG00000232862	1.9859	0.02352	1.762	0.039039	1.9859	0.02352	1.9859	0.02352	0.02352	0.440381732
NA	ENSG00000236414	1.8737	0.030487	0.25238	0.40037	1.8737	0.030487	1.9859	0.023525	0.023525	0.440381732
NA	ENSG00000267193	1.9143	0.027792	1.9222	0.02729	0.85582	0.19605	1.9858	0.023526	0.023526	0.440381732

PARD3	ENSG00000148498	1.9855	0.023545	0.18695	0.42585	0.77599	0.21888	0.6151	0.26924	0.023545	0.440381732
ZFAND2B	ENSG00000158552	1.9854	0.023548	1.4872	0.068482	1.9854	0.023548	1.9854	0.023548	0.023548	0.440381732
DACH1	ENSG00000276644	0.96796	0.16653	1.4787	0.06961	1.9854	0.02355	-0.53447	0.70349	0.02355	0.440381732
RNU6-399P	ENSG00000202034	1.9852	0.023561	1.9852	0.023561	1.9852	0.023561	1.9852	0.023561	0.023561	0.440381732
NOB1	ENSG00000141101	1.0882	0.13825	1.9852	0.023563	0.86583	0.19329	1.5652	0.058766	0.023563	0.440381732
XKR5	ENSG00000275591	-0.74859	0.77295	1.9849	0.023576	1.8238	0.034092	0.49098	0.31172	0.023576	0.440381732
NA	ENSG00000237002	0.88457	0.18819	1.3163	0.094036	1.9849	0.023578	1.3163	0.094036	0.023578	0.440381732
LINC02623	ENSG00000234944	1.8546	0.031824	0.11741	0.45327	0.15205	0.43958	1.9847	0.023592	0.023592	0.440474391
NA	ENSG00000238840	1.9844	0.023606	-0.64685	0.74114	0.7213	0.23536	0.7213	0.23536	0.023606	0.440507278
VDAC3P1	ENSG00000271198	1.4233	0.077325	1.9842	0.023618	-0.28975	0.614	-0.23144	0.59151	0.023618	0.440507278
NA	ENSG00000125385	1.9841	0.023623	-0.33932	0.63282	-0.33932	0.63282	-0.33932	0.63282	0.023623	0.440507278
MPPED2	ENSG00000066382	0.57001	0.28434	0.082017	0.46732	1.9839	0.023634	0.73364	0.23158	0.023634	0.440507278
NA	ENSG00000254551	1.9836	0.023648	1.9836	0.023648	1.9836	0.023648	1.9836	0.023648	0.023648	0.440507278
LINC01176	ENSG00000281404	1.1463	0.12583	1.1463	0.12583	1.9836	0.023648	-1.2411	0.89272	0.023648	0.440507278
NA	ENSG00000200652	1.9833	0.023664	1.9833	0.023664	1.9833	0.023664	1.9833	0.023664	0.023664	0.440616697
NA	ENSG00000254639	-0.39069	0.65199	0.88086	0.1892	-0.16418	0.56521	1.9831	0.023676	0.023676	0.440616697
SLC39A11	ENSG00000133195	-0.51675	0.69733	0.43111	0.33319	1.983	0.023681	0.60105	0.2739	0.023681	0.440616697
RNF2	ENSG00000121481	0.84519	0.199	-0.42706	0.66533	-0.36725	0.64328	1.9827	0.023699	0.023699	0.44065232
ADAMTSL5	ENSG00000185761	1.9827	0.023701	1.9827	0.023701	1.4178	0.078132	-0.058339	0.52326	0.023701	0.44065232
LINC01392	ENSG00000233607	-1.1106	0.86663	-1.1106	0.86663	1.9821	0.023733	-1.1106	0.86663	0.023733	0.440714416
RPS6KA4	ENSG00000162302	1.9821	0.023735	1.1488	0.12533	0.93861	0.17396	-0.30074	0.61819	0.023735	0.440714416
NA	ENSG00000223583	1.982	0.023742	1.982	0.023742	1.4492	0.073642	1.4492	0.073642	0.023742	0.440714416
GNG12	ENSG00000172380	0.50669	0.30619	-0.47956	0.68423	1.8539	0.03188	1.9816	0.023761	0.023761	0.440714416
NA	ENSG00000222031	1.9814	0.023774	0.42136	0.33674	1.9814	0.023774	1.9814	0.023774	0.023774	0.440714416
AMBRA1	ENSG00000110497	1.9813	0.02378	1.4698	0.070812	1.484	0.068907	1.4703	0.070742	0.02378	0.440714416
CRYZ	ENSG00000116791	1.9812	0.023784	1.9812	0.023784	1.9812	0.023784	1.9812	0.023784	0.023784	0.440714416
VWA5A	ENSG00000110002	1.2803	0.10022	1.981	0.023796	1.981	0.023796	1.981	0.023796	0.023796	0.440714416
SLC25A22	ENSG00000177542	0.11602	0.45382	0.11602	0.45382	1.9809	0.023804	1.1179	0.13181	0.023804	0.440714416
PTPRM	ENSG00000173482	0.85224	0.19704	0.68686	0.24609	1.9808	0.023805	-0.27526	0.60844	0.023805	0.440714416
NA	ENSG00000227193	-1.2145	0.88772	0.44509	0.32813	1.9805	0.023825	0.054936	0.47809	0.023825	0.440714416
NA	ENSG00000254844	-0.82789	0.79613	1.9805	0.023826	-0.2721	0.60723	-0.30027	0.61801	0.023826	0.440714416

NA	ENSG00000279891	1.9804	0.023831	1.9804	0.023831	1.9804	0.023831	1.9804	0.023831	0.023831	0.440714416
TLCD2	ENSG00000185561	1.9804	0.023832	1.6481	0.049671	1.9804	0.023832	1.9804	0.023832	0.023832	0.440714416
GPANK1	ENSG00000204438	1.9802	0.02384	0.0042187	0.49832	0.57628	0.28221	0.0042187	0.49832	0.02384	0.440714416
BPIFA2	ENSG00000131050	1.9791	0.023901	1.9438	0.02596	1.5608	0.059289	0.73132	0.23229	0.023901	0.441487522
DOK4	ENSG00000125170	0.12904	0.44866	1.9791	0.023902	0.12904	0.44866	0.92975	0.17625	0.023902	0.441487522
NA	ENSG00000276573	1.979	0.023909	1.979	0.023909	1.979	0.023909	1.979	0.023909	0.023909	0.441487522
TMEM163	ENSG00000152128	1.5996	0.054843	1.9787	0.023923	0.42428	0.33568	1.6504	0.049426	0.023923	0.441578708
BANK1	ENSG00000153064	1.484	0.068902	0.19814	0.42147	-0.90735	0.81789	1.9778	0.023975	0.023975	0.442329803
NA	ENSG00000277319	0.52906	0.29838	1.9776	0.023989	1.2251	0.11027	1.2251	0.11027	0.023989	0.442329803
SLC14A1	ENSG00000141469	1.4273	0.076751	1.556	0.059848	0.5683	0.28491	1.9774	0.023996	0.023996	0.442329803
ELAVL4	ENSG00000162374	1.9774	0.024	1.9197	0.02745	1.9197	0.02745	1.908	0.028195	0.024	0.442329803
NA	ENSG00000259269	1.977	0.024018	-0.021124	0.50843	-0.3204	0.62567	-0.075212	0.52998	0.024018	0.442382211
TXK	ENSG00000074966	1.0836	0.13926	1.977	0.024021	0.98484	0.16235	1.3031	0.096276	0.024021	0.442382211
LINC01089	ENSG00000212694	1.9764	0.024052	1.9764	0.024052	1.9764	0.024052	1.9764	0.024052	0.024052	0.44278578
MMP24	ENSG00000125966	1.5902	0.055895	0.30073	0.38181	1.9763	0.024062	0.10635	0.45765	0.024062	0.442802591
NA	ENSG00000282887	1.976	0.024075	0.50765	0.30585	1.976	0.024075	0.73907	0.22993	0.024075	0.442874575
NA	ENSG00000201442	1.9755	0.024108	1.9755	0.024108	0.90504	0.18272	1.9755	0.024108	0.024108	0.443059977
RCN2	ENSG00000117906	-0.32606	0.62781	0.31203	0.37751	1.9753	0.024114	1.0414	0.14884	0.024114	0.443059977
NA	ENSG00000238158	-1.0312	0.84879	1.9752	0.02412	1.9752	0.02412	-0.50079	0.69174	0.02412	0.443059977
MIR548I1	ENSG00000221737	1.9751	0.02413	1.9751	0.02413	1.0881	0.13827	1.2078	0.11355	0.02413	0.443059977
NA	ENSG00000260058	1.6428	0.050216	1.975	0.024134	1.6428	0.050216	-0.68432	0.75311	0.024134	0.443059977
MZT1P1	ENSG00000271264	1.9747	0.024152	1.9747	0.024152	1.7893	0.036781	1.9747	0.024152	0.024152	0.443059977
AHCYP3	ENSG00000233955	0.92344	0.17789	1.619	0.05272	1.125	0.13029	1.9747	0.024153	0.024153	0.443059977
EXOC7	ENSG00000182473	1.8342	0.033314	1.9744	0.024166	1.4372	0.075326	0.93434	0.17506	0.024166	0.443059977
HSD3BP4	ENSG00000203855	0.041062	0.48362	-1.3799	0.91619	1.9743	0.024174	0.83386	0.20218	0.024174	0.443059977
POM121L3P	ENSG00000167390	-2.4881	0.99358	0.34103	0.36654	1.9743	0.024176	0.87055	0.192	0.024176	0.443059977
NKX2-1-AS1	ENSG00000253563	1.974	0.02419	1.974	0.02419	1.974	0.02419	1.974	0.02419	0.02419	0.443129853
NA	ENSG00000205147	1.9739	0.024198	1.9739	0.024198	1.9739	0.024198	1.9739	0.024198	0.024198	0.443129853
GREB1	ENSG00000196208	1.1177	0.13185	1.2761	0.10096	1.525	0.063633	1.9735	0.024221	0.024221	0.443157858
NA	ENSG00000222883	1.9734	0.024223	1.8528	0.031953	1.9734	0.024223	1.9734	0.024223	0.024223	0.443157858
UQCC2	ENSG00000137288	0.56215	0.28701	1.9733	0.024228	0.36653	0.35699	0.15498	0.43842	0.024228	0.443157858

S100A5	ENSG00000196420	1.9732	0.024237	1.9732	0.024237	1.9526	0.025432	0.37909	0.35231	0.024237	0.443157858
RPS28P1	ENSG00000270912	0.72767	0.23341	0.59372	0.27635	1.2786	0.10052	1.973	0.024245	0.024245	0.443157858
ZNF519	ENSG00000175322	1.6461	0.04987	1.3043	0.096066	1.9722	0.024291	0.86815	0.19266	0.024291	0.443682177
FTH1P9	ENSG00000251395	1.658	0.048658	1.9686	0.024497	1.9722	0.024292	1.7011	0.044463	0.024292	0.443682177
CXXC4-AS1	ENSG00000245384	0.19059	0.42442	0.85062	0.19749	0.47259	0.31825	1.9721	0.024301	0.024301	0.443682177
MIR548H1	ENSG00000221537	1.9711	0.024356	1.9711	0.024356	1.9711	0.024356	1.9711	0.024356	0.024356	0.444519805
NA	ENSG00000228007	1.9562	0.025219	1.8	0.035932	1.9703	0.024401	1.9703	0.024401	0.024401	0.444834245
NA	ENSG00000276633	1.5813	0.056909	1.8173	0.034587	1.4488	0.073697	1.9703	0.024405	0.024405	0.444834245
TRAJ9	ENSG00000211880	1.9701	0.024413	1.9701	0.024413	1.9701	0.024413	1.9701	0.024413	0.024413	0.444834245
TRAJ8	ENSG00000211881	1.9701	0.024413	1.9701	0.024413	1.9701	0.024413	1.9701	0.024413	0.024413	0.444834245
NA	ENSG00000229742	1.9699	0.024426	1.9699	0.024426	1.9699	0.024426	1.9699	0.024426	0.024426	0.444834245
AIDAP3	ENSG00000264189	-0.36236	0.64146	1.9699	0.024428	-0.7678	0.7787	-0.7678	0.7787	0.024428	0.444834245
DEFB124	ENSG00000180383	1.3583	0.087189	1.1591	0.1232	1.9696	0.024442	0.83786	0.20105	0.024442	0.444922921
NA	ENSG00000224550	-0.2247	0.58889	-0.2247	0.58889	-0.2247	0.58889	1.9693	0.024461	0.024461	0.445102513
CEP78	ENSG00000148019	-0.10824	0.5431	-0.71452	0.76255	1.969	0.024478	0.048822	0.48053	0.024478	0.445245592
LINC01254	ENSG00000260913	0.61175	0.27035	1.9688	0.02449	1.0999	0.13568	1.2745	0.10124	0.02449	0.445262687
NA	ENSG00000255045	0.56296	0.28673	1.9683	0.024517	0.56296	0.28673	0.56296	0.28673	0.024517	0.445262687
CDS2	ENSG00000101290	-0.59474	0.72399	1.3777	0.084146	0.21941	0.41317	1.968	0.024532	0.024532	0.445262687
NCAPGP2	ENSG00000259749	-0.036951	0.51474	1.9679	0.024538	1.9679	0.024538	1.9679	0.024538	0.024538	0.445262687
SIM1	ENSG00000112246	0.9989	0.15892	1.2986	0.097045	1.7677	0.038558	1.9678	0.024548	0.024548	0.445262687
USP2	ENSG00000036672	1.9677	0.024551	1.9677	0.024551	0.030423	0.48786	0.59749	0.27509	0.024551	0.445262687
NA	ENSG00000276406	1.9677	0.024551	1.9677	0.024551	1.9677	0.024551	1.9677	0.024551	0.024551	0.445262687
MIR3160-1	ENSG00000265014	1.537	0.062151	1.9673	0.024572	1.537	0.062151	1.537	0.062151	0.024572	0.445262687
RPS6KC1	ENSG00000136643	0.60969	0.27103	1.1422	0.12668	1.2284	0.10965	1.9673	0.024574	0.024574	0.445262687
SOX2	ENSG00000181449	-0.083976	0.53346	1.621	0.052513	1.9669	0.024599	0.76272	0.22282	0.024599	0.445262687
NA	ENSG00000230736	1.9669	0.0246	1.9669	0.0246	1.9669	0.0246	1.9669	0.0246	0.0246	0.445262687
CTAGE12P	ENSG00000215441	0.47972	0.31571	1.9668	0.024604	1.9668	0.024604	1.9668	0.024604	0.024604	0.445262687
HSD17B13	ENSG00000170509	0.48789	0.31282	0.48789	0.31282	1.9666	0.024614	0.029557	0.48821	0.024614	0.445262687
GMPPA	ENSG00000144591	1.6648	0.047979	1.9666	0.024616	1.9666	0.024616	1.9666	0.024616	0.024616	0.445262687
NA	ENSG00000268603	1.9666	0.024616	1.9666	0.024616	1.9666	0.024616	1.9666	0.024616	0.024616	0.445262687
NA	ENSG00000267852	1.9662	0.024639	1.9662	0.024639	1.9662	0.024639	1.9662	0.024639	0.024639	0.445508198

LINC02747	ENSG00000255774	0.13135	0.44775	1.9659	0.024654	-0.44229	0.67086	0.6479	0.25852	0.024654	0.445508198
TMEM145	ENSG00000167619	1.9659	0.024657	0.49715	0.30954	0.94024	0.17355	0.96491	0.1673	0.024657	0.445508198
ARL17A	ENSG00000185829	0.56813	0.28497	1.5989	0.05492	1.9655	0.024676	0.13453	0.44649	0.024676	0.445567498
ERG	ENSG00000157554	1.9653	0.024688	0.5601	0.2877	1.8954	0.029021	0.94309	0.17282	0.024688	0.445567498
RNA5SP467	ENSG00000212576	1.9651	0.0247	1.9651	0.0247	1.9651	0.0247	1.9651	0.0247	0.0247	0.445567498
QRSL1P1	ENSG00000215833	0.22157	0.41232	1.9651	0.024704	1.1317	0.12888	-0.16862	0.56695	0.024704	0.445567498
LINC02641	ENSG00000230131	0.38722	0.3493	-0.21755	0.58611	1.965	0.024706	1.2974	0.097248	0.024706	0.445567498
ZNF620	ENSG00000177842	-0.83711	0.79873	1.9647	0.024723	1.7546	0.039662	-0.26333	0.60385	0.024723	0.445616387
LINC00466	ENSG00000224209	0.28555	0.38761	0.50347	0.30732	0.3501	0.36313	1.9647	0.024727	0.024727	0.445616387
RNU6-308P	ENSG00000212259	1.9641	0.024761	1.9641	0.024761	1.9641	0.024761	1.9641	0.024761	0.024761	0.446014362
CLIC1	ENSG00000213719	1.637	0.05082	1.9638	0.024775	1.9638	0.024775	1.9638	0.024775	0.024775	0.446014362
NA	ENSG00000219992	1.9637	0.024785	1.9637	0.024785	1.9637	0.024785	1.9637	0.024785	0.024785	0.446014362
NA	ENSG00000276702	1.9635	0.024794	1.9635	0.024794	1.9635	0.024794	1.9635	0.024794	0.024794	0.446014362
SATB1-AS1	ENSG00000228956	0.63771	0.26183	0.27479	0.39174	1.1657	0.12187	1.9634	0.024799	0.024799	0.446014362
SPATA45	ENSG00000185523	0.56614	0.28565	0.56614	0.28565	1.9633	0.024804	1.3199	0.09343	0.024804	0.446014362
FARP1	ENSG00000152767	1.2757	0.10104	1.2052	0.11407	1.9631	0.02482	1.2948	0.097697	0.02482	0.446108343
PGAM2	ENSG00000164708	1.9627	0.024838	1.9627	0.024838	1.9627	0.024838	1.9627	0.024838	0.024838	0.446108343
MIR3681HG	ENSG00000224184	1.613	0.053377	-0.012178	0.50486	0.0091753	0.49634	1.9626	0.024845	0.024845	0.446108343
PCDHB2	ENSG00000112852	0.99163	0.16069	1.2483	0.10597	-0.13697	0.55447	1.9626	0.024847	0.024847	0.446108343
NA	ENSG00000274629	0.51102	0.30467	0.51102	0.30467	0.51102	0.30467	1.9625	0.024855	0.024855	0.446108343
NA	ENSG00000242411	1.9621	0.024876	1.9621	0.024876	1.9621	0.024876	1.9621	0.024876	0.024876	0.446131245
MIR124-3	ENSG00000207598	1.962	0.024881	1.1732	0.12035	0.1452	0.44228	0.78431	0.21643	0.024881	0.446131245
NA	ENSG00000249094	1.9619	0.024889	-0.4625	0.67814	-0.4625	0.67814	-0.2913	0.61459	0.024889	0.446131245
HOXB2	ENSG00000173917	1.9613	0.024922	1.9613	0.024922	1.9613	0.024922	1.9613	0.024922	0.024922	0.446131245
MED23	ENSG00000112282	0.15829	0.43711	1.9612	0.024926	0.58461	0.27941	0.20923	0.41713	0.024926	0.446131245
RTCB	ENSG00000100220	0.073166	0.47084	1.9611	0.024936	1.9611	0.024936	1.6571	0.048752	0.024936	0.446131245
COX4I1	ENSG00000131143	1.7438	0.040599	1.2188	0.11147	1.961	0.024942	-0.90947	0.81845	0.024942	0.446131245
GK2	ENSG00000196475	1.7384	0.041074	1.9609	0.024943	1.0149	0.15508	0.4783	0.31622	0.024943	0.446131245
VPS33B	ENSG00000184056	1.2747	0.10121	1.9609	0.024946	1.679	0.046574	1.8374	0.033072	0.024946	0.446131245
CASP7	ENSG00000165806	1.9291	0.026862	1.9608	0.024952	1.6676	0.047701	1.3518	0.088218	0.024952	0.446131245
SMAP1	ENSG00000112305	1.9605	0.02497	0.45232	0.32552	0.26636	0.39498	1.3414	0.089903	0.02497	0.446131245

MIR182	ENSG00000207990	-1.0145	0.84484	1.9602	0.024989	-1.0145	0.84484	-1.0145	0.84484	0.024989	0.446131245
TSEN2	ENSG00000154743	1.914	0.027813	1.96	0.024997	0.21208	0.41602	1.4912	0.067953	0.024997	0.446131245
NA	ENSG00000280434	1.9598	0.025008	1.9598	0.025008	1.9598	0.025008	1.9598	0.025008	0.025008	0.446131245
PDAP1	ENSG00000106244	1.709	0.043726	1.9598	0.02501	1.9329	0.026625	1.7381	0.041093	0.02501	0.446131245
PFN1P11	ENSG00000213061	0.071523	0.47149	1.9596	0.025024	0.070007	0.47209	0.058575	0.47665	0.025024	0.446131245
CLSTN3	ENSG00000139182	1.9595	0.025026	-0.66697	0.7476	0.59034	0.27748	0.33522	0.36873	0.025026	0.446131245
NA	ENSG00000226193	1.9593	0.025036	1.9593	0.025036	1.9593	0.025036	1.9593	0.025036	0.025036	0.446131245
RNY1P13	ENSG00000201900	1.4775	0.069777	1.9592	0.025043	1.9592	0.025043	1.9592	0.025043	0.025043	0.446131245
CAP1	ENSG00000131236	1.9097	0.028085	1.9592	0.025046	0.12105	0.45183	-0.8094	0.79086	0.025046	0.446131245
NA	ENSG00000224946	1.959	0.025054	1.959	0.025054	1.959	0.025054	1.959	0.025054	0.025054	0.446131245
NA	ENSG00000253652	1.9588	0.025067	0.78657	0.21577	-1.0341	0.84946	-1.5805	0.94301	0.025067	0.446131245
DCAF1	ENSG00000145041	-0.43464	0.66809	0.32982	0.37077	1.9587	0.025073	1.8493	0.032209	0.025073	0.446131245
PPM1A	ENSG00000100614	0.54119	0.29419	1.8085	0.035267	1.9587	0.025076	1.429	0.076504	0.025076	0.446131245
NA	ENSG00000205485	0.81517	0.20749	1.9579	0.025121	1.4573	0.072515	0.46613	0.32056	0.025121	0.446143145
RNF157	ENSG00000141576	1.9577	0.025132	0.51637	0.3028	1.2721	0.10166	0.56899	0.28468	0.025132	0.446143145
VINAC1P	ENSG00000283698	1.4386	0.075134	1.9577	0.025132	0.4054	0.34259	0.48064	0.31539	0.025132	0.446143145
NAT14	ENSG00000090971	1.9577	0.025134	1.9577	0.025134	1.9577	0.025134	1.9577	0.025134	0.025134	0.446143145
MLLT3	ENSG00000171843	1.3574	0.087327	-0.50632	0.69368	1.9574	0.025149	0.38704	0.34936	0.025149	0.446143145
RGR	ENSG00000148604	1.9574	0.025152	1.9574	0.025152	-0.32519	0.62748	0.71431	0.23752	0.025152	0.446143145
ADAM5	ENSG00000196115	0.17111	0.43207	1.4006	0.080666	0.99839	0.15905	1.9571	0.025165	0.025165	0.446143145
RNU6-983P	ENSG00000251813	1.5922	0.055675	0.47928	0.31587	1.9571	0.025168	-1.5657	0.94129	0.025168	0.446143145
RGS7BP	ENSG00000186479	0.62636	0.26554	0.48033	0.3155	0.73134	0.23229	1.9569	0.025178	0.025178	0.446143145
NA	ENSG00000253978	1.9569	0.025178	1.9569	0.025178	1.9569	0.025178	1.9569	0.025178	0.025178	0.446143145
OR4K7P	ENSG00000265340	1.1406	0.12701	0.50182	0.3079	0.50182	0.3079	1.9568	0.025188	0.025188	0.446143145
PDIA5	ENSG00000065485	1.9566	0.025196	0.038633	0.48459	1.0643	0.1436	0.077607	0.46907	0.025196	0.446143145
ADIG	ENSG00000182035	1.9564	0.025206	1.0836	0.13926	1.0836	0.13926	1.0836	0.13926	0.025206	0.446143145
MGAT5	ENSG00000152127	1.5194	0.064335	0.95047	0.17094	1.9563	0.025214	0.62705	0.26531	0.025214	0.446143145
NA	ENSG00000266363	1.3853	0.082985	1.2603	0.10378	1.841	0.032812	1.9563	0.025214	0.025214	0.446143145
NA	ENSG00000247970	1.9557	0.02525	1.9557	0.02525	1.252	0.10529	0.50574	0.30652	0.02525	0.446455914
NA	ENSG00000227603	1.9557	0.02525	1.9557	0.02525	1.4884	0.068322	0.24854	0.40186	0.02525	0.446455914
PCSK4	ENSG00000115257	1.0304	0.15141	1.8551	0.031789	1.9434	0.025983	1.9552	0.02528	0.02528	0.44669461

RNU2-62P	ENSG00000222800	-0.70269	0.75888	1.9552	0.025282	-1.3067	0.90435	-0.70269	0.75888	0.025282	0.44669461
ELP4	ENSG00000109911	0.22093	0.41257	1.1123	0.13301	1.955	0.025291	0.55625	0.28902	0.025291	0.44669461
RNU6-835P	ENSG00000252783	1.9547	0.02531	1.9547	0.02531	1.9547	0.02531	1.9547	0.02531	0.02531	0.446868225
RNU6-908P	ENSG00000222092	1.9538	0.025362	0.30581	0.37987	1.9538	0.025362	1.9538	0.025362	0.025362	0.447624143
INTS7	ENSG00000143493	0.9139	0.18038	0.68174	0.2477	1.9532	0.025397	1.3327	0.091322	0.025397	0.448079584
LINC01674	ENSG00000228159	1.104	0.13479	0.15477	0.4385	0.263	0.39627	1.9524	0.025445	0.025445	0.44876397
PIGCP2	ENSG00000229626	1.952	0.025467	1.4013	0.080556	1.4013	0.080556	1.952	0.025467	0.025467	0.448989475
TDRD9	ENSG00000156414	0.91703	0.17956	1.9518	0.025484	0.65516	0.25618	-0.1865	0.57397	0.025484	0.449048623
KCNA7	ENSG00000104848	1.7725	0.038153	1.7725	0.038153	1.9516	0.025493	0.16109	0.43601	0.025493	0.449048623
NA	ENSG00000231724	-0.078759	0.53139	1.148	0.12549	-1.3598	0.91305	1.9515	0.025498	0.025498	0.449048623
NA	ENSG00000235111	-2.0858	0.9815	0.35504	0.36128	1.9514	0.025508	-0.43254	0.66733	0.025508	0.449062442
TTLL7-IT1	ENSG00000233061	0.47685	0.31674	1.9509	0.025534	0.26683	0.3948	0.52264	0.30061	0.025534	0.449170357
NA	ENSG00000231923	0.81791	0.2067	-1.5374	0.9379	1.9508	0.025541	-0.61736	0.7315	0.025541	0.449170357
RPS2P2	ENSG00000177776	-1.3538	0.91209	0.016207	0.49353	-0.38525	0.64997	1.9506	0.025551	0.025551	0.449170357
NA	ENSG00000220702	1.9506	0.025551	1.9506	0.025551	1.7364	0.041247	1.9506	0.025551	0.025551	0.449170357
NA	ENSG00000253198	0.37007	0.35566	-0.40232	0.65628	0.3921	0.34749	1.9503	0.025568	0.025568	0.449268115
NA	ENSG00000225643	-0.70965	0.76104	-0.70965	0.76104	1.9502	0.025575	0.17637	0.43	0.025575	0.449268115
MIS12	ENSG00000167842	0.95331	0.17022	1.3737	0.084761	1.9497	0.025605	1.3737	0.084761	0.025605	0.449433934
FAT3	ENSG00000165323	1.9497	0.025608	1.3388	0.090315	1.7773	0.037763	0.57163	0.28379	0.025608	0.449433934
TBX3	ENSG00000135111	1.9493	0.025631	0.15697	0.43763	0.73092	0.23242	0.44639	0.32766	0.025631	0.449433934
NA	ENSG00000241020	1.949	0.025647	1.949	0.025647	1.949	0.025647	1.949	0.025647	0.025647	0.449433934
NA	ENSG00000259402	1.949	0.025647	1.949	0.025647	1.949	0.025647	1.949	0.025647	0.025647	0.449433934
SMIM4	ENSG00000168273	1.6319	0.051353	1.5371	0.062134	1.949	0.025648	1.949	0.025648	0.025648	0.449433934
BTN1A1	ENSG00000124557	1.8022	0.035759	1.804	0.035616	1.949	0.025649	1.0626	0.14398	0.025649	0.449433934
SLC38A4	ENSG00000139209	1.9483	0.025691	0.5836	0.27974	-0.26539	0.60464	0.95824	0.16897	0.025691	0.449881383
NA	ENSG00000235426	1.6465	0.049827	1.9482	0.025693	1.3525	0.088107	1.1824	0.11853	0.025693	0.449881383
NA	ENSG00000238031	1.0944	0.13689	-0.061889	0.52467	-0.54827	0.70825	1.9476	0.025729	0.025729	0.450205709
SNORA70E	ENSG00000207221	1.1763	0.11975	1.1763	0.11975	1.1763	0.11975	1.9476	0.02573	0.02573	0.450205709
AKAP6	ENSG00000151320	-0.20789	0.58234	1.9474	0.025745	1.0587	0.14486	0.64538	0.25934	0.025745	0.450302268
NA	ENSG00000261012	1.9472	0.025754	1.3567	0.087439	1.9472	0.025754	1.9472	0.025754	0.025754	0.450302268
ITGB4	ENSG00000132470	1.1633	0.12235	1.947	0.025768	-0.038568	0.51538	1.5829	0.056727	0.025768	0.450385452

SLC25A14P1	ENSG00000213608	1.9466	0.025789	1.9466	0.025789	1.9466	0.025789	1.9466	0.025789	0.025789	0.450534176
ANKDD1A	ENSG00000166839	1.1478	0.12552	1.9465	0.025795	0.56162	0.28719	1.2946	0.097728	0.025795	0.450534176
IFITM9P	ENSG00000213275	1.7375	0.04115	0.31388	0.37681	1.9463	0.025809	0.91635	0.17974	0.025809	0.450617187
NA	ENSG00000214942	1.9458	0.025837	1.9458	0.025837	1.9458	0.025837	1.9458	0.025837	0.025837	0.450656575
NA	ENSG00000258125	1.1789	0.11922	1.1789	0.11922	1.9458	0.025838	0.87135	0.19178	0.025838	0.450656575
FAM90A1	ENSG00000171847	1.079	0.14029	1.5406	0.061708	0.21145	0.41627	1.9458	0.025839	0.025839	0.450656575
EEF1A1P28	ENSG00000237709	1.9455	0.025857	-0.17235	0.56842	-0.23364	0.59237	-0.23364	0.59237	0.025857	0.450809163
MYH7	ENSG00000092054	0.95435	0.16995	1.5764	0.057468	1.4424	0.074588	1.9453	0.02587	0.02587	0.450874499
RPL13AP5	ENSG00000236552	1.9447	0.025904	1.7685	0.03849	1.9447	0.025904	1.9447	0.025904	0.025904	0.451282174
NA	ENSG00000232748	1.9443	0.025929	1.7745	0.037994	1.9443	0.025929	1.117	0.13201	0.025929	0.451282174
NA	ENSG00000278745	1.9441	0.025941	1.9441	0.025941	1.9441	0.025941	1.9441	0.025941	0.025941	0.451282174
NA	ENSG00000258386	1.0098	0.15629	-0.60767	0.7283	1.9441	0.025943	1.9441	0.025943	0.025943	0.451282174
NA	ENSG00000254645	1.9439	0.025952	1.1345	0.1283	1.4042	0.080132	1.2104	0.11306	0.025952	0.451282174
RPS29P29	ENSG00000233717	0.95949	0.16866	1.9439	0.025955	1.9439	0.025955	1.9439	0.025955	0.025955	0.451282174
NA	ENSG00000230728	1.0178	0.1544	0.045471	0.48187	1.9437	0.025968	1.9437	0.025968	0.025968	0.451282174
SEPTIN7P7	ENSG00000229897	1.9436	0.025971	1.9436	0.025971	1.9436	0.025971	1.8204	0.034347	0.025971	0.451282174
KIAA0895	ENSG00000164542	-1.3499	0.91148	1.9435	0.025978	-1.3338	0.90886	-0.69196	0.75552	0.025978	0.451282174
NA	ENSG00000278514	1.9434	0.025986	1.9434	0.025986	1.9434	0.025986	1.9434	0.025986	0.025986	0.451282174
TEX101	ENSG00000131126	-0.079334	0.53162	1.4328	0.075954	0.82225	0.20547	1.943	0.02601	0.02601	0.451538048
PPFIBP2	ENSG00000166387	1.9425	0.026038	0.081862	0.46738	0.88198	0.18889	0.43756	0.33085	0.026038	0.451863155
NA	ENSG00000253215	1.9423	0.026048	-1.3696	0.9146	-1.3696	0.9146	-1.3696	0.9146	0.026048	0.451875771
RPS3AP28	ENSG00000234995	-0.085876	0.53422	-0.085876	0.53422	-1.5538	0.93988	1.942	0.026066	0.026066	0.451924226
BBS4	ENSG00000140463	0.37878	0.35243	1.0676	0.14285	1.0394	0.1493	1.9419	0.026075	0.026075	0.451924226
CHDH	ENSG00000016391	1.9416	0.026092	0.87894	0.18972	1.4514	0.073328	0.28342	0.38843	0.026092	0.451924226
NA	ENSG00000266368	0.23383	0.40756	-1.4177	0.92187	1.9414	0.026106	-1.4177	0.92187	0.026106	0.451924226
RPL22P13	ENSG00000242670	1.9413	0.02611	-0.71744	0.76345	1.3095	0.095182	0.8016	0.21139	0.02611	0.451924226
RCC1L	ENSG00000274523	1.9412	0.026119	0.83497	0.20187	0.8767	0.19033	0.3904	0.34812	0.026119	0.451924226
NA	ENSG00000254115	1.9411	0.026126	0.24062	0.40492	0.17531	0.43042	-0.91126	0.81892	0.026126	0.451924226
NA	ENSG00000270705	1.0672	0.14293	1.2888	0.098727	1.0672	0.14293	1.941	0.026129	0.026129	0.451924226
LRRC25	ENSG00000175489	-0.10329	0.54113	1.9408	0.02614	0.10596	0.45781	1.1785	0.1193	0.02614	0.451924226
NA	ENSG00000265815	1.9405	0.026162	1.9405	0.026162	1.9405	0.026162	1.9405	0.026162	0.026162	0.451924226

NA	ENSG00000234509	1.9403	0.02617	1.5979	0.055034	1.9403	0.02617	1.5053	0.066124	0.02617	0.451924226
LINC02383	ENSG00000248143	1.4709	0.070663	1.9403	0.026172	0.0095872	0.49618	0.32896	0.37109	0.026172	0.451924226
NA	ENSG00000238221	1.9403	0.026173	0.1069	0.45743	0.1069	0.45743	-1.1081	0.86608	0.026173	0.451924226
ADAMTS7P1	ENSG00000274376	0.52904	0.29839	-0.44146	0.67056	0.61831	0.26819	1.9401	0.026185	0.026185	0.451924226
CHSY1	ENSG00000131873	1.9398	0.026201	0.72044	0.23563	-0.47553	0.68279	-0.1694	0.56726	0.026201	0.451924226
LINC01423	ENSG00000231231	-0.016471	0.50657	-0.48097	0.68473	-0.621	0.7327	1.9396	0.026217	0.026217	0.451924226
NDUFAF6	ENSG00000156170	1.9395	0.026219	1.5099	0.06553	1.7574	0.039427	1.3978	0.081087	0.026219	0.451924226
NA	ENSG00000254810	1.9395	0.026219	1.9395	0.026219	1.9395	0.026219	1.9395	0.026219	0.026219	0.451924226
NA	ENSG00000274292	1.5496	0.060622	1.9394	0.026227	1.8922	0.029234	1.8922	0.029234	0.026227	0.451924226
NA	ENSG00000265552	1.33	0.09176	1.33	0.09176	1.9389	0.026254	1.33	0.09176	0.026254	0.452229558
NA	ENSG00000257817	0.8805	0.18929	1.294	0.097826	1.9387	0.026271	1.303	0.096294	0.026271	0.452362484
NA	ENSG00000260725	-0.036629	0.51461	-0.65295	0.74311	1.3022	0.096429	1.9384	0.026286	0.026286	0.452387677
RN7SL491P	ENSG00000264400	1.9377	0.026332	1.9377	0.026332	1.9377	0.026332	1.9377	0.026332	0.026332	0.452387677
RNU6-1096P	ENSG00000252898	1.9376	0.026337	-0.31953	0.62534	1.2613	0.1036	-0.31953	0.62534	0.026337	0.452387677
EHD2	ENSG00000024422	1.9375	0.026342	-1.2585	0.8959	-1.4024	0.91961	-0.32958	0.62914	0.026342	0.452387677
FADS6	ENSG00000172782	0.23709	0.40629	0.23709	0.40629	0.23709	0.40629	1.9371	0.026364	0.026364	0.452387677
ARF4	ENSG00000168374	1.9371	0.026369	-0.41153	0.65966	-0.55788	0.71154	-0.73154	0.76777	0.026369	0.452387677
NA	ENSG00000266411	1.937	0.026375	1.0351	0.15031	0.0303	0.48791	1.8394	0.032928	0.026375	0.452387677
TFF1	ENSG00000160182	-0.041885	0.5167	0.39823	0.34523	1.9369	0.026377	-0.012165	0.50485	0.026377	0.452387677
RBBP5	ENSG00000117222	1.858	0.031582	1.9368	0.026386	1.9368	0.026386	1.6406	0.050441	0.026386	0.452387677
SOD1	ENSG00000142168	0.90907	0.18166	1.9368	0.026387	0.056677	0.4774	-1.2792	0.89959	0.026387	0.452387677
SLC22A12	ENSG00000197891	1.4419	0.074671	1.8131	0.034911	1.8763	0.030304	1.9368	0.026387	0.026387	0.452387677
PTPN7	ENSG00000143851	0.29396	0.3844	0.51054	0.30484	-0.45825	0.67661	1.9365	0.026404	0.026404	0.452387677
COLEC12	ENSG00000158270	1.9364	0.026411	1.2792	0.10041	0.64643	0.259	0.82936	0.20345	0.026411	0.452387677
SHE	ENSG00000169291	1.7538	0.039734	1.4431	0.074502	1.9364	0.026412	1.5836	0.056644	0.026412	0.452387677
NA	ENSG00000228361	1.9364	0.026412	0.25317	0.40007	-0.91681	0.82038	-0.91681	0.82038	0.026412	0.452387677
HNF4A-AS1	ENSG00000229005	-1.1128	0.86711	1.9362	0.026421	1.0889	0.13809	0.42192	0.33654	0.026421	0.452387677
COX14	ENSG00000178449	1.6806	0.046417	1.0689	0.14257	1.0689	0.14257	1.9358	0.026446	0.026446	0.452473385
CTSE	ENSG00000196188	-0.97181	0.83443	-0.44568	0.67209	-0.51964	0.69834	1.9356	0.02646	0.02646	0.452473385
C5orf34-AS1	ENSG00000248554	1.9352	0.026482	1.9352	0.026482	1.9352	0.026482	1.9352	0.026482	0.026482	0.452473385
CWF19L1	ENSG00000095485	1.1459	0.12592	1.3337	0.091152	1.3337	0.091152	1.9352	0.026485	0.026485	0.452473385

NA	ENSG00000225243	1.8621	0.031298	-1.0945	0.86313	1.935	0.026493	-0.51276	0.69594	0.026493	0.452473385
DEK	ENSG00000124795	1.7185	0.042856	0.75379	0.22549	0.61124	0.27052	1.9349	0.026501	0.026501	0.452473385
ASB3	ENSG00000115239	0.61874	0.26804	1.9346	0.026517	-0.16257	0.56457	-0.41584	0.66124	0.026517	0.452473385
TCF25	ENSG00000141002	1.2615	0.10357	1.4027	0.08036	1.9344	0.026531	1.5946	0.055401	0.026531	0.452473385
NFAT5	ENSG00000102908	1.4993	0.066904	0.83731	0.20121	1.5652	0.058772	1.9343	0.026536	0.026536	0.452473385
RPL36	ENSG00000130255	-0.48038	0.68452	1.9342	0.026542	-0.51455	0.69657	-0.39075	0.65201	0.026542	0.452473385
NA	ENSG00000264281	1.9342	0.026543	1.9342	0.026543	1.9342	0.026543	1.9342	0.026543	0.026543	0.452473385
NFU1P2	ENSG00000233557	1.9341	0.026552	1.9341	0.026552	1.9341	0.026552	0.9583	0.16896	0.026552	0.452473385
NA	ENSG00000270387	0.014401	0.49426	0.014401	0.49426	1.7591	0.039279	1.934	0.026555	0.026555	0.452473385
NA	ENSG00000241317	1.934	0.026556	1.2058	0.11394	-1.0813	0.86023	-1.0813	0.86023	0.026556	0.452473385
C18orf25	ENSG00000152242	1.6927	0.045253	-0.33033	0.62943	1.9335	0.02659	1.6078	0.053935	0.02659	0.45267427
NISCH	ENSG00000010322	1.9334	0.026591	1.8072	0.035363	1.6824	0.046245	1.4093	0.079366	0.026591	0.45267427
BEND5	ENSG00000162373	1.193	0.11643	1.933	0.026617	1.1053	0.13452	1.1053	0.13452	0.026617	0.45267427
NA	ENSG00000254362	0.84327	0.19954	0.92647	0.1771	1.9327	0.026637	1.4215	0.077589	0.026637	0.45267427
NA	ENSG00000268803	1.9327	0.026637	1.9327	0.026637	1.9327	0.026637	1.9327	0.026637	0.026637	0.45267427
GPX4	ENSG00000167468	1.9327	0.026638	1.9327	0.026638	1.9327	0.026638	1.9327	0.026638	0.026638	0.45267427
VPS50	ENSG00000004766	1.9325	0.026647	1.7996	0.03596	0.34869	0.36366	0.34869	0.36366	0.026647	0.45267427
NA	ENSG00000202248	1.9324	0.026655	1.9324	0.026655	1.9324	0.026655	1.9324	0.026655	0.026655	0.45267427
ABO	ENSG00000175164	1.9322	0.026664	0.71492	0.23733	1.0111	0.15598	1.7354	0.041336	0.026664	0.45267427
NA	ENSG00000280371	-1.4179	0.92189	0.87107	0.19186	1.9321	0.026676	0.34973	0.36327	0.026676	0.45267427
CNTN3	ENSG00000113805	1.9089	0.02814	1.9319	0.026684	-0.52219	0.69923	-1.0936	0.86293	0.026684	0.45267427
TSHR	ENSG00000165409	1.9317	0.026697	1.52	0.064261	0.70909	0.23913	0.90211	0.1835	0.026697	0.45267427
NA	ENSG00000254131	1.0706	0.14218	1.0706	0.14218	1.0706	0.14218	1.9316	0.026706	0.026706	0.45267427
NDUFB4P3	ENSG00000231096	1.9314	0.026716	1.9314	0.026716	1.9314	0.026716	1.9314	0.026716	0.026716	0.45267427
OR7A10	ENSG00000127515	0.372	0.35495	1.9313	0.026726	-0.0029501	0.50118	0.372	0.35495	0.026726	0.45267427
ATG10	ENSG00000152348	0.80216	0.21123	1.0809	0.13986	0.99657	0.15949	1.9312	0.02673	0.02673	0.45267427
UBE2D3	ENSG00000109332	1.6175	0.052888	1.2137	0.11243	1.9311	0.026734	1.4974	0.067139	0.026734	0.45267427
NA	ENSG00000246089	1.3246	0.092647	1.2795	0.10036	1.9311	0.026735	1.9311	0.026735	0.026735	0.45267427
SERPINB6	ENSG00000124570	-0.42011	0.6628	0.29607	0.38359	0.62767	0.26511	1.9309	0.026749	0.026749	0.452754001
NA	ENSG00000279251	1.9303	0.026782	1.9303	0.026782	1.9303	0.026782	1.9303	0.026782	0.026782	0.453043588
NA	ENSG00000244740	1.4612	0.071978	1.4612	0.071978	1.4612	0.071978	1.9301	0.026794	0.026794	0.453043588

RNA5SP460	ENSG00000252598	1.9301	0.026794	1.9301	0.026794	1.9301	0.026794	1.9301	0.026794	0.026794	0.453043588
BRCA2	ENSG00000139618	1.9296	0.026826	0.98387	0.16259	1.8752	0.030382	1.7369	0.041202	0.026826	0.453248118
RPL12P26	ENSG00000236171	0.54225	0.29382	1.9296	0.026829	0.14038	0.44418	0.14038	0.44418	0.026829	0.453248118
NA	ENSG00000230160	1.9295	0.026834	0.43751	0.33087	1.1499	0.1251	1.9295	0.026834	0.026834	0.453248118
SRP68	ENSG00000167881	0.25141	0.40075	1.9292	0.026855	1.9292	0.026855	-0.29577	0.6163	0.026855	0.453445651
RET	ENSG00000165731	-0.9515	0.82933	0.1052	0.45811	1.65	0.049476	1.9285	0.026895	0.026895	0.453627401
NRXN2	ENSG00000110076	1.4374	0.075299	0.93978	0.17366	0.80894	0.20928	1.9285	0.026898	0.026898	0.453627401
ZNF124	ENSG00000196418	0.78987	0.2148	0.54768	0.29196	1.9284	0.026903	1.7033	0.044255	0.026903	0.453627401
LINC01923	ENSG00000236653	0.37499	0.35384	1.2025	0.11459	1.9284	0.026903	1.2078	0.11357	0.026903	0.453627401
TRH	ENSG00000170893	1.9281	0.026922	1.9281	0.026922	1.9281	0.026922	1.9281	0.026922	0.026922	0.453790751
RPL35AP30	ENSG00000226729	1.9273	0.026973	1.9273	0.026973	1.9273	0.026973	1.9273	0.026973	0.026973	0.45401154
NA	ENSG00000225879	0.96556	0.16713	0.72252	0.23499	0.53141	0.29757	1.9272	0.026977	0.026977	0.45401154
LIPG	ENSG00000101670	0.25151	0.40071	1.7255	0.042222	1.9272	0.02698	0.084944	0.46615	0.02698	0.45401154
HELZ2	ENSG00000130589	0.95151	0.17067	0.88289	0.18865	1.9271	0.026981	0.45175	0.32572	0.026981	0.45401154
PBX4	ENSG00000105717	1.9271	0.026985	0.4183	0.33786	1.9271	0.026985	-0.082992	0.53307	0.026985	0.45401154
RNU6-465P	ENSG00000207149	0.70852	0.23931	0.00623	0.49751	1.927	0.026991	1.0353	0.15027	0.026991	0.45401154
NA	ENSG00000252115	1.9264	0.027028	1.9264	0.027028	1.9264	0.027028	1.9264	0.027028	0.027028	0.454136761
NA	ENSG00000257078	1.9263	0.027032	1.7561	0.039534	0.18437	0.42686	1.4705	0.070719	0.027032	0.454136761
SSMEM1	ENSG00000165120	1.9262	0.027041	1.9262	0.027041	1.9262	0.027041	1.9262	0.027041	0.027041	0.454136761
TUBA1A	ENSG00000167552	1.6083	0.053884	1.6083	0.053884	1.1673	0.12155	1.9261	0.027044	0.027044	0.454136761
PHC2-AS1	ENSG00000233246	1.926	0.027051	1.926	0.027051	1.926	0.027051	1.926	0.027051	0.027051	0.454136761
ZXDC	ENSG00000070476	1.9259	0.027056	-0.14862	0.55907	0.044838	0.48212	0.28525	0.38772	0.027056	0.454136761
ARMC2	ENSG00000118690	0.10922	0.45651	1.5656	0.058725	1.9258	0.027065	0.79255	0.21402	0.027065	0.454136761
NA	ENSG00000225208	1.9257	0.027073	1.8498	0.03217	1.9119	0.027943	1.0567	0.14532	0.027073	0.454136761
TMEM239	ENSG00000198326	1.8041	0.035608	1.9254	0.027092	-0.1347	0.55358	1.8041	0.035608	0.027092	0.454299092
CIBAR1P2	ENSG00000230219	1.9249	0.027119	-0.47871	0.68393	0.36146	0.35888	0.81773	0.20676	0.027119	0.454534142
NPIPP1	ENSG00000188599	0.7234	0.23472	-1.7284	0.95804	1.9247	0.027132	0.33579	0.36852	0.027132	0.454534142
MAGEL2	ENSG00000254585	-0.26471	0.60439	-0.23678	0.59359	-1.0526	0.85373	1.9247	0.027134	0.027134	0.454534142
NA	ENSG00000270302	0.27118	0.39313	0.2825	0.38878	0.56642	0.28555	1.9243	0.027158	0.027158	0.454657094
NA	ENSG00000229960	0.67034	0.25132	1.9243	0.02716	0.67034	0.25132	0.67034	0.25132	0.02716	0.454657094
GJB5	ENSG00000189280	1.9237	0.027193	1.6804	0.046442	-0.11866	0.54723	1.1995	0.11517	0.027193	0.454980446

ADGRF5	ENSG00000069122	-1.1032	0.86503	1.9236	0.027204	0.21981	0.41301	-0.99319	0.83969	0.027204	0.454980446
NA	ENSG00000225676	1.9234	0.027216	1.9234	0.027216	0.78457	0.21635	1.9234	0.027216	0.027216	0.454980446
CILK1	ENSG00000112144	0.29387	0.38443	0.47511	0.31735	1.9233	0.027219	1.8116	0.035025	0.027219	0.454980446
FAM181A	ENSG00000140067	1.8516	0.032044	1.8516	0.032044	1.8516	0.032044	1.9232	0.027226	0.027226	0.454980446
NMBR	ENSG00000135577	1.9229	0.027243	-0.68808	0.7543	-0.68808	0.7543	0.59659	0.27539	0.027243	0.455108464
NA	ENSG00000239930	1.9226	0.027263	1.9226	0.027263	1.9226	0.027263	1.9226	0.027263	0.027263	0.455130521
NA	ENSG00000255094	1.9226	0.027263	0.53587	0.29602	0.53587	0.29602	0.53587	0.29602	0.027263	0.455130521
RTKN	ENSG00000114993	0.69971	0.24205	0.69971	0.24205	1.9224	0.027278	0.69971	0.24205	0.027278	0.455224979
C2CD4B	ENSG00000205502	1.9222	0.027293	1.9222	0.027293	1.9222	0.027293	1.9222	0.027293	0.027293	0.455319374
ANKRD30B	ENSG00000180777	1.0426	0.14857	1.9219	0.027311	0.67945	0.24843	1.7681	0.038523	0.027311	0.45540794
ADAMTS3	ENSG00000156140	1.7681	0.038524	0.95818	0.16899	1.9218	0.027317	0.82285	0.2053	0.027317	0.45540794
DPP8	ENSG00000074603	-0.93226	0.8244	-0.86105	0.80539	0.76124	0.22326	1.9212	0.027353	0.027353	0.455710204
NA	ENSG00000272154	1.9211	0.027362	-0.45798	0.67652	0.6956	0.24334	0.6956	0.24334	0.027362	0.455710204
QRFPR	ENSG00000186867	1.6673	0.047732	1.0338	0.1506	1.0338	0.1506	1.921	0.027365	0.027365	0.455710204
PLET1	ENSG00000188771	1.9207	0.027382	-0.4848	0.68609	1.2056	0.11399	0.19247	0.42369	0.027382	0.455710204
PIGF	ENSG00000151665	1.1877	0.11748	0.51273	0.30407	1.9205	0.027399	0.65697	0.2556	0.027399	0.455710204
OR5K1	ENSG00000232382	1.2973	0.097261	-0.0076505	0.50305	1.9204	0.027401	-0.0076505	0.50305	0.027401	0.455710204
MRPL28	ENSG00000086504	1.8253	0.033981	1.9203	0.02741	1.7328	0.041562	1.749	0.040148	0.02741	0.455710204
ITIH4	ENSG00000055955	1.1127	0.13291	0.66523	0.25295	1.9201	0.02742	1.6537	0.049096	0.02742	0.455710204
NA	ENSG00000260202	-0.056822	0.52266	0.11584	0.45389	1.9201	0.027422	1.9201	0.027422	0.027422	0.455710204
NA	ENSG00000227637	1.92	0.027431	1.92	0.027431	1.92	0.027431	1.92	0.027431	0.027431	0.455710204
RAMACL	ENSG00000235272	0.89084	0.18651	1.9198	0.027438	-0.20228	0.58015	-2.2209	0.98682	0.027438	0.455710204
OPCML	ENSG00000183715	0.53635	0.29586	1.7905	0.036686	1.9193	0.027471	-0.61302	0.73007	0.027471	0.456047074
POM121C	ENSG00000272391	0.035003	0.48604	0.75935	0.22382	1.1878	0.11745	1.9192	0.027477	0.027477	0.456047074
SFXN5	ENSG00000144040	1.4819	0.069187	1.7832	0.037274	1.7918	0.036582	1.9191	0.027487	0.027487	0.456057715
NA	ENSG00000278923	1.9187	0.027513	1.8181	0.034522	1.9187	0.027513	1.8704	0.030717	0.027513	0.456166484
NA	ENSG00000272638	1.9186	0.027519	1.9186	0.027519	1.9186	0.027519	1.9186	0.027519	0.027519	0.456166484
DDX39B	ENSG00000198563	0.035317	0.48591	0.29619	0.38354	1.5581	0.059603	1.9185	0.027524	0.027524	0.456166484
ZFP91	ENSG00000186660	1.5977	0.055057	1.1445	0.1262	1.9184	0.027531	1.4021	0.080447	0.027531	0.456166484
NA	ENSG00000250751	1.9181	0.027548	1.9181	0.027548	1.9181	0.027548	1.9181	0.027548	0.027548	0.456293012
NA	ENSG00000227172	0.12776	0.44917	1.2156	0.11208	1.9178	0.02757	1.3906	0.082176	0.02757	0.456324598

TRDV1	ENSG00000211804	1.9176	0.027578	0.52892	0.29843	1.9176	0.027578	1.9176	0.027578	0.027578	0.456324598
OR13A1	ENSG00000256574	1.9123	0.027918	1.2851	0.099375	1.9177	0.027578	1.9177	0.027578	0.027578	0.456324598
PON2	ENSG00000105854	0.21896	0.41334	0.088189	0.46486	1.9172	0.027605	1.221	0.11105	0.027605	0.45661631
NA	ENSG00000233754	1.9165	0.027652	1.9165	0.027652	1.3753	0.084515	1.9165	0.027652	0.027652	0.456693272
PSME3IP1	ENSG00000172775	1.1155	0.13232	1.3098	0.09513	1.4626	0.071791	1.9163	0.027662	0.027662	0.456693272
RAD51C	ENSG00000108384	-0.24366	0.59625	-0.13135	0.55225	0.06857	0.47267	1.9163	0.027663	0.027663	0.456693272
PLAC8L1	ENSG00000173261	1.9162	0.027673	1.9162	0.027673	1.9162	0.027673	1.3964	0.081297	0.027673	0.456693272
RPL31P61	ENSG00000243297	1.9162	0.027673	1.9162	0.027673	1.9162	0.027673	1.9162	0.027673	0.027673	0.456693272
NA	ENSG00000232035	1.9161	0.027676	0.33352	0.36937	0.48872	0.31252	0.33352	0.36937	0.027676	0.456693272
RNU6-1172P	ENSG00000202095	-0.34082	0.63338	-0.73453	0.76869	-0.14948	0.55941	1.9161	0.027679	0.027679	0.456693272
OR5B12	ENSG00000172362	-0.15459	0.56143	1.9159	0.02769	-0.57354	0.71686	-0.2459	0.59712	0.02769	0.456693272
NA	ENSG00000276945	1.9158	0.027694	0.20019	0.42066	1.9158	0.027694	1.9158	0.027694	0.027694	0.456693272
ANK1	ENSG00000029534	-0.037846	0.51509	-0.64826	0.74159	-0.67553	0.75033	1.9156	0.027707	0.027707	0.456753082
ASIC1	ENSG00000110881	1.3831	0.083321	1.9152	0.027733	0.30817	0.37898	0.9626	0.16788	0.027733	0.456954949
FBXL21P	ENSG00000164616	1.4895	0.068179	0.77662	0.21869	1.6114	0.053545	1.9151	0.027738	0.027738	0.456954949
RN7SL507P	ENSG00000239702	1.7164	0.043048	0.26608	0.39509	1.6252	0.052056	1.9149	0.027754	0.027754	0.457064015
ZSWIM4	ENSG00000132003	1.9147	0.027765	0.41374	0.33953	-0.62025	0.73245	-0.070202	0.52798	0.027765	0.457090693
NA	ENSG00000243116	-0.015066	0.50601	-1.3263	0.90763	-0.49722	0.69048	1.9144	0.027783	0.027783	0.457170618
MIR3121	ENSG00000265435	1.9142	0.027797	1.9142	0.027797	1.9142	0.027797	1.9142	0.027797	0.027797	0.457170618
ANKRD10	ENSG00000088448	0.3441	0.36538	1.9142	0.027798	1.3024	0.096393	1.4889	0.068262	0.027798	0.457170618
TPM3P1	ENSG00000213050	0.22022	0.41285	1.9139	0.027817	0.22022	0.41285	0.22022	0.41285	0.027817	0.457328748
GSTA4	ENSG00000170899	1.9134	0.02785	1.9134	0.02785	1.9134	0.02785	1.1166	0.13208	0.02785	0.457716863
TUBB4A	ENSG00000104833	-0.90414	0.81704	-0.91141	0.81896	1.0451	0.14799	1.9126	0.027899	0.027899	0.458367589
RN7SL105P	ENSG00000243704	1.9124	0.027913	1.9124	0.027913	1.9124	0.027913	1.9124	0.027913	0.027913	0.458443037
ACOXL	ENSG00000153093	1.9121	0.02793	1.6057	0.054173	1.7003	0.044539	1.8656	0.031051	0.02793	0.458567689
NA	ENSG00000226041	1.9115	0.027968	1.9115	0.027968	1.9115	0.027968	1.9115	0.027968	0.027968	0.459030037
UBE2CP1	ENSG00000258648	1.9114	0.027977	0.47484	0.31745	0.32421	0.37289	0.53053	0.29787	0.027977	0.459030037
FGFR3P1	ENSG00000230994	1.9107	0.028025	1.9107	0.028025	1.9107	0.028025	1.9107	0.028025	0.028025	0.459559373
KCNQ1-AS1	ENSG00000229414	1.5623	0.059107	1.9104	0.02804	0.30126	0.38161	1.2029	0.11451	0.02804	0.459559373
NA	ENSG00000269786	1.9104	0.028041	1.9104	0.028041	0.25823	0.39811	1.9104	0.028041	0.028041	0.459559373
RNF2P1	ENSG00000231381	-0.18425	0.57309	1.8623	0.031283	0.31075	0.37799	1.9102	0.028053	0.028053	0.459559373

LCE1A	ENSG00000186844	0.81307	0.20809	0.42731	0.33458	1.9097	0.028086	-1.0254	0.84742	0.028086	0.459559373
HDAC4-AS1	ENSG00000222020	1.9096	0.028093	1.9096	0.028093	1.9096	0.028093	1.9096	0.028093	0.028093	0.459559373
NA	ENSG00000228953	-1.4009	0.91938	1.9096	0.028095	-1.4009	0.91938	-1.4009	0.91938	0.028095	0.459559373
LINC02379	ENSG00000251649	1.9095	0.0281	1.3715	0.085116	0.62925	0.26459	1.3715	0.085116	0.0281	0.459559373
XCL2	ENSG00000143185	1.147	0.1257	0.8963	0.18505	1.9094	0.028107	1.5051	0.066154	0.028107	0.459559373
NA	ENSG00000241917	1.6463	0.049849	1.9094	0.028107	1.556	0.059859	1.6676	0.047698	0.028107	0.459559373
CCNG1	ENSG00000113328	1.9093	0.028113	1.9093	0.028113	1.9093	0.028113	1.9093	0.028113	0.028113	0.459559373
GAPDHP66	ENSG00000250933	-0.26461	0.60434	1.909	0.028128	-0.62637	0.73447	0.26452	0.39569	0.028128	0.459650382
NA	ENSG00000283269	1.4864	0.068581	1.9085	0.028165	0.6332	0.2633	1.2695	0.10213	0.028165	0.460100721
TRIB3	ENSG00000101255	0.42413	0.33573	0.73453	0.23131	1.8093	0.035202	1.9078	0.028211	0.028211	0.460439789
SLC7A6OS	ENSG00000103061	1.9077	0.028217	1.9077	0.028217	1.9077	0.028217	0.56671	0.28545	0.028217	0.460439789
OSER1	ENSG00000132823	-0.75812	0.77581	0.36298	0.35831	-0.99051	0.83904	1.9076	0.02822	0.02822	0.460439789
GLUL	ENSG00000135821	-0.28196	0.61101	0.38641	0.3496	-0.22412	0.58867	1.9075	0.028226	0.028226	0.460439789
SLC35F3	ENSG00000183780	1.9074	0.028233	0.94842	0.17146	1.1705	0.12091	-0.17093	0.56786	0.028233	0.460439789
NA	ENSG00000216017	1.1141	0.13261	0.33364	0.36933	1.9069	0.028266	1.9069	0.028266	0.028266	0.460790364
NA	ENSG00000253347	1.9065	0.028295	1.9065	0.028295	1.9065	0.028295	1.9065	0.028295	0.028295	0.460790364
DNAJA2	ENSG00000069345	1.0931	0.13718	1.9062	0.028309	1.7369	0.041205	0.27566	0.3914	0.028309	0.460790364
NA	ENSG00000231754	1.9062	0.028309	-0.97062	0.83413	0.10282	0.45905	1.9062	0.028309	0.028309	0.460790364
PFKL	ENSG00000141959	1.5342	0.062492	-0.57009	0.71569	1.1817	0.11867	1.9059	0.028331	0.028331	0.460790364
NA	ENSG00000270359	-0.089454	0.53564	-0.16792	0.56668	1.9058	0.028338	1.9058	0.028338	0.028338	0.460790364
ZYXP1	ENSG00000274572	-0.95795	0.83096	1.9058	0.02834	-0.3365	0.63175	-0.3365	0.63175	0.02834	0.460790364
TMEM183A	ENSG00000163444	-1.152	0.87533	1.9056	0.028353	-0.92165	0.82164	-0.97088	0.8342	0.028353	0.460790364
DEPDC1	ENSG00000024526	0.86012	0.19486	1.4205	0.07773	1.9051	0.028386	1.9051	0.028386	0.028386	0.460790364
NA	ENSG00000259622	0.96112	0.16824	1.905	0.028393	1.9041	0.028452	1.2695	0.10212	0.028393	0.460790364
PISRT1	ENSG00000281473	-0.5736	0.71688	1.9048	0.028402	-0.5736	0.71688	-0.5736	0.71688	0.028402	0.460790364
PACS1	ENSG00000175115	1.0238	0.15295	0.47039	0.31904	1.2723	0.10164	1.9046	0.028416	0.028416	0.460790364
UGT8	ENSG00000174607	1.9045	0.028422	1.315	0.09425	1.6483	0.049651	0.24425	0.40352	0.028422	0.460790364
PCBP1-AS1	ENSG00000179818	1.8865	0.029611	1.9045	0.028422	1.6798	0.0465	1.6093	0.053771	0.028422	0.460790364
ZRANB1	ENSG00000019995	-1.2921	0.90183	-1.2373	0.89202	-1.4495	0.92641	1.9045	0.028423	0.028423	0.460790364
DNAJC4	ENSG00000110011	0.3391	0.36727	0.3391	0.36727	1.9043	0.028436	-0.32813	0.62859	0.028436	0.460790364
RN7SL26P	ENSG00000242014	1.9043	0.028438	1.9043	0.028438	1.9043	0.028438	1.9043	0.028438	0.028438	0.460790364

NA	ENSG00000240934	1.6843	0.046066	1.4481	0.073791	1.9042	0.02844	0.89082	0.18651	0.02844	0.460790364
THSD4-AS1	ENSG00000259964	1.9039	0.028462	-0.051227	0.52043	-1.6233	0.94773	0.71193	0.23826	0.028462	0.460790364
NA	ENSG00000262633	0.19738	0.42176	-0.3746	0.64602	1.4532	0.073084	1.9038	0.028468	0.028468	0.460790364
NA	ENSG00000272438	1.4762	0.069951	1.1	0.13567	1.9036	0.028482	1.9036	0.028482	0.028482	0.460790364
NOS3	ENSG00000164867	-0.15917	0.56323	1.9032	0.028507	1.9032	0.028507	1.9032	0.028507	0.028507	0.460790364
NA	ENSG00000251088	-0.49109	0.68832	1.9032	0.02851	-0.66099	0.74569	-0.66099	0.74569	0.02851	0.460790364
NA	ENSG00000242012	1.9031	0.028514	1.9031	0.028514	0.78084	0.21745	1.9031	0.028514	0.028514	0.460790364
NA	ENSG00000231200	0.95732	0.1692	0.95732	0.1692	1.9031	0.028517	0.95732	0.1692	0.028517	0.460790364
ASPRV1	ENSG00000244617	1.903	0.028519	1.903	0.028519	1.903	0.028519	1.903	0.028519	0.028519	0.460790364
NA	ENSG00000243780	-0.85312	0.8032	-0.85312	0.8032	-0.85312	0.8032	1.903	0.028522	0.028522	0.460790364
NA	ENSG00000255462	1.9028	0.028534	1.9028	0.028534	1.9028	0.028534	1.9028	0.028534	0.028534	0.460790364
SMIM13	ENSG00000224531	0.72266	0.23494	1.9027	0.028542	-0.91703	0.82044	-0.95853	0.8311	0.028542	0.460790364
SPDYE16	ENSG00000185040	1.9026	0.028546	0.51028	0.30493	0.063028	0.47487	0.63409	0.26301	0.028546	0.460790364
RUSC1-AS1	ENSG00000225855	-0.68552	0.75349	1.9025	0.028551	-1.8302	0.96639	-1.8302	0.96639	0.028551	0.460790364
HMGB1P26	ENSG00000225984	-0.12565	0.55	-0.12565	0.55	1.9022	0.028571	-0.26612	0.60493	0.028571	0.460790364
ING4	ENSG00000111653	-0.067979	0.5271	-0.067979	0.5271	-0.2408	0.59514	1.9022	0.028574	0.028574	0.460790364
MASP1	ENSG00000127241	1.4675	0.071126	1.9022	0.028576	0.2138	0.41535	1.691	0.045422	0.028576	0.460790364
RNU7-61P	ENSG00000251706	1.9017	0.028605	1.9017	0.028605	1.9017	0.028605	1.9017	0.028605	0.028605	0.460938133
RNU6-58P	ENSG00000252952	1.6319	0.051353	1.9015	0.028622	0.67285	0.25052	1.9015	0.028622	0.028622	0.460938133
RNF112	ENSG00000128482	1.9014	0.028623	1.9014	0.028623	1.9014	0.028623	1.9014	0.028623	0.028623	0.460938133
NA	ENSG00000238018	1.3671	0.085793	1.9014	0.028623	1.3398	0.09015	1.7225	0.042487	0.028623	0.460938133
NA	ENSG00000238991	0.15329	0.43908	1.9012	0.02864	0.15329	0.43908	0.15329	0.43908	0.02864	0.461059531
NA	ENSG00000249198	1.2999	0.096812	0.61575	0.26903	1.4478	0.073843	1.901	0.028651	0.028651	0.46108429
RNU1-134P	ENSG00000199805	1.9007	0.028674	1.9007	0.028674	1.2011	0.11486	1.9007	0.028674	0.028674	0.461302086
NA	ENSG00000248128	1.9002	0.028705	0.6135	0.26977	0.28793	0.3867	-0.19737	0.57823	0.028705	0.461601032
NPM1P38	ENSG00000219257	0.60075	0.274	0.27635	0.39114	0.27635	0.39114	1.9	0.028717	0.028717	0.461601032
FAM183DP	ENSG00000205716	-1.0805	0.86004	1.8999	0.028721	-0.98866	0.83858	-0.98866	0.83858	0.028721	0.461601032
AMPD1	ENSG00000116748	1.8998	0.028732	1.8998	0.028732	1.8998	0.028732	0.097956	0.46098	0.028732	0.461625572
NOL8P1	ENSG00000248327	-0.02005	0.508	1.8993	0.02876	0.015672	0.49375	0.39203	0.34752	0.02876	0.461923138
HSPD1P6	ENSG00000230067	1.8991	0.028774	1.8991	0.028774	1.8991	0.028774	1.8991	0.028774	0.028774	0.461923804
CLIC6	ENSG00000159212	1.8991	0.028779	0.62884	0.26473	0.49997	0.30855	1.1374	0.12768	0.028779	0.461923804

RHBDF1	ENSG00000007384	0.27843	0.39034	-0.85474	0.80365	1.8986	0.028805	0.61723	0.26854	0.028805	0.462188887
LINC02228	ENSG00000251273	1.8983	0.028831	-0.33641	0.63172	-1.987	0.97654	-1.8564	0.9683	0.028831	0.46224618
FSTL3	ENSG00000070404	1.8982	0.028833	1.0211	0.15359	1.0604	0.14447	1.6881	0.045698	0.028833	0.46224618
LINC01942	ENSG00000253428	-0.45208	0.6744	1.898	0.028846	0.25492	0.39939	-1.6215	0.94754	0.028846	0.46224618
HID1	ENSG00000167861	0.59608	0.27556	0.59608	0.27556	-0.61109	0.72943	1.8979	0.028854	0.028854	0.46224618
ATP5MF	ENSG00000241468	1.7812	0.037444	1.8979	0.028856	1.8287	0.033724	0.96846	0.16641	0.028856	0.46224618
SYT4	ENSG00000132872	1.8976	0.028877	1.3104	0.095023	0.93541	0.17479	0.89694	0.18488	0.028877	0.462294658
SYT8	ENSG00000149043	0.60747	0.27177	1.355	0.087708	-0.33518	0.63126	1.8975	0.028878	0.028878	0.462294658
NOL4L	ENSG00000197183	1.0626	0.14398	1.1842	0.11816	1.897	0.028917	1.0929	0.13721	0.028917	0.462567769
AURKAP2	ENSG00000230165	1.8967	0.028934	1.8967	0.028934	1.8967	0.028934	1.8967	0.028934	0.028934	0.462567769
SRMS	ENSG00000125508	1.258	0.1042	1.8966	0.028941	-0.21306	0.58436	0.90828	0.18187	0.028941	0.462567769
TSPAN5	ENSG00000168785	-0.87199	0.80839	0.93916	0.17382	1.8964	0.028951	0.63803	0.26173	0.028951	0.462567769
MTCH2	ENSG00000109919	1.8964	0.028957	-0.01106	0.50441	1.6864	0.045863	-0.013128	0.50524	0.028957	0.462567769
ST6GALNAC4	ENSG00000136840	-0.42978	0.66632	1.8963	0.028957	1.8963	0.028957	1.8963	0.028957	0.028957	0.462567769
KLHL12	ENSG00000117153	1.8962	0.028966	0.36232	0.35856	0.61661	0.26875	0.61661	0.26875	0.028966	0.462567769
MKX	ENSG00000150051	0.32185	0.37378	1.6198	0.052641	0.64384	0.25984	1.8961	0.028971	0.028971	0.462567769
RPL29	ENSG00000162244	1.8958	0.028993	1.8958	0.028993	1.8958	0.028993	1.8958	0.028993	0.028993	0.462615878
TAS2R62P	ENSG00000234066	1.8958	0.028993	1.8958	0.028993	1.8958	0.028993	1.8958	0.028993	0.028993	0.462615878
PRKD2	ENSG00000105287	1.8953	0.029023	1.8953	0.029023	1.0418	0.14874	1.838	0.03303	0.029023	0.462831387
LFNG	ENSG00000106003	0.57508	0.28262	0.67739	0.24908	1.8952	0.029031	0.78661	0.21575	0.029031	0.462831387
NA	ENSG00000267109	0.17278	0.43141	0.17278	0.43141	1.8952	0.029035	0.17278	0.43141	0.029035	0.462831387
FAXC	ENSG00000146267	1.7894	0.036773	1.7894	0.036773	1.7894	0.036773	1.8946	0.029075	0.029075	0.463078324
DUBR	ENSG00000243701	1.8946	0.029076	-0.04302	0.51716	0.24412	0.40357	1.114	0.13264	0.029076	0.463078324
TRBV7-6	ENSG00000211727	-0.11912	0.54741	-0.11912	0.54741	1.8945	0.029079	-0.11912	0.54741	0.029079	0.463078324
FOXP1	ENSG00000114861	0.65049	0.25769	0.39339	0.34701	1.8943	0.029094	1.8894	0.02942	0.029094	0.463165835
NA	ENSG00000272308	1.894	0.029113	1.894	0.029113	1.894	0.029113	1.894	0.029113	0.029113	0.463316946
NA	ENSG00000254718	1.7036	0.044229	0.86141	0.19451	1.893	0.029178	1.893	0.029178	0.029178	0.464102804
NA	ENSG00000229832	1.8929	0.029186	1.8929	0.029186	1.8929	0.029186	1.8929	0.029186	0.029186	0.464102804
RNA5SP126	ENSG00000201962	1.8928	0.029194	0.96118	0.16823	0.27705	0.39087	0.13489	0.44635	0.029194	0.464102804
DEGS2	ENSG00000168350	1.407	0.07971	1.407	0.07971	1.407	0.07971	1.8926	0.029203	0.029203	0.464102804
C9orf106	ENSG00000179082	-1.8894	0.97058	1.8925	0.02921	-1.0654	0.85665	-1.7342	0.95856	0.02921	0.464102804

FAT4	ENSG00000196159	1.8924	0.029222	1.6716	0.047298	1.8065	0.035423	0.93631	0.17456	0.029222	0.464142132
MYOM1	ENSG00000101605	0.60257	0.2734	0.81718	0.20691	1.8921	0.029242	0.49074	0.31181	0.029242	0.464308459
NA	ENSG00000271314	-1.1513	0.8752	0.21339	0.41551	1.1713	0.12074	1.8918	0.029256	0.029256	0.46437944
BCL2L14	ENSG00000121380	1.8915	0.029281	-0.34769	0.63596	0.98873	0.1614	1.231	0.10917	0.029281	0.46462492
KCNK18	ENSG00000186795	-1.3546	0.91223	1.8913	0.029292	0.4455	0.32798	-0.54736	0.70793	0.029292	0.464648164
EMCN	ENSG00000164035	-0.081438	0.53245	1.6477	0.049712	-0.35004	0.63685	1.8908	0.029325	0.029325	0.464939665
LINC00467	ENSG00000153363	-0.63072	0.73589	0.015709	0.49373	1.8907	0.029334	0.71578	0.23706	0.029334	0.464939665
GSG1	ENSG00000111305	0.64232	0.26033	0.059237	0.47638	-0.097688	0.53891	1.8906	0.029339	0.029339	0.464939665
GBP1P1	ENSG00000225492	0.15018	0.44031	0.92532	0.1774	1.8902	0.029363	1.2945	0.09775	0.029363	0.465115525
SLITRK6	ENSG00000184564	1.6177	0.052864	1.89	0.029381	1.4995	0.066871	-0.13017	0.55178	0.029381	0.465115525
TTC37	ENSG00000198677	1.4022	0.080423	1.4398	0.074955	1.8898	0.02939	1.4119	0.078992	0.02939	0.465115525
NA	ENSG00000249328	1.8898	0.029393	1.095	0.13677	1.8898	0.029393	1.8898	0.029393	0.029393	0.465115525
BNIP3P28	ENSG00000271095	-1.1443	0.87375	-1.3715	0.9149	1.8896	0.029403	-0.22279	0.58815	0.029403	0.465115525
NA	ENSG00000268628	1.0277	0.15204	1.8895	0.029412	1.0277	0.15204	1.0277	0.15204	0.029412	0.465115525
NA	ENSG00000273211	1.8894	0.029419	1.8894	0.029419	1.8894	0.029419	1.8894	0.029419	0.029419	0.465115525
KDM3A	ENSG00000115548	0.11336	0.45487	1.1308	0.12907	1.8893	0.029427	0.44673	0.32753	0.029427	0.465115525
NA	ENSG00000250166	1.3688	0.08553	1.8891	0.029436	1.3688	0.08553	1.3688	0.08553	0.029436	0.465115525
RNU6ATAC7P	ENSG00000221038	1.8886	0.02947	1.8886	0.02947	1.8886	0.02947	0.08421	0.46644	0.02947	0.465261861
CACNA1C-IT2	ENSG00000256257	1.8886	0.029473	-0.22205	0.58786	0.21862	0.41347	1.655	0.048958	0.029473	0.465261861
SDK1-AS1	ENSG00000236708	0.32765	0.37159	-0.90033	0.81603	-1.7188	0.95717	1.8886	0.029475	0.029475	0.465261861
NA	ENSG00000269296	1.8884	0.029489	-0.73734	0.76954	-0.73734	0.76954	-0.73734	0.76954	0.029489	0.465261861
RN7SL300P	ENSG00000265813	-0.81881	0.79355	-0.54074	0.70565	1.8883	0.029493	-0.54074	0.70565	0.029493	0.465261861
BAIAP2L2	ENSG00000128298	1.8881	0.02951	1.2206	0.11112	1.495	0.067455	0.39403	0.34678	0.02951	0.465379385
CHCHD4P2	ENSG00000237360	1.1011	0.13544	1.8869	0.029587	1.1011	0.13544	-0.23026	0.59106	0.029587	0.46644274
LINC01994	ENSG00000241098	-1.5485	0.93925	0.61212	0.27023	1.8867	0.0296	-1.5485	0.93925	0.0296	0.466496766
CFHR3	ENSG00000116785	1.8863	0.02963	0.96697	0.16678	-0.27372	0.60785	0.25354	0.39993	0.02963	0.466602342
NA	ENSG00000230199	0.84204	0.19988	1.8861	0.029637	0.032111	0.48719	0.19919	0.42106	0.029637	0.466602342
NA	ENSG00000231867	1.8861	0.029641	1.8861	0.029641	1.8861	0.029641	1.8861	0.029641	0.029641	0.466602342
SEC11A	ENSG00000140612	1.886	0.029645	1.7405	0.040885	1.7405	0.040885	1.7405	0.040885	0.029645	0.466602342
NA	ENSG00000234949	1.5718	0.058002	1.7015	0.044422	1.8858	0.029658	1.1807	0.11886	0.029658	0.466606654
RN7SL470P	ENSG00000263669	1.8857	0.02967	1.8857	0.02967	1.8857	0.02967	1.8857	0.02967	0.02967	0.466606654

NA	ENSG00000261744	1.6409	0.050405	0.21761	0.41387	1.4918	0.067878	1.8856	0.029674	0.029674	0.466606654
SYTL1	ENSG00000142765	1.8855	0.029684	1.6736	0.047104	1.348	0.088831	1.1924	0.11655	0.029684	0.466613329
PIGN	ENSG00000197563	0.13804	0.4451	-0.19034	0.57548	-0.1788	0.57095	1.8852	0.029704	0.029704	0.466630947
NA	ENSG00000267756	1.0041	0.15766	0.042603	0.48301	1.8851	0.029707	-0.057566	0.52295	0.029707	0.466630947
PHLDB2	ENSG00000144824	1.8849	0.029723	1.2715	0.10178	1.7568	0.039475	1.1976	0.11553	0.029723	0.466630947
NA	ENSG00000264515	-1.0411	0.85109	-0.24875	0.59822	0.8803	0.18935	1.8848	0.029727	0.029727	0.466630947
RNU6-708P	ENSG00000206944	1.8847	0.029733	1.8847	0.029733	1.8847	0.029733	1.8847	0.029733	0.029733	0.466630947
RNU6-217P	ENSG00000206891	0.45014	0.3263	-0.42502	0.66459	0.82788	0.20387	1.8845	0.029749	0.029749	0.466731735
TAF5L	ENSG00000135801	1.884	0.029781	-0.22824	0.59027	0.10509	0.45815	0.17691	0.42979	0.029781	0.466926829
NA	ENSG00000253273	1.8837	0.029802	1.8837	0.029802	1.8837	0.029802	1.8837	0.029802	0.029802	0.466926829
VN1R13P	ENSG00000216762	1.8836	0.02981	0.507	0.30608	-0.50513	0.69327	-1.5315	0.93718	0.02981	0.466926829
ABRAXAS1	ENSG00000163322	0.60116	0.27387	1.8835	0.029814	1.8835	0.029814	1.7935	0.036446	0.029814	0.466926829
MCHR2	ENSG00000152034	0.32744	0.37167	0.38537	0.34998	0.59502	0.27592	1.8835	0.029816	0.029816	0.466926829
NA	ENSG00000249748	0.85281	0.19688	-0.41234	0.65996	1.8834	0.02982	-1.2405	0.89261	0.02982	0.466926829
ZNF863P	ENSG00000236538	1.8833	0.029832	1.8833	0.029832	1.8833	0.029832	1.8833	0.029832	0.029832	0.466926829
COTL1	ENSG00000103187	1.8832	0.029839	-0.90453	0.81714	-0.0416	0.51659	-2.0143	0.97801	0.029839	0.466926829
TRAPPC2B	ENSG00000256060	1.8829	0.029857	1.8829	0.029857	1.8829	0.029857	1.8829	0.029857	0.029857	0.466926829
MAN2A2	ENSG00000196547	1.8828	0.029867	1.8828	0.029867	1.7924	0.036536	1.2276	0.1098	0.029867	0.466926829
NA	ENSG00000249763	-1.3789	0.91603	-0.48451	0.68599	-0.95282	0.82966	1.8828	0.029867	0.029867	0.466926829
PHBP7	ENSG00000213711	-0.87548	0.80934	-0.87548	0.80934	-0.87548	0.80934	1.8826	0.029877	0.029877	0.466926829
ZGPAT	ENSG00000197114	0.71708	0.23666	0.71708	0.23666	1.6541	0.04905	1.8825	0.029886	0.029886	0.466926829
DYNC2LI1	ENSG00000138036	1.882	0.029916	1.882	0.029916	1.882	0.029916	1.882	0.029916	0.029916	0.467245731
RNA5SP466	ENSG00000252546	1.8817	0.029937	1.8817	0.029937	1.8817	0.029937	1.8817	0.029937	0.029937	0.467423906
LINC02722	ENSG00000255169	0.61759	0.26842	0.61759	0.26842	1.8815	0.029954	0.61759	0.26842	0.029954	0.467539532
GRPEL1	ENSG00000109519	0.15138	0.43984	1.8746	0.030425	1.8811	0.029977	0.92959	0.17629	0.029977	0.467590732
NA	ENSG00000225044	1.8806	0.030013	0.045168	0.48199	-0.78299	0.78318	-0.78299	0.78318	0.030013	0.467590732
CELSR3	ENSG00000008300	1.8805	0.030023	1.6321	0.051326	1.4072	0.079689	1.3382	0.090416	0.030023	0.467590732
SUCLA2P3	ENSG00000229593	1.205	0.11411	1.5296	0.063061	1.5296	0.063061	1.8805	0.030023	0.030023	0.467590732
CAPNS1P1	ENSG00000215874	1.8804	0.030027	1.8804	0.030027	0.77068	0.22045	1.2247	0.11034	0.030027	0.467590732
CACNA1C-AS3	ENSG00000256769	1.8803	0.030032	1.8803	0.030032	1.8803	0.030032	1.8803	0.030032	0.030032	0.467590732
NA	ENSG00000235815	-0.020384	0.50813	-0.23375	0.59241	1.1971	0.11563	1.8801	0.030047	0.030047	0.467590732

NA	ENSG00000266573	-1.3063	0.90428	-0.37427	0.6459	1.88	0.030051	-0.79565	0.78688	0.030051	0.467590732
SNORD18C	ENSG00000199574	1.8797	0.030071	1.8797	0.030071	1.8797	0.030071	1.8797	0.030071	0.030071	0.467590732
RN7SL738P	ENSG00000243339	1.8797	0.030077	1.8797	0.030077	1.8797	0.030077	1.8797	0.030077	0.030077	0.467590732
NA	ENSG00000259033	0.78761	0.21546	1.8796	0.030079	0.78761	0.21546	0.78761	0.21546	0.030079	0.467590732
RNU6-1008P	ENSG00000206887	1.8796	0.030084	1.8796	0.030084	1.8796	0.030084	1.8796	0.030084	0.030084	0.467590732
PLXDC1	ENSG00000161381	1.6921	0.045309	1.8792	0.030109	1.2135	0.11246	0.042105	0.48321	0.030109	0.467590732
BNC2	ENSG00000173068	1.1795	0.11909	0.82449	0.20483	1.8792	0.03011	0.94001	0.17361	0.03011	0.467590732
MIR4746	ENSG00000266437	1.8792	0.030112	1.8792	0.030112	1.8792	0.030112	1.8792	0.030112	0.030112	0.467590732
THAP12P7	ENSG00000253919	1.2557	0.10461	1.8791	0.030113	0.58792	0.27829	0.13425	0.4466	0.030113	0.467590732
KLC4	ENSG00000137171	1.425	0.077074	1.879	0.030121	0.732	0.23208	-0.051054	0.52036	0.030121	0.467590732
TENM2	ENSG00000145934	1.8789	0.03013	1.2798	0.10031	0.42023	0.33716	0.84171	0.19997	0.03013	0.467590732
NA	ENSG00000273965	0.96682	0.16682	1.8781	0.030181	1.8781	0.030181	1.1829	0.11843	0.030181	0.468099574
BASP1-AS1	ENSG00000215196	1.8781	0.030182	0.6224	0.26684	0.48289	0.31459	0.08255	0.4671	0.030182	0.468099574
ADSS1	ENSG00000185100	1.0718	0.14191	1.8777	0.030214	1.5298	0.063033	-0.65122	0.74255	0.030214	0.468446777
RNU4-23P	ENSG00000199709	1.8775	0.030225	1.8775	0.030225	1.8775	0.030225	1.8775	0.030225	0.030225	0.468468273
INTS11	ENSG00000127054	1.8772	0.030246	1.778	0.037705	1.7635	0.038908	1.7754	0.037918	0.030246	0.468563648
VCP	ENSG00000165280	0.23686	0.40638	-0.68314	0.75274	-0.45603	0.67581	1.8771	0.030253	0.030253	0.468563648
NA	ENSG00000228108	-0.42527	0.66468	1.877	0.03026	0.49239	0.31122	1.586	0.056368	0.03026	0.468563648
NICN1	ENSG00000145029	1.8763	0.030306	1.8763	0.030306	1.8763	0.030306	1.8763	0.030306	0.030306	0.468933725
FAR1P1	ENSG00000235879	-0.5372	0.70444	1.8758	0.030339	-0.5372	0.70444	-0.5372	0.70444	0.030339	0.468933725
ATP1A4	ENSG00000132681	1.8758	0.030344	1.6255	0.052023	1.1436	0.12639	1.5978	0.05504	0.030344	0.468933725
NA	ENSG00000235038	-0.0045653	0.50182	1.8755	0.030363	-0.35865	0.64007	-0.0045653	0.50182	0.030363	0.468933725
ARHGAP15-AS1	ENSG00000231758	1.2437	0.1068	1.854	0.03187	1.8754	0.030372	-0.0017194	0.50069	0.030372	0.468933725
NA	ENSG00000199332	-0.026661	0.51063	1.8753	0.030374	1.0871	0.1385	0.70023	0.24189	0.030374	0.468933725
CADPS2	ENSG00000081803	1.8753	0.030377	0.37024	0.3556	0.91367	0.18044	0.13404	0.44668	0.030377	0.468933725
PAX8-AS1	ENSG00000189223	1.8661	0.03101	1.8752	0.030381	1.8661	0.03101	1.8661	0.03101	0.030381	0.468933725
APOC3	ENSG00000110245	1.5504	0.060525	1.5504	0.060525	1.0093	0.15642	1.8751	0.030391	0.030391	0.468933725
NA	ENSG00000233021	1.7337	0.041482	1.875	0.030397	1.875	0.030397	1.875	0.030397	0.030397	0.468933725
CAMTA1-IT1	ENSG00000237402	0.49964	0.30867	0.49119	0.31165	1.8749	0.030401	0.072466	0.47112	0.030401	0.468933725
BEND7	ENSG00000165626	1.8748	0.030408	1.8748	0.030408	1.7491	0.04014	1.6007	0.054717	0.030408	0.468933725
BUD31	ENSG00000106245	1.8748	0.030409	1.8748	0.030409	0.94932	0.17123	1.8392	0.03294	0.030409	0.468933725

MIR648	ENSG00000207780	1.3668	0.085837	1.3668	0.085837	1.8744	0.030439	1.3668	0.085837	0.030439	0.469205122
CYP2AB1P	ENSG00000233441	0.29034	0.38578	1.6973	0.044823	1.874	0.030463	0.24727	0.40235	0.030463	0.469205122
LRRC3	ENSG00000160233	1.874	0.030468	0.87758	0.19009	1.5793	0.057137	-0.23174	0.59163	0.030468	0.469205122
CYP3A54P	ENSG00000261809	-1.3466	0.91095	1.2771	0.10078	1.8739	0.030475	0.21403	0.41526	0.030475	0.469205122
NA	ENSG00000259390	1.1034	0.13493	1.43	0.076352	1.8737	0.030489	0.21257	0.41583	0.030489	0.469205122
ATP5MF-PTCD1	ENSG00000248919	1.8736	0.030494	1.8736	0.030494	1.8736	0.030494	1.8736	0.030494	0.030494	0.469205122
NA	ENSG00000278900	-0.64734	0.7413	1.8736	0.030494	1.842	0.032735	1.0043	0.15761	0.030494	0.469205122
MRGPRX9P	ENSG00000255424	1.8733	0.030511	1.8733	0.030511	1.8733	0.030511	1.8733	0.030511	0.030511	0.469231401
ST3GAL2	ENSG00000157350	1.8731	0.030527	1.7514	0.039935	1.8255	0.03396	0.58856	0.27808	0.030527	0.469231401
SPAG5-AS1	ENSG00000227543	1.8729	0.030543	1.8729	0.030543	1.8729	0.030543	1.8729	0.030543	0.030543	0.469231401
NA	ENSG00000263635	1.2139	0.11239	1.8728	0.030549	1.8728	0.030549	1.8465	0.032412	0.030549	0.469231401
CENPT	ENSG00000102901	-0.70319	0.75903	-0.70319	0.75903	-0.54291	0.70641	1.8726	0.030563	0.030563	0.469231401
NA	ENSG00000203286	0.34573	0.36477	0.97704	0.16428	0.782	0.21711	1.8725	0.030569	0.030569	0.469231401
KNOP1P2	ENSG00000270429	1.8724	0.030575	1.8724	0.030575	1.8724	0.030575	1.8724	0.030575	0.030575	0.469231401
PIK3AP1	ENSG00000155629	-1.3114	0.90514	-1.3114	0.90514	-1.3114	0.90514	1.8724	0.030579	0.030579	0.469231401
NA	ENSG00000218499	1.8601	0.031436	-0.79004	0.78525	1.8722	0.03059	0.3818	0.3513	0.03059	0.469231401
LYAR	ENSG00000145220	1.8722	0.030592	0.9342	0.1751	0.65223	0.25713	1.8722	0.030592	0.030592	0.469231401
NA	ENSG00000237090	1.8718	0.030619	1.8718	0.030619	1.8718	0.030619	1.8718	0.030619	0.030619	0.469376674
AKR1B15	ENSG00000227471	0.0020438	0.49918	1.8716	0.030633	0.37851	0.35252	-0.84274	0.80031	0.030633	0.469376674
NBPF6	ENSG00000186086	0.55205	0.29046	1.8715	0.030639	0.54122	0.29418	0.24112	0.40473	0.030639	0.469376674
B3GALT1	ENSG00000172318	1.8715	0.03064	-0.26392	0.60408	0.010578	0.49578	-1.0923	0.86265	0.03064	0.469376674
CRPPA-AS1	ENSG00000229688	-1.3027	0.90365	1.8712	0.030662	-1.3027	0.90365	-1.3027	0.90365	0.030662	0.469479793
SAP30BP	ENSG00000161526	1.8711	0.030666	1.8711	0.030666	1.8711	0.030666	1.7394	0.040984	0.030666	0.469479793
NA	ENSG00000227748	1.8709	0.030681	1.616	0.05305	1.5463	0.061014	1.3553	0.087667	0.030681	0.469561913
NA	ENSG00000234630	1.8707	0.030696	1.8707	0.030696	0.61377	0.26968	-0.15292	0.56077	0.030696	0.469643981
SFTPA2	ENSG00000185303	0.67902	0.24856	1.8705	0.03071	0.97131	0.1657	0.83994	0.20047	0.03071	0.469710703
RN7SL601P	ENSG00000243346	0.096153	0.4617	0.56091	0.28743	-0.42145	0.66329	1.8702	0.030726	0.030726	0.469721738
NPIPB8	ENSG00000255524	0.74877	0.227	-0.63613	0.73765	1.8702	0.03073	0.18668	0.42596	0.03073	0.469721738
NEDD8-MDP1	ENSG00000255526	1.8694	0.030782	1.8694	0.030782	1.8694	0.030782	1.8694	0.030782	0.030782	0.470369037
ALG14-AS1	ENSG00000230427	0.74132	0.22925	0.84154	0.20002	0.33175	0.37004	1.8692	0.030794	0.030794	0.470392121
TRAPPC11	ENSG00000168538	1.2377	0.10792	1.8689	0.030817	0.50403	0.30712	0.54706	0.29217	0.030817	0.470392121

TNPO1P3	ENSG00000229586	1.8688	0.030828	1.1904	0.11695	1.4078	0.079589	-0.70669	0.76012	0.030828	0.470392121
SLC6A16	ENSG00000063127	1.8687	0.030834	0.89784	0.18464	1.8687	0.030834	1.8687	0.030834	0.030834	0.470392121
NA	ENSG00000244345	-0.65224	0.74288	-1.4451	0.92578	-0.9388	0.82608	1.8686	0.030841	0.030841	0.470392121
NA	ENSG00000231054	1.8682	0.030867	1.8682	0.030867	1.8682	0.030867	1.8682	0.030867	0.030867	0.470392121
DSCAM	ENSG00000171587	0.66859	0.25188	1.8682	0.03087	1.4701	0.070772	0.58379	0.27968	0.03087	0.470392121
HHIP	ENSG00000164161	1.8679	0.030886	-0.15503	0.5616	-0.64574	0.74077	1.4304	0.076298	0.030886	0.470392121
PLEKHO1	ENSG00000023902	1.5314	0.062831	1.4682	0.071026	1.4682	0.071026	1.8679	0.030887	0.030887	0.470392121
TXNIP	ENSG00000265972	1.8351	0.033247	1.7098	0.043649	1.8677	0.0309	1.3832	0.0833	0.0309	0.470392121
SNORD115-1	ENSG00000201831	1.8677	0.030901	1.8677	0.030901	1.8677	0.030901	1.8677	0.030901	0.030901	0.470392121
DMAC1P1	ENSG00000270814	1.8677	0.030901	1.8677	0.030901	1.8677	0.030901	1.8677	0.030901	0.030901	0.470392121
TENM4	ENSG00000149256	0.71927	0.23599	0.067266	0.47319	0.65256	0.25702	1.8676	0.030909	0.030909	0.470392121
DDX19B	ENSG00000157349	0.53492	0.29635	1.8666	0.030981	1.0955	0.13666	1.0955	0.13666	0.030981	0.4705357
ZNF548	ENSG00000188785	0.64949	0.25801	1.2987	0.097019	1.8665	0.030985	1.8665	0.030985	0.030985	0.4705357
TRBV7-7	ENSG00000253291	1.8665	0.030989	-0.55866	0.7118	1.4337	0.075833	1.4337	0.075833	0.030989	0.4705357
SNAI3-AS1	ENSG00000260630	1.8664	0.030992	-0.36526	0.64254	0.91725	0.17951	0.96495	0.16728	0.030992	0.4705357
LINC02837	ENSG00000264464	0.55307	0.29011	0.55307	0.29011	1.1769	0.11961	1.8662	0.031008	0.031008	0.4705357
NA	ENSG00000225144	1.7992	0.035995	0.186	0.42622	1.7992	0.035995	1.8661	0.031015	0.031015	0.4705357
RPL7P1	ENSG00000214485	-0.23593	0.59326	1.866	0.031018	-0.23593	0.59326	-0.23593	0.59326	0.031018	0.4705357
MIR4324	ENSG00000265407	1.8658	0.031032	1.8658	0.031032	1.8658	0.031032	1.8658	0.031032	0.031032	0.4705357
RNU6-928P	ENSG00000252685	1.8655	0.031059	1.8655	0.031059	1.8655	0.031059	1.8655	0.031059	0.031059	0.4705357
NA	ENSG00000266932	1.8655	0.031059	0.46152	0.32221	-0.252	0.59948	-0.252	0.59948	0.031059	0.4705357
DPYSL2	ENSG00000092964	0.86185	0.19439	0.96863	0.16636	1.3869	0.082735	1.8652	0.031077	0.031077	0.4705357
ZNF543	ENSG00000178229	0.55618	0.28904	1.3635	0.086355	1.3635	0.086355	1.8651	0.031085	0.031085	0.4705357
LINC02492	ENSG00000250590	1.865	0.031089	-0.76928	0.77914	0.11067	0.45594	-0.40976	0.65901	0.031089	0.4705357
NA	ENSG00000234735	1.8648	0.031106	1.8648	0.031106	1.8648	0.031106	1.8648	0.031106	0.031106	0.4705357
RPS19	ENSG00000105372	1.8647	0.031109	1.8647	0.031109	1.2103	0.11307	1.8647	0.031109	0.031109	0.4705357
RER1	ENSG00000157916	0.42888	0.33401	1.8647	0.031115	0.24961	0.40144	0.24961	0.40144	0.031115	0.4705357
NA	ENSG00000228120	0.93422	0.17509	1.1889	0.11724	0.39179	0.34761	1.8644	0.031134	0.031134	0.4705357
PIP5K1A	ENSG00000143398	1.6703	0.047433	1.8643	0.031141	1.4004	0.080692	0.90814	0.1819	0.031141	0.4705357
LINC01104	ENSG00000232084	1.5554	0.059922	1.8558	0.031744	1.8642	0.031148	0.97623	0.16448	0.031148	0.4705357
FAM187B	ENSG00000177558	1.8641	0.031153	1.8641	0.031153	1.8641	0.031153	1.8641	0.031153	0.031153	0.4705357

NA	ENSG00000260051	1.8637	0.031179	1.8637	0.031179	1.8637	0.031179	1.8637	0.031179	0.031179	0.4705357
RPL15P3	ENSG00000212802	-0.74321	0.77132	-0.9445	0.82754	1.0859	0.13877	1.8635	0.031193	0.031193	0.4705357
NA	ENSG00000271272	1.8635	0.031197	1.0699	0.14233	1.8635	0.031197	1.448	0.073814	0.031197	0.4705357
NA	ENSG00000259187	1.0482	0.14726	1.0482	0.14726	-1.0344	0.84953	1.8634	0.031202	0.031202	0.4705357
RNA5SP472	ENSG00000200738	0.26539	0.39536	1.8634	0.031204	-0.90564	0.81744	-0.90564	0.81744	0.031204	0.4705357
GPR55	ENSG00000135898	1.8633	0.031209	0.47019	0.31911	-1.1854	0.88207	-1.0808	0.86012	0.031209	0.4705357
RNU6-406P	ENSG00000252279	0.79899	0.21215	-0.076623	0.53054	-0.14493	0.55762	1.863	0.031232	0.031232	0.4705357
DCST1	ENSG00000163357	0.48806	0.31275	0.48806	0.31275	0.48806	0.31275	1.8628	0.031244	0.031244	0.4705357
NA	ENSG00000264422	1.8627	0.031251	0.19871	0.42124	-1.8083	0.96472	1.1394	0.12727	0.031251	0.4705357
LRRC3-DT	ENSG00000229356	1.8625	0.031269	1.3429	0.089659	1.8625	0.031269	1.8625	0.031269	0.031269	0.4705357
RNA5SP305	ENSG00000199535	1.8622	0.031288	1.3279	0.092098	1.8622	0.031288	1.8622	0.031288	0.031288	0.4705357
CBARP	ENSG00000099625	-0.18565	0.57364	-1.562	0.94085	-0.20128	0.57976	1.8621	0.031295	0.031295	0.4705357
NA	ENSG00000231702	-0.47523	0.68269	1.8621	0.031297	-0.47523	0.68269	-0.47523	0.68269	0.031297	0.4705357
RNU1-77P	ENSG00000212170	1.862	0.0313	0.83501	0.20186	1.1475	0.12559	0.83501	0.20186	0.0313	0.4705357
EFHB	ENSG00000163576	1.744	0.040577	1.0658	0.14325	1.8617	0.031323	-0.28872	0.6136	0.031323	0.4705357
RPS3AP14	ENSG00000242229	0.57089	0.28404	0.067572	0.47306	1.8612	0.031358	0.89308	0.18591	0.031358	0.4705357
NA	ENSG00000227912	0.25493	0.39939	0.25493	0.39939	0.25493	0.39939	1.8609	0.031377	0.031377	0.4705357
RPS4XP2	ENSG00000226948	-0.62489	0.73398	1.8608	0.031387	-0.62489	0.73398	0.36821	0.35636	0.031387	0.4705357
RNU11-3P	ENSG00000212413	0.38076	0.35169	1.8607	0.031394	1.8607	0.031394	1.8607	0.031394	0.031394	0.4705357
GUCA2A	ENSG00000197273	1.8605	0.031407	0.69407	0.24382	0.8226	0.20537	1.3131	0.094571	0.031407	0.4705357
NA	ENSG00000202224	1.8603	0.031418	1.8603	0.031418	1.3085	0.09535	-0.28298	0.6114	0.031418	0.4705357
RN7SKP247	ENSG00000223026	0.87919	0.18965	1.3879	0.082588	1.6856	0.045933	1.8603	0.03142	0.03142	0.4705357
RPL17P45	ENSG00000267664	1.8602	0.031428	1.8602	0.031428	1.8602	0.031428	1.8602	0.031428	0.031428	0.4705357
LINC01433	ENSG00000230176	1.86	0.031443	1.86	0.031443	1.86	0.031443	1.86	0.031443	0.031443	0.4705357
ZDBF2	ENSG00000204186	0.68217	0.24756	1.8599	0.031453	1.8599	0.031453	1.8599	0.031453	0.031453	0.4705357
RPS15AP3	ENSG00000214903	1.8595	0.031481	1.1179	0.1318	1.8595	0.031481	1.8595	0.031481	0.031481	0.4705357
LINC01956	ENSG00000258910	0.46875	0.31962	-0.61647	0.73121	1.8594	0.031483	1.8594	0.031483	0.031483	0.4705357
BBOF1	ENSG00000119636	0.77099	0.22036	1.0867	0.13858	1.6833	0.046156	1.8592	0.031498	0.031498	0.4705357
ARHGAP22	ENSG00000128805	-0.62503	0.73402	-0.53844	0.70486	-0.77233	0.78004	1.8587	0.031532	0.031532	0.4705357
LEMD1-DT	ENSG00000224717	1.8585	0.03155	1.8585	0.03155	1.8585	0.03155	1.8585	0.03155	0.03155	0.4705357
NA	ENSG00000237844	0.69982	0.24202	0.34563	0.36481	1.8584	0.031554	-0.1506	0.55985	0.031554	0.4705357

RPS3AP23	ENSG00000218351	0.81529	0.20745	1.8584	0.031555	0.81529	0.20745	0.81529	0.20745	0.031555	0.4705357
TWF2	ENSG00000247596	1.3157	0.094132	1.8583	0.031564	1.8583	0.031564	1.8583	0.031564	0.031564	0.4705357
NA	ENSG00000225936	1.8581	0.031579	1.8581	0.031579	1.8581	0.031579	1.8581	0.031579	0.031579	0.4705357
ZNF791	ENSG00000173875	1.8578	0.031599	0.2159	0.41453	0.2159	0.41453	-0.0090268	0.5036	0.031599	0.4705357
SLC25A20	ENSG00000178537	1.5082	0.065758	1.8578	0.031599	0.55942	0.28794	0.90603	0.18246	0.031599	0.4705357
SDAD1P1	ENSG00000228451	1.8577	0.031603	1.1752	0.11996	1.1752	0.11996	1.1752	0.11996	0.031603	0.4705357
NA	ENSG00000258989	1.8577	0.031605	1.8577	0.031605	1.8577	0.031605	1.8577	0.031605	0.031605	0.4705357
NA	ENSG00000236957	1.8576	0.03161	-0.026625	0.51062	-0.80263	0.78891	1.8576	0.03161	0.03161	0.4705357
NA	ENSG00000252399	0.98373	0.16262	0.9346	0.175	1.8575	0.03162	0.80924	0.20919	0.03162	0.4705357
MYF5	ENSG00000111049	1.8574	0.031629	0.39883	0.34501	0.39883	0.34501	0.39883	0.34501	0.031629	0.4705357
CENPBD1	ENSG00000177946	1.8573	0.031636	1.8573	0.031636	1.8573	0.031636	1.8573	0.031636	0.031636	0.4705357
HHIPL2	ENSG00000143512	1.8571	0.03165	-0.070266	0.52801	1.2178	0.11165	-1.2094	0.88675	0.03165	0.4705357
MBD3L2B	ENSG00000196589	-1.2015	0.88522	-1.2779	0.89936	0.32959	0.37085	1.8569	0.031665	0.031665	0.4705357
RBM26-AS1	ENSG00000227354	1.8567	0.031675	1.4703	0.070736	1.6181	0.052822	1.667	0.047753	0.031675	0.4705357
RAC1P6	ENSG00000226962	1.8565	0.031688	-0.092373	0.5368	-0.092373	0.5368	-0.2403	0.59495	0.031688	0.4705357
MRPS7	ENSG00000125445	1.3265	0.092333	1.3265	0.092333	1.8564	0.031698	0.71255	0.23806	0.031698	0.4705357
H2AC6	ENSG00000180573	1.8564	0.031702	1.0243	0.15285	1.0243	0.15285	0.51824	0.30215	0.031702	0.4705357
VPS33B-DT	ENSG00000214432	0.44211	0.32921	0.5278	0.29882	1.6738	0.047081	1.8562	0.031714	0.031714	0.4705357
SOD1P3	ENSG00000253651	1.856	0.031728	1.856	0.031728	1.856	0.031728	1.856	0.031728	0.031728	0.4705357
MYO18B	ENSG00000133454	-1.3896	0.91767	0.77557	0.219	1.8559	0.031733	0.69373	0.24393	0.031733	0.4705357
MTUS2	ENSG00000132938	-0.79786	0.78752	1.8559	0.031735	-0.55427	0.7103	-0.4596	0.6771	0.031735	0.4705357
SLC16A14	ENSG00000163053	1.6192	0.052704	1.8558	0.031743	0.39575	0.34614	1.4354	0.075585	0.031743	0.4705357
NA	ENSG00000275494	1.8557	0.031747	1.8557	0.031747	1.8557	0.031747	1.8557	0.031747	0.031747	0.4705357
NFATC2IP	ENSG00000176953	1.0274	0.15212	1.0996	0.13575	1.8557	0.03175	0.33528	0.36871	0.03175	0.4705357
NA	ENSG00000276259	1.8554	0.031768	1.8554	0.031768	1.8554	0.031768	1.8554	0.031768	0.031768	0.4705357
RPS16P9	ENSG00000242675	0.28483	0.38789	-0.029443	0.51174	1.8553	0.031773	0.28483	0.38789	0.031773	0.4705357
SLC14A2	ENSG00000132874	1.1254	0.13021	1.6761	0.046855	1.6597	0.048492	1.8551	0.031789	0.031789	0.4705357
LINC00160	ENSG00000230978	-0.55591	0.71086	1.8551	0.031794	1.2403	0.10742	1.0301	0.15148	0.031794	0.4705357
NA	ENSG00000226208	1.855	0.031795	1.855	0.031795	1.855	0.031795	1.855	0.031795	0.031795	0.4705357
NA	ENSG00000224326	1.8549	0.031804	1.8549	0.031804	1.4685	0.070991	0.87524	0.19072	0.031804	0.4705357
CHRNB2	ENSG00000160716	0.084852	0.46619	1.8549	0.031806	1.8349	0.033259	1.8453	0.032494	0.031806	0.4705357

RNU6-392P	ENSG00000201796	1.8549	0.031806	0.81965	0.20621	0.81978	0.20617	1.4853	0.068726	0.031806	0.4705357
DNM1	ENSG00000106976	-0.24081	0.59515	1.8544	0.03184	1.0613	0.14427	1.3856	0.082932	0.03184	0.4705357
NA	ENSG00000272970	0.12291	0.45109	0.60999	0.27093	1.8543	0.031847	-1.4578	0.92755	0.031847	0.4705357
S100A4	ENSG00000196154	1.8543	0.03185	1.8543	0.03185	1.8543	0.03185	1.8543	0.03185	0.03185	0.4705357
LINC02005	ENSG00000242741	0.15098	0.43999	-1.2082	0.88651	1.8542	0.031852	1.6536	0.049104	0.031852	0.4705357
BCL2L11	ENSG00000153094	1.8389	0.032967	1.5204	0.064203	-0.066632	0.52656	1.8542	0.031854	0.031854	0.4705357
NA	ENSG00000236304	1.8542	0.031856	1.8542	0.031856	1.8542	0.031856	1.8542	0.031856	0.031856	0.4705357
ERICD	ENSG00000280303	1.8541	0.03186	1.8541	0.03186	1.8541	0.03186	1.8541	0.03186	0.03186	0.4705357
WDR75	ENSG00000115368	0.92445	0.17763	1.0173	0.15451	1.4719	0.070526	1.854	0.031866	0.031866	0.4705357
NA	ENSG00000241961	-0.48612	0.68656	-0.48612	0.68656	-0.19895	0.57885	1.8539	0.031876	0.031876	0.4705357
PYDC1	ENSG00000169900	1.8538	0.031881	1.8538	0.031881	1.2515	0.10538	0.46862	0.31967	0.031881	0.4705357
TRIM72	ENSG00000177238	1.8538	0.031881	0.32502	0.37258	-0.094066	0.53747	1.2519	0.1053	0.031881	0.4705357
NA	ENSG00000283285	1.0975	0.13621	1.0543	0.14587	1.8538	0.031885	1.8538	0.031885	0.031885	0.4705357
HOXB-AS1	ENSG00000230148	1.8538	0.031886	1.8538	0.031886	1.8538	0.031886	1.8538	0.031886	0.031886	0.4705357
NA	ENSG00000270781	1.8537	0.031888	1.8537	0.031888	1.8537	0.031888	1.8537	0.031888	0.031888	0.4705357
LINC02361	ENSG00000256576	-0.75732	0.77557	0.20128	0.42024	1.8537	0.031892	1.8537	0.031892	0.031892	0.4705357
WDR41	ENSG00000164253	1.8535	0.031905	1.5385	0.061966	0.79689	0.21276	0.44217	0.32918	0.031905	0.4705357
SYBU	ENSG00000147642	1.0579	0.14505	1.8535	0.031907	1.507	0.065901	1.526	0.063505	0.031907	0.4705357
NA	ENSG00000253528	1.8535	0.031908	1.8535	0.031908	1.8535	0.031908	1.8535	0.031908	0.031908	0.4705357
BTN2A2	ENSG00000124508	1.7943	0.036387	0.4186	0.33775	0.44691	0.32747	1.8534	0.03191	0.03191	0.4705357
BTN2A3P	ENSG00000124549	1.3842	0.083154	1.8534	0.031913	1.5561	0.05984	1.442	0.074647	0.031913	0.4705357
NA	ENSG00000266872	1.8531	0.031933	1.8531	0.031933	1.8531	0.031933	1.8531	0.031933	0.031933	0.470546302
TET2	ENSG00000168769	0.9949	0.15989	0.56888	0.28472	0.87574	0.19059	1.853	0.031943	0.031943	0.470546302
TXN2P1	ENSG00000213540	1.8527	0.031961	1.8527	0.031961	1.8527	0.031961	0.92287	0.17804	0.031961	0.470546302
NA	ENSG00000269889	1.8527	0.031961	1.8527	0.031961	1.8527	0.031961	1.8527	0.031961	0.031961	0.470546302
NA	ENSG00000267620	1.8527	0.031962	1.8527	0.031962	1.8527	0.031962	1.8527	0.031962	0.031962	0.470546302
BUB1	ENSG00000169679	1.8523	0.031992	0.62044	0.26748	0.43416	0.33208	0.11473	0.45433	0.031992	0.470587121
NA	ENSG00000271858	1.8523	0.031994	1.8523	0.031994	1.8523	0.031994	1.8523	0.031994	0.031994	0.470587121
RPS29P30	ENSG00000268243	1.8519	0.032022	1.8519	0.032022	1.8519	0.032022	1.8519	0.032022	0.032022	0.470587121
JARID2	ENSG00000008083	1.6367	0.050851	1.2019	0.1147	0.29545	0.38383	1.8517	0.032037	0.032037	0.470587121
NA	ENSG00000239142	0.46727	0.32015	1.8516	0.032042	1.5064	0.065988	1.5064	0.065988	0.032042	0.470587121

NA	ENSG00000227519	1.8516	0.032043	1.8516	0.032043	1.8516	0.032043	1.8516	0.032043	0.032043	0.470587121
CHRM1	ENSG00000168539	-0.50697	0.69391	-0.034821	0.51389	1.8515	0.032047	-0.10361	0.54126	0.032047	0.470587121
WDR59	ENSG00000103091	0.29513	0.38395	0.29513	0.38395	1.8513	0.032061	0.98046	0.16343	0.032061	0.470587121
SALL1	ENSG00000103449	1.6546	0.049006	1.4542	0.072943	0.36666	0.35694	1.8513	0.032064	0.032064	0.470587121
NA	ENSG00000206728	1.8512	0.032068	1.8512	0.032068	1.8512	0.032068	1.8512	0.032068	0.032068	0.470587121
NA	ENSG00000237297	1.3317	0.091475	1.8512	0.032071	-0.40881	0.65866	0.5095	0.3052	0.032071	0.470587121
NA	ENSG00000267782	1.8506	0.032111	1.8506	0.032111	1.8506	0.032111	1.8506	0.032111	0.032111	0.470919798
NA	ENSG00000270577	1.8506	0.032113	1.8506	0.032113	1.8506	0.032113	1.8506	0.032113	0.032113	0.470919798
ZC3H12C	ENSG00000149289	1.5513	0.060419	1.8504	0.032131	1.5513	0.060419	1.5857	0.056404	0.032131	0.470929651
ARMC4	ENSG00000169126	0.089412	0.46438	-0.44573	0.6721	1.8503	0.032133	-0.96517	0.83277	0.032133	0.470929651
TRAJ29	ENSG00000211860	1.8497	0.032176	1.8497	0.032176	1.8497	0.032176	1.8497	0.032176	0.032176	0.470988397
TRAJ28	ENSG00000211861	1.8497	0.032176	1.8497	0.032176	1.8497	0.032176	1.8497	0.032176	0.032176	0.470988397
NA	ENSG00000266450	-0.70171	0.75857	-1.3765	0.91567	1.8496	0.032188	-1.3765	0.91567	0.032188	0.470988397
OR9I2P	ENSG00000186508	1.8495	0.032192	-0.056217	0.52242	1.1718	0.12064	1.1718	0.12064	0.032192	0.470988397
NA	ENSG00000255317	1.8495	0.032194	-0.58962	0.72228	0.84789	0.19825	0.86959	0.19226	0.032194	0.470988397
SEPTIN2	ENSG00000168385	1.8495	0.032195	1.2379	0.10787	1.3563	0.087498	1.3747	0.084618	0.032195	0.470988397
SNORA12	ENSG00000212464	1.8485	0.032266	1.8485	0.032266	1.8485	0.032266	1.8485	0.032266	0.032266	0.471885408
NA	ENSG00000267226	-0.41071	0.65936	1.8482	0.032284	-0.41071	0.65936	-0.41071	0.65936	0.032284	0.472006997
STN1	ENSG00000107960	1.523	0.063873	1.8481	0.032297	-0.10271	0.5409	1.1694	0.12112	0.032297	0.472055432
LINC02619	ENSG00000249382	-0.58437	0.72051	-0.58437	0.72051	-0.58437	0.72051	1.8478	0.032316	0.032316	0.472144737
LINC00114	ENSG00000223806	0.87121	0.19182	1.8477	0.032323	1.2647	0.10299	1.6613	0.048326	0.032323	0.472144737
RNU6-75P	ENSG00000251753	1.8475	0.032338	1.8475	0.032338	1.8475	0.032338	1.8475	0.032338	0.032338	0.472144737
NUP133	ENSG00000069248	-1.3539	0.91211	-0.36716	0.64325	-0.8038	0.78924	1.8473	0.032354	0.032354	0.472144737
UBE3B	ENSG00000151148	0.59025	0.27751	1.1153	0.13235	0.64069	0.26086	1.8472	0.032361	0.032361	0.472144737
TNC	ENSG00000041982	1.847	0.03237	-0.43058	0.66661	0.50165	0.30796	-0.088382	0.53521	0.03237	0.472144737
NA	ENSG00000267360	1.8469	0.032383	1.8469	0.032383	1.8469	0.032383	1.8469	0.032383	0.032383	0.472144737
TFRC	ENSG00000072274	0.46104	0.32238	1.8467	0.032395	-0.74659	0.77234	0.060493	0.47588	0.032395	0.472144737
NA	ENSG00000249317	1.8466	0.032399	1.8466	0.032399	0.47844	0.31617	1.8466	0.032399	0.032399	0.472144737
CLSTN1	ENSG00000171603	1.8466	0.0324	1.7407	0.040865	1.7253	0.042237	0.57382	0.28304	0.0324	0.472144737
IPO5	ENSG00000065150	1.0636	0.14375	0.80401	0.2107	0.60557	0.2724	1.8462	0.032432	0.032432	0.472191953
NA	ENSG00000233099	1.8462	0.032434	1.8462	0.032434	1.8462	0.032434	1.8462	0.032434	0.032434	0.472191953

NA	ENSG00000236194	1.8461	0.032441	1.8461	0.032441	1.8461	0.032441	1.8461	0.032441	0.032441	0.472191953
NA	ENSG00000232034	-0.81064	0.79121	1.8461	0.032442	0.29201	0.38514	-0.73988	0.77031	0.032442	0.472191953
CCNE2	ENSG00000175305	1.8458	0.03246	1.8458	0.03246	1.2439	0.10677	1.2439	0.10677	0.03246	0.472297208
RPL21P37	ENSG00000229503	1.8456	0.032473	1.8456	0.032473	1.8456	0.032473	1.8456	0.032473	0.032473	0.472297208
TMEM159	ENSG00000011638	0.3441	0.36539	1.8454	0.032487	-1.1882	0.88263	-0.95355	0.82985	0.032487	0.472297208
RPL21P47	ENSG00000241344	1.8454	0.032488	1.8454	0.032488	1.8454	0.032488	1.8454	0.032488	0.032488	0.472297208
DOCK10	ENSG00000135905	0.69601	0.24321	1.4072	0.079685	-0.64618	0.74092	1.8451	0.03251	0.03251	0.472476081
NA	ENSG00000260385	1.1819	0.11863	-0.28392	0.61176	1.1055	0.13447	1.8448	0.032531	0.032531	0.472640319
LYRM4	ENSG00000214113	1.5339	0.062529	0.27958	0.3899	1.8445	0.032555	0.042253	0.48315	0.032555	0.472837819
TMEM254	ENSG00000133678	1.1012	0.1354	0.92636	0.17713	1.8444	0.032564	0.20053	0.42053	0.032564	0.472837819
METTL21AP1	ENSG00000229623	1.8442	0.032578	1.8442	0.032578	1.8442	0.032578	1.8442	0.032578	0.032578	0.472900191
TUBGCP2	ENSG00000130640	1.3634	0.086383	-1.3689	0.91448	-0.33395	0.63079	1.844	0.03259	0.03259	0.472933502
SULT1A1	ENSG00000196502	1.8437	0.032611	1.1063	0.1343	0.35782	0.36024	0.31226	0.37742	0.032611	0.472956557
TUBB3	ENSG00000258947	1.0352	0.15028	1.8437	0.032611	-0.075077	0.52992	-0.075077	0.52992	0.032611	0.472956557
NA	ENSG00000273374	1.8435	0.032626	-0.9032	0.81679	-0.43933	0.66979	-0.87041	0.80796	0.032626	0.473033317
IRF2	ENSG00000168310	1.8432	0.032652	1.7069	0.043923	1.0898	0.13789	-0.1756	0.56969	0.032652	0.473068987
DERA	ENSG00000023697	0.1287	0.4488	0.1287	0.4488	1.8431	0.032656	-0.30408	0.61947	0.032656	0.473068987
C1orf127	ENSG00000175262	1.1135	0.13275	0.54348	0.2934	-0.75657	0.77535	1.8429	0.032669	0.032669	0.473068987
LINC01052	ENSG00000234767	1.8429	0.032671	1.8429	0.032671	1.8429	0.032671	1.8429	0.032671	0.032671	0.473068987
NA	ENSG00000258600	1.8428	0.032677	-0.82623	0.79566	-0.82623	0.79566	-0.82623	0.79566	0.032677	0.473068987
NA	ENSG00000207303	-1.3332	0.90876	-1.3332	0.90876	-1.3332	0.90876	1.8423	0.032713	0.032713	0.47311114
HSPA12A	ENSG00000165868	-0.39401	0.65321	0.86579	0.1933	1.8423	0.032717	0.4706	0.31896	0.032717	0.47311114
PCDHGA2	ENSG00000081853	0.3376	0.36783	1.8418	0.032749	0.08021	0.46804	0.08021	0.46804	0.032749	0.47311114
LIN52	ENSG00000205659	1.8418	0.032752	1.7335	0.041505	1.6857	0.045931	0.58862	0.27806	0.032752	0.47311114
NA	ENSG00000267030	1.8417	0.032761	1.8417	0.032761	1.8417	0.032761	1.8417	0.032761	0.032761	0.47311114
NA	ENSG00000280104	-0.37614	0.64659	1.8416	0.032766	0.53051	0.29788	0.57546	0.28249	0.032766	0.47311114
LINC01740	ENSG00000228067	0.99189	0.16063	0.99189	0.16063	0.99189	0.16063	1.8416	0.032767	0.032767	0.47311114
H2AZ1-DT	ENSG00000245322	1.8415	0.032775	1.7671	0.038607	1.3627	0.086488	0.46708	0.32022	0.032775	0.47311114
GLRX3	ENSG00000108010	-1.3972	0.91883	1.8415	0.032776	-0.67016	0.74862	-0.50353	0.6927	0.032776	0.47311114
NA	ENSG00000264040	-1.0682	0.85728	1.8415	0.032777	-0.96601	0.83298	-0.77879	0.78195	0.032777	0.47311114
LRMDA	ENSG00000148655	0.46384	0.32138	1.841	0.032811	1.2317	0.10904	1.4947	0.067501	0.032811	0.473364804

NKAIN2	ENSG00000188580	1.8407	0.03283	0.97296	0.16529	-0.12568	0.55001	0.38377	0.35057	0.03283	0.473364804
LINC02753	ENSG00000248844	0.98965	0.16117	1.8407	0.032831	1.2634	0.10323	0.004109	0.49836	0.032831	0.473364804
SDHAP3	ENSG00000185986	-0.75898	0.77607	-0.72942	0.76713	1.8403	0.032862	1.1221	0.13092	0.032862	0.473364804
OR2F1	ENSG00000213215	1.8403	0.032863	1.8403	0.032863	1.8403	0.032863	1.8403	0.032863	0.032863	0.473364804
ZBTB49	ENSG00000168826	1.8401	0.032876	0.63094	0.26404	0.31012	0.37824	0.63819	0.26167	0.032876	0.473364804
HMGB1P5	ENSG00000132967	0.60313	0.27321	0.60313	0.27321	0.31968	0.37461	1.8401	0.03288	0.03288	0.473364804
NA	ENSG00000279593	1.2183	0.11155	1.8103	0.035127	1.8401	0.03288	0.69	0.2451	0.03288	0.473364804
PSKH1	ENSG00000159792	1.84	0.032882	0.64214	0.26039	1.84	0.032882	1.84	0.032882	0.032882	0.473364804
KY	ENSG00000174611	-0.062979	0.52511	0.91726	0.1795	1.8399	0.032893	-0.57401	0.71702	0.032893	0.473383311
SHANK2-AS3	ENSG00000171671	0.53348	0.29685	0.84119	0.20012	0.83748	0.20116	1.8396	0.032916	0.032916	0.473434675
LBX1-AS1	ENSG00000227128	1.7966	0.036197	1.8396	0.032916	1.5859	0.056381	1.3782	0.084072	0.032916	0.473434675
TATDN3	ENSG00000203705	1.1864	0.11773	1.8394	0.03293	1.1702	0.12095	1.4998	0.066828	0.03293	0.473442855
SNX19P1	ENSG00000228184	1.8393	0.032936	1.8025	0.035732	0.22196	0.41217	0.050086	0.48003	0.032936	0.473442855
SLC25A12	ENSG00000115840	0.86937	0.19232	1.8387	0.032979	0.40968	0.34102	0.43821	0.33062	0.032979	0.473692919
NA	ENSG00000232188	0.69008	0.24507	1.8386	0.032984	1.8386	0.032984	1.8386	0.032984	0.032984	0.473692919
NA	ENSG00000233691	1.8386	0.032984	1.8386	0.032984	1.8386	0.032984	1.8386	0.032984	0.032984	0.473692919
SLC35D2	ENSG00000130958	-0.50197	0.69216	-1.1896	0.88289	-1.1527	0.87549	1.8385	0.032993	0.032993	0.473692919
NA	ENSG00000283064	1.8384	0.033002	1.331	0.091594	1.8384	0.033002	1.8384	0.033002	0.033002	0.473692919
CAVIN2	ENSG00000168497	-1.5741	0.94226	1.8373	0.033084	-1.5741	0.94226	-1.5741	0.94226	0.033084	0.474368414
CCDC195	ENSG00000283428	1.8373	0.033084	1.3398	0.090147	0.56981	0.2844	0.58647	0.27878	0.033084	0.474368414
ILF2P2	ENSG00000257720	1.8373	0.033085	1.8373	0.033085	1.8373	0.033085	1.8373	0.033085	0.033085	0.474368414
MGAM2	ENSG00000257743	-1.4745	0.92983	-1.677	0.95323	-1.6964	0.9551	1.8372	0.033088	0.033088	0.474368414
NA	ENSG00000271973	1.8367	0.033128	0.97719	0.16424	0.97719	0.16424	0.97719	0.16424	0.033128	0.474728659
PRC1	ENSG00000198901	1.6345	0.051075	1.6345	0.051075	1.8366	0.033137	1.4819	0.069186	0.033137	0.474728659
NA	ENSG00000260223	1.2544	0.10485	0.75835	0.22412	1.8365	0.033143	1.2544	0.10485	0.033143	0.474728659
NA	ENSG00000263120	1.8363	0.033153	1.8363	0.033153	1.8363	0.033153	1.8363	0.033153	0.033153	0.474728659
KIAA1671-AS1	ENSG00000203280	1.8359	0.033186	1.8359	0.033186	1.8359	0.033186	1.8359	0.033186	0.033186	0.474728659
DIP2A-IT1	ENSG00000223692	1.2253	0.11023	1.8358	0.033195	1.8358	0.033195	1.8358	0.033195	0.033195	0.474728659
SIGLEC15	ENSG00000197046	-0.74306	0.77128	-0.087759	0.53497	1.8357	0.033198	0.40041	0.34443	0.033198	0.474728659
RNU6-83P	ENSG00000207298	1.751	0.039969	0.6331	0.26333	1.8357	0.033205	0.68565	0.24647	0.033205	0.474728659
ETF1P2	ENSG00000240132	-0.5854	0.72086	1.7637	0.038889	1.8356	0.03321	0.28803	0.38666	0.03321	0.474728659

ZNF773	ENSG00000152439	1.822	0.034229	1.8203	0.034356	1.8354	0.033225	1.8182	0.034518	0.033225	0.474728659
NR3C2	ENSG00000151623	0.88264	0.18872	-0.53221	0.70271	1.8353	0.033231	1.4567	0.072599	0.033231	0.474728659
RNU6-949P	ENSG00000206767	0.076906	0.46935	0.076906	0.46935	0.092355	0.46321	1.8351	0.033245	0.033245	0.474728659
NA	ENSG00000250939	1.835	0.03325	-1.2664	0.89732	0.19461	0.42285	0.19461	0.42285	0.03325	0.474728659
NA	ENSG00000256056	0.60302	0.27325	0.60302	0.27325	0.60302	0.27325	1.8348	0.033264	0.033264	0.474728659
NA	ENSG00000239350	1.8348	0.033269	1.8348	0.033269	1.5773	0.057362	1.8348	0.033269	0.033269	0.474728659
NA	ENSG00000276471	0.61734	0.26851	0.74234	0.22894	1.8348	0.033269	1.8348	0.033269	0.033269	0.474728659
CTDSPL	ENSG00000144677	1.834	0.033323	1.6307	0.05148	1.4888	0.068275	1.6428	0.050211	0.033323	0.475360009
F13B	ENSG00000143278	0.96796	0.16653	0.96796	0.16653	1.8334	0.033373	0.96796	0.16653	0.033373	0.475725918
RPS20P25	ENSG00000228375	1.8332	0.033383	1.8332	0.033383	1.3515	0.088273	1.0981	0.13607	0.033383	0.475725918
UGT1A8	ENSG00000242366	-0.3507	0.63709	1.8332	0.033384	0.37552	0.35364	-0.92379	0.8222	0.033384	0.475725918
C1orf53	ENSG00000203724	-0.34006	0.6331	1.7709	0.038286	-0.34006	0.6331	1.8329	0.033407	0.033407	0.475725918
LINC02301	ENSG00000258743	1.8327	0.033422	0.47281	0.31817	0.61076	0.27068	0.11511	0.45418	0.033422	0.475725918
NA	ENSG00000274584	1.8326	0.033433	0.58106	0.2806	-1.3058	0.90419	-1.3058	0.90419	0.033433	0.475725918
TC2N	ENSG00000165929	1.2361	0.10821	1.8325	0.03344	1.516	0.064762	0.21546	0.4147	0.03344	0.475725918
RNA5SP445	ENSG00000251698	1.8324	0.033445	1.8324	0.033445	1.8324	0.033445	1.8324	0.033445	0.033445	0.475725918
NA	ENSG00000227683	1.8323	0.033456	1.8323	0.033456	0.9744	0.16493	1.8323	0.033456	0.033456	0.475725918
NA	ENSG00000261038	-0.41386	0.66051	-1.0972	0.86372	1.832	0.033473	-0.41386	0.66051	0.033473	0.475725918
NA	ENSG00000272991	1.8319	0.033487	1.8319	0.033487	1.8319	0.033487	1.8319	0.033487	0.033487	0.475725918
MTCO1P57	ENSG00000232975	0.72776	0.23338	-0.80863	0.79064	1.8318	0.033494	0.72776	0.23338	0.033494	0.475725918
MRGPRX11P	ENSG00000254738	1.6298	0.051576	1.0572	0.1452	1.8316	0.033505	1.8316	0.033505	0.033505	0.475725918
NA	ENSG00000250803	0.11419	0.45454	1.8315	0.033513	1.8315	0.033513	1.8315	0.033513	0.033513	0.475725918
ZFP69B	ENSG00000187801	1.4166	0.078293	-0.10375	0.54132	0.62693	0.26535	1.8313	0.033526	0.033526	0.475725918
MUC4	ENSG00000145113	0.30077	0.38179	-0.18516	0.57345	1.8313	0.033532	1.7399	0.040935	0.033532	0.475725918
NA	ENSG00000265781	1.7975	0.036125	-0.68389	0.75298	1.4963	0.067285	1.8312	0.033532	0.033532	0.475725918
EPHA8	ENSG00000070886	1.2543	0.10487	0.83329	0.20234	1.8012	0.035838	1.8308	0.033562	0.033562	0.475725918
RPL34P18	ENSG00000240509	1.8306	0.033577	0.91202	0.18088	-0.22995	0.59093	-0.45314	0.67477	0.033577	0.475725918
NA	ENSG00000200294	0.8708	0.19193	-1.0236	0.84698	1.8305	0.033587	-1.3239	0.90724	0.033587	0.475725918
SEMA3C	ENSG00000075223	1.0842	0.13913	1.8304	0.033593	1.5332	0.062609	1.7335	0.041505	0.033593	0.475725918
NA	ENSG00000280048	1.8304	0.033597	1.8304	0.033597	1.8304	0.033597	1.8304	0.033597	0.033597	0.475725918
SETD1B	ENSG00000139718	1.8149	0.034768	1.8302	0.033607	0.2435	0.40381	1.1766	0.11968	0.033607	0.475725918

HOXA-AS2	ENSG00000253552	1.8302	0.03361	0.8691	0.1924	0.8691	0.1924	0.8691	0.1924	0.03361	0.475725918
MRPS14	ENSG00000120333	0.94541	0.17222	0.94541	0.17222	0.94541	0.17222	1.8302	0.033611	0.033611	0.475725918
ISYNA1	ENSG00000105655	1.8302	0.033613	1.1868	0.11766	1.6943	0.045101	1.6943	0.045101	0.033613	0.475725918
RPSAP51	ENSG00000244131	1.8301	0.033618	-0.44273	0.67102	-0.22942	0.59073	1.2092	0.11329	0.033618	0.475725918
LINC00370	ENSG00000223617	0.6696	0.25156	1.4853	0.068737	1.83	0.033622	0.93997	0.17362	0.033622	0.475725918
VTA1P2	ENSG00000253401	1.1903	0.11696	0.76186	0.22307	1.8298	0.033641	0.28881	0.38636	0.033641	0.475856583
IL2RA	ENSG00000134460	1.0711	0.14206	1.0472	0.14749	0.098867	0.46062	1.8295	0.033662	0.033662	0.475933908
NDUFAB1P1	ENSG00000217767	1.8294	0.033666	1.8294	0.033666	1.8294	0.033666	1.8294	0.033666	0.033666	0.475933908
RIC8B	ENSG00000111785	1.8163	0.034663	1.758	0.039377	1.829	0.033703	1.7498	0.04008	0.033703	0.47594477
INO80D	ENSG00000114933	1.8289	0.033708	1.8289	0.033708	1.8289	0.033708	1.8289	0.033708	0.033708	0.47594477
NA	ENSG00000271682	-1.1005	0.86443	1.4098	0.079292	1.8286	0.03373	0.44261	0.32902	0.03373	0.47594477
SMG6	ENSG00000070366	1.4223	0.077473	1.5062	0.066003	1.3219	0.093093	1.8284	0.033746	0.033746	0.47594477
UTF1	ENSG00000171794	1.8284	0.033748	-0.11152	0.5444	-0.50276	0.69243	-0.57345	0.71683	0.033748	0.47594477
WDFY3-AS2	ENSG00000180769	1.8283	0.03375	1.4809	0.069316	1.4478	0.073839	1.6988	0.044675	0.03375	0.47594477
GCNT1P4	ENSG00000219653	-1.1365	0.87212	1.8281	0.033771	-0.24237	0.59575	-0.24237	0.59575	0.033771	0.47594477
RNU1-131P	ENSG00000238833	-0.36698	0.64318	-0.36698	0.64318	1.8276	0.033801	-1.0406	0.85098	0.033801	0.47594477
RNA5SP66	ENSG00000252987	-1.7797	0.96243	-1.7797	0.96243	1.8277	0.033801	0.091225	0.46366	0.033801	0.47594477
DEPDC4	ENSG00000166153	1.8276	0.033805	1.8276	0.033805	1.4193	0.077912	1.4193	0.077912	0.033805	0.47594477
PPP6R1	ENSG00000105063	1.0243	0.15285	1.0243	0.15285	1.0243	0.15285	1.8274	0.033816	0.033816	0.47594477
NA	ENSG00000235817	-0.67891	0.7514	1.3883	0.08253	1.8274	0.033817	1.8274	0.033817	0.033817	0.47594477
NA	ENSG00000214641	0.76463	0.22225	0.81001	0.20897	1.8273	0.033824	1.0961	0.13652	0.033824	0.47594477
XRCC1	ENSG00000073050	1.7539	0.039722	1.8273	0.03383	0.71526	0.23722	0.57862	0.28142	0.03383	0.47594477
NRON	ENSG00000253079	-0.47474	0.68252	0.63385	0.26309	1.8272	0.033838	0.11862	0.45279	0.033838	0.47594477
NA	ENSG00000231476	1.8271	0.033839	0.20463	0.41893	0.77763	0.21839	1.7673	0.038591	0.033839	0.47594477
GALNT10	ENSG00000164574	1.1744	0.12012	1.2176	0.11168	1.2609	0.10367	1.8271	0.033842	0.033842	0.47594477
CUBNP3	ENSG00000235690	1.8266	0.033878	1.8266	0.033878	1.8266	0.033878	1.8266	0.033878	0.033878	0.47594477
XPR1	ENSG00000143324	1.8262	0.033908	1.3457	0.089194	1.4801	0.069423	1.4725	0.070442	0.033908	0.47594477
MOGAT2	ENSG00000166391	1.0133	0.15547	1.8261	0.033916	1.0196	0.15396	1.5791	0.057152	0.033916	0.47594477
SH3BP2	ENSG00000087266	1.826	0.033922	1.1638	0.12226	0.62935	0.26456	-0.35626	0.63918	0.033922	0.47594477
NPSR1	ENSG00000187258	0.36081	0.35912	1.826	0.033923	0.53345	0.29686	0.71675	0.23676	0.033923	0.47594477
PRB4	ENSG00000230657	1.2059	0.11394	1.8257	0.03395	-0.54939	0.70863	-0.55957	0.71211	0.03395	0.47594477

GTF3AP1	ENSG00000237274	-0.11938	0.54751	1.8256	0.033959	1.8256	0.033959	1.8256	0.033959	0.033959	0.47594477
GABPB2	ENSG00000143458	1.3584	0.087166	1.6898	0.045535	1.8251	0.033994	1.1907	0.1169	0.033994	0.47594477
ROBO3	ENSG00000154134	0.29049	0.38572	1.1503	0.12501	0.64007	0.26106	1.825	0.034	0.034	0.47594477
DBH-AS1	ENSG00000225756	1.8249	0.034007	0.38506	0.3501	1.8249	0.034007	1.8249	0.034007	0.034007	0.47594477
GAPDHP38	ENSG00000249210	0.40542	0.34259	0.40542	0.34259	1.8248	0.034014	0.40542	0.34259	0.034014	0.47594477
RBAK-RBAKDN	ENSG00000272968	1.8246	0.034028	1.8246	0.034028	1.8246	0.034028	1.2124	0.11267	0.034028	0.47594477
PCBP2	ENSG00000197111	1.0001	0.15863	0.33316	0.36951	1.8241	0.034072	1.313	0.094596	0.034072	0.47594477
NECAB3	ENSG00000125967	0.15479	0.4385	-0.6135	0.73023	1.8239	0.034082	0.3738	0.35428	0.034082	0.47594477
ZNF784	ENSG00000179922	1.8238	0.034093	1.8238	0.034093	1.8238	0.034093	1.8238	0.034093	0.034093	0.47594477
NA	ENSG00000207503	1.8237	0.034099	0.40536	0.34261	1.8237	0.034099	1.8237	0.034099	0.034099	0.47594477
LINC01569	ENSG00000262468	0.28828	0.38657	1.8234	0.034119	1.4733	0.070332	1.4733	0.070332	0.034119	0.47594477
TINAG	ENSG00000137251	1.3644	0.086216	1.8233	0.034127	0.65713	0.25555	1.0602	0.14453	0.034127	0.47594477
NA	ENSG00000253295	1.8233	0.034132	0.23536	0.40696	1.1312	0.12898	1.1312	0.12898	0.034132	0.47594477
FKBP1BP1	ENSG00000258536	1.8232	0.034136	1.8232	0.034136	1.8232	0.034136	1.8232	0.034136	0.034136	0.47594477
RAVER2	ENSG00000162437	1.8232	0.034138	0.18412	0.42696	1.3673	0.085764	-0.11995	0.54774	0.034138	0.47594477
ARHGEF33	ENSG00000214694	1.8232	0.034138	1.3853	0.082981	0.8309	0.20302	0.85054	0.19751	0.034138	0.47594477
NA	ENSG00000223070	1.8232	0.03414	1.1559	0.12386	-0.97701	0.83572	-0.55787	0.71153	0.03414	0.47594477
NA	ENSG00000264834	1.8231	0.034142	1.8231	0.034142	1.8231	0.034142	1.8231	0.034142	0.034142	0.47594477
C5orf64-AS1	ENSG00000251575	-0.082937	0.53305	-0.082937	0.53305	1.8229	0.034158	-0.082937	0.53305	0.034158	0.47594477
TOMM20	ENSG00000173726	-0.24143	0.59539	1.094	0.13698	1.8229	0.03416	-0.25284	0.59981	0.03416	0.47594477
NA	ENSG00000249228	1.8225	0.034191	1.8225	0.034191	0.29794	0.38287	0.0080201	0.4968	0.034191	0.47594477
IFNA22P	ENSG00000224416	1.8223	0.034203	-0.032789	0.51308	0.98799	0.16158	1.1546	0.12413	0.034203	0.47594477
TRIM11	ENSG00000154370	1.7894	0.036779	1.6128	0.053389	1.8221	0.03422	1.0618	0.14417	0.03422	0.47594477
C5AR2	ENSG00000134830	-0.31984	0.62545	1.822	0.034226	-0.10916	0.54346	-0.96465	0.83264	0.034226	0.47594477
NPM1P43	ENSG00000259440	1.8218	0.034246	-0.3422	0.6339	-0.3422	0.6339	-1.141	0.87307	0.034246	0.47594477
NA	ENSG00000271840	1.1511	0.12485	1.1511	0.12485	1.1511	0.12485	1.8217	0.034252	0.034252	0.47594477
BMP8B	ENSG00000116985	-0.99432	0.83997	-0.84449	0.8008	0.51794	0.30225	1.8217	0.034254	0.034254	0.47594477
RNU6-1263P	ENSG00000207331	1.8214	0.03427	1.8214	0.03427	1.8214	0.03427	1.8214	0.03427	0.03427	0.47594477
RN7SL76P	ENSG00000241959	1.3506	0.088413	1.8214	0.034271	1.3506	0.088413	1.3506	0.088413	0.034271	0.47594477
NA	ENSG00000253205	0.16555	0.43425	0.4524	0.32549	0.19998	0.42075	1.8213	0.03428	0.03428	0.47594477
STIMATE	ENSG00000213533	1.2681	0.10239	1.8213	0.034282	0.32418	0.3729	0.71143	0.23841	0.034282	0.47594477

TARS2P1	ENSG00000248104	-0.77556	0.781	1.8212	0.034285	1.8212	0.034285	1.8212	0.034285	0.034285	0.47594477
NA	ENSG00000260447	0.62434	0.2662	0.74676	0.22761	-1.1811	0.88123	1.8212	0.034291	0.034291	0.47594477
ZBTB1	ENSG00000126804	1.8211	0.034292	1.8211	0.034292	1.8211	0.034292	1.8211	0.034292	0.034292	0.47594477
METAP2P1	ENSG00000234119	1.8209	0.034308	1.8209	0.034308	1.8209	0.034308	1.8209	0.034308	0.034308	0.47594477
ACTRT3	ENSG00000184378	1.8209	0.034309	1.8209	0.034309	1.8209	0.034309	1.8209	0.034309	0.034309	0.47594477
NA	ENSG00000272247	0.89337	0.18583	0.72334	0.23474	1.8209	0.034313	1.8209	0.034313	0.034313	0.47594477
NA	ENSG00000272944	1.8207	0.034322	1.8207	0.034322	1.8207	0.034322	1.8207	0.034322	0.034322	0.47594477
RCCD1	ENSG00000166965	1.8207	0.034327	1.8207	0.034327	0.45698	0.32384	1.8207	0.034327	0.034327	0.47594477
FCSK	ENSG00000157353	1.4559	0.072714	1.8206	0.034336	0.5982	0.27485	1.1065	0.13425	0.034336	0.47594477
LINC01397	ENSG00000258673	-1.3025	0.90362	-1.1281	0.87037	-1.3025	0.90362	1.8205	0.034338	0.034338	0.47594477
SH3RF2	ENSG00000156463	0.2207	0.41266	1.0794	0.14021	1.8205	0.034342	-0.0033103	0.50132	0.034342	0.47594477
MIR4265	ENSG00000264934	1.8204	0.034349	0.6286	0.26481	0.6286	0.26481	0.6286	0.26481	0.034349	0.47594477
RAP2A	ENSG00000125249	1.7195	0.042761	1.8204	0.034351	1.8204	0.034351	1.3483	0.088775	0.034351	0.47594477
NA	ENSG00000242660	-1.3326	0.90866	-1.3326	0.90866	-1.3326	0.90866	1.8201	0.034369	0.034369	0.47594477
NA	ENSG00000248795	1.3927	0.081856	1.3927	0.081856	1.8199	0.034391	-0.88083	0.8108	0.034391	0.47594477
SPINK8	ENSG00000229453	0.70264	0.24114	0.1504	0.44022	1.8198	0.034395	1.1919	0.11666	0.034395	0.47594477
NA	ENSG00000264697	-0.63449	0.73712	-0.63449	0.73712	-0.63449	0.73712	1.8197	0.034405	0.034405	0.47594477
DLEU2	ENSG00000231607	1.8196	0.034413	-0.05045	0.52012	0.26296	0.39629	0.38681	0.34945	0.034413	0.47594477
MIR1471	ENSG00000222246	0.74561	0.22795	1.8195	0.03442	1.0133	0.15546	-0.19671	0.57797	0.03442	0.47594477
NA	ENSG00000236985	1.8193	0.034431	-0.33483	0.63112	0.19481	0.42277	1.1773	0.11955	0.034431	0.47594477
MX2	ENSG00000183486	-1.1821	0.88142	-1.1821	0.88142	1.6217	0.052431	1.8192	0.034439	0.034439	0.47594477
DHX37	ENSG00000150990	0.60881	0.27133	0.60881	0.27133	0.4768	0.31675	1.8191	0.034448	0.034448	0.47594477
ZAR1L	ENSG00000189167	1.8191	0.03445	1.1802	0.11897	1.1662	0.12177	1.1662	0.12177	0.03445	0.47594477
PRPF4B	ENSG00000112739	-0.47378	0.68217	1.819	0.034452	0.95199	0.17055	-0.090477	0.53605	0.034452	0.47594477
FTLP19	ENSG00000237913	1.819	0.034454	1.819	0.034454	1.819	0.034454	1.819	0.034454	0.034454	0.47594477
MIR4671	ENSG00000264377	-0.35569	0.63896	1.8189	0.034467	0.97185	0.16556	0.97185	0.16556	0.034467	0.47594477
NA	ENSG00000276851	1.8187	0.034475	1.8187	0.034475	1.8187	0.034475	1.8187	0.034475	0.034475	0.47594477
NA	ENSG00000233936	-1.6071	0.94599	1.8185	0.03449	1.4621	0.071851	0.8432	0.19956	0.03449	0.47594477
PINX1	ENSG00000258724	1.8185	0.034494	1.8185	0.034494	1.8185	0.034494	1.8185	0.034494	0.034494	0.47594477
NA	ENSG00000207383	1.8182	0.034514	1.8182	0.034514	1.8182	0.034514	1.8182	0.034514	0.034514	0.47594477
PPIAP64	ENSG00000236259	1.3338	0.091141	-0.25858	0.60202	-0.93111	0.8241	1.8181	0.034525	0.034525	0.47594477

KRR1	ENSG00000111615	1.4083	0.079516	0.83057	0.20311	1.818	0.034528	1.2251	0.11027	0.034528	0.47594477
NA	ENSG00000250256	0.42161	0.33666	1.818	0.034529	1.818	0.034529	1.818	0.034529	0.034529	0.47594477
RNU6-512P	ENSG00000201308	1.8178	0.034549	1.0179	0.15437	1.0179	0.15437	1.0179	0.15437	0.034549	0.47594477
NA	ENSG00000253573	1.8177	0.034553	1.2303	0.10929	-0.18418	0.57306	0.77644	0.21875	0.034553	0.47594477
RN7SL116P	ENSG00000240474	0.04309	0.48281	0.04309	0.48281	0.04309	0.48281	1.8177	0.034555	0.034555	0.47594477
MIR5693	ENSG00000266072	1.8175	0.034567	1.8175	0.034567	1.8175	0.034567	1.8175	0.034567	0.034567	0.47594477
NA	ENSG00000263877	1.8175	0.034568	-1.3718	0.91494	-1.3718	0.91494	-1.3718	0.91494	0.034568	0.47594477
NA	ENSG00000236457	0.90255	0.18338	1.8173	0.034586	1.3861	0.082851	1.5839	0.056606	0.034586	0.47594477
PKD1P6	ENSG00000250251	1.817	0.034609	-0.32715	0.62822	-0.6483	0.7416	-0.6483	0.7416	0.034609	0.47594477
OR1L8	ENSG00000171496	1.817	0.034611	1.817	0.034611	0.39628	0.34595	0.39628	0.34595	0.034611	0.47594477
LRP2	ENSG00000081479	0.21906	0.4133	1.1737	0.12026	1.8166	0.034641	-0.4361	0.66862	0.034641	0.47594477
LINC01485	ENSG00000254211	1.1979	0.11549	1.8165	0.034647	1.5147	0.06492	1.2841	0.099552	0.034647	0.47594477
SPATA7	ENSG00000042317	0.73066	0.23249	1.8165	0.034649	1.3761	0.084402	1.0534	0.14607	0.034649	0.47594477
RARRES2P8	ENSG00000259996	1.8165	0.03465	1.4596	0.072194	1.8165	0.03465	0.51839	0.30209	0.03465	0.47594477
PPY	ENSG00000108849	1.8164	0.034657	-0.026	0.51037	0.51222	0.30425	1.8164	0.034657	0.034657	0.47594477
NA	ENSG00000236021	1.8163	0.034659	0.76174	0.22311	1.8163	0.034659	1.8163	0.034659	0.034659	0.47594477
NA	ENSG00000235047	1.8163	0.034663	1.8163	0.034663	1.8163	0.034663	1.8163	0.034663	0.034663	0.47594477
SMIM23	ENSG00000185662	1.8156	0.034712	-0.71361	0.76227	-0.71361	0.76227	0.27955	0.38991	0.034712	0.476483313
PCCB	ENSG00000114054	0.98589	0.16209	1.8148	0.034777	-0.4124	0.65998	0.10479	0.45827	0.034777	0.477241118
NA	ENSG00000270293	1.8145	0.0348	1.8145	0.0348	1.8145	0.0348	1.8145	0.0348	0.0348	0.477422297
SRRD	ENSG00000100104	1.5605	0.059322	1.5213	0.064095	1.8143	0.034815	1.5754	0.057577	0.034815	0.477474519
NA	ENSG00000179038	1.8141	0.034834	1.3433	0.089582	-0.16267	0.56461	-0.16267	0.56461	0.034834	0.477474519
NA	ENSG00000234647	0.063828	0.47455	1.814	0.034838	0.39885	0.345	0.39885	0.345	0.034838	0.477474519
KCNA1	ENSG00000111262	1.8139	0.034843	0.22147	0.41236	0.41504	0.33906	0.22147	0.41236	0.034843	0.477474519
VGLL3	ENSG00000206538	1.8134	0.034889	1.5977	0.055059	1.4479	0.07382	1.4461	0.074071	0.034889	0.477573019
MRS2	ENSG00000124532	-0.44419	0.67155	1.8131	0.034905	-0.45826	0.67662	-0.35033	0.63695	0.034905	0.477573019
LINC00921	ENSG00000281005	1.8131	0.034906	0.70522	0.24034	1.8131	0.034906	1.8131	0.034906	0.034906	0.477573019
TARS3	ENSG00000185418	1.8131	0.034907	1.8131	0.034907	1.8131	0.034907	1.8131	0.034907	0.034907	0.477573019
TRAJ31	ENSG00000211858	0.083629	0.46668	1.813	0.034914	1.813	0.034914	1.813	0.034914	0.034914	0.477573019
TRAJ30	ENSG00000211859	1.813	0.034914	1.813	0.034914	1.813	0.034914	1.813	0.034914	0.034914	0.477573019
NA	ENSG00000271200	1.8128	0.034932	-0.87582	0.80944	0.43606	0.3314	-0.71308	0.7621	0.034932	0.477573019

LINC02417 ENSG00000257048 0.26551 0.39531 1.8126 0.034943 0.86399
0.1938 0.86399 0.1938 0.034943 0.477573019
IGF2-AS ENSG00000099869 1.8122 0.034977 1.8122 0.034977 1.6074
0.053986 1.8122 0.034977 0.034977 0.477573019
EMX1 ENSG00000135638 1.5223 0.06397 -0.41971 0.66265 1.8119 0.034999
-0.3775 0.6471 0.034999 0.477573019
SDF4 ENSG00000078808 -0.13766 0.55474 1.8119 0.035002 -0.13766 0.55474
-0.076018 0.5303 0.035002 0.477573019
PIEZO1 ENSG00000103335 1.8119 0.035004 1.2894 0.098636 1.6028
0.054486 1.0497 0.14693 0.035004 0.477573019
FBXL4 ENSG00000112234 1.2114 0.11287 1.8118 0.035009 0.29366 0.38451
0.29366 0.38451 0.035009 0.477573019
TWNK ENSG00000107815 1.5235 0.063813 1.8117 0.035018 0.91567 0.17992
0.45866 0.32324 0.035018 0.477573019
LINC00901 ENSG00000242385 0.79702 0.21272 -0.28934 0.61384 0.44665
0.32756 1.8117 0.03502 0.03502 0.477573019
KRT18P42 ENSG00000214794 0.27419 0.39197 0.78631 0.21584 -0.21556
0.58534 1.8115 0.035033 0.035033 0.477573019
DLX2 ENSG00000115844 1.8114 0.03504 0.8179 0.20671 1.4644 0.071548
1.5399 0.061787 0.03504 0.477573019
NUDT9 ENSG00000170502 -0.26018 0.60264 1.3856 0.082927 0.96827 0.16645
1.8113 0.035048 0.035048 0.477573019
CACNA2D2 ENSG00000007402 1.811 0.03507 1.482 0.069175 1.674 0.047065 0.5333
0.29691 0.03507 0.477573019
NA ENSG00000253515 1.8109 0.035075 -0.95486 0.83017 0.63581 0.26245
-0.47434 0.68237 0.035075 0.477573019
LINC02430 ENSG00000250252 0.081717 0.46744 1.8109 0.035082 0.081717
0.46744 0.081717 0.46744 0.035082 0.477573019
RNU6-1275P ENSG00000212389 1.1994 0.11518 1.1344 0.12832 1.8108
0.035086 1.7323 0.041614 0.035086 0.477573019
NA ENSG00000255342 1.1592 0.12318 0.57834 0.28152 -0.10633 0.54234
1.8108 0.035087 0.035087 0.477573019
AATK ENSG00000181409 -0.40955 0.65893 0.032739 0.48694 0.081854 0.46738
1.8108 0.035089 0.035089 0.477573019
SNPH ENSG00000101298 -0.53959 0.70526 -1.2896 0.90141 1.8103 0.035128
-1.503 0.93358 0.035128 0.477573019
RNA5SP188 ENSG00000199786 1.8102 0.035131 1.8102 0.035131 1.8102
0.035131 1.8102 0.035131 0.035131 0.477573019
NA ENSG00000261467 1.8102 0.035131 0.70912 0.23913 -1.1475 0.87442
0.87853 0.18983 0.035131 0.477573019
PKMP5 ENSG00000215057 0.074772 0.4702 1.8102 0.035133 -0.97636 0.83556
0.18965 0.42479 0.035133 0.477573019
NA ENSG00000271399 1.8101 0.035143 1.8101 0.035143 1.8101 0.035143
1.8101 0.035143 0.035143 0.477573019
LINC02520 ENSG00000204110 1.81 0.035145 -0.1572 0.56245 -1.1507 0.87507
-0.1572 0.56245 0.035145 0.477573019
LOXL1-AS1 ENSG00000261801 -0.63122 0.73605 1.8099 0.035154 -0.52708
0.70093 0.1772 0.42968 0.035154 0.477573019
CCDC154 ENSG00000197599 1.8098 0.035164 1.8098 0.035164 1.8098
0.035164 1.4324 0.076015 0.035164 0.47757573
NA ENSG00000258437 -0.17629 0.56997 1.8093 0.035205 1.8093 0.035205
1.8093 0.035205 0.035205 0.477999345
FGF5 ENSG00000138675 0.086284 0.46562 1.809 0.035225 0.28433 0.38808
0.28433 0.38808 0.035225 0.478101315
NA ENSG00000237520 1.8088 0.035241 1.2094 0.11325 1.8088 0.035241
1.8088 0.035241 0.035241 0.478101315
LINC01868 ENSG00000234169 0.64213 0.26039 0.26747 0.39455 1.8088
0.035245 0.84382 0.19938 0.035245 0.478101315
SELENBP1 ENSG00000143416 1.8084 0.035271 1.7745 0.037987 -0.081412
0.53244 1.7745 0.037987 0.035271 0.478101315
CSRNP3 ENSG00000178662 0.97437 0.16494 1.8084 0.035273 0.67612
0.24948 0.86496 0.19353 0.035273 0.478101315

HACD3	ENSG00000074696	1.7792	0.0376	-0.87886	0.81026	1.8082	0.035287	0.077068	0.46928	0.035287	0.478101315
NA	ENSG00000230628	1.6631	0.048146	1.775	0.037948	1.6631	0.048146	1.8082	0.035288	0.035288	0.478101315
RPL23AP12	ENSG00000228861	0.95466	0.16988	1.3893	0.082377	0.52598	0.29945	1.8082	0.035291	0.035291	0.478101315
MIR153-1	ENSG00000207647	1.8079	0.035311	1.8079	0.035311	1.8079	0.035311	1.8079	0.035311	0.035311	0.478239308
CAPN5	ENSG00000149260	1.1979	0.11548	1.5205	0.06419	1.8076	0.035332	1.3364	0.090703	0.035332	0.478390764
TRAJ16	ENSG00000211873	1.8075	0.035343	1.8075	0.035343	1.8075	0.035343	1.8075	0.035343	0.035343	0.478394545
NA	ENSG00000258729	0.16443	0.4347	0.19682	0.42198	1.8069	0.035386	1.8069	0.035386	0.035386	0.478394545
NA	ENSG00000245008	1.8069	0.035387	1.0123	0.1557	1.3505	0.088425	0.18384	0.42707	0.035387	0.478394545
MMD2	ENSG00000136297	-0.44383	0.67142	1.8069	0.035389	-0.39095	0.65208	1.3995	0.080839	0.035389	0.478394545
RNA5SP70	ENSG00000252977	1.8069	0.035392	1.8069	0.035392	1.8069	0.035392	1.8069	0.035392	0.035392	0.478394545
NA	ENSG00000257180	1.2868	0.099073	1.2868	0.099073	1.3921	0.081945	1.8068	0.0354	0.0354	0.478394545
NA	ENSG00000235358	1.0469	0.14756	1.8068	0.035401	0.8147	0.20762	-0.28775	0.61323	0.035401	0.478394545
RCVRN	ENSG00000109047	1.684	0.046091	1.8065	0.035424	1.684	0.046091	1.684	0.046091	0.035424	0.478494024
NUP210P1	ENSG00000198284	0.27496	0.39167	1.8064	0.035428	0.60986	0.27098	0.64103	0.26075	0.035428	0.478494024
NA	ENSG00000199890	1.8062	0.035441	1.8062	0.035441	1.8062	0.035441	1.8062	0.035441	0.035441	0.478536971
LINC02453	ENSG00000245017	1.8055	0.0355	1.8055	0.0355	1.8055	0.0355	1.8055	0.0355	0.0355	0.478863888
NA	ENSG00000278239	1.8054	0.035503	1.8054	0.035503	1.8054	0.035503	1.8054	0.035503	0.035503	0.478863888
TXNDC12-AS1	ENSG00000228369	0.012096	0.49517	0.012096	0.49517	1.8052	0.03552	0.012096	0.49517	0.03552	0.478863888
NA	ENSG00000271409	1.8052	0.035522	1.7226	0.042478	1.6948	0.045061	1.6741	0.047052	0.035522	0.478863888
LINC01441	ENSG00000224008	1.0214	0.15352	1.4969	0.067213	1.8052	0.035523	1.4969	0.067213	0.035523	0.478863888
SCNN1D	ENSG00000162572	1.8052	0.035525	1.1847	0.11808	1.1847	0.11808	1.1847	0.11808	0.035525	0.478863888
CIDEB	ENSG00000136305	1.448	0.073806	1.448	0.073806	1.805	0.035534	0.72342	0.23471	0.035534	0.478863888
NA	ENSG00000242578	0.51142	0.30453	1.8048	0.035556	0.94349	0.17271	0.20167	0.42009	0.035556	0.478994362
NA	ENSG00000261402	1.8045	0.035573	0.55117	0.29076	1.5094	0.065601	-1.4813	0.93074	0.035573	0.478994362
CCDC140	ENSG00000163081	1.8045	0.035575	1.8045	0.035575	1.8045	0.035575	1.8045	0.035575	0.035575	0.478994362
SNORA63D	ENSG00000201229	0.23453	0.40729	0.23453	0.40729	1.0425	0.1486	1.8044	0.035583	0.035583	0.478994362
FAM131A	ENSG00000175182	1.804	0.035617	0.32871	0.37119	-0.35107	0.63723	-0.35107	0.63723	0.035617	0.479213857
GTF2A2	ENSG00000140307	0.14187	0.44359	1.8039	0.035623	0.14187	0.44359	0.14187	0.44359	0.035623	0.479213857
NA	ENSG00000226040	0.42454	0.33559	1.8037	0.035642	0.42454	0.33559	0.401	0.34421	0.035642	0.479213857
RN7SL813P	ENSG00000241074	-1.615	0.94685	-1.615	0.94685	-1.615	0.94685	1.8035	0.035658	0.035658	0.479213857
PRKRA	ENSG00000180228	1.8032	0.035675	1.8032	0.035675	-0.22917	0.59063	0.16295	0.43528	0.035675	0.479213857

GBP1	ENSG00000117228	0.28631	0.38732	0.38672	0.34948	0.25124	0.40082	1.8027	0.035714	0.035714	0.479213857
FAM161B	ENSG00000156050	1.5523	0.060297	1.8027	0.035717	1.3139	0.094437	1.5523	0.060297	0.035717	0.479213857
BTD	ENSG00000169814	1.8026	0.035726	-1.4649	0.92853	-1.4735	0.92969	-0.97042	0.83408	0.035726	0.479213857
NA	ENSG00000271580	1.6653	0.047929	1.8025	0.035735	1.8025	0.035735	1.8025	0.035735	0.035735	0.479213857
NA	ENSG00000258576	-0.74249	0.7711	0.15125	0.43989	-0.31749	0.62456	1.8024	0.035741	0.035741	0.479213857
DIO2	ENSG00000211448	-0.24718	0.59762	-0.016248	0.50648	1.8023	0.035747	0.5978	0.27499	0.035747	0.479213857
SPATA3-AS1	ENSG00000238062	-1.1922	0.88341	1.8023	0.035749	-0.45064	0.67388	-1.4639	0.92839	0.035749	0.479213857
NA	ENSG00000255309	-1.4684	0.92901	-1.149	0.87472	-1.1914	0.88326	1.8023	0.03575	0.03575	0.479213857
SKA2P1	ENSG00000232387	1.802	0.03577	-0.47575	0.68287	-1.3285	0.90799	-1.015	0.84495	0.03577	0.479213857
NA	ENSG00000234446	1.8019	0.035778	1.4767	0.069872	1.4767	0.069872	1.4767	0.069872	0.035778	0.479213857
DMTN	ENSG00000158856	1.8019	0.035782	1.3663	0.085918	-0.42508	0.66461	0.033314	0.48671	0.035782	0.479213857
CDC25A	ENSG00000164045	1.0669	0.143	1.0669	0.143	1.8017	0.035793	1.6457	0.049913	0.035793	0.479213857
NA	ENSG00000225050	1.8017	0.035795	0.78396	0.21653	-0.04013	0.51601	0.91601	0.17983	0.035795	0.479213857
RPS29P8	ENSG00000232238	0.95566	0.16962	0.95566	0.16962	0.95566	0.16962	1.8014	0.035818	0.035818	0.479213857
DCDC1	ENSG00000170959	1.8014	0.035819	1.4442	0.074338	1.6701	0.047455	0.48482	0.3139	0.035819	0.479213857
APOH	ENSG00000091583	0.44782	0.32714	0.21306	0.41564	0.5129	0.30401	1.801	0.035854	0.035854	0.479213857
ZBTB20-AS1	ENSG00000241560	1.1945	0.11615	1.8009	0.035863	0.71151	0.23838	1.1194	0.13148	0.035863	0.479213857
IGLV3-31	ENSG00000253920	0.93598	0.17464	1.8008	0.035865	1.8008	0.035865	1.8008	0.035865	0.035865	0.479213857
LINC02756	ENSG00000255332	0.41987	0.33729	1.8008	0.035871	1.8008	0.035871	1.8008	0.035871	0.035871	0.479213857
NA	ENSG00000249579	1.0029	0.15796	0.92753	0.17683	1.8007	0.035877	1.0029	0.15796	0.035877	0.479213857
NA	ENSG00000236887	-0.60735	0.72819	1.8005	0.035892	0.026376	0.48948	0.026376	0.48948	0.035892	0.479213857
NA	ENSG00000260793	0.58834	0.27815	0.58834	0.27815	1.8003	0.035903	0.010451	0.49583	0.035903	0.479213857
NA	ENSG00000250319	-0.3973	0.65443	1.8003	0.035906	1.8003	0.035906	1.8003	0.035906	0.035906	0.479213857
LINC00456	ENSG00000233124	1.8002	0.035915	1.7504	0.040027	1.8002	0.035915	1.8002	0.035915	0.035915	0.479213857
NA	ENSG00000270911	1.8002	0.035915	1.2359	0.10825	1.8002	0.035915	1.8002	0.035915	0.035915	0.479213857
RNU6-1282P	ENSG00000252544	-0.22256	0.58806	-1.1439	0.87367	-1.0669	0.85699	1.8	0.035928	0.035928	0.479213857
IQCM	ENSG00000234828	1.7999	0.035937	1.4227	0.077406	0.34868	0.36366	1.1526	0.12453	0.035937	0.479213857
NA	ENSG00000236169	-0.34785	0.63602	1.0807	0.13991	1.7999	0.035939	1.7999	0.035939	0.035939	0.479213857
NA	ENSG00000265010	1.7997	0.035955	1.7997	0.035955	1.7997	0.035955	1.7997	0.035955	0.035955	0.479213857
WDFY3	ENSG00000163625	1.526	0.063501	1.7996	0.035961	1.7502	0.040044	1.694	0.045137	0.035961	0.479213857
BCL2L12	ENSG00000126453	1.7995	0.035969	1.7995	0.035969	1.7995	0.035969	1.7995	0.035969	0.035969	0.479213857

IRF3	ENSG00000126456	1.7995	0.035969	1.7995	0.035969	1.7995	0.035969	1.7995	0.035969	0.035969	0.479213857
NA	ENSG00000237087	1.7995	0.035973	1.7995	0.035973	1.7995	0.035973	1.7995	0.035973	0.035973	0.479213857
ZNF862	ENSG00000106479	-0.45861	0.67674	1.799	0.036009	0.0075941	0.49697	-1.7122	0.95657	0.036009	0.479291411
HSD3BP1	ENSG00000187481	0.54222	0.29383	-0.301	0.61829	1.7989	0.036013	1.7989	0.036013	0.036013	0.479291411
NA	ENSG00000234424	0.99542	0.15976	1.5736	0.057795	1.7989	0.036015	1.0835	0.1393	0.036015	0.479291411
TLCD4	ENSG00000152078	0.40517	0.34268	0.40517	0.34268	0.40517	0.34268	1.7988	0.036024	0.036024	0.479291411
NA	ENSG00000264449	-0.31002	0.62173	0.18577	0.42631	1.2987	0.097031	1.7988	0.036028	0.036028	0.479291411
NA	ENSG00000273153	1.7986	0.036042	1.7986	0.036042	1.7986	0.036042	1.7986	0.036042	0.036042	0.479346796
NA	ENSG00000229118	0.0098878	0.49606	1.7984	0.036059	0.267	0.39473	0.88864	0.1871	0.036059	0.47937772
LINC02866	ENSG00000253665	0.75532	0.22503	0.75532	0.22503	1.7983	0.036064	0.045143	0.482	0.036064	0.47937772
NA	ENSG00000234091	1.2757	0.10102	1.7979	0.036095	0.71842	0.23625	1.4633	0.071689	0.036095	0.479636547
LINC01167	ENSG00000224758	-0.40978	0.65902	1.7977	0.03611	-0.36932	0.64405	-0.36932	0.64405	0.03611	0.479636547
NA	ENSG00000284513	0.091077	0.46372	0.14884	0.44084	1.7977	0.036113	0.091077	0.46372	0.036113	0.479636547
PRKCH	ENSG00000027075	1.1242	0.13047	1.4106	0.07918	1.7972	0.036155	1.3605	0.086829	0.036155	0.4800322
GGCTP2	ENSG00000232943	1.7967	0.03619	1.7967	0.03619	1.3945	0.081582	0.81993	0.20613	0.03619	0.4800322
NA	ENSG00000279011	-0.21324	0.58443	-0.21324	0.58443	-0.21324	0.58443	1.7967	0.036193	0.036193	0.4800322
PSMG1	ENSG00000183527	1.7966	0.036199	1.7966	0.036199	1.7966	0.036199	1.5063	0.065991	0.036199	0.4800322
RPS26P15	ENSG00000223416	-0.57781	0.7183	1.7965	0.03621	-0.98865	0.83858	-0.98865	0.83858	0.03621	0.4800322
SESN1	ENSG00000080546	1.6467	0.049813	1.6467	0.049813	1.6467	0.049813	1.7964	0.036219	0.036219	0.4800322
SRF	ENSG00000112658	1.7962	0.036233	1.5559	0.059872	1.5559	0.059872	0.13433	0.44657	0.036233	0.4800322
PNMA1	ENSG00000176903	0.99385	0.16015	1.7961	0.036242	-0.024792	0.50989	-0.024792	0.50989	0.036242	0.4800322
NA	ENSG00000264932	1.7959	0.036253	1.7959	0.036253	1.7959	0.036253	1.7959	0.036253	0.036253	0.4800322
OR52M2P	ENSG00000226616	1.2016	0.11476	0.58107	0.2806	1.7874	0.036936	1.7959	0.036254	0.036254	0.4800322
PCLO	ENSG00000186472	1.0238	0.15296	0.13685	0.44558	-0.14076	0.55597	1.7959	0.036256	0.036256	0.4800322
MRPL54	ENSG00000183617	1.7958	0.036261	1.7958	0.036261	1.7958	0.036261	1.7958	0.036261	0.036261	0.4800322
LINC02653	ENSG00000236373	0.69328	0.24407	1.7954	0.036298	0.54057	0.2944	-0.49151	0.68847	0.036298	0.48039151
NA	ENSG00000236768	1.6009	0.054695	1.7951	0.03632	1.4898	0.068143	0.83276	0.20249	0.03632	0.480552159
NCAPG	ENSG00000109805	0.65661	0.25572	1.7949	0.036335	0.80993	0.20899	0.32933	0.37095	0.036335	0.480620128
NA	ENSG00000223930	-0.42728	0.66541	0.0024215	0.49903	0.067234	0.4732	1.7948	0.036346	0.036346	0.480635164
CCDC85C	ENSG00000205476	1.7946	0.036356	1.7877	0.036913	1.3301	0.091743	1.3594	0.087004	0.036356	0.480636972
RNU4-60P	ENSG00000253048	-1.08	0.85992	1.2652	0.10289	0.48655	0.31329	1.7943	0.036379	0.036379	0.480800905

MCF2L2	ENSG00000053524	0.61274	0.27002	-0.093678	0.53732	0.65229	0.25711	1.7942	0.036391	0.036391	0.480800905
TMEM201	ENSG00000188807	-0.19118	0.57581	1.0374	0.14977	0.60379	0.27299	1.7941	0.036398	0.036398	0.480800905
L3MBTL1	ENSG00000185513	1.7939	0.036417	1.2971	0.097294	1.6102	0.053679	0.61345	0.26979	0.036417	0.480870439
GPATCH3	ENSG00000198746	0.40823	0.34155	0.40823	0.34155	0.40823	0.34155	1.7938	0.036423	0.036423	0.480870439
ZRANB2	ENSG00000132485	1.7936	0.036439	0.48856	0.31258	1.7936	0.036439	0.146	0.44196	0.036439	0.480941619
TMEM231P1	ENSG00000262583	1.7935	0.036449	1.7935	0.036449	1.7935	0.036449	0.6015	0.27375	0.036449	0.480941619
BCL7A	ENSG00000110987	-0.18401	0.573	0.4577	0.32358	0.43462	0.33192	1.7934	0.036458	0.036458	0.480941619
RPS11P1	ENSG00000235544	1.793	0.036485	1.793	0.036485	1.793	0.036485	1.793	0.036485	0.036485	0.481050536
NA	ENSG00000258534	0.04068	0.48378	0.59268	0.2767	1.793	0.036486	1.6323	0.051311	0.036486	0.481050536
SLC7A7	ENSG00000155465	1.6093	0.053776	1.7926	0.036515	1.7926	0.036515	1.6506	0.049412	0.036515	0.481057619
RNU2-13P	ENSG00000251718	1.7926	0.03652	-0.20605	0.58162	1.7926	0.03652	1.7926	0.03652	0.03652	0.481057619
UOX	ENSG00000240520	1.7924	0.036531	1.5258	0.063527	1.7924	0.036531	1.7924	0.036531	0.036531	0.481057619
HNRNPCL4	ENSG00000179412	-0.76232	0.77706	-0.76232	0.77706	-0.76232	0.77706	1.7923	0.036542	0.036542	0.481057619
CEND1P1	ENSG00000213159	1.7922	0.03655	0.20244	0.41979	-0.92395	0.82224	0.097035	0.46135	0.03655	0.481057619
NA	ENSG00000267288	1.7918	0.036584	1.7918	0.036584	1.7918	0.036584	1.7918	0.036584	0.036584	0.481057619
NA	ENSG00000267238	0.4642	0.32125	1.7664	0.038662	1.7918	0.036585	1.7918	0.036585	0.036585	0.481057619
NR2E1	ENSG00000112333	0.90914	0.18164	0.90914	0.18164	1.7917	0.036587	-0.16661	0.56616	0.036587	0.481057619
TMEM182	ENSG00000170417	0.61097	0.27061	0.61097	0.27061	0.33644	0.36827	1.7916	0.036595	0.036595	0.481057619
NA	ENSG00000254914	1.7916	0.036602	1.5705	0.058145	1.7313	0.041698	1.7313	0.041698	0.036602	0.481057619
RNA5SP323	ENSG00000212396	1.7915	0.036605	1.2812	0.10005	1.7915	0.036605	1.7915	0.036605	0.036605	0.481057619
ERCC8-AS1	ENSG00000233847	1.7915	0.036605	1.7915	0.036605	1.7915	0.036605	1.7915	0.036605	0.036605	0.481057619
SOCS7	ENSG00000274211	1.7911	0.03664	1.7214	0.042589	1.7911	0.03664	1.7911	0.03664	0.03664	0.481154584
KXD1	ENSG00000105700	1.5697	0.058237	0.47823	0.31624	1.7911	0.036641	1.4653	0.071422	0.036641	0.481154584
JARID2-AS1	ENSG00000235488	1.7911	0.036642	1.7911	0.036642	1.7911	0.036642	1.7911	0.036642	0.036642	0.481154584
CYCSP12	ENSG00000224263	1.7908	0.036663	-0.0086328	0.50344	1.3605	0.086835	1.0054	0.15735	0.036663	0.481300644
NA	ENSG00000248842	1.7468	0.040338	1.7905	0.036688	0.57867	0.28141	0.57867	0.28141	0.036688	0.48140884
NA	ENSG00000224970	1.7905	0.036691	1.7905	0.036691	1.7905	0.036691	1.6798	0.046499	0.036691	0.48140884
SARM1	ENSG00000004139	-0.13535	0.55383	-0.52617	0.70061	-0.52617	0.70061	1.7899	0.036735	0.036735	0.481665717
CST1	ENSG00000170373	1.7898	0.036745	0.19485	0.42275	-0.59966	0.72563	1.111	0.13328	0.036745	0.481665717
NA	ENSG00000274330	1.7897	0.036752	1.7897	0.036752	1.7897	0.036752	1.7897	0.036752	0.036752	0.481665717
KIAA1614	ENSG00000135835	0.23579	0.4068	1.7896	0.036759	0.23579	0.4068	-0.36338	0.64184	0.036759	0.481665717

NA	ENSG00000253690	-0.50688	0.69388	-0.4311	0.6668	-1.2697	0.89791	1.7896	0.03676	0.03676	0.481665717
SIMC1	ENSG00000170085	1.789	0.036807	1.2436	0.10682	0.56658	0.2855	0.7745	0.21932	0.036807	0.482022335
NA	ENSG00000254424	0.70154	0.24148	-1.2701	0.89798	0.80393	0.21072	1.789	0.036807	0.036807	0.482022335
NA	ENSG00000279554	1.7886	0.036842	1.7886	0.036842	1.7886	0.036842	1.7886	0.036842	0.036842	0.482351064
NA	ENSG00000225489	1.19	0.11701	1.7883	0.036867	1.19	0.11701	1.19	0.11701	0.036867	0.482430895
TMPRSS11BNL	ENSG00000250026	1.7882	0.036876	-0.90804	0.81807	0.65069	0.25762	-0.94102	0.82665	0.036876	0.482430895
RNU105C	ENSG00000199212	1.7881	0.036881	1.7881	0.036881	1.7881	0.036881	1.7881	0.036881	0.036881	0.482430895
IMPDH1	ENSG00000106348	0.53791	0.29532	1.6296	0.05159	1.7879	0.036897	1.5275	0.063322	0.036897	0.482430895
RARB	ENSG00000077092	1.6614	0.048314	1.4386	0.075132	1.7878	0.036908	1.2629	0.10331	0.036908	0.482430895
LINC01354	ENSG00000231768	1.0691	0.14251	1.7044	0.044151	1.7876	0.036923	1.6756	0.046908	0.036923	0.482430895
NA	ENSG00000213386	0.9741	0.165	1.4311	0.076205	1.4983	0.067032	1.7876	0.036924	0.036924	0.482430895
NA	ENSG00000276923	-0.39315	0.6529	1.7875	0.036932	-0.39315	0.6529	-0.39315	0.6529	0.036932	0.482430895
HAO1	ENSG00000101323	0.57634	0.28219	0.45535	0.32443	-2.0485	0.97975	1.7874	0.036938	0.036938	0.482430895
NA	ENSG00000257803	0.12099	0.45185	-0.59851	0.72525	-0.59851	0.72525	1.7873	0.036948	0.036948	0.482430895
IGHV1-3	ENSG00000211935	-0.58833	0.72184	1.787	0.036965	1.787	0.036965	0.75508	0.2251	0.036965	0.482430895
RNU7-141P	ENSG00000238777	1.7868	0.036986	1.7868	0.036986	1.7868	0.036986	1.7868	0.036986	0.036986	0.482430895
KIAA0100	ENSG00000007202	0.52787	0.2988	-0.5115	0.6955	-0.5115	0.6955	1.7866	0.037005	0.037005	0.482430895
NA	ENSG00000201218	1.7865	0.037011	-0.57327	0.71677	0.40761	0.34178	0.31126	0.3778	0.037011	0.482430895
C3	ENSG00000125730	1.3767	0.084298	1.4015	0.080538	0.53232	0.29725	1.786	0.037047	0.037047	0.482430895
NA	ENSG00000257307	1.1615	0.12271	0.4387	0.33044	1.4828	0.069061	1.786	0.037054	0.037054	0.482430895
CTAGE15	ENSG00000271079	-0.87759	0.80992	1.7859	0.037054	-0.066276	0.52642	-1.0724	0.85822	0.037054	0.482430895
NA	ENSG00000249761	-0.90555	0.81741	-0.1721	0.56832	-0.82324	0.79482	1.7859	0.037057	0.037057	0.482430895
LBX1	ENSG00000138136	1.4753	0.070063	1.5741	0.057738	1.7859	0.037061	-0.63727	0.73803	0.037061	0.482430895
SRC	ENSG00000197122	0.81194	0.20841	1.1046	0.13466	0.70592	0.24012	1.7857	0.037075	0.037075	0.482430895
NA	ENSG00000282048	1.7856	0.037079	-0.1247	0.54962	-0.1247	0.54962	-0.1247	0.54962	0.037079	0.482430895
FAM32CP	ENSG00000213525	1.7856	0.037085	1.7856	0.037085	1.7856	0.037085	1.2688	0.10226	0.037085	0.482430895
NA	ENSG00000270832	1.0542	0.14591	0.29321	0.38468	-0.45541	0.67559	1.7854	0.037095	0.037095	0.482430895
STRIP2	ENSG00000128578	1.7853	0.037108	0.36804	0.35642	1.2879	0.098893	1.2446	0.10664	0.037108	0.482430895
NA	ENSG00000270897	1.7851	0.037126	1.7851	0.037126	1.7851	0.037126	1.7851	0.037126	0.037126	0.482430895
RNU6-925P	ENSG00000207359	1.7838	0.037228	1.7849	0.037139	1.7838	0.037228	1.7838	0.037228	0.037139	0.482430895
NA	ENSG00000273329	1.7849	0.037141	1.7849	0.037141	1.7849	0.037141	1.4439	0.074379	0.037141	0.482430895

ATP6V1G1P3	ENSG00000224662	1.7848	0.037144	1.4469	0.073968	1.4469	0.073968	-0.046907	0.51871	0.037144	0.482430895
EIF4BP8	ENSG00000243918	1.2799	0.10029	0.65655	0.25573	1.7847	0.037151	0.66627	0.25262	0.037151	0.482430895
NA	ENSG00000270838	1.4447	0.074271	0.63562	0.26251	1.7847	0.037154	1.5982	0.055	0.037154	0.482430895
NCKIPSD	ENSG00000213672	1.4846	0.068821	1.7847	0.037155	0.74093	0.22937	1.7847	0.037155	0.037155	0.482430895
NA	ENSG00000255972	1.7844	0.037183	1.7844	0.037183	1.7844	0.037183	1.7844	0.037183	0.037183	0.482665847
CYP7A1	ENSG00000167910	1.7839	0.037218	1.7839	0.037218	1.7839	0.037218	1.7839	0.037218	0.037218	0.482991515
HEBP2	ENSG00000051620	0.35835	0.36004	1.7837	0.03724	1.235	0.10841	0.72111	0.23542	0.03724	0.483148349
ST7-OT4	ENSG00000214188	-0.039745	0.51585	-0.039745	0.51585	1.7833	0.037267	-0.039745	0.51585	0.037267	0.483361175
NPAP1P7	ENSG00000277357	1.7831	0.037281	-0.60286	0.7267	-0.60286	0.7267	-0.60286	0.7267	0.037281	0.483361175
LONRF2P5	ENSG00000273692	1.0617	0.14418	0.63975	0.26117	1.7831	0.037289	0.70741	0.23966	0.037289	0.483361175
EPN2	ENSG00000072134	1.4985	0.067004	0.80084	0.21161	1.7829	0.037304	0.11217	0.45534	0.037304	0.483361175
ZDHHC21	ENSG00000175893	0.72081	0.23551	0.76963	0.22076	0.17684	0.42982	1.7828	0.037306	0.037306	0.483361175
PIK3R2	ENSG00000105647	0.33876	0.36739	-0.57332	0.71678	0.68554	0.2465	1.7825	0.037335	0.037335	0.483391246
ZNF71	ENSG00000197951	-0.0052311	0.50209	1.0433	0.14841	1.7825	0.037336	1.0433	0.14841	0.037336	0.483391246
CST4	ENSG00000101441	0.89068	0.18655	0.54403	0.29321	1.7824	0.037344	0.844	0.19933	0.037344	0.483391246
NA	ENSG00000232259	0.091354	0.46361	0.091354	0.46361	1.7823	0.037348	1.3966	0.081264	0.037348	0.483391246
MIR626	ENSG00000207766	1.782	0.037372	1.782	0.037372	1.782	0.037372	1.782	0.037372	0.037372	0.483573436
NA	ENSG00000276704	1.7819	0.037385	1.7819	0.037385	-0.51904	0.69813	-1.084	0.86081	0.037385	0.483592283
RPL34P17	ENSG00000240534	1.7816	0.037404	1.7816	0.037404	1.7816	0.037404	1.7816	0.037404	0.037404	0.483592283
TEMN3-AS1	ENSG00000251336	1.3578	0.087267	1.3578	0.087267	1.7816	0.037406	0.33912	0.36726	0.037406	0.483592283
TM4SF19	ENSG00000145107	1.5238	0.063784	0.66875	0.25183	1.7813	0.037429	1.6973	0.044824	0.037429	0.483592283
RNU6-511P	ENSG00000200366	1.7813	0.037432	1.7813	0.037432	1.7813	0.037432	1.7813	0.037432	0.037432	0.483592283
CNPPD1	ENSG00000115649	0.26704	0.39472	1.6077	0.053948	1.7813	0.037433	1.6077	0.053948	0.037433	0.483592283
PTPN23	ENSG00000076201	-1.4043	0.91988	0.60438	0.27279	1.7809	0.037465	1.2314	0.10908	0.037465	0.483839576
NA	ENSG00000257226	1.7808	0.037472	1.7808	0.037472	1.7808	0.037472	1.7808	0.037472	0.037472	0.483839576
NA	ENSG00000232208	0.61195	0.27029	0.54657	0.29234	1.1121	0.13306	1.7805	0.037496	0.037496	0.484021213
FAM111B	ENSG00000189057	-0.73208	0.76794	0.80509	0.21038	1.6274	0.051829	1.7801	0.037527	0.037527	0.484293091
NA	ENSG00000253183	1.78	0.037541	-0.024476	0.50976	1.0749	0.14121	0.065693	0.47381	0.037541	0.484345494
ZNF484	ENSG00000127081	1.7797	0.037561	0.11251	0.45521	0.30527	0.38008	1.1656	0.12189	0.037561	0.484446384
NA	ENSG00000249112	1.7795	0.037575	0.54479	0.29295	0.54479	0.29295	0.54479	0.29295	0.037575	0.484446384
LINC01613	ENSG00000261432	1.7795	0.037583	1.7795	0.037583	1.7795	0.037583	1.7795	0.037583	0.037583	0.484446384

LINC00958 ENSG00000251381 1.7548 0.039643 0.94277 0.1729 1.5195
0.064324 1.7793 0.037597 0.037597 0.484446384
NA ENSG00000237250 -0.8722 0.80845 -0.38067 0.64828 1.7791 0.037609
0.18912 0.425 0.037609 0.484446384
EIF1P5 ENSG00000266563 1.7789 0.037631 1.7789 0.037631 1.7789
0.037631 1.7789 0.037631 0.037631 0.484446384
NA ENSG00000236025 1.7788 0.037638 0.082525 0.46711 0.082525 0.46711
0.082525 0.46711 0.037638 0.484446384
KCNA10 ENSG00000143105 -1.2985 0.90294 1.7788 0.03764 -0.77324
0.78031 -1.1333 0.87145 0.03764 0.484446384
NA ENSG00000266586 -1.1461 0.87412 -0.12277 0.54885 1.7787 0.037643
0.42129 0.33677 0.037643 0.484446384
RNF103-CHMP3 ENSG00000249884 1.7784 0.037668 -0.13419 0.55337 0.084836
0.4662 0.20493 0.41882 0.037668 0.484446384
NA ENSG00000261434 1.7784 0.037669 1.7784 0.037669 1.7784 0.037669
1.7784 0.037669 0.037669 0.484446384
NA ENSG00000258202 -0.16212 0.56439 -0.64687 0.74114 1.0154 0.15496
1.7783 0.037675 0.037675 0.484446384
RPL11 ENSG00000142676 0.047272 0.48115 1.7782 0.037688 1.7782 0.037688
0.089134 0.46449 0.037688 0.484446384
H2BU1 ENSG00000196890 1.7782 0.037688 1.7782 0.037688 1.7782 0.037688
1.7782 0.037688 0.037688 0.484446384
RNU4ATAC2P ENSG00000221601 1.7779 0.037712 1.7779 0.037712 1.7779
0.037712 1.7779 0.037712 0.037712 0.484467457
C1QTNF3-AMACR ENSG00000273294 -0.15762 0.56262 -0.15762 0.56262 1.7778
0.037715 0.96961 0.16612 0.037715 0.484467457
NA ENSG00000225236 0.24833 0.40194 1.4681 0.071036 1.1539 0.12428
1.7777 0.037723 0.037723 0.484467457
RPS29P5 ENSG00000230777 1.7774 0.03775 0.52117 0.30112 1.6971
0.044841 0.52117 0.30112 0.03775 0.484467457
LINC01600 ENSG00000164385 1.7773 0.037762 1.7773 0.037762 -0.4608
0.67753 1.4483 0.07376 0.037762 0.484467457
RNU6-699P ENSG00000252890 0.38459 0.35027 1.7773 0.037763 1.7773
0.037763 1.7773 0.037763 0.037763 0.484467457
NA ENSG00000232310 1.7771 0.037777 1.7771 0.037777 1.4974 0.067146
0.93311 0.17538 0.037777 0.484467457
RN7SL516P ENSG00000242699 1.7769 0.037792 1.7769 0.037792 1.7769
0.037792 1.7769 0.037792 0.037792 0.484467457
NA ENSG00000248714 0.65888 0.25499 0.65888 0.25499 0.65888 0.25499
1.7769 0.037796 0.037796 0.484467457
RPS10P3 ENSG00000217716 -0.32762 0.6284 0.048914 0.48049 0.42759
0.33447 1.7768 0.037798 0.037798 0.484467457
NA ENSG00000255447 -0.67956 0.75161 -0.67956 0.75161 -0.67956 0.75161
1.7768 0.037799 0.037799 0.484467457
NA ENSG00000279874 1.7764 0.037836 1.7764 0.037836 1.7764 0.037836
1.7764 0.037836 0.037836 0.484574467
NA ENSG00000275178 0.42082 0.33694 0.86595 0.19326 0.42082 0.33694
1.7763 0.037841 0.037841 0.484574467
DMGDH ENSG00000132837 1.7761 0.037856 -1.6666 0.95221 -1.3775 0.91581
-1.8933 0.97084 0.037856 0.484574467
ZNF668 ENSG00000167394 1.4893 0.068206 1.4893 0.068206 0.25113
0.40086 1.7758 0.037882 0.037882 0.484574467
NA ENSG00000258942 1.7757 0.037889 1.7757 0.037889 1.7757 0.037889
1.7757 0.037889 0.037889 0.484574467
PCBP1 ENSG00000169564 1.7756 0.037901 1.7756 0.037901 1.4626 0.071792
1.7756 0.037901 0.037901 0.484574467
RNU6-723P ENSG00000206700 1.7754 0.037912 0.55374 0.28988 0.55374
0.28988 0.55374 0.28988 0.037912 0.484574467
NA ENSG00000252852 1.7751 0.037938 1.7751 0.037938 1.7751 0.037938
1.7751 0.037938 0.037938 0.484574467
TRGVB ENSG00000231202 1.775 0.037945 1.775 0.037945 1.775 0.037945 1.775 0.037945
0.037945 0.484574467

TIAF1	ENSG00000221995	1.775	0.037952	1.775	0.037952	1.775	0.037952	1.775	0.037952	0.037952	0.484574467
PRC1-AS1	ENSG00000258725	1.7749	0.037957	1.7749	0.037957	1.7749	0.037957	1.7749	0.037957	0.037957	0.484574467
LINC01760	ENSG00000228504	0.38474	0.35021	1.7748	0.037965	-0.46066	0.67748	1.7748	0.037965	0.037965	0.484574467
RPL12P36	ENSG00000242423	1.7748	0.037965	1.7748	0.037965	1.7748	0.037965	1.7748	0.037965	0.037965	0.484574467
RNF168	ENSG00000163961	-0.38392	0.64948	1.7748	0.037969	0.37956	0.35214	-1.3431	0.91038	0.037969	0.484574467
LINC01082	ENSG00000269186	1.7748	0.037969	1.7748	0.037969	1.7748	0.037969	1.7748	0.037969	0.037969	0.484574467
TBC1D3P1	ENSG00000238283	0.67113	0.25107	1.7746	0.037982	0.99741	0.15928	0.1961	0.42227	0.037982	0.484574467
NA	ENSG00000259229	1.7746	0.037985	0.32797	0.37147	-0.23539	0.59305	0.32797	0.37147	0.037985	0.484574467
PTPRG-AS1	ENSG00000241472	1.7745	0.03799	1.7745	0.03799	1.7745	0.03799	0.698	0.24259	0.03799	0.484574467
NA	ENSG00000260361	0.31883	0.37493	0.31883	0.37493	1.7743	0.038007	0.31883	0.37493	0.038007	0.484574467
VN1R21P	ENSG00000224752	1.7741	0.038026	1.7741	0.038026	-0.17895	0.57101	1.1172	0.13195	0.038026	0.484574467
NA	ENSG00000241475	1.774	0.038032	1.774	0.038032	1.774	0.038032	1.774	0.038032	0.038032	0.484574467
RN7SKP104	ENSG00000199475	1.2118	0.11279	1.7739	0.038037	-0.21743	0.58606	1.2118	0.11279	0.038037	0.484574467
SLC28A1	ENSG00000156222	1.5921	0.055681	1.7739	0.038042	0.76885	0.22099	1.5323	0.062728	0.038042	0.484574467
CUL9	ENSG00000112659	0.85885	0.19521	0.68128	0.24785	1.7738	0.038049	1.5398	0.061804	0.038049	0.484574467
NA	ENSG00000257253	1.7736	0.038063	-0.55454	0.7104	0.01716	0.49315	0.01716	0.49315	0.038063	0.484574467
NA	ENSG00000228044	1.3153	0.094206	1.2222	0.11082	1.7735	0.038074	1.6256	0.052022	0.038074	0.484574467
NA	ENSG00000271259	1.7734	0.038083	1.7734	0.038083	1.7734	0.038083	1.7734	0.038083	0.038083	0.484574467
LINC01958	ENSG00000283436	1.7733	0.038093	1.2066	0.11379	1.2066	0.11379	1.2808	0.10013	0.038093	0.484574467
RPSAP31	ENSG00000234371	1.7728	0.038135	1.7728	0.038135	1.7728	0.038135	1.7728	0.038135	0.038135	0.484574467
OR5K4	ENSG00000196098	1.2728	0.10155	1.7726	0.038146	-0.40628	0.65773	-0.2783	0.60961	0.038146	0.484574467
WNT3A	ENSG00000154342	-0.7736	0.78042	1.7726	0.03815	-0.48382	0.68574	-0.58079	0.71931	0.03815	0.484574467
NA	ENSG00000234646	1.7725	0.038157	1.7725	0.038157	1.7725	0.038157	1.7725	0.038157	0.038157	0.484574467
NA	ENSG00000274256	1.7724	0.038163	1.7724	0.038163	1.7724	0.038163	1.7724	0.038163	0.038163	0.484574467
NA	ENSG00000229330	-0.5439	0.70674	-0.77188	0.77991	1.7723	0.038175	-0.5243	0.69997	0.038175	0.484574467
SHISA9	ENSG00000237515	0.41688	0.33838	1.1387	0.12741	1.1097	0.13356	1.7723	0.038176	0.038176	0.484574467
PRDM12	ENSG00000130711	1.7722	0.038184	1.3226	0.092983	1.2712	0.10184	-0.28626	0.61266	0.038184	0.484574467
NA	ENSG00000219529	1.3797	0.083833	1.772	0.038198	1.3457	0.089195	1.3457	0.089195	0.038198	0.484574467
DCDC2C	ENSG00000214866	-2.2944	0.98912	-1.4266	0.92315	-0.059574	0.52375	1.772	0.0382	0.0382	0.484574467
CELA2A	ENSG00000142615	1.7719	0.038204	1.7387	0.041044	1.5336	0.062562	-0.15746	0.56256	0.038204	0.484574467
LNCPRESS1	ENSG00000232301	1.7719	0.038208	0.97265	0.16536	1.5316	0.062809	1.04	0.14916	0.038208	0.484574467

NA	ENSG00000237363	0.9008	0.18385	1.7718	0.038216	-0.43	0.6664	0.48846	0.31261	0.038216	0.484574467
NA	ENSG00000248783	1.3969	0.081218	0.77088	0.22039	1.7717	0.038225	0.77088	0.22039	0.038225	0.484574467
LINC00326	ENSG00000231023	1.7715	0.038239	1.7715	0.038239	1.7715	0.038239	1.6486	0.049613	0.038239	0.484601225
NA	ENSG00000223795	0.58053	0.28078	-1.6425	0.94976	-0.95423	0.83002	1.7714	0.038247	0.038247	0.484601225
MYH13	ENSG00000006788	-0.1672	0.56639	0.10184	0.45944	-0.54144	0.7059	1.771	0.038281	0.038281	0.484805247
NA	ENSG00000242888	0.26184	0.39672	0.71225	0.23816	1.771	0.038283	0.51652	0.30275	0.038283	0.484805247
ANGPTL6	ENSG00000130812	1.7707	0.038305	1.7707	0.038305	1.7707	0.038305	1.7707	0.038305	0.038305	0.484884368
ZBTB25	ENSG00000089775	1.7324	0.041599	1.7228	0.042466	1.7705	0.03832	1.5981	0.055008	0.03832	0.484884368
NA	ENSG00000258823	0.36853	0.35624	0.36853	0.35624	0.36853	0.35624	1.7704	0.038333	0.038333	0.484884368
PRRT4	ENSG00000224940	1.7703	0.038337	1.0207	0.1537	0.60701	0.27192	-0.16232	0.56447	0.038337	0.484884368
LINC01867	ENSG00000225444	1.7703	0.038339	1.7703	0.038339	1.7703	0.038339	1.7703	0.038339	0.038339	0.484884368
SLC1A5	ENSG00000105281	1.7701	0.038359	1.1722	0.12056	1.1722	0.12056	0.68868	0.24551	0.038359	0.484948825
CCNJL	ENSG00000135083	1.6203	0.052581	1.77	0.038364	0.75615	0.22478	1.4785	0.069642	0.038364	0.484948825
NA	ENSG00000260488	1.7697	0.038391	1.7697	0.038391	1.7697	0.038391	1.7697	0.038391	0.038391	0.485039069
ITCH	ENSG00000078747	1.7696	0.038399	1.3249	0.092602	1.0125	0.15565	1.1195	0.13146	0.038399	0.485039069
CLIC4P2	ENSG00000269546	1.7695	0.038401	1.7695	0.038401	1.7695	0.038401	1.7695	0.038401	0.038401	0.485039069
NA	ENSG00000259594	0.8156	0.20736	-0.81911	0.79364	1.3752	0.084533	1.7694	0.038411	0.038411	0.485039655
NA	ENSG00000254347	1.7692	0.038431	1.7692	0.038431	1.7692	0.038431	1.7692	0.038431	0.038431	0.485041411
NA	ENSG00000265803	1.7691	0.038436	1.7691	0.038436	0.63797	0.26175	0.2692	0.39389	0.038436	0.485041411
C15orf41	ENSG00000186073	1.2657	0.10281	0.81323	0.20804	1.4627	0.071777	1.7691	0.038441	0.038441	0.485041411
NA	ENSG00000254730	-0.60382	0.72702	1.7688	0.038467	-0.83113	0.79705	0.70137	0.24154	0.038467	0.485243829
NA	ENSG00000231747	1.7683	0.038504	1.0774	0.14066	1.7683	0.038504	-0.7462	0.77223	0.038504	0.485572701
MIR590	ENSG00000207741	1.7682	0.038513	1.7682	0.038513	1.7682	0.038513	1.7682	0.038513	0.038513	0.485572701
NA	ENSG00000266268	0.4525	0.32546	0.69563	0.24333	1.7678	0.03855	-0.57113	0.71604	0.03855	0.485788211
CDYLP1	ENSG00000224039	1.7677	0.038552	1.7677	0.038552	1.7677	0.038552	1.7677	0.038552	0.038552	0.485788211
NA	ENSG00000263489	-0.46181	0.67789	-0.46181	0.67789	1.7676	0.03856	0.034527	0.48623	0.03856	0.485788211
OSGIN1	ENSG00000140961	1.4075	0.079637	1.2108	0.11298	1.7671	0.038606	1.3908	0.082143	0.038606	0.486204519
RSPH1	ENSG00000160188	1.767	0.038613	-0.6151	0.73076	0.30486	0.38024	1.238	0.10786	0.038613	0.486204519
NA	ENSG00000199473	1.7667	0.038637	1.7667	0.038637	1.7667	0.038637	1.7667	0.038637	0.038637	0.486381041
NA	ENSG00000230492	0.62928	0.26458	1.0816	0.13971	1.7666	0.038649	1.7666	0.038649	0.038649	0.486406449
ZNF394	ENSG00000160908	1.4848	0.068795	1.7194	0.042772	1.7663	0.038669	1.6895	0.045562	0.038669	0.486532499

FAM189A1	ENSG00000104059	-0.56644	0.71445	-0.56644	0.71445	1.7662	0.038685	-0.95406	0.82997	0.038685	0.48660817
DUXAP5	ENSG00000255377	1.7659	0.038707	0.36947	0.35589	-0.62225	0.73311	-0.10991	0.54376	0.038707	0.486621274
KBTBD3	ENSG00000182359	1.7658	0.038714	1.3638	0.086314	1.7658	0.038714	1.7658	0.038714	0.038714	0.486621274
PLD1	ENSG00000075651	0.01282	0.49489	-0.32359	0.62687	0.15654	0.4378	1.7658	0.038716	0.038716	0.486621274
RFC4	ENSG00000163918	1.7654	0.038745	-0.60982	0.72901	-0.60982	0.72901	0.97666	0.16437	0.038745	0.486860199
RMRPP5	ENSG00000223088	1.7652	0.038765	0.76048	0.22348	1.4984	0.067016	1.4984	0.067016	0.038765	0.486985937
SLC5A8	ENSG00000256870	0.73283	0.23183	0.31822	0.37516	0.95899	0.16878	1.7648	0.038799	0.038799	0.487274888
NA	ENSG00000230631	1.4338	0.075813	1.7647	0.038808	1.0438	0.14828	1.5557	0.059887	0.038808	0.487274888
NA	ENSG00000223929	1.7644	0.038829	1.7644	0.038829	1.7644	0.038829	1.7644	0.038829	0.038829	0.487412975
VDAC1P4	ENSG00000235060	1.764	0.038867	1.764	0.038867	1.1913	0.11676	1.764	0.038867	0.038867	0.48773867
UBQLN4	ENSG00000160803	1.7638	0.038879	1.7638	0.038879	0.79182	0.21423	0.49551	0.31012	0.038879	0.48773867
NA	ENSG00000266443	1.4303	0.076319	1.4355	0.075575	1.2787	0.10051	1.7637	0.038891	0.038891	0.48773867
RABGGTB	ENSG00000137955	1.7635	0.03891	1.7635	0.03891	1.7635	0.03891	1.7635	0.03891	0.03891	0.48773867
ACE	ENSG00000159640	1.7634	0.038914	1.4854	0.06872	1.7634	0.038914	1.7586	0.039322	0.038914	0.48773867
NA	ENSG00000280035	1.7634	0.038915	1.7634	0.038915	1.7634	0.038915	1.7634	0.038915	0.038915	0.48773867
TBC1D19	ENSG00000109680	-1.6659	0.95214	1.7628	0.038963	-0.34127	0.63355	-0.78734	0.78446	0.038963	0.488088998
MIR3976HG	ENSG00000261738	1.7628	0.038969	-0.089087	0.53549	0.31566	0.37613	0.76663	0.22165	0.038969	0.488088998
NA	ENSG00000259523	1.7627	0.038973	1.7627	0.038973	1.7627	0.038973	1.7627	0.038973	0.038973	0.488088998
SSH3	ENSG00000172830	0.36607	0.35716	1.4244	0.077158	1.7625	0.038989	1.3819	0.0835	0.038989	0.488163918
FBXO28	ENSG00000143756	1.7624	0.039004	1.5797	0.057093	1.4471	0.073932	-0.55025	0.70893	0.039004	0.488226283
VNN1	ENSG00000112299	1.7619	0.039044	0.42529	0.33531	0.66174	0.25407	0.88381	0.1884	0.039044	0.488484886
DNAJB9	ENSG00000128590	1.7618	0.039049	1.7618	0.039049	1.7618	0.039049	1.7618	0.039049	0.039049	0.488484886
IP6K2	ENSG00000068745	1.6762	0.046851	1.7617	0.039056	1.5741	0.057728	1.6138	0.053284	0.039056	0.488484886
LINC02226	ENSG00000245729	1.7616	0.03907	-0.12854	0.55114	-0.12854	0.55114	-0.11608	0.5462	0.03907	0.488484886
SLA2	ENSG00000101082	1.7613	0.039096	1.7613	0.039096	0.6762	0.24946	0.31537	0.37624	0.039096	0.488484886
TRNP1	ENSG00000253368	1.237	0.10804	1.7612	0.0391	-0.20326	0.58053	1.237	0.10804	0.0391	0.488484886
KIF12	ENSG00000136883	1.1261	0.13007	0.14892	0.44081	1.7609	0.039129	1.5346	0.062439	0.039129	0.488484886
NA	ENSG00000254031	1.7607	0.039145	1.4177	0.078136	-1.4305	0.92371	-0.17129	0.568	0.039145	0.488484886
ERC1	ENSG00000082805	-0.19868	0.57874	0.364	0.35793	0.53454	0.29649	1.7607	0.039146	0.039146	0.488484886
OR7E115P	ENSG00000182531	-0.63576	0.73753	1.7606	0.039151	0.66913	0.25171	0.20638	0.41825	0.039151	0.488484886
NA	ENSG00000228391	1.7606	0.039152	0.14454	0.44254	-0.033185	0.51324	1.0182	0.1543	0.039152	0.488484886

NA	ENSG00000279393	1.7606	0.039157	1.7606	0.039157	1.7606	0.039157	1.7606	0.039157	0.039157	0.488484886
WHRN	ENSG00000095397	0.52414	0.30009	0.8038	0.21076	-1.1352	0.87185	1.7605	0.039161	0.039161	0.488484886
BRD8	ENSG00000112983	1.7605	0.039165	-1.2691	0.8978	1.2716	0.10176	1.1509	0.12488	0.039165	0.488484886
PLXNB2	ENSG00000196576	1.7596	0.039238	-0.39466	0.65345	-0.89683	0.81509	-0.28227	0.61113	0.039238	0.48909466
RPL30P12	ENSG00000240631	1.0575	0.14515	1.7595	0.039244	1.7595	0.039244	1.5651	0.058778	0.039244	0.48909466
NA	ENSG00000280239	1.7595	0.039244	1.7595	0.039244	1.7595	0.039244	1.7595	0.039244	0.039244	0.48909466
PYDC2	ENSG00000253548	0.82276	0.20532	1.7592	0.039274	1.3402	0.090095	1.6061	0.054125	0.039274	0.489330417
RAB11FIP3	ENSG00000090565	0.25091	0.40094	1.7591	0.039283	1.1416	0.1268	1.7388	0.041037	0.039283	0.489330417
NA	ENSG00000258084	0.34362	0.36557	-0.1499	0.55958	1.7588	0.039308	0.052608	0.47902	0.039308	0.489352182
TSPYL5	ENSG00000180543	1.7585	0.039328	1.7585	0.039328	1.7585	0.039328	0.34655	0.36446	0.039328	0.489352182
TCHHL1	ENSG00000182898	1.7585	0.039334	0.27843	0.39034	-0.83269	0.79749	0.58436	0.27949	0.039334	0.489352182
RPL7AP69	ENSG00000268483	1.1832	0.11837	1.7584	0.039336	0.063191	0.47481	1.7584	0.039336	0.039336	0.489352182
NA	ENSG00000259106	1.7584	0.039342	0.8294	0.20344	0.8294	0.20344	0.8294	0.20344	0.039342	0.489352182
NA	ENSG00000225761	0.43568	0.33153	1.7583	0.039345	-0.63245	0.73645	1.7583	0.039345	0.039345	0.489352182
ALDH1B1	ENSG00000137124	0.64125	0.26068	-0.24641	0.59732	0.54187	0.29395	1.7579	0.039379	0.039379	0.489650082
AGBL4	ENSG00000186094	1.5761	0.057502	1.3695	0.085419	1.7575	0.039415	1.2952	0.09762	0.039415	0.489921745
RUNX1T1	ENSG00000079102	1.569	0.058324	1.7572	0.039445	1.1189	0.13158	0.58841	0.27813	0.039445	0.489921745
NA	ENSG00000241168	1.7568	0.039474	0.76842	0.22112	0.76842	0.22112	0.30074	0.38181	0.039474	0.489921745
NA	ENSG00000260114	1.6748	0.046988	1.7568	0.039478	1.6748	0.046988	1.6748	0.046988	0.039478	0.489921745
NA	ENSG00000271654	1.7565	0.039504	1.7565	0.039504	1.7565	0.039504	1.7565	0.039504	0.039504	0.489921745
DPEP2	ENSG00000167261	-0.01029	0.5041	0.12218	0.45138	-0.74609	0.77219	1.7564	0.039506	0.039506	0.489921745
NA	ENSG00000262879	1.7564	0.039508	1.5581	0.059599	1.3927	0.081856	0.73148	0.23224	0.039508	0.489921745
TBL1XR1	ENSG00000177565	1.6628	0.048172	0.79108	0.21445	1.7562	0.039525	0.42917	0.3339	0.039525	0.489921745
HSPE1P21	ENSG00000238059	-0.010549	0.50421	1.756	0.039542	-1.7745	0.96201	-1.7745	0.96201	0.039542	0.489921745
SEPTIN7	ENSG00000122545	-0.94911	0.82872	-0.89481	0.81456	0.25969	0.39755	1.756	0.039547	0.039547	0.489921745
LINC02355	ENSG00000248210	0.38932	0.34852	0.76651	0.22169	1.7556	0.039579	0.60377	0.273	0.039579	0.489921745
LINC01910	ENSG00000266278	-0.60594	0.72772	1.7554	0.039598	-0.60594	0.72772	-0.60594	0.72772	0.039598	0.489921745
NA	ENSG00000269391	1.6003	0.054761	1.7553	0.039608	-0.90898	0.81832	-0.90898	0.81832	0.039608	0.489921745
NPAS1	ENSG00000130751	1.2773	0.10075	1.7552	0.039609	1.2517	0.10535	0.095899	0.4618	0.039609	0.489921745
NA	ENSG00000260152	0.85014	0.19762	1.7552	0.039611	0.5001	0.3085	-0.42958	0.66625	0.039611	0.489921745
NA	ENSG00000188646	1.7548	0.039645	1.7548	0.039645	1.7548	0.039645	1.7548	0.039645	0.039645	0.489921745

NA	ENSG00000275265	1.7548	0.039646	1.7548	0.039646	1.7548	0.039646	1.7548	0.039646	0.039646	0.489921745
ZNF418	ENSG00000196724	-1.8233	0.96587	1.7547	0.039654	1.4368	0.075388	-1.8233	0.96587	0.039654	0.489921745
RBM26	ENSG00000139746	0.3481	0.36388	1.6969	0.044862	1.7547	0.039656	1.3395	0.090207	0.039656	0.489921745
NA	ENSG00000274718	0.1795	0.42877	1.3651	0.086107	1.7547	0.039657	1.6246	0.052127	0.039657	0.489921745
DES	ENSG00000175084	0.085862	0.46579	1.7545	0.039675	1.4455	0.074152	1.2802	0.10025	0.039675	0.489921745
THBS1	ENSG00000137801	1.6165	0.052989	0.70863	0.23928	0.78579	0.216	1.7544	0.039677	0.039677	0.489921745
OR2X1P	ENSG00000224830	-1.0815	0.86027	-1.0815	0.86027	-1.651	0.95063	1.7544	0.039683	0.039683	0.489921745
PTPRQ	ENSG00000139304	0.64892	0.2582	1.5853	0.056444	1.7543	0.03969	1.6607	0.048389	0.03969	0.489921745
DPH6	ENSG00000134146	1.3325	0.091355	-0.62635	0.73446	1.754	0.039711	-0.021312	0.5085	0.039711	0.489921745
HIC1	ENSG00000177374	1.2472	0.10616	1.5956	0.055284	0.90672	0.18228	1.7539	0.03972	0.03972	0.489921745
PDXDC2P	ENSG00000255185	0.65467	0.25634	0.81676	0.20703	1.7538	0.03973	1.7538	0.03973	0.03973	0.489921745
NA	ENSG00000260271	1.7538	0.039733	0.5368	0.2957	1.741	0.040842	1.7538	0.039733	0.039733	0.489921745
FALEC	ENSG00000228126	-0.057097	0.52277	1.7537	0.039737	0.049309	0.48034	0.049309	0.48034	0.039737	0.489921745
CYP3A137P	ENSG00000261511	1.7537	0.03974	0.51072	0.30477	0.51072	0.30477	0.51072	0.30477	0.03974	0.489921745
RAB18	ENSG00000099246	1.3277	0.092135	0.42313	0.3361	0.98681	0.16187	1.7536	0.039749	0.039749	0.489921745
NA	ENSG00000278181	-0.16893	0.56707	1.7536	0.039753	0.83494	0.20188	0.83494	0.20188	0.039753	0.489921745
NA	ENSG00000243491	1.7536	0.039754	0.92361	0.17785	1.1411	0.12691	0.24451	0.40342	0.039754	0.489921745
ARGFX	ENSG00000186103	0.046562	0.48143	0.94442	0.17248	0.76188	0.22307	1.7534	0.039768	0.039768	0.489921745
TEX53	ENSG00000230054	0.57762	0.28176	-0.055892	0.52229	-1.3261	0.9076	1.7532	0.039782	0.039782	0.489921745
RNU6-1159P	ENSG00000199803	0.18142	0.42802	1.7531	0.039796	1.7531	0.039796	1.7531	0.039796	0.039796	0.489921745
RNU6-115P	ENSG00000201180	1.7531	0.039796	1.7531	0.039796	1.7531	0.039796	1.7531	0.039796	0.039796	0.489921745
DNAJC10	ENSG00000077232	0.43201	0.33287	0.79411	0.21357	1.753	0.039802	0.98067	0.16338	0.039802	0.489921745
MAP4	ENSG00000047849	0.87045	0.19203	1.1882	0.11737	1.3688	0.085534	1.753	0.039803	0.039803	0.489921745
LINC02814	ENSG00000236975	1.753	0.039803	-0.087895	0.53502	-0.38776	0.6509	-0.38776	0.6509	0.039803	0.489921745
CIDEC	ENSG00000187288	0.15344	0.43903	1.7475	0.040273	1.7527	0.03983	0.83964	0.20056	0.03983	0.490066654
NMRAL2P	ENSG00000171658	0.41305	0.33978	0.11643	0.45366	1.7524	0.039853	1.4148	0.078567	0.039853	0.490066654
SYT16	ENSG00000139973	0.8716	0.19171	1.7524	0.039855	0.75167	0.22612	0.92147	0.1784	0.039855	0.490066654
RN7SL465P	ENSG00000244297	1.7524	0.039855	1.7524	0.039855	1.7524	0.039855	1.7524	0.039855	0.039855	0.490066654
SUMO2P16	ENSG00000253579	1.7519	0.039893	1.7519	0.039893	1.7519	0.039893	1.7519	0.039893	0.039893	0.490222818
NA	ENSG00000224901	1.487	0.068509	1.7519	0.039898	-0.5324	0.70278	1.7519	0.039898	0.039898	0.490222818
NA	ENSG00000248547	0.17893	0.429	1.7519	0.039898	0.17893	0.429	1.1014	0.13536	0.039898	0.490222818

NA	ENSG00000271901	1.7518	0.039908	-0.78623	0.78413	1.7518	0.039908	1.7518	0.039908	0.039908	0.490222818
LSMEM1	ENSG00000181016	-0.32287	0.6266	1.7516	0.039918	-0.54556	0.70732	-0.54556	0.70732	0.039918	0.490222818
NA	ENSG00000223923	1.5535	0.060149	1.2829	0.099756	1.7512	0.039958	1.4124	0.078922	0.039958	0.490575657
PRRG2	ENSG00000126460	1.7511	0.039967	1.7511	0.039967	1.7511	0.039967	1.7269	0.042092	0.039967	0.490575657
HIVEP1	ENSG00000095951	-0.21465	0.58498	-1.064	0.85634	1.7509	0.039983	0.3773	0.35297	0.039983	0.490575657
NAMPTP1	ENSG00000229644	1.7508	0.039987	0.0071559	0.49715	-1.6755	0.95308	1.7508	0.039987	0.039987	0.490575657
MIR4714	ENSG00000264480	-0.51403	0.69638	-0.51403	0.69638	0.5263	0.29934	1.7506	0.04001	0.04001	0.49073169
GRK6	ENSG00000198055	1.3143	0.094366	1.7503	0.040033	0.49219	0.31129	0.79634	0.21292	0.040033	0.49073169
RN7SL632P	ENSG00000241524	1.7503	0.040037	1.7503	0.040037	1.7503	0.040037	-0.12488	0.54969	0.040037	0.49073169
KRT6A	ENSG00000205420	0.69881	0.24234	0.60702	0.27192	1.7502	0.04004	0.70775	0.23955	0.04004	0.49073169
NA	ENSG00000253160	1.75	0.040058	1.75	0.040058	1.75	0.040058	0.11566	0.45396	0.040058	0.490828851
NA	ENSG00000270076	0.84522	0.19899	0.78678	0.2157	1.7495	0.040101	1.6739	0.047072	0.040101	0.49104
TPRX2P	ENSG00000259009	1.7493	0.040116	1.7493	0.040116	1.7493	0.040116	1.1921	0.11661	0.040116	0.49104
TNRC18P1	ENSG00000249661	1.7493	0.040123	1.7493	0.040123	1.7493	0.040123	1.7493	0.040123	0.040123	0.49104
PPP1R2P2	ENSG00000234008	0.2883	0.38656	1.7492	0.040131	0.66876	0.25182	-0.89683	0.8151	0.040131	0.49104
NA	ENSG00000259747	1.749	0.040143	1.6402	0.050485	1.4472	0.073916	0.53293	0.29704	0.040143	0.49104
UBE2Z	ENSG00000159202	1.739	0.041015	1.749	0.040144	1.6931	0.045217	1.4795	0.069503	0.040144	0.49104
NA	ENSG00000241782	1.7489	0.040153	1.7489	0.040153	1.7489	0.040153	0.097804	0.46104	0.040153	0.49104
MRPL44	ENSG00000135900	-0.13784	0.55482	-0.79903	0.78786	1.7489	0.040156	-1.2482	0.89402	0.040156	0.49104
NA	ENSG00000268742	1.7488	0.040167	-0.9742	0.83502	-2.0522	0.97992	1.5081	0.065758	0.040167	0.49104
HYAL1	ENSG00000114378	1.7487	0.040176	1.7181	0.042888	1.6789	0.046582	1.6789	0.046582	0.040176	0.49104
NA	ENSG00000229525	1.7483	0.040203	1.7483	0.040203	1.7483	0.040203	1.7483	0.040203	0.040203	0.491232596
PGBD5	ENSG00000177614	-0.1978	0.5784	1.7482	0.040212	1.2476	0.1061	0.53325	0.29693	0.040212	0.491232596
SCAF11	ENSG00000139218	1.281	0.1001	1.7481	0.040222	0.88442	0.18824	0.15474	0.43851	0.040222	0.491232596
NPW	ENSG00000183971	0.10145	0.4596	-0.51134	0.69544	0.11834	0.4529	1.7478	0.040251	0.040251	0.491463601
NA	ENSG00000238512	1.7475	0.040274	-0.4675	0.67993	-0.17604	0.56987	0.49327	0.31091	0.040274	0.491478598
NA	ENSG00000257634	-0.98834	0.83851	-0.82724	0.79595	-0.36576	0.64273	1.7473	0.040291	0.040291	0.491478598
NA	ENSG00000271977	-0.58997	0.72239	0.73673	0.23064	1.7472	0.040299	1.7472	0.040299	0.040299	0.491478598
NA	ENSG00000267758	1.7472	0.040302	1.2634	0.10322	1.7472	0.040302	1.7472	0.040302	0.040302	0.491478598
SNORC	ENSG00000182600	-0.92582	0.82273	1.7471	0.040308	-0.30691	0.62054	-0.35438	0.63847	0.040308	0.491478598
NME5	ENSG00000112981	1.747	0.04032	1.2428	0.10697	0.88901	0.187	0.81867	0.20649	0.04032	0.491478598

MIRLET7I	ENSG00000199179	1.7467	0.040343	1.7467	0.040343	1.7467	0.040343	1.7467	0.040343	0.040343	0.491478598
LINC01465	ENSG00000221949	1.7467	0.040343	1.7467	0.040343	1.7467	0.040343	1.7467	0.040343	0.040343	0.491478598
NA	ENSG00000275180	1.7467	0.040343	1.7467	0.040343	1.7467	0.040343	1.7467	0.040343	0.040343	0.491478598
C2CD4C	ENSG00000183186	0.28217	0.38891	-0.022535	0.50899	0.59762	0.27505	1.7466	0.040354	0.040354	0.491488686
CISD2	ENSG00000145354	1.7465	0.040364	0.94614	0.17204	1.1106	0.13336	-0.46147	0.67777	0.040364	0.491488686
LINC02052	ENSG00000224406	1.7462	0.040388	0.8511	0.19736	1.13	0.12923	-0.26363	0.60397	0.040388	0.491658066
S100A3	ENSG00000188015	1.5008	0.0667	1.7458	0.040422	1.7458	0.040422	0.66238	0.25386	0.040422	0.491949066
NA	ENSG00000268423	1.7455	0.040447	1.7455	0.040447	1.7455	0.040447	1.0422	0.14865	0.040447	0.491976234
LINC02062	ENSG00000248489	1.7454	0.040455	1.7454	0.040455	0.069088	0.47246	1.7454	0.040455	0.040455	0.491976234
NA	ENSG00000239173	0.41785	0.33803	0.20386	0.41923	1.7452	0.040478	1.0541	0.14592	0.040478	0.491976234
CCL20	ENSG00000115009	-0.39026	0.65183	1.7451	0.040483	0.60202	0.27358	0.89984	0.1841	0.040483	0.491976234
NA	ENSG00000226723	1.7449	0.040502	-1.1159	0.86777	1.1501	0.12505	0.94581	0.17212	0.040502	0.491976234
NA	ENSG00000250523	1.7449	0.040503	1.7449	0.040503	1.7449	0.040503	1.7449	0.040503	0.040503	0.491976234
SOCS5P5	ENSG00000216904	-0.52152	0.699	1.7449	0.040504	-0.47282	0.68183	-0.4845	0.68598	0.040504	0.491976234
NA	ENSG00000242169	0.33231	0.36983	1.7449	0.040505	1.3846	0.083091	1.3846	0.083091	0.040505	0.491976234
NA	ENSG00000257235	1.7444	0.040545	1.7444	0.040545	1.7444	0.040545	1.7444	0.040545	0.040545	0.492029469
LINC01873	ENSG00000232164	-0.095686	0.53811	-1.1394	0.87274	1.7443	0.040551	0.0050739	0.49798	0.040551	0.492029469
SRP14-AS1	ENSG00000248508	0.0096522	0.49615	1.7443	0.040552	0.0096522	0.49615	-0.22848	0.59036	0.040552	0.492029469
LINC02639	ENSG00000230573	1.7441	0.040572	-0.7254	0.7659	0.61993	0.26765	0.32574	0.37231	0.040572	0.492029469
NA	ENSG00000272563	-0.2799	0.61022	1.744	0.040582	1.2607	0.10372	1.2607	0.10372	0.040582	0.492029469
NA	ENSG00000187904	1.7436	0.040618	0.69386	0.24388	0.69386	0.24388	0.69386	0.24388	0.040618	0.492029469
NA	ENSG00000228028	1.7434	0.040629	1.7434	0.040629	1.7434	0.040629	1.7434	0.040629	0.040629	0.492029469
EEF1A1P25	ENSG00000241429	0.92147	0.1784	0.92147	0.1784	1.7434	0.040634	0.40541	0.34259	0.040634	0.492029469
AKR1D1	ENSG00000122787	1.7433	0.040636	0.42229	0.33641	0.38484	0.35018	0.015491	0.49382	0.040636	0.492029469
SNORA70F	ENSG00000206869	1.7431	0.040659	1.7431	0.040659	1.7431	0.040659	1.7431	0.040659	0.040659	0.492029469
RPS16P3	ENSG00000233329	1.743	0.040666	-0.58541	0.72086	-0.58541	0.72086	-0.58541	0.72086	0.040666	0.492029469
TMEM87B	ENSG00000153214	1.3351	0.090921	1.743	0.040667	1.5375	0.062091	1.6722	0.047239	0.040667	0.492029469
COA7	ENSG00000162377	0.82566	0.2045	1.7429	0.040679	0.94156	0.17321	1.0452	0.14796	0.040679	0.492029469
RN7SL254P	ENSG00000240014	1.7427	0.040695	1.7427	0.040695	1.7427	0.040695	1.7427	0.040695	0.040695	0.492029469
RAPGEF3	ENSG00000079337	1.7424	0.040718	-0.40254	0.65636	0.83658	0.20141	0.9029	0.18329	0.040718	0.492029469
DPH3	ENSG00000154813	0.59162	0.27705	1.7424	0.040719	0.25544	0.39919	0.48633	0.31337	0.040719	0.492029469

WNT4	ENSG00000162552	1.7423	0.040732	0.51399	0.30363	0.22561	0.41075	0.18228	0.42768	0.040732	0.492029469
SLC5A9	ENSG00000117834	0.44991	0.32639	1.7422	0.040737	0.56606	0.28568	0.56606	0.28568	0.040737	0.492029469
CDC42-IT1	ENSG00000230068	1.0046	0.15755	0.57567	0.28242	1.742	0.040751	0.98612	0.16204	0.040751	0.492029469
NA	ENSG00000228852	0.49606	0.30993	1.742	0.040754	1.742	0.040754	1.742	0.040754	0.040754	0.492029469
NA	ENSG00000273971	1.7418	0.040771	1.7418	0.040771	1.7418	0.040771	1.7418	0.040771	0.040771	0.492029469
NDUFB4P4	ENSG00000217718	1.596	0.055239	1.7417	0.040779	-0.24756	0.59776	0.055156	0.47801	0.040779	0.492029469
PIP4K2A	ENSG00000150867	1.7416	0.040789	-0.17741	0.57041	1.7416	0.040789	-1.032	0.84896	0.040789	0.492029469
RETREG2	ENSG00000144567	1.7416	0.040792	-0.18904	0.57497	-0.18904	0.57497	-0.18904	0.57497	0.040792	0.492029469
STK32B	ENSG00000152953	0.55887	0.28812	0.37355	0.35437	0.97532	0.1647	1.7413	0.040819	0.040819	0.492029469
OR7E94P	ENSG00000249646	1.7412	0.040824	1.6855	0.045945	1.285	0.099402	-0.39	0.65173	0.040824	0.492029469
RPS2P28	ENSG00000179157	0.79414	0.21356	1.7409	0.040852	-0.91404	0.81965	-0.91404	0.81965	0.040852	0.492029469
BPIFB3	ENSG00000186190	1.0365	0.14998	1.7409	0.040852	0.83824	0.20095	0.67581	0.24958	0.040852	0.492029469
CDCA7	ENSG00000144354	0.78716	0.21559	1.3592	0.087036	0.16794	0.43332	1.7408	0.040856	0.040856	0.492029469
TRAJ10	ENSG00000211879	1.7408	0.040862	1.7408	0.040862	1.7408	0.040862	1.7408	0.040862	0.040862	0.492029469
TYRO3	ENSG00000092445	0.85914	0.19513	-1.8962	0.97103	1.4707	0.070682	1.7406	0.04088	0.04088	0.492029469
PNPT1P1	ENSG00000229241	0.89375	0.18573	0.5024	0.30769	1.7404	0.040899	0.8832	0.18857	0.040899	0.492029469
IRX6	ENSG00000159387	1.7403	0.040907	1.7403	0.040907	1.7403	0.040907	1.7403	0.040907	0.040907	0.492029469
RAB3C	ENSG00000152932	0.44097	0.32962	0.98868	0.16141	1.74	0.040927	0.14779	0.44125	0.040927	0.492029469
NA	ENSG00000233920	0.56729	0.28526	0.46238	0.3219	1.74	0.040932	0.91869	0.17913	0.040932	0.492029469
SALL3	ENSG00000256463	1.5855	0.056429	-0.95762	0.83087	0.42199	0.33652	1.7396	0.040964	0.040964	0.492029469
LAMB3	ENSG00000196878	1.7283	0.041971	1.7395	0.040973	1.3551	0.087693	0.86785	0.19274	0.040973	0.492029469
SLC2A2	ENSG00000163581	-0.28594	0.61254	-0.10699	0.5426	1.7393	0.04099	0.24077	0.40487	0.04099	0.492029469
NA	ENSG00000275716	1.3712	0.08516	1.2953	0.097606	1.7393	0.040991	1.4777	0.06974	0.040991	0.492029469
NA	ENSG00000279348	1.7391	0.041004	1.7391	0.041004	1.7391	0.041004	1.7391	0.041004	0.041004	0.492029469
OSGIN2	ENSG00000164823	0.8898	0.18679	1.6094	0.053766	1.6094	0.053766	1.739	0.041022	0.041022	0.492029469
NA	ENSG00000254095	1.2811	0.10008	1.7389	0.041023	0.38154	0.3514	0.58509	0.27924	0.041023	0.492029469
NRDC	ENSG00000078618	-0.37096	0.64467	0.51085	0.30473	1.7387	0.04104	-1.1261	0.86993	0.04104	0.492029469
SPATA6L	ENSG00000106686	0.12658	0.44963	0.41902	0.3376	-0.10725	0.54271	1.7387	0.041043	0.041043	0.492029469
OR8F1P	ENSG00000239426	1.7386	0.04105	1.7386	0.04105	1.7386	0.04105	1.6631	0.04815	0.04105	0.492029469
NA	ENSG00000273110	1.7386	0.041057	1.7386	0.041057	1.7386	0.041057	1.7386	0.041057	0.041057	0.492029469
QKI	ENSG00000112531	1.7385	0.041061	0.77511	0.21914	0.15689	0.43767	0.84907	0.19792	0.041061	0.492029469

HOXC-AS1	ENSG00000250451	1.7385	0.041064	1.7385	0.041064	1.7385	0.041064	1.7385	0.041064	0.041064	0.492029469
SLC7A9	ENSG00000021488	-0.46252	0.67815	-0.46252	0.67815	-0.40192	0.65613	1.7384	0.041068	0.041068	0.492029469
NA	ENSG00000266642	1.7382	0.041087	1.7382	0.041087	1.7382	0.041087	1.7382	0.041087	0.041087	0.492029469
NA	ENSG00000261273	-0.55369	0.71011	-0.0090524	0.50361	0.018151	0.49276	1.7381	0.041098	0.041098	0.492029469
RNF40	ENSG00000103549	-1.0836	0.86072	1.7378	0.041123	-0.94964	0.82885	-0.94964	0.82885	0.041123	0.492029469
NA	ENSG00000259493	1.7378	0.041124	-0.16053	0.56377	-0.16053	0.56377	-0.16053	0.56377	0.041124	0.492029469
TBCEL	ENSG00000154114	1.3283	0.092033	1.7376	0.041144	0.40412	0.34306	1.7376	0.041144	0.041144	0.492029469
NCKAP1	ENSG00000061676	1.5761	0.057496	1.6899	0.045524	1.7374	0.04116	1.6727	0.047189	0.04116	0.492029469
NA	ENSG00000271870	1.7373	0.041167	1.7373	0.041167	1.7373	0.041167	1.7373	0.041167	0.041167	0.492029469
RNU1-42P	ENSG00000200903	1.7373	0.041169	1.7373	0.041169	1.7373	0.041169	1.7373	0.041169	0.041169	0.492029469
NA	ENSG00000277011	1.7372	0.041175	1.7372	0.041175	1.7372	0.041175	1.7372	0.041175	0.041175	0.492029469
NA	ENSG00000278872	1.7372	0.041175	1.7372	0.041175	1.7372	0.041175	1.7372	0.041175	0.041175	0.492029469
LINC02098	ENSG00000272575	1.6433	0.050163	1.6231	0.052287	1.4868	0.068532	1.7372	0.041177	0.041177	0.492029469
MIR346	ENSG00000199104	1.7371	0.041185	1.5466	0.060977	1.5466	0.060977	-0.44599	0.6722	0.041185	0.492029469
CYP4B1	ENSG00000142973	0.39561	0.3462	1.7369	0.041204	0.82684	0.20416	0.69509	0.2435	0.041204	0.492029469
PPP1R3D	ENSG00000132825	1.7369	0.041205	1.7369	0.041205	1.7369	0.041205	1.7369	0.041205	0.041205	0.492029469
TMED11P	ENSG00000215367	0.39966	0.34471	1.7368	0.041215	1.7368	0.041215	1.4758	0.069997	0.041215	0.492029469
NA	ENSG00000245261	1.7367	0.041224	1.7367	0.041224	1.7367	0.041224	1.7367	0.041224	0.041224	0.492029469
NA	ENSG00000212224	1.7366	0.041226	1.7366	0.041226	1.7366	0.041226	1.7366	0.041226	0.041226	0.492029469
ASS1P9	ENSG00000217791	1.7366	0.041232	1.0789	0.14031	-0.7181	0.76365	1.0789	0.14031	0.041232	0.492029469
TPSP2	ENSG00000260284	-0.1148	0.5457	-0.1148	0.5457	1.7362	0.041261	0.82863	0.20366	0.041261	0.492029469
NA	ENSG00000226669	0.38241	0.35108	0.38241	0.35108	1.7362	0.041268	0.38241	0.35108	0.041268	0.492029469
PPM1B	ENSG00000138032	-0.33926	0.63279	1.7361	0.041276	0.4984	0.3091	0.43296	0.33252	0.041276	0.492029469
SMIM32	ENSG00000271824	1.6088	0.053835	1.4777	0.069745	1.171	0.1208	1.736	0.04128	0.04128	0.492029469
PBRM1	ENSG00000163939	1.2292	0.1095	1.2865	0.099143	1.2365	0.10814	1.736	0.041286	0.041286	0.492029469
MACROH2A1	ENSG00000113648	0.0051849	0.49793	-0.64901	0.74183	-1.5358	0.9377	1.7358	0.0413	0.0413	0.492029469
RNU6-278P	ENSG00000212247	1.7355	0.041327	1.7355	0.041327	1.7355	0.041327	1.7355	0.041327	0.041327	0.492029469
NA	ENSG00000254920	1.7355	0.041328	-2.1039	0.98231	-1.074	0.8586	-1.074	0.8586	0.041328	0.492029469
NA	ENSG00000269185	1.7354	0.041337	1.7354	0.041337	1.7354	0.041337	1.7354	0.041337	0.041337	0.492029469
GPR107	ENSG00000148358	0.37377	0.35429	-0.43756	0.66915	-0.18677	0.57408	1.7349	0.041379	0.041379	0.492029469
GFY	ENSG00000261949	1.7348	0.04139	1.7348	0.04139	1.7348	0.04139	1.7348	0.04139	0.04139	0.492029469

CNOT6L	ENSG00000138767	0.012291	0.4951	1.4121	0.078966	0.40679	0.34208	1.7348	0.041391	0.041391	0.492029469
SNORD116-26	ENSG00000251815	1.7347	0.041394	1.7347	0.041394	1.7347	0.041394	1.7347	0.041394	0.041394	0.492029469
NA	ENSG00000256813	1.7346	0.041408	1.7346	0.041408	1.7346	0.041408	1.7346	0.041408	0.041408	0.492029469
STAT5B	ENSG00000173757	0.66953	0.25158	1.7346	0.041409	1.6603	0.04843	-0.15884	0.5631	0.041409	0.492029469
NRSN2	ENSG00000125841	-0.81041	0.79115	-0.77946	0.78215	-0.076026	0.5303	1.7345	0.04141	0.04141	0.492029469
NA	ENSG00000279250	1.7345	0.041411	-0.28357	0.61163	-0.28357	0.61163	-0.28357	0.61163	0.041411	0.492029469
NDUFAF7	ENSG00000003509	1.7344	0.041421	1.7344	0.041421	1.7344	0.041421	0.63674	0.26215	0.041421	0.492029469
DTWD1	ENSG00000104047	1.7344	0.041426	1.4544	0.072916	1.4544	0.072916	1.4544	0.072916	0.041426	0.492029469
GAL3ST4	ENSG00000197093	1.7343	0.041431	0.56101	0.2874	0.69124	0.24471	1.7343	0.041431	0.041431	0.492029469
RAB35	ENSG00000111737	0.16489	0.43451	0.16489	0.43451	0.16489	0.43451	1.7342	0.041445	0.041445	0.492029469
NDUFB4P9	ENSG00000249754	1.138	0.12756	1.138	0.12756	1.7341	0.04145	1.138	0.12756	0.04145	0.492029469
NA	ENSG00000264705	-0.048512	0.51935	0.38572	0.34985	-0.29583	0.61632	1.7341	0.041453	0.041453	0.492029469
MPP7	ENSG00000150054	1.4337	0.075832	1.7339	0.041471	0.9646	0.16737	1.509	0.06565	0.041471	0.492029469
PAPSS1	ENSG00000138801	0.84281	0.19967	1.7337	0.041484	0.34693	0.36432	-0.47119	0.68125	0.041484	0.492029469
PIF1	ENSG00000140451	0.56967	0.28445	1.6367	0.050842	1.7337	0.041485	1.3481	0.088819	0.041485	0.492029469
ZNF621	ENSG00000172888	0.36992	0.35572	1.7337	0.041486	1.5938	0.055492	1.7337	0.041486	0.041486	0.492029469
RPL35AP4	ENSG00000225644	1.7336	0.041497	1.7336	0.041497	0.97727	0.16422	0.54444	0.29307	0.041497	0.492029469
KIFC1	ENSG00000237649	0.60886	0.27131	0.53253	0.29718	1.7336	0.041497	1.7336	0.041497	0.041497	0.492029469
RNU6-1120P	ENSG00000199858	1.7334	0.041517	1.7334	0.041517	1.7334	0.041517	1.7334	0.041517	0.041517	0.492029469
SLAMF6	ENSG00000162739	0.91291	0.18065	0.47214	0.31841	1.7331	0.041536	1.7331	0.041536	0.041536	0.492029469
RASD1	ENSG00000108551	1.733	0.041551	1.733	0.041551	-0.12749	0.55072	0.57246	0.2835	0.041551	0.492029469
NA	ENSG00000232184	0.084256	0.46643	0.084256	0.46643	1.7329	0.041553	0.26375	0.39599	0.041553	0.492029469
NA	ENSG00000242973	1.7329	0.041556	1.7329	0.041556	1.7329	0.041556	1.7329	0.041556	0.041556	0.492029469
TOPORSLP	ENSG00000242111	1.3528	0.088067	1.7327	0.041573	1.7327	0.041573	1.4634	0.071679	0.041573	0.492029469
NA	ENSG00000262888	0.22429	0.41126	1.0393	0.14934	1.0393	0.14934	1.7327	0.041578	0.041578	0.492029469
ZEB2P1	ENSG00000249506	0.33644	0.36827	0.66682	0.25244	0.12887	0.44873	1.7326	0.041582	0.041582	0.492029469
PDZD7	ENSG00000186862	1.3197	0.093463	0.98265	0.16289	1.3906	0.082169	1.7325	0.041593	0.041593	0.492029469
CENPU	ENSG00000151725	1.7322	0.041615	0.53046	0.2979	0.74022	0.22958	0.24865	0.40182	0.041615	0.492029469
LINC00562	ENSG00000260388	1.7321	0.041626	1.7321	0.041626	1.6026	0.054514	1.7321	0.041626	0.041626	0.492029469
KDM1B	ENSG00000165097	0.8486	0.19805	1.403	0.080304	0.67647	0.24937	1.732	0.04164	0.04164	0.492029469
RAP1AP	ENSG00000258769	1.7317	0.041666	-0.21814	0.58634	1.7317	0.041666	1.7317	0.041666	0.041666	0.492029469

RN7SL664P	ENSG00000265078	1.7317	0.041666	1.7317	0.041666	1.7317	0.041666	1.7317	0.041666	0.041666	0.492029469
LZTS2	ENSG00000107816	1.1388	0.12739	0.94137	0.17326	0.94137	0.17326	1.7316	0.041675	0.041675	0.492029469
RNA5SP105	ENSG00000212425	1.7315	0.041683	0.55043	0.29101	-0.03547	0.51415	1.1467	0.12576	0.041683	0.492029469
AHSA1	ENSG00000100591	1.7314	0.041692	1.7314	0.041692	1.7314	0.041692	-0.33522	0.63127	0.041692	0.492029469
LARS1	ENSG00000133706	1.7312	0.041707	1.7312	0.041707	1.398	0.081051	0.75047	0.22649	0.041707	0.492029469
LINC00905	ENSG00000167459	1.731	0.041724	0.02112	0.49157	1.731	0.041724	1.731	0.041724	0.041724	0.492029469
RN7SKP132	ENSG00000251810	1.731	0.041728	1.731	0.041728	-0.20226	0.58015	1.5574	0.059692	0.041728	0.492029469
PLBD1-AS1	ENSG00000256751	-0.051356	0.52048	0.9468	0.17187	0.9468	0.17187	1.7309	0.041736	0.041736	0.492029469
ALOX5	ENSG00000012779	1.1683	0.12134	1.0538	0.14599	0.2818	0.38905	1.7308	0.04174	0.04174	0.492029469
C1QL4	ENSG00000186897	0.94547	0.17221	-0.10788	0.54295	1.7307	0.041753	1.667	0.047762	0.041753	0.492029469
PSEN1	ENSG00000080815	1.0124	0.15568	1.0928	0.13724	1.3293	0.091878	1.7305	0.041771	0.041771	0.492029469
ACVR2B	ENSG00000114739	1.3851	0.083006	1.7303	0.041784	1.1214	0.13107	0.34454	0.36522	0.041784	0.492029469
ATP2B1	ENSG00000070961	1.7303	0.041789	0.62409	0.26628	0.24053	0.40496	0.24053	0.40496	0.041789	0.492029469
NDUFS5	ENSG00000168653	1.7303	0.04179	1.7303	0.04179	1.5778	0.057306	0.22399	0.41138	0.04179	0.492029469
ATG10-IT1	ENSG00000236447	1.7303	0.041791	1.7303	0.041791	1.7303	0.041791	1.7303	0.041791	0.041791	0.492029469
RNU6-725P	ENSG00000252371	1.4848	0.068803	1.4848	0.068803	1.7302	0.041793	1.4848	0.068803	0.041793	0.492029469
COX6CP4	ENSG00000270500	0.54047	0.29444	0.54047	0.29444	-0.61049	0.72923	1.7302	0.041799	0.041799	0.492029469
MEGF8	ENSG00000105429	0.21962	0.41309	0.0051962	0.49793	1.73	0.041813	-0.36501	0.64245	0.041813	0.492029469
LINC01706	ENSG00000224711	0.39009	0.34824	1.73	0.041818	1.73	0.041818	1.73	0.041818	0.041818	0.492029469
CLDN9	ENSG00000213937	1.7299	0.041822	-0.20592	0.58157	1.7299	0.041822	1.7299	0.041822	0.041822	0.492029469
NA	ENSG00000255376	1.7299	0.041822	1.7299	0.041822	1.7299	0.041822	1.7299	0.041822	0.041822	0.492029469
KPNA3	ENSG00000102753	1.7231	0.042438	0.10946	0.45642	0.048528	0.48065	1.7297	0.041843	0.041843	0.492157709
CDH11	ENSG00000140937	-0.64698	0.74118	-0.47765	0.68355	1.7291	0.041899	-0.039653	0.51582	0.041899	0.492339819
CLEC4A	ENSG00000111729	0.46658	0.3204	0.63719	0.262	1.729	0.041902	-1.2539	0.89506	0.041902	0.492339819
NA	ENSG00000275198	0.43339	0.33237	1.729	0.041905	-0.34678	0.63562	0.64705	0.2588	0.041905	0.492339819
C8orf17	ENSG00000250733	1.4061	0.079847	1.729	0.041906	1.4151	0.078524	1.2473	0.10615	0.041906	0.492339819
ARHGDIB	ENSG00000111348	1.729	0.041909	1.729	0.041909	1.729	0.041909	0.87757	0.19009	0.041909	0.492339819
LRP12	ENSG00000147650	-1.0229	0.84683	1.7287	0.041931	0.98135	0.16321	-0.10931	0.54352	0.041931	0.492479544
NA	ENSG00000245498	0.41152	0.34034	1.7285	0.041949	0.41152	0.34034	0.41152	0.34034	0.041949	0.492572234
RN7SL596P	ENSG00000264519	0.11977	0.45233	-0.60596	0.72773	-1.3151	0.90576	1.7282	0.041977	0.041977	0.492685762
EXT1	ENSG00000182197	-0.96376	0.83242	-1.3303	0.90828	-0.044338	0.51768	1.7281	0.041984	0.041984	0.492685762

GLTPD2	ENSG00000182327	1.7281	0.041989	1.7281	0.041989	1.7281	0.041989	1.7281	0.041989	0.041989	0.492685762
RNU6-331P	ENSG00000207455	1.2837	0.099628	1.567	0.058557	1.7278	0.042016	1.2837	0.099628	0.042016	0.49288389
MANBA	ENSG00000109323	1.727	0.042083	1.6153	0.053126	1.5477	0.060846	1.0155	0.15493	0.042083	0.493383924
SPEF2	ENSG00000152582	1.227	0.10991	1.7269	0.042089	0.86522	0.19346	-0.036495	0.51456	0.042089	0.493383924
NA	ENSG00000230303	1.1529	0.12448	-0.39969	0.65531	1.7269	0.042089	1.1572	0.12359	0.042089	0.493383924
LRRC34	ENSG00000171757	1.4596	0.072205	1.4596	0.072205	1.4596	0.072205	1.7267	0.042112	0.042112	0.493498165
NA	ENSG00000233933	1.7266	0.042119	0.89154	0.18632	1.1099	0.13352	0.031306	0.48751	0.042119	0.493498165
NA	ENSG00000279106	-0.083767	0.53338	1.7263	0.042144	0.48473	0.31393	-0.60754	0.72825	0.042144	0.49362097
DNM1P33	ENSG00000260096	-0.21859	0.58651	-0.44088	0.67035	1.6717	0.047294	1.7262	0.042156	0.042156	0.49362097
NA	ENSG00000278513	0.20383	0.41924	1.7261	0.042165	1.7261	0.042165	0.69661	0.24302	0.042165	0.49362097
NA	ENSG00000224134	0.073228	0.47081	1.4662	0.071297	1.6097	0.053732	1.726	0.04217	0.04217	0.49362097
SIRPA	ENSG00000198053	1.7252	0.042245	0.75331	0.22563	-0.62241	0.73316	0.70781	0.23953	0.042245	0.494102797
NA	ENSG00000254040	-0.043237	0.51724	-0.30007	0.61794	-0.051785	0.52065	1.7251	0.042255	0.042255	0.494102797
MIR550A1	ENSG00000207771	-0.20091	0.57961	1.2138	0.11241	0.047616	0.48101	1.725	0.042259	0.042259	0.494102797
NA	ENSG00000280920	0.28199	0.38898	1.7249	0.04227	0.28199	0.38898	0.28199	0.38898	0.04227	0.494102797
NA	ENSG00000267274	0.12772	0.44919	0.45963	0.32289	1.7249	0.042271	-0.24403	0.5964	0.042271	0.494102797
PELI1	ENSG00000197329	0.3294	0.37093	0.53578	0.29606	0.9381	0.1741	1.7249	0.042272	0.042272	0.494102797
NA	ENSG00000250781	0.76236	0.22292	1.7247	0.042292	1.4842	0.068873	1.0454	0.14792	0.042292	0.494111218
STARD7-AS1	ENSG00000204685	1.2974	0.09725	0.73695	0.23058	1.7247	0.042293	1.2974	0.09725	0.042293	0.494111218
TRAV2	ENSG00000211776	1.7244	0.042314	-0.31629	0.62411	-0.31629	0.62411	-0.31629	0.62411	0.042314	0.494238068
IL12A	ENSG00000168811	1.7243	0.04233	0.43265	0.33263	1.0474	0.14746	-0.20271	0.58032	0.04233	0.494248172
NA	ENSG00000266670	0.2147	0.415	1.7242	0.042338	1.2553	0.10468	0.63029	0.26425	0.042338	0.494248172
NA	ENSG00000124835	1.724	0.042351	-0.10828	0.54312	0.5589	0.28812	-0.84219	0.80016	0.042351	0.494248172
SRP72P1	ENSG00000265182	1.7237	0.042378	1.6388	0.050631	0.86788	0.19273	0.86788	0.19273	0.042378	0.494248172
NA	ENSG00000256041	0.73378	0.23154	0.48951	0.31224	-1.067	0.85701	1.7237	0.042383	0.042383	0.494248172
RNU6-1025P	ENSG00000222561	0.083398	0.46677	-0.12657	0.55036	-0.65498	0.74376	1.7234	0.042408	0.042408	0.494248172
RNU7-125P	ENSG00000238964	1.7233	0.042421	1.7229	0.042453	1.7233	0.042421	1.7233	0.042421	0.042421	0.494248172
RNU7-186P	ENSG00000238419	0.95144	0.17069	1.7231	0.042438	0.95144	0.17069	0.95144	0.17069	0.042438	0.494248172
NA	ENSG00000272108	0.052429	0.47909	1.7229	0.042456	0.87667	0.19033	0.87667	0.19033	0.042456	0.494248172
METRNL	ENSG00000176845	1.7229	0.042457	-0.42269	0.66374	1.6281	0.051754	1.7229	0.042457	0.042457	0.494248172
NA	ENSG00000217801	1.1313	0.12897	1.7227	0.042469	1.1313	0.12897	0.2846	0.38798	0.042469	0.494248172

MIR4319	ENSG00000265957	1.7227	0.042475	1.0088	0.15654	1.7227	0.042475	1.7227	0.042475	0.042475	0.494248172
NA	ENSG00000236132	1.7226	0.04248	1.7226	0.04248	1.7226	0.04248	1.7226	0.04248	0.04248	0.494248172
NA	ENSG00000233574	1.5808	0.056962	1.5808	0.056962	1.7226	0.042483	0.99492	0.15989	0.042483	0.494248172
NA	ENSG00000238012	0.69485	0.24357	1.6303	0.051521	1.4847	0.068813	1.7224	0.042497	0.042497	0.494248172
COPA	ENSG00000122218	1.2519	0.10531	0.91443	0.18025	1.2519	0.10531	1.7223	0.042505	0.042505	0.494248172
HEYL	ENSG00000163909	1.7222	0.042518	1.3922	0.081938	1.0571	0.14522	-0.8606	0.80527	0.042518	0.494248172
INTS10	ENSG00000104613	-1.1767	0.88034	-0.79724	0.78734	-1.1091	0.86631	1.7221	0.042525	0.042525	0.494248172
FRMD6	ENSG00000139926	1.3576	0.087297	1.2063	0.11384	1.6802	0.046459	1.7221	0.042527	0.042527	0.494248172
RNU6-410P	ENSG00000252815	-0.17298	0.56867	1.7221	0.042529	-0.45888	0.67684	-0.636	0.73761	0.042529	0.494248172
NA	ENSG00000177452	1.722	0.042532	1.722	0.042532	1.722	0.042532	1.722	0.042532	0.042532	0.494248172
NA	ENSG00000253712	1.7218	0.042556	0.93946	0.17375	1.7218	0.042556	1.7218	0.042556	0.042556	0.494248172
GJB2	ENSG00000165474	0.84856	0.19806	1.7216	0.042573	-0.27773	0.60939	-0.67843	0.75125	0.042573	0.494248172
CHIC2	ENSG00000109220	1.2329	0.10881	-1.8885	0.97052	-1.2109	0.88703	1.7215	0.042582	0.042582	0.494248172
NA	ENSG00000280167	1.7215	0.042584	1.7215	0.042584	1.7215	0.042584	1.7215	0.042584	0.042584	0.494248172
ZCCHC8	ENSG00000033030	0.80248	0.21114	0.67984	0.2483	0.10487	0.45824	1.7214	0.042589	0.042589	0.494248172
CASP8AP2	ENSG00000118412	-1.0474	0.85253	1.7213	0.042601	-0.20073	0.57955	-0.44382	0.67141	0.042601	0.494248172
LINC00251	ENSG00000280725	1.094	0.13698	1.094	0.13698	1.7212	0.042607	1.094	0.13698	0.042607	0.494248172
NA	ENSG00000282940	1.4811	0.069294	1.7212	0.042609	0.97575	0.16459	1.4811	0.069294	0.042609	0.494248172
NA	ENSG00000259933	1.7207	0.042649	1.7207	0.042649	1.7207	0.042649	1.7207	0.042649	0.042649	0.494297841
ARNILA	ENSG00000235072	0.20254	0.41975	-0.003163	0.50126	1.7207	0.042651	-0.79121	0.78559	0.042651	0.494297841
GGCTP1	ENSG00000251000	1.7207	0.042652	1.5912	0.055784	0.70125	0.24157	1.7207	0.042652	0.042652	0.494297841
STK32C	ENSG00000165752	0.2464	0.40269	0.41154	0.34034	0.58382	0.27967	1.7206	0.042658	0.042658	0.494297841
SAE1	ENSG00000142230	0.16339	0.43511	1.6105	0.053643	1.7206	0.042664	-0.92024	0.82128	0.042664	0.494297841
LTB4R2	ENSG00000213906	1.6217	0.052437	1.7203	0.04269	1.6217	0.052437	1.6217	0.052437	0.04269	0.494350741
LINC01562	ENSG00000203356	0.40031	0.34446	0.40031	0.34446	0.40031	0.34446	1.7202	0.042698	0.042698	0.494350741
SBNO2	ENSG00000064932	1.7202	0.042699	0.94043	0.1735	-0.23946	0.59463	0.10592	0.45782	0.042699	0.494350741
NA	ENSG00000251024	1.082	0.13962	1.082	0.13962	1.7201	0.042711	0.067298	0.47317	0.042711	0.494356438
HIF1A	ENSG00000100644	1.0519	0.14642	1.7199	0.042725	0.87315	0.19129	0.79667	0.21282	0.042725	0.494356438
GAK	ENSG00000178950	1.7196	0.042753	0.43857	0.33048	-0.093736	0.53734	-0.27284	0.60751	0.042753	0.494356438
NA	ENSG00000253917	1.7192	0.042789	1.7192	0.042789	0.41931	0.33749	0.37224	0.35486	0.042789	0.494356438
NA	ENSG00000260362	0.68941	0.24528	1.7192	0.042791	-0.072822	0.52903	0.10018	0.4601	0.042791	0.494356438

PHF5CP	ENSG00000267178	1.719	0.042804	1.719	0.042804	1.719	0.042804	1.719	0.042804	0.042804	0.494356438
SLC25A48	ENSG00000145832	0.099011	0.46056	1.6815	0.046335	1.7188	0.042826	1.3206	0.093321	0.042826	0.494356438
PPIAP81	ENSG00000213548	1.2138	0.11241	1.7187	0.042833	1.306	0.095771	0.92347	0.17788	0.042833	0.494356438
HERC3	ENSG00000138641	0.93755	0.17424	-0.16826	0.56681	-0.13643	0.55426	1.7187	0.042838	0.042838	0.494356438
SNX18P13	ENSG00000230965	-0.43231	0.66724	-0.36614	0.64287	-1.0575	0.85486	1.7185	0.042852	0.042852	0.494356438
MIR3166	ENSG00000266581	1.7184	0.042859	1.7184	0.042859	1.7184	0.042859	1.5397	0.061815	0.042859	0.494356438
ZACN	ENSG00000186919	1.7182	0.042878	1.7182	0.042878	1.7182	0.042878	1.7182	0.042878	0.042878	0.494356438
DEFA3	ENSG00000239839	-0.49426	0.68944	1.7179	0.042909	-0.46598	0.67938	0.68949	0.24526	0.042909	0.494356438
NA	ENSG00000277935	1.7179	0.042909	1.7179	0.042909	1.7179	0.042909	1.7179	0.042909	0.042909	0.494356438
RNA5SP138	ENSG00000201968	-0.062127	0.52477	-0.094268	0.53755	-0.25792	0.60176	1.7177	0.042928	0.042928	0.494356438
NA	ENSG00000232411	1.7148	0.043192	1.1454	0.12603	1.7177	0.042928	1.7177	0.042928	0.042928	0.494356438
CTXND1	ENSG00000259417	-0.60914	0.72878	-0.60914	0.72878	0.79195	0.21419	1.7175	0.042945	0.042945	0.494356438
NA	ENSG00000258811	0.37949	0.35216	0.37949	0.35216	1.717	0.042987	0.37949	0.35216	0.042987	0.494356438
FMO5	ENSG00000131781	1.7167	0.043016	1.3144	0.094355	0.48833	0.31266	1.4614	0.071947	0.043016	0.494356438
NA	ENSG00000264761	1.7162	0.043067	-0.019787	0.50789	0.068121	0.47284	1.7162	0.043067	0.043067	0.494356438
RPGRIP1	ENSG00000092200	1.4187	0.07799	1.6895	0.045559	1.7161	0.043068	1.6342	0.051106	0.043068	0.494356438
MIR1289-2	ENSG00000221287	1.2318	0.10901	1.2318	0.10901	1.7161	0.04307	1.2318	0.10901	0.04307	0.494356438
RPL34P23	ENSG00000243669	-0.72451	0.76562	0.1411	0.4439	1.7161	0.043076	0.48623	0.3134	0.043076	0.494356438
MTCO2P25	ENSG00000230654	1.7158	0.043102	1.7158	0.043102	1.7158	0.043102	1.7158	0.043102	0.043102	0.494356438
IGF2BP1	ENSG00000159217	1.6623	0.048222	1.7157	0.043107	-0.12963	0.55157	-0.12963	0.55157	0.043107	0.494356438
DSEL	ENSG00000171451	1.7157	0.043113	0.82627	0.20432	0.6159	0.26898	0.25715	0.39853	0.043113	0.494356438
RNU6-338P	ENSG00000206615	1.2513	0.10541	0.2817	0.38909	1.7155	0.043124	0.66227	0.2539	0.043124	0.494356438
MTHFD2P1	ENSG00000244681	-0.68039	0.75187	1.3777	0.084151	0.29811	0.38281	1.7154	0.043138	0.043138	0.494356438
TMEM17	ENSG00000186889	1.7152	0.043154	1.5711	0.058077	1.0778	0.14057	0.46971	0.31928	0.043154	0.494356438
NA	ENSG00000219240	-0.79583	0.78693	-0.37487	0.64612	0.032385	0.48708	1.7152	0.043154	0.043154	0.494356438
NA	ENSG00000279016	1.7152	0.043155	1.7152	0.043155	1.7152	0.043155	1.7152	0.043155	0.043155	0.494356438
RPL12P1	ENSG00000204194	1.7151	0.043164	1.7151	0.043164	0.52541	0.29965	1.7151	0.043164	0.043164	0.494356438
LINC01920	ENSG00000236049	-0.94579	0.82787	1.7151	0.043168	0.30683	0.37949	-0.75083	0.77362	0.043168	0.494356438
RD3L	ENSG00000227729	1.715	0.043169	0.17662	0.4299	1.4199	0.07782	0.17662	0.4299	0.043169	0.494356438
CNTN4	ENSG00000144619	1.715	0.043172	1.166	0.1218	-0.7213	0.76464	-0.3163	0.62411	0.043172	0.494356438
MRPL30P1	ENSG00000276576	1.7149	0.043177	1.7149	0.043177	1.7149	0.043177	1.7149	0.043177	0.043177	0.494356438

CHD5 ENSG00000116254 0.51491 0.30331 1.7148 0.043188 0.77984 0.21774
-0.2093 0.58289 0.043188 0.494356438
PDE3B ENSG00000152270 -0.58923 0.72215 -0.5832 0.72012 1.7147 0.043202
-0.41258 0.66004 0.043202 0.494356438
LINC01396 ENSG00000273396 -0.64338 0.74001 1.113 0.13286 1.7145 0.043221
-0.98754 0.83831 0.043221 0.494356438
RDH8 ENSG00000080511 1.4867 0.068549 0.72258 0.23497 0.0035756 0.49857
1.7144 0.043223 0.043223 0.494356438
RUVBL1 ENSG00000175792 1.0658 0.14327 1.7144 0.043228 0.64101
0.26076 0.81336 0.20801 0.043228 0.494356438
UBE2Q2P8 ENSG00000259633 1.7143 0.043235 0.31639 0.37585 -0.47122
0.68126 0.55383 0.28985 0.043235 0.494356438
NA ENSG00000230638 1.7143 0.043238 1.7143 0.043238 -1.1464 0.87419
-0.013208 0.50527 0.043238 0.494356438
RBM44 ENSG00000177483 -0.37876 0.64757 0.84008 0.20043 1.7142 0.043246
0.56076 0.28748 0.043246 0.494356438
CLIP1-AS1 ENSG00000257097 1.3197 0.093465 1.7142 0.043247 0.70637
0.23998 0.5703 0.28424 0.043247 0.494356438
NA ENSG00000284523 1.3555 0.087633 1.714 0.043261 1.3565 0.087469
1.6396 0.050544 0.043261 0.494356438
NA ENSG00000263393 0.7231 0.23481 1.7136 0.043297 0.7231 0.23481
0.7231 0.23481 0.043297 0.494356438
NA ENSG00000231015 1.7135 0.043308 1.7135 0.043308 1.7135 0.043308
1.7135 0.043308 0.043308 0.494356438
POLR2K ENSG00000147669 -0.70659 0.76009 1.7135 0.04331 -1.2701
0.89797 -0.69754 0.75727 0.04331 0.494356438
GP6 ENSG00000088053 0.94604 0.17206 -0.18785 0.5745 1.7135 0.043313
-0.086562 0.53449 0.043313 0.494356438
BRD9 ENSG00000028310 0.64681 0.25888 1.7134 0.043321 1.2221 0.11083
0.46714 0.3202 0.043321 0.494356438
LINC01411 ENSG00000249306 -0.70722 0.76029 0.49111 0.31167 0.22202
0.41215 1.7134 0.043322 0.043322 0.494356438
HNRNPF ENSG00000169813 1.7133 0.043327 1.7133 0.043327 0.50576
0.30651 1.145 0.12611 0.043327 0.494356438
NAIP ENSG00000249437 0.47578 0.31712 1.7133 0.043331 -0.41043 0.65925
0.65359 0.25669 0.043331 0.494356438
VHLL ENSG00000189030 1.7133 0.043333 1.7133 0.043333 1.7133 0.043333
1.7133 0.043333 0.043333 0.494356438
RNU4-41P ENSG00000201296 0.096706 0.46148 1.6144 0.053218 1.6144
0.053218 1.7132 0.043335 0.043335 0.494356438
NA ENSG00000258569 1.6808 0.046398 1.7131 0.04335 0.36053 0.35922
0.79634 0.21292 0.04335 0.494356438
RNU6-123P ENSG00000251972 1.713 0.043357 1.713 0.043357 1.713 0.043357 1.713
0.043357 0.043357 0.494356438
NA ENSG00000224638 1.3808 0.083669 0.83169 0.20279 -0.35315 0.63801
1.713 0.043357 0.043357 0.494356438
NA ENSG00000260441 -0.90716 0.81784 1.7129 0.043364 -0.90716 0.81784
-0.90716 0.81784 0.043364 0.494356438
NA ENSG00000269519 1.7129 0.043368 1.7129 0.043368 1.7129 0.043368
1.7129 0.043368 0.043368 0.494356438
NA ENSG00000259140 1.2415 0.1072 1.0171 0.15456 1.7126 0.043397
0.77369 0.21956 0.043397 0.494356438
NA ENSG00000228569 1.4555 0.072759 1.7125 0.043405 1.3561 0.087532
1.7125 0.043405 0.043405 0.494356438
NA ENSG00000227133 1.7122 0.043431 0.68911 0.24538 0.68911 0.24538
-0.066314 0.52644 0.043431 0.494356438
KRT17P4 ENSG00000205312 -0.82272 0.79467 1.7121 0.043441 0.24553
0.40302 1.011 0.15602 0.043441 0.494356438
NA ENSG00000258982 1.3345 0.091014 0.40294 0.34349 1.3345 0.091014
1.7118 0.043463 0.043463 0.494356438
CHORDC2P ENSG00000259050 1.5984 0.054981 1.7118 0.04347 1.5984
0.054981 1.7118 0.04347 0.04347 0.494356438

C10orf71-AS1	ENSG00000236208	-0.29441	0.61578	1.7117	0.043474	1.7117	0.043474	1.7117	0.043474	0.043474	0.494356438
RNU6-506P	ENSG00000252362	0.66441	0.25322	1.4487	0.073712	0.68722	0.24597	1.7115	0.04349	0.04349	0.494356438
ZNF197-AS1	ENSG00000233509	-1.6083	0.94612	-1.0791	0.85973	-1.0791	0.85973	1.7115	0.043498	0.043498	0.494356438
NMU	ENSG00000109255	-0.57342	0.71682	1.7112	0.043522	-0.77504	0.78084	-1.5215	0.93593	0.043522	0.494356438
NA	ENSG00000254222	1.7111	0.043527	1.1626	0.12249	-0.69495	0.75646	-0.63552	0.73746	0.043527	0.494356438
KRTAP24-1	ENSG00000188694	1.7109	0.043546	1.7109	0.043546	1.7109	0.043546	1.7109	0.043546	0.043546	0.494356438
NA	ENSG00000261707	1.7109	0.043546	1.7109	0.043546	1.7109	0.043546	1.7109	0.043546	0.043546	0.494356438
LINC01139	ENSG00000215808	1.7109	0.043548	1.1362	0.12794	1.4452	0.074198	1.4452	0.074198	0.043548	0.494356438
NA	ENSG00000275674	1.7109	0.043551	-0.24907	0.59835	1.2073	0.11367	-0.43486	0.66817	0.043551	0.494356438
RNA5SP493	ENSG00000200985	1.7107	0.043567	1.7107	0.043567	1.7107	0.043567	1.7107	0.043567	0.043567	0.494356438
NA	ENSG00000279254	1.1341	0.12838	1.7107	0.043567	1.7107	0.043567	0.24118	0.40471	0.043567	0.494356438
C3AR1	ENSG00000171860	1.7106	0.043574	-0.49798	0.69075	1.7106	0.043574	1.7106	0.043574	0.043574	0.494356438
VASH2	ENSG00000143494	1.6099	0.053713	1.4793	0.069536	1.4529	0.07313	1.7105	0.043591	0.043591	0.494356438
GRK2	ENSG00000173020	0.95832	0.16895	0.82752	0.20397	0.58005	0.28094	1.7102	0.043614	0.043614	0.494356438
UNC79	ENSG00000133958	0.50648	0.30626	1.7101	0.043625	1.0534	0.14607	-0.13009	0.55175	0.043625	0.494356438
C18orf32	ENSG00000177576	-0.091544	0.53647	1.4432	0.074475	0.061704	0.4754	1.71	0.043632	0.043632	0.494356438
LITAF	ENSG00000189067	1.71	0.043632	-0.65216	0.74285	-0.057529	0.52294	-0.079963	0.53187	0.043632	0.494356438
TRGJP	ENSG00000211691	0.26142	0.39689	1.71	0.043634	1.71	0.043634	1.71	0.043634	0.043634	0.494356438
CLCA1	ENSG00000016490	-0.36207	0.64135	1.71	0.043636	-0.33912	0.63274	-0.77802	0.78172	0.043636	0.494356438
NA	ENSG00000224260	-0.37456	0.64601	1.6251	0.052075	-0.37729	0.64702	1.7099	0.043647	0.043647	0.494356438
NRBF2P6	ENSG00000251485	1.7098	0.043652	1.7098	0.043652	1.7098	0.043652	1.7098	0.043652	0.043652	0.494356438
NA	ENSG00000229846	1.4725	0.07044	1.3181	0.093727	1.3181	0.093727	1.7097	0.043665	0.043665	0.494356438
ARHGEF7-AS2	ENSG00000235875	-0.30939	0.62149	1.7096	0.043673	1.7096	0.043673	1.7096	0.043673	0.043673	0.494356438
NA	ENSG00000242444	1.7094	0.043686	1.2203	0.11118	-0.2741	0.60799	1.7094	0.043686	0.043686	0.494356438
CYP17A1	ENSG00000148795	0.21101	0.41644	0.67612	0.24948	1.7094	0.043687	1.2808	0.10013	0.043687	0.494356438
ENTPD5	ENSG00000187097	1.683	0.046186	1.7092	0.043703	1.5898	0.055937	1.1542	0.12422	0.043703	0.494356438
OR5AC2	ENSG00000196578	1.7092	0.043705	0.71858	0.2362	0.25812	0.39816	1.7092	0.043705	0.043705	0.494356438
SLC4A4	ENSG00000080493	1.4206	0.077718	1.7091	0.043715	1.0643	0.14361	1.259	0.10401	0.043715	0.494356438
RNU6ATAC33P	ENSG00000221574	-1.3815	0.91644	0.96416	0.16748	-0.28986	0.61404	1.7089	0.043731	0.043731	0.494356438
DCAF4	ENSG00000119599	1.5518	0.060357	0.076146	0.46965	1.7088	0.043746	1.059	0.1448	0.043746	0.494356438
GRB2	ENSG00000177885	1.7088	0.043748	1.6773	0.046743	1.6525	0.049219	1.6931	0.045218	0.043748	0.494356438

CCDC85A	ENSG00000055813	-2.0038	0.97745	-1.7524	0.96015	-1.7027	0.95569	1.7087	0.043753	0.043753	0.494356438
OR11H5P	ENSG00000258625	1.7087	0.043755	0.98305	0.16279	1.7087	0.043755	1.7087	0.043755	0.043755	0.494356438
NA	ENSG00000202198	1.7084	0.043782	1.7084	0.043782	1.7084	0.043782	1.7084	0.043782	0.043782	0.494356438
NA	ENSG00000277746	-0.063141	0.52517	1.7084	0.043782	0.073693	0.47063	0.2987	0.38259	0.043782	0.494356438
RN7SK	ENSG00000283293	1.7084	0.043782	1.7084	0.043782	1.7084	0.043782	1.7084	0.043782	0.043782	0.494356438
TNFRSF17	ENSG00000048462	1.664	0.048053	1.708	0.043814	0.8525	0.19697	1.0558	0.14553	0.043814	0.494356438
NA	ENSG00000248529	1.708	0.043816	1.0471	0.14752	0.8161	0.20722	0.8161	0.20722	0.043816	0.494356438
PLEKHG1	ENSG00000120278	1.708	0.043817	0.53826	0.2952	1.3029	0.096307	1.0645	0.14354	0.043817	0.494356438
NA	ENSG00000260071	0.22355	0.41155	-0.23708	0.5937	-0.037483	0.51495	1.708	0.043818	0.043818	0.494356438
RNA5SP89	ENSG00000222232	1.708	0.04382	1.708	0.04382	1.708	0.04382	1.708	0.04382	0.04382	0.494356438
NA	ENSG00000236885	-0.1712	0.56797	1.708	0.043821	-0.1712	0.56797	-0.1712	0.56797	0.043821	0.494356438
EEF1B2P4	ENSG00000257119	1.6174	0.052891	1.5655	0.058738	1.1804	0.11892	1.7078	0.043833	0.043833	0.494356438
NA	ENSG00000274116	1.7077	0.043845	-2.3303	0.9901	-2.3303	0.9901	-2.3303	0.9901	0.043845	0.494356438
PTRH2	ENSG00000141378	1.7077	0.043847	1.7077	0.043847	1.7077	0.043847	1.7077	0.043847	0.043847	0.494356438
RNU6-573P	ENSG00000206665	1.7077	0.043849	1.7077	0.043849	1.7077	0.043849	1.7077	0.043849	0.043849	0.494356438
NA	ENSG00000237920	0.91373	0.18043	0.73935	0.22985	0.994	0.16011	1.7076	0.043856	0.043856	0.494356438
NA	ENSG00000271366	1.7071	0.043904	1.7071	0.043904	1.7071	0.043904	1.7071	0.043904	0.043904	0.494783053
TMEM268	ENSG00000157693	0.53336	0.29689	1.6937	0.045161	-0.083073	0.5331	1.7068	0.043931	0.043931	0.494972862
STAM	ENSG00000136738	-0.8686	0.80747	-0.5352	0.70374	-0.70562	0.75979	1.7065	0.043957	0.043957	0.495039536
KIF3AP1	ENSG00000235836	1.7065	0.043958	1.7065	0.043958	1.7065	0.043958	-0.7219	0.76482	0.043958	0.495039536
NA	ENSG00000236356	0.28704	0.38704	0.28704	0.38704	1.7064	0.043968	0.28704	0.38704	0.043968	0.495039536
NA	ENSG00000255063	1.706	0.044	-0.67645	0.75062	-0.67645	0.75062	-0.67645	0.75062	0.044	0.495039536
IGLVIV-53	ENSG00000253935	1.706	0.044004	1.706	0.044004	1.706	0.044004	1.706	0.044004	0.044004	0.495039536
LTV1	ENSG00000135521	1.7059	0.044011	-0.49174	0.68855	1.7059	0.044011	0.23556	0.40689	0.044011	0.495039536
NA	ENSG00000197462	0.27326	0.39233	-0.38058	0.64824	1.7051	0.044086	0.58753	0.27842	0.044086	0.495039536
MIR1225	ENSG00000221656	1.7051	0.044087	1.7051	0.044087	1.7051	0.044087	1.7051	0.044087	0.044087	0.495039536
NA	ENSG00000186354	0.47098	0.31883	0.86137	0.19452	0.58923	0.27785	1.7051	0.044091	0.044091	0.495039536
NA	ENSG00000197991	1.2055	0.11401	1.705	0.044102	0.52573	0.29954	0.49605	0.30993	0.044102	0.495039536
NDUFS7	ENSG00000115286	1.7021	0.044372	1.7048	0.044113	1.7048	0.044113	1.1418	0.12677	0.044113	0.495039536
NA	ENSG00000264748	1.7048	0.044113	1.7048	0.044113	1.7048	0.044113	1.7048	0.044113	0.044113	0.495039536
RNU1-84P	ENSG00000202317	-0.70362	0.75916	-0.4122	0.6599	1.4938	0.067611	1.7048	0.044115	0.044115	0.495039536

NA	ENSG00000199715	1.7048	0.044118	0.17239	0.43157	1.7048	0.044118	1.7048	0.044118	0.044118	0.495039536
NA	ENSG00000252473	1.7047	0.044125	1.7047	0.044125	1.7047	0.044125	1.7047	0.044125	0.044125	0.495039536
KCNQ2-AS1	ENSG00000226390	0.34486	0.3651	0.61814	0.26824	1.7046	0.044131	-0.4795	0.68421	0.044131	0.495039536
NA	ENSG00000224307	-0.8098	0.79097	-0.69764	0.7573	1.7045	0.044142	-1.7261	0.95784	0.044142	0.495039536
TNN	ENSG00000120332	1.7045	0.044145	1.4201	0.077784	1.6715	0.047316	1.5624	0.059092	0.044145	0.495039536
SOD1P2	ENSG00000261282	1.7044	0.044154	1.7044	0.044154	1.7044	0.044154	1.605	0.054248	0.044154	0.495039536
LGALS16	ENSG00000249861	0.70418	0.24066	0.79882	0.2122	1.7043	0.044167	1.7043	0.044167	0.044167	0.495039536
HOGA1	ENSG00000241935	1.7041	0.044177	1.6736	0.0471	-0.18306	0.57263	-1.0081	0.8433	0.044177	0.495039536
ABHD16A	ENSG00000204427	0.69436	0.24373	1.704	0.044187	-0.30147	0.61847	-0.30147	0.61847	0.044187	0.495039536
BPI	ENSG00000101425	1.7038	0.044213	-0.53203	0.70265	0.55207	0.29045	0.23979	0.40525	0.044213	0.495039536
NA	ENSG00000215945	0.06509	0.47405	0.74018	0.22959	1.7037	0.044218	0.10269	0.4591	0.044218	0.495039536
NA	ENSG00000249774	1.7036	0.044224	0.049375	0.48031	1.7036	0.044224	1.7036	0.044224	0.044224	0.495039536
ENPP2	ENSG00000136960	0.43368	0.33226	-0.64021	0.73898	1.7036	0.044228	-0.7493	0.77316	0.044228	0.495039536
LINC02253	ENSG00000259485	-0.12567	0.55	1.3317	0.091475	1.7035	0.044236	1.1835	0.11831	0.044236	0.495039536
EXOC6	ENSG00000138190	1.5924	0.05565	1.7035	0.044237	1.0272	0.15217	0.21019	0.41676	0.044237	0.495039536
NA	ENSG00000267976	0.64415	0.25974	-0.50145	0.69197	1.7035	0.044238	0.14791	0.44121	0.044238	0.495039536
NA	ENSG00000253181	-0.86456	0.80636	0.34693	0.36432	1.7034	0.044246	-0.035277	0.51407	0.044246	0.495039536
GIP	ENSG00000159224	1.7032	0.044261	0.99474	0.15993	0.98054	0.16341	0.87645	0.19039	0.044261	0.495039536
NA	ENSG00000263714	1.7032	0.044262	1.7032	0.044262	1.7032	0.044262	1.7032	0.044262	0.044262	0.495039536
COQ6	ENSG00000119723	1.6967	0.044872	1.4909	0.067999	1.7029	0.044293	1.6967	0.044872	0.044293	0.495145697
LINC01952	ENSG00000234183	1.7027	0.044311	1.7027	0.044311	1.7027	0.044311	1.7027	0.044311	0.044311	0.495145697
TRIB1	ENSG00000173334	-0.6553	0.74386	-0.64479	0.74047	1.7024	0.044336	-1.8223	0.96579	0.044336	0.495145697
NA	ENSG00000213579	-0.17202	0.56829	-0.17202	0.56829	-0.91938	0.82105	1.7024	0.044336	0.044336	0.495145697
OR7E33P	ENSG00000226537	1.7023	0.044352	1.5744	0.057695	-0.99856	0.841	1.6032	0.054448	0.044352	0.495145697
NA	ENSG00000248245	1.6519	0.049274	1.1557	0.12391	1.7022	0.044358	1.7022	0.044358	0.044358	0.495145697
NA	ENSG00000249436	1.7019	0.044384	1.7019	0.044384	1.7019	0.044384	1.7019	0.044384	0.044384	0.495145697
MBLAC2	ENSG00000176055	1.7018	0.044396	0.14153	0.44373	1.0302	0.15145	0.13037	0.44814	0.044396	0.495145697
C4orf36	ENSG00000163633	0.96758	0.16663	1.7017	0.044402	0.77832	0.21819	1.6635	0.048108	0.044402	0.495145697
NSFP1	ENSG00000260075	0.45094	0.32602	-0.63216	0.73636	1.7016	0.044417	0.18551	0.42641	0.044417	0.495145697
RNU7-172P	ENSG00000252934	1.7015	0.044423	1.7015	0.044423	0.73199	0.23209	1.7015	0.044423	0.044423	0.495145697
MSH5	ENSG00000204410	1.6836	0.046129	1.7014	0.044435	0.43736	0.33093	-0.36148	0.64113	0.044435	0.495145697

NA	ENSG00000277795	1.7013	0.044446	-0.92941	0.82366	0.2495	0.40149	0.2495	0.40149	0.044446	0.495145697
NA	ENSG00000200298	1.7012	0.044456	1.7012	0.044456	0.037316	0.48512	1.5392	0.061873	0.044456	0.495145697
NA	ENSG00000228711	1.3324	0.091366	0.79078	0.21454	1.7012	0.044456	1.7012	0.044456	0.044456	0.495145697
AP5M1	ENSG00000053770	1.5206	0.06418	1.7011	0.044459	1.4084	0.079509	-0.41484	0.66087	0.044459	0.495145697
NA	ENSG00000221323	1.701	0.044471	1.701	0.044471	1.701	0.044471	1.701	0.044471	0.044471	0.495145697
NA	ENSG00000283535	1.2421	0.1071	1.7009	0.044481	1.2421	0.1071	1.2421	0.1071	0.044481	0.495145697
MED15P8	ENSG00000248796	1.535	0.062386	-1.3125	0.90533	1.7008	0.044488	0.0094201	0.49624	0.044488	0.495145697
NENF	ENSG00000117691	1.4564	0.072639	1.7008	0.044489	1.7008	0.044489	1.1612	0.12278	0.044489	0.495145697
NA	ENSG00000232353	1.7007	0.044498	0.50912	0.30533	0.50912	0.30533	-0.019966	0.50796	0.044498	0.495145697
COL14A1	ENSG00000187955	-0.083532	0.53329	1.7007	0.044501	0.42127	0.33678	-0.17307	0.5687	0.044501	0.495145697
STOML3	ENSG00000133115	0.27965	0.38987	1.7006	0.044513	1.7006	0.044513	1.3025	0.096375	0.044513	0.495145697
MIR1303	ENSG00000221552	-0.034262	0.51367	-0.18097	0.57181	1.7004	0.044532	-0.48831	0.68733	0.044532	0.495145697
NA	ENSG00000231741	1.7002	0.044548	-0.90216	0.81651	-0.90216	0.81651	-0.90216	0.81651	0.044548	0.495145697
PIN1	ENSG00000127445	-0.11894	0.54734	-0.11894	0.54734	-0.11894	0.54734	1.7002	0.044549	0.044549	0.495145697
LINC02861	ENSG00000262185	1.7001	0.044554	1.7001	0.044554	1.2724	0.10161	1.7001	0.044554	0.044554	0.495145697
NA	ENSG00000249639	1.7001	0.044556	0.31147	0.37772	0.59789	0.27496	0.30929	0.37855	0.044556	0.495145697
NA	ENSG00000265189	1.6999	0.044571	-0.0077117	0.50308	1.6999	0.044571	1.6999	0.044571	0.044571	0.49519946
ATP5MGP7	ENSG00000267189	1.6997	0.044594	1.6997	0.044594	0.96353	0.16764	1.6997	0.044594	0.044594	0.495209058
LINC02339	ENSG00000279797	1.6997	0.044596	1.6997	0.044596	1.6997	0.044596	1.6997	0.044596	0.044596	0.495209058
LCP2	ENSG00000043462	-0.51873	0.69803	0.12938	0.44853	1.6995	0.044613	0.043952	0.48247	0.044613	0.495209058
NA	ENSG00000258540	1.652	0.049268	1.6994	0.044618	1.5485	0.060751	1.652	0.049268	0.044618	0.495209058
NA	ENSG00000223570	1.6992	0.044639	1.6992	0.044639	1.6992	0.044639	1.6992	0.044639	0.044639	0.495209058
DDX49	ENSG00000105671	1.6992	0.04464	1.6992	0.04464	1.6992	0.04464	1.6992	0.04464	0.04464	0.495209058
AQP11	ENSG00000178301	0.38006	0.35195	1.0081	0.1567	1.6992	0.044643	0.95993	0.16855	0.044643	0.495209058
PCCA-AS1	ENSG00000234650	0.5351	0.29629	1.6988	0.044677	0.8372	0.20124	0.08228	0.46721	0.044677	0.495473421
CEACAM21	ENSG00000007129	1.6112	0.053564	1.6986	0.044697	1.6986	0.044697	1.552	0.060326	0.044697	0.495569065
FRMD6-AS1	ENSG00000273888	1.5443	0.061254	1.6982	0.044731	0.035509	0.48584	0.035509	0.48584	0.044731	0.495569065
ASB14	ENSG00000239388	0.96323	0.16772	0.96323	0.16772	0.96323	0.16772	1.6982	0.044734	0.044734	0.495569065
NA	ENSG00000273176	1.6982	0.044735	1.6982	0.044735	1.6982	0.044735	1.6982	0.044735	0.044735	0.495569065
LINC01448	ENSG00000238284	0.25721	0.39851	1.6981	0.044741	0.9017	0.18361	-0.36171	0.64121	0.044741	0.495569065
ROBO4	ENSG00000154133	1.6979	0.044761	1.6979	0.044761	1.6979	0.044761	1.6979	0.044761	0.044761	0.495569065

NA	ENSG00000223009	1.6979	0.044764	1.6979	0.044764	0.39992	0.34461	1.6979	0.044764	0.044764	0.495569065
ATP5POP1	ENSG00000159186	1.6976	0.044789	1.6976	0.044789	1.6976	0.044789	1.6976	0.044789	0.044789	0.495569065
NA	ENSG00000222586	-1.5605	0.94068	-1.5605	0.94068	-1.5605	0.94068	1.6976	0.044793	0.044793	0.495569065
RN7SL377P	ENSG00000266569	1.6806	0.046421	1.06	0.14457	0.88452	0.18821	1.6975	0.044797	0.044797	0.495569065
NA	ENSG00000259661	1.6974	0.04481	1.6974	0.04481	1.6974	0.04481	1.6974	0.04481	0.04481	0.495569065
NA	ENSG00000263958	1.6974	0.044813	-0.19942	0.57903	-0.19942	0.57903	-0.19942	0.57903	0.044813	0.495569065
LINC01088	ENSG00000249307	0.87159	0.19172	1.6972	0.044827	1.5805	0.057	0.19273	0.42359	0.044827	0.495569065
EEF1DP4	ENSG00000213640	0.42197	0.33653	0.42197	0.33653	1.6972	0.044828	0.4907	0.31182	0.044828	0.495569065
SYNJ2	ENSG00000078269	1.5007	0.066714	-0.50412	0.69291	1.697	0.044847	-0.22648	0.58959	0.044847	0.495666661
NA	ENSG00000278979	1.6969	0.044858	1.6969	0.044858	1.6969	0.044858	1.6969	0.044858	0.044858	0.495675814
SHISAL2A	ENSG00000182183	-0.22568	0.58927	-0.82438	0.79514	0.53211	0.29732	1.6968	0.044872	0.044872	0.495718105
RNU6-1062P	ENSG00000206635	1.2376	0.10793	1.6146	0.053198	1.485	0.068777	1.6965	0.044897	0.044897	0.495836803
JPT1	ENSG00000189159	-0.37427	0.6459	-0.37427	0.6459	0.90186	0.18357	1.6959	0.044951	0.044951	0.495836803
YBX1P10	ENSG00000213866	1.6958	0.044964	1.1338	0.12843	0.21716	0.41404	1.3534	0.087957	0.044964	0.495836803
NA	ENSG00000241954	1.6956	0.044977	1.6956	0.044977	1.6956	0.044977	1.6956	0.044977	0.044977	0.495836803
PKLR	ENSG00000143627	0.59238	0.2768	1.1285	0.12955	0.59505	0.2759	1.6956	0.044983	0.044983	0.495836803
CARMIL1	ENSG00000079691	1.3341	0.09109	1.1124	0.13298	1.6956	0.044985	0.87573	0.19059	0.044985	0.495836803
OR7D1P	ENSG00000188314	0.58396	0.27962	1.6955	0.044986	0.58396	0.27962	0.58396	0.27962	0.044986	0.495836803
NA	ENSG00000264990	1.6955	0.044986	1.6955	0.044986	1.6955	0.044986	1.6955	0.044986	0.044986	0.495836803
FAM111A-DT	ENSG00000245571	1.6955	0.044989	1.6955	0.044989	1.6955	0.044989	1.6955	0.044989	0.044989	0.495836803
ADRA1B	ENSG00000170214	1.426	0.076938	0.2198	0.41301	1.6955	0.044992	1.3398	0.090153	0.044992	0.495836803
LNPEP	ENSG00000113441	0.95434	0.16995	1.6954	0.044999	0.95434	0.16995	0.95434	0.16995	0.044999	0.495836803
NA	ENSG00000258559	1.695	0.045037	1.695	0.045037	1.695	0.045037	1.695	0.045037	0.045037	0.495836803
RRP12	ENSG00000052749	0.86147	0.19449	1.6949	0.045045	1.692	0.045326	1.6056	0.054185	0.045045	0.495836803
C4orf47	ENSG00000205129	0.20037	0.4206	1.6946	0.045074	1.0655	0.14333	1.6612	0.048338	0.045074	0.495836803
RNA5SP94	ENSG00000252726	1.6946	0.045076	0.98282	0.16285	0.98282	0.16285	-0.70539	0.75972	0.045076	0.495836803
FGF7	ENSG00000140285	1.4506	0.073451	1.1195	0.13146	1.2236	0.11055	1.6946	0.045077	0.045077	0.495836803
NA	ENSG00000280121	1.6945	0.045085	1.6945	0.045085	1.6945	0.045085	1.6945	0.045085	0.045085	0.495836803
NA	ENSG00000258885	-0.11064	0.54405	0.66406	0.25333	1.6943	0.0451	1.6943	0.0451	0.0451	0.495836803
RNVU1-34	ENSG00000200997	0.32281	0.37342	1.6942	0.04511	0.32281	0.37342	0.32281	0.37342	0.04511	0.495836803
ACTBP9	ENSG00000266920	1.6942	0.045114	0.35349	0.36186	0.35349	0.36186	1.198	0.11545	0.045114	0.495836803

NA	ENSG00000258847	0.64716	0.25876	1.694	0.045129	0.94694	0.17183	0.91881	0.1791	0.045129	0.495836803
RNU7-144P	ENSG00000238452	0.19131	0.42414	-1.3893	0.91763	-0.36383	0.64201	1.694	0.04513	0.04513	0.495836803
OAS2	ENSG00000111335	1.6939	0.04514	1.6939	0.04514	1.1938	0.11629	0.15636	0.43788	0.04514	0.495836803
CALM1	ENSG00000198668	1.6939	0.045145	1.2904	0.09846	-1.0421	0.85132	0.77755	0.21842	0.045145	0.495836803
PMPCA	ENSG00000165688	-1.1086	0.86619	-1.1017	0.86471	-1.3795	0.91613	1.6937	0.045156	0.045156	0.495836803
LINC00924	ENSG00000259134	1.6936	0.045171	0.92555	0.17734	-0.73587	0.7691	0.17671	0.42987	0.045171	0.495836803
MICAL3	ENSG00000243156	0.97472	0.16485	1.5104	0.065475	1.419	0.077946	1.6935	0.045179	0.045179	0.495836803
GALNT5	ENSG00000136542	0.11956	0.45241	1.367	0.085809	0.75527	0.22504	1.6935	0.045183	0.045183	0.495836803
RN7SKP270	ENSG00000223229	1.6935	0.045184	0.90663	0.1823	1.6935	0.045184	1.6935	0.045184	0.045184	0.495836803
NA	ENSG00000280092	1.6934	0.045188	1.4565	0.07263	1.4565	0.07263	1.4565	0.07263	0.045188	0.495836803
NA	ENSG00000202216	1.2603	0.10379	1.6933	0.045199	0.54864	0.29163	1.6126	0.053421	0.045199	0.495845851
NA	ENSG00000256312	1.6931	0.045219	1.6931	0.045219	0.059033	0.47646	0.475	0.31739	0.045219	0.495885866
NA	ENSG00000201034	1.6931	0.045223	0.81516	0.20749	0.81516	0.20749	0.81516	0.20749	0.045223	0.495885866
ZNF22	ENSG00000165512	1.6927	0.045259	0.60876	0.27134	-0.2912	0.61455	0.60876	0.27134	0.045259	0.496040512
TUT7	ENSG00000083223	1.0386	0.1495	1.6926	0.04527	1.5741	0.057736	1.3743	0.084672	0.04527	0.496040512
ADCY5	ENSG00000173175	0.20049	0.42055	0.67179	0.25086	1.6925	0.045277	1.5821	0.056811	0.045277	0.496040512
C7orf66	ENSG00000205174	-0.43499	0.66822	-0.43499	0.66822	-0.43499	0.66822	1.6924	0.045287	0.045287	0.496040512
HMGB1P45	ENSG00000229316	1.6924	0.045288	0.50701	0.30608	1.6924	0.045288	1.6924	0.045288	0.045288	0.496040512
NA	ENSG00000275175	1.6922	0.045302	1.1228	0.13076	1.1228	0.13076	1.1228	0.13076	0.045302	0.496082351
NA	ENSG00000277803	-0.49172	0.68854	0.21563	0.41464	0.5025	0.30766	1.6919	0.045332	0.045332	0.496185866
NA	ENSG00000217557	0.8268	0.20418	0.8268	0.20418	0.8268	0.20418	1.6919	0.045336	0.045336	0.496185866
NA	ENSG00000183791	1.6918	0.045342	1.6918	0.045342	-0.31993	0.62549	0.57043	0.28419	0.045342	0.496185866
CPXM2	ENSG00000121898	-0.67985	0.7517	0.20591	0.41843	-0.95793	0.83095	1.6916	0.045357	0.045357	0.496238574
EFNB2	ENSG00000125266	0.95311	0.17027	0.72261	0.23496	0.24596	0.40286	1.6912	0.0454	0.0454	0.496597531
NA	ENSG00000249881	1.1003	0.13561	1.6911	0.045412	1.1003	0.13561	-0.36168	0.6412	0.045412	0.496617316
NA	ENSG00000252235	-0.99369	0.83981	-0.68521	0.75339	-0.29693	0.61674	1.6908	0.045439	0.045439	0.496705185
NA	ENSG00000273654	0.2483	0.40195	0.2483	0.40195	0.2483	0.40195	1.6907	0.045443	0.045443	0.496705185
UNC45B	ENSG00000141161	-0.62726	0.73476	1.4828	0.069063	1.6906	0.045453	-0.95624	0.83052	0.045453	0.496705185
MRPS18C	ENSG00000163319	1.6905	0.045466	1.6905	0.045466	1.6905	0.045466	1.6905	0.045466	0.045466	0.496705185
LAMA1	ENSG00000101680	0.48106	0.31524	0.37815	0.35266	1.6905	0.045471	0.55106	0.2908	0.045471	0.496705185
FBF1	ENSG00000188878	0.18631	0.4261	1.6902	0.045493	0.75224	0.22595	1.2265	0.11	0.045493	0.496755564

LYPD4	ENSG00000273111	1.6902	0.045496	1.6902	0.045496	1.6902	0.045496	1.6902	0.045496	0.045496	0.496755564
NRXN2-AS1	ENSG00000237410	1.6899	0.045527	1.6899	0.045527	1.6899	0.045527	1.6899	0.045527	0.045527	0.496847414
NA	ENSG00000279191	-0.26789	0.60561	0.15343	0.43903	1.5749	0.057638	1.6898	0.045533	0.045533	0.496847414
NA	ENSG00000251828	0.88841	0.18716	1.6898	0.045535	1.0937	0.13704	0.13012	0.44824	0.045535	0.496847414
NA	ENSG00000228064	1.6894	0.045569	1.6894	0.045569	1.6894	0.045569	1.6894	0.045569	0.045569	0.496857031
TRAM1	ENSG00000067167	0.68359	0.24712	0.69564	0.24333	1.6894	0.045573	0.23404	0.40748	0.045573	0.496857031
BNIPL	ENSG00000163141	1.6893	0.045577	1.6212	0.052486	0.47625	0.31695	0.47625	0.31695	0.045577	0.496857031
NA	ENSG00000251461	-1.2342	0.89144	-1.2342	0.89144	1.6892	0.045592	-1.2342	0.89144	0.045592	0.496857031
LHFPL3-AS2	ENSG00000225329	1.689	0.045608	0.24425	0.40352	1.5276	0.063309	-0.050959	0.52032	0.045608	0.496857031
ELF3-AS1	ENSG00000234678	1.19	0.11701	1.6391	0.050601	1.6889	0.045618	1.6889	0.045618	0.045618	0.496857031
ZNF518B	ENSG00000178163	1.6889	0.045622	0.055422	0.4779	1.6889	0.045622	1.6889	0.045622	0.045622	0.496857031
CARD11	ENSG00000198286	-1.3327	0.90868	1.6888	0.04563	0.63752	0.26189	-0.32719	0.62824	0.04563	0.496857031
SCARNA3	ENSG00000252906	1.6883	0.045676	1.6883	0.045676	1.6883	0.045676	1.6883	0.045676	0.045676	0.496857031
VMO1	ENSG00000182853	1.6883	0.045681	1.6883	0.045681	1.6883	0.045681	1.6883	0.045681	0.045681	0.496857031
NA	ENSG00000280254	1.6883	0.045681	1.6883	0.045681	1.6883	0.045681	1.6883	0.045681	0.045681	0.496857031
RPUSD4	ENSG00000165526	1.6882	0.045685	0.16681	0.43376	0.07148	0.47151	0.091106	0.4637	0.045685	0.496857031
SECISBP2	ENSG00000187742	1.6882	0.045685	1.317	0.093926	1.4122	0.078951	1.1673	0.12154	0.045685	0.496857031
NA	ENSG00000273291	1.6881	0.045693	1.6881	0.045693	1.6881	0.045693	1.6881	0.045693	0.045693	0.496857031
SPDEF	ENSG00000124664	-0.81231	0.79169	1.6881	0.045699	-1.3881	0.91745	-0.38489	0.64984	0.045699	0.496857031
PRKAR2A	ENSG00000114302	1.6881	0.045701	1.617	0.052934	1.2802	0.10024	0.82382	0.20502	0.045701	0.496857031
ELOA	ENSG00000011007	1.6878	0.045725	1.0958	0.13658	1.0958	0.13658	1.0958	0.13658	0.045725	0.496857031
TRIP13	ENSG00000071539	1.6877	0.045732	1.217	0.11179	1.217	0.11179	0.71262	0.23804	0.045732	0.496857031
LINC01202	ENSG00000280776	-1.173	0.87961	1.6877	0.045736	-0.5659	0.71427	0.10612	0.45774	0.045736	0.496857031
HOXC9	ENSG00000180806	1.6876	0.045742	1.6876	0.045742	1.1414	0.12686	1.4178	0.078131	0.045742	0.496857031
FAM217B	ENSG00000196227	1.6875	0.04575	0.39557	0.34621	1.4982	0.067044	1.4982	0.067044	0.04575	0.496857031
SLC22A13	ENSG00000172940	1.6872	0.045783	1.6872	0.045783	1.6872	0.045783	1.6872	0.045783	0.045783	0.496928892
SERPINB1	ENSG00000021355	0.10663	0.45754	0.36582	0.35725	1.6869	0.045807	0.83046	0.20314	0.045807	0.496928892
BAHCC1	ENSG00000266074	0.60755	0.27174	1.3833	0.083283	0.86867	0.19251	1.6869	0.045807	0.045807	0.496928892
KCNJ11	ENSG00000187486	1.6868	0.045818	0.189	0.42504	1.378	0.084102	0.67873	0.24866	0.045818	0.496928892
NA	ENSG00000240777	1.6868	0.045821	1.6868	0.045821	1.6868	0.045821	1.6868	0.045821	0.045821	0.496928892
NA	ENSG00000251281	-0.59404	0.72376	-0.42766	0.66555	-0.21534	0.58525	1.6868	0.045825	0.045825	0.496928892

FADS2	ENSG00000134824	0.92382	0.17779	1.6867	0.045828	1.3711	0.085173	0.788	0.21535	0.045828	0.496928892
FITM2	ENSG00000197296	1.6863	0.045865	1.5733	0.057823	1.1183	0.13172	1.2638	0.10316	0.045865	0.497113154
SBF1	ENSG00000100241	-0.81605	0.79276	-2.4918	0.99364	-0.42475	0.66449	1.6862	0.045881	0.045881	0.497113154
GRHL2	ENSG00000083307	0.40285	0.34353	-0.38167	0.64865	-0.36242	0.64148	1.6861	0.045885	0.045885	0.497113154
NA	ENSG00000250674	1.0627	0.14396	0.92289	0.17803	1.6861	0.045889	0.63311	0.26333	0.045889	0.497113154
DELE1	ENSG00000081791	-0.92234	0.82182	1.686	0.045896	-0.010241	0.50409	-0.35573	0.63898	0.045896	0.497113154
PRSS55	ENSG00000184647	1.6858	0.045914	0.0013001	0.49948	0.97571	0.1646	0.1167	0.45355	0.045914	0.497197604
RNU6-232P	ENSG00000252953	0.48769	0.31288	0.31009	0.37824	1.6857	0.04593	-1.616	0.94696	0.04593	0.497236804
CPNE2	ENSG00000140848	0.74446	0.2283	0.69489	0.24356	1.6853	0.045967	0.2855	0.38763	0.045967	0.497236804
ARHGAP11A	ENSG00000198826	-0.79045	0.78537	0.39107	0.34787	1.6851	0.045987	-1.1477	0.87445	0.045987	0.497236804
NA	ENSG00000212249	0.00049479	0.4998	0.51732	0.30247	-0.5733	0.71678	1.6848	0.046009	0.046009	0.497236804
ZBTB47	ENSG00000114853	1.0798	0.14012	1.6845	0.046044	0.77648	0.21873	1.2853	0.099345	0.046044	0.497236804
H3P15	ENSG00000251005	1.6845	0.046046	1.1563	0.12378	1.6845	0.046046	1.4241	0.077209	0.046046	0.497236804
RNU6-1163P	ENSG00000201386	0.99405	0.1601	1.6842	0.046068	0.99405	0.1601	0.99405	0.1601	0.046068	0.497236804
CCNL2	ENSG00000221978	1.6842	0.046072	1.6842	0.046072	1.6807	0.046414	0.86868	0.19251	0.046072	0.497236804
SMARCC1	ENSG00000173473	1.5273	0.063342	1.539	0.061904	1.0015	0.15829	1.6841	0.046077	0.046077	0.497236804
TFAP2A-AS1	ENSG00000229950	1.684	0.046086	1.684	0.046086	1.684	0.046086	1.684	0.046086	0.046086	0.497236804
MCHR2-AS1	ENSG00000229315	0.79979	0.21192	0.51388	0.30367	0.24718	0.40239	1.6837	0.046116	0.046116	0.497236804
WDR81	ENSG00000167716	1.6837	0.046119	1.3596	0.086974	0.85779	0.1955	1.1788	0.11925	0.046119	0.497236804
NA	ENSG00000227072	1.6837	0.046119	1.6837	0.046119	1.6837	0.046119	1.6837	0.046119	0.046119	0.497236804
STAT4	ENSG00000138378	0.7686	0.22107	0.87679	0.1903	0.83292	0.20245	1.6837	0.046121	0.046121	0.497236804
REEP2	ENSG00000132563	1.6834	0.046153	1.343	0.089635	1.0697	0.14238	1.445	0.074226	0.046153	0.497236804
RAET1K	ENSG00000218358	1.6834	0.046153	0.014903	0.49405	1.0038	0.15773	0.99105	0.16083	0.046153	0.497236804
LPP	ENSG00000145012	1.3118	0.094793	0.59552	0.27575	0.68611	0.24632	1.6832	0.046167	0.046167	0.497236804
RNU7-151P	ENSG00000252739	1.6706	0.047399	1.6831	0.046177	1.6831	0.046177	1.6706	0.047399	0.046177	0.497236804
MIR3663HG	ENSG00000234474	1.683	0.046183	-0.39637	0.65408	0.71595	0.23701	1.2686	0.10229	0.046183	0.497236804
KMT2D	ENSG00000167548	1.3017	0.096513	1.683	0.046191	1.1853	0.11795	1.2063	0.11386	0.046191	0.497236804
TPRXL	ENSG00000180438	0.53248	0.2972	1.6828	0.046209	1.0232	0.15311	-0.11534	0.54591	0.046209	0.497236804
GOPC	ENSG00000047932	-1.2536	0.89501	-0.40779	0.65829	1.6827	0.046221	1.6827	0.046221	0.046221	0.497236804
RNU6-1010P	ENSG00000199627	0.43389	0.33219	0.80798	0.20955	1.6825	0.046239	0.45575	0.32429	0.046239	0.497236804
NA	ENSG00000228076	1.4175	0.078174	1.6824	0.046245	1.4175	0.078174	1.4175	0.078174	0.046245	0.497236804

ZNF592	ENSG00000166716	1.5918	0.055718	1.5918	0.055718	1.6823	0.046252	1.454	0.07298	0.046252	0.497236804
BTBD7P1	ENSG00000203414	1.3593	0.087029	-0.37653	0.64674	0.55036	0.29104	1.6823	0.046253	0.046253	0.497236804
PSMC1P8	ENSG00000256896	1.6822	0.046264	1.6822	0.046264	1.6822	0.046264	0.079142	0.46846	0.046264	0.497236804
KRBOX1-AS1	ENSG00000206552	1.6821	0.046274	1.6821	0.046274	-1.3026	0.90365	1.6821	0.046274	0.046274	0.497236804
NA	ENSG00000249605	1.4994	0.066887	0.57035	0.28422	1.4994	0.066887	1.682	0.046281	0.046281	0.497236804
TMEM200C	ENSG00000206432	-1.7576	0.96059	-1.0971	0.8637	1.682	0.046282	-1.0185	0.84578	0.046282	0.497236804
RPL23A	ENSG00000198242	1.682	0.046288	1.682	0.046288	1.682	0.046288	1.682	0.046288	0.046288	0.497236804
RARS1P1	ENSG00000257037	-0.70195	0.75864	1.6819	0.046289	-0.70195	0.75864	-0.70195	0.75864	0.046289	0.497236804
RNU6-46P	ENSG00000206587	1.6819	0.046297	1.6819	0.046297	-0.3461	0.63537	0.66599	0.25271	0.046297	0.497236804
TPPP	ENSG00000171368	0.46173	0.32214	-0.0052952	0.50211	1.6818	0.046309	-0.45433	0.6752	0.046309	0.497236804
CLDN15	ENSG00000106404	1.4755	0.070043	1.6817	0.046312	1.6817	0.046312	1.3948	0.081537	0.046312	0.497236804
FLT4	ENSG00000037280	-0.066752	0.52661	1.6817	0.046314	-0.088542	0.53528	-0.11836	0.54711	0.046314	0.497236804
NA	ENSG00000255285	1.6816	0.046321	1.6816	0.046321	1.6816	0.046321	1.6816	0.046321	0.046321	0.497236804
RNA5SP450	ENSG00000223138	0.8543	0.19647	0.59884	0.27464	1.6449	0.049997	1.6816	0.046326	0.046326	0.497236804
CEND1	ENSG00000184524	1.6815	0.046336	-0.13756	0.55471	-0.33395	0.63079	-0.33395	0.63079	0.046336	0.497236804
RDX	ENSG00000137710	1.2429	0.10695	0.31628	0.3759	1.3897	0.08231	1.6814	0.046339	0.046339	0.497236804
NA	ENSG00000260568	1.6812	0.046362	0.86644	0.19313	0.081878	0.46737	0.081878	0.46737	0.046362	0.497236804
IL1RL2	ENSG00000115598	0.02498	0.49004	1.6812	0.046365	0.78498	0.21623	1.3912	0.08208	0.046365	0.497236804
NA	ENSG00000278694	1.0512	0.14658	1.2193	0.11137	0.67755	0.24903	1.6811	0.046368	0.046368	0.497236804
STAP2	ENSG00000178078	1.0183	0.15427	1.681	0.04638	1.0183	0.15427	1.681	0.04638	0.04638	0.497236804
NA	ENSG00000226252	1.6809	0.046387	1.6809	0.046387	1.6809	0.046387	1.6809	0.046387	0.046387	0.497236804
NA	ENSG00000254641	1.6809	0.046387	1.6809	0.046387	1.6809	0.046387	1.6809	0.046387	0.046387	0.497236804
RNU6-250P	ENSG00000252767	0.5425	0.29374	-0.77487	0.78079	1.6802	0.046464	0.58786	0.27831	0.046464	0.497428071
HSPA8P8	ENSG00000229091	1.5417	0.061575	1.5417	0.061575	0.054657	0.47821	1.6801	0.046471	0.046471	0.497428071
RN7SKP195	ENSG00000252396	1.68	0.046479	1.68	0.046479	1.68	0.046479	1.68	0.046479	0.046479	0.497428071
CGB5	ENSG00000189052	1.2076	0.1136	1.6799	0.046484	0.34653	0.36447	1.673	0.047161	0.046484	0.497428071
LINC02393	ENSG00000256597	0.20668	0.41813	1.5619	0.059158	1.5619	0.059158	1.6799	0.04649	0.04649	0.497428071
NUDT4B	ENSG00000177144	-1.3823	0.91656	1.6798	0.046502	1.04	0.14916	-1.2749	0.89883	0.046502	0.497428071
NA	ENSG00000242282	1.6797	0.046506	0.83042	0.20315	0.87783	0.19002	0.42923	0.33388	0.046506	0.497428071
CDK1	ENSG00000170312	1.5764	0.057466	1.6796	0.04652	0.52542	0.29964	0.52542	0.29964	0.04652	0.497428071
YKT6	ENSG00000106636	0.33152	0.37013	1.6794	0.046538	-1.5342	0.9375	-1.5342	0.9375	0.046538	0.497428071

COG1	ENSG00000166685	1.3014	0.096559	1.6793	0.046543	1.6309	0.051457	1.603	0.054462	0.046543	0.497428071
NA	ENSG00000256004	-0.24795	0.59791	-0.2154	0.58527	0.18577	0.42631	1.6793	0.046548	0.046548	0.497428071
ATP2B2	ENSG00000157087	0.67364	0.25027	0.83278	0.20248	1.6429	0.050197	1.6792	0.046552	0.046552	0.497428071
ADIPOQ	ENSG00000181092	-1.7009	0.95552	-0.55248	0.70969	1.6792	0.046557	0.2566	0.39874	0.046557	0.497428071
NA	ENSG00000229957	1.3363	0.090731	1.6789	0.046586	1.1366	0.12785	1.6789	0.046586	0.046586	0.497428071
NA	ENSG00000225744	-0.16511	0.56557	-0.16511	0.56557	-0.16511	0.56557	1.6789	0.046588	0.046588	0.497428071
NA	ENSG00000235475	0.70189	0.24137	0.70189	0.24137	0.70189	0.24137	1.6789	0.046589	0.046589	0.497428071
NA	ENSG00000225122	0.96343	0.16767	0.96343	0.16767	1.6789	0.04659	0.96343	0.16767	0.04659	0.497428071
NA	ENSG00000253256	1.09	0.13787	1.09	0.13787	1.09	0.13787	1.6788	0.046599	0.046599	0.497428071
C2	ENSG00000166278	0.68529	0.24658	1.6787	0.046608	1.2916	0.098255	0.47706	0.31666	0.046608	0.497428071
LINC02405	ENSG00000249345	1.5283	0.063225	0.23334	0.40775	1.6787	0.046609	0.080104	0.46808	0.046609	0.497428071
LCMT1	ENSG00000205629	1.6785	0.046629	1.6785	0.046629	1.6785	0.046629	1.2654	0.10287	0.046629	0.497444972
C7orf69	ENSG00000136275	1.6784	0.046631	1.6784	0.046631	0.86906	0.19241	-0.75652	0.77533	0.046631	0.497444972
ALB	ENSG00000163631	0.0019183	0.49923	1.6783	0.046645	1.6783	0.046645	1.6783	0.046645	0.046645	0.497485413
NA	ENSG00000261394	1.6779	0.046687	1.6779	0.046687	1.6779	0.046687	1.6779	0.046687	0.046687	0.49776909
NA	ENSG00000267642	1.6777	0.046706	1.6777	0.046706	1.5842	0.056572	1.6318	0.051358	0.046706	0.49776909
NA	ENSG00000224516	1.6774	0.046736	1.6774	0.046736	1.6774	0.046736	1.6774	0.046736	0.046736	0.49776909
NA	ENSG00000213383	1.6773	0.046743	1.6773	0.046743	1.6773	0.046743	1.6773	0.046743	0.046743	0.49776909
NA	ENSG00000236234	1.6771	0.046766	1.6771	0.046766	1.6771	0.046766	1.6771	0.046766	0.046766	0.49776909
NA	ENSG00000226681	1.6767	0.046798	1.6767	0.046798	1.6767	0.046798	1.6767	0.046798	0.046798	0.49776909
RPS3AP51	ENSG00000225971	1.4769	0.069846	1.6765	0.04682	1.4769	0.069846	1.4769	0.069846	0.04682	0.49776909
ZNF814	ENSG00000204514	1.6764	0.04683	0.79508	0.21328	0.38763	0.34915	0.19939	0.42098	0.04683	0.49776909
AK7	ENSG00000140057	-0.13427	0.55341	1.6763	0.046836	1.2264	0.11003	-0.49965	0.69134	0.046836	0.49776909
NA	ENSG00000261757	1.6762	0.046845	1.6762	0.046845	-0.14436	0.55739	1.0156	0.15491	0.046845	0.49776909
C4B	ENSG00000224389	1.6762	0.046848	0.54475	0.29296	-0.76803	0.77876	0.061789	0.47537	0.046848	0.49776909
ZNF345	ENSG00000251247	1.6762	0.046851	1.6762	0.046851	1.6762	0.046851	1.6762	0.046851	0.046851	0.49776909
NA	ENSG00000268199	1.6762	0.046851	1.6762	0.046851	1.6762	0.046851	1.6762	0.046851	0.046851	0.49776909
VMP1	ENSG00000062716	1.6761	0.046855	0.4017	0.34395	0.47711	0.31664	1.4095	0.07934	0.046855	0.49776909
NA	ENSG00000176043	1.6761	0.046864	1.6761	0.046864	1.6761	0.046864	1.6761	0.046864	0.046864	0.49776909
ZNF839	ENSG0000022976	-0.99047	0.83903	-1.034	0.84943	1.2491	0.10581	1.676	0.046867	0.046867	0.49776909
SLC6A2	ENSG00000103546	1.676	0.046873	1.1239	0.13053	1.1438	0.12635	1.1119	0.13309	0.046873	0.49776909

CDH19	ENSG00000071991	-0.17584	0.56979	-0.23278	0.59203	-0.1113	0.54431	1.6759	0.046874	0.046874	0.49776909
RNVU1-32	ENSG00000207110	1.6759	0.046882	1.6759	0.046882	1.6759	0.046882	1.6759	0.046882	0.046882	0.49776909
COMMD4P2	ENSG00000214702	1.6758	0.046892	1.559	0.059499	0.50223	0.30775	1.3452	0.089288	0.046892	0.49776909
NA	ENSG00000271420	1.6757	0.046897	1.6757	0.046897	1.6757	0.046897	1.6757	0.046897	0.046897	0.49776909
KRTAP5-5	ENSG00000185940	1.6753	0.046938	1.6183	0.052797	1.6183	0.052797	1.6753	0.046938	0.046938	0.49776909
NA	ENSG00000212054	0.27596	0.39129	1.6625	0.048204	1.6752	0.04695	1.6293	0.051622	0.04695	0.49776909
ING2	ENSG00000168556	1.6751	0.046953	0.49243	0.31121	-0.23808	0.59409	1.2573	0.10431	0.046953	0.49776909
LTB4R	ENSG00000213903	1.6751	0.046958	1.6751	0.046958	1.6751	0.046958	1.6751	0.046958	0.046958	0.49776909
CPA6	ENSG00000165078	0.48854	0.31259	0.59587	0.27563	1.6751	0.046959	0.16035	0.4363	0.046959	0.49776909
ERCC1	ENSG00000012061	0.25173	0.40062	0.43919	0.33026	1.6751	0.04696	-0.80478	0.78953	0.04696	0.49776909
LINC02844	ENSG00000253679	1.494	0.067587	1.6748	0.046986	1.494	0.067587	1.494	0.067587	0.046986	0.49776909
TRAV14DV4	ENSG00000211792	1.5294	0.063087	1.6747	0.047001	1.6747	0.047001	1.6747	0.047001	0.047001	0.49776909
TMPO	ENSG00000120802	1.6746	0.047006	1.6746	0.047006	1.6746	0.047006	1.2216	0.11093	0.047006	0.49776909
NA	ENSG00000272574	1.6742	0.047045	1.6742	0.047045	1.6742	0.047045	1.6742	0.047045	0.047045	0.49776909
ASIC4	ENSG00000072182	1.6742	0.047046	0.4372	0.33098	-1.3737	0.91523	0.80784	0.20959	0.047046	0.49776909
NA	ENSG00000225457	1.6739	0.047079	0.36971	0.3558	0.36971	0.3558	0.36971	0.3558	0.047079	0.49776909
MED4-AS1	ENSG00000229111	1.6737	0.047091	1.6737	0.047091	1.6737	0.047091	1.6737	0.047091	0.047091	0.49776909
LTBR	ENSG00000111321	1.6736	0.047101	-0.64414	0.74026	0.91977	0.17885	0.17844	0.42919	0.047101	0.49776909
UGT2B24P	ENSG00000249956	0.62976	0.26443	0.74072	0.22943	0.42319	0.33608	1.6736	0.047105	0.047105	0.49776909
CCDC144NL	ENSG00000205212	0.034488	0.48624	-0.13423	0.55339	1.6735	0.047111	-0.74889	0.77304	0.047111	0.49776909
NA	ENSG00000280449	1.6735	0.047118	1.6735	0.047118	1.6735	0.047118	0.27998	0.38975	0.047118	0.49776909
NA	ENSG00000252722	1.0903	0.13779	1.6734	0.047125	1.0903	0.13779	1.6734	0.047125	0.047125	0.49776909
RNU5E-8P	ENSG00000200372	1.6733	0.047138	1.6733	0.047138	1.6733	0.047138	1.6733	0.047138	0.047138	0.49776909
OAS3	ENSG00000111331	0.77633	0.21878	-0.42791	0.66564	-0.19045	0.57552	1.6731	0.047151	0.047151	0.49776909
NA	ENSG00000253500	0.011552	0.49539	1.4965	0.067263	-0.23588	0.59324	1.6731	0.047152	0.047152	0.49776909
COX15	ENSG00000014919	1.673	0.047168	-0.3042	0.61951	0.23727	0.40622	1.0749	0.14122	0.047168	0.49776909
RNU4-61P	ENSG00000223175	1.6729	0.047171	1.6729	0.047171	1.6729	0.047171	1.6729	0.047171	0.047171	0.49776909
NA	ENSG00000222007	0.76655	0.22167	1.2317	0.10903	1.2648	0.10298	1.6726	0.047201	0.047201	0.49776909
NA	ENSG00000257746	1.6726	0.047207	-0.065947	0.52629	0.54487	0.29292	1.3337	0.091156	0.047207	0.49776909
ACP3	ENSG00000014257	1.6724	0.047222	1.1628	0.12246	0.11501	0.45422	0.051966	0.47928	0.047222	0.49776909
DNAJB2	ENSG00000135924	1.6724	0.047223	1.6616	0.048297	1.6616	0.048297	1.6616	0.048297	0.047223	0.49776909

NA	ENSG00000249825	1.0315	0.15114	-0.53334	0.7031	1.1493	0.12521	1.6724	0.047226	0.047226	0.49776909
SYNE3	ENSG00000176438	1.6723	0.047228	-0.45292	0.6747	0.35775	0.36026	0.868	0.1927	0.047228	0.49776909
NA	ENSG00000240219	1.6721	0.047252	1.6721	0.047252	1.6721	0.047252	1.6721	0.047252	0.047252	0.49776909
RNU4-30P	ENSG00000222177	1.672	0.047262	1.672	0.047262	1.672	0.047262	1.672	0.047262	0.047262	0.49776909
LINC01744	ENSG00000236497	1.672	0.047263	1.4943	0.067554	1.4943	0.067554	0.40453	0.34291	0.047263	0.49776909
SP1	ENSG00000185591	1.608	0.053917	1.54	0.061785	1.291	0.098344	1.6719	0.047268	0.047268	0.49776909
FAM83G	ENSG00000188522	1.6717	0.04729	1.6717	0.04729	1.6717	0.04729	1.6717	0.04729	0.04729	0.49776909
NA	ENSG00000232396	1.6717	0.047294	1.6717	0.047294	1.6717	0.047294	1.6717	0.047294	0.047294	0.49776909
NA	ENSG00000239446	1.6717	0.047294	1.6717	0.047294	1.6717	0.047294	1.6717	0.047294	0.047294	0.49776909
HDLBP	ENSG00000115677	1.418	0.078102	1.6716	0.047299	0.30439	0.38041	1.1965	0.11575	0.047299	0.49776909
NA	ENSG00000275002	1.6716	0.047303	1.6716	0.047303	1.6716	0.047303	1.6716	0.047303	0.047303	0.49776909
ARL5AP2	ENSG00000236818	0.32116	0.37404	1.6716	0.047305	1.6716	0.047305	1.6716	0.047305	0.047305	0.49776909
HMGB3P12	ENSG00000237603	1.6715	0.047307	1.6715	0.047307	1.6715	0.047307	0.98865	0.16142	0.047307	0.49776909
RFESDP1	ENSG00000231650	0.37059	0.35547	1.6714	0.047318	-0.27785	0.60944	1.6714	0.047318	0.047318	0.49776909
HSPA8P3	ENSG00000234788	1.6714	0.047318	1.6714	0.047318	1.6714	0.047318	1.6714	0.047318	0.047318	0.49776909
HMGXB4	ENSG00000100281	1.6712	0.047337	0.60842	0.27145	0.98717	0.16178	0.81687	0.207	0.047337	0.49776909
MRPS2	ENSG00000122140	-0.56617	0.71436	-0.56617	0.71436	-0.56617	0.71436	1.6711	0.047349	0.047349	0.49776909
LINC02504	ENSG00000248339	-0.55505	0.71057	1.6711	0.047351	-0.4515	0.67418	-0.82038	0.794	0.047351	0.49776909
KIRREL1	ENSG00000183853	1.5133	0.065099	1.4902	0.06808	1.671	0.047356	1.2283	0.10967	0.047356	0.49776909
HOXA4	ENSG00000197576	1.2865	0.099129	1.6707	0.047386	1.2865	0.099129	1.2865	0.099129	0.047386	0.49776909
NA	ENSG00000256011	1.6707	0.047392	-0.33048	0.62948	-0.33048	0.62948	-0.33048	0.62948	0.047392	0.49776909
NA	ENSG00000254286	1.6706	0.047399	0.95112	0.17077	1.2996	0.096875	-0.11475	0.54568	0.047399	0.49776909
LRRC31	ENSG00000114248	-0.16797	0.5667	-0.16797	0.5667	-0.58464	0.72061	1.6705	0.047413	0.047413	0.49776909
RPL38	ENSG00000172809	1.6704	0.047425	-0.67567	0.75037	-0.13991	0.55563	0.3521	0.36238	0.047425	0.49776909
NA	ENSG00000274080	1.6702	0.047437	1.6702	0.047437	1.6702	0.047437	1.6702	0.047437	0.047437	0.49776909
NA	ENSG00000178193	-0.019358	0.50772	-0.019358	0.50772	1.6702	0.047439	-0.48657	0.68672	0.047439	0.49776909
ZNF557	ENSG00000130544	1.0409	0.14895	-0.13982	0.5556	1.4689	0.070924	1.6701	0.047447	0.047447	0.49776909
SLC34A1	ENSG00000131183	-1.4003	0.91929	1.6701	0.047453	-0.83013	0.79677	-1.125	0.8697	0.047453	0.49776909
POLR3G	ENSG00000113356	1.6699	0.047471	0.42162	0.33665	1.6699	0.047471	1.6181	0.052821	0.047471	0.49776909
FNDC7	ENSG00000143107	-0.12385	0.54928	0.37207	0.35492	-0.12385	0.54928	1.6697	0.047489	0.047489	0.49776909
GPC2	ENSG00000213420	1.3594	0.087011	1.6697	0.047492	1.6697	0.047492	1.6697	0.047492	0.047492	0.49776909

NA	ENSG00000227482	1.6697	0.047493	0.64941	0.25804	-0.032299	0.51288	0.11175	0.45551	0.047493	0.49776909
CHRM4	ENSG00000180720	1.6696	0.047499	1.6696	0.047499	1.6696	0.047499	1.6696	0.047499	0.047499	0.49776909
TLK2	ENSG00000146872	-0.081183	0.53235	1.6693	0.047524	0.21515	0.41483	1.2454	0.1065	0.047524	0.497837915
NA	ENSG00000237760	0.27386	0.3921	0.43311	0.33247	1.1618	0.12266	1.6693	0.047526	0.047526	0.497837915
NA	ENSG00000237356	0.95284	0.17034	1.6692	0.047538	0.77222	0.21999	1.1839	0.11822	0.047538	0.497856596
MRPL45	ENSG00000278845	0.91948	0.17892	1.1425	0.12663	-1.2097	0.8868	1.6689	0.047564	0.047564	0.497925492
MROH7	ENSG00000184313	0.39048	0.34809	0.55674	0.28885	1.3785	0.084019	1.6682	0.047639	0.047639	0.497925492
RNU6-640P	ENSG00000200563	0.91123	0.18109	0.9121	0.18086	1.0584	0.14493	1.6681	0.047643	0.047643	0.497925492
LINC01299	ENSG00000254081	1.6679	0.047666	1.1749	0.12002	1.493	0.067715	1.1894	0.11713	0.047666	0.497925492
NA	ENSG00000248867	1.1599	0.12304	0.89362	0.18576	1.6678	0.047675	0.067723	0.473	0.047675	0.497925492
OR7A19P	ENSG00000272963	1.1934	0.11635	-0.065492	0.52611	1.6678	0.047675	1.1934	0.11635	0.047675	0.497925492
RNU6-1212P	ENSG00000252832	1.0805	0.13996	0.90769	0.18202	0.067454	0.47311	1.6678	0.047679	0.047679	0.497925492
PTPN21	ENSG00000070778	1.6675	0.047705	0.10697	0.45741	1.6675	0.047705	0.10697	0.45741	0.047705	0.497925492
LINC02565	ENSG00000267726	-0.13403	0.55331	1.6675	0.047707	0.55188	0.29052	1.6675	0.047707	0.047707	0.497925492
SERPINA13P	ENSG00000187483	-0.29346	0.61542	0.22309	0.41173	1.6669	0.047762	-0.26368	0.60399	0.047762	0.497925492
SSC4D	ENSG00000146700	1.6669	0.047765	1.4867	0.068552	1.4269	0.076804	1.6301	0.051543	0.047765	0.497925492
NA	ENSG00000260090	1.6669	0.047767	1.6641	0.048044	0.30575	0.3799	0.30575	0.3799	0.047767	0.497925492
NA	ENSG00000251112	-0.18013	0.57147	1.6669	0.04777	-0.18013	0.57147	-0.18013	0.57147	0.04777	0.497925492
NA	ENSG00000266544	1.6668	0.04778	1.6668	0.04778	1.6668	0.04778	1.1889	0.11725	0.04778	0.497925492
SERP2	ENSG00000151778	-1.9231	0.97276	1.6667	0.047787	-1.4654	0.92859	-0.52508	0.70024	0.047787	0.497925492
NA	ENSG00000260661	-1.2226	0.88925	1.6665	0.04781	-1.2226	0.88925	-0.90655	0.81768	0.04781	0.497925492
MIR1229	ENSG00000221394	1.6665	0.047811	1.6665	0.047811	1.6665	0.047811	1.6665	0.047811	0.047811	0.497925492
NA	ENSG00000280129	1.6663	0.047822	1.6663	0.047822	1.6663	0.047822	1.6663	0.047822	0.047822	0.497925492
RPL39P31	ENSG00000242176	1.6663	0.047826	1.6663	0.047826	1.6663	0.047826	1.6663	0.047826	0.047826	0.497925492
MIR7515HG	ENSG00000236172	0.94529	0.17226	1.3802	0.083755	1.1995	0.11517	1.6662	0.047839	0.047839	0.497925492
NA	ENSG00000253369	1.666	0.047852	1.666	0.047852	1.666	0.047852	1.666	0.047852	0.047852	0.497925492
UPF2	ENSG00000151461	1.5492	0.060666	1.5082	0.065751	1.6659	0.047865	1.284	0.099576	0.047865	0.497925492
GOLGA6L4	ENSG00000184206	-0.0043567	0.50174	1.6659	0.047868	0.011925	0.49524	0.15369	0.43893	0.047868	0.497925492
NA	ENSG00000213527	1.5516	0.060375	1.6658	0.047879	1.306	0.095772	1.5516	0.060375	0.047879	0.497925492
RPS12P16	ENSG00000215184	1.1706	0.12087	0.84759	0.19833	1.6656	0.047901	1.1706	0.12087	0.047901	0.497925492
RN7SL350P	ENSG00000243254	1.6656	0.047901	1.6656	0.047901	1.6656	0.047901	0.87149	0.19174	0.047901	0.497925492

FBXW12	ENSG00000164049	1.1289	0.12946	1.0959	0.13657	1.5083	0.065742	1.6655	0.047911	0.047911	0.497925492
NA	ENSG00000279668	0.66995	0.25144	1.6654	0.047917	-0.070272	0.52801	-0.48853	0.68741	0.047917	0.497925492
PRDX3P2	ENSG00000231468	1.5523	0.060295	1.6652	0.047932	1.5523	0.060295	1.5523	0.060295	0.047932	0.497925492
NA	ENSG00000215958	-0.99192	0.83938	-0.99192	0.83938	1.6652	0.047941	-0.963	0.83223	0.047941	0.497925492
NA	ENSG00000240404	1.6651	0.047943	1.6651	0.047943	1.6651	0.047943	1.6651	0.047943	0.047943	0.497925492
SETP6	ENSG00000241319	1.6651	0.047943	1.5109	0.065405	1.6651	0.047943	1.6651	0.047943	0.047943	0.497925492
RPS27AP9	ENSG00000242706	-0.25708	0.60144	0.15491	0.43845	1.6651	0.047943	1.6651	0.047943	0.047943	0.497925492
BSN	ENSG00000164061	0.81083	0.20873	0.44585	0.32785	-0.21622	0.58559	1.6646	0.047998	0.047998	0.497925492
ZNF85	ENSG00000105750	1.6646	0.048001	0.3807	0.35171	1.0355	0.15023	-0.5536	0.71007	0.048001	0.497925492
RASSF5	ENSG00000266094	0.65195	0.25722	1.6645	0.048006	0.70299	0.24103	1.3006	0.096695	0.048006	0.497925492
TBC1D8	ENSG00000204634	1.6644	0.04802	-0.081831	0.53261	0.33527	0.36871	1.0159	0.15485	0.04802	0.497925492
MORC3	ENSG00000159256	0.0071129	0.49716	1.6643	0.048025	0.48934	0.3123	0.18757	0.42561	0.048025	0.497925492
ATP8B4	ENSG00000104043	1.6128	0.053396	1.6642	0.048041	1.4039	0.080176	0.82596	0.20441	0.048041	0.497925492
IFITM2	ENSG00000185201	0.32144	0.37394	0.32144	0.37394	1.6642	0.048041	-0.11414	0.54544	0.048041	0.497925492
SNORA36B	ENSG00000222370	0.17219	0.43164	1.3188	0.093614	1.664	0.04806	1.664	0.04806	0.04806	0.497925492
CCDC167	ENSG00000198937	-0.35016	0.63689	1.6639	0.048063	-0.59769	0.72498	0.18943	0.42488	0.048063	0.497925492
LINC02132	ENSG00000268804	0.55705	0.28875	1.6639	0.048067	1.6639	0.048067	1.4613	0.071972	0.048067	0.497925492
ZFP64P1	ENSG00000258491	1.6638	0.04808	1.0996	0.13576	1.6638	0.04808	1.6638	0.04808	0.04808	0.497925492
NA	ENSG00000235105	1.6637	0.048085	1.1884	0.11733	0.6754	0.24971	0.65319	0.25682	0.048085	0.497925492
TDRD10	ENSG00000163239	1.3402	0.090085	0.69028	0.24501	1.6637	0.048087	1.0268	0.15227	0.048087	0.497925492
NA	ENSG00000257932	0.41951	0.33742	0.17642	0.42998	1.6635	0.048111	-0.85517	0.80377	0.048111	0.497925492
NA	ENSG00000236426	1.6634	0.048121	1.404	0.080153	1.6634	0.048121	1.6634	0.048121	0.048121	0.497925492
NA	ENSG00000236543	0.73675	0.23064	-0.83731	0.79879	-1.3407	0.90999	1.6633	0.048127	0.048127	0.497925492
PRELID3BP2	ENSG00000271242	1.6631	0.048142	1.2089	0.11336	1.2481	0.10599	0.56865	0.2848	0.048142	0.497925492
ARL6IP5	ENSG00000144746	1.5553	0.059932	1.5663	0.058635	1.0485	0.1472	1.663	0.048156	0.048156	0.497925492
NA	ENSG00000276107	1.6629	0.048163	1.6629	0.048163	1.6629	0.048163	1.6629	0.048163	0.048163	0.497925492
BMP1	ENSG00000168487	-0.83879	0.79921	1.6627	0.048187	0.7683	0.22115	-0.11868	0.54723	0.048187	0.497925492
PPDPF	ENSG00000125534	0.44572	0.3279	1.3359	0.090791	1.6626	0.048193	1.6626	0.048193	0.048193	0.497925492
MTPAP	ENSG00000107951	1.6624	0.048212	1.6624	0.048212	0.14284	0.44321	1.5371	0.062135	0.048212	0.497925492
SAMD13	ENSG00000203943	1.5231	0.063863	1.5231	0.063863	1.5231	0.063863	1.6622	0.048236	0.048236	0.497925492
TUBA4B	ENSG00000243910	1.6622	0.048239	1.4093	0.079378	1.6622	0.048239	1.6622	0.048239	0.048239	0.497925492

DLGAP1-AS5	ENSG00000261520	1.6621	0.048244	0.4456	0.32794	-0.35918	0.64027	-0.50028	0.69156	0.048244	0.497925492
STXBP3	ENSG00000116266	1.6621	0.048245	1.2777	0.10068	1.6621	0.048245	1.5097	0.065558	0.048245	0.497925492
RNU6-815P	ENSG00000206807	-0.4091	0.65877	1.6621	0.04825	0.32451	0.37278	-0.4091	0.65877	0.04825	0.497925492
LINC01817	ENSG00000231420	-0.21576	0.58541	1.662	0.048256	1.1547	0.1241	-0.13225	0.55261	0.048256	0.497925492
CPHL1P	ENSG00000240216	1.0885	0.13819	1.2351	0.1084	1.662	0.048257	0.98543	0.16221	0.048257	0.497925492
LINC01565	ENSG00000198685	1.4451	0.074221	1.6619	0.048261	1.022	0.15339	0.22349	0.41158	0.048261	0.497925492
RNA5SP182	ENSG00000251873	1.6619	0.048267	1.5768	0.057424	1.2649	0.10295	1.6619	0.048267	0.048267	0.497925492
RN7SL685P	ENSG00000242315	0.20921	0.41714	0.72443	0.2344	1.3962	0.081329	1.6619	0.048269	0.048269	0.497925492
RNU7-59P	ENSG00000238880	1.6618	0.048273	-0.24658	0.59738	0.67251	0.25063	1.4116	0.079029	0.048273	0.497925492
PTP4A2P2	ENSG00000254481	1.336	0.090767	1.3672	0.085775	1.6143	0.053232	1.6616	0.048298	0.048298	0.497925492
NA	ENSG00000204365	1.6615	0.048303	0.84524	0.19899	0.84524	0.19899	0.84524	0.19899	0.048303	0.497925492
PCDHA9	ENSG00000204961	1.6615	0.048305	-0.3186	0.62499	-0.58458	0.72058	-0.097398	0.53879	0.048305	0.497925492
RBP3	ENSG00000265203	-0.0091515	0.50365	1.6613	0.048323	1.2658	0.10279	1.5854	0.056438	0.048323	0.497925492
PIGQ	ENSG00000007541	1.6613	0.04833	0.18193	0.42782	1.4677	0.071092	1.5551	0.059964	0.04833	0.497925492
NA	ENSG00000278126	1.6613	0.04833	1.6613	0.04833	1.6613	0.04833	1.6613	0.04833	0.04833	0.497925492
RPL27AP	ENSG00000215467	1.6612	0.048336	-0.37792	0.64726	-0.37792	0.64726	-0.37792	0.64726	0.048336	0.497925492
LINC02863	ENSG00000238160	1.6612	0.048336	1.1358	0.12803	1.6087	0.05384	0.7759	0.2189	0.048336	0.497925492
MRPL50P2	ENSG00000238285	1.6611	0.048343	1.6611	0.048343	1.6611	0.048343	1.5381	0.062014	0.048343	0.497925492
GTF2IP4	ENSG00000233369	1.661	0.048357	0.46792	0.31992	0.54845	0.29169	0.73389	0.23151	0.048357	0.497925492
NA	ENSG00000271893	1.1903	0.11696	1.6609	0.048364	1.6061	0.054122	0.84938	0.19783	0.048364	0.497925492
NA	ENSG00000249261	1.6609	0.048368	1.6609	0.048368	1.6609	0.048368	1.6609	0.048368	0.048368	0.497925492
LSMEM2	ENSG00000179564	1.4598	0.072175	1.6606	0.048395	1.5966	0.055182	1.2249	0.1103	0.048395	0.497925492
NA	ENSG00000260042	-0.094545	0.53766	1.6606	0.048398	-0.24469	0.59665	-0.06321	0.5252	0.048398	0.497925492
TIMD4	ENSG00000145850	0.095414	0.46199	1.6605	0.048403	0.095414	0.46199	-0.035981	0.51435	0.048403	0.497925492
NA	ENSG00000273058	1.3695	0.085426	1.3781	0.084087	1.2364	0.10816	1.6604	0.048412	0.048412	0.497925492
RALGAPB	ENSG00000170471	1.6603	0.048426	1.6603	0.048426	1.6603	0.048426	1.6603	0.048426	0.048426	0.497925492
NA	ENSG00000231691	1.6603	0.048431	1.6603	0.048431	1.6603	0.048431	1.6603	0.048431	0.048431	0.497925492
NA	ENSG00000233818	1.5259	0.063513	1.6603	0.048431	1.4573	0.072514	1.1306	0.12911	0.048431	0.497925492
NA	ENSG00000221048	0.56149	0.28723	0.75504	0.22511	-0.33893	0.63267	1.6602	0.048433	0.048433	0.497925492
CCIN	ENSG00000185972	1.6599	0.048464	1.5401	0.061768	0.56516	0.28598	0.79482	0.21336	0.048464	0.497925492
SMAD1-AS2	ENSG00000250582	1.3806	0.083695	1.4777	0.069746	1.6599	0.048466	1.4964	0.067271	0.048466	0.497925492

TPST2P1	ENSG00000260582	1.6599	0.048466	1.6599	0.048466	1.6599	0.048466	1.6599	0.048466	0.048466	0.497925492
VPS25	ENSG00000131475	1.6596	0.048495	1.6596	0.048495	1.6596	0.048495	1.6596	0.048495	0.048495	0.497925492
TRAJ26	ENSG00000211863	1.6596	0.048502	1.6596	0.048502	1.6596	0.048502	1.6596	0.048502	0.048502	0.497925492
TRAJ25	ENSG00000211864	1.6596	0.048502	1.6596	0.048502	1.6596	0.048502	1.6596	0.048502	0.048502	0.497925492
NA	ENSG00000237217	1.6595	0.048509	0.39075	0.34799	1.6595	0.048509	1.6595	0.048509	0.048509	0.497925492
NA	ENSG00000267587	1.6594	0.048515	1.6594	0.048515	1.6594	0.048515	1.6594	0.048515	0.048515	0.497925492
ARL5A	ENSG00000162980	1.6593	0.048531	0.52912	0.29836	0.23682	0.4064	0.29773	0.38296	0.048531	0.497925492
RPS19BP1	ENSG00000187051	-0.29309	0.61527	0.12195	0.45147	-0.50202	0.69217	1.6591	0.048545	0.048545	0.497925492
TPM1	ENSG00000140416	1.6589	0.048563	1.3199	0.093433	0.4048	0.34281	1.0892	0.13802	0.048563	0.497925492
C9orf57	ENSG00000204669	0.86961	0.19226	0.83988	0.20049	1.6588	0.048577	1.1271	0.12985	0.048577	0.497925492
GBX1	ENSG00000164900	1.6039	0.054373	0.27676	0.39098	1.6588	0.048582	1.6039	0.054373	0.048582	0.497925492
MND1	ENSG00000121211	-0.42242	0.66364	-0.85825	0.80462	1.6585	0.048608	0.85504	0.19626	0.048608	0.497925492
GGNBP1	ENSG00000204188	1.6584	0.048621	0.27247	0.39263	1.4089	0.079429	0.81497	0.20754	0.048621	0.497925492
NA	ENSG00000224049	1.3076	0.095499	-0.17525	0.56956	1.6584	0.048623	1.6584	0.048623	0.048623	0.497925492
CLIC5	ENSG00000112782	-0.40259	0.65638	-0.58351	0.72022	0.87506	0.19077	1.6582	0.048636	0.048636	0.497925492
SLC7A1	ENSG00000139514	0.60322	0.27318	0.04193	0.48328	1.1885	0.11731	1.6582	0.048639	0.048639	0.497925492
LINC02694	ENSG00000175779	-0.27096	0.60679	1.6121	0.053475	1.6581	0.048645	0.38775	0.3491	0.048645	0.497925492
LIF	ENSG00000128342	1.4188	0.077983	1.6581	0.048649	1.2083	0.11347	1.2083	0.11347	0.048649	0.497925492
RNU4-10P	ENSG00000201253	1.6579	0.048669	1.6579	0.048669	1.6579	0.048669	1.6579	0.048669	0.048669	0.497925492
NA	ENSG00000205740	-0.23438	0.59265	1.6579	0.04867	-0.23438	0.59265	-0.23438	0.59265	0.04867	0.497925492
PRMT7	ENSG00000132600	0.66612	0.25267	0.20425	0.41908	1.6578	0.048675	1.6578	0.048675	0.048675	0.497925492
RHBG	ENSG00000132677	0.89875	0.18439	1.6578	0.048675	0.82067	0.20592	0.38212	0.35119	0.048675	0.497925492
RGPD8	ENSG00000169629	1.0165	0.15469	1.6577	0.048684	1.098	0.1361	-0.54557	0.70732	0.048684	0.497925492
PRKD3	ENSG00000115825	1.6577	0.048689	0.92061	0.17863	1.4233	0.077321	1.3088	0.095302	0.048689	0.497925492
RNU6-1234P	ENSG00000252686	1.6576	0.048703	1.6576	0.048703	1.6576	0.048703	1.6576	0.048703	0.048703	0.497964161
PMVK	ENSG00000163344	1.6467	0.04981	1.3376	0.090515	1.6569	0.048773	-1.1731	0.87962	0.048773	0.498491139
MOCOS	ENSG00000075643	1.6569	0.048775	1.1834	0.11832	-0.1078	0.54292	-0.15349	0.56099	0.048775	0.498491139
IVD	ENSG00000128928	-0.010821	0.50432	0.63726	0.26198	1.6567	0.048793	0.77975	0.21777	0.048793	0.498560238
MIR4688	ENSG00000264102	1.6563	0.048828	1.6563	0.048828	1.6563	0.048828	1.6563	0.048828	0.048828	0.498560238
LINC02712	ENSG00000273409	0.50311	0.30745	1.656	0.04886	0.92916	0.1764	0.92916	0.1764	0.04886	0.498560238
RNY3P7	ENSG00000206800	1.656	0.048866	1.656	0.048866	1.656	0.048866	1.656	0.048866	0.048866	0.498560238

NA	ENSG00000267144	1.6559	0.048872	1.6559	0.048872	1.6559	0.048872	1.6559	0.048872	0.048872	0.498560238
LINC01912	ENSG00000264699	0.14468	0.44248	0.77146	0.22022	1.6559	0.048876	0.7973	0.21264	0.048876	0.498560238
LINC01950	ENSG00000251027	1.5132	0.065116	0.94507	0.17231	1.5132	0.065116	1.6558	0.048878	0.048878	0.498560238
NA	ENSG00000238999	1.6555	0.048916	1.6555	0.048916	1.6555	0.048916	1.6555	0.048916	0.048916	0.498560238
SNORA50A	ENSG00000206952	1.6554	0.04892	1.6554	0.04892	1.6554	0.04892	1.6554	0.04892	0.04892	0.498560238
SCNN1A	ENSG00000111319	1.6554	0.048921	0.22434	0.41125	-1.8856	0.97033	1.066	0.14321	0.048921	0.498560238
MLX	ENSG00000108788	1.0242	0.15288	1.6554	0.048924	1.0999	0.1357	0.33098	0.37033	0.048924	0.498560238
TSG101	ENSG00000074319	0.98777	0.16163	-1.5664	0.94137	1.6552	0.04894	-0.10766	0.54287	0.04894	0.498560238
NA	ENSG00000262172	-0.14557	0.55787	1.2419	0.10714	1.6551	0.048956	-0.32581	0.62772	0.048956	0.498560238
SLC28A2	ENSG00000137860	1.4259	0.076947	1.4259	0.076947	1.6549	0.048973	1.44	0.07493	0.048973	0.498560238
ZNF222	ENSG00000159885	1.6549	0.048976	1.6549	0.048976	1.6549	0.048976	1.6549	0.048976	0.048976	0.498560238
ERI3-IT1	ENSG00000233602	0.39509	0.34639	1.6548	0.048981	0.39509	0.34639	-0.45927	0.67698	0.048981	0.498560238
MUC12	ENSG00000205277	1.6547	0.048992	0.63763	0.26186	1.6547	0.048992	1.6547	0.048992	0.048992	0.498560238
SPACA7	ENSG00000153498	-0.34696	0.63569	0.79145	0.21434	1.3273	0.092199	1.6547	0.048995	0.048995	0.498560238
WARS2P1	ENSG00000227209	1.6545	0.049013	0.59921	0.27452	1.6545	0.049013	-1.7696	0.9616	0.049013	0.498560238
NA	ENSG00000266891	1.212	0.11276	1.6544	0.049023	0.91359	0.18047	1.6544	0.049023	0.049023	0.498560238
NA	ENSG00000249721	1.6544	0.049025	1.6544	0.049025	1.6544	0.049025	1.6544	0.049025	0.049025	0.498560238
PIM1	ENSG00000137193	0.65937	0.25483	0.35348	0.36186	1.6543	0.04903	1.1594	0.12315	0.04903	0.498560238
NA	ENSG00000230490	1.2804	0.10021	1.6543	0.049033	1.2596	0.10391	0.74461	0.22825	0.049033	0.498560238
RNA5SP275	ENSG00000200806	1.3514	0.088281	1.3268	0.092295	0.53587	0.29602	1.6542	0.04904	0.04904	0.498560238
POC1A	ENSG00000164087	0.90337	0.18316	1.0592	0.14475	1.6542	0.049048	1.3004	0.09673	0.049048	0.498560238
TPD52L1	ENSG00000111907	0.99292	0.16038	0.34594	0.3647	1.6541	0.049058	1.6541	0.049058	0.049058	0.498560238
CCNYL3	ENSG00000216671	0.072652	0.47104	0.23222	0.40819	0.21025	0.41674	1.6541	0.049058	0.049058	0.498560238
LONRF2	ENSG00000170500	1.6538	0.049086	1.0373	0.14979	1.0627	0.14396	1.1288	0.12949	0.049086	0.49874078
SH3BP4	ENSG00000130147	-2.1189	0.98295	-1.0065	0.8429	0.98312	0.16277	1.6535	0.049119	0.049119	0.498759315
RNU6-1289P	ENSG00000207160	1.6534	0.049122	1.6534	0.049122	1.6534	0.049122	1.6534	0.049122	0.049122	0.498759315
RNU6-1226P	ENSG00000207044	1.6534	0.049125	1.6534	0.049125	1.6534	0.049125	1.6534	0.049125	0.049125	0.498759315
RPL17P33	ENSG00000243844	1.6534	0.04913	0.087068	0.46531	1.6534	0.04913	1.6534	0.04913	0.04913	0.498759315
NA	ENSG00000272508	1.6533	0.049139	1.6533	0.049139	1.6533	0.049139	1.6533	0.049139	0.049139	0.498759315
NA	ENSG00000254746	1.6531	0.049151	1.6531	0.049151	1.6531	0.049151	0.35409	0.36164	0.049151	0.498777224
AGO4	ENSG00000134698	1.5334	0.062591	1.3115	0.094838	1.653	0.049169	0.25068	0.40103	0.049169	0.498856

MYLIP	ENSG00000007944	1.6526	0.049209	1.4393	0.075035	1.1086	0.13379	1.1472	0.12564	0.049209	0.499157904
NA	ENSG00000260132	1.6524	0.049226	1.6524	0.049226	1.6524	0.049226	1.6524	0.049226	0.049226	0.499163374
MTND4P18	ENSG00000228694	0.66142	0.25417	0.66142	0.25417	1.6522	0.049245	0.66142	0.25417	0.049245	0.499163374
NPIPB7	ENSG00000233232	0.27544	0.39149	0.85057	0.19751	0.74445	0.2283	1.6521	0.049259	0.049259	0.499163374
CFAP99	ENSG00000206113	0.72491	0.23425	1.522	0.064005	0.69196	0.24448	1.6521	0.049261	0.049261	0.499163374
NA	ENSG00000236920	0.67595	0.24954	0.817	0.20696	1.652	0.049263	1.4855	0.068702	0.049263	0.499163374
NA	ENSG00000188424	1.652	0.049271	-0.88589	0.81216	-1.2053	0.88596	-0.39477	0.65349	0.049271	0.499163374
ELOA2	ENSG00000206181	1.4149	0.078545	1.6518	0.049283	1.5431	0.061398	1.6518	0.049283	0.049283	0.499181166
RN7SL756P	ENSG00000244610	1.6516	0.049311	1.6516	0.049311	1.6516	0.049311	1.6516	0.049311	0.049311	0.499277476
RPS29P32	ENSG00000276703	-0.37259	0.64528	0.91983	0.17883	0.52944	0.29825	1.6515	0.049313	0.049313	0.499277476
NA	ENSG00000264989	1.6265	0.051922	1.6513	0.049344	0.52299	0.30049	1.4024	0.080402	0.049344	0.499487561
NANOS2	ENSG00000188425	1.2354	0.10834	1.6507	0.049405	1.2947	0.097705	0.35391	0.3617	0.049405	0.499713305
NA	ENSG00000212458	1.6505	0.049425	1.6505	0.049425	0.93922	0.17381	1.1842	0.11817	0.049425	0.499713305
NA	ENSG00000224532	0.7304	0.23257	1.4929	0.067735	1.4929	0.067735	1.6504	0.049432	0.049432	0.499713305
NA	ENSG00000234594	0.23807	0.40591	-0.50299	0.69251	1.0346	0.15044	1.6502	0.049446	0.049446	0.499713305
CDC73	ENSG00000134371	0.31691	0.37566	1.65	0.049472	0.31691	0.37566	0.093856	0.46261	0.049472	0.499713305
H3-5	ENSG00000188375	0.70811	0.23944	1.6499	0.049481	1.6499	0.049481	1.6499	0.049481	0.049481	0.499713305
RNY3P13	ENSG00000202412	1.5827	0.056747	1.6498	0.049494	1.3163	0.094033	1.5055	0.066099	0.049494	0.499713305
DCUN1D2	ENSG00000150401	1.0443	0.14816	0.65453	0.25639	1.6496	0.049507	0.91146	0.18103	0.049507	0.499713305
MRPL15	ENSG00000137547	1.3076	0.095502	0.45498	0.32456	1.6496	0.049511	1.5049	0.066169	0.049511	0.499713305
NA	ENSG00000233517	0.14914	0.44072	0.088523	0.46473	1.6496	0.049517	0.14914	0.44072	0.049517	0.499713305
FAM86LP	ENSG00000242731	1.6494	0.049535	1.1645	0.12211	-1.0217	0.84653	-0.27271	0.60746	0.049535	0.499713305
PCNPP2	ENSG00000225856	1.6494	0.049537	1.6494	0.049537	1.6494	0.049537	0.11411	0.45458	0.049537	0.499713305
NOL4L-DT	ENSG00000204393	1.264	0.10311	1.6493	0.049547	1.2259	0.11012	1.6076	0.053966	0.049547	0.499713305
NA	ENSG00000283341	1.649	0.049577	1.649	0.049577	1.649	0.049577	1.649	0.049577	0.049577	0.499713305
NA	ENSG00000249403	0.0053246	0.49788	0.97335	0.16519	1.6489	0.049583	0.0053246	0.49788	0.049583	0.499713305
COL21A1	ENSG00000124749	1.6488	0.049599	0.34932	0.36342	-0.50927	0.69472	-0.36964	0.64417	0.049599	0.499713305
TECPR2	ENSG00000196663	1.63	0.051547	1.6487	0.049604	1.3111	0.094919	1.0621	0.1441	0.049604	0.499713305
GPS2P1	ENSG00000236496	1.6487	0.049606	0.19248	0.42368	-0.69084	0.75517	0.19248	0.42368	0.049606	0.499713305
SSTR2	ENSG00000180616	1.6487	0.049608	1.0661	0.14319	0.081603	0.46748	-1.1393	0.87271	0.049608	0.499713305
UCP2	ENSG00000175567	-0.59061	0.72261	-0.33296	0.63042	1.6486	0.049613	-0.62218	0.73309	0.049613	0.499713305

SMAD5	ENSG00000113658	0.035915	0.48568	1.4624	0.071819	1.6484	0.049631	0.82942	0.20343	0.049631	0.499713305
RNU6-633P	ENSG00000199482	0.32414	0.37292	0.3633	0.35819	0.53567	0.29609	1.6484	0.049637	0.049637	0.499713305
LINC00701	ENSG00000234556	0.28411	0.38816	0.71615	0.23695	1.6483	0.049641	1.6483	0.049641	0.049641	0.499713305
RPL30P1	ENSG00000225713	1.6483	0.049645	1.6483	0.049645	1.6483	0.049645	0.83482	0.20191	0.049645	0.499713305
RNU6-584P	ENSG00000222282	1.3118	0.094796	-0.12017	0.54782	1.6482	0.049652	1.6482	0.049652	0.049652	0.499713305
NA	ENSG00000266944	1.6482	0.049652	1.6482	0.049652	1.6482	0.049652	1.6482	0.049652	0.049652	0.499713305
NA	ENSG00000284328	1.6482	0.049658	1.6482	0.049658	1.6482	0.049658	1.6482	0.049658	0.049658	0.499713305
BRAT1	ENSG00000106009	1.4703	0.070736	1.6482	0.04966	0.85353	0.19668	1.107	0.13415	0.04966	0.499713305
PAX2	ENSG00000075891	1.6478	0.049692	0.12041	0.45208	0.35513	0.36124	0.14615	0.4419	0.049692	0.499713305
DEPDC5	ENSG00000100150	0.05406	0.47844	1.6477	0.049703	0.93604	0.17463	1.43	0.076357	0.049703	0.499713305
RNU6-346P	ENSG00000252097	0.21856	0.4135	1.6477	0.049711	1.3099	0.095118	1.6477	0.049711	0.049711	0.499713305
NA	ENSG00000213041	1.6436	0.050129	1.6476	0.049717	1.1626	0.12249	1.1626	0.12249	0.049717	0.499713305
NA	ENSG00000234977	0.80776	0.20961	-0.18675	0.57407	1.6476	0.049719	1.6476	0.049719	0.049719	0.499713305
MAP3K6	ENSG00000142733	0.28019	0.38967	0.28019	0.38967	1.6474	0.049735	-0.69465	0.75636	0.049735	0.499713305
NA	ENSG00000263984	1.6472	0.049763	-0.39753	0.65451	-0.39753	0.65451	-0.39753	0.65451	0.049763	0.499713305
LAT	ENSG00000213658	1.0009	0.15845	1.0009	0.15845	1.6471	0.049767	1.0009	0.15845	0.049767	0.499713305
APOL2	ENSG00000128335	1.37	0.085341	1.2308	0.1092	1.2308	0.1092	1.6471	0.049769	0.049769	0.499713305
RNF145	ENSG00000145860	-0.45619	0.67587	1.6471	0.04977	0.49562	0.31008	1.5249	0.063636	0.04977	0.499713305
NA	ENSG00000267232	1.647	0.049776	1.647	0.049776	1.647	0.049776	1.647	0.049776	0.049776	0.499713305
PPP2R2C	ENSG00000074211	1.0368	0.14993	1.647	0.049781	1.2821	0.099898	1.3647	0.086173	0.049781	0.499713305
HSP90AA2P	ENSG00000224411	-1.0388	0.85056	1.6469	0.049789	-1.8883	0.97051	-1.0388	0.85056	0.049789	0.499713305
MXD1	ENSG00000059728	1.4821	0.069162	0.68481	0.24673	1.6468	0.049797	0.67796	0.2489	0.049797	0.499713305
ILDR1	ENSG00000145103	0.88216	0.18884	0.88216	0.18884	1.5004	0.066749	1.6467	0.04981	0.04981	0.499738287
RN7SL344P	ENSG00000241395	1.6466	0.04982	1.6466	0.04982	1.6466	0.04982	1.6466	0.04982	0.04982	0.499738287
AFF3	ENSG00000144218	0.010356	0.49587	-0.1416	0.5563	1.6463	0.049848	0.5862	0.27887	0.049848	0.499772322
DEPDC1-AS1	ENSG00000234264	1.0927	0.13726	1.0668	0.14302	1.1534	0.12438	1.6463	0.049856	0.049856	0.499772322
NA	ENSG00000238290	1.6462	0.049857	1.5074	0.065852	0.428	0.33432	-0.65662	0.74429	0.049857	0.499772322
CAMK1	ENSG00000134072	-1.3488	0.9113	-0.54126	0.70583	-0.26664	0.60513	1.6461	0.049877	0.049877	0.499772322
NA	ENSG00000240790	-0.13534	0.55383	1.646	0.049877	0.31457	0.37654	0.40938	0.34113	0.049877	0.499772322
NA	ENSG00000263011	1.6459	0.049891	1.6459	0.049891	1.6459	0.049891	0.20066	0.42048	0.049891	0.499772322
NME7	ENSG00000143156	-0.73204	0.76793	1.6456	0.049923	0.10343	0.45881	-0.17007	0.56752	0.049923	0.499772322

RNU2-59P	ENSG00000222414	1.6455	0.04993	1.4251	0.077069	1.4251	0.077069	1.4251	0.077069	0.04993	0.499772322
IPMKP1	ENSG00000226507	0.7972	0.21267	1.3642	0.086245	1.6455	0.049931	1.3642	0.086245	0.049931	0.499772322
NA	ENSG00000228618	0.69866	0.24238	1.4199	0.077821	1.6454	0.049943	1.6454	0.049943	0.049943	0.499772322
NA	ENSG00000200252	0.39629	0.34595	0.39629	0.34595	1.6454	0.049946	0.39629	0.34595	0.049946	0.499772322
C8orf37	ENSG00000156172	0.14884	0.44084	0.89996	0.18407	1.6451	0.04997	0.23204	0.40825	0.04997	0.499772322
NACAP5	ENSG00000249488	1.645	0.049988	0.36852	0.35624	0.89507	0.18537	-0.49805	0.69078	0.049988	0.499772322
GJA10	ENSG00000135355	-0.087007	0.53467	-0.066906	0.52667	1.6449	0.049991	-0.36453	0.64227	0.049991	0.499772322
MIR629	ENSG00000207965	1.1891	0.1172	1.6449	0.049998	0.54856	0.29165	0.65863	0.25507	0.049998	0.499772322
NA	ENSG00000224865	-1.0159	0.84517	1.6449	0.05	-0.44826	0.67302	0.47558	0.31719	0.05	0.499772322
AP2S1	ENSG00000042753	0.63161	0.26382	1.6448	0.050004	-0.82242	0.79458	-1.4914	0.93208	0.050004	0.499772322
RASGEF1C	ENSG00000146090	-0.29111	0.61452	1.6448	0.050008	-0.23722	0.59376	-0.53497	0.70366	0.050008	0.499772322
MIR3136	ENSG00000265355	1.6443	0.050061	1.6443	0.050061	1.6443	0.050061	1.6443	0.050061	0.050061	0.500146818
NA	ENSG00000254392	1.6442	0.050066	0.51261	0.30411	1.6442	0.050066	0.80197	0.21129	0.050066	0.500146818
DMRTC2	ENSG00000142025	0.93337	0.17532	-0.4988	0.69104	0.20259	0.41973	1.644	0.050088	0.050088	0.500150991
GMNC	ENSG00000205835	-2.0435	0.9795	1.6439	0.050093	-2.0435	0.9795	-2.0435	0.9795	0.050093	0.500150991
GJA9	ENSG00000131233	1.6438	0.050113	1.6438	0.050113	1.6438	0.050113	1.6438	0.050113	0.050113	0.500150991
LINC02846	ENSG00000260193	0.087675	0.46507	-1.5646	0.94116	1.6437	0.050114	-0.38868	0.65124	0.050114	0.500150991
MIR5590	ENSG00000263783	1.6437	0.050118	1.6437	0.050118	1.6437	0.050118	1.6437	0.050118	0.050118	0.500150991
LINC01229	ENSG00000260876	1.6436	0.050128	1.2244	0.1104	1.6436	0.050128	1.6436	0.050128	0.050128	0.500150991
NA	ENSG00000243939	0.31614	0.37595	0.89913	0.18429	1.6435	0.050141	0.93898	0.17387	0.050141	0.500178287
ARHGAP1	ENSG00000175220	1.4099	0.07929	1.2048	0.11413	1.4099	0.07929	1.6432	0.050167	0.050167	0.500246863
FAM204CP	ENSG00000230902	-0.85457	0.8036	1.6431	0.050185	0.93927	0.17379	-0.30715	0.62063	0.050185	0.500246863
NSUN3	ENSG00000178694	-0.54901	0.7085	-0.54901	0.7085	-0.54901	0.7085	1.643	0.050192	0.050192	0.500246863
NA	ENSG00000145965	1.6428	0.050212	1.6428	0.050212	-1.1394	0.87272	1.6428	0.050212	0.050212	0.500246863
SUGCT	ENSG00000175600	-0.31942	0.6253	1.6427	0.05022	-0.54781	0.70809	0.53747	0.29547	0.05022	0.500246863
GRM2	ENSG00000164082	1.6427	0.050221	-1.0919	0.86256	1.5909	0.05582	-0.069606	0.52775	0.050221	0.500246863
NA	ENSG00000261736	0.72307	0.23482	1.6427	0.050222	-0.37968	0.64791	-0.37968	0.64791	0.050222	0.500246863
NA	ENSG00000260101	1.6426	0.05023	1.6426	0.05023	1.6426	0.05023	-0.23444	0.59268	0.05023	0.500246863
NA	ENSG00000266248	1.6425	0.050248	1.6425	0.050248	0.60442	0.27278	1.6425	0.050248	0.050248	0.500323874
ENPP7P14	ENSG00000273963	1.6423	0.050263	0.42265	0.33628	-0.04531	0.51807	0.78115	0.21736	0.050263	0.500329093
NA	ENSG00000272255	-1.0356	0.8498	1.6422	0.050277	-0.72526	0.76585	-0.72526	0.76585	0.050277	0.500329093

NA	ENSG00000277315	1.6421	0.050287	-0.024505	0.50978	-0.024505	0.50978	-0.38321	0.64922	0.050287	0.500329093
NA	ENSG00000283097	0.87651	0.19038	1.642	0.050299	-0.2287	0.59045	-0.2287	0.59045	0.050299	0.500329093
NA	ENSG00000258999	1.6419	0.050306	0.52161	0.30097	1.1112	0.13325	0.69884	0.24233	0.050306	0.500329093
NA	ENSG00000265413	0.6927	0.24425	1.6418	0.05032	0.79464	0.21341	0.79464	0.21341	0.05032	0.500329093
DBNL	ENSG00000136279	1.6415	0.050344	1.5849	0.056489	1.5872	0.056229	-0.30316	0.61911	0.050344	0.500329093
NCMAP	ENSG00000184454	0.34852	0.36372	1.6414	0.050355	0.018759	0.49252	-1.0364	0.84999	0.050355	0.500329093
LINC02273	ENSG00000245954	-1.2996	0.90313	-0.74275	0.77118	1.6414	0.050359	1.3013	0.096585	0.050359	0.500329093
GRIK3	ENSG00000163873	-0.4376	0.66916	0.29621	0.38353	-0.38505	0.6499	1.6412	0.050376	0.050376	0.500329093
NA	ENSG00000225923	1.6412	0.050376	1.6412	0.050376	1.6412	0.050376	1.6412	0.050376	0.050376	0.500329093
NA	ENSG00000258216	0.96872	0.16634	1.6412	0.050377	1.6412	0.050377	1.6412	0.050377	0.050377	0.500329093
HRH4	ENSG00000134489	1.09	0.13786	1.09	0.13786	1.6412	0.050382	0.87363	0.19116	0.050382	0.500329093
CNTF	ENSG00000242689	0.51752	0.3024	0.15875	0.43693	0.15875	0.43693	1.641	0.050395	0.050395	0.500356225
NA	ENSG00000225486	-0.61411	0.73043	0.44992	0.32638	1.6408	0.050423	-0.61411	0.73043	0.050423	0.500509701
NA	ENSG00000260249	0.73112	0.23235	1.3625	0.086522	1.3625	0.086522	1.6407	0.050431	0.050431	0.500509701
MBL1P	ENSG00000242600	-0.059785	0.52384	1.6404	0.050457	0.63774	0.26182	0.63774	0.26182	0.050457	0.500611514
DAPP1	ENSG00000070190	-0.0093806	0.50374	0.40261	0.34362	1.2186	0.11149	1.6402	0.050482	0.050482	0.500611514
NA	ENSG00000247735	1.6402	0.050484	1.6402	0.050484	1.6402	0.050484	1.6402	0.050484	0.050484	0.500611514
LPIN2	ENSG00000101577	-0.97903	0.83622	1.64	0.050505	-0.42857	0.66588	1.1944	0.11617	0.050505	0.500611514
NA	ENSG00000265987	1.6399	0.050508	1.6399	0.050508	1.6399	0.050508	1.5101	0.065507	0.050508	0.500611514
TLR3	ENSG00000164342	1.6399	0.05051	-0.00065845	0.50026	-0.00065845	0.50026	-0.00065845	0.50026	0.05051	0.500611514
COL7A1	ENSG00000114270	1.6397	0.050536	1.0862	0.1387	1.4807	0.069348	0.9094	0.18157	0.050536	0.500611514
LINC01391	ENSG00000244578	1.2226	0.11074	1.6395	0.050551	0.28385	0.38826	-0.13054	0.55193	0.050551	0.500611514
NA	ENSG00000260983	0.48564	0.31361	0.88202	0.18888	1.6395	0.050551	1.6395	0.050551	0.050551	0.500611514
TM2D1	ENSG00000162604	0.39062	0.34804	-0.89745	0.81526	0.20177	0.42005	1.6394	0.050569	0.050569	0.500611514
RASGRF1	ENSG00000058335	1.597	0.055133	1.2081	0.1135	-1.6549	0.95103	1.6393	0.050576	0.050576	0.500611514
ASCC1	ENSG00000138303	0.75581	0.22488	0.455	0.32455	-0.49634	0.69017	1.6393	0.050578	0.050578	0.500611514
KRTAP19-8	ENSG00000206102	1.5264	0.063454	0.52557	0.29959	-0.37434	0.64593	1.6392	0.050584	0.050584	0.500611514
LINC02564	ENSG00000262352	1.6392	0.050586	1.2832	0.099706	-0.80383	0.78925	-0.80383	0.78925	0.050586	0.500611514
RNU6-157P	ENSG00000206684	1.639	0.050603	0.54492	0.2929	0.13024	0.44819	0.13024	0.44819	0.050603	0.500611514
IFIT1B	ENSG00000204010	0.90933	0.18159	0.69688	0.24294	0.4033	0.34336	1.639	0.050606	0.050606	0.500611514
MEA1	ENSG00000124733	1.6388	0.050624	1.6388	0.050624	1.6388	0.050624	1.6388	0.050624	0.050624	0.500611514

NA	ENSG00000249423	-0.065547	0.52613	0.51366	0.30374	1.6385	0.050657	0.30276	0.38104	0.050657	0.500611514
MGAT5B	ENSG00000167889	1.6385	0.05066	1.4227	0.077411	1.5217	0.064048	0.40735	0.34188	0.05066	0.500611514
NA	ENSG00000265645	1.6385	0.050663	1.6385	0.050663	1.6385	0.050663	0.49702	0.30959	0.050663	0.500611514
MAP6	ENSG00000171533	-0.8787	0.81022	1.6383	0.050684	-0.87075	0.80805	-1.1474	0.87438	0.050684	0.500611514
USP6NL	ENSG00000148429	1.4545	0.072904	1.6382	0.050689	1.6207	0.052543	1.5547	0.060006	0.050689	0.500611514
PRRC2A	ENSG00000204469	0.23208	0.40824	1.6382	0.050689	-0.22228	0.58795	0.53614	0.29593	0.050689	0.500611514
RN7SL364P	ENSG00000243560	1.6382	0.050693	1.6382	0.050693	1.6382	0.050693	1.6382	0.050693	0.050693	0.500611514
THSD7B	ENSG00000144229	-1.3347	0.90902	-1.3252	0.90744	0.43977	0.33005	1.6377	0.050737	0.050737	0.500611514
NA	ENSG00000214919	1.6377	0.050737	1.6377	0.050737	1.6377	0.050737	1.6377	0.050737	0.050737	0.500611514
NA	ENSG00000253932	1.6377	0.050747	1.6377	0.050747	1.0052	0.1574	1.6377	0.050747	0.050747	0.500611514
SETD7	ENSG00000145391	1.6375	0.050766	1.0892	0.13802	1.2297	0.10941	1.0597	0.14465	0.050766	0.500611514
RBAK	ENSG00000146587	1.3949	0.081527	0.52584	0.2995	1.6375	0.050767	1.1949	0.11606	0.050767	0.500611514
PDCD5	ENSG00000105185	1.5459	0.061059	1.6374	0.050769	1.5459	0.061059	0.29285	0.38482	0.050769	0.500611514
SNORA75B	ENSG00000206780	0.88993	0.18675	1.6374	0.05077	-0.43062	0.66663	0.024161	0.49036	0.05077	0.500611514
MIR3935	ENSG00000265281	1.6374	0.05077	1.6374	0.05077	1.6374	0.05077	1.6374	0.05077	0.05077	0.500611514
NA	ENSG00000266261	1.6373	0.050784	1.6373	0.050784	1.6373	0.050784	1.6373	0.050784	0.050784	0.500642855
FTH1P26	ENSG00000218213	1.6371	0.050802	1.6371	0.050802	1.6371	0.050802	1.6371	0.050802	0.050802	0.500642855
NA	ENSG00000267404	1.6371	0.050804	1.6371	0.050804	1.6371	0.050804	1.6371	0.050804	0.050804	0.500642855
TRAF2	ENSG00000127191	1.0088	0.15654	0.97789	0.16407	1.6367	0.050848	1.0088	0.15654	0.050848	0.500721398
NA	ENSG00000271656	0.73482	0.23122	0.73482	0.23122	0.63515	0.26266	1.6366	0.05086	0.05086	0.500721398
CCDC197	ENSG00000175699	0.36946	0.35589	1.6365	0.050866	0.82203	0.20553	1.524	0.063753	0.050866	0.500721398
CELA3B	ENSG00000219073	1.6363	0.050891	-0.48554	0.68635	0.45806	0.32346	0.43823	0.33061	0.050891	0.500721398
NA	ENSG00000267551	1.6362	0.050894	1.6362	0.050894	1.6362	0.050894	1.6362	0.050894	0.050894	0.500721398
TMEM176A	ENSG00000002933	0.88136	0.18906	1.6362	0.050902	0.88136	0.18906	1.4561	0.072685	0.050902	0.500721398
RABEP2	ENSG00000177548	0.94531	0.17225	0.80426	0.21062	1.6362	0.050902	0.067289	0.47318	0.050902	0.500721398
SPRY4-AS1	ENSG00000231185	0.50534	0.30666	1.636	0.050915	1.2977	0.097198	0.95113	0.17077	0.050915	0.500721398
FAM71A	ENSG00000162771	0.38722	0.3493	0.38722	0.3493	0.38722	0.3493	1.636	0.050916	0.050916	0.500721398
NA	ENSG00000253891	-0.11595	0.54615	1.636	0.050924	-0.5156	0.69693	-1.0857	0.8612	0.050924	0.500721398
NA	ENSG00000283583	-0.82918	0.7965	1.1605	0.12292	1.636	0.050925	-1.3238	0.90722	0.050925	0.500721398
LINC01482	ENSG00000267659	-0.13904	0.55529	1.6355	0.050971	0.080436	0.46795	0.49744	0.30944	0.050971	0.501059984
AGXT2	ENSG00000113492	-0.19826	0.57858	-0.14052	0.55588	1.6354	0.05098	-0.33294	0.63041	0.05098	0.501059984

BTF3P2 ENSG00000258282 -0.41812 0.66207 -0.41812 0.66207 -0.41812
0.66207 1.6351 0.051013 0.051013 0.50128322
HPS4 ENSG00000100099 1.6348 0.051046 0.95662 0.16938 0.95662 0.16938
1.3966 0.081265 0.051046 0.501478669
RFX4 ENSG00000111783 1.1095 0.13361 1.4061 0.079851 1.3169 0.093943
1.6347 0.051061 0.051061 0.501478669
TRAV23DV6 ENSG00000211803 1.6346 0.051069 1.6346 0.051069 1.6346
0.051069 0.10598 0.4578 0.051069 0.501478669
NA ENSG00000248763 1.6345 0.05108 0.28452 0.38801 0.27425 0.39195
0.32164 0.37386 0.05108 0.501478669
RN7SL504P ENSG00000241789 1.2443 0.10669 1.6343 0.051096 1.6343
0.051096 1.6343 0.051096 0.051096 0.501478669
NA ENSG00000226571 1.6342 0.051108 1.6342 0.051108 1.6342 0.051108
1.6342 0.051108 0.051108 0.501478669
TAC1 ENSG00000006128 0.39383 0.34685 -1.0598 0.85537 1.6341 0.051121
-0.82229 0.79454 0.051121 0.501478669
ATG9B ENSG00000181652 -0.64462 0.74041 -0.871 0.80812 -0.64462 0.74041
1.634 0.051129 0.051129 0.501478669
NA ENSG00000238596 0.60649 0.2721 0.60649 0.2721 0.60649 0.2721
1.634 0.05113 0.05113 0.501478669
HBQ1 ENSG00000086506 -0.90624 0.8176 1.6339 0.051142 -1.0365 0.85001
-1.0365 0.85001 0.051142 0.501478669
NTRK2 ENSG00000148053 0.76022 0.22356 1.6337 0.051164 -0.082893 0.53303
0.50618 0.30636 0.051164 0.501478669
PIP5K1C ENSG00000186111 1.6337 0.051164 0.34274 0.3659 1.228 0.10973
0.88837 0.18717 0.051164 0.501478669
NA ENSG00000235300 1.6335 0.051181 0.17437 0.43079 0.17437 0.43079
0.17437 0.43079 0.051181 0.501478669
TRAJ1 ENSG00000211888 1.6335 0.051185 1.6335 0.051185 1.6335 0.051185
1.6335 0.051185 0.051185 0.501478669
STARD13 ENSG00000133121 1.4044 0.080101 1.2011 0.11486 1.6334
0.051197 -0.33831 0.63244 0.051197 0.501478669
HLX ENSG00000136630 0.60627 0.27217 1.4238 0.077247 1.1705 0.1209
1.6333 0.051206 0.051206 0.501478669
MIR644A ENSG00000207997 1.6332 0.051219 1.6332 0.051219 1.6332
0.051219 1.6332 0.051219 0.051219 0.501478669
DSTYK ENSG00000133059 1.6331 0.051228 1.4918 0.067871 0.96418 0.16748
1.1839 0.11823 0.051228 0.501478669
PPARG ENSG00000132170 0.61955 0.26778 -0.029759 0.51187 1.633 0.051231
-0.032855 0.5131 0.051231 0.501478669
NA ENSG00000237400 1.6329 0.051247 -1.4288 0.92347 -1.0799 0.85992
-1.6924 0.95472 0.051247 0.501478669
BEGAIN ENSG00000183092 0.02279 0.49091 0.38813 0.34896 0.92985
0.17623 1.6329 0.051249 0.051249 0.501478669
NA ENSG00000270135 1.6324 0.051293 1.6324 0.051293 1.6324 0.051293
1.6324 0.051293 0.051293 0.501546709
NA ENSG00000233878 1.5914 0.055764 -0.026593 0.51061 1.6323 0.05131
1.3208 0.093277 0.05131 0.501546709
NA ENSG00000253809 0.61463 0.2694 0.18396 0.42702 1.6322 0.051319
1.6322 0.051319 0.051319 0.501546709
TCIM ENSG00000176907 1.6322 0.051322 0.25039 0.40114 0.27433 0.39192
1.6322 0.051322 0.051322 0.501546709
SNORD60 ENSG00000206630 1.6321 0.051325 1.6321 0.051325 1.6321
0.051325 1.6321 0.051325 0.051325 0.501546709
SNHG19 ENSG00000260260 1.6321 0.051325 1.6321 0.051325 1.6321
0.051325 1.6321 0.051325 0.051325 0.501546709
NA ENSG00000228098 1.6321 0.051328 1.6321 0.051328 1.6321 0.051328
1.6321 0.051328 0.051328 0.501546709
NA ENSG00000217585 1.5008 0.06671 1.6319 0.05135 0.9879 0.1616
1.0325 0.15091 0.05135 0.501589836
RNU7-54P ENSG00000238590 0.98238 0.16296 0.31097 0.37791 1.6319
0.051353 0.56984 0.28439 0.051353 0.501589836

NA ENSG00000271214 1.257 0.10438 1.6316 0.051379 1.257 0.10438 1.1767 0.11965 0.051379 0.501659341
NA ENSG00000248459 1.122 0.13093 -0.61248 0.72989 1.122 0.13093 1.6316 0.051384 0.051384 0.501659341
NA ENSG00000278496 1.3965 0.081289 1.6315 0.051391 1.4309 0.076226 0.74136 0.22924 0.051391 0.501659341
DHX30 ENSG00000132153 1.2457 0.10644 1.1888 0.11727 1.1408 0.12698 1.6314 0.051404 0.051404 0.501685744
ABLIM3 ENSG00000173210 1.631 0.051445 -0.079846 0.53182 0.17097 0.43213 -0.53393 0.70331 0.051445 0.501755463
COPB2 ENSG00000184432 -0.52296 0.6995 -0.52296 0.6995 -0.52296 0.6995 1.6309 0.051458 0.051458 0.501755463
NA ENSG00000257958 -1.366 0.91403 0.40399 0.34311 -1.1846 0.8819 1.6306 0.051485 0.051485 0.501755463
FCN1 ENSG00000085265 0.13338 0.44695 1.6306 0.051486 0.13338 0.44695 0.57661 0.2821 0.051486 0.501755463
SMAD5-AS1 ENSG00000164621 0.95599 0.16954 1.6306 0.051491 1.6306 0.051491 1.6306 0.051491 0.051491 0.501755463
MIR5190 ENSG00000266146 0.79139 0.21436 0.18247 0.42761 0.50414 0.30708 1.6306 0.051492 0.051492 0.501755463
COIL ENSG00000121058 1.6305 0.051497 -1.0562 0.85457 0.21994 0.41296 0.56608 0.28567 0.051497 0.501755463
NA ENSG00000269971 0.73619 0.23081 0.80384 0.21075 1.6304 0.051505 0.73619 0.23081 0.051505 0.501755463
NA ENSG00000258107 0.70162 0.24146 -0.80631 0.78997 1.6303 0.051524 -0.89705 0.81515 0.051524 0.501755463
NA ENSG00000283166 1.6302 0.051528 1.6302 0.051528 1.6302 0.051528 1.6302 0.051528 0.051528 0.501755463
LINC01514 ENSG00000237579 1.3738 0.084759 1.3335 0.091186 1.5944 0.055424 1.6302 0.051535 0.051535 0.501755463
IRF4 ENSG00000137265 -0.29479 0.61592 -0.39978 0.65534 0.86343 0.19395 1.6301 0.051541 0.051541 0.501755463
NA ENSG00000265437 -1.0003 0.84142 -0.89387 0.8143 -0.89387 0.8143 1.6301 0.051545 0.051545 0.501755463
ANKRD46 ENSG00000186106 0.29476 0.38409 1.5705 0.058151 1.6298 0.051575 0.12017 0.45217 0.051575 0.501881089
LINC02399 ENSG00000257725 -0.31131 0.62222 1.6297 0.051583 1.6297 0.051583 1.6297 0.051583 0.051583 0.501881089
NA ENSG00000258699 1.4889 0.068254 1.4889 0.068254 1.6296 0.05159 1.4889 0.068254 0.05159 0.501881089
ITGB2 ENSG00000160255 1.4656 0.071382 1.6293 0.05162 -1.1874 0.88246 -1.3176 0.90619 0.05162 0.501881089
RNU6-253P ENSG00000207461 -0.55214 0.70957 -0.55214 0.70957 -0.73457 0.7687 1.6293 0.051622 0.051622 0.501881089
HTT ENSG00000197386 1.5626 0.059068 0.76591 0.22186 1.1983 0.1154 1.6293 0.051628 0.051628 0.501881089
CDK6-AS1 ENSG00000237819 1.6293 0.05163 1.6293 0.05163 1.6293 0.05163 0.9772 0.16423 0.05163 0.501881089
NA ENSG00000212620 1.6291 0.051645 0.41341 0.33965 0.99097 0.16085 0.99097 0.16085 0.051645 0.501926775
MVK ENSG00000110921 1.6288 0.051673 1.1837 0.11828 1.1797 0.11907 1.1438 0.12635 0.051673 0.502070492
PRRT1 ENSG00000204314 1.6286 0.051696 -0.5626 0.71315 0.78299 0.21682 0.066554 0.47347 0.051696 0.502070492
PPT2 ENSG00000221988 1.6286 0.051696 -0.20787 0.58234 1.6286 0.051696 1.6286 0.051696 0.051696 0.502070492
NA ENSG00000274863 -0.1327 0.55279 -0.31649 0.62418 1.6286 0.051701 0.63225 0.26361 0.051701 0.502070492
NA ENSG00000240354 1.6283 0.051736 1.6283 0.051736 1.6283 0.051736 1.6283 0.051736 0.051736 0.502152803
TIMM9P3 ENSG00000232786 1.6281 0.051752 1.6281 0.051752 1.6281 0.051752 1.6281 0.051752 0.051752 0.502152803

TMEM161A ENSG00000064545 0.26616 0.39506 1.6281 0.051753 1.5828
0.056731 1.0029 0.15796 0.051753 0.502152803
PICK1 ENSG00000100151 0.5752 0.28258 1.628 0.051758 0.46943 0.31938
-0.27381 0.60788 0.051758 0.502152803
NA ENSG00000218672 1.628 0.051761 0.66917 0.25169 0.69022 0.24503
0.74281 0.2288 0.051761 0.502152803
LINC02233 ENSG00000250488 -0.69798 0.75741 1.6278 0.051783 -0.69798
0.75741 -0.69798 0.75741 0.051783 0.50226624
MIR3201 ENSG00000266508 -0.25667 0.60128 1.053 0.14617 1.6274 0.051823
-0.97766 0.83588 0.051823 0.50251237
NA ENSG00000255369 -0.3342 0.63089 0.37466 0.35395 -0.1332 0.55298
1.6274 0.051829 0.051829 0.50251237
HELQ ENSG00000163312 1.6271 0.051862 0.38179 0.35131 1.6271 0.051862
1.6271 0.051862 0.051862 0.502671078
RN7SL862P ENSG00000242651 -0.70027 0.75812 -0.99293 0.83963 -0.70027
0.75812 1.627 0.051866 0.051866 0.502671078
KRT8P5 ENSG00000267573 0.33672 0.36817 1.6269 0.051884 0.33672
0.36817 0.33672 0.36817 0.051884 0.50274554
NA ENSG00000247193 1.6264 0.051929 1.6264 0.051929 1.6264 0.051929
0.44519 0.32809 0.051929 0.502767696
MRPL42P4 ENSG00000242552 0.46848 0.31972 1.6263 0.051946 1.6263
0.051946 1.6263 0.051946 0.051946 0.502767696
MIR181B1 ENSG00000207975 1.6262 0.051948 1.6202 0.052592 -1.173
0.8796 -1.173 0.8796 0.051948 0.502767696
DHX40P1 ENSG00000266992 1.626 0.051973 1.11 0.13349 0.31991 0.37452
1.2574 0.10431 0.051973 0.502767696
DEPDC1B ENSG00000035499 -1.1473 0.87437 -1.0129 0.84445 -0.91287
0.81934 1.626 0.051977 0.051977 0.502767696
ALPP ENSG00000163283 1.2129 0.11258 1.626 0.051979 0.68407 0.24696
0.48486 0.31389 0.051979 0.502767696
FBLN2 ENSG00000163520 1.474 0.070242 0.29904 0.38246 1.1905 0.11693
1.6258 0.051999 0.051999 0.502767696
MUC3A ENSG00000169894 1.2286 0.10962 0.59393 0.27628 1.6257 0.052007
0.93327 0.17534 0.052007 0.502767696
C20orf203 ENSG00000198547 1.6256 0.052013 0.6794 0.24844 0.99442
0.16001 1.2421 0.10711 0.052013 0.502767696
PPIAP63 ENSG00000231570 1.6254 0.052035 -0.62742 0.73481 1.321 0.093252
0.53186 0.29741 0.052035 0.502767696
AZGP1P2 ENSG00000214252 1.6253 0.052052 0.74088 0.22938 0.086117
0.46569 1.2102 0.1131 0.052052 0.502767696
ATL3 ENSG00000184743 0.83358 0.20226 0.83358 0.20226 1.6253 0.052053
1.4274 0.076727 0.052053 0.502767696
SSX2IP ENSG00000117155 1.1596 0.12311 0.89738 0.18476 1.6253
0.052054 0.64913 0.25813 0.052054 0.502767696
TTC1 ENSG00000113312 0.071695 0.47142 0.99762 0.15923 1.6252 0.05206
0.9548 0.16984 0.05206 0.502767696
ZNF511 ENSG00000198546 1.6252 0.052061 -0.89897 0.81567 -1.2981
0.90287 -1.2981 0.90287 0.052061 0.502767696
RASSF1-AS1 ENSG00000281358 1.6252 0.052064 1.6252 0.052064 1.6252
0.052064 1.6252 0.052064 0.052064 0.502767696
IPO7P2 ENSG00000225674 -0.10495 0.54179 -0.10495 0.54179 1.6251
0.052067 1.495 0.067451 0.052067 0.502767696
TANGO2 ENSG00000183597 -0.59167 0.72296 1.6251 0.052072 0.26564
0.39526 0.20515 0.41873 0.052072 0.502767696
CUBNP1 ENSG00000231009 1.625 0.052083 -0.42439 0.66436 1.0055 0.15733
0.67584 0.24957 0.052083 0.502774285
NA ENSG00000207193 1.6248 0.052105 1.6248 0.052105 1.6248 0.052105
1.6248 0.052105 0.052105 0.502797548
NA ENSG00000237735 -0.14656 0.55826 1.6247 0.052117 1.6247 0.052117
1.6247 0.052117 0.052117 0.502797548
NIPA2P1 ENSG00000225843 1.6246 0.052124 1.6246 0.052124 1.6246
0.052124 1.6246 0.052124 0.052124 0.502797548

ZNF410	ENSG00000119725	1.5683	0.058409	1.6245	0.052137	1.5394	0.061851	1.3409	0.089975	0.052137	0.502797548
LINC02172	ENSG00000251632	1.6245	0.052137	-0.22205	0.58786	1.6245	0.052137	1.6245	0.052137	0.052137	0.502797548
PSAT1P2	ENSG00000232144	1.6243	0.052159	0.7377	0.23035	1.3291	0.091902	1.3291	0.091902	0.052159	0.502820336
RHBDF1P1	ENSG00000234123	1.6243	0.05216	1.6243	0.05216	1.6243	0.05216	1.6243	0.05216	0.05216	0.502820336
CLNS1AP1	ENSG00000213335	1.6238	0.052213	-0.39171	0.65236	-0.057985	0.52312	-1.3112	0.9051	0.052213	0.503154829
AOX1	ENSG00000138356	0.54286	0.29361	1.6236	0.052226	0.41878	0.33769	-0.046705	0.51863	0.052226	0.503154829
ZNF417	ENSG00000173480	-0.15281	0.56073	1.6236	0.052229	0.34862	0.36369	-1.7726	0.96185	0.052229	0.503154829
NA	ENSG00000283529	0.65874	0.25503	1.4015	0.080538	0.95157	0.17066	1.6235	0.052236	0.052236	0.503154829
NA	ENSG00000239739	0.96648	0.1669	0.96648	0.1669	1.6232	0.05227	0.20143	0.42018	0.05227	0.503350763
ERICH2	ENSG00000204334	1.6232	0.052277	-0.28555	0.61239	0.56683	0.28541	0.20612	0.41835	0.052277	0.503350763
POTEC	ENSG00000183206	-0.17983	0.57136	-0.17983	0.57136	0.67919	0.24851	1.623	0.05229	0.05229	0.503376472
C1orf229	ENSG00000221953	1.6229	0.052302	1.6229	0.052302	0.62621	0.26559	1.1134	0.13278	0.052302	0.503392546
MYO7A	ENSG00000137474	1.1148	0.13247	1.3322	0.091402	1.346	0.089148	1.6227	0.052331	0.052331	0.503572202
RNU6ATAC8P	ENSG00000221387	0.95564	0.16963	0.95564	0.16963	0.95564	0.16963	1.6224	0.052362	0.052362	0.503674045
SPARC	ENSG00000113140	-1.1693	0.87885	-1.1942	0.8838	-0.85085	0.80257	1.6221	0.052387	0.052387	0.503674045
NA	ENSG00000231866	1.6221	0.052396	1.6221	0.052396	1.6221	0.052396	1.6221	0.052396	0.052396	0.503674045
MIR4491	ENSG00000264032	1.6219	0.052416	1.6219	0.052416	0.21302	0.41566	1.6219	0.052416	0.052416	0.503674045
MTPN	ENSG00000105887	-0.39073	0.652	1.6218	0.052422	-0.045727	0.51824	-0.39073	0.652	0.052422	0.503674045
NA	ENSG00000228679	-0.21442	0.58489	1.6217	0.052434	1.6217	0.052434	1.6217	0.052434	0.052434	0.503674045
DNAJC18	ENSG00000170464	1.6217	0.052435	-0.077606	0.53093	-0.92828	0.82337	-0.28807	0.61335	0.052435	0.503674045
NA	ENSG00000238582	1.6215	0.05245	1.6215	0.05245	1.6215	0.05245	1.6215	0.05245	0.05245	0.503674045
PRKCD	ENSG00000163932	1.6214	0.052462	0.57736	0.28185	0.76816	0.2212	-0.47902	0.68404	0.052462	0.503674045
POLE4	ENSG00000115350	1.6212	0.052491	1.6212	0.052491	1.6212	0.052491	-0.14328	0.55697	0.052491	0.503674045
NA	ENSG00000270927	-1.2635	0.89679	1.6211	0.052495	-0.81389	0.79215	1.0302	0.15146	0.052495	0.503674045
NA	ENSG00000237838	1.6211	0.052499	0.12793	0.4491	1.3193	0.093534	0.11883	0.45271	0.052499	0.503674045
TMEM147	ENSG00000105677	1.621	0.052511	1.621	0.052511	1.621	0.052511	1.621	0.052511	0.052511	0.503674045
NA	ENSG00000249149	0.0075485	0.49699	1.6208	0.052528	-0.17439	0.56922	-0.63167	0.7362	0.052528	0.503674045
RPL21P44	ENSG00000229585	1.1868	0.11764	0.63399	0.26304	1.599	0.054915	1.6207	0.052537	0.052537	0.503674045
THRSP	ENSG00000151365	1.0897	0.13792	1.6207	0.052538	1.0897	0.13792	1.0897	0.13792	0.052538	0.503674045
LINC00574	ENSG00000231690	1.6207	0.052546	0.11409	0.45458	0.9764	0.16443	-1.6946	0.95492	0.052546	0.503674045
NA	ENSG00000248636	-0.39274	0.65274	-1.9513	0.97449	1.6205	0.052565	-1.2931	0.90201	0.052565	0.503674045

RPL12P7	ENSG00000242941	-1.5146	0.93506	-0.37362	0.64566	1.6204	0.052574	-0.5265	0.70073	0.052574	0.503674045
CLSPN	ENSG00000092853	-0.058556	0.52335	1.6204	0.052577	-0.45805	0.67654	1.0647	0.14351	0.052577	0.503674045
CHAC1	ENSG00000128965	-0.098067	0.53906	-0.098067	0.53906	1.6204	0.052577	0.26329	0.39617	0.052577	0.503674045
LINC-PINT	ENSG00000231721	1.5493	0.060649	1.6203	0.052587	0.41911	0.33757	0.10385	0.45864	0.052587	0.503674045
NA	ENSG00000213331	-0.34416	0.63464	1.6201	0.052603	-0.23078	0.59126	-1.3628	0.91353	0.052603	0.503674045
GBX2	ENSG00000168505	1.6201	0.052605	-0.31238	0.62262	1.0315	0.15116	-0.31238	0.62262	0.052605	0.503674045
CEACAM18	ENSG00000213822	1.6198	0.052634	1.6198	0.052634	1.6198	0.052634	1.6198	0.052634	0.052634	0.503674045
MYCBP	ENSG00000214114	0.052862	0.47892	1.6198	0.052642	1.2068	0.11376	1.2068	0.11376	0.052642	0.503674045
SCG5-AS1	ENSG00000241818	-0.0809	0.53224	0.55126	0.29073	-0.7789	0.78198	1.6197	0.052651	0.052651	0.503674045
NA	ENSG00000262803	-0.68462	0.75321	-0.11866	0.54723	-0.68462	0.75321	1.6197	0.052651	0.052651	0.503674045
NA	ENSG00000233358	0.65193	0.25722	0.65193	0.25722	0.10054	0.45996	1.6196	0.052655	0.052655	0.503674045
CNTNAP4	ENSG00000152910	1.6196	0.052656	1.4389	0.075086	0.25351	0.39994	0.26711	0.39469	0.052656	0.503674045
SDC2	ENSG00000169439	0.56381	0.28644	0.28765	0.38681	0.61659	0.26875	1.6196	0.052662	0.052662	0.503674045
GAB1	ENSG00000109458	0.19824	0.42143	1.3589	0.087093	0.78815	0.2153	1.6193	0.05269	0.05269	0.503763096
LLPHP3	ENSG00000233868	1.4915	0.067917	1.4915	0.067917	1.6192	0.052705	1.4915	0.067917	0.052705	0.503763096
NA	ENSG00000271576	1.6191	0.052715	1.6191	0.052715	1.6191	0.052715	1.6191	0.052715	0.052715	0.503763096
NA	ENSG00000260278	-0.34392	0.63455	1.6191	0.052717	-0.34392	0.63455	-0.34392	0.63455	0.052717	0.503763096
RNU1-31P	ENSG00000200745	1.3782	0.084067	1.5715	0.058033	1.2488	0.10586	1.619	0.052723	0.052723	0.503763096
CAP1P2	ENSG00000232004	-0.21568	0.58538	1.6188	0.052741	1.6188	0.052741	1.6188	0.052741	0.052741	0.50378487
ROGDI	ENSG00000067836	1.6186	0.052769	1.6186	0.052769	-0.18679	0.57409	-0.10865	0.54326	0.052769	0.50378487
NA	ENSG00000234311	-0.49058	0.68814	1.0347	0.15039	1.6185	0.052772	-0.054272	0.52164	0.052772	0.50378487
MARCHF5	ENSG00000198060	1.3259	0.092436	1.3259	0.092436	0.12619	0.44979	1.6185	0.052778	0.052778	0.50378487
CYCSP7	ENSG00000269383	1.0861	0.13871	1.6184	0.052791	1.0861	0.13871	1.0861	0.13871	0.052791	0.50378487
RDH13	ENSG00000160439	1.6181	0.052817	0.49284	0.31106	0.95417	0.17	0.95417	0.17	0.052817	0.50378487
NA	ENSG00000282080	-0.080352	0.53202	1.6181	0.052823	-0.080352	0.53202	-0.080352	0.53202	0.052823	0.50378487
LINC01618	ENSG00000250302	0.47629	0.31693	1.618	0.052827	0.27295	0.39245	0.47472	0.31749	0.052827	0.50378487
ZNF578	ENSG00000258405	1.6179	0.052838	-1.251	0.89454	-0.13162	0.55236	-0.79928	0.78794	0.052838	0.50378487
RNF19B	ENSG00000116514	1.6179	0.052839	0.85295	0.19684	1.342	0.089803	0.64933	0.25806	0.052839	0.50378487
LINC00112	ENSG00000232401	1.6179	0.052839	1.6179	0.052839	1.409	0.079425	1.6179	0.052839	0.052839	0.50378487
NA	ENSG00000221720	1.4654	0.071401	-0.14519	0.55772	1.1318	0.12887	1.6178	0.052854	0.052854	0.503816544
TNNC1	ENSG00000114854	0.49023	0.31199	1.6177	0.052863	0.49023	0.31199	0.49023	0.31199	0.052863	0.503816544

GPAT3	ENSG00000138678	0.51959	0.30168	0.72653	0.23376	0.58179	0.28035	1.6174	0.052899	0.052899	0.504029204
TRAV12-2	ENSG00000211789	1.6173	0.052906	1.6173	0.052906	1.6173	0.052906	1.6173	0.052906	0.052906	0.504029204
MTCO1P51	ENSG00000232981	1.617	0.052943	0.62146	0.26715	1.617	0.052943	1.617	0.052943	0.052943	0.50428311
RNU2-43P	ENSG00000222238	0.82777	0.2039	1.3772	0.084225	1.5484	0.060758	1.6168	0.052963	0.052963	0.504375022
MMS22L	ENSG00000146263	1.4773	0.069801	1.6166	0.052985	1.2877	0.098933	1.4101	0.079258	0.052985	0.504485942
LINC01963	ENSG00000260804	1.0519	0.14642	1.0519	0.14642	1.6163	0.053012	1.6163	0.053012	0.053012	0.504602957
NOCT	ENSG00000151014	1.2307	0.10922	1.6163	0.053018	1.3779	0.084123	1.1184	0.13169	0.053018	0.504602957
NA	ENSG00000221690	1.6161	0.053034	1.6161	0.053034	1.6161	0.053034	1.6161	0.053034	0.053034	0.504624742
GPATCH1	ENSG00000076650	-1.0186	0.84581	0.20038	0.42059	-1.1321	0.87121	1.6161	0.053041	0.053041	0.504624742
NA	ENSG00000251244	1.6158	0.053065	1.6158	0.053065	0.70336	0.24092	1.6158	0.053065	0.053065	0.504662142
RPL21P54	ENSG00000241829	1.6158	0.053066	1.6158	0.053066	1.6158	0.053066	1.6158	0.053066	0.053066	0.504662142
NA	ENSG00000274219	-0.12958	0.55155	-0.12958	0.55155	1.6157	0.053076	-0.12958	0.55155	0.053076	0.504662142
RGS17	ENSG00000091844	0.012917	0.49485	-0.36016	0.64064	1.6154	0.053112	-1.039	0.85061	0.053112	0.504704928
NA	ENSG00000264880	-0.038193	0.51523	-0.038193	0.51523	-0.038193	0.51523	1.6154	0.053116	0.053116	0.504704928
LRRC38	ENSG00000162494	-0.28912	0.61375	1.6153	0.053126	0.55897	0.28809	0.43927	0.33023	0.053126	0.504704928
RNU6-1146P	ENSG00000252091	1.6152	0.053129	1.6152	0.053129	0.65899	0.25495	-0.072688	0.52897	0.053129	0.504704928
NA	ENSG00000234758	1.6151	0.053143	1.6151	0.053143	1.6151	0.053143	1.6151	0.053143	0.053143	0.504704928
NFE2L3	ENSG00000050344	-0.49073	0.68819	1.615	0.053152	0.16588	0.43413	-1.2025	0.88542	0.053152	0.504704928
NA	ENSG00000258115	1.615	0.053153	1.615	0.053153	1.615	0.053153	1.615	0.053153	0.053153	0.504704928
MRPS6P4	ENSG00000257820	1.6149	0.053169	1.6149	0.053169	1.6149	0.053169	1.6149	0.053169	0.053169	0.504755097
CKS1B	ENSG00000173207	1.6148	0.053179	0.38712	0.34933	0.74391	0.22847	0.74391	0.22847	0.053179	0.504755097
NA	ENSG00000238861	0.86044	0.19477	1.339	0.090289	1.6147	0.05319	0.64305	0.2601	0.05319	0.504761188
PLXDC2	ENSG00000120594	1.4528	0.073139	1.6144	0.053224	1.003	0.15792	-0.0063092	0.50252	0.053224	0.50480716
NA	ENSG00000255299	0.37262	0.35471	0.28489	0.38787	1.0372	0.14981	1.6143	0.053234	0.053234	0.50480716
NA	ENSG00000283377	1.6142	0.053237	1.6142	0.053237	1.6142	0.053237	1.6142	0.053237	0.053237	0.50480716
LGALS8	ENSG00000116977	1.1159	0.13224	1.0686	0.14263	1.6142	0.053239	1.1763	0.11974	0.053239	0.50480716
TOMM40	ENSG00000130204	1.6141	0.05325	1.6141	0.05325	1.6141	0.05325	1.6141	0.05325	0.05325	0.50480716
RNU6-1051P	ENSG00000202341	-0.18128	0.57192	-0.2949	0.61596	0.83953	0.20059	1.6141	0.053257	0.053257	0.50480716
FGF20	ENSG00000078579	0.43033	0.33348	-0.1255	0.54993	-0.1255	0.54993	1.6137	0.053295	0.053295	0.504981299
PATL1	ENSG00000166889	0.26722	0.39465	1.1168	0.13204	1.6136	0.053302	-0.2846	0.61203	0.053302	0.504981299
EMC10	ENSG00000161671	0.30174	0.38143	-0.25285	0.59981	0.30174	0.38143	1.6135	0.053317	0.053317	0.504981299

GASK1A	ENSG00000144649	1.6135	0.053321	0.60999	0.27094	-0.7593	0.77616	0.65819	0.25521	0.053321	0.504981299
NA	ENSG00000277688	1.6134	0.053328	1.6134	0.053328	1.6134	0.053328	1.6134	0.053328	0.053328	0.504981299
NA	ENSG00000234626	0.82643	0.20428	0.63951	0.26125	1.6132	0.05335	-1.0352	0.84971	0.05335	0.504981299
RCCD1-AS1	ENSG00000258384	1.6132	0.053353	1.6132	0.053353	1.6132	0.053353	1.6132	0.053353	0.053353	0.504981299
EZH2	ENSG00000106462	-0.8869	0.81243	1.6131	0.053365	-0.77422	0.7806	-0.77422	0.7806	0.053365	0.504981299
FAM104A	ENSG00000133193	1.4861	0.068624	1.613	0.053376	1.5453	0.061139	1.613	0.053376	0.053376	0.504981299
NA	ENSG00000266024	-1.6702	0.95256	0.82705	0.20411	1.6129	0.053379	-0.41841	0.66217	0.053379	0.504981299
BUB3P1	ENSG00000227968	1.6128	0.053398	0.60121	0.27385	0.60121	0.27385	0.60121	0.27385	0.053398	0.505021719
H2AC4	ENSG00000278463	1.6127	0.053404	1.6127	0.053404	1.6127	0.053404	1.6127	0.053404	0.053404	0.505021719
SP9	ENSG00000217236	1.3242	0.092721	1.3242	0.092721	0.10983	0.45627	1.6126	0.053415	0.053415	0.505027736
RPL23AP56	ENSG00000244413	1.6124	0.053442	1.6124	0.053442	1.6124	0.053442	1.6124	0.053442	0.053442	0.505184997
SRSF11	ENSG00000116754	1.6122	0.053463	1.4225	0.077436	-1.5858	0.9436	-1.4104	0.92078	0.053463	0.505285491
RAB4A	ENSG00000168118	-0.67662	0.75068	1.612	0.05348	0.46847	0.31973	0.68667	0.24615	0.05348	0.505287964
WDR93	ENSG00000140527	0.98094	0.16331	1.0014	0.15831	1.1878	0.11745	1.612	0.053484	0.053484	0.505287964
NA	ENSG00000252192	1.6116	0.053519	1.6116	0.053519	1.6116	0.053519	1.6116	0.053519	0.053519	0.505488756
RN7SKP115	ENSG00000200047	0.42217	0.33645	0.19201	0.42387	1.6116	0.053526	0.62153	0.26712	0.053526	0.505488756
LINC01149	ENSG00000230174	1.6114	0.053549	1.2184	0.11154	1.1893	0.11716	1.4996	0.066855	0.053549	0.505563132
LINC02013	ENSG00000228730	0.23136	0.40852	-1.3288	0.90805	1.6113	0.053559	-0.023244	0.50927	0.053559	0.505563132
WIPF3	ENSG00000122574	0.24018	0.4051	1.1198	0.1314	0.39405	0.34677	1.6112	0.053565	0.053565	0.505563132
NA	ENSG00000237586	0.9391	0.17384	1.6109	0.053603	0.9391	0.17384	0.9391	0.17384	0.053603	0.505589069
RPS3P2	ENSG00000231307	1.6108	0.053607	1.6108	0.053607	1.6108	0.053607	1.6108	0.053607	0.053607	0.505589069
DENND1B	ENSG00000213047	0.69772	0.24267	1.1741	0.12017	1.6108	0.053609	0.91784	0.17935	0.053609	0.505589069
VSTM5	ENSG00000214376	-0.60825	0.72849	1.6107	0.053622	-0.60825	0.72849	-0.32696	0.62815	0.053622	0.505589069
NA	ENSG00000243583	1.6107	0.053625	1.2341	0.10858	1.6107	0.053625	1.6107	0.053625	0.053625	0.505589069
NA	ENSG00000250333	-0.22428	0.58873	-0.087417	0.53483	-0.087417	0.53483	1.6106	0.05363	0.05363	0.505589069
HMGB3P6	ENSG00000213070	1.6105	0.053645	1.6105	0.053645	1.6105	0.053645	1.6105	0.053645	0.053645	0.50563266
KLHL6-AS1	ENSG00000242522	1.6104	0.053659	0.39256	0.34732	1.0771	0.14072	1.0771	0.14072	0.053659	0.505666809
ZNF587B	ENSG00000269343	1.61	0.053697	1.0344	0.15047	1.0393	0.14934	0.99788	0.15917	0.053697	0.505844525
CENPNP1	ENSG00000233671	1.61	0.053702	0.97572	0.1646	0.91391	0.18038	-1.6345	0.94892	0.053702	0.505844525
NA	ENSG00000250032	0.67282	0.25053	0.67282	0.25053	0.67282	0.25053	1.6099	0.053709	0.053709	0.505844525
MTF1	ENSG00000188786	1.4175	0.078167	0.2566	0.39874	1.6097	0.053733	1.2282	0.10969	0.053733	0.505972771

NA	ENSG00000212529	-0.0041782	0.50167	-0.0041782	0.50167	0.096271	0.46165	1.6093	0.05377	0.05377	0.50621029
NA	ENSG00000273474	1.6093	0.053779	1.6093	0.053779	1.6093	0.053779	1.6093	0.053779	0.053779	0.50621029
RNA5SP371	ENSG00000222302	1.6091	0.053799	0.28861	0.38644	0.40065	0.34434	0.40065	0.34434	0.053799	0.506220255
SMIM17	ENSG00000268182	1.609	0.053811	1.609	0.053811	1.609	0.053811	1.609	0.053811	0.053811	0.506220255
NA	ENSG00000273637	1.6089	0.053817	-0.2642	0.60419	1.2068	0.11376	1.2068	0.11376	0.053817	0.506220255
TRANK1	ENSG00000168016	0.95699	0.16929	1.0075	0.15685	1.6089	0.053824	0.43646	0.33125	0.053824	0.506220255
NA	ENSG00000232089	0.65399	0.25656	1.6088	0.053832	1.2396	0.10756	0.65399	0.25656	0.053832	0.506220255
NA	ENSG00000263279	1.6077	0.053945	1.6077	0.053945	1.6077	0.053945	1.6077	0.053945	0.053945	0.507130012
NA	ENSG00000229309	-0.69023	0.75497	-0.69023	0.75497	0.70004	0.24195	1.6077	0.053955	0.053955	0.507130012
TDRD6	ENSG00000180113	1.4285	0.076571	1.4285	0.076571	1.6074	0.053987	0.91474	0.18017	0.053987	0.507130012
EEF1A1P36	ENSG00000223912	1.6073	0.05399	0.96816	0.16648	1.1327	0.12868	0.96816	0.16648	0.05399	0.507130012
RN7SL726P	ENSG00000272232	-0.58433	0.7205	0.23223	0.40818	0.75084	0.22637	1.6073	0.05399	0.05399	0.507130012
NA	ENSG00000262420	1.6072	0.054001	1.6072	0.054001	1.6072	0.054001	1.6072	0.054001	0.054001	0.507130012
NA	ENSG00000232006	-0.2015	0.57985	-1.0787	0.85964	-1.1206	0.86877	1.6072	0.054002	0.054002	0.507130012
MIR4448	ENSG00000263932	1.6071	0.054012	1.6071	0.054012	1.6071	0.054012	1.6071	0.054012	0.054012	0.507130012
KRTAP10-1	ENSG00000215455	1.607	0.054025	0.20601	0.41839	-0.98132	0.83678	0.20601	0.41839	0.054025	0.507154354
FUS	ENSG00000089280	1.6064	0.054095	0.4559	0.32423	1.6064	0.054095	1.6064	0.054095	0.054095	0.507615896
TUBA1B	ENSG00000123416	0.97157	0.16563	0.28743	0.38689	1.6064	0.054095	1.5476	0.060854	0.054095	0.507615896
NA	ENSG00000279498	1.6059	0.054144	1.6059	0.054144	1.6059	0.054144	1.6059	0.054144	0.054144	0.507917621
NA	ENSG00000218806	0.14196	0.44356	0.96834	0.16644	0.73939	0.22984	1.6059	0.054148	0.054148	0.507917621
RNU6-843P	ENSG00000207183	1.6058	0.054163	1.6058	0.054163	1.6058	0.054163	1.3751	0.084544	0.054163	0.507956569
STMN3	ENSG00000197457	1.4992	0.066916	1.6057	0.054173	1.5714	0.058041	1.288	0.098877	0.054173	0.507956569
ARHGEF37	ENSG00000183111	1.6055	0.054188	1.334	0.091097	1.371	0.085191	0.10366	0.45872	0.054188	0.507999469
ZC3H4	ENSG00000130749	1.5308	0.062908	0.75712	0.22449	0.99249	0.16048	1.6053	0.054214	0.054214	0.508099962
VILL	ENSG00000136059	1.6052	0.054221	1.4771	0.06982	0.42875	0.33405	1.2164	0.11192	0.054221	0.508099962
NA	ENSG00000270094	1.6052	0.05423	1.6052	0.05423	1.6052	0.05423	1.6052	0.05423	0.05423	0.508099962
CYP4V2	ENSG00000145476	1.2697	0.10209	1.2697	0.10209	1.2697	0.10209	1.6049	0.054262	0.054262	0.508302049
ADGRE4P	ENSG00000268758	0.51645	0.30277	0.60126	0.27383	-0.92065	0.82138	1.6048	0.054274	0.054274	0.508316744
NA	ENSG00000259731	1.6045	0.054305	1.2757	0.10103	1.5066	0.065963	1.5113	0.065359	0.054305	0.508421014
NA	ENSG00000237456	0.69298	0.24416	-0.022985	0.50917	1.6045	0.054306	0.12097	0.45186	0.054306	0.508421014
NA	ENSG00000233558	1.4283	0.076599	1.4938	0.067613	0.11044	0.45603	1.6041	0.054349	0.054349	0.508656224

NA	ENSG00000236106	0.38881	0.34871	0.24149	0.40459	1.6041	0.054352	0.28031	0.38962	0.054352	0.508656224
NA	ENSG00000263272	1.6038	0.054384	1.6038	0.054384	1.6038	0.054384	1.6038	0.054384	0.054384	0.508752048
DTHD1	ENSG00000197057	1.6037	0.054389	1.5395	0.061835	1.6037	0.054389	0.47021	0.3191	0.054389	0.508752048
NA	ENSG00000279324	1.6036	0.054398	1.1647	0.12208	0.74025	0.22957	0.74025	0.22957	0.054398	0.508752048
TP53RK	ENSG00000172315	1.6036	0.054404	1.6036	0.054404	1.6036	0.054404	1.0594	0.1447	0.054404	0.508752048
MIR3659	ENSG00000265596	-1.0859	0.86123	-1.2392	0.89237	-1.0859	0.86123	1.603	0.054467	0.054467	0.509164468
IGLJ4	ENSG00000211680	0.028888	0.48848	1.603	0.054469	0.028888	0.48848	0.028888	0.48848	0.054469	0.509164468
NA	ENSG00000252204	0.60047	0.2741	0.60047	0.2741	-0.9873	0.83825	1.6027	0.054499	0.054499	0.509347194
FNDC11	ENSG00000125531	-0.41296	0.66018	1.1471	0.12568	-0.34254	0.63403	1.6025	0.054524	0.054524	0.509483129
CTH	ENSG00000116761	1.6021	0.054565	-0.82781	0.79611	1.6021	0.054565	-0.39746	0.65449	0.054565	0.509680119
SECISBP2L	ENSG00000138593	1.5769	0.057411	1.6021	0.054566	1.6021	0.054566	0.71554	0.23714	0.054566	0.509680119
HOXA3	ENSG00000105997	1.2777	0.10067	1.6017	0.054608	1.6017	0.054608	1.6017	0.054608	0.054608	0.509707057
PARP14	ENSG00000173193	1.3379	0.090459	0.63506	0.26269	1.6013	0.054659	0.85676	0.19579	0.054659	0.509707057
LINC01893	ENSG00000264260	1.6013	0.054661	1.6013	0.054661	1.6013	0.054661	1.6013	0.054661	0.054661	0.509707057
ATP6V1G1	ENSG00000136888	1.6012	0.054664	1.6012	0.054664	1.6012	0.054664	0.048448	0.48068	0.054664	0.509707057
RNU6ATAC29P	ENSG00000221015	1.6012	0.054665	0.66044	0.25449	-1.1666	0.87831	0.33877	0.36739	0.054665	0.509707057
RNU6-668P	ENSG00000201050	1.5805	0.056992	1.5509	0.060461	1.6012	0.05467	1.5805	0.056992	0.05467	0.509707057
NA	ENSG00000269994	1.601	0.05469	0.41705	0.33832	-1.1795	0.88091	-2.4425	0.99271	0.05469	0.509707057
COL4A4	ENSG00000081052	1.601	0.054694	1.0739	0.14144	1.2784	0.10056	0.50576	0.30651	0.054694	0.509707057
OR7E21P	ENSG00000251178	1.2965	0.0974	1.2965	0.0974	-0.45187	0.67432	1.6009	0.054695	0.054695	0.509707057
NA	ENSG00000237320	1.6009	0.054703	-0.40752	0.65819	0.18717	0.42576	-0.024274	0.50968	0.054703	0.509707057
CREB5	ENSG00000146592	0.4886	0.31256	0.60964	0.27105	1.6009	0.054705	-0.13899	0.55527	0.054705	0.509707057
ARMC10P1	ENSG00000178660	-0.84609	0.80125	1.6008	0.054707	-0.84609	0.80125	-0.84609	0.80125	0.054707	0.509707057
NA	ENSG00000282692	-0.36638	0.64296	-0.34223	0.63391	0.38112	0.35156	1.6008	0.054716	0.054716	0.509707057
NT5C1A	ENSG00000116981	-0.23561	0.59313	-1.1106	0.86663	-0.23561	0.59313	1.6006	0.054734	0.054734	0.509707057
TTC39DP	ENSG00000227238	0.13784	0.44518	0.13784	0.44518	0.13784	0.44518	1.6006	0.054734	0.054734	0.509707057
TAAR3P	ENSG00000179073	1.6003	0.054767	0.056229	0.47758	0.6392	0.26135	0.53026	0.29797	0.054767	0.509707057
NMNAT1P2	ENSG00000225719	0.55762	0.28855	1.6003	0.054767	1.318	0.093749	1.318	0.093749	0.054767	0.509707057
PQLC1P1	ENSG00000231763	1.6003	0.05477	1.6003	0.05477	1.6003	0.05477	1.6003	0.05477	0.05477	0.509707057
RCC2P4	ENSG00000249558	0.68219	0.24756	1.6002	0.054774	0.92269	0.17808	1.0159	0.15484	0.054774	0.509707057
MIR5580	ENSG00000266431	0.24037	0.40502	1.6001	0.054783	1.6001	0.054783	1.6001	0.054783	0.054783	0.509707057

NA	ENSG00000207119	-0.42254	0.66369	0.9279	0.17673	-0.055751	0.52223	1.6	0.054798	0.054798	0.509707057
NA	ENSG00000205971	1.6	0.054799	1.6	0.054799	0.48226	0.31481	1.6	0.054799	0.054799	0.509707057
NA	ENSG00000244061	-0.74497	0.77185	1.5998	0.054821	-0.56995	0.71564	-1.0258	0.8475	0.054821	0.509758658
TAF12	ENSG00000120656	1.1491	0.12527	1.5995	0.05485	0.0056723	0.49774	0.32185	0.37378	0.05485	0.509758658
NA	ENSG00000219298	1.5383	0.06199	1.5994	0.054865	1.48	0.069439	1.5383	0.06199	0.054865	0.509758658
RNU6-1178P	ENSG00000252483	1.5993	0.054875	1.5993	0.054875	1.5993	0.054875	1.5993	0.054875	0.054875	0.509758658
NA	ENSG00000254890	1.5992	0.054893	1.5992	0.054893	1.5992	0.054893	1.5992	0.054893	0.054893	0.509758658
NA	ENSG00000267528	0.036056	0.48562	1.453	0.073106	1.5991	0.054899	1.3218	0.09311	0.054899	0.509758658
NA	ENSG00000270632	-0.023857	0.50952	1.599	0.054912	-0.023857	0.50952	-0.38801	0.651	0.054912	0.509758658
MFSD9	ENSG00000135953	1.599	0.054913	1.599	0.054913	1.599	0.054913	1.599	0.054913	0.054913	0.509758658
NDUFAF4P4	ENSG00000231378	1.5989	0.054917	0.58613	0.27889	0.58613	0.27889	0.38749	0.3492	0.054917	0.509758658
NA	ENSG00000242590	1.5989	0.054926	1.5989	0.054926	1.5989	0.054926	1.5989	0.054926	0.054926	0.509758658
NCLP1	ENSG00000213212	1.5988	0.054935	1.5988	0.054935	1.5988	0.054935	1.5988	0.054935	0.054935	0.509758658
APBA3	ENSG00000011132	1.5987	0.054942	0.16754	0.43347	1.5987	0.054942	1.5987	0.054942	0.054942	0.509758658
LINC02823	ENSG00000258100	0.82887	0.20359	1.3921	0.081952	1.1238	0.13055	1.5987	0.054949	0.054949	0.509758658
NA	ENSG00000261404	0.50894	0.3054	0.64604	0.25913	1.5986	0.054951	1.0304	0.15141	0.054951	0.509758658
NA	ENSG00000248733	0.80759	0.20966	1.5983	0.05499	-1.3995	0.91917	-1.3995	0.91917	0.05499	0.509851274
NA	ENSG00000230967	1.4798	0.069468	1.5982	0.054995	0.41316	0.33975	-0.29195	0.61484	0.054995	0.509851274
RNU1-17P	ENSG00000199892	1.5979	0.05503	-0.878	0.81003	-0.37054	0.64451	-1.436	0.9245	0.05503	0.509851274
FAM171B	ENSG00000144369	0.82054	0.20595	-0.018974	0.50757	1.5978	0.055039	1.5978	0.055039	0.055039	0.509851274
NA	ENSG00000213690	1.4001	0.080746	1.5978	0.05504	1.5978	0.05504	1.5978	0.05504	0.05504	0.509851274
AOC2	ENSG00000131480	1.5977	0.055051	1.5977	0.055051	1.5977	0.055051	1.5977	0.055051	0.055051	0.509851274
CCDC28A-AS1	ENSG00000279968	-1.8158	0.9653	1.5977	0.055055	0.45508	0.32453	-0.88324	0.81145	0.055055	0.509851274
SAXO2	ENSG00000188659	-1.2653	0.89712	1.5976	0.055064	-0.34825	0.63618	0.89752	0.18472	0.055064	0.509851274
NA	ENSG00000201965	1.5976	0.055071	1.5976	0.055071	1.5976	0.055071	1.5976	0.055071	0.055071	0.509851274
LINC01695	ENSG00000236532	1.3022	0.096423	1.5975	0.055082	1.4928	0.067742	1.0354	0.15025	0.055082	0.509851274
NA	ENSG00000271734	1.5974	0.055092	1.5974	0.055092	1.5974	0.055092	1.5974	0.055092	0.055092	0.509851274
NA	ENSG00000240419	-0.69644	0.75692	1.5974	0.055093	0.80577	0.21019	1.5974	0.055093	0.055093	0.509851274
SOS1	ENSG00000115904	1.5233	0.063844	1.3971	0.081191	1.5973	0.055097	1.5154	0.064832	0.055097	0.509851274
MITF	ENSG00000187098	0.32049	0.3743	0.60277	0.27333	0.099266	0.46046	1.5968	0.055151	0.055151	0.510024648
LINC02135	ENSG00000268532	-0.4775	0.6835	-1.3168	0.90605	-0.4775	0.6835	1.5968	0.05516	0.05516	0.510024648

TP53BP2	ENSG00000143514	1.5967	0.055162	0.2293	0.40932	0.2293	0.40932	1.1542	0.12422	0.055162	0.510024648
NANOGP1	ENSG00000176654	0.60176	0.27367	1.5967	0.055162	1.5967	0.055162	0.60459	0.27273	0.055162	0.510024648
PPP2R2B-IT1	ENSG00000249553	-0.74282	0.7712	1.5966	0.055178	0.77153	0.2202	-0.26915	0.60609	0.055178	0.510024648
EIF4B	ENSG00000063046	0.45836	0.32335	-0.18467	0.57326	-0.22855	0.59039	1.5966	0.05518	0.05518	0.510024648
TMEM72-AS1	ENSG00000224812	-2.0574	0.98018	1.5965	0.055189	-1.5494	0.93936	-0.64439	0.74034	0.055189	0.510024648
NA	ENSG00000253105	1.1109	0.13331	0.49535	0.31018	0.73875	0.23003	1.5963	0.055212	0.055212	0.510140455
IGLCOR22-2	ENSG00000271093	1.596	0.055241	1.596	0.055241	1.596	0.055241	1.596	0.055241	0.055241	0.510236103
HIF1A-AS1	ENSG00000258777	1.596	0.055246	1.5775	0.057336	0.30142	0.38155	0.30142	0.38155	0.055246	0.510236103
FAM181A-AS1	ENSG00000258584	1.3474	0.088923	0.13264	0.44724	0.50999	0.30503	1.5958	0.055263	0.055263	0.510236103
C7orf65	ENSG00000221845	-1.1711	0.87922	1.5956	0.055287	-1.1711	0.87922	-1.1711	0.87922	0.055287	0.510236103
MRPS18AP1	ENSG00000229759	0.94526	0.17226	0.86806	0.19268	1.5955	0.0553	1.2201	0.11121	0.0553	0.510236103
NA	ENSG00000275936	1.5954	0.055311	1.5954	0.055311	1.5954	0.055311	1.5954	0.055311	0.055311	0.510236103
LINC02476	ENSG00000225546	-0.85983	0.80506	-0.85983	0.80506	1.5953	0.055324	-0.85983	0.80506	0.055324	0.510236103
SPDYE1	ENSG00000136206	-0.5959	0.72438	1.5952	0.055329	-1.4987	0.93302	0.26047	0.39725	0.055329	0.510236103
NUTM2B-AS1	ENSG00000225484	0.64524	0.25939	1.5952	0.055331	0.016532	0.4934	1.1302	0.1292	0.055331	0.510236103
LGALS9DP	ENSG00000266786	0.7481	0.2272	0.95945	0.16867	-0.41657	0.6615	1.5952	0.055333	0.055333	0.510236103
LNCNEF	ENSG00000237396	1.5951	0.055341	-1.0489	0.85288	-0.0032605	0.5013	-1.08	0.85992	0.055341	0.510236103
LINC01234	ENSG00000249550	-0.36338	0.64184	-0.18042	0.57159	1.5951	0.055348	-0.30124	0.61838	0.055348	0.510236103
LINC00582	ENSG00000229228	1.4527	0.07315	1.4423	0.074604	1.2916	0.098254	1.5949	0.055371	0.055371	0.510316248
NA	ENSG00000236961	0.38644	0.34959	1.5947	0.055387	0.47714	0.31663	1.2607	0.1037	0.055387	0.510316248
RNU6-1207P	ENSG00000207071	1.5945	0.055411	1.5945	0.055411	1.5945	0.055411	1.5945	0.055411	0.055411	0.510316248
TUBA4A	ENSG00000127824	1.5944	0.055424	1.5944	0.055424	1.5944	0.055424	1.5944	0.055424	0.055424	0.510316248
PPA1	ENSG00000180817	0.78289	0.21685	1.5944	0.055424	0.71721	0.23662	0.60436	0.2728	0.055424	0.510316248
UBALD2	ENSG00000185262	0.53983	0.29466	1.5944	0.055427	0.53983	0.29466	1.0647	0.1435	0.055427	0.510316248
SERPINB8P1	ENSG00000217707	1.1634	0.12234	1.1634	0.12234	1.5943	0.05543	1.1634	0.12234	0.05543	0.510316248
NA	ENSG00000261526	1.5941	0.055452	1.5941	0.055452	1.5941	0.055452	1.5941	0.055452	0.055452	0.510336167
NA	ENSG00000255853	1.0749	0.1412	1.594	0.05547	1.0749	0.1412	1.0749	0.1412	0.05547	0.510336167
MTCH1	ENSG00000137409	1.248	0.10602	-0.011248	0.50449	1.5017	0.066582	1.594	0.055471	0.055471	0.510336167
SNRPD2P1	ENSG00000228938	1.5939	0.055476	0.65009	0.25782	1.5939	0.055476	0.19319	0.42341	0.055476	0.510336167
NA	ENSG00000227946	1.5938	0.055494	1.5938	0.055494	1.5938	0.055494	1.5938	0.055494	0.055494	0.510336167
FANCD2P2	ENSG00000230342	0.82692	0.20414	-0.26729	0.60538	1.5938	0.055495	-0.4933	0.6891	0.055495	0.510336167

KCTD9	ENSG00000104756	-0.16819	0.56678	0.57314	0.28327	-0.25905	0.6022	1.5935	0.055521	0.055521	0.51047893
NA	ENSG00000251352	-0.16328	0.56485	0.21778	0.4138	-0.69659	0.75697	1.5934	0.055537	0.055537	0.510529713
NA	ENSG00000230649	-0.14402	0.55726	-1.833	0.9666	1.5929	0.055586	1.1164	0.13212	0.055586	0.510783009
SERPINB3	ENSG00000057149	-1.9221	0.97271	0.82715	0.20408	1.5929	0.055595	-2.0909	0.98173	0.055595	0.510783009
NA	ENSG00000267789	1.5929	0.055596	0.54428	0.29313	1.2992	0.096945	1.2992	0.096945	0.055596	0.510783009
NA	ENSG00000200874	1.5926	0.05562	1.5926	0.05562	1.5926	0.05562	1.5926	0.05562	0.05562	0.510907182
ATIC	ENSG00000138363	0.9202	0.17873	0.3391	0.36727	0.38947	0.34847	1.5923	0.055657	0.055657	0.511129254
NAXD	ENSG00000213995	-0.15241	0.56057	0.6122	0.2702	-0.6912	0.75528	1.5922	0.05567	0.05567	0.511129254
B4GALNT4	ENSG00000182272	0.41384	0.33949	-1.8214	0.96573	1.5921	0.055683	-0.33267	0.63031	0.055683	0.511129254
MTHFD2P4	ENSG00000251253	1.5919	0.055699	1.5919	0.055699	1.5919	0.055699	1.5919	0.055699	0.055699	0.511129254
TMEM263	ENSG00000151135	1.3614	0.086689	1.5919	0.055701	1.3614	0.086689	1.378	0.084097	0.055701	0.511129254
NA	ENSG00000255757	0.27878	0.39021	1.5918	0.055717	0.27878	0.39021	0.27878	0.39021	0.055717	0.511129254
LMBR1L	ENSG00000139636	0.39359	0.34694	1.5916	0.055735	0.76686	0.22158	1.3193	0.093542	0.055735	0.511129254
SLC25A2	ENSG00000120329	0.28329	0.38848	0.75219	0.22597	0.28329	0.38848	1.5916	0.055741	0.055741	0.511129254
RPL22P12	ENSG00000216867	1.5914	0.05576	0.53945	0.29479	0.95358	0.17015	0.95358	0.17015	0.05576	0.511129254
LINC02864	ENSG00000263711	0.64848	0.25834	1.5912	0.055777	-0.76008	0.7764	-0.56851	0.71516	0.055777	0.511129254
FOCAD	ENSG00000188352	1.5152	0.064861	1.5912	0.055783	0.80826	0.20947	0.97345	0.16516	0.055783	0.511129254
HSD11B1	ENSG00000117594	0.79787	0.21247	-0.13489	0.55365	1.5911	0.055789	-1.1311	0.87099	0.055789	0.511129254
RFPL3	ENSG00000128276	1.3252	0.092557	1.5911	0.055797	1.5661	0.058663	1.5661	0.058663	0.055797	0.511129254
AHCYL2	ENSG00000158467	1.401	0.080607	0.85915	0.19513	0.43954	0.33013	1.5911	0.055797	0.055797	0.511129254
NA	ENSG00000254153	1.0538	0.14599	0.7556	0.22495	1.591	0.055807	0.9839	0.16258	0.055807	0.511129254
RNY3P3	ENSG00000200686	0.58218	0.28022	0.58218	0.28022	1.5909	0.055812	0.58218	0.28022	0.055812	0.511129254
NA	ENSG00000258355	0.61004	0.27092	1.5908	0.055831	-1.3059	0.9042	-1.3059	0.9042	0.055831	0.511207183
UNK	ENSG00000132478	1.4296	0.076415	0.76607	0.22182	1.5906	0.055852	-0.56115	0.71265	0.055852	0.511266924
PTCSC2	ENSG00000236130	-0.081204	0.53236	-0.025565	0.5102	1.5905	0.055863	1.5905	0.055863	0.055863	0.511266924
HS3ST6	ENSG00000162040	1.5904	0.055869	0.71741	0.23656	-0.33412	0.63086	-0.90798	0.81806	0.055869	0.511266924
NA	ENSG00000263604	-0.067813	0.52703	0.61985	0.26768	1.5901	0.055904	0.49257	0.31116	0.055904	0.51149116
NA	ENSG00000221711	1.5899	0.055928	1.5899	0.055928	1.5899	0.055928	1.5899	0.055928	0.055928	0.511614687
GNL2	ENSG00000134697	1.2768	0.10084	1.5896	0.055964	1.2768	0.10084	1.2	0.11507	0.055964	0.511825025
DHH	ENSG00000139549	1.5895	0.055972	-0.2575	0.6016	1.3482	0.088791	0.62527	0.2659	0.055972	0.511825025
RPS3AP29	ENSG00000237818	1.5894	0.055991	-0.78724	0.78443	1.2871	0.099026	0.16894	0.43292	0.055991	0.511861308

KCTD13	ENSG00000174943	1.589	0.056026	1.589	0.056026	1.589	0.056026	1.3579	0.087253	0.056026	0.511861308
NA	ENSG00000200610	1.589	0.056026	-0.49141	0.68843	1.589	0.056026	1.589	0.056026	0.056026	0.511861308
HAUS1	ENSG00000152240	1.0872	0.13847	1.589	0.056034	0.93261	0.17551	0.44302	0.32888	0.056034	0.511861308
NA	ENSG00000225063	1.4052	0.079981	0.92789	0.17673	1.172	0.12059	1.5889	0.05604	0.05604	0.511861308
NA	ENSG00000226206	0.56298	0.28672	-0.78649	0.78421	-0.31667	0.62425	1.5888	0.056048	0.056048	0.511861308
MRPL20-AS1	ENSG00000224870	1.5887	0.05606	1.5887	0.05606	1.5887	0.05606	1.5887	0.05606	0.05606	0.511861308
NA	ENSG00000252915	1.5887	0.05606	1.5887	0.05606	1.5887	0.05606	1.5887	0.05606	0.05606	0.511861308
CYP4A43P	ENSG00000261196	1.5399	0.061791	1.5402	0.061752	0.04618	0.48158	1.5886	0.056078	0.056078	0.511929738
HDAC2	ENSG00000196591	1.5883	0.056111	1.5883	0.056111	1.5883	0.056111	1.5883	0.056111	0.056111	0.51213505
SLC1A2	ENSG00000110436	-0.068393	0.52726	-0.22105	0.58747	-0.25928	0.60229	1.5879	0.056159	0.056159	0.512426834
ALDH4A1	ENSG00000159423	1.4361	0.075489	1.5878	0.056164	1.1609	0.12283	1.051	0.14663	0.056164	0.512426834
REG1A	ENSG00000115386	1.2027	0.11455	1.5877	0.056179	1.2027	0.11455	1.2027	0.11455	0.056179	0.512467741
MIR548AP	ENSG00000264406	1.5875	0.056198	1.5875	0.056198	1.5875	0.056198	1.5875	0.056198	0.056198	0.512485677
CCDC58P3	ENSG00000267076	1.5875	0.056202	1.248	0.10601	1.138	0.12755	-0.33226	0.63015	0.056202	0.512485677
HLCS	ENSG00000159267	0.047539	0.48104	1.5873	0.056219	0.037089	0.48521	0.11343	0.45485	0.056219	0.51248762
PEMT	ENSG00000133027	-2.1183	0.98292	-1.6338	0.94885	1.5871	0.056242	-0.55631	0.711	0.056242	0.51248762
NA	ENSG00000258017	1.4887	0.06829	1.587	0.056252	1.4893	0.068208	1.3105	0.095005	0.056252	0.51248762
TRBV5-7	ENSG00000211731	-0.72409	0.76549	-0.60027	0.72584	1.587	0.056257	1.3988	0.080929	0.056257	0.51248762
MIR1294	ENSG00000221430	-0.10697	0.54259	-0.10697	0.54259	1.5868	0.056279	-0.10697	0.54259	0.056279	0.51248762
NA	ENSG00000259242	1.5868	0.05628	1.5868	0.05628	1.5868	0.05628	1.5868	0.05628	0.05628	0.51248762
ETV3	ENSG00000117036	-0.086863	0.53461	0.05304	0.47885	0.05304	0.47885	1.5866	0.056298	0.056298	0.51248762
NA	ENSG00000260517	0.95657	0.16939	0.52603	0.29943	0.81242	0.20828	1.5866	0.056307	0.056307	0.51248762
TMPO-AS1	ENSG00000257167	1.5864	0.056322	1.5864	0.056322	1.5864	0.056322	1.5864	0.056322	0.056322	0.51248762
NA	ENSG00000279858	1.5864	0.056322	1.5864	0.056322	1.5864	0.056322	1.5864	0.056322	0.056322	0.51248762
PTPRK	ENSG00000152894	-0.79951	0.788	-1.1279	0.87032	0.88488	0.18811	1.5864	0.056329	0.056329	0.51248762
NA	ENSG00000180211	1.5861	0.056353	-1.6226	0.94767	0.68515	0.24662	0.9332	0.17536	0.056353	0.51248762
MAGI3	ENSG00000081026	1.2883	0.098813	1.5861	0.056354	0.91984	0.17883	1.2111	0.11292	0.056354	0.51248762
ZNF385C	ENSG00000187595	1.1927	0.11649	1.3075	0.095527	1.586	0.05637	1.555	0.059973	0.05637	0.51248762
NQO2-AS1	ENSG00000224846	1.586	0.056373	1.586	0.056373	1.0435	0.14836	1.586	0.056373	0.056373	0.51248762
GPR65	ENSG00000140030	1.2077	0.11359	1.3376	0.090506	1.3102	0.095066	1.586	0.056374	0.056374	0.51248762
FAM133CP	ENSG00000183055	1.5859	0.056381	0.81241	0.20828	0.81241	0.20828	0.81241	0.20828	0.056381	0.51248762

NA	ENSG00000223719	1.5857	0.056403	1.2643	0.10307	1.293	0.098006	1.293	0.098006	0.056403	0.512591979
FEM1A	ENSG00000141965	-0.80489	0.78956	1.3118	0.094788	1.5855	0.05643	1.3504	0.088447	0.05643	0.51274173
NA	ENSG00000268686	1.2327	0.10884	1.2327	0.10884	1.5853	0.056444	1.2327	0.10884	0.056444	0.512773326
LINC02119	ENSG00000248461	0.87912	0.18967	0.90828	0.18186	1.585	0.056488	0.87912	0.18967	0.056488	0.513077398
NA	ENSG00000259762	1.5845	0.056541	1.5845	0.056541	1.5845	0.056541	1.5845	0.056541	0.056541	0.513335328
TSC22D2	ENSG00000196428	-0.29592	0.61635	1.5844	0.056547	-0.18097	0.57181	0.90213	0.18349	0.056547	0.513335328
CHORDC1P5	ENSG00000233020	-1.4367	0.92459	1.5844	0.056548	1.1256	0.13017	1.199	0.11526	0.056548	0.513335328
UFSP2	ENSG00000109775	1.5842	0.056569	0.3402	0.36685	0.92767	0.17679	0.90217	0.18348	0.056569	0.513430317
RPS14P4	ENSG00000226928	0.91003	0.1814	0.91003	0.1814	1.5838	0.056619	0.4895	0.31224	0.056619	0.513473004
MAP2K1	ENSG00000169032	0.059418	0.47631	1.2384	0.10779	1.5838	0.056621	1.0779	0.14054	0.056621	0.513473004
AHDC1	ENSG00000126705	1.5838	0.056622	-0.79546	0.78683	-1.5664	0.94138	-0.95938	0.83132	0.056622	0.513473004
RNU6-1305P	ENSG00000201223	1.5838	0.056622	1.5838	0.056622	1.5838	0.056622	1.5838	0.056622	0.056622	0.513473004
NA	ENSG00000280044	1.2324	0.1089	1.5835	0.056652	1.2324	0.1089	1.2324	0.1089	0.056652	0.513473004
REM1	ENSG00000088320	1.5835	0.056658	1.5835	0.056658	1.3528	0.088063	1.2175	0.1117	0.056658	0.513473004
PDE4C	ENSG00000105650	0.16779	0.43337	0.74892	0.22695	0.81386	0.20786	1.5835	0.056658	0.056658	0.513473004
SUB1P2	ENSG00000258737	1.5835	0.056658	1.5835	0.056658	1.5086	0.065695	1.536	0.062264	0.056658	0.513473004
MRPS36P1	ENSG00000236152	1.5833	0.05668	1.5833	0.05668	1.5833	0.05668	1.5833	0.05668	0.05668	0.513512068
LINC02732	ENSG00000254416	0.14907	0.44075	1.5832	0.056688	0.66578	0.25278	0.50121	0.30811	0.056688	0.513512068
NA	ENSG00000216895	0.72829	0.23322	1.583	0.056709	1.2221	0.11084	1.2209	0.11105	0.056709	0.513512068
NA	ENSG00000272677	1.583	0.056711	1.583	0.056711	1.583	0.056711	1.583	0.056711	0.056711	0.513512068
NA	ENSG00000197149	1.583	0.056715	0.12592	0.4499	1.583	0.056715	1.583	0.056715	0.056715	0.513512068
SNORD19	ENSG00000212493	1.5827	0.056745	1.5827	0.056745	1.5827	0.056745	1.5827	0.056745	0.056745	0.51359284
NA	ENSG00000252787	1.5827	0.056745	1.5827	0.056745	1.5827	0.056745	1.5827	0.056745	0.056745	0.51359284
STAC2	ENSG00000141750	1.5822	0.056797	1.5822	0.056797	1.5822	0.056797	1.5822	0.056797	0.056797	0.513627886
NA	ENSG00000252234	0.5798	0.28103	1.5822	0.056798	-0.81058	0.7912	-0.81219	0.79166	0.056798	0.513627886
ARL2BP	ENSG00000102931	1.5822	0.056799	1.5822	0.056799	1.5822	0.056799	0.1197	0.45236	0.056799	0.513627886
CCNT1	ENSG00000129315	-0.017351	0.50692	1.582	0.056824	0.83161	0.20281	-0.41525	0.66102	0.056824	0.513627886
BTN3A3	ENSG00000111801	1.3293	0.09187	1.5819	0.05684	1.1585	0.12333	0.44792	0.3271	0.05684	0.513627886
DNHD1	ENSG00000179532	0.87164	0.1917	0.29431	0.38426	0.75183	0.22608	1.5819	0.056841	0.056841	0.513627886
NA	ENSG00000228317	-0.71395	0.76237	1.5818	0.056843	1.5818	0.056843	1.5818	0.056843	0.056843	0.513627886
NA	ENSG00000269048	1.5818	0.056852	1.5818	0.056852	1.5818	0.056852	1.5818	0.056852	0.056852	0.513627886

RPS3AP34	ENSG00000242607	0.65887	0.25499	0.8026	0.2111	0.59903	0.27458	1.5817	0.056859	0.056859	0.513627886
RNA5SP353	ENSG00000201476	1.5817	0.056864	-0.36586	0.64277	-0.36586	0.64277	-0.36586	0.64277	0.056864	0.513627886
MYADML	ENSG00000239649	1.3677	0.085697	1.2415	0.10721	1.5816	0.056871	0.50428	0.30703	0.056871	0.513627886
NA	ENSG00000255830	1.5813	0.056908	1.5813	0.056908	0.11156	0.45559	1.2777	0.10067	0.056908	0.513627886
EIF5A	ENSG00000132507	1.5322	0.062731	1.5322	0.062731	1.5322	0.062731	1.5812	0.056918	0.056918	0.513627886
NA	ENSG00000225718	-0.45352	0.67491	-0.45352	0.67491	0.88786	0.18731	1.5812	0.056919	0.056919	0.513627886
IGLVIVOR22-2	ENSG00000243519	1.5811	0.056924	1.5811	0.056924	1.5811	0.056924	1.5811	0.056924	0.056924	0.513627886
REPS1	ENSG00000135597	1.1105	0.13339	1.581	0.056934	0.052416	0.4791	-0.0098595	0.50393	0.056934	0.513627886
H4C9	ENSG00000276180	1.5809	0.056947	1.5809	0.056947	1.5809	0.056947	1.5809	0.056947	0.056947	0.513627886
PRDM1	ENSG00000057657	1.4446	0.074285	-0.064914	0.52588	1.5808	0.056967	0.95395	0.17005	0.056967	0.513627886
MNAT1	ENSG00000020426	-0.63684	0.73789	1.5807	0.056978	0.1991	0.42109	0.072636	0.47105	0.056978	0.513627886
NA	ENSG00000248126	1.5805	0.056992	1.5805	0.056992	1.5805	0.056992	1.5805	0.056992	0.056992	0.513627886
MIR551A	ENSG00000207776	0.43899	0.33033	0.69951	0.24212	0.19636	0.42216	1.5805	0.056997	0.056997	0.513627886
NA	ENSG00000262147	1.5805	0.056999	1.5805	0.056999	1.5805	0.056999	1.5805	0.056999	0.056999	0.513627886
STARD7	ENSG00000084090	1.2765	0.10089	1.5804	0.057007	0.76629	0.22175	1.1666	0.12169	0.057007	0.513627886
TRIM36	ENSG00000152503	1.2716	0.10175	1.5804	0.057012	0.68178	0.24769	0.68178	0.24769	0.057012	0.513627886
NA	ENSG00000257568	1.5803	0.057023	1.5803	0.057023	1.5803	0.057023	1.5803	0.057023	0.057023	0.513627886
HMGB3P5	ENSG00000237642	-1.3186	0.90634	-1.3186	0.90634	1.5801	0.057038	0.65954	0.25478	0.057038	0.513627886
ANKMY2	ENSG00000106524	1.5801	0.057044	1.5801	0.057044	1.5801	0.057044	-0.34175	0.63373	0.057044	0.513627886
KDM3AP1	ENSG00000233996	1.5801	0.057044	0.96539	0.16717	0.96539	0.16717	0.96539	0.16717	0.057044	0.513627886
NA	ENSG00000270522	0.558	0.28842	0.79718	0.21267	1.5798	0.057076	0.96498	0.16728	0.057076	0.513821075
SP100	ENSG00000067066	1.5796	0.0571	0.52966	0.29817	0.55216	0.29042	0.2774	0.39073	0.0571	0.513942187
NA	ENSG00000279394	-0.017147	0.50684	1.5795	0.057112	0.45965	0.32288	1.4014	0.080554	0.057112	0.513950343
NA	ENSG00000253687	1.5794	0.057122	1.5794	0.057122	1.5794	0.057122	1.5794	0.057122	0.057122	0.513950343
AGAP10P	ENSG00000230869	-1.3631	0.91357	1.2253	0.11023	1.5792	0.057147	0.74549	0.22799	0.057147	0.513971559
FAM78B-AS1	ENSG00000203307	1.5792	0.057148	1.3576	0.087299	0.42152	0.33669	0.42152	0.33669	0.057148	0.513971559
RN7SL416P	ENSG00000242101	-1.7088	0.95626	-1.6217	0.94757	1.4123	0.078926	1.5791	0.057156	0.057156	0.513971559
IGLV1-50	ENSG00000211645	0.98365	0.16264	1.1114	0.1332	1.5789	0.057179	1.5789	0.057179	0.057179	0.514052142
MTND6P15	ENSG00000225723	-0.11212	0.54464	-0.11212	0.54464	-0.11212	0.54464	1.5787	0.057207	0.057207	0.514052142
F11R	ENSG00000158769	1.0642	0.14361	1.4273	0.07674	1.427	0.076787	1.5786	0.057211	0.057211	0.514052142
PGAM3P	ENSG00000237256	1.2182	0.11157	1.3126	0.094659	1.3125	0.094679	1.5784	0.057231	0.057231	0.514052142

USP12-AS2	ENSG00000230641	1.5784	0.057241	1.5784	0.057241	-0.49812	0.6908	-0.47282	0.68183	0.057241	0.514052142
RN7SL279P	ENSG00000265648	1.5782	0.057261	1.5782	0.057261	1.5782	0.057261	1.5782	0.057261	0.057261	0.514052142
FECHP1	ENSG00000235534	-0.58029	0.71914	-1.5373	0.93789	1.5782	0.057265	-0.58029	0.71914	0.057265	0.514052142
ZNF589	ENSG00000164048	1.3454	0.089254	1.1855	0.11791	1.2228	0.1107	1.5781	0.057274	0.057274	0.514052142
HOXB4	ENSG00000182742	1.5781	0.057276	1.5781	0.057276	1.5781	0.057276	1.5781	0.057276	0.057276	0.514052142
OR7E159P	ENSG00000258849	1.1714	0.12071	1.1714	0.12071	1.1714	0.12071	1.5781	0.057276	0.057276	0.514052142
PBXIP1	ENSG00000163346	0.069099	0.47246	0.99727	0.15932	1.578	0.057281	0.062728	0.47499	0.057281	0.514052142
LINC00529	ENSG00000236827	1.5779	0.057299	-0.25717	0.60148	1.1099	0.13351	-0.99875	0.84104	0.057299	0.514114059
BOLA3P3	ENSG00000213630	0.87518	0.19074	1.5778	0.057309	0.41557	0.33886	0.8458	0.19883	0.057309	0.514114059
TGIF1	ENSG00000177426	1.5777	0.05732	-0.53534	0.70379	-0.081267	0.53239	-0.26701	0.60527	0.05732	0.514118093
OSTM1	ENSG00000081087	1.0125	0.15565	1.0029	0.15796	0.97827	0.16397	1.5773	0.057358	0.057358	0.514364251
RNU6-522P	ENSG00000201367	1.3752	0.084537	1.3752	0.084537	1.3752	0.084537	1.5771	0.057387	0.057387	0.514529625
LINC01146	ENSG00000258867	1.5769	0.057411	1.2715	0.10178	1.3406	0.09003	1.3406	0.09003	0.057411	0.514650116
CDCP1	ENSG00000163814	0.98659	0.16192	1.2979	0.097156	1.5768	0.057426	-0.081693	0.53255	0.057426	0.514689899
NA	ENSG00000269825	1.5762	0.057493	1.5762	0.057493	1.5762	0.057493	1.5762	0.057493	0.057493	0.515130384
PDE6B	ENSG00000133256	0.70619	0.24003	0.057758	0.47697	0.088632	0.46469	1.5759	0.057523	0.057523	0.515130384
PDIA4	ENSG00000155660	1.5759	0.057525	-0.25327	0.59997	-0.90839	0.81816	-1.454	0.92702	0.057525	0.515130384
NA	ENSG00000241114	1.5759	0.057528	1.5759	0.057528	1.5759	0.057528	0.76198	0.22303	0.057528	0.515130384
ZSCAN2	ENSG00000176371	0.93497	0.1749	-1.2746	0.89878	0.017516	0.49301	1.5759	0.057529	0.057529	0.515130384
SSR2	ENSG00000163479	1.4348	0.075673	1.5758	0.057539	0.93425	0.17509	-0.31944	0.6253	0.057539	0.515130384
SCN1A	ENSG00000144285	0.4155	0.33889	0.63032	0.26424	0.70179	0.2414	1.5755	0.057576	0.057576	0.515130384
C1QC	ENSG00000159189	0.75596	0.22484	1.5753	0.05759	0.1292	0.4486	0.1292	0.4486	0.05759	0.515130384
HMGN1P2	ENSG00000229046	1.5753	0.057594	1.5753	0.057594	1.5753	0.057594	1.5753	0.057594	0.057594	0.515130384
MIR138-1	ENSG00000207954	-1.1567	0.8763	1.5753	0.057595	-1.1567	0.8763	-2.8645	0.99791	0.057595	0.515130384
PDP1	ENSG00000164951	-0.23395	0.59249	1.5752	0.057601	0.31928	0.37476	-0.23395	0.59249	0.057601	0.515130384
ZNF439	ENSG00000171291	0.47498	0.3174	0.40779	0.34171	1.5752	0.057602	1.5716	0.058017	0.057602	0.515130384
NA	ENSG00000275997	1.5749	0.057645	1.5749	0.057645	1.556	0.059857	0.54596	0.29255	0.057645	0.5151837
LINC02583	ENSG00000187600	-0.032433	0.51294	0.30006	0.38207	1.5748	0.05765	1.1943	0.11617	0.05765	0.5151837
NA	ENSG00000200309	1.5748	0.057657	1.5748	0.057657	1.5748	0.057657	1.5748	0.057657	0.057657	0.5151837
ZNF547	ENSG00000152433	1.5747	0.057664	0.67406	0.25014	0.68801	0.24572	1.5747	0.057664	0.057664	0.5151837
NA	ENSG00000251312	1.5747	0.057668	1.2799	0.1003	1.4825	0.06911	0.96267	0.16786	0.057668	0.5151837

SLC25A3P1	ENSG00000236253	1.5745	0.057684	-0.12323	0.54904	0.77389	0.2195	1.1521	0.12464	0.057684	0.5151837
NA	ENSG00000228800	0.81677	0.20703	1.5744	0.057695	1.4489	0.07368	0.63886	0.26146	0.057695	0.5151837
SLC9A9-AS2	ENSG00000244493	1.5743	0.057704	1.5743	0.057704	1.5743	0.057704	1.5743	0.057704	0.057704	0.5151837
NA	ENSG00000279673	0.65303	0.25687	1.5741	0.057732	0.35259	0.3622	0.35259	0.3622	0.057732	0.5151837
H1-12P	ENSG00000216331	-0.61571	0.73096	1.5741	0.057734	-0.62133	0.73281	-0.62133	0.73281	0.057734	0.5151837
ASB9P1	ENSG00000258527	0.016147	0.49356	1.5317	0.062796	0.029161	0.48837	1.574	0.057742	0.057742	0.5151837
NA	ENSG00000228413	1.5733	0.05782	-1.2541	0.8951	-1.2541	0.8951	-1.2541	0.8951	0.05782	0.5151837
UBE2D3-AS1	ENSG00000246560	1.5732	0.057833	1.5732	0.057833	1.5732	0.057833	1.4406	0.074851	0.057833	0.5151837
SPRED2	ENSG00000198369	0.74407	0.22842	1.5732	0.057836	0.78716	0.21559	1.0948	0.13679	0.057836	0.5151837
NA	ENSG00000201827	-0.33004	0.62931	0.31465	0.37651	1.1298	0.12929	1.5732	0.057838	0.057838	0.5151837
NA	ENSG00000278990	1.5732	0.057838	1.5732	0.057838	1.5732	0.057838	1.5732	0.057838	0.057838	0.5151837
AQP2	ENSG00000167580	0.80553	0.21026	1.5731	0.057846	-0.1719	0.56824	-0.26612	0.60493	0.057846	0.5151837
OPA3	ENSG00000125741	0.8937	0.18574	1.573	0.057864	1.1343	0.12834	1.4045	0.08009	0.057864	0.5151837
TMPRSS11B	ENSG00000185873	1.4118	0.079002	1.2089	0.11335	-0.91837	0.82079	1.573	0.057864	0.057864	0.5151837
LTC4S	ENSG00000213316	0.75242	0.2259	1.5729	0.057866	0.75242	0.2259	0.75242	0.2259	0.057866	0.5151837
NA	ENSG00000250614	1.5729	0.057866	1.5729	0.057866	1.5729	0.057866	1.5729	0.057866	0.057866	0.5151837
CDK17	ENSG00000059758	1.5729	0.057875	1.2756	0.10105	1.3529	0.088041	0.93426	0.17509	0.057875	0.5151837
NA	ENSG00000275613	1.5728	0.057883	1.4677	0.071087	0.9845	0.16244	1.4677	0.071087	0.057883	0.5151837
ADCY6-DT	ENSG00000257660	1.5727	0.05789	1.5727	0.05789	1.5727	0.05789	1.5727	0.05789	0.05789	0.5151837
NA	ENSG00000250749	1.5727	0.057892	1.5727	0.057892	0.26623	0.39503	-0.19427	0.57702	0.057892	0.5151837
NA	ENSG00000259155	1.5726	0.057911	1.5726	0.057911	1.5726	0.057911	1.5726	0.057911	0.057911	0.5151837
NA	ENSG00000232682	1.5725	0.05792	1.5725	0.05792	1.5725	0.05792	1.5725	0.05792	0.05792	0.5151837
NA	ENSG00000280294	1.5724	0.057932	0.83817	0.20097	0.83817	0.20097	0.83817	0.20097	0.057932	0.5151837
JAKMIP3	ENSG00000188385	0.2569	0.39863	-0.43122	0.66685	0.2329	0.40792	1.5723	0.057941	0.057941	0.5151837
NA	ENSG00000250820	1.5719	0.057986	0.72336	0.23473	0.42702	0.33468	1.4266	0.076845	0.057986	0.5151837
RNA5SP306	ENSG00000223019	-0.061284	0.52443	-1.0639	0.85632	-0.061284	0.52443	1.5719	0.057987	0.057987	0.5151837
PKD1	ENSG00000008710	1.3579	0.087246	1.5028	0.06644	1.5718	0.057997	1.2271	0.1099	0.057997	0.5151837
OR7E149P	ENSG00000177586	-1.4525	0.92682	1.5718	0.058003	-1.4283	0.92339	-1.1679	0.87858	0.058003	0.5151837
MRAS	ENSG00000158186	0.66655	0.25253	1.1511	0.12484	1.1511	0.12484	1.5717	0.058009	0.058009	0.5151837
CES5A	ENSG00000159398	-0.40913	0.65878	-0.26385	0.60405	1.379	0.08395	1.5716	0.058025	0.058025	0.5151837
PCAT6	ENSG00000228288	-0.91334	0.81947	1.5715	0.058029	-0.91334	0.81947	-0.91334	0.81947	0.058029	0.5151837

C1QTNF7-AS1	ENSG00000249252	-0.32438	0.62717	0.62782	0.26506	1.5715	0.058037	1.4272	0.076758	0.058037	0.5151837
MAP11	ENSG00000146826	1.5712	0.058066	1.5712	0.058066	-0.63654	0.73779	-0.70125	0.75843	0.058066	0.5151837
NA	ENSG00000250547	1.5711	0.058074	1.5711	0.058074	1.5711	0.058074	1.5711	0.058074	0.058074	0.5151837
NA	ENSG00000281772	1.5711	0.058083	1.5711	0.058083	1.5711	0.058083	1.5711	0.058083	0.058083	0.5151837
NA	ENSG00000230732	1.571	0.058092	1.571	0.058092	1.571	0.058092	1.571	0.058092	0.058092	0.5151837
NA	ENSG00000251670	1.571	0.058092	0.038535	0.48463	-0.21596	0.58549	-0.52334	0.69963	0.058092	0.5151837
FKRP	ENSG00000181027	1.5422	0.061515	1.474	0.070242	1.5422	0.061515	1.5709	0.0581	0.0581	0.5151837
PDZRN3	ENSG00000121440	1.5709	0.058106	0.48607	0.31346	1.4775	0.069776	0.44397	0.32853	0.058106	0.5151837
NA	ENSG00000260796	1.5709	0.058107	1.3272	0.092227	0.87743	0.19013	0.87743	0.19013	0.058107	0.5151837
CCL11	ENSG00000172156	1.5707	0.058125	0.51295	0.30399	0.51295	0.30399	0.51295	0.30399	0.058125	0.5151837
RN7SKP144	ENSG00000223040	-0.77731	0.78151	1.5707	0.058125	-0.2826	0.61126	0.33208	0.36991	0.058125	0.5151837
GUF1	ENSG00000151806	1.5707	0.058126	1.5707	0.058126	1.5707	0.058126	1.5707	0.058126	0.058126	0.5151837
NA	ENSG00000265702	1.5707	0.058126	1.5707	0.058126	1.5707	0.058126	1.5707	0.058126	0.058126	0.5151837
NA	ENSG00000254177	-0.59841	0.72522	1.5703	0.058177	-0.59841	0.72522	-0.59841	0.72522	0.058177	0.515436617
NA	ENSG00000278576	-0.7428	0.7712	-0.7428	0.7712	-0.7428	0.7712	1.5702	0.058182	0.058182	0.515436617
PCDHB5	ENSG00000113209	0.3475	0.36411	0.63279	0.26344	1.5702	0.058188	0.3475	0.36411	0.058188	0.515436617
SPATA20	ENSG00000006282	0.14595	0.44198	-0.075567	0.53012	1.57	0.058206	-0.20102	0.57966	0.058206	0.515436617
ELP1	ENSG00000070061	0.7925	0.21404	0.47941	0.31582	1.57	0.058213	0.54695	0.29221	0.058213	0.515436617
LSM3P5	ENSG00000260014	1.5699	0.058218	1.5699	0.058218	1.5699	0.058218	1.5699	0.058218	0.058218	0.515436617
GLIPR1L2	ENSG00000180481	1.5697	0.058248	0.40012	0.34453	-0.12892	0.55129	1.4642	0.071564	0.058248	0.515474382
RNU6-311P	ENSG00000252033	0.70781	0.23953	0.70781	0.23953	0.70781	0.23953	1.5696	0.058251	0.058251	0.515474382
LINC02195	ENSG00000236481	0.22994	0.40907	1.5696	0.058254	-1.251	0.89453	0.18923	0.42496	0.058254	0.515474382
NA	ENSG00000248511	0.28406	0.38818	1.5691	0.058315	1.5691	0.058315	1.5691	0.058315	0.058315	0.51592047
NA	ENSG00000279043	1.5688	0.058352	1.5688	0.058352	1.5688	0.058352	1.5688	0.058352	0.058352	0.515984973
NA	ENSG00000205041	1.5687	0.058353	1.5687	0.058353	1.5687	0.058353	1.5687	0.058353	0.058353	0.515984973
IRAIN	ENSG00000259424	-0.549	0.7085	-0.62332	0.73346	1.5686	0.058366	1.5686	0.058366	0.058366	0.515984973
NA	ENSG00000265128	0.60895	0.27128	1.5686	0.058375	1.5686	0.058375	1.5686	0.058375	0.058375	0.515984973
NA	ENSG00000228235	1.5685	0.058385	0.24605	0.40282	1.1469	0.12571	1.1469	0.12571	0.058385	0.515984973
BORCS8-MEF2B	ENSG00000064489	1.5683	0.058403	0.15376	0.4389	1.5683	0.058403	-0.45674	0.67607	0.058403	0.515984973
NA	ENSG00000275179	-0.17445	0.56924	1.5683	0.058404	1.4006	0.080672	1.4006	0.080672	0.058404	0.515984973
C2CD5	ENSG00000111731	0.060622	0.47583	1.3154	0.094185	1.5683	0.058407	0.9113	0.18107	0.058407	0.515984973

KCNMB1	ENSG00000145936	1.5681	0.058428	1.5681	0.058428	1.4781	0.069685	0.65949	0.25479	0.058428	0.5159943
NA	ENSG00000255353	1.568	0.058435	0.68587	0.2464	1.568	0.058435	1.568	0.058435	0.058435	0.5159943
NA	ENSG00000187229	0.3848	0.35019	-1.7365	0.95876	1.568	0.05844	-2.1861	0.9856	0.05844	0.5159943
RNU6-396P	ENSG00000202239	1.5679	0.058457	1.5679	0.058457	1.5679	0.058457	1.5679	0.058457	0.058457	0.5159943
SERPINB7	ENSG00000166396	0.69931	0.24218	-0.42967	0.66628	0.61536	0.26916	1.5678	0.058461	0.058461	0.5159943
RPSAP7	ENSG00000237032	-0.87408	0.80896	-0.87408	0.80896	1.5676	0.058489	-0.87408	0.80896	0.058489	0.516147948
LRCH1	ENSG00000136141	1.5674	0.058509	-0.075962	0.53028	0.3597	0.35954	0.71919	0.23601	0.058509	0.516230956
CLK3	ENSG00000179335	1.5672	0.058537	0.26356	0.39606	0.09856	0.46074	0.86754	0.19282	0.058537	0.51625869
NA	ENSG00000270990	-0.32078	0.62581	-0.32078	0.62581	1.567	0.058556	-1.1217	0.86901	0.058556	0.51625869
RAB22A	ENSG00000124209	0.61905	0.26794	1.567	0.058559	1.567	0.058559	0.89411	0.18563	0.058559	0.51625869
RNF19A	ENSG00000034677	-0.57412	0.71706	0.47114	0.31877	0.42452	0.33559	1.567	0.058563	0.058563	0.51625869
NKX6-2	ENSG00000148826	0.78359	0.21664	1.5668	0.058583	0.78359	0.21664	0.78359	0.21664	0.058583	0.51625869
TSPO2	ENSG00000112212	0.97761	0.16413	0.97761	0.16413	0.97761	0.16413	1.5667	0.058593	0.058593	0.51625869
RNU6-626P	ENSG00000200224	1.5665	0.058615	1.1233	0.13066	0.70493	0.24043	0.70493	0.24043	0.058615	0.51625869
POLR3C	ENSG00000186141	1.5665	0.058621	0.15584	0.43808	-0.17107	0.56792	1.2898	0.098565	0.058621	0.51625869
NA	ENSG00000255387	0.15676	0.43772	-1.4594	0.92777	-0.93559	0.82526	1.5663	0.058638	0.058638	0.51625869
IDE	ENSG00000119912	-0.85872	0.80475	-1.0894	0.86201	1.5663	0.058644	-0.08087	0.53223	0.058644	0.51625869
LINC00624	ENSG00000278811	0.13395	0.44672	-1.2276	0.8902	1.5662	0.058653	-1.2276	0.8902	0.058653	0.51625869
NA	ENSG00000271989	-0.97635	0.83555	1.5662	0.058654	0.76753	0.22138	0.76753	0.22138	0.058654	0.51625869
RNU6-209P	ENSG00000206845	1.5661	0.058658	1.5661	0.058658	1.5661	0.058658	1.5661	0.058658	0.058658	0.51625869
NA	ENSG00000251588	1.5661	0.058666	0.94142	0.17324	1.0975	0.13621	0.58794	0.27828	0.058666	0.51625869
ANXA5	ENSG00000164111	0.92853	0.17657	1.5659	0.058688	0.10226	0.45928	0.35852	0.35998	0.058688	0.51625869
MYLK2	ENSG00000101306	0.28789	0.38672	0.16739	0.43353	-0.13156	0.55233	1.5658	0.058699	0.058699	0.51625869
BOD1L1	ENSG00000038219	-1.1899	0.88296	-0.020072	0.50801	-1.0498	0.8531	1.5656	0.058718	0.058718	0.51625869
ZNF276	ENSG00000158805	0.78519	0.21617	0.60126	0.27383	1.5655	0.058727	0.81134	0.20859	0.058727	0.51625869
RPS3AP35	ENSG00000244289	-0.57664	0.71791	1.5655	0.058738	0.41005	0.34088	0.2058	0.41847	0.058738	0.51625869
NA	ENSG00000253901	-0.72923	0.76707	1.5654	0.058741	0.54257	0.29371	-0.31747	0.62456	0.058741	0.51625869
ABTB2	ENSG00000166016	0.88855	0.18712	1.5654	0.058748	0.37262	0.35471	-0.12385	0.54928	0.058748	0.51625869
NA	ENSG00000269938	1.5653	0.05876	1.5653	0.05876	1.5653	0.05876	1.5653	0.05876	0.05876	0.51625869
RNA5SP413	ENSG00000252182	1.5653	0.058761	1.5653	0.058761	1.5653	0.058761	1.5653	0.058761	0.058761	0.51625869
TRIM47	ENSG00000132481	1.5652	0.05877	1.5652	0.05877	1.0808	0.13989	1.4825	0.069106	0.05877	0.51625869

NA	ENSG00000237601	1.5651	0.058777	-1.0149	0.84493	-1.0149	0.84493	-1.0149	0.84493	0.058777	0.51625869
RBPJ	ENSG00000168214	1.565	0.058792	0.38264	0.35099	-0.72625	0.76616	-0.050764	0.52024	0.058792	0.516260596
SEPTIN9-DT	ENSG00000263718	-0.77294	0.78022	-0.77294	0.78022	0.76497	0.22214	1.5649	0.058808	0.058808	0.516260596
LINC01815	ENSG00000235519	-0.64854	0.74168	1.5649	0.058809	-0.64854	0.74168	0.051724	0.47937	0.058809	0.516260596
NA	ENSG00000266416	1.5647	0.058823	1.5647	0.058823	1.5647	0.058823	1.5647	0.058823	0.058823	0.516290488
FAM120AOS	ENSG00000188938	1.1334	0.12852	1.5645	0.05885	1.5645	0.05885	1.4365	0.075424	0.05885	0.516341466
NA	ENSG00000267560	1.5645	0.05885	1.5645	0.05885	1.5645	0.05885	1.5645	0.05885	0.05885	0.516341466
NA	ENSG00000275332	1.5643	0.058873	1.5643	0.058873	1.5643	0.058873	1.5643	0.058873	0.058873	0.516409948
MIR1972-2	ENSG00000239118	1.5522	0.060301	0.12611	0.44982	1.5643	0.058879	1.5643	0.058879	0.058879	0.516409948
ELOBP1	ENSG00000234152	-0.51642	0.69722	-0.33784	0.63226	1.5638	0.058929	0.75863	0.22404	0.058929	0.516671147
TRAV8-5	ENSG00000256379	1.5638	0.05893	1.5638	0.05893	1.0482	0.14727	1.5638	0.05893	0.05893	0.516671147
PCSK7	ENSG00000160613	0.39236	0.3474	0.39236	0.3474	1.5636	0.058956	0.12613	0.44982	0.058956	0.516671147
RNU6-231P	ENSG00000252931	1.5635	0.058962	1.5635	0.058962	1.1844	0.11812	1.5635	0.058962	0.058962	0.516671147
SAMD4A	ENSG00000020577	-0.37752	0.64711	0.76056	0.22346	0.24048	0.40498	1.5634	0.058974	0.058974	0.516671147
NA	ENSG00000228634	-1.2754	0.89892	-1.2754	0.89892	1.5634	0.058982	0.7241	0.2345	0.058982	0.516671147
IL10RA	ENSG00000110324	1.5634	0.058983	1.5634	0.058983	0.26403	0.39588	0.86073	0.19469	0.058983	0.516671147
PARP1P2	ENSG00000258995	1.5632	0.058998	1.1005	0.13557	0.89903	0.18432	-0.089066	0.53549	0.058998	0.516709659
TNFAIP8L2	ENSG00000163154	1.2474	0.10613	1.1066	0.13422	1.5625	0.059082	0.46843	0.31974	0.059082	0.517199517
FKTN	ENSG00000106692	1.3898	0.082292	1.5624	0.059099	1.5624	0.059099	1.1045	0.13468	0.059099	0.517199517
LINC02092	ENSG00000234721	1.5623	0.059104	-1.539	0.9381	1.5623	0.059104	1.5623	0.059104	0.059104	0.517199517
NA	ENSG00000243486	-0.41514	0.66098	-0.14209	0.5565	-0.04708	0.51878	1.5623	0.059106	0.059106	0.517199517
EPHX1	ENSG00000143819	-0.63036	0.73577	-0.3795	0.64784	-0.042276	0.51686	1.5623	0.059107	0.059107	0.517199517
NA	ENSG00000263630	0.043928	0.48248	1.5619	0.059151	-0.48595	0.6865	-0.78775	0.78458	0.059151	0.517491603
NA	ENSG00000226619	1.5617	0.059178	1.5617	0.059178	1.5617	0.059178	1.5617	0.059178	0.059178	0.517634884
FUCA1P1	ENSG00000237053	1.5616	0.059196	-1.2586	0.89591	0.11123	0.45572	0.11123	0.45572	0.059196	0.517693949
NA	ENSG00000268439	1.5615	0.059206	1.5615	0.059206	1.5615	0.059206	1.5615	0.059206	0.059206	0.517693949
MSANTD2	ENSG00000120458	0.21109	0.41641	0.21109	0.41641	1.5613	0.059231	0.21109	0.41641	0.059231	0.517819632
TRERF1	ENSG00000124496	1.161	0.12282	1.0237	0.153	1.2619	0.10349	1.5611	0.059251	0.059251	0.517901566
SEMA7A	ENSG00000138623	1.5608	0.059285	0.86271	0.19415	1.1147	0.13248	0.82547	0.20455	0.059285	0.517999638
NA	ENSG00000261662	1.5454	0.061127	1.5607	0.059296	1.5454	0.061127	1.5454	0.061127	0.059296	0.517999638
TEC	ENSG00000135605	0.55667	0.28888	1.5607	0.059301	-0.35396	0.63832	0.024526	0.49022	0.059301	0.517999638

LINC01727	ENSG00000279082	0.7456	0.22796	1.5605	0.059318	0.26906	0.39394	-0.51688	0.69738	0.059318	0.517999638
SPATA41	ENSG00000189419	-1.5621	0.94087	1.1147	0.13249	0.30136	0.38157	1.5605	0.059324	0.059324	0.517999638
PPP4R1L	ENSG00000124224	1.5605	0.059326	1.1775	0.1195	1.403	0.080308	1.159	0.12323	0.059326	0.517999638
S100A16	ENSG00000188643	-0.031745	0.51266	1.5602	0.059362	-0.031745	0.51266	-0.031745	0.51266	0.059362	0.518221114
WDR6	ENSG00000178252	1.5599	0.059397	1.4993	0.066899	1.5599	0.059397	1.5599	0.059397	0.059397	0.518433783
ATG2B	ENSG00000066739	1.5597	0.059411	1.2749	0.10118	0.28616	0.38738	0.255	0.39936	0.059411	0.518463114
NA	ENSG00000276535	0.64434	0.25968	0.64434	0.25968	1.5594	0.059451	0.64434	0.25968	0.059451	0.518658819
DEPP1	ENSG00000165507	1.5594	0.059455	1.1836	0.11828	1.1836	0.11828	1.1836	0.11828	0.059455	0.518658819
RPS27P18	ENSG00000233135	1.5592	0.05947	1.5592	0.05947	1.5592	0.05947	1.3451	0.089296	0.05947	0.518658819
NCKAP5L	ENSG00000167566	1.396	0.081362	-0.14155	0.55628	1.5592	0.059476	-0.55377	0.71013	0.059476	0.518658819
RNF34	ENSG00000170633	0.68067	0.24804	0.7085	0.23932	1.5591	0.059488	1.0479	0.14735	0.059488	0.518665034
TFCP2	ENSG00000135457	0.42436	0.33565	-0.17537	0.5696	1.559	0.059498	1.1805	0.1189	0.059498	0.518665034
NEO1	ENSG00000067141	1.2054	0.11402	1.0109	0.15603	1.5588	0.059523	0.54688	0.29223	0.059523	0.518790161
MIR5197	ENSG00000266478	-0.2931	0.61528	-0.2931	0.61528	1.5586	0.059543	-0.2931	0.61528	0.059543	0.518871672
NA	ENSG00000246575	1.5583	0.05958	1.5583	0.05958	1.5583	0.05958	1.5583	0.05958	0.05958	0.518901353
NOP9	ENSG00000196943	1.5583	0.059586	1.5583	0.059586	1.5583	0.059586	1.5583	0.059586	0.059586	0.518901353
EEF1DP3	ENSG00000229715	-0.36957	0.64415	1.5582	0.059589	0.10884	0.45666	0.48746	0.31297	0.059589	0.518901353
NA	ENSG00000279970	1.4563	0.072656	1.4126	0.078885	1.5582	0.059589	1.5582	0.059589	0.059589	0.518901353
RN7SKP207	ENSG00000223136	1.558	0.059615	1.558	0.059615	1.558	0.059615	1.558	0.059615	0.059615	0.518942292
NA	ENSG00000262048	1.558	0.059615	1.558	0.059615	1.558	0.059615	1.558	0.059615	0.059615	0.518942292
NA	ENSG00000231172	1.4343	0.075743	1.2688	0.10226	1.5576	0.059666	1.4343	0.075743	0.059666	0.519209448
FAM111A	ENSG00000166801	1.5576	0.059667	1.5576	0.059667	0.61569	0.26905	1.5576	0.059667	0.059667	0.519209448
TMEM179	ENSG00000258986	1.5574	0.059689	0.4474	0.32729	0.31493	0.37641	1.1288	0.1295	0.059689	0.519308154
VRK1	ENSG00000100749	1.1934	0.11636	1.1934	0.11636	1.5572	0.059709	1.1934	0.11636	0.059709	0.519348911
NA	ENSG00000231170	1.5572	0.059715	1.5572	0.059715	1.5572	0.059715	1.5572	0.059715	0.059715	0.519348911
RN7SKP78	ENSG00000201581	1.557	0.059729	1.0083	0.15666	0.41717	0.33828	-0.47186	0.68149	0.059729	0.519377975
RNU6-502P	ENSG00000199289	1.5569	0.059742	1.5569	0.059742	1.5569	0.059742	1.5569	0.059742	0.059742	0.519392608
NA	ENSG00000252075	1.2677	0.10246	1.4706	0.070697	0.7643	0.22234	1.5569	0.059752	0.059752	0.519392608
SLC10A7	ENSG00000120519	1.1462	0.12585	1.027	0.15222	0.63861	0.26154	1.5566	0.059785	0.059785	0.519586775
HARBI1	ENSG00000180423	1.5515	0.060393	1.5515	0.060393	1.5515	0.060393	1.5564	0.059802	0.059802	0.519636078
NA	ENSG00000251031	1.1092	0.13368	1.5564	0.059812	0.32794	0.37148	0.32794	0.37148	0.059812	0.519636078

NA	ENSG00000265833	1.556	0.059851	1.556	0.059851	1.556	0.059851	1.556	0.059851	0.059851	0.519882216
MIR3925	ENSG00000263894	0.44113	0.32956	-0.055972	0.52232	-0.50167	0.69205	1.5559	0.059864	0.059864	0.519902463
NA	ENSG00000243171	0.95816	0.16899	1.5557	0.059896	1.5557	0.059896	1.5557	0.059896	0.059896	0.520087684
NA	ENSG00000250698	0.67816	0.24883	1.5554	0.05992	0.57877	0.28137	1.334	0.091101	0.05992	0.520203385
NA	ENSG00000228620	1.5553	0.059941	1.5553	0.059941	1.5553	0.059941	-0.24269	0.59588	0.059941	0.520226145
SERINC2	ENSG00000168528	-0.0010188	0.50041	1.3992	0.080876	1.287	0.099039	1.5552	0.059952	0.059952	0.520226145
MIR4438	ENSG00000265734	0.064925	0.47412	1.5551	0.059956	0.064925	0.47412	0.064925	0.47412	0.059956	0.520226145
CDK6	ENSG00000105810	0.44744	0.32728	0.091967	0.46336	0.64664	0.25893	1.555	0.059972	0.059972	0.520226145
NA	ENSG00000267713	1.555	0.059976	1.555	0.059976	1.555	0.059976	1.555	0.059976	0.059976	0.520226145
MMADHC-DT	ENSG00000231969	1.5549	0.05999	-1.555	0.94003	0.99176	0.16066	0.99176	0.16066	0.05999	0.520234579
NA	ENSG00000239828	0.94156	0.17321	1.3049	0.095965	0.8385	0.20087	1.5547	0.060005	0.060005	0.520234579
NA	ENSG00000248659	1.5546	0.060019	1.5547	0.060009	1.128	0.12965	0.87682	0.19029	0.060009	0.520234579
SPATS1	ENSG00000249481	1.5546	0.06002	0.49465	0.31042	0.87187	0.19164	0.088136	0.46488	0.06002	0.520237389
TCF4-AS1	ENSG00000267028	1.2279	0.10974	0.40645	0.3422	1.5544	0.060043	0.73916	0.2299	0.060043	0.520271801
METAP1D	ENSG00000172878	1.5544	0.060048	1.3849	0.083048	0.83959	0.20057	0.53722	0.29556	0.060048	0.520271801
KIAA2026	ENSG00000183354	0.085389	0.46598	0.085389	0.46598	1.5543	0.060056	-0.44129	0.6705	0.060056	0.520271801
RASSF1	ENSG00000068028	1.554	0.060087	1.4961	0.06732	1.4961	0.06732	1.4961	0.06732	0.060087	0.520447833
ANO4	ENSG00000151572	0.35243	0.36226	0.51477	0.30336	0.3912	0.34782	1.5538	0.060117	0.060117	0.520476215
SNRPE	ENSG00000182004	0.11212	0.45536	1.5538	0.06012	1.1884	0.11735	-0.34873	0.63636	0.06012	0.520476215
DENND2D	ENSG00000162777	1.5537	0.060133	0.86191	0.19437	0.86191	0.19437	0.86191	0.19437	0.060133	0.520476215
NA	ENSG00000269588	1.5537	0.060133	0.87643	0.1904	0.45735	0.32371	0.45735	0.32371	0.060133	0.520476215
EEF1AKMT3	ENSG00000123427	1.5535	0.060152	1.5535	0.060152	1.5535	0.060152	1.5535	0.060152	0.060152	0.520548208
TEX49	ENSG00000257987	1.5533	0.060179	1.2699	0.10206	1.1101	0.13348	1.2699	0.10206	0.060179	0.520689395
RNY3P12	ENSG00000202297	1.0049	0.15748	1.0049	0.15748	1.0049	0.15748	1.5529	0.06022	0.06022	0.520801335
NA	ENSG00000259531	0.4894	0.31228	1.0672	0.14295	1.5529	0.060221	1.5529	0.060221	0.060221	0.520801335
NA	ENSG00000230435	1.0904	0.13777	1.1597	0.12308	1.5529	0.060224	1.5529	0.060224	0.060224	0.520801335
RHEBL1	ENSG00000167550	1.0708	0.14214	1.5527	0.060249	0.98404	0.16255	1.2818	0.099959	0.060249	0.520852653
GAPDHP42	ENSG00000219881	1.5527	0.060253	1.5527	0.060253	1.5527	0.060253	1.5527	0.060253	0.060253	0.520852653
KREMEN1	ENSG00000183762	1.5526	0.060262	0.49369	0.31076	0.49369	0.31076	0.49369	0.31076	0.060262	0.520852653
NA	ENSG00000254536	1.5521	0.060316	1.5521	0.060316	1.5521	0.060316	1.5521	0.060316	0.060316	0.521226934
NA	ENSG00000234116	0.17046	0.43232	-0.65531	0.74387	1.552	0.060333	1.0074	0.15687	0.060333	0.521281399

PCDHGC5	ENSG00000240764	1.5518	0.060349	1.5518	0.060349	1.5518	0.060349	1.5518	0.060349	0.060349	0.521327206
SYCP1	ENSG00000198765	1.5515	0.060387	0.063804	0.47456	0.23237	0.40812	-0.25487	0.60059	0.060387	0.521463639
NA	ENSG00000248294	1.5515	0.060396	0.65084	0.25758	1.5515	0.060396	0.61256	0.27008	0.060396	0.521463639
RNU7-187P	ENSG00000238998	1.1964	0.11577	1.5513	0.060416	1.5513	0.060416	1.5513	0.060416	0.060416	0.521463639
CARMN	ENSG00000249669	0.64798	0.2585	-0.31382	0.62317	1.5513	0.060421	1.4207	0.077698	0.060421	0.521463639
NTN5	ENSG00000142233	1.3516	0.088251	1.5512	0.060426	1.0072	0.15693	1.3516	0.088251	0.060426	0.521463639
NA	ENSG00000251411	0.43948	0.33016	1.5512	0.060429	1.3361	0.09076	1.013	0.15554	0.060429	0.521463639
NA	ENSG00000199912	1.551	0.060447	1.551	0.060447	1.551	0.060447	1.551	0.060447	0.060447	0.521526613
FMC1	ENSG00000164898	1.5508	0.060474	1.5508	0.060474	0.62857	0.26481	1.1034	0.13493	0.060474	0.521587652
NA	ENSG00000258513	0.25421	0.39967	1.2446	0.10663	1.5508	0.06048	0.42367	0.3359	0.06048	0.521587652
KIFBP	ENSG00000198954	1.5507	0.060488	0.044008	0.48245	0.99162	0.16069	0.2537	0.39986	0.060488	0.521587652
NA	ENSG00000254626	0.93829	0.17405	0.93829	0.17405	0.93829	0.17405	1.5506	0.060504	0.060504	0.521587652
NA	ENSG00000228737	-1.045	0.852	1.5504	0.060522	-1.045	0.852	-1.045	0.852	0.060522	0.521587652
FOXP2	ENSG00000128573	0.11057	0.45598	1.4327	0.075968	1.5504	0.060525	0.87804	0.18996	0.060525	0.521587652
DAB2IP	ENSG00000136848	0.39308	0.34713	-0.28888	0.61366	1.5503	0.060529	1.0877	0.13836	0.060529	0.521587652
NA	ENSG00000275312	1.5499	0.060583	1.5499	0.060583	1.5499	0.060583	1.5499	0.060583	0.060583	0.521930064
AGO3	ENSG00000126070	1.4467	0.073991	1.5497	0.060604	1.5357	0.062302	1.5497	0.060604	0.060604	0.521930064
DERL2	ENSG00000072849	1.4034	0.080249	1.4034	0.080249	1.5496	0.060624	1.4079	0.079574	0.060624	0.521930064
TIPIN	ENSG00000075131	1.5495	0.060628	1.2755	0.10106	-0.90649	0.81766	-0.75671	0.77539	0.060628	0.521930064
SLC45A3	ENSG00000158715	-0.15205	0.56043	1.5495	0.060629	1.4181	0.07808	-0.42461	0.66444	0.060629	0.521930064
ATP23	ENSG00000166896	1.5495	0.060633	0.031461	0.48745	-0.16404	0.56515	-0.28306	0.61144	0.060633	0.521930064
NA	ENSG00000276524	1.5493	0.060658	1.5189	0.064389	1.5189	0.064389	1.2849	0.09942	0.060658	0.522053045
ZMIZ1	ENSG00000108175	1.1238	0.13055	0.26642	0.39496	0.87528	0.19071	1.5488	0.06071	0.06071	0.522337556
LINC02881	ENSG00000277288	-0.14242	0.55663	1.5487	0.060728	-1.2376	0.89207	0.7162	0.23693	0.060728	0.522337556
NA	ENSG00000252432	-0.41581	0.66123	-1.0303	0.84857	0.11314	0.45496	1.5487	0.060732	0.060732	0.522337556
IL12B	ENSG00000113302	-1.0235	0.84696	1.328	0.09209	1.0365	0.14999	1.5486	0.060736	0.060736	0.522337556
SNRPCP20	ENSG00000267182	-0.529	0.7016	0.77231	0.21996	1.5485	0.060756	-1.1294	0.87064	0.060756	0.522337556
KAZN-AS1	ENSG00000234593	1.5484	0.060768	-0.26504	0.60451	-0.89311	0.8141	-1.2195	0.88867	0.060768	0.522337556
ZIC2	ENSG00000043355	0.31324	0.37705	-0.10326	0.54112	0.29852	0.38265	1.5482	0.060789	0.060789	0.522337556
FRG2LP	ENSG00000267704	1.5479	0.060822	1.5479	0.060822	1.5479	0.060822	1.5479	0.060822	0.060822	0.522337556
NA	ENSG00000268069	1.5478	0.060834	1.5478	0.060834	1.5478	0.060834	1.5478	0.060834	0.060834	0.522337556

RNU4-77P	ENSG00000252501	1.3706	0.085251	1.3706	0.085251	0.029695	0.48816	1.5478	0.06084	0.06084	0.522337556
NA	ENSG00000258204	0.75083	0.22638	1.5477	0.060843	0.75083	0.22638	0.75083	0.22638	0.060843	0.522337556
BTF3P10	ENSG00000231120	1.5477	0.060851	1.5477	0.060851	1.5477	0.060851	1.5477	0.060851	0.060851	0.522337556
GBP7	ENSG00000213512	0.7512	0.22627	0.42546	0.33525	1.5476	0.060854	1.4967	0.067241	0.060854	0.522337556
RNA5SP42	ENSG00000201148	1.0204	0.15377	1.5476	0.06086	1.0204	0.15377	0.85101	0.19738	0.06086	0.522337556
NA	ENSG00000250080	0.13692	0.44555	0.13692	0.44555	1.5476	0.06086	-0.12768	0.5508	0.06086	0.522337556
LINC02427	ENSG00000271538	-1.4285	0.92343	0.30699	0.37943	1.5476	0.060863	0.62046	0.26748	0.060863	0.522337556
WNT9B	ENSG00000158955	1.5474	0.060879	-0.18208	0.57224	1.3798	0.083824	1.0951	0.13674	0.060879	0.522337556
NA	ENSG00000216781	1.5474	0.060884	0.02687	0.48928	0.02687	0.48928	0.02687	0.48928	0.060884	0.522337556
AMTN	ENSG00000187689	-0.61004	0.72908	1.5472	0.060913	-0.61004	0.72908	-0.61004	0.72908	0.060913	0.522396233
DDIT4L	ENSG00000145358	1.0754	0.14109	1.5471	0.060917	1.5471	0.060917	1.5471	0.060917	0.060917	0.522396233
RNU6-237P	ENSG00000251794	1.5471	0.060923	1.5471	0.060923	1.5471	0.060923	1.5471	0.060923	0.060923	0.522396233
UNC5CL	ENSG00000124602	1.2065	0.11382	1.5466	0.060978	-1.0899	0.86213	0.97546	0.16467	0.060978	0.522709609
NA	ENSG00000235582	1.5466	0.060981	1.5466	0.060981	1.5466	0.060981	1.5466	0.060981	0.060981	0.522709609
NA	ENSG00000232693	1.5465	0.060997	1.244	0.10674	1.5465	0.060997	1.5465	0.060997	0.060997	0.522722863
AMHR2	ENSG00000135409	1.2341	0.10858	1.2341	0.10858	1.5464	0.061004	1.2341	0.10858	0.061004	0.522722863
LINC00362	ENSG00000229483	1.5461	0.061044	0.81248	0.20826	0.80305	0.21097	-0.46931	0.68058	0.061044	0.52297365
NA	ENSG00000279869	-0.19432	0.57704	0.30687	0.37947	1.5456	0.061098	0.46469	0.32108	0.061098	0.523003506
NA	ENSG00000253535	-0.81061	0.7912	0.29217	0.38508	-0.30299	0.61905	1.5456	0.0611	0.0611	0.523003506
NA	ENSG00000243518	1.2642	0.10308	1.5453	0.061139	-0.14787	0.55878	1.1178	0.13182	0.061139	0.523003506
CASQ2	ENSG00000118729	-0.4626	0.67818	0.76009	0.2236	0.58489	0.27931	1.5453	0.061143	0.061143	0.523003506
MBNL2	ENSG00000139793	-0.044731	0.51784	1.1383	0.1275	-0.42885	0.66598	1.5452	0.061144	0.061144	0.523003506
CARD9	ENSG00000187796	-0.66762	0.74781	1.5451	0.061157	-0.18294	0.57258	-1.2577	0.89575	0.061157	0.523003506
DST-AS1	ENSG00000231441	-1.328	0.90791	-1.328	0.90791	1.5451	0.061162	-1.328	0.90791	0.061162	0.523003506
RNU6-198P	ENSG00000206985	-0.38725	0.65072	1.545	0.061174	-0.38725	0.65072	-0.38725	0.65072	0.061174	0.523003506
RNU6-391P	ENSG00000202050	1.545	0.061178	1.545	0.061178	1.545	0.061178	1.545	0.061178	0.061178	0.523003506
ATP2B4	ENSG00000058668	-0.38309	0.64917	-0.19209	0.57616	1.4007	0.080656	1.5449	0.061183	0.061183	0.523003506
EXOSC10-AS1	ENSG00000230337	1.5449	0.061184	0.10924	0.45651	0.0015003	0.4994	0.0015003	0.4994	0.061184	0.523003506
NA	ENSG00000254141	-0.44712	0.67261	-0.18731	0.57429	1.5448	0.061198	1.0196	0.15396	0.061198	0.523003506
TRAJ4	ENSG00000211885	-0.73583	0.76908	-1.5904	0.94413	1.5448	0.061203	-0.73583	0.76908	0.061203	0.523003506
VPS37D	ENSG00000176428	-0.23243	0.5919	1.5002	0.066787	1.5447	0.061207	0.51293	0.304	0.061207	0.523003506

NA	ENSG00000244669	-0.2746	0.60819	-0.2746	0.60819	-0.26345	0.6039	1.5447	0.06121	0.06121	0.523003506
NA	ENSG00000221101	1.5443	0.061262	0.91884	0.17909	-0.29605	0.6164	1.5443	0.061262	0.061262	0.523003506
RGS19	ENSG00000171700	1.5442	0.061271	0.63379	0.26311	0.63379	0.26311	0.63379	0.26311	0.061271	0.523003506
MANEAL	ENSG00000185090	1.3737	0.08477	1.197	0.11566	1.5441	0.061281	-0.26396	0.6041	0.061281	0.523003506
PPP5D1	ENSG00000230510	0.85517	0.19623	1.5441	0.061284	1.2926	0.098074	0.85517	0.19623	0.061284	0.523003506
NA	ENSG00000227935	1.5441	0.061287	1.1258	0.13013	1.5441	0.061287	1.5441	0.061287	0.061287	0.523003506
NA	ENSG00000249792	1.5441	0.061288	0.54031	0.29449	0.54031	0.29449	0.54031	0.29449	0.061288	0.523003506
DDX19A	ENSG00000168872	1.5041	0.066284	0.064417	0.47432	0.29916	0.38241	1.5439	0.061312	0.061312	0.523003506
PLA2G12B	ENSG00000138308	1.5437	0.061325	0.043896	0.48249	1.5437	0.061325	1.4914	0.067928	0.061325	0.523003506
NA	ENSG00000275015	1.5437	0.061325	0.91114	0.18111	1.5437	0.061325	1.5437	0.061325	0.061325	0.523003506
CD84	ENSG00000066294	1.5437	0.06133	1.5437	0.06133	1.5437	0.06133	1.5437	0.06133	0.06133	0.523003506
NA	ENSG00000272249	1.5437	0.061336	-0.52999	0.70194	1.5437	0.061336	1.5437	0.061336	0.061336	0.523003506
NA	ENSG00000199840	1.5436	0.061343	1.5436	0.061343	1.2994	0.096909	1.5436	0.061343	0.061343	0.523003506
CSP2	ENSG00000241218	0.015018	0.49401	1.5436	0.061348	0.015018	0.49401	0.20488	0.41883	0.061348	0.523003506
RPSAP17	ENSG00000226855	0.98503	0.1623	0.98503	0.1623	0.21002	0.41683	1.5433	0.061379	0.061379	0.52315294
PSORS1C3	ENSG00000204528	-1.9393	0.97377	-1.6743	0.95296	1.5432	0.061387	-3.2991	0.99952	0.061387	0.52315294
LINC01119	ENSG00000239332	1.0197	0.15393	1.0197	0.15393	1.0197	0.15393	1.5429	0.061431	0.061431	0.52331305
RN7SL657P	ENSG00000264293	1.5428	0.061442	1.5428	0.061442	1.5428	0.061442	1.5428	0.061442	0.061442	0.52331305
CCL21	ENSG00000137077	1.5427	0.061447	1.2783	0.10057	1.1412	0.1269	0.53158	0.29751	0.061447	0.52331305
NA	ENSG00000264000	1.5427	0.061455	1.2035	0.11438	0.57609	0.28228	1.5427	0.061455	0.061455	0.52331305
MAPK10-AS1	ENSG00000250062	-0.43445	0.66802	0.20068	0.42047	0.31613	0.37595	1.5426	0.061463	0.061463	0.52331305
GOLGA6L5P	ENSG00000230373	1.5425	0.061478	1.13	0.12924	0.01279	0.4949	0.68079	0.248	0.061478	0.52331305
NA	ENSG00000217514	0.38173	0.35133	0.38173	0.35133	0.38173	0.35133	1.5425	0.061482	0.061482	0.52331305
RPL28	ENSG00000108107	1.4417	0.07469	0.96693	0.16679	0.96693	0.16679	1.5421	0.06152	0.06152	0.52331305
PPIAP1	ENSG00000215351	-1.8668	0.96903	1.5421	0.061525	-1.8668	0.96903	-1.8668	0.96903	0.061525	0.52331305
OR4C9P	ENSG00000254925	1.5419	0.061546	0.037134	0.48519	-0.73256	0.76809	-0.45086	0.67395	0.061546	0.52331305
NA	ENSG00000260926	0.5374	0.2955	0.5374	0.2955	0.5374	0.2955	1.5417	0.061574	0.061574	0.52331305
NA	ENSG00000279227	1.5416	0.061587	1.5416	0.061587	1.5416	0.061587	1.5416	0.061587	0.061587	0.52331305
NA	ENSG00000261011	1.5415	0.061601	1.5415	0.061601	1.5415	0.061601	1.5415	0.061601	0.061601	0.52331305
NA	ENSG00000267345	1.5414	0.061605	1.5414	0.061605	1.5414	0.061605	1.5414	0.061605	0.061605	0.52331305
BEND6	ENSG00000151917	0.98308	0.16278	0.75309	0.2257	0.81755	0.20681	1.5414	0.061611	0.061611	0.52331305

SUMO2P17	ENSG00000248278	1.5414	0.061613	0.70761	0.23959	1.3275	0.092177	1.3275	0.092177	0.061613	0.52331305
NA	ENSG00000266061	1.5414	0.061615	0.98832	0.1615	1.5414	0.061615	1.5414	0.061615	0.061615	0.52331305
RNU5B-2P	ENSG00000199906	1.5413	0.061625	1.5413	0.061625	1.5413	0.061625	1.5413	0.061625	0.061625	0.52331305
NA	ENSG00000251838	0.32771	0.37157	0.32771	0.37157	0.32771	0.37157	1.5412	0.061635	0.061635	0.52331305
PHBP2	ENSG00000254512	0.6609	0.25434	0.6609	0.25434	0.6609	0.25434	1.5412	0.061635	0.061635	0.52331305
MTHFD2P5	ENSG00000213530	1.1871	0.11759	0.61505	0.26926	1.541	0.061655	1.0898	0.1379	0.061655	0.52331305
NA	ENSG00000269533	1.5409	0.061668	1.5409	0.061668	1.5409	0.061668	1.5409	0.061668	0.061668	0.52331305
LINC01765	ENSG00000233730	0.18635	0.42608	0.66026	0.25454	0.66026	0.25454	1.5408	0.061686	0.061686	0.52331305
NA	ENSG00000229010	0.40613	0.34232	0.91425	0.18029	1.5407	0.0617	0.74746	0.22739	0.0617	0.52331305
SNX11	ENSG00000002919	1.5406	0.061706	-0.32242	0.62643	-0.29729	0.61688	1.4683	0.071009	0.061706	0.52331305
NA	ENSG00000273123	0.45046	0.32619	1.5405	0.06172	-0.62789	0.73496	0.090349	0.46401	0.06172	0.52331305
NA	ENSG00000231143	-0.36346	0.64187	0.51772	0.30233	-0.31147	0.62228	1.5405	0.061723	0.061723	0.52331305
NA	ENSG00000249878	1.5405	0.061724	1.394	0.081663	-0.89707	0.81516	-0.89707	0.81516	0.061724	0.52331305
YARS1	ENSG00000134684	1.5402	0.061754	1.5402	0.061754	-0.47142	0.68133	0.14152	0.44373	0.061754	0.52331305
NA	ENSG00000272993	1.5402	0.061759	1.5402	0.061759	1.5402	0.061759	1.5402	0.061759	0.061759	0.52331305
CCDC33	ENSG00000140481	1.2643	0.10306	0.026032	0.48962	0.98676	0.16188	1.54	0.061777	0.061777	0.52331305
CLEC2L	ENSG00000236279	-0.92886	0.82352	1.54	0.061784	-0.32597	0.62778	0.3398	0.36701	0.061784	0.52331305
RPL12P33	ENSG00000244585	1.5399	0.061789	1.5399	0.061789	1.5399	0.061789	1.5399	0.061789	0.061789	0.52331305
KRT18P36	ENSG00000226246	0.48229	0.3148	1.5399	0.061793	1.5399	0.061793	-0.49668	0.69029	0.061793	0.52331305
NA	ENSG00000218416	-0.83788	0.79895	1.5399	0.061798	-0.15925	0.56327	-0.75277	0.77421	0.061798	0.52331305
NA	ENSG00000223211	1.5398	0.061804	-1.579	0.94283	-1.579	0.94283	-1.579	0.94283	0.061804	0.52331305
LIMK1	ENSG00000106683	0.27154	0.39299	0.79475	0.21338	1.5397	0.061821	1.1054	0.13449	0.061821	0.52331305
SERPINA1	ENSG00000197249	0.55055	0.29097	1.5397	0.061821	0.55055	0.29097	0.77198	0.22006	0.061821	0.52331305
PARP9	ENSG00000138496	-0.49168	0.68853	1.2991	0.096955	0.66412	0.25331	1.5394	0.061851	0.061851	0.52331305
NA	ENSG00000259020	1.5394	0.061853	1.5394	0.061853	1.5394	0.061853	1.5394	0.061853	0.061853	0.52331305
RAP2B	ENSG00000181467	0.26059	0.3972	0.48361	0.31433	1.5393	0.061861	0.013954	0.49443	0.061861	0.52331305
ATXN2L	ENSG00000168488	0.76279	0.22279	1.5393	0.06187	0.62462	0.26611	1.4421	0.074639	0.06187	0.52331305
MKKS	ENSG00000125863	0.49798	0.30925	1.3499	0.088527	1.5392	0.061873	0.46185	0.3221	0.061873	0.52331305
RPL32P23	ENSG00000214015	0.96477	0.16733	1.5391	0.061889	0.23004	0.40903	0.74557	0.22796	0.061889	0.52331305
NA	ENSG00000225605	-1.1468	0.87427	1.5389	0.061911	-0.32401	0.62703	0.78026	0.21762	0.061911	0.52331305
LINC01736	ENSG00000228058	1.5389	0.061915	1.5389	0.061915	0.60055	0.27407	1.4789	0.069579	0.061915	0.52331305

TRAV8-4	ENSG00000211790	1.0597	0.14464	1.5388	0.061922	0.79995	0.21187	1.3726	0.084938	0.061922	0.52331305
LINC00550	ENSG00000281778	-0.45266	0.6746	-1.5406	0.93829	1.5388	0.061922	-1.5406	0.93829	0.061922	0.52331305
FAM133B	ENSG00000234545	1.1136	0.13272	1.5388	0.061932	1.1361	0.12796	1.1361	0.12796	0.061932	0.52331305
NCAPGP1	ENSG00000250290	1.2577	0.10424	0.3827	0.35097	1.5381	0.062013	1.5381	0.062013	0.062013	0.523906638
NA	ENSG00000230647	1.5379	0.062033	0.09042	0.46398	0.17615	0.43009	0.17615	0.43009	0.062033	0.523984762
CLDN1	ENSG00000163347	1.1779	0.11941	1.1779	0.11941	1.3038	0.096151	1.5378	0.062047	0.062047	0.524011844
TNPO3	ENSG00000064419	-0.16353	0.56495	-0.16353	0.56495	-0.16353	0.56495	1.5376	0.062072	0.062072	0.524011844
SNRPEP4	ENSG00000233270	1.5375	0.062084	-0.11086	0.54414	-0.11086	0.54414	-0.11086	0.54414	0.062084	0.524011844
CETP	ENSG00000087237	-1.6234	0.94775	-1.6234	0.94775	-2.0029	0.97741	1.5375	0.062091	0.062091	0.524011844
LSM6P2	ENSG00000257730	0.076607	0.46947	1.1956	0.11594	1.5373	0.062107	1.1862	0.11778	0.062107	0.524011844
NA	ENSG00000277728	1.5373	0.062114	1.5373	0.062114	1.5373	0.062114	1.5373	0.062114	0.062114	0.524011844
NA	ENSG00000269599	0.79786	0.21247	0.33183	0.37001	1.5372	0.062116	1.4905	0.068045	0.062116	0.524011844
HTR3C2P	ENSG00000233576	1.5372	0.062126	-0.2988	0.61745	1.4247	0.077125	-0.84727	0.80158	0.062126	0.524011844
NA	ENSG00000262171	1.537	0.06215	1.537	0.06215	1.537	0.06215	1.537	0.06215	0.06215	0.524011844
RN7SL66P	ENSG00000264030	0.43058	0.33339	0.046903	0.4813	1.5369	0.062157	0.1018	0.45946	0.062157	0.524011844
GPER1	ENSG00000164850	1.5368	0.062165	1.5368	0.062165	0.82559	0.20452	1.3589	0.08709	0.062165	0.524011844
LINC02315	ENSG00000251363	1.0027	0.15799	0.27226	0.39271	1.5368	0.062169	0.51021	0.30495	0.062169	0.524011844
TAS1R3	ENSG00000169962	1.5368	0.062176	1.5124	0.065217	1.5368	0.062176	1.5368	0.062176	0.062176	0.524011844
ABCC3	ENSG00000108846	0.027831	0.4889	1.3764	0.084355	-0.43383	0.66779	1.5364	0.062219	0.062219	0.524235048
ZNF648	ENSG00000179930	1.5364	0.062224	-0.54617	0.70753	0.63956	0.26123	0.53378	0.29675	0.062224	0.524235048
LINC01861	ENSG00000251183	-2.9168	0.99823	1.536	0.062267	-2.9168	0.99823	-2.9168	0.99823	0.062267	0.524356331
NA	ENSG00000267291	1.536	0.062271	1.536	0.062271	1.536	0.062271	1.536	0.062271	0.062271	0.524356331
MED4	ENSG00000136146	1.5358	0.062292	1.5358	0.062292	1.5358	0.062292	0.46436	0.32119	0.062292	0.524356331
LINC01680	ENSG00000233882	1.5357	0.062307	1.5357	0.062307	1.5357	0.062307	1.5357	0.062307	0.062307	0.524356331
DNAJA3	ENSG00000103423	1.0177	0.15442	1.5355	0.062326	1.5355	0.062326	-0.15508	0.56162	0.062326	0.524356331
NA	ENSG00000248015	1.5355	0.06233	1.5355	0.06233	1.5355	0.06233	1.5355	0.06233	0.06233	0.524356331
H3Y2	ENSG00000268799	0.38857	0.3488	-0.30099	0.61829	1.5354	0.062339	1.5354	0.062339	0.062339	0.524356331
NA	ENSG00000267724	-0.1028	0.54094	1.5354	0.06234	-0.1028	0.54094	-0.1028	0.54094	0.06234	0.524356331
NA	ENSG00000234658	1.5354	0.062344	-1.0347	0.84959	-0.84207	0.80013	-0.84207	0.80013	0.062344	0.524356331
RNF4	ENSG00000063978	1.5354	0.062346	1.0559	0.14551	0.90717	0.18216	1.0614	0.14425	0.062346	0.524356331
LRRC7	ENSG00000033122	0.23853	0.40573	1.5351	0.062381	0.74945	0.22679	-0.5214	0.69896	0.062381	0.52456016

NA	ENSG00000249642	-0.1268	0.55045	1.5347	0.062423	0.32915	0.37102	-0.045347	0.51808	0.062423	0.524612057
RNU6-755P	ENSG00000201270	0.10358	0.45875	1.5347	0.062424	0.10358	0.45875	0.10358	0.45875	0.062424	0.524612057
CBX3P6	ENSG00000228837	1.3445	0.0894	1.5347	0.062429	1.5347	0.062429	1.5347	0.062429	0.062429	0.524612057
THAP9	ENSG00000168152	-0.065168	0.52598	-0.10639	0.54236	1.5346	0.062441	-0.4003	0.65553	0.062441	0.524612057
NA	ENSG00000267121	1.5346	0.062441	0.25779	0.39828	-0.31363	0.6231	1.2746	0.10122	0.062441	0.524612057
PPP1R14B	ENSG00000173457	1.5345	0.062457	1.5345	0.062457	1.5345	0.062457	1.5345	0.062457	0.062457	0.524656027
NA	ENSG00000248758	1.172	0.1206	1.172	0.1206	1.172	0.1206	1.5341	0.062499	0.062499	0.524747809
PLCH2	ENSG00000149527	0.047321	0.48113	-0.35366	0.6382	1.5341	0.0625	-0.91448	0.81977	0.0625	0.524747809
SPHK1	ENSG00000176170	0.89407	0.18564	-0.22919	0.59064	-0.22919	0.59064	1.5341	0.062505	0.062505	0.524747809
NAT2	ENSG00000156006	1.534	0.062511	1.534	0.062511	1.534	0.062511	1.534	0.062511	0.062511	0.524747809
NA	ENSG00000263041	0.95609	0.16951	1.4476	0.073864	1.5339	0.062529	0.2552	0.39929	0.062529	0.524808503
NA	ENSG00000267324	0.24785	0.40212	1.5337	0.062548	0.35781	0.36024	-0.70549	0.75975	0.062548	0.524877568
AGR3	ENSG00000173467	-1.2296	0.89058	-0.80132	0.78853	-1.0413	0.85114	1.5333	0.062602	0.062602	0.525240265
RN7SKP37	ENSG00000201364	0.89991	0.18408	1.5331	0.06262	0.89991	0.18408	0.89991	0.18408	0.06262	0.525300844
LINC02051	ENSG00000226859	1.185	0.11801	1.3066	0.095679	1.5329	0.062654	1.5329	0.062654	0.062654	0.525348304
NR2F6	ENSG00000160113	1.5329	0.062656	1.5329	0.062656	1.5329	0.062656	1.5329	0.062656	0.062656	0.525348304
HMGB3P27	ENSG00000267132	1.382	0.083492	1.1835	0.1183	1.1835	0.1183	1.5328	0.062658	0.062658	0.525348304
GNMT	ENSG00000124713	0.33571	0.36855	0.3229	0.37339	1.5326	0.06269	1.252	0.10529	0.06269	0.525398304
NA	ENSG00000236283	1.5324	0.062708	0.70876	0.23924	1.0984	0.13601	1.2024	0.11461	0.062708	0.525398304
GAPDHP40	ENSG00000248626	1.5323	0.062723	0.54876	0.29159	0.97575	0.16459	0.97575	0.16459	0.062723	0.525398304
EBAG9	ENSG00000147654	0.75124	0.22625	1.3948	0.081539	1.4472	0.073922	1.5321	0.062744	0.062744	0.525398304
AKR1B1P6	ENSG00000225212	1.5321	0.06275	1.5321	0.06275	1.5321	0.06275	1.5321	0.06275	0.06275	0.525398304
NA	ENSG00000240827	-0.68118	0.75212	-0.68118	0.75212	1.5321	0.06275	-0.68118	0.75212	0.06275	0.525398304
NA	ENSG00000235911	1.5147	0.064925	1.5321	0.062754	1.5321	0.062754	0.45524	0.32447	0.062754	0.525398304
NA	ENSG00000227227	-0.98182	0.83691	0.75045	0.22649	0.75045	0.22649	1.532	0.062757	0.062757	0.525398304
NA	ENSG00000235209	1.532	0.062761	-1.3648	0.91384	-1.3648	0.91384	-2.1414	0.98388	0.062761	0.525398304
FPR2	ENSG00000171049	-1.489	0.93176	-1.489	0.93176	0.84593	0.1988	1.5319	0.062776	0.062776	0.525427062
NA	ENSG00000250961	1.4742	0.070214	0.88464	0.18818	1.5318	0.062786	1.3214	0.09318	0.062786	0.525427062
NA	ENSG00000221730	-0.26316	0.60378	1.5313	0.062842	-0.39291	0.65281	-1.7397	0.95904	0.062842	0.525733634
AKR1C6P	ENSG00000151631	-0.53222	0.70271	0.93924	0.1738	1.5313	0.062847	0.33838	0.36754	0.062847	0.525733634
GDF1	ENSG00000130283	1.1282	0.12962	1.5312	0.062855	1.0977	0.13617	0.85658	0.19584	0.062855	0.525733634

NA	ENSG00000278017	1.5308	0.062913	1.5308	0.062913	1.5308	0.062913	1.5308	0.062913	0.062913	0.525956572
LTA	ENSG00000226979	0.14972	0.44049	1.5308	0.062915	1.5308	0.062915	1.0138	0.15533	0.062915	0.525956572
PLUT	ENSG00000247381	0.14072	0.44405	0.66386	0.25339	1.5307	0.062923	0.14072	0.44405	0.062923	0.525956572
RPL35AP31	ENSG00000233460	-0.86237	0.80576	-0.34348	0.63438	1.5306	0.062932	-0.26366	0.60398	0.062932	0.525956572
HCN4	ENSG00000138622	-0.25737	0.60155	-0.36064	0.64082	0.44578	0.32788	1.5306	0.062936	0.062936	0.525956572
TRHDE	ENSG00000072657	0.96148	0.16815	1.5304	0.062958	1.0391	0.14938	-0.23814	0.59411	0.062958	0.525956572
NA	ENSG00000260060	1.5304	0.062963	1.5304	0.062963	1.5304	0.062963	1.5304	0.062963	0.062963	0.525956572
NA	ENSG00000271468	1.5303	0.062968	0.060361	0.47593	0.060361	0.47593	0.060361	0.47593	0.062968	0.525956572
ANKRD34C-AS1	ENSG00000259234	1.3407	0.090014	0.68597	0.24637	1.5302	0.06298	1.239	0.10767	0.06298	0.52596665
RN7SKP264	ENSG00000223012	0.83742	0.20118	1.5298	0.063037	0.35877	0.35988	0.35877	0.35988	0.063037	0.526352469
HEATR4	ENSG00000187105	1.5297	0.063048	0.69797	0.2426	-0.72514	0.76582	1.2789	0.10046	0.063048	0.526354127
PTF1A	ENSG00000168267	1.5296	0.063061	1.1512	0.12482	0.10925	0.4565	-0.61162	0.7296	0.063061	0.526372479
SEH1L	ENSG00000085415	1.386	0.082867	1.5295	0.063073	1.3033	0.096237	0.6721	0.25076	0.063073	0.526382478
NA	ENSG00000251048	0.49031	0.31196	1.5292	0.063106	1.5018	0.066568	0.93067	0.17601	0.063106	0.526407409
RNU6-1298P	ENSG00000199326	1.5292	0.063109	1.5292	0.063109	1.0248	0.15272	1.5078	0.065799	0.063109	0.526407409
KIF26B-AS1	ENSG00000232192	0.48983	0.31213	-1.0483	0.85274	1.5292	0.063112	-0.089697	0.53574	0.063112	0.526407409
BOLA3	ENSG00000163170	-0.90544	0.81739	1.5291	0.063123	-1.3957	0.91859	-1.4656	0.92862	0.063123	0.526407409
UBL5P4	ENSG00000260224	1.529	0.06313	1.529	0.06313	1.529	0.06313	1.529	0.06313	0.06313	0.526407409
MIR577	ENSG00000207931	-0.087724	0.53495	1.5286	0.063176	-0.087724	0.53495	-0.32806	0.62857	0.063176	0.52654091
NBEAL1	ENSG00000144426	1.5284	0.063209	-0.036423	0.51453	0.81069	0.20877	-0.14498	0.55764	0.063209	0.52654091
HS1BP3	ENSG00000118960	1.0221	0.15338	1.5284	0.063212	0.35506	0.36127	1.128	0.12967	0.063212	0.52654091
FSD1L	ENSG00000106701	1.3348	0.090963	0.10621	0.45771	1.5283	0.063218	0.94788	0.17159	0.063218	0.52654091
EPB41L1	ENSG00000088367	0.41021	0.34083	1.5283	0.063223	1.0466	0.14764	-0.072226	0.52879	0.063223	0.52654091
CSPG5	ENSG00000114646	1.5282	0.063229	1.4298	0.076389	1.4724	0.070462	0.5757	0.28241	0.063229	0.52654091
RRN3P2	ENSG00000103472	0.49508	0.31027	1.528	0.063259	1.0002	0.1586	-0.070463	0.52809	0.063259	0.52654091
HMGN3	ENSG00000118418	1.5278	0.063281	1.2502	0.10561	1.2502	0.10561	0.56535	0.28592	0.063281	0.52654091
LINC02681	ENSG00000229649	1.3051	0.095924	1.4906	0.068037	1.5278	0.063281	-0.99483	0.84009	0.063281	0.52654091
NA	ENSG00000224247	0.3756	0.35361	1.5278	0.063285	-1.3042	0.90391	1.0619	0.14414	0.063285	0.52654091
NA	ENSG00000272418	1.5278	0.063287	1.5278	0.063287	1.5278	0.063287	1.5278	0.063287	0.063287	0.52654091
LINC00242	ENSG00000229214	1.5275	0.06332	-0.82906	0.79646	-0.91676	0.82037	-0.50584	0.69352	0.06332	0.52654091
NA	ENSG00000219133	1.3347	0.090988	1.3347	0.090983	1.0558	0.14553	1.5273	0.063342	0.063342	0.52654091

ABR	ENSG00000159842	1.5095	0.065587	1.5273	0.063345	0.065791	0.47377	0.097826	0.46104	0.063345	0.52654091
NA	ENSG00000234455	1.1092	0.13368	-0.93527	0.82518	1.5272	0.063362	1.1489	0.1253	0.063362	0.52654091
CDC42	ENSG00000070831	0.28652	0.38724	1.5271	0.063369	1.304	0.096119	0.28652	0.38724	0.063369	0.52654091
LINC02043	ENSG00000232233	1.5271	0.06337	-0.18894	0.57493	-0.18894	0.57493	-0.18894	0.57493	0.06337	0.52654091
NA	ENSG00000213480	0.2863	0.38733	-0.4259	0.66491	1.5267	0.063412	0.66622	0.25264	0.063412	0.52654091
CPTP	ENSG00000224051	1.5267	0.063423	1.5267	0.063423	1.5267	0.063423	1.5267	0.063423	0.063423	0.52654091
TMEM81	ENSG00000174529	1.5266	0.063427	1.4462	0.074066	1.5266	0.063427	1.5266	0.063427	0.063427	0.52654091
NA	ENSG00000221617	1.5266	0.063432	1.5266	0.063432	0.041339	0.48351	1.5266	0.063432	0.063432	0.52654091
LCMT1-AS1	ENSG00000260448	1.5265	0.06344	1.5265	0.06344	1.5265	0.06344	0.65774	0.25535	0.06344	0.52654091
RNU6-214P	ENSG00000206857	1.5265	0.063441	0.6096	0.27106	-1.1628	0.87754	0.40423	0.34302	0.063441	0.52654091
PTPN11	ENSG00000179295	1.4833	0.068996	1.1399	0.12716	1.5264	0.063457	0.85317	0.19678	0.063457	0.52654091
TMEM242	ENSG00000215712	0.80531	0.21032	-0.2547	0.60052	-0.23712	0.59372	1.5263	0.063464	0.063464	0.52654091
NA	ENSG00000271911	0.24747	0.40227	-0.03026	0.51207	-0.64695	0.74117	1.5263	0.063469	0.063469	0.52654091
BMP2K	ENSG00000138756	0.70904	0.23915	0.6403	0.26099	1.5263	0.06347	-1.1862	0.88224	0.06347	0.52654091
PRSS1	ENSG00000204983	1.5261	0.063496	0.27701	0.39089	-0.52927	0.70169	0.35059	0.36295	0.063496	0.52654091
NA	ENSG00000230226	1.5261	0.063496	1.5261	0.063496	1.5261	0.063496	1.5261	0.063496	0.063496	0.52654091
VPS25P1	ENSG00000226755	1.526	0.0635	1.526	0.0635	1.526	0.0635	1.526	0.0635	0.0635	0.52654091
SS18	ENSG00000141380	1.5259	0.063515	0.72267	0.23494	0.72267	0.23494	0.88159	0.189	0.063515	0.52654091
NPBWR2	ENSG00000125522	1.5259	0.063517	1.5259	0.063517	1.5259	0.063517	1.3726	0.084931	0.063517	0.52654091
ZYG11A	ENSG00000203995	-0.08581	0.53419	1.5259	0.063517	1.0978	0.13615	1.2993	0.096927	0.063517	0.52654091
NA	ENSG00000250167	1.1846	0.11809	1.5259	0.063519	0.23305	0.40786	0.3641	0.35789	0.063519	0.52654091
RNU6-743P	ENSG00000201021	1.1812	0.11875	1.2834	0.09968	1.5258	0.063534	0.88535	0.18799	0.063534	0.52654091
NA	ENSG00000281021	1.5258	0.063535	1.5258	0.063535	1.5258	0.063535	1.5258	0.063535	0.063535	0.52654091
NA	ENSG00000254051	1.5255	0.063568	0.0079586	0.49683	0.0079586	0.49683	0.0079586	0.49683	0.063568	0.526724816
NA	ENSG00000212205	1.5252	0.063603	1.5252	0.063603	1.5252	0.063603	1.1708	0.12085	0.063603	0.526731638
NGEF	ENSG00000066248	1.1039	0.13482	0.53068	0.29782	0.83669	0.20138	1.5252	0.063608	0.063608	0.526731638
NA	ENSG00000225182	1.5251	0.063617	1.5251	0.063617	1.5251	0.063617	1.5251	0.063617	0.063617	0.526731638
YRDCP3	ENSG00000230859	1.525	0.063633	-0.94243	0.82701	-0.94243	0.82701	-0.94243	0.82701	0.063633	0.526731638
PRKAG1	ENSG00000181929	1.5248	0.063652	1.5248	0.063652	1.5248	0.063652	0.38376	0.35058	0.063652	0.526731638
DDN-AS1	ENSG00000257913	1.241	0.1073	1.241	0.1073	1.5248	0.063652	1.5248	0.063652	0.063652	0.526731638
RPL31P49	ENSG00000241680	1.5247	0.063666	1.5247	0.063666	1.5247	0.063666	1.5247	0.063666	0.063666	0.526731638

NA	ENSG00000282742	1.5245	0.063695	0.4756	0.31718	-0.78291	0.78316	-0.78291	0.78316	0.063695	0.526731638
OAS1	ENSG00000089127	1.045	0.14801	0.88678	0.1876	1.5244	0.063702	-0.085684	0.53414	0.063702	0.526731638
CSF2RB	ENSG00000100368	0.03608	0.48561	1.5244	0.063705	0.03608	0.48561	0.03608	0.48561	0.063705	0.526731638
AZGP1	ENSG00000160862	-0.083786	0.53339	1.5243	0.063719	-1.3276	0.90785	0.17867	0.4291	0.063719	0.526731638
CDK5R2	ENSG00000171450	1.5242	0.063729	1.4782	0.069673	0.95824	0.16897	0.91795	0.17932	0.063729	0.526731638
LINC02771	ENSG00000224535	1.3327	0.091322	0.070012	0.47209	0.88193	0.18891	1.5242	0.06373	0.06373	0.526731638
NA	ENSG00000250260	1.5241	0.063743	1.5241	0.063743	1.5241	0.063743	1.5241	0.063743	0.063743	0.526731638
NA	ENSG00000250024	-0.91543	0.82002	-0.12185	0.54849	1.5241	0.063745	1.5241	0.063745	0.063745	0.526731638
CDKN2A	ENSG00000147889	0.0037733	0.49849	1.1409	0.12695	0.0037733	0.49849	1.524	0.063758	0.063758	0.526731638
FMC1-LUC7L2	ENSG00000269955	1.5239	0.063766	1.5239	0.063766	1.5239	0.063766	1.5239	0.063766	0.063766	0.526731638
NA	ENSG00000274840	-0.63757	0.73812	1.5238	0.063778	1.5238	0.063778	1.5238	0.063778	0.063778	0.526731638
KRTAP27-1	ENSG00000206107	1.5238	0.063783	-0.73392	0.7685	-0.13627	0.5542	-1.2481	0.89401	0.063783	0.526731638
RNU6ATAC11P	ENSG00000221468	-0.22568	0.58927	-0.22568	0.58927	-0.40885	0.65868	1.5237	0.063794	0.063794	0.526731638
TEX14	ENSG00000121101	1.1307	0.12909	-0.52356	0.69971	1.5236	0.063801	-0.9354	0.82521	0.063801	0.526731638
NA	ENSG00000251286	0.88771	0.18735	1.5236	0.06381	1.5236	0.06381	0.33798	0.36769	0.06381	0.526731638
NA	ENSG00000271709	1.2987	0.097025	1.5235	0.063822	1.5215	0.064064	-0.22222	0.58793	0.063822	0.526731638
IGLV3-32	ENSG00000211657	1.5234	0.063835	1.5234	0.063835	1.5234	0.063835	1.5234	0.063835	0.063835	0.526731638
AFP	ENSG00000081051	0.28137	0.38921	0.8472	0.19844	1.5233	0.063846	0.38033	0.35185	0.063846	0.526731638
SIRPD	ENSG00000125900	0.52902	0.29839	0.30539	0.38004	1.5232	0.063851	0.073282	0.47079	0.063851	0.526731638
NA	ENSG00000260184	1.5231	0.063866	1.5231	0.063866	1.5231	0.063866	1.5231	0.063866	0.063866	0.526731638
NCF4	ENSG00000100365	1.523	0.063882	-0.28353	0.61162	0.13603	0.4459	-0.33454	0.63102	0.063882	0.526731638
NA	ENSG00000234141	1.5228	0.063908	1.5228	0.063908	1.5228	0.063908	1.5228	0.063908	0.063908	0.526731638
NA	ENSG00000271784	1.5227	0.063911	1.5227	0.063911	1.5227	0.063911	1.5227	0.063911	0.063911	0.526731638
OVCH2	ENSG00000183378	-0.74527	0.77194	0.61348	0.26978	-0.64254	0.73974	1.5227	0.063914	0.063914	0.526731638
NA	ENSG00000219487	-0.80156	0.7886	-0.80156	0.7886	-0.80156	0.7886	1.5227	0.063917	0.063917	0.526731638
RGS4	ENSG00000117152	1.27	0.10204	1.276	0.10098	1.5226	0.063932	1.05	0.14687	0.063932	0.526731638
HARS2	ENSG00000112855	-0.75732	0.77557	-0.75732	0.77557	-0.75732	0.77557	1.5224	0.063952	0.063952	0.526731638
ARIH2OS	ENSG00000221883	1.5223	0.063965	1.5223	0.063965	1.5223	0.063965	1.5223	0.063965	0.063965	0.526731638
CTSH	ENSG00000103811	0.31408	0.37673	0.089786	0.46423	0.49852	0.30906	1.5222	0.063974	0.063974	0.526731638
NA	ENSG00000223539	1.5221	0.063991	0.87418	0.19101	0.87418	0.19101	0.6962	0.24315	0.063991	0.526731638
OBI1-AS1	ENSG00000234377	1.5221	0.063996	0.081656	0.46746	-0.92022	0.82127	1.2581	0.10417	0.063996	0.526731638

NA	ENSG00000228363	0.7273	0.23352	0.7273	0.23352	0.99003	0.16108	1.522	0.064008	0.064008	0.526731638
AK4P2	ENSG00000249410	1.522	0.064008	0.27129	0.39308	1.1565	0.12375	-0.15479	0.56151	0.064008	0.526731638
RNA5SP34	ENSG00000252696	0.19407	0.42306	1.5219	0.064012	0.19407	0.42306	-0.75018	0.77343	0.064012	0.526731638
KLF9	ENSG00000119138	1.2225	0.11075	1.2225	0.11075	1.2225	0.11075	1.5218	0.064023	0.064023	0.526733208
HSPB1	ENSG00000106211	0.91732	0.17949	0.61141	0.27047	1.1783	0.11935	1.5216	0.064049	0.064049	0.526826815
NA	ENSG00000250962	1.1499	0.12509	-0.40427	0.65699	1.5216	0.064056	1.5216	0.064056	0.064056	0.526826815
NA	ENSG00000241180	1.5214	0.064076	1.5214	0.064076	1.5214	0.064076	1.5214	0.064076	0.064076	0.526846364
MON1A	ENSG00000164077	-2.14	0.98382	-1.9671	0.97541	1.5214	0.06408	0.090785	0.46383	0.06408	0.526846364
NA	ENSG00000259093	1.0378	0.14968	1.5212	0.064101	1.5212	0.064101	1.5212	0.064101	0.064101	0.526890046
RNA5SP208	ENSG00000251965	-0.80929	0.79083	1.5211	0.064121	1.5211	0.064121	1.5211	0.064121	0.064121	0.526890046
RN7SL870P	ENSG00000244732	1.339	0.090287	1.1687	0.12126	1.5209	0.064141	1.5209	0.064141	0.064141	0.526890046
PPIAP10	ENSG00000224530	-1.8495	0.96781	1.5208	0.064149	-1.8495	0.96781	-0.69059	0.75509	0.064149	0.526890046
SLC6A15	ENSG00000072041	0.81772	0.20676	1.5208	0.06415	1.4118	0.079008	1.4118	0.079008	0.06415	0.526890046
MIR3616	ENSG00000264901	1.5208	0.06416	1.5208	0.06416	1.5208	0.06416	1.5208	0.06416	0.06416	0.526890046
PRLR	ENSG00000113494	1.0481	0.1473	1.5208	0.064161	0.48109	0.31523	1.0478	0.14737	0.064161	0.526890046
NA	ENSG00000276627	1.5203	0.064216	-0.085352	0.53401	-0.31261	0.62271	-0.31261	0.62271	0.064216	0.527114075
NA	ENSG00000206741	0.60362	0.27305	0.60362	0.27305	1.5203	0.064218	1.2039	0.11432	0.064218	0.527114075
TBCB	ENSG00000105254	-0.20255	0.58026	0.62516	0.26593	-0.20255	0.58026	1.5203	0.064223	0.064223	0.527114075
NA	ENSG00000248802	0.44996	0.32637	0.44996	0.32637	0.44996	0.32637	1.5201	0.064243	0.064243	0.527114075
NA	ENSG00000254434	1.5201	0.064245	-1.7415	0.9592	-1.01	0.84375	-0.62287	0.73332	0.064245	0.527114075
PAIP2B	ENSG00000124374	0.039921	0.48408	1.52	0.064259	1.0381	0.14961	1.093	0.13721	0.064259	0.527114075
NA	ENSG00000254851	1.3236	0.092817	1.5199	0.064264	0.50258	0.30763	1.1104	0.13341	0.064264	0.527114075
NA	ENSG00000267096	1.5197	0.06429	1.5197	0.06429	1.5197	0.06429	1.5197	0.06429	0.06429	0.527231869
CAPZA2	ENSG00000198898	0.76103	0.22332	0.49294	0.31103	0.49294	0.31103	1.5196	0.0643	0.0643	0.527231869
ELOCP21	ENSG00000226186	0.14251	0.44334	1.5192	0.064352	1.5192	0.064352	1.5192	0.064352	0.064352	0.527569475
FBXL5	ENSG00000118564	1.5189	0.064395	0.8833	0.18854	0.6413	0.26067	0.53052	0.29788	0.064395	0.527760816
NA	ENSG00000230941	1.5189	0.064397	1.5189	0.064397	1.5189	0.064397	1.5189	0.064397	0.064397	0.527760816
CABP5	ENSG00000105507	1.5184	0.064463	1.5184	0.064463	1.5184	0.064463	0.50767	0.30584	0.064463	0.528212879
B3GNT5	ENSG00000176597	0.25299	0.40014	1.5181	0.064488	-1.4594	0.92777	1.5146	0.064936	0.064488	0.528237129
RPL41P5	ENSG00000256393	1.5181	0.064498	1.5181	0.064498	1.5181	0.064498	1.5181	0.064498	0.064498	0.528237129
PLXNB1	ENSG00000164050	1.1952	0.11601	1.5179	0.064518	1.0899	0.13788	1.2859	0.099235	0.064518	0.528237129

EFCAB7	ENSG00000203965	0.53332	0.29691	0.94986	0.17109	0.82763	0.20394	1.5178	0.064528	0.064528	0.528237129
GRTP1-AS1	ENSG00000225083	1.5178	0.064529	-0.55124	0.70927	1.5178	0.064529	1.5178	0.064529	0.064529	0.528237129
MIR641	ENSG00000207631	1.5178	0.064531	1.5178	0.064531	1.5178	0.064531	1.5178	0.064531	0.064531	0.528237129
CSNK1A1P3	ENSG00000250162	1.5177	0.064542	-0.79382	0.78635	-0.65219	0.74286	1.3486	0.088726	0.064542	0.528238438
AMIGO1	ENSG00000181754	1.5176	0.064554	1.5176	0.064554	1.5176	0.064554	-0.28973	0.61399	0.064554	0.528247929
NA	ENSG00000227045	1.517	0.064631	1.517	0.064631	1.517	0.064631	1.517	0.064631	0.064631	0.528661946
NA	ENSG00000274154	0.43112	0.33319	0.43112	0.33319	0.43112	0.33319	1.517	0.064632	0.064632	0.528661946
USH1C	ENSG00000006611	1.5166	0.064688	1.4111	0.079107	1.5166	0.064688	0.97744	0.16418	0.064688	0.528661946
GSTM2	ENSG00000213366	-0.50367	0.69275	1.5166	0.064689	0.47115	0.31877	-1.0573	0.8548	0.064689	0.528661946
NA	ENSG00000253214	1.5165	0.064691	1.5165	0.064691	1.5165	0.064691	-1.3128	0.90537	0.064691	0.528661946
NA	ENSG00000213287	1.1313	0.12896	1.5165	0.064699	0.93281	0.17546	1.5165	0.064699	0.064699	0.528661946
ST7	ENSG00000004866	0.55059	0.29096	1.5165	0.0647	1.0265	0.15233	0.63323	0.26329	0.0647	0.528661946
RNU6-61P	ENSG00000202398	-0.6902	0.75497	-1.0507	0.85331	-0.78763	0.78454	1.5164	0.064711	0.064711	0.528661946
ADAMTS1	ENSG00000154734	1.373	0.084879	1.3454	0.089255	0.75967	0.22373	1.5163	0.064716	0.064716	0.528661946
REV1	ENSG00000135945	1.4186	0.078001	1.233	0.10879	0.98955	0.1612	1.5159	0.064772	0.064772	0.528661946
RPS18P13	ENSG00000243455	1.5158	0.064786	0.56835	0.2849	1.3391	0.090265	1.3735	0.084806	0.064786	0.528661946
LINC02362	ENSG00000249096	1.3723	0.084992	0.99074	0.16091	0.38343	0.3507	1.5157	0.064796	0.064796	0.528661946
KAZALD1	ENSG00000107821	-0.72436	0.76558	-0.72436	0.76558	-0.72436	0.76558	1.5156	0.064812	0.064812	0.528661946
CALHM6-AS1	ENSG00000244158	1.5156	0.064812	0.36799	0.35644	1.5156	0.064812	1.5156	0.064812	0.064812	0.528661946
INSR	ENSG00000171105	1.5154	0.064831	1.044	0.14825	0.72492	0.23425	-1.2378	0.8921	0.064831	0.528661946
STXBP2	ENSG00000076944	1.5154	0.064834	1.5154	0.064834	1.5154	0.064834	1.2237	0.11053	0.064834	0.528661946
NA	ENSG00000227734	1.5154	0.064837	1.5154	0.064837	1.5154	0.064837	1.5154	0.064837	0.064837	0.528661946
NA	ENSG00000234425	1.1009	0.13547	1.0209	0.15365	1.1009	0.13547	1.5153	0.064845	0.064845	0.528661946
SLC15A4	ENSG00000139370	1.515	0.064881	1.4338	0.075817	1.133	0.12862	0.3774	0.35294	0.064881	0.528661946
LINC00113	ENSG00000225298	1.515	0.064884	-0.9518	0.8294	-0.57181	0.71627	0.29662	0.38338	0.064884	0.528661946
NA	ENSG00000257277	0.3581	0.36013	1.515	0.064889	1.3452	0.089283	1.515	0.064889	0.064889	0.528661946
KRT18P65	ENSG00000215807	0.38325	0.35077	0.38325	0.35077	0.38325	0.35077	1.515	0.064893	0.064893	0.528661946
STK33	ENSG00000130413	0.52471	0.29989	0.79253	0.21403	1.5149	0.064894	0.38448	0.35031	0.064894	0.528661946
NA	ENSG00000229195	1.5149	0.064904	1.5149	0.064904	1.0673	0.14291	1.3773	0.084203	0.064904	0.528661946
NA	ENSG00000226851	1.2838	0.099599	0.64096	0.26077	1.5148	0.064905	0.87027	0.19208	0.064905	0.528661946
KIF20A	ENSG00000112984	1.5147	0.064929	-1.7886	0.96316	-1.7886	0.96316	-1.7886	0.96316	0.064929	0.528661946

RNU6-452P	ENSG00000207135	0.8089	0.20929	0.8089	0.20929	1.5146	0.064937	1.1045	0.13469	0.064937	0.528661946
FAM91A1	ENSG00000176853	0.38143	0.35144	0.011216	0.49553	1.4171	0.078228	1.5146	0.064941	0.064941	0.528661946
TST	ENSG00000128311	0.50953	0.30519	0.50953	0.30519	0.50953	0.30519	1.5145	0.06495	0.06495	0.528661946
NA	ENSG00000271888	0.29725	0.38314	1.0344	0.15048	1.5145	0.064954	1.5145	0.064954	0.064954	0.528661946
RN7SL311P	ENSG00000265892	1.1976	0.11553	0.47889	0.31601	1.1976	0.11553	1.5144	0.064961	0.064961	0.528661946
BAD	ENSG00000002330	1.5144	0.064962	-0.7085	0.76068	1.482	0.069173	-1.7894	0.96323	0.064962	0.528661946
NA	ENSG00000264825	0.73466	0.23127	0.73466	0.23127	-0.014104	0.50563	1.5143	0.064974	0.064974	0.528661946
HERC6	ENSG00000138642	0.3069	0.37946	1.5143	0.064981	0.70841	0.23934	1.0591	0.14478	0.064981	0.528661946
NA	ENSG00000257390	1.5142	0.064985	1.5142	0.064985	1.5142	0.064985	1.5142	0.064985	0.064985	0.528661946
NA	ENSG00000265472	1.1767	0.11967	1.5141	0.065005	1.1767	0.11967	1.1767	0.11967	0.065005	0.528661946
PI4K2B	ENSG00000038210	0.43647	0.33125	-0.091224	0.53634	0.10815	0.45694	1.5141	0.065006	0.065006	0.528661946
MIR9-3HG	ENSG00000255571	0.25447	0.39957	1.5138	0.065041	0.93929	0.17379	-0.083701	0.53335	0.065041	0.528684788
RPSAP71	ENSG00000254256	0.59324	0.27651	1.5138	0.065045	1.3122	0.094719	1.3122	0.094719	0.065045	0.528684788
RCOR1	ENSG00000089902	1.5137	0.065046	0.93412	0.17512	0.48487	0.31388	0.96912	0.16624	0.065046	0.528684788
DTNB	ENSG00000138101	-0.89521	0.81466	1.5136	0.065062	-0.87778	0.80997	-0.31763	0.62462	0.065062	0.528684788
DMRTA1	ENSG00000176399	1.1809	0.11882	1.5135	0.065075	0.22306	0.41175	1.5135	0.065075	0.065075	0.528684788
NA	ENSG00000274191	1.5135	0.065075	1.5135	0.065075	1.5135	0.065075	1.5135	0.065075	0.065075	0.528684788
TUBG2	ENSG00000037042	1.0392	0.14937	1.3205	0.093329	1.0392	0.14937	1.5133	0.065101	0.065101	0.528684788
NA	ENSG00000228488	0.19694	0.42194	-1.3719	0.91495	1.5132	0.065116	-0.72063	0.76443	0.065116	0.528684788
EPHA2	ENSG00000142627	1.0969	0.13633	0.24895	0.4017	0.50253	0.30765	1.5131	0.065123	0.065123	0.528684788
GNA14	ENSG00000156049	0.46074	0.32249	1.1943	0.11619	0.64569	0.25924	1.5131	0.065132	0.065132	0.528684788
NA	ENSG00000279010	1.513	0.065138	1.4872	0.06848	1.4872	0.06848	1.5117	0.065302	0.065138	0.528684788
PCOTH	ENSG00000205861	1.513	0.065139	1.513	0.065139	1.513	0.065139	1.513	0.065139	0.065139	0.528684788
HMGN2P4	ENSG00000249014	1.2099	0.11316	-0.4808	0.68467	1.5128	0.065165	-0.4808	0.68467	0.065165	0.528807735
KLHL29	ENSG00000119771	-0.18185	0.57215	0.74147	0.2292	1.5126	0.065194	0.14919	0.4407	0.065194	0.528833552
TRIAP1P1	ENSG00000237956	0.53633	0.29586	0.04884	0.48052	1.5125	0.065207	1.2413	0.10725	0.065207	0.528833552
NA	ENSG00000233393	1.5124	0.06521	1.5124	0.06521	0.24997	0.40131	0.27551	0.39146	0.06521	0.528833552
NA	ENSG00000248346	0.92919	0.17639	1.5123	0.065234	1.5123	0.065234	1.5123	0.065234	0.065234	0.528833552
NA	ENSG00000278586	1.5122	0.065237	1.5122	0.065237	1.5122	0.065237	1.5122	0.065237	0.065237	0.528833552
MXRA8	ENSG00000162576	1.485	0.068772	1.5122	0.065238	1.485	0.068772	1.2405	0.1074	0.065238	0.528833552
NKTR	ENSG00000114857	1.5121	0.065252	1.4966	0.067255	1.4737	0.07028	0.96044	0.16842	0.065252	0.528833552

TDRD5	ENSG00000162782	-0.96429	0.83255	1.5121	0.065255	0.55818	0.28836	-0.6025	0.72658	0.065255	0.528833552
CLCF1	ENSG00000175505	1.1571	0.12362	1.5119	0.065274	1.2564	0.10448	1.3616	0.086662	0.065274	0.528883796
LINC02645	ENSG00000234170	1.5119	0.065286	0.37691	0.35312	0.1993	0.42101	1.5119	0.065286	0.065286	0.528883796
MIR2392	ENSG00000266461	-0.63972	0.73882	0.28058	0.38952	1.5118	0.065298	-0.41567	0.66117	0.065298	0.528883796
SNORD19C	ENSG00000222345	1.5117	0.065308	1.5117	0.065308	1.5117	0.065308	1.5117	0.065308	0.065308	0.528883796
C22orf42	ENSG00000205856	0.99385	0.16015	1.5115	0.065328	0.29003	0.38589	-1.3424	0.91027	0.065328	0.528883796
MRPL36P1	ENSG00000225392	1.5114	0.065338	1.5114	0.065338	1.5114	0.065338	1.5114	0.065338	0.065338	0.528883796
SERPINE2	ENSG00000135919	0.75681	0.22458	0.61561	0.26908	0.63843	0.2616	1.5114	0.065341	0.065341	0.528883796
VN1R67P	ENSG00000260722	1.5112	0.065373	-0.45438	0.67522	-0.45438	0.67522	-0.45438	0.67522	0.065373	0.528883796
NA	ENSG00000260466	1.5111	0.06538	1.5111	0.06538	1.5111	0.06538	1.5111	0.06538	0.06538	0.528883796
GYS2	ENSG00000111713	1.5111	0.065382	0.63002	0.26434	0.16045	0.43626	0.90651	0.18233	0.065382	0.528883796
PPIAP86	ENSG00000213873	0.83329	0.20234	-0.21659	0.58574	-1.6009	0.94531	1.511	0.0654	0.0654	0.528883796
LINC00343	ENSG00000226620	1.4267	0.076826	1.2841	0.099546	1.5109	0.065413	1.4127	0.078871	0.065413	0.528883796
VN2R3P	ENSG00000234680	-0.34875	0.63636	1.5108	0.065414	-0.77027	0.77943	1.4668	0.071218	0.065414	0.528883796
IZUMO1	ENSG00000182264	1.5108	0.065417	1.5108	0.065417	1.5108	0.065417	1.5108	0.065417	0.065417	0.528883796
NA	ENSG00000228834	1.5108	0.065424	1.5108	0.065424	1.5108	0.065424	1.5108	0.065424	0.065424	0.528883796
VPS72	ENSG00000163159	-0.57078	0.71593	1.5103	0.065483	-0.57078	0.71593	-0.05216	0.5208	0.065483	0.528929089
QARS1	ENSG00000172053	1.5103	0.065485	0.79296	0.2139	1.1681	0.12139	-0.71668	0.76321	0.065485	0.528929089
NAT10	ENSG00000135372	0.75956	0.22376	1.053	0.14617	0.97665	0.16437	1.5103	0.06549	0.06549	0.528929089
NA	ENSG00000231492	1.5102	0.065493	1.2156	0.11207	0.82588	0.20443	-0.0066749	0.50266	0.065493	0.528929089
RNY4P7	ENSG00000201470	1.5101	0.065512	1.5101	0.065512	1.5101	0.065512	1.5101	0.065512	0.065512	0.528929089
ZNF362	ENSG00000160094	-0.47661	0.68318	0.96943	0.16617	1.51	0.065516	0.26164	0.3968	0.065516	0.528929089
TRBV5-5	ENSG00000211725	1.51	0.065522	1.51	0.065522	1.51	0.065522	1.51	0.065522	0.065522	0.528929089
NA	ENSG00000280349	1.51	0.065526	-0.26564	0.60474	1.51	0.065526	1.51	0.065526	0.065526	0.528929089
NDUFA8P1	ENSG00000271710	1.5099	0.065535	1.5099	0.065535	1.5099	0.065535	1.5099	0.065535	0.065535	0.528929089
ZFYVE27	ENSG00000155256	1.5098	0.065543	-0.66401	0.74666	-0.26892	0.606	-0.68696	0.75395	0.065543	0.528929089
LCT-AS1	ENSG00000226806	1.5098	0.065549	1.5098	0.065549	1.5098	0.065549	1.5098	0.065549	0.065549	0.528929089
MIR3691	ENSG00000265083	1.5095	0.065583	-0.17611	0.56989	-0.80609	0.7899	-0.2871	0.61298	0.065583	0.529013395
NA	ENSG00000243199	1.5093	0.065606	0.28158	0.38913	1.3822	0.08346	-1.5579	0.94038	0.065606	0.529013395
PCDHB8	ENSG00000120322	1.5091	0.065637	1.5091	0.065637	1.5091	0.065637	1.5091	0.065637	0.065637	0.529013395
NA	ENSG00000251367	1.509	0.065646	-0.20001	0.57926	1.509	0.065646	1.509	0.065646	0.065646	0.529013395

NECTIN4	ENSG00000143217	-1.7012	0.95555	-1.6663	0.95217	-1.7012	0.95555	1.509	0.065649	0.065649	0.529013395
ELOVL7	ENSG00000164181	0.8841	0.18832	0.8841	0.18832	1.5089	0.065659	1.4662	0.071293	0.065659	0.529013395
PRSS51	ENSG00000253649	0.086626	0.46548	1.3228	0.092956	1.5089	0.065661	1.1239	0.13053	0.065661	0.529013395
ZNF236	ENSG00000130856	1.0616	0.14422	1.5088	0.065677	1.0745	0.14131	0.013195	0.49474	0.065677	0.529013395
NA	ENSG00000259368	1.5086	0.065698	1.5086	0.065698	1.5086	0.065698	1.5086	0.065698	0.065698	0.529013395
NA	ENSG00000200502	0.15757	0.4374	0.15757	0.4374	0.15757	0.4374	1.5086	0.065705	0.065705	0.529013395
NA	ENSG00000234589	-0.92309	0.82202	1.5085	0.065708	-0.60429	0.72718	-0.60429	0.72718	0.065708	0.529013395
NA	ENSG00000222545	1.3076	0.095498	0.18359	0.42717	1.5085	0.065708	1.3924	0.081904	0.065708	0.529013395
NA	ENSG00000206806	0.87355	0.19118	0.87355	0.19118	-1.0757	0.85898	1.5085	0.065711	0.065711	0.529013395
KCNK4	ENSG00000182450	1.5085	0.065718	0.47579	0.31711	0.54698	0.2922	0.47579	0.31711	0.065718	0.529013395
MIR1-1HG-AS1	ENSG00000174403	-1.586	0.94363	-1.2836	0.90035	0.84515	0.19901	1.5084	0.065725	0.065725	0.529013395
EFHD1	ENSG00000115468	0.1551	0.43837	0.51927	0.30179	1.5083	0.065737	0.5132	0.3039	0.065737	0.529013395
RNU6-1215P	ENSG00000207170	1.5083	0.065744	1.5083	0.065744	1.5083	0.065744	1.5083	0.065744	0.065744	0.529013395
NA	ENSG00000232891	1.1097	0.13356	1.5079	0.065788	-0.54047	0.70556	-0.75251	0.77413	0.065788	0.529037713
MIR4801	ENSG00000264319	-0.10958	0.54363	0.35674	0.36064	1.5079	0.065789	1.5079	0.065789	0.065789	0.529037713
AKR1B1P3	ENSG00000213785	1.5079	0.065793	0.19551	0.4225	0.59659	0.27539	0.59659	0.27539	0.065793	0.529037713
NXPH1	ENSG00000122584	0.97512	0.16475	1.5078	0.065804	0.97512	0.16475	0.97512	0.16475	0.065804	0.529037713
FMN1	ENSG00000248905	1.0733	0.14157	1.2371	0.10803	1.5077	0.06582	1.4924	0.067795	0.06582	0.529037713
PRELID3BP11	ENSG00000231108	1.5077	0.065821	1.2222	0.11082	0.76066	0.22343	-1.6747	0.953	0.065821	0.529037713
SNF8	ENSG00000159210	1.1512	0.12482	-0.00058459	0.50023	1.5075	0.065837	0.63199	0.2637	0.065837	0.529037713
C17orf80	ENSG00000141219	1.1688	0.12124	1.4187	0.077996	1.4667	0.071228	1.5074	0.06585	0.06585	0.529037713
NA	ENSG00000260750	1.5074	0.065859	-1.6228	0.94768	-0.42946	0.66621	0.1769	0.42979	0.065859	0.529037713
LINC01987	ENSG00000267790	1.5071	0.065898	1.5071	0.065898	1.5071	0.065898	1.5071	0.065898	0.065898	0.529037713
DAAM1	ENSG00000100592	1.507	0.065901	-0.53194	0.70262	-1.1522	0.87539	-1.0321	0.84899	0.065901	0.529037713
AGAP3	ENSG00000133612	-1.589	0.94397	1.5069	0.065913	-0.055327	0.52206	1.0653	0.14337	0.065913	0.529037713
RARRES2P6	ENSG00000261010	1.4849	0.06878	1.4242	0.0772	1.5069	0.06592	1.5069	0.06592	0.06592	0.529037713
NA	ENSG00000282393	1.5067	0.065944	0.43584	0.33148	-0.7067	0.76012	0.1702	0.43243	0.065944	0.529037713
H3C6	ENSG00000274750	-0.036093	0.5144	0.78144	0.21727	1.5066	0.06596	0.36587	0.35723	0.06596	0.529037713
RN7SL804P	ENSG00000266185	1.5066	0.065962	1.5066	0.065962	1.5066	0.065962	1.5066	0.065962	0.065962	0.529037713
LINC02548	ENSG00000255411	0.04667	0.48139	1.5065	0.065969	0.11846	0.45285	-0.5137	0.69627	0.065969	0.529037713
FAM124B	ENSG00000124019	1.5065	0.065973	0.504	0.30713	1.4066	0.079772	0.504	0.30713	0.065973	0.529037713

RPL37P12	ENSG00000237916	1.0695	0.14241	1.2059	0.11394	1.5062	0.066008	1.5062	0.066008	0.066008	0.529037713
HMGB1P29	ENSG00000250862	1.1388	0.12738	1.5062	0.066009	1.5062	0.066009	1.0847	0.13902	0.066009	0.529037713
GAPDHP60	ENSG00000248180	1.5061	0.066015	1.5061	0.066015	1.5061	0.066015	1.5061	0.066015	0.066015	0.529037713
LINC01926	ENSG00000267593	-1.2078	0.88643	1.1956	0.11592	0.73442	0.23135	1.506	0.066032	0.066032	0.529037713
NA	ENSG00000279207	1.506	0.066039	1.506	0.066039	1.506	0.066039	1.506	0.066039	0.066039	0.529037713
NA	ENSG00000235204	1.5059	0.066051	1.5059	0.066051	1.5059	0.066051	1.5059	0.066051	0.066051	0.529037713
ABI1P1	ENSG00000271484	1.5058	0.066054	1.5058	0.066054	1.5058	0.066054	1.5058	0.066054	0.066054	0.529037713
RWDD4	ENSG00000182552	1.4239	0.077242	1.3363	0.090718	1.322	0.093081	1.5058	0.06606	0.06606	0.529037713
GLT6D1	ENSG00000204007	0.093813	0.46263	0.41856	0.33777	1.5058	0.06606	-1.518	0.9355	0.06606	0.529037713
EPHA5	ENSG00000145242	-1.5497	0.93939	1.5057	0.066066	-0.42336	0.66398	0.8479	0.19825	0.066066	0.529037713
SEZ6	ENSG00000063015	0.67551	0.24968	1.5057	0.066074	1.3193	0.093527	1.0929	0.13721	0.066074	0.529037713
NA	ENSG00000255608	1.341	0.08996	-1.6846	0.95396	1.5057	0.066078	-0.39392	0.65318	0.066078	0.529037713
NA	ENSG00000228697	1.5055	0.066096	-0.62442	0.73382	0.69437	0.24372	0.63458	0.26285	0.066096	0.529037713
ICA1L	ENSG00000163596	0.47648	0.31687	1.0751	0.14118	0.55687	0.28881	1.5055	0.066103	0.066103	0.529037713
KRTAP23-1	ENSG00000186980	1.5054	0.066111	1.5054	0.066111	1.5054	0.066111	0.78266	0.21691	0.066111	0.529037713
NA	ENSG00000262227	1.5053	0.066122	1.5053	0.066122	1.5053	0.066122	1.5053	0.066122	0.066122	0.529037713
NA	ENSG00000242963	1.5053	0.066127	1.5053	0.066127	1.5053	0.066127	-0.58442	0.72053	0.066127	0.529037713
MEIS1-AS3	ENSG00000226819	1.5051	0.066154	0.18941	0.42489	1.1317	0.12887	1.0841	0.13915	0.066154	0.529079997
GRM7-AS2	ENSG00000237665	-0.46085	0.67755	0.63616	0.26234	1.5051	0.066154	1.5051	0.066154	0.066154	0.529079997
ICAM5	ENSG00000105376	1.5049	0.066173	1.5049	0.066173	0.50925	0.30529	0.50925	0.30529	0.066173	0.529086834
POFUT2	ENSG00000186866	0.0089672	0.49642	0.44581	0.32787	1.5048	0.066183	-0.44131	0.67051	0.066183	0.529086834
NA	ENSG00000269921	1.5047	0.066206	1.5047	0.066206	1.5047	0.066206	1.5047	0.066206	0.066206	0.529086834
NA	ENSG00000232537	-0.18591	0.57374	-0.997	0.84062	1.5046	0.066208	-0.4011	0.65583	0.066208	0.529086834
CWC25	ENSG00000273559	1.5046	0.066211	1.5046	0.066211	1.4464	0.074037	1.1808	0.11884	0.066211	0.529086834
NA	ENSG00000275461	-1.0514	0.85346	-1.0514	0.85346	1.5046	0.06622	-1.1163	0.86786	0.06622	0.529086834
HSCB	ENSG00000100209	1.5043	0.066251	1.5043	0.066251	1.5043	0.066251	0.17292	0.43136	0.066251	0.529114205
SKIL	ENSG00000136603	1.2153	0.11213	-0.96097	0.83172	-1.3546	0.91223	1.5043	0.066254	0.066254	0.529114205
SOCS6	ENSG00000170677	0.82833	0.20374	1.5043	0.066256	0.82833	0.20374	0.43322	0.33243	0.066256	0.529114205
TRPM8	ENSG00000144481	0.48062	0.31539	1.5041	0.066273	-1.7333	0.95848	-1.0238	0.84703	0.066273	0.529163246
NA	ENSG00000242391	1.5038	0.066316	1.5038	0.066316	1.5038	0.066316	1.5038	0.066316	0.066316	0.529233804
CSTF2T	ENSG00000177613	1.144	0.12632	1.5038	0.066317	1.3479	0.088851	1.2506	0.10554	0.066317	0.529233804

NA ENSG00000236723 1.5038 0.066322 -1.2902 0.90152 -1.2902 0.90152
-1.2902 0.90152 0.066322 0.529233804
CFH ENSG00000000971 1.5036 0.066341 0.84878 0.198 0.91063 0.18125
-1.2121 0.88727 0.066341 0.529233804
TFPI2 ENSG00000105825 1.5036 0.066344 -0.98267 0.83711 -0.6773 0.75089
-0.6773 0.75089 0.066344 0.529233804
MS4A6A ENSG00000110077 -0.26934 0.60617 -0.1962 0.57777 1.0921
0.1374 1.5036 0.066347 0.066347 0.529233804
MRPL20 ENSG00000242485 1.5032 0.066392 1.5032 0.066392 1.5032
0.066392 1.5032 0.066392 0.066392 0.529365796
NA ENSG00000233423 1.5032 0.066395 0.28029 0.38963 0.28029 0.38963
0.19018 0.42458 0.066395 0.529365796
NA ENSG00000226490 1.5031 0.066405 0.039552 0.48423 0.85841 0.19533
-0.78126 0.78268 0.066405 0.529365796
NA ENSG00000235586 -0.73504 0.76884 1.5031 0.066407 -0.73504 0.76884
-0.73504 0.76884 0.066407 0.529365796
GGCX ENSG00000115486 0.52022 0.30146 -0.72241 0.76498 0.042749 0.48295
1.5029 0.066431 0.066431 0.529391882
NA ENSG00000229044 -0.97095 0.83421 -0.23869 0.59433 1.5029 0.066432
0.55635 0.28899 0.066432 0.529391882
NA ENSG00000250348 1.5027 0.066458 1.5027 0.066458 1.5027 0.066458
1.5027 0.066458 0.066458 0.529470039
NFYBP1 ENSG00000214380 1.1903 0.11696 1.5026 0.066476 1.3762
0.084378 1.1982 0.11542 0.066476 0.529470039
NA ENSG00000267656 1.1734 0.12032 0.9603 0.16845 1.5025 0.066485
-1.1383 0.87251 0.066485 0.529470039
ALAS1 ENSG00000023330 0.21637 0.41435 1.1241 0.13049 1.0066 0.15706
1.5024 0.066501 0.066501 0.529470039
CEBPG ENSG00000153879 0.76548 0.22199 0.76548 0.22199 0.76548 0.22199
1.5024 0.066502 0.066502 0.529470039
MTCO1P25 ENSG00000229031 1.5023 0.066507 1.5023 0.066507 1.339 0.090285
1.3211 0.093232 0.066507 0.529470039
RNA5SP475 ENSG00000222500 1.502 0.066553 1.502 0.066553 1.502 0.066553 1.502
0.066553 0.066553 0.529710937
SIPA1L1 ENSG00000197555 1.4867 0.068542 1.5019 0.066559 1.2891
0.09869 0.83882 0.20078 0.066559 0.529710937
BZW1P2 ENSG00000198406 1.5017 0.066586 1.0622 0.14407 -0.0073506
0.50293 1.1812 0.11875 0.066586 0.529800516
NA ENSG00000259656 1.5017 0.066592 1.5017 0.066592 1.5017 0.066592
1.5017 0.066592 0.066592 0.529800516
NA ENSG00000229892 1.369 0.085493 1.5013 0.066644 1.3115 0.094845 1.369
0.085493 0.066644 0.530122611
CYP4A26P ENSG00000259832 1.501 0.066672 1.501 0.066672 0.342 0.36618 1.501
0.066672 0.066672 0.530122611
NA ENSG00000279444 1.501 0.066673 1.501 0.066673 1.501 0.066673 1.501 0.066673
0.066673 0.530122611
RPS26P35 ENSG00000242206 0.47614 0.31699 -1.4589 0.92771 1.501 0.066676
1.501 0.066676 0.066676 0.530122611
NA ENSG00000251393 1.5008 0.0667 0.607 0.27193 0.61515 0.26923
1.3689 0.085517 0.0667 0.530190651
NA ENSG00000249959 1.5008 0.066708 1.5008 0.066708 1.5008 0.066708
1.5008 0.066708 0.066708 0.530190651
NA ENSG00000201309 1.5006 0.066734 1.5006 0.066734 1.5006 0.066734
1.0358 0.15015 0.066734 0.530190651
OR7A2P ENSG00000172148 1.1997 0.11512 1.5004 0.066752 1.5004
0.066752 1.5004 0.066752 0.066752 0.530190651
NA ENSG00000278891 1.5003 0.066773 0.89491 0.18542 0.89491 0.18542
0.89491 0.18542 0.066773 0.530190651
KLHL25 ENSG00000183655 1.5003 0.066774 1.5003 0.066774 1.1337
0.12845 1.1864 0.11773 0.066774 0.530190651
RN7SL708P ENSG00000265675 1.5002 0.066783 1.5002 0.066783 1.5002
0.066783 1.5002 0.066783 0.066783 0.530190651

NA ENSG00000273448 -0.14126 0.55617 1.5001 0.066789 -0.0029994 0.5012
-0.22884 0.5905 0.066789 0.530190651
BRF1 ENSG00000185024 1.5001 0.066792 -0.19456 0.57713 -0.37077 0.64459
0.26955 0.39375 0.066792 0.530190651
LINC00963 ENSG00000204054 0.92851 0.17657 -1.0961 0.86349 -1.0961
0.86349 1.4999 0.066823 0.066823 0.530190651
TRMT10A ENSG00000145331 0.62363 0.26644 0.66453 0.25317 0.40396
0.34312 1.4999 0.066825 0.066825 0.530190651
NA ENSG00000233799 1.4999 0.066826 1.4999 0.066826 1.4999 0.066826
1.4999 0.066826 0.066826 0.530190651
PLCH1-AS1 ENSG00000239508 1.4999 0.066826 1.4999 0.066826 1.4999
0.066826 1.4999 0.066826 0.066826 0.530190651
PHIP ENSG00000146247 1.4988 0.066963 0.6913 0.24469 0.64124 0.26068
1.1817 0.11865 0.066963 0.531036522
RNA5SP128 ENSG00000202175 1.1807 0.11887 1.4987 0.06697 1.4987
0.06697 1.4987 0.06697 0.06697 0.531036522
ULK2 ENSG00000083290 0.35558 0.36108 0.222 0.41216 1.4987 0.066973
0.42904 0.33395 0.066973 0.531036522
CLTA ENSG00000122705 -0.94978 0.82889 1.4987 0.066977 0.087013 0.46533
0.087013 0.46533 0.066977 0.531036522
RNU6-844P ENSG00000252984 0.86227 0.19427 0.42997 0.33361 1.4986
0.066994 0.42997 0.33361 0.066994 0.531036522
NA ENSG00000248195 0.35069 0.36291 0.64543 0.25932 1.4985 0.066998
1.4985 0.066998 0.066998 0.531036522
NA ENSG00000228829 0.76996 0.22066 1.4979 0.067086 1.397 0.081201
-0.66976 0.74849 0.067086 0.531121488
MSH4 ENSG00000057468 0.38778 0.34909 0.026968 0.48924 1.4977 0.067107
-0.41205 0.65985 0.067107 0.531121488
NA ENSG00000257086 1.4977 0.067108 1.4977 0.067108 1.4977 0.067108
1.4977 0.067108 0.067108 0.531121488
CHIAP3 ENSG00000234020 -0.96536 0.83282 1.4976 0.067122 -0.96536
0.83282 -0.13526 0.5538 0.067122 0.531121488
NIP7P2 ENSG00000213264 1.4975 0.067133 1.4311 0.076204 -2.0226
0.97844 1.4311 0.076204 0.067133 0.531121488
ARF6 ENSG00000165527 1.4975 0.067136 -0.47674 0.68323 -0.53785 0.70466
-0.85948 0.80496 0.067136 0.531121488
NA ENSG00000273145 1.4817 0.069214 1.4974 0.067148 0.3181 0.37521
1.2622 0.10344 0.067148 0.531121488
NA ENSG00000274212 0.22758 0.40999 -0.60189 0.72638 1.4974 0.06715
0.22758 0.40999 0.06715 0.531121488
HRCT1 ENSG00000196196 1.4971 0.067178 -0.18514 0.57344 -1.0263 0.84763
1.3636 0.086347 0.067178 0.531121488
MRGPRX5P ENSG00000255536 -1.4533 0.92693 -1.4533 0.92693 -1.4533
0.92693 1.4971 0.067186 0.067186 0.531121488
DENR ENSG00000139726 1.497 0.067192 0.04038 0.48389 1.3049 0.09596
0.9471 0.17179 0.067192 0.531121488
NA ENSG00000260981 1.497 0.067195 0.79343 0.21376 0.79343 0.21376
0.79343 0.21376 0.067195 0.531121488
DDX18P1 ENSG00000259165 1.4969 0.067215 -1.23 0.89065 -1.23 0.89065 -1.23
0.89065 0.067215 0.531121488
NA ENSG00000199901 1.4968 0.067218 1.149 0.12527 0.76723 0.22147
1.0456 0.14788 0.067218 0.531121488
NA ENSG00000253110 1.4968 0.067218 -0.44084 0.67034 -0.36746 0.64336
-0.69016 0.75495 0.067218 0.531121488
NA ENSG00000265737 -0.68163 0.75226 -0.68163 0.75226 -0.68163 0.75226
1.4968 0.067229 0.067229 0.531121488
MCAT ENSG00000100294 -1.0591 0.85522 1.4966 0.067242 -1.0591 0.85522
-1.3036 0.90381 0.067242 0.531121488
ZDHHC16 ENSG00000171307 1.4966 0.067247 0.71677 0.23676 -0.33465
0.63106 -0.058522 0.52333 0.067247 0.531121488
EXOSC1 ENSG00000171311 1.4966 0.067247 1.4966 0.067247 1.4966
0.067247 1.3031 0.096273 0.067247 0.531121488

PIP4K2B	ENSG00000276293	0.22207	0.41213	1.4966	0.067247	0.22207	0.41213	0.87919	0.18965	0.067247	0.531121488
HDHD3	ENSG00000119431	1.4966	0.067253	1.4966	0.067253	1.4966	0.067253	1.4966	0.067253	0.067253	0.531121488
NA	ENSG00000253921	1.4964	0.067269	-1.3473	0.91105	-1.3473	0.91105	-1.3473	0.91105	0.067269	0.531121488
TMEM178A	ENSG00000152154	0.82698	0.20413	-0.33546	0.63136	0.14253	0.44333	1.4963	0.067292	0.067292	0.531121488
SNORD45C	ENSG00000206620	1.4963	0.067292	1.4963	0.067292	1.4963	0.067292	1.4963	0.067292	0.067292	0.531121488
RNF38	ENSG00000137075	-1.2551	0.89529	-0.33875	0.6326	-0.014907	0.50595	1.4963	0.067293	0.067293	0.531121488
BCAT1	ENSG00000060982	1.4962	0.067297	-0.0089386	0.50357	0.70567	0.2402	0.47583	0.3171	0.067297	0.531121488
F13A1	ENSG00000124491	0.6059	0.27229	-1.076	0.85905	-0.87766	0.80994	1.4962	0.067303	0.067303	0.531121488
NA	ENSG00000277050	-0.12108	0.54819	1.3055	0.095868	1.4961	0.067319	0.89945	0.18421	0.067319	0.531161734
NA	ENSG00000213987	0.066625	0.47344	0.066625	0.47344	1.4958	0.067349	0.066625	0.47344	0.067349	0.531259341
H2AC20	ENSG00000184260	1.4957	0.067366	1.4957	0.067366	1.4957	0.067366	1.4957	0.067366	0.067366	0.531259341
RN7SL147P	ENSG00000263988	-1.0327	0.84912	-1.0327	0.84912	-1.0327	0.84912	1.4956	0.067375	0.067375	0.531259341
NA	ENSG00000267005	0.57902	0.28129	1.4954	0.067411	0.57902	0.28129	-0.4387	0.66956	0.067411	0.531259341
USP12-AS1	ENSG00000232162	1.4953	0.067415	1.1951	0.11601	1.3356	0.090845	0.85219	0.19706	0.067415	0.531259341
SNORD45B	ENSG00000201487	1.4953	0.067418	1.4953	0.067418	1.4953	0.067418	1.4953	0.067418	0.067418	0.531259341
RNF175	ENSG00000145428	1.4952	0.067437	1.4952	0.067437	0.60731	0.27182	0.91567	0.17992	0.067437	0.531259341
RPS4XP15	ENSG00000240721	1.4951	0.06744	-0.022063	0.5088	-0.064046	0.52553	1.1091	0.13369	0.06744	0.531259341
RNA5SP103	ENSG00000238379	1.4951	0.067444	-0.078804	0.53141	0.55185	0.29053	0.40268	0.34359	0.067444	0.531259341
LINC00460	ENSG00000233532	1.495	0.067457	1.495	0.067457	1.495	0.067457	0.94801	0.17156	0.067457	0.531259341
MAFK	ENSG00000198517	0.83485	0.2019	0.96515	0.16723	1.4949	0.067465	1.2115	0.11285	0.067465	0.531259341
C22orf23	ENSG00000128346	1.4949	0.067476	1.4949	0.067476	1.4949	0.067476	1.4949	0.067476	0.067476	0.531259341
EFCAB14P1	ENSG00000228348	1.4948	0.067483	1.4948	0.067483	1.4948	0.067483	1.4948	0.067483	0.067483	0.531259341
RNA5SP27	ENSG00000200709	1.4948	0.067484	1.4948	0.067484	1.4948	0.067484	1.4948	0.067484	0.067484	0.531259341
NKX2-2	ENSG00000125820	0.90171	0.1836	-0.4351	0.66826	1.4946	0.067511	1.4946	0.067511	0.067511	0.5313
NA	ENSG00000273432	1.4944	0.067535	1.4944	0.067535	1.4944	0.067535	1.4944	0.067535	0.067535	0.5313
NA	ENSG00000215094	1.4944	0.067536	1.4944	0.067536	1.4944	0.067536	1.4944	0.067536	0.067536	0.5313
PIK3CD	ENSG00000171608	0.40117	0.34415	1.1166	0.13207	1.4944	0.06754	0.18102	0.42818	0.06754	0.5313
DDIT4-AS1	ENSG00000269926	1.4942	0.067565	1.4942	0.067565	1.4942	0.067565	1.4942	0.067565	0.067565	0.5313
NA	ENSG00000277466	-1.2385	0.89224	1.494	0.067586	0.53542	0.29618	-0.06259	0.52495	0.067586	0.5313
PDE6D	ENSG00000156973	-1.0158	0.84513	-1.0443	0.85184	-1.4817	0.93079	1.4939	0.067595	0.067595	0.5313
RNASEH1P1	ENSG00000265790	1.4939	0.067598	1.4939	0.067598	1.4939	0.067598	1.4939	0.067598	0.067598	0.5313

NA	ENSG00000235066	0.93489	0.17492	1.31	0.095103	0.98103	0.16329	1.4939	0.067603	0.067603	0.5313
ATF4P3	ENSG00000228218	1.4938	0.06761	1.2885	0.098787	1.2885	0.098787	1.2885	0.098787	0.06761	0.5313
NA	ENSG00000277170	1.4938	0.067614	1.4938	0.067614	1.4938	0.067614	1.4938	0.067614	0.067614	0.5313
NA	ENSG00000267780	1.4938	0.06762	1.2966	0.097377	-0.18566	0.57364	1.2966	0.097377	0.06762	0.5313
TSNAX	ENSG00000116918	-0.0077157	0.50308	1.3889	0.082434	1.4935	0.067654	1.413	0.078825	0.067654	0.531372967
USP25	ENSG00000155313	1.4934	0.067661	0.81304	0.2081	-1.0051	0.84257	-0.84593	0.80121	0.067661	0.531372967
DTX2	ENSG00000091073	1.4934	0.067662	1.0794	0.14021	-0.096315	0.53836	0.099612	0.46033	0.067662	0.531372967
CEP57L1	ENSG00000183137	1.4933	0.067685	1.4933	0.067685	1.4933	0.067685	0.143	0.44315	0.067685	0.531410265
FKBP1AP3	ENSG00000230243	0.32168	0.37385	0.80348	0.21085	-0.074541	0.52971	1.4932	0.067699	0.067699	0.531410265
NA	ENSG00000250360	1.0132	0.15549	-0.078599	0.53132	1.4931	0.067704	1.4931	0.067704	0.067704	0.531410265
DBF4B	ENSG00000161692	1.493	0.067713	1.493	0.067713	0.11249	0.45522	1.1849	0.11804	0.067713	0.531410265
SLC19A3	ENSG00000135917	1.2224	0.11078	1.4929	0.067733	1.4929	0.067733	-0.40536	0.65739	0.067733	0.531410265
FOXC1	ENSG00000054598	-0.97	0.83398	-0.79901	0.78786	1.4929	0.067734	-0.54667	0.7077	0.067734	0.531410265
ALPK3	ENSG00000136383	1.296	0.097488	1.4927	0.067761	1.0369	0.14988	1.0369	0.14988	0.067761	0.531410265
NA	ENSG00000233839	1.4926	0.067768	0.78554	0.21607	0.91691	0.1796	1.0451	0.14798	0.067768	0.531410265
NA	ENSG00000253750	1.4925	0.067778	1.0625	0.14401	0.93823	0.17406	0.39719	0.34561	0.067778	0.531410265
MC2R	ENSG00000185231	-0.0062673	0.5025	1.4925	0.067782	-0.13467	0.55356	0.9365	0.17451	0.067782	0.531410265
BAG6	ENSG00000204463	1.4924	0.067791	1.3775	0.084175	1.2324	0.1089	1.1908	0.11686	0.067791	0.531410265
LINC02746	ENSG00000282916	1.4924	0.0678	1.4924	0.0678	1.4924	0.0678	1.4924	0.0678	0.0678	0.531410265
NA	ENSG00000225867	1.4923	0.067812	1.4923	0.067812	1.4923	0.067812	1.1011	0.13542	0.067812	0.531410265
NA	ENSG00000261018	1.1636	0.12229	-0.51689	0.69738	1.4922	0.067821	-0.68123	0.75214	0.067821	0.531410265
TAPBP	ENSG00000231925	1.4915	0.06792	0.3459	0.36471	-0.43378	0.66778	1.2606	0.10373	0.06792	0.531410265
RNY4P34	ENSG00000201649	-0.4558	0.67573	0.78964	0.21487	1.4914	0.067922	1.4914	0.067922	0.067922	0.531410265
NA	ENSG00000253507	0.54273	0.29366	1.4914	0.067927	1.0383	0.14957	-0.69435	0.75627	0.067927	0.531410265
TNFSF15	ENSG00000181634	1.4914	0.067928	0.64683	0.25887	1.4914	0.067928	1.4914	0.067928	0.067928	0.531410265
DTNBP1	ENSG00000047579	1.1511	0.12484	0.41034	0.34078	1.4912	0.067948	1.2874	0.098985	0.067948	0.531410265
HDDC2	ENSG00000111906	0.91457	0.18021	1.4913	0.067948	0.69354	0.24398	0.68278	0.24737	0.067948	0.531410265
NA	ENSG00000280180	1.3432	0.089599	-0.31662	0.62423	1.4912	0.06795	0.53999	0.2946	0.06795	0.531410265
IL6R	ENSG00000160712	1.4912	0.067956	-0.17565	0.56972	0.21916	0.41326	-0.13935	0.55541	0.067956	0.531410265
MTND5P20	ENSG00000238256	0.35481	0.36137	-0.14401	0.55725	1.4912	0.067956	1.4008	0.08063	0.067956	0.531410265
NA	ENSG00000251652	0.61046	0.27078	1.1044	0.13471	1.1044	0.13471	1.4912	0.067957	0.067957	0.531410265

MKX-AS1	ENSG00000230500	1.2617	0.10352	1.2617	0.10352	0.22431	0.41126	1.4911	0.067971	0.067971	0.531410265
TXNDC11	ENSG00000153066	0.90138	0.18369	1.2925	0.098089	1.491	0.067978	0.35236	0.36228	0.067978	0.531410265
NA	ENSG00000233052	-0.53948	0.70522	1.491	0.067978	-0.58102	0.71939	-0.58102	0.71939	0.067978	0.531410265
RSPRY1	ENSG00000159579	0.76124	0.22326	1.4205	0.077732	1.491	0.067979	1.4205	0.077732	0.067979	0.531410265
HGF	ENSG00000019991	1.491	0.067983	0.23945	0.40538	0.85506	0.19626	0.85506	0.19626	0.067983	0.531410265
NA	ENSG00000255836	0.11757	0.4532	0.088149	0.46488	1.4909	0.067999	1.4909	0.067999	0.067999	0.531450083
HOXB1	ENSG00000120094	1.1675	0.1215	1.1317	0.12888	1.4906	0.068029	1.4906	0.068029	0.068029	0.531553122
ABCA6	ENSG00000154262	0.57331	0.28322	1.4906	0.068034	1.4906	0.068034	0.8443	0.19925	0.068034	0.531553122
PIH1D1	ENSG00000104872	1.4668	0.071211	1.4668	0.071211	0.78195	0.21712	1.4904	0.068054	0.068054	0.531612771
RN7SL353P	ENSG00000263608	1.4902	0.068089	1.4902	0.068089	1.4902	0.068089	1.4902	0.068089	0.068089	0.531612771
LINC00159	ENSG00000230323	1.394	0.081651	1.0198	0.1539	0.70393	0.24074	1.4901	0.068102	0.068102	0.531612771
NA	ENSG00000236948	-0.91058	0.81874	1.4901	0.068105	-0.92538	0.82262	-0.54678	0.70774	0.068105	0.531612771
NA	ENSG00000259137	-0.56229	0.71304	-0.17122	0.56797	1.4901	0.068105	-0.17122	0.56797	0.068105	0.531612771
LINC01242	ENSG00000280683	0.10709	0.45736	1.3444	0.089414	1.1048	0.13462	1.49	0.068113	0.068113	0.531612771
AGO1	ENSG00000092847	1.2314	0.10909	1.2508	0.1055	1.306	0.095769	1.49	0.068118	0.068118	0.531612771
SEC62	ENSG00000008952	1.4897	0.068146	-0.78391	0.78345	-0.96509	0.83275	-0.55336	0.70999	0.068146	0.53174613
NA	ENSG00000279633	1.4896	0.068158	-0.34471	0.63484	1.4896	0.068158	1.4896	0.068158	0.068158	0.531754617
NA	ENSG00000273258	1.1624	0.12254	1.4894	0.068189	1.1624	0.12254	1.1624	0.12254	0.068189	0.531911313
NA	ENSG00000262213	1.4888	0.068267	-0.11937	0.54751	1.4888	0.068267	0.97218	0.16548	0.068267	0.53218269
FAM216A	ENSG00000204856	1.4887	0.06828	-0.059584	0.52376	0.88646	0.18768	0.54523	0.2928	0.06828	0.53218269
HORMAD2-AS1	ENSG00000227117	0.24113	0.40473	0.67863	0.24869	1.0704	0.14221	1.4887	0.068287	0.068287	0.53218269
CDV3P1	ENSG00000176243	1.4886	0.068303	-0.42725	0.6654	-1.2327	0.89115	-0.64863	0.74171	0.068303	0.53218269
NA	ENSG00000275740	1.3247	0.092636	1.4885	0.06831	1.3247	0.092636	1.3247	0.092636	0.06831	0.53218269
FNBP1P1	ENSG00000257800	0.28542	0.38766	1.4885	0.068314	0.83003	0.20326	0.17497	0.43055	0.068314	0.53218269
GMPR	ENSG00000137198	-0.86351	0.80607	-0.62265	0.73324	0.75636	0.22472	1.4885	0.068316	0.068316	0.53218269
NA	ENSG00000274695	1.0909	0.13765	1.4884	0.068323	1.2027	0.11455	0.81155	0.20852	0.068323	0.53218269
TADA2B	ENSG00000173011	-0.27327	0.60768	1.4883	0.06833	-0.7034	0.7591	-0.097348	0.53877	0.06833	0.53218269
PIGH	ENSG00000100564	1.4883	0.068333	1.4883	0.068333	1.4883	0.068333	0.68034	0.24815	0.068333	0.53218269
ZNF154	ENSG00000179909	0.57187	0.28371	0.57187	0.28371	1.488	0.06838	0.0094767	0.49622	0.06838	0.532463631
NA	ENSG00000232518	1.4878	0.068404	1.4878	0.068404	1.4878	0.068404	1.4878	0.068404	0.068404	0.532565413
RNU1-22P	ENSG00000200204	1.4873	0.068464	1.4873	0.068464	1.4873	0.068464	1.4873	0.068464	0.068464	0.532947399

SSPOP	ENSG00000197558	0.37838	0.35257	-1.3736	0.91522	1.0879	0.13832	1.4872	0.068475	0.068475	0.532947892
LINC02134	ENSG00000260450	0.65693	0.25561	0.44812	0.32703	1.4871	0.0685	0.73491	0.2312	0.0685	0.532973194
EEF1DP6	ENSG00000229280	1.1413	0.12688	1.4869	0.068515	-1.4224	0.92254	-1.4224	0.92254	0.068515	0.532973194
NA	ENSG00000240809	1.1273	0.12981	1.4869	0.06852	1.4869	0.06852	1.4869	0.06852	0.06852	0.532973194
RIPK2	ENSG00000104312	1.4869	0.068522	1.0756	0.14106	1.4869	0.068522	0.43147	0.33306	0.068522	0.532973194
NA	ENSG00000232648	1.0582	0.14497	1.4868	0.068539	-0.017798	0.5071	0.43902	0.33032	0.068539	0.533020343
IGSF11-AS1	ENSG00000239877	1.4859	0.068657	1.4859	0.068657	1.4859	0.068657	-0.21811	0.58633	0.068657	0.533261598
XKR8	ENSG00000158156	0.80021	0.21179	1.4858	0.06866	0.069709	0.47221	0.019339	0.49229	0.06866	0.533261598
IGDCC3	ENSG00000174498	0.56592	0.28572	0.25848	0.39802	0.34897	0.36355	1.4858	0.068668	0.068668	0.533261598
NA	ENSG00000232615	1.3742	0.084683	1.3742	0.084683	0.42044	0.33708	1.4857	0.068678	0.068678	0.533261598
NA	ENSG00000279262	1.4857	0.068684	1.4857	0.068684	1.4857	0.068684	1.4857	0.068684	0.068684	0.533261598
RPL4P5	ENSG00000230207	1.4193	0.077913	1.4856	0.068695	1.4193	0.077913	1.4193	0.077913	0.068695	0.533261598
SCGN	ENSG00000079689	0.45241	0.32549	1.4856	0.068698	0.7981	0.21241	0.7981	0.21241	0.068698	0.533261598
NA	ENSG00000226927	1.4856	0.068699	1.4856	0.068699	1.4856	0.068699	1.4856	0.068699	0.068699	0.533261598
NA	ENSG00000280061	1.4854	0.068714	1.4854	0.068714	1.4854	0.068714	1.4854	0.068714	0.068714	0.533261598
NA	ENSG00000273325	1.4854	0.068718	1.1124	0.13299	-0.79837	0.78767	-0.79837	0.78767	0.068718	0.533261598
CXCL1	ENSG00000163739	-1.4751	0.92991	-0.082995	0.53307	1.4853	0.068729	0.44816	0.32702	0.068729	0.533261598
ZWILCH	ENSG00000174442	-0.2332	0.5922	1.4853	0.068731	0.6939	0.24387	-0.27901	0.60988	0.068731	0.533261598
NA	ENSG00000236173	0.040736	0.48375	1.4852	0.068744	-1.9234	0.97278	-0.27342	0.60773	0.068744	0.533261598
LAT2	ENSG00000086730	-0.12489	0.5497	0.36936	0.35593	1.4851	0.068753	-0.11729	0.54668	0.068753	0.533261598
HAR1B	ENSG00000231133	1.4851	0.06876	0.10208	0.45935	-0.58263	0.71993	-0.17132	0.56801	0.06876	0.533261598
NA	ENSG00000270112	0.83422	0.20208	0.83422	0.20208	0.83422	0.20208	1.485	0.068776	0.068776	0.533261598
NA	ENSG00000258891	0.93839	0.17402	1.4848	0.068794	1.2613	0.10359	1.2613	0.10359	0.068794	0.533261598
LINC00615	ENSG00000196243	1.4846	0.068829	1.4343	0.075742	0.27472	0.39177	-0.50625	0.69366	0.068829	0.533261598
NA	ENSG00000213959	-0.23925	0.59454	1.4845	0.068834	-0.21298	0.58433	-0.21298	0.58433	0.068834	0.533261598
KASH5	ENSG00000161609	1.4844	0.06885	1.0802	0.14002	1.0802	0.14002	1.0802	0.14002	0.06885	0.533261598
SFMBT1	ENSG00000163935	0.80919	0.2092	0.020761	0.49172	1.4843	0.068863	0.76068	0.22342	0.068863	0.533261598
MIR1269A	ENSG00000221563	1.4843	0.068868	1.4843	0.068868	0.954	0.17004	1.4843	0.068868	0.068868	0.533261598
NA	ENSG00000248050	1.4842	0.068877	0.69556	0.24335	1.4842	0.068877	1.4842	0.068877	0.068877	0.533261598
NA	ENSG00000223720	1.4841	0.068886	0.057108	0.47723	0.59093	0.27728	1.399	0.080912	0.068886	0.533261598
FKBP1AP1	ENSG00000269304	1.484	0.068902	1.484	0.068902	1.484	0.068902	1.484	0.068902	0.068902	0.533261598

VWC2L ENSG00000174453 1.484 0.068905 0.80449 0.21056 0.49964 0.30866
0.50843 0.30558 0.068905 0.533261598
NHLH2 ENSG00000177551 -0.070458 0.52809 1.484 0.06891 -0.070458 0.52809
-0.64025 0.739 0.06891 0.533261598
NA ENSG00000223492 0.29859 0.38263 1.4838 0.068933 0.57877 0.28137
-1.4062 0.92017 0.068933 0.533261598
MSR1 ENSG00000038945 -0.15022 0.55971 1.4838 0.068935 -1.2896 0.9014
0.006522 0.4974 0.068935 0.533261598
DDIAS ENSG00000165490 1.2203 0.11117 -0.18085 0.57176 1.4837 0.06895
0.51061 0.30481 0.06895 0.533261598
CBY1P1 ENSG00000236832 1.4836 0.068956 1.1119 0.13308 1.1237
0.13058 1.1139 0.13266 0.068956 0.533261598
TCP11L1 ENSG00000176148 1.2983 0.09709 0.65622 0.25584 1.4835
0.068973 0.65622 0.25584 0.068973 0.533261598
BPGM ENSG00000172331 0.10424 0.45849 0.61218 0.27021 0.53313 0.29697
1.4834 0.068979 0.068979 0.533261598
HCG11 ENSG00000228223 0.90432 0.18291 1.2038 0.11434 1.4832 0.069006
1.2655 0.10285 0.069006 0.533261598
TRAPPC12 ENSG00000171853 1.4832 0.069011 0.96062 0.16837 0.71133
0.23844 1.3987 0.08095 0.069011 0.533261598
H2AC21 ENSG00000184270 1.4831 0.069025 1.4831 0.069025 1.4831
0.069025 1.4831 0.069025 0.069025 0.533261598
NA ENSG00000257261 0.20716 0.41794 0.7132 0.23786 0.32769 0.37157
1.483 0.069038 0.069038 0.533261598
EIF2A ENSG00000144895 0.58275 0.28003 0.99366 0.16019 0.99366 0.16019
1.483 0.069039 0.069039 0.533261598
MIR128-1 ENSG00000207654 -0.86778 0.80724 1.483 0.069041 0.66687 0.25243
-0.90554 0.81741 0.069041 0.533261598
CHCHD2P7 ENSG00000249973 1.4829 0.069047 0.48641 0.31334 0.92175
0.17833 0.41609 0.33867 0.069047 0.533261598
NA ENSG00000258465 1.4826 0.06909 1.4826 0.06909 1.4826 0.06909
1.4826 0.06909 0.06909 0.533261598
NA ENSG00000275839 1.4826 0.069096 1.4826 0.069096 1.4826 0.069096
1.4826 0.069096 0.069096 0.533261598
NA ENSG00000219622 -0.11047 0.54398 1.4822 0.069149 0.888 0.18727
0.27629 0.39116 0.069149 0.533261598
NA ENSG00000245651 1.1643 0.12215 1.4742 0.070212 1.3738 0.084755
1.4821 0.069154 0.069154 0.533261598
NA ENSG00000220291 0.70228 0.24125 1.482 0.069172 1.482 0.069172 1.482
0.069172 0.069172 0.533261598
ECSCR ENSG00000249751 0.8174 0.20685 0.20764 0.41776 0.86663 0.19307
1.4818 0.069194 0.069194 0.533261598
SEL1L2 ENSG00000101251 1.4816 0.069224 0.59097 0.27727 1.4816
0.069224 1.0445 0.14812 0.069224 0.533261598
LINC01340 ENSG00000250331 1.4815 0.069231 -0.5188 0.69805 -0.041525
0.51656 -0.025119 0.51002 0.069231 0.533261598
ULBP1 ENSG00000111981 0.76875 0.22102 0.88178 0.18895 1.4814 0.069251
0.35826 0.36008 0.069251 0.533261598
NA ENSG00000250609 -0.072183 0.52877 1.4813 0.069268 -0.072183 0.52877
-0.072183 0.52877 0.069268 0.533261598
WBP1P1 ENSG00000231351 0.60443 0.27278 1.4812 0.069271 0.13853
0.44491 -0.19038 0.5755 0.069271 0.533261598
LINC00271 ENSG00000231028 -0.056262 0.52243 1.481 0.069302 -0.12677 0.55044
0.82748 0.20398 0.069302 0.533261598
SIGLEC11 ENSG00000161640 0.63394 0.26306 0.78039 0.21758 1.481 0.069303
1.481 0.069303 0.069303 0.533261598
NA ENSG00000283217 1.4809 0.06932 1.4809 0.06932 1.4809 0.06932
1.3877 0.082616 0.06932 0.533261598
NFKBIE ENSG00000146232 0.90033 0.18397 1.1845 0.11811 1.4383
0.075173 1.4809 0.069321 0.069321 0.533261598
ZNF17 ENSG00000186272 0.74542 0.22801 1.3899 0.082282 1.4808 0.069333
1.4808 0.069333 0.069333 0.533261598

NA	ENSG00000264689	1.4807	0.069345	1.4807	0.069345	1.4807	0.069345	1.4807	0.069345	0.069345	0.533261598
TOMM5	ENSG00000175768	1.4807	0.069346	1.2432	0.1069	-0.68158	0.75225	-0.64822	0.74158	0.069346	0.533261598
NA	ENSG00000256966	1.4807	0.069346	1.4807	0.069346	1.4807	0.069346	1.4807	0.069346	0.069346	0.533261598
NA	ENSG00000267636	1.4807	0.069349	-0.14157	0.55629	1.4807	0.069349	1.4807	0.069349	0.069349	0.533261598
BAG2	ENSG00000112208	0.16872	0.43301	1.4806	0.069353	0.16872	0.43301	0.78675	0.21571	0.069353	0.533261598
OR7E129P	ENSG00000180770	-0.26048	0.60275	0.7371	0.23053	1.4806	0.069361	1.199	0.11526	0.069361	0.533261598
FAM155A-IT1	ENSG00000227248	1.4805	0.069364	1.1114	0.13321	0.45671	0.32394	1.009	0.15649	0.069364	0.533261598
SFI1	ENSG00000198089	0.26821	0.39427	0.66994	0.25145	0.42378	0.33586	1.4805	0.069365	0.069365	0.533261598
RHOD	ENSG00000173156	1.4805	0.069367	-0.82193	0.79444	-0.29295	0.61522	0.36455	0.35772	0.069367	0.533261598
PACSIN3	ENSG00000165912	1.4805	0.06937	-0.071119	0.52835	0.8205	0.20596	1.4805	0.06937	0.06937	0.533261598
GAPDHP37	ENSG00000231907	1.4804	0.069378	1.4804	0.069378	1.356	0.087552	-0.8321	0.79732	0.069378	0.533261598
TMSB4XP3	ENSG00000223437	0.90954	0.18153	1.1888	0.11726	1.1888	0.11726	1.4804	0.069382	0.069382	0.533261598
ATP6V0A4	ENSG00000105929	0.85032	0.19757	0.8333	0.20234	1.4797	0.069476	1.3535	0.087952	0.069476	0.533261598
RPL23AP26	ENSG00000228884	1.4797	0.069483	-0.34717	0.63577	-0.35031	0.63695	-0.35031	0.63695	0.069483	0.533261598
NA	ENSG00000221741	1.4796	0.069496	1.4796	0.069496	1.4796	0.069496	1.4796	0.069496	0.069496	0.533261598
PCDHGA7	ENSG00000253537	1.4795	0.069499	1.4795	0.069499	1.4795	0.069499	1.4795	0.069499	0.069499	0.533261598
ST13P4	ENSG00000232150	-1.7526	0.96017	1.4794	0.069515	0.52025	0.30144	-0.76057	0.77654	0.069515	0.533261598
KCNG3	ENSG00000171126	1.4794	0.069519	0.094749	0.46226	0.95186	0.17059	-0.15901	0.56317	0.069519	0.533261598
ALOX12	ENSG00000108839	1.4793	0.069524	-0.13019	0.55179	1.0121	0.15576	0.74878	0.22699	0.069524	0.533261598
ADH5	ENSG00000197894	-0.26438	0.60426	1.4793	0.069526	1.4793	0.069526	-0.62599	0.73434	0.069526	0.533261598
KIAA0040	ENSG00000235750	0.52645	0.29929	1.4793	0.069528	-1.0286	0.84817	-0.12028	0.54787	0.069528	0.533261598
NA	ENSG00000272489	1.4793	0.069532	1.4793	0.069532	1.4793	0.069532	1.4793	0.069532	0.069532	0.533261598
NPTXR	ENSG00000221890	1.3704	0.085282	1.4793	0.069534	1.3704	0.085282	0.74526	0.22806	0.069534	0.533261598
SMAD1-AS1	ENSG00000250902	1.187	0.11761	0.11773	0.45314	1.187	0.11761	1.4792	0.069541	0.069541	0.533261598
NA	ENSG00000240731	1.4792	0.069542	1.4792	0.069542	1.4792	0.069542	1.4792	0.069542	0.069542	0.533261598
B3GNT6	ENSG00000198488	1.4792	0.069546	0.6907	0.24488	1.0396	0.14925	1.4428	0.074534	0.069546	0.533261598
OR1L6	ENSG00000171459	1.4792	0.06955	-0.43097	0.66675	1.1817	0.11866	-1.5652	0.94124	0.06955	0.533261598
PPP1R26	ENSG00000196422	1.4791	0.06956	-0.6943	0.75625	-0.84087	0.79979	-0.95257	0.8296	0.06956	0.533261598
NA	ENSG00000255704	0.0080439	0.49679	0.0080439	0.49679	1.479	0.069571	-0.40772	0.65826	0.069571	0.533261598
RNU6-1271P	ENSG00000252858	1.479	0.069574	1.479	0.069574	1.479	0.069574	1.479	0.069574	0.069574	0.533261598
NA	ENSG00000214797	-0.66436	0.74677	1.4789	0.069585	1.4415	0.074718	-0.66436	0.74677	0.069585	0.533261598

LHX4	ENSG00000121454	1.4788	0.069592	-0.69775	0.75733	1.1248	0.13035	0.18693	0.42586	0.069592	0.533261598
NA	ENSG00000270852	0.29487	0.38405	0.0941	0.46251	0.021618	0.49138	1.4788	0.069593	0.069593	0.533261598
C2orf50	ENSG00000150873	1.2718	0.10173	1.4451	0.074213	1.4787	0.069609	1.4787	0.069609	0.069609	0.533261598
CFAP300	ENSG00000137691	-1.5709	0.9419	-1.5709	0.9419	1.4787	0.06961	0.452	0.32564	0.06961	0.533261598
AKR7A3	ENSG00000162482	1.4787	0.069613	0.48435	0.31407	-0.046273	0.51845	0.42213	0.33646	0.069613	0.533261598
NA	ENSG00000226471	1.3113	0.094885	1.4786	0.069622	0.58634	0.27882	0.68286	0.24735	0.069622	0.533261598
ZNF451	ENSG00000112200	1.369	0.0855	1.1919	0.11665	0.65497	0.25624	1.4786	0.069624	0.069624	0.533261598
ARMC1	ENSG00000104442	1.128	0.12967	0.48696	0.31314	0.23133	0.40853	1.4785	0.069638	0.069638	0.533261598
C19orf57	ENSG00000132016	1.4785	0.069643	1.458	0.072418	-0.47884	0.68397	0.72542	0.2341	0.069643	0.533261598
RNF180	ENSG00000164197	1.4784	0.069645	0.17156	0.43189	0.81623	0.20718	-0.39874	0.65496	0.069645	0.533261598
PROS1	ENSG00000184500	0.2714	0.39304	1.3229	0.092936	-0.12358	0.54918	1.4782	0.069671	0.069671	0.533261598
LINC00476	ENSG00000175611	1.4782	0.069682	0.057954	0.47689	-0.23901	0.59445	-0.50923	0.6947	0.069682	0.533261598
NA	ENSG00000224886	1.4782	0.069683	1.4782	0.069683	0.1133	0.4549	1.2019	0.11469	0.069683	0.533261598
SPCS1	ENSG00000114902	1.1585	0.12333	1.4781	0.069694	0.83305	0.20241	0.75596	0.22484	0.069694	0.533261598
HMGN2P5	ENSG00000234664	1.4779	0.069714	1.4779	0.069714	1.4779	0.069714	1.4779	0.069714	0.069714	0.533261598
NA	ENSG00000278893	0.78118	0.21735	1.4779	0.069718	0.78118	0.21735	0.78118	0.21735	0.069718	0.533261598
RNU6-236P	ENSG00000200756	1.4778	0.069727	1.4778	0.069727	1.4778	0.069727	1.4778	0.069727	0.069727	0.533261598
NDUFB8P2	ENSG00000270264	1.4778	0.069729	1.4778	0.069729	1.4778	0.069729	1.4778	0.069729	0.069729	0.533261598
RN7SKP197	ENSG00000252886	1.4778	0.06973	1.4778	0.06973	1.4778	0.06973	1.4778	0.06973	0.06973	0.533261598
GRHPR	ENSG00000137106	1.4775	0.069766	0.17662	0.4299	-0.63833	0.73837	0.21849	0.41352	0.069766	0.53345319
NA	ENSG00000278215	1.4772	0.069809	1.4772	0.069809	1.4772	0.069809	1.4772	0.069809	0.069809	0.533482126
TFEC	ENSG00000105967	-0.4484	0.67307	0.087553	0.46512	-0.41275	0.66011	1.4772	0.069815	0.069815	0.533482126
NA	ENSG00000270071	0.79596	0.21303	0.26378	0.39597	0.76636	0.22173	1.477	0.069831	0.069831	0.533482126
SAFB2	ENSG00000130254	-0.49245	0.6888	-1.7666	0.96135	1.4769	0.069849	-0.31356	0.62307	0.069849	0.533482126
MIR4476	ENSG00000265368	1.4383	0.075174	1.4768	0.069859	1.4383	0.075174	1.4383	0.075174	0.069859	0.533482126
NA	ENSG00000280355	-0.060418	0.52409	1.0087	0.15656	1.4768	0.06986	1.4768	0.06986	0.06986	0.533482126
LCA5	ENSG00000135338	0.30949	0.37848	-0.081344	0.53242	1.4768	0.069866	0.389	0.34864	0.069866	0.533482126
AARS1P1	ENSG00000249038	1.4767	0.069878	1.4767	0.069878	1.4767	0.069878	1.1628	0.12245	0.069878	0.533482126
NA	ENSG00000271857	0.40895	0.34129	-0.46805	0.68013	1.4766	0.069889	1.1257	0.13015	0.069889	0.533482126
NA	ENSG00000259742	-1.718	0.9571	-1.3603	0.91313	-0.81141	0.79144	1.4765	0.069902	0.069902	0.533482126
CDK7P1	ENSG00000213194	0.92991	0.17621	0.53599	0.29598	1.4765	0.069905	1.4765	0.069905	0.069905	0.533482126

NA ENSG00000267194 -0.24358 0.59622 -0.18909 0.57499 0.20144 0.42018
1.4765 0.069905 0.069905 0.533482126
PLAAT4 ENSG00000133321 1.4764 0.069917 -0.76949 0.7792 -0.76949
0.7792 -0.76949 0.7792 0.069917 0.533482126
FUT4 ENSG00000196371 1.4763 0.069926 1.4763 0.069926 1.4763 0.069926
1.4763 0.069926 0.069926 0.533482126
STX17-AS1 ENSG00000255145 0.98399 0.16256 0.98399 0.16256 1.4763
0.069934 0.98399 0.16256 0.069934 0.533482126
NA ENSG00000280159 1.4757 0.07001 1.4757 0.07001 1.4757 0.07001
0.90745 0.18209 0.07001 0.533978291
RGS7 ENSG00000182901 -0.09591 0.5382 0.19681 0.42199 1.4756 0.070029
0.33662 0.3682 0.070029 0.53403962
NA ENSG00000233290 -1.1231 0.8693 1.4754 0.070058 -0.96267 0.83214
-0.60099 0.72608 0.070058 0.534101835
PIGU ENSG00000101464 0.31808 0.37521 1.3367 0.090666 0.029041 0.48842
1.4753 0.070067 0.070067 0.534101835
NA ENSG00000257904 1.4752 0.070075 -0.46632 0.67951 -0.88144 0.81096
-1.1966 0.88428 0.070075 0.534101835
NA ENSG00000237729 1.4752 0.070081 -0.52619 0.70062 1.4752 0.070081
1.4752 0.070081 0.070081 0.534101835
ZNF136 ENSG00000196646 1.47 0.070787 0.40123 0.34413 1.475 0.070101
0.24674 0.40256 0.070101 0.534170716
GSC2 ENSG00000063515 1.4749 0.070125 1.4749 0.070125 1.4749 0.070125
1.4749 0.070125 0.070125 0.534232239
RPL12P2 ENSG00000216412 0.40743 0.34184 1.4748 0.070131 1.4135
0.078761 0.68592 0.24638 0.070131 0.534232239
NA ENSG00000259703 1.1678 0.12145 1.4746 0.070158 1.4746 0.070158
1.4746 0.070158 0.070158 0.534354383
NA ENSG00000265550 1.4745 0.070173 1.4745 0.070173 1.4745 0.070173
1.4745 0.070173 0.070173 0.534385105
UCA1 ENSG00000214049 1.3966 0.081269 1.0175 0.15445 1.4743 0.070204
1.4743 0.070204 0.070204 0.534537644
UGT1A3 ENSG00000243135 1.4739 0.070249 1.4739 0.070249 1.4739
0.070249 1.4739 0.070249 0.070249 0.534796715
PZP ENSG00000126838 1.1002 0.13563 1.4738 0.070271 1.4738 0.070271
-0.27525 0.60844 0.070271 0.534804902
DOCK7 ENSG00000116641 -0.21817 0.58635 1.053 0.14617 1.4737 0.070282
-0.65801 0.74474 0.070282 0.534804902
NA ENSG00000248533 1.4737 0.070283 1.4737 0.070283 1.4737 0.070283
1.4737 0.070283 0.070283 0.534804902
LINC00839 ENSG00000185904 -0.14046 0.55585 1.4733 0.070333 -0.2997
0.6178 1.2435 0.10685 0.070333 0.534965362
NA ENSG00000279803 1.4733 0.070342 1.4733 0.070342 1.4733 0.070342
1.4733 0.070342 0.070342 0.534965362
XRCC6P3 ENSG00000230318 1.4732 0.070348 1.4732 0.070348 0.95056
0.17091 1.4732 0.070348 0.070348 0.534965362
RPL34P21 ENSG00000244451 1.1478 0.12554 1.1478 0.12554 1.4732
0.070348 1.1478 0.12554 0.070348 0.534965362
SSU72P5 ENSG00000284018 1.4729 0.070386 -1.0364 0.84998 -0.018234
0.50727 0.045299 0.48193 0.070386 0.535118449
SNORD58C ENSG00000202093 1.4728 0.070405 1.4728 0.070405 1.4728
0.070405 1.4728 0.070405 0.070405 0.535118449
NUTM2A ENSG00000184923 0.84981 0.19772 1.4727 0.07041 0.43052
0.33341 0.2191 0.41328 0.07041 0.535118449
OR1D2 ENSG00000184166 -0.95729 0.83079 -0.94118 0.82669 1.4726 0.070427
-1.0064 0.84289 0.070427 0.535118449
NA ENSG00000273367 1.4726 0.070429 1.4726 0.070429 1.4726 0.070429
1.4726 0.070429 0.070429 0.535118449
RNU6-900P ENSG00000207227 1.4725 0.070444 1.4725 0.070444 1.4725
0.070444 1.4725 0.070444 0.070444 0.535118449
NA ENSG00000267769 1.4725 0.070445 1.4725 0.070445 1.4725 0.070445
1.4725 0.070445 0.070445 0.535118449

NA	ENSG00000278254	-0.58909	0.7221	1.4723	0.070475	-0.58909	0.7221	-0.79392	0.78638	0.070475	0.535164431
MAP1LC3C	ENSG00000197769	0.76469	0.22223	1.4722	0.07048	0.80949	0.20912	-1.0174	0.84553	0.07048	0.535164431
NA	ENSG00000249200	1.4722	0.070484	1.4722	0.070484	1.4722	0.070484	1.4722	0.070484	0.070484	0.535164431
PTPRR	ENSG00000153233	-0.31861	0.62499	0.88447	0.18822	-0.59383	0.72369	1.472	0.070508	0.070508	0.535249289
DAZAP1	ENSG00000071626	0.32965	0.37083	0.67617	0.24947	0.32965	0.37083	1.4718	0.070536	0.070536	0.535249289
ZMYND8	ENSG00000101040	1.4717	0.070548	1.2131	0.11256	-0.35283	0.63789	1.0667	0.14305	0.070548	0.535249289
SETDB1	ENSG00000143379	1.385	0.083034	1.3296	0.091823	1.4716	0.070566	0.12969	0.44841	0.070566	0.535249289
HNRNPKP3	ENSG00000251557	0.77116	0.22031	0.68529	0.24658	1.4716	0.070566	0.77116	0.22031	0.070566	0.535249289
FAM174B	ENSG00000185442	-0.16717	0.56638	1.4715	0.070571	-0.16717	0.56638	0.37856	0.35251	0.070571	0.535249289
METTL15	ENSG00000169519	0.83164	0.20281	0.0088082	0.49649	1.4713	0.070602	-0.18213	0.57226	0.070602	0.535249289
NA	ENSG00000241411	1.4713	0.070606	-1.2212	0.88899	-1.2212	0.88899	-1.2212	0.88899	0.070606	0.535249289
NA	ENSG00000207384	1.4712	0.070613	1.4712	0.070613	0.53195	0.29738	0.23938	0.40541	0.070613	0.535249289
ZNF420	ENSG00000197050	0.56762	0.28515	1.4712	0.070616	-0.14381	0.55718	0.87981	0.18948	0.070616	0.535249289
TRMT112P3	ENSG00000265262	-0.16599	0.56592	1.4712	0.070616	-0.16599	0.56592	-0.16599	0.56592	0.070616	0.535249289
GNRH1	ENSG00000147437	1.4708	0.070671	-0.71912	0.76397	-0.71912	0.76397	-0.15224	0.5605	0.070671	0.535545124
TRIM40	ENSG00000204614	1.4707	0.07068	1.4707	0.07068	1.4707	0.07068	0.39702	0.34568	0.07068	0.535545124
CCDC169	ENSG00000242715	1.4707	0.070688	0.74423	0.22837	-0.3985	0.65487	-0.53152	0.70247	0.070688	0.535545124
RN7SKP12	ENSG00000252084	0.096163	0.4617	1.4706	0.070701	0.46206	0.32202	0.86984	0.19219	0.070701	0.53556035
ARHGEF7	ENSG00000102606	1.4705	0.070717	-0.58577	0.72098	-0.76773	0.77868	-0.63304	0.73665	0.070717	0.535598292
NA	ENSG00000270713	1.4702	0.070753	-1.0277	0.84796	0.65376	0.25663	-0.8223	0.79455	0.070753	0.535787675
SFMBT2	ENSG00000198879	1.3173	0.09387	1.47	0.070781	1.0795	0.14019	1.2292	0.1095	0.070781	0.535916428
NA	ENSG00000204717	0.74203	0.22903	0.40496	0.34275	1.2635	0.10321	1.4699	0.070798	0.070798	0.535961867
LINC01995	ENSG00000244247	1.3768	0.084291	1.4697	0.070823	0.6325	0.26353	-0.33938	0.63284	0.070823	0.536067846
RPL7AP4	ENSG00000213641	0.78156	0.21724	0.78156	0.21724	1.4696	0.070839	1.4696	0.070839	0.070839	0.536105679
MIR4257	ENSG00000264553	1.4694	0.070858	1.4694	0.070858	1.4694	0.070858	1.4694	0.070858	0.070858	0.536166202
SPRR2F	ENSG00000244094	1.4693	0.070872	-1.3981	0.91896	-0.31316	0.62292	-0.31316	0.62292	0.070872	0.536188878
NA	ENSG00000266814	1.4692	0.070892	-0.40454	0.65709	1.0705	0.14219	-0.49949	0.69128	0.070892	0.536248791
CALM1P2	ENSG00000230564	1.4687	0.070959	1.4687	0.070959	-0.32709	0.6282	0.1787	0.42909	0.070959	0.536248791
ID3	ENSG00000117318	1.4686	0.070974	-0.25442	0.60041	1.4686	0.070974	1.4686	0.070974	0.070974	0.536248791
SEC22C	ENSG00000093183	-0.5521	0.70956	1.4685	0.070982	1.4685	0.070982	1.2516	0.10536	0.070982	0.536248791
NA	ENSG00000271027	1.4684	0.071	1.4684	0.071	1.4684	0.071	1.4684	0.071	0.071	0.536248791

MIR302D	ENSG00000199145	1.4683	0.071007	1.4683	0.071007	1.4683	0.071007	1.4683	0.071007	0.071007	0.536248791
MIR367	ENSG00000199169	1.4683	0.071007	1.4683	0.071007	1.4683	0.071007	1.4683	0.071007	0.071007	0.536248791
RNU6-1144P	ENSG00000206974	1.4683	0.071013	1.4683	0.071013	1.4683	0.071013	1.4683	0.071013	0.071013	0.536248791
RNU1-130P	ENSG00000200800	-0.50516	0.69328	1.4392	0.075049	0.72496	0.23424	1.4683	0.071016	0.071016	0.536248791
KLF4P1	ENSG00000233186	0.55828	0.28833	1.4683	0.071018	0.39006	0.34825	1.4683	0.071018	0.071018	0.536248791
LINC02385	ENSG00000257193	-0.75483	0.77482	-0.24296	0.59598	-0.4583	0.67663	1.4681	0.071045	0.071045	0.536248791
PRODH	ENSG00000100033	1.468	0.071057	0.58897	0.27794	1.0352	0.15029	0.56588	0.28574	0.071057	0.536248791
NA	ENSG00000280878	0.84365	0.19943	1.468	0.071057	0.65159	0.25733	0.65159	0.25733	0.071057	0.536248791
MRPS5P3	ENSG00000214743	0.014998	0.49402	0.014998	0.49402	1.4679	0.071063	-0.081559	0.5325	0.071063	0.536248791
NA	ENSG00000254614	1.4679	0.071063	1.4679	0.071063	1.4679	0.071063	1.4679	0.071063	0.071063	0.536248791
TCP11L2	ENSG00000166046	1.4679	0.071071	1.2599	0.10385	1.4092	0.079381	1.4132	0.078799	0.071071	0.536248791
DPYD-AS2	ENSG00000235777	-0.59661	0.72462	1.4678	0.071075	-0.16274	0.56464	-0.59661	0.72462	0.071075	0.536248791
PPP1R8	ENSG00000117751	1.1869	0.11763	1.3909	0.082125	0.73144	0.23225	1.4678	0.071078	0.071078	0.536248791
NA	ENSG00000221455	1.4677	0.071094	1.2581	0.10418	1.394	0.081658	1.4677	0.071094	0.071094	0.536263554
ASS1	ENSG00000130707	0.8407	0.20026	1.4676	0.071104	0.92623	0.17716	0.78568	0.21603	0.071104	0.536263554
NA	ENSG00000234837	0.63837	0.26162	0.90038	0.18396	1.4674	0.071127	1.4468	0.073976	0.071127	0.536263554
ASPG	ENSG00000166183	0.61055	0.27075	1.4673	0.071149	0.62521	0.26592	1.1673	0.12155	0.071149	0.536263554
LINC00350	ENSG00000225316	1.4673	0.071153	-2.2872	0.98891	-2.2872	0.98891	-0.83607	0.79844	0.071153	0.536263554
MYHAS	ENSG00000272975	1.4672	0.071163	0.40969	0.34102	-0.11891	0.54733	0.86623	0.19318	0.071163	0.536263554
NA	ENSG00000239219	-1.132	0.87119	1.4672	0.071167	-1.132	0.87119	-1.132	0.87119	0.071167	0.536263554
NA	ENSG00000272506	1.4671	0.071178	1.4671	0.071178	1.4671	0.071178	1.4671	0.071178	0.071178	0.536263554
UGT2B11	ENSG00000213759	-0.68111	0.7521	0.50318	0.30742	-0.51394	0.69635	1.4671	0.071179	0.071179	0.536263554
MYH2	ENSG00000125414	0.70393	0.24074	1.1205	0.13126	1.4669	0.071201	1.1205	0.13126	0.071201	0.53631616
RN7SKP237	ENSG00000201533	0.81432	0.20773	-0.4489	0.67325	1.4668	0.071217	0.89106	0.18645	0.071217	0.53631616
ECEL1P1	ENSG00000204121	-0.36552	0.64264	0.55901	0.28808	0.55582	0.28917	1.4668	0.071219	0.071219	0.53631616
NA	ENSG00000253908	1.4658	0.071348	0.22296	0.41178	0.22296	0.41178	0.22296	0.41178	0.071348	0.537106033
SHQ1	ENSG00000144736	1.4658	0.07135	0.49909	0.30886	1.1961	0.11582	0.19699	0.42192	0.07135	0.537106033
CENPS	ENSG00000175279	1.4657	0.071367	0.048084	0.48082	1.206	0.11391	0.24679	0.40254	0.071367	0.537106033
RNA5SP447	ENSG00000200619	0.26866	0.39409	0.26866	0.39409	1.4656	0.071375	0.26866	0.39409	0.071375	0.537106033
CIAO3	ENSG00000103245	-1.1318	0.87114	1.4656	0.071379	-1.4362	0.92453	-0.25633	0.60115	0.071379	0.537106033
DEFA9P	ENSG00000233238	1.4655	0.071397	0.50003	0.30853	0.81091	0.20871	0.81091	0.20871	0.071397	0.537158532

NA	ENSG00000283525	-1.4313	0.92383	0.59043	0.27745	-0.87933	0.81039	1.4653	0.071421	0.071421	0.537180569
NA	ENSG00000241073	1.4653	0.071426	1.4653	0.071426	1.4653	0.071426	1.4653	0.071426	0.071426	0.537180569
PPP1R2P1	ENSG00000234515	-0.18426	0.5731	-0.62841	0.73513	0.47964	0.31574	1.4652	0.071433	0.071433	0.537180569
GMPS	ENSG00000163655	1.2392	0.10763	1.3874	0.082659	1.3874	0.082659	1.4651	0.071448	0.071448	0.537210468
RNA5SP31	ENSG00000223076	1.465	0.071464	0.36812	0.35639	1.465	0.071464	1.465	0.071464	0.071464	0.537247874
NA	ENSG00000237606	0.0081993	0.49673	1.4648	0.071484	-0.12157	0.54838	0.0081993	0.49673	0.071484	0.537277175
NA	ENSG00000254777	-0.63758	0.73813	-0.63758	0.73813	-0.63758	0.73813	1.4647	0.0715	0.0715	0.537277175
EIF4BP2	ENSG00000228753	0.72675	0.23369	1.4646	0.07151	1.4646	0.07151	1.4646	0.07151	0.07151	0.537277175
FAM83A-AS1	ENSG00000204949	1.2202	0.1112	1.4646	0.071512	1.2202	0.1112	1.2202	0.1112	0.071512	0.537277175
NA	ENSG00000248747	0.19279	0.42356	0.62685	0.26538	1.4643	0.071551	0.62685	0.26538	0.071551	0.537419496
H3P2	ENSG00000237279	1.4643	0.071553	1.4643	0.071553	1.4643	0.071553	1.4643	0.071553	0.071553	0.537419496
NAPG	ENSG00000134265	1.386	0.082878	0.78866	0.21515	1.0984	0.13602	1.4642	0.07157	0.07157	0.537437689
CARD19	ENSG00000165233	0.037496	0.48504	-1.7073	0.95612	1.4641	0.071583	-0.21733	0.58602	0.071583	0.537437689
NA	ENSG00000215899	0.66214	0.25394	0.66214	0.25394	1.4639	0.071604	0.66214	0.25394	0.071604	0.537437689
TLR5	ENSG00000187554	1.4637	0.071632	1.304	0.096111	0.51242	0.30418	0.94323	0.17278	0.071632	0.537437689
NA	ENSG00000266135	-0.44875	0.67319	0.71149	0.23839	1.2373	0.10799	1.4637	0.071636	0.071636	0.537437689
DEFB1	ENSG00000164825	1.4469	0.073964	0.28238	0.38882	1.4636	0.071646	1.4116	0.079029	0.071646	0.537437689
OR7E25P	ENSG00000187847	-0.11612	0.54622	0.44484	0.32822	0.39039	0.34812	1.4635	0.071669	0.071669	0.537437689
NA	ENSG00000248288	1.4635	0.071672	1.4635	0.071672	1.4635	0.071672	1.4635	0.071672	0.071672	0.537437689
NA	ENSG00000280936	1.4633	0.07169	1.4633	0.07169	1.4633	0.07169	1.4633	0.07169	0.07169	0.537437689
KIF3A	ENSG00000131437	0.91475	0.18016	1.3463	0.089099	1.4633	0.071693	-1.0553	0.85436	0.071693	0.537437689
NA	ENSG00000235119	-0.20972	0.58306	1.0978	0.13615	1.4632	0.071707	1.4632	0.071707	0.071707	0.537437689
LRRC77P	ENSG00000244227	1.4632	0.071713	-0.49276	0.68891	-1.6164	0.947	-1.1188	0.86838	0.071713	0.537437689
NA	ENSG00000272918	1.463	0.071736	1.463	0.071736	1.463	0.071736	1.463	0.071736	0.071736	0.537437689
POSTN	ENSG00000133110	0.8189	0.20642	1.4629	0.071741	0.33324	0.36948	1.2501	0.10563	0.071741	0.537437689
NA	ENSG00000225563	0.9192	0.17899	1.4629	0.071746	1.4629	0.071746	1.4629	0.071746	0.071746	0.537437689
RN7SL577P	ENSG00000265942	1.4628	0.071757	1.4628	0.071757	1.4628	0.071757	1.4628	0.071757	0.071757	0.537437689
MYL2	ENSG00000111245	1.4628	0.071764	-1.7151	0.95684	0.12794	0.4491	-0.65934	0.74516	0.071764	0.537437689
NA	ENSG00000264634	1.4627	0.071773	1.0218	0.15345	1.0218	0.15345	1.0218	0.15345	0.071773	0.537437689
NA	ENSG00000254467	1.4627	0.071775	0.73566	0.23097	0.58273	0.28004	0.19957	0.42091	0.071775	0.537437689
ZNF890P	ENSG00000159904	-0.71938	0.76405	-1.0644	0.85642	0.59997	0.27426	1.4627	0.071776	0.071776	0.537437689

CLEC16A	ENSG00000038532	1.4623	0.07183	-0.51424	0.69646	-0.47272	0.68179	-1.0474	0.85254	0.07183	0.537638621
NA	ENSG00000270610	1.4622	0.071848	0.25531	0.39924	0.73231	0.23199	0.61863	0.26808	0.071848	0.537638621
SLC49A3	ENSG00000169026	1.4621	0.071852	0.91753	0.17943	1.0712	0.14204	-1.5226	0.93607	0.071852	0.537638621
MIR5089	ENSG00000264999	1.4621	0.071857	1.4621	0.071857	1.4621	0.071857	1.4621	0.071857	0.071857	0.537638621
NA	ENSG00000270178	-0.11356	0.54521	1.4621	0.071858	1.4621	0.071858	1.4621	0.071858	0.071858	0.537638621
MTFMT	ENSG00000103707	1.4619	0.071889	1.1401	0.12712	1.4619	0.071889	-0.093109	0.53709	0.071889	0.53778799
EHBP1	ENSG00000115504	0.46724	0.32016	1.1782	0.11936	0.9752	0.16473	1.4617	0.071916	0.071916	0.537889582
RPL22P19	ENSG00000241129	1.4615	0.071939	1.4615	0.071939	1.4615	0.071939	-0.93966	0.8263	0.071939	0.537889582
RHOC	ENSG00000155366	1.4613	0.071971	0.19217	0.4238	-0.59952	0.72559	1.2636	0.10319	0.071971	0.537889582
RNU7-23P	ENSG00000239099	1.4612	0.071976	-0.41948	0.66257	0.15927	0.43673	0.13023	0.44819	0.071976	0.537889582
EDNRA	ENSG00000151617	-0.29843	0.61731	1.4612	0.071981	0.040748	0.48375	0.3588	0.35987	0.071981	0.537889582
NA	ENSG00000229462	1.4611	0.071993	1.3952	0.081482	0.53163	0.29749	-1.2123	0.8873	0.071993	0.537889582
ZNF775	ENSG00000196456	1.4611	0.071997	1.4611	0.071997	1.4611	0.071997	1.4577	0.072465	0.071997	0.537889582
ELK2BP	ENSG00000234402	0.27292	0.39246	1.4609	0.072019	1.4609	0.072019	0.5384	0.29515	0.072019	0.537889582
KIF9-AS1	ENSG00000227398	-1.3857	0.91707	-1.1149	0.86756	-1.105	0.86542	1.4609	0.072021	0.072021	0.537889582
NA	ENSG00000239891	1.4609	0.072023	1.4609	0.072023	1.4609	0.072023	0.35678	0.36063	0.072023	0.537889582
OBI1	ENSG00000152193	1.4609	0.072024	-0.90264	0.81664	0.25847	0.39802	-0.093161	0.53711	0.072024	0.537889582
GALNT8	ENSG00000130035	1.196	0.11585	0.47546	0.31723	-0.92387	0.82222	1.4608	0.072041	0.072041	0.537934099
LINC02395	ENSG00000257771	0.10768	0.45712	0.47974	0.31571	1.4606	0.072062	-0.57507	0.71738	0.072062	0.537946977
NFKBIB	ENSG00000104825	1.4605	0.072077	1.4605	0.072077	1.4605	0.072077	1.4605	0.072077	0.072077	0.537946977
NA	ENSG00000264388	-0.009195	0.50367	1.4605	0.072083	1.4605	0.072083	1.3071	0.095585	0.072083	0.537946977
IMMP2L	ENSG00000184903	0.60944	0.27112	1.4603	0.07211	-0.34489	0.63491	-0.33014	0.62935	0.07211	0.537946977
NA	ENSG00000238813	1.4602	0.072115	1.2645	0.10303	1.4602	0.072115	1.4602	0.072115	0.072115	0.537946977
KDM4C	ENSG00000107077	1.063	0.14389	0.86882	0.19247	0.35714	0.36049	1.4602	0.072118	0.072118	0.537946977
FCF1	ENSG00000119616	-1.5434	0.93863	-0.57146	0.71615	1.4602	0.07212	0.24351	0.4038	0.07212	0.537946977
LINC00163	ENSG00000234880	-2.1432	0.98395	-0.99095	0.83915	-1.4001	0.91926	1.46	0.07215	0.07215	0.538013198
CNTN5	ENSG00000149972	-0.63621	0.73768	1.4599	0.072155	1.0521	0.14637	0.63614	0.26234	0.072155	0.538013198
SNAP91	ENSG00000065609	0.34616	0.36461	-0.76239	0.77709	0.46386	0.32138	1.4599	0.072162	0.072162	0.538013198
TMEM68	ENSG00000167904	0.1036	0.45874	1.4598	0.072174	0.98706	0.16181	0.98706	0.16181	0.072174	0.538020349
LINC02622	ENSG00000259267	-1.9243	0.97284	-1.5835	0.94334	1.4597	0.072189	-0.082905	0.53304	0.072189	0.538049858
LSP1P1	ENSG00000269099	0.89644	0.18501	1.2796	0.10035	0.8912	0.18641	1.4594	0.072226	0.072226	0.538100764

TBX21	ENSG00000073861	0.14172	0.44365	0.3349	0.36885	0.14172	0.44365	1.4594	0.072234	0.072234	0.538100764
NA	ENSG00000247867	0.91811	0.17928	-0.83584	0.79838	0.078824	0.46859	1.4593	0.072237	0.072237	0.538100764
LDHBP3	ENSG00000248950	1.4593	0.07224	0.88893	0.18702	1.4593	0.07224	1.4593	0.07224	0.07224	0.538100764
NA	ENSG00000271730	1.4592	0.072254	1.3582	0.087202	1.4592	0.072254	1.4592	0.072254	0.072254	0.538122791
HSPA13	ENSG00000155304	0.23358	0.40766	1.459	0.072278	0.29272	0.38487	0.23358	0.40766	0.072278	0.538178104
ZNF662	ENSG00000182983	1.4589	0.0723	1.2287	0.1096	1.2287	0.1096	-0.23256	0.59195	0.0723	0.538178104
PERCC1	ENSG00000284395	0.66659	0.25252	0.37701	0.35308	1.4589	0.0723	1.4589	0.0723	0.0723	0.538178104
NA	ENSG00000271427	1.4588	0.072309	1.4588	0.072309	1.4588	0.072309	1.4588	0.072309	0.072309	0.538178104
SLC13A1	ENSG00000081800	1.4587	0.07233	0.029281	0.48832	0.5291	0.29837	0.032883	0.48688	0.07233	0.538178104
AQP6	ENSG00000086159	-0.58514	0.72077	-0.52898	0.70159	1.4585	0.072355	0.15474	0.43851	0.072355	0.538178104
NA	ENSG00000270890	1.4584	0.072366	1.4584	0.072366	1.4584	0.072366	1.4584	0.072366	0.072366	0.538178104
NA	ENSG00000232663	-0.39113	0.65215	1.4583	0.072381	-0.29197	0.61485	0.54657	0.29234	0.072381	0.538178104
NA	ENSG00000233825	1.4583	0.072383	1.4583	0.072383	1.4583	0.072383	1.4583	0.072383	0.072383	0.538178104
RNU6-182P	ENSG00000252779	1.4583	0.072383	1.4583	0.072383	1.4583	0.072383	1.4583	0.072383	0.072383	0.538178104
NA	ENSG00000279121	1.0812	0.13981	1.4582	0.072397	0.52578	0.29952	1.4582	0.072397	0.072397	0.538178104
MIR571	ENSG00000207642	1.4582	0.0724	0.65095	0.25754	0.65095	0.25754	0.9802	0.16349	0.0724	0.538178104
ULBP2	ENSG00000131015	0.42458	0.33557	-0.6972	0.75716	0.98616	0.16203	1.4581	0.072405	0.072405	0.538178104
NA	ENSG00000161583	1.4578	0.072447	-1.6386	0.94935	-1.8156	0.96528	-0.12537	0.54989	0.072447	0.538254437
ARL4AP4	ENSG00000231395	0.90028	0.18399	1.4576	0.072481	0.84257	0.19974	0.84257	0.19974	0.072481	0.538254437
DDX20	ENSG00000064703	0.58585	0.27899	1.1506	0.12494	1.4575	0.072487	0.19606	0.42228	0.072487	0.538254437
MEGF6	ENSG00000162591	0.52286	0.30054	-0.057085	0.52276	-0.63866	0.73848	1.4575	0.072494	0.072494	0.538254437
LINC00487	ENSG00000205837	0.50471	0.30688	1.4574	0.072501	0.16972	0.43262	0.99689	0.15941	0.072501	0.538254437
CEACAMP6	ENSG00000238092	-0.61205	0.72975	1.4573	0.072518	-0.60409	0.72711	0.26988	0.39363	0.072518	0.538254437
CLEC4GP1	ENSG00000268297	1.4572	0.072526	-0.10977	0.5437	1.4572	0.072526	-0.82489	0.79528	0.072526	0.538254437
WASHC2C	ENSG00000172661	0.49509	0.31027	0.53409	0.29664	1.4572	0.072537	0.55677	0.28884	0.072537	0.538254437
TLE2	ENSG00000065717	1.4571	0.072547	1.2647	0.10298	0.19882	0.4212	-0.97891	0.83619	0.072547	0.538254437
NA	ENSG00000267320	0.48699	0.31313	-0.92841	0.8234	1.457	0.072552	-0.66769	0.74783	0.072552	0.538254437
IFNAR2	ENSG00000159110	-0.39252	0.65266	-0.39252	0.65266	-0.85041	0.80245	1.457	0.072554	0.072554	0.538254437
POGZ	ENSG00000143442	1.4569	0.072571	0.53558	0.29613	0.47794	0.31635	-0.041117	0.5164	0.072571	0.538254437
NA	ENSG00000261532	1.4569	0.072574	1.4569	0.072574	1.4569	0.072574	1.4569	0.072574	0.072574	0.538254437
DAPK3	ENSG00000167657	0.73313	0.23174	-0.40659	0.65784	0.38429	0.35038	1.4568	0.072583	0.072583	0.538254437

NA ENSG00000227958 1.4568 0.072584 0.49657 0.30975 -0.12066 0.54802
-1.6679 0.95233 0.072584 0.538254437
NA ENSG00000267009 1.4568 0.072592 -1.1256 0.86982 -0.83649 0.79856
-0.48276 0.68537 0.072592 0.538254437
ABCC6 ENSG00000091262 1.4566 0.072613 -0.73605 0.76915 0.77212 0.22002
-1.324 0.90724 0.072613 0.538328235
SP7 ENSG00000170374 1.4565 0.072632 1.2308 0.10919 0.35418 0.3616
0.76513 0.2221 0.072632 0.538364593
NA ENSG00000242262 1.4564 0.07264 0.61853 0.26811 1.4564 0.07264
1.4564 0.07264 0.07264 0.538364593
FXYD6P1 ENSG00000270762 1.4563 0.072653 -1.2318 0.89099 -0.0047107
0.50188 0.88269 0.1887 0.072653 0.538379059
NA ENSG00000218631 0.85688 0.19575 1.456 0.07269 0.85688 0.19575
0.68703 0.24603 0.07269 0.53857134
NA ENSG00000240573 0.40691 0.34204 0.40691 0.34204 1.456 0.072702
-0.01698 0.50677 0.072702 0.538578361
CTSS ENSG00000163131 -0.19806 0.5785 -0.21466 0.58498 1.4559 0.072716
1.2885 0.098778 0.072716 0.538584992
NA ENSG00000279342 -0.11655 0.54639 1.4558 0.072725 -0.64664 0.74107
-0.64664 0.74107 0.072725 0.538584992
NA ENSG00000248240 1.4557 0.072738 1.4557 0.072738 1.4557 0.072738
1.4557 0.072738 0.072738 0.538599413
NA ENSG00000273472 1.4554 0.072775 1.4554 0.072775 1.4554 0.072775
1.4554 0.072775 0.072775 0.538664454
CCDC192 ENSG00000230561 0.77267 0.21986 1.1739 0.12021 -0.024791
0.50989 1.4554 0.072781 0.072781 0.538664454
NA ENSG00000279737 1.4553 0.07279 0.59932 0.27448 0.59932 0.27448
0.59932 0.27448 0.07279 0.538664454
FAM187B2P ENSG00000262497 0.88224 0.18882 1.4553 0.072791 0.88224
0.18882 0.88224 0.18882 0.072791 0.538664454
LINC00430 ENSG00000233528 -0.78204 0.78291 0.067362 0.47315 0.067362
0.47315 1.4548 0.072857 0.072857 0.538688799
GATA3-AS1 ENSG00000197308 -0.64397 0.7402 0.97909 0.16377 1.4546
0.072886 1.4546 0.072886 0.072886 0.538688799
NA ENSG00000227741 1.4545 0.072905 1.4545 0.072905 1.4545 0.072905
1.4545 0.072905 0.072905 0.538688799
NA ENSG00000238481 1.4545 0.072909 1.4545 0.072909 1.4545 0.072909
1.4545 0.072909 0.072909 0.538688799
LINC02820 ENSG00000258815 1.4544 0.072912 1.4544 0.072912 1.4544
0.072912 1.0212 0.15357 0.072912 0.538688799
DEFB109F ENSG00000244050 1.4543 0.072933 1.3893 0.082367 0.25031
0.40118 0.25031 0.40118 0.072933 0.538688799
DDIT4 ENSG00000168209 1.4543 0.072938 0.036335 0.48551 -0.23935 0.59458
1.4543 0.072938 0.072938 0.538688799
NA ENSG00000201003 1.4542 0.07294 1.4542 0.07294 1.4542 0.07294
1.4542 0.07294 0.07294 0.538688799
MMAB ENSG00000139428 1.1868 0.11765 1.4542 0.072946 1.4542 0.072946
1.4542 0.072946 0.072946 0.538688799
ELMO1-AS1 ENSG00000224101 -1.2101 0.88688 0.067994 0.4729 1.4541
0.072958 -0.11068 0.54406 0.072958 0.538688799
NA ENSG00000239216 1.4541 0.07296 1.4541 0.07296 1.4541 0.07296
1.4541 0.07296 0.07296 0.538688799
RN7SKP76 ENSG00000201289 1.454 0.07297 1.454 0.07297 1.454 0.07297 1.454
0.07297 0.07297 0.538688799
SEC61A1 ENSG00000058262 0.32492 0.37262 1.3914 0.082047 1.4538 0.073
-0.74099 0.77065 0.073 0.538688799
RNU6-850P ENSG00000252743 1.4535 0.073038 1.4535 0.073038 1.4535
0.073038 1.4535 0.073038 0.073038 0.538688799
LINC01544 ENSG00000260440 1.4534 0.07305 1.4534 0.07305 1.4534
0.07305 1.4534 0.07305 0.07305 0.538688799
NA ENSG00000229459 0.80032 0.21176 -0.35491 0.63867 0.57209 0.28363
1.4534 0.073061 0.073061 0.538688799

NA	ENSG00000280029	1.4534	0.073061	1.4534	0.073061	1.4534	0.073061	1.4534	0.073061	0.073061	0.538688799
KLF2	ENSG00000127528	1.4531	0.073103	1.4531	0.073103	0.049027	0.48045	1.4531	0.073103	0.073103	0.538688799
H2BC21	ENSG00000184678	1.4531	0.073103	1.3841	0.083168	1.4531	0.073103	1.4531	0.073103	0.073103	0.538688799
NA	ENSG00000240441	1.2688	0.10226	1.453	0.073109	0.86174	0.19441	-0.092318	0.53678	0.073109	0.538688799
NA	ENSG00000237617	0.34913	0.3635	0.68695	0.24606	1.3111	0.09491	1.453	0.073114	0.073114	0.538688799
SNORD116-27	ENSG00000251896	1.453	0.073115	1.453	0.073115	1.453	0.073115	1.453	0.073115	0.073115	0.538688799
ABHD17A	ENSG00000129968	0.45917	0.32306	1.4527	0.07315	0.14357	0.44292	0.24555	0.40302	0.07315	0.538688799
NA	ENSG00000260620	-0.38864	0.65123	0.20428	0.41907	-0.11407	0.54541	1.4526	0.07316	0.07316	0.538688799
OR10Y1P	ENSG00000254403	1.4526	0.073163	1.4526	0.073163	1.3346	0.091001	0.87273	0.19141	0.073163	0.538688799
LINC01679	ENSG00000237989	0.024639	0.49017	0.024639	0.49017	0.81678	0.20703	1.4525	0.073183	0.073183	0.538688799
EPHB2	ENSG00000133216	1.3408	0.089986	1.4524	0.073198	-0.1299	0.55168	0.40124	0.34412	0.073198	0.538688799
RNU6-1204P	ENSG00000200818	1.4523	0.073209	1.4523	0.073209	0.46552	0.32078	1.4523	0.073209	0.073209	0.538688799
RN7SL461P	ENSG00000243801	1.4523	0.073209	1.4523	0.073209	1.4523	0.073209	1.4523	0.073209	0.073209	0.538688799
NME9	ENSG00000181322	0.74938	0.22681	-0.03826	0.51526	0.74938	0.22681	1.4523	0.073214	0.073214	0.538688799
NA	ENSG00000232949	1.4521	0.073232	-0.25282	0.5998	0.0086053	0.49657	-1.1324	0.87126	0.073232	0.538688799
NA	ENSG00000223679	1.4521	0.073234	0.17225	0.43162	1.4521	0.073234	1.4521	0.073234	0.073234	0.538688799
PCDH20	ENSG00000280165	-1.5402	0.93824	0.94993	0.17107	1.4521	0.073241	1.4521	0.073241	0.073241	0.538688799
NA	ENSG00000265637	1.4518	0.07328	1.4518	0.07328	1.4518	0.07328	1.4518	0.07328	0.07328	0.538688799
LINC01195	ENSG00000261075	0.19033	0.42453	-0.78152	0.78275	1.4518	0.073284	-0.94404	0.82743	0.073284	0.538688799
RNU6-865P	ENSG00000201642	1.4516	0.073309	0.7264	0.2338	1.4516	0.073309	1.4516	0.073309	0.073309	0.538688799
COL9A3	ENSG00000092758	0.14216	0.44348	0.31262	0.37729	0.50597	0.30644	1.4516	0.073311	0.073311	0.538688799
ATP11B	ENSG00000058063	0.10475	0.45829	1.4516	0.073312	0.2554	0.39921	1.3518	0.088222	0.073312	0.538688799
NA	ENSG00000251665	1.4515	0.073322	1.4515	0.073322	1.4515	0.073322	1.4515	0.073322	0.073322	0.538688799
ERGIC1	ENSG00000113719	1.4193	0.077907	1.4515	0.073324	1.4513	0.073353	0.020684	0.49175	0.073324	0.538688799
NA	ENSG00000251314	-0.2074	0.58215	0.66752	0.25222	1.4515	0.073327	0.634	0.26304	0.073327	0.538688799
NA	ENSG00000200630	1.4514	0.073334	1.4514	0.073334	1.4514	0.073334	1.4514	0.073334	0.073334	0.538688799
PPP3CA	ENSG00000138814	1.4512	0.073364	0.28997	0.38592	0.30048	0.3819	-0.60662	0.72795	0.073364	0.538688799
CHRM3-AS1	ENSG00000234601	1.4511	0.073373	0.71056	0.23868	0.16821	0.43321	0.71056	0.23868	0.073373	0.538688799
LINC02803	ENSG00000227740	1.4511	0.073375	1.4511	0.073375	1.4511	0.073375	1.4511	0.073375	0.073375	0.538688799
NA	ENSG00000272675	1.451	0.073384	1.451	0.073384	1.451	0.073384	1.451	0.073384	0.073384	0.538688799
CEP20	ENSG00000133393	1.3361	0.090755	1.4509	0.073397	1.4158	0.07842	1.3887	0.082456	0.073397	0.538688799

TSPYL1	ENSG00000189241	0.38044	0.35181	1.1399	0.12717	1.4509	0.073407	0.68649	0.2462	0.073407	0.538688799
NA	ENSG00000225106	0.10238	0.45923	0.36578	0.35727	0.36578	0.35727	1.4508	0.073424	0.073424	0.538688799
RN7SL18P	ENSG00000241625	1.4362	0.075479	1.4507	0.073425	1.0886	0.13817	1.4507	0.073425	0.073425	0.538688799
SHANK1	ENSG00000161681	1.4507	0.073429	0.071628	0.47145	1.4507	0.073429	1.421	0.077652	0.073429	0.538688799
COL4A1	ENSG00000187498	0.20535	0.41865	-1.2656	0.89717	1.4507	0.07343	-0.18367	0.57286	0.07343	0.538688799
RNA5SP492	ENSG00000223262	1.4507	0.073432	1.4507	0.073432	1.4507	0.073432	1.4507	0.073432	0.073432	0.538688799
RN7SL530P	ENSG00000239910	1.1354	0.12809	1.4505	0.073453	1.1469	0.12572	0.97411	0.165	0.073453	0.538688799
FAM83H	ENSG00000180921	1.4505	0.073461	1.4119	0.07899	0.13928	0.44461	0.4959	0.30998	0.073461	0.538688799
LYPLA2P3	ENSG00000236604	1.4504	0.073475	1.2347	0.10848	1.4504	0.073475	1.4504	0.073475	0.073475	0.538688799
CENPN	ENSG00000166451	1.0111	0.15599	1.4504	0.073477	1.2614	0.10358	1.4504	0.073477	0.073477	0.538688799
ADD2	ENSG00000075340	1.1545	0.12415	0.45304	0.32526	1.4504	0.073478	1.1158	0.13225	0.073478	0.538688799
BROX	ENSG00000162819	0.39249	0.34735	1.1006	0.13553	1.4503	0.073489	0.39249	0.34735	0.073489	0.538688799
TNS3	ENSG00000136205	1.4502	0.073498	0.17081	0.43219	0.46763	0.32002	0.49378	0.31073	0.073498	0.538688799
RPS18P9	ENSG00000220848	1.4502	0.073505	1.4502	0.073505	1.4502	0.073505	1.4502	0.073505	0.073505	0.538688799
RAPGEF4	ENSG00000091428	1.4501	0.073509	0.94583	0.17212	0.86859	0.19254	0.88905	0.18699	0.073509	0.538688799
NA	ENSG00000226622	1.0217	0.15345	1.4501	0.073518	0.50274	0.30757	0.66012	0.25459	0.073518	0.538688799
DECR1	ENSG00000104325	1.0743	0.14134	0.834	0.20214	1.2549	0.10476	1.45	0.073524	0.073524	0.538688799
NA	ENSG00000207480	1.45	0.073531	1.45	0.073531	1.45	0.073531	1.1894	0.11713	0.073531	0.538688799
RNU6-789P	ENSG00000252914	1.45	0.073531	0.94478	0.17239	1.0033	0.15785	0.88364	0.18844	0.073531	0.538688799
RN7SL65P	ENSG00000243770	1.4499	0.073536	1.4499	0.073536	-0.029278	0.51168	0.69174	0.24455	0.073536	0.538688799
HSD17B1P1	ENSG00000108785	1.0348	0.15039	1.4499	0.073546	0.20049	0.42055	1.2992	0.09694	0.073546	0.538688799
NA	ENSG00000270049	1.4497	0.073578	-0.80488	0.78955	0.30988	0.37832	-0.48832	0.68734	0.073578	0.538790512
NA	ENSG00000255348	1.4496	0.073582	1.4496	0.073582	1.4496	0.073582	1.4496	0.073582	0.073582	0.538790512
MIRLET7C	ENSG00000199030	0.66807	0.25205	0.66807	0.25205	0.66807	0.25205	1.4493	0.073628	0.073628	0.538859059
KCTD1	ENSG00000134504	0.79137	0.21436	0.85037	0.19756	1.4493	0.073631	0.94424	0.17252	0.073631	0.538859059
NA	ENSG00000238297	1.449	0.073664	1.449	0.073664	1.449	0.073664	1.449	0.073664	0.073664	0.538859059
ZNF204P	ENSG00000204789	-0.46421	0.67875	-0.66597	0.74729	1.4489	0.07369	1.4489	0.07369	0.07369	0.538859059
RN7SKP257	ENSG00000201395	1.1248	0.13034	1.4488	0.073693	1.4488	0.073693	0.34828	0.36381	0.073693	0.538859059
CELF5	ENSG00000161082	0.43462	0.33192	1.4488	0.073694	-0.64512	0.74057	-0.55016	0.7089	0.073694	0.538859059
SFTPB	ENSG00000168878	1.4488	0.073697	-0.77248	0.78008	-1.048	0.85269	0.13566	0.44604	0.073697	0.538859059
TTYH1	ENSG00000167614	-0.45323	0.67481	1.4488	0.073699	-0.45323	0.67481	-0.45323	0.67481	0.073699	0.538859059

CBLN3	ENSG00000139899	1.0615	0.14423	1.4487	0.073707	1.0615	0.14423	1.0615	0.14423	0.073707	0.538859059
SGSH	ENSG00000181523	1.4487	0.073707	1.4487	0.073707	1.4487	0.073707	1.4487	0.073707	0.073707	0.538859059
TNFSF4	ENSG00000117586	1.4487	0.073713	1.1806	0.11888	1.2145	0.11229	0.94943	0.1712	0.073713	0.538859059
CCDC172	ENSG00000182645	0.68944	0.24527	1.4484	0.073748	0.48583	0.31354	-0.2417	0.59549	0.073748	0.538982448
HIGD1AP18	ENSG00000253952	0.32585	0.37227	1.4484	0.073752	1.4484	0.073752	1.4484	0.073752	0.073752	0.538982448
ETV6	ENSG00000139083	1.4482	0.073779	-0.42052	0.66295	-0.81202	0.79161	1.2091	0.11332	0.073779	0.539098916
NA	ENSG00000202035	1.1121	0.13305	0.28837	0.38653	1.448	0.073804	-0.59505	0.72409	0.073804	0.539178348
NA	ENSG00000215567	0.3702	0.35562	0.5308	0.29778	1.448	0.073812	0.75985	0.22367	0.073812	0.539178348
KLF17	ENSG00000171872	-0.49593	0.69003	-0.49593	0.69003	1.4478	0.073832	-0.93635	0.82545	0.073832	0.539243609
TFAMP1	ENSG00000230444	1.4477	0.073847	0.23049	0.40885	0.92722	0.17691	0.40912	0.34123	0.073847	0.539272338
ZIC4	ENSG00000174963	1.4475	0.073883	-0.040925	0.51632	0.72341	0.23471	0.57812	0.28159	0.073883	0.53934457
NA	ENSG00000234773	1.4474	0.073892	0.67246	0.25065	0.7417	0.22913	1.4474	0.073892	0.073892	0.53934457
CCDC173	ENSG00000154479	1.4473	0.0739	1.4473	0.0739	1.4473	0.0739	1.4473	0.0739	0.0739	0.53934457
NA	ENSG00000279708	-0.19824	0.57857	0.29733	0.38311	1.4471	0.073933	1.4471	0.073933	0.073933	0.53934457
DNAH2	ENSG00000183914	0.70117	0.2416	0.13028	0.44817	1.4471	0.073937	-1.1659	0.87818	0.073937	0.53934457
MYH4	ENSG00000264424	0.21036	0.41669	1.3556	0.087618	1.4471	0.073938	1.2584	0.10413	0.073938	0.53934457
NA	ENSG00000259362	1.447	0.073944	0.68575	0.24644	1.2938	0.097866	1.4141	0.078669	0.073944	0.53934457
NAGK	ENSG00000124357	0.37883	0.35241	1.4467	0.073985	0.37883	0.35241	1.4102	0.079236	0.073985	0.53934457
NA	ENSG00000254951	0.52604	0.29943	1.4467	0.073986	-0.041784	0.51666	-0.041784	0.51666	0.073986	0.53934457
NUS1P2	ENSG00000234685	1.4467	0.073989	-0.47955	0.68423	-0.13901	0.55528	0.93294	0.17543	0.073989	0.53934457
NA	ENSG00000253526	0.66687	0.25243	0.66687	0.25243	0.58426	0.27952	1.4466	0.07401	0.07401	0.53934457
NA	ENSG00000249335	0.34393	0.36545	-0.92126	0.82154	0.57138	0.28387	1.4465	0.074025	0.074025	0.53934457
SPG21	ENSG00000090487	0.55909	0.28805	1.4463	0.074046	1.4463	0.074046	1.4463	0.074046	0.074046	0.53934457
CXADR	ENSG00000154639	-0.35704	0.63947	1.4463	0.074048	0.19032	0.42453	-0.21909	0.58671	0.074048	0.53934457
LGALS12	ENSG00000133317	1.4463	0.074051	0.7889	0.21509	0.7889	0.21509	-1.1682	0.87863	0.074051	0.53934457
PIAS2	ENSG00000078043	1.385	0.083028	1.2451	0.10654	1.4154	0.078477	1.4462	0.074066	0.074066	0.53934457
NAIPP4	ENSG00000251634	0.10351	0.45878	1.446	0.074091	0.045496	0.48186	0.23242	0.40811	0.074091	0.53934457
CNN2P11	ENSG00000237383	1.446	0.074093	-0.20275	0.58033	0.95805	0.16902	0.93892	0.17389	0.074093	0.53934457
NA	ENSG00000279176	1.4458	0.07412	1.4458	0.07412	1.4458	0.07412	1.4458	0.07412	0.07412	0.53934457
NA	ENSG00000239383	1.4457	0.074128	0.44085	0.32966	0.099584	0.46034	0.16361	0.43502	0.074128	0.53934457
NA	ENSG00000276925	-1.4185	0.92198	1.4456	0.07414	-1.4185	0.92198	-1.4185	0.92198	0.07414	0.53934457

RPL27P8	ENSG00000237041	1.4456	0.074145	1.4456	0.074145	1.4456	0.074145	1.4456	0.074145	0.074145	0.53934457
NA	ENSG00000224525	1.4455	0.074153	1.3806	0.083706	1.3806	0.083706	0.83727	0.20122	0.074153	0.53934457
NA	ENSG00000248858	1.1679	0.12142	0.51168	0.30444	0.43035	0.33347	1.4455	0.074159	0.074159	0.53934457
ZSCAN21	ENSG00000166529	1.1421	0.1267	0.8585	0.19531	-0.02689	0.51073	1.4455	0.074161	0.074161	0.53934457
LINC00513	ENSG00000233559	-0.63292	0.73661	-1.4957	0.93264	-0.63292	0.73661	1.4455	0.074164	0.074164	0.53934457
NA	ENSG00000223565	1.4454	0.074172	1.4454	0.074172	1.4454	0.074172	1.4454	0.074172	0.074172	0.53934457
NA	ENSG00000280274	1.4453	0.074189	0.52962	0.29819	1.4453	0.074189	1.4453	0.074189	0.074189	0.53934457
NA	ENSG00000259946	1.4453	0.074191	1.4453	0.074191	1.4453	0.074191	1.4453	0.074191	0.074191	0.53934457
LINC00410	ENSG00000231674	1.4452	0.074194	1.4452	0.074194	1.4452	0.074194	1.4452	0.074194	0.074194	0.53934457
NA	ENSG00000221104	-0.92046	0.82133	-0.92046	0.82133	-0.92046	0.82133	1.4452	0.0742	0.0742	0.53934457
ZNF286B	ENSG00000249459	1.4451	0.07422	0.60444	0.27277	1.4451	0.07422	0.7826	0.21693	0.07422	0.539409485
NA	ENSG00000262587	1.4445	0.074299	1.4445	0.074299	1.4445	0.074299	-0.28719	0.61302	0.074299	0.539564104
NA	ENSG00000267507	1.1836	0.11829	0.62012	0.26759	1.4444	0.074307	-0.36529	0.64255	0.074307	0.539564104
NA	ENSG00000275734	1.4444	0.074308	1.4444	0.074308	1.4444	0.074308	1.4444	0.074308	0.074308	0.539564104
DIO1	ENSG00000211452	1.3734	0.084817	0.2993	0.38235	1.1436	0.12639	1.4443	0.074322	0.074322	0.539564104
CCNI	ENSG00000118816	0.73346	0.23164	0.27091	0.39323	1.4443	0.07433	-0.43065	0.66664	0.07433	0.539564104
NA	ENSG00000283259	0.48516	0.31378	-0.069214	0.52759	-0.20563	0.58146	1.4443	0.074333	0.074333	0.539564104
NA	ENSG00000274225	1.4442	0.074334	1.4442	0.074334	1.4442	0.074334	1.4442	0.074334	0.074334	0.539564104
NA	ENSG00000270780	1.4442	0.074348	0.41934	0.33749	0.41934	0.33749	0.41934	0.33749	0.074348	0.539564104
FKBP1C	ENSG00000198225	0.80775	0.20962	1.4441	0.074351	1.4441	0.074351	1.4441	0.074351	0.074351	0.539564104
RNU6-659P	ENSG00000252263	1.4441	0.074352	0.91627	0.17976	0.37566	0.35358	0.6875	0.24588	0.074352	0.539564104
FCF1P6	ENSG00000225671	1.4439	0.074378	1.4439	0.074378	1.0403	0.1491	1.4439	0.074378	0.074378	0.539672415
PSMD10P1	ENSG00000228663	1.0716	0.14196	1.1839	0.11823	0.11343	0.45485	1.4437	0.074406	0.074406	0.539753815
RCOR3	ENSG00000117625	0.51543	0.30313	1.0764	0.14087	1.4436	0.074421	0.75587	0.22486	0.074421	0.539753815
THAP6	ENSG00000174796	1.3735	0.0848	1.4435	0.074436	1.3735	0.0848	0.65079	0.25759	0.074436	0.539753815
OR7E163P	ENSG00000251166	-0.49863	0.69098	-1.5639	0.94107	-0.65051	0.74232	1.4433	0.074464	0.074464	0.539753815
PCDH9-AS3	ENSG00000225263	1.4429	0.074529	1.4429	0.074529	1.4429	0.074529	1.4429	0.074529	0.074529	0.539753815
MBP	ENSG00000197971	0.86658	0.19309	1.4426	0.074566	0.14969	0.44051	0.51368	0.30374	0.074566	0.539753815
NA	ENSG00000248172	1.4425	0.074577	1.4425	0.074577	1.4425	0.074577	1.4425	0.074577	0.074577	0.539753815
RNA5SP213	ENSG00000212333	-0.045239	0.51804	1.4424	0.074591	1.4424	0.074591	1.4424	0.074591	0.074591	0.539753815
ATP2A1	ENSG00000196296	-0.085642	0.53412	0.51197	0.30434	0.61635	0.26883	1.4424	0.074597	0.074597	0.539753815

BRWD1-AS1	ENSG00000238141	0.86075	0.19469	1.0474	0.14745	0.86075	0.19469	1.4423	0.074602	0.074602	0.539753815
RNU1-73P	ENSG00000206698	1.4423	0.074609	0.21949	0.41313	1.4423	0.074609	1.4423	0.074609	0.074609	0.539753815
SYT12	ENSG00000173227	1.4422	0.074627	0.40609	0.34234	0.86477	0.19358	-1.2524	0.89479	0.074627	0.539753815
SAMHD1	ENSG00000101347	-0.077027	0.5307	0.42779	0.3344	0.079635	0.46826	1.4421	0.07463	0.07463	0.539753815
GDF11	ENSG00000135414	0.32986	0.37075	1.1059	0.13438	1.4421	0.074632	0.90891	0.1817	0.074632	0.539753815
NA	ENSG00000251239	1.4421	0.074639	1.4421	0.074639	1.4421	0.074639	1.4421	0.074639	0.074639	0.539753815
COX7B2	ENSG00000170516	0.69053	0.24493	1.3325	0.091356	0.638	0.26174	1.4419	0.074663	0.074663	0.539753815
RPL10AP5	ENSG00000235467	1.4419	0.074665	-0.27937	0.61002	0.27684	0.39095	0.010418	0.49584	0.074665	0.539753815
LINS1	ENSG00000140471	-0.96129	0.8318	1.4419	0.074669	-0.84855	0.80193	0.5261	0.29941	0.074669	0.539753815
NDUFAF4P1	ENSG00000259467	1.4418	0.074686	1.4418	0.074686	1.4418	0.074686	1.4418	0.074686	0.074686	0.539753815
MIR1226	ENSG00000221585	1.4417	0.074688	1.4417	0.074688	1.4417	0.074688	1.4417	0.074688	0.074688	0.539753815
RPSAP3	ENSG00000242952	0.37106	0.3553	0.37106	0.3553	1.4416	0.074703	0.37106	0.3553	0.074703	0.539753815
EFL1	ENSG00000140598	1.2184	0.11154	1.4022	0.080424	1.4416	0.074706	0.11883	0.4527	0.074706	0.539753815
RNU6-592P	ENSG00000223265	0.64569	0.25924	-0.55751	0.71141	-0.43588	0.66854	1.4416	0.074706	0.074706	0.539753815
NA	ENSG00000258703	1.3888	0.082447	0.56286	0.28676	0.22657	0.41038	1.4416	0.074709	0.074709	0.539753815
LINC01571	ENSG00000260057	1.0006	0.1585	1.4413	0.074743	1.1321	0.12881	0.45289	0.32531	0.074743	0.539753815
NA	ENSG00000237500	-0.62224	0.73311	1.4413	0.074745	-1.1485	0.87462	-1.1485	0.87462	0.074745	0.539753815
NA	ENSG00000256044	0.28139	0.3892	1.4412	0.07476	-0.21878	0.58659	-0.21878	0.58659	0.07476	0.539753815
NA	ENSG00000229283	0.85915	0.19513	1.4409	0.074803	1.4409	0.074803	1.4409	0.074803	0.074803	0.539753815
NA	ENSG00000266954	1.4409	0.074806	1.4409	0.074806	1.4409	0.074806	1.4409	0.074806	0.074806	0.539753815
JAM3	ENSG00000166086	1.2283	0.10967	0.514	0.30363	1.3484	0.088763	1.4407	0.074834	0.074834	0.539753815
EOGT	ENSG00000163378	1.4406	0.074855	0.022677	0.49095	0.033812	0.48651	0.033812	0.48651	0.074855	0.539753815
NA	ENSG00000224331	1.4405	0.074863	1.4405	0.074863	1.4405	0.074863	1.4405	0.074863	0.074863	0.539753815
VWA3A	ENSG00000175267	1.4405	0.074864	1.203	0.1145	0.28732	0.38693	0.27699	0.39089	0.074864	0.539753815
RN7SKP159	ENSG00000222589	0.51498	0.30328	-0.22644	0.58957	-0.22363	0.58848	1.4405	0.074868	0.074868	0.539753815
POLD3	ENSG00000077514	1.4403	0.074888	0.24258	0.40417	0.6159	0.26898	1.0009	0.15843	0.074888	0.539753815
NA	ENSG00000260177	0.43825	0.3306	0.60411	0.27289	1.4402	0.074904	-0.39409	0.65324	0.074904	0.539753815
FIGN	ENSG00000182263	1.4401	0.074917	0.59598	0.27559	1.258	0.10419	-0.090035	0.53587	0.074917	0.539753815
HTATIP2	ENSG00000109854	1.44	0.074939	0.92693	0.17698	0.895	0.18539	1.44	0.074939	0.074939	0.539753815
NA	ENSG00000260835	1.4399	0.074948	-0.67662	0.75068	-0.053665	0.5214	0.90004	0.18405	0.074948	0.539753815
MUC5B	ENSG00000117983	1.4399	0.074949	0.84754	0.19835	0.96227	0.16796	-0.20322	0.58052	0.074949	0.539753815

NA	ENSG00000270891	0.66975	0.25151	-0.049704	0.51982	1.4399	0.07495	0.94483	0.17237	0.07495	0.539753815
STYXL1	ENSG00000127952	-0.53544	0.70383	-1.2226	0.88925	-0.80207	0.78874	1.4397	0.07497	0.07497	0.539753815
NA	ENSG00000267215	1.4397	0.074978	-0.28666	0.61281	0.95182	0.1706	1.4397	0.074978	0.074978	0.539753815
NA	ENSG00000237072	0.72772	0.23339	0.72772	0.23339	0.72772	0.23339	1.4396	0.07499	0.07499	0.539753815
NA	ENSG00000248134	1.4396	0.07499	-0.45657	0.67601	0.07538	0.46996	0.07538	0.46996	0.07499	0.539753815
NA	ENSG00000267304	1.4396	0.074992	1.4396	0.074992	0.90765	0.18203	0.04424	0.48236	0.074992	0.539753815
NA	ENSG00000207502	1.4394	0.075013	0.70248	0.24119	0.43784	0.33075	0.43784	0.33075	0.075013	0.539753815
NA	ENSG00000256789	1.4394	0.075013	1.4394	0.075013	1.4394	0.075013	1.4394	0.075013	0.075013	0.539753815
LINC01356	ENSG00000215866	-1.0709	0.85789	-0.87327	0.80874	-1.0665	0.85691	1.4394	0.075014	0.075014	0.539753815
BAK1	ENSG00000030110	0.47218	0.3184	0.52301	0.30048	1.4394	0.075023	-0.6433	0.73999	0.075023	0.539753815
NA	ENSG00000280425	1.4392	0.075042	1.4392	0.075042	1.4392	0.075042	1.4392	0.075042	0.075042	0.539753815
NA	ENSG00000250390	1.439	0.075069	1.439	0.075069	1.439	0.075069	1.439	0.075069	0.075069	0.539753815
MIR1-1HG	ENSG00000174407	0.2474	0.4023	1.439	0.075071	0.2474	0.4023	-0.058007	0.52313	0.075071	0.539753815
RNA5SP354	ENSG00000212549	1.439	0.075072	1.439	0.075072	1.439	0.075072	1.439	0.075072	0.075072	0.539753815
NA	ENSG00000233778	-0.003854	0.50154	0.44937	0.32658	1.2293	0.10949	1.4389	0.075087	0.075087	0.539753815
NA	ENSG00000230074	1.1695	0.12111	0.5521	0.29044	0.5521	0.29044	1.4389	0.07509	0.07509	0.539753815
MTND5P33	ENSG00000261904	-0.0067693	0.5027	-0.0067693	0.5027	1.1551	0.12402	1.4388	0.0751	0.0751	0.539753815
NA	ENSG00000223811	-1.3109	0.90506	-1.3109	0.90506	-0.5881	0.72177	1.4388	0.075104	0.075104	0.539753815
LDB3	ENSG00000122367	-0.27483	0.60828	0.21278	0.41575	0.7617	0.22312	1.4384	0.075163	0.075163	0.539753815
PXDC1	ENSG00000168994	0.32828	0.37135	1.4382	0.075183	1.2278	0.10977	0.26479	0.39558	0.075183	0.539753815
NA	ENSG00000231916	1.4382	0.075183	1.4382	0.075183	1.4382	0.075183	1.4382	0.075183	0.075183	0.539753815
LINC00307	ENSG00000227342	0.32748	0.37165	1.4381	0.075198	1.4381	0.075198	1.4381	0.075198	0.075198	0.539753815
MMP20	ENSG00000137674	1.4381	0.075199	1.4381	0.075199	1.4381	0.075199	1.4381	0.075199	0.075199	0.539753815
NA	ENSG00000229863	1.438	0.075212	1.438	0.075212	1.438	0.075212	1.438	0.075212	0.075212	0.539753815
ERVFRD-1	ENSG00000244476	1.438	0.075215	1.2409	0.10732	0.47157	0.31862	0.53989	0.29464	0.075215	0.539753815
PDE4DIP	ENSG00000178104	1.438	0.07522	0.95	0.17106	0.86701	0.19297	1.3771	0.084238	0.07522	0.539753815
LINC01450	ENSG00000232458	1.438	0.075222	-0.25073	0.59899	-0.30597	0.62018	0.92464	0.17758	0.075222	0.539753815
AKAP3	ENSG00000111254	-1.649	0.95043	-1.649	0.95043	-1.649	0.95043	1.4379	0.075237	0.075237	0.539753815
NA	ENSG00000255320	1.4378	0.075241	1.4378	0.075241	1.4378	0.075241	1.4378	0.075241	0.075241	0.539753815
CCDC71	ENSG00000177352	1.2741	0.10132	1.1733	0.12033	1.4378	0.07525	0.27437	0.3919	0.07525	0.539753815
NA	ENSG00000256008	0.63921	0.26134	1.4377	0.075255	1.0672	0.14294	0.76343	0.2226	0.075255	0.539753815

ELP6	ENSG00000163832	1.4377	0.075259	1.1501	0.12505	1.404	0.080165	1.1557	0.1239	0.075259	0.539753815
WFDC12	ENSG00000168703	0.95932	0.1687	1.4377	0.075265	-0.55966	0.71214	1.4377	0.075265	0.075265	0.539753815
OR10J8P	ENSG00000251686	1.4376	0.075267	1.4376	0.075267	1.4376	0.075267	1.4376	0.075267	0.075267	0.539753815
DHFR2	ENSG00000178700	-0.54901	0.7085	1.4375	0.075289	-0.54901	0.7085	-0.54901	0.7085	0.075289	0.539753815
RPS3AP16	ENSG00000243894	1.4374	0.075307	0.6599	0.25466	0.6599	0.25466	-1.462	0.92813	0.075307	0.539753815
NA	ENSG00000279822	1.4373	0.07532	1.3345	0.091015	1.2755	0.10106	1.2755	0.10106	0.07532	0.539753815
FGF18	ENSG00000156427	0.24209	0.40435	1.1253	0.13023	1.437	0.075354	1.1807	0.11886	0.075354	0.539753815
NA	ENSG00000224733	0.92145	0.17841	0.54222	0.29383	1.437	0.075359	0.82485	0.20473	0.075359	0.539753815
RNA5SP125	ENSG00000201713	-0.42619	0.66502	-0.42619	0.66502	-0.42619	0.66502	1.4369	0.07537	0.07537	0.539753815
CIT	ENSG00000122966	1.4368	0.075382	-0.3826	0.64899	-0.55318	0.70993	-0.19425	0.57701	0.075382	0.539753815
TMEM8B	ENSG00000137103	0.14338	0.44299	1.4368	0.075386	1.0313	0.15121	-0.35757	0.63967	0.075386	0.539753815
BTBD11	ENSG00000151136	0.35392	0.3617	0.9915	0.16072	0.62553	0.26581	1.4367	0.075396	0.075396	0.539753815
PPP1R14B-AS1	ENSG00000256940	1.4367	0.075403	1.4367	0.075403	1.4367	0.075403	1.4367	0.075403	0.075403	0.539753815
PDIA6	ENSG00000143870	1.4366	0.075419	-0.1537	0.56107	0.018452	0.49264	0.50102	0.30818	0.075419	0.539753815
NA	ENSG00000257379	1.4365	0.075424	1.4069	0.07973	1.4365	0.075424	1.4365	0.075424	0.075424	0.539753815
NA	ENSG00000234573	1.4365	0.075436	1.4365	0.075436	1.4365	0.075436	1.4365	0.075436	0.075436	0.539753815
NA	ENSG00000232638	1.4363	0.075456	1.4363	0.075456	1.4363	0.075456	1.4363	0.075456	0.075456	0.539753815
CCL19	ENSG00000172724	1.4361	0.075484	-0.23248	0.59192	-0.56546	0.71412	-0.29133	0.6146	0.075484	0.539753815
NA	ENSG00000227726	-0.056099	0.52237	0.77259	0.21988	-2.3503	0.99062	1.4361	0.075487	0.075487	0.539753815
FARP2	ENSG00000006607	1.2458	0.10641	1.4361	0.075489	1.0966	0.13641	1.1599	0.12304	0.075489	0.539753815
SNORD116-25	ENSG00000252326	1.4358	0.075523	1.4358	0.075523	1.4358	0.075523	1.4358	0.075523	0.075523	0.539753815
LINC01087	ENSG00000224559	-0.15779	0.56269	1.4357	0.075544	-0.61729	0.73148	-0.61729	0.73148	0.075544	0.539753815
NA	ENSG00000263508	0.43817	0.33063	1.4356	0.075554	0.54374	0.29331	1.4218	0.077539	0.075554	0.539753815
IL20RB	ENSG00000174564	0.52101	0.30118	1.4356	0.075556	0.8704	0.19204	-1.036	0.84989	0.075556	0.539753815
RNA5SP244	ENSG00000212238	1.4356	0.075558	1.4356	0.075558	1.4356	0.075558	1.4356	0.075558	0.075558	0.539753815
RNU6-1197P	ENSG00000212485	-1.3131	0.90543	-1.0622	0.85594	1.4356	0.075565	-0.0079687	0.50318	0.075565	0.539753815
AIP	ENSG00000110711	-0.87965	0.81048	1.4354	0.075586	-0.87965	0.81048	-0.87965	0.81048	0.075586	0.539753815
NA	ENSG00000251010	-1.2086	0.88659	0.85378	0.19661	1.4353	0.075596	0.69719	0.24284	0.075596	0.539753815
UGT2B7	ENSG00000171234	1.4353	0.075606	1.3679	0.085672	1.4353	0.075606	1.4353	0.075606	0.075606	0.539753815
HOXB8	ENSG00000120068	0.013292	0.4947	1.4352	0.075609	0.62927	0.26459	0.4637	0.32143	0.075609	0.539753815
L3MBTL4	ENSG00000154655	1.4352	0.075616	0.85125	0.19731	0.96129	0.1682	1.0062	0.15715	0.075616	0.539753815

RNU6-474P ENSG00000206970 0.47722 0.3166 0.064929 0.47412 1.4351
0.075632 0.66376 0.25342 0.075632 0.539753815
ZNF670-ZNF695 ENSG00000135747 1.4351 0.075635 -0.26074 0.60285 0.30414
0.38051 -0.21623 0.5856 0.075635 0.539753815
NTM-AS1 ENSG00000224795 -0.024501 0.50977 1.4351 0.075636 -0.24326
0.5961 -0.39859 0.6549 0.075636 0.539753815
FAM71F1 ENSG00000135248 1.3741 0.084703 1.435 0.075643 0.99873 0.15896
1.435 0.075643 0.075643 0.539753815
FLRT3 ENSG00000125848 1.4349 0.075659 -0.50732 0.69404 -0.2503 0.59882
-0.48293 0.68543 0.075659 0.539753815
NA ENSG00000254092 0.98641 0.16197 -0.17034 0.56763 1.4348 0.075668
1.4348 0.075668 0.075668 0.539753815
DTX2P1-UPK3BP1-PMS2P11 ENSG00000265479 1.1327 0.12868 1.4348 0.075672
0.81005 0.20895 1.4348 0.075672 0.075672 0.539753815
NUP205 ENSG00000155561 1.0955 0.13665 -0.024314 0.5097 0.92274
0.17807 1.4347 0.07568 0.07568 0.539753815
SAMD14 ENSG00000167100 1.4347 0.075685 1.4347 0.075685 1.4347
0.075685 1.4347 0.075685 0.075685 0.539753815
RPL21P120 ENSG00000244582 0.084314 0.4664 0.32204 0.37371 -0.2906
0.61432 1.4346 0.075697 0.075697 0.539753815
LINC01707 ENSG00000223883 1.4345 0.075708 -0.95185 0.82941 -0.62697
0.73466 -0.95185 0.82941 0.075708 0.539753815
MEP1B ENSG00000141434 0.80058 0.21169 -0.38233 0.64889 1.4345 0.075722
0.62952 0.2645 0.075722 0.539753815
LINC02612 ENSG00000224048 1.4344 0.075727 0.15342 0.43903 -0.60789
0.72837 -0.10665 0.54246 0.075727 0.539753815
PGAM1P8 ENSG00000255200 1.4344 0.075727 1.4344 0.075727 1.4344
0.075727 1.4344 0.075727 0.075727 0.539753815
SNRPCP10 ENSG00000243794 1.4343 0.075744 1.3446 0.089373 0.38165
0.35136 -0.015997 0.50638 0.075744 0.539753815
NA ENSG00000259079 1.4343 0.075746 -1.0793 0.85977 -1.422 0.92248
-1.3929 0.91817 0.075746 0.539753815
NUP85 ENSG00000125450 1.4343 0.075748 1.3217 0.093137 0.56324 0.28664
0.48155 0.31506 0.075748 0.539753815
LINC02144 ENSG00000248708 -0.029425 0.51174 1.4342 0.07576 -0.76127
0.77675 -0.76127 0.77675 0.07576 0.539753815
TUBB3P1 ENSG00000220418 1.4341 0.075772 1.4341 0.075772 1.4341
0.075772 -0.14504 0.55766 0.075772 0.539753815
NA ENSG00000232124 0.49703 0.30959 1.4341 0.075772 -0.2873 0.61306
-0.20099 0.57965 0.075772 0.539753815
VPREB1 ENSG00000169575 0.25581 0.39905 0.25581 0.39905 0.25581
0.39905 1.434 0.07579 0.07579 0.539753815
PLA2G4B ENSG00000243708 1.434 0.075792 1.0111 0.15599 1.434 0.075792 1.434
0.075792 0.075792 0.539753815
NA ENSG00000258551 1.434 0.075792 1.434 0.075792 1.434 0.075792 1.434 0.075792
0.075792 0.539753815
NA ENSG00000253798 0.74667 0.22763 0.74803 0.22722 1.4339 0.075795
0.52313 0.30044 0.075795 0.539753815
NSRP1P1 ENSG00000235613 1.4339 0.075796 1.4339 0.075796 1.4339
0.075796 1.4339 0.075796 0.075796 0.539753815
RTEL1 ENSG00000258366 1.4339 0.075807 0.57311 0.28328 -0.21122 0.58364
-0.21122 0.58364 0.075807 0.539753815
NA ENSG00000233708 1.4337 0.075831 1.4337 0.075831 1.4337 0.075831
-0.61119 0.72946 0.075831 0.539771349
LPCAT2 ENSG00000087253 -1.2126 0.88736 1.3969 0.081226 1.4336
0.075843 0.16579 0.43416 0.075843 0.539771349
ZBTB20 ENSG00000181722 0.61768 0.26839 0.40776 0.34172 1.201 0.11488
1.4336 0.075844 0.075844 0.539771349
NR2F2-AS1 ENSG00000247809 1.4335 0.075864 0.77997 0.2177 0.93088
0.17596 0.083794 0.46661 0.075864 0.539771349
ITGA7 ENSG00000135424 -0.62315 0.73341 -0.62315 0.73341 1.4334 0.075873
-0.66753 0.74778 0.075873 0.539771349

OR7D4	ENSG00000174667	1.4333	0.075887	1.4333	0.075887	1.4333	0.075887	1.4333	0.075887	0.075887	0.539771349
NA	ENSG00000248155	0.11767	0.45316	0.37774	0.35281	1.4333	0.075887	0.49722	0.30952	0.075887	0.539771349
NDUFB5	ENSG00000136521	0.54535	0.29276	1.226	0.1101	1.4331	0.07592	0.36319	0.35823	0.07592	0.539840808
C11orf91	ENSG00000205177	1.433	0.075926	1.433	0.075926	1.433	0.075926	1.433	0.075926	0.075926	0.539840808
TRPM4	ENSG00000130529	-0.63365	0.73685	-0.50108	0.69184	0.71914	0.23603	1.433	0.07593	0.07593	0.539840808
NA	ENSG00000250696	1.2296	0.10942	1.4328	0.07596	1.4328	0.07596	1.4328	0.07596	0.07596	0.539917314
NIPA2P2	ENSG00000241022	-1.3014	0.90343	1.4328	0.075963	-1.7437	0.9594	0.74897	0.22694	0.075963	0.539917314
NA	ENSG00000225806	-0.049146	0.5196	0.26758	0.39451	1.4327	0.075974	0.39138	0.34776	0.075974	0.539917314
RNU6-1176P	ENSG00000212257	0.28793	0.3867	1.43	0.07636	0.33546	0.36864	1.4326	0.075986	0.075986	0.539923852
TLCD3B	ENSG00000149926	0.8593	0.19509	0.8593	0.19509	1.1757	0.11987	1.4322	0.076048	0.076048	0.540113321
AMZ2	ENSG00000196704	0.79801	0.21243	1.4321	0.076056	1.4044	0.080098	-0.66193	0.74599	0.076056	0.540113321
GNAS-AS1	ENSG00000235590	0.35652	0.36073	1.1171	0.13197	1.3117	0.09481	1.4321	0.076056	0.076056	0.540113321
NA	ENSG00000275956	1.4321	0.076057	0.89471	0.18547	1.2515	0.10537	1.2515	0.10537	0.076057	0.540113321
WSCD1	ENSG00000179314	0.52828	0.29865	0.73136	0.23228	1.432	0.076075	0.42943	0.3338	0.076075	0.540162429
RN7SL700P	ENSG00000244521	-0.045532	0.51816	-0.75887	0.77604	1.1012	0.13539	1.4317	0.076111	0.076111	0.540296093
MIR16-1	ENSG00000208006	1.4317	0.076116	-0.14032	0.5558	-0.36229	0.64143	0.38793	0.34903	0.076116	0.540296093
SNX27	ENSG00000143376	-0.11933	0.54749	1.317	0.093924	1.4315	0.076144	0.31064	0.37804	0.076144	0.540386468
PEX7	ENSG00000112357	-0.0052322	0.50209	-0.66435	0.74677	1.4314	0.076162	-1.2416	0.89281	0.076162	0.540386468
RPL7P20	ENSG00000254252	0.7523	0.22593	1.4314	0.076162	0.7523	0.22593	0.7523	0.22593	0.076162	0.540386468
GRIA4	ENSG00000152578	-1.5606	0.94069	-0.44615	0.67226	0.77434	0.21936	1.4313	0.076178	0.076178	0.540420026
LINC00636	ENSG00000240423	1.4311	0.076199	0.55867	0.28819	0.83837	0.20091	0.26982	0.39365	0.076199	0.540420026
NA	ENSG00000244137	1.4311	0.0762	-0.35905	0.64022	1.4311	0.0762	1.4311	0.0762	0.0762	0.540420026
RPL26P28	ENSG00000223987	-0.4071	0.65803	0.058799	0.47656	0.5781	0.2816	1.4306	0.076268	0.076268	0.540586718
ADNP	ENSG00000101126	1.1523	0.12459	0.41994	0.33726	1.4305	0.076286	1.2385	0.10776	0.076286	0.540586718
NA	ENSG00000243554	1.4305	0.076289	1.1121	0.13305	-0.14352	0.55706	-0.54877	0.70842	0.076289	0.540586718
RNU6ATAC30P	ENSG00000221114	1.4305	0.07629	1.4305	0.07629	1.4305	0.07629	1.4305	0.07629	0.07629	0.540586718
NA	ENSG00000245330	1.4304	0.076295	1.3361	0.090757	0.63471	0.26281	0.63471	0.26281	0.076295	0.540586718
KRT14	ENSG00000186847	-1.4955	0.9326	-0.40334	0.65665	-0.3606	0.6408	1.4303	0.076316	0.076316	0.540586718
DMBT1	ENSG00000187908	0.18385	0.42707	-0.42482	0.66452	0.32493	0.37262	1.4303	0.076321	0.076321	0.540586718
NA	ENSG00000251680	-0.7915	0.78567	0.11001	0.4562	-0.56636	0.71442	1.4302	0.076323	0.076323	0.540586718
NA	ENSG00000276093	1.4302	0.076325	0.30868	0.37878	1.4302	0.076325	-0.027915	0.51114	0.076325	0.540586718

NA	ENSG00000257925	1.43	0.076363	1.43	0.076363	1.43	0.076363	1.43	0.076363	0.076363	0.540586718
RNU6-731P	ENSG00000253022	1.0445	0.14814	1.4299	0.076366	1.0425	0.14859	1.0425	0.14859	0.076366	0.540586718
TNIK	ENSG00000154310	0.43525	0.33169	0.87271	0.19141	0.16913	0.43285	1.4298	0.076384	0.076384	0.540586718
ATP5F1AP4	ENSG00000258877	1.4298	0.076386	1.4298	0.076386	1.4298	0.076386	1.4298	0.076386	0.076386	0.540586718
SKAP1	ENSG00000141293	1.4298	0.076387	1.3609	0.08677	0.87196	0.19161	0.06417	0.47442	0.076387	0.540586718
NA	ENSG00000265545	1.4297	0.076397	-0.30309	0.61909	-2.1459	0.98406	-0.87897	0.81029	0.076397	0.540586718
NA	ENSG00000266708	1.4297	0.076401	1.4297	0.076401	1.4297	0.076401	1.4297	0.076401	0.076401	0.540586718
ZNRF3-AS1	ENSG00000177993	1.4295	0.076425	1.4295	0.076425	1.4295	0.076425	1.4295	0.076425	0.076425	0.540654118
NA	ENSG00000235713	0.78721	0.21558	0.11645	0.45365	1.4294	0.076441	0.30566	0.37993	0.076441	0.540654118
NA	ENSG00000243224	1.4293	0.076455	1.4293	0.076455	1.4293	0.076455	1.4293	0.076455	0.076455	0.540654118
NEK7	ENSG00000151414	1.4293	0.076456	1.294	0.09784	-0.61677	0.73131	-0.61677	0.73131	0.076456	0.540654118
PHBP9	ENSG00000230224	1.4292	0.076466	1.4292	0.076466	1.4292	0.076466	1.4292	0.076466	0.076466	0.540654118
SUN2	ENSG00000100242	-1.2895	0.90138	-0.89984	0.8159	0.072772	0.47099	1.429	0.076504	0.076504	0.540729166
NA	ENSG00000278621	1.4289	0.07651	1.4289	0.07651	1.4289	0.07651	1.4289	0.07651	0.07651	0.540729166
NA	ENSG00000198106	1.4289	0.076517	0.35874	0.3599	1.0772	0.14069	1.3262	0.092385	0.076517	0.540729166
NA	ENSG00000212168	1.4289	0.076521	1.4289	0.076521	-0.637	0.73794	1.4289	0.076521	0.076521	0.540729166
C20orf96	ENSG00000196476	1.4287	0.076547	0.95184	0.17059	1.2937	0.09789	-0.54457	0.70697	0.076547	0.540834466
REG1CP	ENSG00000204787	-0.4663	0.6795	-0.4663	0.6795	-0.4663	0.6795	1.4286	0.076559	0.076559	0.540840834
USP36	ENSG00000055483	-0.62366	0.73357	0.39604	0.34604	1.4281	0.076625	0.59214	0.27688	0.076625	0.540893836
H2AZ2	ENSG00000105968	1.4281	0.076638	0.38841	0.34886	-0.18139	0.57197	1.1709	0.12081	0.076638	0.540893836
NA	ENSG00000201633	1.2176	0.11169	1.3835	0.083254	1.2377	0.10791	1.428	0.076648	0.076648	0.540893836
KNOP1P5	ENSG00000250563	1.4279	0.07666	1.4279	0.07666	1.4279	0.07666	1.4279	0.07666	0.07666	0.540893836
M6PR	ENSG00000003056	1.4279	0.076665	1.4279	0.076665	1.4279	0.076665	1.4279	0.076665	0.076665	0.540893836
MIR548V	ENSG00000265520	1.4278	0.076677	1.4278	0.076677	1.4278	0.076677	1.4278	0.076677	0.076677	0.540893836
ARHGAP42P1	ENSG00000227817	0.28882	0.38636	0.28882	0.38636	1.4278	0.076678	-0.97813	0.836	0.076678	0.540893836
CCNQP1	ENSG00000233827	1.4278	0.07668	1.4278	0.07668	1.4278	0.07668	0.15657	0.43779	0.07668	0.540893836
RNU6-219P	ENSG00000201598	1.4277	0.076696	1.4277	0.076696	0.83378	0.2022	0.77624	0.2188	0.076696	0.540893836
NA	ENSG00000266390	-1.6858	0.95408	0.73808	0.23023	1.4277	0.076696	-0.70748	0.76037	0.076696	0.540893836
NA	ENSG00000283053	1.4276	0.076699	0.67243	0.25066	-0.72553	0.76594	0.68387	0.24703	0.076699	0.540893836
NA	ENSG00000273353	1.4275	0.076713	1.4275	0.076713	1.4275	0.076713	1.4275	0.076713	0.076713	0.540893836
SLC48A1	ENSG00000211584	0.56402	0.28637	1.4275	0.07672	1.2079	0.11355	1.2079	0.11355	0.07672	0.540893836

SBDSP1	ENSG00000225648	1.4275	0.076722	0.86909	0.1924	0.65425	0.25647	0.89804	0.18458	0.076722	0.540893836
FAM135A	ENSG00000082269	0.69612	0.24318	1.2082	0.11348	0.87388	0.19109	1.4274	0.076733	0.076733	0.540893836
NA	ENSG00000254695	1.0362	0.15004	0.87684	0.19029	1.4271	0.07678	0.96773	0.16659	0.07678	0.540931876
ALPG	ENSG00000163286	1.2022	0.11464	0.90551	0.1826	1.4271	0.076782	0.27053	0.39338	0.076782	0.540931876
PTGR2	ENSG00000140043	1.2103	0.11309	1.1325	0.12871	1.1325	0.12871	1.427	0.076792	0.076792	0.540931876
NA	ENSG00000258922	1.2237	0.11053	1.1542	0.1242	1.427	0.076793	1.1416	0.12682	0.076793	0.540931876
NA	ENSG00000280117	1.427	0.076796	1.427	0.076796	1.427	0.076796	1.427	0.076796	0.076796	0.540931876
CCNI2	ENSG00000205089	1.4269	0.076805	1.4269	0.076805	1.4269	0.076805	1.4269	0.076805	0.076805	0.540931876
RN7SKP129	ENSG00000222220	1.4267	0.076835	-0.38665	0.65049	-0.037573	0.51499	-0.67964	0.75164	0.076835	0.541036073
IGF2BP2	ENSG00000073792	1.0009	0.15845	1.1912	0.11678	1.4266	0.076842	1.2383	0.1078	0.076842	0.541036073
PELO	ENSG00000152684	1.4264	0.076874	1.3346	0.091	1.4264	0.076874	0.71036	0.23874	0.076874	0.541180837
NA	ENSG00000268743	1.4263	0.076886	1.4263	0.076886	1.4263	0.076886	1.4263	0.076886	0.076886	0.541180837
NA	ENSG00000270335	1.2262	0.11006	1.4262	0.0769	0.97558	0.16464	0.94101	0.17335	0.0769	0.541180837
TRBV19	ENSG00000211746	-0.53996	0.70539	1.4262	0.076909	-0.97301	0.83473	1.0327	0.15088	0.076909	0.541180837
GNL3	ENSG00000163938	1.426	0.076936	1.426	0.076936	1.426	0.076936	1.426	0.076936	0.076936	0.541180837
FGF14-IT1	ENSG00000243319	-0.20289	0.58039	1.4259	0.076953	1.4259	0.076953	0.73097	0.2324	0.076953	0.541180837
GPR19	ENSG00000183150	0.67614	0.24948	1.4258	0.076969	0.2915	0.38534	0.44917	0.32666	0.076969	0.541180837
NA	ENSG00000232883	1.4256	0.076987	1.4256	0.076987	1.4256	0.076987	1.2732	0.10146	0.076987	0.541180837
MIR567	ENSG00000207940	1.4256	0.076989	-0.24387	0.59633	1.4256	0.076989	1.4256	0.076989	0.076989	0.541180837
DHFRP6	ENSG00000219095	-1.0927	0.86275	-1.0795	0.85983	1.4255	0.077001	-1.2947	0.90228	0.077001	0.541180837
LINC00926	ENSG00000247982	0.26919	0.39389	-0.31171	0.62237	0.26919	0.39389	1.4255	0.07701	0.07701	0.541180837
HSD17B12	ENSG00000149084	1.4254	0.077024	1.0119	0.1558	0.83762	0.20112	0.88255	0.18874	0.077024	0.541180837
ACTRT2	ENSG00000169717	-1.5285	0.93681	1.425	0.077083	0.57184	0.28372	0.33897	0.36732	0.077083	0.541180837
LINC01148	ENSG00000258804	1.4248	0.077101	1.0484	0.14723	-0.18708	0.5742	-0.18708	0.5742	0.077101	0.541180837
LINC02031	ENSG00000238837	1.4248	0.077112	0.69833	0.24249	-0.17558	0.56969	0.15545	0.43823	0.077112	0.541180837
NA	ENSG00000235572	-1.3825	0.9166	1.4247	0.077117	-1.1925	0.88348	-2.2519	0.98784	0.077117	0.541180837
PCAT7	ENSG00000231806	1.4247	0.077118	0.77125	0.22028	-0.47367	0.68213	-0.47367	0.68213	0.077118	0.541180837
FCN2	ENSG00000160339	-1.4996	0.93313	-0.63559	0.73748	-1.2586	0.89591	1.4247	0.077121	0.077121	0.541180837
NA	ENSG00000221007	1.4246	0.077131	-0.14006	0.55569	-0.14006	0.55569	-0.14006	0.55569	0.077131	0.541180837
BOLA2P2	ENSG00000271161	1.4246	0.077141	-0.32405	0.62705	-1.1579	0.87654	-1.1579	0.87654	0.077141	0.541180837
PDCL3P3	ENSG00000214286	1.4245	0.077155	1.4245	0.077155	1.4245	0.077155	1.4245	0.077155	0.077155	0.541180837

NA	ENSG00000206816	1.4244	0.077161	1.4244	0.077161	1.4244	0.077161	1.4244	0.077161	0.077161	0.541180837
NA	ENSG00000271239	-1.4699	0.92921	1.4244	0.077161	-1.4699	0.92921	-1.4699	0.92921	0.077161	0.541180837
CCBE1	ENSG00000183287	0.63324	0.26329	1.4244	0.077163	0.12201	0.45144	-0.99951	0.84123	0.077163	0.541180837
FBXW10	ENSG00000171931	1.4244	0.077168	-1.435	0.92435	0.17147	0.43193	-0.70614	0.75995	0.077168	0.541180837
RASSF10-DT	ENSG00000254670	1.4244	0.07717	1.4244	0.07717	1.4244	0.07717	1.4244	0.07717	0.07717	0.541180837
ENGASE	ENSG00000167280	1.4242	0.077193	-0.28382	0.61173	-0.45294	0.67471	-0.60465	0.7273	0.077193	0.541180837
NA	ENSG00000273306	1.4242	0.077201	1.4242	0.077201	1.4242	0.077201	1.4242	0.077201	0.077201	0.541180837
NA	ENSG00000278595	-0.23114	0.5914	-0.23344	0.59229	1.4241	0.077202	-0.25789	0.60175	0.077202	0.541180837
NA	ENSG00000251811	-1.5128	0.93484	-0.46117	0.67766	0.067828	0.47296	1.4241	0.077212	0.077212	0.541180837
EGLN1	ENSG00000135766	0.31612	0.37596	1.424	0.07722	0.48734	0.31301	-0.90077	0.81614	0.07722	0.541180837
PGM2	ENSG00000169299	0.63822	0.26167	0.63822	0.26167	1.424	0.077223	1.2555	0.10465	0.077223	0.541180837
RAC1P7	ENSG00000236101	0.60161	0.27372	-0.2005	0.57946	1.4238	0.077249	-0.57042	0.71581	0.077249	0.541180837
DEFA5	ENSG00000164816	1.4237	0.077264	1.4237	0.077264	1.4237	0.077264	1.4237	0.077264	0.077264	0.541180837
NOTCH1	ENSG00000148400	-0.70288	0.75893	1.4237	0.077267	0.72591	0.23395	-0.39607	0.65397	0.077267	0.541180837
OR5P2	ENSG00000183303	-2.0186	0.97824	-0.5797	0.71894	1.3892	0.082383	1.4236	0.077279	0.077279	0.541180837
NF2	ENSG00000186575	1.1197	0.13141	1.4236	0.07728	1.1239	0.13053	0.44844	0.32692	0.07728	0.541180837
NA	ENSG00000201451	1.4235	0.077301	1.4235	0.077301	1.4235	0.077301	1.4235	0.077301	0.077301	0.541180837
NA	ENSG00000230064	1.4235	0.077301	1.4235	0.077301	1.4235	0.077301	1.4235	0.077301	0.077301	0.541180837
NA	ENSG00000223060	1.4234	0.077307	1.4234	0.077307	1.4234	0.077307	1.4234	0.077307	0.077307	0.541180837
RNA5SP384	ENSG00000252424	1.4233	0.077325	1.4233	0.077325	1.4233	0.077325	1.1945	0.11613	0.077325	0.541180837
TRAF3IP1	ENSG00000204104	1.4233	0.077329	-0.1268	0.55045	1.0759	0.141	-0.21156	0.58377	0.077329	0.541180837
LINC01989	ENSG00000261156	1.4231	0.077346	0.048152	0.4808	-0.61562	0.73093	-0.34518	0.63502	0.077346	0.541222082
RPL31P57	ENSG00000256358	1.423	0.077364	1.423	0.077364	1.423	0.077364	1.423	0.077364	0.077364	0.541270312
NA	ENSG00000280310	1.4227	0.077407	0.079848	0.46818	-2.1496	0.98421	0.30493	0.38021	0.077407	0.541356676
OR10G1P	ENSG00000273586	1.4225	0.077434	-0.48485	0.68611	-0.56954	0.7155	-0.35341	0.63811	0.077434	0.541356676
NA	ENSG00000254067	1.3926	0.081878	1.4225	0.077435	0.84371	0.19942	0.84414	0.1993	0.077435	0.541356676
PADI1	ENSG00000142623	-0.34887	0.63641	-1.0801	0.85995	1.4225	0.077439	-1.3416	0.91014	0.077439	0.541356676
LRIT1	ENSG00000148602	1.4225	0.077441	-0.28748	0.61313	1.4225	0.077441	-0.54239	0.70622	0.077441	0.541356676
RPL12P30	ENSG00000242353	1.23	0.10936	1.2758	0.10101	1.0057	0.15729	1.4225	0.077443	0.077443	0.541356676
DLGAP2	ENSG00000198010	0.29757	0.38302	0.24308	0.40397	0.84985	0.1977	1.4222	0.077479	0.077479	0.541486363
KIAA1217	ENSG00000120549	0.56741	0.28522	1.2358	0.10826	1.4222	0.077487	-0.34532	0.63507	0.077487	0.541486363

TRMT112P2	ENSG00000249778	-1.1772	0.88043	1.4221	0.077499	1.4221	0.077499	1.4221	0.077499	0.077499	0.541486363
POLR2F	ENSG00000100142	0.62336	0.26652	0.46326	0.32159	0.38761	0.34915	1.4221	0.077506	0.077506	0.541486363
RNU6-549P	ENSG00000207162	1.4218	0.077546	1.4218	0.077546	1.4218	0.077546	1.4218	0.077546	0.077546	0.541555291
ANKRD62	ENSG00000181626	-0.56744	0.71479	1.4214	0.077603	0.83444	0.20202	0.29259	0.38492	0.077603	0.541555291
LAIR1	ENSG00000167613	-0.1154	0.54593	1.4213	0.077616	1.4175	0.078165	0.16362	0.43502	0.077616	0.541555291
KRT8P42	ENSG00000220378	1.4213	0.077618	1.4213	0.077618	1.4213	0.077618	1.2445	0.10666	0.077618	0.541555291
STC1	ENSG00000159167	-1.4616	0.92807	-1.4616	0.92807	-0.45457	0.67529	1.4213	0.077619	0.077619	0.541555291
NA	ENSG00000229855	0.7128	0.23798	1.4213	0.077619	1.4213	0.077619	1.4213	0.077619	0.077619	0.541555291
ZNF544	ENSG00000198131	-1.3098	0.90486	-1.113	0.86714	-0.81109	0.79134	1.4212	0.077623	0.077623	0.541555291
LIX1L-AS1	ENSG00000234222	-0.19441	0.57707	0.81796	0.20669	1.4212	0.077623	0.58433	0.2795	0.077623	0.541555291
WDR19	ENSG00000157796	1.4212	0.077624	0.5201	0.3015	0.55024	0.29108	-0.022636	0.50903	0.077624	0.541555291
IL22RA1	ENSG00000142677	-0.67672	0.75071	0.42048	0.33707	1.4212	0.077627	0.3764	0.35331	0.077627	0.541555291
SBF1P1	ENSG00000248522	1.3564	0.087478	0.93448	0.17503	1.1152	0.13238	1.421	0.077665	0.077665	0.541735044
B4GALT2	ENSG00000117411	1.4209	0.077675	1.4209	0.077675	0.087089	0.4653	1.4209	0.077675	0.077675	0.541735044
NA	ENSG00000262791	1.4207	0.077702	1.4207	0.077702	1.4207	0.077702	1.4207	0.077702	0.077702	0.541845801
NA	ENSG00000271771	0.27733	0.39076	1.4205	0.077736	0.11979	0.45232	0.73404	0.23146	0.077736	0.541883682
PRDM14	ENSG00000147596	-1.8816	0.97006	-1.8816	0.97006	0.48257	0.3147	1.4204	0.077749	0.077749	0.541883682
RN7SKP3	ENSG00000223341	0.4209	0.33692	-0.16604	0.56594	1.4204	0.07775	-0.75503	0.77488	0.07775	0.541883682
RPL5	ENSG00000122406	1.4202	0.077772	1.4202	0.077772	1.0204	0.15378	0.076467	0.46952	0.077772	0.541883682
LINC02219	ENSG00000253787	-0.077506	0.53089	-1.1424	0.87335	-0.61926	0.73213	1.4197	0.077844	0.077844	0.541883682
RNU6-1127P	ENSG00000207310	1.4196	0.077866	1.4196	0.077866	1.4196	0.077866	-0.039725	0.51584	0.077866	0.541883682
DCST2	ENSG00000163354	0.31225	0.37743	1.4194	0.077889	0.71178	0.2383	0.81138	0.20857	0.077889	0.541883682
OR11H6	ENSG00000176219	1.4193	0.0779	1.4193	0.0779	1.4193	0.0779	1.0914	0.13755	0.0779	0.541883682
FA2H	ENSG00000103089	0.37879	0.35242	1.1444	0.12624	0.020771	0.49171	1.4191	0.077935	0.077935	0.541883682
NA	ENSG00000264216	-0.033895	0.51352	-0.0063856	0.50255	1.419	0.077952	0.39453	0.34659	0.077952	0.541883682
LRRN2	ENSG00000170382	0.67921	0.2485	1.419	0.077956	0.037285	0.48513	0.30784	0.3791	0.077956	0.541883682
RN7SL215P	ENSG00000242201	0.12197	0.45146	0.49984	0.30859	0.10679	0.45748	1.4188	0.077972	0.077972	0.541883682
NA	ENSG00000274481	1.4188	0.077978	1.4188	0.077978	1.4188	0.077978	1.4188	0.077978	0.077978	0.541883682
NA	ENSG00000229533	1.4187	0.077993	-0.77026	0.77943	1.4187	0.077993	1.4187	0.077993	0.077993	0.541883682
TMCC1	ENSG00000172765	0.73677	0.23063	1.0862	0.13869	1.1784	0.11932	1.4187	0.077996	0.077996	0.541883682
SLC9C2	ENSG00000162753	1.4187	0.077997	0.15365	0.43894	-0.8332	0.79764	0.97818	0.16399	0.077997	0.541883682

SYCP2	ENSG00000196074	1.4187	0.077999	1.4187	0.077999	1.4187	0.077999	1.4187	0.077999	0.077999	0.541883682
KRT223P	ENSG00000229028	-0.19155	0.57595	-0.84661	0.80139	-1.0837	0.86075	1.4186	0.078004	0.078004	0.541883682
NFRKB	ENSG00000170322	1.4186	0.078006	1.4186	0.078006	0.36348	0.35812	0.6705	0.25127	0.078006	0.541883682
NRG1	ENSG00000157168	1.4186	0.078013	-0.43946	0.66984	0.82644	0.20428	0.28106	0.38933	0.078013	0.541883682
ATP8B2	ENSG00000143515	0.64117	0.26071	1.4185	0.078029	1.2776	0.1007	1.3865	0.082799	0.078029	0.541883682
AMZ2P1	ENSG00000214174	-0.20839	0.58254	1.4184	0.07803	0.18952	0.42484	1.1462	0.12585	0.07803	0.541883682
NA	ENSG00000271368	1.1131	0.13282	1.1131	0.13282	1.4183	0.078055	1.1131	0.13282	0.078055	0.541883682
BTF3P9	ENSG00000224834	1.4183	0.078057	0.31893	0.37489	0.23558	0.40688	0.1865	0.42603	0.078057	0.541883682
CCDC90B	ENSG00000137500	-0.13664	0.55434	1.4181	0.078081	-0.73446	0.76867	0.41167	0.34029	0.078081	0.541883682
KRTAP4-4	ENSG00000171396	1.4181	0.078088	1.4181	0.078088	1.4181	0.078088	1.4181	0.078088	0.078088	0.541883682
NA	ENSG00000235185	0.032294	0.48712	-0.4012	0.65586	1.4181	0.078088	-0.32061	0.62575	0.078088	0.541883682
NA	ENSG00000267070	0.1961	0.42227	1.418	0.07809	-1.3175	0.90617	-0.0027035	0.50108	0.07809	0.541883682
UBE2D4	ENSG00000078967	0.54283	0.29362	1.418	0.078092	0.63333	0.26326	0.61312	0.2699	0.078092	0.541883682
NA	ENSG00000271621	1.267	0.10259	1.418	0.078097	1.0638	0.1437	0.25344	0.39996	0.078097	0.541883682
NCAM1	ENSG00000149294	-0.47506	0.68263	-0.093465	0.53723	0.87346	0.19121	1.4179	0.078114	0.078114	0.541883682
FGFRL1	ENSG00000127418	-0.39204	0.65248	-0.45495	0.67543	1.0387	0.14948	1.4178	0.078119	0.078119	0.541883682
ZSCAN5A	ENSG00000131848	0.3427	0.36591	0.53125	0.29762	0.3427	0.36591	1.4178	0.078124	0.078124	0.541883682
SNORA66	ENSG00000207523	1.4178	0.078129	1.4178	0.078129	1.4178	0.078129	1.4178	0.078129	0.078129	0.541883682
NA	ENSG00000233461	1.4177	0.078134	0.35295	0.36206	0.76756	0.22138	0.65521	0.25617	0.078134	0.541883682
KRT8P13	ENSG00000241416	1.4177	0.07814	1.4177	0.07814	1.4177	0.07814	1.4177	0.07814	0.07814	0.541883682
NA	ENSG00000226886	-0.97918	0.83626	0.63746	0.26191	1.4176	0.07815	-1.2128	0.88739	0.07815	0.541883682
DNMT3B	ENSG00000088305	0.58987	0.27764	1.4176	0.078151	0.93065	0.17602	1.3793	0.083897	0.078151	0.541883682
PARP16	ENSG00000138617	1.4139	0.078696	-1.0454	0.85208	1.4176	0.078155	-0.29718	0.61684	0.078155	0.541883682
HMGB3P18	ENSG00000220184	-2.0374	0.9792	1.4175	0.078164	-2.0374	0.9792	1.1414	0.12685	0.078164	0.541883682
CIBAR1	ENSG00000188343	1.4175	0.078174	0.12499	0.45027	0.33826	0.36758	-0.49478	0.68962	0.078174	0.541883682
DNM3	ENSG00000197959	1.2461	0.10636	1.4172	0.078219	0.17718	0.42968	1.0417	0.14877	0.078219	0.541883682
RTBDN	ENSG00000132026	-0.74127	0.77074	-0.74127	0.77074	-0.74127	0.77074	1.4171	0.078225	0.078225	0.541883682
NA	ENSG00000236839	1.0281	0.15196	1.4171	0.078225	1.4171	0.078225	1.4171	0.078225	0.078225	0.541883682
HMGA1P6	ENSG00000233440	-0.45818	0.67659	-1.0541	0.85408	1.4171	0.078229	-1.235	0.89159	0.078229	0.541883682
MEX3A	ENSG00000254726	1.4171	0.078233	0.436	0.33142	1.4157	0.078433	1.4157	0.078433	0.078233	0.541883682
NA	ENSG00000239054	1.4171	0.078233	1.4171	0.078233	1.4171	0.078233	0.86826	0.19263	0.078233	0.541883682

RNU7-57P	ENSG00000238365	1.4167	0.078287	1.4167	0.078287	1.4167	0.078287	1.4167	0.078287	0.078287	0.541883682
SUCLG2-AS1	ENSG00000241316	-0.664	0.74666	-1.18	0.881	0.012611	0.49497	1.4166	0.078293	0.078293	0.541883682
LINC02437	ENSG00000248517	0.95959	0.16863	0.35963	0.35956	1.4166	0.078305	0.81772	0.20676	0.078305	0.541883682
FTH1P16	ENSG00000227376	1.4165	0.078309	1.4165	0.078309	1.4165	0.078309	1.4165	0.078309	0.078309	0.541883682
CLDN8	ENSG00000156284	-0.014137	0.50564	0.14621	0.44188	1.4165	0.078311	1.4165	0.078311	0.078311	0.541883682
LRFN5	ENSG00000165379	1.1221	0.13092	1.4165	0.078319	0.99527	0.1598	-0.42359	0.66407	0.078319	0.541883682
NA	ENSG00000268240	0.25213	0.40047	1.4163	0.078341	0.90496	0.18274	1.4163	0.078341	0.078341	0.541883682
SDAD1P4	ENSG00000236803	0.15649	0.43782	0.15649	0.43782	-0.68256	0.75256	1.4162	0.078359	0.078359	0.541883682
OSBPL2	ENSG00000130703	-0.32746	0.62834	-0.1287	0.5512	1.4161	0.078367	0.08638	0.46558	0.078367	0.541883682
CACNA1S	ENSG00000081248	1.4161	0.078372	0.29351	0.38457	1.3877	0.082616	0.5847	0.27937	0.078372	0.541883682
LYPD1	ENSG00000150551	1.4161	0.078374	-0.67493	0.75014	-0.63753	0.73811	-1.0645	0.85644	0.078374	0.541883682
NSA2P6	ENSG00000250980	1.2994	0.096909	1.416	0.078385	0.019497	0.49222	1.416	0.078385	0.078385	0.541883682
IGKV1OR2-3	ENSG00000204670	0.55548	0.28928	1.416	0.078388	0.81924	0.20633	0.23272	0.40799	0.078388	0.541883682
SLC26A11	ENSG00000181045	-0.044463	0.51773	1.416	0.078392	0.96938	0.16618	-0.89708	0.81516	0.078392	0.541883682
CPPED1	ENSG00000103381	1.4159	0.078405	1.4159	0.078405	1.3516	0.088251	1.0337	0.15063	0.078405	0.541883682
CLIC1P1	ENSG00000231313	1.238	0.10785	0.35952	0.3596	1.4159	0.078408	0.063414	0.47472	0.078408	0.541883682
NA	ENSG00000240890	1.4154	0.078474	1.3239	0.092762	1.3239	0.092762	1.3239	0.092762	0.078474	0.542198967
CLEC11A	ENSG00000105472	1.4153	0.078487	0.41716	0.33828	1.4153	0.078487	1.4153	0.078487	0.078487	0.542198967
NA	ENSG00000256763	1.4153	0.078487	1.4153	0.078487	-0.20707	0.58202	-0.95724	0.83078	0.078487	0.542198967
NA	ENSG00000250892	1.4152	0.078508	-0.55048	0.709	0.69567	0.24332	0.97079	0.16583	0.078508	0.542267164
NA	ENSG00000227388	1.4148	0.078568	-0.11589	0.54613	0.59297	0.2766	1.222	0.11085	0.078568	0.542434593
HOXC10	ENSG00000180818	-0.87858	0.81019	1.4147	0.078575	-0.87858	0.81019	-0.87858	0.81019	0.078575	0.542434593
NA	ENSG00000249184	1.4146	0.078594	-0.35124	0.63729	0.42751	0.3345	0.42751	0.3345	0.078594	0.542434593
GON7	ENSG00000170270	0.26073	0.39715	1.4146	0.078598	0.48659	0.31328	0.17434	0.4308	0.078598	0.542434593
RPL11P5	ENSG00000242137	0.43086	0.33328	-1.2002	0.88497	1.4144	0.078629	-0.79976	0.78807	0.078629	0.542434593
LRRC37A15P	ENSG00000230069	1.4142	0.078645	1.4142	0.078645	1.4142	0.078645	1.4142	0.078645	0.078645	0.542434593
KRT8P46	ENSG00000248971	1.4142	0.078645	1.4142	0.078645	1.4142	0.078645	1.4142	0.078645	0.078645	0.542434593
NA	ENSG00000265997	-0.16281	0.56467	0.44669	0.32755	1.2265	0.11001	1.4141	0.078673	0.078673	0.542434593
PPIL4	ENSG00000131013	-1.1717	0.87934	1.414	0.078675	-1.1717	0.87934	0.51392	0.30365	0.078675	0.542434593
KATNA1	ENSG00000186625	1.3917	0.082011	1.4139	0.078692	1.3958	0.081392	1.3027	0.096344	0.078692	0.542434593
NA	ENSG00000235886	1.2908	0.098379	1.4139	0.078701	1.2908	0.098379	1.2908	0.098379	0.078701	0.542434593

FN3KRP	ENSG00000141560	-1.1701	0.87903	-0.90558	0.81742	1.4138	0.078715	0.0017119	0.49932	0.078715	0.542434593
NA	ENSG00000261095	1.0543	0.14586	1.0427	0.14855	1.4137	0.078722	-0.32585	0.62773	0.078722	0.542434593
POU2AF1	ENSG00000110777	0.25979	0.39751	1.4137	0.078728	1.4137	0.078728	1.4137	0.078728	0.078728	0.542434593
NA	ENSG00000235594	-0.6806	0.75194	1.4136	0.078735	-1.0132	0.84452	-1.0132	0.84452	0.078735	0.542434593
SLC18B1	ENSG00000146409	1.1746	0.12007	0.73748	0.23041	0.58356	0.27976	1.4136	0.078738	0.078738	0.542434593
NA	ENSG00000255515	1.1514	0.12477	0.92254	0.17812	1.4136	0.078743	0.72513	0.23419	0.078743	0.542434593
PABPC1P7	ENSG00000250651	1.4135	0.078747	1.4135	0.078747	1.4135	0.078747	1.4135	0.078747	0.078747	0.542434593
NA	ENSG00000270993	1.4135	0.078753	1.4135	0.078753	1.4135	0.078753	1.4135	0.078753	0.078753	0.542434593
HNRNPC	ENSG00000092199	1.4135	0.078761	0.43712	0.33101	0.011982	0.49522	0.70499	0.24041	0.078761	0.542434593
NA	ENSG00000284300	1.4134	0.078766	1.4134	0.078766	1.4134	0.078766	1.4134	0.078766	0.078766	0.542434593
NA	ENSG00000261298	0.3816	0.35138	0.94369	0.17267	0.11864	0.45278	1.4132	0.078804	0.078804	0.542619602
MIR215	ENSG00000207590	1.3135	0.094509	1.413	0.078826	1.413	0.078826	1.413	0.078826	0.078826	0.542677815
NA	ENSG00000279237	1.4129	0.07884	0.75894	0.22394	0.69504	0.24352	0.55406	0.28977	0.07884	0.542677815
TMEM82	ENSG00000162460	0.91453	0.18022	-1.5472	0.93909	-0.61503	0.73073	1.4129	0.078846	0.078846	0.542677815
NA	ENSG00000254710	0.8242	0.20491	0.86888	0.19246	1.4128	0.078857	0.8242	0.20491	0.078857	0.542677815
SAP30L-AS1	ENSG00000245275	1.1046	0.13467	0.22844	0.40965	1.3723	0.084992	1.4127	0.07887	0.07887	0.542690638
NA	ENSG00000246465	1.4123	0.078925	0.20237	0.41981	1.1761	0.11978	1.4123	0.078925	0.078925	0.542992411
OVAAL	ENSG00000236719	1.1669	0.12162	1.4122	0.078946	1.074	0.1414	1.4122	0.078946	0.078946	0.543060217
LMNB1-DT	ENSG00000251072	0.53593	0.296	1.412	0.07897	-0.12975	0.55162	-0.031806	0.51269	0.07897	0.543092618
NA	ENSG00000231760	1.412	0.078973	0.54698	0.2922	1.412	0.078973	1.412	0.078973	0.078973	0.543092618
NA	ENSG00000230805	1.4119	0.078989	-0.55383	0.71015	1.4119	0.078989	1.4119	0.078989	0.078989	0.543126001
PTPRT	ENSG00000196090	0.82991	0.20329	0.40789	0.34168	0.47665	0.31681	1.4118	0.079012	0.079012	0.5432075
DST	ENSG00000151914	1.4116	0.079034	0.99656	0.15949	0.90843	0.18183	0.87757	0.19009	0.079034	0.543212348
NA	ENSG00000241499	1.4116	0.079035	-0.25026	0.59881	-0.25026	0.59881	-0.25026	0.59881	0.079035	0.543212348
NA	ENSG00000255434	1.4113	0.079082	1.4113	0.079082	1.4113	0.079082	1.4113	0.079082	0.079082	0.543443941
LINC01326	ENSG00000244321	0.079153	0.46846	0.079153	0.46846	1.4112	0.079091	0.079153	0.46846	0.079091	0.543443941
DBF4P3	ENSG00000271097	-0.066951	0.52669	1.411	0.079127	0.29854	0.38265	1.411	0.079127	0.079127	0.54361465
ZBTB20-AS4	ENSG00000242767	-0.59352	0.72358	1.4108	0.079148	-1.9578	0.97487	0.68561	0.24648	0.079148	0.543672171
LINC02422	ENSG00000255760	1.4107	0.079163	0.60231	0.27348	1.4107	0.079163	0.68326	0.24722	0.079163	0.543672171
RNU6-607P	ENSG00000223215	-1.0153	0.84502	1.4107	0.079171	0.86225	0.19427	-0.38504	0.64989	0.079171	0.543672171
OR1E3	ENSG00000142163	-1.3532	0.91201	1.4106	0.07918	-1.1582	0.8766	-1.8917	0.97073	0.07918	0.543672171

SELL	ENSG00000188404	-1.5026	0.93353	-1.7117	0.95652	-1.7117	0.95652	1.4104	0.079209	0.079209	0.54379467
NA	ENSG00000234273	1.4101	0.079262	1.2651	0.10292	0.057683	0.477	1.2077	0.11357	0.079262	0.544081879
MAP2K7	ENSG00000076984	1.4098	0.079298	0.11007	0.45618	1.4098	0.079298	1.4098	0.079298	0.079298	0.54418941
COL17A1	ENSG00000065618	1.4098	0.0793	1.301	0.096624	1.4098	0.0793	1.4098	0.0793	0.0793	0.54418941
NA	ENSG00000227200	1.4097	0.079312	1.4097	0.079312	1.4097	0.079312	1.4097	0.079312	0.079312	0.544195123
MIDEAS	ENSG00000156030	1.4096	0.079327	-0.20348	0.58062	0.55581	0.28917	0.49615	0.30989	0.079327	0.544221415
MIR31	ENSG00000199177	1.4095	0.079339	1.4095	0.079339	1.4095	0.079339	1.4095	0.079339	0.079339	0.544227121
STAT6	ENSG00000166888	0.48187	0.31495	0.011209	0.49553	-0.25283	0.5998	1.4093	0.079377	0.079377	0.544390371
RPL21P126	ENSG00000242320	1.409	0.079415	1.409	0.079415	1.409	0.079415	1.409	0.079415	0.079415	0.544390371
LINC01619	ENSG00000257242	1.4089	0.079426	-0.11637	0.54632	-0.0049353	0.50197	0.51854	0.30204	0.079426	0.544390371
TAF11L2	ENSG00000284373	1.4089	0.079434	1.4089	0.079434	1.4089	0.079434	1.4089	0.079434	0.079434	0.544390371
NA	ENSG00000231986	1.4089	0.079435	1.4089	0.079435	1.4089	0.079435	1.4089	0.079435	0.079435	0.544390371
GRINA	ENSG00000178719	1.4089	0.079438	1.4089	0.079438	1.4089	0.079438	1.4089	0.079438	0.079438	0.544390371
PRRC2C	ENSG00000117523	0.77934	0.21789	0.40107	0.34418	1.4088	0.079441	0.47948	0.3158	0.079441	0.544390371
NA	ENSG00000259388	1.2121	0.11273	1.4087	0.079458	1.4087	0.079458	1.4087	0.079458	0.079458	0.544430307
NA	ENSG00000251468	1.4085	0.079488	0.58768	0.27837	1.4085	0.079488	1.4085	0.079488	0.079488	0.544559291
ZNF705A	ENSG00000196946	-0.065577	0.52614	1.4083	0.079514	0.34226	0.36608	0.77736	0.21847	0.079514	0.544660841
SYNGAP1	ENSG00000197283	-0.25466	0.60051	0.17111	0.43207	0.65055	0.25767	1.4081	0.079544	0.079544	0.544789757
NA	ENSG00000252993	-0.037728	0.51505	-0.037728	0.51505	-0.037728	0.51505	1.4078	0.079597	0.079597	0.544998292
RN7SKP42	ENSG00000222845	1.4078	0.0796	1.4078	0.0796	1.4078	0.0796	1.4078	0.0796	0.0796	0.544998292
NA	ENSG00000276993	-1.7163	0.95695	1.4077	0.079608	-1.7163	0.95695	-0.759	0.77607	0.079608	0.544998292
NA	ENSG00000252448	1.4076	0.079628	1.4076	0.079628	1.4076	0.079628	1.4076	0.079628	0.079628	0.545058637
RHBDL3	ENSG00000141314	0.63175	0.26378	1.4075	0.079644	1.1067	0.13421	-0.12782	0.55085	0.079644	0.54509159
NA	ENSG00000226438	1.4072	0.079687	1.0346	0.15042	0.090553	0.46392	0.090553	0.46392	0.079687	0.545204706
OLA1	ENSG00000138430	0.55974	0.28783	-0.24194	0.59559	1.407	0.079707	0.50018	0.30847	0.079707	0.545204706
RNU6-943P	ENSG00000252769	1.4069	0.079728	1.2156	0.11206	1.4069	0.079728	0.69437	0.24373	0.079728	0.545204706
ECEL1P2	ENSG00000244280	1.4068	0.079737	0.68283	0.24736	1.4068	0.079737	1.4068	0.079737	0.079737	0.545204706
VASH1-AS1	ENSG00000258301	-0.052559	0.52096	1.4067	0.079753	-0.052559	0.52096	-0.052559	0.52096	0.079753	0.545204706
SLC47A1	ENSG00000142494	-0.74332	0.77136	1.4067	0.07976	-0.1595	0.56336	-0.80796	0.79044	0.07976	0.545204706
UBXN6	ENSG00000167671	1.2893	0.098646	1.1405	0.12704	1.4066	0.079767	0.83161	0.20281	0.079767	0.545204706
NA	ENSG00000229389	0.30629	0.37969	1.4066	0.079767	-1.4768	0.93014	0.37182	0.35501	0.079767	0.545204706

ZNF175	ENSG00000105497	1.4065	0.079789	1.4065	0.079789	1.4065	0.079789	1.4065	0.079789	0.079789	0.545204706
ZNF83	ENSG00000167766	0.38058	0.35176	-1.0835	0.8607	-0.04454	0.51776	1.4064	0.079802	0.079802	0.545204706
NA	ENSG00000226573	1.4064	0.079802	1.4064	0.079802	1.4064	0.079802	1.4064	0.079802	0.079802	0.545204706
NA	ENSG00000275850	1.4063	0.079815	-1.8904	0.97065	-0.16046	0.56374	-0.16046	0.56374	0.079815	0.545204706
DOP1B	ENSG00000142197	0.27712	0.39085	0.10164	0.45952	0.95475	0.16985	1.4062	0.079826	0.079826	0.545204706
NA	ENSG00000276478	-0.50748	0.69409	1.4061	0.07984	-0.50748	0.69409	-0.2199	0.58702	0.07984	0.545204706
ANKRD2	ENSG00000165887	1.4061	0.079842	1.3186	0.093644	1.4061	0.079842	1.4061	0.079842	0.079842	0.545204706
NA	ENSG00000241962	0.58689	0.27864	0.58689	0.27864	1.3335	0.091186	1.4061	0.079848	0.079848	0.545204706
GCNT7	ENSG00000124091	1.1623	0.12256	0.13815	0.44506	1.406	0.079857	0.7243	0.23444	0.079857	0.545204706
RNU6-687P	ENSG00000252252	1.0413	0.14888	1.4059	0.079874	-0.6439	0.74018	-0.86221	0.80571	0.079874	0.545204706
NA	ENSG00000250990	1.4057	0.0799	1.4057	0.0799	1.4057	0.0799	1.4057	0.0799	0.0799	0.545204706
MIR4639	ENSG00000263712	1.4057	0.079911	0.065351	0.47395	-1.5337	0.93744	-1.5337	0.93744	0.079911	0.545204706
ABCB1	ENSG00000085563	0.16947	0.43271	-0.33061	0.62953	-0.44045	0.67019	1.4056	0.079915	0.079915	0.545204706
SSU72	ENSG00000160075	-0.6964	0.75691	-1.0707	0.85785	-0.48552	0.68635	1.4056	0.079923	0.079923	0.545204706
RPL17P3	ENSG00000271705	0.20653	0.41819	1.4055	0.079934	0.20653	0.41819	0.20653	0.41819	0.079934	0.545204706
KLF7	ENSG00000118263	0.46235	0.32192	0.91479	0.18015	1.4053	0.079962	0.22772	0.40993	0.079962	0.545204706
LINC02061	ENSG00000250885	1.2195	0.11133	1.4053	0.079963	1.1934	0.11636	0.29619	0.38354	0.079963	0.545204706
LBH	ENSG00000213626	-0.43884	0.66961	1.4052	0.079974	0.0075808	0.49698	-0.30897	0.62133	0.079974	0.545204706
LLGL2	ENSG00000073350	0.6401	0.26105	0.6401	0.26105	0.062992	0.47489	1.4051	0.079992	0.079992	0.545204706
NA	ENSG00000280225	0.87583	0.19056	0.9542	0.16999	1.4051	0.079997	1.4051	0.079997	0.079997	0.545204706
NA	ENSG00000229817	1.0575	0.14515	1.0575	0.14515	1.405	0.080008	1.0575	0.14515	0.080008	0.545204706
RPL3P3	ENSG00000243388	-0.39878	0.65497	1.4049	0.080025	-0.39878	0.65497	-0.39878	0.65497	0.080025	0.545204706
ESM1	ENSG00000164283	0.45607	0.32417	1.4049	0.080027	0.45607	0.32417	0.45607	0.32417	0.080027	0.545204706
NA	ENSG00000258809	1.3381	0.090438	1.4047	0.080049	1.3381	0.090438	1.3381	0.090438	0.080049	0.545204706
NA	ENSG00000231585	1.4045	0.080086	1.4045	0.080086	1.4045	0.080086	1.4045	0.080086	0.080086	0.545204706
NA	ENSG00000197376	-0.81801	0.79332	-0.36986	0.64426	-0.36986	0.64426	1.4044	0.080093	0.080093	0.545204706
RPAP2P1	ENSG00000270874	0.81413	0.20779	1.4044	0.080093	0.80327	0.21091	0.45029	0.32625	0.080093	0.545204706
NA	ENSG00000201549	0.3284	0.3713	1.4044	0.080104	0.3284	0.3713	0.1625	0.43546	0.080104	0.545204706
NA	ENSG00000279319	1.3867	0.082762	1.4044	0.080105	1.4044	0.080105	1.4044	0.080105	0.080105	0.545204706
NA	ENSG00000279378	-0.27107	0.60683	1.4043	0.080109	1.4043	0.080109	1.4043	0.080109	0.080109	0.545204706
NA	ENSG00000264687	-0.17384	0.569	-0.99439	0.83998	0.63454	0.26286	1.4042	0.080129	0.080129	0.545204706

BZW1-AS1	ENSG00000230408	0.77848	0.21814	1.4042	0.080136	0.77848	0.21814	1.0148	0.15511	0.080136	0.545204706
NA	ENSG00000253220	1.4041	0.08014	0.34504	0.36503	1.4041	0.08014	1.4041	0.08014	0.08014	0.545204706
NA	ENSG00000231873	1.4041	0.080145	-0.64121	0.73931	-1.4984	0.93299	0.47734	0.31656	0.080145	0.545204706
SMG1P2	ENSG00000205534	0.72935	0.23289	1.1777	0.11947	1.4041	0.08015	0.71306	0.2379	0.08015	0.545204706
SNORD91B	ENSG00000275084	1.404	0.080158	1.404	0.080158	1.404	0.080158	1.404	0.080158	0.080158	0.545204706
NA	ENSG00000283579	-0.55006	0.70886	0.87701	0.19024	-0.79564	0.78688	1.404	0.080164	0.080164	0.545204706
ALG1L	ENSG00000189366	-0.71669	0.76322	-0.008203	0.50327	1.4036	0.080213	-1.261	0.89634	0.080213	0.545328736
HSPE1P24	ENSG00000225158	-0.13932	0.5554	-0.13932	0.5554	1.2607	0.10371	1.4036	0.080217	0.080217	0.545328736
ENO1P1	ENSG00000244457	1.4036	0.080226	1.4036	0.080226	1.4036	0.080226	1.4036	0.080226	0.080226	0.545328736
TAS1R1	ENSG00000173662	1.1062	0.13431	1.1062	0.13431	1.4035	0.080227	1.3434	0.089565	0.080227	0.545328736
NA	ENSG00000258082	0.63256	0.26351	0.47744	0.31653	1.3368	0.090638	1.4034	0.080255	0.080255	0.545442978
NA	ENSG00000271573	1.4032	0.080278	1.4032	0.080278	1.4032	0.080278	1.4032	0.080278	0.080278	0.54552321
NA	ENSG00000266535	-0.61162	0.7296	1.403	0.080309	0.33307	0.36954	0.33307	0.36954	0.080309	0.545553203
WNT9A	ENSG00000143816	0.40665	0.34213	1.403	0.080311	0.47854	0.31613	-0.33706	0.63196	0.080311	0.545553203
NA	ENSG00000273461	1.403	0.080316	1.403	0.080316	1.403	0.080316	1.403	0.080316	0.080316	0.545553203
NA	ENSG00000271629	0.80642	0.21	0.80642	0.21	0.80642	0.21	1.4026	0.080363	0.080363	0.545572393
SLC44A1	ENSG00000070214	0.89532	0.18531	1.4026	0.080366	1.1021	0.13521	0.67762	0.24901	0.080366	0.545572393
RNA5SP212	ENSG00000212525	0.72711	0.23358	0.72711	0.23358	0.72711	0.23358	1.4026	0.080373	0.080373	0.545572393
LINC02787	ENSG00000229201	-1.8439	0.9674	-0.9735	0.83485	1.4025	0.080378	1.4025	0.080378	0.080378	0.545572393
NA	ENSG00000212363	1.4025	0.080379	1.0284	0.15189	1.4025	0.080379	-0.66532	0.74708	0.080379	0.545572393
LIMD1	ENSG00000144791	0.66513	0.25298	1.4025	0.080386	-0.50507	0.69325	0.25198	0.40053	0.080386	0.545572393
RGPD1	ENSG00000187627	1.4023	0.080406	-0.11143	0.54436	-0.048164	0.51921	0.91598	0.17984	0.080406	0.545632138
CUL7	ENSG00000044090	1.4018	0.080484	1.3944	0.081605	1.2877	0.09893	1.3528	0.088059	0.080484	0.546085397
LINC00407	ENSG00000237585	1.1963	0.1158	1.4016	0.080513	-0.90664	0.8177	-0.064715	0.5258	0.080513	0.546200447
C3orf35	ENSG00000198590	-0.37495	0.64615	-0.19641	0.57785	0.87041	0.19204	1.4014	0.080541	0.080541	0.546200447
HYI-AS1	ENSG00000229348	1.4014	0.080545	1.4014	0.080545	1.4014	0.080545	1.4014	0.080545	0.080545	0.546200447
RPL21P1	ENSG00000214760	1.4014	0.080551	0.77038	0.22054	1.0161	0.1548	1.0161	0.1548	0.080551	0.546200447
RNA5SP336	ENSG00000201059	1.3577	0.087279	1.4013	0.080557	1.4013	0.080557	1.4013	0.080557	0.080557	0.546200447
BMS1P4	ENSG00000242338	1.4011	0.080588	0.54873	0.2916	1.4011	0.080588	1.4011	0.080588	0.080588	0.546334619
PPP1R14BP5	ENSG00000213133	1.401	0.080603	1.401	0.080603	0.14974	0.44049	0.80765	0.20965	0.080603	0.546360299
KATNBL1P3	ENSG00000235422	1.4009	0.080624	1.4009	0.080624	1.4009	0.080624	1.4009	0.080624	0.080624	0.546425733

GRIN2D	ENSG00000105464	0.34702	0.36429	0.34702	0.36429	1.4008	0.080642	0.34702	0.36429	0.080642	0.546425733
NA	ENSG00000264813	1.4007	0.080651	1.4007	0.080651	1.4007	0.080651	1.4007	0.080651	0.080651	0.546425733
RPL12P20	ENSG00000239627	0.40108	0.34418	0.40085	0.34426	1.4005	0.080677	1.4005	0.080677	0.080677	0.546425733
NA	ENSG00000246323	0.99072	0.16091	0.63564	0.26251	1.4005	0.080685	1.243	0.10694	0.080685	0.546425733
CENPNP2	ENSG00000228615	0.15381	0.43888	1.4004	0.080692	0.15381	0.43888	1.3226	0.092977	0.080692	0.546425733
NA	ENSG00000260817	0.13214	0.44744	1.4004	0.080696	0.3301	0.37066	0.3301	0.37066	0.080696	0.546425733
LINC01716	ENSG00000225657	0.61326	0.26985	-0.38475	0.64979	0.22185	0.41222	1.4004	0.080704	0.080704	0.546425733
TRIM16L	ENSG00000108448	-0.44004	0.67004	1.4002	0.08073	0.72348	0.23469	1.2644	0.10305	0.08073	0.546425733
PDCL3	ENSG00000115539	-1.5435	0.93864	-0.8345	0.798	-2.3646	0.99097	1.4002	0.080734	0.080734	0.546425733
NA	ENSG00000176984	1.4001	0.080736	0.93906	0.17385	0.063218	0.4748	-0.34667	0.63558	0.080736	0.546425733
LINC01711	ENSG00000268941	1.3999	0.080767	-0.56369	0.71352	0.86847	0.19257	1.1405	0.12704	0.080767	0.546517058
NA	ENSG00000216663	1.3998	0.08079	1.3998	0.08079	1.3998	0.08079	1.2201	0.11122	0.08079	0.546517058
NA	ENSG00000257995	1.3997	0.080806	0.23847	0.40576	0.23847	0.40576	0.23847	0.40576	0.080806	0.546517058
CLCA3P	ENSG00000153923	1.3996	0.080813	-0.58267	0.71994	-0.74077	0.77058	-0.6434	0.74002	0.080813	0.546517058
H2AZP2	ENSG00000253173	1.3996	0.080823	0.18867	0.42518	1.2388	0.10771	0.12572	0.44998	0.080823	0.546517058
NA	ENSG00000250132	1.3156	0.094147	1.3995	0.080825	0.69187	0.24451	1.3523	0.088142	0.080825	0.546517058
THAP5	ENSG00000177683	1.3995	0.080828	1.3995	0.080828	1.3995	0.080828	0.87268	0.19142	0.080828	0.546517058
FAM209A	ENSG00000124103	-0.018113	0.50723	0.014307	0.49429	1.3994	0.080843	1.3994	0.080843	0.080843	0.546542646
RASGRP2	ENSG00000068831	-0.27789	0.60945	1.3992	0.080882	0.095863	0.46181	-0.27789	0.60945	0.080882	0.546571317
NA	ENSG00000262801	0.73444	0.23134	-0.40769	0.65825	1.3991	0.080892	1.3991	0.080892	0.080892	0.546571317
IFIT2	ENSG00000119922	-1.3037	0.90383	1.3991	0.080897	-0.22594	0.58938	0.40113	0.34416	0.080897	0.546571317
NA	ENSG00000204187	-1.1516	0.87526	-0.71076	0.76138	1.3988	0.08093	-0.17047	0.56768	0.08093	0.546571317
NA	ENSG00000260853	1.3988	0.080936	1.3988	0.080936	1.3988	0.080936	1.3988	0.080936	0.080936	0.546571317
ALDH1L1	ENSG00000144908	1.3988	0.080937	-0.097801	0.53896	0.10919	0.45652	0.49027	0.31197	0.080937	0.546571317
TECTA	ENSG00000109927	-0.32314	0.62671	1.3986	0.080963	-0.032276	0.51287	-0.18262	0.57245	0.080963	0.546571317
LINC02615	ENSG00000251432	-1.5492	0.93933	-0.39217	0.65254	1.3984	0.080991	-0.96752	0.83336	0.080991	0.546571317
LINC01443	ENSG00000266554	0.87799	0.18998	1.3984	0.081001	1.3984	0.081001	1.3213	0.093195	0.081001	0.546571317
NA	ENSG00000236531	1.3983	0.081013	-0.16879	0.56702	1.3983	0.081013	1.3983	0.081013	0.081013	0.546571317
BMP7-AS1	ENSG00000235032	-1.103	0.86499	1.3982	0.081019	-1.103	0.86499	-1.103	0.86499	0.081019	0.546571317
ZNF777	ENSG00000196453	1.3981	0.081036	-0.79116	0.78558	0.28451	0.38801	0.69135	0.24467	0.081036	0.546571317
NA	ENSG00000231992	1.3981	0.081036	1.3981	0.081036	1.3981	0.081036	1.3981	0.081036	0.081036	0.546571317

NA	ENSG00000238005	1.1159	0.13224	1.3761	0.084396	1.1159	0.13224	1.3981	0.081036	0.081036	0.546571317
NINJ2	ENSG00000171840	-1.1678	0.87855	1.3981	0.08104	-0.40349	0.65671	-0.22733	0.58992	0.08104	0.546571317
POLE3	ENSG00000148229	1.1938	0.11628	1.3981	0.081044	1.3631	0.086419	0.37286	0.35462	0.081044	0.546571317
MAB21L2	ENSG00000181541	1.3981	0.081047	1.3981	0.081047	1.3981	0.081047	1.3981	0.081047	0.081047	0.546571317
EWSAT1	ENSG00000212766	-0.27507	0.60837	1.3085	0.095346	1.3979	0.081072	0.31784	0.3753	0.081072	0.546571317
ATP5MC1P3	ENSG00000242244	1.3979	0.081072	1.3856	0.08294	0.74387	0.22848	-0.39297	0.65283	0.081072	0.546571317
PTCHD3	ENSG00000182077	0.97535	0.16469	-0.15223	0.5605	1.3978	0.081084	-0.87162	0.80829	0.081084	0.546571317
NA	ENSG00000270631	1.2619	0.1035	0.53222	0.29729	1.3978	0.081089	-0.17458	0.56929	0.081089	0.546571317
ZNF75A	ENSG00000162086	-0.26254	0.60355	-0.26254	0.60355	1.3978	0.081094	-0.50009	0.69149	0.081094	0.546571317
RNU2-47P	ENSG00000222581	1.3975	0.081129	0.72693	0.23363	1.0233	0.15308	1.0233	0.15308	0.081129	0.546626652
LINC01208	ENSG00000223715	1.3973	0.081156	1.3973	0.081156	1.3973	0.081156	0.91691	0.1796	0.081156	0.546626652
KLC2	ENSG00000174996	0.21089	0.41648	1.3973	0.081159	0.21089	0.41648	0.36732	0.35669	0.081159	0.546626652
HIGD2AP2	ENSG00000235695	1.3972	0.081174	1.3972	0.081174	1.3972	0.081174	1.3972	0.081174	0.081174	0.546626652
H2BP6	ENSG00000226908	-1.6677	0.95231	0.59501	0.27592	-1.2197	0.88871	1.3972	0.081179	0.081179	0.546626652
NA	ENSG00000259481	1.3971	0.081197	-1.3246	0.90735	1.3971	0.081197	1.3971	0.081197	0.081197	0.546626652
NA	ENSG00000228194	0.3382	0.36761	1.3969	0.08122	1.3969	0.08122	0.66226	0.2539	0.08122	0.546626652
NA	ENSG00000232227	1.3969	0.081222	1.3969	0.081222	1.3969	0.081222	1.3969	0.081222	0.081222	0.546626652
LAMP1	ENSG00000185896	-0.2411	0.59526	-0.17629	0.56997	1.3967	0.081251	-0.24981	0.59863	0.081251	0.546626652
NA	ENSG00000272017	1.3967	0.081253	1.3967	0.081253	1.3967	0.081253	1.3967	0.081253	0.081253	0.546626652
NA	ENSG00000273249	0.66848	0.25192	0.54821	0.29178	1.3967	0.081255	-0.88146	0.81097	0.081255	0.546626652
NA	ENSG00000271155	1.3966	0.081263	-0.084904	0.53383	1.3966	0.081263	1.3966	0.081263	0.081263	0.546626652
ZC3H11B	ENSG00000215817	0.25323	0.40004	0.15241	0.43943	0.74075	0.22942	1.3966	0.081271	0.081271	0.546626652
HRH2	ENSG00000113749	0.28776	0.38677	-1.0523	0.85368	1.3966	0.081272	1.1644	0.12212	0.081272	0.546626652
CAPS2-AS1	ENSG00000254451	0.42775	0.33442	1.3964	0.081292	0.22525	0.41089	0.22525	0.41089	0.081292	0.546626652
PADI4	ENSG00000159339	-0.84271	0.80031	1.3963	0.081308	-1.3835	0.91675	0.50965	0.30515	0.081308	0.546626652
NA	ENSG00000250669	1.3963	0.081318	1.3963	0.081318	1.3963	0.081318	1.3963	0.081318	0.081318	0.546626652
PCDHGC3	ENSG00000240184	1.3962	0.081321	1.3962	0.081321	1.3645	0.086199	1.3962	0.081321	0.081321	0.546626652
MISP	ENSG00000099812	1.3959	0.081372	0.033706	0.48656	-0.13867	0.55514	-0.20734	0.58213	0.081372	0.546626652
NA	ENSG00000236018	1.3958	0.081385	1.3958	0.081385	1.3958	0.081385	1.3958	0.081385	0.081385	0.546626652
SETD6	ENSG00000103037	1.0808	0.13989	1.1503	0.12501	1.3956	0.081419	0.96836	0.16643	0.081419	0.546626652
SLC25A19	ENSG00000125454	0.62938	0.26455	1.3956	0.081419	1.1016	0.13531	1.2883	0.09882	0.081419	0.546626652

GINM1	ENSG00000055211	1.2561	0.10454	1.2572	0.10433	1.3255	0.092499	1.3955	0.08143	0.08143	0.546626652
PCDHB15	ENSG00000113248	1.3954	0.081447	1.3954	0.081447	1.3954	0.081447	1.3954	0.081447	0.081447	0.546626652
MYH14	ENSG00000105357	-0.0066869	0.50267	1.3953	0.081467	0.10914	0.45655	0.36295	0.35832	0.081467	0.546626652
LINC01256	ENSG00000251398	0.21008	0.4168	-0.74315	0.7713	1.3952	0.081478	1.3952	0.081478	0.081478	0.546626652
NA	ENSG00000279885	0.30284	0.381	1.0778	0.14057	0.88067	0.18925	1.395	0.081503	0.081503	0.546626652
NA	ENSG00000235840	-0.12716	0.55059	1.395	0.081515	0.12221	0.45137	0.25922	0.39773	0.081515	0.546626652
NA	ENSG00000253572	-0.95144	0.82931	0.5573	0.28866	-0.59799	0.72508	1.3948	0.081543	0.081543	0.546626652
LINC02843	ENSG00000228189	0.47979	0.31569	0.58717	0.27855	0.47979	0.31569	1.3945	0.081585	0.081585	0.546626652
RFX3-AS1	ENSG00000232104	1.3945	0.081586	-0.86282	0.80588	0.28221	0.38889	-0.35043	0.63699	0.081586	0.546626652
PANX1	ENSG00000110218	0.52127	0.30109	-0.072886	0.52905	-0.072886	0.52905	1.3945	0.08159	0.08159	0.546626652
RUNX2	ENSG00000124813	-0.48503	0.68617	1.3944	0.081599	-0.14406	0.55727	-0.14222	0.55655	0.081599	0.546626652
NA	ENSG00000275406	0.66889	0.25178	0.66889	0.25178	0.66889	0.25178	1.3944	0.081602	0.081602	0.546626652
NPPC	ENSG00000163273	1.3943	0.081615	0.48435	0.31407	0.95599	0.16954	0.60277	0.27333	0.081615	0.546626652
GADD45B	ENSG00000099860	1.3942	0.081629	0.058445	0.4767	1.3942	0.081629	1.3942	0.081629	0.081629	0.546626652
LRPAP1	ENSG00000163956	0.19869	0.42125	0.38982	0.34833	0.75103	0.22632	1.3941	0.081639	0.081639	0.546626652
PRRT1B	ENSG00000283526	-0.54774	0.70806	0.55949	0.28791	1.3941	0.081643	0.68311	0.24727	0.081643	0.546626652
NA	ENSG00000235293	0.89604	0.18512	-0.15919	0.56324	1.3941	0.081648	1.3941	0.081648	0.081648	0.546626652
NA	ENSG00000261251	0.20208	0.41993	1.394	0.081661	-0.40566	0.6575	-0.40566	0.6575	0.081661	0.546626652
NA	ENSG00000223948	0.15548	0.43822	1.3939	0.081679	1.3939	0.081679	1.3939	0.081679	0.081679	0.546626652
PAFAH1B1	ENSG00000007168	1.3937	0.08171	-1.9309	0.97325	0.94462	0.17243	0.5055	0.3066	0.08171	0.546626652
CUL2	ENSG00000108094	1.3936	0.081725	1.3146	0.094316	1.1759	0.11981	0.59961	0.27438	0.081725	0.546626652
NA	ENSG00000230343	1.3935	0.081731	-0.32421	0.62711	0.56472	0.28613	0.2512	0.40083	0.081731	0.546626652
RNA5SP55	ENSG00000252510	1.3935	0.081734	1.3935	0.081734	1.3935	0.081734	1.3935	0.081734	0.081734	0.546626652
CXXC5	ENSG00000171604	-0.046167	0.51841	-0.1509	0.55997	1.3935	0.081739	-0.46631	0.6795	0.081739	0.546626652
RPL7AP57	ENSG00000242248	1.2785	0.10053	1.3934	0.081743	1.0484	0.14723	1.0484	0.14723	0.081743	0.546626652
MS4A8	ENSG00000166959	-1.053	0.85382	-1.053	0.85382	1.3934	0.081746	-0.88713	0.8125	0.081746	0.546626652
NA	ENSG00000271559	1.0908	0.13769	-0.76368	0.77747	1.3933	0.081764	1.3933	0.081764	0.081764	0.546626652
NA	ENSG00000271360	1.3933	0.081767	1.3933	0.081767	1.3933	0.081767	1.3933	0.081767	0.081767	0.546626652
NA	ENSG00000229022	-0.15325	0.5609	1.3933	0.081769	-0.38363	0.64937	1.0124	0.15568	0.081769	0.546626652
NA	ENSG00000263551	1.3931	0.081792	-0.13947	0.55546	-0.93545	0.82522	-0.57214	0.71639	0.081792	0.546626652
LAMA4	ENSG00000112769	-0.95701	0.83072	0.31168	0.37764	-0.51843	0.69792	1.3931	0.081796	0.081796	0.546626652

RN7SKP75	ENSG00000199940	0.42358	0.33594	1.393	0.081808	-0.15016	0.55968	1.3924	0.0819	0.081808	0.546626652
MUC6	ENSG00000184956	1.3456	0.089222	0.5738	0.28305	0.66543	0.25289	1.3928	0.08184	0.08184	0.546626652
NA	ENSG00000241978	-1.0406	0.85096	0.072799	0.47098	-0.14501	0.55765	1.3928	0.08184	0.08184	0.546626652
LSM3	ENSG00000170860	-0.50343	0.69267	-0.29277	0.61515	1.3927	0.081857	-0.22923	0.59065	0.081857	0.546626652
C9orf64	ENSG00000165118	0.25565	0.39911	0.71166	0.23834	1.3926	0.081872	1.0403	0.1491	0.081872	0.546626652
OSBP	ENSG00000110048	1.3926	0.081875	1.2352	0.10837	1.3039	0.096126	1.0261	0.15242	0.081875	0.546626652
NA	ENSG00000234535	1.0973	0.13625	1.0973	0.13625	1.0973	0.13625	1.3925	0.081879	0.081879	0.546626652
RNU6-931P	ENSG00000200720	-1.2335	0.8913	1.3925	0.081882	-0.32577	0.6277	-0.32577	0.6277	0.081882	0.546626652
RPS4XP9	ENSG00000217404	0.61176	0.27035	0.61176	0.27035	1.3924	0.081899	0.61176	0.27035	0.081899	0.546626652
PRELP	ENSG00000188783	-0.2391	0.59449	-0.70404	0.7593	-0.051303	0.52046	1.3924	0.081902	0.081902	0.546626652
NA	ENSG00000268536	1.3923	0.081915	1.3923	0.081915	1.1711	0.12079	1.3923	0.081915	0.081915	0.546626652
PYCR2	ENSG00000143811	0.17452	0.43073	1.3922	0.081936	0.17452	0.43073	-0.49703	0.69042	0.081936	0.546626652
NA	ENSG00000278994	-0.41812	0.66207	1.3922	0.081938	-0.012066	0.50481	0.68259	0.24743	0.081938	0.546626652
NA	ENSG00000261641	1.3921	0.081949	1.3921	0.081949	1.3921	0.081949	1.3921	0.081949	0.081949	0.546626652
SNHG8	ENSG00000269893	0.54111	0.29422	1.392	0.081963	-0.044607	0.51779	0.48895	0.31244	0.081963	0.546626652
CLCC1	ENSG00000121940	0.5905	0.27743	-0.23429	0.59262	1.392	0.081966	-0.29889	0.61749	0.081966	0.546626652
VAPA	ENSG00000101558	1.3919	0.081983	0.65293	0.2569	1.209	0.11332	1.3133	0.09454	0.081983	0.546626652
SNORD58B	ENSG00000271982	1.3918	0.08199	1.3918	0.08199	1.3918	0.08199	1.3918	0.08199	0.08199	0.546626652
TMEM14B	ENSG00000137210	1.2031	0.11446	1.3917	0.082004	1.2031	0.11446	-0.65947	0.7452	0.082004	0.546626652
NA	ENSG00000257872	1.3917	0.082007	1.3917	0.082007	1.3917	0.082007	1.3917	0.082007	0.082007	0.546626652
NEDD8	ENSG00000129559	1.3916	0.082021	1.3916	0.082021	1.3916	0.082021	1.3916	0.082021	0.082021	0.546626652
RN7SKP235	ENSG00000222594	1.3916	0.082024	0.52046	0.30137	0.18702	0.42582	0.33237	0.36981	0.082024	0.546626652
NA	ENSG00000261745	0.22796	0.40984	0.93935	0.17378	1.3916	0.082027	1.3916	0.082027	0.082027	0.546626652
NA	ENSG00000224797	-0.32051	0.62571	0.9065	0.18234	0.89087	0.1865	1.3915	0.082038	0.082038	0.546626652
RPS24P14	ENSG00000223688	-0.58065	0.71926	1.3914	0.082045	-0.58941	0.72221	-0.70342	0.7591	0.082045	0.546626652
NA	ENSG00000268204	1.3914	0.082047	1.3914	0.082047	1.3914	0.082047	1.3914	0.082047	0.082047	0.546626652
NA	ENSG00000231615	1.3343	0.091053	1.3914	0.082052	1.3914	0.082052	1.3914	0.082052	0.082052	0.546626652
LPGAT1-AS1	ENSG00000229258	1.3913	0.082069	1.3913	0.082069	1.3913	0.082069	1.3913	0.082069	0.082069	0.546626652
NA	ENSG00000223703	1.091	0.13764	0.98737	0.16173	0.79433	0.2135	1.3913	0.082073	0.082073	0.546626652
RSU1	ENSG00000148484	-0.47885	0.68398	-0.3301	0.62934	1.1457	0.12596	1.3912	0.08208	0.08208	0.546626652
NA	ENSG00000259579	-0.47592	0.68293	1.3912	0.082084	0.18992	0.42469	-0.73109	0.76764	0.082084	0.546626652

POU5F1P4	ENSG00000237872	1.3911	0.082101	1.0243	0.15284	1.2193	0.11136	1.2193	0.11136	0.082101	0.546626652
OR13Z1P	ENSG00000226653	0.54441	0.29308	1.391	0.082106	1.111	0.13329	1.111	0.13329	0.082106	0.546626652
NA	ENSG00000264739	1.391	0.082107	1.391	0.082107	1.391	0.082107	1.391	0.082107	0.082107	0.546626652
H3-3B	ENSG00000132475	1.391	0.082116	1.3855	0.082943	1.0513	0.14657	1.0513	0.14657	0.082116	0.546626652
MTCO3P24	ENSG00000262158	0.2542	0.39967	1.3909	0.082125	0.59468	0.27603	0.60202	0.27358	0.082125	0.546626652
NA	ENSG00000251456	1.1077	0.13399	-0.18591	0.57374	1.3909	0.082128	0.35199	0.36242	0.082128	0.546626652
SLC15A5	ENSG00000188991	1.3908	0.08214	-0.17955	0.57125	0.79207	0.21416	-1.553	0.93979	0.08214	0.546626652
PRR12	ENSG00000126464	0.061847	0.47534	1.3908	0.082146	1.3908	0.082146	-0.040078	0.51598	0.082146	0.546626652
PDE1B	ENSG00000123360	1.3907	0.082163	0.5108	0.30475	0.21891	0.41336	1.3907	0.082163	0.082163	0.546626652
U2AF2	ENSG00000063244	1.3635	0.086367	1.3906	0.082172	1.3906	0.082172	1.3906	0.082172	0.082172	0.546626652
NA	ENSG00000232465	-1.2082	0.88652	0.77658	0.2187	-0.6577	0.74464	1.3906	0.082177	0.082177	0.546626652
COPS8P2	ENSG00000214552	1.3905	0.082195	0.35695	0.36057	1.0138	0.15535	1.3905	0.082195	0.082195	0.546626652
LLPHP1	ENSG00000270793	0.57041	0.2842	0.57041	0.2842	1.3904	0.082205	0.25772	0.39831	0.082205	0.546626652
DUSP8P5	ENSG00000235316	1.3904	0.082208	1.3904	0.082208	1.3904	0.082208	1.2509	0.10549	0.082208	0.546626652
NA	ENSG00000213488	1.0989	0.1359	0.3208	0.37418	0.94811	0.17154	1.3903	0.082223	0.082223	0.546626652
NA	ENSG00000234996	1.3902	0.082232	1.3902	0.082232	1.3902	0.082232	1.3902	0.082232	0.082232	0.546626652
NA	ENSG00000271071	0.084259	0.46643	0.13967	0.44446	0.58864	0.27805	1.3901	0.082242	0.082242	0.546626652
HPD	ENSG00000158104	0.7769	0.21861	1.3899	0.082281	1.3317	0.091472	0.50669	0.30619	0.082281	0.546626652
NA	ENSG00000279088	1.3898	0.082288	1.3898	0.082288	1.3898	0.082288	1.3898	0.082288	0.082288	0.546626652
C5orf34	ENSG00000172244	0.35579	0.361	-0.71507	0.76272	1.3897	0.082309	-0.1803	0.57154	0.082309	0.546626652
SNORD58A	ENSG00000206602	1.3897	0.082315	1.3897	0.082315	1.3897	0.082315	1.3897	0.082315	0.082315	0.546626652
NA	ENSG00000260401	1.3896	0.082324	-0.56622	0.71438	1.3896	0.082324	1.3896	0.082324	0.082324	0.546626652
NA	ENSG00000251545	0.90841	0.18183	0.90841	0.18183	1.3894	0.082354	0.36844	0.35627	0.082354	0.546626652
RN7SL396P	ENSG00000244642	1.2302	0.10932	1.1784	0.11931	1.3788	0.083979	1.3894	0.08236	0.08236	0.546626652
LINC02557	ENSG00000225413	-0.44249	0.67093	1.3894	0.082361	1.3894	0.082361	1.3894	0.082361	0.082361	0.546626652
LARP4P	ENSG00000261315	1.3893	0.082364	1.3893	0.082364	1.3893	0.082364	1.3893	0.082364	0.082364	0.546626652
TMEM271	ENSG00000273238	1.1259	0.13011	-1.1443	0.87375	1.3893	0.082373	-1.1443	0.87375	0.082373	0.546626652
LMO2	ENSG00000135363	0.56084	0.28745	0.46305	0.32166	-1.2435	0.89316	1.3892	0.082379	0.082379	0.546626652
VNN3	ENSG00000093134	0.96439	0.16742	1.3892	0.08238	0.96439	0.16742	0.56075	0.28748	0.08238	0.546626652
HMGN1P10	ENSG00000244101	1.3892	0.082381	1.3892	0.082381	1.3892	0.082381	1.3892	0.082381	0.082381	0.546626652
SAP30L	ENSG00000164576	0.36358	0.35809	0.36358	0.35809	0.49158	0.31151	1.3889	0.082431	0.082431	0.54679038

SEMA6A	ENSG00000092421	-0.055008	0.52193	-0.060066	0.52395	1.3888	0.082448	-0.21317	0.5844	0.082448	0.54679038
NA	ENSG00000282246	1.3888	0.082449	1.3888	0.082449	1.3888	0.082449	1.3888	0.082449	0.082449	0.54679038
SPHKAP	ENSG00000153820	0.31055	0.37807	1.3887	0.082461	0.98331	0.16273	0.21769	0.41383	0.082461	0.54679038
NA	ENSG00000256372	1.3887	0.082463	1.3887	0.082463	1.3887	0.082463	1.3887	0.082463	0.082463	0.54679038
CFAP251	ENSG00000158023	1.3152	0.094224	1.3886	0.082473	1.2577	0.10425	0.91786	0.17935	0.082473	0.54679038
LARP4B	ENSG00000107929	1.3112	0.094899	0.89198	0.1862	1.388	0.082564	-0.48725	0.68696	0.082564	0.54717548
MGST1	ENSG00000008394	1.388	0.08257	-1.1075	0.86596	-0.0032246	0.50129	1.082	0.13963	0.08257	0.54717548
TMEM207	ENSG00000198398	-0.078976	0.53147	1.388	0.082576	0.91953	0.17891	0.71677	0.23676	0.082576	0.54717548
MTND2P9	ENSG00000225901	1.3879	0.082576	1.3879	0.082576	1.3879	0.082576	1.3879	0.082576	0.082576	0.54717548
NA	ENSG00000236461	1.3878	0.082599	1.3878	0.082599	1.3878	0.082599	1.3878	0.082599	0.082599	0.547192323
NA	ENSG00000202343	1.3878	0.082601	1.3878	0.082601	1.3878	0.082601	1.3878	0.082601	0.082601	0.547192323
LINC01934	ENSG00000234663	0.31812	0.3752	-2.354	0.99071	1.3877	0.082613	-0.93165	0.82424	0.082613	0.54719743
MIR550A2	ENSG00000207573	-0.60639	0.72787	-0.45351	0.67491	-1.5739	0.94224	1.3873	0.082682	0.082682	0.547330861
NA	ENSG00000203498	1.3872	0.082689	1.3872	0.082689	1.3872	0.082689	1.2153	0.11213	0.082689	0.547330861
RPL23P5	ENSG00000230406	1.3871	0.082699	-0.016935	0.50676	1.1952	0.11601	-0.21871	0.58656	0.082699	0.547330861
OR10J5	ENSG00000184155	1.3871	0.082701	-0.66491	0.74694	-0.34311	0.63424	-0.34311	0.63424	0.082701	0.547330861
NA	ENSG00000271851	1.3871	0.082704	1.3871	0.082704	1.3871	0.082704	1.3871	0.082704	0.082704	0.547330861
NA	ENSG00000233258	1.3871	0.082709	1.3871	0.082709	1.3871	0.082709	1.3871	0.082709	0.082709	0.547330861
LINC01426	ENSG00000234380	1.387	0.082722	0.66384	0.2534	0.61934	0.26785	0.094696	0.46228	0.082722	0.547330861
NA	ENSG00000257921	1.387	0.082723	1.387	0.082723	1.387	0.082723	1.387	0.082723	0.082723	0.547330861
NA	ENSG00000257579	0.52937	0.29827	0.63427	0.26295	1.3866	0.082774	1.3866	0.082774	0.082774	0.547593948
NA	ENSG00000273736	1.0633	0.14383	1.3864	0.082815	1.0633	0.14383	1.0822	0.13959	0.082815	0.547790817
CHD1	ENSG00000153922	-0.52604	0.70057	1.386	0.08288	-0.20797	0.58237	0.32207	0.3737	0.08288	0.548091816
RNU6-125P	ENSG00000207234	1.3859	0.082883	1.1008	0.13548	1.1008	0.13548	1.1008	0.13548	0.082883	0.548091816
PPIAP43	ENSG00000255059	1.3857	0.082914	1.3857	0.082914	1.3857	0.082914	1.3857	0.082914	0.082914	0.548167875
RPS3AP19	ENSG00000244004	0.098926	0.4606	-0.76171	0.77688	-0.85531	0.80381	1.3857	0.082917	0.082917	0.548167875
ACADM	ENSG00000117054	0.5354	0.29619	1.3856	0.082939	0.8297	0.20335	-0.080999	0.53228	0.082939	0.548177801
NA	ENSG00000227950	0.48219	0.31484	0.48219	0.31484	1.3856	0.082941	0.48219	0.31484	0.082941	0.548177801
ACOT2	ENSG00000119673	-1.0488	0.85288	1.3855	0.082957	-0.74013	0.77039	-0.74013	0.77039	0.082957	0.548209196
GLRX3P2	ENSG00000216657	0.46469	0.32108	0.038618	0.4846	1.3853	0.082978	-1.2066	0.88621	0.082978	0.548258746
SKOR2	ENSG00000215474	1.3853	0.082987	-0.50387	0.69282	0.44824	0.32699	1.0978	0.13615	0.082987	0.548258746

NA	ENSG00000229774	1.385	0.083024	0.71727	0.2366	0.32971	0.37081	0.61426	0.26952	0.083024	0.548302936
RNU6ATAC31P	ENSG00000221440	1.385	0.083029	1.385	0.083029	1.385	0.083029	1.385	0.083029	0.083029	0.548302936
RNA5SP231	ENSG00000222428	0.8448	0.19911	0.8448	0.19911	0.8448	0.19911	1.3849	0.083039	0.083039	0.548302936
ADCY9	ENSG00000162104	1.3848	0.083063	-0.71531	0.76279	-0.014637	0.50584	0.33241	0.36979	0.083063	0.548302936
CD4	ENSG00000010610	1.3846	0.08309	-0.39609	0.65398	-0.051702	0.52062	-0.083207	0.53316	0.08309	0.548302936
SMARCE1	ENSG00000073584	1.134	0.1284	1.3845	0.083103	-0.035998	0.51436	-0.035998	0.51436	0.083103	0.548302936
CATSPERD	ENSG00000174898	0.070128	0.47205	-0.40298	0.65652	-0.56826	0.71507	1.3844	0.083111	0.083111	0.548302936
TRGJP1	ENSG00000211692	1.3843	0.083136	1.3843	0.083136	1.3843	0.083136	1.3843	0.083136	0.083136	0.548302936
DNAH5	ENSG00000039139	0.23748	0.40614	1.3842	0.083155	-0.59958	0.72561	-0.23591	0.59325	0.083155	0.548302936
NA	ENSG00000221060	0.97048	0.1659	1.3841	0.083168	0.5191	0.30185	0.73591	0.23089	0.083168	0.548302936
DAD1	ENSG00000129562	-1.0531	0.85386	1.384	0.083175	-0.46791	0.68007	1.1121	0.13305	0.083175	0.548302936
RN7SL216P	ENSG00000244404	-1.5875	0.9438	1.384	0.083177	-1.5875	0.9438	-1.5875	0.9438	0.083177	0.548302936
RNU6-1094P	ENSG00000199286	0.98029	0.16347	-0.69346	0.75599	1.3838	0.083208	-0.65364	0.74333	0.083208	0.548302936
LINC01985	ENSG00000227260	1.3838	0.083212	1.3838	0.083212	1.3838	0.083212	1.3838	0.083212	0.083212	0.548302936
NA	ENSG00000230739	1.3838	0.083214	1.1075	0.13403	1.1956	0.11593	-0.041712	0.51664	0.083214	0.548302936
NA	ENSG00000266538	-1.9633	0.97519	-1.5121	0.93475	-1.1421	0.8733	1.3837	0.083218	0.083218	0.548302936
F2	ENSG00000180210	-0.54336	0.70656	0.95643	0.16943	1.3836	0.083247	0.84454	0.19918	0.083247	0.548302936
RPL7P6	ENSG00000227525	1.1328	0.12865	1.3835	0.083257	-0.25869	0.60206	-0.065248	0.52601	0.083257	0.548302936
INPP5B	ENSG00000204084	1.3834	0.083276	0.78529	0.21614	1.073	0.14163	0.74728	0.22745	0.083276	0.548302936
SOX6	ENSG00000110693	0.41422	0.33936	1.366	0.085966	0.45288	0.32532	1.3833	0.083279	0.083279	0.548302936
PDE11A-AS1	ENSG00000229941	0.71635	0.23689	0.43995	0.32999	1.3833	0.083283	0.94056	0.17346	0.083283	0.548302936
ZNF555	ENSG00000186300	-0.28032	0.61039	1.3832	0.083295	1.2101	0.11311	1.2101	0.11311	0.083295	0.548302936
NA	ENSG00000249522	0.0035221	0.49859	0.69988	0.242	0.69988	0.242	1.3832	0.083297	0.083297	0.548302936
PLA2G4C	ENSG00000105499	1.3832	0.083298	0.056829	0.47734	0.23189	0.40831	-0.30434	0.61957	0.083298	0.548302936
TEX29	ENSG00000153495	-0.67576	0.7504	1.3832	0.083298	-0.33236	0.63019	-0.84126	0.7999	0.083298	0.548302936
NUBP1	ENSG00000103274	0.41436	0.33931	1.3832	0.083301	0.41436	0.33931	-0.18372	0.57289	0.083301	0.548302936
NA	ENSG00000224235	1.3831	0.083323	0.57141	0.28386	1.3831	0.083323	1.3831	0.083323	0.083323	0.548302936
VSX2	ENSG00000119614	1.3831	0.083324	1.36	0.086918	1.2423	0.10706	1.1305	0.12914	0.083324	0.548302936
NA	ENSG00000263320	1.3829	0.083351	1.3829	0.083351	1.3829	0.083351	1.3829	0.083351	0.083351	0.548302936
NA	ENSG00000267313	1.0678	0.14281	1.3829	0.083352	1.3829	0.083352	1.3829	0.083352	0.083352	0.548302936
NTN4	ENSG00000074527	0.2275	0.41002	1.3828	0.083356	0.78243	0.21698	-0.51212	0.69572	0.083356	0.548302936

CBX8	ENSG00000141570	0.015989	0.49362	1.0081	0.15671	0.84946	0.19781	1.3828	0.08336	0.08336	0.548302936
CCT6P4	ENSG00000232610	-0.43749	0.66912	1.3828	0.083365	-0.43749	0.66912	-0.43749	0.66912	0.083365	0.548302936
SNORD69	ENSG00000212452	1.3827	0.083385	1.3827	0.083385	1.3827	0.083385	1.3827	0.083385	0.083385	0.548317635
NA	ENSG00000261245	1.3826	0.08339	1.3826	0.08339	1.3826	0.08339	1.3826	0.08339	0.08339	0.548317635
DEFB131B	ENSG00000225805	-0.56682	0.71458	0.10896	0.45662	1.3825	0.083403	-1.2497	0.89429	0.083403	0.548317635
OSBPL11	ENSG00000144909	-0.64995	0.74214	1.3825	0.083415	-0.022869	0.50912	-0.60626	0.72783	0.083415	0.548317635
KDM2A	ENSG00000173120	1.0965	0.13644	0.98391	0.16258	1.2597	0.10388	1.3824	0.083426	0.083426	0.548317635
MIR4425	ENSG00000264371	0.59724	0.27518	1.3823	0.083441	0.59724	0.27518	1.1479	0.1255	0.083441	0.548317635
NA	ENSG00000266769	-0.1935	0.57672	-0.57095	0.71598	0.59192	0.27695	1.3823	0.083446	0.083446	0.548317635
TBC1D31	ENSG00000156787	1.3818	0.083518	0.27281	0.3925	1.1805	0.11891	0.92913	0.17641	0.083518	0.54871504
SERPINA3	ENSG00000196136	-1.3245	0.90734	-1.3245	0.90734	1.3817	0.083529	1.3699	0.085354	0.083529	0.54871504
NA	ENSG00000267801	1.3814	0.083574	1.3814	0.083574	1.3814	0.083574	1.3814	0.083574	0.083574	0.548808526
NA	ENSG00000224174	1.3813	0.083588	1.3813	0.083588	1.3813	0.083588	1.3813	0.083588	0.083588	0.548808526
RNU6ATAC10P	ENSG00000221562	0.031429	0.48746	0.092241	0.46325	1.3812	0.083603	-0.25244	0.59965	0.083603	0.548808526
NA	ENSG00000267607	1.3811	0.08362	1.3811	0.08362	1.3811	0.08362	1.3811	0.08362	0.08362	0.548808526
DEFB128	ENSG00000185982	1.381	0.083645	0.38115	0.35155	0.21984	0.413	0.88254	0.18874	0.083645	0.548808526
NPM1P30	ENSG00000228219	-0.34917	0.63652	1.2828	0.099773	1.3809	0.083648	1.3809	0.083648	0.083648	0.548808526
NA	ENSG00000256694	1.3809	0.08365	1.3809	0.08365	1.3809	0.08365	1.3809	0.08365	0.08365	0.548808526
OR1N1	ENSG00000171505	1.3807	0.083679	1.209	0.11332	0.56486	0.28609	1.209	0.11332	0.083679	0.548808526
SLC6A7	ENSG00000011083	-0.54625	0.70755	-0.16797	0.5667	-0.41271	0.66009	1.3807	0.083686	0.083686	0.548808526
BTBD10P2	ENSG00000219433	1.3806	0.0837	1.3806	0.0837	1.3806	0.0837	1.3806	0.0837	0.0837	0.548808526
NA	ENSG00000254638	1.3806	0.083702	1.3806	0.083702	1.3806	0.083702	1.3806	0.083702	0.083702	0.548808526
MED7	ENSG00000155868	-0.7861	0.7841	1.3805	0.083714	-0.7861	0.7841	-0.54512	0.70716	0.083714	0.548808526
CUX2P1	ENSG00000272436	1.3805	0.083718	1.3592	0.087037	1.3592	0.087037	0.67599	0.24952	0.083718	0.548808526
NA	ENSG00000265533	0.75024	0.22656	0.10627	0.45769	1.3805	0.08372	0.75024	0.22656	0.08372	0.548808526
PRDM16-DT	ENSG00000177133	1.0101	0.15623	-0.6524	0.74293	1.3804	0.08373	-0.36655	0.64302	0.08373	0.548808526
NA	ENSG00000271369	1.3803	0.083753	1.3803	0.083753	1.3803	0.083753	1.3803	0.083753	0.083753	0.548808526
CKAP4	ENSG00000136026	1.0982	0.13606	0.47562	0.31717	1.3802	0.083755	1.3693	0.08546	0.083755	0.548808526
LINC01788	ENSG00000229051	1.3802	0.083764	0.64263	0.26023	1.3802	0.083764	1.3802	0.083764	0.083764	0.548808526
SUN5	ENSG00000167098	0.89583	0.18517	0.89583	0.18517	1.3802	0.083766	1.3802	0.083766	0.083766	0.548808526
NA	ENSG00000277767	1.2428	0.10698	1.3801	0.083775	1.3801	0.083775	1.0295	0.15161	0.083775	0.548808526

FABP7P2	ENSG00000235129	-0.95684	0.83067	1.3801	0.08378	-1.3825	0.91659	-0.48942	0.68773	0.08378	0.548808526
NA	ENSG00000261281	1.38	0.083791	1.38	0.083791	1.38	0.083791	1.38	0.083791	0.083791	0.548808526
ZNF318	ENSG00000171467	0.080772	0.46781	1.3797	0.083839	-0.19634	0.57783	-1.1492	0.87476	0.083839	0.549014626
METTL1	ENSG00000037897	1.3797	0.083845	1.3797	0.083845	0.91847	0.17919	0.91847	0.17919	0.083845	0.549014626
LACTB2-AS1	ENSG00000246366	1.3796	0.083861	0.51962	0.30166	1.3796	0.083861	1.3796	0.083861	0.083861	0.549045617
CRY1	ENSG00000008405	1.2315	0.10906	0.95876	0.16884	1.3794	0.08388	0.20802	0.41761	0.08388	0.549096239
LINC00536	ENSG00000249917	1.1918	0.11668	1.3793	0.083906	0.013766	0.49451	-0.35779	0.63975	0.083906	0.549133261
NA	ENSG00000206998	-0.9084	0.81817	0.0093667	0.49626	1.3792	0.083918	0.38007	0.35195	0.083918	0.549133261
MUC17	ENSG00000169876	1.3791	0.083938	0.99683	0.15942	0.89492	0.18542	0.26164	0.3968	0.083938	0.549133261
EIF5A2	ENSG00000163577	0.45454	0.32472	1.379	0.083942	-0.98716	0.83822	-0.98716	0.83822	0.083942	0.549133261
NA	ENSG00000254452	1.379	0.083942	1.379	0.083942	1.379	0.083942	1.379	0.083942	0.083942	0.549133261
NA	ENSG00000233974	0.50686	0.30613	0.71249	0.23808	1.3788	0.083974	-0.79555	0.78685	0.083974	0.549268862
TOX4P1	ENSG00000248697	0.18036	0.42844	1.3786	0.084014	0.91134	0.18106	1.0471	0.14752	0.084014	0.549456747
NOC2L	ENSG00000188976	1.3784	0.084036	-0.25468	0.60051	0.22325	0.41167	-0.72973	0.76722	0.084036	0.549526876
RPL7P59	ENSG00000225918	-0.0066013	0.50263	-0.63303	0.73664	1.3782	0.084072	1.3782	0.084072	0.084072	0.549688523
ZNF778	ENSG00000170100	-0.44631	0.67231	1.1927	0.1165	1.3778	0.08413	0.05472	0.47818	0.08413	0.549918311
LINC01677	ENSG00000233047	1.2213	0.111	1.3778	0.084137	0.62782	0.26506	1.3778	0.084137	0.084137	0.549918311
NA	ENSG00000242753	0.9098	0.18146	0.98766	0.16166	0.88303	0.18861	1.3777	0.084141	0.084141	0.549918311
RGS18	ENSG00000150681	0.9687	0.16635	0.9687	0.16635	1.3777	0.084153	0.9687	0.16635	0.084153	0.549922984
NA	ENSG00000236817	-1.4718	0.92947	1.3776	0.084169	-0.10155	0.54044	-0.19788	0.57843	0.084169	0.549953791
NA	ENSG00000262777	1.3774	0.084191	1.3774	0.084191	1.3774	0.084191	1.3774	0.084191	0.084191	0.550015379
SNRNP70	ENSG00000104852	1.3774	0.084201	-1.0604	0.85552	1.3774	0.084201	0.1222	0.45137	0.084201	0.550015379
EFCAB9	ENSG00000214360	-0.14457	0.55748	1.3772	0.084227	1.3772	0.084227	0.0016834	0.49933	0.084227	0.550063874
NA	ENSG00000200318	0.48718	0.31306	1.3772	0.084231	0.6298	0.26441	0.6298	0.26441	0.084231	0.550063874
SMC3P1	ENSG00000231822	-0.44005	0.67005	1.3767	0.084309	-0.44005	0.67005	-0.44005	0.67005	0.084309	0.550351238
NA	ENSG00000214259	1.3765	0.084326	0.65677	0.25566	-0.40283	0.65646	0.22124	0.41245	0.084326	0.550351238
NA	ENSG00000236308	1.3765	0.084331	1.3765	0.084331	1.3765	0.084331	1.3765	0.084331	0.084331	0.550351238
PPP1R2P5	ENSG00000229360	-0.90247	0.8166	0.6616	0.25411	1.3765	0.084337	1.3133	0.094535	0.084337	0.550351238
NA	ENSG00000260390	1.3763	0.084364	1.0152	0.15501	1.0152	0.15501	1.0152	0.15501	0.084364	0.550351238
NA	ENSG00000261049	-1.5785	0.94277	-0.64605	0.74088	1.3763	0.084369	-0.87062	0.80802	0.084369	0.550351238
NA	ENSG00000276188	1.3762	0.084379	1.3762	0.084379	1.3762	0.084379	1.3762	0.084379	0.084379	0.550351238

PRUNE1	ENSG00000143363	-0.50311	0.69256	1.0759	0.14098	1.3762	0.084383	1.2586	0.10409	0.084383	0.550351238
EVI5L	ENSG00000142459	1.0412	0.1489	1.0412	0.1489	1.0412	0.1489	1.3762	0.084387	0.084387	0.550351238
ASPH	ENSG00000198363	0.90343	0.18315	-1.1396	0.87277	0.021742	0.49133	1.3761	0.084388	0.084388	0.550351238
OR2L1P	ENSG00000224227	1.3759	0.084427	1.3759	0.084427	1.3759	0.084427	-1.1804	0.88109	0.084427	0.550351238
NA	ENSG00000260651	1.3652	0.086094	1.3758	0.084436	1.3652	0.086094	1.3652	0.086094	0.084436	0.550351238
ADAMTS7P4	ENSG00000218052	0.66038	0.2545	0.94602	0.17207	0.054503	0.47827	1.3758	0.084446	0.084446	0.550351238
IMPDH1P11	ENSG00000261647	-0.74829	0.77286	1.3757	0.084456	-0.74829	0.77286	-0.46758	0.67996	0.084456	0.550351238
NA	ENSG00000231791	1.3757	0.08446	1.3757	0.08446	1.3757	0.08446	1.3757	0.08446	0.08446	0.550351238
CASC6	ENSG00000224944	1.3757	0.084462	0.83017	0.20322	0.83017	0.20322	0.83017	0.20322	0.084462	0.550351238
OR11H7	ENSG00000258806	1.3756	0.084467	1.3756	0.084467	1.3756	0.084467	1.3756	0.084467	0.084467	0.550351238
PADI2	ENSG00000117115	1.0786	0.14038	1.3755	0.084495	0.83343	0.2023	1.1081	0.13391	0.084495	0.55037882
NA	ENSG00000265414	0.1523	0.43948	1.3754	0.084505	0.087835	0.465	1.1137	0.1327	0.084505	0.55037882
CC2D1B	ENSG00000154222	0.14149	0.44374	-1.6206	0.94745	-0.79781	0.78751	1.3754	0.08451	0.08451	0.55037882
MCUR1	ENSG00000050393	0.38384	0.35055	1.1273	0.12981	1.3752	0.084528	0.3481	0.36388	0.084528	0.55037882
DEPTOR	ENSG00000155792	1.1934	0.11635	0.68036	0.24814	1.0916	0.13752	1.3752	0.084536	0.084536	0.55037882
SPTB	ENSG00000070182	0.62043	0.26749	1.3752	0.084539	-0.083172	0.53314	-0.26159	0.60318	0.084539	0.55037882
PAF1	ENSG00000006712	1.375	0.084561	-0.48341	0.6856	-0.48341	0.6856	-0.48341	0.6856	0.084561	0.550397184
MN1	ENSG00000169184	-0.55326	0.70996	1.375	0.084568	0.51342	0.30383	-0.50242	0.69231	0.084568	0.550397184
NA	ENSG00000259444	0.57055	0.28415	1.3749	0.084576	-0.24083	0.59516	-0.24083	0.59516	0.084576	0.550397184
NA	ENSG00000236451	1.3749	0.084587	0.53988	0.29464	-1.0103	0.84382	-0.76818	0.77881	0.084587	0.550397184
GRIN2C	ENSG00000161509	1.2573	0.10432	0.71805	0.23636	0.74296	0.22875	1.3746	0.084623	0.084623	0.550557916
RNU6-39P	ENSG00000207072	0.15505	0.43839	1.3254	0.09252	0.80749	0.20969	1.3745	0.08464	0.08464	0.550595007
NA	ENSG00000176515	1.3742	0.084695	1.0224	0.15329	1.1937	0.11629	0.14384	0.44281	0.084695	0.550776043
BCL2L10	ENSG00000137875	1.3741	0.0847	0.7493	0.22684	0.7493	0.22684	0.7493	0.22684	0.0847	0.550776043
MBD3	ENSG00000071655	-0.27173	0.60708	1.374	0.084717	1.374	0.084717	0.60335	0.27314	0.084717	0.550776043
MBL3P	ENSG00000219430	0.74491	0.22816	1.2582	0.10416	1.3738	0.084744	1.3665	0.085889	0.084744	0.550776043
SSTR4	ENSG00000132671	1.3738	0.084746	1.3738	0.084746	1.3738	0.084746	1.3738	0.084746	0.084746	0.550776043
RILP	ENSG00000167705	-0.55666	0.71112	-0.55666	0.71112	1.3737	0.084771	-0.55666	0.71112	0.084771	0.550776043
SNORD94	ENSG00000208772	1.3734	0.084817	1.3734	0.084817	1.3734	0.084817	1.3734	0.084817	0.084817	0.550776043
MVP	ENSG00000013364	0.65886	0.25499	1.2532	0.10507	0.9085	0.18181	1.3734	0.08482	0.08482	0.550776043
NA	ENSG00000261066	-0.34387	0.63453	1.3734	0.08482	-0.34387	0.63453	-0.34387	0.63453	0.08482	0.550776043

KLHL14	ENSG00000197705	1.2081	0.11351	1.3733	0.084833	0.21811	0.41367	0.64981	0.25791	0.084833	0.550776043
HIPK1	ENSG00000163349	-0.025134	0.51003	1.3732	0.084841	0.89909	0.1843	1.3386	0.090355	0.084841	0.550776043
C5orf60	ENSG00000204661	1.3732	0.084841	0.43106	0.33321	0.23675	0.40642	0.92915	0.17641	0.084841	0.550776043
M1AP	ENSG00000159374	0.66588	0.25274	1.272	0.10168	-0.32993	0.62927	1.3732	0.084844	0.084844	0.550776043
NA	ENSG00000233683	0.60902	0.27126	1.0764	0.14087	1.1609	0.12285	1.3732	0.084849	0.084849	0.550776043
CPNE7	ENSG00000178773	1.373	0.084869	1.0969	0.13634	0.44367	0.32864	0.33644	0.36827	0.084869	0.550776043
NA	ENSG00000271816	1.373	0.084881	1.373	0.084881	1.373	0.084881	1.373	0.084881	0.084881	0.550776043
NA	ENSG00000267022	1.3729	0.084895	1.3729	0.084895	1.3729	0.084895	1.3729	0.084895	0.084895	0.550776043
NA	ENSG00000270306	-0.10613	0.54226	1.3729	0.084896	-0.14227	0.55657	-0.14227	0.55657	0.084896	0.550776043
RPL36P19	ENSG00000232540	-0.37104	0.64469	1.3729	0.084897	1.3729	0.084897	1.3729	0.084897	0.084897	0.550776043
GFRA1	ENSG00000151892	1.0694	0.14245	0.70921	0.2391	1.0182	0.1543	1.3728	0.084915	0.084915	0.550776043
PTGS1	ENSG00000095303	-1.0727	0.8583	-0.17476	0.56936	-0.27529	0.60845	1.3727	0.084924	0.084924	0.550776043
NA	ENSG00000270342	1.3726	0.084937	1.3726	0.084937	1.3726	0.084937	1.3726	0.084937	0.084937	0.550776043
NA	ENSG00000203886	1.3725	0.084953	1.3725	0.084953	1.3725	0.084953	1.3725	0.084953	0.084953	0.550776043
NA	ENSG00000254372	1.3724	0.084964	-1.5069	0.93408	1.3724	0.084964	1.3724	0.084964	0.084964	0.550776043
YJU2	ENSG00000105248	1.3724	0.084968	0.58357	0.27975	-2.4034	0.99188	0.17293	0.43135	0.084968	0.550776043
NA	ENSG00000235683	0.47548	0.31722	1.3724	0.084968	-0.96045	0.83158	-0.0073135	0.50292	0.084968	0.550776043
EPC1	ENSG00000120616	0.95645	0.16942	1.3724	0.084973	-0.86762	0.8072	1.2103	0.11309	0.084973	0.550776043
NA	ENSG00000254328	1.3723	0.08499	1.3723	0.08499	1.3723	0.08499	1.3723	0.08499	0.08499	0.550812967
RPL26P3	ENSG00000240179	1.3721	0.085013	1.3721	0.085013	1.3721	0.085013	1.3721	0.085013	0.085013	0.550888762
ATP11A	ENSG00000068650	1.3633	0.086398	1.2471	0.10618	0.8545	0.19641	1.3719	0.08504	0.08504	0.55094814
ADIRF	ENSG00000148671	1.3717	0.085076	1.3717	0.085076	1.3156	0.094153	1.1536	0.12433	0.085076	0.55094814
NA	ENSG00000277589	1.3717	0.08508	1.3717	0.08508	1.3717	0.08508	1.3717	0.08508	0.08508	0.55094814
EGF	ENSG00000138798	1.3717	0.085081	0.12826	0.44897	0.052538	0.47905	-0.15655	0.5622	0.085081	0.55094814
DPY19L4P2	ENSG00000231839	0.80268	0.21108	0.28104	0.38934	1.3717	0.085081	0.90907	0.18166	0.085081	0.55094814
FAM205C	ENSG00000187791	0.24833	0.40194	1.3716	0.08509	0.24289	0.40405	-0.25159	0.59932	0.08509	0.55094814
NA	ENSG00000280219	1.3713	0.085134	0.21793	0.41374	1.3713	0.085134	1.2163	0.11193	0.085134	0.551118952
AHNAK	ENSG00000124942	-0.55549	0.71072	1.1797	0.11905	1.3713	0.085139	1.228	0.10971	0.085139	0.551118952
NA	ENSG00000253803	0.24103	0.40477	-1.0885	0.8618	0.4218	0.33658	1.3711	0.085169	0.085169	0.551239922
NA	ENSG00000234464	-0.59646	0.72457	-0.27146	0.60698	1.1678	0.12144	1.371	0.085192	0.085192	0.551244867
GABRR1	ENSG00000146276	1.3709	0.085198	1.056	0.14549	1.1385	0.12745	0.58604	0.27892	0.085198	0.551244867

NA	ENSG00000267338	0.67384	0.25021	1.3708	0.085226	0.25633	0.39885	-1.0059	0.84276	0.085226	0.551244867
NA	ENSG00000254477	1.3704	0.085279	1.1026	0.13509	1.3704	0.085279	1.3704	0.085279	0.085279	0.551244867
SYPL2	ENSG00000143028	0.99973	0.15872	0.99973	0.15872	0.99973	0.15872	1.3703	0.085289	0.085289	0.551244867
NA	ENSG00000207027	1.3703	0.085294	1.3703	0.085294	1.3703	0.085294	1.3703	0.085294	0.085294	0.551244867
NA	ENSG00000207419	0.3507	0.36291	1.3702	0.085305	1.1204	0.13126	0.54513	0.29283	0.085305	0.551244867
ADTRP	ENSG00000111863	0.44841	0.32693	-0.036879	0.51471	0.39186	0.34758	1.3702	0.085313	0.085313	0.551244867
SMOC1	ENSG00000198732	-0.756	0.77518	1.3702	0.085317	-0.57567	0.71758	-0.0093527	0.50373	0.085317	0.551244867
MCRIP2P2	ENSG00000270920	1.3701	0.08532	1.3701	0.08532	1.3701	0.08532	1.3701	0.08532	0.08532	0.551244867
MIR548G	ENSG00000221369	1.3701	0.085328	1.3701	0.085328	1.3701	0.085328	1.3701	0.085328	0.085328	0.551244867
NA	ENSG00000278477	-0.40794	0.65834	1.37	0.08535	-0.15863	0.56302	1.37	0.08535	0.08535	0.551244867
NA	ENSG00000264166	1.3698	0.085375	1.3698	0.085375	1.3698	0.085375	1.3698	0.085375	0.085375	0.551244867
NA	ENSG00000280016	-0.47471	0.6825	1.3697	0.085397	1.3697	0.085397	1.3697	0.085397	0.085397	0.551244867
SLC9A3R2	ENSG00000065054	-0.65519	0.74383	-0.65519	0.74383	-0.95898	0.83122	1.3696	0.085414	0.085414	0.551244867
TRAPPC8	ENSG00000153339	1.3695	0.085415	-0.52788	0.70121	1.272	0.10168	-0.76349	0.77741	0.085415	0.551244867
FUT8-AS1	ENSG00000276116	1.3695	0.085426	1.3695	0.085426	1.3695	0.085426	1.3695	0.085426	0.085426	0.551244867
HS3ST5	ENSG00000249853	0.063623	0.47464	0.063623	0.47464	0.063623	0.47464	1.3693	0.085456	0.085456	0.551244867
GLUD1P3	ENSG00000250959	1.3693	0.085457	1.3693	0.085457	1.3693	0.085457	1.3693	0.085457	0.085457	0.551244867
LINC02663	ENSG00000228636	1.3693	0.08546	1.3693	0.08546	1.3693	0.08546	1.3693	0.08546	0.08546	0.551244867
NA	ENSG00000238085	0.20355	0.41935	1.3693	0.08546	0.20355	0.41935	-1.1537	0.87569	0.08546	0.551244867
GAS1	ENSG00000180447	-1.0401	0.85086	1.3692	0.085468	-0.62638	0.73447	-1.5341	0.9375	0.085468	0.551244867
RGPD4-AS1	ENSG00000230651	1.369	0.085495	-0.48907	0.6876	1.0165	0.1547	-0.087193	0.53474	0.085495	0.551244867
UNC93B3	ENSG00000172971	0.82291	0.20528	1.369	0.085499	0.87422	0.191	0.089294	0.46442	0.085499	0.551244867
RPS26P13	ENSG00000227887	0.70841	0.23935	1.369	0.085499	-0.82762	0.79606	0.42202	0.3365	0.085499	0.551244867
MRPS24	ENSG00000062582	1.3689	0.085521	1.3689	0.085521	1.3689	0.085521	-0.45644	0.67596	0.085521	0.551244867
ICOS	ENSG00000163600	1.3689	0.085521	0.33213	0.3699	0.12068	0.45197	0.12068	0.45197	0.085521	0.551244867
TRBV24-1	ENSG00000211750	1.3689	0.085522	-0.45498	0.67544	-0.24637	0.5973	-1.1284	0.87043	0.085522	0.551244867
SNORD45A	ENSG00000207241	1.3687	0.085544	1.3687	0.085544	1.3687	0.085544	1.3687	0.085544	0.085544	0.551244867
RPL36AP6	ENSG00000231293	0.075436	0.46993	0.075436	0.46993	0.075436	0.46993	1.3687	0.085549	0.085549	0.551244867
OR4A42P	ENSG00000254674	1.3686	0.08556	0.016835	0.49328	-0.71372	0.7623	-1.1697	0.87894	0.08556	0.551244867
PCNA	ENSG00000132646	1.3686	0.085565	1.3686	0.085565	0.75229	0.22594	0.75229	0.22594	0.085565	0.551244867
LINC02446	ENSG00000256039	0.47716	0.31663	1.3686	0.08557	-0.065904	0.52627	0.29389	0.38442	0.08557	0.551244867

NA	ENSG00000244652	1.3685	0.085582	-0.86496	0.80647	1.3685	0.085582	1.3685	0.085582	0.085582	0.551244867
MPG	ENSG00000103152	0.17415	0.43088	1.0057	0.15728	1.3685	0.085584	1.3685	0.085584	0.085584	0.551244867
TMEM72	ENSG00000187783	-0.12024	0.54786	0.1366	0.44568	1.3684	0.085599	1.3141	0.094407	0.085599	0.551244867
CFL2	ENSG00000165410	1.3683	0.085602	0.68266	0.24741	0.94434	0.1725	0.31665	0.37575	0.085602	0.551244867
DPY19L2P5	ENSG00000225100	1.3683	0.085609	1.3683	0.085609	1.3683	0.085609	1.3683	0.085609	0.085609	0.551244867
PAPPA-AS2	ENSG00000226604	1.3682	0.085618	1.3682	0.085618	1.3682	0.085618	1.3682	0.085618	0.085618	0.551244867
LUADT1	ENSG00000196634	0.15097	0.44	0.15097	0.44	1.3682	0.085631	0.15097	0.44	0.085631	0.551244867
HCG27	ENSG00000206344	-0.41362	0.66042	0.55351	0.28996	0.5447	0.29298	1.3678	0.08569	0.08569	0.551244867
NA	ENSG00000226181	0.62187	0.26701	1.3677	0.085709	-0.64328	0.73998	-0.64328	0.73998	0.085709	0.551244867
LINC02324	ENSG00000255002	1.2013	0.11481	-0.21252	0.58415	0.31009	0.37825	1.3677	0.08571	0.08571	0.551244867
TRIM50	ENSG00000146755	1.1808	0.11885	1.3676	0.085723	0.10084	0.45984	1.1808	0.11885	0.085723	0.551244867
LINC00987	ENSG00000237248	1.3676	0.085725	1.3676	0.085725	1.3676	0.085725	1.3676	0.085725	0.085725	0.551244867
NA	ENSG00000258426	1.3676	0.085726	1.3676	0.085726	1.3676	0.085726	1.3676	0.085726	0.085726	0.551244867
SH3BGR	ENSG00000185437	1.3675	0.085733	0.58824	0.27818	0.39251	0.34734	0.4024	0.34369	0.085733	0.551244867
SLC25A1P1	ENSG00000254479	1.3674	0.085743	0.041486	0.48345	0.34698	0.3643	0.43739	0.33091	0.085743	0.551244867
RNU6-478P	ENSG00000200774	0.86837	0.1926	0.86837	0.1926	1.3674	0.085744	0.41896	0.33762	0.085744	0.551244867
NA	ENSG00000254165	0.34359	0.36558	1.3674	0.085751	0.34359	0.36558	0.34359	0.36558	0.085751	0.551244867
NA	ENSG00000272149	1.3674	0.085755	1.3674	0.085755	1.3674	0.085755	1.3674	0.085755	0.085755	0.551244867
FBN1	ENSG00000166147	1.3289	0.091934	1.1328	0.12864	1.3673	0.085758	1.2505	0.10556	0.085758	0.551244867
RN7SL665P	ENSG00000264169	1.3671	0.085795	1.3671	0.085795	1.3671	0.085795	1.3671	0.085795	0.085795	0.55134808
DHDDS	ENSG00000117682	1.1389	0.12738	0.62587	0.2657	1.367	0.085806	0.68326	0.24722	0.085806	0.55134808
EPHA4	ENSG00000116106	0.90781	0.18199	1.0693	0.14246	1.367	0.085808	-0.24564	0.59702	0.085808	0.55134808
NA	ENSG00000233906	1.3669	0.085835	1.3669	0.085835	1.3669	0.085835	1.3669	0.085835	0.085835	0.551401855
NA	ENSG00000223398	-0.11745	0.54675	-0.11745	0.54675	1.3668	0.085839	-0.11745	0.54675	0.085839	0.551401855
MIR101-1	ENSG00000199135	0.28309	0.38855	0.28309	0.38855	0.28309	0.38855	1.3666	0.085876	0.085876	0.551566822
TVP23B	ENSG00000171928	1.3664	0.085908	-0.49857	0.69096	-0.0093883	0.50375	-1.0123	0.84431	0.085908	0.551613087
PSMB3P2	ENSG00000235444	1.3664	0.085911	1.3664	0.085911	1.3664	0.085911	1.3664	0.085911	0.085911	0.551613087
SKAP2	ENSG00000005020	-0.2104	0.58332	0.51967	0.30165	1.3661	0.08596	0.021578	0.49139	0.08596	0.551613087
SERPINA9	ENSG00000170054	-0.16258	0.56458	1.366	0.08597	0.66207	0.25396	0.61579	0.26902	0.08597	0.551613087
NA	ENSG00000272239	1.366	0.085976	1.366	0.085976	1.366	0.085976	1.366	0.085976	0.085976	0.551613087
MIR5581	ENSG00000263675	1.3659	0.08598	1.3659	0.08598	1.3659	0.08598	1.3659	0.08598	0.08598	0.551613087

PCBP2P1	ENSG00000235701	0.8589	0.1952	0.076785	0.4694	1.3658	0.085997	1.3658	0.085997	0.085997	0.551613087
MEAF6P1	ENSG00000240286	0.0011025	0.49956	0.36036	0.35929	1.3658	0.085998	1.3658	0.085998	0.085998	0.551613087
LINC02225	ENSG00000249584	0.53811	0.29525	-0.80275	0.78894	1.3658	0.086007	-0.18621	0.57386	0.086007	0.551613087
NA	ENSG00000265496	1.3658	0.086008	1.3658	0.086008	1.3658	0.086008	1.3658	0.086008	0.086008	0.551613087
TAF6	ENSG00000106290	0.033785	0.48652	1.238	0.10787	1.3657	0.086023	1.3431	0.089626	0.086023	0.551613087
NA	ENSG00000232995	1.1377	0.12761	1.3655	0.086042	1.1978	0.11549	0.24433	0.40349	0.086042	0.551613087
RN7SL332P	ENSG00000242989	-0.88498	0.81192	1.3655	0.086052	0.85487	0.19631	-0.13878	0.55519	0.086052	0.551613087
GPR12	ENSG00000132975	1.3655	0.086053	1.3655	0.086053	0.26839	0.3942	1.277	0.1008	0.086053	0.551613087
NA	ENSG00000264472	1.3655	0.086053	1.3655	0.086053	1.3655	0.086053	1.3655	0.086053	0.086053	0.551613087
KRTAP13-1	ENSG00000198390	1.3651	0.086106	1.3651	0.086106	1.3651	0.086106	1.3651	0.086106	0.086106	0.55186757
NA	ENSG00000279642	1.365	0.086125	1.365	0.086125	1.365	0.086125	1.365	0.086125	0.086125	0.55186757
PRICKLE2-DT	ENSG00000244564	0.10927	0.45649	0.91492	0.18012	-0.72311	0.76519	1.3649	0.086137	0.086137	0.55186757
NAMPT	ENSG00000105835	0.65187	0.25724	1.3649	0.086138	0.19565	0.42244	0.94625	0.17201	0.086138	0.55186757
NA	ENSG00000177369	1.3644	0.086218	1.3644	0.086218	1.3097	0.095146	1.3644	0.086218	0.086218	0.552149566
HOXD1	ENSG00000128645	1.3643	0.086232	1.3643	0.086232	1.3643	0.086232	0.36294	0.35832	0.086232	0.552149566
NA	ENSG00000267687	0.98216	0.16301	1.3643	0.086232	0.9043	0.18292	0.48213	0.31486	0.086232	0.552149566
NA	ENSG00000200325	0.65862	0.25507	1.3643	0.086235	-0.39566	0.65382	1.109	0.13371	0.086235	0.552149566
SLC8A3	ENSG00000100678	1.3643	0.08624	0.61952	0.26779	0.18119	0.42811	1.3435	0.089548	0.08624	0.552149566
TLR6	ENSG00000174130	1.3642	0.08625	0.69026	0.24502	1.2398	0.10752	0.0027508	0.4989	0.08625	0.552149566
NA	ENSG00000259610	-2.3074	0.98948	1.3641	0.086263	-1.3521	0.91182	-1.3521	0.91182	0.086263	0.552160251
NA	ENSG00000271074	1.3638	0.086314	0.85382	0.1966	-0.16049	0.56375	1.1522	0.12461	0.086314	0.552192383
PDE6C	ENSG00000095464	1.3638	0.086319	1.0826	0.13948	1.3638	0.086319	-0.30885	0.62128	0.086319	0.552192383
DUTP7	ENSG00000250473	1.0488	0.14714	0.42172	0.33661	0.58673	0.27869	1.3636	0.086344	0.086344	0.552192383
MIPEP	ENSG00000027001	1.3636	0.086348	0.91754	0.17943	1.2937	0.097886	1.2435	0.10684	0.086348	0.552192383
APCDD1L	ENSG00000198768	-1.327	0.90775	1.3635	0.086361	-1.1617	0.87732	-1.327	0.90775	0.086361	0.552192383
CTAGE13P	ENSG00000230711	-0.96404	0.83249	1.3635	0.086365	-0.029193	0.51164	-0.029193	0.51164	0.086365	0.552192383
CSNK1A1L	ENSG00000180138	0.17247	0.43153	1.3634	0.086371	0.29235	0.38501	0.086517	0.46553	0.086371	0.552192383
CYP26A1	ENSG00000095596	-0.71901	0.76393	-0.71901	0.76393	1.3634	0.086379	0.39518	0.34635	0.086379	0.552192383
ZNF521	ENSG00000198795	-0.39815	0.65474	0.0063103	0.49748	1.3633	0.08639	0.37206	0.35492	0.08639	0.552192383
NA	ENSG00000279271	1.3633	0.086398	1.3633	0.086398	1.3633	0.086398	1.3633	0.086398	0.086398	0.552192383
AP1M2	ENSG00000129354	0.47597	0.31705	1.3633	0.0864	0.85004	0.19765	-0.97074	0.83416	0.0864	0.552192383

NA	ENSG00000233919	-0.97636	0.83556	-0.35539	0.63885	-0.9875	0.8383	1.3632	0.086404	0.086404	0.552192383
MRPL3	ENSG00000114686	-0.43167	0.66701	1.3631	0.086431	-0.16916	0.56716	-0.59016	0.72246	0.086431	0.552252045
NA	ENSG00000270797	1.363	0.086444	1.363	0.086444	1.363	0.086444	1.363	0.086444	0.086444	0.552252045
TMPRSS11E	ENSG00000087128	1.363	0.086449	1.2971	0.097291	1.0474	0.14746	1.3088	0.095303	0.086449	0.552252045
NA	ENSG00000263916	1.3628	0.086467	1.3628	0.086467	1.3628	0.086467	1.3628	0.086467	0.086467	0.552252045
NA	ENSG00000227236	1.3628	0.08647	1.3628	0.08647	1.3628	0.08647	1.3628	0.08647	0.08647	0.552252045
NA	ENSG00000266973	-1.4324	0.92399	0.35982	0.35949	-1.4324	0.92399	1.3626	0.086502	0.086502	0.552324413
FNTB	ENSG00000257365	1.3626	0.086504	1.3626	0.086504	1.3626	0.086504	0.66071	0.2544	0.086504	0.552324413
LINC00373	ENSG00000231019	1.3625	0.08652	1.3625	0.08652	1.3625	0.08652	1.3625	0.08652	0.08652	0.552354199
MTND2P32	ENSG00000254090	-1.3363	0.90927	-1.3363	0.90927	1.3622	0.08657	-1.4503	0.92651	0.08657	0.552562482
SLC29A2	ENSG00000174669	0.067742	0.473	0.74311	0.22871	0.69236	0.24436	1.3621	0.086578	0.086578	0.552562482
NA	ENSG00000250908	1.3621	0.086589	1.3621	0.086589	1.3621	0.086589	0.13795	0.44514	0.086589	0.552562482
NEU2	ENSG00000115488	0.41824	0.33789	-0.4458	0.67213	1.3619	0.086618	-1.2068	0.88624	0.086618	0.552562482
PRB3	ENSG00000197870	-1.2119	0.88723	-2.3421	0.99041	1.3619	0.086619	-0.62935	0.73544	0.086619	0.552562482
NA	ENSG00000251572	1.3425	0.089713	1.3424	0.08973	1.3618	0.086631	1.2367	0.10809	0.086631	0.552562482
NA	ENSG00000226636	1.3618	0.086632	1.3618	0.086632	1.3618	0.086632	1.3618	0.086632	0.086632	0.552562482
LINC02740	ENSG00000255109	1.3617	0.086647	-0.99005	0.83892	1.3617	0.086647	1.3617	0.086647	0.086647	0.552581495
CCNE1	ENSG00000105173	1.3616	0.086667	1.3616	0.086667	1.3616	0.086667	1.3616	0.086667	0.086667	0.552581495
MIR1290	ENSG00000221662	1.3616	0.086669	1.1457	0.12597	1.2662	0.10272	1.2662	0.10272	0.086669	0.552581495
NA	ENSG00000228353	-0.55368	0.7101	1.3614	0.086689	0.86294	0.19409	0.86294	0.19409	0.086689	0.552587625
ENPP7P4	ENSG00000241278	-0.043297	0.51727	1.3613	0.086711	0.80095	0.21158	0.26159	0.39682	0.086711	0.552587625
NA	ENSG00000258114	0.27871	0.39023	0.57193	0.28369	1.3612	0.086723	-0.72085	0.7645	0.086723	0.552587625
BIN3	ENSG00000147439	0.76822	0.22118	0.20924	0.41713	1.3612	0.086725	0.13515	0.44625	0.086725	0.552587625
BOC	ENSG00000144857	0.43556	0.33158	0.80496	0.21042	-0.23396	0.59249	1.3612	0.086733	0.086733	0.552587625
CDC42SE1	ENSG00000197622	0.86379	0.19385	0.86379	0.19385	1.3611	0.086738	0.65193	0.25722	0.086738	0.552587625
SLC16A6	ENSG00000108932	1.1224	0.13084	-0.2901	0.61413	0.7777	0.21837	1.3609	0.08677	0.08677	0.552649012
LINGO3	ENSG00000220008	1.3608	0.086785	1.3608	0.086785	1.0467	0.14762	0.87054	0.192	0.086785	0.552649012
NA	ENSG00000267108	1.3608	0.08679	1.3608	0.08679	1.3608	0.08679	1.3608	0.08679	0.08679	0.552649012
LPCAT4	ENSG00000176454	1.3608	0.086793	-0.1714	0.56805	0.18958	0.42482	0.47924	0.31589	0.086793	0.552649012
TTC21B-AS1	ENSG00000224490	1.3606	0.086824	1.3606	0.086824	1.3606	0.086824	1.3606	0.086824	0.086824	0.552665633
PSMG3	ENSG00000157778	1.0374	0.14978	0.73102	0.23238	1.3604	0.086844	0.16354	0.43505	0.086844	0.552665633

NBN	ENSG00000104320	0.56009	0.28771	1.3604	0.086853	0.50038	0.3084	0.96082	0.16832	0.086853	0.552665633
MNX1	ENSG00000130675	0.25334	0.4	1.3604	0.086856	0.57612	0.28227	1.1433	0.12647	0.086856	0.552665633
TMEM161B	ENSG00000164180	-1.3842	0.91686	1.3604	0.086858	-1.5033	0.93362	-1.361	0.91324	0.086858	0.552665633
NA	ENSG00000272065	1.3603	0.086871	1.3603	0.086871	1.3603	0.086871	1.3603	0.086871	0.086871	0.552665633
NA	ENSG00000280125	-0.73267	0.76812	1.3603	0.086875	-0.73267	0.76812	-0.73267	0.76812	0.086875	0.552665633
LINC01717	ENSG00000232812	1.3599	0.086936	1.3599	0.086936	0.37208	0.35492	1.3599	0.086936	0.086936	0.552964359
CHL1	ENSG00000134121	-0.56738	0.71477	1.3598	0.086947	-0.5784	0.7185	-0.017567	0.50701	0.086947	0.552964359
HOXA2	ENSG00000105996	-0.35922	0.64028	-0.18625	0.57387	1.3597	0.086956	-0.18625	0.57387	0.086956	0.552964359
TMEM127	ENSG00000135956	1.2367	0.10809	1.087	0.13852	1.3594	0.087005	0.23514	0.40705	0.087005	0.553124901
CACYBP	ENSG00000116161	1.3594	0.087018	0.28753	0.38685	0.28753	0.38685	0.28753	0.38685	0.087018	0.553124901
HSPE1P10	ENSG00000249768	1.3593	0.087033	1.1303	0.12918	0.17529	0.43042	1.3593	0.087033	0.087033	0.553124901
LINC02774	ENSG00000226828	0.64728	0.25872	1.3592	0.087037	0.10174	0.45948	1.0007	0.15848	0.087037	0.553124901
PRR13	ENSG00000205352	1.3592	0.087038	0.31493	0.37641	0.60677	0.272	0.53336	0.29689	0.087038	0.553124901
ABCE1	ENSG00000164163	0.15208	0.43956	1.3588	0.087111	-0.80611	0.78991	-0.36479	0.64237	0.087111	0.553424502
NA	ENSG00000271579	1.3587	0.087119	0.96069	0.16835	1.3587	0.087119	1.3587	0.087119	0.087119	0.553424502
NA	ENSG00000258636	1.3586	0.087135	1.3586	0.087135	0.7336	0.2316	1.3586	0.087135	0.087135	0.553424502
NA	ENSG00000223455	1.3586	0.087138	1.3586	0.087138	0.34131	0.36644	1.3586	0.087138	0.087138	0.553424502
CRB3P1	ENSG00000232563	-1.2474	0.89387	-0.30304	0.61907	1.3584	0.087166	-0.071306	0.52842	0.087166	0.553424502
CD46	ENSG00000117335	1.3584	0.087174	-0.091499	0.53645	-0.091499	0.53645	-0.091499	0.53645	0.087174	0.553424502
MATN2	ENSG00000132561	-0.6572	0.74447	0.96003	0.16852	-0.39626	0.65404	1.3583	0.087176	0.087176	0.553424502
LINC01603	ENSG00000253479	-0.22076	0.58736	1.3584	0.087176	-0.49869	0.691	0.1534	0.43904	0.087176	0.553424502
NA	ENSG00000229337	0.17464	0.43068	0.46383	0.32138	-0.1245	0.54954	1.3581	0.087224	0.087224	0.553528673
RNU6-450P	ENSG00000201524	0.71397	0.23762	1.358	0.087228	0.71397	0.23762	1.358	0.087228	0.087228	0.553528673
MLLT11	ENSG00000213190	1.358	0.08723	1.1506	0.12494	-0.49191	0.68861	-0.49191	0.68861	0.08723	0.553528673
HNRNPA1P72	ENSG00000254713	1.3578	0.087259	1.21	0.11314	1.1711	0.12078	1.3578	0.087259	0.087259	0.553528673
RN7SL847P	ENSG00000265319	1.1216	0.13101	1.3577	0.087275	1.1216	0.13101	1.1216	0.13101	0.087275	0.553528673
NA	ENSG00000199565	0.77554	0.21901	0.77554	0.21901	0.77554	0.21901	1.3577	0.087281	0.087281	0.553528673
TRAV31	ENSG00000249048	1.0715	0.14196	1.3576	0.087289	1.3576	0.087289	1.3576	0.087289	0.087289	0.553528673
NA	ENSG00000276744	1.3576	0.087289	1.3576	0.087289	1.3576	0.087289	1.3576	0.087289	0.087289	0.553528673
PLCXD2	ENSG00000240891	1.3576	0.087297	1.1137	0.13271	0.4644	0.32118	0.84067	0.20027	0.087297	0.553528673
BMS1P3	ENSG00000240089	1.3575	0.087306	1.3575	0.087306	1.3575	0.087306	1.3575	0.087306	0.087306	0.553528673

NA	ENSG00000205622	1.3455	0.089239	1.3573	0.087344	0.15393	0.43883	0.79286	0.21393	0.087344	0.553560953
KHDRBS3	ENSG00000131773	0.61968	0.26773	1.3573	0.08735	-0.054178	0.5216	-0.31916	0.6252	0.08735	0.553560953
NA	ENSG00000250515	-0.72641	0.76621	0.12941	0.44852	1.3572	0.087364	0.75089	0.22636	0.087364	0.553560953
NA	ENSG00000274776	1.3571	0.087373	1.2534	0.10503	1.2534	0.10503	1.1701	0.12097	0.087373	0.553560953
POM121	ENSG00000196313	0.98891	0.16135	1.0802	0.14003	1.128	0.12966	1.3569	0.087414	0.087414	0.553560953
HP	ENSG00000257017	1.3567	0.087432	1.261	0.10365	1.261	0.10365	1.261	0.10365	0.087432	0.553560953
NA	ENSG00000244076	1.3567	0.087442	0.72467	0.23433	0.72467	0.23433	0.72467	0.23433	0.087442	0.553560953
NA	ENSG00000248525	1.3567	0.087445	0.53943	0.29479	1.3567	0.087445	1.3567	0.087445	0.087445	0.553560953
LINC02810	ENSG00000236648	-1.3647	0.91382	1.3565	0.087463	-0.16378	0.56505	-1.1894	0.88287	0.087463	0.553560953
NA	ENSG00000280338	0.61424	0.26953	1.3565	0.087465	-0.17498	0.56945	0.4558	0.32427	0.087465	0.553560953
BPIFB1	ENSG00000125999	0.63179	0.26376	1.3565	0.087476	0.5324	0.29723	-0.76613	0.7782	0.087476	0.553560953
ZNF148	ENSG00000163848	-0.31078	0.62201	-0.73155	0.76778	-0.0092953	0.50371	1.3563	0.087502	0.087502	0.553560953
RN7SL382P	ENSG00000240723	0.76533	0.22204	0.30694	0.37945	0.46559	0.32076	1.3562	0.087523	0.087523	0.553560953
C1orf167-AS1	ENSG00000177553	1.3561	0.087527	1.3561	0.087527	1.3561	0.087527	1.3561	0.087527	0.087527	0.553560953
NA	ENSG00000260723	1.3561	0.087531	1.3561	0.087531	1.3561	0.087531	1.3561	0.087531	0.087531	0.553560953
NA	ENSG00000213875	0.17998	0.42858	1.356	0.087553	1.356	0.087553	1.356	0.087553	0.087553	0.553560953
PCDH9	ENSG00000184226	-0.057239	0.52282	1.356	0.087554	1.1655	0.12191	1.0929	0.13722	0.087554	0.553560953
EFHD2	ENSG00000142634	1.356	0.087556	1.356	0.087556	0.90923	0.18161	-1.2572	0.89566	0.087556	0.553560953
NA	ENSG00000253725	1.3558	0.087574	-1.7601	0.96081	-0.58676	0.72132	-1.2698	0.89793	0.087574	0.553560953
CACFD1	ENSG00000160325	0.15983	0.43651	1.3558	0.087588	0.069009	0.47249	0.15959	0.4366	0.087588	0.553560953
MYH15	ENSG00000144821	1.1405	0.12705	1.3557	0.087594	1.1877	0.11748	0.85371	0.19663	0.087594	0.553560953
KCND2	ENSG00000184408	-1.0601	0.85545	1.3556	0.087619	-0.36752	0.64339	-1.4054	0.92005	0.087619	0.553560953
IGSF9B	ENSG00000080854	1.3555	0.087632	0.27002	0.39357	1.2554	0.10466	0.14743	0.4414	0.087632	0.553560953
NA	ENSG00000221252	-0.27058	0.60664	1.2518	0.10533	0.93113	0.17589	1.3554	0.087652	0.087652	0.553560953
RPL8P2	ENSG00000225267	0.075719	0.46982	0.20251	0.41976	1.3553	0.087654	1.3553	0.087654	0.087654	0.553560953
MGAT4B	ENSG00000161013	0.98068	0.16338	0.30078	0.38179	0.7337	0.23157	1.3553	0.087666	0.087666	0.553560953
EXD1	ENSG00000178997	-0.69957	0.7579	-0.46946	0.68063	-0.94595	0.82791	1.3551	0.087692	0.087692	0.553560953
ADAM20P1	ENSG00000259158	0.13477	0.4464	0.08351	0.46672	1.3551	0.087692	1.3202	0.093386	0.087692	0.553560953
SDHAP2	ENSG00000215837	0.426	0.33505	0.9243	0.17767	1.3551	0.087694	0.19185	0.42393	0.087694	0.553560953
RPS27AP12	ENSG00000225224	1.355	0.087713	1.355	0.087713	1.355	0.087713	1.355	0.087713	0.087713	0.553560953
DDX21	ENSG00000165732	0.34723	0.36421	1.355	0.087714	1.0782	0.14047	0.0068678	0.49726	0.087714	0.553560953

PITPNC1	ENSG00000154217	0.76308	0.22271	1.3549	0.087719	-0.57634	0.71781	0.30036	0.38195	0.087719	0.553560953
RNU4ATAC15P	ENSG00000252224	-0.24143	0.59539	1.3549	0.08772	1.3549	0.08772	1.3549	0.08772	0.08772	0.553560953
RNF14P3	ENSG00000238109	1.3549	0.087721	1.3549	0.087721	1.3549	0.087721	1.3549	0.087721	0.087721	0.553560953
OVOL3	ENSG00000105261	1.3548	0.087737	1.3548	0.087737	1.3548	0.087737	1.3548	0.087737	0.087737	0.553560953
GTSE1	ENSG00000075218	-0.13151	0.55231	0.87877	0.18976	0.0073333	0.49707	1.3548	0.087739	0.087739	0.553560953
NA	ENSG00000254616	0.66337	0.25355	1.3548	0.087747	1.3548	0.087747	1.3548	0.087747	0.087747	0.553560953
GPX1P2	ENSG00000215326	-0.29758	0.61699	-0.52387	0.69982	1.3546	0.087766	0.51849	0.30206	0.087766	0.553560953
NA	ENSG00000272509	1.3546	0.087769	1.3546	0.087769	1.3546	0.087769	1.3546	0.087769	0.087769	0.553560953
RPL29P29	ENSG00000225289	0.99278	0.16041	0.99278	0.16041	1.3546	0.087772	0.85517	0.19623	0.087772	0.553560953
NA	ENSG00000232759	-0.48522	0.68624	0.98626	0.162	1.3545	0.087783	-0.68081	0.75201	0.087783	0.553560953
ACTBL2	ENSG00000169067	1.3544	0.0878	0.9239	0.17777	0.074784	0.47019	0.074784	0.47019	0.0878	0.553560953
NA	ENSG00000279268	0.6834	0.24718	1.3543	0.087814	0.15775	0.43733	0.68099	0.24794	0.087814	0.553560953
SART3	ENSG00000075856	1.3543	0.087819	0.82216	0.20549	0.47915	0.31592	-0.03669	0.51463	0.087819	0.553560953
NUMB	ENSG00000133961	1.2087	0.11339	1.1104	0.13342	1.3399	0.090135	1.3542	0.087831	0.087831	0.553560953
NA	ENSG00000269130	1.3542	0.087835	1.3542	0.087835	1.3542	0.087835	1.3542	0.087835	0.087835	0.553560953
NA	ENSG00000257268	1.3541	0.087845	1.3541	0.087845	0.35765	0.3603	0.19548	0.42251	0.087845	0.553560953
RPL5P16	ENSG00000240411	1.3539	0.087882	0.52878	0.29848	0.52878	0.29848	0.52878	0.29848	0.087882	0.553722506
NA	ENSG00000258419	1.3537	0.087916	0.80617	0.21007	0.46462	0.3211	1.3537	0.087916	0.087916	0.55375
BCAS2	ENSG00000116752	-0.34913	0.63651	-1.0589	0.85517	-0.34913	0.63651	1.3537	0.087922	0.087922	0.55375
PEX5	ENSG00000139197	1.0921	0.1374	1.3189	0.093597	1.3536	0.087925	0.84594	0.19879	0.087925	0.55375
NA	ENSG00000188002	0.0026266	0.49895	1.3534	0.087961	1.2592	0.10398	1.2626	0.10336	0.087961	0.55375
GZMK	ENSG00000113088	1.3534	0.087965	1.3534	0.087965	1.3534	0.087965	1.3534	0.087965	0.087965	0.55375
LINC02441	ENSG00000256643	1.0033	0.15786	1.3534	0.087971	0.16801	0.43329	0.16801	0.43329	0.087971	0.55375
NA	ENSG00000259143	1.3533	0.087983	1.3533	0.087983	1.3533	0.087983	1.3533	0.087983	0.087983	0.55375
LINC01449	ENSG00000224017	-0.066661	0.52657	-0.17195	0.56826	1.3533	0.087984	1.0468	0.14759	0.087984	0.55375
NA	ENSG00000221181	1.3532	0.087996	-1.4339	0.9242	-0.76271	0.77718	-0.76271	0.77718	0.087996	0.55375
NA	ENSG00000253505	1.0824	0.13955	0.24722	0.40237	0.74199	0.22905	1.3532	0.088	0.088	0.55375
NA	ENSG00000225333	-0.46903	0.68047	1.353	0.088033	-0.9071	0.81782	0.079012	0.46851	0.088033	0.553886132
NA	ENSG00000237643	1.2317	0.10904	-0.84233	0.8002	1.2317	0.10904	1.3527	0.088069	0.088069	0.554025517
RN7SL583P	ENSG00000239504	1.3526	0.088093	0.0026385	0.49895	1.3526	0.088093	1.3526	0.088093	0.088093	0.554025517
NA	ENSG00000223969	1.3525	0.088102	1.3525	0.088102	1.3525	0.088102	1.3525	0.088102	0.088102	0.554025517

STK24-AS1 ENSG00000224418 0.4738 0.31782 0.4738 0.31782 0.4738
0.31782 1.3525 0.088105 0.088105 0.554025517
ZNF581 ENSG00000171425 1.3197 0.093474 1.3525 0.088112 0.54505
0.29286 1.3525 0.088112 0.088112 0.554025517
NA ENSG00000227920 1.3524 0.088128 1.3524 0.088128 -0.80001 0.78815
1 0.15865 0.088128 0.55405463
NA ENSG00000255021 1.2262 0.11007 1.3519 0.0882 0.64389 0.25982
1.1836 0.11829 0.0882 0.554179759
RN7SL760P ENSG00000272469 1.3518 0.088213 1.3518 0.088213 1.3518
0.088213 1.3518 0.088213 0.088213 0.554179759
NA ENSG00000279810 1.3518 0.088221 0.84277 0.19968 0.84277 0.19968
0.84277 0.19968 0.088221 0.554179759
NA ENSG00000279946 1.3518 0.088227 1.3518 0.088227 1.3518 0.088227
1.3518 0.088227 0.088227 0.554179759
VEGFB ENSG00000173511 1.3517 0.088232 1.3517 0.088232 1.3517 0.088232
1.3517 0.088232 0.088232 0.554179759
LINC02327 ENSG00000258038 -0.050493 0.52014 0.2656 0.39527 -0.26518
0.60457 1.3517 0.088233 0.088233 0.554179759
NA ENSG00000276984 1.3516 0.088251 1.1663 0.12175 -0.18074 0.57171
-0.18074 0.57171 0.088251 0.554179759
NA ENSG00000223841 1.3516 0.088255 1.3516 0.088255 1.3516 0.088255
1.3516 0.088255 0.088255 0.554179759
MOGAT3 ENSG00000106384 1.3515 0.088264 0.03304 0.48682 0.82718
0.20407 0.32436 0.37283 0.088264 0.554179759
MCTP1 ENSG00000175471 0.60445 0.27277 -0.044827 0.51788 1.3515 0.088269
-1.6556 0.9511 0.088269 0.554179759
NA ENSG00000255202 1.3515 0.088273 0.72353 0.23468 0.94336 0.17275
0.94336 0.17275 0.088273 0.554179759
NA ENSG00000271500 1.3513 0.088298 1.3513 0.088298 1.3513 0.088298
1.3513 0.088298 0.088298 0.554237848
STX18-AS1 ENSG00000247708 -0.056245 0.52243 1.3513 0.088305 1.2265 0.11
0.23515 0.40705 0.088305 0.554237848
NA ENSG00000253153 1.3511 0.088329 1.3511 0.088329 1.3511 0.088329
1.3511 0.088329 0.088329 0.554317086
NA ENSG00000243655 1.3509 0.088356 1.3509 0.088356 1.3509 0.088356
1.3509 0.088356 0.088356 0.554372755
MIR378D2HG ENSG00000264448 -0.22256 0.58806 1.3509 0.088362 -0.22256
0.58806 -0.22256 0.58806 0.088362 0.554372755
RAB5CP1 ENSG00000213731 1.3509 0.088372 1.0384 0.14953 0.38046
0.3518 1.1067 0.13422 0.088372 0.554372755
NPM1P17 ENSG00000242070 -1.2937 0.90211 1.3506 0.088415 -1.4121
0.92104 1.3506 0.088415 0.088415 0.554396208
UTP11 ENSG00000183520 0.52773 0.29884 1.3505 0.088422 -0.77009 0.77938
1.251 0.10548 0.088422 0.554396208
USP12 ENSG00000152484 1.2157 0.11206 1.3505 0.088433 0.85149 0.19725
1.2783 0.10057 0.088433 0.554396208
NA ENSG00000258747 1.3505 0.088434 1.3505 0.088434 0.64125 0.26068
1.3505 0.088434 0.088434 0.554396208
NA ENSG00000237409 0.88335 0.18852 1.3505 0.088435 0.95374 0.17011
0.66724 0.25231 0.088435 0.554396208
NA ENSG00000254163 1.3504 0.088444 -0.31911 0.62518 0.14115 0.44388
0.64943 0.25803 0.088444 0.554396208
MIR26A1 ENSG00000199075 1.35 0.088502 1.35 0.088502 1.35 0.088502 1.35
0.088502 0.088502 0.554600369
NA ENSG00000272720 1.3499 0.088521 1.3499 0.088521 1.3499 0.088521
1.3499 0.088521 0.088521 0.554600369
CDC5L ENSG00000096401 0.26772 0.39446 0.63439 0.26291 1.3499 0.088522
0.32816 0.3714 0.088522 0.554600369
RNU6-514P ENSG00000206935 1.3498 0.088532 0.77829 0.2182 0.36138
0.35891 1.3135 0.094501 0.088532 0.554600369
NA ENSG00000281469 0.51215 0.30427 1.3498 0.08854 0.33566 0.36856
0.91789 0.17934 0.08854 0.554600369

ATP6V0A1	ENSG00000033627	1.3244	0.092689	1.0988	0.13592	1.3497	0.088559	0.91717	0.17953	0.088559	0.554600369
NA	ENSG00000200344	0.51562	0.30306	1.3496	0.088571	0.19422	0.423	0.19422	0.423	0.088571	0.554600369
RPS2P18	ENSG00000231735	0.90869	0.18176	1.3496	0.088572	1.3496	0.088572	0.86065	0.19472	0.088572	0.554600369
PPP1CC	ENSG00000186298	1.1293	0.12938	1.3496	0.088579	0.92549	0.17736	1.1354	0.12811	0.088579	0.554600369
MYDGF	ENSG00000074842	-0.98001	0.83646	-0.98001	0.83646	-0.98001	0.83646	1.3495	0.088594	0.088594	0.554623024
SUMO1P4	ENSG00000235555	1.3494	0.088607	1.3494	0.088607	1.3494	0.088607	1.3494	0.088607	0.088607	0.554632246
RN7SL411P	ENSG00000265327	1.3009	0.096654	1.3493	0.088627	1.3493	0.088627	1.3493	0.088627	0.088627	0.554632246
LAMA4-AS1	ENSG00000226440	0.038377	0.48469	1.3492	0.088638	0.24878	0.40176	1.0085	0.15661	0.088638	0.554632246
GLT8D1	ENSG00000016864	1.3492	0.088641	1.3492	0.088641	0.8088	0.20932	1.0546	0.1458	0.088641	0.554632246
RPS20P32	ENSG00000218198	1.3489	0.08869	0.56021	0.28767	0.56021	0.28767	-0.072236	0.52879	0.08869	0.554836754
TIPARP-AS1	ENSG00000243926	0.11776	0.45313	1.3488	0.088705	-0.1487	0.55911	-0.11421	0.54547	0.088705	0.554836754
NA	ENSG00000272779	0.18606	0.4262	1.0026	0.15802	-0.92138	0.82157	1.3487	0.088714	0.088714	0.554836754
OXSM	ENSG00000151093	1.3486	0.088725	1.3486	0.088725	1.3486	0.088725	1.3486	0.088725	0.088725	0.554836754
CACHD1	ENSG00000158966	0.51837	0.3021	1.0842	0.13914	1.3486	0.088732	0.33091	0.37036	0.088732	0.554836754
RAD17P2	ENSG00000223724	0.17197	0.43173	0.49308	0.31098	1.3485	0.088742	0.65521	0.25616	0.088742	0.554836754
GBP6	ENSG00000183347	0.55949	0.28791	0.6603	0.25453	0.54881	0.29157	1.3484	0.08876	0.08876	0.554878101
RN7SL23P	ENSG00000240823	-1.244	0.89325	1.3483	0.088787	0.60137	0.2738	0.60137	0.2738	0.088787	0.554975694
NA	ENSG00000253553	0.64173	0.26052	-0.83694	0.79869	1.348	0.088824	0.7974	0.21261	0.088824	0.555054769
NA	ENSG00000255449	1.348	0.088825	1.2199	0.11125	1.348	0.088825	1.348	0.088825	0.088825	0.555054769
NA	ENSG00000257124	1.3478	0.088867	-0.32835	0.62868	-0.32835	0.62868	-0.32835	0.62868	0.088867	0.555054769
OPTN	ENSG00000123240	0.84574	0.19885	1.3477	0.088878	-1.1136	0.86728	-0.1129	0.54495	0.088878	0.555054769
FDPS	ENSG00000160752	0.96441	0.16742	0.34516	0.36499	0.37092	0.35535	1.3476	0.088889	0.088889	0.555054769
KCNMB2	ENSG00000197584	1.3476	0.088891	1.1032	0.13497	0.57722	0.2819	0.4309	0.33327	0.088891	0.555054769
NA	ENSG00000229642	1.3476	0.088897	0.5529	0.29017	1.3476	0.088897	1.3476	0.088897	0.088897	0.555054769
RFPL2	ENSG00000128253	0.2842	0.38813	1.3475	0.088911	-1.1149	0.86755	-0.18746	0.57435	0.088911	0.555054769
CD276	ENSG00000103855	1.1198	0.13139	0.80955	0.2091	1.3471	0.088969	-0.76467	0.77777	0.088969	0.555054769
SLC5A11	ENSG00000158865	1.2169	0.11181	1.2169	0.11181	0.31915	0.37481	1.3471	0.088972	0.088972	0.555054769
NA	ENSG00000270066	1.3471	0.088982	1.3471	0.088982	1.3471	0.088982	1.3471	0.088982	0.088982	0.555054769
LINC01504	ENSG00000225434	0.21782	0.41378	0.46172	0.32214	1.3468	0.089016	0.17255	0.4315	0.089016	0.555054769
NA	ENSG00000232363	1.3468	0.089016	-0.096804	0.53856	1.3468	0.089016	-0.91037	0.81869	0.089016	0.555054769
MTCO2P5	ENSG00000231766	1.3468	0.089018	1.3468	0.089018	1.3468	0.089018	1.3468	0.089018	0.089018	0.555054769

NA	ENSG00000266050	0.95703	0.16928	1.3468	0.089029	-0.44125	0.67048	0.41195	0.34019	0.089029	0.555054769
TMEM151A	ENSG00000179292	-0.24463	0.59663	-0.19199	0.57612	0.60377	0.273	1.3464	0.08909	0.08909	0.555054769
IGHV1OR15-9	ENSG00000188403	1.0688	0.14258	1.0764	0.14088	0.80142	0.21145	1.3463	0.089096	0.089096	0.555054769
PDX1	ENSG00000139515	-0.31652	0.62419	-0.62132	0.73281	-0.31652	0.62419	1.3463	0.089097	0.089097	0.555054769
NA	ENSG00000224618	1.3463	0.089104	-0.54449	0.70695	1.3463	0.089104	1.3463	0.089104	0.089104	0.555054769
GPR150	ENSG00000178015	-0.14095	0.55604	1.3463	0.089106	-0.14095	0.55604	-0.14095	0.55604	0.089106	0.555054769
ARHGEF7-AS1	ENSG00000227352	-0.25828	0.60191	-0.25828	0.60191	1.0927	0.13727	1.3462	0.08912	0.08912	0.555054769
NA	ENSG00000258878	0.90201	0.18353	1.3462	0.089126	0.90201	0.18353	0.90201	0.18353	0.089126	0.555054769
ATP1A3	ENSG00000105409	1.2678	0.10243	0.13306	0.44707	-0.48309	0.68548	1.3461	0.08913	0.08913	0.555054769
SIPA1L2	ENSG00000116991	1.3461	0.089135	-0.74797	0.77276	-0.75776	0.7757	0.030317	0.48791	0.089135	0.555054769
ORAI2	ENSG00000160991	1.3461	0.089139	0.88951	0.18687	1.3461	0.089139	0.26176	0.39675	0.089139	0.555054769
NA	ENSG00000261129	0.70902	0.23916	1.3459	0.089169	1.3459	0.089169	1.3459	0.089169	0.089169	0.555054769
TEX45	ENSG00000198723	0.76167	0.22313	0.76167	0.22313	0.76167	0.22313	1.3459	0.089172	0.089172	0.555054769
COPG2	ENSG00000158623	-1.8064	0.96458	-1.0157	0.84511	0.099989	0.46018	1.3457	0.089204	0.089204	0.555054769
ROPN1B	ENSG00000114547	1.3456	0.08921	-0.013993	0.50558	0.5633	0.28661	-0.10411	0.54146	0.08921	0.555054769
HNRNPA1P63	ENSG00000227453	1.3456	0.089219	1.3456	0.089219	1.3456	0.089219	1.3456	0.089219	0.089219	0.555054769
MTCO1P28	ENSG00000262118	1.3455	0.089225	1.3455	0.089225	1.3455	0.089225	1.3455	0.089225	0.089225	0.555054769
NA	ENSG00000244281	1.3455	0.089228	1.3455	0.089228	1.3455	0.089228	1.3455	0.089228	0.089228	0.555054769
UACA	ENSG00000137831	-0.75911	0.77611	-1.4255	0.92299	-1.351	0.91165	1.3455	0.089236	0.089236	0.555054769
ZDHHC11B	ENSG00000206077	0.77665	0.21868	-0.012655	0.50505	-0.20827	0.58249	1.3454	0.08924	0.08924	0.555054769
MAPRE1P3	ENSG00000233204	1.0666	0.14308	1.2455	0.10647	1.3454	0.089241	1.241	0.10729	0.089241	0.555054769
NA	ENSG00000249593	0.8677	0.19278	1.3453	0.089257	1.2708	0.1019	1.2708	0.1019	0.089257	0.555054769
FGFR1OP2P1	ENSG00000230256	-0.17386	0.56901	-1.877	0.96974	1.3452	0.089279	1.3362	0.090738	0.089279	0.555054769
GRM7-AS3	ENSG00000226258	1.3451	0.089295	1.0186	0.1542	0.19375	0.42318	-0.35482	0.63864	0.089295	0.555054769
ANKRD9	ENSG00000156381	0.61811	0.26825	0.97392	0.16505	1.3451	0.089298	1.2352	0.10837	0.089298	0.555054769
SEPSECS	ENSG00000109618	0.89796	0.1846	1.3451	0.089303	0.56776	0.2851	-0.50811	0.69431	0.089303	0.555054769
OR10J1	ENSG00000196184	0.27228	0.39271	0.27228	0.39271	0.27228	0.39271	1.3451	0.089303	0.089303	0.555054769
CSDE1	ENSG00000009307	-0.24156	0.59544	1.0911	0.13761	1.345	0.089305	1.345	0.089305	0.089305	0.555054769
CDH4	ENSG00000179242	1.345	0.089307	0.71847	0.23623	0.61833	0.26818	1.0736	0.14149	0.089307	0.555054769
PUF60	ENSG00000179950	-0.45037	0.67378	1.345	0.08931	-0.30472	0.61971	-0.51894	0.6981	0.08931	0.555054769
RPL21P75	ENSG00000213860	1.345	0.089319	1.345	0.089319	1.345	0.089319	1.345	0.089319	0.089319	0.555054769

NA	ENSG00000269950	1.3449	0.089328	1.3449	0.089328	0.87302	0.19133	1.3449	0.089328	0.089328	0.555054769
NA	ENSG00000253736	1.0044	0.15758	1.3449	0.089335	0.98834	0.16149	0.98834	0.16149	0.089335	0.555054769
CIC	ENSG00000079432	0.8079	0.20957	1.2993	0.096924	1.0904	0.13776	1.3447	0.089359	0.089359	0.555124458
NA	ENSG00000256001	0.92256	0.17812	0.91789	0.17934	0.89676	0.18492	1.3447	0.089369	0.089369	0.555124458
CBLN4	ENSG00000054803	-0.29011	0.61413	-0.10509	0.54185	1.344	0.089471	-0.30923	0.62143	0.089471	0.555415269
RNU1-75P	ENSG00000194297	1.344	0.089475	1.344	0.089475	1.344	0.089475	1.344	0.089475	0.089475	0.555415269
TMEM132D-AS2	ENSG00000256699	1.3439	0.089491	1.3439	0.089491	1.3439	0.089491	1.3439	0.089491	0.089491	0.555415269
TM9SF2	ENSG00000125304	0.41011	0.34086	1.3439	0.089493	0.41011	0.34086	0.8302	0.20321	0.089493	0.555415269
NA	ENSG00000279846	1.3439	0.089497	1.3439	0.089497	1.3439	0.089497	1.3439	0.089497	0.089497	0.555415269
NA	ENSG00000254877	1.0091	0.15646	0.075294	0.46999	1.3438	0.089504	1.3438	0.089504	0.089504	0.555415269
NHEJ1	ENSG00000187736	0.012538	0.495	0.27029	0.39347	0.31788	0.37529	1.3438	0.089507	0.089507	0.555415269
LINC00920	ENSG00000246898	1.3438	0.089507	-0.57079	0.71593	1.3438	0.089507	1.3438	0.089507	0.089507	0.555415269
TSGA13	ENSG00000213265	-1.0214	0.84646	-0.35554	0.63891	1.3437	0.089529	-0.73778	0.76967	0.089529	0.55548105
MIR1255A	ENSG00000221265	0.13507	0.44628	-0.7059	0.75987	-0.1618	0.56427	1.3435	0.089561	0.089561	0.555608852
NA	ENSG00000237686	0.57828	0.28154	1.3433	0.089586	0.011374	0.49546	0.011374	0.49546	0.089586	0.5556932
ZNF771	ENSG00000179965	0.61958	0.26777	1.1704	0.12091	1.3431	0.089614	-0.39301	0.65285	0.089614	0.555796133
NA	ENSG00000278902	0.91635	0.17974	1.3428	0.089668	1.3428	0.089668	-1.061	0.85565	0.089668	0.556060275
NPIPB1P	ENSG00000257513	-1.4109	0.92087	0.63617	0.26233	1.3427	0.089692	0.415	0.33907	0.089692	0.556138333
NA	ENSG00000280335	-0.33699	0.63194	-0.093488	0.53724	-0.33699	0.63194	1.3421	0.089785	0.089785	0.556210549
PRR35	ENSG00000161992	-0.72405	0.76548	1.3421	0.089787	-0.56467	0.71385	-0.56467	0.71385	0.089787	0.556210549
P4HA2	ENSG00000072682	1.3421	0.089788	-1.2289	0.89045	-0.2315	0.59154	-1.9511	0.97448	0.089788	0.556210549
NA	ENSG00000263293	1.3421	0.089789	1.2019	0.1147	1.3421	0.089789	1.332	0.091433	0.089789	0.556210549
SOCS2	ENSG00000120833	0.21657	0.41427	0.21657	0.41427	-0.026991	0.51077	1.342	0.089797	0.089797	0.556210549
NA	ENSG00000224077	1.0999	0.13569	1.1003	0.1356	1.2235	0.11057	1.3419	0.089819	0.089819	0.556210549
GPR180	ENSG00000152749	0.41871	0.33771	0.36646	0.35701	-0.22903	0.59058	1.3418	0.089828	0.089828	0.556210549
C9orf43	ENSG00000157653	0.98443	0.16245	1.1835	0.1183	1.3418	0.089831	1.3418	0.089831	0.089831	0.556210549
LINC01657	ENSG00000224718	1.3417	0.089843	1.3417	0.089843	1.3417	0.089843	1.3417	0.089843	0.089843	0.556210549
SLC22A8	ENSG00000149452	1.3417	0.089853	-0.34794	0.63606	-0.2936	0.61547	0.00764	0.49695	0.089853	0.556210549
NA	ENSG00000254990	-0.57803	0.71838	0.021716	0.49134	1.3416	0.08987	0.62141	0.26716	0.08987	0.556210549
NA	ENSG00000237742	-0.62135	0.73282	-1.3576	0.9127	0.30888	0.37871	1.3415	0.089873	0.089873	0.556210549
PHACTR3	ENSG00000087495	1.039	0.1494	1.2784	0.10056	1.3414	0.089887	1.0239	0.15294	0.089887	0.556210549

NA	ENSG00000234080	0.79495	0.21332	1.3414	0.089892	0.36429	0.35782	0.36429	0.35782	0.089892	0.556210549
NA	ENSG00000265380	1.3413	0.089907	1.3413	0.089907	1.3413	0.089907	-0.066496	0.52651	0.089907	0.556210549
BCAS4	ENSG00000124243	0.65729	0.2555	0.29371	0.38449	1.25	0.10566	1.3412	0.089928	0.089928	0.556210549
MIR378A	ENSG00000199047	0.92467	0.17757	0.92467	0.17757	0.92467	0.17757	1.3412	0.08993	0.08993	0.556210549
CFAP61	ENSG00000089101	1.0521	0.14637	1.3411	0.089938	0.58106	0.2806	0.5595	0.28791	0.089938	0.556210549
LINC00265	ENSG00000188185	1.0651	0.14342	1.3411	0.089939	0.52965	0.29818	-0.091148	0.53631	0.089939	0.556210549
PLA2G3	ENSG00000100078	1.341	0.089953	-0.10291	0.54098	-0.41938	0.66253	0.4034	0.34333	0.089953	0.556210549
SS18L2	ENSG00000008324	1.341	0.089954	1.341	0.089954	0.92361	0.17784	0.63966	0.2612	0.089954	0.556210549
RPL35AP32	ENSG00000243404	1.3409	0.089982	1.3409	0.089982	1.3409	0.089982	1.3409	0.089982	0.089982	0.556210549
GALR1	ENSG00000166573	1.3409	0.089983	1.0449	0.14803	1.3409	0.089983	1.3409	0.089983	0.089983	0.556210549
NA	ENSG00000265614	-0.75704	0.77549	1.3408	0.089985	-0.75704	0.77549	-1.1927	0.88351	0.089985	0.556210549
NA	ENSG00000224649	1.3408	0.089989	1.3408	0.089989	1.3408	0.089989	1.3408	0.089989	0.089989	0.556210549
NA	ENSG00000214248	1.3407	0.090012	1.3407	0.090012	1.3407	0.090012	1.3407	0.090012	0.090012	0.556282151
NKAIN1	ENSG00000084628	0.33491	0.36885	0.73201	0.23208	0.72969	0.23279	1.3403	0.090079	0.090079	0.556506345
QRICH1	ENSG00000198218	0.89661	0.18496	1.3403	0.090082	0.33439	0.36904	-0.061686	0.52459	0.090082	0.556506345
ABCB11	ENSG00000073734	0.1763	0.43003	0.63465	0.26283	1.3401	0.090101	1.3401	0.090101	0.090101	0.556506345
ZC3H11A	ENSG00000058673	0.80557	0.21025	0.31868	0.37498	1.3401	0.090102	0.39285	0.34721	0.090102	0.556506345
RPS12P31	ENSG00000242325	1.34	0.090118	1.34	0.090118	1.34	0.090118	1.34	0.090118	0.090118	0.556506345
SNORA59B	ENSG00000266079	1.34	0.090118	1.34	0.090118	1.34	0.090118	1.34	0.090118	0.090118	0.556506345
C1orf122	ENSG00000197982	1.3399	0.09014	1.3399	0.09014	1.3399	0.09014	1.3399	0.09014	0.09014	0.556506345
NA	ENSG00000250771	1.0161	0.15478	0.89443	0.18555	1.3397	0.090165	0.89443	0.18555	0.090165	0.556506345
SURF1	ENSG00000148290	0.25549	0.39917	-0.06926	0.52761	1.3397	0.090169	0.11615	0.45377	0.090169	0.556506345
LPXN	ENSG00000110031	1.0788	0.14034	0.19671	0.42203	1.3397	0.090176	0.80901	0.20925	0.090176	0.556506345
NA	ENSG00000225297	1.3397	0.090179	1.3397	0.090179	1.3397	0.090179	1.3397	0.090179	0.090179	0.556506345
MTRNR2L12	ENSG00000269028	0.33948	0.36713	1.3396	0.090192	-0.14627	0.55814	-0.14627	0.55814	0.090192	0.556506345
LINC01797	ENSG00000237179	-0.99226	0.83947	1.3395	0.090203	-0.99226	0.83947	-0.99226	0.83947	0.090203	0.556506345
NA	ENSG00000225146	1.0817	0.13969	1.3394	0.090226	1.0195	0.15399	1.3394	0.090226	0.090226	0.556506345
MIR8485	ENSG00000216191	-0.57986	0.719	0.89542	0.18528	1.3394	0.090228	0.89542	0.18528	0.090228	0.556506345
MIR4644	ENSG00000266245	1.3393	0.090231	1.3393	0.090231	1.3393	0.090231	1.3393	0.090231	0.090231	0.556506345
NA	ENSG00000251574	-0.41696	0.66165	0.81578	0.20731	0.09407	0.46253	1.3392	0.090245	0.090245	0.556522254
SPP1	ENSG00000118785	1.339	0.090287	0.3021	0.38129	-0.17417	0.56914	0.28093	0.38938	0.090287	0.556700534

MIR4744	ENSG00000263849	1.3389	0.090307	1.3389	0.090307	1.3389	0.090307	1.3389	0.090307	0.090307	0.556700534
C1orf52	ENSG00000162642	-0.27067	0.60668	0.87774	0.19004	-0.068923	0.52747	1.3388	0.090315	0.090315	0.556700534
LINC00865	ENSG00000232229	0.40484	0.3428	1.1868	0.11765	0.61593	0.26897	1.3388	0.090324	0.090324	0.556700534
HOXB3	ENSG00000120093	0.77841	0.21816	0.99457	0.15997	1.3387	0.090334	1.3387	0.090334	0.090334	0.556700534
LMNA	ENSG00000160789	0.33285	0.36962	1.068	0.14277	1.3385	0.090374	0.90002	0.18406	0.090374	0.556700534
GALC	ENSG00000054983	1.14	0.12714	1.3383	0.090399	0.97885	0.16383	1.1327	0.12866	0.090399	0.556700534
NA	ENSG00000265425	-0.3587	0.64009	0.45422	0.32484	0.87396	0.19107	1.3383	0.090401	0.090401	0.556700534
NA	ENSG00000258108	-1.113	0.86714	-0.31369	0.62312	1.3383	0.090403	0.59917	0.27453	0.090403	0.556700534
EFNA5	ENSG00000184349	1.3383	0.090406	0.94762	0.17166	1.3383	0.090406	1.3383	0.090406	0.090406	0.556700534
RNA5SP476	ENSG00000252422	1.3382	0.090408	1.3382	0.090408	1.3382	0.090408	1.3382	0.090408	0.090408	0.556700534
NA	ENSG00000235208	1.3382	0.090411	1.3382	0.090411	1.3382	0.090411	1.3382	0.090411	0.090411	0.556700534
PSMC3IP	ENSG00000131470	1.3381	0.090437	1.3381	0.090437	1.3381	0.090437	1.3381	0.090437	0.090437	0.556769183
FAM230F	ENSG00000276095	1.338	0.090445	-0.87986	0.81053	-0.074974	0.52988	0.15988	0.43649	0.090445	0.556769183
NUP43	ENSG00000120253	1.3378	0.090487	1.0598	0.14462	0.27431	0.39192	0.911	0.18115	0.090487	0.556927472
RPL5P32	ENSG00000228992	-0.054819	0.52186	-1.2138	0.88758	1.3377	0.090502	-0.054819	0.52186	0.090502	0.556927472
NA	ENSG00000229582	1.3376	0.090505	-0.37574	0.64645	-0.12282	0.54888	-0.12282	0.54888	0.090505	0.556927472
FKBP1A	ENSG00000088832	1.3374	0.090553	-0.83378	0.7978	-0.94991	0.82892	-0.65504	0.74378	0.090553	0.557152486
COX6A1	ENSG00000111775	0.50764	0.30585	1.3372	0.090573	0.09103	0.46373	-0.26732	0.60539	0.090573	0.557184061
NA	ENSG00000248161	1.1867	0.11768	1.2411	0.10729	1.1024	0.13514	1.3372	0.090581	0.090581	0.557184061
WBP2P1	ENSG00000229248	1.3371	0.090601	0.9619	0.16805	1.3371	0.090601	1.3371	0.090601	0.090601	0.55720332
PCMT1	ENSG00000120265	1.337	0.090607	1.2769	0.10082	1.337	0.090607	0.31958	0.37464	0.090607	0.55720332
NA	ENSG00000279199	1.3366	0.09067	1.3366	0.09067	1.3366	0.09067	1.3366	0.09067	0.09067	0.557520391
ALDH7A1	ENSG00000164904	1.3364	0.090709	-0.47006	0.68084	0.50781	0.30579	0.09784	0.46103	0.090709	0.557536296
NA	ENSG00000251187	1.2023	0.11462	1.3364	0.090711	0.88348	0.18849	0.043946	0.48247	0.090711	0.557536296
DRG1	ENSG00000185721	1.3363	0.090723	1.0426	0.14856	1.042	0.1487	1.3013	0.096573	0.090723	0.557536296
LINC01805	ENSG00000223863	0.79437	0.21349	1.3363	0.090732	0.90916	0.18163	1.135	0.12819	0.090732	0.557536296
IRX2	ENSG00000170561	0.10948	0.45641	1.3361	0.090751	0.28284	0.38865	-0.19312	0.57657	0.090751	0.557536296
CAPS2	ENSG00000180881	0.88196	0.1889	1.0712	0.14204	0.88206	0.18887	1.3361	0.090755	0.090755	0.557536296
RBM48P1	ENSG00000223620	1.299	0.096974	1.3361	0.090758	-1.0779	0.85945	-0.72461	0.76565	0.090758	0.557536296
NA	ENSG00000212229	0.47721	0.3166	1.336	0.090779	1.336	0.090779	1.336	0.090779	0.090779	0.557536296
RLIMP2	ENSG00000237278	1.336	0.09078	1.336	0.09078	1.336	0.09078	1.336	0.09078	0.09078	0.557536296

SUPT20H	ENSG00000102710	0.27224	0.39272	1.2666	0.10264	1.3359	0.090787	1.3359	0.090787	0.090787	0.557536296
SPOCK3	ENSG00000196104	0.47719	0.31661	-0.41584	0.66124	1.3355	0.090861	-1.0372	0.85019	0.090861	0.55789053
NA	ENSG00000251438	0.15033	0.44025	0.61145	0.27045	1.3354	0.090869	0.079181	0.46844	0.090869	0.55789053
RNU6-1113P	ENSG00000207076	-0.30198	0.61867	1.3352	0.090912	-0.30198	0.61867	-0.30198	0.61867	0.090912	0.55789053
NA	ENSG00000257548	-0.36519	0.64252	0.84569	0.19886	0.46859	0.31968	1.3349	0.090961	0.090961	0.55789053
UNC13B	ENSG00000198722	0.43519	0.33171	0.38638	0.34961	0.96597	0.16703	1.3348	0.090967	0.090967	0.55789053
ALDH7A1P3	ENSG00000243853	-0.96179	0.83192	-0.85929	0.80491	-1.1061	0.86565	1.3348	0.090968	0.090968	0.55789053
NA	ENSG00000250957	-0.63171	0.73621	1.3348	0.090975	-0.78457	0.78365	1.3348	0.090975	0.090975	0.55789053
NA	ENSG00000228862	0.6398	0.26115	-0.048264	0.51925	1.3347	0.090993	0.35027	0.36307	0.090993	0.55789053
RPS3AP38	ENSG00000226318	1.3346	0.091008	0.72215	0.2351	1.3346	0.091008	1.3346	0.091008	0.091008	0.55789053
C6orf62	ENSG00000112308	0.65499	0.25624	1.0209	0.15366	0.080793	0.4678	1.3345	0.091012	0.091012	0.55789053
HNRNPA1P55	ENSG00000249920	1.3345	0.091018	1.3345	0.091018	1.3345	0.091018	1.3345	0.091018	0.091018	0.55789053
NA	ENSG00000258696	1.3345	0.091023	1.3345	0.091023	-0.85811	0.80458	1.3345	0.091023	0.091023	0.55789053
NA	ENSG00000238058	-0.17015	0.56755	-0.17015	0.56755	1.3344	0.091034	0.52771	0.29885	0.091034	0.55789053
SIGLEC21P	ENSG00000269253	1.3344	0.091038	1.3344	0.091038	1.3344	0.091038	1.3344	0.091038	0.091038	0.55789053
NA	ENSG00000253381	0.7257	0.23401	0.88788	0.1873	1.3343	0.091047	1.3343	0.091047	0.091047	0.55789053
NPAP1P2	ENSG00000214695	-0.72753	0.76655	0.47104	0.31881	-0.72342	0.76529	1.3343	0.091051	0.091051	0.55789053
SP3	ENSG00000172845	0.90404	0.18299	0.62149	0.26714	1.3154	0.094188	1.3343	0.091057	0.091057	0.55789053
SMIM6	ENSG00000259120	1.3343	0.091057	1.3343	0.091057	1.3343	0.091057	1.3343	0.091057	0.091057	0.55789053
NA	ENSG00000231057	1.3342	0.091075	1.1129	0.13287	1.326	0.092419	1.0901	0.13783	0.091075	0.55789053
ANAPC4	ENSG00000053900	-1.2242	0.88957	-0.84875	0.80199	-0.63817	0.73832	1.3341	0.091079	0.091079	0.55789053
PRR14	ENSG00000156858	-1.1097	0.86644	-1.1097	0.86644	1.2248	0.11032	1.334	0.091101	0.091101	0.55789053
PCDHB17P	ENSG00000255622	1.334	0.091107	1.334	0.091107	1.334	0.091107	1.334	0.091107	0.091107	0.55789053
CAMTA1-AS1	ENSG00000225126	0.34002	0.36692	-0.8983	0.81549	1.334	0.091108	0.92469	0.17756	0.091108	0.55789053
PPP2R2A	ENSG00000221914	1.093	0.13719	1.0847	0.13903	1.1933	0.11637	1.3338	0.091136	0.091136	0.55791627
COPS4	ENSG00000138663	0.21255	0.41584	0.19191	0.42391	1.3338	0.091138	-1.0772	0.8593	0.091138	0.55791627
COL18A1	ENSG00000182871	0.7315	0.23224	0.9111	0.18112	1.3337	0.091155	0.35356	0.36184	0.091155	0.55791627
LINC01480	ENSG00000270164	1.3337	0.091158	1.3337	0.091158	1.3337	0.091158	1.3337	0.091158	0.091158	0.55791627
PAX3	ENSG00000135903	0.74707	0.22751	0.56234	0.28694	-1.2971	0.9027	1.3335	0.091186	0.091186	0.557943004
GUSBP1	ENSG00000183666	0.071231	0.47161	1.077	0.14073	1.3334	0.091202	0.93747	0.17426	0.091202	0.557943004
NA	ENSG00000260814	1.3332	0.091234	1.3332	0.091234	1.3332	0.091234	1.3332	0.091234	0.091234	0.557943004

ZNF384	ENSG00000126746	1.3327	0.091322	-0.51595	0.69705	-0.076424	0.53046	-0.23708	0.5937	0.091322	0.557943004
NA	ENSG00000236572	-0.79049	0.78538	-0.64678	0.74111	1.3326	0.091331	-0.028056	0.51119	0.091331	0.557943004
CICP26	ENSG00000233003	1.3326	0.091332	-0.30883	0.62127	1.0438	0.14829	1.0497	0.14692	0.091332	0.557943004
PSMD9	ENSG00000110801	1.2811	0.10007	0.60766	0.27171	0.089177	0.46447	1.3322	0.091397	0.091397	0.557943004
CRIPT	ENSG00000119878	1.3322	0.0914	0.62509	0.26596	1.3322	0.0914	0.34112	0.36651	0.0914	0.557943004
NA	ENSG00000207022	0.52612	0.2994	0.52612	0.2994	1.3322	0.091401	1.3322	0.091401	0.091401	0.557943004
NA	ENSG00000230690	-0.2653	0.60461	0.044312	0.48233	1.3322	0.091403	1.02	0.15387	0.091403	0.557943004
NA	ENSG00000237027	1.3321	0.09141	0.55889	0.28812	1.1311	0.129	1.1311	0.129	0.09141	0.557943004
RTN3	ENSG00000133318	0.95624	0.16948	1.3321	0.091417	0.53216	0.29731	1.0993	0.13583	0.091417	0.557943004
RGS11	ENSG00000076344	-1.4938	0.93239	1.332	0.091422	-1.3187	0.90637	0.20327	0.41946	0.091422	0.557943004
NEGR1	ENSG00000172260	1.1159	0.13224	0.30544	0.38002	1.2572	0.10435	1.332	0.091424	0.091424	0.557943004
TOP3B	ENSG00000100038	-0.40687	0.65795	1.332	0.091425	-1.4242	0.92281	-0.88911	0.81303	0.091425	0.557943004
DNAL1	ENSG00000119661	0.88736	0.18744	0.93734	0.17429	0.97404	0.16502	1.332	0.09143	0.09143	0.557943004
NA	ENSG00000261392	1.3319	0.09144	1.3319	0.09144	1.3319	0.09144	0.69163	0.24459	0.09144	0.557943004
IFFO2	ENSG00000169991	1.3318	0.091466	0.415	0.33907	1.047	0.14754	1.2846	0.099468	0.091466	0.557943004
GPSM2	ENSG00000121957	0.019424	0.49225	0.019424	0.49225	1.3317	0.091475	0.94789	0.17159	0.091475	0.557943004
STRADB	ENSG00000082146	0.93077	0.17599	0.83585	0.20162	1.3317	0.091478	0.25479	0.39944	0.091478	0.557943004
CFAP74	ENSG00000142609	-0.5809	0.71935	-0.29285	0.61518	1.3317	0.091485	0.69734	0.24279	0.091485	0.557943004
TRAJ11	ENSG00000211878	1.3316	0.09149	1.3316	0.09149	1.3316	0.09149	1.3316	0.09149	0.09149	0.557943004
INTS1	ENSG00000164880	0.043171	0.48278	0.41457	0.33923	0.66136	0.25419	1.3316	0.091503	0.091503	0.557943004
NA	ENSG00000239557	1.3315	0.091506	1.3315	0.091506	1.3315	0.091506	1.3315	0.091506	0.091506	0.557943004
NA	ENSG00000224251	1.3314	0.091534	1.3314	0.091534	1.3314	0.091534	1.3314	0.091534	0.091534	0.557943004
DHRS11	ENSG00000278535	-0.25239	0.59963	1.3313	0.09155	0.86814	0.19266	0.45961	0.3229	0.09155	0.557943004
NA	ENSG00000263164	1.3312	0.091569	1.3312	0.091569	1.3312	0.091569	0.72042	0.23563	0.091569	0.557943004
NA	ENSG00000254587	1.3311	0.091574	0.43853	0.3305	1.3311	0.091574	0.28432	0.38808	0.091574	0.557943004
NA	ENSG00000265053	1.3311	0.091574	1.3311	0.091574	1.3311	0.091574	1.3311	0.091574	0.091574	0.557943004
STAM2	ENSG00000115145	0.70641	0.23997	1.0673	0.14292	1.331	0.091592	1.0404	0.14907	0.091592	0.557943004
CIR1	ENSG00000138433	0.21233	0.41592	1.331	0.091596	1.1575	0.12353	1.1575	0.12353	0.091596	0.557943004
NA	ENSG00000274501	0.76764	0.22135	1.331	0.0916	1.331	0.0916	1.331	0.0916	0.0916	0.557943004
PKN2-AS1	ENSG00000237505	0.27833	0.39038	0.19292	0.42351	1.331	0.091601	0.27833	0.39038	0.091601	0.557943004
NA	ENSG00000277579	1.1177	0.13184	1.0128	0.15559	1.3309	0.091608	1.3309	0.091608	0.091608	0.557943004

SLAMF7 ENSG00000026751 1.3308 0.091625 0.16664 0.43383 -0.65794 0.74471 -0.16077 0.56386 0.091625 0.557943004
NA ENSG00000239300 1.3308 0.091627 1.3308 0.091627 1.3308 0.091627 1.3308 0.091627 0.091627 0.557943004
SLC16A5 ENSG00000170190 0.38764 0.34914 -0.26169 0.60322 1.3307 0.091636 0.73861 0.23007 0.091636 0.557943004
NA ENSG00000224067 1.3307 0.091639 1.3307 0.091639 1.3307 0.091639 1.3307 0.091639 0.091639 0.557943004
NA ENSG00000247134 1.1312 0.12899 1.2844 0.099501 1.3307 0.091646 1.3307 0.091646 0.091646 0.557943004
NA ENSG00000269064 1.3307 0.091646 1.3307 0.091646 1.3307 0.091646 1.3307 0.091646 0.091646 0.557943004
NA ENSG00000235281 1.3306 0.091653 -0.35427 0.63843 0.066583 0.47346 -0.26661 0.60512 0.091653 0.557943004
GNAI2 ENSG00000114353 -0.477 0.68332 -0.477 0.68332 0.87015 0.19211 1.3306 0.091656 0.091656 0.557943004
CAPNS1 ENSG00000126247 -1.5408 0.93832 1.3305 0.091674 -1.5408 0.93832 -1.5408 0.93832 0.091674 0.557943004
RNU4-88P ENSG00000223152 1.3305 0.091684 1.3305 0.091684 0.031963 0.48725 1.3305 0.091684 0.091684 0.557943004
N4BP3 ENSG00000145911 1.0623 0.14405 0.0055637 0.49778 1.0623 0.14405 1.3303 0.091705 0.091705 0.557943004
NA ENSG00000250842 0.88974 0.1868 1.3302 0.091724 1.2527 0.10517 0.41216 0.34011 0.091724 0.557943004
NA ENSG00000224484 0.62871 0.26477 0.62871 0.26477 0.29909 0.38244 1.3302 0.091733 0.091733 0.557943004
SGCG ENSG00000102683 1.2726 0.10157 0.35404 0.36166 -0.366 0.64282 1.3301 0.091743 0.091743 0.557943004
TGOLN2 ENSG00000152291 1.33 0.091753 1.2968 0.097355 1.1165 0.1321 1.33 0.091753 0.091753 0.557943004
TMEM125 ENSG00000179178 -0.031142 0.51242 0.086168 0.46567 1.3298 0.091789 1.3298 0.091789 0.091789 0.557943004
MACROD2 ENSG00000172264 0.066679 0.47342 1.3298 0.091792 0.53292 0.29705 0.5403 0.2945 0.091792 0.557943004
ZIM3 ENSG00000141946 1.3298 0.091795 1.3298 0.091795 1.3298 0.091795 1.3298 0.091795 0.091795 0.557943004
S100A6 ENSG00000197956 1.3298 0.091797 1.3298 0.091797 1.3298 0.091797 1.3298 0.091797 0.091797 0.557943004
NA ENSG00000236439 0.079128 0.46847 0.18324 0.4273 1.2198 0.11127 1.3298 0.091798 0.091798 0.557943004
NA ENSG00000250100 1.3298 0.091799 1.3298 0.091799 1.3298 0.091799 1.0462 0.14773 0.091799 0.557943004
SLMAP ENSG00000163681 -0.10025 0.53993 1.3297 0.091814 -0.011666 0.50465 -0.15963 0.56341 0.091814 0.557943004
NA ENSG00000223646 0.40586 0.34242 1.3297 0.091815 -0.1498 0.55954 -0.96011 0.8315 0.091815 0.557943004
NA ENSG00000227842 1.3295 0.091841 -1.0737 0.85851 0.6812 0.24787 -1.2697 0.8979 0.091841 0.558031413
NA ENSG00000263884 0.3057 0.37992 -0.86156 0.80553 -0.86405 0.80622 1.3291 0.091902 0.091902 0.558178346
PLCL2-AS1 ENSG00000226441 1.1232 0.13068 1.329 0.091922 1.329 0.091922 1.329 0.091922 0.091922 0.558178346
TOMM22P6 ENSG00000241874 -0.73589 0.7691 1.329 0.091924 0.48784 0.31283 0.41452 0.33925 0.091924 0.558178346
ZNF597 ENSG00000167981 0.3885 0.34882 0.094874 0.46221 0.3885 0.34882 1.329 0.091928 0.091928 0.558178346
ANO6 ENSG00000177119 1.2628 0.10333 0.64996 0.25786 1.3289 0.091935 0.83737 0.20119 0.091935 0.558178346
NA ENSG00000248686 1.1606 0.12291 0.24369 0.40373 0.11509 0.45419 1.3289 0.09194 0.09194 0.558178346
MUC20 ENSG00000176945 1.1291 0.12943 0.44799 0.32708 1.3288 0.091961 0.44437 0.32839 0.091961 0.558178346

CLEC2D	ENSG00000069493	-0.26659	0.60511	-0.12753	0.55074	1.3288	0.091964	0.69779	0.24265	0.091964	0.558178346
GABRB3	ENSG00000166206	1.3285	0.092007	0.83504	0.20185	0.45905	0.3231	0.53212	0.29732	0.092007	0.558178346
OR52W1	ENSG00000175485	1.3285	0.09201	1.3285	0.09201	1.3285	0.09201	1.3285	0.09201	0.09201	0.558178346
NA	ENSG00000284500	-0.63988	0.73888	-0.94482	0.82763	0.82926	0.20348	1.3285	0.092013	0.092013	0.558178346
NA	ENSG00000270039	1.3284	0.092023	1.3284	0.092023	1.3284	0.092023	1.3284	0.092023	0.092023	0.558178346
CENPUP1	ENSG00000255075	1.3283	0.092033	-0.85173	0.80282	-0.79033	0.78533	-0.20898	0.58277	0.092033	0.558178346
LINC00606	ENSG00000226567	-0.080095	0.53192	1.0202	0.15382	0.11011	0.45616	1.3283	0.092036	0.092036	0.558178346
NDUFS4	ENSG00000164258	1.2191	0.11141	1.3283	0.092037	0.22265	0.41191	0.079498	0.46832	0.092037	0.558178346
NA	ENSG00000228873	1.3282	0.092062	1.3282	0.092062	1.3282	0.092062	1.3282	0.092062	0.092062	0.558227403
TPTEP2	ENSG00000244627	0.83578	0.20164	1.3281	0.092068	0.38971	0.34838	0.60093	0.27394	0.092068	0.558227403
C1QL3	ENSG00000165985	0.26488	0.39555	-0.53262	0.70285	1.3278	0.092128	-0.53262	0.70285	0.092128	0.558361052
SARS2	ENSG00000104835	0.91161	0.18099	1.3277	0.092131	-0.20886	0.58272	1.0849	0.13898	0.092131	0.558361052
NA	ENSG00000266578	0.84791	0.19824	-0.1035	0.54122	1.0748	0.14124	1.3276	0.092158	0.092158	0.558361052
FCF1P3	ENSG00000239997	1.3276	0.092161	1.3276	0.092161	1.3276	0.092161	1.3276	0.092161	0.092161	0.558361052
NA	ENSG00000274627	0.79527	0.21323	0.79527	0.21323	0.79527	0.21323	1.3276	0.092163	0.092163	0.558361052
NA	ENSG00000259421	0.78727	0.21556	0.21959	0.41309	-0.44316	0.67117	1.3274	0.092196	0.092196	0.558361052
NA	ENSG00000279187	1.3273	0.092198	1.3273	0.092198	1.3273	0.092198	1.3273	0.092198	0.092198	0.558361052
VWFP1	ENSG00000241717	-0.048213	0.51923	1.089	0.13808	-0.44062	0.67026	1.3273	0.092209	0.092209	0.558361052
PMFBP1	ENSG00000118557	1.3272	0.092215	-0.94316	0.8272	-0.21929	0.58679	-0.11564	0.54603	0.092215	0.558361052
NAT8B	ENSG00000204872	0.092411	0.46319	1.3272	0.092218	1.222	0.11085	1.222	0.11085	0.092218	0.558361052
GABRA4	ENSG00000109158	1.3272	0.092229	0.64305	0.26009	0.078261	0.46881	-0.030116	0.51201	0.092229	0.558361052
CRTAP	ENSG00000170275	1.0524	0.14632	1.3271	0.092233	1.1463	0.12584	1.3254	0.092519	0.092233	0.558361052
NA	ENSG00000237502	1.3271	0.092239	1.3271	0.092239	1.3271	0.092239	1.3271	0.092239	0.092239	0.558361052
NA	ENSG00000257654	-0.39749	0.6545	1.327	0.092258	1.327	0.092258	-0.34229	0.63393	0.092258	0.558361557
TCFL5	ENSG00000101190	1.327	0.092262	0.83602	0.20157	0.94268	0.17292	0.58223	0.28021	0.092262	0.558361557
NPFF	ENSG00000139574	1.3266	0.092323	1.3266	0.092323	1.3266	0.092323	1.3266	0.092323	0.092323	0.558604079
MTCYBP4	ENSG00000220113	0.13498	0.44631	0.49499	0.3103	1.3266	0.092325	1.0011	0.1584	0.092325	0.558604079
P2RX5	ENSG00000083454	-0.23261	0.59197	1.3264	0.092346	0.95458	0.16989	0.23501	0.4071	0.092346	0.558641252
MILR1	ENSG00000271605	1.3263	0.092372	1.0759	0.14098	1.0507	0.14669	0.082403	0.46716	0.092372	0.558641252
LINC01816	ENSG00000231327	-0.56524	0.71404	1.3263	0.092375	-0.56524	0.71404	0.99837	0.15905	0.092375	0.558641252
ACTBP2	ENSG00000213763	1.3263	0.092377	1.3263	0.092377	1.3263	0.092377	1.3263	0.092377	0.092377	0.558641252

NLRP7	ENSG00000167634	-0.022281	0.50889	1.3261	0.092401	-0.13576	0.554	0.022524	0.49102	0.092401	0.558653779
TMEM161B-AS1	ENSG00000247828	-1.4835	0.93103	-1.6508	0.95061	1.3261	0.092402	-1.7845	0.96283	0.092402	0.558653779
SUDS3P1	ENSG00000249129	1.3256	0.092479	1.3256	0.092479	1.3256	0.092479	-0.54025	0.70549	0.092479	0.558956788
CYTH4	ENSG00000100055	-0.1619	0.56431	1.3256	0.092491	-0.022185	0.50885	-0.25167	0.59935	0.092491	0.558956788
NA	ENSG00000251488	1.3256	0.092492	1.3256	0.092492	0.51866	0.302	0.55242	0.29033	0.092492	0.558956788
THOC3	ENSG00000051596	0.88639	0.1877	0.62648	0.2655	0.41675	0.33843	1.3255	0.092498	0.092498	0.558956788
YBX1P3	ENSG00000240002	-0.47908	0.68406	1.3254	0.09252	1.3254	0.09252	1.3254	0.09252	0.09252	0.559020409
MUPP	ENSG00000237906	0.67468	0.24994	0.67468	0.24994	1.3252	0.09256	1.3252	0.09256	0.09256	0.559133859
NA	ENSG00000253028	0.30347	0.38077	1.3251	0.092571	0.88576	0.18787	0.88576	0.18787	0.092571	0.559133859
GLI2	ENSG00000074047	1.2176	0.11169	1.2284	0.10964	1.325	0.092584	-1.2153	0.88787	0.092584	0.559133859
CERS4	ENSG00000090661	-0.15931	0.56329	0.46153	0.32221	0.11436	0.45447	1.3249	0.09261	0.09261	0.559133859
NA	ENSG00000263826	0.80766	0.20964	1.3248	0.092616	0.80766	0.20964	0.80766	0.20964	0.092616	0.559133859
GLIPR1L1	ENSG00000173401	1.3248	0.092627	1.3248	0.092627	0.85102	0.19738	0.83312	0.20239	0.092627	0.559133859
RBM27	ENSG00000091009	-0.39006	0.65175	0.2808	0.38943	0.2808	0.38943	1.3246	0.092644	0.092644	0.559133859
NA	ENSG00000224361	1.3246	0.092651	1.3246	0.092651	1.3232	0.092877	0.45356	0.32507	0.092651	0.559133859
RNASEL	ENSG00000135828	1.1175	0.13189	0.62362	0.26644	0.42186	0.33657	1.3246	0.092659	0.092659	0.559133859
IFITM3P6	ENSG00000258352	1.3243	0.092699	1.3243	0.092699	1.3243	0.092699	1.3243	0.092699	0.092699	0.559133859
NA	ENSG00000275343	0.046771	0.48135	0.27122	0.39311	1.3242	0.092711	0.046771	0.48135	0.092711	0.559133859
BASP1	ENSG00000176788	0.42004	0.33723	1.2601	0.10382	1.3242	0.092716	0.36182	0.35874	0.092716	0.559133859
CDK13	ENSG00000065883	0.09824	0.46087	0.74984	0.22668	0.66264	0.25378	1.324	0.092747	0.092747	0.559133859
MDK	ENSG00000110492	0.56602	0.28569	0.56602	0.28569	1.2428	0.10697	1.3239	0.092765	0.092765	0.559133859
NA	ENSG00000279774	1.3239	0.092765	1.3239	0.092765	1.3239	0.092765	1.3239	0.092765	0.092765	0.559133859
NA	ENSG00000268146	1.3239	0.092765	1.3239	0.092765	1.3239	0.092765	1.3239	0.092765	0.092765	0.559133859
EXO1	ENSG00000174371	0.066814	0.47336	-0.066827	0.52664	1.3239	0.092772	0.025531	0.48982	0.092772	0.559133859
DLX2-DT	ENSG00000236651	0.60192	0.27361	-0.24979	0.59863	0.72225	0.23507	1.3238	0.092782	0.092782	0.559133859
CEACAM7	ENSG00000007306	0.12848	0.44889	1.135	0.12818	1.3238	0.092786	1.3238	0.092786	0.092786	0.559133859
NA	ENSG00000237788	-0.15297	0.56079	-0.15297	0.56079	-0.15297	0.56079	1.3238	0.092786	0.092786	0.559133859
GPRC5C	ENSG00000170412	0.10618	0.45772	1.3237	0.092807	-0.51632	0.69719	0.47356	0.31791	0.092807	0.559133859
NA	ENSG00000267101	0.75989	0.22366	0.56736	0.28523	1.3237	0.092808	1.2377	0.10791	0.092808	0.559133859
CTSL3P	ENSG00000188029	0.66713	0.25234	1.0742	0.14137	0.66713	0.25234	1.3236	0.09281	0.09281	0.559133859
RPS12	ENSG00000112306	0.84645	0.19865	1.3235	0.092835	0.1022	0.4593	1.1112	0.13323	0.092835	0.559133859

NA	ENSG00000270040	1.3235	0.092842	1.3235	0.092842	1.3235	0.092842	1.3235	0.092842	0.092842	0.559133859
CTRL	ENSG00000141086	1.3234	0.092851	1.3234	0.092851	1.3234	0.092851	0.62204	0.26696	0.092851	0.559133859
NA	ENSG00000237773	1.3233	0.092863	-0.42205	0.66351	1.3233	0.092863	0.73508	0.23115	0.092863	0.559133859
LINC01687	ENSG00000233215	1.3233	0.092864	0.47345	0.31795	-0.94152	0.82678	-0.94152	0.82678	0.092864	0.559133859
NA	ENSG00000279175	0.66521	0.25296	1.3232	0.092878	-0.13749	0.55468	0.48191	0.31493	0.092878	0.559133859
NA	ENSG00000255996	0.38394	0.35051	1.3232	0.092883	-0.17365	0.56893	-0.80914	0.79078	0.092883	0.559133859
NA	ENSG00000279461	1.3227	0.092961	1.3227	0.092961	1.3227	0.092961	1.3227	0.092961	0.092961	0.55953428
RFPL1	ENSG00000128250	-0.14523	0.55774	1.3226	0.092983	0.47134	0.3187	0.47134	0.3187	0.092983	0.559543624
NA	ENSG00000233178	-1.196	0.88416	-2.0031	0.97741	-0.62185	0.73298	1.3225	0.092997	0.092997	0.559543624
NA	ENSG00000283392	0.65135	0.25741	0.28392	0.38824	-0.79537	0.7868	1.3225	0.092997	0.092997	0.559543624
MIR4698	ENSG00000263838	-0.88862	0.8129	1.3224	0.093012	-0.88862	0.8129	0.36822	0.35635	0.093012	0.559564785
RPS2P7	ENSG00000235508	-0.19218	0.5762	0.52099	0.30119	0.52099	0.30119	1.3222	0.093044	0.093044	0.55963115
LINC00973	ENSG00000240476	0.8477	0.1983	0.61106	0.27058	1.2141	0.11236	1.3222	0.093046	0.093046	0.55963115
NA	ENSG00000201683	1.3221	0.093064	1.3221	0.093064	0.019029	0.49241	0.50244	0.30768	0.093064	0.559667417
NA	ENSG00000249007	1.3221	0.093075	1.3221	0.093075	1.3221	0.093075	1.3221	0.093075	0.093075	0.559667417
LINC01290	ENSG00000260468	1.2408	0.10734	1.2408	0.10734	1.2408	0.10734	1.3219	0.093101	0.093101	0.559754686
HNRNPCP9	ENSG00000232048	1.3217	0.093136	1.3217	0.093136	1.3217	0.093136	1.3217	0.093136	0.093136	0.559863039
STX16	ENSG00000124222	1.3217	0.093142	-0.761	0.77667	-1.0715	0.85803	-0.79312	0.78615	0.093142	0.559863039
NA	ENSG00000236292	1.3215	0.09316	1.3215	0.09316	1.3215	0.09316	1.3215	0.09316	0.09316	0.559875189
RGMB	ENSG00000174136	0.95084	0.17084	-0.69654	0.75695	1.3215	0.093167	-0.081498	0.53248	0.093167	0.559875189
MIR4500HG	ENSG00000228824	-0.56463	0.71384	1.3213	0.093195	-0.56463	0.71384	-0.56463	0.71384	0.093195	0.559875574
NA	ENSG00000224273	-0.23109	0.59138	1.3213	0.093199	0.13494	0.44633	1.1846	0.11808	0.093199	0.559875574
RN7SKP120	ENSG00000222693	0.82194	0.20555	0.82194	0.20555	0.82194	0.20555	1.3212	0.093211	0.093211	0.559875574
NA	ENSG00000258383	1.3212	0.093216	1.3212	0.093216	1.3212	0.093216	1.3212	0.093216	0.093216	0.559875574
KIAA1958	ENSG00000165185	0.84968	0.19775	0.64909	0.25814	1.3211	0.09323	0.78548	0.21609	0.09323	0.559875574
NA	ENSG00000252266	0.16609	0.43404	0.5037	0.30724	1.3211	0.093236	0.038741	0.48455	0.093236	0.559875574
NFASC	ENSG00000163531	-0.059139	0.52358	0.8368	0.20135	1.3209	0.093266	-0.12179	0.54847	0.093266	0.559941963
NA	ENSG00000235869	1.3208	0.09328	0.8513	0.1973	1.3208	0.09328	0.89534	0.1853	0.09328	0.559941963
LRRC6P1	ENSG00000254784	1.3208	0.093292	-0.096795	0.53856	-0.096795	0.53856	-0.096795	0.53856	0.093292	0.559941963
ZNF451-AS1	ENSG00000226803	1.226	0.11009	1.3207	0.093299	1.226	0.11009	1.226	0.11009	0.093299	0.559941963
ASB18	ENSG00000182177	0.84332	0.19952	0.67432	0.25005	0.68499	0.24667	1.3207	0.093309	0.093309	0.559941963

NRBF2P4	ENSG00000253228	0.50573	0.30652	1.3206	0.093316	1.143	0.12651	1.2145	0.11229	0.093316	0.559941963
LINC00482	ENSG00000185168	1.3205	0.093339	1.3205	0.093339	1.3205	0.093339	0.16967	0.43264	0.093339	0.559981119
AGPAT2	ENSG00000169692	-0.18189	0.57217	-0.18189	0.57217	1.3204	0.093352	-0.18189	0.57217	0.093352	0.559981119
VLDLR-AS1	ENSG00000236404	-1.3961	0.91866	-0.40405	0.65691	1.3204	0.093357	0.82032	0.20602	0.093357	0.559981119
NA	ENSG00000224621	1.3201	0.093399	1.3201	0.093399	1.3201	0.093399	1.3201	0.093399	0.093399	0.560077404
TSC2	ENSG00000103197	-1.6687	0.95241	1.32	0.09342	0.75565	0.22493	0.297	0.38323	0.09342	0.560077404
TUBD1	ENSG00000108423	-0.55734	0.71135	1.32	0.093423	0.14268	0.44327	0.51299	0.30398	0.093423	0.560077404
LINC00934	ENSG00000281196	1.3199	0.093433	-0.1943	0.57703	-1.4505	0.92654	0.27759	0.39066	0.093433	0.560077404
NA	ENSG00000227755	-0.47472	0.68251	-0.5351	0.70371	1.3198	0.093456	-0.8859	0.81217	0.093456	0.560077404
NA	ENSG00000175911	0.95039	0.17096	0.054787	0.47815	1.3197	0.09346	-0.63591	0.73758	0.09346	0.560077404
RN7SL334P	ENSG00000244618	1.3197	0.093463	0.27593	0.3913	0.55481	0.28951	0.27593	0.3913	0.093463	0.560077404
NA	ENSG00000227681	0.53076	0.29779	0.64727	0.25873	0.64727	0.25873	1.3197	0.093465	0.093465	0.560077404
NA	ENSG00000222373	0.80468	0.2105	-0.53905	0.70507	0.51457	0.30343	1.3196	0.093485	0.093485	0.560125409
NA	ENSG00000229272	1.2673	0.10252	1.3195	0.093496	1.3195	0.093496	1.3195	0.093496	0.093496	0.560125409
NA	ENSG00000272810	1.3194	0.093513	1.3194	0.093513	1.3194	0.093513	1.3194	0.093513	0.093513	0.560158388
NA	ENSG00000231683	0.66438	0.25323	1.3193	0.093527	1.3193	0.093527	1.2167	0.11186	0.093527	0.560165057
CD74	ENSG00000019582	-0.61344	0.73021	0.6103	0.27083	1.3191	0.093562	-0.61344	0.73021	0.093562	0.560165057
NA	ENSG00000273366	1.3191	0.093564	1.3191	0.093564	1.3191	0.093564	1.3191	0.093564	0.093564	0.560165057
LINC01676	ENSG00000230768	0.78716	0.21559	1.3191	0.093574	-0.38787	0.65094	-1.2921	0.90184	0.093574	0.560165057
HMGN3-AS1	ENSG00000270362	1.3189	0.093594	1.3189	0.093594	1.3189	0.093594	1.3189	0.093594	0.093594	0.560165057
NA	ENSG00000255875	1.3188	0.093616	1.3188	0.093616	1.3188	0.093616	1.3188	0.093616	0.093616	0.560165057
NA	ENSG00000274446	0.028193	0.48875	-1.4725	0.92955	1.3188	0.093626	-0.56213	0.71298	0.093626	0.560165057
NA	ENSG00000255005	1.3187	0.093641	1.3187	0.093641	1.3187	0.093641	1.3187	0.093641	0.093641	0.560165057
OR6C7P	ENSG00000257757	-0.34841	0.63623	0.30841	0.37889	1.3186	0.093644	-0.28328	0.61152	0.093644	0.560165057
MRGPRX3	ENSG00000179826	1.3185	0.093671	-1.5923	0.94434	-0.43716	0.669	0.13226	0.44739	0.093671	0.560165057
NA	ENSG00000257254	1.3185	0.093672	1.3185	0.093672	1.3185	0.093672	1.3185	0.093672	0.093672	0.560165057
SNIP1	ENSG00000163877	0.50124	0.3081	0.50124	0.3081	0.50124	0.3081	1.3184	0.093683	0.093683	0.560165057
TSPAN33	ENSG00000158457	1.0377	0.14971	-0.55957	0.71212	-0.18602	0.57379	1.3183	0.093701	0.093701	0.560165057
CKMT2-AS1	ENSG00000247572	-0.54989	0.7088	1.3183	0.093706	-0.15901	0.56317	-0.15901	0.56317	0.093706	0.560165057
NA	ENSG00000257407	0.87404	0.19105	1.3182	0.093721	1.1557	0.12391	1.1052	0.13454	0.093721	0.560165057
GHDC	ENSG00000167925	-0.030709	0.51225	-0.030709	0.51225	-0.030709	0.51225	1.3181	0.093727	0.093727	0.560165057

NA	ENSG00000273132	1.3181	0.093732	1.3181	0.093732	1.3181	0.093732	1.3181	0.093732	0.093732	0.560165057
CALML6	ENSG00000169885	1.3181	0.093737	1.2786	0.10052	0.30074	0.38181	1.3181	0.093737	0.093737	0.560165057
DLGAP1-AS4	ENSG00000263878	1.318	0.093744	0.20985	0.41689	-0.14934	0.55936	-0.031605	0.51261	0.093744	0.560165057
NA	ENSG00000207292	1.3179	0.093763	1.1754	0.11993	1.1754	0.11993	1.1754	0.11993	0.093763	0.560165057
IGLJ6	ENSG00000211682	0.55475	0.28953	1.3179	0.093772	1.3179	0.093772	0.59348	0.27643	0.093772	0.560165057
NYNRIN	ENSG00000205978	1.1999	0.11509	0.73433	0.23137	1.3178	0.093788	1.2084	0.11344	0.093788	0.560165057
RFPL3S	ENSG00000205853	1.3178	0.09379	1.3178	0.09379	1.3178	0.09379	1.3178	0.09379	0.09379	0.560165057
LINC00989	ENSG00000250334	1.2806	0.10017	0.048829	0.48053	0.11098	0.45582	1.3178	0.09379	0.09379	0.560165057
LINC00957	ENSG00000235314	1.3176	0.093811	1.3176	0.093811	1.3176	0.093811	1.3176	0.093811	0.093811	0.56021001
NA	ENSG00000284263	1.3175	0.093832	1.3175	0.093832	1.3175	0.093832	1.3175	0.093832	0.093832	0.56021001
ZNF619P1	ENSG00000224547	1.3175	0.093842	0.16021	0.43636	1.3175	0.093842	1.3175	0.093842	0.093842	0.56021001
ATG12P2	ENSG00000224121	1.3173	0.093862	1.3173	0.093862	1.3173	0.093862	1.3173	0.093862	0.093862	0.56021001
LINC02561	ENSG00000224034	1.3173	0.093875	1.0144	0.15518	0.3055	0.37999	0.40139	0.34407	0.093875	0.56021001
MTCYBP33	ENSG00000263241	1.3173	0.093875	0.051599	0.47942	-0.062848	0.52506	-0.062848	0.52506	0.093875	0.56021001
LEXM	ENSG00000162398	1.3172	0.093878	-0.65293	0.7431	-0.28773	0.61322	-0.61478	0.73065	0.093878	0.56021001
MIF4GD	ENSG00000125457	1.3171	0.093902	1.3171	0.093902	1.3171	0.093902	1.3171	0.093902	0.093902	0.560227953
CCNC	ENSG00000112237	1.3171	0.093904	1.3171	0.093904	1.3171	0.093904	1.3171	0.093904	0.093904	0.560227953
RN7SKP285	ENSG00000222069	0.19581	0.42238	1.3169	0.093935	-0.27012	0.60646	-0.27012	0.60646	0.093935	0.560344296
SLC39A10	ENSG00000196950	-0.12402	0.54935	1.2092	0.11329	1.3168	0.093957	0.19357	0.42326	0.093957	0.560362255
QSOX1	ENSG00000116260	0.96019	0.16848	0.94851	0.17143	1.3167	0.093966	0.23413	0.40744	0.093966	0.560362255
NA	ENSG00000236741	1.1477	0.12554	1.1477	0.12554	1.1477	0.12554	1.3167	0.093975	0.093975	0.560362255
HPR	ENSG00000261701	1.3164	0.094022	1.3164	0.094022	1.3164	0.094022	1.3164	0.094022	0.094022	0.560362255
STMN1P2	ENSG00000251440	0.5394	0.2948	0.5394	0.2948	0.5394	0.2948	1.3164	0.094023	0.094023	0.560362255
TRGC1	ENSG00000211689	1.2956	0.097554	0.15464	0.43855	1.3164	0.094025	1.3164	0.094025	0.094025	0.560362255
SPRY4	ENSG00000187678	0.39149	0.34772	-1.1143	0.86742	-1.063	0.85612	1.3162	0.094048	0.094048	0.560362255
UBE2J1	ENSG00000198833	1.1466	0.12577	0.8736	0.19117	0.8736	0.19117	1.3161	0.094074	0.094074	0.560362255
NA	ENSG00000262678	1.316	0.094082	1.316	0.094082	1.316	0.094082	1.316	0.094082	0.094082	0.560362255
NA	ENSG00000218027	1.316	0.094084	-0.19654	0.57791	1.316	0.094084	0.59387	0.2763	0.094084	0.560362255
NA	ENSG00000259055	1.0228	0.1532	0.96578	0.16708	-0.3408	0.63337	1.316	0.094088	0.094088	0.560362255
FBN2	ENSG00000138829	0.96878	0.16633	0.60872	0.27135	1.3159	0.094109	-0.16078	0.56387	0.094109	0.560362255
IGSF6	ENSG00000140749	1.3159	0.094111	-0.36171	0.64122	-0.36171	0.64122	-0.36171	0.64122	0.094111	0.560362255

RNU6-48P	ENSG00000206888	0.64456	0.25961	1.0401	0.14915	1.3158	0.09412	1.3158	0.09412	0.09412	0.560362255
ACTG1P20	ENSG00000241547	1.3158	0.09412	1.3158	0.09412	1.3158	0.09412	1.3158	0.09412	0.09412	0.560362255
EPHA7	ENSG00000135333	-0.10651	0.54241	1.3158	0.094122	0.29675	0.38333	-1.072	0.85813	0.094122	0.560362255
GIGYF1	ENSG00000146830	0.54216	0.29385	-1.0817	0.86031	-1.1084	0.86616	1.3156	0.094148	0.094148	0.560448576
NA	ENSG00000253223	1.3155	0.094163	1.3155	0.094163	1.3155	0.094163	1.3155	0.094163	0.094163	0.560469401
RNU7-55P	ENSG00000239078	1.3152	0.094213	-0.89272	0.814	-0.90792	0.81804	-1.0552	0.85433	0.094213	0.560698521
SPATA5L1	ENSG00000171763	0.48212	0.31486	1.3149	0.094268	0.49549	0.31013	-1.4544	0.92709	0.094268	0.560888845
RPS26P55	ENSG00000239210	1.3149	0.094268	1.3149	0.094268	1.3149	0.094268	1.3149	0.094268	0.094268	0.560888845
LINC02584	ENSG00000254417	-0.039562	0.51578	1.3147	0.094298	0.80711	0.2098	0.80711	0.2098	0.094298	0.560915302
NA	ENSG00000268957	1.3147	0.094307	0.1435	0.44295	1.3147	0.094307	0.57186	0.28371	0.094307	0.560915302
RNU7-119P	ENSG00000239119	0.18328	0.42729	0.18328	0.42729	0.18328	0.42729	1.3146	0.094322	0.094322	0.560915302
FTH1P15	ENSG00000218980	-0.18678	0.57408	-0.18678	0.57408	0.48344	0.31439	1.3146	0.09433	0.09433	0.560915302
SLC9A7P1	ENSG00000227825	1.3146	0.09433	1.3146	0.09433	1.0968	0.13637	0.5273	0.29899	0.09433	0.560915302
NA	ENSG00000227210	1.3142	0.094386	0.017161	0.49315	0.017161	0.49315	0.017161	0.49315	0.094386	0.560938027
ABCA10	ENSG00000154263	0.39599	0.34606	1.1256	0.13016	-0.2904	0.61425	1.314	0.09443	0.09443	0.560938027
NA	ENSG00000221670	-0.48362	0.68567	0.75765	0.22433	1.3139	0.094437	0.75765	0.22433	0.094437	0.560938027
POLR3KP2	ENSG00000265469	1.3138	0.094451	1.0949	0.13678	1.0291	0.15171	0.202	0.41996	0.094451	0.560938027
ADAMTS9-AS2	ENSG00000241684	1.3138	0.094462	0.033456	0.48666	-1.6095	0.94624	-0.063535	0.52533	0.094462	0.560938027
NA	ENSG00000260519	1.3137	0.094468	1.3137	0.094468	1.3137	0.094468	1.3137	0.094468	0.094468	0.560938027
CACNG4	ENSG00000075461	-1.631	0.94855	-1.631	0.94855	-1.631	0.94855	1.3137	0.094474	0.094474	0.560938027
LINC01322	ENSG00000244128	0.28454	0.388	0.62132	0.26719	1.3136	0.094483	-0.040739	0.51625	0.094483	0.560938027
NA	ENSG00000275812	1.3136	0.094497	1.3136	0.094497	1.3136	0.094497	1.3136	0.094497	0.094497	0.560938027
DEFB126	ENSG00000125788	0.58956	0.27774	1.3135	0.0945	0.44804	0.32706	0.57883	0.28135	0.0945	0.560938027
RUVBL2	ENSG00000183207	1.3135	0.094507	1.3135	0.094507	1.3135	0.094507	1.3135	0.094507	0.094507	0.560938027
LINC01358	ENSG00000237352	-0.27131	0.60692	1.041	0.14894	1.3135	0.09451	-0.73626	0.76921	0.09451	0.560938027
NA	ENSG00000255158	-0.064024	0.52552	1.3135	0.094511	-0.064024	0.52552	-0.064024	0.52552	0.094511	0.560938027
NA	ENSG00000234147	1.3135	0.094512	1.3135	0.094512	1.0542	0.1459	1.3135	0.094512	0.094512	0.560938027
RPL7AP7	ENSG00000213880	1.3135	0.094515	1.3135	0.094515	1.3135	0.094515	0.83419	0.20209	0.094515	0.560938027
GPAT2P2	ENSG00000188383	0.25103	0.40089	-0.74165	0.77085	1.3134	0.094518	1.3134	0.094518	0.094518	0.560938027
RNU6-948P	ENSG00000252183	0.91775	0.17938	0.36344	0.35814	1.3132	0.094556	-0.31314	0.62291	0.094556	0.561095212
SLC66A2	ENSG00000122490	-1.1108	0.86668	1.313	0.094595	-1.0264	0.84765	0.53693	0.29566	0.094595	0.561258292

UBOX5	ENSG00000185019	1.3129	0.094609	1.3129	0.094609	-1.3075	0.90447	0.26158	0.39682	0.094609	0.561273018
RNU6-1155P	ENSG00000200424	1.3127	0.094634	1.3127	0.094634	1.3127	0.094634	0.39219	0.34746	0.094634	0.561352991
RPL21P129	ENSG00000242636	1.3126	0.094659	1.3126	0.094659	1.3126	0.094659	1.3126	0.094659	0.094659	0.561432944
DISC1-IT1	ENSG00000226758	0.68006	0.24823	0.29766	0.38298	1.3124	0.094696	0.28843	0.38651	0.094696	0.561584043
NA	ENSG00000238033	1.3122	0.094723	1.3122	0.094723	1.3122	0.094723	1.0231	0.15313	0.094723	0.561586573
CD63	ENSG00000135404	0.1742	0.43085	0.1742	0.43085	-0.43052	0.66659	1.3122	0.094724	0.094724	0.561586573
RNU7-147P	ENSG00000238698	1.3122	0.094731	1.3122	0.094731	-0.17117	0.56796	-0.29078	0.61439	0.094731	0.561586573
NA	ENSG00000271196	0.4482	0.327	0.96489	0.1673	1.3119	0.09478	0.78714	0.2156	0.09478	0.561746306
TRIM65	ENSG00000141569	1.3119	0.094781	1.3119	0.094781	0.96497	0.16728	1.0593	0.14473	0.094781	0.561746306
RPL36AP26	ENSG00000235828	-0.20873	0.58267	1.3116	0.094826	-0.20873	0.58267	-0.20873	0.58267	0.094826	0.561926514
SPRY1	ENSG00000164056	0.40668	0.34212	1.3115	0.094843	0.53449	0.2965	0.73783	0.23031	0.094843	0.561926514
PRRT3-AS1	ENSG00000230082	0.52354	0.3003	1.3115	0.094846	-1.1994	0.88482	-1.1994	0.88482	0.094846	0.561926514
FABP5P10	ENSG00000213201	0.38948	0.34846	0.38948	0.34846	0.38948	0.34846	1.3112	0.094891	0.094891	0.562097914
SNORA2C	ENSG00000221491	1.3112	0.094898	1.3112	0.094898	1.3112	0.094898	1.3112	0.094898	0.094898	0.562097914
LINC00919	ENSG00000260268	-0.96774	0.83341	-0.96774	0.83341	-0.54435	0.7069	1.311	0.094924	0.094924	0.562138952
NA	ENSG00000251740	1.311	0.094928	0.53462	0.29646	-0.16736	0.56646	-0.16736	0.56646	0.094928	0.562138952
ZNF691	ENSG00000164011	1.3107	0.094983	1.3107	0.094983	1.3107	0.094983	1.3107	0.094983	0.094983	0.562322442
EEF1A1P37	ENSG00000254124	1.3105	0.095006	1.3105	0.095006	1.3105	0.095006	1.3105	0.095006	0.095006	0.562322442
NA	ENSG00000258592	0.72954	0.23283	1.0921	0.13741	1.3105	0.095006	-0.80328	0.78909	0.095006	0.562322442
RNU7-196P	ENSG00000238913	-0.39533	0.6537	1.3105	0.095015	-1.2566	0.89556	-1.2566	0.89556	0.095015	0.562322442
NA	ENSG00000260367	0.28978	0.38599	0.86572	0.19332	1.3104	0.095029	1.3104	0.095029	0.095029	0.562322442
NA	ENSG00000279597	0.53202	0.29736	-0.20325	0.58053	1.3103	0.095049	0.53202	0.29736	0.095049	0.562322442
NA	ENSG00000272369	-0.33317	0.6305	0.71198	0.23824	1.3102	0.095057	-0.060091	0.52396	0.095057	0.562322442
CDH2	ENSG00000170558	0.17785	0.42942	0.81125	0.20861	1.1577	0.1235	1.3101	0.095074	0.095074	0.562322442
NA	ENSG00000276248	0.86261	0.19417	1.3101	0.095079	1.2002	0.11504	1.2002	0.11504	0.095079	0.562322442
NA	ENSG00000200829	0.16047	0.43626	0.6311	0.26399	0.6311	0.26399	1.3101	0.095084	0.095084	0.562322442
PLXNA4	ENSG00000221866	-0.54703	0.70782	-0.59358	0.7236	0.05058	0.47983	1.3101	0.095089	0.095089	0.562322442
RPS15P4	ENSG00000233762	1.3099	0.095107	1.3099	0.095107	1.3099	0.095107	1.3099	0.095107	0.095107	0.562322442
FAM76B	ENSG00000077458	1.3099	0.095109	-0.45934	0.677	0.78527	0.21615	1.285	0.099388	0.095109	0.562322442
KLHL20	ENSG00000076321	1.3096	0.095164	-0.23558	0.59312	0.5765	0.28214	-0.052021	0.52074	0.095164	0.562490183
YBX1P1	ENSG00000224861	1.3096	0.095171	0.62823	0.26493	1.3096	0.095171	1.3096	0.095171	0.095171	0.562490183

NA	ENSG00000282964	0.71038	0.23873	1.3096	0.095172	0.65883	0.255	-0.10941	0.54356	0.095172	0.562490183
NANOGP11	ENSG00000233464	1.3095	0.095189	1.3095	0.095189	1.3095	0.095189	1.3095	0.095189	0.095189	0.562522431
OR52Y1P	ENSG00000231070	1.3093	0.09521	1.3093	0.09521	-0.74811	0.7728	-0.24348	0.59618	0.09521	0.56257456
RFC2	ENSG00000049541	0.51945	0.30172	1.0141	0.15526	1.3092	0.095233	0.91444	0.18024	0.095233	0.56257456
C2orf66	ENSG00000187944	-1.3344	0.90897	0.81965	0.20621	1.3092	0.095234	0.71483	0.23736	0.095234	0.56257456
PPATP1	ENSG00000241293	1.2108	0.11298	-0.62893	0.7353	1.3091	0.095244	-0.62893	0.7353	0.095244	0.56257456
BTF3L4	ENSG00000134717	-1.9461	0.97418	0.85171	0.19719	1.309	0.095274	0.59703	0.27524	0.095274	0.56261483
NBPF4	ENSG00000196427	-0.18398	0.57299	-1.2196	0.88868	1.3089	0.095276	0.315	0.37638	0.095276	0.56261483
NA	ENSG00000267568	1.01	0.15624	1.3089	0.095286	1.01	0.15624	1.1435	0.12641	0.095286	0.56261483
PADI6	ENSG00000276747	1.3088	0.095297	0.7189	0.2361	0.15436	0.43866	0.61071	0.2707	0.095297	0.56261483
NA	ENSG00000254664	1.3087	0.095316	1.3087	0.095316	1.3087	0.095316	1.3087	0.095316	0.095316	0.562658835
ASS1P1	ENSG00000220517	0.55953	0.2879	0.40791	0.34167	1.3085	0.095353	1.3085	0.095353	0.095353	0.562809071
PTPRD	ENSG00000153707	-0.42297	0.66384	-1.5052	0.93387	1.3083	0.095392	0.92011	0.17876	0.095392	0.562896014
NA	ENSG00000270015	1.3082	0.095408	-0.12816	0.55099	-0.12816	0.55099	-0.12816	0.55099	0.095408	0.562896014
PWP1	ENSG00000136045	1.308	0.095434	0.98888	0.16136	0.98628	0.162	0.87053	0.19201	0.095434	0.562896014
WDR25	ENSG00000176473	1.3078	0.095476	0.64865	0.25828	-0.7229	0.76513	0.79844	0.21231	0.095476	0.562896014
NA	ENSG00000235077	1.3077	0.095484	1.3077	0.095484	1.3077	0.095484	1.3077	0.095484	0.095484	0.562896014
OR2L6P	ENSG00000232215	1.3077	0.095489	1.3077	0.095489	1.3077	0.095489	1.3077	0.095489	0.095489	0.562896014
SMIM7P1	ENSG00000196085	1.3077	0.095491	-0.88901	0.813	-0.92384	0.82222	-0.88901	0.813	0.095491	0.562896014
NDST1	ENSG00000070614	0.32893	0.3711	1.3077	0.095492	0.046841	0.48132	1.2222	0.11081	0.095492	0.562896014
LRRC71	ENSG00000160838	0.21384	0.41534	1.3076	0.095503	1.3076	0.095503	-0.30822	0.62104	0.095503	0.562896014
SAMD11P1	ENSG00000262480	1.3076	0.095507	1.3076	0.095507	1.3076	0.095507	1.3076	0.095507	0.095507	0.562896014
NA	ENSG00000258883	1.0436	0.14834	1.3075	0.095514	0.059135	0.47642	1.0572	0.14521	0.095514	0.562896014
NA	ENSG00000262339	1.2511	0.10544	1.3075	0.095521	1.3075	0.095521	1.3075	0.095521	0.095521	0.562896014
TMEM63A	ENSG00000196187	1.0664	0.14312	0.86707	0.19295	0.90608	0.18245	1.3075	0.095525	0.095525	0.562896014
TRIM36-IT1	ENSG00000250472	1.3074	0.095537	1.3074	0.095537	1.3074	0.095537	1.3074	0.095537	0.095537	0.562896014
SAMMSON	ENSG00000240405	1.3074	0.095541	1.3074	0.095541	1.3074	0.095541	1.3074	0.095541	0.095541	0.562896014
MIR548D2	ENSG00000263690	0.93088	0.17596	0.021353	0.49148	1.3071	0.09559	1.3071	0.09559	0.09559	0.563057761
HPYR1	ENSG00000253521	1.307	0.095603	1.307	0.095603	1.307	0.095603	1.307	0.095603	0.095603	0.563057761
NA	ENSG00000262766	1.3069	0.095627	1.3069	0.095627	1.3069	0.095627	1.3069	0.095627	0.095627	0.563057761
ANKRD26P2	ENSG00000235550	-0.55441	0.71035	1.3068	0.095642	-0.55441	0.71035	0.98568	0.16215	0.095642	0.563057761

NA	ENSG00000261253	1.3068	0.095643	1.2686	0.10229	1.3068	0.095643	1.3068	0.095643	0.095643	0.563057761
NA	ENSG00000233339	-0.67483	0.75011	1.3068	0.095647	-0.0015151	0.5006	-0.0015151	0.5006	0.095647	0.563057761
USP48	ENSG00000090686	0.78363	0.21663	1.3067	0.095662	0.40657	0.34216	0.086134	0.46568	0.095662	0.563057761
SNX18P3	ENSG00000225345	1.3066	0.095666	0.81242	0.20828	-0.78669	0.78427	-0.5451	0.70716	0.095666	0.563057761
LINC01659	ENSG00000234928	1.3065	0.095683	0.042821	0.48292	-0.0069021	0.50275	0.43541	0.33163	0.095683	0.563057761
BAIAP3	ENSG00000007516	1.3065	0.095684	-0.10492	0.54178	-0.2271	0.58983	-0.2271	0.58983	0.095684	0.563057761
NA	ENSG00000260262	1.3065	0.095696	0.43098	0.33324	0.031704	0.48735	0.39581	0.34612	0.095696	0.563060382
MESP1	ENSG00000166823	0.86303	0.19406	1.3062	0.09574	1.1781	0.11939	0.86303	0.19406	0.09574	0.563200916
NA	ENSG00000266541	0.42984	0.33366	0.74495	0.22815	1.3062	0.095743	1.3062	0.095743	0.095743	0.563200916
NA	ENSG00000279315	1.3061	0.095767	1.3061	0.095767	1.3061	0.095767	1.3061	0.095767	0.095767	0.563202841
DCK	ENSG00000156136	-0.75769	0.77568	-0.6768	0.75073	0.99271	0.16043	1.306	0.095778	0.095778	0.563202841
NA	ENSG00000260940	1.306	0.095778	1.306	0.095778	1.306	0.095778	1.306	0.095778	0.095778	0.563202841
ASS1P8	ENSG00000251418	1.3058	0.095811	-0.11949	0.54756	0.33667	0.36818	0.55181	0.29054	0.095811	0.563219672
NA	ENSG00000240919	1.3058	0.095812	1.3058	0.095812	1.3058	0.095812	1.3058	0.095812	0.095812	0.563219672
NA	ENSG00000272703	1.3056	0.095837	1.3056	0.095837	1.3056	0.095837	1.3056	0.095837	0.095837	0.563219672
NA	ENSG00000221681	0.45046	0.32619	1.3054	0.095874	0.88202	0.18888	-1.5203	0.93578	0.095874	0.563219672
SLC2A4RG	ENSG00000125520	1.3054	0.095875	1.3054	0.095875	1.3054	0.095875	1.3054	0.095875	0.095875	0.563219672
TRIM23	ENSG00000113595	0.17634	0.43001	0.16687	0.43374	-0.65763	0.74461	1.3054	0.09588	0.09588	0.563219672
PRKAG2	ENSG00000106617	0.53286	0.29707	1.3054	0.095884	0.63857	0.26155	0.24807	0.40204	0.095884	0.563219672
TTC21B	ENSG00000123607	1.1634	0.12234	1.3053	0.095894	1.2904	0.098453	-0.20443	0.58099	0.095894	0.563219672
CXCR5	ENSG00000160683	1.3053	0.095901	0.76894	0.22096	1.2203	0.11118	0.84798	0.19822	0.095901	0.563219672
NA	ENSG00000252004	0.64697	0.25883	1.3052	0.095905	1.3052	0.095905	1.3052	0.095905	0.095905	0.563219672
MMP23B	ENSG00000189409	0.97569	0.16461	1.3052	0.095908	-0.80607	0.7899	-1.4094	0.92064	0.095908	0.563219672
NA	ENSG00000234282	1.2127	0.11262	-0.30757	0.62079	1.3051	0.095935	1.1152	0.13238	0.095935	0.563239198
NA	ENSG00000276740	-0.81446	0.79231	0.036324	0.48551	0.036324	0.48551	1.305	0.095943	0.095943	0.563239198
TERC	ENSG00000270141	1.305	0.095946	1.305	0.095946	1.305	0.095946	1.305	0.095946	0.095946	0.563239198
BOD1	ENSG00000145919	0.55645	0.28895	-0.44944	0.67344	1.3048	0.095983	0.49644	0.30979	0.095983	0.563361761
NA	ENSG00000260470	-0.25094	0.59907	1.3047	0.09599	0.96186	0.16806	0.96186	0.16806	0.09599	0.563361761
NA	ENSG00000223728	0.43427	0.33205	1.3046	0.096016	-0.28058	0.61048	0.84613	0.19874	0.096016	0.563384517
TMEM9B-AS1	ENSG00000254860	0.50082	0.30825	0.86725	0.1929	0.19552	0.42249	1.3046	0.096017	0.096017	0.563384517
NA	ENSG00000200206	1.3045	0.096037	1.3045	0.096037	1.3045	0.096037	1.3045	0.096037	0.096037	0.563434025

RNASE3	ENSG00000169397	1.1196	0.13143	-0.48126	0.68483	-0.25595	0.60101	1.3044	0.096056	0.096056	0.563477655
NA	ENSG00000271150	1.3041	0.096091	1.3041	0.096091	1.3041	0.096091	1.3041	0.096091	0.096091	0.563615122
NUAK2	ENSG00000163545	0.69312	0.24412	1.3038	0.096157	0.65331	0.25678	-0.065851	0.52625	0.096157	0.563934362
NA	ENSG00000260744	1.3037	0.09617	0.12338	0.4509	-0.42125	0.66321	0.22376	0.41147	0.09617	0.563942732
NA	ENSG00000249418	1.3036	0.096193	1.3036	0.096193	1.3036	0.096193	1.3036	0.096193	0.096193	0.564009733
NA	ENSG00000219445	1.3035	0.09621	-0.14876	0.55913	-0.56116	0.71265	-0.65991	0.74534	0.09621	0.564041542
POLR3K	ENSG00000161980	0.69164	0.24458	1.2978	0.09718	1.3033	0.096228	-0.64885	0.74178	0.096228	0.564079206
INTS8	ENSG00000164941	1.3032	0.096251	1.3032	0.096251	0.23514	0.40705	1.2089	0.11335	0.096251	0.564127685
NA	ENSG00000201788	1.3031	0.096265	1.3031	0.096265	1.3031	0.096265	1.3031	0.096265	0.096265	0.564127685
NA	ENSG00000253410	1.3031	0.096271	1.3031	0.096271	1.3031	0.096271	1.3031	0.096271	0.096271	0.564127685
NA	ENSG00000259907	1.303	0.096285	0.48656	0.31329	0.86727	0.1929	0.34021	0.36685	0.096285	0.564141884
NA	ENSG00000225963	-0.24542	0.59693	0.53452	0.29649	-0.5985	0.72525	1.3029	0.096303	0.096303	0.564179513
AHCTF1	ENSG00000153207	0.2693	0.39385	0.72732	0.23352	-0.068477	0.5273	1.3027	0.096338	0.096338	0.564208245
GRM4	ENSG00000124493	1.3027	0.096345	0.76603	0.22183	-0.49794	0.69074	0.40836	0.3415	0.096345	0.564208245
BANF2	ENSG00000125888	1.3025	0.096367	0.82876	0.20362	0.27187	0.39286	0.96514	0.16724	0.096367	0.564208245
NA	ENSG00000239706	0.1898	0.42473	1.3025	0.096377	0.1898	0.42473	0.1898	0.42473	0.096377	0.564208245
C1orf61	ENSG00000125462	1.3024	0.096393	0.47861	0.31611	0.51838	0.3021	0.25332	0.40001	0.096393	0.564208245
ARIH1	ENSG00000166233	1.057	0.14526	1.3024	0.096397	1.0949	0.13679	0.31684	0.37568	0.096397	0.564208245
SRP54-AS1	ENSG00000258704	1.3022	0.096426	-0.62154	0.73288	-0.35337	0.63809	0.1407	0.44405	0.096426	0.564208245
LGALS9C	ENSG00000171916	1.3021	0.096439	0.021684	0.49135	-0.98903	0.83868	-2.6343	0.99578	0.096439	0.564208245
TECRL	ENSG00000205678	-0.34048	0.63325	1.1016	0.13532	1.3021	0.096445	-1.1204	0.86873	0.096445	0.564208245
LRRTM4-AS1	ENSG00000234653	1.302	0.096451	1.302	0.096451	0.47014	0.31913	1.302	0.096451	0.096451	0.564208245
SPAAR	ENSG00000235387	0.4573	0.32373	1.302	0.096465	0.39201	0.34753	0.47864	0.3161	0.096465	0.564208245
NA	ENSG00000280450	-0.36939	0.64408	-0.57572	0.7176	0.81251	0.20825	1.3019	0.096468	0.096468	0.564208245
PDGFRL	ENSG00000104213	0.070341	0.47196	1.058	0.14503	-1.3666	0.91413	1.3019	0.09647	0.09647	0.564208245
NA	ENSG00000264019	1.3019	0.09647	1.3019	0.09647	1.3019	0.09647	1.3019	0.09647	0.09647	0.564208245
NA	ENSG00000231979	-0.80717	0.79021	1.3018	0.096492	-0.80717	0.79021	-0.80717	0.79021	0.096492	0.56425379
NA	ENSG00000265428	1.2437	0.1068	1.2437	0.1068	1.3017	0.096511	1.2437	0.1068	0.096511	0.56425379
PPP1R14BP3	ENSG00000179967	1.3015	0.096548	1.2264	0.11002	1.2264	0.11002	1.3015	0.096548	0.096548	0.56425379
DNAL4	ENSG00000100246	-0.17876	0.57094	1.3014	0.096557	-0.17876	0.57094	-0.17876	0.57094	0.096557	0.56425379
NA	ENSG00000260329	0.72804	0.23329	1.2909	0.098364	1.3014	0.096562	1.3014	0.096562	0.096562	0.56425379

AGGF1P8	ENSG00000261752	1.3014	0.096568	1.3014	0.096568	1.3014	0.096568	1.3014	0.096568	0.096568	0.56425379
CCDC153	ENSG00000248712	1.3013	0.096574	1.3013	0.096574	1.3013	0.096574	1.3013	0.096574	0.096574	0.56425379
PRDM9	ENSG00000164256	1.3013	0.096582	-1.2727	0.89844	0.11382	0.45469	0.52861	0.29854	0.096582	0.56425379
NA	ENSG00000207173	-0.29801	0.61715	-0.29801	0.61715	-1.0066	0.84295	1.3013	0.096582	0.096582	0.56425379
LINC02743	ENSG00000255258	1.2803	0.10022	0.57878	0.28137	0.54143	0.29411	1.3008	0.096666	0.096666	0.564676838
TMEM220-AS1	ENSG00000263400	0.5264	0.2993	1.3007	0.096679	0.5264	0.2993	0.5264	0.2993	0.096679	0.564685086
SDHAF2	ENSG00000167985	-0.1009	0.54019	-0.45702	0.67617	-0.89889	0.81564	1.3006	0.096695	0.096695	0.564710852
VEGFA	ENSG00000112715	1.0097	0.15632	1.3005	0.096719	-0.0040235	0.50161	1.0658	0.14326	0.096719	0.564783328
RSL24D1P4	ENSG00000215800	-0.76523	0.77793	-0.78032	0.7824	-0.78032	0.7824	1.3003	0.096757	0.096757	0.564901416
LECT2	ENSG00000145826	-0.39726	0.65441	-0.40895	0.65871	0.13638	0.44576	1.3002	0.096763	0.096763	0.564901416
NA	ENSG00000276071	1.3002	0.096774	0.83174	0.20278	1.3002	0.096774	1.1623	0.12255	0.096774	0.564901416
NA	ENSG00000284452	1.2999	0.096813	1.2999	0.096813	1.2999	0.096813	1.2999	0.096813	0.096813	0.565006801
RBM25	ENSG00000119707	1.2998	0.096836	1.2491	0.10581	0.66996	0.25144	0.66841	0.25194	0.096836	0.565006801
RPL34P27	ENSG00000232858	-2.3169	0.98974	-2.3169	0.98974	-2.3169	0.98974	1.2997	0.096851	0.096851	0.565006801
NA	ENSG00000234190	-0.16699	0.56631	1.2996	0.096868	0.24713	0.4024	-0.011504	0.50459	0.096868	0.565006801
TTK	ENSG00000112742	0.80801	0.20954	1.2996	0.096877	1.2996	0.096877	1.2996	0.096877	0.096877	0.565006801
NPIPB14P	ENSG00000226232	1.2995	0.09688	1.2995	0.09688	1.2995	0.09688	1.2995	0.09688	0.09688	0.565006801
HIGD1AP6	ENSG00000253229	1.2995	0.09688	-0.40982	0.65903	1.2995	0.09688	-0.40982	0.65903	0.09688	0.565006801
NA	ENSG00000255422	1.2994	0.096897	1.2994	0.096897	1.2994	0.096897	1.2994	0.096897	0.096897	0.565006801
GAD1	ENSG00000128683	-0.58887	0.72203	-0.27786	0.60944	1.2155	0.11209	1.2994	0.096905	0.096905	0.565006801
NA	ENSG00000204038	-0.94733	0.82827	-0.94733	0.82827	1.2994	0.096908	-1.1229	0.86927	0.096908	0.565006801
LINC02855	ENSG00000254209	1.2993	0.096922	1.1776	0.11947	1.2342	0.10857	1.2993	0.096922	0.096922	0.565020823
ZNF30-AS1	ENSG00000270876	1.2992	0.09694	1.2992	0.09694	1.2992	0.09694	1.2992	0.09694	0.09694	0.565058158
NA	ENSG00000269604	1.2991	0.096955	1.2991	0.096955	1.2991	0.096955	1.2991	0.096955	0.096955	0.565062993
DYNC1I2	ENSG00000077380	1.2989	0.096997	0.66439	0.25322	0.91037	0.18131	0.90319	0.18321	0.096997	0.565062993
SNORA70J	ENSG00000206909	1.2988	0.097004	1.2988	0.097004	1.2988	0.097004	0.26241	0.3965	0.097004	0.565062993
RPL10P8	ENSG00000254012	1.2988	0.097004	1.2988	0.097004	1.2988	0.097004	1.2988	0.097004	0.097004	0.565062993
ZNF554	ENSG00000172006	1.2987	0.097016	1.0735	0.14152	1.0735	0.14152	0.83809	0.20099	0.097016	0.565062993
NA	ENSG00000235337	0.26288	0.39632	0.26288	0.39632	1.2987	0.097021	0.26288	0.39632	0.097021	0.565062993
NA	ENSG00000279160	1.2987	0.097022	1.2987	0.097022	1.2987	0.097022	1.2987	0.097022	0.097022	0.565062993
NA	ENSG00000229327	0.49954	0.3087	1.2985	0.097066	1.2985	0.097066	1.2985	0.097066	0.097066	0.565155107

ELAPOR1	ENSG00000116299	1.0492	0.14705	-0.53449	0.7035	1.0516	0.14649	1.2984	0.097073	0.097073	0.565155107
IFRD2	ENSG00000214706	1.2983	0.097085	0.50168	0.30795	1.2983	0.097085	1.2983	0.097085	0.097085	0.565155107
CAPN15	ENSG00000103326	1.2983	0.097098	-0.53783	0.70465	1.2983	0.097098	-0.40625	0.65772	0.097098	0.565155107
NUP35P1	ENSG00000223928	-0.30774	0.62086	0.45438	0.32478	1.2982	0.09711	0.53549	0.29615	0.09711	0.565155107
PATZ1	ENSG00000100105	-0.63914	0.73863	1.2982	0.097115	-0.63914	0.73863	0.57171	0.28376	0.097115	0.565155107
NA	ENSG00000201860	1.2981	0.097119	1.2981	0.097119	1.2981	0.097119	1.2981	0.097119	0.097119	0.565155107
NA	ENSG00000275989	-0.57529	0.71745	-0.38018	0.64809	1.298	0.097141	-1.7831	0.96272	0.097141	0.565169144
BHMT2	ENSG00000132840	1.298	0.097148	-0.62712	0.73471	-1.2533	0.89495	-0.62712	0.73471	0.097148	0.565169144
OR10V2P	ENSG00000255538	1.2979	0.097166	1.2979	0.097166	1.2979	0.097166	1.2979	0.097166	0.097166	0.565169144
MIR3177	ENSG00000265820	1.2978	0.097172	0.30208	0.38129	0.30208	0.38129	0.30208	0.38129	0.097172	0.565169144
NA	ENSG00000254242	1.2977	0.097189	-0.64274	0.7398	0.14919	0.4407	1.2977	0.097189	0.097189	0.565169144
LIVAR	ENSG00000266304	1.2977	0.097191	1.2977	0.097191	1.2977	0.097191	1.2977	0.097191	0.097191	0.565169144
ODF1	ENSG00000155087	1.2976	0.09722	1.2976	0.09722	1.2976	0.09722	1.2976	0.09722	0.09722	0.565266839
WNT7B	ENSG00000188064	0.73403	0.23147	0.89139	0.18636	0.97676	0.16434	1.2974	0.097255	0.097255	0.565266839
RNU2-11P	ENSG00000239122	1.2973	0.097257	0.47332	0.31799	1.2973	0.097257	1.2973	0.097257	0.097257	0.565266839
DGKG	ENSG00000058866	0.63065	0.26414	1.0302	0.15145	1.2973	0.097263	-0.49564	0.68993	0.097263	0.565266839
ZFPL1	ENSG00000162300	1.2973	0.09727	1.2973	0.09727	1.2973	0.09727	1.2973	0.09727	0.09727	0.565266839
OR10A6	ENSG00000279000	-0.012568	0.50501	1.2971	0.097302	1.2971	0.097302	1.2971	0.097302	0.097302	0.565266839
NA	ENSG00000235263	0.60861	0.27139	-0.16519	0.5656	-0.019315	0.50771	1.2971	0.097304	0.097304	0.565266839
BARX1	ENSG00000131668	-0.71252	0.76193	1.0634	0.1438	1.297	0.097318	-0.98112	0.83673	0.097318	0.565266839
FAF1-AS1	ENSG00000225767	1.297	0.097322	1.297	0.097322	1.297	0.097322	0.41833	0.33785	0.097322	0.565266839
TRIM66	ENSG00000166436	1.2969	0.09733	1.2969	0.09733	1.1002	0.13562	1.0605	0.14446	0.09733	0.565266839
NA	ENSG00000236392	-0.96565	0.83289	1.2969	0.097341	-1.0659	0.85676	-1.0659	0.85676	0.097341	0.565266839
MIR4525	ENSG00000266107	1.2968	0.097347	1.2968	0.097347	1.2968	0.097347	1.2968	0.097347	0.097347	0.565266839
NA	ENSG00000270441	1.0909	0.13765	1.174	0.1202	1.2965	0.097398	-0.24339	0.59615	0.097398	0.565440937
NA	ENSG00000232762	-1.7833	0.96273	-1.4628	0.92824	1.2964	0.097411	-0.69648	0.75694	0.097411	0.565440937
NA	ENSG00000201245	1.2911	0.098341	0.6145	0.26944	0.21316	0.4156	1.2964	0.097424	0.097424	0.565440937
NA	ENSG00000250978	-1.4505	0.92655	-1.1312	0.87102	-1.1312	0.87102	1.2963	0.097428	0.097428	0.565440937
IRF2BP2	ENSG00000168264	-0.016505	0.50658	1.2963	0.097435	0.89756	0.18471	0.12303	0.45104	0.097435	0.565440937
GANAB	ENSG00000089597	1.296	0.097491	0.39356	0.34695	1.296	0.097491	1.296	0.097491	0.097491	0.565637808
FSHR	ENSG00000170820	1.2959	0.097505	0.63808	0.26171	1.2959	0.097505	0.73415	0.23143	0.097505	0.565637808

NA	ENSG00000267646	1.2958	0.097515	1.2958	0.097515	1.2958	0.097515	1.2958	0.097515	0.097515	0.565637808
NA	ENSG00000272279	0.089609	0.4643	0.18013	0.42852	1.2958	0.097521	-0.50064	0.69169	0.097521	0.565637808
RNA5SP453	ENSG00000199373	0.47329	0.318	1.2958	0.09753	-0.22984	0.59089	-0.28789	0.61328	0.09753	0.565637808
RNU6-531P	ENSG00000252503	0.24878	0.40177	1.2956	0.097553	1.2956	0.097553	1.2956	0.097553	0.097553	0.565637808
LMTK2	ENSG00000164715	0.24917	0.40162	0.015313	0.49389	1.2956	0.097565	0.1808	0.42826	0.097565	0.565637808
NA	ENSG00000146677	-0.76336	0.77738	1.2955	0.097573	0.50445	0.30697	-0.92152	0.82161	0.097573	0.565637808
NA	ENSG00000263843	1.2955	0.097575	1.2955	0.097575	1.2955	0.097575	1.2955	0.097575	0.097575	0.565637808
XPO6	ENSG00000169180	1.0623	0.14405	1.0241	0.15289	0.21947	0.41314	1.2954	0.097585	0.097585	0.565637808
SULT1D1P	ENSG00000236959	-0.79566	0.78688	1.2952	0.097626	1.2952	0.097626	1.2952	0.097626	0.097626	0.56569374
NA	ENSG00000249326	0.0062838	0.49749	1.295	0.097652	-0.054696	0.52181	-0.34009	0.6331	0.097652	0.56569374
EBF2	ENSG00000221818	1.295	0.097655	0.74808	0.22721	0.88715	0.1875	-0.094216	0.53753	0.097655	0.56569374
LINC02401	ENSG00000257762	1.2486	0.1059	0.92686	0.177	1.2949	0.09767	0.78525	0.21615	0.09767	0.56569374
MTCO3P13	ENSG00000264316	0.74624	0.22776	1.2949	0.09767	1.2949	0.09767	1.2949	0.09767	0.09767	0.56569374
BCL7B	ENSG00000106635	-0.11504	0.54579	1.2949	0.097673	0.51573	0.30302	0.29394	0.3844	0.097673	0.56569374
CIRBP	ENSG00000099622	1.2949	0.097683	1.2038	0.11433	0.83914	0.2007	0.74217	0.22899	0.097683	0.56569374
RN7SL34P	ENSG00000239868	1.2946	0.097735	1.2946	0.097735	1.2946	0.097735	1.2946	0.097735	0.097735	0.56569374
IGHV3OR16-13	ENSG00000271178	0.52476	0.29987	0.52033	0.30142	-0.60132	0.72619	1.2944	0.097757	0.097757	0.56569374
NA	ENSG00000272679	1.2943	0.097773	1.2943	0.097773	1.2943	0.097773	1.2943	0.097773	0.097773	0.56569374
LINC02832	ENSG00000227308	-0.1554	0.56175	1.2943	0.097784	0.84119	0.20012	0.51107	0.30465	0.097784	0.56569374
HSPE1P23	ENSG00000250356	1.2942	0.097794	-1.1011	0.86458	-0.18546	0.57356	-0.42094	0.6631	0.097794	0.56569374
MRPS11P1	ENSG00000225803	1.2941	0.097815	0.85524	0.19621	1.1084	0.13384	0.80601	0.21012	0.097815	0.56569374
MIR495	ENSG00000207743	0.44111	0.32957	1.294	0.097831	0.44111	0.32957	0.44111	0.32957	0.097831	0.56569374
NA	ENSG00000268505	1.294	0.097835	0.70723	0.23971	1.294	0.097835	1.294	0.097835	0.097835	0.56569374
NA	ENSG00000235776	1.294	0.097838	1.294	0.097838	1.294	0.097838	1.294	0.097838	0.097838	0.56569374
ZNF235	ENSG00000159917	1.2939	0.097845	1.2939	0.097845	1.2939	0.097845	1.2939	0.097845	0.097845	0.56569374
NA	ENSG00000244738	1.2939	0.097847	1.2939	0.097847	1.2939	0.097847	1.2939	0.097847	0.097847	0.56569374
NA	ENSG00000278084	0.83241	0.20259	0.57051	0.28417	1.2939	0.097847	0.54473	0.29297	0.097847	0.56569374
TMCO5A	ENSG00000166069	6.89E-05	0.49997	0.087703	0.46506	-0.15296	0.56079	1.2938	0.097859	0.097859	0.56569374
KCNIP1	ENSG00000182132	0.69457	0.24366	0.32336	0.37321	1.2935	0.097914	0.92362	0.17784	0.097914	0.56569374
SELENOO	ENSG00000073169	-0.38046	0.6482	1.2935	0.097921	-0.30707	0.62061	0.7862	0.21588	0.097921	0.56569374
LINC01715	ENSG00000229388	1.1613	0.12276	1.1613	0.12276	1.2935	0.097921	0.40929	0.34116	0.097921	0.56569374

DSTNP5 ENSG00000236681 -0.51434 0.69649 0.85115 0.19734 1.2935
0.097921 1.0759 0.14098 0.097921 0.56569374
KRTAP5-7 ENSG00000244411 -0.4095 0.65891 -0.7306 0.76749 1.2935
0.097925 0.54775 0.29193 0.097925 0.56569374
DEFB127 ENSG00000088782 0.91315 0.18058 0.88637 0.18771 1.0989
0.1359 1.2934 0.097935 0.097935 0.56569374
DSC1 ENSG00000134765 0.82109 0.2058 -0.37396 0.64578 1.2934 0.097939
-1.0353 0.84974 0.097939 0.56569374
NDUFS5P5 ENSG00000230671 0.28618 0.38737 0.25755 0.39838 1.2933
0.097947 -0.86222 0.80572 0.097947 0.56569374
NA ENSG00000238084 -0.53037 0.70207 -0.83158 0.79718 1.2929 0.098026
1.2929 0.098026 0.098026 0.56569374
RN7SL363P ENSG00000241869 -0.58078 0.71931 0.36015 0.35937 1.2929
0.098031 1.2929 0.098031 0.098031 0.56569374
RPL21P38 ENSG00000233934 1.2195 0.11133 1.2928 0.098038 1.0498
0.1469 1.0827 0.13948 0.098038 0.56569374
SFTPA1 ENSG00000122852 1.2928 0.098045 0.508 0.30573 1.2262 0.11007
1.2262 0.11007 0.098045 0.56569374
ZYG11B ENSG00000162378 1.2927 0.098052 -0.72302 0.76517 1.1515
0.12477 0.68422 0.24692 0.098052 0.56569374
NA ENSG00000255410 1.2927 0.098052 1.2927 0.098052 1.2927 0.098052
1.2927 0.098052 0.098052 0.56569374
C1QB ENSG00000173369 1.0316 0.15112 0.1721 0.43168 0.79965 0.21196
1.2927 0.098053 0.098053 0.56569374
EMC3 ENSG00000125037 1.2927 0.098057 0.39133 0.34778 0.00090097 0.49964
-0.2565 0.60122 0.098057 0.56569374
NA ENSG00000236378 1.2927 0.098064 1.2927 0.098064 1.2927 0.098064
1.2927 0.098064 0.098064 0.56569374
RAC1P3 ENSG00000231404 1.1621 0.1226 1.1621 0.1226 0.43586
0.33147 1.2926 0.09808 0.09808 0.56569374
NA ENSG00000267048 1.2926 0.098081 1.2926 0.098081 1.2926 0.098081
1.2926 0.098081 0.098081 0.56569374
TRGJ2 ENSG00000211687 -0.79923 0.78792 1.2925 0.098097 1.0695 0.14243
0.65815 0.25522 0.098097 0.56569374
MIR186 ENSG00000207721 1.2924 0.098103 1.2924 0.098103 1.2924
0.098103 1.2924 0.098103 0.098103 0.56569374
LINC01233 ENSG00000269364 1.2924 0.098109 1.2924 0.098109 1.2924
0.098109 1.2924 0.098109 0.098109 0.56569374
ALDOA ENSG00000149925 1.2923 0.098118 0.30861 0.37881 1.2923 0.098118
1.2923 0.098118 0.098118 0.56569374
PDPK2P ENSG00000205918 -0.55103 0.70919 1.2923 0.098129 -0.32033
0.62564 -0.63442 0.7371 0.098129 0.56569374
NA ENSG00000283423 1.2922 0.098144 1.2922 0.098144 1.2922 0.098144
1.2922 0.098144 0.098144 0.56569374
NA ENSG00000259275 1.0888 0.13812 1.2921 0.098155 -1.4166 0.9217
-1.4166 0.9217 0.098155 0.56569374
CAND2 ENSG00000144712 -1.0019 0.8418 -0.92226 0.8218 1.2921 0.098159
0.25489 0.3994 0.098159 0.56569374
RN7SKP244 ENSG00000252322 -1.0443 0.85182 -1.0443 0.85182 -1.0443
0.85182 1.292 0.098177 0.098177 0.56569374
NA ENSG00000277227 -0.33334 0.63056 1.2919 0.09819 0.89504 0.18538
0.89504 0.18538 0.09819 0.56569374
ARMC4P1 ENSG00000238021 1.2486 0.10591 1.2919 0.098196 1.1858
0.11784 -0.31736 0.62452 0.098196 0.56569374
NID1 ENSG00000116962 1.2917 0.098222 -1.898 0.97116 -1.898 0.97116
-0.5239 0.69983 0.098222 0.56569374
SPDYE4 ENSG00000183318 0.9148 0.18015 0.54716 0.29213 1.2917
0.098234 -0.98986 0.83888 0.098234 0.56569374
GCNT1P2 ENSG00000249206 1.2916 0.098253 -1.1193 0.8685 -0.33764
0.63218 -1.1193 0.8685 0.098253 0.56569374
NA ENSG00000221412 1.2915 0.098268 -0.43874 0.66958 0.98868 0.16141
0.40306 0.34345 0.098268 0.56569374

HTR7 ENSG00000148680 0.45993 0.32278 0.42725 0.3346 1.2914 0.098283
0.057835 0.47694 0.098283 0.56569374
NA ENSG00000251188 0.31123 0.37781 1.2913 0.098293 0.31123 0.37781
0.31123 0.37781 0.098293 0.56569374
TESC ENSG00000088992 -0.042101 0.51679 -0.13859 0.55511 1.2913 0.098295
1.111 0.13328 0.098295 0.56569374
STX7 ENSG00000079950 -0.15485 0.56153 1.2371 0.10803 1.2913 0.098302
0.40011 0.34454 0.098302 0.56569374
NA ENSG00000201867 0.30882 0.37873 1.2198 0.11128 0.86411 0.19376
1.2913 0.098306 0.098306 0.56569374
NA ENSG00000279403 0.52444 0.29999 1.2913 0.098307 1.2913 0.098307
1.2913 0.098307 0.098307 0.56569374
NA ENSG00000224959 0.71176 0.23831 -1.5278 0.93671 -0.25073 0.59899
1.2912 0.098309 0.098309 0.56569374
NF1P8 ENSG00000236956 1.2912 0.098323 -0.81359 0.79206 -0.67876 0.75135
0.11983 0.45231 0.098323 0.56569374
MST1L ENSG00000186715 0.49074 0.3118 -0.7654 0.77798 1.2911 0.098326
-0.16443 0.56531 0.098326 0.56569374
NA ENSG00000270555 1.2909 0.098372 1.1576 0.1235 1.0457 0.14785
0.71425 0.23754 0.098372 0.565821266
ACP5 ENSG00000102575 1.2909 0.098373 0.19667 0.42204 1.2909 0.098373
1.2909 0.098373 0.098373 0.565821266
RPS14P8 ENSG00000239528 1.2908 0.098383 1.2908 0.098383 -0.7514
0.77379 1.2908 0.098383 0.098383 0.565821266
TMEM132D-AS1 ENSG00000249196 -0.32088 0.62585 1.2906 0.098425 1.2906
0.098425 1.2906 0.098425 0.098425 0.565996017
RPL7L1P8 ENSG00000228766 -1.1869 0.88236 1.2902 0.098488 -1.1869
0.88236 -1.1869 0.88236 0.098488 0.566053005
ANO2 ENSG00000047617 -0.37537 0.64631 0.38767 0.34913 -0.017323 0.50691
1.2901 0.098508 0.098508 0.566053005
NA ENSG00000273066 1.2901 0.098511 1.2901 0.098511 1.2901 0.098511
1.2901 0.098511 0.098511 0.566053005
GLYATL1P4 ENSG00000254399 0.20339 0.41941 0.4601 0.32272 -0.53013
0.70199 1.2899 0.098546 0.098546 0.566053005
NA ENSG00000283175 1.2898 0.098553 0.95808 0.16901 1.2898 0.098553
1.0148 0.15511 0.098553 0.566053005
RNU1-89P ENSG00000207322 -0.56168 0.71283 -0.56168 0.71283 1.2898
0.098561 -0.56168 0.71283 0.098561 0.566053005
EEF1A1P38 ENSG00000261557 0.18277 0.42749 1.2897 0.09857 1.2897
0.09857 1.2897 0.09857 0.09857 0.566053005
ZNF436 ENSG00000125945 1.2897 0.098577 1.2276 0.10979 0.4015
0.34402 0.052775 0.47896 0.098577 0.566053005
NA ENSG00000280393 1.2897 0.098582 1.2897 0.098582 1.2897 0.098582
1.2897 0.098582 0.098582 0.566053005
NA ENSG00000199711 1.2896 0.098591 -0.5796 0.71891 0.93547 0.17477
-0.5796 0.71891 0.098591 0.566053005
RFX7 ENSG00000181827 0.30227 0.38122 0.10226 0.45928 0.30227 0.38122
1.2896 0.098593 0.098593 0.566053005
NA ENSG00000225785 -0.97521 0.83527 -0.45944 0.67704 1.2895 0.098617
1.2895 0.098617 0.098617 0.566053005
TEX41 ENSG00000226674 -0.06359 0.52535 -0.40355 0.65673 -0.06359 0.52535
1.2895 0.098618 0.098618 0.566053005
NA ENSG00000269919 1.2895 0.098618 1.2895 0.098618 1.2895 0.098618
1.2895 0.098618 0.098618 0.566053005
RPL21P18 ENSG00000213343 -0.51651 0.69725 1.2894 0.098621 0.90858
0.18179 -0.63508 0.73731 0.098621 0.566053005
NA ENSG00000240695 0.90971 0.18149 -0.36755 0.6434 0.2383 0.40582
1.2894 0.098625 0.098625 0.566053005
ELFN1-AS1 ENSG00000236081 1.2894 0.098634 1.003 0.15793 0.19661 0.42207
0.62754 0.26515 0.098634 0.566053005
AP2A2 ENSG00000183020 0.38432 0.35037 1.2893 0.098644 0.32196 0.37374
0.96798 0.16653 0.098644 0.566053005

NA	ENSG00000253571	0.091702	0.46347	1.2892	0.098668	-0.37115	0.64474	-0.37115	0.64474	0.098668	0.566068112
NA	ENSG00000269916	1.2891	0.098679	1.2891	0.098679	1.2891	0.098679	1.2891	0.098679	0.098679	0.566068112
HPSE	ENSG00000173083	0.50242	0.30768	0.11031	0.45608	0.83068	0.20308	1.289	0.098703	0.098703	0.566068112
ANKRD11	ENSG00000167522	1.289	0.098707	0.61824	0.26821	0.35876	0.35989	0.13889	0.44477	0.098707	0.566068112
FRG2JP	ENSG00000259897	0.86562	0.19335	1.2888	0.098729	0.76422	0.22237	0.70434	0.24061	0.098729	0.566068112
ATP5PB	ENSG00000116459	1.2887	0.098752	0.66595	0.25272	0.48258	0.3147	0.12916	0.44862	0.098752	0.566068112
STK24	ENSG00000102572	0.61257	0.27008	1.2887	0.09876	0.80212	0.21124	1.1998	0.1151	0.09876	0.566068112
ITFG2	ENSG00000111203	-0.060765	0.52423	0.59778	0.27499	0.59778	0.27499	1.2885	0.098787	0.098787	0.566068112
IL17RD	ENSG00000144730	0.96641	0.16692	1.2884	0.098797	0.41511	0.33903	1.2177	0.11166	0.098797	0.566068112
NA	ENSG00000266602	0.76189	0.22306	1.2884	0.098808	-0.082414	0.53284	-0.50481	0.69315	0.098808	0.566068112
BRMS1L	ENSG00000100916	0.67366	0.25026	-1.2844	0.9005	1.2883	0.098823	0.32171	0.37384	0.098823	0.566068112
GAPDHP44	ENSG00000213352	0.9606	0.16838	1.2881	0.098848	-0.45812	0.67657	-0.35908	0.64023	0.098848	0.566068112
CPEB3	ENSG00000107864	1.2881	0.098852	1.196	0.11584	0.34729	0.36419	0.21282	0.41573	0.098852	0.566068112
LINC00462	ENSG00000233610	-0.94103	0.82666	1.2881	0.098852	0.65783	0.25533	0.3458	0.36475	0.098852	0.566068112
NA	ENSG00000279613	1.2881	0.098856	1.2881	0.098856	1.2881	0.098856	1.2881	0.098856	0.098856	0.566068112
CLTB	ENSG00000175416	0.080144	0.46806	-0.53015	0.70199	1.2881	0.098861	0.094773	0.46225	0.098861	0.566068112
PTMAP4	ENSG00000231503	1.2881	0.098862	1.2881	0.098862	1.2881	0.098862	1.2881	0.098862	0.098862	0.566068112
PRMT5-AS1	ENSG00000237054	1.288	0.098873	0.25922	0.39773	0.48516	0.31378	0.48516	0.31378	0.098873	0.566068112
TOB1	ENSG00000141232	1.2679	0.10242	1.288	0.098879	1.2705	0.10196	0.34381	0.36549	0.098879	0.566068112
FOCAD-AS1	ENSG00000227071	1.239	0.10767	1.1266	0.12995	-0.074972	0.52988	1.2877	0.098918	0.098918	0.566068112
PRSS42P	ENSG00000178055	-0.001362	0.50054	1.2877	0.09892	-1.2475	0.8939	-1.2475	0.8939	0.09892	0.566068112
NA	ENSG00000243831	1.1536	0.12433	1.2877	0.09892	-0.48308	0.68548	1.2863	0.099171	0.09892	0.566068112
NA	ENSG00000229603	0.76753	0.22138	0.21844	0.41354	0.63358	0.26318	1.2876	0.098937	0.098937	0.566068112
NA	ENSG00000270131	1.2876	0.098941	-1.2652	0.89711	-1.1228	0.86924	-1.1228	0.86924	0.098941	0.566068112
PSMD12	ENSG00000197170	0.49263	0.31114	0.92831	0.17662	0.47257	0.31826	1.2874	0.098979	0.098979	0.566068112
DCAF5	ENSG00000139990	-0.26711	0.60531	1.2872	0.099005	-0.26711	0.60531	1.1172	0.13195	0.099005	0.566068112
NA	ENSG00000261257	1.2872	0.099014	1.2872	0.099014	0.62175	0.26705	1.2872	0.099014	0.099014	0.566068112
NA	ENSG00000250330	-0.5856	0.72093	-0.5856	0.72093	-0.5856	0.72093	1.2872	0.099015	0.099015	0.566068112
INPP5A	ENSG00000068383	0.72858	0.23313	1.287	0.09904	1.1208	0.13118	0.018581	0.49259	0.09904	0.566068112
NA	ENSG00000205653	-0.78145	0.78273	1.287	0.09905	-0.14035	0.55581	0.99961	0.15875	0.09905	0.566068112
MIR4462	ENSG00000263926	1.2035	0.11438	1.2869	0.099059	0.15059	0.44015	0.15768	0.43735	0.099059	0.566068112

NA	ENSG00000235871	1.2868	0.099084	1.2868	0.099084	1.2868	0.099084	1.2868	0.099084	0.099084	0.566068112
HOXB7	ENSG00000260027	1.2867	0.099103	1.2867	0.099103	1.2867	0.099103	1.2867	0.099103	0.099103	0.566068112
RNU6-457P	ENSG00000200869	-1.0148	0.8449	-1.0148	0.8449	-1.0148	0.8449	1.2866	0.099122	0.099122	0.566068112
RP9	ENSG00000164610	1.2865	0.099126	1.2865	0.099126	1.2865	0.099126	1.2865	0.099126	0.099126	0.566068112
NA	ENSG00000276620	1.2865	0.099127	1.2865	0.099127	1.2865	0.099127	1.2865	0.099127	0.099127	0.566068112
CALB2	ENSG00000172137	0.67137	0.25099	0.082911	0.46696	1.2865	0.099134	1.2865	0.099134	0.099134	0.566068112
KCND3-IT1	ENSG00000232558	1.2863	0.099172	-0.0715	0.5285	1.2863	0.099172	0.66721	0.25232	0.099172	0.566068112
C9orf62	ENSG00000178243	0.94498	0.17234	1.2862	0.099182	0.32107	0.37408	0.93159	0.17577	0.099182	0.566068112
NA	ENSG00000199224	-0.88283	0.81134	0.85813	0.19541	-0.88283	0.81134	1.286	0.09923	0.09923	0.566068112
RPL30P5	ENSG00000239525	1.2859	0.099241	0.21882	0.4134	1.2736	0.10141	1.2275	0.10982	0.099241	0.566068112
NA	ENSG00000232499	0.10852	0.45679	-0.83609	0.79845	1.2859	0.099245	-0.83609	0.79845	0.099245	0.566068112
TUBA3C	ENSG00000198033	0.74163	0.22916	0.99	0.16109	1.2285	0.10963	1.2857	0.099275	0.099275	0.566068112
SPATA22	ENSG00000141255	0.77325	0.21969	-1.5902	0.9441	1.2856	0.099288	1.0491	0.14706	0.099288	0.566068112
PEX5L-AS1	ENSG00000243799	1.2856	0.099288	1.2856	0.099288	1.2856	0.099288	1.2856	0.099288	0.099288	0.566068112
TROAP	ENSG00000135451	0.48459	0.31398	0.39555	0.34622	0.25224	0.40043	1.2856	0.09929	0.09929	0.566068112
ATN1	ENSG00000111676	1.2856	0.099291	0.81732	0.20687	0.60781	0.27166	0.65536	0.25612	0.099291	0.566068112
MIR194-1	ENSG00000207624	1.2855	0.099305	1.2855	0.099305	1.2855	0.099305	1.2855	0.099305	0.099305	0.566068112
TRIOBP	ENSG00000100106	1.2854	0.099321	0.22045	0.41276	0.15825	0.43713	0.83893	0.20075	0.099321	0.566068112
LINC01269	ENSG00000258689	1.2853	0.099337	0.85086	0.19742	1.2853	0.099337	1.2853	0.099337	0.099337	0.566068112
LINC02057	ENSG00000249279	0.42138	0.33674	1.2851	0.099373	-0.65714	0.74445	0.54096	0.29427	0.099373	0.566068112
NA	ENSG00000281392	1.0972	0.13627	1.1557	0.12391	1.2851	0.099377	0.53641	0.29584	0.099377	0.566068112
LRP11	ENSG00000120256	0.48712	0.31309	1.285	0.099398	1.2717	0.10174	0.7301	0.23266	0.099398	0.566068112
NA	ENSG00000259111	0.36174	0.35877	-0.6519	0.74277	1.285	0.099402	0.72969	0.23279	0.099402	0.566068112
NA	ENSG00000237152	0.11013	0.45615	-0.25687	0.60136	1.2849	0.099412	0.038975	0.48446	0.099412	0.566068112
NA	ENSG00000260035	1.2847	0.09944	1.2847	0.09944	1.2847	0.09944	1.2847	0.09944	0.09944	0.566068112
EYA3	ENSG00000158161	1.0556	0.14558	1.2847	0.099456	0.93326	0.17534	0.28447	0.38803	0.099456	0.566068112
LINC02165	ENSG00000261014	-1.3174	0.90614	1.2845	0.099478	-1.7759	0.96213	-1.7759	0.96213	0.099478	0.566068112
FAM13B	ENSG00000031003	0.65524	0.25616	0.78014	0.21765	1.2752	0.10113	1.2845	0.099479	0.099479	0.566068112
PLCB2	ENSG00000137841	0.81545	0.20741	1.2845	0.09948	0.79387	0.21363	0.1405	0.44413	0.09948	0.566068112
RPS20P2	ENSG00000219274	1.2845	0.099481	1.2845	0.099481	1.2845	0.099481	1.2845	0.099481	0.099481	0.566068112
NA	ENSG00000225365	-0.57124	0.71608	0.97176	0.16558	1.2844	0.099495	0.97176	0.16558	0.099495	0.566068112

KIFC3	ENSG00000140859	0.16796	0.43331	-0.16123	0.56404	-0.16123	0.56404	1.2844	0.099501	0.099501	0.566068112
FAM227B	ENSG00000166262	1.1888	0.11727	0.6076	0.27173	1.2844	0.099502	0.1718	0.4318	0.099502	0.566068112
POM121L6P	ENSG00000243307	0.33688	0.3681	-0.54396	0.70677	-0.46412	0.67872	1.2844	0.099508	0.099508	0.566068112
NA	ENSG00000225594	-1.7807	0.96252	-1.5551	0.94004	1.2843	0.099514	0.15395	0.43883	0.099514	0.566068112
NA	ENSG00000229983	-0.139	0.55527	1.2843	0.099521	-0.139	0.55527	-0.139	0.55527	0.099521	0.566068112
NA	ENSG00000267564	1.1344	0.12832	1.2842	0.099529	0.67342	0.25034	1.1071	0.13413	0.099529	0.566068112
NA	ENSG00000230603	1.2842	0.099535	1.1767	0.11965	1.1767	0.11965	1.1767	0.11965	0.099535	0.566068112
NA	ENSG00000211534	-2.1629	0.98473	1.2841	0.099554	-0.7415	0.7708	-1.2685	0.89769	0.099554	0.566068112
NA	ENSG00000223344	1.2839	0.09958	0.73933	0.22985	1.1076	0.13402	1.1076	0.13402	0.09958	0.566068112
GMEB1	ENSG00000162419	-0.65312	0.74316	0.69928	0.24219	0.75803	0.22422	1.2839	0.099591	0.099591	0.566068112
CLCN7	ENSG00000103249	1.2838	0.099607	0.57528	0.28255	-0.060985	0.52431	0.099822	0.46024	0.099607	0.566068112
PLCB3	ENSG00000149782	-0.64769	0.74141	0.43154	0.33304	1.1207	0.13122	1.2837	0.099623	0.099623	0.566068112
GABRG3-AS1	ENSG00000228740	1.2516	0.10536	1.2837	0.099623	-0.306	0.6202	1.2837	0.099623	0.099623	0.566068112
NA	ENSG00000254000	1.2837	0.099632	0.32762	0.3716	0.42018	0.33718	1.184	0.1182	0.099632	0.566068112
IL4	ENSG00000113520	-1.1458	0.87407	1.2836	0.099642	0.99229	0.16053	-0.67621	0.75055	0.099642	0.566068112
NA	ENSG00000253275	0.6534	0.25675	1.2833	0.099689	1.2833	0.099689	1.2833	0.099689	0.099689	0.566068112
NA	ENSG00000255256	1.2833	0.0997	1.2833	0.0997	1.2833	0.0997	1.2833	0.0997	0.0997	0.566068112
LINC02794	ENSG00000226133	0.22005	0.41291	-0.039375	0.5157	-0.16043	0.56373	1.2832	0.099718	0.099718	0.566068112
ERHP1	ENSG00000254270	-0.39365	0.65308	0.13956	0.4445	0.96018	0.16848	1.2832	0.099719	0.099719	0.566068112
MTRNR2L4	ENSG00000232196	1.2831	0.099731	1.2831	0.099731	1.2124	0.11269	0.99872	0.15896	0.099731	0.566068112
SLC22A18AS	ENSG00000254827	0.53333	0.2969	1.2819	0.099941	1.2831	0.099734	0.70731	0.23969	0.099734	0.566068112
NA	ENSG00000256480	1.283	0.09974	0.8553	0.19619	-0.36832	0.64368	-0.51043	0.69512	0.09974	0.566068112
OR1A2	ENSG00000172150	-0.75993	0.77635	1.283	0.099746	-0.91496	0.81989	0.50498	0.30679	0.099746	0.566068112
RNA5SP285	ENSG00000252428	1.0592	0.14477	1.283	0.099752	0.31776	0.37533	0.78012	0.21766	0.099752	0.566068112
NA	ENSG00000227809	1.283	0.099754	1.1816	0.11868	1.0304	0.15141	1.0304	0.15141	0.099754	0.566068112
DCHS1	ENSG00000166341	1.0642	0.14362	1.0642	0.14362	1.2828	0.099776	1.0642	0.14362	0.099776	0.566068112
ARHGEF3-AS1	ENSG00000240198	1.2828	0.099787	-1.9725	0.97573	0.74146	0.22921	-1.9011	0.97135	0.099787	0.566068112
NA	ENSG00000235653	1.2827	0.099806	1.2827	0.099806	1.2827	0.099806	1.2827	0.099806	0.099806	0.566068112
CYP4F35P	ENSG00000265787	-0.075405	0.53005	1.2826	0.099808	0.13966	0.44446	-0.9733	0.8348	0.099808	0.566068112
HDDC3	ENSG00000184508	0.83958	0.20057	1.2826	0.099811	1.2826	0.099811	1.2826	0.099811	0.099811	0.566068112
ATF7IP2	ENSG00000166669	0.62515	0.26594	0.12866	0.44881	0.6191	0.26792	1.2826	0.099813	0.099813	0.566068112

SF3A3	ENSG00000183431	0.83401	0.20214	1.0184	0.15425	-0.86009	0.80513	1.2826	0.099818	0.099818	0.566068112
NA	ENSG00000263555	1.2826	0.099819	1.2826	0.099819	1.2826	0.099819	1.2826	0.099819	0.099819	0.566068112
NA	ENSG00000267487	1.2826	0.099824	1.2826	0.099824	1.2826	0.099824	1.2826	0.099824	0.099824	0.566068112
NA	ENSG00000199471	1.2825	0.099828	1.2825	0.099828	1.2825	0.099828	1.2825	0.099828	0.099828	0.566068112
RNU6-557P	ENSG00000252651	0.80199	0.21128	0.72139	0.23533	1.0938	0.13702	1.2825	0.09984	0.09984	0.566068112
LINC02086	ENSG00000244649	0.79072	0.21455	1.2822	0.099888	0.79072	0.21455	1.1196	0.13145	0.099888	0.566068112
MFN2	ENSG00000116688	0.46666	0.32037	1.2821	0.099911	-0.031431	0.51254	-0.70499	0.75959	0.099911	0.566068112
NA	ENSG00000259548	1.282	0.099915	-0.4754	0.68275	0.57697	0.28198	0.63924	0.26133	0.099915	0.566068112
NA	ENSG00000254575	-0.38365	0.64938	1.2819	0.099936	1.2819	0.099936	1.2819	0.099936	0.099936	0.566068112
LPCAT1	ENSG00000153395	0.80444	0.21057	1.2819	0.099938	1.115	0.13243	-1.3223	0.90697	0.099938	0.566068112
SNRPEP10	ENSG00000227002	0.97885	0.16383	0.53958	0.29474	0.53958	0.29474	1.2819	0.099944	0.099944	0.566068112
NCK2	ENSG00000071051	0.69015	0.24505	0.13839	0.44497	1.2818	0.099955	0.093465	0.46277	0.099955	0.566068112
NA	ENSG00000253924	0.12436	0.45051	0.50928	0.30528	1.2817	0.099966	1.2817	0.099966	0.099966	0.566068112
RETREG3	ENSG00000141699	1.2817	0.099979	1.0344	0.15048	-0.36711	0.64323	0.70223	0.24127	0.099979	0.566068112
NA	ENSG00000268108	1.2816	0.099983	1.2816	0.099983	1.2816	0.099983	1.2816	0.099983	0.099983	0.566068112
EEF1B2P8	ENSG00000230497	1.2815	0.1	0.9853	0.16224	0.9853	0.16224	0.93774	0.17419	0.1	0.566068112
NA	ENSG00000265378	1.2815	0.1	0.36317	0.35824	1.2815	0.1	0.6151	0.26925	0.1	0.566068112
UBASH3B	ENSG00000154127	1.2815	0.10001	0.82747	0.20399	0.98592	0.16209	0.62035	0.26752	0.10001	0.566068112
PSMD4	ENSG00000159352	1.0844	0.13909	1.2815	0.10001	1.2815	0.10001	1.2815	0.10001	0.10001	0.566068112
MRPS16P1	ENSG00000229182	1.2815	0.10001	0.37122	0.35524	1.2815	0.10001	1.2815	0.10001	0.10001	0.566068112
NA	ENSG00000212266	1.2814	0.10002	1.2814	0.10002	1.2814	0.10002	0.28133	0.38923	0.10002	0.566068112
NA	ENSG00000261543	1.2814	0.10002	0.36762	0.35658	0.79359	0.21372	0.97901	0.16379	0.10002	0.566068112
C16orf78	ENSG00000166152	0.61857	0.2681	1.2813	0.10004	0.55107	0.29079	1.0344	0.15047	0.10004	0.566068112
NA	ENSG00000281028	1.2813	0.10005	1.2813	0.10005	1.2813	0.10005	1.2813	0.10005	0.10005	0.566068112
MIR584	ENSG00000207714	1.2812	0.10007	0.18608	0.42619	0.18608	0.42619	0.18608	0.42619	0.10007	0.566068112
NA	ENSG00000267224	1.2812	0.10007	1.2812	0.10007	1.2812	0.10007	1.2812	0.10007	0.10007	0.566068112
EHF	ENSG00000135373	1.2811	0.10008	1.2811	0.10008	1.2811	0.10008	0.082559	0.4671	0.10008	0.566068112
NA	ENSG00000250866	0.066471	0.4735	1.2811	0.10008	0.066471	0.4735	0.066471	0.4735	0.10008	0.566068112
UGT2B29P	ENSG00000250566	0.5308	0.29778	0.023068	0.4908	1.281	0.1001	0.36736	0.35667	0.1001	0.566068112
NA	ENSG00000255856	1.281	0.1001	1.281	0.1001	1.281	0.1001	1.281	0.1001	0.1001	0.566068112
OR7G3	ENSG00000170920	1.2809	0.10011	-0.26349	0.60391	0.24127	0.40467	0.70539	0.24028	0.10011	0.566068112

LATS1	ENSG00000131023	1.2541	0.1049	1.2748	0.10119	1.2809	0.10012	1.2346	0.10848	0.10012	0.566068112
NA	ENSG00000270611	1.2808	0.10013	-1.288	0.90112	-1.288	0.90112	-1.288	0.90112	0.10013	0.566068112
NXNL2	ENSG00000130045	-0.61204	0.72974	-0.23917	0.59451	-1.031	0.84873	1.2808	0.10014	0.10014	0.566068112
SOCS4	ENSG00000180008	1.2808	0.10014	1.2808	0.10014	1.2808	0.10014	1.2808	0.10014	0.10014	0.566068112
PPFIA2-AS1	ENSG00000257467	-0.89726	0.81521	1.2807	0.10015	0.08007	0.46809	0.08007	0.46809	0.10015	0.566068112
NA	ENSG00000186076	0.20185	0.42002	0.39364	0.34692	-0.76929	0.77914	1.2807	0.10016	0.10016	0.566068112
MCPH1-AS1	ENSG00000249898	0.44806	0.32706	-0.24643	0.59733	0.83102	0.20298	1.2806	0.10017	0.10017	0.566068112
ERVFRD-3	ENSG00000264801	1.2805	0.10018	0.19545	0.42252	0.19545	0.42252	0.19545	0.42252	0.10018	0.566068112
MRPL42P2	ENSG00000216364	1.1942	0.1162	1.2804	0.1002	-0.36097	0.64094	-1.4639	0.92838	0.1002	0.566106341
RN7SL510P	ENSG00000266308	1.2804	0.10021	1.2804	0.10021	1.2804	0.10021	1.2804	0.10021	0.10021	0.566106341
NA	ENSG00000233891	-0.44882	0.67322	0.82981	0.20332	0.71759	0.23651	1.2802	0.10023	0.10023	0.566153692
NACAP8	ENSG00000257210	1.2799	0.10029	1.2799	0.10029	1.2799	0.10029	1.2799	0.10029	0.10029	0.56640861
NA	ENSG00000230092	-1.8304	0.9664	-1.8304	0.9664	-0.88929	0.81308	1.2799	0.1003	0.1003	0.56640861
OR2S2	ENSG00000278889	-0.24344	0.59617	0.41822	0.33789	1.2798	0.10031	0.93239	0.17557	0.10031	0.56640861
SLC25A36	ENSG00000114120	1.2796	0.10035	-0.39691	0.65428	-0.1411	0.5561	-0.39691	0.65428	0.10035	0.566568822
TTL	ENSG00000114999	1.1351	0.12817	1.2793	0.10039	0.94007	0.17359	0.97063	0.16587	0.10039	0.566570175
MTCO1P40	ENSG00000262902	1.2793	0.10039	1.2793	0.10039	1.2793	0.10039	1.2793	0.10039	0.10039	0.566570175
CNPY3	ENSG00000137161	1.2793	0.1004	-0.68786	0.75423	1.2462	0.10635	0.49867	0.30901	0.1004	0.566570175
AASDH	ENSG00000157426	0.26613	0.39507	1.2792	0.10042	0.65121	0.25745	0.19232	0.42374	0.10042	0.566570175
LINC01049	ENSG00000234384	0.6172	0.26855	0.13596	0.44593	0.6172	0.26855	1.2792	0.10042	0.10042	0.566570175
NA	ENSG00000249695	0.69175	0.24455	1.1694	0.12113	1.2792	0.10042	1.2792	0.10042	0.10042	0.566570175
RAB3B	ENSG00000169213	0.58692	0.27863	0.58286	0.27999	1.0862	0.1387	1.279	0.10045	0.10045	0.566673825
TMCC2	ENSG00000133069	1.2788	0.10048	-0.006762	0.5027	1.1364	0.12789	-0.10852	0.54321	0.10048	0.566702662
SP140	ENSG00000079263	-0.039534	0.51577	1.1862	0.11776	1.2787	0.10049	-0.6725	0.74937	0.10049	0.566702662
NA	ENSG00000262136	1.2788	0.10049	1.2788	0.10049	1.2788	0.10049	1.2788	0.10049	0.10049	0.566702662
LINC02164	ENSG00000261811	0.21058	0.41661	-0.46189	0.67792	0.21058	0.41661	1.2787	0.10051	0.10051	0.566741901
NA	ENSG00000230696	1.2785	0.10054	1.2785	0.10054	1.2785	0.10054	1.2785	0.10054	0.10054	0.566741901
NA	ENSG00000279535	-0.46952	0.68065	1.2785	0.10054	1.2785	0.10054	1.2785	0.10054	0.10054	0.566741901
NA	ENSG00000282738	-1.4654	0.92859	0.64317	0.26006	-1.0345	0.84956	1.2784	0.10056	0.10056	0.566741901
KLF6	ENSG00000067082	-0.38197	0.64876	1.2783	0.10057	0.17159	0.43188	0.053398	0.47871	0.10057	0.566741901
RPL7AP22	ENSG00000227331	1.2782	0.10058	0.57192	0.28369	1.2782	0.10058	0.50553	0.30659	0.10058	0.566741901

LRRC3B	ENSG00000179796	0.81301	0.20811	0.011157	0.49555	0.81143	0.20856	1.2782	0.10059	0.10059	0.566741901
NA	ENSG00000238438	0.25281	0.40021	-0.18992	0.57531	-0.18992	0.57531	1.2782	0.10059	0.10059	0.566741901
MIR4755	ENSG00000264616	-0.32679	0.62809	-0.32679	0.62809	1.2781	0.10061	-0.32679	0.62809	0.10061	0.566789052
NME8	ENSG00000086288	1.278	0.10063	0.57888	0.28134	-0.1183	0.54708	1.0471	0.14753	0.10063	0.566836192
NA	ENSG00000231597	-0.79664	0.78717	-0.88257	0.81127	1.2779	0.10065	-1.991	0.97676	0.10065	0.566883322
MYNN	ENSG00000085274	-0.17635	0.56999	1.2777	0.10068	1.2777	0.10068	-0.14055	0.55589	0.10068	0.566921239
OR8G7P	ENSG00000276316	1.2777	0.10068	1.2777	0.10068	1.2777	0.10068	1.2777	0.10068	0.10068	0.566921239
NA	ENSG00000233383	1.2775	0.10072	-0.51262	0.69589	0.017371	0.49307	-0.94244	0.82702	0.10072	0.566940737
SMCO2	ENSG00000165935	0.40417	0.34304	0.39359	0.34694	0.40417	0.34304	1.2774	0.10073	0.10073	0.566940737
RNA5SP302	ENSG00000201766	1.2774	0.10073	1.2774	0.10073	1.2774	0.10073	1.2774	0.10073	0.10073	0.566940737
NA	ENSG00000268421	1.2774	0.10073	0.63303	0.26336	1.2774	0.10073	1.2774	0.10073	0.10073	0.566940737
LSAMP	ENSG00000185565	1.2771	0.10078	-0.42006	0.66278	0.66803	0.25206	0.79555	0.21315	0.10078	0.566980743
LGALS9B	ENSG00000170298	1.2769	0.10081	0.25765	0.39834	-0.59583	0.72436	0.89052	0.18659	0.10081	0.566980743
NA	ENSG00000267433	1.2769	0.10081	1.2769	0.10081	1.1741	0.12017	1.2769	0.10081	0.10081	0.566980743
RITA1	ENSG00000139405	0.95652	0.1694	0.87776	0.19004	0.0050743	0.49798	1.2769	0.10083	0.10083	0.566980743
NA	ENSG00000254364	0.70969	0.23895	1.2768	0.10084	-0.5421	0.70613	-0.11472	0.54567	0.10084	0.566980743
HTR5BP	ENSG00000125631	0.36331	0.35819	0.57746	0.28181	1.2766	0.10086	1.2729	0.10152	0.10086	0.566980743
ATP5MC1	ENSG00000159199	1.2767	0.10086	1.2767	0.10086	1.2767	0.10086	1.2767	0.10086	0.10086	0.566980743
BAZ1B	ENSG00000009954	1.2766	0.10088	1.0154	0.15495	0.12064	0.45199	0.72409	0.23451	0.10088	0.566980743
PDCL	ENSG00000136940	0.69437	0.24372	0.39391	0.34682	1.2766	0.10088	1.081	0.13984	0.10088	0.566980743
MRPL20-DT	ENSG00000272455	1.2766	0.10088	1.2766	0.10088	1.2766	0.10088	1.2766	0.10088	0.10088	0.566980743
C1orf21	ENSG00000116667	0.2642	0.39581	1.0097	0.15633	1.2765	0.10089	0.59145	0.27711	0.10089	0.566980743
COPZ2	ENSG00000005243	1.2765	0.1009	-0.038827	0.51549	0.30249	0.38114	-0.54694	0.70779	0.1009	0.566980743
MTARC1	ENSG00000186205	1.2654	0.10286	1.2396	0.10756	1.2764	0.1009	1.0009	0.15844	0.1009	0.566980743
NA	ENSG00000232063	-1.2114	0.88714	1.2764	0.1009	1.1763	0.11974	0.67588	0.24956	0.1009	0.566980743
RNA5SP62	ENSG00000200327	1.2547	0.10479	-0.87291	0.80864	1.2762	0.10094	1.2762	0.10094	0.10094	0.56700936
RNU6ATAC15P	ENSG00000221206	1.0281	0.15196	1.0281	0.15196	1.0281	0.15196	1.2762	0.10094	0.10094	0.56700936
GAPDHP22	ENSG00000234005	1.2762	0.10094	1.2762	0.10094	1.2762	0.10094	1.2762	0.10094	0.10094	0.56700936
RRAS2P1	ENSG00000236360	1.276	0.10098	1.276	0.10098	1.276	0.10098	1.276	0.10098	0.10098	0.567168672
PCDHGB2	ENSG00000253910	0.78722	0.21558	0.5716	0.2838	0.78722	0.21558	1.2758	0.10101	0.10101	0.567262572
NA	ENSG00000248486	0.71003	0.23884	-0.34764	0.63594	1.2758	0.10102	0.97439	0.16493	0.10102	0.567262572

EXOSC8 ENSG00000120699 1.2756 0.10105 1.2054 0.11402 1.2201 0.11121 1.2201 0.11121 0.10105 0.567356429
NA ENSG00000267624 1.2756 0.10106 1.2756 0.10106 1.2756 0.10106 1.2756 0.10106 0.10106 0.567356429
RNU2-19P ENSG00000253097 1.2753 0.1011 0.37118 0.35525 0.37118 0.35525 0.37118 0.35525 0.1011 0.567375668
RACGAP1P ENSG00000257331 1.2753 0.1011 1.2753 0.1011 1.2753 0.1011 1.2753 0.1011 0.1011 0.567375668
RNY3P11 ENSG00000201023 1.2753 0.10111 1.2753 0.10111 1.2753 0.10111 1.2753 0.10111 0.10111 0.567375668
NA ENSG00000254754 0.66158 0.25412 1.2753 0.10111 -0.022609 0.50902 -0.022609 0.50902 0.10111 0.567375668
TTYH3 ENSG00000136295 0.55353 0.28995 1.275 0.10115 0.036506 0.48544 0.26747 0.39455 0.10115 0.567397209
AURKA ENSG00000087586 -1.0194 0.84599 1.122 0.13094 -1.0194 0.84599 1.2749 0.10116 0.10116 0.567397209
NA ENSG00000280010 1.2749 0.10117 1.2749 0.10117 1.2749 0.10117 1.2749 0.10117 0.10117 0.567397209
RPS20P14 ENSG00000223803 1.2477 0.10607 1.0259 0.15247 1.0259 0.15247 1.2748 0.10118 0.10118 0.567397209
LINC02347 ENSG00000214043 0.33019 0.37063 0.33019 0.37063 1.2745 0.10124 0.33072 0.37043 0.10124 0.567397209
LRRC52 ENSG00000162763 0.16713 0.43363 1.2745 0.10125 0.18426 0.42691 -0.27507 0.60837 0.10125 0.567397209
FABP5P2 ENSG00000236044 -0.43797 0.6693 1.2745 0.10125 1.2745 0.10125 1.2745 0.10125 0.10125 0.567397209
CAP2P1 ENSG00000259151 0.52391 0.30017 1.2744 0.10125 0.52391 0.30017 0.52391 0.30017 0.10125 0.567397209
LINC02284 ENSG00000259719 0.022877 0.49087 1.2745 0.10125 0.58945 0.27778 0.84741 0.19838 0.10125 0.567397209
NA ENSG00000259322 1.2744 0.10126 1.2744 0.10126 1.2744 0.10126 1.2744 0.10126 0.10126 0.567397209
RN7SL775P ENSG00000243370 1.2744 0.10127 1.2744 0.10127 1.2744 0.10127 1.2744 0.10127 0.10127 0.567397209
NA ENSG00000240895 1.2742 0.10129 0.74388 0.22847 1.2742 0.10129 -0.41617 0.66136 0.10129 0.567397209
LINC01724 ENSG00000227421 1.2742 0.1013 1.1796 0.11907 1.0444 0.14816 0.2135 0.41547 0.1013 0.567397209
CSNK1A1P1 ENSG00000223518 1.2741 0.10131 1.2741 0.10131 1.2741 0.10131 1.2741 0.10131 0.10131 0.567397209
ERCC6L2 ENSG00000182150 1.266 0.10276 1.2741 0.10132 0.19919 0.42106 -0.074196 0.52957 0.10132 0.567397209
RN7SL783P ENSG00000265885 0.87673 0.19032 -0.24255 0.59582 1.274 0.10132 1.274 0.10132 0.10132 0.567397209
LINC00240 ENSG00000224843 -0.12037 0.5479 0.096226 0.46167 -1.1377 0.87239 1.274 0.10133 0.10133 0.567397209
IFT140 ENSG00000187535 -0.047366 0.51889 0.1134 0.45486 1.2739 0.10134 0.054526 0.47826 0.10134 0.567397209
NA ENSG00000233835 1.018 0.15435 1.2739 0.10134 1.018 0.15435 0.55646 0.28895 0.10134 0.567397209
WIPI1 ENSG00000070540 1.2305 0.10925 0.66717 0.25233 0.89167 0.18628 1.2738 0.10137 0.10137 0.567397209
CHD6 ENSG00000124177 1.2738 0.10137 0.70631 0.24 1.2738 0.10137 1.2738 0.10137 0.10137 0.567397209
IGHV3-7 ENSG00000211938 1.2738 0.10137 1.2738 0.10137 1.2738 0.10137 -0.34588 0.63528 0.10137 0.567397209
TPRKB ENSG00000144034 1.2737 0.10139 0.33683 0.36812 1.2737 0.10139 0.72598 0.23393 0.10139 0.567443976
SNORA22B ENSG00000206603 1.2736 0.10141 1.2736 0.10141 1.2736 0.10141 1.2736 0.10141 0.10141 0.567481525
NA ENSG00000276863 1.2735 0.10142 1.2735 0.10142 1.2735 0.10142 1.2735 0.10142 0.10142 0.567481525

PPP1R1AP1	ENSG00000255255	0.73677	0.23063	1.2733	0.10146	-0.80319	0.78907	-0.80319	0.78907	0.10146	0.567612499
NA	ENSG00000269906	0.84046	0.20032	0.84046	0.20032	0.84046	0.20032	1.2732	0.10148	0.10148	0.567612499
NPAS3	ENSG00000151322	1.0586	0.14489	-0.050809	0.52026	0.36727	0.35671	1.2731	0.10149	0.10149	0.567612499
NA	ENSG00000266905	-0.26486	0.60444	0.65349	0.25672	1.2731	0.10149	1.1748	0.12003	0.10149	0.567612499
CHRNA2	ENSG00000120903	-0.26533	0.60462	0.33926	0.36721	1.2729	0.10152	1.2594	0.10395	0.10152	0.567649983
NA	ENSG00000254586	1.273	0.10152	1.273	0.10152	-0.85807	0.80457	-0.53456	0.70352	0.10152	0.567649983
XCL1	ENSG00000143184	1.2572	0.10434	1.006	0.1572	0.98091	0.16332	1.2726	0.10157	0.10157	0.567679957
NA	ENSG00000233611	0.83193	0.20272	-0.083936	0.53345	1.2727	0.10157	0.80333	0.21089	0.10157	0.567679957
MAN1C1	ENSG00000117643	1.2726	0.10159	-0.83271	0.79749	1.0242	0.15287	0.04691	0.48129	0.10159	0.567679957
EEF1B2P2	ENSG00000213864	0.99675	0.15944	0.99675	0.15944	0.99675	0.15944	1.2726	0.10159	0.10159	0.567679957
SCEL	ENSG00000136155	1.2724	0.10162	-0.45411	0.67513	-0.72527	0.76586	-0.82127	0.79425	0.10162	0.567679957
MIR4498	ENSG00000266704	1.2722	0.10165	1.2722	0.10165	1.2722	0.10165	1.2722	0.10165	0.10165	0.567679957
NA	ENSG00000271355	-0.73353	0.76838	1.2722	0.10165	-0.73353	0.76838	-0.73353	0.76838	0.10165	0.567679957
PFDN4	ENSG00000101132	0.95951	0.16865	1.2721	0.10166	0.92023	0.17873	1.237	0.10804	0.10166	0.567679957
CDCA8	ENSG00000134690	0.26319	0.3962	0.94813	0.17153	0.94813	0.17153	1.2722	0.10166	0.10166	0.567679957
NA	ENSG00000253147	-0.63017	0.73571	1.2721	0.10166	-0.48861	0.68744	-0.46576	0.67931	0.10166	0.567679957
RIPK3	ENSG00000129465	1.2721	0.10167	1.2721	0.10167	1.2721	0.10167	1.2168	0.11184	0.10167	0.567679957
NA	ENSG00000228943	1.2719	0.1017	0.57078	0.28407	0.93163	0.17576	1.0491	0.14708	0.1017	0.567679957
NA	ENSG00000253842	0.064103	0.47444	0.064103	0.47444	1.2719	0.10171	0.064103	0.47444	0.10171	0.567679957
LMBRD2	ENSG00000164187	0.18402	0.427	-0.095784	0.53815	1.2718	0.10172	0.18599	0.42623	0.10172	0.567679957
ANO3	ENSG00000134343	1.2717	0.10173	-0.20209	0.58008	-0.64447	0.74036	-0.57119	0.71607	0.10173	0.567679957
TRAV26-2	ENSG00000211812	1.0486	0.14718	1.2717	0.10174	1.0486	0.14718	0.6002	0.27419	0.10174	0.567679957
NA	ENSG00000200664	1.2716	0.10175	1.2716	0.10175	1.2716	0.10175	1.2716	0.10175	0.10175	0.567679957
NA	ENSG00000260977	0.055519	0.47786	1.2716	0.10175	-0.22816	0.59024	-0.22816	0.59024	0.10175	0.567679957
FUT1	ENSG00000174951	1.2716	0.10176	1.2716	0.10176	1.2716	0.10176	1.2716	0.10176	0.10176	0.567679957
ACP1	ENSG00000143727	-1.0344	0.84952	0.66227	0.2539	0.66227	0.2539	1.2715	0.10177	0.10177	0.567679957
FAIM	ENSG00000158234	0.085954	0.46575	1.2715	0.10177	0.34752	0.3641	-0.35495	0.63869	0.10177	0.567679957
SFTPA3P	ENSG00000225827	1.0926	0.13729	1.1826	0.11849	1.0926	0.13729	1.2714	0.10179	0.10179	0.567726531
LHX1-DT	ENSG00000277268	0.89067	0.18655	0.95099	0.17081	1.2712	0.10182	1.231	0.10917	0.10182	0.567746004
EI24	ENSG00000149547	-0.087448	0.53484	1.2711	0.10184	0.48948	0.31225	0.54855	0.29166	0.10184	0.567746004
TMEM14EP	ENSG00000221962	1.271	0.10186	0.97536	0.16469	1.271	0.10186	1.271	0.10186	0.10186	0.567746004

CRLF1	ENSG00000006016	-0.31893	0.62511	1.2709	0.10188	-0.31893	0.62511	-0.12585	0.55007	0.10188	0.567746004
ZNF28	ENSG00000198538	1.0689	0.14256	0.31769	0.37536	1.2709	0.10188	1.2709	0.10188	0.10188	0.567746004
RNU6-580P	ENSG00000202029	1.2708	0.10189	1.2708	0.10189	1.2708	0.10189	1.2708	0.10189	0.10189	0.567746004
NA	ENSG00000256951	1.2708	0.1019	1.2708	0.1019	1.2708	0.1019	1.2708	0.1019	0.1019	0.567746004
ARHGAP23	ENSG00000275832	0.8967	0.18494	0.81979	0.20617	1.2708	0.1019	0.96124	0.16822	0.1019	0.567746004
NA	ENSG00000279972	1.2708	0.1019	-0.8171	0.79306	-0.58553	0.72091	1.2708	0.1019	0.1019	0.567746004
ELF2	ENSG00000109381	0.32473	0.37269	1.2707	0.10191	0.63787	0.26178	0.77604	0.21886	0.10191	0.567746004
NA	ENSG00000237897	1.2707	0.10193	1.2707	0.10193	1.2707	0.10193	1.2707	0.10193	0.10193	0.567774114
LINC02593	ENSG00000223764	1.1782	0.11937	1.1782	0.11937	1.1782	0.11937	1.2705	0.10195	0.10195	0.567774114
SCAMP1-AS1	ENSG00000245556	1.2705	0.10195	1.2705	0.10195	0.95011	0.17103	-0.30357	0.61927	0.10195	0.567774114
RPS7P15	ENSG00000237170	0.47965	0.31574	1.2703	0.10198	-1.0556	0.85442	0.19462	0.42284	0.10198	0.567839475
NA	ENSG00000254571	1.0346	0.15042	1.2702	0.102	0.12017	0.45217	0.12017	0.45217	0.102	0.567839475
GLUD1	ENSG00000148672	1.2702	0.10201	0.49441	0.31051	0.49441	0.31051	0.8891	0.18698	0.10201	0.567839475
NA	ENSG00000257653	1.2702	0.10201	1.2702	0.10201	1.2702	0.10201	1.2702	0.10201	0.10201	0.567839475
NA	ENSG00000253420	0.18163	0.42794	1.2701	0.10202	-1.3929	0.91818	-0.50375	0.69278	0.10202	0.567839475
ITGAM	ENSG00000169896	-0.076785	0.5306	1.2136	0.11245	0.96698	0.16678	1.27	0.10204	0.10204	0.56788593
NPEPL1	ENSG00000215440	0.72106	0.23544	1.2699	0.10206	0.62215	0.26692	0.78429	0.21643	0.10206	0.567923167
NA	ENSG00000238272	-0.67587	0.75044	-0.77581	0.78107	1.2698	0.10207	1.2435	0.10683	0.10207	0.567923167
ERLIN1	ENSG00000107566	0.69912	0.24224	0.85647	0.19587	0.85647	0.19587	1.2697	0.1021	0.1021	0.567933174
MEI4	ENSG00000269964	-0.61963	0.73225	0.91751	0.17944	1.2697	0.1021	-0.81514	0.7925	0.1021	0.567933174
MCUR1P1	ENSG00000262402	1.2696	0.10211	-1.2239	0.8895	-1.2239	0.8895	-1.2239	0.8895	0.10211	0.567933174
NA	ENSG00000279674	0.032004	0.48723	0.032004	0.48723	1.2695	0.10212	0.032004	0.48723	0.10212	0.567933174
SNORD12C	ENSG00000209042	1.2693	0.10217	1.2693	0.10217	1.2693	0.10217	1.2693	0.10217	0.10217	0.567933174
NA	ENSG00000243531	0.24332	0.40388	-0.54316	0.70649	0.041407	0.48349	1.2693	0.10218	0.10218	0.567933174
NA	ENSG00000270241	1.2692	0.10218	0.35831	0.36006	0.35831	0.36006	0.35831	0.36006	0.10218	0.567933174
NA	ENSG00000238988	0.72517	0.23417	-0.03382	0.51349	0.19381	0.42316	1.2692	0.10218	0.10218	0.567933174
DAXX	ENSG00000204209	0.95158	0.17066	0.9376	0.17423	1.2692	0.10219	0.47526	0.3173	0.10219	0.567933174
APC	ENSG00000134982	1.2691	0.1022	0.25713	0.39854	0.89648	0.185	0.99666	0.15947	0.1022	0.567933174
RPS7P7	ENSG00000240167	1.2691	0.1022	1.2691	0.1022	1.2691	0.1022	0.60131	0.27382	0.1022	0.567933174
NA	ENSG00000235168	-0.46652	0.67958	1.269	0.10222	-0.46652	0.67958	-0.46652	0.67958	0.10222	0.567979544
HSPA8P17	ENSG00000228557	1.1752	0.11996	1.1065	0.13426	1.2688	0.10225	1.2688	0.10225	0.10225	0.56799901

AP1B1P1	ENSG00000234479	1.2687	0.10228	0.62078	0.26737	0.49667	0.30971	-0.30838	0.6211	0.10228	0.56799901
EMC2	ENSG00000104412	1.2686	0.10229	-0.16605	0.56594	-0.16605	0.56594	0.8838	0.1884	0.10229	0.56799901
HSPA8P11	ENSG00000248159	1.2685	0.1023	0.63487	0.26276	0.084216	0.46644	-0.42782	0.66561	0.1023	0.56799901
LRRC1	ENSG00000137269	1.2685	0.10231	-0.14001	0.55567	-0.83923	0.79933	-1.6128	0.94661	0.10231	0.56799901
NA	ENSG00000244757	1.2685	0.10231	0.61914	0.26791	0.61914	0.26791	0.61914	0.26791	0.10231	0.56799901
STAB1	ENSG00000010327	1.107	0.13414	1.2685	0.10232	1.1635	0.12231	1.1768	0.11964	0.10232	0.56799901
PTPRJ	ENSG00000149177	-0.16802	0.56671	-0.3556	0.63893	1.2684	0.10233	0.36381	0.358	0.10233	0.56799901
MYPOP	ENSG00000176182	1.2683	0.10235	1.2683	0.10235	1.2683	0.10235	0.23641	0.40656	0.10235	0.56799901
NCSTNP1	ENSG00000219592	-0.21258	0.58417	1.2683	0.10235	-1.0368	0.85008	-1.0368	0.85008	0.10235	0.56799901
GNG8	ENSG00000167414	1.2681	0.10237	1.2681	0.10237	1.2681	0.10237	1.2681	0.10237	0.10237	0.56799901
LINC02635	ENSG00000283000	1.2681	0.10238	1.2435	0.10684	0.82962	0.20338	1.2681	0.10238	0.10238	0.56799901
ADAT3	ENSG00000213638	-0.8862	0.81224	-0.34306	0.63422	1.268	0.10239	-0.34306	0.63422	0.10239	0.56799901
MMP19	ENSG00000123342	1.268	0.1024	1.268	0.1024	1.268	0.1024	1.268	0.1024	0.1024	0.56799901
NA	ENSG00000233330	1.2679	0.10241	1.2679	0.10241	1.2679	0.10241	1.2679	0.10241	0.10241	0.56799901
TMC3-AS1	ENSG00000259343	1.2679	0.10241	1.2679	0.10241	1.2679	0.10241	1.2679	0.10241	0.10241	0.56799901
MRPL38	ENSG00000204316	1.2674	0.1025	1.2674	0.1025	1.2674	0.1025	1.2674	0.1025	0.1025	0.56830413
MIR606	ENSG00000207583	1.2675	0.1025	1.2675	0.1025	0.23438	0.40735	-0.47887	0.68399	0.1025	0.56830413
LINC00635	ENSG00000241469	0.8253	0.2046	0.6558	0.25598	1.2674	0.1025	1.2674	0.1025	0.1025	0.56830413
MIR1237	ENSG00000221273	1.2672	0.10254	1.2672	0.10254	1.2672	0.10254	1.2672	0.10254	0.10254	0.568461229
RNU6-856P	ENSG00000252768	1.2669	0.10259	1.2669	0.10259	1.2669	0.10259	1.2669	0.10259	0.10259	0.568664468
NA	ENSG00000257359	-0.2503	0.59882	1.2669	0.1026	-0.2503	0.59882	-0.2503	0.59882	0.1026	0.568664468
NA	ENSG00000178836	1.2667	0.10263	1.2667	0.10263	1.2667	0.10263	0.57501	0.28264	0.10263	0.568766053
RNU6-686P	ENSG00000201709	0.40165	0.34397	0.40165	0.34397	0.40165	0.34397	1.2666	0.10266	0.10266	0.568793668
PKP4P1	ENSG00000229670	1.2666	0.10266	1.2666	0.10266	1.2093	0.11328	1.155	0.12404	0.10266	0.568793668
CLNS1A	ENSG00000074201	0.937	0.17438	1.2665	0.10267	0.55588	0.28915	0.8359	0.2016	0.10267	0.568793668
FAM169A	ENSG00000198780	-1.1545	0.87585	1.2664	0.10269	0.13425	0.4466	0.60099	0.27392	0.10269	0.568820849
LEKR1	ENSG00000197980	1.0728	0.14168	-0.12248	0.54874	1.2663	0.10271	0.69598	0.24322	0.10271	0.568820849
RFPL4AP6	ENSG00000278292	0.80642	0.21	0.8268	0.20417	0.07439	0.47035	1.2662	0.10272	0.10272	0.568820849
DEFB107B	ENSG00000198129	0.015965	0.49363	-0.6679	0.7479	0.62632	0.26555	1.266	0.10275	0.10275	0.568820849
DSCC1	ENSG00000136982	0.56789	0.28505	-0.58329	0.72015	-0.38958	0.65158	1.2659	0.10277	0.10277	0.568820849
GPR139	ENSG00000180269	0.75661	0.22464	1.2659	0.10277	-0.88703	0.81247	0.75661	0.22464	0.10277	0.568820849

NA	ENSG00000214908	-0.415	0.66093	-1.0363	0.84997	1.2658	0.10279	-0.17101	0.56789	0.10279	0.568820849
NA	ENSG00000273893	1.2658	0.1028	0.55242	0.29033	1.2658	0.1028	1.2658	0.1028	0.1028	0.568820849
NA	ENSG00000253805	-0.022246	0.50887	1.2657	0.10281	0.913	0.18062	-0.10913	0.54345	0.10281	0.568820849
TIAM1	ENSG00000156299	1.0149	0.15508	1.2656	0.10282	0.48755	0.31293	0.74835	0.22713	0.10282	0.568820849
NA	ENSG00000248515	0.41087	0.34058	-1.3692	0.91453	-1.3552	0.91233	1.2656	0.10282	0.10282	0.568820849
NA	ENSG00000270218	1.2657	0.10282	1.2657	0.10282	1.2657	0.10282	1.2657	0.10282	0.10282	0.568820849
RNU6-771P	ENSG00000202250	0.23406	0.40747	1.2656	0.10284	-0.40402	0.6569	-0.62027	0.73246	0.10284	0.568820849
NA	ENSG00000250760	0.14808	0.44114	1.2655	0.10284	0.14808	0.44114	0.14808	0.44114	0.10284	0.568820849
NA	ENSG00000228554	1.2655	0.10285	1.2655	0.10285	1.2655	0.10285	1.2655	0.10285	0.10285	0.568820849
WNT16	ENSG00000002745	0.48843	0.31262	0.089804	0.46422	1.2653	0.10287	-0.58999	0.7224	0.10287	0.568829889
NA	ENSG00000277672	1.2653	0.10288	1.2653	0.10288	1.2653	0.10288	1.2653	0.10288	0.10288	0.568829889
SLC43A3	ENSG00000134802	1.2652	0.1029	1.2652	0.1029	0.03103	0.48762	1.2652	0.1029	0.1029	0.568829889
MRPL30	ENSG00000185414	0.87557	0.19063	-1.0389	0.85058	0.58121	0.28055	1.2652	0.10291	0.10291	0.568829889
LINC01583	ENSG00000259518	-1.0453	0.85206	-1.2798	0.89969	1.2652	0.10291	-0.9884	0.83852	0.10291	0.568829889
NA	ENSG00000228950	-1.6891	0.9544	-0.35343	0.63812	0.22602	0.41059	1.2649	0.10295	0.10295	0.568986447
FOXS1	ENSG00000179772	-0.8783	0.81011	0.95898	0.16879	1.2648	0.10297	1.2648	0.10297	0.10297	0.569032445
NA	ENSG00000233783	1.0109	0.15603	1.0149	0.15508	1.2647	0.10299	-0.98684	0.83814	0.10299	0.569059891
TRAV9-2	ENSG00000211793	0.73153	0.23223	1.2646	0.10301	0.77947	0.21785	1.2646	0.10301	0.10301	0.569059891
NA	ENSG00000252433	1.2646	0.10301	1.2646	0.10301	1.2646	0.10301	1.2646	0.10301	0.10301	0.569059891
NA	ENSG00000278899	1.1079	0.13394	1.2644	0.10304	1.1079	0.13394	1.1079	0.13394	0.10304	0.56911351
LINC00708	ENSG00000232170	-0.31538	0.62377	1.2643	0.10305	1.2643	0.10305	1.2643	0.10305	0.10305	0.56911351
ATP5MFP3	ENSG00000232851	-0.90688	0.81776	-0.90688	0.81776	-0.90688	0.81776	1.2643	0.10306	0.10306	0.56911351
NA	ENSG00000251317	1.2641	0.10309	1.2641	0.10309	1.2641	0.10309	1.2641	0.10309	0.10309	0.56911351
NA	ENSG00000251894	1.2639	0.10313	0.23603	0.4067	0.65416	0.2565	-0.46521	0.67911	0.10313	0.56911351
NA	ENSG00000228604	1.2639	0.10314	1.1328	0.12865	0.57836	0.28151	1.1328	0.12865	0.10314	0.56911351
SMAD9	ENSG00000120693	-0.018425	0.50735	1.2638	0.10315	0.062217	0.4752	-0.010871	0.50434	0.10315	0.56911351
USP31	ENSG00000103404	0.50776	0.30581	0.45406	0.32489	1.2637	0.10316	0.98672	0.16189	0.10316	0.56911351
NA	ENSG00000263154	1.2636	0.10318	1.2636	0.10318	1.2636	0.10318	-1.1144	0.86745	0.10318	0.56911351
ACTG1P12	ENSG00000226642	0.060219	0.47599	1.2636	0.10319	1.2636	0.10319	0.65918	0.25489	0.10319	0.56911351
LGALSL	ENSG00000119862	-0.027576	0.511	-0.027576	0.511	1.2635	0.1032	1.2522	0.10526	0.1032	0.56911351
NMT2	ENSG00000152465	0.38915	0.34858	1.2635	0.1032	0.72714	0.23357	1.2635	0.1032	0.1032	0.56911351

SV2C ENSG00000122012 -0.34136 0.63358 0.42361 0.33593 1.2634 0.10322
0.29949 0.38228 0.10322 0.56911351
MACC1 ENSG00000183742 -0.0058545 0.50234 0.22132 0.41242 0.056441 0.4775
1.2634 0.10322 0.10322 0.56911351
LINC01085 ENSG00000248698 1.2634 0.10322 0.61965 0.26774 1.2634
0.10322 0.42548 0.33524 0.10322 0.56911351
NA ENSG00000215953 1.2634 0.10323 -0.80558 0.78976 1.2634 0.10323
1.2634 0.10323 0.10323 0.56911351
ACHE ENSG00000087085 -0.19801 0.57848 1.2633 0.10325 0.014912 0.49405
0.37998 0.35198 0.10325 0.56911351
OR7E41P ENSG00000250640 -0.98552 0.83782 1.2632 0.10325 -1.7031
0.95572 -1.1983 0.8846 0.10325 0.56911351
NA ENSG00000272114 1.2632 0.10326 1.2632 0.10326 1.2632 0.10326
1.2632 0.10326 0.10326 0.56911351
ITPKB ENSG00000143772 0.73224 0.23201 1.0167 0.15464 1.2631 0.10328
0.53138 0.29758 0.10328 0.56911351
CREG1 ENSG00000143162 -2.0146 0.97803 1.263 0.10329 -1.1485 0.87462
-0.15993 0.56353 0.10329 0.56911351
PCDHB11 ENSG00000197479 1.263 0.10329 0.64484 0.25951 1.263 0.10329 1.263
0.10329 0.10329 0.56911351
RNU1-46P ENSG00000207154 1.263 0.10329 1.263 0.10329 1.263 0.10329 0.16493
0.4345 0.10329 0.56911351
NA ENSG00000252458 1.263 0.1033 1.263 0.1033 1.263 0.1033 1.263 0.1033
0.1033 0.56911351
RPL31 ENSG00000071082 -0.3132 0.62293 1.2628 0.10332 -0.61838 0.73184
-1.0161 0.84521 0.10332 0.569150096
LINC02505 ENSG00000248215 1.2628 0.10333 1.0826 0.1395 0.55858
0.28822 0.95003 0.17105 0.10333 0.569150096
OPRD1 ENSG00000116329 0.33522 0.36873 0.87439 0.19095 0.60722 0.27185
1.2627 0.10335 0.10335 0.569168162
TCEA1 ENSG00000187735 1.2626 0.10336 0.96356 0.16763 1.2626 0.10336
-0.60325 0.72683 0.10336 0.569168162
SLFN5 ENSG00000166750 0.28961 0.38606 0.041684 0.48338 1.2625 0.10338
1.2625 0.10338 0.10338 0.569168162
SMARCE1P3 ENSG00000259102 1.2625 0.10338 0.36976 0.35578 0.36976
0.35578 0.36976 0.35578 0.10338 0.569168162
SHC4 ENSG00000185634 0.27215 0.39275 0.35216 0.36236 1.2623 0.10342
1.223 0.11067 0.10342 0.569271621
NA ENSG00000227760 0.24452 0.40341 0.21517 0.41482 0.059558 0.47625
1.2622 0.10343 0.10343 0.569271621
NA ENSG00000232848 -0.49967 0.69135 0.36376 0.35802 1.2621 0.10345
0.2616 0.39682 0.10345 0.569271621
ACTG1P13 ENSG00000241305 0.27089 0.39324 0.27089 0.39324 0.27089
0.39324 1.2621 0.10346 0.10346 0.569271621
NA ENSG00000273047 1.2621 0.10346 1.2621 0.10346 1.2621 0.10346
1.2621 0.10346 0.10346 0.569271621
DISP1 ENSG00000154309 1.2619 0.10349 1.2619 0.10349 0.8135 0.20797
1.2619 0.10349 0.10349 0.569271621
H4C3 ENSG00000197061 1.2619 0.10349 1.2619 0.10349 1.2619 0.10349
1.2619 0.10349 0.10349 0.569271621
NA ENSG00000237754 1.2616 0.10355 1.2616 0.10355 1.2616 0.10355
1.2616 0.10355 0.10355 0.569271621
NA ENSG00000272137 0.13212 0.44744 0.13212 0.44744 1.2616 0.10355
-0.86969 0.80777 0.10355 0.569271621
RPL29P2 ENSG00000240480 -0.032377 0.51291 1.2615 0.10356 0.14986
0.44044 0.80296 0.211 0.10356 0.569271621
EIF4E2P2 ENSG00000244157 -0.4011 0.65583 -0.4011 0.65583 1.2615
0.10356 0.81784 0.20672 0.10356 0.569271621
MIR4443 ENSG00000265483 0.59507 0.2759 1.042 0.14871 1.2615 0.10356
1.2493 0.10577 0.10356 0.569271621
RNU6-1211P ENSG00000238382 1.2615 0.10357 0.84452 0.19919 1.2615
0.10357 1.2615 0.10357 0.10357 0.569271621

GRM8	ENSG00000179603	0.88206	0.18887	0.29976	0.38218	0.78396	0.21653	1.2613	0.1036	0.1036	0.569271621
OR2S1P	ENSG00000228307	-1.9593	0.97496	1.2613	0.10361	-1.9593	0.97496	-1.9593	0.97496	0.10361	0.569271621
CNDP2	ENSG00000133313	0.52542	0.29964	0.102	0.45938	0.52542	0.29964	1.2611	0.10363	0.10363	0.569271621
ECI2-DT	ENSG00000234817	1.2611	0.10364	-0.46806	0.68013	0.059646	0.47622	0.059646	0.47622	0.10364	0.569271621
RNU6-869P	ENSG00000207138	1.261	0.10365	1.261	0.10365	1.261	0.10365	1.261	0.10365	0.10365	0.569271621
NA	ENSG00000253140	1.261	0.10366	0.28416	0.38814	1.261	0.10366	-0.25115	0.59915	0.10366	0.569271621
GUCY2D	ENSG00000132518	1.2608	0.10369	0.014561	0.49419	1.0423	0.14864	0.25406	0.39972	0.10369	0.569271621
PRNT	ENSG00000180259	1.2608	0.10369	0.047027	0.48125	0.49885	0.30894	0.6065	0.27209	0.10369	0.569271621
TXNL1	ENSG00000091164	1.2607	0.1037	1.0889	0.1381	1.1413	0.12687	0.94047	0.17349	0.1037	0.569271621
NA	ENSG00000242072	0.24872	0.40179	1.2605	0.10374	0.24872	0.40179	0.24872	0.40179	0.10374	0.569271621
HGFAC	ENSG00000109758	1.2604	0.10376	1.2604	0.10376	1.2604	0.10376	0.25887	0.39787	0.10376	0.569271621
MIR4642	ENSG00000266619	1.2604	0.10377	1.2604	0.10377	1.2604	0.10377	1.2604	0.10377	0.10377	0.569271621
NA	ENSG00000252210	1.2601	0.10382	1.2601	0.10382	1.2601	0.10382	1.2601	0.10382	0.10382	0.569271621
NA	ENSG00000255027	-0.60956	0.72892	-0.2125	0.58414	1.2601	0.10382	-0.60956	0.72892	0.10382	0.569271621
NA	ENSG00000243012	1.26	0.10383	1.26	0.10383	1.26	0.10383	0.63152	0.26385	0.10383	0.569271621
FAM207BP	ENSG00000228797	0.90225	0.18346	1.26	0.10384	-0.40632	0.65775	-0.14937	0.55937	0.10384	0.569271621
NA	ENSG00000248918	1.26	0.10384	1.26	0.10384	-0.57561	0.71756	0.28754	0.38685	0.10384	0.569271621
NA	ENSG00000260600	1.26	0.10384	1.26	0.10384	1.26	0.10384	1.26	0.10384	0.10384	0.569271621
MRPS15P1	ENSG00000259186	1.2599	0.10385	1.2599	0.10385	1.2599	0.10385	1.2599	0.10385	0.10385	0.569271621
PHOX2B	ENSG00000109132	-0.080779	0.53219	1.2599	0.10386	0.71925	0.23599	-0.4536	0.67494	0.10386	0.569271621
NTF4	ENSG00000225950	1.2599	0.10386	1.2599	0.10386	1.2599	0.10386	1.2599	0.10386	0.10386	0.569271621
NA	ENSG00000268287	1.2599	0.10386	1.2599	0.10386	1.2599	0.10386	1.2599	0.10386	0.10386	0.569271621
SGPP1	ENSG00000126821	0.95175	0.17061	0.95175	0.17061	0.95175	0.17061	1.2597	0.10389	0.10389	0.569271621
NA	ENSG00000255310	1.2596	0.1039	1.2596	0.1039	0.88804	0.18726	1.2596	0.1039	0.1039	0.569271621
ACP7	ENSG00000183760	-0.81387	0.79214	1.2596	0.10391	1.2169	0.11183	0.47167	0.31858	0.10391	0.569271621
NA	ENSG00000249541	0.55222	0.2904	1.0474	0.14746	0.75225	0.22595	1.2596	0.10391	0.10391	0.569271621
RN7SL454P	ENSG00000243426	1.2594	0.10394	1.2594	0.10394	1.2594	0.10394	1.2594	0.10394	0.10394	0.569271621
PPDPFL	ENSG00000168333	0.49672	0.30969	1.2593	0.10395	1.2593	0.10395	1.2593	0.10395	0.10395	0.569271621
NA	ENSG00000264608	1.2593	0.10395	1.2593	0.10395	1.2593	0.10395	1.2593	0.10395	0.10395	0.569271621
LXN	ENSG00000079257	1.2593	0.10396	1.2593	0.10396	1.2593	0.10396	1.2593	0.10396	0.10396	0.569271621
XRCC6P1	ENSG00000237417	0.18172	0.4279	1.2593	0.10396	-0.33161	0.62991	-0.38906	0.65138	0.10396	0.569271621

LINC02125	ENSG00000250514	0.95398	0.17005	0.35665	0.36068	0.83005	0.20326	1.2592	0.10398	0.10398	0.569271621
LINC00933	ENSG00000259728	0.99233	0.16052	0.025075	0.49	-0.13731	0.55461	1.2592	0.10398	0.10398	0.569271621
RPL7AP73	ENSG00000237952	1.259	0.10401	1.259	0.10401	-0.32391	0.627	0.036143	0.48558	0.10401	0.569271621
RNU7-183P	ENSG00000252407	1.259	0.10401	0.87728	0.19017	1.1485	0.12537	1.1485	0.12537	0.10401	0.569271621
NA	ENSG00000253094	1.2563	0.10451	0.77684	0.21863	1.259	0.10401	1.259	0.10401	0.10401	0.569271621
NA	ENSG00000279134	-0.28122	0.61073	1.259	0.10401	1.259	0.10401	1.259	0.10401	0.10401	0.569271621
NA	ENSG00000206249	-0.06561	0.52616	1.259	0.10402	-0.06561	0.52616	-0.06561	0.52616	0.10402	0.569271621
SFTPD	ENSG00000133661	1.2364	0.10815	1.2364	0.10815	1.2589	0.10403	1.1447	0.12618	0.10403	0.569271621
CANX	ENSG00000127022	1.2588	0.10405	1.0921	0.1374	-0.061197	0.5244	0.14639	0.44181	0.10405	0.569271621
NA	ENSG00000254992	1.006	0.1572	-0.91011	0.81862	1.006	0.1572	1.2588	0.10405	0.10405	0.569271621
MIR4431	ENSG00000264975	0.44319	0.32881	0.31513	0.37633	0.11614	0.45377	1.2588	0.10405	0.10405	0.569271621
CIPC	ENSG00000198894	-0.69997	0.75803	-0.69997	0.75803	1.2587	0.10407	-0.6869	0.75393	0.10407	0.569271621
AAAS	ENSG00000094914	-1.1855	0.88209	1.1758	0.11984	0.90155	0.18365	1.2586	0.10408	0.10408	0.569271621
NA	ENSG00000240519	1.2585	0.1041	1.2585	0.1041	1.2585	0.1041	1.2585	0.1041	0.1041	0.569271621
TMEM51-AS1	ENSG00000175147	1.2585	0.10411	0.93339	0.17531	1.0658	0.14326	0.41844	0.33781	0.10411	0.569271621
NA	ENSG00000270008	1.2585	0.10411	1.2585	0.10411	1.2585	0.10411	1.2585	0.10411	0.10411	0.569271621
RNU4-82P	ENSG00000199313	1.2583	0.10414	1.2583	0.10414	1.2583	0.10414	1.2583	0.10414	0.10414	0.569271621
NORAD	ENSG00000260032	0.9438	0.17264	0.87168	0.19169	0.88787	0.1873	1.2583	0.10415	0.10415	0.569271621
THRAP3P3	ENSG00000235920	1.0971	0.13629	1.2581	0.10417	0.20533	0.41866	-0.025922	0.51034	0.10417	0.569271621
NA	ENSG00000253189	-1.0134	0.84456	-0.74056	0.77052	-0.25075	0.59899	1.2581	0.10417	0.10417	0.569271621
LINC02439	ENSG00000255814	-0.055396	0.52209	1.2581	0.10418	-0.81664	0.79293	-0.84342	0.8005	0.10418	0.569271621
PROCR	ENSG00000101000	-0.21935	0.58681	0.66115	0.25426	1.258	0.1042	0.53923	0.29486	0.1042	0.569271621
TRDMT1	ENSG00000107614	1.2579	0.10421	-0.21624	0.5856	0.13509	0.44627	0.47343	0.31795	0.10421	0.569271621
PROK2	ENSG00000163421	0.75798	0.22423	0.75798	0.22423	1.2577	0.10425	1.2041	0.11427	0.10425	0.569271621
NA	ENSG00000201898	1.2576	0.10426	1.2576	0.10426	1.2576	0.10426	1.2576	0.10426	0.10426	0.569271621
FREM1	ENSG00000164946	0.40327	0.34337	0.54511	0.29284	0.33975	0.36702	1.2575	0.10428	0.10428	0.569271621
LINC01945	ENSG00000236485	-0.58963	0.72228	-0.25975	0.60247	1.2575	0.10428	0.70909	0.23913	0.10428	0.569271621
IGLC5	ENSG00000254030	1.2575	0.10428	1.2575	0.10428	1.2575	0.10428	1.2575	0.10428	0.10428	0.569271621
IGHMBP2	ENSG00000132740	-0.045314	0.51807	-1.1614	0.87727	1.2575	0.10429	0.52127	0.30109	0.10429	0.569271621
TGM6	ENSG00000166948	-0.31133	0.62223	-0.71017	0.7612	-0.74897	0.77306	1.2575	0.10429	0.10429	0.569271621
NA	ENSG00000237596	-0.56581	0.71424	0.28003	0.38973	1.2575	0.10429	0.36664	0.35694	0.10429	0.569271621

RNF123	ENSG00000164068	0.14834	0.44104	-0.088019	0.53507	-0.53284	0.70293	1.2574	0.1043	0.1043	0.569271621
UBE2V1P2	ENSG00000214192	1.2574	0.1043	0.64712	0.25878	0.060979	0.47569	0.060979	0.47569	0.1043	0.569271621
OR51M1	ENSG00000184698	1.254	0.10492	1.2574	0.10431	1.2574	0.10431	1.2574	0.10431	0.10431	0.569271621
EHBP1L1	ENSG00000173442	-0.015167	0.50605	0.1374	0.44536	-0.38583	0.65019	1.257	0.10438	0.10438	0.569314865
NA	ENSG00000253880	-0.93134	0.82416	1.257	0.10438	0.019662	0.49216	-1.0819	0.86036	0.10438	0.569314865
NA	ENSG00000279625	0.65954	0.25477	0.31612	0.37596	1.2569	0.10439	0.31612	0.37596	0.10439	0.569314865
C10orf62	ENSG00000203942	-0.67242	0.74934	1.2569	0.1044	-0.67242	0.74934	-0.67242	0.74934	0.1044	0.569314865
MACO1	ENSG00000204178	1.2542	0.10489	1.2542	0.10489	1.2566	0.10445	0.70581	0.24015	0.10445	0.569314865
FAM120A	ENSG00000048828	0.025899	0.48967	-0.044434	0.51772	0.61315	0.26989	1.2565	0.10447	0.10447	0.569314865
CBFA2T3	ENSG00000129993	-0.85515	0.80377	0.48245	0.31474	1.2565	0.10447	0.62481	0.26605	0.10447	0.569314865
SOX10	ENSG00000100146	1.2564	0.10448	1.2564	0.10448	0.21664	0.41424	1.2564	0.10448	0.10448	0.569314865
NA	ENSG00000226757	1.2564	0.10448	1.2564	0.10448	1.2564	0.10448	1.2564	0.10448	0.10448	0.569314865
RPL7P10	ENSG00000229752	1.2564	0.10449	1.2564	0.10449	1.2564	0.10449	1.2564	0.10449	0.10449	0.569314865
NA	ENSG00000256916	1.2564	0.10449	1.2564	0.10449	1.2564	0.10449	1.2564	0.10449	0.10449	0.569314865
RABGGTA	ENSG00000100949	1.2563	0.10451	1.2563	0.10451	1.2563	0.10451	1.2563	0.10451	0.10451	0.569314865
NA	ENSG00000235091	-0.65384	0.74339	1.2563	0.10451	-0.25782	0.60173	-0.18397	0.57298	0.10451	0.569314865
NA	ENSG00000252778	0.23548	0.40692	1.2562	0.10451	0.001139	0.49955	0.31595	0.37602	0.10451	0.569314865
HIPK2	ENSG00000064393	1.2561	0.10454	0.62923	0.2646	-0.30613	0.62025	1.0869	0.13854	0.10454	0.569314865
NA	ENSG00000278913	0.88058	0.18927	0.74729	0.22744	0.88058	0.18927	1.2561	0.10454	0.10454	0.569314865
MIR548H4	ENSG00000221616	-0.18765	0.57442	-0.18765	0.57442	1.256	0.10456	-0.18644	0.57395	0.10456	0.569314865
CCDC58P2	ENSG00000236944	1.2559	0.10457	1.2559	0.10457	1.2559	0.10457	1.2559	0.10457	0.10457	0.569314865
NA	ENSG00000261368	1.2559	0.10457	1.2559	0.10457	1.2559	0.10457	1.2559	0.10457	0.10457	0.569314865
WDR77	ENSG00000116455	1.2559	0.10458	0.62116	0.26725	1.2559	0.10458	0.68287	0.24734	0.10458	0.569314865
CLEC12B	ENSG00000256660	1.2558	0.10459	1.2558	0.10459	1.2558	0.10459	1.2558	0.10459	0.10459	0.569314865
NA	ENSG00000231412	0.41691	0.33837	1.2558	0.1046	1.2055	0.114	1.2558	0.1046	0.1046	0.569314865
NA	ENSG00000250149	0.61517	0.26922	0.93889	0.17389	-0.23421	0.59259	1.2558	0.1046	0.1046	0.569314865
LINC01044	ENSG00000223626	-0.28324	0.6115	0.57229	0.28356	-0.92667	0.82295	1.2557	0.10461	0.10461	0.569314865
NA	ENSG00000257657	1.2557	0.10461	-1.0429	0.85151	-0.98899	0.83867	-0.98899	0.83867	0.10461	0.569314865
NA	ENSG00000253107	1.2556	0.10464	1.2556	0.10464	1.2556	0.10464	1.2556	0.10464	0.10464	0.569332095
ATXN1	ENSG00000124788	0.86595	0.19326	0.72821	0.23324	1.2555	0.10466	-0.23669	0.59355	0.10466	0.569332095
NA	ENSG00000280096	-0.032567	0.51299	0.99757	0.15924	-0.032567	0.51299	1.2553	0.10469	0.10469	0.569332095

DZIP1 ENSG00000134874 0.78513 0.21619 0.70849 0.23932 1.2552 0.1047 0.4694 0.31939 0.1047 0.569332095
SPDYE18 ENSG00000205482 0.52688 0.29914 1.2552 0.1047 0.85851 0.1953 0.45693 0.32386 0.1047 0.569332095
LINC01102 ENSG00000235597 0.09868 0.4607 -0.029349 0.51171 0.17765 0.4295 1.2552 0.10471 0.10471 0.569332095
NA ENSG00000283273 1.2552 0.10471 -1.0041 0.84234 -0.59344 0.72356 -0.59344 0.72356 0.10471 0.569332095
AZGP1P1 ENSG00000214313 0.9309 0.17595 -0.12417 0.54941 0.022809 0.4909 1.2551 0.10472 0.10472 0.569332095
SPATA9 ENSG00000145757 0.23852 0.40574 0.11123 0.45572 0.23852 0.40574 1.2551 0.10473 0.10473 0.569332095
ARHGAP19 ENSG00000213390 0.62636 0.26554 0.39009 0.34824 1.2551 0.10473 0.9562 0.16948 0.10473 0.569332095
NA ENSG00000225335 1.255 0.10475 -0.32468 0.62729 0.93893 0.17388 1.1538 0.1243 0.10475 0.569358994
MAML1 ENSG00000161021 1.0109 0.15604 1.2549 0.10476 0.46389 0.32136 0.29055 0.3857 0.10476 0.569358994
LINC00504 ENSG00000248360 -0.26516 0.60456 -0.80143 0.78856 -0.29212 0.6149 1.2548 0.10477 0.10477 0.569358994
CACNA2D1-AS1 ENSG00000223770 1.1021 0.13521 0.44369 0.32863 0.2452 0.40315 1.2547 0.1048 0.1048 0.569458519
GALNT1 ENSG00000141429 0.17068 0.43224 1.2545 0.10482 -0.35599 0.63908 0.021867 0.49128 0.10482 0.56950369
RNU6-987P ENSG00000207490 1.1303 0.12918 0.10863 0.45675 0.45114 0.32595 1.2544 0.10486 0.10486 0.569648322
APPL1 ENSG00000157500 -0.063085 0.52515 -0.188 0.57456 1.2543 0.10487 -0.18978 0.57526 0.10487 0.569648322
AP1S1 ENSG00000106367 1.2542 0.10489 1.2542 0.10489 0.65208 0.25717 -0.17742 0.57041 0.10489 0.569664382
NA ENSG00000238165 1.2541 0.10491 1.2541 0.10491 1.2541 0.10491 1.2541 0.10491 0.10491 0.569664382
RN7SL123P ENSG00000266863 1.2539 0.10493 -1.0924 0.86267 -0.81183 0.79155 -0.81183 0.79155 0.10493 0.569664382
SOS2 ENSG00000100485 1.2539 0.10494 1.1903 0.11697 1.0479 0.14734 1.2539 0.10494 0.10494 0.569664382
KRT128P ENSG00000258120 1.2538 0.10495 1.2538 0.10495 1.2538 0.10495 1.2538 0.10495 0.10495 0.569664382
NA ENSG00000230532 1.2538 0.10496 1.2538 0.10496 1.2538 0.10496 1.2538 0.10496 0.10496 0.569664382
ALKAL2 ENSG00000189292 0.42432 0.33567 0.91679 0.17963 0.70452 0.24055 1.2537 0.10497 0.10497 0.569664382
NA ENSG00000266065 1.2537 0.10498 -0.25673 0.60131 -0.25673 0.60131 -0.25673 0.60131 0.10498 0.569664382
MIR1288 ENSG00000221355 0.61952 0.26779 1.2536 0.10499 1.2536 0.10499 1.2536 0.10499 0.10499 0.569664382
WDCP ENSG00000163026 1.2535 0.10501 0.16459 0.43463 0.13588 0.44596 -0.12112 0.5482 0.10501 0.569664382
APOBEC3D ENSG00000243811 1.2534 0.10503 -0.49369 0.68924 1.2534 0.10503 -1.1742 0.87984 0.10503 0.569664382
NA ENSG00000284554 1.2534 0.10503 1.2534 0.10503 1.2534 0.10503 1.2534 0.10503 0.10503 0.569664382
LINC00404 ENSG00000229520 -0.15576 0.56189 1.2533 0.10504 -0.034531 0.51377 -0.034531 0.51377 0.10504 0.569664382
NA ENSG00000255899 1.2533 0.10505 1.2533 0.10505 1.2533 0.10505 1.2533 0.10505 0.10505 0.569664382
TBC1D16 ENSG00000167291 0.9327 0.17549 1.2532 0.10506 0.41292 0.33983 0.50232 0.30772 0.10506 0.569664382
MIR4433B ENSG00000264297 1.2532 0.10506 1.2532 0.10506 1.2532 0.10506 1.2532 0.10506 0.10506 0.569664382
HIF1A-AS3 ENSG00000258667 0.44771 0.32718 0.54256 0.29372 1.2531 0.10508 1.2531 0.10508 0.10508 0.569709435

LINC02461	ENSG00000258331	-0.35897	0.64019	1.2529	0.10511	-0.35897	0.64019	-0.35897	0.64019	0.10511	0.569808688
HMGN1P24	ENSG00000226977	1.2528	0.10514	0.022877	0.49087	0.022877	0.49087	1.2528	0.10514	0.10514	0.569862001
NA	ENSG00000267537	1.2527	0.10515	-0.14803	0.55884	-1.1416	0.87318	-1.1416	0.87318	0.10515	0.569862001
NA	ENSG00000254183	0.83259	0.20254	-0.039119	0.5156	1.2527	0.10517	0.13198	0.4475	0.10517	0.569862001
TSEN15	ENSG00000198860	1.2526	0.10518	0.39503	0.34641	0.63794	0.26176	0.83547	0.20173	0.10518	0.569862001
NA	ENSG00000240426	-0.60348	0.7269	1.2525	0.10519	-0.60348	0.7269	-0.60348	0.7269	0.10519	0.569862001
EDIL3-DT	ENSG00000250320	1.2525	0.10519	0.28684	0.38712	1.2525	0.10519	1.1939	0.11626	0.10519	0.569862001
CYSLTR2	ENSG00000152207	1.2522	0.10524	1.0344	0.15047	0.73576	0.23094	1.0424	0.14862	0.10524	0.569906065
NA	ENSG00000250654	0.36156	0.35884	1.0254	0.1526	1.2523	0.10524	1.2523	0.10524	0.10524	0.569906065
KCNQ1DN	ENSG00000237941	1.2522	0.10525	0.93514	0.17486	0.17408	0.4309	0.16339	0.43511	0.10525	0.569906065
CLUL1	ENSG00000079101	-0.75784	0.77573	1.2521	0.10526	-0.50238	0.6923	-1.3612	0.91328	0.10526	0.569906065
ITGAV	ENSG00000138448	0.7429	0.22877	0.23582	0.40678	1.2521	0.10527	0.9626	0.16787	0.10527	0.569906065
LINC00838	ENSG00000261683	-0.8417	0.80002	-0.14657	0.55826	1.2521	0.10527	-0.14657	0.55826	0.10527	0.569906065
IQCD	ENSG00000166578	1.0781	0.1405	1.0781	0.1405	1.2521	0.10528	0.58409	0.27958	0.10528	0.569906065
NA	ENSG00000258665	1.2518	0.10532	1.2518	0.10532	1.2518	0.10532	1.2518	0.10532	0.10532	0.569995957
NA	ENSG00000275929	1.2518	0.10532	1.2518	0.10532	1.2518	0.10532	1.2518	0.10532	0.10532	0.569995957
LINC01584	ENSG00000260477	0.33883	0.36737	0.097095	0.46133	1.2517	0.10534	0.097095	0.46133	0.10534	0.570030692
ADI1	ENSG00000182551	-0.11361	0.54523	1.2516	0.10535	0.88264	0.18872	0.95486	0.16983	0.10535	0.570030692
MTMR6	ENSG00000139505	1.2515	0.10537	0.48039	0.31548	-0.49867	0.69099	0.48039	0.31548	0.10537	0.570030692
ITGB5	ENSG00000082781	1.0712	0.14204	1.2515	0.10538	0.10358	0.45875	0.21346	0.41549	0.10538	0.570030692
GYS1	ENSG00000104812	1.2423	0.10705	1.2514	0.1054	1.2423	0.10705	1.2423	0.10705	0.1054	0.570030692
RNA5SP334	ENSG00000201695	1.2514	0.1054	1.2514	0.1054	1.2514	0.1054	1.2514	0.1054	0.1054	0.570030692
NA	ENSG00000261385	1.2513	0.10541	1.2513	0.10541	1.2513	0.10541	1.2513	0.10541	0.10541	0.570030692
SLC1A3	ENSG00000079215	0.24918	0.40161	1.139	0.12735	0.74792	0.22725	1.2513	0.10542	0.10542	0.570030692
RASAL3	ENSG00000105122	1.2511	0.10544	1.2511	0.10544	1.2511	0.10544	1.2511	0.10544	0.10544	0.57007558
NA	ENSG00000241057	-0.98672	0.83811	-0.24146	0.5954	1.251	0.10546	1.251	0.10546	0.10546	0.57011127
NA	ENSG00000277640	1.251	0.10547	1.251	0.10547	1.251	0.10547	0.17476	0.43064	0.10547	0.57011127
NA	ENSG00000234686	1.2509	0.10549	1.2509	0.10549	1.2509	0.10549	1.2509	0.10549	0.10549	0.570146945
LINC01375	ENSG00000226159	-0.88456	0.8118	0.17521	0.43046	0.13733	0.44539	1.2508	0.1055	0.1055	0.570146945
NA	ENSG00000254630	1.2507	0.10553	1.2507	0.10553	1.2507	0.10553	1.2507	0.10553	0.10553	0.570172313
IL11RA	ENSG00000137070	0.19878	0.42122	0.19878	0.42122	0.19878	0.42122	1.2504	0.10557	0.10557	0.570172313

NA	ENSG00000228658	0.50308	0.30745	1.2504	0.10557	-0.54543	0.70727	0.50308	0.30745	0.10557	0.570172313
USH2A	ENSG00000042781	-0.67164	0.74909	-0.61703	0.73139	-0.07221	0.52878	1.2504	0.10558	0.10558	0.570172313
RNU6-567P	ENSG00000252555	1.2503	0.10559	1.2503	0.10559	1.2503	0.10559	1.2503	0.10559	0.10559	0.570172313
MIR4262	ENSG00000265172	0.4986	0.30903	0.32678	0.37192	1.2503	0.10559	-0.83367	0.79777	0.10559	0.570172313
NA	ENSG00000263729	1.2503	0.1056	1.2503	0.1056	1.2503	0.1056	1.2503	0.1056	0.1056	0.570172313
GRM7-AS1	ENSG00000236202	1.2335	0.10869	0.31012	0.37824	0.72033	0.23566	1.2502	0.10561	0.10561	0.570172313
NA	ENSG00000266014	0.58026	0.28087	0.32782	0.37153	1.2502	0.10561	1.2502	0.10561	0.10561	0.570172313
COL28A1	ENSG00000215018	-0.013286	0.5053	0.69594	0.24323	-0.30874	0.62124	1.25	0.10565	0.10565	0.570234348
TUBBP10	ENSG00000226147	1.25	0.10566	1.25	0.10566	1.25	0.10566	1.25	0.10566	0.10566	0.570234348
LINC02020	ENSG00000234548	0.55993	0.28776	0.55993	0.28776	1.2499	0.10566	0.55993	0.28776	0.10566	0.570234348
PRSS12	ENSG00000164099	1.2499	0.10567	1.147	0.1257	0.87098	0.19188	0.72639	0.2338	0.10567	0.570234348
NA	ENSG00000226829	1.2498	0.10568	-0.11952	0.54757	-0.2925	0.61505	-0.91015	0.81863	0.10568	0.570234348
TRAJ24	ENSG00000211865	1.2497	0.10571	1.2497	0.10571	1.2497	0.10571	1.2497	0.10571	0.10571	0.570269932
TRAJ23	ENSG00000211866	1.2497	0.10571	1.2497	0.10571	1.2497	0.10571	1.2497	0.10571	0.10571	0.570269932
NA	ENSG00000238490	0.92	0.17879	1.2494	0.10576	1.2494	0.10576	1.2494	0.10576	0.10576	0.57041337
NA	ENSG00000250602	1.2494	0.10576	0.20126	0.42025	1.2494	0.10576	1.2494	0.10576	0.10576	0.57041337
RPL12P24	ENSG00000243601	1.2493	0.10578	-0.99497	0.84012	1.0791	0.14026	0.23248	0.40808	0.10578	0.570456813
POU3F2	ENSG00000184486	0.30176	0.38142	0.30176	0.38142	1.2491	0.10581	-0.025179	0.51004	0.10581	0.570456813
SCGB1D4	ENSG00000197745	0.10405	0.45856	1.2491	0.10582	-1.0483	0.85274	-0.31013	0.62177	0.10582	0.570456813
IGFBP4	ENSG00000141753	1.249	0.10583	0.68263	0.24742	1.249	0.10583	1.249	0.10583	0.10583	0.570456813
NA	ENSG00000248387	0.70945	0.23902	0.844	0.19934	1.249	0.10583	0.7831	0.21679	0.10583	0.570456813
LPAR3	ENSG00000171517	1.2489	0.10584	0.41	0.3409	-0.4666	0.67961	0.37727	0.35299	0.10584	0.570456813
ADGRA1-AS1	ENSG00000256925	1.2489	0.10585	1.0186	0.1542	-1.3485	0.91125	-1.3485	0.91125	0.10585	0.570456813
KRBOX1	ENSG00000240747	0.90621	0.18241	1.2487	0.10588	-0.032559	0.51299	0.77595	0.21889	0.10588	0.570555391
RNU6-1006P	ENSG00000252807	0.15876	0.43693	1.2487	0.1059	1.2487	0.1059	1.2487	0.1059	0.1059	0.570600066
HOXC11	ENSG00000123388	1.2047	0.11415	1.2485	0.10593	0.75569	0.22492	0.7742	0.21941	0.10593	0.570635518
NA	ENSG00000250493	1.2485	0.10593	0.9572	0.16923	0.9572	0.16923	0.9572	0.16923	0.10593	0.570635518
NUP88	ENSG00000108559	0.94969	0.17114	0.94969	0.17114	0.94969	0.17114	1.2483	0.10596	0.10596	0.570670955
MIR137HG	ENSG00000225206	1.2483	0.10596	0.38279	0.35094	1.0653	0.14337	0.47097	0.31883	0.10596	0.570670955
WBP1L	ENSG00000166272	0.61953	0.26778	-0.065267	0.52602	1.2482	0.10598	0.60703	0.27192	0.10598	0.570715593
RNA5SP230	ENSG00000252848	1.2478	0.10605	1.2478	0.10605	1.2478	0.10605	1.2478	0.10605	0.10605	0.571029448

RAB36	ENSG00000100228	1.2477	0.10607	0.58558	0.27908	0.32908	0.37105	0.58558	0.27908	0.10607	0.571064781
NA	ENSG00000259407	1.2477	0.10608	1.2477	0.10608	1.2477	0.10608	1.2477	0.10608	0.10608	0.571064781
LINC02358	ENSG00000251256	1.2475	0.1061	0.27602	0.39127	-0.08935	0.5356	-0.08935	0.5356	0.1061	0.571109356
FAM32BP	ENSG00000203416	1.2473	0.10614	1.2473	0.10614	1.2473	0.10614	1.2473	0.10614	0.10614	0.571243021
NA	ENSG00000257183	1.2472	0.10615	-0.032857	0.51311	-0.032857	0.51311	-0.032857	0.51311	0.10615	0.571243021
SNX29	ENSG00000048471	0.83503	0.20185	1.2472	0.10616	0.28476	0.38792	0.49455	0.31046	0.10616	0.571243021
NA	ENSG00000237107	0.96173	0.16809	1.2468	0.10623	1.1664	0.12173	1.0555	0.1456	0.10623	0.571463648
GPR37L1	ENSG00000170075	1.2467	0.10626	0.93857	0.17398	0.93476	0.17496	0.93707	0.17436	0.10626	0.571463648
KCNQ1	ENSG00000053918	1.2466	0.10627	0.66131	0.25421	-0.36149	0.64113	-0.274	0.60796	0.10627	0.571463648
LINC02357	ENSG00000283314	-0.43694	0.66892	-1.5506	0.9395	0.7485	0.22708	1.2466	0.10627	0.10627	0.571463648
SRSF3P4	ENSG00000224061	0.32422	0.37288	0.32422	0.37288	0.32422	0.37288	1.2466	0.10628	0.10628	0.571463648
SPATA18	ENSG00000163071	-0.80784	0.79041	0.28951	0.3861	0.081169	0.46765	1.2465	0.10629	0.10629	0.571463648
COL4A2-AS1	ENSG00000232814	1.2465	0.10629	1.2465	0.10629	1.2465	0.10629	1.2465	0.10629	0.10629	0.571463648
NA	ENSG00000273812	-1.1543	0.87582	-1.1543	0.87582	0.32464	0.37272	1.2463	0.10632	0.10632	0.571463648
NA	ENSG00000277611	1.2463	0.10632	1.2463	0.10632	1.2463	0.10632	1.2463	0.10632	0.10632	0.571463648
MIR662	ENSG00000207579	1.2463	0.10633	1.2463	0.10633	1.2463	0.10633	1.2463	0.10633	0.10633	0.571463648
NA	ENSG00000279136	1.2463	0.10633	1.2463	0.10633	1.2463	0.10633	1.2463	0.10633	0.10633	0.571463648
UBXN7	ENSG00000163960	1.2461	0.10636	1.0991	0.13586	0.54816	0.29179	0.77682	0.21863	0.10636	0.571470977
LGALS9	ENSG00000168961	1.2461	0.10636	-0.59279	0.72334	1.0405	0.14906	-0.25453	0.60046	0.10636	0.571470977
CHMP3	ENSG00000115561	1.0454	0.14792	1.0033	0.15785	1.2461	0.10637	0.93374	0.17522	0.10637	0.571470977
LINC00501	ENSG00000203645	-1.0886	0.86184	1.0463	0.14772	1.246	0.10638	1.1731	0.12038	0.10638	0.571470977
NA	ENSG00000225913	1.2459	0.10639	0.44044	0.32981	0.97341	0.16517	0.44044	0.32981	0.10639	0.571470977
NA	ENSG00000245857	1.2458	0.10642	0.8404	0.20034	-0.41993	0.66273	1.1674	0.12153	0.10642	0.5715671
TMEM260	ENSG00000070269	1.0593	0.14474	0.38945	0.34847	1.2457	0.10644	1.1737	0.12026	0.10644	0.5715671
AK3	ENSG00000147853	1.0794	0.1402	1.0321	0.15102	1.2456	0.10646	-0.623	0.73336	0.10646	0.5715671
NA	ENSG00000237530	1.2456	0.10646	1.0032	0.15789	0.25322	0.40005	0.19931	0.42101	0.10646	0.5715671
RN7SL50P	ENSG00000242985	1.1833	0.11835	1.0531	0.14614	1.2455	0.10647	0.65208	0.25717	0.10647	0.5715671
OLFM2	ENSG00000105088	-0.70334	0.75908	-0.8988	0.81562	-0.7398	0.77029	1.2454	0.10648	0.10648	0.5715671
NA	ENSG00000225541	1.2338	0.10865	1.2454	0.10649	-0.66074	0.74561	1.0672	0.14294	0.10649	0.5715671
NA	ENSG00000259013	1.2453	0.10651	1.2453	0.10651	1.2453	0.10651	1.2453	0.10651	0.10651	0.57159292
PRDM4	ENSG00000110851	1.0063	0.15714	0.97226	0.16546	1.0977	0.13618	1.2453	0.10652	0.10652	0.57159292

PIEZO1P2	ENSG00000237121	1.2452	0.10653	0.41242	0.34002	0.90914	0.18164	0.41242	0.34002	0.10653	0.57159292
BMF-AS1	ENSG00000259409	1.2451	0.10656	1.2451	0.10656	1.2451	0.10656	1.2451	0.10656	0.10656	0.571647589
RPS6P3	ENSG00000237549	0.19835	0.42139	-0.77537	0.78094	1.2447	0.10661	-0.51137	0.69545	0.10661	0.571647589
NA	ENSG00000238371	-1.3664	0.9141	-1.4478	0.92616	0.28053	0.38953	1.2447	0.10661	0.10661	0.571647589
RHOQ	ENSG00000119729	0.0019719	0.49921	0.59456	0.27607	-0.24315	0.59606	1.2447	0.10662	0.10662	0.571647589
LINC01745	ENSG00000225919	1.2446	0.10663	1.2446	0.10663	1.2446	0.10663	1.2446	0.10663	0.10663	0.571647589
ZNF205	ENSG00000122386	1.2446	0.10664	1.2446	0.10664	1.2446	0.10664	-0.6199	0.73234	0.10664	0.571647589
ANKRD20A8P	ENSG00000229089	0.333	0.36957	1.2446	0.10664	-0.082019	0.53268	0.96554	0.16714	0.10664	0.571647589
DDX11	ENSG00000013573	0.14624	0.44187	1.2446	0.10665	0.83043	0.20315	0.99614	0.15959	0.10665	0.571647589
ATP6V0E1P4	ENSG00000227883	1.2444	0.10667	1.2444	0.10667	1.2444	0.10667	1.2444	0.10667	0.10667	0.571647589
NA	ENSG00000256695	1.2444	0.10668	0.10737	0.45725	1.1144	0.13256	0.85853	0.1953	0.10668	0.571647589
TXLNB	ENSG00000164440	0.68318	0.24725	0.68318	0.24725	0.68318	0.24725	1.2443	0.1067	0.1067	0.571647589
ZNF202	ENSG00000166261	1.2441	0.10672	-0.96574	0.83291	-0.96574	0.83291	-0.96574	0.83291	0.10672	0.571647589
NA	ENSG00000254061	1.2442	0.10672	0.31194	0.37754	0.94099	0.17336	0.31194	0.37754	0.10672	0.571647589
NA	ENSG00000265217	0.2261	0.41056	0.45376	0.325	1.2441	0.10674	0.45376	0.325	0.10674	0.571647589
EEF2KMT	ENSG00000118894	-1.0039	0.84227	0.097135	0.46131	1.244	0.10675	-0.65914	0.7451	0.10675	0.571647589
IFT22	ENSG00000128581	0.56417	0.28632	0.44898	0.32672	1.244	0.10675	0.23072	0.40877	0.10675	0.571647589
NA	ENSG00000250740	1.2439	0.10676	1.2439	0.10676	1.2439	0.10676	1.2439	0.10676	0.10676	0.571647589
AATF	ENSG00000275700	1.0839	0.13922	0.075962	0.46972	1.2439	0.10677	-0.25529	0.60075	0.10677	0.571647589
FANCI	ENSG00000140525	0.71697	0.2367	1.2437	0.1068	1.2044	0.11421	0.66035	0.25451	0.1068	0.571647589
UTP23	ENSG00000147679	0.016337	0.49348	1.2437	0.1068	-0.91056	0.81874	0.29498	0.384	0.1068	0.571647589
NA	ENSG00000251638	1.2437	0.1068	1.2437	0.1068	1.2437	0.1068	1.2437	0.1068	0.1068	0.571647589
SLC22A15	ENSG00000163393	1.2437	0.10681	-0.37165	0.64493	-0.31938	0.62528	-0.9135	0.81951	0.10681	0.571647589
SDHD	ENSG00000204370	1.2436	0.10681	-0.69752	0.75726	-0.69752	0.75726	-0.69752	0.75726	0.10681	0.571647589
IFITM3	ENSG00000142089	-1.4275	0.92328	1.2434	0.10686	0.70427	0.24063	-1.4275	0.92328	0.10686	0.571680489
NA	ENSG00000237324	1.2434	0.10686	1.2434	0.10686	1.2434	0.10686	1.2434	0.10686	0.10686	0.571680489
FAM207A	ENSG00000160256	0.18634	0.42609	-0.021715	0.50866	-1.5011	0.93333	1.2433	0.10687	0.10687	0.571680489
PAPSS2	ENSG00000198682	0.29593	0.38364	-0.40478	0.65718	1.2433	0.10688	0.29593	0.38364	0.10688	0.571680489
HMGN2P13	ENSG00000241810	1.2431	0.10691	0.67289	0.25051	0.084101	0.46649	0.478	0.31633	0.10691	0.571680489
NA	ENSG00000250321	1.2431	0.10691	1.2431	0.10691	1.2431	0.10691	1.2431	0.10691	0.10691	0.571680489
NA	ENSG00000253163	0.75205	0.22601	0.75205	0.22601	0.75205	0.22601	1.2431	0.10691	0.10691	0.571680489

NA	ENSG00000269471	0.6146	0.26941	-0.44294	0.6711	-0.74061	0.77054	1.2431	0.10691	0.10691	0.571680489
NA	ENSG00000228925	1.2428	0.10697	-0.12238	0.5487	-0.85841	0.80467	-0.85841	0.80467	0.10697	0.571899956
NA	ENSG00000249877	1.2427	0.10698	-0.62271	0.73326	0.58802	0.27826	0.61982	0.26769	0.10698	0.571899956
IFNE	ENSG00000184995	1.2427	0.10699	-0.060698	0.5242	0.46095	0.32242	0.46095	0.32242	0.10699	0.571899956
DNAJB8	ENSG00000179407	0.78364	0.21663	-0.037088	0.51479	1.2426	0.107	1.2426	0.107	0.107	0.571899956
RNA5SP335	ENSG00000200687	1.2425	0.10702	1.2425	0.10702	1.2425	0.10702	1.2425	0.10702	0.10702	0.571899956
TPT1P10	ENSG00000180221	1.2424	0.10704	1.2424	0.10704	1.2424	0.10704	1.2424	0.10704	0.10704	0.571899956
NA	ENSG00000226541	1.2424	0.10705	1.1186	0.13166	1.1186	0.13166	1.1784	0.11931	0.10705	0.571899956
NA	ENSG00000258026	0.50178	0.30791	1.2423	0.10707	0.50178	0.30791	0.50178	0.30791	0.10707	0.571899956
LINC02217	ENSG00000248455	1.2422	0.10708	1.2422	0.10708	1.1019	0.13526	1.2422	0.10708	0.10708	0.571899956
RNU6-72P	ENSG00000207012	1.2421	0.10709	1.2421	0.10709	1.2421	0.10709	0.80169	0.21137	0.10709	0.571899956
NA	ENSG00000254425	1.242	0.10712	1.242	0.10712	-0.77385	0.78049	1.242	0.10712	0.10712	0.571899956
SEPTIN7P2	ENSG00000214765	0.92672	0.17704	1.2419	0.10713	-0.39122	0.65218	0.34742	0.36414	0.10713	0.571899956
ZFR2	ENSG00000105278	-0.10971	0.54368	1.2419	0.10714	-0.9433	0.82724	-0.16478	0.56544	0.10714	0.571899956
NA	ENSG00000250636	1.1303	0.12919	-0.013725	0.50548	1.1303	0.12919	1.2418	0.10715	0.10715	0.571899956
NA	ENSG00000239715	-1.4323	0.92397	-0.31457	0.62346	-0.14836	0.55897	1.2418	0.10716	0.10716	0.571899956
RNU6-168P	ENSG00000251998	0.26471	0.39562	1.2417	0.10718	0.26471	0.39562	0.26471	0.39562	0.10718	0.571899956
LINC02204	ENSG00000280639	1.2416	0.10718	0.14196	0.44356	0.077095	0.46927	-0.85425	0.80352	0.10718	0.571899956
NA	ENSG00000199241	0.88289	0.18865	1.1898	0.11705	0.73277	0.23185	1.2415	0.10721	0.10721	0.571899956
RNA5SP205	ENSG00000201523	1.2415	0.10721	1.2415	0.10721	1.2415	0.10721	1.2415	0.10721	0.10721	0.571899956
F2RL3	ENSG00000127533	1.2414	0.10722	1.2414	0.10722	1.0713	0.14203	1.2414	0.10722	0.10722	0.571899956
RNU6-927P	ENSG00000252008	1.2414	0.10723	1.2414	0.10723	0.82418	0.20492	0.72139	0.23533	0.10723	0.571899956
HSPBP1	ENSG00000133265	1.0081	0.15669	1.2413	0.10726	1.2413	0.10726	1.2413	0.10726	0.10726	0.571899956
MIR1275	ENSG00000221697	0.61064	0.27072	-0.96741	0.83333	1.2412	0.10726	-0.50812	0.69432	0.10726	0.571899956
HIVEP2	ENSG00000010818	0.16672	0.43379	-0.15901	0.56317	0.80391	0.21072	1.2411	0.10728	0.10728	0.571899956
H3P9	ENSG00000227430	0.58767	0.27838	0.063482	0.47469	1.2411	0.10728	0.025123	0.48998	0.10728	0.571899956
LYPLAL1-DT	ENSG00000228063	-1.0228	0.8468	0.58976	0.27768	1.241	0.1073	0.5207	0.30129	0.1073	0.571899956
NA	ENSG00000237494	1.241	0.1073	1.241	0.1073	1.241	0.1073	1.241	0.1073	0.1073	0.571899956
RPL23AP33	ENSG00000237824	1.241	0.10731	0.81567	0.20734	1.241	0.10731	1.241	0.10731	0.10731	0.571899956
NA	ENSG00000229751	-0.30703	0.62059	0.064647	0.47423	-1.4085	0.92051	1.2409	0.10733	0.10733	0.571899956
LINC02751	ENSG00000254946	1.2408	0.10735	1.2408	0.10735	0.14329	0.44303	0.14329	0.44303	0.10735	0.571899956

NA	ENSG00000276968	1.2408	0.10735	1.2408	0.10735	1.2408	0.10735	1.2408	0.10735	0.10735	0.571899956
ZNF512B	ENSG00000196700	1.2407	0.10736	1.119	0.13156	-1.3246	0.90735	-1.3246	0.90735	0.10736	0.571899956
MIR342	ENSG00000199082	1.2407	0.10736	1.2407	0.10736	1.2407	0.10736	1.2407	0.10736	0.10736	0.571899956
MRPS18CP6	ENSG00000228807	1.2407	0.10736	1.2407	0.10736	1.2407	0.10736	1.2407	0.10736	0.10736	0.571899956
LINC01467	ENSG00000258977	1.2406	0.10737	1.2406	0.10737	1.2406	0.10737	1.2406	0.10737	0.10737	0.571899956
AMMECR1L	ENSG00000144233	0.5289	0.29844	1.2405	0.10739	0.3112	0.37782	0.78809	0.21532	0.10739	0.571899956
NA	ENSG00000273860	1.2404	0.10741	1.2404	0.10741	1.2404	0.10741	1.2404	0.10741	0.10741	0.571899956
NA	ENSG00000179253	-0.042189	0.51683	-0.042189	0.51683	1.2404	0.10742	-0.67027	0.74866	0.10742	0.571899956
IGKV2OR2-1	ENSG00000270748	1.2403	0.10743	0.75549	0.22498	0.13197	0.4475	1.2403	0.10743	0.10743	0.571899956
NA	ENSG00000235024	1.2403	0.10744	1.1548	0.12408	1.2403	0.10744	1.2403	0.10744	0.10744	0.571899956
MICB	ENSG00000204516	1.2402	0.10745	1.034	0.15057	0.88081	0.18921	0.81922	0.20633	0.10745	0.571899956
EIF5B	ENSG00000158417	1.2401	0.10747	0.77082	0.22041	1.055	0.14571	0.80431	0.21061	0.10747	0.571899956
ADAM19	ENSG00000135074	1.24	0.10748	1.2372	0.10801	0.87264	0.19143	1.0647	0.14351	0.10748	0.571899956
RNU6-818P	ENSG00000201707	0.78947	0.21492	0.90132	0.18371	0.78947	0.21492	1.24	0.10749	0.10749	0.571899956
PIGG	ENSG00000174227	0.77061	0.22047	1.24	0.1075	0.71208	0.23821	0.1571	0.43758	0.1075	0.571899956
PJVK	ENSG00000204311	1.24	0.1075	1.24	0.1075	1.24	0.1075	1.24	0.1075	0.1075	0.571899956
NA	ENSG00000199938	-1.3901	0.91775	1.2399	0.10751	-1.4704	0.92927	-1.4704	0.92927	0.10751	0.571899956
PAFAH1B3	ENSG00000079462	0.76582	0.22189	-0.18241	0.57237	1.2398	0.10753	0.99961	0.15875	0.10753	0.571899956
ASAH2B	ENSG00000204147	-1.3271	0.90777	1.2398	0.10753	-0.74627	0.77225	0.68415	0.24694	0.10753	0.571899956
NXPH4	ENSG00000182379	-0.33525	0.63128	0.15782	0.4373	-0.20163	0.5799	1.2397	0.10754	0.10754	0.571899956
IFNA5	ENSG00000147873	-0.46372	0.67858	-0.2101	0.58321	1.004	0.15769	1.2397	0.10755	0.10755	0.571899956
UBLCP1	ENSG00000164332	0.50546	0.30662	0.54272	0.29366	0.81335	0.20801	1.2395	0.10759	0.10759	0.571899956
NA	ENSG00000274944	1.2395	0.10759	1.2395	0.10759	1.2395	0.10759	1.2395	0.10759	0.10759	0.571899956
TRIM13	ENSG00000204977	0.61278	0.27001	1.2394	0.1076	-0.13847	0.55507	0.42454	0.33559	0.1076	0.571899956
CTF2P	ENSG00000230447	1.2394	0.1076	0.48278	0.31463	1.2394	0.1076	1.2394	0.1076	0.1076	0.571899956
LINC00336	ENSG00000197251	-0.36049	0.64076	1.2393	0.10761	-0.18699	0.57417	0.6264	0.26553	0.10761	0.571899956
NA	ENSG00000230234	-0.54457	0.70697	0.3959	0.34609	1.2393	0.10762	1.2393	0.10762	0.10762	0.571899956
MRGPRX4	ENSG00000179817	-0.83355	0.79773	1.239	0.10767	-0.75409	0.7746	-0.75409	0.7746	0.10767	0.571976272
NA	ENSG00000261758	1.1021	0.13522	1.239	0.10768	1.2133	0.11251	1.2133	0.11251	0.10768	0.571976272
MTLN	ENSG00000175701	1.2389	0.1077	0.24278	0.40409	0.11196	0.45543	0.45644	0.32404	0.1077	0.571976272
HCG22	ENSG00000228789	-2.2224	0.98687	-2.7959	0.99741	1.2388	0.1077	0.71101	0.23854	0.1077	0.571976272

NA	ENSG00000260966	0.88011	0.1894	0.8936	0.18577	1.2389	0.1077	0.8936	0.18577	0.1077	0.571976272
BTN3A2	ENSG00000186470	0.86193	0.19436	1.1934	0.11636	1.2387	0.10772	1.1552	0.124	0.10772	0.571976272
CCL2	ENSG00000108691	1.2387	0.10773	-0.80014	0.78819	-0.80014	0.78819	-0.80014	0.78819	0.10773	0.571976272
LINC01620	ENSG00000168746	1.2319	0.109	1.2387	0.10773	1.2319	0.109	-0.80556	0.78975	0.10773	0.571976272
NA	ENSG00000260395	1.2386	0.10774	-0.85087	0.80258	1.2386	0.10774	1.2386	0.10774	0.10774	0.571976272
NA	ENSG00000233508	1.2385	0.10777	-0.029177	0.51164	-0.12693	0.5505	0.55558	0.28925	0.10777	0.572001677
NME6	ENSG00000172113	1.1749	0.12001	1.0977	0.13616	1.2384	0.10778	1.1749	0.12001	0.10778	0.572001677
NA	ENSG00000275963	1.2384	0.10778	0.6938	0.2439	0.91368	0.18044	0.84368	0.19942	0.10778	0.572001677
PAMR1	ENSG00000149090	-1.3079	0.90454	0.16998	0.43251	-0.7255	0.76593	1.2383	0.1078	0.1078	0.572045519
FGR	ENSG00000000938	-0.1143	0.5455	0.62265	0.26676	-0.1248	0.54966	1.2382	0.10782	0.10782	0.572066254
EEF1A2	ENSG00000101210	-0.47851	0.68386	-0.4365	0.66876	0.37201	0.35494	1.238	0.10786	0.10786	0.572066254
IQCE	ENSG00000106012	0.77303	0.21975	-0.11182	0.54452	0.36543	0.35739	1.238	0.10786	0.10786	0.572066254
SLC7A5P1	ENSG00000260727	0.017928	0.49285	0.017928	0.49285	1.238	0.10786	0.55913	0.28804	0.10786	0.572066254
NA	ENSG00000259081	1.2379	0.10787	-0.37114	0.64473	0.37005	0.35567	0.37005	0.35567	0.10787	0.572066254
TMCO2	ENSG00000188800	0.1668	0.43376	0.1668	0.43376	0.1668	0.43376	1.2378	0.10789	0.10789	0.572066254
NA	ENSG00000252550	1.2378	0.1079	-0.71731	0.76341	1.2378	0.1079	-0.29257	0.61507	0.1079	0.572066254
NA	ENSG00000227197	1.2377	0.10791	1.2377	0.10791	1.2377	0.10791	1.2377	0.10791	0.10791	0.572066254
OR8S21P	ENSG00000273434	1.2376	0.10793	1.2376	0.10793	-0.006529	0.5026	-0.79737	0.78738	0.10793	0.572066254
SEC24C	ENSG00000176986	1.2375	0.10795	1.1544	0.12417	1.11	0.1335	1.1544	0.12417	0.10795	0.572066254
NA	ENSG00000261654	0.33776	0.36777	0.48789	0.31281	1.2375	0.10795	0.14502	0.44235	0.10795	0.572066254
FAM204DP	ENSG00000258552	-0.62939	0.73545	0.64579	0.25921	1.2375	0.10796	0.64579	0.25921	0.10796	0.572066254
SNTG2-AS1	ENSG00000235688	0.53569	0.29609	0.16666	0.43382	1.2374	0.10797	1.2374	0.10797	0.10797	0.572066254
NA	ENSG00000234203	1.2374	0.10798	1.2374	0.10798	1.2374	0.10798	1.2374	0.10798	0.10798	0.572066254
RNU6-139P	ENSG00000252449	0.40657	0.34216	1.2374	0.10798	1.2374	0.10798	1.2374	0.10798	0.10798	0.572066254
SMPD4P1	ENSG00000223553	0.66753	0.25222	0.39348	0.34698	0.39348	0.34698	1.2372	0.10801	0.10801	0.572162985
HMGN1	ENSG00000205581	-0.30514	0.61987	1.1353	0.12813	1.2368	0.10807	0.58497	0.27929	0.10807	0.572294186
RPL36AP33	ENSG00000235619	0.15627	0.43791	1.2368	0.10807	1.2368	0.10807	0.3056	0.37995	0.10807	0.572294186
NA	ENSG00000259723	0.67212	0.25075	0.44272	0.32898	1.2369	0.10807	-0.78132	0.78269	0.10807	0.572294186
RNU6-694P	ENSG00000200941	1.2367	0.1081	0.55861	0.28821	0.67532	0.24974	0.84674	0.19857	0.1081	0.572328661
ABCD1P3	ENSG00000261233	0.95704	0.16927	-0.073322	0.52923	1.2367	0.1081	1.2367	0.1081	0.1081	0.572328661
NA	ENSG00000217805	-0.5239	0.69983	1.2366	0.10812	-0.97436	0.83506	1.2366	0.10812	0.10812	0.572372363

RNU6-1114P	ENSG00000251831	1.2364	0.10815	-0.067012	0.52671	0.070242	0.472	0.52707	0.29907	0.10815	0.572459737
OR10H3	ENSG00000171936	-0.056201	0.52241	1.2363	0.10816	-0.056201	0.52241	-0.056201	0.52241	0.10816	0.572459737
MIR4470	ENSG00000264408	1.2362	0.10818	1.2362	0.10818	1.2362	0.10818	1.2362	0.10818	0.10818	0.572484908
MOB4	ENSG00000115540	0.23512	0.40706	0.47077	0.3189	-0.59636	0.72453	1.2362	0.10819	0.10819	0.572484908
FAM192BP	ENSG00000231841	1.1368	0.1278	1.2361	0.1082	0.025263	0.48992	0.94144	0.17324	0.1082	0.572484908
NA	ENSG00000251416	1.2361	0.10822	0.96973	0.16609	1.2361	0.10822	-1.1176	0.86814	0.10822	0.572519312
TRAV8-2	ENSG00000211786	0.062956	0.4749	-0.23951	0.59465	-0.23951	0.59465	1.236	0.10823	0.10823	0.572519312
NA	ENSG00000232952	1.2358	0.10827	1.2358	0.10827	1.2358	0.10827	1.2358	0.10827	0.10827	0.572659475
RNF220	ENSG00000187147	1.2357	0.10828	0.4676	0.32004	0.32552	0.37239	-0.11177	0.5445	0.10828	0.572659475
ANK2	ENSG00000145362	1.2355	0.10832	0.75264	0.22583	1.0669	0.14301	0.69553	0.24336	0.10832	0.572738456
PRDX2P4	ENSG00000213177	1.2354	0.10835	0.37123	0.35523	1.2354	0.10835	1.2354	0.10835	0.10835	0.572738456
NA	ENSG00000279392	1.2353	0.10835	0.54403	0.29321	1.2353	0.10835	1.2353	0.10835	0.10835	0.572738456
LINC01147	ENSG00000258826	1.2353	0.10836	1.2353	0.10836	0.76219	0.22297	0.49787	0.30929	0.10836	0.572738456
UPF1	ENSG00000005007	1.2351	0.10839	1.174	0.12019	1.2236	0.11055	-0.22759	0.59002	0.10839	0.572738456
MATN4	ENSG00000124159	0.97106	0.16576	-0.03077	0.51227	1.2351	0.1084	0.092725	0.46306	0.1084	0.572738456
NA	ENSG00000227930	1.2351	0.1084	-0.6532	0.74318	-0.6532	0.74318	-0.6532	0.74318	0.1084	0.572738456
ANKRD40CL	ENSG00000167117	1.235	0.10841	1.235	0.10841	1.235	0.10841	1.235	0.10841	0.10841	0.572738456
LINC01440	ENSG00000235166	-0.47789	0.68364	1.0622	0.14408	1.2349	0.10843	0.7724	0.21994	0.10843	0.572738456
RBMX2P3	ENSG00000223575	1.0804	0.13999	1.2349	0.10844	0.047265	0.48115	-0.016756	0.50668	0.10844	0.572738456
LINC02328	ENSG00000258733	0.18187	0.42784	0.88772	0.18735	1.2348	0.10846	-0.70642	0.76004	0.10846	0.572738456
PRPF8	ENSG00000174231	0.36357	0.35809	0.046315	0.48153	1.2347	0.10847	-0.72276	0.76508	0.10847	0.572738456
ATP8B5P	ENSG00000179766	1.0427	0.14854	0.44238	0.32911	0.56573	0.28579	1.2347	0.10847	0.10847	0.572738456
DARS2	ENSG00000117593	-0.64661	0.74106	-0.63301	0.73664	1.2347	0.10848	-0.27254	0.6074	0.10848	0.572738456
GOT1	ENSG00000120053	1.2345	0.1085	-0.8193	0.79369	0.14222	0.44345	-0.98684	0.83814	0.1085	0.572738456
RNU6-578P	ENSG00000212282	-1.3973	0.91884	-1.1102	0.86655	-1.7926	0.96348	1.2345	0.1085	0.1085	0.572738456
NA	ENSG00000259178	-1.7723	0.96182	1.2345	0.1085	-1.7723	0.96182	-1.7723	0.96182	0.1085	0.572738456
MYO9B	ENSG00000099331	-0.67998	0.75174	0.2283	0.40971	1.2344	0.10853	-0.048232	0.51923	0.10853	0.572738456
RNU6-1059P	ENSG00000200622	1.2344	0.10853	0.55668	0.28887	0.40827	0.34154	-0.37873	0.64756	0.10853	0.572738456
MTND2P13	ENSG00000263914	1.2344	0.10853	1.2344	0.10853	1.2344	0.10853	1.2344	0.10853	0.10853	0.572738456
MRPS9-AS2	ENSG00000224509	0.59339	0.27646	0.53632	0.29587	0.28854	0.38647	1.2341	0.10857	0.10857	0.572744962
LINC00871	ENSG00000258700	1.2342	0.10857	-1.0591	0.85523	-0.50218	0.69223	-1.3066	0.90432	0.10857	0.572744962

NA	ENSG00000215409	1.2341	0.10858	0.5355	0.29615	0.89698	0.18486	0.89698	0.18486	0.10858	0.572744962
TJP1	ENSG00000104067	-0.68007	0.75177	-0.2975	0.61696	0.36244	0.35851	1.234	0.10859	0.10859	0.572744962
PTEN	ENSG00000171862	1.2341	0.10859	0.93365	0.17524	0.64535	0.25935	1.004	0.15769	0.10859	0.572744962
NA	ENSG00000254854	0.37069	0.35544	0.37069	0.35544	1.234	0.10861	0.052919	0.4789	0.10861	0.572788452
BCAT2	ENSG00000105552	1.2338	0.10864	-0.94092	0.82663	1.2338	0.10864	-0.94092	0.82663	0.10864	0.572794927
NA	ENSG00000250326	0.13834	0.44498	-0.42099	0.66312	0.34437	0.36529	1.2338	0.10864	0.10864	0.572794927
NA	ENSG00000279120	0.48625	0.31339	1.2337	0.10865	-0.55196	0.70951	0.48625	0.31339	0.10865	0.572794927
PARL	ENSG00000175193	1.2337	0.10866	0.32909	0.37104	-0.07167	0.52857	0.8659	0.19327	0.10866	0.572794927
EIF4E2P1	ENSG00000213384	0.54184	0.29396	0.97895	0.1638	1.2337	0.10867	-0.030877	0.51232	0.10867	0.572794927
NA	ENSG00000270096	1.2335	0.1087	1.2335	0.1087	1.2335	0.1087	1.2335	0.1087	0.1087	0.572801395
NA	ENSG00000201370	0.069439	0.47232	0.069439	0.47232	0.65237	0.25708	1.2334	0.10871	0.10871	0.572801395
RSPH4A	ENSG00000111834	1.2333	0.10872	1.2333	0.10872	1.2333	0.10872	1.2333	0.10872	0.10872	0.572801395
NA	ENSG00000249807	-0.060453	0.5241	1.2333	0.10873	1.2333	0.10873	-0.16619	0.56599	0.10873	0.572801395
NA	ENSG00000272084	1.2333	0.10873	0.50141	0.30804	1.1516	0.12475	-0.45635	0.67593	0.10873	0.572801395
CPOX	ENSG00000080819	1.2331	0.10876	1.2331	0.10876	-0.29977	0.61783	0.58865	0.27805	0.10876	0.572897503
NA	ENSG00000270226	1.233	0.10879	-0.90046	0.81606	0.74848	0.22709	0.74848	0.22709	0.10879	0.572944435
NA	ENSG00000253800	0.50661	0.30621	1.1432	0.12648	0.93138	0.17583	1.2329	0.1088	0.1088	0.572944435
GEMIN8P2	ENSG00000260451	1.2329	0.10881	1.2329	0.10881	1.2329	0.10881	0.80585	0.21017	0.10881	0.572944435
MHENCR	ENSG00000232442	1.1112	0.13324	1.1112	0.13324	1.2328	0.10882	1.2236	0.11055	0.10882	0.572944435
RNU6-989P	ENSG00000199626	1.2327	0.10885	1.2327	0.10885	1.2327	0.10885	1.1028	0.13506	0.10885	0.572944435
TEKT4	ENSG00000163060	0.15676	0.43772	0.3078	0.37912	1.2326	0.10886	0.61896	0.26797	0.10886	0.572944435
ZMYND10-AS1	ENSG00000235058	1.2326	0.10887	1.2326	0.10887	1.2326	0.10887	1.2326	0.10887	0.10887	0.572944435
NA	ENSG00000271702	-0.84202	0.80011	-0.018985	0.50757	-0.48373	0.68571	1.2326	0.10887	0.10887	0.572944435
NA	ENSG00000173366	1.0279	0.152	1.0279	0.152	0.95656	0.16939	1.2325	0.10888	0.10888	0.572944435
NANOGP2	ENSG00000228670	1.2324	0.10891	0.086337	0.4656	1.2324	0.10891	1.2324	0.10891	0.10891	0.572944435
LINC01675	ENSG00000234142	-0.24302	0.596	-0.37711	0.64695	-0.19039	0.5755	1.2324	0.10891	0.10891	0.572944435
NA	ENSG00000279903	1.2323	0.10891	1.2323	0.10891	1.2323	0.10891	1.2323	0.10891	0.10891	0.572944435
NA	ENSG00000251176	1.2323	0.10893	1.2323	0.10893	-0.42222	0.66357	1.2323	0.10893	0.10893	0.572975655
SWT1	ENSG00000116668	1.0844	0.13909	0.39384	0.34685	1.2322	0.10895	-0.033768	0.51347	0.10895	0.572975655
LY86-AS1	ENSG00000216863	0.5603	0.28764	1.2321	0.10896	1.1054	0.13449	-1.9584	0.97491	0.10896	0.572975655
SCAMP3	ENSG00000116521	0.75534	0.22502	1.232	0.10898	0.35012	0.36312	0.081467	0.46754	0.10898	0.572975655

PLEKHD1	ENSG00000175985	-0.85835	0.80465	1.2319	0.10899	-1.0951	0.86327	0.72674	0.23369	0.10899	0.572975655
PIK3R4	ENSG00000196455	1.2319	0.10899	1.2319	0.10899	1.2319	0.10899	0.34943	0.36338	0.10899	0.572975655
ZNF419	ENSG00000105136	0.62764	0.26512	1.2319	0.109	0.93716	0.17434	1.1123	0.133	0.109	0.572975655
MTND5P5	ENSG00000251149	-1.0116	0.84413	1.2318	0.10901	-1.0116	0.84413	0.9494	0.17121	0.10901	0.572975655
NA	ENSG00000272428	0.3344	0.36904	1.2317	0.10904	-0.072378	0.52885	0.42613	0.335	0.10904	0.573062278
GNPDA2	ENSG00000163281	1.2316	0.10905	0.60975	0.27101	1.2316	0.10905	0.91267	0.18071	0.10905	0.573062278
HSD17B4	ENSG00000133835	0.98411	0.16253	1.0238	0.15297	0.54732	0.29208	1.2315	0.10908	0.10908	0.573087074
NA	ENSG00000236434	1.2314	0.10908	-1.6077	0.94605	-1.6077	0.94605	-1.6077	0.94605	0.10908	0.573087074
PREX2	ENSG00000046889	0.86885	0.19247	1.0946	0.13685	1.2314	0.10909	0.041537	0.48343	0.10909	0.573087074
EI24P1	ENSG00000236357	-0.88618	0.81224	1.2313	0.10911	0.81977	0.20617	-0.63704	0.73795	0.10911	0.573130355
FAM230I	ENSG00000178248	-1.0192	0.84593	-0.90844	0.81818	0.47271	0.31821	1.2312	0.10913	0.10913	0.573164371
NA	ENSG00000236495	0.98969	0.16116	1.2311	0.10914	-0.58935	0.72219	1.1674	0.12153	0.10914	0.573164371
NA	ENSG00000260963	1.231	0.10916	1.231	0.10916	1.231	0.10916	1.231	0.10916	0.10916	0.573189119
C9orf152	ENSG00000188959	1.2309	0.10918	-0.056762	0.52263	-0.056762	0.52263	-1.0256	0.84745	0.10918	0.573189119
NA	ENSG00000270521	-0.091734	0.53655	-0.1215	0.54835	-0.61361	0.73026	1.2309	0.10918	0.10918	0.573189119
LINC01293	ENSG00000230836	1.2307	0.10922	1.2307	0.10922	1.2307	0.10922	1.2307	0.10922	0.10922	0.573248733
ANKLE1	ENSG00000160117	1.2306	0.10924	1.2306	0.10924	1.2306	0.10924	1.2306	0.10924	0.10924	0.573248733
MYB-AS1	ENSG00000236703	1.2305	0.10925	1.2305	0.10925	1.2305	0.10925	1.2305	0.10925	0.10925	0.573248733
FOXP1-AS1	ENSG00000244203	0.67825	0.24881	1.2305	0.10926	0.67825	0.24881	0.67825	0.24881	0.10926	0.573248733
CCDC9B	ENSG00000188549	1.2304	0.10927	1.2304	0.10927	1.0196	0.15396	1.0664	0.14311	0.10927	0.573248733
ZNF404	ENSG00000176222	1.2304	0.10928	-0.80233	0.78882	-0.53556	0.70387	-0.80233	0.78882	0.10928	0.573248733
AQP9	ENSG00000103569	-0.2083	0.5825	-0.23599	0.59328	-0.20831	0.58251	1.2303	0.10929	0.10929	0.573248733
RICTOR	ENSG00000164327	0.74443	0.22831	1.2302	0.10931	0.44957	0.32651	1.1701	0.12097	0.10931	0.573248733
NA	ENSG00000203565	1.23	0.10935	1.23	0.10935	1.23	0.10935	0.57197	0.28367	0.10935	0.573248733
NA	ENSG00000268649	1.23	0.10935	1.0458	0.14782	1.0458	0.14782	1.0458	0.14782	0.10935	0.573248733
BMPR1A	ENSG00000107779	0.94981	0.1711	0.71158	0.23836	1.1678	0.12144	1.2299	0.10936	0.10936	0.573248733
CACNA1I	ENSG00000100346	0.10907	0.45657	0.34871	0.36365	0.96585	0.16706	1.2298	0.10938	0.10938	0.573248733
NA	ENSG00000226276	1.2298	0.10938	0.24577	0.40293	1.2298	0.10938	1.2298	0.10938	0.10938	0.573248733
CTSLP4	ENSG00000230011	-0.83327	0.79765	-1.0615	0.85577	-0.99197	0.83939	1.2298	0.10938	0.10938	0.573248733
NA	ENSG00000234513	0.14722	0.44148	1.0918	0.13746	1.2299	0.10938	1.0918	0.13746	0.10938	0.573248733
CLGN	ENSG00000153132	-0.84013	0.79958	-2.1618	0.98468	1.2297	0.10941	-0.60879	0.72867	0.10941	0.573248733

RREB1	ENSG00000124782	1.0944	0.13689	1.2296	0.10942	0.90812	0.18191	0.88403	0.18834	0.10942	0.573248733
ACTG2	ENSG00000163017	1.2296	0.10943	-0.012411	0.50495	0.38459	0.35027	-0.30175	0.61858	0.10943	0.573248733
LRTM2	ENSG00000166159	0.7446	0.22826	-0.28525	0.61227	0.73306	0.23176	1.2295	0.10944	0.10944	0.573248733
MRPS21P1	ENSG00000234496	-0.79347	0.78625	0.47067	0.31894	1.2295	0.10944	0.15639	0.43786	0.10944	0.573248733
RNU6-1161P	ENSG00000251985	-1.8234	0.96588	-1.8234	0.96588	1.2294	0.10947	-1.8234	0.96588	0.10947	0.573248733
NA	ENSG00000237336	1.2293	0.10949	0.17948	0.42878	1.2089	0.11335	0.0071313	0.49716	0.10949	0.573248733
NA	ENSG00000271344	-0.14151	0.55626	-0.14151	0.55626	-0.14151	0.55626	1.2292	0.1095	0.1095	0.573248733
NA	ENSG00000223986	1.2108	0.11298	-2.0285	0.97875	1.2291	0.10951	0.11718	0.45336	0.10951	0.573248733
LINC01932	ENSG00000253315	1.2291	0.10952	1.2291	0.10952	1.2291	0.10952	1.2291	0.10952	0.10952	0.573248733
IGLV1-51	ENSG00000211644	1.229	0.10953	1.229	0.10953	1.229	0.10953	1.229	0.10953	0.10953	0.573248733
UHMK1	ENSG00000152332	0.53919	0.29488	0.69014	0.24505	1.229	0.10954	1.229	0.10954	0.10954	0.573248733
LINC02862	ENSG00000237571	1.229	0.10954	0.37406	0.35418	0.11072	0.45592	0.978	0.16404	0.10954	0.573248733
MC3R	ENSG00000124089	1.2289	0.10956	1.2289	0.10956	1.2289	0.10956	0.42909	0.33393	0.10956	0.573248733
UBBP1	ENSG00000230037	1.2288	0.10957	1.2288	0.10957	1.2288	0.10957	1.2288	0.10957	0.10957	0.573248733
NA	ENSG00000198491	-0.18795	0.57454	1.2287	0.10959	0.86105	0.19461	0.41918	0.33754	0.10959	0.573248733
RNU1-18P	ENSG00000199932	1.0893	0.13801	1.2287	0.10959	0.0040099	0.4984	-0.38769	0.65088	0.10959	0.573248733
ZNRF2P2	ENSG00000225264	1.2285	0.10962	1.2285	0.10962	1.2285	0.10962	1.2285	0.10962	0.10962	0.573248733
NA	ENSG00000267545	1.2285	0.10963	1.2285	0.10963	1.2285	0.10963	1.2285	0.10963	0.10963	0.573248733
SPRYD3	ENSG00000167778	-0.16085	0.56389	-0.41706	0.66168	1.2284	0.10964	-0.41919	0.66246	0.10964	0.573248733
NPM1P26	ENSG00000235677	1.2283	0.10968	-0.13832	0.555	-0.13832	0.555	-0.13832	0.555	0.10968	0.573248733
ELOCP20	ENSG00000229101	0.44311	0.32884	-1.9655	0.97532	1.2282	0.10969	-0.23535	0.59303	0.10969	0.573248733
NANOGNBP1	ENSG00000226034	1.2281	0.1097	-1.0262	0.84761	1.2281	0.1097	1.2281	0.1097	0.1097	0.573248733
NA	ENSG00000279379	1.228	0.10972	-0.36411	0.64211	-0.10496	0.54179	0.35122	0.36271	0.10972	0.573248733
GRAMD2B	ENSG00000155324	1.2279	0.10974	-0.49321	0.68907	0.53084	0.29776	-1.035	0.84968	0.10974	0.573248733
S100A12	ENSG00000163221	0.38174	0.35133	1.0293	0.15166	1.2278	0.10976	-0.084731	0.53376	0.10976	0.573248733
C8orf34	ENSG00000165084	-0.14903	0.55924	1.0469	0.14757	0.98143	0.16319	1.2277	0.10977	0.10977	0.573248733
NA	ENSG00000263709	1.2274	0.10983	1.2274	0.10983	1.2274	0.10983	-0.33884	0.63263	0.10983	0.573248733
ABHD12	ENSG00000100997	0.11417	0.45455	1.2274	0.10984	-0.29085	0.61442	0.15962	0.43659	0.10984	0.573248733
RAET1G	ENSG00000203722	0.31771	0.37535	1.2273	0.10985	0.50597	0.30644	0.11741	0.45327	0.10985	0.573248733
NSA2P4	ENSG00000259750	0.23575	0.40681	1.2273	0.10985	0.23575	0.40681	0.23575	0.40681	0.10985	0.573248733
NA	ENSG00000262837	1.2273	0.10985	1.2273	0.10985	1.2273	0.10985	1.2273	0.10985	0.10985	0.573248733

TMEM230	ENSG00000089063	0.75792	0.22425	1.2272	0.10988	-1.224	0.88952	0.33363	0.36933	0.10988	0.573248733
QPRT	ENSG00000103485	1.2272	0.10988	0.11346	0.45483	0.11346	0.45483	0.91575	0.1799	0.10988	0.573248733
NA	ENSG00000265261	1.227	0.10991	-0.73581	0.76908	-0.73581	0.76908	-0.73581	0.76908	0.10991	0.573248733
CHCHD2P8	ENSG00000235115	-0.089104	0.5355	-0.65489	0.74373	1.2269	0.10993	0.89168	0.18628	0.10993	0.573248733
NA	ENSG00000268643	1.2269	0.10993	1.2269	0.10993	1.2269	0.10993	1.2269	0.10993	0.10993	0.573248733
SFRP2	ENSG00000145423	0.48432	0.31408	0.1522	0.43951	0.26867	0.39409	1.2268	0.10994	0.10994	0.573248733
SRGAP3-AS2	ENSG00000228723	0.68119	0.24788	1.2269	0.10994	0.4702	0.3191	1.2269	0.10994	0.10994	0.573248733
LSM11	ENSG00000155858	0.41995	0.33726	-0.068854	0.52745	0.70505	0.24039	1.2268	0.10995	0.10995	0.573248733
DEFA8P	ENSG00000223629	0.30701	0.37942	0.22431	0.41126	1.2268	0.10995	0.14743	0.4414	0.10995	0.573248733
NA	ENSG00000260188	0.52103	0.30117	1.2267	0.10997	-0.50949	0.6948	-0.62598	0.73434	0.10997	0.573248733
RRN3P1	ENSG00000248124	1.2267	0.10998	1.2077	0.11357	0.31817	0.37518	-0.93764	0.82578	0.10998	0.573248733
NA	ENSG00000262052	0.84193	0.19991	0.14125	0.44384	1.2267	0.10998	0.43579	0.33149	0.10998	0.573248733
NA	ENSG00000267579	1.2266	0.10998	0.34038	0.36678	0.78475	0.2163	-0.41538	0.66107	0.10998	0.573248733
LINC02037	ENSG00000238097	-1.2033	0.88557	-1.0395	0.85071	-1.2033	0.88557	1.2265	0.11	0.11	0.573248733
AKR7L	ENSG00000211454	0.76467	0.22223	0.77778	0.21835	1.2265	0.11001	1.2265	0.11001	0.11001	0.573248733
HMGXB3	ENSG00000113716	0.80428	0.21062	-1.0569	0.85471	1.2264	0.11003	0.26337	0.39613	0.11003	0.573248733
NA	ENSG00000233219	-0.8682	0.80736	-1.1416	0.87318	1.2264	0.11003	1.2189	0.11145	0.11003	0.573248733
NA	ENSG00000249685	-0.24666	0.59742	-0.24666	0.59742	1.2263	0.11003	-0.01081	0.50431	0.11003	0.573248733
NA	ENSG00000217060	-0.066838	0.52664	-0.30345	0.61923	1.2262	0.11006	-1.1256	0.86984	0.11006	0.573248733
NA	ENSG00000233017	-1.0569	0.85473	1.2261	0.11009	-1.2311	0.89086	-1.0499	0.85311	0.11009	0.573248733
COPS2	ENSG00000166200	0.59544	0.27577	1.2259	0.11011	-0.56698	0.71463	0.16635	0.43394	0.11011	0.573248733
RRM1	ENSG00000167325	-0.28382	0.61173	-0.018032	0.50719	0.40006	0.34456	1.2259	0.11012	0.11012	0.573248733
DGKA	ENSG00000065357	0.69168	0.24457	1.2258	0.11013	0.6877	0.24582	-0.34581	0.63526	0.11013	0.573248733
PGK2	ENSG00000170950	0.15887	0.43689	1.2259	0.11013	1.2259	0.11013	-0.2776	0.60934	0.11013	0.573248733
SAA1	ENSG00000173432	1.2258	0.11013	1.2258	0.11013	1.2258	0.11013	1.2258	0.11013	0.11013	0.573248733
OR7A3P	ENSG00000269787	0.45997	0.32277	0.45997	0.32277	1.2258	0.11013	1.2258	0.11013	0.11013	0.573248733
NA	ENSG00000278367	1.2259	0.11013	1.2259	0.11013	1.2259	0.11013	1.2259	0.11013	0.11013	0.573248733
RPL12P13	ENSG00000172746	1.2258	0.11014	1.2258	0.11014	-0.074452	0.52967	-0.40747	0.65817	0.11014	0.573248733
SRGAP3-AS4	ENSG00000235830	1.2258	0.11014	1.2258	0.11014	1.2258	0.11014	1.2258	0.11014	0.11014	0.573248733
PCDHB6	ENSG00000113211	0.47441	0.31761	1.2257	0.11016	1.1011	0.13543	1.1011	0.13543	0.11016	0.573248733
NA	ENSG00000251136	0.9494	0.17121	1.0798	0.14011	1.2256	0.11018	0.26349	0.39609	0.11018	0.573248733

NA	ENSG00000253949	0.36907	0.35604	1.2256	0.11018	-0.063836	0.52545	-0.66094	0.74568	0.11018	0.573248733
MIR5707	ENSG00000265598	1.2255	0.11018	1.2255	0.11018	1.2255	0.11018	1.2255	0.11018	0.11018	0.573248733
NA	ENSG00000279930	1.2256	0.11018	1.2256	0.11018	1.2256	0.11018	1.2256	0.11018	0.11018	0.573248733
ATP5IF1	ENSG00000130770	1.2255	0.11019	0.59468	0.27603	0.92774	0.17677	0.93873	0.17393	0.11019	0.573248733
S100A7P1	ENSG00000203781	1.2255	0.11019	0.91737	0.17947	0.91737	0.17947	0.91737	0.17947	0.11019	0.573248733
FKBP15	ENSG00000119321	0.17736	0.42961	1.2255	0.1102	1.2255	0.1102	0.94554	0.17219	0.1102	0.573248733
FAM217A	ENSG00000145975	-1.4866	0.93144	1.2255	0.1102	0.39406	0.34677	0.53039	0.29792	0.1102	0.573248733
PPARGC1A	ENSG00000109819	0.26272	0.39638	0.97172	0.16559	1.2253	0.11022	1.1789	0.11921	0.11022	0.573248733
NA	ENSG00000278829	1.2253	0.11024	1.2253	0.11024	1.2253	0.11024	1.2253	0.11024	0.11024	0.573248733
ARHGAP5-AS1	ENSG00000258655	-1.0344	0.84953	-0.78127	0.78268	1.2252	0.11025	1.2252	0.11025	0.11025	0.573248733
OR9S24P	ENSG00000231278	-1.0365	0.85003	-0.007244	0.50289	-1.0848	0.86101	1.2252	0.11026	0.11026	0.573248733
LLCFC1	ENSG00000165131	1.2251	0.11027	1.2251	0.11027	1.2251	0.11027	1.2251	0.11027	0.11027	0.573248733
MIR4303	ENSG00000263890	0.25262	0.40028	0.25262	0.40028	0.10133	0.45964	1.2249	0.1103	0.1103	0.573248733
RNU6ATAC35P	ENSG00000221571	1.2249	0.11031	1.2249	0.11031	1.2249	0.11031	1.2249	0.11031	0.11031	0.573248733
PAQR5	ENSG00000137819	-0.19011	0.57539	-0.65244	0.74294	0.48413	0.31415	1.2247	0.11034	0.11034	0.573248733
LRGUK	ENSG00000155530	1.0534	0.14608	1.2247	0.11035	-0.15417	0.56126	0.58953	0.27775	0.11035	0.573248733
UNC5C	ENSG00000182168	0.42389	0.33582	0.41896	0.33762	0.61039	0.2708	1.2247	0.11035	0.11035	0.573248733
CYB561	ENSG00000008283	0.097799	0.46105	0.0032185	0.49872	1.2246	0.11036	0.18213	0.42774	0.11036	0.573248733
NA	ENSG00000266763	1.2246	0.11036	1.2246	0.11036	1.2246	0.11036	1.2246	0.11036	0.11036	0.573248733
LINC02110	ENSG00000251518	1.2246	0.11037	1.2246	0.11037	1.2246	0.11037	1.2246	0.11037	0.11037	0.573248733
NA	ENSG00000255345	0.60679	0.27199	-0.45478	0.67537	0.60679	0.27199	1.2244	0.11039	0.11039	0.573248733
KDM4D	ENSG00000186280	-0.24667	0.59742	0.65519	0.25617	1.2244	0.1104	1.2244	0.1104	0.1104	0.573248733
ZC3H14	ENSG00000100722	1.2243	0.11043	0.33809	0.36765	0.084978	0.46614	0.084978	0.46614	0.11043	0.573248733
LCE1D	ENSG00000172155	1.2077	0.11357	1.2077	0.11357	0.99663	0.15947	1.2242	0.11043	0.11043	0.573248733
NA	ENSG00000269121	1.2243	0.11043	1.2243	0.11043	1.2243	0.11043	1.2243	0.11043	0.11043	0.573248733
ZNF846	ENSG00000196605	0.094891	0.4622	1.2241	0.11045	-0.32716	0.62823	-0.11454	0.54559	0.11045	0.573248733
LINC00903	ENSG00000241397	1.2241	0.11047	0.97273	0.16534	0.97273	0.16534	1.1886	0.1173	0.11047	0.573248733
NA	ENSG00000200485	1.224	0.11048	1.224	0.11048	1.224	0.11048	1.224	0.11048	0.11048	0.573248733
CCDC93	ENSG00000125633	1.2238	0.11051	1.1289	0.12947	0.71753	0.23652	0.085181	0.46606	0.11051	0.573248733
NA	ENSG00000251213	-0.68587	0.7536	0.87877	0.18976	1.2236	0.11054	0.33977	0.36701	0.11054	0.573248733
RNFT1	ENSG00000189050	0.62851	0.26484	1.118	0.13179	1.2235	0.11056	0.12822	0.44899	0.11056	0.573248733

RNA5SP225	ENSG00000222806	0.96547	0.16715	0.58707	0.27858	1.2235	0.11056	-0.06666	0.52657	0.11056	0.573248733
NA	ENSG00000225620	1.2235	0.11057	0.27897	0.39013	0.83691	0.20132	0.83691	0.20132	0.11057	0.573248733
RPS3AP10	ENSG00000226292	0.3803	0.35186	1.2235	0.11057	0.48685	0.31318	0.3803	0.35186	0.11057	0.573248733
NA	ENSG00000202533	1.2235	0.11058	1.2235	0.11058	1.2235	0.11058	1.2235	0.11058	0.11058	0.573248733
LIME1	ENSG00000203896	1.1477	0.12554	1.2233	0.11062	1.2233	0.11062	1.2233	0.11062	0.11062	0.573248733
RNA5SP156	ENSG00000222182	1.2233	0.11062	-0.24393	0.59636	0.27067	0.39332	0.16184	0.43572	0.11062	0.573248733
NA	ENSG00000231291	1.2232	0.11062	1.2232	0.11062	1.0197	0.15393	0.88776	0.18734	0.11062	0.573248733
NA	ENSG00000236829	1.2232	0.11062	0.48216	0.31485	0.3367	0.36817	0.3367	0.36817	0.11062	0.573248733
ZNF402P	ENSG00000220248	-0.52265	0.69939	1.2232	0.11064	1.2232	0.11064	0.19654	0.4221	0.11064	0.573248733
LIX1	ENSG00000145721	0.19033	0.42453	1.2231	0.11065	-0.076697	0.53057	0.41376	0.33953	0.11065	0.573248733
ANTXR2	ENSG00000163297	0.43887	0.33038	0.55992	0.28777	1.2229	0.11068	-0.51861	0.69798	0.11068	0.573248733
NEK4	ENSG00000114904	0.82452	0.20482	1.2229	0.11069	0.14701	0.44156	0.83616	0.20153	0.11069	0.573248733
TSHZ1	ENSG00000179981	-0.94741	0.82829	-0.14759	0.55867	0.60002	0.27424	1.2228	0.1107	0.1107	0.573248733
NA	ENSG00000255663	-0.22203	0.58785	-0.22203	0.58785	-0.22203	0.58785	1.2228	0.11071	0.11071	0.573248733
LINC02346	ENSG00000206187	1.2227	0.11073	-0.49795	0.69074	-0.30106	0.61832	1.2227	0.11073	0.11073	0.573248733
NA	ENSG00000279135	1.2226	0.11073	-0.51411	0.69641	1.2226	0.11073	0.13101	0.44788	0.11073	0.573248733
PNPLA3	ENSG00000100344	-0.1362	0.55417	-0.92549	0.82264	1.2226	0.11074	0.25565	0.39911	0.11074	0.573248733
RNU6-1231P	ENSG00000252994	1.0772	0.1407	1.2225	0.11075	1.0263	0.15237	1.0263	0.15237	0.11075	0.573248733
COL25A1	ENSG00000188517	1.0898	0.1379	0.49744	0.30944	1.2225	0.11076	-0.06158	0.52455	0.11076	0.573248733
NA	ENSG00000233359	1.2225	0.11076	0.62086	0.26735	0.017678	0.49295	-0.22207	0.58787	0.11076	0.573248733
RPL5P33	ENSG00000243521	-0.86963	0.80775	-0.78426	0.78356	1.1921	0.11661	1.2225	0.11076	0.11076	0.573248733
NA	ENSG00000261350	1.2225	0.11076	1.2225	0.11076	1.2225	0.11076	1.2225	0.11076	0.11076	0.573248733
NA	ENSG00000273998	1.2225	0.11077	1.2225	0.11077	1.2225	0.11077	1.2225	0.11077	0.11077	0.573248733
LIN28AP1	ENSG00000213120	0.60916	0.27121	0.45293	0.3253	0.44884	0.32677	1.2223	0.11079	0.11079	0.573248733
NA	ENSG00000235629	0.76118	0.22327	0.29922	0.38239	1.2223	0.1108	0.34044	0.36676	0.1108	0.573248733
NA	ENSG00000275830	-0.38975	0.65164	0.16497	0.43448	1.2222	0.11081	0.16497	0.43448	0.11081	0.573248733
FTLP13	ENSG00000263725	1.158	0.12344	1.0507	0.14669	0.64549	0.2593	1.2222	0.11082	0.11082	0.573248733
NA	ENSG00000199472	1.2222	0.11083	1.2222	0.11083	1.2222	0.11083	1.2222	0.11083	0.11083	0.573248733
NDFIP2	ENSG00000102471	-0.096352	0.53838	0.97364	0.16512	-0.12647	0.55032	1.2221	0.11084	0.11084	0.573248733
SNAI1P1	ENSG00000237194	1.2221	0.11084	1.2221	0.11084	1.2221	0.11084	1.2221	0.11084	0.11084	0.573248733
RNA5SP114	ENSG00000200650	-0.40256	0.65637	-0.40256	0.65637	1.222	0.11086	-0.40256	0.65637	0.11086	0.573248733

NA	ENSG00000233682	0.30242	0.38117	0.54489	0.29291	0.55827	0.28833	1.222	0.11086	0.11086	0.573248733
GUCY1A2	ENSG00000152402	0.58531	0.27917	1.2217	0.1109	1.2058	0.11396	0.59263	0.27671	0.1109	0.573248733
NA	ENSG00000222614	1.2218	0.1109	1.2218	0.1109	1.2218	0.1109	1.2218	0.1109	0.1109	0.573248733
KRT18P22	ENSG00000218153	1.2217	0.11091	1.2217	0.11091	1.2217	0.11091	1.2217	0.11091	0.11091	0.573248733
RPSAP9	ENSG00000234618	-0.26813	0.6057	1.0417	0.14878	0.48193	0.31493	1.2217	0.11091	0.11091	0.573248733
NA	ENSG00000265287	1.2217	0.11092	1.2217	0.11092	1.2217	0.11092	1.2217	0.11092	0.11092	0.573248733
RNU4-24P	ENSG00000201435	1.2216	0.11093	1.2216	0.11093	1.2216	0.11093	1.2216	0.11093	0.11093	0.573248733
PXN	ENSG00000089159	0.37452	0.35401	1.2215	0.11095	0.87256	0.19145	1.0786	0.14039	0.11095	0.573248733
UBE2V2P2	ENSG00000267463	1.2215	0.11095	1.2215	0.11095	1.2215	0.11095	1.2215	0.11095	0.11095	0.573248733
LINC01874	ENSG00000223985	0.385	0.35012	1.2213	0.11098	-0.048637	0.5194	1.1816	0.11868	0.11098	0.573248733
NA	ENSG00000256706	1.0339	0.15059	1.2213	0.11098	1.0339	0.15059	1.0339	0.15059	0.11098	0.573248733
NA	ENSG00000279387	0.42369	0.3359	0.18728	0.42572	-0.60246	0.72657	1.2213	0.11098	0.11098	0.573248733
ZNF514	ENSG00000144026	-0.11581	0.5461	0.17596	0.43016	1.2211	0.11103	1.2211	0.11103	0.11103	0.573248733
FOXK2	ENSG00000141568	0.3383	0.36757	-0.56808	0.71501	-0.28162	0.61088	1.221	0.11104	0.11104	0.573248733
NA	ENSG00000223300	1.221	0.11104	0.71359	0.23774	1.221	0.11104	1.221	0.11104	0.11104	0.573248733
FICD	ENSG00000198855	1.2208	0.11108	0.11655	0.45361	-0.3067	0.62047	-0.41807	0.66205	0.11108	0.573248733
NA	ENSG00000233706	1.2208	0.11108	1.2208	0.11108	1.2208	0.11108	1.2208	0.11108	0.11108	0.573248733
NA	ENSG00000199366	1.0584	0.14493	1.2207	0.11109	1.0584	0.14493	1.0584	0.14493	0.11109	0.573248733
NA	ENSG00000234943	0.34109	0.36652	0.34109	0.36652	0.34109	0.36652	1.2208	0.11109	0.11109	0.573248733
NA	ENSG00000258077	0.16496	0.43449	1.1736	0.12027	1.2207	0.1111	-0.037484	0.51495	0.1111	0.573248733
NEURL3	ENSG00000163121	0.73725	0.23048	0.91419	0.18031	1.2206	0.11111	1.2162	0.11195	0.11111	0.573248733
NA	ENSG00000231460	0.030072	0.48801	1.2206	0.11111	-1.1001	0.86436	0.030072	0.48801	0.11111	0.573248733
TMEM144	ENSG00000164124	1.1522	0.12461	1.2206	0.11112	0.96388	0.16755	0.82713	0.20408	0.11112	0.573248733
NARS2	ENSG00000137513	0.97127	0.16571	0.25737	0.39845	1.2205	0.11114	0.2872	0.38698	0.11114	0.573248733
MICA	ENSG00000204520	0.8504	0.19755	1.2204	0.11116	0.24755	0.40224	0.91711	0.17954	0.11116	0.573248733
NA	ENSG00000250540	-0.61717	0.73144	1.2203	0.11117	0.31445	0.37659	0.91673	0.17964	0.11117	0.573248733
NA	ENSG00000260674	1.2202	0.11119	0.33963	0.36707	1.2202	0.11119	1.2202	0.11119	0.11119	0.573248733
RNU6-604P	ENSG00000212219	-0.72653	0.76624	-0.64621	0.74093	1.2201	0.11121	-0.24627	0.59726	0.11121	0.573248733
PLAGL2	ENSG00000126003	1.125	0.1303	0.83026	0.20319	0.83026	0.20319	1.22	0.11123	0.11123	0.573248733
NA	ENSG00000236673	1.22	0.11124	0.53245	0.29721	0.2866	0.38721	0.71334	0.23782	0.11124	0.573248733
PCDHGB3	ENSG00000262209	1.0897	0.13791	1.22	0.11124	1.0897	0.13791	1.0897	0.13791	0.11124	0.573248733

HTR1DP1	ENSG00000275811	0.11503	0.45421	1.2199	0.11125	-0.8737	0.80886	1.2199	0.11125	0.11125	0.573248733
OR7E14P	ENSG00000184669	1.2199	0.11126	-0.079037	0.5315	0.24786	0.40212	0.78903	0.21505	0.11126	0.573248733
UPF3AP2	ENSG00000214832	0.45905	0.3231	-1.0175	0.84555	1.2199	0.11126	-0.38303	0.64915	0.11126	0.573248733
NA	ENSG00000237835	1.2198	0.11127	1.2198	0.11127	1.2198	0.11127	1.2198	0.11127	0.11127	0.573248733
NA	ENSG00000263991	1.2198	0.11127	1.2198	0.11127	1.2198	0.11127	1.2198	0.11127	0.11127	0.573248733
OR51F5P	ENSG00000272634	0.45595	0.32421	1.2198	0.11128	0.45595	0.32421	0.45595	0.32421	0.11128	0.573248733
NA	ENSG00000249977	0.6437	0.25988	0.53287	0.29706	0.6437	0.25988	1.2196	0.11131	0.11131	0.573248733
NA	ENSG00000224745	0.058947	0.4765	1.2195	0.11132	-0.19569	0.57757	-0.19569	0.57757	0.11132	0.573248733
RNU1-35P	ENSG00000199652	0.87417	0.19101	1.2195	0.11134	1.2195	0.11134	0.044716	0.48217	0.11134	0.573248733
ATP5MFP6	ENSG00000261102	1.2193	0.11136	1.2193	0.11136	1.2193	0.11136	1.2193	0.11136	0.11136	0.573248733
RNU6-528P	ENSG00000252735	0.77359	0.21959	0.18561	0.42638	1.2192	0.11138	0.77374	0.21954	0.11138	0.573248733
NEB	ENSG00000183091	0.80013	0.21182	-0.3928	0.65277	1.219	0.11141	0.31306	0.37712	0.11141	0.573248733
RNU6-105P	ENSG00000200779	1.2191	0.11141	1.2191	0.11141	1.2191	0.11141	1.2191	0.11141	0.11141	0.573248733
CEACAM1	ENSG00000079385	1.219	0.11142	0.49382	0.31072	0.25434	0.39962	-0.30003	0.61792	0.11142	0.573248733
ANKRD54	ENSG00000100124	0.89925	0.18426	1.1344	0.12831	0.95287	0.17033	1.219	0.11142	0.11142	0.573248733
ARIH2	ENSG00000177479	0.91206	0.18087	1.219	0.11142	0.9893	0.16126	-0.35826	0.63993	0.11142	0.573248733
ARHGEF19	ENSG00000142632	1.2189	0.11143	-0.95715	0.83075	-1.2198	0.88874	-1.2198	0.88874	0.11143	0.573248733
NA	ENSG00000260331	0.88337	0.18852	0.42423	0.3357	0.2304	0.40889	1.2189	0.11143	0.11143	0.573248733
GSTM3P2	ENSG00000226878	1.2189	0.11144	1.2189	0.11144	1.2189	0.11144	1.2189	0.11144	0.11144	0.573248733
GOLGA8J	ENSG00000179938	0.96744	0.16666	-0.10366	0.54128	1.2189	0.11145	-0.044398	0.51771	0.11145	0.573248733
NA	ENSG00000232855	-0.11042	0.54396	1.2189	0.11145	0.43485	0.33183	0.53848	0.29512	0.11145	0.573248733
CAMTA1-DT	ENSG00000237436	1.2186	0.11149	-0.18782	0.57449	0.2143	0.41516	0.2143	0.41516	0.11149	0.573263874
RNU6-488P	ENSG00000251727	0.50541	0.30664	-0.35919	0.64027	-0.52571	0.70046	1.2187	0.11149	0.11149	0.573263874
OR2T32P	ENSG00000197067	-0.15371	0.56108	-0.83527	0.79822	1.2186	0.1115	-1.5825	0.94324	0.1115	0.573263874
RNU6ATAC32P	ENSG00000251835	1.2186	0.1115	1.2186	0.1115	1.2186	0.1115	1.2186	0.1115	0.1115	0.573263874
NA	ENSG00000253361	0.078038	0.4689	-0.80132	0.78853	-0.42543	0.66474	1.2185	0.11152	0.11152	0.573306214
CNTN2	ENSG00000184144	0.72206	0.23513	0.58873	0.27802	1.2184	0.11154	0.91668	0.17965	0.11154	0.573348544
NA	ENSG00000275383	1.2183	0.11156	1.2183	0.11156	1.2183	0.11156	1.2183	0.11156	0.11156	0.573381787
APOBR	ENSG00000184730	0.26671	0.39484	0.83539	0.20175	1.2182	0.11157	0.8215	0.20568	0.11157	0.573381787
SERPINA6	ENSG00000170099	-0.035147	0.51402	1.2181	0.1116	0.51899	0.30189	1.2181	0.1116	0.1116	0.573466396
COMMD1	ENSG00000173163	-0.75671	0.77539	-0.53526	0.70376	1.218	0.11161	-0.18014	0.57148	0.11161	0.573466396

FTCD-AS1 ENSG00000237338 1.2178 0.11165 1.2178 0.11165 1.2178
0.11165 1.2178 0.11165 0.11165 0.573608218
UBA7 ENSG00000182179 1.2177 0.11167 -0.53093 0.70227 -0.53093 0.70227
-0.53093 0.70227 0.11167 0.573608218
SDCBP ENSG00000137575 1.2176 0.11168 1.2176 0.11168 1.2176 0.11168
1.1607 0.12288 0.11168 0.573608218
CALHM6 ENSG00000188820 1.2175 0.1117 1.2175 0.1117 1.2175
0.1117 -0.36676 0.6431 0.1117 0.573608218
RPP25 ENSG00000178718 1.2175 0.11171 1.2175 0.11171 1.2175 0.11171
1.2175 0.11171 0.11171 0.573608218
GOLGA2P8 ENSG00000214433 0.15861 0.43699 -0.11033 0.54393 1.2175
0.11171 0.89013 0.1867 0.11171 0.573608218
LINC01543 ENSG00000263862 0.87516 0.19074 1.2174 0.11172 0.87516
0.19074 -0.13764 0.55474 0.11172 0.573608218
STIP1 ENSG00000168439 0.1355 0.44611 1.2172 0.11177 0.1355 0.44611
-1.5318 0.93721 0.11177 0.573744032
NA ENSG00000271100 1.2172 0.11177 1.0381 0.14961 1.0998 0.13572
1.0381 0.14961 0.11177 0.573744032
SRRT ENSG00000087087 1.191 0.11683 -0.17517 0.56953 1.217 0.11179 1.217
0.11179 0.11179 0.573777146
LTN1 ENSG00000198862 0.028312 0.48871 0.028312 0.48871 0.028312 0.48871
1.217 0.1118 0.1118 0.573777146
NA ENSG00000204684 0.62529 0.26589 1.2169 0.11182 -0.32232 0.62639
0.15323 0.43911 0.11182 0.573819356
NA ENSG00000241537 -1.3824 0.91657 0.19884 0.42119 1.2167 0.11186
0.48136 0.31513 0.11186 0.57395162
ATP6V1E2 ENSG00000250565 0.26692 0.39477 1.2165 0.1119 -0.42216
0.66355 -0.42216 0.66355 0.1119 0.57395162
LINC01787 ENSG00000231987 1.2165 0.11191 0.78014 0.21765 0.66134
0.2542 1.1476 0.12556 0.11191 0.57395162
SCGB1A1 ENSG00000149021 1.2164 0.11192 0.12006 0.45222 -0.82755
0.79604 0.16947 0.43271 0.11192 0.57395162
KLHL8 ENSG00000145332 -0.56743 0.71479 0.87927 0.18963 0.87927 0.18963
1.2163 0.11193 0.11193 0.57395162
ATF3 ENSG00000162772 -0.24371 0.59627 0.95702 0.16928 -0.24371 0.59627
1.2163 0.11193 0.11193 0.57395162
NA ENSG00000265046 0.3926 0.34731 0.90667 0.18229 0.35838 0.36003
1.2164 0.11193 0.11193 0.57395162
LINC01507 ENSG00000230945 1.2163 0.11194 -0.14066 0.55593 -0.14066
0.55593 -0.14066 0.55593 0.11194 0.57395162
NA ENSG00000215302 0.93007 0.17617 1.2161 0.11197 -0.42204 0.6635
0.45457 0.32471 0.11197 0.574045039
NA ENSG00000272840 1.216 0.11199 -0.91832 0.82077 -0.23743 0.59384
1.2147 0.11224 0.11199 0.574087177
NA ENSG00000223623 1.2158 0.11202 0.20314 0.41951 -0.74084 0.77061
-0.4732 0.68196 0.11202 0.574180562
NA ENSG00000232598 -0.17965 0.57129 0.18272 0.42751 0.018506 0.49262
1.2157 0.11205 0.11205 0.574264781
IPCEF1 ENSG00000074706 1.0486 0.14719 1.2156 0.11206 0.87542
0.19067 0.31657 0.37579 0.11206 0.574264781
TTC28 ENSG00000100154 1.1284 0.12958 0.95641 0.16943 0.58933 0.27782
1.2155 0.11208 0.11208 0.574297729
GNG4 ENSG00000168243 -0.82978 0.79667 -0.82978 0.79667 0.66892 0.25177
1.2155 0.11209 0.11209 0.574297729
RAB2A ENSG00000104388 0.50957 0.30518 -0.028845 0.51151 1.179 0.1192
1.2154 0.11211 0.11211 0.574339813
WDR45B ENSG00000141580 1.2153 0.11213 1.1862 0.11777 -0.034971
0.51395 -0.42733 0.66543 0.11213 0.574381888
ZNF529-AS1 ENSG00000233527 1.2151 0.11217 1.2151 0.11217 1.2151
0.11217 1.2151 0.11217 0.11217 0.574445637
NA ENSG00000259961 1.2151 0.11217 1.2151 0.11217 1.2151 0.11217
1.2151 0.11217 0.11217 0.574445637

DNAI2	ENSG00000171595	-1.4005	0.91932	1.215	0.11219	0.54235	0.29379	0.44954	0.32652	0.11219	0.574445637
SLC9A3-AS1	ENSG00000225138	1.1799	0.11903	1.0735	0.14153	1.2148	0.11222	1.2148	0.11222	0.11222	0.574445637
NA	ENSG00000268723	1.2146	0.11225	1.2146	0.11225	1.2146	0.11225	1.2146	0.11225	0.11225	0.574445637
XAB2	ENSG00000076924	0.96511	0.16724	0.96511	0.16724	0.96511	0.16724	1.2145	0.11228	0.11228	0.574445637
NA	ENSG00000278975	1.2145	0.11228	1.2145	0.11228	1.2145	0.11228	0.65243	0.25706	0.11228	0.574445637
C17orf67	ENSG00000214226	0.37855	0.35251	-0.76997	0.77934	1.2144	0.11231	0.61156	0.27042	0.11231	0.574445637
SPC25	ENSG00000152253	1.2143	0.11232	1.2143	0.11232	1.2143	0.11232	1.2143	0.11232	0.11232	0.574445637
SCN9A	ENSG00000169432	0.39456	0.34658	-0.7744	0.78065	-0.24181	0.59554	1.2143	0.11232	0.11232	0.574445637
NA	ENSG00000257897	1.2143	0.11232	-1.4913	0.93206	-0.19081	0.57566	-0.19081	0.57566	0.11232	0.574445637
RAB24	ENSG00000169228	1.2142	0.11233	-0.0074711	0.50298	-0.8188	0.79355	-0.15242	0.56057	0.11233	0.574445637
EPHB1	ENSG00000154928	0.69453	0.24367	0.14828	0.44106	0.20972	0.41694	1.2142	0.11234	0.11234	0.574445637
NA	ENSG00000224981	1.2142	0.11234	0.20503	0.41877	-0.37534	0.6463	-0.37534	0.6463	0.11234	0.574445637
RNU6-679P	ENSG00000212305	-0.29353	0.61544	1.214	0.11237	1.214	0.11237	1.214	0.11237	0.11237	0.574445637
LURAP1	ENSG00000171357	1.214	0.11238	0.031988	0.48724	0.031988	0.48724	0.031988	0.48724	0.11238	0.574445637
NA	ENSG00000185296	1.214	0.11238	0.82766	0.20393	0.82766	0.20393	0.60859	0.2714	0.11238	0.574445637
UBA52P7	ENSG00000241656	1.214	0.11238	0.6748	0.2499	-1.0441	0.85178	0.29259	0.38492	0.11238	0.574445637
NA	ENSG00000263840	0.6143	0.26951	1.214	0.11238	1.1835	0.1183	1.0188	0.15414	0.11238	0.574445637
PCDHA2	ENSG00000204969	0.74602	0.22783	0.71618	0.23694	1.2139	0.11239	0.060525	0.47587	0.11239	0.574445637
ZNF503-AS1	ENSG00000226051	0.64101	0.26076	0.35882	0.35986	1.2139	0.11239	1.1451	0.12609	0.11239	0.574445637
CCDC63	ENSG00000173093	0.67282	0.25053	1.2137	0.11243	0.88671	0.18762	0.2443	0.4035	0.11243	0.574589816
NA	ENSG00000227803	-0.41277	0.66011	0.41745	0.33817	-0.013472	0.50537	1.2135	0.11247	0.11247	0.574637072
NA	ENSG00000235371	1.1054	0.13449	1.2135	0.11247	1.0359	0.15012	0.69257	0.24429	0.11247	0.574637072
NDUFAF3	ENSG00000178057	1.2133	0.1125	0.084434	0.46636	0.83022	0.20321	0.63238	0.26357	0.1125	0.574637072
NA	ENSG00000267617	-0.042651	0.51701	-0.050854	0.52028	1.2133	0.1125	0.7246	0.23435	0.1125	0.574637072
ZNF300P1	ENSG00000197083	0.64087	0.2608	0.82568	0.20449	0.085167	0.46606	1.2133	0.11251	0.11251	0.574637072
NA	ENSG00000271390	1.1909	0.11684	1.2133	0.11251	0.48022	0.31554	1.1909	0.11684	0.11251	0.574637072
TMEM97P2	ENSG00000253866	1.0063	0.15713	0.082369	0.46718	0.92064	0.17862	1.2132	0.11253	0.11253	0.574669831
NA	ENSG00000274800	1.2131	0.11254	0.65596	0.25593	0.87134	0.19178	1.0741	0.14139	0.11254	0.574669831
NA	ENSG00000269933	0.020753	0.49172	-0.61666	0.73127	1.2129	0.11258	1.2129	0.11258	0.11258	0.574804683
OR2A1-AS1	ENSG00000244479	-0.012946	0.50516	-1.2442	0.89328	-0.983	0.8372	1.2129	0.11259	0.11259	0.574804683
NA	ENSG00000256733	1.2128	0.11261	1.2128	0.11261	1.2128	0.11261	-0.28718	0.61301	0.11261	0.574846564

NA	ENSG00000228436	1.2126	0.11263	1.2126	0.11263	1.2126	0.11263	1.2126	0.11263	0.11263	0.574888436
IQGAP3	ENSG00000183856	0.14217	0.44347	0.60629	0.27216	1.2124	0.11268	0.065139	0.47403	0.11268	0.574962974
MIR422A	ENSG00000199156	0.19617	0.42224	0.19617	0.42224	1.2124	0.11268	1.2124	0.11268	0.11268	0.574962974
NA	ENSG00000278022	1.2124	0.11268	1.2124	0.11268	-1.2506	0.89447	-0.31373	0.62314	0.11268	0.574962974
RNU6-625P	ENSG00000238658	1.2123	0.1127	1.2123	0.1127	1.2123	0.1127	1.2123	0.1127	0.1127	0.574991567
NA	ENSG00000234041	1.2123	0.11271	1.2123	0.11271	1.2123	0.11271	1.2123	0.11271	0.11271	0.574991567
GCSHP2	ENSG00000259525	1.2121	0.11273	0.081752	0.46742	0.26911	0.39392	0.26911	0.39392	0.11273	0.574991567
EIF4A1P11	ENSG00000231564	1.212	0.11275	1.212	0.11275	1.212	0.11275	1.212	0.11275	0.11275	0.574991567
NA	ENSG00000236847	1.212	0.11275	1.212	0.11275	1.212	0.11275	1.212	0.11275	0.11275	0.574991567
NA	ENSG00000272576	1.212	0.11276	1.212	0.11276	1.212	0.11276	1.212	0.11276	0.11276	0.574991567
NA	ENSG00000261555	1.2119	0.11278	1.2119	0.11278	1.2119	0.11278	1.2119	0.11278	0.11278	0.574991567
NA	ENSG00000221347	0.35375	0.36176	-1.5175	0.93543	0.35375	0.36176	1.2119	0.11278	0.11278	0.574991567
HOXB5	ENSG00000120075	0.78626	0.21586	0.8135	0.20796	0.61989	0.26766	1.2118	0.1128	0.1128	0.575005846
SERPINA2	ENSG00000258597	1.2117	0.11282	1.2117	0.11282	1.2117	0.11282	1.2117	0.11282	0.11282	0.575005846
UNCX	ENSG00000164853	0.34989	0.36321	0.23258	0.40804	1.2116	0.11283	0.17711	0.42971	0.11283	0.575005846
NSUN7	ENSG00000179299	-0.96307	0.83224	-0.75013	0.77341	-1.6239	0.9478	1.2116	0.11283	0.11283	0.575005846
ADAMTS17	ENSG00000140470	1.0532	0.14612	-1.3083	0.90461	-0.5861	0.7211	1.2114	0.11286	0.11286	0.575029284
ITGB5-AS1	ENSG00000244286	0.80701	0.20983	0.80701	0.20983	1.1148	0.13247	1.2115	0.11286	0.11286	0.575029284
NA	ENSG00000240995	-1.4126	0.92111	-1.4126	0.92111	-1.1561	0.87618	1.2114	0.11287	0.11287	0.575029284
NA	ENSG00000214955	1.2112	0.11291	0.86158	0.19446	0.86341	0.19396	-1.0325	0.84907	0.11291	0.575112815
NA	ENSG00000229943	1.2112	0.11291	0.93494	0.17491	0.93494	0.17491	0.93494	0.17491	0.11291	0.575112815
RGL3	ENSG00000205517	-0.82313	0.79478	0.24379	0.4037	1.2111	0.11293	0.3846	0.35027	0.11293	0.575122888
ASXL3	ENSG00000141431	0.91278	0.18068	1.2108	0.11298	0.87936	0.1896	1.0474	0.14746	0.11298	0.575122888
NA	ENSG00000200024	1.0914	0.13755	0.9449	0.17235	1.0914	0.13755	1.2108	0.11299	0.11299	0.575122888
NA	ENSG00000257354	0.63501	0.26271	-0.14053	0.55588	1.2108	0.11299	0.96583	0.16706	0.11299	0.575122888
NA	ENSG00000212072	-0.13764	0.55474	1.2108	0.11299	-0.030998	0.51236	-0.36235	0.64145	0.11299	0.575122888
CLDN7	ENSG00000181885	1.2107	0.113	1.2107	0.113	1.2107	0.113	1.2107	0.113	0.113	0.575122888
PPP2R5D	ENSG00000112640	1.2107	0.11301	0.75339	0.22561	1.0007	0.15848	0.73538	0.23105	0.11301	0.575122888
LINC01811	ENSG00000226320	0.41519	0.339	0.91192	0.1809	1.2107	0.11301	-1.8149	0.96523	0.11301	0.575122888
C14orf178	ENSG00000197734	1.2106	0.11302	1.2106	0.11302	1.2106	0.11302	1.2106	0.11302	0.11302	0.575122888
NA	ENSG00000251168	0.21978	0.41302	0.56753	0.28518	1.2106	0.11303	0.21232	0.41593	0.11303	0.575122888

NA	ENSG00000274614 1.2104	0.11306	-0.66348	0.74649	-0.66348	0.74649
-0.66348	0.74649	0.11306	0.575196971
CRIP1P3	ENSG00000227109 1.2102	0.11309	0.23413	0.40744	0.19491
0.42273	0.94523	0.17227	0.11309	0.575196971
NDUFB8P1	ENSG00000258972 -0.050957 0.52032	1.2101	0.11311	-0.050957
0.52032	-0.050957 0.52032	0.11311	0.575196971
RNA5SP496	ENSG00000212542 1.21 0.11314	1.21 0.11314	1.21 0.11314	-0.13797
0.55487	0.11314	0.575196971
NA	ENSG00000253666 1.21 0.11315	1.1386	0.12744	0.38209	0.3512
1.1494	0.1252	0.11315	0.575196971
SUCO	ENSG00000094975 -0.13112	0.55216	1.2098	0.11317	0.20822	0.41753
0.67296	0.25049	0.11317	0.575196971
ITGB3	ENSG00000259207 0.77172	0.22014	0.71471	0.23739	1.2098	0.11317
0.9068	0.18226	0.11317	0.575196971
PPP1R14C	ENSG00000198729 -0.47682	0.68326	1.2097	0.1132	-0.45394
0.67507	0.073456	0.47072	0.1132	0.575196971
RNU6-1069P ENSG00000252421 1.2095	0.11324	1.2095	0.11324	1.2095
0.11324	-0.6941	0.75619	0.11324	0.575196971
NA	ENSG00000227304 1.2093	0.11327	1.2093	0.11327	1.2093	0.11327
1.2093	0.11327	0.11327	0.575196971
PGLS	ENSG00000130313 1.2092	0.1133	1.2092	0.1133	-0.07192	0.52867
-0.27834	0.60962	0.1133	0.575196971
SLC25A30	ENSG00000174032 1.2092	0.1133	1.2092	0.1133	-0.16862
0.56695	-0.1744	0.56922	0.1133	0.575196971
ARL14EP	ENSG00000152219 1.1142	0.13259	1.2051	0.11409	1.2089
0.11334	0.26507	0.39548	0.11334	0.575196971
LINC02406	ENSG00000258144 1.209 0.11334	1.209 0.11334	-0.61152	0.72957
-0.81577	0.79268	0.11334	0.575196971
ABHD13	ENSG00000139826 0.19652	0.4221	1.0965	0.13644	-0.46946
0.68063	1.2089	0.11335	0.11335	0.575196971
RNU6-1260P ENSG00000199814 1.2089	0.11335	0.2159	0.41453	1.2089
0.11335	1.2089	0.11335	0.11335	0.575196971
ACMSD	ENSG00000153086 0.079107	0.46847	0.78218	0.21706	1.2088	0.11336
0.38017	0.35191	0.11336	0.575196971
MTCYBP42	ENSG00000228803 0.16404	0.43485	0.17456	0.43071	1.2088
0.11337	-0.60261	0.72662	0.11337	0.575196971
SPATC1L	ENSG00000160284 1.0046	0.15753	1.2087	0.11339	1.1169
0.13201	0.57589	0.28235	0.11339	0.575196971
RPL21P10	ENSG00000239272 1.2087	0.11339	-0.14 0.55567	-0.14 0.55567	-0.14
0.55567	0.11339	0.575196971
BMP5	ENSG00000112175 0.11729	0.45331	1.2087	0.1134	1.1277	0.12973
-1.1879	0.88257	0.1134	0.575196971
NA	ENSG00000227107 -0.55257	0.70972	-1.0585	0.85509	-1.0337	0.84937
1.2086	0.1134	0.1134	0.575196971
FAM126A	ENSG00000122591 1.1542	0.12422	1.2085	0.11343	0.61942
0.26782	0.43045	0.33343	0.11343	0.575196971
MIR151B	ENSG00000265154 1.2085	0.11343	1.2085	0.11343	1.2085
0.11343	1.2085	0.11343	0.11343	0.575196971
NA	ENSG00000268618 1.2085	0.11343	1.2085	0.11343	1.2085	0.11343
1.2085	0.11343	0.11343	0.575196971
DNAJC14	ENSG00000135392 1.0809	0.13987	1.2084	0.11344	1.0809
0.13987	-0.59057	0.7226	0.11344	0.575196971
NA	ENSG00000234811 -0.016732	0.50667	-0.016732	0.50667	-1.3173	0.90613
1.2084	0.11344	0.11344	0.575196971
SPCS3-AS1	ENSG00000248980 -1.011	0.84399	-0.068256	0.52721	0.32356
0.37313	1.2084	0.11344	0.11344	0.575196971
MIR124-2HG ENSG00000254377 1.2084	0.11344	1.0186	0.15419	0.16432
0.43474	0.16432	0.43474	0.11344	0.575196971
NA	ENSG00000256473 0.39448	0.34661	-0.020053	0.508 1.2084	0.11344
1.2084	0.11344	0.11344	0.575196971
NA	ENSG00000232311 1.2085	0.11344	1.2085	0.11344	1.2085	0.11344
1.2085	0.11344	0.11344	0.575196971

NA	ENSG00000264856	1.2085	0.11344	1.2085	0.11344	1.2085	0.11344	1.2085	0.11344	0.11344	0.575196971
CALCOCO1	ENSG00000012822	-0.94706	0.8282	1.2083	0.11347	0.40469	0.34285	0.63578	0.26246	0.11347	0.575196971
BNIP3P11	ENSG00000271550	1.2083	0.11347	0.144	0.44275	1.2083	0.11347	1.2083	0.11347	0.11347	0.575196971
ST8SIA1	ENSG00000111728	1.1133	0.1328	1.0902	0.13781	1.1598	0.12307	1.2082	0.11348	0.11348	0.575196971
RPL6P21	ENSG00000223445	-1.5963	0.94479	1.2082	0.11348	-1.5963	0.94479	-1.5963	0.94479	0.11348	0.575196971
NA	ENSG00000215156	0.91991	0.17881	-0.60174	0.72633	0.021161	0.49156	1.2081	0.1135	0.1135	0.575196971
NA	ENSG00000273369	1.2081	0.11351	1.2081	0.11351	1.2081	0.11351	1.2081	0.11351	0.11351	0.575196971
ZDHHC19	ENSG00000163958	0.82113	0.20579	0.89688	0.18489	-0.4142	0.66064	1.208	0.11352	0.11352	0.575196971
PEX11A	ENSG00000166821	1.208	0.11353	1.208	0.11353	1.208	0.11353	1.208	0.11353	0.11353	0.575196971
CASTOR3	ENSG00000239521	0.75393	0.22544	1.2078	0.11356	-0.59139	0.72287	-0.10746	0.54279	0.11356	0.575196971
NA	ENSG00000201208	1.2077	0.11357	1.2077	0.11357	1.2077	0.11357	1.2077	0.11357	0.11357	0.575196971
NA	ENSG00000279672	0.85178	0.19717	1.2078	0.11357	0.99654	0.15949	0.99654	0.15949	0.11357	0.575196971
SPTLC1P3	ENSG00000219940	-0.30554	0.62002	1.2077	0.11358	1.2077	0.11358	1.2077	0.11358	0.11358	0.575196971
POLHP1	ENSG00000232970	0.80093	0.21159	1.2077	0.11358	1.2077	0.11358	1.2077	0.11358	0.11358	0.575196971
NA	ENSG00000256973	1.2076	0.1136	1.2076	0.1136	1.2076	0.1136	1.2076	0.1136	0.1136	0.575196971
CDKN2AIPNLP3	ENSG00000224351	0.81542	0.20742	-1.4241	0.9228	-0.30847	0.62114	1.2075	0.11363	0.11363	0.575196971
TPMTP1	ENSG00000267156	1.2074	0.11364	1.2074	0.11364	1.2074	0.11364	1.2074	0.11364	0.11364	0.575196971
COASY	ENSG00000068120	1.2073	0.11366	1.2073	0.11366	-0.78713	0.7844	-0.20186	0.57999	0.11366	0.575196971
SOHLH1	ENSG00000165643	-0.70228	0.75875	-0.097421	0.5388	1.2073	0.11366	-0.32145	0.62606	0.11366	0.575196971
NA	ENSG00000200884	1.207	0.11371	1.207	0.11371	1.207	0.11371	1.207	0.11371	0.11371	0.575196971
MYO19	ENSG00000278259	-0.26418	0.60418	-0.34335	0.63433	1.207	0.11372	0.71542	0.23718	0.11372	0.575196971
NA	ENSG00000253281	1.0301	0.15148	1.2069	0.11373	1.2069	0.11373	1.2069	0.11373	0.11373	0.575196971
NA	ENSG00000254973	1.2069	0.11373	1.2069	0.11373	1.2069	0.11373	0.54802	0.29184	0.11373	0.575196971
NDUFA4P2	ENSG00000236691	0.17196	0.43173	-0.045166	0.51801	1.2068	0.11376	1.2068	0.11376	0.11376	0.575196971
NA	ENSG00000255037	0.15085	0.44005	1.2067	0.11377	0.49159	0.3115	1.1634	0.12234	0.11377	0.575196971
POMZP3	ENSG00000146707	0.76113	0.22329	-0.52037	0.6986	-0.044504	0.51775	1.2067	0.11378	0.11378	0.575196971
PDLIM1	ENSG00000107438	-1.6944	0.9549	1.2066	0.11379	0.18171	0.4279	-0.64827	0.7416	0.11379	0.575196971
SLC25A11	ENSG00000108528	1.2066	0.11379	1.2066	0.11379	1.2066	0.11379	1.2066	0.11379	0.11379	0.575196971
OR6J1	ENSG00000255804	1.1303	0.12917	1.2066	0.11379	1.0577	0.1451	1.1373	0.1277	0.11379	0.575196971
NA	ENSG00000260290	1.0033	0.15787	0.72774	0.23339	1.2066	0.11379	1.2066	0.11379	0.11379	0.575196971
FKBP14	ENSG00000106080	-1.0776	0.85939	-1.0776	0.85939	1.2066	0.1138	-0.65035	0.74227	0.1138	0.575196971

NA	ENSG00000223360	0.50239	0.3077	1.2059	0.11393	0.25232	0.4004	1.2066	0.1138	0.1138	0.575196971
NA	ENSG00000271955	0.19007	0.42463	-0.24624	0.59725	1.2066	0.1138	1.2066	0.1138	0.1138	0.575196971
LINC02749	ENSG00000254480	0.3037	0.38068	0.70071	0.24174	0.69203	0.24446	1.2064	0.11383	0.11383	0.575288934
NA	ENSG00000230325	1.2062	0.11386	1.2062	0.11386	1.2062	0.11386	1.2062	0.11386	0.11386	0.575371744
NA	ENSG00000282432	1.2062	0.11387	1.2062	0.11387	1.2062	0.11387	1.2062	0.11387	0.11387	0.575371744
LGALS3BP	ENSG00000108679	-0.84656	0.80138	-0.86727	0.8071	-0.84656	0.80138	1.206	0.11391	0.11391	0.57545452
ANKRD18A	ENSG00000180071	0.5017	0.30794	1.1122	0.13304	0.12264	0.45119	1.206	0.11391	0.11391	0.57545452
IL16	ENSG00000172349	1.2057	0.11397	1.1335	0.12849	1.186	0.1178	0.89276	0.18599	0.11397	0.575688785
MTFR2P1	ENSG00000233716	1.2056	0.11398	1.2056	0.11398	1.2056	0.11398	1.2056	0.11398	0.11398	0.575688785
NA	ENSG00000245156	1.2055	0.114	1.2055	0.114	1.2055	0.114	1.2055	0.114	0.114	0.575726239
SNORD12B	ENSG00000222365	1.2054	0.11403	1.2054	0.11403	1.2054	0.11403	1.2054	0.11403	0.11403	0.575726239
NA	ENSG00000279650	1.2053	0.11404	0.48106	0.31524	1.2053	0.11404	-0.23881	0.59437	0.11404	0.575726239
PRDM8	ENSG00000152784	-0.56729	0.71474	-0.71441	0.76251	1.2052	0.11406	0.74366	0.22854	0.11406	0.575726239
SLC7A6	ENSG00000103064	1.2051	0.11408	0.92108	0.1785	0.19259	0.42364	-0.021576	0.50861	0.11408	0.575726239
RNU6-783P	ENSG00000252519	0.62915	0.26462	0.9738	0.16508	1.205	0.11409	1.205	0.11409	0.11409	0.575726239
MIR204	ENSG00000207935	1.205	0.1141	1.205	0.1141	1.205	0.1141	1.205	0.1141	0.1141	0.575726239
TRIM3	ENSG00000110171	1.1193	0.13151	-0.077447	0.53087	1.1193	0.13151	1.205	0.11411	0.11411	0.575726239
ZNF354A	ENSG00000169131	-0.21785	0.58623	1.2049	0.11412	-0.21652	0.58571	-0.82347	0.79488	0.11412	0.575726239
EIF3F	ENSG00000175390	0.93027	0.17612	1.0711	0.14206	1.2049	0.11413	1.1735	0.12031	0.11413	0.575726239
NA	ENSG00000270470	1.2048	0.11414	1.2048	0.11414	1.2048	0.11414	0.61004	0.27092	0.11414	0.575726239
NUP210P2	ENSG00000225275	-0.3823	0.64888	0.25666	0.39872	-0.92474	0.82245	1.2046	0.11417	0.11417	0.575726239
NA	ENSG00000260763	0.794	0.2136	1.2047	0.11417	1.0182	0.15428	-0.82683	0.79583	0.11417	0.575726239
RNU6-669P	ENSG00000202445	-0.31223	0.62257	1.0456	0.14788	1.0456	0.14788	1.2045	0.11419	0.11419	0.575726239
KLHL10	ENSG00000161594	-0.95551	0.83034	-0.95551	0.83034	-0.95551	0.83034	1.2045	0.11421	0.11421	0.575726239
CHRNA7	ENSG00000175344	-0.58493	0.7207	0.53023	0.29797	0.14529	0.44224	1.2044	0.11421	0.11421	0.575726239
TOMM22P4	ENSG00000248396	-0.0079974	0.50319	0.15577	0.43811	-0.10546	0.54199	1.2045	0.11421	0.11421	0.575726239
RNA5SP430	ENSG00000199299	1.2043	0.11424	1.2043	0.11424	1.2043	0.11424	1.2043	0.11424	0.11424	0.575726239
NA	ENSG00000261313	0.44573	0.32789	0.69003	0.24509	1.1524	0.12458	1.2043	0.11424	0.11424	0.575726239
INO80E	ENSG00000169592	1.2042	0.11425	1.2042	0.11425	1.2042	0.11425	1.2042	0.11425	0.11425	0.575726239
OR7E110P	ENSG00000235623	0.39241	0.34738	0.9384	0.17402	1.1157	0.13228	1.2042	0.11425	0.11425	0.575726239
NPR3	ENSG00000113389	1.0648	0.14348	1.2041	0.11428	0.48162	0.31504	0.72678	0.23368	0.11428	0.575726239

NA	ENSG00000279019	1.2041	0.11428	1.2041	0.11428	1.2041	0.11428	1.2041	0.11428	0.11428	0.575726239
LINC01487	ENSG00000241336	1.204	0.1143	1.204	0.1143	1.204	0.1143	1.1574	0.12355	0.1143	0.575726239
TRAPPC12-AS1	ENSG00000225234	1.2039	0.11432	1.2039	0.11432	1.2039	0.11432	1.2039	0.11432	0.11432	0.575726239
NA	ENSG00000261187	0.86546	0.19339	1.2036	0.11437	1.2036	0.11437	1.2036	0.11437	0.11437	0.575726239
NA	ENSG00000243687	0.72075	0.23553	0.38868	0.34876	1.2035	0.11439	0.59132	0.27715	0.11439	0.575726239
LINC01499	ENSG00000255171	1.2035	0.11439	-0.66156	0.74587	-1.3625	0.91348	-0.70015	0.75808	0.11439	0.575726239
NA	ENSG00000277595	0.49756	0.3094	1.2035	0.11439	0.70687	0.23982	0.70687	0.23982	0.11439	0.575726239
TANGO6	ENSG00000103047	0.39835	0.34519	-1.1888	0.88274	-0.24624	0.59725	1.2035	0.1144	0.1144	0.575726239
GLRA1	ENSG00000145888	0.18445	0.42683	0.97997	0.16355	1.2035	0.1144	1.1266	0.12995	0.1144	0.575726239
NA	ENSG00000256802	0.81313	0.20807	0.48483	0.3139	0.81313	0.20807	1.2034	0.1144	0.1144	0.575726239
NA	ENSG00000280283	1.2035	0.1144	1.2035	0.1144	1.2035	0.1144	1.0239	0.15295	0.1144	0.575726239
FAM71BP1	ENSG00000270683	0.34461	0.3652	1.2033	0.11442	-1.0006	0.8415	-1.0006	0.8415	0.11442	0.575726239
GJD2	ENSG00000159248	1.2033	0.11443	1.2033	0.11443	1.2033	0.11443	1.2033	0.11443	0.11443	0.575726239
NA	ENSG00000249976	-0.61486	0.73068	-0.57186	0.71629	-1.336	0.90922	1.2033	0.11443	0.11443	0.575726239
A2M-AS1	ENSG00000245105	0.10992	0.45624	1.2032	0.11444	1.2032	0.11444	1.2032	0.11444	0.11444	0.575726239
NA	ENSG00000256987	1.2033	0.11444	1.2033	0.11444	1.2033	0.11444	1.2033	0.11444	0.11444	0.575726239
NA	ENSG00000272097	1.2031	0.11446	1.2031	0.11446	1.2031	0.11446	1.2031	0.11446	0.11446	0.575726239
NA	ENSG00000272162	1.2031	0.11446	1.2031	0.11446	1.2031	0.11446	1.2031	0.11446	0.11446	0.575726239
DCUN1D2-AS	ENSG00000233613	0.27984	0.3898	-0.24677	0.59746	1.2029	0.11451	1.2029	0.11451	0.11451	0.575918289
NA	ENSG00000266549	1.2027	0.11455	1.2027	0.11455	1.2027	0.11455	1.2027	0.11455	0.11455	0.576032539
LASP1	ENSG00000002834	-0.92181	0.82169	-0.93153	0.82421	1.0065	0.15709	1.2026	0.11456	0.11456	0.576032539
NA	ENSG00000241597	1.2026	0.11457	1.2026	0.11457	0.85198	0.19711	1.2026	0.11457	0.11457	0.576032539
BLOC1S3	ENSG00000189114	1.2025	0.11458	1.2025	0.11458	1.2025	0.11458	1.2025	0.11458	0.11458	0.576032539
NA	ENSG00000258294	-0.31637	0.62414	1.2024	0.1146	0.26436	0.39575	-0.31195	0.62246	0.1146	0.576073654
NA	ENSG00000230972	1.2023	0.11463	0.52869	0.29851	0.52869	0.29851	0.52869	0.29851	0.11463	0.576087286
NA	ENSG00000228005	1.2022	0.11464	1.2022	0.11464	1.2022	0.11464	1.2022	0.11464	0.11464	0.576087286
NA	ENSG00000276564	1.0804	0.13999	1.2022	0.11464	1.2022	0.11464	0.5643	0.28628	0.11464	0.576087286
LINC00698	ENSG00000244342	0.48112	0.31522	-1.1085	0.86618	1.2022	0.11465	-0.62381	0.73362	0.11465	0.576087286
NA	ENSG00000201009	1.2021	0.11467	1.2021	0.11467	1.2021	0.11467	1.2021	0.11467	0.11467	0.576128374
GET1	ENSG00000182093	-0.019746	0.50788	-0.019746	0.50788	-0.044509	0.51775	1.2018	0.11473	0.11473	0.576310989
NA	ENSG00000271234	1.2018	0.11473	1.2018	0.11473	1.2018	0.11473	1.2018	0.11473	0.11473	0.576310989

NA	ENSG00000234710	1.2016	0.11476	-0.46856	0.68031	1.2016	0.11476	1.2016	0.11476	0.11476	0.576342863
NA	ENSG00000280486	1.2016	0.11476	1.2016	0.11476	1.2016	0.11476	1.2016	0.11476	0.11476	0.576342863
WNT8B	ENSG00000075290	-0.068189	0.52718	0.25404	0.39973	1.2015	0.11479	0.65587	0.25595	0.11479	0.576374724
CEP57L1P1	ENSG00000224725	1.2014	0.11479	1.2014	0.11479	-0.26126	0.60306	0.48503	0.31383	0.11479	0.576374724
SLC6A21P	ENSG00000269009	1.2013	0.11482	1.2013	0.11482	1.2013	0.11482	1.2013	0.11482	0.11482	0.576379042
RBAKDN	ENSG00000273313	1.2012	0.11483	1.2012	0.11483	1.2012	0.11483	1.2012	0.11483	0.11483	0.576379042
DBIP2	ENSG00000251366	-0.94158	0.8268	1.2012	0.11484	-0.94158	0.8268	-0.94158	0.8268	0.11484	0.576379042
NA	ENSG00000261696	1.2012	0.11484	1.2012	0.11484	1.2012	0.11484	1.2012	0.11484	0.11484	0.576379042
NA	ENSG00000213851	-0.9265	0.82291	1.2011	0.11485	0.53087	0.29776	-2.4209	0.99226	0.11485	0.576379042
PLOD1	ENSG00000083444	0.43016	0.33354	-0.066779	0.52662	1.2008	0.11491	0.21941	0.41317	0.11491	0.576415169
MTND5P15	ENSG00000231888	-2.1165	0.98285	1.2007	0.11493	-2.1165	0.98285	-2.1165	0.98285	0.11493	0.576415169
NA	ENSG00000261035	0.62362	0.26644	1.2007	0.11493	0.048492	0.48066	0.048492	0.48066	0.11493	0.576415169
NA	ENSG00000269547	1.2007	0.11493	1.2007	0.11493	1.2007	0.11493	1.2007	0.11493	0.11493	0.576415169
CLASP2	ENSG00000163539	1.1739	0.12023	0.59159	0.27706	1.2007	0.11494	0.94745	0.17171	0.11494	0.576415169
GAST	ENSG00000184502	1.2007	0.11494	1.2007	0.11494	1.1143	0.13258	1.2007	0.11494	0.11494	0.576415169
SACM1L	ENSG00000211456	-0.30675	0.62048	1.2007	0.11494	1.0976	0.13618	-0.47499	0.6826	0.11494	0.576415169
RPL39P5	ENSG00000214289	0.38886	0.34869	1.2006	0.11496	0.38886	0.34869	0.38886	0.34869	0.11496	0.576419461
NA	ENSG00000261751	0.4218	0.33658	0.11548	0.45403	0.3717	0.35506	1.2005	0.11497	0.11497	0.576419461
FAM171A1	ENSG00000148468	1.2004	0.11499	0.26232	0.39654	-0.54846	0.70831	-0.099471	0.53962	0.11499	0.576419461
C11orf95	ENSG00000188070	1.2004	0.11499	1.2004	0.11499	0.85851	0.19531	-0.24219	0.59568	0.11499	0.576419461
TIGD4	ENSG00000169989	1.2003	0.115	1.2003	0.115	1.2003	0.115	1.2003	0.115	0.115	0.576419461
MIR7-3	ENSG00000207630	1.2002	0.11503	1.2002	0.11503	1.2002	0.11503	1.2002	0.11503	0.11503	0.576500678
LIM2	ENSG00000105370	1.2	0.11506	1.2	0.11506	1.2	0.11506	0.19724	0.42182	0.11506	0.576500678
VPS9D1	ENSG00000075399	0.85293	0.19685	0.98457	0.16242	1.2	0.11507	1.1132	0.13281	0.11507	0.576500678
NA	ENSG00000273443	1.2	0.11507	1.2	0.11507	1.2	0.11507	1.2	0.11507	0.11507	0.576500678
EXOSC9	ENSG00000123737	1.0508	0.14668	1.1093	0.13366	0.35721	0.36047	1.1999	0.11509	0.11509	0.576500678
UPK3BP1	ENSG00000242435	1.1998	0.1151	1.1998	0.1151	1.1998	0.1151	1.1998	0.1151	0.1151	0.576500678
TOP1MT	ENSG00000184428	0.5832	0.27988	-0.61439	0.73052	1.1998	0.11511	0.40642	0.34222	0.11511	0.576500678
LRRIQ1	ENSG00000133640	-0.69354	0.75601	-0.48233	0.68521	1.1997	0.11512	0.61826	0.2682	0.11512	0.576500678
LINC00400	ENSG00000229928	-0.72282	0.7651	1.1997	0.11513	1.1997	0.11513	-0.1663	0.56604	0.11513	0.576500678
C16orf95	ENSG00000260456	0.17583	0.43021	-0.61685	0.73133	-1.7121	0.95656	1.1997	0.11514	0.11514	0.576500678

GDI2P2	ENSG00000233994	1.1538	0.12429	1.1996	0.11515	1.1996	0.11515	1.1996	0.11515	0.11515	0.576500678
OLAH	ENSG00000152463	1.1995	0.11516	-0.26169	0.60322	0.014045	0.4944	-0.13848	0.55507	0.11516	0.576500678
RNF185	ENSG00000138942	1.1995	0.11517	1.1995	0.11517	-0.74486	0.77182	0.075113	0.47006	0.11517	0.576500678
BCO1	ENSG00000135697	-1.4888	0.93173	1.1994	0.1152	1.1994	0.1152	-0.146	0.55804	0.1152	0.576554949
NA	ENSG00000254171	-0.19948	0.57905	1.1993	0.11521	0.59613	0.27554	-0.83693	0.79868	0.11521	0.576554949
MLLT10	ENSG00000078403	0.96196	0.16804	1.1991	0.11524	0.026054	0.48961	0.79058	0.21459	0.11524	0.576554949
AKAP8P1	ENSG00000234146	-0.81683	0.79299	-1.2198	0.88873	0.76675	0.22161	1.1991	0.11524	0.11524	0.576554949
JAZF1-AS1	ENSG00000234336	-0.50458	0.69307	-0.54917	0.70856	1.1072	0.1341	1.1991	0.11524	0.11524	0.576554949
NA	ENSG00000267988	1.1371	0.12774	1.1989	0.11529	1.1371	0.12774	1.1371	0.12774	0.11529	0.576677512
C17orf58	ENSG00000186665	1.1988	0.1153	0.32308	0.37332	0.32308	0.37332	0.32308	0.37332	0.1153	0.576677512
NA	ENSG00000260393	1.1988	0.1153	1.1988	0.1153	1.1988	0.1153	1.1988	0.1153	0.1153	0.576677512
NA	ENSG00000266473	1.1986	0.11533	1.1986	0.11533	1.1986	0.11533	1.1986	0.11533	0.11533	0.57676836
VPS33A	ENSG00000139719	-0.31059	0.62194	0.50117	0.30813	1.1985	0.11536	0.38607	0.34972	0.11536	0.576859189
SFTPC	ENSG00000168484	0.83487	0.2019	1.1983	0.1154	1.1983	0.1154	1.1983	0.1154	0.1154	0.577
RNU7-143P	ENSG00000252590	1.1982	0.11542	1.1982	0.11542	0.8383	0.20093	-0.9785	0.83609	0.11542	0.57701715
RPS4XP19	ENSG00000242058	1.1981	0.11543	-0.064338	0.52565	0.44065	0.32973	0.15982	0.43651	0.11543	0.57701715
NA	ENSG00000279806	1.198	0.11545	0.51356	0.30378	1.198	0.11545	1.198	0.11545	0.11545	0.57701715
PDE8A	ENSG00000073417	-0.8471	0.80153	1.1979	0.11548	-0.18464	0.57324	-0.89386	0.8143	0.11548	0.57701715
LEPR	ENSG00000116678	0.84965	0.19776	-0.46507	0.67906	0.8296	0.20338	1.1979	0.11548	0.11548	0.57701715
CRNKL1	ENSG00000101343	1.0164	0.15473	1.1978	0.11549	1.0736	0.14151	0.99951	0.15877	0.11549	0.57701715
NA	ENSG00000259989	-1.5093	0.93439	0.45217	0.32557	-0.059956	0.5239	1.1978	0.1155	0.1155	0.57701715
NA	ENSG00000230331	1.1977	0.11551	-0.14172	0.55635	-0.14172	0.55635	0.054323	0.47834	0.11551	0.57701715
NA	ENSG00000259356	1.1977	0.11551	1.1977	0.11551	1.1977	0.11551	0.4315	0.33305	0.11551	0.57701715
IKZF1	ENSG00000185811	1.1975	0.11555	0.36297	0.35832	1.1975	0.11555	0.65441	0.25642	0.11555	0.577148591
NA	ENSG00000269761	-1.4803	0.9306	1.1975	0.11556	0.83589	0.20161	0.83589	0.20161	0.11556	0.577148591
NA	ENSG00000201026	-1.087	0.86149	1.1816	0.11868	-0.47296	0.68188	1.1974	0.11558	0.11558	0.577189322
MCM7	ENSG00000166508	0.66028	0.25454	0.9442	0.17253	0.66028	0.25454	1.1973	0.1156	0.1156	0.577211618
NEUROG3	ENSG00000122859	1.1972	0.11561	-0.29916	0.61759	1.1972	0.11561	1.1972	0.11561	0.11561	0.577211618
TCEA1P4	ENSG00000236184	1.1972	0.11562	0.79684	0.21277	1.1972	0.11562	1.1972	0.11562	0.11562	0.577211618
NA	ENSG00000254568	1.197	0.11566	1.197	0.11566	1.197	0.11566	1.197	0.11566	0.11566	0.57730829
SERGEF	ENSG00000129158	0.76216	0.22298	1.1969	0.11568	0.17486	0.43059	-1.5187	0.93559	0.11568	0.57730829

NA	ENSG00000272986 1.1968	0.11569	1.0814	0.13975	1.1968	0.11569
	1.1968	0.11569	0.11569	0.57730829
NA	ENSG00000248373 0.23784	0.406 -0.7719	0.77991	0.74323	0.22867
	1.1967	0.11571	0.11571	0.57730829
NA	ENSG00000259889 -0.054532	0.52174	-1.9888	0.97664	-0.62304	0.73337
	1.1966	0.11573	0.11573	0.57730829
NA	ENSG00000230033 1.1965	0.11576	0.98818	0.16153	1.131 0.12904
	-0.097205	0.53872	0.11576	0.57730829
IL4R	ENSG00000077238 -1.2792	0.89959	0.70929	0.23907	-1.2792	0.89959
	1.1963	0.11578	0.11578	0.57730829
NA	ENSG00000200976 1.1964	0.11578	1.1964	0.11578	1.1964	0.11578
	0.87592	0.19054	0.11578	0.57730829
MIR299	ENSG00000207749 0.54689	0.29223	1.1964	0.11578	1.1964
	0.11578	1.1964	0.11578	0.11578	0.57730829
RNU6-768P	ENSG00000207414 1.1962	0.1158	1.1962	0.1158	1.1962
	0.1158	1.1962	0.1158	0.1158	0.57730829
IGLJ2 ENSG00000211676 -0.1477	0.55871	0.83062	0.20309	1.1962	0.11581
	1.1962	0.11581	0.11581	0.57730829
MNX1-AS2	ENSG00000235029 1.1962	0.11581	1.1962	0.11581	1.1962
	0.11581	1.1962	0.11581	0.11581	0.57730829
GCM1	ENSG00000137270 0.7427	0.22883	0.7427	0.22883	0.069217	0.47241
	1.1961	0.11582	0.11582	0.57730829
NA	ENSG00000259545 1.1962	0.11582	-0.57518	0.71742	1.1962	0.11582
	-0.39265	0.65271	0.11582	0.57730829
LINC02047	ENSG00000239454 1.1961	0.11583	-0.36297	0.64169	-0.96392
	0.83246	-1.3053	0.9041	0.11583	0.57730829
NA	ENSG00000234398 1.196 0.11584	-0.61805	0.73173	1.196 0.11584	1.196
	0.11584	0.11584	0.57730829
PRAMEF35P	ENSG00000236179 1.196 0.11586	1.0931	0.13718	0.51318	0.30391
	-1.093	0.8628	0.11586	0.57730829
ATP5F1EP1	ENSG00000250922 1.1959	0.11586	1.1959	0.11586	1.1959
	0.11586	-0.24749	0.59774	0.11586	0.57730829
KLHDC3	ENSG00000124702 1.1959	0.11587	1.1959	0.11587	1.1959
	0.11587	1.1959	0.11587	0.11587	0.57730829
CYCSP17	ENSG00000219627 1.1958	0.11589	1.1958	0.11589	0.0041322
	0.49835	0.18001	0.42857	0.11589	0.57730829
ZNF230	ENSG00000159882 1.1957	0.1159	1.1957	0.1159	1.1957
	0.1159	1.1957	0.1159	0.1159	0.57730829
CLEC3B	ENSG00000163815 0.65288	0.25692	0.90603	0.18246	0.78226
	0.21703	1.1958	0.1159	0.1159	0.57730829
FAM83D	ENSG00000101447 -0.59186	0.72303	-0.59186	0.72303	-0.29066
	0.61434	1.1956	0.11593	0.11593	0.577332677
NA	ENSG00000260677 -0.65177	0.74273	-0.65177	0.74273	-0.36163	0.64119
	1.1955	0.11594	0.11594	0.577332677
LINC02765	ENSG00000234476 -0.56394	0.7136	-0.65764	0.74461	1.1955
	0.11595	0.33655	0.36823	0.11595	0.577332677
NA	ENSG00000257894 1.1954	0.11596	-0.79811	0.7876	1.1954	0.11596
	1.1954	0.11596	0.11596	0.577332677
C1QTNF7	ENSG00000163145 1.1954	0.11597	0.98605	0.16205	1.1412
	0.1269	0.34741	0.36414	0.11597	0.577332677
NA	ENSG00000256915 1.1952	0.116 1.0269	0.15222	1.0269	0.15222
	1.0269	0.15222	0.116 0.577332677
ELOBP4	ENSG00000234167 -0.039549	0.51577	1.1952	0.11601	1.1952
	0.11601	1.1952	0.11601	0.11601	0.577332677
OTX2-AS1	ENSG00000248550 1.1952	0.11601	-0.64714	0.74123	1.0872
	0.13847	-1.6933	0.9548	0.11601	0.577332677
TCERG1	ENSG00000113649 0.57246	0.28351	1.1211	0.13113	0.20536
	0.41865	1.1951	0.11602	0.11602	0.577332677
QSER1 ENSG00000060749 -1.7872	0.96305	1.195 0.11604	-2.5332	0.99435
	-0.25456	0.60047	0.11604	0.577332677
PTMAP12	ENSG00000282886 -1.8198	0.96561	1.195 0.11604	-0.45219	0.67443
	-0.7449	0.77184	0.11604	0.577332677

CSNK1G2	ENSG00000133275	1.1949	0.11606	-1.1835	0.8817	-0.11246	0.54477	-0.9759	0.83544	0.11606	0.577332677
VXN	ENSG00000169085	0.64306	0.26009	1.1948	0.11608	0.64306	0.26009	0.64306	0.26009	0.11608	0.577332677
NA	ENSG00000280160	1.1948	0.11608	1.1948	0.11608	1.1948	0.11608	1.1948	0.11608	0.11608	0.577332677
SYNGR2	ENSG00000108639	0.45957	0.32291	1.1947	0.11609	0.20608	0.41836	0.20608	0.41836	0.11609	0.577332677
CT62	ENSG00000225362	0.29157	0.38531	0.29157	0.38531	1.1947	0.11611	0.12822	0.44899	0.11611	0.577332677
NA	ENSG00000274569	1.1946	0.11612	1.1946	0.11612	1.1946	0.11612	1.1946	0.11612	0.11612	0.577332677
NA	ENSG00000276272	1.0357	0.15017	0.88434	0.18826	1.1946	0.11612	1.1946	0.11612	0.11612	0.577332677
OR4T1P	ENSG00000258641	1.1946	0.11613	0.99453	0.15998	1.1946	0.11613	1.1946	0.11613	0.11613	0.577332677
CENPX	ENSG00000169689	1.0563	0.14541	1.1943	0.11618	1.0563	0.14541	0.91401	0.18036	0.11618	0.577463423
RNA5SP396	ENSG00000201704	1.1943	0.11618	1.1943	0.11618	1.1943	0.11618	1.1943	0.11618	0.11618	0.577463423
CSNK1G2P1	ENSG00000234534	0.72924	0.23293	0.86531	0.19343	0.86531	0.19343	1.1941	0.11622	0.11622	0.577516821
TM9SF3	ENSG00000077147	-0.46536	0.67916	-0.46536	0.67916	1.194	0.11624	1.0416	0.14879	0.11624	0.577516821
ZNF233	ENSG00000159915	1.194	0.11624	1.194	0.11624	1.194	0.11624	1.194	0.11624	0.11624	0.577516821
NA	ENSG00000270437	0.17504	0.43052	0.16385	0.43493	0.26622	0.39504	1.194	0.11624	0.11624	0.577516821
FKBP6	ENSG00000077800	1.1939	0.11625	1.1939	0.11625	1.1939	0.11625	0.61572	0.26904	0.11625	0.577516821
NA	ENSG00000239180	1.1938	0.11628	1.1938	0.11628	1.1938	0.11628	1.1938	0.11628	0.11628	0.577524198
TEX43	ENSG00000196900	-0.83557	0.7983	-0.34699	0.6357	1.1937	0.11629	0.13094	0.44791	0.11629	0.577524198
NA	ENSG00000258752	1.1938	0.11629	-0.47002	0.68083	0.35778	0.36026	-0.022048	0.5088	0.11629	0.577524198
MIR2113	ENSG00000238367	0.25055	0.40108	1.1937	0.1163	0.68585	0.2464	0.92693	0.17698	0.1163	0.577524198
NA	ENSG00000270558	1.1936	0.11632	1.1936	0.11632	1.1936	0.11632	1.1936	0.11632	0.11632	0.577524198
CCDC80	ENSG00000091986	-0.65079	0.74241	-1.3497	0.91144	1.1715	0.12069	1.1934	0.11635	0.11635	0.577524198
HERC2P9	ENSG00000206149	-0.34941	0.63661	1.1935	0.11635	-0.47982	0.68432	0.52656	0.29925	0.11635	0.577524198
PLAAT1	ENSG00000127252	-0.86618	0.8068	1.1934	0.11636	-1.3657	0.91398	-2.3475	0.99055	0.11636	0.577524198
MIR3910-1	ENSG00000266855	-0.37878	0.64757	-0.11939	0.54752	0.85555	0.19612	1.1934	0.11636	0.11636	0.577524198
NA	ENSG00000278376	1.1934	0.11637	1.1934	0.11637	1.1934	0.11637	1.1934	0.11637	0.11637	0.577524198
NA	ENSG00000163009	-0.2244	0.58878	-0.39598	0.65394	-0.49269	0.68888	1.1932	0.1164	0.1164	0.577614257
PDCD6IPP2	ENSG00000261377	0.68652	0.24619	-0.29473	0.6159	1.1929	0.11646	0.10741	0.45723	0.11646	0.577843932
HOXC-AS2	ENSG00000250133	0.5743	0.28288	1.1928	0.11647	0.21137	0.4163	0.21137	0.4163	0.11647	0.577843932
NA	ENSG00000227101	1.1927	0.1165	0.61106	0.27058	-0.49083	0.68823	0.48142	0.31511	0.1165	0.577875102
NA	ENSG00000264123	0.00070439	0.49972	0.9912	0.16079	0.60056	0.27407	1.1927	0.1165	0.1165	0.577875102
ARHGEF2	ENSG00000116584	0.40396	0.34312	0.59635	0.27547	0.83082	0.20304	1.1925	0.11654	0.11654	0.577928182

OTUD4	ENSG00000164164	0.62241	0.26683	0.030679	0.48776	0.10899	0.4566	1.1924	0.11655	0.11655	0.577928182
SNHG10	ENSG00000247092	-0.65071	0.74238	1.1924	0.11655	-0.54534	0.70724	-0.024632	0.50983	0.11655	0.577928182
DCTN3	ENSG00000137100	-0.51199	0.69567	-0.51199	0.69567	-0.51199	0.69567	1.1923	0.11657	0.11657	0.577928182
NA	ENSG00000279221	-2.1245	0.98319	1.1923	0.11657	0.088168	0.46487	-2.1245	0.98319	0.11657	0.577928182
RSU1P3	ENSG00000213659	1.1922	0.11659	1.1922	0.11659	1.1922	0.11659	0.24856	0.40185	0.11659	0.577968535
FRMPD1	ENSG00000070601	0.279	0.39012	1.192	0.11662	0.76301	0.22273	-0.20059	0.57949	0.11662	0.578058448
DBX2	ENSG00000185610	1.1919	0.11665	0.94331	0.17276	1.1919	0.11665	1.1919	0.11665	0.11665	0.578111397
NCR3	ENSG00000204475	1.1918	0.11666	0.74227	0.22896	1.1101	0.13347	0.85494	0.19629	0.11666	0.578111397
VN1R8P	ENSG00000219280	0.59056	0.27741	-0.45169	0.67425	1.1918	0.11668	-0.11886	0.54731	0.11668	0.578111397
TNFRSF14-AS1	ENSG00000238164	1.1917	0.11668	-0.64479	0.74047	-0.050714	0.52022	-0.050714	0.52022	0.11668	0.578111397
KBTBD11-OT1	ENSG00000253696	1.1917	0.11669	0.82978	0.20333	0.23321	0.4078	0.23321	0.4078	0.11669	0.578111397
MTHFS	ENSG00000136371	0.441	0.32961	0.441	0.32961	1.1916	0.11671	0.88828	0.1872	0.11671	0.578151703
NA	ENSG00000200506	0.5508	0.29089	0.6952	0.24347	0.50733	0.30596	1.1914	0.11674	0.11674	0.578241533
RN7SL741P	ENSG00000242381	1.1631	0.12239	-0.97173	0.83441	0.046866	0.48131	1.1912	0.11678	0.11678	0.578371616
PARPBP	ENSG00000185480	-0.45752	0.67635	1.0585	0.14493	-0.45752	0.67635	1.1912	0.11679	0.11679	0.578371616
FAM131C	ENSG00000185519	-0.0030763	0.50123	-0.183	0.5726	-0.5407	0.70564	1.1909	0.11685	0.11685	0.578609948
NA	ENSG00000272967	1.1907	0.11688	1.1907	0.11688	1.1907	0.11688	1.1907	0.11688	0.11688	0.578699695
LIMK2	ENSG00000182541	0.97483	0.16482	0.97483	0.16482	0.97483	0.16482	1.1906	0.11691	0.11691	0.578721341
OR1P1	ENSG00000262085	0.75764	0.22433	-0.74055	0.77052	-0.80114	0.78847	1.1906	0.11691	0.11691	0.578721341
CPED1	ENSG00000106034	0.055051	0.47805	1.1905	0.11692	0.7733	0.21967	1.0815	0.13973	0.11692	0.578721341
CD93	ENSG00000125810	0.22766	0.40996	0.25327	0.40003	1.1904	0.11694	-0.1315	0.55231	0.11694	0.578761548
FGD3	ENSG00000127084	-0.10624	0.5423	-0.11922	0.54745	1.1903	0.11696	0.63019	0.26429	0.11696	0.578792455
ZNF664	ENSG00000179195	0.74935	0.22682	-0.65725	0.74449	0.3755	0.35364	1.1903	0.11697	0.11697	0.578792455
NA	ENSG00000281365	0.39515	0.34637	0.26583	0.39518	-0.21194	0.58392	1.1901	0.117	0.117	0.578804771
WNT10A	ENSG00000135925	0.91158	0.18099	1.1901	0.11701	0.16581	0.43415	1.1901	0.11701	0.11701	0.578804771
MME	ENSG00000196549	-0.24792	0.5979	1.1901	0.11701	0.52704	0.29908	-0.10241	0.54078	0.11701	0.578804771
MIR941-2	ENSG00000216141	1.19	0.11702	1.19	0.11702	1.19	0.11702	1.19	0.11702	0.11702	0.578804771
NA	ENSG00000240497	0.4782	0.31625	0.4782	0.31625	0.4782	0.31625	1.1899	0.11704	0.11704	0.578844941
S100A15A	ENSG00000224784	0.84505	0.19904	1.1897	0.11709	0.84505	0.19904	0.84505	0.19904	0.11709	0.579005539
SUMO1P3	ENSG00000235082	1.1896	0.11711	1.1896	0.11711	1.1896	0.11711	1.1896	0.11711	0.11711	0.579005539
SINHCAFP2	ENSG00000267742	1.1895	0.11711	1.1895	0.11711	1.1895	0.11711	1.1895	0.11711	0.11711	0.579005539

NA ENSG00000271558 -0.51133 0.69544 1.1895 0.11712 1.1895 0.11712
-0.17555 0.56968 0.11712 0.579005539
NA ENSG00000250375 -0.095758 0.53814 0.76836 0.22114 1.1893 0.11716
1.1279 0.12969 0.11716 0.579017757
NA ENSG00000200120 1.1893 0.11717 -0.074115 0.52954 -0.27231 0.60731
-0.27231 0.60731 0.11717 0.579017757
ACTR3P3 ENSG00000242573 -1.4085 0.92051 1.1893 0.11717 -1.4085
0.92051 -1.4085 0.92051 0.11717 0.579017757
NA ENSG00000280354 1.1892 0.11717 1.1892 0.11717 1.1892 0.11717
1.1892 0.11717 0.11717 0.579017757
DUXAP1 ENSG00000259056 1.1891 0.11719 1.1891 0.11719 1.1891
0.11719 1.1891 0.11719 0.11719 0.579057869
HEMGN ENSG00000136929 1.1891 0.11721 1.1891 0.11721 -0.4837 0.6857
0.33577 0.36852 0.11721 0.579079361
RNU6-938P ENSG00000223217 1.189 0.11722 1.189 0.11722 1.189 0.11722 1.189
0.11722 0.11722 0.579079361
RN7SL184P ENSG00000239279 1.1054 0.13449 1.1889 0.11723 0.60852
0.27142 -1.8889 0.97055 0.11723 0.579079361
SFRP5 ENSG00000120057 0.62999 0.26435 0.62999 0.26435 0.90013 0.18403
1.1888 0.11726 0.11726 0.579140916
DOCK9 ENSG00000088387 1.1574 0.12356 0.73474 0.23125 0.52815 0.2987
1.1887 0.11727 0.11727 0.579140916
PPM1H ENSG00000111110 0.80708 0.20981 1.1887 0.11728 1.1553 0.12399
-0.29501 0.61601 0.11728 0.579140916
NA ENSG00000268652 1.1886 0.11729 1.1886 0.11729 1.1886 0.11729
1.1886 0.11729 0.11729 0.579140916
RNU6-525P ENSG00000200555 -0.86654 0.8069 -0.86654 0.8069 1.1885
0.11732 0.10551 0.45799 0.11732 0.579165202
TFEB ENSG00000112561 1.1884 0.11734 0.074725 0.47022 1.0058 0.15727
-0.88747 0.81259 0.11734 0.579165202
MIR4650-2 ENSG00000264494 0.26099 0.39705 -0.43737 0.66908 1.1884
0.11735 -0.81828 0.7934 0.11735 0.579165202
GOLGA5P1 ENSG00000251215 1.1883 0.11736 -0.574 0.71702 1.1883
0.11736 1.1883 0.11736 0.11736 0.579165202
NA ENSG00000273181 1.1883 0.11736 -1.2071 0.8863 -1.2071 0.8863
-1.2071 0.8863 0.11736 0.579165202
RPL5P11 ENSG00000242882 0.46475 0.32106 1.1883 0.11737 -1.1901 0.883
-1.1901 0.883 0.11737 0.579165202
NA ENSG00000260496 1.0461 0.14776 1.1882 0.11738 -0.54352 0.70661
-0.20267 0.5803 0.11738 0.579165202
NA ENSG00000237227 1.1881 0.11739 1.1881 0.11739 1.1881 0.11739
1.1881 0.11739 0.11739 0.579165202
NA ENSG00000235389 1.188 0.11742 1.188 0.11742 1.188 0.11742 1.188 0.11742
0.11742 0.579216458
NA ENSG00000271778 1.1879 0.11743 1.1668 0.12164 -0.64555 0.74071
-0.64555 0.74071 0.11743 0.579216458
NA ENSG00000271034 1.1878 0.11746 1.1878 0.11746 1.1878 0.11746
1.1878 0.11746 0.11746 0.579216458
NA ENSG00000228683 1.1876 0.1175 -0.41874 0.6623 1.1876 0.1175
-0.26879 0.60595 0.1175 0.579216458
TGS1 ENSG00000137574 1.1875 0.11751 0.31576 0.37609 0.48552 0.31365
0.86085 0.19466 0.11751 0.579216458
MRPS31P2 ENSG00000232894 -2.1363 0.98367 1.1875 0.11751 -1.8589
0.96848 -1.3914 0.91795 0.11751 0.579216458
C3orf22 ENSG00000180697 -2.2254 0.98697 1.1874 0.11753 -1.1117
0.86687 -1.08 0.85992 0.11753 0.579216458
HSP90AB7P ENSG00000235838 1.1874 0.11753 -0.94143 0.82676 1.1874
0.11753 1.1874 0.11753 0.11753 0.579216458
HOXA11 ENSG00000005073 1.1874 0.11754 0.97355 0.16514 1.1843
0.11816 -0.72507 0.7658 0.11754 0.579216458
NA ENSG00000248114 0.29885 0.38253 0.50021 0.30846 1.1874 0.11754
1.1874 0.11754 0.11754 0.579216458

NA	ENSG00000253512	1.1874	0.11754	1.1874	0.11754	1.1874	0.11754	1.0806	0.13993	0.11754	0.579216458
BACH1-IT3	ENSG00000234293	0.37423	0.35412	1.1873	0.11756	0.024855	0.49009	0.24447	0.40343	0.11756	0.579216458
NA	ENSG00000236075	1.1029	0.13504	0.73375	0.23155	0.3211	0.37407	1.1872	0.11758	0.11758	0.579216458
ZNF829	ENSG00000185869	1.1871	0.11759	0.22327	0.41166	1.1871	0.11759	1.1871	0.11759	0.11759	0.579216458
LINC01770	ENSG00000225285	1.1871	0.1176	1.1871	0.1176	1.1871	0.1176	1.1871	0.1176	0.1176	0.579216458
STX1A	ENSG00000106089	-0.83536	0.79824	1.187	0.11761	-0.99949	0.84122	-0.16088	0.56391	0.11761	0.579216458
CIR1P1	ENSG00000253146	1.1599	0.12304	1.1869	0.11763	1.1599	0.12304	1.1599	0.12304	0.11763	0.579216458
NA	ENSG00000284209	1.1869	0.11763	0.87532	0.1907	-0.16975	0.5674	-0.16975	0.5674	0.11763	0.579216458
PRF1	ENSG00000180644	-0.19821	0.57856	1.1869	0.11764	-0.93221	0.82439	-1.3139	0.90557	0.11764	0.579216458
TLX1	ENSG00000107807	1.1868	0.11765	-1.2736	0.8986	-0.13624	0.55419	-0.8797	0.81049	0.11765	0.579216458
NA	ENSG00000275457	1.1868	0.11765	1.1868	0.11765	1.1868	0.11765	1.1868	0.11765	0.11765	0.579216458
NA	ENSG00000279076	0.3612	0.35897	1.1865	0.11771	1.1865	0.11771	1.063	0.14388	0.11771	0.579387805
DUS1L	ENSG00000169718	0.19203	0.42386	1.1864	0.11774	0.99255	0.16046	-1.044	0.85175	0.11774	0.579387805
NA	ENSG00000100101	0.44116	0.32955	-0.56927	0.71541	1.1863	0.11776	-0.59645	0.72456	0.11776	0.579387805
PCDHGA1	ENSG00000204956	1.1863	0.11776	0.74412	0.2284	1.1863	0.11776	1.1863	0.11776	0.11776	0.579387805
RN7SL518P	ENSG00000265486	1.1862	0.11776	1.1862	0.11776	1.1862	0.11776	1.1862	0.11776	0.11776	0.579387805
CCDC144NL-AS1	ENSG00000233098	-0.044488	0.51774	1.1861	0.11779	0.30983	0.37835	1.1594	0.12314	0.11779	0.579387805
GSPT1	ENSG00000103342	0.18908	0.42502	0.71891	0.2361	0.24908	0.40165	1.1859	0.11782	0.11782	0.579387805
EAPP	ENSG00000129518	1.1859	0.11782	0.64326	0.26003	1.1859	0.11782	-0.29198	0.61485	0.11782	0.579387805
NA	ENSG00000222274	0.86882	0.19247	1.186	0.11782	0.75594	0.22484	0.62091	0.26733	0.11782	0.579387805
ERF	ENSG00000105722	1.1859	0.11783	0.81683	0.20701	0.92369	0.17782	0.99227	0.16053	0.11783	0.579387805
SHISA5	ENSG00000164054	0.78251	0.21696	1.1859	0.11783	0.44664	0.32757	0.96608	0.167	0.11783	0.579387805
CRTC1	ENSG00000105662	0.25501	0.39936	-0.0136	0.50543	-0.22835	0.59031	1.1856	0.11788	0.11788	0.579387805
ZNF30	ENSG00000168661	1.1856	0.1179	1.1856	0.1179	1.1856	0.1179	0.53722	0.29556	0.1179	0.579387805
LINC01595	ENSG00000259108	1.1855	0.1179	1.1855	0.1179	1.1855	0.1179	1.1855	0.1179	0.1179	0.579387805
NA	ENSG00000279345	0.24097	0.40479	1.1855	0.1179	0.24097	0.40479	0.24097	0.40479	0.1179	0.579387805
MIR339	ENSG00000199023	1.1855	0.11791	1.1855	0.11791	0.55688	0.28881	1.1855	0.11791	0.11791	0.579387805
NPVF	ENSG00000105954	-1.1754	0.88007	-1.0219	0.84659	1.1855	0.11792	-1.0219	0.84659	0.11792	0.579387805
MTRF1L	ENSG00000112031	0.036061	0.48562	1.1854	0.11793	0.51457	0.30343	1.1854	0.11793	0.11793	0.579387805
NA	ENSG00000252228	-0.38723	0.65071	-0.10582	0.54214	1.1854	0.11794	1.1854	0.11794	0.11794	0.579387805
NA	ENSG00000202269	1.1853	0.11795	1.1853	0.11795	-1.3026	0.90364	-0.69858	0.75759	0.11795	0.579387805

CTNNA1	ENSG00000044115	-0.73079	0.76755	-0.65736	0.74453	0.15369	0.43893	1.1852	0.11797	0.11797	0.579387805
NA	ENSG00000267714	1.1851	0.11799	1.1851	0.11799	1.1851	0.11799	1.1851	0.11799	0.11799	0.579387805
NA	ENSG00000271825	1.185	0.118	1.185	0.118	1.185	0.118	1.185	0.118	0.118	0.579387805
RAET1L	ENSG00000155918	0.81918	0.20634	1.185	0.11801	0.27606	0.39125	-0.3812	0.64847	0.11801	0.579387805
RNU6-1039P	ENSG00000206658	1.1849	0.11803	-0.29998	0.6179	-0.29998	0.6179	-0.29998	0.6179	0.11803	0.579387805
NA	ENSG00000253824	-0.0095135	0.5038	-0.61689	0.73135	1.1712	0.12076	1.1849	0.11803	0.11803	0.579387805
NA	ENSG00000233028	1.1848	0.11804	-0.8983	0.81549	-1.0583	0.85505	0.027444	0.48905	0.11804	0.579387805
RN7SKP33	ENSG00000222874	0.7904	0.21465	1.1848	0.11805	0.64952	0.258	0.75347	0.22558	0.11805	0.579387805
TEX19	ENSG00000182459	0.33708	0.36803	-1.0387	0.85052	-0.83409	0.79788	1.1847	0.11806	0.11806	0.579387805
KCNMA1-AS2	ENSG00000225497	1.1847	0.11806	-0.29642	0.61654	-1.9776	0.97602	-1.0095	0.84363	0.11806	0.579387805
NBEA	ENSG00000172915	-0.36583	0.64276	0.72459	0.23435	1.1846	0.11809	-0.37775	0.64719	0.11809	0.579387805
GPRC5D-AS1	ENSG00000247498	-0.69218	0.75559	1.1845	0.11811	-0.41402	0.66057	-0.41402	0.66057	0.11811	0.579387805
UBBP4	ENSG00000263563	1.1845	0.11811	0.32551	0.3724	-0.3011	0.61833	0.20481	0.41886	0.11811	0.579387805
RNU4-31P	ENSG00000206938	-0.43247	0.6673	0.32466	0.37272	0.32466	0.37272	1.1843	0.11815	0.11815	0.579387805
ZNF790	ENSG00000197863	0.015282	0.4939	1.1843	0.11816	1.1843	0.11816	1.1843	0.11816	0.11816	0.579387805
CNOT6	ENSG00000113300	1.1287	0.12952	1.1842	0.11817	1.0429	0.14849	1.0489	0.1471	0.11817	0.579387805
TCTEX1D2	ENSG00000213123	0.60507	0.27257	1.1842	0.11818	0.024776	0.49012	0.080334	0.46799	0.11818	0.579387805
LCAT	ENSG00000213398	1.1841	0.11819	1.1841	0.11819	1.1841	0.11819	1.1841	0.11819	0.11819	0.579387805
HDAC1	ENSG00000116478	0.44161	0.32938	1.1514	0.12479	0.43824	0.33061	1.1839	0.11823	0.11823	0.579387805
HPS3	ENSG00000163755	1.1839	0.11823	-0.32937	0.62906	0.44915	0.32666	1.113	0.13286	0.11823	0.579387805
MIR2053	ENSG00000238399	1.1838	0.11825	1.1838	0.11825	1.1838	0.11825	1.1838	0.11825	0.11825	0.579387805
ZSCAN5C	ENSG00000204532	1.1837	0.11826	0.2364	0.40656	0.63723	0.26199	0.57002	0.28433	0.11826	0.579387805
SERINC5	ENSG00000164300	-0.31025	0.62182	0.043105	0.48281	-0.69621	0.75685	1.1836	0.11829	0.11829	0.579387805
NA	ENSG00000234259	-0.67469	0.75006	-0.67469	0.75006	0.25962	0.39758	1.1836	0.11829	0.11829	0.579387805
IQCA1L	ENSG00000278685	-0.59311	0.72345	-0.59311	0.72345	-0.59311	0.72345	1.1836	0.11829	0.11829	0.579387805
RNA5SP93	ENSG00000199395	-0.23316	0.59218	1.1835	0.1183	1.1835	0.1183	1.1835	0.1183	0.1183	0.579387805
RPS6P20	ENSG00000226356	-1.3849	0.91697	0.030834	0.4877	1.1834	0.11832	-0.26795	0.60563	0.11832	0.579387805
SNORA2A	ENSG00000206612	-1.3648	0.91384	1.1832	0.11837	1.1832	0.11837	1.1832	0.11837	0.11837	0.579387805
NA	ENSG00000251017	-0.11743	0.54674	0.45637	0.32406	0.13557	0.44608	1.1832	0.11837	0.11837	0.579387805
PHBP11	ENSG00000227621	1.183	0.1184	1.183	0.1184	1.183	0.1184	-0.49029	0.68803	0.1184	0.579387805
NA	ENSG00000279278	0.084863	0.46619	1.1829	0.11842	0.77985	0.21774	0.022336	0.49109	0.11842	0.579387805
CWH43	ENSG00000109182	-0.69359	0.75603	1.1829	0.11843	-0.14488	0.5576				

-0.4149 0.66089 0.11843 0.579387805
APEH ENSG00000164062 -0.75967 0.77627 -1.3559 0.91243 -1.4908 0.932
1.1828 0.11845 0.11845 0.579387805
CENPCP1 ENSG00000226982 1.1827 0.11846 1.1827 0.11846 1.1827
0.11846 1.1827 0.11846 0.11846 0.579387805
BNIP3P41 ENSG00000248302 0.26685 0.39479 0.50811 0.30569 1.1826
0.11847 1.0389 0.14943 0.11847 0.579387805
NA ENSG00000254574 1.1826 0.11848 1.1826 0.11848 1.1826 0.11848
1.1826 0.11848 0.11848 0.579387805
NA ENSG00000279943 1.1825 0.1185 1.1825 0.1185 1.1825 0.1185
1.1825 0.1185 0.1185 0.579387805
CRYGS ENSG00000213139 0.16342 0.43509 0.18176 0.42788 1.1824 0.11852
1.1824 0.11852 0.11852 0.579387805
RN7SL328P ENSG00000240853 -0.012589 0.50502 -0.28809 0.61336 1.1824
0.11853 -0.30342 0.61921 0.11853 0.579387805
NA ENSG00000250848 -0.72934 0.7671 1.1824 0.11853 0.48004 0.3156
0.41806 0.33795 0.11853 0.579387805
RRP36 ENSG00000124541 1.1823 0.11854 1.1823 0.11854 1.094 0.13698 1.094
0.13698 0.11854 0.579387805
THUMPD2 ENSG00000138050 1.1823 0.11854 0.71135 0.23844 0.78655
0.21577 0.78655 0.21577 0.11854 0.579387805
PDE8B ENSG00000113231 -0.558 0.71158 1.1823 0.11855 -1.4634 0.92833
-1.3298 0.90821 0.11855 0.579387805
RNU6-422P ENSG00000207362 1.1822 0.11856 1.1822 0.11856 1.1822
0.11856 1.1822 0.11856 0.11856 0.579387805
KCNK3 ENSG00000171303 1.1822 0.11857 -0.28434 0.61192 -0.1783 0.57076
0.30849 0.37885 0.11857 0.579387805
NA ENSG00000253768 -0.1154 0.54594 1.1822 0.11857 0.60688 0.27197
-0.34928 0.63656 0.11857 0.579387805
LINC02109 ENSG00000259786 0.17096 0.43213 0.67597 0.24953 1.1822
0.11857 0.67597 0.24953 0.11857 0.579387805
NA ENSG00000271302 0.97443 0.16492 1.1821 0.11859 1.1821 0.11859
0.40909 0.34124 0.11859 0.579387805
SEL1L ENSG00000071537 -0.44654 0.6724 -0.34331 0.63432 1.1137 0.1327
1.182 0.11861 0.11861 0.579387805
DIRC3-AS1 ENSG00000233143 1.182 0.11861 1.0867 0.13858 0.46758 0.32004
-0.14072 0.55596 0.11861 0.579387805
ILF3 ENSG00000129351 0.8388 0.20079 0.82211 0.20551 1.1819 0.11862
0.77269 0.21985 0.11862 0.579387805
MIOX ENSG00000100253 1.1818 0.11864 1.1818 0.11864 1.1818 0.11864
1.1818 0.11864 0.11864 0.579387805
RNASEH2C ENSG00000172922 -0.50447 0.69303 -0.50447 0.69303 -0.55845
0.71173 1.1818 0.11864 0.11864 0.579387805
PLAC9P1 ENSG00000214100 1.1538 0.12428 1.1818 0.11864 0.37739
0.35294 0.11137 0.45566 0.11864 0.579387805
PCDHGA12 ENSG00000253159 0.59993 0.27428 -0.08305 0.53309 0.59993
0.27428 1.1818 0.11864 0.11864 0.579387805
NA ENSG00000248478 0.83499 0.20186 -1.1798 0.88097 0.57196 0.28368
1.1818 0.11864 0.11864 0.579387805
NA ENSG00000225304 -0.28297 0.6114 1.1816 0.11869 -0.28297 0.6114
-0.28297 0.6114 0.11869 0.579387805
MEIOC ENSG00000180336 0.97813 0.16401 0.018185 0.49275 1.1815 0.1187
0.94144 0.17324 0.1187 0.579387805
LINC01470 ENSG00000249484 0.22835 0.40969 1.1815 0.1187 -0.16695
0.56629 0.65681 0.25565 0.1187 0.579387805
NA ENSG00000271192 -1.1185 0.86831 0.46218 0.32198 0.81615 0.20721
1.1815 0.1187 0.1187 0.579387805
NA ENSG00000262370 -0.8778 0.80997 0.29769 0.38297 0.97899 0.16379
1.1815 0.11871 0.11871 0.579387805
PLCXD3 ENSG00000182836 1.1814 0.11873 -0.84241 0.80022 0.33846
0.36751 1.1814 0.11873 0.11873 0.579387805
VAV2 ENSG00000160293 0.84585 0.19882 1.1813 0.11874 0.47177 0.31855

1.077 0.14074 0.11874 0.579387805
TMEM151B ENSG00000178233 1.1813 0.11875 0.21328 0.41555 1.0262
0.15239 1.0262 0.15239 0.11875 0.579387805
COX6CP13 ENSG00000234144 1.1813 0.11875 1.1813 0.11875 1.1813
0.11875 1.1813 0.11875 0.11875 0.579387805
NA ENSG00000258719 1.1812 0.11875 1.1812 0.11875 -0.22625 0.5895
1.1812 0.11875 0.11875 0.579387805
NA ENSG00000224888 1.1811 0.11878 1.1811 0.11878 1.1811 0.11878
1.1811 0.11878 0.11878 0.579387805
ZNF57 ENSG00000171970 1.181 0.1188 1.1665 0.12171 1.181 0.1188 1.181
0.1188 0.1188 0.579387805
FANCA ENSG00000187741 0.84018 0.2004 0.66831 0.25197 0.79162 0.21429
1.181 0.1188 0.1188 0.579387805
OR7E16P ENSG00000267775 0.17522 0.43045 1.181 0.1188 0.65429 0.25646
0.14585 0.44202 0.1188 0.579387805
PHF13 ENSG00000116273 1.1809 0.11881 1.1173 0.13192 1.1809 0.11881
1.1809 0.11881 0.11881 0.579387805
ARHGEF19-AS1 ENSG00000234166 -1.1678 0.87856 1.181 0.11881 -1.1678
0.87856 -1.1678 0.87856 0.11881 0.579387805
SERPINB9 ENSG00000170542 0.53851 0.29511 1.1809 0.11882 0.39547
0.34625 0.074881 0.47015 0.11882 0.579387805
NA ENSG00000268655 1.1809 0.11882 1.1809 0.11882 1.1809 0.11882
1.1809 0.11882 0.11882 0.579387805
NA ENSG00000224844 0.41193 0.3402 -2.1932 0.98585 -0.84081 0.79977
1.1808 0.11883 0.11883 0.579387805
OFD1P17 ENSG00000228212 1.1808 0.11883 1.1116 0.13315 1.1116
0.13315 1.1116 0.13315 0.11883 0.579387805
NA ENSG00000265689 -0.3536 0.63818 -2.362 0.99091 -1.068 0.85725
1.1808 0.11884 0.11884 0.579387805
MIR4316 ENSG00000264060 1.1807 0.11885 1.1807 0.11885 1.1807
0.11885 1.1807 0.11885 0.11885 0.579387805
NA ENSG00000275894 1.1807 0.11887 1.1807 0.11887 1.1807 0.11887
1.1807 0.11887 0.11887 0.579411585
NA ENSG00000229052 1.1805 0.11889 1.0666 0.14308 1.1805 0.11889
0.79272 0.21397 0.11889 0.579411585
RN7SL244P ENSG00000242865 1.1805 0.1189 0.96556 0.16713 0.96556
0.16713 0.96556 0.16713 0.1189 0.579411585
ANAPC10 ENSG00000164162 0.98357 0.16266 0.62481 0.26605 1.1804
0.11892 0.66335 0.25355 0.11892 0.579411585
KRTAP5-AS1 ENSG00000233930 1.1804 0.11892 -0.73061 0.76749 -0.81075
0.79124 -0.81075 0.79124 0.11892 0.579411585
NA ENSG00000273550 -0.06149 0.52452 1.1803 0.11894 0.087245 0.46524
0.68079 0.248 0.11894 0.579411585
NA ENSG00000259084 1.1802 0.11895 1.1802 0.11895 1.1802 0.11895
1.1802 0.11895 0.11895 0.579411585
EIF4A3P1 ENSG00000266181 0.42499 0.33542 0.42499 0.33542 0.60987
0.27097 1.1803 0.11895 0.11895 0.579411585
NA ENSG00000199461 0.90578 0.18253 1.1801 0.11897 1.1801 0.11897
1.1801 0.11897 0.11897 0.579451084
ARHGAP35 ENSG00000160007 1.1798 0.11903 0.77211 0.22002 0.49251
0.31118 0.099488 0.46038 0.11903 0.579685379
OR4K16P ENSG00000258822 1.1797 0.11906 -0.32409 0.62706 -0.76709
0.77849 -0.76709 0.77849 0.11906 0.579727357
SMPDL3A ENSG00000172594 1.1797 0.11907 1.1797 0.11907 1.1797
0.11907 1.1797 0.11907 0.11907 0.579727357
ZNF141 ENSG00000131127 1.1796 0.11908 1.1796 0.11908 1.1796
0.11908 -0.0010862 0.50043 0.11908 0.579727357
NA ENSG00000271971 1.1796 0.11909 1.1796 0.11909 1.1796 0.11909
1.1796 0.11909 0.11909 0.579727357
OTX2 ENSG00000165588 0.18857 0.42521 0.18857 0.42521 1.1795 0.11911
0.18857 0.42521 0.11911 0.579727357
LAMC1-AS1 ENSG00000224468 1.1794 0.11911 1.1794 0.11911 1.1794

0.11911 1.1794 0.11911 0.11911 0.579727357
GNLY ENSG00000115523 0.28156 0.38914 0.5635 0.28655 1.1794 0.11913
0.22529 0.41088 0.11913 0.579766793
ALDH8A1 ENSG00000118514 1.1793 0.11915 0.79475 0.21338 -0.65814
0.74477 0.0815 0.46752 0.11915 0.579806221
VPS13A-AS1 ENSG00000232998 1.179 0.1192 0.0037036 0.49852 0.0037036 0.49852
0.0037036 0.49852 0.1192 0.579915213
GRIFIN ENSG00000275572 1.1789 0.11921 0.33024 0.37061 0.1143
0.4545 0.66427 0.25326 0.11921 0.579915213
LINC00272 ENSG00000203729 0.79648 0.21288 -1.3696 0.91459 1.1789
0.11922 -0.62903 0.73533 0.11922 0.579915213
NA ENSG00000235182 1.1789 0.11922 1.1789 0.11922 1.1789 0.11922
1.1789 0.11922 0.11922 0.579915213
PTCD3 ENSG00000132300 -0.071672 0.52857 -0.13099 0.55211 1.1788 0.11924
0.95055 0.17092 0.11924 0.579915639
LINC02434 ENSG00000248370 0.44416 0.32846 0.31669 0.37574 1.1786
0.11928 0.29974 0.38219 0.11928 0.579915639
C10orf55 ENSG00000222047 0.74752 0.22737 0.59307 0.27657 1.1784
0.11931 0.68235 0.24751 0.11931 0.579915639
NA ENSG00000264871 -0.091218 0.53634 0.24344 0.40383 1.1784 0.11932
0.81317 0.20806 0.11932 0.579915639
NA ENSG00000253390 1.1784 0.11933 1.1784 0.11933 1.1784 0.11933
0.63105 0.26401 0.11933 0.579915639
LINC01494 ENSG00000228135 0.85939 0.19506 1.0358 0.15015 1.1783
0.11934 1.0358 0.15015 0.11934 0.579915639
NA ENSG00000268362 1.1782 0.11936 0.83513 0.20182 0.83513 0.20182
0.88101 0.18916 0.11936 0.579915639
PPA2 ENSG00000138777 -0.15592 0.56195 -0.070496 0.5281 0.57166 0.28378
1.1781 0.11937 0.11937 0.579915639
MCCC2 ENSG00000131844 0.15818 0.43716 0.04954 0.48024 1.1781 0.11938
1.0953 0.13669 0.11938 0.579915639
RNA5SP157 ENSG00000251816 -0.36292 0.64167 1.1781 0.11938 1.1781
0.11938 1.1781 0.11938 0.11938 0.579915639
NA ENSG00000266088 -0.68808 0.7543 0.33542 0.36866 -0.56924 0.7154
1.178 0.11939 0.11939 0.579915639
GTF2B ENSG00000137947 1.178 0.1194 0.11428 0.45451 0.11428 0.45451
0.98753 0.16169 0.1194 0.579915639
TRBV30 ENSG00000237254 1.1779 0.11942 1.1779 0.11942 1.1779
0.11942 1.1779 0.11942 0.11942 0.579915639
TRA2B ENSG00000136527 1.1779 0.11943 0.13574 0.44602 -0.17972 0.57132
-0.65374 0.74336 0.11943 0.579915639
AKR1C3 ENSG00000196139 0.62256 0.26679 0.74737 0.22742 0.86507
0.1935 1.1778 0.11943 0.11943 0.579915639
RNU2-31P ENSG00000252763 0.82892 0.20357 -1.3847 0.91693 1.1778
0.11943 -0.57724 0.71811 0.11943 0.579915639
ESRRB ENSG00000119715 1.1778 0.11944 -0.49776 0.69067 0.76986 0.22069
1.1394 0.12727 0.11944 0.579915639
BBOX1 ENSG00000129151 -0.14284 0.55679 -0.79325 0.78618 1.1778 0.11944
-0.10645 0.54239 0.11944 0.579915639
RN7SKP101 ENSG00000223308 1.1777 0.11945 1.1777 0.11945 1.1777
0.11945 1.0165 0.15471 0.11945 0.579915639
RPS15AP1 ENSG00000214535 1.1777 0.11946 1.1777 0.11946 1.1777
0.11946 1.1777 0.11946 0.11946 0.579915639
CNFN ENSG00000105427 0.76742 0.22142 1.1282 0.12962 1.1776 0.11947
1.1776 0.11947 0.11947 0.579915639
STK11 ENSG00000118046 -0.66435 0.74677 -0.68358 0.75288 0.28265 0.38872
1.1775 0.1195 0.1195 0.579976093
TSC22D4 ENSG00000166925 -0.85039 0.80245 1.1774 0.11952 -1.2623
0.89658 -1.2623 0.89658 0.11952 0.579976093
GAPDHP25 ENSG00000213594 1.1774 0.11952 -0.56788 0.71494 0.15627
0.43791 0.15627 0.43791 0.11952 0.579976093
CFAP43 ENSG00000197748 -0.24875 0.59822 1.1773 0.11954 -0.18531

							0.57351	0.11318	0.45494	0.11954	0.579976093
KCNB1	ENSG00000158445	0.11891	0.45268	0.10269	0.45911	0.032061	0.48721	1.1772	0.11956	0.11956	0.579976093
NA	ENSG00000252202	1.1771	0.11957	1.1771	0.11957	1.1771	0.11957	1.1771	0.11957	0.11957	0.579976093
RNU7-27P	ENSG00000238750	1.1771	0.11958	1.1771	0.11958	1.1771	0.11958	1.1771	0.11958	0.11958	0.579976093
NA	ENSG00000272921	1.1771	0.11958	-0.93721	0.82567	-0.93721	0.82567	-0.93721	0.82567	0.11958	0.579976093
RN7SKP95	ENSG00000199730	0.10167	0.45951	1.177	0.11959	-0.56103	0.71261	1.177	0.11959	0.11959	0.579976093
SFRP4	ENSG00000106483	0.022984	0.49083	0.022984	0.49083	0.022984	0.49083	1.1768	0.11964	0.11964	0.579976093
NA	ENSG00000251373	0.073753	0.4706	1.1767	0.11965	1.1767	0.11965	1.1767	0.11965	0.11965	0.579976093
MSANTD1	ENSG00000188981	0.4329	0.33254	0.13289	0.44714	-0.43771	0.6692	1.1767	0.11966	0.11966	0.579976093
HCAR2	ENSG00000182782	0.055271	0.47796	0.055271	0.47796	-0.7035	0.75913	1.1766	0.11967	0.11967	0.579976093
NA	ENSG00000239661	1.1767	0.11967	1.1767	0.11967	1.1767	0.11967	0.030839	0.4877	0.11967	0.579976093
NAA35	ENSG00000135040	-1.4837	0.93105	-1.4837	0.93105	1.1624	0.12254	1.1765	0.11969	0.11969	0.579976093
RNU6-1171P	ENSG00000252515	1.1765	0.11969	1.1765	0.11969	1.1765	0.11969	1.1765	0.11969	0.11969	0.579976093
NA	ENSG00000199862	0.44734	0.32731	1.1765	0.1197	0.44734	0.32731	0.62361	0.26644	0.1197	0.579976093
TAAR5	ENSG00000135569	1.1148	0.13247	1.1763	0.11973	1.1763	0.11973	1.1763	0.11973	0.11973	0.579976093
NA	ENSG00000254522	1.1763	0.11975	1.1763	0.11975	1.1763	0.11975	1.1763	0.11975	0.11975	0.579976093
NA	ENSG00000266378	0.18716	0.42577	-1.2169	0.88818	1.1762	0.11975	0.21299	0.41567	0.11975	0.579976093
SSU72P3	ENSG00000284546	1.1762	0.11975	-0.13649	0.55428	-1.1327	0.87133	0.60838	0.27147	0.11975	0.579976093
LILRB1	ENSG00000104972	0.58425	0.27952	0.40891	0.3413	1.1761	0.11977	0.58425	0.27952	0.11977	0.579976093
DCLRE1CP1	ENSG00000237470	-1.7491	0.95986	1.1761	0.11977	-1.2048	0.88586	-1.2048	0.88586	0.11977	0.579976093
ATP6V1F	ENSG00000128524	1.1761	0.11978	1.1761	0.11978	1.1761	0.11978	1.1761	0.11978	0.11978	0.579976093
EFCAB6	ENSG00000186976	0.035207	0.48596	1.1761	0.11978	0.10041	0.46001	0.22849	0.40963	0.11978	0.579976093
NA	ENSG00000253339	-1.9033	0.9715	1.176	0.1198	-1.9033	0.9715	-0.9013	0.81628	0.1198	0.579996881
PARD3B	ENSG00000116117	0.32617	0.37215	0.45431	0.3248	1.1759	0.11982	0.66975	0.25151	0.11982	0.579996881
MIR4298	ENSG00000264493	1.1759	0.11982	0.41709	0.33831	0.41709	0.33831	0.41709	0.33831	0.11982	0.579996881
FCF1P2	ENSG00000228638	1.1756	0.11988	1.1756	0.11988	1.1756	0.11988	1.1756	0.11988	0.11988	0.580229678
PTPRD-AS2	ENSG00000226717	1.1753	0.11994	0.97738	0.16419	0.88749	0.18741	0.97738	0.16419	0.11994	0.580304631
ZEB1-AS1	ENSG00000237036	-1.2131	0.88745	-1.255	0.89525	0.10054	0.45996	1.1753	0.11995	0.11995	0.580304631
NA	ENSG00000257604	1.0639	0.14369	1.1752	0.11995	0.6485	0.25833	0.27635	0.39114	0.11995	0.580304631
IFT81	ENSG00000122970	1.0817	0.13968	1.0782	0.14047	1.0815	0.13975	1.1751	0.11997	0.11997	0.580304631
NBPF1	ENSG00000219481	1.175	0.11999	0.77242	0.21993	-0.40549	0.65744	1.1505	0.12496	0.11999	0.580304631
NSD2	ENSG00000109685	1.175	0.12001	0.85007	0.19764	0.084457	0.46635				

0.01029 0.49589 0.12001 0.580304631
NA ENSG00000260052 1.1749 0.12002 0.20897 0.41723 -0.32426 0.62713
1.1085 0.13383 0.12002 0.580304631
NA ENSG00000278873 0.18736 0.42569 -0.34516 0.63501 1.1749 0.12002
1.1749 0.12002 0.12002 0.580304631
RPS13P2 ENSG00000228929 1.1745 0.12009 1.1745 0.12009 1.1745
0.12009 1.1745 0.12009 0.12009 0.580304631
LIMA1 ENSG00000050405 1.1744 0.12012 0.96475 0.16733 0.95762 0.16913
0.17212 0.43167 0.12012 0.580304631
GNG13 ENSG00000127588 -0.052223 0.52082 -0.49217 0.6887 1.1744 0.12012
-1.4699 0.92921 0.12012 0.580304631
NA ENSG00000225005 1.1743 0.12013 1.1743 0.12013 1.1743 0.12013
1.1743 0.12013 0.12013 0.580304631
DPPA2P4 ENSG00000261429 1.1743 0.12013 1.1743 0.12013 1.1743
0.12013 1.1743 0.12013 0.12013 0.580304631
NA ENSG00000206669 1.1743 0.12014 1.1743 0.12014 1.1743 0.12014
1.1743 0.12014 0.12014 0.580304631
PPP6R2P1 ENSG00000233442 0.67638 0.2494 1.1743 0.12015 -0.66357
0.74652 -1.4394 0.92498 0.12015 0.580304631
NA ENSG00000238110 1.1743 0.12015 0.33062 0.37047 -0.33467 0.63106
-0.36837 0.6437 0.12015 0.580304631
MED28P2 ENSG00000240511 1.1741 0.12018 1.1741 0.12018 1.1741
0.12018 1.1741 0.12018 0.12018 0.580304631
DNAAF2 ENSG00000165506 0.20188 0.42001 0.3308 0.3704 0.20188
0.42001 1.174 0.12019 0.12019 0.580304631
RN7SL263P ENSG00000240160 1.174 0.1202 1.174 0.1202 1.174 0.1202 1.174
0.1202 0.1202 0.580304631
PCBP2P3 ENSG00000248826 0.5804 0.28082 0.5804 0.28082 0.34007
0.3669 1.174 0.1202 0.1202 0.580304631
NA ENSG00000266962 1.1739 0.12022 1.1739 0.12022 1.1739 0.12022
1.1739 0.12022 0.12022 0.580304631
MOB1A ENSG00000114978 -0.82251 0.79461 -1.7667 0.96136 1.1737 0.12025
-0.27419 0.60803 0.12025 0.580304631
NA ENSG00000269425 1.1737 0.12025 1.1737 0.12025 1.1737 0.12025
1.1737 0.12025 0.12025 0.580304631
NA ENSG00000270669 -1.7548 0.96036 -1.6101 0.94631 -1.6101 0.94631
1.1737 0.12026 0.12026 0.580304631
H2AZ1 ENSG00000164032 1.1736 0.12027 1.1736 0.12027 1.1736 0.12027
1.1736 0.12027 0.12027 0.580304631
RNU1-32P ENSG00000200807 1.1736 0.12027 1.1736 0.12027 1.1736
0.12027 1.1736 0.12027 0.12027 0.580304631
TIPARP ENSG00000163659 0.059029 0.47646 0.83394 0.20216 1.1735
0.12029 0.99023 0.16103 0.12029 0.580304631
LINC01685 ENSG00000238063 -0.5361 0.70405 1.0636 0.14376 -0.40833
0.65849 1.1735 0.12029 0.12029 0.580304631
NA ENSG00000251535 0.58107 0.2806 0.65301 0.25687 1.1735 0.12029
1.1735 0.12029 0.12029 0.580304631
OR8G5 ENSG00000255298 1.1735 0.12031 1.1735 0.12031 1.1735 0.12031
1.1735 0.12031 0.12031 0.580304631
ADH5P3 ENSG00000223694 1.1734 0.12032 1.1734 0.12032 1.1734
0.12032 1.1226 0.13079 0.12032 0.580304631
RNU6-99P ENSG00000201591 1.1733 0.12033 0.71437 0.2375 0.71437
0.2375 0.71437 0.2375 0.12033 0.580304631
NA ENSG00000273920 1.1733 0.12033 1.1733 0.12033 1.1733 0.12033
1.1733 0.12033 0.12033 0.580304631
NA ENSG00000200351 0.88258 0.18873 0.43771 0.3308 0.88258 0.18873
1.1733 0.12034 0.12034 0.580304631
NA ENSG00000266938 1.1733 0.12034 1.03 0.15151 1.1733 0.12034
1.1733 0.12034 0.12034 0.580304631
NA ENSG00000271343 0.32544 0.37242 0.32544 0.37242 0.32544 0.37242
1.1733 0.12035 0.12035 0.580304631
COL6A4P1 ENSG00000230524 0.56645 0.28554 -0.34753 0.6359 1.1732

0.12036 1.0687 0.14261 0.12036 0.580304631
FBLN5 ENSG00000140092 -1.1962 0.88419 1.1731 0.12038 0.76444 0.2223
0.43609 0.33139 0.12038 0.580304631
NA ENSG00000248393 1.1731 0.12038 -0.59566 0.7243 1.1731 0.12038
1.1731 0.12038 0.12038 0.580304631
MIR941-4 ENSG00000216195 1.173 0.1204 1.173 0.1204 1.173 0.1204 1.173
0.1204 0.1204 0.580304631
RPS26P24 ENSG00000243920 1.1729 0.12041 1.1729 0.12041 1.1729
0.12041 1.1729 0.12041 0.12041 0.580304631
NA ENSG00000260675 1.1729 0.12043 1.1729 0.12043 1.1729 0.12043
1.1729 0.12043 0.12043 0.580304631
RGS21 ENSG00000253148 1.1728 0.12044 -1.2568 0.89558 -1.2568 0.89558
-1.2568 0.89558 0.12044 0.580304631
NA ENSG00000231978 0.68503 0.24666 1.1728 0.12045 0.39063 0.34803
0.17982 0.42865 0.12045 0.580304631
NA ENSG00000255016 -2.959 0.99846 1.1727 0.12045 -1.4642 0.92844
-0.2508 0.59901 0.12045 0.580304631
NA ENSG00000215198 0.67547 0.24969 0.97342 0.16517 1.0639 0.14368
1.1727 0.12046 0.12046 0.580304631
MIR3659HG ENSG00000224592 -0.78482 0.78372 -0.14934 0.55936 -1.3094
0.9048 1.1726 0.12047 0.12047 0.580304631
NA ENSG00000232028 1.1726 0.12047 1.1726 0.12047 1.1726 0.12047
1.1726 0.12047 0.12047 0.580304631
NA ENSG00000217512 1.1726 0.12048 1.1726 0.12048 1.1726 0.12048
1.1726 0.12048 0.12048 0.580304631
NA ENSG00000239117 1.1725 0.1205 0.21248 0.41587 1.1499 0.12508
0.21248 0.41587 0.1205 0.580304631
CHPF2 ENSG00000033100 1.1175 0.13188 1.1175 0.13188 1.0627 0.14395
1.1722 0.12056 0.12056 0.580304631
SGK2 ENSG00000101049 1.1721 0.12057 0.96052 0.1684 0.96052 0.1684
0.82278 0.20532 0.12057 0.580304631
LINC00189 ENSG00000215533 0.99515 0.15983 1.1721 0.12057 -0.14974
0.55951 0.88664 0.18764 0.12057 0.580304631
NA ENSG00000259966 1.1722 0.12057 1.1471 0.12568 0.838 0.20102 0.838
0.20102 0.12057 0.580304631
COL6A6 ENSG00000206384 1.0851 0.13894 1.172 0.1206 0.83971 0.20053
-0.78317 0.78324 0.1206 0.580304631
LINC00354 ENSG00000226903 1.0527 0.14623 1.172 0.1206 -1.0505 0.85325
-0.76019 0.77643 0.1206 0.580304631
LINC01513 ENSG00000246016 -1.1186 0.86835 1.172 0.1206 1.1569 0.12366
-1.1186 0.86835 0.1206 0.580304631
PLA2G12A ENSG00000123739 1.1719 0.12061 1.1719 0.12061 -0.10616
0.54227 0.21107 0.41642 0.12061 0.580304631
NA ENSG00000251399 0.84632 0.19869 -0.25748 0.6016 1.0105 0.15612
1.1719 0.12061 0.12061 0.580304631
LINC01668 ENSG00000283051 1.1719 0.12061 1.1719 0.12061 1.1719
0.12061 1.1719 0.12061 0.12061 0.580304631
NA ENSG00000272777 1.1718 0.12064 1.1718 0.12064 1.1718 0.12064
1.1718 0.12064 0.12064 0.580391667
CELF2-DT ENSG00000270111 0.81571 0.20733 1.1717 0.12066 -0.013663
0.50545 0.10756 0.45717 0.12066 0.580430582
GUCY2EP ENSG00000204529 1.1716 0.12068 -0.60978 0.72899 -0.6789
0.7514 0.37923 0.35226 0.12068 0.58046949
GPR137B ENSG00000077585 1.1715 0.1207 0.68631 0.24626 -0.59317
0.72347 -2.0502 0.97983 0.1207 0.580508389
OR2AP1 ENSG00000179615 -0.24087 0.59517 1.1714 0.12072 -0.32188
0.62623 -0.36894 0.64391 0.12072 0.580547281
IGKV6D-21 ENSG00000225523 1.1713 0.12074 1.1713 0.12074 1.1713
0.12074 1.1713 0.12074 0.12074 0.580576961
RNU7-155P ENSG00000238759 1.1713 0.12075 1.1713 0.12075 1.1713
0.12075 1.1713 0.12075 0.12075 0.580576961
NEK2 ENSG00000117650 0.083156 0.46686 0.30733 0.3793 0.083156 0.46686

1.1711 0.12077 0.12077 0.580615835
RPL32P3 ENSG00000251474 1.1711 0.12079 1.1711 0.12079 1.1711
0.12079 1.1711 0.12079 0.12079 0.580651571
CXCL14 ENSG00000145824 -0.40119 0.65586 1.0568 0.14529 1.171 0.12081
0.92979 0.17624 0.12081 0.580651571
NA ENSG00000283189 1.1708 0.12083 1.1708 0.12083 1.1708 0.12083
1.1708 0.12083 0.12083 0.580651571
LINC02563 ENSG00000263520 1.1707 0.12085 -0.05688 0.52268 1.1707
0.12085 1.1707 0.12085 0.12085 0.580651571
SPTLC2 ENSG00000100596 1.1707 0.12086 -1.0997 0.86428 -0.19968
0.57914 0.32061 0.37425 0.12086 0.580651571
CRYZP2 ENSG00000250293 -0.0040481 0.50161 1.1707 0.12086 -0.026595
0.51061 -0.026595 0.51061 0.12086 0.580651571
NA ENSG00000270507 1.1186 0.13166 1.1706 0.12088 0.40891 0.3413
0.86597 0.19325 0.12088 0.580651571
XYLT2 ENSG00000015532 -0.11511 0.54582 -0.11511 0.54582 -0.52703 0.70091
1.1705 0.1209 0.1209 0.580651571
RNY1P4 ENSG00000207325 1.1705 0.1209 1.1705 0.1209 1.1705
0.1209 1.1705 0.1209 0.1209 0.580651571
ICMT ENSG00000116237 1.1705 0.12091 0.1453 0.44224 0.098843 0.46063
0.99273 0.16042 0.12091 0.580651571
AVPR1B ENSG00000198049 -1.1074 0.86595 1.1705 0.12091 -0.60354
0.72693 0.60866 0.27138 0.12091 0.580651571
PEX19 ENSG00000162735 1.1703 0.12094 1.1703 0.12094 1.1703 0.12094
1.1703 0.12094 0.12094 0.580651571
TPT1P8 ENSG00000219491 0.68465 0.24678 1.1703 0.12094 1.1703
0.12094 0.024983 0.49003 0.12094 0.580651571
KRT18P23 ENSG00000215333 1.1703 0.12095 1.1703 0.12095 1.1703
0.12095 1.1703 0.12095 0.12095 0.580651571
NA ENSG00000279153 -0.10697 0.54259 1.1702 0.12096 0.095985 0.46177
0.095985 0.46177 0.12096 0.580651571
RNU6-89P ENSG00000272262 -1.2037 0.88565 0.66769 0.25217 1.1702
0.12097 -0.22001 0.58707 0.12097 0.580651571
NA ENSG00000224676 0.14573 0.44207 0.82227 0.20546 1.1701 0.12098
-1.408 0.92043 0.12098 0.580651571
MIR1913 ENSG00000222958 1.17 0.121 1.17 0.121 1.17 0.121 1.17 0.121 0.121
0.580681172
MIR3200 ENSG00000264661 -0.54029 0.7055 -0.91546 0.82003 -0.54029
0.7055 1.1699 0.12101 0.12101 0.580681172
RN7SKP253 ENSG00000252233 -0.34345 0.63437 -0.34345 0.63437 1.1352
0.12815 1.1698 0.12103 0.12103 0.580719959
NIPAL3 ENSG00000001461 0.95391 0.17006 0.36989 0.35573 0.70982
0.23891 1.1698 0.12105 0.12105 0.580758738
ARL3 ENSG00000138175 -0.3297 0.62919 -1.0904 0.86224 0.41183 0.34023
1.1697 0.12107 0.12107 0.580797509
MANCR ENSG00000231298 1.1695 0.12109 0.086157 0.46567 -0.94074 0.82658
-0.86964 0.80775 0.12109 0.580836273
B9D1 ENSG00000108641 -0.66285 0.74629 -0.66285 0.74629 1.1694 0.12112
0.39733 0.34556 0.12112 0.580922992
ZNF200 ENSG00000010539 0.51157 0.30447 0.19251 0.42367 0.043976
0.48246 1.1693 0.12114 0.12114 0.580961736
SPATA19 ENSG00000166118 1.1692 0.12116 -0.15352 0.561 1.0973 0.13626
0.22347 0.41158 0.12116 0.580982035
NA ENSG00000258542 1.1692 0.12117 1.1692 0.12117 1.1692 0.12117
1.1692 0.12117 0.12117 0.580982035
LINC02429 ENSG00000251266 1.1691 0.12118 0.035165 0.48597 -0.29433
0.61575 0.069981 0.4721 0.12118 0.580982035
CHRFAM7A ENSG00000166664 1.1689 0.12122 0.63647 0.26224 -0.35838
0.63997 0.31885 0.37492 0.12122 0.581015222
NDUFAF5 ENSG00000101247 1.0961 0.13651 1.1688 0.12123 1.0466
0.14764 -0.96119 0.83177 0.12123 0.581015222
SEPHS2 ENSG00000179918 0.57075 0.28409 0.75464 0.22523 1.1688

0.12125 0.91539 0.17999 0.12125 0.581015222
NA ENSG00000257454 -0.28726 0.61304 -1.1159 0.86777 1.1687 0.12126
1.1687 0.12126 0.12126 0.581015222
NA ENSG00000267549 1.1687 0.12126 1.1687 0.12126 1.1687 0.12126
1.1687 0.12126 0.12126 0.581015222
CENPUP2 ENSG00000258546 1.1687 0.12127 1.1687 0.12127 1.1687
0.12127 1.1687 0.12127 0.12127 0.581015222
OSTN ENSG00000188729 -0.42047 0.66293 0.24977 0.40138 1.1686 0.12129
1.1686 0.12129 0.12129 0.581015222
RN7SL854P ENSG00000239319 0.57224 0.28358 0.85827 0.19537 1.1684
0.12131 -0.60021 0.72582 0.12131 0.581015222
OR1F1 ENSG00000168124 1.1684 0.12132 1.1684 0.12132 -0.33094 0.62966
1.1684 0.12132 0.12132 0.581015222
NA ENSG00000260633 0.69801 0.24258 0.69801 0.24258 0.69801 0.24258
1.1684 0.12132 0.12132 0.581015222
TRMT1L ENSG00000121486 1.1684 0.12133 0.28529 0.38771 0.28529
0.38771 0.28529 0.38771 0.12133 0.581015222
NA ENSG00000272905 -0.18479 0.5733 1.1684 0.12133 -0.22256 0.58806
-0.74936 0.77318 0.12133 0.581015222
ERI3 ENSG00000117419 1.1682 0.12136 0.039448 0.48427 0.88805 0.18726
0.56311 0.28668 0.12136 0.581101779
FBXL15 ENSG00000107872 -0.399 0.65505 1.1681 0.12138 1.1681
0.12138 1.1681 0.12138 0.12138 0.581131221
MRPL57P8 ENSG00000282625 -0.12142 0.54832 -0.12142 0.54832 1.1681
0.12139 1.0052 0.1574 0.12139 0.581131221
ACAP1 ENSG00000072818 1.1677 0.12146 0.18337 0.42726 0.53385 0.29672
0.5285 0.29858 0.12146 0.581226418
NA ENSG00000264039 0.3671 0.35677 -1.5137 0.93495 0.73508 0.23115
1.1677 0.12146 0.12146 0.581226418
LINC01091 ENSG00000249464 0.67421 0.25009 1.1676 0.12148 0.80291
0.21101 0.66647 0.25255 0.12148 0.581226418
OR5H5P ENSG00000249321 -0.27488 0.6083 -0.85342 0.80329 1.1675
0.1215 -0.12792 0.55089 0.1215 0.581226418
ADAM2 ENSG00000104755 -1.4595 0.92778 0.7109 0.23857 1.1675 0.12151
1.0055 0.15733 0.12151 0.581226418
TMEM150C ENSG00000249242 -0.935 0.82511 -0.935 0.82511 0.12581
0.44994 1.1674 0.12152 0.12152 0.581226418
RNU6-1111P ENSG00000212420 1.1672 0.12156 1.1672 0.12156 1.1672
0.12156 1.1672 0.12156 0.12156 0.581226418
RAET1E ENSG00000164520 0.35099 0.3628 0.35099 0.3628 0.35099
0.3628 1.1672 0.12157 0.12157 0.581226418
NA ENSG00000214135 -0.14674 0.55833 0.47979 0.31569 0.46361 0.32146
1.1672 0.12157 0.12157 0.581226418
PHF24 ENSG00000122733 -0.2812 0.61072 1.1671 0.12159 -0.26318 0.60379
0.57417 0.28293 0.12159 0.581226418
FAM21FP ENSG00000237840 1.167 0.1216 0.40384 0.34317 0.60006 0.27423
0.76603 0.22183 0.1216 0.581226418
MIR4696 ENSG00000265902 0.27251 0.39261 0.17438 0.43078 1.1669
0.12163 0.17438 0.43078 0.12163 0.581226418
APC2 ENSG00000115266 -0.23948 0.59463 -1.7641 0.96115 1.1668 0.12164
-1.3605 0.91317 0.12164 0.581226418
NA ENSG00000249588 0.79562 0.21313 1.1668 0.12164 0.79562 0.21313
-0.64777 0.74143 0.12164 0.581226418
UBE2MP1 ENSG00000261461 1.1668 0.12164 -0.805 0.78959 0.61065
0.27071 0.15377 0.4389 0.12164 0.581226418
LINC02819 ENSG00000224943 1.1668 0.12165 1.1668 0.12165 1.1668
0.12165 1.1668 0.12165 0.12165 0.581226418
HMGN2P16 ENSG00000231376 0.29945 0.3823 -0.40007 0.65545 -0.78124
0.78267 1.1668 0.12165 0.12165 0.581226418
DUXAP3 ENSG00000270552 1.1668 0.12165 -0.0069314 0.50277 1.1668
0.12165 1.1668 0.12165 0.12165 0.581226418
OR7E91P ENSG00000205847 0.73624 0.23079 0.87675 0.19031 1.1667

0.12166	-0.17353	0.56888	0.12166	0.581226418
IFT122	ENSG00000163913	1.1666	0.12168	0.51608	0.3029	0.9949
0.15989	0.73719	0.2305	0.12168	0.581226418
HIGD2B	ENSG00000175202	-0.20391	0.58079	1.1666	0.12168	-0.20391
0.58079	-0.20391	0.58079	0.12168	0.581226418
NA	ENSG00000278193	0.08905	0.46452	0.08905	0.46452	0.08905	0.46452
1.1666	0.12168	0.12168	0.581226418
METTL2A	ENSG00000087995	0.7496	0.22675	-0.50856	0.69447	1.1666
0.12169	0.72512	0.23419	0.12169	0.581226418
RN7SKP1	ENSG00000202395	0.43639	0.33128	0.10892	0.45663	1.1665
0.12171	0.89543	0.18528	0.12171	0.581226418
SULT1E1	ENSG00000109193	0.35562	0.36106	1.1664	0.12172	-0.72827
0.76678	0.041797	0.48333	0.12172	0.581226418
NA	ENSG00000260368	-0.67545	0.75031	1.1664	0.12172	1.1664	0.12172
1.1664	0.12172	0.12172	0.581226418
NA	ENSG00000255160	1.1661	0.12178	-1.7848	0.96286	0.044542	0.48224
-0.33625	0.63166	0.12178	0.581313014
NA	ENSG00000259072	-0.46641	0.67954	0.59736	0.27513	-0.36978	0.64423
1.1661	0.12178	0.12178	0.581313014
GDI2	ENSG00000057608	0.57759	0.28177	0.78189	0.21714	-0.043119	0.5172
1.1661	0.12179	0.12179	0.581313014
ADCY8	ENSG00000155897	0.90345	0.18314	0.38986	0.34832	1.166	0.1218
0.46593	0.32063	0.1218	0.581313014
CTBP2	ENSG00000175029	-1.2035	0.8856	1.1658	0.12184	0.2912	0.38545
-1.4787	0.93039	0.12184	0.581313014
FAM160B2	ENSG00000158863	1.105	0.13457	-0.035548	0.51418	-0.20475	0.58112
1.1658	0.12185	0.12185	0.581313014
ELK4	ENSG00000158711	0.61942	0.26782	0.89765	0.18469	1.1657	0.12186
1.1204	0.13128	0.12186	0.581313014
NA	ENSG00000279509	0.91388	0.18039	1.1658	0.12186	-0.28243	0.61119
8.45E-05	0.49997	0.12186	0.581313014
SNORD116-23	ENSG00000207375	1.1656	0.12188	1.1656	0.12188	1.1656
0.12188	1.1656	0.12188	0.12188	0.581313014
NA	ENSG00000257319	1.1656	0.12189	1.1656	0.12189	1.1656	0.12189
1.1656	0.12189	0.12189	0.581313014
SPTLC1P1	ENSG00000230397	1.1655	0.12191	-0.12047	0.54794	1.1655
0.12191	1.1655	0.12191	0.12191	0.581313014
NA	ENSG00000240305	0.76157	0.22316	1.1655	0.12191	0.76157	0.22316
0.76157	0.22316	0.12191	0.581313014
EVA1B	ENSG00000142694	1.1651	0.12199	1.1651	0.12199	1.1651	0.12199
1.1651	0.12199	0.12199	0.581313014
ARF1P2	ENSG00000213137	0.4742	0.31768	1.0027	0.158	1.1651	0.12199
1.0508	0.14668	0.12199	0.581313014
PDK2	ENSG00000005882	1.165	0.122	-0.34995	0.63681	1.165	0.122	1.0261	0.15241
0.122	0.581313014
ECE1-AS1	ENSG00000231105	0.33732	0.36794	0.50719	0.30601	0.33732
0.36794	1.165	0.122	0.122	0.581313014
NSMAF	ENSG00000035681	0.27531	0.39154	0.79868	0.21224	0.79868	0.21224
1.165	0.12201	0.12201	0.581313014
OR51F4P	ENSG00000273051	1.165	0.12201	1.165	0.12201	1.165	0.12201	1.165
0.12201	0.12201	0.581313014
NA	ENSG00000263311	1.165	0.12202	1.165	0.12202	1.165	0.12202	1.165	0.12202
0.12202	0.581313014
NA	ENSG00000260443	1.1647	0.12207	1.1647	0.12207	1.1647	0.12207
1.1647	0.12207	0.12207	0.581313014
STPG1	ENSG00000001460	0.27169	0.39293	1.1647	0.12208	-1.0905	0.86225
0.35373	0.36177	0.12208	0.581313014
KRT74	ENSG00000170484	0.82372	0.20505	0.79547	0.21317	0.53441	0.29653
1.1647	0.12208	0.12208	0.581313014
POLD2	ENSG00000106628	0.76995	0.22066	1.1646	0.12209	1.1646	0.12209
1.1646	0.12209	0.12209	0.581313014
MTCYBP19	ENSG00000253504	1.1646	0.12209	1.1646	0.12209	1.1646

0.12209 1.1646 0.12209 0.12209 0.581313014
MTND5P41 ENSG00000253685 1.1646 0.12209 1.1646 0.12209 1.1646
0.12209 1.1646 0.12209 0.12209 0.581313014
NA ENSG00000236325 1.1645 0.1221 1.1645 0.1221 1.1645 0.1221
1.1645 0.1221 0.1221 0.581313014
NA ENSG00000179755 0.47526 0.3173 0.58363 0.27973 0.96706 0.16676
1.1645 0.12211 0.12211 0.581313014
DNAH12 ENSG00000174844 0.80186 0.21132 -1.1234 0.86937 1.1644
0.12212 0.098655 0.46071 0.12212 0.581313014
IMMP1LP1 ENSG00000270269 1.1644 0.12212 1.1644 0.12212 1.1644
0.12212 1.1644 0.12212 0.12212 0.581313014
TNFRSF9 ENSG00000049249 0.28438 0.38806 0.46427 0.32123 1.1644
0.12213 0.46427 0.32123 0.12213 0.581313014
RNA5SP442 ENSG00000201920 1.1644 0.12214 1.1644 0.12214 1.1644
0.12214 1.1644 0.12214 0.12214 0.581313014
RPL12P39 ENSG00000237314 0.0022503 0.4991 1.1644 0.12214 1.1644
0.12214 0.74949 0.22678 0.12214 0.581313014
GTF2F2P2 ENSG00000233910 -0.7318 0.76786 0.14706 0.44154 -0.091399
0.53641 1.1643 0.12215 0.12215 0.581313014
NA ENSG00000259317 1.1643 0.12216 1.1643 0.12216 1.1643 0.12216
1.1643 0.12216 0.12216 0.581313014
TOR3A ENSG00000186283 0.22867 0.40956 0.34925 0.36345 0.58758 0.27841
1.1641 0.12219 0.12219 0.581313014
ADCY7 ENSG00000121281 0.86191 0.19437 -0.0095208 0.5038 1.1641 0.1222
1.0496 0.14694 0.1222 0.581313014
TRIM73 ENSG00000178809 1.164 0.12221 -0.13731 0.55461 -0.60592 0.72771
0.29043 0.38574 0.12221 0.581313014
DOCK11P1 ENSG00000259000 0.96631 0.16695 1.1639 0.12223 0.81055
0.20881 0.81055 0.20881 0.12223 0.581313014
PPP1R16B ENSG00000101445 0.025887 0.48967 0.025887 0.48967 -0.3864
0.6504 1.1638 0.12225 0.12225 0.581313014
MIR1267 ENSG00000221650 1.1638 0.12226 0.37657 0.35325 0.37657
0.35325 0.92624 0.17716 0.12226 0.581313014
NA ENSG00000271931 0.33585 0.36849 1.1638 0.12226 0.33585 0.36849
1.1285 0.12955 0.12226 0.581313014
KLRF1 ENSG00000150045 1.1637 0.12227 1.1637 0.12227 1.1637 0.12227
1.1637 0.12227 0.12227 0.581313014
NA ENSG00000230747 0.9174 0.17947 -0.28996 0.61408 1.0115 0.15589
1.1637 0.12227 0.12227 0.581313014
ACSL1 ENSG00000151726 0.13107 0.44786 1.1636 0.12229 -0.33639 0.63171
-0.71425 0.76246 0.12229 0.581313014
NA ENSG00000283234 1.1636 0.1223 1.0324 0.15095 1.1636 0.1223
1.1636 0.1223 0.1223 0.581313014
NA ENSG00000211553 1.1636 0.1223 -0.19444 0.57709 0.07825 0.46881
-0.13325 0.553 0.1223 0.581313014
RPLP0P11 ENSG00000239780 0.88881 0.18705 0.88881 0.18705 0.88881
0.18705 1.1634 0.12233 0.12233 0.581313014
NA ENSG00000278041 -0.26964 0.60628 0.14859 0.44094 1.1634 0.12233
-0.20139 0.5798 0.12233 0.581313014
MIR2115 ENSG00000252466 1.1634 0.12234 1.1634 0.12234 1.1634
0.12234 1.1634 0.12234 0.12234 0.581313014
LINC02840 ENSG00000224893 1.1633 0.12235 1.1633 0.12235 1.1633
0.12235 1.1633 0.12235 0.12235 0.581313014
HOXC4 ENSG00000198353 1.1633 0.12236 -0.9381 0.8259 -1.4312 0.92381
1.1633 0.12236 0.12236 0.581313014
ST18 ENSG00000147488 1.1632 0.12237 -0.35506 0.63873 0.79015 0.21472
0.16004 0.43642 0.12237 0.581313014
NA ENSG00000235128 1.1631 0.1224 0.29863 0.38261 1.1631 0.1224
1.1631 0.1224 0.1224 0.581313014
ARID5B ENSG00000150347 -1.012 0.84422 -0.33222 0.63014 -0.37214
0.64511 1.163 0.12241 0.12241 0.581313014
ACAD8 ENSG00000151498 1.1628 0.12245 0.32081 0.37418 0.32081 0.37418

0.57161 0.28379 0.12245 0.581313014
OPALIN ENSG00000197430 0.6564 0.25578 0.79615 0.21297 1.1628
0.12245 0.039489 0.48425 0.12245 0.581313014
MACROD2-AS1 ENSG00000235914 1.1211 0.13113 1.1628 0.12245 1.1628
0.12245 1.1628 0.12245 0.12245 0.581313014
RN7SL251P ENSG00000244399 1.1627 0.12247 -0.59039 0.72253 1.1627
0.12247 1.1627 0.12247 0.12247 0.581313014
NA ENSG00000214770 0.23975 0.40526 1.1627 0.12248 0.42858 0.33411
-0.30361 0.61929 0.12248 0.581313014
HTATSF1P1 ENSG00000223457 1.1627 0.12248 0.77346 0.21963 -0.1304
0.55188 0.30975 0.37838 0.12248 0.581313014
RAMP3 ENSG00000122679 0.57931 0.28119 0.39416 0.34673 1.1626 0.12249
0.23045 0.40887 0.12249 0.581313014
NA ENSG00000253608 1.1626 0.12249 -0.26062 0.60281 -0.38682 0.65056
-0.065641 0.52617 0.12249 0.581313014
NA ENSG00000252940 1.1623 0.12255 0.34414 0.36537 0.23134 0.40852
0.14293 0.44317 0.12255 0.581313014
PPP1R12A ENSG00000058272 1.1623 0.12256 -0.19472 0.5772 -0.42089
0.66308 0.27358 0.3922 0.12256 0.581313014
SLC5A3 ENSG00000198743 0.079905 0.46816 0.079905 0.46816 0.079905
0.46816 1.1621 0.12259 0.12259 0.581313014
TSFM ENSG00000123297 0.5358 0.29605 0.5358 0.29605 1.1621 0.1226
1.1621 0.1226 0.1226 0.581313014
PTGIS ENSG00000124212 1.162 0.12262 -0.25016 0.59877 0.61785 0.26834
0.52651 0.29927 0.12262 0.581313014
OSTCP6 ENSG00000219790 0.99747 0.15927 1.162 0.12262 1.162 0.12262 1.162
0.12262 0.12262 0.581313014
BID ENSG00000015475 0.26275 0.39637 0.59782 0.27498 0.65865 0.25506
1.1619 0.12263 0.12263 0.581313014
NDRG2 ENSG00000165795 0.81382 0.20787 0.43199 0.33287 -0.46143 0.67776
1.1619 0.12263 0.12263 0.581313014
CLK2 ENSG00000176444 1.1618 0.12265 0.80729 0.20975 1.1618 0.12265
1.1618 0.12265 0.12265 0.581313014
TSTD1 ENSG00000215845 1.1617 0.12268 1.1617 0.12268 1.1617 0.12268
1.1617 0.12268 0.12268 0.581313014
NA ENSG00000254343 1.1617 0.12268 1.1617 0.12268 1.1617 0.12268
1.1617 0.12268 0.12268 0.581313014
ADAMTS12 ENSG00000151388 0.57153 0.28382 0.50117 0.30812 1.1615
0.12272 -0.63653 0.73779 0.12272 0.581313014
PDXP-DT ENSG00000233360 1.1615 0.12272 1.1615 0.12272 1.1615
0.12272 1.1615 0.12272 0.12272 0.581313014
GAS6-DT ENSG00000272695 1.1615 0.12272 1.1615 0.12272 1.1615
0.12272 1.1615 0.12272 0.12272 0.581313014
NA ENSG00000276958 1.1614 0.12275 1.1614 0.12275 1.1614 0.12275
1.1614 0.12275 0.12275 0.581313014
HNRNPA1P73 ENSG00000236569 1.1613 0.12276 0.31991 0.37452 0.31991
0.37452 0.31991 0.37452 0.12276 0.581313014
NA ENSG00000279443 1.0291 0.15171 1.1333 0.12854 1.1613 0.12277
1.1613 0.12277 0.12277 0.581313014
ZMYM1 ENSG00000197056 0.34767 0.36404 -0.42516 0.66464 1.1611 0.12279
0.62939 0.26455 0.12279 0.581313014
ELK3 ENSG00000111145 1.1611 0.1228 -0.023052 0.5092 -0.38116 0.64846
-0.97843 0.83607 0.1228 0.581313014
LINC02777 ENSG00000232453 -0.40533 0.65738 -0.40533 0.65738 1.161 0.12281
-0.34587 0.63528 0.12281 0.581313014
NA ENSG00000254006 0.98193 0.16307 1.161 0.12282 1.161 0.12282 1.161
0.12282 0.12282 0.581313014
NA ENSG00000259588 1.161 0.12282 1.161 0.12282 1.161 0.12282 1.161 0.12282
0.12282 0.581313014
SGPL1 ENSG00000166224 -0.26803 0.60566 -0.36999 0.6443 1.0108 0.15606
1.1609 0.12283 0.12283 0.581313014
EMG1 ENSG00000126749 1.1609 0.12284 1.1609 0.12284 1.1609 0.12284

1.1609 0.12284 0.12284 0.581313014
INCENP ENSG00000149503 0.30924 0.37857 -0.11791 0.54693 -0.029112
0.51161 1.1609 0.12284 0.12284 0.581313014
MIR3129 ENSG00000264725 0.42077 0.33696 1.1609 0.12284 0.42077
0.33696 0.42077 0.33696 0.12284 0.581313014
RARS1 ENSG00000113643 1.1608 0.12286 -0.074552 0.52971 -0.61404 0.73041
-0.08585 0.53421 0.12286 0.581313014
C4orf17 ENSG00000138813 1.1608 0.12287 0.52984 0.29811 -0.24152
0.59542 -0.24152 0.59542 0.12287 0.581313014
RNU6-380P ENSG00000222139 1.1608 0.12287 1.1608 0.12287 -0.062741
0.52501 0.037958 0.48486 0.12287 0.581313014
MIR411 ENSG00000199109 1.1606 0.12289 1.1606 0.12289 1.1606
0.12289 1.1606 0.12289 0.12289 0.581313014
NA ENSG00000223831 -0.87749 0.80989 -0.87749 0.80989 1.1606 0.12289
-0.33554 0.63139 0.12289 0.581313014
SAMD11 ENSG00000187634 0.8203 0.20602 1.1606 0.1229 -0.057319
0.52285 0.82632 0.20431 0.1229 0.581313014
EPGN ENSG00000182585 -1.0018 0.84179 1.1606 0.12291 -0.135 0.55369
-0.36705 0.64321 0.12291 0.581313014
OR7E136P ENSG00000205897 -0.42284 0.6638 -0.048284 0.51926 1.0234
0.15307 1.1605 0.12291 0.12291 0.581313014
HOXA5 ENSG00000106004 0.69026 0.24501 0.69026 0.24501 0.22605 0.41058
1.1604 0.12293 0.12293 0.581313014
ADAMTSL1 ENSG00000178031 1.1604 0.12294 0.27098 0.3932 0.052699
0.47899 1.0348 0.15038 0.12294 0.581313014
NA ENSG00000257994 1.1604 0.12294 1.1604 0.12294 0.99827 0.15907
1.1604 0.12294 0.12294 0.581313014
VAC14-AS1 ENSG00000214353 1.1604 0.12295 -0.19039 0.5755 -0.19039
0.5755 -0.19039 0.5755 0.12295 0.581313014
NA ENSG00000257681 1.1603 0.12295 1.1603 0.12295 1.1603 0.12295
1.1603 0.12295 0.12295 0.581313014
DNAJC5 ENSG00000101152 0.65377 0.25663 0.65377 0.25663 1.0637
0.14372 1.1602 0.12299 0.12299 0.581313014
ANKRD13A ENSG00000076513 0.40274 0.34357 0.15513 0.43836 0.66899
0.25175 1.1601 0.12301 0.12301 0.581313014
CALB1 ENSG00000104327 1.1601 0.12301 0.10664 0.45754 0.10664 0.45754
0.10664 0.45754 0.12301 0.581313014
NA ENSG00000224441 1.16 0.12303 1.16 0.12303 1.16 0.12303 1.16 0.12303
0.12303 0.581313014
NA ENSG00000249392 0.49421 0.31058 0.07606 0.46969 1.1599 0.12304
0.68373 0.24707 0.12304 0.581313014
OR1I1 ENSG00000094661 0.28509 0.38779 -0.62182 0.73297 1.1599 0.12305
0.016889 0.49326 0.12305 0.581313014
ZRANB2-AS1 ENSG00000235079 -0.507 0.69392 -0.33452 0.63101 1.1599
0.12305 1.1599 0.12305 0.12305 0.581313014
RPL31P60 ENSG00000239988 1.1598 0.12306 1.1598 0.12306 1.1598
0.12306 1.1598 0.12306 0.12306 0.581313014
METTL21C ENSG00000139780 -0.53466 0.70356 0.45384 0.32497 1.1597
0.12308 1.1597 0.12308 0.12308 0.581313014
NA ENSG00000279775 1.1597 0.12309 1.1597 0.12309 -0.19925 0.57897
-0.19925 0.57897 0.12309 0.581313014
ATP5PBP2 ENSG00000214342 1.1596 0.1231 1.1596 0.1231 1.1596
0.1231 1.1596 0.1231 0.1231 0.581313014
TRAV9-1 ENSG00000211783 -0.22647 0.58958 -1.0857 0.8612 1.1596
0.12311 -1.0857 0.8612 0.12311 0.581313014
TRAV24 ENSG00000211805 1.1596 0.12311 1.1596 0.12311 0.18157
0.42796 1.1596 0.12311 0.12311 0.581313014
FABP5P7 ENSG00000234964 1.1596 0.12311 1.1596 0.12311 1.1596
0.12311 1.1596 0.12311 0.12311 0.581313014
TIMELESS ENSG00000111602 0.4629 0.32172 0.79337 0.21378 1.1591
0.1232 -0.88616 0.81223 0.1232 0.581625266
PHB2 ENSG00000215021 1.1591 0.1232 1.1591 0.1232 1.1591 0.1232

1.1591 0.1232 0.1232 0.581625266
GIT2 ENSG00000139436 0.3516 0.36257 0.053472 0.47868 0.12428 0.45055
1.1589 0.12326 0.12326 0.581852155
FCRLA ENSG00000132185 1.1587 0.12328 1.0509 0.14665 1.1587 0.12328
1.1587 0.12328 0.12328 0.581881036
FHL3 ENSG00000183386 -0.94203 0.82691 -0.19667 0.57796 1.1587 0.12329
0.88993 0.18675 0.12329 0.581881036
CDCA5 ENSG00000146670 1.1586 0.12331 1.1586 0.12331 1.1586 0.12331
-0.11012 0.54384 0.12331 0.581919068
NA ENSG00000265717 1.1585 0.12334 1.1585 0.12334 1.1585 0.12334
1.1585 0.12334 0.12334 0.58200428
VIPR1-AS1 ENSG00000232354 0.85372 0.19663 1.1582 0.12339 1.1582
0.12339 1.1582 0.12339 0.12339 0.58218384
NA ENSG00000229191 1.158 0.12342 1.158 0.12342 1.158 0.12342 1.158 0.12342
0.12342 0.582241434
RN7SL165P ENSG00000241188 -0.68019 0.75181 1.158 0.12343 -0.64882 0.74177
-0.68019 0.75181 0.12343 0.582241434
SDC4 ENSG00000124145 1.1272 0.12982 1.158 0.12344 0.41694 0.33836
0.086355 0.46559 0.12344 0.582241434
IGSF21 ENSG00000117154 0.72184 0.2352 1.1579 0.12345 -0.65903
0.74506 -0.50123 0.69189 0.12345 0.582241434
IPO8 ENSG00000133704 1.1577 0.12348 0.42221 0.33644 -0.16692 0.56628
-0.29106 0.6145 0.12348 0.582301363
NA ENSG00000270061 1.1577 0.1235 1.1577 0.1235 1.1577 0.1235
1.1577 0.1235 0.1235 0.582301363
NA ENSG00000231678 1.1576 0.12352 1.1576 0.12352 1.1576 0.12352
1.1576 0.12352 0.12352 0.582301363
NA ENSG00000268858 0.95467 0.16987 1.1576 0.12352 1.1576 0.12352
1.1576 0.12352 0.12352 0.582301363
PRDX5 ENSG00000126432 1.1574 0.12356 1.1574 0.12356 0.61542 0.26914
0.62128 0.26721 0.12356 0.582301363
CHRM2 ENSG00000181072 -0.17803 0.57065 1.1573 0.12357 -0.44596 0.67219
0.28351 0.38839 0.12357 0.582301363
CUL1P1 ENSG00000251032 1.1572 0.12359 -0.65437 0.74356 -0.35393
0.63831 -0.51729 0.69752 0.12359 0.582301363
LINC01572 ENSG00000261008 -0.92667 0.82295 -0.21785 0.58623 -0.92667
0.82295 1.1572 0.12359 0.12359 0.582301363
CHODL ENSG00000154645 0.91495 0.18011 0.64146 0.26061 1.1572 0.1236
-0.71568 0.76291 0.1236 0.582301363
FRMD4A ENSG00000151474 -0.61398 0.73039 0.19983 0.42081 1.1571
0.12361 0.86655 0.19309 0.12361 0.582301363
NA ENSG00000237390 0.44722 0.32736 0.53462 0.29646 1.1571 0.12362
1.1571 0.12362 0.12362 0.582301363
NA ENSG00000258631 1.1571 0.12362 -0.67624 0.75056 1.1571 0.12362
1.1571 0.12362 0.12362 0.582301363
SNRPGP6 ENSG00000205433 1.157 0.12363 -1.9451 0.97412 0.086629 0.46548
-1.9451 0.97412 0.12363 0.582301363
NA ENSG00000224063 0.36335 0.35817 1.157 0.12363 0.48878 0.3125
1.0667 0.14305 0.12363 0.582301363
NA ENSG00000199856 0.71368 0.23771 1.1569 0.12365 0.71368 0.23771
0.71368 0.23771 0.12365 0.58232091
A2M ENSG00000175899 0.50603 0.30642 0.29688 0.38328 1.1569 0.12366
-0.6727 0.74943 0.12366 0.58232091
ARSI ENSG00000183876 1.1568 0.12367 0.9676 0.16662 0.98969 0.16116
0.8339 0.20217 0.12367 0.58232091
LINC02603 ENSG00000230262 0.22616 0.41054 -1.1798 0.88096 1.1568
0.12369 0.60138 0.27379 0.12369 0.582331266
LCEP2 ENSG00000229713 -0.76629 0.77825 -0.422 0.66349 -0.90937 0.81842
1.1566 0.12371 0.12371 0.582331266
NA ENSG00000239674 1.1566 0.12371 1.1566 0.12371 1.1566 0.12371
1.1566 0.12371 0.12371 0.582331266
ASB16 ENSG00000161664 -0.6083 0.72851 -0.6083 0.72851 -0.45093 0.67398

								1.1566	0.12372	0.12372	0.582331266
MMP2-AS1	ENSG00000260135	0.78254	0.21695	0.88067	0.18925	1.1564	0.12376	0.72805	0.23329	0.12376	0.582417347
NA	ENSG00000270557	1.1564	0.12377	1.1564	0.12377	1.1564	0.12377	1.1564	0.12377	0.12377	0.582417347
LAPTM4BP1	ENSG00000237008	1.1055	0.13447	1.1563	0.12378	1.1563	0.12378	0.34273	0.3659	0.12378	0.582417347
NA	ENSG00000256357	0.42607	0.33503	0.42607	0.33503	0.42607	0.33503	1.1562	0.12379	0.12379	0.582417347
NA	ENSG00000273552	-0.64417	0.74027	-0.64417	0.74027	-0.4384	0.66945	1.1562	0.1238	0.1238	0.582417347
SLC18A2	ENSG00000165646	1.1561	0.12381	1.1561	0.12381	1.1561	0.12381	0.19342	0.42331	0.12381	0.582417347
NA	ENSG00000280268	1.156	0.12383	1.156	0.12383	1.156	0.12383	1.156	0.12383	0.12383	0.582455203
NA	ENSG00000282440	0.83835	0.20092	-1.1026	0.86489	1.156	0.12385	-0.28222	0.61111	0.12385	0.582493051
RPL22P8	ENSG00000237745	-0.35027	0.63693	1.1558	0.12387	0.29785	0.38291	0.29785	0.38291	0.12387	0.582530892
NPIPB2	ENSG00000234719	0.55136	0.29069	-0.65762	0.74461	1.1557	0.1239	0.15242	0.43943	0.1239	0.582542224
NA	ENSG00000225142	1.1557	0.12391	1.1557	0.12391	1.1557	0.12391	1.0202	0.15383	0.12391	0.582542224
NA	ENSG00000272130	0.33645	0.36827	1.1555	0.12394	-0.60979	0.729	-0.81701	0.79304	0.12394	0.582542224
TTC29	ENSG00000137473	0.29227	0.38504	1.1555	0.12395	0.56732	0.28525	0.92397	0.17775	0.12395	0.582542224
HNRNPA1P39	ENSG00000237804	1.1555	0.12395	1.1555	0.12395	-0.26346	0.6039	0.17301	0.43132	0.12395	0.582542224
NA	ENSG00000234484	1.1554	0.12396	1.1554	0.12396	1.1554	0.12396	1.1554	0.12396	0.12396	0.582542224
COX6B1P4	ENSG00000253813	1.1554	0.12397	-1.6224	0.94764	-1.6224	0.94764	-1.6224	0.94764	0.12397	0.582542224
NA	ENSG00000258792	0.216	0.41449	0.216	0.41449	1.1554	0.12397	-1.2878	0.90109	0.12397	0.582542224
COX19	ENSG00000240230	1.1553	0.12398	-0.59779	0.72501	0.31563	0.37614	1.0902	0.13782	0.12398	0.582542224
HAUS6	ENSG00000147874	1.1551	0.12402	1.0986	0.13598	0.86211	0.19431	0.2159	0.41453	0.12402	0.582571892
NA	ENSG00000235319	1.1551	0.12402	1.1551	0.12402	1.1551	0.12402	1.1551	0.12402	0.12402	0.582571892
NA	ENSG00000256640	1.1551	0.12403	1.1551	0.12403	1.1551	0.12403	1.1551	0.12403	0.12403	0.582571892
PLEKHG5	ENSG00000171680	1.155	0.12404	0.75217	0.22597	1.0826	0.13949	0.9259	0.17725	0.12404	0.582571892
RNU6-690P	ENSG00000202112	1.1549	0.12407	-0.2593	0.6023	0.16759	0.43345	-0.65264	0.74301	0.12407	0.582571892
NA	ENSG00000237372	0.77652	0.21872	1.1549	0.12407	1.0336	0.15067	0.93582	0.17468	0.12407	0.582571892
TRMT9B	ENSG00000250305	1.1549	0.12407	-0.15092	0.55998	0.88691	0.18756	0.21933	0.4132	0.12407	0.582571892
MRPS5P4	ENSG00000266915	0.51794	0.30225	1.0949	0.13679	1.1548	0.12409	0.14267	0.44328	0.12409	0.582609664
NA	ENSG00000235288	1.1545	0.12414	0.24012	0.40512	0.6507	0.25762	1.1545	0.12414	0.12414	0.582639264
CABYRP1	ENSG00000242983	1.1545	0.12414	0.20943	0.41706	0.20943	0.41706	0.20943	0.41706	0.12414	0.582639264
HINT1	ENSG00000169567	0.97797	0.16405	1.1545	0.12415	0.82213	0.2055	0.77858	0.21811	0.12415	0.582639264
MAN1A2	ENSG00000198162	0.32776	0.37155	0.90086	0.18383	0.86599	0.19325	1.1545	0.12415	0.12415	0.582639264
NA	ENSG00000260126	0.62362	0.26644	0.78853	0.21519	1.1544	0.12417				

-2.5998 0.99534 0.12417 0.582639264
RNU6-135P ENSG00000252578 1.1543 0.12418 -0.196 0.57769 -0.196
0.57769 -0.196 0.57769 0.12418 0.582639264
NA ENSG00000254983 0.54727 0.2921 1.1543 0.12418 -0.33472 0.63108
0.9458 0.17212 0.12418 0.582639264
NA ENSG00000226115 1.1541 0.12422 1.1541 0.12422 -0.46597 0.67938
0.38269 0.35097 0.12422 0.582714729
NA ENSG00000275427 1.1542 0.12422 0.35689 0.36059 0.25495 0.39938
0.081744 0.46743 0.12422 0.582714729
CD70 ENSG00000125726 1.1541 0.12424 -0.26135 0.60309 -1.0071 0.84306
0.18739 0.42568 0.12424 0.582724894
DNM1P51 ENSG00000235370 1.1539 0.12426 1.1539 0.12426 1.1539
0.12426 -0.31551 0.62381 0.12426 0.582724894
RPL23AP72 ENSG00000239840 1.1539 0.12426 -0.056124 0.52238 -0.1297
0.5516 -0.1297 0.5516 0.12426 0.582724894
HMGB1P38 ENSG00000243877 1.1539 0.12427 1.1539 0.12427 1.1539
0.12427 1.1539 0.12427 0.12427 0.582724894
LINC00544 ENSG00000122043 1.1538 0.12429 0.78948 0.21492 1.1538
0.12429 1.1538 0.12429 0.12429 0.582726851
TRAPPC6A ENSG00000007255 1.1537 0.12431 1.1537 0.12431 1.1537
0.12431 0.7742 0.21941 0.12431 0.582726851
NA ENSG00000265393 1.1536 0.12433 1.1536 0.12433 1.1536 0.12433
1.1536 0.12433 0.12433 0.582726851
AOAH ENSG00000136250 -0.84079 0.79977 0.84925 0.19787 1.1535 0.12435
-2.26 0.98809 0.12435 0.582726851
IGLVV-58 ENSG00000253637 1.1535 0.12435 1.1535 0.12435 1.1535
0.12435 1.1535 0.12435 0.12435 0.582726851
SOX14 ENSG00000168875 1.1534 0.12438 -1.0596 0.85534 -0.08935 0.5356
-1.0596 0.85534 0.12438 0.582726851
NA ENSG00000221316 1.1534 0.12438 1.1534 0.12438 0.58906 0.27791
1.1534 0.12438 0.12438 0.582726851
CDH8 ENSG00000150394 0.73231 0.23199 0.70617 0.24004 -0.11956 0.54759
1.1533 0.12439 0.12439 0.582726851
RPL27AP3 ENSG00000236667 0.76462 0.22225 1.1533 0.12439 0.173 0.43133
1.1533 0.12439 0.12439 0.582726851
NA ENSG00000276278 1.1533 0.12439 1.1533 0.12439 1.1533 0.12439
1.1533 0.12439 0.12439 0.582726851
NA ENSG00000198671 1.1532 0.12441 1.1532 0.12441 1.1532 0.12441
1.1532 0.12441 0.12441 0.58276452
NA ENSG00000273433 1.1529 0.12447 1.1529 0.12447 1.1529 0.12447
1.1529 0.12447 0.12447 0.582952757
MIR1293 ENSG00000221604 1.1528 0.12449 1.1528 0.12449 1.1528
0.12449 1.1528 0.12449 0.12449 0.582952757
NA ENSG00000260033 -0.55282 0.70981 1.1528 0.12449 -0.17987 0.57137
1.1528 0.12449 0.12449 0.582952757
NA ENSG00000255655 1.1527 0.12451 1.1527 0.12451 1.1527 0.12451
1.1527 0.12451 0.12451 0.582952757
NA ENSG00000272442 1.1527 0.12451 1.1527 0.12451 1.1527 0.12451
1.1527 0.12451 0.12451 0.582952757
NA ENSG00000240521 -0.001933 0.50077 1.1525 0.12455 -0.8172 0.79309
0.3234 0.3732 0.12455 0.583018807
PLEKHA8P1 ENSG00000134297 1.1525 0.12456 0.75613 0.22478 0.69676
0.24298 0.36995 0.35571 0.12456 0.583018807
FSCN2 ENSG00000186765 1.1525 0.12456 0.36009 0.35939 0.38364 0.35062
1.1525 0.12456 0.12456 0.583018807
MTND1P15 ENSG00000264168 1.1523 0.1246 1.1523 0.1246 1.1523
0.1246 1.1523 0.1246 0.1246 0.583131611
HMGB3P19 ENSG00000217085 0.14726 0.44146 0.84802 0.19821 0.66493
0.25305 1.1522 0.12462 0.12462 0.583131611
NA ENSG00000275542 0.87207 0.19159 0.87207 0.19159 1.1522 0.12462
-0.98198 0.83695 0.12462 0.583131611
C11orf88 ENSG00000183644 1.1521 0.12464 0.82013 0.20607 0.82013

0.20607 0.82013 0.20607 0.12464 0.583150792
SASS6 ENSG00000156876 1.152 0.12466 -0.7247 0.76568 1.152 0.12466 1.152
0.12466 0.12466 0.583150792
LINC02255 ENSG00000277718 0.97253 0.16539 0.81627 0.20717 1.152 0.12466
0.61558 0.26909 0.12466 0.583150792
COL4A2 ENSG00000134871 0.79236 0.21408 -0.84239 0.80021 -0.015305
0.50611 1.1518 0.12471 0.12471 0.583216727
NUDT16P1 ENSG00000246082 1.1517 0.12471 1.1517 0.12471 0.8575
0.19559 1.0186 0.1542 0.12471 0.583216727
NA ENSG00000272733 1.1518 0.12471 1.1518 0.12471 1.1518 0.12471
1.1518 0.12471 0.12471 0.583216727
RIOX1 ENSG00000170468 -0.0094606 0.50377 -0.20197 0.58003 1.1516 0.12473
0.88436 0.18825 0.12473 0.583254285
RN7SL372P ENSG00000242565 1.1515 0.12477 0.82519 0.20463 0.76845
0.22111 0.82519 0.20463 0.12477 0.583385348
MAPK15 ENSG00000181085 1.0424 0.14861 0.8489 0.19797 0.082375
0.46717 1.1511 0.12484 0.12484 0.583508957
EEF1GP1 ENSG00000186676 -0.37682 0.64685 0.50919 0.30531 1.151 0.12486
-0.48497 0.68615 0.12486 0.583508957
FNIP1 ENSG00000217128 0.65775 0.25535 1.1509 0.12488 0.33714 0.368
0.69029 0.24501 0.12488 0.583508957
NA ENSG00000234675 0.10637 0.45765 -0.28972 0.61398 0.10637 0.45765
1.151 0.12488 0.12488 0.583508957
MIR4510 ENSG00000265098 -0.11701 0.54657 1.1509 0.12489 -0.11701
0.54657 -0.11701 0.54657 0.12489 0.583508957
NA ENSG00000253496 0.59497 0.27593 0.22289 0.41181 0.70532 0.24031
1.1509 0.1249 0.1249 0.583508957
NA ENSG00000271714 1.1507 0.12492 1.1507 0.12492 1.1507 0.12492
1.1507 0.12492 0.12492 0.583508957
RNU6-1082P ENSG00000201444 1.1507 0.12494 -0.82714 0.79592 1.1507
0.12494 1.1507 0.12494 0.12494 0.583508957
RPL29P28 ENSG00000232378 1.1506 0.12494 1.1506 0.12494 1.1506
0.12494 -0.3052 0.61989 0.12494 0.583508957
PCYT1A ENSG00000161217 -0.018708 0.50746 0.68801 0.24572 0.85145
0.19726 1.1506 0.12495 0.12495 0.583508957
MIR617 ENSG00000207763 1.1506 0.12495 1.1506 0.12495 1.1506
0.12495 1.1506 0.12495 0.12495 0.583508957
RPL12P35 ENSG00000243007 0.27272 0.39253 0.27272 0.39253 0.27272
0.39253 1.1506 0.12496 0.12496 0.583508957
NA ENSG00000274441 0.89814 0.18456 -0.79829 0.78765 1.1505 0.12497
1.145 0.12611 0.12497 0.583508957
AGPS ENSG00000018510 0.9764 0.16443 1.1504 0.12499 1.0778 0.14055
-0.41834 0.66215 0.12499 0.583508957
H3P36 ENSG00000236534 1.1504 0.12499 0.29434 0.38425 0.29434 0.38425
0.5579 0.28846 0.12499 0.583508957
NA ENSG00000257680 1.1504 0.12499 1.1504 0.12499 1.1504 0.12499
1.1504 0.12499 0.12499 0.583508957
LSP1 ENSG00000130592 -0.25796 0.60178 1.1504 0.125 0.21097 0.41646
0.028422 0.48866 0.125 0.583508957
ZNF585B ENSG00000245680 0.54898 0.29151 0.34322 0.36571 -0.20701 0.582
1.1502 0.12503 0.12503 0.583583871
RHBDD1 ENSG00000144468 -0.47897 0.68402 -1.0255 0.84744 0.72984
0.23275 1.1502 0.12504 0.12504 0.583583871
BOD1L2 ENSG00000228075 1.1499 0.12509 1.1499 0.12509 1.1499
0.12509 1.1499 0.12509 0.12509 0.583724383
NA ENSG00000258803 1.1328 0.12865 1.1014 0.13535 0.91433 0.18027
1.1498 0.1251 0.1251 0.583724383
DNAJC27 ENSG00000115137 1.0255 0.15257 0.69331 0.24406 1.1498
0.12511 0.43261 0.33265 0.12511 0.583724383
H2BU2P ENSG00000181201 1.1497 0.12513 1.1497 0.12513 1.1497
0.12513 1.1497 0.12513 0.12513 0.583724383
NA ENSG00000232821 -0.19591 0.57766 1.1497 0.12513 0.068054 0.47287

0.068054 0.47287 0.12513 0.583724383
RALGAPA1P1 ENSG00000229419 1.1496 0.12515 1.1496 0.12515 -1.0468
0.85241 -0.72392 0.76544 0.12515 0.583743334
ACRBP ENSG00000111644 0.986 0.16207 0.8019 0.2113 1.1496 0.12516
0.89724 0.18479 0.12516 0.583743334
HYAL2 ENSG00000068001 1.1495 0.12517 -1.9366 0.9736 1.141 0.12693
-1.9366 0.9736 0.12517 0.583743334
RPF2P1 ENSG00000225357 0.99445 0.16 0.99445 0.16 1.0731 0.1416
1.1494 0.12519 0.12519 0.583780737
MLIP ENSG00000146147 0.7285 0.23315 1.1493 0.12522 0.25803 0.39819
0.09332 0.46282 0.12522 0.583790434
RN7SL714P ENSG00000241757 -1.3229 0.90707 1.1493 0.12522 -1.3229
0.90707 -1.3229 0.90707 0.12522 0.583790434
MIR3125 ENSG00000264370 1.1492 0.12523 0.99768 0.15922 -0.32852
0.62874 -1.054 0.85407 0.12523 0.583790434
NA ENSG00000270265 -0.19676 0.57799 0.7845 0.21637 1.1492 0.12524
-1.0836 0.86073 0.12524 0.583790434
NA ENSG00000254273 1.149 0.12527 1.149 0.12527 1.149 0.12527 1.149 0.12527
0.12527 0.583874424
DNM1P34 ENSG00000260357 0.12902 0.44867 -0.044565 0.51777 1.1489
0.1253 -0.48056 0.68459 0.1253 0.583902963
IFNA16 ENSG00000147885 1.1488 0.12532 0.59656 0.2754 0.25517
0.39929 0.74444 0.2283 0.12532 0.583902963
RNU6-546P ENSG00000206718 -0.1787 0.57091 0.091118 0.4637 1.1487
0.12533 -0.90866 0.81824 0.12533 0.583902963
LSM1P1 ENSG00000233551 1.1488 0.12533 1.1488 0.12533 1.1488
0.12533 1.1488 0.12533 0.12533 0.583902963
RNU6-348P ENSG00000207114 0.90122 0.18374 0.9701 0.166 0.90122 0.18374
1.1487 0.12534 0.12534 0.583902963
NA ENSG00000237870 1.1487 0.12535 0.25418 0.39968 1.1487 0.12535
0.7229 0.23487 0.12535 0.583902963
C1orf68 ENSG00000198854 1.1486 0.12536 1.1486 0.12536 1.1486
0.12536 1.1486 0.12536 0.12536 0.583902963
TLE4 ENSG00000106829 1.1485 0.12538 -0.10814 0.54306 1.1485 0.12538
-0.11213 0.54464 0.12538 0.583940304
KLK2 ENSG00000167751 1.1482 0.12544 0.12586 0.44992 1.1482 0.12544
1.1482 0.12544 0.12544 0.583972164
HOXD-AS2 ENSG00000237380 -1.3254 0.90747 -0.96289 0.8322 -1.219
0.88858 1.1482 0.12544 0.12544 0.583972164
HMGB1P7 ENSG00000231148 1.1481 0.12546 0.70749 0.23963 1.1481
0.12546 -0.69797 0.7574 0.12546 0.583972164
GJA4 ENSG00000187513 -1.2579 0.89578 -1.2579 0.89578 0.71364 0.23772
1.1481 0.12547 0.12547 0.583972164
NA ENSG00000261060 1.1478 0.12552 1.1478 0.12552 1.1478 0.12552
1.1478 0.12552 0.12552 0.583972164
PDE7A ENSG00000205268 -0.27783 0.60943 1.1478 0.12553 -0.27783 0.60943
-0.92407 0.82227 0.12553 0.583972164
STBD1 ENSG00000118804 0.38788 0.34905 1.1477 0.12555 0.51855 0.30204
0.8897 0.18681 0.12555 0.583972164
NA ENSG00000234997 1.1476 0.12556 1.1476 0.12556 -0.59697 0.72474
-0.41236 0.65996 0.12556 0.583972164
SLC2A7 ENSG00000197241 0.59569 0.27569 0.061969 0.47529 1.1476
0.12557 -0.48659 0.68673 0.12557 0.583972164
NDUFB6 ENSG00000165264 -0.088554 0.53528 1.1475 0.12558 0.65486
0.25628 0.78547 0.21609 0.12558 0.583972164
NA ENSG00000226072 1.1475 0.12559 1.1475 0.12559 1.1475 0.12559
1.1475 0.12559 0.12559 0.583972164
MAPK8 ENSG00000107643 0.47254 0.31827 1.1474 0.1256 1.1474 0.1256
1.1474 0.1256 0.1256 0.583972164
NDUFS5P6 ENSG00000255531 0.56982 0.2844 1.1474 0.12561 0.62093
0.26732 0.19893 0.42116 0.12561 0.583972164
ARG1 ENSG00000118520 -0.41366 0.66044 0.50721 0.306 1.1474 0.12562

1.1474 0.12562 0.12562 0.583972164
SLC13A5 ENSG00000141485 0.87338 0.19123 1.1473 0.12562 -0.46985
0.68077 -0.41464 0.6608 0.12562 0.583972164
ZNF33B ENSG00000196693 1.1473 0.12562 -0.34447 0.63475 0.017973
0.49283 -0.23842 0.59422 0.12562 0.583972164
C1QTNF2 ENSG00000145861 0.86589 0.19328 0.32313 0.3733 1.1473
0.12563 1.0677 0.14282 0.12563 0.583972164
SLC2A9 ENSG00000109667 -0.80276 0.78894 -1.2208 0.88893 1.1472
0.12564 0.61437 0.26949 0.12564 0.583972164
LINC01241 ENSG00000236306 0.67776 0.24896 1.1472 0.12565 -1.1737
0.87975 -2.0897 0.98168 0.12565 0.583972164
HMGB1P13 ENSG00000220557 0.9902 0.16104 0.65788 0.25531 0.92391
0.17777 1.1472 0.12566 0.12566 0.583972164
SEMA4A ENSG00000196189 1.1101 0.13348 1.1471 0.12568 0.7832
0.21675 0.8588 0.19523 0.12568 0.583972164
NA ENSG00000248337 1.1471 0.12568 -0.07744 0.53086 -0.07744 0.53086
-0.07744 0.53086 0.12568 0.583972164
NA ENSG00000279334 1.147 0.12568 1.147 0.12568 1.147 0.12568 1.147 0.12568
0.12568 0.583972164
NA ENSG00000226238 1.147 0.12569 0.34068 0.36667 1.147 0.12569 1.147
0.12569 0.12569 0.583972164
TOLLIP ENSG00000078902 0.57458 0.28279 -2.0007 0.97729 0.28538
0.38768 1.1469 0.12571 0.12571 0.583972164
SUSD4 ENSG00000143502 -0.12605 0.55015 1.1469 0.12571 0.92859 0.17655
0.13985 0.44439 0.12571 0.583972164
PPM1E ENSG00000175175 -0.1822 0.57229 1.1468 0.12573 -0.015923 0.50635
-0.01463 0.50584 0.12573 0.583972164
NA ENSG00000259544 1.1468 0.12574 1.1468 0.12574 -0.017002 0.50678
-0.017002 0.50678 0.12574 0.583972164
HTR3D ENSG00000186090 1.1467 0.12575 1.1467 0.12575 -0.093533 0.53726
1.1467 0.12575 0.12575 0.583972164
PCDHGB9P ENSG00000276545 1.1467 0.12575 0.92788 0.17674 1.1467
0.12575 1.1467 0.12575 0.12575 0.583972164
RASL10A ENSG00000100276 -0.28129 0.61076 1.084 0.13919 -0.28129 0.61076
1.1465 0.1258 0.1258 0.583972164
NKPD1 ENSG00000179846 1.1464 0.12582 1.1464 0.12582 1.1464 0.12582
1.1464 0.12582 0.12582 0.583972164
PBX1 ENSG00000185630 1.1463 0.12584 0.49967 0.30865 -0.6487 0.74174
-0.30042 0.61807 0.12584 0.583972164
NA ENSG00000228779 0.37588 0.3535 0.69663 0.24302 -0.12842 0.55109
1.1463 0.12584 0.12584 0.583972164
NA ENSG00000277206 1.1463 0.12584 1.1463 0.12584 1.1463 0.12584
0.93583 0.17468 0.12584 0.583972164
RN7SL178P ENSG00000263616 0.8037 0.21078 0.0016094 0.49936 1.1462
0.12585 -0.6087 0.72864 0.12585 0.583972164
CLUAP1 ENSG00000103351 0.59739 0.27512 0.30393 0.38059 1.1462
0.12586 0.86911 0.19239 0.12586 0.583972164
BSPH1 ENSG00000188334 0.025103 0.48999 -0.0049276 0.50197 1.1462 0.12586
-1.0503 0.85322 0.12586 0.583972164
NA ENSG00000233805 1.1461 0.12588 1.1461 0.12588 0.87668 0.19033
1.1461 0.12588 0.12588 0.583972164
NA ENSG00000277711 1.1426 0.1266 1.146 0.12589 1.146 0.12589 1.146
0.12589 0.12589 0.583972164
RNU6-281P ENSG00000206939 1.146 0.1259 1.146 0.1259 1.146 0.1259 1.146
0.1259 0.1259 0.583972164
NA ENSG00000257869 0.11985 0.4523 -1.2214 0.88904 0.5292 0.29833
1.146 0.12591 0.12591 0.583972164
KCNH5 ENSG00000140015 0.98675 0.16188 0.57714 0.28192 0.60167 0.2737
1.1459 0.12593 0.12593 0.583972164
RNA5SP433 ENSG00000201361 1.1458 0.12594 1.1458 0.12594 -0.22628
0.58951 -1.4092 0.92062 0.12594 0.583972164
NA ENSG00000260858 1.1458 0.12594 1.1458 0.12594 1.1458 0.12594

								1.1458	0.12594	0.12594	0.583972164
NSUN2	ENSG00000037474	-0.19262	0.57637	0.57572	0.2824	1.1457	0.12595	0.23267	0.40801	0.12595	0.583972164
MORN4	ENSG00000171160	-0.27976	0.61017	-0.27976	0.61017	1.1457	0.12597	0.17311	0.43128	0.12597	0.583972164
TICAM1	ENSG00000127666	0.44964	0.32649	1.1455	0.126	-0.27509	0.60838	-0.52322	0.69959	0.126	0.583972164
NCF4-AS1	ENSG00000183822	1.1455	0.126	1.1241	0.13048	1.1455	0.126	1.1455	0.126	0.126	0.583972164
NA	ENSG00000229294	1.1455	0.126	1.1455	0.126	1.1455	0.126	1.1455	0.126	0.126	0.583972164
CBX3P4	ENSG00000256030	1.1455	0.12601	1.1455	0.12601	1.1455	0.12601	1.1455	0.12601	0.12601	0.583972164
EEF1A1P2	ENSG00000258841	1.1455	0.12601	1.1455	0.12601	1.1455	0.12601	1.1455	0.12601	0.12601	0.583972164
NA	ENSG00000242588	-0.41658	0.66151	0.18344	0.42723	-1.539	0.9381	1.1451	0.12608	0.12608	0.584222571
PTPRN	ENSG00000054356	0.59116	0.27721	0.35351	0.36185	1.145	0.1261	0.7713	0.22027	0.1261	0.584222571
MEOX2	ENSG00000106511	1.145	0.1261	1.145	0.1261	1.145	0.1261	1.145	0.1261	0.1261	0.584222571
NALCN-AS1	ENSG00000233009	1.1449	0.12612	0.089269	0.46443	-0.6821	0.75241	-0.68739	0.75408	0.12612	0.584241251
RNU6-794P	ENSG00000253070	-0.0058881	0.50235	-0.0058881	0.50235	1.1448	0.12614	-0.0058881	0.50235	0.12614	0.584241251
NA	ENSG00000263472	-0.58273	0.71996	-0.067289	0.52682	1.1448	0.12614	-0.43113	0.66681	0.12614	0.584241251
SNORD92	ENSG00000264994	1.1446	0.12618	1.1446	0.12618	1.1446	0.12618	1.1446	0.12618	0.12618	0.584370975
NA	ENSG00000255606	-1.7019	0.95561	1.1445	0.1262	0.52466	0.29991	-1.0091	0.84354	0.1262	0.584398831
NA	ENSG00000279407	1.1445	0.12621	1.1445	0.12621	1.1445	0.12621	1.1445	0.12621	0.12621	0.584398831
RRP1B	ENSG00000160208	0.079336	0.46838	-0.53175	0.70255	-0.58197	0.71971	1.1444	0.12623	0.12623	0.584435905
RAB5CP2	ENSG00000251356	1.1442	0.12628	1.1442	0.12628	1.1442	0.12628	1.1442	0.12628	0.12628	0.584574891
NA	ENSG00000223503	1.0548	0.14577	0.069413	0.47233	1.144	0.12631	-0.038416	0.51532	0.12631	0.584574891
ZC3H7A	ENSG00000122299	-0.051166	0.5204	-0.051166	0.5204	-0.051166	0.5204	1.144	0.12632	0.12632	0.584574891
KRT19P1	ENSG00000218014	1.1439	0.12632	1.1439	0.12632	1.1439	0.12632	1.1439	0.12632	0.12632	0.584574891
TCF24	ENSG00000261787	1.144	0.12632	0.81165	0.2085	0.34786	0.36397	-0.24909	0.59836	0.12632	0.584574891
NA	ENSG00000231856	1.1437	0.12637	0.35479	0.36137	-0.4861	0.68655	-0.1825	0.5724	0.12637	0.584603208
LINC01231	ENSG00000236511	1.1436	0.12639	1.1436	0.12639	-0.079167	0.53155	1.1436	0.12639	0.12639	0.584603208
GPR89P	ENSG00000216915	-0.70301	0.75898	1.1435	0.12642	-0.36176	0.64123	-0.48212	0.68514	0.12642	0.584603208
NAV1	ENSG00000134369	1.1434	0.12643	-0.84471	0.80086	1.0585	0.14491	0.29397	0.38439	0.12643	0.584603208
SLK	ENSG00000065613	0.11754	0.45322	0.52868	0.29851	1.1434	0.12644	0.061199	0.4756	0.12644	0.584603208
NA	ENSG00000277646	1.1433	0.12646	1.1433	0.12646	1.1433	0.12646	1.1433	0.12646	0.12646	0.584603208
NA	ENSG00000230090	1.1432	0.12647	0.59073	0.27735	0.0086508	0.49655	-1.1629	0.87756	0.12647	0.584603208
CCDC18	ENSG00000122483	-0.15947	0.56335	0.27667	0.39102	1.1432	0.12649	0.75266	0.22583	0.12649	0.584603208
LINC02296	ENSG00000258859	-0.3363	0.63168	1.1432	0.12649	0.72018					

0.23571 -0.33272 0.63033 0.12649 0.584603208
CNOT1 ENSG00000125107 0.8788 0.18976 0.90597 0.18248 1.143 0.12653
0.45216 0.32558 0.12653 0.584603208
LINC00491 ENSG00000250682 0.96738 0.16668 0.96738 0.16668 1.1429
0.12653 0.96738 0.16668 0.12653 0.584603208
MRGPRD ENSG00000172938 -1.0898 0.8621 -1.0898 0.8621 1.135 0.12819
1.1428 0.12655 0.12655 0.584603208
ZNF70 ENSG00000187792 1.1426 0.12661 1.1426 0.12661 1.1426 0.12661
1.1426 0.12661 0.12661 0.584603208
ADCY6 ENSG00000174233 0.31747 0.37544 0.84858 0.19806 0.83011 0.20324
1.1425 0.12662 0.12662 0.584603208
POU5F1P5 ENSG00000236375 1.1425 0.12663 0.69754 0.24273 1.1425
0.12663 -1.4765 0.93009 0.12663 0.584603208
H6PD ENSG00000049239 1.1424 0.12664 -1.3423 0.91024 -0.32554 0.62762
-1.0318 0.84892 0.12664 0.584603208
METTL9 ENSG00000197006 -0.88885 0.81296 0.50874 0.30547 -0.88885
0.81296 1.1424 0.12664 0.12664 0.584603208
LRFN3 ENSG00000126243 1.1424 0.12665 1.1424 0.12665 1.1424 0.12665
1.1424 0.12665 0.12665 0.584603208
NA ENSG00000259735 -0.30141 0.61845 -1.1309 0.87095 0.17778 0.42945
1.1424 0.12665 0.12665 0.584603208
NCSTN ENSG00000162736 1.1423 0.12666 0.78317 0.21676 1.1423 0.12666
1.1423 0.12666 0.12666 0.584603208
ARHGAP29 ENSG00000137962 -0.50236 0.69229 -1.4952 0.93257 -1.8455
0.96752 1.1422 0.12668 0.12668 0.584603208
PHKBP2 ENSG00000258467 1.1422 0.12668 0.018579 0.49259 1.1422
0.12668 1.1422 0.12668 0.12668 0.584603208
AKR1C1 ENSG00000187134 1.1421 0.1267 0.70809 0.23944 0.056588
0.47744 0.57862 0.28142 0.1267 0.584603208
NA ENSG00000238570 -1.3797 0.91616 0.699 0.24228 0.699 0.24228 1.1421
0.1267 0.1267 0.584603208
SLC35B4 ENSG00000205060 0.9619 0.16805 1.1421 0.12671 1.1421
0.12671 1.1049 0.1346 0.12671 0.584603208
RNA5SP236 ENSG00000251978 1.1421 0.12671 1.1421 0.12671 1.1421
0.12671 1.1421 0.12671 0.12671 0.584603208
RFT1 ENSG00000163933 1.142 0.12672 1.0562 0.14543 0.90626 0.1824
1.0501 0.14683 0.12672 0.584603208
RPL31P44 ENSG00000213657 0.82052 0.20596 1.142 0.12673 1.142 0.12673 1.142
0.12673 0.12673 0.584603208
NDUFB9P2 ENSG00000236281 1.142 0.12673 0.15679 0.4377 1.142 0.12673
0.0090713 0.49638 0.12673 0.584603208
RCC2P7 ENSG00000220666 0.23751 0.40613 -0.59643 0.72456 1.1419
0.12674 1.1373 0.1277 0.12674 0.584603208
RNU6-887P ENSG00000222592 1.1419 0.12675 1.1419 0.12675 0.78596
0.21595 1.1332 0.12857 0.12675 0.584603208
CYP1D1P ENSG00000228024 -0.53146 0.70245 1.1419 0.12675 -0.53146
0.70245 -0.53146 0.70245 0.12675 0.584603208
NA ENSG00000257243 1.1419 0.12675 1.1419 0.12675 1.1419 0.12675
1.1419 0.12675 0.12675 0.584603208
USP19 ENSG00000172046 1.1418 0.12677 1.1241 0.13049 1.0565 0.14537
0.68033 0.24815 0.12677 0.584603208
RNU11-5P ENSG00000212518 -0.55781 0.71151 0.39172 0.34763 1.1418
0.12677 1.1418 0.12677 0.12677 0.584603208
PPIAP27 ENSG00000234363 0.40833 0.34152 0.55776 0.2885 1.1418
0.12677 1.1418 0.12677 0.12677 0.584603208
NA ENSG00000274353 -1.3611 0.91326 0.60078 0.27399 1.1418 0.12677
-1.1885 0.88268 0.12677 0.584603208
MSTO2P ENSG00000203761 1.1416 0.12681 -0.44378 0.6714 -0.44378
0.6714 -0.44378 0.6714 0.12681 0.584667796
ADAM3A ENSG00000197475 1.1416 0.12682 0.75277 0.22579 -1.1299
0.87074 0.23951 0.40536 0.12682 0.584667796
NA ENSG00000269086 0.93942 0.17376 1.1416 0.12682 0.93942 0.17376

								0.93942	0.17376	0.12682	0.584667796
NA	ENSG00000197846	1.1374	0.12769	0.67963	0.24837	-0.14402	0.55726	1.1415	0.12684	0.12684	0.584695469
LINC01070	ENSG00000260102	0.22021	0.41285	-0.49896	0.69109	-1.2997	0.90316	1.1414	0.12685	0.12685	0.584695469
RNU6-1290P	ENSG00000199827	1.1413	0.12687	-0.53205	0.70265	-0.34424	0.63467	-0.34424	0.63467	0.12687	0.58469652
RN7SKP293	ENSG00000223321	0.21676	0.4142	0.21676	0.4142	1.1413	0.12688	0.21676	0.4142	0.12688	0.58469652
MEPCE	ENSG00000146834	-0.15684	0.56232	-0.88312	0.81141	0.17308	0.4313	1.1412	0.12689	0.12689	0.58469652
RNU1-1	ENSG00000206652	-1.0752	0.85885	-1.7866	0.963	1.1412	0.1269	-0.60335	0.72686	0.1269	0.58469652
PRKCA	ENSG00000154229	0.66304	0.25365	1.1409	0.12695	0.20446	0.419	0.61365	0.26972	0.12695	0.58469652
NA	ENSG00000254492	-0.762	0.77697	0.13627	0.44581	1.1409	0.12695	-0.32858	0.62877	0.12695	0.58469652
MIR5092	ENSG00000264986	0.64464	0.25958	0.49693	0.30962	0.2235	0.41157	1.1409	0.12696	0.12696	0.58469652
NA	ENSG00000261729	1.121	0.13115	0.44177	0.32933	0.77261	0.21988	1.1408	0.12697	0.12697	0.58469652
STIM2	ENSG00000109689	1.1405	0.12703	-1.2358	0.89173	-1.3733	0.91516	-1.2054	0.88597	0.12703	0.58469652
NA	ENSG00000277829	-0.71184	0.76172	1.1405	0.12704	1.1405	0.12704	1.1405	0.12704	0.12704	0.58469652
FAM99B	ENSG00000205865	-0.69554	0.75664	-0.45779	0.67645	1.1404	0.12705	0.20512	0.41874	0.12705	0.58469652
NA	ENSG00000231563	1.1404	0.12706	1.1404	0.12706	1.1404	0.12706	1.1404	0.12706	0.12706	0.58469652
NA	ENSG00000260234	1.1404	0.12707	1.1404	0.12707	1.1404	0.12707	1.1404	0.12707	0.12707	0.58469652
NA	ENSG00000201228	1.1403	0.12708	1.1403	0.12708	1.1403	0.12708	1.1403	0.12708	0.12708	0.58469652
RNU6-1156P	ENSG00000222297	1.1403	0.12708	1.1403	0.12708	1.1403	0.12708	1.1403	0.12708	0.12708	0.58469652
RNU5F-6P	ENSG00000199849	-0.28695	0.61292	0.77503	0.21916	1.1402	0.12711	-0.38184	0.64871	0.12711	0.58469652
RN7SL519P	ENSG00000243075	0.015223	0.49393	1.1401	0.12711	0.45153	0.3258	0.45153	0.3258	0.12711	0.58469652
NA	ENSG00000256922	1.1401	0.12712	0.83559	0.20169	-0.40509	0.65729	-0.40509	0.65729	0.12712	0.58469652
TBC1D9	ENSG00000109436	1.14	0.12714	0.92591	0.17725	1.14	0.12714	-0.081709	0.53256	0.12714	0.58469652
NA	ENSG00000231236	-0.49784	0.6907	1.1399	0.12715	0.901	0.18379	1.0462	0.14773	0.12715	0.58469652
NA	ENSG00000207209	1.1399	0.12716	1.1399	0.12716	1.1399	0.12716	0.37871	0.35245	0.12716	0.58469652
ORAI3	ENSG00000175938	1.1397	0.1272	1.1397	0.1272	1.1397	0.1272	1.1397	0.1272	0.1272	0.58469652
NA	ENSG00000261487	1.1397	0.1272	1.1397	0.1272	1.1397	0.1272	1.1397	0.1272	0.1272	0.58469652
COPS5	ENSG00000121022	1.1397	0.12721	0.34319	0.36573	1.1397	0.12721	0.41605	0.33869	0.12721	0.58469652
RRP1	ENSG00000160214	0.9197	0.17887	1.1396	0.12722	0.74987	0.22667	0.14751	0.44136	0.12722	0.58469652
NA	ENSG00000218565	1.1396	0.12722	-0.70459	0.75947	1.1396	0.12722	1.1396	0.12722	0.12722	0.58469652
CARHSP1	ENSG00000153048	0.74012	0.22961	0.70518	0.24035	0.74012	0.22961	1.1395	0.12724	0.12724	0.58469652
PCA3	ENSG00000225937	0.40441	0.34295	0.95989	0.16855	0.99222	0.16055	1.1395	0.12725	0.12725	0.58469652
NA	ENSG00000275880	1.1395	0.12725	1.1395	0.12725	1.1395	0.12725				

1.1395 0.12725 0.12725 0.58469652
KL ENSG00000133116 -0.014374 0.50573 0.56529 0.28594 -0.014374 0.50573
1.1394 0.12726 0.12726 0.58469652
FBXL7 ENSG00000183580 1.1257 0.13015 0.83121 0.20293 -0.94381 0.82737
1.1394 0.12726 0.12726 0.58469652
NA ENSG00000206711 1.1394 0.12726 -0.53262 0.70285 -0.53262 0.70285
-0.53262 0.70285 0.12726 0.58469652
LINC01416 ENSG00000260930 1.1393 0.1273 -0.49289 0.68895 0.92999
0.17619 -0.15985 0.5635 0.1273 0.58469652
RNF113B ENSG00000139797 1.019 0.1541 1.1392 0.12731 1.0875 0.13841
-0.085788 0.53418 0.12731 0.58469652
RNA5SP88 ENSG00000202430 1.1392 0.12731 1.1392 0.12731 1.1392
0.12731 1.1392 0.12731 0.12731 0.58469652
NA ENSG00000236395 1.1391 0.12733 1.0268 0.15225 0.6128 0.27
1.0268 0.15225 0.12733 0.58469652
NA ENSG00000212479 1.032 0.15104 0.94803 0.17156 1.1389 0.12737
1.1389 0.12737 0.12737 0.58469652
NA ENSG00000234089 0.40209 0.34381 0.40209 0.34381 1.1389 0.12737
-0.062227 0.52481 0.12737 0.58469652
NA ENSG00000280014 1.1388 0.1274 1.1388 0.1274 1.1388 0.1274
1.1388 0.1274 0.1274 0.58469652
SUMO2 ENSG00000188612 -0.36368 0.64195 1.1386 0.12743 -0.95808 0.83099
0.27679 0.39097 0.12743 0.58469652
RPL9P25 ENSG00000240821 -0.68064 0.75195 0.8238 0.20503 1.1386
0.12743 1.1386 0.12743 0.12743 0.58469652
ABCB5 ENSG00000004846 1.1385 0.12745 -0.64284 0.73984 -0.60795 0.72839
-0.26174 0.60324 0.12745 0.58469652
NA ENSG00000261072 1.1384 0.12747 1.1384 0.12747 1.1384 0.12747
1.1384 0.12747 0.12747 0.58469652
LINC02582 ENSG00000261780 -1.0483 0.85275 -0.66901 0.74826 1.1383
0.12751 -1.021 0.84636 0.12751 0.58469652
MIR2681 ENSG00000265164 0.26773 0.39446 -0.13308 0.55293 0.35613
0.36087 1.1381 0.12753 0.12753 0.58469652
NA ENSG00000226750 1.1381 0.12755 -3.0729 0.99894 -0.66654 0.74747
-0.65426 0.74353 0.12755 0.58469652
NA ENSG00000240057 0.65614 0.25587 0.62857 0.26482 1.1381 0.12755
0.067288 0.47318 0.12755 0.58469652
NA ENSG00000223691 1.1379 0.12758 1.1201 0.13134 1.1201 0.13134
0.2908 0.3856 0.12758 0.58469652
NA ENSG00000173862 -0.55412 0.71025 1.1379 0.12759 -0.55412 0.71025
-0.55412 0.71025 0.12759 0.58469652
SCRN3 ENSG00000144306 1.1378 0.1276 1.0487 0.14717 1.0487 0.14717
0.055591 0.47783 0.1276 0.58469652
GAPDHP29 ENSG00000178596 -0.21029 0.58328 1.1378 0.1276 1.1378
0.1276 1.1378 0.1276 0.1276 0.58469652
NA ENSG00000255139 1.1312 0.12898 1.1378 0.12761 1.1312 0.12898
1.1312 0.12898 0.12761 0.58469652
SLC15A1 ENSG00000088386 -0.18444 0.57317 -0.01107 0.50442 0.25791
0.39824 1.1377 0.12762 0.12762 0.58469652
FAM99A ENSG00000205866 0.48118 0.3152 0.48118 0.3152 -1.1191
0.86845 1.1377 0.12762 0.12762 0.58469652
NA ENSG00000264694 1.1377 0.12762 0.785 0.21623 0.50128 0.30809
0.14667 0.4417 0.12762 0.58469652
LINC00445 ENSG00000236036 1.1376 0.12763 1.1376 0.12763 1.1376
0.12763 1.1376 0.12763 0.12763 0.58469652
TERT ENSG00000164362 -0.93236 0.82442 -0.59465 0.72396 -0.27521 0.60842
1.1376 0.12764 0.12764 0.58469652
NA ENSG00000272582 1.1373 0.1277 1.1373 0.1277 1.1373 0.1277
1.1373 0.1277 0.1277 0.58469652
ZNF321P ENSG00000213801 -0.85031 0.80242 0.79907 0.21212 -1.2328
0.89118 1.1373 0.12771 0.12771 0.58469652
TMEM202 ENSG00000187806 -0.1342 0.55338 0.49474 0.31039 1.1372

0.12773 0.28087 0.38941 0.12773 0.58469652
TOMM40P4 ENSG00000213222 1.1372 0.12773 1.1372 0.12773 1.1372
0.12773 1.1372 0.12773 0.12773 0.58469652
KIAA1143P1 ENSG00000259086 1.137 0.12777 0.58818 0.27821 -0.073152 0.52916
-1.7321 0.95837 0.12777 0.58469652
LINC00888 ENSG00000240024 0.20703 0.41799 1.137 0.12778 -0.12339 0.5491
-0.12339 0.5491 0.12778 0.58469652
LINC02160 ENSG00000251443 1.1369 0.12778 -0.37995 0.64801 -0.58084
0.71933 0.75886 0.22397 0.12778 0.58469652
NA ENSG00000249411 0.15142 0.43982 1.1368 0.12781 -0.84954 0.80221
-0.43228 0.66723 0.12781 0.58469652
NA ENSG00000266409 1.1368 0.12782 1.1368 0.12782 1.1368 0.12782
1.1368 0.12782 0.12782 0.58469652
NA ENSG00000268473 1.0258 0.1525 -1.0868 0.86144 1.1367 0.12783
-0.14575 0.55794 0.12783 0.58469652
USP4 ENSG00000114316 0.72788 0.23334 1.1366 0.12785 -0.27813 0.60954
-0.093951 0.53743 0.12785 0.58469652
RNU4-32P ENSG00000200274 1.1364 0.1279 1.1364 0.1279 1.1364
0.1279 1.1364 0.1279 0.1279 0.58469652
MRFAP1L1 ENSG00000178988 0.55676 0.28885 1.1363 0.12791 0.4439
0.32856 0.92667 0.17705 0.12791 0.58469652
GDPD3 ENSG00000102886 1.1362 0.12795 1.1362 0.12795 1.1362 0.12795
0.89132 0.18638 0.12795 0.58469652
NA ENSG00000239344 1.1361 0.12795 0.10068 0.4599 -1.8024 0.96426
-0.18877 0.57486 0.12795 0.58469652
CLIP4 ENSG00000115295 0.60483 0.27265 1.1361 0.12796 0.88366 0.18844
0.97225 0.16546 0.12796 0.58469652
NA ENSG00000233852 1.136 0.12797 0.83768 0.20111 1.136 0.12797 1.136
0.12797 0.12797 0.58469652
SUCLA2 ENSG00000136143 1.136 0.12798 0.91464 0.18019 0.92258 0.17811
0.76075 0.2234 0.12798 0.58469652
NA ENSG00000200011 0.80917 0.20921 0.80917 0.20921 1.136 0.12799 1.136
0.12799 0.12799 0.58469652
NA ENSG00000259015 1.1359 0.128 1.1359 0.128 1.1359 0.128 1.1359 0.128
0.128 0.58469652
TMED9 ENSG00000184840 0.77774 0.21836 1.1359 0.12801 0.49978 0.30861
-0.17873 0.57093 0.12801 0.58469652
LINC02143 ENSG00000253236 1.1358 0.12802 -0.17868 0.57091 -0.73634
0.76924 -0.3462 0.6354 0.12802 0.58469652
NA ENSG00000272161 1.1356 0.12805 1.1356 0.12805 1.1356 0.12805
1.1356 0.12805 0.12805 0.58469652
MIR4508 ENSG00000265673 -0.38525 0.64998 -1.0544 0.85416 -0.78866
0.78484 1.1356 0.12806 0.12806 0.58469652
ISCU ENSG00000136003 1.1356 0.12807 -0.09692 0.5386 0.33905 0.36728
-0.43774 0.66921 0.12807 0.58469652
CYB5R4 ENSG00000065615 0.8246 0.2048 1.1355 0.12809 0.060152
0.47602 0.19168 0.424 0.12809 0.58469652
NA ENSG00000260764 1.1354 0.1281 1.1354 0.1281 1.1354 0.1281
1.1354 0.1281 0.1281 0.58469652
LINC00628 ENSG00000280924 1.1354 0.1281 -0.49861 0.69097 -0.94673
0.82811 1.1354 0.1281 0.1281 0.58469652
RNA5SP224 ENSG00000201939 1.1353 0.12812 1.1353 0.12812 1.1353
0.12812 1.1353 0.12812 0.12812 0.58469652
MIR1207 ENSG00000221176 1.0687 0.14261 1.1353 0.12812 1.1353
0.12812 1.0687 0.14261 0.12812 0.58469652
IMMP1LP3 ENSG00000271098 1.1353 0.12812 1.1353 0.12812 1.1353
0.12812 1.1353 0.12812 0.12812 0.58469652
HMGN2P31 ENSG00000214195 0.4674 0.32011 -0.47761 0.68354 1.1353
0.12813 -0.47761 0.68354 0.12813 0.58469652
LINC02338 ENSG00000277448 1.1353 0.12813 1.1353 0.12813 1.1353
0.12813 1.1353 0.12813 0.12813 0.58469652
NDUFB4P2 ENSG00000251306 1.1352 0.12814 1.1352 0.12814 1.1352

0.12814 1.1352 0.12814 0.12814 0.58469652
RPL36AP43 ENSG00000241362 1.1352 0.12815 1.1352 0.12815 1.1352
0.12815 1.1352 0.12815 0.12815 0.58469652
NA ENSG00000250461 -0.3536 0.63818 -0.3536 0.63818 -0.3536 0.63818
1.1352 0.12815 0.12815 0.58469652
PDGFRB ENSG00000113721 1.1351 0.12816 0.28925 0.3862 0.85853
0.1953 -0.37705 0.64693 0.12816 0.58469652
KRT8P43 ENSG00000218186 1.1351 0.12817 1.1351 0.12817 1.1351
0.12817 1.1351 0.12817 0.12817 0.58469652
STARD13-AS ENSG00000236581 1.1351 0.12818 0.41179 0.34025 0.60867
0.27137 0.09385 0.46261 0.12818 0.58469652
NA ENSG00000254897 0.56986 0.28438 0.70861 0.23928 1.135 0.12818 1.135
0.12818 0.12818 0.58469652
NA ENSG00000260078 0.7605 0.22348 1.1351 0.12818 1.1351 0.12818
1.1351 0.12818 0.12818 0.58469652
SLC2A10 ENSG00000197496 0.18056 0.42836 -0.1764 0.57001 1.135 0.12819
-0.62617 0.7344 0.12819 0.58469652
ZRANB2-AS2 ENSG00000229956 0.92373 0.17781 1.092 0.13741 1.1349 0.12821
0.98319 0.16276 0.12821 0.58469652
NA ENSG00000235530 0.57356 0.28313 1.1348 0.12822 1.1348 0.12822
0.94398 0.17259 0.12822 0.58469652
PPFIA1 ENSG00000131626 -0.59859 0.72528 1.1348 0.12823 -0.48821
0.6873 -0.11586 0.54612 0.12823 0.58469652
MTFR2P2 ENSG00000235647 1.1346 0.12827 1.1346 0.12827 1.1346
0.12827 1.1346 0.12827 0.12827 0.58469652
CFAP100 ENSG00000163885 1.1345 0.12828 0.5552 0.28938 -0.19284
0.57646 1.1345 0.12828 0.12828 0.58469652
NA ENSG00000253854 1.1345 0.1283 1.1345 0.1283 1.1345 0.1283
1.1345 0.1283 0.1283 0.58469652
MIR4289 ENSG00000265873 1.1345 0.1283 1.1345 0.1283 1.1345
0.1283 1.1345 0.1283 0.1283 0.58469652
FDX1 ENSG00000137714 0.96692 0.16679 1.1344 0.12832 0.73208 0.23206
0.094915 0.46219 0.12832 0.58469652
ING5 ENSG00000168395 -0.014954 0.50597 1.1344 0.12832 0.45367 0.32503
-0.19661 0.57793 0.12832 0.58469652
NA ENSG00000235480 0.46728 0.32015 0.50935 0.30525 0.46728 0.32015
1.1344 0.12832 0.12832 0.58469652
NA ENSG00000280007 1.1343 0.12834 1.1343 0.12834 1.1343 0.12834
1.1343 0.12834 0.12834 0.58469652
N4BP2L1 ENSG00000139597 -0.27416 0.60802 -0.27416 0.60802 -0.27416
0.60802 1.1342 0.12835 0.12835 0.58469652
NA ENSG00000263788 -0.19016 0.57541 0.12921 0.44859 1.1342 0.12836
-0.23758 0.5939 0.12836 0.58469652
LINC01931 ENSG00000162947 1.1342 0.12837 1.0621 0.14409 -0.328
0.62855 -0.75176 0.7739 0.12837 0.58469652
RN7SL618P ENSG00000241613 1.1341 0.12837 1.1341 0.12837 1.1341
0.12837 1.1341 0.12837 0.12837 0.58469652
ZNF75BP ENSG00000258212 0.72693 0.23363 1.1341 0.12838 0.34836
0.36379 0.40511 0.3427 0.12838 0.58469652
NA ENSG00000283214 1.1341 0.12838 1.1341 0.12838 1.1341 0.12838
1.1341 0.12838 0.12838 0.58469652
NA ENSG00000278356 1.1341 0.12839 1.1341 0.12839 1.1341 0.12839
1.1341 0.12839 0.12839 0.58469652
ZSWIM6 ENSG00000130449 0.67941 0.24844 0.94929 0.17124 -0.38735
0.65075 1.134 0.1284 0.1284 0.58469652
RNU6-455P ENSG00000207412 1.1339 0.12841 1.1175 0.13189 -0.52108
0.69884 -0.52863 0.70147 0.12841 0.58469652
JSRP1 ENSG00000167476 1.1339 0.12842 1.1339 0.12842 1.1339 0.12842
1.1339 0.12842 0.12842 0.58469652
RNU1-23P ENSG00000200231 0.33392 0.36922 1.1338 0.12844 0.33392
0.36922 0.33392 0.36922 0.12844 0.58469652
RN7SKP13 ENSG00000251742 1.1337 0.12846 1.1337 0.12846 1.1337

0.12846	1.1337	0.12846	0.12846	0.58469652
NA	ENSG00000273259 1.1337	0.12846	0.84381	0.19939	0.43939	0.33019
0.43939	0.33019	0.12846	0.58469652
TNFSF13	ENSG00000161955 0.081293	0.4676	1.1337	0.12847	0.081293
0.4676	-1.0108	0.84394	0.12847	0.58469652
TLX3	ENSG00000164438 -1.4234	0.92269	-1.4234	0.92269	0.107 0.45739
1.1337	0.12847	0.12847	0.58469652
RNU6-169P	ENSG00000202206 1.1336	0.12847	1.1336	0.12847	1.1336
0.12847	1.1336	0.12847	0.12847	0.58469652
RBM17 ENSG00000134453 1.1335	0.1285	0.25479	0.39944	1.051 0.14662
0.56321	0.28665	0.1285	0.58469652
FBXL14	ENSG00000171823 0.50274	0.30757	1.1335	0.1285	0.92425
0.17768	0.27157	0.39297	0.1285	0.58469652
ADCYAP1	ENSG00000141433 1.1335	0.12851	0.018276	0.49271	-0.53018
0.70201	-1.5509	0.93953	0.12851	0.58469652
LINC00559	ENSG00000261446 0.92349	0.17788	1.1334	0.12851	0.81185
0.20844	0.64597	0.25915	0.12851	0.58469652
SLC8B1	ENSG00000089060 1.1333	0.12854	0.49246	0.3112	0.60872
0.27136	0.074044	0.47049	0.12854	0.58469652
KDSR	ENSG00000119537 -0.36563	0.64268	0.96732	0.16669	1.1332	0.12856
-0.51315	0.69608	0.12856	0.58469652
RRN3P3	ENSG00000257122 0.28944	0.38612	-0.1327	0.55278	0.3027
0.38106	1.1332	0.12856	0.12856	0.58469652
CFAP53	ENSG00000172361 1.0554	0.14562	-0.2546	0.60048	-0.2546
0.60048	1.1332	0.12857	0.12857	0.58469652
NA	ENSG00000235934 1.1331	0.1286	0.17132	0.43199	-0.20957	0.583
-0.20957	0.583 0.1286	0.58469652
NA	ENSG00000250407 1.133 0.1286	-0.90452	0.81714	0.5732	0.28325
-0.14838	0.55898	0.1286	0.58469652
NA	ENSG00000273824 1.133 0.1286	1.133 0.1286	1.133 0.1286	1.133 0.1286
0.1286	0.58469652
NA	ENSG00000269676 1.1329	0.12862	-1.4796	0.93051	-1.4796	0.93051
-0.22633	0.58953	0.12862	0.58469652
DISP2 ENSG00000140323 0.90885	0.18171	0.90885	0.18171	0.556 0.2891
1.1329	0.12863	0.12863	0.58469652
LINC00608	ENSG00000236445 1.1329	0.12863	-0.04207	0.51678	1.1329
0.12863	1.1329	0.12863	0.12863	0.58469652
NA	ENSG00000233721 0.5279	0.29878	0.41445	0.33927	-0.88409	0.81168
1.1328	0.12864	0.12864	0.58469652
NA	ENSG00000255182 -0.44109	0.67043	0.024823	0.4901	1.1328	0.12865
1.1328	0.12865	0.12865	0.58469652
MTND4P7	ENSG00000253257 1.1327	0.12867	0.5366	0.29577	1.1327
0.12867	0.24169	0.40451	0.12867	0.58469652
NA	ENSG00000202077 -0.86367	0.80611	-0.86367	0.80611	1.1327	0.12867
-0.062247	0.52482	0.12867	0.58469652
CTCFL ENSG00000124092 0.26085	0.3971	1.1327	0.12868	-0.73049	0.76746
0.52495	0.29981	0.12868	0.58469652
SMIM21	ENSG00000206026 -0.21844	0.58646	-1.0168	0.84537	1.1326
0.12868	0.58972	0.27769	0.12868	0.58469652
NA	ENSG00000226566 1.1327	0.12868	-0.75366	0.77447	0.77776	0.21836
0.77963	0.2178	0.12868	0.58469652
NA	ENSG00000232261 1.1326	0.12868	1.1326	0.12868	1.1326	0.12868
0.63588	0.26243	0.12868	0.58469652
NA	ENSG00000256824 -2.3156	0.98971	0.3112	0.37783	-1.1319	0.87115
1.1325	0.12871	0.12871	0.58469652
SWSAP1	ENSG00000173928 1.1324	0.12873	1.1324	0.12873	1.1324
0.12873	1.1324	0.12873	0.12873	0.58469652
NA	ENSG00000234639 1.1324	0.12873	1.1324	0.12873	0.87979	0.18949
1.1324	0.12873	0.12873	0.58469652
BMS1P19	ENSG00000231099 1.1323	0.12876	1.1323	0.12876	1.1323
0.12876	1.1323	0.12876	0.12876	0.58469652
NA	ENSG00000248268 1.1323	0.12876	0.95017	0.17101	-0.38928	0.65147

-0.38928 0.65147 0.12876 0.58469652
DNASE1L2 ENSG00000167968 1.1322 0.12878 1.1322 0.12878 1.1322
0.12878 1.1322 0.12878 0.12878 0.58469652
NA ENSG00000259780 1.1322 0.12878 1.1322 0.12878 1.1322 0.12878
1.1322 0.12878 0.12878 0.58469652
SLC37A3 ENSG00000157800 -0.15651 0.56218 -0.71225 0.76185 1.1321
0.12879 -0.040983 0.51635 0.12879 0.58469652
NA ENSG00000260413 0.36195 0.3587 0.40167 0.34396 0.55505 0.28943
1.132 0.12881 0.12881 0.58469652
NA ENSG00000233962 1.132 0.12882 1.132 0.12882 1.132 0.12882 1.132 0.12882
0.12882 0.58469652
NA ENSG00000234085 1.132 0.12882 1.132 0.12882 1.132 0.12882 1.132 0.12882
0.12882 0.58469652
NEURL1B ENSG00000214357 0.22762 0.40997 0.52055 0.30134 0.24428
0.40351 1.1319 0.12883 0.12883 0.58469652
SCARNA12 ENSG00000238795 1.1319 0.12883 1.1319 0.12883 1.1319
0.12883 1.1319 0.12883 0.12883 0.58469652
RN7SL369P ENSG00000241542 0.19149 0.42407 0.037435 0.48507 0.19149
0.42407 1.1319 0.12883 0.12883 0.58469652
LRRC8E ENSG00000171017 1.1319 0.12884 1.1319 0.12884 1.1319
0.12884 1.1319 0.12884 0.12884 0.58469652
NA ENSG00000276649 -0.366 0.64282 -0.366 0.64282 1.1319 0.12884
0.22871 0.40955 0.12884 0.58469652
OR5C1 ENSG00000148215 -0.14171 0.55635 1.1317 0.12887 0.59487 0.27596
0.57403 0.28297 0.12887 0.58469652
SIPA1L3 ENSG00000105738 -0.81165 0.7915 -0.060728 0.52421 0.60212
0.27355 1.1317 0.12888 0.12888 0.58469652
COMTD1 ENSG00000165644 1.1317 0.12888 1.1317 0.12888 1.1317
0.12888 1.1317 0.12888 0.12888 0.58469652
NA ENSG00000278830 1.1317 0.12888 0.79948 0.21201 -0.49819 0.69083
-0.49819 0.69083 0.12888 0.58469652
ZFPM1 ENSG00000179588 0.67239 0.25067 -0.72655 0.76625 1.1317 0.12889
0.40249 0.34366 0.12889 0.58469652
C8orf48 ENSG00000164743 0.93829 0.17405 0.93829 0.17405 1.1316
0.1289 0.32929 0.37097 0.1289 0.58469652
EIF2AK1 ENSG00000086232 0.55729 0.28866 1.1315 0.12892 -0.73123
0.76768 -0.24726 0.59765 0.12892 0.58469652
POMGNT2 ENSG00000144647 0.1736 0.43109 1.1314 0.12895 0.081956
0.46734 0.2552 0.39928 0.12895 0.58469652
MCPH1 ENSG00000147316 0.1063 0.45767 0.57704 0.28195 1.1314 0.12895
0.49514 0.31025 0.12895 0.58469652
RNY4P16 ENSG00000201638 1.0572 0.14522 1.0572 0.14522 1.1314
0.12895 1.0572 0.14522 0.12895 0.58469652
LINC01837 ENSG00000267489 0.49632 0.30983 1.1314 0.12895 -0.17415
0.56913 -0.25286 0.59981 0.12895 0.58469652
NA ENSG00000251431 1.1313 0.12897 0.33525 0.36872 -0.70229 0.75875
-0.70229 0.75875 0.12897 0.584732797
PRKD1 ENSG00000184304 0.88601 0.18781 0.1258 0.44995 1.1311 0.12901
0.85428 0.19648 0.12901 0.584859736
SLC12A7 ENSG00000113504 1.0564 0.1454 -0.29305 0.61526 1.131 0.12903
0.50092 0.30821 0.12903 0.584886913
NA ENSG00000261428 0.85659 0.19584 1.131 0.12904 0.81345 0.20798
0.81345 0.20798 0.12904 0.584886913
THORLNC ENSG00000226856 1.1307 0.12908 0.52436 0.30001 -0.091918
0.53662 0.59728 0.27516 0.12908 0.584959397
CYP1B1-AS1 ENSG00000232973 1.1307 0.12908 -1.545 0.93883 -1.0757
0.85897 -1.3965 0.91872 0.12908 0.584959397
TRAPPC9 ENSG00000167632 -0.3036 0.61928 -0.27013 0.60647 1.1306
0.12911 0.63817 0.26168 0.12911 0.584977464
NA ENSG00000223374 1.1306 0.12911 0.20506 0.41876 1.1306 0.12911
1.1306 0.12911 0.12911 0.584977464
FKBP4 ENSG00000004478 1.1306 0.12912 0.47339 0.31797 0.46714 0.3202

0.65558 0.25605 0.12912 0.584977464
ACTR5 ENSG00000101442 1.1304 0.12916 -0.8119 0.79158 -0.58904 0.72208
-0.092377 0.5368 0.12916 0.585095039
CSTB ENSG00000160213 -0.10902 0.54341 0.057846 0.47694 1.1301 0.12921
-0.94323 0.82722 0.12921 0.585095039
RPS25P9 ENSG00000232142 0.51598 0.30293 0.51598 0.30293 1.1301
0.12921 0.51598 0.30293 0.12921 0.585095039
NA ENSG00000206582 1.1301 0.12922 1.1301 0.12922 1.1301 0.12922
1.1301 0.12922 0.12922 0.585095039
CT69 ENSG00000231971 -0.33069 0.62956 -0.34875 0.63636 -0.66319 0.7464
1.1301 0.12922 0.12922 0.585095039
NA ENSG00000228655 1.1301 0.12923 -1.0165 0.8453 -1.3778 0.91587
-1.0165 0.8453 0.12923 0.585095039
MIR4304 ENSG00000265526 1.13 0.12923 1.13 0.12923 1.13 0.12923 1.13
0.12923 0.12923 0.585095039
SPDYE7P ENSG00000179994 0.89194 0.18621 -0.065688 0.52619 1.1295
0.12934 -0.18923 0.57504 0.12934 0.585401362
NA ENSG00000265115 -0.0084902 0.50339 1.1294 0.12937 1.1294 0.12937
1.1294 0.12937 0.12937 0.585401362
NA ENSG00000225058 -0.85049 0.80247 1.1293 0.12938 0.57665 0.28209
0.80894 0.20927 0.12938 0.585401362
CNTROB ENSG00000170037 0.21012 0.41679 0.21012 0.41679 1.1293
0.12939 0.1587 0.43695 0.12939 0.585401362
NA ENSG00000263386 0.74355 0.22857 -0.5082 0.69434 1.1293 0.12939
0.39018 0.3482 0.12939 0.585401362
SPESP1 ENSG00000258484 1.1292 0.1294 -1.1466 0.87423 -0.58357
0.72025 -0.58357 0.72025 0.1294 0.585401362
NA ENSG00000261501 1.1292 0.1294 1.1292 0.1294 1.1292 0.1294
1.1292 0.1294 0.1294 0.585401362
NA ENSG00000273711 1.1292 0.12942 1.1292 0.12942 1.1292 0.12942
1.1292 0.12942 0.12942 0.585401362
ASIP ENSG00000101440 -0.17698 0.57024 0.78626 0.21586 1.1291 0.12943
-1.1389 0.87263 0.12943 0.585401362
CAMK2D ENSG00000145349 -0.44225 0.67084 1.1288 0.12949 0.59524
0.27584 0.48369 0.3143 0.12949 0.585401362
FDX1P1 ENSG00000181741 1.1288 0.12949 1.0018 0.15821 1.1288
0.12949 1.1288 0.12949 0.12949 0.585401362
PGBP ENSG00000227179 0.37385 0.35426 1.1288 0.12949 -0.026383 0.51052
-0.59195 0.72306 0.12949 0.585401362
RNU6-529P ENSG00000200253 1.1288 0.1295 1.1288 0.1295 0.24729
0.40234 0.95236 0.17046 0.1295 0.585401362
OR3A2 ENSG00000221882 1.1286 0.12954 0.85094 0.1974 -1.0314 0.84883
0.70479 0.24047 0.12954 0.585401362
PDCD5P2 ENSG00000251107 1.1286 0.12954 1.1286 0.12954 1.1286
0.12954 1.1286 0.12954 0.12954 0.585401362
NA ENSG00000252170 0.50602 0.30642 0.020857 0.49168 1.1286 0.12954
1.1286 0.12954 0.12954 0.585401362
ADGRL2 ENSG00000117114 1.1285 0.12955 0.78109 0.21737 -0.44542
0.67199 0.53878 0.29502 0.12955 0.585401362
TGFBI ENSG00000120708 -0.20453 0.58103 0.13842 0.44495 -0.14789 0.55879
1.1285 0.12955 0.12955 0.585401362
NA ENSG00000274023 1.1285 0.12955 0.79268 0.21398 0.63674 0.26215
0.81238 0.20829 0.12955 0.585401362
TTC39A ENSG00000085831 0.32133 0.37398 1.1285 0.12956 -0.92529
0.82259 -0.92529 0.82259 0.12956 0.585401362
NA ENSG00000236054 1.1285 0.12956 1.1285 0.12956 1.1285 0.12956
1.1285 0.12956 0.12956 0.585401362
NA ENSG00000241932 0.10386 0.45864 0.79833 0.21234 1.1284 0.12958
0.79833 0.21234 0.12958 0.585401362
NA ENSG00000244593 1.1284 0.12958 1.1284 0.12958 1.1284 0.12958
1.1284 0.12958 0.12958 0.585401362
DOCK5 ENSG00000147459 0.42895 0.33398 0.97899 0.16379 0.16312 0.43521

1.1283 0.1296 0.1296 0.585401362
ZBBX ENSG00000169064 -0.77484 0.78078 0.65924 0.25487 1.1282 0.12961
-1.2733 0.89854 0.12961 0.585401362
NA ENSG00000253695 -0.22448 0.58881 -0.73185 0.76787 -0.12167 0.54842
1.1282 0.12961 0.12961 0.585401362
STMN1 ENSG00000117632 0.35153 0.36259 1.128 0.12966 0.808 0.20954 0.59669
0.27536 0.12966 0.585446211
NA ENSG00000235432 -0.058514 0.52333 1.1279 0.12967 1.1279 0.12967
1.1279 0.12967 0.12967 0.585446211
NA ENSG00000268027 1.128 0.12967 1.128 0.12967 1.128 0.12967 1.128 0.12967
0.12967 0.585446211
GID8 ENSG00000101193 1.1279 0.12968 1.1279 0.12968 1.1279 0.12968
1.1279 0.12968 0.12968 0.585446211
MIR4655 ENSG00000265089 -0.82433 0.79512 1.1279 0.12968 -0.046187
0.51842 -0.046187 0.51842 0.12968 0.585446211
TMEM222 ENSG00000186501 1.1277 0.12971 0.95575 0.1696 -0.13881
0.5552 0.5874 0.27847 0.12971 0.585518303
HSPE1P14 ENSG00000271612 0.94038 0.17351 0.93071 0.176 1.1277 0.12972
1.1277 0.12972 0.12972 0.585518303
SEMA3F-AS1 ENSG00000235016 0.74723 0.22746 0.80941 0.20914 1.1276
0.12975 0.51189 0.30436 0.12975 0.585527046
ZADH2 ENSG00000180011 1.1275 0.12976 -1.1253 0.86976 -0.38904 0.65137
-0.78719 0.78441 0.12976 0.585527046
ALOX15B ENSG00000179593 1.1275 0.12977 0.013907 0.49445 0.013907
0.49445 1.0191 0.15408 0.12977 0.585527046
NA ENSG00000203364 -0.41466 0.6608 1.1275 0.12977 -1.1416 0.87319
0.19672 0.42202 0.12977 0.585527046
NA ENSG00000276362 1.1271 0.12984 0.084373 0.46638 0.20173 0.42006
0.16594 0.4341 0.12984 0.585770416
ASIC4-AS1 ENSG00000227432 1.127 0.12986 1.127 0.12986 1.127 0.12986 1.127
0.12986 0.12986 0.585770416
PRB1 ENSG00000251655 0.14796 0.44119 0.60477 0.27266 1.127 0.12986
0.26365 0.39602 0.12986 0.585770416
ASAP3 ENSG00000088280 0.76456 0.22227 1.127 0.12988 0.67982 0.24831
0.35178 0.3625 0.12988 0.585806405
LINC01217 ENSG00000251219 0.95947 0.16866 -0.27455 0.60817 1.1267
0.12994 0.67145 0.25097 0.12994 0.585958316
SERAC1 ENSG00000122335 0.46683 0.32031 1.1265 0.12998 0.79069
0.21456 0.79069 0.21456 0.12998 0.585958316
NA ENSG00000260328 -1.1247 0.86965 -0.7815 0.78275 0.48884 0.31248
1.1265 0.12998 0.12998 0.585958316
GUSBP2 ENSG00000241549 1.1264 0.12999 0.90422 0.18294 0.5259
0.29948 -0.20258 0.58027 0.12999 0.585958316
ZNF721 ENSG00000182903 1.1264 0.13 0.82125 0.20575 1.0189 0.15413
0.33247 0.36977 0.13 0.585958316
RASGRF2-AS1 ENSG00000251450 1.1264 0.13 1.1264 0.13 1.1264 0.13
1.1264 0.13 0.13 0.585958316
NA ENSG00000254471 -0.044989 0.51794 -0.21234 0.58408 -0.061405 0.52448
1.1263 0.13001 0.13001 0.585958316
CREB3L3 ENSG00000060566 -0.20351 0.58063 -0.20351 0.58063 1.1262
0.13004 -0.77516 0.78088 0.13004 0.585958316
SYNGR3 ENSG00000127561 1.1262 0.13004 -2.1484 0.98416 0.66758
0.2522 -1.7669 0.96137 0.13004 0.585958316
ARL5C ENSG00000141748 0.94325 0.17278 1.1262 0.13005 1.1262 0.13005
1.1262 0.13005 0.13005 0.585958316
TRAV8-3 ENSG00000211787 1.1261 0.13005 1.1261 0.13005 1.1261
0.13005 1.1261 0.13005 0.13005 0.585958316
NMTRS-TGA3-1 ENSG00000241225 1.1261 0.13007 1.1261 0.13007 1.1261
0.13007 1.1261 0.13007 0.13007 0.585958316
MTATP6P3 ENSG00000263483 1.1261 0.13007 1.1261 0.13007 1.1261
0.13007 1.1261 0.13007 0.13007 0.585958316
NA ENSG00000206913 -0.30877 0.62125 -0.30877 0.62125 -0.30877 0.62125

1.1258	0.13012	0.13012	0.586047819
RPL7P35	ENSG00000233518	0.14508	0.44233	1.1258	0.13012	-0.31307
0.62289	-0.35883	0.64014	0.13012	0.586047819
NA	ENSG00000275805	1.1258	0.13013	0.3752	0.35375	1.1258	0.13013
0.14776	0.44127	0.13013	0.586047819
SYPL1	ENSG00000008282	1.1257	0.13015	1.1257	0.13015	1.1257	0.13015
-0.35067	0.63708	0.13015	0.586047819
ATP5F1A	ENSG00000152234	0.56819	0.28495	0.61215	0.27022	1.1257
0.13015	0.33624	0.36835	0.13015	0.586047819
BLOC1S2	ENSG00000196072	1.113	0.13285	1.1255	0.13019	-0.14657	0.55827
0.95292	0.17031	0.13019	0.58614573
QSOX2	ENSG00000165661	0.88206	0.18887	0.29648	0.38343	1.1254	0.13021
0.88206	0.18887	0.13021	0.58614573
NA	ENSG00000217181	1.1252	0.13024	1.1252	0.13024	1.1252	0.13024
1.1252	0.13024	0.13024	0.58614573
NA	ENSG00000268555	1.1253	0.13024	1.1253	0.13024	1.1253	0.13024
1.1253	0.13024	0.13024	0.58614573
C4BPB	ENSG00000123843	-0.36215	0.64138	0.36448	0.35775	-0.51349	0.69619
1.1251	0.13027	0.13027	0.58614573
NA	ENSG00000269506	1.1251	0.13027	1.1251	0.13027	1.1251	0.13027
1.1251	0.13027	0.13027	0.58614573
TMEM205	ENSG00000105518	1.1251	0.13028	1.1251	0.13028	1.1251
0.13028	1.1251	0.13028	0.13028	0.58614573
TUBA1C	ENSG00000167553	1.1251	0.13028	0.3763	0.35335	0.43852
0.33051	0.89426	0.18559	0.13028	0.58614573
ZBTB48	ENSG00000204859	0.77478	0.21924	1.1205	0.13125	1.1251
0.13028	1.1205	0.13125	0.13028	0.58614573
MT1G	ENSG00000125144	1.1248	0.13033	1.1248	0.13033	1.1248	0.13033
1.1248	0.13033	0.13033	0.586188742
NA	ENSG00000248185	1.1247	0.13035	0.10864	0.45675	0.10864	0.45675
0.10864	0.45675	0.13035	0.586188742
NA	ENSG00000201031	1.1246	0.13038	0.93754	0.17424	0.93754	0.17424
-0.024156	0.50964	0.13038	0.586188742
NA	ENSG00000229519	1.1245	0.13039	1.1245	0.13039	1.1245	0.13039
1.1245	0.13039	0.13039	0.586188742
CSTA	ENSG00000121552	0.54853	0.29166	1.1245	0.1304	-0.89292	0.81405
-0.58439	0.72052	0.1304	0.586188742
HNRNPA1P17	ENSG00000243188	1.1245	0.1304	1.1245	0.1304	1.1245
0.1304	1.1245	0.1304	0.1304	0.586188742
MTCO1P30	ENSG00000248729	-0.32676	0.62807	0.65877	0.25502	1.1244
0.13042	-0.37576	0.64645	0.13042	0.586188742
PIGR	ENSG00000162896	-1.3225	0.907	1.1244	0.13043	-1.0641	0.85635
-1.2194	0.88866	0.13043	0.586188742
RNU6-344P	ENSG00000252444	0.70386	0.24076	1.1244	0.13043	0.70386
0.24076	0.70386	0.24076	0.13043	0.586188742
NUP93	ENSG00000102900	0.79781	0.21249	0.77431	0.21937	1.1243	0.13044
0.84825	0.19815	0.13044	0.586188742
NA	ENSG00000271527	0.22624	0.41051	-2.196	0.98596	1.1243	0.13045
0.87269	0.19142	0.13045	0.586188742
MCM6	ENSG00000076003	0.23825	0.40584	0.31667	0.37575	0.81206	0.20838
1.1242	0.13046	0.13046	0.586188742
FGF23	ENSG00000118972	-0.35518	0.63877	1.1242	0.13047	-0.10843	0.54317
0.10727	0.45729	0.13047	0.586188742
GPR31	ENSG00000120436	1.0244	0.15281	1.1242	0.13047	0.34967	0.36329
0.64158	0.26057	0.13047	0.586188742
LIPJ	ENSG00000204022	0.85614	0.19596	0.85614	0.19596	1.1242	0.13047
-0.18579	0.5737	0.13047	0.586188742
ECHDC3	ENSG00000134463	1.1241	0.13049	1.0502	0.14681	0.9777
0.16411	1.0655	0.14334	0.13049	0.586224551
NA	ENSG00000240265	1.1237	0.13056	1.1237	0.13056	-0.2031	0.58047
1.1237	0.13056	0.13056	0.586427055
SPPL3	ENSG00000157837	0.58702	0.2786	0.72624	0.23385	0.54951	0.29133

1.1235	0.1306	0.1306	0.586427055
NA	ENSG00000276688 0.79719	0.21267	0.79719	0.21267	1.1235	0.13061
0.79719	0.21267	0.13061	0.586427055
NA	ENSG00000275725 1.1235	0.13062	-0.61761	0.73158	-0.70061	0.75823
-0.64652	0.74103	0.13062	0.586427055
MEFV	ENSG00000103313 0.21343	0.4155	1.1234	0.13063	0.7453	0.22805
1.0955	0.13664	0.13063	0.586427055
WDFY4	ENSG00000128815 0.19191	0.42391	0.7709	0.22038	1.1234	0.13063
0.465 0.32097	0.13063	0.586427055
NA	ENSG00000259403 -1.3695	0.91458	-1.3695	0.91458	1.1234	0.13064
-1.3695	0.91458	0.13064	0.586427055
MROH6	ENSG00000204839 1.1233	0.13065	-1.432	0.92392	-0.61176	0.72965
-0.61176	0.72965	0.13065	0.586427055
IGKV2OR2-2 ENSG00000271402	1.1233	0.13065	1.1233	0.13065	1.1233
0.13065	1.1233	0.13065	0.13065	0.586427055
ADAM20	ENSG00000134007	1.1232	0.13068	0.26558	0.39528	0.62313
0.2666	0.62313	0.2666	0.13068	0.586427055
SPTLC1	ENSG00000090054	0.12305	0.45103	1.1231	0.13071	0.52034
0.30141	-0.11186	0.54453	0.13071	0.586427055
PNP	ENSG00000198805 1.123 0.13071	0.68719	0.24598	0.68719	0.24598
-0.089133	0.53551	0.13071	0.586427055
NA	ENSG00000259212 1.1229	0.13074	1.1229	0.13074	1.1229	0.13074
1.1229	0.13074	0.13074	0.586427055
SLC23A2	ENSG00000089057	-1.1003	0.86439	-1.5609	0.94073	-1.0742
0.85863	1.1229	0.13075	0.13075	0.586427055
TREM2	ENSG00000095970 0.175 0.43054	0.175 0.43054	0.175 0.43054	1.1229
0.13075	0.13075	0.586427055
NA	ENSG00000279249 0.38319	0.35079	1.1228	0.13075	-0.20536	0.58135
-0.20536	0.58135	0.13075	0.586427055
WDR95P	ENSG00000231894	-1.1398	0.87281	-1.155	0.87595	0.34375
0.36552	1.1227	0.13078	0.13078	0.586427055
CHMP4BP1	ENSG00000258469	1.1226	0.1308	1.1226	0.1308	1.1226
0.1308	1.1226	0.1308	0.1308	0.586427055
NA	ENSG00000250877 1.1226	0.13081	-0.043315	0.51727	-0.13157	0.55234
-0.51302	0.69603	0.13081	0.586427055
WDFY3-AS1	ENSG00000251260	1.1226	0.13081	1.1226	0.13081	1.1226
0.13081	1.1226	0.13081	0.13081	0.586427055
CSPG4P5	ENSG00000272887	1.1225	0.13082	1.1225	0.13082	1.1225
0.13082	1.1225	0.13082	0.13082	0.586427055
PPIE	ENSG00000084072 1.1225	0.13083	1.1225	0.13083	0.38953	0.34844
0.30073	0.38181	0.13083	0.586427055
BBS7	ENSG00000138686 0.0083359	0.49667	1.1225	0.13083	0.079211	0.46843
0.010832	0.49568	0.13083	0.586427055
TMSB10P1	ENSG00000228499	1.1224	0.13084	1.1224	0.13084	1.1224
0.13084	1.1224	0.13084	0.13084	0.586427055
NA	ENSG00000199900 1.1224	0.13084	1.1224	0.13084	1.1224	0.13084
1.1224	0.13084	0.13084	0.586427055
NUCKS1	ENSG00000069275	-0.18107	0.57184	-0.17907	0.57106	-0.15155
0.56023	1.1224	0.13085	0.13085	0.586427055
GRAMD4P8	ENSG00000270930	1.1223	0.13086	0.15121	0.43991	-1.2831
0.90027	-1.2831	0.90027	0.13086	0.586427055
MIR3196	ENSG00000266463	1.122 0.13093	1.1159	0.13222	1.122 0.13093	1.122
0.13093	0.13093	0.586677657
NA	ENSG00000257094 -0.077719	0.53097	0.15993	0.43647	-0.68861	0.75447
1.122 0.13094	0.13094	0.586677657
RNA5SP115	ENSG00000223092	1.1218	0.13098	1.1218	0.13098	1.1218
0.13098	1.1218	0.13098	0.13098	0.586739838
EEF1B2P5	ENSG00000225121	-0.57658	0.71789	1.1218	0.13098	1.1218
0.13098	1.1218	0.13098	0.13098	0.586739838
NPY1R	ENSG00000164128 -0.33738	0.63208	1.1217	0.13099	-0.48009	0.68442
0.055806	0.47775	0.13099	0.586739838
NA	ENSG00000259113 1.1216	0.13101	0.19632	0.42218	1.1216	0.13101

1.1216 0.13101 0.13101 0.586775487
NA ENSG00000242618 1.1214 0.13106 0.80703 0.20982 1.1214 0.13106
-0.090648 0.53611 0.13106 0.586891546
LINC02882 ENSG00000251138 0.50438 0.307 -0.32529 0.62752 0.41289 0.33984
1.1214 0.13106 0.13106 0.586891546
DNAJB3 ENSG00000227802 1.1213 0.13109 1.1213 0.13109 1.1213
0.13109 -0.15627 0.56209 0.13109 0.586935327
RPL12P28 ENSG00000240180 1.1212 0.13111 1.1212 0.13111 1.1212
0.13111 1.1212 0.13111 0.13111 0.586935327
ZSWIM8-AS1 ENSG00000272589 1.1211 0.13113 1.1211 0.13113 1.1211
0.13113 1.1211 0.13113 0.13113 0.586935327
NA ENSG00000277558 1.1211 0.13113 1.1211 0.13113 1.1211 0.13113
1.1211 0.13113 0.13113 0.586935327
NA ENSG00000278937 1.121 0.13113 -0.80962 0.79092 -0.40587 0.65758
0.85288 0.19686 0.13113 0.586935327
AQP12A ENSG00000184945 1.1209 0.13116 -0.99003 0.83892 0.72886
0.23304 0.70631 0.24 0.13116 0.586943476
KRT3 ENSG00000186442 1.0947 0.13682 1.1209 0.13117 1.1209 0.13117
1.1209 0.13117 0.13117 0.586943476
ATP6V0CP2 ENSG00000244142 0.1055 0.45799 0.1055 0.45799 0.1055
0.45799 1.1208 0.13118 0.13118 0.586943476
NA ENSG00000257787 1.1209 0.13118 1.0398 0.14921 1.0398 0.14921
1.0398 0.14921 0.13118 0.586943476
RABEPK ENSG00000136933 0.3544 0.36152 1.1207 0.1312 0.34943
0.36338 -0.031595 0.5126 0.1312 0.586968903
OPCML-IT2 ENSG00000255371 -0.17531 0.56958 1.1205 0.13125 0.4186
0.33775 1.0948 0.13681 0.13125 0.586968903
DOK6 ENSG00000206052 0.247 0.40245 0.63261 0.26349 0.49576 0.31003
1.1205 0.13126 0.13126 0.586968903
PET100 ENSG00000229833 1.1205 0.13126 1.1205 0.13126 1.1205
0.13126 1.1205 0.13126 0.13126 0.586968903
C2orf69P4 ENSG00000260480 1.1205 0.13126 1.1205 0.13126 1.1205
0.13126 1.1205 0.13126 0.13126 0.586968903
NA ENSG00000268400 1.1205 0.13126 1.1205 0.13126 1.1205 0.13126
1.1205 0.13126 0.13126 0.586968903
NA ENSG00000258681 1.1204 0.13127 0.98462 0.16241 1.1204 0.13127
1.1204 0.13127 0.13127 0.586968903
NA ENSG00000238337 -0.43319 0.66756 -0.43319 0.66756 -0.43319 0.66756
1.1203 0.13129 0.13129 0.587004468
PANX3 ENSG00000154143 0.99842 0.15904 1.1201 0.13133 0.61563 0.26907
1.1201 0.13133 0.13133 0.58712944
NA ENSG00000219784 -0.48005 0.6844 -0.16363 0.56499 1.12 0.13136
-0.16363 0.56499 0.13136 0.587209687
LHPP ENSG00000107902 1.1198 0.13139 -0.30601 0.6202 0.22988 0.40909
-0.19487 0.57725 0.13139 0.587253246
RNU6-112P ENSG00000200469 1.1198 0.1314 1.1198 0.1314 1.1198
0.1314 1.1198 0.1314 0.1314 0.587253246
NA ENSG00000242140 0.93948 0.17374 1.1198 0.13141 1.1198 0.13141
0.074352 0.47037 0.13141 0.587253246
NA ENSG00000229143 -1.2537 0.89503 0.49473 0.31039 -1.2537 0.89503
1.1197 0.13143 0.13143 0.587253246
RN7SL743P ENSG00000243954 1.1197 0.13143 1.1197 0.13143 1.1197
0.13143 -0.033122 0.51321 0.13143 0.587253246
NA ENSG00000253931 0.06949 0.4723 -0.21704 0.58591 1.1195 0.13146
-0.22662 0.58964 0.13146 0.587279593
MAFA-AS1 ENSG00000254338 0.069312 0.47237 1.0231 0.15313 0.21639
0.41434 1.1195 0.13146 0.13146 0.587279593
CHGB ENSG00000089199 0.1804 0.42842 1.089 0.13809 1.1191 0.13154
-1.2103 0.88692 0.13154 0.587583115
POLR1B ENSG00000125630 0.99816 0.1591 0.86739 0.19287 1.119 0.13157
1.003 0.15793 0.13157 0.587600202
NA ENSG00000271401 1.119 0.13157 1.119 0.13157 1.119 0.13157 1.119 0.13157

0.13157	0.587600202
SGCB	ENSG00000163069	1.119	0.13158	0.85006	0.19765	0.60895	0.27128	0.66725	0.25231	0.13158	0.587600202
ME1	ENSG00000065833	0.44731	0.32732	-0.15484	0.56153	0.49329	0.3109	1.1188	0.13161	0.13161	0.587615971
HSPA8	ENSG00000109971	1.0504	0.14678	1.0504	0.14678	0.19996	0.42075	1.1188	0.13162	0.13162	0.587615971
VPREB3	ENSG00000128218	1.1186	0.13165	1.1186	0.13165	1.1186	0.13165	1.1186	0.13165	0.13165	0.587615971
NA	ENSG00000265728	-0.87101	0.80813	0.22063	0.41269	1.1186	0.13165	0.37859	0.35249	0.13165	0.587615971
NA	ENSG00000284540	-0.17002	0.5675	-0.52925	0.70168	-0.47025	0.68091	1.1186	0.13165	0.13165	0.587615971
NA	ENSG00000249735	0.050832	0.47973	1.1186	0.13166	0.52841	0.29861	0.97394	0.16504	0.13166	0.587615971
NA	ENSG00000224967	1.1185	0.13167	1.1185	0.13167	1.1185	0.13167	1.1185	0.13167	0.13167	0.587615971
NA	ENSG00000280108	0.13048	0.44809	-0.63095	0.73596	1.1185	0.13168	0.057287	0.47716	0.13168	0.587615971
NA	ENSG00000242421	1.1184	0.1317	1.1184	0.1317	1.1184	0.1317	1.1184	0.1317	0.1317	0.587651406
CEACAM4	ENSG00000105352	0.41182	0.34023	1.1182	0.13173	0.41182	0.34023	0.41182	0.34023	0.13173	0.587659268
NA	ENSG00000260866	1.1182	0.13174	0.22219	0.41208	1.1082	0.13389	0.24196	0.40441	0.13174	0.587659268
LINC02470	ENSG00000225231	0.29318	0.38469	0.29318	0.38469	0.29318	0.38469	1.1182	0.13175	0.13175	0.587659268
NA	ENSG00000260269	0.64589	0.25918	1.1182	0.13175	1.1074	0.13406	0.023197	0.49075	0.13175	0.587659268
H2BC4	ENSG00000180596	-0.086549	0.53449	0.35732	0.36043	0.85113	0.19735	1.118	0.13179	0.13179	0.587774687
NA	ENSG00000254204	1.1179	0.1318	1.1179	0.1318	1.1179	0.1318	1.1179	0.1318	0.1318	0.587774687
RNU7-173P	ENSG00000238950	-0.89817	0.81545	0.63316	0.26331	1.1178	0.13182	0.43386	0.33219	0.13182	0.587800888
LINC01638	ENSG00000233521	1.1178	0.13183	1.1178	0.13183	1.1178	0.13183	1.1178	0.13183	0.13183	0.587800888
NA	ENSG00000228577	1.1177	0.13185	1.1177	0.13185	1.1177	0.13185	1.1177	0.13185	0.13185	0.587805903
NA	ENSG00000201498	1.1174	0.13191	1.1174	0.13191	0.78575	0.21601	0.91947	0.17893	0.13191	0.587805903
EMC1-AS1	ENSG00000230424	1.1173	0.13194	1.1173	0.13194	1.1173	0.13194	1.1173	0.13194	0.13194	0.587805903
LINC00896	ENSG00000236499	-0.82844	0.79629	1.1172	0.13195	0.27982	0.38981	0.47413	0.3177	0.13195	0.587805903
ZBTB20-AS3	ENSG00000239946	0.88566	0.1879	0.93935	0.17378	0.88566	0.1879	1.1172	0.13195	0.13195	0.587805903
LINC02699	ENSG00000254456	-0.20169	0.57992	1.1172	0.13195	0.33002	0.37069	-0.25034	0.59884	0.13195	0.587805903
MYOSLID	ENSG00000229647	1.1172	0.13196	1.1172	0.13196	1.1172	0.13196	1.1172	0.13196	0.13196	0.587805903
NA	ENSG00000250413	1.1172	0.13196	-0.0040648	0.50162	1.1172	0.13196	1.1172	0.13196	0.13196	0.587805903
NA	ENSG00000260563	1.0561	0.14546	1.1172	0.13196	1.1172	0.13196	1.0425	0.14859	0.13196	0.587805903
OR4H12P	ENSG00000176312	1.1171	0.13198	-0.2463	0.59727	-1.7958	0.96374	-0.64759	0.74137	0.13198	0.587805903
NA	ENSG00000273338	1.1171	0.13198	1.1171	0.13198	1.1171	0.13198	1.1171	0.13198	0.13198	0.587805903
CDH22	ENSG00000149654	0.2561	0.39894	-0.54734	0.70793	1.117	0.13199	0.24727	0.40235	0.13199	0.587805903
NA	ENSG00000233737	1.117	0.13199	1.117	0.13199	-0.19028	0.57546	1.117

0.13199	0.13199	0.587805903
RNU6-54P	ENSG00000202433	-0.16115	0.56401	1.117	0.132	1.117	0.132	1.117	0.132	0.132
0.587805903
NA	ENSG00000228484	-0.10079	0.54014	1.1167	0.13206	0.06928	0.47238
-0.084656	0.53373	0.13206	0.587938526
RNA5SP392	ENSG00000252714	1.1167	0.13206	1.1167	0.13206	1.1167
0.13206	1.1167	0.13206	0.13206	0.587938526
PRPSAP2	ENSG00000141127	-1.1599	0.87696	0.11244	0.45524	1.1166
0.13209	-0.67085	0.74884	0.13209	0.587938526
ITGA6	ENSG00000091409	1.1164	0.13212	0.31918	0.37479	0.098492	0.46077
-0.32367	0.62691	0.13212	0.587938526
NA	ENSG00000136315	1.1164	0.13213	1.1164	0.13213	-0.10428	0.54153
-1.0013	0.84166	0.13213	0.587938526
RNU6-905P	ENSG00000207163	0.44528	0.32806	0.38678	0.34946	1.1163
0.13215	0.44528	0.32806	0.13215	0.587938526
RAD1P1	ENSG00000234569	0.064943	0.47411	0.45065	0.32612	1.1162
0.13216	1.1162	0.13216	0.13216	0.587938526
CYP20A1	ENSG00000119004	0.8107	0.20877	0.96915	0.16623	-1.0735
0.85848	1.1162	0.13217	0.13217	0.587938526
EIF4A1P9	ENSG00000262953	1.1162	0.13217	1.1162	0.13217	-0.37526
0.64627	-0.37526	0.64627	0.13217	0.587938526
RNU6-165P	ENSG00000252979	1.1161	0.13219	1.1161	0.13219	1.1161
0.13219	1.1161	0.13219	0.13219	0.587938526
DNAJB4	ENSG00000162616	1.1161	0.1322	-0.33401	0.63081	1.1161
0.1322	1.1161	0.1322	0.1322	0.587938526
ANGPTL5	ENSG00000187151	1.116	0.1322	-1.4233	0.92268	-1.4233	0.92268
-1.4233	0.92268	0.1322	0.587938526
OR51V1	ENSG00000176742	1.116	0.13222	1.116	0.13222	1.116	0.13222	1.116
0.13222	0.13222	0.587938526
RNF13	ENSG00000082996	0.051091	0.47963	1.1159	0.13223	-0.93092	0.82405
-0.81107	0.79134	0.13223	0.587938526
OGG1	ENSG00000114026	0.79425	0.21352	-0.27843	0.60966	-0.1358	0.55401
1.1159	0.13224	0.13224	0.587938526
RNU6-199P	ENSG00000199824	0.95852	0.1689	0.95852	0.1689	1.1158
0.13225	0.20477	0.41888	0.13225	0.587938526
FRG2DP	ENSG00000261711	1.1158	0.13225	0.33094	0.37035	1.1158
0.13225	1.1158	0.13225	0.13225	0.587938526
NA	ENSG00000275719	1.1158	0.13225	1.1158	0.13225	1.1158	0.13225
1.1158	0.13225	0.13225	0.587938526
NA	ENSG00000238528	1.1157	0.13227	1.1157	0.13227	1.1157	0.13227
1.1157	0.13227	0.13227	0.587938526
LINC01362	ENSG00000230817	1.1156	0.13229	-0.69094	0.7552	0.55109
0.29079	-0.64612	0.7409	0.13229	0.587938526
NA	ENSG00000278654	1.1156	0.13229	1.1156	0.13229	1.1156	0.13229
0.97316	0.16524	0.13229	0.587938526
NA	ENSG00000279925	1.1155	0.13232	1.1155	0.13232	1.1155	0.13232
1.1155	0.13232	0.13232	0.587938526
MSH2-OT1	ENSG00000235760	1.1154	0.13233	-1.0535	0.85395	1.0246
0.15278	1.1154	0.13233	0.13233	0.587938526
NA	ENSG00000234120	0.36561	0.35733	-0.45499	0.67544	1.1154	0.13234
0.13221	0.44741	0.13234	0.587938526
SLC25A39P2	ENSG00000257016	0.70616	0.24005	0.29752	0.38304	1.1153
0.13236	1.1153	0.13236	0.13236	0.587938526
GPR89B	ENSG00000188092	0.89477	0.18546	1.1152	0.13238	0.26905
0.39394	0.31873	0.37497	0.13238	0.587938526
ADARB1	ENSG00000197381	0.63996	0.2611	0.70439	0.2406	0.69129
0.24469	1.1152	0.13239	0.13239	0.587938526
MACC1-AS1	ENSG00000228598	1.1152	0.13239	1.1152	0.13239	1.1152
0.13239	1.1152	0.13239	0.13239	0.587938526
TRDJ4	ENSG00000211826	0.12827	0.44897	-0.27356	0.60779	1.1151	0.1324
-0.070061	0.52793	0.1324	0.587938526
NA	ENSG00000267618	1.1151	0.1324	1.1151	0.1324	1.1151	0.1324

1.1151 0.1324 0.1324 0.587938526
NA ENSG00000259677 1.1151 0.13241 0.36097 0.35906 0.36097 0.35906
0.36097 0.35906 0.13241 0.587938526
NA ENSG00000275445 1.115 0.13242 1.115 0.13242 1.115 0.13242 1.115 0.13242
0.13242 0.587938526
NLGN2 ENSG00000169992 0.1784 0.4292 -0.36653 0.64302 -0.36653 0.64302
1.1149 0.13244 0.13244 0.587938526
PEX12P1 ENSG00000215418 1.1149 0.13244 1.1149 0.13244 1.1149
0.13244 1.1149 0.13244 0.13244 0.587938526
MIR7-3HG ENSG00000176840 0.8442 0.19928 0.76399 0.22244 1.1147
0.13248 -0.2021 0.58008 0.13248 0.587981818
EIF1 ENSG00000173812 -0.88167 0.81102 -0.88167 0.81102 1.1146 0.13251
-0.88167 0.81102 0.13251 0.587981818
NA ENSG00000222997 0.19673 0.42202 0.14301 0.44314 1.1144 0.13254
-0.35197 0.63757 0.13254 0.587981818
CNOT11 ENSG00000158435 1.1143 0.13258 -0.044159 0.51761 -0.85721
0.80433 -0.052055 0.52076 0.13258 0.587981818
TRIB2 ENSG00000071575 -1.1083 0.86614 -0.52119 0.69888 -0.09805 0.53905
1.1143 0.13259 0.13259 0.587981818
NA ENSG00000253832 -0.090539 0.53607 1.1142 0.1326 -0.4353 0.66833
1.1142 0.1326 0.1326 0.587981818
NA ENSG00000249729 0.30475 0.38028 0.22672 0.41032 0.77056 0.22048
1.1142 0.13261 0.13261 0.587981818
MIR5002 ENSG00000266383 1.0565 0.14536 -0.67773 0.75103 1.1141
0.13261 -0.67773 0.75103 0.13261 0.587981818
NA ENSG00000276569 0.32174 0.37382 1.1141 0.13261 1.1141 0.13261
1.1141 0.13261 0.13261 0.587981818
FABP5P12 ENSG00000249367 1.114 0.13263 1.114 0.13263 -0.29839 0.6173 1.114
0.13263 0.13263 0.587981818
IFI27L1 ENSG00000165948 -0.37682 0.64685 -0.37682 0.64685 -0.37682
0.64685 1.114 0.13265 0.13265 0.587981818
STX3 ENSG00000166900 0.98954 0.1612 1.114 0.13265 0.3313 0.37021
1.0923 0.13735 0.13265 0.587981818
MIR4664 ENSG00000265660 1.114 0.13265 1.114 0.13265 1.114 0.13265 1.114
0.13265 0.13265 0.587981818
ANKRD61 ENSG00000157999 1.1139 0.13266 1.1139 0.13266 1.1139
0.13266 1.1139 0.13266 0.13266 0.587981818
NA ENSG00000230107 1.0222 0.15335 0.88584 0.18785 0.67159 0.25092
1.1138 0.13269 0.13269 0.587981818
MET ENSG00000105976 1.1137 0.1327 0.38567 0.34987 1.1137 0.1327
0.20774 0.41772 0.1327 0.587981818
SAP25 ENSG00000205307 -1.6689 0.95243 1.1136 0.13272 -1.0195 0.84601
-1.0195 0.84601 0.13272 0.587981818
SSC5D ENSG00000179954 0.81517 0.20749 -0.34611 0.63537 -0.25937 0.60233
1.1135 0.13274 0.13274 0.587981818
NA ENSG00000279557 1.1135 0.13275 1.1135 0.13275 1.1135 0.13275
1.1135 0.13275 0.13275 0.587981818
RPL9P28 ENSG00000242067 0.26538 0.39536 0.26538 0.39536 1.1134
0.13276 -0.80265 0.78891 0.13276 0.587981818
MORN1 ENSG00000116151 1.1134 0.13277 -0.74944 0.7732 0.28371 0.38831
0.86968 0.19224 0.13277 0.587981818
NA ENSG00000218536 1.1134 0.13278 1.1134 0.13278 1.1134 0.13278
1.1134 0.13278 0.13278 0.587981818
ALDOAP1 ENSG00000242849 1.0774 0.14065 1.0774 0.14065 1.1133
0.13279 1.0027 0.15801 0.13279 0.587981818
MINDY1 ENSG00000143409 1.0376 0.14973 1.1133 0.1328 0.25871
0.39793 0.19913 0.42108 0.1328 0.587981818
NEFMP1 ENSG00000224697 1.1133 0.1328 1.1133 0.1328 1.1133
0.1328 1.1133 0.1328 0.1328 0.587981818
RN7SL573P ENSG00000239607 1.1132 0.1328 1.1132 0.1328 1.1132
0.1328 1.1132 0.1328 0.1328 0.587981818
NA ENSG00000274723 -1.1683 0.87865 -0.40591 0.6576 1.1132 0.1328

-0.20457	0.58105	0.1328	0.587981818
TULP2 ENSG00000104804 -0.13644 0.55426 1.1131 0.13283 0.23441 0.40733
0.21625 0.4144 0.13283 0.587981818
TWISTNB ENSG00000105849 1.1131 0.13283 1.0766 0.14082 1.0779
0.14053 1.0999 0.13568 0.13283 0.587981818
NA ENSG00000245648 0.46375 0.32141 1.1131 0.13283 1.1131 0.13283
1.1131 0.13283 0.13283 0.587981818
NA ENSG00000278918 1.1131 0.13283 1.1131 0.13283 1.1131 0.13283
1.1131 0.13283 0.13283 0.587981818
DHPS ENSG00000095059 -1.3082 0.90459 1.113 0.13285 -1.3082 0.90459
-1.3082 0.90459 0.13285 0.587981818
MRGPRX10P ENSG00000254720 -1.6348 0.94896 1.113 0.13285 1.113 0.13285
-1.023 0.84685 0.13285 0.587981818
MAP2K2 ENSG00000126934 1.113 0.13286 0.56366 0.28649 0.32536 0.37246
0.32536 0.37246 0.13286 0.587981818
RPL31P45 ENSG00000233318 1.1128 0.1329 1.1128 0.1329 1.1128
0.1329 0.22336 0.41163 0.1329 0.58810543
ENO1P3 ENSG00000243986 1.1126 0.13293 0.47808 0.3163 -0.043032
0.51716 0.9729 0.1653 0.13293 0.588175613
MIR3149 ENSG00000266712 1.1126 0.13294 1.1126 0.13294 1.1126
0.13294 1.1126 0.13294 0.13294 0.588175613
NA ENSG00000229955 -0.089257 0.53556 1.1125 0.13296 0.16268 0.43539
1.0609 0.14436 0.13296 0.588201534
TXNRD2 ENSG00000184470 -0.081352 0.53242 -0.06379 0.52543 -0.98903
0.83868 1.1125 0.13297 0.13297 0.588201534
NA ENSG00000236905 1.1123 0.13299 1.1123 0.13299 1.0973 0.13627
0.77448 0.21932 0.13299 0.588227446
GAS7 ENSG00000007237 0.8752 0.19073 0.74059 0.22947 1.1123 0.133
0.29358 0.38454 0.133 0.588227446
ELL ENSG00000105656 0.31013 0.37823 0.52848 0.29858 0.54195 0.29393
1.1122 0.13303 0.13303 0.588253348
SLFNL1 ENSG00000171790 1.1122 0.13303 1.1122 0.13303 1.1122
0.13303 1.1122 0.13303 0.13303 0.588253348
OBP2A ENSG00000122136 -0.12459 0.54958 0.47882 0.31603 1.1118 0.13312
1.034 0.15056 0.13312 0.588504386
SCN4A ENSG00000007314 -0.72685 0.76634 -1.1757 0.88015 -1.1757 0.88015
1.1117 0.13313 0.13313 0.588504386
NA ENSG00000274421 1.1117 0.13313 1.1117 0.13313 1.1117 0.13313
1.1117 0.13313 0.13313 0.588504386
ABHD16B ENSG00000183260 1.1116 0.13315 1.1116 0.13315 1.1116
0.13315 1.1116 0.13315 0.13315 0.588504386
IL24 ENSG00000162892 1.1115 0.13318 -0.49321 0.68907 -0.95627 0.83053
-0.34541 0.63511 0.13318 0.588504386
NA ENSG00000277911 1.1115 0.13319 0.94681 0.17187 0.94681 0.17187
0.94681 0.17187 0.13319 0.588504386
MAP3K5-AS1 ENSG00000234263 -0.61598 0.73105 1.1113 0.13321 -0.61598
0.73105 -0.61598 0.73105 0.13321 0.588504386
MRPS31P1 ENSG00000275256 0.18223 0.4277 0.30657 0.37959 0.5054
0.30664 1.1114 0.13321 0.13321 0.588504386
PTPN6 ENSG00000111679 0.53244 0.29721 0.42433 0.33566 1.1113 0.13323
0.42433 0.33566 0.13323 0.588504386
NA ENSG00000228335 1.1113 0.13323 1.1113 0.13323 1.1113 0.13323
1.1113 0.13323 0.13323 0.588504386
PREPL ENSG00000138078 1.1112 0.13325 -1.1208 0.86881 -1.1717 0.87933
-1.1248 0.86966 0.13325 0.588504386
NA ENSG00000239532 0.7032 0.24097 0.95995 0.16854 1.1111 0.13325
1.1111 0.13325 0.13325 0.588504386
LINC01486 ENSG00000256560 1.1112 0.13325 0.19307 0.42345 -0.65511
0.7438 0.19307 0.42345 0.13325 0.588504386
RRAGC ENSG00000116954 -0.40441 0.65704 0.47186 0.31851 0.56041 0.2876
1.1111 0.13326 0.13326 0.588504386
RNA5SP180 ENSG00000251990 -0.33546 0.63136 0.34834 0.36379 1.1111

0.13327	0.40027	0.34448	0.13327	0.588504386
GPALPP1	ENSG00000133114	-0.097174	0.53871	-0.85657	0.80416	-0.46322
0.6784	1.111	0.13328	0.13328	0.588504386
MIR181A1HG	ENSG00000229989	-0.56747	0.7148	1.1109	0.1333	0.24834
0.40193	-0.55628	0.71099	0.1333	0.588539368
RASSF6	ENSG00000169435	0.57146	0.28384	0.3064	0.37965	0.38753
0.34918	1.1109	0.13332	0.13332	0.588574343
NA	ENSG00000265356	0.19648	0.42212	1.1107	0.13335	0.19648	0.42212
-1.5376	0.93793	0.13335	0.588596661
HSP90AA5P	ENSG00000205955	-0.74203	0.77097	-0.34158	0.63367	-0.34158
0.63367	1.1106	0.13337	0.13337	0.588596661
CCT8L1P	ENSG00000020219	1.1106	0.13338	1.1106	0.13338	-0.81103
0.79132	-0.27512	0.60839	0.13338	0.588596661
ARID2	ENSG00000189079	0.12275	0.45115	0.34446	0.36525	0.92	0.17879
1.1106	0.13338	0.13338	0.588596661
DDX52	ENSG00000278053	1.0471	0.14753	0.96875	0.16633	1.1105	0.13339
0.99524	0.15981	0.13339	0.588596661
SLCO4A1	ENSG00000101187	-2.1136	0.98273	1.1104	0.13342	-1.5606
0.9407	-0.18531	0.57351	0.13342	0.588596661
CNTNAP1	ENSG00000108797	0.70175	0.24142	1.1103	0.13343	0.70175
0.24142	0.34564	0.36481	0.13343	0.588596661
RAC2	ENSG00000128340	1.1103	0.13344	1.1103	0.13344	0.11556	0.454
0.15994	0.43646	0.13344	0.588596661
ACE3P	ENSG00000224353	0.87477	0.19085	0.87477	0.19085	0.87477	0.19085
1.1102	0.13345	0.13345	0.588596661
NA	ENSG00000280177	1.1102	0.13346	1.1102	0.13346	1.1102	0.13346
1.1102	0.13346	0.13346	0.588596661
OR10P1	ENSG00000175398	1.1101	0.13347	-1.7238	0.95763	-1.0714	0.858
-0.82249	0.7946	0.13347	0.588596661
SNX18P14	ENSG00000235571	-0.80649	0.79002	-0.85651	0.80414	-0.21611
0.58555	1.1101	0.13347	0.13347	0.588596661
NA	ENSG00000282718	1.1098	0.13353	1.1098	0.13353	1.1098	0.13353
-0.32478	0.62733	0.13353	0.588807972
NA	ENSG00000226134	1.1097	0.13356	0.65665	0.2557	-0.81312	0.79192
-0.99721	0.84067	0.13356	0.588833692
NA	ENSG00000259869	1.1004	0.13557	1.1004	0.13557	1.1097	0.13356
1.1004	0.13557	0.13356	0.588833692
NBPF2P	ENSG00000227001	0.68824	0.24565	1.1095	0.1336	0.099041
0.46055	0.099041	0.46055	0.1336	0.588929176
RN7SKP124	ENSG00000201600	1.1095	0.13362	1.1095	0.13362	1.1095
0.13362	1.1095	0.13362	0.13362	0.588929176
NA	ENSG00000214280	1.1095	0.13362	1.1095	0.13362	1.1095	0.13362
1.1095	0.13362	0.13362	0.588929176
FAM81A	ENSG00000157470	0.68451	0.24683	1.1094	0.13363	1.0901
0.13783	0.31271	0.37725	0.13363	0.588929176
CTNNAL1	ENSG00000119326	-0.31663	0.62424	1.1092	0.13367	-0.11921
0.54745	-0.02017	0.50805	0.13367	0.588980535
FDPSP6	ENSG00000233676	1.1092	0.13367	-0.029639	0.51182	0.41697
0.33835	1.1092	0.13367	0.13367	0.588980535
NA	ENSG00000242150	1.1092	0.13368	1.1092	0.13368	1.1092	0.13368
1.1092	0.13368	0.13368	0.588980535
NA	ENSG00000250111	-0.64363	0.74009	0.2818	0.38905	0.98221	0.163
1.1091	0.13369	0.13369	0.588980535
RN7SL868P	ENSG00000242928	1.109	0.13372	1.109	0.13372	1.109	0.13372	1.109
0.13372	0.13372	0.589041052
NA	ENSG00000250626	1.1089	0.13373	0.18517	0.42655	1.1089	0.13373
0.11876	0.45273	0.13373	0.589041052
CILP	ENSG00000138615	0.60025	0.27417	0.67807	0.24886	0.33863	0.36745
1.1089	0.13374	0.13374	0.589041052
LINC01151	ENSG00000253819	0.70698	0.23979	1.1088	0.13376	-1.513
0.93485	0.060995	0.47568	0.13376	0.589056992
C4orf46	ENSG00000205208	1.1087	0.13379	1.1087	0.13379	1.1087

0.13379	0.92316	0.17796	0.13379	0.589056992
NA	ENSG00000277482	1.1086	0.1338	1.1086	0.1338	1.1086	0.1338
1.1086	0.1338	0.1338	0.589056992
HACL1	ENSG00000131373	-0.02177	0.50868	-1.2023	0.88537	1.1085	0.13381
0.26037	0.39729	0.13381	0.589056992
NA	ENSG00000249240	1.1085	0.13382	1.1085	0.13382	1.1085	0.13382
1.1085	0.13382	0.13382	0.589056992
IFNWP5	ENSG00000232138	1.1085	0.13383	0.86569	0.19333	-0.33778
0.63224	-0.33778	0.63224	0.13383	0.589056992
NA	ENSG00000283573	-0.45189	0.67433	-1.7948	0.96366	1.1085	0.13383
-0.30071	0.61818	0.13383	0.589056992
ST13	ENSG00000100380	1.1082	0.1339	0.43346	0.33234	-0.31068	0.62198
-1.0523	0.85368	0.1339	0.589056992
ST6GAL2	ENSG00000144057	-0.86006	0.80512	-0.85191	0.80287	0.06866
0.47263	1.1081	0.13391	0.13391	0.589056992
FRMD4B	ENSG00000114541	1.1081	0.13392	-0.012087	0.50482	0.28329
0.38848	0.042688	0.48298	0.13392	0.589056992
NA	ENSG00000224478	1.108	0.13392	0.69642	0.24308	0.54385	0.29327
0.68584	0.24641	0.13392	0.589056992
NA	ENSG00000255397	1.1079	0.13395	1.1079	0.13395	1.1079	0.13395
0.59649	0.27542	0.13395	0.589056992
COPRS	ENSG00000172301	-0.66961	0.74845	1.1078	0.13397	-0.13484	0.55363
-0.27267	0.60745	0.13397	0.589056992
CASC11	ENSG00000249375	-0.641	0.73924	1.1077	0.13399	-1.3718
0.91494	-1.5716	0.94198	0.13399	0.589056992
ATP8A1	ENSG00000124406	-0.22034	0.5872	0.60279	0.27333	1.1077	0.134
0.76898	0.22095	0.134	0.589056992
DLX1	ENSG00000144355	-0.71798	0.76362	0.77207	0.22004	1.1077	0.134
1.0891	0.13805	0.134	0.589056992
FEZ2	ENSG00000171055	1.1077	0.134	0.68636	0.24624	-0.58387	0.72035
-0.9426	0.82706	0.134	0.589056992
KHDC4	ENSG00000132680	0.43537	0.33165	1.1076	0.13401	0.43537	0.33165
0.92474	0.17755	0.13401	0.589056992
TMEM155	ENSG00000164112	1.1076	0.13401	1.1076	0.13401	1.1076
0.13401	1.1076	0.13401	0.13401	0.589056992
NA	ENSG00000255415	0.019568	0.49219	-0.1894	0.57511	1.1076	0.13402
-0.19491	0.57727	0.13402	0.589056992
KRT8P19	ENSG00000257528	1.1076	0.13402	-0.49687	0.69036	1.1076
0.13402	1.1076	0.13402	0.13402	0.589056992
MIR4428	ENSG00000266262	1.1076	0.13402	1.1076	0.13402	1.1076
0.13402	1.1076	0.13402	0.13402	0.589056992
SACS-AS1	ENSG00000229558	1.1075	0.13404	0.22444	0.41121	0.9223
0.17819	-0.15758	0.5626	0.13404	0.589056992
NA	ENSG00000206847	0.41017	0.34084	1.1075	0.13405	1.1075	0.13405
1.1075	0.13405	0.13405	0.589056992
MIR586	ENSG00000207769	-1.0905	0.86225	-1.0905	0.86225	-1.0905
0.86225	1.1074	0.13405	0.13405	0.589056992
CALN1	ENSG00000183166	0.59998	0.27426	0.97126	0.16571	0.70276	0.2411
1.1074	0.13406	0.13406	0.589056992
NA	ENSG00000279746	1.1074	0.13407	1.1074	0.13407	1.1074	0.13407
1.1074	0.13407	0.13407	0.589056992
RNU7-126P	ENSG00000238632	1.1072	0.1341	1.1072	0.1341	-0.48505
0.68618	0.14299	0.44315	0.1341	0.589135683
RN7SKP276	ENSG00000252051	0.24031	0.40504	1.1071	0.13413	0.19917
0.42106	1.1071	0.13413	0.13413	0.589142889
MIR2117HG	ENSG00000267151	1.1071	0.13414	1.1071	0.13414	1.1071
0.13414	1.1071	0.13414	0.13414	0.589142889
NA	ENSG00000273210	1.1071	0.13414	0.51359	0.30377	1.1071	0.13414
1.1071	0.13414	0.13414	0.589142889
NA	ENSG00000261324	1.107	0.13415	1.107	0.13415	1.107	0.13415	1.107	0.13415
0.13415	0.589142889
NA	ENSG00000267665	1.1065	0.13426	0.40896	0.34128	0.86314	0.19403

0.029621 0.48818 0.13426 0.589448347
POLQ ENSG00000051341 -0.72107 0.76457 0.54581 0.2926 1.1063 0.1343
0.68532 0.24657 0.1343 0.589448347
DCUN1D4 ENSG00000109184 0.8429 0.19964 1.0747 0.14124 1.1062
0.13431 0.92018 0.17874 0.13431 0.589448347
RPL7L1P7 ENSG00000251137 -0.20301 0.58043 1.1062 0.13431 -0.31333
0.62299 0.068291 0.47278 0.13431 0.589448347
DPP3P2 ENSG00000221844 0.68684 0.24609 1.1062 0.13432 0.024231
0.49033 -0.5361 0.70406 0.13432 0.589448347
ERVMER34-1 ENSG00000226887 -1.7543 0.96031 -1.8674 0.96907 0.0029042
0.49884 1.1061 0.13434 0.13434 0.589448347
NA ENSG00000233060 1.1061 0.13434 1.1061 0.13434 1.1061 0.13434
1.1061 0.13434 0.13434 0.589448347
CBY3 ENSG00000204659 0.84651 0.19863 1.106 0.13436 -0.0172 0.50686
-0.72599 0.76608 0.13436 0.589448347
NA ENSG00000225084 1.1059 0.13438 1.1059 0.13438 1.1059 0.13438
1.1059 0.13438 0.13438 0.589448347
PSMB10 ENSG00000205220 1.1059 0.13439 0.62601 0.26565 1.1059
0.13439 1.1059 0.13439 0.13439 0.589448347
GAPDHP74 ENSG00000232780 0.86379 0.19385 0.74027 0.22957 1.1059
0.13439 1.1059 0.13439 0.13439 0.589448347
ZNF628 ENSG00000197483 1.1058 0.13441 1.07 0.14232 1.0378 0.14967
1.1058 0.13441 0.13441 0.589448347
C1QTNF8 ENSG00000184471 0.50593 0.30645 0.5361 0.29595 1.1057
0.13442 1.1057 0.13442 0.13442 0.589448347
NA ENSG00000255355 0.9928 0.1604 0.92449 0.17761 1.1057 0.13442
1.1057 0.13442 0.13442 0.589448347
NA ENSG00000278896 0.7979 0.21246 0.16032 0.43632 -0.23938 0.5946
1.1056 0.13445 0.13445 0.589448347
SMC5 ENSG00000198887 0.41753 0.33815 0.024038 0.49041 0.68835 0.24562
1.1056 0.13446 0.13446 0.589448347
GKN3P ENSG00000283586 1.1056 0.13446 0.31552 0.37618 0.72492 0.23425
-0.32936 0.62906 0.13446 0.589448347
KSR1P1 ENSG00000229485 -0.39826 0.65478 -0.44165 0.67063 1.1055
0.13447 -0.86156 0.80553 0.13447 0.589448347
RPS16P8 ENSG00000213664 1.1055 0.13448 0.57837 0.28151 0.70617
0.24004 0.60228 0.27349 0.13448 0.589448347
RECQL4 ENSG00000160957 0.12471 0.45037 1.1053 0.13451 1.1053
0.13451 1.1053 0.13451 0.13451 0.589448347
GAMT ENSG00000130005 1.1052 0.13454 1.1052 0.13454 1.1052 0.13454
1.1052 0.13454 0.13454 0.589448347
TAF11 ENSG00000064995 0.31687 0.37567 1.1051 0.13456 1.1051 0.13456
-0.024875 0.50992 0.13456 0.589448347
UBAC2-AS1 ENSG00000228889 0.33787 0.36773 0.36499 0.35756 1.1051
0.13456 0.59989 0.27429 0.13456 0.589448347
SNX6 ENSG00000129515 0.32077 0.37419 0.54441 0.29308 1.105 0.13458
0.4688 0.31961 0.13458 0.589448347
CSNK1G2-AS1 ENSG00000180846 0.38282 0.35093 0.68984 0.24515 -0.99551
0.84026 1.105 0.13458 0.13458 0.589448347
NA ENSG00000236921 1.105 0.13458 1.105 0.13458 0.10064 0.45992 0.070552
0.47188 0.13458 0.589448347
SNX29P1 ENSG00000158482 1.105 0.13459 -0.50718 0.69399 -0.024283 0.50969
-0.59591 0.72438 0.13459 0.589448347
LINC01609 ENSG00000253103 1.0118 0.15583 -0.96302 0.83223 1.1049
0.1346 0.65692 0.25562 0.1346 0.589448347
SLC25A30-AS1 ENSG00000251015 0.2659 0.39516 1.1049 0.13461 1.1049
0.13461 1.1049 0.13461 0.13461 0.589448347
NA ENSG00000235581 0.43895 0.33035 0.29256 0.38493 1.1047 0.13464
0.3787 0.35245 0.13464 0.589448347
RIOK3P1 ENSG00000225944 1.1018 0.13528 1.1018 0.13528 1.1018
0.13528 1.1047 0.13465 0.13465 0.589448347
MTND1P11 ENSG00000228166 1.1046 0.13466 1.1046 0.13466 1.1046

0.13466 1.1046 0.13466 0.13466 0.589448347
KANSL2 ENSG00000139620 -0.42739 0.66545 1.1046 0.13468 1.1046
0.13468 1.1046 0.13468 0.13468 0.589448347
ZNF454 ENSG00000178187 1.069 0.14252 -0.2865 0.61275 -0.2865 0.61275
1.1045 0.13468 0.13468 0.589448347
DLGAP3 ENSG00000116544 0.21637 0.41435 1.1044 0.1347 1.1044
0.1347 0.21672 0.41421 0.1347 0.589448347
SFN ENSG00000175793 0.13484 0.44637 0.13484 0.44637 1.1044 0.13472
0.22054 0.41272 0.13472 0.589448347
CASP4 ENSG00000196954 -0.57288 0.71664 -1.4695 0.92914 1.1043 0.13472
-1.2645 0.89698 0.13472 0.589448347
NA ENSG00000228700 -0.40847 0.65854 -0.61051 0.72924 0.11054 0.45599
1.1044 0.13472 0.13472 0.589448347
CENPW ENSG00000203760 -0.67147 0.74904 0.72747 0.23347 0.57031 0.28423
1.1043 0.13473 0.13473 0.589448347
PDZD2 ENSG00000133401 -0.2778 0.60942 0.12564 0.45001 1.1042 0.13476
-0.65879 0.74499 0.13476 0.589448347
NA ENSG00000196390 -0.56964 0.71554 -0.88608 0.81221 1.1042 0.13476
1.1042 0.13476 0.13476 0.589448347
NA ENSG00000239517 1.1042 0.13476 1.1042 0.13476 1.1042 0.13476
1.1042 0.13476 0.13476 0.589448347
NA ENSG00000259341 1.1042 0.13476 0.17341 0.43116 1.1042 0.13476
0.53473 0.29642 0.13476 0.589448347
NA ENSG00000244176 1.1041 0.13477 1.1041 0.13477 1.1041 0.13477
0.65541 0.2561 0.13477 0.589448347
OR4K11P ENSG00000179381 1.104 0.13479 1.104 0.13479 0.41762 0.33811
-0.029106 0.51161 0.13479 0.589448347
RPS2P44 ENSG00000243609 -0.51537 0.69685 1.1039 0.13483 -0.6704
0.7487 1.1039 0.13483 0.13483 0.589448347
ENOX1 ENSG00000120658 1.1038 0.13485 0.70599 0.2401 0.63539 0.26259
0.63512 0.26267 0.13485 0.589448347
RNU6-1065P ENSG00000207242 1.1036 0.13488 0.77921 0.21793 -0.043804
0.51747 -0.66078 0.74562 0.13488 0.589448347
NA ENSG00000269752 1.1036 0.13489 1.1036 0.13489 1.1036 0.13489
1.1036 0.13489 0.13489 0.589448347
EIF4A1P6 ENSG00000236608 1.1035 0.1349 -0.60812 0.72845 1.1035
0.1349 1.1035 0.1349 0.1349 0.589448347
ACAA2 ENSG00000167315 1.1035 0.13491 1.0985 0.13599 1.0124 0.15567
0.65929 0.25485 0.13491 0.589448347
NA ENSG00000267678 1.1035 0.13491 1.1035 0.13491 1.1035 0.13491
1.1035 0.13491 0.13491 0.589448347
LINC01999 ENSG00000267772 1.1035 0.13491 1.1035 0.13491 1.1035
0.13491 1.1035 0.13491 0.13491 0.589448347
RNU6-349P ENSG00000199272 1.1034 0.13493 0.89927 0.18425 1.1034
0.13493 0.86056 0.19474 0.13493 0.589448347
ADCY2 ENSG00000078295 1.1033 0.13494 0.40609 0.34234 0.71552 0.23714
1.0372 0.14983 0.13494 0.589448347
NIPA2P5 ENSG00000236277 0.61818 0.26823 0.11448 0.45443 1.1033
0.13495 1.1033 0.13495 0.13495 0.589448347
NA ENSG00000260573 0.95899 0.16878 1.1033 0.13496 0.95899 0.16878
0.95899 0.16878 0.13496 0.589448347
WDR49 ENSG00000174776 0.17704 0.42974 1.1032 0.13497 -0.40954 0.65893
1.1032 0.13497 0.13497 0.589448347
NA ENSG00000260467 1.1032 0.13498 1.1032 0.13498 1.1032 0.13498
1.1032 0.13498 0.13498 0.589448347
NA ENSG00000235356 1.1031 0.135 1.1031 0.135 1.1031 0.135 1.1031 0.135
0.135 0.589448347
MIR589 ENSG00000207973 1.103 0.13501 1.103 0.13501 1.103 0.13501 1.103
0.13501 0.13501 0.589448347
CDK3 ENSG00000250506 1.103 0.13501 0.46087 0.32245 0.46087 0.32245
-0.0022489 0.5009 0.13501 0.589448347
NA ENSG00000257169 1.103 0.13501 0.76708 0.22152 0.49406 0.31063

0.85456 0.1964 0.13501 0.589448347
UGT1A11P ENSG00000227846 1.103 0.13502 0.59016 0.27754 0.94024 0.17355
1.0018 0.15821 0.13502 0.589448347
HNRNPA2B1 ENSG00000122566 0.35524 0.36121 1.1029 0.13503 -1.0256
0.84746 -0.89497 0.8146 0.13503 0.589448347
ADH5P2 ENSG00000232676 1.1029 0.13503 -1.0929 0.86278 1.1029
0.13503 1.1029 0.13503 0.13503 0.589448347
NA ENSG00000279918 0.23639 0.40656 0.034493 0.48624 1.1029 0.13503
0.5613 0.2873 0.13503 0.589448347
LINC00649 ENSG00000237945 0.99256 0.16046 1.1028 0.13506 0.065444
0.47391 0.35737 0.36041 0.13506 0.589499033
TECTB ENSG00000119913 0.42162 0.33665 0.45587 0.32424 1.1026 0.13509
0.42162 0.33665 0.13509 0.589499033
H3P26 ENSG00000224447 1.1027 0.13509 1.1027 0.13509 1.1027 0.13509
1.1027 0.13509 0.13509 0.589499033
LINC02306 ENSG00000257986 0.71251 0.23807 0.71251 0.23807 1.1026
0.13509 -0.20455 0.58104 0.13509 0.589499033
ACSM4 ENSG00000215009 -0.15239 0.56056 0.72061 0.23557 1.1025 0.13512
-0.29205 0.61488 0.13512 0.589549682
ATP5MGP4 ENSG00000225176 -0.22891 0.59053 -0.22891 0.59053 1.1024
0.13513 0.77242 0.21993 0.13513 0.589549682
CASP16P ENSG00000228146 1.1024 0.13515 1.1024 0.13515 1.1024
0.13515 1.1024 0.13515 0.13515 0.589549682
NA ENSG00000230084 0.61891 0.26799 1.1024 0.13515 1.1024 0.13515
1.1024 0.13515 0.13515 0.589549682
ACTE1P ENSG00000172900 1.1023 0.13518 0.46083 0.32246 0.13854
0.44491 1.0791 0.14026 0.13518 0.589574993
RNU2-69P ENSG00000251870 -0.077969 0.53107 -0.11915 0.54742 -0.11783
0.5469 1.1023 0.13518 0.13518 0.589574993
EXOC4 ENSG00000131558 0.70922 0.23909 -0.78467 0.78368 1.0094 0.15638
1.1021 0.1352 0.1352 0.589600295
IRX1 ENSG00000170549 -0.48882 0.68752 0.32872 0.37118 0.066141 0.47363
1.1021 0.13521 0.13521 0.589600295
CACNB1 ENSG00000067191 0.58401 0.27961 1.102 0.13523 0.75654 0.22466
0.51688 0.30262 0.13523 0.589616434
ACSF3 ENSG00000176715 0.76054 0.22347 1.1019 0.13525 -0.12835 0.55107
1.0005 0.15854 0.13525 0.589616434
MIR3163 ENSG00000266423 1.1019 0.13525 -0.35246 0.63775 -0.11419
0.54546 -0.2953 0.61612 0.13525 0.589616434
NA ENSG00000252670 1.1018 0.13528 1.1018 0.13528 1.1018 0.13528
1.1018 0.13528 0.13528 0.589694463
NA ENSG00000239381 -1.5153 0.93515 0.63986 0.26113 0.2263 0.41048
1.1017 0.1353 0.1353 0.589728891
LINC01056 ENSG00000237119 0.91465 0.18019 0.65276 0.25695 0.8066
0.20995 1.1015 0.13535 0.13535 0.589770237
NA ENSG00000274383 1.1014 0.13535 1.1014 0.13535 1.1014 0.13535
1.1014 0.13535 0.13535 0.589770237
PLA2G4C-AS1 ENSG00000269420 1.1014 0.13536 1.1014 0.13536 1.1014
0.13536 1.1014 0.13536 0.13536 0.589770237
NA ENSG00000279842 1.1014 0.13536 -0.2074 0.58215 0.60757 0.27174
-0.096021 0.53825 0.13536 0.589770237
CCDC102B ENSG00000150636 0.60728 0.27183 0.17048 0.43232 -0.98276
0.83714 1.1013 0.13537 0.13537 0.589770237
DNTT ENSG00000107447 1.1013 0.13539 -0.113 0.54498 1.1013 0.13539
0.69429 0.24375 0.13539 0.589795477
NA ENSG00000273154 1.1012 0.1354 1.1012 0.1354 1.1012 0.1354
1.1012 0.1354 0.1354 0.589795477
LCLAT1 ENSG00000172954 0.47951 0.31579 0.04931 0.48034 1.1011
0.13543 -0.6907 0.75512 0.13543 0.589820708
ARID3C ENSG00000205143 1.1011 0.13543 1.1011 0.13543 1.1011
0.13543 1.1011 0.13543 0.13543 0.589820708
COX7A2P2 ENSG00000236764 -0.39768 0.65457 1.101 0.13546 -0.076454 0.53047

0.50522 0.3067 0.13546 0.589898642
RPL35P9 ENSG00000174418 1.1008 0.13549 1.1008 0.13549 1.1008
0.13549 1.1008 0.13549 0.13549 0.589976562
IL13 ENSG00000169194 0.23994 0.40519 0.23994 0.40519 0.23994 0.40519
1.1007 0.13552 0.13552 0.590001751
LINC01060 ENSG00000249378 1.1007 0.13552 0.23481 0.40718 -0.35995
0.64056 -0.80003 0.78815 0.13552 0.590001751
OR1M1 ENSG00000170929 1.1006 0.13554 0.18022 0.42849 -1.1255 0.86982
-1.1255 0.86982 0.13554 0.590017756
LGALS3 ENSG00000131981 1.0651 0.14341 0.6009 0.27395 1.0651
0.14341 1.1005 0.13556 0.13556 0.590017756
NA ENSG00000266908 1.1005 0.13556 1.1005 0.13556 1.1005 0.13556
1.1005 0.13556 0.13556 0.590017756
UGT1A5 ENSG00000240224 1.1004 0.13558 0.48447 0.31403 0.48447
0.31403 0.48447 0.31403 0.13558 0.590042927
NNT-AS1 ENSG00000248092 1.1003 0.13559 0.80617 0.21007 1.1003
0.13559 1.1003 0.13559 0.13559 0.590042927
OR8G1 ENSG00000197849 1.1002 0.13562 1.1002 0.13562 1.1002 0.13562
1.1002 0.13562 0.13562 0.59004476
NA ENSG00000231393 1.0656 0.1433 1.1001 0.13564 1.0656 0.1433
1.0656 0.1433 0.13564 0.59004476
ZNF487 ENSG00000243660 0.22547 0.41081 0.2071 0.41797 0.22547
0.41081 1.1 0.13566 0.13566 0.59004476
C3orf84 ENSG00000236980 1.0228 0.1532 1.1 0.13567 -0.33187 0.63001
0.61384 0.26966 0.13567 0.59004476
GPLD1 ENSG00000112293 -0.26039 0.60272 -0.2441 0.59642 -0.80455 0.78946
1.0999 0.13568 0.13568 0.59004476
DYNLRB1 ENSG00000125971 0.05571 0.47779 0.11875 0.45274 1.0998
0.13571 1.0998 0.13571 0.13571 0.59004476
MGAT4D ENSG00000205301 -0.60771 0.72831 -0.60771 0.72831 -0.60771
0.72831 1.0998 0.13572 0.13572 0.59004476
ENPP4 ENSG00000001561 1.0996 0.13575 1.0996 0.13575 1.0996 0.13575
0.020303 0.4919 0.13575 0.59004476
PURG ENSG00000172733 1.0996 0.13575 -0.21957 0.5869 0.71876 0.23615
-0.21957 0.5869 0.13575 0.59004476
NA ENSG00000277501 1.0995 0.13577 -0.19756 0.5783 -0.19756 0.5783
-0.19756 0.5783 0.13577 0.59004476
NA ENSG00000258615 1.0995 0.13578 1.0995 0.13578 1.0995 0.13578
1.0995 0.13578 0.13578 0.59004476
NA ENSG00000261770 1.0995 0.13578 -1.3013 0.90343 -0.24243 0.59578
-0.0030362 0.50121 0.13578 0.59004476
CST2 ENSG00000170369 -0.056811 0.52265 1.0993 0.13581 0.63396 0.26305
0.56095 0.28742 0.13581 0.59004476
NA ENSG00000258525 1.0993 0.13582 1.0993 0.13582 1.0993 0.13582
1.0993 0.13582 0.13582 0.59004476
PPIAP46 ENSG00000260266 -0.075467 0.53008 1.0993 0.13582 -0.34886
0.6364 -0.34886 0.6364 0.13582 0.59004476
C7orf50 ENSG00000146540 0.97521 0.16473 0.041947 0.48327 1.0992
0.13584 0.097751 0.46106 0.13584 0.59004476
POLH ENSG00000170734 0.11985 0.4523 0.36354 0.3581 1.0992 0.13584
-0.68085 0.75202 0.13584 0.59004476
RN7SL634P ENSG00000242894 1.0992 0.13584 0.0043817 0.49825 0.0043817
0.49825 0.0043817 0.49825 0.13584 0.59004476
SH3PXD2B ENSG00000174705 0.65257 0.25702 1.0991 0.13586 0.40139
0.34407 0.56462 0.28617 0.13586 0.59004476
NA ENSG00000279485 -0.2039 0.58078 1.099 0.13588 0.72172 0.23523
0.14052 0.44412 0.13588 0.59004476
NA ENSG00000259707 1.0989 0.1359 1.0989 0.1359 1.0989 0.1359
1.0989 0.1359 0.1359 0.59004476
NA ENSG00000261267 0.77262 0.21987 1.0989 0.1359 1.0989 0.1359
1.0989 0.1359 0.1359 0.59004476
NA ENSG00000267177 1.0989 0.1359 -0.88353 0.81153 0.044173 0.48238

-0.39507 0.65361 0.1359 0.59004476
MT1P3 ENSG00000229230 -0.11766 0.54683 -0.11766 0.54683 1.0988 0.13592
-0.5129 0.69599 0.13592 0.59004476
NA ENSG00000232097 0.66294 0.25369 1.0988 0.13592 0.0035974 0.49856
-0.15661 0.56222 0.13592 0.59004476
UBA3 ENSG00000144744 0.27965 0.38987 0.75244 0.22589 0.64432 0.25968
1.0988 0.13593 0.13593 0.59004476
NA ENSG00000225794 1.0987 0.13595 1.0987 0.13595 1.0987 0.13595
1.0987 0.13595 0.13595 0.59004476
NA ENSG00000260920 -0.56327 0.71338 1.0987 0.13595 1.0987 0.13595
0.99276 0.16041 0.13595 0.59004476
CALCRL ENSG00000064989 0.39403 0.34678 1.0986 0.13596 0.20006
0.42072 0.30472 0.38029 0.13596 0.59004476
RPLP1P6 ENSG00000213433 -0.73355 0.76839 -0.73355 0.76839 -0.73355
0.76839 1.0986 0.13596 0.13596 0.59004476
CD83 ENSG00000112149 -0.40434 0.65702 0.010968 0.49562 1.0986 0.13598
0.64766 0.2586 0.13598 0.59004476
MAPK6P6 ENSG00000226389 1.0986 0.13598 -0.76144 0.7768 0.89355
0.18578 0.28952 0.38609 0.13598 0.59004476
ZKSCAN7 ENSG00000196345 -0.37029 0.64442 1.0985 0.13599 0.54891
0.29153 -0.1984 0.57864 0.13599 0.59004476
NA ENSG00000225537 0.18017 0.42851 -0.171 0.56789 1.0984 0.13601
-0.11086 0.54414 0.13601 0.590069848
NA ENSG00000258975 1.0984 0.13602 1.0984 0.13602 1.0984 0.13602
1.0984 0.13602 0.13602 0.590069848
EEF1A1P33 ENSG00000258846 -0.24573 0.59705 1.0982 0.13606 1.0982
0.13606 1.0982 0.13606 0.13606 0.590190831
CDCA4P4 ENSG00000234219 1.0979 0.13612 1.0979 0.13612 1.0979
0.13612 1.0979 0.13612 0.13612 0.59038019
RN7SL318P ENSG00000242079 1.066 0.14322 1.0979 0.13613 0.80836 0.20944
1.0434 0.14838 0.13613 0.59038019
NA ENSG00000284196 1.0978 0.13614 -0.071388 0.52846 1.0978 0.13614
0.81035 0.20887 0.13614 0.59038019
NT5DC4 ENSG00000144130 -0.6124 0.72987 1.0112 0.15596 1.0976
0.13619 -0.459 0.67688 0.13619 0.590439386
NA ENSG00000236505 0.82684 0.20416 0.2626 0.39643 -0.17691 0.57021
1.0976 0.13619 0.13619 0.590439386
NA ENSG00000256342 0.42526 0.33532 0.42526 0.33532 1.0976 0.13619
0.42526 0.33532 0.13619 0.590439386
NA ENSG00000274261 1.0974 0.13622 -0.80074 0.78836 -0.80074 0.78836
0.47279 0.31818 0.13622 0.59050773
MRGPRE ENSG00000184350 1.0974 0.13623 1.0974 0.13623 0.7511
0.2263 1.0974 0.13623 0.13623 0.59050773
LINC01758 ENSG00000229639 -0.65899 0.74505 1.0971 0.13629 -0.65899
0.74505 0.76331 0.22264 0.13629 0.590715263
LAX1 ENSG00000122188 0.82667 0.20421 1.097 0.13632 0.20347 0.41938
0.24934 0.40155 0.13632 0.590783539
NA ENSG00000214999 1.097 0.13633 1.097 0.13633 1.097 0.13633 1.097 0.13633
0.13633 0.590783539
NA ENSG00000176882 1.0968 0.13636 1.0968 0.13636 1.0968 0.13636
0.83306 0.20241 0.13636 0.590808464
H3P8 ENSG00000270915 0.23499 0.40711 -0.075351 0.53003 1.0968 0.13636
0.45451 0.32473 0.13636 0.590808464
FOXN3 ENSG00000053254 -1.3082 0.90459 -0.61869 0.73194 0.027421 0.48906
1.0966 0.1364 0.1364 0.590914548
NA ENSG00000271248 -0.77452 0.78069 1.0966 0.13641 0.89305 0.18592
0.20639 0.41824 0.13641 0.590914548
RNU6-268P ENSG00000201044 -0.85834 0.80465 -0.85834 0.80465 -2.1536
0.98436 1.0964 0.13645 0.13645 0.590914548
PGAM1P2 ENSG00000225639 -0.017654 0.50704 1.0964 0.13645 0.10498
0.4582 1.0964 0.13645 0.13645 0.590914548
FAM186B ENSG00000135436 1.0963 0.13648 0.032231 0.48714 1.0963

0.13648 0.70651 0.23994 0.13648 0.590914548
HCG4 ENSG00000176998 1.0962 0.1365 0.41054 0.34071 -0.52917 0.70166
0.34969 0.36328 0.1365 0.590914548
NA ENSG00000229727 -1.0549 0.85427 -1.0217 0.84654 1.0962 0.1365
-0.67518 0.75022 0.1365 0.590914548
NA ENSG00000275620 1.0962 0.1365 1.0962 0.1365 1.0962 0.1365
0.71292 0.23795 0.1365 0.590914548
GSK3A ENSG00000105723 0.59591 0.27562 1.0034 0.15783 1.0961 0.13652
0.94552 0.1722 0.13652 0.590914548
NA ENSG00000279539 1.0961 0.13652 1.0961 0.13652 1.0961 0.13652
1.0961 0.13652 0.13652 0.590914548
NA ENSG00000204957 1.0961 0.13652 1.0961 0.13652 1.0961 0.13652
1.0961 0.13652 0.13652 0.590914548
COX6CP16 ENSG00000270313 0.21883 0.41339 0.11592 0.45386 1.096 0.13653
1.096 0.13653 0.13653 0.590914548
HEATR6 ENSG00000068097 -1.4682 0.92897 1.096 0.13655 -1.4682 0.92897
-1.4682 0.92897 0.13655 0.590948623
ITCH-IT1 ENSG00000231795 0.95232 0.17047 1.0958 0.13657 0.95232
0.17047 0.95232 0.17047 0.13657 0.590949583
CYP27B1 ENSG00000111012 1.0958 0.13658 1.0958 0.13658 1.0958
0.13658 1.0958 0.13658 0.13658 0.590949583
NA ENSG00000224884 -0.065096 0.52595 1.0956 0.13663 1.0956 0.13663
0.17986 0.42863 0.13663 0.590949583
SEPTIN4 ENSG00000108387 1.0955 0.13664 -0.13922 0.55536 -0.30294
0.61903 -0.22728 0.5899 0.13664 0.590949583
NA ENSG00000249856 0.69484 0.24358 0.44539 0.32802 1.0955 0.13665
-0.25969 0.60245 0.13665 0.590949583
NA ENSG00000251313 -0.46198 0.67795 1.0955 0.13665 0.31594 0.37602
0.67212 0.25075 0.13665 0.590949583
ANKRD33B ENSG00000164236 1.0954 0.13666 -0.26243 0.6035 -0.4071
0.65803 0.26624 0.39503 0.13666 0.590949583
NA ENSG00000226548 0.7237 0.23463 1.0954 0.13667 0.66761 0.25219
0.040018 0.48404 0.13667 0.590949583
EHMT2 ENSG00000204371 0.75942 0.2238 -0.97132 0.83431 1.0954 0.13668
1.0954 0.13668 0.13668 0.590949583
NA ENSG00000253269 0.25302 0.40013 0.99147 0.16073 1.0954 0.13668
0.25401 0.39974 0.13668 0.590949583
NPIPB3 ENSG00000169246 0.37225 0.35485 -1.3557 0.9124 -0.0092765
0.5037 1.0953 0.13669 0.13669 0.590949583
CYP3A5 ENSG00000106258 1.0952 0.13671 0.026452 0.48945 0.27394
0.39206 0.1525 0.4394 0.13671 0.590949583
TINAGL1 ENSG00000142910 -0.18019 0.5715 -0.038097 0.51519 1.0952
0.13671 -0.10325 0.54112 0.13671 0.590949583
NA ENSG00000235361 1.0952 0.13672 1.0952 0.13672 1.0952 0.13672
1.0952 0.13672 0.13672 0.590949583
PTMA ENSG00000187514 1.0951 0.13675 1.0951 0.13675 -0.99777 0.8408
0.49845 0.30908 0.13675 0.591024051
PHKBP1 ENSG00000226262 1.0949 0.13678 -0.22276 0.58814 -0.50522
0.6933 0.86813 0.19266 0.13678 0.591024051
ABCG8 ENSG00000143921 1.0948 0.13679 -0.40819 0.65843 -0.99344 0.83975
-0.93947 0.82625 0.13679 0.591024051
SNED1 ENSG00000162804 -0.73221 0.76798 -0.67757 0.75098 -1.3483 0.91122
1.0948 0.13681 0.13681 0.591024051
CD200R1 ENSG00000163606 0.93993 0.17363 1.0948 0.13681 0.70662
0.2399 0.9016 0.18363 0.13681 0.591024051
LINC02175 ENSG00000262155 1.0948 0.13681 1.0948 0.13681 0.28443
0.38804 1.0948 0.13681 0.13681 0.591024051
NA ENSG00000250522 0.54181 0.29397 1.0943 0.13691 0.54181 0.29397
0.54181 0.29397 0.13691 0.591394398
ADCY4 ENSG00000129467 -0.47408 0.68228 0.552 0.29047 -0.47154 0.68137
1.0942 0.13692 0.13692 0.591394398
MIR5093 ENSG00000266307 -0.78664 0.78425 1.0941 0.13695 -0.015296

0.5061	-0.78664	0.78425	0.13695	0.59147155
MRRF	ENSG00000148187	1.0304	0.1514	1.094	0.13697	0.096457	0.46158
0.29413	0.38433	0.13697	0.591496269
IGSF5	ENSG00000183067	1.094	0.13698	0.45655	0.324	0.97418	0.16498	0.79857
0.21227	0.13698	0.591496269
SNRNP200	ENSG00000144028	0.34127	0.36645	-0.15897	0.56316	1.0937
0.13704	0.72415	0.23449	0.13704	0.591502787
STXBP5	ENSG00000164506	1.0937	0.13705	0.2946	0.38415	0.66759
0.2522	0.67754	0.24903	0.13705	0.591502787
MRPL11	ENSG00000174547	0.036791	0.48533	0.036791	0.48533	-0.67606
0.7505	1.0935	0.13709	0.13709	0.591502787
RNU6-1031P	ENSG00000207504	-1.3933	0.91823	1.0935	0.13709	0.016155
0.49356	0.21702	0.4141	0.13709	0.591502787
ZNF625-ZNF20	ENSG00000213297	1.0935	0.1371	0.97267	0.16536	0.25134
0.40078	1.0935	0.1371	0.1371	0.591502787
BDH2P1	ENSG00000232699	1.0934	0.1371	1.0934	0.1371	1.0934
0.1371	1.0934	0.1371	0.1371	0.591502787
ZNF625	ENSG00000257591	1.0935	0.1371	1.0935	0.1371	1.0935
0.1371	1.0935	0.1371	0.1371	0.591502787
GALNT12	ENSG00000119514	-0.13564	0.55395	-0.11347	0.54517	1.0934
0.13712	0.85387	0.19659	0.13712	0.591502787
PLPBP	ENSG00000147471	0.73715	0.23051	1.0932	0.13715	0.41824	0.33789
-0.4919	0.6886	0.13715	0.591502787
MRPL21	ENSG00000197345	-0.84871	0.80198	-1.1534	0.87563	1.0932
0.13715	-0.87749	0.80989	0.13715	0.591502787
NA	ENSG00000259839	1.0932	0.13715	-0.20527	0.58132	0.21744	0.41393
0.9422	0.17304	0.13715	0.591502787
QRICH2	ENSG00000129646	1.0931	0.13717	0.17189	0.43176	-0.35301
0.63796	-1.5243	0.93629	0.13717	0.591502787
NA	ENSG00000252239	1.0931	0.13717	0.61052	0.27076	0.46084	0.32246
0.46084	0.32246	0.13717	0.591502787
C1orf112	ENSG00000000460	-0.19705	0.5781	-0.38382	0.64944	-0.38382
0.64944	1.0931	0.13718	0.13718	0.591502787
RNASEH1	ENSG00000171865	0.91616	0.17979	1.0233	0.15308	0.91616
0.17979	1.0931	0.13718	0.13718	0.591502787
LINC02494	ENSG00000250340	1.0931	0.13718	-0.85015	0.80238	0.2884
0.38652	0.2884	0.38652	0.13718	0.591502787
GMDS-DT	ENSG00000250903	0.10248	0.45919	0.47928	0.31587	1.093	0.13719
-0.096345	0.53838	0.13719	0.591502787
NA	ENSG00000284327	1.093	0.1372	0.72769	0.2334	0.48597	0.31349
0.54837	0.29172	0.1372	0.591502787
MTND4P3	ENSG00000225959	1.0884	0.13822	1.0884	0.13822	1.0929
0.13722	1.0884	0.13822	0.13722	0.591518238
MEF2C-AS1	ENSG00000248309	0.599	0.27459	1.0928	0.13724	-1.2533	0.89495
1.0561	0.14546	0.13724	0.591518238
LINC01970	ENSG00000265692	1.0928	0.13724	1.0928	0.13724	0.11326
0.45491	-0.061916	0.52469	0.13724	0.591518238
NA	ENSG00000251284	1.0927	0.13726	-1.1413	0.87313	0.91084	0.18119
0.91084	0.18119	0.13726	0.591533681
UQCRC1	ENSG00000010256	0.5111	0.30464	0.5111	0.30464	1.0926
0.13728	0.61969	0.26773	0.13728	0.591533681
ARHGEF18	ENSG00000104880	0.5437	0.29332	1.0926	0.13728	0.94116
0.17331	1.0436	0.14834	0.13728	0.591533681
NA	ENSG00000258758	0.0037156	0.49852	-0.31363	0.6231	1.0924	0.13733
-0.31363	0.6231	0.13733	0.59167224
NA	ENSG00000271459	1.0922	0.13737	1.0922	0.13737	1.0922	0.13737
1.0922	0.13737	0.13737	0.59167224
NA	ENSG00000206784	-0.64519	0.7406	1.0922	0.13738	0.042779	0.48294
0.042779	0.48294	0.13738	0.59167224
MTND1P8	ENSG00000263177	1.0922	0.13738	0.57332	0.28321	1.0922
0.13738	1.0922	0.13738	0.13738	0.59167224
NA	ENSG00000221418	1.0922	0.13738	1.0922	0.13738	1.0922	0.13738

0.25341 0.39998 0.13738 0.59167224
TMEM181 ENSG00000146433 -0.23273 0.59201 -0.3317 0.62994 0.78304
0.2168 1.092 0.13741 0.13741 0.59167224
IFNA11P ENSG00000231195 1.092 0.13742 1.092 0.13742 1.092 0.13742 0.78369
0.21661 0.13742 0.59167224
NA ENSG00000249803 0.16561 0.43423 1.0919 0.13743 0.85135 0.19729
1.0919 0.13743 0.13743 0.59167224
NA ENSG00000260857 1.0919 0.13744 1.0919 0.13744 1.0919 0.13744
1.0919 0.13744 0.13744 0.59167224
TP53TG3HP ENSG00000269622 1.0919 0.13744 1.0919 0.13744 1.0919
0.13744 1.0919 0.13744 0.13744 0.59167224
LAMC3 ENSG00000050555 -0.49352 0.68918 1.0918 0.13746 -0.25469 0.60052
-0.76264 0.77716 0.13746 0.59167224
RGS6 ENSG00000182732 0.89274 0.186 0.64048 0.26093 0.53141 0.29757
1.0918 0.13746 0.13746 0.59167224
RNA5SP241 ENSG00000212628 1.0918 0.13747 0.47283 0.31817 -0.062693
0.52499 0.33123 0.37023 0.13747 0.59167224
NA ENSG00000222588 1.0917 0.13749 1.0917 0.13749 1.0917 0.13749
1.0917 0.13749 0.13749 0.591706058
CABP1 ENSG00000157782 1.03 0.1515 1.0916 0.13751 0.89481 0.18544
0.33079 0.3704 0.13751 0.591739871
CALM2P4 ENSG00000233662 1.0914 0.13755 0.89333 0.18584 0.82717
0.20407 -0.70099 0.75835 0.13755 0.591742875
NA ENSG00000270140 1.0913 0.13756 1.0913 0.13756 1.0913 0.13756
1.0913 0.13756 0.13756 0.591742875
NPM1P42 ENSG00000259259 1.0912 0.13758 1.0912 0.13758 1.0912
0.13758 1.0912 0.13758 0.13758 0.591742875
LAMTOR3 ENSG00000109270 0.1556 0.43817 1.0912 0.1376 0.2036
0.41933 -0.065014 0.52592 0.1376 0.591742875
OR6A2 ENSG00000184933 0.32345 0.37318 1.0912 0.1376 0.32345 0.37318
0.32345 0.37318 0.1376 0.591742875
OSTC ENSG00000198856 0.14141 0.44377 0.36012 0.35938 1.0911 0.13761
0.86583 0.19329 0.13761 0.591742875
TERF2IP ENSG00000166848 0.79482 0.21336 1.0911 0.13762 1.0911
0.13762 0.15296 0.43922 0.13762 0.591742875
DCHS2 ENSG00000197410 0.88611 0.18778 0.78791 0.21538 0.33781 0.36775
1.0911 0.13762 0.13762 0.591742875
TCN1 ENSG00000134827 1.0908 0.13768 1.0669 0.14301 1.0669 0.14301
0.24952 0.40148 0.13768 0.591742875
CSPP1 ENSG00000104218 1.0907 0.1377 0.91325 0.18056 0.91325 0.18056
-0.86898 0.80757 0.1377 0.591742875
DEFB132 ENSG00000186458 -0.28811 0.61337 1.0906 0.13772 -1.3092
0.90477 -1.2946 0.90228 0.13772 0.591742875
MIR548J ENSG00000221760 1.0906 0.13772 1.0906 0.13772 1.0906
0.13772 1.0906 0.13772 0.13772 0.591742875
NA ENSG00000228814 -0.2787 0.60976 -0.2787 0.60976 0.63498 0.26272
1.0905 0.13774 0.13774 0.591742875
SNORA79 ENSG00000221303 1.0905 0.13775 1.0905 0.13775 1.0905
0.13775 1.0905 0.13775 0.13775 0.591742875
NA ENSG00000269106 1.0903 0.13778 1.0903 0.13778 1.0903 0.13778
1.0903 0.13778 0.13778 0.591742875
NA ENSG00000275197 0.15957 0.43661 -0.33665 0.63181 1.0903 0.13779
1.0903 0.13779 0.13779 0.591742875
THG1L ENSG00000113272 0.59347 0.27643 1.0903 0.1378 0.63359 0.26317
0.73158 0.23221 0.1378 0.591742875
BAZ1A ENSG00000198604 0.1767 0.42987 0.16779 0.43337 -0.23148 0.59153
1.0903 0.1378 0.1378 0.591742875
NA ENSG00000277096 -1.6023 0.94546 -1.7401 0.95908 -1.6023 0.94546
1.0903 0.1378 0.1378 0.591742875
KYNU ENSG00000115919 1.0902 0.13782 0.97022 0.16597 0.57213 0.28362
0.95246 0.17043 0.13782 0.591742875
NINJ1 ENSG00000131669 1.0902 0.13782 -1.1922 0.8834 -0.72115 0.76459

0.9064	0.18236	0.13782	0.591742875
PGD	ENSG00000142657	1.0901	0.13783	-0.6855	0.75348	0.37418	0.35413
0.50721	0.306	0.13783	0.591742875
KCNH7-AS1	ENSG00000237750	-0.50401	0.69287	1.0901	0.13784	-0.50401
0.69287	-1.2905	0.90157	0.13784	0.591742875
C1orf216	ENSG00000142686	0.53939	0.29481	1.09	0.13785	0.54239	0.29378
0.48725	0.31304	0.13785	0.591742875
RN7SL757P	ENSG00000239249	0.5215	0.30101	1.09	0.13785	0.76982	0.2207
0.59855	0.27474	0.13785	0.591742875
PKIA	ENSG00000171033	0.29951	0.38228	0.53655	0.29579	0.93092	0.17595
1.09	0.13786	0.13786	0.591742875
SEPHS1	ENSG00000086475	-0.056789	0.52264	0.95053	0.17092	1.0899
0.13788	-0.10597	0.5422	0.13788	0.591742875
S100A7A	ENSG00000184330	1.0899	0.13789	-0.55576	0.71081	0.55232
0.29037	-0.64709	0.74121	0.13789	0.591742875
KLHL41	ENSG00000239474	1.0898	0.1379	0.43562	0.33156	1.0245
0.15279	1.0245	0.15279	0.1379	0.591742875
DPH6-DT	ENSG00000248079	0.011154	0.49555	1.0898	0.1379	0.9704
0.16592	1.0898	0.1379	0.1379	0.591742875
NA	ENSG00000267222	1.0898	0.1379	1.0898	0.1379	1.0898	0.1379
1.0898	0.1379	0.1379	0.591742875
MIR3147	ENSG00000266168	1.0897	0.13792	1.0897	0.13792	1.0897
0.13792	0.90797	0.18195	0.13792	0.591742875
LCE4A	ENSG00000187170	1.0897	0.13793	0.84884	0.19798	0.84884	0.19798
0.84884	0.19798	0.13793	0.591742875
NA	ENSG00000272988	-1.8299	0.96637	-0.58464	0.72061	1.0896	0.13794
1.0896	0.13794	0.13794	0.591742875
FAM135A-AS1	ENSG00000224349	1.0895	0.13796	1.0895	0.13796	1.0895
0.13796	1.0895	0.13796	0.13796	0.591742875
RPL12P21	ENSG00000242683	1.0895	0.13796	1.0895	0.13796	1.0895
0.13796	1.0895	0.13796	0.13796	0.591742875
NA	ENSG00000236947	1.0895	0.13796	0.64369	0.25989	0.33777	0.36777
-0.31326	0.62296	0.13796	0.591742875
PTBP2	ENSG00000117569	1.0513	0.14655	0.60113	0.27388	1.0894	0.13799
1.0894	0.13799	0.13799	0.59180716
ABCD1P5	ENSG00000214330	1.0893	0.13801	1.0893	0.13801	1.0893
0.13801	1.0893	0.13801	0.13801	0.59180716
CST12P	ENSG00000226344	-0.27267	0.60745	1.025	0.15268	0.90528	0.18266
1.0893	0.13802	0.13802	0.59180716
SLC38A3	ENSG00000188338	0.65371	0.25665	0.65371	0.25665	0.65371
0.25665	1.0892	0.13803	0.13803	0.59180716
NA	ENSG00000224905	0.23273	0.40799	0.58967	0.27771	-0.19199	0.57612
1.0891	0.13805	0.13805	0.59180716
NA	ENSG00000231487	1.0891	0.13806	-0.14532	0.55777	0.55458	0.28959
0.55458	0.28959	0.13806	0.59180716
OR7E46P	ENSG00000234485	0.50978	0.3051	1.0891	0.13806	-0.27324
0.60766	0.76888	0.22098	0.13806	0.59180716
RNU6-271P	ENSG00000200728	1.0889	0.1381	1.0889	0.1381	1.0889
0.1381	1.0889	0.1381	0.1381	0.591851227
NA	ENSG00000170647	1.0889	0.1381	1.0889	0.1381	1.0889	0.1381
1.0889	0.1381	0.1381	0.591851227
HDC	ENSG00000140287	-0.69117	0.75527	-0.69117	0.75527	1.0889	0.13811
-0.69117	0.75527	0.13811	0.591851227
ECM1	ENSG00000143369	0.062782	0.47497	0.062782	0.47497	1.0887	0.13815
0.062782	0.47497	0.13815	0.591851227
NME3	ENSG00000103024	1.0885	0.13819	1.0885	0.13819	1.0885	0.13819
1.0885	0.13819	0.13819	0.591851227
RN7SL130P	ENSG00000244256	1.0883	0.13823	1.0883	0.13823	1.0883
0.13823	1.0883	0.13823	0.13823	0.591851227
CYP4F24P	ENSG00000267594	-1.0096	0.84365	1.0882	0.13825	-1.14	0.87287
0.21181	0.41613	0.13825	0.591851227
TOMM40P3	ENSG00000249360	0.83739	0.20119	1.088	0.1383	-0.68944	0.75473

-0.68944 0.75473 0.1383 0.591851227
MIR4421 ENSG00000265538 1.0879 0.13832 0.15039 0.44023 0.059266
0.47637 0.059266 0.47637 0.13832 0.591851227
CLBA1 ENSG00000140104 0.31277 0.37723 0.095448 0.46198 0.095448 0.46198
1.0878 0.13834 0.13834 0.591851227
NA ENSG00000253471 1.0878 0.13834 1.0878 0.13834 1.0878 0.13834
1.0878 0.13834 0.13834 0.591851227
NA ENSG00000268333 1.0878 0.13835 1.0837 0.13926 0.81862 0.2065
0.81862 0.2065 0.13835 0.591851227
ADAM20P2 ENSG00000248574 -0.48363 0.68567 1.0877 0.13836 -0.17022
0.56758 0.25585 0.39903 0.13836 0.591851227
BMS1P7 ENSG00000270025 -2.1377 0.98373 -0.8059 0.78985 -0.17824
0.57073 1.0877 0.13836 0.13836 0.591851227
PDE1A ENSG00000115252 0.41367 0.33956 0.1696 0.43266 1.0876 0.1384
0.49127 0.31162 0.1384 0.591851227
EMID1 ENSG00000186998 -0.60586 0.7277 1.0875 0.13841 0.13332 0.44697
-1.302 0.90354 0.13841 0.591851227
THAP12P6 ENSG00000227882 -0.20094 0.57963 1.0874 0.13843 -1.1706
0.87911 -1.3297 0.90818 0.13843 0.591851227
NA ENSG00000228863 1.0874 0.13843 0.62834 0.26489 0.33589 0.36848
0.92807 0.17668 0.13843 0.591851227
EIF4G1 ENSG00000114867 1.0872 0.13847 -0.83871 0.79918 0.28894
0.38631 -0.098086 0.53907 0.13847 0.591851227
LINC02260 ENSG00000250456 -0.99931 0.84118 0.18139 0.42803 0.75757
0.22435 1.0872 0.13847 0.13847 0.591851227
NA ENSG00000239065 -1.2542 0.89511 1.0872 0.13848 0.037369 0.4851
0.12837 0.44893 0.13848 0.591851227
NA ENSG00000253204 0.97187 0.16556 1.0871 0.13849 0.41446 0.33927
-0.23617 0.59335 0.13849 0.591851227
SLF2 ENSG00000119906 0.77945 0.21786 0.82658 0.20424 1.087 0.13852
-0.61385 0.73034 0.13852 0.591851227
RNU1-19P ENSG00000200176 1.087 0.13852 0.69185 0.24452 1.087 0.13852 1.087
0.13852 0.13852 0.591851227
IGF1R ENSG00000140443 0.049026 0.48045 1.087 0.13853 -0.94122 0.8267
-1.621 0.94749 0.13853 0.591851227
NA ENSG00000239870 0.47403 0.31774 0.20124 0.42025 0.47403 0.31774
1.087 0.13853 0.13853 0.591851227
NA ENSG00000232597 -0.46528 0.67914 -0.46528 0.67914 0.89069 0.18655
1.0868 0.13855 0.13855 0.591851227
NEK1 ENSG00000137601 0.041331 0.48352 1.0868 0.13856 -0.67962 0.75163
0.095556 0.46194 0.13856 0.591851227
LCE1B ENSG00000196734 1.0868 0.13856 1.0868 0.13856 -0.50731 0.69403
-0.75565 0.77507 0.13856 0.591851227
NA ENSG00000260625 -0.25847 0.60198 -0.25847 0.60198 1.0868 0.13857
-0.54481 0.70706 0.13857 0.591851227
PXN-AS1 ENSG00000255857 1.0867 0.13858 0.49301 0.311 -0.083715 0.53336
-0.083715 0.53336 0.13858 0.591851227
RNU6-239P ENSG00000212292 -1.1687 0.87874 -1.1687 0.87874 0.42257
0.3363 1.0866 0.1386 0.1386 0.591851227
MRPS34 ENSG00000074071 1.0865 0.13862 1.0865 0.13862 1.0865
0.13862 0.57714 0.28192 0.13862 0.591851227
EME2 ENSG00000197774 1.0865 0.13862 1.0865 0.13862 1.0865 0.13862
1.0865 0.13862 0.13862 0.591851227
NA ENSG00000201502 1.0866 0.13862 1.0866 0.13862 1.0866 0.13862
-0.18896 0.57494 0.13862 0.591851227
C1R ENSG00000159403 0.1056 0.45795 1.0865 0.13864 0.1066 0.45755
-0.42336 0.66399 0.13864 0.591851227
GNA15 ENSG00000060558 -1.0648 0.85652 -0.51974 0.69838 0.90463 0.18283
1.0864 0.13865 0.13865 0.591851227
NA ENSG00000227438 1.0864 0.13865 -0.27604 0.60874 1.0864 0.13865
1.0864 0.13865 0.13865 0.591851227
LINC01754 ENSG00000224565 -0.41206 0.65985 1.0863 0.13866 0.41342

0.33965 -0.61194 0.72971 0.13866 0.591851227
CAST ENSG00000153113 1.0863 0.13868 1.0182 0.15428 0.57607 0.28228
0.96481 0.16732 0.13868 0.591851227
NA ENSG00000233757 -1.0325 0.84908 1.0863 0.13868 -0.70897 0.76083
-0.51849 0.69794 0.13868 0.591851227
LINC02775 ENSG00000283518 1.0863 0.13868 0.97353 0.16514 1.079 0.14029
0.34355 0.36559 0.13868 0.591851227
FSTL4 ENSG00000053108 -1.7091 0.95628 1.0861 0.13872 0.80172 0.21136
-0.54776 0.70807 0.13872 0.591851227
NA ENSG00000280414 -0.17774 0.57054 1.086 0.13874 0.99076 0.1609
0.99076 0.1609 0.13874 0.591851227
EGR1 ENSG00000120738 0.65443 0.25642 0.25565 0.39911 1.0859 0.13875
0.90415 0.18296 0.13875 0.591851227
XPOT ENSG00000184575 -0.55696 0.71122 1.086 0.13875 1.086 0.13875 0.80416
0.21065 0.13875 0.591851227
NA ENSG00000274682 0.87185 0.19165 1.0857 0.1388 0.87185 0.19165
0.87185 0.19165 0.1388 0.591851227
PSTPIP2 ENSG00000152229 0.30984 0.37834 0.5762 0.28224 0.30984
0.37834 1.0857 0.13881 0.13881 0.591851227
NA ENSG00000238629 -0.19586 0.57764 1.0251 0.15265 -0.59393 0.72372
1.0857 0.13881 0.13881 0.591851227
BCL2 ENSG00000171791 0.96506 0.16726 0.87388 0.19109 1.0856 0.13882
0.83457 0.20198 0.13882 0.591851227
RFC5P1 ENSG00000233526 1.0856 0.13882 1.0856 0.13882 0.84029
0.20037 -0.25009 0.59874 0.13882 0.591851227
ADAM10 ENSG00000137845 1.0856 0.13883 0.29579 0.38369 0.66752
0.25222 0.73075 0.23247 0.13883 0.591851227
NA ENSG00000234350 0.50182 0.3079 0.74537 0.22803 0.53293 0.29704
1.0852 0.13891 0.13891 0.591851227
SEPTIN2P1 ENSG00000227850 -0.18318 0.57267 1.085 0.13895 0.77927 0.21791
-1.0014 0.84168 0.13895 0.591851227
NA ENSG00000259931 1.0851 0.13895 1.0851 0.13895 1.0851 0.13895
1.0851 0.13895 0.13895 0.591851227
NYAP2 ENSG00000144460 1.0749 0.14121 0.18706 0.42581 1.085 0.13896
0.71848 0.23623 0.13896 0.591851227
NA ENSG00000253986 -0.90394 0.81699 1.085 0.13896 1.0306 0.15136
-0.90394 0.81699 0.13896 0.591851227
NA ENSG00000228590 0.23649 0.40653 0.090103 0.4641 0.23649 0.40653
1.085 0.13897 0.13897 0.591851227
RN7SL271P ENSG00000239250 0.59065 0.27738 0.66085 0.25435 1.085 0.13897
-0.86329 0.80601 0.13897 0.591851227
NA ENSG00000259381 -0.28793 0.6133 1.0849 0.13898 1.0849 0.13898
1.0849 0.13898 0.13898 0.591851227
RN7SL164P ENSG00000242614 0.38116 0.35154 0.14245 0.44336 1.0848 0.139
0.36828 0.35633 0.139 0.591851227
NA ENSG00000282924 0.71671 0.23678 -0.45811 0.67656 1.0848 0.139
-0.069357 0.52765 0.139 0.591851227
NA ENSG00000225637 1.0238 0.15296 1.0238 0.15296 1.0238 0.15296
1.0847 0.13903 0.13903 0.591851227
C20orf144 ENSG00000149609 -0.017219 0.50687 1.0845 0.13906 -0.017219
0.50687 -0.017219 0.50687 0.13906 0.591851227
C2orf27B ENSG00000186825 1.0845 0.13906 0.46805 0.31988 -0.34206
0.63385 0.46805 0.31988 0.13906 0.591851227
NA ENSG00000259287 1.0845 0.13907 1.0845 0.13907 1.0845 0.13907
1.0845 0.13907 0.13907 0.591851227
SLC25A45 ENSG00000162241 -0.15999 0.56355 -0.1797 0.57131 -0.57786
0.71832 1.0845 0.13908 0.13908 0.591851227
RNA5SP265 ENSG00000222955 1.0844 0.13908 1.0844 0.13908 1.0844
0.13908 1.0844 0.13908 0.13908 0.591851227
AAMDC ENSG00000087884 1.0843 0.13911 1.0843 0.13911 0.26861 0.39411
0.3456 0.36482 0.13911 0.591851227
NA ENSG00000233713 1.0841 0.13915 1.0841 0.13915 1.0841 0.13915

1.0841 0.13915 0.13915 0.591851227
RNU6-881P ENSG00000252622 1.0842 0.13915 1.0842 0.13915 1.0842
0.13915 1.0842 0.13915 0.13915 0.591851227
PAK4 ENSG00000130669 1.0841 0.13916 0.37984 0.35203 0.23283 0.40795
1.0841 0.13916 0.13916 0.591851227
PTAR1 ENSG00000188647 0.12772 0.44919 0.094938 0.46218 1.084 0.13918
0.018301 0.4927 0.13918 0.591851227
HNRNPA0 ENSG00000177733 0.74934 0.22683 -0.8117 0.79152 0.73898
0.22996 1.084 0.13919 0.13919 0.591851227
NA ENSG00000239544 1.0133 0.15547 1.0839 0.1392 1.0133 0.15547
1.0133 0.15547 0.1392 0.591851227
PELI3 ENSG00000174516 -0.15839 0.56292 -0.35076 0.63711 -0.42604 0.66496
1.0838 0.13923 0.13923 0.591851227
RN7SL354P ENSG00000243027 1.0838 0.13923 -0.64197 0.73955 1.0838
0.13923 1.0838 0.13923 0.13923 0.591851227
RNA5SP122 ENSG00000207186 1.0837 0.13925 1.0837 0.13925 1.0837
0.13925 1.0837 0.13925 0.13925 0.591851227
DENND1A ENSG00000119522 0.93046 0.17607 0.31622 0.37592 0.25003
0.40128 1.0837 0.13926 0.13926 0.591851227
PEAK1 ENSG00000173517 1.0835 0.13928 0.71904 0.23606 0.87587 0.19055
0.16144 0.43587 0.13928 0.591851227
GTF2IP20 ENSG00000272645 0.80081 0.21162 1.0836 0.13928 -1.1294
0.87064 0.11286 0.45507 0.13928 0.591851227
SLC16A3 ENSG00000141526 -1.5067 0.93405 1.0835 0.1393 -0.94598
0.82792 -0.88851 0.81287 0.1393 0.591851227
NA ENSG00000224064 1.0835 0.1393 1.0835 0.1393 1.0835 0.1393
0.91554 0.17996 0.1393 0.591851227
KLHL18 ENSG00000114648 -1.6485 0.95038 -2.0923 0.9818 0.62637
0.26554 1.0834 0.13931 0.13931 0.591851227
ZNF443 ENSG00000180855 -1.4624 0.92818 0.67907 0.24855 1.0834
0.13932 -0.25975 0.60247 0.13932 0.591851227
LINC01548 ENSG00000229086 1.0832 0.13935 -0.1143 0.5455 1.0832
0.13935 0.13412 0.44665 0.13935 0.591851227
NA ENSG00000250354 1.0832 0.13935 1.0832 0.13935 1.0832 0.13935
1.0832 0.13935 0.13935 0.591851227
HMGCR ENSG00000113161 0.028315 0.48871 1.0832 0.13937 -2.0014 0.97733
0.31336 0.377 0.13937 0.591851227
FOXM1 ENSG00000111206 -0.069561 0.52773 -0.60103 0.72609 1.0831 0.13939
0.35258 0.3622 0.13939 0.591851227
TNIP1 ENSG00000145901 1.021 0.15363 0.061627 0.47543 1.0831 0.13939
0.75516 0.22508 0.13939 0.591851227
NA ENSG00000233990 1.083 0.1394 1.083 0.1394 1.083 0.1394 1.083 0.1394
0.1394 0.591851227
BTN3A1 ENSG00000026950 1.0829 0.13942 0.99732 0.1593 0.90025
0.18399 0.98816 0.16154 0.13942 0.591851227
RPS12P10 ENSG00000225147 -1.1096 0.8664 -1.1096 0.8664 1.0829
0.13943 0.31218 0.37745 0.13943 0.591851227
SLC1A6 ENSG00000105143 0.53966 0.29471 -0.4072 0.65807 1.0828
0.13945 0.53966 0.29471 0.13945 0.591851227
CDC42P1 ENSG00000234933 1.0828 0.13945 1.0049 0.15747 1.0828
0.13945 1.0828 0.13945 0.13945 0.591851227
OR8B5P ENSG00000255030 -0.84537 0.80105 0.50328 0.30738 1.0828
0.13946 1.0828 0.13946 0.13946 0.591851227
NA ENSG00000273597 -0.90098 0.8162 1.0827 0.13946 -0.77681 0.78136
0.71545 0.23717 0.13946 0.591851227
NA ENSG00000253628 0.60474 0.27268 0.42402 0.33578 0.42402 0.33578
1.0827 0.13947 0.13947 0.591851227
DNM1P49 ENSG00000261820 1.0827 0.13947 -1.3285 0.90799 -1.3285
0.90799 -1.3285 0.90799 0.13947 0.591851227
TRIM67 ENSG00000119283 1.0826 0.13948 0.94852 0.17143 1.0306
0.15136 0.11254 0.4552 0.13948 0.591851227
CA10 ENSG00000154975 1.0826 0.13949 -0.64781 0.74145 0.43325 0.33242

0.24508 0.4032 0.13949 0.591851227
NA ENSG00000257004 0.2044 0.41902 -0.21734 0.58603 0.72411 0.2345
1.0826 0.13949 0.13949 0.591851227
CNNM1 ENSG00000119946 -0.28118 0.61071 -0.23649 0.59347 0.92218 0.17822
1.0825 0.13951 0.13951 0.591851227
VPS35P1 ENSG00000260809 1.0825 0.13951 0.11607 0.4538 1.0317
0.1511 0.90429 0.18292 0.13951 0.591851227
RPSAP28 ENSG00000214484 1.0825 0.13952 1.0825 0.13952 1.0825
0.13952 1.0825 0.13952 0.13952 0.591851227
LINC01997 ENSG00000241383 0.5038 0.3072 1.0825 0.13952 0.82119
0.20577 0.82119 0.20577 0.13952 0.591851227
SPOP ENSG00000121067 0.92226 0.1782 1.0825 0.13953 0.66615 0.25266
-0.72505 0.76579 0.13953 0.591851227
CCDC50 ENSG00000152492 -0.68938 0.75471 -0.88423 0.81172 0.28045
0.38957 1.0824 0.13953 0.13953 0.591851227
LINC02371 ENSG00000274659 1.0823 0.13957 -0.062058 0.52474 1.0823
0.13957 0.053303 0.47875 0.13957 0.591851227
CRIP3 ENSG00000146215 1.0821 0.1396 1.0821 0.1396 1.0821 0.1396
1.0821 0.1396 0.1396 0.591851227
RPL12P47 ENSG00000219222 1.0821 0.1396 1.0821 0.1396 1.0821
0.1396 1.0821 0.1396 0.1396 0.591851227
SCAND1 ENSG00000171222 1.0821 0.13961 1.0821 0.13961 0.74378
0.2285 0.057327 0.47714 0.13961 0.591851227
PSEN2 ENSG00000143801 1.082 0.13962 0.54421 0.29315 0.4703 0.31907
0.15585 0.43807 0.13962 0.591851227
HLA-DQB3 ENSG00000226030 0.8542 0.1965 0.96144 0.16816 1.082 0.13962
0.8542 0.1965 0.13962 0.591851227
RNU7-6P ENSG00000252654 1.082 0.13963 1.082 0.13963 1.082 0.13963 1.082
0.13963 0.13963 0.591851227
DHRS7C ENSG00000184544 1.0818 0.13968 0.064892 0.47413 -1.2013
0.88517 -1.1896 0.88289 0.13968 0.591851227
PMS2P1 ENSG00000078319 -0.39079 0.65202 -0.0013781 0.50055 1.0817
0.13969 0.16116 0.43598 0.13969 0.591851227
CLEC1B ENSG00000165682 -0.067424 0.52688 1.0817 0.13969 0.20368
0.4193 0.20368 0.4193 0.13969 0.591851227
KRT18P18 ENSG00000261764 0.48652 0.3133 0.48652 0.3133 0.48652
0.3133 1.0817 0.13969 0.13969 0.591851227
RFK ENSG00000135002 -0.34249 0.63401 -0.84397 0.80066 1.0816 0.13971
-0.16138 0.5641 0.13971 0.591851227
SLC26A2 ENSG00000155850 -1.3575 0.91269 0.28836 0.38654 1.0816
0.13972 0.74681 0.22759 0.13972 0.591851227
PEA15 ENSG00000162734 1.0816 0.13972 1.0248 0.15274 1.0248 0.15274
1.0248 0.15274 0.13972 0.591851227
ABCG1 ENSG00000160179 1.0815 0.13974 0.38198 0.35124 0.34208 0.36615
0.49298 0.31101 0.13974 0.591851227
HOXB-AS3 ENSG00000233101 1.0815 0.13974 1.0815 0.13974 1.0815
0.13974 1.0815 0.13974 0.13974 0.591851227
CHEK2P4 ENSG00000235969 -1.399 0.91909 -0.41373 0.66046 0.31903
0.37485 1.0815 0.13974 0.13974 0.591851227
NA ENSG00000249574 -0.79955 0.78801 0.55885 0.28813 0.795 0.21331
1.0815 0.13974 0.13974 0.591851227
SLCO2A1 ENSG00000174640 1.0814 0.13976 0.16651 0.43388 0.8054
0.21029 -0.68578 0.75357 0.13976 0.591851227
RNU6-517P ENSG00000207090 1.0814 0.13977 1.0814 0.13977 1.0814
0.13977 1.0814 0.13977 0.13977 0.591851227
RN7SKP171 ENSG00000201287 1.0385 0.14951 0.57351 0.28315 1.0813
0.13978 1.0813 0.13978 0.13978 0.591851227
NA ENSG00000283431 1.0813 0.13979 -0.74178 0.77089 -0.82866 0.79635
-0.82866 0.79635 0.13979 0.591851227
LINC00511 ENSG00000227036 -0.77447 0.78067 1.0812 0.13981 0.15795
0.43725 -1.3911 0.9179 0.13981 0.591851227
ACADSB ENSG00000196177 0.042656 0.48299 -0.2204 0.58722 -0.079173

0.53155 1.0811 0.13982 0.13982 0.591851227
LINC02222 ENSG00000248717 0.063818 0.47456 1.0811 0.13983 0.27381
0.39211 -0.91928 0.82103 0.13983 0.591851227
PRCP ENSG00000137509 1.0811 0.13984 0.34456 0.36521 0.2176 0.41387
0.12155 0.45163 0.13984 0.591851227
RPS4XP4 ENSG00000215520 -0.58181 0.71965 -0.038034 0.51517 -0.90494
0.81725 1.0811 0.13984 0.13984 0.591851227
LINC02585 ENSG00000228350 1.081 0.13985 1.081 0.13985 1.081 0.13985 1.081
0.13985 0.13985 0.591851227
PPCDC ENSG00000138621 0.87297 0.19134 0.71309 0.2379 0.29241 0.38499
1.0809 0.13986 0.13986 0.591851227
ATAD3B ENSG00000160072 -0.20597 0.58159 -0.041813 0.51668 1.0809
0.13986 0.25281 0.40021 0.13986 0.591851227
DNAJB14 ENSG00000164031 1.0809 0.13986 0.98627 0.162 0.17963 0.42872
-0.11069 0.54407 0.13986 0.591851227
CCDC185 ENSG00000178395 -0.16479 0.56545 -0.16479 0.56545 -0.16479
0.56545 1.081 0.13986 0.13986 0.591851227
NA ENSG00000200737 1.081 0.13986 1.0266 0.15231 1.081 0.13986 1.081
0.13986 0.13986 0.591851227
VDAC2P4 ENSG00000234682 -0.89491 0.81458 1.0808 0.13989 -0.46119
0.67767 -0.89491 0.81458 0.13989 0.591851227
U2SURP ENSG00000163714 0.33615 0.36838 0.26594 0.39514 1.0807
0.13991 -0.28497 0.61216 0.13991 0.591851227
NA ENSG00000279778 1.0807 0.13991 0.44342 0.32873 0.21611 0.41445
0.44342 0.32873 0.13991 0.591851227
ABHD10 ENSG00000144827 1.0807 0.13992 0.026909 0.48927 1.0807
0.13992 1.0427 0.14855 0.13992 0.591851227
EML1 ENSG00000066629 1.0806 0.13993 -0.036022 0.51437 0.53454 0.29648
-0.03592 0.51433 0.13993 0.591851227
CAMSAP3 ENSG00000076826 1.0736 0.14149 1.0806 0.13993 1.0736
0.14149 -0.16047 0.56374 0.13993 0.591851227
SERPINF1 ENSG00000132386 1.0806 0.13994 -0.049042 0.51956 0.63767
0.26185 -0.049042 0.51956 0.13994 0.591851227
LINC01589 ENSG00000238120 1.0806 0.13994 0.46068 0.32251 -0.57985
0.71899 0.37012 0.35564 0.13994 0.591851227
NA ENSG00000243584 0.318 0.37524 1.0733 0.14157 1.0805 0.13995
-0.12933 0.55145 0.13995 0.591851227
ADAM21 ENSG00000139985 0.75265 0.22583 0.75265 0.22583 1.0805
0.13996 0.75265 0.22583 0.13996 0.591851227
NA ENSG00000229447 1.0805 0.13996 1.0805 0.13996 1.0805 0.13996
1.0805 0.13996 0.13996 0.591851227
KIF27 ENSG00000165115 1.0805 0.13997 0.32893 0.3711 -0.46475 0.67894
-0.85108 0.80264 0.13997 0.591851227
ZNF277 ENSG00000198839 1.0804 0.13997 -0.12394 0.54932 1.0804
0.13997 -0.28911 0.61375 0.13997 0.591851227
LINC01077 ENSG00000233456 0.77505 0.21916 -1.2824 0.90015 0.39149
0.34772 1.0803 0.13999 0.13999 0.591851227
NA ENSG00000279164 -0.021831 0.50871 1.0804 0.13999 1.0804 0.13999
1.0804 0.13999 0.13999 0.591851227
NA ENSG00000225315 0.16838 0.43314 0.16838 0.43314 1.0803 0.14001
0.99606 0.15961 0.14001 0.591851227
ANKRD44-IT1 ENSG00000236977 -0.67848 0.75127 -0.99498 0.84013 -0.93141
0.82418 1.0803 0.14001 0.14001 0.591851227
ESR2 ENSG00000140009 0.8802 0.18938 1.0802 0.14002 1.0612 0.14429
0.80157 0.2114 0.14002 0.591851227
NA ENSG00000252485 1.0802 0.14002 1.0802 0.14002 1.0802 0.14002
1.0802 0.14002 0.14002 0.591851227
NA ENSG00000236168 -0.37898 0.64765 1.0802 0.14003 -0.0080455 0.50321
-0.0080455 0.50321 0.14003 0.591851227
EREG ENSG00000124882 1.0801 0.14005 0.24502 0.40322 0.52356 0.30029
0.0070724 0.49718 0.14005 0.591851227
LINC01043 ENSG00000260343 0.88971 0.18681 0.62199 0.26697 1.0801

0.14005 0.77994 0.21771 0.14005 0.591851227
NA ENSG00000254076 0.95048 0.17093 1.08 0.14007 1.08 0.14007 -0.37749 0.64709 0.14007 0.591884417
SERP1 ENSG00000120742 1.0798 0.14011 1.0798 0.14011 1.0798 0.14011 0.17936 0.42883 0.14011 0.592002107
PMPCAP1 ENSG00000248817 0.59459 0.27606 0.60931 0.27116 1.0797 0.14013 1.0797 0.14013 0.14013 0.592035278
GRPEL2P1 ENSG00000233211 1.0797 0.14015 1.0797 0.14015 1.0797 0.14015 1.0797 0.14015 0.14015 0.592059362
NA ENSG00000229779 0.019912 0.49206 -0.020856 0.50832 1.0796 0.14016 0.30138 0.38156 0.14016 0.592059362
NA ENSG00000275611 1.0795 0.14018 1.0795 0.14018 1.0795 0.14018 1.0795 0.14018 0.14018 0.59209252
NA ENSG00000256209 1.0794 0.1402 -0.3558 0.63901 -1.2633 0.89676 0.95738 0.16919 0.1402 0.592125672
DCTN1 ENSG00000204843 -1.2546 0.89518 -1.2546 0.89518 1.0793 0.14022 -0.62804 0.73501 0.14022 0.592149731
SP140L ENSG00000185404 0.028069 0.4888 0.46812 0.31985 0.024565 0.4902 1.0793 0.14023 0.14023 0.592149731
UQCR10 ENSG00000184076 1.0792 0.14025 1.0792 0.14025 1.0792 0.14025 1.0792 0.14025 0.14025 0.59218287
NA ENSG00000267113 1.0791 0.14028 1.0791 0.14028 1.0791 0.14028 -0.93735 0.82571 0.14028 0.592258222
EEF1E1 ENSG00000124802 1.0788 0.14034 0.75553 0.22496 0.75553 0.22496 0.75553 0.22496 0.14034 0.592297177
SATB1 ENSG00000182568 -0.11187 0.54454 1.0788 0.14034 0.032939 0.48686 1.0633 0.14383 0.14034 0.592297177
NA ENSG00000260769 1.0788 0.14034 1.0788 0.14034 1.0788 0.14034 1.0788 0.14034 0.14034 0.592297177
NA ENSG00000231949 0.3458 0.36475 0.14242 0.44338 0.94675 0.17188 1.0788 0.14035 0.14035 0.592297177
NA ENSG00000250164 1.0787 0.14035 1.0787 0.14035 1.0787 0.14035 1.0787 0.14035 0.14035 0.592297177
PHOSPHO2 ENSG00000144362 0.97345 0.16516 0.97345 0.16516 1.0787 0.14037 1.0787 0.14037 0.14037 0.592330282
TRAJ12 ENSG00000211877 1.0786 0.14039 1.0786 0.14039 1.0786 0.14039 1.0786 0.14039 0.14039 0.592354286
PLEKHA3P1 ENSG00000269800 1.0785 0.1404 0.76948 0.2208 1.0785 0.1404 1.0785 0.1404 0.1404 0.592354286
MIR596 ENSG00000207826 -0.64982 0.7421 -0.24591 0.59713 1.0784 0.14042 -0.64982 0.7421 0.14042 0.592387378
RBMX2P5 ENSG00000227087 1.0783 0.14044 0.18468 0.42674 1.0783 0.14044 1.0783 0.14044 0.14044 0.592420464
SREK1IP1P1 ENSG00000229336 1.0783 0.14046 -0.12026 0.54786 1.0783 0.14046 1.0783 0.14046 0.14046 0.592435344
CCT6B ENSG00000132141 1.0782 0.14048 0.97628 0.16446 1.0185 0.15422 -1.1683 0.87865 0.14048 0.592435344
PCDHGA10 ENSG00000253846 1.0781 0.14048 1.0781 0.14048 1.0781 0.14048 1.0781 0.14048 0.14048 0.592435344
DOCK3 ENSG00000088538 0.92846 0.17658 0.87361 0.19117 0.87667 0.19033 1.0781 0.1405 0.1405 0.592468415
DNAJC3-DT ENSG00000247400 1.078 0.14053 0.60957 0.27107 0.60957 0.27107 0.18379 0.42709 0.14053 0.592522077
UNKL ENSG00000059145 0.11134 0.45567 1.0779 0.14055 0.99704 0.15937 0.89348 0.1858 0.14055 0.592522077
PRSS46P ENSG00000261603 0.79395 0.21361 -0.0058716 0.50234 0.92014 0.17875 1.0777 0.14058 0.14058 0.592522077
RNU6-558P ENSG00000201382 1.0777 0.14059 1.0777 0.14059 0.63122 0.26395 1.0777 0.14059 0.14059 0.592522077
RPL37P13 ENSG00000233702 1.0777 0.14059 1.0777 0.14059 1.0777 0.14059 1.0777 0.14059 0.14059 0.592522077
INSYN1 ENSG00000205363 -2.0102 0.97779 1.0776 0.1406 -1.0943

0.86308 -0.70149 0.7585 0.1406 0.592522077
RFPL4A ENSG00000223638 0.71901 0.23607 -0.14941 0.55939 0.96648
0.1669 1.0776 0.1406 0.1406 0.592522077
CYP2A7P1 ENSG00000213908 0.51945 0.30172 1.0776 0.14061 0.13977
0.44442 0.13977 0.44442 0.14061 0.592522077
RNU7-51P ENSG00000252369 1.0775 0.14064 1.0775 0.14064 1.0775
0.14064 1.0775 0.14064 0.14064 0.592560844
NA ENSG00000234136 1.0774 0.14065 1.0774 0.14065 1.0774 0.14065
1.0774 0.14065 0.14065 0.592560844
GTF2A1 ENSG00000165417 -1.2164 0.88809 0.7856 0.21605 -0.94136
0.82674 1.0773 0.14066 0.14066 0.592560844
RNU6-220P ENSG00000212221 0.44894 0.32674 -0.66555 0.74715 -0.33792
0.63229 1.0773 0.14067 0.14067 0.592560844
RPL9P15 ENSG00000240601 1.0773 0.14068 1.0773 0.14068 1.0773
0.14068 1.0773 0.14068 0.14068 0.592560844
PDCD4-AS1 ENSG00000203497 -1.1656 0.87811 -0.03294 0.51314 -0.06571
0.5262 1.0772 0.1407 0.1407 0.592584764
NA ENSG00000254248 1.0771 0.14071 1.0771 0.14071 1.0771 0.14071
0.58427 0.27952 0.14071 0.592584764
ZNF729 ENSG00000196350 1.077 0.14073 -1.1802 0.88104 -1.4763 0.93006
-0.91907 0.82097 0.14073 0.592599577
SOX21-AS1 ENSG00000227640 1.077 0.14074 1.077 0.14074 1.077 0.14074 1.077
0.14074 0.14074 0.592599577
RN7SKP47 ENSG00000202217 1.0769 0.14075 1.0769 0.14075 1.0769
0.14075 1.0769 0.14075 0.14075 0.592599577
RPSAP44 ENSG00000220773 1.0768 0.14079 0.1189 0.45268 0.1189
0.45268 0.1189 0.45268 0.14079 0.592665575
NA ENSG00000253983 0.80278 0.21105 -0.14588 0.55799 0.80278 0.21105
1.0768 0.14079 0.14079 0.592665575
CYP7B1 ENSG00000172817 0.94584 0.17212 -0.40884 0.65867 1.0766
0.14084 0.0046167 0.49816 0.14084 0.592751972
KBTBD7 ENSG00000120696 1.0764 0.14087 1.0764 0.14087 0.95617
0.16949 0.95617 0.16949 0.14087 0.592751972
MIR4325 ENSG00000266666 -0.7452 0.77193 1.0764 0.14087 -1.2815
0.89999 0.88818 0.18722 0.14087 0.592751972
FAM210A ENSG00000177150 1.0764 0.14088 -0.008797 0.50351 0.13562
0.44606 0.12915 0.44862 0.14088 0.592751972
NA ENSG00000272936 1.0764 0.14088 1.0764 0.14088 1.0764 0.14088
1.0764 0.14088 0.14088 0.592751972
NA ENSG00000227139 1.0763 0.1409 1.0763 0.1409 -0.36133 0.64107
1.0763 0.1409 0.1409 0.592751972
UBE2FP3 ENSG00000233337 1.0763 0.1409 1.0763 0.1409 1.0763
0.1409 1.0763 0.1409 0.1409 0.592751972
NA ENSG00000252612 1.0763 0.14091 1.0763 0.14091 1.0763 0.14091
1.0763 0.14091 0.14091 0.592751972
NA ENSG00000269307 1.0762 0.14092 1.0762 0.14092 1.0762 0.14092
1.0762 0.14092 0.14092 0.592751972
HS6ST1P1 ENSG00000187952 0.099041 0.46055 0.099041 0.46055 1.0761
0.14095 0.55443 0.28964 0.14095 0.592808776
ZNF503 ENSG00000165655 0.20497 0.4188 0.6485 0.25833 -0.40551
0.65745 1.076 0.14097 0.14097 0.592808776
NA ENSG00000238156 -0.59396 0.72373 -0.59396 0.72373 1.076 0.14097
0.076773 0.4694 0.14097 0.592808776
NA ENSG00000255506 1.0759 0.14099 0.99968 0.15873 0.99968 0.15873
0.99968 0.15873 0.14099 0.592841721
RBM12B ENSG00000183808 1.0758 0.14101 1.0758 0.14101 0.66699
0.25239 0.52592 0.29947 0.14101 0.592874659
NA ENSG00000224569 1.0756 0.14104 1.0756 0.14104 -0.96203 0.83198
0.12373 0.45076 0.14104 0.592924364
NOL9 ENSG00000162408 1.006 0.15721 1.0754 0.14109 1.0368 0.1499
0.62402 0.26631 0.14109 0.592924364
CC2D2B ENSG00000188649 -0.055154 0.52199 1.0755 0.14109 0.49601

0.30994 -0.23057 0.59117 0.14109 0.592924364
NA ENSG00000232646 -0.92833 0.82338 1.0754 0.1411 -0.92833 0.82338
-0.92833 0.82338 0.1411 0.592924364
ZNFX1 ENSG00000124201 0.97684 0.16432 1.0753 0.14112 -0.31055 0.62193
0.86492 0.19354 0.14112 0.592924364
NA ENSG00000256663 -0.48056 0.68458 1.0753 0.14112 1.0753 0.14112
1.0753 0.14112 0.14112 0.592924364
KRT8P52 ENSG00000232479 1.0753 0.14113 0.74648 0.22769 1.0753
0.14113 1.0753 0.14113 0.14113 0.592924364
NA ENSG00000227088 -0.23399 0.5925 1.0752 0.14114 -1.064 0.85634
-0.63593 0.73759 0.14114 0.592924364
ATOH8 ENSG00000168874 -0.16655 0.56614 1.0751 0.14116 -0.31121 0.62218
0.63805 0.26172 0.14116 0.592924364
NA ENSG00000237016 0.99912 0.15887 0.93532 0.17481 1.0751 0.14116
0.67712 0.24916 0.14116 0.592924364
NA ENSG00000277235 -0.3566 0.6393 1.0751 0.14116 -0.31289 0.62282
-0.3566 0.6393 0.14116 0.592924364
WT1 ENSG00000184937 1.0151 0.15502 0.20726 0.4179 1.075 0.14118
0.53613 0.29593 0.14118 0.592924364
NA ENSG00000279193 1.0751 0.14118 0.65305 0.25686 1.0751 0.14118
-1.0016 0.84173 0.14118 0.592924364
RCHY1 ENSG00000163743 -0.45819 0.67659 1.0748 0.14122 -0.5856 0.72093
0.23245 0.4081 0.14122 0.593013914
NA ENSG00000199476 1.0552 0.14568 1.0748 0.14124 1.0748 0.14124
1.0748 0.14124 0.14124 0.593013914
NA ENSG00000201680 0.84302 0.19961 0.84302 0.19961 1.0748 0.14124
0.97395 0.16504 0.14124 0.593013914
DUSP6 ENSG00000139318 -0.10215 0.54068 0.55457 0.2896 1.0747 0.14125
0.18158 0.42795 0.14125 0.593013914
KRT18P39 ENSG00000224791 -0.019744 0.50788 1.0746 0.14127 0.7402
0.22959 0.7402 0.22959 0.14127 0.593025011
PIMREGP2 ENSG00000216425 -0.27432 0.60808 1.0745 0.1413 0.0081969
0.49673 -0.61067 0.72929 0.1413 0.593025011
ZNF524 ENSG00000171443 1.0745 0.14131 1.0745 0.14131 0.84684
0.19854 1.0745 0.14131 0.14131 0.593025011
KRT18P50 ENSG00000219941 0.66131 0.25421 0.66131 0.25421 1.0745
0.14131 0.79887 0.21218 0.14131 0.593025011
TLDC2 ENSG00000101342 -0.24877 0.59823 -0.52056 0.69866 0.3112 0.37783
1.0744 0.14133 0.14133 0.593025011
PRKAR1A ENSG00000108946 -0.10592 0.54218 1.0743 0.14134 0.54355
0.29338 -0.0945 0.53764 0.14134 0.593025011
HSPE1P25 ENSG00000232015 1.0743 0.14135 1.0743 0.14135 1.0743
0.14135 1.0743 0.14135 0.14135 0.593025011
LINC01856 ENSG00000237574 0.2281 0.40978 1.0611 0.14432 1.0743
0.14135 0.27817 0.39044 0.14135 0.593025011
SLC7A11 ENSG00000151012 -0.98831 0.8385 -1.3103 0.90495 -1.058
0.85498 1.0742 0.14138 0.14138 0.593048756
EIF5AP3 ENSG00000267346 1.0741 0.14138 1.0741 0.14138 1.0741
0.14138 1.0741 0.14138 0.14138 0.593048756
AP2M1 ENSG00000161203 0.66809 0.25204 0.66809 0.25204 0.66809 0.25204
1.074 0.14142 0.14142 0.593102262
NA ENSG00000225603 1.0738 0.14145 -1.699 0.95535 0.78017 0.21765
-1.5433 0.93863 0.14145 0.593102262
RPL23AP70 ENSG00000241052 1.0738 0.14145 -0.17177 0.56819 -0.59379
0.72367 0.36134 0.35892 0.14145 0.593102262
NA ENSG00000207286 1.0736 0.14149 1.0736 0.14149 1.0736 0.14149
1.0736 0.14149 0.14149 0.593102262
SOX1-OT ENSG00000224243 0.59203 0.27691 -0.034531 0.51377 1.0736
0.14151 -0.64644 0.741 0.14151 0.593102262
TERF1 ENSG00000147601 0.20422 0.41909 -0.89244 0.81392 -0.38057 0.64824
1.0735 0.14152 0.14152 0.593102262
SLC6A6P1 ENSG00000226818 1.0734 0.14154 1.0734 0.14154 1.0734

0.14154 1.0734 0.14154 0.14154 0.593102262
MIR4429 ENSG00000264010 1.0734 0.14154 1.0734 0.14154 1.0734
0.14154 1.0734 0.14154 0.14154 0.593102262
PTBP1 ENSG00000011304 0.89543 0.18528 1.0734 0.14155 1.0734 0.14155
0.84655 0.19862 0.14155 0.593102262
LINC01307 ENSG00000231671 0.16429 0.43475 1.0734 0.14155 0.87094
0.19189 1.0734 0.14155 0.14155 0.593102262
HNRNPH1P1 ENSG00000220305 0.75171 0.22611 1.0733 0.14156 -0.096162
0.5383 0.90573 0.18254 0.14156 0.593102262
OR2C3 ENSG00000196242 1.0732 0.14159 1.0732 0.14159 1.0732 0.14159
1.0732 0.14159 0.14159 0.593102262
CAMSAP1 ENSG00000130559 0.42263 0.33628 1.0731 0.14161 -0.46471
0.67893 1.0425 0.1486 0.14161 0.593102262
LINC02790 ENSG00000237435 0.59409 0.27623 1.0731 0.14162 0.53845
0.29513 1.0041 0.15768 0.14162 0.593102262
NA ENSG00000267248 1.0731 0.14162 0.14288 0.44319 0.82025 0.20604
0.26476 0.3956 0.14162 0.593102262
FCAMR ENSG00000162897 0.54395 0.29324 0.30815 0.37898 1.073 0.14163 1.073
0.14163 0.14163 0.593102262
KRT8P3 ENSG00000254285 0.37639 0.35331 1.073 0.14163 0.58986 0.27764
0.58986 0.27764 0.14163 0.593102262
NA ENSG00000267160 1.073 0.14163 1.073 0.14163 1.073 0.14163 1.073 0.14163
0.14163 0.593102262
MIR1251 ENSG00000221479 1.0727 0.1417 1.0727 0.1417 1.0727
0.1417 1.0727 0.1417 0.1417 0.593102262
PCID2 ENSG00000126226 -1.0757 0.85898 -0.53116 0.70235 0.82829 0.20375
1.0727 0.14171 0.14171 0.593102262
MTND1P28 ENSG00000230158 0.051098 0.47962 -0.091245 0.53635 1.0726
0.14172 -0.87464 0.80912 0.14172 0.593102262
ZNF334 ENSG00000198185 0.44546 0.32799 1.0726 0.14173 0.44546
0.32799 0.99284 0.1604 0.14173 0.593102262
TBC1D8-AS1 ENSG00000272902 0.242 0.40439 0.87582 0.19056 1.0726 0.14173
0.63937 0.26129 0.14173 0.593102262
NA ENSG00000231039 -0.48716 0.68693 1.0725 0.14175 -0.48716 0.68693
-0.48716 0.68693 0.14175 0.593102262
LINC01517 ENSG00000232624 -0.60922 0.72881 -0.60922 0.72881 -0.84785
0.80174 1.0724 0.14176 0.14176 0.593102262
LINC01772 ENSG00000226029 1.0724 0.14177 1.0724 0.14177 1.0724
0.14177 1.0724 0.14177 0.14177 0.593102262
RSPH10B2 ENSG00000169402 0.32477 0.37268 0.75816 0.22418 1.0724
0.14178 -0.7372 0.7695 0.14178 0.593102262
LINC00996 ENSG00000242258 -0.63518 0.73734 1.0723 0.14178 -0.317
0.62438 -0.58475 0.72064 0.14178 0.593102262
NA ENSG00000221807 0.92891 0.17647 1.0723 0.1418 0.79752 0.21258
-0.070215 0.52799 0.1418 0.593102262
RNA5SP51 ENSG00000201620 1.0722 0.14182 1.0722 0.14182 1.0722
0.14182 1.0722 0.14182 0.14182 0.593102262
RNU7-176P ENSG00000238962 1.0722 0.14182 1.0722 0.14182 1.0722
0.14182 1.0722 0.14182 0.14182 0.593102262
ASB4 ENSG00000005981 1.0721 0.14183 -0.79803 0.78757 -0.41074 0.65937
-0.90609 0.81756 0.14183 0.593102262
SNORA19 ENSG00000207468 1.0721 0.14184 1.0721 0.14184 1.0721
0.14184 1.0721 0.14184 0.14184 0.593102262
RNU6ATAC37P ENSG00000221255 1.0721 0.14184 0.73678 0.23063 0.83264
0.20253 1.0721 0.14184 0.14184 0.593102262
WDR46 ENSG00000227057 1.0721 0.14184 -0.27703 0.60912 0.9301 0.17616
0.63599 0.26239 0.14184 0.593102262
PRAMENP ENSG00000197549 1.0721 0.14185 0.98209 0.16303 -0.85748
0.80441 -0.85748 0.80441 0.14185 0.593102262
EYA4 ENSG00000112319 0.60582 0.27232 0.83388 0.20217 1.072 0.14186
0.20388 0.41922 0.14186 0.593102262
NA ENSG00000226782 0.12841 0.44891 0.15658 0.43779 -0.64812 0.74155

1.0717 0.14193 0.14193 0.593102262
NA ENSG00000261418 -1.7149 0.95682 -1.0502 0.85318 1.0717 0.14193
0.7834 0.21669 0.14193 0.593102262
RAPGEF5 ENSG00000136237 -1.5434 0.93864 0.71512 0.23727 -0.59841
0.72522 1.0716 0.14194 0.14194 0.593102262
TMEM184A ENSG00000164855 1.0716 0.14194 -0.034648 0.51382 -0.1306
0.55195 0.0019475 0.49922 0.14194 0.593102262
KRT18P25 ENSG00000249064 -0.67612 0.75052 0.45661 0.32397 -0.41536
0.66106 1.0716 0.14194 0.14194 0.593102262
FTH1P12 ENSG00000213362 1.0716 0.14195 1.0716 0.14195 1.0716
0.14195 1.0716 0.14195 0.14195 0.593102262
NA ENSG00000231892 0.98162 0.16314 1.0716 0.14195 0.76137 0.22322
1.0716 0.14195 0.14195 0.593102262
CNTN6 ENSG00000134115 -2.2015 0.98615 0.56259 0.28686 -0.63176 0.73623
1.0715 0.14196 0.14196 0.593102262
TPTE2P6 ENSG00000205822 1.0715 0.14197 -0.75623 0.77524 0.7029
0.24106 0.58289 0.27998 0.14197 0.593102262
KRTAP13-2 ENSG00000182816 1.0714 0.14199 1.0714 0.14199 1.0714
0.14199 1.0714 0.14199 0.14199 0.593102262
STAG1 ENSG00000118007 0.75133 0.22623 0.070252 0.472 0.10416 0.45852
1.0713 0.14202 0.14202 0.593102262
NA ENSG00000236129 1.0712 0.14204 1.0712 0.14204 1.0712 0.14204
-0.51113 0.69537 0.14204 0.593102262
RNF138 ENSG00000134758 0.69553 0.24336 0.69553 0.24336 1.0711
0.14205 0.35797 0.36018 0.14205 0.593102262
LMO7DN-IT1 ENSG00000223458 0.83346 0.20229 1.0215 0.15351 1.0712
0.14205 1.0712 0.14205 0.14205 0.593102262
NA ENSG00000236109 -0.77752 0.78158 -0.77752 0.78158 1.0712 0.14205
-0.77752 0.78158 0.14205 0.593102262
NA ENSG00000269583 1.0712 0.14205 1.0712 0.14205 1.0712 0.14205
1.0712 0.14205 0.14205 0.593102262
NA ENSG00000271195 1.0712 0.14205 1.0712 0.14205 1.0712 0.14205
1.0712 0.14205 0.14205 0.593102262
ZNF267 ENSG00000185947 0.41632 0.33859 1.0711 0.14207 -0.17056
0.56771 -0.044425 0.51772 0.14207 0.593107752
NA ENSG00000238295 1.071 0.14209 1.071 0.14209 1.071 0.14209 1.071 0.14209
0.14209 0.593107752
UIMC1 ENSG00000087206 -0.68938 0.75471 0.54758 0.29199 0.61787 0.26833
1.0709 0.1421 0.1421 0.593107752
CD164 ENSG00000135535 0.71303 0.23791 1.0709 0.1421 0.5758 0.28238
0.97739 0.16419 0.1421 0.593107752
MDFIC ENSG00000135272 -0.53672 0.70427 1.0708 0.14214 1.0708 0.14214
1.0708 0.14214 0.14214 0.593173092
RN7SL448P ENSG00000264322 1.0708 0.14214 1.0708 0.14214 1.0708
0.14214 1.0708 0.14214 0.14214 0.593173092
RNU1-88P ENSG00000238554 -0.065717 0.5262 0.65824 0.25519 -0.0075275 0.503
1.0706 0.14218 0.14218 0.593280135
NA ENSG00000258683 1.0705 0.14219 0.43814 0.33064 0.43814 0.33064
0.43814 0.33064 0.14219 0.593280135
NA ENSG00000230612 1.0703 0.14224 1.0703 0.14224 1.0703 0.14224
1.0703 0.14224 0.14224 0.593437945
CISD1P1 ENSG00000213403 1.0702 0.14227 0.56161 0.28719 0.56161
0.28719 0.88884 0.18704 0.14227 0.593457765
PCDHGB4 ENSG00000253953 0.95581 0.16958 1.0701 0.14228 0.95581
0.16958 0.95581 0.16958 0.14228 0.593457765
TRAJ13 ENSG00000211876 0.19475 0.4228 1.0701 0.1423 0.68741
0.24591 0.32932 0.37096 0.1423 0.593457765
PCDHGB7 ENSG00000254122 1.07 0.14231 1.07 0.14231 1.07 0.14231 1.07
0.14231 0.14231 0.593457765
PAFAH2 ENSG00000158006 1.0699 0.14233 0.81387 0.20786 0.058966
0.47649 0.38903 0.34863 0.14233 0.593457765
LCORL ENSG00000178177 1.0119 0.1558 1.0699 0.14233 0.49504 0.31029

1.044 0.14825 0.14233 0.593457765
NCOA6 ENSG00000198646 1.0699 0.14233 0.96016 0.16849 -0.24535 0.59691
0.80968 0.20906 0.14233 0.593457765
THBS4 ENSG00000113296 0.077616 0.46907 1.0614 0.14424 1.0695 0.14241
-0.12319 0.54902 0.14241 0.593736689
NA ENSG00000237212 0.050139 0.48001 1.0693 0.14246 0.050139 0.48001
0.050139 0.48001 0.14246 0.593736689
NA ENSG00000244733 -0.22325 0.58833 0.70129 0.24156 0.43986 0.33002
1.0693 0.14246 0.14246 0.593736689
ST6GALNAC2 ENSG00000070731 0.4341 0.33211 0.36807 0.35641 1.0693
0.14247 0.31567 0.37613 0.14247 0.593736689
RNU6-129P ENSG00000212324 1.0693 0.14247 1.0693 0.14247 1.0693
0.14247 1.0693 0.14247 0.14247 0.593736689
NA ENSG00000279098 1.0693 0.14247 1.0693 0.14247 1.0693 0.14247
1.0693 0.14247 0.14247 0.593736689
SRSF5 ENSG00000100650 1.0691 0.14251 -1.7854 0.9629 -0.59467 0.72397
-0.63186 0.73626 0.14251 0.59380706
MAML3 ENSG00000196782 0.37988 0.35202 0.84439 0.19923 0.84542 0.19894
1.069 0.14254 0.14254 0.59380706
AKR1B10P1 ENSG00000213606 -0.38349 0.64932 0.70807 0.23945 1.069 0.14254
0.075504 0.46991 0.14254 0.59380706
NA ENSG00000270058 0.78664 0.21575 0.73795 0.23027 1.0689 0.14255
1.0689 0.14255 0.14255 0.59380706
POU3F1 ENSG00000185668 0.16675 0.43378 1.0689 0.14256 0.15547
0.43823 -1.5626 0.94093 0.14256 0.59380706
NA ENSG00000266335 1.0689 0.14256 -0.13216 0.55257 -0.1092 0.54348
-0.1092 0.54348 0.14256 0.59380706
NA ENSG00000272529 1.0687 0.14259 1.0687 0.14259 1.0687 0.14259
1.0687 0.14259 0.14259 0.593881256
ZC2HC1B ENSG00000118491 1.0686 0.14263 -0.505 0.69322 1.0686
0.14263 -0.88146 0.81097 0.14263 0.593987968
NA ENSG00000272622 -0.71988 0.7642 -0.72437 0.76558 -0.28784 0.61327
1.0685 0.14264 0.14264 0.593987968
FTLP4 ENSG00000214020 1.0683 0.14269 1.0683 0.14269 1.0683 0.14269
1.0683 0.14269 0.14269 0.594094643
NA ENSG00000267506 -1.7471 0.95969 1.0683 0.14269 0.22475 0.41109
-0.25256 0.5997 0.14269 0.594094643
OR7E28P ENSG00000251491 1.0681 0.14274 1.0681 0.14274 1.0681
0.14274 1.0681 0.14274 0.14274 0.594146516
DRAP1 ENSG00000175550 1.0681 0.14275 1.0681 0.14275 0.7371 0.23053
0.87627 0.19044 0.14275 0.594146516
C11orf68 ENSG00000175573 0.11735 0.45329 0.97645 0.16442 1.0681
0.14275 1.0681 0.14275 0.14275 0.594146516
TNRC6C ENSG00000078687 1.068 0.14276 -0.058544 0.52334 0.82808 0.20381
-0.80436 0.78941 0.14276 0.594146516
RBM6 ENSG00000004534 -1.6062 0.94588 -1.6192 0.9473 0.063589 0.47465
1.0679 0.14278 0.14278 0.594146516
NA ENSG00000236468 1.0679 0.14278 1.0679 0.14278 1.0679 0.14278
1.0679 0.14278 0.14278 0.594146516
GPS2P2 ENSG00000230951 1.0679 0.14279 -0.27141 0.60696 -0.27141
0.60696 -0.27141 0.60696 0.14279 0.594146516
RNU2-21P ENSG00000222300 1.0678 0.1428 0.70798 0.23948 1.0678
0.1428 1.0678 0.1428 0.1428 0.594146516
NA ENSG00000283679 1.0675 0.14288 1.0675 0.14288 1.0675 0.14288
1.0675 0.14288 0.14288 0.594428618
NA ENSG00000234173 1.0671 0.14297 1.0671 0.14297 1.0671 0.14297
1.0671 0.14297 0.14297 0.594752271
SLC43A2 ENSG00000167703 1.0668 0.14302 0.075501 0.46991 -1.2274
0.89017 -0.22193 0.58782 0.14302 0.594840348
TFR2 ENSG00000106327 -0.35658 0.6393 0.29684 0.3833 1.0667 0.14304
-0.64141 0.73937 0.14304 0.594840348
NA ENSG00000227055 1.0668 0.14304 0.96129 0.1682 1.0668 0.14304

1.0668 0.14304 0.14304 0.594840348
NA ENSG00000258760 0.26694 0.39476 -0.76722 0.77852 -1.7768 0.9622
1.0668 0.14304 0.14304 0.594840348
TEX9 ENSG00000151575 1.0666 0.14307 0.030476 0.48784 -0.49979 0.69139
0.16138 0.4359 0.14307 0.594863575
SNAPC3 ENSG00000164975 -0.39558 0.65379 1.0666 0.14307 0.6481
0.25846 0.64565 0.25925 0.14307 0.594863575
ETF1 ENSG00000120705 0.83892 0.20076 1.0665 0.1431 0.75701 0.22452
-0.34382 0.63451 0.1431 0.594937548
TNFRSF14 ENSG00000157873 -0.50131 0.69192 -0.2231 0.58827 -0.9691
0.83375 1.0663 0.14315 0.14315 0.595094651
LY6H ENSG00000176956 -0.27932 0.61 1.0661 0.14319 -0.24579 0.59708
-1.1191 0.86846 0.14319 0.595150192
MTND4LP18 ENSG00000254747 0.058552 0.47665 1.0661 0.14319 0.058552
0.47665 0.058552 0.47665 0.14319 0.595150192
FERMT2 ENSG00000073712 1.066 0.1432 0.27039 0.39343 1.066 0.1432
0.57086 0.28405 0.1432 0.595150192
FAM71C ENSG00000180219 0.6361 0.26236 0.67286 0.25052 0.71966
0.23587 1.0659 0.14324 0.14324 0.595205704
LYSMD2 ENSG00000140280 1.0658 0.14325 1.0658 0.14325 1.0658
0.14325 -1.2166 0.88812 0.14325 0.595205704
NA ENSG00000253946 1.0658 0.14325 1.0658 0.14325 1.0658 0.14325
1.0658 0.14325 0.14325 0.595205704
SNORD42B ENSG00000238423 1.0656 0.14331 1.0656 0.14331 1.0656
0.14331 1.0656 0.14331 0.14331 0.595265914
SNORD4A ENSG00000238578 1.0656 0.14331 1.0656 0.14331 1.0656
0.14331 1.0656 0.14331 0.14331 0.595265914
PTP4A1P1 ENSG00000231158 1.0655 0.14332 -0.94105 0.82666 -1.7349
0.95862 -1.7349 0.95862 0.14332 0.595265914
NA ENSG00000260422 0.40844 0.34147 1.0655 0.14332 0.50301 0.30748
0.50301 0.30748 0.14332 0.595265914
PLD5 ENSG00000180287 -0.026029 0.51038 1.0655 0.14333 0.36454 0.35773
0.53719 0.29557 0.14333 0.595265914
GMEB2 ENSG00000101216 1.0654 0.14335 0.55604 0.28909 0.85531 0.19619
0.79643 0.21289 0.14335 0.595265914
NA ENSG00000279555 0.95495 0.1698 0.95495 0.1698 1.0654 0.14335
0.95495 0.1698 0.14335 0.595265914
PWWP2A ENSG00000170234 0.80285 0.21103 -0.77241 0.78006 1.0653
0.14337 0.15739 0.43747 0.14337 0.595270636
LINC01698 ENSG00000231648 -0.37719 0.64698 -0.37719 0.64698 1.0652
0.14339 -0.1658 0.56584 0.14339 0.595270636
HSPA8P14 ENSG00000257539 1.0652 0.1434 0.81309 0.20808 0.62834
0.26489 -0.77007 0.77937 0.1434 0.595270636
NA ENSG00000283142 1.0652 0.1434 0.20276 0.41966 1.0652 0.1434
1.0652 0.1434 0.1434 0.595270636
RPS27P25 ENSG00000239881 1.0646 0.14353 1.0646 0.14353 1.0646
0.14353 1.0646 0.14353 0.14353 0.59557967
TRMT112P5 ENSG00000250161 -0.77662 0.78131 1.0646 0.14353 -1.0301
0.84852 0.60591 0.27229 0.14353 0.59557967
FOXF1 ENSG00000103241 1.0645 0.14355 1.0645 0.14355 1.0645 0.14355
-0.19729 0.5782 0.14355 0.59557967
NA ENSG00000256496 1.0645 0.14355 1.0645 0.14355 1.0645 0.14355
1.0645 0.14355 0.14355 0.59557967
NA ENSG00000279400 0.016653 0.49336 1.0645 0.14355 0.016653 0.49336
0.016653 0.49336 0.14355 0.59557967
NPM2 ENSG00000158806 1.0645 0.14356 0.47953 0.31578 -0.089196 0.53554
-0.13905 0.55529 0.14356 0.59557967
COL6A3 ENSG00000163359 1.0645 0.14356 1.0007 0.15849 1.0308
0.15132 0.5973 0.27515 0.14356 0.59557967
PANTR1 ENSG00000233639 1.0642 0.14362 0.13874 0.44483 -0.71501
0.7627 0.351 0.36279 0.14362 0.595727154
NA ENSG00000252150 1.0642 0.14362 1.0642 0.14362 1.0642 0.14362

1.0642 0.14362 0.14362 0.595727154
NA ENSG00000275371 1.0641 0.14364 1.0641 0.14364 1.0641 0.14364
1.0641 0.14364 0.14364 0.595759401
RBX1P1 ENSG00000226860 1.0638 0.14371 1.0638 0.14371 1.0638
0.14371 1.0638 0.14371 0.14371 0.595896632
NA ENSG00000232879 0.36303 0.35829 1.0638 0.14371 1.0638 0.14371
1.0638 0.14371 0.14371 0.595896632
NA ENSG00000201384 0.20231 0.41984 -1.6426 0.94977 -1.6426 0.94977
1.0637 0.14374 0.14374 0.595896632
LINC01850 ENSG00000227708 1.0637 0.14374 1.0637 0.14374 1.0637
0.14374 1.0637 0.14374 0.14374 0.595896632
DDX59-AS1 ENSG00000260088 -1.0822 0.86041 -1.0822 0.86041 -0.46873
0.68037 1.0636 0.14376 0.14376 0.595896632
ZNF683 ENSG00000176083 0.31413 0.37671 1.0635 0.14377 1.0635
0.14377 1.0635 0.14377 0.14377 0.595896632
NA ENSG00000234271 0.68128 0.24785 1.0635 0.14378 0.68128 0.24785
0.68128 0.24785 0.14378 0.595896632
NA ENSG00000253722 1.0634 0.14379 1.0634 0.14379 1.0634 0.14379
1.0634 0.14379 0.14379 0.595896632
KLHL21 ENSG00000162413 1.0631 0.14386 1.0631 0.14386 0.79982
0.21191 0.90963 0.18151 0.14386 0.595896632
DCUN1D3 ENSG00000188215 0.028773 0.48852 0.028773 0.48852 1.0631
0.14386 0.028773 0.48852 0.14386 0.595896632
NA ENSG00000272895 1.0631 0.14386 1.0631 0.14386 1.0631 0.14386
1.0631 0.14386 0.14386 0.595896632
NA ENSG00000273375 1.0631 0.14386 1.0631 0.14386 1.0631 0.14386
1.0631 0.14386 0.14386 0.595896632
NA ENSG00000276632 1.0631 0.14386 1.0631 0.14386 1.0631 0.14386
0.84936 0.19784 0.14386 0.595896632
FLVCR1-DT ENSG00000198468 1.0631 0.14387 1.0631 0.14387 1.0631
0.14387 1.0631 0.14387 0.14387 0.595896632
NA ENSG00000257202 1.0631 0.14387 1.0631 0.14387 1.0631 0.14387
1.0631 0.14387 0.14387 0.595896632
LINC02108 ENSG00000250127 -0.27385 0.6079 0.1842 0.42693 0.94379
0.17264 1.063 0.14388 0.14388 0.595896632
PACS2 ENSG00000179364 1.0629 0.1439 -1.5789 0.94282 -0.26079 0.60287
0.966 0.16702 0.1439 0.595896632
NA ENSG00000236390 -0.15518 0.56166 0.97473 0.16485 1.0629 0.1439
-0.15518 0.56166 0.1439 0.595896632
RUNDC3B ENSG00000105784 0.57933 0.28118 1.0629 0.14391 -0.61686
0.73134 0.50689 0.30612 0.14391 0.595896632
NTM ENSG00000182667 0.11624 0.45373 0.80738 0.20973 1.0628 0.14394
0.51563 0.30306 0.14394 0.595896632
CCNA2 ENSG00000145386 1.0626 0.14398 0.92374 0.17781 0.92374 0.17781
0.92374 0.17781 0.14398 0.595896632
KLHL23 ENSG00000213160 -0.47858 0.68388 0.81358 0.20794 1.0626
0.14398 0.90284 0.18331 0.14398 0.595896632
ZNF595 ENSG00000272602 0.43064 0.33336 0.42006 0.33722 0.51103
0.30467 1.0626 0.14399 0.14399 0.595896632
CYP11A1 ENSG00000140459 0.52661 0.29923 1.0625 0.144 0.7056 0.24022
0.55796 0.28844 0.144 0.595896632
FAF1 ENSG00000185104 0.89729 0.18478 0.98932 0.16125 1.0625 0.144
0.50285 0.30753 0.144 0.595896632
VPS26C ENSG00000157538 1.0624 0.14402 0.8636 0.1939 0.96344
0.16766 -0.20214 0.58009 0.14402 0.595896632
NA ENSG00000265511 1.0624 0.14402 1.0624 0.14402 1.0624 0.14402
1.0624 0.14402 0.14402 0.595896632
RN7SL435P ENSG00000263944 -0.40037 0.65556 1.0624 0.14403 -0.16975
0.5674 -0.40037 0.65556 0.14403 0.595896632
NA ENSG00000177340 1.0623 0.14405 1.0623 0.14405 1.0623 0.14405
1.0623 0.14405 0.14405 0.595896632
NA ENSG00000253493 0.95726 0.16922 0.95726 0.16922 1.0621 0.14408

0.95726 0.16922 0.14408 0.595896632
NA ENSG00000260431 0.19414 0.42303 1.0622 0.14408 0.19414 0.42303
-0.040766 0.51626 0.14408 0.595896632
PHC1 ENSG00000111752 0.43827 0.3306 -0.51027 0.69507 1.062 0.14411 1.062
0.14411 0.14411 0.595896632
BACH1 ENSG00000156273 1.062 0.14412 0.76794 0.22126 0.40685 0.34206
0.35539 0.36115 0.14412 0.595896632
HDAC4 ENSG00000068024 -0.91389 0.81961 -0.56509 0.714 1.0619 0.14413
0.70985 0.2389 0.14413 0.595896632
SRRM2-AS1 ENSG00000205913 1.0619 0.14413 1.0619 0.14413 1.0619
0.14413 1.0619 0.14413 0.14413 0.595896632
ARHGEF38 ENSG00000236699 0.27905 0.3901 0.71663 0.2368 1.0619
0.14414 1.0619 0.14414 0.14414 0.595896632
NA ENSG00000280351 1.0619 0.14414 1.0619 0.14414 1.0619 0.14414
1.0619 0.14414 0.14414 0.595896632
ELDR ENSG00000280890 0.43448 0.33197 0.52481 0.29986 -0.019176 0.50765
1.0619 0.14414 0.14414 0.595896632
NA ENSG00000237704 1.0618 0.14415 -0.044737 0.51784 1.0618 0.14415
1.0618 0.14415 0.14415 0.595896632
NA ENSG00000282478 1.0616 0.1442 -0.49775 0.69067 0.2827 0.3887
0.2827 0.3887 0.1442 0.595922391
NA ENSG00000206846 0.74534 0.22803 1.0616 0.14421 -0.59263 0.72329
-0.72341 0.76529 0.14421 0.595922391
OSTM1-AS1 ENSG00000225174 -0.75163 0.77386 0.65422 0.25648 1.0614
0.14425 -1.1318 0.87114 0.14425 0.595922391
SMIM11A ENSG00000205670 -0.15298 0.56079 0.031013 0.48763 -0.10485
0.54175 1.0614 0.14426 0.14426 0.595922391
RNU7-110P ENSG00000252700 1.0614 0.14426 1.0614 0.14426 1.0614
0.14426 1.0614 0.14426 0.14426 0.595922391
NA ENSG00000261866 1.0613 0.14427 1.0613 0.14427 1.0613 0.14427
1.0613 0.14427 0.14427 0.595922391
ZNF710 ENSG00000140548 0.75326 0.22565 1.0612 0.14429 -0.83767
0.79889 0.691 0.24478 0.14429 0.595922391
SMCP ENSG00000163206 0.57475 0.28273 1.0611 0.14432 -0.40686 0.65794
0.21229 0.41594 0.14432 0.595922391
OR7A15P ENSG00000176923 1.0611 0.14433 0.0064674 0.49742 1.0611
0.14433 1.0611 0.14433 0.14433 0.595922391
NDE1P2 ENSG00000271337 1.061 0.14434 1.061 0.14434 1.061 0.14434 1.061
0.14434 0.14434 0.595922391
NA ENSG00000228586 -0.1504 0.55978 -0.1504 0.55978 -0.13203 0.55252
1.061 0.14435 0.14435 0.595922391
NA ENSG00000243024 1.0608 0.14438 1.0608 0.14438 0.070232 0.472
1.0608 0.14438 0.14438 0.595922391
FRG2HP ENSG00000260846 1.0608 0.14439 0.60564 0.27238 0.73251
0.23193 0.73251 0.23193 0.14439 0.595922391
H3C7 ENSG00000277775 1.0608 0.14439 0.36338 0.35816 0.36338 0.35816
0.36338 0.35816 0.14439 0.595922391
NCAPH ENSG00000121152 0.22301 0.41176 1.0608 0.1444 0.056618 0.47742
0.7309 0.23242 0.1444 0.595922391
NA ENSG00000201465 0.92279 0.17806 0.799 0.21214 1.0607 0.14442
0.5986 0.27472 0.14442 0.595922391
NA ENSG00000279459 0.46556 0.32077 1.0606 0.14442 0.027094 0.48919
-0.16809 0.56674 0.14442 0.595922391
NA ENSG00000265023 1.0607 0.14442 1.0233 0.15308 1.0607 0.14442
1.0607 0.14442 0.14442 0.595922391
NA ENSG00000264272 1.0606 0.14443 0.44152 0.32942 1.0606 0.14443
1.0606 0.14443 0.14443 0.595922391
NA ENSG00000279154 -0.46533 0.67915 1.0606 0.14444 -0.34726 0.6358
-0.34726 0.6358 0.14444 0.595922391
PCDH10 ENSG00000138650 0.053409 0.4787 1.0606 0.14445 0.32937
0.37094 0.32937 0.37094 0.14445 0.595922391
NA ENSG00000279786 -0.85584 0.80396 1.0605 0.14445 -0.50474 0.69313

0.91682 0.17962 0.14445 0.595922391
ACSL6 ENSG00000164398 1.0604 0.14448 0.19031 0.42453 0.48762 0.31291
0.30268 0.38107 0.14448 0.595922391
RNU6-411P ENSG00000207180 1.0604 0.14449 1.0604 0.14449 0.19626
0.4222 1.0604 0.14449 0.14449 0.595922391
ZBTB7C ENSG00000184828 0.096247 0.46166 1.0602 0.14452 0.38602
0.34974 0.91317 0.18058 0.14452 0.595922391
ADH1C ENSG00000248144 0.32473 0.37269 -0.43611 0.66862 1.0602 0.14452
0.82274 0.20533 0.14452 0.595922391
PDXK ENSG00000160209 -0.0036267 0.50145 1.0601 0.14455 0.29109 0.38549
0.77837 0.21818 0.14455 0.595922391
FZD9 ENSG00000188763 -0.41705 0.66168 -0.41705 0.66168 -0.41705 0.66168
1.06 0.14456 0.14456 0.595922391
RNU6-684P ENSG00000222761 0.71445 0.23747 1.0599 0.14459 0.71445
0.23747 0.71445 0.23747 0.14459 0.595922391
FXNP2 ENSG00000177736 1.0059 0.15723 1.0599 0.1446 1.0059 0.15723
1.0059 0.15723 0.1446 0.595922391
NA ENSG00000278824 -0.16389 0.56509 0.24531 0.40311 0.63423 0.26297
1.0598 0.14461 0.14461 0.595922391
B4GALNT3 ENSG00000139044 0.86693 0.19299 1.0596 0.14466 -0.21295
0.58432 -0.22496 0.58899 0.14466 0.595922391
NA ENSG00000231662 1.0596 0.14466 0.56468 0.28615 0.78928 0.21497
-0.19529 0.57742 0.14466 0.595922391
YDJC ENSG00000161179 1.0595 0.14469 1.0595 0.14469 1.0595 0.14469
1.0595 0.14469 0.14469 0.595922391
HOXA9 ENSG00000078399 1.0594 0.14471 0.40528 0.34264 -0.57016 0.71571
-0.57016 0.71571 0.14471 0.595922391
BRD4 ENSG00000141867 0.05945 0.4763 -0.41747 0.66183 1.0594 0.14471
-0.98412 0.83747 0.14471 0.595922391
ASCL1 ENSG00000139352 0.84896 0.19795 1.0267 0.15227 1.0593 0.14472
-0.36932 0.64406 0.14472 0.595922391
NA ENSG00000223730 1.0593 0.14473 1.0593 0.14473 1.0593 0.14473
1.0593 0.14473 0.14473 0.595922391
USP38 ENSG00000170185 0.45085 0.32605 -0.27685 0.60905 1.0592 0.14474
0.28211 0.38893 0.14474 0.595922391
NA ENSG00000265400 0.37971 0.35208 1.059 0.14479 1.0453 0.14794
-0.25787 0.60174 0.14479 0.595922391
NA ENSG00000284490 1.059 0.1448 1.059 0.1448 -0.020139 0.50803 1.059
0.1448 0.1448 0.595922391
UNC5D ENSG00000156687 0.59292 0.27662 1.0589 0.14483 -0.28686 0.61289
-0.21064 0.58342 0.14483 0.595922391
NA ENSG00000268120 1.0589 0.14483 1.0589 0.14483 1.0589 0.14483
1.0589 0.14483 0.14483 0.595922391
LINC01965 ENSG00000256637 0.0055165 0.4978 0.49567 0.31006 1.0588
0.14485 0.6742 0.25009 0.14485 0.595922391
PDPK1 ENSG00000140992 -0.64199 0.73956 1.0585 0.1449 0.61307 0.26992
0.79224 0.21411 0.1449 0.595922391
RN7SL236P ENSG00000243870 1.0585 0.14491 0.48595 0.3135 0.70781
0.23953 -0.29242 0.61502 0.14491 0.595922391
LGALS14 ENSG00000006659 -0.25046 0.59889 1.0584 0.14493 -0.49546
0.68986 -1.0234 0.84694 0.14493 0.595922391
TAFA5 ENSG00000219438 -1.3406 0.90998 -1.086 0.86125 -0.12041 0.54792
1.0584 0.14494 0.14494 0.595922391
S1PR4 ENSG00000125910 0.39523 0.34634 0.51694 0.3026 1.0583 0.14495
-0.53783 0.70465 0.14495 0.595922391
LINC02286 ENSG00000258098 0.92786 0.17674 1.0583 0.14496 0.042652
0.48299 -0.015489 0.50618 0.14496 0.595922391
NA ENSG00000266765 1.0583 0.14497 1.0583 0.14497 1.0583 0.14497
-0.5453 0.70722 0.14497 0.595922391
NA ENSG00000259920 0.26271 0.39639 1.0582 0.14499 -1.2303 0.89071
-1.2303 0.89071 0.14499 0.595922391
NA ENSG00000254164 1.0581 0.145 1.0581 0.145 1.0581 0.145 1.0581 0.145

0.145 0.595922391
MIR4646 ENSG00000266776 1.0581 0.14501 1.0581 0.14501 1.0581
0.14501 1.0581 0.14501 0.14501 0.595922391
OC90 ENSG00000253117 0.9454 0.17223 1.0579 0.14506 0.27862 0.39027
-0.73714 0.76948 0.14506 0.595922391
ERI2 ENSG00000196678 0.26134 0.39691 0.26134 0.39691 0.26134 0.39691
1.0578 0.14507 0.14507 0.595922391
LINC01287 ENSG00000234722 1.0578 0.14507 0.83732 0.20121 0.10139
0.45962 -0.73579 0.76907 0.14507 0.595922391
NA ENSG00000206738 1.0577 0.1451 1.0577 0.1451 1.0577 0.1451
0.56204 0.28705 0.1451 0.595922391
NA ENSG00000263946 1.0577 0.1451 1.0577 0.1451 1.0577 0.1451
1.0577 0.1451 0.1451 0.595922391
CCDC182 ENSG00000166329 -0.92147 0.8216 -0.14636 0.55818 -1.3346 0.909
1.0576 0.14511 0.14511 0.595922391
BAHD1 ENSG00000140320 1.0576 0.14512 -0.47949 0.68421 -1.0423 0.85137
-0.72258 0.76503 0.14512 0.595922391
ATP4A ENSG00000105675 1.0575 0.14515 1.0575 0.14515 1.0575 0.14515
1.0575 0.14515 0.14515 0.595922391
MON2 ENSG00000061987 -0.034454 0.51374 0.59333 0.27648 1.0574 0.14516
0.4568 0.32391 0.14516 0.595922391
NA ENSG00000279679 -0.59266 0.7233 -0.74588 0.77213 -0.59266 0.7233
1.0574 0.14516 0.14516 0.595922391
GDF7 ENSG00000143869 1.0573 0.14518 -0.26027 0.60267 -0.1866 0.57401
-0.38819 0.65106 0.14518 0.595922391
TULP3 ENSG00000078246 0.9739 0.16505 0.62304 0.26663 -0.35144 0.63737
1.0571 0.14523 0.14523 0.595922391
RPL13P2 ENSG00000213820 0.16948 0.43271 1.0571 0.14523 -0.52525
0.70029 0.2055 0.41859 0.14523 0.595922391
NA ENSG00000255107 -0.15219 0.56048 1.0571 0.14523 0.11966 0.45237
0.10038 0.46002 0.14523 0.595922391
RPL35AP21 ENSG00000229814 1.0571 0.14524 1.0571 0.14524 1.0571
0.14524 1.0571 0.14524 0.14524 0.595922391
NA ENSG00000248705 0.64159 0.26057 0.95761 0.16913 1.057 0.14525 1.057
0.14525 0.14525 0.595922391
SNORD12 ENSG00000212304 1.057 0.14527 1.057 0.14527 1.057 0.14527 1.057
0.14527 0.14527 0.595922391
NA ENSG00000279818 0.78466 0.21633 0.78466 0.21633 0.78466 0.21633
1.0569 0.14528 0.14528 0.595922391
NA ENSG00000224100 1.0568 0.14531 -0.78462 0.78366 -0.3292 0.629
0.38595 0.34977 0.14531 0.595922391
YWHAZP3 ENSG00000229932 0.12399 0.45066 0.12399 0.45066 0.12399
0.45066 1.0568 0.14531 0.14531 0.595922391
NA ENSG00000231829 -0.62072 0.73261 0.39638 0.34591 -0.39984 0.65536
1.0568 0.14531 0.14531 0.595922391
SPCS2P4 ENSG00000228589 1.0567 0.14532 0.19058 0.42443 -0.87981
0.81052 -0.87981 0.81052 0.14532 0.595922391
WSPAR ENSG00000249073 0.66969 0.25153 1.0567 0.14532 0.65828 0.25518
0.78375 0.21659 0.14532 0.595922391
CCDC75P1 ENSG00000182921 1.0566 0.14536 1.0566 0.14536 1.0566
0.14536 1.0566 0.14536 0.14536 0.595922391
STEAP1B ENSG00000105889 1.0565 0.14537 0.52888 0.29844 0.22924
0.40934 0.60753 0.27175 0.14537 0.595922391
ZNF707 ENSG00000181135 0.86802 0.19269 0.76493 0.22216 1.0565
0.14537 1.0433 0.1484 0.14537 0.595922391
NA ENSG00000273481 1.0565 0.14538 1.0565 0.14538 1.0565 0.14538
1.0565 0.14538 0.14538 0.595922391
MIR646 ENSG00000207802 1.0564 0.14539 1.0564 0.14539 0.44439
0.32838 1.0468 0.14759 0.14539 0.595922391
EXTL3-AS1 ENSG00000246339 -1.7163 0.95695 0.70847 0.23933 -0.94786
0.8284 1.0564 0.14539 0.14539 0.595922391
LINC02507 ENSG00000251688 1.0563 0.14541 -0.97051 0.8341 -0.91461

							0.8198	0.1369	0.44556	0.14541	0.595922391
NT5DC1	ENSG00000178425	0.9777	0.16411	0.62421	0.26624	1.0562	0.14543	0.92182	0.17831	0.14543	0.595922391
CAP2	ENSG00000112186	-0.99323	0.8397	-1.2156	0.88793	-0.47356	0.68209	1.0562	0.14544	0.14544	0.595922391
KLHDC8B	ENSG00000185909	1.0562	0.14545	0.62824	0.26492	0.82563	0.20451	0.82563	0.20451	0.14545	0.595922391
NA	ENSG00000223711	1.056	0.14548	1.056	0.14548	1.056	0.14548	1.056	0.14548	0.14548	0.595922391
UBE3AP2	ENSG00000228941	0.50444	0.30697	-0.20497	0.5812	0.50444	0.30697	1.056	0.14548	0.14548	0.595922391
CELSR2	ENSG00000143126	-0.2426	0.59584	0.19152	0.42406	-1.0101	0.84378	1.0559	0.14551	0.14551	0.595922391
NA	ENSG00000282965	-0.89438	0.81444	0.78364	0.21663	0.71642	0.23687	1.0558	0.14552	0.14552	0.595922391
NA	ENSG00000228141	1.0558	0.14553	1.0558	0.14553	0.57733	0.28186	0.088355	0.4648	0.14553	0.595922391
NA	ENSG00000226965	1.0557	0.14556	0.34618	0.3646	0.20953	0.41702	0.00064905	0.49974	0.14556	0.595922391
C19orf84	ENSG00000262874	1.0557	0.14556	1.0557	0.14556	1.0557	0.14556	1.0557	0.14556	0.14556	0.595922391
NA	ENSG00000258810	1.0556	0.14557	1.0556	0.14557	1.0556	0.14557	1.0556	0.14557	0.14557	0.595922391
GOLGA6D	ENSG00000140478	0.54076	0.29434	0.8054	0.21029	1.0556	0.14559	0.46243	0.32189	0.14559	0.595922391
CERS6	ENSG00000172292	-1.2553	0.89531	-0.03159	0.5126	1.0555	0.14559	-0.75957	0.77624	0.14559	0.595922391
AMY2A	ENSG00000243480	-0.033302	0.51328	0.098004	0.46096	1.0555	0.14559	-0.21033	0.58329	0.14559	0.595922391
NA	ENSG00000271187	1.0555	0.14559	1.0555	0.14559	1.0555	0.14559	1.0555	0.14559	0.14559	0.595922391
TUBG1	ENSG00000131462	0.21913	0.41327	0.21913	0.41327	1.0555	0.1456	1.0555	0.1456	0.1456	0.595922391
MINDY4B	ENSG00000214237	1.0555	0.1456	-0.7166	0.76319	1.0555	0.1456	-0.074922	0.52986	0.1456	0.595922391
PSMD12P1	ENSG00000240135	1.0555	0.1456	1.0555	0.1456	1.0555	0.1456	1.0555	0.1456	0.1456	0.595922391
FGFR1	ENSG00000077782	1.0554	0.14562	0.78763	0.21546	0.70761	0.2396	0.32244	0.37356	0.14562	0.595922391
ETV5	ENSG00000244405	-0.02517	0.51004	0.88704	0.18753	0.67961	0.24838	1.0554	0.14562	0.14562	0.595922391
NA	ENSG00000273001	1.0554	0.14562	1.0554	0.14562	1.0554	0.14562	1.0554	0.14562	0.14562	0.595922391
DUTP3	ENSG00000226857	1.0553	0.14563	1.0553	0.14563	1.0553	0.14563	1.0553	0.14563	0.14563	0.595922391
NA	ENSG00000270976	0.81076	0.20875	1.0553	0.14564	1.0553	0.14564	1.0553	0.14564	0.14564	0.595922391
GREM2	ENSG00000180875	0.28779	0.38676	0.59625	0.2755	-0.017516	0.50699	1.0552	0.14566	0.14566	0.595922391
RN7SKP150	ENSG00000222448	1.0552	0.14567	1.0552	0.14567	1.0552	0.14567	1.0552	0.14567	0.14567	0.595922391
RNU6-770P	ENSG00000200799	0.16448	0.43468	0.78084	0.21745	0.31545	0.37621	1.0551	0.14568	0.14568	0.595922391
NA	ENSG00000227602	0.83957	0.20057	1.0551	0.14568	-0.17941	0.57119	1.0551	0.14568	0.14568	0.595922391
LINC00327	ENSG00000232977	-0.078198	0.53116	1.0549	0.14572	0.26468	0.39563	0.63963	0.26121	0.14572	0.595922391
CDC42SE2	ENSG00000158985	1.0548	0.14575	0.78715	0.2156	0.18438	0.42686	0.60336	0.27314	0.14575	0.595922391
NA	ENSG00000276915	1.0548	0.14575	1.0548	0.14575	1.0548	0.14575	1.0548	0.14575	0.14575	0.595922391
NLGN1	ENSG00000169760	0.048555	0.48064	1.0548	0.14577	0.13949	0.44453				

-0.65592 0.74406 0.14577 0.595922391
OPCML-IT1 ENSG00000254896 0.063343 0.47475 0.063343 0.47475 1.0547
0.14578 0.063343 0.47475 0.14578 0.595922391
RPL24P4 ENSG00000181524 1.0546 0.1458 0.016381 0.49347 0.72525
0.23415 0.72525 0.23415 0.1458 0.595922391
CARMIL2 ENSG00000159753 1.0546 0.14581 0.72023 0.23569 1.0546
0.14581 -0.3587 0.64009 0.14581 0.595922391
SQSTM1 ENSG00000161011 1.0546 0.14581 0.70962 0.23897 -0.94198
0.8269 0.27923 0.39004 0.14581 0.595922391
ALDH7A1P2 ENSG00000232292 1.0545 0.14584 0.33708 0.36803 1.0545
0.14584 0.12583 0.44993 0.14584 0.595922391
ATP2A2 ENSG00000174437 0.55495 0.28946 -0.074247 0.52959 1.0543
0.14586 0.53121 0.29764 0.14586 0.595922391
IFNLR1 ENSG00000185436 0.85807 0.19543 0.88687 0.18757 0.51929
0.30178 1.0544 0.14586 0.14586 0.595922391
NA ENSG00000236627 0.032742 0.48694 0.84909 0.19792 1.0543 0.14587
0.032742 0.48694 0.14587 0.595922391
NA ENSG00000237414 -0.23043 0.59112 1.0543 0.14587 1.0543 0.14587
1.0543 0.14587 0.14587 0.595922391
TMEM270 ENSG00000175877 -1.1351 0.87183 -1.3828 0.91664 -0.82627
0.79568 1.0542 0.14589 0.14589 0.595922391
HLFP1 ENSG00000218194 0.97293 0.1653 1.0542 0.14591 1.0469 0.14758
0.75775 0.2243 0.14591 0.595922391
KRT19P4 ENSG00000234102 0.64747 0.25866 1.0541 0.14593 0.64747
0.25866 0.64747 0.25866 0.14593 0.595922391
OR7E7P ENSG00000238228 -0.87395 0.80893 0.81758 0.2068 -0.69616
0.75684 1.0541 0.14593 0.14593 0.595922391
LINC00161 ENSG00000226935 -0.60798 0.7284 -0.63381 0.7369 1.054 0.14595
-0.60798 0.7284 0.14595 0.595922391
ZNF847P ENSG00000215812 -0.093863 0.53739 1.0539 0.14596 0.13188
0.44754 1.0454 0.14791 0.14596 0.595922391
NUP214 ENSG00000126883 -0.12224 0.54865 -0.57069 0.7159 0.17913
0.42892 1.0539 0.14597 0.14597 0.595922391
SBK1 ENSG00000188322 0.44315 0.32883 0.55478 0.28952 1.0539 0.14597
0.94592 0.17209 0.14597 0.595922391
PRAC2 ENSG00000229637 1.0539 0.14597 1.0539 0.14597 1.0539 0.14597
1.0539 0.14597 0.14597 0.595922391
LINC00499 ENSG00000251372 0.36452 0.35773 1.0539 0.14597 0.89242
0.18608 -0.37749 0.64709 0.14597 0.595922391
MREG ENSG00000118242 1.0537 0.146 1.0537 0.146 1.0537 0.146 0.21791
0.41375 0.146 0.595922391
HIGD1B ENSG00000131097 1.0538 0.146 1.0538 0.146 1.0538 0.146 1.0538
0.146 0.146 0.595922391
ADGRG5 ENSG00000159618 0.060567 0.47585 1.0536 0.14603 -0.082203
0.53276 1.0256 0.15254 0.14603 0.595922391
FEZF2 ENSG00000153266 -0.41583 0.66123 -0.41583 0.66123 1.0536 0.14604
0.72321 0.23478 0.14604 0.595922391
OR52K1 ENSG00000196778 1.0536 0.14604 0.12318 0.45098 -0.43633
0.6687 0.66023 0.25455 0.14604 0.595922391
C2-AS1 ENSG00000281756 1.0535 0.14605 1.0535 0.14605 1.0535
0.14605 1.0535 0.14605 0.14605 0.595922391
NA ENSG00000259418 -0.23247 0.59191 -0.30627 0.6203 -0.03942 0.51572
1.0535 0.14607 0.14607 0.595922391
NA ENSG00000259061 1.0531 0.14614 0.79409 0.21357 0.8761 0.19049
0.8761 0.19049 0.14614 0.595922391
PGLYRP3 ENSG00000159527 1.0531 0.14615 0.66752 0.25222 -0.47995
0.68437 0.80043 0.21173 0.14615 0.595922391
TNR-IT1 ENSG00000235628 0.67188 0.25083 0.64804 0.25848 1.0529
0.14619 0.84002 0.20045 0.14619 0.595922391
MIR4791 ENSG00000271898 0.33799 0.36768 0.33799 0.36768 0.33799
0.36768 1.0529 0.14619 0.14619 0.595922391
NA ENSG00000257095 -1.5978 0.94496 0.30649 0.37962 1.0529 0.1462

-1.5978 0.94496 0.1462 0.595922391
NA ENSG00000261218 1.0529 0.1462 0.77162 0.22017 0.17495 0.43056
-0.8129 0.79186 0.1462 0.595922391
LINC02716 ENSG00000205106 -0.13098 0.5521 0.46634 0.32048 1.0528
0.14621 -1.0362 0.84993 0.14621 0.595922391
RPL22P3 ENSG00000226457 1.0528 0.14621 1.0528 0.14621 1.0528
0.14621 1.0528 0.14621 0.14621 0.595922391
NA ENSG00000232751 1.0528 0.14621 0.92044 0.17867 1.0528 0.14621
1.0528 0.14621 0.14621 0.595922391
NA ENSG00000255504 -0.1612 0.56403 0.65781 0.25533 1.0528 0.14621
-0.92829 0.82337 0.14621 0.595922391
CHGA ENSG00000100604 0.41956 0.3374 0.13885 0.44478 1.0527 0.14624
-0.61823 0.73179 0.14624 0.595922391
NA ENSG00000219682 0.60562 0.27238 1.0527 0.14624 -0.67875 0.75135
1.0527 0.14624 0.14624 0.595922391
NA ENSG00000277182 0.35018 0.3631 0.35018 0.3631 0.35018 0.3631
1.0526 0.14626 0.14626 0.595922391
MTMR3 ENSG00000100330 -0.54248 0.70626 0.85059 0.1975 1.0046 0.15754
1.0526 0.14627 0.14627 0.595922391
MIPEPP3 ENSG00000233325 0.25971 0.39754 -0.53116 0.70234 1.0526
0.14627 1.0526 0.14627 0.14627 0.595922391
CRB1 ENSG00000134376 0.30721 0.37934 1.0513 0.14655 1.0524 0.1463
0.74329 0.22865 0.1463 0.595922391
SLN ENSG00000170290 0.075023 0.4701 1.0524 0.1463 -0.33137 0.62982
-0.0049083 0.50196 0.1463 0.595922391
NAT1 ENSG00000171428 1.0286 0.15184 1.0525 0.1463 -0.094946 0.53782
0.993 0.16036 0.1463 0.595922391
PSG9 ENSG00000183668 1.0524 0.14631 0.35946 0.35963 -0.34661 0.63556
0.13971 0.44445 0.14631 0.595922391
CELA3A ENSG00000142789 0.51656 0.30273 -0.91403 0.81965 1.0523
0.14632 0.95936 0.16869 0.14632 0.595922391
RUBCN ENSG00000145016 0.35111 0.36275 1.0523 0.14633 0.45168 0.32575
0.45402 0.32491 0.14633 0.595922391
NA ENSG00000226994 -0.46989 0.68078 -0.98489 0.83766 -1.4382 0.92482
1.0522 0.14636 0.14636 0.595922391
NA ENSG00000280183 1.0522 0.14636 1.0522 0.14636 1.0522 0.14636
0.69194 0.24449 0.14636 0.595922391
OLA1P1 ENSG00000213790 -0.73619 0.76919 0.97295 0.16529 1.0521
0.14638 1.0521 0.14638 0.14638 0.595922391
OR1L3 ENSG00000171481 1.052 0.14639 -2.3786 0.99131 -2.3786 0.99131
-2.3786 0.99131 0.14639 0.595922391
PFN1P10 ENSG00000176378 -0.51316 0.69608 1.0521 0.14639 -1.4153
0.92151 0.11148 0.45562 0.14639 0.595922391
NA ENSG00000260253 -0.58949 0.72223 1.052 0.14639 -0.84561 0.80111
-1.2939 0.90215 0.14639 0.595922391
LSG1 ENSG00000041802 1.052 0.14641 0.099585 0.46034 -0.2965 0.61658
0.45119 0.32593 0.14641 0.595922391
MTR ENSG00000116984 -0.12192 0.54852 -0.032474 0.51295 -0.6375 0.7381
1.052 0.14641 0.14641 0.595922391
NA ENSG00000266114 1.0519 0.14642 1.0519 0.14642 1.0519 0.14642
1.0519 0.14642 0.14642 0.595922391
RHOA-IT1 ENSG00000235908 1.0519 0.14643 1.0519 0.14643 1.0519
0.14643 1.0519 0.14643 0.14643 0.595922391
LAMTOR4 ENSG00000188186 0.59496 0.27593 0.75201 0.22602 1.0518
0.14646 0.84708 0.19848 0.14646 0.595922391
NOTO ENSG00000214513 1.0517 0.14648 0.9358 0.17469 -0.276 0.60873
0.77377 0.21953 0.14648 0.595922391
NA ENSG00000225813 -0.97343 0.83483 -0.97343 0.83483 -0.97343 0.83483
1.0517 0.14648 0.14648 0.595922391
RN7SL597P ENSG00000244264 1.0515 0.14652 1.0515 0.14652 -1.2819
0.90007 0.62533 0.26588 0.14652 0.595922391
NA ENSG00000254941 0.19596 0.42232 0.19596 0.42232 0.19596 0.42232

1.0515 0.14652 0.14652 0.595922391
LINC02739 ENSG00000255008 0.54963 0.29129 0.54963 0.29129 1.0515
0.14652 0.54963 0.29129 0.14652 0.595922391
SRSF8 ENSG00000263465 1.0515 0.14652 -0.76357 0.77744 -0.62086 0.73265
-0.90348 0.81686 0.14652 0.595922391
SNTB1 ENSG00000172164 -0.30955 0.62155 -0.30955 0.62155 -0.30955 0.62155
1.0514 0.14653 0.14653 0.595922391
NA ENSG00000272140 -0.53812 0.70475 0.49102 0.3117 1.0514 0.14653
1.0514 0.14653 0.14653 0.595922391
RNU6-1022P ENSG00000201919 0.88818 0.18722 0.88818 0.18722 1.0513
0.14655 0.63121 0.26395 0.14655 0.595922391
NA ENSG00000271047 1.0513 0.14655 -1.1495 0.87483 -0.43277 0.66741
-0.43277 0.66741 0.14655 0.595922391
RPL17P51 ENSG00000271429 1.0513 0.14655 1.0513 0.14655 1.0513
0.14655 1.0513 0.14655 0.14655 0.595922391
COX6A1P2 ENSG00000226976 -0.72275 0.76508 1.0513 0.14657 0.20166
0.42009 0.39037 0.34813 0.14657 0.595922391
NA ENSG00000237609 1.0512 0.14657 1.0512 0.14657 1.0512 0.14657
1.0512 0.14657 0.14657 0.595922391
NA ENSG00000253207 1.0512 0.14659 0.13262 0.44725 0.13262 0.44725
0.13262 0.44725 0.14659 0.595922391
GPD2 ENSG00000115159 0.4071 0.34197 1.0511 0.1466 0.25128 0.4008
-0.63128 0.73607 0.1466 0.595922391
FSIP2 ENSG00000188738 0.20707 0.41798 0.20707 0.41798 0.11041 0.45604
1.0511 0.1466 0.1466 0.595922391
SYNE2 ENSG00000054654 0.89132 0.18638 1.0511 0.14661 -0.039484 0.51575
-0.10873 0.54329 0.14661 0.595922391
NA ENSG00000253322 1.0511 0.14661 0.6175 0.26845 -0.2561 0.60106
0.57573 0.2824 0.14661 0.595922391
NA ENSG00000269095 1.051 0.14664 1.051 0.14664 1.051 0.14664 1.051 0.14664
0.14664 0.595922391
RCAN1 ENSG00000159200 0.82522 0.20462 0.072783 0.47099 1.0509 0.14665
0.70831 0.23938 0.14665 0.595922391
OPRL1 ENSG00000125510 1.0509 0.14666 -0.018522 0.50739 0.77552 0.21902
0.15472 0.43852 0.14666 0.595922391
ACYP2 ENSG00000170634 1.0508 0.14666 0.46609 0.32057 0.2772 0.39081
0.048336 0.48072 0.14666 0.595922391
AKAP5 ENSG00000179841 1.0508 0.14666 1.0306 0.15137 1.0508 0.14666
1.0508 0.14666 0.14666 0.595922391
NA ENSG00000257433 1.0509 0.14666 0.4048 0.34281 0.69308 0.24413
0.69308 0.24413 0.14666 0.595922391
RNU6-1181P ENSG00000206918 1.0508 0.14667 1.0508 0.14667 1.0508
0.14667 1.0508 0.14667 0.14667 0.595922391
TMEM59L ENSG00000105696 0.70149 0.2415 0.70149 0.2415 1.0508
0.14668 0.43863 0.33047 0.14668 0.595922391
RNA5SP65 ENSG00000200036 1.0508 0.14668 1.0508 0.14668 1.0508
0.14668 1.0508 0.14668 0.14668 0.595922391
NA ENSG00000239395 -0.37019 0.64438 -0.37995 0.64801 1.0507 0.14669
1.0507 0.14669 0.14669 0.595922391
NA ENSG00000280042 1.0507 0.14669 -0.31098 0.62209 1.0507 0.14669
1.0507 0.14669 0.14669 0.595922391
NA ENSG00000245466 -0.39521 0.65366 1.0507 0.1467 -0.39521 0.65366
-0.39521 0.65366 0.1467 0.595922391
NA ENSG00000215288 0.4517 0.32574 1.0507 0.14671 -0.079151 0.53154
-0.19111 0.57578 0.14671 0.595922391
NA ENSG00000265458 1.0506 0.14671 1.0506 0.14671 1.0506 0.14671
1.0506 0.14671 0.14671 0.595922391
NA ENSG00000229722 0.29814 0.3828 0.29814 0.3828 0.29814 0.3828
1.0506 0.14673 0.14673 0.595922391
NA ENSG00000273245 -0.53592 0.70399 -0.36714 0.64324 0.29115 0.38547
1.0505 0.14675 0.14675 0.595922391
AMMECR1LP1 ENSG00000233273 1.0504 0.14676 1.0504 0.14676 1.0504

0.14676 0.70901 0.23916 0.14676 0.595922391
RN7SKP289 ENSG00000253053 0.38867 0.34876 1.0504 0.14676 1.0504
0.14676 1.0504 0.14676 0.14676 0.595922391
ADI1P3 ENSG00000258369 1.0503 0.14678 0.65622 0.25584 -0.47138
0.68132 -0.47138 0.68132 0.14678 0.595922391
PLPP3 ENSG00000162407 -0.8632 0.80599 -0.22005 0.58708 0.3475 0.36411
1.0503 0.14679 0.14679 0.595922391
NA ENSG00000233877 1.0503 0.14679 1.0503 0.14679 1.0503 0.14679
0.62379 0.26638 0.14679 0.595922391
FMN2 ENSG00000155816 0.81243 0.20827 0.81791 0.2067 0.70472 0.24049
1.0503 0.1468 0.1468 0.595922391
HNRNPR ENSG00000125944 0.91983 0.17883 0.52873 0.2985 1.0502
0.14682 0.71879 0.23614 0.14682 0.595922391
LINC00612 ENSG00000214851 1.0502 0.14682 1.0502 0.14682 1.0502
0.14682 1.0502 0.14682 0.14682 0.595922391
PKMP3 ENSG00000220563 1.0501 0.14683 0.17856 0.42914 1.0501 0.14683
1.0501 0.14683 0.14683 0.595922391
NA ENSG00000223660 1.0501 0.14684 1.0501 0.14684 1.0501 0.14684
1.0501 0.14684 0.14684 0.595922391
EEF1B2P7 ENSG00000213055 1.05 0.14685 0.36835 0.3563 -0.36554 0.64265
-0.36554 0.64265 0.14685 0.595922391
GTF2IP2 ENSG00000226930 -0.2787 0.60976 1.05 0.14685 0.33983 0.36699
0.49428 0.31056 0.14685 0.595922391
RN7SL297P ENSG00000240183 1.05 0.14686 1.05 0.14686 1.05 0.14686 1.05
0.14686 0.14686 0.595922391
SNORD3H ENSG00000207215 0.64466 0.25957 0.66757 0.25221 1.05 0.14687
0.60832 0.27149 0.14687 0.595922391
HESX1 ENSG00000163666 1.0499 0.14688 1.0499 0.14688 1.0499 0.14688
0.45127 0.3259 0.14688 0.595922391
NA ENSG00000278963 0.51017 0.30497 0.51017 0.30497 1.0498 0.14689
0.51017 0.30497 0.14689 0.595922391
NA ENSG00000260599 1.0498 0.1469 0.41235 0.34004 0.89706 0.18484
0.90849 0.18181 0.1469 0.595922391
HNRNPA1P65 ENSG00000248444 1.0498 0.14691 1.0498 0.14691 1.0498
0.14691 1.0498 0.14691 0.14691 0.595922391
CPHXL ENSG00000283755 1.0497 0.14692 0.90043 0.18395 0.44525 0.32807
0.53768 0.2954 0.14692 0.595922391
NA ENSG00000199444 -0.74755 0.77263 1.0495 0.14697 1.0495 0.14697
1.0495 0.14697 0.14697 0.596074489
NA ENSG00000257738 0.31723 0.37554 0.31723 0.37554 1.0494 0.14701
0.31723 0.37554 0.14701 0.596074489
GAS5-AS1 ENSG00000270084 1.0492 0.14704 1.0492 0.14704 1.0492
0.14704 1.0492 0.14704 0.14704 0.596074489
MAD1L1 ENSG00000002822 -0.17958 0.57126 1.0491 0.14706 -0.83258
0.79746 0.27052 0.39338 0.14706 0.596074489
NA ENSG00000259530 -0.17758 0.57047 1.0491 0.14707 -0.17758 0.57047
-0.17758 0.57047 0.14707 0.596074489
GALNT13 ENSG00000144278 0.36815 0.35638 0.054721 0.47818 1.0491
0.14708 0.04966 0.4802 0.14708 0.596074489
ERRFI1 ENSG00000116285 0.94406 0.17257 -0.26835 0.60579 1.049 0.14709
0.54067 0.29437 0.14709 0.596074489
NA ENSG00000278002 -0.6201 0.7324 -0.14622 0.55813 1.0489 0.14712
0.70082 0.24171 0.14712 0.596074489
HABP2 ENSG00000148702 -0.75571 0.77509 1.0488 0.14713 -0.29917 0.61759
-0.36639 0.64296 0.14713 0.596074489
NA ENSG00000271919 1.0488 0.14713 1.0488 0.14713 -0.0045142 0.5018
1.0488 0.14713 0.14713 0.596074489
LINC02693 ENSG00000212719 -0.28176 0.61094 0.10441 0.45842 -0.87091
0.8081 1.0488 0.14714 0.14714 0.596074489
RBMXP4 ENSG00000249465 0.73069 0.23248 1.0488 0.14714 0.86082
0.19467 0.86082 0.19467 0.14714 0.596074489
TASOR2 ENSG00000108021 0.41778 0.33805 1.0488 0.14715 -0.44407

0.6715 0.47873 0.31606 0.14715 0.596074489
DNAJC11 ENSG00000007923 1.0487 0.14716 0.72573 0.234 -0.022596 0.50901
-0.15577 0.56189 0.14716 0.596074489
HILPDA ENSG00000135245 0.55896 0.28809 0.55896 0.28809 1.0487
0.14717 0.2533 0.40002 0.14717 0.596074489
NA ENSG00000281657 -0.6295 0.73549 -0.64366 0.7401 1.0487 0.14717
-1.642 0.9497 0.14717 0.596074489
UBE2Q2 ENSG00000140367 0.50685 0.30613 1.0485 0.1472 0.71534
0.2372 -0.8123 0.79169 0.1472 0.596074489
MEIOB ENSG00000162039 0.33087 0.37037 0.65262 0.257 0.85737 0.19562
1.0485 0.1472 0.1472 0.596074489
MIR4290HG ENSG00000227555 1.0485 0.14722 0.37708 0.35306 0.28857
0.38645 -0.029115 0.51161 0.14722 0.596074489
UBE2D1 ENSG00000072401 1.0483 0.14724 1.0483 0.14724 1.0483
0.14724 -1.2077 0.88643 0.14724 0.596074489
GYPE ENSG00000197465 0.23809 0.4059 -0.24837 0.59808 -0.27497 0.60833
1.0482 0.14727 0.14727 0.596074489
LINC01971 ENSG00000278200 1.0482 0.14727 0.85662 0.19583 -0.075183
0.52997 -0.075183 0.52997 0.14727 0.596074489
VDAC2P3 ENSG00000213262 1.0482 0.14728 1.0482 0.14728 1.0482
0.14728 1.0482 0.14728 0.14728 0.596074489
NA ENSG00000229418 1.0482 0.14728 0.90171 0.18361 1.0054 0.15735
0.13313 0.44705 0.14728 0.596074489
SERBP1P3 ENSG00000242142 -0.11055 0.54402 1.0481 0.14729 -0.22068
0.58733 0.094833 0.46222 0.14729 0.596074489
NA ENSG00000260092 0.95863 0.16887 1.0481 0.14729 0.95863 0.16887
0.95863 0.16887 0.14729 0.596074489
OR4C2P ENSG00000182565 0.32723 0.37175 1.0481 0.1473 -0.99818
0.8409 -0.81257 0.79177 0.1473 0.596074489
RN7SKP223 ENSG00000222514 1.0481 0.1473 1.0481 0.1473 1.0481
0.1473 1.0481 0.1473 0.1473 0.596074489
HOXC13-AS ENSG00000249641 1.0477 0.1474 0.90073 0.18387 0.9485
0.17144 -1.2575 0.89572 0.1474 0.596266255
VPS13B-DT ENSG00000253948 0.00066728 0.49973 0.23324 0.40779 1.0477
0.1474 1.0477 0.1474 0.1474 0.596266255
NA ENSG00000254310 0.91935 0.17896 0.91935 0.17896 0.91935 0.17896
1.0476 0.1474 0.1474 0.596266255
PRXL2AP1 ENSG00000257220 1.0476 0.14742 1.0476 0.14742 1.0476
0.14742 1.0476 0.14742 0.14742 0.596266255
ITIH1 ENSG00000055957 0.36835 0.35631 0.8523 0.19702 1.0474 0.14745
0.6798 0.24832 0.14745 0.596266255
TTC28-AS1 ENSG00000235954 1.031 0.15127 1.0475 0.14745 0.86359 0.19391
0.79286 0.21393 0.14745 0.596266255
SNORD116-30 ENSG00000252277 1.0474 0.14745 1.0474 0.14745 1.0474
0.14745 1.0474 0.14745 0.14745 0.596266255
NA ENSG00000243300 0.63308 0.26334 1.0473 0.14747 0.55124 0.29074
0.55124 0.29074 0.14747 0.596266255
NA ENSG00000258232 1.0473 0.14748 1.0473 0.14748 1.0473 0.14748
1.0473 0.14748 0.14748 0.596266255
NA ENSG00000264324 1.0473 0.14748 1.0473 0.14748 1.0473 0.14748
1.0473 0.14748 0.14748 0.596266255
LAYN ENSG00000204381 0.82855 0.20368 1.0472 0.1475 1.0456 0.14788
1.0184 0.15424 0.1475 0.596266255
NDC80 ENSG00000080986 1.0471 0.14752 1.0458 0.14783 1.0471 0.14752
0.79141 0.21435 0.14752 0.596266255
RPL31P11 ENSG00000213075 1.0471 0.14752 0.8061 0.21009 0.27298
0.39243 0.8061 0.21009 0.14752 0.596266255
RNU1-38P ENSG00000199687 0.66512 0.25299 -0.32558 0.62763 0.60136
0.2738 1.047 0.14755 0.14755 0.596266255
RNU6-1027P ENSG00000206725 1.047 0.14756 -0.38329 0.64925 -0.79281 0.78606
-0.98972 0.83885 0.14756 0.596266255
RABEPKP1 ENSG00000225801 1.0231 0.15313 1.0469 0.14758 1.0231

0.15313 0.88809 0.18725 0.14758 0.596266255
PHBP4 ENSG00000230251 0.12271 0.45117 -0.5748 0.71729 0.12271 0.45117
1.0468 0.14759 0.14759 0.596266255
PROZ ENSG00000126231 0.34972 0.36327 -0.17933 0.57116 1.0468 0.1476
0.89196 0.18621 0.1476 0.596266255
SDHA ENSG00000073578 -0.12654 0.55035 0.46708 0.32022 0.20341 0.41941
1.0467 0.14762 0.14762 0.596266255
ZNF799 ENSG00000196466 0.20854 0.4174 1.0467 0.14762 -1.1948
0.88392 0.066822 0.47336 0.14762 0.596266255
TGM1 ENSG00000092295 1.0466 0.14764 1.0466 0.14764 1.0466 0.14764
1.0466 0.14764 0.14764 0.596266255
MTND1P19 ENSG00000251449 1.0466 0.14764 1.0466 0.14764 -0.026273
0.51048 1.0466 0.14764 0.14764 0.596266255
NA ENSG00000260918 0.89501 0.18539 0.51328 0.30388 1.0466 0.14764
1.0466 0.14764 0.14764 0.596266255
ITM2C ENSG00000135916 -0.46047 0.67741 0.33529 0.3687 -0.40698 0.65799
1.0465 0.14766 0.14766 0.596266255
GAPDHP76 ENSG00000249840 -0.96538 0.83282 1.0465 0.14766 -0.96538
0.83282 1.0465 0.14766 0.14766 0.596266255
NECTIN3 ENSG00000177707 0.28543 0.38766 0.28543 0.38766 1.0464
0.14769 0.28543 0.38766 0.14769 0.596266255
NA ENSG00000227908 1.0464 0.14769 1.0464 0.14769 1.0464 0.14769
1.0464 0.14769 0.14769 0.596266255
LINC00330 ENSG00000235097 0.25427 0.39964 -1.3358 0.90919 0.75228
0.22594 1.0464 0.14769 0.14769 0.596266255
SENP5 ENSG00000119231 -0.9949 0.84011 1.0461 0.14776 0.46458 0.32112
0.52024 0.30145 0.14776 0.596296542
NA ENSG00000271397 0.21885 0.41338 0.97123 0.16572 1.046 0.14777 1.046
0.14777 0.14777 0.596296542
ZNF566 ENSG00000186017 0.29365 0.38451 -0.096021 0.53825 1.046 0.14778
-0.096021 0.53825 0.14778 0.596296542
CYFIP2 ENSG00000055163 1.0014 0.15831 -0.22848 0.59036 0.59138
0.27713 1.046 0.14779 0.14779 0.596296542
BNIP3P29 ENSG00000269742 0.3306 0.37047 1.046 0.14779 1.046 0.14779 1.046
0.14779 0.14779 0.596296542
UBASH3A ENSG00000160185 1.0459 0.1478 1.0459 0.1478 0.030872
0.48769 0.8769 0.19027 0.1478 0.596296542
TPCN1 ENSG00000186815 1.0458 0.14782 1.0458 0.14782 0.77251 0.21991
0.96114 0.16824 0.14782 0.596296542
AURKAP1 ENSG00000213033 1.0458 0.14783 0.33248 0.36976 1.0458
0.14783 1.0458 0.14783 0.14783 0.596296542
ENTPD1-AS1 ENSG00000226688 0.64731 0.25871 0.93105 0.17591 1.0457
0.14785 1.0457 0.14785 0.14785 0.596296542
NA ENSG00000256176 1.0457 0.14785 0.91989 0.17881 1.0457 0.14785
1.0457 0.14785 0.14785 0.596296542
NA ENSG00000257759 -0.011178 0.50446 1.0457 0.14785 -1.2724 0.89839
-1.6947 0.95494 0.14785 0.596296542
ITPK1-AS1 ENSG00000258730 1.0333 0.15072 -0.39836 0.65482 1.0457
0.14786 0.82017 0.20606 0.14786 0.596296542
GAD2 ENSG00000136750 -0.44784 0.67287 1.0455 0.14789 -0.14474 0.55754
0.88763 0.18737 0.14789 0.596296542
RN7SKP202 ENSG00000222371 1.0455 0.1479 0.69636 0.2431 1.0455
0.1479 1.0455 0.1479 0.1479 0.596296542
VN1R6P ENSG00000174677 -0.037566 0.51498 1.0454 0.14792 1.0454
0.14792 1.0454 0.14792 0.14792 0.596296542
RNU6-1331P ENSG00000200528 1.0454 0.14792 1.0454 0.14792 1.0454
0.14792 1.0454 0.14792 0.14792 0.596296542
MIR3912 ENSG00000264249 0.41438 0.3393 1.0454 0.14792 0.41438
0.3393 0.41438 0.3393 0.14792 0.596296542
N4BP2L2-IT2 ENSG00000281026 0.10279 0.45906 1.0454 0.14792 -0.32878
0.62884 -0.32878 0.62884 0.14792 0.596296542
DUOX1 ENSG00000137857 1.0454 0.14793 0.40217 0.34378 -0.77245 0.78008

-0.93075 0.82401 0.14793 0.596296542
ARHGAP21 ENSG00000107863 1.0451 0.14799 0.82596 0.20441 0.69231
0.24437 0.6492 0.2581 0.14799 0.596331513
NA ENSG00000224700 1.045 0.14802 1.045 0.14802 1.045 0.14802 1.045 0.14802
0.14802 0.596331513
NA ENSG00000270680 1.045 0.14802 0.15418 0.43873 0.27347 0.39224
-0.024196 0.50965 0.14802 0.596331513
DNAJB8-AS1 ENSG00000242049 1.028 0.15196 1.0449 0.14804 1.0449 0.14804
1.0449 0.14804 0.14804 0.596331513
SELENOP ENSG00000250722 1.0448 0.14806 -0.43834 0.66943 -0.22052
0.58727 -0.3206 0.62574 0.14806 0.596331513
IL15RA ENSG00000134470 0.69722 0.24283 1.0448 0.14807 0.57938
0.28117 0.48519 0.31377 0.14807 0.596331513
NA ENSG00000217746 -0.43046 0.66657 1.0448 0.14807 -0.43046 0.66657
-0.43046 0.66657 0.14807 0.596331513
PLPPR2 ENSG00000105520 -0.035905 0.51432 1.0447 0.14808 -0.035905
0.51432 -0.035905 0.51432 0.14808 0.596331513
RNU6-767P ENSG00000206859 1.0447 0.14808 1.0447 0.14808 1.0447
0.14808 1.0447 0.14808 0.14808 0.596331513
NA ENSG00000254178 1.0447 0.14808 1.0447 0.14808 1.0447 0.14808
1.0447 0.14808 0.14808 0.596331513
TPM3P8 ENSG00000183022 1.0446 0.1481 1.0446 0.1481 -0.074655
0.52976 1.0446 0.1481 0.1481 0.596331513
PAPOLB ENSG00000218823 1.0446 0.1481 -0.34415 0.63463 -0.095799
0.53816 0.11357 0.45479 0.1481 0.596331513
CCT6P2 ENSG00000250526 -1.0097 0.84367 -0.8171 0.79306 -1.0085
0.8434 1.0446 0.1481 0.1481 0.596331513
NA ENSG00000279952 1.0446 0.14811 1.0446 0.14811 1.0446 0.14811
1.0446 0.14811 0.14811 0.596331513
NA ENSG00000226160 -0.82959 0.79661 -0.82959 0.79661 1.0445 0.14814
-0.72504 0.76579 0.14814 0.596403024
R3HDM2P2 ENSG00000216817 1.0442 0.1482 1.0442 0.1482 1.0442
0.1482 1.0442 0.1482 0.1482 0.596586263
ZNF22-AS1 ENSG00000165511 1.0441 0.14821 1.0441 0.14821 1.0441
0.14821 1.0441 0.14821 0.14821 0.596586263
NA ENSG00000252824 1.0439 0.14825 1.0439 0.14825 -0.022391 0.50893
1.0439 0.14825 0.14825 0.596633993
RPL15 ENSG00000174748 -0.54512 0.70716 0.69776 0.24266 0.69776 0.24266
1.0439 0.14827 0.14827 0.596633993
NA ENSG00000266106 1.0439 0.14828 1.0439 0.14828 1.0439 0.14828
1.0439 0.14828 0.14828 0.596633993
CLDN18 ENSG00000066405 0.20648 0.41821 1.0437 0.14831 0.96293
0.16779 0.9724 0.16543 0.14831 0.596633993
FBXL12 ENSG00000127452 1.0437 0.14831 0.62893 0.2647 0.95601
0.16953 0.20957 0.417 0.14831 0.596633993
LCEP4 ENSG00000226947 1.0437 0.14831 -0.43823 0.66939 1.0437 0.14831
1.0437 0.14831 0.14831 0.596633993
NA ENSG00000261541 1.0437 0.14832 0.93485 0.17493 1.0437 0.14832
1.0437 0.14832 0.14832 0.596633993
RNA5SP356 ENSG00000251781 1.0436 0.14833 0.047174 0.48119 -0.72424
0.76554 -0.72424 0.76554 0.14833 0.596633993
NA ENSG00000188242 0.56024 0.28766 1.0436 0.14834 1.0316 0.15113
1.0316 0.15113 0.14834 0.596633993
NA ENSG00000258411 -0.27159 0.60703 -0.27159 0.60703 0.44164 0.32938
1.0435 0.14835 0.14835 0.596633993
LINC02629 ENSG00000223470 1.0435 0.14837 -0.77577 0.78106 1.0435
0.14837 1.0435 0.14837 0.14837 0.596633993
NA ENSG00000248878 1.0434 0.14838 1.0434 0.14838 1.0434 0.14838
-0.76136 0.77678 0.14838 0.596633993
MIR3686 ENSG00000263763 1.0433 0.14839 1.0433 0.14839 1.0433
0.14839 1.0433 0.14839 0.14839 0.596633993
KIF2B ENSG00000141200 1.0433 0.14841 1.0433 0.14841 1.0433 0.14841

1.0433 0.14841 0.14841 0.596633993
ACBD3 ENSG00000182827 1.0433 0.14841 -0.23187 0.59168 1.0433 0.14841
0.19855 0.42131 0.14841 0.596633993
KLF16 ENSG00000129911 -0.81683 0.79299 -0.81683 0.79299 -0.81683 0.79299
1.0432 0.14843 0.14843 0.596633993
ADRA2B ENSG00000274286 1.0432 0.14843 0.82793 0.20385 -0.36873
0.64383 -0.73467 0.76873 0.14843 0.596633993
NA ENSG00000214846 1.043 0.14847 1.043 0.14847 1.043 0.14847 1.043 0.14847
0.14847 0.596669278
SALL4P1 ENSG00000250308 1.0429 0.14849 -0.17361 0.56891 1.0429
0.14849 1.0429 0.14849 0.14849 0.596669278
ZC3H18 ENSG00000158545 -0.84509 0.80097 0.37167 0.35507 1.0429
0.1485 0.5593 0.28798 0.1485 0.596669278
NA ENSG00000243280 1.0429 0.1485 1.0429 0.1485 1.0429 0.1485
1.0429 0.1485 0.1485 0.596669278
NA ENSG00000267666 1.0429 0.1485 1.0429 0.1485 1.0429 0.1485
1.0429 0.1485 0.1485 0.596669278
MIR1538 ENSG00000223109 0.091004 0.46374 1.0428 0.14853 1.0428
0.14853 1.0428 0.14853 0.14853 0.59672257
MANSC1 ENSG00000111261 0.72465 0.23433 0.96412 0.16749 1.0427
0.14855 -0.40828 0.65847 0.14855 0.59672257
RPL12P31 ENSG00000241228 1.0427 0.14855 1.0427 0.14855 1.0427
0.14855 1.0427 0.14855 0.14855 0.59672257
RB1 ENSG00000139687 0.36776 0.35653 1.0425 0.14859 1.0294 0.15164
0.9399 0.17364 0.14859 0.59683405
BTG2 ENSG00000159388 1.0423 0.14863 1.0423 0.14863 0.28868 0.38642
1.0423 0.14863 0.14863 0.596896316
PRR27 ENSG00000187533 1.0423 0.14863 1.0423 0.14863 1.0423 0.14863
-0.22833 0.59031 0.14863 0.596896316
NA ENSG00000242029 1.0423 0.14865 -0.25702 0.60142 0.65433 0.25645
0.98419 0.16251 0.14865 0.596918408
NFIC ENSG00000141905 1.0422 0.14866 -0.77524 0.7809 -1.0956 0.86337
-0.30154 0.6185 0.14866 0.596918408
NA ENSG00000254478 0.53236 0.29724 -0.33735 0.63207 -0.84875 0.80199
1.0421 0.14869 0.14869 0.596931461
EXOC5P1 ENSG00000180673 1.042 0.1487 0.28504 0.38781 0.33961 0.36707
0.33961 0.36707 0.1487 0.596931461
NA ENSG00000263110 0.32702 0.37183 0.84578 0.19884 1.042 0.1487
0.3566 0.3607 0.1487 0.596931461
SMPDL3B ENSG00000130768 0.35689 0.36059 0.89591 0.18515 0.30287
0.38099 1.0418 0.14875 0.14875 0.597033811
NA ENSG00000225394 0.16389 0.43491 1.0418 0.14875 -0.078262 0.53119
1.0418 0.14875 0.14875 0.597033811
STYK1 ENSG00000060140 0.54094 0.29427 1.0417 0.14878 1.0417 0.14878
-0.88223 0.81117 0.14878 0.59710504
MIR638 ENSG00000207972 1.0415 0.14883 -0.036435 0.51453 1.0415
0.14883 1.0415 0.14883 0.14883 0.597151904
ZNF687-AS1 ENSG00000232671 1.0414 0.14884 1.0414 0.14884 1.0414
0.14884 1.0414 0.14884 0.14884 0.597151904
NA ENSG00000207300 1.0412 0.14888 1.0412 0.14888 -0.45233 0.67449
1.0412 0.14888 0.14888 0.597151904
AARS2 ENSG00000124608 0.93874 0.17393 1.0412 0.14889 0.97982 0.16359
1.0412 0.14889 0.14889 0.597151904
NA ENSG00000249678 0.77522 0.2191 -0.008449 0.50337 0.77522 0.2191
1.0412 0.14889 0.14889 0.597151904
NA ENSG00000262714 1.0412 0.14889 1.0412 0.14889 1.0412 0.14889
1.0412 0.14889 0.14889 0.597151904
NA ENSG00000283205 1.0412 0.14889 -1.4559 0.9273 0.53294 0.29704
0.37596 0.35347 0.14889 0.597151904
MESTP4 ENSG00000215004 1.0411 0.14891 0.32225 0.37363 -0.96487
0.8327 -0.96487 0.8327 0.14891 0.597151904
NA ENSG00000258964 1.0411 0.14892 1.0411 0.14892 1.0411 0.14892

1.0411 0.14892 0.14892 0.597151904
FLII ENSG00000177731 0.45475 0.32464 0.25356 0.39992 1.041 0.14893 1.041 0.14893 0.14893 0.597151904
NA ENSG00000234393 1.041 0.14894 0.29011 0.38587 0.29011 0.38587 0.29011 0.38587 0.14894 0.597151904
NA ENSG00000260660 0.10154 0.45956 1.041 0.14895 -0.97311 0.83475 -0.73354 0.76839 0.14895 0.597151904
RN7SL310P ENSG00000240663 1.0409 0.14897 -0.78293 0.78317 1.0409 0.14897 1.0409 0.14897 0.14897 0.597151904
NA ENSG00000215922 1.0408 0.14898 -0.66589 0.74726 -0.43205 0.66715 -0.43205 0.66715 0.14898 0.597151904
NA ENSG00000249738 0.55261 0.29027 0.73455 0.23131 -0.36839 0.64371 1.0408 0.14899 0.14899 0.597151904
GCNT2 ENSG00000111846 0.52043 0.30138 0.71314 0.23788 1.0407 0.149 0.08972 0.46425 0.149 0.597151904
NA ENSG00000231397 -2.2829 0.98878 -0.58924 0.72215 -0.26972 0.60631 1.0407 0.149 0.149 0.597151904
IHO1 ENSG00000173421 1.0406 0.14903 0.69983 0.24202 0.95114 0.17077 -0.51758 0.69762 0.14903 0.597223018
RNA5SP30 ENSG00000202422 1.0405 0.14906 0.99457 0.15997 0.99457 0.15997 0.99457 0.15997 0.14906 0.597245009
NA ENSG00000239106 1.0405 0.14906 -0.15325 0.5609 -0.15325 0.5609 0.64827 0.25841 0.14906 0.597245009
SPHK2 ENSG00000063176 -0.079942 0.53186 1.0403 0.1491 -0.079942 0.53186 -0.079942 0.53186 0.1491 0.597257953
NA ENSG00000206961 0.78039 0.21758 0.78039 0.21758 0.78039 0.21758 1.0403 0.1491 0.1491 0.597257953
NA ENSG00000279061 0.64997 0.25786 1.0403 0.1491 -0.029527 0.51178 -0.029527 0.51178 0.1491 0.597257953
TMEM231 ENSG00000205084 0.92788 0.17673 0.92788 0.17673 0.92788 0.17673 1.0401 0.14916 0.14916 0.597440133
NA ENSG00000233251 -0.27873 0.60978 1.04 0.14917 0.29869 0.38259 -1.4115 0.92095 0.14917 0.597440133
RPL17P25 ENSG00000219547 1.0398 0.14921 -0.074908 0.52986 1.0398 0.14921 1.0398 0.14921 0.14921 0.597542165
DDX17 ENSG00000100201 1.0223 0.15331 1.0223 0.15331 1.0398 0.14922 1.0317 0.1511 0.14922 0.597542165
DCAF8 ENSG00000132716 0.078404 0.46875 0.8264 0.20429 -0.39277 0.65275 1.0396 0.14925 0.14925 0.597613188
MTCO2P4 ENSG00000253977 1.0394 0.14931 1.0394 0.14931 1.0394 0.14931 1.0394 0.14931 0.14931 0.597804314
MYO15B ENSG00000266714 1.0393 0.14933 0.98395 0.16257 0.72839 0.23319 0.96385 0.16756 0.14933 0.597835269
ST13P14 ENSG00000240959 0.80324 0.21092 1.0389 0.14942 0.84208 0.19987 0.83176 0.20277 0.14942 0.598039085
NA ENSG00000269667 1.0389 0.14942 -1.0002 0.84139 1.0389 0.14942 1.0389 0.14942 0.14942 0.598039085
PPM1M ENSG00000164088 0.68379 0.24705 1.0389 0.14943 0.9058 0.18252 0.68379 0.24705 0.14943 0.598039085
TUFM ENSG00000178952 0.55982 0.2878 0.80824 0.20947 1.0389 0.14943 1.0389 0.14943 0.14943 0.598039085
SLC9A3 ENSG00000066230 -3.2855 0.99949 1.0388 0.14946 -0.5367 0.70426 -0.99655 0.84051 0.14946 0.598110027
NA ENSG00000243402 1.0386 0.14949 1.0386 0.14949 1.0386 0.14949 1.0386 0.14949 0.14949 0.598180957
NA ENSG00000282804 1.0385 0.14952 1.0385 0.14952 1.0385 0.14952 1.0385 0.14952 0.14952 0.598251876
NA ENSG00000199404 1.0384 0.14955 1.0384 0.14955 1.0384 0.14955 1.0384 0.14955 0.14955 0.598264554
QRSL1P2 ENSG00000227613 1.0383 0.14956 -0.67124 0.74897 1.0383 0.14956 1.0383 0.14956 0.14956 0.598264554
NA ENSG00000267243 -0.04897 0.51953 -0.1156 0.54601 1.0383 0.14956

1.0383 0.14956 0.14956 0.598264554
NA ENSG00000275827 0.63568 0.26249 -0.26809 0.60568 1.0382 0.1496
0.4102 0.34083 0.1496 0.598342523
RAX ENSG00000134438 -0.10257 0.54085 1.0381 0.14961 -0.74571 0.77208
0.11795 0.45305 0.14961 0.598342523
CARMIL3 ENSG00000186648 1.038 0.14963 1.038 0.14963 1.038 0.14963 -0.50519
0.69329 0.14963 0.598342523
NA ENSG00000231781 1.038 0.14963 0.88788 0.1873 1.038 0.14963 1.038
0.14963 0.14963 0.598342523
BMS1P21 ENSG00000283101 1.0379 0.14966 1.0379 0.14966 1.0379
0.14966 0.74611 0.2278 0.14966 0.598342523
CD36 ENSG00000135218 1.0379 0.14967 -0.085868 0.53421 0.54959 0.2913
0.1124 0.45526 0.14967 0.598342523
NA ENSG00000279399 1.0378 0.14967 1.0378 0.14967 1.0378 0.14967
1.0378 0.14967 0.14967 0.598342523
PAM16 ENSG00000217930 -0.34156 0.63366 1.0377 0.14969 0.41805 0.33795
0.41805 0.33795 0.14969 0.598342523
NA ENSG00000226798 1.0378 0.14969 1.0378 0.14969 1.0378 0.14969
1.0378 0.14969 0.14969 0.598342523
JUN ENSG00000177606 -0.005087 0.50203 -0.099398 0.53959 1.0376 0.14972
0.049596 0.48022 0.14972 0.598413353
NA ENSG00000226304 1.0376 0.14974 1.0376 0.14974 1.0376 0.14974
0.28155 0.38914 0.14974 0.598435091
LINC02682 ENSG00000254661 -0.85714 0.80432 1.0375 0.14975 0.36459
0.35771 0.78096 0.21741 0.14975 0.598435091
RPS15AP6 ENSG00000233072 1.0373 0.1498 0.3635 0.35812 1.0337
0.15065 0.23541 0.40695 0.1498 0.598494633
EXOSC7 ENSG00000075914 0.84485 0.1991 1.0372 0.14982 0.47586
0.31709 0.70145 0.24151 0.14982 0.598494633
BORCS8 ENSG00000254901 1.0371 0.14984 1.0371 0.14984 1.0371
0.14984 1.0371 0.14984 0.14984 0.598494633
RPL31P50 ENSG00000231788 -0.38163 0.64863 1.037 0.14987 0.32557 0.37237
0.36947 0.35589 0.14987 0.598494633
NA ENSG00000232999 -0.62636 0.73446 1.037 0.14987 -0.62636 0.73446
-0.62636 0.73446 0.14987 0.598494633
NA ENSG00000253955 -0.3218 0.6262 1.037 0.14987 0.17276 0.43142
-0.259 0.60218 0.14987 0.598494633
RPGRIP1L ENSG00000103494 0.26449 0.3957 1.0369 0.14989 0.26449
0.3957 0.26449 0.3957 0.14989 0.598494633
CHST15 ENSG00000182022 0.030507 0.48783 -1.045 0.85198 -0.79726
0.78735 1.0369 0.14989 0.14989 0.598494633
NECAP1 ENSG00000089818 0.45258 0.32543 0.42302 0.33614 -0.11439
0.54553 1.0368 0.1499 0.1499 0.598494633
CDK18 ENSG00000117266 -0.11722 0.54666 0.62204 0.26696 0.31887 0.37491
1.0369 0.1499 0.1499 0.598494633
NA ENSG00000229911 -1.2442 0.89329 1.0369 0.1499 0.1903 0.42454
-1.2442 0.89329 0.1499 0.598494633
MTRNR2L11 ENSG00000270188 1.0368 0.14992 1.0368 0.14992 1.0368
0.14992 1.0368 0.14992 0.14992 0.598525447
NA ENSG00000214301 0.26754 0.39453 1.0365 0.14998 0.26754 0.39453
0.26754 0.39453 0.14998 0.598666891
JAKMIP2-AS1 ENSG00000280780 0.073136 0.47085 1.0365 0.14998 -0.71639
0.76312 -0.18389 0.57295 0.14998 0.598666891
HNRNPA3 ENSG00000170144 -0.39391 0.65318 1.0364 0.15001 -1.3982
0.91897 0.19014 0.4246 0.15001 0.598711329
NA ENSG00000251498 1.0364 0.15002 -0.36985 0.64425 -0.54098 0.70574
-0.59899 0.72541 0.15002 0.598711329
NUPR1 ENSG00000176046 0.10647 0.45761 0.90176 0.18359 0.58374 0.2797
1.0362 0.15004 0.15004 0.598711329
NA ENSG00000231521 1.0362 0.15006 1.0362 0.15006 1.0362 0.15006
1.0362 0.15006 0.15006 0.598711329
NA ENSG00000261821 1.0361 0.15007 0.79503 0.2133 1.0306 0.15137

1.0306 0.15137 0.15007 0.598711329
ASXL1 ENSG00000171456 0.98109 0.16328 1.036 0.15011 0.88136 0.18906
0.62877 0.26475 0.15011 0.598711329
SMCR2 ENSG00000223979 1.036 0.15011 0.5477 0.29195 0.52697 0.29911
0.5477 0.29195 0.15011 0.598711329
ZNF213-AS1 ENSG00000263072 1.036 0.15011 0.53306 0.29699 1.036 0.15011 1.036
0.15011 0.15011 0.598711329
COL6A2 ENSG00000142173 -0.41507 0.66096 0.21068 0.41657 0.32239
0.37358 1.0359 0.15012 0.15012 0.598711329
NA ENSG00000272916 1.0359 0.15013 1.0359 0.15013 1.0359 0.15013
1.0359 0.15013 0.15013 0.598711329
MORN3 ENSG00000139714 -0.54794 0.70813 -0.4827 0.68535 1.0355 0.15022
1.0358 0.15014 0.15014 0.598711329
FLAD1 ENSG00000160688 1.0358 0.15016 1.0358 0.15016 1.0358 0.15016
0.90986 0.18145 0.15016 0.598711329
NA ENSG00000279460 0.80812 0.20951 1.0357 0.15017 0.13488 0.44635
1.0357 0.15017 0.15017 0.598711329
ASPHD2 ENSG00000128203 0.31725 0.37553 1.0356 0.15019 1.0356
0.15019 1.0356 0.15019 0.15019 0.598711329
SOAT2 ENSG00000167780 -0.71619 0.76306 -0.21404 0.58474 0.3259 0.37225
1.0356 0.15019 0.15019 0.598711329
RBM39 ENSG00000131051 0.84023 0.20039 0.95051 0.17093 0.61601 0.26894
1.0356 0.1502 0.1502 0.598711329
ZNF876P ENSG00000198155 0.25763 0.39835 0.68421 0.24692 1.0356
0.1502 0.714 0.23761 0.1502 0.598711329
NA ENSG00000257647 1.0355 0.15022 1.0355 0.15022 1.0355 0.15022
1.0355 0.15022 0.15022 0.598742074
NA ENSG00000279662 0.32714 0.37178 1.0354 0.15024 1.0354 0.15024
0.92718 0.17692 0.15024 0.598772814
RNU1-104P ENSG00000253089 1.0353 0.15026 1.0353 0.15026 1.0353
0.15026 1.0353 0.15026 0.15026 0.598785315
GRWD1 ENSG00000105447 1.0353 0.15028 -0.67449 0.75 -0.63617 0.73767
-0.38078 0.64832 0.15028 0.598785315
NA ENSG00000235122 1.0352 0.15028 0.48532 0.31372 0.086516 0.46553
0.48532 0.31372 0.15028 0.598785315
PCNPP3 ENSG00000213344 1.0351 0.15031 0.41298 0.33981 1.0351
0.15031 1.0351 0.15031 0.15031 0.598855883
NA ENSG00000264212 0.63638 0.26226 0.60923 0.27119 1.0349 0.15035
0.28439 0.38805 0.15035 0.598929773
DLGAP5 ENSG00000126787 0.34644 0.36451 0.85702 0.19572 1.0348
0.15037 0.16613 0.43403 0.15037 0.598929773
NA ENSG00000227745 1.0348 0.15039 0.63741 0.26193 0.36183 0.35874
0.36183 0.35874 0.15039 0.598929773
NA ENSG00000233064 1.0347 0.15039 0.63752 0.26189 -0.018908 0.50754
0.35284 0.3621 0.15039 0.598929773
NA ENSG00000278071 1.0348 0.15039 1.0348 0.15039 1.0348 0.15039
1.0348 0.15039 0.15039 0.598929773
COQ8A ENSG00000163050 0.8566 0.19583 0.46858 0.31969 -0.48312 0.6855
1.0345 0.15045 0.15045 0.598954672
LINC01068 ENSG00000227676 -0.63803 0.73827 -0.61262 0.72994 1.0344
0.15046 -0.36617 0.64288 0.15046 0.598954672
NA ENSG00000228982 1.0345 0.15046 1.0345 0.15046 1.0345 0.15046
0.40951 0.34108 0.15046 0.598954672
TRAV37 ENSG00000259068 1.0345 0.15046 1.008 0.15674 1.008 0.15674 1.008
0.15674 0.15046 0.598954672
NA ENSG00000279326 1.0344 0.15047 1.0344 0.15047 1.0344 0.15047
1.0344 0.15047 0.15047 0.598954672
NA ENSG00000239475 0.5225 0.30066 -0.6191 0.73208 1.0344 0.15047
0.70343 0.24089 0.15047 0.598954672
RPS2 ENSG00000140988 -1.4881 0.93164 -1.4881 0.93164 1.0343 0.1505
-1.4881 0.93164 0.1505 0.598976233
MIR1976 ENSG00000238705 1.0343 0.1505 1.0343 0.1505 1.0343

0.1505 1.0343 0.1505 0.1505 0.598976233
ZNF462 ENSG00000148143 0.030273 0.48792 1.034 0.15056 -0.16325 0.56484 -0.73005 0.76732 0.15056 0.59905912
LINC02282 ENSG00000257056 0.4828 0.31462 0.4828 0.31462 1.034 0.15056 1.034 0.15056 0.15056 0.59905912
NA ENSG00000214559 -0.3342 0.63089 -0.3342 0.63089 -0.3342 0.63089 1.034 0.15057 0.15057 0.59905912
LINC02063 ENSG00000250921 1.034 0.15057 0.20986 0.41689 0.2291 0.40939 -0.67128 0.74898 0.15057 0.59905912
ZNF805 ENSG00000204524 1.0339 0.15059 -0.28251 0.61122 -0.28251 0.61122 -0.28251 0.61122 0.15059 0.599089779
YIPF7 ENSG00000177752 0.00094783 0.49962 -0.21976 0.58697 0.64058 0.2609 1.0338 0.15061 0.15061 0.599102179
NA ENSG00000282024 -1.4147 0.92142 1.0338 0.15062 0.62649 0.2655 0.62649 0.2655 0.15062 0.599102179
NSMCE2 ENSG00000156831 1.0337 0.15063 -0.62721 0.73474 0.58437 0.27949 -0.019881 0.50793 0.15063 0.599102179
NA ENSG00000267637 -0.087111 0.53471 0.72864 0.23311 1.0335 0.15068 1.0335 0.15068 0.15068 0.599252134
NA ENSG00000281938 1.0334 0.1507 1.0334 0.1507 1.0334 0.1507 1.0334 0.1507 0.1507 0.599282765
TTC13 ENSG00000143643 1.0332 0.15075 -0.51025 0.69506 -0.71582 0.76295 0.53823 0.29521 0.15075 0.599316592
MYB ENSG00000118513 1.0332 0.15076 1.0332 0.15076 1.0332 0.15076 1.0332 0.15076 0.15076 0.599316592
HIGD2A ENSG00000146066 1.0332 0.15076 1.0332 0.15076 1.0332 0.15076 1.0332 0.15076 0.15076 0.599316592
NA ENSG00000277692 1.0332 0.15076 1.0332 0.15076 1.0332 0.15076 1.0332 0.15076 0.15076 0.599316592
GNAQ ENSG00000156052 0.99112 0.16081 -0.26864 0.6059 1.0332 0.15077 0.46598 0.32061 0.15077 0.599316592
NA ENSG00000271664 1.0093 0.15641 1.0331 0.15079 1.0093 0.15641 1.0093 0.15641 0.15079 0.599347202
LINC01005 ENSG00000228564 1.0326 0.15089 -0.94838 0.82853 -0.81908 0.79363 0.15529 0.4383 0.15089 0.599656325
GSTA9P ENSG00000243236 0.91556 0.17995 1.0325 0.15093 1.0325 0.15093 1.0325 0.15093 0.15093 0.599656325
NA ENSG00000255275 0.95907 0.16876 1.0324 0.15093 0.99663 0.15947 0.99663 0.15947 0.15093 0.599656325
ALMS1P1 ENSG00000163016 0.27893 0.39015 0.087733 0.46504 0.87215 0.19156 1.0324 0.15094 0.15094 0.599656325
PRKAR2B-AS1 ENSG00000243193 -0.017756 0.50708 -0.68575 0.75357 -1.2929 0.90197 1.0324 0.15094 0.15094 0.599656325
NA ENSG00000260605 1.0322 0.15098 -0.26532 0.60462 -0.67607 0.7505 0.88989 0.18676 0.15098 0.599656325
HTR6 ENSG00000158748 0.63395 0.26306 1.0322 0.15099 0.74646 0.22769 1.0043 0.15761 0.15099 0.599656325
EMX2 ENSG00000170370 -0.15985 0.5635 0.95593 0.16955 -1.5213 0.93591 1.0322 0.15099 0.15099 0.599656325
LINC02344 ENSG00000227911 -0.5279 0.70122 1.0321 0.15102 -0.5279 0.70122 -0.33537 0.63133 0.15102 0.599656325
HMGN1P32 ENSG00000237589 1.032 0.15103 1.032 0.15103 1.032 0.15103 1.032 0.15103 0.15103 0.599656325
NA ENSG00000200579 1.032 0.15104 1.032 0.15104 1.032 0.15104 1.032 0.15104 0.15104 0.599656325
NA ENSG00000231912 -0.033186 0.51324 0.46181 0.32211 1.0319 0.15105 0.55977 0.28782 0.15105 0.599656325
NA ENSG00000261693 1.0318 0.15108 1.0318 0.15108 1.0318 0.15108 1.0318 0.15108 0.15108 0.599656325
C1QBPP2 ENSG00000213409 1.0318 0.15109 1.0318 0.15109 1.0318 0.15109 1.0318 0.15109 0.15109 0.599656325
TVP23A ENSG00000166676 0.91655 0.17969 0.91655 0.17969 1.0317

0.1511 -0.19094 0.57571 0.1511 0.599656325
COPS6 ENSG00000168090 0.71579 0.23706 -0.46628 0.67949 1.0317 0.1511
0.59706 0.27523 0.1511 0.599656325
NA ENSG00000200496 -1.5329 0.93735 -0.38859 0.65121 1.0317 0.1511
-0.39605 0.65397 0.1511 0.599656325
DBR1 ENSG00000138231 -2.0123 0.97791 -2.0123 0.97791 -1.1427 0.87342
1.0317 0.15111 0.15111 0.599656325
MIR4520-1 ENSG00000264468 1.0316 0.15112 1.0316 0.15112 1.0316
0.15112 1.0316 0.15112 0.15112 0.599656325
NA ENSG00000216516 0.18933 0.42492 0.18933 0.42492 1.0316 0.15113
0.18933 0.42492 0.15113 0.599656325
ARFGAP1 ENSG00000101199 0.41714 0.33829 1.0315 0.15115 0.36707
0.35678 -0.40438 0.65703 0.15115 0.599656325
NA ENSG00000274299 0.47518 0.31733 1.0315 0.15116 0.40727 0.34191
0.66251 0.25382 0.15116 0.599656325
C12orf76 ENSG00000174456 0.93014 0.17615 0.79368 0.21369 1.0313
0.1512 0.54769 0.29195 0.1512 0.599656325
RPL26P31 ENSG00000242807 1.0313 0.15121 -0.057897 0.52308 1.0313
0.15121 1.0313 0.15121 0.15121 0.599656325
SNHG7 ENSG00000233016 0.5945 0.27609 0.5945 0.27609 0.5945 0.27609
1.0312 0.15122 0.15122 0.599656325
DIS3L2P1 ENSG00000223788 0.48472 0.31394 0.8451 0.19903 1.0169
0.1546 1.0311 0.15124 0.15124 0.599656325
NA ENSG00000237655 0.96404 0.16751 0.96404 0.16751 1.0311 0.15124
0.96404 0.16751 0.15124 0.599656325
NA ENSG00000250650 1.0311 0.15124 -1.124 0.8695 0.040464 0.48386
0.040464 0.48386 0.15124 0.599656325
NA ENSG00000267509 1.0311 0.15125 0.43658 0.33121 -0.52126 0.69891
-0.52126 0.69891 0.15125 0.599656325
RRS1-AS1 ENSG00000246145 1.031 0.15126 0.10547 0.458 0.70038 0.24185
-0.20985 0.58311 0.15126 0.599656325
SLC36A4 ENSG00000180773 0.71894 0.23609 0.45675 0.32392 1.031 0.15128
0.98446 0.16244 0.15128 0.599656325
C17orf75 ENSG00000108666 1.0309 0.1513 -0.139 0.55528 -1.0577
0.85491 -0.139 0.55528 0.1513 0.599656325
KRT18P57 ENSG00000215867 0.117 0.45343 1.0308 0.15131 0.117 0.45343 0.117
0.45343 0.15131 0.599656325
NA ENSG00000259601 0.4047 0.34285 1.0308 0.15131 1.0308 0.15131
1.0308 0.15131 0.15131 0.599656325
NA ENSG00000227330 1.0307 0.15133 1.0307 0.15133 1.0307 0.15133
1.0307 0.15133 0.15133 0.599656325
GAPDHP41 ENSG00000277172 0.65314 0.25683 1.0307 0.15133 -0.67886
0.75139 -0.62344 0.7335 0.15133 0.599656325
RNU4ATAC18P ENSG00000251988 0.037934 0.48487 1.0307 0.15134 0.037934
0.48487 0.037934 0.48487 0.15134 0.599656325
SYT6 ENSG00000134207 1.0307 0.15135 -1.3802 0.91623 -1.3802 0.91623
-1.3802 0.91623 0.15135 0.599656325
GSX2 ENSG00000180613 1.0306 0.15136 -0.64354 0.74006 -0.64354 0.74006
-0.64354 0.74006 0.15136 0.599656325
NA ENSG00000255381 1.0306 0.15136 0.2839 0.38824 1.0306 0.15136
1.0306 0.15136 0.15136 0.599656325
SEPTIN8 ENSG00000164402 1.0134 0.15544 1.0306 0.15138 0.83188
0.20274 0.3888 0.34871 0.15138 0.599686806
CDIP1 ENSG00000089486 1.0302 0.15147 1.0302 0.15147 -0.0023279 0.50093
0.48359 0.31434 0.15147 0.599994562
LINC02685 ENSG00000254654 1.03 0.15151 1.03 0.15151 1.03 0.15151 1.03
0.15151 0.15151 0.600104227
NA ENSG00000213514 -0.64004 0.73893 -0.73137 0.76772 -0.73137 0.76772
1.0299 0.15153 0.15153 0.600134664
TANC2 ENSG00000170921 0.28193 0.389 1.0298 0.15155 0.1312 0.44781
0.1499 0.44042 0.15155 0.600165095
NA ENSG00000222509 0.60075 0.274 0.60075 0.274 1.0297 0.15158 0.040173

0.48398 0.15158 0.600235121
RNU6-486P ENSG00000199986 -0.59744 0.72489 -0.18457 0.57322 -0.9833
0.83727 1.0296 0.1516 0.1516 0.600247185
NA ENSG00000202251 1.0295 0.15162 -0.015308 0.50611 1.0295 0.15162
1.0295 0.15162 0.15162 0.600247185
RN7SL531P ENSG00000264071 1.0295 0.15162 1.0295 0.15162 0.83196
0.20272 1.0295 0.15162 0.15162 0.600247185
SLC7A15P ENSG00000232800 1.0294 0.15164 0.93852 0.17399 -0.34313
0.63425 0.69132 0.24468 0.15164 0.600252951
ASPSCR1 ENSG00000169696 -0.44703 0.67257 -0.38055 0.64823 -0.31005
0.62174 1.0293 0.15166 0.15166 0.600252951
NA ENSG00000248564 1.0293 0.15167 1.0293 0.15167 1.0293 0.15167
1.0293 0.15167 0.15167 0.600252951
NA ENSG00000269489 1.0292 0.15169 -0.8837 0.81157 -0.8837 0.81157
-0.8837 0.81157 0.15169 0.600252951
LINC02857 ENSG00000230852 1.0292 0.1517 1.0292 0.1517 1.0292
0.1517 0.83797 0.20102 0.1517 0.600252951
TACC2 ENSG00000138162 1.0291 0.15171 1.0291 0.15171 0.027438 0.48906
0.11276 0.45511 0.15171 0.600252951
MIR29B2CHG ENSG00000203709 0.29817 0.38279 -0.46683 0.67969 -0.46683
0.67969 1.0291 0.15172 0.15172 0.600252951
RPS12P26 ENSG00000225193 1.0291 0.15172 -0.60009 0.72578 -1.7753
0.96208 -0.17454 0.56928 0.15172 0.600252951
ABCD3 ENSG00000117528 0.43912 0.33029 0.81337 0.208 1.0289 0.15175
0.75512 0.22509 0.15175 0.600313137
RNU7-128P ENSG00000253056 1.0288 0.15178 0.83387 0.20218 0.83387
0.20218 0.83387 0.20218 0.15178 0.600313137
NA ENSG00000202332 -0.25192 0.59945 1.0287 0.1518 -0.25192 0.59945
-0.25192 0.59945 0.1518 0.600313137
TNS1 ENSG00000079308 0.75586 0.22487 0.91705 0.17956 0.75483 0.22517
1.0287 0.15181 0.15181 0.600313137
RPL27AP5 ENSG00000182383 1.0287 0.15182 1.0287 0.15182 1.0222
0.15333 1.0287 0.15182 0.15182 0.600313137
NA ENSG00000231982 -0.69356 0.75602 -0.69356 0.75602 -0.936 0.82536
1.0287 0.15182 0.15182 0.600313137
NA ENSG00000260030 1.0286 0.15184 1.0286 0.15184 -0.59335 0.72353
-0.85954 0.80498 0.15184 0.600313137
NA ENSG00000201088 1.0285 0.15185 0.30402 0.38056 1.0285 0.15185
1.0285 0.15185 0.15185 0.600313137
NA ENSG00000271565 0.54285 0.29362 1.0285 0.15186 1.0285 0.15186
1.0285 0.15186 0.15186 0.600313137
RPS16P2 ENSG00000213729 0.51233 0.30421 0.51233 0.30421 1.0284
0.15187 0.51233 0.30421 0.15187 0.600313137
ZDHHC18 ENSG00000204160 1.0284 0.15188 0.53591 0.29601 0.93723
0.17432 0.5011 0.30815 0.15188 0.600313137
NA ENSG00000257823 1.0283 0.1519 0.34345 0.36563 1.0283 0.1519
1.0283 0.1519 0.1519 0.600313137
NA ENSG00000243160 1.0282 0.15192 -0.16538 0.56568 -0.88483 0.81188
-0.88483 0.81188 0.15192 0.600313137
AACSP1 ENSG00000250420 -1.6005 0.94526 -1.6526 0.9508 1.0282
0.15192 -0.42386 0.66417 0.15192 0.600313137
NA ENSG00000273759 0.64718 0.25876 1.0282 0.15192 1.0282 0.15192
1.0282 0.15192 0.15192 0.600313137
PHAX ENSG00000164902 0.0019258 0.49923 1.0281 0.15195 1.005 0.15746
0.95866 0.16886 0.15195 0.600373828
LINC02644 ENSG00000230720 0.75247 0.22589 1.0281 0.15196 1.0031
0.1579 -1.4251 0.92293 0.15196 0.600373828
MIR582 ENSG00000202601 0.6279 0.26503 1.0277 0.15203 0.10909
0.45656 -0.58722 0.72147 0.15203 0.600437318
NA ENSG00000250999 1.0278 0.15203 0.33406 0.36917 0.2748 0.39173
0.78906 0.21504 0.15203 0.600437318
CYP39A1 ENSG00000146233 0.037947 0.48486 0.86593 0.19326 0.44508

							0.32813	1.0277	0.15204	0.15204	0.600437318
LINC02112	ENSG00000249781	0.47206	0.31844	1.0277	0.15204	-0.20997	0.58315	0.51087	0.30472	0.15204	0.600437318
IRF5P1	ENSG00000251347	1.0277	0.15204	0.78894	0.21507	0.98361	0.16265	0.98361	0.16265	0.15204	0.600437318
CACNA2D4	ENSG00000151062	0.89597	0.18513	1.0277	0.15205	0.65273	0.25696	0.49908	0.30886	0.15205	0.600437318
PRKCE	ENSG00000171132	0.60215	0.27354	0.58234	0.28017	0.64352	0.25994	1.0276	0.15208	0.15208	0.600475287
MICALL1	ENSG00000100139	0.19919	0.42106	1.0274	0.15211	0.19919	0.42106	1.0224	0.1533	0.15211	0.600475287
SFXN4	ENSG00000183605	0.99048	0.16097	0.91067	0.18124	1.0274	0.15211	-0.26019	0.60264	0.15211	0.600475287
RN7SKP52	ENSG00000252028	0.13539	0.44615	1.0274	0.15212	0.13539	0.44615	0.13539	0.44615	0.15212	0.600475287
NA	ENSG00000225325	1.0272	0.15217	0.51745	0.30242	0.15924	0.43674	1.0106	0.1561	0.15217	0.600475287
RN7SKP180	ENSG00000200783	1.0271	0.15219	1.0271	0.15219	1.0271	0.15219	1.0271	0.15219	0.15219	0.600475287
RPL36AP48	ENSG00000213180	1.0271	0.15219	1.0271	0.15219	0.26612	0.39507	1.0271	0.15219	0.15219	0.600475287
NA	ENSG00000264421	1.0271	0.15219	1.0271	0.15219	1.0271	0.15219	1.0271	0.15219	0.15219	0.600475287
LINC00659	ENSG00000228705	0.77908	0.21797	0.77164	0.22016	0.85078	0.19744	1.0271	0.1522	0.1522	0.600475287
NA	ENSG00000229797	-0.44629	0.6723	-0.64014	0.73896	1.027	0.1522	1.027	0.1522	0.1522	0.600475287
PPP1R42	ENSG00000178125	-0.19089	0.5757	-0.18616	0.57384	-0.28846	0.6135	1.027	0.15221	0.15221	0.600475287
ARF1P1	ENSG00000219807	1.027	0.15222	1.027	0.15222	1.027	0.15222	1.027	0.15222	0.15222	0.600475287
SLC43A1	ENSG00000149150	0.70004	0.24195	-0.52032	0.69858	0.79921	0.21208	1.0269	0.15223	0.15223	0.600475287
NA	ENSG00000228876	0.10183	0.45945	1.0268	0.15225	0.38481	0.35019	-0.60067	0.72597	0.15225	0.600475287
MAFTRR	ENSG00000261390	-0.65413	0.74348	1.0268	0.15225	0.34052	0.36673	1.0268	0.15225	0.15225	0.600475287
MIR548H5	ENSG00000266807	0.17757	0.42953	-0.21366	0.58459	0.17757	0.42953	1.0267	0.15227	0.15227	0.600475287
NA	ENSG00000266743	0.72619	0.23386	1.0267	0.15229	0.54246	0.29375	1.0267	0.15229	0.15229	0.600475287
YPEL3	ENSG00000090238	1.0265	0.15233	1.0265	0.15233	-0.32854	0.62875	1.0265	0.15233	0.15233	0.600475287
GPN2	ENSG00000142751	0.54777	0.29192	1.0265	0.15233	0.49081	0.31178	0.65243	0.25706	0.15233	0.600475287
NA	ENSG00000250616	1.0265	0.15233	1.0265	0.15233	1.0265	0.15233	1.0265	0.15233	0.15233	0.600475287
NA	ENSG00000283029	1.0265	0.15233	1.0265	0.15233	1.0265	0.15233	1.0265	0.15233	0.15233	0.600475287
NA	ENSG00000270127	1.0264	0.15234	-0.18413	0.57304	-0.31441	0.62339	-0.31441	0.62339	0.15234	0.600475287
NA	ENSG00000238502	1.0264	0.15235	1.0264	0.15235	1.0264	0.15235	1.0264	0.15235	0.15235	0.600475287
RAB33B	ENSG00000172007	0.68948	0.24526	0.68948	0.24526	0.68948	0.24526	1.0264	0.15236	0.15236	0.600475287
UBE2E2	ENSG00000182247	0.63223	0.26362	0.50177	0.30792	-0.32884	0.62886	1.0263	0.15238	0.15238	0.600475287
NA	ENSG00000270710	-0.79619	0.78704	-0.79619	0.78704	1.0263	0.15238	0.19714	0.42186	0.15238	0.600475287
TMEM104	ENSG00000109066	0.90656	0.18232	0.85632	0.19591	1.0003	0.15859	1.0258	0.15248	0.15248	0.600763012
LINC01728	ENSG00000233277	1.0258	0.15249	1.0258	0.15249	1.0258					

0.15249 1.0258 0.15249 0.15249 0.600763012
PNMA8C ENSG00000277531 -0.69076 0.75514 -0.45402 0.67509 1.0258
0.15249 1.0258 0.15249 0.15249 0.600763012
TRIML2 ENSG00000179046 1.0256 0.15253 0.80071 0.21165 1.0256
0.15253 0.53331 0.29691 0.15253 0.600792142
LINC02192 ENSG00000261325 -0.77409 0.78056 1.0256 0.15255 -0.33492
0.63116 -0.19446 0.57709 0.15255 0.600792142
GOLGA8A ENSG00000175265 1.0254 0.15258 -0.65645 0.74423 -0.49783
0.6907 -0.24796 0.59792 0.15258 0.600792142
NA ENSG00000236712 -0.37815 0.64734 1.0254 0.15258 1.0254 0.15258
1.0254 0.15258 0.15258 0.600792142
ARHGEF15 ENSG00000198844 0.68076 0.24801 1.0254 0.15259 0.96335
0.16768 0.64896 0.25818 0.15259 0.600792142
NA ENSG00000261362 1.0254 0.15259 0.85111 0.19735 -1.3918 0.918
-0.51377 0.69629 0.15259 0.600792142
NA ENSG00000271137 1.0254 0.1526 1.0254 0.1526 1.0254 0.1526
1.0254 0.1526 0.1526 0.600792142
LINC01977 ENSG00000262772 0.69217 0.24442 0.69217 0.24442 0.84909
0.19792 1.0253 0.15262 0.15262 0.600792142
SCMH1 ENSG00000010803 0.96244 0.16792 1.0252 0.15265 0.7145 0.23746
0.76111 0.2233 0.15265 0.600792142
TRIM77 ENSG00000214414 -0.89401 0.81434 -1.0888 0.86189 -0.80292
0.78899 1.0251 0.15266 0.15266 0.600792142
NA ENSG00000235495 1.0251 0.15266 0.71091 0.23857 1.0251 0.15266
1.0251 0.15266 0.15266 0.600792142
RNA5SP268 ENSG00000252637 1.0251 0.15266 1.0251 0.15266 1.0251
0.15266 1.0251 0.15266 0.15266 0.600792142
NA ENSG00000261261 1.0251 0.15266 0.90739 0.1821 1.0251 0.15266
1.0251 0.15266 0.15266 0.600792142
RNA5SP309 ENSG00000252479 -0.37629 0.64665 -1.343 0.91036 1.0251
0.15267 1.0251 0.15267 0.15267 0.600792142
PPP2R3C ENSG00000092020 1.0249 0.15271 0.77801 0.21828 0.29207
0.38512 -0.45366 0.67496 0.15271 0.600836119
VTRNA1-3 ENSG00000202515 1.0247 0.15275 -0.8681 0.80733 -0.8981
0.81543 -0.53765 0.70459 0.15275 0.600836119
OR7E59P ENSG00000250561 1.0247 0.15275 -0.31421 0.62332 0.77122
0.22029 -0.95284 0.82966 0.15275 0.600836119
NA ENSG00000199392 -1.5359 0.93771 1.0246 0.15279 0.092174 0.46328
0.059477 0.47629 0.15279 0.600836119
RLBP1 ENSG00000140522 -0.40043 0.65558 -0.40043 0.65558 -0.40043 0.65558
1.0245 0.1528 0.1528 0.600836119
MAB21L4 ENSG00000172478 0.23783 0.40601 0.56564 0.28582 1.0245
0.1528 0.15341 0.43904 0.1528 0.600836119
RAD52 ENSG00000002016 -0.083066 0.5331 0.38416 0.35043 0.73641 0.23074
1.0245 0.15281 0.15281 0.600836119
DRAIC ENSG00000245750 0.82065 0.20592 0.54824 0.29176 1.0244 0.15282
0.38357 0.35065 0.15282 0.600836119
SNRPCP8 ENSG00000250580 -0.11657 0.5464 1.0243 0.15284 0.49515
0.31025 -0.28359 0.61164 0.15284 0.600836119
NA ENSG00000257831 1.0243 0.15285 1.0243 0.15285 1.0243 0.15285
-0.70262 0.75885 0.15285 0.600836119
NA ENSG00000226785 0.451 0.326 -0.61276 0.72998 1.0242 0.15286 -0.14867
0.55909 0.15286 0.600836119
ANP32B ENSG00000136938 0.029204 0.48835 -1.2862 0.90081 -1.2862
0.90081 1.0242 0.15287 0.15287 0.600836119
RNU6-1037P ENSG00000238680 1.0242 0.15287 1.0242 0.15287 1.0242
0.15287 1.0242 0.15287 0.15287 0.600836119
PROX1 ENSG00000117707 -0.79385 0.78636 1.0242 0.15288 0.76589 0.22187
-0.08433 0.5336 0.15288 0.600836119
ZNF329 ENSG00000181894 1.0241 0.15289 0.72628 0.23383 0.97869
0.16387 -0.44285 0.67106 0.15289 0.600836119
DPPA3 ENSG00000187569 0.60896 0.27128 -0.16255 0.56456 1.0241 0.1529

0.99589 0.15965 0.1529 0.600836119
RNU6-911P ENSG00000252332 -0.13882 0.55521 1.0241 0.1529 -0.13882
0.55521 -0.13882 0.55521 0.1529 0.600836119
PEX6 ENSG00000124587 0.71708 0.23666 0.71443 0.23748 1.0239 0.15294
0.51091 0.30471 0.15294 0.600836119
GOLGA6L3 ENSG00000188388 0.078157 0.46885 -0.30473 0.61972 1.0239
0.15294 -0.14183 0.55639 0.15294 0.600836119
NA ENSG00000277200 1.0239 0.15295 1.0239 0.15295 1.0239 0.15295
1.0239 0.15295 0.15295 0.600836119
CHMP6 ENSG00000176108 -0.41501 0.66093 -0.41501 0.66093 -0.46461 0.67889
1.0237 0.15298 0.15298 0.600836119
HMGB4 ENSG00000176256 -0.31945 0.62531 0.98128 0.16323 0.81371 0.2079
1.0237 0.15299 0.15299 0.600836119
TAB1 ENSG00000100324 -0.29988 0.61787 1.0236 0.15301 1.0236 0.15301
-0.0020066 0.5008 0.15301 0.600836119
CHCHD2P9 ENSG00000186940 0.39642 0.3459 0.13108 0.44786 0.55468
0.28956 1.0235 0.15304 0.15304 0.600836119
VDAC2 ENSG00000165637 0.16562 0.43423 1.0234 0.15305 0.16562 0.43423
0.66538 0.2529 0.15305 0.600836119
MYOM3 ENSG00000142661 1.0234 0.15306 -1.1973 0.88441 0.78329 0.21673
0.076392 0.46955 0.15306 0.600836119
TALDO1 ENSG00000177156 0.23861 0.40571 0.28175 0.38907 -0.098087
0.53907 1.0234 0.15306 0.15306 0.600836119
RPS7P6 ENSG00000215278 1.0234 0.15307 -0.028507 0.51137 -0.23708
0.5937 -0.040935 0.51633 0.15307 0.600836119
MRPL37 ENSG00000116221 1.0233 0.15308 1.0233 0.15308 -0.24634
0.59729 -0.99815 0.8409 0.15308 0.600836119
PRKCB ENSG00000166501 0.38694 0.3494 1.0233 0.15308 -0.21966 0.58693
0.44333 0.32876 0.15308 0.600836119
TMEM9B ENSG00000175348 0.7127 0.23802 0.7127 0.23802 1.0233
0.15309 0.7127 0.23802 0.15309 0.600836119
NA ENSG00000249316 0.55506 0.28943 0.3822 0.35116 1.0232 0.15311
0.64324 0.26003 0.15311 0.600836119
TMEM256P2 ENSG00000226726 -1.3206 0.90668 0.82606 0.20438 -1.3206
0.90668 1.0232 0.15312 0.15312 0.600836119
RPL23AP63 ENSG00000243721 1.0111 0.15599 1.0232 0.15312 1.0232
0.15312 1.0232 0.15312 0.15312 0.600836119
CHTF8 ENSG00000168802 1.023 0.15316 -1.549 0.9393 -1.549 0.9393
-1.549 0.9393 0.15316 0.600836119
DDX54 ENSG00000123064 0.93269 0.17549 0.93269 0.17549 0.64283 0.26017
1.0229 0.15317 0.15317 0.600836119
NA ENSG00000270330 1.0229 0.15318 1.0229 0.15318 1.0229 0.15318
1.0229 0.15318 0.15318 0.600836119
KLF7P1 ENSG00000240704 1.0228 0.15319 0.58421 0.27954 0.075415
0.46994 0.65882 0.25501 0.15319 0.600836119
RNU6-1232P ENSG00000252108 0.67891 0.2486 0.37604 0.35344 1.0228
0.1532 1.0228 0.1532 0.1532 0.600836119
GDPD4 ENSG00000178795 -0.15358 0.56103 -0.10059 0.54006 1.0226 0.15324
-0.15358 0.56103 0.15324 0.600836119
TOGARAM2 ENSG00000189350 0.092079 0.46332 1.0226 0.15324 -0.3907
0.65199 1.0223 0.15331 0.15324 0.600836119
NA ENSG00000254869 0.08581 0.46581 1.0226 0.15324 1.0226 0.15324
-0.14289 0.55681 0.15324 0.600836119
MTCO2P27 ENSG00000230321 -0.625 0.73401 1.0226 0.15325 -0.625
0.73401 -0.625 0.73401 0.15325 0.600836119
SPEM2 ENSG00000184560 1.0225 0.15326 1.0225 0.15326 1.0225 0.15326
1.0225 0.15326 0.15326 0.600836119
RNA5SP227 ENSG00000251993 1.0226 0.15326 1.0226 0.15326 1.0226
0.15326 1.0226 0.15326 0.15326 0.600836119
NA ENSG00000269189 1.0225 0.15328 1.0225 0.15328 1.0225 0.15328
1.0225 0.15328 0.15328 0.600836119
MIR5699 ENSG00000263511 -0.26218 0.60341 -2.5098 0.99396 -0.27415

0.60802 1.0224 0.15329 0.15329 0.600836119
RNU6-590P ENSG00000201909 0.011266 0.49551 1.0224 0.1533 0.011266
0.49551 0.011266 0.49551 0.1533 0.600836119
RTRAF ENSG00000087302 1.0224 0.15331 -0.21798 0.58628 -0.21798 0.58628
-0.058773 0.52343 0.15331 0.600836119
C8orf74 ENSG00000171060 1.0223 0.15331 0.34631 0.36455 0.70242
0.24121 -0.31003 0.62173 0.15331 0.600836119
LINC01818 ENSG00000230645 -0.35817 0.63989 1.0223 0.15331 -0.16347
0.56493 0.64121 0.26069 0.15331 0.600836119
GOLT1A ENSG00000174567 1.0223 0.15333 -0.56863 0.7152 1.0223
0.15333 0.058232 0.47678 0.15333 0.600857044
NXF1 ENSG00000162231 -0.4291 0.66608 -0.4291 0.66608 -0.4291 0.66608
1.0222 0.15334 0.15334 0.600857044
RPS10P21 ENSG00000232881 -0.20675 0.5819 0.5365 0.2958 1.0221
0.15336 1.0221 0.15336 0.15336 0.600887095
YBX3P1 ENSG00000261614 0.93887 0.1739 0.85121 0.19733 1.022 0.15338
0.85121 0.19733 0.15338 0.600908007
NA ENSG00000258196 -1.787 0.96303 -1.1522 0.87537 1.022 0.15339 1.022
0.15339 0.15339 0.600908007
NA ENSG00000236669 1.0217 0.15345 0.7354 0.23105 -0.77748 0.78156
0.63214 0.26365 0.15345 0.601060749
ZSCAN32 ENSG00000140987 0.89584 0.18517 0.38853 0.34881 0.62224
0.26689 1.0217 0.15346 0.15346 0.601060749
LINC01578 ENSG00000272888 0.72204 0.23514 0.83432 0.20205 0.82691
0.20414 1.0217 0.15347 0.15347 0.601060749
GLYATL1B ENSG00000255151 0.37895 0.35236 0.82551 0.20454 0.66247
0.25384 1.0216 0.15349 0.15349 0.601060749
PCSK1 ENSG00000175426 0.43418 0.33208 1.0215 0.15351 -0.45006 0.67367
-0.0132 0.50527 0.15351 0.601060749
RUNDC3A ENSG00000108309 0.5718 0.28373 0.75159 0.22615 0.74128
0.22926 1.0215 0.15352 0.15352 0.601060749
PLEKHH3 ENSG00000068137 -1.4114 0.92094 -1.4114 0.92094 -1.4114
0.92094 1.0214 0.15353 0.15353 0.601060749
CYP2AC1P ENSG00000216998 0.92942 0.17633 1.0214 0.15353 0.23362
0.40764 -0.28409 0.61183 0.15353 0.601060749
NA ENSG00000265946 1.0213 0.15354 1.0213 0.15354 0.63523 0.26264
1.0213 0.15354 0.15354 0.601060749
NKD1 ENSG00000140807 1.0213 0.15357 -0.63248 0.73646 0.25186 0.40057
0.66174 0.25407 0.15357 0.601129898
FAM227A ENSG00000184949 0.99031 0.16101 1.0211 0.15361 0.58404
0.2796 0.62217 0.26692 0.15361 0.601133593
NA ENSG00000282890 1.021 0.15363 0.44136 0.32948 0.29843 0.38269 1.021
0.15363 0.15363 0.601133593
NA ENSG00000237445 1.0209 0.15364 0.68175 0.2477 0.70546 0.24026
0.12179 0.45153 0.15364 0.601133593
AIRE ENSG00000160224 0.51036 0.3049 1.0209 0.15366 0.5324 0.29723
0.57772 0.28173 0.15366 0.601133593
LRRC61 ENSG00000127399 0.25707 0.39856 1.0208 0.15367 0.23367
0.40762 0.33509 0.36878 0.15367 0.601133593
PTX3 ENSG00000163661 -0.5167 0.69732 -0.3272 0.62824 -0.22895 0.59054
1.0208 0.15367 0.15367 0.601133593
NABP1 ENSG00000173559 1.0208 0.15368 -0.17585 0.56979 1.0208 0.15368
-0.031243 0.51246 0.15368 0.601133593
YPEL1 ENSG00000100027 0.39185 0.34758 -1.6839 0.9539 1.0208 0.15369
-0.12744 0.55071 0.15369 0.601133593
RMI2 ENSG00000175643 -1.7683 0.96149 -1.444 0.92563 1.0207 0.15369
-2.3862 0.99149 0.15369 0.601133593
FGF10 ENSG00000070193 0.89184 0.18624 -0.26683 0.6052 1.0206 0.15372
0.9797 0.16362 0.15372 0.601133593
QDPR ENSG00000151552 0.43458 0.33193 -0.19399 0.57691 1.0205 0.15375
-1.1734 0.87969 0.15375 0.601133593
RN7SL265P ENSG00000241709 0.090992 0.46375 1.0204 0.15376 0.090992

0.46375 0.090992 0.46375 0.15376 0.601133593
PLGLA ENSG00000240935 -0.99736 0.84071 1.0204 0.15377 -1.1802 0.88104
-1.1802 0.88104 0.15377 0.601133593
FOXP4-AS1 ENSG00000234753 0.80068 0.21166 0.72399 0.23454 1.0203
0.15378 -0.10022 0.53991 0.15378 0.601133593
LINC01558 ENSG00000146521 0.51061 0.30481 1.0203 0.15379 -0.01441
0.50575 -0.81279 0.79183 0.15379 0.601133593
SLC27A2 ENSG00000140284 0.66849 0.25191 -0.74217 0.77101 1.0202
0.15381 0.16811 0.43325 0.15381 0.601133593
JCAD ENSG00000165757 0.49582 0.31001 -0.44213 0.6708 0.45095 0.32601
1.0202 0.15381 0.15381 0.601133593
NA ENSG00000260878 0.79763 0.21254 0.79763 0.21254 1.0202 0.15381
-0.13026 0.55182 0.15381 0.601133593
COPS5P1 ENSG00000213187 1.0202 0.15382 1.0202 0.15382 1.0202
0.15382 1.0202 0.15382 0.15382 0.601133593
NA ENSG00000242615 1.0202 0.15382 1.0202 0.15382 1.0202 0.15382
1.0202 0.15382 0.15382 0.601133593
NA ENSG00000255977 1.0201 0.15383 1.0201 0.15383 -1.091 0.86237
-0.32736 0.6283 0.15383 0.601133593
NA ENSG00000234206 1.0201 0.15385 1.0201 0.15385 1.0201 0.15385
1.0201 0.15385 0.15385 0.601163539
SV2B ENSG00000185518 -0.05438 0.52168 0.26437 0.39575 1.02 0.15387
-0.18073 0.57171 0.15387 0.601193481
POLR2C ENSG00000102978 -0.73807 0.76977 -0.73807 0.76977 -0.48532
0.68628 1.0198 0.15392 0.15392 0.601275074
NA ENSG00000267693 1.0198 0.15392 1.0198 0.15392 1.0198 0.15392
1.0198 0.15392 0.15392 0.601275074
MIR4787 ENSG00000272543 0.95335 0.17021 1.0196 0.15396 0.62654
0.26548 0.62654 0.26548 0.15396 0.601275074
NA ENSG00000218048 1.0195 0.15397 0.63935 0.2613 -0.83731 0.79879
-0.021246 0.50848 0.15397 0.601275074
NA ENSG00000249262 -0.40815 0.65842 -1.3816 0.91645 -0.40815 0.65842
1.0195 0.15397 0.15397 0.601275074
GNB1 ENSG00000078369 0.71448 0.23747 0.49181 0.31143 1.0195 0.15398
-0.55051 0.70901 0.15398 0.601275074
HMGN2P24 ENSG00000229072 1.0194 0.154 0.96633 0.16694 0.67354 0.2503
-0.083096 0.53311 0.154 0.601275074
NA ENSG00000275115 0.10841 0.45684 1.0194 0.154 1.0194 0.154 1.0194
0.154 0.154 0.601275074
NA ENSG00000229853 -2.1946 0.9859 0.81728 0.20689 1.0193 0.15403
1.0193 0.15403 0.15403 0.601275074
NA ENSG00000280400 1.0193 0.15404 1.0193 0.15404 1.0193 0.15404
1.0193 0.15404 0.15404 0.601275074
NA ENSG00000233884 -0.0064871 0.50259 -0.0064871 0.50259 -0.0064871 0.50259
1.0192 0.15406 0.15406 0.601275074
PDXP ENSG00000241360 1.0192 0.15406 1.0192 0.15406 1.0192 0.15406
1.0192 0.15406 0.15406 0.601275074
SERPINA12 ENSG00000165953 -0.64751 0.74135 0.86658 0.19309 1.0191
0.15408 1.0191 0.15408 0.15408 0.601275074
NA ENSG00000271882 -0.60284 0.72669 0.25691 0.39862 1.0191 0.15408
-0.60284 0.72669 0.15408 0.601275074
KIZ-AS1 ENSG00000232712 1.0191 0.15409 1.0191 0.15409 1.0191
0.15409 1.0191 0.15409 0.15409 0.601275074
NA ENSG00000273584 1.0191 0.15409 0.8848 0.18813 1.0191 0.15409
1.0191 0.15409 0.15409 0.601275074
LINC02153 ENSG00000253199 1.0189 0.15412 0.59332 0.27648 -0.37221
0.64513 0.039287 0.48433 0.15412 0.601275074
REX1BD ENSG00000006015 1.0189 0.15413 -0.29408 0.61565 -0.29408
0.61565 0.82091 0.20585 0.15413 0.601275074
NA ENSG00000260611 0.7593 0.22384 0.75033 0.22653 0.67169 0.25089
1.0188 0.15414 0.15414 0.601275074
MTATP6P24 ENSG00000262381 1.0188 0.15415 1.0188 0.15415 1.0188

0.15415	1.0188	0.15415	0.15415	0.601275074
NA	ENSG00000272328 1.0188	0.15415	1.0188	0.15415	1.0188	0.15415
1.0188	0.15415	0.15415	0.601275074
MEP1A ENSG00000112818 -0.73548	0.76898	1.0187	0.15418	-0.55569	0.71079
-0.38424	0.6496	0.15418	0.601289216
RNU7-24P	ENSG00000239081 1.0185	0.15421	-1.9258	0.97294	-1.9258
0.97294	-1.164	0.8778	0.15421	0.601289216
PSMD7 ENSG00000103035 0.060185	0.476 1.0185	0.15423	-1.4744	0.92981
-0.94772	0.82836	0.15423	0.601289216
DPH3P1	ENSG00000233838 1.0184	0.15423	1.0184	0.15423	1.0184
0.15423	1.0184	0.15423	0.15423	0.601289216
RNA5SP262	ENSG00000252261 -1.6157	0.94692	1.0184	0.15423	0.71942
0.23594	-0.46423	0.67876	0.15423	0.601289216
RN7SL488P	ENSG00000264271 1.0184	0.15424	1.0184	0.15424	1.0184
0.15424	1.0184	0.15424	0.15424	0.601289216
MIR3922	ENSG00000264295 1.0184	0.15424	1.0184	0.15424	1.0184
0.15424	0.27694	0.39091	0.15424	0.601289216
NA	ENSG00000259655 0.4727	0.31821	-1.486	0.93136	-0.3352	0.63126
1.0183	0.15426	0.15426	0.60130084
NA	ENSG00000258510 0.4568	0.32391	0.4568	0.32391	0.4568	0.32391
1.0183	0.15427	0.15427	0.60130084
NA	ENSG00000213940 1.0183	0.15428	0.062013	0.47528	-0.77004	0.77936
0.062013	0.47528	0.15428	0.60130084
NA	ENSG00000238176 0.060899	0.47572	0.028726	0.48854	-0.1257	0.55001
1.0181	0.15431	0.15431	0.601360544
CMAHP ENSG00000168405 0.69387	0.24388	1.0181	0.15432	-0.34871	0.63635
0.68073	0.24802	0.15432	0.601360544
NA	ENSG00000217576 1.0179	0.15435	0.21946	0.41315	0.44646	0.32763
0.075554	0.46989	0.15435	0.601385301
PRR36 ENSG00000183248 1.0178	0.15438	0.62685	0.26538	1.0178	0.15438
0.010134	0.49596	0.15438	0.601385301
NA	ENSG00000279015 1.0178	0.15438	0.432 0.33287	0.7347	0.23126
0.7347	0.23126	0.15438	0.601385301
NA	ENSG00000260715 -0.53428	0.70343	1.0177	0.15441	0.0837	0.46665
1.0177	0.15441	0.15441	0.601385301
TTC22 ENSG00000006555 0.39711	0.34564	-0.76489	0.77783	1.0176	0.15443
-0.57545	0.71751	0.15443	0.601385301
NA	ENSG00000260148 1.0176	0.15443	0.66672	0.25248	0.66672	0.25248
0.66672	0.25248	0.15443	0.601385301
NA	ENSG00000260975 -1.0596	0.85533	0.32982	0.37077	-1.0596	0.85533
1.0176	0.15443	0.15443	0.601385301
NA	ENSG00000267558 1.0175	0.15445	1.0175	0.15445	1.0175	0.15445
1.0175	0.15445	0.15445	0.601385301
FOXO6 ENSG00000204060 0.22392	0.41141	1.0175	0.15447	0.42243	0.33636
0.038364	0.4847	0.15447	0.601385301
NA	ENSG00000280387 1.0174	0.15449	1.0174	0.15449	1.0174	0.15449
1.0174	0.15449	0.15449	0.601385301
MIR550A3	ENSG00000212024 -0.22787	0.59013	-0.4663	0.6795	-0.016072
0.50641	1.0173	0.15451	0.15451	0.601385301
PCBD2 ENSG00000132570 -0.43438	0.66799	-0.3383	0.63243	-1.5048	0.93381
1.0173	0.15452	0.15452	0.601385301
RN7SKP137	ENSG00000223006 1.0172	0.15453	-0.55482	0.71049	0.43783
0.33076	0.20141	0.42019	0.15453	0.601385301
NA	ENSG00000201830 1.0171	0.15454	1.0171	0.15454	1.0171	0.15454
1.0171	0.15454	0.15454	0.601385301
ISCA2P1	ENSG00000226912 1.0171	0.15454	1.0171	0.15454	1.0171
0.15454	1.0171	0.15454	0.15454	0.601385301
NA	ENSG00000252021 -0.80165	0.78862	1.0171	0.15454	0.27943	0.38996
1.0171	0.15454	0.15454	0.601385301
ITLN2 ENSG00000158764 1.0171	0.15455	1.0171	0.15455	0.79855	0.21227
0.30751	0.37923	0.15455	0.601385301
DLG4	ENSG00000132535 0.19754	0.4217	0.19754	0.4217	0.42737	0.33455

1.017 0.15457 0.15457 0.601385301
DEFA7P ENSG00000206042 1.017 0.15457 1.017 0.15457 1.017 0.15457 1.017
0.15457 0.15457 0.601385301
NA ENSG00000277270 0.40123 0.34412 0.46993 0.3192 1.017 0.15458
0.59168 0.27703 0.15458 0.601385301
NA ENSG00000220110 -0.4495 0.67346 1.0168 0.15463 0.57372 0.28308
0.38947 0.34846 0.15463 0.601385301
NA ENSG00000255273 -0.95337 0.8298 -0.29087 0.61442 0.21592 0.41452
1.0168 0.15463 0.15463 0.601385301
PTPRG ENSG00000144724 0.74155 0.22918 0.62377 0.26639 0.72359 0.23466
1.0167 0.15464 0.15464 0.601385301
EGFLAM ENSG00000164318 0.35091 0.36283 0.28178 0.38906 1.0168
0.15464 0.51229 0.30422 0.15464 0.601385301
TCEA2 ENSG00000171703 0.61526 0.26919 1.0166 0.15466 0.91166 0.18097
-0.77302 0.78025 0.15466 0.601385301
ECE1 ENSG00000117298 0.39348 0.34698 0.8308 0.20304 1.0165 0.15469
0.82888 0.20359 0.15469 0.601385301
POLK ENSG00000122008 -1.6122 0.94654 -1.6122 0.94654 -1.6122 0.94654
1.0165 0.1547 0.1547 0.601385301
NA ENSG00000255127 1.0165 0.1547 1.0165 0.1547 1.0165 0.1547
1.0165 0.1547 0.1547 0.601385301
RAI14 ENSG00000039560 1.0164 0.15471 0.27533 0.39153 0.16598 0.43409
0.43486 0.33183 0.15471 0.601385301
RPL3L ENSG00000140986 -1.2002 0.88497 -1.2002 0.88497 -1.2002 0.88497
1.0164 0.15472 0.15472 0.601385301
LINC00315 ENSG00000184274 -0.69049 0.75506 0.77815 0.21824 0.362 0.35868
1.0163 0.15474 0.15474 0.601385301
CDS1 ENSG00000163624 0.9449 0.17235 1.0163 0.15475 0.9449 0.17235
0.3224 0.37357 0.15475 0.601385301
HHLA2 ENSG00000114455 1.0162 0.15477 0.068548 0.47267 0.29467 0.38412
0.36984 0.35575 0.15477 0.601385301
NPR1 ENSG00000169418 -0.50477 0.69314 0.76714 0.2215 0.48177 0.31498
1.0162 0.15477 0.15477 0.601385301
NA ENSG00000280154 1.0162 0.15477 1.0162 0.15477 1.0162 0.15477
1.0162 0.15477 0.15477 0.601385301
IDH2-DT ENSG00000259685 1.0161 0.15479 1.0161 0.15479 1.0161
0.15479 0.74674 0.22761 0.15479 0.601385301
RNU7-25P ENSG00000252353 1.0161 0.1548 1.0161 0.1548 1.0161
0.1548 1.0161 0.1548 0.1548 0.601385301
CEACAMP7 ENSG00000227349 1.002 0.15816 1.002 0.15816 1.016 0.15481 0.37734
0.35296 0.15481 0.601385301
HNRNPFP1 ENSG00000227538 1.016 0.15482 0.114 0.45462 0.619 0.26796 0.28485
0.38788 0.15482 0.601385301
NA ENSG00000269439 1.016 0.15482 1.016 0.15482 1.016 0.15482 1.016 0.15482
0.15482 0.601385301
ST7-AS2 ENSG00000226367 1.0159 0.15484 0.91081 0.1812 1.0093
0.15641 1.0093 0.15641 0.15484 0.601415049
ZNF213 ENSG00000085644 1.0158 0.15486 0.6654 0.2529 1.0158
0.15486 1.0158 0.15486 0.15486 0.601444792
ANKRD16 ENSG00000134461 1.0155 0.15494 0.6325 0.26353 0.65554
0.25606 0.60495 0.27261 0.15494 0.601689307
RPL7P9 ENSG00000137970 1.0154 0.15496 0.51443 0.30347 0.407 0.34201
1.0154 0.15496 0.15496 0.601689307
HSPBAP1 ENSG00000169087 -0.39194 0.65245 1.0154 0.15496 1.0154
0.15496 1.0154 0.15496 0.15496 0.601689307
NA ENSG00000257157 0.46583 0.32067 -0.14636 0.55818 1.0153 0.15499
0.73425 0.2314 0.15499 0.601742546
RPL39P36 ENSG00000239912 1.015 0.15504 1.015 0.15504 1.015 0.15504 1.015
0.15504 0.15504 0.601742546
NA ENSG00000252236 1.015 0.15504 0.42699 0.33469 0.16472 0.43458
0.16472 0.43458 0.15504 0.601742546
ASH1L ENSG00000116539 0.5035 0.30731 0.6423 0.26034 0.6765 0.24936

1.015 0.15506 0.15506 0.601742546
SCN7A ENSG00000136546 -0.95349 0.82983 1.015 0.15506 -1.5102 0.93451
-0.96737 0.83332 0.15506 0.601742546
RNA5SP304 ENSG00000223131 1.0149 0.15508 -0.24861 0.59817 -0.32291
0.62662 -0.32291 0.62662 0.15508 0.601742546
CYP4F11 ENSG00000171903 -0.60042 0.72589 -0.27139 0.60695 1.0149
0.15509 -1.1292 0.87059 0.15509 0.601742546
ATP8A2P2 ENSG00000229800 1.0148 0.1551 1.0148 0.1551 1.0148
0.1551 0.4713 0.31871 0.1551 0.601742546
NA ENSG00000240888 1.0147 0.15513 1.0147 0.15513 1.0147 0.15513
1.0147 0.15513 0.15513 0.601742546
RNU6-1294P ENSG00000251804 1.0146 0.15515 1.0146 0.15515 1.0146
0.15515 1.0146 0.15515 0.15515 0.601742546
NA ENSG00000265712 1.0146 0.15515 0.34234 0.36605 0.18151 0.42798
0.15666 0.43776 0.15515 0.601742546
NA ENSG00000226919 1.0146 0.15516 0.45429 0.32481 0.5334 0.29688
-0.3658 0.64274 0.15516 0.601742546
AKT3 ENSG00000117020 0.26539 0.39536 0.017648 0.49296 1.0144 0.15519
0.47498 0.3174 0.15519 0.601742546
NA ENSG00000259683 -0.08091 0.53224 0.35851 0.35998 1.0144 0.1552
1.0144 0.1552 0.1552 0.601742546
SNORD53 ENSG00000265145 1.0143 0.15522 1.0143 0.15522 1.0143
0.15522 1.0143 0.15522 0.15522 0.601742546
SLC22A2 ENSG00000112499 0.52912 0.29836 -1.3937 0.9183 0.84253
0.19975 1.0142 0.15524 0.15524 0.601742546
NA ENSG00000276916 1.0142 0.15524 1.0142 0.15524 1.0142 0.15524
1.0142 0.15524 0.15524 0.601742546
DND1P1 ENSG00000264070 -0.034624 0.51381 -0.16851 0.56691 1.0141
0.15528 -1.3326 0.90866 0.15528 0.601742546
NA ENSG00000201724 -0.019656 0.50784 -0.20467 0.58109 1.014 0.1553
0.088274 0.46483 0.1553 0.601742546
RN7SL560P ENSG00000264438 1.014 0.1553 -0.47326 0.68199 1.014 0.1553 1.014
0.1553 0.1553 0.601742546
SCAP ENSG00000114650 0.005522 0.4978 -0.081568 0.5325 1.0139 0.15531
-0.24501 0.59678 0.15531 0.601742546
HACD1 ENSG00000165996 1.0139 0.15531 -0.51013 0.69502 -0.56583 0.71424
-1.0176 0.84557 0.15531 0.601742546
NA ENSG00000230936 1.0139 0.15532 1.0139 0.15532 1.0139 0.15532
0.72244 0.23501 0.15532 0.601742546
NA ENSG00000253991 -0.17813 0.57069 -0.17813 0.57069 -0.17813 0.57069
1.0139 0.15533 0.15533 0.601742546
NA ENSG00000267255 1.0138 0.15533 1.0138 0.15533 1.0138 0.15533
1.0138 0.15533 0.15533 0.601742546
C3orf14 ENSG00000114405 1.0138 0.15534 -0.09258 0.53688 0.76788
0.22128 -0.18018 0.57149 0.15534 0.601742546
NA ENSG00000271350 -1.6483 0.95036 0.60005 0.27424 1.0138 0.15534
1.0138 0.15534 0.15534 0.601742546
KLHL9 ENSG00000198642 0.73926 0.22987 0.73926 0.22987 1.0137 0.15536
0.71111 0.23851 0.15536 0.601742546
LINC00470 ENSG00000132204 -0.25823 0.60188 0.24343 0.40384 1.0137
0.15537 0.59955 0.2744 0.15537 0.601742546
C17orf98 ENSG00000275489 1.0136 0.15538 1.0136 0.15538 1.0136
0.15538 1.0136 0.15538 0.15538 0.601742546
C8orf58 ENSG00000241852 0.055135 0.47802 1.0135 0.15542 0.62618
0.2656 0.62618 0.2656 0.15542 0.601742546
NA ENSG00000276668 1.0135 0.15542 1.0135 0.15542 1.0135 0.15542
1.0135 0.15542 0.15542 0.601742546
NA ENSG00000270457 0.44173 0.32934 1.0134 0.15544 -0.65632 0.74419
-0.67637 0.7506 0.15544 0.601742546
NA ENSG00000270699 1.0134 0.15544 1.0134 0.15544 1.0134 0.15544
1.0134 0.15544 0.15544 0.601742546
APTX ENSG00000137074 0.32161 0.37388 -0.37397 0.64579 1.0133 0.15545

0.25147 0.40072 0.15545 0.601742546
IL6-AS1 ENSG00000179428 0.66443 0.25321 1.0133 0.15547 0.66443
0.25321 0.66443 0.25321 0.15547 0.601742546
HOTAIR ENSG00000228630 1.0132 0.15547 1.0132 0.15547 1.0132
0.15547 1.0132 0.15547 0.15547 0.601742546
NA ENSG00000271179 -1.0379 0.85033 -1.5475 0.93913 1.0132 0.15547
0.98698 0.16183 0.15547 0.601742546
RCAN2 ENSG00000172348 0.4786 0.31611 -0.55007 0.70887 -0.41798 0.66202
1.0132 0.15548 0.15548 0.601742546
OR2T1 ENSG00000175143 1.0132 0.15548 -1.0278 0.84798 -1.1131 0.86717
0.23452 0.40729 0.15548 0.601742546
ZNF461 ENSG00000197808 0.25453 0.39954 1.0132 0.15548 1.0132
0.15548 1.0132 0.15548 0.15548 0.601742546
CFB ENSG00000243649 1.0129 0.15555 1.0129 0.15555 1.0129 0.15555
1.0129 0.15555 0.15555 0.60175453
NA ENSG00000269302 1.0129 0.15555 1.0129 0.15555 1.0129 0.15555
1.0129 0.15555 0.15555 0.60175453
NA ENSG00000279209 -0.33323 0.63052 -0.33323 0.63052 1.0129 0.15556
0.47549 0.31722 0.15556 0.60175453
NA ENSG00000279304 1.0129 0.15556 1.0129 0.15556 1.0129 0.15556
-0.3486 0.63631 0.15556 0.60175453
CYP2U1 ENSG00000155016 -1.776 0.96213 -1.776 0.96213 -0.53865
0.70494 1.0128 0.15557 0.15557 0.60175453
NA ENSG00000236347 -0.067262 0.52681 1.0128 0.15557 -0.31359 0.62308
0.096761 0.46146 0.15557 0.60175453
NIFKP6 ENSG00000268144 1.0128 0.15558 1.0128 0.15558 1.0128
0.15558 1.0128 0.15558 0.15558 0.60175453
APBA1 ENSG00000107282 0.67718 0.24915 1.0127 0.1556 0.50755 0.30589
0.34005 0.36691 0.1556 0.60175453
SULT1C2 ENSG00000198203 -0.9329 0.82456 -0.66766 0.74783 -0.8846
0.81181 1.0127 0.15561 0.15561 0.60175453
NA ENSG00000200270 1.0125 0.15565 1.0125 0.15565 -0.92336 0.82209
0.4979 0.30928 0.15565 0.60175453
KRT8P37 ENSG00000213771 1.0124 0.15566 1.0124 0.15566 1.0124
0.15566 1.0124 0.15566 0.15566 0.60175453
RNF217-AS1 ENSG00000236548 -0.26534 0.60463 1.0125 0.15566 -0.26534
0.60463 -0.26534 0.60463 0.15566 0.60175453
SRD5A3-AS1 ENSG00000249700 -0.0047501 0.50189 0.004408 0.49824 0.55337
0.29001 1.0123 0.15569 0.15569 0.60175453
NA ENSG00000263821 -0.63791 0.73823 -0.63791 0.73823 1.0124 0.15569
-0.63791 0.73823 0.15569 0.60175453
LINC00355 ENSG00000227674 -0.58955 0.72225 1.0122 0.15571 0.84234
0.1998 -1.4355 0.92443 0.15571 0.60175453
RHAG ENSG00000112077 1.0122 0.15573 0.5979 0.27495 -1.2831 0.90026
0.012391 0.49506 0.15573 0.60175453
RMND5A ENSG00000153561 1.0122 0.15573 0.054509 0.47826 0.66976
0.2515 -0.015635 0.50624 0.15573 0.60175453
C2orf91 ENSG00000205086 0.11663 0.45358 1.0122 0.15573 0.38565
0.34988 -0.12953 0.55153 0.15573 0.60175453
TIAL1 ENSG00000151923 0.30341 0.38079 1.0121 0.15575 0.078948 0.46854
0.048878 0.48051 0.15575 0.60175453
PNKP ENSG00000039650 1.012 0.15577 -0.65322 0.74319 -0.65322 0.74319
-0.65322 0.74319 0.15577 0.60175453
FSTL5 ENSG00000168843 -1.433 0.92406 -0.8282 0.79622 1.012 0.15578
-0.7945 0.78655 0.15578 0.60175453
DDX39B-AS1 ENSG00000234006 1.0119 0.1558 1.0119 0.1558 1.0119
0.1558 1.0119 0.1558 0.1558 0.60175453
NA ENSG00000236335 1.0119 0.1558 1.0119 0.1558 1.0119 0.1558
1.0119 0.1558 0.1558 0.60175453
NA ENSG00000260711 1.0119 0.1558 1.0119 0.1558 1.0119 0.1558
1.0119 0.1558 0.1558 0.60175453
SNORD84 ENSG00000265236 1.0119 0.1558 1.0119 0.1558 1.0119

0.1558 1.0119 0.1558 0.1558 0.60175453
RPAIN ENSG00000129197 1.0117 0.15584 1.0117 0.15584 1.0117 0.15584
1.0117 0.15584 0.15584 0.60175453
PPP6C ENSG00000119414 0.68621 0.24629 0.78217 0.21706 1.0117 0.15585
-1.1614 0.87726 0.15585 0.60175453
ANKRD19P ENSG00000187984 0.83868 0.20082 0.51181 0.30439 1.0115
0.15588 0.20105 0.42033 0.15588 0.60175453
PPIAP68 ENSG00000213739 1.0115 0.15588 0.41529 0.33896 1.0115
0.15588 1.0115 0.15588 0.15588 0.60175453
NA ENSG00000278408 1.0116 0.15588 1.0116 0.15588 1.0116 0.15588
1.0116 0.15588 0.15588 0.60175453
OR7G1 ENSG00000161807 1.0114 0.1559 1.0114 0.1559 1.0114 0.1559
1.0114 0.1559 0.1559 0.60175453
RN7SKP54 ENSG00000222636 1.0115 0.1559 1.0115 0.1559 1.0115
0.1559 1.0115 0.1559 0.1559 0.60175453
DAP3P1 ENSG00000225082 0.8618 0.1944 0.77317 0.21971 1.0115
0.1559 0.71916 0.23602 0.1559 0.60175453
NA ENSG00000248281 1.0114 0.15591 -0.48475 0.68607 -1.2765 0.89912
-0.55987 0.71221 0.15591 0.60175453
KIF23 ENSG00000137807 -0.79706 0.78729 -0.23306 0.59214 -0.15853 0.56298
1.0114 0.15592 0.15592 0.60175453
OR2BH1P ENSG00000272882 -0.21092 0.58353 0.3187 0.37498 1.0113
0.15593 1.0113 0.15593 0.15593 0.60175453
NA ENSG00000206895 1.0113 0.15594 -0.67911 0.75147 -0.62107 0.73272
-0.62107 0.73272 0.15594 0.60175453
ANO9 ENSG00000185101 1.0112 0.15596 0.39234 0.3474 0.54516 0.29282
0.42944 0.3338 0.15596 0.601774988
NA ENSG00000222374 1.0111 0.15597 1.0111 0.15597 1.0111 0.15597
1.0111 0.15597 0.15597 0.601774988
NA ENSG00000260510 1.0111 0.15599 1.0111 0.15599 1.0111 0.15599
1.0111 0.15599 0.15599 0.601804505
LRP1B ENSG00000168702 0.11958 0.45241 -0.63145 0.73613 -0.70013 0.75808
1.0109 0.15603 0.15603 0.601863524
KCNK9 ENSG00000169427 0.29476 0.38409 -0.41969 0.66265 -0.071568 0.52853
1.0109 0.15603 0.15603 0.601863524
STING1 ENSG00000184584 0.30444 0.3804 0.30444 0.3804 1.0108
0.15605 0.30444 0.3804 0.15605 0.601893027
BICD1P1 ENSG00000224346 1.0106 0.1561 1.0106 0.1561 1.0106
0.1561 1.0106 0.1561 0.1561 0.602029147
KRT19P3 ENSG00000249681 1.0106 0.15611 1.0106 0.15611 1.0106
0.15611 -0.3517 0.63747 0.15611 0.602029147
CNTNAP3 ENSG00000106714 1.0105 0.15613 -0.42077 0.66304 -1.4658
0.92865 0.53573 0.29607 0.15613 0.602058629
NA ENSG00000255790 1.0103 0.15616 0.692 0.24447 0.36128 0.35894
-0.38317 0.6492 0.15616 0.602126666
HNRNPKP1 ENSG00000250859 1.0103 0.15618 -0.37692 0.64688 -0.017064
0.50681 -0.11901 0.54736 0.15618 0.602151463
CYCSP27 ENSG00000255444 1.0102 0.1562 1.0102 0.1562 1.0102
0.1562 1.0102 0.1562 0.1562 0.602151463
TOX3 ENSG00000103460 1.0101 0.15622 0.8728 0.19139 0.8728 0.19139
0.8728 0.19139 0.15622 0.602151463
RNU6-1080P ENSG00000206709 0.66438 0.25322 1.01 0.15624 1.01 0.15624 1.01
0.15624 0.15624 0.602151463
FAM185BP ENSG00000214439 1.01 0.15624 0.79538 0.2132 -0.11497 0.54577
0.17342 0.43116 0.15624 0.602151463
RN7SL30P ENSG00000243845 1.0099 0.15626 1.0099 0.15626 0.089317
0.46442 1.0099 0.15626 0.15626 0.602151463
RAB3GAP2 ENSG00000118873 -0.10838 0.54315 0.15387 0.43885 -0.0050372
0.50201 1.0099 0.15628 0.15628 0.602151463
SYT9 ENSG00000170743 -0.64591 0.74083 0.53248 0.2972 0.81019 0.20891
1.0099 0.15628 0.15628 0.602151463
GPR149 ENSG00000174948 -0.73998 0.77035 1.0098 0.15629 0.12031

0.45212 -0.0061426 0.50245 0.15629 0.602151463
NA ENSG00000234104 1.0098 0.15629 -0.19653 0.5779 -0.19653 0.5779
-0.19653 0.5779 0.15629 0.602151463
EXOC5 ENSG00000070367 -0.70624 0.75998 -1.0954 0.86334 1.0097 0.15631
0.38518 0.35005 0.15631 0.602171826
NDUFB9P1 ENSG00000271045 -1.4086 0.92052 1.0097 0.15632 -1.4086
0.92052 -1.4086 0.92052 0.15632 0.602171826
ITGA9 ENSG00000144668 -0.29254 0.61506 0.32366 0.3731 -0.91394 0.81963
1.0094 0.15638 0.15638 0.602355339
MFFP1 ENSG00000215720 -0.30941 0.62149 -0.30941 0.62149 -0.3629 0.64166
1.0093 0.1564 0.1564 0.602384761
NA ENSG00000235728 1.0093 0.15642 1.0093 0.15642 1.0093 0.15642
1.0093 0.15642 0.15642 0.602398112
TRAV12-1 ENSG00000211785 1.0093 0.15643 0.17255 0.4315 0.17255
0.4315 0.17255 0.4315 0.15643 0.602398112
LINC02264 ENSG00000229565 1.0091 0.15645 -0.7673 0.77855 -0.86959
0.80774 -0.62515 0.73406 0.15645 0.602398112
ANKRD10-IT1 ENSG00000229152 1.0091 0.15647 1.0091 0.15647 1.0091
0.15647 1.0091 0.15647 0.15647 0.602398112
NA ENSG00000232245 -1.3063 0.90427 -1.3063 0.90427 -0.83546 0.79827
1.009 0.15648 0.15648 0.602398112
STAT5A ENSG00000126561 1.009 0.15649 0.045348 0.48191 0.13123 0.4478
0.62399 0.26632 0.15649 0.602398112
SAMD9 ENSG00000205413 -0.81994 0.79387 -0.94239 0.827 1.009 0.15649 -1.3497
0.91145 0.15649 0.602398112
MIR4479 ENSG00000266507 1.0088 0.15654 1.0088 0.15654 1.0088
0.15654 1.0088 0.15654 0.15654 0.602413567
MINDY2 ENSG00000128923 0.99122 0.16079 0.44446 0.32836 0.32301
0.37334 1.0087 0.15656 0.15656 0.602413567
NA ENSG00000253208 0.68459 0.2468 0.38986 0.34832 1.0087 0.15656
0.68459 0.2468 0.15656 0.602413567
RPL23AP75 ENSG00000240125 1.0087 0.15657 1.0087 0.15657 1.0087
0.15657 1.0087 0.15657 0.15657 0.602413567
FXR1 ENSG00000114416 -0.032639 0.51302 0.1323 0.44738 1.0086 0.15658
0.047078 0.48123 0.15658 0.602413567
ARL17B ENSG00000228696 0.3047 0.3803 0.12186 0.45151 1.0086
0.15659 0.49744 0.30944 0.15659 0.602413567
NA ENSG00000221753 -0.19303 0.57653 -0.19303 0.57653 -0.19303 0.57653
1.0085 0.1566 0.1566 0.602413567
FAM153CP ENSG00000204677 0.32542 0.37243 -0.23614 0.59334 1.0085
0.15661 -1.3687 0.91445 0.15661 0.602413567
PYGL ENSG00000100504 0.2822 0.38889 1.0085 0.15662 -0.004199 0.50168
-0.40017 0.65549 0.15662 0.602413567
VSTM2B ENSG00000187135 -0.4298 0.66633 0.94624 0.17201 -0.16083
0.56389 1.0084 0.15663 0.15663 0.602413567
PITPNM2-AS1 ENSG00000251497 1.0084 0.15663 1.0084 0.15663 1.0084
0.15663 1.0084 0.15663 0.15663 0.602413567
PTPN14 ENSG00000152104 -0.81866 0.79351 -1.5114 0.93465 1.0082
0.15668 -1.7019 0.95561 0.15668 0.602558314
NA ENSG00000228289 0.43533 0.33166 -1.1013 0.86462 1.008 0.15672
0.89864 0.18442 0.15672 0.602646378
NA ENSG00000238019 0.85406 0.19653 0.98186 0.16308 1.008 0.15673
-0.34863 0.63632 0.15673 0.602646378
RPL32P26 ENSG00000241941 1.0079 0.15674 1.0079 0.15674 1.0079
0.15674 0.16793 0.43332 0.15674 0.602646378
PTGFRN ENSG00000134247 1.0079 0.15676 0.87119 0.19183 0.7923
0.21409 -0.45153 0.6742 0.15676 0.602675724
NA ENSG00000213661 1.0077 0.15679 -0.25441 0.60041 0.22253 0.41195
-0.45324 0.67481 0.15679 0.602743507
NA ENSG00000223727 1.0074 0.15686 -0.50365 0.69275 0.39037 0.34813
-0.15657 0.56221 0.15686 0.602965039
BICD2 ENSG00000185963 0.69509 0.2435 0.3775 0.3529 0.69509 0.2435

								1.0074	0.15688	0.15688	0.602976104
RWDD1	ENSG00000111832	-0.088036	0.53508	1.0073	0.15689	1.0073	0.15689	1.0073	0.15689	0.15689	0.602976104
RNU7-123P	ENSG00000251720	1.0073	0.1569	0.68481	0.24673	0.33939	0.36716	0.3772	0.35301	0.1569	0.602976104
CCDC69	ENSG00000198624	1.0071	0.15693	1.0071	0.15693	1.0071	0.15693	0.76212	0.22299	0.15693	0.602996286
NA	ENSG00000225386	0.0049366	0.49803	1.0071	0.15693	0.0049366	0.49803	0.0049366	0.49803	0.15693	0.602996286
NKX2-8	ENSG00000136327	1.0069	0.157	1.0069	0.157	1.0069	0.157	0.72622	0.23385	0.157	0.60303855
NA	ENSG00000201314	0.90924	0.18161	0.14678	0.44165	0.14678	0.44165	1.0068	0.15701	0.15701	0.60303855
LSR	ENSG00000105699	0.96099	0.16828	0.96099	0.16828	0.96099	0.16828	1.0068	0.15703	0.15703	0.60303855
NOMO2	ENSG00000185164	0.66238	0.25386	1.0067	0.15703	-0.27395	0.60794	-0.49767	0.69064	0.15703	0.60303855
NA	ENSG00000228528	0.69763	0.2427	0.69763	0.2427	1.0067	0.15703	0.69763	0.2427	0.15703	0.60303855
NA	ENSG00000258435	1.0067	0.15703	-0.25992	0.60254	0.88272	0.18869	0.97774	0.1641	0.15703	0.60303855
NIPSNAP1	ENSG00000184117	-0.57981	0.71898	-1.2461	0.89363	1.0067	0.15704	-0.57981	0.71898	0.15704	0.60303855
NA	ENSG00000252461	1.0067	0.15704	1.0067	0.15704	1.0067	0.15704	1.0067	0.15704	0.15704	0.60303855
BHLHE22	ENSG00000180828	0.047454	0.48108	0.16848	0.4331	0.21009	0.4168	1.0066	0.15706	0.15706	0.603067828
CYP3A43	ENSG00000021461	0.21462	0.41503	-0.037816	0.51508	1.0065	0.15709	0.8359	0.20161	0.15709	0.603087978
NA	ENSG00000132832	0.57783	0.28169	-1.7397	0.95904	1.0065	0.15709	-0.56527	0.71405	0.15709	0.603087978
NA	ENSG00000223834	1.0062	0.15715	1.0062	0.15715	1.0062	0.15715	1.0062	0.15715	0.15715	0.603270797
APOL4	ENSG00000100336	1.0061	0.15718	-0.24561	0.59701	0.028301	0.48871	-0.10601	0.54221	0.15718	0.603338432
LSINCT5	ENSG00000281560	-1.3614	0.91331	1.006	0.15722	0.48634	0.31336	0.15344	0.43903	0.15722	0.603426146
RNA5SP144	ENSG00000222778	1.0059	0.15723	0.61514	0.26923	0.61514	0.26923	0.61514	0.26923	0.15723	0.603426146
RN7SL128P	ENSG00000240869	-0.13552	0.5539	-0.13552	0.5539	-0.13552	0.5539	1.0059	0.15724	0.15724	0.603426146
CHRD	ENSG00000090539	-0.32455	0.62724	1.0058	0.15726	-0.90157	0.81636	-1.7384	0.95893	0.15726	0.603455374
NA	ENSG00000207283	-0.65983	0.74532	-0.65983	0.74532	1.0056	0.1573	-0.65983	0.74532	0.1573	0.603552185
NA	ENSG00000231562	1.0056	0.15731	1.0056	0.15731	1.0056	0.15731	1.0056	0.15731	0.15731	0.603552185
NA	ENSG00000270243	0.24114	0.40472	1.0055	0.15733	0.68367	0.24709	-0.52546	0.70037	0.15733	0.603581397
HEY1	ENSG00000164683	0.51199	0.30433	1.0012	0.15836	0.86439	0.19369	1.0054	0.15736	0.15736	0.603648965
YY1	ENSG00000100811	1.0053	0.15738	-0.53327	0.70308	0.34376	0.36551	0.89754	0.18472	0.15738	0.603678164
MAP3K5	ENSG00000197442	0.090104	0.4641	-0.22092	0.58742	1.0051	0.15742	-0.026322	0.5105	0.15742	0.603784069
LINC02237	ENSG00000253434	1.0048	0.15749	-0.093414	0.53721	1.0048	0.15749	0.93261	0.17551	0.15749	0.603979084
IGKV1D-42	ENSG00000211633	0.57036	0.28422	1.0046	0.15754	1.0046	0.15754	1.0046	0.15754	0.15754	0.603979084
NA	ENSG00000226207	0.7667	0.22163	1.0046	0.15754	1.0046	0.15754	-0.41431	0.66067	0.15754	0.603979084
NA	ENSG00000278133	1.0046	0.15754	1.0046	0.15754	1.0046	0.15754				

1.0046 0.15754 0.15754 0.603979084
MTATP6P11 ENSG00000228097 0.15438 0.43865 1.0046 0.15755 1.0046
0.15755 0.26546 0.39533 0.15755 0.603979084
NA ENSG00000228886 1.0046 0.15756 0.93216 0.17563 1.0046 0.15756
1.0046 0.15756 0.15756 0.603979084
NA ENSG00000237193 0.11623 0.45373 -0.039158 0.51562 1.0045 0.15756
-0.17532 0.56959 0.15756 0.603979084
RPL35AP19 ENSG00000239872 -0.45378 0.67501 -0.45378 0.67501 1.0045
0.15757 -0.45378 0.67501 0.15757 0.603979084
APRT ENSG00000198931 1.0044 0.15759 1.0044 0.15759 1.0044 0.15759
1.0044 0.15759 0.15759 0.603999056
NA ENSG00000235140 -0.86634 0.80685 -0.90358 0.81689 1.0044 0.1576
0.35524 0.36121 0.1576 0.603999056
TGM4 ENSG00000163810 1.0042 0.15764 -0.32991 0.62926 0.35372 0.36177
-0.34734 0.63583 0.15764 0.604104844
NA ENSG00000266222 1.0039 0.15771 1.0039 0.15771 1.0039 0.15771
1.0039 0.15771 0.15771 0.604259647
IGLJ5 ENSG00000211681 1.0039 0.15772 1.0039 0.15772 1.0039 0.15772
1.0039 0.15772 0.15772 0.604259647
HAT1 ENSG00000128708 1.0038 0.15773 0.2878 0.38675 0.70894 0.23918
0.38832 0.34889 0.15773 0.604259647
SNRPFP4 ENSG00000235627 1.0038 0.15773 1.0038 0.15773 1.0038
0.15773 1.0038 0.15773 0.15773 0.604259647
LINC01063 ENSG00000232065 0.77628 0.21879 1.0036 0.15778 0.77628
0.21879 0.77628 0.21879 0.15778 0.604259647
NA ENSG00000254989 0.37134 0.35519 -1.0531 0.85386 0.23464 0.40724
1.0036 0.15779 0.15779 0.604259647
SYT17 ENSG00000103528 1.0035 0.1578 0.59655 0.2754 -0.91105 0.81887
1.0035 0.1578 0.1578 0.604259647
NA ENSG00000226058 1.0035 0.1578 0.98309 0.16278 1.0035 0.1578
1.0035 0.1578 0.1578 0.604259647
WDR33 ENSG00000136709 0.66586 0.25275 1.0035 0.15782 0.7766 0.2187
0.62165 0.26709 0.15782 0.604259647
PKMP4 ENSG00000254023 -0.78884 0.7849 0.83246 0.20257 -0.78884 0.7849
1.0035 0.15782 0.15782 0.604259647
LRRIQ4 ENSG00000188306 1.0033 0.15785 1.0033 0.15785 1.0033
0.15785 1.0033 0.15785 0.15785 0.604259647
SLC35A3 ENSG00000117620 1.0033 0.15786 -0.24351 0.5962 0.94806
0.17155 0.46322 0.32161 0.15786 0.604259647
NA ENSG00000249971 1.0033 0.15786 1.0033 0.15786 1.0033 0.15786
1.0033 0.15786 0.15786 0.604259647
NA ENSG00000250667 0.97993 0.16356 0.97993 0.16356 1.0033 0.15786
0.1521 0.43955 0.15786 0.604259647
NA ENSG00000224342 0.6993 0.24218 0.6993 0.24218 1.0033 0.15787
0.6993 0.24218 0.15787 0.604259647
NA ENSG00000280333 1.003 0.15793 1.003 0.15793 1.003 0.15793 1.003 0.15793
0.15793 0.604259647
NA ENSG00000233787 1.003 0.15794 1.003 0.15794 1.003 0.15794 1.003 0.15794
0.15794 0.604259647
NA ENSG00000261786 -1.9278 0.97306 1.003 0.15794 -1.101 0.86454
-0.21573 0.5854 0.15794 0.604259647
SIRPB3P ENSG00000223750 0.62119 0.26724 -0.19237 0.57627 0.1853
0.4265 1.0029 0.15795 0.15795 0.604259647
RN7SKP7 ENSG00000222472 0.2383 0.40582 0.2383 0.40582 1.0028
0.15797 0.35952 0.3596 0.15797 0.604259647
NA ENSG00000279982 -0.12602 0.55014 0.98339 0.16271 1.0028 0.15797
0.98339 0.16271 0.15797 0.604259647
PSTPIP1 ENSG00000140368 0.7568 0.22458 1.0028 0.15798 0.66013
0.25459 0.922 0.17826 0.15798 0.604259647
NA ENSG00000236658 1.0027 0.15799 0.97282 0.16532 1.0027 0.15799
0.53095 0.29773 0.15799 0.604259647
NA ENSG00000228480 1.0027 0.158 1.0027 0.158 0.72611 0.23389 1.0027

									0.158	0.158	0.604259647
AKAP13	ENSG00000170776	1.0027	0.15801	-0.23301	0.59212	0.038603	0.4846	0.12819	0.449	0.15801	0.604259647
LINC01015	ENSG00000224582	1.0026	0.15802	1.0026	0.15802	1.0026	0.15802	1.0026	0.15802	0.15802	0.604259647
KBTBD11	ENSG00000176595	1.0025	0.15804	0.097013	0.46136	0.70026	0.24188	-0.38382	0.64945	0.15804	0.604259647
RNU5B-1	ENSG00000200156	1.0026	0.15804	1.0026	0.15804	0.72958	0.23282	1.0026	0.15804	0.15804	0.604259647
NA	ENSG00000272799	0.467	0.32025	1.0025	0.15804	0.29136	0.38539	0.29136	0.38539	0.15804	0.604259647
NA	ENSG00000253175	0.5508	0.29089	0.30131	0.38159	1.0025	0.15806	0.59629	0.27549	0.15806	0.604270348
SRMP1	ENSG00000215448	0.074714	0.47022	0.80717	0.20978	0.074714	0.47022	1.0024	0.15808	0.15808	0.604270348
RN7SL111P	ENSG00000243510	1.0024	0.15808	1.0024	0.15808	1.0024	0.15808	1.0024	0.15808	0.15808	0.604270348
NA	ENSG00000246528	-0.28551	0.61237	1.0023	0.1581	-0.28551	0.61237	0.11391	0.45465	0.1581	0.60429022
ENG	ENSG00000106991	-0.58011	0.71908	-1.3762	0.91562	0.22377	0.41147	1.0023	0.15811	0.15811	0.60429022
NA	ENSG00000279684	-0.31044	0.62189	0.69367	0.24394	1.0021	0.15814	1.0021	0.15814	0.15814	0.604357478
RRN3	ENSG00000085721	0.4724	0.31832	0.61397	0.26962	0.26426	0.39579	1.002	0.15817	0.15817	0.604424726
NA	ENSG00000201596	-0.58199	0.71971	1.0018	0.15821	-0.93688	0.82559	0.83874	0.20081	0.15821	0.604502603
NA	ENSG00000272128	0.93154	0.17579	0.93154	0.17579	1.0018	0.15822	0.20054	0.42053	0.15822	0.604502603
PTPN18	ENSG00000072135	1.0017	0.15824	0.58953	0.27775	0.95467	0.16987	0.96229	0.16795	0.15824	0.604502603
NA	ENSG00000254606	0.72233	0.23504	-0.75311	0.77431	1.0017	0.15824	0.78724	0.21557	0.15824	0.604502603
NA	ENSG00000228079	-0.27427	0.60806	1.0017	0.15826	-0.27427	0.60806	-0.27427	0.60806	0.15826	0.604531614
NEK4P3	ENSG00000270754	-0.078305	0.53121	-0.078305	0.53121	-0.078305	0.53121	1.0015	0.15829	0.15829	0.604598816
LINC00092	ENSG00000225194	0.82959	0.20338	1.0014	0.15831	0.82168	0.20563	0.82168	0.20563	0.15831	0.604618621
MT1H	ENSG00000205358	1.0014	0.15832	1.0014	0.15832	1.0014	0.15832	1.0014	0.15832	0.15832	0.604618621
ACACA	ENSG00000278540	0.20118	0.42028	0.32804	0.37144	0.97859	0.16389	1.0011	0.15839	0.15839	0.604747883
NA	ENSG00000230694	1.0011	0.1584	1.0011	0.1584	1.0011	0.1584	-1.1806	0.88111	0.1584	0.604747883
RIC1	ENSG00000107036	0.48307	0.31452	0.24396	0.40363	-0.52386	0.69981	1.001	0.15842	0.15842	0.604747883
COX7A2	ENSG00000112695	-0.89107	0.81355	-0.32088	0.62585	1.0009	0.15843	-0.41715	0.66171	0.15843	0.604747883
H3C9P	ENSG00000220875	0.87019	0.1921	0.68917	0.24536	1.0009	0.15843	0.94003	0.1736	0.15843	0.604747883
MIR483	ENSG00000207805	1.0009	0.15844	1.0009	0.15844	1.0009	0.15844	1.0009	0.15844	0.15844	0.604747883
NA	ENSG00000261552	-1.1963	0.88422	1.0009	0.15845	1.0009	0.15845	1.0009	0.15845	0.15845	0.604747883
HIC2	ENSG00000169635	0.10907	0.45657	-0.32999	0.6293	1.0008	0.15847	-0.11686	0.54652	0.15847	0.604747883
CLYBL-AS2	ENSG00000227659	1.0008	0.15847	1.0008	0.15847	0.70532	0.24031	1.0008	0.15847	0.15847	0.604747883
CASC9	ENSG00000249395	1.0007	0.1585	-0.046987	0.51874	-0.18948	0.57514	-1.2709	0.89812	0.1585	0.604747883
NA	ENSG00000250284	0.42135	0.33675	0.88554	0.18793	1.0007	0.1585				

-0.99576 0.84032 0.1585 0.604747883
ZC3H3 ENSG00000014164 0.84069 0.20026 -0.24676 0.59745 1.0005 0.15852
0.25754 0.39838 0.15852 0.604747883
PLA2G7 ENSG00000146070 0.098592 0.46073 1.0005 0.15854 0.098592
0.46073 0.7282 0.23325 0.15854 0.604747883
NA ENSG00000253844 -0.54687 0.70777 -0.54687 0.70777 1.0005 0.15854
-0.20152 0.57985 0.15854 0.604747883
NA ENSG00000258843 1.0005 0.15854 1.0005 0.15854 1.0005 0.15854
1.0005 0.15854 0.15854 0.604747883
DHRS7B ENSG00000109016 -0.4608 0.67753 0.14059 0.4441 0.44193
0.32927 1.0003 0.15858 0.15858 0.60479658
NA ENSG00000254290 1.0003 0.15858 1.0003 0.15858 0.60971 0.27103
1.0003 0.15858 0.15858 0.60479658
SRP54 ENSG00000100883 0.97135 0.16569 0.94189 0.17312 1.0003 0.15859
0.81529 0.20745 0.15859 0.60479658
NA ENSG00000266216 0.27692 0.39092 -0.43542 0.66837 -0.68 0.75175
1.0002 0.15861 0.15861 0.604797935
HNF1A ENSG00000135100 1.0001 0.15863 0.19908 0.4211 0.83043 0.20315
0.607 0.27193 0.15863 0.604797935
NA ENSG00000233870 1 0.15864 1 0.15864 1 0.15864 1 0.15864
0.15864 0.604797935
NA ENSG00000238888 1 0.15864 -1.4787 0.93038 -0.90221 0.81653
-1.0969 0.86366 0.15864 0.604797935
RN7SKP218 ENSG00000222154 0.99985 0.15869 0.99985 0.15869 -0.69019
0.75496 0.5183 0.30212 0.15869 0.604941227
RNU6-1198P ENSG00000252713 0.99971 0.15873 0.99971 0.15873 0.99971
0.15873 0.99971 0.15873 0.15873 0.605046378
NA ENSG00000229263 -0.42249 0.66367 0.97319 0.16523 -0.81929 0.79369
0.99958 0.15876 0.15876 0.605094971
SVIL2P ENSG00000234814 0.045043 0.48204 0.99955 0.15877 -0.68005
0.75176 0.99556 0.15973 0.15877 0.605094971
RPL7AP34 ENSG00000213312 -0.16 0.56356 0.99951 0.15878 -0.82157 0.79434
0.99951 0.15878 0.15878 0.605094971
NA ENSG00000279071 0.99928 0.15883 0.99928 0.15883 0.99928 0.15883
0.99928 0.15883 0.15883 0.605238184
NA ENSG00000240874 -0.0043968 0.50175 -0.0043968 0.50175 -0.0043968 0.50175
0.99916 0.15886 0.15886 0.605277494
RNU6-227P ENSG00000199735 0.99907 0.15888 -0.075906 0.53025 -0.075906
0.53025 0.073335 0.47077 0.15888 0.605277494
NA ENSG00000280037 0.99906 0.15888 0.99906 0.15888 0.99906 0.15888
0.99906 0.15888 0.15888 0.605277494
NA ENSG00000273901 0.99901 0.15889 0.99901 0.15889 0.99901 0.15889
0.99901 0.15889 0.15889 0.605277494
LAMP3 ENSG00000078081 0.99869 0.15897 -0.19928 0.57898 -0.59223 0.72315
0.44311 0.32884 0.15897 0.605525668
NPM1P35 ENSG00000255213 0.34623 0.36458 0.34623 0.36458 0.34623
0.36458 0.99865 0.15898 0.15898 0.605525668
ADAM33 ENSG00000149451 0.99852 0.15901 -0.69512 0.75651 -0.88209
0.81114 -1.2905 0.90156 0.15901 0.605592599
OR7E12P ENSG00000189398 -2.1432 0.98395 -0.75477 0.77481 0.99846
0.15903 -0.42982 0.66634 0.15903 0.605621436
RNU6-228P ENSG00000199570 0.99823 0.15908 -0.31999 0.62551 0.75864
0.22403 0.30883 0.37872 0.15908 0.605745996
SEMA3F ENSG00000001617 -0.90521 0.81732 0.99816 0.1591 0.39409
0.34676 0.17428 0.43082 0.1591 0.605745996
LINC00620 ENSG00000224514 0.44857 0.32687 0.99816 0.1591 -0.11564
0.54603 -0.53669 0.70426 0.1591 0.605745996
LAMC1 ENSG00000135862 0.71685 0.23673 0.78816 0.2153 0.99785 0.15918
0.71608 0.23697 0.15918 0.606003234
RIOK2 ENSG00000058729 0.99757 0.15924 0.36968 0.35581 0.26839 0.3942
0.64089 0.2608 0.15924 0.606007132
LNC-LBCS ENSG00000228412 0.68555 0.2465 0.43899 0.33034 0.9975

0.15926 0.20687 0.41805 0.15926 0.606007132
OR6C76 ENSG00000185821 0.99745 0.15927 -0.18759 0.5744 -0.25819
0.60187 -0.18759 0.5744 0.15927 0.606007132
NA ENSG00000275454 0.99746 0.15927 0.99746 0.15927 0.99746 0.15927
0.99746 0.15927 0.15927 0.606007132
RBFOX1 ENSG00000078328 0.99743 0.15928 0.086048 0.46571 0.45175
0.32572 -0.018906 0.50754 0.15928 0.606007132
TMEM65 ENSG00000164983 -1.4516 0.92669 0.99739 0.15929 -0.63933
0.73869 0.01265 0.49495 0.15929 0.606007132
B4GALT1-AS1 ENSG00000233554 0.99737 0.15929 0.99737 0.15929 0.99737
0.15929 0.99737 0.15929 0.15929 0.606007132
FOXO1B ENSG00000214295 -0.60832 0.72851 0.99728 0.15931 -0.84962
0.80223 -0.022135 0.50883 0.15931 0.606007132
FAM66C ENSG00000226711 0.99728 0.15931 0.74893 0.22695 0.05331
0.47874 0.2102 0.41676 0.15931 0.606007132
RIMKLB ENSG00000166532 0.49569 0.31006 -0.72115 0.76459 0.43631
0.33131 0.99721 0.15933 0.15933 0.606007132
CCN6 ENSG00000112761 -0.61108 0.72943 -1.1041 0.86522 -1.1795 0.8809
0.99718 0.15934 0.15934 0.606007132
RNA5SP96 ENSG00000251850 0.99713 0.15935 0.99713 0.15935 0.99713
0.15935 0.99713 0.15935 0.15935 0.606007132
CADM3-AS1 ENSG00000225670 0.99711 0.15936 0.99711 0.15936 0.99711
0.15936 0.99711 0.15936 0.15936 0.606007132
NA ENSG00000256481 0.49198 0.31137 0.81806 0.20666 0.99708 0.15936
0.83805 0.201 0.15936 0.606007132
CHST5 ENSG00000135702 0.997 0.15938 0.997 0.15938 0.997 0.15938 0.54735
0.29207 0.15938 0.606007132
NDUFA3P2 ENSG00000254856 0.997 0.15938 -0.52276 0.69943 0.997 0.15938 0.997
0.15938 0.15938 0.606007132
CCDC27 ENSG00000162592 0.74814 0.22719 0.99688 0.15941 0.99688
0.15941 0.70952 0.239 0.15941 0.60607391
KLF3P2 ENSG00000241879 0.99675 0.15944 -0.36113 0.641 0.99675 0.15944
0.99675 0.15944 0.15944 0.606093393
NA ENSG00000270317 -1.0271 0.84781 0.65689 0.25563 0.99676 0.15944
0.65689 0.25563 0.15944 0.606093393
NA ENSG00000270248 0.99668 0.15946 0.99668 0.15946 0.99668 0.15946
0.99668 0.15946 0.15946 0.606094338
SLC10A1 ENSG00000100652 -0.65905 0.74507 -0.82164 0.79436 0.78249
0.21696 0.99662 0.15947 0.15947 0.606094338
FSIP1 ENSG00000150667 0.99655 0.15949 -0.76257 0.77714 0.75057 0.22645
0.58451 0.27944 0.15949 0.606094338
NA ENSG00000232389 -0.20378 0.58074 -0.20378 0.58074 0.99655 0.15949
-0.44085 0.67034 0.15949 0.606094338
SUMO1P1 ENSG00000241721 0.56617 0.28564 0.99644 0.15952 0.3726
0.35472 -0.27813 0.60955 0.15952 0.606161073
KLHDC7A ENSG00000179023 0.46946 0.31937 0.9963 0.15955 0.11801
0.45303 0.8377 0.2011 0.15955 0.606220124
MIR4309 ENSG00000266015 0.9962 0.15958 -0.44742 0.67271 0.59042
0.27746 0.43266 0.33263 0.15958 0.606220124
BCLAF1 ENSG00000029363 -1.3302 0.90827 -1.3302 0.90827 -0.84749
0.80164 0.99615 0.15959 0.15959 0.606220124
DUSP1 ENSG00000120129 0.97179 0.16558 0.97179 0.16558 0.96295 0.16779
0.99605 0.15961 0.15961 0.606220124
LINC00111 ENSG00000227702 -0.76847 0.7789 -0.96769 0.8334 -0.76847
0.7789 0.99607 0.15961 0.15961 0.606220124
OR6C75 ENSG00000187857 -0.52641 0.7007 0.21506 0.41486 0.99593
0.15964 0.21506 0.41486 0.15964 0.606220124
CYP4F32P ENSG00000223564 0.76543 0.22201 -0.6879 0.75424 0.86543
0.1934 0.9959 0.15965 0.15965 0.606220124
GNRHR2 ENSG00000211451 0.99586 0.15966 0.99586 0.15966 0.99586
0.15966 0.99586 0.15966 0.15966 0.606220124
ZDHHC5 ENSG00000156599 0.61314 0.26989 0.99579 0.15968 0.63331

0.26326 0.47593 0.31706 0.15968 0.606220124
LAMP5-AS1 ENSG00000225988 0.65007 0.25782 0.99573 0.15969 0.65007
0.25782 0.65007 0.25782 0.15969 0.606220124
CCNG1P1 ENSG00000218991 0.9957 0.1597 -0.29421 0.6157 -0.29421
0.6157 -0.29421 0.6157 0.1597 0.606220124
RNU6ATAC14P ENSG00000252027 0.99569 0.1597 0.99569 0.1597 0.99569
0.1597 0.99569 0.1597 0.1597 0.606220124
OSBPL9P1 ENSG00000271389 0.99564 0.15971 0.99564 0.15971 0.99564
0.15971 0.99564 0.15971 0.15971 0.606220124
C1S ENSG00000182326 0.46001 0.32275 0.43243 0.33271 0.25783 0.39827
0.99555 0.15973 0.15973 0.606220124
TOP2B ENSG00000077097 0.91648 0.17971 0.99554 0.15974 -0.063818 0.52544
0.45465 0.32468 0.15974 0.606220124
TMEM9 ENSG00000116857 0.7587 0.22402 0.11346 0.45483 0.99543 0.15976
0.11346 0.45483 0.15976 0.606220124
STX12 ENSG00000117758 0.86572 0.19332 0.94551 0.1722 0.99546 0.15976
0.86711 0.19294 0.15976 0.606220124
SLC45A1 ENSG00000162426 0.91771 0.17938 0.35607 0.36089 0.99546
0.15976 0.89903 0.18432 0.15976 0.606220124
C15orf40 ENSG00000169609 0.71581 0.23705 0.9953 0.15979 0.076696
0.46943 0.092276 0.46324 0.15979 0.606220124
NA ENSG00000254927 0.99532 0.15979 0.99532 0.15979 0.99532 0.15979
-0.087481 0.53486 0.15979 0.606220124
FBP1 ENSG00000165140 0.99522 0.15981 -0.63529 0.73738 -1.1361 0.87204
-1.3238 0.90722 0.15981 0.606220124
EIF4A3 ENSG00000141543 -0.39684 0.65426 0.99519 0.15982 -0.76557
0.77803 0.66089 0.25434 0.15982 0.606220124
NA ENSG00000272568 0.99513 0.15984 0.99513 0.15984 0.8092 0.2092
0.99513 0.15984 0.15984 0.606220124
FOXB1 ENSG00000171956 -0.2288 0.59049 -0.2288 0.59049 -0.2288 0.59049
0.99507 0.15985 0.15985 0.606220124
P4HA1 ENSG00000122884 0.43951 0.33014 -0.38382 0.64944 0.99504 0.15986
-1.1775 0.88049 0.15986 0.606220124
BPIFB9P ENSG00000125997 -0.80954 0.7909 0.995 0.15987 0.995 0.15987 0.995
0.15987 0.15987 0.606220124
FTLP1 ENSG00000235525 0.99496 0.15988 0.99496 0.15988 0.99496 0.15988
0.99496 0.15988 0.15988 0.606220124
OR5AH1P ENSG00000268067 0.99491 0.15989 -0.035313 0.51409 -0.035313
0.51409 -0.035313 0.51409 0.15989 0.606220124
ACTB ENSG00000075624 0.99482 0.15991 0.050274 0.47995 -0.23293 0.59209
0.22135 0.41241 0.15991 0.606220124
ATP2B1-AS1 ENSG00000271614 0.99477 0.15992 0.99477 0.15992 0.99477
0.15992 0.99477 0.15992 0.15992 0.606220124
SPATA31C2 ENSG00000177910 -0.10929 0.54351 -0.15914 0.56322 0.99472
0.15994 0.81062 0.20879 0.15994 0.606220124
CFAP77 ENSG00000188523 0.99467 0.15995 -0.3032 0.61913 -2.0465
0.97965 0.27913 0.39007 0.15995 0.606220124
RNU6-1066P ENSG00000200443 0.99467 0.15995 -0.29015 0.61415 0.99467
0.15995 0.99467 0.15995 0.15995 0.606220124
ARHGEF12 ENSG00000196914 0.71538 0.23719 -0.089884 0.53581 0.99461
0.15996 -1.024 0.84709 0.15996 0.606220124
NIFKP3 ENSG00000256331 -0.17526 0.56956 0.024806 0.4901 0.9945
0.15999 0.9945 0.15999 0.15999 0.606220124
NA ENSG00000224504 0.99446 0.16 0.79521 0.21324 0.99446 0.16 0.31964
0.37462 0.16 0.606220124
NA ENSG00000280059 0.99442 0.16001 0.31768 0.37536 0.99442 0.16001
0.22593 0.41063 0.16001 0.606220124
IL20 ENSG00000162891 -1.4629 0.92826 0.99433 0.16003 -1.4629 0.92826
0.61264 0.27006 0.16003 0.606220124
MTND5P25 ENSG00000227348 0.99427 0.16005 0.99427 0.16005 0.99427
0.16005 0.99427 0.16005 0.16005 0.606220124
CLUHP11 ENSG00000278138 0.99419 0.16006 0.99419 0.16006 0.99419

0.16006 0.99419 0.16006 0.16006 0.606220124
SERPINB12 ENSG00000166634 -3.0305 0.99878 -3.0305 0.99878 0.99416
0.16007 -2.3714 0.99114 0.16007 0.606220124
WDR12 ENSG00000138442 0.95439 0.16994 0.9941 0.16009 0.71808 0.23635
0.38538 0.34998 0.16009 0.606220124
RHOA ENSG00000067560 0.994 0.16011 0.60695 0.27194 -0.2497 0.59859
0.69738 0.24278 0.16011 0.606220124
SEC63P2 ENSG00000250416 0.99398 0.16012 0.99398 0.16012 0.99398
0.16012 0.99398 0.16012 0.16012 0.606220124
RNU6-213P ENSG00000206778 0.9939 0.16014 0.47485 0.31745 0.9939
0.16014 0.9939 0.16014 0.16014 0.606220124
GMIP ENSG00000089639 -0.33762 0.63217 -0.050803 0.52026 0.079636 0.46826
0.99384 0.16015 0.16015 0.606220124
PDE7B ENSG00000171408 0.99385 0.16015 0.5636 0.28651 0.13774 0.44522
0.0059064 0.49764 0.16015 0.606220124
CFL1P1 ENSG00000223820 0.36009 0.35939 0.19647 0.42212 -0.19395
0.57689 0.99384 0.16015 0.16015 0.606220124
SESN2 ENSG00000130766 0.65935 0.25484 0.43291 0.33254 0.99379 0.16016
0.18098 0.42819 0.16016 0.606220124
NA ENSG00000272692 0.99375 0.16017 0.36072 0.35915 0.15868 0.43696
0.15868 0.43696 0.16017 0.606220124
NA ENSG00000272814 0.99376 0.16017 -0.9402 0.82644 -0.9402 0.82644
-0.9402 0.82644 0.16017 0.606220124
RPS23P9 ENSG00000231586 -0.070922 0.52827 0.99369 0.16019 0.38166
0.35136 -0.28971 0.61398 0.16019 0.606248734
ERGIC3 ENSG00000125991 0.17769 0.42948 0.99351 0.16023 -0.3183
0.62487 0.47476 0.31748 0.16023 0.60634378
AKAP8L ENSG00000011243 0.38461 0.35026 0.99348 0.16024 0.99348
0.16024 0.99348 0.16024 0.16024 0.60634378
NA ENSG00000230355 -0.85591 0.80398 -0.85591 0.80398 0.99337 0.16027
-0.85591 0.80398 0.16027 0.606400963
MFSD12 ENSG00000161091 0.99332 0.16028 -0.32174 0.62617 -0.32174
0.62617 -0.32174 0.62617 0.16028 0.606400963
NPIPB15 ENSG00000196436 0.99296 0.16036 0.80737 0.20973 0.39508
0.34639 -0.63078 0.73591 0.16036 0.606656533
KNCN ENSG00000162456 -0.16491 0.56549 -0.16491 0.56549 -0.16491 0.56549
0.99271 0.16042 0.16042 0.606827129
FAM66A ENSG00000227888 0.88126 0.18909 -0.23526 0.593 0.99271 0.16043
0.34866 0.36367 0.16043 0.606827129
ZNF285B ENSG00000176761 0.76738 0.22143 0.94473 0.1724 0.99261
0.16045 0.99261 0.16045 0.16045 0.606855674
MCUB ENSG00000005059 -0.48995 0.68791 0.99243 0.16049 -0.59082 0.72268
-1.0904 0.86224 0.16049 0.606894188
TNFSF14 ENSG00000125735 -1.2228 0.8893 -1.2228 0.8893 -1.2228
0.8893 0.99246 0.16049 0.16049 0.606894188
NA ENSG00000277704 0.88379 0.18841 -0.34686 0.63565 -0.43222 0.66721
0.99241 0.1605 0.1605 0.606894188
RN7SKP113 ENSG00000200761 -1.0535 0.85394 0.99235 0.16051 -0.36334
0.64183 -1.049 0.85292 0.16051 0.606894188
NA ENSG00000206717 0.033694 0.48656 0.99227 0.16053 0.033694 0.48656
0.033694 0.48656 0.16053 0.606904158
KRT18P64 ENSG00000213211 0.99224 0.16054 0.99224 0.16054 0.99224
0.16054 0.99224 0.16054 0.16054 0.606904158
UBTFL2 ENSG00000234376 0.9922 0.16055 0.9922 0.16055 -1.7537
0.96026 -1.4978 0.93291 0.16055 0.606904158
NA ENSG00000226375 0.99207 0.16058 0.99207 0.16058 0.99207 0.16058
0.99207 0.16058 0.16058 0.606948039
NR3C1 ENSG00000113580 0.99195 0.16061 0.70211 0.2413 0.14795 0.44119
0.75802 0.22422 0.16061 0.606948039
NA ENSG00000255313 -0.94824 0.8285 -0.42975 0.66631 0.99193 0.16062
-1.0633 0.85619 0.16062 0.606948039
NA ENSG00000235308 0.99189 0.16063 -0.85019 0.80239 -0.85019 0.80239

-1.5552 0.94005 0.16063 0.606948039
NA ENSG00000271600 0.99182 0.16064 0.99182 0.16064 0.99182 0.16064
0.99182 0.16064 0.16064 0.606948039
PIK3CD-AS2 ENSG00000231789 0.99179 0.16065 -0.031802 0.51269 0.76715
0.2215 0.46696 0.32027 0.16065 0.606948039
NA ENSG00000275833 0.10788 0.45704 -0.84026 0.79962 0.99175 0.16066
0.99175 0.16066 0.16066 0.606948039
AKR1C2 ENSG00000151632 0.71585 0.23704 0.90795 0.18195 0.95148
0.17068 0.99161 0.16069 0.16069 0.606948039
TNFAIP2 ENSG00000185215 0.62279 0.26671 0.95806 0.16902 0.99163
0.16069 0.70937 0.23905 0.16069 0.606948039
GMCL2 ENSG00000244234 0.99145 0.16073 0.99145 0.16073 0.99145 0.16073
0.99145 0.16073 0.16073 0.606948039
RNU6-431P ENSG00000206601 0.99135 0.16076 0.99135 0.16076 0.99135
0.16076 0.99135 0.16076 0.16076 0.606948039
CTBP2P5 ENSG00000214602 0.99134 0.16076 0.99134 0.16076 0.99134
0.16076 0.99134 0.16076 0.16076 0.606948039
LYPD2 ENSG00000197353 -0.93649 0.82549 -0.93649 0.82549 -0.11731 0.54669
0.99131 0.16077 0.16077 0.606948039
NA ENSG00000277566 0.9913 0.16077 0.9913 0.16077 0.9913 0.16077
0.9913 0.16077 0.16077 0.606948039
NA ENSG00000280198 0.99126 0.16078 0.99126 0.16078 0.99126 0.16078
0.99126 0.16078 0.16078 0.606948039
DSCAM-IT1 ENSG00000233756 -0.10562 0.54206 0.99113 0.16081 0.59412
0.27622 -0.16169 0.56423 0.16081 0.606948039
NA ENSG00000255923 0.99109 0.16082 0.99109 0.16082 0.99109 0.16082
0.99109 0.16082 0.16082 0.606948039
NA ENSG00000254502 -0.26527 0.6046 -0.26527 0.6046 -0.26527 0.6046
0.99106 0.16083 0.16083 0.606948039
NA ENSG00000283384 0.32693 0.37186 0.99106 0.16083 0.27779 0.39059
-0.57169 0.71623 0.16083 0.606948039
RNU6-1214P ENSG00000200520 0.25881 0.39789 0.58148 0.28046 0.99092
0.16086 0.99092 0.16086 0.16086 0.606948039
NA ENSG00000252802 0.99094 0.16086 0.99094 0.16086 0.99094 0.16086
0.99094 0.16086 0.16086 0.606948039
GSTK1 ENSG00000197448 0.99089 0.16087 0.99089 0.16087 0.99089 0.16087
0.99089 0.16087 0.16087 0.606948039
POU2F3 ENSG00000137709 -0.54734 0.70793 0.19464 0.42284 0.99085
0.16088 -0.34429 0.63469 0.16088 0.606948039
SNRNP27 ENSG00000124380 0.36393 0.35796 0.5906 0.27739 0.99077
0.1609 0.90426 0.18293 0.1609 0.606948039
IGFL4 ENSG00000204869 0.99077 0.1609 -0.81695 0.79302 -1.1187 0.86836
0.29995 0.38211 0.1609 0.606948039
DIAPH3-AS2 ENSG00000223815 0.99076 0.1609 0.99076 0.1609 0.99076
0.1609 0.99076 0.1609 0.1609 0.606948039
NA ENSG00000258653 0.99069 0.16092 0.99069 0.16092 0.99069 0.16092
0.99069 0.16092 0.16092 0.606948039
NA ENSG00000248560 0.99066 0.16093 0.8505 0.19752 0.99066 0.16093
0.99066 0.16093 0.16093 0.606948039
NANOGP5 ENSG00000231697 -0.18474 0.57328 -0.18474 0.57328 -0.18474
0.57328 0.9906 0.16094 0.16094 0.606948039
GRTP1 ENSG00000139835 -1.5373 0.93789 0.99058 0.16095 -0.19468 0.57718
-1.0108 0.84395 0.16095 0.606948039
NA ENSG00000243018 0.99051 0.16096 0.34042 0.36677 0.99051 0.16096
0.99051 0.16096 0.16096 0.606948039
NA ENSG00000279288 0.99039 0.16099 -0.847 0.8015 -1.6211 0.9475
-1.3247 0.90737 0.16099 0.606948039
DCDC2 ENSG00000146038 0.99036 0.161 0.6814 0.24781 -0.25946 0.60236
0.73539 0.23105 0.161 0.606948039
LUC7L2 ENSG00000146963 0.42886 0.33401 0.82653 0.20425 0.99035 0.161
0.43406 0.33212 0.161 0.606948039
NA ENSG00000268582 0.30831 0.37892 0.99035 0.161 0.99035 0.161 0.99035

0.161 0.161 0.606948039
NA ENSG00000279123 -0.32082 0.62583 0.99031 0.16101 0.99031 0.16101
0.99031 0.16101 0.16101 0.606948039
HIPK3 ENSG00000110422 -0.21482 0.58505 0.51329 0.30387 -0.25285 0.59981
0.99021 0.16104 0.16104 0.606967888
CEP192 ENSG00000101639 0.56439 0.28624 0.99012 0.16106 -0.034489
0.51376 -0.19825 0.57858 0.16106 0.606967888
JPH1 ENSG00000104369 -0.53656 0.70421 -0.12796 0.55091 -1.003 0.84206
0.99002 0.16108 0.16108 0.606967888
CHST12 ENSG00000136213 -0.22592 0.58937 0.49936 0.30876 0.99003
0.16108 -0.99633 0.84045 0.16108 0.606967888
CBX6 ENSG00000183741 -0.62988 0.73561 0.036202 0.48556 0.98997 0.16109
-0.20881 0.5827 0.16109 0.606967888
NA ENSG00000202001 0.98998 0.16109 0.98998 0.16109 0.98998 0.16109
-0.62488 0.73397 0.16109 0.606967888
RNU6-4P ENSG00000206932 0.16839 0.43314 -0.024834 0.50991 0.9898
0.16114 -1.1048 0.86537 0.16114 0.606969303
NA ENSG00000260072 0.98975 0.16115 -0.54198 0.70608 -0.18982 0.57528
0.45022 0.32628 0.16115 0.606969303
NA ENSG00000249746 0.98966 0.16117 0.98966 0.16117 0.98966 0.16117
0.98966 0.16117 0.16117 0.606969303
TEKT5 ENSG00000153060 0.040925 0.48368 0.98949 0.16121 0.20567 0.41853
0.66128 0.25422 0.16121 0.606969303
MTCYBP13 ENSG00000266529 0.98946 0.16122 0.80014 0.21182 0.80014
0.21182 0.80014 0.21182 0.16122 0.606969303
MSANTD2P1 ENSG00000231058 0.98937 0.16124 0.98937 0.16124 0.98937
0.16124 0.98937 0.16124 0.16124 0.606969303
RPS7P3 ENSG00000231940 0.55104 0.2908 0.55104 0.2908 0.55104
0.2908 0.98932 0.16125 0.16125 0.606969303
NA ENSG00000275720 -0.32792 0.62851 0.033355 0.4867 0.98935 0.16125
-0.70847 0.76067 0.16125 0.606969303
NA ENSG00000267250 -0.0058991 0.50235 -0.88914 0.81304 0.98922 0.16128
-1.2432 0.89311 0.16128 0.606969303
H1-10-AS1 ENSG00000206417 -1.0059 0.84276 -1.0059 0.84276 -1.0059
0.84276 0.98919 0.16129 0.16129 0.606969303
PIPSL ENSG00000180764 -1.9418 0.97392 -0.99992 0.84133 -0.26525 0.60459
0.98914 0.1613 0.1613 0.606969303
NA ENSG00000272719 -0.25314 0.59992 -0.25314 0.59992 0.98911 0.1613
-1.0153 0.84502 0.1613 0.606969303
NA ENSG00000226836 0.98903 0.16132 0.98903 0.16132 0.98903 0.16132
-0.16126 0.56406 0.16132 0.606969303
NA ENSG00000267466 -0.24741 0.59771 -0.37545 0.64634 -0.19305 0.57654
0.98904 0.16132 0.16132 0.606969303
RNA5SP162 ENSG00000251823 0.98902 0.16133 0.98902 0.16133 0.98902
0.16133 0.98902 0.16133 0.16133 0.606969303
NA ENSG00000224739 0.98893 0.16135 0.46071 0.3225 -0.48962 0.6878
-0.61895 0.73203 0.16135 0.606969303
RN7SL617P ENSG00000241162 0.98892 0.16135 0.98892 0.16135 0.98892
0.16135 0.98892 0.16135 0.16135 0.606969303
NDUFC1 ENSG00000109390 0.4006 0.34436 0.28817 0.38661 0.98886
0.16137 0.74145 0.22921 0.16137 0.606969303
RPL5P20 ENSG00000218643 -0.60066 0.72597 -0.60066 0.72597 -0.60066
0.72597 0.98884 0.16137 0.16137 0.606969303
RNA5SP402 ENSG00000252957 0.23562 0.40686 -0.44105 0.67041 0.67971
0.24834 0.98871 0.1614 0.1614 0.606969303
PLB1 ENSG00000163803 -0.089397 0.53562 0.899 0.18433 -0.021557 0.5086
0.98868 0.16141 0.16141 0.606969303
TRAV38-1 ENSG00000211816 0.98862 0.16143 0.98862 0.16143 0.98862
0.16143 0.98862 0.16143 0.16143 0.606969303
MRFAP1 ENSG00000179010 -0.27636 0.60886 0.98845 0.16147 -1.0971
0.8637 -1.0971 0.8637 0.16147 0.606969303
BCL7C ENSG00000099385 0.98832 0.1615 0.11865 0.45278 0.59134 0.27715

0.49365 0.31078 0.1615 0.606969303
CD2AP ENSG00000198087 0.98829 0.1615 -0.053112 0.52118 -0.64804 0.74152
-0.097863 0.53898 0.1615 0.606969303
LINC01497 ENSG00000237560 0.98829 0.1615 0.98829 0.1615 0.98829
0.1615 0.98829 0.1615 0.1615 0.606969303
NA ENSG00000273478 0.98833 0.1615 0.98554 0.16218 0.82809 0.20381
0.82809 0.20381 0.1615 0.606969303
PCYOX1 ENSG00000116005 0.35769 0.36029 0.35769 0.36029 0.094937
0.46218 0.98827 0.16151 0.16151 0.606969303
GPR160P2 ENSG00000265863 0.98818 0.16153 0.98818 0.16153 0.98818
0.16153 0.98818 0.16153 0.16153 0.606969303
NA ENSG00000273151 0.53379 0.29674 0.98815 0.16154 0.5531 0.2901
0.5531 0.2901 0.16154 0.606969303
DSG1 ENSG00000134760 0.98813 0.16155 0.98813 0.16155 0.98813 0.16155
0.7946 0.21342 0.16155 0.606969303
FAM193B ENSG00000146067 -0.59815 0.72513 0.9881 0.16155 0.79028
0.21468 -0.60288 0.72671 0.16155 0.606969303
RPL31P17 ENSG00000235225 0.98809 0.16155 0.12141 0.45168 -0.9097
0.81851 0.12141 0.45168 0.16155 0.606969303
NA ENSG00000257355 0.98808 0.16156 0.31686 0.37567 0.31686 0.37567
0.31686 0.37567 0.16156 0.606969303
MYO1G ENSG00000136286 0.98802 0.16157 0.8133 0.20802 0.28011 0.3897
-1.2236 0.88944 0.16157 0.606969303
RFFL ENSG00000092871 -1.729 0.9581 -0.067113 0.52675 0.98797 0.16158
-1.516 0.93524 0.16158 0.606969303
FLYWCH1 ENSG00000059122 -0.32014 0.62557 0.98791 0.1616 0.93354
0.17527 0.22807 0.4098 0.1616 0.606969303
ACVR2A ENSG00000121989 -0.19237 0.57627 -0.13364 0.55316 0.98788
0.16161 0.75143 0.2262 0.16161 0.606969303
FKBP2 ENSG00000173486 0.86362 0.1939 0.86362 0.1939 0.98785 0.16161
0.98785 0.16161 0.16161 0.606969303
A1CF ENSG00000148584 0.4795 0.31579 0.6125 0.2701 0.6125 0.2701
0.98782 0.16162 0.16162 0.606969303
NA ENSG00000227907 0.98776 0.16164 0.094633 0.4623 0.55263 0.29026
0.55263 0.29026 0.16164 0.606969303
HNRNPA3P13 ENSG00000250826 0.98772 0.16164 -1.2978 0.90282 0.35396
0.36168 0.39076 0.34799 0.16164 0.606969303
DDB1 ENSG00000167986 -0.0025422 0.50101 0.43228 0.33277 0.98772 0.16165
-0.0051952 0.50207 0.16165 0.606969303
NA ENSG00000279125 0.98766 0.16166 0.18523 0.42652 0.18523 0.42652
0.59903 0.27458 0.16166 0.606969303
SIRPG ENSG00000089012 -0.045751 0.51825 0.21624 0.4144 0.98753 0.16169
-0.25464 0.6005 0.16169 0.606969303
DLGAP4 ENSG00000080845 -0.38242 0.64892 -0.32284 0.62659 -0.87957
0.81045 0.98751 0.1617 0.1617 0.606969303
MPPE1 ENSG00000154889 -0.09838 0.53918 0.98746 0.16171 -0.39735 0.65445
-0.13822 0.55497 0.16171 0.606969303
MTCO3P1 ENSG00000235040 0.78993 0.21479 0.96383 0.16757 0.94753
0.17169 0.98741 0.16172 0.16172 0.606969303
NA ENSG00000248738 0.98729 0.16175 0.61006 0.27091 0.50994 0.30505
0.6641 0.25331 0.16175 0.606969303
IL25 ENSG00000166090 0.554 0.28979 0.81518 0.20748 0.98727 0.16176
-0.25482 0.60057 0.16176 0.606969303
SETP9 ENSG00000235101 0.79765 0.21254 0.98725 0.16176 0.93893 0.17388
0.95008 0.17103 0.16176 0.606969303
NWD2 ENSG00000174145 0.98719 0.16177 0.29459 0.38415 0.15085 0.44005
0.76901 0.22094 0.16177 0.606969303
ADH7 ENSG00000196344 -0.2819 0.61099 0.75228 0.22594 0.93581 0.17469
0.98721 0.16177 0.16177 0.606969303
NA ENSG00000230928 0.98716 0.16178 0.98716 0.16178 0.98716 0.16178
0.98716 0.16178 0.16178 0.606969303
CGNL1 ENSG00000128849 0.40444 0.34294 0.27543 0.39149 0.98705 0.16181

-0.80119 0.78849 0.16181 0.606969303
CRTC3 ENSG00000140577 0.27229 0.3927 -0.40728 0.6581 0.98705 0.16181
0.29456 0.38416 0.16181 0.606969303
NA ENSG00000266840 0.98688 0.16185 0.77549 0.21902 0.29684 0.3833
0.78325 0.21674 0.16185 0.606969303
OR6E1P ENSG00000235213 0.98684 0.16186 0.69977 0.24204 0.72878
0.23307 0.72878 0.23307 0.16186 0.606969303
HMGN2P9 ENSG00000180150 0.98681 0.16187 0.34458 0.36521 -0.86635
0.80685 0.34458 0.36521 0.16187 0.606969303
NA ENSG00000281706 0.98679 0.16187 -0.48256 0.68529 -0.35194 0.63756
0.98679 0.16187 0.16187 0.606969303
LY6E ENSG00000160932 0.63576 0.26246 0.63576 0.26246 0.98677 0.16188
0.74816 0.22718 0.16188 0.606969303
LHFPL5 ENSG00000197753 -0.038195 0.51523 0.53524 0.29624 0.98672
0.16189 0.80438 0.21059 0.16189 0.606969303
NA ENSG00000250315 0.98672 0.16189 0.21596 0.41451 0.21596 0.41451
0.21596 0.41451 0.16189 0.606969303
NA ENSG00000229878 0.9867 0.1619 0.9867 0.1619 0.9867 0.1619
0.9867 0.1619 0.1619 0.606969303
PCAT4 ENSG00000251321 0.98668 0.1619 0.4185 0.33779 0.31726 0.37552
0.31726 0.37552 0.1619 0.606969303
NA ENSG00000224469 -1.37 0.91466 0.98657 0.16193 0.98034 0.16346 -1.37
0.91466 0.16193 0.606988377
NA ENSG00000256540 -0.28966 0.61396 0.98656 0.16193 0.98656 0.16193
0.98656 0.16193 0.16193 0.606988377
RABIF ENSG00000183155 0.98637 0.16197 0.98637 0.16197 -0.43285 0.66744
0.19685 0.42197 0.16197 0.607035715
MIR599 ENSG00000207804 0.98635 0.16198 0.98635 0.16198 0.98635
0.16198 0.98635 0.16198 0.16198 0.607035715
MIR875 ENSG00000216069 0.98635 0.16198 0.98635 0.16198 0.98635
0.16198 0.98635 0.16198 0.16198 0.607035715
NA ENSG00000176349 0.2263 0.41048 0.57435 0.28287 0.98628 0.162
0.7496 0.22675 0.162 0.607045564
HERC2P4 ENSG00000230267 0.78997 0.21477 0.85641 0.19589 0.87687
0.19028 0.98625 0.16201 0.16201 0.607045564
NA ENSG00000227155 0.60853 0.27142 0.60853 0.27142 0.60853 0.27142
0.98621 0.16202 0.16202 0.607045564
NA ENSG00000266400 0.92146 0.1784 0.98603 0.16206 -1.1529 0.87552
-1.3783 0.91595 0.16206 0.607139537
XIRP2-AS1 ENSG00000254552 0.98599 0.16207 -1.2744 0.89874 -0.95951
0.83135 -0.95951 0.83135 0.16207 0.607139537
TAL1 ENSG00000162367 0.75807 0.2242 0.75807 0.2242 0.98571 0.16214
-0.53708 0.70439 0.16214 0.607234063
NA ENSG00000231675 0.98569 0.16214 0.15588 0.43806 0.98569 0.16214
0.98569 0.16214 0.16214 0.607234063
NLRP3 ENSG00000162711 0.11275 0.45511 -0.13193 0.55248 0.77833 0.21819
0.98559 0.16217 0.16217 0.607234063
RNU6-302P ENSG00000202119 0.98557 0.16217 0.98557 0.16217 0.98557
0.16217 0.98557 0.16217 0.16217 0.607234063
LINC02106 ENSG00000251573 0.98557 0.16217 0.98557 0.16217 0.98557
0.16217 0.98557 0.16217 0.16217 0.607234063
BRWD1P2 ENSG00000258290 -0.46069 0.67749 0.80334 0.21089 0.98557
0.16217 0.98557 0.16217 0.16217 0.607234063
CEMIP2 ENSG00000135048 -0.55828 0.71167 0.98551 0.16219 0.44695
0.32745 0.876 0.19051 0.16219 0.607238702
NA ENSG00000269779 -1.4275 0.92329 -1.3614 0.91331 -0.13277 0.55281
0.98541 0.16221 0.16221 0.607238702
MIR10B ENSG00000207744 0.9321 0.17564 -0.133 0.5529 0.9853
0.16224 0.9853 0.16224 0.16224 0.607238702
AJUBA ENSG00000129474 0.28993 0.38593 0.22191 0.41219 0.98524 0.16225
0.3846 0.35027 0.16225 0.607238702
TEX46 ENSG00000227868 0.60912 0.27122 0.98516 0.16227 0.64207 0.26041

0.64207	0.26041	0.16227	0.607238702
ELMOD1	ENSG00000110675	0.76462	0.22225	0.027773	0.48892	0.98506
0.1623	0.55527	0.28935	0.1623	0.607238702
NA	ENSG00000236391	-0.24398	0.59638	-0.033737	0.51346	0.98493	0.16233
-0.13533	0.55382	0.16233	0.607238702
FABP5P6	ENSG00000249919	0.98493	0.16233	0.98493	0.16233	0.98493
0.16233	0.98493	0.16233	0.16233	0.607238702
GRIP2	ENSG00000144596	0.15796	0.43724	-1.4267	0.92317	0.67929	0.24848
0.98489	0.16234	0.16234	0.607238702
ATP11AUN	ENSG00000197595	0.9849	0.16234	-0.40907	0.65876	-0.39353
0.65303	0.0027182	0.49892	0.16234	0.607238702
TOM1	ENSG00000100284	0.98483	0.16235	-0.44635	0.67233	0.7663	0.22175
0.60187	0.27363	0.16235	0.607238702
ADA2	ENSG00000093072	0.25206	0.4005	0.45708	0.32381	-0.74635	0.77227
0.9848	0.16236	0.16236	0.607238702
NA	ENSG00000250762	0.98481	0.16236	0.98481	0.16236	0.98481	0.16236
0.98481	0.16236	0.16236	0.607238702
MTCO1P11	ENSG00000224083	0.98477	0.16237	0.98477	0.16237	0.98477
0.16237	0.98477	0.16237	0.16237	0.607238702
NA	ENSG00000251293	0.98473	0.16238	0.98473	0.16238	0.98473	0.16238
-0.70623	0.75998	0.16238	0.607238702
PIEZO1P1	ENSG00000233686	0.32439	0.37282	0.46518	0.3209	0.98465
0.1624	0.46518	0.3209	0.1624	0.607238702
RN7SL750P	ENSG00000263672	0.98463	0.1624	0.98463	0.1624	0.98463
0.1624	0.98463	0.1624	0.1624	0.607238702
DIPK1A	ENSG00000154511	0.94908	0.17129	0.98444	0.16245	0.43039
0.33346	0.80036	0.21175	0.16245	0.607238702
FGF14	ENSG00000102466	0.98439	0.16246	-0.091704	0.53653	0.60448	0.27276
0.50827	0.30563	0.16246	0.607238702
KDM8	ENSG00000155666	-1.5487	0.93927	-2.405	0.99191	-1.5487	0.93927
0.98441	0.16246	0.16246	0.607238702
C1QTNF1-AS1	ENSG00000265096	0.98437	0.16247	0.98437	0.16247	0.98437
0.16247	0.98437	0.16247	0.16247	0.607238702
PLEKHN1	ENSG00000187583	0.98432	0.16248	-0.10705	0.54263	0.23415
0.40743	-0.27828	0.6096	0.16248	0.607238702
NA	ENSG00000276790	0.98432	0.16248	0.98432	0.16248	0.98432	0.16248
0.98432	0.16248	0.16248	0.607238702
SUCLG1	ENSG00000163541	0.02113	0.49157	0.98428	0.16249	-0.08414
0.53353	-1.254	0.89508	0.16249	0.607238702
RPS9	ENSG00000170889	0.98429	0.16249	0.98429	0.16249	0.98429	0.16249
0.98429	0.16249	0.16249	0.607238702
MTCO1P2	ENSG00000259045	-0.079228	0.53157	0.52158	0.30098	-0.079228
0.53157	0.98419	0.16251	0.16251	0.607238702
NA	ENSG00000261337	0.98406	0.16254	0.98406	0.16254	0.98406	0.16254
0.98406	0.16254	0.16254	0.607238702
ZCCHC17	ENSG00000121766	0.48004	0.3156	-0.24187	0.59556	0.98403
0.16255	-0.16668	0.56619	0.16255	0.607238702
NA	ENSG00000250869	0.98399	0.16256	0.98399	0.16256	0.98399	0.16256
0.98399	0.16256	0.16256	0.607238702
NA	ENSG00000261482	0.98399	0.16256	0.98399	0.16256	0.98399	0.16256
0.98399	0.16256	0.16256	0.607238702
ANKRD60	ENSG00000124227	0.090132	0.46409	-0.48252	0.68528	-0.71391
0.76236	0.98393	0.16257	0.16257	0.607238702
RNU6-804P	ENSG00000207013	0.63553	0.26254	-0.55877	0.71184	0.98394
0.16257	0.63553	0.26254	0.16257	0.607238702
RNA5SP465	ENSG00000212497	0.6812	0.24787	0.89269	0.18601	0.27297
0.39244	0.98375	0.16262	0.16262	0.607360503
NA	ENSG00000250383	0.98367	0.16264	0.98367	0.16264	0.60705	0.27191
0.98367	0.16264	0.16264	0.607360503
NA	ENSG00000254971	0.98365	0.16264	0.98365	0.16264	0.98365	0.16264
0.98365	0.16264	0.16264	0.607360503
LINC02770	ENSG00000228215	0.9835	0.16268	0.26265	0.39641	0.26265

0.39641 0.26265 0.39641 0.16268 0.607361584
SIGLEC22P ENSG00000268849 0.88549 0.18795 0.98346 0.16269 0.88549
0.18795 0.88549 0.18795 0.16269 0.607361584
PAXIP1-AS2 ENSG00000214106 0.73327 0.2317 0.98338 0.16271 0.67472
0.24993 0.16639 0.43392 0.16271 0.607361584
NA ENSG00000259351 -0.37803 0.6473 0.98338 0.16271 0.98338 0.16271
0.98338 0.16271 0.16271 0.607361584
TULP4 ENSG00000130338 0.91255 0.18074 0.98334 0.16272 0.62426 0.26623
0.43617 0.33136 0.16272 0.607361584
BOLA3P2 ENSG00000267332 0.21407 0.41525 -0.40219 0.65623 0.98334
0.16272 0.38275 0.35095 0.16272 0.607361584
NA ENSG00000253508 -0.25732 0.60154 -0.37209 0.64509 0.9833 0.16273
0.9833 0.16273 0.16273 0.607361584
BST1 ENSG00000109743 0.98327 0.16274 0.073051 0.47088 0.54912 0.29146
-0.16833 0.56684 0.16274 0.607361584
CSTF3-DT ENSG00000247151 0.97531 0.1647 0.98296 0.16281 0.98296
0.16281 0.98296 0.16281 0.16281 0.607548672
NA ENSG00000253288 -0.39733 0.65444 -0.71584 0.76296 -1.9053 0.97163
0.98288 0.16283 0.16283 0.607548672
C11orf97 ENSG00000257057 -0.017086 0.50682 0.98286 0.16284 0.98286
0.16284 -0.11498 0.54577 0.16284 0.607548672
VN1R92P ENSG00000268908 0.98286 0.16284 0.80503 0.2104 0.52197
0.30085 0.98286 0.16284 0.16284 0.607548672
SNHG26 ENSG00000228649 0.98272 0.16287 0.71291 0.23795 0.00025766
0.4999 0.00025766 0.4999 0.16287 0.60759566
YTHDF2 ENSG00000198492 0.86334 0.19397 0.98265 0.16289 0.061987
0.47529 0.0052361 0.49791 0.16289 0.60759566
NA ENSG00000261028 0.98264 0.16289 0.43168 0.33299 0.43168 0.33299
0.43168 0.33299 0.16289 0.60759566
HPGD ENSG00000164120 0.35516 0.36124 0.07996 0.46813 0.98253 0.16292
0.07996 0.46813 0.16292 0.607614542
NA ENSG00000259116 0.64048 0.26093 0.98251 0.16292 0.64048 0.26093
0.64048 0.26093 0.16292 0.607614542
RPL23AP27 ENSG00000225669 -0.89572 0.8148 -0.89572 0.8148 -0.89572
0.8148 0.98239 0.16295 0.16295 0.607671152
HCN3 ENSG00000143630 0.98234 0.16297 0.8026 0.2111 0.59072 0.27735
0.89844 0.18447 0.16297 0.607671152
CCER1 ENSG00000197651 0.98228 0.16298 0.98228 0.16298 0.98228 0.16298
0.98228 0.16298 0.16298 0.607671152
SPO11 ENSG00000054796 0.0073095 0.49708 -0.99048 0.83903 0.98224 0.16299
-0.59046 0.72256 0.16299 0.607671152
LINC01393 ENSG00000225535 0.98214 0.16301 0.98214 0.16301 0.98214
0.16301 0.98214 0.16301 0.16301 0.607671152
NA ENSG00000283393 0.98218 0.16301 0.98218 0.16301 0.98218 0.16301
0.98218 0.16301 0.16301 0.607671152
NA ENSG00000272644 -0.56795 0.71497 -0.56795 0.71497 -0.56795 0.71497
0.98202 0.16304 0.16304 0.607727283
DHRS12 ENSG00000102796 0.97632 0.16445 0.98199 0.16305 0.07212
0.47125 -1.1108 0.86668 0.16305 0.607727283
RNU6-1034P ENSG00000202205 0.98192 0.16307 0.98192 0.16307 0.98192
0.16307 0.98192 0.16307 0.16307 0.60773692
HOMER2 ENSG00000103942 0.2969 0.38327 0.98183 0.16309 -0.30214
0.61873 0.6324 0.26356 0.16309 0.60773692
RPS15 ENSG00000115268 0.85334 0.19673 0.24752 0.40225 0.98182 0.16309
-0.30086 0.61824 0.16309 0.60773692
ZBTB20-AS2 ENSG00000241295 0.98175 0.16311 0.98175 0.16311 0.98175
0.16311 0.98175 0.16311 0.16311 0.607739463
PPP1R3G ENSG00000219607 0.25332 0.40001 0.98163 0.16314 0.089575
0.46431 0.089575 0.46431 0.16314 0.607739463
NA ENSG00000274691 0.32804 0.37144 0.98163 0.16314 0.39015 0.34821
0.39015 0.34821 0.16314 0.607739463
CNIH2 ENSG00000174871 0.32551 0.3724 0.98152 0.16317 0.98152 0.16317

0.98152	0.16317	0.16317	0.607739463
RPS26P3	ENSG00000212829	0.98153	0.16317	0.85359	0.19667	-0.20938
0.58292	-0.74507	0.77189	0.16317	0.607739463
B4GAT1	ENSG00000174684	0.98144	0.16319	0.98144	0.16319	0.98144
0.16319	0.98144	0.16319	0.16319	0.607739463
MROCKI	ENSG00000227502	0.98141	0.1632	0.27305	0.39241	0.054311
0.47834	-0.35012	0.63688	0.1632	0.607739463
NA	ENSG00000218073	-0.32745	0.62833	0.98126	0.16323	-0.06576	0.52622
-0.06576	0.52622	0.16323	0.607739463
IVL	ENSG00000163207	0.4073	0.34189	0.98121	0.16324	0.73059	0.23252
0.98121	0.16324	0.16324	0.607739463
LINC01515	ENSG00000228065	0.39304	0.34714	0.98123	0.16324	0.68	0.24825
-0.31639	0.62415	0.16324	0.607739463
RPS19P3	ENSG00000240463	0.98123	0.16324	0.98123	0.16324	0.98123
0.16324	0.98123	0.16324	0.16324	0.607739463
NA	ENSG00000266149	0.37727	0.35299	0.50696	0.30609	0.098273	0.46086
0.9812	0.16325	0.16325	0.607739463
NA	ENSG00000199934	0.98101	0.16329	0.98101	0.16329	-0.52553	0.70039
-0.91109	0.81888	0.16329	0.607739463
NA	ENSG00000264148	0.48808	0.31275	0.011581	0.49538	0.52241	0.30069
0.98084	0.16334	0.16334	0.607739463
NA	ENSG00000225487	0.6314	0.26389	0.98078	0.16335	0.6314	0.26389
0.6314	0.26389	0.16335	0.607739463
NA	ENSG00000266929	0.98079	0.16335	0.98079	0.16335	0.98079	0.16335
0.98079	0.16335	0.16335	0.607739463
ZNF366	ENSG00000178175	0.20074	0.42045	-0.11968	0.54763	0.98076
0.16336	-0.40794	0.65834	0.16336	0.607739463
NA	ENSG00000237003	0.98075	0.16336	0.98075	0.16336	0.98075	0.16336
0.98075	0.16336	0.16336	0.607739463
RGS22	ENSG00000132554	0.98071	0.16337	0.42726	0.3346	0.58693	0.27863
0.64489	0.2595	0.16337	0.607739463
SPNS2	ENSG00000183018	0.98064	0.16338	0.98064	0.16338	0.98064	0.16338
-0.56625	0.71439	0.16338	0.607739463
ZNF652P1	ENSG00000235278	0.9017	0.18361	0.81178	0.20846	0.65425
0.25648	0.9806	0.16339	0.16339	0.607739463
FOXD1	ENSG00000251493	0.98062	0.16339	0.059661	0.47621	0.16391	0.4349
0.16391	0.4349	0.16339	0.607739463
RNU6-264P	ENSG00000252943	0.98062	0.16339	0.98062	0.16339	0.98062
0.16339	0.98062	0.16339	0.16339	0.607739463
OR2AG1	ENSG00000279486	0.98062	0.16339	-0.031054	0.51239	0.57892
0.28132	0.57892	0.28132	0.16339	0.607739463
TCF3	ENSG00000071564	-0.19744	0.57826	0.16621	0.434	-0.18096	0.5718
0.98056	0.16341	0.16341	0.607739886
NA	ENSG00000263004	0.95436	0.16995	0.95436	0.16995	-0.14139	0.55622
0.9805	0.16342	0.16342	0.607739886
TBC1D32	ENSG00000146350	0.48401	0.31419	0.07341	0.47074	0.0078885
0.49685	0.98047	0.16343	0.16343	0.607739886
NA	ENSG00000230118	0.9804	0.16344	0.9804	0.16344	0.9804	0.16344
0.9804	0.16344	0.16344	0.607739886
NSMCE3	ENSG00000185115	0.98035	0.16346	0.37068	0.35544	0.37068
0.35544	0.35729	0.36044	0.16346	0.607767877
NA	ENSG00000248624	0.98017	0.1635	0.98017	0.1635	0.98017	0.1635
0.85716	0.19568	0.1635	0.607787896
RN7SL19P	ENSG00000243532	0.98012	0.16351	0.98012	0.16351	0.98012
0.16351	0.98012	0.16351	0.16351	0.607787896
EIF3LP1	ENSG00000258501	0.98011	0.16352	0.98011	0.16352	0.98011
0.16352	0.98011	0.16352	0.16352	0.607787896
NA	ENSG00000228667	0.43326	0.33241	0.43326	0.33241	0.98003	0.16354
0.67666	0.24931	0.16354	0.607787896
ASCC2	ENSG00000100325	0.97998	0.16355	0.97998	0.16355	0.96119	0.16823
-1.0632	0.85615	0.16355	0.607787896
PHACTR3-AS1	ENSG00000238194	0.9747	0.16486	0.97993	0.16356	0.9747

0.16486 0.9747 0.16486 0.16356 0.607787896
RN7SL376P ENSG00000265814 0.97995 0.16356 0.97995 0.16356 0.97995
0.16356 0.97995 0.16356 0.16356 0.607787896
PRSS57 ENSG00000185198 0.97986 0.16358 0.73526 0.23109 0.13593
0.44594 0.81039 0.20886 0.16358 0.607787896
NA ENSG00000270773 -0.13878 0.55519 0.97987 0.16358 0.31211 0.37748
0.033093 0.4868 0.16358 0.607787896
WDTC1 ENSG00000142784 0.46688 0.32029 0.64394 0.25981 0.97979 0.1636
0.39725 0.34559 0.1636 0.607787896
RNU7-66P ENSG00000238949 0.15678 0.43771 0.15678 0.43771 0.97968
0.16362 0.15678 0.43771 0.16362 0.607787896
NA ENSG00000273100 -0.0079466 0.50317 0.9797 0.16362 0.81605 0.20724
0.16458 0.43464 0.16362 0.607787896
ZNF20 ENSG00000132010 0.84764 0.19832 0.2527 0.40025 0.84764 0.19832
0.97965 0.16363 0.16363 0.607787896
NA ENSG00000248975 -1.6099 0.94629 0.97962 0.16364 -0.64502 0.74054
-0.40221 0.65624 0.16364 0.607787896
GGT8P ENSG00000236969 0.97948 0.16367 0.97948 0.16367 0.31437 0.37662
0.091971 0.46336 0.16367 0.607790744
NA ENSG00000254559 0.97944 0.16368 0.97944 0.16368 0.97944 0.16368
0.97944 0.16368 0.16368 0.607790744
LINC02860 ENSG00000222004 0.086252 0.46563 0.36861 0.35621 -1.3467
0.91095 0.97935 0.1637 0.1637 0.607790744
NA ENSG00000224790 0.97927 0.16372 0.97927 0.16372 0.97927 0.16372
0.97927 0.16372 0.16372 0.607790744
ATP5PBP3 ENSG00000214003 0.97919 0.16374 0.97919 0.16374 0.97919
0.16374 0.97919 0.16374 0.16374 0.607790744
NA ENSG00000207417 -0.43209 0.66716 -0.16136 0.5641 0.97909 0.16377
0.13626 0.44581 0.16377 0.607790744
UTP14C ENSG00000253797 0.97895 0.1638 0.97895 0.1638 0.97895
0.1638 0.97895 0.1638 0.1638 0.607790744
NA ENSG00000267461 -1.1169 0.86799 -1.4368 0.92461 0.9789 0.16381
-1.4368 0.92461 0.16381 0.607790744
LINC02390 ENSG00000256582 0.97889 0.16382 -0.0097582 0.50389 0.0080153
0.4968 0.0080153 0.4968 0.16382 0.607790744
CC2D2A ENSG00000048342 0.97886 0.16383 -0.035474 0.51415 -0.090073
0.53589 0.78834 0.21525 0.16383 0.607790744
TMA7 ENSG00000232112 0.97885 0.16383 0.97885 0.16383 0.97885 0.16383
0.97885 0.16383 0.16383 0.607790744
LINC01902 ENSG00000283503 0.97877 0.16385 0.97877 0.16385 0.97877
0.16385 -0.079601 0.53172 0.16385 0.607790744
ATXN7L1 ENSG00000146776 -1.7718 0.96178 -0.92935 0.82365 -1.3252
0.90745 0.97864 0.16388 0.16388 0.607790744
LINC02550 ENSG00000271584 0.19446 0.42291 0.97865 0.16388 -0.011867
0.50473 0.73031 0.2326 0.16388 0.607790744
AZI2 ENSG00000163512 0.9786 0.16389 0.59784 0.27497 -0.61976 0.73229
-0.48976 0.68785 0.16389 0.607790744
LINC01376 ENSG00000236204 0.028153 0.48877 0.035683 0.48577 -0.29753
0.61697 0.97858 0.16389 0.16389 0.607790744
SNCA-AS1 ENSG00000247775 0.61609 0.26892 0.97856 0.1639 -0.68965
0.75479 0.86683 0.19302 0.1639 0.607790744
LINC01128 ENSG00000228794 -0.35179 0.6375 0.17675 0.42985 -0.48808
0.68725 0.97851 0.16391 0.16391 0.607790744
CYP1A2 ENSG00000140505 0.72543 0.23409 -0.49306 0.68902 0.97839
0.16394 0.7993 0.21206 0.16394 0.607790744
ZCCHC7 ENSG00000147905 -0.34346 0.63437 0.78694 0.21566 0.97837
0.16395 -0.31638 0.62414 0.16395 0.607790744
NA ENSG00000258401 0.97833 0.16395 0.97833 0.16395 0.97833 0.16395
0.97833 0.16395 0.16395 0.607790744
MIR4472-1 ENSG00000265247 -1.2428 0.89303 -1.2289 0.89044 -0.87429
0.80902 0.97834 0.16395 0.16395 0.607790744
FBXW11 ENSG00000072803 0.46515 0.32091 0.83813 0.20098 0.53862

							0.29508	0.97831	0.16396	0.16396	0.607790744
TTLL3	ENSG00000214021	0.70762	0.23959	-0.053981	0.52152	0.97829	0.16396	-0.81426	0.79225	0.16396	0.607790744
NA	ENSG00000240881	-0.24451	0.59658	-0.90012	0.81597	0.97828	0.16397	0.97828	0.16397	0.16397	0.607790744
NA	ENSG00000258793	0.97828	0.16397	0.97828	0.16397	0.97828	0.16397	0.97828	0.16397	0.16397	0.607790744
NA	ENSG00000183663	0.97819	0.16399	-0.39132	0.65222	0.10023	0.46008	-0.043021	0.51716	0.16399	0.607790744
GDAP2	ENSG00000196505	0.62355	0.26646	0.9782	0.16399	0.90381	0.18305	-0.074136	0.52955	0.16399	0.607790744
ZNF638	ENSG00000075292	0.97807	0.16402	0.76995	0.22066	0.56261	0.28685	0.66494	0.25304	0.16402	0.607846532
RPS3AP26	ENSG00000214389	-0.50058	0.69167	-0.50058	0.69167	-0.50058	0.69167	0.97803	0.16403	0.16403	0.607846532
SLAMF9	ENSG00000162723	0.12759	0.44924	0.97796	0.16405	0.12759	0.44924	0.12759	0.44924	0.16405	0.60787442
NA	ENSG00000251600	0.97746	0.16417	0.5186	0.30202	-0.4657	0.67929	0.2315	0.40846	0.16417	0.608143586
RPL21P92	ENSG00000225922	0.97744	0.16418	0.97744	0.16418	0.97744	0.16418	0.97744	0.16418	0.16418	0.608143586
SETD3	ENSG00000183576	0.97738	0.16419	0.97738	0.16419	0.97738	0.16419	0.97738	0.16419	0.16419	0.608143586
DUSP27	ENSG00000198842	0.9774	0.16419	0.94854	0.17143	0.9774	0.16419	0.7907	0.21456	0.16419	0.608143586
ZNF808	ENSG00000198482	0.97735	0.1642	-0.92013	0.82125	-0.58868	0.72196	0.78513	0.21619	0.1642	0.608143586
RN7SKP246	ENSG00000201423	0.97733	0.1642	0.71822	0.23631	0.71822	0.23631	0.71822	0.23631	0.1642	0.608143586
ARHGAP32	ENSG00000134909	0.9773	0.16421	0.1634	0.4351	0.57966	0.28107	-0.099419	0.5396	0.16421	0.608143586
GINS2	ENSG00000131153	0.58242	0.28014	0.084484	0.46634	0.084484	0.46634	0.97724	0.16423	0.16423	0.608162249
PSMC1P11	ENSG00000217385	0.15107	0.43996	0.97718	0.16424	0.97718	0.16424	0.97718	0.16424	0.16424	0.608162249
LINC02695	ENSG00000254699	-0.26527	0.6046	0.97705	0.16427	-0.26527	0.6046	-0.26527	0.6046	0.16427	0.608227118
DNASE2	ENSG00000105612	0.33854	0.36748	0.97692	0.1643	0.97692	0.1643	0.97692	0.1643	0.1643	0.608255218
RNU6-287P	ENSG00000252039	0.9769	0.16431	0.9769	0.16431	0.9769	0.16431	0.9769	0.16431	0.16431	0.608255218
RNU6-603P	ENSG00000223335	0.41685	0.33839	0.97687	0.16432	0.41685	0.33839	0.41685	0.33839	0.16432	0.608255218
CLCN3P1	ENSG00000232000	0.4724	0.31832	0.59155	0.27708	0.77005	0.22064	0.97682	0.16433	0.16433	0.608255218
NA	ENSG00000267585	0.97678	0.16434	0.94858	0.17142	0.94858	0.17142	0.94858	0.17142	0.16434	0.608255218
ZMYND15	ENSG00000141497	0.97659	0.16439	0.061397	0.47552	0.061397	0.47552	0.66987	0.25147	0.16439	0.608329484
PLAG1	ENSG00000181690	-0.71808	0.76365	0.97653	0.1644	0.97653	0.1644	0.97653	0.1644	0.1644	0.608329484
IL1B	ENSG00000125538	0.245	0.40323	0.97649	0.16441	0.97649	0.16441	-0.49078	0.68821	0.16441	0.608329484
NAT8	ENSG00000144035	0.6453	0.25937	0.97647	0.16441	0.83266	0.20252	0.83266	0.20252	0.16441	0.608329484
RAVER1	ENSG00000161847	-0.11841	0.54713	-0.11841	0.54713	0.9763	0.16446	0.9763	0.16446	0.16446	0.608459087
TREML3P	ENSG00000184106	0.94366	0.17267	0.97624	0.16447	0.97624	0.16447	0.97624	0.16447	0.16447	0.608459087
LINC02223	ENSG00000249937	-0.11237	0.54474	0.01859	0.49258	-0.11237	0.54474	0.97606	0.16452	0.16452	0.608496464
MAU2	ENSG00000129933	0.97601	0.16453	0.66109	0.25428	0.97601	0.16453				

0.97601 0.16453 0.16453 0.608496464
EFR3B ENSG00000084710 0.45756 0.32363 0.71233 0.23813 0.97587 0.16456
-0.23928 0.59456 0.16456 0.608496464
CACNB4 ENSG0000182389 0.97587 0.16456 -0.28568 0.61244 -0.33867
0.63257 -0.064175 0.52558 0.16456 0.608496464
NA ENSG00000224653 0.97587 0.16457 0.97587 0.16457 0.97587 0.16457
0.97587 0.16457 0.16457 0.608496464
NA ENSG00000226345 0.97585 0.16457 0.28921 0.38621 0.97585 0.16457
0.97585 0.16457 0.16457 0.608496464
NLRP2 ENSG00000022556 0.97583 0.16458 -0.14212 0.55651 0.078998 0.46852
-0.67845 0.75126 0.16458 0.608496464
LCE2C ENSG00000187180 0.18887 0.4251 0.85832 0.19536 0.78305 0.2168
0.97581 0.16458 0.16458 0.608496464
HNRNPCP6 ENSG00000213305 -0.37234 0.64518 0.97571 0.1646 -0.37234
0.64518 0.92328 0.17793 0.1646 0.608524239
NA ENSG00000248378 0.47424 0.31766 0.47424 0.31766 -1.5385 0.93804
0.97557 0.16464 0.16464 0.608533796
RNA5SP360 ENSG00000252364 0.97552 0.16465 0.20329 0.41946 0.16075
0.43615 0.48306 0.31453 0.16465 0.608533796
UCN3 ENSG00000178473 0.97549 0.16466 0.71984 0.23581 -1.0971 0.8637
-1.0971 0.8637 0.16466 0.608533796
NA ENSG00000230828 0.97543 0.16467 0.97543 0.16467 0.97543 0.16467
0.97543 0.16467 0.16467 0.608533796
LINC02574 ENSG00000233975 0.21386 0.41533 -0.14543 0.55781 0.75487
0.22516 0.97543 0.16467 0.16467 0.608533796
RPS17P15 ENSG00000239354 0.97538 0.16469 0.97538 0.16469 0.97538
0.16469 0.97538 0.16469 0.16469 0.608533796
NA ENSG00000254325 0.97535 0.16469 0.97535 0.16469 0.97535 0.16469
0.54135 0.29413 0.16469 0.608533796
CYB5AP2 ENSG00000214369 -0.48308 0.68548 0.90641 0.18236 0.97503
0.16477 0.97503 0.16477 0.16477 0.60857192
EBPL ENSG00000123179 0.19655 0.42209 0.96296 0.16778 0.97501 0.16478
0.65489 0.25627 0.16478 0.60857192
RNU6-1105P ENSG00000207505 0.97495 0.16479 -1.4564 0.92735 -0.27141
0.60696 -0.27141 0.60696 0.16479 0.60857192
FANK1 ENSG00000203780 -1.2031 0.88554 -1.7082 0.9562 0.97486 0.16482
-0.43931 0.66978 0.16482 0.60857192
ZNF720P1 ENSG00000260010 0.73121 0.23233 0.56079 0.28747 0.97484
0.16482 0.56079 0.28747 0.16482 0.60857192
MIR3135A ENSG00000266745 0.97482 0.16482 0.97482 0.16482 0.97482
0.16482 0.97482 0.16482 0.16482 0.60857192
DOCK9-DT ENSG00000260992 0.97462 0.16488 0.97462 0.16488 0.97462
0.16488 0.97462 0.16488 0.16488 0.60857192
DNAJC12 ENSG00000108176 0.41027 0.3408 0.41027 0.3408 0.41027
0.3408 0.97456 0.16489 0.16489 0.60857192
BBS9 ENSG00000122507 0.57226 0.28357 -0.56324 0.71336 0.97455 0.16489
-1.2851 0.90062 0.16489 0.60857192
OR6C68 ENSG00000205327 -1.9934 0.97689 0.97456 0.16489 -0.75982
0.77632 -0.75982 0.77632 0.16489 0.60857192
UQCC1 ENSG00000101019 -0.27703 0.60912 -0.26054 0.60278 -0.018156 0.50724
0.97453 0.1649 0.1649 0.60857192
AP4M1 ENSG00000221838 0.97446 0.16491 0.97446 0.16491 0.97446 0.16491
0.97446 0.16491 0.16491 0.60857192
NA ENSG00000255230 0.97445 0.16492 -0.44072 0.67029 -0.97683 0.83567
-0.78301 0.78319 0.16492 0.60857192
TMPRSS7 ENSG00000176040 -2.2618 0.98815 -0.87675 0.80969 0.97438
0.16493 -1.0355 0.84979 0.16493 0.60857192
TRPS1 ENSG00000104447 -0.082021 0.53269 -0.88295 0.81137 0.97437 0.16494
0.80354 0.21083 0.16494 0.60857192
HLA-DMA ENSG00000204257 -0.69225 0.75561 -0.30074 0.61819 0.16441
0.43471 0.97434 0.16494 0.16494 0.60857192
GPIHBP1 ENSG00000277494 -0.51083 0.69526 -0.37338 0.64557 -0.75004

0.77339 0.97434 0.16494 0.16494 0.60857192
CCDC73 ENSG00000186714 -0.010478 0.50418 0.30835 0.37891 0.5759
0.28234 0.97428 0.16496 0.16496 0.60857192
NA ENSG00000277020 0.47957 0.31577 0.4702 0.31911 -0.63242 0.73644
0.9742 0.16498 0.16498 0.60857192
TFAP4 ENSG00000090447 0.97413 0.165 0.021323 0.49149 -0.15457 0.56142
0.55636 0.28898 0.165 0.60857192
RABGAP1L-DT ENSG00000227373 -0.056614 0.52257 0.97413 0.165 0.7942
0.21354 0.97413 0.165 0.165 0.60857192
NA ENSG00000250400 -0.70069 0.75825 0.9741 0.165 0.9741 0.165 0.9741
0.165 0.165 0.60857192
NTF6A ENSG00000267019 0.97406 0.16501 0.97406 0.16501 0.97406 0.16501
0.97406 0.16501 0.16501 0.60857192
ATP5PBP6 ENSG00000219806 0.76817 0.22119 0.97398 0.16503 -0.89663
0.81504 -0.89663 0.81504 0.16503 0.60857192
HOMEZ ENSG00000215271 0.13112 0.44784 -0.036148 0.51442 0.97394 0.16504
0.24544 0.40306 0.16504 0.60857192
NA ENSG00000258039 0.97394 0.16504 -0.69341 0.75597 0.39727 0.34559
-0.58016 0.7191 0.16504 0.60857192
MPND ENSG00000008382 0.72188 0.23518 0.45916 0.32306 0.28293 0.38862
0.97393 0.16505 0.16505 0.60857192
NA ENSG00000250031 0.45146 0.32583 -0.25211 0.59952 -0.090362 0.536
0.97392 0.16505 0.16505 0.60857192
NA ENSG00000239121 0.25493 0.39939 0.25493 0.39939 0.97381 0.16508
0.25493 0.39939 0.16508 0.608627304
SPRED1 ENSG00000166068 0.58344 0.2798 0.86045 0.19477 0.97375
0.16509 0.17379 0.43101 0.16509 0.608627304
GRK5 ENSG00000198873 0.45948 0.32294 0.97364 0.16512 -0.2508 0.59901
-0.339 0.63269 0.16512 0.608627454
NA ENSG00000257531 0.91479 0.18015 0.89563 0.18522 0.97364 0.16512
0.97364 0.16512 0.16512 0.608627454
HNRNPA1P48 ENSG00000224578 0.29132 0.3854 0.97358 0.16513 0.34833
0.3638 0.29132 0.3854 0.16513 0.608627454
TXNP6 ENSG00000234036 0.97356 0.16514 0.97356 0.16514 0.97356 0.16514
0.97356 0.16514 0.16514 0.608627454
ZSWIM8 ENSG00000214655 0.97339 0.16518 0.6431 0.26008 0.6431
0.26008 -0.1658 0.56584 0.16518 0.608719654
NA ENSG00000270236 0.0066592 0.49734 0.97333 0.16519 0.97333 0.16519
-0.85756 0.80443 0.16519 0.608719654
NA ENSG00000266839 0.97314 0.16524 0.97314 0.16524 0.97314 0.16524
0.97314 0.16524 0.16524 0.608729195
SEPTIN14P12 ENSG00000235748 0.97311 0.16525 0.97311 0.16525 0.97311
0.16525 0.97311 0.16525 0.16525 0.608729195
RPL23AP71 ENSG00000237450 -0.66191 0.74598 -0.41239 0.65997 0.97309
0.16525 0.92824 0.17664 0.16525 0.608729195
PBLD ENSG00000108187 0.50592 0.30645 -0.17174 0.56818 0.97308 0.16526
0.24917 0.40162 0.16526 0.608729195
LINC01160 ENSG00000231346 -0.21584 0.58544 -0.21584 0.58544 -0.21584
0.58544 0.97301 0.16527 0.16527 0.608729195
KMT5AP2 ENSG00000232531 0.97295 0.16529 0.97295 0.16529 0.97295
0.16529 0.97295 0.16529 0.16529 0.608729195
MRPL57P6 ENSG00000249449 0.97291 0.1653 0.97291 0.1653 0.97291
0.1653 0.97291 0.1653 0.1653 0.608729195
PRR15L ENSG00000167183 0.94379 0.17264 0.94379 0.17264 0.94379
0.17264 0.97286 0.16531 0.16531 0.608729195
NA ENSG00000228082 0.96798 0.16653 0.96798 0.16653 -0.03932 0.51568
0.97284 0.16532 0.16532 0.608729195
CADM3 ENSG00000162706 0.9728 0.16533 -1.4963 0.93271 0.95723 0.16923
0.76629 0.22175 0.16533 0.608729195
P4HTM ENSG00000178467 0.97279 0.16533 0.39901 0.34494 0.50195 0.30785
-0.57417 0.71707 0.16533 0.608729195
NA ENSG00000279368 0.97267 0.16536 0.97267 0.16536 0.97267 0.16536

0.97267 0.16536 0.16536 0.608766123
NA ENSG00000280272 -0.19573 0.57759 0.65947 0.2548 0.97264 0.16537
-1.0688 0.85741 0.16537 0.608766123
NDST2 ENSG00000166507 -0.29368 0.6155 0.97254 0.16539 0.97254 0.16539
0.97254 0.16539 0.16539 0.608766123
UBE2V1P15 ENSG00000217680 0.97254 0.16539 0.66743 0.25225 0.97254
0.16539 0.97254 0.16539 0.16539 0.608766123
EIF4EP2 ENSG00000229944 -1.3105 0.90499 0.97244 0.16542 -0.82585
0.79556 -0.82585 0.79556 0.16542 0.608784578
CLEC5A ENSG00000258227 0.97242 0.16542 0.77244 0.21993 -2.369
0.99108 -1.4428 0.92546 0.16542 0.608784578
UGT3A1 ENSG00000145626 -0.46576 0.67931 0.97228 0.16546 -0.81481
0.79241 -0.58713 0.72144 0.16546 0.608867442
NA ENSG00000244932 -0.26781 0.60558 0.49599 0.30995 0.97223 0.16547
0.43305 0.33249 0.16547 0.608867442
NA ENSG00000272320 0.3079 0.37908 0.3079 0.37908 0.3079 0.37908
0.97217 0.16548 0.16548 0.608867442
LINC02167 ENSG00000261122 0.85022 0.1976 0.97209 0.1655 0.85022
0.1976 0.85022 0.1976 0.1655 0.608895055
SNX20 ENSG00000167208 0.41209 0.34014 0.41209 0.34014 0.33826 0.36758
0.97192 0.16555 0.16555 0.608922908
NA ENSG00000264327 -1.2135 0.88753 0.24441 0.40346 -1.7817 0.9626
0.97191 0.16555 0.16555 0.608922908
RPS2P4 ENSG00000196183 0.97186 0.16556 -0.3085 0.62115 0.56847
0.28486 0.25735 0.39845 0.16556 0.608922908
ANKRD26 ENSG00000107890 -0.49106 0.68831 0.97177 0.16558 -1.1159
0.86777 -1.3345 0.90899 0.16558 0.608922908
RNA5SP53 ENSG00000212601 -1.3887 0.91753 -1.3887 0.91753 0.97164
0.16561 -1.3887 0.91753 0.16561 0.608922908
NA ENSG00000236710 0.19098 0.42427 -0.3304 0.62945 0.97167 0.16561
0.97167 0.16561 0.16561 0.608922908
RNU6-464P ENSG00000253026 -0.50541 0.69337 0.036702 0.48536 0.97157
0.16563 0.97157 0.16563 0.16563 0.608922908
SLCO1B3 ENSG00000111700 0.34191 0.36621 0.97152 0.16564 0.11966
0.45238 0.57964 0.28108 0.16564 0.608922908
SCGB3A1 ENSG00000161055 0.97153 0.16564 0.46074 0.32249 -0.69656
0.75696 -0.4874 0.68701 0.16564 0.608922908
LINC02869 ENSG00000228208 -0.11326 0.54509 -0.11326 0.54509 -0.21479
0.58503 0.97152 0.16564 0.16564 0.608922908
MRPS18A ENSG00000096080 -0.40999 0.65909 -0.40999 0.65909 0.52031
0.30142 0.97146 0.16566 0.16566 0.608922908
IFI6 ENSG00000126709 0.16213 0.4356 0.16213 0.4356 0.16213 0.4356
0.97142 0.16567 0.16567 0.608922908
RNA5SP449 ENSG00000252680 0.80076 0.21164 0.97131 0.1657 0.97131
0.1657 0.97131 0.1657 0.1657 0.608922908
NA ENSG00000258177 -0.16982 0.56742 -0.71772 0.76353 0.97128 0.1657
0.97128 0.1657 0.1657 0.608922908
MAPK1IP1L ENSG00000168175 0.97125 0.16571 0.94512 0.1723 0.97125
0.16571 0.97125 0.16571 0.16571 0.608922908
NA ENSG00000233755 0.97124 0.16571 -0.64664 0.74107 0.97124 0.16571
-1.1529 0.87552 0.16571 0.608922908
RPL31P54 ENSG00000229865 0.97123 0.16572 0.86756 0.19282 0.86756
0.19282 0.86756 0.19282 0.16572 0.608922908
RBISP1 ENSG00000226209 0.97104 0.16576 0.97104 0.16576 0.97104
0.16576 0.97104 0.16576 0.16576 0.608941363
NA ENSG00000274677 0.13354 0.44688 0.041199 0.48357 0.97108 0.16576
0.97108 0.16576 0.16576 0.608941363
STKLD1 ENSG00000198870 0.72871 0.23309 0.052607 0.47902 0.97102
0.16577 0.046699 0.48138 0.16577 0.608941363
LAGE3P1 ENSG00000225693 0.97092 0.16579 0.97092 0.16579 0.73334
0.23168 0.34966 0.3633 0.16579 0.608941363
NA ENSG00000213240 -1.0802 0.85997 0.45866 0.32324 0.97092 0.16579

0.84067 0.20027 0.16579 0.608941363
NA ENSG00000279914 0.97089 0.1658 0.97089 0.1658 0.97089 0.1658
0.97089 0.1658 0.1658 0.608941363
CCKBR ENSG00000110148 -0.59214 0.72312 -0.59214 0.72312 0.97084 0.16582
-0.59214 0.72312 0.16582 0.60896892
NA ENSG00000201619 0.9706 0.16587 0.68978 0.24517 0.9706 0.16587
0.9706 0.16587 0.16587 0.609079108
TUBB2BP1 ENSG00000216819 0.97055 0.16589 -1.4476 0.92614 0.18579
0.42631 0.65157 0.25734 0.16589 0.609079108
NA ENSG00000231098 0.97053 0.16589 0.97053 0.16589 0.97053 0.16589
0.97053 0.16589 0.16589 0.609079108
NA ENSG00000257323 0.7753 0.21908 0.9705 0.1659 0.9705 0.1659
0.9705 0.1659 0.1659 0.609079108
C2orf27A ENSG00000197927 0.4459 0.32783 0.97032 0.16594 0.019696
0.49214 0.75233 0.22593 0.16594 0.609125
NA ENSG00000236389 -0.66691 0.74759 0.14266 0.44328 0.97029 0.16595
0.14266 0.44328 0.16595 0.609125
NA ENSG00000249409 0.9703 0.16595 0.9703 0.16595 0.49669 0.30971
0.9703 0.16595 0.16595 0.609125
UBR4 ENSG00000127481 0.85048 0.19753 0.97023 0.16597 0.70325 0.24095
0.8251 0.20466 0.16597 0.609152502
NA ENSG00000240393 0.9701 0.166 0.9701 0.166 0.9701 0.166 0.9701 0.166
0.166 0.609152502
NA ENSG00000265643 0.24335 0.40387 0.52254 0.30065 0.97005 0.16601
-0.30662 0.62044 0.16601 0.609152502
PITPNM1 ENSG00000110697 -0.12164 0.54841 0.96994 0.16604 -0.46644
0.67955 0.08473 0.46624 0.16604 0.609152502
PARVG ENSG00000138964 0.03321 0.48675 0.96995 0.16604 -0.65626 0.74417
0.13574 0.44601 0.16604 0.609152502
IFNA6 ENSG00000120235 0.96991 0.16605 -0.3061 0.62024 0.96991 0.16605
0.88593 0.18783 0.16605 0.609152502
STAMBPL1 ENSG00000138134 -0.22577 0.58931 -0.54801 0.70816 -1.0697
0.85762 0.96983 0.16607 0.16607 0.609152502
IL9 ENSG00000145839 0.96979 0.16608 0.67628 0.24943 0.2308 0.40873
-0.67795 0.7511 0.16608 0.609152502
MYL10 ENSG00000106436 0.21391 0.41531 0.96974 0.16609 0.17294 0.43135
-0.50599 0.69357 0.16609 0.609152502
NA ENSG00000253489 0.2377 0.40606 0.96974 0.16609 0.96974 0.16609
0.96974 0.16609 0.16609 0.609152502
NA ENSG00000217477 -0.036626 0.51461 0.96968 0.1661 0.96968 0.1661
-0.77221 0.78001 0.1661 0.609152502
RAP1A ENSG00000116473 -0.40023 0.65551 -0.59943 0.72556 -0.59943 0.72556
0.96961 0.16612 0.16612 0.609152502
HNRNPA1P59 ENSG00000230280 0.96962 0.16612 0.96962 0.16612 0.96962
0.16612 0.96962 0.16612 0.16612 0.609152502
NA ENSG00000212187 0.96951 0.16615 0.96951 0.16615 0.96951 0.16615
0.96951 0.16615 0.16615 0.609216667
NA ENSG00000221322 -0.56436 0.71375 0.9694 0.16617 -0.56436 0.71375
-0.56436 0.71375 0.16617 0.609244158
ITGB1BP1 ENSG00000119185 0.96919 0.16623 0.20182 0.42003 0.17711
0.42971 0.61544 0.26913 0.16623 0.609409103
NA ENSG00000257286 0.96912 0.16624 0.96912 0.16624 0.96912 0.16624
0.96912 0.16624 0.16624 0.609409103
NA ENSG00000216718 -0.56766 0.71487 -0.56766 0.71487 0.96895 0.16628
-0.39433 0.65333 0.16628 0.609463733
NA ENSG00000258851 0.96898 0.16628 0.96898 0.16628 0.96898 0.16628
0.96898 0.16628 0.16628 0.609463733
MTCO1P42 ENSG00000230327 0.9689 0.1663 0.9689 0.1663 0.9689
0.1663 0.9689 0.1663 0.1663 0.609463733
IRF8 ENSG00000140968 0.5502 0.29109 0.96884 0.16631 0.41215 0.34011
0.41215 0.34011 0.16631 0.609463733
CYP4F29P ENSG00000228314 0.64156 0.26058 0.027879 0.48888 0.96874

0.16634 0.30099 0.38171 0.16634 0.609463733
LINC00618 ENSG00000225163 0.67809 0.24886 0.7463 0.22774 0.67809
0.24886 0.96869 0.16635 0.16635 0.609463733
NA ENSG00000260235 0.96868 0.16635 0.96868 0.16635 0.96868 0.16635
0.96868 0.16635 0.16635 0.609463733
FBXO21 ENSG00000135108 0.96855 0.16638 -0.1575 0.56258 0.90174
0.1836 -0.79562 0.78687 0.16638 0.609463733
KRT87P ENSG00000135477 -0.12007 0.54779 0.04754 0.48104 -0.90038
0.81604 0.96858 0.16638 0.16638 0.609463733
MYL12A ENSG00000101608 -1.5709 0.9419 -0.69517 0.75653 -2.2569
0.98799 0.96855 0.16639 0.16639 0.609463733
DPH1 ENSG00000108963 0.96845 0.16641 0.94557 0.17218 -0.024422 0.50974
-0.54849 0.70832 0.16641 0.609463733
CAPN12 ENSG00000182472 0.32644 0.37205 0.15537 0.43826 0.96846
0.16641 -0.16584 0.56586 0.16641 0.609463733
PDRG1 ENSG00000088356 0.23985 0.40522 0.23985 0.40522 -0.49509 0.68973
0.96838 0.16643 0.16643 0.609463733
CWC22 ENSG00000163510 0.96837 0.16643 0.96837 0.16643 0.72031 0.23567
0.95081 0.17085 0.16643 0.609463733
MAN2B1 ENSG00000104774 0.96822 0.16647 -0.56151 0.71277 -1.241
0.8927 0.046661 0.48139 0.16647 0.60950024
PSMB7 ENSG00000136930 -0.33141 0.62983 0.96818 0.16648 -0.28843 0.61349
0.08781 0.46501 0.16648 0.60950024
NA ENSG00000259252 0.5947 0.27602 -0.19124 0.57583 0.96812 0.16649
0.5947 0.27602 0.16649 0.60950024
NA ENSG00000272079 0.96811 0.16649 0.96811 0.16649 0.96811 0.16649
0.96811 0.16649 0.16649 0.60950024
NPFFR2 ENSG00000056291 -0.17388 0.56902 0.9679 0.16655 0.18463
0.42676 0.2015 0.42016 0.16655 0.609674091
NR6A1 ENSG00000148200 -0.18762 0.57441 0.26934 0.39383 0.19246 0.42369
0.96781 0.16657 0.16657 0.609701502
NA ENSG00000284591 0.96769 0.1666 0.96769 0.1666 0.96769 0.1666
0.96769 0.1666 0.1666 0.60976551
NA ENSG00000256950 0.96752 0.16664 0.96752 0.16664 0.96752 0.16664
0.96752 0.16664 0.16664 0.609847698
AP3B1 ENSG00000132842 0.77169 0.22015 0.96749 0.16665 0.77434 0.21936
0.63658 0.2622 0.16665 0.609847698
HSP90AB1 ENSG00000096384 0.38282 0.35093 0.31737 0.37548 0.96744
0.16666 0.4983 0.30914 0.16666 0.609847698
NA ENSG00000229635 -0.65329 0.74322 0.2247 0.41111 0.96712 0.16674
0.96712 0.16674 0.16674 0.610094624
LINC01537 ENSG00000227467 0.96701 0.16677 -0.24194 0.59559 0.96701
0.16677 0.96701 0.16677 0.16677 0.610120496
RNU6-7 ENSG00000201654 -0.53115 0.70234 0.37066 0.35544 -0.30652
0.6204 0.96692 0.16679 0.16679 0.610120496
NA ENSG00000250905 0.68842 0.24559 0.29535 0.38386 0.96692 0.16679
-0.25259 0.59971 0.16679 0.610120496
RAB25 ENSG00000132698 0.44051 0.32979 0.96681 0.16682 0.61015 0.27088
0.94458 0.17244 0.16682 0.610120496
STX17 ENSG00000136874 0.068866 0.47255 0.36111 0.35901 0.65963 0.25475
0.96682 0.16682 0.16682 0.610120496
GPR85 ENSG00000164604 -1.3405 0.90996 -1.3405 0.90996 0.96677 0.16683
-1.3405 0.90996 0.16683 0.610120496
PSMC1P2 ENSG00000213781 -0.015525 0.50619 0.96666 0.16686 -0.015525
0.50619 -0.015525 0.50619 0.16686 0.610120496
PACRG-AS3 ENSG00000225683 -0.04538 0.5181 -0.40326 0.65662 0.96653
0.16689 -0.65787 0.74469 0.16689 0.610120496
LATS2-AS1 ENSG00000233851 0.91338 0.18052 0.96653 0.16689 0.96653
0.16689 0.86649 0.19311 0.16689 0.610120496
NA ENSG00000266199 0.96652 0.16689 0.96652 0.16689 -0.56395 0.71361
-0.99654 0.84051 0.16689 0.610120496
RPS23P2 ENSG00000213448 0.9665 0.1669 0.9665 0.1669 0.9665

0.1669 0.9665 0.1669 0.1669 0.610120496
AGRN ENSG00000188157 0.92814 0.17667 0.12259 0.45122 0.96635 0.16694
0.2792 0.39005 0.16694 0.610120496
NA ENSG00000283010 0.96631 0.16695 0.79258 0.21401 0.55675 0.28885
0.4157 0.33881 0.16695 0.610120496
ARID5A ENSG00000196843 0.38773 0.34911 -0.070441 0.52808 -0.21147
0.58374 0.96624 0.16696 0.16696 0.610120496
RNU6-400P ENSG00000212475 -1.9025 0.97145 -0.35804 0.63985 -0.68418
0.75307 0.96627 0.16696 0.16696 0.610120496
SLC35E1P1 ENSG00000238286 0.96625 0.16696 0.21607 0.41447 0.96625
0.16696 0.96625 0.16696 0.16696 0.610120496
OR7A11P ENSG00000198367 0.96617 0.16698 0.96617 0.16698 0.96617
0.16698 0.96617 0.16698 0.16698 0.610120496
RPL7AP33 ENSG00000254200 0.96616 0.16698 0.96616 0.16698 0.96616
0.16698 0.96616 0.16698 0.16698 0.610120496
MRPL50P4 ENSG00000235469 0.96615 0.16699 0.96615 0.16699 0.96615
0.16699 -0.15146 0.56019 0.16699 0.610120496
CHD4 ENSG00000111642 0.10201 0.45938 0.9661 0.167 -0.6655 0.74713
-0.099561 0.53965 0.167 0.610120496
LINC01876 ENSG00000226383 0.35153 0.36259 -0.79789 0.78753 0.42245
0.33635 0.96605 0.16701 0.16701 0.610120496
NA ENSG00000240729 0.96594 0.16704 -0.31459 0.62346 -0.31459 0.62346
-0.31459 0.62346 0.16704 0.61013861
ANKRD20A18P ENSG00000249493 0.23033 0.40892 -0.74153 0.77081 0.96595
0.16704 0.51734 0.30246 0.16704 0.61013861
NA ENSG00000251135 0.96583 0.16706 0.43141 0.33309 0.96583 0.16706
0.96583 0.16706 0.16706 0.610147512
HMGA2-AS1 ENSG00000197301 0.83647 0.20145 0.56441 0.28624 0.96576
0.16708 0.96576 0.16708 0.16708 0.610147512
NA ENSG00000238153 0.96578 0.16708 0.96578 0.16708 0.96578 0.16708
0.96578 0.16708 0.16708 0.610147512
NA ENSG00000273844 0.96563 0.16712 0.96563 0.16712 0.96563 0.16712
0.96563 0.16712 0.16712 0.610238641
DYNLT3P2 ENSG00000229758 0.96555 0.16713 0.96555 0.16713 0.96555
0.16713 0.96555 0.16713 0.16713 0.610238641
RNU6-14P ENSG00000207360 0.96542 0.16717 0.96542 0.16717 0.96542
0.16717 0.96542 0.16717 0.16717 0.610274465
RPL5P25 ENSG00000225251 0.69529 0.24344 -0.048417 0.51931 0.96542
0.16717 0.18361 0.42716 0.16717 0.610274465
MIR196A1 ENSG00000210741 0.96527 0.1672 0.96527 0.1672 0.96527
0.1672 0.96527 0.1672 0.1672 0.610274465
OR52B2 ENSG00000255307 0.96528 0.1672 -0.37518 0.64624 -0.37518
0.64624 -0.37518 0.64624 0.1672 0.610274465
SLC22A18 ENSG00000110628 -1.0184 0.84575 -0.52391 0.69983 0.96526
0.16721 0.31854 0.37504 0.16721 0.610274465
NA ENSG00000278012 0.96522 0.16722 0.96522 0.16722 0.96522 0.16722
0.96522 0.16722 0.16722 0.610274465
NDUFB8 ENSG00000166136 0.080444 0.46794 0.25222 0.40044 0.080444
0.46794 0.96516 0.16723 0.16723 0.610274465
NA ENSG00000232519 0.96514 0.16724 0.96514 0.16724 0.96514 0.16724
0.96514 0.16724 0.16724 0.610274465
LINC00366 ENSG00000229437 -1.0803 0.85999 -0.11641 0.54634 0.96503
0.16727 0.74161 0.22916 0.16727 0.610338233
RGS9BP ENSG00000186326 0.9648 0.16732 0.9648 0.16732 0.9648
0.16732 0.9648 0.16732 0.16732 0.610465339
OR10D1P ENSG00000196403 0.62696 0.26534 0.62696 0.26534 0.96473
0.16734 0.62696 0.26534 0.16734 0.610465339
NA ENSG00000264108 0.28701 0.38705 0.35108 0.36276 0.96469 0.16735
-0.090946 0.53623 0.16735 0.610465339
NA ENSG00000264488 -0.51631 0.69718 -1.1599 0.87696 0.96465 0.16736
-1.0173 0.84549 0.16736 0.610465339
KRT18P33 ENSG00000214533 0.14126 0.44383 0.49678 0.30967 0.2352

							0.40703	0.96457	0.16738	0.16738	0.610465339
NA	ENSG00000268592	0.96455	0.16738	0.96455	0.16738	0.96455	0.16738	0.96455	0.16738	0.16738	0.610465339
RNU6ATAC16P	ENSG00000221518	0.9644	0.16742	0.9644	0.16742	0.9644	0.16742	0.9644	0.16742	0.16742	0.610492182
PICALM	ENSG00000073921	-0.61227	0.72982	0.96438	0.16743	0.92348	0.17788	0.35153	0.3626	0.16743	0.610492182
TCTN1	ENSG00000204852	0.9643	0.16745	0.49778	0.30932	0.82308	0.20523	0.37708	0.35306	0.16745	0.610492182
NA	ENSG00000243273	0.96428	0.16745	0.96428	0.16745	0.96428	0.16745	0.96428	0.16745	0.16745	0.610492182
NA	ENSG00000249815	0.45697	0.32385	-0.59259	0.72327	-0.52584	0.7005	0.96429	0.16745	0.16745	0.610492182
NA	ENSG00000204055	-0.82809	0.79619	-0.072293	0.52882	0.96421	0.16747	0.7298	0.23276	0.16747	0.610519421
NA	ENSG00000226780	0.10394	0.45861	-0.33349	0.63062	0.96403	0.16752	-0.085961	0.53425	0.16752	0.610610337
NA	ENSG00000279910	0.84803	0.19821	0.96401	0.16752	0.76356	0.22257	0.76356	0.22257	0.16752	0.610610337
RNU6-110P	ENSG00000207237	0.17648	0.42996	0.96392	0.16754	0.96392	0.16754	0.96392	0.16754	0.16754	0.610637562
MRPS33P2	ENSG00000249649	-0.083745	0.53337	0.40424	0.34302	0.96386	0.16756	-0.083745	0.53337	0.16756	0.610664782
LINC02380	ENSG00000248505	0.44059	0.32976	-0.64425	0.74029	0.96379	0.16758	0.96379	0.16758	0.16758	0.610691998
MRPS12	ENSG00000128626	0.96369	0.1676	0.96369	0.1676	0.96369	0.1676	0.96369	0.1676	0.1676	0.61071921
NRBF2P1	ENSG00000267797	-1.4079	0.92042	0.25531	0.39924	0.96352	0.16764	-1.7066	0.95605	0.16764	0.610819291
NA	ENSG00000223428	0.96324	0.16771	0.96324	0.16771	0.96324	0.16771	0.15931	0.43671	0.16771	0.610955243
KRT18P29	ENSG00000213958	-0.26595	0.60486	-0.26595	0.60486	-0.26595	0.60486	0.96321	0.16772	0.16772	0.610955243
NA	ENSG00000274340	-0.99819	0.84091	0.9632	0.16772	0.31606	0.37598	0.65642	0.25578	0.16772	0.610955243
RNA5SP184	ENSG00000223003	0.27182	0.39288	0.27182	0.39288	0.96313	0.16774	0.27182	0.39288	0.16774	0.610955243
NA	ENSG00000274310	0.47508	0.31737	0.47508	0.31737	0.96312	0.16774	0.47508	0.31737	0.16774	0.610955243
TP73-AS1	ENSG00000227372	0.95428	0.16997	0.96307	0.16776	0.96307	0.16776	0.96307	0.16776	0.16776	0.610982422
RNU6-1299P	ENSG00000252125	-0.14044	0.55585	-1.0175	0.84555	0.33337	0.36943	0.9628	0.16782	0.16782	0.611155265
MARK2P8	ENSG00000239503	-0.22318	0.5883	-0.66987	0.74853	0.96272	0.16785	-1.0879	0.86168	0.16785	0.611218839
OR4D7P	ENSG00000254770	0.69642	0.24308	0.96253	0.16789	0.80119	0.21151	-0.15183	0.56034	0.16789	0.611263877
NA	ENSG00000283879	0.96252	0.16789	0.96252	0.16789	0.96252	0.16789	0.96252	0.16789	0.16789	0.611263877
IGLV4-60	ENSG00000211639	0.86102	0.19461	0.86102	0.19461	0.86102	0.19461	0.96248	0.1679	0.1679	0.611263877
MSLNL	ENSG00000162006	-0.49727	0.6905	0.96225	0.16796	0.19264	0.42362	-0.064425	0.52568	0.16796	0.611333391
DOK7	ENSG00000175920	0.12781	0.44915	0.44158	0.3294	0.96211	0.168	-0.018032	0.50719	0.168	0.611333391
NA	ENSG00000279068	0.96209	0.168	0.96209	0.168	0.96209	0.168	0.96209	0.168	0.168	0.611333391
RPL39P25	ENSG00000227271	0.96205	0.16801	0.96205	0.16801	0.96205	0.16801	0.96205	0.16801	0.16801	0.611333391
NA	ENSG00000229969	-0.45677	0.67608	0.96199	0.16803	-0.10608	0.54224	0.63454	0.26287	0.16803	0.611333391
NA	ENSG00000239696	0.96196	0.16804	0.57737	0.28184	-0.19175	0.57603				

-0.19175 0.57603 0.16804 0.611333391
NUBPL ENSG00000151413 0.83943 0.20061 0.96191 0.16805 0.80879 0.20932
0.57439 0.28285 0.16805 0.611333391
NA ENSG00000273382 0.96191 0.16805 0.96191 0.16805 0.96191 0.16805
0.96191 0.16805 0.16805 0.611333391
PAPLN ENSG00000100767 0.96186 0.16806 0.68035 0.24814 0.32052 0.37429
0.82474 0.20476 0.16806 0.611333391
NA ENSG00000226649 0.89684 0.1849 0.89684 0.1849 0.89684 0.1849
0.96181 0.16807 0.16807 0.611333391
NA ENSG00000254020 0.96172 0.16809 0.96172 0.16809 0.96172 0.16809
0.96172 0.16809 0.16809 0.611333391
NA ENSG00000274387 -0.43395 0.66784 0.96174 0.16809 0.080568 0.46789
0.080568 0.46789 0.16809 0.611333391
CD200R1L-AS1 ENSG00000242770 0.96171 0.1681 0.010647 0.49575 0.96171
0.1681 0.96171 0.1681 0.1681 0.611333391
SLC20A1 ENSG00000144136 -0.013143 0.50524 -0.013143 0.50524 -0.34764
0.63594 0.96166 0.16811 0.16811 0.611333391
RNU6-729P ENSG00000207128 -0.22901 0.59057 -0.22901 0.59057 0.96166
0.16811 0.085602 0.46589 0.16811 0.611333391
NA ENSG00000239983 0.38112 0.35156 -0.4305 0.66659 0.96159 0.16813
0.96159 0.16813 0.16813 0.611333391
ATG16L1 ENSG00000085978 0.96152 0.16815 -0.39036 0.65186 -0.27056
0.60664 -0.3502 0.6369 0.16815 0.611333391
TRMT6 ENSG00000089195 0.96152 0.16815 -0.25117 0.59916 -1.018 0.84566
-0.25819 0.60187 0.16815 0.611333391
NA ENSG00000245688 -0.13982 0.5556 0.96146 0.16816 -0.4305 0.66658
-0.4305 0.66658 0.16816 0.611333391
NDRG4 ENSG00000103034 -0.56658 0.7145 0.96143 0.16817 0.94007 0.17359
0.16731 0.43356 0.16817 0.611333391
CXXC1 ENSG00000154832 0.96129 0.1682 0.96129 0.1682 0.96129 0.1682
-0.13908 0.55531 0.1682 0.611351234
C1orf226 ENSG00000239887 0.31591 0.37603 0.96131 0.1682 0.96131
0.1682 0.33683 0.36812 0.1682 0.611351234
NA ENSG00000218730 0.96111 0.16825 0.96111 0.16825 0.96111 0.16825
0.96111 0.16825 0.16825 0.611487358
NA ENSG00000202335 0.96085 0.16831 0.96085 0.16831 0.96085 0.16831
0.96085 0.16831 0.16831 0.611609744
NA ENSG00000254205 0.65099 0.25753 0.56654 0.28551 0.65099 0.25753
0.96082 0.16832 0.16832 0.611609744
LINC02669 ENSG00000233321 -0.69835 0.75752 0.9226 0.17811 0.96074
0.16834 -0.33379 0.63073 0.16834 0.611609744
NA ENSG00000269415 -0.01738 0.50693 0.96076 0.16834 -1.6502 0.95055
-1.6502 0.95055 0.16834 0.611609744
KLF3 ENSG00000109787 0.96067 0.16836 0.87262 0.19143 0.56882 0.28474
0.92526 0.17742 0.16836 0.611609744
SLC6A3 ENSG00000142319 0.34807 0.3639 0.2853 0.38771 0.85845
0.19532 0.96059 0.16838 0.16838 0.611609744
RNA5SP207 ENSG00000212176 0.96058 0.16838 0.96058 0.16838 0.96058
0.16838 0.96058 0.16838 0.16838 0.611609744
NA ENSG00000260387 -0.86813 0.80734 0.96037 0.16843 -1.3541 0.91215
0.12078 0.45193 0.16843 0.611609744
NRG4 ENSG00000169752 -0.72334 0.76527 0.8901 0.18671 -0.5868 0.72133
0.9603 0.16845 0.16845 0.611609744
MALRD1 ENSG00000204740 0.96018 0.16848 -0.64524 0.74061 -0.85782
0.8045 -0.045785 0.51826 0.16848 0.611609744
NA ENSG00000230387 0.96019 0.16848 0.96019 0.16848 0.62607 0.26564
0.86004 0.19488 0.16848 0.611609744
B3GLCT ENSG00000187676 0.96009 0.1685 0.073651 0.47064 0.6573
0.25549 -0.029753 0.51187 0.1685 0.611609744
GOLIM4 ENSG00000173905 -1.769 0.96155 0.20125 0.42025 0.96008
0.16851 -0.053254 0.52124 0.16851 0.611609744
NA ENSG00000213153 0.96008 0.16851 0.96008 0.16851 0.69842 0.24246

0.49142 0.31156 0.16851 0.611609744
VN1R66P ENSG00000237185 0.18062 0.42833 0.29006 0.38589 0.96008
0.16851 0.51691 0.30261 0.16851 0.611609744
PEBP1P1 ENSG00000259059 0.9272 0.17691 -0.029493 0.51176 0.96005
0.16851 0.91674 0.17964 0.16851 0.611609744
NA ENSG00000263271 0.96008 0.16851 0.69347 0.24401 0.69347 0.24401
0.69347 0.24401 0.16851 0.611609744
LINC01262 ENSG00000250739 0.96002 0.16852 -0.16933 0.56723 -0.36948
0.64411 -0.0067174 0.50268 0.16852 0.611609744
NA ENSG00000260635 0.95998 0.16853 0.95998 0.16853 0.95998 0.16853
0.95998 0.16853 0.16853 0.611609744
ACTG1P15 ENSG00000259904 0.95994 0.16854 0.95994 0.16854 0.92637
0.17713 0.92637 0.17713 0.16854 0.611609744
APOA5 ENSG00000110243 0.43405 0.33213 -0.18276 0.57251 0.95988 0.16856
0.12347 0.45087 0.16856 0.611609744
NA ENSG00000182376 0.043689 0.48258 0.95985 0.16857 0.043689 0.48258
0.043689 0.48258 0.16857 0.611609744
GJA3 ENSG00000121743 -0.93272 0.82452 -0.57608 0.71772 0.9598 0.16858
-0.048073 0.51917 0.16858 0.611609744
BSND ENSG00000162399 0.62647 0.2655 0.95977 0.16859 0.62647 0.2655
0.95089 0.17083 0.16859 0.611609744
RERGL ENSG00000111404 0.9597 0.1686 0.9597 0.1686 0.9597 0.1686
0.9597 0.1686 0.1686 0.611609744
MGARP ENSG00000137463 0.33502 0.3688 0.36649 0.357 0.95968 0.16861
0.16375 0.43497 0.16861 0.611609744
NA ENSG00000231794 0.95948 0.16866 0.95948 0.16866 0.95948 0.16866
0.95948 0.16866 0.16866 0.611672275
NA ENSG00000272100 0.95944 0.16867 0.95944 0.16867 0.95944 0.16867
0.95944 0.16867 0.16867 0.611672275
PLA2G2D ENSG00000117215 -0.66692 0.74759 0.45427 0.32482 -0.50645
0.69373 0.95939 0.16868 0.16868 0.611672275
LINC01974 ENSG00000262031 0.82235 0.20544 0.83706 0.20128 0.95941
0.16868 0.95941 0.16868 0.16868 0.611672275
MAP2K4 ENSG00000065559 0.95934 0.16869 0.4437 0.32863 -0.29727
0.61687 -0.061011 0.52432 0.16869 0.611672275
NA ENSG00000240052 -0.29388 0.61557 -0.55782 0.71152 -0.23852 0.59426
0.95922 0.16872 0.16872 0.611726278
NA ENSG00000204752 0.95918 0.16873 0.95918 0.16873 0.95918 0.16873
0.95918 0.16873 0.16873 0.611726278
NA ENSG00000276612 0.9591 0.16875 0.9591 0.16875 0.9591 0.16875
0.9591 0.16875 0.16875 0.611752491
TMCC3 ENSG00000057704 -1.5467 0.93904 0.959 0.16878 -0.16516 0.56559
-0.14733 0.55856 0.16878 0.611752491
ZNF705E ENSG00000214534 0.018085 0.49279 0.21876 0.41342 0.95898
0.16878 -0.71842 0.76375 0.16878 0.611752491
RNU7-96P ENSG00000238719 0.95892 0.1688 0.95892 0.1688 0.95892
0.1688 0.95892 0.1688 0.1688 0.611752491
NA ENSG00000211732 0.56292 0.28674 -0.23345 0.5923 0.95894 0.1688
0.10927 0.45649 0.1688 0.611752491
ZNF585A ENSG00000196967 0.37591 0.35349 0.95879 0.16883 0.7801
0.21767 0.77838 0.21817 0.16883 0.611770219
CPEB2-DT ENSG00000247624 0.47745 0.31652 -0.89934 0.81577 0.95882
0.16883 -0.24873 0.59822 0.16883 0.611770219
CCDC58P1 ENSG00000264311 0.95868 0.16886 0.95868 0.16886 0.95868
0.16886 0.95868 0.16886 0.16886 0.61183343
ECI2 ENSG00000198721 0.95857 0.16889 0.85016 0.19762 0.85016 0.19762
0.85016 0.19762 0.16889 0.611896632
SAMD3 ENSG00000164483 0.95827 0.16896 0.85811 0.19542 0.90719 0.18215
-0.25476 0.60055 0.16896 0.612059233
RN7SKP49 ENSG00000252484 -0.84221 0.80016 -0.84221 0.80016 0.95829
0.16896 -0.84221 0.80016 0.16896 0.612059233
NA ENSG00000228510 -1.2941 0.90219 0.95821 0.16898 0.26799 0.39435

0.015796 0.4937 0.16898 0.612076906
NA ENSG00000228702 0.67807 0.24886 0.95816 0.16899 -1.3855 0.91704
-1.3855 0.91704 0.16899 0.612076906
NA ENSG00000250195 0.78834 0.21525 0.087203 0.46526 0.9581 0.16901
0.55363 0.28992 0.16901 0.61210385
RSF1-IT2 ENSG00000254985 0.95793 0.16905 0.95793 0.16905 0.95793
0.16905 0.95793 0.16905 0.16905 0.612184656
OR7E8P ENSG00000177400 0.95788 0.16906 0.60666 0.27204 0.14515
0.44229 0.82862 0.20366 0.16906 0.612184656
TFAP2E ENSG00000116819 0.95784 0.16907 0.016256 0.49351 -0.13264
0.55276 0.55162 0.2906 0.16907 0.612184656
NA ENSG00000282418 -0.65834 0.74484 0.95767 0.16911 0.61088 0.27064
-0.14451 0.55745 0.16911 0.612246862
INO80B ENSG00000115274 0.95761 0.16913 0.90426 0.18293 0.95761
0.16913 0.94978 0.17111 0.16913 0.612246862
LRRC66 ENSG00000188993 0.95759 0.16913 0.90165 0.18362 0.74756
0.22736 0.74756 0.22736 0.16913 0.612246862
NA ENSG00000224887 0.95754 0.16915 0.95754 0.16915 0.95754 0.16915
0.95754 0.16915 0.16915 0.612246862
NA ENSG00000277684 0.50634 0.30631 -0.05744 0.5229 0.95754 0.16915
0.95754 0.16915 0.16915 0.612246862
ZNRF2 ENSG00000180233 0.32648 0.37203 0.95745 0.16917 -0.72306 0.76518
0.64606 0.25912 0.16917 0.612273775
TTC39C-AS1 ENSG00000264745 0.20626 0.41829 -1.5626 0.94093 0.95735
0.1692 0.051131 0.47961 0.1692 0.612282119
POLR3B ENSG00000013503 0.8876 0.18738 0.70206 0.24132 0.0033298
0.49867 0.9573 0.16921 0.16921 0.612282119
NA ENSG00000257700 0.95731 0.16921 0.95731 0.16921 0.95731 0.16921
0.95731 0.16921 0.16921 0.612282119
HMGB1P10 ENSG00000213707 0.95721 0.16923 0.95721 0.16923 0.95721
0.16923 0.95721 0.16923 0.16923 0.61229974
RHOBTB3 ENSG00000164292 0.95719 0.16924 -1.329 0.90808 0.037284
0.48513 -0.54453 0.70696 0.16924 0.61229974
NA ENSG00000250421 -0.78983 0.78519 -1.1615 0.87729 0.033256 0.48674
0.95692 0.1693 0.1693 0.612471344
GPRIN1 ENSG00000169258 0.47873 0.31606 0.11009 0.45617 0.9568
0.16933 -0.52647 0.70072 0.16933 0.612488933
RIPPLY2 ENSG00000203877 0.62173 0.26706 0.95682 0.16933 0.62173
0.26706 0.62173 0.26706 0.16933 0.612488933
NA ENSG00000279703 0.95667 0.16937 0.95667 0.16937 0.95667 0.16937
-1.2569 0.8956 0.16937 0.612545028
TNF ENSG00000232810 0.95653 0.1694 0.95653 0.1694 0.95653 0.1694
0.95653 0.1694 0.1694 0.612545028
NA ENSG00000271498 0.95648 0.16941 0.95648 0.16941 0.95648 0.16941
0.95648 0.16941 0.16941 0.612545028
OOEP-AS1 ENSG00000231332 0.95641 0.16943 0.95641 0.16943 0.95641
0.16943 0.62967 0.26446 0.16943 0.612545028
RNA5SP297 ENSG00000212331 0.95639 0.16944 0.085587 0.4659 -1.4219
0.92248 -0.50255 0.69236 0.16944 0.612545028
WRAP73 ENSG00000116213 0.95634 0.16945 0.95634 0.16945 0.95634
0.16945 0.95634 0.16945 0.16945 0.612545028
MIR4689 ENSG00000264101 0.020268 0.49191 0.95631 0.16946 0.95631
0.16946 0.95631 0.16946 0.16946 0.612545028
BIVM ENSG00000134897 0.15261 0.43935 0.75879 0.22399 0.95609 0.16951
0.32551 0.3724 0.16951 0.612545028
TSPY26P ENSG00000235217 0.95611 0.16951 0.95611 0.16951 0.95611
0.16951 0.95611 0.16951 0.16951 0.612545028
NA ENSG00000236408 0.29335 0.38463 0.95607 0.16952 0.2064 0.41824
0.2064 0.41824 0.16952 0.612545028
HNRNPA1P18 ENSG00000229910 0.95598 0.16954 -1.0235 0.84695 0.95598
0.16954 0.95598 0.16954 0.16954 0.612545028
NFKBIL1 ENSG00000204498 0.047197 0.48118 0.44377 0.3286 0.95588

0.16957	-0.20488	0.58117	0.16957	0.612545028
FBLN1	ENSG00000077942	0.95572	0.16961	-0.46423	0.67876	0.80083	0.21161
0.80948	0.20912	0.16961	0.612545028
NA	ENSG00000220548	0.83496	0.20187	0.44193	0.32927	0.24587	0.40289
0.95569	0.16962	0.16962	0.612545028
PSD4	ENSG00000125637	-0.24573	0.59705	0.95565	0.16963	-0.95491	0.83019
-0.24573	0.59705	0.16963	0.612545028
NA	ENSG00000227360	0.19877	0.42122	0.95558	0.16964	-0.30175	0.61858
0.19877	0.42122	0.16964	0.612545028
LINC02097	ENSG00000226101	0.18966	0.42479	-1.0192	0.84596	0.56102
0.28739	0.95555	0.16965	0.16965	0.612545028
ZNF792	ENSG00000180884	0.9555	0.16966	0.59188	0.27697	0.59188
0.27697	0.59188	0.27697	0.16966	0.612545028
DCBLD2	ENSG00000057019	-0.10913	0.54345	-0.094456	0.53763	0.22222
0.41207	0.95544	0.16968	0.16968	0.612545028
NA	ENSG00000267529	0.95544	0.16968	0.95544	0.16968	0.95544	0.16968
0.95544	0.16968	0.16968	0.612545028
RNF185-AS1	ENSG00000254835	0.95538	0.16969	0.95538	0.16969	0.95538
0.16969	0.95538	0.16969	0.16969	0.612545028
NA	ENSG00000269066	-0.10884	0.54334	0.95527	0.16972	-0.10884	0.54334
-1.4221	0.92251	0.16972	0.612545028
NF1P3	ENSG00000183249	-0.21454	0.58494	0.64282	0.26017	0.95522	0.16973
0.038578	0.48461	0.16973	0.612545028
MED28P1	ENSG00000214875	0.34463	0.36519	0.95514	0.16975	0.80507
0.21039	0.80507	0.21039	0.16975	0.612545028
HOXC13	ENSG00000123364	0.88583	0.18785	0.95507	0.16977	0.69939
0.24215	-1.786	0.96295	0.16977	0.612545028
NA	ENSG00000268889	0.95502	0.16978	0.95502	0.16978	0.95502	0.16978
0.95502	0.16978	0.16978	0.612545028
CD244	ENSG00000122223	0.31756	0.37541	0.31756	0.37541	0.31756	0.37541
0.9549	0.16981	0.16981	0.612545028
DACT1	ENSG00000165617	-0.024579	0.5098	0.176	0.43015	0.95491	0.16981
0.5401	0.29456	0.16981	0.612545028
NA	ENSG00000173966	0.95487	0.16982	0.95487	0.16982	0.95487	0.16982
0.95487	0.16982	0.16982	0.612545028
NA	ENSG00000243696	0.95483	0.16983	0.95483	0.16983	0.95483	0.16983
0.95483	0.16983	0.16983	0.612545028
C1orf141	ENSG00000203963	0.9548	0.16984	0.46537	0.32083	0.9548
0.16984	0.17912	0.42892	0.16984	0.612545028
NA	ENSG00000241525	0.71187	0.23827	-0.0082915	0.50331	0.95468	0.16987
0.31594	0.37602	0.16987	0.612545028
RNU7-129P	ENSG00000251937	-1.1905	0.88308	-0.75874	0.77599	0.95466
0.16988	0.95466	0.16988	0.16988	0.612545028
RPAP1	ENSG00000103932	0.13609	0.44588	0.52544	0.29964	0.47295	0.31812
0.95462	0.16989	0.16989	0.612545028
SOCS1	ENSG00000185338	-0.41036	0.65923	-1.5395	0.93816	-1.2578	0.89576
0.9546	0.16989	0.16989	0.612545028
SRFBP1	ENSG00000151304	0.95457	0.1699	-0.60602	0.72775	0.95457
0.1699	0.95457	0.1699	0.1699	0.612545028
NA	ENSG00000279217	0.31863	0.375	0.030251	0.48793	0.95449	0.16992
-0.66571	0.7472	0.16992	0.612545028
AQP12B	ENSG00000185176	-0.10502	0.54182	-0.076698	0.53057	0.95441
0.16994	-0.31518	0.62369	0.16994	0.612545028
NA	ENSG00000229224	0.95438	0.16995	-0.62133	0.73281	-0.31148	0.62228
-0.31148	0.62228	0.16995	0.612545028
WDR63	ENSG00000162643	-0.84623	0.80129	0.042965	0.48286	-0.90371	0.81692
0.95429	0.16997	0.16997	0.612545028
BLZF2P	ENSG00000258565	0.91847	0.17919	0.95427	0.16997	0.41774
0.33807	0.052331	0.47913	0.16997	0.612545028
CRYGGP	ENSG00000223710	0.95426	0.16998	-0.48917	0.68764	-0.48917
0.68764	-0.48917	0.68764	0.16998	0.612545028
CD9	ENSG00000010278	-0.65955	0.74523	0.9542	0.16999	0.0049158	0.49804

-0.59002 0.72241 0.16999 0.612545028
LINC01216 ENSG00000250223 0.95421 0.16999 0.95421 0.16999 0.95421
0.16999 0.95421 0.16999 0.16999 0.612545028
NA ENSG00000199636 0.95413 0.17001 0.95413 0.17001 0.95413 0.17001
0.95413 0.17001 0.17001 0.612545028
RNU6-752P ENSG00000252185 0.95413 0.17001 -0.6939 0.75613 0.95413
0.17001 0.95413 0.17001 0.17001 0.612545028
RN7SKP213 ENSG00000222678 0.94652 0.17194 0.94652 0.17194 0.94652
0.17194 0.95408 0.17002 0.17002 0.612545028
RPL31P47 ENSG00000233541 0.95409 0.17002 0.95409 0.17002 0.95409
0.17002 0.95409 0.17002 0.17002 0.612545028
PSG10P ENSG00000248257 -0.22121 0.58754 0.20255 0.41975 0.9541
0.17002 0.11309 0.45498 0.17002 0.612545028
HSPB2-C11orf52 ENSG00000254445 0.95408 0.17002 0.95408 0.17002 0.95408
0.17002 0.95408 0.17002 0.17002 0.612545028
AS3MT ENSG00000214435 0.31426 0.37666 -0.79796 0.78755 0.38952 0.34845
0.95402 0.17004 0.17004 0.612545028
ATP2A3 ENSG00000074370 -0.74682 0.77241 0.95397 0.17005 -0.8498
0.80228 -0.23595 0.59326 0.17005 0.612545028
GOLGA6C ENSG00000167195 0.12699 0.44948 -0.14688 0.55839 0.95393
0.17006 -0.17541 0.56962 0.17006 0.612545028
MAGEF1 ENSG00000177383 0.051743 0.47937 -0.55546 0.71071 0.95394
0.17006 0.94645 0.17196 0.17006 0.612545028
DFFBP1 ENSG00000232303 0.37104 0.3553 0.38686 0.34943 0.38686
0.34943 0.95393 0.17006 0.17006 0.612545028
SPART ENSG00000133104 0.88902 0.187 0.61318 0.26988 0.3572 0.36047
0.95387 0.17007 0.17007 0.612545028
CBX4 ENSG00000141582 -0.43468 0.6681 -0.34007 0.6331 0.9539 0.17007
0.3184 0.37509 0.17007 0.612545028
SUSD5 ENSG00000173705 0.90713 0.18217 0.95385 0.17008 0.95385 0.17008
0.95385 0.17008 0.17008 0.612545028
CDK5RAP1 ENSG00000101391 0.24817 0.402 0.95381 0.17009 -0.3059 0.62016
-0.27191 0.60715 0.17009 0.612545028
NA ENSG00000231557 0.5436 0.29336 0.95374 0.17011 0.77477 0.21924
-0.22906 0.59059 0.17011 0.612545028
LINC02343 ENSG00000271850 0.95368 0.17012 0.95368 0.17012 -0.44711
0.6726 0.95368 0.17012 0.17012 0.612545028
IGKV1D-17 ENSG00000242766 0.95367 0.17013 0.95367 0.17013 0.95367
0.17013 0.95367 0.17013 0.17013 0.612545028
ZFP91-CNTF ENSG00000255073 0.95359 0.17014 0.95359 0.17014 0.95359
0.17014 0.95359 0.17014 0.17014 0.612545028
INMT-MINDY4 ENSG00000254959 -2.0612 0.98036 -2.0612 0.98036 0.34706
0.36427 0.95356 0.17015 0.17015 0.612545028
NA ENSG00000282906 0.95348 0.17017 0.12955 0.44846 0.95348 0.17017
0.85167 0.1972 0.17017 0.612571774
NA ENSG00000225069 -0.10547 0.542 0.95341 0.17019 -0.81129 0.7914
-0.13817 0.55495 0.17019 0.612589261
NA ENSG00000278698 0.95339 0.1702 0.95339 0.1702 0.95339 0.1702
0.95339 0.1702 0.1702 0.612589261
LMBRD1 ENSG00000168216 -0.98121 0.83676 -1.088 0.86169 0.95328
0.17022 0.45444 0.32476 0.17022 0.612613936
NUDT7 ENSG00000140876 0.36977 0.35578 0.39886 0.345 0.39886 0.345 0.9532
0.17024 0.17024 0.612613936
GPR33 ENSG00000214943 0.95315 0.17026 -0.44256 0.67096 -1.414 0.92131
0.27507 0.39163 0.17026 0.612613936
NA ENSG00000250049 0.95314 0.17026 -1.0518 0.85355 -1.0518 0.85355
-1.0518 0.85355 0.17026 0.612613936
FANCF ENSG00000183161 0.062457 0.4751 0.95299 0.1703 0.16082 0.43612
0.95299 0.1703 0.1703 0.612613936
TTC25 ENSG00000204815 0.47482 0.31746 0.47482 0.31746 0.95298 0.1703
-1.033 0.84921 0.1703 0.612613936
ELF3 ENSG00000163435 0.53185 0.29742 0.50633 0.30631 0.78594 0.21595

0.95293 0.17031 0.17031 0.612613936
UPF3AP1 ENSG00000226478 0.95291 0.17032 -1.3792 0.91608 -0.52289
0.69947 -0.12514 0.54979 0.17032 0.612613936
RPS3AP49 ENSG00000242060 0.19134 0.42413 0.95289 0.17032 0.19134
0.42413 -0.97427 0.83504 0.17032 0.612613936
NA ENSG00000279833 0.9528 0.17034 0.9528 0.17034 0.9528 0.17034
0.9528 0.17034 0.17034 0.61262009
NA ENSG00000234210 -0.42446 0.66439 -0.10392 0.54138 0.9527 0.17037
0.083102 0.46689 0.17037 0.61262009
NA ENSG00000248648 0.95254 0.17041 0.95254 0.17041 0.95254 0.17041
0.95254 0.17041 0.17041 0.61262009
TUSC3 ENSG00000104723 0.15218 0.43952 0.95244 0.17044 0.3668 0.35689
-0.25969 0.60245 0.17044 0.61262009
SURF6 ENSG00000148296 0.12097 0.45186 0.91324 0.18056 0.95241 0.17044
-0.10223 0.54071 0.17044 0.61262009
TAF11L13 ENSG00000283776 0.95242 0.17044 -0.34862 0.63631 -0.63005
0.73567 -0.63005 0.73567 0.17044 0.61262009
ELK1P1 ENSG00000234359 0.41299 0.33981 -0.95032 0.82903 0.9524
0.17045 0.9524 0.17045 0.17045 0.61262009
STK16 ENSG00000115661 0.91422 0.1803 0.91422 0.1803 0.91422 0.1803
0.95236 0.17046 0.17046 0.61262009
RSPH3 ENSG00000130363 0.76287 0.22277 0.66876 0.25183 0.95234 0.17046
0.36668 0.35693 0.17046 0.61262009
RNU6-376P ENSG00000207462 0.43178 0.33295 0.90681 0.18225 0.91896
0.17906 0.95234 0.17046 0.17046 0.61262009
NA ENSG00000269481 0.95235 0.17046 0.95235 0.17046 0.95235 0.17046
0.95235 0.17046 0.17046 0.61262009
U2AF1L5 ENSG00000275895 0.95217 0.1705 0.95217 0.1705 0.95217
0.1705 0.95217 0.1705 0.1705 0.612709409
LINC01107 ENSG00000225493 0.46281 0.32175 0.50194 0.30786 0.74852
0.22707 0.95214 0.17051 0.17051 0.612709409
NA ENSG00000265490 0.95193 0.17056 0.95193 0.17056 0.95193 0.17056
0.95193 0.17056 0.17056 0.612778045
DGCR2 ENSG00000070413 0.29581 0.38369 0.29581 0.38369 0.67415 0.25011
0.95191 0.17057 0.17057 0.612778045
RNA5SP190 ENSG00000202092 0.49316 0.31095 0.95178 0.1706 -0.87755
0.80991 -0.49111 0.68833 0.1706 0.612778045
NA ENSG00000234361 -0.25044 0.59888 0.95179 0.1706 -0.25044 0.59888
-0.067676 0.52698 0.1706 0.612778045
NA ENSG00000276007 -0.17544 0.56963 0.95181 0.1706 -0.17544 0.56963
-0.17544 0.56963 0.1706 0.612778045
CGB3 ENSG00000104827 0.95175 0.17061 0.95175 0.17061 0.95175 0.17061
0.95175 0.17061 0.17061 0.612778045
SNORD38C ENSG00000207109 0.9517 0.17063 0.9517 0.17063 0.9517
0.17063 0.9517 0.17063 0.17063 0.612778045
NA ENSG00000270585 0.2263 0.41048 -0.40427 0.65699 0.95168 0.17063
0.95168 0.17063 0.17063 0.612778045
BZW1 ENSG00000082153 0.95153 0.17067 0.42219 0.33644 0.95153 0.17067
0.13369 0.44683 0.17067 0.612778045
UBB ENSG00000170315 0.95153 0.17067 0.54509 0.29285 0.95153 0.17067
0.95153 0.17067 0.17067 0.612778045
NA ENSG00000230607 0.95154 0.17067 0.95154 0.17067 0.95154 0.17067
0.95154 0.17067 0.17067 0.612778045
NA ENSG00000256321 -0.82576 0.79553 0.95148 0.17068 -0.82576 0.79553
-0.82576 0.79553 0.17068 0.612778045
RBFOX3 ENSG00000167281 -0.43932 0.66979 0.044167 0.48239 0.73041
0.23257 0.9513 0.17073 0.17073 0.612866146
NA ENSG00000249022 0.78242 0.21698 0.18656 0.426 0.85262 0.19693
0.9513 0.17073 0.17073 0.612866146
NA ENSG00000255480 -0.080371 0.53203 0.9512 0.17075 -0.080371 0.53203
-0.080371 0.53203 0.17075 0.612866146
EIPR1-IT1 ENSG00000224885 -0.61078 0.72933 -0.8824 0.81122 -0.88154

0.81099 0.95115 0.17076 0.17076 0.612866146
ARHGEF4 ENSG00000136002 0.30315 0.38089 0.95113 0.17077 -0.71956
0.7641 -0.38798 0.65099 0.17077 0.612866146
UBTD1 ENSG00000165886 0.82583 0.20445 0.95109 0.17078 0.8954 0.18529
-0.94121 0.8267 0.17078 0.612866146
FAM219A ENSG00000164970 -1.1041 0.86522 -0.66437 0.74677 0.95101
0.1708 -0.63541 0.73742 0.1708 0.612892784
NA ENSG00000224136 0.95085 0.17084 0.95085 0.17084 -0.55166 0.70941
0.95085 0.17084 0.17084 0.612991179
PHKG2 ENSG00000156873 0.95076 0.17086 0.22254 0.41195 0.31088 0.37795
0.23189 0.40831 0.17086 0.613017803
PELI2 ENSG00000139946 0.1978 0.4216 0.036527 0.48543 0.95051 0.17093
0.31807 0.37522 0.17093 0.613223802
TUT4 ENSG00000134744 0.95039 0.17096 -1.3197 0.90653 -0.0309 0.51233
-0.17983 0.57136 0.17096 0.613238711
NA ENSG00000234318 -0.076376 0.53044 0.95036 0.17096 -0.076376 0.53044
-0.076376 0.53044 0.17096 0.613238711
MIR134 ENSG00000207993 0.95026 0.17099 0.95026 0.17099 0.95026
0.17099 0.95026 0.17099 0.17099 0.613238711
NA ENSG00000226291 0.95018 0.17101 0.95018 0.17101 0.95018 0.17101
0.95018 0.17101 0.17101 0.613238711
PGGHG ENSG00000142102 0.95015 0.17102 -1.5427 0.93855 -1.5427 0.93855
-1.5427 0.93855 0.17102 0.613238711
ATF6B ENSG00000213676 0.95016 0.17102 0.16854 0.43308 -0.15482 0.56152
-0.71442 0.76252 0.17102 0.613238711
ZNF787 ENSG00000142409 0.95009 0.17103 0.95009 0.17103 0.95009
0.17103 0.95009 0.17103 0.17103 0.613238711
SDS ENSG00000135094 0.7981 0.21241 0.95007 0.17104 -0.78595 0.78405
-0.11701 0.54657 0.17104 0.613238711
GSR ENSG00000104687 0.48077 0.31534 -1.5489 0.9393 0.95004 0.17105
-0.85515 0.80377 0.17105 0.613238711
NA ENSG00000103200 0.27764 0.39064 0.94999 0.17106 0.94999 0.17106
0.67775 0.24896 0.17106 0.613238711
NA ENSG00000216307 0.94985 0.17109 -0.062873 0.52507 0.46312 0.32164
0.59921 0.27452 0.17109 0.61330114
PUDPP3 ENSG00000214245 0.94972 0.17113 0.94972 0.17113 0.94972
0.17113 0.94972 0.17113 0.17113 0.613399404
NA ENSG00000230003 0.94964 0.17115 0.94964 0.17115 -0.64614 0.74091
-0.90637 0.81763 0.17115 0.613415433
RPS3AP27 ENSG00000226205 0.94957 0.17117 0.47254 0.31827 0.47254
0.31827 0.40029 0.34447 0.17117 0.613415433
CCDC42 ENSG00000161973 0.19272 0.42359 0.54416 0.29316 0.89913
0.18429 0.94947 0.17119 0.17119 0.613415433
SOWAHA ENSG00000198944 -0.28234 0.61116 -0.066573 0.52654 0.56491
0.28607 0.94942 0.1712 0.1712 0.613415433
NA ENSG00000266460 0.70786 0.23952 -0.12436 0.54949 -0.10048 0.54002
0.94939 0.17121 0.17121 0.613415433
NA ENSG00000279791 0.94938 0.17121 0.94938 0.17121 0.94938 0.17121
0.94938 0.17121 0.17121 0.613415433
ADGRD2 ENSG00000180264 -1.058 0.85497 -1.9766 0.97595 -0.88264
0.81128 0.94915 0.17127 0.17127 0.613585289
TRBV6-5 ENSG00000211721 0.94881 0.17136 0.94881 0.17136 0.94881
0.17136 -0.24686 0.59749 0.17136 0.613770982
NA ENSG00000253523 0.94879 0.17136 0.45581 0.32426 0.45581 0.32426
0.45581 0.32426 0.17136 0.613770982
NECTIN1-AS1 ENSG00000255247 0.94881 0.17136 0.9341 0.17513 -0.39997
0.65541 0.94881 0.17136 0.17136 0.613770982
CUL3 ENSG00000036257 0.27123 0.39311 0.28302 0.38858 0.2562 0.3989
0.94866 0.1714 0.1714 0.613770982
NA ENSG00000199566 0.89462 0.1855 0.89462 0.1855 0.89462 0.1855
0.94863 0.1714 0.1714 0.613770982
IL1R1-AS1 ENSG00000226925 0.94862 0.17141 0.94862 0.17141 0.94862

0.17141 0.94862 0.17141 0.17141 0.613770982
NA ENSG00000273071 0.94863 0.17141 0.94863 0.17141 0.94863 0.17141
0.94863 0.17141 0.17141 0.613770982
NA ENSG00000279416 -0.40745 0.65816 0.94838 0.17147 0.42478 0.3355
-0.01063 0.50424 0.17147 0.613912804
RPL17P13 ENSG00000223604 0.94835 0.17148 0.94835 0.17148 0.94835
0.17148 0.94835 0.17148 0.17148 0.613912804
SCAI ENSG00000173611 0.73566 0.23097 0.94828 0.17149 0.47886 0.31602
0.10286 0.45904 0.17149 0.613912804
KMT2E ENSG00000005483 -0.38084 0.64834 0.34882 0.36361 0.94824 0.1715
-1.0901 0.86216 0.1715 0.613912804
RNU6-117P ENSG00000202285 0.94811 0.17154 0.94811 0.17154 0.94811
0.17154 0.94811 0.17154 0.17154 0.613992274
LINC02508 ENSG00000251619 0.94805 0.17155 0.94805 0.17155 0.94805
0.17155 0.94805 0.17155 0.17155 0.613992274
SLC5A4 ENSG00000100191 0.94803 0.17156 0.94803 0.17156 0.94803
0.17156 -0.52843 0.7014 0.17156 0.613992274
NA ENSG00000259636 0.9479 0.17159 0.16598 0.43408 0.046643 0.4814
-0.45296 0.67471 0.17159 0.614015883
ATP5MFP5 ENSG00000256103 0.94784 0.17161 0.076974 0.46932 -0.47214
0.68159 0.68108 0.24791 0.17161 0.614015883
MIR99AHG ENSG00000215386 -0.40987 0.65905 0.94776 0.17162 0.4506
0.32614 -0.16048 0.56375 0.17162 0.614015883
NA ENSG00000213663 0.94773 0.17163 -0.88296 0.81137 -0.88296 0.81137
-0.88296 0.81137 0.17163 0.614015883
NA ENSG00000257470 -0.21712 0.58594 0.65358 0.25669 0.94772 0.17163
-0.21712 0.58594 0.17163 0.614015883
NA ENSG00000260300 0.94765 0.17165 0.94765 0.17165 0.94765 0.17165
0.94765 0.17165 0.17165 0.614015883
DBX1 ENSG00000109851 -0.63224 0.73639 0.94755 0.17168 -0.086354 0.53441
-0.63224 0.73639 0.17168 0.614015883
RNU5E-10P ENSG00000200376 0.94756 0.17168 0.94756 0.17168 0.94756
0.17168 0.94756 0.17168 0.17168 0.614015883
NA ENSG00000231880 0.94756 0.17168 0.94756 0.17168 0.94756 0.17168
0.94756 0.17168 0.17168 0.614015883
ASTN2 ENSG00000148219 0.2015 0.42016 0.94745 0.1717 -0.8256 0.79548
-1.0792 0.85975 0.1717 0.614022308
MCMBP ENSG00000197771 -0.43601 0.66859 0.94726 0.17175 0.56789 0.28506
-0.81921 0.79367 0.17175 0.614022308
MBNL1-AS1 ENSG00000229619 0.94726 0.17175 0.94726 0.17175 0.94726
0.17175 0.94726 0.17175 0.17175 0.614022308
GOLGA2P4 ENSG00000229608 0.94723 0.17176 0.56082 0.28746 0.56082
0.28746 -0.045955 0.51833 0.17176 0.614022308
CORO7-PAM16 ENSG00000103426 0.61181 0.27033 0.84587 0.19881 0.94721
0.17177 0.24329 0.40389 0.17177 0.614022308
NA ENSG00000234810 0.94721 0.17177 0.75844 0.22409 0.32723 0.37175
-0.82413 0.79507 0.17177 0.614022308
PHBP21 ENSG00000260326 0.44898 0.32672 0.94721 0.17177 0.81978
0.20617 0.77595 0.21889 0.17177 0.614022308
NA ENSG00000243779 -0.59034 0.72252 0.23441 0.40733 0.94703 0.17181
0.94703 0.17181 0.17181 0.614038019
NA ENSG00000256948 0.947 0.17182 0.58605 0.27892 -0.62899 0.73532
-0.42966 0.66628 0.17182 0.614038019
RPS21P8 ENSG00000223782 0.26774 0.39445 0.78702 0.21563 0.94696
0.17183 -0.63533 0.73739 0.17183 0.614038019
PBX1-AS1 ENSG00000233693 -0.64488 0.7405 -0.76669 0.77837 0.94695
0.17183 -0.65026 0.74224 0.17183 0.614038019
RNASEH1-AS1 ENSG00000234171 0.94686 0.17185 0.94686 0.17185 0.94686
0.17185 0.94686 0.17185 0.17185 0.614038019
NA ENSG00000258738 0.94687 0.17185 0.94687 0.17185 0.94687 0.17185
0.94687 0.17185 0.17185 0.614038019
PLBD1 ENSG00000121316 0.9468 0.17187 0.9468 0.17187 -0.14734 0.55857

0.10004 0.46016 0.17187 0.614053716
NLRP14 ENSG00000158077 0.94674 0.17188 -0.69047 0.75505 0.93454
0.17501 0.53947 0.29478 0.17188 0.614053716
NA ENSG00000260142 0.78575 0.21601 0.94665 0.17191 0.78575 0.21601
0.78575 0.21601 0.17191 0.614053716
RPL31P53 ENSG00000225352 0.94661 0.17192 0.94661 0.17192 0.94661
0.17192 0.94661 0.17192 0.17192 0.614053716
ALG1L3P ENSG00000251087 -2.3245 0.98995 0.64032 0.26098 -1.0532
0.85388 0.9466 0.17192 0.17192 0.614053716
WFDC11 ENSG00000180083 -0.51808 0.6978 0.94656 0.17193 -0.82795
0.79615 -1.1382 0.87249 0.17193 0.614053716
HNRNPLL ENSG00000143889 -1.1456 0.87401 0.94651 0.17195 0.7104
0.23873 -0.29536 0.61614 0.17195 0.614080139
CACNG2 ENSG00000166862 0.94608 0.17205 0.26335 0.39614 -0.79526
0.78677 0.031051 0.48761 0.17205 0.614181348
NA ENSG00000258772 -0.29582 0.61631 0.49972 0.30864 -0.21591 0.58547
0.94611 0.17205 0.17205 0.614181348
NA ENSG00000268203 0.94607 0.17206 0.94607 0.17206 0.94607 0.17206
0.94607 0.17206 0.17206 0.614181348
DUSP16 ENSG00000111266 0.94603 0.17207 0.762 0.22303 0.65288 0.25692
0.52751 0.29892 0.17207 0.614181348
NA ENSG00000275995 -0.11421 0.54546 0.60612 0.27222 0.946 0.17208
-0.11421 0.54546 0.17208 0.614181348
NA ENSG00000279091 0.94598 0.17208 0.94598 0.17208 0.94598 0.17208
0.94598 0.17208 0.17208 0.614181348
RN7SL290P ENSG00000264613 -0.58197 0.71971 0.94595 0.17209 -0.0034921
0.50139 -0.58197 0.71971 0.17209 0.614181348
MIR640 ENSG00000207821 0.94591 0.1721 0.94591 0.1721 0.94591
0.1721 0.94591 0.1721 0.1721 0.614181348
ATG10-AS1 ENSG00000248192 0.94584 0.17211 0.94584 0.17211 0.94584
0.17211 0.94584 0.17211 0.17211 0.614181348
NAMPTP3 ENSG00000261053 0.16811 0.43325 0.55597 0.28912 0.94584
0.17211 0.94584 0.17211 0.17211 0.614181348
NA ENSG00000239005 0.94576 0.17214 0.94576 0.17214 0.94576 0.17214
0.94576 0.17214 0.17214 0.614181348
MIR4518 ENSG00000266305 0.94573 0.17214 0.94573 0.17214 0.94573
0.17214 0.94573 0.17214 0.17214 0.614181348
C21orf58 ENSG00000160298 0.18472 0.42673 0.062649 0.47502 -0.14355
0.55707 0.94572 0.17215 0.17215 0.614181348
EPHB3 ENSG00000182580 0.31041 0.37812 0.3258 0.37229 0.18888 0.4251
0.94561 0.17217 0.17217 0.614181348
ZNF716 ENSG00000182111 0.23941 0.4054 0.94558 0.17218 0.64459
0.2596 -0.49841 0.6909 0.17218 0.614181348
NA ENSG00000238207 -0.73976 0.77028 -0.36308 0.64173 0.63885 0.26146
0.94557 0.17218 0.17218 0.614181348
BMS1 ENSG00000165733 -0.1744 0.56922 0.36123 0.35896 0.94551 0.1722
0.17223 0.43163 0.1722 0.614207729
MARK1 ENSG00000116141 0.94535 0.17224 0.22446 0.4112 0.867 0.19297
-1.8427 0.96731 0.17224 0.614238906
EDARADD ENSG00000186197 0.94525 0.17227 0.94525 0.17227 0.60959
0.27107 0.34243 0.36602 0.17227 0.614238906
MITD1 ENSG00000158411 0.83822 0.20095 0.65754 0.25542 0.65754 0.25542
0.94516 0.17229 0.17229 0.614238906
NA ENSG00000224079 0.050978 0.47967 0.13745 0.44534 0.050978 0.47967
0.94515 0.17229 0.17229 0.614238906
NA ENSG00000259560 0.45563 0.32433 -0.44069 0.67028 0.94517 0.17229
-0.60364 0.72696 0.17229 0.614238906
NA ENSG00000235574 0.61018 0.27087 0.94511 0.1723 0.94511 0.1723
0.94511 0.1723 0.1723 0.614238906
EIF4BP3 ENSG00000224546 0.2928 0.38484 -0.70036 0.75815 -0.81554
0.79262 0.94502 0.17232 0.17232 0.614238906
OR5BD1P ENSG00000254749 0.26234 0.39653 0.94501 0.17233 -2.0358

0.97912 -2.0358 0.97912 0.17233 0.614238906
PSMA2P2 ENSG00000251234 0.94497 0.17234 0.94497 0.17234 -1.0055
0.84268 -1.4737 0.92972 0.17234 0.614238906
NA ENSG00000221458 -1.1212 0.8689 0.94493 0.17235 -0.84103 0.79983
0.68123 0.24786 0.17235 0.614238906
RN7SKP291 ENSG00000199831 0.94489 0.17236 -0.84001 0.79955 -1.3197
0.90653 0.94489 0.17236 0.17236 0.614238906
NA ENSG00000261813 0.94488 0.17236 0.25889 0.39786 0.35893 0.35982
0.25363 0.39989 0.17236 0.614238906
NA ENSG00000262408 0.94479 0.17238 0.94479 0.17238 0.94479 0.17238
0.94479 0.17238 0.17238 0.614265258
PDK4 ENSG00000004799 0.9446 0.17243 0.39658 0.34584 0.9446 0.17243
-0.42068 0.663 0.17243 0.614398501
DUSP11 ENSG00000144048 0.94454 0.17245 -0.014189 0.50566 -0.56411
0.71366 -0.80446 0.78943 0.17245 0.614424837
C4orf45 ENSG00000164123 0.40658 0.34216 -1.2102 0.88689 -0.46811
0.68015 0.94432 0.1725 0.1725 0.614513122
KCNQ1OT1 ENSG00000269821 0.30332 0.38082 0.82514 0.20465 0.94432
0.1725 0.53456 0.29648 0.1725 0.614513122
RBM11 ENSG00000185272 0.94421 0.17253 -0.65878 0.74498 -1.69 0.95449
-1.0781 0.85952 0.17253 0.614575066
ZNF354C ENSG00000177932 -0.32344 0.62682 -0.9181 0.82072 0.81027
0.20889 0.9441 0.17256 0.17256 0.614592077
LINC00446 ENSG00000229249 0.94409 0.17256 0.34746 0.36412 0.94409
0.17256 0.94409 0.17256 0.17256 0.614592077
NA ENSG00000226838 -0.48775 0.68714 0.37207 0.35492 0.94401 0.17258
0.85863 0.19527 0.17258 0.614618388
NA ENSG00000254321 0.94386 0.17262 0.85262 0.19693 0.78242 0.21698
0.019017 0.49241 0.17262 0.614715916
STK10 ENSG00000072786 0.85485 0.19632 0.94367 0.17267 0.47235 0.31834
0.62412 0.26627 0.17267 0.614729626
SMYD4 ENSG00000186532 0.94366 0.17267 0.80795 0.20956 0.94366 0.17267
0.94366 0.17267 0.17267 0.614729626
RPL23P4 ENSG00000223903 0.94368 0.17267 0.94368 0.17267 0.94368
0.17267 0.94368 0.17267 0.17267 0.614729626
LSM2 ENSG00000204392 0.94361 0.17269 0.94361 0.17269 0.94361 0.17269
-0.60599 0.72774 0.17269 0.614729626
NA ENSG00000226442 -0.24165 0.59547 -0.44156 0.6706 0.94347 0.17272
0.94347 0.17272 0.17272 0.614729626
MOB1AP1 ENSG00000228241 0.94342 0.17273 0.76402 0.22243 0.94342
0.17273 0.94342 0.17273 0.17273 0.614729626
TWIST2 ENSG00000233608 0.94341 0.17274 -0.20133 0.57978 -0.38457
0.64972 0.24406 0.40359 0.17274 0.614729626
NA ENSG00000264238 0.16975 0.4326 0.94339 0.17274 0.63157 0.26384
0.031014 0.48763 0.17274 0.614729626
DDX10P1 ENSG00000237135 0.73056 0.23252 0.94337 0.17275 0.54693
0.29221 0.058507 0.47667 0.17275 0.614729626
NA ENSG00000250948 -0.93908 0.82616 -0.93908 0.82616 -0.93908 0.82616
0.94337 0.17275 0.17275 0.614729626
MTCO2P11 ENSG00000235917 0.94329 0.17277 0.94329 0.17277 0.94329
0.17277 0.94329 0.17277 0.17277 0.614755904
H1-3 ENSG00000124575 -1.4969 0.93279 -1.4969 0.93279 0.94315 0.1728
-0.65396 0.74343 0.1728 0.614806788
SH3D19 ENSG00000109686 0.94308 0.17282 -0.70137 0.75846 -0.28024
0.61035 -0.54881 0.70843 0.17282 0.614806788
FBH1 ENSG00000134452 0.943 0.17284 0.943 0.17284 0.943 0.17284 0.41518 0.339
0.17284 0.614806788
IFT52 ENSG00000101052 0.94297 0.17285 0.79934 0.21205 0.73627 0.23078
0.93755 0.17424 0.17285 0.614806788
KLHL32 ENSG00000186231 0.26615 0.39506 0.94294 0.17286 -0.64569
0.74076 -0.92459 0.82241 0.17286 0.614806788
RNU6-44P ENSG00000206772 0.94294 0.17286 0.94294 0.17286 0.94294

0.17286 0.94294 0.17286 0.17286 0.614806788
OR1S2 ENSG00000197887 0.57969 0.28106 0.94281 0.17289 0.94281 0.17289
-0.55274 0.70978 0.17289 0.61483138
SOAT1 ENSG00000057252 0.41592 0.33873 0.52361 0.30027 0.94271 0.17291
-0.57757 0.71822 0.17291 0.61483138
GSTM3P1 ENSG00000227693 -1.0094 0.84361 -1.0094 0.84361 0.94264
0.17293 0.80023 0.21179 0.17293 0.61483138
NA ENSG00000253775 -1.0135 0.84459 -0.92406 0.82227 0.94266 0.17293
-0.085493 0.53407 0.17293 0.61483138
NA ENSG00000268209 0.69928 0.24219 -0.18328 0.57271 0.94266 0.17293
0.94266 0.17293 0.17293 0.61483138
RNY1P6 ENSG00000206920 0.9425 0.17297 0.9425 0.17297 0.9425
0.17297 0.9425 0.17297 0.17297 0.61492873
NA ENSG00000261117 0.94242 0.17299 0.81285 0.20815 0.37301 0.35457
0.37301 0.35457 0.17299 0.614954968
PM20D2 ENSG00000146281 0.84887 0.19798 0.94236 0.17301 0.93728
0.17431 0.93728 0.17431 0.17301 0.614962577
CDKN2AIPNLP1 ENSG00000177770 0.94225 0.17303 -1.7684 0.9615 -1.7684
0.9615 -1.3739 0.91526 0.17303 0.614962577
NA ENSG00000243302 0.096898 0.4614 -0.59114 0.72279 0.94227 0.17303
-0.93626 0.82543 0.17303 0.614962577
GRK1 ENSG00000185974 0.54977 0.29124 0.94211 0.17307 -1.5909 0.94418
-1.5558 0.94012 0.17307 0.61504711
NA ENSG00000249887 0.94197 0.1731 0.94197 0.1731 0.94197 0.1731
0.94197 0.1731 0.1731 0.61504711
NA ENSG00000261229 0.94196 0.17311 0.72275 0.23492 -0.89701 0.81514
-0.89701 0.81514 0.17311 0.61504711
NA ENSG00000264754 0.94195 0.17311 0.94195 0.17311 0.94195 0.17311
0.94195 0.17311 0.17311 0.61504711
ETS1-AS1 ENSG00000254588 0.94187 0.17313 0.94187 0.17313 0.94187
0.17313 0.33189 0.36998 0.17313 0.61504711
SLC25A24 ENSG00000085491 -0.70806 0.76055 0.38948 0.34846 0.94177
0.17316 -0.48595 0.6865 0.17316 0.61504711
MIXL1 ENSG00000185155 0.94177 0.17316 0.94177 0.17316 0.94177 0.17316
0.94177 0.17316 0.17316 0.61504711
MEF2B ENSG00000213999 0.94172 0.17317 0.94172 0.17317 0.94172 0.17317
0.13845 0.44494 0.17317 0.61504711
KLHL3 ENSG00000146021 0.60351 0.27308 0.75797 0.22424 0.94168 0.17318
0.63903 0.2614 0.17318 0.61504711
NA ENSG00000236079 -2.8526 0.99783 0.72849 0.23316 -2.8526 0.99783
0.94166 0.17318 0.17318 0.61504711
TPTE2P2 ENSG00000272281 0.45605 0.32418 0.31102 0.37789 0.55502
0.28944 0.94158 0.1732 0.1732 0.615064002
DELEC1 ENSG00000173077 0.48036 0.31549 0.82746 0.20399 0.87915
0.18966 0.94154 0.17321 0.17321 0.615064002
MRPS23 ENSG00000181610 0.9185 0.17918 0.88574 0.18788 0.94145
0.17324 -0.18414 0.57305 0.17324 0.615125707
NA ENSG00000225832 0.9412 0.1733 0.40077 0.3443 0.9412 0.1733
0.9412 0.1733 0.1733 0.615249089
YWHAZP1 ENSG00000259148 0.94119 0.1733 0.94119 0.1733 0.94119
0.1733 0.94119 0.1733 0.1733 0.615249089
SPAG16 ENSG00000144451 0.32126 0.37401 0.94108 0.17333 0.88295
0.18863 -0.17738 0.57039 0.17333 0.615290323
NA ENSG00000199291 -0.02301 0.50918 0.42284 0.33621 0.42284 0.33621
0.94102 0.17335 0.17335 0.615290323
NA ENSG00000249157 -0.52031 0.69858 0.74064 0.22946 0.94095 0.17336
0.94095 0.17336 0.17336 0.615290323
NA ENSG00000236045 0.94091 0.17338 -0.26073 0.60285 -0.26073 0.60285
-0.26073 0.60285 0.17338 0.615290323
C1orf162 ENSG00000143110 0.11857 0.45281 0.94086 0.17339 0.08362
0.46668 0.073829 0.47057 0.17339 0.615290323
LINC01845 ENSG00000253298 0.9408 0.1734 0.40379 0.34318 0.40379

0.34318 -0.47265 0.68177 0.1734 0.615290323
NA ENSG00000280852 0.57603 0.2823 0.94083 0.1734 0.64641 0.25901
-0.78281 0.78313 0.1734 0.615290323
SHPRH ENSG00000146414 0.28918 0.38622 0.69406 0.24382 0.032052 0.48722
0.9407 0.17343 0.17343 0.615307164
NA ENSG00000267478 0.78977 0.21483 0.94071 0.17343 0.94071 0.17343
0.94071 0.17343 0.17343 0.615307164
FHAD1-AS1 ENSG00000233485 0.94047 0.17349 0.19638 0.42216 0.94047
0.17349 0.94047 0.17349 0.17349 0.615475226
LAMB2 ENSG00000172037 0.58901 0.27793 0.26112 0.397 0.94038 0.17351
-1.4414 0.92526 0.17351 0.615482706
PPIL6 ENSG00000185250 -0.20807 0.58241 0.9403 0.17353 0.92116 0.17848
-0.65294 0.7431 0.17353 0.615482706
TMEM273 ENSG00000204161 -1.0816 0.86029 0.31761 0.37539 0.9403
0.17353 -0.098703 0.53931 0.17353 0.615482706
RN7SL40P ENSG00000242696 0.4221 0.33647 0.8456 0.19889 0.94019
0.17356 0.79985 0.2119 0.17356 0.615506985
NA ENSG00000278859 0.94015 0.17357 0.94015 0.17357 0.94015 0.17357
0.94015 0.17357 0.17357 0.615506985
ANTKMT ENSG00000103254 -1.4989 0.93304 0.94012 0.17358 -1.9277
0.97305 -1.9277 0.97305 0.17358 0.615506985
SLC17A2 ENSG00000112337 0.94007 0.17359 0.72146 0.23531 -0.17737
0.57039 0.15116 0.43992 0.17359 0.615506985
NA ENSG00000266290 0.94005 0.1736 0.94005 0.1736 0.94005 0.1736
0.94005 0.1736 0.1736 0.615506985
MIR512-2 ENSG00000207644 0.93989 0.17364 0.93989 0.17364 0.93989
0.17364 0.93989 0.17364 0.17364 0.615604016
HOXD4 ENSG00000170166 0.93978 0.17367 0.93978 0.17367 0.43634 0.3313
-0.7139 0.76236 0.17367 0.615624338
NA ENSG00000265094 0.93974 0.17368 0.93974 0.17368 0.93974 0.17368
0.93974 0.17368 0.17368 0.615624338
FEM1AP3 ENSG00000217770 0.27907 0.3901 0.9396 0.17371 0.9396
0.17371 0.9396 0.17371 0.17371 0.615624338
NA ENSG00000249870 0.9396 0.17371 0.51853 0.30204 0.51853 0.30204
0.51853 0.30204 0.17371 0.615624338
PPIAP50 ENSG00000261330 0.9395 0.17374 0.9395 0.17374 0.9395
0.17374 0.8404 0.20034 0.17374 0.615624338
NA ENSG00000231464 0.93933 0.17378 -0.097231 0.53873 -0.032525 0.51297
-0.22002 0.58707 0.17378 0.615624338
RUFY1-AS1 ENSG00000244945 0.93934 0.17378 0.17261 0.43148 0.1669
0.43372 0.1669 0.43372 0.17378 0.615624338
DSCAM-AS1 ENSG00000235123 0.93931 0.17379 0.93931 0.17379 -0.20415
0.58088 -0.20415 0.58088 0.17379 0.615624338
LINC00426 ENSG00000238121 0.80223 0.21121 0.024261 0.49032 0.93928
0.17379 0.33082 0.37039 0.17379 0.615624338
NA ENSG00000253266 0.93009 0.17616 0.076899 0.46935 0.93928 0.17379
0.269 0.39397 0.17379 0.615624338
RTF1 ENSG00000137815 0.86958 0.19227 -0.49689 0.69037 0.93927 0.1738
0.76186 0.22307 0.1738 0.615624338
NA ENSG00000267234 -0.25549 0.60083 0.81853 0.20653 0.8682 0.19264
0.93922 0.17381 0.17381 0.615624338
CTSA ENSG00000064601 -1.1977 0.88448 -1.1977 0.88448 0.67002 0.25142
0.9391 0.17384 0.17384 0.615624338
NUP155 ENSG00000113569 0.83695 0.20131 0.17054 0.43229 0.93907
0.17385 0.12432 0.45053 0.17385 0.615624338
NA ENSG00000252374 0.93908 0.17385 -0.86791 0.80728 -0.86791 0.80728
-0.86791 0.80728 0.17385 0.615624338
PSMC1P9 ENSG00000255993 0.93903 0.17386 0.93903 0.17386 0.93903
0.17386 0.93903 0.17386 0.17386 0.615624338
KRTAP11-1 ENSG00000182591 0.93898 0.17387 0.93898 0.17387 0.93898
0.17387 0.93898 0.17387 0.17387 0.615624338
NA ENSG00000176183 0.93881 0.17391 -1.1904 0.88306 -0.56491 0.71393

-1.665 0.95205 0.17391 0.615624338
NA ENSG00000255284 0.93877 0.17392 0.93877 0.17392 0.93877 0.17392
0.93877 0.17392 0.17392 0.615624338
MTCO3P11 ENSG00000237711 0.93874 0.17393 0.93874 0.17393 0.93874
0.17393 0.93874 0.17393 0.17393 0.615624338
SPDYE6 ENSG00000260097 0.93876 0.17393 0.51885 0.30193 -0.23447
0.59269 -0.23447 0.59269 0.17393 0.615624338
RPSAP6 ENSG00000214629 0.9387 0.17394 0.71209 0.23821 0.1138
0.4547 0.78308 0.21679 0.17394 0.615624338
NA ENSG00000211515 0.9387 0.17394 0.9387 0.17394 0.9387 0.17394
0.9387 0.17394 0.17394 0.615624338
USP40 ENSG00000085982 0.44967 0.32648 0.93859 0.17397 0.29331 0.38464
0.746 0.22784 0.17397 0.615624338
NA ENSG00000258380 0.39089 0.34794 0.93858 0.17397 -0.071944 0.52868
0.6017 0.27369 0.17397 0.615624338
PPIG ENSG00000138398 0.33252 0.36975 0.34069 0.36667 0.93842 0.17401
0.76234 0.22293 0.17401 0.615624338
IGLC6 ENSG00000222037 0.93842 0.17401 0.93842 0.17401 0.93842 0.17401
0.93842 0.17401 0.17401 0.615624338
NA ENSG00000270347 0.93842 0.17402 0.93842 0.17402 0.93842 0.17402
0.93842 0.17402 0.17402 0.615624338
NA ENSG00000213865 -1.1775 0.88051 0.9384 0.17402 -1.1775 0.88051
0.19763 0.42167 0.17402 0.615624338
LINC02576 ENSG00000232613 -0.21597 0.5855 -1.2433 0.89312 -0.87266
0.80858 0.93835 0.17403 0.17403 0.615624338
CHST11 ENSG00000171310 0.93833 0.17404 0.16693 0.43371 -0.052734
0.52103 0.16693 0.43371 0.17404 0.615624338
FCER1A ENSG00000179639 0.34594 0.36469 0.14474 0.44246 0.14474
0.44246 0.9383 0.17405 0.17405 0.615624338
RPS27P21 ENSG00000240759 0.93821 0.17407 0.93821 0.17407 0.93821
0.17407 0.93821 0.17407 0.17407 0.615650392
MVB12B ENSG00000196814 -0.32242 0.62643 -0.06196 0.5247 -0.50836
0.6944 0.93804 0.17411 0.17411 0.615700355
MIR653 ENSG00000208014 0.93806 0.17411 0.93806 0.17411 0.93806
0.17411 0.93806 0.17411 0.17411 0.615700355
VTRNA2-2P ENSG00000251900 0.42269 0.33626 0.93786 0.17416 0.93786
0.17416 -0.16816 0.56677 0.17416 0.615700355
CYP4F27P ENSG00000248313 0.93782 0.17417 0.93782 0.17417 0.93782
0.17417 0.93782 0.17417 0.17417 0.615700355
RPL7AP25 ENSG00000235294 0.93778 0.17418 0.93778 0.17418 0.93778
0.17418 0.93778 0.17418 0.17418 0.615700355
NA ENSG00000270584 0.93773 0.17419 0.93773 0.17419 0.12761 0.44923
0.93773 0.17419 0.17419 0.615700355
MRPL45P1 ENSG00000232508 0.83566 0.20167 0.93769 0.1742 0.6978
0.24265 0.83566 0.20167 0.1742 0.615700355
ENTPD4 ENSG00000197217 0.69804 0.24258 0.93765 0.17421 0.91212
0.18085 0.50994 0.30505 0.17421 0.615700355
NA ENSG00000231207 0.9375 0.17425 -0.78633 0.78416 -0.78633 0.78416
-0.78633 0.78416 0.17425 0.615700355
NA ENSG00000236073 0.61939 0.26783 0.93751 0.17425 0.91636 0.17974
-0.50208 0.69219 0.17425 0.615700355
CCR7 ENSG00000126353 0.57499 0.28265 0.93739 0.17428 -0.70311 0.75901
0.10667 0.45752 0.17428 0.615700355
CPNE4 ENSG00000196353 -0.4791 0.68407 -0.32151 0.62609 0.030634 0.48778
0.9374 0.17428 0.17428 0.615700355
C20orf202 ENSG00000215595 -0.75453 0.77473 -0.75453 0.77473 -0.75453
0.77473 0.9374 0.17428 0.17428 0.615700355
POMT2 ENSG00000009830 0.7125 0.23808 0.72633 0.23382 0.25027 0.40119
0.93729 0.1743 0.1743 0.615700355
NA ENSG00000237280 -0.3811 0.64844 0.15994 0.43647 -0.23842 0.59422
0.93729 0.1743 0.1743 0.615700355
NA ENSG00000280082 0.93732 0.1743 0.93732 0.1743 0.93732 0.1743

0.93732 0.1743 0.1743 0.615700355
EVL ENSG00000196405 0.12728 0.44936 0.104 0.45858 0.20496 0.4188
0.93718 0.17433 0.17433 0.615700355
RN7SL789P ENSG00000242436 0.93718 0.17433 0.93718 0.17433 0.93718
0.17433 0.93718 0.17433 0.17433 0.615700355
PNMA8B ENSG00000204851 0.44259 0.32903 0.9371 0.17435 0.80216
0.21123 0.9371 0.17435 0.17435 0.615700355
NRXN1 ENSG00000179915 0.30632 0.37968 -1.0239 0.84705 0.93708 0.17436
0.16546 0.43429 0.17436 0.615700355
DNAH9 ENSG00000007174 0.73686 0.2306 0.93699 0.17438 0.86288 0.1941
-1.4194 0.9221 0.17438 0.615700355
RBBP4P6 ENSG00000248674 0.93698 0.17438 0.93698 0.17438 0.93698
0.17438 0.93698 0.17438 0.17438 0.615700355
DLEU2L ENSG00000116652 0.93696 0.17439 0.93696 0.17439 0.81467
0.20763 0.93696 0.17439 0.17439 0.615700355
LINC00643 ENSG00000186369 0.11172 0.45552 0.45296 0.32529 0.93691
0.1744 0.28515 0.38776 0.1744 0.615700355
PCNAP1 ENSG00000249065 0.93691 0.1744 -0.71389 0.76235 -0.71389
0.76235 -0.71389 0.76235 0.1744 0.615700355
CCT3 ENSG00000163468 0.93683 0.17442 0.46688 0.32029 0.46688 0.32029
0.22144 0.41238 0.17442 0.615726355
FIS1 ENSG00000214253 0.91333 0.18053 0.93668 0.17446 0.93668 0.17446
0.93668 0.17446 0.17446 0.61574113
HCP5 ENSG00000206337 -0.6103 0.72917 0.34034 0.3668 0.34034 0.3668
0.93666 0.17447 0.17447 0.61574113
SAMM50 ENSG00000100347 -0.66921 0.74832 -1.0986 0.86403 -0.75212
0.77401 0.93652 0.1745 0.1745 0.61574113
HPCAL4 ENSG00000116983 -1.1849 0.88197 -0.78477 0.78371 0.1704
0.43235 0.93651 0.1745 0.1745 0.61574113
FAM242C ENSG00000250432 -0.33667 0.63182 0.5273 0.29899 0.13532
0.44618 0.93652 0.1745 0.1745 0.61574113
NA ENSG00000272545 0.93653 0.1745 0.93653 0.1745 0.93653 0.1745
0.93653 0.1745 0.1745 0.61574113
EEF1G ENSG00000254772 0.93631 0.17456 0.11502 0.45421 0.11502 0.45421
0.11502 0.45421 0.17456 0.615908247
RNA5SP36 ENSG00000222129 0.93621 0.17458 -0.63069 0.73588 -0.54191
0.70606 -0.91828 0.82076 0.17458 0.615924904
FGFR3P5 ENSG00000260984 0.16231 0.43553 0.5443 0.29312 0.93616
0.17459 -0.45635 0.67593 0.17459 0.615924904
SMCO4 ENSG00000166002 0.80826 0.20947 0.93609 0.17461 0.68085 0.24798
0.70632 0.24 0.17461 0.61593564
OR8G3P ENSG00000255425 0.93602 0.17463 0.93602 0.17463 0.93602
0.17463 0.93602 0.17463 0.17463 0.61593564
GHRH ENSG00000118702 0.71726 0.23661 0.93592 0.17466 0.6207 0.2674
0.53065 0.29783 0.17466 0.61593564
ZNRF3 ENSG00000183579 0.18415 0.42695 0.24844 0.4019 0.93592 0.17466
0.56485 0.28609 0.17466 0.61593564
IFNA17 ENSG00000234829 0.93569 0.17472 0.18412 0.42696 -0.66595
0.74728 -0.21138 0.5837 0.17472 0.61593564
NA ENSG00000267644 0.79398 0.2136 0.79398 0.2136 0.79398 0.2136
0.93568 0.17472 0.17472 0.61593564
NA ENSG00000231705 0.9356 0.17474 0.9356 0.17474 0.9356 0.17474
0.9356 0.17474 0.17474 0.61593564
MIR633 ENSG00000207552 -0.017024 0.50679 -0.14279 0.55677 0.93556
0.17475 -0.0643 0.52563 0.17475 0.61593564
CLEC18B ENSG00000140839 -1.4549 0.92715 0.93553 0.17476 -0.6132
0.73013 0.31975 0.37458 0.17476 0.61593564
NA ENSG00000201196 0.93553 0.17476 0.92886 0.17648 0.93553 0.17476
0.93553 0.17476 0.17476 0.61593564
MS4A13 ENSG00000204979 0.79242 0.21406 -1.1028 0.86493 0.93553
0.17476 0.6585 0.25511 0.17476 0.61593564
CACNA1G ENSG00000006283 -0.79383 0.78635 0.93547 0.17477 -0.63078

0.73591 -0.94032 0.82647 0.17477 0.61593564
FHDC1 ENSG00000137460 0.53147 0.29755 0.41318 0.33974 0.7613 0.22324
0.93548 0.17477 0.17477 0.61593564
GAPDHP24 ENSG00000185221 0.9355 0.17477 0.9355 0.17477 0.9355
0.17477 0.9355 0.17477 0.17477 0.61593564
NA ENSG00000221612 -0.058745 0.52342 0.29026 0.38581 0.93533 0.17481
-0.039222 0.51564 0.17481 0.615998172
ZNF44 ENSG00000197857 0.40148 0.34403 0.93514 0.17486 0.40148 0.34403
0.40148 0.34403 0.17486 0.615998172
NA ENSG00000271032 0.93513 0.17486 0.93513 0.17486 0.93513 0.17486
0.93513 0.17486 0.17486 0.615998172
KCTD5P1 ENSG00000224232 0.93505 0.17488 0.93505 0.17488 0.93505
0.17488 0.93505 0.17488 0.17488 0.615998172
NA ENSG00000207525 0.93495 0.17491 0.93495 0.17491 0.93495 0.17491
0.93495 0.17491 0.17491 0.615998172
MDGA1 ENSG00000112139 -1.0728 0.85832 -1.2663 0.89729 -0.50576 0.69349
0.93489 0.17492 0.17492 0.615998172
NA ENSG00000226842 0.93489 0.17492 0.93489 0.17492 0.93489 0.17492
0.93489 0.17492 0.17492 0.615998172
PLPP4 ENSG00000203805 0.93485 0.17493 -0.71593 0.76298 -0.55203 0.70954
-0.71593 0.76298 0.17493 0.615998172
NA ENSG00000240207 0.54948 0.29134 0.54948 0.29134 0.93484 0.17494
0.82397 0.20498 0.17494 0.615998172
RPL12P46 ENSG00000255903 -0.17551 0.56966 0.93483 0.17494 -0.26067
0.60283 -0.94751 0.82831 0.17494 0.615998172
OR52R1 ENSG00000279270 0.93482 0.17494 0.26904 0.39395 0.93482
0.17494 0.93482 0.17494 0.17494 0.615998172
RNA5SP258 ENSG00000199620 0.38394 0.35051 0.77375 0.21954 0.61098
0.27061 0.93476 0.17496 0.17496 0.615998172
NA ENSG00000282946 0.93474 0.17496 0.93474 0.17496 0.93474 0.17496
0.93474 0.17496 0.17496 0.615998172
MIR548T ENSG00000221296 0.93472 0.17497 0.75012 0.22659 0.50971
0.30513 -0.2797 0.61014 0.17497 0.615998172
RNU6-295P ENSG00000252710 -0.32384 0.62697 0.93467 0.17498 -0.30481
0.61975 -0.65522 0.74384 0.17498 0.615998172
MAN2C1 ENSG00000140400 -0.70475 0.75952 0.93463 0.17499 -0.79668
0.78718 0.7088 0.23922 0.17499 0.615998172
DCPS ENSG00000110063 -1.2725 0.8984 -1.1447 0.87382 -1.2725 0.8984
0.93456 0.17501 0.17501 0.616024075
NA ENSG00000215972 0.93447 0.17503 0.93447 0.17503 0.93447 0.17503
0.93447 0.17503 0.17503 0.616040676
IGKV3D-11 ENSG00000211632 0.93442 0.17504 0.93442 0.17504 0.93442
0.17504 0.93442 0.17504 0.17504 0.616040676
NA ENSG00000233234 -0.67651 0.75064 0.93429 0.17508 0.71401 0.23761
-0.72536 0.76589 0.17508 0.616136954
NSG2 ENSG00000170091 -0.78638 0.78418 0.93412 0.17512 -1.6005 0.94526
-1.3005 0.90328 0.17512 0.616233218
NA ENSG00000235023 0.93398 0.17516 0.48015 0.31556 0.93398 0.17516
-0.69732 0.7572 0.17516 0.616266344
PPP2R5A ENSG00000066027 0.7959 0.21305 0.90516 0.18269 0.93394
0.17517 0.90653 0.18233 0.17517 0.616266344
NA ENSG00000238090 0.070918 0.47173 0.93394 0.17517 0.93394 0.17517
0.93394 0.17517 0.17517 0.616266344
NA ENSG00000249142 -0.60279 0.72668 -0.68331 0.75279 -0.76715 0.7785
0.93389 0.17518 0.17518 0.616266344
NA ENSG00000254429 -0.82367 0.79494 -0.37733 0.64703 0.93345 0.17529
0.93345 0.17529 0.17529 0.616587979
RNU6-1148P ENSG00000222924 0.81515 0.20749 0.93337 0.17531 0.93337
0.17531 0.93337 0.17531 0.17531 0.616587979
PPIAP23 ENSG00000214178 0.93336 0.17532 0.93336 0.17532 0.93336
0.17532 0.93336 0.17532 0.17532 0.616587979
NA ENSG00000260603 0.93328 0.17534 0.93328 0.17534 0.93328 0.17534

0.93328 0.17534 0.17534 0.616587979
DCBLD1 ENSG00000164465 0.93322 0.17535 -0.37034 0.64444 -0.40185
0.6561 -0.37183 0.64499 0.17535 0.616587979
TPM1-AS ENSG00000259498 0.93324 0.17535 0.53523 0.29624 0.93324
0.17535 0.93324 0.17535 0.17535 0.616587979
NA ENSG00000268953 -0.29119 0.61455 0.93319 0.17536 0.93319 0.17536
0.93319 0.17536 0.17536 0.616587979
CLP1 ENSG00000172409 0.93292 0.17543 0.93292 0.17543 0.12854 0.44886
0.13809 0.44509 0.17543 0.616723178
GALNT16 ENSG00000100626 -0.42568 0.66483 0.9329 0.17544 -1.2691
0.89781 -0.67899 0.75143 0.17544 0.616723178
C9orf135 ENSG00000204711 0.3298 0.37078 0.67675 0.24928 -0.82602
0.7956 0.93289 0.17544 0.17544 0.616723178
RN7SL307P ENSG00000243338 0.93284 0.17545 0.26793 0.39438 0.93284
0.17545 0.93284 0.17545 0.17545 0.616723178
LINC01519 ENSG00000237267 0.9326 0.17551 -0.75169 0.77388 -0.41536
0.66106 -0.41536 0.66106 0.17551 0.616723178
YEATS2 ENSG00000163872 0.47053 0.31899 -0.059085 0.52356 -0.40649
0.65781 0.93256 0.17552 0.17552 0.616723178
RRAS2P2 ENSG00000233751 0.14754 0.44135 -0.14738 0.55858 -0.44445
0.67164 0.93257 0.17552 0.17552 0.616723178
NA ENSG00000273980 0.93249 0.17554 -1.1045 0.86531 -1.37 0.91466 -1.37
0.91466 0.17554 0.616723178
FNDC3A ENSG00000102531 -0.31949 0.62532 -0.31705 0.6244 0.039475
0.48426 0.93219 0.17562 0.17562 0.616723178
MCTP1-AS1 ENSG00000249545 0.9322 0.17562 -0.85368 0.80336 -0.35001
0.63683 -0.33583 0.6315 0.17562 0.616723178
NA ENSG00000253416 0.26037 0.39729 0.93218 0.17562 0.93218 0.17562
0.012406 0.49505 0.17562 0.616723178
UBE3C ENSG00000009335 0.70411 0.24068 0.91255 0.18074 0.8514 0.19727
0.93209 0.17564 0.17564 0.616723178
CLIC4 ENSG00000169504 -0.3246 0.62726 -0.67531 0.75026 0.93209 0.17564
0.30679 0.3795 0.17564 0.616723178
ARMC6 ENSG00000105676 -1.5773 0.94264 0.93209 0.17565 -1.0289 0.84823
-1.0289 0.84823 0.17565 0.616723178
BNIP3P33 ENSG00000268717 -0.26863 0.60589 0.93206 0.17565 -0.26863
0.60589 -0.26863 0.60589 0.17565 0.616723178
ARMC3 ENSG00000165309 0.45184 0.32569 0.65657 0.25573 0.93203 0.17566
0.5761 0.28228 0.17566 0.616723178
PITPNB ENSG00000180957 0.93198 0.17567 0.63791 0.26177 0.42876
0.33405 0.73217 0.23203 0.17567 0.616723178
PKD2L2 ENSG00000078795 0.93192 0.17569 -0.22263 0.58809 0.27886
0.39018 0.68655 0.24618 0.17569 0.616723178
STXBP6 ENSG00000168952 0.49655 0.30975 0.28682 0.38712 0.58677
0.27868 0.93192 0.17569 0.17569 0.616723178
NA ENSG00000270165 0.9319 0.17569 0.9319 0.17569 0.9319 0.17569
0.9319 0.17569 0.17569 0.616723178
TXNDC9 ENSG00000115514 0.39735 0.34555 -0.23347 0.5923 0.20008
0.42071 0.93179 0.17572 0.17572 0.616723178
KCTD2 ENSG00000180901 -0.095887 0.53819 -0.092815 0.53697 0.93173 0.17574
0.30252 0.38113 0.17574 0.616723178
RNU6-475P ENSG00000251882 0.9317 0.17575 -1.0305 0.8486 0.3 0.38209
-0.21606 0.58553 0.17575 0.616723178
RNU7-197P ENSG00000239168 0.93159 0.17577 0.93159 0.17577 0.93159
0.17577 0.65148 0.25737 0.17577 0.616723178
TRIM6-TRIM34 ENSG00000258588 0.93148 0.1758 0.93148 0.1758 0.93148
0.1758 0.93148 0.1758 0.1758 0.616723178
AGAP12P ENSG00000265018 -0.32197 0.62626 0.65058 0.25766 0.93147
0.1758 0.064913 0.47412 0.1758 0.616723178
FIZ1 ENSG00000179943 0.93146 0.17581 0.93146 0.17581 0.93146 0.17581
0.93146 0.17581 0.17581 0.616723178
NA ENSG00000232286 0.93144 0.17581 0.93144 0.17581 0.93144 0.17581

0.93144 0.17581 0.17581 0.616723178
BNIP3P32 ENSG00000268830 0.93144 0.17581 -0.77009 0.77938 0.31768
0.37537 0.31768 0.37537 0.17581 0.616723178
SCGB2B3P ENSG00000269811 0.93144 0.17581 0.93144 0.17581 0.93144
0.17581 0.93144 0.17581 0.17581 0.616723178
NA ENSG00000279971 0.93144 0.17581 0.93144 0.17581 0.93144 0.17581
0.93144 0.17581 0.17581 0.616723178
CATSPERB ENSG00000133962 0.56828 0.28492 -0.42433 0.66434 0.41143
0.34038 0.93137 0.17583 0.17583 0.616723178
NA ENSG00000237747 -1.6449 0.95 0.93138 0.17583 -2.0684 0.9807
-2.0684 0.9807 0.17583 0.616723178
NA ENSG00000224579 0.93126 0.17586 0.93126 0.17586 0.93126 0.17586
0.93126 0.17586 0.17586 0.616723178
ABHD17C ENSG00000136379 0.93124 0.17587 0.93124 0.17587 0.20987
0.41688 0.43445 0.33198 0.17587 0.616723178
RNU6-653P ENSG00000199944 -0.19698 0.57808 -0.19698 0.57808 0.34728
0.36419 0.93118 0.17588 0.17588 0.616723178
RPS26P43 ENSG00000244641 0.20187 0.42001 0.93117 0.17588 0.93117
0.17588 0.93117 0.17588 0.17588 0.616723178
FARS2 ENSG00000145982 0.77324 0.21969 0.36724 0.35672 0.93104 0.17592
0.68652 0.24619 0.17592 0.616723178
ZNF491 ENSG00000177599 0.93105 0.17592 0.91038 0.18131 0.13816
0.44506 0.63681 0.26212 0.17592 0.616723178
NA ENSG00000256625 0.93103 0.17592 -1.902 0.97142 -1.6556 0.9511
-1.1573 0.87643 0.17592 0.616723178
NA ENSG00000279162 0.93103 0.17592 0.11553 0.45401 0.93103 0.17592
0.93103 0.17592 0.17592 0.616723178
VASH1 ENSG00000071246 -0.083056 0.5331 0.39412 0.34675 -0.083056 0.5331
0.93099 0.17593 0.17593 0.616723178
ARHGAP23P1 ENSG00000260781 0.93091 0.17595 0.62019 0.26757 0.005232
0.49791 -0.72669 0.76629 0.17595 0.61673961
PLCL1 ENSG00000115896 -0.81766 0.79322 0.93088 0.17596 -0.25073 0.59899
0.43446 0.33198 0.17596 0.61673961
NA ENSG00000264976 -0.22717 0.58985 -0.22717 0.58985 0.93071 0.176
-0.22717 0.58985 0.176 0.61675876
TCP11 ENSG00000124678 0.048315 0.48073 0.91872 0.17912 0.38223 0.35114
0.9307 0.17601 0.17601 0.61675876
PCDHGA4 ENSG00000262576 0.93066 0.17601 0.93066 0.17601 0.93066
0.17601 0.93066 0.17601 0.17601 0.61675876
VCL ENSG00000035403 0.84562 0.19888 0.93051 0.17605 -0.042797 0.51707
0.54551 0.2927 0.17605 0.61675876
DIO2-AS1 ENSG00000258766 -1.0647 0.85649 0.93054 0.17605 0.47566
0.31716 0.35436 0.36153 0.17605 0.61675876
NA ENSG00000250546 0.153 0.4392 0.93049 0.17606 0.7547 0.22521
0.74975 0.2267 0.17606 0.61675876
NA ENSG00000248125 0.33138 0.37018 0.12546 0.45008 0.93043 0.17607
0.56789 0.28506 0.17607 0.61675876
USP43 ENSG00000154914 -0.044536 0.51776 0.93038 0.17609 0.34373 0.36552
-0.17726 0.57035 0.17609 0.61675876
RAD54L ENSG00000085999 0.2668 0.39481 0.77085 0.2204 0.93032
0.1761 -0.45947 0.67705 0.1761 0.61675876
SALRNA1 ENSG00000258952 0.93031 0.1761 -0.022162 0.50884 -0.8619
0.80563 -0.39764 0.65455 0.1761 0.61675876
GCNT1 ENSG00000187210 0.30892 0.37869 0.16404 0.43485 -0.084003 0.53347
0.93021 0.17613 0.17613 0.61675876
NA ENSG00000229019 -0.0083663 0.50334 0.28746 0.38688 -0.257 0.60141
0.9302 0.17613 0.17613 0.61675876
NA ENSG00000280665 -0.16092 0.56392 0.93021 0.17613 0.45433 0.3248
0.033007 0.48683 0.17613 0.61675876
IQCA1-AS1 ENSG00000232893 0.024153 0.49037 0.83831 0.20093 0.038105
0.4848 0.93013 0.17615 0.17615 0.616768579
RN7SL807P ENSG00000243125 0.93001 0.17618 0.93001 0.17618 0.93001

0.17618 0.93001 0.17618 0.17618 0.616768579
DBIP1 ENSG00000218748 0.93 0.17619 0.93 0.17619 0.93 0.17619 0.93 0.17619
0.17619 0.616768579
NA ENSG00000261845 0.92995 0.1762 0.92995 0.1762 0.92995 0.1762
0.92995 0.1762 0.1762 0.616768579
TMEM106B ENSG00000106460 0.77766 0.21838 0.80333 0.21089 -0.03911
0.5156 0.92986 0.17622 0.17622 0.616768579
NA ENSG00000261398 0.92982 0.17623 0.92982 0.17623 0.92982 0.17623
0.92982 0.17623 0.17623 0.616768579
OCLN ENSG00000197822 0.92971 0.17626 -0.25797 0.60178 0.58739 0.27847
0.73728 0.23048 0.17626 0.616768579
EID2 ENSG00000176396 -0.023949 0.50955 0.28525 0.38773 0.28525 0.38773
0.92966 0.17627 0.17627 0.616768579
NA ENSG00000279962 0.45845 0.32332 0.92969 0.17627 0.92969 0.17627
0.92969 0.17627 0.17627 0.616768579
TNFRSF1A ENSG00000067182 -0.57373 0.71693 -0.45624 0.67589 -0.060884
0.52427 0.92955 0.1763 0.1763 0.616768579
GSTA12P ENSG00000217228 -0.6992 0.75779 0.92957 0.1763 -0.81193
0.79158 -1.3705 0.91473 0.1763 0.616768579
NA ENSG00000237821 0.073849 0.47057 0.92954 0.1763 0.92954 0.1763
0.92954 0.1763 0.1763 0.616768579
NA ENSG00000255745 -0.97639 0.83556 0.47811 0.31629 0.92954 0.1763
-2.1975 0.98601 0.1763 0.616768579
SLC38A2 ENSG00000134294 -0.38123 0.64848 0.31866 0.37499 0.92952
0.17631 0.68401 0.24698 0.17631 0.616768579
NA ENSG00000237982 0.92934 0.17636 0.92934 0.17636 0.92934 0.17636
0.92934 0.17636 0.17636 0.616899203
ENPP7P1 ENSG00000249188 -0.25185 0.59942 -0.18948 0.57514 -0.58438
0.72052 0.92919 0.1764 0.1764 0.616976216
N4BP2L2 ENSG00000244754 -0.35091 0.63717 0.42664 0.33482 0.92914
0.17641 0.22626 0.4105 0.17641 0.616976216
ALDH7A1P4 ENSG00000234489 0.92911 0.17642 0.92911 0.17642 0.92911
0.17642 0.92911 0.17642 0.17642 0.616976216
MLXIPL ENSG00000009950 0.92903 0.17644 -0.15078 0.55993 0.40856
0.34143 0.59281 0.27666 0.17644 0.616983268
NA ENSG00000272732 0.92897 0.17645 0.26389 0.39593 -0.23385 0.59245
-0.10759 0.54284 0.17645 0.616983268
NA ENSG00000272444 0.92892 0.17646 0.92892 0.17646 0.92892 0.17646
0.92892 0.17646 0.17646 0.616983268
PTAFR ENSG00000169403 0.76571 0.22192 0.47901 0.31597 0.92883 0.17649
0.37233 0.35483 0.17649 0.61699962
NA ENSG00000240842 0.92883 0.17649 0.88911 0.18697 0.16819 0.43322
0.88911 0.18697 0.17649 0.61699962
NA ENSG00000231769 0.044415 0.48229 0.92871 0.17652 0.29015 0.38585
0.59842 0.27478 0.17652 0.617023006
ANKRD62P1-PARP4P3 ENSG00000189295 0.72287 0.23488 -0.42634 0.66507
-0.62895 0.73531 0.92869 0.17653 0.17653 0.617023006
DZANK1 ENSG00000089091 0.92859 0.17655 0.92859 0.17655 0.92859
0.17655 -0.78547 0.78391 0.17655 0.617023006
NA ENSG00000267265 0.92861 0.17655 0.92861 0.17655 0.92861 0.17655
0.92861 0.17655 0.17655 0.617023006
NMD3P1 ENSG00000226342 0.92857 0.17656 0.63158 0.26383 -1.0717
0.85808 -1.4516 0.92669 0.17656 0.617023006
TMPRSS4 ENSG00000137648 0.92841 0.1766 0.32068 0.37423 0.92841
0.1766 0.39872 0.34505 0.1766 0.617109228
NA ENSG00000223716 -0.27285 0.60752 0.44533 0.32804 0.26255 0.39645
0.92838 0.17661 0.17661 0.617109228
RNU6-597P ENSG00000202490 0.92819 0.17665 0.92819 0.17665 0.92819
0.17665 -0.66496 0.74696 0.17665 0.617160489
NA ENSG00000238713 -0.64308 0.73991 0.59032 0.27749 0.92822 0.17665
0.92822 0.17665 0.17665 0.617160489
LINC01982 ENSG00000263317 0.92809 0.17668 0.83826 0.20094 0.71632

0.2369 0.71632 0.2369 0.17668 0.617176803
NA ENSG00000269693 0.9281 0.17668 0.9281 0.17668 0.9281 0.17668
0.9281 0.17668 0.17668 0.617176803
NA ENSG00000234132 0.92797 0.17671 0.92797 0.17671 0.92797 0.17671
0.92797 0.17671 0.17671 0.617197898
RNU6-483P ENSG00000206815 0.92794 0.17672 0.92794 0.17672 0.92794
0.17672 0.92794 0.17672 0.17672 0.617197898
NA ENSG00000236986 0.92789 0.17673 0.091655 0.46349 0.63274 0.26345
0.63274 0.26345 0.17673 0.617197898
RN7SL28P ENSG00000244512 -0.46102 0.67761 0.52484 0.29985 0.75374
0.2255 0.9278 0.17675 0.17675 0.617197898
RNU7-62P ENSG00000238653 0.10831 0.45688 0.9277 0.17678 0.74682
0.22758 0.9277 0.17678 0.17678 0.617197898
MOB2 ENSG00000182208 -0.55088 0.70914 -0.014953 0.50597 0.92768 0.17679
-0.36049 0.64076 0.17679 0.617197898
RNU6-374P ENSG00000201016 0.92767 0.17679 -0.36717 0.64326 0.92767
0.17679 0.92767 0.17679 0.17679 0.617197898
LINC02156 ENSG00000257906 0.92763 0.1768 0.56514 0.28599 0.92763
0.1768 0.15271 0.43931 0.1768 0.617197898
ST3GAL4 ENSG00000110080 0.84972 0.19774 0.84972 0.19774 0.50466
0.3069 0.9276 0.17681 0.17681 0.617197898
FAM153A ENSG00000170074 0.1606 0.4362 0.8998 0.18411 -1.2043
0.88576 0.92754 0.17682 0.17682 0.617197898
CMC1 ENSG00000187118 -0.32069 0.62578 -0.092016 0.53666 0.86414 0.19376
0.92746 0.17685 0.17685 0.617197898
TUBE1 ENSG00000074935 0.89479 0.18545 -0.18677 0.57408 0.89479 0.18545
0.9274 0.17686 0.17686 0.617197898
ZNF438 ENSG00000183621 0.64361 0.25992 0.15075 0.44009 0.92736
0.17687 -0.033449 0.51334 0.17687 0.617197898
MRPS21P2 ENSG00000227425 0.92724 0.1769 0.92724 0.1769 0.92724
0.1769 0.92724 0.1769 0.1769 0.617197898
NA ENSG00000261292 -0.89059 0.81343 0.92718 0.17692 -0.89059 0.81343
-0.89059 0.81343 0.17692 0.617197898
ASH2L ENSG00000129691 0.7767 0.21867 0.92709 0.17694 0.10326 0.45888
0.7767 0.21867 0.17694 0.617197898
PPIAP26 ENSG00000150276 0.92708 0.17694 0.92708 0.17694 0.92708
0.17694 -0.14278 0.55677 0.17694 0.617197898
NAXE ENSG00000163382 0.92706 0.17695 0.68853 0.24556 0.68853 0.24556
0.68853 0.24556 0.17695 0.617197898
NA ENSG00000203644 0.92699 0.17697 0.92699 0.17697 0.18051 0.42837
0.92699 0.17697 0.17697 0.617197898
APTR ENSG00000214293 0.62306 0.26662 0.82531 0.2046 0.42922 0.33388
0.92699 0.17697 0.17697 0.617197898
NA ENSG00000273143 0.33297 0.36958 0.92694 0.17698 0.60752 0.27175
-1.6209 0.94748 0.17698 0.617197898
VASP ENSG00000125753 0.92691 0.17699 0.32276 0.37344 0.92691 0.17699
0.92691 0.17699 0.17699 0.617197898
NA ENSG00000240197 -0.57412 0.71706 0.9269 0.17699 0.3248 0.37267
0.32276 0.37344 0.17699 0.617197898
NA ENSG00000243613 0.9268 0.17702 0.9268 0.17702 0.9268 0.17702
0.9268 0.17702 0.17702 0.617197898
TSKU ENSG00000182704 -0.68027 0.75183 0.92669 0.17704 0.66569 0.25281
0.042184 0.48318 0.17704 0.617197898
NUDCD1 ENSG00000120526 0.92668 0.17705 0.73011 0.23266 -0.072175
0.52877 0.57009 0.28431 0.17705 0.617197898
PLEKHH1 ENSG00000054690 -0.67682 0.75074 -0.40521 0.65734 0.92651
0.17709 -1.1974 0.88442 0.17709 0.617197898
PARVA ENSG00000197702 -0.37863 0.64752 0.9265 0.17709 -0.12183 0.54848
-1.1073 0.86591 0.17709 0.617197898
ZNF441 ENSG00000197044 0.92647 0.1771 -0.97876 0.83615 -1.2805
0.89981 -1.3908 0.91786 0.1771 0.617197898
NA ENSG00000260255 0.64264 0.26023 0.92648 0.1771 0.75619 0.22477

0.75619 0.22477 0.1771 0.617197898
NA ENSG00000250509 -0.27416 0.60802 0.17717 0.42969 -0.9697 0.8339
0.92645 0.17711 0.17711 0.617197898
NA ENSG00000266772 -0.33918 0.63276 -0.72856 0.76687 -1.5211 0.93588
0.92645 0.17711 0.17711 0.617197898
NA ENSG00000227757 0.52138 0.30105 0.92639 0.17712 -0.47894 0.68401
0.52138 0.30105 0.17712 0.617197898
ABI2 ENSG00000138443 0.38045 0.35181 0.90254 0.18339 0.87651 0.19038
0.92635 0.17713 0.17713 0.617197898
NA ENSG00000249295 0.92635 0.17713 0.92635 0.17713 -0.43503 0.66823
0.92635 0.17713 0.17713 0.617197898
NA ENSG00000260066 0.92627 0.17715 0.10907 0.45657 -0.79972 0.78806
0.80724 0.20976 0.17715 0.617197898
C14orf180 ENSG00000184601 0.67167 0.2509 0.1878 0.42551 0.92619
0.17717 0.63952 0.26124 0.17717 0.617197898
MOSMO ENSG00000185716 0.92617 0.17718 0.92617 0.17718 0.92617 0.17718
0.25944 0.39765 0.17718 0.617197898
NA ENSG00000272632 0.92616 0.17718 -0.34101 0.63345 -0.82532 0.79541
-0.82532 0.79541 0.17718 0.617197898
ABITRAM ENSG00000119328 0.92604 0.17721 0.92604 0.17721 0.92604
0.17721 0.92604 0.17721 0.17721 0.617258277
CNMD ENSG00000136110 0.42195 0.33653 -1.2541 0.8951 0.92597 0.17723
0.62811 0.26497 0.17723 0.61726524
VPS13A ENSG00000197969 0.06684 0.47335 0.92588 0.17725 0.49751
0.30941 0.39588 0.3461 0.17725 0.61726524
NA ENSG00000212445 0.80634 0.21002 0.92591 0.17725 0.80634 0.21002
0.80634 0.21002 0.17725 0.61726524
NA ENSG00000257703 0.92582 0.17727 -0.6117 0.72963 -0.6117 0.72963
-0.6117 0.72963 0.17727 0.617281486
NA ENSG00000202385 0.92576 0.17728 0.92576 0.17728 0.92576 0.17728
0.92576 0.17728 0.17728 0.617281486
NA ENSG00000265995 0.92562 0.17732 0.45132 0.32588 0.68343 0.24717
0.011884 0.49526 0.17732 0.617346426
HMGB1P28 ENSG00000249745 0.92556 0.17734 0.92556 0.17734 0.92556
0.17734 0.92556 0.17734 0.17734 0.617346426
RNU6-1187P ENSG00000201641 0.57643 0.28216 0.92543 0.17737 0.80977
0.20904 0.80977 0.20904 0.17737 0.617346426
MIR592 ENSG00000207692 0.47238 0.31833 0.92537 0.17739 0.92537
0.17739 0.92537 0.17739 0.17739 0.617346426
RNA5SP76 ENSG00000252086 0.77958 0.21782 0.92536 0.17739 0.92536
0.17739 0.92536 0.17739 0.17739 0.617346426
RN7SL188P ENSG00000241943 0.92532 0.1774 -0.26348 0.60391 0.50133
0.30807 -0.6911 0.75525 0.1774 0.617346426
MIR378D1 ENSG00000263631 0.061536 0.47547 0.92532 0.1774 0.16606
0.43405 0.13544 0.44613 0.1774 0.617346426
NA ENSG00000279541 0.92532 0.1774 0.92532 0.1774 0.92532 0.1774
0.92532 0.1774 0.1774 0.617346426
ABCC10 ENSG00000124574 0.92514 0.17745 0.55239 0.29034 0.55167
0.29059 -0.22687 0.58974 0.17745 0.617388155
LINC02789 ENSG00000231718 0.92512 0.17745 0.92512 0.17745 0.7763
0.21879 0.92512 0.17745 0.17745 0.617388155
NA ENSG00000279589 0.92511 0.17745 0.92511 0.17745 0.92511 0.17745
0.92511 0.17745 0.17745 0.617388155
NA ENSG00000235410 0.49594 0.30997 0.15675 0.43772 0.925 0.17748
0.49594 0.30997 0.17748 0.617411291
CNGA3 ENSG00000144191 -0.3269 0.62813 -0.27977 0.61017 0.92497 0.17749
-0.0049135 0.50196 0.17749 0.617411291
CADM1 ENSG00000182985 0.51245 0.30417 -0.053166 0.5212 0.92492 0.1775
0.31151 0.37771 0.1775 0.617411291
TRGVA ENSG00000225992 0.018868 0.49247 0.92491 0.17751 0.018868 0.49247
0.018868 0.49247 0.17751 0.617411291
OR4X1 ENSG00000176567 -0.23626 0.59338 -0.77393 0.78051 0.92486 0.17752

0.21902	0.41332	0.17752	0.617411291
NA	ENSG00000238472	-0.1754	0.56962	0.38356	0.35065	0.45336	0.32515
0.92473	0.17755	0.17755	0.617443696
ZSCAN18	ENSG00000121413	0.92465	0.17757	0.92465	0.17757	0.92465
0.17757	0.92465	0.17757	0.17757	0.617443696
TTYH2	ENSG00000141540	0.92461	0.17758	0.7664	0.22172	-1.0855	0.86114
0.6657	0.2528	0.17758	0.617443696
BEST1	ENSG00000167995	0.34929	0.36344	-0.33979	0.63299	0.17104	0.43209
0.92461	0.17758	0.17758	0.617443696
RAC1P2	ENSG00000249936	-1.6913	0.95461	-2.1467	0.98409	0.28285
0.38865	0.92447	0.17762	0.17762	0.617538713
RNA5SP294	ENSG00000223238	0.92429	0.17767	0.92429	0.17767	0.92429
0.17767	0.92429	0.17767	0.17767	0.617659181
DSN1	ENSG00000149636	-0.69493	0.75645	-1.4737	0.92973	-0.69493	0.75645
0.92423	0.17768	0.17768	0.617659181
AK5	ENSG00000154027	-0.20659	0.58183	-0.36723	0.64328	-0.46657	0.6796
0.92412	0.17771	0.17771	0.6177101
RPL19P1	ENSG00000226217	0.8203	0.20602	0.92411	0.17772	0.8203
0.20602	0.8203	0.20602	0.17772	0.6177101
SYT1	ENSG00000067715	-1.0842	0.86086	-0.12653	0.55034	0.92388	0.17777
-0.18786	0.57451	0.17777	0.617777146
NA	ENSG00000250997	0.92387	0.17778	0.92387	0.17778	0.92387	0.17778
0.92387	0.17778	0.17778	0.617777146
NA	ENSG00000203396	-0.86769	0.80722	0.92383	0.17779	0.46246	0.32188
0.46246	0.32188	0.17779	0.617777146
NA	ENSG00000270571	0.92381	0.17779	0.92381	0.17779	0.92381	0.17779
0.92381	0.17779	0.17779	0.617777146
CBX7	ENSG00000100307	0.92369	0.17782	-0.04326	0.51725	0.35827	0.36007
0.15681	0.4377	0.17782	0.617837333
NA	ENSG00000266101	0.9236	0.17785	0.9236	0.17785	0.9236	0.17785
0.9236	0.17785	0.17785	0.617878892
ANKRD26P4	ENSG00000229427	0.16308	0.43523	0.6169	0.26865	0.92356
0.17786	0.11195	0.45543	0.17786	0.617878892
GPT	ENSG00000167701	0.21359	0.41543	0.35911	0.35976	0.92352	0.17787
0.92352	0.17787	0.17787	0.617878892
NA	ENSG00000261997	0.92341	0.1779	0.92341	0.1779	0.92341	0.1779
0.92341	0.1779	0.1779	0.617939055
UPB1	ENSG00000100024	-0.014359	0.50573	-0.13645	0.55427	0.10935	0.45646
0.92329	0.17793	0.17793	0.617999209
CACNA1C	ENSG00000151067	0.31846	0.37507	0.65396	0.25657	0.47306
0.31808	0.92318	0.17796	0.17796	0.618015308
CST2P1	ENSG00000232595	0.23545	0.40693	0.92315	0.17796	0.2513
0.40079	-0.18176	0.57211	0.17796	0.618015308
NA	ENSG00000267606	-0.65157	0.74266	-0.65157	0.74266	0.92299	0.17801
-0.65157	0.74266	0.17801	0.618132057
NA	ENSG00000250560	0.83802	0.20101	-0.010518	0.5042	0.83802	0.20101
0.92285	0.17804	0.17804	0.618132057
RPL5P8	ENSG00000213917	0.92276	0.17807	0.024123	0.49038	0.024123
0.49038	0.024123	0.49038	0.17807	0.618132057
CPO	ENSG00000144410	-0.48821	0.6873	0.44093	0.32963	-0.43381	0.66779
0.92263	0.1781	0.1781	0.618132057
SLC16A1P1	ENSG00000242280	0.92253	0.17813	0.92253	0.17813	0.92253
0.17813	0.92253	0.17813	0.17813	0.618132057
NA	ENSG00000248935	-0.20866	0.58264	0.42377	0.33587	0.9225	0.17813
0.42377	0.33587	0.17813	0.618132057
NUDT5	ENSG00000165609	0.89755	0.18471	0.69059	0.24491	0.92249	0.17814
0.72205	0.23513	0.17814	0.618132057
ATP2A1-AS1	ENSG00000260442	0.92247	0.17814	0.92247	0.17814	0.92247
0.17814	0.92247	0.17814	0.17814	0.618132057
SOD2	ENSG00000112096	0.92244	0.17815	0.89999	0.18406	0.87579	0.19057
0.36186	0.35873	0.17815	0.618132057
MIR5692C2	ENSG00000266668	0.92238	0.17816	0.92238	0.17816	0.92238

0.17816 0.92238 0.17816 0.17816 0.618132057
NA ENSG00000234087 -0.32984 0.62924 0.92235 0.17817 0.92235 0.17817
0.92235 0.17817 0.17817 0.618132057
LINC02621 ENSG00000234756 0.92236 0.17817 0.019695 0.49214 0.92236
0.17817 0.92236 0.17817 0.17817 0.618132057
NA ENSG00000235181 0.72366 0.23464 0.92237 0.17817 0.72366 0.23464
0.72366 0.23464 0.17817 0.618132057
PDE4D ENSG00000113448 -0.81061 0.79121 0.29828 0.38274 0.14887 0.44083
0.92226 0.1782 0.1782 0.618132057
LINC01729 ENSG00000225640 0.92221 0.17821 0.75385 0.22547 0.018168
0.49275 -0.25841 0.60195 0.17821 0.618132057
NA ENSG00000240562 0.92218 0.17822 0.92218 0.17822 0.92218 0.17822
0.92218 0.17822 0.17822 0.618132057
NA ENSG00000260570 0.92218 0.17822 0.92218 0.17822 0.92218 0.17822
0.92218 0.17822 0.17822 0.618132057
LRCH3 ENSG00000186001 0.77923 0.21792 0.61751 0.26845 0.66827 0.25198
0.92214 0.17823 0.17823 0.618132057
TMCO3 ENSG00000150403 0.81789 0.20671 0.92207 0.17825 -0.53028 0.70204
0.43615 0.33137 0.17825 0.618132057
MIR494 ENSG00000194717 -0.046174 0.51841 -0.046174 0.51841 -0.046174
0.51841 0.92201 0.17826 0.17826 0.618132057
NA ENSG00000230929 0.922 0.17826 0.922 0.17826 0.922 0.17826 0.922 0.17826
0.17826 0.618132057
NA ENSG00000272166 0.21608 0.41446 0.92193 0.17828 0.77847 0.21815
0.77847 0.21815 0.17828 0.618157421
KLC3 ENSG00000104892 0.92172 0.17834 0.92172 0.17834 0.92172 0.17834
0.92172 0.17834 0.17834 0.618265557
TRPM3 ENSG00000083067 0.39792 0.34534 0.72275 0.23492 0.17615 0.43009
0.92163 0.17836 0.17836 0.618265557
NA ENSG00000240440 0.92152 0.17839 0.92152 0.17839 0.92152 0.17839
0.92152 0.17839 0.17839 0.618265557
FBXO36P1 ENSG00000266117 0.92151 0.17839 -1.1077 0.866 -1.6796 0.95348
-1.6796 0.95348 0.17839 0.618265557
SEC14L5 ENSG00000103184 -1.0866 0.86139 -0.97368 0.83489 -0.97368
0.83489 0.92147 0.1784 0.1784 0.618265557
AIDA ENSG00000186063 0.92147 0.1784 -0.51079 0.69525 -0.51079 0.69525
-0.51079 0.69525 0.1784 0.618265557
PHBP13 ENSG00000234835 0.10468 0.45831 -0.024816 0.5099 0.92147
0.1784 0.10468 0.45831 0.1784 0.618265557
RNA5SP477 ENSG00000199509 0.92139 0.17842 0.92139 0.17842 0.92139
0.17842 0.92139 0.17842 0.17842 0.61827227
CCR10 ENSG00000184451 0.92136 0.17843 0.92136 0.17843 0.92136 0.17843
0.92136 0.17843 0.17843 0.61827227
ADGRG6 ENSG00000112414 0.10205 0.45936 0.90866 0.18176 0.92133
0.17844 0.83442 0.20202 0.17844 0.61827227
KLRK1 ENSG00000213809 0.92107 0.17851 -0.057477 0.52292 0.58987 0.27764
0.58987 0.27764 0.17851 0.618283082
NA ENSG00000278332 -0.63198 0.7363 0.92102 0.17852 -0.99172 0.83933
-0.99172 0.83933 0.17852 0.618283082
ESS2 ENSG00000100056 0.92099 0.17853 0.86326 0.194 0.6193 0.26786
0.6105 0.27076 0.17853 0.618283082
THAP2 ENSG00000173451 0.92097 0.17853 0.92097 0.17853 0.92097 0.17853
-1.1747 0.87995 0.17853 0.618283082
LEPROT ENSG00000213625 0.92094 0.17854 0.88013 0.18939 0.88013
0.18939 0.88013 0.18939 0.17854 0.618283082
TSHZ3 ENSG00000121297 0.57253 0.28348 -0.19833 0.57861 0.9209 0.17855
0.9005 0.18393 0.17855 0.618283082
NA ENSG00000272909 0.92091 0.17855 0.92091 0.17855 0.92091 0.17855
0.92091 0.17855 0.17855 0.618283082
NA ENSG00000199899 0.92086 0.17856 0.92086 0.17856 0.92086 0.17856
0.92086 0.17856 0.17856 0.618283082
ERMN ENSG00000136541 0.92081 0.17857 0.92081 0.17857 0.92081 0.17857

0.92081 0.17857 0.17857 0.618283082
LRRC34P1 ENSG00000256371 0.92083 0.17857 0.92083 0.17857 0.92083
0.17857 0.92083 0.17857 0.17857 0.618283082
ANKRD13C ENSG00000118454 0.92072 0.1786 -1.9611 0.97506 -1.9611
0.97506 -1.9611 0.97506 0.1786 0.61834302
ATP13A1 ENSG00000105726 -0.030021 0.51197 -0.28115 0.6107 0.60215
0.27354 0.92056 0.17864 0.17864 0.618393635
NA ENSG00000256988 0.92055 0.17864 -0.52219 0.69923 0.92055 0.17864
0.92055 0.17864 0.17864 0.618393635
NA ENSG00000252789 -1.1159 0.86778 0.92047 0.17866 0.55255 0.29029
0.48214 0.31485 0.17866 0.618418937
WFS1 ENSG00000109501 -0.92517 0.82256 -0.7628 0.77721 0.9009 0.18382
0.92027 0.17872 0.17872 0.618582683
NA ENSG00000260861 0.92004 0.17878 0.92004 0.17878 0.92004 0.17878
0.92004 0.17878 0.17878 0.618637173
CPT1C ENSG00000169169 0.91994 0.1788 0.91994 0.1788 -0.90458 0.81716
0.91994 0.1788 0.1788 0.618637173
MGST2 ENSG00000085871 0.81142 0.20856 0.9199 0.17881 0.79484 0.21335
0.88241 0.18878 0.17881 0.618637173
AKIRIN2P1 ENSG00000251482 0.25667 0.39872 0.11086 0.45586 0.91991
0.17881 0.91991 0.17881 0.17881 0.618637173
TRNAU1AP ENSG00000180098 0.37322 0.35449 0.37322 0.35449 0.91987
0.17882 -0.29709 0.6168 0.17882 0.618637173
NA ENSG00000267292 0.91988 0.17882 0.62383 0.26637 0.62383 0.26637
0.62383 0.26637 0.17882 0.618637173
NA ENSG00000248567 -0.41328 0.6603 -0.64158 0.73943 0.39268 0.34728
0.91983 0.17883 0.17883 0.618637173
NA ENSG00000219681 0.57396 0.283 0.23862 0.4057 0.14439 0.4426
0.91975 0.17885 0.17885 0.618637173
NA ENSG00000230975 -0.33322 0.63051 0.91975 0.17885 0.91975 0.17885
-1.1499 0.87491 0.17885 0.618637173
ABL1 ENSG00000097007 0.91954 0.17891 0.64213 0.26039 0.86714 0.19293
0.4422 0.32917 0.17891 0.618747541
NA ENSG00000236907 0.88935 0.18691 0.91953 0.17891 0.91953 0.17891
0.91953 0.17891 0.17891 0.618747541
C1orf195 ENSG00000204464 0.91947 0.17892 -1.4235 0.92271 0.039284
0.48433 -0.88804 0.81274 0.17892 0.618747541
NA ENSG00000212565 0.6134 0.26981 0.91931 0.17897 -0.29207 0.61488
-0.27218 0.60726 0.17897 0.618776644
NA ENSG00000229668 0.91928 0.17897 0.5801 0.28092 0.58691 0.27863
-0.52728 0.701 0.17897 0.618776644
NA ENSG00000248895 0.91931 0.17897 0.91931 0.17897 0.91931 0.17897
0.91931 0.17897 0.17897 0.618776644
CEP89 ENSG00000121289 0.91923 0.17899 -0.24401 0.59639 0.63934 0.2613
0.52756 0.2989 0.17899 0.618776644
RNF115 ENSG00000265491 0.91913 0.17901 0.69607 0.24319 -0.878
0.81003 0.52598 0.29945 0.17901 0.618776644
NA ENSG00000258538 -1.3172 0.90611 -0.59862 0.72529 -1.3172 0.90611
0.9191 0.17902 0.17902 0.618776644
HNRNPA1P52 ENSG00000269119 -0.64012 0.73895 0.26189 0.3967 0.9191
0.17902 0.26189 0.3967 0.17902 0.618776644
RASSF10 ENSG00000189431 0.91907 0.17903 0.91907 0.17903 0.91907
0.17903 0.91907 0.17903 0.17903 0.618776644
FUT8 ENSG00000033170 -0.36452 0.64226 -0.10147 0.54041 0.058512 0.47667
0.9189 0.17907 0.17907 0.618787072
MRPL13 ENSG00000172172 0.84769 0.1983 0.34985 0.36323 -0.17107
0.56791 0.91892 0.17907 0.17907 0.618787072
OR7E116P ENSG00000225072 0.25478 0.39945 -0.03062 0.51221 -0.49825
0.69085 0.91887 0.17908 0.17908 0.618787072
NA ENSG00000274235 0.25426 0.39965 0.91885 0.17909 0.69383 0.24389
-1.4832 0.93098 0.17909 0.618787072
NA ENSG00000254935 0.91878 0.17911 0.22441 0.41122 0.91064 0.18124

0.63377 0.26312 0.17911 0.618787072
NA ENSG00000271537 -0.9215 0.8216 -0.9215 0.8216 -1.093 0.8628
0.91871 0.17912 0.17912 0.618787072
NA ENSG00000258210 0.91869 0.17913 0.91869 0.17913 0.91869 0.17913
0.91869 0.17913 0.17913 0.618787072
NA ENSG00000269895 0.91863 0.17915 0.91863 0.17915 0.91863 0.17915
0.91863 0.17915 0.17915 0.618787072
NA ENSG00000176075 -0.11238 0.54474 0.9186 0.17915 -0.11238 0.54474
0.9186 0.17915 0.17915 0.618787072
SECTM1 ENSG00000141574 0.36254 0.35847 -0.16717 0.56638 -0.18257
0.57243 0.91857 0.17916 0.17916 0.618787072
NA ENSG00000257497 0.91843 0.1792 0.27955 0.38991 0.27955 0.38991
0.27955 0.38991 0.1792 0.618881361
NA ENSG00000229102 0.91823 0.17925 -0.13151 0.55231 -0.87065 0.80803
-0.12814 0.55098 0.17925 0.619010169
RNY3P9 ENSG00000207458 0.62043 0.26749 0.62043 0.26749 0.62043
0.26749 0.91793 0.17933 0.17933 0.619126783
MIR30A ENSG00000207827 -1.926 0.97295 0.91792 0.17933 -1.926
0.97295 -1.926 0.97295 0.17933 0.619126783
NA ENSG00000243929 0.48921 0.31235 0.81566 0.20735 0.91792 0.17933
0.41708 0.33831 0.17933 0.619126783
FSIP2-AS1 ENSG00000231646 0.9179 0.17934 0.9179 0.17934 0.9179
0.17934 0.9179 0.17934 0.17934 0.619126783
NA ENSG00000230248 0.91782 0.17936 0.91782 0.17936 0.91782 0.17936
0.91782 0.17936 0.17936 0.619126783
NA ENSG00000254192 0.6342 0.26298 -1.2115 0.88715 0.9178 0.17936
0.9178 0.17936 0.17936 0.619126783
SLC25A32 ENSG00000164933 0.13641 0.44575 -1.5437 0.93867 -0.51582
0.69701 0.91752 0.17943 0.17943 0.619187267
NA ENSG00000226335 0.3214 0.37395 0.91754 0.17943 0.8625 0.19421
0.87957 0.18955 0.17943 0.619187267
NA ENSG00000200314 0.58035 0.28084 0.91747 0.17945 0.91747 0.17945
0.91747 0.17945 0.17945 0.619187267
NA ENSG00000270120 0.91734 0.17948 0.91734 0.17948 0.91734 0.17948
0.91734 0.17948 0.17948 0.619187267
TPGS2 ENSG00000134779 -0.29249 0.61504 -0.094523 0.53765 -0.61149 0.72956
0.9173 0.17949 0.17949 0.619187267
ARNT ENSG00000143437 -0.39842 0.65484 0.91731 0.17949 -0.81843 0.79345
0.26451 0.3957 0.17949 0.619187267
PINX1 ENSG00000254093 0.70393 0.24074 0.22727 0.41011 0.6945 0.24369
0.91732 0.17949 0.17949 0.619187267
GUK1 ENSG00000143774 -0.7574 0.77559 -0.49711 0.69045 0.91727 0.1795
-0.73063 0.7675 0.1795 0.619187267
NA ENSG00000235277 0.91726 0.1795 0.91726 0.1795 -0.8029 0.78898
0.91726 0.1795 0.1795 0.619187267
NA ENSG00000267350 0.91723 0.17951 0.91723 0.17951 0.91723 0.17951
0.91723 0.17951 0.17951 0.619187267
LINC01344 ENSG00000228918 0.28514 0.38777 0.04567 0.48179 0.15016
0.44032 0.91719 0.17952 0.17952 0.619187267
EBLN1 ENSG00000223601 0.91715 0.17953 0.91715 0.17953 0.91715 0.17953
0.91715 0.17953 0.17953 0.619187267
DPYD ENSG00000188641 0.91696 0.17958 0.43506 0.33176 0.10561 0.45794
0.55169 0.29058 0.17958 0.619306539
HEY2 ENSG00000135547 0.34761 0.36407 0.91692 0.17959 -0.0010099 0.5004
-0.31406 0.62326 0.17959 0.619306539
BIN1 ENSG00000136717 0.47471 0.3175 0.30262 0.38109 0.91677 0.17963
0.38872 0.34874 0.17963 0.619400644
NA ENSG00000268568 0.91665 0.17966 0.91665 0.17966 0.91665 0.17966
0.91665 0.17966 0.17966 0.619460256
POLE2 ENSG00000100479 0.91651 0.1797 0.61981 0.26769 0.91651 0.1797
-0.12302 0.54895 0.1797 0.619535625
NA ENSG00000279159 0.91647 0.17971 0.91647 0.17971 0.91647 0.17971

0.91647 0.17971 0.17971 0.619535625
NA ENSG00000278266 0.91644 0.17972 0.91644 0.17972 0.91644 0.17972
0.91644 0.17972 0.17972 0.619535625
CYP2C56P ENSG00000229641 0.10517 0.45812 -0.3475 0.63589 0.91631
0.17975 -0.3475 0.63589 0.17975 0.619542033
NA ENSG00000271976 0.91632 0.17975 0.91632 0.17975 0.91632 0.17975
0.91632 0.17975 0.17975 0.619542033
NA ENSG00000280352 0.91629 0.17976 0.91629 0.17976 0.91629 0.17976
0.91629 0.17976 0.17976 0.619542033
NPRL3 ENSG00000103148 0.16607 0.43405 -0.35602 0.63909 -0.33804 0.63233
0.91582 0.17988 0.17988 0.619858556
ARMT1 ENSG00000146476 -0.72345 0.7653 0.21672 0.41421 0.91582 0.17988
-1.0607 0.85558 0.17988 0.619858556
NA ENSG00000227485 0.91578 0.17989 0.91578 0.17989 0.66988 0.25147
0.29881 0.38254 0.17989 0.619858556
NA ENSG00000227175 0.9156 0.17994 0.618 0.26829 0.71235 0.23812
-0.08504 0.53389 0.17994 0.619906126
NA ENSG00000265100 0.91558 0.17994 0.91558 0.17994 0.91558 0.17994
0.91558 0.17994 0.17994 0.619906126
NA ENSG00000273680 0.91546 0.17997 0.91546 0.17997 0.91546 0.17997
0.91546 0.17997 0.17997 0.619906126
SPSB1 ENSG00000171621 -0.18972 0.57524 -0.067648 0.52697 0.91545 0.17998
-0.17159 0.56812 0.17998 0.619906126
NA ENSG00000253177 0.91539 0.17999 0.91539 0.17999 0.91539 0.17999
0.91539 0.17999 0.17999 0.619906126
MIR5705 ENSG00000263981 0.91532 0.18001 0.91532 0.18001 0.91532
0.18001 0.91532 0.18001 0.18001 0.619906126
NA ENSG00000230030 0.051943 0.47929 0.051943 0.47929 0.9153 0.18002
0.1737 0.43105 0.18002 0.619906126
MAP2K6 ENSG00000108984 -0.86944 0.8077 -0.84925 0.80213 -1.1319
0.87115 0.91508 0.18008 0.18008 0.619906126
RBISP2 ENSG00000271378 0.50137 0.30806 0.91505 0.18008 0.91505
0.18008 0.91505 0.18008 0.18008 0.619906126
PIWIL4 ENSG00000134627 0.91502 0.18009 -0.1191 0.5474 -0.6607
0.7456 0.029835 0.4881 0.18009 0.619906126
NA ENSG00000229229 -0.33272 0.63033 0.91501 0.18009 -0.14668 0.55831
-0.14668 0.55831 0.18009 0.619906126
IARS1 ENSG00000196305 -0.87751 0.80989 0.40077 0.3443 0.91497 0.1801
0.1763 0.43003 0.1801 0.619906126
RNU6-1074P ENSG00000206722 0.45035 0.32623 0.89003 0.18672 0.91495
0.18011 0.89003 0.18672 0.18011 0.619906126
ROMO1 ENSG00000125995 0.61802 0.26828 0.61802 0.26828 0.9149 0.18012
0.61802 0.26828 0.18012 0.619906126
NA ENSG00000256967 0.91485 0.18013 0.91485 0.18013 0.91485 0.18013
0.91485 0.18013 0.18013 0.619906126
MIR3680-2 ENSG00000266758 0.91483 0.18014 0.21665 0.41424 0.33322
0.36949 0.33322 0.36949 0.18014 0.619906126
NA ENSG00000228140 0.91481 0.18015 0.91481 0.18015 0.19847 0.42134
0.19847 0.42134 0.18015 0.619906126
KRTAP19-6 ENSG00000186925 0.91463 0.18019 0.91463 0.18019 0.91463
0.18019 0.91463 0.18019 0.18019 0.619906126
NA ENSG00000261468 -0.24445 0.59656 -0.25055 0.59892 0.91462 0.1802
-0.25055 0.59892 0.1802 0.619906126
RNU7-48P ENSG00000238386 -1.1375 0.87234 0.91458 0.18021 -1.1375
0.87234 -1.1375 0.87234 0.18021 0.619906126
ECPAS ENSG00000136813 -0.46666 0.67963 0.91454 0.18022 -0.28399 0.61179
-1.5039 0.93369 0.18022 0.619906126
SLC6A1-AS1 ENSG00000232287 0.18383 0.42707 0.18383 0.42707 0.18383
0.42707 0.91453 0.18022 0.18022 0.619906126
CCDC28A ENSG00000024862 0.9145 0.18023 -0.57445 0.71717 -1.4433
0.92554 -0.57445 0.71717 0.18023 0.619906126
NA ENSG00000276672 0.46972 0.31928 0.91448 0.18023 0.91448 0.18023

0.91448	0.18023	0.18023	0.619906126
KDM1A	ENSG00000004487	0.63567	0.26249	0.50386	0.30718	0.40708	0.34197
0.91446	0.18024	0.18024	0.619906126
RNU6-622P	ENSG00000206999	0.9144	0.18025	-0.28428	0.6119	-0.14286
0.5568	-1.6031	0.94554	0.18025	0.619906126
NA	ENSG00000212585	-0.89015	0.81331	0.9144	0.18025	0.89953	0.18418
0.89953	0.18418	0.18025	0.619906126
TGDS	ENSG00000088451	0.32032	0.37436	0.91437	0.18026	0.32032	0.37436
-1.1527	0.87548	0.18026	0.619906126
NA	ENSG00000272416	0.6829	0.24733	0.91429	0.18028	0.6829	0.24733
0.6829	0.24733	0.18028	0.619921797
RSPO4	ENSG00000101282	0.91427	0.18029	0.43073	0.33333	-0.81826	0.7934
-0.141	0.55606	0.18029	0.619921797
FMO3	ENSG00000007933	-0.63284	0.73658	-0.13613	0.55414	-0.5436	0.70664
0.91413	0.18032	0.18032	0.619937463
PSMA2P1	ENSG00000254582	0.0745	0.47031	-0.17839	0.57079	0.91414
0.18032	0.91414	0.18032	0.18032	0.619937463
MRC2	ENSG00000011028	0.51459	0.30342	0.041449	0.48347	0.037971	0.48486
0.91398	0.18036	0.18036	0.620003153
TMC1	ENSG00000165091	0.58743	0.27846	0.78907	0.21503	0.91395	0.18037
0.828	0.20383	0.18037	0.620003153
CSTF3	ENSG00000176102	0.91391	0.18038	0.7914	0.21435	-0.27298	0.60757
0.63747	0.26191	0.18038	0.620003153
NA	ENSG00000279631	0.91389	0.18039	-0.57162	0.71621	0.91389	0.18039
0.91389	0.18039	0.18039	0.620003153
ALG1L5P	ENSG00000226943	0.0091711	0.49634	0.0087857	0.4965	-0.14588
0.55799	0.91367	0.18044	0.18044	0.620131265
C11orf53	ENSG00000150750	-1.0092	0.84356	0.28237	0.38883	0.42201
0.33651	0.91361	0.18046	0.18046	0.620156262
NA	ENSG00000250068	0.91348	0.18049	0.91348	0.18049	0.91348	0.18049
0.91348	0.18049	0.18049	0.620215619
CAV1	ENSG00000105974	-0.20564	0.58146	0.91329	0.18055	-0.48247	0.68526
-0.24803	0.59794	0.18055	0.620334309
TSACC	ENSG00000163467	0.91326	0.18055	0.91326	0.18055	0.6924	0.24434
0.078005	0.46891	0.18055	0.620334309
PRR7	ENSG00000131188	0.9132	0.18057	-0.09841	0.5392	-0.09841	0.5392
-0.09841	0.5392	0.18057	0.620359285
CINP	ENSG00000100865	-1.1221	0.8691	-1.1221	0.8691	-1.1004	0.86441
0.91311	0.18059	0.18059	0.620384258
ODF3L1	ENSG00000182950	-1.6758	0.95311	0.91299	0.18062	-0.7363
0.76923	0.31665	0.37576	0.18062	0.620415427
ADK	ENSG00000156110	0.56402	0.28637	0.85222	0.19705	0.91293	0.18064
0.52018	0.30147	0.18064	0.620415427
RPL9P6	ENSG00000241891	0.91288	0.18065	0.91288	0.18065	0.91288
0.18065	0.91288	0.18065	0.18065	0.620415427
NA	ENSG00000255445	0.91288	0.18065	0.91288	0.18065	0.91288	0.18065
0.91288	0.18065	0.18065	0.620415427
SLC17A4	ENSG00000146039	0.91278	0.18068	-0.98209	0.83697	0.41869
0.33772	-0.83496	0.79813	0.18068	0.620446579
PHBP20	ENSG00000271072	0.91269	0.1807	0.91269	0.1807	0.91269
0.1807	0.91269	0.1807	0.1807	0.620446579
HAUS1P3	ENSG00000253226	0.91266	0.18071	0.91266	0.18071	0.91266
0.18071	0.91266	0.18071	0.18071	0.620446579
VN1R9P	ENSG00000276400	0.91268	0.18071	-0.54277	0.70636	0.54459
0.29302	0.91268	0.18071	0.18071	0.620446579
LINC02423	ENSG00000256750	0.91246	0.18076	-1.2708	0.89811	-0.63219
0.73637	-1.3749	0.91542	0.18076	0.620449577
LINC00703	ENSG00000224382	0.71162	0.23835	0.9124	0.18078	0.69369
0.24394	0.66969	0.25153	0.18078	0.620449577
NA	ENSG00000227747	0.80352	0.21084	-0.41198	0.65982	0.59732	0.27515
0.91241	0.18078	0.18078	0.620449577
DENND5B-AS1	ENSG00000255867	-0.2907	0.61436	0.47423	0.31767	0.9124

0.18078 0.9124 0.18078 0.18078 0.620449577
ERFE ENSG00000178752 0.91236 0.18079 0.91236 0.18079 0.91236 0.18079
0.91236 0.18079 0.18079 0.620449577
RN7SL426P ENSG00000263815 0.91234 0.18079 0.91234 0.18079 0.91234
0.18079 0.91234 0.18079 0.18079 0.620449577
NA ENSG00000249086 0.91233 0.1808 0.91233 0.1808 0.91233 0.1808
0.91233 0.1808 0.1808 0.620449577
RPS3P1 ENSG00000230935 0.91222 0.18083 0.91222 0.18083 0.91222
0.18083 0.91222 0.18083 0.18083 0.620465139
RNU5F-8P ENSG00000251750 0.9122 0.18083 -0.086574 0.53449 -1.8831
0.97016 -1.8831 0.97016 0.18083 0.620465139
NA ENSG00000232835 -1.0329 0.84917 -1.0329 0.84917 0.91213 0.18085
0.91213 0.18085 0.18085 0.620490073
BTRC ENSG00000166167 0.43925 0.33024 0.28473 0.38793 0.91207 0.18087
0.55152 0.29064 0.18087 0.620507876
ALDH1L1-AS1 ENSG00000250218 0.9118 0.18094 0.9118 0.18094 0.9118
0.18094 0.9118 0.18094 0.18094 0.620507876
DEDD2 ENSG00000160570 0.35104 0.36278 0.57459 0.28278 0.65047 0.2577
0.91173 0.18095 0.18095 0.620507876
NA ENSG00000273013 -0.30297 0.61904 0.91173 0.18095 -0.30297 0.61904
-0.30297 0.61904 0.18095 0.620507876
RYR2 ENSG00000198626 0.91162 0.18098 0.034124 0.48639 0.40119 0.34414
-1.5032 0.93361 0.18098 0.620507876
NA ENSG00000206682 0.91164 0.18098 0.91164 0.18098 0.91164 0.18098
0.91164 0.18098 0.18098 0.620507876
PPP2R3A ENSG00000073711 0.41917 0.33754 0.2784 0.39035 0.9116
0.18099 -0.36418 0.64214 0.18099 0.620507876
MSX1 ENSG00000163132 -1.1217 0.869 -1.5012 0.93335 -1.5389 0.93808
0.9116 0.18099 0.18099 0.620507876
NA ENSG00000248447 -0.10481 0.54174 0.91157 0.181 0.91157 0.181 0.91157
0.181 0.181 0.620507876
ASB17 ENSG00000154007 0.91153 0.18101 0.91153 0.18101 0.91153 0.18101
0.91153 0.18101 0.18101 0.620507876
ZNRF2P3 ENSG00000235924 -0.6859 0.75361 0.91147 0.18102 0.91147
0.18102 0.91147 0.18102 0.18102 0.620507876
GNL3LP1 ENSG00000215032 0.91145 0.18103 0.91145 0.18103 0.91145
0.18103 0.91145 0.18103 0.18103 0.620507876
ABCC1 ENSG00000103222 -0.22664 0.58965 0.21621 0.41441 0.84321 0.19956
0.91139 0.18105 0.18105 0.620507876
NA ENSG00000250914 -0.18311 0.57264 -0.18311 0.57264 0.91132 0.18106
0.84729 0.19842 0.18106 0.620507876
RNU6-704P ENSG00000201032 0.91119 0.1811 0.91119 0.1811 0.91119
0.1811 0.88168 0.18898 0.1811 0.620507876
PLCD3 ENSG00000161714 0.91111 0.18112 -0.27251 0.60738 -0.021587 0.50861
-0.75551 0.77503 0.18112 0.620507876
OR51F3P ENSG00000272559 0.91108 0.18113 0.91108 0.18113 0.91108
0.18113 -0.29614 0.61644 0.18113 0.620507876
RNU6ATAC38P ENSG00000221046 0.91103 0.18114 0.91103 0.18114 0.91103
0.18114 0.91103 0.18114 0.18114 0.620507876
VN1R11P ENSG00000219770 -0.38784 0.65093 0.91092 0.18117 -0.70124
0.75842 -0.70124 0.75842 0.18117 0.620507876
CCDC127 ENSG00000164366 0.23283 0.40795 0.18842 0.42527 0.91084
0.18119 0.78636 0.21583 0.18119 0.620507876
RPS2P39 ENSG00000240698 -0.16463 0.56538 -0.22999 0.59095 0.91085
0.18119 0.91085 0.18119 0.18119 0.620507876
SLC3A2 ENSG00000168003 0.91081 0.1812 -1.5763 0.94253 -0.57968
0.71894 -0.51816 0.69783 0.1812 0.620507876
RNU6-280P ENSG00000201015 0.9108 0.1812 0.68311 0.24727 0.9108
0.1812 0.9108 0.1812 0.1812 0.620507876
NA ENSG00000223837 0.9108 0.1812 0.9108 0.1812 0.9108 0.1812
0.9108 0.1812 0.1812 0.620507876
RPL7P17 ENSG00000214062 -0.41266 0.66007 0.14452 0.44254 -0.046712

0.51863 0.91075 0.18121 0.18121 0.620507876
LINC01832 ENSG00000224231 0.91076 0.18121 0.91076 0.18121 0.91076
0.18121 0.91076 0.18121 0.18121 0.620507876
CRISP3 ENSG00000096006 0.91074 0.18122 0.91074 0.18122 0.91074
0.18122 0.91074 0.18122 0.18122 0.620507876
RPL21P109 ENSG00000237816 0.91072 0.18122 0.91072 0.18122 0.91072
0.18122 0.91072 0.18122 0.18122 0.620507876
CPEB2 ENSG00000137449 -0.038837 0.51549 0.91065 0.18124 0.21346 0.41548
0.22032 0.41281 0.18124 0.620507876
LINC00626 ENSG00000225826 0.16388 0.43491 0.80442 0.21058 0.16388
0.43491 0.91057 0.18126 0.18126 0.620507876
RN7SKP96 ENSG00000252757 -0.39767 0.65456 -0.39767 0.65456 -0.39767
0.65456 0.91046 0.18129 0.18129 0.620507876
AHSG ENSG00000145192 0.87219 0.19155 0.91039 0.18131 -0.58768 0.72163
0.017731 0.49293 0.18131 0.620507876
RSPO2 ENSG00000147655 0.9103 0.18133 0.73471 0.23126 0.6481 0.25846
0.32374 0.37307 0.18133 0.620507876
LINC02688 ENSG00000254872 0.44484 0.32822 0.032244 0.48714 0.91032
0.18133 -0.13644 0.55426 0.18133 0.620507876
PCDHB16 ENSG00000272674 0.91031 0.18133 0.91031 0.18133 0.91031
0.18133 0.91031 0.18133 0.18133 0.620507876
UBL3 ENSG00000122042 0.24756 0.40224 0.7272 0.23355 -0.35157 0.63742
0.91028 0.18134 0.18134 0.620507876
NA ENSG00000226063 0.91022 0.18135 0.91022 0.18135 0.91022 0.18135
0.91022 0.18135 0.18135 0.620507876
NA ENSG00000182625 0.91019 0.18136 0.91019 0.18136 0.91019 0.18136
0.91019 0.18136 0.18136 0.620507876
MARK2P17 ENSG00000244065 0.9102 0.18136 -1.98 0.97615 -1.98 0.97615 -1.98
0.97615 0.18136 0.620507876
NA ENSG00000225017 0.9101 0.18138 0.68777 0.2458 0.81304 0.2081
0.9101 0.18138 0.18138 0.620507876
C19orf18 ENSG00000177025 0.91006 0.18139 -0.74136 0.77076 -0.74136
0.77076 -0.058024 0.52314 0.18139 0.620507876
CCDC159 ENSG00000183401 0.9101 0.18139 0.9101 0.18139 0.9101
0.18139 0.9101 0.18139 0.18139 0.620507876
NXPE1 ENSG00000095110 -0.70636 0.76002 0.40715 0.34195 0.90991 0.18143
-0.082324 0.53281 0.18143 0.620520181
NA ENSG00000250644 0.90991 0.18143 0.028167 0.48876 0.18953 0.42484
0.18953 0.42484 0.18143 0.620520181
NFS1 ENSG00000244005 -0.20096 0.57964 0.90986 0.18145 0.57539 0.28251
0.51076 0.30476 0.18145 0.620520181
RN7SL703P ENSG00000266317 -1.2027 0.88545 -1.2027 0.88545 -1.4932
0.93231 0.90987 0.18145 0.18145 0.620520181
RNU6-441P ENSG00000207275 0.14256 0.44332 -0.53952 0.70523 -0.37556
0.64638 0.90983 0.18146 0.18146 0.620520181
TMEM63B ENSG00000137216 0.90976 0.18147 0.1411 0.4439 0.70549
0.24025 -0.12597 0.55012 0.18147 0.620520181
NA ENSG00000248652 0.90959 0.18152 0.90959 0.18152 0.90959 0.18152
0.90959 0.18152 0.18152 0.620600835
NA ENSG00000279677 -0.86799 0.8073 0.35095 0.36281 -0.86799 0.8073
0.90959 0.18152 0.18152 0.620600835
NA ENSG00000282021 0.23272 0.40799 -2.2393 0.98743 -2.2393 0.98743
0.90952 0.18154 0.18154 0.620600835
ENPP7P3 ENSG00000248618 0.90947 0.18155 -0.14997 0.55961 -0.023039
0.50919 0.48031 0.3155 0.18155 0.620600835
UVRAG ENSG00000198382 -0.72739 0.76651 -0.19149 0.57593 0.90938 0.18157
-1.0758 0.85899 0.18157 0.620600835
LINC01734 ENSG00000230324 0.15893 0.43686 0.78482 0.21628 0.15893
0.43686 0.90938 0.18157 0.18157 0.620600835
BEST4 ENSG00000142959 -0.11757 0.5468 -1.4429 0.92548 0.90925 0.18161
-1.1165 0.86789 0.18161 0.620675309
NUTM2HP ENSG00000232706 -1.5417 0.93843 0.90921 0.18162 -0.13574

0.55399 -1.0354 0.84976 0.18162 0.620675309
MTG2 ENSG00000101181 0.002653 0.49894 0.56632 0.28559 0.56632 0.28559
0.90916 0.18163 0.18163 0.620675309
NA ENSG00000235056 0.79719 0.21267 0.6104 0.2708 0.90909 0.18165
0.90909 0.18165 0.18165 0.62067818
KIF26A ENSG00000066735 -0.542 0.70609 -0.35076 0.63712 0.909 0.18167
-1.2648 0.89703 0.18167 0.62067818
NA ENSG00000279174 0.90903 0.18167 0.90903 0.18167 0.90903 0.18167
0.90903 0.18167 0.18167 0.62067818
COMMD8 ENSG00000169019 0.90895 0.18169 0.16815 0.43323 0.90895
0.18169 0.16815 0.43323 0.18169 0.62067818
FBXW2 ENSG00000119402 0.90892 0.1817 0.50894 0.3054 0.69116 0.24473
0.24286 0.40406 0.1817 0.62067818
NA ENSG00000253693 0.90889 0.18171 0.90889 0.18171 0.90889 0.18171
0.90889 0.18171 0.18171 0.62067818
NEMF ENSG00000165525 0.90883 0.18172 0.49846 0.30908 -0.49933 0.69123
-0.51008 0.695 0.18172 0.62067818
RPL5P31 ENSG00000214878 0.90876 0.18174 0.72096 0.23547 0.53518
0.29626 0.65401 0.25655 0.18174 0.620702986
FABP7P1 ENSG00000226766 0.90859 0.18178 0.90859 0.18178 0.90859
0.18178 -0.41876 0.6623 0.18178 0.620724536
KRT18P61 ENSG00000267083 0.90857 0.18179 0.90857 0.18179 0.90857
0.18179 0.90857 0.18179 0.18179 0.620724536
INKA2 ENSG00000197852 0.90853 0.1818 0.058033 0.47686 0.058033 0.47686
0.59663 0.27538 0.1818 0.620724536
HERC2P2 ENSG00000276550 0.10114 0.45972 0.90853 0.1818 -1.5576
0.94033 0.19845 0.42134 0.1818 0.620724536
NA ENSG00000254102 0.90848 0.18181 0.90848 0.18181 0.90848 0.18181
0.90848 0.18181 0.18181 0.620724536
ENPP7 ENSG00000182156 -0.30521 0.6199 -0.74813 0.77281 -1.2017 0.88526
0.9084 0.18183 0.18183 0.620730223
C1orf100 ENSG00000173728 -0.95848 0.83109 -0.95848 0.83109 -0.95848
0.83109 0.90836 0.18185 0.18185 0.620730223
MUC20-OT1 ENSG00000242086 0.90831 0.18186 0.61643 0.2688 0.80316
0.21094 0.47375 0.31784 0.18186 0.620730223
PI4K2A ENSG00000155252 0.60705 0.27191 -0.29737 0.61691 0.6626
0.25379 0.90826 0.18187 0.18187 0.620730223
DDX50P2 ENSG00000240048 -0.73929 0.77013 -0.12928 0.55143 -0.31413
0.62329 0.90821 0.18188 0.18188 0.620730223
NA ENSG00000272994 0.19672 0.42202 0.0085779 0.49658 0.90809 0.18191
0.90809 0.18191 0.18191 0.620730223
NA ENSG00000235070 0.33899 0.36731 0.90807 0.18192 -0.47433 0.68237
-0.47433 0.68237 0.18192 0.620730223
FOXO3B ENSG00000240445 0.90802 0.18194 -0.67467 0.75006 0.50124
0.3081 0.24922 0.40159 0.18194 0.620730223
MIR548AG1 ENSG00000265829 0.90792 0.18196 0.90792 0.18196 0.90792
0.18196 0.90792 0.18196 0.18196 0.620730223
IPPK ENSG00000127080 -0.17604 0.56987 -0.25095 0.59907 0.0089064 0.49645
0.90787 0.18197 0.18197 0.620730223
RN7SKP250 ENSG00000200794 0.90786 0.18198 -0.18264 0.57246 -1.2645
0.89697 0.80564 0.21022 0.18198 0.620730223
ANP32AP1 ENSG00000259516 -0.35927 0.6403 0.90784 0.18198 -0.35927
0.6403 -0.35927 0.6403 0.18198 0.620730223
NA ENSG00000258188 0.90785 0.18198 0.091624 0.4635 0.61835 0.26817
-0.7431 0.77129 0.18198 0.620730223
RPL23AP59 ENSG00000215002 -0.10068 0.5401 0.90781 0.18199 0.90781
0.18199 0.90781 0.18199 0.18199 0.620730223
NA ENSG00000207302 -0.17166 0.56815 -0.17166 0.56815 -0.17166 0.56815
0.90771 0.18201 0.18201 0.62075499
LINC02139 ENSG00000278214 0.34014 0.36687 0.53461 0.29646 0.60294
0.27327 0.90765 0.18203 0.18203 0.620779754
NA ENSG00000183729 0.90752 0.18207 -0.08748 0.53485 0.10893 0.45663

0.31863 0.375 0.18207 0.620872715
NA ENSG00000270708 0.25202 0.40051 0.90744 0.18209 0.90744 0.18209
0.90744 0.18209 0.18209 0.620897467
GGT3P ENSG00000197421 -1.3841 0.91684 0.57901 0.28129 0.90732 0.18212
-0.95613 0.8305 0.18212 0.62094696
HNRNPA1P23 ENSG00000240236 0.40245 0.34368 -0.62377 0.73361 -0.55702
0.71124 0.90726 0.18213 0.18213 0.62094696
FER1L5 ENSG00000249715 0.90721 0.18215 -0.23392 0.59247 -0.73812
0.76978 -0.059615 0.52377 0.18215 0.620971701
NA ENSG00000213587 0.71462 0.23742 0.89856 0.18444 0.90711 0.18217
0.4875 0.31295 0.18217 0.620974421
RNY3P14 ENSG00000207036 0.90708 0.18218 0.90708 0.18218 0.56668
0.28546 0.90708 0.18218 0.18218 0.620974421
FABP5P5 ENSG00000213716 0.907 0.1822 0.907 0.1822 0.907 0.1822 0.907
0.1822 0.1822 0.620974421
NA ENSG00000230746 0.90699 0.18221 0.05311 0.47882 0.05311 0.47882
0.05311 0.47882 0.18221 0.620974421
CHST6 ENSG00000183196 0.47461 0.31753 0.90694 0.18222 0.48469 0.31395
0.75334 0.22562 0.18222 0.620974421
NA ENSG00000254740 -0.52302 0.69952 0.90688 0.18223 -0.52302 0.69952
-0.52302 0.69952 0.18223 0.620974421
NA ENSG00000229387 0.90687 0.18224 0.90687 0.18224 0.90687 0.18224
0.90687 0.18224 0.18224 0.620974421
LINC02883 ENSG00000259416 0.90676 0.18227 0.90676 0.18227 0.90676
0.18227 0.90676 0.18227 0.18227 0.620989799
NA ENSG00000259932 0.90675 0.18227 0.90675 0.18227 0.90675 0.18227
0.90675 0.18227 0.18227 0.620989799
NA ENSG00000266950 -0.045028 0.51796 0.37301 0.35457 0.90667 0.18229
0.046838 0.48132 0.18229 0.62101452
UBP1 ENSG00000153560 -0.16993 0.56747 0.9065 0.18233 0.55113 0.29077
-0.077163 0.53075 0.18233 0.621045254
NA ENSG00000236319 -0.25951 0.60238 -0.19578 0.57761 -0.7467 0.77238
0.9065 0.18234 0.18234 0.621045254
ANGEL1 ENSG00000013523 0.6716 0.25092 0.6716 0.25092 0.90645
0.18235 0.41396 0.33945 0.18235 0.621045254
RN7SL269P ENSG00000243051 0.90643 0.18235 0.90643 0.18235 0.90643
0.18235 0.90643 0.18235 0.18235 0.621045254
SLAMF1 ENSG00000117090 -0.077625 0.53094 0.90635 0.18238 0.14674
0.44167 -1.1336 0.87151 0.18238 0.621104018
NA ENSG00000228505 -1.3056 0.90415 -0.2849 0.61214 0.90618 0.18242
-0.25579 0.60094 0.18242 0.62114071
FOXH1 ENSG00000160973 0.069494 0.4723 -0.36647 0.64299 0.90612 0.18244
0.63903 0.2614 0.18244 0.62114071
RN7SL387P ENSG00000263537 -0.17769 0.57052 0.90605 0.18245 0.90605
0.18245 0.90605 0.18245 0.18245 0.62114071
DTL ENSG00000143476 0.649 0.25817 0.71101 0.23854 0.55916 0.28802 0.906
0.18247 0.18247 0.62114071
NA ENSG00000254143 0.906 0.18247 0.70322 0.24096 -0.98443 0.83755
-0.46438 0.67881 0.18247 0.62114071
ITGB1 ENSG00000150093 -1.2169 0.88818 -0.96859 0.83363 0.90597 0.18248
-0.72269 0.76507 0.18248 0.62114071
H2AC5P ENSG00000234816 -0.9033 0.81682 -0.9033 0.81682 0.049574
0.48023 0.90594 0.18248 0.18248 0.62114071
NA ENSG00000279428 0.90588 0.1825 0.90588 0.1825 0.90588 0.1825
0.90588 0.1825 0.1825 0.621165398
RNU6-667P ENSG00000252995 0.69034 0.24499 -0.66236 0.74613 0.90569
0.18255 -0.52986 0.70189 0.18255 0.621292185
VN1R28P ENSG00000227182 0.53981 0.29466 0.72151 0.2353 0.15448
0.43861 0.90546 0.18261 0.18261 0.621409588
NA ENSG00000248491 0.90545 0.18261 -0.6196 0.73224 0.43275 0.3326
-1.3264 0.90764 0.18261 0.621409588
NA ENSG00000265644 -0.63217 0.73636 -0.096222 0.53833 0.90537 0.18263

										-0.86532	0.80657	0.18263	0.621424885
CREB3L2	ENSG00000182158	-0.79548	0.78683	-0.47809	0.68371	-0.14734	0.55857	0.90535	0.18264	0.18264	0.621424885		
KCNT1	ENSG00000107147	-0.19884	0.57881	0.90529	0.18266	-0.32586	0.62773	-0.78078	0.78253	0.18266	0.621449543		
NA	ENSG00000253106	0.90521	0.18268	0.90521	0.18268	0.90521	0.18268	0.90521	0.18268	0.18268	0.621464831		
NA	ENSG00000259434	0.90518	0.18269	0.90518	0.18269	0.73946	0.22981	0.90518	0.18269	0.18269	0.621464831		
RN7SKP186	ENSG00000202241	0.053299	0.47875	0.905	0.18273	-0.25207	0.5995	-0.77256	0.78011	0.18273	0.621491942		
NA	ENSG00000260144	-0.60035	0.72586	0.90497	0.18274	0.094203	0.46247	0.094203	0.46247	0.18274	0.621491942		
SNORA2B	ENSG00000207313	-1.8408	0.96718	-0.64473	0.74045	0.9049	0.18276	0.9049	0.18276	0.18276	0.621491942		
CCER2	ENSG00000262484	0.9049	0.18276	0.9049	0.18276	0.9049	0.18276	0.9049	0.18276	0.18276	0.621491942		
NA	ENSG00000257256	0.6744	0.25003	0.49267	0.31112	0.90477	0.18279	0.90477	0.18279	0.18279	0.621491942		
GUCA2B	ENSG00000044012	0.48057	0.31541	-0.047761	0.51905	0.21055	0.41662	0.90475	0.1828	0.1828	0.621491942		
SHLD2	ENSG00000122376	0.063642	0.47463	0.90476	0.1828	0.63847	0.26158	0.18711	0.42579	0.1828	0.621491942		
VANGL1	ENSG00000173218	-0.30567	0.62007	0.90473	0.1828	0.50768	0.30584	-0.25406	0.60028	0.1828	0.621491942		
NA	ENSG00000229839	0.27376	0.39214	0.86135	0.19452	0.87905	0.18969	0.90459	0.18284	0.18284	0.621577618		
FGFR4	ENSG00000160867	0.83702	0.20129	0.35794	0.3602	0.90449	0.18287	0.60196	0.2736	0.18287	0.621577618		
NA	ENSG00000250038	0.88548	0.18795	-0.31437	0.62338	-0.31437	0.62338	0.90441	0.18289	0.18289	0.621577618		
NA	ENSG00000272572	0.68186	0.24766	0.90431	0.18291	0.71438	0.2375	0.71438	0.2375	0.18291	0.621577618		
RN7SKP208	ENSG00000202344	0.7466	0.22765	0.59202	0.27692	0.90427	0.18292	-0.67123	0.74896	0.18292	0.621577618		
RNU6ATAC18P	ENSG00000221340	0.77253	0.2199	0.90431	0.18292	0.77253	0.2199	0.77253	0.2199	0.18292	0.621577618		
ZNF669	ENSG00000188295	0.53961	0.29473	-0.25454	0.60046	-0.51967	0.69835	0.90427	0.18293	0.18293	0.621577618		
ZUP1	ENSG00000153975	-0.72554	0.76594	-1.587	0.94374	0.90423	0.18294	0.033582	0.48661	0.18294	0.621577618		
NA	ENSG00000272301	0.90422	0.18294	0.16263	0.43541	0.90422	0.18294	0.90422	0.18294	0.18294	0.621577618		
PRICKLE2	ENSG00000163637	-0.052804	0.52106	-0.20811	0.58243	0.90399	0.183	0.47558	0.31719	0.183	0.621597681		
NA	ENSG00000237551	0.90391	0.18302	-0.65752	0.74458	-0.65752	0.74458	-0.30569	0.62008	0.18302	0.621597681		
PTPRZ1	ENSG00000106278	0.14698	0.44157	0.54841	0.29171	0.9038	0.18305	0.71617	0.23694	0.18305	0.621597681		
HELB	ENSG00000127311	0.9038	0.18305	0.9038	0.18305	0.9038	0.18305	0.9038	0.18305	0.18305	0.621597681		
CCDC116	ENSG00000161180	0.9038	0.18305	0.9038	0.18305	0.9038	0.18305	0.9038	0.18305	0.18305	0.621597681		
NA	ENSG00000267142	-0.59695	0.72473	0.90381	0.18305	0.19786	0.42158	0.19786	0.42158	0.18305	0.621597681		
NA	ENSG00000270174	-0.5819	0.71968	-0.43705	0.66896	0.9038	0.18305	0.28136	0.38922	0.18305	0.621597681		
LRRN1	ENSG00000175928	-0.19863	0.57872	-0.22267	0.5881	-0.15775	0.56267	0.90369	0.18308	0.18308	0.621597681		
LINC00412	ENSG00000234772	-0.072948	0.52908	0.90371	0.18308	-0.072948	0.52908	-0.072948	0.52908	0.18308	0.621597681		
NA	ENSG00000274351	0.90359	0.18311	0.13302	0.44709	0.90359	0.18311						

0.90359 0.18311 0.18311 0.621597681
COL9A1 ENSG00000112280 -0.047708 0.51903 -0.21228 0.58405 -0.33975
0.63298 0.90353 0.18312 0.18312 0.621597681
GNG7 ENSG00000176533 -0.37275 0.64533 0.9035 0.18313 -0.079687 0.53176
-0.47293 0.68187 0.18313 0.621597681
SNORA7B ENSG00000207088 0.90349 0.18313 0.90349 0.18313 0.90349
0.18313 0.84605 0.19876 0.18313 0.621597681
NA ENSG00000239568 0.88329 0.18854 0.88329 0.18854 0.90348 0.18314
-0.4603 0.67735 0.18314 0.621597681
ZNF460 ENSG00000197714 0.90342 0.18315 0.28806 0.38665 0.28806
0.38665 0.28806 0.38665 0.18315 0.621597681
NA ENSG00000236856 0.90341 0.18315 0.059353 0.47634 -0.25693 0.60138
-0.7065 0.76006 0.18315 0.621597681
NA ENSG00000228573 0.61675 0.2687 0.90335 0.18317 0.42519 0.33535
-0.84788 0.80175 0.18317 0.621622265
NA ENSG00000259644 0.90325 0.1832 0.90325 0.1832 0.90325 0.1832
0.90325 0.1832 0.1832 0.621652712
INCA1 ENSG00000196388 0.90316 0.18322 0.90316 0.18322 0.90316 0.18322
0.90316 0.18322 0.18322 0.621652712
PDS5B ENSG00000083642 0.68938 0.24529 0.45104 0.32598 -0.10295 0.541
0.90312 0.18323 0.18323 0.621652712
RN7SKP62 ENSG00000207269 0.90313 0.18323 -0.0040711 0.50162 0.90313
0.18323 0.90313 0.18323 0.18323 0.621652712
OR13C6P ENSG00000179443 0.90301 0.18326 -0.24818 0.598 -1.074 0.85859
-0.31496 0.62361 0.18326 0.621683142
CEACAMP4 ENSG00000230681 0.90292 0.18328 0.20767 0.41774 0.90292
0.18328 0.90292 0.18328 0.18328 0.621683142
NA ENSG00000251410 0.90294 0.18328 0.26337 0.39613 0.26337 0.39613
0.26337 0.39613 0.18328 0.621683142
NA ENSG00000264693 -1.3822 0.91655 -0.52098 0.69881 0.90289 0.18329
-0.52098 0.69881 0.18329 0.621683142
NA ENSG00000279558 -0.70017 0.75809 0.90277 0.18332 -0.37145 0.64485
0.28611 0.3874 0.18332 0.62174162
CAB39 ENSG00000135932 0.89997 0.18407 0.31411 0.37672 0.9027 0.18334
-0.03651 0.51456 0.18334 0.621766177
LRRC20 ENSG00000172731 0.25509 0.39933 0.90261 0.18337 0.88943
0.18689 0.14097 0.44395 0.18337 0.621781372
MRPL42P3 ENSG00000219297 -1.1437 0.87362 0.90259 0.18337 -1.1437
0.87362 -0.29497 0.61599 0.18337 0.621781372
LINC01522 ENSG00000237423 0.90245 0.18341 0.42067 0.337 -1.4516 0.92669
-0.22647 0.58958 0.18341 0.621873734
IGKV1-6 ENSG00000239855 0.90214 0.18349 0.90214 0.18349 0.48065
0.31538 0.90214 0.18349 0.18349 0.622049043
GNAT1 ENSG00000114349 0.90211 0.1835 0.49289 0.31104 0.24993 0.40132
0.90211 0.1835 0.1835 0.622049043
NA ENSG00000226900 -0.5101 0.69501 0.90212 0.1835 0.24773 0.40217
0.23512 0.40706 0.1835 0.622049043
NA ENSG00000248518 0.90198 0.18353 0.90198 0.18353 0.90198 0.18353
-0.16565 0.56578 0.18353 0.622064193
NA ENSG00000257509 0.90198 0.18353 0.90198 0.18353 0.90198 0.18353
0.90198 0.18353 0.18353 0.622064193
WWP1 ENSG00000123124 0.90174 0.1836 0.74266 0.22884 0.90174 0.1836
0.3252 0.37251 0.1836 0.622107391
NA ENSG00000269155 0.90165 0.18362 0.90165 0.18362 0.90165 0.18362
0.90165 0.18362 0.18362 0.622107391
NA ENSG00000258458 -0.056549 0.52255 -0.40035 0.65555 0.90163 0.18363
0.90163 0.18363 0.18363 0.622107391
PDLIM4 ENSG00000131435 0.66065 0.25442 0.16068 0.43617 0.66065
0.25442 0.90142 0.18368 0.18368 0.622107391
NA ENSG00000246225 0.052036 0.47925 0.9014 0.18369 0.9014 0.18369
0.9014 0.18369 0.18369 0.622107391
ELSPBP1 ENSG00000169393 0.79016 0.21472 0.90136 0.1837 0.90136

0.1837 0.90136 0.1837 0.1837 0.622107391
NA ENSG00000250280 -0.9085 0.81819 0.90135 0.1837 -1.0228 0.84679
-0.37593 0.64652 0.1837 0.622107391
NA ENSG00000253519 0.90137 0.1837 0.90137 0.1837 0.90137 0.1837
0.90137 0.1837 0.1837 0.622107391
SNRPGP16 ENSG00000255287 0.90134 0.1837 0.68039 0.24813 0.38528
0.35002 -0.26206 0.60336 0.1837 0.622107391
HYI ENSG00000178922 0.90124 0.18373 0.90124 0.18373 0.90124 0.18373
0.069348 0.47236 0.18373 0.622107391
NPM1P36 ENSG00000214604 0.77822 0.21822 -1.337 0.90939 -0.060814
0.52425 0.90111 0.18376 0.18376 0.622107391
SLC25A51P3 ENSG00000254181 0.90111 0.18376 -0.11393 0.54535 0.71029
0.23876 0.71029 0.23876 0.18376 0.622107391
SRPK1 ENSG00000096063 0.90111 0.18377 0.73612 0.23083 0.52045 0.30137
-1.2212 0.88899 0.18377 0.622107391
MAPK1 ENSG00000100030 0.82642 0.20428 0.21534 0.41475 0.9011 0.18377
0.36121 0.35897 0.18377 0.622107391
SUGP1 ENSG00000105705 0.13327 0.44699 0.2614 0.39689 0.13327 0.44699
0.90101 0.18379 0.18379 0.622107391
RPS7P11 ENSG00000213326 0.90102 0.18379 0.90102 0.18379 0.90102
0.18379 0.90102 0.18379 0.18379 0.622107391
CACNA1G-AS1 ENSG00000250107 0.90093 0.18381 0.90093 0.18381 0.90093
0.18381 0.90093 0.18381 0.18381 0.622107391
LMBR1 ENSG00000105983 0.11819 0.45296 -0.99151 0.83928 0.90086 0.18383
-0.18772 0.57445 0.18383 0.622107391
C9 ENSG00000113600 -1.1144 0.86744 -0.66651 0.74746 -0.99517 0.84017
0.90087 0.18383 0.18383 0.622107391
MBTPS1 ENSG00000140943 -0.62995 0.73564 0.90086 0.18383 -0.62045
0.73252 0.27284 0.39249 0.18383 0.622107391
LIG4 ENSG00000174405 0.7411 0.22932 0.89338 0.18583 0.90087 0.18383
-0.88646 0.81232 0.18383 0.622107391
KRT16 ENSG00000186832 0.63523 0.26264 0.079861 0.46817 0.18726 0.42573
0.90083 0.18384 0.18384 0.622107391
ENKUR ENSG00000151023 0.26472 0.39561 0.90078 0.18385 0.19465 0.42283
0.20654 0.41818 0.18385 0.622107391
NA ENSG00000249923 -0.979 0.83621 0.90079 0.18385 -0.78618 0.78412
-0.77616 0.78117 0.18385 0.622107391
IARS2 ENSG00000067704 -0.17877 0.57094 0.90069 0.18388 0.10003 0.46016
0.57947 0.28114 0.18388 0.622107391
INTS4 ENSG00000149262 0.39823 0.34523 0.63451 0.26288 0.90053 0.18392
0.22346 0.41159 0.18392 0.622107391
RPL7AP23 ENSG00000241367 -0.16704 0.56633 0.82471 0.20477 0.90051
0.18392 0.90051 0.18392 0.18392 0.622107391
MGAT3-AS1 ENSG00000227188 0.90044 0.18394 0.90044 0.18394 0.90044
0.18394 0.90044 0.18394 0.18394 0.622107391
NA ENSG00000226277 0.90042 0.18395 0.90042 0.18395 0.31931 0.37475
0.19825 0.42142 0.18395 0.622107391
SUCLA2-AS1 ENSG00000227848 0.90043 0.18395 0.90043 0.18395 0.90043
0.18395 0.90043 0.18395 0.18395 0.622107391
MIR3678 ENSG00000264511 0.90035 0.18397 0.90035 0.18397 0.90035
0.18397 0.90035 0.18397 0.18397 0.622107391
MPV17L ENSG00000156968 -2.0804 0.98125 0.90032 0.18398 -1.768
0.96147 -0.19935 0.57901 0.18398 0.622107391
NA ENSG00000237385 0.90022 0.184 -0.10354 0.54123 0.90022 0.184 0.90022
0.184 0.184 0.622107391
NA ENSG00000255866 0.90006 0.18404 0.90006 0.18404 0.90006 0.18404
0.90006 0.18404 0.18404 0.622107391
H3P42 ENSG00000271626 0.90007 0.18404 0.90007 0.18404 0.90007 0.18404
0.90007 0.18404 0.18404 0.622107391
NA ENSG00000231316 0.9 0.18406 0.9 0.18406 0.9 0.18406 -1.3474
0.91107 0.18406 0.622107391
NA ENSG00000221123 0.90002 0.18406 -0.32446 0.62721 -1.2055 0.886

0.12743 0.4493 0.18406 0.622107391
NA ENSG00000207922 -0.91652 0.8203 0.89997 0.18407 0.70471 0.2405
0.70471 0.2405 0.18407 0.622107391
RPL22 ENSG00000116251 0.47406 0.31773 0.89994 0.18408 0.031828 0.4873
-0.44597 0.67219 0.18408 0.622107391
GPR18 ENSG00000125245 0.89994 0.18408 0.25426 0.39965 0.016154 0.49356
0.26048 0.39725 0.18408 0.622107391
GRHL3 ENSG00000158055 -1.1278 0.8703 -1.2497 0.8943 0.89982 0.18411
-0.89719 0.81519 0.18411 0.622107391
SPZ1 ENSG00000164299 0.89981 0.18411 0.89981 0.18411 0.89981 0.18411
0.89981 0.18411 0.18411 0.622107391
SNORD70B ENSG00000212309 0.89982 0.18411 0.89982 0.18411 0.89982
0.18411 0.89982 0.18411 0.18411 0.622107391
FBXO22 ENSG00000167196 0.89976 0.18412 0.89976 0.18412 0.89976
0.18412 0.89976 0.18412 0.18412 0.622107391
UBE2F ENSG00000184182 -0.63397 0.73695 -0.1838 0.57291 -1.1114 0.86681
0.89978 0.18412 0.18412 0.622107391
NA ENSG00000234716 0.89973 0.18413 0.67004 0.25142 0.53145 0.29755
-0.029368 0.51171 0.18413 0.622107391
RNU6-57P ENSG00000223280 0.28464 0.38796 -0.13656 0.55431 0.89961
0.18416 0.89961 0.18416 0.18416 0.622113136
LARS2 ENSG00000011376 0.8996 0.18417 -0.10976 0.5437 0.47299 0.31811
0.48967 0.31218 0.18417 0.622113136
NA ENSG00000238316 0.89957 0.18417 0.89957 0.18417 0.89957 0.18417
0.89957 0.18417 0.18417 0.622113136
AAR2 ENSG00000131043 0.89928 0.18425 0.082965 0.46694 0.71085 0.23859
-0.032441 0.51294 0.18425 0.622204164
PPIAP69 ENSG00000228337 0.55471 0.28955 0.47994 0.31563 0.86707
0.19295 0.89925 0.18426 0.18426 0.622204164
FBXO41 ENSG00000163013 -0.28742 0.61311 0.89919 0.18427 -1.5808
0.94304 -0.72457 0.76564 0.18427 0.622204164
UGGT1 ENSG00000136731 0.22526 0.41089 -0.51942 0.69827 0.80627 0.21004
0.89916 0.18428 0.18428 0.622204164
SGCD ENSG00000170624 -0.40674 0.6579 0.89918 0.18428 -0.56062 0.71247
0.51944 0.30173 0.18428 0.622204164
LHCGR ENSG00000138039 0.025009 0.49002 0.0058824 0.49765 -0.24131 0.59534
0.89914 0.18429 0.18429 0.622204164
NA ENSG00000279505 0.89914 0.18429 0.89914 0.18429 0.89914 0.18429
0.89914 0.18429 0.18429 0.622204164
BECN1P2 ENSG00000219669 -0.36285 0.64164 0.89906 0.18431 -0.36285
0.64164 -0.36285 0.64164 0.18431 0.622204164
LINC01568 ENSG00000258779 -0.34719 0.63578 0.89879 0.18438 0.59047
0.27744 0.59047 0.27744 0.18438 0.622204164
NA ENSG00000260276 0.89881 0.18438 0.8285 0.20369 0.54601 0.29253
0.24214 0.40434 0.18438 0.622204164
NA ENSG00000272512 -0.3555 0.63889 0.89877 0.18439 -0.3555 0.63889
-0.3555 0.63889 0.18439 0.622204164
PTGR1 ENSG00000106853 0.89865 0.18442 0.89865 0.18442 -0.40479 0.65718
-0.2837 0.61168 0.18442 0.622204164
CDC42EP5 ENSG00000167617 0.45175 0.32572 0.45175 0.32572 0.41895
0.33763 0.89865 0.18442 0.18442 0.622204164
RN7SL754P ENSG00000239822 0.14994 0.44041 0.8986 0.18443 0.82026
0.20604 0.76713 0.2215 0.18443 0.622204164
INTS3 ENSG00000143624 -0.14731 0.55856 0.44441 0.32837 0.71196 0.23825
0.89856 0.18444 0.18444 0.622204164
NA ENSG00000241722 -0.41345 0.66036 0.14201 0.44353 0.89854 0.18445
-0.39815 0.65474 0.18445 0.622204164
PFN2 ENSG00000070087 -0.073823 0.52942 -0.23767 0.59393 0.89847 0.18447
0.28857 0.38646 0.18447 0.622204164
EIF1B ENSG00000114784 0.098802 0.46065 0.098802 0.46065 -0.58849 0.7219
0.89848 0.18447 0.18447 0.622204164
TRAV39 ENSG00000211818 0.89847 0.18447 0.47862 0.3161 0.89847

0.18447 0.89847 0.18447 0.18447 0.622204164
NA ENSG00000281167 0.89845 0.18447 -0.35377 0.63824 0.89845 0.18447
0.34363 0.36556 0.18447 0.622204164
LHX5 ENSG00000089116 0.34101 0.36655 0.45795 0.3235 -0.5755 0.71752
0.89826 0.18452 0.18452 0.622204164
NA ENSG00000249249 0.32051 0.37429 0.89827 0.18452 0.89827 0.18452
0.89827 0.18452 0.18452 0.622204164
RNA5SP494 ENSG00000222585 0.89822 0.18453 0.89822 0.18453 0.89822
0.18453 0.89822 0.18453 0.18453 0.622204164
RNU6-608P ENSG00000206654 0.60417 0.27286 0.65195 0.25722 0.89818
0.18454 0.4563 0.32409 0.18454 0.622204164
CCNB1IP1 ENSG00000100814 -1.8554 0.96823 -0.96222 0.83203 0.89816
0.18455 -0.72849 0.76684 0.18455 0.622204164
RPS6KA1 ENSG00000117676 0.55609 0.28907 0.69207 0.24445 0.4863
0.31338 0.89818 0.18455 0.18455 0.622204164
PIGM ENSG00000143315 -0.11311 0.54503 -0.11311 0.54503 0.18188 0.42784
0.89812 0.18456 0.18456 0.622204164
MIR323A ENSG00000199069 0.89806 0.18458 0.89806 0.18458 0.89806
0.18458 0.89806 0.18458 0.18458 0.622204164
MIR1197 ENSG00000221745 0.89806 0.18458 0.89806 0.18458 0.89806
0.18458 0.89806 0.18458 0.18458 0.622204164
RNU6-1052P ENSG00000223330 -1.2148 0.88777 0.89803 0.18458 0.014377
0.49426 -0.35092 0.63717 0.18458 0.622204164
STON1 ENSG00000243244 -0.08345 0.53325 -0.38521 0.64996 -0.08345 0.53325
0.89792 0.18461 0.18461 0.622262247
CXCL17 ENSG00000189377 0.023554 0.4906 0.89774 0.18466 0.023554
0.4906 0.023554 0.4906 0.18466 0.622298002
RNA5SP194 ENSG00000201532 0.66012 0.25459 -0.51992 0.69844 0.89774
0.18466 -0.19213 0.57618 0.18466 0.622298002
LINC02518 ENSG00000232316 0.10992 0.45623 0.89777 0.18466 0.10992
0.45623 0.10992 0.45623 0.18466 0.622298002
NA ENSG00000257045 -0.23254 0.59194 0.5248 0.29986 0.89767 0.18468
0.89767 0.18468 0.18468 0.622298002
ZDHHC8P1 ENSG00000133519 0.89759 0.1847 0.3878 0.34908 -0.41505
0.66095 -1.1325 0.87128 0.1847 0.622298002
DIPK2A ENSG00000181744 0.11494 0.45425 0.83315 0.20238 0.89756
0.18471 0.51369 0.30374 0.18471 0.622298002
MIR1205 ENSG00000221771 0.89757 0.18471 0.74938 0.22681 0.74938
0.22681 -0.76049 0.77652 0.18471 0.622298002
NA ENSG00000260877 0.89724 0.18479 0.89724 0.18479 0.89724 0.18479
0.89724 0.18479 0.18479 0.622425408
LTK ENSG00000062524 -0.35498 0.6387 0.12434 0.45052 0.8972 0.18481
-0.27816 0.60955 0.18481 0.622425408
SPATS2 ENSG00000123352 0.77769 0.21837 0.89716 0.18482 0.71723
0.23662 0.19569 0.42243 0.18482 0.622425408
SNUPN ENSG00000169371 0.73295 0.2318 0.50297 0.30749 0.89717 0.18482
0.8805 0.18929 0.18482 0.622425408
RPL7AP50 ENSG00000213609 0.89711 0.18483 0.89711 0.18483 0.54641
0.29239 -0.11011 0.54384 0.18483 0.622425408
PSMD7P1 ENSG00000266707 0.47859 0.31612 0.8971 0.18483 0.27507
0.39163 -0.05934 0.52366 0.18483 0.622425408
ST6GALNAC5 ENSG00000117069 0.89704 0.18485 0.17842 0.4292 -0.74809
0.7728 -0.79021 0.7853 0.18485 0.622425408
NA ENSG00000227562 0.33828 0.36758 0.89703 0.18485 0.29909 0.38243
0.89703 0.18485 0.18485 0.622425408
AMFR ENSG00000159461 0.89689 0.18489 -1.4974 0.93286 -1.1202 0.86868
-1.8014 0.96418 0.18489 0.622431067
BHLHA9 ENSG00000205899 -1.6127 0.94659 0.70361 0.24084 0.89687
0.18489 0.35313 0.362 0.18489 0.622431067
NA ENSG00000267173 0.27688 0.39094 0.89688 0.18489 0.17841 0.4292
0.3575 0.36036 0.18489 0.622431067
NA ENSG00000231811 0.20552 0.41858 0.20552 0.41858 0.20552 0.41858

0.89676 0.18492 0.18492 0.622489058
FAM238B ENSG00000231976 0.89661 0.18496 -0.067714 0.52699 -0.68075
0.75198 -0.68075 0.75198 0.18496 0.6225807
RPL23P2 ENSG00000176054 0.83565 0.20168 0.88775 0.18734 0.83565
0.20168 0.89646 0.185 0.185 0.622608805
NA ENSG00000248699 0.89639 0.18502 0.38971 0.34837 -1.7103 0.9564
-0.23148 0.59153 0.18502 0.622608805
CRLS1 ENSG00000088766 0.40787 0.34168 0.051499 0.47946 -1.0545 0.85418
0.89635 0.18503 0.18503 0.622608805
NA ENSG00000242488 0.011987 0.49522 0.011987 0.49522 0.011987 0.49522
0.89625 0.18506 0.18506 0.622608805
RNU6-703P ENSG00000252755 -0.41383 0.6605 0.89625 0.18506 0.89625
0.18506 -0.73635 0.76924 0.18506 0.622608805
NA ENSG00000270393 -1.8011 0.96415 -0.97943 0.83632 -1.8011 0.96415
0.89626 0.18506 0.18506 0.622608805
LINC01520 ENSG00000230962 0.89616 0.18508 0.76634 0.22174 0.29631
0.3835 0.45399 0.32492 0.18508 0.622608805
NA ENSG00000263622 0.53369 0.29678 0.89606 0.18511 0.63044 0.2642
0.07212 0.47125 0.18511 0.622608805
NA ENSG00000227680 0.89599 0.18513 0.89599 0.18513 0.89599 0.18513
0.89599 0.18513 0.18513 0.622608805
OR8B10P ENSG00000254646 0.896 0.18513 0.896 0.18513 0.896 0.18513 0.896
0.18513 0.18513 0.622608805
NA ENSG00000275120 0.896 0.18513 0.8327 0.20251 0.61244 0.27012
0.61244 0.27012 0.18513 0.622608805
NA ENSG00000259505 0.30612 0.37976 0.30612 0.37976 0.30612 0.37976
0.89596 0.18514 0.18514 0.622608805
FAM87A ENSG00000182366 0.69192 0.24449 0.33361 0.36934 0.48118
0.31519 0.89593 0.18515 0.18515 0.622608805
RPL23P10 ENSG00000244708 -2.135 0.98362 0.89593 0.18515 -2.135
0.98362 -2.135 0.98362 0.18515 0.622608805
NA ENSG00000266625 0.89588 0.18516 0.89588 0.18516 0.89588 0.18516
0.89588 0.18516 0.18516 0.622608805
RNA5SP277 ENSG00000202356 -0.21446 0.58491 0.89574 0.1852 -0.21446
0.58491 -0.21446 0.58491 0.1852 0.622648037
SIKE1 ENSG00000052723 0.8957 0.18521 0.23883 0.40562 0.8957 0.18521
0.8957 0.18521 0.18521 0.622648037
NA ENSG00000265188 0.89569 0.18521 0.89569 0.18521 0.89569 0.18521
0.89569 0.18521 0.18521 0.622648037
IFIT5 ENSG00000152778 -1.6229 0.94769 0.89547 0.18527 -0.56939 0.71546
-1.6229 0.94769 0.18527 0.622806781
RPL7P48 ENSG00000187504 0.89532 0.18531 0.75877 0.224 0.89532 0.18531
0.89532 0.18531 0.18531 0.622860872
RNU6ATAC6P ENSG00000221059 0.28705 0.38704 -1.2893 0.90135 -1.1004
0.86442 0.89529 0.18532 0.18532 0.622860872
RPL10AP6 ENSG00000226360 0.8952 0.18534 0.8952 0.18534 0.8952
0.18534 0.8952 0.18534 0.18534 0.622860872
NA ENSG00000272668 0.89521 0.18534 0.89521 0.18534 0.89521 0.18534
0.89521 0.18534 0.18534 0.622860872
ARRB1 ENSG00000137486 0.27778 0.39059 -0.2507 0.59898 -0.97499 0.83522
0.89515 0.18535 0.18535 0.622860872
NA ENSG00000254629 0.31286 0.37719 0.89485 0.18543 0.30697 0.37943
0.33249 0.36976 0.18543 0.622944707
NA ENSG00000280093 -0.45544 0.6756 0.89488 0.18543 -0.45544 0.6756
-0.45544 0.6756 0.18543 0.622944707
NA ENSG00000233235 0.89478 0.18545 0.078516 0.46871 0.75629 0.22474
0.79007 0.21474 0.18545 0.622944707
RNU6-1128P ENSG00000199695 0.8947 0.18547 0.8947 0.18547 0.8947
0.18547 0.8947 0.18547 0.18547 0.622944707
SLC16A11 ENSG00000174326 0.27089 0.39324 0.89467 0.18548 -0.26616
0.60494 0.22648 0.41041 0.18548 0.622944707
SNORD86 ENSG00000212498 0.8947 0.18548 0.8947 0.18548 0.8947

0.18548 0.8947 0.18548 0.18548 0.622944707
NA ENSG00000260095 0.89467 0.18548 0.89467 0.18548 0.89467 0.18548
0.89467 0.18548 0.18548 0.622944707
SNORA51 ENSG00000271798 0.8947 0.18548 0.8947 0.18548 0.8947
0.18548 0.8947 0.18548 0.18548 0.622944707
NA ENSG00000216085 0.89463 0.18549 0.89463 0.18549 0.89463 0.18549
0.89463 0.18549 0.18549 0.622944707
NA ENSG00000250007 0.89453 0.18552 0.89453 0.18552 0.89453 0.18552
0.89453 0.18552 0.18552 0.623002522
NA ENSG00000260774 0.65391 0.25658 0.89419 0.18561 0.50631 0.30632
0.50631 0.30632 0.18561 0.623233703
SMIM19 ENSG00000176209 0.066261 0.47358 0.053297 0.47875 0.89416
0.18562 0.06879 0.47258 0.18562 0.623233703
SPATA31B1P ENSG00000231649 0.66427 0.25326 0.033272 0.48673 -0.2726
0.60742 0.89412 0.18563 0.18563 0.623233703
NA ENSG00000226849 -0.86351 0.80607 0.8941 0.18564 -0.86351 0.80607
-0.86351 0.80607 0.18564 0.623233703
C1DP4 ENSG00000227515 0.89399 0.18566 0.89399 0.18566 0.89399 0.18566
0.89399 0.18566 0.18566 0.623257909
TAS2R9 ENSG00000121381 -1.0155 0.84507 -0.22773 0.59007 0.8938
0.18571 0.8938 0.18571 0.18571 0.623339875
RNU6ATAC40P ENSG00000221507 0.89382 0.18571 0.89382 0.18571 0.89382
0.18571 0.89382 0.18571 0.18571 0.623339875
RNU1-78P ENSG00000212550 -0.57981 0.71898 0.53799 0.29529 0.89358
0.18577 -0.38101 0.6484 0.18577 0.623359281
NA ENSG00000276097 0.89355 0.18578 0.89355 0.18578 0.89355 0.18578
0.89355 0.18578 0.18578 0.623359281
RABGAP1L ENSG00000152061 0.89347 0.1858 0.86042 0.19478 0.69478
0.2436 0.85923 0.19511 0.1858 0.623359281
NA ENSG00000199273 0.89345 0.18581 0.89345 0.18581 0.89345 0.18581
0.89345 0.18581 0.18581 0.623359281
VN1R85P ENSG00000269345 -1.7777 0.96227 -0.95296 0.8297 -1.0585
0.85509 0.89323 0.18587 0.18587 0.623359281
NA ENSG00000226396 -1.0921 0.86261 0.89319 0.18588 -0.092226 0.53674
-0.15578 0.5619 0.18588 0.623359281
NA ENSG00000249309 0.89316 0.18589 0.89316 0.18589 0.89316 0.18589
0.89316 0.18589 0.18589 0.623359281
NA ENSG00000261710 -0.40021 0.6555 -0.90947 0.81845 0.89312 0.1859
-0.41953 0.66259 0.1859 0.623359281
BCL2L2 ENSG00000129473 0.68178 0.24769 0.89306 0.18591 0.46093
0.32242 0.20962 0.41698 0.18591 0.623359281
OR14K1 ENSG00000153230 -0.91269 0.8193 -0.29495 0.61598 0.89308
0.18591 0.15033 0.44025 0.18591 0.623359281
METAP1 ENSG00000164024 -1.392 0.91803 -0.71282 0.76202 -1.1072
0.86589 0.89305 0.18591 0.18591 0.623359281
NR4A2 ENSG00000153234 0.37473 0.35393 0.84768 0.19831 0.89305 0.18592
0.43159 0.33302 0.18592 0.623359281
RNU6-765P ENSG00000252725 0.40092 0.34424 0.89299 0.18593 0.40092
0.34424 -0.29284 0.61518 0.18593 0.623359281
NA ENSG00000251445 0.89296 0.18594 0.89296 0.18594 0.89296 0.18594
0.89296 0.18594 0.18594 0.623359281
FOXJ2 ENSG00000065970 -0.4612 0.67767 0.52301 0.30048 0.7493 0.22684
0.89289 0.18596 0.18596 0.623359281
ASB5 ENSG00000164122 -1.4093 0.92062 -1.4093 0.92062 -0.34428 0.63468
0.89289 0.18596 0.18596 0.623359281
NA ENSG00000253841 0.89285 0.18597 0.89285 0.18597 0.89285 0.18597
0.89285 0.18597 0.18597 0.623359281
NA ENSG00000270688 0.89282 0.18598 0.89282 0.18598 0.89282 0.18598
0.89282 0.18598 0.18598 0.623359281
CCDC168 ENSG00000175820 0.56559 0.28584 0.89278 0.18599 0.89278
0.18599 0.89278 0.18599 0.18599 0.623359281
LINC02363 ENSG00000180712 0.11712 0.45338 -0.60305 0.72676 -0.27438

0.6081 0.89279 0.18599 0.18599 0.623359281
ZNF717 ENSG00000227124 0.89277 0.18599 -0.37545 0.64634 0.42325
0.33606 -0.56196 0.71293 0.18599 0.623359281
PDHX ENSG00000110435 -0.057534 0.52294 -1.1772 0.88044 0.89268 0.18601
0.014245 0.49432 0.18601 0.623359281
NA ENSG00000271936 0.89271 0.18601 0.89271 0.18601 0.89271 0.18601
0.89271 0.18601 0.18601 0.623359281
PCCA ENSG00000175198 0.013653 0.49455 0.89251 0.18606 0.77542 0.21905
0.26891 0.394 0.18606 0.623431731
NA ENSG00000206990 0.89251 0.18606 0.89251 0.18606 0.89251 0.18606
0.89251 0.18606 0.18606 0.623431731
NA ENSG00000228033 -0.3678 0.64349 0.89248 0.18607 0.59685 0.2753
0.59685 0.2753 0.18607 0.623431731
NA ENSG00000238323 0.89227 0.18612 0.27267 0.39255 -0.64051 0.73908
0.69436 0.24373 0.18612 0.62349084
IGF1 ENSG00000017427 0.89213 0.18616 0.49786 0.30929 0.21479 0.41496
0.53402 0.29666 0.18616 0.62349084
NA ENSG00000213963 0.37231 0.35483 0.68522 0.2466 0.7618 0.22309
0.89214 0.18616 0.18616 0.62349084
SKP2 ENSG00000145604 0.89209 0.18617 -0.53808 0.70474 -0.36671 0.64308
0.36294 0.35833 0.18617 0.62349084
LINC01629 ENSG00000258602 0.89204 0.18618 0.63423 0.26297 0.49966
0.30866 -1.845 0.96748 0.18618 0.62349084
H3C8 ENSG00000273983 0.89206 0.18618 0.037039 0.48523 -0.31566 0.62387
0.18889 0.42509 0.18618 0.62349084
CALM2 ENSG00000143933 -0.40423 0.65698 -0.93156 0.82422 0.89202 0.18619
-0.9479 0.82841 0.18619 0.62349084
CELP ENSG00000170827 -0.91 0.81859 -0.32754 0.62837 0.89204 0.18619
0.10796 0.45701 0.18619 0.62349084
RCC2P8 ENSG00000250185 -0.085768 0.53417 0.89183 0.18624 0.53513
0.29628 -0.97143 0.83433 0.18624 0.623577962
NA ENSG00000256975 -0.72341 0.76529 0.89179 0.18625 0.61089 0.27064
0.2987 0.38258 0.18625 0.623577962
GABRB1 ENSG00000163288 0.14233 0.44341 -0.12817 0.55099 0.89174
0.18627 0.43873 0.33043 0.18627 0.623577962
MAP3K11 ENSG00000173327 -0.65785 0.74468 0.030998 0.48764 0.89173
0.18627 -0.97047 0.83409 0.18627 0.623577962
HIF1AN ENSG00000166135 0.13867 0.44485 -0.098396 0.53919 0.20682
0.41807 0.8917 0.18628 0.18628 0.623577962
NA ENSG00000253623 -0.97562 0.83537 -0.50223 0.69225 -0.50223 0.69225
0.89161 0.1863 0.1863 0.623602074
CCDC162P ENSG00000203799 0.036957 0.48526 0.010884 0.49566 0.15274
0.4393 0.89143 0.18635 0.18635 0.623726595
DCN ENSG00000011465 0.89125 0.1864 0.89125 0.1864 0.89125 0.1864
0.49428 0.31055 0.1864 0.623841721
LINC02244 ENSG00000259590 0.8912 0.18641 0.8912 0.18641 0.8912
0.18641 0.8912 0.18641 0.18641 0.623841721
LINC02878 ENSG00000181097 0.89113 0.18643 0.77902 0.21798 0.89113
0.18643 0.67301 0.25047 0.18643 0.623865808
WDPCP ENSG00000143951 0.89096 0.18647 0.23557 0.40688 0.5692 0.28461
0.66071 0.2544 0.18647 0.623885838
F5 ENSG00000198734 0.26123 0.39696 0.89094 0.18648 0.82642 0.20428
0.14868 0.4409 0.18648 0.623885838
MEPE ENSG00000152595 0.75487 0.22516 0.89092 0.18649 0.48604 0.31347
0.048002 0.48086 0.18649 0.623885838
ARRDC5 ENSG00000205784 0.4918 0.31143 0.4918 0.31143 -0.74498
0.77186 0.89086 0.1865 0.1865 0.623885838
NA ENSG00000220447 0.89086 0.1865 0.89086 0.1865 0.89086 0.1865
0.89086 0.1865 0.1865 0.623885838
NA ENSG00000229985 0.77674 0.21866 0.50528 0.30668 0.89079 0.18652
0.2627 0.39639 0.18652 0.623909912
NA ENSG00000269458 -0.07543 0.53006 0.89073 0.18654 -0.69231 0.75563

-0.26351 0.60392 0.18654 0.623933983
AGTPBP1 ENSG00000135049 0.89064 0.18656 -0.3863 0.65036 -0.24645
0.59733 0.14231 0.44342 0.18656 0.623958051
NA ENSG00000279588 0.52365 0.30026 0.47512 0.31735 -0.4678 0.68004
0.89054 0.18659 0.18659 0.623992715
LINC00342 ENSG00000232931 0.42054 0.33704 0.89042 0.18662 0.33286
0.36962 -0.50852 0.69446 0.18662 0.623992715
BCYRN1 ENSG00000236824 0.89038 0.18663 0.89038 0.18663 0.89038
0.18663 0.89038 0.18663 0.18663 0.623992715
NA ENSG00000227606 -0.64975 0.74207 -0.12426 0.54944 -0.15031 0.55974
0.89034 0.18664 0.18664 0.623992715
NA ENSG00000237101 0.89033 0.18664 0.89033 0.18664 0.89033 0.18664
0.89033 0.18664 0.18664 0.623992715
NA ENSG00000258399 0.89031 0.18665 0.89031 0.18665 0.89031 0.18665
0.89031 0.18665 0.18665 0.623992715
MIR141 ENSG00000207708 0.89028 0.18666 0.89028 0.18666 0.89028
0.18666 0.89028 0.18666 0.18666 0.623992715
RPA1 ENSG00000132383 -1.0133 0.84453 -1.0133 0.84453 0.099082 0.46054
0.89015 0.18669 0.18669 0.624027345
BMP10 ENSG00000163217 0.89008 0.18671 -1.1424 0.87336 -1.1424 0.87336
-1.1424 0.87336 0.18671 0.624027345
NA ENSG00000261215 0.89003 0.18672 0.89003 0.18672 0.89003 0.18672
0.89003 0.18672 0.18672 0.624027345
GDF10 ENSG00000266524 0.16744 0.43351 0.41146 0.34037 0.89001 0.18673
0.40819 0.34157 0.18673 0.624027345
LINC01090 ENSG00000231689 0.52484 0.29985 0.88998 0.18674 -1.2211
0.88899 0.72584 0.23397 0.18674 0.624027345
EDNRB ENSG00000136160 0.32252 0.37353 0.88991 0.18676 0.88991 0.18676
0.88991 0.18676 0.18676 0.624027345
NA ENSG00000283973 0.88989 0.18676 0.88989 0.18676 0.88989 0.18676
0.88989 0.18676 0.18676 0.624027345
ARPP19P2 ENSG00000204637 0.8895 0.18687 0.8895 0.18687 -0.37119
0.64475 -0.091305 0.53637 0.18687 0.624352081
NA ENSG00000258887 0.50222 0.30775 0.88942 0.18689 -0.096209 0.53832
0.095653 0.4619 0.18689 0.624376094
SELENOWP1 ENSG00000215900 0.28043 0.38957 0.28043 0.38957 0.88922
0.18694 0.28079 0.38944 0.18694 0.624448112
RBM7P1 ENSG00000223427 0.88922 0.18694 0.88922 0.18694 0.88922
0.18694 -0.72776 0.76662 0.18694 0.624448112
RPS5P2 ENSG00000224598 0.88919 0.18695 0.88919 0.18695 0.88919
0.18695 0.88919 0.18695 0.18695 0.624448112
DHRS9 ENSG00000073737 0.64056 0.26091 0.88911 0.18697 0.88911 0.18697
0.88911 0.18697 0.18697 0.624472111
CTU2 ENSG00000174177 -0.30985 0.62166 0.88893 0.18702 -0.30985 0.62166
-0.30985 0.62166 0.18702 0.624549267
RPL23AP57 ENSG00000206147 0.88894 0.18702 0.88894 0.18702 0.88894
0.18702 0.88894 0.18702 0.18702 0.624549267
RASEF ENSG00000165105 -1.0369 0.85012 -0.92518 0.82256 0.88886 0.18704
-0.36643 0.64298 0.18704 0.624549267
RERE-AS1 ENSG00000232912 -0.19322 0.57661 0.66383 0.2534 0.88877
0.18706 0.82131 0.20573 0.18706 0.624549267
NA ENSG00000264334 -0.72804 0.7667 -0.72804 0.7667 0.88877 0.18706
-0.72804 0.7667 0.18706 0.624549267
COX5BP8 ENSG00000223794 0.88875 0.18707 0.88875 0.18707 0.88875
0.18707 0.88875 0.18707 0.18707 0.624549267
MIR541 ENSG00000216179 0.88858 0.18711 0.88858 0.18711 0.88858
0.18711 0.88858 0.18711 0.18711 0.624640015
NSD1 ENSG00000165671 0.686 0.24636 0.57939 0.28116 0.57199 0.28367
0.88838 0.18717 0.18717 0.624778678
CD34 ENSG00000174059 0.88835 0.18718 0.49865 0.30901 0.72565 0.23403
0.31173 0.37762 0.18718 0.624778678
GUSBP12 ENSG00000169325 0.76279 0.2228 0.88829 0.18719 -0.11148

0.54438 -0.068276 0.52722 0.18719 0.624778678
CSNKA2IP ENSG00000283434 -0.064056 0.52554 0.88818 0.18722 0.18819
0.42536 -0.9513 0.82927 0.18722 0.624784641
NA ENSG00000181514 -0.44936 0.67341 0.34437 0.36528 0.12557 0.45003
0.88815 0.18723 0.18723 0.624784641
NA ENSG00000262362 0.53221 0.29729 0.53221 0.29729 0.53221 0.29729
0.88799 0.18727 0.18727 0.624784641
EDDM13 ENSG00000267710 0.88799 0.18727 0.88799 0.18727 0.88799
0.18727 0.88799 0.18727 0.18727 0.624784641
NA ENSG00000212371 0.88795 0.18728 0.88795 0.18728 0.88795 0.18728
0.88795 0.18728 0.18728 0.624784641
RNU6-539P ENSG00000252474 0.88798 0.18728 0.88798 0.18728 0.88798
0.18728 0.88798 0.18728 0.18728 0.624784641
GLYATL3 ENSG00000203972 -0.98515 0.83773 0.14438 0.4426 0.88791
0.1873 -0.16563 0.56578 0.1873 0.624784641
APOBEC3A ENSG00000128383 -1.0683 0.8573 0.015229 0.49392 -1.2026
0.88543 0.88784 0.18731 0.18731 0.624784641
NA ENSG00000199713 -1.7227 0.95753 -1.7227 0.95753 0.88787 0.18731
-0.51254 0.69586 0.18731 0.624784641
KCNE1 ENSG00000180509 -0.7842 0.78354 0.063096 0.47485 0.88783 0.18732
-0.23912 0.59449 0.18732 0.624784641
NA ENSG00000229986 0.69862 0.2424 0.69862 0.2424 0.4545 0.32473
0.88775 0.18734 0.18734 0.624799172
RNA5SP26 ENSG00000199276 -0.010093 0.50403 0.15505 0.43839 0.88769
0.18735 0.52755 0.2989 0.18735 0.624799172
OR5E1P ENSG00000170688 0.88764 0.18737 0.88764 0.18737 0.046609
0.48141 0.88764 0.18737 0.18737 0.624813699
NA ENSG00000227270 0.88759 0.18738 -1.309 0.90473 -0.94123 0.82671
-0.30898 0.62133 0.18738 0.624813699
NA ENSG00000225000 0.74447 0.2283 0.74447 0.2283 0.88751 0.1874
-0.19738 0.57823 0.1874 0.624828222
SCD5 ENSG00000145284 0.88748 0.18741 0.72578 0.23399 -0.057538 0.52294
-0.69817 0.75747 0.18741 0.624828222
RBM33 ENSG00000184863 0.47594 0.31706 0.56408 0.28635 0.7536 0.22554
0.88725 0.18747 0.18747 0.624924687
NA ENSG00000252891 0.88722 0.18748 0.88722 0.18748 0.88722 0.18748
0.88722 0.18748 0.18748 0.624924687
MIR655 ENSG00000207646 0.88709 0.18751 0.88709 0.18751 0.88709
0.18751 0.88709 0.18751 0.18751 0.624924687
NA ENSG00000214215 0.88709 0.18752 0.88709 0.18752 0.88709 0.18752
0.79256 0.21402 0.18752 0.624924687
COX6B1P6 ENSG00000253349 0.4788 0.31604 0.4788 0.31604 0.4788
0.31604 0.88703 0.18753 0.18753 0.624924687
RNA5SP41 ENSG00000222952 0.88697 0.18755 0.88697 0.18755 0.88697
0.18755 0.88697 0.18755 0.18755 0.624924687
LINC02853 ENSG00000259245 0.88692 0.18756 0.54808 0.29182 0.88692
0.18756 0.88692 0.18756 0.18756 0.624924687
RNU6-1134P ENSG00000202026 0.32636 0.37207 0.32636 0.37207 0.32636
0.37207 0.88687 0.18757 0.18757 0.624924687
FGF4 ENSG00000075388 0.673 0.25047 0.88685 0.18758 0.26503 0.39549
0.49808 0.30921 0.18758 0.624924687
EMSY ENSG00000158636 -0.17542 0.56963 0.3911 0.34786 0.88684 0.18758
-0.1947 0.57719 0.18758 0.624924687
NA ENSG00000223530 0.88685 0.18758 0.88685 0.18758 0.88685 0.18758
0.88685 0.18758 0.18758 0.624924687
RN7SL277P ENSG00000240490 0.88668 0.18763 0.88668 0.18763 0.88668
0.18763 0.88668 0.18763 0.18763 0.625005804
TNFRSF10A-AS1 ENSG00000253930 0.88665 0.18763 0.88665 0.18763 0.88665
0.18763 0.88665 0.18763 0.18763 0.625005804
RPL23AP41 ENSG00000224085 -1.6657 0.95211 0.8324 0.20259 0.88638
0.18771 0.67647 0.24937 0.18771 0.625191848
NA ENSG00000280241 0.88631 0.18772 0.12162 0.4516 0.13653 0.4457

0.13653	0.4457	0.18772	0.625191848
ZNF587	ENSG00000198466	0.88626	0.18774	0.88626	0.18774	0.1571
0.43758	0.79817	0.21239	0.18774	0.625191848
GNL1	ENSG00000204590	-0.33284	0.63037	-0.52143	0.69897	-0.2385	0.59425
0.88622	0.18775	0.18775	0.625191848
NA	ENSG00000272175	0.88622	0.18775	0.88622	0.18775	0.88622	0.18775
0.88622	0.18775	0.18775	0.625191848
LINC01880	ENSG00000232002	-0.16289	0.5647	0.88616	0.18777	-0.16289
0.5647	-0.16289	0.5647	0.18777	0.625215723
PIK3C2A	ENSG00000011405	0.16688	0.43373	-0.11826	0.54707	-0.11826
0.54707	0.88592	0.18783	0.18783	0.625368333
MBOAT1	ENSG00000172197	-0.79953	0.78801	-0.79953	0.78801	0.88585
0.18785	-0.86655	0.80691	0.18785	0.625368333
FAM184B	ENSG00000047662	0.88576	0.18787	0.21276	0.41576	-0.95579
0.83041	0.22718	0.41014	0.18787	0.625368333
DEUP1	ENSG00000165325	0.51714	0.30253	0.11419	0.45454	0.88576	0.18787
0.31047	0.3781	0.18787	0.625368333
CGB1	ENSG00000267631	0.88574	0.18788	0.88574	0.18788	0.88574	0.18788
0.88574	0.18788	0.18788	0.625368333
HSD3B1	ENSG00000203857	-1.6798	0.9535	0.3165	0.37581	0.8855
0.18794	0.8855	0.18794	0.18794	0.625525319
SEPSECS-AS1	ENSG00000281501	0.88536	0.18798	0.69447	0.24369	0.69447
0.24369	0.69447	0.24369	0.18798	0.625606276
TICRR	ENSG00000140534	0.88533	0.18799	0.44909	0.32668	-0.040335	0.51609
-0.20949	0.58297	0.18799	0.625606276
CHST4	ENSG00000140835	0.88526	0.18801	0.88526	0.18801	-0.32365	0.6269
0.38635	0.34962	0.18801	0.625630108
UPP2-IT1	ENSG00000237327	0.88517	0.18803	0.81435	0.20772	0.81435
0.20772	0.81435	0.20772	0.18803	0.625653936
CDH26	ENSG00000124215	-1.0047	0.84248	0.85342	0.19671	0.6471	0.25878
0.885	0.18808	0.18808	0.625763579
MIR574	ENSG00000207944	0.88486	0.18812	0.88486	0.18812	0.88486
0.18812	0.88486	0.18812	0.18812	0.625763579
NA	ENSG00000225177	0.88485	0.18812	0.88485	0.18812	0.88485	0.18812
0.61579	0.26902	0.18812	0.625763579
TRDC	ENSG00000211829	0.056828	0.47734	0.056828	0.47734	-0.62707	0.73469
0.88481	0.18813	0.18813	0.625763579
NA	ENSG00000251987	0.8848	0.18813	0.47464	0.31752	-0.39294	0.65282
0.47464	0.31752	0.18813	0.625763579
NA	ENSG00000237901	0.88476	0.18814	-0.25805	0.60182	0.88476	0.18814
0.88476	0.18814	0.18814	0.625763579
NA	ENSG00000248870	-1.0094	0.84361	0.65969	0.25473	0.88468	0.18816
0.81614	0.20721	0.18816	0.625787387
EIF4A2P2	ENSG00000227382	-0.50887	0.69458	0.88463	0.18818	-0.50887
0.69458	-0.50887	0.69458	0.18818	0.625792289
RRAGD	ENSG00000025039	0.10366	0.45872	0.88454	0.1882	0.19709	0.42188
0.7381	0.23023	0.1882	0.625792289
NA	ENSG00000279172	0.88455	0.1882	0.88455	0.1882	0.88455	0.1882
0.88455	0.1882	0.1882	0.625792289
LINC01493	ENSG00000254562	-0.17866	0.5709	-0.50104	0.69183	0.78935
0.21495	0.88429	0.18827	0.18827	0.625982335
TLX2	ENSG00000115297	0.88401	0.18835	0.66688	0.25242	0.88401	0.18835
0.88401	0.18835	0.18835	0.626205601
PARP11-AS1	ENSG00000256862	-2.6545	0.99603	0.29324	0.38467	0.88385
0.18839	-0.86698	0.80702	0.18839	0.626286493
HCRTR2	ENSG00000137252	-0.29192	0.61482	-0.82073	0.7941	-1.4199
0.92218	0.88374	0.18842	0.18842	0.626286493
KIRREL3-AS1	ENSG00000257271	0.71895	0.23609	-0.31308	0.62289	0.88375
0.18842	-0.84062	0.79972	0.18842	0.626286493
NA	ENSG00000248103	-1.3244	0.90731	-0.24096	0.59521	-1.3244	0.90731
0.8837	0.18843	0.18843	0.626286493
NA	ENSG00000225612	0.024849	0.49009	0.024849	0.49009	0.88366	0.18844

-0.083004 0.53308 0.18844 0.626286493
C9orf163 ENSG00000196366 0.55024 0.29108 0.828 0.20384 0.88356 0.18847
0.88356 0.18847 0.18847 0.626286493
TPBG ENSG00000146242 0.12024 0.45214 0.8835 0.18848 -1.0332 0.84924
0.12024 0.45214 0.18848 0.626286493
RUFY1 ENSG00000176783 -0.63134 0.73609 0.31619 0.37593 0.88348 0.18849
0.59954 0.27441 0.18849 0.626286493
NA ENSG00000229305 0.88349 0.18849 0.81041 0.20885 -0.17863 0.57088
0.24387 0.40367 0.18849 0.626286493
CMC2 ENSG00000103121 0.85149 0.19725 0.88338 0.18851 0.88338 0.18851
0.871 0.19188 0.18851 0.626305555
RNU1-133P ENSG00000201737 0.88329 0.18854 0.73723 0.23049 0.88329
0.18854 0.88329 0.18854 0.18854 0.626305555
MIR302C ENSG00000199102 0.88324 0.18855 0.88324 0.18855 0.88324
0.18855 0.88324 0.18855 0.18855 0.626305555
MIR302A ENSG00000207927 0.88324 0.18855 0.88324 0.18855 0.88324
0.18855 0.88324 0.18855 0.18855 0.626305555
NA ENSG00000276505 0.88323 0.18856 0.88323 0.18856 0.88323 0.18856
0.88323 0.18856 0.18856 0.626305555
LINC01846 ENSG00000248901 0.74159 0.22917 0.52489 0.29983 0.74753
0.22737 0.88291 0.18864 0.18864 0.626462177
NA ENSG00000235782 0.26398 0.3959 0.8829 0.18865 0.8829 0.18865
0.8829 0.18865 0.18865 0.626462177
LINC00437 ENSG00000236354 0.35872 0.3599 0.75907 0.22391 0.85699
0.19573 0.88288 0.18865 0.18865 0.626462177
PPIAP45 ENSG00000258116 0.88277 0.18868 0.25072 0.40101 0.88277
0.18868 0.88277 0.18868 0.18868 0.626462177
CCNB1IP1P1 ENSG00000229815 -0.033308 0.51329 0.8827 0.1887 0.1939
0.42313 -0.38287 0.64909 0.1887 0.626462177
NA ENSG00000267426 0.88271 0.1887 0.88271 0.1887 0.88271 0.1887
0.88271 0.1887 0.1887 0.626462177
TBL3 ENSG00000183751 0.15677 0.43771 0.88265 0.18871 -0.15951 0.56337
0.16599 0.43408 0.18871 0.626462177
BICRA-AS1 ENSG00000269806 0.88267 0.18871 0.88267 0.18871 0.88267
0.18871 0.88267 0.18871 0.18871 0.626462177
ALPK1 ENSG00000073331 0.33753 0.36786 0.424 0.33578 -0.33428 0.63091
0.8826 0.18873 0.18873 0.626485892
TRMT112P7 ENSG00000270275 -0.69791 0.75738 -0.69791 0.75738 -0.69791
0.75738 0.88244 0.18877 0.18877 0.626533313
NA ENSG00000280053 -0.19911 0.57891 0.22816 0.40976 -0.48896 0.68757
0.88243 0.18877 0.18877 0.626533313
TMEM97P1 ENSG00000234030 0.88224 0.18882 0.39473 0.34652 -0.88756
0.81261 -0.88756 0.81261 0.18882 0.626552259
SAP18P1 ENSG00000249777 0.88217 0.18884 -0.88388 0.81162 0.34978
0.36325 0.34978 0.36325 0.18884 0.626552259
MIR3150BHG ENSG00000245080 0.88215 0.18885 0.88215 0.18885 0.88215
0.18885 0.88215 0.18885 0.18885 0.626552259
NA ENSG00000263571 0.88207 0.18887 0.88207 0.18887 0.88207 0.18887
0.88207 0.18887 0.18887 0.626552259
SNRPCP6 ENSG00000254683 0.28662 0.3872 0.14618 0.44189 0.88201
0.18888 -0.50115 0.69187 0.18888 0.626552259
TTLL11-IT1 ENSG00000237548 0.018453 0.49264 0.88199 0.18889 0.6482
0.25843 -0.91679 0.82037 0.18889 0.626552259
NA ENSG00000206912 -0.79288 0.78608 0.88194 0.1889 0.1559 0.43806
-0.94226 0.82697 0.1889 0.626552259
RNA5SP232 ENSG00000201014 0.78764 0.21545 0.88194 0.18891 0.70065
0.24176 0.74566 0.22794 0.18891 0.626552259
CAPN2 ENSG00000162909 0.26556 0.39529 0.47482 0.31746 0.42905 0.33394
0.88187 0.18892 0.18892 0.626552259
LINC00222 ENSG00000203801 0.88186 0.18893 0.47882 0.31603 0.29556
0.38378 0.7041 0.24069 0.18893 0.626552259
MTND4LP19 ENSG00000219087 0.88184 0.18893 0.88184 0.18893 0.88184

0.18893 0.88184 0.18893 0.18893 0.626552259
MTATP6P25 ENSG00000262095 0.88184 0.18893 -1.076 0.85905 0.88184
0.18893 0.88184 0.18893 0.18893 0.626552259
RN7SL385P ENSG00000241333 0.88175 0.18895 0.88175 0.18895 0.88175
0.18895 0.85086 0.19742 0.18895 0.626566467
NA ENSG00000283999 0.88174 0.18896 0.88174 0.18896 0.88174 0.18896
0.88174 0.18896 0.18896 0.626566467
NA ENSG00000249994 -0.43301 0.6675 -0.43301 0.6675 0.88161 0.18899
-0.43301 0.6675 0.18899 0.626623304
PLAU ENSG00000122861 -0.86146 0.80551 -0.86146 0.80551 0.88153 0.18902
-0.38862 0.65122 0.18902 0.62662802
RPL23AP40 ENSG00000242321 -0.108 0.543 -0.108 0.543 -0.108 0.543 0.8815
0.18902 0.18902 0.62662802
RNU6-613P ENSG00000222344 0.69309 0.24413 0.76021 0.22357 0.58286
0.27999 0.88146 0.18903 0.18903 0.62662802
SCGB1D5P ENSG00000248287 0.88138 0.18906 -0.46201 0.67796 0.35221
0.36234 -0.92666 0.82295 0.18906 0.626684838
NA ENSG00000242034 -0.13677 0.55439 0.88124 0.18909 -0.42033 0.66288
-0.42033 0.66288 0.18909 0.626741647
NA ENSG00000213172 0.88109 0.18914 0.53642 0.29583 0.78422 0.21646
0.37611 0.35342 0.18914 0.626793642
EFEMP2 ENSG00000172638 0.88104 0.18915 0.88104 0.18915 0.64136
0.26064 0.88104 0.18915 0.18915 0.626793642
FOSL1 ENSG00000175592 0.88101 0.18916 -0.025067 0.51 0.78263 0.21692
0.78263 0.21692 0.18916 0.626793642
NA ENSG00000279083 0.73289 0.23181 0.881 0.18916 0.73289 0.23181
0.73289 0.23181 0.18916 0.626793642
NTF6G ENSG00000267099 0.88096 0.18917 0.88096 0.18917 0.88096 0.18917
0.88096 0.18917 0.18917 0.626793642
NA ENSG00000279591 0.88081 0.18921 0.88081 0.18921 0.88081 0.18921
0.88081 0.18921 0.18921 0.626855088
NA ENSG00000223675 0.88074 0.18923 0.88074 0.18923 0.88074 0.18923
0.88074 0.18923 0.18923 0.626855088
NA ENSG00000231996 0.88071 0.18924 0.88071 0.18924 -0.02826 0.51127
0.88071 0.18924 0.18924 0.626855088
NA ENSG00000257415 -0.08231 0.5328 0.88071 0.18924 0.25553 0.39916
-0.25209 0.59951 0.18924 0.626855088
IQCF1 ENSG00000173389 0.1694 0.43274 0.25595 0.39899 0.59251 0.27675
0.88052 0.18929 0.18929 0.626968599
NA ENSG00000255132 0.44485 0.32821 -0.38748 0.6508 0.88047 0.1893
0.88047 0.1893 0.1893 0.626968599
PLA2G4E-AS1 ENSG00000246740 -1.3818 0.91648 -1.2878 0.90109 0.88036
0.18933 0.88036 0.18933 0.18933 0.627025343
NARF ENSG00000141562 0.76927 0.22087 0.88013 0.18939 -0.17103 0.5679
0.048586 0.48062 0.18939 0.627091312
NA ENSG00000262786 -1.3694 0.91456 0.88014 0.18939 0.78074 0.21748
0.64154 0.26059 0.18939 0.627091312
SPAG16-DT ENSG00000196096 0.88007 0.18941 0.88007 0.18941 0.88007
0.18941 0.88007 0.18941 0.18941 0.627091312
RN7SKP173 ENSG00000199691 0.88007 0.18941 0.11205 0.45539 0.11205
0.45539 0.11205 0.45539 0.18941 0.627091312
NA ENSG00000236119 0.88001 0.18943 0.88001 0.18943 0.88001 0.18943
0.64886 0.25821 0.18943 0.627091312
NA ENSG00000243144 0.79243 0.21405 0.88001 0.18943 0.76603 0.22183
0.22338 0.41162 0.18943 0.627091312
ZKSCAN1 ENSG00000106261 0.87997 0.18944 0.50037 0.30841 -0.023832
0.50951 0.59917 0.27453 0.18944 0.627091312
RAD21-AS1 ENSG00000253327 0.87988 0.18946 0.87988 0.18946 0.87988
0.18946 0.87988 0.18946 0.18946 0.62710542
NA ENSG00000224876 0.87984 0.18947 -0.67505 0.75018 0.87984 0.18947
0.87984 0.18947 0.18947 0.62710542
LRRC45 ENSG00000169683 -0.25575 0.60093 0.87971 0.18951 0.10525

0.45809 -0.16685 0.56625 0.18951 0.627180497
CIZ1 ENSG00000148337 0.87955 0.18955 -0.84112 0.79986 -0.35177 0.63749
-1.5571 0.94028 0.18955 0.627180497
NA ENSG00000201548 0.87957 0.18955 0.87957 0.18955 0.87957 0.18955
0.87957 0.18955 0.18955 0.627180497
KCNQ5-AS1 ENSG00000229154 0.87953 0.18956 -0.68642 0.75378 -0.63114
0.73602 -0.37677 0.64683 0.18956 0.627180497
NA ENSG00000280144 -0.54625 0.70755 0.87943 0.18958 0.39393 0.34682
-1.8487 0.96775 0.18958 0.627180497
NA ENSG00000218834 0.17376 0.43103 -0.59767 0.72497 0.87936 0.1896
-0.60481 0.72735 0.1896 0.627180497
KRT8P29 ENSG00000236430 0.8793 0.18962 0.076568 0.46948 0.30386
0.38062 0.82911 0.20352 0.18962 0.627180497
VNN2 ENSG00000112303 0.029411 0.48827 0.029411 0.48827 0.87926 0.18963
0.80764 0.20965 0.18963 0.627180497
LINC02184 ENSG00000260854 -0.21379 0.58465 0.87928 0.18963 0.64934
0.25806 0.36565 0.35731 0.18963 0.627180497
BIN2P1 ENSG00000234568 0.18999 0.42466 -0.20749 0.58219 0.8792
0.18965 0.71293 0.23794 0.18965 0.627180497
NA ENSG00000270116 0.87917 0.18965 0.87917 0.18965 0.87917 0.18965
0.87917 0.18965 0.18965 0.627180497
HLA-G ENSG00000204632 0.64964 0.25796 0.87915 0.18966 -0.3178 0.62468
0.69402 0.24383 0.18966 0.627180497
NA ENSG00000269987 0.87914 0.18966 0.87914 0.18966 0.87914 0.18966
0.87914 0.18966 0.18966 0.627180497
NA ENSG00000265239 0.60418 0.27286 0.879 0.1897 0.60418 0.27286
-0.073528 0.52931 0.1897 0.627270204
SVEP1 ENSG00000165124 0.87886 0.18974 -0.3839 0.64947 0.26298 0.39628
-0.18788 0.57452 0.18974 0.6273599
GPR148 ENSG00000173302 -0.12445 0.54952 -0.42579 0.66487 0.87879
0.18976 0.12517 0.45019 0.18976 0.627383459
CLEC4C ENSG00000198178 0.87864 0.1898 -0.247 0.59754 0.39821
0.34524 0.74844 0.2271 0.1898 0.627392553
NA ENSG00000253513 -0.22962 0.59081 0.87863 0.1898 0.49952 0.30871
0.87863 0.1898 0.1898 0.627392553
NA ENSG00000259006 0.87859 0.18981 0.87859 0.18981 0.87859 0.18981
0.87859 0.18981 0.18981 0.627392553
KRT86 ENSG00000170442 0.87855 0.18982 -0.72789 0.76666 -1.1914 0.88325
-1.9833 0.97633 0.18982 0.627392553
NA ENSG00000229532 0.87852 0.18983 0.87852 0.18983 0.87852 0.18983
0.87852 0.18983 0.18983 0.627392553
SNRPCP11 ENSG00000241346 0.25775 0.3983 0.22289 0.41181 0.43733
0.33094 0.87848 0.18984 0.18984 0.627392553
COX5A ENSG00000178741 -1.6202 0.94741 0.3445 0.36524 0.044803 0.48213
0.87843 0.18986 0.18986 0.627396697
KCNQ2 ENSG00000075043 0.87838 0.18987 0.22409 0.41134 0.41847 0.3378
-0.14204 0.55647 0.18987 0.627396697
LCE2D ENSG00000187223 0.60841 0.27146 -0.41198 0.65982 0.15811 0.43718
0.87827 0.1899 0.1899 0.627396697
JPH4 ENSG00000092051 0.87816 0.18993 -0.73447 0.76867 0.7159 0.23703
-0.23674 0.59357 0.18993 0.627396697
SCG3 ENSG00000104112 -0.60563 0.72762 -0.60563 0.72762 0.1017 0.4595
0.87815 0.18993 0.18993 0.627396697
GAPDHP62 ENSG00000251013 0.87812 0.18994 -0.96413 0.83251 -1.3504
0.91155 -0.50304 0.69253 0.18994 0.627396697
NA ENSG00000254554 0.87811 0.18994 -0.17143 0.56806 -0.0098487 0.50393
-0.0098487 0.50393 0.18994 0.627396697
CRIP1P2 ENSG00000238225 0.87803 0.18996 -0.463 0.67832 -0.463
0.67832 -0.463 0.67832 0.18996 0.627396697
SPON1-AS1 ENSG00000254418 -1.9297 0.97318 0.8779 0.19 0.8779 0.19
0.73531 0.23107 0.19 0.627396697
NA ENSG00000259998 0.87785 0.19001 0.74819 0.22717 0.87785 0.19001

0.87785	0.19001	0.19001	0.627396697
NA	ENSG00000228751 0.87781	0.19002	0.87781	0.19002	0.87781	0.19002
0.87781	0.19002	0.19002	0.627396697
LINC00665	ENSG00000232677 0.87783	0.19002	0.48522	0.31376	-0.14939
0.55938	-0.15791	0.56274	0.19002	0.627396697
OR52X1P	ENSG00000234895 -1.1485	0.87461	0.87781	0.19002	-1.1422
0.87332	0.46343	0.32153	0.19002	0.627396697
ILF2	ENSG00000143621 0.59059	0.2774	0.18586	0.42628	-0.13238	0.55266
0.87766	0.19006	0.19006	0.627396697
LRRC37A	ENSG00000176681 0.87757	0.19009	0.56284	0.28677	-0.27356
0.60779	0.58279	0.28002	0.19009	0.627396697
MRPL16	ENSG00000166902 0.33888	0.36735	0.3971	0.34565	0.87753
0.1901	0.3971	0.34565	0.1901	0.627396697
HVCN1	ENSG00000122986 0.78644	0.21581	0.87748	0.19011	0.60671	0.27202
0.62405	0.2663	0.19011	0.627396697
ELL2	ENSG00000118985 0.87742	0.19013	-0.52674	0.70081	0.87742	0.19013
0.51619	0.30286	0.19013	0.627396697
EDRF1	ENSG00000107938 0.87736	0.19014	-0.47894	0.68401	-0.06887	0.52745
-0.86321	0.80599	0.19014	0.627396697
PDCD6IPP1	ENSG00000275325 0.87733	0.19015	-1.0533	0.85389	-0.12985
0.55166	-0.79609	0.78701	0.19015	0.627396697
LUZP1	ENSG00000169641 0.36787	0.35649	0.75079	0.22639	0.87729	0.19016
-0.052834	0.52107	0.19016	0.627396697
FGF22	ENSG00000070388 -0.82407	0.79505	0.87726	0.19017	-0.66367	0.74655
0.0036442	0.49855	0.19017	0.627396697
RHBDD3	ENSG00000100263 -1.5951	0.94466	-0.24218	0.59568	0.87727
0.19017	0.51829	0.30213	0.19017	0.627396697
NA	ENSG00000228137 0.87722	0.19018	0.87722	0.19018	0.87722	0.19018
0.87722	0.19018	0.19018	0.627396697
TMEM238L	ENSG00000263429 0.65354	0.25671	0.8771	0.19022	0.65354
0.25671	0.65354	0.25671	0.19022	0.627396697
NA	ENSG00000273017 0.87708	0.19022	0.77243	0.21993	0.77243	0.21993
0.77243	0.21993	0.19022	0.627396697
LIN28A	ENSG00000131914 0.12538	0.45011	0.87703	0.19024	0.87703
0.19024	0.87703	0.19024	0.19024	0.627396697
B4GALNT2P1 ENSG00000248883 0.87702	0.19024	0.87702	0.19024	0.87702
0.19024	0.87702	0.19024	0.19024	0.627396697
NA	ENSG00000230063 0.82402	0.20496	0.87693	0.19026	0.37943	0.35218
-0.4925	0.68882	0.19026	0.627396697
NA	ENSG00000200897 0.87691	0.19027	0.87691	0.19027	0.30969	0.3784
0.30969	0.3784	0.19027	0.627396697
FOXP1-IT1	ENSG00000242094 0.79088	0.21451	-0.086472	0.53445	0.87679
0.1903	0.028728	0.48854	0.1903	0.627396697
SRD5A1	ENSG00000145545 0.87677	0.19031	0.87677	0.19031	0.87677
0.19031	0.20331	0.41945	0.19031	0.627396697
RPA2P3	ENSG00000255547 0.87674	0.19031	-0.92024	0.82128	0.87674
0.19031	0.87674	0.19031	0.19031	0.627396697
HERC2P10	ENSG00000259845 -0.26899	0.60603	-0.22882	0.5905	0.87672
0.19032	-1.0164	0.84527	0.19032	0.627396697
NA	ENSG00000279276 0.8767	0.19033	0.8767	0.19033	0.8767	0.19033
0.8767	0.19033	0.19033	0.627396697
NA	ENSG00000233942 -0.44934	0.67341	0.87661	0.19035	-0.065767	0.52622
-0.59538	0.72421	0.19035	0.627396697
NOP16	ENSG00000048162 0.87657	0.19036	0.87657	0.19036	0.87657	0.19036
-0.28266	0.61128	0.19036	0.627396697
HEPN1	ENSG00000221932 0.87635	0.19042	0.87635	0.19042	0.87635	0.19042
0.87635	0.19042	0.19042	0.627396697
NA	ENSG00000279261 -0.22073	0.58735	-1.1071	0.86587	-0.22073	0.58735
0.87633	0.19042	0.19042	0.627396697
MIR548AC	ENSG00000264419 0.20498	0.41879	-1.2868	0.90092	0.7614
0.22321	0.87633	0.19043	0.19043	0.627396697
RNU6-33P	ENSG00000207524 0.87628	0.19044	0.87628	0.19044	0.87628

0.19044 0.87628 0.19044 0.19044 0.627396697
IMPACT ENSG00000154059 0.66567 0.25281 0.59098 0.27727 -0.34456
0.63479 0.87625 0.19045 0.19045 0.627396697
NA ENSG00000271257 0.87621 0.19046 0.87621 0.19046 0.87621 0.19046
0.87621 0.19046 0.19046 0.627396697
DDR1-DT ENSG00000237775 0.87616 0.19047 -0.37189 0.64501 -0.37189
0.64501 -0.37189 0.64501 0.19047 0.627396697
NA ENSG00000278102 0.87617 0.19047 0.87617 0.19047 0.87617 0.19047
0.87617 0.19047 0.19047 0.627396697
NA ENSG00000199677 0.70089 0.24168 0.70089 0.24168 0.87606 0.1905
-0.013453 0.50537 0.1905 0.627396697
FAM210CP ENSG00000213770 0.87605 0.1905 0.61352 0.26976 -0.86445
0.80633 -0.86445 0.80633 0.1905 0.627396697
NA ENSG00000251668 -0.78551 0.78392 -1.6538 0.95092 0.87605 0.1905
-0.78551 0.78392 0.1905 0.627396697
SMN1 ENSG00000172062 0.876 0.19052 0.65185 0.25725 -0.17418 0.56914
-0.031089 0.5124 0.19052 0.627396697
NA ENSG00000258965 0.87598 0.19052 -0.25724 0.6015 0.87598 0.19052
0.87598 0.19052 0.19052 0.627396697
PAH ENSG00000171759 0.3792 0.35227 0.87594 0.19053 0.67866 0.24868
0.69868 0.24238 0.19053 0.627396697
MIR381 ENSG00000199020 0.87585 0.19056 0.87585 0.19056 0.87585
0.19056 0.87585 0.19056 0.19056 0.627396697
MIR487B ENSG00000207754 0.87585 0.19056 0.87585 0.19056 0.87585
0.19056 0.87585 0.19056 0.19056 0.627396697
OR2F2 ENSG00000221910 0.87583 0.19056 0.52654 0.29926 0.52654 0.29926
0.52654 0.29926 0.19056 0.627396697
NA ENSG00000254789 0.82765 0.20393 -0.70183 0.75861 0.87583 0.19056
0.63717 0.26201 0.19056 0.627396697
RNU6-1245P ENSG00000223062 0.87581 0.19057 0.87581 0.19057 0.87581
0.19057 0.87581 0.19057 0.19057 0.627396697
SLC25A6P2 ENSG00000232203 0.87571 0.19059 -0.40239 0.6563 -0.81426
0.79225 -0.38664 0.65049 0.19059 0.627396697
LINC02271 ENSG00000240152 -0.97709 0.83574 -0.97709 0.83574 0.87573
0.19059 -0.97709 0.83574 0.19059 0.627396697
CDKN2B ENSG00000147883 -0.16503 0.56554 0.6831 0.24727 0.87557
0.19063 0.87557 0.19063 0.19063 0.627396697
NA ENSG00000224448 0.87555 0.19064 0.87555 0.19064 0.87555 0.19064
0.87555 0.19064 0.19064 0.627396697
RASIP1 ENSG00000105538 0.87552 0.19065 0.87552 0.19065 0.092513
0.46315 0.13999 0.44433 0.19065 0.627396697
NA ENSG00000216084 0.87535 0.19069 0.48727 0.31303 0.87535 0.19069
0.87535 0.19069 0.19069 0.627396697
LINC02340 ENSG00000275294 0.59698 0.27526 -0.88366 0.81156 0.44711
0.3274 0.87531 0.1907 0.1907 0.627396697
NA ENSG00000227723 -0.83817 0.79903 0.87521 0.19073 0.30265 0.38108
-0.97965 0.83637 0.19073 0.627396697
MIR539 ENSG00000202560 0.87511 0.19076 0.87511 0.19076 0.87511
0.19076 0.87511 0.19076 0.19076 0.627396697
RNA5SP64 ENSG00000207341 0.51726 0.30249 0.87511 0.19076 0.87511
0.19076 -0.18564 0.57364 0.19076 0.627396697
NA ENSG00000213067 0.049205 0.48038 0.74881 0.22699 0.8751 0.19076
0.483 0.31455 0.19076 0.627396697
MIR889 ENSG00000216099 0.87511 0.19076 0.87511 0.19076 0.87511
0.19076 0.87511 0.19076 0.19076 0.627396697
NA ENSG00000229169 0.87511 0.19076 0.87511 0.19076 0.016733 0.49332
0.016733 0.49332 0.19076 0.627396697
NA ENSG00000260808 -0.23268 0.592 0.8751 0.19076 -0.78657 0.78423
-0.78657 0.78423 0.19076 0.627396697
MIR4478 ENSG00000266583 -1.4949 0.93253 -0.77489 0.7808 0.35752
0.36035 0.87507 0.19077 0.19077 0.627396697
NA ENSG00000225303 -1.4276 0.92329 -0.9682 0.83353 -1.4276 0.92329

0.87498 0.19079 0.19079 0.627396697
RNA5SP139 ENSG00000201210 0.87494 0.1908 0.87494 0.1908 0.87494
0.1908 0.87494 0.1908 0.1908 0.627396697
C16orf82 ENSG00000234186 -0.59456 0.72393 0.029248 0.48833 0.48433
0.31408 0.87484 0.19083 0.19083 0.627396697
SLC4A1APP1 ENSG00000232060 0.8747 0.19087 0.71402 0.23761 0.71402
0.23761 0.71402 0.23761 0.19087 0.627396697
ELOCP19 ENSG00000241975 0.8747 0.19087 0.8747 0.19087 0.8747
0.19087 0.8747 0.19087 0.19087 0.627396697
MTRNR2L1 ENSG00000256618 0.87464 0.19089 0.87464 0.19089 0.87464
0.19089 0.87464 0.19089 0.19089 0.627396697
NDC1 ENSG00000058804 0.017792 0.4929 -0.35711 0.6395 0.2709 0.39323
0.87458 0.1909 0.1909 0.627396697
MFSD14A ENSG00000156875 0.7882 0.21529 0.7882 0.21529 0.7882
0.21529 0.87459 0.1909 0.1909 0.627396697
TTC30B ENSG00000196659 0.87446 0.19093 0.87446 0.19093 0.87446
0.19093 0.87446 0.19093 0.19093 0.627396697
POM121L13P ENSG00000236339 0.87442 0.19094 0.87442 0.19094 -0.40571
0.65752 -0.78055 0.78247 0.19094 0.627396697
NA ENSG00000225026 -0.3864 0.6504 -0.3864 0.6504 0.87439 0.19095
-0.3864 0.6504 0.19095 0.627396697
KRT18P46 ENSG00000235716 0.87432 0.19097 0.67442 0.25002 0.87432
0.19097 -0.15614 0.56204 0.19097 0.627396697
NA ENSG00000187186 0.87429 0.19098 0.87429 0.19098 0.87429 0.19098
0.87429 0.19098 0.19098 0.627396697
NA ENSG00000242861 0.87429 0.19098 0.87429 0.19098 0.87429 0.19098
0.87429 0.19098 0.19098 0.627396697
CDCP2 ENSG00000157211 0.6508 0.25759 0.87426 0.19099 0.064715 0.4742
0.18961 0.42481 0.19099 0.627396697
FBXO34 ENSG00000178974 0.65996 0.25464 0.87427 0.19099 0.65996
0.25464 0.58252 0.28011 0.19099 0.627396697
EIF4A1P1 ENSG00000233300 0.68886 0.24546 0.87426 0.19099 -0.3827
0.64903 -0.3827 0.64903 0.19099 0.627396697
LRRC37A17P ENSG00000263142 0.87426 0.19099 0.87426 0.19099 0.87426
0.19099 0.87426 0.19099 0.19099 0.627396697
NA ENSG00000230291 0.87422 0.191 0.87422 0.191 0.87422 0.191 0.87422 0.191
0.191 0.627396697
NA ENSG00000258464 0.87407 0.19104 -0.021861 0.50872 -0.046817 0.51867
0.29394 0.3844 0.19104 0.627485791
NA ENSG00000279622 0.31034 0.37815 0.87401 0.19106 -1.1879 0.88256
-0.27015 0.60648 0.19106 0.627509186
NPAS2 ENSG00000170485 0.44909 0.32668 0.87378 0.19112 0.012264 0.49511
-0.46326 0.67841 0.19112 0.627621646
NA ENSG00000215246 0.87377 0.19112 -1.2676 0.89753 -1.2676 0.89753
0.42651 0.33487 0.19112 0.627621646
SUGT1 ENSG00000165416 -0.3814 0.64855 -0.20115 0.57971 0.32566 0.37234
0.8737 0.19114 0.19114 0.627626115
NA ENSG00000237633 0.60743 0.27178 -0.1346 0.55353 0.87366 0.19115
-0.86067 0.80529 0.19115 0.627626115
NA ENSG00000253395 0.87362 0.19116 0.29592 0.38365 0.13882 0.4448
-2.0699 0.98077 0.19116 0.627626115
GGACT ENSG00000134864 0.78939 0.21494 0.78939 0.21494 -0.54374 0.70669
0.87353 0.19119 0.19119 0.627644501
NA ENSG00000229607 0.69748 0.24275 0.87344 0.19121 0.059796 0.47616
0.0055048 0.4978 0.19121 0.627644501
NA ENSG00000236968 0.71774 0.23646 0.87343 0.19121 -1.5927 0.94439
-2.1122 0.98267 0.19121 0.627644501
LYN ENSG00000254087 -0.6117 0.72963 0.72526 0.23415 -0.82867 0.79636
0.87341 0.19122 0.19122 0.627644501
COL5A2 ENSG00000204262 0.65768 0.25537 0.59443 0.27611 0.16636
0.43394 0.87338 0.19123 0.19123 0.627644501
POU6F1 ENSG00000184271 0.87329 0.19125 0.16997 0.43252 -0.15192

0.56037 0.18374 0.42711 0.19125 0.627667868
NA ENSG00000215795 0.044908 0.48209 0.76255 0.22287 0.87316 0.19129
0.51245 0.30417 0.19129 0.627756866
BIRC7 ENSG00000101197 0.76429 0.22235 0.67244 0.25065 0.50322 0.30741
0.87307 0.19131 0.19131 0.627780222
RPS8P3 ENSG00000215457 0.77089 0.22038 0.87296 0.19134 0.87296
0.19134 0.87296 0.19134 0.19134 0.627794115
NA ENSG00000279738 0.77667 0.21868 0.87298 0.19134 0.76699 0.22154
0.77667 0.21868 0.19134 0.627794115
KCND3 ENSG00000171385 0.38665 0.34951 -0.2124 0.5841 0.8727 0.19141
0.49889 0.30893 0.19141 0.627953096
SRSF6 ENSG00000124193 0.83866 0.20083 0.1004 0.46001 0.87263 0.19143
-0.21871 0.58656 0.19143 0.627953096
KCNG2 ENSG00000178342 0.80025 0.21178 -0.16849 0.5669 0.8726 0.19144
-0.3043 0.61955 0.19144 0.627953096
NA ENSG00000215805 0.8726 0.19144 0.8726 0.19144 0.8726 0.19144
0.8726 0.19144 0.19144 0.627953096
PFN1P9 ENSG00000227205 -1.0813 0.86022 0.39331 0.34705 -1.6896
0.95445 0.87252 0.19146 0.19146 0.627976429
FERMT3 ENSG00000149781 0.87229 0.19153 -0.29736 0.6169 -0.18966
0.57521 0.62203 0.26696 0.19153 0.628121799
TRAV27 ENSG00000211809 0.87215 0.19156 0.87215 0.19156 0.87215
0.19156 0.87215 0.19156 0.19156 0.628121799
NA ENSG00000253123 0.10829 0.45688 0.87214 0.19157 0.10829 0.45688
0.51314 0.30393 0.19157 0.628121799
NA ENSG00000279900 0.76362 0.22255 0.76362 0.22255 0.76362 0.22255
0.87208 0.19158 0.19158 0.628121799
ATP5F1D ENSG00000099624 -0.3265 0.62798 -0.3265 0.62798 -0.3265
0.62798 0.87206 0.19159 0.19159 0.628121799
MSRB1 ENSG00000198736 0.8325 0.20256 0.87201 0.1916 0.8325 0.20256
0.62973 0.26444 0.1916 0.628121799
OR2T34 ENSG00000183310 0.095503 0.46196 0.22395 0.4114 0.87195
0.19162 -0.66464 0.74686 0.19162 0.628121799
NDUFA6-DT ENSG00000237037 0.79604 0.213 0.87193 0.19162 0.87193 0.19162
0.87193 0.19162 0.19162 0.628121799
MED6P1 ENSG00000227905 0.87192 0.19163 0.87192 0.19163 0.87192
0.19163 0.87192 0.19163 0.19163 0.628121799
KRT26 ENSG00000186393 0.87185 0.19165 -0.77279 0.78018 0.87185 0.19165
0.87185 0.19165 0.19165 0.628121799
IFNNP1 ENSG00000230208 0.8718 0.19166 -0.58492 0.7207 -0.58492
0.7207 -0.58492 0.7207 0.19166 0.628121799
MUC2 ENSG00000198788 -0.13759 0.55472 0.76292 0.22276 0.87176 0.19167
-1.0264 0.84765 0.19167 0.628121799
NA ENSG00000263437 -0.78816 0.7847 -0.78816 0.7847 -0.78816 0.7847
0.87173 0.19168 0.19168 0.628121799
NA ENSG00000271358 0.87165 0.1917 -1.2429 0.89305 -0.1375 0.55468
-1.3154 0.90582 0.1917 0.628121799
NA ENSG00000270988 0.87162 0.19171 0.87162 0.19171 0.87162 0.19171
0.87162 0.19171 0.19171 0.628121799
MTFR1 ENSG00000066855 0.13927 0.44462 0.28108 0.38932 0.87146 0.19175
0.24172 0.4045 0.19175 0.628121799
NA ENSG00000258677 0.87148 0.19175 0.87148 0.19175 0.87148 0.19175
0.87148 0.19175 0.19175 0.628121799
LINC02530 ENSG00000234015 -0.74876 0.773 0.87132 0.19179 -1.041 0.85107
-1.0114 0.8441 0.19179 0.628121799
NA ENSG00000264514 -1.8708 0.96932 0.87129 0.1918 -1.8708 0.96932
-1.8708 0.96932 0.1918 0.628121799
OPTC ENSG00000188770 -1.0562 0.85456 0.87126 0.19181 0.51617 0.30287
0.81821 0.20662 0.19181 0.628121799
RNA5SP264 ENSG00000199985 0.87122 0.19182 0.87122 0.19182 0.11615
0.45377 0.87122 0.19182 0.19182 0.628121799
FAM86DP ENSG00000244026 0.87119 0.19182 0.053786 0.47855 -0.065234

0.52601 0.2246 0.41115 0.19182 0.628121799
NA ENSG00000273119 0.87114 0.19184 -0.31685 0.62432 -0.31685 0.62432
-0.87679 0.8097 0.19184 0.628121799
LINC02293 ENSG00000257185 0.03254 0.48702 -0.37617 0.6466 0.8711
0.19185 -0.58665 0.72128 0.19185 0.628121799
NA ENSG00000250865 0.7869 0.21567 0.7869 0.21567 0.7869 0.21567
0.87106 0.19186 0.19186 0.628121799
MIR4774 ENSG00000265694 -1.1253 0.86977 0.87105 0.19186 -0.58291
0.72002 -0.39817 0.65475 0.19186 0.628121799
RPS6P23 ENSG00000230871 -0.10338 0.54117 -0.050526 0.52015 0.87093
0.1919 0.26488 0.39555 0.1919 0.628121799
NA ENSG00000249135 -0.0079663 0.50318 0.87091 0.1919 -1.255 0.89525
-0.47058 0.68103 0.1919 0.628121799
GATAD2A ENSG00000167491 0.87072 0.19195 0.75936 0.22382 0.088608
0.4647 0.82847 0.2037 0.19195 0.628121799
OR6K2 ENSG00000196171 0.87055 0.192 0.48821 0.3127 0.48821 0.3127
0.48821 0.3127 0.192 0.628121799
NA ENSG00000270691 -0.95653 0.8306 0.87052 0.19201 -0.95653 0.8306
-0.95653 0.8306 0.19201 0.628121799
ETNPPL ENSG00000164089 0.63696 0.26207 0.78198 0.21711 0.74563
0.22794 0.87046 0.19202 0.19202 0.628121799
IDH3B ENSG00000101365 0.63606 0.26237 0.63606 0.26237 0.87039 0.19204
-1.3793 0.91609 0.19204 0.628121799
MIR3156-1 ENSG00000263476 0.87029 0.19207 0.87029 0.19207 0.87029
0.19207 0.87029 0.19207 0.19207 0.628121799
MRPL48P1 ENSG00000238076 0.87022 0.19209 0.043956 0.48247 0.87022
0.19209 0.87022 0.19209 0.19209 0.628121799
SETD4 ENSG00000185917 0.0017772 0.49929 -0.3503 0.63694 -0.091589 0.53649
0.87015 0.19211 0.19211 0.628121799
NA ENSG00000226211 0.87001 0.19215 0.87001 0.19215 0.87001 0.19215
0.87001 0.19215 0.19215 0.628121799
OR7E121P ENSG00000244222 0.38681 0.34945 0.33916 0.36724 0.86995
0.19216 -0.12375 0.54924 0.19216 0.628121799
TAF3 ENSG00000165632 -0.14878 0.55913 0.86992 0.19217 -0.49447 0.68951
0.86992 0.19217 0.19217 0.628121799
NA ENSG00000240499 0.86992 0.19217 0.11612 0.45378 0.11612 0.45378
0.11612 0.45378 0.19217 0.628121799
LINC02481 ENSG00000246526 0.14267 0.44327 0.45797 0.32349 -0.1928
0.57644 0.86994 0.19217 0.19217 0.628121799
TRIL ENSG00000255690 0.86993 0.19217 -0.25059 0.59894 0.23007 0.40902
0.23007 0.40902 0.19217 0.628121799
GLIPR1 ENSG00000139278 0.8698 0.19221 0.8698 0.19221 0.8698
0.19221 0.8698 0.19221 0.19221 0.628121799
NA ENSG00000213028 0.86976 0.19221 -0.47537 0.68274 0.64659 0.25895
0.64659 0.25895 0.19221 0.628121799
NA ENSG00000231132 0.25335 0.4 0.25335 0.4 0.86978 0.19221 -0.25665
0.60128 0.19221 0.628121799
NA ENSG00000267765 0.86977 0.19221 0.86977 0.19221 0.86977 0.19221
0.86977 0.19221 0.19221 0.628121799
NA ENSG00000275119 -0.080349 0.53202 0.0010503 0.49958 0.86969 0.19223
-0.82357 0.79491 0.19223 0.628121799
ATG4C ENSG00000125703 0.86969 0.19224 0.60336 0.27314 0.82495 0.2047
0.33148 0.37014 0.19224 0.628121799
NA ENSG00000267581 0.86959 0.19226 0.86959 0.19226 0.86959 0.19226
0.86959 0.19226 0.19226 0.628121799
NA ENSG00000228513 0.86934 0.19233 0.86934 0.19233 0.86934 0.19233
0.86934 0.19233 0.19233 0.628121799
NA ENSG00000228351 0.86923 0.19236 -0.48213 0.68514 -0.24985 0.59865
0.13831 0.445 0.19236 0.628121799
NA ENSG00000253878 0.86922 0.19236 0.86922 0.19236 0.86922 0.19236
0.86922 0.19236 0.19236 0.628121799
NA ENSG00000259350 0.86924 0.19236 0.86924 0.19236 -0.021666 0.50864

-0.4441 0.67152 0.19236 0.628121799
NA ENSG00000223869 0.86919 0.19237 0.86919 0.19237 0.86919 0.19237
0.86919 0.19237 0.19237 0.628121799
NA ENSG00000280302 0.86914 0.19238 0.86914 0.19238 0.86914 0.19238
0.86914 0.19238 0.19238 0.628121799
OXNAD1 ENSG00000154814 0.86912 0.19239 0.54028 0.2945 0.67348
0.25032 0.60541 0.27245 0.19239 0.628121799
AIDAP2 ENSG00000251429 0.007179 0.49714 0.17283 0.43139 0.86913
0.19239 0.76199 0.22303 0.19239 0.628121799
KRTAP5-14P ENSG00000249387 0.86908 0.1924 0.86908 0.1924 0.86908
0.1924 0.86908 0.1924 0.1924 0.628121799
LINC00518 ENSG00000183674 0.53974 0.29469 0.53974 0.29469 0.53974
0.29469 0.86905 0.19241 0.19241 0.628121799
NA ENSG00000213144 0.86896 0.19243 0.86896 0.19243 0.86896 0.19243
0.86896 0.19243 0.19243 0.628121799
NA ENSG00000230597 -0.29457 0.61584 -0.29457 0.61584 0.86897 0.19243
0.37649 0.35328 0.19243 0.628121799
LINC01602 ENSG00000205293 0.5935 0.27642 0.86896 0.19244 -0.33097
0.62967 0.86896 0.19244 0.19244 0.628121799
HNRNPU ENSG00000153187 0.8689 0.19245 -0.96159 0.83187 -0.23664
0.59353 -0.18357 0.57283 0.19245 0.628121799
NA ENSG00000273175 0.8689 0.19245 0.8689 0.19245 0.8689 0.19245
0.8689 0.19245 0.19245 0.628121799
LTV1P1 ENSG00000250219 0.78389 0.21655 -0.2708 0.60673 0.84267
0.19971 0.86875 0.19249 0.19249 0.628121799
PPIAP71 ENSG00000244183 0.86868 0.19251 0.86868 0.19251 0.86868
0.19251 0.86868 0.19251 0.19251 0.628121799
SNORA79B ENSG00000222489 0.86859 0.19253 -0.80271 0.78893 -0.80271
0.78893 -0.80271 0.78893 0.19253 0.628121799
PLSCR3 ENSG00000187838 0.86859 0.19254 0.86859 0.19254 0.86859
0.19254 0.86859 0.19254 0.19254 0.628121799
NA ENSG00000266680 0.86856 0.19254 -0.8375 0.79884 0.86856 0.19254
0.86856 0.19254 0.19254 0.628121799
NA ENSG00000267421 0.86857 0.19254 0.86857 0.19254 0.86857 0.19254
0.86857 0.19254 0.19254 0.628121799
DERL1 ENSG00000136986 0.44906 0.32669 0.86855 0.19255 0.59941 0.27445
0.038373 0.4847 0.19255 0.628121799
SLC24A4 ENSG00000140090 0.86844 0.19258 0.32971 0.37081 0.61393
0.26963 -0.63581 0.73755 0.19258 0.628121799
NA ENSG00000279472 0.86841 0.19258 0.86841 0.19258 0.86841 0.19258
0.86841 0.19258 0.19258 0.628121799
ZBTB46 ENSG00000130584 0.22909 0.4094 0.05752 0.47707 0.83204
0.20269 0.86832 0.19261 0.19261 0.628121799
SEC24B-AS1 ENSG00000247950 0.35772 0.36027 -0.38254 0.64897 0.56212
0.28702 0.86811 0.19267 0.19267 0.628121799
NA ENSG00000207379 0.74214 0.229 0.74214 0.229 0.86805 0.19268 0.74214
0.229 0.19268 0.628121799
KLLN ENSG00000227268 -0.14191 0.55642 0.25327 0.40003 0.86804 0.19269
0.21326 0.41556 0.19269 0.628121799
LINC00421 ENSG00000236834 -1.4808 0.93067 0.86802 0.19269 -0.25876
0.60209 -0.017213 0.50687 0.19269 0.628121799
FAUP2 ENSG00000237806 0.86799 0.1927 0.86799 0.1927 0.86799 0.1927
0.86799 0.1927 0.1927 0.628121799
ACSBG2 ENSG00000130377 -0.47843 0.68383 0.78544 0.2161 0.86797
0.19271 -1.0819 0.86036 0.19271 0.628121799
LINC01338 ENSG00000281327 0.59138 0.27713 -0.068783 0.52742 0.86795
0.19271 -0.068783 0.52742 0.19271 0.628121799
NA ENSG00000201151 0.86792 0.19272 0.86792 0.19272 0.86792 0.19272
0.86792 0.19272 0.19272 0.628121799
DPPA5 ENSG00000203909 0.86792 0.19272 0.86792 0.19272 0.86792 0.19272
0.86792 0.19272 0.19272 0.628121799
EPB41L2 ENSG00000079819 0.7149 0.23734 -0.64087 0.7392 0.86787

0.19273 -0.89582 0.81483 0.19273 0.628121799
TPT1P4 ENSG00000217027 -0.44111 0.67043 0.86789 0.19273 -0.44111
0.67043 -1.0483 0.85275 0.19273 0.628121799
RPL34-DT ENSG00000234492 0.86788 0.19273 0.55049 0.29099 0.69679
0.24297 0.63925 0.26133 0.19273 0.628121799
ARHGEF40 ENSG00000165801 0.34696 0.36431 -0.032728 0.51305 0.86784
0.19274 0.34696 0.36431 0.19274 0.628121799
RNU6-936P ENSG00000206732 0.86784 0.19274 0.86784 0.19274 0.86784
0.19274 0.86784 0.19274 0.19274 0.628121799
NA ENSG00000252011 0.86784 0.19274 0.86784 0.19274 0.86784 0.19274
0.86784 0.19274 0.19274 0.628121799
RN7SL120P ENSG00000244169 0.29677 0.38332 -0.37436 0.64593 0.022672
0.49096 0.86782 0.19275 0.19275 0.628121799
SH3PXD2A ENSG00000107957 -0.58465 0.72061 0.48351 0.31437 0.86761
0.1928 0.21623 0.4144 0.1928 0.628121799
WDR91 ENSG00000105875 0.07365 0.47064 0.86759 0.19281 0.71234 0.23813
0.71234 0.23813 0.19281 0.628121799
RNF167 ENSG00000108523 0.86756 0.19282 0.86756 0.19282 0.86756
0.19282 0.86756 0.19282 0.19282 0.628121799
CATSPER4 ENSG00000188782 -1.4199 0.92218 -1.4199 0.92218 -1.4199
0.92218 0.86743 0.19285 0.19285 0.628121799
CASC3 ENSG00000108349 0.45951 0.32293 0.86737 0.19287 0.41534 0.33895
0.45951 0.32293 0.19287 0.628121799
RPS27P1 ENSG00000223876 0.20423 0.41909 0.20423 0.41909 0.20423
0.41909 0.86736 0.19287 0.19287 0.628121799
CTAGE3P ENSG00000232872 0.20303 0.41956 0.86733 0.19288 0.789 0.21506
0.15143 0.43982 0.19288 0.628121799
NA ENSG00000240131 0.62949 0.26452 0.62949 0.26452 0.86725 0.1929
0.21638 0.41435 0.1929 0.628121799
HSPH1 ENSG00000120694 0.85472 0.19635 -0.16407 0.56516 0.86723 0.19291
-0.063168 0.52518 0.19291 0.628121799
CLN5 ENSG00000102805 0.86714 0.19293 0.86714 0.19293 0.86714 0.19293
0.34906 0.36352 0.19293 0.628121799
TRBJ2-1 ENSG00000211764 0.86715 0.19293 0.86715 0.19293 -0.72409
0.76549 -0.78063 0.78249 0.19293 0.628121799
TRBD1 ENSG00000282431 -1.2512 0.89456 0.86715 0.19293 0.86715 0.19293
0.86715 0.19293 0.19293 0.628121799
TDRD7 ENSG00000196116 -0.47927 0.68413 0.14737 0.44142 0.14737 0.44142
0.86713 0.19294 0.19294 0.628121799
ARHGAP42P2 ENSG00000231147 0.86708 0.19295 -0.81494 0.79245 0.3198
0.37456 0.088974 0.46455 0.19295 0.628121799
NA ENSG00000237955 0.86708 0.19295 0.776 0.21887 0.86708 0.19295
0.86708 0.19295 0.19295 0.628121799
AKIRIN1 ENSG00000174574 -0.39799 0.65468 -0.39799 0.65468 0.86705
0.19296 -1.526 0.93649 0.19296 0.628121799
NA ENSG00000237699 0.86701 0.19297 0.86701 0.19297 0.86701 0.19297
0.86701 0.19297 0.19297 0.628121799
SNORD115-34 ENSG00000199311 -1.6866 0.95416 -1.6866 0.95416 -1.6866
0.95416 0.86696 0.19298 0.19298 0.628121799
ZNF510 ENSG00000081386 0.044015 0.48245 0.73647 0.23072 0.86693
0.19299 -0.41031 0.65921 0.19299 0.628121799
ROBO1 ENSG00000169855 -0.25584 0.60096 0.86694 0.19299 -0.2031 0.58047
0.79681 0.21278 0.19299 0.628121799
C5orf64 ENSG00000178722 0.17636 0.43001 0.28682 0.38713 -0.13205
0.55253 0.86685 0.19301 0.19301 0.628121799
NA ENSG00000227914 0.20628 0.41829 0.20628 0.41829 0.20628 0.41829
0.86684 0.19301 0.19301 0.628121799
NA ENSG00000233262 0.8668 0.19302 0.8668 0.19302 0.8668 0.19302
0.8668 0.19302 0.19302 0.628121799
DHRS4L2 ENSG00000187630 0.6793 0.24847 0.86676 0.19304 0.10554
0.45798 0.23466 0.40723 0.19304 0.628121799
NA ENSG00000233926 0.86673 0.19304 0.86673 0.19304 0.50545 0.30662

0.76069 0.22342 0.19304 0.628121799
SPRYD7 ENSG00000123178 -0.82452 0.79518 0.86669 0.19306 -0.85595
0.80399 -1.0233 0.84691 0.19306 0.628121799
NA ENSG00000260580 -0.11952 0.54757 0.86668 0.19306 0.067915 0.47293
-0.86309 0.80596 0.19306 0.628121799
ZNF749 ENSG00000186230 0.53425 0.29659 0.86659 0.19308 -0.32905
0.62894 0.51846 0.30207 0.19308 0.628121799
CSRP3-AS1 ENSG00000255308 0.8666 0.19308 -0.3004 0.61806 0.77777
0.21835 0.13454 0.44649 0.19308 0.628121799
NRAS ENSG00000213281 0.86648 0.19311 0.86648 0.19311 0.86648 0.19311
0.86648 0.19311 0.19311 0.628121799
NA ENSG00000275438 0.8665 0.19311 0.15134 0.43985 0.49463 0.31043
-0.25478 0.60056 0.19311 0.628121799
NA ENSG00000233862 0.031092 0.4876 0.86643 0.19313 0.57167 0.28377
0.58853 0.27809 0.19313 0.628121799
UGT2B17 ENSG00000197888 -0.93859 0.82603 0.82905 0.20354 0.86637
0.19314 -0.18043 0.57159 0.19314 0.628121799
ZNF549 ENSG00000121406 0.06474 0.47419 -0.45376 0.675 -0.01713 0.50683
0.86633 0.19315 0.19315 0.628121799
RPL29P19 ENSG00000224594 -0.12346 0.54913 0.8663 0.19316 -1.3263
0.90763 0.40736 0.34187 0.19316 0.628121799
NA ENSG00000238045 0.86631 0.19316 0.86631 0.19316 0.86631 0.19316
0.86631 0.19316 0.19316 0.628121799
NA ENSG00000272051 -0.19666 0.57796 0.47235 0.31834 0.8663 0.19316
0.61654 0.26877 0.19316 0.628121799
RNU6-244P ENSG00000212330 0.86627 0.19317 0.86627 0.19317 0.86627
0.19317 0.86627 0.19317 0.19317 0.628121799
NA ENSG00000199591 0.84037 0.20035 0.86624 0.19318 0.86624 0.19318
0.86624 0.19318 0.19318 0.628121799
NA ENSG00000244471 0.86622 0.19318 -0.2352 0.59297 -0.16422 0.56522
0.48132 0.31514 0.19318 0.628121799
NA ENSG00000256552 -0.76956 0.77922 0.86608 0.19322 -0.086038 0.53428
-0.086038 0.53428 0.19322 0.628128207
PRR20A ENSG00000204919 -1.1012 0.8646 -0.714 0.76239 -0.714
0.76239 0.86597 0.19325 0.19325 0.628128207
UBR7 ENSG00000012963 -0.38836 0.65112 0.019371 0.49227 -1.0951 0.86325
0.86588 0.19328 0.19328 0.628128207
NA ENSG00000227186 0.86585 0.19329 0.86585 0.19329 0.86585 0.19329
0.86585 0.19329 0.19329 0.628128207
NA ENSG00000235485 0.86582 0.19329 -0.04505 0.51797 0.86582 0.19329
0.86582 0.19329 0.19329 0.628128207
RBM22P12 ENSG00000260631 0.57786 0.28168 0.86582 0.19329 0.1032
0.4589 -0.025245 0.51007 0.19329 0.628128207
KCTD14 ENSG00000151364 0.54307 0.29354 0.86575 0.19331 0.54307
0.29354 0.54307 0.29354 0.19331 0.628128207
C6orf120 ENSG00000185127 0.86574 0.19332 0.86574 0.19332 0.86574
0.19332 0.86574 0.19332 0.19332 0.628128207
FTH1P4 ENSG00000242992 -0.95575 0.8304 -0.99933 0.84118 -0.55446
0.71037 0.86575 0.19332 0.19332 0.628128207
LINC01951 ENSG00000204754 0.42772 0.33443 -0.68191 0.75235 0.86564
0.19334 -0.53023 0.70203 0.19334 0.628128207
VPS29 ENSG00000111237 0.16115 0.43599 0.40913 0.34122 0.71083 0.23859
0.8656 0.19335 0.19335 0.628128207
AP5Z1 ENSG00000242802 -0.37868 0.64754 0.29635 0.38348 -0.19971 0.57915
0.86553 0.19337 0.19337 0.628128207
NA ENSG00000200142 0.86544 0.1934 0.86544 0.1934 0.86544 0.1934
0.86544 0.1934 0.1934 0.628128207
MIR4450 ENSG00000263445 0.12231 0.45133 0.12231 0.45133 0.86544
0.1934 0.12231 0.45133 0.1934 0.628128207
IGDCC4 ENSG00000103742 0.26721 0.39465 0.13567 0.44604 0.86528
0.19344 0.26721 0.39465 0.19344 0.628128207
ARSK ENSG00000164291 0.55716 0.28871 -0.421 0.66312 0.55716 0.28871

0.86528 0.19344 0.19344 0.628128207
AADAT ENSG00000109576 -0.49999 0.69146 0.86525 0.19345 0.86525 0.19345
0.86525 0.19345 0.19345 0.628128207
GAL3ST2 ENSG00000154252 -0.56252 0.71312 0.86517 0.19347 -0.56252
0.71312 -0.56252 0.71312 0.19347 0.628128207
NA ENSG00000274341 0.86517 0.19347 0.86517 0.19347 0.86517 0.19347
0.86517 0.19347 0.19347 0.628128207
NMD3P2 ENSG00000231504 0.42733 0.33457 -0.79049 0.78538 0.74351
0.22859 0.86512 0.19349 0.19349 0.628128207
NA ENSG00000241002 -0.53353 0.70317 -0.53353 0.70317 -0.53353 0.70317
0.86512 0.19349 0.19349 0.628128207
MRM3 ENSG00000171861 0.38517 0.35006 0.84764 0.19832 0.86508 0.1935
-0.48938 0.68771 0.1935 0.628128207
NA ENSG00000233031 0.86508 0.1935 0.86508 0.1935 0.86508 0.1935
0.86508 0.1935 0.1935 0.628128207
ERFL ENSG00000268041 0.86505 0.19351 -0.58749 0.72156 -0.58749 0.72156
-0.58749 0.72156 0.19351 0.628128207
DBT ENSG00000137992 0.24127 0.40467 -0.10618 0.54228 0.86498 0.19352
-0.59426 0.72383 0.19352 0.628128207
NA ENSG00000273733 0.86501 0.19352 0.86501 0.19352 0.86501 0.19352
0.86501 0.19352 0.19352 0.628128207
NA ENSG00000266346 0.86497 0.19353 0.86497 0.19353 0.86497 0.19353
0.86497 0.19353 0.19353 0.628128207
FAM76A ENSG00000009780 0.14314 0.44309 0.31092 0.37793 -0.20303
0.58044 0.86483 0.19357 0.19357 0.628192344
INTS6-AS1 ENSG00000236778 0.56173 0.28715 -0.46539 0.67917 0.86475
0.19359 0.84589 0.19881 0.19359 0.628192344
NA ENSG00000259415 0.86473 0.19359 0.86473 0.19359 0.86473 0.19359
0.86473 0.19359 0.19359 0.628192344
ERVW-1 ENSG00000242950 -0.51961 0.69833 0.49084 0.31177 0.86469
0.19361 -0.37372 0.6457 0.19361 0.628192344
PLXNA1 ENSG00000114554 0.46248 0.32187 0.23202 0.40826 0.86458
0.19363 0.56094 0.28742 0.19363 0.628192344
NA ENSG00000281195 0.86461 0.19363 0.86461 0.19363 0.86461 0.19363
0.86461 0.19363 0.19363 0.628192344
DNAJC3 ENSG00000102580 0.86455 0.19364 0.54256 0.29372 0.86455
0.19364 0.47779 0.3164 0.19364 0.628192344
CBWD3 ENSG00000196873 0.86445 0.19367 -1.5514 0.93959 -1.9872 0.97655
-0.14163 0.55631 0.19367 0.628196645
ARF4P4 ENSG00000231222 0.86446 0.19367 0.86446 0.19367 -0.1915
0.57593 0.86446 0.19367 0.19367 0.628196645
LINC01399 ENSG00000233080 -0.71121 0.76152 -1.3751 0.91544 0.86442
0.19368 -0.2943 0.61573 0.19368 0.628196645
NA ENSG00000248419 0.86435 0.1937 0.86435 0.1937 0.86435 0.1937
0.86435 0.1937 0.1937 0.628210322
NA ENSG00000222335 0.86429 0.19371 0.86429 0.19371 0.86429 0.19371
0.86429 0.19371 0.19371 0.628210322
RPL35 ENSG00000136942 0.86398 0.1938 0.86398 0.1938 0.86398 0.1938
0.86398 0.1938 0.1938 0.628441586
RHBDD2 ENSG00000005486 0.04872 0.48057 -0.21869 0.58655 0.86395
0.19381 -0.0059838 0.50239 0.19381 0.628441586
NA ENSG00000262061 0.8639 0.19382 0.8639 0.19382 0.8639 0.19382
0.8639 0.19382 0.19382 0.628441586
H2BC9 ENSG00000275713 0.2431 0.40396 0.2431 0.40396 -0.63528 0.73738
0.86371 0.19387 0.19387 0.628561883
PPP2CB ENSG00000104695 -0.4527 0.67462 -0.65163 0.74268 0.86362
0.1939 -0.73628 0.76922 0.1939 0.628575506
HTR5A ENSG00000157219 0.86362 0.1939 0.86362 0.1939 0.86362 0.1939
0.86362 0.1939 0.1939 0.628575506
KATNB1 ENSG00000140854 -0.56181 0.71288 0.86352 0.19393 0.11079
0.45589 0.30953 0.37846 0.19393 0.628579727
C9orf135-DT ENSG00000225626 0.86349 0.19393 0.86349 0.19393 0.86349

0.19393 0.86349 0.19393 0.19393 0.628579727
RCBTB2 ENSG00000136161 0.86345 0.19394 -0.091639 0.53651 0.2699
0.39362 0.2134 0.41551 0.19394 0.628579727
IFNA7 ENSG00000214042 -0.058448 0.5233 0.86336 0.19397 -0.94938 0.82879
-0.94938 0.82879 0.19397 0.628587208
ALG1L7P ENSG00000251271 -0.942 0.8269 -0.72553 0.76594 0.86318
0.19402 0.47554 0.3172 0.19402 0.628587208
FPGT-TNNI3K ENSG00000259030 0.86318 0.19402 0.78203 0.2171 0.86318
0.19402 0.86318 0.19402 0.19402 0.628587208
TNRC18 ENSG00000182095 0.65092 0.25755 0.35698 0.36055 -0.95001
0.82895 0.86312 0.19404 0.19404 0.628587208
LINC01438 ENSG00000249519 0.53534 0.29621 0.53534 0.29621 0.86311
0.19404 0.7045 0.24056 0.19404 0.628587208
VN1R12P ENSG00000220581 -1.0668 0.85697 0.84469 0.19914 0.86308
0.19405 0.84469 0.19914 0.19405 0.628587208
NA ENSG00000214144 0.33154 0.37012 0.86303 0.19406 0.86303 0.19406
-0.30387 0.61938 0.19406 0.628587208
MRPL15P1 ENSG00000259441 0.86303 0.19406 0.86303 0.19406 0.86303
0.19406 0.86303 0.19406 0.19406 0.628587208
AP3M1 ENSG00000185009 0.4187 0.33772 0.15968 0.43657 0.86297 0.19408
0.5788 0.28136 0.19408 0.628587208
NA ENSG00000271370 0.86291 0.19409 0.86291 0.19409 0.86291 0.19409
0.86291 0.19409 0.19409 0.628587208
SELENON ENSG00000162430 -1.8803 0.96997 -1.8803 0.96997 -1.8803
0.96997 0.86287 0.1941 0.1941 0.628587208
HSPA14 ENSG00000187522 -0.39084 0.65204 -1.3528 0.91193 -0.39084
0.65204 0.86284 0.19411 0.19411 0.628587208
NA ENSG00000261367 0.86284 0.19411 0.86284 0.19411 0.86284 0.19411
0.86284 0.19411 0.19411 0.628587208
MIR200B ENSG00000207730 0.8627 0.19415 0.8627 0.19415 0.8627
0.19415 0.8627 0.19415 0.19415 0.628651492
MRTFB ENSG00000186260 0.86265 0.19417 0.16687 0.43374 0.56224 0.28698
0.27796 0.39052 0.19417 0.628651492
JRKL ENSG00000183340 0.86247 0.19421 0.86247 0.19421 0.86247 0.19421
0.86247 0.19421 0.19421 0.628651492
NA ENSG00000200026 0.86245 0.19422 0.86245 0.19422 0.087451 0.46516
-0.35141 0.63736 0.19422 0.628651492
NA ENSG00000267116 0.19764 0.42166 -0.66758 0.7478 0.86237 0.19424
-1.251 0.89452 0.19424 0.628651492
RAB31 ENSG00000168461 0.86222 0.19428 0.50033 0.30842 0.2713 0.39308
-0.29641 0.61654 0.19428 0.628651492
FRG1-DT ENSG00000245685 0.15814 0.43717 0.43753 0.33087 0.86219
0.19429 0.43482 0.33185 0.19429 0.628651492
NA ENSG00000229065 0.86211 0.19431 0.86211 0.19431 0.86211 0.19431
0.86211 0.19431 0.19431 0.628651492
RNA5SP155 ENSG00000223086 0.86204 0.19433 -0.65396 0.74343 -0.65396
0.74343 -0.65396 0.74343 0.19433 0.628651492
NA ENSG00000258702 0.3115 0.37771 0.86207 0.19433 0.39168 0.34765
0.43926 0.33024 0.19433 0.628651492
KRTAP9-3 ENSG00000204873 0.86201 0.19434 0.86201 0.19434 0.47835
0.3162 -1.9734 0.97577 0.19434 0.628651492
NA ENSG00000224400 0.86195 0.19436 0.23268 0.40801 -0.47634 0.68308
-0.47634 0.68308 0.19436 0.628651492
ZNF317 ENSG00000130803 0.082298 0.4672 0.21191 0.41609 0.74984
0.22668 0.86185 0.19438 0.19438 0.628651492
NA ENSG00000219039 -0.069433 0.52768 0.86188 0.19438 0.86188 0.19438
0.75214 0.22598 0.19438 0.628651492
LINC01127 ENSG00000281162 0.17824 0.42927 0.73121 0.23232 0.86186
0.19438 0.47541 0.31725 0.19438 0.628651492
IL2 ENSG00000109471 0.84519 0.199 0.86181 0.1944 0.84519 0.199 0.84519
0.199 0.1944 0.628651492
NA ENSG00000228923 0.86176 0.19441 0.86176 0.19441 0.86176 0.19441

0.86176 0.19441 0.19441 0.628651492
NA ENSG00000267327 -0.038476 0.51535 0.77936 0.21788 0.86178 0.19441
-0.74971 0.77329 0.19441 0.628651492
CFAP52 ENSG00000166596 0.86172 0.19442 -1.5828 0.94327 -0.56343
0.71343 -0.30546 0.61999 0.19442 0.628651492
NA ENSG00000256626 0.86169 0.19443 0.86169 0.19443 0.86169 0.19443
0.86169 0.19443 0.19443 0.628651492
NA ENSG00000249984 -0.048677 0.51941 0.58315 0.2799 -0.34765 0.63595
0.86157 0.19446 0.19446 0.628651492
NA ENSG00000255281 -0.093347 0.53719 0.86146 0.19449 -0.49519 0.68977
-0.49519 0.68977 0.19449 0.628651492
AKT1 ENSG00000142208 -0.65775 0.74465 0.3788 0.35242 -0.21063 0.58341
0.86125 0.19455 0.19455 0.628651492
RPSAP54 ENSG00000213621 0.86125 0.19455 0.86125 0.19455 0.86125
0.19455 -0.26268 0.6036 0.19455 0.628651492
NA ENSG00000274173 0.13823 0.44503 -0.37565 0.64641 0.86125 0.19455
-0.50195 0.69215 0.19455 0.628651492
NA ENSG00000248305 -0.13885 0.55522 -0.13885 0.55522 -0.13885 0.55522
0.86121 0.19456 0.19456 0.628651492
FAM218BP ENSG00000221891 0.86117 0.19457 0.096095 0.46172 0.86117
0.19457 0.86117 0.19457 0.19457 0.628651492
NA ENSG00000257105 0.86118 0.19457 -0.43542 0.66837 -0.42909 0.66607
0.085217 0.46604 0.19457 0.628651492
RNU7-106P ENSG00000238842 0.86114 0.19458 0.86114 0.19458 0.86114
0.19458 0.86114 0.19458 0.19458 0.628651492
NA ENSG00000222412 -0.9361 0.82539 0.86111 0.19459 -0.046198 0.51842
-0.43871 0.66956 0.19459 0.628651492
FCRL1 ENSG00000163534 0.75406 0.22541 -0.12643 0.5503 0.86103 0.19461
-0.95795 0.83096 0.19461 0.628651492
NA ENSG00000250577 0.86104 0.19461 0.86104 0.19461 0.86104 0.19461
0.86104 0.19461 0.19461 0.628651492
LINC01097 ENSG00000281202 0.86093 0.19464 -0.2445 0.59658 -1.1286
0.87046 -1.1561 0.87618 0.19464 0.628651492
MYH1 ENSG00000109061 0.39181 0.3476 0.86089 0.19465 0.86089 0.19465
0.86089 0.19465 0.19465 0.628651492
PTP4A2P1 ENSG00000267185 0.86077 0.19468 0.86077 0.19468 0.86077
0.19468 0.86077 0.19468 0.19468 0.628651492
NA ENSG00000277435 0.86079 0.19468 0.33369 0.36931 0.33369 0.36931
0.33369 0.36931 0.19468 0.628651492
NA ENSG00000267455 0.051779 0.47935 0.86074 0.19469 0.77699 0.21858
-0.3034 0.61921 0.19469 0.628651492
CKS2 ENSG00000123975 0.86067 0.19471 0.67464 0.24995 0.84353 0.19947
0.74561 0.22795 0.19471 0.628651492
MIR5692A1 ENSG00000266318 -0.8794 0.81041 -1.0439 0.85173 0.86053
0.19475 0.86053 0.19475 0.19475 0.628651492
OTOP3 ENSG00000182938 0.58081 0.28069 0.58081 0.28069 0.58081 0.28069
0.8605 0.19476 0.19476 0.628651492
MYH10 ENSG00000133026 -1.2573 0.89568 0.86047 0.19477 -1.0483 0.85276
-0.34613 0.63538 0.19477 0.628651492
EDEM2 ENSG00000088298 0.52882 0.29846 0.52882 0.29846 0.8604 0.19478
0.56096 0.28741 0.19478 0.628651492
SCARNA17 ENSG00000251992 0.86042 0.19478 0.86042 0.19478 0.86042
0.19478 0.86042 0.19478 0.19478 0.628651492
DGCR8 ENSG00000128191 -0.84547 0.80108 -0.24476 0.59668 -0.65691 0.74438
0.86037 0.19479 0.19479 0.628651492
RPL21P6 ENSG00000239199 0.86039 0.19479 0.86039 0.19479 0.86039
0.19479 0.86039 0.19479 0.19479 0.628651492
NA ENSG00000221704 -1.1509 0.87512 0.86039 0.19479 0.85054 0.19751
-1.123 0.86929 0.19479 0.628651492
SAR1A ENSG00000079332 0.86032 0.19481 0.11066 0.45594 0.7889 0.21508
0.33621 0.36836 0.19481 0.628651492
NA ENSG00000256070 -1.1897 0.88291 -2.1589 0.98457 0.86031 0.19481

0.30375 0.38066 0.19481 0.628651492
STUB1P1 ENSG00000226273 -0.31949 0.62532 -0.31949 0.62532 -0.64366
0.7401 0.86027 0.19482 0.19482 0.628651492
FAM177A1 ENSG00000151327 0.24315 0.40395 0.30467 0.38031 0.86019
0.19484 0.7188 0.23613 0.19484 0.628651492
SLC17A3 ENSG00000124564 -0.098366 0.53918 -0.098366 0.53918 0.27292
0.39246 0.86015 0.19485 0.19485 0.628651492
NA ENSG00000240484 0.86013 0.19486 0.38122 0.35152 -0.26888 0.60599
0.40183 0.3439 0.19486 0.628651492
OSMR-AS1 ENSG00000249740 0.86014 0.19486 0.72539 0.23411 -0.29372
0.61551 -0.99031 0.83899 0.19486 0.628651492
RNU6-1141P ENSG00000201390 0.86007 0.19487 0.86007 0.19487 0.86007
0.19487 0.86007 0.19487 0.19487 0.628651492
RNU6-143P ENSG00000252751 -0.79786 0.78753 -1.2625 0.89662 0.86011
0.19487 0.37694 0.35311 0.19487 0.628651492
VN2R19P ENSG00000235974 -0.20462 0.58107 -0.20462 0.58107 -0.44868
0.67317 0.85999 0.1949 0.1949 0.628651492
MSTN ENSG00000138379 0.31556 0.37617 -0.16032 0.56368 0.8599 0.19492
-0.12433 0.54947 0.19492 0.628651492
OR8B8 ENSG00000197125 0.85991 0.19492 0.85991 0.19492 -0.26682 0.6052
0.52898 0.29841 0.19492 0.628651492
ZNF587P1 ENSG00000223965 0.24661 0.40261 0.85991 0.19492 -0.52646
0.70072 0.272 0.39281 0.19492 0.628651492
NA ENSG00000250657 0.14638 0.44181 0.14638 0.44181 0.85989 0.19492
0.14638 0.44181 0.19492 0.628651492
RNA5SP193 ENSG00000222285 0.022603 0.49098 0.022603 0.49098 -0.29472
0.6159 0.85978 0.19495 0.19495 0.628651492
RN7SKP109 ENSG00000222524 0.85978 0.19495 0.85978 0.19495 0.85978
0.19495 0.85978 0.19495 0.19495 0.628651492
RPS10P7 ENSG00000223396 0.23241 0.40811 0.004931 0.49803 -0.56826
0.71507 0.85975 0.19496 0.19496 0.628651492
UNG ENSG00000076248 -0.22912 0.59061 0.85969 0.19498 -0.55081 0.70912
-0.19506 0.57733 0.19498 0.628651492
PRR5-ARHGAP8 ENSG00000248405 0.85962 0.195 0.85962 0.195 0.85962 0.195
0.85962 0.195 0.195 0.628651492
CENPS-CORT ENSG00000251503 0.85961 0.195 0.85961 0.195 0.85961 0.195 0.85961
0.195 0.195 0.628651492
NA ENSG00000240086 -0.38612 0.65029 0.85958 0.19501 -0.72765 0.76659
0.77365 0.21957 0.19501 0.628651492
NA ENSG00000253582 0.85958 0.19501 -2.0352 0.97909 -2.0352 0.97909
-2.0352 0.97909 0.19501 0.628651492
LINC00605 ENSG00000251533 0.85954 0.19502 0.85954 0.19502 0.61668
0.26872 0.85954 0.19502 0.19502 0.628651492
TIMP4 ENSG00000157150 0.75559 0.22495 0.85943 0.19505 0.75559 0.22495
0.75559 0.22495 0.19505 0.628697255
NA ENSG00000276949 0.12872 0.44879 0.8594 0.19506 -1.4359 0.92448
-1.4359 0.92448 0.19506 0.628697255
MAPKAP1 ENSG00000119487 0.63067 0.26413 0.43033 0.33348 0.8592
0.19512 -0.36227 0.64142 0.19512 0.62874717
NRBP2 ENSG00000185189 0.85917 0.19512 -1.139 0.87264 -0.72596 0.76607
-0.72596 0.76607 0.19512 0.62874717
SVOP ENSG00000166111 -0.97004 0.83399 0.85912 0.19514 -0.73365 0.76842
-0.098647 0.53929 0.19514 0.62874717
KRT18P51 ENSG00000250504 0.85912 0.19514 0.68817 0.24567 -0.85418
0.8035 -0.22498 0.589 0.19514 0.62874717
MIR548AO ENSG00000263372 0.8591 0.19514 0.8591 0.19514 0.8591
0.19514 0.8591 0.19514 0.19514 0.62874717
NA ENSG00000255451 0.014488 0.49422 0.51126 0.30459 0.24619 0.40277
0.85905 0.19516 0.19516 0.628770036
NA ENSG00000235010 0.0029922 0.49881 0.85875 0.19524 0.85875 0.19524
0.85875 0.19524 0.19524 0.628869746
NA ENSG00000272604 0.85871 0.19525 0.85871 0.19525 0.85871 0.19525

0.85871 0.19525 0.19525 0.628869746
DYNLL2 ENSG00000264364 0.34042 0.36677 0.85864 0.19527 0.85864
0.19527 0.39245 0.34736 0.19527 0.628869746
HSPD1P16 ENSG00000218792 0.85856 0.19529 0.095991 0.46176 0.095991
0.46176 0.095991 0.46176 0.19529 0.628869746
PIEZO2 ENSG00000154864 -1.1447 0.87384 -0.9078 0.81801 -0.57011
0.7157 0.85852 0.1953 0.1953 0.628869746
CALM2P2 ENSG00000229097 0.85852 0.1953 0.85852 0.1953 0.85852
0.1953 0.85852 0.1953 0.1953 0.628869746
NA ENSG00000237501 0.34845 0.36375 0.85855 0.1953 0.34845 0.36375
0.34845 0.36375 0.1953 0.628869746
NA ENSG00000279114 0.19362 0.42324 0.85851 0.19531 0.19362 0.42324
0.19362 0.42324 0.19531 0.628869746
CAMK2G ENSG00000148660 0.60079 0.27399 -0.057535 0.52294 0.85844
0.19532 0.32615 0.37216 0.19532 0.628869746
NA ENSG00000249053 -0.0017015 0.50068 -0.0017015 0.50068 -0.0017015 0.50068
0.85847 0.19532 0.19532 0.628869746
NA ENSG00000202078 0.85835 0.19535 0.85835 0.19535 0.85835 0.19535
0.85835 0.19535 0.19535 0.628906071
RFC3 ENSG00000133119 -0.69769 0.75731 -0.10152 0.54043 0.28762 0.38682
0.85832 0.19536 0.19536 0.628906071
FBXO45 ENSG00000174013 -0.54812 0.7082 -1.084 0.86083 0.85827
0.19537 -1.5436 0.93865 0.19537 0.628906071
LILRB5 ENSG00000105609 0.85819 0.19539 0.85819 0.19539 0.85819
0.19539 0.142 0.44354 0.19539 0.628928905
NA ENSG00000279117 0.8581 0.19542 0.8581 0.19542 0.8581 0.19542
0.8581 0.19542 0.19542 0.628983923
LYPLA1 ENSG00000120992 0.43472 0.33188 0.43472 0.33188 0.85796
0.19546 0.13778 0.44521 0.19546 0.629071118
NA ENSG00000233635 0.8578 0.1955 0.8578 0.1955 0.8578 0.1955
0.8578 0.1955 0.1955 0.629158301
RPS4XP11 ENSG00000234335 -0.8733 0.80875 -0.51304 0.69604 0.85773
0.19552 0.85773 0.19552 0.19552 0.629181113
NA ENSG00000265229 0.85762 0.19555 0.75407 0.2254 -0.085236 0.53396
-0.085236 0.53396 0.19555 0.6292361
PARP12 ENSG00000059378 0.85744 0.1956 0.85744 0.1956 0.85744
0.1956 0.12798 0.44908 0.1956 0.629355431
NA ENSG00000223866 0.85734 0.19563 0.85734 0.19563 0.85734 0.19563
0.85734 0.19563 0.19563 0.629362192
MED16 ENSG00000175221 -0.079918 0.53185 0.85727 0.19565 0.55247 0.29031
-0.75744 0.77561 0.19565 0.629362192
NA ENSG00000221734 0.85725 0.19565 0.85725 0.19565 0.85725 0.19565
0.85725 0.19565 0.19565 0.629362192
ZNF77 ENSG00000175691 0.24419 0.40354 -0.60559 0.72761 0.85716 0.19568
0.44058 0.32976 0.19568 0.629362192
MEOX1 ENSG00000005102 0.63211 0.26366 0.50025 0.30845 0.48209 0.31487
0.85707 0.1957 0.1957 0.629362192
ATP4B ENSG00000186009 0.052029 0.47925 -1.5933 0.94445 0.85704 0.19571
-1.3238 0.90722 0.19571 0.629362192
GPRC5D ENSG00000111291 0.85697 0.19573 0.85697 0.19573 0.85697
0.19573 0.85697 0.19573 0.19573 0.629362192
RAE1 ENSG00000101146 0.53274 0.29711 0.40371 0.34321 0.85695 0.19574
-1.5217 0.93595 0.19574 0.629362192
H3P28 ENSG00000220347 -0.32289 0.62661 -0.15768 0.56264 -0.46261 0.67818
0.85693 0.19574 0.19574 0.629362192
SUCLG2P4 ENSG00000249226 0.027798 0.48891 0.8569 0.19575 -0.23563
0.59314 -0.14177 0.55637 0.19575 0.629362192
NA ENSG00000212712 0.85685 0.19576 0.85685 0.19576 0.79743 0.2126
0.85685 0.19576 0.19576 0.629362192
FAM78B ENSG00000188859 0.069395 0.47234 -1.4697 0.92918 -0.31265
0.62273 0.85685 0.19577 0.19577 0.629362192
NA ENSG00000250969 0.85684 0.19577 -0.31943 0.6253 0.85684 0.19577

0.18202 0.42778 0.19577 0.629362192
KLHDC8A ENSG00000162873 -0.20851 0.58258 0.14097 0.44395 -0.7061
0.75994 0.85676 0.19579 0.19579 0.629384966
HAUS6P1 ENSG00000227344 0.85662 0.19583 0.85662 0.19583 0.85662
0.19583 0.75157 0.22615 0.19583 0.629472025
NA ENSG00000279214 0.85649 0.19586 0.85649 0.19586 0.85649 0.19586
0.85649 0.19586 0.19586 0.629514793
RNU6-1242P ENSG00000212354 0.85643 0.19588 0.85643 0.19588 0.85643
0.19588 0.85643 0.19588 0.19588 0.629514793
MIR30E ENSG00000198974 0.85633 0.19591 0.80652 0.20997 0.80652
0.20997 0.80652 0.20997 0.19591 0.629514793
HNRNPRP1 ENSG00000223984 0.85633 0.19591 0.85633 0.19591 0.85633
0.19591 0.85633 0.19591 0.19591 0.629514793
NA ENSG00000267683 0.43653 0.33123 0.26704 0.39472 0.85631 0.19591
0.85631 0.19591 0.19591 0.629514793
BRD3 ENSG00000169925 0.50896 0.30539 -0.11433 0.54551 0.44183 0.32931
0.85609 0.19597 0.19597 0.629514793
GRIA1 ENSG00000155511 0.84359 0.19945 -0.048543 0.51936 0.85608 0.19598
0.16501 0.43447 0.19598 0.629514793
RORB ENSG00000198963 0.85605 0.19599 -0.52132 0.69893 -0.73062 0.76749
-0.62737 0.73479 0.19599 0.629514793
NA ENSG00000237188 0.85598 0.196 0.85598 0.196 0.85598 0.196 0.85598 0.196
0.196 0.629514793
NA ENSG00000248506 0.85594 0.19601 0.63291 0.2634 -1.0591 0.85522
0.75349 0.22558 0.19601 0.629514793
NA ENSG00000279758 0.85595 0.19601 0.85595 0.19601 0.85595 0.19601
0.85595 0.19601 0.19601 0.629514793
CEP112 ENSG00000154240 -0.15585 0.56192 0.85591 0.19602 0.37962
0.35212 0.3718 0.35502 0.19602 0.629514793
ZNF426-DT ENSG00000278611 0.79699 0.21273 0.85593 0.19602 0.48813
0.31273 0.48813 0.31273 0.19602 0.629514793
DIP2A ENSG00000160305 0.85588 0.19603 0.26248 0.39647 0.39478 0.3465
0.39478 0.3465 0.19603 0.629514793
TRAPPC10 ENSG00000160218 0.40036 0.34444 0.77916 0.21794 0.85585
0.19604 0.72254 0.23498 0.19604 0.629514793
FOXO6-AS1 ENSG00000229901 0.8558 0.19605 0.29856 0.38264 0.23067
0.40878 0.23067 0.40878 0.19605 0.629514793
ADAD1P2 ENSG00000267511 0.85573 0.19607 0.8506 0.1975 0.60315
0.2732 0.60315 0.2732 0.19607 0.62953753
NA ENSG00000270941 0.85568 0.19609 0.85568 0.19609 0.85568 0.19609
0.34297 0.36581 0.19609 0.629540133
IGLC2 ENSG00000211677 0.85555 0.19612 0.85555 0.19612 0.85555 0.19612
0.85555 0.19612 0.19612 0.629540133
LINC00283 ENSG00000231633 0.85553 0.19613 0.85553 0.19613 0.85553
0.19613 0.85553 0.19613 0.19613 0.629540133
SERPINB5 ENSG00000206075 -1.255 0.89525 0.85542 0.19616 -0.98013
0.83649 -0.39029 0.65184 0.19616 0.629540133
DAB1 ENSG00000173406 -0.19692 0.57806 -0.04739 0.5189 0.59481 0.27598
0.85539 0.19617 0.19617 0.629540133
TMF1 ENSG00000144747 0.42411 0.33574 0.52099 0.30119 0.42411 0.33574
0.85535 0.19618 0.19618 0.629540133
NA ENSG00000267984 0.85532 0.19619 0.85532 0.19619 0.85532 0.19619
0.85532 0.19619 0.19619 0.629540133
C3orf70 ENSG00000187068 0.85528 0.1962 -0.57039 0.71579 0.33961
0.36707 -0.13358 0.55313 0.1962 0.629540133
NA ENSG00000255188 0.044003 0.48245 0.85528 0.1962 -1.2209 0.88894
-1.2209 0.88894 0.1962 0.629540133
NA ENSG00000258632 0.85529 0.1962 -1.3471 0.91103 -3.2688 0.99946
-3.2688 0.99946 0.1962 0.629540133
RGS3 ENSG00000138835 0.66337 0.25355 0.56393 0.2864 0.85509 0.19625
0.61545 0.26913 0.19625 0.629659106
RPSAP26 ENSG00000242735 0.85504 0.19627 0.85504 0.19627 0.85504

0.19627 0.85504 0.19627 0.19627 0.629681816
QRFP ENSG00000188710 0.8549 0.1963 0.05075 0.47976 0.05075 0.47976
0.05075 0.47976 0.1963 0.629708478
TMEM79 ENSG00000163472 0.85489 0.19631 0.85489 0.19631 0.85489
0.19631 0.85489 0.19631 0.19631 0.629708478
DENND3 ENSG00000105339 0.14067 0.44406 0.85482 0.19633 0.41788
0.33802 0.1529 0.43924 0.19633 0.629708478
NA ENSG00000261804 0.85479 0.19633 0.85479 0.19633 0.65554 0.25606
0.5571 0.28873 0.19633 0.629708478
FRY-AS1 ENSG00000237637 -0.34333 0.63432 0.85474 0.19635 0.30366
0.38069 0.34377 0.36551 0.19635 0.629724281
NA ENSG00000273035 0.85463 0.19638 0.85463 0.19638 0.85463 0.19638
0.85463 0.19638 0.19638 0.629724281
COL18A1-AS1 ENSG00000183535 -0.68177 0.75231 -1.2245 0.88963 0.68161
0.24774 0.85459 0.19639 0.19639 0.629724281
NA ENSG00000258580 -0.29123 0.61456 0.85454 0.1964 -0.29123 0.61456
-0.16867 0.56697 0.1964 0.629724281
RPS9P1 ENSG00000214889 0.85448 0.19642 0.85448 0.19642 0.85448
0.19642 0.85448 0.19642 0.19642 0.629724281
NA ENSG00000279755 0.13846 0.44494 0.13846 0.44494 0.13846 0.44494
0.85437 0.19645 0.19645 0.629724281
NA ENSG00000225792 0.85435 0.19646 0.85435 0.19646 0.85435 0.19646
0.85435 0.19646 0.19646 0.629724281
TRAJ14 ENSG00000211875 0.85431 0.19647 0.85431 0.19647 0.85431
0.19647 0.85431 0.19647 0.19647 0.629724281
OR52P2P ENSG00000171999 0.85426 0.19648 0.85426 0.19648 0.85426
0.19648 0.85426 0.19648 0.19648 0.629724281
UBE2V1P11 ENSG00000231124 0.85427 0.19648 0.85427 0.19648 0.85427
0.19648 0.85427 0.19648 0.19648 0.629724281
TRIM38 ENSG00000112343 0.37957 0.35213 0.70394 0.24073 0.85423
0.19649 0.56578 0.28577 0.19649 0.629724281
NA ENSG00000244756 0.32911 0.37104 0.85421 0.19649 -0.14086 0.55601
-0.14086 0.55601 0.19649 0.629724281
NA ENSG00000267764 0.85409 0.19653 -0.057143 0.52278 -0.70643 0.76004
-0.39626 0.65404 0.19653 0.62976421
C12orf42 ENSG00000179088 0.85405 0.19654 0.026301 0.48951 -0.010439
0.50416 0.80714 0.20979 0.19654 0.62976421
ST13P5 ENSG00000212789 0.85399 0.19656 0.34516 0.36499 0.85399
0.19656 0.85399 0.19656 0.19656 0.62976421
NA ENSG00000232754 0.85398 0.19656 0.85398 0.19656 0.85398 0.19656
0.85398 0.19656 0.19656 0.62976421
OR10H4 ENSG00000176231 0.85389 0.19658 0.15239 0.43944 -1.6819
0.9537 -1.6819 0.9537 0.19658 0.62976421
GSTP1P1 ENSG00000257569 0.85392 0.19658 0.50072 0.30828 0.85392
0.19658 0.85392 0.19658 0.19658 0.62976421
NPM1P46 ENSG00000213104 -0.38749 0.6508 -0.1607 0.56383 -0.11311
0.54503 0.85378 0.19661 0.19661 0.629772084
ZNF480 ENSG00000198464 0.023255 0.49072 0.60863 0.27139 0.25733
0.39846 0.85365 0.19665 0.19665 0.629772084
NDRG1 ENSG00000104419 0.85363 0.19666 0.85363 0.19666 0.85363 0.19666
0.47481 0.31746 0.19666 0.629772084
PRDX3 ENSG00000165672 -0.48912 0.68762 -0.7501 0.7734 0.85361 0.19666
-0.13889 0.55523 0.19666 0.629772084
CLDN6 ENSG00000184697 -0.87493 0.8092 0.85362 0.19666 0.11847 0.45285
0.11847 0.45285 0.19666 0.629772084
NA ENSG00000271335 0.8536 0.19666 0.8536 0.19666 0.42847 0.33416
-0.51574 0.69698 0.19666 0.629772084
CLU ENSG00000120885 -0.40068 0.65567 -0.698 0.75741 0.85347 0.1967
-1.5638 0.94107 0.1967 0.629808035
LIG3 ENSG00000005156 -0.42791 0.66564 -0.40736 0.65813 0.85344 0.19671
-0.48975 0.68785 0.19671 0.629808035
NA ENSG00000251243 -0.80183 0.78867 -1.647 0.95022 -1.647 0.95022

0.85342 0.19671 0.19671 0.629808035
IBA57 ENSG00000181873 -0.59067 0.72263 -1.3329 0.90872 0.85329 0.19675
-0.28462 0.61203 0.19675 0.62988535
LINC02875 ENSG00000187013 0.25947 0.39764 0.85326 0.19676 -1.3094
0.9048 0.46479 0.32104 0.19676 0.62988535
ZCCHC24 ENSG00000165424 0.85309 0.1968 -0.63449 0.73712 -0.22362
0.58847 -0.090345 0.53599 0.1968 0.629930636
CD55 ENSG00000196352 0.8531 0.1968 0.20738 0.41786 -0.12663 0.55038
0.20738 0.41786 0.1968 0.629930636
ERVE-1 ENSG00000267259 0.5474 0.29205 0.72827 0.23323 0.85303
0.19682 0.16848 0.4331 0.19682 0.629943905
LINC01825 ENSG00000223466 -0.78229 0.78298 0.85301 0.19683 0.85301
0.19683 0.13297 0.44711 0.19683 0.629943905
RPS29P33 ENSG00000226470 0.21529 0.41477 0.15137 0.43984 0.85285
0.19687 0.21529 0.41477 0.19687 0.630030544
NA ENSG00000267044 0.079937 0.46814 0.079937 0.46814 0.85279 0.19689
0.079937 0.46814 0.19689 0.630053172
SERPINF2 ENSG00000167711 0.85259 0.19694 0.23615 0.40666 0.70019
0.24191 0.70019 0.24191 0.19694 0.630166264
NA ENSG00000266806 0.85249 0.19697 0.85249 0.19697 0.39113 0.34785
-0.25974 0.60247 0.19697 0.630166264
SPART-AS1 ENSG00000120664 0.69131 0.24468 0.85247 0.19698 0.84143
0.20005 -0.29862 0.61739 0.19698 0.630166264
FRA10AC1 ENSG00000148690 0.85245 0.19698 -1.5965 0.94482 -1.44 0.92507
-0.50976 0.69489 0.19698 0.630166264
MRTO4 ENSG00000053372 0.85241 0.19699 0.56401 0.28637 0.48984 0.31212
0.25107 0.40088 0.19699 0.630166264
NA ENSG00000280083 0.85236 0.19701 -0.29448 0.6158 0.25005 0.40127
0.62887 0.26472 0.19701 0.630188874
CFHR2 ENSG00000080910 0.7449 0.22817 0.85219 0.19705 0.21565 0.41463
-0.40928 0.65883 0.19705 0.630266067
RPL21P136 ENSG00000271215 0.85216 0.19706 0.85216 0.19706 0.85216
0.19706 0.85216 0.19706 0.19706 0.630266067
MRPS22 ENSG00000175110 -1.1645 0.87789 -0.39664 0.65418 -1.7487
0.95983 0.85202 0.1971 0.1971 0.630300144
NA ENSG00000253845 -0.37457 0.64601 0.85194 0.19712 -0.37457 0.64601
-0.37457 0.64601 0.19712 0.630300144
TAF13 ENSG00000197780 0.037587 0.48501 0.037587 0.48501 0.85184 0.19715
-1.0317 0.84889 0.19715 0.630300144
NA ENSG00000253271 0.85181 0.19716 0.85181 0.19716 0.85181 0.19716
0.85181 0.19716 0.19716 0.630300144
NA ENSG00000261663 0.85182 0.19716 0.85182 0.19716 0.85182 0.19716
0.85182 0.19716 0.19716 0.630300144
NA ENSG00000266196 0.85182 0.19716 0.85182 0.19716 0.85182 0.19716
0.85182 0.19716 0.19716 0.630300144
MORC2-AS1 ENSG00000235989 -2.0545 0.98004 -1.5156 0.93519 0.85171
0.19719 -0.5552 0.71062 0.19719 0.630300144
RILPL2 ENSG00000150977 -1.1899 0.88297 -1.0232 0.8469 0.85168
0.1972 -0.55783 0.71152 0.1972 0.630300144
TMEM229B ENSG00000198133 0.85165 0.1972 0.61898 0.26796 0.1781
0.42932 -0.24716 0.59761 0.1972 0.630300144
NA ENSG00000279333 0.85167 0.1972 0.85167 0.1972 0.85167 0.1972
0.85167 0.1972 0.1972 0.630300144
SENP2 ENSG00000163904 0.14246 0.44336 -0.2941 0.61566 0.8516 0.19722
0.45775 0.32357 0.19722 0.630322726
SERPINI1 ENSG00000163536 0.8514 0.19727 0.8514 0.19727 0.05089
0.47971 0.8514 0.19727 0.19727 0.630371689
RPL35P8 ENSG00000226142 0.85137 0.19728 0.22615 0.41054 0.85137
0.19728 0.84631 0.19869 0.19728 0.630371689
MIR466 ENSG00000265376 0.85137 0.19728 0.85137 0.19728 0.85137
0.19728 0.85137 0.19728 0.19728 0.630371689
PSMD5 ENSG00000095261 -0.67979 0.75168 -1.6264 0.94806 0.85131 0.1973

-0.18151 0.57202 0.1973 0.630371689
TRAV8-1 ENSG00000211782 0.38254 0.35103 0.38254 0.35103 0.85132
0.1973 0.38254 0.35103 0.1973 0.630371689
MIR623 ENSG00000207719 0.25732 0.39847 0.85123 0.19732 0.25732
0.39847 0.25732 0.39847 0.19732 0.630394257
LRRC69 ENSG00000214954 0.70628 0.24001 0.85112 0.19735 0.85112
0.19735 0.10127 0.45967 0.19735 0.630448768
DMRT3 ENSG00000064218 0.851 0.19738 -0.98815 0.83846 0.73256 0.23191
-1.249 0.89417 0.19738 0.630461943
BCAS2P3 ENSG00000226670 -0.55819 0.71164 0.85103 0.19738 -1.66 0.95154
-0.088501 0.53526 0.19738 0.630461943
ALMS1 ENSG00000116127 0.73741 0.23044 0.8509 0.19741 0.16085 0.4361
-0.27636 0.60886 0.19741 0.630507052
FAXDC2 ENSG00000170271 0.27466 0.39179 0.23751 0.40613 -0.073729
0.52939 0.85087 0.19742 0.19742 0.630507052
NA ENSG00000249199 0.10199 0.45938 0.49213 0.31131 0.85077 0.19745
0.10091 0.45981 0.19745 0.630561537
ABCA5 ENSG00000154265 0.85068 0.19747 -0.18039 0.57158 0.37131 0.3552
0.85068 0.19747 0.19747 0.630584083
NA ENSG00000225087 0.85058 0.1975 -0.12365 0.54921 -0.36238 0.64147
0.73978 0.22972 0.1975 0.630638556
LRRC18 ENSG00000165383 -0.71963 0.76412 -0.44707 0.67259 -0.43825
0.6694 0.85052 0.19752 0.19752 0.63065545
NA ENSG00000258957 -1.4132 0.92121 -1.4132 0.92121 -0.57423 0.71709
0.85044 0.19754 0.19754 0.63065545
NA ENSG00000230537 0.85037 0.19756 0.85037 0.19756 0.85037 0.19756
0.85037 0.19756 0.19756 0.63065545
NA ENSG00000260364 -0.76742 0.77858 0.11366 0.45475 -0.48285 0.6854
0.85032 0.19757 0.19757 0.63065545
NA ENSG00000279423 0.18542 0.42645 0.10313 0.45893 0.85034 0.19757
0.85034 0.19757 0.19757 0.63065545
ZBTB37 ENSG00000185278 0.35715 0.36049 0.35715 0.36049 0.85028
0.19759 0.35715 0.36049 0.19759 0.630677979
SPRR2B ENSG00000196805 -0.55362 0.71008 -0.55362 0.71008 -0.94498
0.82766 0.8501 0.19763 0.19763 0.630685465
ADI1P2 ENSG00000250684 0.85008 0.19764 0.85008 0.19764 0.85008
0.19764 0.85008 0.19764 0.19764 0.630685465
TSPAN15 ENSG00000099282 -1.043 0.85153 0.85004 0.19765 0.249 0.40168
-0.67489 0.75013 0.19765 0.630685465
NA ENSG00000229652 0.85006 0.19765 0.85006 0.19765 0.85006 0.19765
0.85006 0.19765 0.19765 0.630685465
RPL7P36 ENSG00000234109 0.42382 0.33585 0.85002 0.19766 0.85002
0.19766 -0.2546 0.60048 0.19766 0.630685465
MROH7-TTC4 ENSG00000271723 0.84999 0.19767 0.84999 0.19767 0.84999
0.19767 0.84999 0.19767 0.19767 0.630685465
OR9K1P ENSG00000196534 0.036193 0.48556 -0.27311 0.60762 0.8498
0.19772 -0.27311 0.60762 0.19772 0.630724838
NA ENSG00000277491 0.8498 0.19772 0.8498 0.19772 0.8498 0.19772
0.8498 0.19772 0.19772 0.630724838
NLRP1 ENSG00000091592 0.75566 0.22493 -0.013805 0.50551 -0.90612 0.81756
0.84974 0.19773 0.19773 0.630724838
RN7SL678P ENSG00000263969 0.84971 0.19774 0.84971 0.19774 0.84971
0.19774 0.84971 0.19774 0.19774 0.630724838
RPL31P12 ENSG00000227207 0.79826 0.21236 0.84967 0.19775 0.6696
0.25155 0.84967 0.19775 0.19775 0.630724838
MIR770 ENSG00000211574 0.84966 0.19776 0.84966 0.19776 0.84966
0.19776 0.84966 0.19776 0.19776 0.630724838
OR1K1 ENSG00000165204 0.3783 0.3526 0.21796 0.41373 0.21205 0.41603
0.84942 0.19782 0.19782 0.630758519
NA ENSG00000233648 -1.3287 0.90803 -1.3287 0.90803 -0.90144 0.81632
0.84941 0.19783 0.19783 0.630758519
NA ENSG00000269901 0.84937 0.19784 -0.032708 0.51305 0.31564 0.37614

0.66132 0.2542 0.19784 0.630758519
PRR26 ENSG00000180525 -0.13734 0.55462 -0.62887 0.73528 0.8493 0.19786
-1.6242 0.94783 0.19786 0.630758519
OR2M3 ENSG00000228198 -0.66723 0.74769 -0.66723 0.74769 0.84931 0.19786
-0.47857 0.68388 0.19786 0.630758519
CAPN8 ENSG00000203697 0.053045 0.47885 0.84924 0.19787 0.075285 0.46999
0.68601 0.24635 0.19787 0.630758519
DENND10 ENSG00000119979 -0.5948 0.72401 0.24948 0.4015 0.81723
0.2069 0.84922 0.19788 0.19788 0.630758519
VAV1 ENSG00000141968 0.3172 0.37555 -0.15599 0.56198 -1.3269 0.90773
0.84924 0.19788 0.19788 0.630758519
NEK6 ENSG00000119408 -1.0104 0.84386 0.84918 0.19789 0.70729 0.23969
0.4702 0.31911 0.19789 0.630758519
NSMCE1-DT ENSG00000245888 0.52837 0.29862 0.52837 0.29862 0.39597
0.34606 0.84913 0.1979 0.1979 0.630758519
USHBP1 ENSG00000130307 0.76155 0.22316 0.84897 0.19795 -0.1942
0.57699 0.026256 0.48953 0.19795 0.630825974
KPNA7 ENSG00000185467 -1.1775 0.8805 -0.69124 0.75529 -0.38013 0.64808
0.84898 0.19795 0.19795 0.630825974
ANO1 ENSG00000131620 0.81175 0.20847 0.84892 0.19796 -0.20145 0.57983
-0.33227 0.63016 0.19796 0.630825974
KRTAP21-4P ENSG00000236400 0.84868 0.19803 0.84868 0.19803 0.84868
0.19803 0.84868 0.19803 0.19803 0.630932638
NA ENSG00000262833 0.68314 0.24726 0.84869 0.19803 0.72895 0.23302
-0.26346 0.6039 0.19803 0.630932638
IGFBP3 ENSG00000146674 0.29272 0.38487 0.84862 0.19805 -0.069353
0.52765 0.29466 0.38413 0.19805 0.630932638
NA ENSG00000248988 0.84859 0.19806 0.84859 0.19806 0.84859 0.19806
0.84859 0.19806 0.19806 0.630932638
NA ENSG00000231403 -1.1254 0.86979 0.84851 0.19808 -1.1254 0.86979
-0.23677 0.59358 0.19808 0.630932638
NA ENSG00000257398 0.31181 0.37759 0.70339 0.24091 0.84841 0.1981
0.84841 0.1981 0.1981 0.630932638
TRIM9 ENSG00000100505 0.50741 0.30593 0.02361 0.49058 0.84838 0.19811
0.5583 0.28832 0.19811 0.630932638
CEPT1 ENSG00000134255 0.19324 0.42339 0.84837 0.19811 0.84837 0.19811
0.84837 0.19811 0.19811 0.630932638
NA ENSG00000224935 0.84839 0.19811 0.021124 0.49157 0.014987 0.49402
0.69255 0.2443 0.19811 0.630932638
ZNF785 ENSG00000197162 0.84826 0.19815 0.84826 0.19815 0.84826
0.19815 0.84826 0.19815 0.19815 0.630977553
NA ENSG00000239791 0.84826 0.19815 0.84826 0.19815 0.84826 0.19815
0.84826 0.19815 0.19815 0.630977553
TUBB1 ENSG00000101162 0.84814 0.19818 0.10891 0.45664 0.84814 0.19818
-0.46237 0.67809 0.19818 0.630984103
NA ENSG00000254680 0.84796 0.19823 0.84796 0.19823 0.84796 0.19823
0.84796 0.19823 0.19823 0.630984103
ILKAP ENSG00000132323 0.84783 0.19827 0.40785 0.34169 0.51087 0.30472
0.27833 0.39038 0.19827 0.630984103
NA ENSG00000212197 0.84782 0.19827 0.84782 0.19827 0.84782 0.19827
0.84782 0.19827 0.19827 0.630984103
NA ENSG00000255365 0.8478 0.19828 0.8478 0.19828 0.8478 0.19828
0.8478 0.19828 0.19828 0.630984103
ZMYND11 ENSG00000015171 -0.44266 0.67099 0.082868 0.46698 0.84772
0.1983 0.14916 0.44071 0.1983 0.630984103
CASC17 ENSG00000260785 0.84759 0.19833 -0.21251 0.58414 0.27647
0.39109 0.46642 0.32046 0.19833 0.630984103
KRT18P66 ENSG00000235626 0.84746 0.19837 0.84746 0.19837 0.84746
0.19837 0.84746 0.19837 0.19837 0.630984103
SLC23A1 ENSG00000170482 0.68217 0.24756 0.5144 0.30349 0.84741
0.19838 -0.27295 0.60755 0.19838 0.630984103
NA ENSG00000226425 -0.36355 0.6419 0.84741 0.19838 -0.36355 0.6419

-0.36355 0.6419 0.19838 0.630984103
NA ENSG00000261044 -1.0308 0.84869 -0.35385 0.63828 -0.95095 0.82919
0.84741 0.19838 0.19838 0.630984103
NA ENSG00000255767 0.84742 0.19838 0.84742 0.19838 0.84742 0.19838
0.84742 0.19838 0.19838 0.630984103
TRIM32 ENSG00000119401 0.8474 0.19839 0.8474 0.19839 0.8474
0.19839 0.8474 0.19839 0.19839 0.630984103
GRIK5 ENSG00000105737 -0.91185 0.81908 0.29496 0.38401 -0.17347 0.56886
0.84736 0.1984 0.1984 0.630984103
PWWP2B ENSG00000171813 0.84735 0.1984 -0.43717 0.66901 -0.63694
0.73792 -0.29682 0.6167 0.1984 0.630984103
NA ENSG00000272848 -1.7668 0.96137 0.84736 0.1984 -0.29737 0.61691
0.73932 0.22986 0.1984 0.630984103
UGT1A2P ENSG00000228445 0.84732 0.19841 0.84732 0.19841 0.84732
0.19841 0.84732 0.19841 0.19841 0.630984103
RGS5 ENSG00000143248 -0.48556 0.68636 0.50248 0.30767 0.44153 0.32942
0.84723 0.19843 0.19843 0.630984103
NA ENSG00000261996 0.65599 0.25592 0.84721 0.19844 0.56108 0.28737
0.82806 0.20382 0.19844 0.630984103
CALML3 ENSG00000178363 0.84716 0.19845 0.84716 0.19845 0.84716
0.19845 0.84716 0.19845 0.19845 0.630984103
BRD2 ENSG00000204256 0.80893 0.20928 0.847 0.1985 0.81971 0.20619
0.73237 0.23197 0.1985 0.630984103
RPL31P13 ENSG00000228646 0.84691 0.19852 0.84691 0.19852 0.76731
0.22145 0.84691 0.19852 0.19852 0.630984103
NA ENSG00000255115 0.84684 0.19854 0.84684 0.19854 0.84684 0.19854
0.84684 0.19854 0.19854 0.630984103
NA ENSG00000283045 0.84684 0.19854 0.14867 0.44091 -0.021769 0.50868
0.14867 0.44091 0.19854 0.630984103
RPS26P54 ENSG00000224161 0.84682 0.19855 -0.66702 0.74762 0.13331
0.44697 -1.0004 0.84143 0.19855 0.630984103
RPSAP22 ENSG00000229648 0.84681 0.19855 0.5223 0.30073 -0.90572
0.81746 -1.0665 0.8569 0.19855 0.630984103
RBMS3-AS1 ENSG00000235593 0.84677 0.19856 0.84677 0.19856 0.84677
0.19856 0.84677 0.19856 0.19856 0.630984103
LMTK3 ENSG00000142235 0.67032 0.25133 0.67032 0.25133 0.67032 0.25133
0.84667 0.19859 0.19859 0.630984103
CEACAMP8 ENSG00000236932 -1.2565 0.89552 -1.2565 0.89552 -1.2565
0.89552 0.84668 0.19859 0.19859 0.630984103
NA ENSG00000250448 0.84664 0.1986 0.84664 0.1986 0.84664 0.1986
0.84664 0.1986 0.1986 0.630984103
NA ENSG00000273507 0.84664 0.1986 0.84664 0.1986 -0.79384 0.78636
-0.67216 0.74926 0.1986 0.630984103
EEF1A1P6 ENSG00000233476 0.67559 0.24965 0.84661 0.19861 0.75148
0.22618 0.084238 0.46643 0.19861 0.630984103
NA ENSG00000278500 0.84661 0.19861 0.84661 0.19861 0.84661 0.19861
0.84661 0.19861 0.19861 0.630984103
NA ENSG00000276434 0.84655 0.19862 0.84655 0.19862 0.84655 0.19862
0.84655 0.19862 0.19862 0.630984103
TLL2 ENSG00000095587 0.66012 0.25459 0.64732 0.25871 -0.058379 0.52328
0.84647 0.19864 0.19864 0.630984103
CLRN1 ENSG00000163646 0.70454 0.24055 0.84648 0.19864 0.70454 0.24055
0.70454 0.24055 0.19864 0.630984103
NA ENSG00000273102 0.84647 0.19865 0.84647 0.19865 0.84647 0.19865
0.84647 0.19865 0.19865 0.630984103
VN1R51P ENSG00000228038 -0.24378 0.5963 -0.16778 0.56662 0.84641
0.19866 0.2266 0.41037 0.19866 0.630984103
NDUFA5P5 ENSG00000242365 -0.56486 0.71392 -0.56486 0.71392 0.84641
0.19866 -0.56486 0.71392 0.19866 0.630984103
UBE2V1P8 ENSG00000233118 0.8464 0.19867 -0.28023 0.61035 0.8464
0.19867 -0.0565 0.52253 0.19867 0.630984103
NA ENSG00000244310 -0.95644 0.83058 -1.0212 0.84642 0.84626 0.1987

-0.60989 0.72903 0.1987 0.631038256
PABPC1P10 ENSG00000239443 0.84615 0.19873 -0.10035 0.53997 -0.614
0.73039 0.29041 0.38575 0.19873 0.631092401
NA ENSG00000261568 0.84592 0.1988 0.84592 0.1988 0.5377 0.29539
0.84592 0.1988 0.1988 0.631223045
NA ENSG00000234431 0.84587 0.19881 0.84587 0.19881 0.84587 0.19881
0.84587 0.19881 0.19881 0.631223045
NA ENSG00000283117 0.67609 0.24949 0.84587 0.19881 -0.10564 0.54207
-0.57562 0.71756 0.19881 0.631223045
NA ENSG00000267213 0.84566 0.19887 0.84566 0.19887 0.84566 0.19887
0.84566 0.19887 0.19887 0.631233893
FAHD2B ENSG00000144199 0.81875 0.20646 0.26537 0.39536 0.84562
0.19888 0.24135 0.40464 0.19888 0.631233893
NA ENSG00000241008 0.32912 0.37103 0.82132 0.20573 0.84564 0.19888
0.27438 0.3919 0.19888 0.631233893
NA ENSG00000271536 0.84563 0.19888 0.84563 0.19888 0.84563 0.19888
0.84563 0.19888 0.19888 0.631233893
MGAT4EP ENSG00000184774 -0.08691 0.53463 -0.08691 0.53463 -0.08691
0.53463 0.84554 0.19891 0.19891 0.631233893
NA ENSG00000275130 -0.089502 0.53566 0.84552 0.19891 -1.4339 0.9242
-0.50929 0.69472 0.19891 0.631233893
DRD5 ENSG00000169676 0.84543 0.19893 0.56196 0.28707 0.80559 0.21024
0.75586 0.22487 0.19893 0.631233893
RNU6-183P ENSG00000200331 -0.072074 0.52873 -0.27778 0.60941 0.22712
0.41017 0.84546 0.19893 0.19893 0.631233893
NA ENSG00000206714 0.84546 0.19893 0.84546 0.19893 0.84546 0.19893
0.84546 0.19893 0.19893 0.631233893
NA ENSG00000228318 0.84521 0.199 0.84521 0.199 0.84521 0.199 0.84521 0.199
0.199 0.631272837
PPP1R11P1 ENSG00000236216 -0.59193 0.72305 -0.83321 0.79764 -0.35472
0.6386 0.84519 0.199 0.199 0.631272837
LILRB3 ENSG00000204577 0.84515 0.19901 -0.43999 0.67003 -0.43999
0.67003 0.68435 0.24688 0.19901 0.631272837
NA ENSG00000249101 0.54865 0.29162 0.81487 0.20757 0.84516 0.19901
0.66384 0.2534 0.19901 0.631272837
NA ENSG00000253749 0.57719 0.28191 0.84512 0.19902 0.57719 0.28191
0.66194 0.25401 0.19902 0.631272837
NA ENSG00000272456 0.84514 0.19902 0.84514 0.19902 0.29268 0.38488
-0.10011 0.53987 0.19902 0.631272837
RPL10P7 ENSG00000242052 0.84505 0.19904 0.027382 0.48908 0.84505
0.19904 -0.28441 0.61195 0.19904 0.631295184
MPTX1 ENSG00000215846 0.84497 0.19906 0.7341 0.23144 0.7341 0.23144
0.7341 0.23144 0.19906 0.631317527
LINC02232 ENSG00000250125 0.036809 0.48532 0.74485 0.22818 0.78931
0.21497 0.84484 0.1991 0.1991 0.631403293
RNU1-64P ENSG00000202313 0.73297 0.23179 -1.3126 0.90535 -0.54097
0.70574 0.84472 0.19913 0.19913 0.631457337
CALCOCO2 ENSG00000136436 0.016641 0.49336 -0.56552 0.71414 -0.25318
0.59994 0.84462 0.19916 0.19916 0.631480973
NA ENSG00000226332 0.84442 0.19922 -0.40345 0.65669 0.28935 0.38616
0.28935 0.38616 0.19922 0.631480973
NA ENSG00000255250 0.84442 0.19922 0.84442 0.19922 0.84442 0.19922
0.84442 0.19922 0.19922 0.631480973
APOBEC3F ENSG00000128394 -0.2923 0.61497 0.038716 0.48456 0.84438
0.19923 0.1933 0.42336 0.19923 0.631480973
NA ENSG00000260685 0.84434 0.19924 0.84434 0.19924 0.84434 0.19924
0.84434 0.19924 0.19924 0.631480973
TMEM274P ENSG00000283611 -0.98905 0.83868 0.84435 0.19924 0.84435
0.19924 0.84435 0.19924 0.19924 0.631480973
RNU7-18P ENSG00000252174 0.8443 0.19925 0.8443 0.19925 0.8443
0.19925 0.8443 0.19925 0.19925 0.631480973
NA ENSG00000246541 -0.48022 0.68446 0.84428 0.19926 -0.4374 0.66909

0.34885 0.3636 0.19926 0.631480973
MKNK1-AS1 ENSG00000269956 0.84428 0.19926 -1.2744 0.89874 0.84428
0.19926 0.44262 0.32902 0.19926 0.631480973
MYL6P1 ENSG00000226543 0.84423 0.19927 -0.1365 0.55429 0.38648
0.34957 -1.4809 0.93068 0.19927 0.631480973
CISD1 ENSG00000122873 0.73809 0.23023 0.71679 0.23675 0.71679 0.23675
0.8442 0.19928 0.19928 0.631480973
ZNF225 ENSG00000256294 0.44699 0.32744 0.84402 0.19933 0.84402
0.19933 0.84402 0.19933 0.19933 0.631598342
NA ENSG00000222072 0.26621 0.39504 0.84383 0.19938 0.81927 0.20632
0.79355 0.21373 0.19938 0.631696918
NA ENSG00000275576 0.84382 0.19939 0.070602 0.47186 0.84382 0.19939
0.84382 0.19939 0.19939 0.631696918
TMPRSS12 ENSG00000186452 0.84375 0.1994 0.82711 0.20409 0.49964
0.30867 -0.78133 0.7827 0.1994 0.631696918
NA ENSG00000248605 -0.049923 0.51991 0.84368 0.19943 -1.014 0.84472
-0.069757 0.52781 0.19943 0.631741498
MGAM ENSG00000257335 0.84361 0.19944 0.011434 0.49544 -0.87895 0.81029
0.1269 0.44951 0.19944 0.631741498
NA ENSG00000270997 -0.59424 0.72383 0.60333 0.27315 -1.5007 0.93328
0.84354 0.19946 0.19946 0.631763783
NA ENSG00000273073 -0.16619 0.566 -0.22107 0.58748 -0.01095 0.50437
0.84344 0.19949 0.19949 0.631817737
RPSAP40 ENSG00000231368 0.042566 0.48302 -0.025566 0.5102 0.8433
0.19953 0.8433 0.19953 0.19953 0.631834111
RN7SL547P ENSG00000240584 0.84329 0.19953 0.84329 0.19953 0.84329
0.19953 0.84329 0.19953 0.19953 0.631834111
NA ENSG00000282987 0.023815 0.4905 0.49965 0.30866 0.84324 0.19955
0.49965 0.30866 0.19955 0.631834111
NA ENSG00000225140 0.84318 0.19956 -1.451 0.92661 -0.53903 0.70507
0.44926 0.32662 0.19956 0.631834111
NA ENSG00000232896 -1.0238 0.84704 0.84321 0.19956 -1.1425 0.87338
-0.20043 0.57943 0.19956 0.631834111
NXPE2P1 ENSG00000255982 0.84287 0.19965 0.84287 0.19965 0.84287
0.19965 0.84287 0.19965 0.19965 0.631958287
EEF1E1-BLOC1S5 ENSG00000265818 0.84287 0.19965 0.84287 0.19965 0.84287
0.19965 0.84287 0.19965 0.19965 0.631958287
NA ENSG00000258717 -1.4297 0.9236 0.84283 0.19966 -0.83538 0.79825
-0.83538 0.79825 0.19966 0.631958287
RNU6-363P ENSG00000199765 -0.074165 0.52956 0.75929 0.22384 -0.044431
0.51772 0.84277 0.19968 0.19968 0.631958287
TPT1P5 ENSG00000214108 -0.070598 0.52814 -0.3255 0.6276 -0.070598
0.52814 0.84277 0.19968 0.19968 0.631958287
BPIFB4 ENSG00000186191 0.11727 0.45332 -0.62513 0.73406 0.84272
0.19969 -0.62513 0.73406 0.19969 0.631958287
RTP2 ENSG00000198471 0.84273 0.19969 -0.90338 0.81684 -0.55809 0.71161
-0.92204 0.82175 0.19969 0.631958287
PIK3CA ENSG00000121879 0.24488 0.40328 -0.19953 0.57907 0.84262
0.19972 -0.66116 0.74575 0.19972 0.632012183
NA ENSG00000235733 0.84242 0.19978 0.60797 0.2716 0.37425 0.35411
-0.22924 0.59066 0.19978 0.63209952
SPDYE3 ENSG00000214300 0.84236 0.19979 -0.34721 0.63579 0.51328
0.30388 -1.8129 0.96508 0.19979 0.63209952
NA ENSG00000244337 0.089388 0.46439 -0.14046 0.55585 0.84234 0.1998
0.001954 0.49922 0.1998 0.63209952
RNU4-5P ENSG00000272160 0.84232 0.19981 0.84232 0.19981 0.84232
0.19981 0.84232 0.19981 0.19981 0.63209952
COX5BP1 ENSG00000248448 0.51373 0.30372 0.84224 0.19983 0.51373
0.30372 -0.3218 0.6262 0.19983 0.63209952
NA ENSG00000223436 -0.78641 0.78418 0.21821 0.41363 0.84204 0.19988
-0.35129 0.63731 0.19988 0.63209952
GUSBP4 ENSG00000239650 -1.0344 0.84952 0.84202 0.19989 0.098955

0.46059	-1.0255	0.84743	0.19989	0.63209952
NA	ENSG00000254676 0.842 0.19989	0.842 0.19989	0.842 0.19989	0.842 0.19989
0.19989	0.63209952
CENPV ENSG00000166582 0.66253	0.25381	0.80141	0.21145	0.039636	0.48419
0.84191	0.19992	0.19992	0.63209952
ZDHHC24	ENSG00000174165 -0.40495	0.65724	-0.50087	0.69177	0.84181
0.19995	0.12033	0.45211	0.19995	0.63209952
MIR412	ENSG00000199012 0.84178	0.19996	0.84178	0.19996	0.13086
0.44794	0.84178	0.19996	0.19996	0.63209952
MIR369	ENSG00000199025 0.84178	0.19996	0.84178	0.19996	0.84178
0.19996	0.84178	0.19996	0.19996	0.63209952
MIR409	ENSG00000199107 0.84178	0.19996	0.84178	0.19996	0.84178
0.19996	0.84178	0.19996	0.19996	0.63209952
NA	ENSG00000267130 0.31136	0.37776	0.84168	0.19998	-0.61303	0.73007
0.31136	0.37776	0.19998	0.63209952
ASB10 ENSG00000146926 -1.8753	0.96963	-1.8753	0.96963	-1.8753	0.96963
0.84165	0.19999	0.19999	0.63209952
TMEM94	ENSG00000177728 0.079891	0.46816	0.78074	0.21748	0.079891
0.46816	0.8416	0.2	0.2	0.63209952
RBM43P1	ENSG00000239263 0.84162	0.2	-0.75472	0.77479	-0.14729	0.55855
-0.13199	0.5525	0.2	0.63209952
CEP72 ENSG00000112877 0.84159	0.20001	0.76088	0.22336	0.70552	0.24024
0.55166	0.29059	0.20001	0.63209952
EEF1A1P24	ENSG00000223668 -0.13511	0.55374	0.43645	0.33125	0.84156
0.20002	-0.063873	0.52546	0.20002	0.63209952
LINC00639	ENSG00000259070 0.84155	0.20002	-1.0962	0.86351	-0.33591
0.63153	-0.2362	0.59336	0.20002	0.63209952
NA	ENSG00000261832 0.84154	0.20002	0.84154	0.20002	0.84154	0.20002
0.84154	0.20002	0.20002	0.63209952
RPL5P17	ENSG00000243859 -0.39379	0.65313	-0.27045	0.60659	0.23116
0.40859	0.84139	0.20006	0.20006	0.632119192
NA	ENSG00000244358 -0.9125	0.81925	0.8414	0.20006	-0.9125	0.81925
-0.9125	0.81925	0.20006	0.632119192
CEP55 ENSG00000138180 -0.26372	0.604 0.84136	0.20007	0.84136	0.20007
-0.91047	0.81871	0.20007	0.632119192
NA	ENSG00000254373 0.84133	0.20008	0.84133	0.20008	0.84133	0.20008
0.84133	0.20008	0.20008	0.632119192
NA	ENSG00000275807 0.84118	0.20012	0.84118	0.20012	0.84118	0.20012
0.84118	0.20012	0.20012	0.632119192
MCM8	ENSG00000125885 -0.36142	0.64111	-0.63538	0.73741	-0.63538	0.73741
0.84116	0.20013	0.20013	0.632119192
TCTE3 ENSG00000184786 0.84116	0.20013	-1.7671	0.96139	-1.0603	0.8555
-0.93005	0.82383	0.20013	0.632119192
LINC01863	ENSG00000253959 0.023794	0.49051	-0.89859	0.81557	-0.30428
0.61954	0.84114	0.20013	0.20013	0.632119192
DHRS4 ENSG00000157326 0.84099	0.20018	0.39856	0.34511	0.79716	0.21268
0.60643	0.27212	0.20018	0.632132882
RPL17-C18orf32	ENSG00000215472 0.84098	0.20018	0.84098	0.20018	0.84098
0.20018	0.84098	0.20018	0.20018	0.632132882
RPL17 ENSG00000265681 0.84098	0.20018	0.84098	0.20018	0.84098	0.20018
0.84098	0.20018	0.20018	0.632132882
TRAV40	ENSG00000211819 0.84091	0.2002	0.78416	0.21647	0.78416
0.21647	0.71727	0.2366	0.2002	0.632132882
AKAP8 ENSG00000105127 -1.1308	0.87093	0.84084	0.20022	0.3404	0.36678
0.84084	0.20022	0.20022	0.632132882
LINC02575	ENSG00000228709 0.84083	0.20022	0.84083	0.20022	0.84083
0.20022	-0.10192	0.54059	0.20022	0.632132882
FUT10 ENSG00000172728 0.84081	0.20023	0.56702	0.28535	-0.080396	0.53204
0.45392	0.32494	0.20023	0.632132882
NA	ENSG00000267166 0.45328	0.32517	0.84078	0.20024	0.013252	0.49471
-0.61232	0.72984	0.20024	0.632132882
LINC01966	ENSG00000268580 0.70584	0.24014	0.8407	0.20026	0.8407

							0.20026	0.8407	0.20026	0.20026	0.632132882
NIP7P1	ENSG00000232805	0.84066	0.20027	-0.06586	0.52626	-0.06586	0.52626	-0.06586	0.52626	0.20027	0.632132882
CREBBP	ENSG00000005339	-0.25081	0.59902	0.8406	0.20029	-0.91688	0.8204	0.73826	0.23018	0.20029	0.632132882
LINC02006	ENSG00000238755	0.84057	0.20029	0.51208	0.3043	0.50764	0.30585	0.16469	0.4346	0.20029	0.632132882
DYNC1H1	ENSG00000197102	0.61831	0.26818	0.51522	0.3032	0.70172	0.24143	0.84051	0.20031	0.20031	0.632145681
NRBF2	ENSG00000148572	-1.4279	0.92334	-0.20854	0.5826	0.11311	0.45497	0.84047	0.20032	0.20032	0.632145681
NA	ENSG00000260777	0.30807	0.37901	0.30807	0.37901	0.30807	0.37901	0.84028	0.20037	0.20037	0.63222404
NA	ENSG00000262621	0.5434	0.29343	-0.18675	0.57407	0.84026	0.20038	0.84026	0.20038	0.20038	0.63222404
MYL12AP1	ENSG00000254387	0.84021	0.2004	0.84021	0.2004	0.84021	0.2004	-0.79303	0.78612	0.2004	0.63222404
NA	ENSG00000253363	0.84011	0.20042	0.84011	0.20042	0.21421	0.41519	0.84011	0.20042	0.20042	0.63222404
CREBL2	ENSG00000111269	0.5671	0.28532	0.84004	0.20044	0.64976	0.25792	0.33169	0.37006	0.20044	0.63222404
KATNAL2	ENSG00000167216	0.33649	0.36825	0.1411	0.4439	0.044865	0.48211	0.84003	0.20045	0.20045	0.63222404
RNU7-157P	ENSG00000238431	-0.53927	0.70515	0.8399	0.20048	0.79449	0.21346	0.0036692	0.49854	0.20048	0.63222404
GLIPR2	ENSG00000122694	0.60743	0.27178	0.66703	0.25238	0.83986	0.20049	-0.89833	0.8155	0.20049	0.63222404
NA	ENSG00000271618	0.83983	0.2005	0.83983	0.2005	0.83983	0.2005	-0.18671	0.57406	0.2005	0.63222404
RNY4P29	ENSG00000222733	0.11393	0.45465	0.83975	0.20052	-0.77125	0.77972	-0.77125	0.77972	0.20052	0.63222404
BNIP3P31	ENSG00000270191	0.83978	0.20052	0.83978	0.20052	0.83978	0.20052	0.83978	0.20052	0.20052	0.63222404
TYMSP2	ENSG00000267190	0.83972	0.20053	0.83972	0.20053	0.83972	0.20053	0.83972	0.20053	0.20053	0.63222404
RNA5SP164	ENSG00000252342	0.83971	0.20054	-0.32579	0.62771	-0.32579	0.62771	-0.32579	0.62771	0.20054	0.63222404
WDR34	ENSG00000119333	0.8039	0.21073	0.83959	0.20057	0.026745	0.48933	0.25474	0.39946	0.20057	0.63222404
PHF23	ENSG00000040633	-0.16695	0.5663	0.83952	0.20059	-0.79988	0.78811	0.83952	0.20059	0.20059	0.63222404
FAM174C	ENSG00000228300	0.83953	0.20059	0.83953	0.20059	0.83953	0.20059	0.83953	0.20059	0.20059	0.63222404
NA	ENSG00000251417	0.83946	0.2006	0.088584	0.46471	0.83946	0.2006	0.38387	0.35054	0.2006	0.63222404
BDKRB1	ENSG00000100739	0.83945	0.20061	-0.34432	0.6347	-0.028151	0.51123	-1.3047	0.90401	0.20061	0.63222404
TMED5	ENSG00000117500	0.83933	0.20064	-0.32377	0.62694	0.83933	0.20064	0.3378	0.36776	0.20064	0.63222404
ROPN1L	ENSG00000145491	0.79191	0.21421	-0.53345	0.70314	0.40438	0.34297	0.8393	0.20065	0.20065	0.63222404
WDR27	ENSG00000184465	0.16557	0.43425	-0.57486	0.71731	-0.78354	0.78334	0.83927	0.20066	0.20066	0.63222404
OR9K2	ENSG00000170605	-0.28951	0.6139	-0.28951	0.6139	-1.1712	0.87924	0.83923	0.20067	0.20067	0.63222404
HCG4B	ENSG00000227262	-0.073886	0.52945	-1.9203	0.97259	0.8392	0.20068	-1.364	0.91372	0.20068	0.63222404
FTH1P5	ENSG00000230204	-0.13014	0.55177	-0.39431	0.65332	-0.10695	0.54259	0.83917	0.20069	0.20069	0.63222404
MUCL1	ENSG00000172551	-0.20428	0.58093	0.82519	0.20463	0.83912	0.2007	0.82519	0.20463	0.2007	0.63222404
G6PC	ENSG00000131482	-0.53038	0.70208	-0.53038	0.70208	-0.53038	0.70208				

0.83905 0.20072 0.20072 0.63222404
TSHZ2 ENSG00000182463 0.83905 0.20072 0.52228 0.30074 -0.7 0.75804
-1.0869 0.86146 0.20072 0.63222404
NA ENSG00000199571 0.83904 0.20072 0.83904 0.20072 0.83904 0.20072
0.83904 0.20072 0.20072 0.63222404
MPHOSPH6P1 ENSG00000271428 0.83902 0.20073 0.83902 0.20073 0.83902
0.20073 0.83902 0.20073 0.20073 0.63222404
ZNF322 ENSG00000181315 0.83896 0.20074 0.77852 0.21813 0.45584
0.32425 0.4856 0.31362 0.20074 0.63222404
NA ENSG00000237594 -0.16953 0.56731 0.8389 0.20076 -0.81811 0.79335
0.80881 0.20931 0.20076 0.63222404
NA ENSG00000249106 -1.035 0.84965 0.010262 0.49591 0.83891 0.20076
-0.21323 0.58443 0.20076 0.63222404
NA ENSG00000255669 0.83886 0.20078 0.83886 0.20078 0.83886 0.20078
-0.1877 0.57445 0.20078 0.632246165
EGR4 ENSG00000135625 -0.29214 0.61491 -0.59073 0.72265 -0.45247 0.67453
0.83876 0.2008 0.2008 0.63225892
NA ENSG00000258387 0.36456 0.35772 -0.96914 0.83376 0.83872 0.20081
-0.8963 0.81495 0.20081 0.63225892
KAZN ENSG00000189337 -0.5677 0.71488 -0.33734 0.63207 0.83858 0.20085
-0.42272 0.66375 0.20085 0.632334634
MEMO1P1 ENSG00000226054 0.83854 0.20086 0.27065 0.39333 0.83854
0.20086 0.83854 0.20086 0.20086 0.632334634
TAC4 ENSG00000176358 0.83845 0.20089 -1.0245 0.8472 -1.8523 0.96801
-1.0245 0.8472 0.20089 0.632388224
NA ENSG00000234105 0.83823 0.20095 0.83823 0.20095 0.83823 0.20095
0.83823 0.20095 0.20095 0.632467252
NA ENSG00000262668 0.51449 0.30345 0.059319 0.47635 0.83823 0.20095
0.51449 0.30345 0.20095 0.632467252
NA ENSG00000252040 0.83814 0.20097 -0.45904 0.6769 0.83814 0.20097
0.83814 0.20097 0.20097 0.632467252
RNU4-22P ENSG00000222990 0.70754 0.23962 0.83811 0.20098 0.83811
0.20098 0.83811 0.20098 0.20098 0.632467252
NA ENSG00000282022 0.83813 0.20098 0.83813 0.20098 0.83813 0.20098
0.83813 0.20098 0.20098 0.632467252
DBH ENSG00000123454 0.21729 0.41399 -0.18227 0.57231 0.67402 0.25015
0.83802 0.20101 0.20101 0.632511435
KRT77 ENSG00000189182 0.83797 0.20102 -0.5926 0.72328 0.48512 0.31379
-0.72266 0.76505 0.20102 0.632511435
MIR1302-4 ENSG00000221628 0.21983 0.413 0.21983 0.413 0.8379 0.20104
0.21983 0.413 0.20104 0.632533523
MIR4439 ENSG00000263828 0.83777 0.20108 0.14996 0.4404 0.83777
0.20108 0.83777 0.20108 0.20108 0.632568311
TAF6L ENSG00000162227 -0.020936 0.50835 -0.80215 0.78877 0.035755 0.48574
0.83773 0.20109 0.20109 0.632568311
NA ENSG00000255886 0.83773 0.20109 -0.26658 0.6051 0.83773 0.20109
0.83773 0.20109 0.20109 0.632568311
PLCL2 ENSG00000154822 0.61522 0.26921 0.019781 0.49211 0.83763 0.20112
0.38585 0.34981 0.20112 0.632621844
DDX39AP1 ENSG00000225777 0.47454 0.31756 -0.63989 0.73888 0.83746
0.20117 0.54359 0.29336 0.20117 0.632637846
LINC01935 ENSG00000224095 0.83743 0.20118 0.83743 0.20118 0.83743
0.20118 0.83743 0.20118 0.20118 0.632637846
OR1D4 ENSG00000255095 -0.023503 0.50938 0.83742 0.20118 0.45745 0.32367
-0.61792 0.73169 0.20118 0.632637846
LINC02335 ENSG00000278630 0.83741 0.20118 -1.0683 0.8573 0.83741
0.20118 -0.27819 0.60956 0.20118 0.632637846
NA ENSG00000283342 0.73637 0.23075 -0.69666 0.75699 0.83737 0.20119
-0.3911 0.65214 0.20119 0.632637846
NA ENSG00000268735 -0.021406 0.50854 -0.021406 0.50854 0.83729 0.20121
0.022902 0.49086 0.20121 0.632659911
NA ENSG00000225123 0.83718 0.20125 0.83718 0.20125 -0.3415 0.63364

-1.0859 0.86124 0.20125 0.632744855
LINC00348 ENSG00000226846 0.71805 0.23636 0.71805 0.23636 -0.1015
0.54042 0.83691 0.20132 0.20132 0.632814286
RN7SL323P ENSG00000241997 0.11326 0.45491 0.11326 0.45491 0.3521
0.36238 0.8369 0.20132 0.20132 0.632814286
ARHGAP20 ENSG00000137727 0.49286 0.31106 0.83683 0.20134 -0.39423
0.65329 0.54297 0.29357 0.20134 0.632814286
NA ENSG00000254821 -0.52516 0.70026 0.83685 0.20134 -0.57091 0.71597
0.83685 0.20134 0.20134 0.632814286
NA ENSG00000278977 0.83684 0.20134 0.83684 0.20134 0.83684 0.20134
0.83684 0.20134 0.20134 0.632814286
CRABP2 ENSG00000143320 0.83681 0.20135 0.83681 0.20135 0.12383
0.45073 -0.75411 0.77461 0.20135 0.632814286
LINC00052 ENSG00000259527 -0.61107 0.72942 0.8367 0.20138 -0.50197
0.69216 -0.50197 0.69216 0.20138 0.632867754
NCAPH2 ENSG00000025770 -0.73833 0.76984 -0.43227 0.66723 -1.1734
0.87968 0.83654 0.20143 0.20143 0.632890179
OOSP1P1 ENSG00000238037 0.83651 0.20143 0.83651 0.20143 0.83651
0.20143 0.83651 0.20143 0.20143 0.632890179
NA ENSG00000249865 -0.52726 0.70099 0.83641 0.20146 -0.52726 0.70099
-0.52726 0.70099 0.20146 0.632890179
HDGFL1 ENSG00000112273 -0.66017 0.74543 0.83636 0.20148 -0.66017
0.74543 -0.66017 0.74543 0.20148 0.632890179
NA ENSG00000257524 0.83632 0.20149 0.83632 0.20149 0.83632 0.20149
0.83632 0.20149 0.20149 0.632890179
EIF4A2 ENSG00000156976 0.83628 0.2015 0.83628 0.2015 0.83628
0.2015 0.83628 0.2015 0.2015 0.632890179
CCT8P1 ENSG00000226015 0.83627 0.2015 0.83627 0.2015 0.83627
0.2015 0.83627 0.2015 0.2015 0.632890179
NA ENSG00000248115 -1.2414 0.89277 0.39065 0.34803 -0.15712 0.56243
0.83628 0.2015 0.2015 0.632890179
RPL7P18 ENSG00000243806 0.83626 0.20151 0.83626 0.20151 0.83626
0.20151 0.83626 0.20151 0.20151 0.632890179
NA ENSG00000253940 0.83622 0.20152 0.13089 0.44793 0.83622 0.20152
0.83622 0.20152 0.20152 0.632890179
ZNF37BP ENSG00000234420 0.5776 0.28177 0.16543 0.4343 0.16942
0.43273 0.83616 0.20153 0.20153 0.632890179
RANBP10 ENSG00000141084 0.82532 0.20459 0.83602 0.20157 -0.66369
0.74655 -0.39172 0.65237 0.20157 0.632965616
NA ENSG00000223036 -0.92781 0.82325 -0.92781 0.82325 0.836 0.20158
0.78941 0.21494 0.20158 0.632965616
NA ENSG00000247877 0.83564 0.20168 -0.060155 0.52398 0.63208 0.26367
-0.7065 0.76006 0.20168 0.633238814
OR8D2 ENSG00000279116 -0.40477 0.65718 -0.80609 0.7899 0.83551 0.20172
0.83551 0.20172 0.20172 0.633257773
NA ENSG00000279756 0.83546 0.20173 0.83546 0.20173 0.83546 0.20173
0.83546 0.20173 0.20173 0.633257773
NA ENSG00000254003 0.83538 0.20175 0.83538 0.20175 0.83538 0.20175
0.83538 0.20175 0.20175 0.633257773
LIMCH1 ENSG00000064042 0.83531 0.20177 0.037205 0.48516 0.65109
0.25749 0.081534 0.46751 0.20177 0.633257773
MIB2 ENSG00000197530 0.83526 0.20178 0.42962 0.33374 0.83526 0.20178
0.56489 0.28608 0.20178 0.633257773
RNU2-54P ENSG00000222231 0.83529 0.20178 0.81481 0.20759 0.83529
0.20178 0.83529 0.20178 0.20178 0.633257773
NA ENSG00000227163 -0.9313 0.82415 -0.9313 0.82415 0.83523 0.20179
-0.9313 0.82415 0.20179 0.633257773
NA ENSG00000254295 0.83525 0.20179 0.83525 0.20179 0.83525 0.20179
0.83525 0.20179 0.20179 0.633257773
NA ENSG00000249627 0.83509 0.20183 0.83509 0.20183 0.83509 0.20183
0.83509 0.20183 0.20183 0.633292331
CORO7 ENSG00000262246 0.83511 0.20183 0.83511 0.20183 0.83511 0.20183

0.83511 0.20183 0.20183 0.633292331
S100A8 ENSG00000143546 0.83505 0.20184 0.83505 0.20184 0.7458
0.22789 0.5319 0.2974 0.20184 0.633292331
RN7SL728P ENSG00000241144 0.83496 0.20187 0.83496 0.20187 0.83496
0.20187 0.64251 0.26027 0.20187 0.63334568
ZNF236-DT ENSG00000264278 0.83488 0.20189 0.419 0.33761 0.060272 0.47597
0.77767 0.21838 0.20189 0.63336765
MIR2355 ENSG00000253008 0.42041 0.33709 0.42041 0.33709 0.83452
0.20199 0.65055 0.25767 0.20199 0.633640576
NA ENSG00000236118 0.83438 0.20203 0.61001 0.27093 0.61001 0.27093
0.61001 0.27093 0.20203 0.633678147
RNU6-150P ENSG00000252046 0.27449 0.39185 0.27449 0.39185 0.043164
0.48279 0.83436 0.20204 0.20204 0.633678147
NA ENSG00000253051 0.83432 0.20205 0.83432 0.20205 0.83432 0.20205
0.83432 0.20205 0.20205 0.633678147
BHLHE40-AS1 ENSG00000235831 0.83425 0.20207 -0.55483 0.7105 0.33157
0.37011 -0.14391 0.55721 0.20207 0.633678147
LINC01230 ENSG00000281769 0.18782 0.42551 -0.29596 0.61637 0.83425
0.20207 0.83425 0.20207 0.20207 0.633678147
OR1D3P ENSG00000262106 -1.3551 0.91231 -0.12672 0.55042 0.37384
0.35426 0.83423 0.20208 0.20208 0.633678147
REXO1L5P ENSG00000277436 0.83413 0.2021 -1.0616 0.8558 -0.58024
0.71912 -1.0515 0.85347 0.2021 0.633700084
NA ENSG00000200547 -0.98016 0.8365 0.83408 0.20212 -0.73442 0.76865
-0.73442 0.76865 0.20212 0.633722018
MACF1 ENSG00000127603 0.25879 0.3979 0.3295 0.37089 0.83389 0.20217
0.4515 0.32581 0.20217 0.633756284
NDUFB1P1 ENSG00000231884 0.83387 0.20218 0.83387 0.20218 0.83387
0.20218 0.83387 0.20218 0.20218 0.633756284
GPR171 ENSG00000174946 0.83382 0.20219 0.83382 0.20219 0.83382
0.20219 0.83382 0.20219 0.20219 0.633756284
NA ENSG00000230920 0.83383 0.20219 0.15729 0.43751 -0.72344 0.76529
-0.23939 0.5946 0.20219 0.633756284
CCDC198 ENSG00000100557 0.8338 0.2022 0.041819 0.48332 -0.50226
0.69226 -1.1971 0.88437 0.2022 0.633756284
NA ENSG00000254684 0.83371 0.20222 -0.49233 0.68876 -0.93449 0.82497
-0.49233 0.68876 0.20222 0.633756284
MPHOSPH10 ENSG00000124383 -0.023703 0.50946 0.4884 0.31263 0.83364
0.20224 0.4884 0.31263 0.20224 0.633756284
NA ENSG00000272327 0.8336 0.20225 0.8336 0.20225 0.8336 0.20225
0.8336 0.20225 0.20225 0.633756284
TIGIT ENSG00000181847 0.1485 0.44097 0.83357 0.20226 0.1485 0.44097
0.53045 0.2979 0.20226 0.633756284
ZNF570 ENSG00000171827 0.83349 0.20228 -1.4467 0.92601 -1.4467
0.92601 -1.4467 0.92601 0.20228 0.633756284
ARFGEF1 ENSG00000066777 0.83348 0.20229 -1.2067 0.88622 -1.2067
0.88622 -0.18855 0.57478 0.20229 0.633756284
CHCHD4P5 ENSG00000225222 0.67462 0.24996 0.83348 0.20229 0.83348
0.20229 0.83348 0.20229 0.20229 0.633756284
YIPF4 ENSG00000119820 0.83343 0.2023 0.42486 0.33547 0.36983 0.35575
0.1316 0.44765 0.2023 0.633756284
SSUH2 ENSG00000125046 -0.74919 0.77313 -1.6947 0.95493 0.83329 0.20234
-1.8906 0.97066 0.20234 0.633784346
TMED8 ENSG00000100580 0.83321 0.20236 0.83321 0.20236 0.83321 0.20236
-0.17371 0.56895 0.20236 0.633784346
NA ENSG00000267503 0.35348 0.36186 -0.0090134 0.5036 0.68508 0.24665
0.83318 0.20237 0.20237 0.633784346
MRI1 ENSG00000037757 0.40191 0.34387 0.83313 0.20239 0.78022 0.21763
-0.01653 0.50659 0.20239 0.633784346
NA ENSG00000249808 0.64071 0.26086 0.14322 0.44306 0.83313 0.20239
-0.72401 0.76547 0.20239 0.633784346
RN7SL843P ENSG00000239547 0.83307 0.2024 0.41708 0.33831 -0.69347

0.75599 0.41708 0.33831 0.2024 0.633784346
NA ENSG00000266100 0.83309 0.2024 0.83309 0.2024 0.83309 0.2024
0.83309 0.2024 0.2024 0.633784346
NOM1 ENSG00000146909 0.70771 0.23956 0.8327 0.20251 0.31105 0.37788
0.45734 0.32371 0.20251 0.633940624
ZNF525 ENSG00000203326 0.64822 0.25842 -0.10021 0.53991 -0.10021
0.53991 0.83265 0.20252 0.20252 0.633940624
NA ENSG00000218577 0.83267 0.20252 -0.73856 0.76991 0.70953 0.239
-1.4494 0.92638 0.20252 0.633940624
NA ENSG00000223970 0.83258 0.20254 0.83258 0.20254 0.83258 0.20254
0.83258 0.20254 0.20254 0.633940624
PDYN-AS1 ENSG00000233896 -0.31653 0.6242 0.17196 0.43174 -0.31653
0.6242 0.83258 0.20254 0.20254 0.633940624
SRRM4 ENSG00000139767 0.73425 0.2314 0.1653 0.43436 -0.7854 0.78389
0.83249 0.20257 0.20257 0.633940624
NA ENSG00000258424 0.83249 0.20257 0.83249 0.20257 0.83249 0.20257
0.83249 0.20257 0.20257 0.633940624
HCFC1R1 ENSG00000103145 0.83243 0.20258 0.83243 0.20258 0.83243
0.20258 0.83243 0.20258 0.20258 0.633940624
THOC6 ENSG00000131652 0.83243 0.20258 0.83243 0.20258 0.83243 0.20258
0.83243 0.20258 0.20258 0.633940624
CYFIP1 ENSG00000273749 -1.2747 0.89878 -1.1857 0.88213 -0.12987
0.55166 0.83244 0.20258 0.20258 0.633940624
NA ENSG00000253525 -0.28741 0.6131 -0.382 0.64877 0.83238 0.2026
-0.83564 0.79832 0.2026 0.633943666
NA ENSG00000258716 0.83233 0.20261 0.83233 0.20261 0.83233 0.20261
0.83233 0.20261 0.20261 0.633943666
NA ENSG00000227711 0.68904 0.2454 0.33363 0.36933 0.83229 0.20262
0.6412 0.2607 0.20262 0.633943666
ERN1 ENSG00000178607 0.11587 0.45388 0.8322 0.20265 0.15209 0.43956
0.11587 0.45388 0.20265 0.633996822
HMGA1P4 ENSG00000234705 0.39596 0.34607 0.83189 0.20274 -1.2039
0.88569 0.83189 0.20274 0.20274 0.634209377
LARP7 ENSG00000174720 0.8318 0.20276 -1.2797 0.89967 -0.82092 0.79415
-0.80467 0.78949 0.20276 0.634209377
DLK1 ENSG00000185559 -0.33481 0.63112 0.83179 0.20276 -0.19885 0.57881
-0.51546 0.69688 0.20276 0.634209377
NA ENSG00000253197 -0.10561 0.54206 0.83175 0.20277 0.83175 0.20277
0.75577 0.22489 0.20277 0.634209377
KIR2DL1 ENSG00000125498 0.38026 0.35187 0.82629 0.20432 0.83171
0.20279 0.6542 0.25649 0.20279 0.634212366
SDF2 ENSG00000132581 0.83163 0.20281 0.83163 0.20281 0.83163 0.20281
0.83163 0.20281 0.20281 0.634212366
MIR3609 ENSG00000266019 0.83163 0.20281 0.83163 0.20281 0.83163
0.20281 0.83163 0.20281 0.20281 0.634212366
NA ENSG00000225216 0.8315 0.20285 0.3278 0.37153 0.8315 0.20285
0.8315 0.20285 0.20285 0.634256047
NA ENSG00000249380 -0.10255 0.54084 0.56021 0.28767 0.83147 0.20285
0.48169 0.31501 0.20285 0.634256047
RLN2 ENSG00000107014 -1.3493 0.91137 0.29119 0.38545 -0.37622 0.64662
0.83132 0.2029 0.2029 0.634330981
KCNE1B ENSG00000276289 0.8313 0.2029 0.8313 0.2029 0.8313
0.2029 0.8313 0.2029 0.2029 0.634330981
OR13C5 ENSG00000277556 -0.25632 0.60115 0.83122 0.20292 -0.25959
0.60241 -0.25959 0.60241 0.20292 0.634343377
KANSL1L ENSG00000144445 -0.79324 0.78618 -1.0458 0.85217 0.83119
0.20293 -0.85953 0.80498 0.20293 0.634343377
LYZL2 ENSG00000151033 0.03444 0.48626 -0.33457 0.63103 0.83108 0.20296
0.82886 0.20359 0.20296 0.634360569
ENOX1-AS1 ENSG00000233821 0.83083 0.20303 0.83083 0.20303 0.83083
0.20303 0.83083 0.20303 0.20303 0.634360569
RNU6-775P ENSG00000238619 0.8308 0.20304 0.62181 0.26703 0.62181

0.26703 0.7094 0.23904 0.20304 0.634360569
RSU1P1 ENSG00000230425 -0.57848 0.71853 -0.34468 0.63483 0.0084398
0.49663 0.83077 0.20305 0.20305 0.634360569
NA ENSG00000233987 0.83064 0.20309 0.73399 0.23148 0.67888 0.24861
-1.0235 0.84697 0.20309 0.634360569
LINC01219 ENSG00000232987 0.83055 0.20311 0.83055 0.20311 0.83055
0.20311 0.83055 0.20311 0.20311 0.634360569
MSI1 ENSG00000135097 0.046517 0.48145 -0.2084 0.58254 -0.033604 0.5134
0.83053 0.20312 0.20312 0.634360569
RPL23AP18 ENSG00000225338 0.83054 0.20312 0.83054 0.20312 0.83054
0.20312 0.83054 0.20312 0.20312 0.634360569
NA ENSG00000242279 0.83043 0.20315 0.83043 0.20315 0.83043 0.20315
0.83043 0.20315 0.20315 0.634360569
NA ENSG00000279184 0.43379 0.33222 0.83042 0.20315 0.78748 0.2155
0.78748 0.2155 0.20315 0.634360569
SELENOH ENSG00000211450 0.8304 0.20316 0.1423 0.44342 0.44496
0.32818 0.016623 0.49337 0.20316 0.634360569
KANSL3 ENSG00000114982 0.27819 0.39043 0.83036 0.20317 0.023651
0.49057 0.074374 0.47036 0.20317 0.634360569
SLC38A10 ENSG00000157637 -0.062915 0.52508 -0.028524 0.51138 -0.23699
0.59367 0.83028 0.20319 0.20319 0.634360569
NA ENSG00000223542 0.83023 0.20321 -0.75968 0.77628 -0.45172 0.67426
-0.75968 0.77628 0.20321 0.634360569
NFIB ENSG00000147862 -0.21043 0.58333 0.83017 0.20322 -0.48965 0.68781
0.31319 0.37707 0.20322 0.634360569
OTUD7A ENSG00000169918 -1.0404 0.85091 -0.030738 0.51226 0.25302
0.40013 0.83018 0.20322 0.20322 0.634360569
NA ENSG00000260461 0.042471 0.48306 0.33098 0.37033 0.83018 0.20322
0.33098 0.37033 0.20322 0.634360569
NA ENSG00000280266 0.83019 0.20322 0.83019 0.20322 0.83019 0.20322
0.83019 0.20322 0.20322 0.634360569
RNU6-356P ENSG00000206867 0.83014 0.20323 -0.50425 0.69296 -1.0517
0.85352 -0.96775 0.83341 0.20323 0.634360569
NA ENSG00000203472 0.83009 0.20325 0.83009 0.20325 0.83009 0.20325
0.83009 0.20325 0.20325 0.634360569
PWP2 ENSG00000241945 0.63684 0.26212 0.37909 0.35231 0.50506 0.30676
0.83009 0.20325 0.20325 0.634360569
NA ENSG00000275769 0.83 0.20327 0.83 0.20327 0.83 0.20327 0.83 0.20327
0.20327 0.634360569
ZSCAN5B ENSG00000197213 0.82995 0.20328 0.82995 0.20328 0.82995
0.20328 0.82995 0.20328 0.20328 0.634360569
NA ENSG00000207244 0.82998 0.20328 0.82998 0.20328 0.82998 0.20328
0.82998 0.20328 0.20328 0.634360569
MPDZ ENSG00000107186 0.82993 0.20329 0.82993 0.20329 -0.015445 0.50616
0.82993 0.20329 0.20329 0.634360569
BRWD1-AS2 ENSG00000255568 0.82992 0.20329 0.82992 0.20329 0.82992
0.20329 0.82992 0.20329 0.20329 0.634360569
NA ENSG00000260015 0.82992 0.20329 0.82992 0.20329 0.82992 0.20329
0.82992 0.20329 0.20329 0.634360569
SAG ENSG00000130561 0.8299 0.2033 -0.19524 0.5774 -0.55578 0.71082
-0.93841 0.82598 0.2033 0.634360569
NA ENSG00000254867 -0.80838 0.79056 0.82978 0.20333 -0.80838 0.79056
-0.80838 0.79056 0.20333 0.634413555
NA ENSG00000252831 0.78078 0.21747 0.60358 0.27306 -0.11313 0.54504
0.82966 0.20336 0.20336 0.634457113
RPL7P24 ENSG00000240003 0.82963 0.20337 0.82963 0.20337 0.82963
0.20337 0.82963 0.20337 0.20337 0.634457113
NA ENSG00000250017 0.059316 0.47635 -0.001588 0.50063 -0.42529 0.66469
0.82955 0.2034 0.2034 0.634510083
NA ENSG00000250839 0.82934 0.20345 0.82934 0.20345 -0.28671 0.61283
0.82934 0.20345 0.20345 0.634537019
C3orf18 ENSG00000088543 -0.27444 0.60813 0.82933 0.20346 -0.34486

0.6349	0.21659	0.41426	0.20346	0.634537019
B3GAT2	ENSG00000112309	0.82934	0.20346	0.82934	0.20346	0.78971
0.21485	0.58035	0.28084	0.20346	0.634537019
NA	ENSG00000258660	0.82921	0.20349	0.82921	0.20349	0.82921	0.20349
0.82921	0.20349	0.20349	0.634537019
NA	ENSG00000237251	0.26212	0.39662	0.82918	0.2035	0.29664	0.38337
0.29664	0.38337	0.2035	0.634537019
NA	ENSG00000269124	0.8292	0.2035	0.8292	0.2035	0.8292	0.2035
0.8292	0.2035	0.2035	0.634537019
KRTAP21-2	ENSG00000187026	0.82915	0.20351	0.82915	0.20351	0.82915
0.20351	0.82915	0.20351	0.20351	0.634537019
NA	ENSG00000284418	-0.076508	0.53049	0.20015	0.42068	0.82906	0.20353
-0.066147	0.52637	0.20353	0.634537019
NA	ENSG00000265037	0.82906	0.20353	0.82906	0.20353	0.82906	0.20353
0.82906	0.20353	0.20353	0.634537019
CCT8	ENSG00000156261	0.82898	0.20356	0.82898	0.20356	0.82898	0.20356
0.82898	0.20356	0.20356	0.634537019
RNU6-1335P	ENSG00000202296	0.82892	0.20357	-0.65491	0.74374	0.23515
0.40705	0.017986	0.49283	0.20357	0.634537019
IGHV1OR15-2	ENSG00000259337	0.8289	0.20358	0.49268	0.31112	0.8289
0.20358	0.8289	0.20358	0.20358	0.634537019
EXTL2	ENSG00000162694	0.82882	0.2036	0.37714	0.35303	-0.26386	0.60406
-0.57303	0.71669	0.2036	0.634537019
NA	ENSG00000228622	0.82882	0.2036	0.82882	0.2036	0.82882	0.2036
0.82882	0.2036	0.2036	0.634537019
BHLHE40	ENSG00000134107	0.4512	0.32592	0.80637	0.21001	0.37132
0.3552	0.82874	0.20362	0.20362	0.634537019
RNU6-898P	ENSG00000202497	-0.57202	0.71635	-0.57202	0.71635	0.82872
0.20363	-0.14482	0.55757	0.20363	0.634537019
NME1P1	ENSG00000237175	-0.71722	0.76338	0.82873	0.20363	-1.2765
0.89912	-0.71722	0.76338	0.20363	0.634537019
LINC00443	ENSG00000230156	0.82858	0.20367	0.82858	0.20367	0.053875
0.47852	0.82858	0.20367	0.20367	0.63459573
NA	ENSG00000243995	-0.30539	0.61997	-0.84177	0.80004	0.82854	0.20368
-0.84177	0.80004	0.20368	0.63459573
NA	ENSG00000228962	0.33214	0.36989	0.4352	0.33171	-0.5379	0.70468
0.8285	0.20369	0.20369	0.63459573
RPL35P6	ENSG00000244018	0.046018	0.48165	0.82839	0.20373	-0.61764
0.73159	0.82839	0.20373	0.20373	0.63459573
SDAD1P3	ENSG00000227707	0.82834	0.20374	-0.7576	0.77566	-0.7576
0.77566	-0.7576	0.77566	0.20374	0.63459573
NA	ENSG00000270951	-1.0148	0.8449	0.82834	0.20374	0.15075	0.44009
0.15075	0.44009	0.20374	0.63459573
NA	ENSG00000266577	-0.27725	0.60921	0.03883	0.48451	-0.092307	0.53677
0.82834	0.20374	0.20374	0.63459573
NA	ENSG00000261026	-0.13858	0.55511	0.82825	0.20376	0.0026254	0.49895
-0.96804	0.83349	0.20376	0.634617461
MAB21L1	ENSG00000180660	0.82809	0.20381	0.82809	0.20381	0.82809
0.20381	0.82809	0.20381	0.20381	0.63467322
BARD1	ENSG00000138376	-0.22356	0.58845	0.14053	0.44412	-0.12298	0.54894
0.82806	0.20382	0.20382	0.63467322
LINC01802	ENSG00000225064	0.82801	0.20383	0.63378	0.26311	-0.35482
0.63864	0.63378	0.26311	0.20383	0.63467322
NA	ENSG00000234174	-0.546	0.70747	-1.1275	0.87024	0.82801	0.20383
-0.546	0.70747	0.20383	0.63467322
PRMT2	ENSG00000160310	0.82788	0.20387	0.82788	0.20387	0.82788	0.20387
0.82788	0.20387	0.20387	0.63475721
LINC02285	ENSG00000259004	0.8191	0.20636	0.82774	0.20391	0.8191
0.20636	0.59621	0.27552	0.20391	0.63484119
UBTF	ENSG00000108312	0.40912	0.34123	0.82767	0.20393	0.19339	0.42333
0.17395	0.43095	0.20393	0.634862895
SNORD31B	ENSG00000201847	0.20233	0.41983	0.20233	0.41983	0.20233

0.41983 0.82759 0.20395 0.20395 0.634884598
NA ENSG00000212538 0.82754 0.20397 0.82754 0.20397 0.82754 0.20397
0.82754 0.20397 0.20397 0.634906298
ZNF300 ENSG00000145908 -0.82786 0.79612 0.72409 0.2345 0.82741 0.204
-0.82786 0.79612 0.204 0.634916849
ZNF223 ENSG00000178386 0.13941 0.44456 0.82738 0.20401 -0.0026625
0.50106 0.13941 0.44456 0.20401 0.634916849
LDHAL6DP ENSG00000250257 0.56425 0.28629 0.46239 0.3219 0.82726
0.20405 0.71499 0.23731 0.20405 0.634916849
RPL39P ENSG00000228601 0.82722 0.20406 0.82722 0.20406 0.82722
0.20406 0.82722 0.20406 0.20406 0.634916849
CAVIN3 ENSG00000170955 -0.14745 0.55861 -0.14745 0.55861 -0.43238
0.66727 0.827 0.20412 0.20412 0.634916849
TUSC8 ENSG00000237361 0.827 0.20412 -0.092078 0.53668 -0.3605 0.64076
-1.3542 0.91216 0.20412 0.634916849
NA ENSG00000283125 0.82696 0.20413 0.58912 0.27789 0.82696 0.20413
0.82696 0.20413 0.20413 0.634916849
RNU6-1210P ENSG00000200645 0.82694 0.20414 0.82694 0.20414 0.82694
0.20414 0.82694 0.20414 0.20414 0.634916849
NA ENSG00000231020 0.034482 0.48625 0.17704 0.42974 0.82693 0.20414
0.82693 0.20414 0.20414 0.634916849
SLC11A2 ENSG00000110911 0.8269 0.20415 -1.3921 0.91805 -8.12E-05
0.50003 -1.0978 0.86386 0.20415 0.634916849
LINC02571 ENSG00000256166 -0.53563 0.70389 -0.54603 0.70748 0.26225
0.39657 0.82679 0.20418 0.20418 0.634916849
NA ENSG00000213080 0.3494 0.36339 0.82672 0.2042 0.71122 0.23847
0.3494 0.36339 0.2042 0.634916849
ITGAD ENSG00000156886 0.74033 0.22955 0.13606 0.44589 -0.75163 0.77386
0.82661 0.20423 0.20423 0.634916849
NA ENSG00000256306 0.2208 0.41263 0.2208 0.41263 0.82661 0.20423
-0.13914 0.55533 0.20423 0.634916849
GABARAPL2 ENSG00000034713 0.48565 0.31361 0.82657 0.20424 0.82657
0.20424 0.82657 0.20424 0.20424 0.634916849
NA ENSG00000220734 -0.028767 0.51147 -0.028767 0.51147 0.29141 0.38537
0.82659 0.20424 0.20424 0.634916849
NA ENSG00000224984 -0.58406 0.72041 -0.58406 0.72041 0.82656 0.20424
-0.58406 0.72041 0.20424 0.634916849
NA ENSG00000235564 0.82658 0.20424 0.31765 0.37538 0.31765 0.37538
0.31765 0.37538 0.20424 0.634916849
NA ENSG00000188850 -0.00048293 0.50019 -0.38096 0.64838 0.79078
0.21453 0.82644 0.20428 0.20428 0.634916849
NA ENSG00000267786 0.82637 0.2043 0.82637 0.2043 0.82637 0.2043
0.82637 0.2043 0.2043 0.634916849
NA ENSG00000268805 0.82638 0.2043 0.82638 0.2043 -1.2703 0.89801
-2.0098 0.97778 0.2043 0.634916849
NA ENSG00000226403 0.8262 0.20434 0.8262 0.20434 0.8262 0.20434
0.8262 0.20434 0.20434 0.634916849
COQ10BP2 ENSG00000251552 -0.38352 0.64933 0.82623 0.20434 -0.38352
0.64933 -0.96375 0.83241 0.20434 0.634916849
NA ENSG00000277901 0.82623 0.20434 -0.30428 0.61954 -0.54022 0.70548
0.52322 0.30041 0.20434 0.634916849
NA ENSG00000207231 0.82611 0.20437 0.82611 0.20437 0.82611 0.20437
0.82611 0.20437 0.20437 0.634916849
NA ENSG00000270149 0.8261 0.20437 0.8261 0.20437 0.8261 0.20437
0.8261 0.20437 0.20437 0.634916849
NA ENSG00000253985 -0.60145 0.72623 0.28132 0.38923 -0.59772 0.72499
0.82603 0.20439 0.20439 0.634916849
ICOSLG ENSG00000160223 -0.28911 0.61375 0.82599 0.2044 0.82599
0.2044 0.1577 0.43735 0.2044 0.634916849
NA ENSG00000229494 -0.40654 0.65783 -1.2752 0.89888 0.82589 0.20443
-0.40654 0.65783 0.20443 0.634916849
CDKN1C ENSG00000129757 0.82586 0.20444 -0.87536 0.80931 0.40719

0.34193 -0.87536 0.80931 0.20444 0.634916849
NA ENSG00000201807 0.82581 0.20446 0.82581 0.20446 0.82581 0.20446
0.50223 0.30775 0.20446 0.634916849
HDAC11-AS1 ENSG00000244502 -0.041803 0.51667 0.82575 0.20447 0.29779
0.38293 0.52794 0.29877 0.20447 0.634916849
SLC9A1 ENSG00000090020 0.37619 0.35339 0.82567 0.20449 0.15345
0.43902 0.17739 0.4296 0.20449 0.634916849
NA ENSG00000199962 0.82569 0.20449 0.82569 0.20449 0.82569 0.20449
0.82569 0.20449 0.20449 0.634916849
RUNX3 ENSG00000020633 0.80185 0.21132 0.61667 0.26873 0.82566 0.2045
0.76529 0.22205 0.2045 0.634916849
BFAR ENSG00000103429 -0.057018 0.52273 0.82567 0.2045 0.75974 0.22371
0.6585 0.25511 0.2045 0.634916849
NA ENSG00000255483 0.82561 0.20451 0.82561 0.20451 0.82561 0.20451
0.82561 0.20451 0.20451 0.634916849
NA ENSG00000241667 -0.16487 0.56548 0.8256 0.20452 0.8256 0.20452
0.8256 0.20452 0.20452 0.634916849
KRT18P45 ENSG00000249438 0.35578 0.361 0.35578 0.361 0.57967 0.28107
0.82557 0.20452 0.20452 0.634916849
RIPOR2 ENSG00000111913 0.8197 0.20619 0.82551 0.20454 0.3094
0.37851 0.16561 0.43423 0.20454 0.634916849
LRRIQ3 ENSG00000162620 -0.2193 0.58679 0.82551 0.20454 0.61361
0.26974 0.61361 0.26974 0.20454 0.634916849
LINC02796 ENSG00000227016 0.82551 0.20454 0.82551 0.20454 0.82551
0.20454 0.82551 0.20454 0.20454 0.634916849
TRMT2A ENSG00000099899 0.82547 0.20455 -1.392 0.91803 -0.44379
0.6714 -0.18666 0.57404 0.20455 0.634916849
SKIDA1 ENSG00000180592 0.42136 0.33675 0.64867 0.25827 0.82547
0.20455 0.082179 0.46725 0.20455 0.634916849
ANGEL2 ENSG00000174606 0.74689 0.22756 0.74689 0.22756 0.74689
0.22756 0.82544 0.20456 0.20456 0.634916849
AHRR ENSG00000063438 0.20346 0.41939 0.47959 0.31576 0.82531 0.2046
0.44218 0.32918 0.2046 0.634916849
NA ENSG00000258091 0.81269 0.2082 -0.43613 0.66863 0.81269 0.2082
0.82528 0.20461 0.20461 0.634916849
NA ENSG00000258375 0.82523 0.20462 0.82523 0.20462 0.82523 0.20462
0.82523 0.20462 0.20462 0.634916849
LINC01628 ENSG00000232688 0.8252 0.20463 0.8252 0.20463 0.8252
0.20463 0.8252 0.20463 0.20463 0.634916849
RBM28 ENSG00000106344 -0.74045 0.77049 0.43362 0.33228 -0.38242 0.64892
0.82515 0.20464 0.20464 0.634916849
NA ENSG00000257863 0.82509 0.20466 0.82509 0.20466 0.82509 0.20466
0.82509 0.20466 0.20466 0.634916849
RPL18AP3 ENSG00000213442 0.46216 0.32198 0.46216 0.32198 0.62943
0.26453 0.82505 0.20467 0.20467 0.634916849
TMEM143 ENSG00000161558 -0.0089461 0.50357 0.19541 0.42253 -1.2066
0.88621 0.82499 0.20469 0.20469 0.634916849
RPL4P3 ENSG00000230364 0.82499 0.20469 0.82499 0.20469 0.82499
0.20469 0.82499 0.20469 0.20469 0.634916849
NA ENSG00000279637 -1.001 0.84157 -0.66297 0.74633 0.82497 0.20469
-0.59317 0.72347 0.20469 0.634916849
ASF1B ENSG00000105011 0.82477 0.20475 -1.3762 0.91562 0.040556 0.48383
-1.5718 0.942 0.20475 0.634995772
NIPAL2 ENSG00000104361 0.82475 0.20476 0.093162 0.46289 0.57207
0.28364 0.19104 0.42425 0.20476 0.634995772
RPL23AP80 ENSG00000213035 0.82474 0.20476 0.82474 0.20476 0.82474
0.20476 0.82474 0.20476 0.20476 0.634995772
NA ENSG00000279411 0.82459 0.2048 0.82459 0.2048 0.82459 0.2048
0.82459 0.2048 0.2048 0.634995772
THY1 ENSG00000154096 -0.59069 0.72264 -1.4345 0.92429 -1.12 0.86865
0.82445 0.20484 0.20484 0.634995772
SEPTIN10 ENSG00000186522 0.65107 0.2575 0.82445 0.20484 0.14807

0.44114 -0.12295 0.54893 0.20484 0.634995772
RNU6-84P ENSG00000272445 -1.9003 0.9713 0.30125 0.38161 0.82446
0.20484 0.82446 0.20484 0.20484 0.634995772
NA ENSG00000199894 -0.80661 0.79005 -1.004 0.8423 -0.26444 0.60428
0.82443 0.20485 0.20485 0.634995772
NA ENSG00000270799 0.82432 0.20488 0.82432 0.20488 0.32489 0.37263
0.82432 0.20488 0.20488 0.634995772
RNY3P4 ENSG00000207157 0.82428 0.20489 -1.3094 0.9048 -1.3094
0.9048 -1.3094 0.9048 0.20489 0.634995772
CYP4Z1 ENSG00000186160 -1.3492 0.91137 -0.72934 0.7671 0.8242
0.20491 -0.65014 0.7422 0.20491 0.634995772
LHFPL3 ENSG00000187416 0.82423 0.20491 0.0017792 0.49929 0.060338
0.47594 -0.2004 0.57942 0.20491 0.634995772
NA ENSG00000270207 0.065346 0.47395 0.70948 0.23901 0.82423 0.20491
-0.17547 0.56964 0.20491 0.634995772
NA ENSG00000245482 -2.0736 0.98094 -0.94628 0.828 -1.9719 0.97569
0.82414 0.20493 0.20493 0.634995772
UBE2W ENSG00000104343 0.59812 0.27488 -0.72961 0.76719 0.82411 0.20494
0.54075 0.29434 0.20494 0.634995772
NA ENSG00000216754 -0.18052 0.57163 0.40707 0.34198 -0.5993 0.72551
0.82406 0.20495 0.20495 0.634995772
NA ENSG00000276742 0.82407 0.20495 0.82407 0.20495 0.82407 0.20495
0.82407 0.20495 0.20495 0.634995772
NA ENSG00000283982 -0.90779 0.818 0.82407 0.20495 0.27668 0.39101
0.25096 0.40092 0.20495 0.634995772
NA ENSG00000254461 0.82395 0.20499 0.82395 0.20499 0.82395 0.20499
0.82395 0.20499 0.20499 0.635079325
MPLKIP ENSG00000168303 0.82387 0.20501 0.82387 0.20501 0.82387
0.20501 0.82387 0.20501 0.20501 0.635080098
CDYL ENSG00000153046 0.82383 0.20502 -0.85593 0.80398 -0.10568 0.54208
-0.45546 0.67561 0.20502 0.635080098
NA ENSG00000268390 0.82377 0.20504 0.82377 0.20504 0.82377 0.20504
0.82377 0.20504 0.20504 0.635080098
NA ENSG00000259273 0.69087 0.24482 -0.18635 0.57392 -0.18635 0.57392
0.82356 0.20509 0.20509 0.635080098
SERPING1 ENSG00000149131 0.10676 0.45749 0.82349 0.20511 0.10676
0.45749 0.129 0.44868 0.20511 0.635080098
NA ENSG00000223624 0.8235 0.20511 0.8235 0.20511 0.8235 0.20511
0.8235 0.20511 0.20511 0.635080098
NA ENSG00000254111 0.82351 0.20511 -0.72822 0.76676 -0.72822 0.76676
-1.1317 0.87112 0.20511 0.635080098
NA ENSG00000279779 0.82331 0.20517 0.82331 0.20517 0.82331 0.20517
0.82331 0.20517 0.20517 0.635080098
NA ENSG00000266233 0.8233 0.20517 0.8233 0.20517 0.8233 0.20517
0.8233 0.20517 0.20517 0.635080098
SLAIN2 ENSG00000109171 -0.53085 0.70224 -0.1227 0.54883 -0.86764
0.8072 0.82322 0.20519 0.20519 0.635080098
GAPDHP59 ENSG00000231739 0.82316 0.20521 -0.0030463 0.50122 -1.6263
0.94805 -1.6263 0.94805 0.20521 0.635080098
PRPF31 ENSG00000105618 -0.70548 0.75974 -0.70548 0.75974 0.82312
0.20522 -0.80694 0.79015 0.20522 0.635080098
FABP7 ENSG00000164434 -1.293 0.90199 -1.293 0.90199 0.82313 0.20522
-1.293 0.90199 0.20522 0.635080098
RPL21P32 ENSG00000224019 0.82305 0.20524 0.82305 0.20524 0.82305
0.20524 0.82305 0.20524 0.20524 0.635080098
NA ENSG00000260038 0.82306 0.20524 0.82306 0.20524 0.82306 0.20524
0.82306 0.20524 0.20524 0.635080098
MIR548AK ENSG00000265653 0.82303 0.20525 0.82303 0.20525 0.82303
0.20525 0.82303 0.20525 0.20525 0.635080098
MTSS2 ENSG00000132613 -0.95274 0.82964 0.82296 0.20526 0.79398 0.2136
0.10409 0.45855 0.20526 0.635080098
NA ENSG00000227498 0.34037 0.36679 0.823 0.20526 -0.4164 0.66144

-0.1661 0.56596 0.20526 0.635080098
OLFML3 ENSG00000116774 0.7877 0.21544 0.7877 0.21544 0.82295
0.20527 0.81053 0.20882 0.20527 0.635080098
GDNF ENSG00000168621 0.82286 0.20529 0.15836 0.43709 0.82286 0.20529
0.42069 0.33699 0.20529 0.635080098
RPL34P19 ENSG00000230240 0.82286 0.20529 0.82286 0.20529 -0.75071
0.77359 -0.56486 0.71391 0.20529 0.635080098
NA ENSG00000235993 0.82287 0.20529 -0.13908 0.55531 -0.13908 0.55531
-0.46391 0.67864 0.20529 0.635080098
NA ENSG00000271413 0.82287 0.20529 0.82287 0.20529 0.82287 0.20529
0.82287 0.20529 0.20529 0.635080098
SH3BP1 ENSG00000100092 -0.1824 0.57237 -0.071114 0.52835 0.35106
0.36277 0.82279 0.20531 0.20531 0.635081878
NA ENSG00000250155 -0.061811 0.52464 -0.48119 0.68481 0.077595 0.46907
0.8227 0.20534 0.20534 0.635081878
NA ENSG00000238520 0.82262 0.20536 0.82262 0.20536 0.82262 0.20536
0.40174 0.34394 0.20536 0.635081878
SLC11A1 ENSG00000018280 -0.66283 0.74628 -0.55753 0.71142 -0.34317
0.63427 0.82259 0.20537 0.20537 0.635081878
PLD6 ENSG00000179598 -0.12056 0.54798 0.82253 0.20539 0.82253 0.20539
-0.088587 0.5353 0.20539 0.635081878
DPP3 ENSG00000254986 -0.52177 0.69909 -0.39348 0.65302 -0.91105 0.81887
0.82253 0.20539 0.20539 0.635081878
UNC93B6 ENSG00000255562 0.82249 0.2054 0.82249 0.2054 0.82249
0.2054 0.82249 0.2054 0.2054 0.635081878
SMAP2 ENSG00000084070 0.29901 0.38247 0.82241 0.20542 0.0014251 0.49943
-0.079415 0.53165 0.20542 0.635081878
NA ENSG00000259753 0.82242 0.20542 0.82242 0.20542 0.82242 0.20542
0.82242 0.20542 0.20542 0.635081878
NDUFA11 ENSG00000174886 0.52397 0.30015 0.82232 0.20545 0.52397
0.30015 -0.49208 0.68867 0.20545 0.635081878
CNN2P10 ENSG00000232223 0.8223 0.20545 0.8223 0.20545 0.8223
0.20545 0.8223 0.20545 0.20545 0.635081878
NA ENSG00000249433 0.8223 0.20545 0.8223 0.20545 0.8223 0.20545
-0.72065 0.76444 0.20545 0.635081878
MEX3C ENSG00000176624 0.82227 0.20546 0.82227 0.20546 0.82227 0.20546
-0.50499 0.69322 0.20546 0.635081878
KIF25 ENSG00000125337 -0.81956 0.79377 0.82216 0.20549 -1.3497 0.91144
0.2783 0.39039 0.20549 0.635094038
C1orf131 ENSG00000143633 0.23875 0.40565 0.82219 0.20549 0.82219
0.20549 0.57565 0.28243 0.20549 0.635094038
DAP ENSG00000112977 -0.15126 0.56011 -0.51293 0.696 0.2798 0.38982
0.82204 0.20553 0.20553 0.635107952
C10orf99 ENSG00000188373 -0.57004 0.71567 0.12246 0.45127 -0.86232
0.80574 0.82187 0.20558 0.20558 0.635107952
ADH1A ENSG00000187758 -1.1035 0.86509 -0.94572 0.82785 0.16066 0.43618
0.82183 0.20559 0.20559 0.635107952
NA ENSG00000232959 0.82183 0.20559 0.13542 0.44614 0.13542 0.44614
0.13542 0.44614 0.20559 0.635107952
NA ENSG00000264303 -0.55067 0.70907 0.042382 0.4831 0.82182 0.20559
0.082058 0.4673 0.20559 0.635107952
ZBTB2 ENSG00000181472 0.82178 0.2056 0.82178 0.2056 0.66706 0.25237
-0.44761 0.67278 0.2056 0.635107952
NA ENSG00000256927 0.62474 0.26607 0.82179 0.2056 -1.8589 0.96848
-1.8589 0.96848 0.2056 0.635107952
TMEM160 ENSG00000130748 -0.34333 0.63432 -0.39763 0.65455 -0.34333
0.63432 0.82172 0.20562 0.20562 0.635107952
NA ENSG00000270694 0.51712 0.30254 0.50586 0.30648 0.39403 0.34678
0.82169 0.20563 0.20563 0.635107952
NA ENSG00000201980 0.82164 0.20564 0.82164 0.20564 0.82164 0.20564
0.82164 0.20564 0.20564 0.635107952
FOXE3 ENSG00000186790 -0.083541 0.53329 -0.083541 0.53329 -0.083541 0.53329

								0.82162	0.20565	0.20565	0.635107952
PHTF1	ENSG00000116793	0.82157	0.20566	-1.329	0.90808	-0.69421	0.75622	-1.1942	0.8838	0.20566	0.635107952
FNDC1-IT1	ENSG00000235086	0.82151	0.20568	0.82151	0.20568	0.82151	0.20568	0.82151	0.20568	0.20568	0.635107952
NA	ENSG00000264051	0.82151	0.20568	0.82151	0.20568	0.82151	0.20568	0.82151	0.20568	0.20568	0.635107952
NA	ENSG00000249429	0.82148	0.20569	0.82148	0.20569	0.81918	0.20634	0.17229	0.43161	0.20569	0.635107952
NA	ENSG00000243305	0.82125	0.20575	0.82125	0.20575	0.82125	0.20575	0.82125	0.20575	0.20575	0.635212715
NA	ENSG00000261393	0.82127	0.20575	0.82127	0.20575	0.82127	0.20575	0.82127	0.20575	0.20575	0.635212715
NA	ENSG00000229395	0.7777	0.21837	-0.52132	0.69893	0.82118	0.20577	-0.43848	0.66948	0.20577	0.635234216
FAM245A	ENSG00000228055	0.21812	0.41367	0.82093	0.20584	-0.91719	0.82048	0.046641	0.4814	0.20584	0.635288189
NA	ENSG00000241157	0.82094	0.20584	0.82094	0.20584	0.82094	0.20584	0.82094	0.20584	0.20584	0.635288189
ZNF92P2	ENSG00000269505	0.82089	0.20586	0.82089	0.20586	0.82089	0.20586	0.82089	0.20586	0.20586	0.635288189
CECR2	ENSG00000099954	-0.7764	0.78124	0.82082	0.20587	0.74407	0.22842	-0.87897	0.81029	0.20587	0.635288189
EAF2	ENSG00000145088	0.82079	0.20588	-0.9644	0.83258	-0.15809	0.56281	-0.9644	0.83258	0.20588	0.635288189
OR2C1	ENSG00000168158	0.81357	0.20794	0.69625	0.24314	0.58275	0.28003	0.82079	0.20588	0.20588	0.635288189
NA	ENSG00000257964	0.82077	0.20589	-0.36394	0.64205	0.82077	0.20589	0.82077	0.20589	0.20589	0.635288189
NA	ENSG00000230908	-0.19081	0.57566	0.82061	0.20593	-0.16942	0.56727	-0.048321	0.51927	0.20593	0.635288189
KRTAP5-6	ENSG00000205864	0.36145	0.35888	0.36145	0.35888	-1.3979	0.91893	0.8206	0.20594	0.20594	0.635288189
NA	ENSG00000247810	0.25197	0.40053	0.093727	0.46266	0.093727	0.46266	0.82054	0.20595	0.20595	0.635288189
NA	ENSG00000277608	0.82052	0.20596	0.37903	0.35233	0.37903	0.35233	0.37903	0.35233	0.20596	0.635288189
NA	ENSG00000251506	-0.7576	0.77565	0.82048	0.20597	0.18287	0.42745	0.18287	0.42745	0.20597	0.635288189
RFPL4AP7	ENSG00000254268	0.4938	0.31073	0.4938	0.31073	0.8205	0.20597	0.4938	0.31073	0.20597	0.635288189
NA	ENSG00000255468	0.82049	0.20597	0.46718	0.32018	0.82049	0.20597	0.82049	0.20597	0.20597	0.635288189
DNAJC5G	ENSG00000163793	0.82036	0.20601	0.82036	0.20601	0.82036	0.20601	0.82036	0.20601	0.20601	0.635371348
RPSAP45	ENSG00000219773	0.82027	0.20603	0.82027	0.20603	0.82027	0.20603	0.19191	0.42391	0.20603	0.635392817
TKT	ENSG00000163931	-0.36027	0.64068	0.11213	0.45536	0.82022	0.20605	0.21038	0.41669	0.20605	0.63540491
CDC34	ENSG00000099804	0.50309	0.30745	-0.010897	0.50435	0.060343	0.47594	0.82018	0.20606	0.20606	0.63540491
NA	ENSG00000241690	0.81997	0.20612	0.81997	0.20612	0.81997	0.20612	0.81997	0.20612	0.20612	0.635413066
OTOA	ENSG00000155719	-0.12071	0.54804	0.81992	0.20613	-0.956	0.83046	-0.31059	0.62194	0.20613	0.635413066
RABEP1	ENSG00000029725	0.78471	0.21631	0.81986	0.20615	0.81986	0.20615	0.75221	0.22596	0.20615	0.635413066
DHFRP1	ENSG00000188985	0.10289	0.45903	0.81987	0.20615	0.10289	0.45903	0.10289	0.45903	0.20615	0.635413066
SRSF3P5	ENSG00000235325	0.35147	0.36262	0.35147	0.36262	-0.15307	0.56083	0.81984	0.20615	0.20615	0.635413066
NA	ENSG00000251229	0.81986	0.20615	0.81986	0.20615	0.81986	0.20615				

0.81986 0.20615 0.20615 0.635413066
NFKB2 ENSG00000077150 0.20346 0.41939 0.8198 0.20617 0.8198 0.20617
0.8198 0.20617 0.20617 0.635413066
LINC02075 ENSG00000249497 0.81979 0.20617 -0.37013 0.64436 0.81979
0.20617 0.81979 0.20617 0.20617 0.635413066
PI4KB ENSG00000143393 0.077468 0.46913 -0.32145 0.62606 0.81976 0.20618
0.54086 0.2943 0.20618 0.635413066
CXCL1P1 ENSG00000250339 0.58664 0.27872 -0.44617 0.67226 0.81966
0.20621 0.80172 0.21136 0.20621 0.635465332
EPB41L3 ENSG00000082397 -1.8612 0.96864 0.81917 0.20634 0.61617
0.26889 -1.1695 0.87889 0.20634 0.635825737
TACSTD2 ENSG00000184292 0.8191 0.20636 0.8191 0.20636 0.8191
0.20636 0.8191 0.20636 0.20636 0.635837766
OR13Z2P ENSG00000272443 0.81908 0.20637 0.81908 0.20637 0.80083
0.21161 0.32842 0.3713 0.20637 0.635837766
CRADD ENSG00000169372 0.81903 0.20639 0.81903 0.20639 0.81903 0.20639
0.38268 0.35098 0.20639 0.635859183
GATD3A ENSG00000160221 0.57214 0.28361 0.81886 0.20643 0.13447
0.44651 0.46555 0.32077 0.20643 0.635879088
NA ENSG00000121388 0.81881 0.20645 0.22317 0.4117 0.0036531 0.49854
0.76123 0.22326 0.20645 0.635879088
NLRC3 ENSG00000167984 0.81877 0.20646 0.33706 0.36804 0.67419 0.2501
0.55115 0.29077 0.20646 0.635879088
NA ENSG00000227965 0.81878 0.20646 0.81878 0.20646 0.81878 0.20646
0.81878 0.20646 0.20646 0.635879088
NA ENSG00000244255 0.81875 0.20647 0.81875 0.20647 0.81875 0.20647
0.81875 0.20647 0.20647 0.635879088
NA ENSG00000279620 0.73048 0.23255 0.81868 0.20648 -0.13446 0.55348
0.81868 0.20648 0.20648 0.635879088
NA ENSG00000200891 -0.37832 0.6474 0.81855 0.20652 -0.37832 0.6474
-0.37832 0.6474 0.20652 0.635879088
RPLP0 ENSG00000089157 0.81847 0.20654 0.81847 0.20654 0.81847 0.20654
0.81847 0.20654 0.20654 0.635879088
CAB39L ENSG00000102547 0.50546 0.30662 0.50402 0.30712 0.8185
0.20654 0.11558 0.45399 0.20654 0.635879088
SLC27A1 ENSG00000130304 -0.57371 0.71692 -0.064838 0.52585 0.33566
0.36857 0.81847 0.20654 0.20654 0.635879088
NA ENSG00000279093 0.8185 0.20654 0.8185 0.20654 0.8185 0.20654
0.8185 0.20654 0.20654 0.635879088
CPA2 ENSG00000158516 0.81835 0.20658 -0.50566 0.69345 -0.0429 0.51711
0.44987 0.3264 0.20658 0.635962057
SMARCA5 ENSG00000153147 -0.1342 0.55338 0.10281 0.45906 0.81822
0.20662 0.0054885 0.49781 0.20662 0.636004839
VTI1BP2 ENSG00000213434 0.73367 0.23158 0.20969 0.41696 0.81822
0.20662 -0.55234 0.70964 0.20662 0.636004839
GTF2IP6 ENSG00000266736 -0.59028 0.7225 0.11878 0.45273 -0.52138
0.69895 0.81815 0.20664 0.20664 0.636026225
NA ENSG00000262434 0.81805 0.20666 0.81805 0.20666 0.81805 0.20666
0.81805 0.20666 0.20666 0.636038215
MYO1H ENSG00000174527 0.25885 0.39788 -0.10485 0.54175 0.70765 0.23958
0.81802 0.20667 0.20667 0.636038215
RN7SL205P ENSG00000241392 0.81796 0.20669 -0.97989 0.83643 -0.68354
0.75287 -0.17313 0.56872 0.20669 0.636040809
NA ENSG00000278532 0.81793 0.2067 -0.80706 0.79018 -1.4994 0.93311
-1.4994 0.93311 0.2067 0.636040809
NA ENSG00000248148 0.72196 0.23516 0.4952 0.31023 0.8179 0.20671
0.6698 0.25149 0.20671 0.636040809
NA ENSG00000276663 0.81772 0.20676 0.81772 0.20676 0.81772 0.20676
0.81772 0.20676 0.20676 0.636135694
SMAD6 ENSG00000137834 0.062559 0.47506 0.13407 0.44667 -0.89938 0.81578
0.81765 0.20678 0.20678 0.636135694
NA ENSG00000230333 0.55063 0.29094 0.81765 0.20678 0.45699 0.32384

-0.20586 0.58155 0.20678 0.636135694
NA ENSG00000282375 0.81757 0.2068 -0.10381 0.54134 -0.40507 0.65729
-0.012622 0.50504 0.2068 0.636157061
ATP6V1E1P1 ENSG00000225099 0.8174 0.20685 0.8174 0.20685 -0.23851
0.59426 -1.6404 0.94954 0.20685 0.636270704
NOS2P3 ENSG00000230528 -2.1182 0.98292 -1.5469 0.93905 0.81732
0.20687 -1.8363 0.96684 0.20687 0.63629206
NA ENSG00000272397 -0.098554 0.53925 -0.098554 0.53925 -0.098554 0.53925
0.81727 0.20689 0.20689 0.636313412
NA ENSG00000254007 -0.3176 0.6246 -1.5013 0.93336 0.81717 0.20692
0.18068 0.42831 0.20692 0.636346703
MIR1915HG ENSG00000204682 0.21449 0.41508 0.78874 0.21513 0.81712
0.20693 0.43159 0.33302 0.20693 0.636346703
UGT2B15 ENSG00000196620 -1.8672 0.96906 -0.44596 0.67219 -0.69897
0.75772 0.81708 0.20694 0.20694 0.636346703
NA ENSG00000261723 0.81694 0.20698 0.81694 0.20698 0.81694 0.20698
0.81694 0.20698 0.20698 0.636429543
SVOPL ENSG00000157703 0.058779 0.47656 -1.4725 0.92956 0.81679 0.20702
-0.5173 0.69753 0.20702 0.636512373
NRG2 ENSG00000158458 -0.24452 0.59659 0.81673 0.20704 -1.0144 0.8448
-1.7207 0.95735 0.20704 0.636526788
NA ENSG00000257262 -0.40974 0.659 -0.13899 0.55527 -0.34634 0.63546
0.81663 0.20707 0.20707 0.636526788
PHETA2 ENSG00000177096 0.43286 0.33256 0.81659 0.20708 0.37902
0.35234 0.54754 0.292 0.20708 0.636526788
GGT1 ENSG00000100031 0.59502 0.27591 -0.98616 0.83797 0.81655 0.20709
-0.0604 0.52408 0.20709 0.636526788
NA ENSG00000238871 0.81657 0.20709 0.70841 0.23935 0.62401 0.26631
0.62401 0.26631 0.20709 0.636526788
LINC01967 ENSG00000235493 -0.88112 0.81087 0.8164 0.20714 0.51754
0.30239 0.8164 0.20714 0.20714 0.636640315
RNU6-560P ENSG00000200877 -0.51512 0.69677 -0.51512 0.69677 -0.51512
0.69677 0.81627 0.20717 0.20717 0.63665221
EFCAB8 ENSG00000215529 0.81627 0.20717 0.64939 0.25804 0.096975
0.46137 -0.28994 0.61407 0.20717 0.63665221
TBC1D22A-AS1 ENSG00000280080 0.81618 0.2072 0.81618 0.2072 0.81618
0.2072 0.81618 0.2072 0.2072 0.636654685
ABL2 ENSG00000143322 0.02978 0.48812 0.65845 0.25512 0.78459 0.21635
0.81614 0.20721 0.20721 0.636654685
NA ENSG00000212428 0.81613 0.20721 0.81613 0.20721 0.81613 0.20721
0.81613 0.20721 0.20721 0.636654685
NA ENSG00000280222 0.81603 0.20724 0.59288 0.27663 0.81603 0.20724
0.81603 0.20724 0.20724 0.636706715
RPL7L1P12 ENSG00000231981 0.81592 0.20727 0.5053 0.30667 0.81592
0.20727 0.81592 0.20727 0.20727 0.636718597
NA ENSG00000236544 0.81591 0.20727 0.81591 0.20727 0.67552 0.24967
0.48859 0.31256 0.20727 0.636718597
NA ENSG00000243781 0.81568 0.20734 0.81568 0.20734 0.81568 0.20734
0.81568 0.20734 0.20734 0.636843912
NA ENSG00000221565 0.81567 0.20734 0.81567 0.20734 0.81567 0.20734
0.81567 0.20734 0.20734 0.636843912
ADIRF-AS1 ENSG00000272734 0.80914 0.20922 0.81566 0.20735 0.81566
0.20735 0.81566 0.20735 0.20735 0.636843912
DTD1 ENSG00000125821 -1.8918 0.97074 -1.8547 0.96818 0.8155 0.20739
-0.094782 0.53776 0.20739 0.636926621
RHOG ENSG00000177105 0.81542 0.20742 -1.0873 0.86154 -1.7108 0.95644
-0.56176 0.71286 0.20742 0.636978611
C1orf94 ENSG00000142698 0.81534 0.20744 0.41147 0.34036 -0.88035
0.81066 -1.057 0.85474 0.20744 0.636999887
ABRACL ENSG00000146386 0.81486 0.20758 0.81486 0.20758 0.81486
0.20758 0.81486 0.20758 0.20758 0.637361261
NA ENSG00000253796 -0.039243 0.51565 0.81482 0.20759 0.27805 0.39049

0.09182 0.46342 0.20759 0.637361261
CPXM1 ENSG00000088882 0.81474 0.20761 0.81474 0.20761 0.81474 0.20761
0.81474 0.20761 0.20761 0.637361261
LINC00547 ENSG0000275226 0.68114 0.24789 0.43013 0.33355 0.52703
0.29909 0.81475 0.20761 0.20761 0.637361261
NA ENSG00000231335 -1.2954 0.90242 -1.2569 0.89561 -1.263 0.89671
0.81463 0.20764 0.20764 0.637413204
NA ENSG00000259541 -0.55745 0.71139 -1.0257 0.84749 0.81449 0.20768
0.81449 0.20768 0.20768 0.637464955
LINC02245 ENSG00000237638 -0.12635 0.55027 0.8144 0.20771 -1.0897
0.86209 0.72321 0.23478 0.20771 0.637464955
EGLN1P1 ENSG00000254779 0.8144 0.20771 0.8144 0.20771 0.8144
0.20771 0.8144 0.20771 0.20771 0.637464955
BLCAP ENSG00000166619 -0.18341 0.57276 0.81422 0.20776 0.174 0.43093 0.174
0.43093 0.20776 0.637464955
NA ENSG00000207407 -0.66181 0.74595 0.81421 0.20776 -0.02585 0.51031
0.15819 0.43715 0.20776 0.637464955
NA ENSG00000278305 -0.18071 0.5717 0.81421 0.20776 -0.10851 0.5432
0.17611 0.4301 0.20776 0.637464955
EIF4A1P8 ENSG00000213484 0.089326 0.46441 -0.94019 0.82644 0.52886
0.29845 0.81414 0.20778 0.20778 0.637464955
NA ENSG00000231977 0.19209 0.42384 0.41545 0.3389 0.81415 0.20778
0.22371 0.41149 0.20778 0.637464955
NA ENSG00000264921 0.81409 0.2078 0.81409 0.2078 0.81409 0.2078
0.81409 0.2078 0.2078 0.637464955
NDUFA6 ENSG00000184983 0.81399 0.20782 0.81399 0.20782 0.81399
0.20782 0.81399 0.20782 0.20782 0.637464955
NA ENSG00000261884 0.81402 0.20782 0.81402 0.20782 0.81402 0.20782
0.81402 0.20782 0.20782 0.637464955
PRR33 ENSG00000283787 0.50569 0.30654 0.81398 0.20783 0.50569 0.30654
0.50569 0.30654 0.20783 0.637464955
FAM83A ENSG00000147689 0.45351 0.32509 0.70846 0.23933 0.81392
0.20784 0.81169 0.20848 0.20784 0.637464955
IGKV1OR2-6 ENSG00000271569 0.81395 0.20784 -0.67906 0.75145 -0.67906
0.75145 -0.83883 0.79922 0.20784 0.637464955
FMO11P ENSG00000232148 -0.12013 0.54781 -0.6034 0.72688 0.0052056
0.49792 0.81382 0.20787 0.20787 0.637467271
SEC63 ENSG00000025796 0.81379 0.20788 0.6939 0.24387 -0.31751 0.62457
-0.024546 0.50979 0.20788 0.637467271
NA ENSG00000225588 0.15238 0.43944 0.68815 0.24568 0.81379 0.20788
0.60404 0.27291 0.20788 0.637467271
OR8Q1P ENSG00000255341 -0.88974 0.8132 -0.041356 0.51649 -0.9412
0.8267 0.81364 0.20793 0.20793 0.637556707
NA ENSG00000260536 0.81354 0.20795 -0.50079 0.69174 -0.50079 0.69174
-0.50079 0.69174 0.20795 0.637556707
NPM1P37 ENSG00000219085 0.81345 0.20798 0.81345 0.20798 0.81345
0.20798 0.81345 0.20798 0.20798 0.637556707
NA ENSG00000234703 0.24019 0.40509 0.45422 0.32484 0.24019 0.40509
0.81346 0.20798 0.20798 0.637556707
OLA1P3 ENSG00000257966 0.81338 0.208 0.72325 0.23476 0.72325 0.23476
0.72325 0.23476 0.208 0.637556707
NA ENSG00000235612 0.81335 0.20801 -0.65652 0.74425 0.81335 0.20801
0.81335 0.20801 0.20801 0.637556707
KDM4B ENSG00000127663 -0.61892 0.73202 -0.64534 0.74065 0.81332 0.20802
-1.511 0.93461 0.20802 0.637556707
ITIH2 ENSG00000151655 0.68798 0.24573 0.68798 0.24573 0.81327 0.20803
-0.39658 0.65416 0.20803 0.637556707
NA ENSG00000230794 -0.014116 0.50563 0.29056 0.38569 0.38822 0.34893
0.81327 0.20803 0.20803 0.637556707
NA ENSG00000244468 0.64051 0.26092 0.81323 0.20804 0.64218 0.26038
0.64218 0.26038 0.20804 0.637556707
CIRBP-AS1 ENSG00000267493 0.81297 0.20812 0.81297 0.20812 0.81297

0.20812 0.81297 0.20812 0.20812 0.637761766
NA ENSG00000271156 0.22709 0.41018 0.37567 0.35358 0.81289 0.20814
0.81289 0.20814 0.20814 0.637782947
LINC01840 ENSG00000230215 -0.055474 0.52212 0.15329 0.43908 0.49866
0.30901 0.81275 0.20818 0.20818 0.637828345
NA ENSG00000272984 0.81266 0.20821 0.81266 0.20821 0.81266 0.20821
0.81266 0.20821 0.20821 0.637828345
FAM89B ENSG00000176973 0.5513 0.29071 0.67361 0.25028 0.81263
0.20822 0.81263 0.20822 0.20822 0.637828345
MIEF1 ENSG00000100335 -0.90786 0.81802 -1.1565 0.87626 -0.56185 0.71289
0.81254 0.20824 0.20824 0.637828345
POLR2KP1 ENSG00000258284 0.8125 0.20825 0.8125 0.20825 0.8125
0.20825 0.8125 0.20825 0.20825 0.637828345
LINC02633 ENSG00000224265 0.10078 0.45986 0.10078 0.45986 0.81238
0.20829 -1.0783 0.85955 0.20829 0.637828345
LINC02473 ENSG00000261121 -0.064817 0.52584 -0.064817 0.52584 0.81234
0.2083 0.13159 0.44765 0.2083 0.637828345
NA ENSG00000283328 0.81232 0.2083 0.81232 0.2083 0.81232 0.2083
0.81232 0.2083 0.2083 0.637828345
RPL23AP38 ENSG00000184423 -0.33579 0.63149 0.57585 0.28236 0.81224
0.20833 -0.23811 0.5941 0.20833 0.637828345
NA ENSG00000283761 0.8122 0.20834 0.8122 0.20834 0.8122 0.20834
0.8122 0.20834 0.20834 0.637828345
TSHB ENSG00000134200 0.81217 0.20835 0.81217 0.20835 0.81217 0.20835
0.81217 0.20835 0.20835 0.637828345
PABPC4 ENSG00000090621 -1.2754 0.89892 0.81214 0.20836 0.010781
0.4957 -1.1709 0.87918 0.20836 0.637828345
DHX36 ENSG00000174953 0.68213 0.24758 0.090384 0.46399 0.27269 0.39255
0.81197 0.20841 0.20841 0.637828345
MIR4633 ENSG00000264563 0.81194 0.20841 0.81194 0.20841 0.81194
0.20841 0.81194 0.20841 0.20841 0.637828345
NA ENSG00000268293 0.81196 0.20841 0.81196 0.20841 0.81196 0.20841
0.81196 0.20841 0.20841 0.637828345
CHD8 ENSG00000100888 0.81187 0.20843 -0.76195 0.77695 0.70968 0.23895
-0.69344 0.75598 0.20843 0.637828345
AIMP1P1 ENSG00000234187 0.81185 0.20844 0.81185 0.20844 0.81185
0.20844 0.81185 0.20844 0.20844 0.637828345
CACNA1A ENSG00000141837 -0.75238 0.77409 -0.50665 0.6938 -0.042587
0.51698 0.8118 0.20845 0.20845 0.637828345
PCF11 ENSG00000165494 0.47477 0.31748 0.81182 0.20845 0.47477 0.31748
0.61747 0.26846 0.20845 0.637828345
NA ENSG00000266774 -1.2856 0.90072 0.54442 0.29308 -1.2856 0.90072
0.81182 0.20845 0.20845 0.637828345
EIF2S2 ENSG00000125977 0.8117 0.20848 0.53919 0.29488 -0.51049
0.69515 -0.13575 0.55399 0.20848 0.637828345
RPS2P17 ENSG00000213399 0.81168 0.20849 -0.38898 0.65135 -0.38898
0.65135 -0.38898 0.65135 0.20849 0.637828345
PPTC7 ENSG00000196850 0.81156 0.20852 0.40938 0.34113 -0.56224 0.71302
-0.4704 0.68096 0.20852 0.637828345
CYP2J2 ENSG00000134716 0.8115 0.20854 -0.15361 0.56104 0.8115
0.20854 0.8115 0.20854 0.20854 0.637828345
NA ENSG00000233271 0.81145 0.20855 0.81145 0.20855 0.81145 0.20855
0.81145 0.20855 0.20855 0.637828345
HSD3B2 ENSG00000203859 -0.78306 0.7832 -0.61969 0.73227 0.54507
0.29285 0.81139 0.20857 0.20857 0.637828345
C22orf39 ENSG00000242259 0.81135 0.20858 0.81135 0.20858 0.81135
0.20858 0.81135 0.20858 0.20858 0.637828345
MAS1 ENSG00000130368 -0.10268 0.54089 0.2423 0.40427 0.81127 0.2086
-0.47137 0.68131 0.2086 0.637828345
LINC01280 ENSG00000224391 0.81126 0.20861 -0.82397 0.79502 -0.61816
0.73177 -1.1446 0.87382 0.20861 0.637828345
CHST13 ENSG00000180767 0.14794 0.4412 -0.49858 0.69096 0.79412

0.21356 0.8112 0.20862 0.20862 0.637828345
MIR613 ENSG00000207983 0.81123 0.20862 0.54682 0.29225 0.81123
0.20862 0.0025769 0.49897 0.20862 0.637828345
TMEM11 ENSG00000178307 -0.25586 0.60097 0.81119 0.20863 0.56215
0.28701 0.28603 0.38743 0.20863 0.637828345
NA ENSG00000236213 0.81114 0.20864 -0.27181 0.60712 0.025721 0.48974
0.81114 0.20864 0.20864 0.637828345
PTMAP8 ENSG00000243014 0.81113 0.20864 0.56477 0.28612 -0.54225
0.70618 -1.0599 0.8554 0.20864 0.637828345
NA ENSG00000251172 0.81115 0.20864 0.81115 0.20864 0.81115 0.20864
0.81115 0.20864 0.20864 0.637828345
HIGD1AP8 ENSG00000237303 0.81105 0.20867 0.81105 0.20867 0.81105
0.20867 -0.95213 0.82949 0.20867 0.637828345
NT5DC3 ENSG00000111696 0.54309 0.29353 0.54309 0.29353 0.54309
0.29353 0.81102 0.20868 0.20868 0.637828345
NA ENSG00000231266 0.81101 0.20868 0.81101 0.20868 0.81101 0.20868
0.81101 0.20868 0.20868 0.637828345
ANXA3 ENSG00000138772 0.81091 0.20871 0.12034 0.45211 -0.45654 0.676
-0.13367 0.55317 0.20871 0.637828345
MIR518A2 ENSG00000207699 0.81089 0.20871 -0.47626 0.68305 0.28045
0.38957 0.34688 0.36434 0.20871 0.637828345
NA ENSG00000272299 0.81086 0.20872 0.78159 0.21723 0.37151 0.35513
0.37151 0.35513 0.20872 0.637828345
RTN3P1 ENSG00000251333 0.34689 0.36434 -1.2849 0.90059 0.81083
0.20873 -0.25856 0.60201 0.20873 0.637828345
RNU6-176P ENSG00000253005 0.81085 0.20873 0.65382 0.25661 0.81085
0.20873 0.81085 0.20873 0.20873 0.637828345
NA ENSG00000275348 -0.70215 0.75871 -0.49682 0.69034 0.81081 0.20874
-0.49682 0.69034 0.20874 0.637828345
SMIM15-AS1 ENSG00000251279 -0.011663 0.50465 -1.0451 0.85201 0.81076
0.20875 -0.53013 0.70199 0.20875 0.637828345
INF2 ENSG00000203485 -0.94721 0.82824 -1.113 0.86715 -1.3334 0.90881
0.81073 0.20876 0.20876 0.637828345
NA ENSG00000226599 0.81069 0.20877 0.81069 0.20877 0.81069 0.20877
0.81069 0.20877 0.20877 0.637828345
RNU6-726P ENSG00000201550 0.81053 0.20882 -1.1129 0.86712 0.016591
0.49338 0.81053 0.20882 0.20882 0.637941107
RPL7P38 ENSG00000239473 0.81043 0.20885 0.81043 0.20885 0.81043
0.20885 0.81043 0.20885 0.20885 0.63799276
NA ENSG00000267672 0.8103 0.20888 0.8103 0.20888 0.8103 0.20888
0.8103 0.20888 0.20888 0.638044405
NA ENSG00000273449 0.81025 0.2089 0.81025 0.2089 0.81025 0.2089
0.81025 0.2089 0.2089 0.638065501
NR1I3 ENSG00000143257 0.81019 0.20892 0.81019 0.20892 0.43881 0.3304
-0.20186 0.57999 0.20892 0.638086594
NRN1 ENSG00000124785 -0.47346 0.68206 0.11532 0.4541 0.80998 0.20898
0.59131 0.27716 0.20898 0.638210929
NA ENSG00000280374 0.80992 0.20899 0.80992 0.20899 0.80992 0.20899
0.80992 0.20899 0.20899 0.638210929
NA ENSG00000215146 -0.84009 0.79957 -0.28289 0.61137 -0.7977 0.78748
0.8099 0.209 0.209 0.638210929
ZC2HC1A ENSG00000104427 -1.1609 0.87716 0.16281 0.43533 -1.3469 0.911
0.8098 0.20903 0.20903 0.638213094
MIR199A2 ENSG00000208024 0.80979 0.20903 0.80979 0.20903 0.80979
0.20903 0.80979 0.20903 0.20903 0.638213094
NA ENSG00000280604 0.80976 0.20904 0.80976 0.20904 0.80976 0.20904
0.80976 0.20904 0.20904 0.638213094
NA ENSG00000249513 0.80968 0.20906 0.80968 0.20906 0.80968 0.20906
0.80968 0.20906 0.20906 0.638224713
NA ENSG00000258595 -0.036488 0.51455 -0.075245 0.52999 0.24126 0.40468
0.80965 0.20907 0.20907 0.638224713
LINC01194 ENSG00000248131 0.80953 0.2091 0.16176 0.43575 0.16176

0.43575 -0.54475 0.70704 0.2091 0.638238489
CPLX1 ENSG00000168993 0.80951 0.20911 -0.023109 0.50922 -0.040095 0.51599
-0.26143 0.60312 0.20911 0.638238489
CFAP97 ENSG00000164323 -0.53027 0.70204 -0.29264 0.6151 0.80946
0.20913 -0.40335 0.65666 0.20913 0.638238489
LINC00211 ENSG00000237803 -0.095286 0.53796 0.80945 0.20913 0.14132
0.44381 -0.6544 0.74357 0.20913 0.638238489
GRXCR1 ENSG00000215203 -0.35435 0.63846 -0.73689 0.7694 0.48786
0.31282 0.80942 0.20914 0.20914 0.638238489
PCDHGB1 ENSG00000254221 0.80926 0.20918 0.80926 0.20918 0.80926
0.20918 0.80926 0.20918 0.20918 0.638320584
RNU5A-4P ENSG00000252397 0.80919 0.2092 0.80919 0.2092 0.80919
0.2092 0.80919 0.2092 0.2092 0.638336465
NA ENSG00000257890 -1.5941 0.94455 -0.93905 0.82615 -1.5406 0.93829
0.80907 0.20924 0.20924 0.638336465
NA ENSG00000262526 0.80901 0.20925 0.80901 0.20925 0.80901 0.20925
0.80901 0.20925 0.20925 0.638336465
NA ENSG00000259280 -0.44432 0.67159 -0.14008 0.5557 -0.4011 0.65583
0.80895 0.20927 0.20927 0.638336465
TREML4 ENSG00000188056 0.69598 0.24322 0.49478 0.31038 0.4232
0.33607 0.80893 0.20928 0.20928 0.638336465
ZNF137P ENSG00000123870 0.48664 0.31326 0.80889 0.20929 0.48664
0.31326 0.48664 0.31326 0.20929 0.638336465
CHCHD3P1 ENSG00000236420 0.8089 0.20929 0.8089 0.20929 0.8089
0.20929 0.8089 0.20929 0.20929 0.638336465
REELD1 ENSG00000250673 -0.93867 0.82605 -0.028375 0.51132 0.80889
0.20929 0.56578 0.28577 0.20929 0.638336465
NA ENSG00000257797 0.80883 0.20931 0.80883 0.20931 0.80883 0.20931
0.80883 0.20931 0.20931 0.638348057
GNGT1 ENSG00000127928 -0.53132 0.7024 -0.74154 0.77082 -0.25657 0.60125
0.8088 0.20932 0.20932 0.638348057
RALGDS ENSG00000160271 0.30294 0.38097 0.69216 0.24442 0.80858
0.20938 0.4671 0.32021 0.20938 0.638481616
NA ENSG00000260322 -1.4455 0.92585 0.80855 0.20939 -1.4455 0.92585
-1.4455 0.92585 0.20939 0.638481616
MTND4LP9 ENSG00000227795 0.80847 0.20941 0.80847 0.20941 -0.7054
0.75972 0.80847 0.20941 0.20941 0.638502647
TRGJP2 ENSG00000211688 0.80837 0.20944 0.80837 0.20944 0.80837
0.20944 0.80837 0.20944 0.20944 0.638554164
OR11A1 ENSG00000204694 0.80816 0.2095 0.80816 0.2095 0.80816
0.2095 0.80816 0.2095 0.2095 0.638697135
NA ENSG00000254581 0.11836 0.45289 0.80796 0.20956 0.31403 0.37675
0.31403 0.37675 0.20956 0.638781973
NA ENSG00000254084 0.58956 0.27774 0.58956 0.27774 0.58956 0.27774
0.80792 0.20957 0.20957 0.638781973
GJE1 ENSG00000203733 0.80785 0.20959 0.80785 0.20959 0.80785 0.20959
0.80785 0.20959 0.20959 0.638781973
SLC35A5 ENSG00000138459 -0.13348 0.55309 -0.36713 0.64324 -0.15806
0.56279 0.80782 0.2096 0.2096 0.638781973
ABHD14A ENSG00000248487 0.80782 0.2096 0.80782 0.2096 0.80782
0.2096 0.80782 0.2096 0.2096 0.638781973
SNORD2 ENSG00000238942 0.80766 0.20964 0.80766 0.20964 0.80766
0.20964 0.80766 0.20964 0.20964 0.638781973
NA ENSG00000236267 0.80762 0.20965 0.80762 0.20965 0.80762 0.20965
-0.12728 0.55064 0.20965 0.638781973
NA ENSG00000255491 0.37477 0.35392 0.80765 0.20965 0.52528 0.2997
-0.40667 0.65787 0.20965 0.638781973
MIR548AE1 ENSG00000266808 -0.26886 0.60598 0.70671 0.23987 0.80758
0.20967 0.80758 -0.20967 0.20967 0.638781973
USP14 ENSG00000101557 -1.3025 0.90362 -1.2002 0.88497 -1.2576 0.89574
0.80752 0.20968 0.20968 0.638781973
RAD23B ENSG00000119318 0.32612 0.37217 -0.93091 0.82405 -0.93091

0.82405 0.80745 0.2097 0.2097 0.638781973
NA ENSG00000250344 0.80746 0.2097 0.26654 0.39491 0.025383 0.48987
-0.97097 0.83422 0.2097 0.638781973
NVL ENSG00000143748 0.80739 0.20972 0.50967 0.30514 0.48541 0.31369
0.60604 0.27224 0.20972 0.638781973
NA ENSG00000232935 0.80732 0.20974 0.80732 0.20974 0.80732 0.20974
0.80732 0.20974 0.20974 0.638781973
NA ENSG00000236777 0.80732 0.20974 0.80732 0.20974 0.80732 0.20974
0.80732 0.20974 0.20974 0.638781973
PLAAT3 ENSG00000176485 -0.62269 0.73325 -1.2853 0.90066 0.80729
0.20975 -1.4286 0.92343 0.20975 0.638781973
FAM117B ENSG00000138439 0.80725 0.20976 -0.953 0.82971 0.077025
0.4693 0.18433 0.42688 0.20976 0.638781973
PTGES2 ENSG00000148334 0.80719 0.20978 0.80719 0.20978 0.80719
0.20978 0.80719 0.20978 0.20978 0.638781973
NA ENSG00000232850 0.80719 0.20978 0.80719 0.20978 0.80719 0.20978
0.80719 0.20978 0.20978 0.638781973
RNU7-165P ENSG00000238380 -0.92494 0.8225 0.80715 0.20979 -1.0836
0.86073 0.32326 0.37325 0.20979 0.638781973
NA ENSG00000261067 0.8071 0.20981 0.8071 0.20981 0.8071 0.20981
0.8071 0.20981 0.20981 0.63879349
NA ENSG00000270447 0.79407 0.21358 0.80705 0.20982 0.79407 0.21358
0.79407 0.21358 0.20982 0.63879349
NA ENSG00000236583 0.80695 0.20985 0.76283 0.22278 0.28944 0.38612
0.60523 0.27251 0.20985 0.638844912
HKDC1 ENSG00000156510 -0.18205 0.57223 0.16203 0.43564 -0.98527 0.83775
0.80689 0.20987 0.20987 0.638865886
NKG7 ENSG00000105374 0.80677 0.2099 0.80677 0.2099 0.80677 0.2099
0.80677 0.2099 0.2099 0.638867922
NA ENSG00000240687 0.80676 0.2099 0.42052 0.33705 0.013474 0.49462
0.013474 0.49462 0.2099 0.638867922
NA ENSG00000267630 -0.54481 0.70706 0.80673 0.20991 0.80673 0.20991
0.80673 0.20991 0.20991 0.638867922
RNU6-830P ENSG00000252162 0.80649 0.20998 -0.40852 0.65856 0.80649
0.20998 0.80649 0.20998 0.20998 0.638974749
CRYGFP ENSG00000232799 0.28416 0.38814 -1.6794 0.95346 -0.356
0.63908 0.80644 0.20999 0.20999 0.638974749
NA ENSG00000266153 0.45032 0.32624 0.12973 0.44839 0.80642 0.21
-0.18397 0.57298 0.21 0.638974749
RAB28 ENSG00000157869 -0.34878 0.63637 0.80632 0.21003 -0.20719 0.58207
-1.0714 0.858 0.21003 0.638974749
NA ENSG00000249253 0.28815 0.38662 0.80629 0.21004 -0.13519 0.55377
-0.86483 0.80643 0.21004 0.638974749
RNU7-10P ENSG00000271841 0.80628 0.21004 0.80628 0.21004 0.80628
0.21004 0.80628 0.21004 0.21004 0.638974749
BTG4 ENSG00000137707 0.65979 0.25469 0.65979 0.25469 -0.63697 0.73793
0.80624 0.21005 0.21005 0.638974749
NA ENSG00000221011 -0.41789 0.66199 0.80626 0.21005 -0.60533 0.72752
-0.0087023 0.50347 0.21005 0.638974749
C1orf147 ENSG00000162888 0.80609 0.2101 0.80609 0.2101 -0.71427
0.76247 0.80609 0.2101 0.2101 0.639037592
WHSC1L2P ENSG00000267253 0.80607 0.2101 0.80607 0.2101 0.80607
0.2101 0.017758 0.49292 0.2101 0.639037592
MTND5P17 ENSG00000236626 -0.028541 0.51138 -1.4255 0.92299 0.80603
0.21011 0.80603 0.21011 0.21011 0.639037592
MAT2A ENSG00000168906 -0.2942 0.6157 -0.0091811 0.50366 0.26461 0.39566
0.8059 0.21015 0.21015 0.63911936
NA ENSG00000252409 0.80578 0.21018 0.2578 0.39828 0.2578 0.39828
0.80578 0.21018 0.21018 0.639161229
TMEM213 ENSG00000214128 0.80576 0.21019 -0.20433 0.58095 -0.20433
0.58095 -0.20433 0.58095 0.21019 0.639161229
CNN3 ENSG00000117519 0.44224 0.32916 0.80569 0.21021 0.44224 0.32916

0.44224 0.32916 0.21021 0.63918216
NA ENSG00000254569 0.047707 0.48097 -0.53198 0.70263 -0.31633 0.62412
0.80555 0.21025 0.21025 0.639263898
LINC00411 ENSG0000229599 0.80546 0.21028 -0.40352 0.65672 -1.2981
0.90287 -0.40352 0.65672 0.21028 0.63930574
MTRNR2L9 ENSG00000255633 0.80542 0.21029 -0.28847 0.61351 -1.2657
0.89719 0.14279 0.44323 0.21029 0.63930574
TRIM21 ENSG00000132109 -0.15185 0.56035 0.80535 0.21031 -0.27605
0.60875 0.56724 0.28528 0.21031 0.639307691
NA ENSG00000221031 -0.048556 0.51936 0.80532 0.21032 -0.94098 0.82664
-0.2892 0.61379 0.21032 0.639307691
FAM3B ENSG00000183844 0.53865 0.29506 0.80528 0.21033 0.74896 0.22694
-0.2708 0.60673 0.21033 0.639307691
NA ENSG00000216802 0.80521 0.21035 0.80521 0.21035 0.80521 0.21035
0.80521 0.21035 0.21035 0.639309641
ABLIM1 ENSG00000099204 -0.4848 0.68609 -0.28456 0.61201 0.55373
0.28988 0.80518 0.21036 0.21036 0.639309641
ALDH1L1-AS2 ENSG00000246022 -0.24608 0.59719 0.80513 0.21037 -0.4455
0.67202 -0.4455 0.67202 0.21037 0.639309641
NA ENSG00000279474 0.80502 0.2104 0.80502 0.2104 0.80502 0.2104
0.80502 0.2104 0.2104 0.639323017
NA ENSG00000234352 0.80493 0.21043 0.047407 0.48109 -0.7376 0.76962
0.35933 0.35968 0.21043 0.639323017
BBC3 ENSG00000105327 -0.77194 0.77993 -1.2935 0.90207 0.8049 0.21044
-1.2935 0.90207 0.21044 0.639323017
NA ENSG00000219273 0.8049 0.21044 0.8049 0.21044 0.8049 0.21044
0.8049 0.21044 0.21044 0.639323017
RN7SL446P ENSG00000244544 0.80491 0.21044 0.80491 0.21044 0.80491
0.21044 0.80491 0.21044 0.21044 0.639323017
RNU6-642P ENSG00000200029 0.19126 0.42416 0.75674 0.2246 0.80477
0.21048 0.80477 0.21048 0.21048 0.639347803
LINC01712 ENSG00000231364 -1.384 0.91682 -0.30612 0.62024 0.80477
0.21048 0.57879 0.28136 0.21048 0.639347803
LINC01738 ENSG00000227947 -0.15076 0.55992 -3.0665 0.99892 -3.0665
0.99892 0.80463 0.21052 0.21052 0.639347803
NA ENSG00000243062 -0.40698 0.65799 -0.40698 0.65799 -0.40698 0.65799
0.80461 0.21052 0.21052 0.639347803
NA ENSG00000265890 0.80462 0.21052 0.80462 0.21052 0.80462 0.21052
0.80462 0.21052 0.21052 0.639347803
IL1R1 ENSG00000115594 -0.42306 0.66388 0.63819 0.26167 0.80453 0.21054
0.19807 0.4215 0.21054 0.639347803
NA ENSG00000282827 0.80455 0.21054 0.80455 0.21054 0.80455 0.21054
0.80455 0.21054 0.21054 0.639347803
NA ENSG00000226286 0.2334 0.40772 0.80433 0.2106 0.18058 0.42835
0.3415 0.36636 0.2106 0.639382041
WDR48 ENSG00000114742 -1.3836 0.91676 -1.3836 0.91676 0.80431 0.21061
-0.46267 0.6782 0.21061 0.639382041
FCER1G ENSG00000158869 0.80431 0.21061 0.80431 0.21061 0.80431
0.21061 0.80431 0.21061 0.21061 0.639382041
FOXF2 ENSG00000137273 -0.67416 0.7499 0.80426 0.21062 -0.1509 0.55997
-0.885 0.81192 0.21062 0.639382041
ATP6V0CP3 ENSG00000213435 0.80428 0.21062 0.80428 0.21062 0.80428
0.21062 0.80428 0.21062 0.21062 0.639382041
TNRC6C-AS1 ENSG00000204282 0.80425 0.21063 0.80425 0.21063 0.80425
0.21063 0.80425 0.21063 0.21063 0.639382041
TIMM8BP1 ENSG00000236258 -0.45255 0.67456 0.15112 0.43994 0.8041
0.21067 0.8041 0.21067 0.21067 0.639463629
NA ENSG00000254573 -0.31999 0.62551 0.80386 0.21074 0.16263 0.43541
0.082663 0.46706 0.21074 0.639636263
NA ENSG00000273320 0.80368 0.21079 0.80368 0.21079 0.80368 0.21079
0.80368 0.21079 0.21079 0.639748175
CYCSP55 ENSG00000214810 -1.04 0.85083 0.80353 0.21083 -1.3604 0.91315

-0.0072751 0.5029 0.21083 0.639755632
NA ENSG00000264227 0.80355 0.21083 0.80355 0.21083 0.78853 0.21519
0.80355 0.21083 0.21083 0.639755632
HAUS4 ENSG00000092036 0.80347 0.21085 0.64606 0.25912 0.80347 0.21085
0.67672 0.24929 0.21085 0.639755632
SH2B1 ENSG00000178188 0.51371 0.30373 0.78279 0.21688 0.80343 0.21086
0.75404 0.22541 0.21086 0.639755632
LCN9 ENSG00000148386 0.35282 0.36211 -0.74002 0.77036 0.13463 0.44645
0.80335 0.21089 0.21089 0.639755632
SMAD3 ENSG00000166949 0.80322 0.21092 0.51001 0.30502 0.80322 0.21092
0.56937 0.28455 0.21092 0.639755632
MCHR1 ENSG00000128285 0.80318 0.21093 0.80318 0.21093 0.80318 0.21093
0.80318 0.21093 0.21093 0.639755632
IGLL3P ENSG00000206066 0.76186 0.22307 0.55822 0.28835 0.052969
0.47888 0.80319 0.21093 0.21093 0.639755632
MIR570 ENSG00000207650 0.80316 0.21094 0.80316 0.21094 0.80316
0.21094 0.80316 0.21094 0.21094 0.639755632
NA ENSG00000226509 0.80317 0.21094 -0.48052 0.68457 -0.58351 0.72022
-1.0664 0.85687 0.21094 0.639755632
NA ENSG00000274653 0.80317 0.21094 0.80317 0.21094 0.80317 0.21094
0.80317 0.21094 0.21094 0.639755632
HIGD1C ENSG00000214511 -0.36446 0.64224 -0.10962 0.54365 0.80315
0.21095 0.02724 0.48913 0.21095 0.639755632
PTGES ENSG00000148344 0.80289 0.21102 -0.014884 0.50594 0.80289 0.21102
-0.30307 0.61908 0.21102 0.639899608
PEF1-AS1 ENSG00000235790 0.80284 0.21103 0.80284 0.21103 0.80284
0.21103 0.80284 0.21103 0.21103 0.639899608
CD37 ENSG00000104894 0.80279 0.21105 0.80279 0.21105 0.80279 0.21105
0.61144 0.27046 0.21105 0.639899608
TMEM198B ENSG00000182796 0.80277 0.21105 0.70312 0.24099 0.70312
0.24099 0.70312 0.24099 0.21105 0.639899608
ZNF580 ENSG00000213015 0.80263 0.21109 0.80263 0.21109 0.80263
0.21109 0.80263 0.21109 0.21109 0.639981068
NEFHP1 ENSG00000223489 0.80249 0.21113 0.65487 0.25628 -0.53514
0.70372 0.29609 0.38358 0.21113 0.640062517
NA ENSG00000250081 -0.73164 0.76781 -0.57978 0.71897 -0.083677 0.53334
0.80218 0.21123 0.21123 0.64028601
NA ENSG00000272462 0.80216 0.21123 0.36821 0.35636 0.36821 0.35636
0.68771 0.24582 0.21123 0.64028601
MTCO1P14 ENSG00000232551 -0.79905 0.78787 -0.79905 0.78787 0.30516
0.38012 0.80207 0.21126 0.21126 0.640337115
NA ENSG00000240015 0.80195 0.21129 -1.0615 0.85577 0.80195 0.21129
0.80195 0.21129 0.21129 0.640388214
MIR3928 ENSG00000264141 0.80174 0.21135 0.80174 0.21135 0.80174
0.21135 0.80174 0.21135 0.21135 0.640524133
TNS4 ENSG00000131746 0.80164 0.21138 0.57753 0.28179 0.80164 0.21138
0.80164 0.21138 0.21138 0.640524133
NA ENSG00000214772 0.80157 0.2114 -0.58074 0.71929 0.80157 0.2114
0.80157 0.2114 0.2114 0.640524133
ZMYND19P1 ENSG00000258917 0.80156 0.2114 0.80156 0.2114 0.80156
0.2114 0.80156 0.2114 0.2114 0.640524133
NA ENSG00000253764 0.80154 0.21141 0.80154 0.21141 0.80154 0.21141
0.80154 0.21141 0.21141 0.640524133
SERF2 ENSG00000140264 0.80148 0.21143 0.80148 0.21143 0.80148 0.21143
-0.38172 0.64866 0.21143 0.640524133
RPS3AP54 ENSG00000229057 -0.60477 0.72733 -0.60477 0.72733 0.80146
0.21143 -0.60477 0.72733 0.21143 0.640524133
NA ENSG00000259950 0.80144 0.21144 0.80144 0.21144 0.80144 0.21144
0.80144 0.21144 0.21144 0.640524133
SERTAD2 ENSG00000179833 -0.88327 0.81146 0.80134 0.21147 -0.096328
0.53837 -0.32711 0.62821 0.21147 0.640535374
BPIFA4P ENSG00000183566 0.80134 0.21147 -0.076195 0.53037 -0.78231

							0.78298	-0.076195	0.53037	0.21147	0.640535374
RNF10	ENSG00000022840	0.80125	0.21149	0.48114	0.31521	-0.41085	0.65941	-0.40674	0.6579	0.21149	0.640546613
SLCO1B3-SLCO1B7	ENSG00000257046	0.80124	0.2115	0.18886	0.4251	0.18886	0.4251	0.18886	0.4251	0.2115	0.640546613
NA	ENSG00000225726	0.55334	0.29001	0.75689	0.22456	0.8011	0.21154	-0.69771	0.75732	0.21154	0.640627941
NA	ENSG00000259681	0.80094	0.21158	0.80094	0.21158	0.80094	0.21158	0.80094	0.21158	0.21158	0.640666677
MAP4K3	ENSG00000011566	0.56997	0.28435	0.8009	0.2116	0.64326	0.26003	0.54097	0.29427	0.2116	0.640666677
KRTAP21-1	ENSG00000187005	0.80082	0.21162	0.80082	0.21162	0.68435	0.24688	0.80082	0.21162	0.21162	0.640666677
CUTC	ENSG00000119929	-1.4481	0.9262	0.15393	0.43883	0.15393	0.43883	0.80076	0.21163	0.21163	0.640666677
AMT	ENSG00000145020	0.80076	0.21164	0.80076	0.21164	0.80076	0.21164	-0.75878	0.77601	0.21164	0.640666677
MICAL2	ENSG00000133816	0.80057	0.21169	-0.59764	0.72496	-0.56613	0.71435	-0.84578	0.80116	0.21169	0.640666677
TSPAN2	ENSG00000134198	0.6322	0.26363	0.80056	0.21169	0.76969	0.22074	0.76969	0.22074	0.21169	0.640666677
FAM168B	ENSG00000152102	0.80054	0.2117	-1.4441	0.92565	-0.0090543	0.50361	0.16491	0.43451	0.2117	0.640666677
LARP1BP1	ENSG00000231442	0.62015	0.26758	0.71955	0.2359	0.71955	0.2359	0.80055	0.2117	0.2117	0.640666677
RPS15AP16	ENSG00000239483	0.8005	0.21171	0.8005	0.21171	0.8005	0.21171	0.8005	0.21171	0.21171	0.640666677
ZNF883	ENSG00000228623	-0.2552	0.60072	-0.70408	0.75931	0.32992	0.37073	0.80042	0.21173	0.21173	0.640666677
NA	ENSG00000248762	0.80045	0.21173	-0.092882	0.537	0.21128	0.41633	0.21128	0.41633	0.21173	0.640666677
TTC30A	ENSG00000197557	0.8004	0.21174	0.8004	0.21174	0.8004	0.21174	0.8004	0.21174	0.21174	0.640666677
TAPBPL	ENSG00000139192	0.80038	0.21175	-0.77438	0.78065	0.6674	0.25226	-1.6685	0.95239	0.21175	0.640666677
NA	ENSG00000232056	-0.42937	0.66617	0.80035	0.21175	0.11765	0.45317	0.44207	0.32922	0.21175	0.640666677
NA	ENSG00000230968	0.22067	0.41267	0.80028	0.21177	-0.26605	0.6049	0.080421	0.46795	0.21177	0.640687409
NA	ENSG00000279649	0.80019	0.2118	0.80019	0.2118	0.80019	0.2118	0.80019	0.2118	0.2118	0.640738391
NA	ENSG00000280089	0.80011	0.21182	0.80011	0.21182	0.80011	0.21182	0.80011	0.21182	0.21182	0.640759116
NA	ENSG00000225881	-0.23949	0.59464	-0.23949	0.59464	-0.23949	0.59464	0.79999	0.21186	0.21186	0.640827909
GLP2R	ENSG00000065325	0.79987	0.21189	0.23751	0.40613	0.57092	0.28403	0.55032	0.29105	0.21189	0.640827909
SFXN2	ENSG00000156398	-0.3985	0.65487	0.51427	0.30353	-0.23964	0.59469	0.79988	0.21189	0.21189	0.640827909
NA	ENSG00000267275	0.79973	0.21193	0.79973	0.21193	0.24661	0.4026	0.24661	0.4026	0.21193	0.640827909
NA	ENSG00000214669	-0.85119	0.80267	0.59962	0.27438	0.79971	0.21194	0.79971	0.21194	0.21194	0.640827909
GPX2	ENSG00000176153	0.79968	0.21195	0.79968	0.21195	0.79968	0.21195	0.79968	0.21195	0.21195	0.640827909
GAPDHP46	ENSG00000224678	-1.6996	0.95539	0.79966	0.21195	-0.59267	0.7233	-0.59267	0.7233	0.21195	0.640827909
TSN	ENSG00000211460	0.79952	0.21199	0.79952	0.21199	0.79952	0.21199	-0.6437	0.74011	0.21199	0.640827909
RPL12P8	ENSG00000219932	0.74396	0.22845	0.79955	0.21199	0.74396	0.22845	0.69591	0.24324	0.21199	0.640827909
AKR1A1	ENSG00000117448	0.79949	0.212	0.0021702	0.49913	0.0021702	0.49913				

-1.2465 0.89371 0.212 0.640827909
MEX3B ENSG00000183496 0.57465 0.28276 0.79947 0.21201 0.09776 0.46106
-0.2932 0.61532 0.21201 0.640827909
BRD1 ENSG00000100425 0.79942 0.21202 -0.65098 0.74247 0.31581 0.37608
0.28132 0.38923 0.21202 0.640827909
ELN ENSG00000049540 0.22141 0.41239 0.25312 0.40009 -1.3169 0.90606
0.79939 0.21203 0.21203 0.640827909
CHST9 ENSG00000154080 -1.2855 0.90068 0.056575 0.47744 0.79941 0.21203
0.19944 0.42096 0.21203 0.640827909
NA ENSG00000257726 0.79935 0.21204 0.47167 0.31858 0.40745 0.34184
0.019792 0.4921 0.21204 0.640827909
TRDN-AS1 ENSG00000235535 0.48954 0.31223 0.79925 0.21207 -0.089186
0.53553 0.53463 0.29645 0.21207 0.640839086
NA ENSG00000251409 0.79924 0.21207 0.79924 0.21207 0.79924 0.21207
0.79924 0.21207 0.21207 0.640839086
LINC01079 ENSG00000229609 0.60437 0.2728 0.036154 0.48558 -0.82464
0.79521 0.79919 0.21209 0.21209 0.64085026
NA ENSG00000271947 0.79917 0.2121 -0.10115 0.54029 -0.31061 0.62195
-0.38987 0.65168 0.2121 0.64085026
TRPV5 ENSG00000127412 0.69974 0.24204 0.69974 0.24204 0.69974 0.24204
0.79879 0.21221 0.21221 0.641077737
NA ENSG00000253848 0.79873 0.21222 0.79873 0.21222 0.79873 0.21222
0.79873 0.21222 0.21222 0.641077737
RN7SKP59 ENSG00000222452 0.7987 0.21223 -0.12317 0.54902 0.62096
0.26731 -0.12317 0.54902 0.21223 0.641077737
NA ENSG00000246203 0.79867 0.21224 0.79867 0.21224 0.79867 0.21224
0.79867 0.21224 0.21224 0.641077737
MISP3 ENSG00000141854 -0.29902 0.61754 0.79859 0.21226 -0.23887 0.5944
0.79859 0.21226 0.21226 0.641077737
B3GAT3P1 ENSG00000244009 0.79852 0.21228 0.79852 0.21228 0.79852
0.21228 0.5144 0.30349 0.21228 0.641077737
NA ENSG00000251332 -0.51619 0.69714 -0.51619 0.69714 0.79852 0.21228
0.79852 0.21228 0.21228 0.641077737
TCEA3 ENSG00000204219 0.55361 0.28992 0.18567 0.42635 0.7985 0.21229
-0.43629 0.66869 0.21229 0.641077737
LINC02070 ENSG00000241328 -0.31229 0.62259 -0.31229 0.62259 -0.31229
0.62259 0.79848 0.2123 0.2123 0.641077737
NA ENSG00000202283 0.79839 0.21232 -1.0221 0.84664 -0.14094 0.55604
-0.14094 0.55604 0.21232 0.641077737
VTRNA2-1 ENSG00000270123 0.064872 0.47414 0.79838 0.21232 -0.33867
0.63257 -0.54653 0.70765 0.21232 0.641077737
NA ENSG00000207069 0.58753 0.27842 0.53542 0.29618 0.59044 0.27745
0.79833 0.21234 0.21234 0.641098401
HSD3BP2 ENSG00000203858 -0.27176 0.6071 0.76983 0.2207 0.79825
0.21236 -0.60075 0.726 0.21236 0.641119063
DENND2B ENSG00000166444 -0.99242 0.8395 -0.77717 0.78147 0.79816
0.21239 -1.3452 0.91072 0.21239 0.641169911
RERG-AS1 ENSG00000255660 0.79808 0.21241 0.79808 0.21241 0.25256
0.4003 0.2075 0.41781 0.21241 0.641190566
S100A14 ENSG00000189334 0.79796 0.21245 0.79796 0.21245 0.79796
0.21245 0.79796 0.21245 0.21245 0.641271587
NOL8 ENSG00000198000 0.10733 0.45726 0.22176 0.41225 -0.4916 0.6885
0.79782 0.21249 0.21249 0.641312876
LINC01947 ENSG00000254130 0.79782 0.21249 0.79782 0.21249 0.79782
0.21249 -0.45607 0.67583 0.21249 0.641312876
LINC01425 ENSG00000233997 0.79769 0.21252 -0.62628 0.73443 0.79769
0.21252 0.66971 0.25152 0.21252 0.641363696
NA ENSG00000235523 0.79756 0.21256 0.11223 0.45532 0.75343 0.2256
-0.069405 0.52767 0.21256 0.641425598
ENTR1 ENSG00000165689 0.62963 0.26447 0.79753 0.21257 -0.38023 0.64811
-0.38023 0.64811 0.21257 0.641425598
KRT18P15 ENSG00000234737 0.57125 0.28392 0.3925 0.34734 0.7975

0.21258 -0.0081991 0.50327 0.21258 0.641425598
FAM229B ENSG00000203778 0.79739 0.21261 0.79739 0.21261 -0.16023
0.56365 0.79739 0.21261 0.21261 0.64142714
NA ENSG00000201340 0.79737 0.21262 0.79737 0.21262 0.79737 0.21262
0.79737 0.21262 0.21262 0.64142714
RNA5SP339 ENSG00000212289 0.79735 0.21262 0.79735 0.21262 0.79735
0.21262 0.79735 0.21262 0.21262 0.64142714
TFAP2A ENSG00000137203 0.65 0.25785 -1.2586 0.89591 0.79711 0.21269
0.45129 0.32589 0.21269 0.641579494
MAP3K8 ENSG00000107968 -0.14185 0.5564 -0.62747 0.73482 -0.14185
0.5564 0.79707 0.2127 0.2127 0.641579494
RNU1-11P ENSG00000206702 0.79704 0.21271 0.79704 0.21271 -1.1371
0.87225 0.76515 0.22209 0.21271 0.641579494
RAD17P1 ENSG00000232400 0.79687 0.21276 0.79687 0.21276 0.79687
0.21276 0.79687 0.21276 0.21276 0.641671477
CHD1L ENSG00000131778 0.58284 0.28 0.58073 0.28071 0.3629 0.35834
0.79684 0.21277 0.21277 0.641671477
NA ENSG00000232355 -0.14687 0.55838 0.79682 0.21278 -1.571 0.94191
-0.00054275 0.50022 0.21278 0.641671477
SDSL ENSG00000139410 -0.55284 0.70981 0.79669 0.21281 -0.25402 0.60026
-0.96427 0.83254 0.21281 0.641682526
RNU6-970P ENSG00000252121 0.79671 0.21281 -1.204 0.8857 -1.204
0.8857 -1.204 0.8857 0.21281 0.641682526
NA ENSG00000267615 0.79659 0.21284 0.79659 0.21284 0.79659 0.21284
0.79659 0.21284 0.21284 0.641733276
NPHS2 ENSG00000116218 0.49293 0.31103 0.25324 0.40004 0.79642 0.21289
0.48775 0.31286 0.21289 0.641826664
GABPA ENSG00000154727 0.78053 0.21754 -0.91768 0.82061 0.79639 0.2129
-0.72071 0.76445 0.2129 0.641826664
ALOXE3 ENSG00000179148 0.7963 0.21293 0.7963 0.21293 0.7963
0.21293 0.7963 0.21293 0.21293 0.641826664
FAM214B ENSG00000005238 -0.22929 0.59068 -0.173 0.56867 0.79626
0.21294 0.024531 0.49021 0.21294 0.641826664
RNU7-92P ENSG00000238785 0.1554 0.43825 -0.059496 0.52372 0.79626
0.21294 -0.75383 0.77452 0.21294 0.641826664
NA ENSG00000259819 -0.2662 0.60496 0.79623 0.21295 -1.6066 0.94593
-1.6066 0.94593 0.21295 0.641826664
FUT5 ENSG00000130383 0.79617 0.21297 0.32742 0.37167 -0.12159 0.54839
-0.12159 0.54839 0.21297 0.641840616
TRDD3 ENSG00000228985 0.79608 0.21299 0.79608 0.21299 0.79608 0.21299
0.79608 0.21299 0.21299 0.641840616
NA ENSG00000256810 0.79602 0.21301 -1.0177 0.84559 -0.23042 0.59112
0.57706 0.28195 0.21301 0.641840616
NA ENSG00000281016 -0.53747 0.70453 0.79601 0.21301 0.79601 0.21301
0.79601 0.21301 0.21301 0.641840616
RNU6-680P ENSG00000207333 0.79587 0.21305 0.79587 0.21305 0.79587
0.21305 0.79587 0.21305 0.21305 0.641840616
LINC00519 ENSG00000258955 0.10314 0.45892 0.79587 0.21305 0.06352
0.47468 -0.97192 0.83445 0.21305 0.641840616
RXFP4 ENSG00000173080 0.79586 0.21306 0.66926 0.25167 0.66926 0.25167
0.66926 0.25167 0.21306 0.641840616
UMODL1 ENSG00000177398 0.79586 0.21306 -0.64367 0.74011 -1.5869
0.94373 -0.75766 0.77567 0.21306 0.641840616
MIR544A ENSG00000207587 0.79578 0.21308 0.79578 0.21308 0.79578
0.21308 0.79578 0.21308 0.21308 0.641858933
NA ENSG00000283415 0.24235 0.40425 -0.35968 0.64046 0.29093 0.38555
0.79573 0.2131 0.2131 0.641858933
NA ENSG00000267146 0.37909 0.35231 0.7956 0.21313 -0.73099 0.76761
-0.99301 0.83965 0.21313 0.641858933
PRR13P2 ENSG00000254884 0.7955 0.21316 0.7955 0.21316 0.7955
0.21316 0.7955 0.21316 0.21316 0.641858933
CIR1P2 ENSG00000250215 0.79547 0.21317 0.79547 0.21317 0.79547

0.21317 0.79547 0.21317 0.21317 0.641858933
NA ENSG00000275785 0.38641 0.3496 0.79546 0.21317 0.22546 0.41081
-0.018527 0.50739 0.21317 0.641858933
MIR3185 ENSG00000263602 0.79536 0.2132 0.79536 0.2132 0.79536
0.2132 0.79536 0.2132 0.2132 0.641858933
PA2G4P6 ENSG00000231799 0.79534 0.21321 -1.2748 0.89881 0.79534
0.21321 0.79534 0.21321 0.21321 0.641858933
FSHB ENSG00000131808 0.50158 0.30798 0.50158 0.30798 0.031061 0.48761
0.79531 0.21322 0.21322 0.641858933
CAMK1G ENSG00000008118 0.061749 0.47538 -0.40053 0.65562 -0.11672
0.54646 0.79528 0.21323 0.21323 0.641858933
CFAP206 ENSG00000272514 0.79522 0.21324 0.79522 0.21324 0.79522
0.21324 0.79522 0.21324 0.21324 0.641858933
LINC01957 ENSG00000251542 0.79518 0.21325 0.025436 0.48985 0.79518
0.21325 0.79518 0.21325 0.21325 0.641858933
BUB3 ENSG00000154473 -0.26372 0.604 -1.5243 0.93628 0.79513 0.21327
-0.26372 0.604 0.21327 0.641858933
PMF1 ENSG00000160783 -0.076735 0.53058 -0.13069 0.55199 -0.13069 0.55199
0.79511 0.21327 0.21327 0.641858933
NA ENSG00000228714 0.49663 0.30973 0.36499 0.35756 0.79506 0.21329
0.79506 0.21329 0.21329 0.641858933
HSPA8P9 ENSG00000241478 0.20117 0.42028 0.67484 0.24989 0.79506
0.21329 -0.77475 0.78076 0.21329 0.641858933
RNA5SP444 ENSG00000252653 -0.93542 0.82521 0.79507 0.21329 -0.15705
0.5624 0.41673 0.33844 0.21329 0.641858933
ARL6IP1P2 ENSG00000225355 -0.4468 0.67249 -0.82721 0.79594 0.79497
0.21332 -0.25546 0.60082 0.21332 0.641880935
RPS14 ENSG00000164587 0.79488 0.21334 -0.13185 0.55245 0.010421 0.49584
0.13536 0.44616 0.21334 0.641880935
NA ENSG00000240458 -0.34774 0.63598 0.34897 0.36355 -2.1995 0.98608
0.79489 0.21334 0.21334 0.641880935
NA ENSG00000248778 0.79484 0.21335 0.79484 0.21335 0.79484 0.21335
0.79484 0.21335 0.21335 0.641880935
SLC22A16 ENSG00000004809 -0.17909 0.57107 0.65644 0.25577 0.79468
0.2134 0.024625 0.49018 0.2134 0.641991727
MIR4436A ENSG00000265510 0.7946 0.21342 0.7946 0.21342 0.7946
0.21342 0.7946 0.21342 0.21342 0.64201226
C14orf132 ENSG00000227051 0.77187 0.22009 -0.81024 0.7911 0.79441
0.21348 -1.1426 0.8734 0.21348 0.642153111
NA ENSG00000250418 -0.39998 0.65542 0.63227 0.26361 -1.259 0.89599
0.79435 0.2135 0.2135 0.642154515
MUC15 ENSG00000169550 0.79428 0.21352 0.79428 0.21352 0.79428 0.21352
-0.54875 0.70841 0.21352 0.642154515
NA ENSG00000256304 0.79426 0.21352 0.79426 0.21352 0.79426 0.21352
0.79426 0.21352 0.21352 0.642154515
RSL24D1P6 ENSG00000224452 0.79416 0.21355 -0.011568 0.50462 0.60937
0.27114 0.79416 0.21355 0.21355 0.642195545
RPS15AP13 ENSG00000228568 0.79414 0.21356 0.79414 0.21356 0.79414
0.21356 0.67321 0.25041 0.21356 0.642195545
LINC02707 ENSG00000255427 0.13728 0.4454 0.794 0.2136 0.061473 0.47549
0.13728 0.4454 0.2136 0.642199729
PLA2G6 ENSG00000184381 0.17964 0.42872 0.17964 0.42872 0.14039
0.44417 0.79394 0.21361 0.21361 0.642199729
LHX6 ENSG00000106852 -0.43102 0.66677 -0.96544 0.83284 0.79388 0.21363
-0.96544 0.83284 0.21363 0.642199729
TMEM255B ENSG00000184497 0.67909 0.24854 0.79385 0.21364 0.72461
0.23435 -0.50019 0.69153 0.21364 0.642199729
BORCS6 ENSG00000196544 0.79382 0.21365 -0.063247 0.52521 0.49517
0.31024 0.16058 0.43621 0.21365 0.642199729
NA ENSG00000233457 0.79382 0.21365 0.79382 0.21365 0.65122 0.25745
0.79382 0.21365 0.21365 0.642199729
YRDCP2 ENSG00000236745 0.79378 0.21366 0.79378 0.21366 0.79378

0.21366 0.79378 0.21366 0.21366 0.642199729
NA ENSG00000251537 -1.7613 0.9609 0.14545 0.44218 -1.0695 0.85758
0.79376 0.21367 0.21367 0.642199729
LINC01646 ENSG00000232596 0.79371 0.21368 -0.26564 0.60474 0.66664
0.2525 0.020404 0.49186 0.21368 0.642199729
FOXN4 ENSG00000139445 -0.73704 0.76945 -0.73704 0.76945 -1.1833 0.88166
0.79362 0.21371 0.21371 0.642201122
FAM72A ENSG00000196550 0.34941 0.36339 -0.68453 0.75318 -0.5667
0.71454 0.79359 0.21372 0.21372 0.642201122
PCDHGC4 ENSG00000242419 0.79359 0.21372 0.0292 0.48835 0.79359
0.21372 0.79359 0.21372 0.21372 0.642201122
SLC6A1 ENSG00000157103 0.20064 0.42049 0.79345 0.21376 0.75924
0.22385 0.42404 0.33577 0.21376 0.642213457
NA ENSG00000270868 0.79343 0.21376 -0.46938 0.6806 0.51607 0.3029
-0.075099 0.52993 0.21376 0.642213457
XXYLT1-AS2 ENSG00000230266 0.79338 0.21378 0.59358 0.2764 -0.42222
0.66357 0.79338 0.21378 0.21378 0.642213457
RN7SL36P ENSG00000244314 0.61787 0.26833 -0.077571 0.53092 0.79338
0.21378 0.79338 0.21378 0.21378 0.642213457
NA ENSG00000231139 0.79334 0.21379 0.79334 0.21379 -0.12496 0.54972
0.53246 0.2972 0.21379 0.642213457
ADCY10P1 ENSG00000161912 -0.094744 0.53774 -0.46163 0.67783 0.79326
0.21381 -1.9062 0.97169 0.21381 0.642233946
NA ENSG00000216184 0.79321 0.21383 -0.96575 0.83292 0.11991 0.45228
-0.062077 0.52475 0.21383 0.642254432
SMG7 ENSG00000116698 -0.068239 0.5272 0.79315 0.21385 -0.78397 0.78347
-0.11615 0.54623 0.21385 0.642274915
LINC02577 ENSG00000228742 -0.27677 0.60902 0.79292 0.21391 -0.44915
0.67334 -0.77165 0.77984 0.21391 0.642339085
HELLPAR ENSG00000281344 0.79288 0.21392 0.61256 0.27008 0.73041
0.23257 0.67624 0.24945 0.21392 0.642339085
EXOC2 ENSG00000112685 -0.56054 0.71244 -0.19304 0.57653 0.019509 0.49222
0.79284 0.21394 0.21394 0.642339085
HSPB2 ENSG00000170276 0.79281 0.21394 0.79281 0.21394 0.79281 0.21394
0.79281 0.21394 0.21394 0.642339085
GPR157 ENSG00000180758 -0.94562 0.82783 0.6691 0.25171 -1.0714 0.858
0.79283 0.21394 0.21394 0.642339085
DPPA2 ENSG00000163530 -0.54492 0.7071 -0.54492 0.7071 0.79268 0.21398
-1.5659 0.94131 0.21398 0.642339085
NA ENSG00000250503 0.60526 0.2725 0.79267 0.21398 0.36972 0.35579
0.34124 0.36646 0.21398 0.642339085
NA ENSG00000254811 0.79268 0.21398 0.054274 0.47836 0.41471 0.33918
0.55592 0.28913 0.21398 0.642339085
SEPTIN7P1 ENSG00000259090 0.79267 0.21399 -0.15995 0.56354 0.16409
0.43483 -0.42791 0.66564 0.21399 0.642339085
NA ENSG00000250159 -0.48921 0.68765 0.26991 0.39361 0.58003 0.28095
0.79248 0.21404 0.21404 0.642446773
NA ENSG00000249710 0.79241 0.21406 0.79241 0.21406 0.79241 0.21406
0.79241 0.21406 0.21406 0.642446773
CPA4 ENSG00000128510 0.79236 0.21407 0.26826 0.39425 -0.883 0.81138
0.032748 0.48694 0.21407 0.642446773
RN7SL268P ENSG00000244425 0.79234 0.21408 0.79234 0.21408 0.79234
0.21408 0.79234 0.21408 0.21408 0.642446773
VAMP2 ENSG00000220205 0.79223 0.21411 0.79223 0.21411 0.79223 0.21411
0.78307 0.21679 0.21411 0.642446773
NA ENSG00000255029 -0.036212 0.51444 0.79226 0.21411 0.79226 0.21411
0.79226 0.21411 0.21411 0.642446773
LINC00320 ENSG00000224924 0.10192 0.45941 0.32799 0.37146 0.79219
0.21412 -0.81578 0.79269 0.21412 0.642446773
PUSL1 ENSG00000169972 0.79214 0.21414 0.79214 0.21414 0.79214 0.21414
0.79214 0.21414 0.21414 0.642446773
PSKH2 ENSG00000147613 -1.2022 0.88536 0.79202 0.21417 -0.45562 0.67567

-0.49185 0.68859 0.21417 0.642446773
NA ENSG00000269918 0.79203 0.21417 0.79203 0.21417 0.79203 0.21417
0.79203 0.21417 0.21417 0.642446773
NA ENSG00000267674 0.74674 0.22761 0.60999 0.27093 0.79192 0.2142
-0.25969 0.60245 0.2142 0.642446773
SEC13 ENSG00000157020 0.79185 0.21422 0.12332 0.45093 -0.23381 0.59243
0.66812 0.25203 0.21422 0.642446773
NA ENSG00000271781 0.79186 0.21422 0.12804 0.44906 -0.95315 0.82974
-0.95315 0.82974 0.21422 0.642446773
GNB1L ENSG00000185838 0.79184 0.21423 0.6627 0.25376 0.79184 0.21423
-0.68749 0.75411 0.21423 0.642446773
RNU6-586P ENSG00000253074 -0.097605 0.53888 0.79184 0.21423 0.52759
0.29889 0.52759 0.29889 0.21423 0.642446773
ANKRD30BP3 ENSG00000230501 0.7918 0.21424 0.76781 0.2213 -0.64638
0.74098 0.76781 0.2213 0.21424 0.642446773
C20orf197 ENSG00000176659 -0.40494 0.65724 -0.07325 0.5292 0.022565 0.491
0.79178 0.21425 0.21425 0.642446773
ADAM8 ENSG00000151651 -1.2858 0.90074 0.79168 0.21427 -1.2858 0.90074
-1.2858 0.90074 0.21427 0.642457663
PRR16 ENSG00000184838 0.29562 0.38376 -0.29291 0.6152 -0.17917 0.5711
0.79165 0.21428 0.21428 0.642457663
CHAF1B ENSG00000159259 0.4814 0.31512 0.79154 0.21431 -0.43749
0.66912 0.21621 0.41441 0.21431 0.642488983
AKR1B1P7 ENSG00000266899 -0.10749 0.5428 0.79151 0.21432 0.79151
0.21432 0.79151 0.21432 0.21432 0.642488983
RELN ENSG00000189056 -0.41016 0.65916 -1.2037 0.88565 0.7915 0.21433
-0.61938 0.73217 0.21433 0.642488983
NA ENSG00000233256 -0.3741 0.64583 0.79143 0.21435 -0.3741 0.64583
0.69866 0.24238 0.21435 0.642509411
NA ENSG00000224018 0.59835 0.2748 0.79131 0.21438 0.79131 0.21438
0.035532 0.48583 0.21438 0.642559811
NA ENSG00000261538 0.79124 0.2144 0.3599 0.35946 0.79124 0.2144
0.31542 0.37622 0.2144 0.642570683
RNU6-324P ENSG00000200827 0.79121 0.21441 0.79121 0.21441 0.79121
0.21441 0.79121 0.21441 0.21441 0.642570683
RN7SKP283 ENSG00000253006 0.79102 0.21447 -0.33442 0.63097 0.3492
0.36347 0.54635 0.29241 0.21447 0.642701414
PNPLA8 ENSG00000135241 0.37142 0.35516 0.79097 0.21448 0.40204
0.34383 -0.12644 0.55031 0.21448 0.642701414
MIR876 ENSG00000215966 0.79086 0.21451 0.79086 0.21451 0.79086
0.21451 0.79086 0.21451 0.21451 0.642742381
NECAB1 ENSG00000123119 0.79079 0.21453 -0.0194 0.50774 0.71394
0.23763 0.58904 0.27792 0.21453 0.642742381
LINC00960 ENSG00000242516 0.79075 0.21454 -0.13213 0.55256 -0.19463
0.57716 0.030875 0.48768 0.21454 0.642742381
MIR4252 ENSG00000265392 0.79065 0.21457 0.50791 0.30576 -0.13949
0.55547 -0.28605 0.61258 0.21457 0.642742381
R3HDML ENSG00000101074 -2.0406 0.97936 0.57891 0.28132 -2.0501
0.97982 0.79064 0.21458 0.21458 0.642742381
EVC ENSG00000072840 -0.20931 0.5829 0.79051 0.21462 -1.0537 0.85398
-2.338 0.99031 0.21462 0.642742381
NA ENSG00000261430 0.79037 0.21465 0.79037 0.21465 0.79037 0.21465
0.79037 0.21465 0.21465 0.642742381
AKAP1 ENSG00000121057 0.54322 0.29349 0.37277 0.35466 0.7903 0.21468
0.16687 0.43373 0.21468 0.642742381
SCIMP ENSG00000161929 0.59928 0.27449 -0.0051104 0.50204 -0.0051104 0.50204
0.7902 0.21471 0.21471 0.642742381
RNU6-377P ENSG00000251774 0.79015 0.21472 0.79015 0.21472 0.79015
0.21472 0.79015 0.21472 0.21472 0.642742381
MPRIP ENSG00000133030 0.66085 0.25435 0.72546 0.23408 0.79012 0.21473
0.1545 0.43861 0.21473 0.642742381
NA ENSG00000264151 0.79013 0.21473 0.40127 0.34411 -0.58301 0.72006

0.1726 0.43148 0.21473 0.642742381
MIR519C ENSG00000207788 0.79002 0.21476 0.79002 0.21476 0.79002
0.21476 0.79002 0.21476 0.21476 0.642742381
GOLGA8UP ENSG00000103832 0.098977 0.46058 -0.42655 0.66515 0.78996
0.21477 0.1316 0.44765 0.21477 0.642742381
LINC01896 ENSG00000263146 0.17246 0.43154 -0.21233 0.58408 0.78999
0.21477 -0.95522 0.83027 0.21477 0.642742381
NA ENSG00000240040 0.78988 0.2148 -0.6447 0.74044 0.78988 0.2148
0.78988 0.2148 0.2148 0.642742381
LINC02392 ENSG00000258183 0.78987 0.2148 0.74346 0.2286 0.78987
0.2148 0.78987 0.2148 0.2148 0.642742381
SLC25A10 ENSG00000183048 -1.0705 0.85781 -0.94586 0.82789 -0.73655
0.7693 0.78981 0.21482 0.21482 0.642742381
PTP4A1P4 ENSG00000250359 -0.22017 0.58713 0.7898 0.21482 0.49949
0.30872 0.49949 0.30872 0.21482 0.642742381
OTOGL ENSG00000165899 0.31944 0.3747 0.71815 0.23633 0.78978 0.21483
0.49609 0.30992 0.21483 0.642742381
GPR27 ENSG00000170837 0.78978 0.21483 0.78978 0.21483 0.78978 0.21483
0.78978 0.21483 0.21483 0.642742381
ENSAP3 ENSG00000177261 0.61856 0.2681 0.61856 0.2681 0.61856
0.2681 0.78974 0.21484 0.21484 0.642742381
NA ENSG00000251458 0.1594 0.43668 0.78965 0.21487 -0.84021 0.79961
0.12603 0.44986 0.21487 0.642742381
C5orf24 ENSG00000181904 0.78952 0.2149 0.088462 0.46475 0.78952
0.2149 0.27142 0.39304 0.2149 0.642742381
NA ENSG00000279110 0.78951 0.21491 0.73424 0.2314 0.78951 0.21491
0.78951 0.21491 0.21491 0.642742381
PGM3 ENSG00000013375 -0.22983 0.59089 0.7894 0.21494 -0.22983 0.59089
0.015389 0.49386 0.21494 0.642742381
EPYC ENSG00000083782 0.40422 0.34303 0.34072 0.36666 0.7893 0.21497
0.38531 0.35 0.21497 0.642742381
ACTR2 ENSG00000138071 0.099342 0.46043 0.78914 0.21501 0.48799 0.31278
0.057793 0.47696 0.21501 0.642742381
NA ENSG00000220130 0.7891 0.21503 0.70293 0.24105 0.46327 0.32159
0.7891 0.21503 0.21503 0.642742381
NA ENSG00000272088 -0.54515 0.70717 0.78908 0.21503 0.072803 0.47098
0.072803 0.47098 0.21503 0.642742381
PPP1R2P10 ENSG00000213232 -0.16761 0.56656 -1.0348 0.84961 0.78905
0.21504 0.029659 0.48817 0.21504 0.642742381
OR52B4 ENSG00000221996 -0.35336 0.63809 -0.48905 0.6876 -1.2303
0.89071 0.78905 0.21504 0.21504 0.642742381
[truncated: 443,576 more chars]
